# Fermentative Spirochaetes mediate necromass recycling in anoxic hydrocarbon-contaminated habitats

**DOI:** 10.1038/s41396-018-0148-3

**Published:** 2018-05-30

**Authors:** Xiyang Dong, Chris Greening, Thomas Brüls, Ralf Conrad, Kun Guo, Svenja Blaskowski, Farnusch Kaschani, Markus Kaiser, Nidal Abu Laban, Rainer U. Meckenstock

**Affiliations:** 1 University of Duisburg-Essen, Biofilm Centre, Universitätsstrasse 5, 45141 Essen, Germany; 2 Helmholtz Zentrum München, Institute of Groundwater Ecology, Ingolstädter Landstrasse 1, 85764 Neuherberg, Germany; 3 Monash University, Centre for Geometric Biology, School of Biological Sciences, Clayton, VIC 3800 Australia; 4 CEA, DRF, IG, Genoscope, Evry, 91057 France; 5 CNRS-UMR8030 and Université Paris-Saclay, UEVE, Evry, 91000 France; 6 Department of Biogeochemistry, Max Planck Institute for Terrestrial Microbiology, Karl-von-Frisch-Strasse 10, D-35043 Marburg, Germany; 7 Ghent University, Center for Microbial Ecology and Technology, Coupure Links 653, B-9000 Ghent, Belgium; 8 University of Duisburg-Essen, Centre of Medical Biotechnology, Universitätsstrasse 2, 45117 Essen, Germany

## Abstract

Spirochaetes are frequently detected in anoxic hydrocarbon- and organohalide-polluted groundwater, but their role in such ecosystems has remained unclear. To address this, we studied a sulfate-reducing, naphthalene-degrading enrichment culture, mainly comprising the sulfate reducer *Desulfobacterium* N47 and the rod-shaped Spirochete *Rectinema cohabitans* HM. Genome sequencing and proteome analysis suggested that the Spirochete is an obligate fermenter that catabolizes proteins and carbohydrates, resulting in acetate, ethanol, and molecular hydrogen (H_2_) production. Physiological experiments inferred that hydrogen is an important link between the two bacteria in the enrichment culture, with H_2_ derived from fermentation by *R. cohabitans* used as reductant for sulfate reduction by *Desulfobacterium* N47. Differential proteomics and physiological experiments showed that *R. cohabitans* utilizes biomass (proteins and carbohydrates) released from dead cells of *Desulfobacterium* N47. Further comparative and community genome analyses indicated that other *Rectinema* phylotypes are widespread in contaminated environments and may perform a hydrogenogenic fermentative lifestyle similar to *R. cohabitans*. Together, these findings indicate that environmental Spirochaetes scavenge detrital biomass and in turn drive necromass recycling at anoxic hydrocarbon-contaminated sites and potentially other habitats.

## Introduction

The bacterial phylum Spirochaetes is well known for its commensal and pathogenic species, among them the causative agents of syphilis and Lyme disease [1]. However, 16S rRNA gene sequences of environmental Spirochaetes are also frequently detected in anoxic environments, including sites contaminated with organic pollutants [2, 3]. Environmental Spirochaetes are also present in many enrichment cultures that degrade hydrocarbons or chlorinated compounds under anoxic conditions, including toluene [4], naphthalene [5], alkanes [2], terephthalate [6], and trichloroethene [7, 8]. For example, the relative abundances of Spirochaetes range from 1.4 to 20.8% in 10 reported sulfate-reducing or methanogenic hydrocarbon-degrading enrichment cultures originating from hydrocarbon-contaminated groundwater, aquifer sediments, and oil sands tailings ponds [2, 9]. In these cultures, sulfate-reducing or fermenting bacteria primarily mediate the anaerobic transformation of hydrocarbon compounds [10]. In contrast, the functional role of the Spirochaetes has remained unresolved.

The recent isolation of *Sphaerochaeta pleomorpha* and *Sphaerochaeta globosa* from trichloroethene-dechlorinating enrichment cultures provided evidence that some environmental Spirochaetes are morphologically and metabolically distinct from host-associated representatives from the phylum [8]. Contrary to other Spirochaetes, which are motile and spiral-shaped, the two strains are rod-shaped, non-motile microorganisms that lack flagella [7, 8]. It was proposed that these environmental Spirochaetes might serve as autotrophic acetogens [11, 12], but homoacetogenic growth with H_2_–CO_2_ was not observed [8]. Genome-based inferences of the metabolic capacity of these isolates suggested a fermentative and saccharolytic lifestyle [7]. Nevertheless, their functional role was untested experimentally and remains unclear.

We have shown that, under sulfate-reducing conditions, naphthalene can be anaerobically mineralized to CO_2_ by the stable enrichment culture N47 derived from sediments of a contaminated aquifer [5, 13]. Based on 16S rRNA gene sequence analysis, ~93% of the culture comprised a hitherto uncultured sulfate reducer *Desulfobacterium* strain N47 that has been shown to be responsible for naphthalene degradation [5, 14]. The remainder of the enrichment culture mainly comprised a Spirochete, *Rectinema cohabitans* strain HM, which is neither able to degrade naphthalene nor reduce sulfate or other sulfur compounds in pure culture [15]. Other possible species might exist in this enrichment with unknown functions, but were not detected by terminal restriction fragment length polymorphism fingerprinting [15] because they represented less than 1% of the total DNA [16].

Here, we intended to elucidate the functional role of environmental Spirochaetes using the enrichment culture N47 as an example. By means of physiological, proteogenomic, and comparative genome analyses, we show that environmental Spirochaetes are involved in necromass recycling and provide hydrogen and nutrients to associated hydrocarbon degraders. Through reconstructing the genomes of two other putative *Rectinema* species, we also provide evidence that environmental Spirochaetes may generally contribute to necromass recycling in anoxic hydrocarbon- and organohalide-contaminated environments as well as other anoxic habitats.

## Materials and methods

### Bacterial cultures and cultivation

The naphthalene-degrading, sulfate-reducing enrichment culture N47 was cultivated in bicarbonate-buffered freshwater medium as described previously [5]. After autoclaving, 3 mL of 1.5% (w/v) naphthalene dissolved in 2,2,4,4,6,8,8-heptamethylnonane (Sigma-Aldrich, Steinheim, Germany) were added to each cultivation bottle. Enrichment culture N47 mainly contained a sulfate reducer, candidate *Desulfobacterium* strain N47, and a spirochete, *Rectinema cohabitans* [15].

Attempts to isolate *Desulfobacterium* strain N47 in pure culture by serial dilution in agar roll tubes resulted in a highly enriched *Desulfobacterium* culture that lacked the Spirochete *R. cohabitans* and was 96% dominated by *Desulfobacterium* strain N47 (Figure S2, details in Supporting Information). The culture performed sulfate reduction and naphthalene oxidation and was cultivated in the same way as the original enrichment culture. This culture is described as “highly enriched *Desulfobacterium* culture” hereafter. *R. cohabitans* strain HM [15] was isolated from the enrichment culture N47 and cultivated in bicarbonate-buffered freshwater medium (pH 7.0), which was reduced with a final concentration of 0.5 mM sodium sulfide and amended with 10 mM glucose and 0.1% yeast extract. All culture bottles were incubated stationary at 30 °C in the dark.

To test whether *R. cohabitans* can utilize dead biomass of *Desulfobacterium* N47 as a carbon source, cells of the highly enriched *Desulfobacterium* culture were harvested from 150 mL cultures by centrifugation at 3214 × *g* (30 min). The supernatant, together with the heptamethylnonane phase containing the naphthalene, was discarded. The cell pellets were suspended in 2.5 mL anoxic fresh medium (without substrate or yeast extract), and autoclaved three times in a closed 100 mL serum bottle at 121 °C for 20 min. Thereafter 0.5 mL dead biomass was transferred to serum bottles filled with 30 mL fresh medium and the bottles were inoculated with 3 mL cultures of *R. cohabitans*. Due to the carryover from the previous cultures, glucose, yeast extract, and fermentation products could have been transferred with the inoculum. Thus, fresh media with only starter cultures of *R. cohabitans* or only dead biomass were incubated as control experiments. To investigate whether *R. cohabitans* can utilize putative carbon sources from the supernatant of the highly enriched *Desulfobacterium* culture, 30 mL centrifuged supernatant were filtered through 0.22 μm pore-size membranes (Sarstedt, Germany), transferred to fresh serum bottles, and inoculated with 3 mL starter cultures of *R. cohabitans*. Non-inoculated bottles served as controls.

To further test whether *R. cohabitans* has the capability to ferment necromass, different substrates relevant to subsurface habitats were added to 50 mL bicarbonate-buffered freshwater medium and inoculated with 5 mL starter cultures of *R. cohabitans*. These substrates included 5 mM sucrose, 5 mM valine, 5 mM pimelic acid, or 0.1% tryptone. Furthermore, living cells of the highly enriched *Desulfobacterium* culture grown in 75 mL cultures were harvested by centrifugation and added as substrate as described above. Fresh media with only starter cultures of *R. cohabitans* were incubated as carryover controls.

### Gas chromatography

Hydrogen was measured with a Shimadzu GC-8A Gas Chromatograph [17] or a Compact GC (Global Analyser Solutions, Breda, The Netherlands) [18]. For the former, high concentrations of hydrogen (>100 parts per million by volume (ppmv)) were measured with a thermal conductivity detector, whereas low concentrations (<100 ppmv) were measured with a reduction gas detector (RGD2 detector, Trace Analytical, Stanford, CA, USA). The measurements were calibrated with standard H_2_ gases (Messer Schweiz) (2 and 50 ppmv for samples lower than 100 ppmv, 1000 ppmv for samples higher than 100 ppmv). For the latter, the GC was equipped with a Molsieve 5A pre-column and Porabond column (CH_4_, O_2_, H_2_, and N_2_). The carrier gases were N_2_ for the H_2_ channel and He for the rest of the gases (CH_4_, O_2_ and N_2_). The concentrations (>100 ppmv) were determined using a thermal conductivity detector.

### Sequencing and reconstruction of Spirochete genomes

For comparative genomic analyses, three high-quality draft genomes were obtained, namely those of *R. cohabitans*, uncultured Spirochete bacterium bdmA 4, and uncultured Spirochete bacterium SA-8. The latter two genomes contained 16S rRNA gene sequences with 95% identity to *R. cohabitans*. The draft genome of the uncultured Spirochete bacterium SA-8 was derived from the metagenome of the terephthalate-degrading community TA, which is available on IMG/M (Integrated Microbial Genomes & Microbiomes) as taxon object ID 3300001095 [6]. The draft genome of uncultured Spirochete bacterium bdmA 4 was reconstructed from the metagenome of another sulfate-reducing, naphthalene-degrading enrichment culture (designated Sob). Culture Sob was enriched from a creosote-contaminated wood preservation facility located in Sobĕslav, south of Bohemia (Czech Republic), as described by Kummel et al. [9]. Information on the shotgun sequencing, assembly, and binning processes [19] for the genome of *R. cohabitans* and the metagenome of enrichment culture Sob are described in the Supporting Information. The draft genomes of *R. cohabitans*, uncultured Spirochete bacterium bdmA 4, and uncultured Spirochete bacterium SA-8 were loaded into the MicroScope genome annotation platform and automatically annotated [20]. The annotations were manually curated and edited using the Magnifying Genome (MaGe) environment [21], and metabolic pathways were predicted using the integrated pathway tools of MaGe that are based on the KEGG (Kyoto Encyclopedia of Genes and Genomes) and MicroCyc databases [22, 23]. The presence of glycoside hydrolases and extracellular peptidases was evaluated using dbCAN (E < 1e^−10^ and cover fraction >0.4) [24] and MEROPS (E < 1e^−10^) [25], respectively.

### Phylogenetic analysis

Potential genes encoding hydrogenase catalytic subunits were identified in the genomes of *R. cohabitans* and *Desulfobacterium* N47 by screening translated sequences through the web database HydDB [26]. Sequences were validated as hydrogenases if they encoded cysteine-containing motifs characteristic of hydrogenases [27] and branched with hydrogenase reference sequences on phylogenetic trees. To infer evolutionary relationships, the source and reference sequences were aligned using the MUSCLE algorithm [28] and visualized on neighbor-joining phylogenetic trees [29] constructed with MEGA7 [30] and bootstrapped with 500 replicates. For genetic organization maps, protein/domain function was predicted using the CDD (Conserved Domain Database) [31] and iron–sulfur clusters were predicted by identifying conserved cysteine residues as previously described [27].

The phylogeny of the Spirochaetes was inferred using 16S rRNA gene sequences retrieved from the NCBI (National Center for Biotechnology Information) Genome Database. Sequences were aligned using the MUSCLE algorithm included in MEGA7 and trimmed to uniform length [30]. A maximum likelihood phylogenetic tree was constructed in MEGA7 using a general time reversible substitution model and uniform rates among sites. The tree was bootstrapped with 500 replicates.

### Mass spectrometry-based proteome analyses

For proteomics, the enrichment culture N47 and the highly enriched *Desulfobacterium* culture (without *R. cohabitans*) were grown with naphthalene (four parallel cultures each). In addition, four cultures of *R. cohabitans* were grown with 10 mM glucose and 0.1% yeast extract. The cultures were harvested after 6 weeks for the enrichment culture or 10 days for *R. cohabitans* and then immediately frozen at −20 °C. The frozen cell pellets were molten at room temperature and taken up in 1 mL phosphate-buffered saline (3 mM Na_2_HPO_4_, 1.1 mM KH_2_PO_4_, and 155.2 mM NaCl; pH 7.4). The samples were lysed by ultrasonification in a Bioruptor® (Diagenode) for 5 min to break the cells, release the proteome, and shear the genomic DNA (experimental settings: water bath temperature, 4 °C; power setting, H position (High); sonication cycle, 30 sec ON, 30 sec OFF; total sonication time, 5 min). The turbid solution was cleared by centrifugation (12,000 × g, 4 °C, 5 min) and the supernatant was transferred to a fresh 1.5 mL Eppendorf tube. The protein concentration was determined by using a modified Bradford assay (RotiNanoquant; Roth) following the manufacturer’s instructions. A detailed protocol for the subsequent sample preparation for Liquid chromatography tandem-mass spectrometry, as well as the settings during data acquisition and data analysis, can be found in the Supporting Information. The mass spectrometry proteomics data have been deposited to the ProteomeXchange Consortium via the PRIDE [32] partner repository (https://www.ebi.ac.uk/pride/archive/) with the dataset identifier PXD005624.

## Results

### The genome of *R. cohabitans* HM suggests it is an obligate fermenter and hydrogen producer

**Fig. 1:**
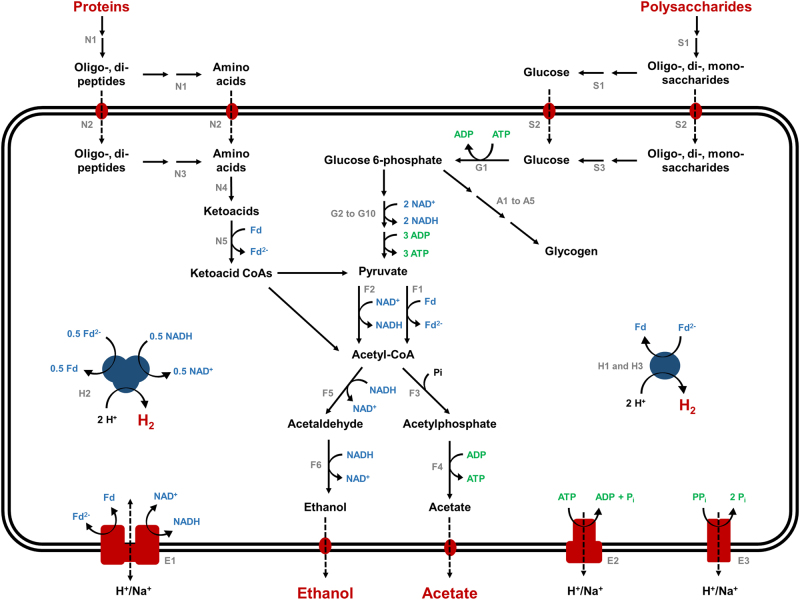
Metabolic pathway reconstruction for Spirochaetes in hydrocarbon- and organohalide-contaminated environments. Genes for the illustrated pathways were detected in the genomes of *R. cohabitans*, uncultured Spirochete bacterium bdmA 4, and uncultured Spirochete bacterium SA-8. The predicted functions of the putative proteins indicated by the numbers in the figure can be found in Table S1

We sequenced and assembled a draft genome of *R. cohabitans* HM (2.82 Mb, estimated to be 98% complete, see Supporting Information) to develop hypotheses about its ecophysiological role in the enrichment culture N47. Analysis of the metabolic capacity from the genome suggests that *R. cohabitans* performs a fermentative lifestyle. It is predicted to be capable of degrading proteins and carbohydrates, resulting in the production of the endproducts ethanol, acetate, and H_2_ (Fig. 1 and Supporting Information). In contrast, key genetic determinants of aerobic and anaerobic respiration, the tricarboxylic acid cycle, and carbon fixation via the reductive acetyl-CoA pathway are absent from the genome. This is consistent with previous substrate utilization tests [15].

**Fig. 2:**
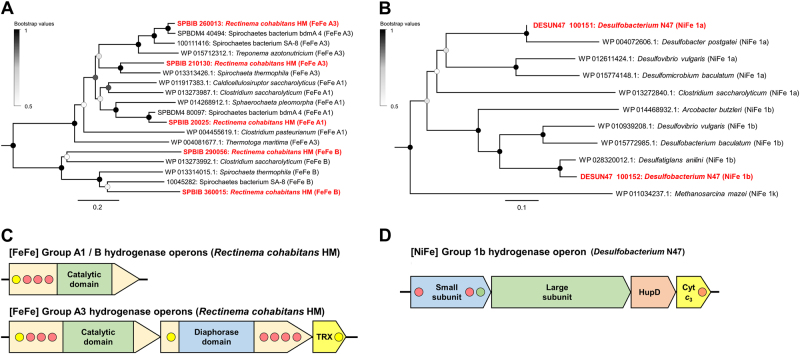
Determinants of H_2_ metabolism in Spirochaetes in hydrocarbon- and organohalide-contaminated environments. **a** Phylogenetic tree of the [FeFe]-hydrogenase catalytic subunit sequences detected in *R. cohabitans*, uncultured Spirochete bacterium bdmA 4, and uncultured Spirochete bacterium SA-8. **b** Phylogenetic tree of the [NiFe]-hydrogenase catalytic subunit sequences detected in *Desulfobacterium* N47. **c** Genetic organization of hydrogenases from *R. cohabitan*s. **d** Genetic organization of the hydrogenase operon detected in *Desulfobacterium* N47. The illustrated tree for Figure 2a is condensed, with the full tree including all hydrogenases in Figure S1. The genetic organization of the Group 1a [NiFe] hydrogenase gene is not shown due to incomplete sequence coverage. Genetic organization diagrams are shown to scale and genes/domains are color-coded as follows: green, catalytic site; blue, secondary subunit; yellow, electron acceptor or donor; light orange, maturation factor. Redox-active centers are shown in circles, where yellow indicates [2Fe2S] cluster, green [3Fe4S] cluster, and red [4Fe4S] cluster

Furthermore, we identified putative hydrogenase genes in the genomes of both *R. cohabitans* and *Desulfobacterium* N47. To gain insight into their potential ecophysiological roles, we classified them based on their amino acid sequence phylogeny and genetic organization according to a recently developed hydrogenase classification scheme [26, 27]. Seven putative [FeFe]-hydrogenase genes were identified in the genome of the Spirochete. Three are monomeric [FeFe] Group A1 or B hydrogenases (Fig. 2a, c), which are known to couple oxidation of reduced ferredoxin to evolution of H_2_ during carbohydrate and protein fermentation in diverse bacteria [27, 33]. Two others encode trimeric electron-bifurcating [FeFe] Group A3 hydrogenases (Fig. 2a, c), which reversibly couple the oxidation of ferredoxin and NADH to the evolution of H_2_, thereby providing a novel mechanism to balance redox state and adenosine triphosphate demand [34]. The genome also encodes [FeFe] Group C1 and C3 hydrogenases (Figure S1), which are candidate subgroups involved in H_2_ sensing [26].

Re-analysis of the genome of the sulfate-reducing *Desulfobacterium* N47 [14] revealed the presence of two Group 1a and Group 1b [NiFe]-hydrogenases (Fig. 2b). These are known to couple H_2_ oxidation to cytochrome *c*_3_ reduction in the respiratory chains of sulfate-reducing deltaproteobacteria. Whereas the former sequence is incomplete due to sequence coverage gaps, a complete operon encoding the 1b enzyme and its cytochrome *c*_3_ partner was detected (Fig. 2d) [27].

The genome-based predictions indicate that the association of the two predominant bacteria in the enrichment culture may be driven by the transfer of H_2_; we predict that H_2_ is produced during fermentation by *R. cohabitans* using [FeFe]-hydrogenases and subsequently consumed by the sulfate-reducing *Desulfobacterium* N47 using [NiFe]-hydrogenases.

### Hydrogen is exchanged between *R. cohabitans* and *Desulfobacterium* N47

**Fig. 3:**
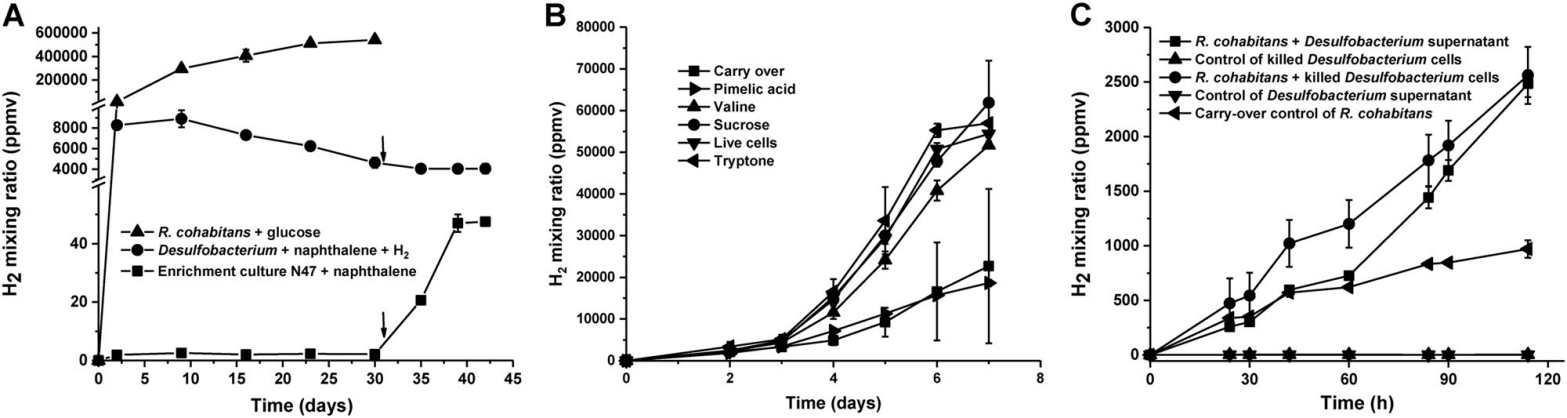
H_2_ oxidation and evolution under different growth conditions. **a** Interspecies hydrogen exchange between *R. cohabitans* and *Desulfobacterium* N47. Arrows indicate injection of sodium molybdate. **b** Hydrogen evolution with various substrates as carbon sources for *R. cohabitans*. **c** Hydrogen evolution with dead biomass and metabolites as carbon sources for *R. cohabitans*. *Desulfobacterium* in the figure represents biomass from the highly enriched *Desulfobacterium* culture as a substrate. Values are means of two or three individual incubations. Error bars indicate SD of biological replicates

To confirm that *R. cohabitans* is a fermentative H_2_ producer, we performed pure culture growth experiments with 10 mM glucose as the electron and carbon source. Gas chromatography demonstrated that H_2_ rapidly accumulated in the headspace during cultivation with glucose, resulting in H_2_ concentrations up to 400,000 ppmv after 2 weeks (Fig. 3a). Proteomic analysis of cells grown with glucose confirmed the expression of all hydrogenase-related genes with high peptide abundances (Table S2). Additionally, when grown with valine, sucrose, and tryptone, respectively, *R. cohabitans* produced almost threefold more hydrogen compared to controls after 1 week (Fig. 3b). This further confirms that *R. cohabitans* performs a proteolytic and saccharolytic lifestyle that results in hydrogen production.

The highly enriched *Desulfobacterium* culture was cultivated under sulfate-reducing conditions with naphthalene as carbon source. Meanwhile, the headspace was supplemented with a limited amount of H_2_ gas (~8285 ppmv) as an additional electron donor for the reduction of sulfate (20 mM). Consistent with genome-based predictions of hydrogenases for *Desulfobacterium* N47, we observed a gradual decrease in H_2_ concentration (Fig. 3a). Consumption of H_2_ by *Desulfobacterium* N47 was inhibited when 5 mM sodium molybdate (Na_2_MoO_4_), a potent inhibitor of sulfate reduction [35], was added at day 31 (Fig. 3a). This suggests that, in common with classical sulfate-reducing isolates [36], *Desulfobacterium* N47 directly couples H_2_ oxidation to sulfate reduction through an anaerobic respiratory chain.

Finally, in order to reveal a potential relationship between the two members, we monitored H_2_ concentrations in the enrichment culture N47 (*R. cohabitans* +* Desulfobacterium* N47) with naphthalene and sulfate. The concentration of H_2_ remained constant (~2 ppmv) for 1 month (Fig. 3a). However, a gradual increase in H_2_ concentration was observed when sulfate reduction by *Desulfobacterium* N47 was inhibited by the addition of sodium molybdate. This suggests that there is a tight coupling between Spirochete-mediated fermentative H_2_ evolution and Deltaproteobacterium-mediated respiratory H_2_ consumption during the growth of *Desulfobacterium* with naphthalene as the sole supplemented carbon source and sulfate as the electron acceptor.

### Organic carbon derived from necromass fuels fermentation by *R. cohabitans*

While the above results demonstrate that H_2_ is an important link between *R. cohabitans* and *Desulfobacterium* N47, the question remained about the origin of the reducing equivalents (i.e., electron sources) used for H_2_ production by *R. cohabitans*. Considering that *R. cohabitans* cannot utilize naphthalene and no other exogenous organic sources were added, the potential carbon sources for *R. cohabitans* include metabolites formed during naphthalene degradation and/or dead biomass from the deltaproteobacterium.

**Fig. 4:**
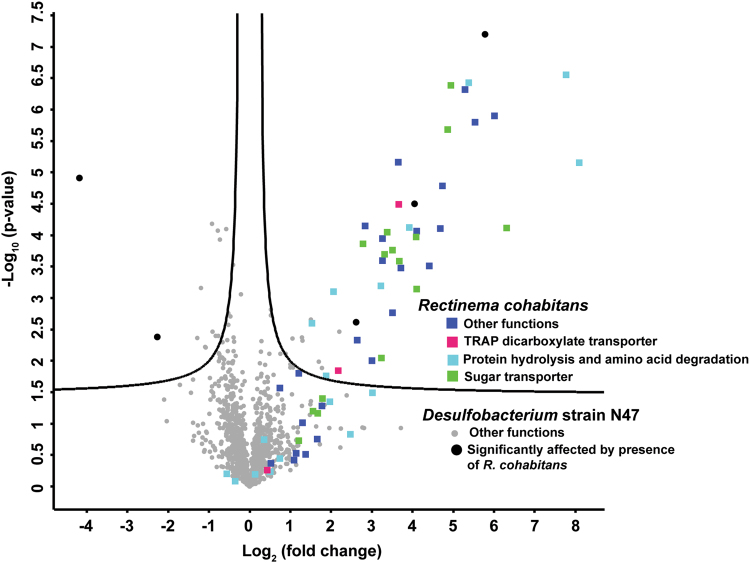
Quantitative proteomic analysis of the highly enriched *Desulfobacterium* culture (without *R. cohabitans*) and enrichment culture N47 grown in the presence of naphthalene. The volcano plot was generated using the PERSEUS software. The calculated Log2 (fold change) (*x*-axis) was then plotted against the corresponding *p* values (*y*-axis). Proteins to the left and above the significance line are significantly depleted in enrichment culture N47 and proteins to the right and above the significance line are significantly enriched in enrichment culture N47. The filled squares indicate proteins from *R. cohabitans*; the filled circles represent proteins from *Desulfobacterium* N47. The different colors highlight functional groups (for details see legend within figure)

To address this question, we compared the proteomes of the highly enriched *Desulfobacterium* culture (without *R. cohabitans*, Figure S2) and the enrichment culture N47. The two proteomes revealed that only 5 out of 1266 proteins detected in total and assigned to *Desulfobacterium* N47 were differentially expressed between the two cultures, none of which could be assigned to a distinct ecophysiological function (Fig. 4). However, 53 proteins belonging to *R. cohabitans* were identified only in the enrichment culture N47 but not in the highly enriched *Desulfobacterium* culture (Table S3). Only a few proteins of *R. cohabitans* were detected in enrichment culture N47, most likely due to its relatively low abundance compared to the *Desulfobacterium*. Most of the *R. cohabitans* proteins expressed in the enrichment culture N47 were related to transporters for oligopeptides, dipeptides, amino acids, sugars, and dicarboxylic acids (Fig. 4 and Table S3). Other enriched enzymes include protease M41, glutamate dehydrogenase (amino acid catabolism), pyruvate-ferredoxin oxidoreductase (ferredoxin reduction), and components of the Sec translocon (secretion of hydrolases, insertion of transporters) (Table S3). This further supports that the Spirochaete performed fermentative degradation of polysaccharides and proteins in the enrichment culture N47.

As we did not add complex carbohydrates or peptides to the medium, these compounds are likely to originate from dead biomass of *Desulfobacterium* N47. We verified that *R. cohabitans* could use organic carbon sources derived from *Desulfobacterium* N47 to support fermentative H_2_ production by cultivating the Spirochaete with biomass of the highly enriched *Desulfobacterium* culture. Initially, the biomass was firstly autoclaved to produce artificial necromass and then added to culture bottles of *R. cohabitans*. After 3 days, the H_2_ production by *R. cohabitans* was twofold higher in fresh media incubations supplemented with either heat-killed cells or filtered supernatant of the highly enriched *Desulfobacterium* culture (Fig. 3c). Given that autoclaved biomass does not reflect the biomass present in the natural environment, we tested if biomass without any treatment could support growth of *R. cohabitans*. As *Desulfobacterium* N47 cannot survive under the defined growth conditions of *R. cohabitans*, it will die automatically and thus produce necromass due to lack of energy. After 7 days, the H_2_ production by *R. cohabitans* was 2.4 times higher compared to a control with inoculum only and no substrate (Fig. 3b). The two experiments show that *R. cohabitans* can use necromass and potentially exudates in the supernatant derived from *Desulfobacterium* N47 to support its obligately fermentative lifestyle.

Pimelate and other dicarboxylic acids are important downstream metabolites of anaerobic naphthalene degradation and have been detected in the supernatant of the enrichment culture N47 [10]. However, the genomic search did not support that *R. cohabitans* could utilize them as carbon sources, as we could not find genes for the β-oxidation of dicarboxylic acids [37]. Further substantiating this, substrate testing showed that pimelate does not stimulate hydrogen production (Fig. 3b). Together, the genome predictions (Fig. 1), substrate tests (Fig. 3), and proteomic data (Fig. 4) suggest that *R. cohabitans* primarily feeds on proteins and sugars derived from detrital biomass in the enrichment culture N47.

### Spirochaetes may generally mediate necromass recycling in anoxic hydrocarbon- and organohalide-contaminated habitats

To further illuminate the ecological niche of Spirochaetes in polluted habitats, we compared the genome of *R. cohabitans* with the draft genomes of two uncultured environmental Spirochaetes that were reconstructed from metagenomics sequences: uncultured Spirochete bacterium SA-8, originating from a terephthalate-degrading, methane-producing bioreactor [6], and uncultured Spirochete bacterium bdmA 4, derived from a naphthalene-degrading, sulfate-reducing culture enriched from a creosote spill [9].

Genome-based reconstructions suggest that, akin to *R. cohabitans*, both organisms are non-spiral Spirochaetes and obligate fermenters (Fig. 1 and Table S1). In addition, both genomes encode [FeFe] Groups A and B hydrogenases that are phylogenetically related to those of *R. cohabitans* (Fig. 2a and S1), suggesting that they also fermentatively produce H_2_, potentially sustaining associated hydrogenotrophic methanogens and sulfate reducers. Previously reported transcriptomic analysis supports that the uncultured Spirochete bacterium SA-8 participates in proteolytic amino acid degradation similar to *R. cohabitans* [6]. Revisiting the published genomes of the nonspiral Spirochaetes *S. pleomorpha* and *S. globosa* from trichloroethene-degrading cultures also indicated a potential for H_2_ production through saccharolytic mixed-acid fermentation (Fig. 2a and S1) [7]. In these cases, H_2_ probably serves as an electron donor to enhance reductive dechlorination activities of organohalide-respiring bacteria.

**Fig. 5:**
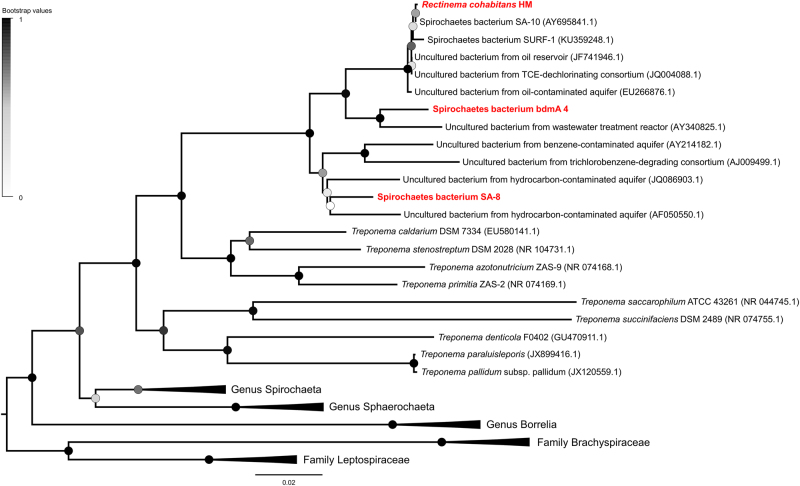
Condensed maximum likelihood tree of partial 16S rRNA gene sequences of Spirochaetes showing the phylogenetic affiliation of *R. cohabitans*, uncultured Spirochete bacterium bdmA 4, and uncultured Spirochete bacterium SA-8. Phylogenetic trees were constructed by the maximum likelihood method and are bootstrapped with 500 replicates. The expanded tree is shown in Figure S3

The 16S rRNA gene sequences of *R. cohabitans*, uncultured Spirochete bacterium bdmA 4, and uncultured Spirochete bacterium SA-8 are 95% identical to each other, and form a common clade distinct from *Treponema* species in the 16S rRNA-based phylogenetic tree (Fig. 5 and S3). In the same clade, we could also identify numerous 16S rRNA gene sequences derived from contaminated aquifers or bioreactors containing hydrocarbons or organohalides as well as oil reservoirs. A small selection is shown in Figure 5. This indicates that *Rectinema* phylotypes are widely distributed in hydrocarbon- and organohalide-contaminated anoxic sites. The two strains *S. globosa* and *S. pleomorpha* are only distantly related to the three members of the genus *Rectinema* [15], suggesting that nonspiral morphology occurs in several lineages and that a similar metabolism is present in distinct groups of environmental Spirochaetes (Figure S3). Altogether, comparative genomic analyses suggest that these environmental nonspiral Spirochaetes fulfill ecophysiological functions similar to *R. cohabitans* in their communities: recycling necromass and providing hydrogen for hydrogenotrophic degraders of organohalides and hydrocarbons in these communities.

## Discussion

**Fig. 6:**
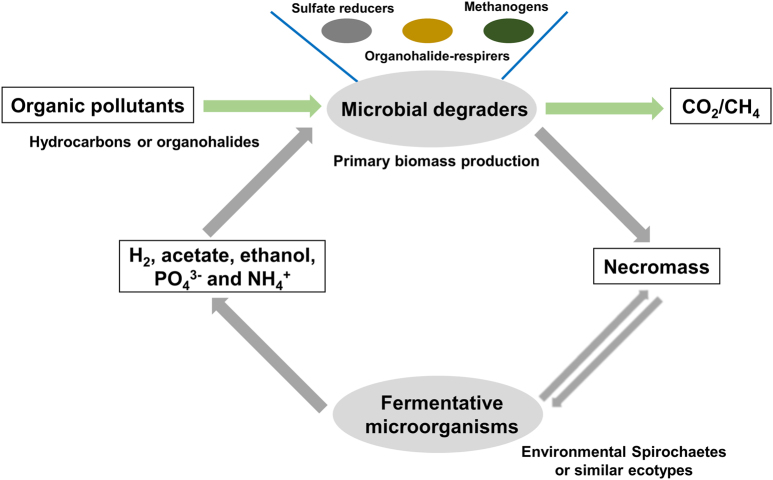
A proposed conceptual model of a subsurface microbial loop for nutrient recycling at anoxic hydrocarbon- and organohalide-contaminated habitats. Hydrocarbons and organohalides serve as exogenous energy, electron, and carbon inputs, leading to primary biomass production in the absence of sunlight. Bacteria like environmental Spirochaetes use extracellular hydrolases and fermentative pathways to recycle biomass. In turn, they release usable electron donors such as hydrogen and potentially nutrients like phosphorus and nitrogen to the system. This in turn may stimulate microbial degradation processes performed by key players, e.g., sulfate-reducing bacteria and organohalide-respiring bacteria, and other community members like methanogenic archaea

Anaerobic degradation by indigenous microorganisms is the most important process that contributes to removal of anthropogenic organic pollutants in subsurface sites (e.g. contaminated groundwater) [38]. For some time, research on anaerobic degradation processes mostly focused on key players that are responsible for initial degradation of pollutants and/or reduction of electron acceptors (Fig. 6). However, it has become clear in recent years that the complete oxidation of organic pollutants to CO_2_ is based on labor sharing: participation of both key players for initial degradation of pollutants and secondary degraders for complete mineralization of catabolic by-products [6, 39, 40]. Additionally, molecular surveys of anaerobic enrichment cultures or contaminated sites have revealed that many microbial species are not clearly correlated to degradation processes. They seem to be neither key players nor secondary degraders and are therefore unlikely to be involved in direct degradation of pollutants [6, 41]. Reflecting this, environmental Spirochaetes are common and abundant in contaminated sites and in enrichment cultures that degrade hydrocarbons and organohalides, but there are no indications that they are linked to degradation of either these compounds or by-products [15]. Our study of the enrichment culture N47 now provides experimental proof of one functional trait to these frequently detected organisms.

In the enrichment culture N47, the chemo-organo-heterotrophic *Desulfobacterium* strain N47 degrades naphthalene as energy and carbon source with sulfate as electron acceptor, leading to primary biomass production in the absence of sunlight. Under nutrient-rich conditions, if there is sufficient sulfate and naphthalene in the aqueous system (e.g., >~50 μM) [42], the sulfate reducer can live alone without the need of *R. cohabitans* to supply additional electron donors. In contrast, *R. cohabitans* relies on organic carbon sources derived from detrital biomass of *Desulfobacterium* N47 to sustain its growth. In turn, necromass degradation via hydrolysis and fermentation results in production of hydrogen and potentially short-chain fatty acids and alcohols. These products are then utilized by *Desulfobacterium* N47, thereby resulting in the recycling of necromass.

*R. cohabitans* is only one example out of many fermentative microorganisms that might mediate equivalent functions in necromass recycling. Even sulfate reducers themselves are quite versatile microorganisms and some perform hydrogenogenic fermentation when lacking electron acceptors such as sulfate or nitrate [43]. However, a fermenter that is more specialized for proteolysis and can secrete extracellular proteases and sacharolytic enzymes will most likely outcompete such sulfate reducers or other fermenters for necromass degradation. Consistently, the genomes of environmental Spirochaetes like *R. cohabitans* have genes encoding several extracellular peptidases (Merops family M23B, S8A, S26A, and S33) and carbohydrate-converting enzymes including hydrolases (Fig. 1 and Supporting Information). This is because necromass usually occurs in the form of biopolymers that must be hydrolyzed outside the cell before metabolizing by microorganisms. Hence, exoenzymes play an essential role in initial processing by hydrolyzing complex organic matter derived from detrital compounds of other community members (e.g., sulfate reducers and fermenters) in the system [44]. Similar physiological properties are also well known for other organisms, such as Clostridia, that fulfill similar ecological roles in anoxic habitats.

In anoxic hydrocarbon- and organohalide-contaminated habitats, the biomass turnover rates are potentially high [45] because hydrocarbons (e.g., naphthalene and other aromatic compounds) or organohalides (e.g., trichloroethene) are usually present in relatively high concentrations in such habitats compared to pristine sites [38]. Their presence reshapes the abundances and interactions of indigenous microbes. In addition, they provide exogenous energy and carbon inputs, allowing for the buildup of significant biomass especially at contaminant plume fringes [38, 45]. For example, in the terephthalate-degrading bioreactor mentioned above, up to 10% of the terephthalate-derived carbon was converted to biomass which is a typical yield for anaerobic cultures [6]. In some benzene and toluene-degrading enrichment cultures, heterotrophic CO_2_ assimilation is also supposed to contribute greatly to biomass accumulation as evidenced from protein-based stable isotope probing (protein-SIP) and DNA-SIP experiments [41, 46]. Furthermore, at hydrocarbon- and organohalide-contaminated sites, high concentrations of organic solvents could challenge the integrity of cell membranes, inducing cell leakage or lysis [40, 47, 48].

Given the potential high biomass turnover, recycling of necromass is therefore important for ecosystem function and prevents loss of nutrients in contaminated ecosystems (Fig. 6). Most notably, it may contribute to enhancing biodegradation rates at contaminant plume fringes in groundwater or oil reservoirs where electron donors (e.g., hydrocarbons) and acceptors (e.g., sulfate or nitrate) meet in opposing gradients (Fig. 6) [38]. Firstly, during necromass recycling, the production of H_2_ and other substrates such as acetate and ethanol has frequently been reported to stimulate microbial blooms (the so-called “priming”) [49] or serve as secondary substrates for co-metabolism, enhancing biodegradation rates [50]. Secondly, environmental Spirochaetes or similar ecotypes remineralize the necromass, which may also be important for recycling of nutrients such as nitrogen, phosphorus, and trace elements within a nutrient-poor subsurface ecosystem, increasing overall rates of biodegradation [51, 52].

At this point, we can only speculate on the importance of necromass recycling in the subsurface because quantitative data are not available. In our enrichment culture N47, *R. cohabitans* constituted ~7% of the microbial population. Typical growth yields for anaerobic microorganisms are around 5–10% of the consumed substrates, as exemplified by the terephthalate-degrading culture mentioned above [6]. If necromass is the only electron and carbon source for *R. cohabitans*, at least a tenfold number of *Desulfobacterium* cells (i.e., 70% of total cell numbers) must have died to form necromass and serve as substrate for *R. cohabitans* to produce the observed 7% of the total cell numbers. Considering the actual proportion of 93% *Desulfobacterium* cells, this means that 163% of the total *Desulfobacterium* cell numbers (93% living cells plus 70% necromass) must have grown. Thus, approximately 43% of all *Desulfobacterium* cells that have grown in the culture died and were recycled by *R. cohabitans*. The necessary difference in growth rate to account for the necromass recycling is quite small here. For example, if the culture grows exponentially from a 10% parent inoculum to full density (i.e., the cell numbers increased tenfold) in 6 weeks, the doubling time is approximately 13 days. Taken the described necromass recycling calculated above into consideration, it only shortens to ~11 days for the *Desulfobacterium* cells (i.e., the actual cell numbers increased 16.3-fold in 6 weeks). However, if such values of cell death and necromass recycling are transferable to subsurface habitats, and assuming that cell death is much higher under non-optimal conditions in the environment, we may speculate that fermentative organisms like *R. cohabitans* might constitute a subsurface microbial loop [53, 54] that can explain biomass recycling in the underground without a food chain (Fig. 6).

## Electronic supplementary material

Supporting Information

Supplementary Table S1

Supplementary Table S2

Supplementary Table S3

Supplementary Table S4

Supplementary Table S5

Supplementary Table S6

Supplementary Database 1

Supplementary Database 2

Supplementary Database 3

Supplementary Database 4
