# Supplementary material for: Fermentative Spirochaetes mediate necromass recycling in anoxic hydrocarbon-contaminated habitats
Source: ISME J. 2018 May 30;12(8):2039–50. doi: 10.1038/s41396-018-0148-3 (PMC6052044; doi:10.1038/s41396-018-0148-3)
Supplement: Supplementary file 1 — Supporting Information [file 41396_2018_148_MOESM1_ESM.docx]

Supporting Information

**Fermentative Spirochaetes mediate necromass recycling in anoxic hydrocarbon-contaminated habitats**

Xiyang Dong ^1, 2^, Chris Greening ^3^, Thomas Brüls ^4, 5^, Ralf Conrad ^6^, Kun Guo ^7^, Svenja Blaskowski ^8^, Farnusch Kaschani ^8^, Markus Kaiser ^8^, Nidal Abu Laban ^1^, Rainer U. Meckenstock ^1, *^

^1^ University of Duisburg-Essen, Biofilm Centre, Universitätsstrasse 5, 45141 Essen, Germany

^2^ Helmholtz Zentrum München, Institute of Groundwater Ecology, Ingolstädter Landstrasse 1, 85764 Neuherberg, Germany

^3^ Monash University, Centre for Geometric Biology, School of Biological Sciences, Clayton, VIC 3800, Australia

^4^ CEA, DRF, IG, Genoscope, Evry 91057, France

^5^ CNRS-UMR8030 and Université Paris-Saclay, UEVE, Evry 91000, France

^6^ Max Planck Institute for Terrestrial Microbiology, Department of Biogeochemistry, Karl-von-Frisch-Strasse 10, D-35043 Marburg, Germany

^7^ Ghent University, Center for Microbial Ecology and Technology, Coupure Links 653, B-9000 Ghent, Belgium

^8^ University of Duisburg-Essen, Centre of Medical Biotechnology, Universitätsstrasse 2, 45117 Essen, Germany.

* Corresponding author. E-mail: rainer.meckenstock@uni-due.de. Telephone: +49 (201) 183-6601. Fax: +49 (201) 183-6603.

**List of content**

**Materials and methods**

**Supporting text**

**References**

**Figures S1-S3**

**Captions for external Tables S1 to S6**

Table S1 List of genes identified in the genomes and used for metabolic pathway reconstruction for Spirochaetes in hydrocarbon- and organohalide-contaminated environments shown in Figure 1. Genes for the illustrated pathways were detected in the genomes of *Rectinema cohabitans* HM, uncultured Spirochaete bacterium bdmA 4, and uncultured Spirochaete bacterium SA-8.

Table S2 Proteomic analysis of *R. cohabitans* grown on glucose. Four cultures of *R. cohabitans* strain HM were grown with 10 mM glucose and 0.1% yeast extract.

Table S3 List of identified proteins for *R. cohabitans* HM as shown in Figure 4.

Table S4 The MEROPS server was used to identify the presence of various extracellular peptidases enzymes in the genomes of *Rectinema cohabitans* HM, uncultured Spirochaete bacterium bdmA 4, and uncultured Spirochaete bacterium SA-8.

Table S5 List of relative genes encoding transporters for branched amino acids, dipeptides and oligopeptides. The genes were identified in the genome of *Rectinema cohabitans* HM using the MicroScope genome annotation platform.

Table S6 The dbCAN server was used to identify the presence of various glycoside hydrolases enzymes in in the genomes of *Rectinema cohabitans* HM, uncultured Spirochaete bacterium bdmA 4, and uncultured Spirochaete bacterium SA-8.

**Captions for external Databases 1 to 4**

Database 1 FASTA Amino Acid format for protein coding genes in the genome of *R. cohabitans* HM.

Database 2 FASTA Amino Acid format for protein coding genes in the genome of uncultured Spirochaete bacterium SA-8.

Database 3 FASTA Amino Acid format for protein coding genes in the genome of uncultured Spirochaete bacterium bdmA 4.

Database 4 Amino Acid format for hydrogenase sequences used for Figures 2 and S1.

**Materials and methods**

**Sequencing and reconstruction of Spirochaete genomes**

*Construction and sequencing of paired-end DNA libraries*. DNA (30 to 250 ng) was sonicated to a 100- to 800-bp size range on the E210 Covaris instrument (Covaris, Inc., USA). Fragments were end-repaired, then 3'-adenylated, and Illumina adapters were added by using a NEBNext Sample Reagent Set (New England Biolabs). Ligation products were purified by Ampure XP (Beckmann Coulter) and DNA fragments (>200 bp) were PCR-amplified using Illumina adapter-specific primers and Platinum Pfx DNA polymerase (Invitrogen). The amplified library fragments were size selected on 3% agarose gel at around 300 bp. After library profile analysis by Agilent 2100 Bioanalyzer (Agilent Technologies, USA) and qPCR quantification (MxPro, Agilent Technologies, USA), the library was sequenced using 101 base-length read chemistry in a paired-end flow cell V3 on the Illumina Hiseq2000 sequencer (RTA version 1.13.48) (Illumina, USA) in order to obtain overlapping reads and generate meta-reads of 180 bp.

*Nextera mate-paired library preparation and sequencing.* Three mate pair libraries were prepared for each sample following the Nextera protocol (Nextera Mate Pair sample preparation kit, Illumina). Briefly, genomic DNA was simultaneously enzymatically fragmented and tagged with a biotinylated adaptor. Fragments were size selected (3-5 Kb, 5-8 Kb and 8-11Kb) through regular gel electrophoresis, and circularized overnight with a ligase. Linear, non-circularized fragments were digested, and circularized DNA was fragmented to 300-1000-bp size range using Covaris E210. Biotinylated DNA was immobilized on streptavidin beads, end-repaired, then 3'-adenylated, and Illumina adapters were added. DNA fragments were PCR-amplified using Illumina adapter-specific primers and then purified. Finally, libraries were quantified by qPCR and libraries profiles were evaluated using an Agilent 2100 bioanalyzer (Agilent Technologies, USA). For enrichment culture Sob, 3-5kb, 5-8kb and 8-11Kb libraries were sequenced using 101 base length read chemistry (v1) in a paired-end flow cell on the Illumina HISEQ2500 sequencer (rapid mode, RTA version 1.17.21.3) (Illumina, USA). For *R. cohabitans*, the 8-11kb library was sequenced using 151 base-length read chemistry (v1) on a paired-end flow cell on the Illumina Miseq platform (RTA version 1.18.54) (Illumina, USA).

*Assembly of the R. cohabitans genome.* Reads from the paired-end library were down-sampled before being combined with sequences from the largest (8-11 kbp) mate-paired library and assembled with the ALLPATHS engine (Ribeiro et al. 2012).

*Reconstruction of the genome for uncultured Spirochaete bacterium bdmA 4.* Although the enrichment culture Sob was dominated by *D. naphthalenivorans*, with about 30% of the raw reads (from either the paired-end or mate-paired libraries) mapping to its genome, the metagenome was endowed with significant phylogenetic diversity and was estimated to harbor about 30-50 distinct genomes. A combination of assembly-free (binning) and targeted assembly techniques were used in order to direct assembly efforts towards lower complexity read partitions. This was achieved by using sequence clustering methods extracting coverage and/or compositional signals from the reads to partition the sequences in k-mer space in an overlap and alignment independent way (Gkanogiannis et al. 2016). The resulting partitions were then assembled independently, leading to the reconstruction of several dozens of megabase sized genomic fragments, including the nearly complete genome for uncultured Spirochaete bacterium bdmA 4 (estimated 97% complete based on the distribution of 141 lineage specific marker genes compiled by the checkM software) (Parks et al. 2015).

**Purity analysis of highly enriched *Desulfobacterium* cultures**

*DNA extraction.* The DNA extraction was carried out from triplicate 10 ml samples of highly enriched *Desulfobacterium* cultures. The samples were centrifuged at 4000 × g for 30 min at 4 °C. The billeted cells were used for extraction using a PowerLyzer® PowerSoil® DNA Isolation Kit according manufacturer’s instruction (MO BIO Laboratories, Inc., USA). The samples were eluated with 50 μl of EB buffer and DNA concentrations were measured using Qubit™ dsDNA HS Assay Kit (Thermo Scientific).

*PCR Amplification, Barcoding, and DNA Sequencing.* Miseq Libraries prepared using 2 step PCR reactions. The first stage triplicate PCR for targeting the V3-V4 region 16S rRNA genes using the Illumina platform and overhang adaptors attached with target primer set Pro341F/805R (Takahashi et al. 2014). A total of 25 μl reaction contained 5-10 ng of microbial DNA, 2 μM of primers, 12.5 μl 2x KAPA HiFi Hot Start (Kapa Biosystems, Boston, USA). A thermocycler was programmed for a hot start PCR and 30 repetition of each cycle: 95 °C for 30 sec, 56 °C for 45 sec, 72 °C for 60 sec, and elongation step 72 °C for 4 min. Triplicate products (~65 μl) were pooled and purified using AMPure XP beads with ratio 4:5 (bead: PCR pro according manufacture’s instruction (Beckman Coulter, USA). The second stage indexed PCR with overhang adapters attached using 5 μl of amplified DNA and Illumina index primers S5XX and N7XX as indicated in 16S metagenomic sequencing library preparation guide (part no. 1504422315044223 Rev. B, Illumina, Inc., USA). Libraries were normalized and pooled for sequencing on the MiSeq Sequencer (Illumina, San Diego, CA) using the 2 × 300 bp MiSeq Reagent Kit v3.

*Bioinformatics anaysis.* Paired ends (250 bp) sequence data generated from triplicate amplicon libraries were analyzed in the MetaAmp pipeline (Dong et al. 2017) (<http://ebg.ucalgary.ca/metaamp>) using rRNA Gene the marker gene type, 0.99 similarity cutoff and 35 bp minimum length of overlap. The reuslts are shown as Figure S2.

**Mass spectrometry-based proteome analyses**

*Reduction/alkylation and tryptic digestion.* An aliquot corresponding to 25 µg total protein content was removed from each sample. Sodium dodecyl sulfate (SDS) and dithiothreitol were added to a final concentration of 2% and 5 mM respectively. The protein solution was incubated at 90 °C for 5 min. After the samples had cooled down to room temperature, 20 mM iodoacetamide was added and the samples were incubated for 30 min at ambient temperature. A chloroform/methanol precipitation was performed in order to remove the reducing and alkylating agents and to remove the SDS (Wessel and Flügge 1984). The obtained protein pellet was then dissolved in 25 µL of 8M urea and 100 mM ammonium bicarbonate and extensively vortexed. The solutions were cleared by centrifugation and the supernatants transferred to 96 well plates (Eppendorf). To start the protein digestion we added 833 ng Lys‑C (1:30; Wako Laboratory Chemicals) and incubated the samples for 3 h at 37 °C. Next the samples were diluted to 25 mM ammonium bicarbonate, supplemented with 1.0 M urea, and 1.25 µg sequencing grade Trypsin (1/20; Promega), and incubated overnight at 37 °C with shaking. On the next morning, the samples were acidified by adding formic acid (final 0.5% v/v).

*Sample clean-up for LC-MS.* Acidified tryptic digests were desalted on custom-made C18 StageTips as described (Rappsilber et al. 2007). Approximately 15 µg of peptides (based on the initial protein concentration) were loaded to each to two disc StageTip. After elution from the StageTips, samples were dried using a vacuum concentrator (Eppendorf) and the peptides were taken up in 10 µL 0.1 % formic acid solution.

*LC-MS/MS*. Experiments were performed on an Orbitrap Elite instrument (Thermo) (Michalski et al. 2012) that was coupled to an EASY-nLC 1000 liquid chromatography (LC) system (Thermo). The LC was operated in the one-column mode. The analytical column was a fused silica capillary (75 µm × 30 cm) with an integrated PicoFrit emitter (New Objective) packed in-house with Reprosil-Pur 120 C18-AQ 1.9 µm resin (Dr. Maisch). The analytical column was encased by a column oven (Sonation) and attached to a nanospray flex ion source (Thermo). The column oven temperature was adjusted to 45 °C during data acquisition and in all other modi at 30 °C. The LC was equipped with two mobile phases: solvent A (0.1% formic acid, FA, in water) and solvent B (0.1% FA in acetonitrile, ACN). All solvents were of UHPLC (ultra high performance liquid chromatography) grade (Sigma). Peptides were directly loaded onto the analytical column with a maximum flow rate that would not exceed the set pressure limit of 980 bar (usually around 0.5 – 0.8 µL/min). Peptides were subsequently separated on the analytical column by running a 300 min gradient of solvent A and solvent B (start with 7% B; gradient 7% to 35% B for 280 min; gradient 35% to 100% B for 10 min and 100% B for 10 min) at a flow rate of 300 nl/min. The mass spectrometer was operated using Xcalibur software (version 2.2 SP1.48). The mass spectrometer was set in the positive ion mode. Precursor ion scanning was performed in the Orbitrap analyzer (FTMS; Fourier Transform Mass Spectrometry) in the scan range of *m/z* 300-1800 and at a resolution of 60,000 with the internal lock mass option turned on (lock mass was 445.120025 *m/z*, polysiloxane) (Olsen et al. 2005). Product ion spectra were recorded in a data dependent fashion in the ion trap (ITMS; Ion Trap Mass Spectrometry) in a variable scan range and at a rapid scan rate. The ionization potential (spray voltage) was set to 1.8 kV. Peptides were analyzed using a repeating cycle consisting of a full precursor ion scan (1.0 × 10^6^ ions or 30 ms) followed by 15 product ion scans (1.0 × 10^4^ ions or 50 ms) where peptides are isolated based on their intensity in the full survey scan (threshold of 500 counts) for tandem mass spectrum (MS2) generation that permits peptide sequencing and identification. CID (collision-induced dissociation) collision energy was set to 35% for the generation of MS2 spectra. During MS2 data acquisition dynamic ion exclusion was set to 60 seconds with a maximum list of excluded ions consisting of 500 members and a repeat count of one. Ion injection time prediction, preview mode for the FTMS, monoisotopic precursor selection and charge state screening were enabled. Only charge states higher than 1 were considered for fragmentation.

*Peptide and Protein Identification using MaxQuant*. RAW spectra were submitted to an Andromeda (Cox et al. 2011) search in MaxQuant (version 1.5.0.25) using the default settings (Cox and Mann 2008). Label-free quantification and match-between-runs was activated (Cox et al. 2014). MS/MS spectra data were searched against the in-house generated protein sequences of *Desulfobacterium naphthalenivorans* N47 (5297 entries) and *Rectinema* *cohabilitans* HM (2580 entries). All searches included a contaminants database (as implemented in MaxQuant, 267 sequences). The contaminants database contains known MS contaminants and was included to estimate the level of contamination. Andromeda searches allowed oxidation of methionine residues (16 Da), acetylation of protein N-terminus (42 Da), Carbamylation (K) and Deamidation (NQ) as dynamic modification and the static modification of cysteine (57 Da, alkylation with iodoacetamide). Enzyme specificity was set to “Trypsin/P”. The instrument type in Andromeda searches was set to Orbitrap and the precursor mass tolerance was set to ±20 ppm (first search) and ±4.5 ppm (main search). The MS/MS match tolerance was set to ±0.5 Da. The peptide spectrum match FDR and the protein FDR were set to 0.01 (based on target-decoy approach). Minimum peptide length was seven amino acids. For protein quantification unique and razor peptides were allowed. Modified peptides were allowed for quantification. The minimum score for modified peptides was 40.

*Data Analysis*: Initial data analysis was performed by using the PERSEUS computational platform (version 1.5.5.3) (Tyanova et al. 2016).

**Supporting text**

**The genome and proteome of *R. cohabilitans* suggest it is an obligate fermenter**

The 2.82 Mb genome comprises 41 contigs and, based on CheckM analysis (Parks et al. 2015), is estimated to be 98% complete with no detectable strain-level heterogeneity. The organism encodes two 16S-23S-5S ribosomal RNA (rRNA) operons, 46 transfer RNA (tRNA) genes for all 20 amino acids, and 2583 potential protein-coding sequences. As in other non-spiral Spirochaetes, genes associated with motility and chemotaxis are absent (Caro-Quintero et al. 2012).

The genome suggests that *R. cohabitans* degrades proteins, peptides, and carbohydrates. For example, genes encoding several extracellular peptidases (Merops family M23B, S8A, S26A, and S33) may mediate decomposition of bacterial proteins and cell wall constituents (Figure 1, Tables S1 and S4). These enzymes may be secreted by the predicted Type II secretory system encoded in the genome (Table S1). The genome also contains a diverse suite of genes encoding transporters for branched amino-acids, dipeptides, and oligopeptides, suggesting that *R. cohabitans* has the capacity to import hydrolysis products into the cell (Figure 1 and Table S5). Also identified were numerous genes encoding intracellular peptidases, aminotransferases, and four ketoacid-ferredoxin oxidoreductases (Figure 1 and Tables S1). The genome also encodes 92 predicted carbohydrate-active enzymes, including hydrolases, extracellular binding proteins and ABC transporters involved in carbohydrate acquisition and breakdown (Figure 1 and Table S6), consistent with its ability to utilize saccharides as carbon sources (Koelschbach et al. 2017).

For central metabolism, the genome encodes a complete Embden-Meyerhof-Parnas glycolysis pathway for oxidizing glucose to pyruvate concomitant with ATP and NADH production (Figure 1 and Table S1). The determinants of the classical pentose phosphate pathway involved in NADPH and pentose synthesis were also found (Table S1). The resulting pyruvate from glycolysis can be converted to acetyl-CoA through ferredoxin-dependent (pyruvate-ferredoxin oxidoreductase) or NAD-dependent (pyruvate dehydrogenase complex) enzymes. Like many other fermenting bacteria, *R. cohabitans* is predicted to convert acetyl-CoA to the fermentation product acetate with concomitant ATP production by phosphate acetyltransferase and acetate kinase (James et al. 2016). The presence of genes for aldehyde and alcohol dehydrogenases suggests that acetyl-CoA can also be reduced to ethanol in two steps using NADH as a reductant. Proteomic analysis of *R. cohabitans* grown on glucose revealed that genes related to mixed-acid fermentation were expressed at high peptide abundances (Table S2).

Furthermore, a set of genes are also present in the genome encoding a membrane-bound RnfABCDGE-type redox complex (Figure 1 and Table S1). This complex is frequently found in the genomes of anaerobic organisms, including other obligately fermentative bacteria (Dodsworth et al. 2013, Sorokin et al. 2014), and was recently shown to link the oxidation of reduced ferredoxin and reduction of NAD^+^ to electrogenic pumping of protons or sodium ions across the cell membrane (Biegel et al. 2011, Tremblay et al. 2012). This complex is likely to have dual roles in maintaining the redox balance and generating membrane potential to fuel ATP synthesis and active transport. A V-type ATPase and a membrane-associated pyrophosphatase are also likely to support the membrane potential maintenance *via* ATP and pyrophosphate hydrolysis. Proteomic analysis revealed that genes related to the V-type ATPase and pyrophosphatase were expressed at high peptide abundances (Table S2).

**References**

Biegel E, Schmidt S, Gonzalez JM, Muller V (2011). Biochemistry, evolution and physiological function of the Rnf complex, a novel ion-motive electron transport complex in prokaryotes. *Cell Mol Life Sci* **68:** 613-634.

Caro-Quintero A, Ritalahti KM, Cusick KD, Loffler FE, Konstantinidis KT (2012). The chimeric genome of Sphaerochaeta: nonspiral spirochetes that break with the prevalent dogma in spirochete biology. *MBio* **3**.

Cox J, Mann M (2008). MaxQuant enables high peptide identification rates, individualized p.p.b.-range mass accuracies and proteome-wide protein quantification. *Nat Biotechnol* **26:** 1367-1372.

Cox J, Neuhauser N, Michalski A, Scheltema RA, Olsen JV, Mann M (2011). Andromeda: a peptide search engine integrated into the MaxQuant environment. *J Proteome Res* **10:** 1794-1805.

Cox J, Hein MY, Luber CA, Paron I, Nagaraj N, Mann M (2014). Accurate proteome-wide label-free quantification by delayed normalization and maximal peptide ratio extraction, termed MaxLFQ. *Mol Cell Proteomics* **13:** 2513-2526.

Dodsworth JA, Blainey PC, Murugapiran SK, Swingley WD, Ross CA, Tringe SG *et al* (2013). Single-cell and metagenomic analyses indicate a fermentative and saccharolytic lifestyle for members of the OP9 lineage. *Nat Commun* **4:** 1854.

Dong X, Kleiner M, Sharp CE, Thorson E, Li C, Liu D *et al* (2017). Fast and simple analysis of MiSeq amplicon sequencing data with MetaAmp. *Front Microbiol* **8**.

Gkanogiannis A, Gazut S, Salanoubat M, Kanj S, Brüls T (2016). A scalable assembly-free variable selection algorithm for biomarker discovery from metagenomes. *BMC Bioinformatics* **17:** 311.

James KL, Ríos-Hernández LA, Wofford NQ, Mouttaki H, Sieber JR, Sheik CS *et al* (2016). Pyrophosphate-dependent ATP formation from acetyl Coenzyme A in *Syntrophus aciditrophicus*, a new twist on ATP formation. *mBio* **7**.

Koelschbach JS, Mouttaki H, Pickl C, Heipieper HJ, Rachel R, Lawson PA *et al* (2017). *Rectinema cohabitans* gen. nov., sp. nov., a rod-shaped spirochaete isolated from an anaerobic naphthalene-degrading enrichment culture. *Int J Syst Evol Microbiol* **67:** 1288-1295.

Michalski A, Damoc E, Lange O, Denisov E, Nolting D, Muller M *et al* (2012). Ultra high resolution linear ion trap Orbitrap mass spectrometer (Orbitrap Elite) facilitates top down LC MS/MS and versatile peptide fragmentation modes. *Mol Cell Proteomics* **11:** O111 013698.

Olsen JV, de Godoy LM, Li G, Macek B, Mortensen P, Pesch R *et al* (2005). Parts per million mass accuracy on an Orbitrap mass spectrometer via lock mass injection into a C-trap. *Mol Cell Proteomics* **4:** 2010-2021.

Parks DH, Imelfort M, Skennerton CT, Hugenholtz P, Tyson GW (2015). CheckM: assessing the quality of microbial genomes recovered from isolates, single cells, and metagenomes. *Genome Res* **25:** 1043-1055.

Rappsilber J, Mann M, Ishihama Y (2007). Protocol for micro-purification, enrichment, pre-fractionation and storage of peptides for proteomics using StageTips. *Nat Protoc* **2:** 1896-1906.

Ribeiro FJ, Przybylski D, Yin S, Sharpe T, Gnerre S, Abouelleil A *et al* (2012). Finished bacterial genomes from shotgun sequence data. *Genome Res* **22:** 2270-2277.

Sorokin DY, Gumerov VM, Rakitin AL, Beletsky AV, Damsté JSS, Muyzer G *et al* (2014). Genome analysis of *Chitinivibrio alkaliphilus* gen. nov., sp. nov., a novel extremely haloalkaliphilic anaerobic chitinolytic bacterium from the candidate phylum Termite Group 3. *Environ Microbiol* **16:** 1549-1565.

Takahashi S, Tomita J, Nishioka K, Hisada T, Nishijima M (2014). Development of a prokaryotic universal primer for simultaneous analysis of bacteria and archaea using next-generation sequencing. *PLOS ONE* **9:** e105592.

Tremblay PL, Zhang T, Dar SA, Leang C, Lovley DR (2012). The Rnf complex of *Clostridium ljungdahlii* is a proton-translocating ferredoxin:NAD^+^ oxidoreductase essential for autotrophic growth. *MBio* **4:** e00406-00412.

Tyanova S, Temu T, Sinitcyn P, Carlson A, Hein MY, Geiger T *et al* (2016). The Perseus computational platform for comprehensive analysis of (prote)omics data. *Nat Methods* **13:** 731-740.

Wessel D, Flügge UI (1984). A method for the quantitative recovery of protein in dilute-solution in the presence of detergents and lipids. *Anal Biochem* **138:** 141-143.


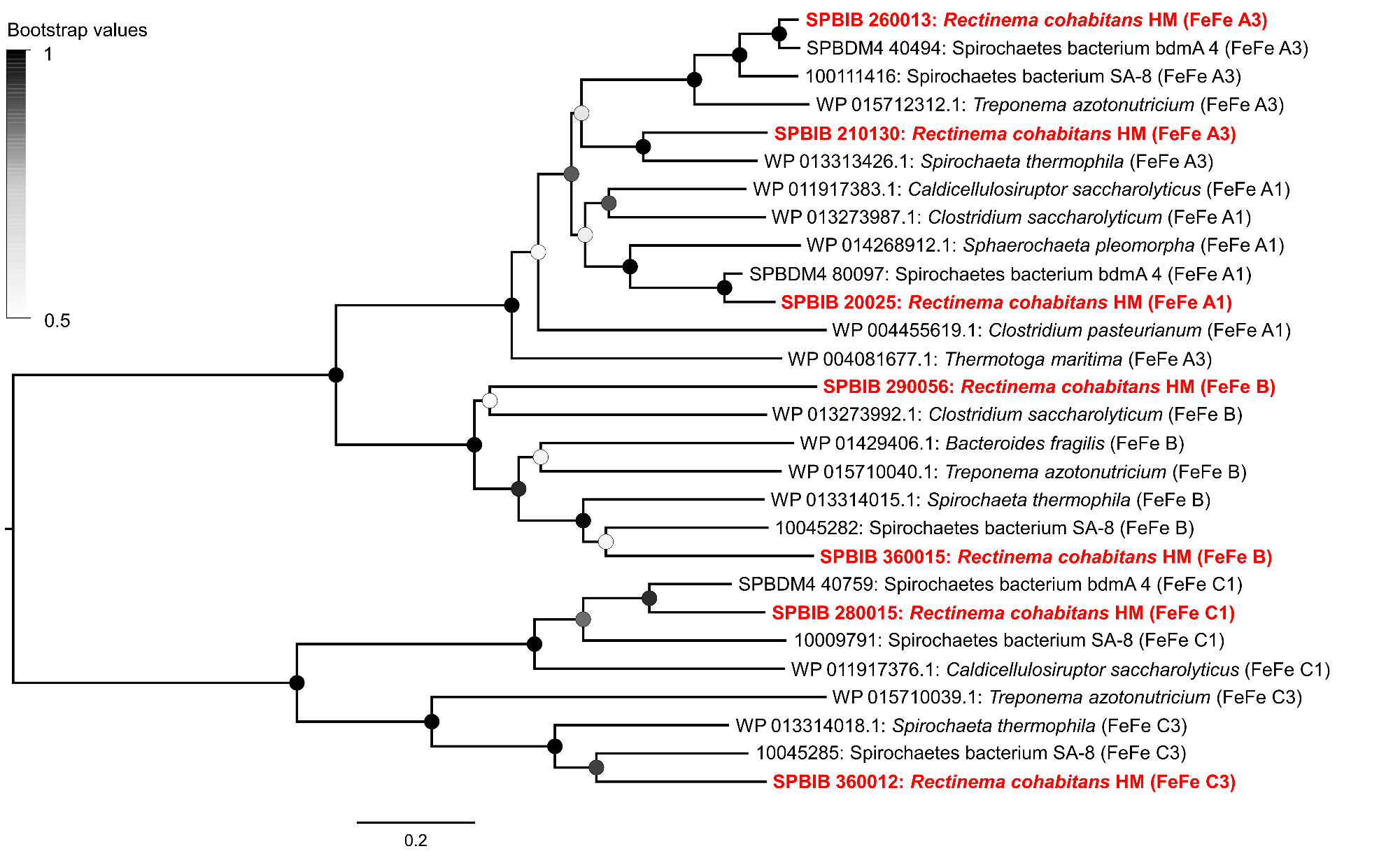


Figure S1 Full phylogenetic tree showing the phylogeny of the [FeFe]-hydrogenase catalytic subunit sequences detected in *R. cohabitans* HM, uncultured Spirochaete bacterium bdmA 4, and uncultured Spirochaete bacterium SA-8.

Figure S2 Purity analysis of highly enriched *Desulfobacterium* cultures in triplicate samples. Total microbial community at Class/Family were identified at ≤ 0.01 distances by Miseq Illumina sequencing (400 bp; assembled paired ends) of 16S rRNA genes and analysis using MetaAmp pipeline.


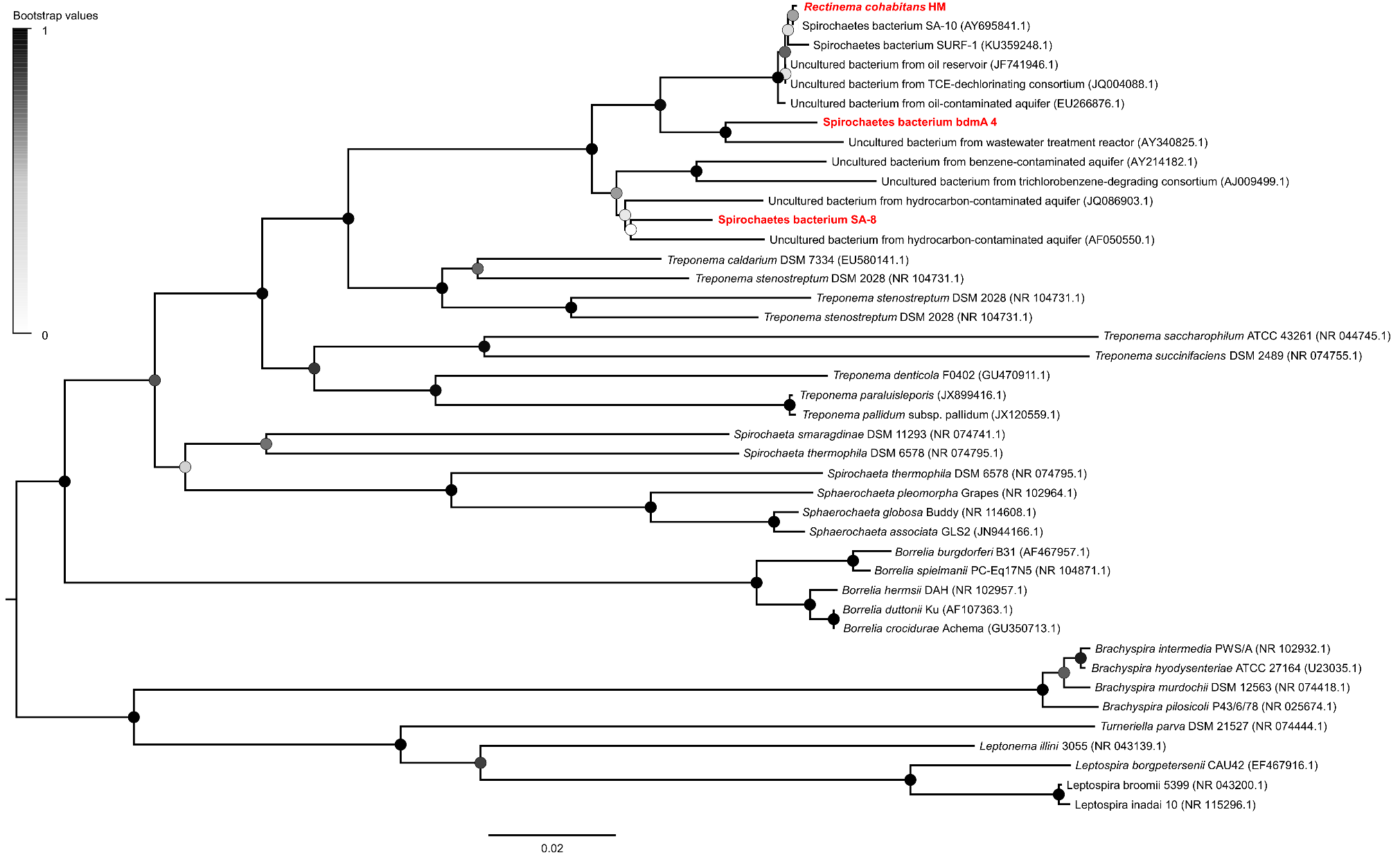


Figure S3 Expanded maximum likelihood tree of partial 16S rRNA gene sequences of Spirochaetes. This shows the phylogenetic affiliation of *R. cohabitans*, uncultured Spirochaete bacterium bdmA 4, and uncultured Spirochaete bacterium SA-8.
