# Supplementary material for: Fermentative Spirochaetes mediate necromass recycling in anoxic hydrocarbon-contaminated habitats
Source: ISME J. 2018 May 30;12(8):2039–50. doi: 10.1038/s41396-018-0148-3 (PMC6052044; doi:10.1038/s41396-018-0148-3)
Supplement: Supplementary file 2 — Supplementary Table S1 [file 41396_2018_148_MOESM2_ESM.docx]

**Supplementary Table S1** List of genes identified in the genomes and used for metabolic pathway reconstruction for Spirochaetes in hydrocarbon- and organohalide-contaminated environments shown in Figure 1. Genes for the illustrated pathways were detected in the genomes of *Rectinema cohabitans* HM, uncultured Spirochaete bacterium bdmA 4, and uncultured Spirochaete bacterium SA-8.

| **Glycolysis/Gluconeogenesis** | | | | | |
| --- | --- | --- | --- | --- | --- |
| No. | Enzyme | EC Number(s) | Spirochaete Bin 1 SA-8 | uncultured Spirochaete bacterium bdmA 4 | *R. cohabitans* |
| G1 | glucokinase | 2.7.1.2 | SPSA8_v1_410004 | SPBDM4_v1_70210 | SPBIB_v1_120011 |
| G2 | glucose-6-phosphate isomerase | 5.3.1.9 | SPSA8_v1_690007_pgi | SPBDM4_v1_50976 - pgi, SPBDM4_v1_40538 - pgiA | SPBIB_v1_130044 - pgi |
| G3 | 6-phosphofructokinase | 2.7.1.11 | not identified | SPBDM4_v1_40290 | SPBIB_v1_350003 |
| G4 | fructose-bisphosphate aldolase | 4.1.2.13 | SPSA8_v1_230024_fba | SPBDM4_v1_50273, SPBDM4_v1_50942 - fba, SPBDM4_v1_40557 - fbaA | SPBIB_v1_140014 - fba |
| G5 | triose-phosphate isomerase | 5.3.1.1 | SPSA8_v1_560006 | SPBDM4_v1_51054 - tpiA | SPBIB_v1_100039 - tpiA |
| G6 | glyceraldehyde-3-phosphate dehydrogenase (phosphorylating) | 1.2.1.12 | SPSA8_v1_560002 | SPBDM4_v1_51050 - gapA | SPBIB_v1_100034 - gapA |
| G7 | phosphoglycerate kinase | 2.7.2.3 | SPSA8_v1_560003 | SPBDM4_v1_51051 - pgk, SPBDM4_v1_41029 | SPBIB_v1_100035 - pgk, SPBIB_v1_210059, SPBIB_v1_290179 |
| G8 | phosphoglycerate mutase | 5.4.2.1 | SPSA8_v1_410010_gpmI | SPBDM4_v1_40326, SPBDM4_v1_70215 - gpmI, SPBDM4_v1_40696 - gpmA | SPBIB_v1_90032 - gpmI, SPBIB_v1_270010 - gpmA |
| G9 | phosphopyruvate hydratase | 4.2.1.11 | not identified | SPBDM4_v1_40824 - eno | SPBIB_v1_280034 - eno |
| G10 | pyruvate kinase | 2.7.1.40 | SPSA8_v1_280005 | SPBDM4_v1_50076 - pyk | SPBIB_v1_290204 - pyk |
| **Pentose phosphate pathway** | | | | | |
| No. | Enzyme | EC Number(s) | Spirochaete Bin 1 SA-8 | uncultured Spirochaete bacterium bdmA 4 | *R. cohabitans* |
| P1 | Glucose-6-phosphate dehydrogenase (NADP(+)) | 1.1.1.49 | SPSA8_v1_290001 | SPBDM4_v1_50483 - zwf | SPBIB_v1_210196 - zwf |
| P2 | 6-phosphogluconolactonase | 3.1.1.31 | SPSA8_v1_290002 | SPBDM4_v1_50484 | SPBIB_v1_210195 |
| P3 | Phosphogluconate dehydrogenase (decarboxylating) | 1.1.1.44 | SPSA8_v1_640011 | SPBDM4_v1_70232 - gnd | SPBIB_v1_90014 - gnd |
| P4 | D-glyceraldehyde-3-phosphate glycolaldehyde transferase | 2.2.1.1 | SPSA8_v1_280002 | SPBDM4_v1_40259, SPBDM4_v1_40260, SPBDM4_v1_41032 - tkt, SPBDM4_v1_50175 - tkt | SPBIB_v1_10035, SPBIB_v1_10036, SPBIB_v1_250033 - tktB |
| P5 | ribulose-phosphate 3-epimerase | 5.1.3.1 | SPSA8_v1_330009 | SPBDM4_v1_70027 - rpe | SPBIB_v1_100090 - rpe |
| P6 | ribose-5-phosphate isomerase | 5.3.1.6 | SPSA8_v1_780001 | not identified | SPBIB_v1_10034 - rpiB, SPBIB_v1_410042 - rpiB |
| P7 | ribose-5-phosphate diphosphotransferase | 2.7.6.1 | not identified | SPBDM4_v1_40813 | SPBIB_v1_280022 |
| **Starch and sucrose metabolism pathway** | | | | | |
| No. | Enzyme | EC Number(s) | Spirochaete Bin 1 SA-8 | uncultured Spirochaete bacterium bdmA 4 | *R. cohabitans* |
| A1 | Glucose-1-phosphate adenylyltransferase | 2.7.7.27 | not identified | SPBDM4_v1_51063 - glgC | SPBIB_v1_100048 - glgC |
| A2 | Glycogen synthase | 2.4.1.21 | SPSA8_v1_500008 | SPBDM4_v1_80030 - glgA | SPBIB_v1_50016 - glgA |
| A3 | 1,4-alpha-glucan branching enzyme | 2.4.1.18 | not identified | not identified | not identified |
| A4 | Glycogen phosphorylase | 2.4.1.1 | SPSA8_v1_570005-6 | SPBDM4_v1_50116 - glgP | SPBIB_v1_250079 - glgP |
| A5 | Phosphoglucomutase | 5.4.2.2 | SPSA8_v1_410001 | SPBDM4_v1_70206 - pgcA | SPBIB_v1_120008 - pgcA |
| **Fermentation** | | | | | |
| No. | Enzyme | EC Number(s) | Spirochaete Bin 1 SA-8 | uncultured Spirochaete bacterium bdmA 4 | *R. cohabitans* |
| F1 | pyruvate ferredoxin oxidoreductase | 1.2.7.1 | SPSA8_v1_240015_ydbK | SPBDM4_v1_40344 - ydbK | SPBIB_v1_340021 - ydbK |
| F2 | multi-enzyme complex pyruvate dehydrogenase | 1.2.4.1, 2.3.1.12, 1.8.1.4 | SPSA8_v1_670003-6 | SPBDM4_v1_40897-9 | SPBIB_v1_150091-3 |
| F3 | phosphate acetyltransferase (annotated as Phosphate butyryltransferase in the platform) | 2.3.1.8 | SPSA8_v1_50027 | SPBDM4_v1_50824 | SPBIB_v1_150125 |
| F4 | acetate kinase | 2.7.2.1 | SPSA8_v1_50028 | SPBDM4_v1_40312 - ackA | SPBIB_v1_340053 - ackA |
| F5 | Acetaldehyde dehydrogenase (acetylating) | 1.2.1.10 | not identified | SPBDM4_v1_50365 - alkH, SPBDM4_v1_30004 - aldA | SPBIB_v1_10033, SPBIB_v1_200018, SPBIB_v1_250096 |
| F6 | alcohol dehydrogenase | 1.1.1.1 | SPSA8_v1_730004 | SPBDM4_v1_50015, SPBDM4_v1_41037, SPBDM4_v1_41043 | SPBIB_v1_290156 |
| **Membrane energization** | | | | | |
| No. | Enzyme | EC Number(s) | Spirochaete Bin 1 SA-8 | uncultured Spirochaete bacterium bdmA 4 | *R. cohabitans* |
| E1 | Electron transport complex, RnfABCDGE-type |  | SPSA8_v1_380010-15 | SPBDM4_v1_40884-9 | SPBIB_v1_290012-7 |
| E2 | V-type ATP synthase | 3.6.1.14 | SPSA8_v1_40015-21 | SPBDM4_v1_40178-84, SPBDM4_v1_70188-94 | SPBIB_v1_370004-10, SPBIB_v1_110048-54 |
| E3 | Pyrophosphate-energized proton pump |  | SPSA8_v1_180025 | SPBDM4_v1_40486 | SPBIB_v1_260006 |
| **Protein degradation** | | | | | |
| N1 | extracellular peptidases (M23B, S8A, S26A, S33) |  | table S4 | table S4 | table S4 |
| N2 | oligo/dipeptide and amino acid transoprters |  | table S4 | table S4 | table S4 |
| N3 | intracellular peptidases |  | table S4 | table S4 | table S4 |
| N4 | Aminotranferases |  | table S4 | table S4 | table S4 |
| N5 | Indolepyruvate ferredoxin oxidoreductase | 1.2.7.8 | SPSA8_v1_30002-3 | SPBDM4_v1_40271-2 | SPBIB_v1_350033-4 |
| N5 | pyruvate/ketoisovalerate oxidoreductase | 1.2.7.7 | SPSA8_v1_50024-6 | SPBDM4_v1_50825-8 | SPBIB_v1_150121-4 |
| N5 | 2-oxoglutarate synthase subunit | 1.2.7.3 | SPSA8_v1_220016-9 | SPBDM4_v1_50767-70 | SPBIB_v1_150177-80 |
| N5 | Tungsten-containing aldehyde ferredoxin oxidoreductase | 1.2.7.5 | SPSA8_v1_700003-4 | not identified | not identified |
| N5 | pyruvate ferredoxin oxidoreductase | 1.2.7.1 | SPSA8_v1_240015_ydbK | SPBDM4_v1_40344 - ydbK | SPBIB_v1_340021 - ydbK |
| **Hydrogen metabolism** | | | | | |
| No. | Enzyme | EC Number(s) | Spirochaete Bin 1 SA-8 | uncultured Spirochaete bacterium bdmA 4 | *R. cohabitans* |
| H1 | FeFe A1 hydrogenase |  | not identified | SPBDM4_v1_80097 | SPBIB_v1_20025 |
| H2 | FeFe A3 hydrogenase |  | SPSA8_v1_300018 | SPBDM4_v1_40494 | SPBIB_v1_260013, SPBIB_v1_210130 |
| H3 | FeFe B hydrogenase |  | SPSA8_v1_810002 | not identified | SPBIB_v1_290056, SPBIB_v1_360015 |
| H4 | FeFe C hydrogenase |  | SPSA8_v1_270001, SPSA8_v1_810005 | SPBDM4_v1_40759 | SPBIB_v1_360012, SPBIB_v1_280015 |
| **Secretion systems** | | | | | |
| No. | Enzyme |  |  |  |  |
|  | Preprotein translocase, SecG subunit |  |  |  | SPBIB_v1_100040 |
|  | Protein translocase subunit SecA |  |  |  | SPBIB_v1_110017 |
|  | Protein-export membrane protein SecF |  |  |  | SPBIB_v1_210207 |
|  | Protein translocase subunit SecD |  |  |  | SPBIB_v1_210208 |
|  | putative SEC-C motif domain protein |  |  |  | SPBIB_v1_280056 |
|  | Protein translocase subunit SecE |  |  |  | SPBIB_v1_310045 |
|  | preprotein translocase membrane subunit |  |  |  | SPBIB_v1_370006 |
| **Carbohydrate-active enzymes (polysaccharide degradation)** | | | | | |
| S1 | hydrolases and extracelluar binding proteins |  |  |  | table S6 |
| S2 | ABC tranporters |  |  |  | table S6 |
| S3 | Hydrolases |  |  |  | table S6 |
