# Supplementary material for: Fermentative Spirochaetes mediate necromass recycling in anoxic hydrocarbon-contaminated habitats
Source: ISME J. 2018 May 30;12(8):2039–50. doi: 10.1038/s41396-018-0148-3 (PMC6052044; doi:10.1038/s41396-018-0148-3)
Supplement: Supplementary file 3 — Supplementary Table S2 [file 41396_2018_148_MOESM3_ESM.docx]

**Supplementary Table S2** Proteomic analysis of *R. cohabitans* grown on glucose. Four cultures of *R. cohabitans* strain HM were grown with 10 mM glucose and 0.1% yeast extract.

| **Peptides** | **Sequence coverage**  **[%]** | **MS/MS Count** | **Protein IDs** | **Function** |
| --- | --- | --- | --- | --- |
| 8 | 12.3 | 16 | SPBIB_v1_100001 | putative Radical SAM domain protein |
| 10 | 34 | 32 | SPBIB_v1_100002 | putative DEAD/DEAH box helicase domain protein |
| 8 | 25.2 | 22 | SPBIB_v1_100003 | Ribonuclease Z |
| 11 | 38.5 | 36 | SPBIB_v1_100004 | exported protein of unknown function |
| 7 | 28.3 | 16 | SPBIB_v1_100005 | conserved protein of unknown function |
| 24 | 72.6 | 100 | SPBIB_v1_100006 | UDP-N-acetylmuramoylalanine--D-glutamate ligase |
| 23 | 44.9 | 79 | SPBIB_v1_100007 | Pyruvate/2-oxoglutarate dehydrogenase complex, dihydrolipoamide dehydrogenase component |
| 9 | 32.8 | 28 | SPBIB_v1_100008 | putative oxidoreductase |
| 15 | 47 | 64 | SPBIB_v1_100010 | cysteine desulfurase (tRNA sulfurtransferase), PLP-dependent |
| 8 | 50 | 25 | SPBIB_v1_100011 | IscU protein |
| 18 | 71.8 | 103 | SPBIB_v1_100013 | TRAP dicarboxylate transporter, DctP subunit |
| 9 | 29.4 | 13 | SPBIB_v1_100016 | D-alanyl-D-alanine carboxypeptidase |
| 26 | 57.9 | 127 | SPBIB_v1_100017 | asparaginyl tRNA synthetase |
| 3 | 24.7 | 5 | SPBIB_v1_100018 | TM2 domain protein |
| 3 | 17.9 | 5 | SPBIB_v1_100019 | putative Peptidase C14 caspase catalytic subunit p20 |
| 4 | 12.6 | 8 | SPBIB_v1_410077;SPBIB_v1_10002;SPBIB_v1_350045 | transposase;transposase;transposase (fragment) |
| 6 | 17.6 | 12 | SPBIB_v1_100022 | protein of unknown function |
| 12 | 43.2 | 35 | SPBIB_v1_100023 | FolC bifunctional protein |
| 12 | 24.1 | 30 | SPBIB_v1_100025 | AAA ATPase central domain protein |
| 2 | 5.5 | 3 | SPBIB_v1_100026 | protein of unknown function |
| 2 | 11.6 | 5 | SPBIB_v1_100027 | exported protein of unknown function |
| 21 | 77.7 | 93 | SPBIB_v1_100029 | conserved protein of unknown function |
| 4 | 37.7 | 7 | SPBIB_v1_410078;SPBIB_v1_10003 | toxin of the ChpA-ChpR toxin-antitoxin system, endoribonuclease;toxin of the ChpA-ChpR toxin-antitoxin system, endoribonuclease |
| 6 | 16.8 | 15 | SPBIB_v1_100030 | exported protein of unknown function |
| 7 | 48 | 12 | SPBIB_v1_100031 | protein of unknown function |
| 18 | 30.5 | 37 | SPBIB_v1_100033 | Penicillin-binding protein, 1A family |
| 49 | 97.5 | 559 | SPBIB_v1_100034 | glyceraldehyde-3-phosphate dehydrogenase A |
| 41 | 92.1 | 339 | SPBIB_v1_100035 | phosphoglycerate kinase |
| 16 | 83.3 | 125 | SPBIB_v1_100037 | cysteine synthase A, O-acetylserine sulfhydrolase A subunit |
| 19 | 77.1 | 119 | SPBIB_v1_100039 | Triosephosphate isomerase |
| 4 | 18 | 7 | SPBIB_v1_100042 | putative Chaperone protein DnaJ |
| 24 | 66.8 | 107 | SPBIB_v1_100043 | putative PpiC-type peptidyl-prolyl cis-trans isomerase |
| 4 | 32.7 | 8 | SPBIB_v1_100044 | N utilization substance protein B homolog |
| 4 | 16.1 | 8 | SPBIB_v1_100045 | Methionine aminopeptidase |
| 20 | 59.3 | 115 | SPBIB_v1_100046 | Protease Do |
| 9 | 69.5 | 29 | SPBIB_v1_100047 | Phosphoribosyl transferase domain protein |
| 22 | 68.6 | 103 | SPBIB_v1_100048 | Glucose-1-phosphate adenylyltransferase |
| 16 | 27.9 | 24 | SPBIB_v1_100049 | Uncharacterized ATP-dependent helicase YprA |
| 11 | 49.3 | 40 | SPBIB_v1_100051 | putative dual-specificity RNA methyltransferase RlmN |
| 22 | 65.9 | 103 | SPBIB_v1_100052 | Tetratricopeptide TPR_2 repeat-containing protein |
| 19 | 56.7 | 56 | SPBIB_v1_100055 | putative PEGA domain protein |
| 7 | 34.3 | 20 | SPBIB_v1_100056 | Signal peptidase I |
| 10 | 42.5 | 32 | SPBIB_v1_100058 | putative Oxygen-independent coproporphyrinogen-III oxidase-like protein YggW |
| 22 | 75.8 | 128 | SPBIB_v1_100059;REV__SPBIB_v1_290077 | conserved protein of unknown function |
| 8 | 67.3 | 20 | SPBIB_v1_100060 | Metallo-beta-lactamase domain protein |
| 6 | 26.6 | 14 | SPBIB_v1_100061 | putative Pentapeptide repeat protein |
| 6 | 35.4 | 14 | SPBIB_v1_100062 | putative Glutamate racemase |
| 16 | 29.4 | 30 | SPBIB_v1_100064 | Endonuclease MutS2 |
| 7 | 36.6 | 34 | SPBIB_v1_100065 | conserved hypothetical protein |
| 8 | 51.5 | 34 | SPBIB_v1_100067 | Holliday junction ATP-dependent DNA helicase RuvA |
| 12 | 38.6 | 35 | SPBIB_v1_100068 | ATP-dependent DNA helicase, component of RuvABC resolvasome |
| 11 | 42.8 | 16 | SPBIB_v1_100069 | S-adenosylmethionine:tRNA ribosyltransferase-isomerase |
| 10 | 54.2 | 36 | SPBIB_v1_100070 | Thymidylate kinase |
| 10 | 9 | 15 | SPBIB_v1_100071 | exported protein of unknown function |
| 17 | 22.6 | 58 | SPBIB_v1_100072 | Outer membrane protein assembly complex, YaeT protein |
| 20 | 39.6 | 39 | SPBIB_v1_100073 | DNA mismatch repair protein MutS |
| 2 | 19.2 | 8 | SPBIB_v1_100075 | putative Ribosome maturation factor RimP |
| 24 | 51.9 | 84 | SPBIB_v1_100076 | Transcription termination/antitermination protein NusA |
| 28 | 47.2 | 89 | SPBIB_v1_100077 | translation initiation factor IF-2 |
| 5 | 44.8 | 15 | SPBIB_v1_100078 | Ribosome-binding factor A |
| 8 | 39 | 17 | SPBIB_v1_100079 | tRNA pseudouridine synthase B (fragment) |
| 8 | 43 | 26 | SPBIB_v1_100080 | putative riboflavin biosynthesis protein |
| 6 | 48.3 | 33 | SPBIB_v1_100081 | 30S ribosomal subunit protein S15 |
| 41 | 59.2 | 187 | SPBIB_v1_100082 | polynucleotide phosphorylase/polyadenylase |
| 3 | 24.5 | 14 | SPBIB_v1_100084 | Deoxyuridine 5-triphosphate nucleotidohydrolase |
| 15 | 61.6 | 49 | SPBIB_v1_100087 | Queuine tRNA-ribosyltransferase |
| 54 | 65.4 | 310 | SPBIB_v1_100088 | Transcription elongation factor |
| 6 | 25.3 | 20 | SPBIB_v1_100089 | Tetratricopeptide TPR_2 repeat-containing protein |
| 4 | 18.2 | 8 | SPBIB_v1_100091 | exported protein of unknown function |
| 9 | 33.6 | 28 | SPBIB_v1_100092 | exported protein of unknown function |
| 5 | 35.9 | 15 | SPBIB_v1_100093 | D-tyrosyl-tRNA(Tyr) deacylase |
| 72 | 75.4 | 557 | SPBIB_v1_100094 | Pyruvate, phosphate dikinase |
| 6 | 46.5 | 25 | SPBIB_v1_100095 | exported protein of unknown function |
| 38 | 61.4 | 181 | SPBIB_v1_100097 | Methionine--tRNA ligase |
| 4 | 12.2 | 12 | SPBIB_v1_100098 | Methicillin resistance protein |
| 6 | 44.4 | 13 | SPBIB_v1_100099 | conserved protein of unknown function |
| 16 | 45.5 | 77 | SPBIB_v1_100102 | putative galactokinase (Galactose kinase) |
| 7 | 57.1 | 24 | SPBIB_v1_100103 | 5-nucleotidase SurE |
| 15 | 25.5 | 18 | SPBIB_v1_100105 | DNA primase |
| 21 | 45.4 | 75 | SPBIB_v1_100106 | RNA polymerase sigma factor RpoD |
| 19 | 55.4 | 91 | SPBIB_v1_100107 | conserved protein of unknown function |
| 19 | 64 | 58 | SPBIB_v1_100109 | cell wall structural complex MreBCD, actin-like component MreB |
| 2 | 8.2 | 5 | SPBIB_v1_100110 | putative Cell shape-determining protein MreC |
| 6 | 11.8 | 9 | SPBIB_v1_100112 | Penicillin-binding protein 2 |
| 19 | 31.1 | 47 | SPBIB_v1_100114 | Radical SAM domain protein |
| 2 | 9.9 | 7 | SPBIB_v1_100115 | exported protein of unknown function |
| 12 | 52.9 | 64 | SPBIB_v1_100116 | conserved protein of unknown function |
| 22 | 54 | 98 | SPBIB_v1_100117 | Transcriptional regulator, NifA subfamily, Fis Family |
| 37 | 70.4 | 197 | SPBIB_v1_100118 | 30S ribosomal protein S1 |
| 9 | 50.6 | 33 | SPBIB_v1_100119 | Cytidylate kinase |
| 6 | 51.4 | 12 | SPBIB_v1_410087;SPBIB_v1_10012 | Plasmid stabilization system protein;Plasmid stabilization system protein |
| 8 | 46.5 | 23 | SPBIB_v1_100121 | Segregation and condensation protein B |
| 6 | 37.7 | 17 | SPBIB_v1_100122 | ScpA/B protein |
| 2 | 24.7 | 7 | SPBIB_v1_100123 | 2-amino-4-hydroxy-6-hydroxymethyldihydropteridine pyrophosphokinase |
| 25 | 68.7 | 98 | SPBIB_v1_100124 | DNA strand exchange and recombination protein with protease and nuclease activity |
| 7 | 40.5 | 25 | SPBIB_v1_100126 | outer membrane-specific lipoprotein transporter subunit;ATP-binding component of ABC superfamily |
| 12 | 53.1 | 39 | SPBIB_v1_100128 | Signal recognition particle receptor FtsY |
| 8 | 25.1 | 19 | SPBIB_v1_100130 | Peptide chain release factor 2 |
| 13 | 54.2 | 42 | SPBIB_v1_100131 | Aldose 1-epimerase |
| 5 | 23.5 | 25 | SPBIB_v1_100132 | Short chain dehydrogenase/reductase family oxidoreductase |
| 7 | 44.2 | 41 | SPBIB_v1_100133 | Cytidylate kinase |
| 12 | 53.1 | 52 | SPBIB_v1_100134 | conserved protein of unknown function |
| 17 | 51.8 | 70 | SPBIB_v1_100135 | UDP-N-acetylmuramate--L-alanine ligase |
| 18 | 38.8 | 57 | SPBIB_v1_100136 | putative 4-alpha-glucanotransferase |
| 3 | 13 | 8 | SPBIB_v1_100138 | Pseudouridine synthase, RluA family |
| 14 | 56.2 | 66 | SPBIB_v1_100139 | Protein HflK |
| 3 | 27.1 | 12 | SPBIB_v1_410089;SPBIB_v1_10023;SPBIB_v1_10014 | conserved protein of unknown function;conserved protein of unknown function;conserved protein of unknown function |
| 16 | 54.4 | 44 | SPBIB_v1_100140 | Protein HflC |
| 6 | 38.5 | 12 | SPBIB_v1_100141 | putative Metallophosphoesterase |
| 11 | 41.1 | 49 | SPBIB_v1_100142 | conserved protein of unknown function |
| 4 | 12.6 | 11 | SPBIB_v1_100145 | Glucose import ATP-binding protein TsgD13 |
| 17 | 75.1 | 179 | SPBIB_v1_100146 | putative basic membrane lipoprotein |
| 7 | 38.9 | 30 | SPBIB_v1_100148 | Purine nucleoside phosphorylase DeoD-type |
| 15 | 45.1 | 59 | SPBIB_v1_100149 | Tyrosine--tRNA ligase |
| 17 | 86.9 | 102 | SPBIB_v1_100150 | DNA-binding protein HRL18 |
| 33 | 71.6 | 210 | SPBIB_v1_100151 | Glycine--tRNA ligase |
| 39 | 63.8 | 217 | SPBIB_v1_100152 | glutamyl-tRNA synthetase |
| 30 | 68.1 | 128 | SPBIB_v1_100153 | Glutamate--tRNA ligase |
| 6 | 45.1 | 19 | SPBIB_v1_100155 | Histidine triad (HIT) protein |
| 11 | 65.7 | 25 | SPBIB_v1_100156 | Thiamine diphosphokinase |
| 9 | 28.6 | 25 | SPBIB_v1_100157 | Polynucleotide adenylyltransferase/metal dependent phosphohydrolase |
| 5 | 21 | 20 | SPBIB_v1_100158 | Ribonuclease 3 |
| 9 | 94.9 | 43 | SPBIB_v1_100159 | acyl carrier protein (ACP) |
| 4 | 10.4 | 6 | SPBIB_v1_10016 | Kinase domain protein |
| 9 | 49.4 | 33 | SPBIB_v1_100160 | Phosphopantetheine adenylyltransferase |
| 2 | 6.9 | 4 | SPBIB_v1_100161 | Apolipoprotein N-acyltransferase |
| 7 | 60 | 28 | SPBIB_v1_100162 | conserved protein of unknown function |
| 4 | 16.5 | 7 | SPBIB_v1_100163 | Radical SAM domain protein (fragment) |
| 28 | 60.3 | 143 | SPBIB_v1_100166 | Trigger factor |
| 7 | 40.9 | 27 | SPBIB_v1_100167 | proteolytic subunit of ClpA-ClpP and ClpX-ClpP ATP-dependent serine proteases |
| 17 | 48.7 | 50 | SPBIB_v1_100168 | ATPase and specificity subunit of ClpX-ClpP ATP-dependent serine protease |
| 17 | 63.1 | 86 | SPBIB_v1_100169 | Thiamine-binding periplasmic protein |
| 7 | 53.1 | 19 | SPBIB_v1_100172 | Shikimate kinase |
| 16 | 66.3 | 80 | SPBIB_v1_100173 | Membrane dipeptidase |
| 27 | 51 | 73 | SPBIB_v1_100174 | excinulease of nucleotide excision repair, DNA damage recognition component |
| 13 | 44.8 | 34 | SPBIB_v1_100175 | Peptidase S1 and S6 chymotrypsin/Hap |
| 7 | 57.2 | 40 | SPBIB_v1_100176 | Cob(I)yrinic acid a,c-diamide adenosyltransferase |
| 2 | 13.8 | 3 | SPBIB_v1_100177 | putative Arginyltransferase |
| 23 | 58.9 | 93 | SPBIB_v1_100178 | putative PpiC-type peptidyl-prolyl cis-trans isomerase |
| 23 | 53.3 | 76 | SPBIB_v1_100179 | GTP-binding membrane protein |
| 6 | 61 | 18 | SPBIB_v1_100180 | Glutaredoxin |
| 4 | 40 | 23 | SPBIB_v1_100181 | Ferredoxin thioredoxin reductase beta chain |
| 10 | 46.7 | 20 | SPBIB_v1_100182 | Aminotransferase class V |
| 10 | 33.7 | 24 | SPBIB_v1_100183 | putative tRNA sulfurtransferase |
| 19 | 49.8 | 55 | SPBIB_v1_100184 | RelA/SpoT domain protein |
| 7 | 28 | 9 | SPBIB_v1_100186 | Cell wall hydrolase/autolysin |
| 44 | 57.8 | 218 | SPBIB_v1_100187 | Vitamin B12-dependent ribonucleotide reductase |
| 13 | 41.1 | 45 | SPBIB_v1_100188 | Arginine deiminase |
| 25 | 65.7 | 100 | SPBIB_v1_100189 | conserved protein of unknown function |
| 4 | 7.4 | 4 | SPBIB_v1_100192 | ABC-type multidrug/protein/lipid transport system, ATPase component |
| 5 | 11.2 | 9 | SPBIB_v1_100193 | ABC transporter related protein |
| 4 | 28.6 | 13 | SPBIB_v1_100194 | Ribonuclease H |
| 3 | 7.4 | 3 | SPBIB_v1_100195 | conserved exported protein of unknown function |
| 9 | 40.1 | 39 | SPBIB_v1_100196 | conserved protein of unknown function |
| 7 | 22.7 | 15 | SPBIB_v1_100197 | conserved exported protein of unknown function |
| 2 | 7.5 | 6 | SPBIB_v1_100198 | conserved membrane protein of unknown function |
| 7 | 43.9 | 31 | SPBIB_v1_100199 | putative Maf-like protein TDE_2348 |
| 18 | 78.5 | 82 | SPBIB_v1_100200 | 30S ribosomal protein S2 |
| 22 | 79.2 | 119 | SPBIB_v1_100201 | Elongation factor Ts |
| 23 | 78.8 | 93 | SPBIB_v1_100202 | ribosome recycling factor |
| 6 | 42.6 | 12 | SPBIB_v1_100203 | undecaprenyl pyrophosphate synthase |
| 5 | 13.4 | 10 | SPBIB_v1_100205 | 1-deoxy-D-xylulose 5-phosphate reductoisomerase |
| 5 | 63.6 | 33 | SPBIB_v1_10038 | Thioredoxin-1 |
| 5 | 9.3 | 12 | SPBIB_v1_10040 | Beta-mannosidase |
| 23 | 60.6 | 94 | SPBIB_v1_10042 | Extracellular solute-binding protein family 1 |
| 11 | 31.2 | 34 | SPBIB_v1_10045 | Beta-glucosidase |
| 2 | 21.9 | 5 | SPBIB_v1_10047 | putative Transcriptional regulator, MarR family protein |
| 7 | 11.3 | 18 | SPBIB_v1_10048 | Xenobiotic-transporting ATPase |
| 8 | 20.5 | 11 | SPBIB_v1_10049 | conserved membrane protein of unknown function |
| 55 | 85.9 | 686 | SPBIB_v1_10050 | Cpn60 chaperonin GroEL, large subunit of GroESL |
| 9 | 56.4 | 34 | SPBIB_v1_10051 | RluA family pseudouridine synthase |
| 22 | 70 | 92 | SPBIB_v1_10053 | Aminotransferase class I and II |
| 16 | 47.4 | 67 | SPBIB_v1_10054 | GTPase HflX |
| 16 | 44.8 | 71 | SPBIB_v1_10055 | 2-dehydropantoate 2-reductase |
| 6 | 24.6 | 14 | SPBIB_v1_10056 | putative Biotin-- |
| 23 | 48.8 | 80 | SPBIB_v1_10057 | Glutamine--fructose-6-phosphate aminotransferase |
| 3 | 10.8 | 2 | SPBIB_v1_10059 | Signal transduction histidine kinase, LytS |
| 4 | 12.9 | 9 | SPBIB_v1_10060 | Two component transcriptional regulator, AraC family |
| 4 | 13.8 | 6 | SPBIB_v1_10061 | D-xylose transporter subunit;periplasmic-binding component of ABC superfamily |
| 51 | 90.3 | 1083 | SPBIB_v1_10062 | Periplasmic sugar-binding protein |
| 26 | 59.4 | 69 | SPBIB_v1_10063 | fused D-xylose transporter subunits of ABC superfamily: ATP-binding components |
| 1 | 5.1 | 5 | SPBIB_v1_10064 | Xylose transport system permease protein XylH |
| 9 | 41.4 | 13 | SPBIB_v1_110002 | Replicative DNA helicase |
| 13 | 56.3 | 62 | SPBIB_v1_110003 | 50S ribosomal protein L9 |
| 4 | 38.1 | 22 | SPBIB_v1_110005 | 30S ribosomal protein S18 |
| 6 | 40.7 | 24 | SPBIB_v1_110006 | Single-stranded DNA-binding protein |
| 7 | 81.7 | 58 | SPBIB_v1_110007 | 30S ribosomal protein S6 |
| 13 | 27.3 | 28 | SPBIB_v1_70009;SPBIB_v1_400029;SPBIB_v1_360036;SPBIB_v1_110008 | transposase;transposase;transposase;transposase |
| 20 | 59.1 | 54 | SPBIB_v1_110009 | Histidine--tRNA ligase |
| 6 | 36.2 | 13 | SPBIB_v1_110010 | tRNA (guanine-N(7)-)-methyltransferase |
| 35 | 44.7 | 103 | SPBIB_v1_110011 | Helicase-associated domain protein |
| 19 | 57.5 | 50 | SPBIB_v1_110012 | Exodeoxyribonuclease 7 large subunit |
| 5 | 66.2 | 14 | SPBIB_v1_110013 | Exodeoxyribonuclease 7 small subunit |
| 2 | 26.4 | 8 | SPBIB_v1_110014 | CTP pyrophosphohydrolase (fragment) |
| 13 | 36.3 | 33 | SPBIB_v1_110015 | protein of unknown function |
| 2 | 8.9 | 4 | SPBIB_v1_110016 | exported protein of unknown function |
| 48 | 60.6 | 193 | SPBIB_v1_110017 | Protein translocase subunit SecA |
| 10 | 23.9 | 25 | SPBIB_v1_110018 | putative Fibronectin-binding A domain protein |
| 14 | 63.7 | 116 | SPBIB_v1_110019 | conserved exported protein of unknown function |
| 11 | 23.5 | 24 | SPBIB_v1_110022 | putative Multi-sensor hybrid histidine kinase |
| 5 | 14.3 | 5 | SPBIB_v1_110023 | exported protein of unknown function |
| 15 | 32.1 | 32 | SPBIB_v1_110024 | protease, ATP-dependent zinc-metallo |
| 4 | 9 | 3 | SPBIB_v1_110025 | putative tRNA(Ile)-lysidine synthase |
| 16 | 84.3 | 84 | SPBIB_v1_110026 | 50S ribosomal protein L25 |
| 4 | 39.4 | 24 | SPBIB_v1_110027 | putative septation protein SpoVG |
| 5 | 28.7 | 14 | SPBIB_v1_110028 | 4-diphosphocytidyl-2-C-methyl-D-erythritol kinase |
| 3 | 28.1 | 4 | SPBIB_v1_110030 | putative Ribosomal silencing factor RsfS |
| 5 | 14.4 | 9 | SPBIB_v1_110031 | conserved protein of unknown function |
| 5 | 29.4 | 13 | SPBIB_v1_110032 | conserved protein of unknown function |
| 8 | 53.6 | 25 | SPBIB_v1_110033 | putative nicotinate-nucleotide adenylyltransferase |
| 8 | 32.2 | 30 | SPBIB_v1_110034 | GTPase involved in cell partioning and DNA repair |
| 5 | 69.1 | 29 | SPBIB_v1_110035 | 50S ribosomal protein L27 |
| 5 | 55.3 | 28 | SPBIB_v1_110036 | 50S ribosomal subunit protein L21 |
| 32 | 72.4 | 109 | SPBIB_v1_110037 | Arginine--tRNA ligase |
| 20 | 39.7 | 66 | SPBIB_v1_110039 | DNA ligase |
| 7 | 14.9 | 16 | SPBIB_v1_110041 | protein of unknown function |
| 8 | 38.7 | 17 | SPBIB_v1_110042 | putative FAD binding domain in molybdopterin dehydrogenase |
| 14 | 17.9 | 32 | SPBIB_v1_110044 | Transcription-repair-coupling factor |
| 10 | 76.9 | 38 | SPBIB_v1_110046 | Regulatory protein ArsR |
| 14 | 48.4 | 59 | SPBIB_v1_110047 | 4-hydroxy-3-methylbut-2-en-1-yl diphosphate synthase |
| 13 | 29.3 | 45 | SPBIB_v1_110049 | putative V-type ATP synthase subunit I 1 |
| 9 | 40.7 | 29 | SPBIB_v1_110050 | V-type ATP synthase subunit D 1 |
| 20 | 58.8 | 95 | SPBIB_v1_110051 | V-type ATP synthase beta chain |
| 32 | 69.8 | 152 | SPBIB_v1_110052 | V-type ATP synthase alpha chain |
| 4 | 41 | 12 | SPBIB_v1_110053 | conserved exported protein of unknown function |
| 19 | 71.7 | 77 | SPBIB_v1_110054 | putative V-type ATP synthase subunit E |
| 8 | 14.4 | 25 | SPBIB_v1_120001 | Magnesium-transporting ATPase, P-type 1 |
| 11 | 61.1 | 50 | SPBIB_v1_120002 | putative PTS IIA-like nitrogen-regulatory protein PtsN |
| 49 | 79.4 | 317 | SPBIB_v1_120003 | Translation elongation factor G |
| 5 | 38.1 | 13 | SPBIB_v1_120004 | Transcriptional repressor NrdR |
| 10 | 42.1 | 29 | SPBIB_v1_120005 | SPFH domain, Band 7 family protein |
| 9 | 70.4 | 35 | SPBIB_v1_120006 | conserved protein of unknown function |
| 6 | 45.3 | 13 | SPBIB_v1_120007 | HAD-superfamily hydrolase, subfamily IA, variant 3 |
| 37 | 76.1 | 185 | SPBIB_v1_120008 | Phosphoglucomutase |
| 5 | 29.8 | 8 | SPBIB_v1_120009 | Exonuclease, DNA polymerase III, epsilon subunit family (modular protein) |
| 14 | 56.1 | 48 | SPBIB_v1_120011 | putative Glucokinase |
| 10 | 25.6 | 25 | SPBIB_v1_120012 | UDP-glucose pyrophosphorylase |
| 4 | 8.2 | 8 | SPBIB_v1_120013 | putative Trimethylamine-N-oxide reductase |
| 43 | 62.7 | 171 | SPBIB_v1_120014 | Lon protease |
| 4 | 40.2 | 8 | SPBIB_v1_130001 | 6-carboxy-5,6,7,8-tetrahydropterin synthase |
| 18 | 50.3 | 59 | SPBIB_v1_130002 | UDP-N-acetylmuramyl-tripeptide synthetase |
| 21 | 66.1 | 97 | SPBIB_v1_130003 | D-alanine--D-alanine ligase |
| 19 | 61.9 | 140 | SPBIB_v1_130004 | Ornithine aminotransferase |
| 7 | 19.4 | 11 | SPBIB_v1_130005 | exported protein of unknown function |
| 7 | 28.8 | 16 | SPBIB_v1_130006 | exported protein of unknown function |
| 12 | 41.3 | 35 | SPBIB_v1_130007 | (Dimethylallyl)adenosine tRNA methylthiotransferase MiaB |
| 12 | 45.3 | 41 | SPBIB_v1_130009 | GTP cyclohydrolase FolE2 |
| 14 | 64.4 | 68 | SPBIB_v1_130010 | Bifunctional protein FolD |
| 14 | 48.6 | 34 | SPBIB_v1_130011 | conserved protein of unknown function |
| 4 | 11.8 | 5 | SPBIB_v1_130012 | CoA-disulfide reductase |
| 2 | 26.1 | 12 | SPBIB_v1_130013 | Rhodanese-like protein |
| 10 | 32.8 | 28 | SPBIB_v1_130014 | Extracellular solute-binding protein family 1 |
| 4 | 15.2 | 7 | SPBIB_v1_130017 | polyamine transporter subunit;ATP-binding component of ABC superfamily |
| 26 | 53.8 | 91 | SPBIB_v1_130018;REV__SPBIB_v1_310102 | Glutamate formiminotransferase |
| 16 | 73.7 | 97 | SPBIB_v1_130023 | Redox-sensing transcriptional repressor Rex |
| 3 | 14.6 | 6 | SPBIB_v1_130024 | protein of unknown function |
| 4 | 37.2 | 11 | SPBIB_v1_130025 | HTH-type transcriptional regulator IscR |
| 23 | 54.7 | 91 | SPBIB_v1_130026 | Orotidine 5-phosphate decarboxylase |
| 14 | 39 | 45 | SPBIB_v1_130027 | conserved exported protein of unknown function |
| 18 | 36.3 | 29 | SPBIB_v1_130028 | putative DNA mismatch repair protein MutL |
| 6 | 27.2 | 23 | SPBIB_v1_130030 | conserved protein of unknown function |
| 4 | 29.2 | 12 | SPBIB_v1_130032 | 30S ribosomal protein S20 |
| 10 | 72.8 | 66 | SPBIB_v1_130033 | DNA-binding protein HU |
| 19 | 41.2 | 57 | SPBIB_v1_130035 | Transcription termination factor Rho |
| 9 | 80.1 | 33 | SPBIB_v1_130037 | Non-canonical purine NTP pyrophosphatase |
| 17 | 50.3 | 65 | SPBIB_v1_130040 | DHH superfamily protein, subfamily 1 |
| 23 | 81.5 | 123 | SPBIB_v1_130041 | Ornithine carbamoyltransferase |
| 22 | 61 | 105 | SPBIB_v1_130044 | Glucose-6-phosphate isomerase |
| 12 | 43.7 | 35 | SPBIB_v1_130045 | UDP-N-acetylglucosamine--N-acetylmuramyl-(pentapeptide) pyrophosphoryl-undecaprenol N-acetylglucosamine transferase |
| 18 | 56.9 | 65 | SPBIB_v1_130047 | putative DNA polymerase III domain-containing protein |
| 12 | 37.3 | 26 | SPBIB_v1_130048 | PAS/PAC sensor signal transduction histidine kinase |
| 16 | 45.9 | 55 | SPBIB_v1_130049 | fused response regulator of ato opeon, in two-component system with AtoS: response regulator;sigma54 interaction protein |
| 25 | 57.3 | 118 | SPBIB_v1_130051 | molecular chaperone and ATPase component of HslUV protease |
| 5 | 34.2 | 25 | SPBIB_v1_130052 | peptidase component of the HslUV protease |
| 46 | 64.3 | 223 | SPBIB_v1_130054 | DNA topoisomerase 1 |
| 23 | 70.4 | 67 | SPBIB_v1_130057 | GTP-binding tubulin-like cell division protein |
| 13 | 40 | 44 | SPBIB_v1_130058 | Cell division protein FtsA |
| 13 | 34.3 | 54 | SPBIB_v1_130062 | UDP-N-acetylmuramoyl-tripeptide--D-alanyl-D-alanine ligase |
| 8 | 27.7 | 35 | SPBIB_v1_130064 | Ribosomal RNA small subunit methyltransferase H |
| 6 | 49 | 16 | SPBIB_v1_130065 | Protein MraZ |
| 14 | 44.1 | 42 | SPBIB_v1_140002 | conserved protein of unknown function |
| 9 | 17.2 | 11 | SPBIB_v1_140004 | UvrD/REP helicase |
| 10 | 42.9 | 32 | SPBIB_v1_140007 | tRNA dimethylallyltransferase 1 |
| 7 | 62.5 | 21 | SPBIB_v1_140009 | protein of unknown function |
| 13 | 44 | 54 | SPBIB_v1_140010 | protein of unknown function |
| 5 | 34.5 | 24 | SPBIB_v1_140011 | 50S ribosomal subunit protein L20 |
| 3 | 33.3 | 9 | SPBIB_v1_140012 | 50S ribosomal subunit protein L35 |
| 12 | 62.1 | 66 | SPBIB_v1_140013 | protein chain initiation factor IF-3 |
| 34 | 89.2 | 241 | SPBIB_v1_140014 | Fructose-bisphosphate aldolase |
| 7 | 26.3 | 24 | SPBIB_v1_140017 | conserved protein of unknown function |
| 32 | 44.8 | 100 | SPBIB_v1_140019 | protein disaggregation chaperone |
| 4 | 37.6 | 18 | SPBIB_v1_140020 | conserved protein of unknown function |
| 8 | 61.4 | 43 | SPBIB_v1_150001 | Arginine repressor |
| 7 | 27 | 15 | SPBIB_v1_150002 | putative L-lysine 2,3-aminomutase |
| 17 | 39.2 | 73 | SPBIB_v1_150003 | GMP synthase |
| 22 | 63.7 | 104 | SPBIB_v1_150004 | Adenylosuccinate synthetase |
| 19 | 45.8 | 61 | SPBIB_v1_150005 | conserved protein of unknown function |
| 8 | 38.4 | 23 | SPBIB_v1_150006 | conserved protein of unknown function |
| 36 | 78.9 | 338 | SPBIB_v1_150007 | Formate--tetrahydrofolate ligase |
| 40 | 76.5 | 292 | SPBIB_v1_150009 | Dipeptide-binding ABC transporter, periplasmic substrate-binding component |
| 10 | 51.6 | 43 | SPBIB_v1_150010 | D-ala-D-ala transporter subunit;ATP-binding component of ABC superfamily (fragment) |
| 18 | 59.6 | 82 | SPBIB_v1_150011 | dipeptide transporter;ATP-binding component of ABC superfamily |
| 2 | 8.4 | 9 | SPBIB_v1_150012 | Glutathione ABC transporter, permease protein GsiC |
| 8 | 41.1 | 20 | SPBIB_v1_150014 | conserved protein of unknown function |
| 5 | 37.7 | 18 | SPBIB_v1_150016 | putative tRNA threonylcarbamoyladenosine biosynthesis protein TsaB |
| 17 | 60.2 | 43 | SPBIB_v1_150017 | conserved protein of unknown function |
| 9 | 41.6 | 38 | SPBIB_v1_150021 | Lipoyl synthase |
| 5 | 82.5 | 20 | SPBIB_v1_150022 | conserved protein of unknown function |
| 7 | 34.5 | 27 | SPBIB_v1_150023 | Biotin/lipoate A/B protein ligase |
| 5 | 12.1 | 6 | SPBIB_v1_150024 | putative Histidine kinase |
| 20 | 24.2 | 63 | SPBIB_v1_150026 | protein of unknown function |
| 21 | 72.9 | 80 | SPBIB_v1_150028 | Periplasmic binding protein/LacI transcriptional regulator |
| 5 | 12.3 | 6 | SPBIB_v1_150030 | fused D-ribose transporter subunits of ABC superfamily: ATP-binding components |
| 17 | 50.9 | 59 | SPBIB_v1_150031 | L-ribulokinase |
| 12 | 29.8 | 37 | SPBIB_v1_150032 | L-arabinose isomerase |
| 5 | 41.3 | 17 | SPBIB_v1_150033 | L-ribulose-5-phosphate 4-epimerase |
| 7 | 56.6 | 14 | SPBIB_v1_150034 | protein of unknown function |
| 26 | 76.6 | 196 | SPBIB_v1_150035 | Extracellular solute-binding protein |
| 12 | 53 | 38 | SPBIB_v1_150038 | putative dehydrogenase |
| 5 | 25.9 | 14 | SPBIB_v1_150039 | Oxidoreductase domain-containing protein |
| 17 | 49.3 | 59 | SPBIB_v1_150040 | conserved protein of unknown function |
| 18 | 79 | 92 | SPBIB_v1_150042 | conserved exported protein of unknown function |
| 12 | 21.9 | 24 | SPBIB_v1_150047 | DNA topoisomerase 4 subunit B |
| 18 | 29.1 | 41 | SPBIB_v1_150048 | DNA topoisomerase (ATP-hydrolyzing) |
| 16 | 59.8 | 70 | SPBIB_v1_150052 | conserved protein of unknown function |
| 15 | 69.1 | 77 | SPBIB_v1_150053 | conserved hypothetical protein |
| 8 | 55.3 | 18 | SPBIB_v1_150055 | putative Heat shock protein DnaJ domain protein |
| 40 | 57.9 | 150 | SPBIB_v1_150056 | DNA polymerase I |
| 10 | 42.9 | 36 | SPBIB_v1_150058 | putative Sporulation repeat domain protein |
| 23 | 77.9 | 123 | SPBIB_v1_150059 | Purine-binding protein BAB2_0673 |
| 11 | 24.2 | 22 | SPBIB_v1_150060 | Uncharacterized ABC transporter ATP-binding protein YufO |
| 22 | 53.6 | 86 | SPBIB_v1_150063 | Adenine deaminase |
| 28 | 74.5 | 127 | SPBIB_v1_150064 | xanthine dehydrogenase, Fe-S binding subunit (modular protein) |
| 40 | 72.4 | 254 | SPBIB_v1_150065 | Xanthine dehydrogenase, molybdenum binding subunit |
| 24 | 65.8 | 132 | SPBIB_v1_150066 | putative Allantoinase |
| 49 | 62.8 | 262 | SPBIB_v1_150067 | putative oxidoreductase, Fe-S subunit |
| 27 | 75.5 | 145 | SPBIB_v1_150068 | putative chlorohydrolase/aminohydrolase |
| 29 | 81.6 | 211 | SPBIB_v1_150069 | putative peptidase |
| 17 | 58.9 | 63 | SPBIB_v1_150070 | putative threonine synthase |
| 37 | 81.2 | 231 | SPBIB_v1_150072 | Pyridoxal-5-phosphate-dependent protein beta subunit |
| 22 | 63.7 | 95 | SPBIB_v1_150073 | 8-oxoguanine deaminase |
| 39 | 89.4 | 296 | SPBIB_v1_150074 | conserved hypothetical protein |
| 12 | 48.8 | 39 | SPBIB_v1_150078 | putative Periplasmic binding protein |
| 23 | 45.9 | 94 | SPBIB_v1_150082 | L-fucose isomerase |
| 4 | 14.6 | 5 | SPBIB_v1_150083 | Transcriptional regulator, LacI family |
| 7 | 71 | 22 | SPBIB_v1_150085 | L-fucose mutarotase |
| 7 | 30 | 18 | SPBIB_v1_150086 | PfkB domain protein |
| 16 | 69.6 | 151 | SPBIB_v1_150087 | ABC sugar transporter, periplasmic ligand binding protein |
| 10 | 42.6 | 29 | SPBIB_v1_150090 | Transcriptional regulator, DeoR family |
| 16 | 46.7 | 73 | SPBIB_v1_150091 | TPP-dependent acetoin dehydrogenase complex |
| 17 | 43.7 | 62 | SPBIB_v1_150092 | Dihydrolipoyl dehydrogenase |
| 45 | 72.4 | 208 | SPBIB_v1_150093 | Pyruvate dehydrogenase (Acetyl-transferring) |
| 17 | 46.4 | 67 | SPBIB_v1_150094 | conserved protein of unknown function |
| 11 | 43.3 | 53 | SPBIB_v1_150095 | putative Sorbitol-6-phosphate 2-dehydrogenase |
| 19 | 49.8 | 62 | SPBIB_v1_150096 | Carbohydrate kinase, FGGY |
| 21 | 61.7 | 105 | SPBIB_v1_150097 | putative dehydrogenase |
| 22 | 77.6 | 116 | SPBIB_v1_150098 | putative NADH-dependent butanol dehydrogenase 1 |
| 7 | 23 | 10 | SPBIB_v1_150099 | Argininosuccinate lyase |
| 12 | 41.6 | 37 | SPBIB_v1_150100 | Argininosuccinate synthase |
| 15 | 45.4 | 42 | SPBIB_v1_150102 | putative tagatose-6-phosphate kinase (Phosphotagatokinase) |
| 6 | 21.1 | 21 | SPBIB_v1_150103 | Sodium:dicarboxylate symporter |
| 12 | 60.6 | 48 | SPBIB_v1_150104 | Aspartate racemase |
| 2 | 18.3 | 11 | SPBIB_v1_150105 | Cupin 2 conserved barrel domain protein |
| 6 | 28.7 | 10 | SPBIB_v1_150106 | Ring-hydroxylating dioxygenase, large terminal subunit |
| 6 | 22.8 | 10 | SPBIB_v1_150107 | conserved protein of unknown function |
| 23 | 63.8 | 96 | SPBIB_v1_150110 | glycine C-acetyltransferase |
| 6 | 39.6 | 24 | SPBIB_v1_150112 | Exodeoxyribonuclease |
| 6 | 20.4 | 12 | SPBIB_v1_150113 | putative ROK family protein |
| 11 | 22.8 | 28 | SPBIB_v1_150115 | putative ATP-dependent helicase YoaA |
| 5 | 10.2 | 8 | SPBIB_v1_150116 | protein of unknown function |
| 8 | 39.6 | 24 | SPBIB_v1_150120 | protein of unknown function |
| 9 | 50.3 | 43 | SPBIB_v1_150121 | Pyruvate/ketoisovalerate oxidoreductase, gamma subunit |
| 13 | 71 | 90 | SPBIB_v1_150122 | 2-oxoglutarate synthase |
| 18 | 74.6 | 100 | SPBIB_v1_150123 | Ketoisovalerate oxidoreductase subunit VorB |
| 3 | 36.6 | 14 | SPBIB_v1_150124 | conserved protein of unknown function |
| 12 | 54.2 | 42 | SPBIB_v1_150125 | Phosphate butyryltransferase |
| 14 | 47.1 | 49 | SPBIB_v1_150126 | Butyrate kinase 2 |
| 22 | 50.5 | 74 | SPBIB_v1_150129 | Phosphoglucomutase/phosphomannomutase alpha/beta/alpha domain I |
| 7 | 58.5 | 24 | SPBIB_v1_150130 | Ribonuclease HII |
| 12 | 63.6 | 58 | SPBIB_v1_150132 | Thioredoxin reductase |
| 11 | 42.9 | 40 | SPBIB_v1_150133 | protein of unknown function |
| 12 | 59.7 | 52 | SPBIB_v1_150134 | Histidinol phosphate phosphatase, HisJ |
| 9 | 72.9 | 29 | SPBIB_v1_150135 | Transcriptional regulator, TraR/DksA family |
| 2 | 34.7 | 5 | SPBIB_v1_150136 | translation initiation factor IF-1 |
| 2 | 4.7 | 7 | SPBIB_v1_150138 | Na+/glutamate symporter-like protein |
| 12 | 48.6 | 35 | SPBIB_v1_150141 | putative uracil phosphoribosyltransferase |
| 9 | 53.9 | 47 | SPBIB_v1_150142 | Phosphate propanoyltransferase |
| 8 | 38.9 | 24 | SPBIB_v1_150143 | DhnA-type fructose-1,6-bisphosphate aldolase-like enzyme |
| 10 | 30.1 | 21 | SPBIB_v1_150144 | putative Pentulose/hexulose kinase |
| 13 | 43.4 | 28 | SPBIB_v1_150145 | Theronine dehydrogenase-like Zn-dependent dehydrogenase |
| 18 | 63.2 | 78 | SPBIB_v1_150147 | Membrane lipoprotein TpN38(b) |
| 9 | 54.2 | 43 | SPBIB_v1_150151 | Pyrimidine 5-nucleotidase |
| 29 | 66.1 | 133 | SPBIB_v1_150154 | Pyrimidine-nucleoside phosphorylase |
| 30 | 51.8 | 99 | SPBIB_v1_150155 | Single-stranded-DNA-specific exonuclease RecJ |
| 4 | 13.5 | 9 | SPBIB_v1_150157 | exported protein of unknown function |
| 11 | 66.9 | 45 | SPBIB_v1_150160 | Transcription factor |
| 4 | 17 | 8 | SPBIB_v1_150161 | Bifunctional enzyme IspD/IspF |
| 6 | 26.9 | 14 | SPBIB_v1_150163 | protein of unknown function |
| 31 | 89.5 | 332 | SPBIB_v1_150165 | 31 kDa immunogenic protein |
| 3 | 5 | 11 | SPBIB_v1_150167 | TRAP transporter, 4TM/12TM fusion protein |
| 15 | 22.8 | 37 | SPBIB_v1_150168 | Aspartate kinase |
| 3 | 27.3 | 14 | SPBIB_v1_150170 | Glutaconyl-CoA decarboxylase subunit gamma |
| 5 | 59 | 20 | SPBIB_v1_150171 | protein of unknown function |
| 24 | 57.6 | 108 | SPBIB_v1_150172 | Propionyl-CoA carboxylase beta chain |
| 6 | 67.2 | 54 | SPBIB_v1_150173 | conserved protein of unknown function |
| 13 | 50.4 | 56 | SPBIB_v1_150174 | putative enzyme |
| 6 | 71.6 | 39 | SPBIB_v1_150175 | fragment of methylmalonyl-CoA mutase (part 2) |
| 36 | 81.8 | 183 | SPBIB_v1_150176 | fragment of methylmalonyl-CoA mutase (part 1) |
| 12 | 78.8 | 84 | SPBIB_v1_150177 | 2-oxoglutarate synthase subunit KorC |
| 11 | 54.5 | 65 | SPBIB_v1_150178 | 2-oxoglutarate synthase subunit KorB |
| 14 | 49.9 | 98 | SPBIB_v1_150179 | 2-oxoglutarate synthase subunit KorA |
| 13 | 25.6 | 32 | SPBIB_v1_150180 | conserved protein of unknown function |
| 12 | 31.4 | 22 | SPBIB_v1_150182 | tRNA-splicing ligase RtcB |
| 13 | 61.2 | 68 | SPBIB_v1_150183 | putative Glutamate racemase |
| 4 | 24.7 | 16 | SPBIB_v1_150184 | conserved exported protein of unknown function |
| 4 | 31.7 | 6 | SPBIB_v1_150186 | protein of unknown function |
| 3 | 16.4 | 8 | SPBIB_v1_150189 | conserved exported protein of unknown function |
| 34 | 69.8 | 134 | SPBIB_v1_160002 | putative Soluble ligand binding domain protein |
| 11 | 36.4 | 64 | SPBIB_v1_170001 | protein of unknown function |
| 14 | 39.4 | 39 | SPBIB_v1_170002 | conserved protein of unknown function |
| 10 | 31.1 | 29 | SPBIB_v1_170004 | Mannose-1-phosphate guanylyltransferase |
| 6 | 26.4 | 12 | SPBIB_v1_170005 | conserved protein of unknown function |
| 3 | 8.5 | 5 | SPBIB_v1_170006 | conserved protein of unknown function |
| 3 | 43.4 | 6 | SPBIB_v1_170008 | Ribbon-helix-helix domain protein |
| 12 | 33.9 | 41 | SPBIB_v1_170009 | putative ATPase (AAA+ superfamily) |
| 2 | 8.3 | 7 | SPBIB_v1_170012 | conserved protein of unknown function |
| 11 | 41.8 | 29 | SPBIB_v1_170013 | conserved protein of unknown function |
| 14 | 51.6 | 46 | SPBIB_v1_170016 | dTDP-glucose 4,6 dehydratase, NAD(P)-binding |
| 8 | 27.5 | 14 | SPBIB_v1_170019 | putative Glycosyl transferase group 1 |
| 21 | 68.4 | 87 | SPBIB_v1_170020 | putative UDP-N-acetylglucosamine 2-epimerase |
| 15 | 48.1 | 46 | SPBIB_v1_170021 | putative NAD-dependent epimerase/dehydratase |
| 21 | 59.4 | 108 | SPBIB_v1_170022 | UDP-glucose 4-epimerase |
| 5 | 11 | 12 | SPBIB_v1_170024 | putative glycosyltransferase EpsF |
| 23 | 55.8 | 115 | SPBIB_v1_170027 | UDP-N-acetyl-D-glucosamine 6-dehydrogenase |
| 7 | 46 | 24 | SPBIB_v1_170028 | UDP-2-acetamido-3-amino-2,3-dideoxy-D-glucuronate N-acetyltransferase |
| 4 | 44.6 | 8 | SPBIB_v1_170030 | protein of unknown function |
| 15 | 87.7 | 59 | SPBIB_v1_170031 | conserved protein of unknown function |
| 25 | 68.7 | 108 | SPBIB_v1_170032 | Pleiotropic regulatory protein |
| 16 | 66.8 | 46 | SPBIB_v1_170033 | UDP-2,3-diacetamido-2,3-dideoxy-D-glucuronate 2-epimerase |
| 8 | 22.9 | 20 | SPBIB_v1_170034 | conserved protein of unknown function |
| 5 | 11.2 | 8 | SPBIB_v1_170035 | conserved protein of unknown function |
| 4 | 12.8 | 10 | SPBIB_v1_180002 | protein of unknown function |
| 21 | 56.9 | 81 | SPBIB_v1_180004 | CDP-paratose 2-epimerase |
| 25 | 74.4 | 89 | SPBIB_v1_180005 | NAD-dependent epimerase/dehydratase |
| 15 | 55.2 | 47 | SPBIB_v1_180006 | CDP-glucose 4,6-dehydratase |
| 18 | 79.5 | 80 | SPBIB_v1_180007 | Glucose-1-phosphate cytidylyltransferase |
| 18 | 44.1 | 80 | SPBIB_v1_180008 | Lipopolysaccharide biosynthesis protein RfbH |
| 6 | 33.1 | 13 | SPBIB_v1_180009 | putative Nitric oxide dioxygenase |
| 11 | 32.6 | 18 | SPBIB_v1_180011 | conserved protein of unknown function |
| 3 | 9.4 | 3 | SPBIB_v1_180012 | conserved protein of unknown function |
| 9 | 46.4 | 20 | SPBIB_v1_180013 | conserved protein of unknown function |
| 3 | 14.7 | 4 | SPBIB_v1_180017 | conserved protein of unknown function |
| 5 | 12.3 | 7 | SPBIB_v1_180018 | conserved protein of unknown function |
| 10 | 26.4 | 28 | SPBIB_v1_180019 | AAA ATPase |
| 12 | 57.5 | 41 | SPBIB_v1_180022 | protein of unknown function |
| 13 | 14.3 | 20 | SPBIB_v1_180023 | protein of unknown function |
| 3 | 16.6 | 12 | SPBIB_v1_180037 | conserved protein of unknown function |
| 21 | 53.3 | 94 | SPBIB_v1_180039 | protein of unknown function |
| 11 | 49.8 | 36 | SPBIB_v1_180040 | conserved protein of unknown function |
| 21 | 51 | 65 | SPBIB_v1_180041 | Ribonuclease Y |
| 7 | 85.6 | 99 | SPBIB_v1_180042 | putative RNA-binding protein RbpE |
| 7 | 23.2 | 12 | SPBIB_v1_180043 | conserved protein of unknown function |
| 6 | 16.8 | 13 | SPBIB_v1_180044 | protein of unknown function |
| 8 | 33.7 | 23 | SPBIB_v1_180046 | tRNA pseudouridine synthase A |
| 5 | 54.4 | 21 | SPBIB_v1_180047 | Holo- |
| 4 | 25.3 | 5 | SPBIB_v1_180048 | putative YbbR family protein |
| 12 | 57.3 | 50 | SPBIB_v1_180050 | Dihydropteroate synthase |
| 31 | 61.6 | 139 | SPBIB_v1_190001 | Alanine--tRNA ligase |
| 2 | 9.3 | 7 | SPBIB_v1_190002 | protein of unknown function |
| 6 | 37.9 | 12 | SPBIB_v1_190004 | protein of unknown function |
| 6 | 45.6 | 39 | SPBIB_v1_190005 | Asparaginase/glutaminase |
| 7 | 32.3 | 21 | SPBIB_v1_190008 | Transcriptional regulator, TetR family |
| 49 | 56.2 | 197 | SPBIB_v1_190009 | Alpha amylase catalytic region |
| 14 | 21.1 | 23 | SPBIB_v1_190010 | protein of unknown function |
| 24 | 29.7 | 57 | SPBIB_v1_190011 | protein of unknown function |
| 10 | 39.5 | 25 | SPBIB_v1_190014 | conserved protein of unknown function |
| 11 | 42.4 | 35 | SPBIB_v1_190015 | putative ABC transporter, permease protein |
| 25 | 72.2 | 221 | SPBIB_v1_190016 | Peptidase T |
| 5 | 41.9 | 23 | SPBIB_v1_190024 | acetyl-CoA:acetoacetyl-CoA transferase, beta subunit |
| 7 | 38.1 | 27 | SPBIB_v1_190025 | acetyl-CoA:acetoacetyl-CoA transferase, alpha subunit |
| 6 | 7.3 | 14 | SPBIB_v1_190026 | Na+/H+ antiporter NhaC-like protein |
| 6 | 47.6 | 24 | SPBIB_v1_190027 | D-lysine 5,6-aminomutase beta subunit |
| 23 | 64.2 | 89 | SPBIB_v1_190028 | D-lysine 5,6-aminomutase alpha subunit |
| 23 | 47 | 75 | SPBIB_v1_190029 | protein of unknown function |
| 13 | 46.7 | 31 | SPBIB_v1_190030 | exported protein of unknown function |
| 21 | 66.7 | 121 | SPBIB_v1_190031 | L-lysine 2,3-aminomutase |
| 11 | 59 | 35 | SPBIB_v1_190032 | 3-keto-5-aminohexanoate cleavage enzyme |
| 30 | 70 | 156 | SPBIB_v1_190033 | 3-aminobutyryl-CoA aminotransferase |
| 21 | 76 | 118 | SPBIB_v1_190034 | L-erythro-3,5-diaminohexanoate dehydrogenase |
| 13 | 59.8 | 31 | SPBIB_v1_190035 | protein of unknown function |
| 16 | 64.7 | 71 | SPBIB_v1_190037 | putative ABC transporter substrate-binding lipoprotein YvgL |
| 8 | 50.5 | 35 | SPBIB_v1_190038 | Molybdenum cofactor synthesis domain protein |
| 4 | 16.1 | 9 | SPBIB_v1_190039 | Molybdenum cofactor biosynthesis enzyme |
| 5 | 40.3 | 14 | SPBIB_v1_190040 | molybdopterin biosynthesis, protein C |
| 16 | 47.1 | 56 | SPBIB_v1_190041 | Molybdopterin biosynthesis enzyme |
| 7 | 71.1 | 26 | SPBIB_v1_190042 | Molybdenum-binding protein |
| 35 | 79.1 | 208 | SPBIB_v1_190044 | IMP dehydrogenase |
| 36 | 80.6 | 210 | SPBIB_v1_190045 | Bifunctional purine biosynthesis protein PurH |
| 11 | 64.1 | 33 | SPBIB_v1_190046 | Phosphoribosylglycinamide formyltransferase |
| 33 | 57.2 | 133 | SPBIB_v1_190047 | phosphoribosylglycinamide synthetase phosphoribosylamine-glycine ligase (modular protein) |
| 18 | 57 | 71 | SPBIB_v1_190048 | Amidophosphoribosyltransferase |
| 9 | 84 | 59 | SPBIB_v1_190049 | N5-carboxyaminoimidazole ribonucleotide mutase |
| 22 | 69.8 | 103 | SPBIB_v1_190050 | putative phosphoribosylaminoimidazole-succinocarboxamide synthase 2 |
| 7 | 43.9 | 25 | SPBIB_v1_190051 | Phosphoribosylformylglycinamidine synthase 1 |
| 48 | 62 | 218 | SPBIB_v1_190052 | Phosphoribosylformylglycinamidine synthase 2 |
| 11 | 49.1 | 33 | SPBIB_v1_200002 | putative Branched-chain-amino-acid aminotransferase |
| 37 | 73.9 | 426 | SPBIB_v1_20001 | Extracellular solute-binding protein family 1 |
| 3 | 21.3 | 13 | SPBIB_v1_200010 | Sensory transduction protein regX3 |
| 4 | 11.3 | 4 | SPBIB_v1_200011 | putative Integral membrane sensor signal transduction histidine kinase |
| 7 | 28.2 | 24 | SPBIB_v1_200016 | conserved exported protein of unknown function |
| 4 | 21 | 12 | SPBIB_v1_200018 | acetaldehyde-CoA dehydrogenase II, NAD-binding |
| 5 | 27.2 | 17 | SPBIB_v1_200020 | 2-hydroxy-6-oxo-2,4-heptadienoate hydrolase |
| 14 | 49 | 46 | SPBIB_v1_200021 | Transcriptional regulator, IclR family |
| 2 | 26 | 6 | SPBIB_v1_200028 | ThiamineS protein |
| 5 | 35.1 | 16 | SPBIB_v1_200029 | Thiamine biosynthesis protein ThiF, family 2 (fragment) |
| 44 | 68.4 | 239 | SPBIB_v1_200030 | conserved exported protein of unknown function |
| 23 | 66.1 | 122 | SPBIB_v1_200031 | Oxidoreductase, 2-nitropropane dioxygenase family |
| 25 | 69.8 | 109 | SPBIB_v1_200032 | Ketol-acid reductoisomerase |
| 6 | 30 | 9 | SPBIB_v1_200033 | exported protein of unknown function |
| 3 | 35.6 | 11 | SPBIB_v1_200035 | 3-dehydroquinate dehydratase |
| 11 | 57.4 | 28 | SPBIB_v1_200038 | putative Membrane-bound serine protease (ClpP class) |
| 7 | 32.2 | 13 | SPBIB_v1_20004 | Transcriptional regulator, LacI family |
| 3 | 13.5 | 4 | SPBIB_v1_200043;SPBIB_v1_330001 | transposase (fragment) |
| 21 | 29.7 | 53 | SPBIB_v1_20005 | Beta-phosphoglucomutase |
| 4 | 11.1 | 8 | SPBIB_v1_20006 | conserved protein of unknown function |
| 33 | 68 | 234 | SPBIB_v1_20007 | Basic membrane lipoprotein |
| 5 | 11 | 11 | SPBIB_v1_20008 | conserved protein of unknown function |
| 23 | 74.9 | 143 | SPBIB_v1_20011 | Branched-chain amino acid ABC transporter, amino acid-binding protein (TC 3.A.1.4.1) |
| 4 | 28.5 | 7 | SPBIB_v1_20014 | leucine/isoleucine/valine transporter subunit;ATP-binding component of ABC superfamily |
| 3 | 17.6 | 5 | SPBIB_v1_20015 | leucine/isoleucine/valine transporter subunit;ATP-binding component of ABC superfamily |
| 5 | 19.5 | 10 | SPBIB_v1_20016 | Peptidase M20 |
| 4 | 19.6 | 9 | SPBIB_v1_20017 | exported protein of unknown function |
| 13 | 49.4 | 69 | SPBIB_v1_20018 | putative XshC-Cox1-family protein |
| 52 | 81.7 | 384 | SPBIB_v1_20019 | Aldehyde oxidase and xanthine dehydrogenase molybdopterin binding protein |
| 12 | 74.8 | 40 | SPBIB_v1_20020 | Xanthine dehydrogenase iron-sulfur-binding subunit |
| 19 | 86.6 | 141 | SPBIB_v1_20021 | Molybdopterin dehydrogenase FAD-binding protein |
| 46 | 47.9 | 129 | SPBIB_v1_20022 | DNA polymerase III subunit alpha |
| 9 | 37.4 | 24 | SPBIB_v1_20023 | putative Ribonuclease Z |
| 4 | 24.9 | 10 | SPBIB_v1_20024 | putative hydrolase of the HAD superfamily |
| 32 | 62 | 170 | SPBIB_v1_20025 | NADP-reducing hydrogenase subunit HndC |
| 53 | 67.8 | 273 | SPBIB_v1_20026 | NADH-quinone oxidoreductase subunit F 2 |
| 9 | 50.3 | 38 | SPBIB_v1_20027 | NADH:ubiquinone oxidoreductase 24 kD subunit |
| 13 | 80.3 | 47 | SPBIB_v1_20030 | adenylate kinase |
| 6 | 15 | 18 | SPBIB_v1_20031 | exported protein of unknown function |
| 5 | 24.3 | 10 | SPBIB_v1_20032 | protein of unknown function |
| 4 | 17.6 | 12 | SPBIB_v1_20033 | conserved protein of unknown function |
| 8 | 61.7 | 33 | SPBIB_v1_20034 | Nucleoside diphosphate kinase |
| 30 | 64.1 | 163 | SPBIB_v1_20035 | Oligoendopeptidase, pepF/M3 family |
| 31 | 62.3 | 151 | SPBIB_v1_20036 | Lysine--tRNA ligase |
| 9 | 40.5 | 30 | SPBIB_v1_20037 | conserved protein of unknown function |
| 5 | 23.3 | 12 | SPBIB_v1_20038 | O-sialoglycoprotein endopeptidase |
| 4 | 9.3 | 6 | SPBIB_v1_20043 | Peptidoglycan glycosyltransferase |
| 13 | 78.6 | 55 | SPBIB_v1_20044 | Metal dependent phosphohydrolase |
| 16 | 45.9 | 36 | SPBIB_v1_20045 | Signal recognition particle protein |
| 3 | 39.5 | 4 | SPBIB_v1_210003 | conserved protein of unknown function |
| 7 | 34.3 | 15 | SPBIB_v1_210005 | Mannitol-1-phosphate 5-dehydrogenase |
| 8 | 28.2 | 11 | SPBIB_v1_210006 | PfkB domain protein |
| 21 | 75.2 | 88 | SPBIB_v1_210012 | putative amino acid ABC transporter, substrate-binding protein |
| 8 | 25.4 | 15 | SPBIB_v1_210016 | conserved protein of unknown function |
| 12 | 56.3 | 48 | SPBIB_v1_210025 | ABC transporter substrate binding protein |
| 22 | 76.7 | 129 | SPBIB_v1_210026 | Leucine-, isoleucine-, valine-, threonine-, and alanine-binding protein |
| 4 | 19.9 | 5 | SPBIB_v1_210029 | leucine/isoleucine/valine transporter subunit;ATP-binding component of ABC superfamily |
| 3 | 13.9 | 7 | SPBIB_v1_210030 | leucine/isoleucine/valine transporter subunit;ATP-binding component of ABC superfamily |
| 11 | 55.1 | 47 | SPBIB_v1_210031 | putative signal transduction protein with CBS domains |
| 4 | 22.3 | 8 | SPBIB_v1_210032 | Resolvase domain-containing protein (fragment) |
| 13 | 33 | 33 | SPBIB_v1_210044 | putative type I restriction enzyme HindVIIP M protein |
| 3 | 20.2 | 9 | SPBIB_v1_210045 | conserved protein of unknown function |
| 10 | 37.1 | 21 | SPBIB_v1_210046 | protein of unknown function |
| 12 | 35.5 | 37 | SPBIB_v1_210048 | conserved protein of unknown function |
| 13 | 25.8 | 28 | SPBIB_v1_210049 | ATPase-like |
| 9 | 12.6 | 9 | SPBIB_v1_210050 | putative type I restriction enzyme HindVIIP R protein |
| 7 | 22.9 | 22 | SPBIB_v1_210052 | Aminopentol aminotransferase |
| 7 | 27.4 | 22 | SPBIB_v1_210057 | exported protein of unknown function |
| 23 | 68.4 | 72 | SPBIB_v1_210059;SPBIB_v1_290179 | Phosphoglycerate kinase;Phosphoglycerate kinase |
| 17 | 55.7 | 98 | SPBIB_v1_210060;SPBIB_v1_290180 | Glyceraldehyde-3-phosphate dehydrogenase;Glyceraldehyde-3-phosphate dehydrogenase |
| 45 | 70.8 | 166 | SPBIB_v1_210061 | putative NADH oxidase |
| 9 | 31.2 | 25 | SPBIB_v1_210063 | Xylose isomerase domain-containing protein TIM barrel |
| 21 | 79.9 | 126 | SPBIB_v1_210067 | putative Periplasmic sugar binding protein-like protein |
| 14 | 45.1 | 41 | SPBIB_v1_210068 | conserved protein of unknown function |
| 9 | 37.3 | 30 | SPBIB_v1_210069 | putative dipeptidase YkvY |
| 17 | 64.1 | 70 | SPBIB_v1_210070 | Glucose 1-dehydrogenase |
| 10 | 44.4 | 29 | SPBIB_v1_210071 | Uncharacterized oxidoreductase HI_0048 |
| 29 | 84.7 | 138 | SPBIB_v1_210072 | Xylose isomerase domain-containing protein TIM barrel |
| 25 | 72.8 | 103 | SPBIB_v1_210073 | Oxidoreductase domain protein |
| 10 | 31.2 | 21 | SPBIB_v1_210074 | D-xylose isomerase |
| 3 | 9.3 | 4 | SPBIB_v1_210075 | Xylulose kinase |
| 4 | 40 | 16 | SPBIB_v1_210081 | conserved protein of unknown function |
| 9 | 60.4 | 41 | SPBIB_v1_210082 | conserved protein of unknown function |
| 15 | 62 | 62 | SPBIB_v1_210083 | L-allo-threonine aldolase |
| 5 | 46.7 | 14 | SPBIB_v1_210084 | conserved protein of unknown function |
| 3 | 13.7 | 5 | SPBIB_v1_210088 | conserved protein of unknown function |
| 3 | 15.7 | 10 | SPBIB_v1_210089 | SufBD protein |
| 14 | 52.5 | 60 | SPBIB_v1_210090 | dipeptide transporter;ATP-binding component of ABC superfamily |
| 7 | 32.2 | 24 | SPBIB_v1_210091 | oligopeptide transporter subunit;ATP-binding component of ABC superfamily |
| 5 | 12.2 | 12 | SPBIB_v1_210092 | ABC-type transporter, integral membrane subunit |
| 72 | 82.1 | 451 | SPBIB_v1_210094 | ABC-type transporter, periplasmic subunit |
| 22 | 61.6 | 85 | SPBIB_v1_210096 | conserved exported protein of unknown function |
| 10 | 51.4 | 59 | SPBIB_v1_210097 | Ribokinase |
| 9 | 19.7 | 13 | SPBIB_v1_210099 | protein of unknown function |
| 10 | 46.7 | 32 | SPBIB_v1_210102 | HAD-superfamily hydrolase, subfamily IA, variant 3 |
| 5 | 56 | 19 | SPBIB_v1_210103 | Cupin 2, conserved barrel domain protein |
| 5 | 61.5 | 10 | SPBIB_v1_210107 | Regulatory protein ArsR |
| 5 | 38.9 | 19 | SPBIB_v1_210109 | putative NADH dehydrogenase/NAD(P)H nitroreductase AF_2267 |
| 14 | 81.9 | 88 | SPBIB_v1_210110 | Protein ArsC |
| 12 | 35.3 | 35 | SPBIB_v1_210111 | Dihydrolipoyl dehydrogenase |
| 21 | 77.4 | 121 | SPBIB_v1_210113 | exported protein of unknown function |
| 7 | 39.2 | 15 | SPBIB_v1_210114 | Spermidine synthase |
| 6 | 31.3 | 18 | SPBIB_v1_210115 | S-adenosylmethionine decarboxylase |
| 9 | 37 | 19 | SPBIB_v1_210116 | Spermidine/putrescine import ATP-binding protein PotA |
| 27 | 88.1 | 204 | SPBIB_v1_210118 | conserved exported protein of unknown function |
| 28 | 75 | 123 | SPBIB_v1_210125 | ABC-type transporter, periplasmic subunit |
| 15 | 54 | 62 | SPBIB_v1_210126 | Anhydro-N-acetylmuramic acid kinase |
| 4 | 38.4 | 19 | SPBIB_v1_210127 | putative RNA-binding protein RbpA |
| 3 | 23.4 | 13 | SPBIB_v1_210128 | NADH-quinone oxidoreductase, E subunit (modular protein) |
| 17 | 72.8 | 88 | SPBIB_v1_210129 | NADH:ubiquinone oxidoreductase, chain F |
| 24 | 42.8 | 60 | SPBIB_v1_210130 | NADP-reducing hydrogenase subunit HndC |
| 3 | 28 | 13 | SPBIB_v1_210132 | PilT protein domain protein |
| 2 | 25 | 7 | SPBIB_v1_210133 | conserved protein of unknown function |
| 8 | 64.5 | 20 | SPBIB_v1_210135 | conserved protein of unknown function |
| 15 | 61.1 | 55 | SPBIB_v1_210139 | ABC-type Fe3+ transport system, periplasmic component |
| 5 | 11.6 | 7 | SPBIB_v1_210142 | conserved membrane protein of unknown function |
| 2 | 12.2 | 3 | SPBIB_v1_210153 | conserved membrane protein of unknown function |
| 20 | 81.9 | 97 | SPBIB_v1_210155 | Phosphoglycerate dehydrogenase |
| 17 | 56.9 | 43 | SPBIB_v1_210156 | conserved protein of unknown function |
| 14 | 81.6 | 74 | SPBIB_v1_210157 | putative 2-dehydro-3-deoxy-6-phosphogalactonate aldolase |
| 16 | 78.2 | 76 | SPBIB_v1_210158 | Putative KHG/KDPG aldolase |
| 21 | 59.9 | 62 | SPBIB_v1_210159 | conserved protein of unknown function |
| 18 | 58.9 | 131 | SPBIB_v1_210161 | conserved exported protein of unknown function |
| 15 | 67.7 | 92 | SPBIB_v1_210164 | conserved protein of unknown function |
| 12 | 40.7 | 39 | SPBIB_v1_210165 | conserved protein of unknown function |
| 12 | 48.1 | 43 | SPBIB_v1_210166 | Phosphoglycerate dehydrogenase |
| 4 | 32.6 | 10 | SPBIB_v1_210172 | conserved exported protein of unknown function |
| 12 | 22 | 23 | SPBIB_v1_210174 | ABC transporter related protein |
| 7 | 21.3 | 15 | SPBIB_v1_210175 | protein of unknown function |
| 14 | 32.4 | 32 | SPBIB_v1_210176 | Ppx/GppA phosphatase |
| 17 | 33.3 | 31 | SPBIB_v1_210177 | Polyphosphate kinase |
| 4 | 30.6 | 13 | SPBIB_v1_210178 | Ribonucleotide monophosphatase NagD |
| 15 | 49.1 | 49 | SPBIB_v1_210179 | Alpha amylase catalytic region |
| 11 | 57.6 | 65 | SPBIB_v1_210182 | Outer membrane lipoprotein 3 |
| 2 | 19.4 | 6 | SPBIB_v1_210185 | putative Positive regulator of sigma(E) RseC/MucC |
| 9 | 50.9 | 17 | SPBIB_v1_210186 | Phosphoesterase family protein |
| 2 | 16.8 | 4 | SPBIB_v1_210187 | CMP/dCMP deaminase, zinc-binding protein |
| 13 | 57.1 | 84 | SPBIB_v1_210188 | Peptidyl-prolyl cis-trans isomerase |
| 12 | 85.5 | 68 | SPBIB_v1_210191 | OsmC family protein |
| 38 | 61.3 | 211 | SPBIB_v1_210192 | Elongation factor G 2 |
| 11 | 49.6 | 63 | SPBIB_v1_210193 | conserved protein of unknown function |
| 7 | 36 | 18 | SPBIB_v1_210194 | conserved exported protein of unknown function |
| 12 | 56.3 | 33 | SPBIB_v1_210195 | putative 6-phosphogluconolactonase |
| 20 | 44.5 | 62 | SPBIB_v1_210196 | Glucose-6-phosphate 1-dehydrogenase |
| 6 | 15 | 9 | SPBIB_v1_210197 | putative RNA methyltransferase YpsC |
| 14 | 50 | 36 | SPBIB_v1_210198 | Iron-containing alcohol dehydrogenase |
| 12 | 65.7 | 45 | SPBIB_v1_210200 | HAD-superfamily hydrolase, subfamily IIB |
| 6 | 24.7 | 11 | SPBIB_v1_210202 | Phospholipid/glycerol acyltransferase |
| 4 | 38.9 | 24 | SPBIB_v1_210204 | conserved exported protein of unknown function |
| 5 | 12.5 | 4 | SPBIB_v1_210205 | protein of unknown function |
| 2 | 22.5 | 8 | SPBIB_v1_50084;SPBIB_v1_210206 | putative Spore Coat Protein U domain protein;exported protein of unknown function |
| 9 | 26.4 | 29 | SPBIB_v1_210207 | Protein-export membrane protein SecF |
| 17 | 38.1 | 79 | SPBIB_v1_210208 | Protein translocase subunit SecD |
| 5 | 40.5 | 28 | SPBIB_v1_210209 | Preprotein translocase, YajC subunit |
| 15 | 57.3 | 75 | SPBIB_v1_210210 | amino acid (carbamate) kinase |
| 4 | 29 | 11 | SPBIB_v1_210211 | 4-hydroxy-3-methylbut-2-enyl diphosphate reductase |
| 12 | 53.4 | 54 | SPBIB_v1_210212 | Nitroreductase |
| 10 | 67.3 | 45 | SPBIB_v1_210213 | Uridine kinase |
| 3 | 28.1 | 7 | SPBIB_v1_210214 | Peptide methionine sulfoxide reductase MsrA |
| 26 | 85.5 | 144 | SPBIB_v1_210218 | putative ABC-type transport system, periplasmic component/surface lipoprotein |
| 7 | 23.5 | 13 | SPBIB_v1_210219 | PP-loop domain protein |
| 4 | 25.7 | 11 | SPBIB_v1_210220 | putative 2-5-RNA ligase |
| 9 | 27 | 16 | SPBIB_v1_210221 | putative Diacylglycerol kinase catalytic region |
| 12 | 15.7 | 29 | SPBIB_v1_210222 | Multi-sensor signal transduction histidine kinase |
| 16 | 60.9 | 50 | SPBIB_v1_210224 | Response regulator containing a CheY-like receiver domain and an HD-GYP domain |
| 6 | 23.5 | 9 | SPBIB_v1_220001 | conserved protein of unknown function |
| 4 | 32.6 | 14 | SPBIB_v1_220002 | conserved protein of unknown function |
| 6 | 37.3 | 22 | SPBIB_v1_220003 | putative tolQ-type transport protein |
| 29 | 76.4 | 115 | SPBIB_v1_220004 | Adenylosuccinate lyase |
| 12 | 56.2 | 35 | SPBIB_v1_220006 | putative HTH-type transcriptional repressor AllR |
| 15 | 24.8 | 35 | SPBIB_v1_220007 | Peptidase U32 |
| 29 | 62 | 160 | SPBIB_v1_220008 | Elongation factor G 1 |
| 11 | 59.2 | 54 | SPBIB_v1_220010 | conserved protein of unknown function |
| 17 | 22 | 36 | SPBIB_v1_220011 | conserved protein of unknown function |
| 21 | 34.3 | 54 | SPBIB_v1_230001 | Urocanate hydratase |
| 4 | 52.5 | 9 | SPBIB_v1_230002 | Thioredoxin |
| 27 | 83.7 | 137 | SPBIB_v1_230003 | Malate dehydrogenase |
| 5 | 7.1 | 5 | SPBIB_v1_240002 | conserved membrane protein of unknown function |
| 34 | 86 | 213 | SPBIB_v1_240005 | Phosphoserine aminotransferase |
| 20 | 79 | 145 | SPBIB_v1_240006 | D-isomer specific 2-hydroxyacid dehydrogenase NAD-binding protein |
| 33 | 81.5 | 167 | SPBIB_v1_240007 | conserved protein of unknown function |
| 5 | 41.3 | 27 | SPBIB_v1_240010 | putative GTP cyclohydrolase 1 type 2 |
| 2 | 18.1 | 6 | SPBIB_v1_240011 | protein of unknown function |
| 4 | 17.8 | 7 | SPBIB_v1_240012 | protein of unknown function |
| 13 | 56.1 | 69 | SPBIB_v1_240013 | Pyrroline-5-carboxylate reductase |
| 2 | 11.6 | 5 | SPBIB_v1_240014 | 2,3,4,5-tetrahydropyridine-2,6-dicarboxylate N-succinyltransferase |
| 11 | 51.7 | 51 | SPBIB_v1_240015 | 4-hydroxy-tetrahydrodipicolinate synthase |
| 5 | 40.3 | 11 | SPBIB_v1_240016 | putative 4-hydroxy-tetrahydrodipicolinate reductase |
| 5 | 10.7 | 6 | SPBIB_v1_240017 | Aspartokinase |
| 9 | 39.7 | 18 | SPBIB_v1_240018 | Aspartate-semialdehyde dehydrogenase |
| 12 | 38.5 | 46 | SPBIB_v1_240019 | protein of unknown function |
| 39 | 76.4 | 141 | SPBIB_v1_240020 | Glutamine synthetase |
| 4 | 24.6 | 10 | SPBIB_v1_240022 | leucine/isoleucine/valine transporter subunit;ATP-binding component of ABC superfamily |
| 4 | 26.3 | 8 | SPBIB_v1_240023 | leucine/isoleucine/valine transporter subunit;ATP-binding component of ABC superfamily |
| 23 | 75.7 | 120 | SPBIB_v1_240026 | Extracellular ligand-binding receptor |
| 11 | 34.8 | 24 | SPBIB_v1_240027 | conserved protein of unknown function |
| 17 | 51.5 | 44 | SPBIB_v1_240028 | conserved protein of unknown function |
| 27 | 80.7 | 116 | SPBIB_v1_240029 | protein of unknown function |
| 5 | 39.6 | 15 | SPBIB_v1_240031 | Biotin transporter BioY |
| 3 | 7.3 | 5 | SPBIB_v1_240034 | putative Adenylate/guanylate cyclase with Chase sensor |
| 2 | 16.7 | 7 | SPBIB_v1_240035 | putative Peptidase, M48 family |
| 13 | 36.4 | 28 | SPBIB_v1_240037 | Exonuclease I |
| 3 | 53.5 | 13 | SPBIB_v1_240038 | conserved protein of unknown function |
| 28 | 71.7 | 151 | SPBIB_v1_240040 | NAD-dependent aldehyde dehydrogenase |
| 25 | 84.5 | 214 | SPBIB_v1_240041 | conserved protein of unknown function |
| 4 | 23 | 12 | SPBIB_v1_240042 | Regulatory protein TetR |
| 13 | 44.3 | 44 | SPBIB_v1_240043 | conserved protein of unknown function |
| 6 | 41.3 | 17 | SPBIB_v1_240044 | putative Methyltransferase type 11 |
| 15 | 68.7 | 97 | SPBIB_v1_240045 | ATPase-like, ParA/MinD |
| 5 | 32.8 | 27 | SPBIB_v1_240046 | Dinitrogenase iron-molybdenum cofactor biosynthesis protein (modular protein) |
| 10 | 50.8 | 64 | SPBIB_v1_240047 | Serine--glyoxylate aminotransferase protein |
| 7 | 35.9 | 17 | SPBIB_v1_240048 | conserved protein of unknown function |
| 7 | 81.3 | 26 | SPBIB_v1_240050 | Glyoxalase/bleomycin resistance protein/dioxygenase |
| 8 | 49.6 | 32 | SPBIB_v1_240052 | Glucose 1-dehydrogenase |
| 21 | 78.8 | 139 | SPBIB_v1_240055 | Basic membrane lipoprotein |
| 8 | 27.8 | 22 | SPBIB_v1_240059 | Methylthioribose-1-phosphate isomerase 1 |
| 8 | 35.7 | 23 | SPBIB_v1_240060 | Transcriptional regulator |
| 5 | 36.8 | 22 | SPBIB_v1_240061 | Class II aldolase/adducin family protein (fragment) |
| 17 | 41.3 | 50 | SPBIB_v1_240064 | exported protein of unknown function |
| 5 | 21.8 | 8 | SPBIB_v1_240065 | conserved exported protein of unknown function |
| 27 | 83.6 | 165 | SPBIB_v1_240067 | Acetylornithine deacetylase / succinyl-diaminopimelate desuccinylase |
| 23 | 79.3 | 82 | SPBIB_v1_240068 | UDP-glucose 4-epimerase |
| 16 | 65.3 | 82 | SPBIB_v1_250001 | conserved exported protein of unknown function |
| 6 | 29.4 | 21 | SPBIB_v1_250003 | exported protein of unknown function |
| 8 | 22.4 | 28 | SPBIB_v1_250004 | 4-alpha-glucanotransferase |
| 9 | 39.6 | 35 | SPBIB_v1_250008 | conserved exported protein of unknown function |
| 3 | 29.2 | 19 | SPBIB_v1_250009 | exported protein of unknown function |
| 4 | 9.6 | 9 | SPBIB_v1_250010 | Threonylcarbamoyladenosine tRNA methylthiotransferase MtaB |
| 6 | 56.3 | 30 | SPBIB_v1_250016 | protein of unknown function |
| 6 | 29.6 | 17 | SPBIB_v1_250017 | Methyltransferase type 11 |
| 35 | 51.1 | 144 | SPBIB_v1_250018 | exported protein of unknown function |
| 7 | 38.4 | 19 | SPBIB_v1_250019 | Threonylcarbamoyl-AMP synthase |
| 5 | 28.6 | 17 | SPBIB_v1_250020 | B3/4 domain protein |
| 15 | 34.9 | 34 | SPBIB_v1_250023 | exported protein of unknown function |
| 9 | 32.5 | 31 | SPBIB_v1_250024 | conserved protein of unknown function |
| 4 | 15 | 8 | SPBIB_v1_250025 | conserved protein of unknown function |
| 15 | 40.9 | 37 | SPBIB_v1_250027 | Gamma-glutamyltransferase |
| 5 | 52.9 | 25 | SPBIB_v1_250029 | Cupin 2 conserved barrel domain protein |
| 16 | 55.9 | 87 | SPBIB_v1_250030 | putative enzyme |
| 34 | 73.1 | 192 | SPBIB_v1_250033 | transketolase 2, thiamin-binding |
| 21 | 44.2 | 74 | SPBIB_v1_250037 | fused putative transporter subunits of ABC superfamily: ATP-binding components |
| 2 | 28.7 | 6 | SPBIB_v1_250038 | Methylated-DNA--protein-cysteine methyltransferase-related protein |
| 10 | 55.5 | 48 | SPBIB_v1_250039 | glucosamine-6-phosphate deaminase |
| 12 | 55.3 | 39 | SPBIB_v1_250040 | N-acetylglucosamine-6-phosphate deacetylase |
| 23 | 70.5 | 175 | SPBIB_v1_250041 | exported protein of unknown function |
| 3 | 15.8 | 7 | SPBIB_v1_250043 | Deoxynucleoside kinase |
| 20 | 57.5 | 65 | SPBIB_v1_250044 | conserved protein of unknown function |
| 7 | 32.5 | 23 | SPBIB_v1_250045 | conserved exported protein of unknown function |
| 10 | 47.2 | 29 | SPBIB_v1_250047 | putative UspA domain-containing protein |
| 14 | 83.4 | 70 | SPBIB_v1_250048 | lipid hydroperoxide peroxidase |
| 4 | 12.7 | 11 | SPBIB_v1_250049 | protein of unknown function |
| 40 | 65.6 | 178 | SPBIB_v1_250050 | putative glutamine-fructose-6-phosphate transaminase |
| 16 | 53.7 | 70 | SPBIB_v1_250051 | UTP--glucose-1-phosphate uridylyltransferase (modular protein) |
| 20 | 54.8 | 91 | SPBIB_v1_250055 | Proline--tRNA ligase |
| 5 | 29.4 | 14 | SPBIB_v1_250058 | exported protein of unknown function |
| 7 | 54 | 15 | SPBIB_v1_250059 | exported protein of unknown function |
| 4 | 27.6 | 7 | SPBIB_v1_250061 | protein of unknown function |
| 1 | 5.8 | 3 | SPBIB_v1_250064 | exported protein of unknown function |
| 37 | 50.9 | 157 | SPBIB_v1_250066 | Peptidase M16 inactive domain protein |
| 25 | 72.6 | 107 | SPBIB_v1_250067 | Peptidase M16 domain protein |
| 23 | 57.3 | 91 | SPBIB_v1_250068 | Peptidase M16 domain protein |
| 6 | 43.2 | 23 | SPBIB_v1_250069 | Phosphoribosyltransferase |
| 2 | 16.3 | 3 | SPBIB_v1_250070 | Fe2+/Zn2+ uptake regulation proteins |
| 4 | 8.9 | 8 | SPBIB_v1_250072 | Ferrous iron transport protein B |
| 10 | 30.1 | 31 | SPBIB_v1_250073 | conserved exported protein of unknown function |
| 38 | 85.5 | 315 | SPBIB_v1_250074 | 2,3-cyclic-nucleotide 2-phosphodiesterase |
| 8 | 52.4 | 49 | SPBIB_v1_250075 | Heat shock protein Hsp20 |
| 3 | 14.1 | 10 | SPBIB_v1_250076 | conserved protein of unknown function |
| 13 | 39.4 | 68 | SPBIB_v1_250077 | FAD-dependent pyridine nucleotide-disulfide oxidoreductase |
| 21 | 61.7 | 108 | SPBIB_v1_250078 | FAD dependent oxidoreductase |
| 41 | 57.4 | 201 | SPBIB_v1_250079 | Glycogen phosphorylase |
| 6 | 12.7 | 10 | SPBIB_v1_250080 | protein of unknown function |
| 25 | 40.6 | 109 | SPBIB_v1_250081 | NADH dehydrogenase |
| 11 | 41.5 | 26 | SPBIB_v1_250082 | Trans-hexaprenyltranstransferase |
| 15 | 66.4 | 61 | SPBIB_v1_250083 | Bifunctional protein: zinc-containing alcohol dehydrogenase quinone oxidoreductase ( NADPH:quinone reductase) Similar to arginate lyase |
| 17 | 46.1 | 52 | SPBIB_v1_250085 | FAD-dependent pyridine nucleotide-disulfide oxidoreductase |
| 2 | 34.8 | 8 | SPBIB_v1_250087 | fragment of copper transporter (part 2) |
| 10 | 28 | 32 | SPBIB_v1_250088 | chaperone Hsp40, co-chaperone with DnaK |
| 41 | 73.1 | 252 | SPBIB_v1_250089 | chaperone Hsp70, co-chaperone with DnaJ |
| 11 | 57.9 | 42 | SPBIB_v1_250090 | Protein GrpE |
| 4 | 70.8 | 11 | SPBIB_v1_250091 | Carbon dioxide concentrating mechanism protein CcmL |
| 6 | 21.4 | 12 | SPBIB_v1_250092 | reactivating factor for ethanolamine ammonia lyase |
| 6 | 20 | 6 | SPBIB_v1_250093 | ethanolamine ammonia-lyase, large subunit, heavy chain |
| 16 | 40.5 | 51 | SPBIB_v1_250096 | Acetaldehyde dehydrogenase |
| 6 | 50.5 | 48 | SPBIB_v1_250097 | putative carboxysome-like ethanolaminosome structural protein, ethanolamine utilization protein |
| 10 | 60.3 | 43 | SPBIB_v1_250098 | conserved protein of unknown function |
| 3 | 23.2 | 7 | SPBIB_v1_250099 | protein of unknown function |
| 41 | 84.5 | 305 | SPBIB_v1_250100 | ABC transporter substrate binding protein |
| 17 | 62.5 | 61 | SPBIB_v1_250103 | Spermidine/putrescine import ATP-binding protein PotA |
| 12 | 36.5 | 30 | SPBIB_v1_250104;SPBIB_v1_250034 | RNA polymerase sigma-54 factor |
| 5 | 57.7 | 29 | SPBIB_v1_250105 | Ribosomal subunit interface protein |
| 12 | 47 | 36 | SPBIB_v1_250106 | HPr kinase/phosphorylase |
| 23 | 61.8 | 85 | SPBIB_v1_250111 | Extracellular solute-binding protein family 1 |
| 4 | 53.3 | 13 | SPBIB_v1_250115 | Phosphocarrier protein HPr |
| 20 | 74.2 | 112 | SPBIB_v1_260001 | Acetylornithine aminotransferase |
| 18 | 80.8 | 151 | SPBIB_v1_260002 | Acetylglutamate kinase |
| 19 | 66.4 | 80 | SPBIB_v1_260003 | Arginine biosynthesis bifunctional protein ArgJ |
| 17 | 76.5 | 74 | SPBIB_v1_260004 | N-acetyl-gamma-glutamyl-phosphate reductase |
| 7 | 12.9 | 22 | SPBIB_v1_260006 | putative K(+)-stimulated pyrophosphate-energized sodium pump |
| 13 | 68.7 | 60 | SPBIB_v1_260007 | conserved protein of unknown function |
| 6 | 59.4 | 26 | SPBIB_v1_260008 | conserved protein of unknown function |
| 22 | 72.7 | 125 | SPBIB_v1_260010 | Tryptophan--tRNA ligase |
| 8 | 23.9 | 23 | SPBIB_v1_260011 | putative Diguanylate cyclase |
| 4 | 13.2 | 6 | SPBIB_v1_260012 | protein of unknown function |
| 38 | 79 | 249 | SPBIB_v1_260013 | NADP-reducing hydrogenase subunit HndC |
| 34 | 62.2 | 190 | SPBIB_v1_260014 | NADP-reducing hydrogenase subunit HndC |
| 5 | 48.9 | 39 | SPBIB_v1_260015 | NAD(P)-dependent iron-only hydrogenase iron-sulfur protein |
| 7 | 59.8 | 29 | SPBIB_v1_260016 | Histidine kinase |
| 8 | 51.9 | 62 | SPBIB_v1_260017 | NADP-reducing hydrogenase subunit HndA |
| 8 | 68.3 | 35 | SPBIB_v1_260018 | putative Tetratricopeptide TPR_1 repeat-containing protein |
| 11 | 36.5 | 29 | SPBIB_v1_260019 | putative Protein kinase family protein |
| 4 | 16.2 | 4 | SPBIB_v1_260020 | Transcriptional regulator |
| 17 | 57.6 | 64 | SPBIB_v1_260025 | Sugar ABC transporter substrate-binding protein |
| 3 | 30.8 | 7 | SPBIB_v1_260026 | L-rhamnose mutarotase |
| 6 | 21.1 | 21 | SPBIB_v1_260031 | putative opine dehydrogenase |
| 25 | 45.8 | 58 | SPBIB_v1_260032 | Cobalamin B12-binding domain protein |
| 13 | 48.6 | 46 | SPBIB_v1_260033 | putative D-alanine--D-alanine ligase |
| 13 | 43.7 | 47 | SPBIB_v1_260038 | 5-methylthioadenosine/S-adenosylhomocysteine deaminase |
| 11 | 65.5 | 46 | SPBIB_v1_260039 | Purine nucleoside phosphorylase 1 |
| 17 | 55.4 | 58 | SPBIB_v1_260045 | putative amidase |
| 10 | 38.1 | 14 | SPBIB_v1_260046 | Transcriptional regulator, RpiR family |
| 14 | 46.1 | 46 | SPBIB_v1_260047 | Isoaspartyl dipeptidase |
| 35 | 79.8 | 192 | SPBIB_v1_260049 | DppA2 |
| 13 | 51.5 | 51 | SPBIB_v1_260052 | exported protein of unknown function |
| 2 | 19.8 | 8 | SPBIB_v1_260053 | protein of unknown function |
| 13 | 64.3 | 63 | SPBIB_v1_260054 | putative 2-hydroxy-3-oxopropionate reductase |
| 6 | 33.6 | 15 | SPBIB_v1_260055 | Dak phosphatase |
| 14 | 53.6 | 67 | SPBIB_v1_260056 | PTS-dependent dihydroxyacetone kinase, dihydroxyacetone-binding subunit DhaK |
| 19 | 74.8 | 123 | SPBIB_v1_260060 | ABC sugar transporter, periplasmic ligand binding protein |
| 10 | 37.6 | 21 | SPBIB_v1_260061 | Regulatory protein LacI |
| 10 | 28.1 | 33 | SPBIB_v1_260062 | Xylose isomerase |
| 3 | 15.6 | 8 | SPBIB_v1_260063 | D-tagatose 3-epimerase |
| 12 | 28.4 | 27 | SPBIB_v1_260064 | putative Carbohydrate kinase, FGGY |
| 9 | 36.9 | 25 | SPBIB_v1_260065 | NAD-dependent epimerase/dehydratase |
| 3 | 31 | 8 | SPBIB_v1_260066 | putative dual use protein Tyr:Ser/Thr phosphatase |
| 2 | 8.7 | 4 | SPBIB_v1_260067 | exported protein of unknown function |
| 19 | 67.6 | 191 | SPBIB_v1_260070 | TRAP transporter solute receptor, TAXI family |
| 3 | 13.4 | 4 | SPBIB_v1_260071 | Transcriptional regulator, IclR family |
| 22 | 72.9 | 95 | SPBIB_v1_260072 | DctP family TRAP transporter solute receptor |
| 4 | 12.6 | 9 | SPBIB_v1_260075 | Dihydrodipicolinate synthase |
| 6 | 29.8 | 15 | SPBIB_v1_260078 | NYC1 chlorophyll b reductase |
| 7 | 38 | 14 | SPBIB_v1_260079 | conserved protein of unknown function |
| 4 | 57.6 | 12 | SPBIB_v1_260081 | conserved protein of unknown function |
| 25 | 78.9 | 169 | SPBIB_v1_260082 | putative cation ABC transporter, periplasmic binding protein |
| 7 | 33.5 | 14 | SPBIB_v1_260083 | putative Zinc import ATP-binding protein ZnuC |
| 11 | 22.5 | 25 | SPBIB_v1_260088 | membrane protein of unknown function |
| 9 | 68.8 | 53 | SPBIB_v1_260089 | FKBP-type peptidyl-prolyl cis-trans isomerase SlyD |
| 34 | 65.7 | 128 | SPBIB_v1_260092 | Leucine--tRNA ligase |
| 5 | 27.6 | 16 | SPBIB_v1_270005 | conserved exported protein of unknown function |
| 6 | 28.5 | 48 | SPBIB_v1_270006 | conserved exported protein of unknown function |
| 31 | 61.7 | 161 | SPBIB_v1_270007 | Gamma-glutamyltranspeptidase |
| 13 | 53.9 | 52 | SPBIB_v1_270008 | conserved exported protein of unknown function |
| 14 | 26.8 | 33 | SPBIB_v1_270009 | CoA-disulfide reductase |
| 4 | 18.1 | 5 | SPBIB_v1_270010 | phosphoglyceromutase 1 |
| 3 | 21.3 | 6 | SPBIB_v1_270011 | conserved protein of unknown function |
| 11 | 27.1 | 23 | SPBIB_v1_270014 | Monosaccharide-transporting ATPase |
| 26 | 72.5 | 292 | SPBIB_v1_270015 | conserved exported protein of unknown function |
| 15 | 44.2 | 55 | SPBIB_v1_270017 | exported protein of unknown function |
| 2 | 22 | 11 | SPBIB_v1_270019 | conserved protein of unknown function |
| 5 | 19.1 | 18 | SPBIB_v1_270020 | ABC transporter, ATP-binding protein (modular protein) |
| 27 | 53.3 | 120 | SPBIB_v1_270023 | Phosphoenolpyruvate carboxykinase |
| 4 | 8.8 | 4 | SPBIB_v1_270025 | conserved protein of unknown function |
| 20 | 65.8 | 174 | SPBIB_v1_270030 | conserved exported protein of unknown function |
| 10 | 53.4 | 49 | SPBIB_v1_270031 | Dihydrodipicolinate synthase |
| 17 | 68 | 98 | SPBIB_v1_270032 | 4-hydroxy-tetrahydrodipicolinate synthase |
| 6 | 33.1 | 17 | SPBIB_v1_270033 | GntR domain protein |
| 10 | 33.2 | 30 | SPBIB_v1_270034 | 4-hydroxy-tetrahydrodipicolinate synthase |
| 16 | 73 | 78 | SPBIB_v1_270038 | TRAP dicarboxylate transporter, DctP subunit |
| 7 | 29.8 | 22 | SPBIB_v1_270041 | 3-dehydroquinate synthase |
| 22 | 45.8 | 60 | SPBIB_v1_270042 | Electron transfer flavoprotein subunit alpha (modular protein) |
| 15 | 47.2 | 46 | SPBIB_v1_270043 | FAD linked oxidase domain protein |
| 13 | 43.1 | 54 | SPBIB_v1_270047 | putative dTDP-4-dehydrorhamnose reductase |
| 25 | 81.7 | 176 | SPBIB_v1_270048 | Pyridoxal biosynthesis lyase PdxS |
| 9 | 76.7 | 59 | SPBIB_v1_270049 | Glutamine amidotransferase subunit PdxT |
| 14 | 78.5 | 66 | SPBIB_v1_270054 | Ferroxidase |
| 22 | 57.5 | 75 | SPBIB_v1_270055 | Threonine synthase |
| 13 | 58.5 | 41 | SPBIB_v1_270056 | putative Homoserine kinase |
| 4 | 25.2 | 9 | SPBIB_v1_270057 | putative Methylated-DNA-- |
| 3 | 20.8 | 6 | SPBIB_v1_270058 | conserved protein of unknown function |
| 3 | 15.4 | 4 | SPBIB_v1_280001 | Ribosomal RNA small subunit methyltransferase G |
| 24 | 52.9 | 88 | SPBIB_v1_280002 | CTP synthetase |
| 11 | 50 | 30 | SPBIB_v1_280004 | exported protein of unknown function |
| 12 | 59.1 | 62 | SPBIB_v1_280006 | Hydrolase, TatD family |
| 4 | 19.8 | 9 | SPBIB_v1_280007 | putative tRNA-dihydrouridine synthase |
| 3 | 8.7 | 3 | SPBIB_v1_280008 | protein of unknown function |
| 3 | 11.7 | 6 | SPBIB_v1_280009 | Radical SAM protein |
| 27 | 43.4 | 96 | SPBIB_v1_280010 | DNA polymerase III, gamma and tau subunit |
| 6 | 80.3 | 24 | SPBIB_v1_280011 | Nucleoid-associated protein Spica_0202 |
| 9 | 63 | 36 | SPBIB_v1_280012 | gap repair protein |
| 6 | 43.3 | 29 | SPBIB_v1_280013 | conserved protein of unknown function |
| 6 | 47.5 | 25 | SPBIB_v1_280014 | Anti-sigma regulator |
| 12 | 33.4 | 36 | SPBIB_v1_280015 | Fe-S cluster domain protein |
| 16 | 41.7 | 36 | SPBIB_v1_280016 | conserved protein of unknown function |
| 5 | 35.7 | 14 | SPBIB_v1_280017 | protein of unknown function |
| 9 | 50.6 | 28 | SPBIB_v1_280018 | conserved protein of unknown function |
| 22 | 56.4 | 64 | SPBIB_v1_280019 | putative Peptidase M20 |
| 11 | 24.3 | 32 | SPBIB_v1_280020 | AMP-dependent synthetase and ligase |
| 25 | 65.8 | 125 | SPBIB_v1_280022 | Ribose-phosphate pyrophosphokinase |
| 6 | 41.7 | 19 | SPBIB_v1_280023 | putative hydrolase (HAD superfamily) |
| 3 | 11.7 | 8 | SPBIB_v1_280024 | conserved protein of unknown function |
| 7 | 37.8 | 13 | SPBIB_v1_280025 | 2-dehydro-3-deoxyphosphogluconate aldolase/4-hydroxy-2-oxoglutarate aldolase |
| 5 | 45.4 | 18 | SPBIB_v1_280026 | putative beta-D-galactosidase |
| 4 | 34 | 9 | SPBIB_v1_280027 | 2-deoxy-D-gluconate 3-dehydrogenase |
| 8 | 38.4 | 21 | SPBIB_v1_280028 | 5-keto 4-deoxyuronate isomerase |
| 3 | 14.4 | 8 | SPBIB_v1_280029 | Transcriptional regulator |
| 6 | 97.3 | 27 | SPBIB_v1_280030 | Nitrogen-fixing NifU domain-containing protein |
| 6 | 32.7 | 15 | SPBIB_v1_280031 | Protein Soj homolog |
| 18 | 73.8 | 58 | SPBIB_v1_280032 | Stage 0 sporulation protein J |
| 8 | 30.1 | 27 | SPBIB_v1_280033 | exported protein of unknown function |
| 29 | 85.4 | 276 | SPBIB_v1_280034 | enolase |
| 11 | 77.5 | 50 | SPBIB_v1_280036 | DJ-1 family protein |
| 8 | 85.8 | 45 | SPBIB_v1_280037 | Enamine/imine deaminase |
| 16 | 56.5 | 59 | SPBIB_v1_280038 | Cystathionine beta-lyase PatB |
| 8 | 59.1 | 23 | SPBIB_v1_280040 | Flavin reductase domain protein FMN-binding protein |
| 8 | 43.1 | 19 | SPBIB_v1_280041 | exported protein of unknown function |
| 9 | 59.8 | 36 | SPBIB_v1_280042 | conserved exported protein of unknown function |
| 14 | 48.7 | 54 | SPBIB_v1_280044 | Thiamine biosynthesis lipoprotein ApbE |
| 2 | 18.3 | 8 | SPBIB_v1_280045 | putative enzyme |
| 5 | 25.8 | 12 | SPBIB_v1_280046 | protein of unknown function |
| 17 | 52.6 | 42 | SPBIB_v1_280047 | 3-dehydroquinate dehydratase |
| 13 | 48.8 | 42 | SPBIB_v1_280048 | GTPase Era |
| 5 | 42.5 | 8 | SPBIB_v1_280049 | Peptidyl-tRNA hydrolase |
| 7 | 25.8 | 16 | SPBIB_v1_280050 | PHP domain protein |
| 6 | 20.9 | 17 | SPBIB_v1_280051 | protein of unknown function |
| 12 | 59.2 | 91 | SPBIB_v1_280052 | Malonyl CoA-acyl carrier protein transacylase |
| 11 | 58.5 | 48 | SPBIB_v1_280053 | 3-oxoacyl- |
| 14 | 56.3 | 73 | SPBIB_v1_280054 | 3-oxoacyl- |
| 19 | 44.4 | 60 | SPBIB_v1_280055 | Glycogen operon protein GlgX homolog |
| 6 | 24 | 11 | SPBIB_v1_280057 | RNA polymerase sigma factor |
| 9 | 38.7 | 26 | SPBIB_v1_280058 | putative Polyprenyl synthetase |
| 26 | 42 | 81 | SPBIB_v1_290001 | DNA gyrase subunit A |
| 27 | 52.2 | 89 | SPBIB_v1_290002 | DNA gyrase subunit B |
| 9 | 26.8 | 24 | SPBIB_v1_290003 | Chromosomal replication initiator protein DnaA |
| 29 | 79 | 174 | SPBIB_v1_290004 | DNA polymerase III subunit beta |
| 9 | 32.5 | 22 | SPBIB_v1_290005 | DNA replication and repair protein RecF |
| 15 | 27.9 | 33 | SPBIB_v1_290010 | Membrane protein insertase YidC |
| 11 | 48.1 | 48 | SPBIB_v1_290011 | putative RNA-binding protein |
| 11 | 30.8 | 33 | SPBIB_v1_290012 | Electron transport complex protein RnfC |
| 3 | 22.6 | 8 | SPBIB_v1_290014 | Electron transport complex, rnfabcdGe type, g subunit |
| 3 | 10.2 | 10 | SPBIB_v1_290017 | Electron transport complex, RnfABCDGE type, B subunit |
| 5 | 76.5 | 15 | SPBIB_v1_290019 | antitoxin of the YoeB-YefM toxin-antitoxin system |
| 3 | 37.2 | 8 | SPBIB_v1_290020 | toxin of the YoeB-YefM toxin-antitoxin system |
| 9 | 11.1 | 14 | SPBIB_v1_290025 | DNA and RNA helicase |
| 26 | 79.5 | 137 | SPBIB_v1_290031 | methionine adenosyltransferase 1 |
| 22 | 56.3 | 92 | SPBIB_v1_290032 | putative Zn-dependent protease-like protein |
| 11 | 36.3 | 50 | SPBIB_v1_290033 | Peptidase U62 modulator of DNA gyrase |
| 3 | 27.8 | 20 | SPBIB_v1_290034 | putative redox protein |
| 5 | 33.3 | 7 | SPBIB_v1_290035 | glutamate and aspartate transporter subunit;ATP-binding component of ABC superfamily |
| 13 | 77.5 | 53 | SPBIB_v1_290037 | ABC-type transporter, periplasmic subunit family 3 |
| 5 | 13.2 | 9 | SPBIB_v1_290039 | conserved protein of unknown function |
| 3 | 35.8 | 7 | SPBIB_v1_290040 | putative peroxiredoxin |
| 12 | 39.2 | 31 | SPBIB_v1_290042 | regulator with hipB |
| 4 | 10.9 | 7 | SPBIB_v1_290044 | Signal transduction histidine kinase |
| 5 | 32.8 | 12 | SPBIB_v1_290045 | Sensory transduction protein regX3 |
| 5 | 54.9 | 24 | SPBIB_v1_290046 | fragment of copper transporter (part 2) |
| 17 | 29 | 41 | SPBIB_v1_290047 | copper transporter |
| 7 | 12.3 | 8 | SPBIB_v1_290051 | fragment of copper transporter (part 2) |
| 12 | 69.4 | 110 | SPBIB_v1_290053 | putative Nitroreductase |
| 9 | 65.2 | 20 | SPBIB_v1_290054 | Amino acid-binding ACT domain protein |
| 11 | 35.2 | 19 | SPBIB_v1_290056 | Hydrogenase large subunit domain protein |
| 2 | 28.6 | 9 | SPBIB_v1_290057 | conserved protein of unknown function |
| 5 | 25 | 16 | SPBIB_v1_290058 | conserved exported protein of unknown function |
| 5 | 79.7 | 14 | SPBIB_v1_290061 | putative redox protein, regulator of disulfide bond formation |
| 6 | 11.9 | 10 | SPBIB_v1_290064 | Methylenetetrahydrofolate reductase |
| 13 | 14.9 | 19 | SPBIB_v1_290065 | homocysteine-N5-methyltetrahydrofolate transmethylase, B12-dependent |
| 2 | 22.8 | 7 | SPBIB_v1_290067 | NIL domain-containing protein |
| 11 | 35.2 | 31 | SPBIB_v1_290068 | conserved protein of unknown function |
| 7 | 23.9 | 18 | SPBIB_v1_290069 | Small GTP-binding protein |
| 7 | 25.6 | 24 | SPBIB_v1_290070 | Aspartate ammonia-lyase |
| 11 | 34.5 | 34 | SPBIB_v1_290072 | Biotin and thiamin synthesis associated |
| 4 | 65.3 | 10 | SPBIB_v1_290073 | conserved protein of unknown function |
| 11 | 69.9 | 42 | SPBIB_v1_290075 | NADPH-dependent 7-cyano-7-deazaguanine reductase |
| 7 | 22.8 | 13 | SPBIB_v1_290076 | fused diaminohydroxyphosphoribosylaminopyrimidine deaminase;5-amino-6-(5-phosphoribosylamino) uracil reductase |
| 2 | 15.7 | 5 | SPBIB_v1_290077 | Riboflavin synthase |
| 13 | 37 | 39 | SPBIB_v1_290078 | Riboflavin biosynthesis protein RibBA |
| 9 | 78.7 | 67 | SPBIB_v1_290079 | 6,7-dimethyl-8-ribityllumazine synthase (Lumazine synthase)(riboflavin synthase beta chain) |
| 8 | 20.8 | 14 | SPBIB_v1_290080 | Phosphomethylpyrimidine synthase |
| 7 | 28.5 | 20 | SPBIB_v1_290082 | hydoxyethylthiazole kinase |
| 9 | 20.6 | 21 | SPBIB_v1_290085 | conserved exported protein of unknown function |
| 8 | 67.2 | 24 | SPBIB_v1_290088 | conserved protein of unknown function |
| 7 | 14.2 | 13 | SPBIB_v1_290089 | conserved membrane protein of unknown function |
| 18 | 61.1 | 90 | SPBIB_v1_290090 | Alpha-glucosidase |
| 4 | 11.9 | 8 | SPBIB_v1_290091 | Transcriptional regulator, LacI family |
| 20 | 59.8 | 87 | SPBIB_v1_290093 | putative Sugar-binding periplasmic proteins/domains |
| 9 | 41.2 | 29 | SPBIB_v1_290101 | protein of unknown function |
| 17 | 53.4 | 58 | SPBIB_v1_290102 | isocitrate dehydrogenase, specific for NADP+;e14 prophage |
| 19 | 36 | 58 | SPBIB_v1_290103 | Aconitate hydratase |
| 5 | 20.5 | 9 | SPBIB_v1_290106 | putative xylanase/chitin deacetylase |
| 28 | 60.7 | 130 | SPBIB_v1_290108 | Extracellular solute-binding protein family 5 |
| 5 | 15.7 | 7 | SPBIB_v1_290114 | putative Beta-lactamase |
| 6 | 30.1 | 11 | SPBIB_v1_290119 | NAD-dependent protein deacetylase (modular protein) |
| 6 | 23.7 | 16 | SPBIB_v1_290126 | Alcohol dehydrogenase GroES-like protein |
| 11 | 59.5 | 46 | SPBIB_v1_290128 | Response regulator receiver protein |
| 48 | 63.6 | 209 | SPBIB_v1_290129 | conserved protein of unknown function |
| 2 | 23.8 | 7 | SPBIB_v1_290130 | Prevent-host-death family protein |
| 4 | 43.2 | 16 | SPBIB_v1_290131 | PilT protein domain protein |
| 3 | 13 | 6 | SPBIB_v1_290133 | conserved protein of unknown function |
| 9 | 28.6 | 13 | SPBIB_v1_290134 | exported protein of unknown function |
| 15 | 71.9 | 83 | SPBIB_v1_290135 | conserved exported protein of unknown function |
| 6 | 35.2 | 27 | SPBIB_v1_290140 | Phosphorylase |
| 3 | 16.8 | 6 | SPBIB_v1_290141 | putative Regulatory protein GntR, HTH |
| 9 | 41.3 | 27 | SPBIB_v1_290144 | exported protein of unknown function |
| 9 | 27.7 | 20 | SPBIB_v1_290145 | conserved protein of unknown function |
| 32 | 68.1 | 158 | SPBIB_v1_290147 | Gamma-glutamyltranspeptidase |
| 8 | 49.1 | 46 | SPBIB_v1_290148 | conserved exported protein of unknown function |
| 6 | 20.6 | 9 | SPBIB_v1_290151 | Succinylglutamate desuccinylase/aspartoacylase family protein |
| 20 | 74.5 | 105 | SPBIB_v1_290153 | NADP-dependent oxidoreductase domain protein |
| 5 | 21.1 | 13 | SPBIB_v1_290154 | putative DNA repair exonuclease, SbcD |
| 22 | 29.4 | 47 | SPBIB_v1_290155 | protein of unknown function |
| 15 | 50 | 53 | SPBIB_v1_290156 | Alcohol dehydrogenase |
| 48 | 85.5 | 699 | SPBIB_v1_290158 | oligopeptide transporter subunit;periplasmic-binding component of ABC superfamily |
| 9 | 27.3 | 24 | SPBIB_v1_290159 | oligopeptide transporter subunit;membrane component of ABC superfamily |
| 6 | 15.7 | 12 | SPBIB_v1_290160 | ABC-type transporter, integral membrane subunit |
| 15 | 54.2 | 64 | SPBIB_v1_290161 | oligopeptide transporter subunit;ATP-binding component of ABC superfamily |
| 12 | 41.3 | 38 | SPBIB_v1_290162;SPBIB_v1_290112;SPBIB_v1_210121 | oligopeptide transporter subunit;ATP-binding component of ABC superfamily |
| 4 | 41.7 | 14 | SPBIB_v1_290178 | Transcription factor/trp operon repressor (modular protein) |
| 10 | 44.7 | 38 | SPBIB_v1_290181 | Inosose dehydratase |
| 18 | 43.6 | 50 | SPBIB_v1_290182 | 3D-(3,5/4)-trihydroxycyclohexane-1,2-dione hydrolase |
| 6 | 35 | 17 | SPBIB_v1_290183 | Myo-inositol catabolism IolB domain protein |
| 23 | 74.6 | 79 | SPBIB_v1_290184 | Uncharacterized oxidoreductase YrbE |
| 9 | 48 | 27 | SPBIB_v1_290185 | Xylose isomerase domain protein TIM barrel |
| 11 | 47.2 | 26 | SPBIB_v1_290188 | Periplasmic binding protein/LacI transcriptional regulator |
| 10 | 34.3 | 32 | SPBIB_v1_290192 | Transcriptional regulator |
| 4 | 39.6 | 8 | SPBIB_v1_290193 | protein of unknown function |
| 11 | 44.9 | 38 | SPBIB_v1_290194 | ABC-type transport system, periplasmic component |
| 5 | 36.2 | 11 | SPBIB_v1_290199 | FMN-binding domain protein |
| 4 | 16.9 | 7 | SPBIB_v1_290202 | Ferredoxin--NAD(+) reductase |
| 6 | 52.6 | 28 | SPBIB_v1_290203 | conserved protein of unknown function |
| 24 | 64 | 109 | SPBIB_v1_290204 | Pyruvate kinase |
| 16 | 55.3 | 61 | SPBIB_v1_290206 | Extracellular solute-binding protein family 1 |
| 3 | 10.7 | 5 | SPBIB_v1_290209 | Fe(3+) ions import ATP-binding protein FbpC |
| 4 | 20.7 | 8 | SPBIB_v1_290211 | putative HTH-type transcriptional regulator DegA |
| 24 | 66.9 | 98 | SPBIB_v1_290215 | glycerol kinase |
| 3 | 11.9 | 10 | SPBIB_v1_290216 | SagB-type dehydrogenase domain protein |
| 5 | 43.5 | 23 | SPBIB_v1_290217 | conserved protein of unknown function |
| 11 | 37.7 | 45 | SPBIB_v1_290220 | Periplasmic binding protein (fragment) |
| 17 | 58 | 48 | SPBIB_v1_290221 | Glutamate 5-kinase |
| 18 | 56 | 61 | SPBIB_v1_290222 | Gamma-glutamyl phosphate reductase |
| 7 | 37.7 | 25 | SPBIB_v1_290223 | Uncharacterized oxidoreductase YkwC |
| 15 | 49 | 52 | SPBIB_v1_290225 | Thymidylate synthase ThyX |
| 15 | 38.5 | 42 | SPBIB_v1_290227 | Inorganic diphosphatase |
| 5 | 14.8 | 16 | SPBIB_v1_290228 | TraB family protein |
| 9 | 37.9 | 27 | SPBIB_v1_290229 | putative DNA polymerase III, delta subunit |
| 3 | 22.5 | 6 | SPBIB_v1_290230 | LexA repressor |
| 18 | 74.9 | 58 | SPBIB_v1_300002 | Xaa-Pro aminopeptidase |
| 9 | 26.9 | 26 | SPBIB_v1_300003 | Dihydroorotase |
| 23 | 60.9 | 107 | SPBIB_v1_300004 | UDP-N-acetylglucosamine 1-carboxyvinyltransferase |
| 15 | 57.4 | 90 | SPBIB_v1_300005 | conserved protein of unknown function |
| 6 | 51.1 | 51 | SPBIB_v1_300006 | conserved protein of unknown function |
| 2 | 11.2 | 6 | SPBIB_v1_300011 | putative Ribosomal RNA small subunit methyltransferase E |
| 28 | 58.3 | 111 | SPBIB_v1_300012 | Carboxyl-terminal protease |
| 5 | 16.3 | 11 | SPBIB_v1_300014 | Prolipoprotein diacylglyceryl transferase |
| 25 | 53.4 | 98 | SPBIB_v1_300015 | putative glutamine-dependent NAD(+) synthetase |
| 5 | 26 | 4 | SPBIB_v1_300016 | Glycerophosphoryl diester phosphodiesterase |
| 5 | 51.8 | 18 | SPBIB_v1_300017 | protein of unknown function |
| 3 | 11.6 | 4 | SPBIB_v1_30002 | conserved protein of unknown function |
| 98 | 62.1 | 352 | SPBIB_v1_30003 | exported protein of unknown function |
| 3 | 6.2 | 9 | SPBIB_v1_30004 | membrane protein of unknown function |
| 7 | 33.8 | 14 | SPBIB_v1_30005 | UDP-N-acetylenolpyruvoylglucosamine reductase |
| 33 | 79.5 | 143 | SPBIB_v1_30006 | Cysteine--tRNA ligase |
| 2 | 9.1 | 5 | SPBIB_v1_30007 | RNA polymerase sigma-24 factor |
| 4 | 44.7 | 10 | SPBIB_v1_30008 | conserved protein of unknown function |
| 8 | 66 | 20 | SPBIB_v1_30009 | exported protein of unknown function |
| 29 | 73.8 | 185 | SPBIB_v1_30011 | serine hydroxymethyltransferase |
| 3 | 8.1 | 6 | SPBIB_v1_30012 | protein of unknown function |
| 2 | 27.7 | 5 | SPBIB_v1_30014 | Prevent-host-death family protein |
| 5 | 39.6 | 21 | SPBIB_v1_30017 | Elongation factor P |
| 2 | 20.9 | 6 | SPBIB_v1_30019 | conserved protein of unknown function |
| 4 | 7 | 4 | SPBIB_v1_30020 | ATP-dependent DNA helicase RecG |
| 33 | 68.3 | 194 | SPBIB_v1_30021 | Thermostable carboxypeptidase 1 |
| 10 | 27.3 | 25 | SPBIB_v1_30022 | Histidinol dehydrogenase |
| 18 | 46 | 52 | SPBIB_v1_30023 | conserved protein of unknown function |
| 10 | 56.9 | 30 | SPBIB_v1_30024 | imidazole glycerol phosphate synthase, catalytic subunit with HisH |
| 7 | 41.6 | 19 | SPBIB_v1_30025 | Imidazole glycerol phosphate synthase subunit HisH |
| 5 | 28.5 | 13 | SPBIB_v1_30026 | Imidazoleglycerol-phosphate dehydratase |
| 15 | 59.5 | 63 | SPBIB_v1_30027 | putative Histidinol-phosphate aminotransferase |
| 12 | 55.9 | 37 | SPBIB_v1_30028 | ATP phosphoribosyltransferase |
| 3 | 14 | 19 | SPBIB_v1_30031 | conserved membrane protein of unknown function |
| 3 | 21.2 | 3 | SPBIB_v1_30032 | Ribosomal RNA small subunit methyltransferase I |
| 5 | 24.3 | 9 | SPBIB_v1_30033 | tRNA/rRNA methyltransferase (SpoU) |
| 11 | 31.1 | 29 | SPBIB_v1_310003 | tRNA modification GTPase MnmE |
| 9 | 18 | 15 | SPBIB_v1_310004 | tRNA uridine 5-carboxymethylaminomethyl modification enzyme MnmG |
| 10 | 57.5 | 51 | SPBIB_v1_310005 | conserved protein of unknown function |
| 3 | 8.5 | 5 | SPBIB_v1_310008 | acetolactate synthase III, large subunit |
| 11 | 31.8 | 29 | SPBIB_v1_310013 | putative TrkA-N domain protein |
| 9 | 45.1 | 22 | SPBIB_v1_310015 | putative TrkA N-terminal domain protein |
| 26 | 85.5 | 201 | SPBIB_v1_310016 | 8-amino-7-oxononanoate synthase |
| 15 | 84.8 | 138 | SPBIB_v1_310018 | Rubrerythrin |
| 11 | 65.3 | 47 | SPBIB_v1_310019 | Desulfoferrodoxin |
| 6 | 60.4 | 25 | SPBIB_v1_310020 | conserved protein of unknown function |
| 17 | 36.7 | 59 | SPBIB_v1_310021 | putative SAM-dependent methyltransferase |
| 3 | 26.9 | 11 | SPBIB_v1_310022 | conserved protein of unknown function |
| 10 | 32.3 | 21 | SPBIB_v1_310027 | DNA repair protein RadA homolog |
| 27 | 48.4 | 109 | SPBIB_v1_310028 | putative 4-alpha-glucanotransferase (Amylomaltase) |
| 2 | 20.2 | 6 | SPBIB_v1_310031 | conserved protein of unknown function |
| 7 | 49 | 38 | SPBIB_v1_310034 | ATP-dependent Clp protease proteolytic subunit 2 |
| 3 | 15.9 | 5 | SPBIB_v1_310037 | putative Tetratricopeptide TPR_1 repeat-containing protein |
| 14 | 43.4 | 83 | SPBIB_v1_310038 | putative hydroxypyruvate reductase |
| 10 | 55 | 33 | SPBIB_v1_310039 | conserved protein of unknown function |
| 10 | 58 | 45 | SPBIB_v1_310041 | conserved exported protein of unknown function |
| 12 | 48.2 | 30 | SPBIB_v1_310042 | Cof-like hydrolase |
| 1 | 17.2 | 7 | SPBIB_v1_310044 | 50S ribosomal protein L33 |
| 10 | 73.5 | 49 | SPBIB_v1_310046 | transcription termination factor |
| 11 | 80.4 | 51 | SPBIB_v1_310047 | 50S ribosomal protein L11 |
| 14 | 61 | 73 | SPBIB_v1_310048 | 50S ribosomal subunit protein L1 |
| 12 | 72 | 58 | SPBIB_v1_310049 | 50S ribosomal protein L10 |
| 10 | 85.3 | 74 | SPBIB_v1_310050 | 50S ribosomal subunit protein L7/L12 |
| 74 | 67.4 | 349 | SPBIB_v1_310051 | DNA-directed RNA polymerase subunit beta |
| 87 | 63.1 | 416 | SPBIB_v1_310052 | RNA polymerase, beta prime subunit |
| 5 | 43.5 | 22 | SPBIB_v1_310053 | 30S ribosomal protein S12 |
| 9 | 62.8 | 45 | SPBIB_v1_310054 | 30S ribosomal subunit protein S7 |
| 11 | 28.2 | 29 | SPBIB_v1_310055 | putative Elongation factor G |
| 38 | 88.1 | 360 | SPBIB_v1_310056 | protein chain elongation factor EF-Tu (duplicate of tufB) |
| 7 | 55.4 | 32 | SPBIB_v1_310058 | 30S ribosomal subunit protein S10 |
| 16 | 68.6 | 83 | SPBIB_v1_310059 | 50S ribosomal subunit protein L3 |
| 11 | 67 | 50 | SPBIB_v1_310060 | 50S ribosomal protein L4 |
| 5 | 57.4 | 32 | SPBIB_v1_310061 | 50S ribosomal protein L23 |
| 15 | 62.7 | 78 | SPBIB_v1_310062 | 50S ribosomal subunit protein L2 |
| 5 | 60.2 | 20 | SPBIB_v1_310063 | 30S ribosomal subunit protein S19 |
| 8 | 56.6 | 40 | SPBIB_v1_310064 | 50S ribosomal subunit protein L22 |
| 15 | 48.2 | 51 | SPBIB_v1_310065 | 30S ribosomal subunit protein S3 |
| 6 | 37 | 25 | SPBIB_v1_310066 | 50S ribosomal subunit protein L16 |
| 5 | 64.4 | 18 | SPBIB_v1_310068 | 30S ribosomal subunit protein S17 |
| 7 | 53.3 | 30 | SPBIB_v1_310069 | 50S ribosomal protein L14 |
| 4 | 21.7 | 10 | SPBIB_v1_310070 | 50S ribosomal subunit protein L24 |
| 9 | 55.4 | 48 | SPBIB_v1_310071 | 50S ribosomal subunit protein L5 |
| 7 | 47 | 32 | SPBIB_v1_310072 | 30S ribosomal subunit protein S8 |
| 11 | 56.1 | 57 | SPBIB_v1_310073 | 50S ribosomal protein L6 |
| 6 | 59.5 | 33 | SPBIB_v1_310074 | 50S ribosomal protein L18 |
| 14 | 74.7 | 62 | SPBIB_v1_310075 | 30S ribosomal subunit protein S5 |
| 3 | 60 | 18 | SPBIB_v1_310076 | 50S ribosomal subunit protein L30 |
| 12 | 63.7 | 44 | SPBIB_v1_310077 | 50S ribosomal subunit protein L15 |
| 6 | 10.9 | 15 | SPBIB_v1_310078 | preprotein translocase membrane subunit |
| 13 | 64.2 | 56 | SPBIB_v1_310079 | 30S ribosomal subunit protein S13 |
| 5 | 29.5 | 18 | SPBIB_v1_310080 | 30S ribosomal subunit protein S11 |
| 16 | 66.4 | 84 | SPBIB_v1_310081 | 30S ribosomal subunit protein S4 |
| 19 | 64.1 | 127 | SPBIB_v1_310082 | DNA-directed RNA polymerase subunit alpha |
| 3 | 23.6 | 15 | SPBIB_v1_310083 | 50S ribosomal subunit protein L17 |
| 6 | 24.6 | 16 | SPBIB_v1_310088 | Branched-chain amino acid ABC transporter substrate-binding protein |
| 7 | 22 | 16 | SPBIB_v1_310090 | Imidazolonepropionase |
| 64 | 67 | 301 | SPBIB_v1_310091 | conserved protein of unknown function |
| 15 | 37.9 | 58 | SPBIB_v1_310092 | Histidine ammonia-lyase |
| 18 | 77.4 | 72 | SPBIB_v1_310095 | putative Transcriptional regulators of NagC/XylR (ROK) family, sugar kinase |
| 3 | 11.3 | 11 | SPBIB_v1_310096 | putative Beta-lactamase |
| 23 | 71.2 | 135 | SPBIB_v1_310100 | Extracellular solute-binding protein family 1 |
| 7 | 27.2 | 11 | SPBIB_v1_310101 | putative PTS component;possibly regulatory |
| 12 | 43.4 | 30 | SPBIB_v1_310104 | Isoform II |
| 7 | 38.2 | 14 | SPBIB_v1_320002 | conserved exported protein of unknown function |
| 2 | 18.6 | 7 | SPBIB_v1_320003 | DGC domain protein |
| 5 | 28.6 | 7 | SPBIB_v1_320004 | Two component transcriptional regulator, winged helix family |
| 9 | 43.5 | 45 | SPBIB_v1_330005 | exported protein of unknown function |
| 10 | 36.4 | 38 | SPBIB_v1_330006 | Cna protein B-type domain (modular protein) |
| 3 | 11.6 | 9 | SPBIB_v1_330007 | putative Subtilisin-like serine protease |
| 12 | 31.6 | 18 | SPBIB_v1_330009 | NAD-dependent dehydrogenase subunit |
| 8 | 29 | 14 | SPBIB_v1_330014 | DNA-binding protein, excisionase family |
| 2 | 6.8 | 6 | SPBIB_v1_330017 | Oligopeptide transport ATP-binding protein AppF |
| 28 | 65 | 191 | SPBIB_v1_330021 | ABC-type dipeptide transport system, periplasmic component |
| 16 | 56.7 | 82 | SPBIB_v1_340004 | CRISPR-associated protein Cas7/Csd2, subtype I-C/DVULG |
| 14 | 30.6 | 34 | SPBIB_v1_340005 | Csd1 family CRISPR-associated protein |
| 9 | 19.4 | 14 | SPBIB_v1_340009 | Sodium/hydrogen exchanger |
| 3 | 22.6 | 7 | SPBIB_v1_340011 | protein of unknown function |
| 3 | 41.2 | 11 | SPBIB_v1_340014 | conserved protein of unknown function |
| 7 | 18.6 | 16 | SPBIB_v1_340016 | Major facilitator superfamily MFS_1 |
| 12 | 51 | 45 | SPBIB_v1_340018 | MazG family protein |
| 90 | 74.9 | 688 | SPBIB_v1_340021 | putative 2-oxoacid-flavodoxin fused oxidoreductase:conserved protein;4Fe-4S cluster binding protein |
| 6 | 32 | 8 | SPBIB_v1_340022 | dTDP-glucose pyrophosphorylase |
| 7 | 32.1 | 25 | SPBIB_v1_340024 | conserved protein of unknown function |
| 10 | 42.5 | 40 | SPBIB_v1_340025 | Sulfide dehydrogenase subunit beta |
| 26 | 73 | 121 | SPBIB_v1_340026 | Sulfide dehydrogenase subunit alpha |
| 3 | 16.7 | 5 | SPBIB_v1_340027 | putative Tetratricopeptide TPR_1 repeat-containing protein |
| 8 | 21.2 | 26 | SPBIB_v1_340028 | putative p-aminobenzoyl-glutamate hydrolase subunit B |
| 3 | 8.7 | 4 | SPBIB_v1_340029 | 5-methylthioadenosine/S-adenosylhomocysteine deaminase |
| 29 | 81.3 | 152 | SPBIB_v1_340033 | putative CD4+ T-cell-stimulating antigen |
| 11 | 33.3 | 36 | SPBIB_v1_340034 | Peptidase M20 |
| 6 | 36 | 18 | SPBIB_v1_340035 | conserved protein of unknown function |
| 22 | 76.9 | 166 | SPBIB_v1_340036 | Iron ABC transporter, substrate binding protein |
| 10 | 49.2 | 32 | SPBIB_v1_340037 | Fe(3+) ions import ATP-binding protein FbpC |
| 9 | 56 | 39 | SPBIB_v1_340039 | Fructose-2,6-bisphosphatase (modular protein) |
| 3 | 14.9 | 8 | SPBIB_v1_340041 | exported protein of unknown function |
| 12 | 77.8 | 50 | SPBIB_v1_340042 | Purine nucleoside phosphorylase DeoD-type |
| 22 | 55.4 | 77 | SPBIB_v1_340043 | protein of unknown function |
| 2 | 31 | 6 | SPBIB_v1_340044 | 30S ribosomal protein S21 |
| 2 | 13.8 | 6 | SPBIB_v1_340045 | conserved exported protein of unknown function |
| 7 | 50.6 | 22 | SPBIB_v1_340046 | peptide deformylase |
| 12 | 61.5 | 36 | SPBIB_v1_340047 | Methionyl-tRNA formyltransferase |
| 7 | 22.1 | 13 | SPBIB_v1_340048 | PASTA domain containing protein |
| 6 | 25.2 | 8 | SPBIB_v1_340049 | conserved protein of unknown function |
| 6 | 12.2 | 8 | SPBIB_v1_340050 | putative Beta-galactosidase |
| 5 | 36.7 | 11 | SPBIB_v1_340051 | protein of unknown function |
| 9 | 21.5 | 17 | SPBIB_v1_340052 | Primosomal protein N |
| 34 | 85.6 | 276 | SPBIB_v1_340053 | Acetate kinase |
| 18 | 71.8 | 699 | SPBIB_v1_340054 | exported protein of unknown function |
| 2 | 10.1 | 4 | SPBIB_v1_340055 | exported protein of unknown function |
| 3 | 46.4 | 11 | SPBIB_v1_340056 | conserved protein of unknown function |
| 33 | 62.4 | 131 | SPBIB_v1_340058 | Chaperone protein HtpG |
| 3 | 14.4 | 6 | SPBIB_v1_340059 | putative Peptidase M23 |
| 10 | 30 | 20 | SPBIB_v1_340061 | Stage 0 sporulation protein YaaT (modular protein) |
| 11 | 57 | 57 | SPBIB_v1_340064 | Split soret cytochrome c |
| 40 | 65.1 | 199 | SPBIB_v1_340065 | Aspartate--tRNA ligase |
| 3 | 17.9 | 7 | SPBIB_v1_340067 | GntR domain protein |
| 17 | 70.8 | 93 | SPBIB_v1_340068 | TRAP dicarboxylate transporter, DctP subunit |
| 4 | 12.7 | 6 | SPBIB_v1_340071 | Dihydroxy-acid dehydratase |
| 15 | 40.2 | 48 | SPBIB_v1_350003 | 6-phosphofructokinase |
| 26 | 69.7 | 99 | SPBIB_v1_350008 | Hydroxylamine reductase |
| 13 | 64.8 | 46 | SPBIB_v1_350010 | conserved protein of unknown function |
| 19 | 27.2 | 44 | SPBIB_v1_350012 | putative two-component system hybrid sensor and regulator |
| 5 | 18.9 | 18 | SPBIB_v1_350015 | Metallophosphoesterase |
| 11 | 37.8 | 26 | SPBIB_v1_350021 | putative Extracellular solute-binding protein family 1 |
| 34 | 77.6 | 201 | SPBIB_v1_350024 | 5-nucleotidase/2,3-cyclic phosphodiesterase-like hydrolase |
| 10 | 45.9 | 27 | SPBIB_v1_350028 | Extracellular solute-binding protein family 1 |
| 7 | 9.7 | 11 | SPBIB_v1_350031 | putative DegV family protein |
| 29 | 56.3 | 111 | SPBIB_v1_350032 | putative CoA-binding domain-containing protein |
| 11 | 68.2 | 39 | SPBIB_v1_350033 | Indolepyruvate ferredoxin oxidoreductase |
| 19 | 44.8 | 63 | SPBIB_v1_350034 | Indolepyruvate ferredoxin oxidoreductase |
| 19 | 68.5 | 80 | SPBIB_v1_350035 | Glutamine synthetase, type I |
| 2 | 6.7 | 5 | SPBIB_v1_350043;SPBIB_v1_210154 | transposase;transposase |
| 9 | 13.3 | 20 | SPBIB_v1_350048 | Helicase domain protein |
| 5 | 12.9 | 4 | SPBIB_v1_350049 | ATPase AAA |
| 24 | 31.8 | 50 | SPBIB_v1_350050 | conserved protein of unknown function |
| 9 | 18.8 | 18 | SPBIB_v1_350051 | SMC domain protein |
| 4 | 26.2 | 3 | SPBIB_v1_350052 | conserved protein of unknown function |
| 43 | 63.1 | 153 | SPBIB_v1_350053 | conserved protein of unknown function |
| 4 | 43.6 | 7 | SPBIB_v1_350056 | conserved protein of unknown function |
| 2 | 19.1 | 8 | SPBIB_v1_350057 | DNA polymerase, beta domain protein region |
| 3 | 30.6 | 16 | SPBIB_v1_350058 | conserved protein of unknown function |
| 34 | 61.3 | 139 | SPBIB_v1_350059 | Glycoside hydrolase family 13 domain protein |
| 3 | 4.8 | 3 | SPBIB_v1_350060 | Pullulanase, type I (fragment) |
| 28 | 78.3 | 147 | SPBIB_v1_350061 | Alpha amylase catalytic region |
| 36 | 86 | 302 | SPBIB_v1_350064 | conserved exported protein of unknown function |
| 11 | 28.9 | 33 | SPBIB_v1_350065 | Neopullulanase |
| 37 | 72.9 | 218 | SPBIB_v1_350066 | conserved exported protein of unknown function |
| 4 | 27 | 21 | SPBIB_v1_350067 | conserved exported protein of unknown function |
| 4 | 28.4 | 9 | SPBIB_v1_350069 | conserved protein of unknown function |
| 21 | 50.5 | 61 | SPBIB_v1_360001 | 2-isopropylmalate synthase |
| 11 | 30.7 | 39 | SPBIB_v1_360002 | 3-isopropylmalate dehydratase large subunit |
| 6 | 45.4 | 24 | SPBIB_v1_360003 | 3-isopropylmalate dehydratase small subunit 1 (modular protein) |
| 12 | 41.2 | 46 | SPBIB_v1_360004 | 3-isopropylmalate dehydrogenase |
| 5 | 41.6 | 18 | SPBIB_v1_360007 | putative acetyltransferase |
| 3 | 40.4 | 10 | SPBIB_v1_360008 | putative alkylmercury lyase |
| 12 | 85.9 | 76 | SPBIB_v1_360009 | putative reductase |
| 8 | 66.9 | 56 | SPBIB_v1_360010 | peptidyl-prolyl cis-trans isomerase B (rotamase B) |
| 4 | 61.3 | 21 | SPBIB_v1_360011 | conserved exported protein of unknown function |
| 20 | 50.1 | 58 | SPBIB_v1_360012 | conserved protein of unknown function |
| 14 | 47.7 | 46 | SPBIB_v1_360013 | Stage II sporulation protein E |
| 7 | 65.5 | 39 | SPBIB_v1_360014 | conserved protein of unknown function |
| 21 | 56.8 | 109 | SPBIB_v1_360015 | Hydrogenase large subunit domain protein |
| 13 | 60.4 | 52 | SPBIB_v1_360019 | conserved protein of unknown function |
| 3 | 14.8 | 10 | SPBIB_v1_360021 | Sel1 repeat protein (fragment) |
| 5 | 12.5 | 7 | SPBIB_v1_360030 | Radical SAM domain protein |
| 3 | 11.2 | 7 | SPBIB_v1_360033 | exported protein of unknown function |
| 12 | 48.2 | 37 | SPBIB_v1_360034 | conserved protein of unknown function |
| 3 | 12.3 | 8 | SPBIB_v1_360035 | Undecaprenyl-diphosphatase |
| 5 | 51.9 | 24 | SPBIB_v1_370001 | PTS system fructose-specific EIIABC component |
| 1 | 17.6 | 8 | SPBIB_v1_370002 | protein of unknown function |
| 4 | 18.5 | 9 | SPBIB_v1_370007 | putative ATP synthase (E/31 kDa) subunit |
| 11 | 24.3 | 32 | SPBIB_v1_370008 | V-type ATP synthase alpha chain 2 |
| 9 | 23 | 24 | SPBIB_v1_370009 | V-type ATP synthase beta chain 2 |
| 4 | 23.8 | 13 | SPBIB_v1_370011 | UspA domain-containing protein |
| 5 | 23 | 13 | SPBIB_v1_370012 | protein of unknown function |
| 7 | 49.4 | 28 | SPBIB_v1_370013 | putative signal transduction protein with CBS domains |
| 20 | 42.1 | 54 | SPBIB_v1_370014 | AMP-dependent synthetase and ligase |
| 27 | 68.7 | 96 | SPBIB_v1_370015 | Response regulator receiver modulated diguanylate cyclase |
| 10 | 50.7 | 22 | SPBIB_v1_370016 | Chemotaxis response regulator protein-glutamate methylesterase 2 |
| 16 | 28.4 | 25 | SPBIB_v1_370017 | putative Chemotaxis protein |
| 16 | 36.8 | 63 | SPBIB_v1_370018 | conserved exported protein of unknown function |
| 4 | 22.5 | 10 | SPBIB_v1_370019 | protein of unknown function |
| 14 | 34.4 | 24 | SPBIB_v1_370020 | putative MCP methyltransferase, CheR-type |
| 3 | 27.5 | 8 | SPBIB_v1_370021 | protein of unknown function |
| 4 | 48.9 | 9 | SPBIB_v1_370022 | conserved protein of unknown function |
| 7 | 31.1 | 16 | SPBIB_v1_370023 | conserved protein of unknown function |
| 13 | 51 | 59 | SPBIB_v1_370024 | Arsenite-transporting ATPase |
| 9 | 14.2 | 43 | SPBIB_v1_370026 | Carbon starvation protein CstA |
| 12 | 30.9 | 17 | SPBIB_v1_370027 | protein of unknown function |
| 4 | 4.4 | 7 | SPBIB_v1_370028 | Glycosyl transferase |
| 9 | 14.7 | 18 | SPBIB_v1_370029 | conserved exported protein of unknown function |
| 14 | 41.1 | 72 | SPBIB_v1_370030 | Peptidase M29 aminopeptidase II |
| 6 | 58 | 16 | SPBIB_v1_380002 | Spore protein SP21 family protein |
| 34 | 84 | 193 | SPBIB_v1_380003 | Extracellular solute-binding protein family 1 |
| 16 | 68.8 | 67 | SPBIB_v1_380005 | Fe(3+) ions import ATP-binding protein FbpC |
| 5 | 12 | 8 | SPBIB_v1_380006 | taurine transporter subunit;ATP-binding component of ABC superfamily (modular protein) |
| 23 | 69.5 | 200 | SPBIB_v1_380007 | ABC-type nitrate/sulfonate/bicarbonate transport system, periplasmic component |
| 3 | 34.8 | 8 | SPBIB_v1_380008 | putative aromatic acid decarboxylase |
| 15 | 34.8 | 37 | SPBIB_v1_380010 | conserved protein of unknown function |
| 9 | 41.4 | 23 | SPBIB_v1_380014 | conserved exported protein of unknown function |
| 21 | 68.9 | 88 | SPBIB_v1_380018 | conserved protein of unknown function |
| 24 | 81.4 | 116 | SPBIB_v1_380019 | conserved protein of unknown function |
| 15 | 61.2 | 70 | SPBIB_v1_380022 | TRAP dicarboxylate transporter, DctP subunit |
| 12 | 52.7 | 44 | SPBIB_v1_380025 | 3-oxoacyl-(Acyl-carrier-protein) reductase |
| 16 | 49.3 | 51 | SPBIB_v1_380026 | Uronate isomerase |
| 2 | 12.7 | 10 | SPBIB_v1_380027 | glycerol-3-phosphate transporter subunit;membrane component of ABC superfamily |
| 45 | 88 | 663 | SPBIB_v1_380029 | glycerol-3-phosphate transporter subunit;periplasmic-binding component of ABC superfamily |
| 17 | 70.3 | 93 | SPBIB_v1_380030 | Glycerophosphoryl diester phosphodiesterase |
| 10 | 73.5 | 32 | SPBIB_v1_380031 | MutT/NUDIX family protein |
| 19 | 58.5 | 37 | SPBIB_v1_380032 | Fumarate hydratase class I, aerobic |
| 14 | 49.9 | 48 | SPBIB_v1_380033 | conserved protein of unknown function |
| 23 | 58.8 | 94 | SPBIB_v1_380035 | Phosphoenolpyruvate-protein phosphotransferase |
| 20 | 69 | 79 | SPBIB_v1_380038 | M18 family aminopeptidase |
| 76 | 74.4 | 499 | SPBIB_v1_380039 | exported protein of unknown function |
| 41 | 51.4 | 225 | SPBIB_v1_380041 | Isoleucine--tRNA ligase |
| 7 | 30.2 | 15 | SPBIB_v1_380043 | exported protein of unknown function |
| 4 | 28.9 | 5 | SPBIB_v1_380046 | conserved protein of unknown function |
| 13 | 52.9 | 59 | SPBIB_v1_380049 | protein of unknown function |
| 33 | 66.4 | 179 | SPBIB_v1_380050 | Long-chain-fatty-acid--CoA ligase |
| 5 | 17.9 | 11 | SPBIB_v1_380051 | putative tRNA-dihydrouridine synthase |
| 4 | 45.8 | 25 | SPBIB_v1_380052 | 30S ribosomal subunit protein S16 |
| 4 | 45.5 | 16 | SPBIB_v1_380053 | conserved protein of unknown function |
| 2 | 18.3 | 5 | SPBIB_v1_380054 | putative Ribosome maturation factor RimM |
| 5 | 25.8 | 16 | SPBIB_v1_380055 | tRNA (guanine-1-)-methyltransferase |
| 6 | 46.3 | 30 | SPBIB_v1_380056 | 50S ribosomal subunit protein L19 |
| 17 | 50.2 | 82 | SPBIB_v1_380060 | Serine--tRNA ligase |
| 7 | 22.8 | 23 | SPBIB_v1_380061 | peptide chain release factor RF-1 |
| 4 | 26.1 | 8 | SPBIB_v1_380062 | putative Release factor glutamine methyltransferase |
| 10 | 17 | 21 | SPBIB_v1_380063 | (P)ppGpp synthetase I, SpoT/RelA |
| 16 | 53.1 | 50 | SPBIB_v1_390001 | exported protein of unknown function |
| 8 | 66.9 | 26 | SPBIB_v1_390002 | protein of unknown function |
| 13 | 43.9 | 51 | SPBIB_v1_390003 | Peptidase M24 |
| 6 | 48.3 | 19 | SPBIB_v1_390007 | protein of unknown function |
| 17 | 61.7 | 76 | SPBIB_v1_390009 | Amidohydrolase 2 |
| 9 | 35.1 | 32 | SPBIB_v1_390010 | Aminotransferase class I and II |
| 6 | 33.2 | 14 | SPBIB_v1_390011 | putative tRNA threonylcarbamoyladenosine dehydratase |
| 14 | 62.8 | 54 | SPBIB_v1_390015 | protein of unknown function |
| 17 | 62.3 | 117 | SPBIB_v1_390016 | 4-hydroxybutyrate coenzyme A transferase |
| 19 | 64.2 | 96 | SPBIB_v1_390017 | 3-oxoacyl- |
| 13 | 42.9 | 48 | SPBIB_v1_390018 | putative Transcriptional regulator |
| 12 | 47.9 | 50 | SPBIB_v1_390019 | Beta-ketoacyl-acyl-carrier-protein synthase III |
| 39 | 89 | 348 | SPBIB_v1_390020 | Leucine-, isoleucine-, valine-, threonine-, and alanine-binding protein |
| 6 | 42.1 | 16 | SPBIB_v1_390023 | Branched-chain amino acid transport ATP-binding protein LivG |
| 9 | 53.2 | 32 | SPBIB_v1_390024 | leucine/isoleucine/valine transporter subunit;ATP-binding component of ABC superfamily |
| 11 | 64.3 | 44 | SPBIB_v1_390025 | putative hydrolase |
| 11 | 55.2 | 65 | SPBIB_v1_390026 | Putative hydrolase or acyltransferase of alpha/beta superfamily (fragment) |
| 26 | 79.9 | 161 | SPBIB_v1_390027 | 3-ketoacyl-CoA thiolase |
| 6 | 12.4 | 15 | SPBIB_v1_390028 | conserved membrane protein of unknown function |
| 25 | 54.3 | 101 | SPBIB_v1_400001 | Long-chain-fatty-acid--CoA ligase |
| 30 | 44.7 | 118 | SPBIB_v1_400002 | Valine--tRNA ligase |
| 2 | 13.7 | 5 | SPBIB_v1_400003 | conserved protein of unknown function |
| 14 | 62.3 | 81 | SPBIB_v1_400004 | Deoxyribose-phosphate aldolase |
| 4 | 17.8 | 9 | SPBIB_v1_400007 | GGDEF domain protein |
| 5 | 53 | 18 | SPBIB_v1_400008 | Cyclic nucleotide-binding protein |
| 30 | 78.6 | 150 | SPBIB_v1_400009 | Extracellular solute-binding protein family 1 |
| 17 | 66.8 | 95 | SPBIB_v1_40001 | putative sugar transporter subunit: ATP-binding component of ABC superfamily transporter |
| 8 | 48.9 | 42 | SPBIB_v1_400017 | conserved protein of unknown function |
| 15 | 45.4 | 52 | SPBIB_v1_400018 | Carbohydrate ABC transporter substrate-binding protein, CUT1 family |
| 3 | 7.2 | 8 | SPBIB_v1_40002 | Major facilitator superfamily MFS_1 |
| 13 | 43.4 | 35 | SPBIB_v1_400022 | putative N-acetylglucosamine-6-phosphate deacetylase |
| 11 | 45.7 | 34 | SPBIB_v1_400023 | Alanine racemase |
| 19 | 78.2 | 67 | SPBIB_v1_400024 | Diaminopimelate epimerase 1 |
| 12 | 43.3 | 47 | SPBIB_v1_400025 | Amidohydrolase |
| 8 | 43 | 18 | SPBIB_v1_400027 | Peptidase C26 |
| 23 | 56.9 | 92 | SPBIB_v1_400028 | Diaminopimelate decarboxylase |
| 15 | 44.3 | 65 | SPBIB_v1_40003 | conserved protein of unknown function |
| 2 | 19.6 | 7 | SPBIB_v1_400030 | exported protein of unknown function |
| 7 | 26.3 | 15 | SPBIB_v1_400038 | conserved protein of unknown function |
| 3 | 22.6 | 7 | SPBIB_v1_40004 | conserved protein of unknown function |
| 5 | 55.5 | 7 | SPBIB_v1_410003 | protein of unknown function |
| 7 | 42.1 | 22 | SPBIB_v1_410005 | conserved protein of unknown function |
| 4 | 39.1 | 25 | SPBIB_v1_410016 | conserved protein of unknown function |
| 15 | 39.9 | 33 | SPBIB_v1_410019 | protein of unknown function |
| 4 | 28.7 | 16 | SPBIB_v1_410027 | exported protein of unknown function |
| 4 | 27.7 | 19 | SPBIB_v1_410028 | Exonuclease (modular protein) |
| 2 | 54 | 6 | SPBIB_v1_410029 | conserved protein of unknown function |
| 6 | 26.5 | 14 | SPBIB_v1_410030 | Phage P1-related protein in restrction modification operon RflA |
| 11 | 39.3 | 42 | SPBIB_v1_410035 | threonine 3-dehydrogenase, NAD(P)-binding |
| 19 | 85 | 86 | SPBIB_v1_410037 | Transport-associated protein |
| 3 | 14.1 | 6 | SPBIB_v1_410038 | RNA methyltransferase, TrmH family, group 3 |
| 27 | 60.1 | 118 | SPBIB_v1_410039 | Protein PyrBI |
| 13 | 42.2 | 36 | SPBIB_v1_410040 | putative Uncharacterized RNA methyltransferase TDE_2619 |
| 22 | 68.1 | 105 | SPBIB_v1_410041 | Beta-lactamase domain protein |
| 7 | 69.4 | 59 | SPBIB_v1_410042 | Ribose-5-phosphate isomerase B |
| 25 | 54.9 | 94 | SPBIB_v1_410056 | putative Extracellular solute-binding protein family 5 |
| 8 | 19.1 | 18 | SPBIB_v1_410059 | conserved protein of unknown function |
| 16 | 52.8 | 81 | SPBIB_v1_410066 | Extracellular solute-binding protein family 1 |
| 2 | 16.4 | 14 | SPBIB_v1_410075 | exported protein of unknown function |
| 6 | 17.7 | 7 | SPBIB_v1_410076;SPBIB_v1_410044;SPBIB_v1_10001 | transposase |
| 19 | 65.3 | 86 | SPBIB_v1_50001 | conserved exported protein of unknown function |
| 20 | 60.3 | 105 | SPBIB_v1_50002 | Aminotransferase class I and II |
| 20 | 58.9 | 93 | SPBIB_v1_50004 | Orn/DAP/Arg decarboxylase 2 |
| 4 | 10.9 | 6 | SPBIB_v1_50005 | conserved membrane protein of unknown function |
| 17 | 56.4 | 53 | SPBIB_v1_50006 | conserved protein of unknown function |
| 4 | 17 | 9 | SPBIB_v1_50007 | putative Calcineurin-like phosphoesterase |
| 5 | 19.5 | 12 | SPBIB_v1_50008 | conserved exported protein of unknown function |
| 26 | 89.1 | 169 | SPBIB_v1_50010 | Aminomethyltransferase |
| 5 | 61.4 | 32 | SPBIB_v1_50011 | glycine cleavage complex lipoylprotein |
| 20 | 71.8 | 138 | SPBIB_v1_50012 | putative glycine dehydrogenase (decarboxylating) subunit 1 |
| 26 | 80.2 | 130 | SPBIB_v1_50013 | putative glycine dehydrogenase (decarboxylating) subunit 2 |
| 15 | 48.4 | 42 | SPBIB_v1_50016 | Glycogen synthase |
| 7 | 22.6 | 14 | SPBIB_v1_50017 | conserved membrane protein of unknown function |
| 20 | 35.5 | 39 | SPBIB_v1_50019 | UvrD/REP helicase |
| 14 | 47.6 | 59 | SPBIB_v1_50020 | Ribosomal protein S12 methylthiotransferase RimO |
| 10 | 29.9 | 26 | SPBIB_v1_50021 | protein of unknown function |
| 12 | 66.2 | 37 | SPBIB_v1_50022 | Outer membrane lipoprotein carrier protein LolA |
| 34 | 72.3 | 174 | SPBIB_v1_50023 | Pyrophosphate-dependent phosphofructokinase |
| 11 | 39.7 | 38 | SPBIB_v1_50024 | putative LysM domain/M23/M37 peptidase domain protein |
| 3 | 12.9 | 5 | SPBIB_v1_50025 | RNA polymerase sigma factor |
| 46 | 71.5 | 202 | SPBIB_v1_50026 | putative ankyrin |
| 19 | 85.5 | 78 | SPBIB_v1_50027 | D-isomer specific 2-hydroxyacid dehydrogenase NAD-binding protein |
| 23 | 54.5 | 90 | SPBIB_v1_50028 | protein of unknown function |
| 5 | 27.9 | 6 | SPBIB_v1_50029 | Leucyl/phenylalanyl-tRNA--protein transferase |
| 40 | 64.7 | 157 | SPBIB_v1_50030 | ATPase and specificity subunit of ClpA-ClpP ATP-dependent serine protease, chaperone activity |
| 4 | 51.4 | 15 | SPBIB_v1_50031 | ATP-dependent Clp protease adapter protein ClpS (modular protein) |
| 37 | 66.7 | 152 | SPBIB_v1_50032 | Threonine--tRNA ligase |
| 18 | 80.8 | 116 | SPBIB_v1_50033 | putative dihydropyrimidine dehydrogenase |
| 35 | 81 | 212 | SPBIB_v1_50034 | putative NAD-dependent malic enzyme 4 |
| 7 | 42 | 25 | SPBIB_v1_50035 | putative amidohydrolase |
| 60 | 73.5 | 330 | SPBIB_v1_50038 | Oligopeptide-binding protein AliB |
| 11 | 26.1 | 31 | SPBIB_v1_50039 | ABC transporter, permease protein |
| 13 | 45.4 | 44 | SPBIB_v1_50041 | oligopeptide transporter subunit;ATP-binding component of ABC superfamily |
| 18 | 61.4 | 72 | SPBIB_v1_50042 | oligopeptide transporter subunit;ATP-binding component of ABC superfamily |
| 17 | 39.4 | 62 | SPBIB_v1_50043 | AAA ATPase |
| 12 | 64.3 | 31 | SPBIB_v1_50044 | Transcriptional regulator, LacI family |
| 27 | 55.9 | 122 | SPBIB_v1_50045 | Oligoendopeptidase F homolog |
| 9 | 59.8 | 38 | SPBIB_v1_50046 | putative Ribosomal RNA small subunit methyltransferase E 1 |
| 26 | 77.5 | 125 | SPBIB_v1_50047 | putative reductase TDE_0597 |
| 6 | 48.2 | 47 | SPBIB_v1_50048 | 3-hydroxyacyl- |
| 21 | 77.1 | 246 | SPBIB_v1_50049 | 3-oxoacyl- |
| 21 | 76.6 | 110 | SPBIB_v1_50050 | 3-oxoacyl- |
| 21 | 39.6 | 67 | SPBIB_v1_50051 | ATP-dependent zinc metalloprotease FtsH |
| 11 | 100 | 79 | SPBIB_v1_50052 | 10 kDa chaperonin |
| 10 | 77.3 | 55 | SPBIB_v1_50053 | putative PTS IIA-like nitrogen-regulatory protein PtsN |
| 3 | 8.7 | 4 | SPBIB_v1_50055 | protein of unknown function |
| 3 | 12.6 | 5 | SPBIB_v1_50072 | P22 coat protein |
| 6 | 20.2 | 12 | SPBIB_v1_50076 | conserved protein of unknown function |
| 8 | 62.4 | 24 | SPBIB_v1_50077 | Response regulator receiver protein |
| 32 | 45.6 | 70 | SPBIB_v1_50078 | putative Histidine kinase |
| 11 | 24.1 | 41 | SPBIB_v1_50081 | Conserved membrane-spanning protein |
| 6 | 21.2 | 18 | SPBIB_v1_50096 | protein of unknown function |
| 10 | 36.6 | 21 | SPBIB_v1_50097 | TPR repeat-containing protein (fragment) |
| 12 | 37.8 | 32 | SPBIB_v1_60001 | Methyltransferase |
| 15 | 50.6 | 55 | SPBIB_v1_60002 | Type-2 restriction enzyme DdeI |
| 7 | 42.8 | 13 | SPBIB_v1_60007 | conserved protein of unknown function |
| 7 | 19.6 | 13 | SPBIB_v1_60008 | AAA ATPase |
| 34 | 74.4 | 384 | SPBIB_v1_60010 | Extracellular solute-binding protein family 1 |
| 4 | 36.6 | 9 | SPBIB_v1_60015 | protein of unknown function |
| 21 | 51.9 | 73 | SPBIB_v1_60016 | putative enzyme |
| 9 | 69.7 | 62 | SPBIB_v1_60018 | 50S ribosomal subunit protein L13 |
| 4 | 33.3 | 20 | SPBIB_v1_60019 | 30S ribosomal subunit protein S9 |
| 4 | 14.5 | 6 | SPBIB_v1_60020 | protein of unknown function |
| 17 | 66.8 | 104 | SPBIB_v1_60021 | putative PTS IIA-like nitrogen-regulatory protein PtsN |
| 35 | 42.8 | 96 | SPBIB_v1_60022 | Chromosome partition protein Smc |
| 17 | 50.6 | 60 | SPBIB_v1_60023 | putative Threonyl/alanyl tRNA synthetase SAD |
| 17 | 50.1 | 55 | SPBIB_v1_60024 | Thymidine kinase |
| 17 | 47.7 | 65 | SPBIB_v1_60025 | ABC-type transporter, periplasmic subunit |
| 6 | 43.4 | 18 | SPBIB_v1_60026 | GTP-binding protein |
| 16 | 47.9 | 50 | SPBIB_v1_70001 | putative Histidine kinase |
| 12 | 59.4 | 71 | SPBIB_v1_70002 | Two component transcriptional regulator, winged helix family |
| 15 | 82.1 | 92 | SPBIB_v1_70003 | Phosphate-specific transport system accessory protein PhoU |
| 21 | 86.8 | 136 | SPBIB_v1_70004 | phosphate transporter subunit;ATP-binding component of ABC superfamily |
| 5 | 31.5 | 21 | SPBIB_v1_70005 | Dihydroorotate dehydrogenase B (NAD(+)), catalytic subunit |
| 8 | 49.8 | 13 | SPBIB_v1_70006 | putative Dihydroorotate dehydrogenase B (NAD(+)), electron transfer subunit |
| 7 | 52.7 | 24 | SPBIB_v1_80005 | Pyrazinamidase/nicotinamidase |
| 24 | 54.4 | 114 | SPBIB_v1_80006 | Nicotinate phosphoribosyltransferase |
| 13 | 65.7 | 47 | SPBIB_v1_80008 | Shikimate dehydrogenase |
| 8 | 30.7 | 21 | SPBIB_v1_80011 | conserved protein of unknown function |
| 23 | 73.8 | 83 | SPBIB_v1_80012 | conserved protein of unknown function |
| 3 | 8.3 | 6 | SPBIB_v1_80013 | putative HTH-type transcriptional regulator DegA |
| 22 | 78.1 | 160 | SPBIB_v1_80014 | D-ribose-binding protein |
| 5 | 10.8 | 9 | SPBIB_v1_80015 | fused D-ribose transporter subunits of ABC superfamily: ATP-binding components |
| 2 | 7.4 | 3 | SPBIB_v1_80016 | D-ribose transporter subunit;membrane component of ABC superfamily |
| 13 | 50.5 | 48 | SPBIB_v1_80017 | conserved protein of unknown function |
| 16 | 54 | 50 | SPBIB_v1_80019 | tRNA-specific 2-thiouridylase MnmA |
| 18 | 68.6 | 92 | SPBIB_v1_80022 | Beta-lactamase domain protein |
| 9 | 25.1 | 25 | SPBIB_v1_80023 | conserved exported protein of unknown function |
| 15 | 62.6 | 50 | SPBIB_v1_80024 | conserved exported protein of unknown function |
| 6 | 20.9 | 9 | SPBIB_v1_80025 | putative ABC transporter, permease protein |
| 5 | 13.4 | 12 | SPBIB_v1_80026 | conserved membrane protein of unknown function |
| 5 | 35.9 | 14 | SPBIB_v1_80027 | ABC transporter, ATP-binding protein |
| 18 | 55.7 | 93 | SPBIB_v1_80028 | putative enzyme |
| 17 | 57.3 | 61 | SPBIB_v1_80029 | putative GTP-binding protein |
| 28 | 44.8 | 75 | SPBIB_v1_80031 | ATPase and DNA damage recognition protein of nucleotide excision repair excinuclease UvrABC |
| 6 | 27.9 | 9 | SPBIB_v1_80033 | putative enzyme with nucleoside triphosphate hydrolase domain |
| 5 | 9.8 | 9 | SPBIB_v1_80034 | putative 7TM receptor with intracellular metal dependent phosphohydrolase |
| 5 | 45.1 | 18 | SPBIB_v1_80035 | Endoribonuclease YbeY |
| 8 | 40.9 | 29 | SPBIB_v1_80036 | conserved protein of unknown function |
| 48 | 90.8 | 414 | SPBIB_v1_80037 | glutamate dehydrogenase, NADP-specific |
| 10 | 36.8 | 51 | SPBIB_v1_80040 | 3-phosphoshikimate 1-carboxyvinyltransferase |
| 12 | 47.7 | 27 | SPBIB_v1_90002 | Prephenate dehydrogenase |
| 19 | 63.9 | 50 | SPBIB_v1_90003 | Prephenate dehydratase |
| 7 | 23.2 | 11 | SPBIB_v1_90004 | Chorismate synthase |
| 7 | 28.1 | 22 | SPBIB_v1_90005 | 3-dehydroquinate synthase (modular protein) |
| 5 | 23.2 | 12 | SPBIB_v1_90006 | 3-deoxy-7-phosphoheptulonate synthase |
| 20 | 29.2 | 51 | SPBIB_v1_90007 | conserved protein of unknown function |
| 33 | 74.2 | 153 | SPBIB_v1_90008 | Phenylalanine--tRNA ligase alpha subunit |
| 27 | 55.8 | 118 | SPBIB_v1_90009 | Phenylalanine--tRNA ligase beta subunit |
| 7 | 24.6 | 17 | SPBIB_v1_90011 | Oxidoreductase domain protein |
| 8 | 37.8 | 18 | SPBIB_v1_90012 | Alcohol dehydrogenase GroES domain protein |
| 15 | 64.3 | 43 | SPBIB_v1_90013 | PfkB domain protein |
| 14 | 46.4 | 34 | SPBIB_v1_90014 | gluconate-6-phosphate dehydrogenase, decarboxylating |
| 25 | 78.8 | 154 | SPBIB_v1_90016 | Extracellular solute-binding protein family 1 |
| 13 | 52.5 | 40 | SPBIB_v1_90023 | putative DegT/DnrJ/EryC1/StrS aminotransferase family protein |
| 9 | 48.6 | 33 | SPBIB_v1_90024 | putative inorganic polyphosphate/ATP-NAD kinase |
| 12 | 30.4 | 48 | SPBIB_v1_90025 | DNA repair protein RecN |
| 5 | 48.6 | 10 | SPBIB_v1_90026 | Cytidine deaminase |
| 10 | 48.1 | 38 | SPBIB_v1_90029 | Pyridoxal phosphate enzyme, YggS family |
| 7 | 35.5 | 20 | SPBIB_v1_90031 | Adenine phosphoribosyltransferase |
| 28 | 60 | 131 | SPBIB_v1_90032 | putative 2,3-bisphosphoglycerate-independent phosphoglycerate mutase |
| 4 | 26.3 | 7 | SPBIB_v1_90033 | putative Adenylyl cyclase CyaB |
| 3 | 20.4 | 8 | SPBIB_v1_90037 | exported protein of unknown function |
| 2 | 20.8 | 6 | SPBIB_v1_90040 | Ribonuclease VapC |
