# Supplementary material for: Fermentative Spirochaetes mediate necromass recycling in anoxic hydrocarbon-contaminated habitats
Source: ISME J. 2018 May 30;12(8):2039–50. doi: 10.1038/s41396-018-0148-3 (PMC6052044; doi:10.1038/s41396-018-0148-3)
Supplement: Supplementary file 4 — Supplementary Table S3 [file 41396_2018_148_MOESM4_ESM.docx]

**Supplementary Table S3** List of identified proteins for *R. cohabitans* HM as shown in Figure 4.

| **Gene** | **Function** | **Classification** |
| --- | --- | --- |
| SPBIB_v1_250074 | 2,3-cyclic-nucleotide 2-phosphodiesterase | purine metabolism and pyrimidine metabolism. |
| SPBIB_v1_150165 | 31 kDa immunogenic protein |  |
| SPBIB_v1_350024 | 5-nucleotidase/2,3-cyclic phosphodiesterase-like hydrolase | purine metabolism and pyrimidine metabolism. |
| SPBIB_v1_150087 | ABC sugar transporter, periplasmic ligand binding protein | sugar metabolism |
| SPBIB_v1_260060 | ABC sugar transporter, periplasmic ligand binding protein | sugar metabolism |
| SPBIB_v1_250100 | ABC transporter substrate binding protein | malto-oligosaccharides, multiple oligosaccharides, glycerol 3-phosphate, and iron |
| SPBIB_v1_330021 | ABC-type dipeptide transport system, periplasmic component | oligo/dipeptide and amino acid transoprters |
| SPBIB_v1_210094 | ABC-type transporter, periplasmic subunit |  |
| SPBIB_v1_210125 | ABC-type transporter, periplasmic subunit | oligo/dipeptide and amino acid transoprters |
| SPBIB_v1_290103 | Aconitate hydratase |  |
| SPBIB_v1_20007 | Basic membrane lipoprotein |  |
| SPBIB_v1_240055 | Basic membrane lipoprotein |  |
| SPBIB_v1_310088 | Branched-chain amino acid ABC transporter substrate-binding protein | oligo/dipeptide and amino acid transoprters |
| SPBIB_v1_150042 | conserved exported protein of unknown function |  |
| SPBIB_v1_210118 | conserved exported protein of unknown function |  |
| SPBIB_v1_210161 | conserved exported protein of unknown function |  |
| SPBIB_v1_270015 | conserved exported protein of unknown function |  |
| SPBIB_v1_350064 | conserved exported protein of unknown function |  |
| SPBIB_v1_310019 | Desulfoferrodoxin |  |
| SPBIB_v1_100150 | DNA-binding protein HRL18 |  |
| SPBIB_v1_80014 | D-ribose-binding protein |  |
| SPBIB_v1_250059 | exported protein of unknown function |  |
| SPBIB_v1_340054 | exported protein of unknown function |  |
| SPBIB_v1_380039 | exported protein of unknown function |  |
| SPBIB_v1_240026 | Extracellular ligand-binding receptor | oligo/dipeptide and amino acid transoprters |
| SPBIB_v1_150035 | Extracellular solute-binding protein |  |
| SPBIB_v1_10042 | Extracellular solute-binding protein family 1 | malto-oligosaccharides, multiple oligosaccharides, glycerol 3-phosphate, and iron |
| SPBIB_v1_380003 | Extracellular solute-binding protein family 1 | malto-oligosaccharides, multiple oligosaccharides, glycerol 3-phosphate, and iron |
| SPBIB_v1_400009 | Extracellular solute-binding protein family 1 | malto-oligosaccharides, multiple oligosaccharides, glycerol 3-phosphate, and iron |
| SPBIB_v1_60010 | Extracellular solute-binding protein family 1 | malto-oligosaccharides, multiple oligosaccharides, glycerol 3-phosphate, and iron |
| SPBIB_v1_290108 | Extracellular solute-binding protein family 5 | oligo/dipeptide and amino acid transoprters |
| SPBIB_v1_250085 | FAD-dependent pyridine nucleotide-disulfide oxidoreductase |  |
| SPBIB_v1_270007 | Gamma-glutamyltranspeptidase | intracellular peptidases |
| SPBIB_v1_290147 | Gamma-glutamyltranspeptidase | intracellular peptidases |
| SPBIB_v1_80037 | glutamate dehydrogenase, NADP-specific | Amino acid degradation |
| SPBIB_v1_100034 | glyceraldehyde-3-phosphate dehydrogenase A | glycerol fermentation |
| SPBIB_v1_380029 | glycerol-3-phosphate transporter subunit;periplasmic-binding component of ABC superfamily |  |
| SPBIB_v1_340036 | Iron ABC transporter, substrate binding protein |  |
| SPBIB_v1_210026 | Leucine-, isoleucine-, valine-, threonine-, and alanine-binding protein | oligo/dipeptide and amino acid transoprters |
| SPBIB_v1_290158 | oligopeptide transporter subunit;periplasmic-binding component of ABC superfamily | oligo/dipeptide and amino acid transoprters |
| SPBIB_v1_50038 | Oligopeptide-binding protein AliB | oligo/dipeptide and amino acid transoprters |
| SPBIB_v1_150028 | Periplasmic binding protein/LacI transcriptional regulator |  |
| SPBIB_v1_10062 | Periplasmic sugar-binding protein |  |
| SPBIB_v1_410087;SPBIB_v1_10012 | Plasmid stabilization system protein;Plasmid stabilization system protein |  |
| SPBIB_v1_110024 | protease, ATP-dependent zinc-metallo (M41) | Type II sectroy system |
| SPBIB_v1_310056 | protein chain elongation factor EF-Tu (duplicate of tufB) |  |
| SPBIB_v1_110017 | Protein translocase subunit SecA | Protease |
| SPBIB_v1_150059 | Purine-binding protein BAB2_0673 |  |
| SPBIB_v1_340021 | putative 2-oxoacid-flavodoxin fused oxidoreductase:conserved protein;4Fe-4S cluster binding protein | Amino acid degradation |
| SPBIB_v1_210218 | putative ABC-type transport system, periplasmic component/surface lipoprotein |  |
| SPBIB_v1_100146 | putative basic membrane lipoprotein |  |
| SPBIB_v1_260082 | putative cation ABC transporter, periplasmic binding protein |  |
| SPBIB_v1_310055 | putative Elongation factor G |  |
| SPBIB_v1_350021 | putative Extracellular solute-binding protein family 1 | malto-oligosaccharides, multiple oligosaccharides, glycerol 3-phosphate, and iron |
| SPBIB_v1_210067 | putative Periplasmic sugar binding protein-like protein |  |
| SPBIB_v1_340064 | Split soret cytochrome c |  |
| SPBIB_v1_260025 | Sugar ABC transporter substrate-binding protein |  |
| SPBIB_v1_100013 | TRAP dicarboxylate transporter, DctP subunit |  |
| SPBIB_v1_380022 | TRAP dicarboxylate transporter, DctP subunit |  |
