# Supplementary material for: Fermentative Spirochaetes mediate necromass recycling in anoxic hydrocarbon-contaminated habitats
Source: ISME J. 2018 May 30;12(8):2039–50. doi: 10.1038/s41396-018-0148-3 (PMC6052044; doi:10.1038/s41396-018-0148-3)
Supplement: Supplementary file 5 — Supplementary Table S4 [file 41396_2018_148_MOESM5_ESM.docx]

**Supplementary Table S4** The MEROPS server was used to identify the presence of various extracellular peptidases enzymes in the genomes of *Rectinema cohabitans* HM, uncultured Spirochaete bacterium bdmA 4, and uncultured Spirochaete bacterium SA-8.

***Rectinema cohabitans* HM**

| query name | family | query start-query end | predcited active site residues | predicted metal ligands | hit name | hit start-hit end | e-value |
| --- | --- | --- | --- | --- | --- | --- | --- |
| SPBIB_v1_100019\|ID:27162170\| | C14B | 69-215 | H152, C205 |  | MER0039458 | 3-149 | 6.10E-10 |
| SPBIB_v1_250023\|ID:27163222\| | C14B | 30-160 | H107, C159 |  | MER0019325 | 340-465 | 2.10E-17 |
| SPBIB_v1_30025\|ID:27161897\|hisH\| | C26 | 8-153 | C86, >H, >E |  | MER0065588 | 2-149 | 2.80E-29 |
| SPBIB_v1_150003\|ID:27162515\|guaA\| | C26 | 2-233 | C80, H174, E176 |  | MER0045886 | 12-236 | 3.40E-45 |
| SPBIB_v1_270049\|ID:27163456\|pdxT\| | C26 | 5-191 | C84, H176, E178 |  | MER0066916 | 3-194 | 4.10E-43 |
| SPBIB_v1_280002\|ID:27163468\|pyrG\| | C26 | 194-529 | C382, H508, E510 |  | MER0437468 | 204-551 | 4.40E-72 |
| SPBIB_v1_360017\|ID:27164073\|carA\| | C26 | 10-357 |  |  | MER0060647 | 4-354 | 4.20E-100 |
| SPBIB_v1_360026\|ID:27164082\|trpGD\| | C26 | 27-186 | C80, H169, E171 |  | MER0043394 | 26-185 | 9.70E-51 |
| SPBIB_v1_400027\|ID:27164242\| | C26 | 28-229 | C110, H213, E215 |  | MER0031288 | 3-200 | 3.70E-31 |
| SPBIB_v1_290018\|ID:27163543\| | C40 | 95-162 | C119, >H, >Q/E/H/N |  | MER0003986 | 169-237 | 7.40E-12 |
| SPBIB_v1_290018\|ID:27163543\| | C40 | 187-232 | <C, H191, H203 |  | MER0004035 | 141-183 | 2.50E-07 |
| SPBIB_v1_10057\|ID:27161819\|glmS\| | C44 | 56-288 | C56 |  | MER0003327 | 2-233 | 2.80E-49 |
| SPBIB_v1_190048\|ID:27162842\|purF\| | C44 | 43-263 | C43 |  | MER0011806 | 87-309 | 6.40E-50 |
| SPBIB_v1_250050\|ID:27163249\| | C44 | 2-138 | C2 |  | MER0003327 | 2-132 | 2.00E-19 |
| SPBIB_v1_260044\|ID:27163358\|gltB\| | C44 | 68-452 | C68 |  | MER0198917 | 37-408 | 1.30E-81 |
| SPBIB_v1_280036\|ID:27163502\| | C56 | 28-135 | A74E, C101, A102H |  | MER0010992 | 30-139 | 4.30E-15 |
| SPBIB_v1_290145\|ID:27163670\| | C69 | 2-178 | C2 |  | MER0064576 | 6-190 | 3.40E-29 |
| SPBIB_v1_100139\|ID:27162290\|hflK\| | I87 | 110-307 |  |  | MER0192051 | 149-346 | 1.30E-37 |
| SPBIB_v1_100140\|ID:27162291\|hflC\| | I87 | 25-325 |  |  | MER0191412 | 20-330 | 1.20E-38 |
| SPBIB_v1_120005\|ID:27162417\| | I87 | 74-210 |  |  | MER0192051 | 100-236 | 2.50E-08 |
| SPBIB_v1_20035\|ID:27161862\| | M03B | 291-529 | E391 | H390, H394, E417 | MER0004419 | 306-540 | 3.50E-49 |
| SPBIB_v1_50045\|ID:27161956\|yjbG\| | M03B | 291-574 | E391 | H390, H394, E418 | MER0001163 | 290-571 | 1.60E-63 |
| SPBIB_v1_310041\|ID:27163816\| | M15B | 150-250 | E236 | H186, D193, H239 | MER0014983 | 131-232 | 2.00E-21 |
| SPBIB_v1_100083\|ID:27162234\| | M16B | 10-202 | E49, E119 | H46, H50, E126 | MER0003448 | 13-205 | 7.30E-33 |
| SPBIB_v1_250067\|ID:27163266\| | M16B | 74-220 | L90E, A160E | N87H, K91H, S167E | MER0390687 | 20-166 | 9.80E-06 |
| SPBIB_v1_250068\|ID:27163267\| | M16B | 52-228 | E94, E166 | H91, H95, N172E | MER0195774 | 38-213 | 4.00E-20 |
| SPBIB_v1_250066\|ID:27163265\| | M16C | 50-451 | E61, E134 | H58, H62, E156 | MER0014056 | 154-564 | 2.10E-131 |
| SPBIB_v1_380038\|ID:27164161\|apeB\| | M18 | 6-435 | D83, E273 | H81, D243, E274, D319, H413 | MER0003374 | 17-460 | 2.20E-71 |
| SPBIB_v1_100173\|ID:27162324\| | M19 | 10-322 |  | H14, D16, E119, H186, H207 | MER0013425 | 3-303 | 3.50E-42 |
| SPBIB_v1_20016\|ID:27161843\| | M20A | 5-337 | D78, E143 | H76, D109, E144, D170, >H | MER0001269 | 92-444 | 2.00E-43 |
| SPBIB_v1_20016\|ID:27161843\| | M20A | 361-439 | <D, <E | <H, <D, <E, <D/E, >H | MER0033183 | 259-338 | 4.50E-12 |
| SPBIB_v1_150069\|ID:27162581\|ygeY\| | M20A | 4-177 | D79, E144 | H77, D110, E145, E170, >H | MER0003369 | 8-181 | 2.40E-45 |
| SPBIB_v1_150069\|ID:27162581\|ygeY\| | M20A | 289-395 | <D, <E | <H, <D, <E, <D/E, >H | MER0003369 | 181-287 | 2.00E-38 |
| SPBIB_v1_240067\|ID:27163197\| | M20A | 75-245 | D97, E162 | H95, D128, E163, X193D/E, >H | MER0003798 | 83-246 | 3.20E-12 |
| SPBIB_v1_190016\|ID:27162810\|pepT\| | M20B | 26-418 | D93, E186 | H91, D152, E187, D209, H391 | MER0001421 | 14-406 | 2.50E-87 |
| SPBIB_v1_340028\|ID:27163943\| | M20D | 18-311 | D89, E151 | E87, H116, E152, H175, >H | MER0014418 | 10-292 | 1.10E-34 |
| SPBIB_v1_340034\|ID:27163949\| | M20D | 50-343 | D112, E164 | D110, H130, E165, H206, >H | MER0002659 | 52-335 | 6.50E-17 |
| SPBIB_v1_400025\|ID:27164240\| | M20D | 15-359 | D77, E135 | C100, H102, E136, H162, K352H | MER0002655 | 10-357 | 1.10E-66 |
| SPBIB_v1_280019\|ID:27163485\| | M20X | 11-541 | D87, E178 | H85, D145, E179, D210, H522 | MER0002961 | 16-542 | 7.40E-51 |
| SPBIB_v1_20041\|ID:27161868\| | M23B | 354-465 | H451 | H372, D376, H453 | MER0069741 | 277-387 | 3.20E-26 |
| SPBIB_v1_50024\|ID:27161935\| | M23B | 206-312 | I297H | M222D, Y299H | MER0005300 | 293-406 | 1.20E-15 |
| SPBIB_v1_120010\|ID:27162422\| | M23B | 265-327 | H322 | <H, <D, H324 | MER0013541 | 325-381 | 1.20E-06 |
| SPBIB_v1_340059\|ID:27163974\| | M23B | 235-336 | H321 | H241, D245, H323 | MER0003380 | 308-408 | 3.30E-24 |
| SPBIB_v1_380034\|ID:27164157\| | M23B | 190-319 | H303 | H224, D228, H305 | MER0069741 | 261-389 | 7.20E-24 |
| SPBIB_v1_400034\|ID:27164249\| | M23B | 140-174 | H169 | <H, <D, H171 | MER0013541 | 347-381 | 1.70E-05 |
| SPBIB_v1_100045\|ID:27162196\|map\| | M24A | 2-246 | H77 | D94, D105, H168, E201, E232 | MER0001243 | 3-249 | 3.60E-61 |
| SPBIB_v1_210069\|ID:27162961\| | M24B | 133-369 | F213H, H301, H312 | E230D, D241, H305, E335, E349 | MER0004931 | 121-347 | 5.00E-35 |
| SPBIB_v1_300002\|ID:27163757\| | M24B | 142-367 | H216, H304, H315 | D235, D246, H308, E339, E353 | MER0004931 | 120-341 | 4.90E-33 |
| SPBIB_v1_390003\|ID:27164189\| | M24B | 237-454 | H240, H348, H359 | D257, D269, H352, E401, E441 | MER0001248 | 252-465 | 1.10E-37 |
| SPBIB_v1_370030\|ID:27164122\| | M29 | 181-350 | Y316 | E217, E282, H309, M334H, D336 | MER0001285 | 205-392 | 2.20E-13 |
| SPBIB_v1_370030\|ID:27164122\| | M29 | 4-165 | >Y | >E, >E, >H, >H, >D | MER0014416 | 6-182 | 4.30E-05 |
| SPBIB_v1_30021\|ID:27161893\| | M32 | 8-499 | E262 | H261, H265, E293 | MER0001186 | 10-511 | 1.50E-110 |
| SPBIB_v1_150066\|ID:27162578\| | M38 | 33-398 | D321 | H68, H70, C160K, S210H, H253 | MER0030136 | 31-404 | 1.50E-22 |
| SPBIB_v1_150068\|ID:27162580\|ssnA\| | M38 | 174-316 | D316 | <H, <H, K183, R222H, H231 | MER0037714 | 187-330 | 4.50E-09 |
| SPBIB_v1_150073\|ID:27162585\| | M38 | 66-330 | D330 | H73, H75, H200K, R233H, H242 | MER0037714 | 75-330 | 1.80E-11 |
| SPBIB_v1_250040\|ID:27163239\| | M38 | 30-374 | D317 | H71, H73, V177K, H206, H227 | MER0066192 | 26-370 | 2.10E-48 |
| SPBIB_v1_260042\|ID:27163356\| | M38 | Aug-90 | >D | <H, <H, K90, >H, >H | MER0066192 | 91-173 | 2.50E-11 |
| SPBIB_v1_260047\|ID:27163361\|iadA\| | M38 | 3-360 | D284 | X61H, X63H, K155, H194, H223 | MER0001495 | 11-359 | 4.00E-60 |
| SPBIB_v1_290139\|ID:27163664\| | M38 | 60-303 | D301 | H67, H69, A170K, Q204H, H213 | MER0037714 | 75-332 | 5.10E-18 |
| SPBIB_v1_300003\|ID:27163758\|pyrC\| | M38 | 10-302 | D247 | H14, H16, K102, H139, H174 | MER0061068 | 13-306 | 3.20E-49 |
| SPBIB_v1_310090\|ID:27163865\|hutI\| | M38 | 72-386 | P339D | H83, H85, D223K, K251H, K258H | MER0033186 | 76-392 | 4.50E-55 |
| SPBIB_v1_340029\|ID:27163944\|mtaD\| | M38 | 72-313 | D313 | H79, H81, K184, E217H, H226 | MER0037714 | 75-330 | 1.20E-16 |
| SPBIB_v1_400022\|ID:27164237\| | M38 | 31-376 | D316 | H76, H78, K180, H209, H228 | MER0066192 | 26-370 | 2.90E-35 |
| SPBIB_v1_50051\|ID:27161962\|ftsH\| | M41 | 422-632 | E461 | H460, H464, D537 | MER0005466 | 385-592 | 1.00E-53 |
| SPBIB_v1_110024\|ID:27162382\|ftsH\| | M41 | 382-615 | E435 | H434, H438, D510 | MER0001620 | 365-600 | 2.10E-61 |
| SPBIB_v1_150069\|ID:27162581\|ygeY\| | M42 | 19-106 | D79, >D, >E | H77, >H, >D, >E, >D/E, >H | MER0005160 | Jun-95 | 1.10E-05 |
| SPBIB_v1_240035\|ID:27163165\| | M48C | 103-285 | E167 | H166, H170, E232 | MER0042387 | 64-237 | 3.40E-17 |
| SPBIB_v1_100206\|ID:27162357\| | M50B | 18-199 | E19 | H18, H22, >D | MER0004469 | 21-204 | 2.50E-16 |
| SPBIB_v1_100206\|ID:27162357\| | M50B | 281-409 | <E | <H, <H, >D | MER0004480 | 203-335 | 3.90E-11 |
| SPBIB_v1_370013\|ID:27164105\| | M50B | 70-132 | <E | <H, <H, <D | MER0004466 | 218-280 | 1.40E-06 |
| SPBIB_v1_100046\|ID:27162197\| | S01C | 127-469 | H147, D177, S253 |  | MER0001372 | 91-422 | 8.60E-71 |
| SPBIB_v1_100175\|ID:27162326\| | S01C | 143-364 | H151, D181, S260 |  | MER0001372 | 101-313 | 4.30E-51 |
| SPBIB_v1_100206\|ID:27162357\| | S01C | 156-252 | <H, <D, <S |  | MER0001372 | 306-418 | 3.80E-05 |
| SPBIB_v1_110023\|ID:27162381\| | S01C | 284-432 | H291, D331, S402 |  | MER0063616 | 197-345 | 2.10E-35 |
| SPBIB_v1_330007\|ID:27163896\| | S08A | 210-567 | D266, H300, N391, L455S |  | MER0002565 | 395-757 | 3.40E-51 |
| SPBIB_v1_50081\|ID:27161992\| | S09C | 65-263 | S145, P237D, >H |  | MER0017401 | 379-574 | 4.40E-06 |
| SPBIB_v1_260009\|ID:27163323\| | S09C | 93-342 | S196, D290, H323 |  | MER0066184 | 60-297 | 7.70E-28 |
| SPBIB_v1_150119\|ID:27162631\| | S09X | 35-153 | S139, >D, >H |  | MER0031551 | 47-167 | 8.10E-07 |
| SPBIB_v1_150185\|ID:27162697\| | S09X | 67-221 | S157, >D, >H |  | MER0033274 | 627-789 | 1.70E-10 |
| SPBIB_v1_100016\|ID:27162167\| | S11 | 56-417 | S83, K86, S147 |  | MER0000454 | 37-382 | 3.60E-46 |
| SPBIB_v1_210119\|ID:27163011\| | S12 | 171-398 | <S, <K, Y197 |  | MER0003800 | 182-412 | 9.30E-15 |
| SPBIB_v1_210119\|ID:27163011\| | S12 | 25-88 | S67, K70, >Y |  | MER0003800 | 64-133 | 2.10E-05 |
| SPBIB_v1_240021\|ID:27163151\| | S12 | 35-348 | P85S, E88K, R183Y |  | MER0026262 | 7-331 | 2.60E-19 |
| SPBIB_v1_290114\|ID:27163639\| | S12 | 123-283 | <S, <K, Y185 |  | MER0026262 | 98-248 | 6.10E-34 |
| SPBIB_v1_290114\|ID:27163639\| | S12 | 13-103 | S66, K69, >Y |  | MER0026262 | Jun-96 | 4.20E-22 |
| SPBIB_v1_290114\|ID:27163639\| | S12 | 312-404 | <S, <K, <Y |  | MER0026262 | 258-350 | 1.50E-12 |
| SPBIB_v1_310096\|ID:27163871\| | S12 | 99-387 | S112, K115, Y204 |  | MER0003800 | 98-404 | 9.30E-28 |
| SPBIB_v1_100167\|ID:27162318\|clpP\| | S14 | 4-198 | S103, H128, D177 |  | MER0000474 | 13-206 | 3.80E-69 |
| SPBIB_v1_310034\|ID:27163809\|clpP\| | S14 | 23-193 | X97S, H122, D171 |  | MER0000474 | 36-207 | 1.20E-40 |
| SPBIB_v1_410010\|ID:27164265\| | S14 | 40-174 | X85S, H108, E157D |  | MER0002211 | 108-244 | 7.70E-12 |
| SPBIB_v1_90010\|ID:27162094\| | S16 | 74-172 | A77S, A121K/R |  | MER0017402 | 907-1008 | 2.30E-06 |
| SPBIB_v1_120014\|ID:27162426\|lon\| | S16 | 544-780 | S688, K731 |  | MER0000485 | 537-771 | 4.10E-60 |
| SPBIB_v1_310027\|ID:27163802\|radA\| | S16 | 178-422 | A359S, R402 |  | MER0014135 | 207-451 | 1.40E-41 |
| SPBIB_v1_290230\|ID:27163755\|lexA\| | S24 | 114-186 | S124, K161 |  | MER0006172 | Apr-77 | 4.30E-15 |
| SPBIB_v1_100056\|ID:27162207\| | S26A | 58-166 | S69, K156 |  | MER0004537 | 74-171 | 1.20E-10 |
| SPBIB_v1_100056\|ID:27162207\| | S26A | 277-320 | <S, <K |  | MER0055964 | 234-277 | 5.70E-07 |
| SPBIB_v1_150014\|ID:27162526\| | S33 | 7-140 | S109, >D, >H |  | MER0031618 | 25-153 | 1.90E-13 |
| SPBIB_v1_200020\|ID:27162868\|todF\| | S33 | 20-262 | S108, D229, H257 |  | MER0037236 | 23-270 | 5.90E-38 |
| SPBIB_v1_390025\|ID:27164211\| | S33 | 92-250 | S101, D222, H250 |  | MER0037236 | 103-265 | 1.90E-10 |
| SPBIB_v1_390026\|ID:27164212\| | S33 | 52-281 | S121, E209D, H263 |  | MER0031610 | 69-314 | 6.70E-15 |
| SPBIB_v1_300012\|ID:27163767\| | S41A | 234-403 | S334, K359 |  | MER0004196 | 213-382 | 7.50E-40 |
| SPBIB_v1_150111\|ID:27162623\| | S54 | 52-180 | S117, H168 |  | MER0015453 | 122-262 | 7.00E-07 |
| SPBIB_v1_150152\|ID:27162664\| | S54 | 1-206 | S132, H197 |  | MER0017194 | 1-205 | 9.60E-32 |
| SPBIB_v1_210203\|ID:27163095\| | S54 | 19-191 | X126S, H184 |  | MER0017194 | 17-203 | 5.90E-07 |
| SPBIB_v1_130052\|ID:27162478\|hslV\| | T01B | 6-123 | T6 |  | MER0001627 | 2-119 | 6.10E-44 |
| SPBIB_v1_130052\|ID:27162478\|hslV\| | T01B | 168-196 | <S/T |  | MER0001627 | 144-172 | 8.00E-05 |
| SPBIB_v1_250027\|ID:27163226\|ggt\| | T03 | 146-561 | T387 |  | MER0223926 | 105-519 | 7.90E-78 |
| SPBIB_v1_270007\|ID:27163414\|ggt\| | T03 | 51-434 | T383 |  | MER0001978 | 44-444 | 7.20E-83 |
| SPBIB_v1_290147\|ID:27163672\|ggt\| | T03 | 40-557 | T382 |  | MER0001978 | 49-571 | 3.80E-124 |
| SPBIB_v1_260003\|ID:27163317\|argJ\| | T05 | 188-405 | T188 |  | MER0011829 | 215-441 | 5.70E-51 |
| SPBIB_v1_220007\|ID:27163123\| | U32 | 75-343 |  |  | MER0013876 | 31-302 | 3.90E-41 |
| SPBIB_v1_290033\|ID:27163558\| | U62 | 255-465 |  |  | MER0016301 | 246-456 | 2.20E-34 |

**uncultured Spirochaete bacterium bdmA 4**

| query name | family | query start-query end | predcited active site residues | predicted metal ligands | hit name | hit start-hit end | e-value |
| --- | --- | --- | --- | --- | --- | --- | --- |
| SPBDM4_v1_50419\|ID:27158782\| | A08 | 49-126 | D114, >D |  | MER0001313 | 50-125 | 9.60E-07 |
| SPBDM4_v1_40515\|ID:27157820\| | C110 | 197-308 | C252, H292, D307 | | MER0472834 | 171-283 | 3.30E-14 |
| SPBDM4_v1_50263\|ID:27158626\| | C14B | 43-170 | H117, C169 | | MER0019325 | 343-465 | 9.50E-17 |
| SPBDM4_v1_51035\|ID:27159398\| | C14B | 56-189 | H134, C182 | | MER0039457 | 3-146 | 1.60E-07 |
| SPBDM4_v1_40051\|ID:27157356\| | C26 | 28-232 | C110, H213, E215 | | MER0031288 | 3-203 | 4.20E-30 |
| SPBDM4_v1_40199\|ID:27157504\|carA\| | C26 | 10-357 |  |  | MER0060647 | 4-354 | 2.80E-99 |
| SPBDM4_v1_40731\|ID:27158036\|pdxT\| | C26 | 5-194 | C84, H176, E178 | | MER0066916 | 3-197 | 4.60E-42 |
| SPBDM4_v1_40746\|ID:27158051\|pyrG\| | C26 | 194-529 | C382, H508, E510 | | MER0437468 | 204-551 | 8.60E-71 |
| SPBDM4_v1_50930\|ID:27159293\|guaA\| | C26 | 2-233 | C80, H174, E176 | | MER0045886 | 12-236 | 3.60E-44 |
| SPBDM4_v1_80055\|ID:27159831\| | C26 | 7-149 | C84, >H, >E | | MER0065588 | 3-147 | 7.20E-30 |
| SPBDM4_v1_40601\|ID:27157906\| | C39 | 33-151 | <Q, C33, H117, D133 | | MER0014664 | 16-126 | 1.10E-08 |
| SPBDM4_v1_40890\|ID:27158195\| | C40 | 67-160 | C91, S144H, Q146 | | MER0003986 | 169-273 | 6.60E-15 |
| SPBDM4_v1_30046\|ID:27157281\|purF\| | C44 | 31-222 | <C |  | MER0011781 | 100-291 | 5.20E-41 |
| SPBDM4_v1_40523\|ID:27157828\|gltB\| | C44 | 31-417 | C31 |  | MER0198917 | 37-408 | 1.80E-84 |
| SPBDM4_v1_50156\|ID:27158519\| | C44 | 5-141 | C5 |  | MER0003327 | 2-132 | 4.00E-18 |
| SPBDM4_v1_70314\|ID:27159772\|asnB\| | C44 | 2-197 | C2 |  | MER0034539 | 2-196 | 2.30E-58 |
| SPBDM4_v1_80005\|ID:27159781\|asnB\| | C44 | 2-197 | C2 |  | MER0034539 | 2-196 | 7.90E-59 |
| SPBDM4_v1_80129\|ID:27159905\|glmS\| | C44 | 2-226 | C2 |  | MER0003327 | 2-225 | 2.60E-43 |
| SPBDM4_v1_30050\|ID:27157285\|purQ\| | C56 | 46-113 | G58E, C93, N94H | | MER0014721 | 211-269 | 1.30E-05 |
| SPBDM4_v1_40826\|ID:27158131\| | C56 | 31-153 | A74E, C101, A102H | | MER0002455 | 30-152 | 1.50E-15 |
| SPBDM4_v1_41011\|ID:27158316\| | C69 | 126-400 | C126 |  | MER0064573 | 12-281 | 4.80E-27 |
| SPBDM4_v1_50005\|ID:27158368\| | C69 | 2-178 | C2 |  | MER0064576 | 6-190 | 5.30E-32 |
| SPBDM4_v1_50648\|ID:27159011\| | I87 | 17-282 |  |  | MER0192051 | 95-355 | 1.30E-13 |
| SPBDM4_v1_70079\|ID:27159537\|hflK\| | I87 | 110-307 |  |  | MER0192051 | 149-346 | 1.70E-37 |
| SPBDM4_v1_70080\|ID:27159538\|hflC\| | I87 | 25-325 |  |  | MER0191412 | 20-330 | 4.50E-38 |
| SPBDM4_v1_70202\|ID:27159660\| | I87 | 74-210 |  |  | MER0192051 | 100-236 | 7.10E-08 |
| SPBDM4_v1_70316\|ID:27159774\|pepF\| | M03B | 291-574 | E391 | H390, H394, E418 | MER0001163 | 290-571 | 6.30E-65 |
| SPBDM4_v1_80007\|ID:27159783\|pepF\| | M03B | 291-574 | E391 | H390, H394, E418 | MER0001163 | 290-571 | 6.30E-65 |
| SPBDM4_v1_80088\|ID:27159864\| | M03B | 291-529 | E391 | H390, H394, E417 | MER0004419 | 306-540 | 3.00E-49 |
| SPBDM4_v1_40413\|ID:27157718\| | M15B | 123-241 | E227 | H177, D184, H230 | MER0014983 | 112-232 | 1.60E-18 |
| SPBDM4_v1_40097\|ID:27157402\| | M16B | 71-279 | E113, T194E | K110H, Y114H, E201 | MER0001233 | 40-249 | 4.00E-07 |
| SPBDM4_v1_50128\|ID:27158491\| | M16B | 77-253 | E119, E191 | H116, H120, N197E | MER0195774 | 38-213 | 3.40E-20 |
| SPBDM4_v1_50129\|ID:27158492\| | M16B | 104-228 | <E, T169E | <H, <H, A176E | MER0390687 | 41-165 | 2.50E-07 |
| SPBDM4_v1_50130\|ID:27158493\| | M16C | 50-451 | E61, E134 | H58, H62, E156 | MER0014056 | 154-564 | 3.10E-132 |
| SPBDM4_v1_40123\|ID:27157428\| | M18 | 145-431 | <D, E269 | <H, D239, E270, X315D, H409 | MER0003374 | 158-460 | 2.90E-38 |
| SPBDM4_v1_70108\|ID:27159566\| | M19 | 10-322 |  | H14, D16, E119, H186, H207 | MER0013425 | 3-303 | 2.50E-49 |
| SPBDM4_v1_40667\|ID:27157972\| | M20A | 66-413 | D87, E152 | H85, D118, E153, E179, H389 | MER0003798 | 81-418 | 3.50E-27 |
| SPBDM4_v1_50359\|ID:27158722\| | M20A | 75-245 | D97, E162 | H95, D128, E163, D190, >H | MER0003798 | 83-246 | 1.30E-14 |
| SPBDM4_v1_50882\|ID:27159245\|ygeY\| | M20A | 4-177 | D79, E144 | H77, D110, E145, E170, >H | MER0003369 | 8-181 | 5.20E-39 |
| SPBDM4_v1_50882\|ID:27159245\|ygeY\| | M20A | 289-395 | <D, <E | <H, <D, <E, <D/E, >H | MER0003369 | 181-287 | 2.60E-36 |
| SPBDM4_v1_80109\|ID:27159885\| | M20A | 5-328 | D78, E143 | H76, D109, E144, D170, >H | MER0001269 | 92-431 | 3.10E-42 |
| SPBDM4_v1_80109\|ID:27159885\| | M20A | 361-433 | <D, <E | <H, <D, <E, <D/E, >H | MER0033183 | 259-332 | 8.40E-12 |
| SPBDM4_v1_40995\|ID:27158300\| | M20B | 4-350 | D71, E128 | H69, D99, E129, D152, H332 | MER0028941 | 6-363 | 3.60E-46 |
| SPBDM4_v1_50696\|ID:27159059\|pepT\| | M20B | 10-402 | D77, E170 | H75, D136, E171, D193, H375 | MER0001421 | 14-406 | 1.00E-77 |
| SPBDM4_v1_40053\|ID:27157358\| | M20D | 3-391 | D81, E139 | D79, D107, E140, H166, H366 | MER0002007 | 2-390 | 9.00E-77 |
| SPBDM4_v1_50029\|ID:27158392\| | M20D | 15-382 | D92, E138 | Q90C/D/E, H103, E139, H163, H350 | MER0014418 | 10-373 | 1.80E-60 |
| SPBDM4_v1_40763\|ID:27158068\| | M20X | 11-550 | D87, E178 | H85, D145, E179, D210, H522 | MER0002961 | 16-551 | 2.20E-47 |
| SPBDM4_v1_40127\|ID:27157432\| | M23B | 178-307 | H291 | H212, D216, H293 | MER0069741 | 261-389 | 1.10E-21 |
| SPBDM4_v1_40299\|ID:27157604\| | M23B | 231-336 | H321 | H241, D245, H323 | MER0003380 | 305-408 | 2.60E-23 |
| SPBDM4_v1_70209\|ID:27159667\| | M23B | 118-180 | H175 | <H, <D, H177 | MER0013541 | 325-381 | 1.30E-08 |
| SPBDM4_v1_80022\|ID:27159798\| | M23B | 239-347 | I332H | M257D, Y334H | MER0005300 | 291-406 | 6.70E-15 |
| SPBDM4_v1_80082\|ID:27159858\| | M23B | 308-419 | H405 | H326, D330, H407 | MER0069741 | 277-387 | 1.20E-26 |
| SPBDM4_v1_51060\|ID:27159423\|map\| | M24A | 2-246 | H77 | D94, D105, H168, E201, E232 | MER0001243 | 3-249 | 3.60E-61 |
| SPBDM4_v1_40095\|ID:27157400\| | M24B | 262-479 | H265, H373, H384 | D282, D294, H377, E426, E466 | MER0001248 | 252-465 | 1.60E-38 |
| SPBDM4_v1_40481\|ID:27157786\| | M24B | 142-367 | H216, H304, H315 | D235, D246, H308, E339, E353 | MER0004931 | 120-341 | 1.30E-33 |
| SPBDM4_v1_40653\|ID:27157958\| | M24B | 159-379 | I231H, Y319H, V330H | N248D, S259D, H323, D352E, E365 | MER0005462 | 129-350 | 4.50E-21 |
| SPBDM4_v1_40789\|ID:27158094\| | M24B | 153-376 | H222, R307H, H320 | D239, D250, H313, E342, E356 | MER0004931 | 122-347 | 2.20E-35 |
| SPBDM4_v1_50031\|ID:27158394\| | M24B | 135-360 | H204, H293, H304 | D222, D233, H297, E326, E340 | MER0005462 | 130-356 | 3.10E-41 |
| SPBDM4_v1_50280\|ID:27158643\| | M24B | 157-385 | V228H, C317H, A328H | G245D, V256D, H321, D350E, E365 | MER0004931 | 121-347 | 2.30E-23 |
| SPBDM4_v1_40164\|ID:27157469\| | M29 | 170-350 | Y316 | E217, E282, H309, H328, D330 | MER0001285 | 192-392 | 2.90E-12 |
| SPBDM4_v1_80059\|ID:27159835\| | M32 | 8-499 | E262 | H261, H265, E293 | MER0001186 | 10-511 | 7.60E-108 |
| SPBDM4_v1_40056\|ID:27157361\| | M38 | 67-379 | D319 | H79, H81, K183, H212, H231 | MER0066192 | 51-370 | 1.50E-33 |
| SPBDM4_v1_40371\|ID:27157676\|hutI\| | M38 | 79-393 | P346D | H90, H92, D230K, K258H, D265H | MER0033186 | 76-392 | 1.90E-51 |
| SPBDM4_v1_40478\|ID:27157783\|pyrC\| | M38 | Oct-51 | >D | H17, H19, >K, >H, >H | MER0061068 | Oct-51 | 2.00E-08 |
| SPBDM4_v1_40480\|ID:27157785\|pyrC\| | M38 | 37-156 | D101 | <H, <H, <K, <H, <H | MER0061068 | 187-306 | 1.20E-20 |
| SPBDM4_v1_40527\|ID:27157832\|iadA\| | M38 | 3-360 | D284 | X61H, X63H, K155, H194, H223 | MER0001495 | 11-359 | 1.20E-57 |
| SPBDM4_v1_50167\|ID:27158530\| | M38 | 59-370 | D313 | H67, H69, V173K, H202, H223 | MER0066192 | 55-370 | 1.10E-49 |
| SPBDM4_v1_50334\|ID:27158697\| | M38 | 4-399 | D319 | H64, H66, K156, -200H, H243 | MER0015112 | 8-405 | 2.00E-53 |
| SPBDM4_v1_50335\|ID:27158698\| | M38 | 34-395 | D320 | H60, H62, K154, R211H, H251 | MER0015112 | 46-405 | 4.10E-27 |
| SPBDM4_v1_50877\|ID:27159240\| | M38 | 225-346 | D346 | <H, <H, <K, R249H, H258 | MER0037714 | 207-330 | 1.80E-12 |
| SPBDM4_v1_50877\|ID:27159240\| | M38 | 44-105 | >D | H89, H91, >K, >H, >H | MER0030136 | 21-81 | 3.10E-05 |
| SPBDM4_v1_50883\|ID:27159246\|ssnA\| | M38 | 142-316 | D316 | <H, <H, K183, E219H, H231 | MER0037714 | 153-330 | 1.80E-09 |
| SPBDM4_v1_50885\|ID:27159248\| | M38 | 20-401 | D324 | H71, H73, S163K, I214H, N251H | MER0030136 | 27-404 | 6.90E-17 |
| SPBDM4_v1_70163\|ID:27159621\|ftsH\| | M41 | 382-612 | E435 | H434, H438, D510 | MER0001620 | 365-597 | 1.40E-58 |
| SPBDM4_v1_70306\|ID:27159764\|ftsH\| | M41 | 422-643 | E461 | H460, H464, D537 | MER0005466 | 385-603 | 3.10E-55 |
| SPBDM4_v1_50394\|ID:27158757\| | M48C | 100-282 | E164 | H163, H167, E229 | MER0042387 | 64-237 | 4.70E-18 |
| SPBDM4_v1_40176\|ID:27157481\| | M50B | 69-131 | <E | <H, <H, <D | MER0004466 | 218-280 | 2.10E-07 |
| SPBDM4_v1_70141\|ID:27159599\| | M50B | 18-256 | E19 | H18, H22, >D | MER0004469 | 21-271 | 1.00E-15 |
| SPBDM4_v1_70141\|ID:27159599\| | M50B | 283-409 | <E | <H, <H, >D | MER0004480 | 205-335 | 1.50E-09 |
| SPBDM4_v1_40593\|ID:27157898\| | M79 | 143-224 |  |  | MER0059868 | 138-213 | 1.30E-05 |
| SPBDM4_v1_40597\|ID:27157902\| | M79 | 138-224 |  |  | MER0059868 | 133-213 | 6.10E-08 |
| SPBDM4_v1_50274\|ID:27158637\| | M81 | 49-186 |  |  | MER0167712 | 88-226 | 3.50E-08 |
| SPBDM4_v1_51061\|ID:27159424\| | S01C | 127-465 | H147, D177, S253 | | MER0001372 | 91-418 | 3.80E-71 |
| SPBDM4_v1_70110\|ID:27159568\| | S01C | 137-356 | H145, D175, S254 | | MER0001372 | 101-311 | 8.50E-52 |
| SPBDM4_v1_70141\|ID:27159599\| | S01C | 137-252 | <H, <D, <S | | MER0001372 | 286-418 | 1.60E-05 |
| SPBDM4_v1_70162\|ID:27159620\| | S01C | 276-432 | H291, D331, S402 | | MER0063616 | 187-345 | 2.40E-36 |
| SPBDM4_v1_40489\|ID:27157794\| | S09C | 60-305 | S159, D253, H286 | | MER0066184 | 64-297 | 2.20E-31 |
| SPBDM4_v1_40983\|ID:27158288\| | S09C | 427-658 | S531, D612, H644 | | MER0017401 | 363-593 | 1.60E-47 |
| SPBDM4_v1_50519\|ID:27158882\| | S09C | 89-170 | S137, >D, >H | | MER0031556 | 112-190 | 8.20E-07 |
| SPBDM4_v1_41012\|ID:27158317\| | S09X | 59-263 | S141, D231, H261 | | MER0036038 | 38-240 | 1.40E-13 |
| SPBDM4_v1_50764\|ID:27159127\| | S09X | 84-221 | S157, >D, >H | | MER0033274 | 647-789 | 1.30E-08 |
| SPBDM4_v1_51032\|ID:27159395\| | S11 | 52-426 | S83, K86, S147 | | MER0000450 | 38-399 | 4.10E-45 |
| SPBDM4_v1_40364\|ID:27157669\| | S12 | 11-351 | S76, K79, Y168 | | MER0003800 | 52-404 | 5.30E-28 |
| SPBDM4_v1_40594\|ID:27157899\| | S12 | 86-372 | E93S, G96K, E187Y | | MER0028284 | 88-366 | 1.30E-12 |
| SPBDM4_v1_40611\|ID:27157916\| | S12 | 86-270 | E93S, G96K, E187Y | | MER0028284 | 88-270 | 6.90E-12 |
| SPBDM4_v1_40926\|ID:27158231\| | S12 | 13-370 | S66, K69, Y167 | | MER0026262 | 6-352 | 6.90E-77 |
| SPBDM4_v1_50408\|ID:27158771\| | S12 | 35-348 | P85S, E88K, R183Y | | MER0026262 | 7-331 | 1.90E-16 |
| SPBDM4_v1_40419\|ID:27157724\| | S14 | Mar-66 | <S, <H, D44 | | MER0000474 | 144-207 | 4.40E-17 |
| SPBDM4_v1_40420\|ID:27157725\| | S14 | 23-87 | >S, >H, >D | | MER0004857 | 39-103 | 1.20E-12 |
| SPBDM4_v1_70102\|ID:27159560\|clpP\| | S14 | 4-198 | S103, H128, D177 | | MER0000474 | 13-206 | 1.70E-69 |
| SPBDM4_v1_40433\|ID:27157738\|radA\| | S16 | 178-422 | A359S, R402 | | MER0014135 | 207-451 | 3.50E-42 |
| SPBDM4_v1_70212\|ID:27159670\|lon\| | S16 | 571-780 | S688, K731 | | MER0011056 | 709-920 | 3.30E-61 |
| SPBDM4_v1_70236\|ID:27159694\| | S16 | 74-175 | A77S, A121K/R | | MER0003859 | 521-626 | 5.20E-07 |
| SPBDM4_v1_50097\|ID:27158460\|lexA\| | S24 | 114-186 | S124, K161 | | MER0006172 | Apr-77 | 2.20E-14 |
| SPBDM4_v1_51071\|ID:27159434\| | S26A | 58-181 | S69, K156 |  | MER0004537 | 74-186 | 3.70E-09 |
| SPBDM4_v1_51071\|ID:27159434\| | S26A | 277-320 | <S, <K |  | MER0055964 | 234-277 | 5.70E-07 |
| SPBDM4_v1_80081\|ID:27159857\|lepB\| | S26A | 30-104 | S43, K96 |  | MER0000589 | 78-154 | 1.80E-05 |
| SPBDM4_v1_40073\|ID:27157378\| | S33 | 24-253 | S93, E181D, H235 | | MER0031610 | 69-314 | 1.50E-12 |
| SPBDM4_v1_40074\|ID:27157379\| | S33 | 2-248 | S99, D220, H248 | | MER0033259 | 42-272 | 2.00E-11 |
| SPBDM4_v1_41012\|ID:27158317\| | S33 | 53-278 | S141, D231, H261 | | MER0033259 | 57-287 | 1.90E-05 |
| SPBDM4_v1_50919\|ID:27159282\| | S33 | 19-134 | S109, >D, >H | | MER0031618 | 37-147 | 1.40E-12 |
| SPBDM4_v1_40468\|ID:27157773\| | S41A | 216-383 | S314, K339 | | MER0004196 | 215-382 | 1.40E-41 |
| SPBDM4_v1_70102\|ID:27159560\|clpP\| | S49C | 56-194 | M104S, R124K | | MER0042954 | 47-185 | 1.90E-05 |
| SPBDM4_v1_50456\|ID:27158819\| | S54 | 19-191 | X126S, H184 | | MER0017194 | 17-203 | 3.20E-13 |
| SPBDM4_v1_50798\|ID:27159161\| | S54 | 1-201 | S132, H197 | | MER0017194 | 1-200 | 2.90E-33 |
| SPBDM4_v1_50836\|ID:27159199\| | S54 | 52-180 | S117, H168 | | MER0015453 | 122-262 | 1.10E-06 |
| SPBDM4_v1_50968\|ID:27159331\|hslV\| | T01B | 6-123 | T6 |  | MER0001627 | 2-119 | 1.40E-43 |
| SPBDM4_v1_50968\|ID:27159331\|hslV\| | T01B | 152-196 | <S/T |  | MER0001627 | 132-172 | 4.40E-05 |
| SPBDM4_v1_50007\|ID:27158370\|ggt\| | T03 | 45-553 | T378 |  | MER0001978 | 58-571 | 4.30E-121 |
| SPBDM4_v1_50180\|ID:27158543\| | T03 | 135-550 | T376 |  | MER0223926 | 105-519 | 1.50E-74 |
| SPBDM4_v1_80119\|ID:27159895\|ggt\| | T03 | 43-426 | T375 |  | MER0001978 | 44-444 | 2.10E-82 |
| SPBDM4_v1_40484\|ID:27157789\|argJ\| | T05 | 188-405 | T188 |  | MER0011829 | 215-441 | 2.10E-48 |
| SPBDM4_v1_50432\|ID:27158795\| | U32 | 27-289 |  |  | MER0013876 | 31-288 | 6.10E-41 |
| SPBDM4_v1_40893\|ID:27158198\| | U62 | 231-449 |  |  | MER0016301 | 238-456 | 1.60E-30 |

**uncultured Spirochaete bacterium SA-8.**

| query name | family | query start-query end | predcited active site residues | predicted metal ligands | hit name | hit start-hit end | e-value |
| --- | --- | --- | --- | --- | --- | --- | --- |
| SPSA8_v1_10032\|ID:41144801\|hflK\| | I87 | 110-310 |  |  | MER0192051 | 149-349 | 2.20E-34 |
| SPSA8_v1_10033\|ID:41144802\|hflC\| | I87 | 25-324 |  |  | MER0191412 | 21-330 | 1.50E-36 |
| SPSA8_v1_10059\|ID:41144828\|clpP\| | S14 | 8-198 | X103S, H128, D177 |  | MER0000474 | 16-206 | 5.50E-64 |
| SPSA8_v1_20025\|ID:41144854\| | S12 | 38-333 | S61, K64, Y153 |  | MER0004090 | 78-395 | 2.40E-19 |
| SPSA8_v1_30014\|ID:41144898\| | M29 | 190-350 | Y316 | E217, E282, H309, H328, D330 | MER0001285 | 214-392 | 4.70E-14 |
| SPSA8_v1_40031\|ID:41144963\| | M38 | 28-372 | S316D | H65, H67, R170K, H199, H220 | MER0066192 | 26-366 | 3.40E-38 |
| SPSA8_v1_40037\|ID:41144969\| | M50B | 75-124 | <E | <H, <H, <D | MER0004466 | 224-273 | 1.60E-05 |
| SPSA8_v1_50004\|ID:41144984\| | C26 | 8-184 | C85, >H, >E |  | MER0065588 | 3-168 | 5.30E-24 |
| SPSA8_v1_50008\|ID:41144988\|ypwA\| | M32 | 23-494 | E262 | H261, H265, E293 | MER0002064 | 27-498 | 2.20E-105 |
| SPSA8_v1_70016\|ID:41145097\| | U32 | 68-344 |  |  | MER0013876 | 31-309 | 1.20E-42 |
| SPSA8_v1_70021\|ID:41145102\|purQ\| | C56 | 12-105 | A22E, F57C, S58H |  | MER0042827 | 1072-1168 | 3.10E-07 |
| SPSA8_v1_70024\|ID:41145105\|purF\| | C44 | 24-245 | C24 |  | MER0011806 | 87-308 | 5.70E-51 |
| SPSA8_v1_80002\|ID:41145120\| | S33 | 8-118 | S94, >D, >H |  | MER0014065 | 108-221 | 5.90E-12 |
| SPSA8_v1_80002\|ID:41145120\| | S33 | 198-261 | <S, D215, H243 |  | MER0000437 | 231-293 | 1.60E-05 |
| SPSA8_v1_80027\|ID:41145145\| | M24B | 233-448 | H235, H343, H354 | D252, D264, H347, E395, E435 | MER0001248 | 253-465 | 2.10E-30 |
| SPSA8_v1_80029\|ID:41145147\| | M16A | 46-147 | E88, >E | K85H, H89, >E | MER0002423 | 31-131 | 4.70E-06 |
| SPSA8_v1_90006\|ID:41145172\| | M19 | 12-324 |  | H16, D18, E122, H189, H210 | MER0013425 | 3-301 | 1.10E-45 |
| SPSA8_v1_90008\|ID:41145174\| | S01C | 120-339 | H128, D158, S237 |  | MER0001372 | 101-311 | 2.20E-50 |
| SPSA8_v1_90034\|ID:41145200\| | I87 | 77-211 |  |  | MER0192051 | 102-236 | 2.00E-06 |
| SPSA8_v1_100022\|ID:41145225\| | U32 | 33-273 |  |  | MER0013876 | 32-265 | 1.40E-33 |
| SPSA8_v1_110002\|ID:41145240\| | M38 | 48-392 | D315 | H62, H64, T154K, E203H, H247 | MER0030151 | 59-411 | 1.10E-22 |
| SPSA8_v1_120011\|ID:41145277\|ftsH\| | M41 | 421-642 | E460 | H459, H463, D536 | MER0005466 | 385-604 | 6.50E-56 |
| SPSA8_v1_120018\|ID:41145284\|yjbG\| | M03B | 290-573 | E390 | H389, H393, E417 | MER0001163 | 290-571 | 9.20E-62 |
| SPSA8_v1_130033\|ID:41145331\| | M15B | 146-243 | E232 | H182, D189, H235 | MER0014983 | 131-229 | 5.20E-21 |
| SPSA8_v1_150020\|ID:41145392\| | S54 | 42-169 | S117, H168 |  | MER0015455 | 158-294 | 4.10E-13 |
| SPSA8_v1_160015\|ID:41145415\| | M50B | 9-206 | E19 | H18, H22, >D | MER0004469 | 12-210 | 1.90E-23 |
| SPSA8_v1_160015\|ID:41145415\| | M50B | 316-409 | <E | <H, <H, >D | MER0004480 | 245-330 | 1.90E-07 |
| SPSA8_v1_180008\|ID:41145466\| | S41A | 221-382 | S317, K342 |  | MER0004196 | 217-378 | 2.10E-44 |
| SPSA8_v1_180019\|ID:41145477\|pyrC\| | M38 | 4-303 | D248 | H12, H14, K103, H140, H175 | MER0061068 | 9-306 | 9.20E-62 |
| SPSA8_v1_180020\|ID:41145478\| | M24B | 145-371 | H218, H306, H317 | D237, D248, H310, E341, E355 | MER0004931 | 121-343 | 1.10E-36 |
| SPSA8_v1_180023\|ID:41145481\|argJ\| | T05 | 188-405 | T188 |  | MER0011829 | 215-441 | 2.80E-48 |
| SPSA8_v1_190020\|ID:41145506\| | M16C | 50-444 | E61, E134 | H58, H62, E156 | MER0014056 | 154-560 | 1.70E-126 |
| SPSA8_v1_200015\|ID:41145529\| | S33 | 11-138 | S113, >D, >H |  | MER0031618 | 25-147 | 6.30E-13 |
| SPSA8_v1_200020\|ID:41145534\|guaA\| | C26 | 2-228 | C80, H169, E171 |  | MER0045886 | 12-236 | 4.20E-47 |
| SPSA8_v1_220024\|ID:41145586\| | S09C | 82-175 | S159, >D, >H |  | MER0034548 | 83-181 | 4.70E-07 |
| SPSA8_v1_250011\|ID:41145643\| | M18 | 1-428 | D83, E283 | H81, D253, E284, D329, H423 | MER0003374 | 12-443 | 7.50E-70 |
| SPSA8_v1_250015\|ID:41145647\| | M23B | 203-307 | H293 | H214, D218, H295 | MER0003380 | 303-407 | 1.40E-26 |
| SPSA8_v1_270007\|ID:41145675\| | M20X | 10-541 | D87, E178 | H85, D145, E179, D210, H522 | MER0002961 | 15-542 | 8.80E-56 |
| SPSA8_v1_280011\|ID:41145697\| | M50B | 17-76 | <E | <H, <H, <D | MER0004466 | 211-271 | 2.40E-05 |
| SPSA8_v1_290011\|ID:41145717\| | S54 | 38-194 | X128S, H184 |  | MER0015473 | 105-268 | 1.60E-09 |
| SPSA8_v1_320016\|ID:41145786\| | M20D | 9-385 | D78, E136 | D76, D104, E137, H163, H363 | MER0002007 | 11-387 | 4.40E-72 |
| SPSA8_v1_320019\|ID:41145789\| | M38 | 47-318 | T312D | H69, H71, A173K, H202, H221 | MER0066192 | 41-317 | 3.00E-29 |
| SPSA8_v1_330012\|ID:41145802\| | C40 | 82-195 | C103, H154, H166 |  | MER0004035 | 73-183 | 2.90E-18 |
| SPSA8_v1_330017\|ID:41145807\| | M16B | 6-192 | E49, E119 | H46, H50, E126 | MER0003885 | 19-205 | 1.60E-25 |
| SPSA8_v1_340005\|ID:41145813\|pepT\| | M20B | 28-416 | D95, E188 | H93, D154, E189, D211, H393 | MER0001421 | 14-402 | 1.10E-79 |
| SPSA8_v1_350012\|ID:41145839\|lspA\| | A08 | 54-173 | D119, D151 |  | MER0001313 | 50-163 | 3.00E-10 |
| SPSA8_v1_380016\|ID:41145893\| | C40 | 95-179 | <C, H138, H150 |  | MER0004035 | 109-183 | 8.40E-13 |
| SPSA8_v1_380016\|ID:41145893\| | C40 | 41-80 | C65, >H, >E/H/N/Q |  | MER0002450 | 223-262 | 9.80E-05 |
| SPSA8_v1_400020\|ID:41145929\|hutI\| | M38 | 80-393 | P346D | H90, H92, D230K, K258H, V265H | MER0033186 | 77-392 | 2.90E-52 |
| SPSA8_v1_410003\|ID:41145934\| | M23B | 232-331 | H320 | <H, <D, H322 | MER0003906 | 229-308 | 3.80E-08 |
| SPSA8_v1_410007\|ID:41145938\|lon\| | S16 | 544-779 | S688, K731 |  | MER0000485 | 537-770 | 1.40E-61 |
| SPSA8_v1_420015\|ID:41145960\| | M48C | 94-273 | E158 | H157, H161, E223 | MER0031491 | 75-253 | 5.00E-19 |
| SPSA8_v1_430002\|ID:41145963\|glmS\| | C44 | 2-227 | C2 |  | MER0003327 | 2-225 | 2.40E-42 |
| SPSA8_v1_470009\|ID:41146032\| | S16 | 56-158 | S77, A121K/R |  | MER0026213 | 1075-1181 | 2.50E-07 |
| SPSA8_v1_480012\|ID:41146047\| | U62 | 232-450 |  |  | MER0016301 | 238-456 | 3.50E-29 |
| SPSA8_v1_490003\|ID:41146052\| | S01C | 263-420 | H279, D319, S390 |  | MER0063616 | 187-345 | 9.90E-43 |
| SPSA8_v1_490004\|ID:41146053\|ftsH\| | M41 | 385-583 | E437 | H436, H440, D512 | MER0005466 | 372-571 | 6.90E-60 |
| SPSA8_v1_510007\|ID:41146082\| | S26A | 279-336 | <S, <K |  | MER0055964 | 225-276 | 2.50E-11 |
| SPSA8_v1_510007\|ID:41146082\| | S26A | 75-184 | S86, K173 |  | MER0004537 | 74-172 | 7.40E-09 |
| SPSA8_v1_530007\|ID:41146107\| | M23B | 203-309 | I294H | M219D, Y296H | MER0005300 | 293-406 | 8.80E-14 |
| SPSA8_v1_620011\|ID:41146215\|pdxT\| | C26 | 1-201 | C90, H187, E189 |  | MER0066916 | 2-193 | 1.20E-38 |
| SPSA8_v1_630006\|ID:41146222\| | M23B | 285-370 | H365 | H285, D289, H367 | MER0013541 | 297-381 | 8.30E-16 |
| SPSA8_v1_660005\|ID:41146251\| | U32 | 27-309 |  |  | MER0003855 | 30-307 | 8.00E-44 |
| SPSA8_v1_700007\|ID:41146294\| | M16B | 40-254 | E82, E154 | H79, H83, E161 | MER0003448 | 10-221 | 4.60E-19 |
| SPSA8_v1_700008\|ID:41146295\| | M16B | 51-252 | F93E, A163E | L90H, K94H, N170E | MER0015090 | 53-253 | 2.30E-06 |
| SPSA8_v1_750003\|ID:41146329\| | M38 | 213-334 | D334 | <H, <H, <K, R237H, H246 | MER0037714 | 207-330 | 1.30E-14 |
| SPSA8_v1_750003\|ID:41146329\| | M38 | 38-83 | >D | H77, H79, >K, >H, >H | MER0030136 | 27-72 | 5.60E-05 |
| SPSA8_v1_750006\|ID:41146332\|ygeY\| | M20A | 4-177 | D79, E144 | H77, D110, E145, E170, >H | MER0003369 | 8-181 | 9.40E-47 |
| SPSA8_v1_750006\|ID:41146332\|ygeY\| | M20A | 289-395 | <D, <E | <H, <D, <E, <D/E, >H | MER0003369 | 181-287 | 4.50E-38 |
| SPSA8_v1_750006\|ID:41146332\|ygeY\| | M42 | 19-106 | D79, >D, >E | H77, >H, >D, >E, >D/E, >H | MER0005160 | Jun-95 | 7.60E-05 |
| SPSA8_v1_750007\|ID:41146333\|ssnA\| | M38 | 145-317 | D317 | <H, <H, K184, Q223H, H232 | MER0037714 | 152-330 | 1.20E-06 |
| SPSA8_v1_820002\|ID:41146381\| | S09C | 58-306 | S164, D254, H287 |  | MER0066184 | 60-297 | 1.40E-30 |
| SPSA8_v1_820002\|ID:41146381\| | S33 | 57-185 | S164, >D, >H |  | MER0036115 | 22-139 | 9.20E-06 |
| SPSA8_v1_1020002\|ID:41146474\| | C110 | Feb-98 | C42, H82, D97 |  | MER0472834 | 185-283 | 3.30E-12 |
