# Supplementary material for: Fermentative Spirochaetes mediate necromass recycling in anoxic hydrocarbon-contaminated habitats
Source: ISME J. 2018 May 30;12(8):2039–50. doi: 10.1038/s41396-018-0148-3 (PMC6052044; doi:10.1038/s41396-018-0148-3)
Supplement: Supplementary file 6 — Supplementary Table S5 [file 41396_2018_148_MOESM6_ESM.docx]

**Supplementary Table S5** List of relative genes encoding transporters for branched amino acid, dipeptide and oligopeptides. The genes were identified in the genome of *Rectinema cohabitans* HM using the MicroScope genome annotation platform.

| SPBIB_v1_100008 | _ | putative oxidoreductase |
| --- | --- | --- |
| SPBIB_v1_10001 | _ | protein of unknown function |
| SPBIB_v1_100010 | iscS | cysteine desulfurase (tRNA sulfurtransferase), PLP-dependent |
| SPBIB_v1_100037 | cysK | cysteine synthase A, O-acetylserine sulfhydrolase A subunit |
| SPBIB_v1_100038 | cysE | Serine acetyltransferase |
| SPBIB_v1_100171 | _ | putative Fe(3+)-transporting ATPase |
| SPBIB_v1_100172 | aroK | Shikimate kinase |
| SPBIB_v1_100173 | _ | Membrane dipeptidase |
| SPBIB_v1_100182 | _ | Aminotransferase class V |
| SPBIB_v1_100188 | _ | Arginine deiminase |
| SPBIB_v1_10053 | _ | Aminotransferase class I and II |
| SPBIB_v1_10065 | _ | Major facilitator superfamily MFS_1 |
| SPBIB_v1_130004 | rocD | Ornithine aminotransferase |
| SPBIB_v1_130008 | _ | membrane protein of unknown function |
| SPBIB_v1_130011 | _ | conserved protein of unknown function |
| SPBIB_v1_130014 | _ | Extracellular solute-binding protein family 1 |
| SPBIB_v1_130015 | potC | polyamine transporter subunit ; membrane component of ABC superfamily |
| SPBIB_v1_130016 | _ | ABC-type transporter, integral membrane subunit |
| SPBIB_v1_130017 | potA | polyamine transporter subunit ; ATP-binding component of ABC superfamily |
| SPBIB_v1_130018 | _ | Glutamate formiminotransferase |
| SPBIB_v1_130041 | argF | Ornithine carbamoyltransferase |
| SPBIB_v1_150002 | _ | putative L-lysine 2,3-aminomutase |
| SPBIB_v1_150008 | appC | Oligopeptide transport system permease protein AppC |
| SPBIB_v1_150009 | _ | Dipeptide-binding ABC transporter, periplasmic substrate-binding component |
| SPBIB_v1_150010 | _ | D-ala-D-ala transporter subunit ; ATP-binding component of ABC superfamily (fragment) |
| SPBIB_v1_150011 | dppD | dipeptide transporter ; ATP-binding component of ABC superfamily |
| SPBIB_v1_150012 | gsiC | Glutathione ABC transporter, permease protein GsiC |
| SPBIB_v1_150019 | _ | Cystathionine beta-lyase |
| SPBIB_v1_150043 | leuD | 3-isopropylmalate dehydratase small subunit 1 |
| SPBIB_v1_150045 | leuC | 3-isopropylmalate dehydratase large subunit 1 |
| SPBIB_v1_150067 | ygfK | putative oxidoreductase, Fe-S subunit |
| SPBIB_v1_150069 | ygeY | putative peptidase |
| SPBIB_v1_150070 | _ | putative threonine synthase |
| SPBIB_v1_150072 | _ | Pyridoxal-5'-phosphate-dependent protein beta subunit |
| SPBIB_v1_150074 | ygeW | conserved hypothetical protein |
| SPBIB_v1_150097 | yggP | putative dehydrogenase |
| SPBIB_v1_150099 | _ | Argininosuccinate lyase |
| SPBIB_v1_150100 | argG | Argininosuccinate synthase |
| SPBIB_v1_150118 | _ | putative Major facilitator superfamily MFS_1 |
| SPBIB_v1_150119 | _ | conserved membrane protein of unknown function |
| SPBIB_v1_150134 | _ | Histidinol phosphate phosphatase, HisJ |
| SPBIB_v1_150138 | _ | Na+/glutamate symporter-like protein |
| SPBIB_v1_150145 | _ | Theronine dehydrogenase-like Zn-dependent dehydrogenase |
| SPBIB_v1_150162 | _ | membrane protein of unknown function |
| SPBIB_v1_150168 | _ | Aspartate kinase |
| SPBIB_v1_150173 | _ | conserved protein of unknown function |
| SPBIB_v1_150174 | _ | putative enzyme |
| SPBIB_v1_180030 | _ | protein of unknown function |
| SPBIB_v1_180031 | _ | membrane protein of unknown function |
| SPBIB_v1_190005 | _ | Asparaginase/glutaminase |
| SPBIB_v1_190016 | pepT | Peptidase T |
| SPBIB_v1_190022 | sufS | selenocysteine lyase, PLP-dependent |
| SPBIB_v1_190031 | kamA | L-lysine 2,3-aminomutase |
| SPBIB_v1_190034 | kdd | L-erythro-3,5-diaminohexanoate dehydrogenase |
| SPBIB_v1_200002 | _ | putative Branched-chain-amino-acid aminotransferase |
| SPBIB_v1_200017 | mhpE | 4-hyroxy-2-oxovalerate/4-hydroxy-2-oxopentanoic acid aldolase, class I |
| SPBIB_v1_200032 | _ | Ketol-acid reductoisomerase |
| SPBIB_v1_200035 | aroQ | 3-dehydroquinate dehydratase |
| SPBIB_v1_20011 | _ | Branched-chain amino acid ABC transporter, amino acid-binding protein (TC 3.A.1.4.1) |
| SPBIB_v1_20012 | _ | High-affinity branched-chain amino acid transport system permease protein LivH (TC 3.A.1.4.1) |
| SPBIB_v1_20013 | _ | Inner-membrane translocator |
| SPBIB_v1_20014 | livG | leucine/isoleucine/valine transporter subunit ; ATP-binding component of ABC superfamily |
| SPBIB_v1_20015 | livF | leucine/isoleucine/valine transporter subunit ; ATP-binding component of ABC superfamily |
| SPBIB_v1_20016 | _ | Peptidase M20 |
| SPBIB_v1_20026 | _ | NADH-quinone oxidoreductase subunit F 2 |
| SPBIB_v1_20035 | _ | Oligoendopeptidase, pepF/M3 family |
| SPBIB_v1_20037 | _ | conserved protein of unknown function |
| SPBIB_v1_210018 | _ | Major facilitator superfamily MFS_1 |
| SPBIB_v1_210026 | braC | Leucine-, isoleucine-, valine-, threonine-, and alanine-binding protein |
| SPBIB_v1_210027 | livH | leucine/isoleucine/valine transporter subunit ; membrane component of ABC superfamily |
| SPBIB_v1_210028 | _ | ABC-type transporter, integral membrane subunit |
| SPBIB_v1_210029 | livG | leucine/isoleucine/valine transporter subunit ; ATP-binding component of ABC superfamily |
| SPBIB_v1_210030 | livF | leucine/isoleucine/valine transporter subunit ; ATP-binding component of ABC superfamily |
| SPBIB_v1_210058 | _ | Major facilitator superfamily MFS_1 |
| SPBIB_v1_210069 | _ | putative dipeptidase YkvY |
| SPBIB_v1_210083 | ltaA | L-allo-threonine aldolase |
| SPBIB_v1_210090 | dppF | dipeptide transporter ; ATP-binding component of ABC superfamily |
| SPBIB_v1_210091 | oppD | oligopeptide transporter subunit ; ATP-binding component of ABC superfamily |
| SPBIB_v1_210092 | _ | ABC-type transporter, integral membrane subunit |
| SPBIB_v1_210093 | _ | ABC-type transporter, integral membrane subunit |
| SPBIB_v1_210101 | _ | conserved membrane protein of unknown function |
| SPBIB_v1_210114 | speE | Spermidine synthase |
| SPBIB_v1_210115 | speD | S-adenosylmethionine decarboxylase |
| SPBIB_v1_210116 | potA | Spermidine/putrescine import ATP-binding protein PotA |
| SPBIB_v1_210121 | oppF | oligopeptide transporter subunit ; ATP-binding component of ABC superfamily |
| SPBIB_v1_210122 | oppD | oligopeptide transporter subunit ; ATP-binding component of ABC superfamily |
| SPBIB_v1_210123 | yliD | putative peptide transporter permease subunit: membrane component of ABC superfamily |
| SPBIB_v1_210124 | nikB | nickel transporter subunit ; membrane component of ABC superfamily |
| SPBIB_v1_210125 | _ | ABC-type transporter, periplasmic subunit |
| SPBIB_v1_210155 | _ | Phosphoglycerate dehydrogenase |
| SPBIB_v1_210166 | _ | Phosphoglycerate dehydrogenase |
| SPBIB_v1_210210 | yqeA | amino acid (carbamate) kinase |
| SPBIB_v1_230001 | _ | Urocanate hydratase |
| SPBIB_v1_240005 | serC | Phosphoserine aminotransferase |
| SPBIB_v1_240006 | _ | D-isomer specific 2-hydroxyacid dehydrogenase NAD-binding protein |
| SPBIB_v1_240013 | proC | Pyrroline-5-carboxylate reductase |
| SPBIB_v1_240014 | dapD | 2,3,4,5-tetrahydropyridine-2,6-dicarboxylate N-succinyltransferase |
| SPBIB_v1_240015 | dapA | 4-hydroxy-tetrahydrodipicolinate synthase |
| SPBIB_v1_240016 | _ | putative 4-hydroxy-tetrahydrodipicolinate reductase |
| SPBIB_v1_240017 | _ | Aspartokinase |
| SPBIB_v1_240018 | asd | Aspartate-semialdehyde dehydrogenase |
| SPBIB_v1_240022 | livF | leucine/isoleucine/valine transporter subunit ; ATP-binding component of ABC superfamily |
| SPBIB_v1_240023 | livG | leucine/isoleucine/valine transporter subunit ; ATP-binding component of ABC superfamily |
| SPBIB_v1_240024 | _ | ABC-type transporter, integral membrane subunit |
| SPBIB_v1_240025 | livH | leucine/isoleucine/valine transporter subunit ; membrane component of ABC superfamily |
| SPBIB_v1_240026 | _ | Extracellular ligand-binding receptor |
| SPBIB_v1_240039 | _ | conserved membrane protein of unknown function |
| SPBIB_v1_240047 | _ | Serine--glyoxylate aminotransferase protein |
| SPBIB_v1_240050 | _ | Glyoxalase/bleomycin resistance protein/dioxygenase |
| SPBIB_v1_240067 | _ | Acetylornithine deacetylase / succinyl-diaminopimelate desuccinylase |
| SPBIB_v1_250026 | _ | Putative oligopeptide transport ATP-binding protein YkfD (fragment) |
| SPBIB_v1_250027 | ggt | Gamma-glutamyltransferase |
| SPBIB_v1_250028 | _ | Major facilitator superfamily MFS_1 |
| SPBIB_v1_250036 | _ | conserved membrane protein of unknown function |
| SPBIB_v1_250052 | _ | Major facilitator superfamily MFS_1 |
| SPBIB_v1_250054 | _ | membrane protein of unknown function |
| SPBIB_v1_250092 | eutA | reactivating factor for ethanolamine ammonia lyase |
| SPBIB_v1_250093 | eutB | ethanolamine ammonia-lyase, large subunit, heavy chain |
| SPBIB_v1_250094 | eutC | ethanolamine ammonia-lyase, small subunit (light chain) |
| SPBIB_v1_250095 | eutL | putative carboxysome-related structural protein with putative role in ethanolamine utilization |
| SPBIB_v1_250102 | _ | ABC transporter permease protein |
| SPBIB_v1_250103 | potA | Spermidine/putrescine import ATP-binding protein PotA |
| SPBIB_v1_260001 | argD | Acetylornithine aminotransferase |
| SPBIB_v1_260002 | argB | Acetylglutamate kinase |
| SPBIB_v1_260003 | argJ | Arginine biosynthesis bifunctional protein ArgJ [Includes: Glutamate N-acetyltransferase ; Amino-acid acetyltransferase] |
| SPBIB_v1_260004 | argC | N-acetyl-gamma-glutamyl-phosphate reductase |
| SPBIB_v1_260031 | _ | putative opine dehydrogenase |
| SPBIB_v1_260044 | gltB | Ferredoxin-dependent glutamate synthase 1 |
| SPBIB_v1_260049 | dppA | DppA2 |
| SPBIB_v1_260050 | yliC | putative peptide transporter permease subunit: membrane component of ABC superfamily |
| SPBIB_v1_260051 | yliD | putative peptide transporter permease subunit: membrane component of ABC superfamily |
| SPBIB_v1_260075 | _ | Dihydrodipicolinate synthase |
| SPBIB_v1_270002 | _ | CapC protein |
| SPBIB_v1_270007 | ggt | Gamma-glutamyltranspeptidase |
| SPBIB_v1_270026 | nanA | N-acetylneuraminate lyase 1 |
| SPBIB_v1_270031 | _ | Dihydrodipicolinate synthase |
| SPBIB_v1_270032 | dapA | 4-hydroxy-tetrahydrodipicolinate synthase |
| SPBIB_v1_270034 | dapA | 4-hydroxy-tetrahydrodipicolinate synthase |
| SPBIB_v1_270055 | thrC | Threonine synthase |
| SPBIB_v1_270056 | _ | putative Homoserine kinase |
| SPBIB_v1_280019 | _ | putative Peptidase M20 |
| SPBIB_v1_280022 | _ | Ribose-phosphate pyrophosphokinase |
| SPBIB_v1_280038 | patB | Cystathionine beta-lyase PatB |
| SPBIB_v1_280047 | _ | 3-dehydroquinate dehydratase |
| SPBIB_v1_290035 | gltL | glutamate and aspartate transporter subunit ; ATP-binding component of ABC superfamily |
| SPBIB_v1_290036 | _ | Polar amino acid ABC transporter, inner membrane subunit |
| SPBIB_v1_290037 | _ | ABC-type transporter, periplasmic subunit family 3 |
| SPBIB_v1_290064 | _ | Methylenetetrahydrofolate reductase |
| SPBIB_v1_290065 | metH | homocysteine-N5-methyltetrahydrofolate transmethylase, B12-dependent |
| SPBIB_v1_290070 | _ | Aspartate ammonia-lyase |
| SPBIB_v1_290108 | _ | Extracellular solute-binding protein family 5 |
| SPBIB_v1_290109 | ddpB | D-ala-D-ala transporter subunit ; membrane component of ABC superfamily |
| SPBIB_v1_290110 | ddpC | D-ala-D-ala transporter subunit ; membrane component of ABC superfamily |
| SPBIB_v1_290111 | oppD | oligopeptide transporter subunit ; ATP-binding component of ABC superfamily |
| SPBIB_v1_290112 | dppF | dipeptide transporter ; ATP-binding component of ABC superfamily |
| SPBIB_v1_290115 | _ | putative Branched-chain amino acid transport system carrier protein BraB |
| SPBIB_v1_290125 | _ | Helix-turn-helix domain protein |
| SPBIB_v1_290147 | ggt | Gamma-glutamyltranspeptidase |
| SPBIB_v1_290158 | oppA | oligopeptide transporter subunit ; periplasmic-binding component of ABC superfamily |
| SPBIB_v1_290159 | oppB | oligopeptide transporter subunit ; membrane component of ABC superfamily |
| SPBIB_v1_290160 | _ | ABC-type transporter, integral membrane subunit |
| SPBIB_v1_290161 | oppD | oligopeptide transporter subunit ; ATP-binding component of ABC superfamily |
| SPBIB_v1_290162 | oppF | oligopeptide transporter subunit ; ATP-binding component of ABC superfamily |
| SPBIB_v1_290176 | _ | 4Fe-4S ferredoxin iron-sulfur binding domain protein (modular protein) |
| SPBIB_v1_290182 | iolD | 3D-(3,5/4)-trihydroxycyclohexane-1,2-dione hydrolase |
| SPBIB_v1_290190 | _ | conserved membrane protein of unknown function |
| SPBIB_v1_290207 | _ | Binding-protein-dependent transport systems inner membrane component |
| SPBIB_v1_290208 | _ | Binding-protein-dependent transport systems inner membrane component |
| SPBIB_v1_290209 | fbpC | Fe(3+) ions import ATP-binding protein FbpC |
| SPBIB_v1_290212 | _ | putative Major facilitator superfamily MFS_1 |
| SPBIB_v1_290221 | proB | Glutamate 5-kinase |
| SPBIB_v1_290222 | proA | Gamma-glutamyl phosphate reductase |
| SPBIB_v1_300002 | _ | Xaa-Pro aminopeptidase |
| SPBIB_v1_30010 | _ | exported protein of unknown function |
| SPBIB_v1_30011 | glyA | serine hydroxymethyltransferase |
| SPBIB_v1_30021 | _ | Thermostable carboxypeptidase 1 |
| SPBIB_v1_30022 | hisD | Histidinol dehydrogenase |
| SPBIB_v1_30023 | _ | conserved protein of unknown function |
| SPBIB_v1_30024 | hisF | imidazole glycerol phosphate synthase, catalytic subunit with HisH |
| SPBIB_v1_30025 | hisH | Imidazole glycerol phosphate synthase subunit HisH |
| SPBIB_v1_30026 | hisB | Imidazoleglycerol-phosphate dehydratase |
| SPBIB_v1_30027 | _ | putative Histidinol-phosphate aminotransferase |
| SPBIB_v1_30028 | hisG | ATP phosphoribosyltransferase |
| SPBIB_v1_310006 | _ | CDP-alcohol phosphatidyltransferase |
| SPBIB_v1_310008 | ilvI | acetolactate synthase III, large subunit |
| SPBIB_v1_310009 | _ | putative enzyme |
| SPBIB_v1_310010 | ilvH | acetolactate synthase III, thiamin-dependent, small subunit |
| SPBIB_v1_310036 | _ | Gluconate transporter |
| SPBIB_v1_310084 | livF | leucine/isoleucine/valine transporter subunit ; ATP-binding component of ABC superfamily |
| SPBIB_v1_310085 | livG | leucine/isoleucine/valine transporter subunit ; ATP-binding component of ABC superfamily |
| SPBIB_v1_310086 | _ | Branched-chain amino acid ABC transporter permease protein |
| SPBIB_v1_310087 | livH | leucine/isoleucine/valine transporter subunit ; membrane component of ABC superfamily |
| SPBIB_v1_310088 | _ | Branched-chain amino acid ABC transporter substrate-binding protein |
| SPBIB_v1_310092 | hutH | Histidine ammonia-lyase |
| SPBIB_v1_310093 | _ | conserved membrane protein of unknown function |
| SPBIB_v1_320006 | _ | conserved membrane protein of unknown function |
| SPBIB_v1_330017 | appF | Oligopeptide transport ATP-binding protein AppF |
| SPBIB_v1_330018 | oppD | Oligopeptide transport ATP-binding protein OppD |
| SPBIB_v1_330019 | _ | ABC-type dipeptide/oligopeptide/nickel transport system, permease component |
| SPBIB_v1_330020 | _ | Binding-protein-dependent transport systems inner membrane component |
| SPBIB_v1_330021 | _ | ABC-type dipeptide transport system, periplasmic component |
| SPBIB_v1_340016 | _ | Major facilitator superfamily MFS_1 |
| SPBIB_v1_340026 | sudA | Sulfide dehydrogenase subunit alpha |
| SPBIB_v1_340037 | fbpC | Fe(3+) ions import ATP-binding protein FbpC |
| SPBIB_v1_340071 | ilvD | Dihydroxy-acid dehydratase |
| SPBIB_v1_350026 | fbpC | Fe(3+) ions import ATP-binding protein FbpC |
| SPBIB_v1_350035 | _ | Glutamine synthetase, type I |
| SPBIB_v1_350060 | _ | Pullulanase, type I (fragment) |
| SPBIB_v1_350068 | _ | putative Lipolytic protein G-D-S-L family |
| SPBIB_v1_360001 | leuA | 2-isopropylmalate synthase |
| SPBIB_v1_360002 | leuC | 3-isopropylmalate dehydratase large subunit |
| SPBIB_v1_360003 | _ | 3-isopropylmalate dehydratase small subunit 1 (modular protein) |
| SPBIB_v1_360004 | leuB | 3-isopropylmalate dehydrogenase |
| SPBIB_v1_360005 | _ | AzlC family protein |
| SPBIB_v1_360006 | _ | Branched-chain amino acid transport |
| SPBIB_v1_360016 | carB | carbamoyl-phosphate synthase large subunit |
| SPBIB_v1_360017 | carA | Carbamoyl-phosphate synthase small chain |
| SPBIB_v1_360025 | trpE | Anthranilate synthase component 1 |
| SPBIB_v1_360026 | trpGD | Bifunctional protein TrpGD [Includes: Anthranilate synthase component 2 ; Anthranilate phosphoribosyltransferase] |
| SPBIB_v1_360027 | trpCF | Indole-3-glycerol phosphate synthase/N-(5'-phosphoribosyl)anthranilate isomerase |
| SPBIB_v1_360028 | trpB | tryptophan synthase, beta subunit |
| SPBIB_v1_360029 | trpA | Tryptophan synthase alpha chain |
| SPBIB_v1_370030 | _ | Peptidase M29 aminopeptidase II |
| SPBIB_v1_380005 | fbpC | Fe(3+) ions import ATP-binding protein FbpC |
| SPBIB_v1_380038 | apeB | M18 family aminopeptidase |
| SPBIB_v1_380048 | _ | membrane protein of unknown function |
| SPBIB_v1_390003 | _ | Peptidase M24 |
| SPBIB_v1_390008 | _ | conserved membrane protein of unknown function |
| SPBIB_v1_390010 | _ | Aminotransferase class I and II |
| SPBIB_v1_390020 | _ | Leucine-, isoleucine-, valine-, threonine-, and alanine-binding protein |
| SPBIB_v1_390021 | _ | High-affinity branched-chain amino acid transport system permease protein LivH |
| SPBIB_v1_390022 | _ | Inner-membrane translocator |
| SPBIB_v1_390023 | _ | Branched-chain amino acid transport ATP-binding protein LivG |
| SPBIB_v1_390024 | livF | leucine/isoleucine/valine transporter subunit ; ATP-binding component of ABC superfamily |
| SPBIB_v1_390028 | _ | conserved membrane protein of unknown function |
| SPBIB_v1_400016 | _ | Fumarate lyase |
| SPBIB_v1_40002 | _ | Major facilitator superfamily MFS_1 |
| SPBIB_v1_400024 | dapF | Diaminopimelate epimerase 1 |
| SPBIB_v1_400028 | lysA | Diaminopimelate decarboxylase |
| SPBIB_v1_400033 | _ | exported protein of unknown function |
| SPBIB_v1_410017 | _ | protein of unknown function |
| SPBIB_v1_410035 | tdh | threonine 3-dehydrogenase, NAD(P)-binding |
| SPBIB_v1_410036 | _ | conserved membrane protein of unknown function |
| SPBIB_v1_410054 | dppF | dipeptide transporter ; ATP-binding component of ABC superfamily |
| SPBIB_v1_410055 | dppD | dipeptide transporter ; ATP-binding component of ABC superfamily |
| SPBIB_v1_410056 | _ | putative Extracellular solute-binding protein family 5 |
| SPBIB_v1_410057 | _ | ABC-type dipeptide/oligopeptide/nickel transport system, permease component |
| SPBIB_v1_410058 | _ | Binding-protein-dependent transport systems inner membrane component |
| SPBIB_v1_410061 | _ | Lipolytic protein G-D-S-L family |
| SPBIB_v1_50002 | _ | Aminotransferase class I and II |
| SPBIB_v1_50004 | _ | Orn/DAP/Arg decarboxylase 2 |
| SPBIB_v1_50010 | gcvT | Aminomethyltransferase |
| SPBIB_v1_50011 | gcvH | glycine cleavage complex lipoylprotein |
| SPBIB_v1_50012 | gcvPA | putative glycine dehydrogenase (decarboxylating) subunit 1 |
| SPBIB_v1_50013 | gcvPB | putative glycine dehydrogenase (decarboxylating) subunit 2 |
| SPBIB_v1_50018 | _ | protein of unknown function |
| SPBIB_v1_50027 | _ | D-isomer specific 2-hydroxyacid dehydrogenase NAD-binding protein |
| SPBIB_v1_50038 | aliB | Oligopeptide-binding protein AliB |
| SPBIB_v1_50039 | _ | ABC transporter, permease protein |
| SPBIB_v1_50040 | amiD | Oligopeptide transport system permease protein AmiD |
| SPBIB_v1_50041 | oppD | oligopeptide transporter subunit ; ATP-binding component of ABC superfamily |
| SPBIB_v1_50042 | oppF | oligopeptide transporter subunit ; ATP-binding component of ABC superfamily |
| SPBIB_v1_50045 | yjbG | Oligoendopeptidase F homolog |
| SPBIB_v1_60025 | _ | ABC-type transporter, periplasmic subunit |
| SPBIB_v1_80008 | aroE | Shikimate dehydrogenase |
| SPBIB_v1_80017 | _ | conserved protein of unknown function |
| SPBIB_v1_80021 | _ | protein of unknown function |
| SPBIB_v1_80028 | _ | putative enzyme |
| SPBIB_v1_80037 | gdhA | glutamate dehydrogenase, NADP-specific |
| SPBIB_v1_80040 | aroA | 3-phosphoshikimate 1-carboxyvinyltransferase |
| SPBIB_v1_90002 | _ | Prephenate dehydrogenase |
| SPBIB_v1_90003 | _ | Prephenate dehydratase |
| SPBIB_v1_90004 | aroC | Chorismate synthase |
| SPBIB_v1_90005 | _ | 3-dehydroquinate synthase (modular protein) |
| SPBIB_v1_90006 | aroF | 3-deoxy-7-phosphoheptulonate synthase |
| SPBIB_v1_90012 | _ | Alcohol dehydrogenase GroES domain protein |
