# Supplementary material for: Fermentative Spirochaetes mediate necromass recycling in anoxic hydrocarbon-contaminated habitats
Source: ISME J. 2018 May 30;12(8):2039–50. doi: 10.1038/s41396-018-0148-3 (PMC6052044; doi:10.1038/s41396-018-0148-3)
Supplement: Supplementary file 7 — Supplementary Table S6 [file 41396_2018_148_MOESM7_ESM.docx]

**Supplementary Table S6** The dbCAN server was used to identify the presence of various glycoside hydrolases enzymes in in the genomes of *Rectinema cohabitans* HM, uncultured Spirochaete bacterium bdmA 4, and uncultured Spirochaete bacterium SA-8.

***Rectinema cohabitans* HM**

| Query Gene | Subject | E-value | Subject-Start | Subject-End | Covered fraction |
| --- | --- | --- | --- | --- | --- |
| SPBIB_v1_270043\|ID:27163450\| | AA4 | 5.10E-22 | 14 | 193 | 0.342911877 |
| SPBIB_v1_360009\|ID:27164065\| | AA6 | 1.10E-05 | 1 | 116 | 0.58974359 |
| SPBIB_v1_80022\|ID:27162066\| | AA6 | 5.10E-05 | 2 | 99 | 0.497435897 |
| SPBIB_v1_350066\|ID:27164052\| | CBM20 | 0.00015 | 11 | 63 | 0.577777778 |
| SPBIB_v1_350065\|ID:27164051\|nplT\| | CBM34 | 2.50E-24 | 1 | 119 | 0.983333333 |
| SPBIB_v1_350059\|ID:27164045\| | CBM41 | 8.20E-11 | 2 | 100 | 0.960784314 |
| SPBIB_v1_250062\|ID:27163261\| | CBM48 | 1.60E-10 | 4 | 67 | 0.828947368 |
| SPBIB_v1_280055\|ID:27163521\|glgX\| | CBM48 | 4.40E-11 | 5 | 70 | 0.855263158 |
| SPBIB_v1_350059\|ID:27164045\| | CBM48 | 6.80E-11 | 2 | 71 | 0.907894737 |
| SPBIB_v1_20041\|ID:27161868\| | CBM50 | 9.90E-14 | 1 | 40 | 0.975 |
| SPBIB_v1_20041\|ID:27161868\| | CBM50 | 7.70E-11 | 1 | 40 | 0.975 |
| SPBIB_v1_240066\|ID:27163196\| | CBM50 | 2.80E-13 | 1 | 40 | 0.975 |
| SPBIB_v1_240066\|ID:27163196\| | CBM50 | 3.10E-17 | 1 | 40 | 0.975 |
| SPBIB_v1_380034\|ID:27164157\| | CBM50 | 4.00E-12 | 1 | 40 | 0.975 |
| SPBIB_v1_380034\|ID:27164157\| | CBM50 | 2.20E-05 | 8 | 40 | 0.8 |
| SPBIB_v1_380039\|ID:27164162\| | CBM50 | 6.30E-07 | 6 | 40 | 0.85 |
| SPBIB_v1_50024\|ID:27161935\| | CBM50 | 9.20E-12 | 1 | 40 | 0.975 |
| SPBIB_v1_50024\|ID:27161935\| | CBM50 | 3.30E-12 | 1 | 40 | 0.975 |
| SPBIB_v1_250112\|ID:27163311\| | CBM66 | 7.80E-10 | 7 | 148 | 0.909677419 |
| SPBIB_v1_150119\|ID:27162631\| | CE1 | 2.80E-09 | 5 | 132 | 0.559471366 |
| SPBIB_v1_200020\|ID:27162868\|todF\| | CE1 | 2.10E-06 | 29 | 140 | 0.488986784 |
| SPBIB_v1_310104\|ID:27163879\| | CE1 | 3.20E-06 | 16 | 119 | 0.453744493 |
| SPBIB_v1_390025\|ID:27164211\| | CE1 | 0.0001 | 55 | 143 | 0.387665198 |
| SPBIB_v1_150185\|ID:27162697\| | CE10 | 8.80E-36 | 78 | 331 | 0.741935484 |
| SPBIB_v1_210096\|ID:27162988\| | CE10 | 2.60E-08 | 147 | 315 | 0.492668622 |
| SPBIB_v1_350068\|ID:27164054\| | CE12 | 1.10E-06 | 68 | 157 | 0.423809524 |
| SPBIB_v1_290096\|ID:27163621\| | CE14 | 8.50E-21 | 1 | 124 | 0.991935484 |
| SPBIB_v1_410061\|ID:27164316\| | CE3 | 1.10E-13 | 53 | 194 | 0.726804124 |
| SPBIB_v1_170035\|ID:27162740\| | CE4 | 1.90E-06 | 14 | 124 | 0.846153846 |
| SPBIB_v1_290106\|ID:27163631\| | CE4 | 1.40E-27 | 4 | 122 | 0.907692308 |
| SPBIB_v1_250040\|ID:27163239\| | CE9 | 2.80E-118 | 1 | 373 | 0.997319035 |
| SPBIB_v1_400022\|ID:27164237\| | CE9 | 4.50E-82 | 2 | 366 | 0.975871314 |
| SPBIB_v1_10045\|ID:27161807\|bglA\| | GH1 | 1.20E-159 | 3 | 425 | 0.983682984 |
| SPBIB_v1_150038\|ID:27162550\| | GH109 | 5.80E-09 | 2 | 117 | 0.912698413 |
| SPBIB_v1_150039\|ID:27162551\| | GH109 | 6.80E-10 | 61 | 121 | 0.476190476 |
| SPBIB_v1_170034\|ID:27162739\| | GH109 | 1.80E-19 | 3 | 124 | 0.96031746 |
| SPBIB_v1_210073\|ID:27162965\| | GH109 | 7.00E-22 | 2 | 120 | 0.936507937 |
| SPBIB_v1_250030\|ID:27163229\| | GH109 | 3.40E-09 | 59 | 126 | 0.531746032 |
| SPBIB_v1_290184\|ID:27163709\|yrbE\| | GH109 | 7.60E-10 | 2 | 113 | 0.880952381 |
| SPBIB_v1_290189\|ID:27163714\| | GH109 | 1.10E-16 | 1 | 114 | 0.896825397 |
| SPBIB_v1_350022\|ID:27164008\| | GH109 | 9.00E-07 | 5 | 115 | 0.873015873 |
| SPBIB_v1_90011\|ID:27162095\| | GH109 | 1.40E-11 | 1 | 123 | 0.968253968 |
| SPBIB_v1_260040\|ID:27163354\| | GH113 | 3.70E-52 | 3 | 306 | 0.990196078 |
| SPBIB_v1_190009\|ID:27162803\| | GH13 | 4.80E-24 | 19 | 291 | 0.909698997 |
| SPBIB_v1_210179\|ID:27163071\| | GH13 | 7.70E-52 | 8 | 290 | 0.943143813 |
| SPBIB_v1_280055\|ID:27163521\|glgX\| | GH13 | 1.20E-46 | 11 | 296 | 0.953177258 |
| SPBIB_v1_350016\|ID:27164002\| | GH13 | 2.30E-21 | 29 | 283 | 0.849498328 |
| SPBIB_v1_350059\|ID:27164045\| | GH13 | 7.30E-40 | 12 | 292 | 0.936454849 |
| SPBIB_v1_350061\|ID:27164047\| | GH13 | 8.90E-76 | 13 | 294 | 0.939799331 |
| SPBIB_v1_350065\|ID:27164051\|nplT\| | GH13 | 1.00E-73 | 12 | 296 | 0.949832776 |
| SPBIB_v1_400006\|ID:27164221\|ams\| | GH13 | 3.60E-41 | 12 | 294 | 0.943143813 |
| SPBIB_v1_210194\|ID:27163086\| | GH16 | 9.30E-08 | 14 | 148 | 0.708994709 |
| SPBIB_v1_10040\|ID:27161802\| | GH2 | 5.30E-97 | 29 | 492 | 0.615691489 |
| SPBIB_v1_340050\|ID:27163965\| | GH2 | 5.90E-86 | 88 | 487 | 0.530585106 |
| SPBIB_v1_410069\|ID:27164324\| | GH2 | 2.90E-90 | 21 | 485 | 0.617021277 |
| SPBIB_v1_10046\|ID:27161808\| | GH20 | 4.20E-113 | 9 | 336 | 0.970326409 |
| SPBIB_v1_190007\|ID:27162801\| | GH23 | 2.00E-14 | 11 | 103 | 0.681481481 |
| SPBIB_v1_240066\|ID:27163196\| | GH23 | 8.10E-19 | 3 | 124 | 0.896296296 |
| SPBIB_v1_250018\|ID:27163217\| | GH23 | 2.10E-24 | 15 | 123 | 0.8 |
| SPBIB_v1_330022\|ID:27163911\| | GH3 | 8.40E-64 | 7 | 214 | 0.958333333 |
| SPBIB_v1_290092\|ID:27163617\| | GH31 | 7.00E-109 | 1 | 427 | 0.99765808 |
| SPBIB_v1_400021\|ID:27164236\|yihQ\| | GH31 | 1.80E-97 | 5 | 425 | 0.983606557 |
| SPBIB_v1_130057\|ID:27162483\|ftsZ\| | GH4 | 0.00094 | 6 | 86 | 0.446927374 |
| SPBIB_v1_290090\|ID:27163615\|palH\| | GH4 | 1.10E-56 | 1 | 175 | 0.972067039 |
| SPBIB_v1_210095\|ID:27162987\| | GH5 | 4.70E-30 | 2 | 237 | 0.854545455 |
| SPBIB_v1_100136\|ID:27162287\| | GH57 | 3.00E-36 | 17 | 379 | 0.945169713 |
| SPBIB_v1_150005\|ID:27162517\| | GH57 | 6.70E-67 | 1 | 308 | 0.80156658 |
| SPBIB_v1_20006\|ID:27161833\| | GH63 | 9.80E-47 | 331 | 567 | 0.414035088 |
| SPBIB_v1_350017\|ID:27164003\| | GH63 | 1.20E-47 | 358 | 566 | 0.364912281 |
| SPBIB_v1_20005\|ID:27161832\| | GH65 | 3.10E-141 | 1 | 368 | 0.98655914 |
| SPBIB_v1_100026\|ID:27162177\| | GH74 | 1.90E-07 | 21 | 115 | 0.403433476 |
| SPBIB_v1_50063\|ID:27161974\| | GH74 | 2.50E-10 | 36 | 113 | 0.330472103 |
| SPBIB_v1_50063\|ID:27161974\| | GH74 | 1.60E-10 | 43 | 114 | 0.30472103 |
| SPBIB_v1_250004\|ID:27163203\|malQ\| | GH77 | 1.40E-172 | 1 | 492 | 0.993927126 |
| SPBIB_v1_310028\|ID:27163803\| | GH77 | 5.50E-125 | 1 | 489 | 0.987854251 |
| SPBIB_v1_280016\|ID:27163482\| | GH78 | 1.20E-12 | 155 | 352 | 0.390873016 |
| SPBIB_v1_350060\|ID:27164046\| | GH78 | 2.30E-19 | 148 | 325 | 0.351190476 |
| SPBIB_v1_180001\|ID:27162742\| | GT2 | 1.90E-22 | 1 | 137 | 0.80952381 |
| SPBIB_v1_180002\|ID:27162743\| | GT2 | 3.30E-14 | 2 | 120 | 0.702380952 |
| SPBIB_v1_290143\|ID:27163668\| | GT2 | 5.70E-26 | 2 | 168 | 0.988095238 |
| SPBIB_v1_370028\|ID:27164120\| | GT2 | 1.80E-19 | 1 | 163 | 0.964285714 |
| SPBIB_v1_300010\|ID:27163765\| | GT26 | 2.80E-23 | 28 | 141 | 0.660818713 |
| SPBIB_v1_130045\|ID:27162471\|murG\| | GT28 | 1.30E-40 | 2 | 154 | 0.968152866 |
| SPBIB_v1_250079\|ID:27163278\|glgP\| | GT35 | 1.90E-134 | 22 | 550 | 0.783382789 |
| SPBIB_v1_170019\|ID:27162724\| | GT4 | 4.30E-13 | 5 | 160 | 0.96875 |
| SPBIB_v1_170023\|ID:27162728\| | GT4 | 1.10E-23 | 7 | 154 | 0.91875 |
| SPBIB_v1_170024\|ID:27162729\| | GT4 | 4.80E-27 | 8 | 124 | 0.725 |
| SPBIB_v1_170026\|ID:27162731\| | GT4 | 1.10E-07 | 6 | 151 | 0.90625 |
| SPBIB_v1_250044\|ID:27163243\| | GT4 | 1.00E-29 | 11 | 154 | 0.89375 |
| SPBIB_v1_50016\|ID:27161927\|glgA\| | GT5 | 2.30E-178 | 1 | 471 | 0.995762712 |
| SPBIB_v1_100033\|ID:27162184\| | GT51 | 1.40E-56 | 2 | 177 | 0.988700565 |
| SPBIB_v1_150117\|ID:27162629\| | GT51 | 4.80E-48 | 6 | 174 | 0.949152542 |

**uncultured Spirochaete bacterium bdmA 4**

| Query Gene | Subject | E-value | Subject-Start | Subject-End | Covered fraction |
| --- | --- | --- | --- | --- | --- |
| SPBDM4_v1_40056\|ID:27157361\| | CE9 | 3.50E-79 | 3 | 366 | 0.97319 |
| SPBDM4_v1_40060\|ID:27157365\| | GH13 | 2.70E-31 | 12 | 171 | 0.531773 |
| SPBDM4_v1_40074\|ID:27157379\| | CE1 | 0.00016 | 56 | 143 | 0.38326 |
| SPBDM4_v1_40122\|ID:27157427\| | CBM50 | 2.30E-07 | 4 | 40 | 0.9 |
| SPBDM4_v1_40127\|ID:27157432\| | CBM50 | 6.60E-10 | 1 | 40 | 0.975 |
| SPBDM4_v1_40127\|ID:27157432\| | CBM50 | 0.00072 | 7 | 40 | 0.825 |
| SPBDM4_v1_40166\|ID:27157471\| | GT2 | 3.00E-19 | 1 | 163 | 0.964286 |
| SPBDM4_v1_40194\|ID:27157499\|palH\| | GH4 | 4.20E-56 | 1 | 175 | 0.972067 |
| SPBDM4_v1_40208\|ID:27157513\| | CE12 | 2.20E-08 | 67 | 158 | 0.433333 |
| SPBDM4_v1_40217\|ID:27157522\| | GH105 | 4.40E-97 | 12 | 330 | 0.957831 |
| SPBDM4_v1_40222\|ID:27157527\| | GH28 | 1.90E-87 | 7 | 308 | 0.926154 |
| SPBDM4_v1_40315\|ID:27157620\|lacZ\| | GH2 | 7.50E-85 | 90 | 493 | 0.535904 |
| SPBDM4_v1_40432\|ID:27157737\| | GH77 | 1.60E-121 | 1 | 490 | 0.989879 |
| SPBDM4_v1_40444\|ID:27157749\| | GH65 | 4.30E-145 | 1 | 370 | 0.991935 |
| SPBDM4_v1_40471\|ID:27157776\| | GT26 | 6.00E-21 | 37 | 142 | 0.614035 |
| SPBDM4_v1_40489\|ID:27157794\| | CE7 | 0.00097 | 177 | 274 | 0.309904 |
| SPBDM4_v1_40624\|ID:27157929\| | GH4 | 4.10E-55 | 2 | 178 | 0.98324 |
| SPBDM4_v1_40631\|ID:27157936\| | GH43 | 1.50E-44 | 5 | 237 | 0.935484 |
| SPBDM4_v1_40632\|ID:27157937\| | GH95 | 4.10E-12 | 312 | 631 | 0.441828 |
| SPBDM4_v1_40654\|ID:27157959\| | CE4 | 3.40E-17 | 21 | 125 | 0.8 |
| SPBDM4_v1_40728\|ID:27158033\| | AA4 | 1.70E-23 | 14 | 193 | 0.342912 |
| SPBDM4_v1_40870\|ID:27158175\|glgX\| | CBM48 | 1.70E-10 | 5 | 70 | 0.855263 |
| SPBDM4_v1_40870\|ID:27158175\|glgX\| | GH13 | 7.70E-46 | 11 | 296 | 0.953177 |
| SPBDM4_v1_40914\|ID:27158219\| | GH109 | 5.80E-08 | 4 | 117 | 0.896825 |
| SPBDM4_v1_40919\|ID:27158224\| | GH63 | 1.60E-46 | 358 | 565 | 0.363158 |
| SPBDM4_v1_40920\|ID:27158225\| | GH13 | 7.90E-22 | 22 | 290 | 0.896321 |
| SPBDM4_v1_40981\|ID:27158286\| | GT2 | 1.10E-32 | 2 | 167 | 0.982143 |
| SPBDM4_v1_40983\|ID:27158288\| | CE10 | 6.80E-23 | 80 | 337 | 0.753666 |
| SPBDM4_v1_41006\|ID:27158311\| | AA7 | 1.30E-20 | 10 | 205 | 0.425764 |
| SPBDM4_v1_41012\|ID:27158317\| | CE1 | 5.90E-15 | 5 | 218 | 0.938326 |
| SPBDM4_v1_50003\|ID:27158366\| | GT2 | 2.60E-24 | 2 | 167 | 0.982143 |
| SPBDM4_v1_50059\|ID:27158422\|yrbE\| | GH109 | 7.30E-10 | 2 | 113 | 0.880952 |
| SPBDM4_v1_50078\|ID:27158441\| | CE4 | 1.70E-25 | 5 | 123 | 0.907692 |
| SPBDM4_v1_50116\|ID:27158479\|glgP\| | GT35 | 1.60E-134 | 22 | 552 | 0.78635 |
| SPBDM4_v1_50122\|ID:27158485\| | AA6 | 1.50E-05 | 1 | 116 | 0.589744 |
| SPBDM4_v1_50133\|ID:27158496\| | CBM48 | 2.90E-11 | 7 | 71 | 0.842105 |
| SPBDM4_v1_50162\|ID:27158525\| | GT4 | 6.30E-27 | 13 | 156 | 0.89375 |
| SPBDM4_v1_50167\|ID:27158530\| | CE9 | 2.40E-117 | 1 | 373 | 0.997319 |
| SPBDM4_v1_50178\|ID:27158541\| | GH109 | 1.00E-09 | 60 | 126 | 0.52381 |
| SPBDM4_v1_50219\|ID:27158582\| | GH109 | 2.80E-17 | 4 | 124 | 0.952381 |
| SPBDM4_v1_50230\|ID:27158593\| | GT4 | 6.50E-06 | 11 | 144 | 0.83125 |
| SPBDM4_v1_50231\|ID:27158594\|rffE\| | GT19 | 1.40E-05 | 155 | 280 | 0.353107 |
| SPBDM4_v1_50232\|ID:27158595\| | GT4 | 5.00E-14 | 9 | 153 | 0.9 |
| SPBDM4_v1_50234\|ID:27158597\| | GT4 | 2.00E-17 | 9 | 156 | 0.91875 |
| SPBDM4_v1_50235\|ID:27158598\| | GT4 | 1.30E-10 | 9 | 153 | 0.9 |
| SPBDM4_v1_50240\|ID:27158603\| | GT2 | 6.90E-23 | 1 | 167 | 0.988095 |
| SPBDM4_v1_50287\|ID:27158650\| | GH3 | 7.20E-63 | 3 | 215 | 0.981481 |
| SPBDM4_v1_50293\|ID:27158656\| | CE4 | 7.70E-24 | 6 | 123 | 0.9 |
| SPBDM4_v1_50298\|ID:27158661\| | GH109 | 1.50E-19 | 2 | 119 | 0.928571 |
| SPBDM4_v1_50304\|ID:27158667\| | GT1 | 7.10E-48 | 75 | 380 | 0.798429 |
| SPBDM4_v1_50316\|ID:27158679\| | GH63 | 3.80E-35 | 355 | 566 | 0.370175 |
| SPBDM4_v1_50339\|ID:27158702\| | GH23 | 1.50E-22 | 16 | 121 | 0.777778 |
| SPBDM4_v1_50353\|ID:27158716\|malQ\| | GH77 | 7.60E-140 | 90 | 491 | 0.811741 |
| SPBDM4_v1_50360\|ID:27158723\| | GH23 | 1.40E-19 | 3 | 126 | 0.911111 |
| SPBDM4_v1_50360\|ID:27158723\| | CBM50 | 1.70E-13 | 1 | 40 | 0.975 |
| SPBDM4_v1_50360\|ID:27158723\| | CBM50 | 8.60E-15 | 1 | 40 | 0.975 |
| SPBDM4_v1_50370\|ID:27158733\| | GH43 | 2.90E-63 | 1 | 247 | 0.991935 |
| SPBDM4_v1_50373\|ID:27158736\| | GH52 | 1.60E-114 | 2 | 415 | 0.995181 |
| SPBDM4_v1_50455\|ID:27158818\| | CBM66 | 0.00041 | 39 | 114 | 0.483871 |
| SPBDM4_v1_50500\|ID:27158863\| | GH13 | 3.10E-36 | 6 | 190 | 0.615385 |
| SPBDM4_v1_50547\|ID:27158910\| | CE10 | 1.00E-07 | 147 | 315 | 0.492669 |
| SPBDM4_v1_50548\|ID:27158911\| | GH5 | 1.50E-30 | 3 | 237 | 0.850909 |
| SPBDM4_v1_50569\|ID:27158932\| | GH109 | 1.20E-05 | 51 | 117 | 0.52381 |
| SPBDM4_v1_50605\|ID:27158968\| | GT78 | 0.00044 | 32 | 84 | 0.38806 |
| SPBDM4_v1_50645\|ID:27159008\| | GH31 | 8.30E-112 | 1 | 427 | 0.997658 |
| SPBDM4_v1_50695\|ID:27159058\| | AA6 | 0.00093 | 15 | 91 | 0.389744 |
| SPBDM4_v1_50703\|ID:27159066\| | GH13 | 1.50E-22 | 22 | 291 | 0.899666 |
| SPBDM4_v1_50705\|ID:27159068\| | GH23 | 2.50E-16 | 8 | 116 | 0.8 |
| SPBDM4_v1_50741\|ID:27159104\| | GT4 | 3.00E-21 | 7 | 159 | 0.95 |
| SPBDM4_v1_50742\|ID:27159105\| | GT11 | 1.20E-64 | 2 | 275 | 0.98913 |
| SPBDM4_v1_50744\|ID:27159107\| | GT25 | 4.20E-31 | 3 | 178 | 0.966851 |
| SPBDM4_v1_50745\|ID:27159108\| | GT4 | 5.80E-24 | 6 | 157 | 0.94375 |
| SPBDM4_v1_50746\|ID:27159109\| | GT4 | 1.30E-11 | 69 | 137 | 0.425 |
| SPBDM4_v1_50747\|ID:27159110\| | GT27 | 2.10E-14 | 1 | 229 | 0.772881 |
| SPBDM4_v1_50752\|ID:27159115\| | GT2 | 1.20E-23 | 1 | 165 | 0.97619 |
| SPBDM4_v1_50752\|ID:27159115\| | GT27 | 8.10E-18 | 2 | 225 | 0.755932 |
| SPBDM4_v1_50752\|ID:27159115\| | GT4 | 3.10E-24 | 8 | 157 | 0.93125 |
| SPBDM4_v1_50757\|ID:27159120\| | GT2 | 5.40E-35 | 1 | 118 | 0.696429 |
| SPBDM4_v1_50758\|ID:27159121\| | GT4 | 3.10E-36 | 10 | 159 | 0.93125 |
| SPBDM4_v1_50764\|ID:27159127\| | CE10 | 8.40E-34 | 58 | 330 | 0.797654 |
| SPBDM4_v1_50829\|ID:27159192\| | GT51 | 3.30E-48 | 9 | 174 | 0.932203 |
| SPBDM4_v1_50928\|ID:27159291\| | GH57 | 1.80E-68 | 1 | 308 | 0.801567 |
| SPBDM4_v1_50947\|ID:27159310\| | GT32 | 0.00099 | 22 | 66 | 0.488889 |
| SPBDM4_v1_50962\|ID:27159325\|ftsZ\| | GH4 | 0.00091 | 6 | 86 | 0.446927 |
| SPBDM4_v1_50975\|ID:27159338\|murG\| | GT28 | 1.80E-37 | 2 | 142 | 0.89172 |
| SPBDM4_v1_51049\|ID:27159412\| | GT51 | 2.50E-58 | 2 | 177 | 0.988701 |
| SPBDM4_v1_70076\|ID:27159534\| | GH57 | 1.40E-36 | 7 | 361 | 0.924282 |
| SPBDM4_v1_70235\|ID:27159693\| | GH109 | 6.00E-12 | 1 | 118 | 0.928571 |
| SPBDM4_v1_70301\|ID:27159759\| | GT84 | 2.70E-86 | 1 | 214 | 0.990698 |
| SPBDM4_v1_70301\|ID:27159759\| | GH94 | 0 | 2 | 1034 | 0.996139 |
| SPBDM4_v1_80022\|ID:27159798\| | CBM50 | 3.20E-10 | 1 | 40 | 0.975 |
| SPBDM4_v1_80022\|ID:27159798\| | CBM50 | 1.10E-12 | 1 | 40 | 0.975 |
| SPBDM4_v1_80030\|ID:27159806\|glgA\| | GT5 | 1.60E-176 | 1 | 471 | 0.995763 |
| SPBDM4_v1_80082\|ID:27159858\| | CBM50 | 2.10E-13 | 1 | 40 | 0.975 |
| SPBDM4_v1_80082\|ID:27159858\| | CBM50 | 3.80E-12 | 1 | 40 | 0.975 |
| SPBDM4_v1_80141\|ID:27159917\| | GH20 | 4.20E-109 | 9 | 336 | 0.970326 |
| SPBDM4_v1_80142\|ID:27159918\|bglA\| | GH1 | 6.80E-155 | 2 | 424 | 0.983683 |
| SPBDM4_v1_80147\|ID:27159923\| | GH2 | 4.50E-91 | 30 | 492 | 0.614362 |

**uncultured Spirochaete bacterium SA-8**

| Query Gene | Subject | E-value | Subject-Start | Subject-End | Covered fraction |
| --- | --- | --- | --- | --- | --- |
| SPSA8_v1_100025\|ID:41145228\| | GH13.hmm | 6.90E-06 | 59 | 201 | 453 |
| SPSA8_v1_10029\|ID:41144798\| | GH57.hmm | 9.60E-38 | 21 | 369 | 22 |
| SPSA8_v1_150009\|ID:41145381\| | CE7.hmm | 7.40E-08 | 56 | 202 | 157 |
| SPSA8_v1_150028\|ID:41145400\| | GT51.hmm | 1.70E-42 | 32 | 175 | 1 |
| SPSA8_v1_180010\|ID:41145468\| | GT26.hmm | 5.00E-17 | 33 | 127 | 124 |
| SPSA8_v1_200018\|ID:41145532\| | GH57.hmm | 2.20E-71 | 1 | 303 | 9 |
| SPSA8_v1_210013\|ID:41145553\|ams\| | GH13.hmm | 1.80E-36 | 12 | 292 | 103 |
| SPSA8_v1_220002\|ID:41145564\| | GH31.hmm | 1.10E-22 | 278 | 425 | 330 |
| SPSA8_v1_220024\|ID:41145586\| | CE10.hmm | 7.20E-36 | 80 | 334 | 51 |
| SPSA8_v1_250010\|ID:41145642\| | CBM50.hmm | 1.90E-07 | 6 | 40 | 927 |
| SPSA8_v1_250015\|ID:41145647\| | CBM50.hmm | 1.00E-09 | 1 | 40 | 79 |
| SPSA8_v1_250015\|ID:41145647\| | CBM50.hmm | 1.80E-06 | 1 | 27 | 129 |
| SPSA8_v1_270011\|ID:41145679\| | GH28.hmm | 4.80E-89 | 10 | 309 | 33 |
| SPSA8_v1_270016\|ID:41145684\| | GH105.hmm | 1.80E-95 | 8 | 330 | 27 |
| SPSA8_v1_30011\|ID:41144895\| | GT2.hmm | 1.70E-20 | 1 | 165 | 592 |
| SPSA8_v1_320019\|ID:41145789\| | CE9.hmm | 2.00E-71 | 2 | 365 | 7 |
| SPSA8_v1_320020\|ID:41145790\| | GH31.hmm | 1.90E-99 | 11 | 424 | 3 |
| SPSA8_v1_360003\|ID:41145849\|bglA\| | GH1.hmm | ####### | 3 | 425 | 5 |
| SPSA8_v1_40024\|ID:41144956\| | GH88.hmm | 4.80E-66 | 2 | 324 | 51 |
| SPSA8_v1_40031\|ID:41144963\| | CE9.hmm | ####### | 1 | 373 | 9 |
| SPSA8_v1_40033\|ID:41144965\| | GH109.hmm | 1.20E-13 | 1 | 120 | 1 |
| SPSA8_v1_420008\|ID:41145953\| | GH16.hmm | 2.10E-11 | 13 | 175 | 40 |
| SPSA8_v1_450001\|ID:41145994\| | GH3.hmm | 3.30E-55 | 44 | 214 | 16 |
| SPSA8_v1_450009\|ID:41146002\| | GH109.hmm | 3.00E-08 | 61 | 121 | 52 |
| SPSA8_v1_450010\|ID:41146003\| | GH109.hmm | 7.90E-10 | 2 | 116 | 2 |
| SPSA8_v1_500008\|ID:41146072\|glgA\| | GT5.hmm | ####### | 1 | 471 | 7 |
| SPSA8_v1_530007\|ID:41146107\| | CBM50.hmm | 1.30E-13 | 1 | 40 | 30 |
| SPSA8_v1_530007\|ID:41146107\| | CBM50.hmm | 2.50E-10 | 1 | 40 | 76 |
| SPSA8_v1_550012\|ID:41146134\| | GH109.hmm | 9.00E-15 | 1 | 121 | 4 |
| SPSA8_v1_560001\|ID:41146137\| | GT51.hmm | 1.60E-26 | 2 | 97 | 59 |
| SPSA8_v1_570005\|ID:41146156\|glgP\| | GT35.hmm | ####### | 278 | 673 | 4 |
| SPSA8_v1_570006\|ID:41146157\|glgP\| | GT35.hmm | ####### | 1 | 269 | 111 |
| SPSA8_v1_580010\|ID:41146174\| | GH31.hmm | 4.50E-44 | 260 | 425 | 1 |
| SPSA8_v1_590004\|ID:41146178\| | CE12.hmm | 9.50E-08 | 2 | 156 | 6 |
| SPSA8_v1_600004\|ID:41146189\| | CE4.hmm | 3.90E-25 | 5 | 123 | 137 |
| SPSA8_v1_60046\|ID:41145076\|glcD\| | AA4.hmm | 4.10E-27 | 16 | 247 | 11 |
| SPSA8_v1_670009\|ID:41146265\| | CE4.hmm | 1.30E-22 | 6 | 109 | 93 |
| SPSA8_v1_680008\|ID:41146275\| | GH23.hmm | 3.40E-19 | 16 | 126 | 104 |
| SPSA8_v1_690006\|ID:41146284\|murG\| | GT28.hmm | 4.50E-35 | 3 | 152 | 217 |
| SPSA8_v1_70035\|ID:41145116\| | GH109.hmm | 9.90E-16 | 1 | 120 | 4 |
| SPSA8_v1_720001\|ID:41146309\| | GH13.hmm | 1.60E-22 | 18 | 291 | 9 |
| SPSA8_v1_800005\|ID:41146369\|ykcC\| | GT2.hmm | 3.00E-27 | 1 | 164 | 4 |
