# Supplementary material for: Fermentative Spirochaetes mediate necromass recycling in anoxic hydrocarbon-contaminated habitats
Source: ISME J. 2018 May 30;12(8):2039–50. doi: 10.1038/s41396-018-0148-3 (PMC6052044; doi:10.1038/s41396-018-0148-3)
Supplement: Supplementary file 8 — Supplementary Database 1 [file 41396_2018_148_MOESM8_ESM.pdf]

>SPBIB_v1_10001|ID:27161763| protein of unknown function [Uncultured spirochete bib]
VVDIRPVYHHLDDRIRAHVLICFIAMVLVRVAERTLSMSWRDIAYTLTDIRVGHTKGPDG
ELWLTSPLSEMQRTLFTMLKVKHPPKVWDFKKSKKAPIGV*
>SPBIB_v1_10002|ID:27161764| transposase [Uncultured spirochete bib]
MGKVTKAEVSAILQGMQDRELYEKLLGLKEPWSVENVTLDLPSATVTVAISHPKGAKFPC
PVCGTERPIYDHQKRRWRHLDTCGFTTILEAEVPRIQCPEHGVKQVNVPWGEPGSRFTAL
FEAIAISLLKVASFSDVARHLRISWDAASGIMERAVRRGLARREAQPLRRIGIDETSFQK
RHEYVTVVFDQERSCVVDVLDGRKKETLKTWLAANQNALTALESVSMDMWDAYIGAVREA
HPDGEQKICFDRFHVAQYFNKAVDKVRAEEHREFKTRGEQSPLTRTKHAWLRKGAPDAAF
SSLARSNLKTARAWRIKEAAAELLRTKSHEEAARDWRKLLSWMMRSRLGPVVKVAAMIRQ
YLWGILNAARLGATNAKNEAVNATIQKLKVRACGFRNRSRFKMVILSHLGGLSLLPEVMT
*
>SPBIB_v1_10003|ID:27161765|chpA| toxin of the ChpA-ChpR toxin-antitoxin system, endoribonuclease [Uncultured spirochete bib]
MVKPKKYVPERGDIVWLDFNPQLGHEQKGRRPALVISFKEYNEKIGLALFCPITSKVKGY
PFETEVKGKIINGCVLSDQVKNLDWTVRNVEFIEKIEDEKLNDIIDNILLLIEK*
>SPBIB_v1_10004|ID:27161766| Transcriptional regulator/antitoxin, MazE [Uncultured spirochete bib]
MQTVVQKWGNSLGIRIPSVYAKEFNLKHGNSVEIVKEGGKIIIIPPKKTLEEMLSKVTKD
NIHAPIETGSSLGNEEW*
>SPBIB_v1_10005|ID:27161767| PilT protein domain protein [Uncultured spirochete bib]
MLNSILIDTGPLIALFDKDDTYHNKVKDFIKNAKYRFITTTAVITEVSHMLDFNINAQID
FFEWIMKEGVILQEISQKDISRIIELTKEYSDRPMDFADATLVIAAEKTGIKKIISIDSD
FDIYRLPGKVKIENVFHK*
>SPBIB_v1_10006|ID:27161768| CopG-like domain-containing protein DNA-binding [Uncultured spirochete bib]
MTTVRLPIEIEQRLEILARKKHKSKTDLIREALEKLFIQEESEKDSYELGEEYFGKYGSG
DGTLSVTYKDKLKDKINAKLNSH*
>SPBIB_v1_10007|ID:27161769| Phospholipiddiacylglycerol acyltransferase protein [Uncultured spirochete bib]
MPISKSTNWGRKEKMITYTESLRGVSHYSLDGFCEGWQQPLTPLQLRSILKHSTYRILAM
DTEHTRIVGIITALSDKIHWAFIPYLEVIPAYQKQGIGKRLMELMIEKTKGIVCIDLTCD
TEMQAFYEQFGMVASHGMILRRYMDEK*
>SPBIB_v1_10008|ID:27161770| conserved protein of unknown function [Uncultured spirochete bib]
LANFKIAETETFEKKIQSSQYKFLYKKIQHYIYPLLRENPYFGPNIKKLKGEYKEIYRFR
IGDYRLFYTISEETVIIFILDIENRKDAYK*
>SPBIB_v1_10009|ID:27161771| conserved protein of unknown function [Uncultured spirochete bib]
MAKTITVRVEDTTYDIFKKAAEGQKRTISNYLEFAALNYTVNETVVDDSEMKEILAFEND
IKKGLADISAGRYKVIG*
>SPBIB_v1_10010|ID:27161772| Ribbon-helix-helix protein, CopG family [Uncultured spirochete bib]
MKKTNTITLRVPEELKERIENMATLQGVSMNQFALYAFTKELSELENSQYFKKYLKGKKK
EEIIKGFDDVFSKVKPRNVDQWDTL*
>SPBIB_v1_10011|ID:27161773| Toxin-antitoxin system toxin component, PIN family [Uncultured spirochete bib]
MKVVLDTSVLFQALYSSTGASHAILKLIREGALQLPISIPVFEEYREVLLRQSSLDLFEL
STNDVQKIIDFIALIGVKTDIRFLLRPNLRDENDNIFIELAFASGAHYVITKNVNDFKYD
ADLRFNEITIATPAEFMKIWRNTYEKD*
>SPBIB_v1_10012|ID:27161774| Plasmid stabilization system protein [Uncultured spirochete bib]
MSAKYTIVFSRYAEDDLSEIIKYYAEKNSQYALKLLDTLETRVQELRELPARGRIVPELA
QQNILEYRELIEGNYRIIYVIQDSMVVIHAILDSRRNLEELLMQKLMRFYS*
>SPBIB_v1_10013|ID:27161775| Prevent-host-death family protein [Uncultured spirochete bib]
MNVNLKDTIKPISYIKTNAADMMKFINDNKSSIIITQNGEAKAVLVDIDSYQEMRDAFSL
LKIIKLSEKDIAAGNYKESDAVFSNLRTSLSK*
>SPBIB_v1_10014|ID:27161776| conserved protein of unknown function [Uncultured spirochete bib]
MNKKVFIDSDVILDLLSKRQPFYPAAAEIFTFGDYGQLDLYTSSLVFSNVFYILRKSVGN
EKAKELLRKLRILIRIIPIVEKNIDMALNSTFTDLEDAIQYYSSCSFGIDVIVTRNTSDY
KNSEELLIQSPEEFINIIKSIEKE*

>SPBIB_v1_10015|ID:27161777| conserved protein of unknown function [Uncultured spirochete bib]
METKLTLKLDQAIIQRAKQYAEEHNRSVSKLVEDYFRNLIVNRKDKTHYSPLVEELSGVI
TPDDINNSDYTSYLEAKYE*
>SPBIB_v1_10016|ID:27161778| Kinase domain protein [Uncultured spirochete bib]
MENDVIKFIRQKDYTFIRNIGRGATGAAILLKDELIDQLFVCKKYQPAMGIEPAEYYSKF
VNEIKMMYLLNNRNVVRIYNYYLYPKQYTGYILMEYIDGNSIGDYISKNPEKIDDIFIQT
VNGFYELQENGILHRDIRDSNIMVSSEGIVKIIDFGFGKKIEFDSDYDKSISLNWWCQTP
NEFNDGRYDFKTEIYFVGKLFEKLLLDNGLSNFKYSDQLKKMIAPDPENRISSFKEIFSE
AILKNDMENIFEEDEIEIYRSFANGLSAIYAKIENSAKYEKDVELIISKMEECYKKNMLE
QEIIDSNDLAKIFVQGAYTYYKKRTIYLSCLKNFLQLLKGASSEKRRIIMLNIENRLDAI
EHYRKPEDNFTDDIPF*
>SPBIB_v1_10017|ID:27161779| protein of unknown function [Uncultured spirochete bib]
LAYELDYVIFHFTLTREKSI*
>SPBIB_v1_10018|ID:27161780| membrane protein of unknown function [Uncultured spirochete bib]
MAIKEKKQRYFEYFTYLKTSINLAYRPSKLLDYSLKHSFTTLLFNSITLISIFSLTIVIV
LALFNSEYSIENQIKISFAMFLISLLYLPGVLTSILFKNKILKTALKASFFICINIIVFY
LSIPIFSLIIFFKSEIYFFYYAYLFLCIIIFFIYIIILPILYSKGNNHRIINILLSIVIA
LSFNISVSFIIKNQNNNIAESIDPIFYELRSNNLLERSRYDYLQDTGNIIINSVNKYLKN
DNIKYLEELGLEEKPLLLLKESLLELEKKIHFRTNKQLITTSIEAADILRNIISQTSRIT
ELSDELPQSIQKGIAYINDGKELIEKNQKIVNEDMTKINTILDKINNHLKDITTTEYNQM
LKESTKLNNDIININLDIKKIDALLKNADAELETTDQYIEYLNKKNSVIKEITILIEEYE
KNINDRIKAVKKIEQIYKLKNVFYFFP*
>SPBIB_v1_10019|ID:27161781| protein of unknown function [Uncultured spirochete bib]
MIDIYDLEKVLVSKISFKNLIDLRTKKYLMDINYPSLYVFWFNNYDGAIKNLKRNFVIKK
PGKFENNLC*
>SPBIB_v1_10020|ID:27161782| protein of unknown function [Uncultured spirochete bib]
VMIVASFLGFAWGWVLFLFVKMPGEKTPSEISEIDEEKTKKARISIILFFQKVRS*
>SPBIB_v1_10021|ID:27161783| IstB domain protein ATP-binding protein [Uncultured spirochete bib]
MIRTPQERERTRITIASMSRKLMLSSRVVELCESEATPRQEEFLLKVLSEEIDRRERGKK
ARLLNRAGFPVFKSFEDYDFSEIRFPPALSKEELLRADFIPEKKNLVLYGGVGTGKTHMA
IALGIAACEKGLSVRFLTVTELVLKLTEAYKAGTLERLIRDLKQLDLLILDEWGYVPVDR
EGSQLLFRIIADSYESKSLILTTNLEFSKWGGIFTDEQMAAAMIDRLVHHGHLLLFEAKS
YRMTHALMRQPGPGTAKPKTTVEAGSRLGEGGA*
>SPBIB_v1_10022|ID:27161784| Integrase catalytic region [Uncultured spirochete bib]
VINMPQIQGIREQYREGSSVAELARIYGVDPKTVRKYLKQDDFSPKPPEKMTKESILDPY
KPLIDSWLLEDQGRWHKQRHTAKRIHDRLISEVAGYACSYNTVQRYVKRVLQEQRTVRAS
MELVWHPGESQADFGEADFLERGTMVRKKYLTLSFPYSNDSFTQIFGGETAECVCQGLKD
IFAYIGGVPPLVVFDNATGVGRRIGEIIHEAELFSRMRAHYGFSVRFCNPESGHEKGNVE
AKIGYTRRNLFVPEPAFDDIEDYNRTLLSLHASKAQESHYKKLLPIKDLFEEDTRALLPL
PRTAFDPVRYDYLKADGYGKVRIDSRHYYSTSPEYAGQEVLVAIRAHSIDILDDHKRLVV
RHSRVYGERRSDSSDYRTSLAVLMNNPGAWKNSGIRELIPDPLKSLMDRQQRTQLHATLK
TMHTLSSEYGFEIAVQALEEGVQRSRTSFHDAAILAARIAGYGLNMAPERGQDLHVYDEF
LEGVQV*
>SPBIB_v1_10023|ID:27161785| conserved protein of unknown function [Uncultured spirochete bib]
MNKKVFIDSDVILDLLSKRQPFYPAAAEIFTFGDYGQLDLYTSSLVFSNVFYILRKSVGN
EKAKELLRKLRILIRIIPIVEKNIDMALNSTFTDLEDAIQYYSSCSFGIDVIVTRNTSDY
KNSEELLIQSPEEFINIIKSIEKE*
>SPBIB_v1_10024|ID:27161786| conserved protein of unknown function [Uncultured spirochete bib]
METKLTLKLDQAIIQRAKQYAEEHNRSVSKLVEDYFRNLIVNRKDKTHYSPLVEELSGVI
TPDDINNSDYTSYLEAKYE*
>SPBIB_v1_10025|ID:27161787| protein of unknown function [Uncultured spirochete bib]
MIYGILVFVLWCIIGYIFSLITEIPLGYVGPIAILLGIISMNIIRKKMKK*
>SPBIB_v1_10026|ID:27161788| protein of unknown function [Uncultured spirochete bib]
MKSIIISHNKLFTICLHNMLNYLVKDNKNIIDNFVLIVIDATSIDSNIILQKLLLKYSDS

IYRILLIHDHVDDLTEKIVYNSRVVAVYNPRASNSTKFGHLLQHVLDGGVMLSKEKLFEL
RHTESIHTKIVFTKREEEINQLLHNGDSIKVISSKLHISQNTVKVYISRIYKKLGIHSRA
EYYINYDVLSGNMEVKA*
>SPBIB_v1_10027|ID:27161789| exported protein of unknown function [Uncultured spirochete bib]
MKKIFACVLIVLLVVSVPAFARPTARENADFVVDAIIGISLGIVKRAGTALAVVYTAADF
ITYAVTGKTIGGYANDGFEKSSKIIYADDNKPYRGWMGLGMQ*
>SPBIB_v1_10028|ID:27161790| Integron integrase [Uncultured spirochete bib]
MGSIIFDKAAFMNYLMAKCNFSAKGTEWACIWVEKFLRFYPDWSENREKAIAGFSEALSR
RKPANIVNLALRSVQLFIAFIDFELGERPLARSTQSGEDATGSRSKTCSISASSEENPGE
SKDREWQEFASRAMKQVRELIRLKHRSLRTEKTYLGWTRRFLDFIRVRRLANFVDGAPKI
TADHLRAYLSYLAVDVRVSASMQEQALNALLVLFRMVLHIEIEGLSSVLRAKKRKRLPMV
LSREEVSALLAQLRQPYRLMASLMYGCGLRLEECLSLRVKDINFENETIEVRAGKGGKDR
LTMLPGVLKRVLHEHLRELRALWEEMRRRDLPGIFLPEALDRKYPGLSKEWSWFWLFPAK
SPCANVRTGEIAFWHVHPSVIQKKIRLAIQAAGIQKLASAHTLRHSFATNLIEDGYDIRT
IQELLGHSNVQTTMIYTHVAMRNKRGVRSPLENL*
>SPBIB_v1_10029|ID:27161791| protein of unknown function [Uncultured spirochete bib]
MSQCGTRGACAVLWRICEQGSRVLRHSNLFSDSDGESGRRNRFTFQQIAFYHILLKSIVN
RSEEVVNLILEKELEAK*
>SPBIB_v1_10030|ID:27161792| Anion transporter [Uncultured spirochete bib]
MNKNSAARKLAAIGIAAILFAVLAFLPLPDSVRRAGDALLDPKGQRAIAVLAGALVIWVL
EALPFHITGLLAMVMLALVGAGDFATIVKSGFGDDVVLFFIGVLALAAAIVRSGLGKRVS
LLVLSITGNSTRRIIFGFLAAGAFLSMWVTAIASSAIIMPLALAILTEEGAKPGESRFGR
ALMLAVAWGALIGAVGTPAGSGSNPLVVKFMGSLAHVDISFVRWMTIGLPIVALLLPSAW
GVLVLFSPPEMTHLQKSRDDIKHEFSNQSRMSHDEKATLIVFAITVLIWVASPLFQSFWH
IKIPISMGAVAAVVLLFVPGMTSFKWKDLQKEIDWSGIILIAAGISLGMTLYQTGAAAWL
SVVLLGGIGNLPLFVRLVLIVLAVLTVKIAFSSNTLTGTIIVPLVLALGPLLGTGAAGLA
LAAGFTANLAIILVTTSPVNIIPYTTGYFSIKDMAIAGLILTPLAALIIALVFSILGPAM
GIL*
>SPBIB_v1_10031|ID:27161793|sucD| succinyl-CoA synthetase, NAD(P)-binding, alpha subunit [Uncultured spirochete bib]
MSILVDQSTRTIVMGATGSEGTFWTEHMVNLGTHIIAAVTPGKEGEHVGDIPVYHSVHRA
LDASALGSRSAAGAPADTYAPADAAMLFVPPHFTKDAVFECLDAGIKLIVIVADGIPLHD
ALQIRAASRTEGALVVGGNTSGIISPGKAMLGMFPYWIERVYKPGRIGVMTRSGSLTNEV
TAMVVRAGFGVSTLVGVGGDPVPGTRFAEILARFQEDSETDAVVMIGELGGTMEEEAAEM
IASGAFSKPLIAFIGGRHAPKDKRMGHAGAIISGGKGSALDKMRALEEAGALTAERASQV
GILLAETLKSRGQAVTSAKSARSPLSADSAPGASTRGSHNE*
>SPBIB_v1_10032|ID:27161794|sucC| Succinyl-CoA ligase [ADP-forming] subunit beta [Uncultured spirochete bib]
MKLHEYQAKALFREFGIPAPQGELADTVEAAVNSAEHLGWPAALKAQVLRGGRGKAGLVK
IVQTPEEACTAAQAIFASDWKVRKILVEQAIAHERELYVSFSADSRTGAHLLLASAEGGV
EIEDLARTAPEKILKEHIDSIRGILPYQARTAAYDLGFAPSLAKGFASIIEKLYRLYVQK
DCELAEINPLFVTLEGNFIAADAKISIDDNALFRHPEFEQDPADFDSDIAREAAAEGIPY
LQFDGDISLMCAGAGLTTAVYDLVVDFGGTVANYLEFGGPNYRKSVRAMKLCIKNNPKVI
LIVTFGTIARADIMAEGIVEAIRCLRPDCPIVTCIRGTGEDKARETLLSAGLEPLTDTEE
AVKKAVELAHIARSTATERPTPAAASKQGGQP*
>SPBIB_v1_10033|ID:27161795|sauS| Sulfoacetaldehyde dehydrogenase (acylating) [Uncultured spirochete bib]
MNGQSILSEQASSEQASAGQTSSSAPKTDSADGQAFIAEIVARARAAQKIAATWSQERVD
EVCVAVGWSVYNDENIRKLAELAVAETGMGRVEDKITKHKNKVMGVLRDIRGVRTVGLME
EDPARGLRKYAKPVGVVGALAPVTNPTATPASNGLSILKGANAVIFAPHPKAKKSTTLAC
TFMREALRKVGAPEDLVQVIEEPSIELTQELMRQVDLIVATGGSAMVKAAYSSGKPAYGV
GPGNAVQLLCEDADPEDAAKKIATSKSFDNATSCSSENSAVVHRAVWDRFLAGLQKEGGW
LCTAEQKKALRHYLWVTGKNGQENLNPAIIAQDADKIARGAGFDVTKGTRFLIVQGEQPI
ESDRFFWEKISPVLTVLPCDSFEEGLRFVERITDLCGTGHSSGIFTFREDYIDRMGLYMR
SSRIMVRQPMVSGNGGTFFNGMPSTVTLGCGTWGNNITTENIHWKHFINVTWLAMPIELN
RPKDEDIFGDYWKRFGK*

>SPBIB_v1_10034|ID:27161796|rpiB| Ribose-5-phosphate isomerase B [Uncultured spirochete bib]
MPEKIVIAIASDLSGFPLKREIVKHLRENHPELEVLDFGIESEDAPKPYYIQAPKVAKAI
QDGRAQKGIAICGTGQGMAIVCNKHKGIYACVADDIFSGERGRIINNANVLTMGGWITAP
FLGCQIVDAWLSVAFTQKMEFKKDFLTNAFNNVQKIEEENFK*
>SPBIB_v1_10035|ID:27161797| putative transketolase C-terminal section [Uncultured spirochete bib]
MSDQKEMRAVYAETLISLVEEGKDIVVLEADLMRATGTTAFAKKYPDRAVNVGVAEANLV
GIASGLSAAGKVPFAATFACFASRRAYDQFFLSANYAQLNVKLIGTDPGVTAAFNGGTHM
PFEDLTLMRVIPRLTIAEPSDPISLAAITRLGANLKGSFYMRLQRKPAPVIYRENESFSI
GKAKVLREGGDVTLVALGTVMVNEALKAADMLAQEGIKATIIDALWLSPLDEETILAHSG
CGCIVTCENHRVTGGLGSAVAEVIAEKAPGVRLARIGVAADLFGEVGTLDWLAERFALTA
PHIADAARTLIQQK*
>SPBIB_v1_10036|ID:27161798| putative transketolase N-terminal section [Uncultured spirochete bib]
MNTHEIQALERKAREIRGLTIEEIGYLGVGHIGGALSIVDLLVLLYHKHMNINPKEPGWP
ERDMLVLSKGHAGPALYATLADKGYFPLDWLLTLNKGGTNLPSHCDRTKTPGIDMTTGSL
GQGLSAAAGLALGRAMDRKPGFVYCIIGDGESNEGQVWEAAMFAAHRKLSNLIAFADWNG
MQIDGLTKEIIDMDDIVAKWTAFGWHARMVDGHDFAAMDKAIEEAKRRAAPGIERPSMIV
LRTIKGKGASFCEGQVTNHNMSYGIPQTKEALAALGVPARLIPELVHGA*
>SPBIB_v1_10037|ID:27161799| protein of unknown function [Uncultured spirochete bib]
MSKERAMGRMRVRVKRAAGMARLFTRALVMGLAMWVVTGCSPPAIESAVLGDIRPPMVKN
ARLESGYEFILEFDEPVRVPKDSFSAEPKRLAAQAESSENVVSVLFEPQPSPGEVVTLAG
TVQDMCGNSTRVQVQFKGYNDRPAGLVITEIQTAKNTSKKAPHRDYIEFLVKKAGNLGGI
YVQWASSTKTMRFDFPACEVRAGEVIVLHLAPEGVAEEKNETGSELGLSGGIDATAQGRD
FWSDAGGLPDASGAITVHEREGALPIDGIFYADSSKSGKLESAKLIALARELADAGLWKL
DVPPLWENALLWKPSTSRPLVRTSTSPYGASAWGIGESGSQSPGIVN*
>SPBIB_v1_10038|ID:27161800|trxA| Thioredoxin-1 [Uncultured spirochete bib]
MSAEVTLTSENFKKEVLESKIPVLVDFWAEWCMPCKMIAPSVAQIAESYKDRIKVGKLNV
DEQSDLASQYGIISIPTLIVFKDGQVARQKVGAMPRHEIEKLFLDLI*
>SPBIB_v1_10039|ID:27161801| MATE efflux family protein [Uncultured spirochete bib]
MIGKQTEKPMPNTTASPFARSLSLLGPWTFYREALALAIPVMLQSLITGLVSLVDNFMVA
GLGDAKMAGVNIANQVNFVFMVIINVVTIAGGIFLSQHRGAKNQEGMIQAFRFKLVMGTG
LSTAYFLVCVLAPEALLSLMVSNNAQGAVIVAEGARYMRAVSFSFIPIALSGAIGSSFRD
IGEPKVPLLVSTAAAIANTIGNWFLIYGNFGAPRLEVVGAATATVIARGLEITAFIVMLK
ARKPAFAFRLKTIFRINGKFFADVLSRSAMMFFSETAWVVSETIITALYNRRGGPETVAG
MAAGWSVANLFFLVFPAIHTATNVIVGSTLGAGKLDEGRAKARWIMAGSVFFGLAAGLFA
ALSTAVVPLVFGNLSDQARIVTRSIIFVIAIYLPLWCLLNAQFGVSRAGGDTLMGLFVDV
GVTYVLFIPAAFVIARYTSWGPVALFGIAKLSDIPKALVAGWWLAKERWVRNLTVASVFK
AVL*
>SPBIB_v1_10040|ID:27161802| Beta-mannosidase [Uncultured spirochete bib]
VEIINLAGQWRLVRLADGSARPMEVPGDIYSALIQSGELKDPYYGESELKAQWPGREDWK
IERSFTIPEHFLQLETIRLQADVIDTISEVYINGSLAGTSNNMFRSFEANPKPLLHPGEN
FIAVIIRSPEQAAIREAARLHYPIPASLYPVSSPHRNLIRKAQCMAGWDWGPCLMTGGIY
DSIKLIATDGPVIKYVQTRMKALGPFPATEEEPASGPDFSLDIFADIEVPEEMDIETEFF
LVGRHVSHDCHLVSNYHLAAGTHRISATMLVKKPDLWWPNGHGKPALYDLYLKTGSDPLS
EISAEVHKKIGFRELRVVSEEDDIGRSFKFVVNGKEIFAKGANWIPADALPSRWTRARIA
GLLDSAVEAHMNCLRVWGGGRYESDDFYELCDERGIMIWQDCMFSCALYPSSPEFLSNVE
AEITHQVKRLSDHPSIALWCGNNEALGAITWYEESKKNPARYIVDYDRLTEGVLGRVIRA
LDPDRCFWPSSPSAGPNDFSDNWHSDERGDMHFWSVWHEGKPFSEYLTVQPRFCSEFGFQ
AFPSMRTIESFAPSDERNISAPALEAHQKHPRGNSLILDTMLRYFRLPKGFWELVYLSQV
QQAMAIRTAVEYWRSAMPRCMGTLYWQLNDVWPAVSWSSLNYDGTWKLLHYEARRFYAPV
ILALFIKDGIAQAHIVSEIPGEHTATITLQLLDFHGEPRGVLCTNSSTITNLTSQIVWHM
PVSELPYKPEEAYIEAALDVPDLGISTIAILFLTEPKRCKLADPGLDVTLATNEKGQIEA
TVHASEAPAFYVALELEGLPGQFEDAGFYCKKGARKKVAFISEETATTRKHASIATQRAH
AEASAISMANASLQARLRVFHLQNSFEH*
>SPBIB_v1_10041|ID:27161803| ROK family protein [Uncultured spirochete bib]

VSSRSDQWTRTATRIFRTIWRNPNISRVDLAKKLGLDKSTVTKQVADLLENGLIEEKEEG
EASARGGRKPIYLAVNKEYGRFIGIELQLGFFEAVVVDLAGDILETARGEVRITQENFAA
TVADIVDTMQKRFCSDSLLLGVGVGTAGLVDSKKGRIKYSVPLGIQKTLDFAKAIAGKLK
APAFIENDANCCAWGELAFNRDEELKDFLFALVEYRQDETSLIRYGGIGVGFGVVLGGRV
HTGAHGYSGEFRSAFCDGPGELQFSLSREELMCLRGDKNLLHRTVDELARNMAMLINTMD
FQHVYIGGDIEALDIDLPAILRHRLEENWMYPSPKDVEIRYSSLGDKAVAFGAAGMVFER
LMAERSMPGLAVELKNLDQQIGA*
>SPBIB_v1_10042|ID:27161804| Extracellular solute-binding protein family 1 [Uncultured spirochete bib]
MKKGLFVFLALIIALGSVMAQEKVTLNVLNYQQADQAGYQEDVAIWKRFQELNPDITLNM
EVLFNEPYHQKLQAYAAAGTLPDVFYVWPTARSAVIHEKKLAKDLSKLLGPDFLKDFSGA
ATDPNNQLGKYMAMLPQSFTYTSVMYVNKKLLADNGFDLPKTYDDLKKMVPKLKAKGINV
IMLPDKDGWPMQSCLFSTVLGRMAGNSFVDAILAGKAKFTDKPFVDSLKVIDNLYKDGII
AREDNQVGYGEAPGLFVSGKATIYIDGDWRVGAYITDKASGKALIPPAAQESDFALLPFP
AIPGEVNPGVLSAIAGTGWAISASIPAGSAKEKAAVRLIKYLYSDEVQAIRYSTGAYVPT
RKNVKANVEPLVAKVPLFYAANPKTCYVFDGMFDPAVYNVVNDGLIAIGLGTSTPEKVAA
DVQKAYETWKASQK*
>SPBIB_v1_10043|ID:27161805| ABC-type transporter, integral membrane subunit [Uncultured spirochete bib]
MVHLNRVHLEEKRAYWAMVMPAFALYLLVMAFPIILSIMLSVSNYSGGKMFGGEKWGFAG
FSAYARVFTDPWFWNALKNNLYIVLISVFGQLPLGFIFAYFIYSKIVRAPSFWQGVLYVP
NIISVIVVGLLWQVIFSPHGPIAEIVNSIHASSFQGQLKAIFDGAGGFSLSDDVIKKILH
LAGPSGQAMFSDPVPELRDLLASYNGQPISEIYTALSNLFVQKWSPAFLTKTDIAMLPVL
FVILWMYTGMYLILFLANMQKIDAQIIESARIDGANEGQVMRYIILPALSGTIVNSAILA
ISGSLSSFALIFAMTGGGPSRVTEILSIYMYNNAFLGRPNFPLANAISLIMVLISVVLIV
LTKAVEKRYGGKEE*
>SPBIB_v1_10044|ID:27161806| ABC-type transporter, integral membrane subunit [Uncultured spirochete bib]
MSEMIKETKLQKALAAVGRGAVYIVMSLFALMTLYPIFWLIMNSFKTTREFQVSQLAFPR
APTLQNYVEAWKMGDFGLLFPNSVLYTLGATAGIIFLSLMAGFAFAKLKSRATKLIYNSF
VIGILLTTQTLMIPLFLEVNLLGLYNTRLAVLLVYIGAGLPIGIYLATEYIKAIPSAVVE
SARIDGAGFFTIFLKIIVPMSVPVATTLAMLNITGLWNEFALINILVSKTELKSLPLGIY
KFSGSLSTDYGKQFAALTIGMVPMLVFYMIFRKQITKGVAAGAIKG*
>SPBIB_v1_10045|ID:27161807|bglA| Beta-glucosidase [Uncultured spirochete bib]
MKRQDGFPEDFVWGCSTSSYQIEGAASEDGRGPSIWDTFSHTPGKVVQGHTGDIACDSYH
RWREDVALLKELNAGAYRFSVSWTRIQPDGKGKPNQKGLDYYSRLVDGLREAGIEPWLEL
FHWDLPQALEDAGGWRNRDTAFRFEEYARIMYKAIGDRVEHWTSMNEPWCAAFLGHLTGE
HAPGLRDREKTSRAVHHLLLAHGLAARAYREGGFKGEYGLVINAAKPRPATLRPEDVEAS
ERASIERTSLWLDPVFGRGYPELFMQHFGSQMPIEPGDMDIIAAPIDFIGVNYYNEDAVR
AALLSEENPYGFEYVPTWQRKTEMGWDIEAQGLRRILMHIARNWPAKVLYVTENGAAFAD
APSPDGVIRDYDRIEYLREHLAACRQVIADEVPLKGYFVWSLMDNFEWSFGYTRKFGLVS
IDHITKARRPKLSFYYYRDAIAGFGL*
>SPBIB_v1_10046|ID:27161808| Beta-N-acetylhexosaminidase [Uncultured spirochete bib]
MGCDLVPIPVLCEAKEGVFELGPGMSVQVSAGLEKEAALLAEWLSGIEGVGQVGISRAPV
ANAATSISLSLDPGMQRAEEYELGIAPQGIRIAAKDAAGIVRGASSLYQLALSQSRALRS
LRIQDWPRFAWRGFMLDCARNFFRVEFIEKLLDLAALHKLNVFHWHLTDDQAWRLEIASK
PELTGRGAFRQDMRYEIEWEKGGFYTRADVARVVEYAAARHIMVVPEIETPGHSTALLAS
HPEFSCRGASDPSVLFKPEDHYGIFEDILCAGNDRVLAFIDEVLEEVCAMFPGEYVHMGG
DEAPKARWLSCPACRSRMDVLGMRKGGGQYEPERLQAWFMGRMAEMLTRCGKRMIGWDEV
LEGGIQKDTLIMSWRGAEPGVRAASLGYDVVMCPQTKACYLDHKQFDVPEEPGHLGVCTL
KDSYNFDPVPDGLRKEEAAHILGGQANLWSELVYFGRQAEYMLFPRLCALSEVFWSPREN
RDFNDFSTRLATHTQRLDMLDVLYCRGKYF*
>SPBIB_v1_10047|ID:27161809| putative Transcriptional regulator, MarR family protein [Uncultured spirochete bib]
MSEHSELENALKAWISQIMRLSMRGFITFATEIGLSMPQIAVLFRLNGDKRCAVTELGDE
LGVSGAAASQMVDKLVQLGLVDRLEDPHDRRVRRLLLTQKGKTIIERSIEARQEWIGHFC
SSLSEEKAKEAAGLFNQFAQIAEQLETALHTPSSH*
>SPBIB_v1_10048|ID:27161810| Xenobiotic-transporting ATPase [Uncultured spirochete bib]

MRTLFRYLKPYWLSIVLVIALLFIQANADLSLPDYLSKIVNIGIQQGGIEPDLPQVIRIS
SFEALGRLLQAEGAGEKFAALQKVYTHVTPGSDQAKTLSKKWPLANSEPVMSLDLSDKAA
VDAARQVFLAEYPKLVVMSQLPNAAAMGGAGALGGAVPSAGPSAGTLPSAGAVPGAAAPN
MPAPSGQVPSGQASGAPAALSPQAILSQIDSLDPLAKSQLVVRGLQKEFEALGVDTVVLQ
TGYILSIGAIMLIITLISVASTIMVGFLGSRVAAGTARDLRRAVFTKVEDFSLAEFDSFS
TASLITRSTNDVTQVQMMIMMGTRMLFYAPIIGIGGVIRALGKASGMWWIIAAAVGVLIG
IIGLVFALVVPRFRTVQKLVDRLNLVVRENLSGMMVIRAFNRQDHEVQRFDKANRDLTST
MLYVTRVMVVLMPIMTVIMNVVSVSIIWVGSHEVSAGSIRVGDMMAFMQYSMQIFFAFLM
MSMMFIMLPRASVSADRIAEVVNTPVSIKDPEAPKLLPKPTRGEIEFRNVRFRYPGAQED
VLHDISFTAKPGTTTAIIGTTGSGKSTLVSLIPRLYDVTEGAVLLDGTDIRTLALSDLRG
AIGFVPQKSMLFSGSIADNVRYGKDSAAEEEVKEALELSQIWSLVEESPEGLERQISQGG
MNVSGGQRQRLAIARALVRRAPVYIFDESFSALDYRTERAIRAALKTYAKDSTVFLVSQR
VAPIRHAEQIIVLDDGHIVGIGKHEELMKTCDVYRDIALSQLKQEELA*
>SPBIB_v1_10049|ID:27161811| conserved membrane protein of unknown function [Uncultured spirochete bib]
MNGTQKPENIQKSASPAQTSHANSGMEPAKGGAPSSAPSSTQGTAKIRPGAMMGRGGPGA
LMPGEKSKDFKGTMKKLIAYLGPYRWAILAVMILATIATILGIMGPKVLGTATTTLFDGV
VKKLSRTGDIDFAKIGSILLTVLGLYLSSAILQYIQGWVVSGIAIKITYNLRKDILAKIN
RMPFRAFDNTNHGEILSRMTNDVDTVNQTLSQSLSQMITSVMTLIGALVMMLTISWQMTL
VAMVMIPLSLVLVRFIVKRSQKYFKTQQEYLGHLNGHIEENFGAHTVVKAYGGESRAIKT
FDSLNSTLYQSAWKSQFLSSIMMPLMNFVGNLGYVAVAVSGGYLAAKKMVTVGDIQAFLQ
YVRSFTQPITQVANISNVLQQTAAAAERIFEFLAREEESPDSEASATLVPARIEGKVEFR
HVHFGYVPGEPIIKDFSATVLPGQKVAIVGPTGAGKTTLVKLLMRFYEPDSGEILVDGIP
IQDLPRSVVRSWFGMVLQDTWLFSGTVKENIAYGKHDAAEEELVESAKAAHVDHFVRAFP
EGYNLVLNEETTNISQGQKQLLTIARAMLAHPPMLILDEATSNVDTHTELLIQKAMNTLM
QGRTSFVIAHRLSTIRDADLILVMDHGDIVEQGTHEELLAKCGFYAELYNSQFDETGEAQ
VACS*
>SPBIB_v1_10050|ID:27161812|groL| Cpn60 chaperonin GroEL, large subunit of GroESL [Uncultured spirochete bib]
MAKQLLFSEDARRKLLVGVETISKAVKVTLGPKGRNVLLDKKFGAPTVTKDGVSVAKEVE
LEDPYENMGAQLLKEVATKTNDIAGDGTTTATVLAYSIVKEGLKSVAAGIDPMALKRGID
QAVTIAVEEIRKNSKEIKEKEEIAHVASVSANNDIEIGNQIADAMEKVGKDGVITVEESK
TMDTTIEYVEGMQFDRGYTSPYFVTNRDSMTTVFENPLILIHDKKISNMKDLLPVLEKIA
QTGKPLLIIAEDVEGEALATLIVNHLRGTLNVCAVKAPGFGDRRKAMLEDIAILTGGEVI
SDELGLKLENTSLNQLGTAKTVKVDKDNTTIINGGGKQKDIQDRIAQIKKQIEETTSDYD
REKLQERLAKLAGGVAVINVGAATEVEMKEKKHRVEDALSATRAAIEEGIVSGGGIALIQ
AALALDKADISKLSDDEKVGYKIVKRALEEPIRQIAENAGIDGSIIADKAKHEKKGIGFD
AAKMEWVDMMKAGIIDPAKVTRSALQNAASVAALLLTTECAITDLPEKEKPMANPGGGMG
GMGGMDY*
>SPBIB_v1_10051|ID:27161813| RluA family pseudouridine synthase [Uncultured spirochete bib]
VKAQFPILYLDDSIIAIDKPAGVLAIPDHWDPEVPVAQQMLAKEYGTLLPVHRIDKDTTG
VLLYARTQDAHRALNERFSTRQVEKVYLAIVSGEPEHDEWEIDAPLRADGDRMHRTIIDM
SKGKPALTRFEVVERFRGFALVRALPETGRTHQIRVHLAASRLPILADALYGDAEPLMLS
KLKRRWKGDAFEEKPVIARSALHASKVTFLHPTSGMRLEIEAPLPRDFRAALNQLRKLRA
I*
>SPBIB_v1_10052|ID:27161814| protein of unknown function [Uncultured spirochete bib]
MQSMHSRDSGGERMEKGERSAFSMGERDIQAQKVPFGLLMFDIDHFKKVNDTYGHLAGDI
VLKMVAGTISGALRLLDTAARFGGEEFAVLVPNCNEEYLFLQGGSF*
>SPBIB_v1_10053|ID:27161815| Aminotransferase class I and II [Uncultured spirochete bib]
MPLQISDLSDAVLHTHYAVRGPIVARAQELEHLGREIIYCNIGNPQALGQKPLTYVRDVL
ALCEQPALLERAPHAFPDDVMETARTILGQSKYGLGAYSESKGMRFVRKAIADFIAARDS
TGGVMQTSNPEHIYLTDGASKGVQAALRLLIASHKDGILIPIPQYPLYSATITLYGGRQI
GYYLDEDSGWSLNEQMLEEAMHEAIKEGIRARAIVVINPGNPTGAVLTEENIEMVIRFAK
RHNLAILADEVYQENIYKPGARFLSFAHVMTKLSEHEVSLFSFHSTSKGFFGECGQRGGY
MEVRNVPEEVIAQITKLQSVALCANLPGQVMTYLMVNPPKPADPSYKRYVEERSHILSEL
AQRAKILEEGLNRIPGIHCQPIQGAMYAYPSISLPAGKTDEEYCMALLEQTGVCVVPGTG

FGQKPGTAHFRTTILPPTRQIEAVIDAVDAFQRQWG*
>SPBIB_v1_10054|ID:27161816|hflX| GTPase HflX [Uncultured spirochete bib]
MTSNTAQKNQNESVERAFLVGMATDKIRKAEARELLDELHGLARTLGLDIAGEMLIQLRD
PTPSLLVGSGKADEIAAAAEEAHADSIIFDHILSPVQQRNWEKLSSKKVYDRAELIIKIF
ASRALTKEASLQVELAQLQYALPRLAHSYDDLMRQRGGRYGTKGSGEQKIELDRREIERR
IHEIQSELEAVRKERAVQRRRRERASVPRAAIVGYTNAGKSSLLNALTAASVRAEDKLFA
TLDPTTRRLMLTRGQTLLLTDTVGFIRNLPHGLVEAFHSTLEEASQADILIHVADASDSR
VDAHIATTIQVLSEIGAQDVPRILVLNKIDLAEPDVVQSFLARYPGSLAVSAKTGEGLDA
LIEAIQESLTKDMKTCTLRIPHADYWIVSLVMREGTVLEERSDDAYTWLRCRVPQRMESK
ILQYIAAEDPEVGKARESEGE*
>SPBIB_v1_10055|ID:27161817| 2-dehydropantoate 2-reductase [Uncultured spirochete bib]
MDRISSVVIVGAGAIGASVAAMLSDSGHAEVQVCASGERQERYRKEGFVVNGTRYFFPLA
GKDTSRKADLVILAVKNYSLDEAIDEMKPFVGNDTIILSLLNGITAVPRLRDEFGADKVP
FAMILGIDAHRHANEVQFTSKGQIFFGFEKERIAAGESKLRVLGDFLGVCNVPYRIPEDI
VKEVWFKFMMNVAVNQWSALLRASYSLFHRSRHARALLEQTMAEVVNLSKKFGTGLSDDD
IVRMLSILDGLPSFGRTSMLQDVEAGRRTEVEAFAGTMVRLSKEAGIRCPYNEMLYQAIC
ALEESYAS*
>SPBIB_v1_10056|ID:27161818| putative Biotin--[acetyl-CoA-carboxylase] ligase [Uncultured spirochete bib]
MFSLPVVSSTMDEARRIAEEGFPGAALVRADTQSAGRGRLAGRQWVDAPGSSLLVTMLMP
SDFRTVEALPLRAGLGILRALESDTGKSLLLKWPNDVLAPPRWGKLCGILCENSKGRVLI
GFGINIKKSAHARANPSFPAASIEELCGFVPSAFSDLDAAAQRVARSIMKALQDSGWHEA
YESCLWARGSSVQFDAGHPDAPKRIEGILEGVDDEGKLILRVAGQRKTFASGELSHLRSI
*
>SPBIB_v1_10057|ID:27161819|glmS| Glutamine--fructose-6-phosphate aminotransferase [isomerizing] [Uncultured spirochete bib]
MNEKARALQAAYTVTNLAYAVPDSFCSAPLAANPLYDTIELQSCKNCIERKERSMCGIIG
YTGPRKTAKILLEGLRRLEYRGYDSAGIAVGRENAEPRLEIIKSVGKIVELAKKMPEDID
GSWGIGHTRWATHGGVTEANAHPHTDMSGKIVVVHNGIIENHKTLRTMLEKKGVVFKSET
DTEVIPHLIASYYEGDLLKAVLAALQHLEGTYGIACIHANEPGRIVGARNGSPLIVGVGN
DEMFLASDITAMVAYTNRVIYLNDGEVVDITRDSYTITDRHSNMLDKQVDEITWELGAIE
KSGFMHYMEKEIFEQPDSIARAMSGRIDEENATAKLGGLNLSRRQLADVHRVRIIAAGTS
WHAGMTGSYLLEQAARIPAQAELASELRYRNPVVENDSLWFVVSQSGETADSLYAMREVQ
RKGATVLGICNVVGSTIARESDGGVYVHSGPEIAVASTKAFTSQLTAFYLFTLLMARMRD
MSREEGQKFTRSLLAVPDMVRNALAQRDHIQAIAKKYCRAKDFLYLGRGILYPIALEGAL
KLKEISYIHAEGYSAGEMKHGPIALISPEVPSVFLVSDDYLHEKTISNIREIKARGGPII
AVGVEGDTEAMALADDFIAVPKADPRFYPFSMVVPLQLFAYFCALELGRDVDQPRNLAKS
VTVE*
>SPBIB_v1_10058|ID:27161820| conserved exported protein of unknown function [Uncultured spirochete bib]
MKNPAWRMLPIVVFAVAMIGALAFNIALVSRSRSALASTILRNPATVRTARFHIAAIIPD
AVDPFYGHLVEGLREEADRQNAALQVFYYSASAIGEGSNPSEEVWRWFEIALRSKTDGII
LFQSKGMETSRFAAEAESAAMPFVPLAMDAPAQWTRSGVSGDSFSQGKEASSLVLGLLGS
AARIGIILSADTSQGYAQAEEPFYRGVVEMIKNRPGAAIVAAVREEESILGGEDACARML
SSHPDINAILCIDAKATIGAAQVIIDRGIVGQVVIIGSDENSEVNRLIEKGVVHASIVRD
AAAMGRSAVALAVGQRIGIRKPERISVGYHVKPERGTTP*
>SPBIB_v1_10059|ID:27161821| Signal transduction histidine kinase, LytS [Uncultured spirochete bib]
MIDLVSLARPWKPRRQGSLRNRIIVNSIIILLVLVIATSYTAFASFDLARSVELLFRNSI
SMEELRATLRETQESLTGYLSAKNSESLKDFIRTSTSLSNMKLRLNKENKVDDVFLLEKD
LAFLVDSYLVSAEGAVQAKRGRNVTQYVSLYEEARRTASLIEFLSAKMDTIHLGQSLKGF
SSYRANISLALVSNAVLLLAAFLLSIALIARYSYTITEPLARLSVAAEAVGRGEYDHPLP
PYENEDEIRTLHDAFVNMQDSVQKAFAELTRKAELERSLMEERMRVLTYQHRLKDAELLA
LQTQINPHFLYNTLSAGWQLALAEGNEKTAEFLEKLAAFIRYALKPSTRFVLVSEEIECV
RQYIWLLKLRFGERYRFRIEVQDEALGNETPALVLQPLVENAIAHGLRDIEEGGDVIVSA
RIEGETIRLSVSDSGKGMAKDDIARALSAADPEDTTPQRGIGLHNVVRRVALATGGLGKV
EIESEPGHGTRITIVLPVGKPI*

>SPBIB_v1_10060|ID:27161822| Two component transcriptional regulator, AraC family [Uncultured spirochete bib]
MKRVLVVDDEMPIVNGLLLLFKRYFQTEYTVVGVAQSGREAIEKAKELAPDIILMDVQMP
GITGLDAIRELSRQKSATAFILVTAYERFDIAREALSMGVCDYLLKPVSRERLEIALRVA
SDYLDRSRLFDKRELEFRDRQQRLVPLIRTAFFCSVRWQQELKKNLGLVKEMLKINEDAG
VLGIASFSPMDGNAGALYERFCSLIQYKTVALVGPLEDNRYCAWFLPMKKAALESESIAA
SEISAFLGILRGAFSSELATGEIVLAHGQPEILENLERSWSAAVQVFAGSTLIPKKGSDI
AGISAAVGSAIVQPQVNLEIQLSEWEKNHASCALDAQFSEEIMEGQYAMAGQSLEKMLMQ
IDCSHTAARDSLFRIIAALSFAALKLAGGGILSEQVYREFMDFSDIEQLWNQAACHLFAA
KVRERFASLQKYAVAAGSHSPFVVRAIQYIENHYQEPITLESAAEAIGISAGHLTRLMSD
ELKKGFARTLIDYRLQKAKEMLKKPNVSVRDVSRLCGYSDANYFARLFRRMTGVSPSKYS
ARKSGGEESDA*
>SPBIB_v1_10061|ID:27161823|xylF| D-xylose transporter subunit ; periplasmic-binding component of ABC superfamily [Uncultured spirochete bib]
MHNAKHMLQHGPKHVHQFGNFYFSLLIILFAASLSLNLVSCSKARKPIGDRAPIVGFSLD
SLVVERWRRDVDSFTKAAHDLGAEVVLRVANQDANTQISQVKELLNQGIDALVIIPNDAE
KLTEVCREAKRRGVPVMSYDRLVHNADVDLYISFDNEKVGSLQAQAASEAVPSGTYIIVN
GAITDNNAFMINKGFHTVLDPLIQSGRIRLAGEIWPSDWISDEVRTRFETLLQPGQKIDA
VLCGNDMLAETVISVLSENRMVGKTIVTGQDAELAACQRIAEGSQFATIYKPIDALALKA
AGFAVMMARGEKVRYDNKIDDGHYKVPYIALEPILVTAKTLGSTVIKDGFHTREDVYRNV
KR*
>SPBIB_v1_10062|ID:27161824| Periplasmic sugar-binding protein [Uncultured spirochete bib]
MKRTLFIALLFTVVAGFVFADTQVGIVLPTKDEPRWIQDQTRFLDALKSAGFSAKVLFSQ
GDSAKEKANVEALISEKIKVLIICPQDGTAAAAAADLAHKAGVKVISYDRLIRDTTSVDY
YVTFDSFMVGKAWGDYLNSKVPAGSKGNNLYLYAGAASDNNAFIFFEGAWSALQPKIADG
TYIVRNSDKAVALKNKAKLTRDEEAQIIGQVTTNWNFNDAKNKAEANLTAAPKEAKGTVY
ICAPNDGTARAIADAFAADKDVKTYYITGQDAEIASIQYIIDGKQSMTVLKDVRTLVNDA
INAAVAYMKGQTPPVTSYYNNGKKDVPAKPTAIVTVTKDNVKKEIVDSGYWPADKFTGLK
*
>SPBIB_v1_10063|ID:27161825|xylG| fused D-xylose transporter subunits of ABC superfamily: ATP-binding components [Uncultured spirochete bib]
MSDNILEMRHITKTFPGVRALNDVNFVVRRGEIHCLVGENGAGKSTLMKVLSGVYPHTEF
EGEIILNGKKVAFSGIHDSEKAGIGIIYQELALVPEMMVYENLMLGHEIRRGITIDTPEM
IRKAEELLKRVRLSVNPSAKIKDLGIGKQQLVEIAKALSRNVSLLILDEPTAALNEDDCD
NLLDILRLLKSQGVTCIMISHKLKEVLEIAETITVLRDGQTICTLDRQKDEVNEQVLIKH
MVGRSIENIYPPRTTKPSDEILLEVHNWSAFDVNLGRQVLHNVNFHVRKGEVVGLSGLVG
SGRTELARSLFGNPDGYITTGEMIFKGKKQVFRHPRHAIRAGLAYATEDRKRNGLVLIQS
IQHNISLANLQGVSKNGIVNEELDKQNAVNFVRSLSIKTPGIHQLVMNLSGGNQQKVSVA
KWLFVGPDLLIFDEPTRGIDVGAKYEIYTIINDLVAKGMSILMISSELPEILGMSDRIYV
VANGTITGELDAKTATQEAVMRLATQY*
>SPBIB_v1_10064|ID:27161826|xylH| Xylose transport system permease protein XylH [Uncultured spirochete bib]
MNELLTVLKKNVREYGMYIALAVIMAVFTILSKGLFISSRNIANLLNQTGYIAVLAVGMT
LVIVIRHIDLSVGFLSGFLGAVAAIALTQWNWPVWLTILLVLLLGTIAGLVTAFPVAQLG
IPAFVASLAGWLIYRGALMLVTAATGTIIVPNESFNAIGNGYIPDLFASASFLPNVHKTT
LLLGAVAIVLFIIGQISDRRRKLQYNFEVLSTGLFTAKLLFISVVLGFITWILAGYNGMS
WTVVIMVAVVLVYYFITQRTVIGRHIYAVGGNPEAAELSGIDVKRITMAVFASMGFLAAL
SGILFTARLKSATPQAGQLFEMDAIAASYIGGVSAAGGIGSVTGSLVGALVYMSLMNGMN
LLGTDISLQYIIRGLVLLFAVIFDVMTRKRKS*
>SPBIB_v1_10065|ID:27161827| Major facilitator superfamily MFS_1 [Uncultured spirochete bib]
MKEKTKEIAVKGARSTFWIMFCFMLLHQADKLLIGPLTTQIMDTFKISRTQMGAVTTGAL
IVGAIFYPIWGWLYDKFSRPKLIALASFLWGASTWLNAIAPTYPIFLATRAGTGIDDSSY
PGLYSLISDYYPPKKRGKIYGFLQVAQPFGYLVGMILALAFGAVYGWRNVFYLTGSLGIV
VSFIIFLFVKDRPRGSTEPELSSVEHLERFKFSWKTVGGLFRKKSLIILFVQGFIGVFPW
NVITYWFFDYLKTERGYSDTQTLLTMVPAVLILAAGYPLGGALGDRLFKKTPKGRVIVGA
AGVAIGAVLLWITMNIPLAAHLPFGIMLCATALFIPFASPNVLSSFYDVTEPEIRSTTNA

VQNFIEAAGSALAPLMAGIIADKSTLGNAILLICTVAWAACFAFFIFAGRFIPKDIADLK
EKLAARAAAGSV*
>SPBIB_v1_20001|ID:27161828| Extracellular solute-binding protein family 1 [Uncultured spirochete bib]
MKKNLAIVAVLMLCGAMLFAAPAKKITVLGVWGGQELDVFNAMCKAFTDKTGIQVDFEGT
RDLDAVITTRVEAGNPPDVAGLTGPGKMIELANKGKLADLSKILDMNAFSKNYAQGWKDL
GSVNGKLYGIFMKAAVKGLVWYNPKAMKAAGLEEPKNGWTWDDMLAASAKIQAMGKTPWA
VGIESGSASGWVGTDWIENIFLRQNGPQMYKDWYEGKVSWTSPEIKKAWQTFGQIVADPK
MAYGGAAYINSTNFGNAAAPLFQEPPQAYFHMQASFIQSFIMNQFPNAKPVTDFNFVGFP
SINTKYSKAMEVAADLFVAFRSTPEVKEFMNYIASAEAQAFWAAGTGGLGTNRNVALTFY
PDALTKRAASLLNDTEIVVFDASDMMKPEMNNAFWSAVVSYVNKPSDLDSILANLEKVRK
DAYKN*
>SPBIB_v1_20002|ID:27161829|aglF| Alpha-glucoside transport system permease protein AglF [Uncultured spirochete bib]
MNSQTILMLAVVVLGVPLLLVGYITLSERLLGSAAHKRSSSVRAIFWIAPAVVLLVVFLV
YPIITTIILSLKDAASRGYVGLANYKYVFTDRNMLLVLRNNLLWLVLFTLVTLTFGLLIA
LLADRVKYEAVAKSIVFLPMAISFVAAGIIWKFMYDFQPAGAPQIGTVNAVFSAVIPGYQ
PQAWLFNTATNNIALIVVGIWMWTGFAMVILSAGIKGIPTDVLEAARIDGANEFQIFRFV
TMPLLAPTITVVATTLIIDVLKIFDIVYVMTNGNLNTEVIANRMYKEMFNYHNYGRASAI
AVILLVAIIPVMIMNIKRFRQGGANA*
>SPBIB_v1_20003|ID:27161830| ABC-type transporter, integral membrane subunit [Uncultured spirochete bib]
MHKHTSSKRPLSRFILHLILIVICAGWLVPSLGLLVSSFRPRNLISTTGWWTALLPPFKF
TLSNYAYVLTRGHMGISFINSLIIAIPSTVMPILIAAFAAYAFAWMRFPFKETIFLVIIG
LLVVPLQLTLIPVLKLFNALGMTGSFVAIWLAHTGYGLPFAIYLLRNFMQDLPGSVLESA
YLDGATHPQIFFRLIIPMSVPAIASLAIFQFMWVWNDLLIALIYLGGTQSVAPMTVTMSN
MVNSYGGGWEYLTAAAFISMLLPMLVFFTLQKYFVRGILAGSVKG*
>SPBIB_v1_20004|ID:27161831| Transcriptional regulator, LacI family [Uncultured spirochete bib]
MGNRKRKSISQEEIARIAGVSRTTVSFVLNNTRGKSISEETRQRVIVAAKEFGYMPDMSA
VETATSRDGSICLIVCHSESAFSDAYIMRLIEGMSRPIHKERYDLRVLHFRVSRHDYLET
ARKGSFDGAILLNTHADDPGIAELEASNLPFVIIGALPDKGLIQIDIDNSEAAAGVARYL
LSLGHRNIGVIAHAPETFFAVRARLAGFCGEMRRNGVEMCDSNIRFANFTEESGYEAARD
LLSSKQPLTAIFGTNDVVAYGIMRAVEDAGLAIPDDISVVGFDDDYLSLYTNPPLTTVTL
PAEGIGRTAAETLLRLIRGAPVHRGLRLLPTNLTIRQSCRKV*
>SPBIB_v1_20005|ID:27161832| Beta-phosphoglucomutase [Uncultured spirochete bib]
MARCIAQTSFDPARAALYESLFMVGNGHFGIRGSDEERRHSVYRGTFINGFFEKKPIQYG
EWAYGYARNHQTILNVIDAASIELEVDGSPLDFSSGLKTYERCLDLDAGKLVRRLEWESP
SGVLIRVNSERLASFEKAEIAAMRYEVSPDAACSIKLVSFLDGSVRNRPAEAGDPRVGSH
IDANPILWCKVEARDGALLLAGTTVRSGLGLAVAVRHVVSFSGSSILRAEHSAGGETLFS
SYCADCAGGERFRLDKFVAVVNGSADAIKRLADEARAAAEDAANRGYDEIAALQADYLRT
FWAGADIQIQGDPKMEEGLRFNIFHLLQSAGRDGRTSIAAKGLSGEGYEGHYFWDTEIYA
LPFFDYEAPAIARSLVRYRIGILGKARERARELSLPGVLFPWRTIDGEETSAYYPAGTAQ
FHIDADIAYALMRYVEATGERGILLEGGAELLFETARLWMGLGFFNPRKEKKFCIPCVTG
PDEYTALVDNNAYTNLMAEFNLRHAWKVATELQDQHPEEFSALAERIGLSPSEIGEWRKA
ADMMYLPFDKETGIVPQDDQFMDRPAWNLAETPREQFPLLLHYHPLMIYRRQVLKQPDTV
LAMFLRHERFSLAEKMRNFRFYEPLTTGDSSLSHCIQSVAAAECGETGKAYEYFAKTARM
DLDDVHGNSRDGVHIAAMAGSWISVVYGFAGMREGRDCLSFRPMLPPAWKRLAFSIGWRG
SRIACEYTAETSTYSLVWGTEIDIEHEGKRYHLASGAPVCIDERPKPLVWIFDLDGVIAD
TAVLHTRAWMRLADELGIPFRPETGELVKGVSRMASLCIVLGDHAAEYDAESLAELADRK
NCYYRELVEHVGPSDILPGMADLLASLKNDGRMLVLASASRNAPAIIKQLGLEGTFDGIV
DAAAVSMPKPDPEIFIRAAEMAGARLKDCVAFEDAQAGIDAIRAAGIFSVGIGTKLVGAD
IRFDSTSLVDRKAIEDAFYKRG*
>SPBIB_v1_20006|ID:27161833| conserved protein of unknown function [Uncultured spirochete bib]
VVKSVVSTVHFDVMDLPFSRRGSWLAISWIKASDPTRHVPEGLYLRTVRDRGDYQQLFRI
DLPADGGREIRAEASPSIMHLFPGGTKSERSGVVDVCIPTTSSVRFRGRGTLLRLSSIPG
PELSYAIPRGEGSWIVNMLGAGLRLMLTPLRGKLHVDAPWQAQGSQPMRFEMHPDIAGEF

DIVIEEFAQALTPKRLHPPFEDEAAAVEADFLAFAARHGGEGPFSETADIARYILWSCIV
NPEGAITRPTVLATKDRLTGIWSWDHCFTALALCPGDPALAWNQIMTIFDHQDEFGALPD
AVFDRRVVQNFTKPPVHGWAIGRMLKLGCLDRAMKEEAYLHLARWTDFWFSYRDYDGDGV
PQYDHGNDSGWDNASPFIFRPPLEAPDLATFLILQMDTLAVLADELERSKEAIAWREKST
AFLRLATSHLTRRGRMGFVASGTHEEIPNESLLPYLQLLLGNRLTESTRTSLLKSLEEEG
YITQWGIATESTLSQNYSSDGYWLGPIWAPSTFLMVDALFRSGENNLAKEIGSRFLDLVE
TSGFAENFDAITGAPLRDRAFAWTASTYLILARDISARP*
>SPBIB_v1_20007|ID:27161834| Basic membrane lipoprotein [Uncultured spirochete bib]
MRNISRFFLLMLIAIVAGSAAFAQSAPPAAGPNLTIGLVMVGPYNDHGWSQANYDGVQYV
LAKVPGTKLVYIDKANSADRPGTTVSQLGESLVAQGAKLVIFSSDDMTDEAIKFAQDHKD
IFVILTSGSQQWKEGKDYKPLPNMINIMGRMEYMKMVAGVAAAMTTKTGKIGFLGPLIND
ETRRLAASAYLGARYAWVNYLKKDPAKLTFKVTWIGFWFNIPGVTSDPTQVADDFFNSGF
DVVISGIDTTEAVAEAAKYTAQGKQVWAIPYDYSKAIDEGKGVSLGVPYFNWGPAYANAA
KAAMNGTWKNHFEWNGPDWKNINNPDTSAVGFNKGTLSKEASAAIDKFIDELGKGLNLWT
GPLNYQDGSVFLKKGEKATDQQIWYLPQLLQGMEGQSVSK*
>SPBIB_v1_20008|ID:27161835| conserved protein of unknown function [Uncultured spirochete bib]
MERGRKKVAMHIELRGITKCFGALRANDNISFAVPPSSIHGILGENGAGKSTLMKILSGF
YSPDSGDILLDGKKVRIASPADAIRHGIGMLHQDPLDFPPLKVIDDFILGSKGNLIPNHK
SATREFRELAARFGFSIDPESYVDTLTVGERQQLEILRLLWLGARALILDEPTTGISLPQ
KEKLFATLKKLASEGMTILFVSHKLEDVEALCNKVSVLRQGKLVGSVDPPFATGALVEMM
FGKEIALEPKAQLRFGSNVLHIKHVDLEGIRIQIKGLDLQVRAGEVIGLAGMEGSGQTLF
LGACAGLAQPVGGSFCLDEDEKDMTGRTYHEFRKHGVAYLPAARLEQALVPGLTLTEHFI
LSEGMKGLFIDRKKAEALAKSRIQVFNIKGRPDSTVESLSGGNQQRALLALMRDPLKLIL
VEHPTRGLDIESTIYIWNKLKERCARGTSIIFISADLDEILKYSDRVLVFFAGRVSPPLE
ASTLSVERLGRLIGGKDWETLETEAAHA*
>SPBIB_v1_20009|ID:27161836| ABC-type uncharacterized transport system, permease component [Uncultured spirochete bib]
MRDSRATRLLFKVGAILFALIVTTGVLFLAKAQPFAAYKYITLGALGSWDVVTNVFVSWV
PLLLATSGLLVTFTVGLWNIGIEGQITLGAIFTTWAMRLLEHSGWNPTLILMLAVLAGML
GGALWAMLAGALKTFGGVNEIFGGLGLNFVATALNIWLIFGPWKRPGVASMSGTIPFDRS
LWMPIAQGSSRLTPATLVMAIVGILVVYAMIEGTYFGLRLKAVGKNARSAHILGIPTWQY
MMSSFAVCGIFAGVAGAVQVTAVYHRLIPSISSGYGYLGLMVAMLVGYRASLAAPVALFF
AALNVGSIQLPIVLKLDSSLAGVLQGTLVLFVVLGQGVRSRILKKTKVKS*
>SPBIB_v1_20010|ID:27161837| Inner-membrane translocator [Uncultured spirochete bib]
MNNSALFVSLGGVLASAAPVLFAVIGETISERAGVLNLSMNGMILLSAMGSFAVALSTGS
VVLGFLAGALIGALVSLVVVFASLTLLQSQVAVGFVLALLCRDLSYFLGSPIMGQVGPRI
SQLPIPGLEQIPVLGTILFRQDMMTYLSFILVILAWFFIFKTRPGLTLRGIGERPAAAFV
RGANVNRLRYLYAVLGGAIAGLAGPIYSLSVKAGWKGTISGLDGIGWIVLSITIFGGWKP
FRAAFGAYLFAFLQWLGLVLQPSLPSVPSQVLQVAPFPLMILTLLFVNIGNAEWVERSLA
RLPENVRRDIAKVLRAMNATPPAALGVPFEKE*
>SPBIB_v1_20011|ID:27161838| Branched-chain amino acid ABC transporter, amino acid-binding protein (TC 3.A.1.4.1) [Uncultured spirochete bib]
MKKLLFAALAIALVLAGCSSGGKSDVIKVGWLGALTGDQAVWGENELNTVKMLFEEYNAA
GGIDVGGKKYKLEVIGYDNKGDPQEAVNVTKRLTGQDKVVAIIGPNSSGNAIPMAPILEK
AKVPDIATVATNPKVTVQDGKVKPYNFRVCFIDPYQGAVAAGFAYDRLGARTAALLYDVG
DDYSQGLREFFKLNFEKKGGKIVADESFNSGDVDFRPQLSKIKAANPDVIFMPYFFKEVA
LSANQARDLGIKQVLMGGDGWPSDQLISMGGKAVEGSYFVNHLDYADPAVQDFKTRYKAK
YGKETELNGFLAHDAVLLLVEGLKKAGKVNGEALAKAFEGIDVQGITGRIHISPETHNPE
GKDAAIIKIVDGQYVFQEKYAAQ*
>SPBIB_v1_20012|ID:27161839| High-affinity branched-chain amino acid transport system permease protein LivH (TC 3.A.1.4.1) [Uncultured spirochete bib]
MVQFLQQLINGLSIGSVYALMAVGYSLVYSIMNFSNFAHGGVIMLGAYFGFFSLTLLKLP
FVVAFLLASVGTLAVAIIVERIAYKPLRERKAPFLYFIISAMGASILLENIVIATIGPTF
RTYPEVFSKVPITLGPLAIGRLDLIIFAISAVCLTLLILFIEKTKIGKAIQAASYNVKAC

ALMGVNTDRVILTVFGLGGFLAGVAGVFFGMKYTVYPQIGNITTKSFIAAVFGGLGSLPG
AVLGSVILGTIETFVAGYLSSQFRDLIAFVILIAVLVLRPTGIMGKVTEDKA*
>SPBIB_v1_20013|ID:27161840| Inner-membrane translocator [Uncultured spirochete bib]
MSWTYIQGILMLAGINMIAVLGLSLLTGFTGLFSFGHAGFMAIGAYVGAWISASPASTPA
GLGLPFILAIVGGGAAAGIVSYFIGRISLNLKGDYFCIATLGFGEAIRLIFNNVNRFGGA
RGWPGVPGRSTFLVILIADVIAILFLANLVKSRHGRNMIAVREEELAAQIAGINVFRYKM
ISLIISAVYAGVAGVFFAHYMTFIQPKMFSLTKSTELTIIVIFGGLGSISGSVLGALVLT
ALPELLRAFEIWRLVFYGAAVILIMVSRPKGLMGGMELTPSGIRKVLAERNALREAKTKL
LADAQSGEED*
>SPBIB_v1_20014|ID:27161841|livG| leucine/isoleucine/valine transporter subunit ; ATP-binding component of ABC superfamily [Uncultured spirochete bib]
MAPILSIRNLTKKFGGLTAVGDVSFDVPEGSIVGLIGPNGAGKTTIFNLITGVYKPTSGT
IEFREKSILGLEPYRIADMGITRTFQNIRLFKNLTVFENVLTACHMNASYSFAEAILRLP
RFRTQEKALVAKAENLLEIMGLMGYRDLVANNLPYGLQRRLEIVRALALNPVLLLLDEPA
AGMNPDETEQLMRLIGRIRNDFSLSVLVIEHHMDLIMGVCEHIVVLNFGEKIAEGTADEV
SRSPQVIEAYLGTSLEAADA*
>SPBIB_v1_20015|ID:27161842|livF| leucine/isoleucine/valine transporter subunit ; ATP-binding component of ABC superfamily [Uncultured spirochete bib]
MLEVQNLHVAYGGIRALKGVSLSVQKGEIVTIIGANGAGKSTLLNTISGFLKPVRGSVLY
NGSALPRRPDLIVKSGICHVPEGRLIFANLSVQDNLMLGAYLRNDKKAIQADLEKVFAIF
PRLKEREEQRAGTLSGGEQQMLAMGRALMTNGEFILMDEPSLGLAPLLVQTVFEIIERFR
SMGKTILLVEQNAFKALQVADRAYILEQGKIVKEGSARELARDPAVKEAYLGSRSRGGG*
>SPBIB_v1_20016|ID:27161843| Peptidase M20 [Uncultured spirochete bib]
MNIAEKLSQLVKFRTVSSFDPEEEDDGPFAALIAALPELFPLAHARLERICIGSRGIVYR
WQGTDPSLKPAIFCAHFDVVPASETDAWDEPPFSGAIRNGYVWGRGTQDIKVQLACILES
AEQLAANGFQPQRTLYFAFGGDEEVGGTRGAGAIAKWFSEQHIHASWVIDEGSPVGQGLV
GFVRKPLALIGIAEKGYADIIIEAQGQGGHASMPPRHTALGAVAHALAAIEDNPFPARLT
KTTDGFLAALAPHAIAPYKQIFALRRILSPFILKAFSAMPSTNAMVRTTCAATMAQASPK
ENVLPTLAQAAVNVRMIPGMSSAEVVEHFNLLASPFGAKAYIKFPEHMVEASEESSTSSE
GWIAIAKAVSEAFPEAVPAPFLFTASTDTKHYRALTDDIYRFTPLIQTQEDIAAVHNVNE
KVSIENLERCVRFYRSLMQGC*
>SPBIB_v1_20017|ID:27161844| exported protein of unknown function [Uncultured spirochete bib]
MNSTRFTNPAAVILAAGRSSRMGATKPLMLIDDMPMLMYAANAFRAAGIDYIIVVTGFDA
ERVAALAREGALHPVHNSNFDKGMFTSVCAGIEAVPDEFDSAFVLPVDIPFVSPETLKRL
QTSANREHGASAKERGAPSAGGIDGMPAAARSRPIVIPRHTGKPGHPPLIHRSIFGQLRG
WRGPHGLGGFLKAQSSLVAYLDVEDPFILRDIDTPDDMQKIISDRNLHGVAMPHS*
>SPBIB_v1_20018|ID:27161845| putative XshC-Cox1-family protein [Uncultured spirochete bib]
MSELRKWIAARLRRGETVVLAAVTRVSGSTSRGTDALLAMDSTGAMNGTVGGGFTEGQTI
EAARRMLGLGGSQAGAQTTTAQPPGASQDLDFDLTPETNQTQMICGGRLRIHLEKLEPQS
AAARALLACIDSVENGRPGAFVVVKTHCAQYSFALDNRGNDTAVIPAPLLASLRANMPGF
EGAAEFEIEDPSNAVHETAHVFWLGLLPDPVVYIFGAGHVGKAVCDFASLAGFRVIVTDD
RPEMLTTERFPHASDLRLIHSFEDALTDASGAPAVSIGPQDCALVLTRKPDIDKAMLAQV
LHTNAGYIGLIGSKSKRDGVFAALKAEGFADTDIARVHAPIGLAIGARTPEEIAVSILAE
IIAVRAGIPATASISPHSGAQAPASTKVASR*
>SPBIB_v1_20019|ID:27161846| Aldehyde oxidase and xanthine dehydrogenase molybdopterin binding protein [Uncultured spirochete bib]
MRDYHTSTPPASTKELDATQKFNVVAHNISKVDGEGLVLGRPAYTDDLAPQNALYVKLVR
SPHAFARILSIDASEALKMPGVACVLTWKDCPRIPITRAGQGNPEPSPHDRFILDEYVRY
VGDEVAVVAAENEATAEKAASLIKVDYQVLEPILDFEKALDNPIIIHPEPEIHEMFPIGF
EPKRNIAAAYHMEIGDVEKELATSPVTVETTVHTQAQQHVALEPHTAFSYLDIQGRLVIV
TSTQNPWHTRRLLGLAFQMPLRQIRIVKPRIGGGFGGKQHIHIEPYVAMVTMKTGKPARL
ALTRREVFEATFTRHEMRVKVRLGADPDGALRAIDMQVLSNTGAYGEHALTTFMVAGSKT
LPLYNKVKAVRFGGHVVYTNKVSAGAYRGYGAIQGLTGLESAMDELAHKLGMDPVELRRK
NMLHEGETSEVFRIMGEGTEGVAMIIESCKLEECIRRGKELIGWDPTHLVQEVAPSKVRA

KGMAIAMQGSGIPLVDMGSARIELQDGGFFKLHVGATDLGTGSDTILAQIAAEELGVDMK
DIVIHSSDTDHTPFDVGAYASSTTYVSGSAVLRAARSLKQKLIEAVSLKFGVRPQDLTFE
DKVFRTIDGSKELSLNDFSYDTLYHDGAKMKTIEATESFSGDKSPPPYLAAFVEIELDKE
TGKVDVVNYVAVADVGTPINPNLAKIQIEGGLLQGIGMALYEDVRYSDKGHMLSHNMMTY
AIPSREDVGRITVELVDSYEPSGPFGAKSAGEIGIDTPPAAIANAIRNATGVRLTQYPFT
PERVLMAIRQAEKGQKGQT*
>SPBIB_v1_20020|ID:27161847|xdhC| Xanthine dehydrogenase iron-sulfur-binding subunit [Uncultured spirochete bib]
MIITLYINGKKESVECQPGEMLTEVLRRAGYVEVKKGCDTGNCGVCTVLLEGKPVLSCSY
LAIRADGKQITTIQGAAKEAEEFAHFLTAEGADQCGFCAPGFALTVLAMKKELPNPSEGE
IRHYLAGNLCRCSGYEGQIRAIKKYLEAEHA*
>SPBIB_v1_20021|ID:27161848| Molybdopterin dehydrogenase FAD-binding protein [Uncultured spirochete bib]
MEILEFASPRTLDEALHLVRDRHGTAIGGAAWLRMNTKTMPLGVDLSELGLDYIREANGS
IEIGAMTTYRELETSPLLEARFGALFRNTVSHIVGIQLRNIITVGGTLAGRYGFSDLNTT
LCALGAKVVFYPGKIADVASFIQDGEEWPFLIEKVLLPVSARASYQQMRITANDFPIINA
AAAWTGSSWRIAVGSRPAASRLCKKAMVLLGNEPYPSDEKIGQAAVAAAEELQFGSDIRA
SAEYRREILPALMRRALMEVRG*
>SPBIB_v1_20022|ID:27161849|dnaE| DNA polymerase III subunit alpha [Uncultured spirochete bib]
MLLAEVKNHLLPVFRIIAQVSKMGKLGYTGIMAEFVHLHNHTDYSLLDGAAPVQKLIKKA
KEFGMPALAITDHGNMFGVMAFQDECRKAGIKPIIGCEFYVAGGSRLEKTGTENGNKYWH
LILIAENQTGYHNLLKLTSRSFTEGFYYKPRIDDELLAAHSEGLIASTACLAGEIPSLIL
AGKIEQAAKKIGYYKEIFGPDHFYLELQDHGLDEQRIVNKELIRLSKLLDAPLVATNDLH
YVEREDAVAQDILLCIGTNRKRNDPSRMRFPNDQFYLKSPDEMAALFSEVPEALANTLKI
NEMAQLEIEFPGPLLPDYQVPESFSDPDDYLRHIAREGLAKRYPNPSPEATARLEYELDV
ITKMGFTGYFLIVWDFIHWAKEHGIPVGPGRGSGAGSIVAYAMRITDIDPLKYDLLFERF
LNPERVSMPDFDVDFDFERRGEVIEYVAQKYGQNRVAQIITFGTLKAKAVIKDVARALDI
PFEEANQIAKLVPDDPKMTLAKAFKMEPKLAALAENPSYAELFSIAARLENLHRHSSLHA
AGIVISKSELTDYVPLYKDPKTGLVATQYTMEYLENCGLVKMDFLGLKTLTLIRNTLDIL
RRRGIDIDEEKIPDNDPKTFKMLSEGKSTSIFQFESQGMQSILKRAKPSSIEDLIALNAL
YRPGPMDFIDQFINSKNGKMAIEYPHPSLEKYLKGTYGVIVYQEQVMQVAREVAGYSLGR
ADLLRRAMGKKKPEILQKEEGPFIEGAISRGYSREDAKRIFEILTPFAGYGFNKSHAAAY
SVVAYRTAYLKANYPAEFMAANLTNEISNTDKLTEYISEARAMGLSVLPPDINRSDANFS
VDHGQIIYGFLGIKGVGEGIAKAIQLEREQNGRFSDFIDFLSRMSPAGLNRKTLESLILA
GCFDSMKHKRRELVQNLERAIEYVEGKRAFEASRQGSLFELEDMGPYPAFQFEPADEFSN
QEMLQAEKELLGFFFSAHPMDEYRTIWERSSTLDLGRLDQASANREYTVIGLLKEFRTHT
TQNGKRMAFGKLEDYHGSIDIVIFPDMLEKNEQAFIKDRVLCVSGMYDNSRRAPSLKIQA
ILDPESLRKASWRELHIRLANSVRATGQAGFTDRAEPSQASAIFDETSLYQLRDAIYSLH
GQCKVLFHVPLIKGGEAVIEAGPHTTCSANDSDLEFLKGQPAVAEVWRS*
>SPBIB_v1_20023|ID:27161850| putative Ribonuclease Z [Uncultured spirochete bib]
MKIRILGNGGFYNEGLPYNAIAIDGHVLIETPPDILQSLNHSDMRPSQIDTVFISHIHGD
HCFGFPFFFFNWLYAGEADSHYEKGSMLAIIGPAGLREHLLALLRLAIPPEHQYIRDFSE
RAKLVEVDEGDIIAVKGNIWFSFMRTKHSLPTLSLIAGATAGPPPSPKRALKEALFAYSS
DTAMFEGIHTLLESGAKLILCDTNGEKESDVHMSPAQLIAAARVHGLAGNSGRLLLGTHM
SRRIVSQENLRFAQDGEEFSVI*
>SPBIB_v1_20024|ID:27161851| putative hydrolase of the HAD superfamily [Uncultured spirochete bib]
LYIFLMQQRVRFRAVVFDVDGTLYPASALYLRCIDIFVRHPRLVEGFSVVRKELRTLQMR
SDYTPRDAEELHQLQASLLAQRMHIPQDQVRKLMEFFFYATLPERFASIRPFSGVRNAIE
TIRRSGIAVAALSDLPPTEKIEALGLSDLLEHALCAEEFGVLKPHPWVFAALIERLGYAP
SEILYVGNNPVYDIQGAKNAGMAAARCGRPTALADFSFTKWKNLADWVLTNRA*
>SPBIB_v1_20025|ID:27161852|hndD| NADP-reducing hydrogenase subunit HndC [Uncultured spirochete bib]
MEEQLIEITIDGQNVRVDPSANIVEACAHAGVKIPTLCYLKGISQNASCGVCVVEVEGAK
SLVRSCVQKPVPGMKIRTSSPRVIRARKTAVELLLANHPDDCLSCIRSDTCELHTIANIL
EVRADRFPGYKKYPMPDTTSEGIVRDDSKCILCGRCVAVCEETQGVHAIAFSGRGARTRV
STFLDRGLAQSACVQCGQCSVVCPTGAITEKDESREVFSAIEDPRLTVVVQTAPAIRASL

GEALGLPAGSLVTGQMVAALRRLGFNRVFDTQFTADLTIMEEGSELLERLSKGGTLPMIT
SCSPGWINFIEGFYPDLLSHLSTCKSPQQMFGSVAKTYYAKKAGLTPDHMRVVSIMPCTA
KKYEARRKEMDGAWGWWKEQDPGKVPARPFFDVDWALTTRELARMIKLAGIDISRLPEEE
FDDPLGQSTGAGTIFGTTGGVMEAALRTVYELVEKKPLENIEFTKVRGFEAIKTAEVVVG
GSPVRVAVAHGLANARVLLDEIRAGKSPYHFIEIMSCPGGCVGGGGQPVLADLEKKLARS
QALYEEDRKLAIRKSHENPAVRALYQEFLGKPLGHLSHELLHTSYKARVF*
>SPBIB_v1_20026|ID:27161853| NADH-quinone oxidoreductase subunit F 2 [Uncultured spirochete bib]
MKNASDLGFLAQTGLARIHGDKPWISVGMGTCGIGSGADKVWEILNQKAKASEILVRRVG
CFGFCAAEPAVMTWMPGKPVLFFTDVDEQKTRRILGGLENSASYDKIAKLAEAKIEEWDF
RTSKIEFGHGYAQLPAWKDLNFFKGQEKIVLRDAGLIDPESIEDYIAVGGYGSLVRALTS
MPPESIIEEVRKSGLRGRGGAGFPTWKKWSIMRQSLLDNPGEGYIICNADEGDPGAYMNR
NEIESDPHMLLEGMILGAYAMGATRGVVYVRAEYPLAVYRLTKAIEDAREHGLLGINILG
TKFSFDIDIVTGAGAFVCGEETALIASIEGNAGRPRPRPPFPVQKGLYGRPTSINNVETW
CNIPVIIARGGEWFAGFGTPTSTGTKVFSFVGKVRNTGLVELQLGSTLESVIYGICGGMG
PKKKIKAVQCGGPSGGCVPASLFKTPIDYEHLAELGAIMGSGGMVVMDQDNCMVDVARYF
TGFVVGESCGKCTPCREGTSQMLHILQKVSDGEATESDLDTLQDLALTVKDSSLCGLGQT
SANPVLTTLRYFRDEYIQHIKGKRCPAGVCENLYIALCESSCPLHMNIPGYLQLLKENRI
EDAFELTLRENPLPGTIGRICHFHCRMRCRRDMLDEPVSQGEIHRYLADTMYKMGREKQI
YANIIKEKFPSTGKRIAIIGAGPAGLTAASYLVRLGHEVTVYEASDEPGGVLRWGIPAYR
LPKDVLKKEVSFIEKLGVRFVYNTRIAEPEQWRRLSDSNDAIIVAAGASAELELGIPGED
AQGVYKACNFLGMLAKKEKVHTGGEVVVIGGGNSAIDAARSALRLGATVTVVYRRSKIDM
PANEEELKGALEEGIDLICMASPVEIVTKQKEGKKTVKAVRIQRMKAGPVDSSGRPTPIP
TDKIDEISCSTLIVAIGEKVEIPGIESLGVERLKNGRIKADPFSLVTSNPKVYAIGDATL
GPATAAEAMGQAKTVAEIIDQALTGKKRFDQLFRRFDYRMEIPLKISKEKMTRAGMLPVN
ARKSNFMEINLGYTGEQARIEANRCLRCDVREHRREPRGTLVRE*
>SPBIB_v1_20027|ID:27161854| NADH:ubiquinone oxidoreductase 24 kD subunit [Uncultured spirochete bib]
MTKTASNTKMPDIDSIVDSCKEKPGSLLSALEQAQQNDPRKYLSEATLRRISAKLGVPLS
KVYSVATFYSFFSLKPQGEHVIVVCRGTACHTRGSLALLNESLSRLGIKEFKEEEENSAT
SSDSFATIRTVACFGQCALAPVIMVDGKVLSRMTVGKLVSLIEKMKKGGEK*
>SPBIB_v1_20028|ID:27161855| protein of unknown function [Uncultured spirochete bib]
MVPPFRNRRMVRPLIIILLHVAVFVKKKLSIKLYQAIFI*
>SPBIB_v1_20029|ID:27161856| conserved protein of unknown function [Uncultured spirochete bib]
MASFEWDTPGKPCSRPRWPSRFSHASVDIVARKLKKVFDQNANTGYFSCAEIRDYSYYEN
LLIQGLDGIDRKMTSGLLVPVDLSEYPKIIRADRGKIRACIYIGSFDPFQLTHLTVAVRF
LASEMSTSDFVIVVPEGSPDPAKPLKTEYAFRLSIARMQVEGILDPFIKVLDLGMQADTI
EIVRRFIGMHSGLSLELTHLIGSDVLPIAAKYISEDMRIWKKEAQESGVDWRHCIHVVQR
DEESAARPYLEKIEAENVCAMLDPHIVATPSSTDFRTKQAFTIVLPTASIRDKMEIIFRY
RMHKSWSSDQE*
>SPBIB_v1_20030|ID:27161857|adk| adenylate kinase [Uncultured spirochete bib]
MKLVFLGPPGAGKGTISRMVSERLGIPQISTGDIFRTAVKEKTELGLKVQAILAAGGLVP
DELTIAIVKDRLSWPDAQRSYILDGFPRTIAQAEALAGFSSLDAAVDFEVKDELILFRLT
GRRVCKVCGAIYHVVTKPPKVEGICDLCSGELYIRNDDKEETVKARLNAYHEETEPLIGW
YGNRSLLLTIDGSPDAETVYNAFISEIGRFLRK*
>SPBIB_v1_20031|ID:27161858| exported protein of unknown function [Uncultured spirochete bib]
MIPKSSLAIRFLTIFLFLAAGAGGAVCAQMSSASQISPVPIAEIPAPEIPASETPVAAVP
SFEAPAAQADTSAKIAPGTWAGKLILQPVQTGQSGNYSFDFVIRILSAGRGMLVDIPEQG
MFSYPVDRYSLNADSLSLVLDATGAEEELAFSGSYSGTFAPQGSAKKGGIVGTVRGRSWK
GSFYVQKQEAQLKPGEMPFEVPAEGGLLPATITFPVHAAAELNPAAFASFPLVILVAGAG
KTDRNGNNFEVPGKTDTLLQLAEGLRARNVGSFRYDRRGAAEAYMLEKSGHMTSFAQHVK
DLAAVIRAAAALPREGRLILAGMNEGAWMALAALNQLGQDADLVDGLIMLDSSGQSPMEA
LAGSLEDQDEEIRNTAMEAAQTLIKTGALIPVPEILADFFSPSRKEWLASWLAFDPVKEL
KKVPVPVLLVYGENDMQVSREAFAKLADAKPSAAIKIVPGMNYVLKEVHGEDENYAAFTD
PLFKVPAILVDLIAAFAKAQPAPQGLLPWSSH*
>SPBIB_v1_20032|ID:27161859| protein of unknown function [Uncultured spirochete bib]

MMTFDSRNQHNQQDGEERSAEQRKYRIFALLFALTAFSLVFSSCMSVPRDVAVSGLFDHF
ERTPQVLARAESAFLRDMAASFDDSTIQALISMTSENPDNGQKPINRDRLDKTLARAEAA
GIGISWNSKESPSIEAVFAGNFPSLFTSLSFSLDGNWKRIEGGYASKSGKLYVRDPYAGK
LHFTTWAPEQLNNSISAASSIAERSGLLASASDLAIYLDAKSALVSQLPILDGVTLPFDG
ILLTAERDRTSPKGANPHARYTAIFKIQMKDEQTARTYRPIMRVMWALVSGKLTALGIPV
SPETSIEQQGSLFATTPISMSAREIVNVFLALSNMNGGKAASGLVAAPRQ*
>SPBIB_v1_20033|ID:27161860| conserved protein of unknown function [Uncultured spirochete bib]
MILRMNREFHYYVVCYLARKAGFPPEHAETIALSSQMVDDSKLPWKIEGEQNSGHPHTLP
ADQSASFMTEVTQNYLFWDPKIARDVYLPFHFIPGDPQLAAMKRKDRKLHPLAVTADSQN
ARALLVDALRSGNLFRIGIALHSYADTWAHQNFTGTADPFNELEARNILPAVGHLQAGTT
PDQPQALWRDMRLAASYEDISNSARFLEAARMIYRFLSTAQHKKFEDEAFVTGPLEELWL
NRRTSKNDSLAAASDYVIYFDVPPYDPDKWFRSLEARQFDTRELLKAISAEGFGTEWLGA
FSGQSMVRGTISISNYSDSMFAQWNAAASAHRDAFMKLMHTKGIQVL*
>SPBIB_v1_20034|ID:27161861|ndk| Nucleoside diphosphate kinase [Uncultured spirochete bib]
MAIERTFAMLKPGVLSRRIAGEIISRIERKGFDIIGLKIMRITRELAEKHYAEHIGKPFF
NELVAYITSSPVVAMVLEGDQAIKILRMMCGSTKPEEANPGTIRGDYAMHTNINIIHASD
SPEAAAREISLFFKPEELMDWSDGNQHWI*
>SPBIB_v1_20035|ID:27161862| Oligoendopeptidase, pepF/M3 family [Uncultured spirochete bib]
MAETLNVPTWNLDDVFPGFESEKYREAKVRIKAMLEKAKNYLVESSPPVGDAAFADWLWA
LLAELDQIEVLADTLSAYVYARFSTETKNPVVIAELNKIEELLVPATTVLVQFRNMLAQH
EKQLKAAIASDARFAPYAFVLEEALFFQKHQMAPELEDLAADLGRSGADAWSRLQESILA
NTSAVWDEATGERKTMVELRNLAYDPDRAVREKAYRLELGIWKSVEMPVAAALNGVKGTV
STLNKRRGWESALEASLAQARISRATLDALIGAMEVSLPMWRRYLKAKARLLGLERVAFY
DLFGPVERQGAMLPTFQWNEARDFIVRQFGTFDPDMAAFAAHAFEHFWIDAKPREGKSGG
AYCTHFPAARQPRVFCNFDGSFSSLGTIAHELGHAWHYETIKDLPMLLSQYPMTLAETAS
IFSETLVSNAAMAELDQSARLPLIEMHLQDACQVIVDILSRFYFEKAVFEERANGEITAD
RLCALMLDAQNRTYGDAMEPELKHPYMWAVKGHYYIPSLSFYNFPYAFGQLFGVGLYQIY
KEQGSSFARRYRELLRATGSLPAVEVARKAGFDIETPDFWLRAMSTFEEDVRFLEDEAQK
SARE*
>SPBIB_v1_20036|ID:27161863|lysS| Lysine--tRNA ligase [Uncultured spirochete bib]
MEQKKAIHWADQTAEKIIQTWGEQERYTCASGITPSGTVHVGNFREMISVELVVRALRRL
GKNVRFIYSWDDYDVFRKVPLNMPNPEMLEKYLRFPITMVPDPWGRDESYARHHEVDVET
ILPEVGIYPEFIYQANRYRAGTYAEGMRRALEMREHLKTILDKYRDEDHKIQGEWWPVAV
FCSNCNRDTTEIDGWDGDWGLTYHCESCGHKETGDLRTLKGAKLVWRVDWPMRWAYEKVD
FEPAGKDHHSQGGSFDTSKHVVEDVYGRKPPVTFRYDFIGIKGSPGKMSSSKGKVVDLPD
LLRVYQPEIVRYLFAGTRPNTEFVISFDLDVIKIYEDYDRTERIYWGTEKAKNEEIEALE
RRIYELSQVESVPEEMPYQIPFRHLCNMLQIHQGNIDSVIALLPDLKPSQEARVRRRAQC
AWYWITECAPEDFRFFLRKPGEKAELSELEEAAVCRLRDEVVAKLDQFAQENEVAEAIYA
VAQASGIEPKQLFRASYQALIGKDQGPRLAGFLKAIGKEKVLSILEQY*
>SPBIB_v1_20037|ID:27161864| conserved protein of unknown function [Uncultured spirochete bib]
MMPASMNIEKISLEEAARIALAECIELKKQENVLIIANPDTEQSEIAKAFFEQAEEMGAS
PRLLFQPVKTQADYAEDEVIQAIEAAPPVILSISTEKLGKDRKRLSSPLKAPDGRSFDHI
FHYLLHGISAIRAIWTPGITKDMFIRTVPVDYLKMRAAADRLSALLTQASVVMVKSPGGT
DISFSIQGRKAMRDDGDFRSAGKGGNLPAGEVFISPAIKSAEGTIVFDGSISDIEGDIVI
QKPIACTVRGGYVMGIEGGEEADRLEKALMRGLNMASRLVHQQGMAPETALSYGTNARHI
GEFGIGLNPAARIGGNMLEDEKVLGTIHFAIGSNYDEDAPALIHLDGLVKLPTVVLLMPT
GEEITIMEHGALSMVAALTE*
>SPBIB_v1_20038|ID:27161865|gcp| O-sialoglycoprotein endopeptidase [Uncultured spirochete bib]
MRILGIETSCDECAAAVVEDGRHILSNVVMTQIPLHERYQGVVPEIASRAHIEWILGAVR
RALAEAQLDIDDIDGVAATAKPGLIGSLAVGLSFAKAFAWARGLPFKGINHMLAHLYAPQ
LEETIEYPFLGLLVSGGHTVICRADDFDSIAVLGTTIDDAAGEAFDKVAKYYGFGYPGGL
AIDQLAERGDENAFKFPLPSLHKGEHRYDVSYSGLKTAVIHHLHSYLRPGASISPENIAA
SFRKTAIDTLLSRLYRAVRDTGIRTIVAGGGVAANAYLRRELASKKDLRVFFPPLSLCGD
NGAMVAGIAWHYFCRGEADGWDLAPSSRVMDFKWRQNHP*

>SPBIB_v1_20039|ID:27161866| Divergent polysaccharide deacetylase family [Uncultured spirochete bib]
MNGARRGTIHSVGTTQKRHTQKYSLVIACAAFAVFALFALINACTPKDSSKKQAQSSRYG
TPEYVIELPPGYEDLKVIPESQKEPLTQPPANPESLSGSQPGAPGAGASVARLARPEGTV
PSGAPVLIIVIDDVGYNNGELKPFLELPFPITFAVLPQLPHSADSALSIQKAGKEMILHQ
PMEALGGNDPGPHAVYLSMDEATIAKTVAENIDSFPYQPKGMNNHMGSAVTRDRNAMTPV
LKLVKERGMYYLDSLTTPGTAISELCRELGTPYMERNVFLDNKSDRESILAAIEEGKRIA
RKRGAAVMIGHVWSTNLAATLMEIYPELVEQGYSLSTISQYMQLQAEENSGHADSWN*
>SPBIB_v1_20040|ID:27161867| RNA polymerase sigma factor [Uncultured spirochete bib]
MTITDNDESLSLYFDSIRKIPLVTRQEESILASRIARGDKNALKKLIEGNLRLVVRIAKS
MWMPPHSLMDLIQEGNIGLVKAAERYDASKNVKFSTYAVWWIRQAISRSLVNTGRQIRIP
HRKEDALRRLNAIQMRKSLEISRAPTEKELADELGCSEAEVSNIMHLGDQPIALEQSGDE
ELSILDIYEDWRYNPEREIERASISWQTAWLLSNLPSREREVLSRRYGFTEGRTESLKAV
GAVVGCSAETVRHIESRALKSLWHTAARVGLTA*
>SPBIB_v1_20041|ID:27161868| protein of unknown function [Uncultured spirochete bib]
MNLQIQTDDAILEAEDILHKPLVSVMSGENFVVTTPAAPRYRSNKERPHYVPQNVPTSKT
PKARAKNRIDFRTLFRLNEKKNHAPRSGKPQRNNNAGAINRFSVQPAMFPRTIVDVPSEL
GTGVPGADKHRPFELGLVRRRPVARSTMRTLLQGKMLHAFAKIRARWIIALALVLAAAGT
GFLAASIISNSHPIISLPNENSAQDALIAMLEPEPSAVQNSDTALPPLPSLLVERSYTVR
RGDTLQTIARQFGLREDTIISANSLNSKTQLQVGKTLKIPNMNGVYHTVKRNESLSTISR
AYGIEMVRIVDANNIASSALRAGDRLFIPNAKLDSSVLRNFYGDTFIWPVRGPISSPFGY
RTNPFSGERTYHAAIDIVVNRGTSVKATREGKVADTGYNAVFGNYVIIRHTDGYQSLYAH
LDAILARKGARVNQGEVIGRSGNTGQSTGPHLHFSIFRNGQAVDPRKYVK*
>SPBIB_v1_20042|ID:27161869| putative Signal peptidase I [Uncultured spirochete bib]
MWKRPPASAKSFDSTLITIVLVVIVAILLRFFVADAAIVQGKSMLPHYRNGEVVFIFKAA
YGIRLPSGRYIVRWGRPVRGEVVAALRPGTNEIVIKRIGEIREQQPMPVYFLIGDNGIES
IDSRDFGLVTFDAFVGKVIPQR*
>SPBIB_v1_20043|ID:27161870| Peptidoglycan glycosyltransferase [Uncultured spirochete bib]
MENHAQARFNRRLRLLIFVLFTFAAAVLVRYAVLAIEGPDKTEKSTNTVTERGRILDRNG
NVLAFDIPKFNIAIRKNEIDPFRIHEDLRIVASALNTSADALEQKIRDSSQNFVYLAKRL
DIDTVKPLLSQADKGQLAGFLIEEISGRIYPEGRLASHLIGFTGEGNRGLEGVEYKYDTE
LSGAGSENVRGSDIYLTIDAHLQYALEQTARKAFNENHAESLFLMAMDVNTGEVLAYVQM
PDFDPNNFGAFKEAEREDRMSVYSYEPGSVFKIFSMASVLDAGLITPKTQFNCDGAYRKT
LPSGEQIIIKDLHSYGMLDLAGILAKSSNAGAGYASDRMSEAEFYSRLINFGFTQKTGIG
SPGENPGSLREPAKWSARTKPTVAIGQEIRVTALQMITAASAVANGGILLKPETIQRIEQ
ADGKILYNHKPVALRKVIAPETSRAIITAMESAASLEGTGWRAKVPDVRMAVKTGTAQMI
DPKTRAYSETDFIASTLGILPADNPKVAIYVAIVKPKGDSYLGGQIAAPVLREAAESAIS
ILGLPRGKSQNAMHDGLVTIQEPAPAVIGAKMPDLGGFSKRQLLPLLLRSDITVIINGDG
YVVSQDPPPGTAIEAGARIVLRLQ*
>SPBIB_v1_20044|ID:27161871| Metal dependent phosphohydrolase [Uncultured spirochete bib]
MTRDQAVALWRQWNNDESLWRHALSVESVMRHFAHRYGENEEYWGLVGLLHDIDYQKYPE
EHLRHAREILAPAGFDEAFIRAVESHGWGICSDVEPAHVMEKVLFATDELTGFIAACAYV
RPSRSVLDMEVKSVKKKWGSAAFAAGVRRSVIEQGAQMLDMPLEELIGETIVALRSVAAD
IGLKGNL*
>SPBIB_v1_20045|ID:27161872|ffh| Signal recognition particle protein [Uncultured spirochete bib]
MLEKISETFSDVFRTISGKASISEKNVEDALDRIKIALLEADVNVRVVRRFINGALEEAK
GEKVLRSVTPGQQFIKIVFDRMVALLGDERQDLALKGTDTQSVILLLGLQGSGKTTTAAK
LANMLKNKGRKVLLAACDLVRPAAVEQLAVLAGQVGVDIHREDSNDAVQVARNALARARR
EGHDVIIVDTAGRLQIDEPLMQELVRIRDAMSPVESLLVADAMTGQAAVDIAKAFDEKIG
VTGVILTKFDSDTRGGAALSLKSITGKAIKFIGVSERIEGLEPFYPDRIANRILGMGDIV
SLVEKAQEVYDQKEALDLERKMSQETFTLEDYLDQIHKMKKMGSVKSMLEMIPGLAGQID
EDRIDLQEMKWEEAILLSMTKKERQNHLIIGPSRRSRIAKGSGTSVAEVNRLLKKFEKSR
SMMKKMVKNKGAMAKMFGGGNP*
>SPBIB_v1_30001|ID:27161873| conserved protein of unknown function [Uncultured spirochete bib]
VHWFAFCPNPNCPYHHEAPASPWAYAIGWYATKAFGKVRRFRCTRCGKSFSTQTFSTHYY

LKRIVSYRDVLYXX
>SPBIB_v1_30002|ID:27161874| conserved protein of unknown function [Uncultured spirochete bib]
MPAIGHDAQYRCVCEKKAAFEGGFFFCRTVDTELQPHALNADKIKNECVQKGIPMNRGKH
IILALVVIVVLISIFPITVEAVEPVPYRLFPALYKKENFSRIDQTMSLVNYQAGGGFLWD
RVPLIANNTIVAYYGSPLSDRMGILGRFPKEKIAQMVKETAQQYDEVNGKDGVIPAFYII
YGTCWPGGEIGYLDEKVLLDYIKYAQSEGMLVFVDHQIGKYSVREAMDRILPFLRYPNVH
LAIDPEWRTLVPMKEIGSITAKELNDAQDYMDAYIRENNIPGIRMLVVHQFADKMIQSRQ
EVQAHRDRVILIHTSDGFGVPQVKKATYQRNANAANMPVKGFKLFFKSDFPLAGFDMPLM
SPAEVMQLDPRPSLILYQ*
>SPBIB_v1_30003|ID:27161875| exported protein of unknown function [Uncultured spirochete bib]
MNRARKANIAILLLCAALSTVSAYDPPKGASLLGRIYSSWSMGGGMSLTEQGSGAWAFAF
NPASTAFEELPGLEFSYTGIADMSTGSPQGWGSAAQADFALPKAYGVWSGAVRLFSTPGA
MTSMPLGTFVTVGGGFSKKISGRMSVGASAWLAMGDNGSFGWGVWADLGVIQELGDVGFL
KNARLGFVVSGLGKEFNYQTPPVGIAPGSLAATGFPQAFTPGIGFSADLLNEYDLRMRVS
VDLRAPSFSDIEAETSLALSFRNIVSLRLAMATSLYDIQQKSGRSVWPSLTLSGRIPLGK
KSANNAGVSSVAPAFGLMPLYDSLDAFSLGTRLSFGQADKSPPKVKAQLPVSTSGLPAAY
ISPNADGAQDWLEIPITVSDDQRTVAGGVFKIEDRSSGKVVRTITEQASPPETIKSFADL
KEGFGFSRHNAFIPDALRWDGKDDQGNLVPEGTYIVSFHAWDDLGNTNMDYDSCMNVVVD
RTPPSVNAWVLGDEQLYQGTDQLIFSPDGDNSKDTISFRTQGSIENNWKYEILDANDKAV
RTKEIRERSAPRDFVWDGTDDAGKRVPDGYYRFRLSAADPAGNVGSKTIGVAPDKSDWIV
VDTSRPAVRVAADRKAFSPIKEGKAGTIDVSFSLESLKNLVSWKEFIQDQSGKTLWSSSG
DSSNPPLTSFAFNGLDPSGKPFADGQYKAGIEMKYRNGYSPVVYSDPFILDATPPSAQIV
LDDVRAIFSPDGDGSRDALAFKFAGSKEDLWNLSIRNEQGEVVYSQEYRDGLANRFVWNG
LDTTGRRVDDGRYFLHLESKDIAENAFSTEYGPIVVDTRTPRASFALSRDAFSPNDDGVA
DVLKVGIMLESIEGLRAWKLDVASSSAAQIAEKNAAISTLLSGDGSNPPPATWQFDGKTD
RGQLPEGAYRFELTIEYDNGWKTTVSSPEFVLDNTAPKAQVSRSRPYFNPRGSALQSQVI
ISQLGSKENLWTGELRDAQGALKRKWEFKNSEPGEILWDGRTADGALLPDGLYSYRLFCV
DAAGNSFASEPIHIGIDTAAKQSSLSANYMAISPNGDGIQDAMALAISVTSPESVQNWRL
AILKGQETVMEWKGAGNVPQSITWDGTSLTRLPVPDGSYTAFFHADYPNGDATEASLGPL
VVDRIAPQAEVKIARAIFSPNGDGVNDTLPIQQSSVPGDVWQGSILDVDGKVVRSWTWDG
TVQSVEWDGRDNAGNVAADGKYRYKLESKDAAGNAFSYISPAFEIETEKKAVRLTVSDKA
FSPNNDGIKDVQVLSAAIVAPEKVKEFVLQIVAQDGPAALSAVRTWKGETPLSRYEWRGE
TDAQIPAPDGHYAASMRVVYRNGDEAESATGTFLLDRQYPQIDVKVQGSIFSPDGDGRSD
TITIAQSSMPGDDWKGTMKDSAGNVVRSWAWQAQASDFVWDGRNTNGSVVSDGFYTYTVE
SEDAAGNRTMAGPFRIQVETGKRAVQLRLSDKAFSPNGDGIKDELVINVEADARDRIKQY
SLIIRGKDGTAVRIWTGSAGLAREYRWNGTNDAGAAVPDGEYSVGIEVLYLNDAFAKDGP
IPVTVDRIAPQASVRLSRSIFSPNGDGRADTLEIAQSSVYGDRWNGQIISEVGKIVRTWE
WYPMIADIVWDGKDQNGKIVPDGAYYYELRSIDEAGNSFILPRQRIIVDAAQKNVSFRVE
PSAFSPNDNGVKDITYINISAPKPETLLSYAVSIFSGTKAVQGQAPVRSWKGSTDIKTQY
VWDGLTDAGLKVPDGNYSVYLMLEYANGDLFNIGPVPVVVDTVPPKISISADPMLFSPNG
DGIKDYVTITQNSDPGDDWTGRIRSASGAVVRSYTWKGQAKTFNWDGTDANGRLVPNGAY
SYEVVSVDAAGNSASASIKGLTVDSTKPKVYVTASDTGISPNGDGIRDDVSFSLTVENRE
GVESWRFSLIDSKGVERSFFGGSGSDVPTRLVWDGRDLQGQVIEDTYTGKLVVRYLKGDV
AEASSAKIIVKIAPPKVDVTVKPEYFSPDGDGVDDTLTFGIATEKNAGITEWKLEILETA
VVESASMQEAKPTRSFKIWTGKGNPPAQITWNGKSDKGELVESATDYPFEFTCWDALGNQ
TKVKGIIAVDVLVIRDGDRLKIKVPSIVFRANHADFIGLDAETVARNQRVVARIAQILNK
FPDYRIRIEGHGNNVGKMLGYSASRIQQEEINELIPLSTERAEVVRKMLVDNGVDSRRLT
VNGLGSSEPVVPFTDVENRWKNRRVEFVLIKNQ*
>SPBIB_v1_30004|ID:27161876| membrane protein of unknown function [Uncultured spirochete bib]
MHTIHDPAGVFAVLLAALLFSAWLSDLLPMHIAAILPVLGIILGPEVLGIIEGGTVLQIL
GSIGVMYIFFSAGLSANIASRTARRSIVPTSVILKQVFIWTIMPSMAGIAVGLAAGASFI
RACAIGLFFASAGARSALKLNKAKFQEQASYGMPAFVPIFSALCLLVLDTLMSGADARSF
MLVLAFSFIGGGLVWIVFPRLAALFLRHVKIKGSIEVWFLLFLVFGAAYAGTFFSIPAWF
TAFMTGVALSAAAAPSADSELNRIPVRDDIFIPAALLFMGVSARISGILPTGNWILVAVI

FVIGGLAARILIVFLSKRFALVTEPLIGLAVPFASFSLAISWILYGSGLFDSVLFLSALA
LAIISSIISGISMKQGSSGAEAPSAAGGDKSRLAIPNRILIALSKPSSIAQLVELSGILH
GTANSSPIFPLVVRMPEETASQQSADSETLLATAVMQLSQMQKSALPLQIEALNAGLGII
DSAIQKNADTIIIGWNKPPRLSHAFFGNVIDQVVSGTSQMVLVARAQFPWKQIKKIYVVA
PVLVNRHPGFAAALQCIERLAEATYAHIHFIVAEENAEEVQKIGKSLASFNSAQTSHFHS
WRDIPSLIQVSSGSHAMIALISARPGEASWNPAFERLPHVLAEKNSAANVLMLYMPAFLP
EEAQASEYQQPLPEPLAESSAEALLAEAVRAGRIRVDMQTSAIADGIYELMFAALPHTEK
NVLQSVADHFIEMVQRQPIEIEPGVVLLHDRIENISYPIVCFGAHKKGFRLSTLDVPVQV
IVIILVPHNQGAEEHLRFLAEIAALFHSHDLRERLIAANMPENLLSQH*
>SPBIB_v1_30005|ID:27161877|murB| UDP-N-acetylenolpyruvoylglucosamine reductase [Uncultured spirochete bib]
METLWQIAQKINTEQCSVTFDEPMSAHTTFRIGGPADLFLKPRSVEALASVLTLLRSARI
PVFLLGGGANILVADQGIRGAVIDTCLLSSVRSDASNSNIIYAECGASVSTLCTEALVQG
LSGLENFYGMPGSIGGAIYMNARCYEDDISLHINEITAISPTGELVSLSARTMQWSYKRS
PFMRKGAFAGYIVAAGRFSLEPGEPLQIAAHMRSRMEDRMHKHHFDYPSAGSMFKNNRNF
GKPTGKILHELGLRGYRIGNAAISPWHANIFINLGGATARDMRALILHAQAVVLAATGWK
LEPEVLFVGEF*
>SPBIB_v1_30006|ID:27161878|cysS| Cysteine--tRNA ligase [Uncultured spirochete bib]
MHDMYLFNTMGRSLQRFEPLHDHKVGFYGCGPTVYNFAHIGNLRTYIFEDILVRTLRRFG
YDVTHVMNITDVGHLTGDDDTGEDKMVRSARERGSSVLEIAQFYTDAFFVDTQRLNINRP
TIVCKATDHIQDMIDLIQRIEANGYTYFAGGNLYFDVSKFEHYGELAGLQLDQLKAGARI
EIDPNKRNPFDFVLWFTKSKFENQALLWDSPWGRGYPGWHIECSAMSMKYLGEQFDIHAG
GVDHIAVHHTNEIAQSEAATGHKWVNYWMHAEFLVLDKGKMSKSSGSFLTLQTLVDKGYD
PLDYRYFCLGGHYRSQLSFSWEGLDQARAARLALVDRVVALKEAAGQPAHSLARPEQIGA
AAKAYLQAFDDALSDDLAMPRALAQVWTMLKDTEIPPADALACILDMDRVLGLSLDAAEK
REASVDAETVRWIEDMIRRRAAAKKARDYAAADAIRNELKAKGIQLEDRPDGTSWKLTN*
>SPBIB_v1_30007|ID:27161879| RNA polymerase sigma-24 factor [Uncultured spirochete bib]
LFQRKDDSLSSDFDRIFAQNIDLLLRIAVRITNDWESAEDVVQDAFGKVLEKRLQFPSDE
DARYWLIRVVKNGAINWSKRKLREFKAYEHWWKAETTQAKSSSSAASNGEEQNQDAGTEQ
SAEHELLMKESAEEVREALAKLPENLRVVLILKEYEGMNYKEIAKVLGISEGNVKVRAFR
ARQALLSLLSEGGTHVP*
>SPBIB_v1_30008|ID:27161880| conserved protein of unknown function [Uncultured spirochete bib]
MCPDEILLTAFVDNEVPSPWKERIELHLEQCERCRTRVETYRELRVRLRSADSIDALQLA
HAVQRIRLSLEETEKAASMKGARRLVARYPILSALSSRRVSVPLPLLVASALLIVFFAGL
AFGIFGAGRYANQALALSTRLPATTNANIESLVSTLSQTDPSQVVTIRAPGTMLAPLSSA
APVYVIYNIGEQKPTVMAVPAQGEAK*
>SPBIB_v1_30009|ID:27161881| exported protein of unknown function [Uncultured spirochete bib]
MRIRTLVVIAFLLAGAGCLFAQAPSAQPAAQGTGIAPSQNPGASLPLPVFQFRLRAAFML
TPVMVAAIQSAAIHSQNSKVNNAQPLDKDAVPAWDEPILRDVSLTSPLGIKIQGDQLIAM
IVFMPVELVKKDLTMLVQNQIWAKSPDNSIQMNTSVHTIRVPLGALFYYYPLGGDNKQGA
PVAIEILVNQK*
>SPBIB_v1_30010|ID:27161882| exported protein of unknown function [Uncultured spirochete bib]
MKRRAIIIVCMFVLMLAAGAIAFYVVSRPVLIAIDPVSSKPNEVVSLFGRNFGEQPGHVL
FDAAPLPSPAVLYWSPQLISIRMPHGVDTAIVRIQTRFGLSNPLMLADASKIPEISKSAP
IPSIHPVISSMQPTTNAQIGKPLIVSGDHFGDPEEGSAVFFTRIASVSTLALDDPANFIE
VNSVDKLVEVWTENKITVRVPDGAENGFVFIRTKRGTSNVYPVSISRAKGRTWKGETAKY
SIEQRIAIHVAASLPEGQLSLFLAKPPNQANQNAQIEVKSGAEYLVADENNWQEFRLGQA
DASKGKIEIVRGLQVDVSEMRADLASSSLQGIDSPWPAFLASSLAADTLVPSDDPTIQAA
ALAIQKKEKNPFRQIVLVSQWVSSNVKLNDKSQGLSDEALVALKHKAGGTRALALLNCAM
LRALGIPAVPVAGFLATGDNRLIPHFWGEYYLTGMGWIPFDPLLASGYVPQQFSGGFSDR
LIYYRGIDNRHIAMSRGYRLLPAVSREVQKKQKVSWSFFEYDELSLGLSYSSTWDIAMIS
WNANP*
>SPBIB_v1_30011|ID:27161883|glyA| serine hydroxymethyltransferase [Uncultured spirochete bib]
MSYIQSVDPELHAAMLAEMDRQKNKLELIASENYTSRAVLEAAGSVLTNKYAEGYPGKRY
YGGCEFVDIAENLARDRAKKLFGADHANVQPHSGSQANMGVLFAALNPGDTIMGMSLAHG

GHLTHGAPVSFSGKLYKVVSYGVNPDTETIDYDQVAYLAREHKPKLIIAGASAYSRIIDF
DAFRRVADEVGAYLMVDMAHIAGIVAVGRHPSPIPVADFTTTTTHKTLRGPRGGMILVGR
DKENNRGIMTPKGDALRKWSEVIDSMIMPGIQGGPLMHIIAGKAVALKEALEPSFAEYID
RVLLNAKAMAKALSAGGARIVSGGTDNHLMLVDLTPLEITGRDAEKWLDEANITVNKNGI
PFDKKSPFVTSGIRVGTPAITTRGMGPAEMERIAGFIMDVLKSKGDAQVITRVKQEVLAL
TKRFPLP*
>SPBIB_v1_30012|ID:27161884| protein of unknown function [Uncultured spirochete bib]
MVLEGGVGRWYAEALLRSAASQHFFSFAILRKEEWVRVFRVQGVAEEEGGAHVDEGAPES
GAQKSQNAPDSGSASQNSENLWARYQLDSAGAMLVVALRYAPDVEPESDEERAVRGLPPL
PIAKEGRPAAAVGRFARANWYRETIARLADCVKSISDEARARGLPVFAPREWHRFSNSRF
PEKALAVAAGLGTIGRNGLLIAQRTGNAEASDSGCAETSSWPLWSSAVVLGLMLLPFDIG
KEAGTLHDEPIMPLALCGTCQRCIDACPTGALALAELPRYERARCIQNYTSGAGPLPEDI
EAAWGNQLYGCDICLEACPYFKPNSEACTALGRIGGAFDAAFIAAMSEAELRAVFKGSAL
DQKWIAPEALRRNVAIVASLFIGKSSDVPSRLL*
>SPBIB_v1_30013|ID:27161885| conserved protein of unknown function [Uncultured spirochete bib]
MYTLIYTASYEEKVRRFLKKHPEIEKQYIKTLKLLELNPFHPSLRLHHFKTSNFEGYSIS
INLMYRISLEFIMNGKEILLIDIGDHKAIYGKE*
>SPBIB_v1_30014|ID:27161886| Prevent-host-death family protein [Uncultured spirochete bib]
MAILVSANDLKVKGIRAIEENSSNDREVIITVRGKEKYVILPIETYNQLREYELDAAILE
AQKDYAEGRYHTGTIEEHLQRVH*
>SPBIB_v1_30015|ID:27161887| protein of unknown function [Uncultured spirochete bib]
MAESWKCANVQIQILYLQQKHFSIFVSEIDTKGLFCTCIRYIVLRVIRKEHSTKAKTVGE
KLFFADPAFYPALGGDLGTSREAIVAFFLEQAGWNVEALADEQAGDFAISRTGPDTRTER
YTLEVGGRSKKPKQADFVIRDDIDYPAGNAIPLWLAGFMF*
>SPBIB_v1_30016|ID:27161888| Lysine--tRNA ligase [Uncultured spirochete bib]
MEVSILQARSRMVTATRDFFLSRGYLETDTPALAPSLIPESCLEVFATEYVHPTKGRRPL
YLIPSPEIWMKKVIAETGRSVFQLCKSWRNAESVSRIHNPEFTMLEYYGVGLSSSDNIRL
TEELFAALATDETPPQARPPFRVMSMKEAWHEFAHTDLDALAEPEAMREACRERGLLVPQ
DAPWEDAFNILFLSFVEPALPQDQPLVLNEYPAGIECLAMDIPGTTYKERWELYVSGIEI
ANCFTEMASSDAVRAYFASQSEKKRQALVPHHIDDSYASIFEHFPPCSGVAVGFDRLLMV
LLGKRSLAEVMLFPFESFPETEQASLETLRNAAMHDSW*
>SPBIB_v1_30017|ID:27161889|efp| Elongation factor P [Uncultured spirochete bib]
MVRGGEIGIGTCLLINGTPHIVVEREFVSPGKGSAFARVRAKNLKNGNVITQTIKTADFV
EDAQVDLVDCQYQYNDGEFFHFMNNETFDQFEVPVAAHEDRGPYLKEGETYTIVMWEGEP
IDIKIPYKMVFTVVESENYVRGDTVSGATKPVTTETGLVVRVPLFIKQGEKILVNTESNE
YVERVNE*
>SPBIB_v1_30018|ID:27161890| conserved protein of unknown function [Uncultured spirochete bib]
VLDAGCGTGVIGISLAASCPDMEVVMRDRDFRAVAFAARNASRNGLAVKLQGLGGTELAQ
CAKRPFAKIKVVQRNAALIAAPGLLCEPDPNAPYEAVVANLPAKAGPALLSQYFVAVGRE
LLREGGTFAFVIVAPLAEQARAWCMEAGFEIQRTVATKNHMVCIARTGERRAGENAVKEA
LAELGRGKADQWFKAYVRSHVKKSIAGASLVWDGIQGLPEFDEPSFATLCALALGRQVFP
GSLVRRALVIEPGVGIVPLWCKGVLGSSEITLKSHDVLALAASGWNLRRAGFDNSVRILE
PAFAKKENSAGEPVLDSAERGMEVLAPFSIDAAILFPEDIPKFDSASYYWPLLSRALKRG
AVAVIASSATYIGRFVRQKPSGFSKLPQGEHRKGWEAVAFRRE*
>SPBIB_v1_30019|ID:27161891| conserved protein of unknown function [Uncultured spirochete bib]
MKKSEKLIIESATPDEYVTNSLKSRLKPAEKARLARMWMERTGYTRDDIIRARNRNAYWR
KRKMEGAAERTKRRMQEHDYSEGTAIEWTRERIEEFITLNRKDAYGRYIHRDWELAQHFG
TSIPSIQYMRRKYNKIRKMLGPGAKRDKVIDYMSCSELVLQHGGPKSRKRSR*
>SPBIB_v1_30020|ID:27161892|recG| ATP-dependent DNA helicase RecG [Uncultured spirochete bib]
VFLRELTQDVRILKGAGPATMRALANLGIHTVADLLLHLPRDYEDKTKIVPLSRFADAKV
FTTARVIRHEWFGFGRMRTLKILIEDESSEAALLCFNRPFLEQMAPVGSMIQVVYGKFQFK
YGEIQSSSFEIKRIDGELAAEKALSPVYPLTEHLNQMAMRKMVTLALNKFASHIEDELPA
YLLDRRQFLRKTDAIRAIHFPSSWEDAKRAHDALAYEELFYFQLGIALRIRARRAKTIER
KQSPGVLARRLRERLSYSLTDDQQAVLQEIVDDMHKPYPMARLLQGDVGCGKTIVALLAA

LYAVERGGQVAIMAPTELLARQHATIAAHLVEPLGVRLAFVTGSVSNAARPPLLSALKNG
DIDIAIGTHALFSEDVAFKNLELVIIDEQQRFGVLQRIALYKKGHVPDLLMMTATPIPRS
LALTFFGDLQVSTIKNLPPGRRPVVTHLAREDRCDRVYEFARKKLKEGRQAYFVYPVISE
SDRLGLRDAESMAAHLAKEVFPEFKVGLLHSRLGDDAKMEAMNQFAAGHIQVLVATTVVE
VGVDVPNATIMVIEHAERFGLSALHQLRGRIGRGTYQGYCFLIYSKELTEEGRMRLKALY
DTNDGFAIAEEDLKIRGPGDLLGIEQAGELNLRIADMRTDFDLLKEARKDAFAVIEKDPL
LAGPEHEAIRQVLTRANPLRER*
>SPBIB_v1_30021|ID:27161893| Thermostable carboxypeptidase 1 [Uncultured spirochete bib]
MHKETARLLELDREHSLVMHIGAILGWDQETYMPPKAIEERSSQLALLEGLAHQKAVNPE
IGELLAALEAKSDLSEDEKAYVRVVRRDYDRETKLPEAFVTEYAKEASLSQAAWADAKKN
NDFAAFKPHLSRMVELNKKRAHYLNPNAKPYDVLLDLFEPGSTQESVAAVFAKMKSYLVE
ILTRIRERPQIEDKCSGRRVDKHTQERISQYFMKVLGYERDRGRLDVSAHPFTTTLGADD
VRITTRYVEDYFPSSLFSTIHESGHALYEMGIDPNPDYRGTKLAEAVSMAVHESQSRLWE
NIVGRSPAFWERHFPALSALLGEVGEGLDLESFVKSINRVSPSLIRTEADEVTYGLHVIA
RFELESALFDGSLNVEEVPEAWRAKYRELLGIEAPDDRQGCLQDVHWSMGAFGYFPSYAL
GNLYGAQFWAALKKEIPDVEARISGGDTSAVLAWLRKNVHVQGSRYTPGELVKKVTGQAL
DPVWFEKYLREKYSKIYGF*
>SPBIB_v1_30022|ID:27161894|hisD| Histidinol dehydrogenase [Uncultured spirochete bib]
MEKYTLKRITIEEIPEPSMRLFDPEVDAVAIKAIEAVRSGGEVALRRWAQEFDGLAKGAP
LVIAREEMKAAYDSLPEETAALLGRAKGRVAAFAAAQRACLAPLDVPALGGRHGHEFVPV
ARAGCYVPGGAFPLPSSALMTVIPAKAAGVPEVWCAGPKPTRETLAAAWLAGADGFLQCG
GAHAIAALAFGIAVPRCDVIIGPGGKYVAAAKRLLYGIVGTEAPAGPSELLVIADESADP
DIVAADLLAQAEHSPDAMPALIATHSHIADQIDRILRAQLAALPEPNRSIAQRSLQNGFC
CIEPDPARIADGANHCAPEHLEIATSEPRALAKRIKNAGAIFLGSGSAEVFGDYGAGPNH
TLPTGGASRFAAGLSVLHFLRARTWLAIDDPAGLADDTAAFARLEGLEAHARAALIRTRR
SSSTSLAGTSRTRPGPAPAR*
>SPBIB_v1_30023|ID:27161895| conserved protein of unknown function [Uncultured spirochete bib]
MIVPSIDIMNGRAVQLRGGKQPPLDAGNPLELAERYSRVGEFAVVDLDAAMGKGSNRELV
LSMCKRWPVRVGGGIRTQKLALEYLNAGARYIMLGTAATPEFCAQLPRERLIAALDSRND
TIMVEGWTKPREGSVEEAIQKLAPYVAGFLVTFIETEGDQCGIDIERAKRLVKLAPDRKF
TFAGGAAGGERGLADITALDAIGADIQAGTSLVLGDLSLADAFAAPLHTDRPDGLWPTLV
CDESGKVLGLVYSNRESLKHAIESGKGAYHSRSRGLWVKGESSGNGQTLIRADLDCDRDT
IRFTVRQEGVGFCHLKRRNCFDDGYGLEKLNRTISKRMKDAPQGSYTRRLFSDEALLASK
LREEAQELAQSKTKDEAVAEAADVMYFALVKTISMGGSLSDIEAELERRSLRVTRRGGNA
KPEFEGSEGKNEWKSIH*
>SPBIB_v1_30024|ID:27161896|hisF| imidazole glycerol phosphate synthase, catalytic subunit with HisH [Uncultured spirochete bib]
MSGLAVRIIPCLDFKDGRVVKGVRFENLVDSGDPLERALLYEAQGADELAFLDISATVEG
RKTMAKEVARIRKAIAIPITVGGGIASMEDAARLLDSGADRVSVNSAAVREPAIIEAIAR
RFGVQCAVVAIDATKSPAMPSGYEVKIAAGAVATGLDAIEWASEAAQRGAGEILLTSIDR
DGTNLGYECNLIHLVSRAVPIPVVASGGASKPEHFLEGYRNGALALLAAGIFHRSEVSIS
EIKQYLSAHNVEVRL*
>SPBIB_v1_30025|ID:27161897|hisH| Imidazole glycerol phosphate synthase subunit HisH [Uncultured spirochete bib]
MSQIRTPSLVIIETGVANLASVRALAERLGLQPIVTSDPEEVVRAPFVLLPGVGAFGPAM
HKLRERGLDAAIRSRVAEGRPIAGICLGMQLFFEASEESEGVQGLRLLPGKVKRLSGGLP
LPQLGWNRIEPEPGSRLLLAGWVYFANSYGVGIEQKFNDGTADTSKAAFPRFDSISIATT
TYGSAFISAIEAWRDDRPVLLLCQFHPELSGSFGKNLFARWLELASERRRV*
>SPBIB_v1_30026|ID:27161898|hisB| Imidazoleglycerol-phosphate dehydratase [Uncultured spirochete bib]
MSEQSIQNEKGGQSQIARLRRTTRETDVTIALSFLPGAIDIKTGIGFFDHMLTQLAFHAG
WSLELSCKGDLDVDDHHSVEDCAIALGKAFAKLAPSGAGRQRFGYAYAPMDEALARAVID
ISGRSYCVLSGTFTSPSIGALSTSNIAHFFQSFAANANLTLHLDILRGENDHHKAEALFK
AAALALKSALEPRAQAPSASTKGETTFSTPEEGK*
>SPBIB_v1_30027|ID:27161899| putative Histidinol-phosphate aminotransferase [Uncultured spirochete bib]
MLRLDANEGRCLLSEEDLKEILSPEIARRYPVRSLLEEPLAEMLGLPVSCVLATAGADDA

IDRTVRSLAGPGGLVMSTSPGFVEFLEAAKRSSAMFESIPKDPLGAFPVAEICEAVKSRS
PQLLILASPDNPSGCVLAKSELEAVANACREAGTILIFDATYSDFSLRTAKPVDAIAFPN
VLVSGSFSKSRGLAGFRIGYIAGGEEAAPLIARLADAGPPFSLSSAAIEAGRRALSLDSS
TVQAFVDEVRREVALLTAQLRGLGFVVSDSEANFVLLKSSASARVAEMLRERGILVRTWK
GKTAYEGLVRITVPGEKSEFEKLANAIHTLARFKEAAE*
>SPBIB_v1_30028|ID:27161900|hisG| ATP phosphoribosyltransferase [Uncultured spirochete bib]
MREKKNERTELRIGLPKGRMQAKVIELFNDAGIPVSVDEREYRPRVGNGKPQALISKGIA
YEAKLLKPQNIVEMLATGSRDIGFAGADWIRELGVDLVELLDTALDPVTIVAAAPEAVAE
AYGKCKGNIEVVKKVCGRTPIVASEYEGLAKEWIAKNLPGAAFVRSYGATEVFPPEDADI
IIDNCATGTTLRANKLSIIDTLMSSSTRFYASKEAMQDPVKREAADSLILLFRSVLEARR
RVMLEVNISAELLDQLVAILPCLRAPTVSPLGKGDAFAVKVAAPRDTLASLIPEIKRRGG
TDIVVTQMSQLVP*
>SPBIB_v1_30029|ID:27161901| protein of unknown function [Uncultured spirochete bib]
MTDTRHPGFLTPERHQRGEDAASIIYPVVSRRARGLSLGINLFPDSKHCTYNCPYCEVQP
FSNPHARLSEGMIEAALREFFDKDWPAYAKSFALEDISISGNGEPTCSPFLEEALYAAAK
VLSEQVSLDAKLFHVPIVLITNSTGFLSPQICELLHRFSQEARFEIWAKLDGGSSELHSI
LSRSNYAFDRIVGDIAHFAAITPVKIQTMICRDACSGRVLFDAERYCGALMSLIGQGARI
NAIQLYTTARPPAEPWIASIDDIEMAMLASIVRRCLPSEIRLECFGRSGELSIPNSGQEQ
WPK*
>SPBIB_v1_30030|ID:27161902| PHP domain protein [Uncultured spirochete bib]
MAQVDLHTHSSASDGELSPSELMALAHKLGLSTIALTDHDTVAGLAEAAQAAHAHGIECI
PGIEIEIDFEPGEFHLLGYGIDPQSAPLLDATQRLGNARHERNEAILELFANAGFQLDTI
KISELSRESYIGRPHLADLLVKARCVRTRQEAFDRYLGKGRPFYIKKSCLPLEEALSVIK
AAGGVPVVAHPYSLFVSKSMLGTLFSRWKDMGIEGLEAYHPTAKFGQCVILERMARERGM
FVTAGSDFHGKARPECGLGRTAGNMAIDSCYRDALVSALSHPAAQK*
>SPBIB_v1_30031|ID:27161903| conserved membrane protein of unknown function [Uncultured spirochete bib]
MVVLIYPEITKTIIISTMKTLNPRKIAFLLFFLLLFLLVARLFYPFLTVILWSGLLYVFL
EPLHKKLSGSVRVNGRKSFGSHLSALVLSVLGVLILLVPMAFLAVAVTRQLADLLKSAVH
FFETNMDKLRIDPQSQLGATIQSFLGDSFDIRTLDPIRELQSLLAAGANQALRLSSSLIK
NVFRFVIAILFIMFTLYYLLMDGNSLGETFVSMMPIDPEHTRLFMRKLRETGRQLVKGYF
LVALFQGLMMFILSLIFGFRNNLVLAVLTAIASFVPMVGTSLVWFPMGIFIAASGNIGLA
LVFLITAGILVSTLDNFIRPVVLGGQLKVHPLFLFFSIAGGLVVFGFNGIILGPLVLMLF
IAAGDLYRSISVEEDEGAKKSTELTSAPPDEKAR*
>SPBIB_v1_30032|ID:27161904|rsmI| Ribosomal RNA small subunit methyltransferase I [Uncultured spirochete bib]
MQYKTVSTLYMVATPIGNLGDITLRGLETLKAVDVVACEDTRHTLKLLSHFDIHKPLVSC
HANDEQRGAERVAALLDEGKNVAYCSDAGTPGLSDPGAVLVREARSRGHVIVPIPGPSAF
ATLISAAGISGRTFLFDGFLSPKPGKRRARLKELLARGESFVLYESPFRVGKLLDDIASI
EPERCICIGRELTKIHEEIVRGTASELAPRFPADSAKGEFCLIVEGRGSEQPDSKRGKDS
*
>SPBIB_v1_30033|ID:27161905| tRNA/rRNA methyltransferase (SpoU) [Uncultured spirochete bib]
MMDLAHLLNLHGRARVRKTVLILERIEHESTESIHAFPLLYLRELAVMLKESPESPDSLV
QAAGSFLQAIDREQLSSEGRDNSDTRSMLRALNQFRHALMRISGQTPADWDFVNAPRSSS
GFAQRKAGMRVYLEDVRSPFNIGSIFRTAEALGFEEIILSPECADPMHPRARRSAMGTIE
RMAWRRAPVSSLAEMEGVFALEVGGSALGEYTFPLPGVMVLGSEELGISSDALQYCTAGR
VEIPLVGAKASLNVATAFGIAAFAWFS*
>SPBIB_v1_40001|ID:27161906|ycjV| putative sugar transporter subunit: ATP-binding component of ABC superfamily transporter [Uncultured spirochete bib]
MAKVELKGMGKVYEGNVRAVTNANITINDKEFVVFVGPSGCGKTTTLRMIAGLEDITEGE
LYIDGKLVNDVPPKDRDIAMVFQNYALYPHMTVYENMAFGLKIRKLPKDEIDARVKEAAR
ILDIEKLLDRKPKQLSGGQRQRVAVGRAIVRKPKVFLFDEPLSNLDAKLRVQMRAELIEL
HDRLQATMIYVTHDQVEAMTMGDKIVVMKDGLVQQIGSPLYLYNNPINKFVAGFIGSPPM
NFMTVNVVEEGGQIFIDEGSFRLAPDEKQAGLLKPYVGKSVYFGIRPEDLPVADAAHRGQ
KFDAKVTVVEPLGAEIHLQATTPTQVMVARIPPHHLYKHGDTITFAPVMAKAIYFDKETE
KSILPVKWNEQEE*

>SPBIB_v1_40002|ID:27161907| Major facilitator superfamily MFS_1 [Uncultured spirochete bib]
MDRKKGFGPYLGITFLIGFGFFTMGLMDPLYDTYVPIFLSKYIGSMSLVGFIMTIDNILA
IFLIPIVSAWSDRTNTRIGRRMPYILVLLPLTAIFFGAIPYAAGASLAALIAILLLLNVT
KQSVRGPVVALMPDTIPADYRSEANGVINTMGGIASIVGTIGLARLMDLDTKLPLLGNTK
DRLPFPLAGLFVVLAIILLFAFIREKKPDQTETSEQKIPMIESFRNVAAQKDKSALYILI
SLFLWFLGYQGVLPFIGKYSVDVLKTSSGTAALAAGMVGIAYAIFALPSGYVAHRVGRKK
TIRASLLVISILTAILFAHPWLTAAMPGSLKLASFWAIMFLFGIFWVSIITNSFPMLWQM
AEWGTIGIYTGLYYTASQAAAILAPILTGLIIDVFGYSGIFLFCTVCMLAARFVMGKVTK
GEPAESPAEA*
>SPBIB_v1_40003|ID:27161908| conserved protein of unknown function [Uncultured spirochete bib]
MISQKIPHIRRADLSVTAQKNLDARLRRLVSRLPGSIPGKMTGSQAELHYFSSPGRTELG
GNHTDHNHGCVLAASVDLDMLAVARARADRIVTVTSEGYDPIIVDFSDSAARMHEQGTPA
AIIRGLAAWLERRSTPLRRGFDIAIDSEVPAGSGLSSSAAFELLIAAIFDDFGGLGLSPV
EWAIAGQFAENEYFGKPCGLMDQIACAVGGIVSIDFAEPAQPLISSIAYDFASRNLALAI
VDTGSNHEDLTREYAAIPNEMKEVAAIFGKQTMREVEKADLVARAAKIRAQCGDRAFLRA
WHFVHETARPALMQDALERNDLASYLSLVRESGRSSWMYLQNIHAGDPRQQSLAVALALS
EDLLGSEGAWRVHGGGFAGTIQVYVPEPEFPAFSGRMEAVFGRGCVRRLNIRPFGMCRII
ED*
>SPBIB_v1_40004|ID:27161909| conserved protein of unknown function [Uncultured spirochete bib]
MSKEYLANIHKTPNTEKAAYISPHAVIRGAVTLGQDVSVWHGAVLRGDMAPIEVGARSNI
QDGAVLHVAENMPCIVGEEVTIGHGAIVHACTVGNRCLVGMGAIVLNGTVVGDECIIGAG
TVIPGGKNIPPRSMVLGNPGKVTRAITDEEAIHLREQAYTYIALARETAIEERKGSL*
>SPBIB_v1_40005|ID:27161910| protein of unknown function [Uncultured spirochete bib]
MMRXSLYLLGHNYLKPFRIRAXKGXHPRXHAEAAGIPXXLVQHFVQALXGXIXAFLSRCX
LSETMXRTWEKRWKTPGKTKAEYIPKYALA*
>SPBIB_v1_40006|ID:27161911| exported protein of unknown function [Uncultured spirochete bib]
VLHKXXRNPRCLSMXTRMXSFXCANTKGLEVVVAKEVQAXAHHAVCDIAVETDCFPVVVR
EVFAQLPVDVVGRKKGIAEX
>SPBIB_v1_50001|ID:27161912| conserved exported protein of unknown function [Uncultured spirochete bib]
MKKILLLAVALIAVSGIAFAQVDLSKVKDGVYFAQDNGFSSSGWKEQVILEVSKGKIVRA
VWNGVSNLPGATDKKTYAASGKYGMIKASKIKAEWDTQAKTVEDYLVKTQDINFSKFDAK
GHTDAISGATLTVKGFFELAQEALKSSPVEKGSYKDGWYYAEDLNFDKSGWKNSVLVTVV
NGTIVDVVWNGISKDPQAKSKYVQSQLGTYKMNAKNGEWYVQADRVAQAIVKAGDPAKIP
VKADGKTDAVSGVSITVQGITLAIEALKAAK*
>SPBIB_v1_50002|ID:27161913| Aminotransferase class I and II [Uncultured spirochete bib]
MNQLADELNALLSGTVADRLLSDMGRRMYFPKGILSQGAEASERAYRFNATIGMAYEHGQ
PMMLNALRKELPGLTPTEAVAYASTGGVQKLRSVWKDSLYKKNPSLAGKRISLPVVVPGL
TAAVSYICDLFVEPGDTVIVPDLHWPNYRLIVEERKTAAGITYPLFSGEGYNVEALLDGV
RQAGQRRGKAIIILNFPNNPTGYSLTLEEADRLAQGLLEIAQSGTDILAITDDAYFGLQY
EPNLLRESMFARLTDLHPNILAVKADGPTKEDYVWGFRIGFVTFGGCGLSDEQYDALFKK
LAGIIRSSVSSSSSLSQNLLLKALQDSGYDAQKEVYRATLERRYRAMKNKLSTMQLPEGL
VPMPFNSGYFMSFHCQGFSAEALRKKLLDEYGIGTVSLLDRYLRVAFSSVEETDVDELCE
AIAKAAASLISKG*
>SPBIB_v1_50003|ID:27161914| exported protein of unknown function [Uncultured spirochete bib]
MKCKAIPRFALAAGAALFLWLSCQSACAQSAHAESIPQAPPQAALRVATWNVHDCSASNK
KTGQTFLLHAQIARTLHELKIDIIALQEIQVEGRKGADIQLLQNALEHEGWPMSYAAWAK
SPQADDLAILSRFPIAQNQVVLEPSKAPWPRPVLEAHINVAGRELVLYNAHFKAFDDAKS
LAARRAQASALASLLRAQYGDRIKGAAIILAGDFNTIMPEDMAPNTGTLDMLQLRDNEDP
SDDFLSASLKWRYPEPTFISARYSSITDHLIISPALAAHAGKNDIAIVDAPDSGYGFPLS
DHKILILSLLISDLPLLSR*
>SPBIB_v1_50004|ID:27161915| Orn/DAP/Arg decarboxylase 2 [Uncultured spirochete bib]
VKTAYSFPLENFMSREKFERIKEFAKDKETPCLIIDLDVIRKNYENLQTYLPWATIYYAV
KANPDDAVVSLLRDLGSNFDVASRYELDQLLRLGVSPDRMSFGNTIKKEKDIAYFYEKGV
RLFVTDSIADIDKLSRAAPGSKVFFRLLTEGLGADWPLSKKFGSHPDLARQLMKTAMRFG

LEPYGISFHPGSQQRDVGQWSSALAIVAQLFNWARHDLKVDLKMINMGGGFPANYLEPTD
SLEQYAQDIKRFLDNSFGLVWPEKIVIEPGRSMAGDAGVIVSEIINIAKKSVHERYPWVF
LDIGKFGGLIETLDESIKYPIYFEGQGSVEEVILAGPTCDSMDILYERTPYFMPSSAKIG
DRVYILTTGAYTQSYSSVYFNGFPPLKSYILPPA*
>SPBIB_v1_50005|ID:27161916| conserved membrane protein of unknown function [Uncultured spirochete bib]
MIGFFIKKSFFDGWDNLYLLAALNGIFLLVLLLFFAVPVALGAPMWLIIIAVACAIAVLS
IWDVAVSNAMYAIADGKSVHFADIKAAIPRSAKLGLVMGGINIIYGVALTVALPFYLSQK
AMWGVFAGGVLFWTMILAMLILQYVPAIFARDGGTLRQIFRTSLYLFVDNPGFSIFLLLW
RIVTLAISALTMLLAPGPAGSALASAVAVRLRMKKYRYIKDNPGADRRRIPWNELLLEEK
ELVGKRTLKGMIFPWKD*
>SPBIB_v1_50006|ID:27161917| conserved protein of unknown function [Uncultured spirochete bib]
MIEKSRREPGMKPPARGKGHAALNPQKKVRSAEHGENSSTRPLLEAFEERKKLALSSIVF
IRFDGKAAQSHIPEGIDPSIPYPVQLQAPMVKLNPASITPESLLTGMLRVLAWDPDNANG
ATYRAYVRAVRPELFEELIAAGVQKAESKEWLVAEEIFFAASGLDPERPEPFINLALMHE
EHAKQLSDAGDEAQAEKEDELAHKYYQHLLAAKQAFPPAYYHAAFFFLRKHNYDRTVSLL
TSYIGMNDDEERTNKAKSILEKLSSMGYLDTLFKEAYDFIQMGEEQKGLERAKQFVDKFP
SVWNGWFLVGWAHRRLGNWSEGAQAFATALEKGAEGADVFNELAICQMEMGDLEAARSNL
ERALRLEPENVKIIVNLGALSYRQGRYKEAEGFFRAALEFDPEDRIAKEWLKRFDSAGGS
AESTGEQNQ*
>SPBIB_v1_50007|ID:27161918| putative Calcineurin-like phosphoesterase [Uncultured spirochete bib]
MDHDEESSNGTLPASQELEKALEAVLAAAGTIPSSIRPRAPKGSYPGGLIQLPSDKPCVL
VPDVHARPDFVESLLLSVFPGIEGTLDAALGKGLATLVFLGDIPHAEGELAARRWNRAYE
RIVREHDAHAILCPEMDEEMGLSLAALLEVIDLQCRYPSSVFCLKGNHDNMTNAADHGDL
PFYKYADEGRMGALWLEMRYGEKIARLMRRYELSLPVVACSKNFCASHAEPAFPLDKERV
ISFREHSEVVQALIWTANDEAKPGSVAKSLEALLDSMEKAQHSVWFSGHRPVRDNFAIRA
NGQLVQIHNPDLWHIVFLSGNAKQADFYAVQRSGGEAAHVCSLILDPERRRK*
>SPBIB_v1_50008|ID:27161920| conserved exported protein of unknown function [Uncultured spirochete bib]
MAVRKKKRTSAKTGCLLWAVAFLVLLVLFLVKFNDIRSAVQKTGFLDALNHAVSKPASQE
KPVQPSAPASPPSAVTQPPGSQPAITQGLPKDAEKPGQTPPQSAPQPEQKPEAQTSPPSP
AEPGQQIQKTRSAVLYFVHIGEDGTISSERVKRVLPLTDSPIQDTLETLLKGPTESELRT
NLISLIPSGAKLRGISMRGSTVLVDFSDGFTYNRYGKEGYLAQLKQVVFTLTEFQNITDV
QFLIEGKMRPFISEGVPLDKPYSRASF*
>SPBIB_v1_50009|ID:27161919| protein of unknown function [Uncultured spirochete bib]
MFVLPVSVQAEERSEEVSAQVFPHLSVALELLQAVNPVAESQQMAAKLVRTAVLVFPERQ
VSKQRDSMHPENRFSVPRSEYH*
>SPBIB_v1_50010|ID:27161921|gcvT| Aminomethyltransferase [Uncultured spirochete bib]
MAPFAGYDMPITYPTGAIEEHLVTRRSVGLFDIDHMGQLEISGAGADAFVSRMVTAKVSD
LVPPMARYSLLLDEKGGVIDDLFIYRLPQSWWIVVNASNRAKDFEWFKAHAPEDVAVVDR
SDDTYMIAVQGPRAIELIDKVANMAVSSIQRFNWGNISIDGVPVLFGRTGYTGEDGGELF
FPAAEAQHVWELLLAKGEALSIETRPIGLAARDSLRFEAGMPLHGHEISTEINPIEAGFK
WACDFEKEFIGKEALLAIIDKGVSRKLVGIEVFGGVPREGYEVTSPDGKDIGHCVAGMFC
PTVKKYAANAFVLPDYAKVGTEASVVIRGQPRKAVVVKRPLYLPAYRR*
>SPBIB_v1_50011|ID:27161922|gcvH| glycine cleavage complex lipoylprotein [Uncultured spirochete bib]
MAIDKNARYLESHEYAKPEGGNFVIGISDHAQAELGDVVFVELPAVGKTIAKGATFGTIE
SVKAASDLYMPVSGTVVEVNEAVKNDPALVNKDCFGAGWLIKVAPSNPAEFDSLLDASAY
GKAIGEE*
>SPBIB_v1_50012|ID:27161923|gcvPA| putative glycine dehydrogenase (decarboxylating) subunit 1 [Uncultured spirochete bib]
VPFIPNTDAERTEMLNTIGVESVEELFSDIPAHLRIQKLDLPDGVSEMEALAALDALAER
NAQTTKMDWFVGAGAYNSYIPSLVPALASRGEFLTAYTPYQPEVSQGTLQAIFEYQSMAA
KLLGMPVVNASHYDGATALAEAVLMAWKAKEGRNRILLPADLHPEYAKVIATYLTSFDIE
LARYDGDPERASLDERTAAVVVAYPGFSGEVYPVEKAAARAHAAGALCIVQADPVMCAIF
KSPGEQGADIVTAEGQSLGNPLNFGGPYLGMLGTTEALLRRMPGRIAGETKDKEGRRGFV
LTLSTREQHIRREKAVSNICSNQGLSMLQTCIYLAALGKQGLRHVAKQSYDKAHYAAELI

GRIPGFRVATKQFFREFLVQTPKPAADIARALAKKGIVPGLPVSRYFPDKPNELLVCVTE
MNSRPQIERLAIALKEASK*
>SPBIB_v1_50013|ID:27161924|gcvPB| putative glycine dehydrogenase (decarboxylating) subunit 2 [Uncultured spirochete bib]
MNTMNMRDEPLVYELSRPGRIGCTLPRSDVPEYALPEGLVRTDLDLPELDELTVVRHFTR
LSQKNFSIDTEFYPLGSCTMKYNPKANEAAAAHSGWKNLHPLVEPEHAQGALELLWRLQE
ALQKIGGFAAASLQPAAGAHGEFSGVLMIHAALKDRGELGRKKMLIPDSAHGTNPASCTM
AGFVTQTIPSGPDGNIDIAALRAALDDTVAGIMITNPNTLGLFETNIEEICRLVHEAGGY
VYGDGANMNALTGVFKPGSSGIDVMHFNLHKTFSTPHGGGGPGAGMIAANEALAPYLPGP
VAVKRGESYRLEMPAKSIGQVKAFYGNFGVLVRAYTYLLMTGGNGLRRVAENAVLNANYL
KALVEGVYPVPYKRHCMHEFVAKGTIVPGVHTLDIAKRLIDYGFHPPTIYFPLIVQEALM
IEPTETESKETLDQYADAMLKIAKEAAEQPELLHDAPHNAPVARLDEVMAARCPILCYRG
*
>SPBIB_v1_50014|ID:27161925| putative undecaprenyl-phosphate N-acetylglucosaminyl 1-phosphate transferase [Uncultured spirochete bib]
MLWLSLATAVVLSSVLMPLIIQFCHRHQLYDRLNDRKVHSDSKMPRLGGIGIALAFILSI
VIIRIFQIDEFHQLYDGYRLWPIIAGASIIFLTGLLDDLIDLTAWQKLTLQSAAAIMVML
AGYRFKFLVVPFGSGVWNFGLFSYPLTFLWIVGITNAINLIDGIDGLAGGISGLAALAFA
LFFMFTSNVLPAEICLALVGSIIGFLIYNLPPAKIFMGDSGSLFLGYVLAIIPLLSQYKG
FQGTDIGVLSAATVLSIPIFDTLMAMYRRFRAHKSFFSADRKHLHHQLLDRGFHTSEILL
ILYSLTVVLAMVSLSMLFIPLSWSFVLNVAMFGAMLLYFLRFSRPDREKNMHGPL*
>SPBIB_v1_50015|ID:27161926| protein of unknown function [Uncultured spirochete bib]
MLIIVRSHQILQCSLNILLKKPRAQRCRFVSPNLKQSGQSRATFYVRRERMSAIITHRRL
LQVFLRLAILGLVLASMAACAIEAPKSQGTLRIQCILPGEFTHYAAKGIPKGGAPESAAH
SSAALRLIGPQSGWQIASYRLTCTSPEGEIITKTLGRASGDITLKVGGWNILAEGLAADG
SIIVENQTQIDVSAGRTINLPISLHLAKGKGSVQITFSTTQTPNASWKYSLSLEFLGLPG
DATAEGPPAYTAEFAATEQSFNAADLPSGYYRLTVQLKDNTQATLAGSVETILVLPGRIS
AGNCDIVLADPSIDISLSVPLLELSAKAAIGVDRFLNKNKKMLVPLAVAEENANLGIDWF
ANGEAVGGGLETGNQILTGYRLHFGTIDAPGAFSFLKMDALLTDPDTKLAQVVTQKSTNV
SPPLSAHAEWIQSMDYRAALTKALHDNGDPANQGTGVPSDAKWVTASTNGFIAIAGLDKS
SAVHLFFSTAGQEATDSNGIPVVLPAESGWIRLWRDKVVIEKSERSPDRVSLSANGLLLA
AGSSSSNWLRVYVLDTAGAIVAKKDIVSGKNGAPAFANIKALKFSPDTKKLFVLTNSPEK
ILVFNVENLTAGEPVLEAEFLFASCFENPPSSSLGMEDLALLSDGWIAACSSNIARIFLV
HYSDNQFSSATTYASGANGESLGDPKSIVFDEQNTLMYVLGYSKKLHVFSKIDGYTGYQP
VSTISLTNEFDKARSLALARIYPSSSFLVAGGGQSMGIIALDAAGQPIAQSSLASVDANY
AGIASIMNIAALGDTIVAAAGTSGLASVFNIF*
>SPBIB_v1_50016|ID:27161927|glgA| Glycogen synthase [Uncultured spirochete bib]
VKILFVSSEVTPFAKSGGLGDAVSSLATALSRAGHDVRILLPRYYFISKNTLRRTNGLLE
IETAREHYHAFLYTTAMPNSQVKIYFLDCEPLYGRNGIYGYSSAQEFPDNPARFSVLSKA
AFAACRSLGWIPDIMHANDWPTSLVPVYLNTTEKNTEFSDTAGVLTIHNLGYQGIYSRDH
FPELGLGWEYFYGAGFEYYGNVNFLKAGIVSAECITTVSPTYAREIQTPNGGFGLDGLLR
QRSRDLVGILNGIDVDIWNPAADPYIPAHYSYKDMSGKAACKAALQKELGLPSDPNVPVI
GMVTRLVEQKGISEVFGRTYGCMRKILETIHVQVAVLGSGDAWAEEAIMNYSAQYPQFKG
VVGYNERLAHLIEAGADFFLMPSRYEPCGLNQMYSLVYGTLPIVHRTGGLADTVVNYNQE
LGEGTGFMFDDLTPQAVYDTVGWAMWAWYNKLQHIQNMRVRAMQQDFSWARSADEYVRLY
EHAISLRKAR*
>SPBIB_v1_50017|ID:27161928| conserved membrane protein of unknown function [Uncultured spirochete bib]
MSERKTLDYNKNEVRERLLRTFQSGTKEWAAADLARATGLPLAQINAEMPAIADEYRGRL
KVSDKGDILYSFPNGLKSRYRGFGPFMRKLGRALGRGAAEAGKALFKIWILVTLFGYFFL
FIALALVAFFGSIAVQQGGGSRDRGDRRGGGGLGGLWLTTSLFDSMIRLWFYSELFKSSE
ARYRDSFARRERHPLHKAVFSHVFGDGDPNADWDTVLKKAFVAFVQTHKGVITLPEFMAI
SGLKPQEAEDRITRFLVEFEGSPEVTESGALYFFFPSLLAKAGNVSAAAASTFPLKKLKS
FSSNNKKMDRTFRWVNIVNLLFGSYYAWNAMNIGTDVIVRTSQGLSLRGGLTFLYSYTVY
LASQLGAAKPAVVIGWLLGAAPLAFSVLFFAIPLIRSWRLKRENEMLKRENMRKMLYSGV

LSSPQGFDHRAVALPTEEVRPANPKSVEEEIQHLAAWSSAEIRADGKWFFKDVEFVQKEV
EHVRASIKESDYAADRTIFDTNAPV*
>SPBIB_v1_50018|ID:27161929| protein of unknown function [Uncultured spirochete bib]
MRNIMLFQAMQKIGARNTSIISTIEPVFTNVFSMMLLGEILTGQRIVGGVLILAGVIVLD
RLGYSPDSTISATSSIGRSSPISKSPGTDEGKS*
>SPBIB_v1_50019|ID:27161930| UvrD/REP helicase [Uncultured spirochete bib]
MMADRIPDYLQGLNPEQLEAVRFDGTRLLILAGAGTGKTRVITTKIAYLVREKGIPPESI
LAVTFTNKAANEMRERAMAIEPACERAIIRTFHSFGAWFMRRNAQAFGLRSEFTIYDADD
SAELVHSVFPAFSRRDCSDYAFLIAKAKDYCLAPDSPTLGFISQHPEFRRIYAAYEERLR
ATGNVDFGDLILLPALLLEKDEIIRNRTRQRFRVILVDEYQDSNVAQFRLLQQLASPEGM
LCVVGDDDQSIYRFRGAEVRNILNFQTIFPETKIIKLERNYRSHQRILDLAHSIVVRNMS
RLGKKLLATRPGGTKPKLAILDDQDQEIEFCASIIRQHVQNGGHWKDIAILYRTNAQSLG
FERLFPKFDIPYRIVGALRFYEREEIKDMLAFLALLANPRDEIAFKRIINKPSRGIGASA
ISAIVEYAMQKQIDLLEASRDRQAQAHGKGKEGLKYFVGLMDSARSMLMSGLQDENHPKD
LGALISDLAQRSGLIEFYRSKDRIAGTQKEENIEELVNAASEFPLSEEGLLEFLDTIMLD
QSQQNGDSNADSVTLITMHNTKGLEFPIVIVTGLEQGLFPRNDEEGDDLEEQRRLFYVAV
TRAKEHLYLSACRWRRLHGRLFETMPSRFLTEIDPELYELWGASRRFAGAQRQPDLPSAG
SETGRRGYGQSMYVQSAANEYRSQEPAPKPEKPEWEPGMSVYHDEYGSGTIIKVTPSQSA
GLLVIVRFDSGKIAQFFPKYTKKLEKVEH*
>SPBIB_v1_50020|ID:27161931|rimO| Ribosomal protein S12 methylthiotransferase RimO [Uncultured spirochete bib]
MPAFFIDQHGCAKNQVDGEEIASRLVDAGFVAVDSPENAEVIIVNTCGFIEDAKKESIQA
IIEAKRQWPEKKVVVAGCLSQRYPDALFQDLVDADGIVGNADLSLIPDSIQKVVEGERVL
VCPPQQASLPSAYYPRKALFDFPGTAHIKITEGCSNHCTYCAIPLIRGELRSRTIEDIVH
EARTLLERGIFELVLIGQDLGNFGRDLKGRCLLPDLLEALARLEGNFRVRILYIHPDHFP
ETILPIIREDRRFAPYFDLPFQHASEPVLRRMNRKGSATAYLELLASIRESLPDAMIRST
FLVGFPGETEDDFEELLQFQKKAQLDWLGAFEYSREENTPAYTMKNRVPKSIAKKRKALI
EEVQTAITGAKLQRFIGSNDTFVVEEVFDHDDLCIARGWMQAPDVDGVTLIHSRQNPGSA
VHARIISVNGVDFNAILDYTAEP*
>SPBIB_v1_50021|ID:27161932| protein of unknown function [Uncultured spirochete bib]
VVMGKVGNIFRDTRLGKGLTLDQVADETNISKRFLQGIENDNFDGFPGEVYIIGFIRNYA
EFLGLNPAETVARFKAAEPEPEPPSQDKSPEKPSSETPEAIEPIAKSPDTQTSAETKPAR
KEPEVPLLFTEESEEAEAKASTLSSEHAKSGKMPRTSKKQAVAKHPSEPEKAPPAAGPAS
VTESEAPLAKAQPSLTDSQKEKIALSHQPFFPARKNQKVHAAPIQLGHFLLYALAILIII
VGVVSMLPRLKLPSRASRTPADYRAEGLPFEQRLYPQDRVYLPLGNDFISVTLKSVKDKV
VLDTPYGEFTLGLNEETVINPTADKDRLIVSIIDYAPNEPQNGALAHFDVKEALSQQETS
SDITIPASAAAPAASKAEPAPIVLFKSASGPHPFYVNVSFVSPVMFRYEADRKEWVEKYY
RKGESITVNASNSITFWTANAQAVKVSVFQSAGKSTELMMGGPGEISVQRLSWSNSQGGW
ALVAAPLD*
>SPBIB_v1_50022|ID:27161933| Outer membrane lipoprotein carrier protein LolA [Uncultured spirochete bib]
VKRKHFFVLMFIVGFYMALSNIGAQTLLTADQFFSKLADRYASVTDYQADVQITAGRQPM
TGKLIFKSPTLLRIDFSQPADQVIVFDGKTLLVYLPQYRAVLRQDASDQGISLGAATLAS
KEGLSLLRRNYTIGWERSPNEEPLDPGSTEQVYRLMLSRKSVSEGYKNIRLSVSGDTMLI
RRLEGWTVSNDKITFDFQNVLLNQGIRDDRFVYDAPASANVYNNFLFQ*
>SPBIB_v1_50023|ID:27161934| Pyrophosphate-dependent phosphofructokinase [Uncultured spirochete bib]
MLERSPLHTARYAYTPKVPEIFLEPLPHIKALPSEVIEPDKDKELLKSIFPNSFGKPAVK
FVKGEGVPSEKAFTVGVILSGGPAAGGHNVIAGLFDGIKKANPKSKLIGFKGGPSGLIDG
KYLEINADMLEQYRNTGGFDIIGSGRTKIETPQQLEKSFENAIKLGLDALVVIGGDDSNT
NAALLSEYFIQKGAPVTVVGVPKTIDGDLKNEWIEISFGFDTATKVYSELIGNICRDASS
GRKYWHFIKLMGRSASHIALECAFQTRPNIALISEEIEAKQMSLAQIVATIADAIAARAA
KGENFGVIIVPEGLIEFIPEIKALIAQINEIIAAAGSEYESLPRGEAQRSFILSKLPAET
AALFSQLPESIQTQLCWDRDPHGNVQVSRIETEKLLVEMVSRRLKEMSAEGTYTGTFDYQ
THFFGYEGRCAFPSNFDADYCYSLGYSACLLLILHGLTGYIAAIRNLAAPPKEWIPGGIPI
VSMMNIERRHGKEKPVIKKALVDLEGKPFKAFAQVRDKWALSSDYRIPGSIQYFGPPSIT
DVPAIILQLEQNS*

>SPBIB_v1_50024|ID:27161935| putative LysM domain/M23/M37 peptidase domain protein [Uncultured spirochete bib]
MARRAVVIAISLLSLFFMPLGLCGAEQEYHVIQSGETLYSIAKSYSVPYEMLASINGITD
PSKIRPGTVLLIPLVHVVAKGETFFGIAKKYDVSIQELKTANSLSDSYVLRVGDVLVIPD
KGVAAATPSQAAPAQPAPVAASPADTAPPSSAKPTSATPAASIPAAPPSAPAAASPADTA
PPSSSNSNSKNASAASRTPDALSIWPVEGKGQYMSGKLEGIMFQTTRGAPVKAVASGTVV
SAGPSRGFGDVVFIQSKSGFVYVYGGNESILVKTGENVEPGKTIGRVGIDAKDGTAIAYF
FVFRNGQPIDPSLAPRD*
>SPBIB_v1_50025|ID:27161936| RNA polymerase sigma factor [Uncultured spirochete bib]
MSDLENCNTGSSSERPTRKNEDSMADLDPLSLYLRQISKYPLLDASREKEIGAHLADLGA
QLTQIDLAIAEKPDNAELERERNRIAADIRATRELLITSNLRLVVSIAKGYQMRGVNLLD
LIDEGNIGLIEAVSRFDYKRGYRFSTYASWWIRQAIIKCLVDQSRVIRLPIHMLNTIRRC
YASAKQLVQELGRDPNMLELAEKMGIPQNKVESAMQLAQGTSSLDAGLDEERNGSIGDSV
KDEKAPDPFNQAFAATIRELLRYVMCSLSEREQIVLQLRYGLNGEGPKTLEETGHALGIT
RERVRQIQEQALEKIKQRQELSDCRT*
>SPBIB_v1_50026|ID:27161937| putative ankyrin [Uncultured spirochete bib]
MQKHTLRPTRLLGFMILALLLIAGCATSPASNKEVTLSDLVIKGDIEGIRKFYANQEQLN
KADSQGLYPLHYAISRGDAQIAEILIVLGAKPDNKDLSGKTALRYAVDRYPTEPAKQKEI
IKMLVDRGADPFLPDVAGTSPAQVALKSDINILDAMFNSKNINNTAADGRTVLHYAADAL
MTKEVELLLSKGASVQIKDKADRTALDLVLLYPDKLEAAQIAEKLILKGANPSFPEFVWF
AQTVRAPDYNAVRFANGNSPLHEAISRYQYGFASFLLLNKADPNNKNLNGDAPLHLAVKA
GYVEGAKLLLSMNANQDILDANHNTPLLLPIPAARRLEMIKLLLNFKANPALADNQGNTP
LHKAVIQSYAPEIVETLIQAGAPVNAQNANGDTPLILCVRSGRYEYAKPLIEAKADIFKM
NQAGENALKLAISKGYQAVDSIVLSANVNQSDDNSNSVLMTAVSLKAPTDVLKLILAKGA
DPNARNKALDSALHIAVRQNYAEQGIVLLDANADIFQYNSKQENPLFLALTAKPAPLEWF
FRPNVIAARDANGDSVVHHAARKNLPDGIAYLVKKGADINVLNNAEETPLHTAAKYDAVD
AIAYLARAGSSLEAADIKGDIPLQSAVLAGAARATQALLVAGSPIDNQNYSGETALHQAA
KNNISAIVILLAANGAQTEILDSRGFTPLAAAAANGAFESANELLKAKAQVDTRDNSGST
PLFQAVSGEHIATARLLLANGADMHALNARGLSPFKLSLMKNAQVTAEFFAPWLINKPDS
NGDAILHMLAGSSAGPDLIEAALKNGANPNARNARGDTPLMVALKKNDIVTAAQLINAGS
SVFASNADNISALSMIFGMDSGSRTKLLSAILSSIKAPKTDFAGESLLHYAVRANNKEAV
ADLLALRADKSIRNRKGEAPLDIAQSKGFQDIVSLLTNN*
>SPBIB_v1_50027|ID:27161938| D-isomer specific 2-hydroxyacid dehydrogenase NAD-binding protein [Uncultured spirochete bib]
MKIGSFMPLNSVWGPAIKQLRDEFPQHAWLEGLKPESSEIGSLDAMIAGRIPLEVFERAH
SLKTLFQPFTGVNHLPASLLLERNVEVYNVHSNAFDVAERALAMTLAFYGRLIEYHNDLR
NEIWHGFWVNRGAEDNWDSIYGKTCAILGTGAIGVALAKLLKAFDCTVYGWRKSSTATVP
EHFDAIFPTMREAIDAAEIVFIALPATAETEGLFSKEILAGMKGKFLVNVGRGSIVNEEG
LYEALKNGILKGAAIDTWYTYPKTGVIGAPSRYPIHTLSNVILSPHVGGSTNQATRRAVD
DTLKNIRAFLRTGKGIWRADLSRMY*
>SPBIB_v1_50028|ID:27161939| protein of unknown function [Uncultured spirochete bib]
MKLLFVQLPSQMPDWSSAPANVPLAAGYLAAYAESKGILARREWSILEPEIANYGSDSSI
VASIAARЕPDIVSFTLYSWNLERSLFIAERLASLLPHARLVAGGPEVVEHMPVTVRTPFH
SLVFGEGEEAFVDLLRDVQQHRPLAPSYSANALADLEKVPNPYLAGTLRFDPSTQVHLET
MRGCSAKCAYCYYGKNYNTLRRFPHKHVLDVIRAASEAGVPEIYIMDPNFQTGPDFAGRL
RDIAYANHARTAIHTELRLEGITEEIAGLLKEAGIASVEAGLQSTNPKALEAVHRTFDRK
AFERGAELLQKQKILVKTGLILGLPFDGYEQVIETFDFLGMQGLGQEAELYPLSLLPGTE
IRERADEYGMSAMEIPPYLVTSTHWISYDDMVDAIAAFEESFDVEWAMPPAPHFQLFKEG
FVSFIDTRRPENIDWMRLNPEKLSNSMTLLVDSDDPEILVRIVRAARDLRKDNPFTLYQV
VLTSETRIPSEKLVERVREAFLHPEHYYELANSFSPDPQTSYQTRMFFATKNFALAYRAL
EEAQDMETMVVLNAKGGYNAERLAELLPYVVFDKQTLPFDRLYELISIYADFPHMLIEAP
EGLF*
>SPBIB_v1_50029|ID:27161940|aat| Leucyl/phenylalanyl-tRNA--protein transferase [Uncultured spirochete bib]
MRWITMPAYLDEAEEYRFPPASGASRNGVVCYGGNLSPGVLLSAYRQGIFPWPSTPDTLR

WCSPDPRFVIPRGFLYITESARKALKKALRRGSPYTLTLDKAFIDVISNCAAIPRPSQPG
TWIFPNLVNAYTELHGRGYAHSVEVWKNGALVGGLYGVSIGAAFFGESMFSTESNASKIG
FLALAITLFEQGFDFIDCQVYTPYLELMGGIDVPRELYLEMLQGALQKPTIKGDWSRYFQ
NFPEVNLIIAAKRNIETITHNA*
>SPBIB_v1_50030|ID:27161941|clpA| ATPase and specificity subunit of ClpA-ClpP ATP-dependent serine protease, chaperone activity [Uncultured spirochete bib]
MKVSPEVQAIFNAAYNEAKLRNHEYLMPEHILFASLSFEKVRNILENCDADLEQMRHSIE
AYFEQKMPIVRNAEPIQSAGFQAVIERAVMQSQSAGKEEVQISDLVVSLYDEERTYAGYL
MRKLGIKRLQLLEVLSHGMSDEDIEAESLTEEETEEHPEEGGKERRAARPGTLERFATDL
TALAAAGKLEPVIGREAEIERTIQVLCRRLKNNPIHVGDAGVGKTAITEGLAQRIAAGKV
PPKLQGYTIWSLDMGALLAGTKFRGDFEERVKKVIDLLLKKEKSILFIDEIHTIIGAGAV
TGSTLDASNLLKPALTSGKLRCIGSTTYDEYNKIFEKDRALSRRFQKIDIVEPTIPETVE
ILKGLRPKYEEYHDVRYSDETLEIAAKLAAQFITERKLPDKAIDVIDEAGALARIQAYKQ
NQDAQVKTIELTPHDIEVVIAKIARIPERTVTSSEKDKLATLESTLKLEVFGQDQAIEAV
VRAVKRSRAGFRAPDKPIANFLFVGPTGVGKTELARQLAKHLAISLHRFDMSEYQEKHTV
SRLIGSPPGYVGYEEGGLLTDAIRKTPNAVVLLDEIEKAHPDIYNILLQIMDYATLTDNQ
GRKADFRNVILIMTSNAGARDIGKPLIGFGDQAVSVSALDEAVERAFSPEFRNRLDAVVH
FNNLPMDIIERIVRKAIDEFRVQLAEKNVTLEVDDEVVHFLAERGYSREFGARNINRLIE
DQIKTVFVDEVLFGKLEHGGTAVAHIEGDKIAFDITPHVS*
>SPBIB_v1_50031|ID:27161942| ATP-dependent Clp protease adapter protein ClpS (modular protein) [Uncultured spirochete bib]
MGTRVTNATGTQELTQHDLETNEPEEYRVYLINDDFTTMEFVISILMTIFHKSLAEATKL
TLEVHRKGRGMAGVFPYDIATTKIQQVHAMARQRGFPLRCIMEKA*
>SPBIB_v1_50032|ID:27161943|thrS| Threonine--tRNA ligase [Uncultured spirochete bib]
MSADIDRIRHSLAHVMAEAVLHLHPGVQFGIGPAIQDGFYYDFLFPQSIANEDLPAIEKE
MRRIIAQNQTFVREEVSKAQAREIFRDQPFKLELIDGLEEGTISIYRQGDFVDLCRGPHV
ESTKELRPDAFKLKTIAGAYWRGDETRPMLTRIYAYAFATKAELEAYLRMLEEAERRDHR
KLGKELDLFSTHEEAGPGLIYWHPKGGRVRVELEKWWREEHYKNGYEILFTPHIGKSWLW
ETSGHLGFYKANMYAPMTIDEDDYYIKPMNCPFHIMIYKTTSHSYRDLPLRWAELGTVYR
YERSGVLHGLMRVRGFTQDDAHIICTPEQIEDEILEVLRFSLSMWKTLGFSKIKAYLATR
PADSVGDPERWDQALVSLKKAIEKEGLPYEMDEGGGAFYGPKIDLKIQDAIGREWQMTTI
QFDFNLPERFDMTYVDKDGRQKQPYMVHRALLGSIERFFGVFVEHYAGAFPVWLMPDQVA
VIPVAPTFDEYAKEVTAFLRGKGVRAKAMLSEERMNAKIRDAQNQKIPYMVILGQREKDE
RTVSLRLRTGSQENGIPLETFASRVLGKIEAKALDL*
>SPBIB_v1_50033|ID:27161944| putative dihydropyrimidine dehydrogenase [NADP+], similar to dihydroorotate dehydrogenase [Uncultured spirochete bib]
MADLGTWLMGLRLRNPIIVGASGLTSLPDGVEKAAHSGAGAIVLKSLFEEQILAVLGQEI
AGLDIDSYPDAEAFISRTAWEESTDEYLKLIDESKKRTSGVPIIASINCVGPGNWASFAK
RIEEAGADALELNIAYVPFSPHISGQEIEHKVLSTVKEARLATKLPIMVKLGNNYTSLPH
TVHGLSKEGANAVVLFNRFFRFDFNLDTMRLTGVQPLSSETEYHESLRWTAILYKRAGVE
IVSSTGIHTADAAVKCILGGASAVEVCSIIYQKGWKAITAILEEMDSIVESMGYSSLDAI
QGKLSAANAERPEEYLRLQYIKALTGVY*
>SPBIB_v1_50034|ID:27161945|ytsJ| putative NAD-dependent malic enzyme 4 [Uncultured spirochete bib]
MKLNRSLDNLSEAFPDDLTPDQRARAQTLFLKALSEKAHRFYEGKIQVVPKAGLYGFNWF
NVWYTPGVSKISTAIRDNNDQSFALSNRGNLVAVVSDSTRVLGDGDCTPPGGLGVMEGKS
FLMKYLGGVDAIALCINSRGKDGKPDADKIIDFVKMLEPSVGAVNLEDISQPNCFKVLDE
LREACDIPVWHDDAQGTACVTLAGLLNALELAGKKLSDAKIVLLGAGASNTTIARLIMAD
GGKGENMIIFDSKGGLHRNRKDIEQDTRYYRKWELCQATNPQCIETEAEALKGADVLIAL
STPGPDTVKREWVRSMAPKSIVFACANPVPEIWPYAAKEEGAYIVATGRGDFPNQVNNSI
CFPGLLKGALLVRARKITDNMAIRCAHSIADFAKSRGINTENIIATMEETEVFAREAADV
AMAAIEEGVARVTDTWENVYKKAKEDILESRSLTKDLMDRGHIAPIPEEMLKEAFDYAVA
EVRKK*
>SPBIB_v1_50035|ID:27161946| putative amidohydrolase [Uncultured spirochete bib]
MKIALAQFRPEFGESDRNIERLLRLCKENPADLYILPELAYTGYQFVSQEEVHALADSVH

SRRIEAFRNAAKELDACLIFGFPELAGAVIYNSALAVLSDGREYLYRKTHLFYKETLYFS
PGDTGFPVFEYRGARIGMAICFDWFFPESFRTLALKGADIIAHCSNLVLPYCQTADFAAA
VQNRVFIATVNRVGAEQREDEQLAFTGESVLVSPKGEYLLRGPIEDEAVLVAEIDPEAAR
NKSINPYNEVFKMRRPGMYTLQ*
>SPBIB_v1_50036|ID:27161947| membrane protein of unknown function [Uncultured spirochete bib]
MRLFPRPQNGWLKRALILLGAMAFLSVFATIDTSEHFRTWRTARMWTSEQKVTAQAEDAA
AMFAIASASVIFNTAIVAFSAVGALALGISVALDNRKWLSVSWAIGLCASACGILYSFTV
IIYKIRIGERHILKGPIFDYNLALQPPIVIAIVGYILVFILWIALMRSARANQAGQGGQ*
>SPBIB_v1_50037|ID:27161948| protein of unknown function [Uncultured spirochete bib]
MNEEKGTQMADIQKKKPHHLKRPVLTACMATIFALTIAVLTDASHFRNRSSDILFIEAIA
LVIGAWASYLKSRGLSFLVMHPFRRKNLAESWIDRVPEKFVGSAQEMDAEARSLQIQRSR
EFRRDLAIASGILLATGLIVQYI*
>SPBIB_v1_50038|ID:27161949|aliB| Oligopeptide-binding protein AliB [Uncultured spirochete bib]
MYKKIALALVALVLLSAATLGAATPKSKDYVYVFSTDPRSFNYLNDQRATNTQHITNFVD
ALLEHDRYGILRPALAESWKTNDDFTVWTFNIRKGVKWVTADLEPYADVKAQDWVDALKY
MLDNKSTLTYLIDGFVKNAGAYLQGKITDFSQVGVKAKGDYVLEYTLEKPTPYFDTMLTY
NAYYPVNGEFLKTKGKDFGKVDKNGILYNGAYILSNYTSKSVIEYDANPTYWDKGNVSIQ
HVKFVYFDGKDPDSLFNNFDAGVYVAAPVYTDNEALFARAQAKYKDYIFRARQDSTTFVY
AFNYDRNAFASPADPTKGKSPKSDKAKEDTKKAILNRNFRKAIFFGIDRPTILAQRNGDV
NKLAAIRNSYTAPELSFDIAGKDYVKYVEDALKSRNPADFPASFKLDDAQDPYYNPTKAK
AYMAKAKAELTKQGVKFPIELDVAADVSYTKGMKMDQSLKAGLEALFGTDTIKVNVVEMD
SDNYDASTYYAETGAQSNYDIGNTTGWGPDYGDPYTFLQTLEPVVGALLTPIGLDPVDEG
SDKAAATAIGLYEYAKKVEAGNAEYKDYSKRFKLFADAEAQLLDDAIILPYMSFGGAFQV
SRVIPYTAPRAAYGADEYKFKGVIVSDKVVSLAEREKYRQEWEKQRQAEYKKLNAGK*
>SPBIB_v1_50039|ID:27161950| ABC transporter, permease protein [Uncultured spirochete bib]
MRGFMKGYVLKRILQSLVSVFLVSTLTVFLVFSCIPRQLVFKGDQMLQKLANRPDEYQDY
KFRTWESLGYLDYITMKEYAQSIYGNDSDQVSTALQSGSSFYERFRAELQAKRYTVEAQP
VTGYLYAYKDIPTMTRVLRWYANLVQVDHPWRVKDPNNPDLKRGYYFTKDWSGMPALAAS
GTLYKYQIWIDGSFPFLHQNIIHLYMGRSYPTYDGIPVLEVIGMGQGRTARREVTTKVGG
QEVTFYTSIDEHTLKYKPDLDRLDKNKFEDNYADGNNIYQDPSMLGISFRIGIMSLILSL
GIGLTIGVVAAMRKDKMFDKVTMGYIVFISSIPTLLYIALFARFGMKILGLPDKFPFLGA
QNFLSYILPTLSLSLGGIAGEALWIRRYMVDQMNADYVKFARSKGLTQGEVFFRHIVRNA
LIPIVHSIPMAVIGTLAGALITESFYAVPGMGKMFPSSISDYNNAMIIALTFIFTFVSVF
AIFLGDILVTIVDPRISLAARKEAR*
>SPBIB_v1_50040|ID:27161951|amiD| Oligopeptide transport system permease protein AmiD [Uncultured spirochete bib]
MTDKTAEKFTYATFDILQSEHIAGPKYSYWKSVFQTFFKKKWSVFWLVLLLVMTLMAFIQ
PLLSGYDPAIAPNINKPETWYLKPSAQHWFGTDDRGNDMWNVVWAGTRMSLSIALIAAAI
NIGVGILVGAIWGYSKRIDPILLGIYNVVSNVPGILRSMMLMYIFGRGFWQIVLAMTITG
WLGVAFFIRTQVMIIRDREYNLASRCLGTPLKRMVTRNILPYMVSVIATLIYQEIPGLIN
TETVLSYLGIGLPTTYPSLGRMIDTYWSFVDTYPHMIVFPGIVLGLITISFYIVGQLFAD
ASDPRTHR*
>SPBIB_v1_50041|ID:27161952|oppD| oligopeptide transporter subunit ; ATP-binding component of ABC superfamily [Uncultured spirochete bib]
MDTPNIILSAKDLVVKFRVRNKELTAIRHISLDLFEGETLAIVGESGSGKTVLTKTFTGM
LESNGRIDNGSIIFEGQELTKLKTDKQWETIRGAKISTVFQDPMTSLNPLKRIGIQITEV
IEKHQKLPSAEAKKIALNLMERVGIKNAEQRFTDYPFQYSGGMRQRIVIAIALACKPRIL
ICDEPTTALDVTIQAQILDLIRNLQREYGFTTIYITHDLGVVANVADRVAVMYAGQIVEY
GTVREIFYDPKHPYTWALISSLPTLAEKDKPLYAIPGTPPTLFSDIKGDAFAPRNRYALK
IDFEEEPPFYQVSPTHFAKTWLLDPRAPQAEPPDAVKIMRERGRKNTKQTSEAQQ*
>SPBIB_v1_50042|ID:27161953|oppF| oligopeptide transporter subunit ; ATP-binding component of ABC superfamily [Uncultured spirochete bib]
MNAAVNAAANARNNEPLLSVKNLEVTFKNGNTSFTAVRDVSFDIMEGESFALVGESGSGK
TTIGRTIIRINPASKGEIYFKGKRISGIIDKETDSWVIRNIQMIFQDPMASLNERATIDY

IVSEGLYNFNLFSSEEERREKAFRALRDVGLLEEHAERYPHEFSGGQRQRICIARVLVME
PNFIIADEPISALDVSIRAQVLNLLKDLKKERGLSYLFIAHDLTVVRYFADRIAVIHQGE
LKELARTEELFSRPLHPYTRSLLSAVPLPDPDLEKNKKLLVYDPATMHDYSKGLPSWREI
FPGHFVLANDKEAAEEYQEMYR*
>SPBIB_v1_50043|ID:27161954| AAA ATPase [Uncultured spirochete bib]
MDTVSVNVDGYGEVAVPAGTSAELVLEQANIEAASRPKKNAFPPLAVLINNELAPLSAPI
GAPCSIKPVYPDSPMGTEVYRRSLCFILALAAREIVPSRRLTVSMAIGNGYYHYFDDNEP
VSAQLLEALSRRMRELVLSDLPIRIVTRAWEEALEYFKTSNQSDTLALMEYINDPFIQMN
ECNGFRDFHIAPLVPRTGLLSVWELVPYRRGMLLRFPHTKTPYEMDPFSDVPVLYDIAEE
YEHKARILNAESVGALNRINQSGNIQDFILVAEALQNKKLALIADRIAEKSDKTKVVLIA
GPSSSGKTTTAKKLAIQLKVLGFRPIHIELDNYFVDRSRTPLDKDGKPDFECLEALDIEY
LNQQLLDLFDGKEVELPLYDFKSGTRKASGRKISLSNNEILILEGIHGLNDRLTPHIPAE
NKLKIYVSALTQLNIDDHNRVRTTDYRLLRRMVRDYNFRGHSAQATLGMWSSVQRGERLY
IFPFQGSADIAFNSALDYELGVLKVFAEPLLQAVKPQHDEYPDARRIQAFISRISPISPQ
YVPSDSILREFIGGSVFKY*
>SPBIB_v1_50044|ID:27161955| Transcriptional regulator, LacI family [Uncultured spirochete bib]
MSKVTIKDIAQRAGVSKTAVSFALNYPGRISKKTYEKIMAIVEELGYVPNPFARTLTTKR
LGAIGLLLPQKIGDIFGNPHMAQIISGIGEECERREFSLAILPLIRGKIIEAARKSYVDA
LITIGVGPDHEVVDLLKKNHIPFVTIDGEESENTINIGIDHKNAAKEIMLHVLQQGHRDI
AILALEADTKPIESTHTSIVLQRRFQGFQEALEEFSISLAEPSVQILNCKSSMQAGHESV
QILLSNAKRPTAIVAMSDIAAIGALVAIKENGLRVPGDIAVAGFDDIPESSLVSPALTTI
FQPGRQKGTEAARMVLNMLDGAQGTHIKLPYKLIVRESTLRA*
>SPBIB_v1_50045|ID:27161956|yjbG| Oligoendopeptidase F homolog [Uncultured spirochete bib]
MAKAIPARSEVPAEHKWNLSSLFKDDAAWEEGLAALGAMLQDAEACKKAGIGHSAEAFLA
ALSAYEQYLILEERLGYYSHLRVTEDEGNSTARGMFARFVGVSSQGQAAWSWFSPAIQVL
DDAFLQACLADPKFAGYRVFLEKLRRFKPHVLSEAEERLLALQAEPAQTAQEAFSVLTNV
DLNFGTIDTPEGPKPLTQSTFASFMRMPDRSIRKTAYLQFYTQFESHQNTLGSLYAGSVK
LDKYQAQVRKYPSARAMALFPDNVPESVYDNLIATLGKNLPSLHRYYKLRKKILGLDELR
HYDVYVPLVRESRATHSYEEAVDIVTEALKPLGEDYISVLRKGLTSGWVDRYENKGKRSG
AFSAGTYTGEPYILLNYKDDVLHDLFTIAHEGGHSMHSWYSSRSNPFMCYNYTIFEAEVA
STFNEQLVFSYLYQHSTDAMQKAFLIANRIDDTLATLFRQTMFAEFEMRAHEIVEAGQAL
TVDVLRKEYRALLEKYFGPELVFESVSDLEGLRIPHFYNAFYVYKYATGLSASIALSERV
MKGGKEERDAYLGFLKSGGSKFPIEALKLAGVDMSTPQPIEAACTRFADDVNELARLLSI
SLD*
>SPBIB_v1_50046|ID:27161957| putative Ribosomal RNA small subunit methyltransferase E 1 [Uncultured spirochete bib]
MNLCILDSGDEFQALPVSDRRAQHILRVLEKRKGDRIRAGTSDGRLGYAIIDAIGNDEIT
LHFFPESLAPPLRPLTVLLGTVRPIQGARIVKDLATLGVASIVFFPTALGEKSYTQSNFY
KRKEYIQHALDGAEQAGNPRLPEISIAWSLKKALEAIEHAEGTKLVCHPDIAALPLSQIQ
VEQTPVILAIGTERGWTQDELALFERANFSLCALGDRILKTETAAIAAVSVILSKLNFL*
>SPBIB_v1_50047|ID:27161958| putative reductase TDE_0597 [Uncultured spirochete bib]
MILKPMVRNNICINAHPQGCRAEVRAQVERAKAFRDRLAGFGKNGSNVTKPFHVLVIGCS
SGYGLASRIVAAMAYGADTVGFSYEKAPSALKPATPGWYFNNEFDRLAAQAGLFARTFSD
VDAFSYEAKTKAIEVARERNFRYDLVIYSLASPVRTDPDTGELYRSVIKPIGRQYAGRSL
DVFTGKISEATVQPATDEEIAQTVKVMGGEDWQLWIDALNKANVLAPGAITLAYSYIGPS
FSWPIYHDGTIGQAKLHLEKTAHALTSCYQNENGLRAFVSINKAVVTRASAVIPVIPLYV
ATLFKVMKERNIHEDCVDQMLRLFSERLYRKDAAQIPTDQEKRIRIDDLEMGSSVQSEVS
ARISRIDESNLYLLADPEGFKSDFLRAHGFDVPGVDYDQEVSSFE*
>SPBIB_v1_50048|ID:27161959|fabZ| 3-hydroxyacyl-[acyl-carrier-protein] dehydratase FabZ [Uncultured spirochete bib]
VNHIETYLPHRPPFLFVDDAHIEGETIIATHTFHDDDWFFKGHFPSFPIVPGVLLVESMA
QAGGVGAKLMGIYPKSLFMFAKIKEARFKRPVRPGDTLKMEILNVRASSVFLHQKGVGKV
GDEIAVEAEWISISSGVPE*
>SPBIB_v1_50049|ID:27161960|fabF| 3-oxoacyl-[acyl-carrier-protein] synthase II [Uncultured spirochete bib]

MKEEVAVTGLGIISPIGNSIEEFWKNCVNGVSGIGPITHFDASNVESKIAAEVKNFDASL
WIDRKEARKMALFSQYAVAAAVQAWNDAGLPATMDSQSAEKSASIDPYRIAIVIGNGIGG
LEVFTESHAKMLQDGPDRIPPMTIPLMIANEAAANIAMRFGIHGPALTQVTACASGTDAL
GQALDLIRSGRADIVIAGGTEAAITEFAIGAFCRLKALSTSYNDRPELASRPFDRDRDGF
VMGEGAGILVLERMESAKARQAPIHAVFAGYGATCDAYHLTAPQPEGLFGAKAIEIALKD
AALAPEDIGYYNAHGTGTELNDQMETRMLKTAFGPLAQSLQISSTKSMTGHCVAAAGAIE
AIVCIQALKTGILPPTIHLDNPDLENGCDLDYIPNSARTHKINAAMSASLGFGGHNGVVI
FAQP*
>SPBIB_v1_50050|ID:27161961|fabH| 3-oxoacyl-[acyl-carrier-protein] synthase 3 [Uncultured spirochete bib]
MSVLIRAISAYVPPIRISNEELASRIDTTDEWIRSHTGIGARHIAPDGVQTSDMAVSAAK
SALEKAKISIHEIDYIIVATATPDYFGFPATACIVQDKLGAYGASAFDVTAGCTGFIYAL
NIASSMLETSHGRNALVIGAEGLSRITDWNDRSTAVLFGDGAGAAVLSRIDETGRGCLSF
ILGADGSGAKDLYLVQPERAKAFERQPPLVPVISMNGKKVYDFAVKSITVVIERLLHKTA
YRLEDFAWIIPHQANARIVQAAAKRFSIPMDKIFLNIEEYANTSAASIPLALSEMDEKGL
LKPNDLIMLVGFGAGLTYGAAVIRW*
>SPBIB_v1_50051|ID:27161962|ftsH| ATP-dependent zinc metalloprotease FtsH [Uncultured spirochete bib]
MPSKGKNSWSPNQQNKPDKNQGDPNKKPGMPEGFKFRFSLGYVLIALIAVSFFNYFLFRS
DNTVVPYSTFKDKIRTGEIKRVEIDINYYTGYTDARSSEPASPLPATAKPGVVYKTVPVT
DPEFTALMDQKGVVYSASPREGSAILSLLLNWVLPFAIMFFLWRTVMSRFMGSNSNVLAF
GQNRATVVAEGDIKTRFTDVAGVDEAKAELVEVVDFLKNPQKYTEIGGKIPKGVLLVGPP
GTGKTLLARAVAGEANVPFFKMSGAEFVEMFVGVGAARVRDLFKQAREKAPCIIFIDEID
ALGKSRMSGMIGGNDEREQTLNQLLVEMDGFDSTSGLILVAATNRPDVLDPALLRPGRFD
RQVLVDRPDMIGREEILKIHTKTIKMDPSVDLSKIARSTPGFVGADLANVVNEAALLAVR
AGRKKVKEEDFQNAIEKVVAGLEKKNRMINPKEKQIVAVHETGHAITAAFTPGADPVRKI
SIVPRGFGALGYTLQMPLEDRYLVTKEELLGQIDVLLGGRAAEELVFHSISTGAANDIAR
STDIARRMITDYGMSEKFRNVALTKRGGSIPGMPQIADPYATKEYSEDTQRYIDEEIASI
VDERYKHVQNLLIQKKDMLDRISALLLEKEVIEETEFAALTAELTSPSAAAASSKA*
>SPBIB_v1_50052|ID:27161963|groS| 10 kDa chaperonin [Uncultured spirochete bib]
MTVKPLGDRVLVKIKESETKTAGGIIIPQTAQEKTQTGVVVAVGTDSDVIKVKVGDEVMY
DKYAGTQIKIDGAEHLIVKMSDILAILE*
>SPBIB_v1_50053|ID:27161964| putative PTS IIA-like nitrogen-regulatory protein PtsN [Uncultured spirochete bib]
MDLKNLLKPELVSLDLKGKNKEAIIRELVELAFRSGKVLDKEEAIRSVFEREDRMSTGMK
HGIAIPHGKTTAVRELVACIGISPEEIDFDALDRKGCRIFIMTLSPIDKTGPHLQFLAEV
GMLFRSEEKRQALLAAKTPEEVVSILVGNS*
>SPBIB_v1_50054|ID:27161965| putative transporter [Uncultured spirochete bib]
MLLGTMKLWLKYAFGIAMGAILYIALPPSMVRGTAAITFISEISLKIGGYALVFMLASGI
PVSVFRLSEAHRFWKIFSQSFFLLVGSLIVAAGLGLAVALIFKSAPLPLITDSGLTPTID
PAEMVRSTFPPSIFSTFAGSGTWLLPLLVFGFAFGLAVAHDPVMARPLIPVLDVISRTAY
LINTFISEILGILLIAVSLNLFLVLRNTGLPVEYRGILFSAGVAAVVVLFGVFPLAYRIL
GGKANPYVLLYGMLGPLLAAAGSGSIFFSSGSAIRHLSESLGVKRDTNATIFPLALMTGR
AGSAFVVASAFIAMFLSYSRNGPGLAQLLLLLVLVPFSVFVASAGIRSDISVMLSLVCML
FGQGFQNGASLLVPVAFPLSILAAILDSAWMMFAVALIGDRQGERTTKRARNFI*
>SPBIB_v1_50055|ID:27161966| protein of unknown function [Uncultured spirochete bib]
MKDFYAEKRGKVYYIRFKDPLTGKILSARSSGQRNRDLAIKWAALEYEKMKSQAGMPTGK
FGDWANLFFKEGCPHITRLLNQGKSYAESTRRDNRRYVDMFLLKDPISRMKLGEIGRGDI
IDLQDRIVKAYGRTRSAQRIYQAFHIILNEAVIRGKLSANPSNGIAKISYQPKVRKPLTK
AELDRFLDPKHWPNRTHWLMTMMARYTGLRAGEIRALYWEHLNPEEGSIMVLQNLPKNVS
LEALRPPKWGKTRKAVYPKQLQVLLEKERKPTGLVFQEKDGSAVDYWLWHDSVKHARKAS
NITHAGIHALRHSLDTILAENGINQEVRKAIFGWTNAKTEQIYNHPEMYDVGKLSKKIQR
VLKSGNSTNGELSKAGSD*
>SPBIB_v1_50056|ID:27161967| conserved protein of unknown function [Uncultured spirochete bib]
MNACIRFGTCSWNYPSWVGLVYSEPQRRAAAYLREYSQKYDTVEIDSWFYKIPNPREVAD
YLAQVPPSFRFTCKVPQELTLTHLRGKAGAAIGVVNPSFLSPELFEQFLEVIEPMLPKID
AIMFEFEYLNRDKMPSLEAFLKKLDDFLAAMGERGKHLPLAIESRNKNYLTAAYFSFLRE

RHLIPIFSEKLYLPHIYDLYRQYKDSIDTDVVIRLLGGDRTEIENKTNKQWNTIVEPKPD
KEAIVTMARDITHRGHMVTINVNNHYEGSAPLTIKAMQKMLH*
>SPBIB_v1_50057|ID:27161968| conserved exported protein of unknown function [Uncultured spirochete bib]
MKKLTVIGIVVALVFGGCTLFPTVTFDDAKGEWDFPDTTFNNSTITSIHLSVMGENQAAC
RIDLSWNNFNNFYYGDGTMNGNVFTGEYNVGGNSSDTTTYSITVTFSLSGSTLKAVFNGQ
GPLNGLILEHGVKAAT*
>SPBIB_v1_50058|ID:27161969| exported protein of unknown function [Uncultured spirochete bib]
MWKRRLGCGLFLLFFLRQPSALPSDWRQLDTKDLLQEAALAIERLQALNETLTQQAAASE
KQSKELAAQLETLTKEKEKLLNDIERLQAALATLQADWTESERMRKALEHTLTSLQASLQ
SCREEAKKTTRRAGIVGLAVGVGVGMMIGIVSRR*
>SPBIB_v1_50059|ID:27161970| protein of unknown function [Uncultured spirochete bib]
MKQPSIFQEQDGAFSMRRTLALLYALSSNGCLWLATLSGSMAGVWAGLAALVAVLILTGY
TTIEGLKGLAAAIKGTECGKED*
>SPBIB_v1_50060|ID:27161971| conserved protein of unknown function [Uncultured spirochete bib]
MRQGIQTLFAELGEAGCLALCICELGKPGISEGEAVGLILEGIARGFILYDENDRQNPNN
FFVQDRDGFMALVTGQKGWSSTKESSGYTPKPGERIIECWKWDETLAGNVVSHQHFRLPA
WDPIKEARTVRYGQLESYRVFRKAA*
>SPBIB_v1_50061|ID:27161972| protein of unknown function [Uncultured spirochete bib]
MQSIVDKLLDAAAGIAAAAITGTLWLNLRWLFSSKRSMEKVLSEVVGMRSHIAVLYTLQG
PILLGMKASLEALRDGTCNGNVDEALRLIAEEKKKFDEHLLSAITGDTEERAK*
>SPBIB_v1_50062|ID:27161973| putative Phage related protein [Uncultured spirochete bib]
MTMNVPSLRILTNTLELVADLDRYAELYYTRSLSEPGDFAFSLPIEADSEGAVAEGNLLL
IGNDGRRIGIIEEVERKALDKGSEWLIARGHEAKAIFARRIILPPAGAARIDMDAPAETV
MKALVESQCGPSAETRRRFPALKIAADLGRGCRYSLSSGYASLLSELCSIAKASGLGFSL
SFDPGGQKLIFELIEGIDRSAGQKENPRALFASEYDTLSEARLIQGYGRHASILYVLGAK
LAEGRPVALAFDGTEPEGFGRFERTLDAPSFDTLERLFAYGQARLSAYPTTFFLEAVLPE
GSSLVPDRDFALGDLCTVHAYGQWYTVPIHSIEEHWTKDGFQIRLGFGRPSPGAFSASLH
VTNELLEAFRAR*
>SPBIB_v1_50063|ID:27161974| protein of unknown function [Uncultured spirochete bib]
MSEESGFFTSVAGDRKYTAQFMNEKLHEAMQRAEGVVRHADGELMVSTDGSLSVDVAAGV
ALKGGVYYKNPAGLHLALAAPTLGTQRWDRLAVRIDRMRRTMQAVVIQGSEGSDPVVPAY
NPSDDIPLAKVLVNRVEDPPVVTVADEREFRPLFLTDRNSLDDLGEGEVYGRVLKEKAEA
LNAGQAGLSFRQFGFVSRPWSAAQIECALLPLGSGTLFVGRSDTTKLSYSTDSGISWADV
AGITIDNKIMSIVFTGSSYLAAGGSPARIFRGSPMGTWSKVFEDTTQEFVSALVVLSPLQ
ILAACGDKIYASTNGGSSWSQRGTLPGSYLMTAMANLGNGVLLAAGFSTDKIWRSTDSGA
TWTAVKTTDCYEKRPGFIQQVGNGVVLLGYNDGGLLYRSTDWGLTWDSGRQVAEGGLFTG
ILADGEMLYLPQGKALYESADFGQSWSLKYPCANYDSIVALGKDSDGNIITLGYQSHLYW
GYPVAA*
>SPBIB_v1_50064|ID:27161975| putative Phage-related tail protein [Uncultured spirochete bib]
VTLITYISAEDNELAIGTGRLTLLSAEGLDAPPVHLSIAKGPGVDGAIAAHLQLEPRRIT
LEALLDLEGLDEAGMAEERAGICTTMCPAKEGGSLRVTRGTRTRSIEAVPAAAPAFAKKR
WNEAWQTFRLEFVCLKPSFKSTEPVISSVRYYASLTEFGEEGIEFGEEGFECSSIEHSGE
RTTTIVNDGTAPAPVRIRFTGPMVNPFIRNRTTGEIIRITQMIRAGEYLEIDTEPGRRQI
RLWKAGTEQNGMHYLDLASSFFQLAPGENEIELGDESAGEGSEAFFECYGHYLEA*
>SPBIB_v1_50065|ID:27161976| protein of unknown function [Uncultured spirochete bib]
MSEESLQGANSRDDVEQILSVDEEELPGLLEEKRRREELSRQIAFGEGL*
>SPBIB_v1_50066|ID:27161977| protein of unknown function [Uncultured spirochete bib]
MSEGARIAALLDADEELSALLGPGTHILQGSLAAQPPYPLPLVLVSANENIPLLENEDGV
VAEGCRCTIGILAEGGLEALESRTKEVMEEAGYRCEMRKRLPAGRPEWHCCAMSFSGARL
RI*
>SPBIB_v1_50067|ID:27161978| protein of unknown function [Uncultured spirochete bib]
MKRRQIVKLFKRGLTLDGNFALSASWEDGGSFEASLWPARKTGQREGQRYLLVAKAGIGL
EPYDRIETAQGLFRVLWVRIFPKHTEALVEKEVV*
>SPBIB_v1_50068|ID:27161979| conserved protein of unknown function [Uncultured spirochete bib]

MIIEDGTGLSGATSYIGLEEADGYHGERGNGRWTEAGEAEKSAALVRACDWLERAYGRLW
DGTKRVATQRLSFPREEMDGVGEGEIPWWLKEAQCEAALLELSEPGVLAFDASEGGGLQR
EREGEIERFFAPGPGSVRKFPEIYRLVAGHLRSPAQVGIGRA*
>SPBIB_v1_50069|ID:27161980| protein of unknown function [Uncultured spirochete bib]
MGRRAKGISVGPPAAGLAMAVHG*
>SPBIB_v1_50070|ID:27161981| protein of unknown function [Uncultured spirochete bib]
VAFDSEKAGILDRLSPDGQKALLGQELLRLGQEQASLQGALVALGAAVSGLETAVGSLEA
ALARRQRFFARTGVLQAALAGTRVAILSESELGAGEKAYPLGFFLALGGEVAWSGGTGSR
LYLADSGTDLVYRFASVEARVLAPGNFIGVGAEGVALEAEFGLGLGGRAGKGIDIVADGN
FAEGSDLAVTVYGYIG*
>SPBIB_v1_50071|ID:27161982| protein of unknown function [Uncultured spirochete bib]
VAMGVGVGRAVALDVGVKRAVTLLLG*
>SPBIB_v1_50072|ID:27161983| P22 coat protein [Uncultured spirochete bib]
MANLFTALAPILFSAAQEVSAEDTGALQAITLNFDDKQVAKDDIVAVPIASGGTPVDFVP
GNVSPQGDSDTASTVDVRITASKKRSWHLTGEQRRSLENAGTDKEWLRLKLMQEMRSLRN
AAEADCVAAIVQGASRATGTAGTTPFSSSLDELIAVRRILKDNGCPMADAQFVCDSAAET
NLLKLSAIQQAYAAGSDEERRSGVIKRQFGFEIRTSAQITQHQKGTGTNYLTNATTPMAK
GDTATTLSGASGSGTILEGDILSFEGDPNLYVSHAGISAAQQVLRIGRPGVRQAIGTSTP
ISIGNSYTPCLGFDRGAVVGIMRPPVMPENPTMQQALISDMRGLTYLLLEIAQYGQITWE
LHLAWGFKVVMHEHVALLLG*
>SPBIB_v1_50073|ID:27161984| protein of unknown function [Uncultured spirochete bib]
MTSLNDIISHLKFELGKEGPLSKETILPLVEKIEPLAARDWARIKKLERKVEAFSGKSPE
ELETSMKELETLKAELESTKKLLGEKDELLKTLTKERAEKEAALAKSLSEEKAAVARLLL
DAGLTAELSKANVKPSLLGAAKALIREKGILDVDGEGEVRTAVARIRRDGVEQTMSLADW
VREFVASDEGKEFIAAKENAGSGAGEHRASASTASANSIPAERFWSLPAKDRSAFILKGG
TVTED*
>SPBIB_v1_50074|ID:27161985| protein of unknown function [Uncultured spirochete bib]
LGSDPWGFTHCPCMIYYTLVGVRADRTRTGGVTPARALLS*
>SPBIB_v1_50075|ID:27161986| conserved protein of unknown function [Uncultured spirochete bib]
MVDYLLHIYRKGSRPFQTLSDLPEAMALQIMEQLYIEGAVFWERFKEPRSYQSFRKQVEQ
TMRAAFKNKGGKPINKHPIYLIVGRPKWMDIVSDEKTLQTTEILRVPLSMIKRESVSFAY
PDSMVSALMAAEQNPDYYEPEYHGKVFTFDEIMDIIEKKGLPGEGWETRMPKHYAHYIEA
QVWDRSILESIG*
>SPBIB_v1_50076|ID:27161987| conserved protein of unknown function [Uncultured spirochete bib]
MEQDIRGALPKSQQGKRKRILILEDSEVDADLIIRILKKNNLDFEFLRIENEAEFLRQMD
AFAPDLILSDINLPGFSGFKALEIVKQAYPELPFIFVSGMVGEDLAIKMFNEGAVDCINK
SSLSRLPGSVVRALEDSSQKKKRRLAELALEKNLRMTHEILIASIKALATAMEMRDPYTA
GHQRRVADLAVQIAKRRGFSEDKIEGVSLAAAVHDIGKIYVPAEILMRPTRLTSAEYELI
QVHSSAGYEILKDIEFPWPIAKAVYQHHERLDGSGCPRGLKNEEIIVEARIIMVADVVEA
MASHRPYRPALGLAAALEEIRKDAGTRFDPDIVEACASLFADGYQFPQRTS*
>SPBIB_v1_50077|ID:27161988| Response regulator receiver protein [Uncultured spirochete bib]
MLEHEQINLLLIEDNPDDADLALRALRKRHLLNTIKVLSDGEEAIHYLLRRGPYEGTELP
SSIKLILLDLKLPKLNGFEVLHAIRNDERTKRIPVVILTSSQEDPDIQKAYALGANSYVV
KPVDFDNFSKCVSDLGMYWLFLNKPPSHG*
>SPBIB_v1_50078|ID:27161989| putative Histidine kinase [Uncultured spirochete bib]
MKKEARMQDVAERYLSIAAEVILSLDAHGNITMLNESGHRLLGYAQGELIRKNWFDECVP
VDVRNKLKTVFAKLMAGEGSDVSTYENQVITKSGETLDLLWHNTVLRDPDGHITGTLSSA
EDITQRKKMEEELRNSEEQYRMLFTGMDIGVFFFKPGGTISYANPAALDILGVTEEEITS
LVPTDSRWKNIQEDGSEYPDDLLPSTLAMHTGQAVRDKVIGIYNGREKRYRWVIGSAIPR
FHTGEKAPSLVYYVTMMDITERKMLEKEVAHMASFPAQNPSPVLEVGTDGAVRFANAAVMA
TLERLGLPPDARQFLPGTPEELALLRSQCEQSHQVRELRLGGATFLEIISAPEQHSLRVY
ITDITEQKKAEELQKSLFLQVQEEKERLDLLIASMSDEVWFADTAGHFTLENPAACKAFG
LEKPGDIDIQSFAQSLEVFRPDGSPRPVEEAPPLRALRGQTTIEQEEIVRLPKSGQLRYR
LVTSNPVRDFEGNITGCISVVHDITDKKQAELRIEREKAFFDHLIEMAPEGIAITDKTGI

ILKSNAEFCRMFGYSQEEALGKQIDDLIAPPEREAEARNLTASLMHGGTVSVDTQRRRKD
GSLLDVWVIGAPIIVSGREEAVFAIYRDISEKKKLEREREKETARLKILVSILQHQSDSL
QEFLNYALDQAICLTDSKIGYIYHYNEEKQHFTLNTWSRDVMPACTVIDQVKEYDLRDTG
FWGEVVRQRKPIINNDFAAPNPYKKGTPEGHVPLSKFMSIPVFSDGKIVGVVGLANKNED
YTETDILQTSLLMETVWKVVDRLKAEDALKKLNDELEKRVSQRTAAIESANNELESFSYS
VSHDLRAPLRAIDGYVQIFLENYQKNLDDEAKRIFSRISENARRMGQLIDDLLRFSRTAK
LLIARELIDMTALVKQVVEEILAPIKDINYDIEISALEPATGDISLIRQVWMNLIGNAVK
FSRKAEKPHIRIFSERVEGKVVYTVEDNGAGFNMEYADKLFGVFQRLHGQNEFEGTGVGL
ALVKRILTRHSGNIWAEGQVGQGAKFHFSIGEEASDA*
>SPBIB_v1_50079|ID:27161990| protein of unknown function [Uncultured spirochete bib]
LRTFIVAIMQEFIFALQEALHELACAEDGLAAFRNVELAHKSLRERKAIKR*
>SPBIB_v1_50080|ID:27161991| protein of unknown function [Uncultured spirochete bib]
LLEASSQRSTPWCIIIEKNSFKSCPDSVIRFAIDPFVMHGVMNVHATSPLDSFALPQRFV
S*
>SPBIB_v1_50081|ID:27161992| Conserved membrane-spanning protein [Uncultured spirochete bib]
MRKRSAKFWLGLSLIVCLISMLGANFVQTGGYSITVKDLRWETSAGHMMSALLFIPKNAS
VKNPAPAIITSHGWYNNREMQDMNFVEYARRGYVVMSIDMYGHGNSDTLVNSQVQYHATG
MTDAVELMATFPFVDKSRIGVTGHSNGGRAANWAVDDDNLKSTPLIKSVLLVAYDPTYTD
KDGKYINKYGNRDVGVVAAKYDEFFFRVKNKDGSKTAPRDYINQATAQSFLNFGINPEEG
EKRTSSVFYTKVIDGKEAIRAIFTPTQIHPWNTISNKVATFSLDFFNKALGAPNPIPSTN
LVWYWKEIFNVIGLIGFAMFVFAFAKILLQTKYFGVLKTKEEPTPLLASGSKAKVWFWIS
LVLGAFVSFISYLYLPNIVNKPGFRPTFFVQAPVFFIGTWAVVNGLFTLVVMLVTWYFFD
GKSISLKERGIAISGTNLWRTILLSLSVVSAAFFIVFFSDFIFKVDFRLWVIPVKAFTAD
KIPIIILYLPFFLIFYVLHSVSVNGFSFIKQGKEWVNVALLAFFTDLGALMYVIIQYGIF
FARGLSWTEKMNPAISNIYGIWLFPILVYFPLAVILDRKLFKLTKNPYLGGIIFALIMTI
MACTNTLTAVP*
>SPBIB_v1_50082|ID:27161993| Integrase domain protein [Uncultured spirochete bib]
MGLSMQERHRIIAETAVRYRAATKLEKGRILDELTALTGYNRKYALHLLTWWGKTVERVV
GGTRLKLNIGIPRQRKKRTGKKKYSQELYEALRCIWATFDCMCGKRLAVFIRENITFLAR
HEEYAITDTLRAQLTAISPATIDRLLAKEKQSPWFLKRHYQANLTANHYKTKIPVRTFYG
SDEQRPGYLEIDTVFHSGPSAEHEFCCTLNATDTMTGWVELRALPNRAQRWVKEALVEIR
KTLPFRLIAIDSDNGSEFLNKQVYDWCMREHILFTRSRSYHKNDNPFVEQKNSQYVRQFV
GYARYDTPEELAALSEVYRVLCPLLNLFYPSTKLIAKHREQATLRKTYDTPQTPFSRVLA
SPFLPLKAKEQLSALKAHYDPVQLRYELDMALEKLRHAHSHTTRVEISYEES*
>SPBIB_v1_50083|ID:27161994| protein of unknown function [Uncultured spirochete bib]
MRASADGLRHLAPTWVRRRNGTPENPVPGPRYREREARVARAPAPAGDLQTRQLHF*
>SPBIB_v1_50084|ID:27161995| putative Spore Coat Protein U domain protein [Uncultured spirochete bib]
MKKFLLLVLAGLLVGLPVFAADSVSTTLQATVGAALSITTTIPGTKALDPTQTSAALGSV
TITSNLTNWKIVIHSANGGKMVRTGSTDVYPYQLTFGTTTGIDLATDYEIVKSAPQSAVT
TNVSVTYQTAATLGISAGTYEDVLTISLVAL*
>SPBIB_v1_50085|ID:27161996| conserved protein of unknown function [Uncultured spirochete bib]
MNMEESILHPQYRRYAPLWKRCRDVIEGSDAVKKARELYLKRLPGQSQEAYEHYLERAIF
FNVAGKTLELYLSLIFSKAAAIHGIPEESPLLADCDLKGNTLEEFMEETTAETLTVGRCG
VLVDYSGTIEAGMSLADAERGEARPYLVRYPAESILTWRQGRHRGKTLLDRVVLKETTSN
EDETDDSLQYRELLLDNETYTVRIWKKNADDQWTAQKTIIPLLHGMPIGHIPFYFIDPLS
GTPDCKKPPLLDLVDINLGHYRTMADLEHGRFHSGLPTPIFAGFNFQEGETIKLGSTEGI
CSNMPEAKAYYLEFSGKGLEALEKAAQQKEAWMIQLGAGLIDSYQKTQEAAQTLMIRRSG
ANATIGRIAMAVSESMTKALKFLCAWAGMPSEEVKIQLTTEYLPESVNPQEIAILLQAVQ
SGNYRRIDWLYRLKNAGIIGQDAKPEKIDAELEKSSGQVENKIGF*
>SPBIB_v1_50086|ID:27161997| conserved protein of unknown function [Uncultured spirochete bib]
MQLNLTPKQTEVLFSPATEILFGGAAGPGKSHLLRVAAIIWCNDIPGLQVYFLRRTYPEL
MSNHMDGPKSFRALLAPWIEIKKVKIVDNDIRFQHNGAAIHLRHCQYEQDMYNFQGAEIH
CLMIDELTTFTYAIYSFLRSRVRMTGIKLPPQWKDRFPRILCGSNPGNIGHTWVKASFVD
HGTKMHRTPPEEGGMIRQFIPACMEDNPYLLADDPGYRERLKGLGDPGLVRAMEAGDWDI

VAGGAVSDLWDRNRHVIQAFQIPKGWYIDRSFDWGSSKPFSLGWWAESDGSPAILPNGQQ
KTWPKGTLFRIAEWYGWNGKPNEGCKLEDTAIGKRMREMEVAMRETLGIEGVNAGPADGM
IFEAQPGKPSIAQGIEDGYGRKDLFYPADKRPGSRIKRLAIFRRMLAASEHEPLEEPGIF
FFDSCVYGAIRTIPTLPRDPRNPEDIDTRAEDHAWDDIGYRLTTVRPIAKSIQIEAA*
>SPBIB_v1_50087|ID:27161998| conserved protein of unknown function [Uncultured spirochete bib]
MTNKGQFKKGVSGNPKGRPKTGEAITELFKEYLEGIDETTKLERKRLLVEELYARAMGKK
TISKRGKQIQLPGSDELLKYIVNRLDGMPKQAVDLEAFVDGEETLTVFIERPSGAPASRA
DERREGTKDAEDQPA*
>SPBIB_v1_50088|ID:27161999| putative Tyrosine recombinase XerC [Uncultured spirochete bib]
MTNEENKYQWYAAMLDELRARKYSTSTRKRYLAICLQIAQAYPDRDLRSLKKGELESFLA
AMERGGASASTINQAISAATFLWRNVFELPFPIKVRPLKDRQLPTVITRYQVMQLIAAAN
TSKTRLALALAYSAGLRVSEIASLQIGDINRERGVIHIRAGKGRKDRVVPLSQLVSDMLD
KYLAQHPTKRWIFRGTTGGSHVNVRSLQNAMAAARAKAGLSSDVTMHTLRHSFATHLVER
GENLVVVKELLGHSSLSTVQQYVHIAKTGILATKSPLDSPPLY*
>SPBIB_v1_50089|ID:27162000| protein of unknown function [Uncultured spirochete bib]
MARRMRHGLDYFPLDTSWDLSMRLLKAKYGLEGLGTVIQLMQMIYREGYYIEWSSETRQL
FCAENQIDEPKLNAILEFCLGHGLFNQDLFKQYSVLTSQAIQRQWIKICLYSKHKNLGID
ARLNLCPENSDAYGQKREYLDSTSGKNAQNSESLRDNFGDFPDNDGDFPDNCGKFPQNPR
NMQEIGTEIKEKENKEKKSTLAEFCAQPVENKREIQALIKALAQKKSAFPNGPPASK*
>SPBIB_v1_50090|ID:27162001| protein of unknown function [Uncultured spirochete bib]
MSAIDKCLCMVPEQEAFLQFHYKHPNIYLELKALANKDRARGEKSLDIQKLLARIHDPAI
ERLRQERTDAAIILCHYRALLESDFCLWKMFRSGG*
>SPBIB_v1_50091|ID:27162002| protein of unknown function [Uncultured spirochete bib]
MKILHTADLHARRESSREFFISYDSIRSAAMRHDVAMIAIAGDIWHGPVQNSAGSLFPDF
IEAIRSLGDIAPVAMIYGTPSHDVEGSLEIFETQECSHGIKILRPGTAYVLKKGKIEELN
GGNEEEAELLISGIPEPSKRWIISAASEPGSRDADLAANEAFRMLCMATGCMRERYPRLP
SLVLAHGQVEGATTGHGRMLGTGDGLHFTKDNLKSLKAEYIALGDIHQPQHIEGTRAWYA
GSAYPLDFGETHRAGCWIVDIHEPGKPVDVVRENFPHPTNRHLISHASCAMEIPTMHNQK
VWYEVQGTKQELAPLDADIILSRLLAHGAAKGSKVTFDITDSDPVRASEIRTKKSLEEKL
STWAQVSGETLTESIIEKARSLERETAARNAAAGNARYRIDRLILRGATGLWAKSRKDEI
DLDLSSRGPGVIALIGANGAGKTTILENLHPWPRLLTREGPLRDHFRLADSFRDLYLTDE
ATSCKYRCLIRMRADIPSGTTEYWLFRDAGQGYVPLPGINGRLEPYQEWIERLFGSLALY
QRTAFTAQKNSKSCPDLSAATKGERKELFSELCGIDWLEAYREAAKEKEDALSESLKSLE
AKHSILAGSQARCAALQKEIEEHAAYADEKSREEKEIVRKLEEAKDELAKIEKMNQERTR
LIREREEARMRMLELTKKENECMGSIESLRASLRLKPEMQSIINRAREIENRREALAAEK
AAHDARQKQEMKDYLLAMTSYTTQRNDLVAAMNKIKVEIATLKERAHTIEERLAMPLGEN
CPACGQKLPPEQLARQKELRIADEASLESIYAKLIDMLASKKETEQKLQNLVLPSYPAQM
EYPGTEELKSLSKEFAAIDLVRAYDIVQRAEIAEGTIAHLRIELKKLEEEIGKVNRIDED
SKAKLDRMPPKHEEEKLMEAISTLAEELTNTKLDIARAQTRREEAEKQLAEAKRNLEEYE
RLGEQLKALTQEICEWALLGRATGKDGIQALELDALAPSISAIASRLLAASGNEGSIAIQ
TLRLAGKGSRQHAIEDFEIMYISARGDEQEISTLSGGEAVWVRKAIYDAFELIRAQNTGI
QFRTVILDEADGALDHESRLRYLRMIDAAHRESGRYQTIIVTHSLELQEMADMSIAIADL
KPHADRQNAKDIAIPA*
>SPBIB_v1_50092|ID:27162003| protein of unknown function [Uncultured spirochete bib]
MTKSQALEIIKRYPDAKNYIKQSHLEQLSPLVEVAIEAITCKKEDFHCLPGNTYMPRKET
IDRFAMAAGISFNPLEESTRKENDCYIGRSQAMVMGPDGKYCYGDVCEYEYDVTIRHEEE
MLIDRNSRTPRLHAGAKPVEDRARLAYLTLRKTARQRANTGARSRAILSILGMQTGFKDL
FPPDAPPTAEKTFLFSRIIVNTKNEMVLHRMLDSLTAPAKALYGSDARNLEAQPLHLALP
EQPEPEEDPDPATDEHEEAIASIQESLDSGLLGPRATLAARDALLNHQSDDAYLADIAAR
LKAAIENRKHTQQNNQ*
>SPBIB_v1_50093|ID:27162005| protein of unknown function [Uncultured spirochete bib]
LLDVVLGVGVALDDLKRLAFGHFLSLLCYWQGMALGAVLFLQGRLVLLFDPGVEVCDAEE
DAGADAHVGDSAVTGFPVDVGF*
>SPBIB_v1_50094|ID:27162004| protein of unknown function [Uncultured spirochete bib]

MESKIPVNNPMPFPRRERILISVNEAAQMLNLSKSYIYRETRNRTIPHVRIGSRILFRVT
DLNAWIEQQNQPALQEQDCA*
>SPBIB_v1_50095|ID:27162006| protein of unknown function [Uncultured spirochete bib]
MKNSLLKRLALTILKIKIYGGYYYSLWHTHEYPIGEDKRGRRNRFVLVAIAFFLCFQKKW
*
>SPBIB_v1_50096|ID:27162007| protein of unknown function [Uncultured spirochete bib]
MDSNRYYLTTYGIDGNPLVEVDFSIIDQIKKSMNILAIIRTIEEQYDIVVGNYKELLKEI
FEVTIDNNLYSPSDIIFLHEFSRKINIRLLNFLNSAKMYQDHLHASLLKIDNTKGRSYIQ
RVGQLKKQNISICILEALRNHIQHYGIPTFTSEGTKRITRNTEDIIVHYLLIIIEVEYFK
KDKKVRDTIPKEILSQNIELNSHIKEYFSYLSNEIEEVRQLFIEEYSSSTILIDRMIEMY
SKVYPEIMEKWQKILCIIRNEENSYMVEFNRNNIDLIEKLRSRNKKGINLINRFFTDFDT
LALNRL*
>SPBIB_v1_50097|ID:27162008| TPR repeat-containing protein (fragment) [Uncultured spirochete bib]
MKRKIRVFFASPSDLVDEHKIFGEAITQLNVGFGDGANVEFEPFGWEYSLASTGRRAQSV
MNDKIDICDVFILALYRRWGQDAPDASPYSSYTEEEFYRALDRWERTSAPEIFVFFKRVD
AESEADPGPQLRKVLDFRKHLEETHQVLYKYFDSPQSFIDLVDCHLRAFAKDELPKPDKR
RDVVILPMSILQQVEKAKEIALQKTEEAKKAKDAAEQARHQLEALQLEIADDASQFSKEG
KIEFAREKLTKLVTETTNLHILYLGFDFFYRTGDLDSAIKVLKKWLSLSGDENKTEVTAM
AFEMLGILYQTRGELDRAEEMYQKALAIEEALGLKEGMAADYXX
>SPBIB_v1_60001|ID:27162009| Methyltransferase [Uncultured spirochete bib]
MENYKSIYLTADFEIDDGTLKIVNDYLAKQPEPRPFVKPNSLGYVFYFHTLDVSLLRRNI
EALQSLDLFSGNGSNNSSKAGEILRIIYDEVSKIGSYGVKSGKRQYVDFNLERKVQNRKD
KEKNRSQYYYAQKNNFSETNNPLPENLTNKIICGDSLEILRVIPDNSIDLIFTSPPYNFG
LEYDSQDDAHKWELYFEKLFAIFDECIRVLKFSGRIVVNIQPLFSDYIPSHHIISNFFIN
RRMIWKGEILWEKNNYNCKYTAWGSWKSPSNPYLKYTWEFVEIFAKGTLKKSGDPQNADI
TPDEFKEWVVAKWSIAPERKMKEFGHPAMFPEKLAERVIKLFSFVGDTVLDPFNGVGTTT
AVAQKLGRKFVGIDISQEYCDVAQKRVKSMLL*
>SPBIB_v1_60002|ID:27162010|ddeIR| Type-2 restriction enzyme DdeI [Uncultured spirochete bib]
MPYNIGEIQKAYNILVGGIDDKANAENDEGSRAYGGVVRSAKGILVEGIAKNLVEIAWNE
LGGHPSRLSFQKETIRIPLKPEYLKRVRPAEVAEYIKAHIQQYFYGHKTDVHVSIDGQFV
MGIECKAYTENAMLKRILVDFTLLKQVVPNLKCVLLQLESQLTGDYSQPLKPIIYGSPSS
HTLLSYFNVDLNIVTLLEGERKVDEPIHKKPYFKPMTEQALIKAVETLKKLLSEFVVRK*
>SPBIB_v1_60003|ID:27162011| Integrase domain protein (fragment) [Uncultured spirochete bib]
VEQKNSQYVRQFVGYARYDTTEEFEALREVYRVLCPLLNLFYPSTKLIAKHRENATIHKT
YDTPQTPFSRVLASPFVSLTAKEQLSALKAGYDPVVLRYNLDTALDKLHHAHSNKTLIEV
LYEKE*
>SPBIB_v1_60004|ID:27162012| Integrase domain protein (fragment) [Uncultured spirochete bib]
MGLSMKERHRVIAETAGRYRAASKKEKSRILNELTALTCYNRLYAMHLLTWWGKTVQRVI
GGTRLKIVIGSPRVRKKRNGKKKYSEALYESLKRIWATFDCMCGKRLAVSIRENLAFLAL
HEEYAITDAVRAELAAISPATIDRLLAKEKQTXWXXKRHSTTSEAANHYKTKIPIRTYYG
SEEQRPGYLEIDTVFHSGVTVHDEFCCTLDATDTMTGWVELRALPNRAQRWVKEALVDIK
ETLPFRLIAIDSDNGSEFLRQAGL*
>SPBIB_v1_60005|ID:27162013| protein of unknown function [Uncultured spirochete bib]
MDSVNRALDTIIVERFWRSLKYEDIYIKDYRTLAELKLGVSRYIRFYNSAHCLIENLSET
IPMPHHKI*
>SPBIB_v1_60006|ID:27162014| protein of unknown function [Uncultured spirochete bib]
VALEALKGEKTLQELATIYAVHPNMIALWKKQLVERASMIFEKEGKDKEAEAAERTQDEL
FRQIGQLQVENEFLKKLQTAVWERTEAIEPEHPELSIGRQCELLGVSRSTFYYRPEEAIA
RYGVPGIFNTVKGVSSPLMRFSQSWKPMEYRSVWIV*
>SPBIB_v1_60007|ID:27162015| conserved protein of unknown function [Uncultured spirochete bib]
LNNEEVVGHTPSNRMNPEVDAYLRDTKKWQEELAKLRTIVLDCHLTEELKWGVPCYMFQD
RNIVLIHTFKDYCALLFFKGALLKDDKGILIQQTGNVQAGRQIRFTNVREIVEMEPILKA
YINEAIEAEKAGLKVPFKKTAEFIIPEEFQKKLDEIPALKTAFDALTPGRQRAYILYFSA
PKQSKTRALRVEKCIQQILNGKGLNEGE*

>SPBIB_v1_60008|ID:27162016| AAA ATPase [Uncultured spirochete bib]
MAAYNKYMFKREYEPLDQYLSPNKVLVIYGPRRVGKTTLLQNYLKQTPLKYKLDSGDNIR
TQQILSSQDFAQILSYVEGYELLAIDEAQNIPNIGMGLKIIVDQIPGIKVIVTGSSSFEL
TGQIGEPLTGRKTTLNLYAMAQSELLSVYNRFELREKLEDFLIFGTYPEVLQAPNQRTRI
DILTEIANAYLLKDILALDRIKNSRTLLDLLKLLAFQIGSEVALTELATQLSVDVKTVKR
YLDLLEKAFVIHRLNGFSRNLRQEVTNKSKYYFLDNGIRNAVIAQFNSLDQRNDQGQLWE
NFMLVERLKYRTYYSLYANMYFWRNYRQQEIDLIEEHSGNLFGYEFKWSHNKAVRPPATW
TENYPDATFTVIHPANYLEFILP*
>SPBIB_v1_60009|ID:27162017| protein of unknown function [Uncultured spirochete bib]
LNMYLLYAAMGYLSNAGNMADYFDVKY*
>SPBIB_v1_60010|ID:27162018| Extracellular solute-binding protein family 1 [Uncultured spirochete bib]
MKRIALAFMVLMAATAAFGAGKLTLMQNKPEIDAQIKAYAAEWGKKNNVTVVIKSIGGTS
GGMGPQLKADYAAGDMPDIFAFDGLESYKEWEGVILDLSKEPWVSKTSVAFKYNGKVYGF
PVAVEGWGMAYNADLLAKAGIDPKTLTNYDAYKKAFEKLDSMKKELGINSVVSMAASVEM
GWVTAHHNFNSLLSNGLPYGDLSVVNDLLAGKVDMQRLQEYADWVELLFKYADKTVLLTG
NYDAQVGAFATGKAVFLHQGNWVDPNLKAANATFKMAFAPHGSMKKTTDGIFVAAPSFYA
INKDSKNVALAKKFLNDLVTTPEGHTYMVKDAGMIPAFSGINLNPEGQLSKSVQQWSAAG
KVYSWNQYYFSGDFRDKVLTPIYNQFAAGSITKAQFVDLMAKAFKDNAKK*
>SPBIB_v1_60011|ID:27162019|amyD| putative starch degradation products transport system permease protein AmyD [Uncultured spirochete bib]
MTKKTESSLVFWVFLAPVLFAFIMVMVIPFFLGSYYAFTNWSSSARVDGGLRFVGLQNFA
ESFRDPAFLYSFGITFAYTILNMIAINVVAFALALLVTGELRLKNVYRVGFFVPNLIGGL
ILGYIWQFIFNNAIPSLGRIIPVLGFLANPGNLMLSKNTSALAAMIVVGTWQYAGYIMMI
YVAAIENIPQELLEAAKIDGATPWIRLKSITIPLCAQAFTVTMFLTLVNSFKQFDVNVSL
TSGGPSTMLMGQPILGTELLALNIYNTAFISNKLSVAQARAFVFFFVLAIISIIQVYVNK
KREIEL*
>SPBIB_v1_60012|ID:27162020| ABC-type transporter, integral membrane subunit [Uncultured spirochete bib]
MKQLRSRKPGMIVAEIVTALLFILFLFPFVLVLLNSAKTSFEVTQYPLAWPSRWGNIIDN
VVKIWTSESVRYPSSLLTSTIVTVVSLVLINLFSAQAGWVLVRTKSRISSIIFFIFVASM
VIPFQIVMFPLLSWFRTVTVATGIRLLRTYQGIILAYIGFGAPLSIFMFHGFIKSIPLEL
EEAATIDGCKKYQIFYRIIFPILTPIQATVLVLNGIWIWNDYLLPLLVLGKGNDIMTIPL
AVSNFAGAFVKQWDLILTAILMAMVPVIIFFLFAQKYIVKGMVAGAIK*
>SPBIB_v1_60013|ID:27162021| putative integral membrane protein [Uncultured spirochete bib]
MRPMKVLQPKHKIEHATPAFIFLSVIYATLLIISNITVVKLVKVGPFLLTAAFFTYPAVY
VISDIMTEIYGYRLSMKAIWANFAAQALMSAVLAFATWLSGTDNAINDAMKTLFSSTWRI
VIGSLAAYWVGDWMNSAILSKMKVAQKGKWFFLRAMGSSLPAHFVDTTLFNIVAFLGVWT
TGDIVRNALSESSLATVYELALFPVTFLVVKLWKRIEGIDVFDEGISYAPF*
>SPBIB_v1_60014|ID:27162022| exported protein of unknown function [Uncultured spirochete bib]
MPGRLRRFFESLGPAAASSSAISAKNAAAMGQYAAVYNQAASDAETCLAAFKKISPDELP
EAEKSAHACMDQIRLLATTLNELDGLVASMPLRELEKDRAAIEAKKRTAPPALAADYDKS
ISEIDAQRQAHQSLLERKESLEIKLHSMSNQFRQLSLDLASAHAVDAQTKLDSQHAALAT
LSKRAEEIRASIEDLRTGSDDWLSMEIEKLSQNGA*
>SPBIB_v1_60015|ID:27162023| protein of unknown function [Uncultured spirochete bib]
MSGEEIRLCAIMHAEIAKPDALLALGKEEAGAAFAQFRETIRAAAEEKSGRFLESSHGGA
VVAFDTSAAAISAALLIQQNLKPQIKAKIGINVGDVLFTSSGGAIGSAADIAEELVRVLP
AGGICVSAAAWHASPSVDARTIHVQLELPSGTKLDAYRIETGENSLQKCPDA*
>SPBIB_v1_60016|ID:27162024| putative enzyme [Uncultured spirochete bib]
MLAETKYITSVPAKQCILWSTIVYRHFPDYKQVMLPINSLVMYKGRPAIVVAADLDRISI
RTIDAEEKRVRPKDVAMAHTGPISRFPENRAPDEVMEALELLQTEHPEGIPLISWKDFAE
LAWSDPKPEHIVIAWHELSNNPAVETLDEGIRIRSEEEQHKIRDKLQKKKEAAEAKAAFL
VAFRAAWKKKDSSPIEGNSAFQPFLEELSRYARGLADDSPIARELGIKLAPQAVHEALLS
SGYWNLAANPWPERNGCIMSEPEAPSALEERKELAIDRLDLRALPSYAIDNAWSKDPDDA
ISVEGENVWIHIADPASIVGPFSRLDAEAMERGSTLYLPEKIIPMLPDALVQKMGLGLSA
ESKALSFRITLRGDGKIADISILPSMVAVQRLTYEQADTMLEDNPVLQKLSKIASVRAEL

RRRQNAVDIDFPEVAVHVVDGLPTFLPVPQTRSARIVQELMILAGEAAARWAFEKGIPFP
FATQDPPVSENLAGCGAGPSSSLAENFMRRRSMRAALVSSTCSAHAGLGLSFYSQVTSPL
RRYQDLLAHYQIHAVLAAANKLDRNNAPSEPDLLISADTMNERLFRYSNQAAKNRQAERD
SRAHWTLVYLSMNPDWRGEGIVLDASAETGQIFIPQFGYEFQTRIPRGIHVDSRVSLALR
RVSVPDLFASFDILPQGT*
>SPBIB_v1_60017|ID:27162025| conserved protein of unknown function [Uncultured spirochete bib]
MTALLVGIGLIIISVLMVLPGVLGWWNDVLAFLRGAIPVFAVLIGALAVFIGIADIKDKI
EEKKEEEKSKNAENEQETKEEKKD*
>SPBIB_v1_60018|ID:27162026|rplM| 50S ribosomal subunit protein L13 [Uncultured spirochete bib]
MKTVFVTAATAERKWYIIDAAGKPLGRVAVKVASILRGKNKPTFTPSQETGDYVIVINAD
KVAVTGRKRQNKMYYHHTGFPGGLKDYSFDELIDRNPVSPMEIAIRGMLPKGPLGRKLFK
NVKVYAGPNHPHAAQMPIAIDL*
>SPBIB_v1_60019|ID:27162027|rpsI| 30S ribosomal subunit protein S9 [Uncultured spirochete bib]
MRKGNEMVKNINLGIGTGRRKCAIARVFLREGTGKIIINQKELKEYFPIEDLVAKVRKPF
LVTDSENKFDAFITVVGGGPTGQAGACAHGLARALAQIDPANHVSLKSNGLLTRDPRMVE
RKKYGQRGARRRFQFSKR*
>SPBIB_v1_60020|ID:27162028| protein of unknown function [Uncultured spirochete bib]
MALRIESVREKASGTLELALSGGLLFHFDSTDVRLCGMRFDSSSRMLITDDGSRLEFAPE
AEVENEMLVSLRRLDQLHAARKVALGLVARAEQASIQLYEKLAKKGFTKETARIAVQWMC
ENGYVDDRRYVRLLLQSHLVRRGQGPERLKAIAWPRIGLFENPRIIFAEAFSSIEEENLL
EAMRRSTENLLKRGKIPAGYRRTILDDENAENPAAPLSRSRKLAFLRSWFRQEGFPNYAI
DRFLESWEIENKDES*
>SPBIB_v1_60021|ID:27162029| putative PTS IIA-like nitrogen-regulatory protein PtsN [Uncultured spirochete bib]
MDDDILTIEEVAKYLRVSERTVYDWAQKGEIPSGKIGTVWRFKKSEIERWVNERLSSNRP
VALISSVRIQNIISPDRILFLNYPSKRDALMALAENLASAPQIKNKQELISEILRREELM
STAIGKGIAIPHVRLNSVTDLVVSIGISRCDIQDFQTLDDVPVRLLFMIAAAYNQHAYYL
QTLSFFSARLKVVELRDGLLSCKSAQEAYDLLIRQDE*
>SPBIB_v1_60022|ID:27162030|smc| Chromosome partition protein Smc [Uncultured spirochete bib]
MASLRVLFILQERNDFVFLKSLEIFGFKSFADRVRIEFSPGISALLGPNGCGKSNIVDAI
KWVVGEQSAKSLRAESMEDIIFNGTENRRPLSVAEVAITLANDGEVLPLDVPEIEIKRRL
YRSGENEYFINGKQARLKEVRELFWDTGVGKSAYSVLEQGRIDQILSSKPEDRRYLFEEA
AGITKHKVRAREAEQKLAKTEENMRQIESIVAEVKRSYENLKSQADKTIKYRLLKEKIFE
TELDLYLVRLRQHVRERDRNSALFDQKKKEREELVQRIERMSEAMSQGLDLVNELEAKLV
EMQKLLYGLAVEKNGKEKQKILLAERVRELKAKIEQLEGRDRAIASKIESLRDEEGEKEA
EYAGYRARVREVDANIHTFDENINAAALSVKANEETIRQNASESAEIAQKTSQLRQELDA
ITERIAELVDERLRQSESQIEARQNLEAQIHRAILEISASTAARAQRLEDLAKTLPAMNA
QERAQQIDTISADLRALSLLSKSLEQDFTRYIAIAPTFLNELVAPEGVMTQKRRIDAAIL
AHAVRLKEIEAENQALSSRNGDLSRKIETYRKTLEEARLEKAKIQAQMEAAQEALSVLRR
EIAGQEAYRHEIASELAGEQKRQQELQEEIESLEEELAEIEQKGRRLSEEMAELEASISQ
RTSDLAERRKESSELEQKMQALQQELENLHMAVAQSETEIRNLKDTFTELYSRDLLSFES
RMYEIRKPISEIREELTSIKATLAALGSVNLMAPEEFEEVKQRYDFLSGQYEDLVHARTD
LVRITDEIRTESAQLFIDTYNRIKKNFHNMFRRLFGGGRAELRLLDTDHVLESGIEIYAQ
PPGKKLENISLLSGGERSLTAIALLFATYMVKPSPFCFLDEIDAALDEGNIIRFVNLLRE
FGTASQFIVITHNKKTVAGADTLLGVTMEESGVTKAIAIRVQNDNGVVRPVYLPDEPFQE
EEVEYEDGRQLDDQAKSANAPA*
>SPBIB_v1_60023|ID:27162031| putative Threonyl/alanyl tRNA synthetase SAD [Uncultured spirochete bib]
MNVHALSERLFYQNPWLQEADAVIKAIEYGAPDVSADASAAKSGVETLRVLLDITVFYPE
GGGQPPDTGFIDDLRVIDVQEIDGHIWHFVELPGNPSSPLKPHDPLKPGDRVHLRIDWQR
RLDHMQQHTGQHLLSAVLEQEYGIHTLSFHLGTEYSTIDVSAKNPEELPLPDIEAKIEDW
IGHDVSVLVHYCPPEDIAAFKLRKKPPANEAVIRVVEIEGYDWSPCGGTHVERTGQLRAI
KILSLERYKGNVRVYFAAGARAVRLLSATYEETKKTASALGVGIGGISARVFDILGKIAA
LERALKQSTQTWAAAEAKLAASQAAPHEVLEFRLDEEGADSAAELAKAAAELGRAAIAIS
LSDKTIIIQVPQVAGFLSLAGMLKPKLAEFGGKGGGGPAFFRASFSSESELARFAEEAKQ
ALAALSD*

>SPBIB_v1_60024|ID:27162032| Thymidine kinase [Uncultured spirochete bib]
MQPNDETGEFLKSLGFPQLNIHSMSRHHDFESAGKRVLVIGPMGSGKTEFSARIWRDSRV
ALKKKGAAAQLTSSGVVDRRDIFVVRSALDKSRFPDYPDDALAYRGGYERCGDRIGAAHD
SFSLEMLIAENPQVGTWIIDEAAFFDERIAYLMKDEAESRGLSFILPTLVLNFRREIFNQ
TARLLLETATDIIPLTAYCEHEECLKDSLYTYRYYLIDGEECPALYFDPLIIIGGDRTKH
DGKEPNYCTRCDEHHYLPGKEYTFFTLKPFGELAARGNIAPLLAELNALVGNIEESRLYA
SISSRCVEGPGREVQMNALRVPCIAERALVYLYAEQNLLSEEQMRYFIDELNLDREYLSQ
RLADNRRPLAL*
>SPBIB_v1_60025|ID:27162033| ABC-type transporter, periplasmic subunit [Uncultured spirochete bib]
MNSRLALAVFAIAALLAPIGAQETEFVTVYGAQLPQLDPQRALFSNEAQIHTALYEGLFT
YDPQTLEPVRALAQSWSRSIDGKVYRFSIRENAQWSDGTAITAEDFVRSWLRMLDLNAEY
ATFFDIIAGAREYRLGTDRVADHVGIKAIDPMTLEVTLVRPAAYFTRLLCHQSFALIHPS
MIGVKDWRAAIPYPVNGPYRPISMTDSELVLEKNEKYWDAASVEIPTLRMMFTDDDNLAT
SLFNTGHVHWLDGPGNYEQVLLQTAIQVFPIYSTHYWFFNCEFAPWNDQRVRRALALLLP
WDTIRSSDTYRLPAKTLVLPLPGYSKTKGIEKADKEEAKKLLADAGYPNGQGLPEIVIAF
ADYKQPRAIATTFKTEWEKALDLHVTLKPMAPSSYYDSITNRADTTSFTLAHETWIGDFA
DPEAFLQMWTTGAPLNVAGYEDKEFMDYMNQSYAANDDTRMQLLSKAETELLQGAACLPI
YHNFAASIIDAEYVQGWYQNALDIHPYKYLHFGTPSVSPNVADANMPAKARPVSALQ*
>SPBIB_v1_60026|ID:27162034| GTP-binding protein [Uncultured spirochete bib]
MGLEKAFVPERLVVGVLSSDEAAENAALAEMIERYGQLCFHSAKELFLWTRYYCPEMGDT
ILRSYWAFEQLVDPSMLAAIKRQTNAIELKLARNGKRTVNLDPGMLGSARFCLATTKDHS
HRIPLSDGIYAELTLMFEHGEFRALPWTYPDWASQPVRQMLSELRTALLADLKRLTSALN
LV*
>SPBIB_v1_60027|ID:27162035| protein of unknown function [Uncultured spirochete bib]
MGGGSSAFRPADTVGLXVVVPHKVGPEAQHAVCDIAVEADTLTMVVRQVLSELAIGVVRR
KEX
>SPBIB_v1_70001|ID:27162036| putative Histidine kinase [Uncultured spirochete bib]
MTNLGSSVRKRSGRIFAVSAIALAVAALLCFFIAKNYNPAVGVTILAIFAIFLFAFTFRS
IVSMYQMPLDAISQQADEIAKNLQSRTQEVAELTTKLQSIFDTTEEAIAVIRAPNELLSA
NTSAYRLFGLSPGAGYTTESFFFGSPAILGLIETCLKEGKASIEQFSMLKDKNEIILSAR
AQRFAVSQSDAAVIVISDITSSKRTELTKKNFVANVSHELRTPVQIVRGYAEMLNTADIP
EENKSWAEIILHQSLRMERIVSDLLMLAKLEHDPASWIVRERFPIKPILEEAAQTVKLQY
PEISRISIDCPDELEIEASPGLIEQAAFNLISNAAQHSGSHDKIIVGAMQENEDFVLRVR
DYGAGIPPKDLAHIFERFYRADKSRSQSSGKSGSSGGSGLGLAIVKHIAFAHGGTVHAES
WAGEGALFEFRIPARGSEYCSQPCVKT*
>SPBIB_v1_70002|ID:27162037| Two component transcriptional regulator, winged helix family [Uncultured spirochete bib]
MDTILLVEDDRDIAQMISTSLGRAGYRVIVMPDAEHAHAFLKDGAVSAILLDLMLPGMDG
FAFIRKLKKNPALSAIPVIITSAKDDDTDVVAGLELGAEDYIVKPFSLKVLEARLRAVLR
RSDASYINEGTSRRVQKSGILLDSARHEVRCQDIPVDLSATEFAILEVLLKNPGMVFSRD
RMISEIRGGDVAVTERSIDVHILSIRRKLGEKGSLIETVRGVGYRFRDE*
>SPBIB_v1_70003|ID:27162038| Phosphate-specific transport system accessory protein PhoU [Uncultured spirochete bib]
MPREAFERSIEEINKRIISMGAASVEALQKAMHAFRERELDAAKKIKKADADIDAQQLQI
EDLVATTMATQQPVATDLRLLLCSIKMAAELERSADYAAHLAKATKFFAGEPQWRQTEMI
EQMVQIGATMITGTVQAFISRSALQARQVALMDDQIDHIHKSLIKEMVMLLGSRPEDAEK
VAKFIQVSGYLERLGDHMTNACESIIYMVEGIHMELNL*
>SPBIB_v1_70004|ID:27162039|pstB| phosphate transporter subunit ; ATP-binding component of ABC superfamily [Uncultured spirochete bib]
MPEETKSPDSFIIETKDLSITYGDGHEAVKHVSLGFPERNVTAIIGPSGCGKSTLLRALN
RMNELIPNTRTSGEVIYRAQNIYNSSIDPVIIRRQIGMVFQKPNPFPKSVYQNIAWAAKV
HGYAGDMDDLVEMSLRKAALWEEVKDKLKKNALALSGGQQQRLCIARAIALQPEVILMDE
PTSALDPIASSYIEDLISELRKDYTIVIVTHNMQQAGRISDYTAFMLMGDLVEVGPTRKL
FISPSDERTERYLTGRFG*

>SPBIB_v1_70005|ID:27162040|pyrD| Dihydroorotate dehydrogenase B (NAD(+)), catalytic subunit [Uncultured spirochete bib]
MKNPEQEQSKKNEILAGPPPSVLNLGYDLNLSVRIGSLIMPNPVGVASGTFGYGEEYDEL
VHIDALGALYTKAVTLEPREGNPPPRLVETPMGLINSIGLANPGVEKFLREKLPSLRTRL
CPIIVNVAGSTEDDYIQVIERIEAHLASTDSGRPGIDGYEINVSCPNVQKGGMSFGIDPR
LVERLTHSLRQKTSRPLIIKLSPNVTDIAEIARAAEAGGADAISCINTVVGMVIDTEKKK
PAIAMGTGGLSGPAIRPIGVAATYKVGKAVRIPVIGLGGITNASDAIQYLLAGALAVQVG
TALFSDPRAPLKVLDGIIEWMKRHSVHSVSDIRFMLR*
>SPBIB_v1_70006|ID:27162041| putative Dihydroorotate dehydrogenase B (NAD(+)), electron transfer subunit [Uncultured spirochete bib]
MRQFNATVAINRAIAPDWMHMAFEWPSDLPAPKPGQFFTALPPVVELRAGTILRRPFAFA
GFEEAQIEKDSSMLSRPAYAHSIYQVRGPGTRALASMQPGSSLDIIAPLGNAFPYPERGE
RAIIAGGGIGIGPMLFLAEQLGAGTYASSAILVLGFRSALLIPFSGSFSAAGLPSWKKLL
EKAFFSTDDGSAGTPGTVRDALEALWLQASDLKGSGTKNLAQKKWHLYGCGPGPMLASLA
QFAAQNHMHAHFSAEQWMACGVGACHGCVLPAASGGFLRVCADGPVFDARTINWEANVQ*
>SPBIB_v1_70007|ID:27162042| conserved membrane protein of unknown function [Uncultured spirochete bib]
MLRMIQILVLLAFLFGGALAASIRLAPKPRVVEVSITAALWLLLFSMGFRIGNNPELVHS
IGTIGLLGFASAVFTIAGSCIAVVLVSHFAPAVGSVRKIGTAAQKENGFSAASSGPADSE
FVVGASYSVLARLKPPAILLSVVVAGFIAGIALPRLAFDPGLITEWTLNALLFLIGMQFR
QSQVPLASMLRSPAVVALPLATAVGSMAAGLLLVPFFSLRVGKALALASGFGWYSLSGVL
ISNLGDPVLGSSAFLANMIRESLGLILIPVLGQTRVPTMAISVAGATSMDVSLPLIEQTL
GPEAVPLSFISGAFLSALVPVLVPLFMKL*
>SPBIB_v1_70008|ID:27162043| conserved protein of unknown function [Uncultured spirochete bib]
MNQDRVHEILEAIEPAPAPFTLVFSGRTNKKVNGLYKPAKAEIILHNRNFDSDNQLIYTA
IHEYAHHLIFVRHGGLPMPRPHTQEFWALFHELLEKAEQKGFYTSVFDSVPEFASLTMEI
KNRCLAGNGSLLLELGRLLVQAEELCRIHKARFEDYVERVLGLPKRTASTAIQAQTLNLD
PSLGWDGLALVASIRDPEARSKAAEALSNGATPLIAKRVAMPPQDDSGDPVETLLAEHKK
LERSIASMTERLQRIEDKLRTMGISEDEISS*
>SPBIB_v1_70009|ID:27162044| transposase [Uncultured spirochete bib]
MFVQTLFEKKTGRTLLFYYTARRVKGKIVKTKVKRIGYLDEFLDAYPDPLTHFRQEAKRL
TQEAQLKTLTVTFSMDEHFSFGAGFAATEDAAVEKADRTFHYGVLPLLQLYRELKIDAFL
RIKAQYTKVDFNHNHLFQMLVFGRILFPESKLATWRDRTRILQHSDFSDDAVYRALPFFA
QIKDALVQHLHEQVQRQYHRDTTLLYYDVTNYYWEVDREDELRKRGVSKEHRPEPIVQLG
LCMDNSGLPVTYGLFPGNTNDVATMRPMMQHLAESLGTKHLIYVADKGMMGGMNIAQIIL
EHNGYVISSSVRKADAELRRYILDHEGYTELAGGSFKYKSRLVPCTLYVDTPDGRKKQIR
INERQVVFWSEDYWKKARHDRDMAIAKAMARAGYGENTVLNNHAGNRFIKKEIFDPDTRK
EVDHPEFSFALDQELLDSEEELDGYYLIRSNVVGVREGDAPFNQPYRWHAKDNLFELNRP
VVDLDIIDMYRGLWRIEESFKITKSQLKARPAFVHRQDSIEAHFLSCFVALLLLRLLEKR
TGEKIPVATIVESLRKAQLVQLEDETYVNACCDNVIEAIGQALELDLTKKYYTKGELKAL
RGKTAKSR*
>SPBIB_v1_80001|ID:27162045| conserved protein of unknown function [Uncultured spirochete bib]
VHWFAFCPNPNCPYHYEAPASSWAYAIGWYATKAFGKVRRFRCTRCGKSFSTQTYSTHYY
LKRIVSYRDVLYXX
>SPBIB_v1_80002|ID:27162046| Uncharacterized glutaredoxin-like 8.6 kDa protein in rubredoxin operon [Uncultured spirochete bib]
MSVAIYTTPSCGYCRMAKDWFRQNGIPFTEYNVAADMRRAEEMVRKSGQMGVPVIDVNGR
IIVGFNKPEIERALHR*
>SPBIB_v1_80003|ID:27162048| protein of unknown function [Uncultured spirochete bib]
MKNNIYKVVRQDIERQKMKQASNCLRLVNVGIKEYCLISAWVVAFTCGSGCLGLGGRISL
SASTSSTRTYFQYELSPAFQIQAAIPLAIRNEVLTQSVFAESAFAAKGKIAGAEFAAGKL
AINDAAGFLAKADAISNRGSSTLSVSSPGSVNDPWGLSVGAGKWALFAAKGPLYSDAAFQ
YVLADRPGRLAVAAGFLMDAQQEIGTALRHASPWIAISAGYFHHSSSILARIQMLPNFQS
ARQIQADLDWAKGAAARLDFSFRKGNSSVEGFAYAEAGDFISASGDVAAYDALIYSHYDA
VFPGSPIIKSVSLRAGIYSKQGAVSPKTGSLPAWGQYPDPLLLRYWPDKADINFKINNTE

RRFNIFNSPGVLDSAFSAGISIEPSDWRARLQMDLHTRHAGKTYPQYGINLAIQGSFALP
EDKANAASEPENAVENAEENAADGNFYEYPEQQGRAISYLDRFLGRMQISLSFLFEKFSS
RLAVAIPMNAEESRTYSCKIQIGLKTEPLHLEGSLTLEYNPEAMIISLPYARVFASFAL*
>SPBIB_v1_80004|ID:27162047| protein of unknown function [Uncultured spirochete bib]
VRVLLVEALSEILPPRPRHPEPHVNATTHAEIKQYSLIPTFTRRKQLLACFIFWRSIS*
>SPBIB_v1_80005|ID:27162049|pncA| Pyrazinamidase/nicotinamidase [Uncultured spirochete bib]
VESALIVVDIQNDFCPGGVLAVPDGDAIIPAANSLLAAYPISVLTQDWHPLNHCSFASAK
SLPPFSLDTSAEPPNVLWPDHCVAGTKGADFHPALQSWKARFIIRKGTRKELDSYSAFFE
NDGVTPTGLSGLLSSLGIQRVLVCGLATDYCVKATALDARRVGFKVVIVEDAIKGIDANP
GDIDKAKTQMRDAGCVFAKTHELLAKI*
>SPBIB_v1_80006|ID:27162050| Nicotinate phosphoribosyltransferase [Uncultured spirochete bib]
MNCLYQSALFTDFYELTMAQGYWKRRMTMPVVFDYFFRRHPFGGGYSVFAGLATLIEALE
DFAFSREDLDYLATLGLFEKEFLEYLSSFRFKGTIYAAREGEIVFPQEPLLRVEADLITA
QIVEGLVLNVLNFQSLIATKAARVWNASRRGAIMEFGLRRAQGADGALSASRAAFIGGAV
GTSNTLAGKEYGIPVLGTMAHSWVMSYASELEAFNAYADIYPKNTVFLIDTYNSLESGIV
NAIEAGKKLKEKGLSFGVRLDSGDIDYLSRMIRSRLDEAGLQDVKIVVSNELNEEIIESL
VDDKAPIDVWGVGTNLVTGGDEAAFTGVYKLSAIDPEGEKRPVMKFSDNPEKSTNPGVKN
LWRMYDEKGSARLDLISCSEEEIQEGTEYIVHHPSADWRQLKIVPARIEPLLFKVMDHGV
RVLDLPDIKECQAFMKERIKSFDSTYLRLLNPHIYKVSITDRLYDLKVSLINAFLKQKLS
KS*
>SPBIB_v1_80007|ID:27162051| conserved exported protein of unknown function [Uncultured spirochete bib]
MSKNLSGTDSMHTAPRSRKTLKICVFCGSSMGNSPVYADTAARLGRTIAGQGMTLVYGGG
NVGLMGIVAESAMKTGGKVIGIIPERLFALVEQQELTELLVVKDMHERKALMQKKADAFI
SLPGGIGTMEELFEVWSWRYIGYHQKPVALLNIEHYYDQLLSLLRHMVNEGFLHKEIYDD
LIVDTDMDRLIESLVAKIGKGAAVPFFKKPERRS*
>SPBIB_v1_80008|ID:27162052|aroE| Shikimate dehydrogenase [Uncultured spirochete bib]
MPNRNYKAELVGVFGYPVAENPTVVMMEAAFRALGLNWRYLTIEVKPDNLADAIRGLVAF
GMRGINLTIPHKIEVMKYLDEIAGDARLIGAVNTVVAENGRLRGENTDGKGFLRSLKEQG
STRIAGSRVFLLGAGGAARSIAVELALAGAGHIVVANRTRTRGEDLVRTLNEATQCHADF
LLWDRQLAIPEDTDILVNATSIGLFPDIEAVPDIDYRTIRPDLVVCDVIPNPPHTAFLKK
AEARGAKTIDGLGMLVNQGVIGFKLWTGLEAPREVMMEALSKEFAG*
>SPBIB_v1_80009|ID:27162053| Carbohydrate kinase FGGY family protein [Uncultured spirochete bib]
MQHYFLGIDNGGTVAKAALFDETGAEIAIASETVPFSAPKPGFAERDMLELWQANCRIIR
EVLQKSRIPSKLIAGVACTGHGKGLYLWGKNERPAYPGIVSTDTRAWEYPLRWEKDGTAQ
TVYAKTFQKILACQPVSLVAWLKDHASEVIERTKWVFEAKDYVRFMLTGEAYAEITDYSG
SGLLNIRDRSFDSALLEAYGLGDLYDKLPPLVHSTDLCGKISASAAQATGLVEGTPVAGG
MFDIDACAVASGVIDEQYFCVVAGTWSINEYLSRRPVTDGNIMMNSLFCMPEYYLIEESS
PTSAGNYEWFINFFLDKEKNEAAEAGLSIFEFAERMARHIAPEDCDLVFLPFLFGSNYNP
EARACFAGFSARHTRAHMIRAVLEGIVFSHKTHIDKLLATRTPPLAVRLTGGAARSREWV
QMFADVIGLPVETVLASEPGALGCAMSAAVASGTYRDLREAAAHMVKLGSRFEPDSAKAG
MYLKKYKLYKDIADSLDHSWKGFSPQTPSTELPS*
>SPBIB_v1_80010|ID:27162054|ulaE| L-ribulose-5-phosphate 3-epimerase UlaE [Uncultured spirochete bib]
MKHESSRNQYSLGIYEKAMPPSLILSEKLLAAARHGYDFMELSIDESDEKLARLEASAQW
KRELVNAAQDAGIRILTMCLSGHRKYPIGSADPKTRARGMEIMEGAILLAADLGIRIIQI
AGYDAYYEPSTETTQALFAENLAKSVAFAARYGVCLAFETMETPFMNTVEKALRFVRKID
SPFLQIYPDVGNVTNAFEGDITRISQDILGGAGHLVAVHLKETQPGIFREIPYGKGHVNF
DTCVEAAFSAGARIFTSEFWYQKGADWELDIGRAHAFLRKKLDRISSGE*
>SPBIB_v1_80011|ID:27162055| conserved protein of unknown function [Uncultured spirochete bib]
MMHTAADVERIAYFGRNSVLLSNEKVRAIVDDLGGMMPEFSLKKGKGAVNAHWIPDFRGN
SGLPWSSHEHGQYWKANILYILAGDFPCSPNFGPDCTVDGVGLPAHGWTANERWNLASSG
AIQDGNAAYAKFTLESPDVRMPLSYTKYDVVQAGKPAYYSAMTIENHGDAPVSINLARHN
TLGSPFLQAGCRIYVSAERCMTAPKGTEFDDTGRLAQGAEFDSLLKAPGRDGKPVDLSIV
PGMIGYTDFVTGAIPAHKALGWSCVVNPVLKLAYVTFFPGPRGLPNDEIALGFNDLWMQY
GGRNFTPWALHEGGADRTFCLGTENAVGAFANGLAYSRAHPELLGTPTMVTIPAKSARTL

NYGTALVELEDALAAEAITDIEAEGDMLVLKTARTYQRVSLDASFSGLRELVSRLLK*
>SPBIB_v1_80012|ID:27162056| conserved protein of unknown function [Uncultured spirochete bib]
MAHEKQLVEKALNEGNGVFRLEPAWVPRSFCIPGRRLKLHPNDYYAYGANRGGIDERWFA
STTKADNGPLTTPFEGLSFIWVDEKQHVLLKDAIELAGREILGDAVMKKHGGWRAYSKFF
DNQEPLPHHVHHTDKMAANIGQMGKPESYYFPKQLNNHGGWFPYTFFGLNPGTTREQIKQ
CLADWEKKDNNILALSRAYLLQPGTGWDVPPGILHAPGSLLTYEPQRASDVFAMFQNIVW
NSYTPKELLQKDIPEDKKDDLDYYVELLDWELNTDPLFYEHRFMPPKPAKPLEDMRAEGY
EEHDIVYKSEYFSAKELTILPGRSALIKDKGPYGAIVIQGHGKFGSLDIESPAMIRFGQM
THDELFVTERAAREGVLITNTSDTDDLVMLKHFGPEA*
>SPBIB_v1_80013|ID:27162057| putative HTH-type transcriptional regulator DegA [Uncultured spirochete bib]
MPSLKDVARLAGVSTSTVSRAINGTIPVSEETRLRVEKAVRDMGYKPNLVAQSLRIKSTR
LLGLVVPEMQHETFISFIRFTEEAAEAKGYNLIIGSTNSDPDREERFIENLIRRNIDGII
FSRVSDKSHVLKILDRTKIPVVIIDRTLEREDIPSVVMDNYESGKLVAEHLLSLGHRAFA
CITGPLDIAINRDRLAGFRDTVLAGGGTLEDKCIYEGNFKFESGKKGIAYLLDTGARFTA
LWAQNDYMAVGVLNLLSDRGIAVPGEISVAGLDNIQQSWMMRPSLTTVAQPFREMCTHAV
DIIIDRAGREDDNREKIRVMLKPELIVRETTARPPEGSFAAERKEVERK*
>SPBIB_v1_80014|ID:27162058|rbsB| D-ribose-binding protein [Uncultured spirochete bib]
MKKVILVALVVIALVPVSVFAQKQIVIGMTVPGLQFPFFVTMKQEAEAAAAKLGVKINFI
DAQNASDKQMAAIETFISQKVDGILISPMTEDSLVPAIEAAVKAGIPVATVDRKANTDKV
LIHVGADNVEGGRAAARFIIEKLGNKGTVIELEGTPGSSAAIDRKKGFDEVMKASNVKIL
VSQTADFTRSKAQSVMENLMQVYPKFDAVFGANDEMIIGAIEAMLAAGIKPATKVTIGFD
ATTDAFTYMKEGKLNATIDQFPGKQAAMALEALVNKIKNNVNPPQKVVFISPLPVTK*
>SPBIB_v1_80015|ID:27162059|rbsA| fused D-ribose transporter subunits of ABC superfamily: ATP-binding components [Uncultured spirochete bib]
MDDIVLSMKGISKSFPGVQALKSVDFDLKKGEVHALVGENGAGKSTLMKILSGLYRADEG
EIWLKGKKITTRGIKAMINAGVSVIYQELNLIRQLSVAENIFIGREPMLPGGFIDWKKMY
SDVHTLLKPFNVDINPRTKVYMLSPAYQQVVEIAKALSLKSDILVMDEPTAPLTGNEVDK
LFEIIHNLKKSEVSIIYISHRLEEIPKVAERVTILRDGEKILTKPLAELTTAEIIKHMVG
RTLTEQYPKISIPIGEEVLRIEGLSKKGYCTDVSFSVRKGEIVGFTGLVGAGRTEIMQTI
YGRMKKDAGRIYIDGKEEHIRGVCDAVRKGIGLIPEERKHQGLVLGLSVQDNATMTILDK
ESVFGFLKQKKLSALVGKMIETMNIKTPSARQLVRYLSGGNQQKVILAKWFLHNCKVYIF
DEPTRGIDVGAKVEIYKLMQNLAKDGAGVVMVSSELPEVLNMSDRIEVVFGGRIVKEFRR
DEADSETVMEYALGLRENASQQTPLEAGAES*
>SPBIB_v1_80016|ID:27162060|rbsC| D-ribose transporter subunit ; membrane component of ABC superfamily [Uncultured spirochete bib]
MKVKFVETLKNYGIILAFFIILVVLSIFSPVFLTVNNIINVIRQTSIYGIMAVGMTFVIL
TGGIDLSVGSVLGVSGAICAGMLKAGNPIILVIIATLGVGIACGFASGIFITKARITPFV
VTLGMMSIARGLTLIYTKGYPISGFSPAFRLIGGGYVLGLPIPIIIFLVIVIIAYIVLTQ
TKLGRYTYAIGGNEETVRLSGINSNFYKTLVYVISGATAGFSALILTSRLNSAEPVAGTG
YELDVIAAVVIGGASLNGGRGSVWGTFIGSLLIGVINNGMNLLGISPYFQQVVKGLIIIG
AVWLDQLRAKED*
>SPBIB_v1_80017|ID:27162061| conserved protein of unknown function [Uncultured spirochete bib]
MKTEALWYLGERSIELRKMEIPEPKAHEVLVEMEICGMCSWDVLAFAGKFGKFHPYPFAA
GHEGVGRVIKVGDNVASIKVGQRVACHEVPIGTPGGALMARHAIRTEDKATVIPENPIPL
KYWVVEPVVCIVNGLVYAGIQPGDSVALVGAGYMGLIFAQGLAKTLAREVVAFDVDEKRL
SLAREFGVTRTVKIEGDAIPEDYRKHFDVIIETAGKPSSMQLALAVAKTGAIIENFAWHH
HMHEFDLDAWHINAWRILNIQPGVNSHFSDLFQRTIDLMIAGTFSNEKLITHWAPVEQAK
EIFTTALERRDGYMKGAILFS*
>SPBIB_v1_80018|ID:27162062| protein of unknown function [Uncultured spirochete bib]
MVLSIMGVHWFRPVRFVVCAAGGVATLLKVAKPLSANNNGAFALAA*
>SPBIB_v1_80019|ID:27162063|mnmA| tRNA-specific 2-thiouridylase MnmA [Uncultured spirochete bib]
MEAMEAMKAINTAIVAVGLSGGVDSSLAAYLLKEQGYAVIGLTMKIWKGAYKIQEDLKHA
CFGPGEEEDIAACERLCARLDIPYHVIDLSEEYERFVIEYFRKEYLVGRTPNPCIICNRE
IKFGFLIERAHQAGLEFDYFATGHYVRKATIDGITYLKTARDAGKDQSYFLYGLDSERLS

HIMFPLGEMTKEQTRAAARTHGLEVAEKPESQDFVGGGDYAPLFENDKPEAGDIVDIKGN
VLGRHRGLPFYTIGQRRGLGISIGTEPLYVLALDAKLNRVIVGHGKGLFSSGLISNSFRL
QNPCDLGKSFACRVKIRQNHKPAPATVLVQSNGEARIEFEIPQRAVAPGQSAVLYSDDGL
VLGGGIIDEAIPDEDIEE*
>SPBIB_v1_80020|ID:27162064|gcdB| Glutaconyl-CoA decarboxylase subunit beta [Uncultured spirochete bib]
MAAVEYIQSMQDVFIGFTSITWKMLVMYGIGGLLIWLAIKKQYEPMLLLPIGFGSILVNL
PLAIAWEHEGVPGFLHILYDAGIANELFPLLIFIAVGAMIDFGPLFRNPLMIFFGAAAQF
GIFATMIIATLLGFDLKTAASIGIIGAADGPTAIYVANKFARDYLAPISVAAYSYMSLVP
IIQPPVIRLLTTKQERGIHMALHEKPAPHWLRILFPIVVTMTAGIVAPISVPLVGSLMFG
NLIRESGVLERLSQFAQNELAYLVTLLLGITIGGSMSAEKFLNWQTLLILAMGLVAFVFD
TAGGVLFAKFVNLFLPHERKINPMIGACGISAFPMSARVIQKMANDEERGNIILMQAIGA
NVSGQLGSIIAGGLVLALVPLLVK*
>SPBIB_v1_80021|ID:27162065| protein of unknown function [Uncultured spirochete bib]
MENLIETLKNASPAVQAIGVSAGGLIGVFATIAFFFVLIVAADRFGKR*
>SPBIB_v1_80022|ID:27162066| Beta-lactamase domain protein [Uncultured spirochete bib]
MKVHSITPRIYAIHADIRSDDLFEGIWPIPYGVSLNSYLVKGEKIALIDLVRDWVGAPGE
LAGQLAAARTSLSDIDYLILNHLEPDHTGWLGEFLEINKKAIILATAKGIELVNNFYFEH
ERVRAVKNGETLDLGAGQVLQFFETPNVHWPETMMTWALDGGVLFSCDGFGSFGALGDRV
FDDEFSAEEHEFFEAESLRYYANIVASFGLFVKRAVDKLSNLQIKVVAPSHGIIWREHPE
MIIERYLRYADYLEGSREKEIAIVWGSMYGNTENGLQHVIEGIEEEQVPYTIHRVPNDNV
SWVLADAYKSEGIVIAMPTYEYKMFPPMAYVLDIFERKHVWYRKALRIGSFGWVGGAKKE
YEAKIAQLKWDSLEPVEWAGAPDEETAELLRERGRELARLVKGA*
>SPBIB_v1_80023|ID:27162067| conserved exported protein of unknown function [Uncultured spirochete bib]
MTKRFIAIAGIVLFLSMAVQFVSAQEGGTDLDSLFGEEVVVPENPAGQTAQPDQTGQGGS
AIAANPLQGLLKTEAVRIGGSITGTVGISGTWNDPWTKGFDITAPDAQQLSPALSSLVYF
DARPDEEFRVYGSVKTAWPFSSKYSVLNSATLVNSTTLSTTSTSITVPDVSIFELFSDFQ
LGDKAYFRFGKATVKWGVGYFFSPADIINLEQINLFDPTAQREGPLQFRVLMPFGPSQNT
VSFYTIFDTSNPDFSTTALAGKAEFVLGRYELGVSGYYRDDTAERAALTLTGPLGKFDVF
AEGVISRGSPKTFYSFSTSAPYYSTSASKDHRTTLYPSATAGFLYNDQNNNITAIAQYYY
NGEGYADSERSSSISALNALLSMPLPDATKTALTGLSKLFAYGSGRHYGAASISFSEIGG
SDFSASLLGLANFSDLSGLVQPSVSWQVADRLKLTGSALFFFGASDTEYGILRPNNPMTL
SLSLSAGTGNF*
>SPBIB_v1_80024|ID:27162068| conserved exported protein of unknown function [Uncultured spirochete bib]
MNVFFRKRNPKFVKAASLAVMMLAGLLAAVPTVFAQAKPDPVALLKELDQLSDFSGKDFS
AVFTIVTQKPGEKDSVTQARIFRRDTKKQFLILILLPEVNKGQGYLREEDNVWFYDPTSR
KFSHSSVKENLQNTKAKNSDFTLSSFADDYAVTSMTEGTLGKFPVWILDLKAKTNEVSYE
RVVLYIRKDRTMLLKREDYSVNGRLMRTTAYPKYIELDGKLLSTQILILDEINKGEKSQI
TMAEQSVAPLPDKVFTKAFLEQVSR*
>SPBIB_v1_80025|ID:27162069| putative ABC transporter, permease protein [Uncultured spirochete bib]
MNMVLPRIAARNLLRQKKRTILLAGAIAFGIMIVTLINGFAGSFIQNVSENFAYLMGGHV
FVSGSEYTPSGKRISVIRDDSVIMKALEDSGQKWASIAKTSQATASLIFEGKSVSQNLTG
IDITHSKMLKERLVLVKGSWEDASQPDAIIISQSVANKLNVLPGDKIVAQLQTVTGQNNV
GEFRVAAISVDSSIIGSRMTYVQLEYLNNLIGLGKGEYMSLGIMLDRLDLSDGFATSLYT
SMKGNGIQLFDRNAKQESTSTTPFQAMISEQNSETWSGTKYRVYTIDDVLSQARQIVVAL
DTASVAILIVLFAIVMIGISNTFRMSMYERIREIGTMRAVGVQRGEIRAMFLYEALFLAL
AGAIAGIVLALVIMGILGLFSFDPQSAIFLILKRGHLSFYLPPLRAIGNIAIIAVLTLVA
VYAPANAAAKLAPAEALRTVK*
>SPBIB_v1_80026|ID:27162070| conserved membrane protein of unknown function [Uncultured spirochete bib]
MAVILRMAFRNIREHKSKSLIIGILLALGALILVVGTAFIDASQAGIRSTFSDVYTGDIF
ISGISSEGPVSLFGVSSPGGLAATPIIPNYEKVLSIVKSTPHIAGHSSLATGFAQLTRED
STPQQANALKSTIQSQDRFLFLFGVDAASYWNLFNQVEIVQGERLQPGQPGLMISESQRQ
KFSDWLGKPLSIGDTLLIQGFSSAGMRLRELPIVGIFQLKEQGASPEQLAYIDIESLRVM
SGMTVGANEPIQLKPEETSMLSTDNADSLFGEDVTVTQGSSTGIDVNAIAAQIAQESAAK
PLQADEGAWQFIVAKADSPRNVQRTIAALNASFAKEGIPVVAGDWQKAAGPYGQSIDVVR

IVFAAAIIILSIVAIIIIMNTFVISVIERTSEIGTMRAIGAGKGFVRGLFTAEATALALV
SSVVGAGLGIAVTSILRALHIEATNQFFLILFGGKYMNPIVSAGNMITAIVVMVVVGFIA
HLYPVSLALKIQPVRAMQEE*
>SPBIB_v1_80027|ID:27162071| ABC transporter, ATP-binding protein [Uncultured spirochete bib]
MQIIELRNAKKDYLLGKVTVPAIKGVSFAIERGDFVSIAGPSGSGKTTILNMIGLIDIPT
SGEVIIDGKNTSGLSDQELTRFRHEVLGFIFQSFNLIPVLNVWENIEFPLLLGRTRVPKN
EWKDWIDFLIGEVGLGDWRDHKPNELSGGQRQRVAIARALVTKPSIVLADEPTANLDSAT
GEQIIDLMKKINREIQTTFIFSTHDATIVSIADHVIRLRDGTIIENRRNGEDINGSGAVG
APVED*
>SPBIB_v1_80028|ID:27162072| putative enzyme [Uncultured spirochete bib]
MQDFSKYLSANANNMKKSVIRELLKLTNQPDIISFAGGLPAPETFPVEELREASDRVFRK
YGDKILQYGTTEGDNDLKAQLVAYESAQGIKLGPENLLITSASQQALDMLPKLFLDPGDY
VIAGRPTYVGAIQAIQSYQGNVLGIPFSTADDGFDMVELEKRYERAISAKKRIKYIYVIP
DFQNPSGICWSLEKRKALLEFAYREGLFIVEDAPYREIRFMGEPVPSIYQLDQQMQNRGI
VINLKTFSKILAPGTRIGWIIAREDLIQKMVIAKQAMDLCTSVLTQKMIAEFMATGKLKD
IVARTCGIYRNKRNFMLEMFEKYMPKRFDLAWTKPEGGLFLWLSLPRYIDTDKMFYKAVE
KKVAYVVGSAFYFDEPETNSMRINFSYSSFEQIEEGTKRLANVISEEIEAHEAGPRGQTS
PEDI*
>SPBIB_v1_80029|ID:27162073|ychF| putative GTP-binding protein [Uncultured spirochete bib]
MPLNCGIVGLPNVGKSTIFSALTSAKAEAANYPFCTIDPNVGIVPLPDKRLDRLCEIFKP
RRKVPAVVEFVDIAGLVRGASKGEGLGNQFLSHIREVGMIAHVVRCFDDPDIIHVAGKVD
PADDIDVIHTELALADLDSIDRRLDKLNRQLKVQDPNLKKDTEKEIAVLQKVRPALQEGK
PAFTVDLEPEEIEFARRSFLMTMKKELFVCNVDESGVHGNKYVDIVKEIAEKQGSEAVVI
CGKFEAELADIEDPAERAEFLAGIGLEAPGLASLAHAAYRLLGLRTFFTAGEDECRAWTI
LAGDKAPAAAGVIHTDFEKGFIKAEVYHYDDLIQYGSEAAVRAAGKMRQEGREYVVQDGD
IMFFKFNV*
>SPBIB_v1_80030|ID:27162074| conserved protein of unknown function [Uncultured spirochete bib]
MSEPLEKGEPVGQGSIEQGFSPAGDESAPRLLALGSFPSIRSKEKGEYYGHPRNHFWPIL
AAFAMERGIVLPPGSLDNYSAKIALASSLGLLIWDMVQSCRRQTSADGELEIVALNDIRD
LLGQYPSIKWVGLNGALAASLFMRHVVSPENRLLARKALRMAGGRISIEIGGQMRSIMYL
PSTSPVPSRNFRSTIDKQHIWFEFLGA*
>SPBIB_v1_80031|ID:27162075|uvrA| ATPase and DNA damage recognition protein of nucleotide excision repair excinuclease UvrABC [Uncultured spirochete bib]
MDKLIIKGAREHNLKNIDLELPRNKLIVISGLSGSGKSSLAFDTIFAEGQRRYVESLSAY
ARQFLGRMDKPDVDYIEGLSPAISIEQKTTTRNPRSTVGTVTEIYDYLRLLFARIGVPHC
PSCGREIREQSVDQILDVILSWPSETKLQILAPVIRAQKGEHRKVLEDALKQGFLRARVD
GQFIELEVPPALEKQKKHTIELVVDRIRLSEESRRRVAEAVETALGIADGIVTVIREGSG
AQEGQLKEEFFSQKGACPVCGISLPEMEPRLFSFNAPQGACPACAGLGMNMEFDPDLIIP
DKSLSFNEGGCIPYNPTSAWNRSRFEALAHHYHFSLDTPFESLPEKVMHAILYGSDEDIH
VRYDNRDRTGHFEYQSKFPGILADLRRRYMESTSDEIKDWLESFMIEKECEVCHGKRLRP
EALAVTIGGKNIYDITCMPVGEAVQFFESLKLTPTEAQISKLILKEIVARLGFLRNVGLD
YLTLERKAATLSGGEAQRIRLATQIGSSLVGVLYILDEPSIGLHQRDNQRLIDTLTYLRD
LGNTLIVVEHDEQTLRTADYLVDLGPGAGEHGGYVVAQGTVPEVIAHPDSLTGQYLAGTL
RIDVPKVRRQGNGAYITVKGASEHNLKHIDVKIPLGKFVCITGVSGSGKSTLLSDVLYPA
ISNRIMRSSHREGAFEAIHGLEHIDKIINIDQSPIGRTPRSNPATYVGVFTPIRELFASL
PESRARGWKPGRFSFNVKGGRCEHCQGDGTIKIEMNFLPDVYITCEVCHGKRFNADTLDV
RFKGKNIADVLNMTVEEASGFFAHIPSIAHRLKTMLDVGLGYIRLGQSALTLSGGEAQRV
KLSLELAKRATGRTLYFLDEPTTGLHFADVRQLLEVIHRLVDSGNTVVMIEHNLDIIKQA
DWVIDLGPEGGDRGGMIVAEGTPEDIARCKQSYTGQYLGPMLEISGPR*
>SPBIB_v1_80032|ID:27162076| exported protein of unknown function [Uncultured spirochete bib]
MRRILCIFGLLFILILFLPAQEAVNQSSPAEESTAQQTDTQSIPTAISASPVPEDFARKT
LRFRIAAATIYELRDIAAEYGLSADGTADELRARLFAHFGFEPIPKVKGDVSMTIEKAGN
VQYFTIENGTKEIRVNGPLEIRFADSQGTIHRISAQYLVFNRDTNEVQAAGNVEYTRETK
TRTDIYKGQSIAVNLDESSGVFVDGSFNMEPTGAESRTLIVHFGTLISKSEEVVSLANGS

LTACDAIDPHYILRAKKIWLFGSGDWAVVNATLYVGSIPVLWLPFFYYPSKASVFHPVVG
FRSRQGGFVQTTTYVAGTQGAEARQSSAFSLQQGATGSFGTYVSRTQTTTEQSDTSSLAV
LVDAYSSLGAFAGLRGKSASRLPVNLSWLVGVGLSRSVFLESTGYYSPYDWAGNYKSVWN
DWKFGSIALPVRFVTSFEASSRRNASGLSWKVSLPFYSDPFIDQDFLDRKESYDFFSIFG
GASSTVSERTSFVQKASLSWSWRAKNTQKPLTFNLSNLSSSLSWKSKYASTSGMTAAQLR
LNAVNPQRHFFYPENARVVDTTFSASGIIARSNAASLGWTTSNAMYIEDRFYSFAWQKPQ
DIDFKSWYWLLGSRNNASLNSQLSVEKAALDFQFSSGVSGQVQYRPYLYDERVSPTTVHP
FKLADYGYNTASWNAGTSIIWSPLRNIDMLAASKLQYSLSGKIAMVSYEGLDGSGVNANP
VYKLNWLSWDDTMIRDHSILAELAAKIGSTSERLSFKIALPPLLENYTFSLSSAAGIASF
GAAYIISRQSSLADLKSTSLAGNVSLQPLKQLRFAANAAWDFDANAPLSVSADITAWSFN
ARFTAQKANGYTFKSGSWIQDGTQYFRPSTVSLSWKPVLMMKPPQDISDKLVWYFEFGSA
LSLSQNLIQYTNAIFGSDFRISLKNSGGLSLDLSVSSINKSFWRYYAGLLPVAGDLDPEI
YKRNFFNDLLDSLSIWDSARLQRTLFKLQKLSLTLAVDAHDWDLAGSIAAGPTLITPDTG
RPYYQMDVSFSIAVTWKDISAIKSSINYSEGAFRE*
>SPBIB_v1_80033|ID:27162077|ybeZ| putative enzyme with nucleoside triphosphate hydrolase domain [Uncultured spirochete bib]
LSPSHIVLEDTRLLAELCGANDYNLSVLSSLLGARVLSRGNELFIESENDEVVGLFSQLV
SAIGTSIEDGVPATPELIIALHAELTPEGKQPDDRGDQSAPEENESGISKFLDTCIQIPN
GFGKVFPRSSGQSLYLQGIAMHDLVFAIGPAGTGKTYLAMAYALRELLAKTRRKLILTRP
VVEAGESLGYLPGDLEQKINPYLRPLYDAMEALVPYETIRRLEESRAIEVAPLAYMRGRS
LTNCIVILDEGQNTTKEQMKMFLTRLGDHTKAIVTGDITQIDLPKKNESGLVHAVTLLEH
IDEVFVSRLDARDVVRSPLVRKIIEAYEKE*
>SPBIB_v1_80034|ID:27162078| putative 7TM receptor with intracellular metal dependent phosphohydrolase [Uncultured spirochete bib]
MKKNNDELFFSSLLRQLASMAPSMWYFLAAIVIAAVGLVFLGPSALGSRNSLRSIQPGKV
ADSDVYAGKDTVYVDKEATQRKILAEERLVLAVFVLDDKITSAVREKAEAFASYYRQLAG
EPGGVDALSLLLRSRFPDLFQPELYTVLVRAKLNAQAFVYVSDIVESMLGKGVVSLPDGV
FDQFNPNYYELVRTLGTRQESEQLPVNGMITLANLGNRLDDEMDARHVPESMKQYVRLLG
KALLRENVFFDQGLSEKRIAAARARVEPVTRFVSRNEILVRKGELVTPELYQQIKTIRSA
ILVSDVGVLMTGLGLLLVASLLGFLLARVKDLSDFPSDRSSLLFLLSSLLILLYWILVIQ
NVIDSKSLSRGALFVPASLFAGLVSLLYGPRTGIFFSLISFFLTGAATNLNAQFMIGILL
AGISATLTMRTARTRIMLARAAIFQAIIQALLAIILLLNQKPNAAELIQSMGLGALNGFA
GGSFILLLLPLFERLLDRATQFRLMELADINAPILKEMLSNAPGTYAHSIQVAHLAEAAA
KDIGANPLLARIGAYYHDIGKIEHPEYFTENQKGVNRHDDINPSLSASIIRRHVKDGVER
GREIGLPKEVIEIIGQHHGNSVMEAIISKVKDSVSEADVQSFSYQGEHPKSKEAAIVMLA
DTVEAASRSLKNPSSAKLEQFVHQLILHKLNTGQLEDSELTMRDIRITEEAFSRILQSQM
HTRIEYPGQEQQ*
>SPBIB_v1_80035|ID:27162079|ybeY| Endoribonuclease YbeY [Uncultured spirochete bib]
MNGNEVFYSVIGTDEPAWAQRAANYALSVLDALKKQNWQISLTFCDDATMQSLNRDYRNI
DAPTDVLSFTLGELEHVNDGEDVFIAGDVVISVPALYRNAEDFGVSADEELRRLIIHGIL
HLSGMNHEDNEPDQPMMHLQEQLLGQLGGNSIL*
>SPBIB_v1_80036|ID:27162080| conserved protein of unknown function [Uncultured spirochete bib]
MSLLSQIFKPRAKTELSSNESVEEQREMIEGVEHLPEKIVKDVMVPRTDTVCVDNTTSID
EILAILVESGHSRIPVYSDTIDNIVGILYAKDVLAALVKKEPLELKRLIRPPYFVPETKR
IDSLLKEFKRRHVHIAIAVDEYGGTAGIVCLEDIIEEIVGEIQDEFDEETEQVIKVSENT
WRCDARIRLEDLNEAIGAQLPIGEYDSLAGYVFDLFERIPATGEQVTAGDLQFTVEAMEG
HRLTIVRIDRILVS*
>SPBIB_v1_80037|ID:27162081|gdhA| glutamate dehydrogenase, NADP-specific [Uncultured spirochete bib]
MNSYIKEVLDGLRARYPWESEFLQATEEVLESITPLIEAEPKYRQQKILERIVEPERVVM
FRVPWVDDKGEYHVNRGYRVQFNSAIGPYKGGIRFHPKVTLSTLKFLAFEQIFKNSLTGL
PMGGGKGGSDFDPNGKSDNEIMRFCQSFVTELFRHIGPDTDVPAGDIGVGGREVGYMFGQ
YKRLANQFSGVFTGKGLSFGGSLIRPEATGFGAVYFAEEMLKLRGETLEGKTVSVSGFGN
VAWGACIKASQLGAKVVTISGPDGYVVYDPDGVGTQEKWDYLEEMLIIDRNKVETYVKKFP
KAVFYKGKKPWEQKVDIAMPCAIQNELNGEDAKMLIANGVKTVVEVSNMGCTPEAWKLFI

EKEIPFGPGKAANAGGVATSGLEMSQNSMRLAWSAEEVDHRLHEIMTNIHKSCVETAAKY
GFEGNYIVGANIAGFKKVADAMIAQGLV*
>SPBIB_v1_80038|ID:27162082| RNA-directed DNA polymerase and maturase, group II intron origin (fragment) [Uncultured spirochete bib]
VTRTEETNPETTKLIERLVERGTMREAYARVVGNKGAAGVDKMTVEALKPWLQAHWVEVK
GRLLRGTYRPEVVRGVEIPKPNGGKRQLGIPTVVDRFIQQALHQILSPIFEPEFSANSYG
FRPGRGAHDAIRKAKEYQLAGKRWVVDIDLAKFFDEVNHDLLMARIAKKVRDKKVLRLIR
RYLQAGIMKDGVVWDRDKGTPQGGPLSPLLSNIMLDALDKELEKRGLSFCRYADDCNIYV
GSERAGMRVMESITRFIEGTLKLKVNREKSAVARPWERKYLGYSFTNERKVRIQVSASSI
ERFKEKVKALFRGAHGRNLGRFIRETINPFVRGWIQYYSLADTKQFAEELDGWLRRKLRC
NLWRQWKRPWTRLKRLMERGLPEETAVRSAPLTSAARGGTLAPST*
>SPBIB_v1_80039|ID:27162083| protein of unknown function [Uncultured spirochete bib]
LDSVLQSRRYKAVCGRAGWMAPQETTVQPLAAVEETVDAPETADGAWLARRNGSPFRAFN
QRGPWWNSGAQHMNYAFPKKYFDSMGLVSMIDRLCLSR*
>SPBIB_v1_80040|ID:27162084|aroA| 3-phosphoshikimate 1-carboxyvinyltransferase [Uncultured spirochete bib]
MENRIVYSSKVGGTIRAPASKSALQRAIACAVLAQGKSRIIGDPLCDDAQAALHIAEGLG
ATVHVSDGAIEIEGSEQFLQEDGTGTTSLEQQEPLMLSCGESGLCMRMFAPVAALLDRPV
QMEAEGSLRKRPMAMVESALHAFGADCAAQEGLPPLFVRGPLQPKLATLDAKGSSQLVTG
LLISLPVLHGDSELGVENLVSAGYLDLTLEICSQFGVHIDKVSSDGRTARFLIKGNQSYR
PASLRVEGDWSGAAFLAVAAAIAGKPEGLRIQGLKNDSLQPDRAIVDVLALAGARVQFDG
SDVIVQPGDCLPFEFDATDCPDLFPPLAVLASVAKGISSIQGVHRLSVKESNRALALQDM
LANIGIHSKIVDDALQISGGHFSGGRVESHNDHRIAMAAAIAGLAAANPVAIEGAECVAK
SWPGFFEDLQSISSAPNLVPELK*
>SPBIB_v1_90001|ID:27162085| putative membrane protein [Uncultured spirochete bib]
MTGTKQDAKVSQKSAIKIGKKAFLLSAGIILVLMLVSGILTIVLPSGEYQRVIQDGKTLV
VNGTYHEVPKPNYPAWRWLTAPVEILFAQDNVALITIIIFIFCVGGSISILEGAGIMEEL
VKTLVQRFSKRKYLLIAVIIFFFMAISSFIGVYEGMVPMIIFIVPLAIFLGWDSLTGLGL
SLLPLAFGFASAVTNPFTIAVAQRIADLPLFSGSWLRIIFFIIVYGIVTTFMIRHAKKVE
RNPASSLTFKEDEAIRASLAAGEAEAAHNTAAPNGGKHTDTASSRAKTKGLIWFASCVGA
AMAIVLVTARMPGLSDLAFPVMALLFLIGGIGGGLLAKVRPGNIGATFIRGAGNLLPGIV
LILMAYSVKHIIISGKIMDTILHGAATLISQAPPLSAAFLVYLTTLVMNFFIGSASAKAF
LMMPLLTPLADLVGITRQTAVLAFDFGDGFSNMIFPTNALLLIALSFTVVSYPRWMRWTW
KLQAIILVVTSIFLAIAVGIRFGPF*
>SPBIB_v1_90002|ID:27162086| Prephenate dehydrogenase [Uncultured spirochete bib]
MRIAILGAGRMGAWLTEELCWHHDVMVHDTDLLKMKYFIKVHRALSIEEFAEFEPELFIN
CVPLGYTLEAFDKTLPYLPKTCIISDIASVKTGFKEYYEQSGRAFVSSHPMFGPTLANIR
DLHEESAIIISESSKEGAAFFRNFYAGLKIRIFEYSFEEHDKTVAYSLATPFASTMVFAA
CMKKQDAPGTNFKRHLSIAKGLLSEDDRLLTEIMFNPYTIRQLELINSKLAYLTHIIRQR
DYEEMKKFLDSLRRNIGE*
>SPBIB_v1_90003|ID:27162087| Prephenate dehydratase [Uncultured spirochete bib]
MNLDDIRTDIDRVDAKILSLLNERMEKAILARRFKTSTLDTAREQAILDKVRRSSQCLLD
PAFSVKMYEQIMAESRRIQEAGLQTVAFQGEHGAYSEVALRVLMPNAATIPCREFSDVFE
GVEKGIYDYGIVPVENTLGGIVGPVNSILIYTSLKIVAAIDMPIRHCLLTLPGADHRELR
TVWSHTQALAQCRNFLYRNHLDPEPYYDTAGAAKALAESRPKGIAAVASKFAADLYGLEI
IKEDVQDTPHNRTRFFVISTKESDAEGDKCSAVFTAGNKAGSLFAVLRVFADEKINLTRI
ESVPDTPGKYAIFIDFEGALLSAAVQKAISKVSALAEGFKILGCYKEMRVEE*
>SPBIB_v1_90004|ID:27162088|aroC| Chorismate synthase [Uncultured spirochete bib]
MNTGGTIFRVSLYGESHGPAVGVIIDGCPAGISLSENDFAADLGRRKSGAEGTTPRVESD
APLLLSGVFRGRTTGAPIHIEFRNENTQSEDYADFMHIPRPGHADYSAMMKYAGWHDPRG
SGHFSGRITIGLVAAGVIAKKILAHCAPISFDTRIIAAGGSEQIAETIRAAKEAGDSVGA
LVEIRIAGVPAGWGEPFFDAAESVIAHFIFAVPAVRGIEFGDGFAAAAMKGSEHNDPFID
QNGKTARNGAGGINGGITNGNELVIRVAVKPASSIAKPQRTLDFSRNEPTELEIKGRHDA
CIALRSAVVLEAAAACALADLALVARARKSWESNIPWRKS*
>SPBIB_v1_90005|ID:27162089| 3-dehydroquinate synthase (modular protein) [Uncultured spirochete bib]

MQTDRTEILFGPFSSLMEIVPLDKAIFITSRALRQRAEALLASSNATHAAASLKDMPHAA
QKESGLRWIEVPDGEPCKTFPVLESVYHALLNLDANRDTVLVAIGGGSVSDLAGFVAHTW
MRGIRLVLAPTTLLAMIDASIGGKNAIDVGYAKNIVGSFHMPSHILCDVAWLHSLSPQDL
ASGMAEAIKHALLDSEDHVEFLERIAMSGKALPELEAGAFKELVQRSQQVKLRYVQADFL
DAHARHVLNYGHTFGHAIELLTGLPHGFAVSAGMTAANRFAVQRGALSLAAAERIERLLA
RFGLPTSMQAAFNLADRPIDKKALFELMRADKKRRSDIVDFVMPHAIGDLRIEEVTLPQL
EAALEWLTKSASEDSTTRSAL*
>SPBIB_v1_90006|ID:27162090|aroF| 3-deoxy-7-phosphoheptulonate synthase [Uncultured spirochete bib]
VIKNIKLAARKPGQKTTIIDVNGIRVGEGLVIIAGPCSVETERQTIETAIAVKNAGAHML
RGGAFKPRTSPYAFQGLGLKGLKILDKARKETGLPVVTEVVDTRDVSWVGEYADVLQIGA
RNMQNFSLLREAGKSGKPILLKRGMYSTLEEWLNCAEYILAEGNPNVILCERGIRTFETY
TRNTLDLSMIPAVKRESHLPIIIDPSHGTGILSFIEPMSLAAVAAGANGLEIEVHIDPAS
ALSDKDQQLTVPMFESLMNKLRKLRAFMDEPEMSSALQENMDTPAGKSAVQENNAQNAAP
PLPGAFSEAVD*
>SPBIB_v1_90007|ID:27162091| conserved protein of unknown function [Uncultured spirochete bib]
MYFNELVSAYDRQRRERNIYHDLMRDRVREVLLVGSLYDSFVVESDGVLTEQIYGEYFKL
NLNTIPRVTCAYTEESALDLFREGRFDLVIIMASLDFDMPLRLAALMRSIWPDIPLLLMV
TNNSSLAMLDMERPELSAFNRIFVWNGYSKLFVGMIKYIEDWRNVEADTRACEVRVILLI
EDSVRYYSRYLPVLYKVVLRQTQALVEEEHITETYKLMSIRARPKILLASTYEEALRIFE
TYKPYLLTVITDIRFNRNGACDENAGFDFVRFAKQELPDLPVLVQSSEPNVREKAFAIGA
SFIDKNSESLEMELASFLQTNLGFGNFIFRLPDGREITRARNMSEFVQKLNEIPIESLLY
HAEHNHFSAWLMARGEIHVAKILRPYKITDFSSPVQLRQFIVRMIDQIRSTRSRGMVPYF
DPEMVEYQRYLCKIADGSVGGKGRGLIFIHSLLENLDFSQYIQGVRVSMPNTVFVGIDEF
ERFLELNGLWAWAYYGDSASEVRKVFVEKSLTLQLQDRLRQFLAVSTKPLAVRSSGLFED
MLMVPFSGVYDTYILPNNHPDPEVRLKQLCDAIKLIYASLFSKEARAYFEAANYNLEEER
MAVVIQELVGSPHGGYFYPHAAGVAQSYNYYPVSYVKPEDGLCIAALGLGTYVVGGGSAY
RFCPKYPKLDVLSPDHALESTQRYFHALSLEDEAPDLLKGEMASLSELPVSAAEKDRYFP
MLASTWDSADQRFVPGVQAKGSRVIDFANMLKYDAYPFAKAIEVVLDVASKSMGTPVEIE
YAFNFDAGKEEPVLYLLQLKPLIHIEDRIEIDPASILPEVCFILSRNCMGNGRDVSIRNI
IWVDPRTFDRSETLEIAAEIDELDAIARQESFNYLLIGPGRWGTRDRWLGIPVSFSQISH
AKAIVEADLPGFAVESSQGSHFFHNLTTMRIKYIKVTQSSADNFVDWEWLYQMPTRIRTK
HCALTELEAPMDLRFDGRSGIGAVIKSAGLSNNGSSR*
>SPBIB_v1_90008|ID:27162092|pheS| Phenylalanine--tRNA ligase alpha subunit [Uncultured spirochete bib]
MIDQSAARALHPLEVKILLAFKVGDTIDSALVREKLGFREGQDQQAFSWLMAKGVLEEAD
RQTEVFYELTPLGQEWLEKGTPIRRIFSLMKEIGPMSLPDIAQALGFDQKTVGSAFGSLS
KEGVCAMDEARRAILTKSALPQPLLDIEALLKAAAANAGSLPEKSLTQQQKALMAQIAKK
RGAQDAPFRIAERAKVLFRLTSLAEDYRQAVTALGMTGEEFGAITPDMLEKGTWKLGTFR
PYNVQIPPARVIPGRRNPYVEFLDSVKDKLVSLGFEEFDGNMVETEFWNSDALFMPQFHS
ARDIHDVYYIDEPTHAKFIEEPHLSRVAAAHENGGNTGSRGWQYQFDRDFTRRLILRSQG
TVLSARQLPKAKVPGKYFGIVRCFRYDRVDATHLSDFYQTEGIVLGEDVNLRTLLGFLEM
FAVEVAGAKEVKYVPGYFPFTEPSIEVHIKHPVLGWFELGGSGIFRPEVTRSLGIDVPVL
AWGIGIDRMALMALGLNDLRELFSPDLESVRMRRSQYKIQ*
>SPBIB_v1_90009|ID:27162093|pheT| Phenylalanine--tRNA ligase beta subunit [Uncultured spirochete bib]
MPKIEVNEELFFRLAGKTWSDKEAFEQDLTVAKAELDEWDTSLPADSERTIKIELNDTNR
PDLWSTAGLARQLRIYRTNNIPSYPFFASQKKKIAAPYRVVVERSVKEVRPWLAGFVAKG
PAISDALLRDMIQTQEKLAWNFGRKRKGVSIGIYRISLIEWPVRYFGVDPHKVSFVPLQE
TRRMTLNQILEEHPKGIEYASILKGKPIHPLLTDSKGRVLSYPPIINSADLGAVQVGDTE
VFIEVTGSDYPSVALSSSIMACDLADMGFEIKNVQVDYEYDTPFGSSIVFPYYFQGEISV
SIEEANKLLGSGLDVAQAIDALTRMGLCAYSEDGRTIRVAPPEYRNDFLHPVDIVEDIMI
GHGLENFPPERPRDFTIGRLTPIERFSRKAKSIMVGLGYQEMIYNYLGSGKDYAEKMQIP
ASALVKIANPMTENYEYVRNSPLPGLLQTESVSSKASYPHRTFEVGKVALKDVDANYGIV
TRQYIGFLTSHAQADYNEIASHVAALMYFLGKEYTVREASDPRFVPGRQVEVLAGGKRAG
VFGEVHPAVLEAFGIMMPCAASELDLDMLIEE*
>SPBIB_v1_90010|ID:27162094| conserved protein of unknown function [Uncultured spirochete bib]

MEIYAHEPLGFDGFLVRVEADIRNGIPAVEIVGLASTSVREARERARIAVRNAGFEFPQD
RILVNLSPADLPKEGSAYDFPIALKILSQSGQIADVPFPVLAMGELTLEGEIQPVRGILP
AARAAVEKGIGTCIVPIENASEAMVVSSMQVWPISHLSQARQILIALREGEKPGMFLDGI
LLGHGFGHDLQADSAPDSMPDSHMNYSPGAWEHEQSLDFRDFKGDEKFIRALVVAAAGGH
NMFLAGPPGAGKTMAANRFPSLLPDLEATEALDTASLYSLWGQKQARFTKRPPFRAPHHS
ASLEGILGGSKPLRPGEVSLAHHGVLFLDECPEFRRDVLQALREPVERGYVDIVRAGRVI
RFPSDFQLIMAANPCPCGNLGIPGKTCLCTPEEIRRYWKKLGGPLLDRIDMRVAVVPPGP
SRLLRAQSISHSDLQKKVLNARIQQRERLQRADARNARTNARIPPGHISELCKVTGNAEK
LFLSGVSSYGFSARAGHSILKVARTIADLDGRQEIGESEIEEAIEYRQLGDGDAIWPG*
>SPBIB_v1_90011|ID:27162095| Oxidoreductase domain protein [Uncultured spirochete bib]
MNKKLRFGLIGYGKVAALHARALAAAPHCELVSVSGHNKEKRDAFASQWKLASRDGVHEM
VQKDGVEAVLITTPHPRHYFDAMDAFSAGCHVLVEKPLSLSVSEAEEMMRQAQATGRFLS
VISQRRWYPACIRIREAIDQGLLGSPLLGQLTILGWRDEEYYRSDPWRGSWEHEGGGVIV
NQAPHQFDLLCWYMGEVAEVYGAWANVNHPYIEVDDSAVATVRFKSGGLSSVFISNSQKP
GIYAKVHIHGSSGASAGVQTDGGAMFIAGRSGVLEPPYNDLWTVPGQEALREQWHKEDKA
FFAGIDATWYFFSLQEEDFARAILEGRTPAVSGRDGLQVARIIEGIYRSNKEGKPVRY*
>SPBIB_v1_90012|ID:27162096| Alcohol dehydrogenase GroES domain protein [Uncultured spirochete bib]
MKALVLEDYMRLEVRDIPQPTISAPDDVLIQIKAAAICGSDVHGIDGSTGRRRPPLVMGH
EAAGVVAACGSGVAHFVPGDRVTFDSTVWCGECYFCRRGEVNLCDDRRVLGVSCEEYKRD
GTFAEYVVVPERILYHLPNSVSFEEAALTEPISVAMHAFHITNMQPGERAAVVGTGLIGL
LLIQIIRAYSPHLILAFDTDPDRRAAALQSGADIALDPADPLSTSMVLKATDGRGVDRAF
EAVGASASIATAISVARKGGSVTLIGNISPKVEIPLQSVVTREISLLGSCAISGEYPASL
ELIASRKINPRSVISAVAPLEEGPVWFERLYKREKRLLKVVLIP*
>SPBIB_v1_90013|ID:27162097| PfkB domain protein [Uncultured spirochete bib]
MSALQIMPAEKAELDFLSVGGLVMRMDPGVVPFAFADRYDVHVSGGEYNVAANLARCFGK
QTAIASAMVDYPLGEKVEASVRSMGVRGIYKRFKYDGVRGPNIALVWSDRGQGVRPPVVF
YNRANEAAALLKPGDFDFRSIFEKGVRWFHSGGIFSALSATTPELVIEGMKAARAHGSIV
SFDLNYRAKLWAAADTGKKPSDILERIVEHVDVLLGNEEDLQMGLGLPGPDIHSASKLDP
ASFLGTIEQVHAKWPNVKVVATTLREVKSTNRHLWSAVLWIESQHWLAPTMELDVYDRVG
GGDGFAAGLIYGMLEGLDPEECLRLGWAHGALITTYPGDTTMATLEQVKTLAKGGSARIQ
R*
>SPBIB_v1_90014|ID:27162098|gnd| gluconate-6-phosphate dehydrogenase, decarboxylating [Uncultured spirochete bib]
MKADIGLVGLAVMGENLVLNMESHGFTIAVYNRTVEKVDAFTSGRGAGKHIIGAHSPKEL
VAVLKKPRIVMIMVRAGSAVDDTIAQIAPLLEPGDIIVDGGNSNYQDTMRRLAELEGKGL
LYVGTGVSGGEEGALNGPSLMPGGSEAAWPHIKPIFQAIAAKVDDGSPCCDWVGPGGAGH
FVKMVHNGIEYGDMQLIAEVYHMMRDQLGMTPDEMADVFEAWNKGELDSYLVAITRDILR
FKDEDGLPIVDKILDAAGQKGTGKWTGITALDFSVPVTLIVEAVFARCLSAMKDERVRAV
KILGKPSGARFKGSRGAFIEDLGKALYAAKIISYAQGFMLLREAAAEYGWKLNYGSIALM
WRGGCIIRSRFLGRIKEAFDAEPSLSNLVFAPFFAAQVLENESALRNVVGEAVASGIPVP
ALSSALEWFDGLRTERLPANLLQAQRDYFGAHTYERIDKPRGEFFHTNWTGHGGSTAAST
YVV*
>SPBIB_v1_90015|ID:27162099| Transcriptional regulator, RpiR family [Uncultured spirochete bib]
MEKPKSSNCLYLIHSLMEDFSDRERRIADYILANPAKAVHPSIEELSESIGVSVSTLVRF
VKKLGFKGYQQFRISLASEALAPEAKIYETIVDRNDDPVQIAFSSAQKALELTSSMIDRS
ALAALASRIIEAGCVHLFGLGGSAIVAKDAVHKLIRTGIHCVNAEDYHLQLMIASQLGEK
DTAIVISHTGVNKDTLRIAEIAKNAGAFLCVITTYPRSPLARMSDRLFISASSGSQMISE
AFSARIAQLAMIDSLYIAIMEQLGECGIQKVEKMRAAIAKRRM*
>SPBIB_v1_90016|ID:27162100| Extracellular solute-binding protein family 1 [Uncultured spirochete bib]
MKSKLMLVILVVLTVLSVGAQQKEIRVLLANHPYGDLLKAAIPEYEKATGVKVNVESLQE
SQLTTKLTTEFATRSSTVDVFMTRPLQEAKMFYKNGWYEPLADYDFSDYPKNALDVATFG
NKAYLVPLVTEWEVLYYRVDLFKKAGLSVPTNFTELEMAAKKLNSADIAGFASRGKGAAA
VTQLSSYVVYNYGGTYLDKGKAVFNSKEALDAIRFYGKMLANYGPAGITNMSWENIMPLFQ
AGKVAMWTDASVFYGQIVDPTKSQIPAENVGIANFPAGPKGNTPYIVVSWGMAIAKQSKN

KALAADFIKWATSKELAKRGMLANITMARNSAWKDSEVRAKIHPGLIATQEYAAKNGIPY
DRPYMSAVGEARDLIGEVIIESINTGGSSAKLESLARDKVAAVNSLLEDTGEYGVY*
>SPBIB_v1_90017|ID:27162101| Permease component of ABC-type sugar transporter [Uncultured spirochete bib]
MAIKRPKGSFLEKNLHIIFPLPAVVFVIVMMLFPVLYTLFLSFTNWNLTSGMPLSIVFLK
SYQKVLREPRFIAALGRTFSFTALAVAVETILGMIVALILNREFRGKGFTKLIMLLPLVS
TPVAIGIVFNLFYDPTIGLANFVLTRFGLPRQAWTGSAATVIPSLAIVDIWQWTPMIALI
LLSGLASLSSEPYESARVDGASEWQIFWRITLPMVTPTLLTALILRTIDALKTFDIIYAM
TGGGPGYASETLNVMAFKYSFEYFRMGQASVILVFLFMLVLFMSLLIVRMRKSLEL*
>SPBIB_v1_90018|ID:27162102| Binding-protein-dependent transport systems inner membrane component [Uncultured spirochete bib]
MRTALIRKILFFLVILAIVVPVLFPFLWMLTSSFKTQVDIVAWPPRLVFSPTLQNYERVF
REQNFLKYFTNSSIIGISAVSLSLLMGLPAAYSIARYKQKRLSMFILIARLMPGISFLMP
WYIIFSRLKLMDSYIALILSHMLITLPIIVWIMSSYFETIPIEMEESAMVDGATRQRAFL
SVVLPIAGPGIVTSVTLAFIFSWNNFMFSQVLSMERTRTLPIAIYNFVSYAEVDWGAVMA
AAVAIMAPAIILTMIFQKYVVKGLTMGAVKG*
>SPBIB_v1_90019|ID:27162103|trpB| Tryptophan synthase beta chain 2 [Uncultured spirochete bib]
MRIKATLSDSELPRQWYNLAADLPETPNPPLAPDGSSLRPEQLSALFPEPLIEQEVSSDR
WIDIPEEVLEILARWRPTPLVRARNLEKALGTPARIYYKNESLSPAGSHKPNTAVPQAYY
NKITGTKRLTTETGAGQWGSALAFASAQFGLECQVFMVRSSYEKKPYRKVMMQTWGASCT
PSPSQLTRAGRAVLAEHSDSPGSLGIAISEAVEAALEDRTGKTHYALGSVLNHVMLHQTV
IGLEAKRQLALFGEKAPDTIIACVGGGSNFAGLSFPFVKDKIDGANIDIVPVEPASCPSM
TKGLFAYDYGDTAGMTPLLAMYTLGHDFMPSPSHAGGLRYHGMAPLVSHAARHGLLRPVS
VPQLECFSAAVLFARTEGIIVAPETSHAVAQVIREAIQAREEQKEKVILFGLSGHGLLDL
QGYEEYFAGELGDLSLSEEELARSLASLPKAGPAK*
>SPBIB_v1_90020|ID:27162104| Cation diffusion facilitator family transporter [Uncultured spirochete bib]
MQESFRKLGYAEGIASAIINTILFGLKIWVGIKASSIAMTADAWHTLSDTLTSLVVILGF
WISGRPKDEEHPFGHGRAEVIAAVVIATLLAVVGASFFRDSIRQLIERKNAAFSTLSLII
FSISVLVKEALARFSLWAGKKTQSQSLVADGWHHRSDALASLMIVVGAVVGKYVWWIDGV
LGIGVSALILYAAYDIAKSAFHALMGESAGAFLSGEIRRIAAETSPQLKDIHHIHVHRYG
DHLEITLHARMNGETNIQDAHNLSSSLERELKKELNADTTVHIEPEKHA*
>SPBIB_v1_90023|ID:27162107| putative DegT/DnrJ/EryC1/StrS aminotransferase family protein [Uncultured spirochete bib]
MIRVFSSYITRKDMDLVLSRMVEDAVGPGDFNSRFAKAIKDQFGFEYAVALRSPYTAMQR
AFKICGLGQGSKVAISALAPSWHLAAVEDAGYVPIVLDVEEETLHPSKEAIESANPSTVL
LFDALGKMPPSSLLQSLGFPVIEDISQVSGNIAESTSSNTFAYFAVWGLENDSPIATGGG
ALLCARGKRDGQILRSMEESLPSELRMTDYNAALGVSQLKSVSQMVERRKAIQEILSRQL
ARTRHAGLKIAESEATPIYAFAVFAEGSSKEIIEYAKKHGVQAELAFSSSPAIMGDDAAA
CFPVARSIALRCVLFPMHHKLSNQQVDQIGKIVATLP*
>SPBIB_v1_90024|ID:27162108|ppnK| putative inorganic polyphosphate/ATP-NAD kinase [Uncultured spirochete bib]
MQESKSALIFANLNKDDASNAALVIKKGLLERGWQADILGFRSSIGERPDFSRYALLVSL
GGDGTLLYAASFAAPLGLPILPINLGTLGFIAANKIDTWAATFDGWCSGSIEVSKRLMLR
VRAYRENELLYNGIALNDIVVSSEGNARMIRMCLYVNNDRFGHYRADGLIVSTPTGSTAY
NLAAGGPAVHPEISAIIINPICPFTLASRPLVIPGKLPVEIVIDETRKSGALLTVDGHDM
TPLKKGDVAKIEKYEKHALLVVPPGNAFFSALRTKLGWSGDNDA*
>SPBIB_v1_90025|ID:27162109|recN| DNA repair protein RecN [Uncultured spirochete bib]
MLEQLSVQNFAIIEQVDIDLSHGMTVFSGETGAGKSLIVDALGFLLGAKADNSIIREGAA
DCTVSGLFSIRDNPEVQRWLEERGISDESESSILIRRTLKQNGRSLAWIQNRQVSRAELV
EFTQFLVDIHGQHEHQRLIDPATHIEMLDAYASLDQDLKAYQKIYQEWRDSVKTYQSLLE
EKAKRAQEMDYLEFVIKDIASAKPRAGEDIELAAEEKILSQHEKLFAAISEASSSMAAGD
STDALHLLKRARADLETARAIDARLGPFADRLAAAYYELEDISESIAEYRSGLRFDPARL
EAIQNRLAELQRLKRKYGPELADVLQRFDRARSTLDTLAHADENALELEKRTKALKEKVL
SAAAEISEKRMAAAATLSSSMEAIVRRLGMPDARCIVRVARIVDDRGSYRPGPFGFDEVE
FYIAPNPGEPDRPLSRIASGGELSRFALALKAVLAAHDVVDTLVFDEIDTGIGGQVGVAV

GEYLKQLSEYRQVLCVTHLATIAARADQQCKVMKYISEGRTSTEIRYLSRDEREAEIARM
LSGSAESDVSRSHAAELLSKAQIQQ*
>SPBIB_v1_90026|ID:27162110|cdd| Cytidine deaminase [Uncultured spirochete bib]
MQPEYSSLFDLALEACEHSYAPYSKFRVGAALLCEDGSVITGVNVENRSYGLTICAERNA
IAAAITAGKKKFKALVIATPDADYPVSPCGACRQVISEFMPPSAPVVFGNSQNSYMTSSV
GELLPHDALHELSGELGD*
>SPBIB_v1_90027|ID:27162111| exported protein of unknown function [Uncultured spirochete bib]
MHHRNEMKKRRFHIFAFIVAAVLFVLLSVRGTKKQEQISEAIFLPFSSALDPASISRQNP
SPSLISFLGDGYLGYIDSANNQLYAAQFSDRAAIDSSGWISYDRLATALQAFSLGSKPST
IAGYGYPWFKSSRRFLIRADQMGIAEMHADGSIVWEIEFPMPITAIDATPTTLGIGFLDG
SIRFLGKKAENLYSEPHDSQQDSQHIRTVYGIALSGDGRSAAVLRGMSPQKIESYRRAGT
GFVKSSERELPSGAPLQATMCYAEDGSHVVIAQGEQLIYYNTKKNYVRKLANGAALGNVA
SGAFVQHFVLGPTGGNTIAVLQVQSQNPPTTKVLILKHGIIEREEPGAVSASAGKGALII
VFRDGVEIVKGWQQ*
>SPBIB_v1_90028|ID:27162112| exported protein of unknown function [Uncultured spirochete bib]
MKSTKLIAICFLFSVAFLGPLGAIDFQVPAATDIAGFGTILATQGSSNAKELQKTKPGFV
PRSDIALKTEYPEIKAPSSGTVIYSQRESPVASVFSFPLGGAVAILHPESYISLVSGLAL
DTVIFNQAGRGQENSVKKGTSMGQAIGSGIYPERYFGLRLFDLKNSLWINPLFLASWLQD
RTAPVIQNVWLNNENMRGNTPIELKTTAPKGKYIECQQATYEVSVAAIDSIIPGSRSFSA
PYKIVIMLDGKTSIDTSFIAATCKSEGLSFLGNPAPSSKAILENGAYRVGRIPLQRGQHE
IEVQVSDYAGNQSILKASLMVN*
>SPBIB_v1_90029|ID:27162113| Pyridoxal phosphate enzyme, YggS family [Uncultured spirochete bib]
MPMNDSQIADNIARIQERILRAAERAGRAPQSIALMAVTKFHPAEAILDAYHAGIRLFGE
NRVQEAQTKYPPLRDSLVGSRLHMIGTLQKNKINKALGIFDALEGIDSVEVLEAILARLS
SRTEPIELFFELHTGEESKAGFPSLDELLRACDLLASFAAENREEGRLVVLRGLMTMAPF
TTDERAIRHSFSMLAAAKAEVAQRFDFVHFTELSMGMSNDFEIAIEEGSTLVRIGTALFG
ERA*
>SPBIB_v1_90030|ID:27162114| membrane protein of unknown function [Uncultured spirochete bib]
MRWPLLRCIAFLVIVFFVGSAFSFAQDMQEVLPEDQTNPTQETIPQTEDTTGAFPQTEGG
QAEGTQAKPPAVTLPSFSYKTTKESMAGRALILSIGVFPFSYFYTGFVLDVTRFVSNNFD
TAYAPWPFKTQNSVALTNSEMWLKLGISAGLSIGFGILSAILK*
>SPBIB_v1_90031|ID:27162115|apt| Adenine phosphoribosyltransferase [Uncultured spirochete bib]
MANDSAIHEEDEGFNLDDAIRKVSDFPKPGILFYDITSILANPDAFQYCVDSMLKLYKDM
EIDAVAAIESRGFVFAAPFCYKRGIPLLLVRKKGKLPGKTISCSYELEYGSAILEMHVED
IQPGARFLIVDDLIATGGTINATCDMITRSGAVPVRAFSVVGLPFLNYGEKIKDIGVDTL
IEYFGE*
>SPBIB_v1_90032|ID:27162116|gpmI| putative 2,3-bisphosphoglycerate-independent phosphoglycerate mutase [Uncultured spirochete bib]
MVAALKKNPQWKGRRGPLVLVIMDGVGYGEYKEGDAVQAADMLHFRKLEASCPVTKLKAH
GTAVGLPSDDDMGNSEVGHNAIGCGRVFAQGARLVNLSIETGAMFQGRVWRELVANVKNS
RGALHFIGLFSDGNVHSHIEHLKAMLIQAQKEGVQRARIHILLDGRDVGSQSALDYVIPF
EAFLAELRTKGVDYRIASGGGRQYITMDRYGANWKQVKRGWDCHVLGLGRRFSSAEEAIT
TYRKEIPGVIDQDVPEFVIADEKGPVGTIEDGDSVIFFNFRGDRAIEITAAFEQDNFDKF
DRVRRPKVYYAGMMQYDGDLMVPKNYLVSPPAIDRTVGEYIAATGLKTLAISETQKFGHV
TYFFNGNRSGKFSDELEDYVEIKSDRVPFEQRPWMKCAEIADYVLDAIGSGKYSFIRLNF
PNGDMVGHTGVFQAVVCGLEGMDLQIGRIKEAVEKAGGILILTADHGNSDDMFEHDKKTG
KVTLDANGEPKVKTSHSLNPVPCVIYDPEYKGEYKAALRSGLGISSLAATCIELLGYEPP
ADYDPSVLEM*
>SPBIB_v1_90033|ID:27162117| putative Adenylyl cyclase CyaB [Uncultured spirochete bib]
MDQPDRAHMVEIELKARIADRKAVEAQLASFMRFAGDIDKRDEYWEVSGWNPLAPAAFRF
RVRAEPARTTITFKEKTFDGDIEINRESEFSVDNEAVFRKFMEKLEARFVYTKRKIGTRW
ESAEGLVAEVVEVEPLGLFLEVECVCEQLDSEALDEAKCRLYEVIDRCGIPRVNLEPRPY
SQMLGY*
>SPBIB_v1_90034|ID:27162118| exported protein of unknown function [Uncultured spirochete bib]

MIPFAKKLISSFKFRIFLFVLAAIMPAFLLSFLQLHYERNRELSEMRYTTHQIVELIAQQ
ESDVFDGTRQLLEAVAAMPEIRNAAYKEIAIYLRKLASAFHRYRNFFLLDAKGTILANAF
PADDAKTDDKIPALAEIAASKSPVMSGFHIDPDDHSPSVYFGYPLIDRSGYVSGALAAVI
DLSYLNVFEQAIRSKLQPEAVLTKIDREGIVLIREPDPEAYIGKKYQEQAVLMQIGTQQD
GVLLQTDTQGQKSVFAFSRMPSKIYASDIYVIVSIPEHSAFAGINQRFYRELLILGIGMI
LISLLTMLSLDPFLMHDVQELIAAAKKMQKGDLTARARHFKYSSIELQQLVRAFNTMADS
MQKSQQQLLDSYEATLEGWVRILDIRDNETSGHSKRVTDLALQFAREAHVKEDEMPNIRR
GALSRSAVQIRQRP*
>SPBIB_v1_90035|ID:27162119| protein of unknown function [Uncultured spirochete bib]
LKLEMSFFAKGIIVSRASEPDYTICESNYKESLVSDYQIKPKGALNDSRRKKTEVLQMYD
PYYKEGLGTPIDAPAFPRKCVRVSSCGTLQVKRKAGGTCAR*
>SPBIB_v1_90036|ID:27162120| conserved protein of unknown function [Uncultured spirochete bib]
VGLVYSEPQRRAAAYLREYSRKYDTVEIDSWFYKISGPEEVADYLAQVPPAFRFTCKVPQ
ELTLTHLRGNAGASMGVPNPSL*
>SPBIB_v1_90037|ID:27162121| exported protein of unknown function [Uncultured spirochete bib]
MRKAFFLILAVLLIFSSCNLINPVKKPSDIDTVAEINSSDIQSSGATNVPTTESEIVAII
SGIGSNASSDQVIQDLGNSFAEKKLSAAKILKAAKSLSSSFQQQIDKIQQDFDNFPTTKK
IDETISLSGENIGTYFALTTGEAAFSLNAVTTDGNPIDMQGASNLSSLAGEGTLKIAINP
TSALSSLQADASAIKDFKFRLNAGGSASISTKAGAGMERIPDKITLDYAESMAIAFSARV
DTNGGKFSFKIDSKYSGTIDYSNIQSATDPEEVLNTLVPSITITVKVYDDSGNVTFNKTY
TSIEAFVAAFTPAT*
>SPBIB_v1_90038|ID:27162122| exported protein of unknown function [Uncultured spirochete bib]
MKKYALVALFAVAVFGMAFAATTQSGPASISGLAAEVFSLTVPGTYMGTIANGSTAETWN
LGIITVNSNVRNWQLSVSSANSGYLVNTADNTEKIAYTITVAYLDITDWSLSRVAVSPPQ
PRTSKTGNIYPVQLKFGPSADYYQAGTYGDTLTVTISHN*
>SPBIB_v1_90039|ID:27162123| conserved protein of unknown function [Uncultured spirochete bib]
MRTNIVIDDSLMNKAKSISGYKTKKQTIEEALKLLIAQKEQSEIRKLRGKLNWEGNLEEM
RLDR*
>SPBIB_v1_90040|ID:27162124|vapC| Ribonuclease VapC [Uncultured spirochete bib]
MIIVDTSVWIDYVRGIDTPQTDLLDYELLHDRVATGDLIIVEFLQGFREEKDIKIAKQIM
DRLEYYDFLGKEMAIISAENYRKLRKHGVTVRKTIDVIIGTFCLEKGFELLHNDRDFDPM
EQYLGLKVKR*
>SPBIB_v1_90041|ID:27162125| protein of unknown function [Uncultured spirochete bib]
LKDEKAPEQAPSNRMNPKVDAFLRNAKNWQEELAKLRTIVLDCGLTEGSIVITSNRPPED
WFSIFPDQIVGGAIMDRLVSGAVKLIVTSGRSFRKEGTLRNQISEPAA*
>SPBIB_v1_90042|ID:27162126| exported protein of unknown function [Uncultured spirochete bib]
MITYKTKRIVSLLILTAIFLITFGCSEPIVYRTILLYLTSNYGLFSTGEKTDAKIYSISD
GYITTLKPGDMANYTTYVGDRIIAIYSKKVNNTWVDVSKTMIVGEDSSVFYLD*
>SPBIB_v1_90043|ID:27162127| transposase (fragment) [Uncultured spirochete bib]
MYFKKDRAIHLVAGMTDMRKQINGLAQIANEKKADRMFSGDYFVFLGKTRKVMKVLYWDR
TGFCLWHKRLEEETYAAPVKVGLNFPVLV*
>SPBIB_v1_90044|ID:27162128| protein of unknown function [Uncultured spirochete bib]
MKYRKAERGSMKYPLELKQKVLKDYFEGKDGIRGLERTYGIQHQLILSWIKTCQQPSYRM
RQGKVPKEAALHLNEPPVFNDPRKELEYLRAENAYLREMLMLSGVRKNGRKKKALPPSND
SAHEATP*
>SPBIB_v1_90045|ID:27162129| transposase [Uncultured spirochete bib]
VKLLCSIAQVSRSGYYRYLLITQKPNKDARLAEKVREVQEAVCYSYGAKRMAHVLSEQEQ
VPINHKRIARIMQKYLLGARIRLRRHPEYWYRQRHKVRLSDRQCAPNILNRDFLAPCPLK
KLVTDVTWISCADGTLYLSAVMDLFNRQIISYTLSRRNTTATALSPILELVGSYNLQGTL
VHSDRGPAYRSEEYQALLRKHGIVTSYSRAGNCWDNALMECFFGHMKCELGFVTGSQKKQ
RVAQVSQKIHEYLVFYNKTRIQKNLGWVSPMGYKMLYTSSIESNNVST*
>SPBIB_v1_90046|ID:27162130| protein of unknown function [Uncultured spirochete bib]
MGLVQTKTGKLSPTVTVVDRLAHHGHILLFEGENYCMKHALMRQKDQIKLPMA*
>SPBIB_v1_90047|ID:27162131| protein of unknown function [Uncultured spirochete bib]

MPRRIAMIELREIILQLRRGCGIKHIHRTTGHHRTVIRALKAIAEAKDWLNPQKPPPDEA
AVHAAWEATITSKKPHQLDGIQDQLLRSHHEGISFVVMHRLIAGQVSCSESTLRRYIQKM
CPPTQ*
>SPBIB_v1_90048|ID:27162132| transposase [Uncultured spirochete bib]
VIDRLYIPGDCMDVDFWLLGLVDDEQDGRMRKAWVFSARLRYSRKAYRTIVFSQHQDSFL
ACHIEAFEWFGGVPATVVLDNLKAGIVKASWEDPLVTRAYHALAEQYGFTISACRPSTPK
HKGGVENDMHYLKRSFWPEIRAREAQKGHAVPRLAKVVAYLSEWNATVSEVRLVAKVGKT
VTALFAEEKPYLQPLPSTRWEPERWAFATVQADWQIQFDKAFYSVPYRFIGQRVLVQATR
TTVRIFHESDEIAVHERAEHHWQHRVVVDHGPPQAAEYLATSTKGLLMAAETIGPATGRF
AKAIYADRAVDSIRPLWALVHLSERYPCDAIEQQAERLLAYGLASYTSLKNELKFAKEKA
TRFSPSFRFARDPAYYRDAAGVCHG*
>SPBIB_v1_90049|ID:27162133| conserved protein of unknown function [Uncultured spirochete bib]
MADLAVLKPKLTRLKLSGMLDSLQQRFDEAVAQKWSFSDFLEHLLQDEVERRDSKQLAYR
LTKSGLDPHKTFESFDFSFNPKICEPAIREIASCQFLNDKENVFFVGPSGVGKSHLAQAI
GHEAVRRGHEVLFRRTAPLFRWIAAGHGDGSYSRRLKSVIAVPLLIFDDFGLQTLSDEQQ
NDLYEVLCERYESASTIITSNRDFGEWPSVFSNPLMSSAAMDRLVHHAVKFVIEGKSYRV
ESFSSRQQRLTAGN*
>SPBIB_v1_90050|ID:27162134| protein of unknown function [Uncultured spirochete bib]
MNSSILFLSFIIIIIIVFVILLLVKNRNRKKQYVIPSSNISHTREAEDLQLDPASSYSAS
TKTIVKQDIPELIEIKNSPSPVDSQMIDESSINDNKTMENLVNPRLESSIYFTSIYFIRN
DSQKTRKHTLEPIKRGGRPRDPNRDPKYLSLRQTLNYIAKPEIVCWEKGSEWKVGVEIPD
EILANQDISILQNGIYLDRDSNIDNCWNLAAISGEIIIQWSNNEINNKIVITNENTYLIF
RLVGEDQKRGYLVSNPSMGSYLIIAPEIWEYSNISTVNRIALPEPSSLVGYLAHFCEFKA
DKENSVAFKTSENKIITIKPVKPKFELVGNSLEDSSENIGPLFGDSMPQIKAITKNTWND
ISVIIIGEEGRAKKRWRKEFIPDLSQNPQYIPDSFYEKGSGWYYVRIYNKENELVESMNF
RYIELLKKINIPKHLHPAQNDGYQTAKIELIHSPGFVIKSANRRDNIQIEQESSEKTIIK
IPADPDYDNSHWLVEIDNNKFIPLTISLKRIWWALGEENKTPLEWIDKAILLQRSDFTAL
SNKALWIRCPQDFKSKSISISMGENSRDRHYKISDSLAMIPIREFSDVTELMLQNNLEIF
ASLPQDNIRIKICEVLRKLQCKFCDFTSLNEQDIIAHAIANHLDQMFIPLNYDELKANDP
NLPHRIYQCIYCGFYVTSDDIENPTSKIIHHINNDCKKAPRNDGPPRTDFKIISNVDEIR
ENIIAELPNIEKCYFCGKTFERATDNKKKTHLKECHMSLLYKLL*
>SPBIB_v1_90051|ID:27162135| IstB domain protein ATP-binding protein [Uncultured spirochete bib]
MRRPRTNRTLLREEISQLLKKMAFSQTAVQLCEEEGTAPMEQFLLNVLQSEMASREQARR
ARFLREAAFPVYKTLEGFDFTSVSLPPALSRDELLSMQFVAEKKNLVMYGPVGTGKTHLA
TALGVEACNRSMRTKFFTAAELVVRLSEAHKEGMLDKLLKAVLRADLLIIDEWGYVPVDH
QGAQLLFRVIADSYEQRSLILTTNLEFSKWGSIFTDDQMAAAMIDRLAHHGHILLFEGES
YRMKHALMRQKDQIKLPMA*
>SPBIB_v1_90052|ID:27162136| Integrase catalytic region [Uncultured spirochete bib]
MNQIDQIKELQRQGYGPGEIASRLRIDRKTVRTYMKREDFNESLEAHTTWPSKLDRWKPL
IDEWLAEDQRMRFKQRHTAKRIHQRLCGEHAGEYDCSYPLVQRYVKAKKVAQRQMDGFLE
LVWAPGEAQADFGEAEVMEAGVRKTIKYLTLSFPYSNAGFTQVFEGETAECVAQGLQDIF
GYLGGVPRRIVFDNATGVGRKIQQHVALNQLFLRFKCHYGFSVSFCNPNAGHEKGNVENK
IGYTRRNFFVPLPVVDRLVDLNMQLFKKALQDHARKHYKKGQTIAALFAEEQTALSPLPA
RPFNVERYERLRTDGYGKFCLDGKHWYSSAPEYASGEVTVGIKAREIVVYGPDGEVRGEH
RRIYGEERSDSIDWHTSIAALMHKPSAWQNSHFRAATSPSVRQALDALPRDRLRDVLKGL
VQSSERFGFEVALASLEEAVSVARLDSYSVHAVAARHVYDGLYGIPAAGPDLGVYDRAFI
GEKEHTP*
>SPBIB_v1_90053|ID:27162137| protein of unknown function [Uncultured spirochete bib]
VLADIPLPDEILYGKSYMALMDRIQ*
>SPBIB_v1_90054|ID:27162138| protein of unknown function [Uncultured spirochete bib]
VLAYDGSSMKRIDAAGILGMAMSGTTATLLARRWESALLVNVDNDTLRRLMSNEEAMRAL
MGIVSVNFTPLGKNRKRKFHTVGQKSV*
>SPBIB_v1_90055|ID:27162139| transposase [Uncultured spirochete bib]
METALSELPDDVESLKKLVVEKARRAHELEATSKQLKEENAALQLKLLEVTDKYETLQQK

FFGSSSEKRKKEDDNPKQALLFNEAETYAEAPPKPEKSIPVKSHERKVRGRKPLPANLER
REFVYELSEAERTCPSCGAVRPEIGQEVREELEFIPARFVVNAHILKKYGPCQCASCSHP
IVQAEGPAKLIPGSSFSNTTIAFFLTSKFVDSQPFYRMEGILSRWGIDTSRASLCKVAVS
AGRAIGELLDELRKDLAASPVLQMDETVVQVLHEKNRSAQAKSYMWVARGYAEGKPVVFF
HYHPSRAKEIVQKFLHGYHGFVQTDGYAGYNEVGFSPGITHVGCLAHVRRKFFEAEQQGS
TEASAFLERIAELYHAEKLVRKKFEEGVLTTEEFLAARTKEQGPRLSEMKAWLIAKQGQS
PPSLSFGKAVHYALGQWDRIEKYLQHELLTPDNNAVENAIRPFVIGRKNWLFSNTPLGAH
ASAGIYSMIETAKANGHEPYKYLCYLFNALPKARSLEEKRALLPYKLAPNSY*
>SPBIB_v1_90056|ID:27162140| conserved protein of unknown function [Uncultured spirochete bib]
MYFKKDRAIHLVAGVTDMRKQINGLAQIANEKKSDRVFSGDYFIFLGKTHKVMKVLYWDR
TGFCLWVKRLEEETFPWTRKQKGIITLKREKLKLLLKGIDIFREHTEKRYVSVL*
>SPBIB_v1_90057|ID:27162141| transposase [Uncultured spirochete bib]
METALSELPDDVESLKKLVVEKARRAHELEATSKQLKEENAALQLKLLEVTDKYETLQQK
FFGSSSEKRKKEDDNPKQALLFNEAETYAEAPPKPEKSIPVKSHERKVRGRKPLPANLER
REFVYELSEAERTCPSCGAVRPEIGQEVREELEFIPARFVVNAHILKKYGPCQCASCSHP
IVQAEGPAKLIPGSSFSNTTIAFFLTSKFVDSQPFYRMEGILSRWGIDTSRASLCKVAVS
AGRAIGELLDELRKDLAASPVLQMDETVVQVLHEKNRSAQAKSYMWVARGYAEGKPVVFF
HYHPSRAKEIVQKFLHGYHGFVQTDGYAGYNEVGFSPGITHVGCLAHVRRKFFEAEQQGS
TEASAFLERIAELYHAEKLVRKKFEEGVLTTEEFLAARTKEQGPRLSEMKAWLIAKQGQS
PPSLSFGKAVHYALGQWDRIEKYLQHELLTPDNNAVENAIRPFVIGRKNWLFSNTPLGAH
ASAGIYSMIETAKANGHEPYKYLCYLFNALPKARSLEEKRALLPYKLAPNSY*
>SPBIB_v1_90058|ID:27162142| conserved protein of unknown function [Uncultured spirochete bib]
MYFKKDRAIHLVAGVTDMRKQINGLAQIANEKKSDRVFSGDYFIFLGKTHKVMKVLYWDR
TGFCLWVKRLEEETFPWTRKQKGIITLKREKLKLLLKGIDIFREHTEKRYVSVL*
>SPBIB_v1_90059|ID:27162143| protein of unknown function [Uncultured spirochete bib]
LRLLGIEGFRSLNQDIETIINKFEAVKKLKREANDRQLAKEEKLVLSEEEKEYKSKRKQI
QEKLIKFAIRIPAFMYLTDYRERPLKDVITQLELGLFKKVTGLTVKDFELLVSLGFFNNV
SRSV*
>SPBIB_v1_90060|ID:27162144| protein of unknown function [Uncultured spirochete bib]
VPDATLRKLTGHLTPAMTDHYDHTTIDDLRLLAKAQDEKLLGTIEATKEE*
>SPBIB_v1_90061|ID:27162145| IstB domain protein ATP-binding protein [Uncultured spirochete bib]
MIRTPQERERTRITIASMSRKLMLSSRVVELCESEATPRQEEFLLKVLSEEIDRRERGKK
ARLLNRAGFPVFKSFEDYDFSEIRFPPALSKEELLRADFIPEKKNLVLYGGVGTGKTHMA
IALGIAACEKGLSVRFLTVTELVLKLTEAYKAGTLERLIRDLKQLDLLILDEWGYVPVDR
EGSQLLFRIIADSYESKSLILTTNLEFSKWGGIFTDEQMAAAMIDRLVHHGHLLLFEAKS
YRMTHALMRQPGPGTAKPKTTVEAGSRLGEGGA*
>SPBIB_v1_90062|ID:27162146| Integrase catalytic region [Uncultured spirochete bib]
VINMPQIQGIREQYREGSSVAELARIYGVDPKTVRKYLKQDDFSPKPPEKMTKESILDPY
KPLIDSWLLEDQGRWHKQRHTAKRIHDRLISEVAGYACSYNTVQRYVKRVLQEQRTVRAS
MELVWHPGESQADFGEADFLERGTMVRKKYLTLSFPYSNDSFTQIFGGETAECVCQGLKD
IFAYIGGVPPLVVFDNATGVGRRIGEIIHEAELFSRMRAHYGFSVRFCNPESGHEKGNVE
AKIGYTRRNLFVPEPAFDDIEDYNRTLLSLHASKAQESXYKKLLPIKDLFEEDTRALLPL
PRTAFDPVRYDYLKADGYGKVRIDSRHYYSTSPEYAGQEVLVAIRAHSIDILDDHKRLVV
RHSRVYGERRSDSSDYRTSLAVLMNNPGAWKNSGIRELIPDPLKSLMDRQQRMQLHATLK
TMHTLSSEYGFEIAVQALEEGVQRSRTSFHDAAILAARIAGYGLNMAPERGQDLHVYDEF
LEGVQV*
>SPBIB_v1_90063|ID:27162147| protein of unknown function [Uncultured spirochete bib]
VLELGASNPHGSEGFLIYGANPGARLDRAAIERAFDKALIKPSLEGRYDTSSKEEKESVF
NIS*
>SPBIB_v1_90064|ID:27162148| protein of unknown function [Uncultured spirochete bib]
MKRKQEKIDFRVFSRTVWGTKKKYYIRFLFCGKAFLTRATDAPTAAKAGAIAAKIMATED
LEAVAKAKEQKKVIKLDEIDRLAELPVIEFLRLFWNPSRSPYLQDMAAAGRPLSGSYIRD
NARNANTYWAIAPLDSLPMQDLKLKHIDSRIRSMRQKGKSRYTISAAIDCIRTPCSWLSS
RGVMEKIDFSAIVLPKAAVKERGILTNEELDRIVHLEVVGPWQDSNLKTHISVRPRNRMP

GGEKNEGMAPVGWREKLIILLLAYTGARVGEARALMWKNVDLEKGLIRIEINYTDTDGLK
EPKAKSRRTVPIAATLEPFLLEARRVAQEIGTANPDGFVLLNAADPIRPIAMTTVKRAWE
RVLRAIGISYEEQKARNLVIHGLRHLYATRLVDAGLTPIEAAKLTGHRVIATLGRYSDHT
QPETLKKSREILDGIKN*
>SPBIB_v1_90065|ID:27162149| putative Adenosine kinase [Uncultured spirochete bib]
VKAESRGFLFGIGHAMCDVSARLDDEGWAAFEKVFGFSASCRPHHIDIEAALKALAFIEK
LAGEGHADLVYSAGGSALNAVRAASMLGAKASFAGCIGADALGDIIVNDIEARGVRSLVE
RREGEHTGVFCTVRHGDRSEDSKPEAPPIILASPAGARKIREMPLASFIPEGSGVVHAEG
LLADREGLLEEVFQSAKAKGLAVSIDLVSAEMARRYRAPILNLIERFVDYVFCTKAEFEA
LGADIARMRRDIAWIVKADKEGVDCYFQGGSVHEEAPRGPVVDDLGAGDAFAGAFLYGRT
LGWPLEACIKLGSAAAACALRAKGSVPDAVCMRSLQA*
>SPBIB_v1_90066|ID:27162150| protein of unknown function [Uncultured spirochete bib]
MLNRFCVYMAWHNYAKPFRIKANRKARMTHAEAAGIPRELVATGRAWMFRERAFLSRLSL
DLLDQKLWKRAFSTPLKTSAEYLPRYALA*
>SPBIB_v1_90067|ID:27162151| protein of unknown function [Uncultured spirochete bib]
MGHTRLSVRFDPEGLCVVVPGHVDAEPIEHIGDIAGKAXX
>SPBIB_v1_100001|ID:27162152| putative Radical SAM domain protein [Uncultured spirochete bib]
MMHEGKIVTAAVAADMANPIFELASAKILIVRLSPFRDIEVSYSHLVLFDEARRALPDAF
IDFAFLPTAPDRRLLNAKCGAWFFGRASGRSPSEFDIILVSCSFTLELVNLPWLFAQSGI
PTSRAERLRNNTIPFVFLGGSSAVTSGALLQIREGLPVDSLVDAIFFGEGEGRIAEIVRT
ASEGQKQNAAKQVILSEIAAQVKGFWPCSPEWKTERSICEDRPAVLTSPLVLNGEYAGHA
KLAITAGCTGHCSFCLEGWDRRPFREKPFLELSNAASALKKTTGASDVELFSYNFNMHHE
ILNIVPVMGRYFMHVSLMSQRIDLLARIPHLLAAEFAAGKRSFTLGIEGISARMRQYYHK
GISDEQIADAISAILEGNARELKLFFILSGYEKPSDFDELHTLFDAIGRFKEQTRASTRI
IISAGYLVRLPFTPLQFAPLACDRTDLERISATIESLCNERKLEFRLAASFEDYWTDQLL
SIAGPQAHDWLCACAESGFVYDTHISRQAAASLDAFLRRKKEFSALLDEKTSDYRPCFSF
LESDAHWNLLRAHYERAASYLEFSHAPKNQPKLGKRRTQSALQSTSTHSALSIAAEKTQA
VSTIAALMEAKSHFQPLVVRIREDPALAFATLAYESSWLIRAISTLVPNAERALFECRQI
VPGNAWDKVFSRAGIEYGLSGEKWFALYGPDIRTLQKIVDAASRKIKASLQSGQSTAALP
SAYLLDIEPQDPEITPEECVLSFSLPQEYNSFVGRIIEIWLEKQDIHFTGKKIENGYEYL
VAHSSLNKRILLNAEYHTSSSGSFVTMRIGKRADMLLLSDYLHKEASDHELIFNIEYWE*
>SPBIB_v1_100002|ID:27162153| putative DEAD/DEAH box helicase domain protein [Uncultured spirochete bib]
MEAFISLGVDIRLTKALDAFGFVAPSEVQKRAIPVLAKGQDLIMQSETGTGKTFAYLLPI
FSRILGSAARLSGLDWPSALIICPTQELAVQVARQAALLAQSASVPIKSLALLGGTHFSH
QKEDLKAHPHIITGTPGRLADLVRMRFFDISCIEFLVLDEADRLFSKEYIEPVEYLLSKT
PASCVRALASATIPEKTRKKAYPWMHNPVVVDLSGEGILTDAIEHWVFYAEHRKKIDLLK
RIIAAVRPQRCLIFASDTYRVQRATERLQASGLKCHSILSRMEKQSRHSAIDQFRQGIIP
FLVTTDLGARGLDISDISHVISLDLPEDSNAYVHRAGRTGRAGKKGISILVADGLELERA
SRAAVKYGFVFRTKWLESGQVIEPEVEEFFERVQTMEDERSRRLKS*
>SPBIB_v1_100003|ID:27162154|rnz| Ribonuclease Z [Uncultured spirochete bib]
MNLEAFVLGSGGMMPLPYRQLTSMLVRREGELFLFDAGEGTQVSIRRLNLRWKKITAIFI
SHMHADHVTGLPGILMLSSQVDREEPLYIFGPPRLAEYIEQNRRLLDMYINYDIVVREIS
DREVVWQGEDYSIRAFPLKHTKMCFGYALEESPRPGIFYPDKAMSQGVPRGPLWSELQSG
KTVTLSDGREVRPSEVMGEPRSGRKFSYVTDSLYFPEIAKEVSNSDLFICEGMFEHGLVD
SAIEKRHMTARQAAQIAQDAGNVKQLGLIHYSPRYADRELKVLLEEAREVFPNTVLTKDR
MRFPIEFID*
>SPBIB_v1_100004|ID:27162155| exported protein of unknown function [Uncultured spirochete bib]
MKQIFRIILFVATLVLIAASVMYVAMKIGEIRLNGEQSASAEYTILRNAVAPITSEQELG
DQFIRDRLKSLYNASAHLLAVQVLDRNGLVLWKMPDESAYFGSPATTPGSVFRAPGLSTV
IYMTPLPDGMKMMALYSILSQRDIANTLLLPIIALAVWIVLLVILQFVLKEKPAAYAALA
AAPPVAEPQAQSEAPPEGETESASQAEMPAPPSEIAQPDEEIIAENEEIPATKEKPSQEE
APHYPEPEEQGQVSEEAEVPEMMPEPELQPEPEEEPIRPRIAVQEPEYDLEHAEFVSPPP
SQQEEEKILHDFPPSIASGALERALESQLQTRQDEEISLMLIHCVLAGEGDPSALALGVT
IREYFASDTLVFELAKGCHAAILPGVDTGSCLKLAVDLDDVLTTTASLYKDLSAEPPFYF

GISARYGRTVSPARLYKEAYAALQKARESESRILAFKPVNQE*
>SPBIB_v1_100005|ID:27162156| conserved protein of unknown function [Uncultured spirochete bib]
MSVGMKIRKRLLPAGWYPENADEIRSLVAEWTKLQKSLSAYAAVAPHAGWYFSGDLAARA
VWSLRECDTVVVLGGHLGYGDPVLYASEDSFDCTVRIARNDTVLLDAVKKELIDVGINEF
ALDAGMDNSVEIILPLASLRFPEAKIVWLRVPPDFKARELGSALARAASSCGRSLSVIAS
TDLTHYGPNYGFMPHGVGESACDWVRNENDRGLINAALSMDAERVLSHARERFSACSAGA
VAAAISYSLDSGAKRGILIGHKLSYDVHPDRSFVGYAGIAYVK*
>SPBIB_v1_100006|ID:27162157|murD| UDP-N-acetylmuramoylalanine--D-glutamate ligase [Uncultured spirochete bib]
MSITEDITEFRSKKVTIMGLGLHGGGISSARFFARAGAHVTVTDLRDQNTLAPAIEQLQG
LDIRFVLGAHREQDFSDADIVIKNPAVRRDSPYLKVAKRIETDISIFLRYSQSPLIAVTG
SKGKSTVASAIWHVLSQAGKTALLGGNITVSPLDFLDKTAPDVPVVLELSSWQLGDLRGM
GLLKPQIAVLTTILPDHLNYYGSMDAYVADKRVIYQAQDETCYTICNADQNWGRSFAQET
RATVLWYSEKPLEEAIGAGARGGWLALDILSQTKEKNAEAGAPHSGHVLDPKEHPELYAG
YGTFGTDYSHELLVPQHIVVPGLHQKKNLLAAAVALCAFGLSAKEIAPALGSFPGVPHRL
EYVAEIDGVRWYNDSTATIPDAAIAAINSFSCPVVLIAGGSDKSSDFSAFAEKARSLKDI
ILLAGSGTDRIIALLEKEKISYKGPYNSMRDAVDSAAQSAARSDVVLLSPGCASFGLFLH
EFERGDLFKKEVFEISARAHSARLSPAEQSEQSK*
>SPBIB_v1_100007|ID:27162158| Pyruvate/2-oxoglutarate dehydrogenase complex, dihydrolipoamide dehydrogenase component [Uncultured spirochete bib]
MNRIENHPILQSRKDRRQIRFYFDGDEIAGFEGEPVASALIASGHHVFSHHHKDGAPQGL
FCANGQCSQCTMLIDGIPKKSCMTPLSEGMDVRTLYGLPHLPAEDEPLVQAERRSVRTDI
FIIGAGPAGLAAATELGRMGYQVLVADDKAQAGGKLVLQTHKFFGSEADCYAGTRGIDIA
HILEDEAKRHPSVTILTNAPVVGIYKDRKAGIYLNYESYLLVEFTALVVAAGAREKALHF
PGWDLPGVIGAGAFQTLVNRDLVKAYRKILIIGSGNVGLIAAYHALQAGITVAGIIEVAG
RVNGYQVHADKIRRMGVPISLNSTILKAEGRDKVERAIVAAVDEKGQPVAGTARAYEVDA
VLIAAGLSPCNEFLEQARRYGIMAVAAGDAAEIAEASSAMYGGKTAAMDLASMLGKKIIV
NPEWETTRKVLASRPGDSYERSPVVPGSEWRPVFFCSEEIPCNPCATVCPVGAIQLRPMH
GNIMDLPYFEGNRCTGCGSCVAVCPGLAITLVRRIPGEKALADKASGDKASGDRAQVVLP
WEFNADFDIGTELDLVDQKGDFVERAPVVSKRRVSSRNTWLLTFHVDIAHASKIAGIRVQ
PQEASAFQSAEIPADGAADTVLCRCERVTLGEIVEFIRNNKVRDINQLKSLRVGMGACGG
KTCSQLVGKAFRLAGTDPAEVEPATQRPLFMEMPMGAAVNEGLHKLGGKEAGARGAP*
>SPBIB_v1_100008|ID:27162159| putative oxidoreductase [Uncultured spirochete bib]
MKHFDVVIVGAGSVGVPLAWQLAARGLKVAVIDQEASWGRGQNRAAIGGVRATHSDPAKI
RICLESIEIFSSLKSKYGLDVEWQPGGYLYVAYDEATEMQFRSLLKKQKTAGLEIDWISP
EKIARLAPGIRTEGLLGGTFSPYDGYASPLMAATAFHKLALDAGVLFFFNRRVESIRKAD
NTIISLTAGQEEFSAEFFVNAAGAEAADIAKIAGFDIPVFPDCHEAGVTEPVERFMQPMI
VDIRSDAESGNYYFYQARTGQVVFCITPRPQIWGRDKDSTSTFLPICVRRMIELYPRLRN
LRVRRTWRGMYPMTPDGLPIIGYPDMAINFLQAVGMCGQGFMMGPGLGHILAQTIMAGGS
HANPAGATEYNFIFDEFSINRSFDHAELLK*
>SPBIB_v1_100009|ID:27162160| Rrf2 family protein [Uncultured spirochete bib]
MFQVPTKTQYAIRALVHLVHAGSASVATIAEAEHIPAKYLEAILSQLKSAGLVISDRGRS
GGYRLARAASIIQMSEVVQATEGEIRPVECVDNATICVVSEGCLPRRFWLGLKKTVDEYL
ASVTLAEIAEPGLSDSLSAFRKRPAPARMNNRSN*
>SPBIB_v1_100010|ID:27162161|iscS| cysteine desulfurase (tRNA sulfurtransferase), PLP-dependent [Uncultured spirochete bib]
MNARYVYMDYNATTPLRPEVRDLMVRTLDVYGNASSMHELGRVARAEIEEARAHVAALIG
AQNPSRVYFTSGGSESNNTVFNTMFLIGRNQKRRKIITTSIEHPCVLNSAAHLRDEGFPV
VFLPVDRDGRVDMDAYCAELSPDVLLVSVMAANNEIGTIEDIKTIAKLAKEQGALVHTDA
TQAAGKIPVSVEDWGVDYLTLSCHKIYGPKGIGALYVRERAPIEPLIRGGHQEDGVRAGT
YNNLGIMGFGEAARLARAELAEYGQNVSRLRAMLRGLILERVPNVRINGHPTEVLPNTLN
VSFPGAEGESILLSLDLEGIEVSTGSACASGSLDPSHVLMAIGLGPELAHGSIRFSLGKY
TTEDDVRYVADKLPPIIARLRRMSTVPAEEIIGEIRTEGAHPEGAK*
>SPBIB_v1_100011|ID:27162162| IscU protein [Uncultured spirochete bib]

MDAFQWLYSDIVKDHFTNPRNVFDPDKDKDFKPDAEGMVGNIKCGDQMMFMLKIQNDIIA
DVRWKTYGCASAIASTSMLSETIRGMNIRDAYHIKPADIASRLGGLPENKIHCSVLGDKA
LRAAIDVYLEQHGRAGEFKGEDAQIICKCLNITDKDIEEAVRQGAHDWHSLQEATKIGTV
CGSCKTKAEELLHEFVHIYS*
>SPBIB_v1_100012|ID:27162163| Endonuclease III family protein [Uncultured spirochete bib]
MNLSISTAEDIAGARTFILEKGREHYRDLPWRKTSSPWAILVSEVMLQQTQVPRVSKIFP
LWMERFPVPSALAAVPLSEVLRAWSGLGYNRRALNLQKAARILALEYKDAVPPSEEALRS
LPGVGVYTARAVLAFAFNIASVFLETNIRTVFIRHFSAGLSRETDGTVSDRDLEKIGQRL
LDRQNPRAWYSALMDYGAWLKQNEGNFGKQAKKYRPQPPFNGSMRQIRGAILKSLLETSP
LPVDIVAERLLTDPERIYSCALQLAQEGFLEIQTTESAEGPESARWPESAAADSILLNLP
*
>SPBIB_v1_100013|ID:27162164| TRAP dicarboxylate transporter, DctP subunit [Uncultured spirochete bib]
MKRTVYTVLALMLLLVPSAVFGQITLKLAHLNPQQPYDVASAAMAAVFKSEVESKSNGQI
KVELYPNGVLGKEAETLVQVKSGVVQSFISSSGGMAQFYPLIDVTNMPFAFSSYNVGYKV
YDGDFGKELAADIEKKAGFHVLGFGESGGFFAITNSKKPIKSPADMKGIKLRTMALPLHQ
AIVKALGASPTTIAWAEVYTSLQTGVVDGQMNPISIIAMAKLQEVQKYITLTNHLYAPYV
WVINPKFYNSLSDELKNVVDDAARTAIVAGRGLSRIIDSTEKGLPTLAEKLQVYVPTKAE
MKLFRDATVPAAREFMLSQYKDEGKIWVDKFFDAIDKAEKELGY*
>SPBIB_v1_100014|ID:27162165| membrane protein of unknown function [Uncultured spirochete bib]
MENEKPVSRSVRRISALIDKIVSFLCAIVFGAMTIDVLLGVFFRYVLNSPLNFTEELARY
LMIWGASLAISLGVSAGEHVGLTVILDSMKKPNARKALAMLVNFFVFAFLVFMSVYSAGA
TIEAKSQMTQALGISMVLPKLAVPLAMILATIQIILVSIMILADPNGRLTTSTTGYIDI*
>SPBIB_v1_100015|ID:27162166| TRAP dicarboxylate transporter, DctM subunit [Uncultured spirochete bib]
MIVSVLIVFFAMLVIGVPIGFTLGIAGVVGLLLMGSGVGLLSMAPLQFFTGLDMFTLMAM
PFFILAGELMNKAGITRRLVHFANVLVGHWRGGLAHANILASVFFAGMTGAAVSDTAAIG
TMLIPAMVEDGYDVDFSAAVTAASSIIGPTIPPSNMMVIYGSLMNVSIAGLFAAGFMPGF
LLAALLMVMSAYLSFKRGYPKGPKTTFKEKLIATKDAIIPLLMPIIILGGILSGLFTPTE
AASVAVFYALIIGFFVLKSIKLRDIAPMLVKTARVTGTVFLIIGTASILSWVLAINQVPQ
AIAAFLISTTRDPKLVMLLILLLMLVVGMFMDIAAALIILGPILHPIAVSLGYQPIHFGI
IMVLSLNIALMTPPVGACLFVACSISKISLAKLSKAIFPFIIMEVIALFIVAYIPEISLF
LPRLLGFVK*
>SPBIB_v1_100016|ID:27162167| D-alanyl-D-alanine carboxypeptidase [Uncultured spirochete bib]
MKYFRSKRLDGVRKFRQFIAFLFFFELFAIANASALSLDEYSQFGVEIARPPSLNARSAI
LIDAATGTVLYEKNADIALPPASLTKLVTLHLAMEEIKAGRLSPDEMVEIDARDCSPYIP
YGSSLMYLRPGMKVSIRDLMLGAAVVSGNDAAYALARRIAGSNERFAEMMNKAVAEMGFD
RMTFVEPSGLSEKNLVTARQFALFSKRYIELHPEALKELHSVKSIKFPRPEHATTEYHPD
GIIIQYNRNPLIFSYDGADGLKTGYIIEVGYNMAATAQRNGTRFIAVLLGGSSTPYANGT
AVRTKDTKALLDWAFSNFSTARPGYIAPKPLRVWFGKTIQLLPEPNGEAAITVPSSLKDR
VWARIELPKTVRAPIQKGQKIGRIVYEIDKKEIGSVDLVAPTEIQRGGFFRILIDTVLRF
FAFILGKV*
>SPBIB_v1_100017|ID:27162168|asnS| asparaginyl tRNA synthetase [Uncultured spirochete bib]
MTLLPTIKQLLAMEPIGQEVTIRGWVRTKRETKQSTFIEVNDGSCLSNIQCVVESALLEN
EALKSMLARIATGASVEIAGKLVPSPAKGQKAEVSVIRVKLIGEAPADRYPLQKKAHSLE
FLRELAHLRVRTNTFGAVARVRNRMAFAIHQFFQEHGFYYVHTPIITASDAEGAGAMFQV
TTLDLDALAKSGGPVDYDKDFFGKRAYLTVSGQLNVETYCQALSRVYTFGPTFRAENSNT
TRHLAEFWMIEPEMAFAELSDNIALATEFIQYLVAVALKDCAEDIAFFDERIQPGLRESL
EKVAQTPFAHMTYTDAVRELESANTQFEFKPYWGCDLQSEHEKYLTEKVVGGPVVVTDYP
KEIKAFYMKQNEDGKTVAAMDVLVPRFGEIIGGSEREWRLDVLEKRIEELGLSKEVYSWY
LDLRRFGSTPHSGFGLGFERLLLYVTGMTNIRDVIPFPRAPRQAEF*
>SPBIB_v1_100018|ID:27162169| TM2 domain protein [Uncultured spirochete bib]
VRYSLSTAYLLWFISGFGALGFHRFYLGKTGTGLLWLLTGGLGGVGCIYDLFTLPNQVRE
ANIAYAAREALGYDAYGAGSGFIARVRPAETPERVILRIAKANNGMVTAGEVAIEANISI
EEAQKQLDSLAKKGIAQVRVRSSGVLVYFFPEFSKENADFID*
>SPBIB_v1_100019|ID:27162170| putative Peptidase C14 caspase catalytic subunit p20 [Uncultured spirochete bib]

MYFESGKALEQHRLKPVREKGALTMQRLRAKRHLSGIGSLVPLLVLSVILMLTGCELFPK
SASSSPGVKYAIIVGINDYIYLPYGRNNDLSYCVADANSMKTMLEEAGWTVKLITAESNE
STNRFATKSAIESALNNVPSDAESFMFYYSGHGSGGFDPGEAYIIPSDWDPRAFNFTDRM
ISTSEFASLLEKVPAKNKIVILDSCYSGGFVNPAESSDAVPGNVSSQQISSSIDMFLKFG
ELLALNAQYRSADASLAPLTISAAGWDEESWEQGSPIYHGLFTYYLLKAAEVNVEGRMPG
DADGDNVLSCLEAYNYAKKALEKTNYRYLPHISGGLRDFALIDNRGN*
>SPBIB_v1_100020|ID:27162171| exported protein of unknown function [Uncultured spirochete bib]
MQRFMGKTRKAGLVAAFIILLFYANAMNAEAQPTLEAQFLYANAWTWTSATAFSRHTGYA
FSLDAQWLFPLRLSSSAQEENRGISLMFAAGVHAQHVNMGASVWLADGSRYRAWNALGFG
PEVGVGIQFAGLSVGRKQIFALSAGPIANFANYTSTTLYNAYWSGYLKLSWEAEIDRHWA
LDVSLPVEIGSRSDGTSVIAALGAGVQYVF*
>SPBIB_v1_100021|ID:27162172| exported protein of unknown function [Uncultured spirochete bib]
VKKIAAILSLIFASSLLLAGMAACKPRIRLPEDGSITGSAKWLVISSLYCQMKAEPASAS
LDKGILRKGTVLKIEESRFSTAENEQGTLWFKVQNAGQSGWVSIRDAHTYPTETQARNAA
RRME*
>SPBIB_v1_100022|ID:27162173| protein of unknown function [Uncultured spirochete bib]
MDFIRSWIIPPLIGAIIGYFTNWLAIKMLFRPYKEIRIGGIKVPFTPGILPRERERLAES
LGDTVAQELLTPKVITDRIHSPQIQKTAAKAVSIAIKGFLDQDAERLFLMRHSEQISSED
AEAALEDSAAGSSLAHQIIESLRRLVASADIQTAIQHIFAEFITRLGRLKFQSLVSKQQF
VAGIMKAAGSIGSQSVDSDIVHVQSTDSENESGYSRLRPILAALLQLPPDATIRVATDSL
VPPAYALFLPELKQFLRSKEFRSRLEVEGRSFVKRALDRLGPVQRLFVSLAGYETRISQS
MPETIEDLIKTIELLLDDPEVPERLAEAACATIVSRRAKTGPASTAASMQRENIASAIGQ
SLKESSDELKLRAERVYDDIADLSLEELAGGRMNAEDISGFVLSAFVRAIDSEKEISSST
IGTLFIDVLKESAKGKTIAEFFGIQEQDIEQISYTLANALLQLIETRMPLLVEAMDIRGM
VAERIDSLDMKEVERIVLQVVSHELAWITWLGGILGAMIGFIQSLIALL*
>SPBIB_v1_100023|ID:27162174| FolC bifunctional protein [Uncultured spirochete bib]
VNQLHFKSIDEVYDFVLGYVNVEKGQATEFKLDRMNWMASQLGDPHLGRMTIHVAGSKGK
GSVATFIASALERSGIPTGLYTSPHIISWKERITRAGKELPDNIILRAADEVFELVEGKT
ADNFFGGELPTYFELTTLIAFCAFRMAGFQAQVIEVGLGGRLDSTNIVSPDVCVITPIEL
EHTQFLGSTIRLIAGEKAGIIKKKVPVCTIQPKAEALEVIEAKAAAMEAPCLVVGRDIHV
SRVEVDTEGTHCVLEADQNAPSAVHNFLGGRPCPVQTQLVGSIYAGNMALAGLALSQLPV
SIHSEHMQEGFAKASMLARFEIASRNPFIVLDGAHTPESVRTILSTFLELAPSPRILLFG
CAYDKRHDEMADLLAPSFEDIIITRPGSFKQSTPEIVFESFHKRKTDSVIIEDTALAIIE
AVRRTQEKGGSLLVTGSFYLCAEYKKLLSKS*
>SPBIB_v1_100024|ID:27162175| protein of unknown function [Uncultured spirochete bib]
VRIIHAAEIGPSAKPSGRRRNRGTAFSQKPPSQAEESVRVDQPLHLHDKQIIVIIESEGA
VFDIHRYWHETAYLPSFIGCFGGNVDPSLSGEVWRTVALKSSLRGEPPLVILLAALRVLN
TLSPSMQRAVVIKTLEKYSLSESSTEDLLRLDEKHPVEMMVIDWLSMAEGLIQETGRVPH
FSTAEEFLRSVSYIAPQSQVLVYSTLAESSAMRMWQSSGLGECFFRIAGKERGKPGEYLR
AAMSAGFERSSIVAIGASAAMFRAAQLAGIRFFPIVPEQEEESWKILAEQWFPAYLRGEA
WKIDMQAERFYEQMCHEFNVRETAEEIGRKFGLQQSKPKFV*
>SPBIB_v1_100025|ID:27162176| AAA ATPase central domain protein [Uncultured spirochete bib]
VAGQPNSMPSHPLFSPGAVAHSPESGEPLASRMRPRTLDEFVGQEHIVGEGRLLRRAIQK
DQLSSVIFAGPPGTGKTTLARIIANTTKSHFITLNAVLSGVADLREAIEQAKTYRDMYGR
KTILFIDEVHRWNKSQQDALLPWIENGTAILIGATTENPFFEVNRALLSRSRVFVLKQLT
RDDLFRVAQMTLGDKERGYGLYTVEFEEGALEHLVDTADGDARSLLNALELAVETSTPQW
PPPEGSKIFVSMSAAEESIQRRAVLYDKDGDYHFDTISAFIKSVRGSDPDASLYWLARMV
YAGEDPDFILRRLLILASEDIGLADPQAINVVSACALAFDRVGLPEGQFHLAQATLYCAL
APKSNSTLGYFDALQAVQSEQAEVPDHLKDANRDKAAFGHGEGYQYPHAYRDHWVAQQYL
PASLAGKLFYFPSQIGFEGTRRAEVLVRREAQLAVLPSDIENESSSQSLVWSKEGESRTR
WRIRSEFSATERLLAIRKAVFDFVLPAPVDNILITDPRLGFYAMEAIRRSSEGKVFAVLN
DESARMQLENLSKAITEILQPEILLYDECNNGSEGPKKISAFLEEAQPAFILVCEVERIP
LSVIETLAFQAKQAASKTARFFAFDIDASRSSMLSGALSAVANLGKEETKFLERLREFEL
ERQGGPTYAAKPHTQQNRSNWRDVLHPHFPEAILESITLNGAFTRPIDDEELAQWLDPGT

AYGKVFRSIFNQKDIKMMEGIVQRYNRSPSWPIFVSLYELRL*
>SPBIB_v1_100026|ID:27162177| protein of unknown function [Uncultured spirochete bib]
MNIFGYSIKLDKTLATGRAKQKNAALSRKGSDIAGLYAALALTLLVLFAGCDNSFSIFKS
ISQETKQVGVDLFKNVTVRAMANDAANYYALLGQVVWRPIDGSASWNVLSVNGSTDYFAA
GLASDGAKVYVAKANSNNVLVDVYKTIDSGATWTAMNAAASIGSSAAVDWLKCANGTLFV
AFHASGTYSLYYYDGAAFKSAGIANISRPLIDIVWDGSEYWAISDSAAYHGSAGAMTEDT
APALAQGLIGLATDSAGRVLISRSDGLVYSLQSGAWTQITIKASTKLGPLFLLTQPAATK
TIMVGKGIATYGYYEYDETGTGSLKENGSNYISTSSSVYLSTMLSKHVIGFWQPSNDANI
LYVMLAAGGTDSYALYKNIFDPTANAGAGAWSGWTAE*
>SPBIB_v1_100027|ID:27162178| exported protein of unknown function [Uncultured spirochete bib]
MKKKLVAAAIVLLVLCIGMVWAQDSADPFAANRKKGSQSIEISPGGFLPLFVLDSNFAMI
STPNMSPGAAFSVLYRYMLGKNLGVGGSIAGSFVTTVGGRTLFLAPVCVSSTWVFGTDPM
EFDISAEIGMNVMRLSGNGIISPIAKLGGGISRYISESWSIGGKLMWWFVPELHLGSYAN
LNSYASFLEFSIGAQYLF*
>SPBIB_v1_100028|ID:27162179| protein of unknown function [Uncultured spirochete bib]
MLLVRPHGNLIQQIRALKRLAMNWETDPLPMALPEGFICGWFDSQLSQARAISNETFSEE
GYPGAGSKASNEQFFQQALVQHAQEICAALPDVFRFESVVQRGGEIILEFSRDIDSGKLQ
EAMHQFAHDAGLVCRESPAGTYNGIWLGSGSLPELQSPLSFKKYELVLYLAELPESPLNG
YYFKTIARVHRKMPSHARSRKE*
>SPBIB_v1_100029|ID:27162180| conserved protein of unknown function [Uncultured spirochete bib]
MKRKTEELAAFLIDTISRWSGVDCICVDLRSQNDELDPHYALVFDVYYRRGVPSAEKREQ
LFKNPGAFESAPGGMKDRFFLDEIPIRVEYKHIPVVQDMVERPLHHIKLLKNTGTYPLYR
LLKFPLVFSRSDWIDRMRAQLNSFPEEAWKGLFDSFSAKMEHYLSDFGAAAVSENQFFRL
MSRAGFLRFAGASIFMFNHAFEPSHAEYEAQLKDLTNLPANLASLWDAISGERNDLTEFR
KFELARLLAREIFELQ*
>SPBIB_v1_100030|ID:27162181| exported protein of unknown function [Uncultured spirochete bib]
MSIPVEAHRTSSFALFAALFFVLAMIAGCKSGEVKSFAWLDEKGLRLSFRSTAPYAESAG
SFTKQGEKQRYILSRSIAIPDGFAFRLKLAAQSSSVDFQVSYGDEKGKNASVLCSMPQTG
QVAFILPFEQLKNLQWLEIKLQKFDAQKTSIAEKQPASQNGPSTLLQLQEIRLVPQMRGF
RKDADILEISPQFSFQAGKSANRYEIRQPFSSGSEAEKLNQNARLEVVLEGDAGLATLFW
GEQKIIYRHSAGASQLVIPFALFGYDPAKVSLEIPVSMQAKVFCADVYPKDNSLFSLDPG
LLLYHAPLTEQNYFLARWDLMPDVLLFLFKDYATQDRYLKRLAFFVEKIGFVGRLAPDSE
IVNLHGWNAHDYRPEDIARFFDAAASQNFPLAAEEIELRELLLKQGILIKSGSMYLPGKG
AIVSITQESPSYLQSKFLVHELSHALFFTDNRYRDLALSLYQNLNDEEKWFLIQYFRWMR
YNIDSAYLMANEMQAYLVQQPTRDLEKYFSVTLGERLAQEHPELKQNIESYMQNHLAALV
RAANQLNSYLHTAYGFEAGMLFRVR*
>SPBIB_v1_100031|ID:27162182| protein of unknown function [Uncultured spirochete bib]
MEKDKNKSPSANYSDDVPEGLFISPLDDSESFRKMEAEEAKILSPPRAIPANSNTSDEFA
PSAARNNVEQEDATHLPPPKSAEATPEQLIFRFAGELKSIKQDLLSIKQHFEALKKPQPA
AALQPATLQAPAAQQAAAVTQPAAAGQADPDTIALLNDVKKLLLYLDRLLESLPEEKIEE
FANSEYFSLYRNIFEHLGLS*
>SPBIB_v1_100032|ID:27162183| exported protein of unknown function [Uncultured spirochete bib]
MRLPRINIRKGKVLVTSLAYGFFSLLIAYCCVSAIAGKAGVLAYQDLLIQKQEIQKAIDY
LQAQNLQKSRAIEDLKNNSVVAAEGAAVLGYVRQGEMLIVLPDSWRASVKDGEGELRLPV
VMGDSTGLPDAIIRFMAAITGLFALLAIQLFHFNPEAKERKHISLEKQE*
>SPBIB_v1_100033|ID:27162184| Penicillin-binding protein, 1A family [Uncultured spirochete bib]
MRSHKVRAVYIFIAVILMFTAILGAGLGLALSGTANAIRTENFTEFESALPTKIYDINGR
LITEFFAEEQRIPVFIKDLPNYLVEAFITREDQAFYSHRGFSLRSILRAAIGQILGKNLG
GGSTITQQLAGDLYADRKVISLQRKLKELWWALQIERRFTKEEILEMYLNRTIMGPAVYG
VEAASRYFFGHPAKDCTPAEAAILAIQLSSPSRYNPFRNPMLARDRSKEILDQMVARHVI
SRAEADESFDAYWASFDYSRVAVSAFYNREDKAPWFSEYVRRQLEDLFYGSIDIYSDGLS
VYTTLDLDKQAAADLYMKRGIETANASFKATNSQRLTEAENTYVPIVELLGLAFDLPTLY
APQSRSETDSQKYFLNVINPVLDATSLLLNIASLKPYVGAASAKIKTELEKTTVEGALVT
IDNSTGYILALVGGSNYSQANQLIRATQAKLMPGSTFKPLYYSAAIDSKKITEGTYIVDE

PTTFYNEDGTPYSPQNFKGEWKGSVLAWQALANSMNVPSVKVLQTVGFDAAISRAAALLD
ITDPIEIRKTFPRYYPLALGVIGVTPLQMARAFSVLANGGKAVTPIAIRYIEDRYGNVIA
EPEKDALQALRQKSPQVVSPQNSAIMIDMLKRVVSAGTLAGATQSGSIFKQTSPDGKQYV
IPIIGKTGTTQNWADAWTVGSSPYYTTAIWLGFDRPGNSLGVSQTGATVAAPVWANYMRD
IHLGLPYKNFSKPESGLVSVTVCAKSGQLPTEYCTDGTVNLMYLEGTQPTEVCTLHGPSV
APEPVSPPGPSIARPEESPIFQNR*
>SPBIB_v1_100034|ID:27162185|gapA| glyceraldehyde-3-phosphate dehydrogenase A [Uncultured spirochete bib]
MKVAINGFGRIGRLVFQSIVDQNLLGKGKDKIDVVAVVDIVTDASYFAYQLKYDSVQGKM
KAEITSKKSDPSKAEDDILVVNGHEIACIMAEKELKNLPWGKLGVEYVIESTGLFTDEKS
WGHLEAGAKKVIITAPAKSKAEDKKIPILLMGVNQDKYNPAKDHVVSNASCTTNCLAPLV
YVLLKEGIGIETGLMTTIHSYTATQKVVDGVSKKDWRGGRAAAINIIPSTTGAAKAVGEV
LPEIKGKLTGMSFRVPTPTGSVVDLTFRSVKETSIQEIDALMKKASETYLKGILGVTDEE
LVSTDFIHDNRSSIYDSLATLQNNLPGEKRFFKVVSWYDNEWGYSNRVVDLLRYMASKDN
F*
>SPBIB_v1_100035|ID:27162186|pgk| phosphoglycerate kinase [Uncultured spirochete bib]
MIKTVRDISLAGKRIIMRVDFNVPMKDGKVQDDTRIVASLPTIKYILDQKPRSLVLMSHL
GDPDKDAAKAKEKAEKEGKPFDLEAYLSAKHRMKPVAEYLAKLLGKNVVFLPSCFGQKAA
IDALPNGAVAMLENTRFHKEETAKDPAAQEVLARELASYGDIYVNDAFGTAHRAHASTAT
IAKFMNFRVGGFLMEKEVANLEPMLHNPPKPMVAIIGGAKVSSKIAVLENLLKNASSLVI
GGGMAYTFLKAQGIAVGKSMVEDDFLDTARQLLDHAKAQGVEVVLPVDHIAADHFAPDAV
PVAVNAQAVPDALMGLDVGPKTLELYAKVLAGAKSVLWNGPVGVFEFDAFAKGTEKVAQL
VAEATARGALTVVGGGDSVAAVNKFGLASKMSHVSTGGGASLEYLEGKELSGIACLEQK*
>SPBIB_v1_100036|ID:27162187| protein of unknown function [Uncultured spirochete bib]
MREKGRPKGRPFSFDVRVFCMRATVRKGDATGANGTQAPGKDVAYRAKFCDFIL*
>SPBIB_v1_100037|ID:27162188|cysK| cysteine synthase A, O-acetylserine sulfhydrolase A subunit [Uncultured spirochete bib]
LRIYNSILDLIGNTPMVYLNKLAASLLARIAVKLEFQNPGHSVKDRAALSMILEAERTGL
LQPGMTIVEPTSGNTGISLAMIAAARGYRCIIVMPESMSLERRTIMKAYGAELLLTPKSK
GMAGAIEEARALLSKDPAHYFMPMQFENPANPQAHRLGTAQEILSDTEGQVDIFVAGVGT
GGTLTGVGEALKTQKPSVRIVAVEPSASPVLSGGKAGPHGIQGIGAGFVPSILNTKIYDE
IIAISDVDAYAMARRAAKEEGLLVGISSGAVIHAAVQLASRPENKGKLIVAIAASSGERY
LSTPLFADLQDK*
>SPBIB_v1_100038|ID:27162189|cysE| Serine acetyltransferase [Uncultured spirochete bib]
MHMEKREKNNTPKERASAGPLSLFKSDIEAILQRDPAARTKIEAVLVYPGLHAVWLHRLA
HHLWNHGSKLTARLISNISRRLTGIEIHPAARIGQGLFIDHGMGVVIGETAIVGNNVTLY
HGVTLGGTSLDKKKRHPTVGDRVTIGAGAKILGDIHIGADSRIGANAVVVKNVPPNSVVV
GIPGQVVHRHIPHTASDEPDLHHEKIPDVLGQRVHELSARVAALEEVIARMRAGCEDGTR
SDAAL*
>SPBIB_v1_100039|ID:27162190|tpiA| Triosephosphate isomerase [Uncultured spirochete bib]
MKRYFIAGNWKMHKTIAEAVALASELKDKLADCKEKLMIAPPFTALQAVSKIVEGSNILL
GAQNMGPEESGAHTGEISVLMLKDLGVRVVILGHSERRHTYGENDELINKKVRLALSHKL
EVILCVGETLEEREHGMLENVIRRQLSEGLKGVELSALKDVTIAYEPVWAIGTGKTATPE
DADAVHAFCRQVIAEMYGKDAAKAIVIQYGGSVKADNAAALMSKENIDGALVGGASLKAE
AFVPIAKFR*
>SPBIB_v1_100040|ID:27162191| Preprotein translocase, SecG subunit [Uncultured spirochete bib]
MGFFGVLLLVVFVIVCLLLIFLVIIQDEDSDSIGGIFASGSQSAFGSRSSNVVIRITYVL
GTLFFITAFALAVVNKSSTGNVEKVVEQNSAQTATSEWWNNQTQTDQTSQTQPATEGQTP
QTGQPAAPASK*
>SPBIB_v1_100041|ID:27162192| protein of unknown function [Uncultured spirochete bib]
MVRRSFAEMGHEAAIVSDSPYGLSAYDFLVFITEPKSMFGGIASSLPQTLAKSEGLIGKR
CLALVRKSGFRSGNTLRKFMEALEHEGLVVVQGEIVSDASSAAAVARGTPLNRR*
>SPBIB_v1_100042|ID:27162193| putative Chaperone protein DnaJ [Uncultured spirochete bib]
MKNYYEILGVHPDSSPQDIKSAFRKQAKRLHPDMHYSAENARPRESSATLRESAMRLILE
AYKILSDAEKRRAYDRELRRRERDNKGFDYHEFLQQRSDDPESQAKLIVYDLLHDLDEEA

LAIYERSKTFADFRLERWLERGEAMDAEYCIAEEYEKRGKYIKAYQIYKKLIRMELEKPW
FRYYFEVVALKFRFLILQKMPGRVDEEDYLDRLDEAIELKIAPRETAQYLRKKVEILLHR
GEAEAAEEALNQISLIYPKLAGFAALRSRVQRAVDQPLAQNKIS*
>SPBIB_v1_100043|ID:27162194| putative PpiC-type peptidyl-prolyl cis-trans isomerase [Uncultured spirochete bib]
MKRIASWLILFVFAAFPLIAQTSIDKPAATFKLTKQEVISVRQLRADVDRLENATGAKLT
VEQRKDVLDARINSMLFLQFCEREKISVSDAQVNSALAQLKAQLGANATDADLEKSLRAS
GVFVDPKVYVKQRLLFEAYVQTKRLDDMKANLKPPTADEILKAYDLAKASLVRPDTIRLS
VIYTDTRGKSDADIAKTKDLMNSISAALKANPSKFDEYVLRAGDAAGYKAIPSLYLEKTQ
QSKSIFGAEFFDAAFQAKVGEITSVIQTPYGFRIVRVNEFIPQKQLTLSDTVPGNQNLTV
QEFLAMQLASEKEQAFFNKVEAEVIDSLRKEATIKIYTENLSW*
>SPBIB_v1_100044|ID:27162195|nusB| N utilization substance protein B homolog [Uncultured spirochete bib]
VASRRKARILAVQALYAWDLAGHSLPELLSFAWLDEEKKLHYDAEILDFSRLMITGTIEE
IDRIDAIIKKHLEHWAFERLRKVDLAILRVGCYSLLYQKDIPAGISIDEAIEIAKEYGSD
DSYRFINGVLDGIQKEIVGGLHDSNKE*
>SPBIB_v1_100045|ID:27162196|map| Methionine aminopeptidase [Uncultured spirochete bib]
MIRIKNKKQIEGIRASCQMLSALYDALKPLVQPGATPKDLDRFAYDFIVKNGGKPAFLGY
EGYPATLCISVNDVVIHGIPGSRPLEEGDIVGIDSGIDLDGFFSDAAFTLPVGKITTEAQ
KLLDVTKECLDLAIAQIKPHSRISDISRAVFSHATKNGFKVVRQYCGHGVGLEIHEDPQI
PNYVSAGPNPRIMPGMVLAVEPMINVGTSDVRVLDDDWTVVTMDGSLSAHFEHTVLVTDS
GCEVLTRW*
>SPBIB_v1_100046|ID:27162197| Protease Do [Uncultured spirochete bib]
MSFGKKLYSKNFFIANLVILGIVIGFALAFMFRANPSASSTALPIVKAETPPMVAGSDTE
AAISQAEAVQEAFRNIAKTVLPAVVELDVVEGAQKNQPQQQTPQFPFDFFFGPDTPSPDQ
FPPQEGLGSGVIVRRSGKTVYVLTNHHVAGNATKITVKLSDGREYEGKLVGTDERKDVAL
VKFETDDSDIVIAKLGDSSKLQVGDWAIALGSPFGYVSSVTTGIISALGRRGGPDGNIND
FIQTDAAINKGNSGGALVNIRGEVIGINTWIASPTGGSVGLGFAIPINNIKSAIDDFIVH
GTVKYGWLGVSLSNITQDKASAKELGLDGKKGAFIGHVFMNGPADKAGLLPGDFVTAVDG
KSVASTDDLVRMIGDIKAGTAAKLDIIRRGSQMTLSAKIELRDATVASNNANIFPGVTVI
SLKSDSVDQKQVPAGLRGALVVDVLAKSPAATMGLRTGDVITKINGKNVSDLKQFYETLA
KEGRNKLVFTINRDGQTVETLAYVGK*
>SPBIB_v1_100047|ID:27162198| Phosphoribosyl transferase domain protein [Uncultured spirochete bib]
MHKEFVPYDMIRNNAIKLAYRIYKDGFIPDVIYVSLRGGAYMGNVISEYFKFVKGNSRPV
FYAAVVARSYSGFDQQENIKIDGWTYNPDFLRHGDKVLLVDDVFDSGRTINHLAKVIMDR
GLPRSDVKIAVHDYKVREYLPQHLPITPDYWCRKIEVKSKEDELWIHYMSHELVGLTEAE
VAQHYLNDDPDLAEAMRLVTSRN*
>SPBIB_v1_100048|ID:27162199|glgC| Glucose-1-phosphate adenylyltransferase [Uncultured spirochete bib]
MTEILSIVLGGGKGTRLFPLTKERSKPAVPFGGKYRIVDIPISNCINSGFKKIYILTQFN
SASLHLHISRSYNFDHFSKGFVEILAAEQTPAHSGWYEGTADAVRKNLIHFRPHKPSHYL
IVSGDQLYRMDLADMFRKHTESACRLTIAGTLVSREAASSLGIIKIRPDGCIDSFLEKPG
PTKDIRDFAVPEELKPKDADPNKPYLASMGIYIFDAPLIEDALQMDANDFGKEIIPAIVA
KEKVNTYVFGGYWEDIGTIRSFYEANIELTDINPQFNFYDEQMPIYTHARNLPASKLNYC
TLNQALASDGCIITNASITRSIVGIRTVIESGASLDGVVCMGADYYETEAEKAENRRKHI
PNIGIGGGSIIRHAIIDKNGRIGSNCRIGIDPLPREDGEYKTHYIVDGIIVIPKNQIIPD
GTVI*
>SPBIB_v1_100049|ID:27162200|yprA| Uncharacterized ATP-dependent helicase YprA [Uncultured spirochete bib]
MNSETANAICDELASWLSERSEIAWSTKLPARAPDFVPLPQDLDHVLSESLAARGIHALY
RHQKRSYELARAKRDFIVVTPTASGKTLCYNLPVMQTLLEEPEGRALYLFPTKALSQDQQ
SALGEIMLAGSIGMAINTYDGDTPTEIRAKARTSGRIIISNPDMLHSGILPNHAKWVKFF
SNLKYVVVDELHAYRGVFGSHVASVLRRLIRIAKFYGSEPVFIFSSATIANPQELAERYI
EREVALIEESGAGSGEKVLFCVNPPLVDPVQGIRRSSALEAESVMLWLLKKGVRTILFSR
SRLQVELLSSYLNKKLENAYNRNFGLVVKPYRSGLLPSERREIEKGLRDGRIHGVVSTNA
LELGIDIGGLDAAVITGYPGSIASFWQQAGRAGRGLGVSLAVYISSSAPLDQYFAAHPEY
FLSRAPEHAHIDAYNPYIFTDHLKCAAFELPFAESEPFSPDPSNQKAAELTKEALDYLEE
EGIVRHTAGRYFWSAEGYPGEKISLRSATTENVVIVDATNGRHDVIGEMDRPSAKELLFD

KAVYIHLGTQYQVKSLDLEKRVCLVERSDVDYWTDSIVKRDIEVLTIDSQAEHSECTMML
GDILVRSQVEKYKKLRFNTHENIGYGEIWLPPDEMQTRSLIITIAQESESGKLLGALVPE
KADAVLSGVARLMRQLAPASVLCDVHDIGVASRARDLFFKNPALYFYDMYPGGTGIAEAL
YANVQEIFAAALERLGNCSCKAGCPSCIGVESADKDSKAISLQFLKLLQGACAK*
>SPBIB_v1_100050|ID:27162201| protein of unknown function [Uncultured spirochete bib]
MNLKERLALIKNEKTKLKPSAPRKRFIPEGWQEIAPLVWAREVSRPFRQVPETLSPSIMR
PLVPAYDEAMHVEPGSVPAGYIAFFDLETTGLSGGAGTIAFLCTTATFEGSSLFLKQIYL
EDYPGEPEFLKKVIENLSAAKWIASYNGTAFDIPLLQVRCVLNRIAMPQLRHIDLLHDCR
RFWGRSVQSCALQSMEAFLLGKERESDIPGALVPKVWLDLVKSELPSEEQKALMVLVWKH
NLEDVVSLAELFLLAEMAYRDPVSGVARYAIDPAAIASRLLKTGRTEEARKLLLMVRDQN
SALDCQGEKRMRALRVLASLARREKNHRLFAETILAMDNSAYGCVAKAKLFEHVYKDPQA
ALEWAKSAREILASQKELSKELWEGQEPRQLPYLTIESLQHRIARLERKAAKQKGL*
>SPBIB_v1_100051|ID:27162202|rlmN| putative dual-specificity RNA methyltransferase RlmN [Uncultured spirochete bib]
MSGPALSNHMKVYPSGLSLAETAALLPDQPPYRARQVYEWFSKGIASFDAMTNLPQSLRE
RLSGLYGGQVRSSSIEKELIDEDGSLKLQIRLRDGAAVECVLLEDIEGRKTACLSSQVGC
PMGCAFCKTGTLGFLRNLGPDEIIEQFHFLRDRRGFISNVVFMGMGEPLLNLENVRKAIV
VLSSPEGIGMSLRKITISTSGIIPGILDIAQSGPKIRLAISLTSAEPNLRTRLMPVNKTW
PLDELKSALLQFQQATRDRITLEVVLIGNMNASETDAEALAAWITPLKAQVNLIAWNPIQ
GMPFEQPSPRQMRIFSEILERKGIVTVQRMSRGRGVMGACGQLGDTLQTTNPPSA*
>SPBIB_v1_100052|ID:27162203| Tetratricopeptide TPR_2 repeat-containing protein [Uncultured spirochete bib]
MSDMPSLPYNDDFFEVPEEIQSENTEIAETSKKGYQLLKENRFDEAVECFIQILEKDKEN
NYALVGMGDSARKQGRFKEAADYYRRCLVFHPGNSYALFGLADCYKALNQFQKAIEIWEQ
YLLHDNTNITVLTRVADAYRKVHDFRKSKAIYHRVLELNPDNHYALIGLGHLHYDFKEYK
EALSYWQRMVELEGDNVDIRVLTAIGNCYRKLKHFDKGIPYFEKALEKEPENFYALFGIA
DCYRGVGKQNMSLVYWNKILEKDPKNKVILTRAGDAYRNMGDYDKAVEYYQNALNIEFDI
YAVLGLAVIARQQGKYEEAIASLRSLLQNDPKNYRIYIELAQTYLMNNQRQQAIDVLTEF
QHMGIKNPIIQEALAKLTGKA*
>SPBIB_v1_100053|ID:27162204| putative Methyltransferase [Uncultured spirochete bib]
MRITGGLYSGRIVKMPEGDLKIRPAMDRMRESVFAVLGDLTGFSFCDLFSGSGIIGLEAA
SRGAKPVVCIEKDRKKFPVLLQNATIAEPPLFCRAMPAETFIMRNKTAFDIVFLDPPFDY
KFKLQLLERLDCSASLHEGSLVLIHFPHEDRLPEQVGALKSIDTRIFGRSTVRFYRKGPA
*
>SPBIB_v1_100054|ID:27162205|smpB| SsrA-binding protein [Uncultured spirochete bib]
MRSQVPIKIIAQNRRARFDYEIEENIECGIALQGSEVKSIKEGRVSFGDSFAEIKGEEVW
LNNLHIAEYAQASMFSHDPDRPKKLLLHRQEIKRIDRRVREKGYTLVPLSIYLKHGLVKL
ELGLCRGKKEFDKRADIKARDIDREIRRDFRLKDW*
>SPBIB_v1_100055|ID:27162206| putative PEGA domain protein [Uncultured spirochete bib]
MKDADKITEQEIAQAKVTLKPVLGVRPRVYVPVLYSIAILVVLFFLLVNPGLSHPGARLQ
FSGHPDAAAVYIDGAYAGNTQDGAHAKPGPHQIEIRKQGFSSKVLQTTVPNRIFATLIFP
PAVHLGYALEPQSQEAIMLPAFKDFASWALSGKPSAIYQLPMTLSEASRDLASIQNIDRA
ALSDALLAAGISLSMNAASLRDTVYASTAIAAPAGSPLGVLCTARNLAGMLASSKNSAAM
VMETIPDKASDAIRNAAAALKQETAALRINPLQANGLRSVGPHSFIMFKGGLLLDLVSSTP
EGSKTLFQSTLPEFGLASTEVTQREFARFLAENPDWKPENKAVLIEKGLADSSYLKDFDL
AAADNRPITGVSWYAANAYCQWLNRQAPAGYEVVLPTEAMWEAAAAVSSRSISSLGIFKN
HASTGPLPVGSAGTDSLSFSDLFGNVWEWTSDGFRPYAWIQKDSSAFDELIGAISQKTVK
GGSWANSPDQISIDSRGPVPAAHGSEFLGFRPALRKK*
>SPBIB_v1_100056|ID:27162207| Signal peptidase I [Uncultured spirochete bib]
MKKTDFFDRLVAFSESFLTRRKQKSLRAKMKQQKRHPVVDWIYAILWAACVVLVINQYLF
QAYRIPSGSMEKTLLVGDMIFVDKLSYGPELLPGVLKTPGVSKPERGKVIIFENPSYLSR
GPLYTIFQQMLYMVTFTLVDIDREPTGEPRVHYLIKRAIGVGGDTIHVVNGEVYIKPRGS
SEFMSEKSLMQGLGLAVKTQRLVSQSEYAEITNVGIASAYSELNLAFPASLGTPSVQSAN
KDAFHYDMSRVTTLRDANPSEARNAQIAQRYENGWYIADSRVFPMGDNRDNSRDARYFGP
VAEKKVLGHALFIYFPFSRMGSIR*

>SPBIB_v1_100057|ID:27162208| protein of unknown function [Uncultured spirochete bib]
MFARRPVRYAEKVERKKNRLRAIWFVVVFIILSSFIRGVVIQSWQIRDQTMNPSLVVGDV
VLAVPFWSIFSSAPFSFSGPLHQGDLVLIDDGASEVTPPALLTADAVLRFFTLQRFSILE
AQYGRNFGVPEVMRIKSIIRENRENKRVILYTLEYDARRMSADAPADKEVQASRIRAKLV
FRIWPIRRVGPLT*
>SPBIB_v1_100058|ID:27162209| putative Oxygen-independent coproporphyrinogen-III oxidase-like protein YggW [Uncultured spirochete bib]
MSAEHFTDKKPFPTRSLYVHIPFCAGRCAYCDFFSVPRGSCSPSTLASYVSAVLEQAAEW
TKIFEAGEFATIYIGGGTPTVLGTELLGTLVRGLEPYAANECEWTIEANPESLDEEAVQM
LAETKVSRISLGVQTLSQNQWPILGRVGSVEDSLRAIELLGSSRFEVSVDLLAGIPYRAS
DPAGAPEYIEAQLLQSLSALSGKVPHISMYDLTIEEGTVLQRRIVDGELVLPDTDSLARA
RESADGLLAAHGYIRYEVSNYALPGHECRHNLAYWNMDPYLGLGSGAVSTLAYPADAHQR
MLGSMVRLNGTKNLKHFIARPAEIPPEVEIIDKPTAAFEVLMMGFRTARGVDATRFSALF
GIPILELIPSSLERWGGEIIQKDNHIAIHPRNFDILNRFLVECFEEMDENRAMLKT*
>SPBIB_v1_100059|ID:27162210| conserved protein of unknown function [Uncultured spirochete bib]
MTLFQYAVVASILHDEKQKQELLSKFEQALIEAGGELQSVGTHTLCPRNTNLPLFIFVLT
GGTEAEAMQLIQKEKMIEQGTPVVLLAHPFQNSLPAAMEILAKVKQDSGKGLILQADASG
VFDAAALGNIIAVSQSMRTLQASRIGVVGKPSDWLIASSQHPDIAKKAWGLTLIDIPFAE
LMHDIEAIRAKPERAPDMPGLRAKAEFFSEADEADMRKSAEILQALKGIVARYRLTALTL
RCFDLVLQDSSTGCLALSALADEGIDAGCEGDIPSIIALHWMRTLTGMTGWMANPSRISI
DKEKGTAQALIAHCTAPRSILSSYGLRSHFESGLGVAVAGITPEGPVTLVRIGGKNLERV
WFSNASVIGSPRQEGLCRTQALLELPEARAEELLTNPLGNHLVMIRGDWIKRIEAFIRLW
QS*
>SPBIB_v1_100060|ID:27162211| Metallo-beta-lactamase domain protein [Uncultured spirochete bib]
MKIYQHYSIFGFSNSYMIGNEKLGKAIAVDPAELTPTMIEKIEQNHFDLCAIFITHNHTH
HIRGLSTIMKIYSPIIYASNAKILEFQCKKVRDGESFEEAGFSVRAIAVPGHSQDSIVYL
VDESFLFTGDVLHAGLIGKTSSTFNTNALAARIQQKLFSLPQETVIFPGHGPPSTIFSET
RANISYQEGFAEHIKSNYDFFV*
>SPBIB_v1_100061|ID:27162212| putative Pentapeptide repeat protein [Uncultured spirochete bib]
MFKVVPCSVEGCHRPSFSLETLCFDHLKNDAERMRKSLEELRSQTLIMNRVFDDATIMGM
DFSRYRFVGCSFRNVKMHHAMFTGASFHLSFLDGSEFISCDFSGTDIDFCSFGSSNFLDC
SFENSELIQTNFNGSHFSDCTFSGSNLYNSRFMLCDLDTVNFDNCDFKRAFYIPAKESNV
TFVGSNIAEAVRDLEHLYL*
>SPBIB_v1_100062|ID:27162213| putative Glutamate racemase [Uncultured spirochete bib]
MQSNNDALHVCVFDSGIGGLPFLKAIHQEWRGVTISYVADDAGFPYGTKSPEAIRDILFE
RVRRIRARLDPDILVISCVTAVQIGLKDLQAAHRPMKIIGAIPPIAQAARETQSHRIAVL
TTARAAEDAFLDDMIARDAPEIDVFRIPAQDLVDYVEQHLPFAEQESAEAAVEPYIAYAL
EKGADRLVLASSHFVFLEEAIRHVITARGMDDVHCLDSREHVLSALRRFLFSEPAAEAAN
EGGFYLTGDRKPCPSYIAWANRFGTAVPELL*
>SPBIB_v1_100063|ID:27162214|rsgA| putative ribosome biogenesis GTPase RsgA [Uncultured spirochete bib]
VGTSMLGIVLSFSNHYARVRCEDNTVRLCTIKGKRIRSLDGWYNALAAGDRVSVQIISDG
EGVIEKLLPRRNVFGRFNEKGGADQAFAANIDMVVCVTSANSPPFRPRFIDRVSVITEQC
GVPLLILFNKIDLGSDEHAEARLSVFESLGYKVLRCSALLQQGLNALRESILGKTAVFVG
QSGTGKSSIINVLMPGIDRKVAAISEKYNRGKHTTTLAELFFSPEGDIAVIDTPGFRRLA
MRNIEPDDLSAYFPDMMPFIGKCAFGASCSHTHESGCAIMDAVQNGIIHYDRYESYLRIR
SELERSRQWKKEETGKFRKTPKFRLEDEEGW*
>SPBIB_v1_100064|ID:27162215|mutS| Endonuclease MutS2 [Uncultured spirochete bib]
MVMHEHSLARLDFFRLRERLEGYCLSYEGRALMRNTLPCSNPESVRRLKRDLYLLSEGLR
EREIPALPFPDLDVVVQKAKKEGLALDVDELFALGIWATSYEQIVGLVRSILEASDRAKR
AHQPEENVPMTAFDSSADTEEWAMLGIETAVFSAPSLALLRERIFAILTPEGEIRDLPRI
RALKRAIHTAQLENQRLVSQYLSDPDLQDALQSNAATERDNRTVLAIKANFKGRVKGIVH
EFSSTGQTVFIEPLELIERNNRLLELETRLNEEIRAILRETTEAIRPLVPSFERARQHLA
AIDARLARAIQLRREDLVNATLLDEGLHLYQARHPLLGKKAVPIDVDVPDGTRVLIITGP
NTGGKTVSLKTIGLLALLNQYGAGIPALSTSGFAVFDTVFADIGDEQSIDQSLSTFSGHM

RVISDIVRAATGRSLVLLDELGAGTDPEEGCAIAMALLDFFLERKSLTIATTHHGILKNY
GYTKPGCLNASMEFDAQSLSPTYRMCMGIPGESRALEIARQTGLSDEIVATATSYLDDER
TDYSALMRSLGEKQRELDRLEQEKRKQLKGAQESRRNADLQNLKVRQRELELRRQGVAEL
NRLLSESRRTFENLVREIRESGRKVEEASSAREFLNELSEEAKRENDELSRFEAETKTLA
RSLSDGNAPSRRIDPASASVGEKVLYAGREAIIVRKLDEKRVLIQAGSLRIPVAMEDLSI
PPLHKKPDSETKLVYQVELSSPGEGSSKPLPEIDVRGMRLAQALETLAHQIDAASLGGLG
VFSVIHGTGEGILGKGIHEWLKTQPSVADYYFARPEDGGFGKTWVHLKP*
>SPBIB_v1_100065|ID:27162216|yebC| conserved hypothetical protein [Uncultured spirochete bib]
MGTSETIKDLQLTPIYAAWYRYAAFLSAKGVFMSGHSKWATIKHKKGALDAKRGQLFTKL
IKEISIAARMGGGDPEGNPRLRTAVLKARAANMPKDNIDRAIKKGTGELEGTIYEELFYE
AYAPGGGAMLIEVLTDNKNRAAAEIRNIVTRSGANLGTAGSVAYLFKRKGVLTYDGEKYT
EDQILEAALDAGADDVVNEDGSIVVFTDPASFEDVLNAMNAKGFETLGAEISMVPDTYIS
VDAETAAKIQRLIDKLEENEDVQNVYHNIEMPEIEE*
>SPBIB_v1_100066|ID:27162217|ruvC| Crossover junction endodeoxyribonuclease RuvC [Uncultured spirochete bib]
MAARRAIVAIGIDPGIAAVGFGVVRTREGSLELIAHGCIQTESSVPMGKRLAHIFNEIKR
LLQQYAPDVGGIEELYFFRNVSSAFPVAEARGVIRLAFEEANIELMQHTPNAIKKSVTGS
ARADKLQVQEMVRILLGMKEIPKPDHAADALAAAICTLHSIGPAGYTL*
>SPBIB_v1_100067|ID:27162218|ruvA| Holliday junction ATP-dependent DNA helicase RuvA [Uncultured spirochete bib]
MFNRIRGILNGHADDALHVATGAIEWEIFVPVRDPAFFSRVGEEIELYTWLHHYEDGMRL
YGFISAPERQLFLDLQKVEGIGPRQAFKILQGIAPRALATMLDTSDLVSLQKIPGIGPKT
AQKMMLALKGKLIALEGEVGVPAPAVQGRFRDVIRALIEMGFDRREVERVVAQLGADVQQ
GPESEKELFRKALLELSTGAGA*
>SPBIB_v1_100068|ID:27162219|ruvB| ATP-dependent DNA helicase, component of RuvABC resolvasome [Uncultured spirochete bib]
MNSEIPDDESIIHSPMNPGYDPSEDEKEATLRPRYLSEFQGQERIKDNLLIFIRAARERA
EALDHLFLMGPPGLGKTTLALVVASEMGVDFKPTSAPAIEKPKDLAGILTNLRPNAVLFI
DEIHRLKPAIEEMLYIAMEDFELDWVIGQGPAARTVRIPLPPFTLVGATTKAGMVSAPLS
SRFGISLRFDYYSAEELERVVRRSSRILDIKIDEDAVKLLAGTSRGTPRIANRLLRRMRD
FAQVKGAGVITKDIVIDGLARLEVDFLGLERLDREILKAIILRFGGGPVGAETLAVSVSE
SVESLEDYYEPYLIQIGMLARTPRGRIVTPAAYRHLGLEQMNENG*
>SPBIB_v1_100069|ID:27162220|queA| S-adenosylmethionine:tRNA ribosyltransferase-isomerase [Uncultured spirochete bib]
MKTDDFNFELPERLIAQYPPEHRGESRLMVLDRATEKTFDAMVADIASFIEPGTLMIFND
SRVRKARLYGICEQTGSHVEFVFLSPADEAEFDEQQFRGSDHAAQALNMPGSDKPVVPKH
KLPSSSQRWLVVCSKAKRQKIGRKYLFPGELAGTIISDRVDEKIIEFSAPIGDEYFDRYG
HVPLPPYIKREDTKADEQRYQTVYAKRTGSAASPTAGLHFTPEILSSLRDRGIEIAHVTL
HVGLGTFLPVRTENIEDHRMHEEWFEISPEIANRVNAAKKEGRAILAVGTTSLRALESAW
YDGALHSGSYSTRIFIYPGFEFHVVDQLFTNFHTPKSTLLMLVSAFAGRERILEAYRSAI
EKQYRFFSYGDAMLIR*
>SPBIB_v1_100070|ID:27162221|tmk| Thymidylate kinase [Uncultured spirochete bib]
MHSINTDELKKRFIVIEGIDGAGTTTQMNLLAGSLRARGLSCVTTAEPTTSPIGMLIRSV
LSGKNEAAPSTVAHLFAADRNEHLYGKEGIAQKVEEGALVISDRYVLSSLAYQGVTCGPE
IPWQLNSRFPAPGLTLLFEVDPEVCIKRIDTRSNKEIYETLEFQKRVHDMYAVMADRLEQ
QGWHIERINADGDIETVRQRIEAVVFAFLGVPSCGITGSASRLHS*
>SPBIB_v1_100071|ID:27162222| exported protein of unknown function [Uncultured spirochete bib]
MRTKRPAIAIALISLLFAIYSTIVIVNLLRLEVQQAVGRSLDSVQSVVDQATGLSLDYDY
LSIDAVNRFTMHGIRLVRKDAPDVSPVQVRSVSLRISYWALITGRSTEVLKEVSASDLQV
DLVLPEDEFIVQKLYKILFEGPLIVLPRFQLNIEPVHVHLKQNEPLADGTIHIESFQLST
LGGFPEIVAPSISADLGGFFGLPKDLSSKIGIDGSLAADFSTFDFSVNISAGAPSVLLLP
QQFVVSKRGSRIEARRRGKGLELVGWYEKGRWGLQGDMLGYRVSGDMEGKGEVWEVVKGV
RGDGHIEAWGEGKVLGGYRVDVKGAVDEGVRWGKYALGGLAVVLKGEGDSAGYRGVVEGV
HAGYGVRYEGEVGYASWAVKGSYTVSGAGVRVRGTVEGSGDRYGVEVAEGNVRGLVLGEG
AGQVKLTGEGYEVGYRGGVGAGRVSGEGSIGKDGGFEGVVEVEKVDVASVVAATGLKLQG

VEGVVSGRVYGLVRGGKVSWALNGGEYEGRVQGVGVKVGLEGVGDEAGYTLKRAQGVVGG
IAVGLEGKGMYRGEVFRGQVTVGGIGYGVQVGVKEGEVGITVGDTAEGKVVFGGSRIEGQ
VRLKGFGVGVGGGVVWLKGGARGWYEKDAWGLELTNVGVRYEGEGVYPQLEVSGTVDAKG
AKLTIDYFEYNARKLKGELTITYASIESIIRDFDASYTLSSIDYERAKIEGTIKSKDGII
DAAITANALPIEQFIPSSLKIAGDLQLKGKASASLNGDKFSLANFSLLEVQFKMQKGEIN
GLPFSASGTADLKDSVLSIKDGSFAYLNHKIDAISANYDIKSGSAHFGAQVRSVIAEKLF
TAYFNGDGKIAQDNPWNDLQFSGQIAKAIYNSRNIDDIGYVAAFKNGNIDLNLNQANGSN
AHASITSMSEFEVSLNNFFKLTGTAKGSVKENAVEADVSLNKFDLAALQMFIPENYIKDM
SGSASCSLKISGDLNDPAIDGNIALKAVSFSSNIYLLEKVGPFDADMKVSEGIIELGPAI
IKVGTESISLAATASLSRWEIGDIKAFISTQETAALRFKGTIAGLTAKDIRAKTDLTATL
SQSGLEIKGNIFLDNGTLEVNPAGFVVSPETAKATVPLSLNIALTLGKNVELYLPGQDFP
LVSGMSSPNSALNILYDQASGAFSVTGKVELRSGYVLYLLRNFFIKQCTIEFSENQTKFN
PLINAVAELRESSKDGMIIVTLSADQTPFENFKPKLSSIPPKSEIELLALLGGGLALSET
SDNNPLTIREAMIASSEFLTQNSLFRSFEQRVQKALGLDVLYVRSSFIQKWLLDITGQST
KGLANYLSGTELFVGKYITDSAFAHFILRMAQNPLEETGVLQLDSELGLEFQSPFGLLEW
GLSFGKEGTPLNNQKISLSWRINY*
>SPBIB_v1_100072|ID:27162223| Outer membrane protein assembly complex, YaeT protein [Uncultured spirochete bib]
MRLHRALFAALILFFAFQTTMAQDTPDEWFWNKPIAGFQWEGLHYANRNDLDSMLRSYID
VAFTDTVWTDIQAKLYALDWFDSIEPVALPAGDSKDKVIVKFIVTEKPSISSIRIIGNSA
LRTSELLDAASSKNGAIFNADKAKSDTVAMQKLYLEKGYPDAKVEFETAPSPQDASRIIL
TFKVEEGTAVIVRKILFSGNSVVSAQTLKSQVTLKEAGLFQKGAFQESKLAESRSAIEDY
YSSKGYVDAKVVDILRTYTKEEKTSKNYLDITFVISEGKQWVFSGVSFDGNTIFSTERLS
SFISLKPGDPLNLKRLLSDKQKIDDLYYESGYIFNSISMSQTRDEQAGSISFKISIIERD
RAHIGSISFKGNTKTKDYVIAREIPLEVGEVFSKTKIIDGLRNLYNLQYFSSIDPEIHQG
SVENLMDLVINVEEMSTAQIQFGLTLTGLGQSNTFPISGFVKWNDSNLGGTGKNLQVNMT
LSPDEQSLDTSFGQNWLLNKRISQSLSFTIKHAMSQTAQDSIAPIFSSEDIPDPYTALGS
GANEWNGLLSSVPDNYLMKYDDYTFSLGYSLGYVYKIPRGDIGLSGGVSSGLGMSSYDAS
KYRPYEKEMRDYNGQWILKNSIFGRVYLNRLDYWYNPGKGYFLSQRFTYTGFLSNERQFY
IRSDTKAELYATLFSIPFSPSWVFSPVVGLHSGVQTLLAQPWTPLSVTKDWVYLDGTFSV
RGWKSLYGSKGTLVWENWLELHLPIVPQVVSLDGFLDAGAMQTQNGWVNMALDSPSAEGE
WNLQWKHFAFSTGLGVRFVIAQFPFRFYFAKRFVFDGSAVTWKTAAGSFDFVLSITQPLY
*
>SPBIB_v1_100073|ID:27162224|mutS| DNA mismatch repair protein MutS [Uncultured spirochete bib]
MPESSTMLEQYRRIKSQHRGEILFFRLGDFYEMFNEDAQEASSILDLTLTKRQGQPMCGI
PYHAAKTYIARLLKAGKKVAICEQKTIQGRRGLMDRDVVEVITPGTVVEDDFLEQSANNY
LADICLLHGRLCFAYLDVSTGEFRAHSLPFDDRSKEVLRAELYRLSPRELLVQQSLLDQA
NFLQVIRESAAMIEPQPDWTYDVFHSADMLKRRFGVASLKGFGFEDNDPALAAAGNLLEY
VQEMVHQSCPHIRAILRFEQKDYVLLDEATKRNLELVKNLSDSSRRDTLIEILDKTKTAG
GARMLRQWLFQPLRNKARIEARHEAVSFLYHNQILLGDARKILASVLDVERLISRLAMDK
AHAKDLRALRDSVEAGLALFQLIEAEHPPMALIPRVSAEDKAECNQVIATIDSAIQDDPS
VLLTEGGIIRDGYNSELDELRNLHKNAQAVLESYLEEEKQHTGIQNLRIRYNRVIGYYLE
VTKGKLSNVPSHFIRRQSLVGGERYTTDRLAELESKINGAQERIIELEKRLFLEVRENIK
GNVAALLECAHAISEIDCLASLAQAATENGYTRPVMCEEPVIDIVNGRHPVVEMCLPYGD
FVPNSISLSRDNQWFALITGPNMAGKSTILRQTALIVLLAHMGSFVPAEAARIGLVDKIF
CRVGAQDNLARGESTFLVEMHEAAFILNTATANSLVIMDEVGRGTGTLDGVSIAWAVSEY
LVERVGCRTLFATHYHELTTMGLSGLRNMRMAVIEHDGTVSFPKRLEDGASSGSYGIHVA
RLAGLPDGVISRASEIEQHFHQLERGLGQEQGQVEENDAVPGRIPAYSFSPKPSKEAYIP
DAESKKEKRAQMRRTGELFSPEDLVLSELRSINPNEITPLGALQILSELVEKLKQ*
>SPBIB_v1_100074|ID:27162225| conserved protein of unknown function [Uncultured spirochete bib]
MGMRFFHCVADCVDAIFPVRCVLCGSLVSKSPWWPAPLCTSCEKTLECIGGLRCERCGRP
LISEQGLCAQCKNEQYILMHILPVFAYKGNAARLVQCYKIEERASLAKYFSLRLAEMVPL
FAETSSDIAIVPVPPPRPEKIWAGKFDQVGILANYLSRYGYRIWKGLVRRKGGAEQKTLAR
ADRLKNAYQSYMLRAGSMDIPEKALLLDDVCTTGSTLDACAEILAGKGTTILGAIVLAAD

*
>SPBIB_v1_100075|ID:27162226| putative Ribosome maturation factor RimP [Uncultured spirochete bib]
MVEMNGKELEKEIDDLLRAAGLDLLEFSVTRHRGKTHVRAVIYSREGTGTDECAKAHRLI
LPRVQMRLGDQEPYIEVFSPGIDRIIRTEREWQAFTGSYIMILTSDVPEWRKGRLVNFGK
GVVQIEMNETVVDIPIASIQKAKLDSSHEGEKTHGI*
>SPBIB_v1_100076|ID:27162227|nusA| Transcription termination/antitermination protein NusA [Uncultured spirochete bib]
MASEMAEAIRQLISEKGIPEELIIKTLEDSILAAYKKKFGTSENAIIRFKENYEGIEIYA
KKTIVSDDDFEDEVLEIPLEEARKLAEEAEIGDILEIPVDPSEFDFQSVMLAKQTARQSL
RDISRDTLYAEFKSKVGEIIIGYYQRERNGTIFVDLGKVEGIFPRKYQSPRESYHVGDRI
KALIVEVEKTKTGFQVVLSRTHAEFVRKLLELEIPEIYDKTIEIFKIVREPGYRTKVAVY
TKRTDIDPVGACVGPKGMRSQLISQELEGEKIDFIRYDINPKEFIRNALAPAQIKEVYIV
DDARHMALAVVEEHELSIAIGKSGQNVKLANRLCDWNIDVMTEEQYLQDSRNVELKKAAD
SLFSAEEEQGEELLLKDLPGMKPEWILALESAGIQSIEQFLEAEESEMPQRTSIAAEDLA
RIREIIEENVEIVQEEAEEEETEAYSCPECGAPITMDMDHCPSCGVALSFEVDEGRDE*
>SPBIB_v1_100077|ID:27162228|infB| translation initiation factor IF-2 [Uncultured spirochete bib]
MTDSTDKDPKKVHLIKQAKQASQEQPIEPVAAPQPDQGTEKKKVVVVVKKKVVTAKKVQA
KVISPAGSAQGGAQEETNKEPAASASAQVEAHAKLEIARPTPESSAHIASHKGQEPGVSA
GAVASLATQESSGKSEESSAEKKAAETKPDAPRSPAHTSPSQQSHRPDSSYNPQRRYSSD
GRAGNLAQGRPAPTSRYNQDRNESRPPAGTYPTRTSYGQQPSRSYPSTQTRPGQGSPSAY
NRGPRPSGGSQYPGSSQYPGAPGGANRGYPSARSGQKPGSRPGGYAGSRPSGAGGYGGSR
PGGSGGYGARPAPAPAGENRPSTRRQPAKKKGTFGKREEEIELEKQIQLKKKAEAKLAAV
PKSVDIMENISISDLAKKMNLKPADLISKLMSLGVMATINQKIDADTAAILAAEYGCEVR
VVSLYDETVIEKAADKPEELKPRPPIVTVMGHVDHGKTKLLDAIRKTDVVSQEFGGITQH
IGAYMVDTPRGKISFLDTPGHAAFTKMRARGANLTDIVILVVSAVEGVMPQTKEAIDHAK
AADVPIIVAINKIDLPEANPDRVKTQLSELGLIPEEWGGTTQFVEVSALQKKGIPELFDA
ILLQAEMLDLKANFDRLAEGKVIESRIDQGRGIVSSIMIQNGTLRIGDPFVAGIYPGKVR
ALFDDKGQRVEEATPAMPVEVLGFEGMPEAGDPFEAVEDEKFARQISAKRQELKKYEEGR
NVKKVTLDNLYETISQGEIKELKVIIKGDVHGSVEALKGMLEKLSTSEVHLNVIRAAAGA
ITEDDVMMASASNAIIIGFNVRPTPSAKQLAEREKVDVRKYNIIYRAQEEIKLAMEGLLA
PELKEQEVGKAEVRSIFRVPKVGIVAGCQVTEGIVKRNCQVRVIRDNIEIFQGSLSSLKR
FKDDVKEVAAGYECGIGIENCNDLQEGDILEFYETVEVARTLESGNNNGSDTKASS*
>SPBIB_v1_100078|ID:27162229|rbfA| Ribosome-binding factor A [Uncultured spirochete bib]
MDQIRRRRLEELIREEISRMITFGEIKDPRVNSFLSVTRVEAAQDGSHAKVWVSSLEDEE
SRLDEAITGLSHAAGFIQALLAKRVRLRLTPVLTFFPDRGIKEGFEISEKLKDLLK*
>SPBIB_v1_100079|ID:27162230| tRNA pseudouridine synthase B (fragment) [Uncultured spirochete bib]
MSCSGFFLVDKPAGPTSNRVLQEIKRAFSKAYPTLNVKFGHAGTLDSFASGLLIVLTGKC
TRLTPWFMHQTKEYEAVFRFGEETDTLDPLGRIIGHAELPSKDMLEQAIPRFQGDILQIP
PSYSAVHIGGKRSYKIARDGETPDLAPRPVMIEALRMHSYDGEEAHFSIRCSSGTYIRAL
ARDIAQACGSRAFVKSLRRTRIGPFAVDAALRPDMCSAKTMMHFTSEVAISLGLGAGLLN
PAYFDRFLHGSDLPLSAIESRGKALDLFAGRSLADSPLALFSQSDAFLGIVQKSESGWAV
KVMAADEAKE*
>SPBIB_v1_100080|ID:27162231| putative riboflavin biosynthesis protein [Uncultured spirochete bib]
MKVLTWQDFLENVPHARIAATVGVFDGLHLGHQALIETVRKEAPQLKPCVITFRENPKKI
LHPASYRGSLLTTEQKLSAIEASGIEYCVLIDFSGKFATLSGREFLAALYVANVRFVAVG
ENFQFGHRLDTNAVKLEVLSEEIGLRPAIVKNVMYRGHPVSSSRIRHAVQEGRLEEAADM
LGRPYQIIVQRGAEPGVMVAEDDLLLPPDGEYEGIANDGGAMQYCRVEIDHRNLKFGLRM
QSEVVRFAFVDTATQEKENLVWL*
>SPBIB_v1_100081|ID:27162232|rpsO| 30S ribosomal subunit protein S15 [Uncultured spirochete bib]
MALSKEDKAQVVLEYGKDPKNTGAIETQIALISSRISYLTEHFKTHKKDTNSRRGLLKLV
GQRRKLLKYLQRTNLESYREIVEKLQIRK*
>SPBIB_v1_100082|ID:27162233|pnp| polynucleotide phosphorylase/polyadenylase [Uncultured spirochete bib]
MINKLECQIGNETLVFETGRMAKQANGAVFATYGGSAIIATACCSSTPTEGLDFVPLTVE
YSEKYYAAGKIPGGFIKRETRPKDREILVSRIIDRPMRPLFHKEFGREIQVVPTCISADQ

INPPDVIAANAASAAVHISDIPFEGPIGCVRVALVDGKYIVNPTYEQIEHAKLEIVVAGT
AKGITMVEGGSHEASEEEMIEAIETAKEPIARICATIEELKHLAGKEKLPLAPLQVELNN
KEEIRAYAHELLSKALFTKVKQERAKAVAQAIAAVKDKFKDALTDDIQAKLFSKLMDDLQ
YEILRSSILDKQLRVDGRGLEDIRPITCEVGVLPRTHGSAIFTRGETQVLAVTTLGTVFD
EQIFDDIEGDRRERFMLHYNFPPFSVGEVGRLGTGRREIGHGDLARRAIEPMLPPKDKFP
YTVRVVAEVLESNGSSSMATVCSSSMSLMHAGVPVAKPVAGIAMGLITDETRYAVLSDIL
GDEDHLGDMDFKVAGTKEGITGFQMDIKISSVSTEILRKALEQARRGRLHILSIMEKTIS
APQSEISPFAPQVISARIDPEKIGLVIGPSGKNIKALSEKYDVQINIEEDGSVTVYGKDQ
KSAFNARDAILGMVEEPEVGKIYIGTVKRIMDFGAFIEIIPGREGLCHISRLAKGHVEKV
SDILKEGDTIQVKLMEIDHLGRLNLAAIDSLDENGEARERPARSDSRSSDRPRDSRPSRR
DFHRDR*
>SPBIB_v1_100083|ID:27162234| putative Peptidase, M16 family [Uncultured spirochete bib]
MPLLLKSAAGTMLAADSMPNASSVSIGLWLPYGSRNEKPQNRGFFHFIEHMVFKGTISKN
AQALARTFERTGGFVNAFTERSTTCFHCTIPAKEWKYACTTLLEMAFLSIFPEDEFEKEK
IVILSEILQTEDDPEELAHEHFFRLFWKDQPIGLPIAGELADIQTISREKLFSFYLEQFK
PEISFISIAGAIDENEAARFVEAELTRITSLRQKLFGTIGLAAENCEIIAPVSAVPHIFS
AYEKNHASLSYVIQCMQVPPPQNVRRYLLNALMNEIIGGSTISRLFQSLREKEGLCYTVF
SSFETETLEALWIIHLQTGGKQLTRALDVLDDEVKKLSKGYPSAMEFEDARSRVAGLLQL
ASEDTDFRQRRMARSYFAHGTTEGIEQELALVQSIEYDEVCSAASDFGSLNRARFVFGTA
KEKELLKRGYLEHE*
>SPBIB_v1_100084|ID:27162235|dut| Deoxyuridine 5'-triphosphate nucleotidohydrolase [Uncultured spirochete bib]
MSDSFAKQIEVSISLSGNAQMPNYATAQSAGADLCASLSEPVVIQPFARALIPTGVRIAL
PEGYEAQVRPRSGLAFKHGVSCLNTPGTIDADYRGEIQVLLINLGDKPFTVTNGDRIAQL
VIAPCSRARFSQVAHLDETGRGTGGFGSTGL*
>SPBIB_v1_100085|ID:27162236| putative Permease YjgP/YjgQ family protein [Uncultured spirochete bib]
MKEKPPLTFWRMMFSEMMLSFLAAFFFFFIVFLINQVLLFAEDILSRGADLVSVAKLLFY
SLPTIIAITVPFSVLAATLMTSSRQHADNEFLASSTLGIRPLWIYIPFLIVGLGIAVGSF
YANDWFIPRAANGFKTVYAELIRKSAKVELSPYTISKYGDKLLVTGASNAALIQNLLIVD
QKQGADANVVTADDVSIEFSQDSMDAILKMNNITEEKKLPGGEPGDFSMTTARQGDIRIQ
IREQIPSYSSTAPSEMSIASLARQIREKESRLASRLREKESQKATALDRLRLSYAQSSAA
SSALASFKSLASQKTEDTSLQIYRLEYQKKFVIPSACFFFAFLAFPLGIGSRRAGRTAGF
GLALLLAVIYWALLFAGQTFGYRQGFDPALSMWMPNFVMFAAACLIWISRKIFTGHFL*
>SPBIB_v1_100086|ID:27162237| conserved membrane protein of unknown function [Uncultured spirochete bib]
MNEELVKSRFASLDLSQGPGKPFLLWKELTSKVLLWTAGGTLFLSLVISLAELFSMLWKF
LARNASFIDILKWIGFGIPKHIVDAAPVAFLFAIVFILSEWHANNELEAVFSAGISLQRF
VLPLLLVSVLLCATEYFVTDQIAIPFLRERSTLQSGILRESDSRYAVPALMADKGKKVYT
YRFYDEKKGRIYDISVIERDDSGALLRKISAKYASFENELWVFSDAIIYKKSRDVWEFAE
APRFSDPAFDEPPASFMRPTMDVRFMNNAELNGHIAFLKSAGLPAEDAEVEQQRRISFAF
TPLVVVGLAAAFAGRFRKSIFLLSLLSSLSSATLYYVAQMLASLAAKARMSSAGLAIWSV
MLLFAAASTVSYFKART*
>SPBIB_v1_100087|ID:27162238|tgt| Queuine tRNA-ribosyltransferase [Uncultured spirochete bib]
MANSIFIEKHRDPSCRARTGTLFLPHGEVETPTFMPVGTNATVKAVEPADLDAMGFNIIL
ANTYHLFLRPGPDIIAKAGGLHGFSGWKKNFLTDSGGFQVFSLSQFRKILQEGVHFKSHI
DGSSHVLTPEKVVDVQVAFNSDIQMALDICAPWGETEKKAFRAAMLTYDWAKRAKSQWIM
HRENGYQGNLFGIVQGNFYEELRKLSAEQISELDLPGIAIGGLSIGEPKEAYIEYLELTS
ALLPLDKPHYLMGIGTPDYIIEAVRNGIDIFDCVYPTRTARNGLLFTSKGQITIKKAIFK
EDFSPIDPECSCHVCKTYSRAYLHHLFRNNEILYSMLASQHNLHFTADFVGKIRRAIEQD
RFEAFARAFMSGYSEAAAAE*
>SPBIB_v1_100088|ID:27162239| Transcription elongation factor [Uncultured spirochete bib]
MAESPLNEKLQLMLNEEKWTRTLMANYSVASLKELDRLVEDAVQQGVTDDIIKTCTDHLS
QTKNSVIALYVAGILNIMRQPADESYLVQLMDLFADSKKTNIVEYICNRMLDYGENRIAL
MRLAECYAEDDKIEQRYAVWERLVRIDTEEAELAQLIAEWKEKNGRTEEAIDFYKKALIR
YINKSLFANIKDIWLKLIDLSPEDLDFFLHAQKKIAKQISPEKAAMLLMDLYAWYKKNEK
WETALDILKLSIEYDERNPALRKEFLDCYTAIYSGHSMFDECVRLSNLTQTYRSLHEAIA

DFEKRIAFDKGNFVFHRTWSIGRISNVTNDEIVIDFSKSRNHHMSLKMGFDSLTTLSKDH
IWVLKATWPKEKLRDKVKADPAWAIKVIIKSFDNRADMKRIKAELVPSILSTSEWNSWSS
RAKEILKTDPMFGNASDDISTFIVRDRPLSFEEKIYAQFKAEKNFFARAQYFRDYIEMSD
IDSDYFEDMLSYFVSFLKIGQVNEYVVGSYLLLKELSTRYPRLQQHVTIRFPDLFAQIKD
SIVPIYLGLKDTELKNQFIQNIKTLIPNWPDIYIQLLPYARSNKMLEALENADQEEKLKR
LAMYIVENYKDMREAFIWLAKDLKDKPWLKELGIVREKIILTLLHILDITFKEIDSHKDT
TENRKINKLVQNILFKEKDLEEFLEDAPQQVTERVYSMLADIKEVDPAIKLNIKKKIGER
FPDIKFYDGNERLTVSHGLMVTASKYEEKKRLLSHIMEIDVPANQKEIAFALSLGDLREN
AEYKAAKEKQDELNSKVGKLKNELDRAQIIGPEHVDTTSISFGTKVTLKNLASGQEELFT
ILGPWESDPEKQVISYLSPLGKHLLNHKVGETLNFTINERKFSYKVLKITKAEF*
>SPBIB_v1_100089|ID:27162240| Tetratricopeptide TPR_2 repeat-containing protein [Uncultured spirochete bib]
MAIQPQIKNASAASDLLIAYESLKNADIQSADLFLERALKTDFEHPEVLFAMKCMQFWKE
QLLNMPDTKNLLDRGDFLCASWKKFMVFLGHIGESFETTRYAFKRFIFGLALDQYLALPD
EQKESLGAAIDLRLGRSRKATGDIETAIVHLERAAQSQRNDAAILSELADAYAMAGEARL
SKALFREAFFIDPQAVEIDFLESEFILKIIENVRNLGYSGNDIAEWIPVYAEIWGVFNLK
RELSTAEYNRISASARQIEIELRESPQRRSSLLPRLLNRYFWMADYLKAQGDEAGWHSVS
LKIKILDQSIHALYVG*
>SPBIB_v1_100090|ID:27162241|rpe| Ribulose-phosphate 3-epimerase [Uncultured spirochete bib]
VKIGLAKAADLHYSVDCMKMPIIAPSILSADFLEIGAAVESIESSGAEWLHLDVMDGHFV
PPITFGNKMVSDIRKRTGLFLDTHLMIEEPERHIHAFLDAGSDAITFHAEACIHSHRLLQ
TIRHSGKKAGISIVPSTPVSQILPLLQAVDQVLVMTVNPGYGGQKLLPFCLSKVDELRDI
RKKEGLDFLIAVDGGTNLSTISQVAIHEPDILVIGSAFFAAQDKQAFACSIRSAWNE*
>SPBIB_v1_100091|ID:27162242| exported protein of unknown function [Uncultured spirochete bib]
MKKYILGLLLLLAGISLSLHAQGNDEIFAKFSSAFDLFKKENFQKAGSEFALLYETWSES
SLASDSCFMAAQSYFNAGDYPTSYLYCVQFIQNYSDHPNIPDMKFQLGRIFFKTGRFKDA
IIAFDDFLKAYPGSPLFASALFWKAESYYQLGDIARSFPLFNELKEKWPDSDKASLVSWR
LNVMGLEAREAKFSRLMAYETEKAAQMEIGGMQKEALQEQQYIRVYYLLKTLRVRNELPG
SSKLPLYVQYTPQNAPEVDSNLKSRLNKLNVLLEAKKRILELLLAKIDAYMREISP*
>SPBIB_v1_100092|ID:27162243| exported protein of unknown function [Uncultured spirochete bib]
MKRSFALLAVIFCFTSAATALDLRSGLVRIATDDLTLRPMLYRLVDIAGKKKYEPIWYSD
DPRTSFIVINVDGRIYRMGSSQEYQTSQKQIANGIEIEYRSIINRVTQRIQFASLAGSRV
ANGFLVEIEVENFTSRDMKIMLKEVCDTWLGEKSGNHIALASNPKVIDEMYLTPDSQEPY
IVSPGEYASVALLFDSEPRPDAVVIANWKRLSDSMFAYDSSLMRGFTLSPYSINDSALGL
YWNERIVPAKGVIKVSSRWLTGGPGNEFLAWLADNYPPKAEQQTTEAQQNSTEQMSLPAL
DVEAIRSLLARIDSAIQNVDVLSDEDLQQILSELDSLEQGVANPIPQN*
>SPBIB_v1_100093|ID:27162244|dtd| D-tyrosyl-tRNA(Tyr) deacylase [Uncultured spirochete bib]
MRAVIQRVSEASVTMQPEDNSGERLCGRISNGILVYLGVGVDDTDEDASYLAEKIAHLRI
FMDQQQKMNLSVLDLGYEALVISQFTLYADSRKGRRPSYSQAAEPARARYLYELFCKKLR
NQGLHVQTGMFQEIMRVRYTNEGPVTILLDTKN*
>SPBIB_v1_100094|ID:27162245|ppdK| Pyruvate, phosphate dikinase [Uncultured spirochete bib]
MPKKKFVYSFGAGVAEGDGTMKELLGGKGAGLHEMTKIGLPVPAGFTITTEVCDLFFKSG
KKWPEGLEKEVQKHLKQLEKTTGKTLGDPRDPLLVSVRSGAPVSMPGMMETILNLGLTDA
SVEGLAAKTGNRRFALDAYRRFIMMYGSTAMGIEREEFDKAFDEIKAKRTRARLGIPADQ
KVNDTDVNEEELDELIKMFKKIYREHIKVDFPQDPWEQLKGAIDAVFNSWMADKAVTYRK
VEKLVGIKGTAVNVVQMVFGNKGETSGTGVCFTRDPNSGENVFYGDYLVNAQGEDVVAGI
RTPIRLAELDKRDPKVYKQLVDIRRKLEKHYRDMQDLEFTVEEGKLYMLQCRTGKRSPIA
AFRIAVDMVKEGLISKEEAILRIKASDIEGIFYPMIDKSKPEELKKAFLVQGIDAVPGAA
TGKVVFNAKDAEEWAAKGEKVILVRKETSPEDVGGMHAAQGILTATGGKTSHAAVVARGW
GKTCIVGCEKLDIDYEKGIFAVGETVIKEGDYITLDGGTGNVYKGQLKLVKPEPPKAYTT
LMSWVDKIRTLKVRTNADTPYDAEKARQLGAEGIGLCRTEHMFFDSEERILAIREMIIAD
DLESRKKALAKLLPFQTKDFEGIFKAMDGLPVTIRLIDPPLHEFVPHDEEGQRALAKAVG
VSFEKVVQRVEQLHEANPMLGHRGCRLAITYPEILDMQVTAIITAACNMTKKGIKVLPEI
MIPLVIDDKELKILETRTREVADAIIAKSGIKLSYMVGTMIEVPRAALLADRIATVAEFF
SFGTNDLTQMTLGLSRDDAGKFLPDYVDEKKAGVFKADPFQSLDQDGVGMLIRMGIEKGR

SVRPKLKVGICGEHGGDPASVKFCHRVGMNYVSSSPFRVPISRLAAAQAVVEETASAKKP
GKNRGRPAKKASAVAPKKTSTRGRKPASAVRAVSASSAASAAPKRRGRPLGSKNKNK*
>SPBIB_v1_100095|ID:27162246| exported protein of unknown function [Uncultured spirochete bib]
MSRIPGVAKAFLFAAVISIMLASCIGIEAATKMDAKGSGTLSMEYQISNEFAQIGALETT
PSLPLPLSREDIEKSLQRIEGVHLKSYSQNSRANNIIISFTLLFDSPSHLAYYLDPAGKF
VQYSQEDGISHLKLTLGDAIQPLDKQMKDALYEKVKPYNFKFTFEASQAAPEISMQNADF
FKTATSGKKVTLESSMADLLTSVEPPRIDISWR*
>SPBIB_v1_100096|ID:27162247| conserved protein of unknown function [Uncultured spirochete bib]
MLRMVLRLGEASTIKDKRRVVLAIKEKLQHRYKLSCAEVDLLDSVMFAEIGAALVSNSPQ
HGETVLRDAVTFVEDHYPVELYDVQTHYEIFG*
>SPBIB_v1_100097|ID:27162248|metG| Methionine--tRNA ligase [Uncultured spirochete bib]
MKRRLITSALPYVNNVPHLGNLIQVLSADVFARFCRLRGYETLYVCGTDEYGTATETKAL
EENLSPRELCDKFHALHRDIYSWFEIAFDTFGRTSTPDHTEVTQSIFLDLDRNGYITSQS
IEQLYCDSCHRFLADRYVRGTCPYCGYEGARGDQCEMCGKLLDPMELINPKCATCGATPK
PRTTTHLYIDLPAILPKLEAWMKEASIKGFWANNAIQMTQAWIRDGLHPRAITRDLKWGI
PVPKKGYEDKVFYVWFDAPIGYISMTVSLAKEKNFDWKSWWQNPDKVELFQFIGKDNIPF
HTVIFPSSLLGSGKPWTMLHHMSSTEYLNYEAGKFSKSKGIGVFGTDAMETGISADVWRF
YIFWNRPETSDYTFTWADFQEKVNGELIGNLGNLVNRTLTFVSRYYNGVIPEGEPDAAFW
KEVLKLEENVAEHLERAELRDAFRSIFAISDLANKRFQDEAPWKARVEEPAKAASLMRDL
CYVLKDLAIMTYPYMPKAAENLAGFLGLSFGAQGLSWRELGPAGLPGTVRKPEMLFQKLE
DDLISALRERYSGSQKERQERDAQENAEGPGVQGPAAPPSAPPSEPPEQRFFKLVDLRVA
KIVKIERHPKADKLYIETLDDGSGKERTIVSGLVPFYKEDELLGKNIVLVNNLKPAKLRG
VESAGMLLAASVSAPDGTESVEVLAAPNASPGDRVILEKTEGEPASSEIPSAIIDVDAFF
SIPIVAKDGSAWVGTKRLVVNGEPIALRRVLNGEIG*
>SPBIB_v1_100098|ID:27162249| Methicillin resistance protein [Uncultured spirochete bib]
MNVLPHVAFTPISSASDCIPEFEYSSFLQSKFWGLFKARTGWQAYSCSYDFEDDGPHGNL
LVLRRKIAKFLSFLYVPHGGGELSRLPDRWNALSMLGRALSESMGKSDAFVRFDLPWERM
LDEKTEFELTSGPTGSKRLILHKGTDVQVADTVVLDLAKSTEELLSGMKPKWRYNIRLSE
KKGIQVTKHGKEGLPLFMKLYEETAKRDKIAIHPESYYATLFDTVLEVSDLMKEQGIPQQ
DRPILSLYIAHHESDALAGIIVLKHGPVATYMYGASSNLKRNMMPAYALQWHAIQESKEQ
GARMYDFFGIPPQGDDASHAMSGLYLFKTGFGGKIIHRYGAWDVPIHPWLYAIFRSAEKM
RAFWHKKVKKSIRKISPTASAAHPSAGERDDSEKTNPSNP*
>SPBIB_v1_100099|ID:27162250| conserved protein of unknown function [Uncultured spirochete bib]
MKFSIDIDDQKALLKYARKVIQAALEHEPEPEPPELSTTLRCGAFVTLREDASLRGCIGR
MQSNDPLARTIAEMALAAAFEDPRFTSVAAEELPRLEIEITLLSPLKPILPEQVVVGKHG
LLISALGRSGVLLPQVPTEYGWDRETFLEQLCRKAGLAAGTWKSSLAQLYGFEGFVFSES
SLSPAEG*
>SPBIB_v1_100100|ID:27162251| putative MEMO1 family protein Asulf_00361 [Uncultured spirochete bib]
MHTDKLIRSALLPGIFYPADPEQLKSEVRMLAMQTEQMPHSGCAIISPHGSFRYSGLLAA
KAWGSLVGANPSIIILAGPAHLPYEEGVFLPESSQFDIPGAVLNVDVSFAEYLLARIPEL
KKNDLVHLEEHSIEMQLPFAEHFFPGVPILPFIVSGRDEKTVATVEQLFEEIRVCVENQG
VLILSSDLAVSNTPEECDKLSNEFIAALSCGSTASFSAHDVSRHSFCGEPAIRAFRHAYP
ESKSRLLAYANSAPFREGSDELVVGYGAISFSR*
>SPBIB_v1_100101|ID:27162252| conserved protein of unknown function [Uncultured spirochete bib]
MRISRILIWYRMESVIQALLYEEKMLHNRLQRLENECASRPKGSIILKRRFHMVYAYLQW
REGEKVVSRYLGKSDSWKCRSMQAKIIERRKYEQEMREVQNKLKKIHHLLEEAKKLG*
>SPBIB_v1_100102|ID:27162253| putative galactokinase (Galactose kinase) [Uncultured spirochete bib]
MNSMKDIFAIHLSEYETAPDCTVAAPAIVKILGEHTDFTDGLVLAAALSFEVRVAVSFRK
DNSLRFYAGDFNERKRANIATLKYKREDRWANHIKSICDYFLRVFELEPKGLNVTIQSSV
PLGLGLGISSSLNMAAAFIFKTIYNLDIKPDELAAHACKAQSVFFEKDFPITNYLAITAP
AGRSFSVVDLRTRKRRGIQFLPEPWEMVLTDSKVPRLSVEAELEQRTSDCNACLSVLTPR
GNRSIRDIAPRELDELLGIVPERKRRRCLHVLEEVRRVSEAEDALARQDYAGFARIVNKS
HASLRSLYEISCPEIDWLAKRALELDGVLCSRLVGQGFGGSTLTILNNDVKEAYRHRLEE
YERIFGFRPVVYEVSPGSGLRILEH*

>SPBIB_v1_100103|ID:27162254|surE| 5'-nucleotidase SurE [Uncultured spirochete bib]
MRILLTNDDGIQSDGLFALVDSLGSLKNDRGEQLHEIWVVAPEHERSGVSHAMTLKKPTK
LRKLGEHRYSCSGTPADCVIVAGLGVLHEAPDVVISGINKGPNLGTDIIYSGTCGAARQA
ALEGIPAIAVSCASFGDNLEYGGLTSFVASNLEKLIALWKPDSFININGPSTSCTDLCAV
WANPGKNRYLDNLKCFDGADGYTYCFLAEGRQERTPDIYSDHHAVSQGFIALSLVEIHPR
ALHDTALNGTSVFEAPNLHSGSEVKH*
>SPBIB_v1_100104|ID:27162255| conserved exported protein of unknown function [Uncultured spirochete bib]
MEQMMKMPNLRKIMQFSLLSIGLLVMAFSILMVVAPFRRAPAEVKIKNGATLSSVSRDLR
AANVIPSSKIFALYMRLAGAERAIKPGTYRFEPSLPPYEVARKLIRGDTIALKVMIPEGR
TARQIAQILESQKIVDASAFLAAVNDPVLTEKLGIPAKSSEGYLFPDTYFLEPGSDAEQI
VVQMVANFRAAMKKIAGEMQDTLNAQALYEKVILASMIEREYRVPEDAPLIASVFVNRLS
KNMPLQSCATVVYVLTEHLGRPHPSIVYYNDLRVKDPYNTYLNRGLPPGPISNPGEVSLK
AALFPSKTDFLYFRLDDSSSGKHRFSRTLQEHNEAGIIPKGL*
>SPBIB_v1_100105|ID:27162256| DNA primase [Uncultured spirochete bib]
MARIPPQTRQEILDKTDYLSIYQEHVRLQRKGASYWGLCPFHSEKTPSFSVSADTGLFYC
FGCHKGGSVIQFLMDIDKLSYTEAMEELAQRAGVTVHFEESEKAGEAERERQALLELYDR
LSRTFHWFLMQHPSGHRALEILQKRGIPDDLIERFSLGYAPASRSWLFSFLKAKGYSDNF
LKRSGLFSRNDVAWPLFADRIMFPISDAKGRIIAFGGRAMDQDGPKYINSPETALFRKQD
TLFAFAQALESIKKTDEAIVCEGYMDVLSFHAAGLTNAVAPLGTAFTSYQAMALRRKASK
VVLCFDTDEAGLRAAERACSIATAVGLETSVLLLEGGKDASEILERQGAGELTRMAKNTI
NSGLFLLTRASQLFDISTMEGKAKAVAFLFPFLDAMDSDVRRQEYMKEIARTLGVSAHAV
EMDYAKAKASGSYHQRDSANPSVPMGSSGSARTADLVFIAAVILSNNPFLPSVLQLDSED
IEDARAKDLIAALKEAEKEGIRDIEGILSFCPDDAAVRFVHEIDASGELSEGLEKIIEDG
LARKRKKALEKERSRLVASLQAKCADEDRMQEKSILEKIMRIDEELKKTGGDLDE*
>SPBIB_v1_100106|ID:27162257|rpoD| RNA polymerase sigma factor RpoD [Uncultured spirochete bib]
MSDLELDPAILKLLEYAKEKKTISFDELSDMLPEQILNSDKMEAIINLLVSSNVKIEEEV
QIEEEPLPKIVAIPSKNKMVANDKEIASDDPVRLYLRDIGRENLLTAEQEVELSRTMEQG
ENIIKNTLRKSGMLIPEFYQIIVKATRKEPKDLSLQKKENTEKIAERRRLNQFYKDLIDS
CAADLKAYIDLKKWVIAKGGDVFEDAQLKEIRERLYPIINEADIHPEEITAFSEKFIAAA
KKIIRNRKEQDRIEGILQVSSAKDLRALGRNLTIKEKREEIEQRLGLSAEEIKDRIRQLQ
VNEKKLKDIENNFETPIDRIISMAREVNRGRKMMKSAKDKLIKANLRLVVSIAKKYTNRG
MQFFDLVQEGNIGLIKAVEKFEYRKGYKFSTYATWWIRQAITRSISDQARTIRVPVHMIE
QINKVVRESRQLMQKLGREPTDEEVAEQLGWEIDRVKAVKNVAREPVSLDTPIGEDEDSL
LQDFIEDKEVENPAVRTDYNLLQEQLRMVLATLPKREQEVLRMRFGLDEGYSLTLEEVGL
YFNVTRERIRQIEAKALKKLRQFKRSQKLRDYIDH*
>SPBIB_v1_100107|ID:27162258| conserved protein of unknown function [Uncultured spirochete bib]
MPMRMDEVFETLRKYQELLSQRVELESQLEAMPKTIDIQNQMLSRERKSWAEAKEQYEIS
ESNLRELRQELEEAEMKRENAEKQMDGITTQREYDAVNKEIREASEKEQELRRRIQEEER
RFAEIDDRLHRIEISVSTLEAEIAQKRTEIEAKTKIVLDEAEKIKSEETSLTSELDEDLK
FKFERIIRSKSGMGIVPIHGVVCTGCHMILPANFVNEVRQADKIVFCPYCSRILYFEEAE
TDESIEEILAGSLVDLNDEEAEESDESESNDESEETVSKAKRPSSRKKKNQSEPLIDNDF
IIDNMDE*
>SPBIB_v1_100108|ID:27162259| putative Tetratricopeptide repeat domain protein [Uncultured spirochete bib]
MFNAFFRKISTRRFRRPLWIAAIVFVLIGCISTGLFFILKNSDAKGVISHREIAKLWNEG
NIEKTIAESRKATEAFPFDEYYLSMRGISAYYSALDAQDEETRQQLLEESVISLRKALVI
GVPSGMKAQIFYVLAKAYYQKGEPWFDLAERYFLLARNSGSKEKDLPQYLAVVYAGKKDY
EHAIEWFELSLKGDTSDILTLSAAISYKNIGKNDKARELLASLETHATDAKIRLKAKLLL
AQDSYESGDFSTALQRYEEILKEDPLNADAWYGLGLVYAKKNDQLGARAAFRKVVQIDPN
HADARKRLAEKL*
>SPBIB_v1_100109|ID:27162260|mreB| cell wall structural complex MreBCD, actin-like component MreB
[Uncultured spirochete bib]
VGILDMFSPDIGIDLGTCNTLIYVKGKGIVLNEPSVVAVERGTNRIVAVGTEAKNMLSRT
PTDIIAKRPLRDGVIADPDTTEKMIKYFIQRVFPKNLFFKPRVVVGVPTCITKVEENAVI
DSTYKAGARQVKVIAESLAAAIGAGIPITEPAGHMICDIGGGTTEISVISLKGMVVTSAI

RIGGDEFDDAIIKHIRNIHNLIIGDQTAERLKIEIGNASPERTIEKMEIKGTDAITGLPR
RFEVDSVEIREALMDSVNIIVEEIKKTLGKTPPELAADIVERGIVLSGGGALLKGLPKLI
AKETGVPVILAEKPMECVAIGAGKYFEIMQRNIGSSSVYESLNL*
>SPBIB_v1_100110|ID:27162261| putative Cell shape-determining protein MreC [Uncultured spirochete bib]
MKNNGFHRPSRTVHRKAALFILLSLSILFVSTSTVFRVPDLFHGYVIGNIQKAFAAVSSF
VSRTVGAVSELRNIKKDYEALLAKMQEYELKEREFTSLIEENKRLKDLLGLTSFPDAKKI
AAHIIARDPGNTYSSFVIDKGYNEGIRKYMPVIAFQDGVEGLVGKIIDVKASTSTLQPLY
DQRFFAAARLAKTRAEGLANGRGFRDAPMTLLYIPKTEMDTLKQGDIVVTSGLDQVFPQD
IIIGRVGSYQLNEFSSSLVIEVQPAIDLSRVEYVFAIENQGALTPYAGGKQ*
>SPBIB_v1_100111|ID:27162262| putative Rod shape-determining protein MreD [Uncultured spirochete bib]
MRSLFISLSASILMLFIQSTWLSHGIVFGIIPDLAMSIVLFSSFINKDGQGIIVAFIAGL
IADMLSAAPLGYYAFLYVSCAYLATLLSYVAEKDIFIIPFLLGTGAAIARGILTRFVSWI
FAANINTYQIFSAEFGIELLADGFFTVLVFFILTFFQPLLENSPRKALP*
>SPBIB_v1_100112|ID:27162263| Penicillin-binding protein 2 [Uncultured spirochete bib]
MAVLKKKITNRIRILRALVVVLLAIYALNLFSMQILRKQLFTSEAERISLQSTRIPAPRG
EIYDRTGRVVLAGNIEAYSVYITPSELPATKRESVILLLSKLLNISAEDIESKLPTGRLT
SYGRVAVAKNASLSAISEIAAHIDEFPGVSWTSQPLRDYADLRSLSNTIGYVGSITRDEY
KLFYNKGYTLEDLTGKAGIELIYEDLLKGKDGWIAKAVDVHGKDISRNKARIEAPEPGKR
LILSIDSNIQKIAEDALGKRQGSVVVLKPATGEILAMATYPYYDSRVFMSDNAGQEYLKL
LNDPQKPMLNRAYQSSYPPASTFKTVLSTAILEEGFISPDKTIYCSGEINYGGRSWSCWI
KKPGHGAVDLKNALAQSCDIYYWTVGRDYIGVENIVSYAQDYGYGKLTGIDLPGENPGLL
PTPTWKEEKYNDSWTPGDTMNLSIGQGYMLASPLQVANMMAMIVNGGVIYKPHVLKEIRD
PGTGALINEVQPEVLQKSSISEKTFETMRSLLRGVIVYGTARAPISTKAVQIAGKTGTGE
IGLKDRWHSWFASFGPYDAPAKDQIVVVTMIEASNPWEWWGPYAANVIYQAIYAGQDAKT
AAATVGVKLDGIAVGGRRE*
>SPBIB_v1_100113|ID:27162264|mrdB| Rod shape-determining protein RodA [Uncultured spirochete bib]
MNLQSLRRLFGNIDYYLLIATILLCAIGIAAIYSSGVDAEGRLVSNEYVKQLVWFVIGFF
LLAGAALFDFSRFKDTSWIFFAIAIALLVLTRVAGRVVNGARAWIGIGGFGIQPAEFAKI
ATVLMLGRYLDSAEYDSSFKKLVMTGLILSLPLMLILIQPDFGSALVFFPAALIMLTLAD
VDFRYILFGVISILGIFLFLSLPLYAANHYSQDNILNVIIAKTDILVLIFVIILFIAVIA
GVGWLKFKKRYYYWLSYAFAILAFSIAGAFAAHKLLKPYQIERLLVFIDPSIDPRGSGWN
ILQSITAIGSGGFWGKGYLQGSHSHARYIPQQSTDFIFSIIAEEFGFFGCLVLFALYAII
FIRCFVLIETSKDRFSQYIVGGILGIFAFHFMINVGMAMGIMPVTGIPLYFISYGGSAMW
SSLISIGILTGISARRYSA*
>SPBIB_v1_100114|ID:27162265| Radical SAM domain protein [Uncultured spirochete bib]
MRKSFIDPLRDFGDALFHIQKPARYVGGECDAVPAIDQDDQRLRIAICFPDLYEIGMSNN
AIRILYSMLNEKRSSLICERVFAPAPDFENLLREKNIPLYTLESGIPLSECDILAFSIGY
ELLATNVLTVMERGGVPIESIERSDKDPIVLAGGPAVTNPLPFERFFDAVWIGEAEAGFV
ELMDQVAELKKSGAQRTDILRHISRHPSVWMSSASCKALGIEKKKRVVRAIYPDFPKTAY
QPSFPYPVLNPVHSHGTVEIMRGCPNGCRFCHAGYYYRPQRSKYPGIIQKEVEDLVMQKG
YREITLSSLSSGDYPGIVELFETLNSKWSAKKISFQLPSLKVDSFTLPLLEKISEVRKSG
LTFAVETPLDEWQCSINKRVPLEKIELILREAKAKGFRSAKFYFMIGLPVSGRGMNEAEA
IVEYIKKIAAVERIAIHVNVGTFIPKAHTPYEREEQLSEEEALECINYIRRNFRYMRNID
VTYHPPFLSVLEGIISRGDEAVGDLIIDAYRSGARLDAWEEFMNRDVWRAALKKFNETRG
EGAWQAYLHKRSLREELPWHTISLKVSQQWLKHEKEKAENGIITAACNEICTNPCGICDK
YINIVSNSILVEPIFDNIEFVGSSNKKIRKIDFEWAKAKEDNRIRMKLVFTWSKRDLAVL
YPMHEVANAFSRAFQILGIPMQYTEGFNPQPKLELSPPLPLGVESERDILGVFISVDKDA
QQRLFREISESSLAVLQSINENLPKGLAVDSIRLAQGKHTIGSLFSAAEWKYAFIDQESY
SIACAALCSNSADFISWKSFDDSRELFVLENFEPASGKIKNFYKTLKTFSESSKGKEIKF
RACRTKCFAKDEAGNLLPLEEAL*
>SPBIB_v1_100115|ID:27162266| exported protein of unknown function [Uncultured spirochete bib]
MRGRKKYFFDNPRIVAAGAKRKSYIFRALLFASAAISSFLMFSCGTPSAEYLYHPIDFTS
ETGILRLLPNPSNIDDSYVAALFKGYEIYYHVFDTNDAALNSLSLLQSLSSYDPSYFMSI
ATSSTYNYYPLLHRFDYLIDPHRPLIVVSNTTNNTYLEFDLNMEINKSWSITSGTDIIVD

SVVRNLANNTSADFFVSAYYNSKDQDYTGSDSPKTVYIVFFAVSYGISEASLGNALYSEP
VIIDAPVEYTPNS*
>SPBIB_v1_100116|ID:27162267| conserved protein of unknown function [Uncultured spirochete bib]
VNQQEKQPRKDMNVLNKNEKKAFSDKLADFISANRILFIGIAIGIVVAIAAVGIYTAVSE
NVATASSRAMELAEQKLQQWSQETDEQKKADSEKALLVDLDTIAKKWPHTIAAQRALLRK
AAILNQKKEYADAEKTALDALARNKNSYAAPIALELAAVSAEEAGNKDAAIAHYTQLAKD
YLKDNPVAPHALFNLGRLQEEKSDYKAAMASYNQLVSAFGGSDWALLAKNRIIYLKAKGL
AE*
>SPBIB_v1_100117|ID:27162268| Transcriptional regulator, NifA subfamily, Fis Family [Uncultured spirochete bib]
MTAMVSAIDPDKFRTLIDINARLNSSYTNFRSLLKAIVESAARLVGAEAASLALFDAATE
TLRFEIALGPRGLELQGKSIPATEGIAGWVYQNSKSIIVNDAAHDQRFSPTISKEIAYST
NSILAVPLKVKDKTEGVIELINKTSHGLFMADDLEWVEIFAVQAGIAFENAKQFERTEHE
LTYLQQKVQEEQGWHPLVFSSKVMQERLDLVKRVAPSDASVLILGESGVGKELIAEQLHL
LSRRAEKPFVRVNCAALPENLLESELFGHVKGAFTDAISNRAGRFEAADGGTIFLDEIAE
LPLRLQAKLLRVIQQKTFERVGSSETIKVDVRIIAATNRDIEKLVQNQEFRSDLYYRLNV
LPIHIPPLRERLDDIPPLAEHFLLKYARETNRGIMHFSDSAVQCMMGYSWPGNIRELENA
IERAVLIAKGNLITADDLMIGNKAEPSYKQFEGKDLKEAIILFKRHYIQASLEQNRWNQT
ETARHLQIQRTYLSRLIKELNILQNKE*
>SPBIB_v1_100118|ID:27162269| 30S ribosomal protein S1 [Uncultured spirochete bib]
MAENKEVEMDVTEPSRDDIQTQLQEQYLKSLEGLEEGDLIDGKVVQVAGDTVFIDVGYKS
EGKLSLEEFGDNPPKAGDIVKVILIRKETHSGDIVISKKKADEKLFWKSIANAFKDHLPV
EGTITKEVKGGFEVNLGHGLTGFLPLSKADIQRLEKGSELVGTQAKFYLERLYSDKKINI
ILNRRKLMEEEIEQHRASFFQNTQIGDTVKGIVKSFTSFGAFIDLGGFDGLLHINDMSWG
HVTRPKDFVRKGQEIELKVIRLDPEEKRINLSLKHFTPDPWNSFEEKYHVGDVVKGKVTK
LTDYGAFIEIEEGIEGLAHISEFSWVKKVRKPEDMLKAGDIVDCMILGYDIPAGKVSLGL
KQVQPNPWDTIDERYPAGTRLKRKIVKVTNAGAFVELEEGIDGFLHIDDYSWTQKIKNPS
SVLHEGDEIEVIVLESNAEDRNVRLGVKQLSEDPWRSFAKTYKFGAIVEGTVSSVTDFGV
FVKVPGEIEGLIKKQDLVSDRSESYDDALKKYQPGMQVKAVVIDLNPDRQKLGLSVKDLI
QKQQREEISRFIQEDEGEAGYTLGDLLKAKDKEND*
>SPBIB_v1_100119|ID:27162270|cmk| Cytidylate kinase [Uncultured spirochete bib]
MKVAIDGPAGCGKSTIARMIADNLGFLYINSGNLYRAVAYLALREGVSWQDGQALTALMR
QHRFDYKPDGSVLVDGERLLAELRTPQVDAIVAQVSAIPSIRVEVNAIIRAISEGKDVIS
EGRDITTVVFPDAEVKFYLDASSQVRAERRFRERQEARSHAFSQPDSQQAPRDMIAQTIP
APQSAPAIEEIRKNIEMRDQIDTQKSVGALKIAKDAEYLDTSDLTIQQVYEKVYNKIISA
RKAHGRK*
>SPBIB_v1_100120|ID:27162271| Pseudouridine synthase [Uncultured spirochete bib]
MSDLQAKPIRLHAFLAAAGIASRRACEELIKQGRVEVDGQPASIGQKVYGTEDIRVNGQP
IPVLSQKRFRYILLNKPKGYLSSIKDPQGRPCAIDLIAPSIPDRLYNIGRLDQWSSGLLL
FTNDGNFAAQLMHPSSEIDKEYFVQTDQPIPPGFAASFMRGIILEGVRYRAHAVRVVAPD
KAHIVLIEGKNREIRRVLEHFGLRAKILERIRIGPLSIGELSPGQWRDLTEQEIYALKAY
MSSRPSNKGPRK*
>SPBIB_v1_100121|ID:27162272| Segregation and condensation protein B [Uncultured spirochete bib]
MNEQLDAEAALVGAILFLENEPVSEERLAKISGLTDEQISLALQRLCAEYELPVHGFAPT
QSAGGWILAPKLSLWEQLKDHYGKKNEMKLSRAAMETLAIIAYSQPITRAEIEAIRGVNV
DGMIRLLLSRNLIAEIGKRDAPGKPMQYGTTQDFLKYFKLNSIADLPKLDEIENARFSKP
ETPEG*
>SPBIB_v1_100122|ID:27162273| ScpA/B protein [Uncultured spirochete bib]
MMQDFDNDANGEPISVSSTGRTFHLSDFEGPLDLLLYLIKKNEMNIYDIHISSITEQFLQ
CLNSDPSASLDELSDFYQMAATLLYIKSRMLLPNSQDELDEIDDPRRMLIDQLIEYHRYK
KLSELMEQREMEVEWFVERSSSQRTLPFADNPDDEPWIPADSWDLLRTFASMIRHFDSER
IIDLYEEVSINEKIALIHELLDKKGNFRFDDLISRHSSALDLACAFLAVLDSTKNRIISI
RQHRLFGDILIIPYSSPEAPHERTA*
>SPBIB_v1_100123|ID:27162274|folK| 2-amino-4-hydroxy-6-hydroxymethyldihydropteridine pyrophosphokinase [Uncultured spirochete bib]

MEKVWLGLGSNMGDSAQILELAVQDLSKMLSDMHVSGLWRSRARYYEEQEDFYNMVVCGM
TELNPRQLLDSIHDIEACYGRNRSREIKKGPRTLDIDILLYGGKIITEPDLIIPHPGLAE
RKFALLPLLEIDPSLIHPVLGVPLKMLAASLPPQGIYPASESQYDEPYPE*
>SPBIB_v1_100124|ID:27162275|recA| DNA strand exchange and recombination protein with protease and nuclease activity [Uncultured spirochete bib]
MAKRQIRAAEETVSEREAKFSQESNAASKQDSGIDPEIQRERDQKLKALEAAKLQIEKQF
GQGSLMKLGAHVIAQGIEVVSSGSILLDEALGIGGYPRGRIIEIYGPESSGKTTLALHAI
AEAQKKGGIAAFIDAEHALDPVYARNLGVDINELYISQPDNGEQALDITESLIRSGAVDI
IVIDSVAALTPQAEIEGEMGDAHVGLQARLMSQALRKLAGTLSKSKTILIFINQIRMKIG
VMFGNPETTTGGNALKFYASVRLEVRKIETIERGQDEEAIGNKVRVKVVKNKVAPPFRKA
ELEIIFGKGISWSASLLDAAVKCNVIEKKGAWYTWGAEKIGQGRENAKAYLEQFTEEAKK
IESQVREILFHPEKASNANPDQGASSEAFIAVSADELPSADTASDTGRIRAEDLF*
>SPBIB_v1_100125|ID:27162276| conserved membrane protein of unknown function [Uncultured spirochete bib]
MHQVKNPNIRPLTFVGLRLLFGRPANEQYNDLYRARHSVFWGAVLGIGISLVPLYIVLFV
SNGMIQGITDRYLETKTSHLQMSLPFSFDATQRNALTQDVSRMPGIISTSFEVDGIGLAA
SQNGSTSAQIRGLDGAVLQDKGFNRFISVDEGQMYPNRQNEVVLGRYLARKLEVKTGDSV
TLITLRSNDSTNYLPKLAVFRVAGIISSGYRELDANWFIIQSDAALRLLNSATAYAFMGI
KISKPYGSDLDRIASEVSRAGQKYGLTSEQGARVRTWREIEKSLFQSFSSTRSVLVLIMV
IAIIVAALNLASALTTFVIEHRAEIAIIRSFGVSRTQAALIFLLGGTATGAIGCIAGSLA
GIAISIFINQIISGMQAILGFASRLLSADRQTIQLLNPDYYLEHIPITMDWRFVVLILLS
GIALSAIASILPAMKSTSIAPAELIRHE*
>SPBIB_v1_100126|ID:27162277|lolD| outer membrane-specific lipoprotein transporter subunit ; ATP-binding component of ABC superfamily [Uncultured spirochete bib]
VSEPIIFCENLTKTFSSSAEVLRILDNADFVLEKGKACTILGPSGSGKSTFLAILGSLER
FDSGTVKIAGYDLGQAQEKMLHIFRREVIGFVFQFHFLLNDFTALENVAIPAYMAGVPRA
ESWDRAKKLLDLVGLNNRATHFPSQLSGGERQRVAIARALINQPSVVLADEPTGNLDPAS
ASAVKELLKSLPEIARTTLVLVTHDPELAGIGDSSYLLKGGHFECIR*
>SPBIB_v1_100127|ID:27162278| conserved membrane protein of unknown function [Uncultured spirochete bib]
MAANEHGTFEPLQTRKRAGFMALFRIAIRWTITGRKTSHRTSFLFSVIGIAAGIVALIVV
MSVMNGFQRQYIDSLLETSSFHVRIMPESSDPSILAQSLRENSLVRSVTPFIETNLLAEG
AEGRQNVVRAMWISPQDLGADKGFCNALHISERAAEDGIRQGIMIGTETARLLGMYNGQN
LVLRGAVVNPDEGVQQYEIALPVGGLFKSGYYEIDAGLVILPMASASKLKTLPASLVLGI
KLKNPEKVDQFIASLAHTPGIGRTESWRDYNRSFFSALRTEKVVMFILVSIIFAVVAINI
HYAMRRNIARKSRELAILAAIGTDRRSIGSIFMLEGLMAGILGALGGIGIGIPLAKNIDH
VINFAVGIAEAVVSFLYRVGIAKSVPDLSLFSPTIFYIESIPSRVYASDIGFIAFFAILF
PALAVYAAYRRFRNASPLEVLRSE*
>SPBIB_v1_100128|ID:27162279|ftsY| Signal recognition particle receptor FtsY [Uncultured spirochete bib]
MKFAEKIKALFRRNKLSEETFEDLADLLIEGDMGAALAFEIVDSLKAACKKEGISDPDAA
KAKLKELLRPYIKTATFELDPGKLNIVLILGVNGVGKTTSCAKLAQWAGRQYPGNNVILA
AGDTFRAAAIDQLKIHGERLGIRVVAQQHGSDPGAVLWDAIDAAAAAGAQLVIADTAGRM
HTRSDLVKELAKLDRVVSQRADQANYRKLLVLDATTGQNAMRQAETFHEAVKVDGVIMTK
YDSTSKGGMIIAVSRQFGLPTLFLGTGEKYENLQPFNIDAYLEDFVGE*
>SPBIB_v1_100129|ID:27162280| membrane protein of unknown function [Uncultured spirochete bib]
MKTFKGSALLAALVFLFLTSNVFCQTVPANIALAFPVRIEMSALNHLIAENSSEWDIWAS
VRQGLASNQTLSNGSPLPEAGSFVRLLKESFQTRIETRSIAVLDLDAMKVLGEKSNSTLL
EAGELKGAQFFVCTVYAVEGSAFRFGMALLEKSGKGSTYFSDPVDVFSISKAVQAATESM
ILRGTFSAKTVIAPSMANASANLPKTEPWILKSRYEKANNTFKFAIGGVSISLSAAFLSA
GLWQTYQEAAIRNSAFNSAVTISGIAAGASIAATAVFLTAAIWNAVTILQTTR*
>SPBIB_v1_100130|ID:27162281|prfB| Peptide chain release factor 2 [Uncultured spirochete bib]
VLAEIKTLKERLESWKKLHDDILGLEEMYELVRDEGHQDMEGELLANLNDIRNRFDKALI
LELLSGEADRNDAFLTIHAGSGGTEACDWASMLYRMYTRWAERHDFKVETLDFLEAEGGI
KSVTIQISGEYAYGYLKGETGVHRLVRISPFDANARRHTSFASVSVMPVLDDSIEIDIRP
EDIRIDTYRAGGAGGQKVNKTDSAVRITHLATGIVVTCQNERSQYKNKDVAMSILKSRLY
EYYKAERDKETAKFAGEKKEIAWGSQIRSYVFQPYTMVKDHRNKFSVGNVQAVMDGDIDP

FLDAYLHWQWKGGTIVEGEDEDI*
>SPBIB_v1_100131|ID:27162282|mro| Aldose 1-epimerase [Uncultured spirochete bib]
MGIEKKHFGTLASGEDVSLYVLKAGDFLAAFTDYGATWVSFIMPDAHGSFDDILLGFSTL
SGYAGKHPYFGSTVGRFANRIGGARFELDGKEYKLWANNNRNHLHGGRRGFDKYLWNGEL
SSISGDPALMLTRESPDGEEGYPGTLAVELTVSLSPSGSLRLRYQAKTDKKTLVSLTNHA
YFNLAGEGNGTILDHELLMHSSMYLPVDSELIPTGAIASVDGTPLDFRTMKPIGKEIRNA
NDGFDFCFIIDKEYSPLKPFAEIREPKSGRIMTAATTLPAVQFYTGNNLNGIAGKRGSVY
DRHSAFCLETEMYPDSPHHPNFPSAFLLPGEKWEHETIYNFSVMK*
>SPBIB_v1_100132|ID:27162283| Short chain dehydrogenase/reductase family oxidoreductase [Uncultured spirochete bib]
MEKSDDRPVALVTGATSGLGLAIAQSFIDAGFIVFGAGRRQNPENLPAGLIYVRTDVTSN
DSVEECRNYILSNAGRIDVLVCGAGSGISGSIEDTPISEAQAQLDVNFFGVVRTVQAFLP
AMRQQKSGKIIVIGSIAGRIGMPFQAFYSASKFALEGFVESLRHEIRAFNIQACIVEPGD
FRTGFTSARRKLVRPGSAYTSQFEKAIGVQEHDELHGALPALAGKRIVQLASARSLPVRV
TIGPLFERFAVWVRRILPDSWFEAFYKIYYKL*
>SPBIB_v1_100133|ID:27162284|cmk| Cytidylate kinase [Uncultured spirochete bib]
MGIIAISGKSGCGNTTVSRMVADRLGYTPVNYTFRTMAAEMGMPLEELFRKAQESLEYDR
KLDEHQTMLARKGNTVIGSRLAMWMVPEADLKVYLKASPSVRVSRIFAREGGDRKEIERF
TLERDARDRQRYLALYNIDIDDVSVAHLVIDTERWSASQVADIIIDAYNISLYGEQHGKI
R*
>SPBIB_v1_100134|ID:27162285| conserved protein of unknown function [Uncultured spirochete bib]
MKSMTGFAHRTISAKGFLGSITLKSYNNRFFDLSLSIPASASGIEPNIREFLGSRISRGK
IECTLRLKKTEATLDDITMNFQSAKTLTEQLRNLALACGLNDTPSLDVLTRFPGVIELNT
DIDESELWQILFPTMEETWREFEESRIREGLATQQNITNQMNRFTSKLDEIEQNAGQLEQ
LVHDQITARFKELLGDNYDESRVLQELAVQLVRLTINEEIQRLKAHISAFKAIAAEPSCG
KKLDFLCQEMNREVNTIGSKNMLVAVSLAVVEMKDALENIREQLRNVE*
>SPBIB_v1_100135|ID:27162286|murC| UDP-N-acetylmuramate--L-alanine ligase [Uncultured spirochete bib]
MELKQFPSNLYGLQIHLVGAKGTGMTALAEILHSRGALLTGSDVPDVFYTDSILRALNVK
LFENFDAKHIPDACDLVIYSAAYSRDANPELKAAAAHNIPIYSYPEVLGALSLHSTSAGI
AGVHGKTTTTALTGILMEALSMPATVLAGSAISNFNNRCTLIQGDTYFIAETCEYRKHFL
NFKPSWIVLTSVESDHQDYFPTYESIRDAFVEYVLSLPSGGTLIFCADEKGATEVADIAL
QQRKDINFVEYGFSAQGKWKIRSLRTLAGTNTFITGNYPVDISLHVPGQHLVLDAVAALA
LADSIFKAETSRDIEPHEWKMMANALSSFRGSKRRSEVVGEYGGILIIDDYGHHPTAIAA
TIEGIKKFWPHRRLVVDFMSHTYTRTIALQDAFVAALDQADAVVMHKIYASAREQPIQDF
DGQKLFQKLCERRKDLDLLVPDDSHLAATSIGHGESTLRARTGFALYTEEPLDALPMLEK
KLKPGDIFLTMGAGDNWKLGRALAELLENKKAIKHGPERKHTE*
>SPBIB_v1_100136|ID:27162287| putative 4-alpha-glucanotransferase [Uncultured spirochete bib]
MQSFRVVLGMYNSLPYNASDTLYEQAYQGAWRPFLSGLYKFPSIKAVIHFPGNIFAWIEQ
NHPEYLYLLIEMVRKGQIELLGGGFYSPLAPLVSTQDMTGQVEALSAFIRKTIGKRPSGA
WLYEYSWTSSLPAILQNSKIQYTFLPAAQFKEIAPENLSCQPVASEDHRKMVLIYPAFES
HSDDSAFVPYEATLLKLQQDNPQISAFVIMADGNAISSTWERSGLESPDVLFERSFAWFQ
KNCLEYDTITASQLYKSSKAARTIYFAQCYSRRFRDYCERIIQKADIPRFECVQISKQSV
VDHPLVHLLYQKLNFVSAMTNLFRGDKSRKKASLDDIWRAQSGELFWVSPTGGILRSEAR
LGAYASLIEAEKTIRQNRFHHFMAYDDLDFDGIKEVLYQSSVYNCYLKSDSASVAELDSL
RTGRNYACGWNAEHFSTGCFKDEIRAAGTFETRLFPDYEAWNLVENAKESLNATFYREFH
GKIDNLGAFFGCRKSYQFENDYFSINYELTNKGSNPFPIRFCTISEIIALPSLKDHKVQT
LAHRQSRQLPADQSFAADAIDGVEIGDVLGAEKMIIRSDNPFSLDGVPNFAESPKDGRFI
TEKEAQDARIFEGFTLTIGWDFSLPPEGTVLFSVSVHIEH*
>SPBIB_v1_100137|ID:27162288| Acid phosphatase/vanadium-dependent haloperoxidase related protein (modular protein) [Uncultured spirochete bib]
MHFRLDMRGLFPHTFSVEASSHNLPSLLANPIFVSGALSIFLAQVLKAILAFLKRHKLKA
KEIAFIMLWKTGGMPSSHSALVVSLSAAIAFVEGFDSLFVLSFFLGLIVIRDALGVRRSA
GLQSKALNILGKQMSERFNIAFTPVKEIHGHRWQEVVVGAVLGMAVSILVCWNYIFHAVS
SSLQ*

>SPBIB_v1_100138|ID:27162289| Pseudouridine synthase, RluA family [Uncultured spirochete bib]
MLKAPETKIGFARRDGRLCDDASTLKVCGKSPRMSSLKCIADCLRIDLKSLWHYVVNVHE
VKKFSLGADDDGRRADRIVRKILKNIPLSLIYRLFREGKIKKNGLTAEASERVHSGDFME
IIGLDDTAFKKNPPRNKEFSARSIDEQPSVIWQNEHLIAMHKPRGMLTHDGEASLDAFVY
SLLKDNLSPSVSFLPGPLHRLDRNTSGIIIFSKTRLGAEKFSSAIRRREVQKLYLALVEG
TMDGPLRLQDTIERNRKAHVSVLNQDGKGGRLAVMDAIPLFARERYTLVLVDLHTGITHQ
IRAQLATHGFPLAGDTKYGGSPLPLQTGYFLHAYSLRMEHPVFEDMPNSIIAPLPELFGK
TVASIFELDYHDLYKTMQRLTEDI*
>SPBIB_v1_100139|ID:27162290|hflK| Protein HflK [Uncultured spirochete bib]
MANSTIFKKPPNITIRWPLVLIVLIAIGVIIFLSTSFIIVDQTEKAVITTFGKYTKTLGA
GLHYKLPFGIQKAYMVKTQVIQTETFGFRTIKPGIVTQYSDKKYPEESTMLTGDLNIVDV
EWIIQYRIANPEAWLFAVTDREKTIRDTSQTVLNMLIGDRAILGVIGPDRQTIQDSAVVM
MNELFTKYGLGIVVTQVQLQNIVPPEGVQAAFEDVNKAIQDMNRFINEGREAYNAEIPKV
QGQADQILEVAQGYAAERVNVAKGDVARFNAVYDEYRKAPDITRKRLYYEMMEEIFKDQK
NIDLIDKSLQNILPIKNLTQTTQPQEATK*
>SPBIB_v1_100140|ID:27162291|hflC| Protein HflC [Uncultured spirochete bib]
MKKIPIIIAIVAVLAVLFIVIFGPLFVLNEGEQAVVVRFGKIVAVHTEAGLKWRSPFVDT
VMKYPKKLMSWDGEPQRIPTKENQFIYVDTTARWKIVDPVKFYETVGSIDVAYGKLDDVI
DSAVRTIISSNYLREAVRSSDEILNATKIETFQTGEVENSSTLQQLAITPTQYEKIEKGR
RLLADEMRRLVSNIVPTFGIEVVDILPRQIKYSDELTESVYQRMIKERNQIAQAFRAFGE
GKKAEWLGKLENEKRSLLSSAYAKGEIIKGKADAEASKIYADSFSKDPSFFEFWKAIESY
RKTMPAFSKTLSTGMDYFKYLYSPTGR*
>SPBIB_v1_100141|ID:27162292| putative Metallophosphoesterase [Uncultured spirochete bib]
MSTLAIAIADIHGEIEVLEKILEEHPNAQYILIAGDLTNFGREKEAKKVLAALERRFPQS
NLYFVAGNCDTVSARNVFSRHPGYIEGKCAQILEGRAIASNASNPLYVIGCGGGLVHTGL
TPFERKDEEMASSLQAGFLQCNFSNPSAEQKEGGPKPLIVLTHTPPNGTYADMRHMRHVG
SSSFISFLYEYKPLLWICGHIHEGRSIQAEDETLVINPGPAAHGSYALISFEQREGSYHA
VAELRGI*
>SPBIB_v1_100142|ID:27162293| conserved protein of unknown function [Uncultured spirochete bib]
MSKKAWQIDRALRLTAIPLDRRVHTSNWYTSETIFPTGDDLIQLFWDSKVPGSGAPEIPY
VEMVQAMGNKGYDVSAAEELLPEGIELARKGERADLRVLTARLLHAIHTAPVDPAHTYWT
YRHPERWDDVRAAMPAHADDKPRTIPRDIEEKILEGWTGQLAGGAFGTAIEGYHTDQIEK
VYGRIDSYVTEPETVNDDVVYELVFLDVFEQRGRALSSRDIGLEWVRQIPFGWSAEWIAL
HNMADGILPPESGSWHNPYSNWIGAQMRGMVCGLVAPSWPMEAARLAHIDGLVSHAENGV
YGEMYAAALTSLAFAMDNPRELVEQAAGFVPAKSEYAAVLEKCLRIVKEHKSADAAWRVL
DDHFQKYNWIHAYPNIAAVIVALWYGQGDFTESFRILAHAGLDVDCNAGLVGTVLGVMRG
VPSKWADPLGGILETYIAGKEKLSIRELAARTARLAQRRA*
>SPBIB_v1_100143|ID:27162294| putative ABC transporter permease protein [Uncultured spirochete bib]
MNVLIGLITGMISGATPILLAALGGTFTFYAGVFNIAMEGMMLMAAFFAVLGSFFFHSWV
IGLAFGVLGSLTLALIFILFSVKLETDEFVTGIALNLFALGITTYFLRQIFKVKGVFTSK
SIVPVPQLHIPFIEKIPFVGPVLSGRNLIVYIAVIATVFCYYLIFKTRFGLRLRAAGYNA
DSLDSSGVRTSRIRMQALLLCALFCGLGGAFLSLGYVTLFAENMTAGRGWISLAAIILVD
GHPYGIAAISLLFGFFDGLGLFLQNYGVASQFTAMIPYIATLFALYVVYAQRRREKKRKRV
*
>SPBIB_v1_100144|ID:27162295| putative ABC transporter permease protein [Uncultured spirochete bib]
MKRRLWSGLVGVVVALAVGALIMLAQGYKPLSTYVALFQFSLGGIGPFTTTLKNSVPLVL
TGLSAAVAFASGPVNLGQPGQFVFGALAATLGGLYIRLPAVLEIPLLLLLAIAGGALWSS
IAAFLRRAFEMSEFIVTLMLNMIADFFTAWVIAFPLMDRKAFSPMTPQIAKSGWMPDLGL
LSSSTLVMLGAVVLVWFVFHRWRAGYEWRMTGQNSVFARLGGCDIHRNFLAVMLVTGALA
GLAGGLVVMAGPHRFIKGLGANYAWDGIMIAIMANNSIVGTLVYGVFIAAIQTGALGMEL
ITNVPSEIAQVLQAVLVLIIVATREYTNVLLDRLSARRQAKERAA*
>SPBIB_v1_100145|ID:27162296|tsgD| Glucose import ATP-binding protein TsgD13 [Uncultured spirochete bib]
MNDLIALHNIVKVYPPNVLAVDDVSVSFCKGEIHSIVGENGAGKSTLMKLLYGLVPFDSG
RIEFGGVNVRFRKPGDAIAMGIGMVHQEIVLIPQYSVWENVVLGAEPVRTLGRIDSRTAI

DAVRSKIEEFQFNLDPLAIVDDISVAARQKVEILKLLFRNVSVLILDEPTAVLTPQEIPQ
LFAELKRLRDRGHTILFISHRLDEVLELSDRITIMRKGRKVATVEASETNKESLATMMVG
RQVIFTSKRTPHQRGELVFEAQHVDWERGDGLKLLDDVSFSVHAGEIVGIAGVEGNGQFE
LVNSIMGLEQPTRGSFLVKGKDITSLSILERRKLISFVPQDRGKMGASLSASILDNAIMT
HHRLEKRFSRWHGLVLDYRKARDFVDMLKERFSVQMASRQSDFRSLSGGNQQKVILAREL
LLATPFFLMDQPTRGLDVGSIEYVHGEILAMREADRAVLLISADLEELFLVADRILVMHR
GCIVADLMTEKTTIEEVGAYMLGGRASA*
>SPBIB_v1_100146|ID:27162297| putative basic membrane lipoprotein [Uncultured spirochete bib]
MKGRKYVVLLGALFLLGAALTAYSQTPKINTNRVVYLINGALGDNAFYDSGEAGIKNIGR
TYKVETRTIECNFDAGKFQPALDAAVQYADIIFVISYGFEDQLKAIADKYPNKIFVNIDT
VVENPKKTITSVFFLAEQSSYLAGMTAALLTTTTSLKGVNKDKIIGVIGGDTDPIVSAFV
FGFQNGARAVDPGVKVLTKSLGGAWDDSAKGKQAALQLYDQKADVIYQVAAAAGIGVLQA
AAERGLYAIGVDTNQNDLEPGHVVASAVKNVGATIEKVFKTIKDGTYKPGQVINSGLADG
GVDLDFTAKQQVLPKSIIDKVMQARQQIIDGKLTVKLYNGENVWQ*
>SPBIB_v1_100147|ID:27162298| protein of unknown function [Uncultured spirochete bib]
VGSAFQNTSQMRAILKGYRKIRGKGNPGWAIRNASSSGIIGRSMLTARE*
>SPBIB_v1_100148|ID:27162299|deoD| Purine nucleoside phosphorylase DeoD-type [Uncultured spirochete bib]
MHEKEIHEKNLANYRNGTPHNAAKAGEIAPVVLMPGDPLRAKRIAETMLSDVHQFNAIRN
MFGYTGMYQGKRISVMGSGMGAPSMGIYSYELYSFYEVQAIIRIGTCGGLAPDVEVGDLV
IAMSASTDSNYAHQYELNGTFSPCCSYELLEPAVQLARARNMRFHVGNVFSSDVFSLYSA
LPPERGWQKWGRMGCKATDMECYALYCNAAWTNKQALTLLTCSDSNITHKEMTPEARQNS
LDAMIQIALDIA*
>SPBIB_v1_100149|ID:27162300|tyrS| Tyrosine--tRNA ligase [Uncultured spirochete bib]
MNRALEVLQERGFVQQCTDFEGLSRLMDEGPVTFYIGVDPTGPSLHIGHMVPFFALRHLR
DAGHVGIALLGGGTARIGDPSGKTEMRKLISYEEIDENTRRFIKQLDRFVGFDGVHAFTA
NNKDWLADLNYIEFLREIGRHFSVNRMLSFEAYKQRMEAGLSFIEFNYQLLQSYDFLQLY
RRHNCRLQIGGDDQWGNIVAGIELIRRVEGAECYGLTFPLVTTSDGKKMGKTEKGALFLD
PNMTSPYDFFQYFRDVSDSDVERFLLMFTFLPSEECRRLGSLKDAELNQAKERLAWEVTA
LIHGKEEADKALAAARAAFRGDGDAAQLPTIAIKRARLEAGIGVLDLFVEAGLVPSKNEA
RRLVQQGGASVNGQKVENEKQVFTIAALQDGAMMLRAGKKRVARVISKDN*
>SPBIB_v1_100150|ID:27162301| DNA-binding protein HRL18 [Uncultured spirochete bib]
MPSTGKPLTKVEIINALAKKTGFTKKDVGLVLDELAKLSYSEVKKSKKFTLPGIAIMKVV
KRKAKVGRNPATGQQITIPARDVIKISVAKPCKDAVLGK*
>SPBIB_v1_100151|ID:27162302|glyQS| Glycine--tRNA ligase [Uncultured spirochete bib]
MADTTVTMDKIVSLAKRRGFVFQSSEIYGGLSGAWDYGPQGIELKNRIARFWWKEMTQLH
DNIVGIDAAILMHPRVWEASGHVENFTDPLVDCKKCKTRFRADQIPPENLAARKCPECGG
ELTETRKFNLMFKTTLGPVEDESGLVYLRPETAQGIYVNFKNVQQSNRMKIPFGIAQIGK
AFRNEIVTKNFIFRTCEFEQMEMQFFVKPGTDEEWFEYWRDQRWNYYRKLGIDMNKLRWH
RHGPDELAHYAKDAYDIEFEFPMGFKELEGVHNRSNYDLSRHQQYSGKDLQYIDQDNGNE
RYIPFIIETSAGLTRQLLMLLCDAYEEQKVADKGNEDDWRTVLHFHPDIAPITAAVLPLM
KKDGLAELAQDIRAELKEDFVTDYDQAGAIGRRYRRQDEIGTPFCVTIDYQSKEDGTVTV
RDRDTMLQERVSRSHLSEYIRAKIKSYKRAERIS*
>SPBIB_v1_100152|ID:27162303|glnS| glutamyl-tRNA synthetase [Uncultured spirochete bib]
MPEVNEKTEYMDFIREAVAEDLRTGRFKEVHTRFPPEPNGWLHIGHAKALFVDFGVAQDF
GGKCNLRMDDTNPEKEDMEYVEAIQRDIKWLGFDWEDRLFFASDYYETLYELACKLIRKG
LAYVDDLDEEQIKEYRGTNVPDKNNITYTPPGKNSPWRDRSIEENLDLFARMRAGEFPDG
SKTLRAKIDMAHPNLLMRDPVMYRIRRMPHYRTGNTWCIYPMYDFAHPLSDAIEGITHSL
CSLEYEIHRPLYDWFVSNCEVFPSRQIEFARLNITHTVLSKRWLLQLVREKKVSGWDDPR
MPTIAGMRRRGYSAAGIRDFLGRIGIAKTDSMVDIQLLEHCIREDLNASAQRYMAVLNPV
KLVIENWPQGRVEWLDAVNNPENPQAGMRKIPFSGTLYIERDDFRENPPPKYYRLYPGNQ
VRLRYGYIITCTGFDKDPATGEVTEIRCTYDPATKGGDAPDNRKVKGTIHWVSADHAVPF
EARLYDYLFEAERPMEVSEGKTFIDNLSDHSLTVEKRAVAEPALRELAVGQTVQFERLGY
FCKDPDSTNERAVFNRTVTLKDSWTKLEKKLLANG*
>SPBIB_v1_100153|ID:27162304|gltX| Glutamate--tRNA ligase [Uncultured spirochete bib]

MSVRVRYAPSPTGNQHIGGVRTALINYLFAKSQGGSFILRLEDTDRTRYSEEYVQNLYDT
FKWLGFYWDEGPDIGGPVGPYVQSERLDLYKKYAEDLVRMGKAYYCFCDSERLEKIRQEQ
IAAKKDEIGYDRHCRYLPKEELERNIAEGKPYVVRLKIPLEGVTVFHDRLLGTIEWKNED
ISPDPVLLKSDGFPTYHLANVIDDHLMGITHVMRAQEWIPSAPLHKIMYDAFGWEMPELC
HLPMVLGQDGHKLSKRHGATAVNEFRKAGYLPQALINYIALLGCSYEDGRDIYSLDELVS
LFKIERLNKSPAVFDYQKLEWFNGQYIRQTSDSDLAALVRPYLVDAGLRKAQDPDMDRLE
LAAMPLVKERLKFLSDAPVIMSYLYKRLELPSVETFLPKKADAKETAGFLEESKKLLSEY
GLDDIPAVEEKFRSRAAEIGRKLGDLLMPLRVAITYTRVSPPLFESMKILGLKECIERID
EAIRYLRGY*
>SPBIB_v1_100154|ID:27162305| membrane protein of unknown function [Uncultured spirochete bib]
MKGESGKTEKSLFTKIQKFAEILIFAVWILAIVLLILDGSEFFRLSMEILSIALLGVGLF
NVCMMVGFSAAKRSHFQFGLFFIRIAYILLSAASLAISLFVAGIRIR*
>SPBIB_v1_100155|ID:27162306| Histidine triad (HIT) protein [Uncultured spirochete bib]
MEYLFNFNKIAYLKGKRPQGCILCLIRDGSQDVENLVVYQTERFMVSLNLYPYNPGHLLV
FPKRHIVDIRQYTEGEKAELDALVPACLDVLDAFGNPSAYNIGYNMGLSAGASIEHLHLH
IIPRYPREIGIVELIGGSRVLVQDPKDTYAKLKELFNISAGGRN*
>SPBIB_v1_100156|ID:27162307| Thiamine diphosphokinase [Uncultured spirochete bib]
MKALIITGGGCPSSGIIRSLSQGAGLVIAADSGLQPCLSADLDPDYVIGDFDSVETALLA
KIPEERIMQYPEDKDYTDTELAIDLARKKGADRIVLAGGGAGRLDHLLAVRALFERTEPI
HEWHTGAESAFLLPAGEKLKFSSSIGTVVSVFPLSKGAEGMSSEGLKWPLDGLRWDCGNF
GVSNKTIAPNAAISSGRNPILVILPLGIQALISSAS*
>SPBIB_v1_100157|ID:27162308| Polynucleotide adenylyltransferase/metal dependent phosphohydrolase [Uncultured spirochete bib]
MTLRYTPLAGMRIPRIDPELRKIADSLLRAGKKAYLVGGAVRDTFLNRQVTDFDIATNAT
PEECMQIFPRAIPTGIRHGTITVIPRSRRFKVEITTFRTEGSYSDGRRPDSVAFIDDIAM
DLSRRDFTINAMAFDLSNNHFVDPYGGRKDLAAKVIRAVGNPSERFAEDGLRTLRAIRFA
AQLGFAIEPATLKAIEECHEKLSCVSRERIRDEFSKIICAPKPSCGIRLLVDTELILDIL
PEILSCKSVPQPKNHHHLDVFEHLLATVDAVNPDNLSEERLLVLRLAALLHDIGKPCTLN
ISEDDTISFYRHEIESEAIARKTLQDLKYPNEIIDRVCHLVRHHMFNYEPGWTDAAVRRF
VARVGQHAIPDLFALRLADTIATTGEPVSWPLLSEFQTRIDRILEEKQAISLRDLAVNGE
DLASIGIPRNREMGALLSELLEAVLEDQSLNTRAKLLEIAKAKYFNVAR*
>SPBIB_v1_100158|ID:27162309|rnc| Ribonuclease 3 [Uncultured spirochete bib]
VAGGVKRSKSIGSTHTATSTSRLLSLDSFQRRLGISFQNPELLDIALTHSSFSNEIHINE
PNNERLEFLGDSVLGLCVAHILYERFPNRQEGDLARMKSVLVSEASLGGIAQALGLAEYL
LLGKGEEISGGRQKQAILADAMEALLGAYYLDSDFENVKSFIQQLMAPKIEELSVTSGKD
YKSIIQEYAQKQGMNLPKYELVKTEGPEHARRFFVTCTLAGEIFGPCEGLTKKAAEQNAA
QYTFEKLHARGGELARMLDVIAAPLPQSTSLH*
>SPBIB_v1_100159|ID:27162310|acpP| acyl carrier protein (ACP) [Uncultured spirochete bib]
MDELFEKIKKIIAEKLEVDESKITMDASFRQDLGADSLDTYELVYGIEEELGIQIPDEKA
NEFETVKDAYEFIKTQVK*
>SPBIB_v1_100160|ID:27162311|coaD| Phosphopantetheine adenylyltransferase [Uncultured spirochete bib]
MLLLTIAFAYSKVCHMSTAVFAGSFDPPTAGHLDVIEGALGIFDTLYVVVAMNPEKDGMF
TVEERVAMLKAVNTYGDRMKIVTWKGLVTDFAREHNCSVLVRGIRNAAELPYESTMAYMN
RRLDPSIKTVFFLYDAKHVDISSSLVRDLVSHGRLPDGIVPDGVAAVLKDILHARGQPLS
*
>SPBIB_v1_100161|ID:27162312|lnt| Apolipoprotein N-acyltransferase [Uncultured spirochete bib]
VRLAFIVYSNYMREQMNTSNTYLSEIERGPSAGFSQRILWVAAAAVLSALSQPNELFLYG
NWLAGLVCLVPLYFALTNTKKSGEASVLGAVFGALHHALTSYWLFFYKDFAFWTLGTTTI
AYAVVYGAAIMYGWFMLQHSGSMKPIVFALAWAAFEYAKSTGFLGYPWGLLPYSFTAVPI
MLQTADIWGVYGISAFLALSSAACAEMISSLQVRQRSRGNYRWVAVAVGYGLVLLAYGAW
AVAHPWPVRSTVRLLLAQQNTDPWISGENAALESNIELARKALDLNRQSGGRKPDLMVFS
ETSLRRPFTEFRSWFEKNPPTSPLIPFLQQEDIPLLTGLPVVKDWNTYEASNSVGLISPN
GVLLETYAKMHPVPFAEAIPFWEFKWFRSFVQNVIGLESGWVMGDKPVLFKLTLQNRGSV
CTDIHVLPSVQFASPICFEDAFAGLSRDVIIRGADLLINLTNDSWSRTKSAQIQHYAVAR

FRAIESRKTLVRSTNSGVTCVVGADGKNIAELEQFKADSIIVDVPIYAVPMTFYIRFGDW
FALLSASFSALVFIWGIRTEKLKKRIL*
>SPBIB_v1_100162|ID:27162313| conserved protein of unknown function [Uncultured spirochete bib]
LSISNYVSADFFETLEEAGNIVKYREHPAFDAVPFLGSIKKHPYDQEKCLVLLLQPQKRM
PWFKGGEIIELKTRDVQALDELPSAIDLTGTAISVFRIWVKRGAIAVRFEPFEVTDEEYR
PFDTDSISRLLAQRD*
>SPBIB_v1_100163|ID:27162314| Radical SAM domain protein (fragment) [Uncultured spirochete bib]
MSASEIARYWRKMPQKKIQCFLCSHACIIEDGASGHCSARLNRDGEMSLPFYGWISSLAI
DPVEKKPLRRFMPGTWTYSVGFWHCTMNCPFCQNWEIAHPLRIVQEIISPEKLIELAVES
GCPSISFTYSEPTLHIEYVIEAMQRAHARGLKTILVTNGNVLDQPAHEILAHTDATNVDF
KTADAGIYKNILGGDIKVVQNFIHVAYELCHVEVTSLAVPGILDSASQIHSIAQTLAAIS
DEIPLHITHYRPAWKYHKPPLPSEMIQSMAEAARKHLKHVYAYI*
>SPBIB_v1_100164|ID:27162315| protein of unknown function [Uncultured spirochete bib]
LTDFADFGIVSIADCTVGVAQVVECQTVDLVVVGSIPITHPSPSPLSGLGTEPERVRSSA
G*
>SPBIB_v1_100165|ID:27162316| protein of unknown function [Uncultured spirochete bib]
MGNFSNGPLAQLAEQQTLNLRVEGSNPSRLISVKALKMLQALTKRRCSATMRHA*
>SPBIB_v1_100166|ID:27162317|tig| Trigger factor [Uncultured spirochete bib]
MEITEKKIEDLEQSRVRMTLTIPSRDVRAEYDAMMNEYAKNARIDGFRKGHVPVFVLERK
FGDSLKTEAMGRVIEKAVEEGLKDATRQPLTYEPPAVEGEPQFNLDSDFTFSVTYDAYPA
FELPSLEGIEIEVPAVSVSDEDIARELEEIRQRNAIVVEKDGPAETGDIVTADFAEIAED
GTEIPGTARKDFAFELGKQLNIYKFDDEIVGLKAGDEKTFTKTFPADFEYSEYAGKTVTI
KVKVTKVKRQDLPELDDELAQDVSEKYKTLEDLKAAVRNQLNEALEARLRSTKENAIVDE
LLKRTSVSIPRSMLAAELAMRWESLKQQMGVDNDERLEMLLRISGKSRDQLLQDWAPLAE
KALASRILLDKLVEAGSYTATDEDVDAEIAKEAAHTTMSPAEIKAEYEKRGTLEYLKDDI
KVRKLFDAILASAQVKEGTAVSYLDFMKENQ*
>SPBIB_v1_100167|ID:27162318|clpP| proteolytic subunit of ClpA-ClpP and ClpX-ClpP ATP-dependent serine proteases [Uncultured spirochete bib]
MSEHMSSLVPIVIEQTGLGERSYDIYSRLLKDRIVFIDGEINDLTADLVIAQLLFLESQD
PSRDISIYINSPGGMVTAGLALYDTIQYIKPEVQTICIGQASSMAALLLACGTKGKRSAL
PSSRILIHQPWGGVSGQASDISLQAREIIRLKKLTIDYFAQHSGQALEKVAQDMERDFYM
SSQEALEYGIIDTIMEPRKHGKK*
>SPBIB_v1_100168|ID:27162319|clpX| ATPase and specificity subunit of ClpX-ClpP ATP-dependent serine protease [Uncultured spirochete bib]
MAKNKQFDLEQETYCSFCGKSSSYVRKMIAGPGVNICDDCVRICSQILDEEEQKVTAEFL
TEVPKPREIKEFLDQYVVGQEYAKRVLSVAVYNHYKRLLHKVSATAPDVEIEKSNVLLIG
PTGTGKTLLARTLARKLKVPFAMADATTLTEAGYVGEDVENILLKLIQNAGGNIQAAERG
IVYIDEIDKISRKSENPSITRDVSGEGVQQALLKIIEGTIANVPPQGGRKHPNQEYLRID
TTNILFICGGAFVGLDKIIESRVSSHPMGFGADVRAKHEKNLSQLFAELQPDDLIKFGLI
PEFVGRLPIKVALDDLSKEELKRIIIEPRNALLKQYKASMKLDNAELEFEEEAIDAIAEK
AIAQKTGARGLRAIVEKIMIDIMYSLPSDSNVKKVILTRDAVEGNGSCIIEYRQKTA*
>SPBIB_v1_100169|ID:27162320|thiB| Thiamine-binding periplasmic protein [Uncultured spirochete bib]
MMRRKPLIAIFLGVGAIFFASMSLWGQTQPSAGAGAPELVVWTYDSFVSEWGPAAQIAEG
FQKQSGAKVRFVSKGDGGALFSSLLDIAKKPNADIVIGLDDRQLEKALTSDLFAPLKLVN
LADIQKDLLLDPSNRLVPFDFGQFAFIWDSESNIEPPRSLEDLTKPIYRKKVIIMDPRTS
TPGLGFLAWTQAVYKAGWRDYWKRLSPSILAMTPSWDTGYGLFTKGEAPLVLSYSTSPAY
HKAYENTERYKALKFSDGHPQQIEFAGVLSTSQNKLLAQKFVDYLLSQECQSFLPETQWM
FPANRSAKLPASFSVVSKYPSVNAKVQDIDRDPSDAANILISSH*
>SPBIB_v1_100170|ID:27162321| putative ABC-type transporter, integral membrane subunit [Uncultured spirochete bib]
MQSGNGSFEQRLGKTEIILSIALIAILSFSLAPLVAFAKNAIVGAGHASLLASLGSISVP
VIVRSLQFTLLQAFASTALAIGIGLPGAWLAARFRFPGRRALLALAAVPFCFPPILVILA
FILYYGKEGYFSAFLALVMGQKNEYRGLLYSFWGLVLVHAFYNFPIAVHQISALWMRIPE
SQKEAAKTLGANRIMAFRTGTLPWLLPGIAQAAGLIFLYCFFSFTTVLVFGGRTGSTLEV

SIYQALRYQANAGLALLFSVIETGFALLGIWFITGMTQKKQHALRDFGRRRELSSPKGSQ
RVLLAIYGILLLIFFIGPLVSIVVEAFRVPASLGGASHFGFGNFERLLKGFQAPLLKAIR
ATLTLSGSAALLATIAGVIAGISVFIGKKAGKLHFTNRIVEALQWLPLAVSPAVFAYGWL
FLSPDRTISAALIAAQAVIAWPFVSRAVSASLQSLDPRMREAGRTLGASPLRAFLTIDLR
VIAPSVAAAAAFAFSITAGDVNVPLMLGMSEIETLPLLLFRLTAAYRFNEACAAGLVLGL
MTGIVFFLKEKAIDVA*
>SPBIB_v1_100171|ID:27162322| putative Fe(3+)-transporting ATPase [Uncultured spirochete bib]
MSLEVRNLEVVRDGFVLKADFALQDNKVLVLLGPSGCGKTTLLRAIAGLEPIKQGEILLK
GKRIDALPTEKRHIGFVFQDLALFEQMKVRDNIAYSLQIRRENKAHIKERIDYLAMRFRI
DHLLERYPSELSGGERQRVALARALASDPSLILLDEPLSALDAPLRREMRRFMRVQLTQG
YLTAIHVTHDVEEAIDLADEIIVMRNGNMIARGTISDLENSPGSGWLARFMNFGLTLHVD
AVSRKKGANLVMAHCKAGDILCSAHDTAQSAQIESGASYCVYVPFSSLSALDSKKVLARA
PGGILHATIVRCIGILSSTMKLLLSLSGENEQFFEMTLAMGGGILVPKEGSEIELEVDPA
KCRLLPEEPRLPKA*
>SPBIB_v1_100172|ID:27162323|aroK| Shikimate kinase [Uncultured spirochete bib]
MKIALIGMMGSGKTKIGQLLAAHYGIDFLDLDHIIEQRVGLSISEIFAQKGETEFRKIEE
STLAEVVGLGKPLVLACGGGVVLMPSNRELLESECITVWLDVPLRELARRLAGEKDSRPL
LASSNWQETLKEIYDARHMLYKETASIRYVWKENCSIDTSIQRIAQLIDSKKAEFQRTS*
>SPBIB_v1_100173|ID:27162324| Membrane dipeptidase [Uncultured spirochete bib]
MTEQSEDFFVIDAHCDTLMALNGRSMNKKETDPRDFFADNPDAHIDLPKLLRGNVRCQVM
AIFLEDNQLANAPEETMHMIDIFDEVCKKSNGAVCLATNAAEVRRAINEKKLSALLSIEG
AEALGSSLDMLDTYYGRGVRAIGITWNRRNAFGRGLRGEGEGGLTPLGKQLVEKMQVLHM
LIDVAHLSEEGFWEVADIAKGPFIASHANARAVLDHPRNLTDAQIRAIADHGGAIGCVFV
PSFIAKDPNDSNLEAFLLHVDQVVKAGGIESCAIGSDFDGFVPIDASVIRNTSEFPLLYE
GLRKRGYSHGDAAKILGENWLRVFDEVLS*
>SPBIB_v1_100174|ID:27162325|uvrB| excinulease of nucleotide excision repair, DNA damage recognition component [Uncultured spirochete bib]
VKPFKVVAPYGPAGDQGQAIESIVNALQNGERYVTLKGVTGSGKTFTMAKVIEAIQKPAL
VISHNKTLSAQLYREFKGFFPENAVEYFVSYYDYYQPEAYVPSKDLYIEKDSDINEEIDR
MRLAATAALMERRDVVIVATVSCIYGLGSPDLYRDMRLYMDKGSTVDLDEIKRTLVSLQY
ERNDMVLERGRFRIRGDVLEVFPAYLEEAYRIELDFDTVVRIRRLNPVTGEAHDELEEAV
IYPAKHFVMPENMVQRAVERIKAELEERHRFFLEHGKYLEAERIKARTEYDIEMLEEMGY
CTGIENYSAPLSGRKPGERPGVLLDYFPKDFITFIDESHVTLPQIGAMYAGDRSRKLSLV
EYGFRLPCALDNRPLTIAEFESIVPQTVYVSATPGEIEFKRSKTVAEQIIRPTGLVDPEI
QVRPTEGQMEDIYARVRERAQIGERSLIITLTKKMAEELSEYLSGLGLRVRYIHSEIETI
ERVEILTQLRAGEFDVLVGINLLREGIDLPEVSFIAILDADKIGFLRSATSLIQIIGRAA
RNAAGLVVMYADTMSDAMRKAIAETERRRAMQTQYNKEHGITPQTIHKKVQDILERHREE
KIEASQDELALLKRTHNIFVPEQRKAMIREMEKLMFEHAKNLEFEQAALIRDEIAKLKGN
RDV*
>SPBIB_v1_100175|ID:27162326| Peptidase S1 and S6 chymotrypsin/Hap [Uncultured spirochete bib]
MKLYSRTQIIIAVIVSVAITSLSIAGIVLLLNQPSRPKVVSSSSSAAGAINMPAAQQGAA
QNGTASGLTAISPVPASAEEAKSANLTQYLSGYSPEETENISIYEKYNESVVNITTEVLG
INWFLEPVPQSGGSGSGSIIDERGYVLTNNHVVEKAYKLYVSLSDGSRYEAKLVGADPES
DLAVIKFTPEPGKKLKPVPFGSAKNLKVGQKVLAIGNPFGLERTLTTGIVSGLGRPIQKD
ETTILQNMIQTDASINPGNSGGPLFNTKGEMIGINTMIYSPSGGSVGIGFAIPVDTAVRI
VPQLIKDGKVRRGWIDMQAIQLFPSLVDYLKQSGKGAPVESGLLVSTVTQGGNADRAGLK
GGNMAVRYGSTVFYIGGDIVVSVDGKPVTSIAQLYSALEDSSPGQQVVVEFYRGTKKMST
TITLSERKK*
>SPBIB_v1_100176|ID:27162327|yvqK| Cob(I)yrinic acid a,c-diamide adenosyltransferase [Uncultured spirochete bib]
MSIVTKTGDDGSTGLWSGERIGKDSLRVEAYGTIDELSSALGMARHLCFQDNVLYAIEYI
QRLLFRVAGELASLGTSFDRPITEEDEKTIEAKTSEIEARIPLRGFVVPGMTQGSAALDI
ARTIARRAERRVVALSRTDEISPVLLRTLNRLSDFIYMLAREEEAAQGKLTFV*
>SPBIB_v1_100177|ID:27162328| putative Arginyltransferase [Uncultured spirochete bib]
MYNEFKAGIRYASRCKPKTKAYKETSMLSSRQSPIQFQTRSICQYLPDHRPSLSDHMIFA

PSVDPALYGSLISHGWRRYGRELYRMACPGCNICIPLRINAEKIRLSSSLKKILRLNSDL
QIVVKPPSFNAEHFELWKSYSKLKHFSGPGDLTEEVYCGLFEPWSLILEYREMAAQNRLV
AVSHLDPLPDGISSVYFSFAPEAKDRSLGFFSIMAEAYIATSAAHLLNGKLVSIETAAVV
SYYSNRENTRKNSGWYYLGFWVPGSPKMEYKARIAPFEIALPEWHEFGSRLEALEYLRDV
RMPGLGPFLQI*
>SPBIB_v1_100178|ID:27162329| putative PpiC-type peptidyl-prolyl cis-trans isomerase [Uncultured spirochete bib]
MASSENKKQTTNNGKSPIEKKAENKKTESSLKRNFRNPFVYIGTIVILVLTIIAFVLIPG
VSTGTSSGGQAPSFGTWNGKRIEYSSDSYFADQVSQINDYLKQQGMDQSQSQLYAYQVWR
LAFQNTVVRTALLDSAKRAGMKVSEQTIDEEIAKDVQFQVDGKFSLEKYNKTPLATRLSI
RNRLRDDMLIQNYYGDLMAVAPSTAEIEFVSSMAKPQRSIQYVDISYANFPEDKTIEWAK
ANESLFRSIGLSKITITSSQKDAEKVLSQIKANSLSFEDAAKSHSKDSYADKGGDMGTRI
FYDLKNEFQNEKDADAVMALKKGEISPVYKISNNAWAIYRVNSESSAPDFSKQSVLDEVK
SYLFDREKSVMESWALAKADEFAAEAPKSSFAQAAKKTGLEAKTAGPFILNYGKPSFYFY
GQQVGLFQEPYRSLDNDLVGAEENETFLTTLFSTPKGAISKPVVLDASVVVMTVTGDTEG
TEQETSYTKFAYPYFFQTALESQIRNTILSSKKLKDEFNTTFARLFTPAAK*
>SPBIB_v1_100179|ID:27162330|lepA| GTP-binding membrane protein [Uncultured spirochete bib]
MIEPSRIRNFCIIAHIDHGKSTLADRFIEKAHLVDLRKFQDQMLDNMDIERERGITIKSQ
AVSLPYTAKDGQTYMLNLVDTPGHVDFSYEVSRAIAACEGAILVIDATQGVEAQTLSNMY
MALEHNLEIIPAINKIDMPAADVEGVRRQIEKDLGLDGDSALAVSAKLGIGIDELFEAIV
ERIPAPEGKPEDSLQALIFDSHYDPYRGAVVHLRLKQGTIKKGMHIRFMSNGIVHEVEEV
GHFIITATPSEMLIAGEVGYCIAGIKTVKEVHVGDTLTDDEHPCAHPLPGFREVKPVVFS
SIYPIDSADYEELRDSLEKLVLNDASLIFEKDHSLALGFGFRCGFLGLLHLEVVQERLER
EYNQSIIMTAPSVRYKILLKNGTTVYIDNPSEYPDPARIESAEEPYIRAQIITPVDYVGN
VISLCLSKRGTQQSFTHLDEKRVELVFEMPLAEVLFDFYDKLKSTSRGYASFDYDITGYK
PTELAKLDFLINGEPLDALAMLVYKQSAYERARLICERLRNNIPRQQFKVPIQGAIGGQI
IARETVNPVRKDVLAKCYGGDITRKRKLLEKQKEGKKKLKRIGNVELGQDVFLSVLRASE
EEER*
>SPBIB_v1_100180|ID:27162331| Glutaredoxin [Uncultured spirochete bib]
MNLQNLPVVAKDGSRADHEIMVYALSTCGFCKRALAFLDAHQFAYRYLYVDLIPIDTKNA
IKQELKQRFKENVAFPFAVIDDKKYLIGFIEADWKMTLGV*
>SPBIB_v1_100181|ID:27162332| Ferredoxin thioredoxin reductase beta chain [Uncultured spirochete bib]
MANQKTREQTIIFTQMVAKKQGWVLNPDTAFYNSLVDGLTQNYNRYGYYHCPCRDSEGSR
ELDAPAICPCVWSKEDILQYGHCYCALYLSKEFAASGKEPQAIPDRRFKK*
>SPBIB_v1_100182|ID:27162333| Aminotransferase class V [Uncultured spirochete bib]
MTQPVYLDWAATTPADPELMRRALELSLAAYGNPSSSHWAGKQANSILEESRTRLLGALG
MKQGSLIFTASGTEADHIPMLALLKHFNRVASQGKKIHIIISGIEHAAIDCQARMLQKTG
IEISWVAPDTYGHIVPEAVASHIRKETALISVMAVNNETGAIQDIRAVSTLIDDAARTLK
IKKPWFHVDAVQMLSKLPVAEYCMGADSIALSAHKIRGPKGVGALWLGRPLESFVCGGGQ
EKGIRPGTENLFGALAFSLAGEKAQQLLETYVAHAQSLELRLLEGVARIKGAVVVPERKP
KDNGFVPSIVSLSFPGVGGETMVRALSDRGIAISTGSACSSRHKEKGRRILKAMGVPEDI
SFSSIRVSTGILTSEHEIDIFLEQAEDLYRTLKT*
>SPBIB_v1_100183|ID:27162334|thiI| putative tRNA sulfurtransferase [Uncultured spirochete bib]
MRTFFLIKPGEIELKLGNRREFVHRLKEQIQKRLHGIHFDLEEYPGRFFLSVEEKDTDFA
LFVLQHCPGVNGIAQAIKSEKRTERILKAAIEIASKEAARGARRFKVETRRSDKSFPLDS
YGMSAAAGDAVTQALSSLSVDVHNPDFIIAIEIRERAYIYASTSPGPRGLPVGSQGKGVL
LLSGGIDSPVAGYMMARRGLAIESVYFHTYPFTSEEAQKKVEQLARRIAIWTGSMHAWIV
SFTEVQLKIKKGGREEANTLMLRMAMMKAADVIASRIKARAIVTGESLGQVASQTAENLR
LSQSMTSLPVLRPLIGIDKEETIATARRIGTYTISILPYEDCCVLFSPKHPVLKPDFEDL
RAYFSSLELESEILKAVDKAEEKFFSFGDALREYGLDAYAEFWNAGGPRA*
>SPBIB_v1_100184|ID:27162335| RelA/SpoT domain protein [Uncultured spirochete bib]
MSDIENSIPDKAALAEIYANNRANYEEALARAVHDISKLLQRRGIHPTIKSRIKDFESLF
AKKIRLLKTAWDEHRDPLPVNDVLAMRIICPFLRDLDEVENALAEHYEIIEIERKGQDRS
FREFGYESIHVLVHIPPELQPLCKGLERDVIEIQLRTILQEAWAEVEHELVYKAEFTPFD
EPLKRKLAALNATLTLSDIIFQEILDYQKKLNDALKKRREAFYGKIEQASAKVFGIEEEL

SSTIPRPVRLKKTSGKAIPEARSNQDRWASLDIDTLLLSALDAHNASDFDAAISIYTEIL
SRKPEATIAAVVYKHRGMAFFAQSDYPDALSDFTSCIMLDPECYKAFYYRGVVKSIQGEL
QQAIDDFNKALEIHPYHFFSRYRRALCWWQLGDYVQAHADCEIALRIKPENKLAQELQKK
IGEHLARDEI*
>SPBIB_v1_100185|ID:27162336| protein of unknown function [Uncultured spirochete bib]
MSTDSTRRHAERYVIAALILGAVIFILLRGPILQFNGMRLYFPDEKGQNLKLEMRHIAPI
GSLEEKASDVIRELLLGPLTRNLQPLVHSDISLLRVVAGKNSIYLDFSTVSLEEISASYK
LFKEAIEKSLHETIPGNYHVYIYINSILTR*
>SPBIB_v1_100186|ID:27162337| Cell wall hydrolase/autolysin [Uncultured spirochete bib]
MIVARRLRLKGRGLWQTRRALVVLILSVTWFINPAFAAEPAEKDITLSSLAQSLNAMLEY
DPLTRSGTLEKNGKTARFALDTPYVLFDWTALKTLPPPYEDQDSIRVKKEFDIALRQFFD
TKQAPLSKYSVKAIVIDPGHGGKDPGAIGEFENFKIQEKDVNLAIAHRLAELLRIRYPER
TIQLTRTDDTYPSLEERVDMANKIKLAETEAIIYISIHANASFNKNTHGFEVWYLNPSYR
RTVVDEQTAKEKGEDIAPIINTMLEEEFTTESIILAQKVYQRMGKMIGDQSPARGIRAEE
WFVVRNAKMPSILIEVGFVTNKNEATLLSQAGYLRRIADAIYNGVCDFIEHFEY*
>SPBIB_v1_100187|ID:27162338|nrdJ| Vitamin B12-dependent ribonucleotide reductase [Uncultured spirochete bib]
MKIKRLFTKAGRGPYEGIEWEKRRSEIRNPNGTSVFSMDSVIVPSFWSPIASDIIAQKYF
RKAGVPKDKALDWTKFVPSSQTTLSEADVGDDSEHDARQVFHRLAYTWCLWGRQANYFDT
DEDEKAFYDEICYMLAHQIAAPNSPQWFNTGLHAVYGIEGPAQGHYYVDPATNQVVKSES
AYQRPQPHACFILDVQDDLVNEGGIMDLVTREARLFKYGSGTGSNFSRLRGANEKLSGGG
VSSGLLSFLKIADRSAGAIKSGGTTRRAAKMVILDADHPDVSEFVRWKTEEEYKVACLAA
GSAMLNHYADEMVSAIEDMGADDENSYLLDRNILLRKVVKQALDAGVPSSWIHQVLTFYS
QTGKTPRLPLYNTAWESEAYNTVSGQSSNNSIRVSNAFMQAVIDDLDWDLIARTTGSPFR
KVKARELWNEIAEAAWLCADPGLQFHTTVNEWHTCIADGEIRGSNPCSEYMFLDDTACNL
ASLNLISFYDEKTNTFDDAAYRHAIRLWTIVLEISVAMAQFPSKEIARKSYDYRTLGLGY
ANIGSLLMVMGLPYDSDKGRTVAAALSAILTGESYAQSARMAKEFGPFARYNPNKDHMLR
VIRNHRRAAYNAAPQEYESLSVLPKGIEAHLCPENLVKAARQSWDEALELGEKYGFRNAQ
VTAIAPTGTIGLLMDCDTTGIEPDFALVKFKKLAGGGYFKIINTSVPPALRSLGYSEDKI
GDIIAYALGRGTLEGAPGISRESLLEKGIPESALLKAEKALQNSISLEGCFSPYVIGYDV
LEKLGISKEEADVPGFSLLARLGYSENDIEAAELYACGTMGLEGAPHLAEKDLAVFDTAT
PSGKRGTRSISWQAHIAMMAAVQPFITGGISKTINMPNNVSIDDVKGAYMLAWRSMLKSI
ALYRDGSKLSQPLAALAPGSDLIADSIVALQTNQLSESAASQAADNTAGSTIPARLQTAP
EPELPGMPFAPRGIRRALPNRRKGYTQKAKIGGHSIFLRTGEYEDGALGEIFLDMHKEGA
AFRSILNSFAIAVSLGLQYGVPLEEYVDAFTFTRFEPNGMVNGHDYLKMATSVLDFIFRD
LAISYLHRYDLGQVKPEDLISTSTQSILDKGSQAFESGKKQTPAKSENVVSEPRPKKPSA
AAAPAPAKDQTVLLIRAARQKGYEGDPCPVCGNLTLVRNGTCLKCETCGSTTGCS*
>SPBIB_v1_100188|ID:27162339| Arginine deiminase [Uncultured spirochete bib]
MESVRIDSEIGKLRRVIIHNPGSEIEAMTPREAERDLYNDIIPLFAVQREYAKLRDFLHK
VTKTYELADLLAECLANENDKLEFLKEYSHINPIAHIVDQLALLPAQQLAQSLLAGIIAP
KESLAQHLNPHSFIANPLPNAYFMRDSVAIIGNQVISAAFTFDVRMAEAYITRFIFKKHP
AFRNNGLLIDGPAERNRLFTIEGGDIHVLSSDVLAIGISERTTAFAIDRIAQRIARAEAR
PMTIFAIDLPKERATIHLDMVFTMIDRNAALAYKPVMLGAGRAQVYKLDVQANGSVRYSE
EKSLFEGLRGVGIDLEPILCGNGHSVFQEREQWLSGANSFAFGPGKILMYSCNAYTLEAL
NAKGFAILPAQDFLDGKANPDDYARLAVAFDGIELARGGGGARCMTLPVERDSL*
>SPBIB_v1_100189|ID:27162340| conserved protein of unknown function [Uncultured spirochete bib]
MKKRKIIIIGAAGRDFHNFNTRFRNDESVEVIAFTAAQIPNIDGRKYPAELAGPLYPKGI
PIFAESDLPNLIKNYEVDECVFSYSDVPYSHVMKLSAIVNAAGASFTLLGPKDTQIKSSK
PVIAICAVRTGSGKSQTSRKIVQMLMKRGLKVVAIRHPMPYGDLVKQKVQRFATIEDLAR
HNCTIEEMEEYEPHIARGNVIYAGVDYEAILREAEKEADVILWDGGNNDFSFYVPDLMIT
VADPLRAGNEVSFYPGEVSLRTADVVIINKIDSASPEQIQIVRENIARTNPAALVIDAAS
PISVDHPELIRGKRVLCVEDGPTLTHGMMKYGAGIVAARKFQAAQIIDPRPYISGELIKT
FQTYPEIGTLLPAMGYSTTQTRDLEQTIDRTDCDAVIIATPIDLNRVVKIRKPTVRVGYD
LQEIGKPDLNEVLDDFCARHKLV*
>SPBIB_v1_100190|ID:27162341| conserved protein of unknown function [Uncultured spirochete bib]

MAQQQFCSNKEGAMHIEFSFPVKSDIKPRAILSLKDAGDMTYKPKSESPSRLAFFDRFGI
EPTRIAGIELKHSRNVAFVESIQELSHACAANRDGFDGIITTNPWLVPSITVADCMPIWL
FCKECGAFGVLHSGWKGTGILKTAVDGMTNRYRCNPNDISVIFGPAIGSCCYLVDEKRAR
AFKNEFGSQAVDEHMRNGTRVFYLDLLAANLAIAEKIGIGSFFASGSCTSCDGRFGSYRR
EGASSFTRMIALCIFGNAEAENRQRSM*
>SPBIB_v1_100191|ID:27162342| protein of unknown function [Uncultured spirochete bib]
MPFSEQKEPSIITIAHGAIAVFKPAGWHSVFQGVELKSPRALNERSAADFEDMVSWLSSH
ADLLPASELKDAAKAWQARFAGLQAQVQPKAADRFLSELGMLYRLDRETSGIMLFALDRA
TMESMRAAQNAFMLKKRYILMSEASDIELAGSLPRSRSAEREVLLSALYEGNTMPIVSYF
RSYGPRGGLVSCIEPSFRLKAKKKITKDRYQTDLSAAKPMQWPAAGAICIEAEIRKGFRH
QIRAHCAWLGLPIIGDLQYGGGSSKRLWLEAFSVSLMDGRQCVAEWKLHDGPTAILQQ*
>SPBIB_v1_100192|ID:27162343| ABC-type multidrug/protein/lipid transport system, ATPase component [Uncultured spirochete bib]
VAEFFDAEPVTKGYDSQITRRILAYLKPYRLFALIVLITLSVSTAGELLSPTLIRQAVDE
ALVREWYGLDPSAASRFRSKNGSREIVINNRIYVRTSQLAGITEKERTAMEKQGLLDASP
SYIFPLEPDREKKEALALRYSQITISGVWGIVPYSLLTSLSPDDAYLLRSQDSPVLGHYA
LLLLLVLSIVLLSTFAMTWYTNHIGTLIMKDMRLQLYRHVIEQSLAYLSRQPVGRLVTRL
TSDIEVISQFFTDVLSAFIKDATIMLGSLVVLFVLNWKLGLVVFATVPLIFVAAAIARVK
ARDAFRNQRYWTSKVNSFLSEHISGIDIVKLFVQEKRVSEKFGENNRNLLKANIAEMYVY
ATFRPFVDFMSTIASALAIFFGAILFLRLDISIGTLIAFINLISMFYSPIKDLSEKYILL
QSAMASGERVFGLLDSDDRLPEQLPVLADKEAPSVIRGHIELSNVWFAYKNEEWVLKNIS
FTVNPGEKIAIVGYTGAGKSTIANLLARFWDIQKGAILIDGVPIGQYPLKRIRKFIQPVP
QDVFLFQGSIRENIALGLDLSQEQLETAAKAVYAHEFIMALPKGYDTLLSEGGLNLSLGQ
RQLISFARVLAHEPSIIILDEATSSIDTETEKLLQKGIEGLLKDHTSIVIAHRLSTIRDA
DRIIVLGQGHVVETGTHSELIERRGLYWNLYRLQNREME*
>SPBIB_v1_100193|ID:27162344| ABC transporter related protein [Uncultured spirochete bib]
MFKEYATLLPHLKQYRFRYIAGILCLIAVDASQVLIPRYMKTAIDTIVSGSFQLREIVQP
LLIMIALAVLISVGRFFWRYFINVASRRIEAEMRDRLFAHILNMSGGFFRTNTTGDLMAR
ATNDISTIRQATGMGFVALVDGVFMTAMILVAMFANNARVAAWIILPLPLITALILLFGR
IVGKLFKKIQDIYGRLSNIAQESISGIRVVKSFVKEDYFFGRFAQANSEYKNAIMDLVKT
SGFFFPFITFLAGLSTVLLVLFGGNASIRNKMTPGSIIAMLSYLEMLVWPMMSAGFTVNI
VQRGAASLKRINEILNTKPEIPESKNLVNGEPLGDIEIRSLSYAYPGSSRMALENVSVHI
PHASMLGILGKVGSGKSTLLKMLPRMLDPGEGHIFIGGTDICSFKLDTLRAAFGMVPQES
FLFSDSIRANILFAAPGLDSARFEEITRIAGLDRDVSLFPHGWDTIVGERGITLSGGQKQ
RIALARALAANPSILLLDDALSAVDGETEERILSALLEERAGKTTLVVSHRISTLRNADQ
IIVLDGGRITQKGTHEELLEDSEGFYAKIAALQQLEQESCASCGDAGSGEALGEEAAGG*
>SPBIB_v1_100194|ID:27162345|rnhA| Ribonuclease H [Uncultured spirochete bib]
MQEKSIVVYTDGGCKGNPGPGGWAYIMRFGERYREAWGGELQTTNNRMELTAVIRALSFL
KERIDAARASCIRRYIPLPAWISTPIHIFTDSMYVKNGISGWIHDWKKRGWKTAAKKPVM
NRDLWETLDILCAELNPQFEWVEGHAGNPDNEHCDTLVGQAVEELLQHVAPAVYHESEKE
EDLATDAPL*
>SPBIB_v1_100195|ID:27162346| conserved exported protein of unknown function [Uncultured spirochete bib]
MKKITFAGALVVALIVSSCASTPSIPEWVSQKPAPDARYTYFVGSSSAPDSATAANDATA
SLIAGIMQYMGVSVSVSSSAEARASLDDYQAQITQTVKTESKGRLAGFEVVEKYIQKDPK
TGQYTVHVLARYETKELQKEKARIEAIFQEERDAVAIPEKKGDQAATEGRLMDAIRSYAE
AMSAAGGSNLENAKIKLERNAKKASELAASLKLTIVSESSLSVALGSKLPASKVALISNS
GGMKQRVAGAPLLVTYPKKLASGRVGTGTMQVFTDTNGVTAFEVPPIDIAGNYRVAIQLD
FSSISDLLSSLPSWALPYSDAVENELSGIVAYLNYKVISTAKNVPMAVAAYLKDTESARN
ADPAVFLSGLKETLLKEGFSLSDAKLPSGYQTPIDLSVLRAIAPADAQRFALASLDVKSI
TKDGSYFIATAEGSLGVFELASGRTLYSATKSAQGMGLSESEAVSNALKTLGSQAFGKDL
LSALP*
>SPBIB_v1_100196|ID:27162347| conserved protein of unknown function [Uncultured spirochete bib]
MRVGKFLLALMAVVLVISCGTKPPLRKGEVLIEDKGTAYGIKTPKWVELAIIGGYRDIEK
LPDYKDKVVFIAQFEAQNLQSAQLLAERMQADTEIARYLSTRVKDAFKGANVADADSKNF

GAYGERFVASVGEAKFSGYRMEADWWVKVQTYTPENKPDKQIYRVIQLWAIEKGMLQKQF
DILFSQLAGTAQPTPEQKRAIDLVQNTVTKDFFGENK*
>SPBIB_v1_100197|ID:27162348| conserved exported protein of unknown function [Uncultured spirochete bib]
MSDNTNVMKKKKYFALVLAAYAIAALLIGCASKPASPAVSAESQQAKNIVQSTSTPDMPA
TPPAPSPSAQDPSAAAKSVVGSQPMPGQAQAPGAVPGTPQAGTASYEALTPDESHFLQNY
LGRLSYLVYYNEASGLDPQLAKAAVSQANRYLIEKLGLSVIDFDQIQKNKKDQMSAYQAE
TGGSIDIITYIAQKLNADVYVEIDAKTSYSGGPGNWSGSAQGSMKIFDASTAALLGSISF
MSPQTFSPVSADAAMMNAIAGIVWQSMPKVTEQSKALMSASVSRGVRYELVLQNTPDAKA
ISQFEKNLSKKVREIERLSYAPGETRFALYAFMPASRIQEAIYDAAAAAGYPDCYLLYMR
GRSYTFNTGL*
>SPBIB_v1_100198|ID:27162349| conserved membrane protein of unknown function [Uncultured spirochete bib]
MRRQKTSYIIVAGIILFSFHAAPFVSPQNASRPEMQGTYFTETGCNHCDSFLYIQKPRLE
ETYGIHIALETHDILSTKGYELCVEMLEQRGLKFTVFPVLFIGSNVYRGSNAIEENMPKE
IEYFLSHGSYMPTIRQTDQSALAAKNSNAAFAASALPTIFAGLLDGINPCAFSTMLFFLS
FMALKRKDQRSLLWVGLAFIVSVFIAYFLIGLGLLGALRKYLSENRFSIYINIFISAIAA
IFAVLNVRDAIAASRGRAFDSVLQMPTFFKQLNHRFIRYFNQLPLYILGAAISGFLVSFI
ELACTGQIYLPTLAYMNQSMRSSKSILLLLMYNLAFILPLSLVFVLFFFGLRHEKIRHWY
GSHLALVRLLSAVFFVALAVLVWAS*
>SPBIB_v1_100199|ID:27162350| putative Maf-like protein TDE_2348 [Uncultured spirochete bib]
MYGDPILLASESPRRASILESLHIPFITVVPRIDEQIFDHLDPGERVVALAATKARRGKE
RWIEGKKLYSDPLPEPMEKPAEPRFALGADTLVAFRTPEGWHTIGKPIDEQDAYAMLTME
AGNRQYVFSGLCLLDMENDVPHVALSVSEVQFSPMSAADIAWYINLGEWKGAAGAYRIQG
AGSYFIEDIKGSFSGVMGLPIRELYGILRQSGYLSSEEIQG*
>SPBIB_v1_100200|ID:27162351|rpsB| 30S ribosomal protein S2 [Uncultured spirochete bib]
VAVVTMKNLLESGVHFGHQVKRWDPRMKKYIFAERNGIHIIDLQKTIQAIKEAYDAVQKV
VSSGKTVLFVGTKKQAQSAIQKEAERCEQFYVNSRWLGGMLTNFSTIRKSIQRLKKIERM
EVDGTFSSLTKKEIASLLKEKTKLEKNLGGIKEMTSLPGILFVVDTRKEAIAVAEAQRLG
IPIVAIVDTNCNPEGITYPIPGNDDAIRSISLFTQIIANAVIEAGASEGLKIIETLPAEE
EQELAPPAKTEEDEVEIDLETYSNVPIAQEKEPEEPAEVEAPIIDEDRLYEEN*
>SPBIB_v1_100201|ID:27162352|tsf| Elongation factor Ts [Uncultured spirochete bib]
VEIKASDVKALRERTGAGMMDCKKALTESNGNFEAAEKLLREWGMAGVEKRAGRATNEGR
IFVAQNESELSLVELACETDFVARNKDFIDAGTKIAATALGQKASSQNESLESMVKDIAS
LIKENIALKRVAYFNAGMNEHLHSYLHGEGRIGVVVKFRASDPNAFKNEKVASFVHDVAL
HVAAFNPMFLDESKVPASWLAEQKDIFQKQVELDEKMKSKPAKVIEGILAGKVKKLLSEV
CLMNQGFVRDEKVSVADALAQISKETGFSLAIVDYYFAKVGQN*
>SPBIB_v1_100202|ID:27162353|frr| ribosome recycling factor [Uncultured spirochete bib]
MESIKAAYEERMKKTVSALHDEFNAIRTGRASPALLDKVRVDYYGQKTPLSQVATISVPE
ARLIVIQPWDRSLFSEIEKAILKSDLGLNPSNDGKVLRIAIPPLTEQRRKELVKTARNIA
EQSRVAIRNIRRDGLEELKKLQSAGGVAEDAVKKEETELQKLTDAYIAQIGQILETKERE
IMEV*
>SPBIB_v1_100203|ID:27162354|ispU| undecaprenyl pyrophosphate synthase [Uncultured spirochete bib]
MNENRVPSHVAIIMDGNGRWAKARGLPRTEGHQKGLEAAKRIVLAARKEGVKFLSLYTFS
TENWKRTTQEVGFLMSLIRVHLTKELDFYRENEIRIVHSGDRAGLPGEVIIELDKAIEDT
KHHHALTVNLAINYGGRDEIVRSVQRLCRTDSPHIDENAIQQSLDHPEIPDPDLIIRTGG
EMRLSNFLLWQSAYSELYFSPKYWPDFMPEDFHAALADYAHRERRFGAAH*
>SPBIB_v1_100204|ID:27162355| conserved membrane protein of unknown function [Uncultured spirochete bib]
LNANTRSRLLLFFGGIPLFALVLFFLPFGHYALLALLVLAVQYLSSLELRAMLRKSERAD
PGNFVTIVGLVQSLAVYIACVAGARPLEAAAVMFLISILCLMIVLSPIAFSKKESFPLLL
STAGSISLAHFYTGILPGFLMLIVAGFPDAKNAIITFALLTFGNDSLAWLFGKLFGKRRN
VVDVSPNKSVAGFIGGAIGSIAGAFLGLGPLAGSWKPEGSRYLVLSLILGAGMAFFVIAG
DLFESALKRSAGMKDSGVIVPGRGGVLDSFDSLYFSAPFFVAFSYLARLFFL*
>SPBIB_v1_100205|ID:27162356|dxr| 1-deoxy-D-xylulose 5-phosphate reductoisomerase [Uncultured spirochete bib]
MPKRIIILGAAGSIGRQSIEVIRQSAATEEPLVLAGFSVHHNAKVLLSLKNEFPHARAAY
TGDAASAPTGADWAGPEALAQLLSAVQADIVVNGIAGAAGLSASILALQNRMHLALANKE

SIVMGYRLLKQLADDNDLSIIPVDSEHAALFQLTQKIGMHAIAELTITASGGPFRTLPLE
RLAEMTADDACKHPVWKMGRKISIDSATMANKGLELIEASKLFDMPQEHIRVLIHPQSYV
HAMVRTFDGALYAQISEPDMRLPIHAALHWPHTYPSPFGHAELAGKTLEFFEPERNRYPL
VWLARNAMDAGDAACIAYNAANEIAVARFDAARIRFTQIAQVVSETLAHSWNLPVSSFED
IFDIDMKARQISEELVEHIAW*
>SPBIB_v1_100206|ID:27162357| Membrane-associated zinc metalloprotease [Uncultured spirochete bib]
MVTIILGLIGLSLVVIVHEFGHFLIARTVGVDVEAFSVGWGPALLTHKGKKTEWRLGVLP
IGGYCKLKGEDGFRAAIEQKLDFIPADKGSFYAAHPLKRIAIALAGPLFNMIFAVLVFTF
VVAIGTTIRTAPNRIVLSSETGTTMQAGSSNPADMAGLQTGDILQSIDGRTIRDYSDLQE
IIASNPGKALSVQVLRNGSIQNLVLTPRLDANTGAGVIGVYPWIDPLVSSVKEQSPAAIA
DLKPGDKILSANGKPIRNTIDLMNSFKAANGPVELTLERNGQNLSTTIIAKSLEETGIAF
VSITRTDKASSLPDAIAKGAKETMSTFALTIRSIGLLFRGVNIFKAVSGPARITYLIGTA
ASEQIKVEGLAGLVPVLSFLAFLSVGLAIMNLLPLPVLDGGLILVFFIELARRRPLSAKT
LYRYQFIGAAFVLVLFVVATMSDIFFFAAR*
>SPBIB_v1_100207|ID:27162358| protein of unknown function [Uncultured spirochete bib]
LRLQREEVFMHHFHFCPNPACAYHQHAPKERWFVAAGFYVTKTFGKVQRFRCQACXX
>SPBIB_v1_110001|ID:27162359| protein of unknown function [Uncultured spirochete bib]
MFRERAFVSRLSLDLLDQKLWKRAFSTPLKTSAEYLPRYALA*
>SPBIB_v1_110002|ID:27162360|dnaB| Replicative DNA helicase [Uncultured spirochete bib]
MAGPSLKDKLPLYNADAEQACLGALLIDPESMNTVLRYLRPESFYEPSNQETFEALISMH
EKGQKPDLITLSEELRSRGSLDRIGGTAYIASLASFTPSAANVEYYARIVQEMATRRRLI
QLSAEMAAMAHEETMDIDRVLDELQAKIFEISQNRRTIDYHSAKEIVTETMLLIEKLSTN
PEAFTGVPTGITDLDAMTSGLQNSEFVVIGARPSVGKTALALTIAAHASIDLKIPTAFFS
LEMSESAIMLRLISSEARIPAERIRTGRIRTTDYDSLMEAAARIYEAPLYIVDMPNMKLL
ELRTMARRLVLERGVKILFIDYLTLVTHENADLPRWEQISAISRSLKALARELNIPVVAL
SQLKREAEGKQPTLADLRESGSIEQDADLILFLHREREMNKAHDQQSDQVETDLIIAKQR
NGPIGKTSVWFKSSYAKFVNMERHEHK*
>SPBIB_v1_110003|ID:27162361|rplI| 50S ribosomal protein L9 [Uncultured spirochete bib]
MKVILNQDIPNLGEIGDIKEVAAGYARNYLLPKKLVMVYNEKSAAMLQKRQAEILAIKEQ
KRLASRSLKEKLEADTLVISMPAGSNGKLYGAVTNHTIADELLKKGIEVDRKKIEVPGRS
IKSVGNYKVAVKLYEKDEAALRVSIEAQAVASAEEKKAGEGEAKKHRPRRQRTETEPATA
EEQAAAFEAAVNRGQLS*
>SPBIB_v1_110004|ID:27162362| membrane protein of unknown function [Uncultured spirochete bib]
METQNSMDPILVRANRRKIPVWVSAGVLSGLFYASGFIAVAFLVPVQFAFAKDGKKEGLL
SMLASLAVIGIGNAMRLSSLGFMQFSVLVQTFLPPLLLLCGIGALNMLAVDSWKKVIAVS
VILAGVFGFLLKASIGTQEVQQAIASIILQLLGSSGTQLPDAATIARDYVAPAVEVILDC
YGALLFLMLAASWSIGNRLGEKGTGADGTMQKGSIRSIVVPRWLLWPSILAWGLLLLVLY
GHKQGALAIVAWNASLAAASWYALQGIGLISYFFESKGMHRTTGLMLVLLILLILLDRKV
GLAVAILMPVLGVSEVWLQYRIRKGA*
>SPBIB_v1_110005|ID:27162363|rpsR| 30S ribosomal protein S18 [Uncultured spirochete bib]
MDEMKDIEDVKVSRPEADQQDSRRDQDDRNGPRKGKGFFRKKVCRFCTQKVVIDYKDADM
LRRYTTERGKILPRRITGTCSKHQRELAVNIKRARALALLPYVVK*
>SPBIB_v1_110006|ID:27162364|ssb| Single-stranded DNA-binding protein [Uncultured spirochete bib]
VSDINVVVLVGRLTRDSELKYTPSGYAICRFSIAVNRSKKQDEGWVDEPHYFDIEFYGKS
AEGLSKYLLKGRQVAVQGELRQDRWEKDGQQRSKVVVVASTIRPLGSPPQGTDASNRRYA
PDSVPAKPEPSNGQASIPPGVDEFTDDIPF*
>SPBIB_v1_110007|ID:27162365|rpsF| 30S ribosomal protein S6 [Uncultured spirochete bib]
MRQYELVTVLNSEEDQFKAGKQAVADLLAQFKAIDVKEEDMGDRPLAYPVKKKMRAHYVL
YRMSIEPASIVALERAIMLSPAILKHLVVKVED*
>SPBIB_v1_110008|ID:27162366| transposase [Uncultured spirochete bib]
MFVQTLFEKKTGRTLLFYYTARRVKGKIVKTKVKRIGYLDEFLDAYPDPLTHFRQEAKRL
TQEAQLKTLTVTFSMDEHFSFGAGFAATEDAAVEKADRTFHYGVLPLLQLYRELKIDAFL
RIKAQYTKVDFNHNHLFQMLVFGRILFPESKLATWRDRTRILQHSDFSDDAVYRALPFFA
QIKDALVQHLHEQVQRQYHRDTTLLYYDVTNYYWEVDREDELRKRGVSKEHRPEPIVQLG

LCMDNSGLPVTYGLFPGNTNDVATMRPMMQHLAESLGTKHLIYVADKGMMGGMNIAQIIL
EHNGYVISSSVRKADAELRRYILDHEGYTELAGGSFKYKSRLVPCTLYVDTPDGRKKQIR
INERQVVFWSEDYWKKARHDRDMAIAKAMARAGYGENTVLNNHAGNRFIKKEIFDPDTRK
EVDHPEFSFALDQELLDSEEELDGYYLIRSNVVGVREGDAPFNQPYRWHAKDNLFELNRP
VVDLDIIDMYRGLWRIEESFKITKSQLKARPAFVHRQDSIEAHFLSCFVALLLLRLLEKR
TGEKIPVATIVESLRKAQLVQLEDETYVNACCDNVIEAIGQALELDLTKKYYTKGELKAL
RGKTAKSR*
>SPBIB_v1_110009|ID:27162367|hisS| Histidine--tRNA ligase [Uncultured spirochete bib]
MPTITPRVLKGFRDFLPDAEMARASLVKKLEQVFASFGFVPIDTPVLEYAEILLGKGGGE
TDKQVYRFLDHGERDVAMRFDLTVPFARFMAEHVDELYLPFRRYHIAKVWRGENTQRGRY
REFMQCDFDIVGTDSASADADIVLTAAQAMTKLQVGDFSLRVNHRALFNRFLAHIDASDK
SVDILRAVDKLEKIGQQATANALSSLVGSPLASDILEFIRKEDSFEATLDKMHRFCAQKD
QAADMAKDRMLKIMECVSAAGFENRMVLDPSITRGLDYYTGMVLETTLAALPEIGSVCSG
GRYDELASLYTDKKLPGVGASVGLDRLMAALEAIGKPVVSDESTQILIINQDDIQLPEMH
RLANLLRADGFAVEVFPEPKKVVAQYTYAERKHIPLALFLGNASKPYTLRVLGRRENIEI
ADFSAIGDAARKELDGSR*
>SPBIB_v1_110010|ID:27162368|trmB| tRNA (guanine-N(7)-)-methyltransferase [Uncultured spirochete bib]
MARDDAKPLEGDQLHNSSPLEGSQLLDGSVRSFVVRSGRLTEAQRRAIEVWGPTYIIPFT
PVPLDFAKLFDEQRPVILEIGFGMGQATWQIARDRPNYNYLGIEIHAPGVGRLIMDLREH
NIANVRIIQHDALEVLRTAIPPESLAGLHIFYPDPWPKKRHHKRRLMQKAVICLMAEKLA
SGGYLYFVTDIEEYGEFAKNALESCKLLANRYEGFAPRQDWRPVTKFEGHANESGRNAFE
LMFIKS*
>SPBIB_v1_110011|ID:27162369| Helicase-associated domain protein [Uncultured spirochete bib]
MMNPHDLPIYQQRAKILDALEKNQVIVVESPTGSGKTTQLPIILYEAGYAARGIIGVTQP
RRIATLSVSDFIARQLGSAIPGIIGYKMRFEDATASNTVIKIMTDGILLQEMKLDPFLSR
YGVIMVDEAHERSLNIDFILGLLKKVLEARSEFRVIVSSATINAEIFSEYFSECPIVRID
APMFPVRVVYDAIKPVSDLPQPAGEHEAGYYRSSDDALYDKILSIVGRVLDGDAGAEPGD
ILIFLPGEKTIKGCLQQLIASRWAAKLYCIPLYARLGKDEQERVFDDPPPGKIKVVIATN
IAETSVTIDGITVVIDSGLAKINYYNPKTFTASLIETPISKASCNQRKGRAGRTRPGICY
RLYSIKDFESRPLYPTEEIHRTDLSEVLLRMAELGITDFEHFDFITRPPKSAIRGAIEVL
NLLDSLNPDRTLTRIGEMMCAFPLLPKLSRMIVEAILKYPDVIAATLIASAFLSTTSPYI
LPPGEETEARAAHHQFRDPMGDFASYLRLYEAFVAARDKTRFCERNYLDERTLHEIVRIR
EQLELIVSDLGVPIGSGGAIADYICAVSRGLIQFVCARQGRGMFRSLTAERIQIHPGSVM
FRQDADFIVAGEIVRTTRMYAMSVSPLSKAQLYRISPELADQLIRLRQKTSMPPRSRERD
AYRSRNHKESQRGGRKSEISSHDLPQKEKLESLVLGGEVFPIEGKKPSLRTVVLDWNRLK
RIASQIGPADEDLGKGLKAKIEWGKYQLFGNEKFSTVLKALKWMDIEKDLSSGWPKHKNY
SFEADKSESEEDSLEKRRRTLEELADSLGSVLHVTKVGKTGKLGFIGLYSDEAGNFWFQP
TRSFSEALNQSLASAELLVDYAKDLGLEAELKDKIGLLYRRLNDFFSSL*
>SPBIB_v1_110012|ID:27162370|xseA| Exodeoxyribonuclease 7 large subunit [Uncultured spirochete bib]
MHADDWLADSVQSSSPLSVSQLTLLIKNDLENKYPDVTVEGEISNCKVAASGHLYFSLKD
DQAVLQAVMFRRDMLALSFVPRDGMKVWARGGISVYAGRGQYQLIARTMRAAGLGDILAM
LEERKQKFAKEGLFDQDRKKQIPAFPERIAVVTSATGAAIRDIIHVLRRRNPKAHIILLP
AAVQGEEAAASIAARIRQANQWSIADVLIVGRGGGSIEDLLPFSEEIVVRAIAESRIPVI
SAVGHETDWALSDYAADLRAPTPSAAAEMASKDLGLVQKEIEHFETVLAQSMRQIIEHAR
QRLSLVSPRSMESMLVRRHMLLMQRFDTATEAIEQGMRSKVDAFSHRIALAASAIELSNP
KAIMKRGFSIVRKSAEIIRSAEMIKPGEEVHISFYRGEANASILSAQAEDARSKNDEGL*
>SPBIB_v1_110013|ID:27162371|xseB| Exodeoxyribonuclease 7 small subunit [Uncultured spirochete bib]
MKDFEKKIARLEQLAEQMRDTDLPLEKSFEIFEEGITLARSLKKELDELQGKVEVLLNSL
EEDESPRTAAFEDQAQNIQP*
>SPBIB_v1_110014|ID:27162372| CTP pyrophosphohydrolase (fragment) [Uncultured spirochete bib]
MSYRWEFPGGKVEETETDEEALEREFLEEFGARIQVVRYLGETMFAHRGRMRILAAWEIR
MQPKDVAMLNEHSEAAWLPFESITKMDLADSDRSLLPLVQAAFSALQQKM*
>SPBIB_v1_110015|ID:27162373| protein of unknown function [Uncultured spirochete bib]
MRSLVSMSQSLLLDASTRELFNFSADGLMEVASLRLWYALQQEMLSLGALTGKSKDTIAI

AALCGKGDNAGDALAMLRHAKMDGFQRLSVFIPDPATMKENARLNLCRAERCGIDIIRYD
LAPDELEKKLTAQDILLDAVLGTGVKGAAKEPAAALISALASAKKAAVSEKSHFPYLFSI
DVPSGLSDEWEPGFPCVQADATLCIEPMKEALFMHAARPYAGKIIPVGGIFPLWPNEAHP
RVWEIESADLPEFLPPISPWAHKMQRGRVAIFAGSAAGAGAAMHCVRGAAASGAGYIALY
CDEELYSAYLSALGDYAIVRVYSDDTFSAEEWDALVIGPGWGIGEKREGQLARLLQSKTP
IILDADGVRLFAHLAEQHSRNGTLRFFKAPLVLTPHPGEFSALQKFISQEEAAQFQSSGM
VNQVCSLAQKFGIVVALRASTTHIAFPDESCAICDGSTPGLGVAGSGDVLSGLAGGFLAR
WRAARELRAASELNEGDHGMWKDPLSAAIISAVLVHAAAGRSLFDAQGWFNPEELARTCA
RISSWQLDCLKAQ*
>SPBIB_v1_110016|ID:27162374| exported protein of unknown function [Uncultured spirochete bib]
MNRRCTRTLLLLGVLLVLASCKVSSDEAKTHALFDIGYGKTDFTLDLSASEKNNIGLSIN
KGIFHILSRGEAKILSISSYGQTLAMWYDPARGSAPMILKDVKIDAGSSKEDMGRFATQV
SFVDPWPMAADSRQTLYVADRKSNQVSANGTENSAYGAEPDRIVRRFDANGKELGPLGQE
GIAGSPFPEIMRLEITENDTLAVICASETETLVYFYDRNGVFLHLLKLKDNTLPIPAQIL
KKNPEGKGLRIIPSLESIIPAYVAGKTEVILKINYYLENYDVGTGVTLSIDQLGGWILQI
DASAGSILNAFPLQSGMSETGVNLQLVAAKKDSFLTIRWADQYLGGELLKFDSSGKVTGK
LSIRCPDEAAAFVAMTVGDDGYLYLLASLPSALRMYAWKIPGM*
>SPBIB_v1_110017|ID:27162375|secA| Protein translocase subunit SecA [Uncultured spirochete bib]
MSGNILAALLGSKKEKDIKAVLPLLHRINELEPWALSLSDADFPKMTTKFKDRLSKGETL
DDILPEAFALVREAARRRLGERPFDVQLIGGIVLHQGKIVEMKTGEGKTLSSVTAAYLNA
LTGQGVHVITVNDYLAERDSQWMGQIYTFLGLSVGCILSSMDNVARKTSYACDITYGTNN
EFGFDYLRDNMVWSLDQKVQRGHAYCIIDEIDSILIDEARTPLIISGPADDDTYKVNEVN
RLALSLVEAKKNPETGEYPDESKGEVLEGDFKIDEKSKRVMFTSEGMNHIESLLQKRGLI
KGSLFDPANFEFVHYFTQAVRAQRLFNKDVDYVVQDNQVQIVDEFTGRILHGRRYSEGLH
EAIEAKERIKIARRNRTLATITFQNYFRLYKKLAGMTGTADTESEEFNKIYNLDVVVIPT
NRPVVRRDENDLVYLNETEKFDAILNEIKTLHTKGQPILVGTVSIEKSEKLSAMLTKNGI
RHEVLNAKNHAREALIISEAGAKGAVTIATNMAGRGTDIKLGGNPEFRARRKAGTEAPEE
KYREALLHEYDLWARDYEEVKSLGGLCVIGTERHESRRIDNQLRGRSGRQGDPGRSIFFI
SLDDDLMRLFGGENFKQLMSKAGMKPGEPIYHPLLNKSIESAQKKVEERNFEIRKHLLEY
DDVLNKQRNFIYEQRNAILADTDLIERVRTSATEMLEDRSDLLDQSIKENQNKAIADFCS
WLFDTFGIDLSPKQVADLHRSGTIIQEIQKLLDQDLSAKIESAGAESLNFFIRFSYLQEI
DEKWLDHLESMEALREAVYLRSYAQKNPLLEYKLEGSDIFESLILSIRHNIASKVFRVRI
QQKVEQTATIPSRAPRIEVAQTSHQSVGSFAGESEAVRQESAIAAAAAPPAATVIRQGQK
IGRNDPCPCGSGKKYKHCHGR*
>SPBIB_v1_110018|ID:27162376| putative Fibronectin-binding A domain protein [Uncultured spirochete bib]
MSLNCEEIELILSEEPLEGMKIQNIFQPSYDAIVLELYGRGNRLFYLISIAHNACRIHPL
TAPPAKNERPLRFMECLRSRIKGGTIIHAEQISKDRIIKIEITKADEKGSPAKLFLYARL
WSGAGNVLLVSEEGIIIDALRRLPSRQEISGAAFALPAPRDAASQSAKPYTIRDLQGPGP
FWQRIEHFYSEHTGQLSRDALIAQVREIFSRKYSSLENRLSALSAKQNEYQQAERFRQIG
DILIGGYAIEMQGTKTFARALDFYENREILIEIEPSATPAQNAARYYEKYHKAKTGIDDI
AAELERCRTSMSQLKTWLSKLEAEPDPIVIAQALRKGGAARTKEKMRFPCQHIEYKGWTI
LIGKSGKENDEILRHIARGSDLWLHARDYSGSYVFIRAIKNKSFPPEVIQTAARLAIYYS
KARKNLGGDVHTTYVKNLRRAKDGPVGLVIPYLEKNIYISFTEAQIKDILDESLSGEEIE
Q*
>SPBIB_v1_110019|ID:27162377| conserved exported protein of unknown function [Uncultured spirochete bib]
MKRAGSVFCVLLVLAFACTAPAIAQQKSKVVVSVLKGPSGLSSAWMMSELLKTSPDEFGF
ITVASADMVVAKLLNGEIDAGVLPVNIAAKLFNAGAPIRALAVVGNGMVKFLTTDPDLAS
LADVKGKTIYIAGQKATPDFLFQYLCAAYGLNAGSDYTPVYNLAYPEIAAGLAAGKIRYA
VLPEPFASQAILKNASIRAAVDLKHEWQLRTNQEDYPMSLFVARKELVESNPEKARILLD
SYRASLRKAKEDPSGTGKLAESLDLGVSAQVAASAIPLSNFVFIEAPAARHSIETLLSVF
LQFEPASVGGKLPDSAFYAIIR*
>SPBIB_v1_110020|ID:27162378| Binding-protein-dependent transport systems inner membrane component [Uncultured spirochete bib]
VENCLIVLSMQSSARPSWTPLAWRLLGIACFLVAWQAASSIVKSELILPGPPIVLKALAN

LSTTAKFWSAVSGSLARVLESFIISIAIGLLSGFISGISQPAKAFLSPFITGVRATPVLA
LILLAMFWFPSSQVPVFSAVLMAFPVMHTSAEAGIRSVDQNLLQMSRLFHVPKRVMLWRL
RVPSALPYILSGAKNALGLCWKVVVAGEVLSQPRMALGSAMQESRLMLETADVFAWALVT
ILLCGLSEYLFGLLSARANLVYLSVAPQVSRQSSI*
>SPBIB_v1_110021|ID:27162379| ABC transporter related protein [Uncultured spirochete bib]
MSMAIQNLRKRFGDLLVLDSVSFEMQASSITAILGPSGCGKTTLLNILSGILAADAGAMA
GLEDKRFSYCFQEPRLLAWLIAEDNMRYAMASLQDTNTIEQRISRFLEEAGLTEFRHFRP
HQLSGGMQKRLSLARAFAFPSDILLLDEAFSAVDLKQKIELMQAFLKLWKDERPTAVIVT
HDIHDALFLADQVVVLSQRPARVRGILQINTPHEARLFGSNELAAYEQQLYSLLGFSTLD
QDANMPAN*
>SPBIB_v1_110022|ID:27162380| putative Multi-sensor hybrid histidine kinase [Uncultured spirochete bib]
MPKIAVLEADAVIALDIAKSIEQANLASVDVFESFGALEPELTEQHFNLLVIDIDDNREE
DIQTATRVYRDFGIHCIVISDHIASASLVKLREAEPLGMLVKPFSSRELIANVETGLYRV
SMEQKLRDSERRYRNLFAYSLSARCISAFDGAILERNNAFETQFGLPQKIRNIQELFTEE
SIWNRVLDSLEKGQVLQEELRTRDSGEGARDVLSSFSYFQDEQNETRVLCEFIDITESKR
LRDELFQSQKLEAIGRLAGGVAHDLNNFLTSMIGSLEMIKLDAGTSKEISEDIAGMEHVI
QKTSALTRQLLGFSRRKSFSPAVLDLREVLSDSHKMLKRLIPEKIYISLSLPDSPCPVIA
DAGHIEQILLNLVVNARDALETADNPHISILLSHTAVKREKAGKSLVPEGNYALIEVKDN
GIGIEPAVMEKIFEPFFTTKGEGKGTGLGLSIVSSLVDLNGGYIGAESTPSAGTTFRIWL
PLAKGSQTSEVSEASSVLPTENIYNHYASELKGKRILIVDDDESVLATCKRTLERAGCLT
ETCVNAGEALLLLEHHTFDLLLVDIVLPGVYGTELWQRLKKGNSVVSCLYMTGYEPHSLG
VETEEPILLKPFAPAALIEACAKAIKRGRA*
>SPBIB_v1_110023|ID:27162381| exported protein of unknown function [Uncultured spirochete bib]
MKREKRFIISLIPVLVLAFLFQSCLTADEASIAPPEPSEAMAGNIEQLAANDPEASIAML
SDLFEKQREKGEPLENSIIGGKAVELFGKSASTMLGLFRKALQEGDIVTAKRLSFSAHTI
SRYASLYESLKNVLPADFSAMGEPAVRLQLIFLEAEAKFKKNGYVAGKAVLAAALSAEPS
LLSIRDVPSAISKEIDSAQEPALFFLSWAERAKANNDIEAAAWFGAMAGQIKFEAVSPQG
EKEDWIASAVSGVVTVYVDRGFKIQGGYSVPDRVLGTGFQIAPGLYLTNHHVVQSEVDPS
YKGYSRLSIRPSDNPQARVPAKVIGWDEEMDLALLQSEEKLQSVIYIPSSMKFQAGDRVF
AVGSPIGLENSVTAGVVSSLSRKIISYGEAAQIDVPVNQGNSGSPLFLANGLLVGMVFAG
LPSFQNINFALPVQWIVSSLPALFSGTSVVHPRLGIVLGKGSGQNPVVLSDLDKVSRAFR
AGDVLKEINGVPAKDIASIQLQLAAVPPDALCMVEAQRDFQPVRRLRRIQNTGGASLIPA
WDTIKRTYILEGILGARLEQLEDSSKVGGLYSVSWVCPAEAADETGIAENDTINIHKIQL
DRKNKVFILEFSAKSRLSGYFERTIRLELPAESAAII*
>SPBIB_v1_110024|ID:27162382|ftsH| protease, ATP-dependent zinc-metallo [Uncultured spirochete bib]
MSDQNDNSNDKRPELPPAVKGNRAALAVFFSLIVLFGVYLFFGQKENSREIPYSSFLSYL
DLGEVKSVRIIDQRDIDGYLIGKNGAEMSFTTKIPYFDSELMARLQNKGVSVTGAVSGTS
PFQILFELTPWIFGFILIWIMMRQMQGNNKAFSFGKSKAKLYNDSEKRVTFDDVAGQKEA
KYELMEVIDYLKNPKKFVKMGAKIPKGVLLVGMPGTGKTLIAKATAGEANVPFFHMSGSD
FVEMFVGVGASRVRDLFEQGRKHAPCIIFIDELDAVGRTRGSGLGGGHDEREQTLNQMLV
EMDGFDTKDGVIVLAATNRPDVLDPALLRPGRFDRQVVVAMPDVQERKDILKIHMSKIPV
AENVDVERLARATPGTSGADLANMVNEAALFAIRKSKDIVEMEDFEDARDKILMGVARKS
LVIADEEKRSTAIHESGHALLHYFLPNSDPLHKVTIVPRGRALGLALSLPEKDTYSRTYG
WLYDRIVISYGGYAAEKIVYGETTTGAAQDIKQATEIARKMVREWGMSDAIGPVSLGDEE
EPIFLGREIAQHKDYSEETARKIDAAIQSLLSSALEKAMSILTREKDRLLALADRLMQIE
TLEDSDVRTLLGLQDGKFVEEGAVKC*
>SPBIB_v1_110025|ID:27162383| putative tRNA(Ile)-lysidine synthase [Uncultured spirochete bib]
MRGEAVDRLELRMRRILLALGIKPETPLALAFSGGLDSVVLLNLLVRCGLRNIRAVHVCH
NIRPADELAREKVLIKETCMKMHVNLTIVNVKEGAIDQYASKARCGIEAAARHFRYRALV
RSAQRFGIRTVATAHHADDQVETFLLGMVRGGSLRALSGIEPVRMLSASQGIYIIRPLLS
FERVELFQYAMRKGIVWSEDSTNADTAFMRNKIRHVLIPLLNAEFPSWKTSVLSYVEQIR
EEHEFVERSAQKILHRLLAQVEGEELLDIELLKKERRLMRLAVIKLYLARLGGGIAASRE
AVEELDQAISSNKKRIEAGGFEFDLAEGFLKFCGKTRGSSAQAVRAISPLLDSYEKDQYF
LMVPAAGTYRCGPYIIQISEYEHGSTLHDTPPATANSLVCAFFPFILRNRRAGDTVLGPA

GQKAVDTYLKEIKIAARLRHAVPVIEDKDGIAAVIPSAFGNAASARPLLRDPALCQNKKM
KYILLSMKGDSTINVRSKR*
>SPBIB_v1_110026|ID:27162384|rplY| 50S ribosomal protein L25 [Uncultured spirochete bib]
MEHIELKVSPRGKLTKGELNKARKEGKVPAQLYGKEISPVSIFIDREEFGSAARRITESM
IIDLNLEGKKYPSLMKEIQKANISGDLLHIDFNLIERGHKIHVKVPLHLTGSAKGVREGG
ILEHAIHDIEVECDPDMLPEKIEVDITDLEANHALHLRDIAIPEGVKVLTNPETVIAIIK
FARAEVEKVEAPEAAEAEAAAAAAQPAAEGAAETAEK*
>SPBIB_v1_110027|ID:27162385|spoVG| putative septation protein SpoVG [Uncultured spirochete bib]
MEVTDIRIRKVSSEGKLKAYVTVTFDECFVVHNVKIIEGKSGMFIAMPSRRTKTGEYKDI
AHPICPDFRSKLQEKILAAYNSDKANDTTVPEFD*
>SPBIB_v1_110028|ID:27162386|ispE| 4-diphosphocytidyl-2-C-methyl-D-erythritol kinase [Uncultured spirochete bib]
MILLTKNPFAYTVWLMEETNITAFAKINIGLKIFDRRKSGYHDIESLFQNVDIADSLRIR
PNNVGKIVIEGDIGCAPEQSTIYHAAKHFCMEFEDKSPITGVTIKVEKGIPSGAGLGGAG
ADAAATLFGMNELFEKRLSRAELARIGSHVASDVAFFLYGGAAIVRGRGEYVFPIEPRTD
FGLVILQPTWASSTPAAYAALDNYRAIRGIEHACTSEDAAGYSINLPNEQLEKIYRSPIQ
KWGFENDFLPVLAEKKKQYSEMLSLLQEAGAAYVNLTGSGSCVFGVFDNAEAAQKASCTC
SSLLFGEGDRAQSFAAHIIAAKPLARSMIVSYIQDCNEDTRSDKERPCYGSY*
>SPBIB_v1_110029|ID:27162387| protein of unknown function [Uncultured spirochete bib]
MNHKTTMTADPQEDFEIITLTHGNSANTIYSLRQTVRFDGAYDDEFDYDSDDDFEDEEDL
EDDEDFEDDEDFDDDEDLDDDFEDDDEDLDEDEDLDEDDFEDDDFDYEDDTDE*
>SPBIB_v1_110030|ID:27162388| putative Ribosomal silencing factor RsfS [Uncultured spirochete bib]
LDSKAFAYQSAEILARHNALDVLVMDIHDTAGWADYFVIATSTSAAHMRGLERHLEEFFA
NAEISLLNKPEVTDDQSWLLLDGGNVIIHIMTNEARAFYDLESLWFNAPRFSVTFNGKKE
S*
>SPBIB_v1_110031|ID:27162389| conserved protein of unknown function [Uncultured spirochete bib]
MNELQPGIFKRSVLPLLLILVLAAISAFIVVRLTQKGTVEAVIKKDKAMGILFIFEQEKK
PVSNQLLIWYPSRRKAAIMDIPGSMGIILKSANRMSSIDSVYDSRNPARYVKEISDYLRF
PIDGWLTYDERGLSKTVDLLEGIQIFLPEPAMGNNALPDVSLPGGSVLLDGEKASQYLTY
ACSADSYTEEISRKQRLLVSLLSQLSQKSGVISSSKGLRLIASKPKSNFSLETREEIFRH
FVQIDVEMTITQFISGSFRTFEGRKLLFPYYDGELARDIVSQTAKALGTENSTNLQKSAV
TIEILNGTGSKGVAGAASVLFESYGYKVVSVGNASSFDYAKTVMYDNEGDKSALQQVADV
IGCKSFGNPDDLPGPRKANVTIILGKDFNGRYCVGQ*
>SPBIB_v1_110032|ID:27162390| conserved protein of unknown function [Uncultured spirochete bib]
MDYIGISEILSQVEKTVEIELSAKRLAHCRSTAETSAFLCERFGLDVEKGRLAGIAHDMC
RELVYEEQERIIAQFGSCLDRFHASPSLQALLSDPVYRKKMIHGPAAACMLCHEFGVDDA
DILEAIALHSIADNGASDLAKVVYIADKLEPLRQRPSDAEEMLHTLNLDALFVYTLANVV
RWFQESGKSLSPYTAELYNRMLQP*
>SPBIB_v1_110033|ID:27162391|nadD| putative nicotinate-nucleotide adenylyltransferase [Uncultured spirochete bib]
MRIALLGGSFNPPHIGHIILAEEILGTLDYDRVLFIPANIPPHKEPQGDPGTDMRLAMLH
ATLEGWSEFAIEPCELKRPGISYTIDTLREISRKYSFDGKPGLVIGDDLAPDFLKVWKDP
ELILEYADIIVAHREHAEELSLPYAHRYIKNLLIPVSSTLVRERIANHGAWRSLVSPGVQ
EIIETYGLYRNI*
>SPBIB_v1_110034|ID:27162392|obgE| GTPase involved in cell partioning and DNA repair [Uncultured spirochete bib]
VIKFADEAIITVASGKGGDGCVAFRREKFIPFGGPAGGDGGRGGDVIFVVKRNLRTLAYL
RYRQNFRAQNGQPGMGKNMHGRDGEDAIIEVPPGTIIRDAETGEILKDFSARTWKGALQE
GERWVFLKGGKGGWGNTHYKNSVNQAPHFAQPGQPGQERKLKIELSLIADIGLVGFPNAG
KSSLLDYFTNARPKIAPYPFTTKIPNLGVLTVNDRDIILADIPGIIEGAHEGAGLGIRFL
KHIARTACLAFLIDLSDDNWRSAFPTLLHELEMFSPELAKKPRILLGTKLDLPEAQEHSK
DFYAAFPEEQAYCISVFSGAGLDQLKQVFFEKVEAHEASELHSAAETESLFETIDFDDEE
LPE*
>SPBIB_v1_110035|ID:27162393|rpmA| 50S ribosomal protein L27 [Uncultured spirochete bib]
MPHKGVNGRDSNPQHLGVKAYGNQHITAGTIIVRQRGSRINPGKNVGMGKDDTLFALADG
MVSFREHKGKKFVDVEPVTNA*

>SPBIB_v1_110036|ID:27162394|rplU| 50S ribosomal subunit protein L21 [Uncultured spirochete bib]
MYAVVEINGKQYRAENGKNLLVDRLDAAPGESVVFEKVVLLAGENTAFGTPYVKGASVKA
TVNEEVKGDKVVVFKYMPKKHYRRTHGHRQQYTVLTVNEIVGA*
>SPBIB_v1_110037|ID:27162395|argS| Arginine--tRNA ligase [Uncultured spirochete bib]
MEHYIDQWKTIIAAHLRTIADKKGIVDFSLTLNDIIAEKPPKPELGDIGFPMFAFAKQFR
LSPAQIASEVAQSEQLLKDENEKRDWPGNLKAVGPYLNVYLERTRAVTFLLSNAANPDWG
SSAVFSGRKVMIEFSCPNTNKPLHLGHLRNNVLGESLSRIMKAAGAEVKKVNLINDRGVH
ICKSMLAYLAYGEGRTPEDEGLKSDHFVGKYYVLFNKLKEEDPQAEQKAQELLQKWEAGD
PEVIALWKKMNDWAVDGIMETYRRQQVSFDEYHFEHETYKLGKKEVLEGLERGVFYRGED
GAIWIDLEDLGLGKKVLLRKDGTSIYITQDIGTAIMRHESWPFDQLIYVVASEQQYHFKV
LFEVLKRLGYDWASSLYHLSYGLVNLPTGRMKTREGTVVDADDLIDELARLAAVEIAEKG
RADAVGDIQSVAGQIALGALHYYLLQISPAKDMLYNPEQSLSFTGDTGPYIQYMGARASS
ILRKHDQGEGNAAQGIPKANLLSSDADWPLVRTLMELPSIIEESAKGKDPSILAGYAHAV
ASEFSAWYRDNPVLINEDPNLSASRLALVRAVKSVLMRASELLCVPFLEAM*
>SPBIB_v1_110038|ID:27162396| conserved protein of unknown function [Uncultured spirochete bib]
MTRMPGAISREKFAQIAPAVHQEKEGFVVSVTVIPRAPKNEIAGFRNGSLLVKVTVAPEK
GKANEAVLAAIAEFLDIAPSNLRILRGHTCRNKMVLLQNIG*
>SPBIB_v1_110039|ID:27162397|ligA| DNA ligase [Uncultured spirochete bib]
MPDTSRLSESEKNARVNELEAEIKRHQKLYYNGEPEISDEEFDALWDELESLDPGNPLLK
AVGTDRSERWPKSPHRMTMGSLSKATDPESFLAWAAKVQYPLYLVQYKLDGASMELQYDE
GHFVRGVTRGDGVIGDDITPNVAKMNGVPKELPEPFTGAVRGEVLMSRRMHDAKYADKAN
CRNAANGLMKRKDGVGAEDLDIICYDAVSSLATGTPFDTERKKIAWLERMGFKVVPTVEC
ESPEAVIAYRAKIMDIRPDLEYDIDGLVVKGDRIDREDASKLRPELQIAFKFSPEEAVTT
LREVEWSESGATVTPIGIVEPVRLAGTTVQRANLANPDMIRSMDLRIGSKVVITKRGEII
PKIESLVENPPDAVQIEIPTHCSLCSAELIDEGTSLYCPNPACPGKAYHRLEKWLSVIDI
KDLGSALLKRLFDSGKVTRIPDLYRLSVQDLLAFERMGEKSAQKIIRNVHARNEVSLSDF
IAGFDIEGIGVLMADKLIAAGIDTLDKLFAASPSDFEKIDGFAEITARSLYDGLRVVEKD
MRELAEQGYVRILPAARSSGEEAAGVAGKSFCFTGELESIKRSEAEKLVRAAGGTVKSSV
TKDLDYLVTNDPTSGSEKNRKAQEFSIPVIDETQFLALLGRK*
>SPBIB_v1_110040|ID:27162398| conserved protein of unknown function [Uncultured spirochete bib]
MRYFAIQVATRHEDDWLDRIQDQIEHVKFHKIMKKMYIRKRGKTKLEEVPLFPGYIFFEH
PEEELPLEVLSKIRHSRFFIRFLPSNEAPRPLDRHDSDIIRHFVNFGSLIPPSLVKFDED
QKIKVVQGPLQGIEGFIVKVDRRKHRAKVRLTIAESVMILDLSFEVMESDSTEAEAKGSR
HAEKRVHSV*
>SPBIB_v1_110041|ID:27162399| protein of unknown function [Uncultured spirochete bib]
MQKNEYIPFDQLEVPGMLYACMIRATAQIGHILAIRPPALPSGYSILTADQLPLRNELKI
SSVSIPVFASDTVSYAGETVGLIIGPDPLLVEEFASSTAVECDGDSVHRDWRMFPSSRIA
ARLNLQRGNMPEVSQNDSPLVFNSYVSIEPGEFSFNSGIGALAEWDYDKLKLACPTLWPE
HVRSSLASILEASPSDIELLPVLMNDSSEFYFWYPSLLAARAAFAAWTLKRPVKLIVSIK
RERQYLPRVQGISLWFKSIWSSKTSRLLGIECRFSIPTGTYSVFANKILRKTAHLAADIV
PGLPVSITGFAIRTNAVPMGAIESPASAAIYAMLQAHIAQAARALDRDVFELSQSTLSRM
DTSKKAGALGIAEIPFAKIAKPLLEKTDFMRKYAAYELVHKRNPGGKEALLRAISFSLAY
QGASDFLPESARKSEVRLSLDRNLKAHVESDAAFASERLKAALTTLIADNLKIPPAHIFF
AMQKSPHPDSVPLMSSSGIAMLGDIVRRASARVQRLRFREGLPISAHAFSSVRAQENSTN
VLSKIERPSLGAAIIEVEYDTRSGIFQFIRIHISVNAGKILSPLNAKSAIRAASINAMRS
CLVSTSPNAAERNDMYDHILSRSTIAIELLDDEKSTIPRPVGDIVHSLVLSCFLGIMQQL
NETNRFILPFRPVSATIAEGAQQ*
>SPBIB_v1_110042|ID:27162400| putative FAD binding domain in molybdopterin dehydrogenase [Uncultured spirochete bib]
MNLSEIHFPQTIPELLTLISREKNIQLIGGATTFGYLQPTRYLSFPNTIAITSRIPELHA
IHKTERMISYGAACTLSELEHVYPFEHPQSASLLRTVATSAVRNVATIGGHLMYDCSFLS
LWPLFACLDAELEFRAASQSHIKNIWYLVDENGRHCAAGNELLARIRIPLLSIDHIYIKR
IGGNIFPGGDGAHLVSLTSVDRRSINFFRLVLAGARAFRDFEAEQRIISASHPLSPKIAQ
TVLHMYAESLRKTNFWNADLILPLIGQVLEDLQR*

>SPBIB_v1_110043|ID:27162401| putative Tetrapyrrole methylase family protein [Uncultured spirochete bib]
MENKKKESGSLYLIPTPIVPYDPALWKPALLRAMISPAILDRLFRMTHFIVESEKSALRL
LSRILPADRLNELVFMLLDEHSTSNDLNAPLAALKNGNDCALLSEAGMPCIADPGSALVA
AAHAENLRVVPMGGESSLMLALAGSGLNGQKFCFLGYLPVREQDLRKMLVAEGTKASSDG
AARVFIETPYRNAKTFKACIESLPERLHFSFACAFGTQNSLIKSMQVARWRNFLFEPPSQ
PAVFCFGLPAVLPQPQQHRGCRA*
>SPBIB_v1_110044|ID:27162402|mfd| Transcription-repair-coupling factor [Uncultured spirochete bib]
MKVHTLQALFDRISRNAALKEIRVHLELGRLPLHLQNSENSALAMSLALLSRQTKRRFIV
VLPSDQETEEFAQDLALTDAKVLNFPSWPGAPYRAIPVRSRIFSERTSALAKLAENDFEI
MVVSARTLAIAVPPKSYMLAHTLKLEERQLFDPTKISERLAEFGYLRVPSVSLPGEYALR
GEVLDFSMPGEETAIRVHFDFDRIEKITRFDPATQSGHERIQQIILRPLKEVIWDTSTIA
RLKEKAVELARTDARMEQIIEALASGQEIQGEEFWFPFAFDSMHSIVDYADDHTVFVFVA
RERIDALAENIRKEYASMYRLALREMLVPPPDHLIQNLDSLISSISRALFCYALKGVDES
SSRIALGAEPPRSYFGNIRLFREELAGFEKDGYQTWIFASSEPQAERIASLIQDDSILIV
HGPISSGFIFPELRLRAVSEHELFGRRKHVPRSLGKTKSAAIESFIELSPGDYVVHVNYG
IGRFSGIERLTVLGLERDYIRIDYAGEEKVFVPIEQANLVQRYIGNEGEAPHLDSLGSRA
WENRKRRVRKSVEELAERLIRIYARRKAAKGFAFPPDTDWQMQFEATFPFEETEDQIRCI
EEVKRDMESPKPMDRLVCGDVGFGKTEIAMRACFKAITAGKQVAFLAPTTILAEQHYENF
LERVGEFPVRVELLSRLIDKKNQMKILKGLADGSIDMVIGTHRILQKDVRFKDLGLLVID
EEQRFGVKDKERLKEMKAGVDCLTLTATPIPRTLHMSLLKIRDMSVLQTPPMERQSIQTF
VEEFSPELVARAIRNEIARGGQIFYLHNRIETLPEVEQFIRQLVPEALVESAHGQIDPRD
LESIMHRFIHGAFHVLVSTTIIENGIDIPNVNTIIIDRADNYGVSQLYQLKGRVGRSDRQ
AYAYLLYPDKRALSELAMKRLQIISDFTELGSGFKIALKDLEVRGAGNLLGREQSGDIYA
VGFDLYLKLLDEAVSRLSGVEGEEEEPYLELEYSGFIPDSYISVPMIKMEIYKRIASVSN
QDEIDSLHMDLEERFGPIPEEALSLLALAEMRVLCRKLAISSLRERNGLVTVEFSKVSKI
SVEKILRLIRESSGAIRLSPDKPNAIQIQTKAIGLKEKSAYLKERLSFLAG*
>SPBIB_v1_110045|ID:27162403| protein of unknown function [Uncultured spirochete bib]
MFALILIEDTSVADLVLRDLQESGISAISYRDPIKVIDHIAEIKPDVLIIRQKDFPLHIP
LIAAMVRFSESLQRCRIVVIGDEVPSFMQCWQITEEKLQKDASSLARLVFSGAVSPGHRG
SRLVAKAQRMVKE*
>SPBIB_v1_110046|ID:27162404| Regulatory protein ArsR [Uncultured spirochete bib]
MDTTPKPCNEEQLTPYAQKFKAIGHPVRLKILCLIVHQEVPCVGDIWRCLDQPQPVVSQH
LAILKRYGIVSSEVKKTRRIYSISDPFIKNLIDSMICEIGTSAPEPKK*
>SPBIB_v1_110047|ID:27162405|ispG| 4-hydroxy-3-methylbut-2-en-1-yl diphosphate synthase [Uncultured spirochete bib]
MAANISGGVPAERKAVKVGDVLMGQGWPVSIQTMWKDPLPAFSSPDDERLQPILKRLVAL
KAIGCQIIRFAVPDIAAAEALGMLAGFSPMPVVADIHFDWRIALRCMDFPIAKVRINPGN
IGAEWKVREVAAKALDKGIPIRIGINGGSLPKDLDGEPDLALAALKAAAREIEVLESMHF
SDIVVSLKLNEPETVIRANELFAAQYPYPLHLGVTEAGPLVAGVVRNTAALVPLLKKDIG
ATIRVSLSDTMEQEVLAGKEILACAGKKMNGARIVSCPRCGRATFDTHAFTARWMERLYS
IGVDISVAVMGCVVNGPGEARHADIGITGSGNSVLIFKHGEIVRRIAKEEADKAFGEELD
ALVREKSM*
>SPBIB_v1_110048|ID:27162406| conserved membrane protein of unknown function [Uncultured spirochete bib]
MNIALIGAACVLGLAATGSGIGAGIAGMAAIGSWKRSYLNNKTPSFLLVAFAGAPLTQTI
YGFILMGRLMNSSKDPLLLLAAGVMSGLAIGMSAVAQGQAAAAGCDAFGETGKGFANYIT
VVGLCETVALFVLAFTFSAV*
>SPBIB_v1_110049|ID:27162407| putative V-type ATP synthase subunit I 1 [Uncultured spirochete bib]
MIVPMKKFYLIVLDKDRESVPERLRKLGVAHVEELPGSGETYQALERGKSEAETVYYLLS
NYEDKKSKSKHPAREGKGLSQIEAAKLLVQEVSALKHEFDTLNERASQLAREMDRIATWG
EVSVSQLAEVSEKTGLSIHFFEAPSKEAAHIPEELEYVRLAAPKGKIRLGVLIEEGKKLP
ELPAIFQEFLPPEMPLSSMRQEAASIDRRKKEIEAELRARTVHASVLKSYLDELESNITL
ERLRSGMPSQAQFAYLQGFVPARDCDRFKKMVARYGWAAAFDDPSDEELPPTLVENPPAI
RIIDPVFEFLGTVPNYREYDISLWFLMFFSLFFAMIFGDGGYGSLIVLASLVSIVLGKRK
GQKASDAQKLFLLLGAVTVVWGALTASWFGIQYNHLPYILQNISLPLINGQNPNSESNIK

VFCFIIGLVQLSIAHIKNIKRDFPNLKFMSQIGSLLLLAGMFNAALNLVIDAQRFPIRSW
ALICIASGFSLVFVFGNWNGKIVPSLVESLKGLIPTFLGTVSVFADIVSYIRLWAVGLAG
LAISQTVNGMAAGILGGAAGFLLGFILKLLIAVMLLAVSHSLNFVLTVLSVVVHGIRLNM
LEFSGHLGMEWSGYKYNPLREAKSSPAPMQSTLEETGV*
>SPBIB_v1_110050|ID:27162408|atpD| V-type ATP synthase subunit D 1 [Uncultured spirochete bib]
MAKIKLTKNELKKKKDALKMYQRYLPTLQLKKQQLQVEVRSVQTRLEQLLARKATLEKEY
DPWIAVFSEQEKARKSNGEPLLRIVSVRTDEGNIAGVTIPVFQDIEFERSVYDLYAAPVW
IDSALTLMEQLLRTQMELEIVTRQRELLAHELRITTQRVNLFEKVKIPETIADIRKIRIY
LGDQQTAQVVRGKIAKRKVEGAGR*
>SPBIB_v1_110051|ID:27162409|atpB| V-type ATP synthase beta chain [Uncultured spirochete bib]
MNKIYSRIESIVGNVIAVKAQDVAYGELAQVKTRYGMSLAETIRLDKEMVFLQVFAGGRG
ISTGDEVRFLGHPMQVSFSDNLLGRIFDGAGEPRDGGPMLSDNLITIAGPSVNPYKRIVP
REMIRTGIPMIDVFNTLVKSQKLPIFSVSGEPYNQLLARIAMQAEVDLIVLGGMGLKYDD
YLFFRDSLEQGGALSKTIMFVHTAADPIVECLMVPDISLAVAERFALKGKNVLVLLTDMT
NFADALKEIAITQEQVPSNRGYPGDLYSQLAARYEKAVDFSDAGSITILAVTTMPGDDVT
HPVPDNTGYITEGQFYLHGGHIEPFGSLSRLKQMVNGKTREDHRSIMDAMIKLYAAYRDT
LEKKSMGFQMSVWDRKLLKYGELFEQRMMDLSVNIPLEKALDQCWETLAECFEPRETGLR
SDLIQKFWVGRK*
>SPBIB_v1_110052|ID:27162410|atpA| V-type ATP synthase alpha chain [Uncultured spirochete bib]
MTEGIVTAVNGNMVSVETDGNVTMNEVAYIETGTRSLKSEIIRIRGNSVQVQVYEITKGI
KVGNKVRFTNELLSVELGPGLLGQIYDGLQNPLPEIAEKVGYFLEPGLYLKALSRETRWH
FTPLAKVGDTLQRGMAIGWVPELHFRHIIMVPFDLYGEWKLADILPEGDYTIDENVAHVV
DAKGNTKPIKMWFSWPVKRAVDCYSERLKPSEPLVTKTRIIDTFFPVAKGGTYCIPGPFG
AGKTVLQQVTSRNAEVDIVLIAACGERAGEVVETLKEFPEIIDPKTGRSLMERTVIICNT
SSMPVAAREASVYTAVTIAEYYRQMGLDVLLLADSTSRWAQAMREMSGRLEEIPGEEAFP
AYLESVIAAFYERAGIVQLYNGQKGSVTIGGTVSPAGGNFEEPVTQATLKVVGAFHGLSR
ERSDARKYPAIHPLDSWSKYEGIMPQRAVQYAHSFLERGAEVGAMMKVVGEEGTSLEDYV
VYLKSEFLDAVYLQQNSFDPVDAAVPPDRQKHMFSLVLKILASQVKYKDKEDAREFFYDL
RQTMLDLNGTPEDSEMYDSLYAKVEQALADRNPEFENRGLKAISALE*
>SPBIB_v1_110053|ID:27162411| conserved exported protein of unknown function [Uncultured spirochete bib]
MKRYWYFASTLPSFPFGAPPPFSAQEFDALCERLVDKSDRELIQAAGAAVRGEYTGTIEK
SPFLKAFFEWERGTRNALAMLRAREFKWDPEPWIRPGSTNTDALQAAQAIHSASDPLQAE
LTFERERWIAVERLSALSAFELDYILAYKIKLLIATRCISFQRDRGAEGFRIFYQDIVDA
AADVASSALDTGVAT*
>SPBIB_v1_110054|ID:27162412| putative V-type ATP synthase subunit E [Uncultured spirochete bib]
MEIQVQELLERIRNEGIETAKKQAEEIIEKAQAQADEIIARAKKEAEEALTEAERRIASM
DAASRESLLQASRDTMIALKQSVRMFVESALNADIEAAFDAKMAAQVIPEIVRILASSQS
GDIEVLLPPTLEEKIDASIAARLSKELARGITFKPYPAIDAGFRVAVEGSAVQFDFTSES
IAQILSVRVNRLLAEYLKEASGSLT*
>SPBIB_v1_120001|ID:27162413|mgtB| Magnesium-transporting ATPase, P-type 1 [Uncultured spirochete bib]
MTKLPLLLPFLKKRGSSANGGISSSDEYRLISLCSSSVQSTLSELETSARGLSEKEVDKR
ISEFGRNELSQLKKLSFWEDMFERFKSPLVIQLLIIATVSAIIGEATSSIIVGAMILLSV
GLSYILDSRSNKEVEALGKRVQSRTYVLRDGAETEIRMSEIVPGDIVLLQAGAIVPADVR
VISAKDFFVSESALTGESMPVEKTANPPAEPVKSAMELPNACFMGTSVTSGTARAVVVTT
GAHTLFGAISKKLIERREETSFDKGVRSFTWLMIRFMLVMVSVVFLIVGITKGNWLEALL
FALSVAVGLTPEMLPMIVTVNLAKGALAMAKKKVIVKKLPSIQNLGSINILCTDKTGTLT
QDKVVLEHHVDIIGNKSDEVLNYAYLNSYFQTGLKNLLDRAVIEHVDLNVDECHLVDELP
FDFQRRRMSVVVEYEGDNVLICKGAVEEIYSCCTHYQIDEEIYPLIDMIRADLFEEVQKL
NQEGFRVLGIAYREFPRTKTTFTVQDESQLILLGYIAFMDPPKESATEAIKLLAKAGVGV
KVLTGDNGLVTEKVCRDVGIPFTSAITGAELGALDAAAFSEKVRSCEVFVKLTPSQKEQI
VKELRRQGNIVGYMGDGINDATALKAADVGISVDSAVDVAKESADIVLLEKSLLVLEEGI
MEGRRIFANIIKYIRMGASSNFGNMFSVLGASYLLPFLPMQPLQILTNNLLYDFSQTGIP
MDNVDSELITKPVKWDINNIKRFMIFIGPISSIFDYATFALMWFFFKASAYIDPGIAAAQ
KDAIARLFQTGWFVESLLTQTLIVHIIRTRRIPFFQSRASGPMLLTTLAVMAIGAWLPYS

PFAAMLGLVPLPGVYWLWIAGFLITYSVLTHKVKRWFMRRFEGA*
>SPBIB_v1_120002|ID:27162414| putative PTS IIA-like nitrogen-regulatory protein PtsN [Uncultured spirochete bib]
MDSILDALQEGRLFELPENDKNHALQFLAHIIEAFPQIPTGTDIVGNVMAREKSMNTALG
KGWACPHARVDFEEDLMCVVGWSPSGIDYGAPDGQPVSIITMYLVPSNQRNHYLREISIM
AKVLKSSPEIDKLSSIKELNDVRNYLLDLISASKETVGPDARARMIRLQSKAALATQPVA
DLSNLIVEPVSIIAGTGFKPLALTQNLELLNFVESTAGLAERLDSDGTYQNGIWRIVRRS
ATAYQGGRTVFDCIAIMPAKASQKA*
>SPBIB_v1_120003|ID:27162415| Translation elongation factor G [Uncultured spirochete bib]
MSYTTEKIRNIAIIGHGGTGKTTLLEHILFQGGMISKPETVDSGKTVSDYGEDEIARKIS
VRSSLTHVNTNDCKINLIDAPGAGDFVGEAILAIRATETAMLVIDGKSGVQIETVKLWRI
LERHQKPRMVFVTRLDEDRASFASALSDVKEKFKAIPVPVTIPMGEGTAFKGVIDVLNRK
AYPRPASHDQKEEATEVPPEFSEAVEAARAQLFEAAAEGTDELMEKYLLEGELTQEETLQ
GLQKALAMGKIVPAFAGSGIANSGTAAFISFAVQSVPSPLLRTPEKALDPEGNEIEVSID
PSKQASGFVFKTQIDQFSGRLCYVKVMTGTFQPDMDMVITREAHKERIGKLYTLQGKKLE
EVPFLPAGDIGILAKLVSLKTNDTISQFDQLISYIPLRLPSPVHMLAISAVNKKEEDKLS
ELLYKAAEEDLTFRVNYNAETKEMVIAGMGEQQINMILDKIKAQAKIAAETRVPRVAYRE
TITKKAGAEYTHKKQTGGHGQYGRVVFEIEPLERGKGYEFENRIFGGAVSKGFMPGIEKG
IHQAMEAGVLAGYPVVDVKTAIVDGKEHPVDSSEMAFKLAARGAFREAMKQANPVILEPI
MNLSVFVEEKYLGDVMSDLSGRRGKISGQNPIGGGIVQIDAQVPQAELLRYAIDLRSMTS
GTGSFEIEFSHYAPISGKIAEDVIKAAQAFKTQEAEEE*
>SPBIB_v1_120004|ID:27162416|nrdR| Transcriptional repressor NrdR [Uncultured spirochete bib]
MKCPYCGSLEDKVIDSRTLANGEAIRRRRECLACGLRFTSYERIEEKPLMVVKRDGRREP
FERQKIERGLVRALEKRPVSQMSIENLINEIEDEAAEQAKASNEISSEDLGRMVLRKLYA
LDKVAYIRFASVYRKYDTLNEFIHEIERLEKGQTE*
>SPBIB_v1_120005|ID:27162417| SPFH domain, Band 7 family protein [Uncultured spirochete bib]
MSYIFVGFAILAAWWIVFIIVGIARKQTPIAVFLIILFFVWAVPEVLLLLSNPEFASVLF
IVPFILLVQMMRVIPEYQRGVLFRLGRMQKVIQPGFNLVLPFGIDILRKVDMRTFTIDVS
KQEVITRDNVPVIVDAVVYFNVFDPVLAVVKVADYTKSTSLLAQTILRSVLGQHELDEML
SKRAELGQILQRLLDDATDPWGIKVTAVEIKAVELAETMKRAMARQAEAERERRAKVIAA
EGELQASEKLAQAADVLAQSPASLQLRYLQTLTEIAVEKNSTIIFPLPMDFLKALTKLAG
KDQE*
>SPBIB_v1_120006|ID:27162418| conserved protein of unknown function [Uncultured spirochete bib]
MTFSEKLKKFMETSVDASKEFLEKAADQAQVWGEMGKLKIEILQLRSKAQSLTAKLGAEV
YNLLIEKNEPMIGASTPEIEPLIKELEQLGSLIDEKEALYRSKGGKDSDLNEKEK*
>SPBIB_v1_120007|ID:27162419| HAD-superfamily hydrolase, subfamily IA, variant 3 [Uncultured spirochete bib]
MQRYTPALIVFDLDGTLADTIKDIADSVNRIIAMYGFEPHNVDAYKRMVGDGFLSLIEKA
IPNDAARNSELVQEILARAVQEYAAHSLDATKPFDGAKETLKLLSDRGIQLAVLSNKPQD
LSASIVGALFRDIPFIAVWGNLPDRPRKPNPAAVLQICELANIAPQNCAFVGDSSIDMKT
AKASGMTAVGALYGYRSREELLDAGADFLIASPQDLIALFGAE*
>SPBIB_v1_120008|ID:27162420|pgcA| Phosphoglucomutase [Uncultured spirochete bib]
MNHEEILARAKEYISFETDQFFSDEVKALLEKGDWKELEDRFYRDLEFGTGGLRGVIGGG
FNRMNTLVVTRATQGLCDYLKEQFPDKPLSACIAYDSRRKSKEFSLATALVFAANGIKAY
LFSSLRPTPELSFAIRKLGADTGVVVTASHNPPQYNGYKAYWNDGSQVVPPHDTGIIQKV
LKVRDVKLMPESEARAKGLLVNIDKDIDEAYVAMVKSHLLRPELFSKMADSVKVIYTPLH
GTGAMLFERIMKDLGLNVLTVPEQREPNGEFPTVSYPNPEEAAALSMAIEFGKKTHADVV
MATDPDADRLGIAVPDGSGGFTLVTGNQLGSLHLDYIALTLKELGRMPPRPASIRSIVTT
EFQKAIAEKHGIASFECLTGFKWIADLMRQFESNGYDFVYATEESYGHLIEQEVRDKDGI
SAAALTAEMTLYWRSQGKSLLDRLEDLYREFGYFEEKGLSAYFEGEKGMQIMSGIMDAYR
REQPKQFGGIPVVWTRDIKAGTAWDRAGKISSISLPKSDVIQWRLEEGTLLTVRPSGTEP
KIKYYILCHSVGEDLAKTKAETKRKIAAIEADIKAVIDAHRK*
>SPBIB_v1_120009|ID:27162421| Exonuclease, DNA polymerase III, epsilon subunit family (modular protein) [Uncultured spirochete bib]
MTENNGAPLWASLFSDAQPKHEPPSPWYRKRNGAWEGVSWPDPVCGGEPWEMRTYVALDV
ETTGLNAATERIVEFALVPFAFDAEGALIEIQRFSALVNPGIPIPLQASRIHGIRAEDVA

NEPLFGEVAPAVLELCKNRVIVGHNVMFDIGFLEGELGRAGYALETEECADSFGMAKIAF
PAMQSYNLGKLAFALGLPSDATHRALGDALTCMRLFAAAARTLANRCV*
>SPBIB_v1_120010|ID:27162422| protein of unknown function [Uncultured spirochete bib]
MRFAAKCALFGIAGILAALLLRFTMKMIAPYPTISGVVLPETSALVESTIAQSKPALNFF
EELASLDIDRMQNASVAEVRARVRDFISTGLALGMEPEALAAELIPSSGHIIAMLIDSGD
SEEFSQIDGVSLPAKLSYQRIAALNPLMASLRSQFEAIFDSAYAILKKYGRITPFRWLEP
EAARWKGGITLPGKFSLPRISDLDYSHTYALDIFLQDVQYLPGDIQRGPVIHSLSDGIVV
AAETGWIGFPRTSEGLSFLAGGISPKSGNGIIIYSPDEHKYYSYFHLYDVFVRKGQLVRA
GQPLGHGGNSGINARKQGGGEHLHLEVFDANAGRFLRNTQLIALLKASIKKRPAVPQE*
>SPBIB_v1_120011|ID:27162423| putative Glucokinase [Uncultured spirochete bib]
MSKTFLVADIGGTNTNLALARHDGKTIELVFKERYSTQDERSLLEPVQRFLKAASSLGFS
EAIDFCCVSGAGPVQADETIQLTNAPWSISASELSALLGVPVRLINDFTALSYAVTLLNY
KNEAEITRLPHLDGSLGEPADGLMLIVGAGTGLGVGFVDRHGKSVTAYPSEGGHSELPCF
DALSHYFSEWLSERYGYPAGAELAISGQGIANIFEFVCTEKLDARMIAAEYGIPQPGSGS
GLGSGLDAGLEASLDAGPSELSKGILKTPRTAWPAAIAANSRTDAHCALTMELFVRLYAL
KAANLVSILLPRGGVWLAGGISSKNETWLLENHRFMRWFEKNYAPHIREFLSKTPVLIVK
NYDISLLGAAEAAHQFSEHG*
>SPBIB_v1_120012|ID:27162424| UDP-glucose pyrophosphorylase [Uncultured spirochete bib]
MMKEAQLHQEIHPDLRADMLAKHIDIELALSILDRYNRGEFDHVEKIVPAGVPPVDGIHV
VDVRNRQANTTLFKFPKREALQNLAERNVKLPEGIPETTESGAAYLLLDAAKLEAIGRQI
VPSCAYGVLNGGSATSYADLRKNRAIDEALFSALKPAFERYSGLCRDLPKGLTPAYINPD
GSPGASFLELKMRARLLAANRAGFAASDALVVAPENGSAPRSSMLSTEARAGQPERLFMP
LYQMTSMGNHDQLLSAYAELSSSPFLSTLSLRTGLDAAQWLSGKQPLIAAYTHSSEGMPK
RIFDRAYGKEHSSLALPGGHGQCFMVLADTFRALQQQGIRYAMLSNVDNLGAFIDPVELA
ILAISGRPAGFDFAYRTPIDVKGGILVRTQDGTKNVVDIGPAIDIKEVETLEKQGEAILF
NCATGIFDLEWLVPNLENIAHSLPVRFTDQVKDAGSYSQAEQVTWEIVSLLPDFVAFAVN
KERRFLAAKMLAEMLLTSGFGNDVPSVPEVLRQVGAALHDGQSWLLQNVYGLELVNGCWV
PPEIFKN*
>SPBIB_v1_120013|ID:27162425| putative Trimethylamine-N-oxide reductase [Uncultured spirochete bib]
MGFLPYFCGKDCGGDACPLLAEVEAGRVVGMHHNPAAGQWIRACGKGMRAHIEHYSPHRI
TKPLVRRGPRGSGDFVEASWDEALALVARRLSEIRAEGGPGAVMSIASAGSTGALHNTEV
LALRFLNAIGGAVHVSGNYSSNAANYALARIFGASVGETGFDPASTAFSNLIVLWGANPL
EARLGAELPSRLVEAKKRGSRIVAIDPRYSRSARGLGAEWIPVRPGTDPALGYALLYEIV
NSPQYDHEYVAERTEGFDALVRFVLGEIDGIPKSPQWAAPICGIEESMIRRLVHLWWQDQ
PVMLIPGYSIQRVEYGEEAFRLTVAIQLATKNSGKLGASSGSINNRLPGITIAKMREIPE
SIPSETVYSAKVPVLRWADAVLNPQQYGLGKIRMLYSAGGNFLNQGANIQKNIEAFEAVD
FAVCHELFLTPTARYSDVVLPAADPFEKEDIGIPWAGDYVLYKPKIFEPEGMARSDFQIF
SELAERLGAKEIFTGGKNESQWIAYLMAESGIPDIEAFKQSGFYAMPQRLRAGLDAFFAD
PGSNPLQTKTGKIEFSSPLWTYEMARFWEQRNGAKEDNAAKEDDTAPKGNADPKAPKKGQ
GELSAGSSFKLLTPKVAEYVHSQRGAFPDSAKKARVHMHPEDLALIGATEGAVLCIRNDY
GAVLAVASLDANIRRGTVWLEEGAWAEPENGLDPNGSANMLTSDRGTLESTSCIMHGIEV
WIELPPGARS*
>SPBIB_v1_120014|ID:27162426|lon| Lon protease [Uncultured spirochete bib]
MSTSELIPIDQILPNKLPIITLVGKPIFPGIFTPIMIGKEPDIQLVEQAISTDGMIGLVL
QKDDTDEPSAGNLYQVGTAAKVIKKINLPDGGINIFISTFKRFKVKKYMSKEQPIIAAVT
YLDDTNYDTDEVKALTRALISEMKQLSENNPLFSEEMRLNMINIDNPGKIADFIASILNI
EKKEQQEILETLDVRERMEKVLIHISKEKELLRIQKKVQAEINEKIEKSQRDYFLREELK
AIKQELGMAGDARSSDYQRFREKIDSFNLDGEIKEIVEQELEKFNLMEPSSSEYMVTRNW
LDLVCSLPWNAPLSADFDIKHAQKVLEEDHYGLKDVKDRIIEYLAVRKLKRDSRGTILCL
VGPPGVGKTSVGRSVARALGKQFFRFSVGGMRDEAEIKGHRRTYVGALPGKIIQGLKLTK
SRDPVFMIDEIDKMGISYQGDPSSALLEALDPEQNFSFRDHYLDLPFDISNIFFIVTANT
LDTIPRPLLDRMEVIQLPGYVDVEKIEIARHYLIPRSLERNGLAKNQVKYTRDALLAMID
GYAREAGVRNLEKALDKIHRKIAKAIVIENKQEERFVIDKHAVEKYLGKPIFRDDELKRA
TRPGMAVGLAWTSMGGDTLIIEAVANPGKEGFKLTGQMGSVMQESAGIAYTYVRNIAAQK

YNIDAQYFESRQIHLHIPEGATPKDGPSAGITMATALLSLVLGKRIKDRLAMTGELSLTG
QVLPIGGLREKTVAAKRNKIKEIIIPAANEKDLDEIPEHVKKGLVFHPVTRMEEVIDIAI
GS*
>SPBIB_v1_130001|ID:27162427|queD| 6-carboxy-5,6,7,8-tetrahydropterin synthase [Uncultured spirochete bib]
MYHIRVEAEFAAAHRIVHYNGKCERLHGHNYKVRVWVSGATLGDGGMLIDFAVVKNALKS
LIAEKLDHRDLNEIREFEDDPSAERIAKYIFEQLSKALPDVPLSAIDVFETDASMARYVP
DR*
>SPBIB_v1_130002|ID:27162428|murE| UDP-N-acetylmuramyl-tripeptide synthetase [Uncultured spirochete bib]
MAKFLSDLIAGLDILEIRGKVERPVSGVAYDSRDCKPGFLFFALPGIHTDGTKYIGDAIR
NGAIAVVHEGALKSCPADITTLRVADCRWAMSAISSTFYENPSSSLCVIGVTGTEGKSTT
VFLIYQLLNLTGFRAGFFSTVMSDTGEGEKPNPQHQTTPEATAVHEMLANMRDNGLRYAV
VEASSHGLSLRTARLAHVAFDIGLVTNVTHEHLEFHGTWEQYRSDKANLFRHLGENSSKK
QLVGLSCAPYGIVCADDPSASYFIEQSAVPCKTYSSKGFAADLIAADIKSDLEGSDFSVE
GTVNSQRTRIRARINLPGAFNVQNALGAILATSAATRLHWSAFIPYLPKLKPVRGRMQRI
ITGQPFDVIIDYAHTPSSFKEILPPLRSTTKGNIICVFGSGGERDRAKRPQQGRIAADYC
DIVILSDEDPRGEDPRALLEEIAAGCPELPRGERLFLIPDRPTGIRKAFSLAKAGDLVLL
LGKGHENSIIYADRTIPYDEEATALSILAEMGFCRKGKT*
>SPBIB_v1_130003|ID:27162429|ddl| D-alanine--D-alanine ligase [Uncultured spirochete bib]
MMKTIAILYGGRSGEHEVSLISASSIIAYLEKSKYRVLPVGITKKGEWYLQHLPEWAFVP
PEKGIMRSLPAIEPGERVVAVPGDGLWVEQAKGLKKLDIDIVFPVLHGTFGEDGTVQGLL
ETADLPYVGADVLGSAVGMDKEASKRLWLSAGLPVVDYISVGEEDISDGNLQALTRRIET
RFGWPCFIKPSCSGSSVGTSKVSQPEMLKNAVASALRWSERALVEAYVEAREIECAVLGN
EHPVAFPPGEVVPSHEFYDYEAKYKDPNGATLLIPAPLAEETRTRIMSLAVAAYKYASIK
GMARVDFFVEKLTGAVFLNEINTIPGFTAISMYPQMCIQGGLAYPDLLDKLIELGLENYS
KKRSLEYDFSKS*
>SPBIB_v1_130004|ID:27162430|rocD| Ornithine aminotransferase [Uncultured spirochete bib]
MNSQDYITLDNRYGAHNYHPIPVVISKARGCKVWDPEGREYYDFLSAYSAVNQGHLHPHI
VAAAKAQLELVTLTSRAFHNDKMGLFLKKLCDYTGYEKALPMNTGVEAVETALKAARRWG
AEIKGIENGKQNIICAAGNFHGRTLTAVSMSSDPESYVNYGPYVPGFVRVPFGDAEAARA
AIDKNTVAILVEPIQGEAGVVVPPDGYLSALRKICTENGILLIFDEIQTGFCRTGRRFAW
QYEGARPDIMCLGKALGGGIMPISAIVADHEIMDVFTPGTHGSTFGGNPLADAVGVAAIE
VLEQEHLEENAYRLGEHFRKAVRAVSSPKIRLVRGKGLLNAVVFEENFDAYPACLALKEN
GILAKQTHGNIIRFAPPLVITEDELEDALARITGVLASV*
>SPBIB_v1_130005|ID:27162431| exported protein of unknown function [Uncultured spirochete bib]
MLKRLYIAAFLVAFALAAWLVALQRQETLQASIPIENRTVMLESSNEKNQVQQQSAVQQR
ARIAPVPEEIFLKTIDLNLDDDEDFEQVIISRKSTSSQALSIVIAKFSPALGIYFRYFDG
EIAATKSDSIIVQPEDVTGDGIVDLVVQGLDASNNQTLTIFRRLSDRGYAKVFSGRGASV
VLQENTETEKPRAASIILETREIGSNVLLQSKYIWNNRLSAFEKTSEIALDQKARPIFAG
PSGVDVGAFLSWMDRLWTNSDNTAGARFLYLDAKNQELIFSDQQIQQRWIMKSSERNGNR
LYITCSTSESSDLDRVVVIEARAENEIAVSVIDQQIATFRRDEGWSSIYRTASMPNAVAT
EHPKMPQFDFSQLFGRYLGDEGSVLVLSPVKSILVIKGKYREGIARLYEYDGRQVLDFLQ
IQQNGLASERYFYLISIEQSKDGSVERLHLEPATMSADGVSISYLSPYIFKKTS*
>SPBIB_v1_130006|ID:27162432| exported protein of unknown function [Uncultured spirochete bib]
VSMRGKARNGTGDFWRPRAYLAFLFLFIASSVWGQTYRNLSQARQAASSAKTADSLTTVL
KTAITQLPVRDSIALCEEFEPKVPAAFKAELRGTVGGLYLLLGQINDAKSWYAKAAGLDK
KYTMQALRLAIMAGDQKTVLQLLKSESLSRESQEIAAIWISLLDGEYSKASATANQALSS
TTDPQSRREILFLKYIADFGLYGTANSSILKEFPLSIEADMIQGKVFSSAPLILSVGLSW
LGSPTILADLAQKNLAKQDNNKSDSGQWLQVGYFSAKENAERLSRNLSAKGYQTRIVETK
NSNGDSRWAVHVAAKDDWQKTQSMLKDQGYESYLVQP*
>SPBIB_v1_130007|ID:27162433|miaB| (Dimethylallyl)adenosine tRNA methylthiotransferase MiaB [Uncultured spirochete bib]
MKQYHIETYGCEMNKAESAAMEATLREHGWKRVSEQDADLVILNTCTVRTTAENRAWNRI
SQLSARKKERAFALAVVGCMAEQHKGAIQKKAPGVDYVLGTFQKQSFGLMLDLVAKGEKI
DVLEETPTYVFGASYHEPGAFRSFVPIMHGCNNFCSYCIVPYVRGREISRNPSNILKEID

QLEDLGVREITLLGQNVNSYRWEEQGHVLDFPGLLKLIARHLHDRQSSDAHGRIGWIRFL
TSHPKDLSDSLIDTIAEDPIFCRHIHLPVQSGSNSVLARMNRKYTREYYLSLVDRLKAAM
PDLTLSTDILVGFPGETDDDAEQTLDLMRQVRFAYSFMYHFNARGGTPAASMPDKVPDKT
KKQRLARVIALQKEITSALMRERLGQTDEVLIEGVSRRSKKEVLARTARDEMVVLPAAQS
RIGQFARVKLVSISGNTFRGEEV*
>SPBIB_v1_130008|ID:27162434| membrane protein of unknown function [Uncultured spirochete bib]
MESNLPVDPKNLTDKGTKAVMSTAAGLGMLGVNALLGIPVVGTVISAGLLGLGAIGLFGK
SKTDKASGTVLAAAGIAGLTTLFIPGVAHSLLSLGGIGLLGFGVFNLISFLKGLKSKK*
>SPBIB_v1_130009|ID:27162435|folE| GTP cyclohydrolase FolE2 [Uncultured spirochete bib]
MVDVQSFQDDRHIAIQKVGVKGLRYPITLLDKTEKYQHTTAVVNLYANLPRELKGTHMSR
FIEVFDEYRHDLSMPNVIRMLRKIRTELDAQSAYTDIHFPYFINKAAPVSGQTAIMSYDC
FYEASCSEKGHRFIAGVIVPVQTVCPCSKAISDGGAHNQRGLVTLQVSLGPFFWFEDLIR
IVEESGSSELFTLLKREDEKFITEKAYSQPRFVEDVVREVYLKVDALNRFPWFSVEAENF
ESIHNHSAYAYVEKSERSKIEPVHEGHGKIDTFMLD*
>SPBIB_v1_130010|ID:27162436|folD| Bifunctional protein FolD [Includes: Methylenetetrahydrofolate dehydrogenase ; Methenyltetrahydrofolate cyclohydrolase] [Uncultured spirochete bib]
MSAVLIDGKKIAEEIRSELAGKVSALRTAGVVPGLAVILVGENPASVSYVTAKEKACEEI
GIQSFETRFPENVAESVLLEKIAACNRDPKVHGILVQLPLPRHIDERRVIGAIDPSKDVD
GFTPINLGRMLLEEPCYIPCTPLGIIELLKRSGVPTNGARAVVVGRSNIVGKPLANLLIR
KSVNATVTVCHTGTRDLAAHVKEADIVIACAGKPGLVSIDMIKPGACIIDVGVNRVPDPT
KKKGYRLCGDVDFEEASKVAGWITPVPGGVGPMTITMLLSNTIDAAARISDIALQ*
>SPBIB_v1_130011|ID:27162437| conserved protein of unknown function [Uncultured spirochete bib]
MGETEPNELAKIRAQYLGNHETIKELSQSNPTKVGLKRSSEFYRLDAEGNCIYNPEPQGL
TAAREALSMHFAEKGRGISPDNLFFCASTSEGYAWLFKLLCDPGDVVLIPKPGYPLFEHL
AMLEFVRTSAYPLEYSHPSGWHIDIAAIARYLETQQGRRAKAIIVINPNNPTGSYIRASE
RAAILELCERYELALIADEVFFDFPLSDSIECASFIGEERVLTFVLDGLSKRLGMPQMKL
GWICVSGPSMLESRAKKRLELIADTFLSAGTPIMNALPRLLSSEDSFLEEMRRRIRENYA
IYRSILEFNESPHRVLACQGGWTALVESPSYMDEEKTAALLLLQKGIAAQPGYFFDMEKG
IHFAFSLILPQDSAAAWSEDYRDFFENLEAR*
>SPBIB_v1_130012|ID:27162438| CoA-disulfide reductase [Uncultured spirochete bib]
MRLVIIGGVAAGATAAARARRLDENAEITILEKGPYVSFANCGLPYRISGDIQKRGHLIL
QTAEGFFARYRVNVMLNTEAIGIDRQNKNVRVRTKDGESEVPYDKLILAQGGSPIRPQIE
GLDSPNVFNLWTIPDTDKIEAFIKEHAPKHAIVVGGGFIGLEAAEAFQKRGISTTIVELM
NQLMPPADPEFGAQIAEALAEHGVDSITGKSVVRIDWNARTATLNDGSTLPADIILLAVG
VRPNLELAKQAGLEIGSSGGLVVDEFLRTSDPEIYAAGDMIEVIRKPDGAKVRIPLAGPA
NRQGRIAATNALGGSMTYSGALGTSVVKVMDYTFSMTGLSEKAAAAAKIDARAVTIHKAH
HATYYPGYEDLSLKIVYRREDGKVLGAQAFGKEGVEKRIDVLAVAVYAGLTLQNVAELDL
SYAPPYSSANDPMQMASFAALNDMQGFSKFVSAQEAIALIKQGSAKVLDVRTYVEYLNGH
IKDSMHIPLDELRDRIEEIPDENLLIVSKAGFEGHLAYRQLSQHGRNAIRYITGGYMSLR
LLKEAQNIIEEGE*
>SPBIB_v1_130013|ID:27162439| Rhodanese-like protein [Uncultured spirochete bib]
MALNPVEELIKNGAIVVDVRTEEEFEEEHYPNAKCIPVNEIMQRANEIGPKDKPVVLYCA
SGARSAYAARILKSLGFAKVINAGGLYDMPNY*
>SPBIB_v1_130014|ID:27162440| Extracellular solute-binding protein family 1 [Uncultured spirochete bib]
MERLRKGLLDFLGFALVSILLVLSGCGGGSKSKTLYIFNWTYYTPDSVIQKFEKEYGVKV
VYDTFASNEEMFAKLKAGGSNYDITFPSGDYVSIMIKEGMLEKIDKSKLKNYGNIDPAVL
ALCDFDPGNQYSIPYYMGAAGVAVNKTKVQNYDKSWSIFARKDLANRMIMLDDMREVMGD
ALKYLGYSVNTVDQKQIDEARDLINNVWKPNLLKFDAEAFAKSFAAGEVWVAQGYAESIF
AEVDKANWKDVDFFIPKEGGPSYLDSMVILKGSKNKDLALKFIDFIHRPEIYAEFCDYFG
FPSTANVPARALKKGESWYRPEDLLNCELKKDVGADLEKYNAAWQAIRVGK*
>SPBIB_v1_130015|ID:27162441|potC| polyamine transporter subunit ; membrane component of ABC superfamily [Uncultured spirochete bib]
MHALKKPKAIRIAILWRTRRMLRKGGAAARKFMPARSSLSNVIYWVVIGFLFLPLFVLVL
YSFNSGRQATWQGFSFEWYQKLVMASPELWRAFLNSVIIAFGSAMMSTIIGTLAAVGTAR

YSFKLKSFVSTMSFVPMILPEIVVGVSLLIFFAGVGLKLGLATVWIAHTTFNLPFVYLLV
SARLEESDPSIVEAARDLGANEMQILFRIILPMALPGILSAFLTAVTLSLEDFVITFFVT
GPGGTTLPLYIYSMIRFGVSPVINALSAVMVAGTVLLIYPMRNFLKVFAAR*
>SPBIB_v1_130016|ID:27162442| ABC-type transporter, integral membrane subunit [Uncultured spirochete bib]
MKKQVGLYYSLPQIMWLLVFFAAPLGIIIAYSFLKKGLYGGVEPQFSLEAYTAMANPNIL
IVTWRTLKMSVIATILTLLIALPCGYSIARSQNQAMRLFLVIVPFWTNFLVRIYAWIAIL
GNEGFLNDIIRALHLRSESVQFLYNQTAVILVLVYMYLPYAILPLFSTIDKFDFTLLDAA
RDLGASRFQSYVRILLPNIKSGLVTAGLFTFVPIFGAYAVPLLIGGKDSYMLGNIIADQL
TKTRNWPLASSISMTVTILTTGLVFLIAMRKPREQKQVFDADPGLITPVASGGR*
>SPBIB_v1_130017|ID:27162443|potA| polyamine transporter subunit ; ATP-binding component of ABC superfamily [Uncultured spirochete bib]
MKGADVDIVGVSKFFGSFQALKNVSLSISKGEFFSLLGPSGCGKTTVLRLIGGFEEPDSG
TIAIEGKNVIGLPPDKRHCNTVFQSYALFPHLSVFENVAFPLRIRKIPQQLVREKVMRYL
SLVQLEMHAAKKPSQLSGGQRQRVAIARALINEPSILLLDEPLSALDAKLRQHMLMELDA
IHDKVGITFIYVTHDQQEALSVSDRIAVMNMGEVLQIGTPRQIYENPATEFVARFIGETN
VFSLKILSIDGTKIYGEVDGLGPMFVDDETDAKPGETVLATIRPEKIRISADLPNTNGGR
INVLHGIVAEPIYSGFQTKYVVQLDTGMMVTVYRQHANWSEGIPDIEWKDEVYLSFSASD
MVIVEMHEQ*
>SPBIB_v1_130018|ID:27162444| Glutamate formiminotransferase [Uncultured spirochete bib]
MGKPLVECVPNFSEGNDRAKIEAIANVIRSVPGVSLLDVDPGADTNRTVYTFVGSPDAVK
NAALEAARAARELIDMRMHKGAHPRIGALDVCPFVPVSGISMEECVALARDFGQKLAEEF
DVPVYLYEKAATRPERQSLADIRAGEYEGLSAKLSDPAWQPDFGPARFDPRWGATVTGAR
EFLVAYNINLNTKDKKIANDIALAIREAGRTTKDPNGNTIKVPGRLAAVRAIGWYIDTYQ
CTQVSINLLDFRKTPLHLVFDTVREEAEARGVYVTGSELVGLIPLEAIRACGQYYRKKMG
KSPGLADHELVEIAVQTLGLRSVQPFEPEKKIIEWAMSEISPLVTMKVASFVDTVSSDSP
APGGGSVAALAGALGAALAAMDANLTVGKKGYENVFEQLSALAVRAQELKSALLEIVDED
TKAFNAVMDAMRLPKSSDSQKKMRDVAIEAAGKQAALVPLHTARLCLDTMKLSLEAARSG
NANSATDAATGCNMAHAGLQSAIMNVRVNIKTLADEKFKSQLAQECVALEEQAEAEKAEL
DRFMTSVLAG*
>SPBIB_v1_130019|ID:27162445|tatC| Sec-independent protein translocase protein TatC [Uncultured spirochete bib]
MTYIEHLAELRKRLIVVIVFFLGFSILCFGWSKQLATFLVSPAQPVDFVFLSPPDLFMTY
LSLSLYAGFILSLPIILYEVGMFIWPGLEKRERRAIFLSLLLGALLFIGGAAFGFFVMLP
YMLRFFLGFGTEGIKPMISIRDYLGFVGQIVLSFGLAFELPVVTTALAGLGIITSAQLKS
ARKIAVLLIFIVAAILTPPDVISQLLLALPMLVLFEGSIVLASLVEKKSKSKVQEAA*
>SPBIB_v1_130020|ID:27162446|tatA| Sec-independent protein translocase protein TatA [Uncultured spirochete bib]
MFGRIGPMELILILVIALVIFGPKKLPEIGKAIGDAFKAFKKTQEDVTKEVDKITSDSPA
KSSDAKESVQKDADKNETAKS*
>SPBIB_v1_130021|ID:27162447| protein of unknown function [Uncultured spirochete bib]
MEGKEKLAFSAVILNGGQGVRFGGIDKQLLEIDGVPIGFALAKTLVQSCEEVLIVGKKNA
IYKPLEVQQIEDAVPGLGPAGGLLAGMMKARADWIYLCAVDMPFFSYSLSKYMYGRALSK
EADIIVCQYKGELQPFFAFYRKSLAGALASFVTSGVKPSLRRFISSRSYDLVSLKELQDI
GEQEAIFCNINNSGSLDAARQIALEHGLFIDTRL*
>SPBIB_v1_130022|ID:27162448| protein of unknown function [Uncultured spirochete bib]
MPSLSTFMQILKWADWDSPPEQMNCAMPFGEILQTIAQLLPVLDAAPERMDTISPDQAKD
LLDSAIRLYILAPSIVNVLLNYKICVEHGLPLHPTVYYELKEARKYRISHSLGEIQRANE
LFWKSIDVARACCRLDQRACQDLANFASELPFSVKSFIYTAVQDTYTWIGSQPSLLLALA
DKIRTSYTPHLVVAAAHGSIMPALVLAELLQVPLYFVRFSMFKRHDEEPIVSLSDQAWLF
DFRDKEVILYDEDVAGGRTLESFLKRLSPLFRQSKTACSIRHAGSSIQPDFFGKIWWE*
>SPBIB_v1_130023|ID:27162449|rex| Redox-sensing transcriptional repressor Rex [Uncultured spirochete bib]
MKMKLSYAPSIRRLPSYLHIIRTFQRTGEPYISGTWIANELNLEPIQVRKDLAITGIVGK
PKKGYPVEQLISAIEHYLAWDVEQKAIIVGAGNLGSALTGYQEFRNHGLHIVAAFDSDPA
KVGKSVHQIQIYKMDQLKEIVEAMNVTLAILTVPSPYAQSSCDALVAAGIRAIWNFTNVK
LKVPDDVLVQREDLSSGYAILSVMMRTRSEHLS*
>SPBIB_v1_130024|ID:27162450| protein of unknown function [Uncultured spirochete bib]

MRILVISGQRGAGKSSLCRYIAEYAKARSIACYGCIETSLRGKNGVPYQIGLADLASGQT
YLAAERPRDRTDVPFQFHTEAFAHIQASHEEFLHRTSASIQTSAPERFGQSHEKEICIID
EIGPLELERHLGHADFLKMVLDNRRCDVLVLTVRSSLQNALLAQLNENIPQYDVLIIEID
KKSRRAAQNAALSYIGLA*
>SPBIB_v1_130025|ID:27162451|iscR| HTH-type transcriptional regulator IscR [Uncultured spirochete bib]
MIEKEVPKMRATTRGLYALKAILTLAKTSDGQTPVSLHQIASFEGLSPEFLQQIFYRMRK
AGIIQATRGPGGGFYLSKKPEEISVYEILLAAGETLEIVPCAPERSRRRACEQFSSCDAG
KFWSKMEILIIEYARSKHLSDLMTAGGI*
>SPBIB_v1_130026|ID:27162452| Orotidine 5'-phosphate decarboxylase [Uncultured spirochete bib]
MDFFERLESIIETRDSVLCIGLDPAFSAEEIAAKGKHACADQALERNVRIIEATADCVAA
YKPNIAFYEALGDAGMAALRKTLDSIPKEIPIIIDAKRGDISSTAAAYAHALFGELHADA
VTLNPYLGLDTLDPFLEWKGKGIFVLCRTSNPGAGFLQDIIIEGKPLYIEVAKRCAALQK
KVGLVVAGNDLEALRKVRAVAPSQWFLSPGIGAQGGQADLAFAAGARDDGKGILVVAARS
IADASDPAKAAVALRDAMRTARDTMLKQNKTENIQVAAPAAKAAQKAIADLKKAFVDALL
STGCFRLGEFTLKSGKKSPFYIDLRKLVSDPRAMKIAAQAYASLASECEYDRIAGIPAAG
LPLATAASIEIGKPMIWPRMPVKEHGTGNRVEGEFRAGEHILLLDDLITTGASKIEAIDI
LRSEGLIVQDLVVLIERGAQGRQDMGSAGVNLRAFIHVKELFDVLLQNGTIDSRKYEQLM
EYVAQE*
>SPBIB_v1_130027|ID:27162453| conserved exported protein of unknown function [Uncultured spirochete bib]
VRRIALFLVLLTILTPVFAQDNQSKGFDLGIVLGTDLLPDPKDTTIPPAMDSWTKVGFRP
EVAFGKLAVGLDLTFRFKFASGSTTSFEIYEPDWIPQPGKTTILDVYLPKLLYVRYGFQG
IDPFYFKAGSISDFNLGNGLIMDNYSNMLFLPQRRIFGMQVGVDGNLFGFPYVGIEALSG
NLAKFDVIGGRVYIRPLAFMNKSLFGRLQVGAIGVYDRDPLMYADDTAYGTSFGTSSPTY
VIGVDVTLPILQNPLFSLTTFAEGAMEVNKAMGAMTGVRGRLLGFISYGLQLRYLQDNFI
PAYFDTNYDLYRAERFAFIRDNPAGTAYTPSWLASAGVDIFKSLINVRATVDAPFKALPS
PATSNPADYPHLLGRLTLNKGLIPNFSVAARYEKYFLGKKSGNIFSDLIDPTDASIGMTV
SYWAGAAVVSLDYIYSWNPTKNDFDVSSGLSIGFQL*
>SPBIB_v1_130028|ID:27162454| putative DNA mismatch repair protein MutL [Uncultured spirochete bib]
MMGVIRILPQETSRRIAAGEVIDRPASALRELLDNAIDADARDIAISIAQGGIEEITVTD
NGFGMTKEDLELSVLEHATSKIYEPDDILKARTLGFRGEALASIAAVSRLEIASRPRGSN
EGWLLRSVPLQPPAIDAFPCKEGTRVTVRGLFESYPARKQFLKRPQSEALLCRTTFIERA
LSHPHLTFRWQSANDAEVFLPGTHAERIARCYPDLAHAPLFDAVSEAQGLHIHIVYADIS
VYRRDRKYIQVFVNRRKVPEWGVLSLVEYEFAKYLPGGAHACAFLFLEIDPGLADFNIHP
AKKEVRLKNAQEVHAAIHALLSSELKARYSGSTQDLSARFSDMQEPEFFAADRSQPQLNF
AASAFAETRRQEWGARWPSFSEAQNQKRAASNSLPADFWQKIRANNENGPRYIGRGPGPF
LLFELDGILFIMDQHAAHERILYDRLKAKEGISQSLLVPYLIDPEDNEHFLEEAAQALEF
IGYRISKEESAWAVEAVPAIAASQALEALVEWLHQPSVESTPLDSIAAALACKAAVKDGD
ILDQAAAERLIAEALALKEPRCPHGRPVLIAFPREKLDAMFGRTLA*
>SPBIB_v1_130029|ID:27162455| Metallophosphoesterase [Uncultured spirochete bib]
MKKKLRILCVADEVDLLVYSSQINERFADIDLVLSAGDLPDEYLEFIASMLNRPLVSVAG
NHDRSDSPRDRESLRYISDQQRPGLGRIRFSIKKESGISILGLPGSIRYNNGQNQYSDAW
MTCKIICMLPRLLIRRLLFGRSVDIILAHSPPRGIHDGGDPAHTGFSAYRWLIRVAKPYY
FIHGHVHLYDLQSLRELNVGDTAVVNVYGHSVITLLKEDRDAG*
>SPBIB_v1_130030|ID:27162456| conserved protein of unknown function [Uncultured spirochete bib]
MPDDFHTQAEMDFYRAKTKALTARIIGMLKPSVTDLMPFEEAKQLLKPQSETYRGITTVP
LDKIIGSEGRYRDFTRFFFPKKEHLKARWTGIDTLHYKDIVLPPIILYEMGGVYFVRDGN
HRVSVARSLGQEYIDAEVISLQSEIRLNADMSIDDIKHAVIEYEKKRFYQETNYPHIVGT
DDLDFSEPGRFDTIREHVYVHKYYLNQHMTEEIPFHQALYSWHENVYQPLGQAIEAENLL
SLFPGRTVSDLYIFLVAHWDDLKRKYGRYVEIEEAAESFKSQARSARKKPAGMLKALILN
FAKKVKNFFEKS*
>SPBIB_v1_130031|ID:27162457| putative 1-acylglycerol-3-phosphate O-acyltransferase [Uncultured spirochete bib]
MSMLINSAIVIGALLTLFALEFSLVLEYIFAPRSRKNALCSRYEQYGAKLLFGLFRIFRG
FNVDIRNPRKLHIPKHCLVIANHQSLLDIIVLIYMLGFERMPRFVAKKELQFGIPLVSFT
LRKGGHCLIKRRGAPIDTMRSISKMARSCKLEQASPVIFPEGTRSRDGRLGVFYAAGVRK

ILEIEAIPVAAIAIDGGWRVATIRDFLRRFGKEPYVVEIVEIYEAPRGKHEIARVLDDAH
KKIGMSLETMRSLI*
>SPBIB_v1_130032|ID:27162458|rpsT| 30S ribosomal protein S20 [Uncultured spirochete bib]
LSARNLSAEKRQRQNEKRRLRNKSAKTAIRSAAKKVVVAATEKKDTTAAKEALLQMIKLI
DSAAHKGIVKKNTAARKKSRMQKLVNRLG*
>SPBIB_v1_130033|ID:27162459|hup| DNA-binding protein HU [Uncultured spirochete bib]
MADKLTKAELIDALYESLSPSSRTTRKEIHELIDGLFSEIKSAILEGKIVELRGFGTFEV
KLRKGRSKARNPKTGEIVSVSDHGVATFRPGRELKKASWEMNTDRIPKSFRKRD*
>SPBIB_v1_130034|ID:27162460| protein of unknown function [Uncultured spirochete bib]
MKVLSFEPPEPFATQIGQSVILNGKLRSNLPLSLRCTLRGSIEGTFVRIERSAHIEANSC
TVQNAVIAGCFTGRIKAVDSLVFLPSSHISADIYTARLQIIEGATFEGGIEMPGFSATDD
VTGTSH*
>SPBIB_v1_130035|ID:27162461|rho| Transcription termination factor Rho [Uncultured spirochete bib]
MAIYRRKKSAPAQPESPDTTNYDSSLTKEDRIMNESQVEEQFSANNGTLFDEHTVQPPED
KDQNHTDVHSTDSTRNQTQKARRPVAKIRLKKKPSNTHIEQEESQIVEETEPESAEISST
NGFVVASLPSDDHQHQKFAEASRLELESRADQRNETRARLSINDLSKMGIVELRELGAKN
GINHDLLITLKKQELIFNILKAHTEHGGIIYAYGSLEILPDGYGFLRSPQNSYLPGTDDI
YISPSQIRLFNLKTGDTVYGQIRSPKEGERFFAMLRIEQINFNEPSVAQNRIPFENLTPL
YPTQKLNLETATEEISTRIINLFCPIGKGQRALIVSPPRTGKTILLQKIANAITANHPEV
YLIVLLIDERPEEVTDMERTVQAEVISSTFDEQATRHVQVAEMVLEKAKRLVEHKRDVVI
LLDSITRLARAYNQTVPTSGKILSGGVDSNALHKPKRFFGAARNIEQGGSLTIIATALID
TGSRMDEVIFEEFKGTGNMEINLDRRLSDRRLFPAINIKKSGTRKEELLLTEEELQKIWV
LRKVINPMDDIEIIELLIDRMMKTKNNDAFLKSMNTPYQGMD*
>SPBIB_v1_130036|ID:27162462|rpmE| 50S ribosomal subunit protein L31 [Uncultured spirochete bib]
MKQGIHPKYELTTITCACGNVFETRSTVKNIQVEICSACHPFFTGKQKLVDTAGRIERFN
KKYGIKSSENK*
>SPBIB_v1_130037|ID:27162463| Non-canonical purine NTP pyrophosphatase [Uncultured spirochete bib]
MELIIATNNHYKAHELKPLFPRHALLLPADLGIADFNPEENGTSFFENARIKAEALYALV
HKPVLADDSGLCVDALDGKPGIESARYGSVNGHLLSADEKNSLLLSQLAGIRNRQCAFVC
CLVLYLGKQRFIAVEETLEGQIGDAPQGSHGFGYDPIVFLPSYGKTVAELSAQEKNRISH
RGKAAQRMAAILDALEGNLLD*
>SPBIB_v1_130038|ID:27162464| protein of unknown function [Uncultured spirochete bib]
MDSKNYIELVRRMYAHPRLYGFFSEDEISEALSRYRSRIETILDRAEKESITREAYLLSS
MRFVAKSVHRHYYSSNLSENAYVYSHFSEDAVLEVPADAFFDRNRRRIQDADTSGIGFSP
QVFIGKLAPERRRLLYLVIKCAWDIDEELLGKCSYALGMPEQYLFNLIELVKRRTEASRS
QIHQLNEKLNILWIRMRVLELRLDTAIAKSDREEVRSCLERCRKRYRQLLEKRSHRKSSI
SNEFISDLLNVPKGSVDSGLYYLRKNAQSRKPLAEYQELG*
>SPBIB_v1_130039|ID:27162465| protein of unknown function [Uncultured spirochete bib]
LCDRVEQKSFCVVLSDPVQALSVAEDSRICPPNIMPFPFIAKLEPYYCRVRAEISLFRP*
>SPBIB_v1_130040|ID:27162466| DHH superfamily protein, subfamily 1 [Uncultured spirochete bib]
MKPAPKSLLEFIEGHDCFYILGHREPDGDCIGSQLALASMLQSMGKRAHVLSSGPFNRIE
ILPFEARFKSEVPAERDFERTAALVLDCSSMSRIGNIAERMPDIPAAFIDHHATAGAVGP
YDWLDESAPAVSAMILLLMEAMNHAPTKEEAELLFFGLSTDTGFFRHLDEHSSETFRIAA
RLVDAGVSPKRVFMAINGGRTLASRRMLGELLLRIQPYYEGRLLVSWVTIDDQQKYGMSS
RDSDLLYQLMMGIVDCEVCFVVKQETDDMCTVGLRSRDSVNVAKIAEKYGGGGHRLAAGL
SMNGKVNQVVHTLVDAFSSVFSEDTEGK*
>SPBIB_v1_130041|ID:27162467|argF| Ornithine carbamoyltransferase [Uncultured spirochete bib]
MNPSALKGRSLLTWIDFNPEEIRYFLELSKKVKEEAKKGVRKQRFKGKTIALLFEKRSTR
TRAAFETAFGEEGGHPVFLSNQDIQLGSKESIEDTARVLGRMFNAIEFRGFKQEYVEILA
KYSGVPVYNGLTDLYHPTQALADVLTIEESFGSCKGKQLCFVGDGRNNVARSLMVISAKL
GMHFTIVSPKELWPDNELKNLCETYAKDSGSKLKITDDIEEGVKGADAIYTDVWVSMGEE
ALKAERIKLLSSYQVNAELMAKTGNPNCIFLHCLPAVKGEEVTFDVIEGPQSRVFDQAEN
RKHTIKAIMLATI*
>SPBIB_v1_130042|ID:27162468| Endonuclease III (modular protein) [Uncultured spirochete bib]

LCALACCNMAIVDNLMDSTQNSRPSKILASYSVPALLDEVYGRLVPIWPDAHPLLHYRSC
FELLVAVILSAQTTDEQVNSVTDELFNRYPDAHSLAHAEISEVERIIHPVGFFHVKARHI
IEAAQMIEARFDGKLPPSFDQLLELPGVGRKTANLVASACHEVPGIIVDTHVLRVLCRLG
ISPKKDPSLAESIVRAHLAPEKHTHFSYSVNRHGKFTCTARKPACTQNGAPCPLEDICPK
IGITSLKNCDAE*
>SPBIB_v1_130043|ID:27162469| protein of unknown function [Uncultured spirochete bib]
MESEYPESSEEIQEIPPDPVAKLARERFKIDHLAPLQRFVIANILDADTENLLPYRQLVL
FPTGFGKSVCFQLPSLVLEGLTVVVYPLLALMNDQKRRLDEAGIPCALFRGGLADEEWRA
QETAVSSKKARIVIANPEILATSRLRQFLSRCRIAHFVIDEAHCISEWGETFRPTYLTLG
ESAEKLAPHVMSAFTATAGPDIVSSIEKRLFRGNAYRLVTSEADRPNIHYAVVQTLSPTR
TLRRLLSSCQKPAIIFERSRPGTRMKAEFLRSVGFAATRFYHAGLSRAERNEIESWFQKA
EDAVLVSTNAYGMGVDKKNIRTVIHTGLPDSAEAYIQEAGRGGRDGKDSFAVLIHNIAER
AGPVGSDLPLQEQRKARFAPYPLIETCRREFLLHLLGETETPVCGACDNCELEMKMARSF
SSDGSPPRWRDMSIHQRGMPRCMPPHWRLLLQAEGFLETLLLVAANQRRWTMSECIRMLG
PHGIGRGNYTGGLYGWSRTEREEMVSSLLALSIIEIPRRGIWKGSICLSKEGREFLSVFK
RFAARSAHHSWRAELEV*
>SPBIB_v1_130044|ID:27162470|pgi| Glucose-6-phosphate isomerase [Uncultured spirochete bib]
MVTYKNLDRCSSFDSLKKIQAIKLSEKLSAQRIASFWIRHAAAMSYCYAFAPVDDQIIEK
LQALTDEQELVEKYRLLVEGEIMNTGEKRMVLHHMARGRLGKTVRWEGQDMEAFYSQEKQ
RFYEFARKVRDGSIRSSVGTPFAQVVQIGIGGSDLGPRAACIALSRWAVSHKRARLAPWF
ISNVDPDDADSVLSHIDLSESLFVLVSKSGTTQETLANETLVRERLRASGLNPDLHMVAV
TSKTSPLAGNPHYLDSFYIDDFIGGRYSTSSAVGGVIISLAYGPETFEEFLQGAHEADKL
AFEPSLRHNPVLMDAMIGVYERNVLGWPCTAILPYAEPLSRFPAHLQQLDMESNGKSVSR
TGEPLSYSTGPVVFGEPGTNGQHSFYQLLHQGTDIVPLQFIGFLQNQISPDMHSEGSTSR
QKLLANLVAQMLAFAIGKSDSDPNKMFPGNRPSTLIIGDELGPRQLGALFSHFENKVMFQ
GFAWNVNSFDQEGVQLGKVLAKKALGAISKGESTFDARLDALISAALHTSLKAQMQPDTA
GKE*
>SPBIB_v1_130045|ID:27162471|murG| UDP-N-acetylglucosamine--N-acetylmuramyl-(pentapeptide) pyrophosphoryl-undecaprenol N-acetylglucosamine transferase [Uncultured spirochete bib]
MMSAEIAAQPGETQHAATQAGANAQPCIVFTGGGTGGHIYPGLAVIDELRRLGFTGRIAW
IGSEKELDRSIVASHGIEYFAIPSGKFRREISFQNLTDLGRIVAGYFRAKSVLKMLRPAL
LFSKGGYVSVPPCRAATALGIPVFTHESDASPGLATRLNSAHAERILTSWEDTARCFPQS
LRSRIIRTGNPTRPDLFEGDKARGLKLLGFSDDRPILFVLGGSQGAKEVNRLVLSSLSRL
CPTVQIVHQTGQAHYEDVLHAIPDDPAIRAAYRPLAYIGKEIADIYAASDIVAGRAGAGT
VWEAASLGKPMILIPLSGTGTRGDQVENAALASKAGAAIVLEGDKATPEAFVAAVQSFLN
PEAYSAALNACRKLAQVPHDFADPNLQSLDVANSINNAGSQYATEFIARLILARIGWRAK
ETQ*
>SPBIB_v1_130046|ID:27162472| putative Colicin V production protein [Uncultured spirochete bib]
MSTMDWVFSAVVVILAARCFVRGFVHEVLSVASVAVGFLAGLLLSNTVIVQVLPKVGANS
LPYQAQYIIAFVLCFIAGFIIMKIIERMIREGLEASSLDIFDRVLGLALGIIEGFIIVGL
FIVILQIQPFFDTKALLAGSLYAKLLGPIIEPALGGSITPLLKNGDLPGVIQNLKGK*
>SPBIB_v1_130047|ID:27162473| putative DNA polymerase III domain-containing protein [Uncultured spirochete bib]
VFENLLFQDKAKEQLLAMIASGTVPPALLFAGAEGSGKLTAALEFARVLSCEKEALWNCE
CAQCAHHRSLTHPDLLLFGPRSFPQEPSAAADYFLRTPNATSYYTFVRAVRKLLKRFDPA
LWSGEEARLSKAASSIEAIEEQLQDISSMLASGHTQKLGDTANRIVETALSLEPLVPDGI
PVFMVRNMAAWASLTPAGKRKTIILQNADTANESARNAMLKILEEPPESVRFVLTASRRA
TVIATILSRSRLISFDARTSEQAQQIVSRLFRTQEKVEDVSEFFRKKSPFPPDRAESAAA
LFVGALLADAMRGDEAISGELASALVSKARESRVSVLDVLRSVSEETGGFGLKNAKFADA
PSVFLRAVARQLARISSDASSSAALVMLVDRLSSKLRDVAVQNRTYNRSPELLLEAFANI
FGVSHESSV*
>SPBIB_v1_130048|ID:27162474| PAS/PAC sensor signal transduction histidine kinase [Uncultured spirochete bib]
MKAVYENALRRIDRIPPEGLKQIINNLSAENSIYEAALNSTLDAIIVCDLEHKPLFINRT
AEKILKLPSWTGEFPIWEQLKDDEIVGFFRKTLLADENILNREISLNRPGGTRVLSISIS
ALLSNGRISGNLVHMEDITEKRRREAQLRRAESLAALTTLAAGVAHEIKNPLASIRIQLG

IIRRILEKNCNKKTESIFHNISLVEQEIDRLNAIVVDFLFAVRPMDITLIHDRADEVVRE
VAELMSKEAESQNIRVITKFDADLPNVMIDRKYLKQALLNIVKNAIAAMPEGGTLTISVS
VRNDELLIAVSDTGIGIPEELLAKIFEPYFTTKESGTGLGLTITFKIIKEHNGEITVESS
PGIGSTFTIHLPIPQPEQKSLPPWEDGVKIDGSHTED*
>SPBIB_v1_130049|ID:27162475|atoC| fused response regulator of ato opeon, in two-component system with AtoS: response regulator ; sigma54 interaction protein [Uncultured spirochete bib]
MRATLLVVDDETNIREGLAQLFSMEGYDVRTAKDGLEAKTIVDREDIDIIITDYKMGRMS
GLDLLQYVVSTHPGIPVIILTAHGSVDKAVEAMRQGAFDFVAKPPNTDHLVNLVRRALET
RELYRTNIELKAEVESQRARSYIIGQAPSIKRIFDLIRKVAPTKASVLITGESGVGKELV
ADAIHNLSPRKDKPFIKVHCAALAESLLESELFGHEKGAFTGAAARKRGRFEMADGGTLF
LDEIGEINQNVQIKILRVLQERKFERVGGEETIEVDVRLIAATNRDLKKEIAEGRFREDL
YYRLNVVNIHVPPLRERREDIPLLAMAFLKEFSEENGKQIEGFDPKARQALYTYDWPGNV
RELRNCVESAVVMASGKLITIDDLPPGPRSSGQTRNICIPALSSMEDAERILIAETLALV
GGNKSKAAEILKIGRKTLYQKIEQYGIGSQEAPASSASGE*
>SPBIB_v1_130050|ID:27162476|whiG| RNA polymerase sigma factor WhiG [Uncultured spirochete bib]
MDYASLEENRLWELYRRTADIHIRDELVLRYCPLVKYVAGKVSVTLPSMVEYRDLVGYGN
FGLLDAIEKFDPKKHVKFKTYAVTRIRGAIYDHLRELDWVPRSVRRKTKQIERAIAKLES
HLGRPATDEEIAAEMGIERKELMKIMAKVASSSILSLQEIWPMGAESEGGTIGDTLESLH
SANPDVTLEREEIRRVIVEAIQELPDKEKKVLVLYYYENLTLREIGRILEVTESRVSQLH
TKAILRLRAKLSNRRKGIL*
>SPBIB_v1_130051|ID:27162477|hslU| molecular chaperone and ATPase component of HslUV protease [Uncultured spirochete bib]
MAETDTLTPHRIVEELDRYIVGQKKAKKAVAIALRNRIRRQRLPEEIREEIAPKNILMIG
PTGVGKTEIARRLAKLSGAPFIKVEATKFTEVGYVGRDVESMVRDLMASAVSMVKEEMET
QVAEEAKRRAEDRLLEILLPGIASSDEPVGESQATVIVPSSGTETREKFRTLLREGKLDS
REIEITVPAQGPQIELFAGTQFEEMNIALGGLQNLFGGKKKKKTTTVAEALPLLEAEERE
RLVDQEKIVQEARRRTQESGIIFVDEMDKIANREGKGASGIDVSREGVQRDLLPIVEGTT
VNTKWGPVDTTHILFIGAGAFNVAKPQDLIPELQGRFPIRVELDALTGEDFVRILTEPKN
ALVTQYTQLLGTEDVTLEFTEDAIARIAEIAAAANRQSENIGARRLQTVMERLLEDVSFE
ADKYAGTKVTIDKAYVDEHFKDYVQQQDLSKYIL*
>SPBIB_v1_130052|ID:27162478|hslV| peptidase component of the HslUV protease [Uncultured spirochete bib]
MEIHATTVLAVRKDGHVAMAGDGQVTMNNTVLKSNARKIRTIYNGKVLCGFAGATADAFT
LFEHFEAKVQEYGGDLTRASVELAKDWRTDRILRKLEAMLLVADQTKMFLLSGTGDVVEP
SEDAIAIGSGGTYAYAAAIAYLDASRRVEEAAGAQGVSDLTSIPGYFSAREIAQKSLEIA
SSICIFTNDRIVVEEI*
>SPBIB_v1_130053|ID:27162479|xerC| Tyrosine recombinase XerC [Uncultured spirochete bib]
MDARLVDYLEYLEAVRGLSPKTVEVYRRDLAQYEVFLDGKNVDAADATDIRAFAGSLVMD
KRAPASVNRALSAVRGFYKYRVRFHQNEADPAREVENVPAGRPLPSFLFEEETKTLLDSL
DGNAFRDVRDRALLEVLYSTGSRVSEVCGMELPRLNLSAGSVRVKGKGSKERLVFLCETA
RAALKRYLPYRAALMMRRGIEEHDRLFVNAKGLPLGPRGAEKIVERRRVQAGIKKHLTPH
TFRHSFATHLVAAGADMRVVQEMLGHSSISTTQVYAHVDMERLRRVYEQAHPHGSKGK*
>SPBIB_v1_130054|ID:27162480|topA| DNA topoisomerase 1 [Uncultured spirochete bib]
MAASKKEMAGVSAKPAKQKTLVIVESPAKARTIEKYLGTHYRVLASNGQVIDLPKSRMAV
DVEHDFEPEYITVRGKAAKLKELTDEAKRSTAIILASDPDREGEAIAYQIGKYLKEKTKD
TPIRRVTFNEITRPAVQEAMAQPRDIHMSLVEAQKARRVIDRLVGYTLSPLLWKKVKSGL
SAGRVQSVALKLICDREQEVESFIPEEYWTIDAHLRAHQHLVRAELALFDGEKPSIRNES
QANSIIAALSGKPAVVTDIQYTDRSIKPKPPFTTSKLQQTAANRLGFTSTKTMKVAQQLY
EGIDMGHQRLGLITYMRTDSTRISESALRETHEWLAQYFPAQTPDSPIRYSVSNAAQDAH
EAIRPTRVDITPDEAARYIKGDHLKLYTLIWERFVASQMKPAIIRTYTADIQVGEGLFRA
SASSFLEEGFYKVIRLGASKEERTSHQLPLEKGQTLVAEKIQGLQHFTQGPSRYTDATIV
RALEELGIGRPSTYAPTIETLIERYYVQRDKRQLVPTALGKIINEILSKNFPEVINTSFT
ARMESMLDKVEEQKVDWVSELKKFYFPLKDKVADVMHALEDMHGTLDEATNETCPKCGKP
LVKKLGRYGYFLSCSGFPECTFTKSVPLAKCPKCGGDIVPRVSTKGRRKKFYGCSNYPEC
DFMTLYKPTNATCPKCGWFLVEKYDKKKGMHKACINPDCDYLHSSDDGKEGA*

>SPBIB_v1_130055|ID:27162481| DNA protecting protein DprA (modular protein) [Uncultured spirochete bib]
MKAACAELRVNLHGGISKMAHRKPRVEMRSSLVHAELERIAENIPLLDAVELPLKLALHR
LHFLSWQERLLILENIHCKEDFLSLGLAEIESFLYRSIRAREPDMARIWEQAERDAEQLV
RMGAKFVCVTDKEYPPLLREIHRAPFGLFVRGDSRCLGLQAVTMVGTRMPTWAGVRAAVH
LAREAADAGLCVVSGLARGIDAAAHRGALQSAGKATIAVLPCGLDRVYPPSNRPLAAGII
AAGGCLVTEYPPGVSLDRYRFPERNRILAGFSRLTLVIEAPEHSGALITAEFALNEGRDV
AVAAECSGSSRNAGADALSKEGACQVRSGAEIMAIMSGSGAWDWANGRSGIIGRD*
>SPBIB_v1_130056|ID:27162482|xerD| site-specific tyrosine recombinase [Uncultured spirochete bib]
VSILVRRFQAYLLSTRRRSILTADVYIREIEMLETFLLKYGKTPLDASGEDLLTYLVYRS
QSGLQRTTMARIVASIHSFYRFCLNEKFRTDDPSIQIRTPRQDRNLPDVLAPESVEYVLD
SIDISTPNGLRDRALFELIYSCGLRISEAAGLTFQQLYLEEKLLRVLGKRHKERLVPFGD
EALYWLKKYLAEARPLLEKNGKSDFVFLNQEGRGISRKGIWKRFSQIRAKSGIKAKVHTF
RHSFATHLLAGGADLRTVQELLGHTDIATTQIYTHIDEASLGMYHKEYFPRK*
>SPBIB_v1_130057|ID:27162483|ftsZ| GTP-binding tubulin-like cell division protein [Uncultured spirochete bib]
MAIELVDDNGIGSPTVIKVIGTGGGGSNAINRMIMAGLKNVQFIAVNTDLQDLGRSKAEI
RLGIGSKITKGLGAGGKPEIGEKAAVEDRDKIEQALRGADMVFVTAGLGGGTGTGSAPII
AQTARDLGALTVAVVTKPFTFEGRIVNRIAEEGFSKLKQAADTVIVIPNQNLLKIVDKKT
PLKEAFRIADEVLRQGVQGISDLITLAGDINIDFADVRTVMEGQGDAIMGIGVGNGENRA
QDAAEKAVNNPLLDDARIEGARNILVNVTCGEDFSLTEYQDVIDLITSKAAEDAHIIAGV
VSDSSLKDEVRVTVIATGFGAARQKFSDKTPSKSGISMPLGSARPSEFLGPNEWPLREDR
PGQDRDRPAADRDRPAPKENFGKISFDPESDNDELDIPTALRNKRLMSEDFGGRR*
>SPBIB_v1_130058|ID:27162484|ftsA| Cell division protein FtsA [Uncultured spirochete bib]
LIFNQALWCIKQRRPSLADTSIVALDIGTSVTKAIVGAFDENGEFEIIGIGTALSNGLRR
GVVLNIEGTLNSITSALDAAELMSGADIRAVSLALSGANVESFNTNGQVAITGRGREITQ
EDIVRVHEAARAVSISMDREILHVIPRSYTVDEQKGVRNPLHMTGVRLECGVHIITGSVS
TVQNMFKCVTRAGYRVEQSYLGILGSARCVLTDDERDLGCLLIDIGGGTTDFMVFADSEP
VFTSAIPAGGSQITGDISIMLSIPVDAAERLKKESGASWADAIDPEETVIVPGFGAREPV
ELERRKLVSIVQPRVEEIFEMVNERLVKNGLKEYIKAGVVLTGGSALLPHIDDCARAVFG
VPVRIGTPLAIQGLDAEYRSPAFSAVVGVALLEAERTGAVSESSGRKKVRKGKQGSFSLF
KWIKDRFF*
>SPBIB_v1_130059|ID:27162485| protein of unknown function [Uncultured spirochete bib]
MTEIEMRERQLAEKQTTQTERTPTRQPQAKQPQAKQPQASQSQNERSLLSVSDTYSVNIL
HSTTRRETSMPGTAASKGIANKLAAEPARGASGRAAFPAGRVAPEPGPVAPASGRISFPS
GRVAPASVKLTESADRLSAALTRAKKAKKPSGLRILAALALFCVLAAVASVALPRLTKIE
SIRISGLETLTENAIVSAIGAIGDESLLTLNLKEIKARIEQNPRVAQARVFRMLPSALGV
DVRERSAVAVIMISSDQGSKLALIDGEGTAFALVEAEDSKSPDLPVISGVHFEQFQPGQR
LPDMLGPLFADLQKIRNESPELLLAFSEIRIVRISEYSAELLMYPVHMKTAVRMPLRLSA
DALRNSLVVLDILRSRGYGNQPSEIDFQSGTVVYQTKEAVSG*
>SPBIB_v1_130060|ID:27162486| putative cell division protein FtsW [Uncultured spirochete bib]
MQKESRTDRQDLQQRQTDWQMIAAIIVLSMIGLFALWSGSIGFAIRNGGPNSIIGKQAIL
YAVSLVFMALVAVLPVRFLRGVLPFVVAIGIVGLALPVFSPLGVMINNARRWIRIGSFTL
QPSELWKPVLVLYLASFLEKRKEHIVESGMATFPALVLVGLSAFLIYLQQDFSTSILVVA
IALAMLYLAGTPLVFIASLGFFSLLYGGLMIFSSQYRIERLVGFLMPDHHAHTVNYQMYS
ALRAIRAGGLWGKGIGLGSLKISSIPEVQSDFIFAAFVEEAGFIGVTVVLALWAFIMVRA
GKGLARTDYFSKLAGFGLITLLSSQMLLNLAVVTGIVPTTGLALPFFSSGGSAALMTSIT
CGVLINLSGSHERIAHSEMSFVMKGAAHD*
>SPBIB_v1_130061|ID:27162487|mraY| phospho-N-acetylmuramoyl-pentapeptide transferase [Uncultured spirochete bib]
MLREIFLPLVKYFTPFNIFRYLTFRSAYAAVTALVICYLIGPWMIEKLRVLKFGQSIRTD
GPQTHLAKTGTPTMGGILIILAVVVSVVLWIDVKSGYSWIALLSLVGFGLIGAADDLLKI
KKHTSDGLSPIQKLALQFVVSGAAAYAIYRITGPTATKLYVPFFKVHVIDLGAFWLPIAM
IYVTAWSNAVNITDGLDGLASGLVIFAILAFSILTYITGRADWSQYLGIPYIKQASELTV
FNFALLGACVGFLWFNAHPAEVFMGDAGSLSLGGVLGVLSLMVKKELLLLVIGGVFVMEL
GSVMLQVLYFKMTKGKRLFRMAPLHHHFELLGLKETKVVVRFWILGGIFALIALSTLKIQ

*
>SPBIB_v1_130062|ID:27162488| UDP-N-acetylmuramoyl-tripeptide--D-alanyl-D-alanine ligase [Uncultured spirochete bib]
MANPDLPTAELPVLFRAGELAGLIGGACKGFLDAPIRSVVADSRAAKLDSMFVALKGERT
DGHLYIKQAIENGARVVMAEFSRRDMALDAIHASNMGEKICLILVDSTMKGIQQAACEYR
RRNSLLRIGVTGSSGKTTTKECIAAILSACYPPESVAMSPGNLNSDIGLALALFDLRPQH
TIGVFEMGINRKGEMDELASMYEPDIAVITNIGTAHIGMFGSRDEIAKEKGKIFSHFDGH
QKALLWEDDQYRQFLASLVRGTVHYYGLKSTEGIEKIENLGLKGWRIGWKQQTIEFGFPG
QHNLLNACAALSIANMLELDPAQASRGLSSVKPLFGRSEIREGRITVLQDCYNANPDSIA
AAIEFFSALPSDNRKILVLGSMLELGEESERAHENVGLMAGNSGAEAIFLFGQEMAAAEA
ALKGRSFKGMLMLTDSMNELIEAVSGFVQPGDLLLVKGSRGMALERLTKALEEKGFLSPS
GKASGGSHAS*
>SPBIB_v1_130063|ID:27162489| putative cell division protein FtsL [Uncultured spirochete bib]
MNARRFWILAIITCLFIGLLVFQTSRYAALKSNVISLQRMEMDLIKTGKELDANIAKLVS
LERIEKAALKNGMQIAAPEQRIIVTDIQAGNKQEASTNGKP*
>SPBIB_v1_130064|ID:27162490|rsmH| Ribosomal RNA small subunit methyltransferase H [Uncultured spirochete bib]
MPEQIEGKAAELFLHVPVMLEEVQGLAAKIQPLSLYIDCTLGAGGHAEALLSMYPDLRCV
GIDADPDACERAFGRLSRFAERLSIINAYYDDALADLCVCSSAGKVLGANLSDLCNSSHA
PRASFILFDLGLSSYQLGASGRGFSYSADEPLDMRFSPDAPASAEELIHQLSEAKLADLI
YAYGEERYSRRIAHAIVAARKKESIHSAARLASIIADAVPAQYRHGRIHPATRTFQALRI
AVNDELGRIERALVSALELLAPGGICAVISFHSLEDRIVKHFFAEKAKTGRFSLEWKKPR
GPSLEEIKANPPSRSAKFRAIRAVAMRSEQAQGASA*
>SPBIB_v1_130065|ID:27162491|mraZ| Protein MraZ [Uncultured spirochete bib]
MKLLTGEFKNMLDEKGRLSLPARLRSDLPSSLVLTQGVDTCLWLFAPEEWEELSQRILAS
TSLFQARSRLIQRRIIAPAQEVEVDKLGRISIPQSLRDWAGLSKECIILGISRYVEIWDS
QRYQAYLAENQSEFLSAAEELGGLTLS*
>SPBIB_v1_140001|ID:27162492| putative PP-loop domain protein [Uncultured spirochete bib]
MNAILAGKREPWFERLLLRMQAAITQYSMIGREDGILVGLSGGKDSLALLALLAVWNRGV
RPLRACYVENRQFPQNKGSLEALGTFCALLRVPFSIREIETDVRLGCFPCSHARRKALAE
EAVERGCTRIAIGHTRTDLVQTAILNLAHHGTLQGLLPIRQYFENAFAVVRPLIFLDETD
IWRIVRRLDLPVQKSSCPLADNSERAKAAAILKALESMYPGAAANIARAANSALNLV*
>SPBIB_v1_140002|ID:27162493| conserved protein of unknown function [Uncultured spirochete bib]
MSEISLLNALVHHFDVVVRDPVWGDVHMDAPLHALFQSKSFMVLDRIRQLGPVALVYPGA
THTRKAHSLGVYYIARRMALALVEKGEIDFVSKTGLRSFLVAALCHDIGHFPYAHSLKDL
PLSRHEALAGEMIVSEPLRQYVSSCGADPEQTAAIIDPDRSAGESRETLLFRSFLSGVLD
PDKIDYLTRDAFYCGVPYGIQDADYIMRRLVVHDDRLAIDLKGEMSIEALIFAKYQMYRA
VYWHPTVRAATAMVRKAVYLGLAEGVLQPNSLYGLDDTSFGRLMNHSKFAPFKLAREAEE
KHIFRLADEHAFDEENPVHADLLDISKRIDAESTLAQAAELPETDVVIDIPERIKLETDL
MVKIDDHFEAFDQRSTLFQPETVAKVAGALRTIRVYLRPQSNPDLKKIQALVQALLG*
>SPBIB_v1_140003|ID:27162494| Methyltransferase [Uncultured spirochete bib]
VLTSRLDFSDYEHVDFVSGYIFHAADGFLGHLLEEVPAVTRVDGELVSSWNKNGKAPFDL
ASHPVFWTRNVWVEPFLLEFGSISEAARTLKAIQRNWSSVPVRFARRTVLISEQLPHIPT
KPRRFPFELPSAPMGAFTLLDEHLILGSAKCSNPFPGGAFEFEENKNDPPSRAYRKLWEA
LILAGKMPRPGERCLDAGASPGGWTWVLASLGAHVLSVDRAPLEPRIAQMPNVEWMKHDA
FTLKPEDIGRVDWLCSDVICYPEALWQWISKWLASGLAKNYICTIKMQGNTYDRATTLKF
ASVPGSRVVHLWHNKHELTWIKLDAARTQTELSATQQA*
>SPBIB_v1_140004|ID:27162495| UvrD/REP helicase [Uncultured spirochete bib]
MDIDFRSELDPEQYAAVTAAEGPVLIIAGAGSGKTRVITYRIAYLLARGVPQEAILALTF
TNKAAKEMVERAHSLTGLALKNLMVSTFHSFGAWMLRKEAAVLGYRPNFSIYDEQDRTRA
IRECAHELGIAAENLDPVRLSQLFSALRSGYGKPGELSQRELELYALYRKTLKIYNAMDF
DDLIALPLELLQEHEEVREKYRSRFRYIMIDEFQDTSLQQYEFIQQLESGNICVVGDDDQ
SIYSWRGADYRNIERFETDHPGFVEIKLERNYRSTGSILTAANTVIAHNVRRKKKELWSP
QGTHGTPISFFEAEDDDAEAEKIIEKIRELRFSEHADWENFGILVRTNALADAIEDSLVE

HNIPYRVAGGPSFYQRREIKDIIAYLKAAANPDDDVSVLRVLNVPRRGIGRATVEYLSNI
AKAHSTSLHHAAEAARQAVFAPKESHMQKALAEVFSFFDYLGELRSALLEKGILVSRAIR
TIVEDVGYWQYLLDEHKKNDKAAVWKFRNIELLAASAERWEKDPDTFEKGIFAYLARISL
TVRDDANEEEGRLSLLTIHAAKGLEFDYVFIPGCENGILPHARSLEEGGGDIEEERRLFY
VAVTRAKKQLFLSRALHRKKNGSLQETLPSPFLSELPQNLMVMADAQKPAVSEEDLKKEF
FSRLKSRLGA*
>SPBIB_v1_140005|ID:27162496| Small multi-drug export protein [Uncultured spirochete bib]
MNAPILMTIFFSILPISELRGAIPYAVYQGLPILAAAAISIAANICVPLIAFLFLNSIHK
LLYKFSPYRSFFDRVVERTRKKVHAKVEKYGYWGLLLFVAIPFPVTGAWTGALGAWILGL
SYKKAFFAIAGGVLVAGVIVSILVALWGAGTQTIFFKTMH*
>SPBIB_v1_140006|ID:27162497| protein of unknown function [Uncultured spirochete bib]
LYKSLWMLFKNKKAMSGTHIFAAIEMAAAARIGRPWYTAYGIAPLSSEIGKMEKKIVMRI
GAFIEATIARDSLDCLGKAEMC*
>SPBIB_v1_140007|ID:27162498|miaA| tRNA dimethylallyltransferase 1 [Uncultured spirochete bib]
LTKPLATNCLPVDAVILAGPTASGKTDLLDSMFGAGVAEWLPRLRDAWSIDLQSVHVLSA
DSMQAYRFMDIGTAKPDSGLKKRLVHHLIDIKNPDEQYTAGEFVARADALCTELTEQHIL
PVVSGGTGFYLMNFICGLPSAPPSLPEIRAQVAQELEHAGTAALRAELARADPEIAAKIH
EHDVYRLTRAVEILRSSGKPPSFFAPQKVPRQGKRFVVLGVTRPRDELLLRIHARVEAMM
KAGLAEEVAQLVARGYSREDPGMQAIGYREFFELSGRPLETISEAIELHTRQYAKRQMTF
FRALPGIQWIAPEPEQLFRALAETAN*
>SPBIB_v1_140008|ID:27162499|rpoZ| DNA-directed RNA polymerase subunit omega [Uncultured spirochete bib]
MIMPLEALINYSGNSYELAAAVSMRAYQLAVLQTPEVEKNNGKVVSMATQEILDKTIGYK
LLAG*
>SPBIB_v1_140009|ID:27162500| protein of unknown function [Uncultured spirochete bib]
MSSISSDQRMHIEILGQSFSIKTDDSPEYIQSLVSELKQRYAVISTRMGVADPLRVAILA
GLFLLDDLKKTQGQKLSGAEARENTSARSTLSELDEEQILAQLDKRLGELGI*
>SPBIB_v1_140010|ID:27162501| protein of unknown function [Uncultured spirochete bib]
MMLNLEQIRALESRVEKAVVLITKLRQENADLEARLADVLRSAELLKNQKEELERQLAAQ
TRAANESSSRLEGLIARAKEAEEKAANAELKAAEAEERAAAMERKAQAADDEISHYRERA
LTAERRVAELESKAEELRTEQERIEQGLMHALSKLDSFEDMVLEMSLGAGARSESAVKPE
AQHIAAPLQDVGQESDEQAASDISEPDSGGGEKEEPDDNSSPDLKSRDAPFRGGENELDI
F*
>SPBIB_v1_140011|ID:27162502|rplT| 50S ribosomal subunit protein L20 [Uncultured spirochete bib]
MPRAVDGTRRKDHRKKILKHAEGFWGRRHSNYKVAKDAVAKALSDAYKDRRDKKGVFRRL
WIARINAACRMRDISYSRFIEGLYKADVCIDRKMLADIAVRDPAAFDAVVEKAKAALQG*
>SPBIB_v1_140012|ID:27162503|rpmI| 50S ribosomal subunit protein L35 [Uncultured spirochete bib]
MPKMKTKKAAAKRFTLTASGKVKYKKMNLRHILTKKTTKRKRNLRKAGFLDVGPVYQIRK
KLLPYG*
>SPBIB_v1_140013|ID:27162504|infC| protein chain initiation factor IF-3 [Uncultured spirochete bib]
LAEKDLRINEQIRVREVRLIDDKGEQRGIVSTLEALRIAREAGLDLVEVAPQSVPPVCRL
LNYGKYKFEQEKKVKDAKKRAKVTELKEIRMQPKIAEHDLDFKANHVREFLEDGNKVKVT
IRFRGRELAHTEIGEEILKRILAKLEGAFVLERPPLMEGRFMSMVLQPKAGAVKKQPEGD
TVNTASGPEH*
>SPBIB_v1_140014|ID:27162505|fba| Fructose-bisphosphate aldolase [Uncultured spirochete bib]
MTSYKELGLVNSKELFRKAMEGGYAIPAYNFNNMEQLQAIIQACVETKSPVILQVSSGAR
KYANATMLRYMAKGAVEYSKELGYAIPIVLHLDHGDSFELCKDCIENGFSSVMIDGSHLP
YEENVALTRKVCEFAHAQKDYVTVEGELGVLAGVEDDVSAEHSHYTRPEEVVDFVSKTGV
DSLAISIGTSHGRAKFRPEQCTRTADGVLIPPPLRFDILEEIEKRIPGFPIVLHGSSSVP
VEYVRMIEQYGGKLPDSVGIPEEQLRKAARSAVCKINIDSDGRLAMTAMIRKVLFEHPEE
FDPRKYLGPARDELKKLYMHKNSEVLGSANRA*
>SPBIB_v1_140015|ID:27162506| exported protein of unknown function [Uncultured spirochete bib]
MKSYACFRRSAFAALCFILAVAGSLGLLSGCSHPPVSAMLDEAFALVYPDLSARLVREFP
VPSSAAKTAAKATSPYLSYPVQPGKIESLAQSIAQSWPSGSTSSSAHIFVASPAVAHAFP
VKLLDEGSTCVSLFSRAEPALPEIAQLAESPSGRTLAIMWDSEWAYRQIGLIAGYRIGIE

RKNGNTEVNAAILFSSGLGRSEADLDAFVEAFRTAMETAGARDSDNGQALLPFNIDHMNL
PGDKLEQALSALAQIKDKKPSLLVIAAGSRTALEKAQEMKDIELAADLRGLGQGIPTRNL
FAAIGENNSAIIAAIRNAAQLIEKKDVMPPIIYVKPALQFSKEAARIDAILKRTAGQ*
>SPBIB_v1_140016|ID:27162507| conserved protein of unknown function [Uncultured spirochete bib]
MSTIIFYISKILRPVVASPLFIAIIFSTIALFLLRAKSRLQRVIKALALVFLLLVAVISK
PFVATGLARLWEYPRSDLDDCLSKGPYAAIVVLGGSLDPVASKPGAIEGNDSFERLVAAA
ELYRKGAAPIVIASGGTGSLSYADRREAPFMAEFLELMGVRRNAVVIEDKSRNTFENAVY
SKAYLEKREGSGNAKPGRIILVTSAWHQRRASAIFRKAGIDFYPYSVDSLVAPFMLPSDL
FPDAWALTRSTRILIEMAGFVAYRIMGRL*
>SPBIB_v1_140017|ID:27162508| conserved protein of unknown function [Uncultured spirochete bib]
MRLVTLVENVVYNTQLSAEHGLSLYIEYGGHRILFDTGQGDRFLKNAEVLGIDISLVDAL
VLSHGHYDHAGGLETFCRANKNARIYIKAGFFEPKFNKDRKFIGIHYNESLFAGRLITVK
SRTEIFPGLYIMTDIPITNAWDTHFEDLYVQGEHGLVLDQFNDEQFLVLKENGAISVITG
CAHRGISNIVRAAKSAFDLPVRLVLGGFHLSKSSDDMADRLIQELSTFDIEKLGCCHCTG
VEKYARIKSFFGERAFYNCTGTDYAGTDYKGAEYKSAAPLQ*
>SPBIB_v1_140018|ID:27162509| putative Uncharacterized deoxyribonuclease YcfH [Uncultured spirochete bib]
MSAVFTDSHAHLEIVSQRLGKEVLEEVLAAYGRAFGAGARAEEEPAIVLDVGTEPGDLEM
RRALVRQCASQRSAQFVKFSAGVWPGLEALADPDCAMSLLERDIERGVDAVGECGLDYYH
MAASPQAQKVLFSAQIELALRHRLPLVVHSREAFEDTHSLVAPAASRIPVIIHCFGYGPE
EARAFLESGCFISFAGNMTYPKASRLREALALVPPDRLLLETDAPYMNPVPLRGKASSPL
DIARTYALASELLHIDIEELRKRITKNLNRILGNSQ*
>SPBIB_v1_140019|ID:27162510|clpB| protein disaggregation chaperone [Uncultured spirochete bib]
MNYEQFTLKAQEALRDAAAIAQKNDHGSIEPEHLLSALLEQEGGVVSSILERLGADRGRL
DRGLQDLLSHLPKAYGEATQVYFSPALQKVVAKAEGESKAMKDEYVASEHLFLAMLSVES
RAASLLKSAGITREGVLSVLKSIRGTQSASDENAEEKYQVLEKYTRDLTALARSGKLDPV
IGRDEEIRRVMQVLSRRTKNNPVLIGEPGVGKTAIVEGLAQRIISGDVPDSLKDKKLLAL
DLGSLVAGSKFRGEFEERLKAVISEIVKSEGKIILFIDELHTLVGAGAAEGAMDASNLLK
PALARGELRAIGATTLDEYRKHIEKDAALERRFQPVFCAPPSVEDTIAILRGLKERYEVH
HGVRIRDDALIAAAVLSDRYITSRFLPDKAIDLVDEAASRLKMEIESQPTELDQIQRKLI
QLRMEADALKKETDPASVERRGKLEKEIADLSEKRDAMNARWQDEKRLIEEIRQLKQRIE
EYKVEVDQLVRDGQLAKASEIKYGKLVDTQRKLEELSKKVQEEKDKPRLLREEVSDEDIA
RVVSLWTGIPVSKMLSSEMQKYVELESQLAKRVIGQERALRAVADAIRRNRAGLSDPRRP
LGSFLFLGPTGVGKTELAKALAEFLFNDERALTRIDMSEYMEKHSVSRLIGAPPGYVGYE
EGGQLTEAVRRRPYSVVLFDEVEKAHPEVFNVLLQMLDDGRLTDGQGRVVDFRNAIIIMT
SNIGSEFILEMKSMDEIRSRIDELLKTYFKPEFLNRIDEVVVFDRLDETMIRKITDLRLA
ELAQRLAARSIALEVHEAAKDYIAKEGFDRQFGARPLKRAIQNLLENPIAKKLLAGEILE
GDTVTVSLAGRELAISTKRPA*
>SPBIB_v1_140020|ID:27162511| conserved protein of unknown function [Uncultured spirochete bib]
MSQKEKEQELDQLLPAIPEDFRAVIMYGMTKEEALAIMRAVKSVGPSMQEVAFAMSTETN
IQWPLGQLVAELSEEHRMMKEYRAAHGKESIVS*
>SPBIB_v1_140021|ID:27162512| protein of unknown function [Uncultured spirochete bib]
MHHFHFCPNPACAYHQHAPKERWFVAAGFYVTKTFGKVQRFRCQACXX
>SPBIB_v1_150001|ID:27162513|argR| Arginine repressor [Uncultured spirochete bib]
MKERAERLKTIKAIIKENRVDSQEKLLQLLEDHGYSVTQATLSRDLKFLKVGKLSDGRSG
YYYSIPSEEERRESERHYIQDFLRGYVSIDWNQSLVVVRTFSGHSDSVALAIDNMGFDEV
IGTISGRDNVVFIALKQGISGDEFIRSLKAKIPDFEEE*
>SPBIB_v1_150002|ID:27162514| putative L-lysine 2,3-aminomutase [Uncultured spirochete bib]
MHDVIAQGGLPFRATRFWFSLANEPPENCKKNENGVPVDPILAQAIPSPSEALVTSWELR
DPLGESSHTIFEHAVRQYHSRVLVRATGQCFLFCRHCFRRSLLPSERSFMDSKAIKALAA
YLAVHQEVREVLISGGDPLTAPDASLASLFEAVRSVPRPILIRLCTRTPVVLPQRLTSDL
IALLSKMRPLQMVLHINHPKELSQEFMEKAELLLRAGIPLHSQTVLLRGINDAASVLIEL
FSALNRAGIHPYYLFQGDLAAGTAHFRVPLSKGLALYENLRKELSGLELPRYAVDAPGGG
GKIYLPEGIAERRADLWILRAPDGSLHEYPEEG*
>SPBIB_v1_150003|ID:27162515|guaA| GMP synthase [glutamine-hydrolyzing] [Uncultured spirochete bib]

MDKIVILDFGSQYTQLIGRRIRELGVYSEIVPGERKPDSALLEGCKGIVLSGSPYSAWQE
GSPAPDLAMLGTGLPILAICYGIQWLTMHAGGVVERCETREYGSMRVRFLPAVQDSGRAA
TLLEGLPDSFLSWMSHGDSIVRPGEEWDVVGISESGVPAILAHRARPWFGLQFHPEVSHC
EHGMDILANFVFRICGAERGWSMEAYLEQEARLLRERVGKEPVLLLISGGVDSTVAGAFL
LKSLDPDQVYLLYIDTGLMRKAENEEVMHNLSRLGARHLEAVDAEAQFLSALKGVADPET
KRHIIGDMFVKVQQDAVVRLGIPDAFLAQGTLYTDLIESGKGVGNKAQVIKSHHNVRSPL
IEKKRAEGRVIEPLDRLYKDEVRALGRVLGLPEEIVGRHPFPGPGLGVRILGDITKEKCD
ILREADSIFIAELRKRGLYDRIWQAFCVLLSVRSVGVAGDERAYKYVVALRAIQSEDGMT
AQPFAFEPKDLFEISAAITNRVQQIGRVVYDISSKPPATIEWE*
>SPBIB_v1_150004|ID:27162516|purA| Adenylosuccinate synthetase [Uncultured spirochete bib]
MNVVIIGAQWGDEGKGKIVDFLASDAQIVVRFSGGANAGHTIVLGGEKYALHLVPSGILY
PDKIVILGSGMVIDPEALFQELAGLEQKGIDWKGRVFISDRAHIVFPRYRELDKQAEAGR
ARPIGTTGRGIGIAYSLKASRDGIRLADIDDDERLVNLPGDDRAWLSIWKERLLPLRIDL
AAYLRQHRNSWRLFEGAQGALLDIDTGTYPFVSSGMSCAAGAAAQGGIGPRHIDRVLGVF
KAYTTRVGNGPFPTEFADDSPGSLSQYIRNVGNEYGTTTGRPRRCGYLDLVALRYACTVN
SIDRLVLTHIDVYDGLEEFEACVGYKIGDKVIEDFPASVRDLTGARPVLRKFSGWKTPLG
KCRTYEELPLRAREYIEFIEEFTETSISIISVGSDRKQTIVRESPWIK*
>SPBIB_v1_150005|ID:27162517| conserved protein of unknown function [Uncultured spirochete bib]
MKHSPSIALVLNAHLPFVRKPQYAQFYEERWLFEVISETYLPLLRMFRKLELEGVPFKLG
MVFSPTLLSMLSDPLLCDRYVAYLDSQIELALKEKRRLGSDAAFAPLADMYYEMYRRSRD
EFENLHGRNIIRAFDYYSKKGYIELMTTAATHAFLPLYSDIPEVINAQIETAIIAHRSEF
GKNPAGFWLPQLGWYKGLAPRLRAYNINYSIVTTKGALLGSPLPFYGSFSPVMTPAGLAA
FIRDAGATKAVWSETEGYPAHPVYRDFYRDIGYDLDTGYLAPHLSGVERGYTGFKYWAVT
GKTDLKRPYEAAAASAQAVAHAHEYLSSRKVQARAASFWMKDKPPIIVCPYDAELFGHWW
FEGIQFLEAFFRAASRRDEEAIRLVTLSEYLAENSELPESEPEFSSWAEGGYAEVWLDGR
NDWVHRHTRKAAERMRELTVRFPNETGLRERILNQAAREVLLSMSSDWALLLRSGKAADF
ASRQIRESIYNFNHIYEMLSAHTVETEWITSLEKRHNIFPHMNYRVFSPKQ*
>SPBIB_v1_150006|ID:27162518| conserved protein of unknown function [Uncultured spirochete bib]
VPVRILTSGALLSHYSAVDPEKLSLLSDDELRLLAEKMGIYVPDELERVFLIEEIIDAFE
EDTSEKVFSHDTPGHVEEKKLAGHGFIPSQSEEIVIPDHYNETYISAIVRDPLWIFAYWD
IAASLREKILAEFEEPKLLLRVSEIADSEKAFHYDVSVSFDDRKWYINVPYPKRSYRIDL
CIAKTQKAKVIARSNVVRMPAQYMDMPSKLPQMTKSLLILSGSEDLNLIEPREENSLRIL
NMDGE*
>SPBIB_v1_150007|ID:27162519|fhs| Formate--tetrahydrofolate ligase [Uncultured spirochete bib]
MSVQRINPVPQDVDIAQSANIQPISEIAARYSLDEKYLEHYGVDKAKIHLEFLADPPKQA
KRAKYIDVTAITPTPLGEGKTTTTIGLAQGLGYIGKKSIATIRQPSMGPTFGIKGGAAGG
GYSQIVPMTDFNLHLTGDIHAVSAAHNLCAAALDARMYHESRWADSYFEKLGLKKLNIDP
YAVLWRRVVDMNDRTLRSIIIGMGGLENGPLRETGFDISVASEVMAVLALATSLDDLRKR
LGRIVVALDKSGNAITTEDIGVAGAMTILMKDALKPNALQTLEEQLAFVHAGPFANIAHG
NSSIVADLIASQIADYVVTESGFGSDMGFEKFMDIKCRASGLMPDAVVLVATVRALKMHG
GGPKVTPGKPLASSYTQENLELLQKGLCNLQEHIRIIRTYGLPVVVSINAFPTDTEAEHA
LIKDAALKAGAFDAVISKSWALGGEGAADLAMAVDKAASMPHEPKLLYPLDMSLKDKIEI
IARQIYGAGSIVYTPEANAALEKYTALGYGNFPICMAKTQYSLSHDPALKGAPKGFEFPV
REVRLSAGAGFIVPITGEITTMPGLPSKPAYIGMDIDTATGRITGLS*
>SPBIB_v1_150008|ID:27162520|appC| Oligopeptide transport system permease protein AppC [Uncultured spirochete bib]
MIARRSQKIVFSLAVLYLLMLVAAAIAAPLLSPYSPSEQYLDRRFHAPSLDNLLGTDNLG
RDILTRLLYGARSALIVGLVAVSIAFSLGGLVGLLAGMGPKHIDSVLMLLMDSLLSFPTI
LLAITVVSFMGYGLVQVMVALGVISSPVFARLVRAETLSVKTEGYVEAARALGTPPAKIV
VKHIIPNIMGKVIVQCSLTFAQAVVIESSLSYLGVGTQPPAASWGLMLKDARNYLAQAPW
MAMYPGLCLALTVLAFNIIGDSLSERFNPRLG*
>SPBIB_v1_150009|ID:27162521| Dipeptide-binding ABC transporter, periplasmic substrate-binding component [Uncultured spirochete bib]
MKKVPRIASLCILFAFSAASLVVAQTKGTLVFGLSGNPDTLDPQKTAGTLTFQVVKSFYD

TLVEPNTDGTIVPALAESWTVSPDGLIWTFKLRRNVVFHNGQPFTSKDVRASLERIKDEA
TASPKRSEFAAIKEIRTPDAATVVIVLSQPYAPLLASLASGWGAILPSGLIASGHNFGAE
PVGTGPFKFEKWIRDNKITMVRNDTYWMKGLPKLAKVEFQIIPERAVQVQGLSAGVIDAV
EFIDPEDLPLFSSNPKVSIKKELTSLIMVMAMNCSREPLNDLRVRQAVNYAIDKQVVLDV
AYGGGKVGSTFLDTGNAYYTDFSYLYPYNPDKARQLLKAAGVDTREFTITVPQNYPLHVK
AAELIQQMLEKVGMHVKLQLVDWSTWLSSVYNGGNYDFTVIGHTGKLDPDGTLAGYGTGK
YVKWINATAAQKIKEAAAISDFAARKKLYTEVLEIMAKEVPFVYLGTSYRYVGTRSNVVD
FRMTPNLDTFDFRWTELK*
>SPBIB_v1_150010|ID:27162522| D-ala-D-ala transporter subunit ; ATP-binding component of ABC superfamily (fragment) [Uncultured spirochete bib]
MQCRNIVKIHRGRGVEGERHGFRAVDNVSIEIERGRIFGLVGESGCGKTTLARAILYLDP
PTSGSVLFDGVELGSLKPPMLRKYRKRMQIIFQDPHSALDPRMTVYDSLSEGLINIGVDK
ARRDKKIRELLDRVGISSTYSRRFPHEFSTGQRQRIVIARALTMDPEFLILDEPVSNLDV
SIQAQIINLLMDLKKDLNLTYLFISHDLNLVSYMCDVVAVMFGGRIVEQAPTEALLAHPG
HPYTRRLLASIPGGAGRF*
>SPBIB_v1_150011|ID:27162523|dppD| dipeptide transporter ; ATP-binding component of ABC superfamily [Uncultured spirochete bib]
MDESAPLLKIENLNVRFSHRGKSVHALRGIDISFYKDEIHGLVGESGSGKTVTATSIMGL
LPRPSAEIISGSVFYKGKNLLLMPEDELRTYRGRAISMVFQEPAKYLNPAFKIGGQIEEM
VMLHMGLSKVAAKARARELLKLVGLGRDGRVLDAYPHELSSGMKQRAMIAIAISCNPDFL
IADEPTTALDVTLQKQILKLIRTLKNIENMGVLFISHDLGVIREIADRISVIYAGKIVES
AGKESLFNKPMHPYTRLLMLSIPDARHRGRKLATIPGQVLDATSTPEGCLFAPRCPLAEQ
RCFSLTPELVEHEKGHLAACHFAGKSWNL*
>SPBIB_v1_150012|ID:27162524|gsiC| Glutathione ABC transporter, permease protein GsiC [Uncultured spirochete bib]
MDLARFIAKRLLRLVLTVLLISTIVFFVIRVIPGDPALLIAGIDATPSDIAAIRAKLGTD
RPLSAQYFDFLWKVAHLDFGNSMISEEPVTALILQRFPLTLALAVLGMVIGTVIAIPIGI
LSAVKPWSAWDYVGLVMSQLGMAIPSFWLGIFLLLVFAVKIPIFPLFGAETLASLVLPAF
SLGFARAAILLRHTRASMSEELSKEYIIAAKAKGLTDRMIKYKHALKNALLPIITVAGIE
FGYMLGGAIIIEQVFSLPGLGRLFLYAIYQRDFPLIQGGVVFIAIVFSLVNFAADVLYSV
VNPKIRLE*
>SPBIB_v1_150013|ID:27162525|pcnB| Poly(A) polymerase [Uncultured spirochete bib]
VRIRYFRNTKGGLQAKARVYDNTEHNIDPSLVDRDASAIVKRLVSHGFTAYIVGGAVRDL
LLGKRPKDFDIVTDALPNRIRKIFSNSRIIGRRFKLVHIYANHKIFEVSTFRSLSTGTIG
NEYGTIDEDVQRRDFTINALYYDPLTFELLDFLGGLEDLRANRLKAIIPLEKIFREDPVR
MIRGVKYAAVNNFSFSFALRFAIHRDAALLKEVSSSRLAEEFYKIIASGKSAIIIQWLDR
YKLLQYFLPEVSNSLRRSKDFKDTFFQHLESLDALARTPEDASLLNNASAAEIVQTDRRL
ISPFVAWFVRQRLRDFARREDVPKEPPAFRQAAFADIREFLAPLNLPRVALEEAMEWVLQ
EDSLMPAPPVPKRRKRSPRRKKSKQRAEAAHIEISDANLQ*
>SPBIB_v1_150014|ID:27162526| conserved protein of unknown function [Uncultured spirochete bib]
MKKFQASDGKELAYLRYDPKEKPKAVVLIGHGMNDHKERFIPLAEALTPIGISCWIPDLR
GHGDTDPDVNKGYLADSDGFERVVQDLVELGDYASKEMGGLPLFYFGHSFGALVGMALIA
VNGKYFDGAILSAPPAQPNPFLDKIGGLIVSIGAAIRGLHAPAKLANQMTFGDYAKTVPN
ARTRFDWLTRDASIVDAYIADPKCNFICTYGFYRDLIHGLRKVYGEGFLESIPPTLPLFL
FCGSADPVIGMQAGCEKLAQQFRSLGIIDFETKCYEGGRHESINEINRMEVLNDIADWFS
RHIR*
>SPBIB_v1_150015|ID:27162527| protein of unknown function [Uncultured spirochete bib]
VHIDAYRLADEEEFFQTGGEELLGAPGTLSLIEWSERIAHILPPESQIITITVEENGARL
IMLEGDWIEALRWGHFTIPACKETSL*
>SPBIB_v1_150016|ID:27162528| putative tRNA threonylcarbamoyladenosine biosynthesis protein TsaB [Uncultured spirochete bib]
MTILAIDTSTPILKIGVYKGCLIQATIVQPPLTHAEQIIPAIEETFRKAGIAPANAGLIA
VAGGPGSFTGLRIGIATAKGLSLALGIPLVLVPTLDIYGFAWREMGGIVVPVIDARKHRI
YCARYSHGQNMGELMDIEPDQLLPKIDAEEEVHFIGPDADLLESICLERPGWIIHEPDAE

KEVAAIAELGLRIFEEKGPADDAARPIYLREPEIGIPLAR*
>SPBIB_v1_150017|ID:27162529| conserved protein of unknown function [Uncultured spirochete bib]
MAVEIRKVESGKDLRQFIDFPVRLYWDEPNYVPAPRLDEMRTLRKDKNPAFEFCEAEYWT
AWKDSKMAGRIAGIINHRYIEKWGNKYARFGWIDFIDDPEVSKSLLETVERWAASKGMKA
VHGPLGFTDLDREGMLVEGFDQMATLATIYNYPYYPKHLEALGYRKDIDWLQYQIFTPKE
IPEKVQRVTELLAKRSGVHLYEWTDKRVIVQKFGKELFKLIDETYSGLYGTTPLTERQVE
TYIKQYLGFVEPRFTKILVDEEEHLIGFGISMPSLSEAFYKSRGRLFPFGWYYILNALKH
PKVLDLYLVSVKPEYQARGVLAIIMNAFQRSALEAGVEYAETNLELEDNVKVQSIWKDYP
KRQHKRNRAYIKEL*
>SPBIB_v1_150018|ID:27162530| conserved protein of unknown function [Uncultured spirochete bib]
MAEWHEIKYQEIFEGTLTGLKRRREVDPVFSIEDAERQLSHLYILDGNDWLGRGELGDIV
SQATIAAYELFIQEWKVEIGGEHGG*
>SPBIB_v1_150019|ID:27162531| Cystathionine beta-lyase [Uncultured spirochete bib]
MLHKSATIRQTDIALNPIDVSYIPHHMNEDALSEHAVSPPIFQTSNFCFSSFVELKAALE
DEVGSCLYTRGNNPTVNLVESKIAALEHGEKAKLLGAGVAAISNAVMAFVRQGDHIICVA
DSYGWMQRLLTYLERFGVSHTIVDGTRTEDIERAIRPETRIIYLESPTSLTFFIQDIPAI
TAIAKAHGIRTIMDNSWATPIFCNPIDFGVDLVVHSLSKYLGGNSDIIGGVIVGKAEDID
HIVWQESLQMGAVADPFMAWLVMRGMRTLHIRMPVHYRSALAVAQFLENHPRVASVNYPM
LASHPQHELAQKLFRGGSGLFSFRLKTHSVEDVARFVDSLSLFKRAVSWGGYESLVFPEA
AHYAGSVPADRLNLIRLHIGLEDASLLVQDLAQALEGV*
>SPBIB_v1_150020|ID:27162532| conserved protein of unknown function [Uncultured spirochete bib]
MDRLETLRILSQAADWDAAAPSTRIEQSSCAPLDSAHTKAPSCPASEASRAGSSSPPDSA
VREDAAYGICRVKRLGGGSVPILKVLLSNACSYNCAYCVNRRSSNVRRASFQPDELARTV
SDFDARGRIHGAFISSGVLGTPDETMEQLIWTARSLRERYGYQGYLHLKVIPGASPDLVR
LAMRYATRVSVNIELPSEESLVRIAPDKSPDAILSPMSLIAEHMRELQLNKADKPASDSL
STIAASPLTAGQTTQMIVGASPEPDSTILALARHLYQSFNVRRVYYSAFRPEGTDPSLPH
PAAPPYTREFRLYQADMLFRWYGFEPDELFEQSEYLDEDLDPKAAWALRHPGLFPVELMT
ADFLQLVRVPGIGPTAARRILEARKKGGLDFQHLQKMRIRLRNAAWFITLRGKTPFMEAG
FDAFLPDDPGFGVFAPGFAVSRALDHPEILREIIKDKKPPVPPTQQELDFLS*
>SPBIB_v1_150021|ID:27162533|lipA| Lipoyl synthase [Uncultured spirochete bib]
MTIPKPSWLKVQLPHGREWRHVEEVLASKGLHTVCDEARCPNKGECWGAGTATFMIMGDT
CTRGCRFCAVKTAHQGQPLDPAEPLHLAEAVKALGLKYAVITSVDRDDLPDRGAAHFAAC
IRAIRELSPGTLVEVLIPDYVGTELSTVLDAKPDVLAHNVETVRRLQSVRDRRASFDKSL
QTLREAYAAGIPTKSSLLLGLGETEDEVLDALRELRSTGVSIVVMGQYLRPTPQEIPVTE
YITPTRFAEYAEKARALGFASVISAPFARTSYHAREAWGAVDKSARKTGSGPRRGHEAET
EGNTQGGSKS*
>SPBIB_v1_150022|ID:27162534| conserved protein of unknown function [Uncultured spirochete bib]
MKLDLIGKPGGCKLLRMTLEVEPPLSPASRLQRISIRGDFFAVPEEAFEEVEHELEGTAL
DELGARFDALVLEKGIQCAGIGGAGLAELVQNLLVQKEQQHGT*
>SPBIB_v1_150023|ID:27162535| Biotin/lipoate A/B protein ligase [Uncultured spirochete bib]
MAHEFRLLETGFHTAAFNMGLDEALLGSVAEGKSLPTLRFYGWKPPAVSIGYFQGLHEEV
DVDACRAAGVDVVRRITGGGAVFHHYEVTYSIVVPLSHQLARPNILDSYRLLLGGIIDGL
ALLGIEAEFAPINDIVTGGKKISGNAQTRKRGCILQHGTILLDVEVDLMFSLLKVPQEKA
RGKLIEDVKERVTSVQHFFKNRGQTPIFKFLYEETVAALKQGFASALDLSLVGSSPDEAE
LAEAQRLADEKFASRHWTELR*
>SPBIB_v1_150024|ID:27162536| putative Histidine kinase [Uncultured spirochete bib]
VLTALFQNITILISISVLYHFISRSLKGTPWAVSVLNGVLLGVTAILTMLTPFEIEKGLF
YDARSVVIGIAGFFGGPLTAAIAATIALAYRIPRGGAGVIPGSLSIVFPAILSIIAWQAR
QRYPANPQGRKPHMVLETWFLGLIIHASVVLAQLALPGGRWHQIIPLMLFPFLVVFPLVF
SLICLLFFDNERITKAALDLAESEARYRSLFENHHTVMLIIDPSTGRIVDANPAAVEFYG
WDRATLISMRIQDINTLSPEEVEAEMARARMRDKNVFYFKHRRANADPVDVEVFSGPITI
KGTELLYSIVHDNSLRIRAERELQALTQSLEQQVLDRTVLLEAQSRRLQELNKEYEAFAY
SVSHDLRAPLRAIAGFSAILGESLQAQGILKPAPDQKSAGSEANTIEAPDFGHLLDRIQS
NVSRMQQLIDDMLMLSRAGTRKMNPQPIDFTQMAREILGELTEQQKGRHFECTVQDGMTA

IADPDLARILLVNLISNAIKFTKDRDIAHIEVGIEQNSDVPRFFVRDNGVGFDVAAAGDR
LFAPFQRFHSEASFEGTGIGLSIVKRVVARHNGRVSAESVPGQGATFYFDFGEVKQT*
>SPBIB_v1_150025|ID:27162537| Response regulator with CheY-like receiver domain and winged-helix DNA-binding domain [Uncultured spirochete bib]
MTATTSHESILIVEDNPDDAELVVIALRQKGINLPYLIARDGEEALDIAFRRKPLPDQKP
IEPGLILLDLKLPGKNGIEVLKELRSSQRTRYVPVVVLTTSMLEEDMIQSYENGANSFVR
KPVDFATFQHYIELICHYWLHTNLHPALAISQRPTS*
>SPBIB_v1_150026|ID:27162538| protein of unknown function [Uncultured spirochete bib]
MTIVPQETDLNILIVEDNRDDVDLLMYRLRKSFKSVSYTHVSTLEQYLEAVRHLANGKLD
RTSYNIIISDWAVPGYDGFVALQALQDVDETIPFIVVSGTIGEPTAVALIKAGAYDFVLK
DQSAHLPYVITKALEWADQRRIEQEQRRQIEMQTKALSCSPIAMAILDNEGTFEWINPAY
EVLTGWTLAELKGHHLWEFTALASQSDCSQVFEELEHKEAHQCEGMETRKDGSRYHELRQ
LAKLLDTNTTSQRYLLIRQDITFLKEYRTRLEIDAELPQILANCQTLHALYQQAAAYIRR
TFDIERAGFIRLDEQGTSVPPWYGDDMSQSLSLGIIQGTRFPVIVSGTTKALCVVEWKPS
FMEGSALLLSYALEKMEQPLARLEAQENAREWMKRLSLLDVFREYFGSGRNFGAAISKVI
RLIRETLRADSITLYISNNDGTFACRNYDGFRTDLIKGAIIQAGQKNVGLAVKELRIVAA
DDLAQDSSYVPQFRELIAREHFVSQYCVPVILFNEVQAVLELFFRHPFKPDETWLAFAQA
AAYQTGLALEFQSVIEELDGAYQELQSANESIIEGLSSALEFRDEETEGHTLRVTALFMS
FASRFIQDENELKKLRIGSLLHDIGKIGIPDAILNKPSPLTPEERAVMQKHPLISKEILS
RIPSLGDCVDIPLYHHEKYDGTGYPYGLKGEEIPLAARIFAVIDVYEALTSDRPYRKAWS
KEKAIEYIRDNAGTHFDPEIALRFMELNL*
>SPBIB_v1_150027|ID:27162539| Transcriptional regulatory protein [Uncultured spirochete bib]
MTVRGIAEKAGVSIGTVDRVLHGRGRVSPETKERILSIVRESGYTPNPIARHLKLNKKHT
FAIVMPDLDEDSGYWRIAYSGVQKAQKELAAFGVNIKRFEFNRYNRSSFAKAMAAFDPGL
FSGLLIAPVIPEESLLLLRRMPTDFPVVFFDAQIPSFTPLTRIGQNAFQSGMLAGRLLEA
FSKRKGPYIIVSTHSEDYHIQKRIEGFQAYFALAPHTIVLRECFDIEHKEKREFFVRQLL
SEFPSIDGIFVTNASVHGIAARINSRAESPHVAIVGYDLVPENVRQLQAGTIDCIVSQRQ
DHQGYQGLYQLYRKVVLGQDVEAEIEMPIDIYLKENLLQQALPEPQKIISSPAPAIAPSA
RLQGI*
>SPBIB_v1_150028|ID:27162540| Periplasmic binding protein/LacI transcriptional regulator [Uncultured spirochete bib]
MKKLLLVVLVLSLLVPIAVFGQGAKKPVFVMVPKGVHPYYEPCYEGFKAAAAKYGVTVQK
VDPQKFELPLQVKVIEDLIAQRVDGIAISALDDAGLVPVIADAIKAGIKVITFDAPAPSS
AALTYIGTDNETAGYEAGKKMADLMNKEGEIIILQGGLGAANLNLRTKGFKRALAEVAPK
IKVLDVVDVQGDFAVATNKTEAILQTYPNLKAIFAVSAEGAPAAANVLRQQKKSGKIILA
GFDDLKDTLEGIKDGTVSFCLVQKTYKMGWLSVEKLLDAMAGKQIPKVIDTGVLFVTKSN
VNTYMDEMKKEFNP*
>SPBIB_v1_150029|ID:27162541|rbsC| D-ribose transporter subunit ; membrane component of ABC superfamily [Uncultured spirochete bib]
MVAQEKKSILATLFSYRESAIFLALIILMATVTIFAPNFMSGSNLYLVSRQISFVAVVAF
GELFVILTGGIDLSVGSVMGLSGVVAAGAMAGGQSIILSVLLGLATGIALGLINGLLISY
VRIAPFIATLGMLSFARGVILIVTKGWPITNIPKPFLIVGQGDFLSLPIPLWVMIVLAAV
AHFLLSRTAFGRRTYAIGGNEQATFLSGINVKKIKVFLYMISGFMASVVGIILIARFNSA
QADTGTGWELDAIAAAVIGGTSLSGGSGSIFGVIIGAAIMGVIRNGLVLMRVSPYWQTAV
IGIIIVLAAVLDRIKNK*
>SPBIB_v1_150030|ID:27162542|rbsA| fused D-ribose transporter subunits of ABC superfamily: ATP-binding components [Uncultured spirochete bib]
MSEPLLALKHISKSFPGVKALDDIDFSVQKGEVVGLLGENGAGKSTLMKIISGVYTPDSG
DIFWQDKKIKLKSIMDAQEKGIAIIFQELNNCPNLSALENLFLGHEIRTSANFVDFRAMK
KKAKEIFSYLDVQIPLERPVGKLSVALQQMIEIGKALLADAKLIIMDEPTSSLTDKEVQK
LFQIVKELKERGIAVIFISHKLEEVFIITDRIVVLRDGKHVGELKTSAATQDELVALMVG
REMHSFFSKRTKEASDETILKIENFSGPPYIRGVSFYLRKGEILGLAGLIGAGRTELALL
MIGAEQKTSGNMVLHGKEIDVRSPWQAIQHGIAYLSEDRKMKSLVLQMPVRANITMAIHS
AVTGALGMLDRKKELEITNSFIRLLDIKTSGPEQTVNNLSGGNQQKVVLAKWLATKPSIL

ILDEPTRGIDVHAKAEVHKIITDLADKGTSIILISSELPEIVALSDRVVVMHEGQVKSVL
EKKDISQENIMSTVFSA*
>SPBIB_v1_150031|ID:27162543|araB| L-ribulokinase [Uncultured spirochete bib]
MEAVHNPQYVIGIDYGTDSCRAVITDASNGHEMASAVAPYPRWSKGLYCDPAKNQFRQHP
LDYIESLETVMQKLAAQTGTELLPHVKAMAIDTTGSTPCLVTEHIEPLAMLPEYSDNPDA
MFVLWKDHTATDEAFQINELARKWKVDFTMYEGGVYSSEWFWAKLLHIAKAAPDVIRAGF
SAIEHCDWMPALLTGTRSLSQVKRSRCAMGHKAMWHESFGGYPGDEFLGALHPELVRIKK
TLGTETFTSDTVFGALTGEWALRLHLSAGIPVAVGAYDAHMGAVGGGVEEGVLVKVMGTS
TCDMIVGPKPAEENRAGKPSEEEHAGGARAGGGRATGDHVGEAQTEGGRAAAGESEHLVK
GICGQVDGSIVPGMLGYEAGQSAFGDVYAWFKNLLMWPLRELGTEVDQGTEVDAAQARAF
IEELDSRIMLMLEKAASQINPGESGVLALDWLNGRRTPDANQKLKGALTGLTLGTTAPMI
YRALIEATAFGTRAIVERFELEGVQIRKIRAIGGVARKSPLVMQIVADVLGRPIEIVASD
QSVALGAAMFAAVVARIYTSVPHAQAAMKPNIEAVVTPNAANTAIYDGLYRQYLKLGSFI
EKELT*
>SPBIB_v1_150032|ID:27162544|araA| L-arabinose isomerase [Uncultured spirochete bib]
MKAFDSYAFWFVVGSQTLYGPKVLEQVNAHALRIAEFLNQNAAIPATVVPAVVTSPEEIT
SLFRKANADPSCAGIITWMHTFSPSKMWIQGLSENAKPLLHLHTQFNRDIPWDSIDMDFM
NLNQSAHGDREHGFIHTRLKTLRKVVVGYWQDELVLNRIAAWMRAAVAFADGKTAKVARL
GDNMREVAVTEGNKVSAQIQFGWQVNGYGIGEIARRYAEASPRDIDAVLEQYQSRYVILD
SVRHDSSQFKQVREQAAIEVALRQFLADTRAGAFTTTFEDLAGLPQLPGLAVQNIMADGY
GFGAEGDWKTAALVRAMKTMARGLEGGTSFMEDYTYHLEPGNELVLGAHMLEVCPTIAAV
KPQIEVHPLSIGGKDDPARLVFEAKPGMAVAATIIDKGDSFRLILNEVEAVALPRAMPKL
PVARALWKPLPNFKSALELWILAGGAHHTSFSYVVTSEMLEDYARMAGIEVVVIGKETSV
PLLRKELEKDFLR*
>SPBIB_v1_150033|ID:27162545|araD| L-ribulose-5-phosphate 4-epimerase [Uncultured spirochete bib]
MLESLRKEVLQANLELSDRGLALFTWGNASAIDRARGLIVIKPSGVPYERMTAGDMVIVD
LDGKVVEGTLKPSSDLPTHLVLYKAFSSAMGIVHTHSTYATAWAQAGQDLPAEGTTHADH
FYGSVPCTRLLTDEEILGDYELETGKVIVETFKSRAIDPSSVPAALVHSHGPFVWGTSAL
EAVHNAAVLEEVAKIAILSRMIAPPSPMQASLLARHFERKHGKNAYYGQK*
>SPBIB_v1_150034|ID:27162546| protein of unknown function [Uncultured spirochete bib]
MNIKIILNGGLKTCCKSYSKEYIHEAVKSWLTDKDVLEVVDVREQAYKLDELAAYAKQFF
QENTFPIVYIDDRLIAIGQIPDKNSLFEVSAKLDEYQITREAIYKAAKEYELVPAEQEAE
EV*
>SPBIB_v1_150035|ID:27162547| Extracellular solute-binding protein [Uncultured spirochete bib]
MKRVLLAAMIALVALSAFAQQKVTLTIESWRNDDIQIWQDQIIPAFEKKYPNIHVVFAPT
PPAEYNGVLNSKLEGGTAGDLITARPFDASLALYNKGYLADLTNLPGMNNFSNVAKSAWI
TDDGKHVFAVPMASVIHGFFYNKDIFKKLNLKVPQTKAEFMQVLEAIKKDGRYIPLAMGT
ADMWEAATMGFQNIGPNNWKGEEGRLALLAGKQKYTDEPYIKTWKELASWAPYLPKGYQA
VKYPDAQQLFTLGKAAIYPTGSWEIWLFEKDAAFELGIFPPPVEKKGDTVYISDHTDIAL
AMNAKTKYPEEAKKFLEWMTTAEFASLYSNALPGFFTLSNHKITLKDPLANEFLSWRATC
KSTIRNSYQILSRGDQYGAPNLENELWQVSAAVINGTMTPEEAGKRVQAGLDKWYKPKK*
>SPBIB_v1_150036|ID:27162548| Sugar ABC transporter, permease protein [Uncultured spirochete bib]
VLDIRGSRKFPFFLIVFLGPAAICYTLFMIYPLAESLILSFFSVQPDNSYVFSGFANFVK
LFTDSDTAPRFWGALKNNVLFFIIHMLVQNPIGLLLASLLVTKGKARGFYRTVLFMPTVL
SVVIIGFIWQLILNPLWGVAESMMRAVGLGKLFQPWLGLPQYALPAVALISVWQFIGIPM
ILFYTALIGIPDELIEAAVVDGCTETQAFWRIKFPLILPTIGIVSILTFVGNFNAFDLIY
TMKGAFAGPAYSTDIMGTLFYRTFFGQQLQLGNPTMGATVATMMFLIILTGVLIYMFLFQ
RRVKTYQL*
>SPBIB_v1_150037|ID:27162549| Carbohydrate ABC transporter membrane protein 2, CUT1 family [Uncultured spirochete bib]
MMKRRASRASRDLARQRFRGLLQHIILVFFSLIALFPILLILMNSFKLKKYIFAAPFALP
NAKTFSLIGYDTVLQRSNLIVNFGNSIIVTVASLFLILLFGSMAAFALSGYKFRGNAFLG
FYLSIGIMIPIRLGTVSLLKLMASLGLVNTLTGLILIYIAQGLPLTIFILTSFMSQIPDE
LKDAGRIDGASEYRIYALVLPLVRPALGAIGIFTMIPIWNDLWFPLIVAPSAKTATITLG

VQQFLGQYVSDWNAVLSALVLAMVPVLLLYLVFSRQMIRSVTAGAIKT*
>SPBIB_v1_150038|ID:27162550| putative dehydrogenase [Uncultured spirochete bib]
MKIGIIGTGFMGSTHAGAWKKTGAKIAGFLADPPSEAEQIAREYGAHVFSSLEELIESVD
VVDICSPTHLHSDMAIAAARAGRHIVCEKPLARTVEQGEAILDACRIAGVRLFVAHVVRY
FPEYALAAARVHAGDIGKVGTAQYRRLSYRPKKPVGNWFLDEEKSGGILLDLMIHDFDIA
RWIAGDVVSVYAKKVSSFRKESPADYGMVILTHASGALSHVAGAWAYPPPVFRTGFEVCG
DAGVIQHDSDSESPIEALLKQEKSEAPDVGLPASPLAESPYDLEIADFYRCLIEGGEPRV
SAADGLAALKIACAAIESAKTGQVIALDSRREEARQAELELTGELATSKGGQR*
>SPBIB_v1_150039|ID:27162551| Oxidoreductase domain-containing protein [Uncultured spirochete bib]
MKIGIMSAAHVHADAYIGCLRSIPEVDIVGIADHDNERGALFAQQYGLHYFASYEALLGQ
KLDGVIITSENAFHIELAKKAAQTGVHILCEKPLATNVRDAKEIVDAAKKASVILMTAFP
MRFSVPVRTVKEELVSGKLGALLCFNASNQGQLPPRDRAWFTDPVLAGGGAIADHVVHLA
DIMRWYVGTEVVEVYAAANRIFHADEVQVETGGMVSMQFENGIFATIDCSWSRPDSWPSW
GGLSFDLITDKGAVRVDAFRQNLTIYPAPKASASSANAKTAPADTTGVASGAGADHDWTT
AHSHGDRGFGPRWLYWGSDANQAMIEEFISAIQEGRQPAVTGEDGLAAVRIIEAAYKSIQ
EGKAIMV*
>SPBIB_v1_150040|ID:27162552| conserved protein of unknown function [Uncultured spirochete bib]
MHFNLKYGSGTAAVDLDDSRIIDVVLPKPRKPLADALESVRRVLAKPEGTPPLAEMLAAR
KPKKLVIVVNDITRPTPYSVLMPPLLEVIQKAGIPDSHVTLVTATGIHDPHTPEQNILVY
GEEICRRFRVISHNATDPAGLVYKGKLPSGYDFWLNKLVDEADFLITIGVVMPHYFAGFS
GGRKSILPGVAGKETVQKNHARMVELMDNLPPIRENPVSLEMIHAARLAGVDFILNVVVD
DNEQVVEVVAGDFEKAWYKAVDVSESMYMIPIKEQADITVSAASGFPRDINLYQSQKALD
HADRATKKGGTIILVAECREGYGEKVFEEFMNRGWTPQHIMAEIKAHFVMGGHKAYGFAK
VAAEKNIIMVSSLPEKIVASCFARKAGSVQEAVEMALKEQGTEARFIIMPEGSVTVPVIA
AKDG*
>SPBIB_v1_150041|ID:27162553| conserved membrane protein of unknown function [Uncultured spirochete bib]
MTYEVLMQSLTSIFTVQNLLYMIIAIVLGIMAGALPGFTATMAVALMIPFTFTMSPVSGL
ITIGALYCATIFGGSFSAILLNTPGTPSSIGTTFDGYPMAKKGRGMEAIYTATIGSGVGG
ILGTFGLILFAVPLAKAALKFGPPEFFWVAIFGLTIISSISEGSLLKGITAGVLGILVSM
IGNAPVGGDMRFDFGLVSLQGGVEIVSVLIGFFCIPEIFRMAMNPSSKYTDENVVSLEKG
SLKRAFHHVFGHPLATLRASIIGLFVGILPGAGGNIANLIAYNEAKRASKHPETFGTGEP
QGVVATETSNNAVVEGAMVPLLALGIPGSPPAAIIYGALLLQGLSPGPQLFVRNASLVYA
FMLSFFVANILMVIIGFFAGKGMYKTVIKVPVRVLIPSILFLTILGSYAIRNNAMDVIIM
FLSGIIGYILKDLGFNTGAFVLGLILGPIAERGFVQGFLMGNMVSVKMPWIIFFTRPLSI
VLIVLSIISGAWPIFRAAMKKRKDRSAIAEALAFSTSGSGTDVDGSQKKTINSDYIGAAI
VFGISALFYLQIGKWSKYAIMAPRSILILLIALGVALLVKGRIKPEKVIPSFASINRSLV
FLVVVGLLWVFLMEKVGFVVTSMAAYTAILIAYYPGKGARRVIESLLIAGVIVFGMYLLF
EKLLNVTLPLGILI*
>SPBIB_v1_150042|ID:27162554| conserved exported protein of unknown function [Uncultured spirochete bib]
MKMVKRVAMVLIAVLVLAGSAFAQFPQKPLSYVIPFNPGGESDIFARAQQPLLEKILGQK
VVVSYKIGGGGAVGWAELVRTKPDGYTFMGYNLPHIILQPLSLANAGYKTNEIVPVYTFM
STPNILAVSANSPFKTLDDFVKFAKANPGAVTVGGSASASANEIGTVLFNKLAGIKTTYI
PFTGSGDAIPALLGGHVSALMTYTTMGNQYEGQIRVLAVASEKRVSSLPNVPTFRELGYD
LVEGAYRSAAVPPNTPEGVKKVLADAFDKINKDPEFAKKMEANGFKLEFYGPKESEKLVE
EKIKVYTPLVSELGK*
>SPBIB_v1_150043|ID:27162555|leuD| 3-isopropylmalate dehydratase small subunit 1 [Uncultured spirochete bib]
MKITSSLVYKFGNNIDTDQIYPGRYLELTDHEEIGRHAMEGADPTFPSRFKKGGIIVAGT
NFGCGSSREHAVITLKNIGVAAVLAKSFARIFYRNCINLALPAIMIPELDSFGLEEGQSI
DLDLARGVLTSNGVSHHFVPLPEDILELIDRGGVFALYKS*
>SPBIB_v1_150044|ID:27162556| protein of unknown function [Uncultured spirochete bib]
MRVSLYFDKFERGSVIVPLKADAAAVAGETT*
>SPBIB_v1_150045|ID:27162557|leuC| 3-isopropylmalate dehydratase large subunit 1 [Uncultured spirochete bib]
MHAIEKILALASGKSQVSAGEIVTAEIDVAEVNDLYLQVVKSFYELGGTEVKFPDRTTFV
FDHYSPAPTIKAADNHRQMREFCKKMGIKALFDIGKGVCHQVLIESGFVSPGSLVVETDS

HTTTLGALGAFGTGVGATDMALILRNGSLWFRVPEVMKIVLDGELKPGVMAKDLILHVIG
KLKQDCAIYKCVEFAGSAVDAMSIDERLVLCNMSVEMGAKAAYIQPDEKTYAYLARYGIQ
HAKREVFTDPDYRYSAEYHYDVGTIEPSTALPGSVDNLALVREQEGIHIDQIFIGTCTGG
RLNDIETAARILRGKKVAQGTRLVVIPASEVVFKQALAKGYVQTLVEAGAVFSTPGCGPC
LGAHEGVLASGETCLTTSSRNFPGRMGSTEAKIYVVSPATAAASALSGTITDPRSNLSK*
>SPBIB_v1_150046|ID:27162558| Sigma54 specific transcriptional regulator, Fis family [Uncultured spirochete bib]
MAIVFIAPDGHIERTVEKLSGASREKIIVAQGLLENAISVARKYQEEAEVFISRGGTVLV
LRQADLNIPIVEMQVTARDLAVALKKAEKLVKKEKFRIGVVSFPNMIQQLKDFLPFFSLD
ITCYELSIGEDVEKKLKLAMNDGMDVLLGGVTTYAFAKRNGLPVVLVESGETSVKYALEE
AKRIVAAQRLKERRAEELRIITESISEAIIAIDEKGAISRMNNAARQLLSKSQYSFRDHG
TRGSSSSPRMGTEDASLPAALRNVIHEKKESRKIIELGNKRLLVNVIPVTVKGAVASSVA
TLQDVSHIQAMEEEIRREMYFKGYTAKFHFDDIVAHDPITQKTIDLAKRFASTNAAILIH
GESGVGKEVFAQSIHSASMRSSRPFVAMNCAAIPESLIESELFGYVDGAFTGARKKGKPG
LFELAHGGTIFLDEISEMPFHLQARLLRVLQEHQVMRLGDDKIIPVDVRIIAATNRDLPM
LIKSGQFRNDLYWRLNVLNLYLPPLRERKKDILPLIHHMLHESDGCGLAPQISDDCKLYL
ESYPWPGNVRELKNFCERISAICDEPILTTALAASLLQADNRAAERSEQKMGTELGSTFP
PQIPKDIFSSSESLRNLVDSFGSIQAAAHYLGIHRTTLWRHLSKPDLAKRKQSGVSQ*
>SPBIB_v1_150047|ID:27162559|parE| DNA topoisomerase 4 subunit B [Uncultured spirochete bib]
MQSKTWRISGSKAKLAVRGSFGDGGFGQGLHIIKNKNTNGIHNAQRFWGQRLKSPVPRPP
AHVSIYNVGKNPVYDESKIKTLSSLEHIRLRTGMYIGRLGDGSSPDDGIYILLKEVIDNA
VDEFIMGAGDHILVQIDGSRARIRDFGRGIPLGKVIECVSVINTGAKYDDDVFQFSVGLN
GVGTKAVNALSKEFRVVSYREGRFFEARFARGILIDTREGGSREKTGTLVEFIPDEEIFG
EYSFIFDFIEKRLWNYACLNPGLILSLNGKEFHAEKGLFDLLSAELGESPLYDIGWFRGD
RIEFAFTHTKEYGEEHFSFVNGQYTSDGGTHLSAFREGFLKAINDFFQASYRGEDVREGL
AAAVAIRIKNPIFESQTKNKLGNSDIRPWILQEVKKGVDEILRRNPQAAKILQEKILTNE
KLRTELNIVRKEAKEAARKIELKIPNLKDCKAHLGDGGLGERSTIFITEGQSAAGSMVST
RDVHTQAIFSLRGKPENMFDRPRSAIYRNEELYNLMMALGIENNVENIRYARIVIATDAD
YDGFHIRNLLLTFFLTYFEELIVKGRVYLLETPLFRVRTKKQTRYCYSVRERDEAIKELG
PGTEVTRFKGLGEISPAEFGQFIGKDMRLVKVDIQTLSSVPELLGFFMGKNSPERRKFIM
ENLVTEL*
>SPBIB_v1_150048|ID:27162560| DNA topoisomerase (ATP-hydrolyzing) [Uncultured spirochete bib]
VAYIKKLLTDNFLYYASYVIMDRAIPELDDGLKPVQRRILHSLFEMDDGKFHKVANVVGH
CMKYHPHGDASIASALVYLANRGMFIETQGNFGNPITGDEASAPRYIECRCTQFAKEIFY
RPKITRYVDSYDGRNKEPVVFPAKIPVVLMLGAEGIAVGMSTKILPHNPVEVLKAEIACL
QGKPFALLPDFPTGGIVDCSEYAEGTGKVRVRALLDTSDPKKIVIKEIPFGSTTESLIAS
IEEATRLGHLKIASISDFTTDKVEIEIKLARGVYAQDIVDALYAFTECERSISCNCLVIR
DEKPTLTTVSEIVRYHAGKLLNLLRQELELERDELRAEIFARTLERIFIEERIYKSIESK
RTSDSVIKAVIDGFKPFSKELKQAIGTEDVEHLLKIPIRRISLYDIERAQNEMEKIQQQL
SEIDKHLNNLVEFAIDVLKTYIKMLEANWPRRTKIKTYKKIEARAVARHDIALRYDAEAG
YVGTEVSGEKVLEVSAYDKILVLRKNGIYTVLPLPDRIFVGQELLWIAIANKELLSEQPI
TMIYKLLPDKGAYIKRTIIDSWLTGKDYSLVPENAEIIGFSTEKEFSFQLEYKPKPRIKK
IKEIFNASRYPLRGKQAQGIKLTDREIANFIIIPSSIGKSNSLEPENLLPINLCMSQNKE
KQNTGTEDNSLIQIDDSRRESRKKQAKEQSPSQKSSLKEKRNQTIQSKKEQPAIISVKRA
KSKKMTTKMIEKNPSESQSIQQNEAKVSQLELGFSTSVPKNSSESKEQTRTDNAASHSRG
LLEAALKKHKNDR*
>SPBIB_v1_150049|ID:27162561| protein of unknown function [Uncultured spirochete bib]
MVRVKIAITTYEPALYTDLYPVFEDSPHLIIVDEYNRIQKYSPEITAKGTIRGKTEWIIS
RGAKVLVTGSIEDEHYEKLTKAGIAIDWVGFGNAKDLVERARKSAAFLVDKLTKVKVVRT
RFDKRLRPKSYTTCYEIPYIGNDPQFIEELERKAKRQGKKRLLKPKEEEDLDEGFDEEDD
DLFQ*
>SPBIB_v1_150050|ID:27162562| protein of unknown function [Uncultured spirochete bib]
MRKPRFTLPGARYHIIARANRGEFIFQRREIKDLFLALIRKAKKRYKFAIETFCIMDSHI
HLIIRPLEGESISRIMQWLLSVFALRFNRRFGLIGHVWMDRFKSFILFTDAQRMKAHAYI
ANNPVSAGLVHSPYHYSHSAAKFILAGDFSIIEKPPLNLLEFYCNLEMTYHS*

>SPBIB_v1_150051|ID:27162563| putative Malate permease [Uncultured spirochete bib]
MTLSRVVSIFLLLVVGFIAQKRKILDSDGIKTISNFVLRVFLPFTIISSFDKSISLSAAG
DLLKVAIFAIGVHALAIFISKLAYSRFPDPKRKILTYITVFSNCGFMGFPVAESVFGKIG
LMYTSIYVMVFNIFVWTYGIALLSEGKEKKNIWHSILLNPGNIAFAIGFVLWVLPFGLPE
TLNYAVLLLGNCTTPLSMIVVGATLANLGIKGLFSGTEVWIGSIMRLFVVPALVLLLFIL
IGIRSDAAKVANFLAAMPAAAQTVIFAERYDADVSLASRIVFISTVLSAITIPLASGIIA
G*
>SPBIB_v1_150052|ID:27162564| conserved protein of unknown function [Uncultured spirochete bib]
MRLIRCEYDGKVYHGRIEKDEVILFEYFPQIEMKDGQKRILLAEEKGRVPLSVVHLKAPC
VPGKAVCVGLNYRDHAKEFGLAIPASPVLFIKPSTSLLDPDEKILYPSISSRVDYEAELV
AVIGAKARNIEKKDALNYILGYTCGNDVTARDLQPKDGQWTVAKSFDTFMPLGPWIETEL
DPSNLRIQAILNGEVRQLSSTENLIFNVADLVAYVSQIMTLEPGDVIMTGTPSGVGPMQK
GDRIAIEIQGIGRLENIVA*
>SPBIB_v1_150053|ID:27162565|yeiN| conserved hypothetical protein [Uncultured spirochete bib]
MNPYLDITEEVADALHAGLPVVALESTIISHGMPWPRNLETALAVEDAIRKEGAVPATIA
IINGRLKVGLSREELTLFSNSSLHPAKVSRRDLPIVVAQNGNGATTVASTMIIASMAGIR
FFATGGIGGVHRGAELTWDISADLDEFARTDVAVVCAGAKAILDLAKTMEYLETKGVPVI
GYRTSELPAFYSRESGVMLSQRADDPMEIARLLHAKWSLGLSGGAVIANPVPIEYSMTKN
EIDAAIEQALEELAEKRIKGAAVTPYLLARVAEITGGDSLETNIALVINNAHLAARIAIA
YEQMSEK*
>SPBIB_v1_150054|ID:27162566| protein of unknown function [Uncultured spirochete bib]
VHRIEESSEPYCVVIGGINIDIQGFCNAAHIEKDSNPGVITRSLGGVGRNIAENLVRLGV
RTELITVLGNSPAWEALISRIEHLGIGVSHSPRLADVPLPMYLCILERDGGLVSAVADMR
AIERLQIEHLENQDSLLRKAACIIIDGNLPKACIEWIAQQYHAANSQNKKTIPAALSSTE
RENVFVSRDFADPAGKDQNGDRGFQNANVQSQIAVSQHPILIADPVSSSKAVKFRDCFGL
FDIAKPNLAEASTIANFGPTATPSQIIAALKAKSILPNELYLSLGEKGMLVIEDDSIHEI
SLPASNLRPPAANRSGAGDAACAALAWVSIRSMFSERLKNGQFSNLRPSAKAKFALSAAL
LASASKRPVNPKLDPRMLCEKIKFWYPELSDLVEYMMQNGGII*
>SPBIB_v1_150055|ID:27162567| putative Heat shock protein DnaJ domain protein [Uncultured spirochete bib]
VDQIFDRLERLVRAWTSSAFGEGFGRNNFGSSDIFKDQDLADAWEELESYLDPSKTESER
EAEMRRRSEFRSQASNDQDPRKAIERQAVQDAYRFLGLEPYAPFAEVKARYKELLKKHHP
DRHTASPEDMKTATAVSAKINAAYQLIETWEEARAQKAR*
>SPBIB_v1_150056|ID:27162568|polA| DNA polymerase I [Uncultured spirochete bib]
MNEKDPLYVLDVYAFIYRSYFAFIRKPLRNSLGENVSAIFGFFRFLFSLFEQRKPILFAA
ALDSIGPTFRHEQFLTYKATRQKTPEDLIAQIPKIESILRALEVPTIRCEGYEADDVIAT
LATQCSREKRACFIVSGDKDLLQLVGGFTKALRPSEGFSFREINENGVLKEWGVLPDQIR
DYLSLTGDASDNIPGVKGIGDKTAQKLISQFGSLDSIYQHLNEIEPESLRKKLEAGREDA
FLSKDLISLCDNVPSCPSDVASLRASLNMAAAAPLLLREEMKSLVPESLRNKKGVQTSVP
KASTRTSAEADKNEIQTSVPNLSNNLKPEVKHGEGIASIEAQASQQSPSLFSRTDNVPVY
QAILEEEKLEEIIDAALAHGVCAFDTETNSLDSHHARIVGFSLCYEAGKAWYIPLLSPDS
KCIPHKKALAALSRLVSATQMLLVGHNLKFDINVLAAGGTEVTSPCFDTMVAAWVLDAEA
PSFSLSSVAARFLGLSGLEYDQVVPKGATFADVPLATAVQYAAEDADFSFRLYQRLSKEI
DSAQLHNVFYDIEMPLLPILAEMERTGIRVDADKLRAYGVELENELAKIQSEVFHLVGHD
FNLGSPKQLADVLFVERKLPVQKRTKTGFSTDTSVLEELAPLDPVPQLILRHRLLTKLQN
TYIEPLAALAETSGRVHTTYVQTGAATGRLSSRDPNLQNIPIRDEEGRRIREAFIAEPGH
KLISADYSQIELVVMAHLSKDPNLKKAFKEKVDIHKRTASFIFGIPEDQVDASQRRIAKT
INFGVIYGMSAFRLARDLGISNALAKEFIDSYFSTYSGIAEFIKRTIEEAQETGITSTLF
GRKRRILGINSRNKTEQQAAHRVAVNTPIQGTAADIVKIAMIKIHRALATQMPEVRMLLQ
VHDELIFEAPESSVPAAIDLIKEEMEHAVQLDVPLRVSVESAISWGDMHL*
>SPBIB_v1_150057|ID:27162569|coaE| Dephospho-CoA kinase [Uncultured spirochete bib]
MHIIGLTGGYCAGKNEVAKILSAYGWEVVDVDKLGHAALSLSSLAIKHAFGSHVMNEDGS
VDRTKLGQIVFSNAQQMQILESLVHPAMLSLLDREIQRAREQKKEKFCINAALLYRFPQL
SLCEAVIEVQAPLFLRVSRAKKRDNLSATRALKRIFSQRELWKLRPRKGAKVFILLNSGK
KEQLENKTKKLIEKLSQDLQR*

>SPBIB_v1_150058|ID:27162570| putative Sporulation repeat domain protein [Uncultured spirochete bib]
MSDRSKKILWIAVAVSGFALVVFLAAFLLFPPSEASESRPFDLTRRSPSKPLSPNDFSAF
PSIVVQPSGESSQLPTDSQANSTSEKKSSSTVIVVPAPAANQTNMNASQTEGESAVASSA
PKPASSSVSGQSAVKTPQTSVPAAKPPATSVAPVPAAKPASPPSPPPSTKAASSAEYWIQ
VGSFSAKGTADKLRDEFTARGMTSIIAVKEIGGRNYYQVKVGPYPSADEAKKWLSTVKAV
QGASQDAFVTTR*
>SPBIB_v1_150059|ID:27162571| Purine-binding protein BAB2_0673 [Uncultured spirochete bib]
MRKIVLGLIAILMLVSLVLPATAQAKKPLKVAFVYIGPPGDLGWTYEHERGRLAAQKYFG
DKIETKYIENVPEGPDAERVIRQYAIQGYDVIFTTSFGYMDPTYAVAQEFPKVIFEHCSG
YKTAPNMATYFGRIEEARYLTGIIAGRMTKSNKIGYVAAFPIPEVVRGINGFTLGVRSVN
PNAKVQVVWTNTWYDPVKEREAAVALLDAGCDIIAQHQDTTEPQKAAQERGKLSIGYDSD
MGKFVGDTVLASAVWNWETYYIATIQNILNGTWKTHEFWGGLKDNIAKLSDLSPRVPANV
RKEVEDAQKKILSGWKIFSGPIKDQSGKVVYKAGEVVPDDKQLSMDWFVEGVVGKVQ*
>SPBIB_v1_150060|ID:27162572|yufO| Uncharacterized ABC transporter ATP-binding protein YufO [Uncultured spirochete bib]
MSETVIPKVEMRGISKSFPGVLANDCVDLMLHRSEVLALLGENGAGKSTLMNVLCGLYRP
DKGEIYINGSYADIHSPRDAQKYGIGMVHQNFKLVDSMSVLENIILGLKDEPFIPDFKRV
RQRLVDLAAQYHLKVDPNANIWQLSVGEQQRVEILKLLYRNAEILILDEPTAVLTPQESH
ELSRVVHAMKQEGKSAVFITHKMEEVMEFSDRVMVLRKGKVVAEARTADTTPKELARMMV
GREVLFQIEKKPFCPGEVVLELSNIDALGDRGLLALNNVSFQLRAGEILGIAGVAGNGQR
ELAEVVTGLRRSTKGALRIGNKDMTNKSPLEIIRQGVAHIPQDRSSVGAVGDLSVASNLA
MKQYRSRPLAAGIFLLPKRIVDLARALIEKFHIATPSPQTQVKFLSGGNVQKTILAREIG
AARTVMVAVYPSRGLDVGATETVRRQLINQRDGGLGVLFFSEDLDELLQVSDRIAVLFEG
RIMGIFDADKADIEQIGMLMAGMSKEATA*
>SPBIB_v1_150061|ID:27162573| Inner-membrane translocator [Uncultured spirochete bib]
MKFVFEKRKSTSVPALILVPLISFAVSLVLTALLLAIFGADPFKTYAAMAVGAFGTSHGF
AETLVKAIPLMMTGLGVAIAFRLKFWNIGAEGQLTLGGVAAAGVALFMERYFPGRSLLFA
ALFGGLLAGALWAGIPAILKTTLKVDETLVTLMMNYIAILYSEFLYYGPWRDPKGYGFPG
SRMFSANAWLPRIMGRAHVGIWVGIALAIILWLVLKRTRWGFELQIIGASQKAARYQGIA
VERNIILAVMLSGALCGLAGAFEITGISHRLQQGLSIGYGYTAIIVAWMSQLNPLAVPFV
AIALAGLSVGGDQVQMVMGLPAAMGVVMQGLILFPMLAGSLFTEYRFLVVKPEPRAACPE
PSQE*
>SPBIB_v1_150062|ID:27162574| Inner-membrane translocator [Uncultured spirochete bib]
MDFLTSILTITIRAGTSLTIATIGEILTERSGILNLGVEGIMLMGALSAFTAVFYSHSIA
IGLLVAIGVGGLLALLHAFLTTTMRANQVVSGLSITLFGTGFSSFLGQRLGPVANNFRLV
GLRAERIVPLAPEWVKQIPIISAFFNQDIITYLVYILLPLAWFFLYKTRPGLWLRSVGED
PQTADAMGIDVTKTRYLYTILGGMMIALGGAHLSLSYTPGWSENITGGRGWIVIALVIFS
MWNPARAVWGALLFGGINAVQFRLQASGTSIPANFLNMMPYIGTIIVLVVMTWWEALSKK
VGAPAALGTSYMREDK*
>SPBIB_v1_150063|ID:27162575|ade| Adenine deaminase [Uncultured spirochete bib]
MNRDVIDAATGKTICDVNYTNCTVVDVLSGRFLPNSIVSVKGGFIAGINDGLDAKESINL
EGMFLAPGLIDAHVHIESSLLTPAEYARVVLPRGTTTVVADPHEIANVMGYDGMRYMLNV
SRDIPLDVYFMVPSCVPATDFDTAGAALYASDMHQFLQEPRVLGLGEVMNYPGVLARNPQ
LMDKIALFQSNGRPIDGHSPGLRGASLSAYVVAGIGSDHECTTPEEALEKVEKGMYIMLR
EGSTAKDLRPLLPAITRENASRFMLCSDDRHCNDLRDEGHMDFSLRMMLEGGIDPIDAIR
IASSNAARWFGIPGQGAIAPGYKADFVAFPSFEYFEAKIVVKNGVIVARNGALVQEFATV
GTPIRDSVNIKWLTIDDFVIPDKGKPVRVIEVNRESLLTGNGISRLEGIRGRLSADIERD
ILKIFVIERHTGSGNIGKGFIHGLGLKRGAIGSTISHDSHNMIIAGVDDVSIFKAARHLN
KIKGGLVYAVGDEILLDLPLPVAGLMSDKPADFVIERLTAFEKLFRQEGLTATSPLMTLS
FMALPVIPSLKITDMGLVDVDRFERVSLYVD*
>SPBIB_v1_150064|ID:27162576| xanthine dehydrogenase, Fe-S binding subunit (modular protein) [Uncultured spirochete bib]
MALLYIKVNNAWRGLASAEGMPVLDVARGELGLTSTKEGCREGDCGACAVLVGEQIANTV
RYRAVPSCLLALGELKGKHLLSLEGLIEGTEDGLTPVMRAFIEENASQCGFCTPGFIIAL

TSWLAEPQVPDLAGALRAVDGNLCRCTGYAAIRRAAARLVEQFRDLPLEPIARLKALVSA
QVLPQSVLKFIEESKSSSLQTSVPNGATIGTGLDMAGHDNKKAVASTLDPAVIVGGGTDF
YVRNPDPDEGFSPLLTRMIPEFALVEYRDEPQGQWLEIGAAVTVRDFFVSPLVQREIPGI
GRFETMFASTLIRNLATVGGNIVNASPVADITSMLMALGAQLVIVPEARAYEPQAPIRLC
PLEQFFLGYKKLDLRPGEVLKAIRIPAGANRKFSFEKASKRKNLDIAAVNTAIAFKIEEK
HFKDVRISLGGVAPVPMLGTAAMEVLEGAPCNPADRKTLASLAMKTAQAASASISPISDV
RGSADYRRRMVHQLMLAHFVRFFEASGIAEELFP*
>SPBIB_v1_150065|ID:27162577|xdhA [H| Xanthine dehydrogenase, molybdenum binding subunit [Uncultured spirochete bib]
MKEADMKVDKKSMAFLDEADARSGTAHHEAISIPDAASVIPESLPNVLGQTQYIDDIPRP
AGCLQAAVRLSDSAHARILAIHTEEALSLDPSVRVILAKDIPGTNQIGFNKPDEPLLPEE
EWEYWGQPIAIVVAKTRSLARKAAGLLRVESEELPAVIDPREAAAKGDFILPPRTIACGN
VEEAFAKCAYVVEGRVDSGGQEHVYLETQGAIAQVIDGRRMHVISSTQGPTGVQRAVAQV
LGLPMNMVEVEARRLGGAFGGKEDQAANWASMAAVASWVCGKPVELYLNRKDDMRATGKR
HPYTTDFRVGADADGKLLAFEADYYQNSGSTCDLSAAILARTLLHATGAYQIPNVRVTGY
MCHTNLPSFTAFRGFGAPQAFFVIEAAMDALAQKMGTEVVELKRRNLFREGDRTYYGMAL
ERVRASESVERLLAKVDYPALSQRIKTFNATHRLQKKGLGFIPVAFGISFTKLQMNQAGA
LVHVYTDGSVVVSTGAIEMGQQVARKIALVTARTLGVPVSYVTVQRTTTLTVANTVPTAA
STGADLNGMAAKIACQEIRGRLVQKAADLLKAAPEKIDIREGIILCDGKPASLTWKQLVE
AMHAARQDLSAHGFYATPFLEYDMKAERGRPFAYHVYGAALVEATVDILRGTSTLDSVSI
VHDIGDSIDPAVDRGQIEGALAQGLGWSLLEDLRFGPDGKPLSDTLSTYKVPDVTFMPEN
IDIEFLPPIENPTAPYNSKAVGEPPLQYGIAGYFAVLDAIRAAIGKPIVYYNLPLIPEKI
SDLLAEMGSDPLEPAGKRDTALQAH*
>SPBIB_v1_150066|ID:27162578| putative Allantoinase [Uncultured spirochete bib]
MIIANVAAALPGERDFHLVDILIKEGKIAWIKEAGSFQRDSRLANEYAAEEMLDAKGLLA
FPGAIDPHVHFDEPGFTHREDFLHGSAEAARGGVTTVIDMPCTSIPPVTTPHALEGKLSI
VRDRALVDYAFFGGINGTMLPEEISDVVQSLANRVVGFKCYFISGMDTFPAVNDTQFAAA
AKACAKEGRPLLLHAEDSNVIAEASQERQSLRGSQAPSWADYYTSRPMEAEIAAVHKALK
LAGQYTKYLHIVHVGTGKAAIAASEKGASCETCAHYLAFDETDFARFGAALKTAPPVKSP
EQKAILWRLLAEGRLSFVASDHAGAPEYEKFTDNPLTAYGGIPGTGTLFPYLLSEGLFAK
RLSLERFLEATSGGAARRYGLWQGKGSLMPGKDADFVLVDPDHTTYLDPSHMMSKSSITP
FAGMRLSGRIEGTFVRGSYVYASVRLAAALRHQANPAFSNEVGMILAKPGFGKFLTWGYR
*
>SPBIB_v1_150067|ID:27162579|ygfK| putative oxidoreductase, Fe-S subunit [Uncultured spirochete bib]
MSKVMKTTSLPALLTRMAGEYLAKRTIFEIPEATWLDVFAQESESPGITVMSANVSIPLG
PAAGPHTQIAPNLVAAYLSGARVFELKTVQENDHLDIDKPCIDALDEGHNVEWSTELSLD
QAREEYINAWIAINLLARIFSRKPGDFMFNMSVGYTLDGIKSEKMDAFIEGMRRPEATEY
WEKAIDQLKAFIEEPIFAEAFGQAALERARPIAEHMPVRPVHSVTLSTMHGCPPDEIERI
GRYLIEEKGFDTYIKLNPTLLGYNRARVILNQLGWTDIILKPESFSHDLQFDAALALIQS
LTETAAARGRRFGIKLSNTLANINDGRTLPGAERYMSGRALFPITIHLAAKLARALPDFP
ARFSYCGGVSAFNAADLLKAGLGPLTIATDLLKPGGYQRMAHMVKDVLGALTSVPERPDA
DALDQLAKDALEAPYYRKEWREGTAAIGRQLPLFDCFAAPCIEACPVHQKAPAYIAATGS
GDADRGMSVILSDNPLPAITGVLCDHVCQEHCSRVDYEGAVRIRDVKLATVRASEAALAA
EVAPAWPHAPKGKTAVIGAGPAGLACAWYLAQYGQKAVVFDTSSVPGGVPANFIPSFRIA
REDIAADIARLEKLGVEFRFGVDVRSAEKLKAEGFERIVIASGAHTAREMHLKGSGVRVV
HALEFLGVCMAKGAEHFAGAKNVLVVGGGNTACDAVRMATRIPGVKSVLWSYRRTRKEMP
ADLEELTNAIEEAKALQHNKAAALFNGGTSNILVELTLPEVMQPGSITLRRMKLAEKDAS
GRRAPVPTEETFDLPCDLMITAVGEKPDPTFLAAFGIEVGKDGLPAVDEGTMETSVPGIY
VCGDARRGPSSIIASEADGRAAAMAILKNLGIEAASADYRAPAWSSEARASRGKILPSLD
PGDPGFAAREAERCLACDTACLRCVEVCPNRANMVIDTDRLFDQPAQILHIDRLCNECGN
CGLFCPWNGEPYSGKPTLFDSKDDLAHSQNAGFAFAGNKARPFLVLRAEKGGAVLELPYL
AWSGAISVPAQRPMIALARTIWADHRYLVEVHE*
>SPBIB_v1_150068|ID:27162580|ssnA| putative chlorohydrolase/aminohydrolase [Uncultured spirochete bib]
MILFHNVRILELEPPSVSEPTDVAVYESGEGDRAGTVAAIGPNLAVQYPTAKVAGEGGYL

SAGLVCGHTHLYSALARGMLVDIKPSKDFAQLLDHLWWRLDRAIDPVILRSSALVGCSDA
LKAGVTSLVDHHASPECIDGSLSIIREAYEAVGVRGILCYETTDRNGMAGARASVAENVR
FAKEIDAIRASGAKPLVEASIGAHASFTVGNETLEALAEAVRSTGRGIHIHLAEDKFDAV
DARHRFGKDPVERIDAVGALNDRSIIGHGVWLSPSEVEIMNARGAFLAHNARSNMNNSVG
YNILLPTFANVVLGTDGMGADMLEEFKFAVFRHRESQGPWWPGDFLKVLHRGNALIERHF
AQDFAPGTTRASTPAVAPTSTLVTSGEGGFPGFGAVQVGAPADLVLWDYDSPTPLVGDNI
AGHFAFGMSSRSARTVMVAGNVRILDHRPLYDDATIQAEAREQALRLWKRMEER*
>SPBIB_v1_150069|ID:27162581|ygeY| putative peptidase [Uncultured spirochete bib]
MDSKKILEKAREYRDYTAQNLSKIIQVPAFSTTEKERIYLLKQLSEEAGMEDLWIDGLGS
LLGRVGKGSKKIVFDAHIDTVGVGDETQWKLPPFSGLIKDGLVHGRGASDQLGGAASMIT
AARILKELGYSGDYEVWFSFTAIEEDCDGMCWKYLIEEEKFVPDLAVSTEPTSCRLYRGH
RGRMEIQIDIKGISCHGSAPERGDSAAYKAARAALAIEKLNERLQPDDDKFLGKGTIVVS
QIDVHGPSQCAVPDQAMLYCDRRLTWGETDEIAIKQVEDALREAGVNNFTVHMPEYRKPA
WTGTEYHQELYFPTWKIPADHMLVQSGADAYKSLFGKEPVVDKWTFSTNGVAICGRHKIP
VIGFGPGDEAQAHAPNETTRIDDLEIAAAFYAALPYMLEAHK*
>SPBIB_v1_150070|ID:27162582| putative threonine synthase [Uncultured spirochete bib]
MQWLYRCPACGRTYPIEPGRYLCDHCAKEQRPDEPLRGVLECTWEGGNPEPGSVPLPVEE
RFFPPIPVGQTPLWAPERLRAELSMPNLWLKDDTCNPSGSYKDRASWLVAAFARKFGIKE
IVLASTGNAASSMACIGAAAGIKIKVYVPKSAPIAKRVQILQYGAELIEVDGTYDLAFDQ
SLAYSNATGMLSRNTAYNPLTIEGKKTASFEIARDLAAGASRSAPRYLAPDHVFVPTGDG
VIIAGVIRGFEDLVRLGWIDKMPTIWAAQAEGSSAIARALATGRFEAVPSNTIADSISVD
IPRNGYFALDKLRRHSGRAVVVSDAEILEAQRYLSRASGLFAEPSSACAFAGFLKARAEL
DPNAQVVVMLTGSGLKDIKSAALGVGLKI*
>SPBIB_v1_150071|ID:27162583| conserved protein of unknown function [Uncultured spirochete bib]
MKSHIEKILLTEEKETLLVPLWSKAMESRRQNPIFFDRTSKEILDRIEYDFANLKIPRKT
AVMLCIRAKKMDDYVKEFLASHPTSIVIHLGCGLDSRYMRVDNGEVEWYDLDMPEVIELR
RKFYEETSRYRMISSSVNNLGWISAISAQGRLAMVIAEGLFMYLKEEEVKALILALKEAF
PGCLLVFDAYSVLTARSVREHPSIKKTGAVIHWGIDDASAIEQLSEGIRLKEEWYFTQAD
DIKKLGFGFRLAFGIAGLFSAAKKAHRILYYSL*
>SPBIB_v1_150072|ID:27162584| Pyridoxal-5'-phosphate-dependent protein beta subunit [Uncultured spirochete bib]
MIDLNVNESQRKKNIERCRQKGILLPTFAQMRDPAKIPESIKKELSNIGLWDVHPRNLFR
VTWHNEPKEFGGGYGPVNYIEIPRAITGTKARIVGLAGKWFPTGAHKVGAAYACLAPELV
TGRFDPTTKKAVWPSTGNYCRGGAYISRLLACPSVAILPAEMSRERFEWLKTIAEEVIAT
PGCESNVKEIFDKCIELQRTRHDVVIFNQFDQLPNHLWHYAITGPAMEEVFRAIGGQNAH
VGGVVLSSGSAGTLGSGSYIKEKFPGAKLAVGEALQCPTILENGFGGHRIEGIGDKHIPW
IHNFRDTDAAVGVDDELPMRFIRLFNEPAGRKLLIDAGADPTVVNKLDWLGISGVGNLIA
AIKFAKYYELGEDDIVFTMFTDSMAMYQSRLAELTAERGPYDQRQADRDYDRLQGLSVDY
VFEMTHIDKRRAHNLKYFTWIEQLGKDLSELRAQWDDYRTYWGGLHQQADALDRLIEDFN
AEVLK*
>SPBIB_v1_150073|ID:27162585| 8-oxoguanine deaminase [Uncultured spirochete bib]
VILLKDCFAVAVPADAGADGANSRRRFSLAHGVDILLDGNRIAKIAPNIEAPEGATVIDA
SRHVVVPGLVNTHHHFYQTLTRNLPAVQDAKLFDWLVYLYEIWKYLDPEAVYWSSMLAMA
ELAKTGCTLTTDHHYLYPAGFGGDIPSLQFRAASDIGLRFAPTRGSMSRSKKDGGLPPDT
TVQDEDTILAHSEETLRRFHDPAPDAMRKVALAPCSPFSVSERLMKDSAVLARKYGARLH
THLAETSDEDDYCVQVYGRRPLQLMQDCDFVGPDVWYAHGIFFNDEELDFLAKTGTGVAH
CPSSNMRLGSGICRVHEMLDRGVPVGLAVDGSASNDTSDMLGEARQALLLQRIRYGSAGL
TAGEAMYIATEGGARILGFDQAGTIAEGRLADIALFDVMKLEYAGALSDPLAALLFSGYN
HEVDHLIVGGRQVVRHGMLVSADEEMIRRNALSACERMYRKAGIV*
>SPBIB_v1_150074|ID:27162586|ygeW| conserved hypothetical protein [Uncultured spirochete bib]
MDKIHSMIEQLRAKKIDMAGKDFLLTWEKSSDEIEATFLVADILRAMREQNISPRVFDSG
IAVSNFRDNSTRTRFSFASACDLLGLYVQDLDEGKSQIAHGETVRETANMISFLTEAIGI
RDDMYLGEGDRYQREVGKALDEGVKAGVLPSRPTVVNLQSDIDHPTQSMADFLHLAHYFG
GLDKLKGKKIVMSWAYSPSYGKPLSVPQGVIGLASRFGMDIVLAHPKGYDLIPDVVELSK
KQAQASGGSFTYTDSMDEAFKDADIVYPKSWAPYDVMLRRVPLLHAGDKDGLKALEKEAL

ANNAKFQDWECTEEKMKLTKDGKALYMHCLPADITDVSCKAGEVAASVFDRYRDDTYREA
SYKPYIIAAMILLGKFRNPASMLESLTARGKQRVYGI*
>SPBIB_v1_150075|ID:27162587| Cation diffusion facilitator family transporter [Uncultured spirochete bib]
MQERIRTIRLASWIALVGNFILAVTKVLVGFISGSLSVLSDGIDSSTDVLIALMTLFAAR
ISAKPGDKEHPYGHGRAETVASAVIAFVVFFAGSQVLVSAIKAILSGERAVMPAPIALWV
TVFSITGKLALSYSQFHFGKKAGSNMLIANGKNMRGDVVTSVAVLVGLGLAFLTGIPVLD
KILAILVALWIIKNAIGIFLEANTELMDGTADRGPYLDLFNAISSVPAAGNPHRVRLRRI
GSMLVADLDIEVDPHMTVAQGHDIALAVEAAIKDAMPEIYDVIVHIEPNGNVEKERYGLT
ASDAGISE*
>SPBIB_v1_150076|ID:27162588| putative Hemin import ATP-binding protein HmuV [Uncultured spirochete bib]
MKTKLQPIGGIADSATGTATGTEDQATPILQASELSFGWSAERQVLDSISLEIARGQTLA
ILGPNGAGKTTLLSILTGKLAPLRGQVVLEGHPLSDYSARERAWRIAFLPQLEKLPFNYR
VLDFVLMGRTPHMEALALPGTEDEKAARDALDSLGMASFEERNIGELSGGEFQLVRIARC
LAQGASILVLDEPVSMLDPAHARQIADALAGLATVGKTIIYTTHDIGLGIFLGGRALILA
QGRVQWEGESSGLRDAEMLARAFGITFSMREMPSVF*
>SPBIB_v1_150077|ID:27162589| Transport system permease protein [Uncultured spirochete bib]
VSSSKVEKEPKGAQGSEGTKESEGAQRSGQAQLAKGATRSDWALRSGGTARSAWAHRHLS
ALLVGLLLIVFALALFIGRYPKPGFLNPAALVSDPLARQIVLNSRLPRMLGAVLLGLVLG
GAGASLQAVFGNPLVDAGFLGVSQGAAFGAAVALVLGLRAYSLIAVLAFGMAVLALALSL
WLAERFRYGGQILRLILAGLAVSAFFSSLLAMMKYVADPLSQLPDIVFWTMGSMVPMGWK
RLESSAPIALAALAVLYAMRWRVNLLSLDDTVSHSLGIRPGVERKIVSVAAAAGVGVMTA
VCGVVGWVGLVVPQIVRAIDGPDGRSVLPRSMLGGAIFVLVSDTLARSLFSGEIPLGIVT
SLFGALSFAILLTMRKVELAR*
>SPBIB_v1_150078|ID:27162590| putative Periplasmic binding protein [Uncultured spirochete bib]
MKRYKLFLLTALFLVAAFGAFAQQRIVLGGRAVPMLADAVYLFPKTSSRVVAFASSDQGL
GIFMSAIDPDFKSKERFDKSAGAEVYASFKPDLVILKSMMKNQLKAPLDALGIPQLYLNL
ETPEQYYEDIATLGKVLGNEKRASEVVSWFASHEASIVSRTSKIEPAKRPKVLIMQLAVS
GESVWQVPPDSWIQTIMAERAGGQAVWKGANPGSGWATVSIEQIAAWNPDFVFIISYSGN
SSGAAEAFKKDLRLSALKAVRNGSVYGFPLDFYSWDQPDTRWILGFTWLAKRVHPELFAD
ISVARTTREFFSFMYGFDDAMFRTIIQPKIAGDLGEQF*
>SPBIB_v1_150079|ID:27162591| putative ABC transporter, transmembrane region:ABC transporter related [Uncultured spirochete bib]
MNASKRTQGRGQAAGKGLQGSATKAKKARANRSNTAASPSTAKSGPLSHIRWIREVWREK
QGLIWLLLFLTLLSSAVAVAFPLLTKQLFDLLERAIDTHMNKGAAMAQVKRIALYFVAIG
LAGFVAGTFPGIRGALNMVFDYIVRKRYFSEVMQKDIRFFSYFRSGDVITRLTSDISDFP
KLSWFLCSGIFRAVESASKVTFCIAAMLSLDWRLALASLASLPIMLLVFTRTQSAIYDRV
NKNEQAISAINNQLEMSFSGVKVIKAFASEEKYTRFFDDALARRFETEMGVIKLETILQL
IYQYIDYVAQVALVFVGGLMVTRRTISIGTFYAFYNYLNMLIYPVLDIPQLFVFGKRAFV
NIDRLEEMRRYATESAAAEGAVPEGSLQQKSTLAETEMEGAQNPPSQHASKPAPLPVSPA
AWLTRLQSLRFEKATVYYERKHKPAIAEIGVEVRAGDKILVIGPVGSGKTTLIKTALGLL
EPRSGRVLLNGRPVRELGPLERRGLVGYVPQDPLLFSGTIRENILLGLKEGQTLSQLEME
RILEVSRLSDELAAFPEGLETRLGQRGTSVSGGQKQRIAIARALAGYPSLLLLDDMTASL
DTNNEEALWKSLAEMPEFTRAAVIAVSHRLSSIQYVDRVLFLKNGRIAGFGTHAELMAEN
AGYREFVAEHVQMPGA*
>SPBIB_v1_150080|ID:27162592| Xenobiotic-transporting ATPase [Uncultured spirochete bib]
MANQLMTNQPAKNDYLRTILSYLSRYRMLALPGIGVMILIAMGQLAGPYILKQIIDVAVP
KEDTSLLLGYAFAFVAIVSVTGALSYVGMMLLARLGLSIVTLIKQDIFSHLLKLPASFFD
SHPVGELMSRTETDTERVRDMFSNLGANLIVNVLTMLGIFAVTFALVPKLALIMFGVSVT
LLAVMIVFFSKIFPMYEKARSLYAGIAAKVAEFVQGIEVLKAYGRTGWAEAQLDAAAKQK
VALDVRISLIEYSMMSALNALIGPLFIVALLLLYAPKVLAGTMTLGMILLFVEYGARLLR
PIAEIAESLRSMQQARTSLSRIRKLMATEEEPNRGTGKAPSLQHEIRFDHVWFAYNDEDW
VLRDLTFTIPKGSFAAIVGASGSGKSTTIGLICGFMHPQKGCIRIDGTPLDEIDIVAWRK
KIGLVLQEAYLFPGSVLENTRLYHDEIDEKTVWSAISMVHANEAIDSLSEGLSTNLWERG
GNLSSGERQLISFARALTMNPELILLDEATSNVDMATEKRIKESMEVLRKGRTMVVVAHR

LSSILKANTIFFLSEGRLIAQGSHESLYENLLEYRQLVDQQFPAGKGGRA*
>SPBIB_v1_150081|ID:27162593| protein of unknown function [Uncultured spirochete bib]
LPECRKARYNISVWHFRTFYRRYAKNYTEKLGLLTFYGQSTYDQSTSKK*
>SPBIB_v1_150082|ID:27162594|fucI| L-fucose isomerase [Uncultured spirochete bib]
MAENKDFEMQQARNRLKGSAPKIGIRPTIDGRLGGVRESLEAVTMGMAKAAAKLISENLR
HPTGEKVECVIADTTIGGVAEAAACAEKFAREGVAVTLTVTPCWCYGSETMDTDPLTIKA
VWGFNGTDRPGAVYLAAVLAAHSQMGLPAFGIYGHDVQDLTDTDKIPADVAEKILRFAKA
GLAAAWMRGKSYLSVGSVSMGIAGSIVNPDFFREYLGMRNEYVDMSEITRRIEEKIYSEA
EFRRAMEWVKTYCKEGPDNNPPEKQHSRARKDRVWEMSVKMAMIVRDLMVGNPDLRRKGL
GEEALGHNAILAGFQGQRQWTDHFPNGDFLETILNSSFDWNGIRQPYLVATENDSLNGVS
MLFGHLLTGSAQIFSDVRTYWSPEAVQRVTGWKPTGLAEHGFIHLINSGATCLDGTGCQR
LDGKPAIKPWWQVEANEAKACLDATQWRYANLGYFRGGGYSSDFLTEGGMPVTMTRINLV
KGLGPMIQIAEGYTATLPQEVHDTLDLRTDPTWPTTWFVPRLTGTGPFKDVYSVMASWGA
NHGAISYGHIGADLISLAAALRIPVAMHNIPQEKIFRPSYWNAFGMDPEGADYRACAALG
PLYR*
>SPBIB_v1_150083|ID:27162595| Transcriptional regulator, LacI family [Uncultured spirochete bib]
MARETVTIRNVAQRANVSTATVSRVLNNDPRVRYETKQVVLEAMESLGYKLNIVARSLKT
SRTHTIGVLAPDLAGDFFMFIAESMNRELTLHGYSLVVSTSRDSEEEEAKRIRHLAERLV
DGIVLIPVGSSSAYLSQVSALHIPIVFVDRALEGFESDQVLVDNEGGAYEAVCALIGEGH
RRIGFLGGKPEVTTAKERFAGYCRAMEEAGLEIEQKYVRFGHPTLPFGYRTMEEIIKDPD
SPDTWFIVNLFAHLGATSYLVTEGGERAQHITFAAFDEMPYSPLLRYCRYAVQQPIAEMG
KAAANLIIDRIEGKGPPEPQILRFRTRLIQHPLVR*
>SPBIB_v1_150084|ID:27162596| Auxin Efflux Carrier [Uncultured spirochete bib]
MEQASGSLFSIAILMTAGYMLAKRGWLGERAIDGLKKIITNLTLPLLLLRAFLRLTPDGR
NVILALGVFASCAIMGLAGALLAGIARLPRPETRLLFQGFEAGMLGYALFAGFHTAERLP
AFAALDMGQVIYVFTVLMVQMSMRPEGEQGASKTAVFPWWDIAKSKVLWAIGAGIVLSLL
TPGFADMLAVRSGFAGAVFDTVGGLTTPLVCLVIGSSLSTGIVFDRDLLRVVAFRAVLGL
GLGLVFAYLIVPALGFSEWHSRAAIVLFVLPPPFVIPVYHKGKANFVSSVLTLSTVVSIA
LIAVLAIAGVA*
>SPBIB_v1_150085|ID:27162597|fucU| L-fucose mutarotase [Uncultured spirochete bib]
MLIGIKPCISAELLSVLYRMGHGDEIVLADAFFGGDALNARVIRADGIRIPDLLDGILRL
INLDSYVEAPVIMMQPSVGDVLDPEVERHYREVIDRYWPDTPPIARLERFAFYERTKKAF
AVVMTGETVKYANIILKKGVPPVSD*
>SPBIB_v1_150086|ID:27162598| PfkB domain protein [Uncultured spirochete bib]
MIVACGESLIDMVPAEHGSKLFEACPGGCPYNSAIAAARLGAPTWFLGKTSRDFLGDTIV
SKLVDSGVDVSLLARSDQAVTLAFVERDQAGNAKYAFYSADAADRFFLPSDIPAKLPDSA
VFLLVGSISLVQEPSCSTILALIEREHERKLISFDPNVRPNLISSKSEYRARFEWICRRS
AIVKASDSDLEWLYECPANEAANKVLGLGPELVALTRGEHGSLALTKQVRIELAAAKVAV
VDTIGAGDSFHAGLLAALGWLKVRDRKDLASLSEQNLSAALRLATSVAALDCTKRGAEPP
TLHELAVFDPEAVLIAPGIVPQFNAKERIC*
>SPBIB_v1_150087|ID:27162599| ABC sugar transporter, periplasmic ligand binding protein [Uncultured spirochete bib]
MKKRTVILALALMLVAAASLAAQTYYIPLISKGFQHQFWQAVKEGALKAAKDFNVNITFE
GPESEAMVDKQIDMLAAALAKKPNALGFAALDSKAAIPYLQQAKAAGIPVIAFDSGVDSD
IPLTTCATDNVAAAAYAADKMAELIGGAGEVAVIVHDQTSRTGIDRRDGFVNRIKSKYPK
ITIVSVQYGAGDHLKSTDLAKAIIQAHPNLKGFFGANEGSAIGVLNGVKEMGKIGKIVVI
GYDSGAQQIAAIRSGEMAGAITQNPVGIGYKTVEYAVKALKGEKFPKFIDTGFYWYDKTN
IDDPKIKAVLYQ*
>SPBIB_v1_150088|ID:27162600|rbsC| D-ribose transporter subunit ; membrane component of ABC superfamily [Uncultured spirochete bib]
MADKSNGTPAIASIAKITAGTKGAAARQKILAFSSLVLLIIAFSLLSPNFFQPYNLIAIM
LATAVNGVLAIGVTFVIITGGIDLSVGTMMTFTAVMAAQTVAVWNLPVILGVLMALFVGT
ICGLASGVMIAKFKLPPFIATLGMSMLTKGLSLIIAKSKPIYFTNAPSFAKISMGSLFPF
MKDFQIPNAVIILFGLAILASFVLNSTIFGRYTFAIGSNEEAVRLSGINTDRWKIAVYAV

NGLICGIAGILIASRLNSAQPALGQGYELDAIAAAVIGGTSLSGGEGTILGTIIGAFIMS
TLTNGLRILSVPQEWQIVTTGIIIIFAVYVDMLRKRR*
>SPBIB_v1_150089|ID:27162601|rbsA| fused D-ribose transporter subunits of ABC superfamily: ATP-binding components [Uncultured spirochete bib]
VAEPIIVVRNLSKAFPGVQALKDVQFDLYPGEVHTLVGENGAGKSTLMKILSGVYQRDTG
EILINGKPAEIHNPRAAQKLGISIIHQELNLMNHLTAAQNIYIGREPRKPGGFVLDEKAL
NAQARKLFETLRMDFDPRVKVGELTIAKQQMVEIVKALSFNARVLIMDEPTAALNETEVE
ELFDIIRHLRSQGVGIIYISHRMDELFRISDRITVLRDGQYMGTFRASETNLDQIIPLMV
GRKIDETARRIPEGKLGEVVLEVRNLNRGRLVRNVSFNVRKGEILGFAGLMGAGRTETAR
AVFGADPIDSGEIIVKGKKVVIRHPRDAVRHGIGYLSEDRKRYGLAVGMDVLDNICMTDL
PTFMNAMGVLRPKKMQAAAEKQVETLSIKTPSLMQKVKFLSGGNQQKVVVAKWLVKNCDV
LFFDEPTRGIDVGAKQEIYLLLNKLASLGKAVVMISSELPEILRMSDRIIVMCEGRITGE
LDGARATEEEIMKLATQRETMVKTA*
>SPBIB_v1_150090|ID:27162602| Transcriptional regulator, DeoR family [Uncultured spirochete bib]
MLYDLSDRERAILELLADRSYLSVSEFSSILGVSEVTIRGDLSALEEKGYLLRSRGGAIP
AIHRNIIERQKLHVAEKQKIARKAAELVHDGDRIMIEAGTTTALITRYLVGKQDVQIVTN
SMLAFSYARVNPQINLILTGGSFRRETESLVGPVAVQSLGSFNARLAFVGTDGFSLARGM
TTQLTEGAEIVRAMSKRAEITWLIADSSKFDKIGFVSVLPLQAVHGIITDAGLSQEAVQA
LQAEGLEILLA*
>SPBIB_v1_150091|ID:27162603|acoC| TPP-dependent acetoin dehydrogenase complex [Uncultured spirochete bib]
MATTIIMPKLGNTVESSVILSWKVKPGDAVKADTVLCEIETDKATMDVPAGLEGTVLALL
HKEGDDVPVLEPIAVIGAPGEPIGEPTPEKKSSESEPAAQTARPAQPSLADHSAPTAEPA
VAEKHASPRARQAMFQLGVDIEAIPLGSGPQGRIIERDVIAASFAEPKAADGIASQTTQA
AAQEAQPATAAQPAAQPAPAGQSPTQPAQEYETISLIGIRKRIANRMRESIQTTAQYTEH
GSADAERLLALRARLKAQKNPELSGITIGDLVLAAVIKVLPEFPEFNAHLEEGTLKLYKP
IHLGVAVDTPKGLMVPVVRNAQALSLSALSKEVKRLAQACQNGTIDPDSLSGSTFTVTNL
GAYGIEQFTPVINAPETAILGVCAIRPALIQKEKGIETRMRIGLSLTADHQVIDGAYAAR
FLARLAEVITDIDFLFMSF*
>SPBIB_v1_150092|ID:27162604|lpdA| Dihydrolipoyl dehydrogenase [Uncultured spirochete bib]
MAHYDVAILGGGPGGYLAAERLAEAHLRVALIEKGDIGGTCLNIGCIPTKTLLNSAKLYT
HAKESSPFGIETEGVRVNWQRTLEWKNEVVTRLRRSLELTLKKAGVEIVRGSGMLTGAKT
IEVRPETKNQKALSEPAGTASANLASADLAPTGPLQLEADQIIIATGSSPILPPIPGTAG
NPAIKDSTGLLSLSEIPQKLCIIGGGVIGVEFASLFSALGSMVTVIEMMDEIVPVMDPQM
AMMLRNALKQVTFLRSTKVTAVNGNKVEYQTKTGEQGSVEADIVLMSVGRKANIEGWGAQ
EAGLEIKNRSIATDEHMRTNLPGVWAIGDVTGKSQLAHAAYRMAEVAVADILARRTGQHG
PQIFVPETVPWALYSMPEAAGVGYTESEAKAKGYEIEVIRSPYGVSGRFIAENGFSAPGS
IKIIMEKDSQRLLGIHLLGPYASEHIWGLALALERRLPWSALQNMVFPHPTVSEVIREAA
WNAHK*
>SPBIB_v1_150093|ID:27162605| Pyruvate dehydrogenase (Acetyl-transferring) [Uncultured spirochete bib]
MPKSLPVEPSQVRKSGTLKIPEIPLNQYTRNIDVERARFGNEGLRAILHDMIIVREFETM
LNSLKTMGAWEGIEYNHKGPAHLSIGQEAAVVGQAAALDPEDFIFGSHRSHGEILSKCLS
AARKLDEPRLQSVMTSWLDGETLEFAKRMPHSDTFSLARNFIVFGTLAEIFARKAGFNRG
MGGSMHAFFTPFGSMPNNAIVGGSADIALGSALFKRINKKPGIVIANIGDASMGCGPVWE
AMMMASMDQYRTLWPESAGGAPPILFNFFNNFYGMGGQTYGETMGFGVLARAGAGVNPEA
MHAERVDGLDPLAVADAVLRKKKILLEGKGPVLLDTITYRISGHSPSDASSYRTKEEIEE
WQKNDSIAELSQYLLKHSIISESDLAAMRNEIHSTIFEAVKLATNESACPRADGKFIESV
MYSNKKILKLEDREPEVLQAGEDNPRVQAIARRARYAFDADGKALPKNKVYQFRDGIFEA
LLYHFKADPTLAAWGEENRDWGGAFAVYRGLTEALPYPRLFNSSISEGAIVGSGVGYALS
GGRAVVELMYCDFLGRAGDEIFNQASKWQAMSAGVLKMPLVIRVSIGNKYGAQHSQDWSA
LVAHIPGLKVYFPATPYDAKGMMHLALSGTDPVVFLESQLLYDIGEQFEKDGVPQGYYEL
LEGKPAVRKPGKDLTIATYGATLYRALEAARILEEKYGISTEVMDLRFLAPFDYEPLIES
VRKTGRLVLASDAVERGSFLHTIASNVQTLAFDWLDAPVAVVGSRNWITPAAEMESIYFP
QPQWIVDTVHERVLPLPGHTPSSVQTAQDIARRYRAGV*
>SPBIB_v1_150094|ID:27162606| conserved protein of unknown function [Uncultured spirochete bib]

VQPDYPRIDIKIAESIIADPARSPGERLEALSAIARAMGLRLQTGPARRPPARSGESNNH
IHTCYSFSPYTPAGAALAARRAGLDVAGSVDHDSYAAAGEMRAACAMLDLGVVTGFELRV
SLKAAAHEFPEATARLLTERKLNNPDSAGIIYMTIQGVPASARAEVEAFLEPVRSARYAR
TERMVERANALLSSFGLPSINFKNDIVDRSKFAEGGTITERHLLAAVSRSMLSAMQPGPA
LVDWLEAKLGLALSASQRRLLSDPENPFIMYDLLGSLKAGFLDRIFIQPDEECVDVRDAI
ALARRIGAIAAYAYLGDVAESPTGDKKAEKFEDEVLDELMPALKELGFPAITYMPPRNTK
AQLSRLQKLCAQYDFMEISGVDINQPRQSFRCPELLEPEFRHLDDATWAMVAHEALSSID
RRFAFFANDNPFARLPIKERISLYSKAGREIDFADPYSLERITNSLLKEKFL*
>SPBIB_v1_150095|ID:27162607| putative Sorbitol-6-phosphate 2-dehydrogenase [Uncultured spirochete bib]
MKIKVTPSAIAPIVRGLMLSRASFSCMVKTRPESFACPGERTNESADGPIDRRPEERAEE
HIRLVLDGILPQSEIEDAELAQALQHSLTQPQAQAIRRQLRIGDVCLDIMGAAGASIALL
DLVPLEPLDRRYQMMVQSPGDASLLSSASRASVVRGKIALVTGGAQGFGAEIARGLAHSG
AVVWIADINLPAAQSFAQTLSSETGTPSAFAIDIDVSSEASVQRAFEIIALTTGGLDLVI
SNAGILKAGSLLTQPVQEFRLVNEVNYVGFFNIAQHAARLLRLQWLGAPDWFTDIIQINS
KSGLQGSNKNSAYAGSKFGSIGLVQSFALELVEYRIKVNAICPGNFFDGPLWSDPQRGLF
VQYLNSGKVPGARTIEDVRAFYESKVPMGRGCYGPDVLKAIYYLVEQVYETGQALPVTGG
QVMLN*
>SPBIB_v1_150096|ID:27162608| Carbohydrate kinase, FGGY [Uncultured spirochete bib]
MTDAIAVLDIGMTNKKVVLYSTELALLDEKKRVFAPLMLDGLETHDLAAMERWFLSTLRE
YSQRFHIKAIAVSTHGATFVCTDSEGNPVAPCIYYTHEPSQDFHERFFALAGERKMLQAT
TGTPDFSALINPAKGLFFLNERYPRKFTSARWVLSYPQYWGMRLTGKPSAEGTYIGCHTF
LFDWERGTYSSVADALGIRDKLSLPIGQAWEVLGTIKPEIAQQTGLPPDTIVTLGIHDSN
ASILPHLASAAGRDFVLNSTGTWCVLMHPVEKYGFEPDELGKVVFFNRSAWNTPIKTAIF
LGGKEYEAWIQIIASLQGHASKDLPAPTQEDYQRVLKEQRYFILPEIVPGSGQFPGSIAR
AVENGKVFLLSEIEQGRDVPVFLKEPRIGQAVLNLSIVLQTEVALQRTGLAEGAEILTEG
GFRNNSDYNRLLASAFPENSIYLTDLKEATSFGTAMTALAALNHIDPKELTRFIHIEKAL
VAPMLPSETLGAYRNAWHARMQKI*
>SPBIB_v1_150097|ID:27162609|yggP| putative dehydrogenase [Uncultured spirochete bib]
MKTKAVRLYGVNDLRLEEFDLPPIKDDEILARVVSDSICMSSHKLAMQGDKHKRVRAPLA
QNPVIIGHEFCGEIVEVGKKWAHKFKPGMPFAIQPALNYKGTLWAPGYSYQYIGGDATYI
IIPNEVMEMDCLLEYKADAFFMGSLSEPVSCIIGAYHAQYHTKPGSYVHEMGIREGGSLA
LLASVGPMGLGAIDYAIHGGRKPARIVVTDIDQARLDRAQTLITVESARQKGIELIYINT
KDMADPVAALRELNGGKLFDDVFVFAPVRSLVEMADKLLANDGCLNFFAGPTDPNFSAML
NFYNVHYESHHLVGTSGGNTDDIRESLALMAQGLLRPEAMITHVGGLNAVAETTINLDKI
PGGKKLIYTHKKLDLVAIADFAERGKTEPFYAALAEITARHQGLWCKEAEDYLLANAPEI
*
>SPBIB_v1_150098|ID:27162610|yugJ| putative NADH-dependent butanol dehydrogenase 1 [Uncultured spirochete bib]
MDEFVFHNPTTIYFGKRYEAEVGRIAAQYGKRVLLHFGGGSAERTGLLPAIRKALTEAGI
SYFELGGVQPNPRLSLVYKGIELCRKNSVDLILAVGGGSAIDSAKAIAIGTVYEGDVWDF
YTGKAFPTKALPVGTVLTIPAAGSEASPGSVITKEEGLLKRAVNADCIFPRFSILNPERA
FTLPPAQIANGVTDIMCHLMERYFTNSKPVEFTDRLIEATLRTVIAAAPRVLANPTSYDD
WAELMWAGTVAHNELLNTGRVGDWASHDIEHELSAIYDIAHGAGLAIVTPAWMKYTLKHD
VARFAQYAVRVWGVEDNYFDPERVAREGIARLEVFWRSIGQKVRLSEIGIDDSRIAEMAR
KCTDDDAHTVGHFVPLRSKDIEAIYRLAL*
>SPBIB_v1_150099|ID:27162611| Argininosuccinate lyase [Uncultured spirochete bib]
MATLWQVGGGEGLDPAVDRFLSSLAVDARLVFEDIECSQAHAIMLGEQGIIPQVDASALV
DELAKIRKDLQSGALAVDQSAEDVHSFLEAELTRRLGDIGKSIHAGRSRNDQVAAAFKLH
VRNACRQTRARVLDAISACLYVAERNIETLMPGYTHLQRAQHVTLAHHVLAWCAALERDA
DRFADAATRANESPLGAGALAGSGLSVDRERTAELIGFSRVSMNTMDAVADRDAAIEYAA
SAATLMMHLSRACEDIVLWASSEYSFVKISPRASTGSSIMPQKRNPDPAELIRGKAGRVF
GNLQALLVMEKGLPYAYNRDLQEDKALFFEIEETINGALEAFKVLVQSIEPDAGRMRAAL
DEGYLEATDVAEFLVKAGVPFRTAYQAAKLLVQRCVDEQKSLRAITQADLAVHEAFEKAG
ANAAELAEYLEPQACVARRMQTGGPAPLRTREQIDRLRAWLHLATR*

>SPBIB_v1_150100|ID:27162612|argG| Argininosuccinate synthase [Uncultured spirochete bib]
MKKKIVLAYSGGLDTTVIVPWLKENYDCEVIAVCGDVGQEADFDAIGKRAIASGASKFIK
LDQKEEFVKEYLWALVKAGTPYEKKYLLGTSAARPLLAKGLAEVALAEGADAVAHGCTGK
GNDQVRFELGVKAFAPEMEVIAPWRIWDIQSREEEIAYLEARGIPVPMKKSDSYSRDDNL
WHISHEGLDLEDPANEPHFEGMLKMCVTPEKAPDKPEYVTIGFQNGVPVSVNGEKLDPVS
IVSKLNKIGGANGIGIADMVENRVVGMKSRGVYETPGGTIIMEAHDRLEMLCLDKKTLSF
KMTVAQRFAEILYEGEWFSPLCKALCAFVDSTQSTVTGQVTLKLYKGNIIFAGATSPYSL
YDASLASFTTGPLFSHKDSTGFINLFGLPTKVRARLNERIKKDGLAAGPDVVKPGSSGYT
APAGD*
>SPBIB_v1_150101|ID:27162613| protein of unknown function [Uncultured spirochete bib]
MKNTISCDFMQVYLNYYVIKQRAFKKERPGLVSWSCGPVSLAGQRLFLLFFAFLS*
>SPBIB_v1_150102|ID:27162614| putative tagatose-6-phosphate kinase (Phosphotagatokinase) [Uncultured spirochete bib]
MLQNSNISRTSIEAGGQNQDNQNQESHNQNSQIQDGHSSAGPREPSFLCVCLNPTIQKTL
VFSKIHAGEVNRTAQWRIDVAGKGIIPTRIFTQLGERAVHLTQLGGPNRDWFLSMCAEDQ
IPIEWVESDAPIRFCTTLIEESEGHATELVEESHPVAPGTSEAIIARFSELVQKTSAVLL
SGTVATGIVPGTMARLAEIASKAGARLYLDIKKQDLLECLEFHPLCVKPNLEELAQTLGI
PYDEVREETTARRLVAETGRKFYERFGTYLVVTRGAKSTLYWDGRQLCEQPVNPVNVRNP
IGSGDAFGAGLARILERGGSIHDAVKEGTRLGGLNAAQLKPGSIFP*
>SPBIB_v1_150103|ID:27162615| Sodium:dicarboxylate symporter [Uncultured spirochete bib]
MVEEKKKGVLDWYFKTNLLARILIGLILGAIVGIILGFFPSSVKPFVDNSKFFGDLFIRL
LKMIVVPVVLFSLVAGAASIAPSRLGRVGVKILVYYLLTSAFAVLIGLIFANIFQPGAGF
NVVGDAAIKGKEATAPTLVQILLNIVPTNPFESLMKGDVLPIIFFAVVFGIGLSYIKDSK
QQALAKSGTVLLDGVNAAAETMYKVVRGIMQYAPIGVFVLIAQVFAQQGPKAIGPLLMVT
LAVYVALIVHLVVTYGGLLSVYKLGFWKFLKGANEAMITAFVSRSSSATLPVTMRCSEEN
LGIPRSISSFTLPLGATINMDGTAIYLGVCAMFIGFATNQPLTINQQLTVIITATLASIG
TAGVPGAGAIMLLMVLESIGLKVTEGSAVAAAYAMILGIDALLDMGRTSLNVTGDMVGSA
IVAKTEKELDMAKWK*
>SPBIB_v1_150104|ID:27162616| Aspartate racemase [Uncultured spirochete bib]
MKRGTIGILGGMGPDATAAFFSMLVRLDVAPRDQDHLHIIVDSDPSIPDRTRHLLEGGEN
PLPVMLSSARRLLAAGADVAGMPCMTAHAFLPNLRRNCTLRILSAFEETSKALDSFTPPI
RALGILGTIGTKQTRLFETAMPDKTILWPDEDTHRAKVMEAIYGKNGIKAGNLGEEPHQL
LIQAAHRLIAQGADAIVAGCTEVPLVLSQRDFEMPFIDPMEYLARALIVAAKGVYSDHQS
K*
>SPBIB_v1_150105|ID:27162617| Cupin 2 conserved barrel domain protein [Uncultured spirochete bib]
VIIRANERTFETRHEMRGGKGDVSLAMLSNEALAPHLRLFSELTIPPEAGIGTHTHSGET
EYFFIIEGNATVNDNGVTTIVHPGDVLITGGGALHNIENHTAEPVRLVAVIVTEA*
>SPBIB_v1_150106|ID:27162618| Ring-hydroxylating dioxygenase, large terminal subunit [Uncultured spirochete bib]
MIPNQWYVIASSNEVGIKPVGMTRFGEKLVLYRTASGFLVCLSDMCAHRGAALSLGKVCN
GDQIQCPFHGLEYDPSGKCMVIPANGRSTPVPSNFRVRSWPVYEAHGFIWVWYGSEPPTP
EIPEFFDDIPKDAKYATVADHWKAHYSRVIENQLDCVHLPFVHYNTIGRGNRTLVNGPAV
EWVSDHKFFMYVYNETDNGQKPKKPDEVPMPSPNGYKIEFLFPNLWENRIADKVRVLAAF
VPVDEENTLLYLRFYQAFARIPIIGTLIARAAMPFNVYVAHQDRRVVQTQVPKASGLKIG
ENLFQGDLPILEYRKKRQQLQESARPR*
>SPBIB_v1_150107|ID:27162619| conserved protein of unknown function [Uncultured spirochete bib]
MLFRDRCDGKRITGQHALNALMPYMMRGRNESAVYYGREINIENALAYLKLIKKAQADTE
AQERFTLFSLILTAALKTIALYPRLNRFVHRRALYQRKHIAFSFIVKQRFDHDAPEVNAK
VFFDPEDSFETVSMKINAAILDAREHGEGDGERFANIFHRIPGGKALIMGVYRILEYFNI
APWSLIRMDPLYSSIYIANLGSIGLNAPFHHLYEWGNTSIFMVIGKIFSKELWHGATRTR
QRYIDLRVTLDERIADGLIFAEAASVFSRLLSSPDLLNLSIEELKQALS*
>SPBIB_v1_150108|ID:27162620| Type I antifreeze protein (modular protein) [Uncultured spirochete bib]
VRLFTFLRFIAIFCIYFIYIFSLREVIAMPTYEYECRVCSYTFEAFQSINDDPIKTCPLC
GGEVKRLIGGGTGVIFKGSGFYITDSKKSSSASTSAKKSASSSESSGSSAGCGAGCACSP
AKESA*

>SPBIB_v1_150109|ID:27162621| protein of unknown function [Uncultured spirochete bib]
LAGEQAHPAPQPALLPELSLEDADFFALVDAEEDFFESVI*
>SPBIB_v1_150110|ID:27162622|kbl| glycine C-acetyltransferase [Uncultured spirochete bib]
MYGTFKDELAKKLEAIKAEGLYKDERVIITPQGAAIRVADGKEVLNFCANNYLGLSNDPR
IRAAAVEAMERFGYGLSSVRFICGTQSQHKDLERKIADFLGMEDAILFSSCFDANGAVFE
PLLDEESAIITDSLNHASIIDGVRLCKAKRWIYKHADMRDVEEIDPETGKPLKGLERCLK
EAQGSHVIMIATDGVFSMDGDIANLNAICDLAEKYGALVMVDDSHATGFMGVHGRGTWEY
CNVAGRVDIITTTFGKALGGASGGVIAARKEIVEYMRQKGRPYLFSNTLAPAIVGGTLKA
IEILTESTELRDRLESNTALFRRLMTEAGFDIRPGEHPICPVMLYDEKLAHRMADALLDE
GIYVIGFSFPVVPRGKARIRVQISAGHSEAQIRKAVDAFKKIGKRLGVI*
>SPBIB_v1_150111|ID:27162623| Rhomboid family protein [Uncultured spirochete bib]
MRIRYNAPVTLTFTLLAAIVLVLSQTIAPSLIPTVFSTPAPFHSNAFVDYLKLFTHVLGH
ANIQHFIGNFTMILLLGPILESVYGSGFLLLSILITALATGLANTILFPNVVLLGASGVV
FMMIMLSSITNFSKGEVPLTFILIMIVYLGGQVWDALTKQDNISQFSHIIGGLVGSFLGF
YRKK*
>SPBIB_v1_150112|ID:27162624|exoA| Exodeoxyribonuclease [Uncultured spirochete bib]
MVKIYSWNVNGIRACAQKGFLEWIAASDADFVCVQEIKLQGDQLTEELRHPVAGSGKRYH
SFWAFAERKGYSGTGIYALREPVSVSGMGGQEFDSEGRTVVADYGGFVLVSAYFPNSQEA
GMRLDYKLRYCDSMLDFCERMRARGKHVIVAGDYNIAHKPIDLARPEQNEGNAGYLPEER
AWMDRFTAAGYIDTFRWFCKEPGHYSWWSYRAPKAREKNIGWRLDYHCVDPEFAPALSAA
GIHPEVMGSDHCPVSLFLDV*
>SPBIB_v1_150113|ID:27162625| putative ROK family protein [Uncultured spirochete bib]
MTHEEQHPERAHLIRERNQRLVLRLIFRTGILSQSKAVQRTGLKAPTVFRIFSDLEREGL
IEAVSEPSSRSPKNRKKGRRPVNYRIRSSAAYVVGIDFWARSAAAIIQDFAGNLVAERGV
CFASPPSAEDALDIIANLIRDMLVESSIDRSKLLGIGVGAPGSVSVSSGVVQFYARIPGM
NEFPLGERLRSLFSVPVIVTNNASIVALAEERYGQARGVSSLFLFLVRAGVGGAFIQNGK
LVSDRSRTAFEVGHLSIDPSGLACSCGNRGCLELYLNEETVCTALSQIEPCAGIEDAERI
LSANNPEVDAALKPILEISAHAVRDIRRLLAPEAVLIVTRSKSLSEHIAQAASADFERND
QRFGPPGARIIASEYNPVLACKAACDLVYEEYFAHGFGEGSGFPGGGFMGGSQQRSAGQQ
PSGAQAE*
>SPBIB_v1_150114|ID:27162626| protein of unknown function [Uncultured spirochete bib]
MNQEFEKNSALFFGSTRYVQPRWRAMPAQQKFSHSLNEESDRYVHALAKLEQEDEEIPLP
SDRGPIVFRDGLFQIDMSQIEEKGDIDIELKKLIDSIIGE*
>SPBIB_v1_150115|ID:27162627| putative ATP-dependent helicase YoaA [Uncultured spirochete bib]
MGKKQTAIVDAVQRFTTQAQQELKNAISDMEGREVFAIGALDSRGLIKKLEILARGSEGA
VPAPFRDRDSAQVLIHNHPSGELFPSDADVLVAAEAAADGLGSYIVDNAVAKVLVVAEPV
RAKLIRPLNPDEIAAVLDDGGKLSLKMPEFEPRTSQVDMARDVAALISDGGILIAEAGTG
VGKSFAYLIPSLAWAIGNNERVVVSTATINLQQQIFKKDFPVVSSLFKKPAKAAVVKGRG
NYLCKRRLYEAIEEDALFSDTSIKLRQILEWDNNGGSGDKSDLSLPDDDPIWNRVCSESD
YCLSSRCPYHEKCHVVRVRLEAASAQLIIVNHHVLLADLEAKRTREGVLNTILPSYKALV
LDEAHAIESSATSLFSESFSKRSIQRLLGRLVRKKKRTQTGILPSLRRLPDIPAALLDTA
SKQADAAAAAADAFNASALASYPEKESSLLIKNLESFSRSAFLSALQSLERDLAMLVTRL
GEILEAMPPEFEDEESVVELKLTSRSIEEAAALVPRFKNPDAEPQTIFWMQIDRKNPREA
VVICTATPLEVAPLLAERLFSKVRTCICTSATLTINNSFDWWRNRVGLPTKKDGETSKAM
STMGATAGTTEGAIDAPIRNRQYPSPFPYRRNALLAIDTSAPPPDGTQFQAYLNTAVARL
LHASRGRALVLFTSHKALRDTYDYVAPLLEKEGILALRQGQKDRYSLLHTFINDISSVLF
ATESFWEGVDAPGETLSLVIITKLPFRVPTDPIQASRAAAIDARGGNSFAEMSIPEAVIL
FKQGFGRLIRHSNDRGVVAVLDVRIAKKAYGNLFISSLPECKLETGPLDQLCTSVAKFLD
SEG*
>SPBIB_v1_150116|ID:27162628| protein of unknown function [Uncultured spirochete bib]
MTDMEATLPCAAFLENPVQQYAWGRTEGIAPFIEIPPLNGLPAGEVWMGSHHRAPSKAIF
DKGAVPLDELVRAAPEHWLGSKAARHYGDLPFLFKVLAAGAPLSLQLHPDAKAAKQGFAK
EEAAGIPLLAPDRSFKDPNHKPELAVALTPFKALAGFRPLEHIAQFLGPELCRALSWNSI
ARTEDLRLFMRRLFEARGAGFMPLENMLESRAQTLAASTASEEREAGLLALDLKARYPGD

PGQFAPFIFNILSLEPGEGLFVPAGVIHAYLTGSILEIMACSDNVIRAGLTIKHIDIELL
CDILDPDAKPLVVEPYITHAEGVEHAVWNTPADEFRLERLDLDSSANFAYAPEGPEILLC
TQGKAHIQAGSAFDLKARSSLFVAGSCEQVAVKGPATVWRAIGPAPKAADAAGNSLTIWV
DGDSLPRDLRPLLVKRVLSPRKYGEVLVNVHFVAARALSGVPAHCMTLVEPGEGAADRCI
ESLAQPGDIVITRDIPFAERLLAKNIHVINDRGNVFTRDSIAERRSIRDVMAELRAVGIA
QSSPKGSQRTSAETKRFADALERTIVKAARLKKAAAR*
>SPBIB_v1_150117|ID:27162629| Glycosyl transferase family 51 (modular protein) [Uncultured spirochete bib]
MSDRPQGERPHKERARGKNRKPRSLGVRIVRVVVLAVLIAHLAYIGITSALILVYKFANP
PTTVLMIYRSIISHWKVQKPIPLPLANIPLSVRRMLVSVEDGKFYEHHGIDMEAFKRAKA
LNEKIGKPMYGGSTITMQVARTLFLVPDKSYVRKYLEVIAALELEFILSKNRILELYLGY
AEWGKGIFGIERAARVYYGTSVRNISADQAARLIALLSSPIKYTPDTLYRSLILRERYAY
LVQKYVSPIAETKSEAEAPPPAGIEPSADIEPSADVSAEVESAGPPSETAVPAEPAAPSE
PAEIVAPEAPAGDTP*
>SPBIB_v1_150118|ID:27162630| putative Major facilitator superfamily MFS_1 [Uncultured spirochete bib]
MSDQHQRSAKSGMSVSGKHYSIILLAVITRFLQWGILGILIPVSNLLRMSKGLTLPELGF
AAAITAGIVVALELPTGVIADRIGRKRTYLASLAFMAASCAALLFASGFAMVSLAFALYG
VSRALSSGSLEALMIDRYIEANGDSTLHRLMSFVSAADTAGLALGCIAGGYIPGLWTAIA
LNSNRYQGNLLVMLAFLFLLTMLVVFSVAEEKPDPQKQATLRRFMAESLSFIRFSPALLA
FLGAGAAWGIAFSAIETYWQPQVASIVGSAGSDRLNGFLSAGYFLTALIGSLAATPLLEK
TKKNPGLLLLILRLLMVALALQTSAGGFAALYLSMFFWNGMASPPEATVLNRALPSDKRA
SMLSAVSLAVQLGGLVGSVAFGLVVKSSGIRVAWFIAAGVLAVSSFLFPFAASHYGGQTK
NSSQSTPGV*
>SPBIB_v1_150119|ID:27162631| conserved membrane protein of unknown function [Uncultured spirochete bib]
MKKFTWLACLGLAIVLVAGIIGFSVQTAGGKVQIRDVRFVGSNGTVMSALLYVPKNATAK
TPAPGILAVHGYINARETQSGFAIELARRGYVVLALDETGHGYSDSPAFANGFGGPDGLA
FLRSLDIVDKNNIGMEGHSMGGWTILAAAATMPDAYKSVVLEGSSTGKPYAAEGSTTWPR
NLAVVFSTMDEFSVLMWGVPRGKDAPKSPKLQAVFGTTDPIEPGKIYGSIEDGTARALYQ
PVTTHPGDHLSNAAIGYAIDWFAKTLSGGNPIPSSNQIWFWKELMTLAGFIGFVLFILGV
GGLLLSVPVFAPVAKVPPAGKPAKGAGLWIGALIATAIPALTFFTFMGWGAKWIKAGSFW
PQNITSQLAIWALLNGVISIVLFLLWHFVLNRKNGAKAEDYGIGLTWSDAWRTLLYGFAV
IVCGVFLLGVADYLFKIDFRFWVLALKFPSQIQARIALAYAPVFFLYYLVASLALNSQLK
LGGKGGAARYWFAIGTASAGFLVFLLLEYIPLFAGGTLLSAAQPLNTIIAIQFLPLMIVT
SLFSTWFFEKTGRIYAGALINALFISWYIAAGQATHFLVR*
>SPBIB_v1_150120|ID:27162632| protein of unknown function [Uncultured spirochete bib]
MNTKVPALEKGLKIIEFMLTASEPITMSQIAERLGYKVSEVQRTVTYLSAEQYLIRNGAG
SYMPGPKIYQFADQNRETILINRAEGPMRSFAEKTAASVHLSVLVEEMLHVIFEIESKEI
VRISIRPGLYKAGSTASGRLLLAYAQSPILAATERQKIMELGYAFKDIDCVQGVYALAVP
ICTQSGSCIAALASPYVLPRGAKEAFRQELVEPLKRAANDIASLL*
>SPBIB_v1_150121|ID:27162633| Pyruvate/ketoisovalerate oxidoreductase, gamma subunit [Uncultured spirochete bib]
MTEKTFIAGFGGQGVISLGQLWVYSAMKEGLKVTFFPFYGAEKRGGIARASVIVSNDEIT
SPLVTSADSVVVMNQDSVEIGEKVCKEGGTIFVNSSLVKKDPARPDANIVHVPCNDIALK
LGDVRIANMVMMGALSKVTGAVKLDNLETVLKSFFPASKHSLIALNIAAVEEGKKAV*
>SPBIB_v1_150122|ID:27162634| 2-oxoglutarate synthase [Uncultured spirochete bib]
MELAYGHPKSLKPIQTKYCPGCGHGVIHRLIAEVVDEMGLQTKAIITNPVGCSIWADLYF
DFDSVQPAHGRTPAAATGIKRMLPDHLVICYQGDGDLAAIGTAEIIHAANRGEKFTTIFV
NNAIYGMTGGQMAPTTLVGQHATTAPEGRDPGVAGMGYPIRVCELLATLEGTKYLARGSV
NNMVNIRKTKNYIRKAFEAQMRGEGFTMVEILSQCPTNWQMNPVQSVEWLEKNMITYYPL
GEIKNTLNATAGAPATSPAKAN*
>SPBIB_v1_150123|ID:27162635|vorB| Ketoisovalerate oxidoreductase subunit VorB [Uncultured spirochete bib]
MAEEIRLMKGNEAIGEAAVRAGCRAYFGYPITPQNELTAYMAKHMLAKGRTFIQAESEVA
AINMVYGAASTGARAMTSSSSPGVSLKQEGISYLCGADLPAVIVNVARSGPGLGGISPSQ
GDYFQATRGGGHGDYYTIVLAPKGVQDAADLTYEAFSISEKWRVPVLILADGLIGQMMEG
VVLPEPIDPATLPRKPWAVGHAVEMGRPYNHVTSMNLVPDELEAANHARYARYERIKKDI

NRYEEIDVEDADVVFVAYGTSARVSHGALQMARAKGMKVGLFRPISLWPFPYARLHELAE
KGKRFLCVEMSMGQMIEDVRLAVNGKAPVYFYGRCGGNIPSQEEVFAEVARLMGK*
>SPBIB_v1_150124|ID:27162636| conserved protein of unknown function [Uncultured spirochete bib]
MAIKGSVEIDRERCKGCQLCIRACPTKVLGVDTEPNSWGYFPAKVIAADKCIACGNCFAV
CPDVAITVYKL*
>SPBIB_v1_150125|ID:27162637|ptb| Phosphate butyryltransferase [Uncultured spirochete bib]
MKHISEIIEKAKTFGRRKLAVASAQESSVLEAVVDAYNEGIAEPILVGDPAAIRAAAKEA
NGGKGVDISRFELIEEANLSAAAARAVALVRSGEAAFLMKGIIDTSLLLRAALNKESGIN
AGRLASHVAVMEVQTYHKLLVLTDAALNIAPDLPAFVDIINSAVVVANALEVKTPKVALL
AAVEKVNPDKMPCTVTASILTQMNRRGQIKNCIIDGPLALDNAISAESARIKKIQSDVAG
DADILVAPNIEAGNILYKCLLDLAGAKGAAIVMGAAVPIVLTSRADTAETKLASIALASL
LGSTSLRA*
>SPBIB_v1_150126|ID:27162638|buk| Butyrate kinase 2 [Uncultured spirochete bib]
MSLAILVINPGSTSTKFAVFEGGTRILDQSISHSAEEIARFPSIAAQYEFREKAIQDVLL
QKNFDAKKLGAVVGRGGLLHPIPGGVYRINEGMKADLLAAKYGEHASNLGALIADAFARP
LGIPAFIADPVVVDELDDVARVSGNKLFKRNSIFHALNQKAVARRFAKERGRSYEELNLI
VAHMGGGVSIGLHRQGRVVDVNQALNGEGPFSPERSGTLPAGDLAKLCFSGRYTQQEVLK
MITGKGGMVSFLGTNDMREVERMREEGNKEADLYYRAFVYQVGKYIGSLAAAACGKVDGI
ILTGGIAFWKELTDRITEMVSFIAPVVVYPGEGELEALALAGQMALSGEIEIKEYVP*
>SPBIB_v1_150127|ID:27162639| protein of unknown function [Uncultured spirochete bib]
MREPGVFCDALRTLAKVSIIEMAFQNLQISLAFLHISGILHILLYISGVAACHWLSSS*
>SPBIB_v1_150128|ID:27162640| conserved protein of unknown function [Uncultured spirochete bib]
MIGFFEHILFLIRGVKTYALVGSSGTGKSFRAKLVAQKYGIDLIIDDGLLIRGDQIIAGR
SAKKDPTYLGAVKTALFDEREHREQVSRALQHERFRKVLVIGTSERMVQKICERLQLPHP
VKVIKIEEIATKAEIEKAVQSRKVEGKHVIPVPALEIKRNYPSIFYDSVRVFLKRSFGVG
ATLPKLYEKSVVRPEYAKRGRVAISEAALSQMVVHCVNEFDPNVRIMRLAIRNDTQGYRI
TVVLEVPFGTRLASKIYSLQEYIVDSIEKFTGILVAEVNIVIDRLAVRPQKEQKWGRAID
ARRQSV*
>SPBIB_v1_150129|ID:27162641| Phosphoglucomutase/phosphomannomutase alpha/beta/alpha domain I [Uncultured spirochete bib]
MSRIIHPILGLPIADPNKIPLSRENLPSSEDIQNAARSLILSASGWRKVFADPSTEDTYA
QWAYDRSAENSLSRTISSADAVIVAGMALVFASFIKEQCKGRAPIVLLGIDTRPTGPAIA
DVFARVLIGEGCSVRYLFIVPAPEIMAFAAQTTSLPLDNEAHADAFAYISASHNPPGHNG
LKFGIGGGVLTGNQIAPMIAALKSFIADPDSPSRALAAISKADPRALAQCYESVAHWKRF
SQSAYMLFAHRVFTDEDSLERQEEVLSAIATACEKRPLGVVAELNGSARTQSIDQDWLSA
LSLQTKMLNVEPGIFAHRIVPENDSLEDCRAALAEAHQENPAFMLGYVPDCDGDRGNLVY
HSRMLQSAVPLEAQQVFALACLSELAYLKWKGETRPVAVVVNDATSMRIEAIAALFGAQV
FRAETGEANVVACAEKLRSEGWVVRILGEGSNGGNITHPSKVRDPLSTLGSIVRLLRLGD
ANSGKTCFNLWLDAIGASDRYRPDYELEDIIDSLPQWATTSAFEPYAALKIASNDKVALK
NAYQRLFLEEWPRMQPQLAARFGIAAWKALATIGANEQEIGSDFGASGNGGLRIVLFSHA
GEARAFLWMRGSGTEPVFRIAVDIKNGSSEDEAWLRSWHTRLVMQADLLVSKAQDIVG*
>SPBIB_v1_150130|ID:27162642|rnhB| Ribonuclease HII [Uncultured spirochete bib]
MKKSTNYCTFETVLKIPVHLVCGIDEAGRGPLAGPVAAAAAILSPDFPMEILNDSKRMSK
ARRENAFQVITEKALDWSVGWATVEEIEQRNILGATLLAMERAFQGLRKMPALVVVDGIF
TPTLMADGAAVEAATMPKADGVVPAVMAASILAKVARDHCMDCLDQTMPGYGFRVHKGYP
TRLHREAIAALGPSRYHRLSFQLLAQESPLLFDPSGE*
>SPBIB_v1_150131|ID:27162643| protein of unknown function [Uncultured spirochete bib]
MWTSARALLMEESILSFLRTREYECVQAGEAVQSARSVPAGPPLFQIYVHRHSRTAAFEA
IDGIAVQWLDGAVHLILPPSPHDDAGLVALLKDTSNIRRIAGTKHAVMRITGKMPSMNWN
HALFLLMQLPHAQSSHSQQPHSQLLRQKHVSDTPRVDGEPVRCVDASFEDLNALVSLHLE
YEREELALYSADASETEMRMRSLLANQIVCMAWAGNEAIGKVNTNARGIYCDQIGGFYVK
KEWRSMGIGTNLLLYLLKKIEAEGRHAVLFVRHDNLAALRVYQHLGFLAVGEYAISVARQ
HI*
>SPBIB_v1_150132|ID:27162644|trxB| Thioredoxin reductase [Uncultured spirochete bib]

MSQPYDFAIIGAGAAGLAAAQYGARANLKTVVIEEMAPGGQALLIDALENYPGIEEPISG
YDLAERMRVQAERFGASFIDASVSNVRKENGRFIVETSNGPVESLTVLVATGAKHRQLEI
PGEQEFMGKGISYCATCDGPFFKGKRMLVVGGGDAACDEATYLSKLSDQIVMIHRRDRFR
AQKALAQRVLSNPSIQVRFNTVAKEIRGDTKIRSVLLENVLTHEITEEPFEAVFIFIGSI
PQTEFLKDTGVALDETGYIITDCTMQSSIPGLFAAGDVRATPFRQVITAAADGAIAAHAA
ANYIDEQRGQAYR*
>SPBIB_v1_150133|ID:27162645| protein of unknown function [Uncultured spirochete bib]
MNDPDSRSLLEMARGQVRHLLSSLPDIVYILDEQGHFVFLNEAAALLGYEPSALIGKHFS
VIIHPEDRPNISRDIVVEKIRQANKFPEIAPKLFDERRSGERMTRELEVRLVHRDGHIIY
GLVNAYGERDIDVPLLADLVGNAHTIGVIHDISAMHLYQQSLEESLAAKEQLLREIHHRV
KTNLQLVASLAHLKQLDSARRDTSEVLRELEAQVKSIALVHEALYNSEQIDRISAGKFFA
QFCHAAEEALESVGSTVHLRFSSTDCSLDPDRLVPLALATLEILGGAYRFAFEKRAETEI
RLDYRCLENGEQVLELEGEGMLSAADSMVLHALLRQANARLETEVKEGARQRCRMVLLNH
SEKDAAENREQGRAE*
>SPBIB_v1_150134|ID:27162646| Histidinol phosphate phosphatase, HisJ [Uncultured spirochete bib]
MAFSFHTHSTFCDGKADARTMAEAAFAHHYTHLGFSAHAPVPFKTRWNLPWERAQSYVET
VRALAQEFEPKGMKIFLGLETDYAPGITMPDNPAYDVFGLDYRIGSVHYITAPGEEPFTV
DEPQEEFARNVLRWEPDGDYRRIWKRYWQYMSEMIARGGFDIIGHFDLVKKNNPDGRWFD
EQDPAYLDAAFQAVELAAEKQLVAEINTGGIARGKHHEAYPSVRILKRMHERGLRLTLGD
DAHAPSHIGTYQHAAIDAARRAGYQSLWYLDAPGVWKEISIDEAARSR*
>SPBIB_v1_150135|ID:27162647| Transcriptional regulator, TraR/DksA family [Uncultured spirochete bib]
MDNAFIERMKNLLLTQRQEILDTIAESNEQLRTVLSETDPKDSADIASDDIDRIMIEALI
SQDLKRYRAIEAALLRISQGRYGLCAKCGKKIPQERLEAIPYAVLCVECQKSDERRNR*
>SPBIB_v1_150136|ID:27162648|infA| translation initiation factor IF-1 [Uncultured spirochete bib]
VAKEEAIEVEGIVKEALPNTMFRVELANGHIILAHLSGKMRKHYIRIVPGDKVKVGLSPY
DLNRGRIIYREK*
>SPBIB_v1_150137|ID:27162649| putative 1-acyl-sn-glycerol-3-phosphate acyltransferase [Uncultured spirochete bib]
MIHARRYHPTNFKFFTWLMRYTYGLWLRRAYRIVAINAELFKELKPPFILVGNHTTLLDP
FIANAFVPFPIHWVASDGNMRSPIMRFLLIKLVGSIPKSKAIPDIETVNWIVDIIRRKHG
VVGMYPEGQSTWTGTPFPAFYSSAKLLRLLRVPVVLAKTQGGYLTKPRWSHVRRPSKVEI
AFSVLFTPEQLRTTPIADIDAALNNALAYDDTEWCKREAIRFESARGAESLELALYICPK
CGAKASLHSNGNRFACDRCDFSVEYGADGSFALPPGSADPVFLPPFSSEPFLESIAHWDK
LQSAFMQAELAEWLRAARWDPIFSDENIQLKKGKRIDSMKTMLRGRIDLFIDRLEFRDAH
KPGGATGPLIFPLDTVEAEGVLKWNFFEFYQGMNVYRVVFGDPKASGRKYADAIGLLHEL
SHSHLPGR*
>SPBIB_v1_150138|ID:27162650| Na+/glutamate symporter-like protein [Uncultured spirochete bib]
MDFSWKFVIDAGLISIALVFATFLRSKITFLQKYLVPNALTAGFLLLPIYNYLLPSIGYA
TNRLGDLVYHLLNISFISMSLRSSPPKIKGSRSNGGVLGMSAVILFGYASQAILGLLLTL
FFLPKIHPAFGLHLPLGFALGPGQAYAIGKGWESMGFEGGASVGLTFAAIGYLWACFGGM
LLIHKGIRKGWMKSDQLTAMSDKAMLTGIIPREAEKPIGAQLTTDSEAIDSFSYHAALVV
FVYFLSFLFLKGLTLVLGFAGKAGMELATNLWGINFIFSAIIAIIVRKIIDALKLGYVLD
DNSLTRISGMAVDYMVAGALAAISLVFVGKYWFPIVLMSTLCGFMVYYTLPWVCSRIFRD
FRFERMLMLYGVSTGTLSTGLALLRVMDPEFKTKVSSDYMLSAGLTFVLALPFILAINLP
AKAYTSGSMTPFWIFIAIAAAYLVFVAVVYWILARKRRFAMPSDNFYRPGK*
>SPBIB_v1_150139|ID:27162651| putative Transcriptional regulator, Crp/Fnr family [Uncultured spirochete bib]
MNDREIAKLATCNLFRNVSIERFADFMKAARYREHEFRKGELILLQGCMYASLYILLDGS
AYAEMTDDEGRCMRVETFAPVEALASAVLFSPKPVLPVTVGAQSDCRIISFPKEEIIKMC
MSEKAILEGFLADSGARVQFLSERLRLTRFATLRQRIADWLVRKAQAQHTMLRDSSETAQ
SPSPAAQNPVSIRVEPSFEKMADLMGVARPSLSRELSHMRSDGLIDMEGRTIILEDIEAL
KNIRNSKSRQP*
>SPBIB_v1_150140|ID:27162652| putative NAD(+) diphosphatase [Uncultured spirochete bib]
MKKDPQRFMLFRGQDCLVPAQAAGQLQSMAELADSTAASGAHFPNFPEISPGSVSWPAGA
IPLSLFSAKPDGYSYSVFVFQGVVYGAVLLKDDDEPFAEVASLGAFVPFPSRQLALSFSS
HLGRFVLLARAHAQWAAVSRFCGACGAPLVDANGNEDVREPLDDHAYGTRFCPRCKRLFF

PRMSPVVIVLVRRGKEILLEHNVRFPGNRHSLVAGFVEIGETLEEAAAREIREEAGIEVK
NLKYVRSQPWPFPDSLMLGFVADWAGGEARPDGKEIDHLDWYTADNLPELPMKGSIARWI
IDTFMAGGFSNF*
>SPBIB_v1_150141|ID:27162653|upp| putative uracil phosphoribosyltransferase [Uncultured spirochete bib]
MEQRVSKIILKAEDLDGYLSAKDLDYLSQMDSLYRHAMVSFNILATSTSESQKHREEQTL
IDLYNRMGSLMQEICAAEPRIQVYSFVSPQETHGEVSRLIAKLRDVSTGRQEFVYYIQRA
FELLFTLAFGGSGRTNKNYLIVRTPVTIPVQNFAVHKIPNVDDAVHDTVMCVMLRGALLP
SMILSKEIQEYSSTGYITPFALFKIKRDDTKSETTMEYVLDLDHSYFDLAQLDGKHLLFA
DPMNATGGSLVTVVKYLLEQGVKPASVKFFNVISALKGSLRVVRAIDNITIYTLWMDPSL
NERAYIMPGLGDAGDRINGSDEEGHPRDMLRLVADYGTNITGLYRSQLRIIEETVLRHRS
*
>SPBIB_v1_150142|ID:27162654|pduL| Phosphate propanoyltransferase [Uncultured spirochete bib]
MLETKMIVNLSNRHIHLSHEDVEALFGKGYQLTKTKDLMQPGQFACAETVTIKGPKGQFE
GVRILGPERKETQCEILASDVFKLGVPGCPVRESGQLEGSFPFEIIGPKGSVKKEKGLII
AKRHIHFDPESAKRFGVVDKQLVALKVGEERGAILLNVVCRVHPTYALECHLDFDEGNAL
GIGSGVTGEIVQV*
>SPBIB_v1_150143|ID:27162655| DhnA-type fructose-1,6-bisphosphate aldolase-like enzyme [Uncultured spirochete bib]
MNEGAILRMQHIFREDGRTVIVAADHGAIAGPLKGIESPRELARLCAEGGADAILAHRGF
VRAGLEAWKRQLGLVLRASGGFTTLGGRFEEELIAGAEDAVRWAADAVAITVKFGHAREG
EFIKNAAGLISACEPCSLPVMIEAMAFEKGEFSTRADALAIAVRAAEEIGAAFIKAAMPQ
SIDDFVQIARGTHVPIVLLGGERTDSLPALFESIARAMDLGASGVAMGRNIWGQDRPREI
LEAVVGLVHGGWDIQKVIAHCEHTH*
>SPBIB_v1_150144|ID:27162656| putative Pentulose/hexulose kinase [Uncultured spirochete bib]
VIFAIDFGTSRAKGAIFDALGACLGMAEVELTEDSRSSGLIHEIDARGWLTAMAEIAGRL
LDPLDQTARATLKALVICGNGPTILPIDEHGEPLANAITWLDRRAFFESEEASAALGYSL
DAAFNLPKILWIRRHMLRLYEASRYFVSCPEFVAGRLTGEWTTCLPNRGYTKIIWDEAAL
RALALDERKFPPSIGIGGIVGHVQAQAAEAYGVPAGLPVVMGGPDFIASLLGTATVAPGR
TCDKGGTSEGINLCAATDLANAAPSASRAASSGLLVMPHIIEPYYNISGVISTSGKALAW
FKDRFLPEESFGAIYSRAERAKPGASGLVFLPYLAGERSPHWDPDASGVFLGLRLEHDLD
AMVRAVMESTAFAMRDVISVMEDAGAHVEDLRSTGMPAQSAIWNQIKADITGKPVKVPAF
SEPELAGCLAIGRFALGEEKSLAQAAEHVFAPRAIFEPRREYAALYDELFSVYRETYRRL
ADLFPLLRRAGHTAGSAQEEKP*
>SPBIB_v1_150145|ID:27162657| Theronine dehydrogenase-like Zn-dependent dehydrogenase [Uncultured spirochete bib]
MKAAVLVAPGRFEIEERPRPEPKENEVLVKLEACGICTLEQRLFTGAMRLPLPLVPGHEA
AGLIAAMGSKVIEELSPGQKVALDLVERCGECYYCRIGKSNLCLNRYKNPARMLGGFSEY
IVVRPNQIFPVPDSVSYEEASFAEPLACCIHSLKRLKVAMTEDLLIIGAGTMGLLHVLAA
RSMGLRITVSEPDAVRRDTAKALGADFVIDPAKTDIVEFGREITDGIGFSSCVITSPSPA
ALEPAIRAMAKAARINIYTAYEDVMQIPLDLNTIHRNELLITGTEGRVQEDFFQAVRLLS
FGKIDVKPLISARTSFSTIEEGFRTALSGKAYRVVLMHSLP*
>SPBIB_v1_150146|ID:27162658| putative Mercuric resistance operon regulatory protein [Uncultured spirochete bib]
LTERLLKIGELASLFGVTSRTIRYYEELGLIEASNRTEGLHRRYPADVIIRLKRIEELKR
LGLTLGQIREFFMLYAEDPSGEKCRLLMLQIYEDQKKEEEAKILEARRHIAQIEENITAI
KEKKSFFSCPGDECKDCSFDGFCDESVYNQLKE*
>SPBIB_v1_150147|ID:27162659|tpn| Membrane lipoprotein TpN38(b) [Uncultured spirochete bib]
MRRFLVAFVLAIVAGTALWAQAPRIGVFIPGVREGSPIYDTMAKGAERLAAEISGASIKI
FEAGFNQAEWEEKLTSFVASGKFDIVITSNPSMPELVNNVSKSFPKQKFICLDGYLPGNP
NVYSALYNQLEQGYVTGYLAGLVSISGMKGTNPDKKIGMIIGQNYPVMDKMIIPGFMQGL
KAADPAFQLDIRVLGNWYDAAKAADLSRSLYAAGADIILPICGSASQGAVKVAQETGKYL
VFFDDNEFARAPDNILGCAVLHQEDLAYRSLKAAIEGKLPFGKADVVGMKEGYIEFLNTN
PAYIKNVPEPIRKKVDAAIQSIKSGQLTFQVPSL*
>SPBIB_v1_150148|ID:27162660| putative Uncharacterized ABC transporter ATP-binding protein YufO [Uncultured spirochete bib]

MQPMIAVRMRGISKRYFPSGIRANDEATLEVERGSIHAIVGENGAGKTTLMKILAGLELP
DAGTIEIEGKPVHIQSPAHARELGIGMVHQHFLMFDDLTTAENIFFGLEPLRVSKIPGVL
GILDRQSLVEQTRRIAADYGFVLDPMAQAGDLSISARQQVEILRQLARNLHILILDEPTS
VLTEQETTALFEKLAEIRRMGHTIIIVTHKIDEVMRIADRVTVMRQGKTVGTYDIAATTA
NELACLIMGTNVCEEISPRTEPAAQGPVVFQIEGLSARGHHHGETGVTDLHFAVHAGEVL
GVCALSGNGLAELEDALGGFLVPSKGFCIFDGSRLDIRRIHEYRQLLKKGAIGYLPSDRM
RRSMALSLTVRDNFMAVSRTRYFQRGWLESTLATRQTQDALHSFDISAQPEQKTVELSGG
NIQKLAIARLFSIQLPKLLILCEPTWGLDIKSTENVHRRILDAKAAGSAILILSSDVDEI
LALADSIMVLYRGRAVLLASNSGELNRERIGEYLLGARSE*
>SPBIB_v1_150149|ID:27162661| putative ABC-type transporter, integral membrane subunit [Uncultured spirochete bib]
MHSERELPAGARIALAVALSITLAAAVLALFTKDFSPALGQFFFEPFANRYFFGNYIADS
VPLMIAGLGVLIAFRSRNFNLGGEGQIYAGAIAAGMLALSLPERIPPAQAWLFAALAGMV
AGGLIGALSGILKALWHVSEMISSFLISAIVLNVGDYLITGPFQDPASNFQTTRQISSQL
LLPRILAPSKLDISVFIGLALIAVLAIMSKHTRPGFELRVCGENEEFARYCGIPVALYQS
AAMAISGALYGLAGALIVMGPQARVMRGFSSGLGWSAISVALLAQSSEAGILPSALFIAY
LKAGSDHVMIGSGVPSEIISILQAAAMFFITARHFPVRKRRRP*
>SPBIB_v1_150150|ID:27162662| putative ABC-type transport system, permease component [Uncultured spirochete bib]
MSTPITILADILKYTIAAATPLLFASIGGLFSQLAGMLNIALEGLMGLGAFFGMAAAGAS
GSLVVGAMAGVAAAVVGSWLMGIITIKFKANLFITGLAVNLLAVGITAVLSQSWFHTKAV
VAFDIPRVMLAFPAFLGRIPFIGPVLFSQNILSASAWLWVIAAAFIIRNTRHGVRIRATG
MNEEAVNALGYRPDRYHMAALIWSGVGSGLGGFALGVSISAFVPNITAGRGWIALVAMYL
GRGKPLWIAASCLLFALAESLSNYVQGFSAIPPLLVLALPYAVTLVSLVAAAAGKKD*
>SPBIB_v1_150151|ID:27162663| Pyrimidine 5'-nucleotidase [Uncultured spirochete bib]
MIRALLFDLDNTLYSEATGLETGVLQRMNQFVADIFNLPFSEAGKFRREHAKPYGTTLEW
LMREKGFAEPERYFSYIHPEGEEDCLEPDHVLRLMLNSIPLPKAVLTNAPREHAERILAK
LGIAECFIGVYDIWFNELLGKPNPKAYLRALDASGFALQETLFVDDLPKYVKGYADLGGP
AVLKDEMNRFPDLPCRRIQTIYELPEVLDEIGMKAS*
>SPBIB_v1_150152|ID:27162664| Peptidase S54, rhomboid domain protein [Uncultured spirochete bib]
MIPIGDDNSGRRGKAVVNWILIGLNVFVFVFIQKFALDDYATLALAAIPEEILSGHRLFT
LITSQFTHAGFAHILGNMIFLAVFGDNVECRIGKLRYILLYLLSGTFGMLLQILLALMAG
GAALQMPLVGASAAISGVLASYLVLFPGNKVVVLLFYFIPTALSAWVVIGFWFVLQVLGG
LSGLASIQSGGTAYFAHIGGFVSAYLWSRGYKKKELERILAWRKRRLAGDSDGFHWWIVD
D*
>SPBIB_v1_150153|ID:27162665| protein of unknown function [Uncultured spirochete bib]
MAERNERYRGLFLHAKDTARGDRIVTFLCKEGILSLFLFGGPKSSLRSVATPYILADIEV
YHDRRNDFSKLTGATILETFDSLHTSFSHMQAAAAAAEFVIRTSAFGGEYGLALQMITTL
LRELPSIEEDSAPYAMYAFLWNALAPQGLAPDISACEHCGKRFTEEGALPGRLLAGERSF
LCARCAEERRREGFPASAMILIDAMVLQCLREILDVSYSQAARCMSVNPSLLHIEAIIES
LAESAAEGPLQSLRLFH*
>SPBIB_v1_150154|ID:27162666|pdp| Pyrimidine-nucleoside phosphorylase [Uncultured spirochete bib]
MRAVDILMKKRAGETLTEAEIRFIIDGYVKGEIPDYQISALLMAIFFRGMTAKETAILTE
TMLDSGDRMDLSGIPGPFVDKHSTGGVGDKISLPLAPMVAACGVKVPMMSGRALGHTGGT
LDKLESIPGYRTGLELSEFRKGLIEDGFAMTGQTARVVPADKKLYALRDVTATVESIPLI
TASILSKKVAEGADGIVFDVKCGSGAFMKTYNDAKALASSLVATGTAMGKRVVAVLTDMS
QPLGYKVGNFLEIEETLDCLEGKGPQDVMELTFRLGAWMLVLGGAAETIEEGRARCEEAI
SSGAALERFYRNVVRQGGNVGQMLKRRGSWRSDFARELKAENDGYIESIDALDIGLAGVY
LGVGRNRTEDNVSPTAGFIFEKKRGDLIKKGDRIAIAYGKDEASLDPAMALARKAVSISK
EAPQPLSLVIEEITAP*
>SPBIB_v1_150155|ID:27162667| Single-stranded-DNA-specific exonuclease RecJ [Uncultured spirochete bib]
MKWTKKEIDPTLVREIARRYQIDTLTASILVRRNITEPEQLRFYLEDDLQLLHNPFLFAS
MEDAIDRLLMAHDEDEKVLVFGDSDTDGITSTALMTELLRDFGLEVFHKVPEGEEPYGLS
VSAIDFAAENNISLIVTVDCGISNHDEVAYAQRKGIDVIIADHHHLQAPAPPEAIAVLDP

KLPDCGYPFRDLSGCGVALKLAHALAIARLGMYKEPFALLYAGSALSLDEAAGLADDAGG
ADSAMVADSVSALTIEAVRLDNLIETSRLKIRSGSDGVFPSDTLEKLEKFLRGRVIIAWN
KKEIHSFFREHFGNAAELDVMDLGQLSAGFWPAMARSPFASLVRASRLKKYAGSAHSAID
TLKGIFSAYVLQTLQSQNLLTERMFQLAALGTIADLMPLKDENRIIVRKGVESINASPSN
GIRELKSGLGLAKPLGAIEIAWQITPTINAAGRLGTPKLALDLLQAKTVESAITAASALI
QANSERRRLGTEAWENIREALNQSLEKSGGKYAVVGSSEIKPGITGLLASKAANIMKVPV
IVAVFKADGTCTGSIRGGANFPLTRLLAHCADLFLDYGGHDSAAGFTLKTERWQSFLERL
SEFMAQTEYNAEEPELSIDAELPHAYVTPELVNLCRLFEPFGEENDPLVFCSRQVSMIDA
QVVGKNGKNHLKLTLDFGTYKWPAMLWDGAERLERDFSFRKGDRVDILYKVTMNYWNGEE
RPQLELYDIRRAEG*
>SPBIB_v1_150156|ID:27162668| exported protein of unknown function [Uncultured spirochete bib]
MRSTCPVKFLLLGLLLGSASVPRAFSQTVELPAVIIEETLPFDLLAPSAGTTIIEEHAIQ
TSAAPDLPSLLSSVPGVILSPTGSEGAQASVSLRGSTSNQVLVLVDGVRVTDPATGLTDF
SRLGIPLDQIVQIEVQRGGLSAQYGADAVGGVIHIHTRRGSKNLSARISLENTSFLPSSI
TIGNGLSAITVPFVGSALIDGQSLSLAAENQWLSAWAKISRAANGYPYYDTNGERRRREN
AGLLAASGGLQTRFPLSAGEISSSAKVSYRSVGIPGSLDIPTPEAHQEDWNANLTMQFRT
DALLAGALALELSPYAQAGGIRYQQSQTSAVDSHISYRAGLDSSLSWLPPWNGEVKAGAS
FRYDRLESSVVKGAGGSAPERFSGGIFAEPSFGFGTWTAVPALRFDMTNDFPSGVSASFG
LIHDFSKQTAIRATISSAYRAPSFDDLYWPASGGAEGNPSLKPESAISGDISLTHKEDAC
TWSVGTFARYARDVILWQPDDAGIWKPSNFGNALYPGIEAESSWKAGSWNLGANYTFLYS
YVLSGNLSLSDDRRVPYVPVHAASLSASRTTKAFKSAFLFTYQSLRFTTTGNRAFLPATL
IANAKLTWELGPRSDFELSFQNLFDERYEAVKGYPMPGFSLSATVTLRFEGARPGQN*
>SPBIB_v1_150157|ID:27162669| exported protein of unknown function [Uncultured spirochete bib]
MNKLWQRIGRPVLFAGMIAVLLAGLAGCDLFLSPQGSSAVVFAVDSKNGNVYEIDADKAE
SAAVPLVSTQQNATGKMIISGTKAFLAVASWQNTSPGLYWFDLSSKTPAVAQIGPKISAQ
YICIASSTKGYVSSADWAGTYANAVYPFNPSSPSAGFGTAISGFDAGFYPQDVAYVDDGN
GTGRVFVADNGNGKVYRLNAEGTLVEKSFAASAGGTTGLLAGEFDSNGDGVAEAGVFVAN
SGGYDANWNPLPGSIDFIPLGASSDADVVVVQSDLSATALAFLDATHLAATSYGHTWLID
LSKLASDASRLTEIKTSSGASFGSMDIAVREGYAYVPDGAQTVYRFGLNSPTTAIPTGKS
GEMITNIAVRQ*
>SPBIB_v1_150158|ID:27162670| protein of unknown function [Uncultured spirochete bib]
MRQRRNQLFVVYRAAGLALSLAIHALIIFAPIGMRAVRQPFVVSLNIDELREADVLSREAP
KVIQDPIGQIKQIKQELALKQTEAPETTQQPFEKEAVLPQAEAPETTQETFGKKAAPPQT
ETPKIQVPDKSAVSETIAAKEPQQERLSAQAEIAREIPEPSALEEAPAPARAMSEMPIEA
SATLPAAPAVPIAESEVQQFAPAEPPAKPAPQAAQAFAQAAPQAARSTEPTLSDVPADAL
QPTPSQALSVPQAEKPLPAKPKPILSREGLASSLIALLQSISGDVPAGQKQAKGAISRSS
VSTAQSSAAIAPQEGVFHDDAEEIRAEHVNDFLAKRKSGSLAAFQGEQKPESALVPASLH
ESQLQPIAQGSAEASSAGSLEARAEGSASLTNQHAIQNDSGSANAVVEVREQSRADGALA
RSDASEPSGIDAPESPKSAVSASDLDTESPLILEKPLERDPLEPARAEPVQAEPARSKSA
SMESAPASYDTVESLAAAIAGKLAAQKQYPAAALKRKSEGTVRIALQVAPDGTLASLAIQ
SRSGSAILDEAALQLVRSIFPLHIRLETAVSLLIPVEYRIPR*
>SPBIB_v1_150159|ID:27162671| conserved membrane protein of unknown function [Uncultured spirochete bib]
MSNKKEQKKQASLQSGALQPHVSQPEAQKKQPEPQGKQAPGLSSFAFPILLVLFVVAGFL
LRGALAPLLSKKEALREWVLAFGAFGWLVFISLQILQVVIFIIPGEVMQISGGFIFGFWG
GLALTIAGIGIGSAINFLLGKALGPQFLMAILSREQYEKLQRYAKDARTFAGLILLFLIP
GIPKDVLCYFAGAGEREFLYFIVASMLARMPGIIGTTLAGSAVYKDKLGLVVVLAVVTAI
AMILGIFFRNKIEAAIKKLLHRKKA*
>SPBIB_v1_150160|ID:27162672| Transcription factor [Uncultured spirochete bib]
MPNDQTTDFQIDQRVVYPSQGVGRIMEIREKAFNGQKTLYYIIYLEFSDMTVMVPVDKVE
ALGIRPLVSREEAETALEYISQAASTIPSDWKLRYQMNLDLLKKGSIMDIAAVVRSLYQR
SKIKELPILERKLYESALNLLQDEIAFALDRPKEEIVKIIRANLDK*
>SPBIB_v1_150161|ID:27162673|ispDF| Bifunctional enzyme IspD/IspF [Includes: 2-C-methyl-D-erythritol 4-phosphate cytidylyltransferase ; 2-C-methyl-D-erythritol 2,4-cyclodiphosphate synthase] [Uncultured spirochete bib]
MSTSLSKPIPAPSLPLDGRVFALITAAGSSTRFGGQKKELEQFEGCTVLERAIEPFIETC

SGIVVTCPPGARAEFEAFFAQSALSPVIARLERGFAIVEGGSTRQDSVLFGLGALKELGC
DRDIVLIHDGARPWISADLVRTTIEQTRMHGACLPLAPLVETPKIVKGQVVHTHPSRAHV
MTAQTPQAFSFPEILAAHELAAREGYVATDDAMIWDHYVGPVFWIEGERQNRKITYREDL
FGSPAMPEAKPAAAPTTRTGIGYDIHPLIADRPLLLAGVQIESDRGEAGYSDGDVLWHAL
IDALLGAAALGDIGTHFPPGKPEWKNADSTALARKVAAMLHRQDWHIVNIDCTVILEKPR
LGPYREQICSAIARALNIPQGSVSFKAKTKEGFDAVGRGEAIEAHAIVLIAR*
>SPBIB_v1_150162|ID:27162674| membrane protein of unknown function [Uncultured spirochete bib]
MTSLAAYFLALFGNFLLSLGFSLQKKYVSWLSAKRRGVRIKRGEIIGWLSGFTLMNLQPI
FNYLALGKLAPNIVAAIGGSNIVFTIILSYFLLGERIPAGKIPWIALMAGSLALAGFVGQ
ESSKQFQVEAFWTAFFIPTGFAMLVLLANRKMTPAQVGIFLGSAAGALGGFMVLALEALR
MTRGSDFMSWIFSPYLYVYIFCGISSFSIKQVAFERGRMAAVAPSFYGLLVLYPSIATYF
VSSVPLHPQQILAFLGISVSIILISL*
>SPBIB_v1_150163|ID:27162675| protein of unknown function [Uncultured spirochete bib]
LISFENWKEGFLVTVAGQRLLWHSAKRPAVFVHRAGEMRSAWRSAGTCLPTMASGRSALF
RFSGGLVMREELRDARSVAAFADYSSAYSTAIFSLYFSCLDKEIDSVRLRFHDMPKAHWN
GLGPREQHLARGTIALPLGARSAAFAMDGRFILCSSSGERKLRFSEAEMLFDLDLPAEVH
FGYARTPAEACRLWRYSFGSLDSFFENPDESYRGAHSGKSQTLIAAEARAHSVVIPQEAA
GLLGSLSFSGAGPEDYQQLNMAVAQAVREGSIWKYANLMLCQPDIALPAQFRREIGNAFI
SLGDILPARLKNRYAKIRAGLEKYWEFCRMRWTEDGIPALLHPSLLYPQDVALESRDDLL
MAGADMVFAPNLKEKGELASLVLPEGEWVHFWTSRIYPSGRVTVHAPAGAPALFYRAESE
YSWLFDSVRQVASRL*
>SPBIB_v1_150164|ID:27162676| L-fucose isomerase [Uncultured spirochete bib]
MKETITLGVVCLARTTFDYAAAKELWDGIRSNLEAIENVRIVAPHALVFEVDEAKAAASQ
LARAQVDGLVIISGTFHLGHLALELVKTVHAPVLLWGLPELPYNGGKIRLNSVCGVNLNA
SNLYKSGVQNYHVTIGEHIDMNWVDAIRVLKVLSRSHIGILGYRAHGFFNLSVADLALYR
QTGILIDHYELSEVWNFPASDEAVQARATQLRGIFDVSGITAEQLHKVAMLAVKLQGFLD
KYALDALAIRCWPEFAAEYGISPCAAMSLLQSEHRILACEGDVEGGLSMLMHRAVGAETP
FLFDFSQVDFEKDFALFWHCGVAPCNLWDGRCVRSLDTYFAGGKGVTADFVLKEGELSVL
RLDSSEGKYRVFLQRARAIPMEKELKGTYLKAKFDVPVKQVLDKVIGKGVAHHASVVYGN
YIEPFRIAAKIAGWEVIE*
>SPBIB_v1_150165|ID:27162677|bcsP| 31 kDa immunogenic protein [Uncultured spirochete bib]
MKKIFAIALCLVLATSVFAQQRFLTIATGGTAGTYFPLGGALADIWNKNIPKMNATAQST
GASIANINLLKGGNVDVIFTQNDVANYSYNGTELLKDQAYKDLRGMVCLYNETIQLVALE
SSGIKTLADLKGKRVSVGAAGSGTEANARQILEAAGLTYNDIKVQYLSFGESANNMKDGN
IDAAFNTAGIPTAAIQDLSVSKKMVLVPVDKEAASKLMAKYPFYAVQVIPAGTYQGQTQN
VTTVAVKSMLAVSSKMPADLVYELLKTMFDHTDRLIAAHAQGKNVKLETALEGMSIPLHP
GAEKFYKEKGILK*
>SPBIB_v1_150166|ID:27162678| protein of unknown function [Uncultured spirochete bib]
MNDGAGQSVPRRLLDKAGMERTRKRSRWFILAAVILGTAIAGAALLAFLFNVRLVSALII
KDSTGAIIVRLPLQDGQFVHHYIHSIHKTPVDEYFEVHGSSLDLTKVKYDTYGVGMPSDA
GEGFSIENGRFVVVLHRSFSSIPIRVSIVPDHGVIIGGTLFPFTRWVPQESAITLSAGRM
LIFRNGRISPK*
>SPBIB_v1_150167|ID:27162679| TRAP transporter, 4TM/12TM fusion protein [Uncultured spirochete bib]
MSDSRKTVPEENAAVDAQEILKKFDKEANYRTYVGFFAKVISAIAISFSVFQLYTAIFGV
LDAMIQRSIHLSFGLTLIFLLYPTSKKWSRSKLHPVDAVLAVIGALAPMYIIVNYQKLVL
RAGTATPLDIVFGVIGLLLVLEAARRVVGVPMVVIALVFIAYAFAGPYIPGKLAHRGASF
ETLIQHLYFTTEGVFGIPLGVSSTFIFLFILFGAFLERTGLGQLFIDLANAVAGWAAGGP
AKVAVLSSALMGTVSGSSVANVVGTGSFTIPMMKRLGYKPEFAGAVEATASTGGQLMPPI
MGAAAFLMAEFTNIPYARIIGAAVVPAILYYFGVWAGVHFEAKKSGLRGMNRDELPKLKN
IILERGHLIIPLVAIIYLLVTGFTPMKAALWAIILSIASSWLKKSTRIPPIEIVRGLEAG
ARAALGVLAATACAGIIIGVVTLTGLGLKLGSTLVELAGGRIVPTLFFTMITSLILGMGV
PTTANYVITSTIAAPAVIMLLSKQAGLDPYAVAPASIILPAHMFAFYFGIIADVTPPVAL
AAFAGAGIAKANPMKTGINASKLAIAAFLVPYIFVLNPQMLLFNVNAITFIWMLITSLVG
IIAIAAAVNGWLITTTLWYERLIGFAGGILLIYPGLTTDLMGLALVAVMAISQIVRARAA

RALKAR*
>SPBIB_v1_150168|ID:27162680| Aspartate kinase [Uncultured spirochete bib]
MKILKFGGTSVGSPDAVRALVGIVKDSGERIIVVSAFSGITDSLLEAAKLASGGGEWRAI
FETIANRHHSMIDALLEGSFSEPATAYVRSLLDELSSLLYGISLVRDLSARTLDLVASFG
ERLSAFIVAQALSQAGIAAEPVDARQFIITDARFGSAHWLQAETERRALAFFSAFPRVAV
VTGFIAATADGITTTLGRGGSDFTAGILGACLDAEEIQIWTDVDGILTADPRAVPDAFVI
PEISYLEAMEMSHFGAKVLHPPTILPAMAKGIPVRILNTFNPGSPGTKIVREAAPSSWPV
RGIAAIPSISLLLLQGPGLPGVTGIAGRMFAALASAAVNVILITQGSSELSICCAVLPQD
AENGARALKEEFKYEISTGTIQKPAIEKDLSIIAVVGEMMKHRVGISGRVFKALGRNGIN
VVAIAQGSSELNISIVVSAQDRGKAMSAIHDAFFLAGVRTVNVFLVGTGLIGGTLLAQVK
AQHERLFKDHSIRVRVAGIANSRLMLIDANGIDVSDWKTALAERGEPTHLETFVRQLTHL
NLPNACFCDCTASDAPIRYYETLLAASISIVTPNKRANSGPLVRYQRLMRVAREMDVPYR
YETTVGAGLPVIGTIQDLVASGDEIFKIEAVLSGTISFIFNNLGSDPEFKFSALVRQAKE
LGYTEPDPRDDLSARDIVRKTIILAREAGFPLEEAGVRIEPLISKRIAQAPTVETCMVLL
PEMDAEIEARRSNAARHGTVLRYVSAITKEGASLSLSEYGPESPFFSLTGTDNMVVITSK
RYSQNPLVIRGPGAGADVTAGGVFADILKTAESYL*
>SPBIB_v1_150169|ID:27162681|gcdB| Glutaconyl-CoA decarboxylase subunit beta [Uncultured spirochete bib]
MEAFFSHFISSTGFIAMTWGQLIMLTVSCILLYLGIVKEYEPLLLVPIAFGMLLANLPAS
GITDGPKDGLVGGLIYYLYQGVKLGIYPPLIFLGIGAMTDFGPLIANPWSLLLGAAAQLG
IFIPFILAYYLGFDLKSCGSIAIIGGADGPTAIYVTSRLKPELLGPIAIAAYSYMALVPL
IQPPIMKALTTKKERLVKMAQLREVSKAERVVFPILVTILIGLLLPAAVPLVGSLMLGNL
FRESGVTERLSDTAQHALINTLTIFLGVSVGSTASAVRFLNPQTLMIIGLGLFAFSCGTA
GGVIFGKIMYWITKGKVNPLIGSAGVSAVPMAARVSQVVGSKEDPTNFLLMHAMGPNVAG
VIGSAVAAGVLLTVLG*
>SPBIB_v1_150170|ID:27162682|gcdC| Glutaconyl-CoA decarboxylase subunit gamma [Uncultured spirochete bib]
MKHYRITVNGKTYEVTVEDLNRPGHGSGAAAVSQAPAAVSSAASLPAAPSAQPAAAPSNG
ETVVTVKSPMPGTVISFKVIAGQQVKRGDVLLLLEAMKMENEIVAPQDGTVVALRVPASA
SVNTGDPLVDLA*
>SPBIB_v1_150171|ID:27162683| protein of unknown function [Uncultured spirochete bib]
MTNPWMIMLVGVITVFVALFLIILFLLGFPKFFSALKKKEHKKLAPLPEIVEVTKKKAQA
GLLSQSPAEDDEELIAVLTAAVAAARGSELGSFAIAQVKQAAAEGGGFNTPVWGRVERLS
RK*
>SPBIB_v1_150172|ID:27162684|pccB| Propionyl-CoA carboxylase beta chain [Uncultured spirochete bib]
MEEKLRELEERQAKVRAGGGPKRVAAQHDKGKMTARERIEMLLDPGTFVELDAFVEHRAT
ELGMDKIDAPGEGVVTGYGLVNGRTVCVFAQDFTVIGGSLGEMHASKICKIMDLAVKIGC
PCIGINDSGGARIQEGVDALSGYGEIFYRNTLASGVVPQISVIMGPCAGGAVYSPALTDF
VFMTEGTSNMFITGPQVIKAVTGEEVSSEDLGGARVHSEVSGVAHFSFPDEQSTIEAIRK
LLSYLPQNNVEDPPVAETGDDPNRIDEGLADLMPDSPNKPYDVRDIIARLVDAADFFEVQ
PNYAKNIVTGFARLAGRAIGIIANQPKYLAGVLDINASDKATRFIRFCDSFNIPLLTLTD
TAGYLPGVGQEHGGVIRHGAKLLYAYSEASVPKLTVILRKAYGGAYIAMCSRHLGADQVF
AWPTAEIAVMGPEGAANIIFKKEIEEAPDPAAARKDKIEEYKRSFANPYKAAARGYVDDV
IDPAHTRMRLCNALNMLLGKRQSMPSRKHGNIPV*
>SPBIB_v1_150173|ID:27162685| conserved protein of unknown function [Uncultured spirochete bib]
MNTLKLDHIGIAVEKIDAALPVWEGILSLPLHGIEEVADQKVKTAFMPIGESEIELLEST
DSEGPIGKFLAAKGQGVHHLAFRVANIEAALAELKAKGVRLIDETPRYGAGGARIAFIHP
KATGGVLVELCERA*
>SPBIB_v1_150174|ID:27162686| putative enzyme [Uncultured spirochete bib]
MYDLIRRCIEGDVRAVARLITLLESGDREAYAALYRLKDHAGRAQVIGITGPPGAGKSTL
ADKLIAQFRSRGLKVGVLAVDPSSPFSGGAILGDRLRMQGHATDPGVFIRSLAARGSLGG
ISKATHAAIRVLDAAGYDIVLVETVGVGQSEIDIVRVADMVVLVSVPGLGDDIQVIKAGI
MEIGDIFVVNKADRDGADRVVREIRAMLELQAQLKRGQTPEGGPDTLAARAPEFLHHTGL
AEGWQPKAGNESGAPGEKESITTPKPGVLPEAGTVSAALVNQEAGTISAALIIPPVLKTI
AETGDGVKELADAIFSHFETLSRTGELERKRLESIRYQLGQFVSNEIVHLMSLPELAAAQ
DELAHKVFSRKMDVYSAGIALFHTIIEQGASHEHPQA*

>SPBIB_v1_150175|ID:27162687|yliK| fragment of methylmalonyl-CoA mutase (part 2) [Uncultured spirochete bib]
MERKIRVLIAKPGLDGHDRGAKVIARALRDAGMEVIYTGLRQTPEQIVSAAIQEDVDVIG
LSILSGAHNHLFPRVVELLKQSGAENVLVFGGGVIPEEDIPFLKSKGIAEVFGPGTPTSK
TIEFIRANLKRPLN*
>SPBIB_v1_150176|ID:27162688|yliK| fragment of methylmalonyl-CoA mutase (part 1) [Uncultured spirochete bib]
MTDLEKLEQDFNTWTTKVEKANEKYPERKKQFVTGSNAPVKRVYTPLDQKDFDYEKKLGL
PGEFPYTRGVQPTMYRGRLWTMRQYAGFGTAEESNARYKYLLEQGQTGLSVAFDLPTQIG
YDSDHPLSEGEVGKVGVAIDSLADMETLFGGIPLDKVSTSMTINAPAAVLLAMYIAVAEK
QGVSADKLNGTIQNDILKEYIARGTYIFPPAPSMRLITDIFEYCAKNVPNWNTISISGYH
IREAGSTAVQEVAFTLADGIAYVEAAIKAGLDVDEFGPRLSFFFNAHNDLFEEVAKFRAA
RRVWAHIMRDRFGAKNPKSWMLRFHTQTGGSTLTAQQPDNNIIRVTLQALAAVLGGTQSL
HTNSRDEALALPTEEAVRIALRTQQIIAYESGVAETVDPLAGSYYVESLTDRIETEVLQY
IKRIDDLGGAVKAIEQGYIQQEIQDSAYAWQMDVEKNERIIVGLNKFQVKENPPKGLLRV
DPAVGERQKQKLAALRSSRDQGAVNATLAALRNAARSADNLMFPILEAVKAYATLGEICG
VLREEFGEYKPNVMF*
>SPBIB_v1_150177|ID:27162689|korC| 2-oxoglutarate synthase subunit KorC [Uncultured spirochete bib]
MRVEFRLSGSGGQGLLLAGIVLADAAIQDGKNAVQTQSYGPEARGGSSKAEVVISDGDID
YPKATEPDFLLALTLDAFRTYGPLMKKGIIITDTSVAADLPSAGTRLEALTAGVPVITLP
ILETASKKLGRAVVANMVALGVLGGLSGVASPKALREAVRSRVPKGTEDLNLAALEAGLA
LAEEAKAQLAASGVRPRA*
>SPBIB_v1_150178|ID:27162690|korB| 2-oxoglutarate synthase subunit KorB [Uncultured spirochete bib]
MAEFLSYFRQDRLPHIWCPGCGNGTVTGALVRAIDKLGIDKNNIVMVSGIGCSSRSVGYL
DFDTLHTTHGRAIAFATGVKLARPRLKVIVITGDGDSAAIGGNHFIHAARRNIDITTIII
NNDTYGMTSGQASPMTPHGMLGTTAPYGNVEYNFDLCELAKAAGATYVARATTYHYVLLE
ELISKAIMHKGFSVVEALSQCPTYFGRKNKIGGPVETLNWIKDQTINVKAAAALPPEKRE
GKILIGELHNVEKPEFTQQYLEVIAKVQRANPTYARNKGVPGCELNSD*
>SPBIB_v1_150179|ID:27162691|korA| 2-oxoglutarate synthase subunit KorA [Uncultured spirochete bib]
MAEKAKLMQGNEACTMAAIAAGLEFFAGYPITPSTEIAEMCAEELPKVGGKFIQMEDEIG
SISACIGASLTGAKAMTATSGPGFSLMQEAIGYASLCEIPVVIINVQRVGPSTGMPTSPA
QGDLMQARWGTHGDHPVIALVPGNVKECYELTFRAFELSEKYRVPVIVLSDEVIGHMREK
VVLPDPANYKIPVRAMPASPAGYKPYAADPNGGVPLMAPFGMGYRWHCTGLFHDENGAPS
SKPEVASTLMRRLEHKVGSRAEELADYFAEGLDDCELAVVSFGSSAMAALSAVRRARSEG
HKVGMLRLKTVWPFPTKTVRGLLSSAKKIIVPEMNLGQLALEVERAAGCDRPVIRLGKVN
GELFHPDEVFAAIKEAL*
>SPBIB_v1_150180|ID:27162692| conserved protein of unknown function [Uncultured spirochete bib]
MAKYIKIRDVTLRDGQQSQFATRMNNEQVKAVLDDYRASHFYAMEVWGGAVPDSVMRYLN
EDPWHRLGTIKQGIGDASKLTALSRGRNLFGYNPYPDSVIEGFCKNAIASGVDIMRIFDA
LNDIDNMKSSIRFVKEAGGLADCAVCYTVDPHFSFRDRVNALFSGKKIPPKIFTVDYYVK
KAKELAALGADMITIKDMAGLIDPVAAAQLIKALKNELEIPVDLHTHCTPGFGVASLLAA
MVNGVDIVDTAVLSFSGGPAAPAYEIIRVFADRLGIATGVDNAAVARIDRRLREIRKELS
AYDQYKKLPPELDLSKDSLPPQVSKLFDDALEAAVGGKYQKSLAICQEIEKAFNYPEPDE
IVRHAQIPGGMYTNMMAQLKEAKLEQHLNEVLHIVPRVRLDSGLPPLVTPTSQIVGVQAV
NCVIAINQGKDLYTNVSKNFAELIAGSYGKTPWPVDPEFRFKICGTREETPYDTSKYKPQ
PNPPVAEAGGALLAMNEKEQLLLELFPSVATKFLRNKRIEEWKAAHPEEAAKAEASAAVR
AAGMAAGSAPGGQEDSVLSIYESADYEPADGESPEFWEEAPGWAGG*
>SPBIB_v1_150181|ID:27162693| conserved protein of unknown function [Uncultured spirochete bib]
MQQFPFCPNPRCRWHKEAPKGKWAKAKGFYTTKAFGRIQRFQCRACHTTFSSQTFSLSYY
VKRPIPLPDVLGRITSGESLRAISRNLSVSLRLVTNRIDRLARQAIGLHAQALSARVSTD
DICIDGFVSFDGSQYFPSEIPIAITAHSQFVLDFSHASRRRSGSMTPAQKEKASLLYARC
PLERGAIAQSFRETLESALALQPPSPHRPFVLITDEKPDYRRVLHRLEAFRNQTEERRLV
HWTIWSKLPRTIHNPLFSSNYIDRELRKDLAHHHRETVCFNRNVANGMMHLALYLLAHNY
LKPFRIRAPRGSRPGSHAEAAGIPASWVRQFAHSLTGGMRMFLSRLRLSATLKRTWEKGW
KTPGKEKTEYLPKYAFM*
>SPBIB_v1_150182|ID:27162694|rtcB| tRNA-splicing ligase RtcB [Uncultured spirochete bib]

MQIERIDPFTCLIPAHDGMRVPARLYVSDQLFSLLKQDKALDQLVNVARLPGIVSYALGM
PDMHQGFAFPIGGVAAFDVDEGVVSPGGVGFDINCGVRVILTSLREEEVRPKLDAIGKKL
FARVPSGLGSKGLIRLSAKEARAAMAEGVPWALGKGFAEPADLRAIESHGCLEGSDPLAV
SDEAVNRGKEEFGTLGAGNHFLEIDRVEQIFDPETAQKFSLFEGQVLVWIHTGSRGLGHQ
IATDYIARMRPKMAQYKLELFEHDFVSIPLRDPLAGEYLAAMAAAANFAWINRQMITHAV
REVFSEEFGKPADSLGMRLLYDVAHNIAKFEDHIVGKSESQTRGTISAALTSRRLLVHRK
GATRAFPAGHPELLDLAHDAGQPVLLPGDMLRGSYILVGTERAMRESFGSVAHGAGRKMS
RHAAAKEVSFEALSRQMGQQKVRLLAADKRLACEEAPAAYKDVDAVVETVTGSGLARVVA
RTRPLLVIKG*
>SPBIB_v1_150183|ID:27162695| putative Glutamate racemase [Uncultured spirochete bib]
MGGLTVVRSLCLAGVPLDIVYVGDNANVPYGNRPLTEIVALTERMLDVLDRRGVEAVAIA
CNTISATVDMLESRSKIPLVDIIRPAARAMAGLSELHVGLFATEFTVQSGLHAKLVRELN
PSVEVHGVASARLAALIDSVPWDEAAIREEIASMIAKMRRVGPVRTVLLGCTHYPIVQNV
FEELAPDMRFIDPAGLQANEVLRAVGVATAPAKSPQLASEESAISLEVITSGDPAAYRQM
LDKLGIPPAQRFTQL*
>SPBIB_v1_150184|ID:27162696| conserved exported protein of unknown function [Uncultured spirochete bib]
MKTFKKRGQTPIFYFMIAALMLTGVQGAWAQMPGSPAPQLPSSPSAVEKRSIRVQGQASM
SVEPDIAMLTLGVETQSDTAAKAQGQLADRASKVLAALTGAGIDRAKIKTSYYRVSPVYS
TKQDKQNVIIGYKAETTIQVEISDIPAMAGLVDLTMNAGANAVRSLNFDRKNMEAFKAQL
IEAACKDAQLKAQAALRGLAGNGAGLRLGAPISVDVQDSFSARNAGEAMMFKAAASPDFV
SAGELEISVAVAVEFEILVNSSH*
>SPBIB_v1_150185|ID:27162697| putative Esterase/lipase [Uncultured spirochete bib]
MIFIHRQPQSARSMLLERAFRRAGVKESSDSEQPRFITRQYAKSLLSAEPPLHIRARLGV
QSIAVAGSRVHVLTPRFEGCARCILYLHGGSYVATFTRQHWNFLAKLAERTKSVIIAPDY
PLAPEHRWADAYRMLFALWESIGLALPHKDVVLMGDSAGGGLALGFVQALRDAHLTMPAS
VIMLSPWLDVTMENPHIQTLDEVDPFLSVEALRKAGAAWAGGSNPRRMEISPIYGHFEGL
PPMSLFIGTKDILWADCRRLRDICLVTGAHLDYYEYEHMMHVWMLMPIRESKQALDQIAG
ILTTQPECASHDAVQKFKTWGQTPS*
>SPBIB_v1_150186|ID:27162698| protein of unknown function [Uncultured spirochete bib]
MSRERFFDDRQRIIDAAVSLISEIGYENFSTRRLAAKLGISPMTLYNYFENKEEIVQATI
ADAYGKALSAIQNELKEYFEGDVACPLKGFVEIGRKLKDFSVQYPKMYALLFMMDLRPYT
DHPSIQSRYEYAFRKTAERLLDKSIEEELHRHIYLYEVLAGALVRNFHSGCGPVDEQTFE
AHLALAYDRLLKPFEQYFAECEKAR*
>SPBIB_v1_150187|ID:27162699| YbaK/prolyl-tRNA synthetase associated region [Uncultured spirochete bib]
MIPEKVQKILDRHNLKALEFEPGSTPTAETAAQRIGVQTGQIAKSLLFKGKSGRVAMIVC
AGDARISSRKMKALFGEKMSMTDADETFALTGFRPGGVCPFGIENIEIFIDESLARWDTI
YPAAGNDATGVPITVQILIQATNGQPCDLCE*
>SPBIB_v1_150188|ID:27162700| conserved membrane protein of unknown function [Uncultured spirochete bib]
MQYSVVVTSAMLSALTMPFILNFAHSRKLYDIAGGRKNHNGNIPRLGGLGMFLAFATMVA
AFSIVQTGPVSRELWSRGDKLWPFAVGAVLMHTIGLMDDLKAQPAPLKLGVQVLAALVVA
LAGYRFRGFGFRADVLTESWSWLSIVLSVGWIVGVANAINFIDGLDGLAGSISFIAAMAF
GIFYYQSGDASSSFLCLSVAGAAVGFLFFNFPAPRAKIFMGDSGSLFLGFCLAVMPFLGQ
AQAGAVGIGAGTQGAQAGLGLIPSITLLGLPVFDALRVIALRVREGVSPMKADRQHIHYL
FADSGYRPLAVIGVLDVAAIMLVAAVFLAASMPQSLGYLLMLISIGILFILFRYAMSLKP
DKAS*
>SPBIB_v1_150189|ID:27162701| conserved exported protein of unknown function [Uncultured spirochete bib]
MRRRITQAAVFLALIAILAACQISSPVVPPTKISLKPFLSLIVGESETLTPVVEPANASV
EDLDWESDDSSIATVDSTGKVTAIAEGDTVIRVATQYGLKAECDVYVATVSVRDVTLDKT
SLTLKESHMYTLVATVLPSNATNKNVTWSTSNASVVKLVKNDAGKEDGTIFANAEGTAVI
TVTTVDGNKTATCTVTVIKEQ*
>SPBIB_v1_150190|ID:27162702| protein of unknown function [Uncultured spirochete bib]
MRLKKGLRLIFVGGTTGLEIWHAANIAIKARKTAACVILLRMVFALLIEIFYLLNKYIGT
LRISS*
>SPBIB_v1_150191|ID:27162703| protein of unknown function [Uncultured spirochete bib]

LGKNQYELGASLYGNNARFSSKLLXX
>SPBIB_v1_160001|ID:27162704| protein of unknown function [Uncultured spirochete bib]
MKSTLHESEYSLLEAIYASIAQKEASGALSQRELAQASGLSLGMTNALLKRFVERGWIKL
SHLNKRKMLYALTAEGIEEISRRVVDYFSRASHNAALYRKRIDAFVRAVAEQGYTALELV
GPDELDFLFDYACEKNGLSFLKDRKGRHFSFASDQKLVIVMTGDSEIESAALPAGTLAVE
FSEILALSEQDS*
>SPBIB_v1_160002|ID:27162705| putative Soluble ligand binding domain protein [Uncultured spirochete bib]
VVEVSKAVRIVSIEGEVRRPGRYQLLPGEGVKELVSYYGDGALESGKTDLVVLTRKATSQ
KPESESVVFDLGGKGLPELMDGDRVRIPSREEYLPIVYIEGAVAGDQLALSGANTAQTLS
SNNQTLSNKNPTLSTTDQTVSGTDQTTTSKDQSLSSANQTLSSSNQNAVQATYAIIRAPF
RKGLSISSLLRPMKDKILSSADLAHAFIIRKGNQDQNYIDLERLLYANDLRYDFELQPED
RIVIPFGSMSVFVTGEVTKSSWVTITGLTRLRDVIQPFLTRYSSIRDVTVKNQDGIEKTY
DLFKADRYGDLSQDPLLRPGDEIRILPFKNLITIQGEVKRPGTYQLLPGEGLKELVERYA
DGFTEKANLSRLTIVHYLSDASPVGEKAQFDYTKNPNMPLRLYDEITVPSMQELLPVVWF
EGAVGVGSTGSTPETSQRTPYTFFPGETVSQAALANRKLFSAVSDLSNAYLLRSDGSRTK
VNLARFIYDYDLAGDVALQPGDTIIVPFRQFFVSVSGAVRYPGRYPYIPDREWSYYIGLA
GGFDTEKNSGQKITIYDAKSQKVDQANRMIQPEDNIVAASNSFTYQLMRISTILSTVLSL
VAIIVNLTGLKL*
>SPBIB_v1_170001|ID:27162706| protein of unknown function [Uncultured spirochete bib]
MHSRHLGGLFPCDSRRLHRRIDRQNPQGPRDGGEVQSDQLRKGEYVMKKFLILIMALAVA
VSAWGQFTLPSGTTSTGTTSTGSTTSQSALGGQSTQGIDTTQLLQAAGASSSTSQKTSLS
VTVPESQVRAMLAMSTPEYPVTPGDVYALSYLRATQQDTISLLVEGDGSINAGFLGRIQT
SGMTFRALKEMLEKKVIASYPSSNPSLVIVSTGLFSVNIRGEVLSSQIFTAWGLTRLSEA
VKGLATPYASLRDVIVRGRDGASKAYDLFKADRFGDPSQDPLLRPGDVIEVKKADRIILL
EGQVRRPGIYQLLPGEGVGELVRVYGDGTLVSAKTDTIVLTHKASAEKPASESIVFDLIG
SSLPALVDGDTVRVVSREEYLPVVXVEGAVQAGTVLISESAVSQAGTGGTSVAQGTXX
>SPBIB_v1_170002|ID:27162707| conserved protein of unknown function [Uncultured spirochete bib]
MTEQNECMDNAMNRAPAAQPAPSLQSPEDDDEISLVDLAAVLWKRKWLIVGITVTAVIGV
LAYAIGSLMLPPEKSYLPNVYTAKATMLIQTSSSSSLAAALSSSGLASLAGLNASAGNTN
GALAEVLATSNSTLDALNAKFNFAERNKARKPKTKDEKPPLISDIRESIKKNLSAKLDAK
TNLFVVSYTDIDPAFAKEVVDEVVRLLSERFAVLGGNKALEQKALLEKKLAEVDTAVKNL
EAQVKAFQNKYGVIQVEALATEQITILARLRSELIMKEMEIANYQKISNINDPVMTQLKN
ERDGILAKIKEIETGVGSGSRVMPSQKELPTIAFEYARLQRDLAVQTEVFKMLTQQYELA
KLNASGQEPVFQILEMAEVPDKKSGPSRGMICIVATLAAFFLAILVAFIAESIDKIRKDP
ETVARFKAISSGKESM*
>SPBIB_v1_170003|ID:27162708| protein of unknown function [Uncultured spirochete bib]
MSILTRLFFVLLADERIELFELFDFIFGADEGGVWGVDDDDVLAAEKGDEVRGVGGDGEV
AGTFDEEGFGSVLGGWCALGVPGAARRGEHGAVALRIGVREGAVGVP*
>SPBIB_v1_170004|ID:27162709|manC| Mannose-1-phosphate guanylyltransferase [Uncultured spirochete bib]
MSRYAVILAGGSGVRLWPLSRESMPKQLIPVLKGKSLLEAAFERLEGVIPPERRWACGGA
RYETVVRTKIPAIGRYIGEPEGRDTLAAIALSCALVRKEAPDAAVAFLTSDHVIEPVERF
REALRAAFATAEAHPDTLMTFGVAPTFAATGYGYLELGEQLRKRGSDPFLQMAAEEAGAN
AAIRRVRRFREKPDLATAERFVAEGPAHYLWNSGMFVFKASRLLELLAKYEPAIAEAIER
IARAESSEEQAALMRDIYPTITKKSVDYGIMEPASQDSDVTIACVPLDLEWKDIGSWNAY
GSLADPDPQGNGAMLAAARSPRHAEGTPTAQDRPETLFVESTGNLAVTTDPSHLIAFLGC
KDIVVVHTPDATLVCPKNKVEELKKLYPFVSQQHKE*
>SPBIB_v1_170005|ID:27162710| conserved protein of unknown function [Uncultured spirochete bib]
MTTLIPRPALMERIENAFAITPIVTLLGARQCGKTTIARIIAQKTQAPIFDLEDPADDAA
LSHPMLALSDKRGLIVIDEVQRRPELFPILRVLVDRPDNAARFLLLGSASPHLVRGVSES
LAGRTSFVDMQGFSLDEVGSEAWQKLWLRGGFPRSWLADSDAASLEWRKGFIRTFLERDI
PQLGISIPAAGLSRFWTMIAHYHGQVWNSSVLARSLGISDKTVTRYLEILEGTFLVRRLP
PWYENLGKRLVKSPKVYLRDSGILHALLGIADMDGLLSHPSVGASWEGYALETLLSRFPG
AQASFYATYSGAELDLVLDLGTTRYGFEMKLNQAPTLSRSMTIALEDLGLSHLYVVVYPGT
KTYPLAESITAVPLQSVPEIAE*

>SPBIB_v1_170006|ID:27162711| conserved protein of unknown function [Uncultured spirochete bib]
MIPRIELASLLDDLSVFPAVALLGARQAGKTTLAMEVQKLRDALYLDLESERDRAKLAEP
ELYLSEHLDRLVILDEIHRVPQLFPVLRGLIDQRRRAGRNAGLFLLLGSQSPELLRQTGE
SLAGRIAYRELTPFWVLEVPKGQDDRLWVRGGFPESFLAESSARSLRWRQDFIRTYLERD
IRQFDVRLMPETLRRLWVMLAYLQGSMVNVAQLARNVDVDVKTINRYLDLLTDLFLLRRL
QPWHVNIGKRLTKAPKLYIRDSGLSHALLGIEDREALLSHPVVGPSWEGFVIENLLTAAG
ANYQSYYYRTASGAEIDLVLVPPDQRLWAVEIKRSLTPKPERGFYIACEDLKPAQRFVVY
PGDERFPLGHDTEAISLRELAGLLRGG*
>SPBIB_v1_170007|ID:27162712|mazF| mRNA interferase MazF6 [Uncultured spirochete bib]
MMRGELWWADFGLPFGSEPGFRRPVVILQDDAFNTSNIHTTIIVPLTTNMALAEAPGNVV
LPKDESSLSKDSVLVVSQLSAVDKQRLIERIGTLKRQTLEEVEEGIKLILNLT*
>SPBIB_v1_170008|ID:27162713| Ribbon-helix-helix domain protein [Uncultured spirochete bib]
MKTAISLPEGLYKEAEKTAKSLGIPRSQLFAKALQEFITRHAKDNITEDLNKVYSNQKQD
VHNLNVENASIESIRELTKNDAW*
>SPBIB_v1_170009|ID:27162714| putative ATPase (AAA+ superfamily) [Uncultured spirochete bib]
MIQRPLYLEKIRPFMNTDVVKVLTGIRRCGKSVMLELIQQELVQNGIAPSQIIAINFESQ
ALPFPRDIQGAYTYIKAAVAKAAVTATAANGEATRSGRKIYLFLDEIQELEGWERLVNSC
MIDFDADIYITGSNATMLSGELATYLGGRYVEIKVYPFSFREVLDMLSAGGSQLSEAEAF
RRYLVFGGMPFLYRYAFDDASTLQYLNDLFDSIILKDIAQRNHIRDIDKLKRLILYLVAT
TGNTFSARSVMDYLKNEKRSISTETIYNYIEYCKEACLIHMVPREDLIGKRLLSTQEKIY
LVDHGIREALYGNNQRDINQTLENIVFMELVRRGYEVTIGKQRSLEVDFCARKGGTKVYL
QVAYLLADDATVEREFSALETIPDNYPKFVITMDEIDRSRNGIQHYNIRRFLLEAR*
>SPBIB_v1_170010|ID:27162715| ATPase (fragment) [Uncultured spirochete bib]
MELRRLTSDIYYFFDNAAECDFVVNAHREKPLCMQVCSHLNHDNEAREIKGLMKALSFFD
LAEGYIITAAARDEIITQGKKLHVIPAWEDLAATCGLSR*
>SPBIB_v1_170011|ID:27162716| protein of unknown function [Uncultured spirochete bib]
MSIDNKVAFSRVVKKIIDVAGEAAQFHEYEIFMSFISLLLEYMFQ*
>SPBIB_v1_170012|ID:27162717| conserved protein of unknown function [Uncultured spirochete bib]
MPKVRPNPGTRSVQWRLLVLCEGAKDKSESAYFKALINDRRNPDHRLEVKVVDTKKNTAR
ELVREAKKLKATNKDQVWVVFDKDGYTLHAQAFDQAAANGIKIAFSSICFEYWLLLHFGY
TTRPFANCSELIAYIRTEYGYDYDKSSPLTYAKTKHLLAQAKTNAQRCRKYQLQSNPADF
PIYEMNPYTNVDELVTAIEQFSVEEA*
>SPBIB_v1_170013|ID:27162718| conserved protein of unknown function [Uncultured spirochete bib]
MLIDFTLENYLSFKDKATISLVVPGWKEPEHTRYVLEVERGKYALSPFAAIYGQNAAGKS
NVIKALMDFVNLVLYSHTLGIDAPIPSYKPYKLDISRQSAPILFETEFIAEGIRYLLNVI
FDRNSILQEELVYYPQGRRALLYSRKKGESIRFGTGFKGTKKGLEQFLRPNALFLSTAVN
LSNEQLRPVYKYFQTKYRFHVAMDSSEHPIMGTTLTLLAEDPIKREGYKKAILSFLNAAD
ISVKDVVIRKDETLIKNIVFPPDIPQQVKDKFIENLVNRPFLGHTLYKGAESMGENVLFD
LGKEESGGTIKMYELAGEVIESLKSGGVLLIDEFSSGLHPQLCEFIVGLFRDRSINTKGA
QLIVTSHDTNLMARDDIYRDELWLVDRDSHGTSELYSINDFSKDEVRKGTNLEKWYLDGR
FKAVPSIDPSAFKLEVSD*
>SPBIB_v1_170014|ID:27162719| conserved protein of unknown function [Uncultured spirochete bib]
MPTLQVRDLPEDVYYVQISYLAEKEHRSLAQESIVLLKEGIAAKLGNKERRRKLLEKMSDL
TVDGKAFPDPVDLIREDRER*
>SPBIB_v1_170015|ID:27162720| conserved protein of unknown function [Uncultured spirochete bib]
MIIVLDASAAVEIALNRAHSTQLREILRKADLVLAPDTFPSEITNVFWKYASFSAMPIDQ
CEKGIDYCIDLIDEYREIKTMCREVFSESLNNNHPSYDLFYLVLARRFNASILTIDKKMK
ERAQAMKIKLAYEDS*
>SPBIB_v1_170016|ID:27162721|rmlB| dTDP-glucose 4,6 dehydratase, NAD(P)-binding [Uncultured spirochete bib]
MRSLSTILVTGGAGFIGSNFIHWLFAQPEFAGRVINLDALTYAGNLENLADIEARYGKGA
GGLGAVPRYVFVQGDICDRPLIERLFAEYGIDTVVHFAAESHVDRSILGPEAFVRTNVLG
TYTLLDVARQAWCAPTGGMREDVLFHHVSTDEVFGSLGEEGYFTESTPYDPRSPYSASKA
ASDHLAKAYHHTYGLPLTLSNCSNNYGPYQFPEKLIPLMILNMLEGKPLPVYGDGRNIRD
WLYVEDHNEAVWTIMRKGQSGRTYNIGGENEWENIRLLERLIELVAEEARLDIARLRGLI

TYVKDRPGHDRRYAIDCTRLKTELGWHQRATFDEGLRATVRWYLDHREWVEHVRSGAYRA
WLEQNYARRV*
>SPBIB_v1_170017|ID:27162722| conserved protein of unknown function [Uncultured spirochete bib]
MATNLAIDDRLLNEALEIGGYKSKKDTVNAALEEFIKRRKTNDLINIFGTIEYDSSYDYK
KMRSR*
>SPBIB_v1_170018|ID:27162723| conserved protein of unknown function [Uncultured spirochete bib]
MKRVLVDTSVWSLALRKKEWTKEEQLVVDHLSKLIRNLNVVLIGPIRQEILSGISQKEKF
DLLKEKLSIFEDHAIDMHDYELAAEFYNECRHHGIQGSHIDYLLCAFAVNHDLTIFTLDK
DFQNYKEYLNIQLENFV*
>SPBIB_v1_170019|ID:27162724| putative Glycosyl transferase group 1 [Uncultured spirochete bib]
MKIAFLTQYYPVSDSTRSLYSDLMDEISSFGHEITVFRSEEERVFGRPEVSSRKNITIVS
VPTGRVTKTNIMIKTANILLTEPRYLGAMRMHYAGSIPDMIIVTTPPITFCSAASYYRKK
GSMVYLLLKDIFPQNAVDLGMMKKESLVYSFFRAKEKKLYKNADVIGCMSEENKKYLLHH
NLFLRNKKIEINPNSIKPSTFQIDAKQGDELLREYKIPREKIKIIYGGNLGKPQGIDFLL
EVLDKSKEIENVHFIICGSGTEYSKLEKFVKTHPDHPITLIKYLKNSRYRLLLSQMDIGL
IFLDRRFTIPNFPQRVLDYFDFLLPVLAATDKNTDFGKMLINNKCGLWLESGDIDAFMKN
LEYLVSNPEIRISMGKNGRALLDSEFSIKKSAQLIIDKFNLLSDPIS*
>SPBIB_v1_170020|ID:27162725| putative UDP-N-acetylglucosamine 2-epimerase [Uncultured spirochete bib]
MKTKLKVVTVIGTRPEIIRLSEVIRACDKAFDHILVHTGQNWDYTLNEIFFKDLELRAPD
RYLNVVGADLGETMGNIIAASYKMLKEIQPDALLVLGDTNSALCAIPAKRLKIPIFHMEA
GNRCFDQNVPEEINRKIVDHIADINLPYTEHSRRYLLAEGFRKEHIFVTGSPMPEVIHRN
MEKIKSSKALETYGIERGNYFVLSMHREENIDIEYKFNGLVKALDAIAETYGLPIIFSTH
PRTWKRLEAAGIKLNPLIKQVKPLSFTDYCRLQVDAKCVISDSGTLSEESAILRFPAVLL
RNSTERPEAVDKGSIVIGGYTAESLVQSIALAIDFFDRKDHRSADYVDEDVSAKVVKIIQ
GYTPIIKKVIWNK*
>SPBIB_v1_170021|ID:27162726| putative NAD-dependent epimerase/dehydratase [Uncultured spirochete bib]
VRILVTGANGFIGKNLLARLGREKDFEILPFDVENREEDLYEYLKNADRIVHLAGINRPL
TPEEYMQGNFGLTSRIAEFLEKQNKQIPVIFSSSIHAERDNEYARSKRAAEDRLKLYAER
TGASVSVFRFANVFGKWCRPNYNSAVATFCHNIAHELPITINDPSASLRLIYIDDVVDAI
VRELEHPYEGFAFVDAGPVYETTVGKVAEQICMLHEYRPKGFVPDFSDEFLKKLNTTYLS
YVETDKLALEPEIKSDNRGWLFELMKSPHAGQIFVSTTRPGFIRGNHYHDTKVEKFCLVK
GRARILLRKIDAHEKIIYDVDDSRIRIVDIPPGYTHSIENTGSEDCIVLFWANEIFDPSR
PDTYVLEV*
>SPBIB_v1_170022|ID:27162727|capD| UDP-glucose 4-epimerase [Uncultured spirochete bib]
MKASSTLMITGGTGSFGNAVLRKYLKTDIKEIRIFSRDEKKQDDMRHLYKDPRVKYYIGD
VRDIESVRSAIKGVDYIFHAAALKQVPSCEFYPVEAVKTNVLGANNVLTAAIEEGVKRVI
VLSTDKAVYPINAMGMSKALMEKVMIAKSRELDNGKQVFCGTRYGNVMASRGSVIPLFIE
QIMQGKPITITDPEMTRFLLHLDEAVELVEYAFEHGKNGDIFVKKAPASTLADLATALKE
LLHADNEVKIIGTRHGEKLYETLVNREEMAKAEDLGDYYRIPADTRDLNYDKYFSNGDRK
VQEVVEYTSHNTTRLDVEGVKKLLLTLDCVQDAMKERGLL*
>SPBIB_v1_170023|ID:27162728| conserved protein of unknown function [Uncultured spirochete bib]
MKILQINSVFNYGSTGKIVEQLNSLIMKQGWTSYVAYGRHFHNSILKKFKFSSTLEVIIN
ILETRIFDNHGFGTQHATKKLVSYIEKIKPDVVHIHNLHGYYINIQELFKYLKNSSLPVI
ITLHDCWTFTGHCAHFDYIGCEKWKEHCMKCPQIRSYPASFFIDRSYKNFELKRELFNSI
SNLTIVPVSEWINGLVQQSFLASKSRVVIKNGVNLDLFKPLNSRQSIINKYNLQGRYIIL
AVASIWSARKGLNDILCIADNIGNEYLFIIVGDRINKVASKRSNVKIIRKTEDQEELVEL
YSAADLFINPTYEDTLPTTNIEALACGTPVLTYNTGGSPETIDSETGFIVPKGDIDAMIQ
VIRSFSQKGKDSMTVACRKRAEKYFNKDERFMDYIKLYERILKGEK*
>SPBIB_v1_170024|ID:27162729| putative glycosyltransferase EpsF [Uncultured spirochete bib]
MINKIKILHIVSDLNSGGVENMLYNYINYIDNSKFDIAFVSHSYGGMINDQLLKAGFRVF
YVTPRKKNFIKNILDLWKYMHSDNWDIVHSHINQMSAIPILLARISGIRVRIAHAHGSKI
YNDKIKKIIYNILSKIILYNANYLMACGTDAAVRVFGKRIVRQKKVIYLKNAIDLEKFSY
NKKSAQYLRSNLGINERYIILHVARLSEEKNHIFSIEVFCKLIEKNPNMRLLIIGEGPLK
EKLKQKVLLLGLDNKVSFLGARKDVSSFMSLADCLLLPSFHEGLPLVPIEAQANGLQCYL

SKNVPEEVVLTEKACRLDLSLGPAVWAEYIYNNLSKERISDKKIIQKAGYDIKLAAKKLE
NFYIVSATKMGKILYLSTLFNC*
>SPBIB_v1_170025|ID:27162730| membrane protein of unknown function [Uncultured spirochete bib]
MSNIYNIKKEKISYYLSILCMITIFNTDSIRVFGNLSIFYIILILYLFYELFSGKIFLKH
INKYINIIGISSVGFIINILISDLFAKELNIFVIIGNIYPLIGTIIFLFLINEDNKKYFV
DYSVYTNIFYISCQIHAIYGLLQAILFIFFGIRTNNVRGSILNLRVSGLFPDANIYSFSV
LMALSFLLSKENKNSNIITIIFRKILIIINLIVIFLTGSRTAFIVAILIIILNFYFKIKN
IKYKIMFIFFLLFLIIININNIINLIDYYTYNILGRNIDVESDIRISILWKTALEIFFKH
PLIGAGTDAVMRQTGTLAHNMILALLSDRGIIGTLIYLFPLIYLFLKAKKGNKENILMVI
SYLLASMGISNWNLRIPIILLLFNIWELEINRK*
>SPBIB_v1_170026|ID:27162731| conserved protein of unknown function [Uncultured spirochete bib]
MKVLLISYFFAPQNTIAAVRTTKIAKYLIRLGYDVEVVCGPPTFIDPILKSDLKEIKKLN
VVHGLKMYEKKSKEFINNNNVYKNSILKKILRKIKSILYKVLPNPPPVYLAFLNSLIWVY
NARKILTNKIFYYDIIISSYGPIGSHILGLYLKLRHKKIFWIADYRDPMIDDKQKSTIRF
IYSIYQYLSLKYADISLCVSKGLKEELASLQANAKLYIITNGYDNEDMSFLNQEQLNKNK
LIFSYAGSMYAGKRDLSPLFSILNELAIEKKIDIKLIKILYMGNEFSTIKEMAKPYELDE
ILVDLGFVSRDKALNILNNSDVLLLASWNTKKSQGILTGKIYEMILLKKPILAFINGYAK
NSELKEIIVKLNGGFVYEEGDKDSYNSIKDKIASLYKQKFESFFISQEYNMEFRAKYDYK
NIVKTLNNIIHDNKK*
>SPBIB_v1_170027|ID:27162732|wbpA| UDP-N-acetyl-D-glucosamine 6-dehydrogenase [Uncultured spirochete bib]
MTLSEQLLEKIEKNTVKVGIIGLGYVGLPLAVSFARKGISVLGFEKSEKKAQAVNEGRNY
IGDVDDTELARLVQEGHLSATIDFTRIAECDAVIICVPTPLDKFKKPDMSYIERACVDIG
RNMHRGTFISLESTTYPTTTEEFMKPIIERESGMKEGVDFWLCFSPERVDPGNKTYKTDN
TPKVVGGLGEEAQKIALALYGKAIQYLYPVSSPRAAEMVKILENTYRLVNISLINELALL
AGKMQINIWEVIEAAKTKPYGFQAFYPGPGIGGHCIPLDPFYLEYVAKGLNFDLTMIDAA
GKINNLQPYRMMNKIAFALNRHKKPMNGSTILFLGVAYKPDIDDARESPALEVMSLVAHK
GAKVQYHDPYIPEVTDEHGRHWQGVELSDEILASADCVVFTTNHSCFDIEHIVEKANLVV
DMRNAVERVNIENEKVFKL*
>SPBIB_v1_170028|ID:27162733|wbpD| UDP-2-acetamido-3-amino-2,3-dideoxy-D-glucuronate N-acetyltransferase [Uncultured spirochete bib]
MRNNELIEMPAAGGGIYYRHESSYVDEGAQVGEGTKIWHFSHVMAGARIGARCSLGQNVN
VGGRAVLGNTVKVQNNVSIYDDVIIEDEVFCGPSMVFTNVINPRAFVERKHEYQETRVKR
GASIGANATIVCGVTLGEYCFVGAGSVVTKDVPAYGLVYGSPARQHGWVCRCGVKLGEDL
VCPECGKRFAKNEKGLEERV*
>SPBIB_v1_170029|ID:27162734| putative nucleic-acid-binding protein [Uncultured spirochete bib]
MAKRVYLDANVLMAMFRGTHDIAHKAFEVINDNEIEIVCSDALWLELMPKPLFNKNQEEV
EFYQAIFDQSEYAKWDLDVLNDAKNIAKSYGIAAMDAIHISTAINKKVDVFYTNERDTKP
MFRVSDIKIISLATA*
>SPBIB_v1_170030|ID:27162735| protein of unknown function [Uncultured spirochete bib]
MNAVSYKEKDMSSFFDTSRIYAYAFHRNKEVSKVYHFVYDDAFDDLSTNATSIRYILSKN
VVNRESNIPTSIVPKTELVRKLLAIRKKYMENGGKLLSLEEIAEEIQNRRGGLNNGQASI
P*
>SPBIB_v1_170031|ID:27162736| conserved protein of unknown function [Uncultured spirochete bib]
MEVKDLPAYLERSPLVEAIWEIRFNVNPALSIGDLLPGIVFVHLDAHSKGYVLNQLPFVQ
IPRDIIDTNENFKFQAKYRIESTKSPYLYHIGEHMVSLNCRKPYEGWGAFKNKIIELIGI
LKRAPFEFIEVKPSLRYINFLPQNVIPSLSPWLRIKLNIGDFLITMTPLQIRAELPETEC
THTVQIVHPAQLVMHEADAENKGIIVDIDSVLNNTQHSLDDTVAQIEMLHAKNKEFFFHQ
ILTEDAIEKFGPKYTEEASV*
>SPBIB_v1_170032|ID:27162737|degT| Pleiotropic regulatory protein [Uncultured spirochete bib]
MNVPFYTSTREYQNRKAEFDAAVRGVMERGDFILGEEVAAFEREAAEFLGARYAVGVASG
SDALVIGSDILDFKDGAEVLTPTFTFFASTSCVARLGGKPILVDMDPKTLNMDLADAERK
VTKKTVGIIPVHLFVQPTPMQKVMDFARAHGLKVLEDAAEAWGMESRVDGSWKKAGTIGD
IGIYSFFPTKTLGAYGDAGLMVTNDEELYKKIKSYRVHGSSVKYHHDYIGYNSRLDTLQA
AILRVKLQRTKEAIAARARHAAHYTERLSRLPEVRIPRVAGGNRGVYYVYNILVPQRDDL

AAFLKEKGIGTSIYYPLPLHLQKCFSYLGYKEGDFPVAERVCKEILALPIYPELRDDEVD
YVCDAIASFYK*
>SPBIB_v1_170033|ID:27162738|wbpI| UDP-2,3-diacetamido-2,3-dideoxy-D-glucuronate 2-epimerase [Uncultured spirochete bib]
MLEIMTIVGARPQFVKAAVFSRQVRARPYADKIRETIVHTGQHYDENMSDIFFREMEIPE
PDVNLGIGSGTHGAVTGAMLAGIERLILERKPDLVLVYGDTNSTLAGALAASKLHVPVAH
VEAGLRSYMMIMPEEQNRRLADHLATWLFCPTNTAVENLAKEGIVHHGLAHPSADNKAVL
QCGDIMYEASRYYRPKAMEQIEARLRALPDRFALLTLHRAENTDDPTRISAIVRALNRFD
SIEFVFPVHPRTRKILAQYGLELKSHIHAIEPVGYFEMLALEDKCSFVVTDSGGVQKEAY
FFQKPCITLRDATEWRELVEHGWNTLTGADETAILAALASMPKYGDDVTLYGDGTTAARI
ADALLSWQKVR*
>SPBIB_v1_170034|ID:27162739| conserved protein of unknown function [Uncultured spirochete bib]
MIKKKNSTSKSWIVRNMAEIMKSIAIIGCGRISFKHIEAAIKHADRISLVAVCDPIIERA
EQRKTEYQKAFPGAQVAVYADYRQMLEQEKPDICAIATESGYHPRIAIDCLEAGAHVICE
KPMALSTQDADAMIASSRRTGKKLAVCFQNRFNAPVQKARRALEAGRFGRLLHGSIQVRW
NRNESYYAQAPWRGTWELDGGTLMNQCTHGIDLLQWMMGEDAVRVQAVTRRFLRPIEAED
FGAAIVEFASGAVGIIEGSADIYPANLNETLSLFGERGSVVIGGLAVNRIETWRFADAPQ
VGDTEEAVLNPDEKDPPTVYGFGHSALYADFLDAIEQDHEPLVSGEKGKKALEIILAIYK
SQKTGRPVELPVDFFTLEMKGTFDA*
>SPBIB_v1_170035|ID:27162740| conserved protein of unknown function [Uncultured spirochete bib]
MITIYIPNNNKKECEYIINIFFREIFDIEYCINYIEYSDYYEILIDNDRKIYIKDSFFSK
FLKEKEYLDKKYIPLDIKFAESNLFHTEKLPVIYGEDLIRIGDDNCTIYCDIDIFASAFF
MLTRWEEYVNKARDQHDRFPGIESIAYKYGFLDRPAVNEYAELLWSMLKFLGYNRPRKEH
QYQMMLTHDVDEPFEIAYKPLGLILKNIAGDVIKRKEYAIAVKKAMDLFEKTENKLSFDN
FFSFDYIMEQSEKRGLESAFYFLPSGSPEMQFRPKIENPTMKNLLKSIHQRGHEIGIHGH
YETYRNEKAFLDDVELLKKALNENRIEYQIKGGRQHYLRWEMPTTYYNYQNAGLEYDTTL
SYADVAGFRCGICYEFTPFDFLKREKLKIKERPLIAMECSIIDERYMNMGYTERALDYFK
KLKDQCRKYNGNFVLLWHNSRLRNDQEKKFYEQVLDC*
>SPBIB_v1_170036|ID:27162741| protein of unknown function [Uncultured spirochete bib]
LGKNQYELGASLYGNNARFSSKLLXX
>SPBIB_v1_180001|ID:27162742| Glycosyl transferase [Uncultured spirochete bib]
MNTNNPALSIIIPYYNSKEFIEELLSSIPRNKQNEIEVIIVDDKSDNDHVLYLEKLILKY
NNYLNINLYFNKGVKSAGTCRNIGLDFAKGKWVLFADADDYFKKDFYIIVSRYFQSDYDI
IFFTPDSFNIRTRKRGIRHISYENLIWEYLKNPNKENTLKLKYYFRTPTSKMIKMKVITD
NKIRFDEVIVCNDDMFSVKLGKTIKKICATKESIYIITEMDNTLSKRRGKNYYLAFLDVN
IRVYDYLKRNLLENEFKILGYDSIAINILIIAYRIYKINIYLLIYTLKRLKENNIPLFKP
IVQKIKKYFFRALPQ*
>SPBIB_v1_180002|ID:27162743| protein of unknown function [Uncultured spirochete bib]
MSYENLLEILIPTYNRKRYLEKNLKILSNIIRNINAQNEISILISDNSSSDGTKEMLEIF
KINNSDIKINIYIQKENIGLKNNSLFVLKKAIAKYVMYLGDDDYISEEYLLKIKNIISNT
NCVTCIVPSNIAIDLQGNIIPKRGRDFKKSTKIYKKGYLTASQFMWRGHQLSGIVFKRKD
TLGNYYKNCEDNIYLFMYFVGFNAIRGDLIHLTEYPVKVTAAPQELKDWNYGKDGLVSER
FKNSYCLFKNKLIYKFIAEYYLTMNNNRGFLYKILKNDIFSFMIYFSNYLKCKYVSIIGK
IFFIMLTIYDSCRIILNKIIKEVIKRK*
>SPBIB_v1_180003|ID:27162744| putative O-unit flippase [Uncultured spirochete bib]
MREKINLSNINKLFTKDVISTYLNQIWRIISGPLSLILLPMFITPEIQGFWYTFSSISAL
AVFADLGFTTIILQFSAHEFAFLQFSESLDLLGPEEYKKRLASLFRFVLKWSTSIVIIAF
PVIFIIGYILFIQKANIFVWLAPWMIYSIGAAIGFYTNVISSFIQGCDQVSNVQKLSLQT
SIVGTLSMFLSLVLHFGLYSISISLLLANLYNLGEILIKYQKFLKTLWKTEPDKTNWKHD
ILGLLWKYALSWSSSYLIFKIYTPLMFQFHGPVEAGKVGISISLITSMTSLANVWISANI
PKINILIAKKDWIQLDRNFKKILILIVVTYIFEIFVLIFIIMIFKQNIFFIKIFSRFVSI
VPMTMLITGWFFQIIISCLAAYLRGHKQEPYVLLSITHGIIIALSTYVSAKYLPVNLYFS
GFFLSYLIIFPWAIYIFIKKRKEWHL*
>SPBIB_v1_180004|ID:27162745|rfbE| CDP-paratose 2-epimerase [Uncultured spirochete bib]

MTYLITGGCGFLGSNIAAYYINRGEKVIVFDNLYRFGSEKNLEWLHSLNGYFKYIKGDIR
NIYEIEPVIKENKPDIIFHFAGQVAMTTSIENPYLDFEINVKGTINVLESVRKYIPETII
CFSSTNKVYGDLKYIEFEEQDLRYIAKKYTNGFDETMPLDFHSPYGCSKGAADQYMLDYS
RIYGLNTIVFRHSSIFGDRQFSTKDQGWVGWFCQKAYEIKNGILKEPFTISGTGKQVRDI
LFNSDLIECYNLAIQNIERTRGQVYNIGGGMENSFSILELFKVLENDLNIKMIFKKLPWR
ESDQKVFVADISKAKRDFNWFPKIDKYEGIKRMYKWVEAINGQ*
>SPBIB_v1_180005|ID:27162746| NAD-dependent epimerase/dehydratase [Uncultured spirochete bib]
MDKLLIVGGFGFIGKNLVEYLYEKYNIVIIDKKIDKEFFIKFKKELKFYQYDFITEEMFK
LHKIIEKENPNYIINLISIVTTERNLEIFEEMIKINLNVLLKLFNCSKKLSSLKLFIQFG
SGEEYGNILPPFKETDRENPNSPYALAKQLTTNSSIMLNKNYDFPIVVVRPGNLFGKYQN
ANKFIPYTISQLLKNEEINITLGEQKRDFIYTLDFSRGIELLMENYKYFIGEIVNLSSGK
SISLKEIVLFSKEYIKSNSKINFGAISYRENEIKNFLLDISKFEKGVKQKFEIDIMQRIK
EYIDYLKGD*
>SPBIB_v1_180006|ID:27162747|rfbG| CDP-glucose 4,6-dehydratase [Uncultured spirochete bib]
MIDHDFWKGKRVFLTGHTGFKGSWLSIWLNELGAGVYGYALEPPTDPSLFVAADLENKIH
SVKGDIRNFENLKKAVLSAQPEIVIHMAAQPLVRLSYEQPLLTYETNIMGTANLLEAMRG
CASVRSVVIITTDKCYDNKEWLWGYRENDTLGGYDPYSSSKACAELVVGAYRNSFFNPKD
YGRTHHVAIATARAGNVIGGGDWAKDRLVPDIVKAISEGRKVLIRNPEAIRPWQHVLEPL
SGYLILAERLYTKGTEYAEAWNFGPYDADAKPVRWIVEKLCSMWPDAAGFEIDIAPKPHE
ASYLKLDCSKSMNRLGWKPTWNLETSLSKIMDWNLAFLRSEDIYEVSIKQISDFEADNTN
NINLKT*
>SPBIB_v1_180007|ID:27162748|rfbF| Glucose-1-phosphate cytidylyltransferase [Uncultured spirochete bib]
MKVVILAGGYGTRISEESDLKPKPMIEIGGKPILWHIMKIYSHYGFNDFIICCGYKGYVI
KDYFHNYYLHQTDITIDLGKNQIEYHDSSSEPWRVTLVDTGLNTMTGGRIKRIQPFVGDE
PFMLTYGDGVSDIDILALLNFHKQHKRLATLTAVQPSGKFGALGIDSQNAVIQFQEKPKG
DGAWINGGFFVCEPGIFDYIRSGDETIWERQPLEDLSRDGQLSAYKHENFWQPMDTLRDK
NELERLWATSNAPWKVWE*
>SPBIB_v1_180008|ID:27162749|rfbH| Lipopolysaccharide biosynthesis protein RfbH [Uncultured spirochete bib]
MAKTEKELRAQIAELVKEYYDIKFAPKPFEPGKSMVRYAGRVFDEKELQSLVDASLDFWL
TSGRYSEAFESAFCAFTGAEYAFLVNSGSSANLVALASLTSPLLGDRRLKRGDEVIAVAA
GFPTTVNPIIQYGMVPVFVDVEIGTYNIDVRQLEKAISPKTKAIFLAHTLGNPYNLDAIM
KLVEKYNLWLIEDCCDALGSTYKGRLAGSFGHIATCSFYPAHHITMGEGGCVFTSDETLA
RAARSIRDWGRDCYCAPGESNTCGKRFSATYGTLPKGYDHKYVYSHIGYNLKVTDMQAAI
GVEQLKKLPKFIEARKANFKAWTEGFKKFGDAFILPKATEGSDPSWFAFPVTVSENAGFT
RTELTDYLNNHGVETRNLFAGNLLRQPAYIGIEHRVAPGGLERTDRIMNDTFFLGTYPGL
KKEHIEYTLGIVEEFLKSKGVA*
>SPBIB_v1_180009|ID:27162750| putative Nitric oxide dioxygenase [Uncultured spirochete bib]
MSNPVKLKAEVLFIKSFGPDTYSVKFKPASNPPRFKAGQFLHLAIDDYDPAGGFWPESRV
FSIASAPGMPELEIVYSVKGRYTRRMADHLSPGREVWLKLPYGNFNIDTSIAPGQDVVMI
AGGTGISPFLPYFHGLLANNQKGRTVRLYYGIRENSMLLAAELIEHCARAGLIEASLFVE
NEKPNLHLPSFIRQESGRLDIRQIHAESSNLNNSAFFISGPPAMITAFKDALSQAGISPE
NIKIDEWE*
>SPBIB_v1_180010|ID:27162751| protein of unknown function [Uncultured spirochete bib]
VRARLPDIPLRSFTLDEIHELADYTTLYGSLLKDAYAEYLTFGGYPGPVLAPAPLRLDIL
KELLSTYLHKDVAGYQRVEHIGGFNNMVRLLSAQIGSQLNKSELGSTLRLNAETVNRYLD
ILEGTFVFSLVPPWFSNPRKEVSKMPKVYYVNDPGILLATGARPAIRSPYDLLDGHAVENA
AFLTLARRFERIRYWRTTGGAEIDFIVETNRGPLPIEVKFSAATSTEPVALRNFRSTYPD
ARQGIIVSRDAISSKGVPLIPAYVMDFVEV*
>SPBIB_v1_180011|ID:27162752| conserved protein of unknown function [Uncultured spirochete bib]
MQRTLMMKLLEWKARPSRMPLLLYGARQVGKTYLLQEFGRTCFQDTIYINFETDSRLASE
FSGDIDPHRLIGVLELYFNRKINPDSTLIILDEIQACERALTSLKYFCERAPQYPIVAAG
SVLGVTVHRNQHSFPVGKVEMLTLYPMDFEEFLRALGQEALIDVIREAYANHAPLPGALH
EKALELFKTYLMVGGMPGAVLAYLKDGRMLDALSVQSLILSSYVADMSKYASPAETTRIL
ACFDSIPAQLAKENRKFQYKVVKKGGSATIFGPSIDWLVAAGVVLKCDRIEHPFIPLAAH

RDLSAFKLYMADTGLLLCKSGVPASIMFSGWDNSTFSGAIAENYVAASLKAKGYPLYYWE
SKGTAEIDFILPRGEEVIPIEVKAGVHTKSRSLGVYRAQYKPDHVIRISHRNFGVENGIQ
AVPMYAVFCM*
>SPBIB_v1_180012|ID:27162753| conserved protein of unknown function [Uncultured spirochete bib]
MKNRKIGSSILEIADKPGSRILVITGARQTGKTTLARHLFPEYTFLSIEDPVLRSSYANL
TALQWKDLYPHAILDEVQKEPRLIESIKSVYDQWEEPRYVLSGSSQLLLLEKVRESLAGR
CSIIDLYPLTIPELETESWNDPVADSPFQSMLRFPEEIPARIQSLLPDFRLDPNHARIMH
AWNHYVRFGGYPALTNPDMEDEDRYRWLANYVRTYLERDVRDLASFRDLEPFVKLQRVIA
LQTASLVNHSAIGQQVGLSSKTVQRYLRYLEMSYQVVVLPAWSGNERKRLVKSPKIHMLD
YGVLQAVVQKRGSPTGLEFESMVVAELYKQANTILSDARFTYLRTLDGREVDLLIELDAG
YLAFEIKTTEHASTADIRHLRDLGDILDKPLLHAFVLSNDPTTKTLAPGMTAVHAAYLLG
SETG*
>SPBIB_v1_180013|ID:27162754| conserved protein of unknown function [Uncultured spirochete bib]
MRTISFDVPEIDYLQLMEKLKGYAYPRDKVTKLLRSGELVRIRKGLYLSTTTPVPYSKEI
LANLIYGPSYVSLEYALQRHGLIPEAVRIITSVTTGRKKRYSTPVGIFDYVHLPERCFAP
GMQYESLDRRRSYLIASPAKALFDTLFLRYPAVRSAEIESHLFENMRIDEEEFSRIDFSG
IELLLEQCHRPSIRALRQLVQRRKPHG*
>SPBIB_v1_180014|ID:27162755| protein of unknown function [Uncultured spirochete bib]
MADTLSVLVEKRHPQSLDEYDSAEVMYRIREGMDGMGVRRDDGIGQDKRDGFWGRFLK*
>SPBIB_v1_180015|ID:27162756| conserved protein of unknown function [Uncultured spirochete bib]
MMDNEEITRKIIACAFQVHNSLGGGFLESVYRNALLIELSGAGLSVIKEKPISVFYQNHL
VGSFYADIIVEDSVVLELKAIEALAKVHEVQLVNYLKATGYDLGLLINFGPLRVDVKRKT
RVFMPGLDRARRDEQDGTG*
>SPBIB_v1_180016|ID:27162757| conserved protein of unknown function [Uncultured spirochete bib]
MMKFETLLALFGDQVIFDFTSVLVLCGESRESTRVALHRMKKAGKIIELKRGLYAFAAPY
RRVPLNAAALANLLYAPSYLSERWALSWYGVIPEKTAIYTSVTTRPTRSFENAMGVFQYR
TIQTTLFGPYRTDTIMGQQVSIAPPEKALFDLWYLEHGEWTQARMEAYRFEPRAVDTAAL
RALIAASDLPRLARAMRVWEDYAREAAQGVII*
>SPBIB_v1_180017|ID:27162758| conserved protein of unknown function [Uncultured spirochete bib]
MKNDALGLVHSIADPTDRLNVLREYIQALILRSLHESQAFTSIAFFGDTALRFLFNLPRF
SEDLDFSLELEKEYRPVVVWMQKIKRDLQFQGYEVSISWNDRSAVHVAWVGIPHLLKEAGL
SGHSEEKLSVKLEFDTNPPKGAVCASTLINRHTLFAVRHYDLPSLMAGKVNALLTRSFCK
GRDWYDLVWYRSRRPPVEPNLELLRNALAQQGVHLDGDWRTLVRDRIKHTEFEVITKDVA
PFLESHEDAKLLRPEYITALLEEGLG*
>SPBIB_v1_180018|ID:27162759| conserved protein of unknown function [Uncultured spirochete bib]
MIKDMILRQKYEKEKIVKGNYVERKERSTAKKWLESDLIKVITGPRRAGKSVFAFMLLKE
SPFMYFNFDDESIRLRNEFNTDELIKSLHEVYGQSKIVLFDEIQNLPQWELFVNRLQRQG
YNLIITGSNSKLLSKELSTALTGRHIPIEILPFNFREFLSAKHFDFKEEFLQIPQKKGEL
LRLAEEYLLTGGFPEIVTKDLELKEYLRTLFDSIILKDVVQRYRVKYTSEISDIGTFLVN
NFASLLSFRKIQNALSLRSITTTKKYINYLEESYIFFELSQFSTKAIERMKSPRKMYIVD
NGYITAKSVRTSFDRGKLLENLVFIELVKQKKEPNSDFFYYKTRNGREVDFVIRENLHIS
ELIQVSYDTSNIETTQREIKSLIEAREELNAQELEVITWDEEKEIEKNGKTITFKPILKW
LLHS*
>SPBIB_v1_180019|ID:27162760| AAA ATPase [Uncultured spirochete bib]
MKRYALEMLRKWKNEPNRKPLVIRGARQVGKTELVRIFAKEEFDTLIEINFDEHPGKASL
FADEDILSVIRYIEIDSNMKISPGTTLLFLDEIQAAPSVLSRLRYFYERIPQLHVICAGS
LLDFALSEPTFSMPVGRIEFLYLGPMTFGEFLLARGQGELEDYLESYSLTRTIPEAIHAK
LRSSLRDYFLTGGLPGVVKASVQSNCDLEDVAREQHSILQTYYADFGKYKQKVNVPFLQG
IFRNIPAQVGKTVKYTSLNSQARAAQVKENLELLEKARLIYRVFHSDGNGIPLGAEINPS
YFKLIFLDTGLLSAFLGLRLTDFLPNAEYTLIHSGAVAEQFVGQHLLYSAEPWAEPSLYY
WNRQSPGSTAEVDYLMQCRGQVIPIEVKAGSRGRLRSLHMFVQEKQVPLAVRFNTDLPSI
HSTHTAIAGKEARPFVLLSLPLYLVEQTQRLVEGMGDWTGLQDGTG*
>SPBIB_v1_180020|ID:27162761| protein of unknown function [Uncultured spirochete bib]
MEFGLFASIRWTQRIRDSSLLVIRLLSTIDEFHYSQGLGMLEDGMYIFDADKTVQDEFRV

ELDVPVENEYTFG*
>SPBIB_v1_180021|ID:27162762| putative DNA polymerase subunit beta [Uncultured spirochete bib]
MNSSAVRSKEETIARLREYCGAQDDILLLILHGSAAHDQLRAESDIDIALALRHPISIDE
KLDYILIFSRICGREIDLSDLRTIGGLFLHQVLSKGILILNRDIELYTKLVLNSLICIED
IHPILQHAQTL*
>SPBIB_v1_180022|ID:27162763| protein of unknown function [Uncultured spirochete bib]
VVYATTTMSTSKKRYEVFENFAKEMGATFSTNLSDLSEGERSLLEEFARHIHEINTVDDP
LFTPGERFRLLADTYNLSVDEGDIELFLTVELSRLFDKTTLTKKTLASDTAMRDQATERA
LNRVLTGIQYTGISEPHQRELRESGAEAASDILDAFKPSILKNHTVNILREDLMDIYERF
LLWMRMTRDGMLALSTRDRHRVEALLKKMIDLLNLAYVLNEPTPSSDPLVQRLIASSDAR
MEQTVVYTSALKELESIMPSRQKSDDNWDWLDI*
>SPBIB_v1_180023|ID:27162764| protein of unknown function [Uncultured spirochete bib]
MQTQYRVFLNQRVTARVAQMQPAERRRFAEKIRFLALGIWEGGVLVKKLSGTTRKVLFEA
RLTKGDRLLFTLGKIDDQSAVYLWALVKHDDVTRAARAGFPDNAPFLDFPIDTEETIEEL
YLDTAPEQWHTQEGIEEKVVADYGPQKWNVLDDDEWERLLAAHTLSELDFHLYLSNAQYA
ILARDPPLLVSGTAGSGKTTIALYYLSRKEFLGASRLFVTYSPYLRDEASKLYRGLVKGN
PLEDRPPFPDFLTIEELQRQNIDPHSPLQNPATFVGLNEFTSLIAAHPLAKNYDPELLWE
EIRAILKGAMPPYSLKRLETLARELGSGSICLSHRQELAAELARYRQLSFGGRLDAICAA
SSAFSTMEDFSNALGMRPQEGEFQVVSRVLEFLKKKKEFLTRPLLSLEEYEFLGKKRAPN
FIYDRKELYALATYYEKKREEQGLYDEIDLAKRALIERKRMQDPRVWDLVICDEIQDMTD
VHLELLFDLCKNRDHVLFAGDERQTINPSGFRWEAVRARFFESGARVPEISRLSLNFRST
GAIVELGNALLDLKKTFVGAGKFEARESWKFTGKPPVVIEGIEEARILHIVRQSGTNRSI
LVRSPEEKRRLIAQLGTELVFTIQEAKGLEFDTVLLWKFIEDETSNALWSTMTHGATHGA
PEALLPHLSHELNLLYTAIMRARSILTIYDGLKPSAVWGAGRLSSLVITTNDTSLLEESW
SKASSPEEWNRQGEMFFARGYYAAAAECYRNAAHREKEKEARGWAAYQAHDYLHAAPLFE
QSGDIARAAWCFEQSGELATAMALYTQLGDKTSHTRLSILALEREGRYAEAGRVRLKMGE
VDHALEDWRRGGEYELLADYYKKKKDYHHCAHFYEKAGNYEKAVFYYKKNHEEAHAFELI
LARATGPGADPAAVEFLRKEADRCMATRRFAQAATLYVSLNAHQEAAEAYRMAGAHERAA
LEFSRAKMYHEAAHEYSLVPNIERALAEWKAYVPETHDQRLAKLQGIDTLQWKLIARNPS
PEKAYNQAMANALFDEALQTLERGEYLNALALFIFFKEQKQMLECLRHIDDDMLALYTVC
RVAHFTLWSQYRAHKARVVLDARKAIGYLSKIYMSSELTGAHALEMARGLVLLLEDILDA
ADRSMLPQLKNEMLFSLSMLADFCRSSVENLVNAGKDLLRLLARLRDYSRISTAGLDWMS
RFKETPAMRSVVGYLDELALSLNDPLLTVCSSLVRKASFDPEILATQAVDESNWQFLSAI
PETKDKVVAYLLAQHRIQDAAATLRFNRAHLEAAELLESNGYFKDAAQIYEYLNEWEHAF
AIYTRIGDQHRIARMYEHKGDYLAAIELWKQLGFPTQVQRLTRKYQKTIANSHQTHLDFG
D*
>SPBIB_v1_180024|ID:27162765| conserved exported protein of unknown function [Uncultured spirochete bib]
MMKKIILALGIASLCIACTVNPKDATITEKFVISDPCVKLTDQLASIHAEDLQGANDAET
AQNILNWQHEYMKYADPGTQEDASYAMRWNYFLPGIFPVSEMISERTMQEDGKTKIYGVC
WDFAAVFISVAKSYQLTTRMTAWKEYMPGVTGGQNGMGPTEYNALKPKLLAHGVDFTQDQ
INAAAHETWKHYRAEVFIDGTWKAYDGTDPTGDYVNDANYTLVSWDEGAKAALTTKE*
>SPBIB_v1_180025|ID:27162766| conserved protein of unknown function [Uncultured spirochete bib]
MLFSCIMERKSILDLQIWQKKSDRKPLILHGARQVGKTWLLKEFGKRCYKNVAYINFEEN
ERMAALFASNLDVGRLLTGLRIEAGCPIEPASTLLIFDEVQANPRALTSLKYFAENAPEY
HIAAAGSLLGIAMHAGTSFPVGKVEFLDLYPLSFEEFLWALGEQELAEMLGGRDWQFIEA
FREKLIDYLKYYYVVGGMPECVSTFVATGDLNKARELQERLLNAYEQDFSKYADPAMVPR
IRAVWNSIPSQLAREQRKFMYGLVREGARARDYELAIQWFCDAGLLHKVYRVSKPGIPLR
AYQEDNAFKLFMVDTGLLAAHAHIPISTMLRGSDLFEEAKGALTEQYVAQELRLRNDIDV
FYWSSDTSRAKVDFLIQCGESLYPLEVKAAENLQAKSLRVYRDKFAPPRCYRTSLSPYRE
ESWLTNIPLYALAGLRFCQ*
>SPBIB_v1_180026|ID:27162767| protein of unknown function [Uncultured spirochete bib]
LFSTRHIMKSTRAELQSAKVAQGESGCTVQISWLMLGAQSAREAAWPYILECAARQPACP
TGPTCLTCLQLPAHRSFEGGEVVAADGDGEAAQVGLAGAEEVKEGGEVLFWRQGGFGGPS
PAQFADAARLMQSHPTGVAPPLRSSRTRRMASSRRSFMTGFKI*

>SPBIB_v1_180027|ID:27162768| protein of unknown function [Uncultured spirochete bib]
VRLHQAGCVRELRRGGAPKAALPPEKDLASLLDFFRARQSYLSRLTIPISRDYLAAFKTA
MGWEL*
>SPBIB_v1_180028|ID:27162769| protein of unknown function [Uncultured spirochete bib]
MNGIETAAQLPPGTAIIFITAYDRYAIKAFELHAFDYILKPVMKDRLEEAIRRVRELRRG
GATPVGCDCIRRAASANCAGEGPPKPPCRQKRTSPPSLTSSAPASPT*
>SPBIB_v1_180029|ID:27162770| protein of unknown function [Uncultured spirochete bib]
VGTGKRTGPLLDHIVPAVRVLAFNAVAAAATNKAEFKKLNPGVKVYIINPGGTEAMIKKL
EAEKGSPQADIMHSGDSSNYLYAKKQGLLLPSKHIFRRASLPAQKGFHAISPQGSRLGTA
LNTIIPSFSSNPTSSSSMSKCPK*
>SPBIB_v1_180030|ID:27162771| protein of unknown function [Uncultured spirochete bib]
MGNKTAGIGRWRIAVMGTLLQLCLGTVYAWSYFQKPIMERFGWSNSQTAWTFSIAIGALG
LAAAWSGMNLAKYGPRGALEKLWARKVQRDRARADGVRLTRVWRLPARAQSRRCSVPGKF
GARFSPRDSCLCGSCFSSTSRRASCSSAFSHP*
>SPBIB_v1_180031|ID:27162772| membrane protein of unknown function [Uncultured spirochete bib]
MELFPEAHNGAIRLVQQSDGVDVQHRDRGAWARRSLERDEPCKIRPARGLGKALGSEGSA
GQSAGGRGAANQGVAPAGQSAEPALLSSREIRGAIFSARFVLMWLVFFLNITAGIMFIGF
QSPMIQDMVHAQDPGKSAAELAGVGATLIAISSMFNGFGRLFWGGLSDRIGRTRVFRIIL
GSQVLVFVELLFVRSPMVFGMLVCYVLLCYGGGFGAMPSYIAIVFGPRMMAVVYGAMLTA
WSAGGIVGSQIVAFIKDRWPGGAVAASAAQAGAKVAQVAGGAAAGVNTGAAAAGAGPGVR
VAAGAANVAAAGPAAMTAASWIFLIGAALLALGFGLSFLLSDGENLRKGV*
>SPBIB_v1_180032|ID:27162773| conserved protein of unknown function [Uncultured spirochete bib]
MSIEKEYDARIDKKKRLTIRGARYSNYHVCEYENGTVVLEPRELVPPFEVSKHTLEMMDS
AMMNFKNGKASAPVDLSAFSEK*
>SPBIB_v1_180033|ID:27162774| conserved protein of unknown function [Uncultured spirochete bib]
MFSIRLGVPEMEALWAELSSKAKMGTLGKNEAKLYIKMGKAMGLLSNNPKHPALHSHEIE
ALSRRYGQKVWQSYLENKKSGAGRMYWVYGPEQNEITIIGLEPHPEDKKSSGYEKIRLSA
TGQKEA*
>SPBIB_v1_180034|ID:27162775| conserved protein of unknown function [Uncultured spirochete bib]
MKNRKSALSIMARKDKPGSRIIVLTGARQTGKTTLARHLFPEYQFLSIEDPVLRNAYASL
TASQWKELYPKAILDEVQKEPRLIESIKSVYDQWEEPRYVLSGSSQLLLLEKIKESLAGR
CTIIDLYPLTIPELETESWDDTIADSPFQIMLSHPGDISRQLESMLPDFRLDANYAKKMK
AWNHYVRFGGYPALTSPQLNDEDRFEWLKQYVRTYLERDVRDLAMFRDLEPFVKLQRYIA
LQTATILNYSAVGHNIGITTKTVQRYVQYLELSYQVLILPSWSRTEGKRLVKSPKIHFLD
YGVLQAVVQKRGSPTGLEFESMVIAELYKQAKNILSDARFWYLRTFDGKEVDLLVELSQG
YLAFEIKTSEKVSIADARHLRDLSDVLDKPLLHSFVLSNDTATKNLAPDITAIHPAYMLG
*
>SPBIB_v1_180035|ID:27162776| Toxin-antitoxin system, toxin component, RelE family [Uncultured spirochete bib]
MAYIVRISKQADKTIAKAPKAVQQKFALLLDDLEKYGPIRKDWPNFSALGNGLYHCHLSY
SWVAVWRNEKASVLVEVEYAGSRESAPY*
>SPBIB_v1_180036|ID:27162777| protein of unknown function [Uncultured spirochete bib]
MRVVEKAHHIEDSKKLIAVSAEEAGKILEYAKQIDPHAHFVEETPLSDGFISPHESGWYQ
DVKRSWHPGITLRIRRENAGLTQAQLSRMTGLAVANISAIENGRRSMGLNVAKKLATALK
RPVSEFIEKS*
>SPBIB_v1_180037|ID:27162778| conserved protein of unknown function [Uncultured spirochete bib]
MSGYEKFYEESLYPLQNGILRTLAPVCGNVLSLTGGTALSRIYFHHRYSDDLDLFGNSVE
HFNEIVEASLSALENAGYQFVKGSIIRHEAYTQAVVRNGGTNLKLDFVNDVAAHFGECIA
HPLYPRIDSVRNILSNKLTALYRLEPKDIADLYFIACSYQFDWAVALQEAESKEAGLDAI
TIADIISTFPRDMFKAIKWRITPDPGDFFKSLTIMTRDILHASPNSLAM*
>SPBIB_v1_180038|ID:27162779| protein of unknown function [Uncultured spirochete bib]
MFTRERKLELLAGIVWDYDTDPETLLAVLEGRQEKAGGVDKSAIYRRLLRALPWQNIVGL
IGADEAIAMLTTENIAHLWPRELKEHYERLRKILRGEPVSPSEWDSPNACARLRQRTFSH
RWYRAQ*
>SPBIB_v1_180039|ID:27162780| protein of unknown function [Uncultured spirochete bib]

MSQSSNSPKAPTSKPPAHVPEGASFLVRLDPLECAKKVLIPSHRFEPFHSYRISIPRLEI
RLPDASPLPKKNIVLPYNEILRFHNLLNFMDIFYMLEEMYPGNFPADLAQASQAFDRLIR
LSVFDLEPLFPGGIQQGSYVRLSVQRYQKGIFTAAPVTPEEASRLDREAWFSTMDAAFAR
AFDELKFPRRNDTLLSLVYRYGGEALYGNPAAALIDYLDAKRAAEIIYFGGENLIWKKGV
DPEHIVLSGLDAYGTGSRKPGKASPAARTTSTPTSAAPARAENSAAPAKTSNSASAAPPK
TAPAQTNGAPSEAARPIAPVRAPAKPRKGRKPSLKARLNRFFEAYEFAFNADEIKALMLD
MAYRGTTLQDLWDRFFESSPQWTVFPEAADELIDLIEDYCDEALANYDPERDGFGEMRAA
LVDIYANYIAWMRRLDTKLRSPEDLPIEKFTELANLISSLNEFMLIMNSPAKRGPGDPSE
REVLEQFFDSVDYMRQAVTDLEHEIETIALRKKKGV*
>SPBIB_v1_180040|ID:27162781| conserved protein of unknown function [Uncultured spirochete bib]
MSSVTANVLMIGDIVGAPGLRALFTFLPSLIKKTEADLVIANGENALKGFGIGEEEIAAM
RSYGVDVITSGNHVWERKEAADLLEAEPALLRPANYPRSLPGRGVAHIEGQSAPGLPAPT
RASGASGHFEWLVVNLQGRRELYDIDCPFAKADQILANAARENPGALIVVDFHAESNEEK
EALAWYLDGRASVVAGTHTHVPTADERTLPKGTGYLTDLGMTGPVDSVIGMNGDICVRRF
LTQIPYKMETAEGSAAIMGALFRIDPESRRCVGIERVYKSL*
>SPBIB_v1_180041|ID:27162782|rny| Ribonuclease Y [Uncultured spirochete bib]
MAPFVWYIVLPLAGLILGWMIRWLYARFQLSSAEREAEHVLQDAIKEAEAKKKELLVEAK
DQIIRERNQQERELRDRRIELQKYERRVLLKEENLDAKLASIERIEANLKSRENAIAEKE
AFLAGQEERYAAELERVSGFTAEEAKKLIIQNMENEARHDAQVLINKIDQEAQLTADKKA
KDILLTTIQRIATESTAESTVSSVSLPSDEMKGRIIGREGRNIRTLETLTGVDIIIDDTP
EAVVISCFDPIQREIAKISLERLIADGRIHPARIEEMVQKVTREITQKLYDEGEKVVFDL
GLHNMAPELIRAVGRLYYRTSYGQNVLMHSKEVAVIAGLLAAELGLNREIAKRGALLHDI
GKGIVTDSDMNHAEIGTEIAKKFGEDPRVINAIAAHHNDVEPTCPESVIVQIADAISAAR
PGARRETLDNYIKRLEDLEAVAESFSGVEKAFAIQAGRELRIIVNNEQISDDQAKDLCKS
IAKKIESDLRYPGRIKVTIIRETRIVEYAR*
>SPBIB_v1_180042|ID:27162783|rbpE| putative RNA-binding protein RbpE [Uncultured spirochete bib]
MAKKIYVGNMNYNTSERQLQDLFAQYGEVSTVNIIVDRFTGKAKGFGFVEMENAEAADAA
IAALNGQEFMGRQLRVNEAQEKPRYEKEDAGYRSRRY*
>SPBIB_v1_180043|ID:27162784| conserved protein of unknown function [Uncultured spirochete bib]
MSMEHLIEQFRHKVARTPMHFVRSIHDSIQWNARLIGIRGARGVGKTTLLLQHIKQTFAQ
DLNKVLYVSLDNLWFSDHSLLELADSFAKKGGTHLFLDEVHRYPSWSQILKNLYDDYPEL
FITFTGSSLLHLLDAKADLSRRAVVYTMQGLSFREYLALAANMAFPAFSLEKILSEHEAV
SAEIVSQFKPFQFFPAYLQTGYYPYFAEEPDTYPLRLEETINMTLELELPLLRRLDIAYI
PKLKQLLSIIAESAPFIPNISKLSEKIGLNRQTMLTYLGYLSEAKLIRPLYRDAHGVSAL
QKPNKLFLENTNLMYLFRKQSVDIGNARETFLANQLACGHELSFADTGDFIVDGSITIEV
SGKNKTRKQIKDIPDSYVAADNIEYGFGRKIPLWLFGFLY*
>SPBIB_v1_180044|ID:27162785| protein of unknown function [Uncultured spirochete bib]
MVKKVKKRGQTPFLKSDGHQLSVWEGTASLSEPVRADKYLSETLALMSRSQLKARGACLF
CNGKEVKLSHKIKNGDHLRLEWTEAPSEEITPEPVPLTILYRDADVFVIDKPQGMVTHPA
AGNWSGTLANGILWLSLHGEDARATGLQTENEAEAKNELELNRESRAISESKSEPEPEPN
STSKREPENEPIPFYTSFRTLPLSPPPRAGIVHRLDKDTSGVIIVARTARAHEFLCRQFH
DREARKEYLAIVRGAPPTTEGRIDTWLARSPRDRKKFAVSPSGRGKHALTLYKVRAIWEI
PARAESATRAALSRPNTASRAAASCAESAVPTATPPGRPITASRAAAASTRPGTACASEA
PQSLSRRSPSKKATSPQRYTLVALYPRTGRTHQLRVHMAHLGCPILGDPIYSKKDTLFPD
ATLMLHARRLKIRLPADNEFHIFAAPLPEHFKHMIAMLDAQGIRRRAST*
>SPBIB_v1_180045|ID:27162786| protein of unknown function [Uncultured spirochete bib]
MLEVLGKGRGKDVEFVIGGQADFKAPGVEHEGGIGKESVFFAVDWVAKNGAAEVGHVDAQ
LMGAACAGIEGDEGVALGRSGLLGGAAPRKALGRFGGAGGPGAGAGCGGAGSGDGAARRR
GGWDRRFGTGRGSAGSGIGAR*
>SPBIB_v1_180046|ID:27162787|truA| tRNA pseudouridine synthase A [Uncultured spirochete bib]
MQQNPDFQQPPVPKASARPDSMPSRASTLRTIKLVLAYDGTDFSGWQRQKNGRSVQEELE
KALSKMHGHEIRVNGAGRTDAGVHAMGQVASFYTDIQSIPADRFTLALNKLMPRDVRVLS
SEEAPADFHARFDASLRRYRYFLVCGGTPDPFALRCAHFVPYYPRVAVLNAMAAVVLGEH
DFSTFASAQDKSPSRSRHISESVFFFEGEKLVYQIAGNAFLWKQVRSLVGTMLELERIAE

SPAHGESLMRELLDSRDRSKAGATAPACGLFLWNVEYGQRVHGHPRRHSEEAYG*
>SPBIB_v1_180047|ID:27162788|acpS| Holo-[acyl-carrier-protein] synthase [Uncultured spirochete bib]
MISGIGIDIVHIDRIRRWMENPGILVRFFHPAEIETARSRSKGMALSLAARFAAKEAFGK
ALGSGLAHFALKEVAVVNDSAGRPYMELEGNARREFERHGGGTIHISMTHEGDNAVAVVI
IEKGN*
>SPBIB_v1_180048|ID:27162789| putative YbbR family protein [Uncultured spirochete bib]
MSLDARISKLLENWPAKVISLVIAIFIMFLYNLTRLDQRLITVQLNISEGRSFVPSTDYP
KTVRVVIRGERDQIYRIREADIVASLDLTRYQSEGVFRVPVKLERRGEARDIDPLELRAD
PAEIPIGFERLAAKRVAISPTFKGFLEQGYELISYEIVPSEIAIEGPSNLIANTKDISTD
VIELSGKTGDFSLTVPLSKPSDLINLIDVNTVRFTARIQKQERRITLNNVPIKAVNLDPA
LGFAETMPTGKMTLLPRQGQDAAAAADAHLQADFKGITKPGQYTVPLTPVVPDGYDVEAY
DPLVISVHVRTAQSVQSVLPGLLAPSGSSGLPAFPAPAAPSTQP*
>SPBIB_v1_180049|ID:27162790| conserved membrane protein of unknown function [Uncultured spirochete bib]
MNAPALVNFLVTYVRPVLDVAILAFLIYKTYQLLVMTEAVYLIRGLLILVAIYGAAFFLN
LSTVTWIMNILAPGLVVSLAIILQPELRRIFIQLGRQSIIKQKAAARTSWIDAAVSAAVY
LAEKRRGALIVIARKVGLSSYIEKGIALDALVSTSLLIAVFEHDNPLHDGAAIISQGRLA
SAGCFLPLSDQQDIRKTFGSRHRAALGISEESDAVVLIASEETGALSLAYDSKLYYDLSA
DEILERLRMLFSESRSRSMTSEADYEP*
>SPBIB_v1_180050|ID:27162791|folP| Dihydropteroate synthase [Uncultured spirochete bib]
MSDSPASAHCQNEQTELTRTILKLPRQRSLLYGAQPLIMGIVNVTPDSFFEGSRKYEPQA
AVEAALAMVSHGAHIIDFGAESTRPGSIEIDPKEERARLLPVIERFRAASDAVISVDTRH
PEVARAALEAGADILNDIEALAAPGMAEIAAKHHAAVVLMHMQGTPATMQIAPAYADCLE
EVYTFLRAVTKGAIAAGVSAESIILDPGIGFGKLKEHNLALLRGIGRLRSLGFPVLIGLS
RKRLVGELTGREIPERLAGSIGGALAAWMAGADIIRVHDVAETVDAFRVFTAIFPARPSG
KEFSREVSL*
>SPBIB_v1_180051|ID:27162792|dxs| 1-deoxy-D-xylulose-5-phosphate synthase [Uncultured spirochete bib]
MVQYIDMALLDHISGPADLKELSLAELRRLADEIRSRIVETVQLNGGHLASNLGVVELTI
ALHRVFHSPQDAIVWDVGHQCYPHKILTGRAERFHTLRRQGGISGFPKREESEHDIFDTG
HSSTAISSALGLLAARTQTGKQGRVIAVVGDGVLTSGMAMEGLSNAGQLGLPLILVLNDN
RMSISRSVGSISRYLSKLSASMRYQSFRAHIDAAVLKIPRIGKPLSEFINRSKRAVKAIF
FKENLFVDYGFEYVGPIDGHSITAMIDAFAHVRNLNRPVVVHVITKKGKGLERAEEDPES
FHGLGPSCPDIPGAGESSHGESFSSCFANSLIELAEHDARVMAITAAMASGTGVAKMLER
WPQRVFDVGIAEQHAVTFAAGLAQGGLRPVVAIYSTFMQRAVDQVFQDVSLANVPVLFAL
DRAGAVGEDGETHQGIYDIALFKAMPNLIFFAPADGDELAAFMKLSLSLDAPCMMRYPKA
FSPCANGAMRPLEVGKGTMMRERAGARILVCAVGPLAYSAADASDALEKKAVLADVYNVR
FLSHIDEEYLASLCARYDAILTAEDGVRVGGFGETLAAILARRGVRAGIRSLGFENTPLA
QASRDALLAQAGLNTKGIQKALEELQRSLGPESKKNAYAASVAAATIARATPIPVPGEVQ
HVR*
>SPBIB_v1_180052|ID:27162793| protein of unknown function [Uncultured spirochete bib]
MHMLIDIKILHLHRPIYYEKIELVSDEIQNNAISALAARYGECLLVFRNDNLLILSDDGP
HLASKLVPSTAYRIAVRDGGKGSTDTASAIPDESQGNIVGRSAPRQLSADYSIARFTLSA
ADYAFFQWRAESAPQPSIMFEEFAREIWWERLETEGAWFLRLVLEDGKTSVQALRLVLST
LCKRGH*
>SPBIB_v1_180053|ID:27162794| protein of unknown function [Uncultured spirochete bib]
LGRYSAFSFPGVRHSFFQLLAMVGLKGLRVRNARSSKKLLYXX
>SPBIB_v1_190001|ID:27162795|alaS| Alanine--tRNA ligase [Uncultured spirochete bib]
MITIHELRRKYIEFFKARGHSEISGKSLIPENDPTVLFTTAGMHPLVPYLLGEPHPAGKR
LTDYQKCIRTGDIDAVGDPSHLTFFEMLGNWSLGDYFKKEAIRWSYEFLTSPEYLGIDPA
KLSVTVFTGDEGVPRDEESASIWRSLGIPEERIYFLPREDNWWGPAGETGPCGPDTEMFV
DTGKPACGPDCRPGCHCGKYFEIWNDVFMQYNKKNDGSYEPLAHRCVDTGMGIERTVAML
QGKKSVYETEAFTPILAVLESITGKRYGALDNNPETDRSFRIIADHIRAATFILGDPKAV
LPSNIGAGYVLRRLIRRAVRHGRKLGIDGIFLAKPAEAVIEIYKEPYPELLENRARILEE
LEREERKFLETLQKGEHEFEKMLPNLLRNPEKIMPGRLAFRLYDTYGFPIELTEELASEN
GLAVDRAGFDEAFKKHQELSRAGSEQVFKGGLADHSEVVTRYHTATHLLHKALRIVLGEH

VAQKGSNLTAERLRFDFSHPAPMTQEQIAEVERIVNEQIARDLPVSMTMMPLEEAKASGA
IALFGEKYEPVVKVYTIGDFSKEVCGGPHVTHTGELGKFKITKEQSSSAGVRRIYAILE*
>SPBIB_v1_190002|ID:27162796| protein of unknown function [Uncultured spirochete bib]
LKCIFITQVRSRAEKAHARLLIDSIRAFGGPMRDCEIWVFATKPANEPCDDLEGLQVKVI
PLSVQRKFMDYPFGDKVFTCATAEAMAASDTGSLIWLDPECLVVQPPLLFDLGGEFAAAL
RPVHVHNVGLLVSEPLDPFWKGIFRTVGIEDIEFAVESFVDRQAIRAYFNSHGLAVRPGN
GLFRKWLKSFERLVSDREFQLEACCDNFRRLFLFQAVFSAVVVSDVEREKIRILPETYNY
PYNLHEQIPAAWRAAALDDLVCLTYEGRTLAPCRVTDIEIHEPLRSWLETRKGQAFETAH
*
>SPBIB_v1_190003|ID:27162797| conserved protein of unknown function [Uncultured spirochete bib]
MRVFVSGGFGNVGISTVRALLSAGHEVCVFEHPSAKEKRSARLRELLKASGSKLKVFFGD
ITDEAALSEAMACFGIDGSSEGLDAVIHLAGIIPPLADRDPELAYRINVGGTRALLAVCS
ILPRPPRFVFASSIALYGDRLASPWIAATDPLLPNDTYSKTKQECEALIKSAGLDWVILR
LSYVVSSDWLPFDPMLFEVPPATRFEVLHTEDAGRAFAAACQNPDAAGRIFNIGGGAACR
TTYRAYLDRLMRLFGLGSSSFLPDELFAKCNFHCGWFVDSDEAEAVLHFRSKTLEDYYSE
VQWRTRFLRPLASIAAFVVKPWMRSLSPYRVSPGRVRSPQLV*
>SPBIB_v1_190004|ID:27162798| protein of unknown function [Uncultured spirochete bib]
MPRPKTEEKRKLILDTAKRLFALEGVGVSMNEMAEAIGIPVGSIYTYFDSKQVLVETIIE
EGWNEYRLWLEEGIAAIQLGNQNADARLIALKILSFLVNKALPKLFSDVELIMLLLAEAG
TTARLEEKLQYFSSMITKLVSECQVSGAAENDPRQYNTGITVLLLGALETLRLSSKTDLK
LGADDIQAFFRLIVENALGQGLPDTD*
>SPBIB_v1_190005|ID:27162799| Asparaginase/glutaminase [Uncultured spirochete bib]
MLEYLCAAWYYTVIMNPEAIRIIVTGGTFDKHYDEIKGELTFTETHVPEVLKRARVSVPV
QVELNQLIDSLYMQEANRQSVLDACKRAPERHIIITHGTDTMAETAKLIGSARLDKTIVL
TGAMIPYKILDSDALFNFGTAFAAVQLLPAGVYISMNGRIFDWNRVRKNKTLGVFEEIQE
*
>SPBIB_v1_190006|ID:27162800| protein of unknown function [Uncultured spirochete bib]
MLEFLFFVLAKILESNNVTEDEIRKNEAIQKEAAITSLAAFFLVKNTDFFSDCTLKILPK
KPKYSQ*
>SPBIB_v1_190007|ID:27162801| exported protein of unknown function [Uncultured spirochete bib]
VQSEKKSVFLTRKKAARLVIAASFCIASFFLISSSVTLFDSSIFASTKNKNSSIMFAVTL
KNNGSYSDPVSRLYINDKTKNQVLKFFSAITNSMPVAKAILDNSIAHNVRPSLAFAVAYV
ESRYNPKARSVNTSGSLNSGLFQLNSSVFDKISEKQVFDPYHNARIGISHLKEYLILSED
EFTALAAYNAGITRIAEQGIPGVTFKYISEVSIMERKIMDLFVASMALSGNPL*
>SPBIB_v1_190008|ID:27162802| Transcriptional regulator, TetR family [Uncultured spirochete bib]
MMYEKWGIWYNNISTEGVILSGQVAHEKRRQEILEKALDVFIEEGYEDTTFQKIAERCGI
TRTILYLYFENKKQIFAESLKRFLGSVEIEIAVVAHDESMNSEEKILKIGEMVVEACEKE
SKLLSVVLDYLLRLKAAGGNPDEKVRRRTVRMRHILADIVIEGKKRGEFSETSSVKGTVE
IFFALVEATVFRLVVLGRKEATGLAEALTPFVQSMRP*
>SPBIB_v1_190009|ID:27162803| Alpha amylase catalytic region [Uncultured spirochete bib]
MALQISRSLRSVNVPPKHLWMPSGAIAIPDSVSARVVAQKFQLPASELRALSLIDDASRI
IIDLYRQQLSKMLRHITESAMGTEKDRSALADVLKELALEFPPPEVFDGIADASDWLESS
SQAKDGKMLSHREAALEQLILVRLFNENPACVSYRPLFDDGVSPAGTAAAGSLAAKTPYL
AVFLKLEEALKALPGLSYEKGKALDLISFLREPAKRAPQSLKAQLQWIVDNWGELLGDFR
LALLAGIDMINEETAPRFPAGPGPAHAYHYRSSLHEYEKFSADRNWMPSLVLLAKNTLVW
LHQLSVIYGREISRLDQIPDEELITMAERGINGLWLIGIWQRSPASEKIKKLCGNPEAAA
SAYSLFDYEISPELGGWEALDAFREQCRQFGIRLAADMVPNHTGIDSFWVRTKPELFMSV
PYCPFPSYTFNGPDLSSDPSVGIWLEDHYYSRTDAAVVFKRLDRHTGEVRYIYHGNDGTG
MPWNDTAQIDFLNPAAREAVKERILHVASHFSIIRFDAAMVLAKQHIRRLWYPAPGAGGA
IPSRSEHAMSDEAFDKAIPNEFWREVVDLCAEKAPDTLLLAEAFWLMEGYFVRTLGMHRV
YNSAFMNMLKEEKNSLYRLTIKNTQEFDRDILKRFVNFMSNPDEETAVAQFGKGDKYFGV
ATMLATMPGLPMIAHGQIEGFTEKYGMEFKRSYKDETPDRDFIARHEREIFPLLRTRPLF
SDIEHFFLYDYMRADGTIDENVFAYTNGQGEQRALILYNNCWERTSGRIHNSCAFTQKSP
SGKKHLETITLAKALGVEPSPDNYLIMHEIRSGLWFIYRSEEIASSGLQVALEGYQSKVF

LEISSVHDTEGRYARLYEIVDSKGIADLDDALLEADQPDLFRALHNAIVSLNGVEAVESL
SKSEAIARAAIFSEMFFSRLCATAGCEYTHPASRAESIRRAVSWMSSVLSGLYNPEILDE
APLAAYRIGAPRKRLTLLVYAFISALAKSFAADGLSEDIGRVVEEYRITKKLREFAVALS
RTIPDEPENSIDPSAEAEIAIAWALRADAKLFAASAVSNSGKKESAVGASPSAHDILEWA
FSNALMRAALGVNHYRGTEYFNRERFEAFIGLLPAFGWIDSITQIFPEGDVDAVEFWHLR
AELLAAHEADSGYLTKTLLENVLAHASP*
>SPBIB_v1_190010|ID:27162804| protein of unknown function [Uncultured spirochete bib]
MDRRAARYAELLKTPIGSDIERYVRDNTVIFAFPSQDAADSWARALLASKVFKAVALDRF
LGFEPLLRTIGLDDERERESERAGTRMAQHADRWAWAIRALESLKGSEAQHSLVLRRILP
TLPGGESYSISHLLRLVRLVPALYEIDAYARQSRKEPLGSAEKQRQFAFLREELEDLKAL
ARHYRDFLNDQNIIDYHFLAQDQSLSSGHHVRAYGLKRDFARLGLGIEHEQSRLYFPESA
LPDFSEIRLPPYIQFDSAIDEIEYVFASISEEIEQGLEPEDIAISVCRLNPQKAAWIRQI
AADCGVPVSIRSGSPLSSTPFGRLLRAIQQASREGLTLDALDSLAAFTSIQNRDPEAWRQ
LRITAARAHIPSPSPNAAYVHGLWKESAQIGMSVLNLVQKYDALWKDITEVAQSKNFSGL
YLAILRFLERWVDTTKLSAESYTDRSMRMALDELQAFVEREDAFQTSHFSPFEVYLAALS
TKNYIPVMAENAVRVFDFGTTAGLAAISQYIIGASQDGLAAYFENQTALRSELASLLGLD
GTYRIEELVALHSLGDAHFSFARESFDGYEVAHPFFGAALAENANAMRNGTASVLSRVPR
RVERAIWQSTTMLASDTPLALTKGQKQRFSEGFSAFPGHLAFDTRTYAHPAPLDAPIAKR
FLKATDSFDFDRDFAIFSPHSLRDRMRCGFRWFASRIGAADMYALDDLSMILGNFLHTTY
QRIIRALMQHASASTSSEVTDDIFLDIFKQAAKTTAEEMFASKGPGIRPALMTYFDRARH
RLKTLQEFEALAFQPYRREGFELPFEHVFDSEKAILRGRIDCIFSRIDESAGNAQCLVII
DYKKNSIPKASAMRPRGQESSGVVDDSDDIEDTASSMAVQELQVPLYALAEELDGKLVEG
ALYWSIEKAEGVAYIAPPALPPGYGLAKAFKTASETADVRAVLRQTLAAASMAVRNGELI
DLALDRASCGDCPFRALCRYWYFLES*
>SPBIB_v1_190011|ID:27162805| protein of unknown function [Uncultured spirochete bib]
MNKLDLDDAQRLAACTKSTAVVTAGAGAGKTRALVGRFLYLVLEEHVEPESILALTFTRK
AAAEMFQRVYEALGRPENARPDLASRLSNAHIQTLDSFCREIVAGCATLYGYTPDFVIDE
TACAEVAHKTAYRYVLEQMQADGLRELLGAFGFDAVVSDLFASFGSANVEPHWIGQHVCH
RSFEIGMREFEQTGTSVVQQMQAIAARILDEAGKGTISDFKEGSAKTVRVAQVLLEALDT
DASKDGASPEPGTGSMQQFSRLKAFLDYIDKPENSVNFQRVGKKDIEIAIKEQGQLLLRD
KKLLKKLRQIIDFELFLPQYRAAMERLDEYADALCEEKRRANIMNYKDLGALAVDILSRQ
KDIREYWASRFRYILIDEFQDNNAIQKRLLLLLSQEPGIEPGPASAESSTGAGRPAKIAE
GKLFFVGDEKQSIYLFRGADVSVFKALASELAAERFALARNYRSAKALIDFFNEVFAVVL
QPADPAQPQAFEAIYERMEASPEPEPPGFVPRIEYHLVSDEVPLEGTSLQEEAGPGSSAA
TSSGAAARVAKLGSALSARESMAFRMAMWIRNAVESSSPLYIRCKDSLRKAEYSDIAILM
RTTSRQYEIEKYLRMLAVPFVTETTASLFTEAPVNDLYFILRLLLDSHDLFATAAVLRSP
LCRITNDGYVSILTEKLSLAELRARSPSGALAGLSAEDQAAVQRMVFFYDAFSAIADHLP
LMEIISFAWERAFIGLSILSDPQRHPYLEHWNAIRSIAAETEAQGGHVPEFLRTLRAFID
QKRLFDSSTIQKEEAQGVRLMTIHKSKGLEFPIVLIPWMEAGTNKSGGSDLWGVLDAPPD
TNQDRYFTIDIGFHDRADNGSNILQTQARTLRVEKEHAETKRLFYVACTRAIDHLILFGA
APSRSYDTDSFHALLFQSTYKLHPSAAGYPEAERQQTENPQEERPAAEHAEATSSRHLAR
IKLSFEPLAFEQEVHRERRATQKPDLAQVAAMYERARIVDLKRPARKLTVSAVKAHVVRS
EAPVPAFSPRIIQETCFEPASEVFEKKRAAQEGTSDPAAFGTLVHELFAHLASGKPFETF
SPSAMYRTARNSLRADTAGIASVSRNPTGTELQKKPSDEIARAYRELVPLLNSDCFKTLL
QGNTVSIEYPFLLSIPPWMLEGRIDMLIEQSSEVIILDLKTDARFAPIDYALQLGIYQKA
IRELFPDKRVRAGLVYLRFGTFAWIEGELEESAIARYCEALAAAQE*
>SPBIB_v1_190012|ID:27162806| Chromate transporter [Uncultured spirochete bib]
MRMPARAEDITKTEPTPWHIFLVLAQINTLTLGGGYVIVPAIGNAFEKQGWIEEEEFYRI
FSRAQVFPGPIALSTAFLASYRIAGVPGAIAAVLGTVLPPFFALILVGGFISLYGNTELF
KRFLGGAGAVVPGLVASMLWKTTKNRKWSVRAVIEVVGLAILLVVFPSAAFFLLLGGIAV
FYIGRLVWKS*
>SPBIB_v1_190013|ID:27162807| Chromate transporter [Uncultured spirochete bib]
MEILSVSLAFFKIGIVAFGGGWSVVGLIKHEIVPRWMNAEAFNSLIAIAQSTPGPIALNA
ATLVGWQKAGFLGALAATFSVILFPLLAMSSALAFAKYIPLKRSLADESLRSGSMAMILM

TLWTLLPRTALPMPWIFALAAFGITAFTKLNAVYVILGAGALNMLIGALH*
>SPBIB_v1_190014|ID:27162808| conserved protein of unknown function [Uncultured spirochete bib]
MTEMANTHDIAKTAGTAGAPNAARAASAANAINAAPLFEAFSMPESPVDESMRRALFVQL
TAQLLRSAFAGRALSITFLAGAGTRWKASLLKARENPGAWPAGEVAASFPLDAPRGLFPV
PDFIHGEKGEGRIAMAAYAVEAVRAIKEHILVVRGWEREIEAQVLNPLEMPHSRIAFFTQ
KEGPDGQVSGHGDAALQCMSLWQNARYVVTNFAGDANSPLTVELALRAFAAFDAKGIEIG
VIIPVAMTEKPAYPVFLEENGLPAGFWHEKLAGSKPNAAPTSLTNVGIRVYRADWLRRAL
LLLKERYYVEGQGPDAGWHIPGNDPSKRECALDNVDNLLAEMRLARVMPVSLPRELTPLK
SLGEYPAFVQAVKEVQREWREIRRE*
>SPBIB_v1_190015|ID:27162809| putative ABC transporter, permease protein [Uncultured spirochete bib]
MIPTYPEFAPLELDAREALYPNLNLLPDGISEFTFAGLYLFRHTYNYRISRLPDGNLVIS
GHRREKSFFYLPCCFPSVDLFDKLMHQYDYMRHMSETQANQHRIELEARGYLVFEDRDNF
DYLYNREDLAMLNGRAYHKKRNLVNGFVSSYSCEQRAFTKARIKDALAVLEEWRTAKGVD
GDYAAARDALEHFEVLGLRGAVYYIENEPVGWCLGEPLAKGKMFAIHFEKACDRFKGIYQ
FINQAFAQSLAKHYRYINREQDLGDEGLRQAKMTYRPVGFVKKYMVFHPDRADFAPQPLE
PPAECGPGTVHECSDD*
>SPBIB_v1_190016|ID:27162810|pepT| Peptidase T [Uncultured spirochete bib]
MNRTIEFLSRPYADEVMNRFVSYAKIDTQSNRHAEETPTTKGQWDLARTLERELREMGVP
DVELDDRCYLIARLPASPGKEQAPCIGLMAHMDTASDVSGSGVKPRIIRDYDGKAVQLSE
QYILDPAEFPDLAEHVDDTIIVTDGSTLLGADDKAGVAEIMTAIAWLIKHPELEHGPIDI
YFTPDEETGKGMDHFPLKKARAVACYTLDGGKAPEIEAECFNAYAVKAEFFGKVIHIGAA
RGKLANAVAMAASFIGMLPRSESPEATDSWYGYYCPIEVSGTIDHAWTEVYLRDFSSDRM
QERIAALKAFAAAVEVQFPGGQVKLAITQQYLNMKQKLDAQPAVLEKLERAIKTAGFEPV
MKPIRGGTDGSRLTEMGIPTPNLFTGGYNYHSRFEWASVSEMALAVETIINLVLIWAE*
>SPBIB_v1_190017|ID:27162811| NAD(P)H dehydrogenase (Quinone) [Uncultured spirochete bib]
MNALVILAQPKKQSFNSAIAAEIVRSLGQQGYDPVGLIDLYADAFNPVMPQEELPRKFSF
DETTLRYQERIQAADRVVFVYPDWWGGPPAIMKGFLDRVFRPGIAYGFREADFKNADAPG
LFSQKRFDVFITTDAREPESGILNDWPPVMVWKENVLTFCGVTEADVHVFWNLRHSTYAQ
RKAWLDSIHSMLAIQPRSAQD*
>SPBIB_v1_190018|ID:27162812| conserved protein of unknown function [Uncultured spirochete bib]
MAEKTEKFLEAALLEMLKAVKDPELGYSIVDLGLVYRAEQTEDGIEVDFTLTYIGCPLEA
QLRRDIERTLQKKTGIRPVRARLVWNPPWTIDRASDEIRLNMGYAIW*
>SPBIB_v1_190019|ID:27162813|sufC| component of SufBCD complex, ATP-binding component of ABC superfamily [Uncultured spirochete bib]
MQEGLIIRGLSAGLENKRIIDALHLEVPDGEVHALMGPNGHGKSTLANILMGHPGYAVEE
GEVLFDGENLLDLEVWERARKGLFLAFQYPAEIPGVTVSKFLKRIADLRRNPPRNTSVFL
SELKENLRMLDIEPAFVNRYLNDGFSGGEKKRMEILQLLTIQPRFAIFDETDSGLDIDAL
KVVAAGINHMRGPGFSALVITHYRRLLDLVKPDKVHILEKGHIVACGGFELVELLERDGY
DGIRRIVANPANPEVREAVYEH*
>SPBIB_v1_190020|ID:27162814|sufB| component of SufBCD complex [Uncultured spirochete bib]
MSTESAALQPDTSIRAIGEGYAERFGFSMPNRSIVKTSQGLTEETVRAISKTKDEPEWML
RFRLRALHLFQTLPMPTWGANLSQLEFGSMTYYSKPTESAKDSWDALPADIKSTYERLGV
PEAERKFLAGVGAQYDSEMVYHNIREDLKKKGILFSDVETAVREHPGLVKRFFGTVVPPD
DNKFAALNSAVWSGGSFVYVPPGVHVDIPLQAYFRVNSQAFGQFERTLIIADEGSFVHYI
EGCTAPVFSKDSLHSAVVEIIALPNSRIRYSTVQNWSSNMYNLVTKRAFAYDNAIMEWVD
GNIGSKVTMKYPAIFLMGPGARGEVLSIAFAGKGQEQDTGAKIFHFASNTSSVITSKSIS
KDGGTASYRGLVKVEAGLTNIKSKVECDALILDPHSRSNTYPTMDIRSEQATMEHEAKVS
KISEEQLFYLMSRGLSEEEAAIMIVNGFIDPLVKELPMEYAVELNRLIELEMEGSVG*
>SPBIB_v1_190021|ID:27162815| SufBD protein (modular protein) [Uncultured spirochete bib]
MSGTILDTLKVSRREKANDAAALLFPADSKSSGLKRLDIAISSPASGALSGAPQEPKEPA
EYEQYREASIALGAGAEAAVLVRFVAAGAASQDRAADEPARMQDARLARAENPETPGSLR
LTTSLHIRLAAHARLNLYVLSNLPASFSREALSHAALSEGAVLTWTNVVFDEGNGLYTTR
IELEGEGSELDFAGAYGARCTTEGEHALSIHHTAPHSKSRTILKSALKDSAHLAFRGLIH
VENTARGTDAYLANRNLVLEDGARAESFPQLKIETDEVACSHGATTGGPRQEELFYLMSR

GLDRAAAKNMLVLGHLGSVLNRLPPDLAEEMEDIAASALGLNAQGSCI*
>SPBIB_v1_190022|ID:27162816|sufS| selenocysteine lyase, PLP-dependent [Uncultured spirochete bib]
MKTTFSALAEGADISINAAVARELKKEYPIFRTLPNLVYLDNAATTQKPLSVLDAERDFY
MQSCANVHRAIHTIGEEATARYEKARSSMARFIGAAPKELIFTRGTTESINLVARTFGET
LREGDEIILSVMEHHANMVPWQQLAERRGVVLKFIPVTDLGELDLAEYEKLVSPRTRLVA
VTMVSNVLGTINPIDRIVATARSAGVPVLLDAAQAAPSMPLDVKALGADFVAFSGHKMYG
PFGIGILWGSETMLDRMPPFLGGGDMISEVRLEGFSVNELPYKFEAGTPPIAQAIGLEAA
AEWLSSVGLEALGEYESALARRFLDAIKDIPGLRVMGSARQRAGIVAFTLENAHAHDVAA
YLDRRGIAVRAGHHCAHPLARRFGVVSSARASFGAYNLPEDADAAARALADAQEAL*
>SPBIB_v1_190023|ID:27162817| SUF system FeS assembly protein, NifU family [Uncultured spirochete bib]
MSDPYQAIYEEIIHEHYKKPKYRRILEGLPYAENPSCGDRVRISIQVGDDGHIASAAFDG
SGCSISMSSADILAEDLIGKTPEEARAQVERFLAVLRGELDVDELDAYGDAAAFKGIARL
PVRVKCAALAWRAALAQLDAIKSKGNKAAAS*
>SPBIB_v1_190024|ID:27162818|atoA| acetyl-CoA:acetoacetyl-CoA transferase, beta subunit [Uncultured spirochete bib]
MPEYIENKEIIAQRIARFFKTGDVVNLGIGLPTLVGNYIPKEVTVILQSENGLIGLGPEP
APGGEDKDLTNAGGKAVTILPGGCFFDSATSFGIIRGGHVDYTVLGVLEVDQEGNLANYK
IPGKMVPGMGGAMDLVAGSRHVIAATTHFEKSGASKLKRRCSLPLTAAHEVDYVVTDLGF
FVVSGHSFILKEVFAPWTAEWIIAHTEADVELAEDILLWEPA*
>SPBIB_v1_190025|ID:27162819|atoD| acetyl-CoA:acetoacetyl-CoA transferase, alpha subunit [Uncultured spirochete bib]
MIAKPVISAREAAALIQPGSVVHIGGFLGCGSPDAIIAALCEAHIGELTIVCNDTAIYDP
KTGRTTGLAPLILNHLVRKVITSHIGTNAETQKLMSSGEIEVELVPQGTLAERIRAAGFG
LGGILTPTGAGTEVEQGKHVFEVNSRKYLLELPLPGDVAIIKAKKGDRAGNLVYAKTARN
FNPIMAPACSLVIAEVEELVEIGTIDPDHVHTPSIFVDYLVTA*
>SPBIB_v1_190026|ID:27162820| Na+/H+ antiporter NhaC-like protein [Uncultured spirochete bib]
MENFGILALLPPVLTVLLAIWTKDVIVSLFLGIFAGAMTVAGGNPLQALVRLTDLLAEML
ADGWNIRIILFCALLGALVGMWHKTGAAYAFGSWAGKKAKSKTSVLLLTWIFGIIIFIDD
YFNSLTIGACMRPVTDEKKISRAKLAYILDSTAAPVCIIAPISSWVITVMSYTKGSEGFD
KLGISEFTYFIRTIPNNLYALFAILMVAFVAFKGRDFGPMARSEARAEKGLGLFDEKKYG
IVAGKLDQKAATTNATWFDFLIPLLLLIVCAVVFFPVTTWMGSIDGENIHNMGEAMASMS
LGEAFNNTDASKALFYSIMFAIVFSYIYFLLRRLLNISQSAEAIVDGIKSMVPAIVILSL
AWSIGFIIKKSPEDGGVGLANYLAQAVRGGNFPLWLLPLIVFLISCVISFSTGTSWGTMA
IMIPIAMPIAVALGEKMGLSGNALINITVYSTASVMGGAVFGDHCSPISDTTILSSTGAN
CPHLEHVATQMPYAVFVAICAAFGIVTLGITDSTILALLVTAVLFAVGIAVLPKVWGVQK
YKLED*
>SPBIB_v1_190027|ID:27162821|kamE| D-lysine 5,6-aminomutase beta subunit [Uncultured spirochete bib]
MAHIIRPYGDTMDDGKVQLSFSLPVPAGSRALEAARRIALAWGFHEAEVVHMAPMSDEFT
FFVVYAATQHGIDWDTVEGDRDDSEKPMTMDEINAFIRAHIGRKVVVVGACTGFDAHTVG
IDAIMNMKGYNHHYGLERYEMIEAHNLGAQVPNETLIDYAEAHNADAILVSQVVTQKNSH
VHNLTEFIELLEAKGERGRFICIAGGPRISNKLAAKIGFDAGFGHGTYAEDVATFIVKKM
VQEGRK*
>SPBIB_v1_190028|ID:27162822|kamD| D-lysine 5,6-aminomutase alpha subunit [Uncultured spirochete bib]
MVTNKLNLPKEKIDRARELAKNICAPVLDFIDAHTTVTVERATLRLLGADGVSTEGAPVP
NLVVDALRDKMSEGAAVWYANALVQTGLEQGELDRRIAEGFNIATLPMGDRQKAKELAAA
LARRQFSRIDANRAYRESKLEEFKGRSHSPLLYLIVATGNIYEDVKQAQAAALAGADVIA
VIRTTAQSLLDYVPYGPTTEGFGGTYATQENFRIMRQALDEVMEREQRYIYQTNYASGLC
MPEIAAMGALERLDMMLNDSMYGILFRDINMYRTFVDQRFSRMINAYSGIIINTGEDNYL
TTSDAFEKAYTVLASQFLNERFAYAAGLKPWQMGLGHAFEMDPSIENGILYELAQAQMAR
EIFPEHPLKYMPPTKHMSGDIFKGLVMDNMFNFVSKATGQGIHLLGMLTEAIHTPFMMDR
HIAVETALYVMNGIESIMDEIEFRPDGLIVRRAHEVLDRTLGFMEEVERIGLMESIEKAM
FADISRSRTGGKGFEGIVEKGEGYFNPVESMLERSLGLA*
>SPBIB_v1_190029|ID:27162823| protein of unknown function [Uncultured spirochete bib]
MKKPLPVPPHVLSFSQADSLFARLAPLSPYGKERVAEIGFFTDFEALESFYDDIEAAGAA

LDSLEHSARRDKLFWHLSRLTRIPESLAGTSGNSAPGSSGAANFSASNSAAKKISASSPS
NADAWGLMELFLVKKFLVHYKGIFELLDDEARTRFGFFFSTQELLDVLSAGGSDAESFSI
SERHEPALAAVRARIARLSETALERRRKLQEAARTAYGIDFAGREMLILPVDKALDAMGK
GRLGQGPLLAADPYDSRSMMIRLVPDTELLEAEEARRKARDEEKLLEQAAIVRLSALVVA
AKDALQSYCRAVKRFDYSLAQWKLAQELGMVRPRLRARGTACIQCHGARFIPLEEECRER
GVAYMPLDFELDTPVGILSGSNMGGKTCVLQTLVFLQLLAQCGMFVPAAAFETVVFGWID
VIGAAGESRGRGLSAYGFEIRRLIEVLHAAKNEPGFAVFDEFAHTTSAKEAQALMQAAVE
QLASLSGTVALFATHIECSLPEGKGTAYRMAGLDSERARSIIESTPAQSERSLDALLAQI
NSLMQYKVIRTGKASAHAKSDALAVARLLGLDKDLVDRAQELLAESDTRRAPPAQDKQKE
RGAE*
>SPBIB_v1_190030|ID:27162824| exported protein of unknown function [Uncultured spirochete bib]
MSLNAALFAGRVNFIAGFAAASGKTMLAKAILALLREKGLKCALISAGYEGGGREPAGAP
ALKMPSLEMPTFEVEKGEVFSTAANALPFLDCMPELIAGPVGSSALGPIVLARAERSGSA
ILAGPARSEALATLVEAARHLRGIDAVIIDGTLDRLTQISVLPDPQLYCAVRADRADCQQ
IAKRMATFYQLVHLPKWDASRAREGEALIFEGALSSATLEKHKSARDIPILIKDITRVFL
RPDEIASLSARGRLFIQQEISFRGFVVALRDIEPEEFMFYLPHDVADSILSWNPYEHEEG
SRFAQQAQAADIGRSARYKRGDV*
>SPBIB_v1_190031|ID:27162825|kamA| L-lysine 2,3-aminomutase [Uncultured spirochete bib]
MQEFPSFDWRRIPLYKEVTAKEWYDWHWQMAHNIRDIDTLAQVVPLTDKERHDISEVLKV
FRMAITPYYASLIDPANPVCPVRLQAIPRIQETHFLASDQVDPLHEDVDSPVPGLTHRYP
DRVLLLATNICSMNCRHCTRRRFVGHEDAHQSEELIDAAVEYIRKTPIIRDVLISGGDPF
VMSTERLERIIAKVRAIPTVEIIRIGTRTPVVMPMRVTDELVNMLKKYHPIYVNTHFNHP
KELTKEAKEACEKLADAGIPLGNQSVLLRDINDCPVLMKKLVQKLLVIRVKPYYIYQCDM
SLGISHFRTTVSKGIEIIENLRGHTSGMAVPTFVIDAPGGGGKIPVMPDYLITSNEKRVI
LRNYEGVITSYEEPETYHEDCGQCRICYDEPWLNPTCGVAKLLSGEEKVLEPKNLKRGKC
*
>SPBIB_v1_190032|ID:27162826|kce| 3-keto-5-aminohexanoate cleavage enzyme [Uncultured spirochete bib]
MDRKMVITCACTGAETTKAQNPALPVTPEEIADAAYDAWRAGAAILHLHVRDGKGNPTMS
PRIFKKTMELIRRKCDIVIEITTGGAVGDSPEARLAPLVELEPEMASLDCGTVNFGNDYI
VNPLPVMRRFAEAFRKSRVRPTLECFDVGHVYASRILIDEGLVEPPFHYGFVLNVPGSVP
YSIENLLAFQHALPSGSFFTVMGIGRASLPAQYGAIAAGGWIRVGFEDNVYYAKGQLAAS
NAELVERAARIAKEAQFGIATSDDVRSMLKLRR*
>SPBIB_v1_190033|ID:27162827|kat| 3-aminobutyryl-CoA aminotransferase [Uncultured spirochete bib]
MAAKEYPRLKLDRSMQMYTEAKEICPGGIMGIRRPYNFVEGEYPIFLERGYAGHIIDVDG
NDYIDMLCAYGPIILGYNEPEITQAVQAQLEKGFCFSLVQPIQNELERRLVSLIPCAEQA
ILVKTGSDATSLAVRIARGYTDRKYILRCGYHGWHDWCVEVQGGVPEEISSLTIEFEYGK
LDDLEEKLAAHKGEVAGVIITPVGHPNAKPVMAPPPGYLEAVKELVHAYGAVLIFDEVRT
GFRVSMGGAQERYGVTPDLGTFGKALANGYAISAVVGKREVMQVAEKKVFVSSTFFPNSL
EMVAAMKCLDILEREKVPDSLWKRGTQFLARLREIAERSGMPVTVSGIPPMPYMTFNPHP
ADKEKTYRARREYFYTQTIRRGLFIQPYHHWYIAHRHTDADLEKALAAIEEALELTARRF
PIGS*
>SPBIB_v1_190034|ID:27162828|kdd| L-erythro-3,5-diaminohexanoate dehydrogenase [Uncultured spirochete bib]
MPMGDKYGTHRVIEPAGAMPQTAQKLDNDMSKLFDNEILVDVIALNIDSASFTQIENEAA
GDEEKIKAIIMNIVSTRGKQQNPVTGSGGMFIGRVAKIGPALASRDLKPGDKIASLVSLS
LTPLKIDRILAVHKDIDRVEIEGQAILFESGIYAKLPDDMDERLALAALDVAGAPAQTAK
LVKPGDKVLVLGAGGKSGLLCCYEAMKRVGPTGRVVANIHSERSRTYIQDMGLAHEIVVA
DATKPVEFLTQVLSANKGHEFDIAISCVNIQGTEMAAILPVRDGGIVYFFSMATSFTRAA
LGAEGVGKDVLMIIGNGYTKDHAEITLWELRENPKLRAFFESQYVK*
>SPBIB_v1_190035|ID:27162829| protein of unknown function [Uncultured spirochete bib]
MHDDLEKGRRMLSSFNFKEYETVTANSNAFKGVEIDLMIEILKDWKQSPGDPYTLLELRD
GKTLVAFALIHRVSGRNFTFDIRFIVLDQDYRSSSAIQHLFDMIDAELLKKIPFAVIRVE
ISSVKRDSLGEQALENAGYSLIGHIPTYYGENDDFYFYIKAIFRNPPNFIKISKPFEDQA
ASAAPLEEIHTLEE*
>SPBIB_v1_190036|ID:27162830|yvgM| putative molybdenum transport system permease protein YvgM [Uncultured

spirochete bib]
MFDLSPLYISLRAATLSSAIVFVLGTVVARLCYTMQGRKATIVDTILTLPLVLPPTVLGF
FLLVLFGKNGPFGILLAKIGQRGILFSWKASIIAATVVSFPLMYRSALGAFRQIDQELLW
AGRTLGMSEWKLFVHVMVPEAWPGLIAGLALSFSRSLGEFGATLMIAGNIPGKTQTIPMA
IYFATAGGDMQTAWIWVGIIVAISCVSLALTTHFDSRRKG*
>SPBIB_v1_190037|ID:27162831|yvgL| putative ABC transporter substrate-binding lipoprotein YvgL [Uncultured spirochete bib]
MKKRFFVCLFVLLSVVAGFAQGSSRQKTELLISAAASLMDCMNELKAVYMAKNPSIIIRC
NYGSSGALQQQIEQGAPADLFFSAGLKQMQALIDKGLMDTSTVRNILENYVVLVVPKNGI
KLSSFDDLRNPSVTKIGLGDPKSVPAGQYAVQVFRNLGLSDAIAGKLVLAKDVREVLFWV
ETGNVDAGVVYSTDAQISDKVTVSAIAPENSHQKIVYPVGVVKDSAHPKEAKAFEDFLFC
DEAARIFAKYGFSVI*
>SPBIB_v1_190038|ID:27162832| Molybdenum cofactor synthesis domain protein [Uncultured spirochete bib]
VQRGMIKAICASSEKGTPKRPMKEATLKANWGIEGDAHAGTWHRQISLLSADTVDAFNRQ
GANVADGDFGENLLAYGLDFPCLPVGTALVCGDVVLRMTQIGKVCHSGCDIQKRMGKCIM
PTEGAFARVLHGGILRPGMPIEAYTAQRVFILCASDKGYAGERKDESTPALHHLVTEEGY
EVVGTALLPDDREQLSALMARICDSYTADLLLTTGGTGLSLRDVTPEATIDIAERMVPGL
AELMRLRSLSVTARASLSRAVCATRKQTLIVNLPGSPKAAVENLQAILESLPHGLAILQG
TQGECAVDSHI*
>SPBIB_v1_190039|ID:27162833| Molybdenum cofactor biosynthesis enzyme [Uncultured spirochete bib]
MTDGDAGLRDSFGRIHTYLRLSVTEACNYRCRYCGPGVVHRGLSDDQLVFLCSLFRSMGI
RALRITGGEPTVRPGLIPLIARLSSLGFERLALTTNGTSLVRDARALKEAGIQSVNVSLD
AVDADLYASLTGGFPVRPVLEGIAAALSEGLAVKLNCVLLADTYRNQVRQLMAFARKLGI
PLRFIELMPFGDGARWEGVSTATLVAFLGEEYGEEASPRGPKGSEPWGSGPAEYRRFGGV
DVGFIGALTSCFCSQCQRLRLTNEGQLKTCLYHPDRLDIRQLLERGSPPEEICRRIEAFV
LRKQLRHAFQTGPVEWPLSSVGG*
>SPBIB_v1_190040|ID:27162834|moaC| molybdopterin biosynthesis, protein C [Uncultured spirochete bib]
MNGAMTHVDEDGKAIMVDVSAKVDTKRTAIASGRIFMSRQAFDAIAGGTVPKGDVLAAAR
IAGIMATKQTSSLIPLCHNLLLTSAQLDFRLLSEECAIEAVCTVASTGKTGVEMEALTGA
TISLLTIYDFCKAIDRSMHVEDVHLQFKDGGKSGCYDRR*
>SPBIB_v1_190041|ID:27162835| Molybdopterin biosynthesis enzyme [Uncultured spirochete bib]
VKLIRTQDAVGHILCHDMTQIIVGVTKDARFRKGHVVREEDIPVLLSMGKENLYVWEKQE
GMLHENEAAEILCNATKNDHMERSEVKEGKIELKATCEGVLQIDLERLLRINSLGDVIIA
TRHNYARVKKGDKLAGMRVIPLVIDAAKMDQVAAIAGVSNPVLSIKPYVIRRCTVLVTGN
EVKKGIIEDTFSRVVEKKLAEVGVSVSRKILTGDDEVYISSCIKQAVADGEEMVLCTGGM
SVDPDDRTPKAIRNSGARIVSYGAPVLPGAMFLVSYIGDVPVLGLPGCVMYEDRTIFDIL
LPRFVARIPVTAHDIASLGNGGLCLKCRTCTFPNCGFGNGGI*
>SPBIB_v1_190042|ID:27162836| Molybdenum-binding protein [Uncultured spirochete bib]
MAFSFYIRIPFTSDGSFFGPGVNELLHNIDTAKSLSAAANMMGMSYSKAWKIIHVAEKKL
GYPLTVKSIGGAAGGGSSLTEKGRSFMERYDAFVADSYKAVEACFQRYFPEAQQ*
>SPBIB_v1_190043|ID:27162837| UbiA prenyltransferase [Uncultured spirochete bib]
VTFRQFSGIVELRTKIVSASTYSIGLLYALAVKGSVDPLKALLLGVAGLLVDMGTTGFNT
YFDWYRNVDDPRFNREEAKVLIHEGVSPGSALGISLICFGLAGAIGFALTWMVGLPLMVL
GLCSMLVGFFYSAGPKPISSTPFGELFAGGFLGSVYFCICLYVLTGTISGRFLLVSLPQT
LAIAAILSANNACDIEGDSAAGRWTLAVVLGKALAPLLIYVEMGSALLLLMIFGFSGILP
LYSAWCAMLGGIGVVFILNRMHRIGYSHETKGPIMGKISLAFMVQSAAQIAALSLMLIFS
KGFLN*
>SPBIB_v1_190044|ID:27162838|guaB| IMP dehydrogenase [Uncultured spirochete bib]
MGFESFSGGAEGLTFDDVLIVPGYSEVLPSEVDISAELVPGIRLKAPVLSAAMDTVTDSR
LAIAIARIGGIGIIHRNMSPEAQAAEVYKVKRSESGMISDPVWLPETATLAEAEHLMETY
RISGIPVVDPQTERLVGIITNRDRRFCGPEDMQKPISEFMTKQNLVTAPAGTSIEEAKAI
LRQHKIEKLPLVDSEGRLKGLITIKDIFKRDEYPDAATDSRGRLLAGAAVGVGADLEERV
ALLASRGVDVLVVDTAHGHSKKVIDAIRRIRSAAPHIPVIAGNVVTAEGTRALIEAGASA
VKVGVGAGSICTTRIISGAGMPQLSAIYECAKAARAYGIPVIADGGIRYSGDIVKAIAAG

AETVMLGSLLAGLEEAPGELVLYEGRQFKSYRGMGSVGALQGYGRDRYGSGQSNGKLVPE
GVEGMVPYRGKLSDYMVQMLGGLRSGMGYAGARTFTELRKARMVRITAAAYAESHPHSIV
ITKEAPNYQKRD*
>SPBIB_v1_190045|ID:27162839|purH| Bifunctional purine biosynthesis protein PurH [Includes: Phosphoribosylaminoimidazolecarboxamide formyltransferase ; IMP cyclohydrolase] [Uncultured spirochete bib]
MPLALISVYDKKGLEQFAQRLARLGWTFLASGGTSAALRGAGIEPVDIAEYTGSSELLSG
RVKTLHPAIHAGILARPEPADLEELRAHGYKPIDLVVVNLYPFEKTAAQANASEAEIIEQ
IDIGGVALIRAAAKNFARVCVICDPEDYERIALEAESGGIGAETRRELAAKAFAHTAAYD
RAIAEWFAQKAGGAAEREAATGAALASGILPASFEFRGIVERTLRYGENPHQKAFFVIPV
GDWGGSFVSMRKEGSFASEGGSAVSPIVESGGPSPGQPQVRGSIGGSSESIQPIVRGPLG
GRSDSIQPKVRGPLGGQVLGGKELSYNNLLDLDAAWRAVLSFEQPAAVIVKHLSPCGAAE
AENLKDAYEAALACDPVSAFGGIVALNRPLDVSTAEALRELFLECVAAPSFEEDARDVLK
AKKNLRLVEADPLVFSTLCKELRSAAGGLLVQEPDRGDPIDTTWRVVSARQPTEAEMESL
RFAWKLVQHVKSNAIVLAKGHASVGIGGGQTNRVDAVRQACERAGARAKGAVMASDAFFP
FADGIEAAAEAGVTAVVHPGGSVRDVEVLAAADRLGLAVVHTGVRHFRH*
>SPBIB_v1_190046|ID:27162840|purN| Phosphoribosylglycinamide formyltransferase [Uncultured spirochete bib]
MSNVLRLVVLASGEGSNFQALLDAIESGSIRGARIEGLVCDVPGAGCVQRAVQHGIPSFI
LPGPARAKRGSPERLAYDERLAVIVRSWRPDYVLLLGWMRLLSQAFLQHFPERVINLHPA
LPGTFPGTHAIERAFEAFLAGSIEKTGVMLHLVPDEGVDAGPVLRFAEVPIYKEDTLEIL
TARIHSVEHREVVAMVEDLARSKSINYFYEKEIANASRADFGL*
>SPBIB_v1_190047|ID:27162841| phosphoribosylglycinamide synthetase phosphoribosylamine-glycine ligase (modular protein) [Uncultured spirochete bib]
MNVLLVGSGAREHALAMAIAASPLCEKLVIAPGNPGMADVGELAAVALGDIEGLVSLAVA
MQADLVVIGPEAPLAAGLADRVREQGILCFGPSAAAARIESSKAFAKAFMRRHGIPTAEG
KSFSDAEQAKRWARNFVRPVVVKASGLAAGKGVIVPSSLEETEAAIDTLLELGGDIVIEE
KLEGEELSLIALCDGTSYKVLPAACDHKRLEDGDKGPNTGGMGAFAPACTIEEAEQLARI
VIEPAMRGLAEEGTPFVGALYAGLMLTEEGPKVLEYNARFGDPETQAILPLIRSDVLVLL
EACARGAIRVTPLDLRVSSAAVCIVLASKGYPERPVTEKPIRIGVLPERAFCLHAGTKKP
GTKKPGDELVSAGGRVLSVVGLGASLEEARAVAYETVTQIHFDGMQYRSDIGNRADRVAK
AICAALGRVESHDSEGAESAYAKAGVNIDAGNRAVELMKEAVRSTYGPEVVAGIGAFGGQ
FDASVLKGFVKPVLVASTDGVGTKTSLGLALGRLEGLGMDIVNHSINDILVQGARPLFFM
DYIAAGKLDPEHVAEVVSGMAQACREAGCALLGGETAEMPGTYQAGEVDIAGTIVGAAEA
EKLLPRADLSEDDILLGLASSGLHTNGYSLARAVIAGMDLEEVQPELGESLADALLRPHR
SYLPLLERALEAQPSPVKALAHITGGGMVENLPRILPPHLDARIRADSWTWPPLFMLLQA
WGSVSDEEMRRVFNLGIGMVVIVGANETDRMRALLPEPAFVIGKLVPGSGKVLFV*
>SPBIB_v1_190048|ID:27162842|purF| Amidophosphoribosyltransferase [Uncultured spirochete bib]
MNLDCERFSHEPRHCGGSANGWRSGKPRPGEPYPDDSQPRESCGIVAVIADEAARVAFFG
LFALQHRGQESAGIATLDKGSIHMHKDVGLVSQVFRDQNLSALAGSIAIGHTRYSTTGKS
SARNAQPFVIDTQYGPLALGHNGNIANAPALRKTLLSRGLGLMTGSDSELLAMMLAGTQG
SDWIERIEAAMKEWVGAYSLVILTAEGVYAVRDPWGFRPLAWGRIDAGGFAVASETSALR
VMGCSFYEEISPGEILHFDTKGLVQRARADIDVSHAACSFEYVYFSRPDSVWNGKNIHAV
RRRLGEILAEEAPAAADIVIPVPDSSIAAAIGYARQSGIPFGEGLVKNRYIGRTFIEPTK
ALRRQGVALKFSPLRETLEGARIVLVDDSIVRGTTTAPLVKLCRQAGAREVHLRIASPRI
LHPCYMGVDMGTPNELIAVGKGEDEIARLLGADSLAYLSIEGLSEAIGVKGLCRACFDGQ
YPIPVDEGFTKESFETERSAEASGHPETIGRGQV*
>SPBIB_v1_190049|ID:27162843|purE| N5-carboxyaminoimidazole ribonucleotide mutase [Uncultured spirochete bib]
MEAEMVQGRVVILAGSPADMAHVDAIQRALGSFGIMSLVRIASAHKTPCRLLDMIGRYEA
EEIPTVYITVAGRSNALSGLVDAATPYPVIACPPPSEQWAMVDIWSSLRMPSGVAPALIL
DPANAALFVAKLFALENTELRGRIASFQKQNADRLIEEDEKVLKAGRAE*
>SPBIB_v1_190050|ID:27162844|purC| putative phosphoribosylaminoimidazole-succinocarboxamide synthase 2 [Uncultured spirochete bib]
MVSENLIQMLLPKAFNSTGRAEGEGKVREWWGLEGGTRFIATTDRLSAFDRVLARVPFKG
QVLNELSAFWFRQTADIIDNHLISVPDPHCSVVREARPLPVEVVVRGYITGVTSTALWRR
YELGERVIYGQRFPEGMRKNQKLPQPIITPTTKGGPTGHDERLEPREVVEKGYLDARTWS

AVQDAAIALFERGTEVAVRAGLILVDTKYEFGLNSDGTLMLIDEVHTPDSSRFWLASSYA
ERFEAGQEPESRDKEFVRLAYAEKGYRGDGEPPLMPDALWVQASLVYQELYERLTGMDFV
PSAYPVEGRVLEALARERAVRRGVEAGAGKSRRG*
>SPBIB_v1_190051|ID:27162845|purQ| Phosphoribosylformylglycinamidine synthase 1 [Uncultured spirochete bib]
MKALVLKAPGTNRDYDVAEAIEYAGGTTVLLPLSELRERPALLLDYGMLVIPGGFSYGDA
LGAGKLLALDFECFFAEAVMRFVEEKRPVLGICNGFQALIKTGILPGAPFERHTFTLAQN
ARGNFECRWVRLRASTSNSIWTRGIEGFSCPVAHGEGRFASSLPNVVESLRVAGCIALEY
ANERGEPAQGSYPDNPNGSIGDIAGICNSDGNVLGLMPHPENAVFPWQSASMPGWAEDGA
LRLFRNGIEYARS*
>SPBIB_v1_190052|ID:27162846|purL| Phosphoribosylformylglycinamidine synthase 2 [Uncultured spirochete bib]
MDMATRNFLYRIEVRNKGSGGPRIEALQADVRALSMGTLRSARQIALYFVEGEIAPEEMN
LLGLFLFSDPVEQEFRWEVVGQGRSEAIDAGGARTGDFGGAAAAEIVSLAPAEIAGAGAS
SPRVVEICRKPGVTDPVAIEIVRAAQELGVKGLKRAATGVRWELEADWLDEQSVARITKM
LLANPVIERWEFGEIRPVFPQESARPVPVETYDIAAMDDAALIALSAERRLALDLEEMCA
IRTWFAGAGRAATDVELEMIAQTWSEHCVHKTFKADVEVRGELWQKDAAAGAAGKLENAA
QPHSPYPPVVHGIFSTYIKKTTEEIAAPWVRSAFVDNAGIVSFDGQWDISFKVETHNHPS
AIEPFGGANTGVGGVIRDIMGVSARPIAVMDILCFGPPDMPEEELPQGSLHPRMIARGVV
AGVEDYGNKMGIPTVNGAIHFHPGYAANPLVYCGCVGLAPAGVHHHEPKAGDRVIVLGGR
TGRDGIRGATFSSMRMDGSTGDVAGASVQIGDPIVQKRTLDVLLAARDAGLYDAVTDCGA
GGLSSAVGEMASELGADIELEKIGTKYPGLAPWEIWLSEAQERMVLAVPEVSVGAFAELC
SRFGSEFWDIGSFRGDGRLVIRMRGEVVLDLPMEFVHKGIPQKRLVAEAPVKNRGQTTSL
PKGAPQKVKKRGLTPFFKNVSCERIVRRYDYEVQGGTVVKPFAGPEGDAPQDAAVLKPQG
TGGAWGIALSNALRPDYAEIDPYRAAWAVVDEAVRNAVAVGSDPERIAILDNFCMGDPND
PAVMWALLESARGLRDAALAFGTPIISGKDSFYNEYLGPDGRRHAVPPSLLVSALGFVPD
VSKAVTSFLKSPGDSIWLVGRFEPSFLDKDAAGAEADLGAVPGVDPGAQVVYKALFEAMQ
AGEVAAAHDLSEGGLTLALAEMCMGGRKGADIVLPPSQEPAGRAMKECAASPLGLHGMVR
DLLLFGESAGCLLVEVRAGQDEAFGRHFGEEQAFRIGAVSQMPVMRIHDGDSEILSVSIS
DMLKAWKDSAGEVLP*
>SPBIB_v1_190053|ID:27162847| Citrate transporter [Uncultured spirochete bib]
MGRQDGAYASGLCEPFTGTSETVREWPCGLFLCTAEFRIANMGVRAVNVFILVVLVLMYA
GIIAMQDKKAWVSLGAGLVLVIGGATSASHAISSLINWNILLIYLGSLVLAELFIYSRVP
AFLAERIVERSPSVGVAVAIILLLTGLVSAFVENVATVLLVAPVMIELARRTKKNLAQLM
IGLAVMANLQGTATLIGDPPSMIFADFAHYSFNDFFFRAGRPSIFFAIQLASIVGALYFF
FYFRSMEGDGRSLPAERIVSWVPAGLLGLMIAGLAGLSALSAGGGIHSASGIFVVSLAGV
GLGWLGWVRKERAEARNMVRHLDWGTLAFLVGIFVVVGTVSESGLLDGLGIALAKWTGGS
AALGFSLIIAFSVLVSGFVDNVPYIAAMLPVAAKFAATMGGRPELFMFGLLIGSCIGGNL
TPFGASANIVAVSLSEKNGAKVNVWSWLKIAGSFTVLTTVAAGLFVWFVWR*
>SPBIB_v1_190054|ID:27162848| conserved protein of unknown function [Uncultured spirochete bib]
LRLQGEEVFMHHFHFCPNPACAYHQHAPKERWFVAAGFYVTKTFGKVQRFRCSFCGKYFS
TQTFSLDYFAKRKIAYPQIETLLSSSMSIRALSRFFSASCGTIQNRIDRLAXX
>SPBIB_v1_200001|ID:27162849| ABC transporter related protein [Uncultured spirochete bib]
MNILSLKNISLSLKSGPLFESVRMDIDERDHIGLIGKNGAGKTSLLRLIAGLVEPDRGTI
SRTKNFTFSYLAQNTEFDPSLSLREFLYQGESTEIRMLVQGPTLHGRDSKRDHEAQPPIS
VENRYFALLRELGFSDFDAPMQHLSGGELKKAALARALAPYSNFLILDEPTNHLDVETIE
WLESRLRSAAQAFILVTHDRWFLDATVNRIAEIERHTLTLYPGSYAKYLEKKSVELASLS
RSENKRLANLKIELEWLNRGARARATKSERRKKEIEAMRASLIEKPAAAYTFMSQEVRLG
KKVCVLKDVSLAYGDRALFRGFSHEFLPGSKTAIIGPNGSGKTSLLSLIAGLRKPTAGSI
TLGETVRLSFFRQTNEAVNPEMSILDYIQEHADHFRLPDGSSLDAVLLLERFGFERTFQM
QKLRTLSGGEIRRLMLVRLLAESPNFLLLDEPTNDLDIETIERLEEYLSDFSGSLIVVSH
DRLLVDRLAQDMLIITGKGTIERFTGTYFEWNIRKQQEAETQFESKQKESALQKQKQPSP
KSQGQQFTLQSRKPQSTPARTKLNYKEQKELEHLTEHIEQMEQRKKALEEAFQGPPKPGS
SLAHAKKEYDELCRLIESDLLRWEELADRL*
>SPBIB_v1_200002|ID:27162850| putative Branched-chain-amino-acid aminotransferase [Uncultured spirochete bib]
MTTDKENKGSRFESMCVPGSLEPADLASLDWSKLGFSVTVTPYMGGSVAGPDGVFEPCAV

VKTGVLRVPPQACALNYGQSIFEGMKARRGVDGKIRLFRPEANAARFARSAERFMMPAVP
QDLFIETVTKVVKANRDYIPPAEKGSLYIRPVLFGIGKTLQPAPADTTMFVVYVQPVGVY
FKGMATISIKADDTFQRAAARGTGWVKAAGNYAPCFLPAYEAKHEGFSDVLYLDHDGVNV
EEVGSANFAMVKGGKLYVADSPSILKGITRDSVMRIAMEMLGIEVVFAPLELERVLGLGK
YASEGPADEAFCTGTAAAISPIGSMKWKGQSYTFGGGAIGPITQKLFDAIDGIQTGRRPD
PYGWTVVVE*
>SPBIB_v1_200003|ID:27162851| protein of unknown function [Uncultured spirochete bib]
MPRSAKAIRWEIYSRPNAKQGKPVYYYVRAVDIQTKKALLTRSTGSDSRKAARELIAELSA
KMDFTRLAAAKTGNVDAASASQMENMPLSEYFVLFWNKEKSFYLLARADSGKPLSSAYVA
AQYANVKTHAEPFEGFKTTALRDANLFLIETYIRHLRAKGVSGNVVGDCLNAIRTPLSWA
MKRGLVLEPFSFSGIIRPKETYRTRGMITREELARIIALDVADTIQPRPRLKKGETHETP
GPVDLRIKVGVLLAHLGAMRAGEIRALRWAAIDFEQNRIHIEQNFVEGDGLKAPKRESYG
IVPLAEPLREPLQALRTLAFKLGRYNINEYVLFSLTTPNKPISIKGLEHGYSRLLEWIGI
PPEAQKARRLTLHGGWHFAASLLADEIGPALARKITRHRSLQAFKGYDHESAEALDAARK
ALQFEPKNEKAAE*
>SPBIB_v1_200004|ID:27162852| transposase [Uncultured spirochete bib]
MYRTKEKVPEFEKFDMVFGGKLNRQNRWVILANLIPWDRVEEKYAALFVSNNGRPALPVR
VALGALIIKEKSKLADEELVEYIRESPYLQYFLGFEGYKDELPFDPSMMVHFRKRLSGNI
LKEINAMIIERQKEEAEKSHDDQEPPEGGNKGTLIVDATCAPIDIRFPHDVTLLDEARRK
TEQIIDTLHEQAPTGYEKPRTYRKIARKEFLRFIRNRKPREQTIRKALKKQLQYIERNLR
IINDYKGLVGLGGLSRKQYSDLVVIHELVWQQRHLYTKKSHSIEGRILSISRPHVRPIAR
GKARGMYKFGAKLSVSLVNGLVEVHRLSWEAYNESQDLKGQIEQYKRRYGHYPEVVCADK
IYRTRENLQYCQEYGIRLSGPKLGRPFAESEKNRAILREQRRIEREDESTRIAVEGKFGE
GKRRYSLDRIGTKFRKTSESAIMLVYLVMNLMVLYRKKAKAFFVSLLDGLFKIVREHLED
NTGCLLRLDTVKRGFFRNPYVQLYA*
>SPBIB_v1_200005|ID:27162853| conserved protein of unknown function [Uncultured spirochete bib]
MAIIRLEKVKKTYPLGKTVVPAVKGVDFEIQKSDFISLAGPSGSGKTTILNMIGCIDTPT
EGVVEIDGVKTSDLNDKEITRLRHRTIGFIFQSFNLIPVLNVYENIEFPLLIGDTGISQK
EQKKWIQFLIEEVGLGQWQKHRPNELSGGQRQRVAIARALVTKPKIVLADEPTANLDSAT
GETIINLMKKMNKELGTTFLFSTHDQKIVDLADHVIRLKDGLVSENYRPKAV*
>SPBIB_v1_200006|ID:27162854| putative ABC-type transport system, involved in lipoprotein release, permease component [Uncultured spirochete bib]
MVILKIAFRNLLEHKVKTAIVGTLIALAVMFMVTGNSILDTIRTGMKQSYSANYTGDLIV
HGTSKDSFSLLASGPGAASGSDIPQIPEFQTVRSSIEQMPQVQSVLPLLSGSASISLNEE
TAGFTMLWGANFDEYALMFPDSLEFTQGGFPEEPGPYILLSESVREAAEKETGKPLHIGD
SITLAGFGASGSRLREATIAGFFRFKRGNEQLNLVSLIDANILRSLKGMTRNPMGVDIAS
SSLGANGTTEAATTDDELFGNGGSLVQTIQPVQSGALVNYDAILGDTSIRNRYTELDPNA
WNFLLIRLKDSASIPATQESIQTMLTTGSIDAQVSDWSWGAGTVATLAMALQFIFNIIIA
IILVVAIIIIMNTLVISVTERIPEIGTIRAIGAGKGFVRRMILWETLTLSVVAGLVGLLI
GSVLLLVVNRVGIASSNMFFAILFGGSTLRPVLSMGSLVWSLLAAVAIGLVSSLYPASIA
LKIAPVKAMQKI*
>SPBIB_v1_200007|ID:27162855| putative ABC-type transport system, involved in lipoprotein release, permease component [Uncultured spirochete bib]
MLQMIRIALRNISRQKKRSILLGSAIAFGFLVITLVNGFTGGLLKTTKENLSHLFGGHIY
ISGSIVSPLGSELPMIQDSFAAEEAIKAISDRVASVQYRSKTTGTLYFGNKEQTQKLEGV
DFAAEQSFRTNIDISKGSLADIDKPGSLILPEATAKKLGVEIGETLLYRASTVTGQQNIG
EFTLIATTTSSSVMVMDTGYTSKAYLNTLIGLDEGDYQSINIFLKDVAEVQTVADRLYSN
LASNAPVSPRISADSTSSPRNMSAMRSSLMGMNQIVQVAKEEQWKGTKFSVTTIEDLMSP
ILSLLSIIQAVAFLVFIILLVIIMVGIMNSYRMVMIERTEEIGTMRALGVQKSGIRTIFF
TEALSEAVLGTASGFVLALLIGGILSLINFGSGSFLSLFLARGHFVLQFSIPEALKNMLI
ICVMSIMAVYLPARSAANLEPAQALRATY*
>SPBIB_v1_200008|ID:27162856| conserved exported protein of unknown function [Uncultured spirochete bib]
MRKRYALVSLVIVLLLVPMSGIFAQAASAVPDFKAMLKTIDERSNFTGRDFSAKIKMVSE
DPEKGTTTRVAKTFRRDRDDSFLILFMEPADQLGQGYLKLGDNMWFYDPQSRKFTHSSLK

ENVQGTDAKNSDFRQSTYSVDYTVTSWTEGKLGAYDVWILELQGATNEVTYPYKKIYVTK
KDQLLLKSEDYSLSKRLLRTSYFTSYAKIGTSYIANSVMYVDALVQGKRTSITFTDISLD
NLPDSVFTKAYLERVNR*
>SPBIB_v1_200009|ID:27162857| exported protein of unknown function [Uncultured spirochete bib]
MNMRTLFYSIVSAGLLMAGLFPLQAQTDDELFGSSESLVTEAPLVKPVESSASDISTGLL
KTETVKIGGAFTFSLAGTAAGDSTIFSDYPTPSVVSYADLYADARPADNFRFFIKGRFSY
PYTSDSSGNNPFSLREAYADIEPLSGINARLGKQTANWGVGYFFSPGNLLDFGTIDPENP
TEERTGPLAVKFQRSQRTSNYYLYLLLDSAYSGGPIGLAPKAEWVLGSSELSLGAYWQND
KPWAVTGSITFPMGPLDMFVEGAVKGNIDKNFLVLNGPALTAETRQDKIFGQGTIGFSWS
MDDSEGRYSLSLRGQYYYNGLGYENSDLFIAYPMQVASLLQQKTITVQDLKERGQHYGAG
SLGISKILSSDFGLSFFWLGNLSDSSGKTSATLSWSGLDKLNCSIAYSYAYGQEGTEFRP
VGSVASSITLKVSLSMATF*
>SPBIB_v1_200010|ID:27162858|regX| Sensory transduction protein regX3 [Uncultured spirochete bib]
MKARVLVIEDDQALADLMVLYLEREGLECHIAPSAEAALPIMEQNEIDLILLDINLPGMD
GFEFLQELRTHCTVPIIIVSARETEEDIITGLSMGADEYITKPVAPRVLVARVRALLRRM
HMQNNDEAGRRETQFGPYTIDFDACMLMKGSERVPLSAREFDVLAFLVENAGKTYAPEEI
YTQVWGQQYGDITTIGVYIQRLRKKIEANPAEPRYLITVKGKGYLFSYDN*
>SPBIB_v1_200011|ID:27162859| putative Integral membrane sensor signal transduction histidine kinase [Uncultured spirochete bib]
MTIKTKLLVIVLVIVLLPLISGGLLIAYQRFTAGTSYSFLLMESVQNVSRRVQLFLDSGN
YDAFSKLPDGTEIVIKSDDGRILFQNPTTLDISAFGKDTKQDYHMFRFSSKSGSGTVYLS
YPSSDVKTRDLYGLPFKIVFAIIILFIIGTALGILKGLDVSIRKLENATKKIASGDLDFP
ADEFITSDLASLGQALDQMRIQLKEDRERRDRFIMGVSHDLKTPLAGIQGYTAALRDGMA
DTPEKQQAYYKIIADKASILEGRLGHLINLAKVTTNEWYQTLEEQDLNSFLEETGRSLAE
YASFHGFFLEIKNELPKPCPLLFDKDMIRRVFENLVSNALAYGDKTIPVLIHGRRVSDSE
TLEIIVENGGTPIPPEHRDKLFEPFFRGDRSRNSSGFGLGLASVKSIIESHGWTINLDTQ
AMNRTRFVIQIP*
>SPBIB_v1_200012|ID:27162860| Alkylhydroperoxidase like protein, AhpD family [Uncultured spirochete bib]
MAQQFYKKMYSLPEGYIILYQALRTMKYMVKGKKRKEISPEFIERIMLAVTEVNGCEVCT
YGHTKMALEQGMSNEEIQKLLAGVTDGIPDDEVKAVFFAQHYADTRGHPSAASWQQIVDT
YGTTKALGILGAIRAIMFGNAYGIVLSAFRNRIKGKPVEKSNLLYEISMMISIVVFLPIA
AVHALVSSLLGVPIIKGE*
>SPBIB_v1_200013|ID:27162861| conserved membrane protein of unknown function [Uncultured spirochete bib]
VKTFKGILLIFGILGLTLLIWILATPSIEIIPLDRLSHILAGLSLSGLFLVFILSTRNKR
IETWFGGLQNVYVFHKYLAIFSVALIFIHGRLSESVAEISNGGEKTLSAAFGSFGQILFI
VLVLIALFSKKMKYENWRIFHRLLIIPYALGVYHTYFSSRYNLFEFSPLSIWVGLTSLMG
LSAGLYMLFFYQKAQFRYKGKITDIKRVSASSIEIAIKLETTLVYQSGQYVFLKIFQKGI
EKAPHPFSISGGDGSIIYLTIKGLGDYTKQLYSELKEGTAISLDGPYGHLDFEKGRDKQL
WIAGGIGITPFISYLKKHTADKTITLFYSFRGEEEAVYKDFLEKYQKDNPRFSVHFVDTA
KSDRLHFDGYELEAGTDVYICGPEKMIKQHSRYFKRHFKNARIFYEGFKFK*
>SPBIB_v1_200014|ID:27162862| TRAP transporter, DctM subunit [Uncultured spirochete bib]
MTLVLFGSVLVLLAVGAPIATAMAGSGLITILLSGLFNPENAINPVQAVQSFFSSIDSVA
LLAIPFFILAGDLMNKGGLAKRLVNFAGMFVGRITGGLGMTAVLSCMLFAAISGSGIATA
AAIGGVMLAGFKDHSYPKSYATAIVASASPVGIIIPPSIAFILYGTLSKASVGSLYKVGF
PSGILIIIALLVTTYLTAKKHKIPRPVRDLGNKSVKEVVIDTLVALGTPVIIVGGVFLGI
FTPTESAIAAVIYSIIVGVFVFKEIKLKDLPSILMQSAISSAGIMIIIAAASLFAWVMIY
LQIPQKAFEAVLSLGMNKYTLLFAVNITVLFAGMFLESSAIQVIIVPLLLPILEALHIDL
VHFGIILTTNLAIGMVTPPFGLTLLTSSRVLGASLKDSIAEVWPFLLSLIVVLFIITYFP
SVIMWVL*
>SPBIB_v1_200015|ID:27162863| putative TRAP transporter, DctQ-like membrane protein [Uncultured spirochete bib]
MKLLFKISDTIDRICGWAIVSLFSIMIISFALQVILRYFIGTGFKWTEELTRYANVWAVM
IGFAMIAKRRNHINISVLEEILKGRNKKWLIIVQQLISLVIFTIMFIISFRLIKLAGSQL
TTNMRLPKRWVYWIYPPAFSVFVFQTFIGILQGIQDVKMTSEVQA*
>SPBIB_v1_200016|ID:27162864| conserved exported protein of unknown function [Uncultured spirochete bib]

MKKRLIGGMIVATMLFVVVGGAFAQKEIKLGHIAAPNTAYDNWAKEFKRQVEEASNNKYK
IIIYGGGQLGGSTALMEGLQAGNIQLAVITTSDINQFVRELDVLDLPFLFRNWDHVEKFL
ASDVNKKLLTFGDQYGLHFLSNMPRGYRHVTTSAKVGPITKPEDLKGVKIRVAESSLYID
TFKALGANAQAMAWGEVYTALQQGTVDAHENTIVTTRDYKINEVQKYMSETGHVFAFATL
MAGYDWFRKLPADDQIIFQKAANDSAIKLGIIQKNDEAKAKADLISKGMIFNSVDTAPFI
EKVKPVIDKYAKGEIKKLYDEIVAIR*
>SPBIB_v1_200017|ID:27162865|mhpE| 4-hyroxy-2-oxovalerate/4-hydroxy-2-oxopentanoic acid aldolase, class I [Uncultured spirochete bib]
MSRISITDTSLRDGSHSVSHQYTSSDVEKVVAALDDAGIDIIEVTHGDGLGGSSLTYGYS
VENELDLVSTAVKVAKKAKIAVLLVPGIGTLELLKEAHKRGASVVRVATHITEADVSEEY
IKAAKQMGMFVVGFLMMAHMADIDKLVEQARIMESYGADVIYATDSAGALLPDDVYARVA
ALKNALRVPVGHHAHDNLGCAIANSLAAAKAGATYLDGSLGGMGAGAGNAGTEMLVAALK
KSGYEIGADLFKTMDAAEKVLLPLVVEKGKQMPLHDSNSLIMGYAGVYSSFMLHARKAAE
RFGIDVRNVLIEVGKRKAVGGQEDWIIQVAYDLAKATGKAI*
>SPBIB_v1_200018|ID:27162866|mhpF| acetaldehyde-CoA dehydrogenase II, NAD-binding [Uncultured spirochete bib]
MAKKIKSAIIGPGNIGTDLLFKLQRSPLLEVAYVVGVKESPGIEIARKFGIPTTIKGIDE
MLAVDEIKIVFDATGAIPHLQHAPKLKAAGKIAIDLTPAAVGPYVVPSVNLDESIFALDN
VNLVTCAGQATTPIVYAINRVAEVYYAEVVSSVSSKSAGPGTRQNLDEFIETTTKALQTI
GGADSAKTIIVLNPAEPPILMRNTIHTRVRKPDLAAIRASIDDIIKKLRIYVPGYRFLVE
PILQDDVVTTVIEVEGLGDFLPKYAGNLDIINAAAVFIGEQFAQRILEGRR*
>SPBIB_v1_200019|ID:27162867|mhpD| 2-keto-4-pentenoate hydratase [Uncultured spirochete bib]
MALSKYEIDAAANDLHNAWVTANPVTRLSDRFPNLDVDSAYEIQMLNVERALAAGKKISG
KKIGLTSKAMQNMLNVDTPDFGHLFSDMEVKDGRIERRRMIQPKVEAEIAFVLSRDLDMP
GEILVEDVLESTEYVIAALEIVDTRIKDWKISLVDTVADNASSGMYILGNVKKKIYEIDL
KAEKMDLFKNGEKMNSGSGTDVLGDPAFCVAWLANKMRNYGTLLKKGEVILSGALSAAIA
AEKGDTFKATYSSLGEVEVTFY*
>SPBIB_v1_200020|ID:27162868|todF| 2-hydroxy-6-oxo-2,4-heptadienoate hydrolase [Uncultured spirochete bib]
MEEKNQANPEIGESIITGGFKTNYHDVGSGDPVVLIHGSGPGVSAWANWRLVIPKLAARR
RVIAPDMVGFGYTERPENIDFNMDMWGKQLVDFLDALNLEQVDLVGNSFGGALALWMAIY
HPDRIHKLVLMGSMGTEFKITKGLDHVWGYQPSLEAMRVAINSFVYDKAIATEDLVKMRY
EASIRPGYQETFGRMFPAPRQKGVDMMASKYADIAAIRHETLIIHGREDEIIPIETSMTL
FQLIPNAQLHMFGKCGHWTQIEQNLRFVAVVEAFLAGEF*
>SPBIB_v1_200021|ID:27162869| Transcriptional regulator, IclR family [Uncultured spirochete bib]
MRRLSELCSALYNHGMPRKPKALSTASYKEDKSTDATPTVTQEPNRAFTPRPSGYIQSVD
RALSILEIFSSRKPEIGVSEIARLLNLNKSTVFGLISTLERRGYIEQNPDNGLYRLGLKT
IELSQNKLSAFSTAEIAHPILRKLVDTVKETVHLAIYDRGEVVYIDKVECDNALSIASFI
GKRNPAYCTGVGKCLLAFQTDEEIERVLRRPLEKRTPKTITEIKRLKDELATIRKSKIAY
DDEEFSIGLKCFAAPVIDARGAVCAAVSISIPTIRLDVERESFLETSVRGAAKAISEALG
YQNQ*
>SPBIB_v1_200022|ID:27162870| protein of unknown function [Uncultured spirochete bib]
MRFPGSEDKFSYVLGMPFGTEYIKAITSTKPFATMEADFSDLQGPAAAAITRGLSVVASD
STRAEALAVYAIMP*
>SPBIB_v1_200023|ID:27162871| conserved protein of unknown function [Uncultured spirochete bib]
VPSLRISFQRTDEQIQPGSEIKINWNQIFASDDQILQNPSTLLLRPKYTVAMFMGEKELY
SHEATIIIAFSVIDQSAFEKAWENDEARKIFHEKQIMKTLWPILRQQVLDGMTRLGLPGI
PLPWII*
>SPBIB_v1_200024|ID:27162872| Acetyltransferase [Uncultured spirochete bib]
MNITIRNETPEDFRRVEEITREAFWNLYVPGCVEHYLAHVMRSHEDFMRELDFVALADGR
LVGNIMYTRSFVENEQGGRLETITFGPVSVLPEYQRKGIGSALIRHTIQKARESGHAAII
IYGSPYNYCKHGFKSGRDFNIGNAEGRYPFSLLVLELKEGVFAGHAWKYHESVVFGIDPA
AAEEFDRTFEPKEKGWRYTQEEFSIACRH*
>SPBIB_v1_200025|ID:27162873| conserved protein of unknown function [Uncultured spirochete bib]
VRYFISIERIKLVRVSWLLYIQVDVDEHPDERGRYIITGSHQLHIRAKVSQSLAGRTALL

ELLPLSIEELNNAGIAMDRDEYLYTGFLPRIYQEGLDPSMLYRNYYQTYVERDVRQIASI
RNLLAFETFLKLLAGRVGQILNLSDLANSTGVSSTTLSEWLSILEASFIVYRLKPYFRNF
GKRLIKAPKLYFLEPGLVNYLLGIRDVQQIPTHPLLGGMFENLVVSEAIKACLNARMNPN
LYFLRDSKGLECDLLVERENRLMPIEIKASRTFSADFCKKFSAIRKLDPAFETGVIVYGG
DRSFEYAACKVIGFADTAQLFGGKTPADQQVIMK*
>SPBIB_v1_200026|ID:27162874| protein of unknown function [Uncultured spirochete bib]
VNIHTAGSSTSIQVKEMYIFYQRIFSILTLHQMD*
>SPBIB_v1_200027|ID:27162875| putative Metal dependent phosphohydrolase [Uncultured spirochete bib]
MRFPQLNYAPKKIQGFYIFLFMLVLFTSLYGFYRLKSHDFSMYIESIQHSSQIIVEQTAN
IVSYGFFQWDQMRHAIATDNIQFYQEQFDKIKELEPKYIETVTIQKEALNVEQMYQIAKQ
GNALAIRFRIFDDLGQNPLEGVYVLAEFNYKKLLSQLDSRHVLKIADQGYPLPFGFNAAA
SKSLLGIGHYLSAISVALLVVLLMTTFEQQSIRNHYHEDGLYRIISLFERKDSYTANHSR
KVALISEFIGKKAGLRGAALRNLTIAALLHDIGKIGVPEHIIDKQDKLTDEEFKIMKKHA
NLGAEIFRLYPEISHLSDIVLHHHERIDGSGYPEGLKGDEIPFAARIIAIADVYEALTAD
RPYHKATSPEQAVRTMASMPLDPMLLEIVKNDFQELEAMLRKKQEEA*
>SPBIB_v1_200028|ID:27162876| ThiamineS protein [Uncultured spirochete bib]
MKITLEYIGFLKIEGIKSGTVVEAPNGSTAASILDMCKLTGSYRKYIVPIINGERSSHDR
VLKDGDRMFIYLPVGGG*
>SPBIB_v1_200029|ID:27162877| Thiamine biosynthesis protein ThiF, family 2 (fragment) [Uncultured spirochete bib]
MVAANMHGPKTAKPGGQAKGADFLARNPPALEHIAGKRVGIIGLGGLGSNIAMMLARAGI
TSFRLADFDTVSLENLNRQHYFSAHIGLAKTDALAAQMKALNPGIELELWPTRIEEEDLD
AFAQDCDVLVEAVDDAATKAMIYAWFRQEKRALWLVTASGLGGLEPANTIRTMHLAGKLI
ACGDFETSSENGNGVCAPRVMLVAAHQALAVLRILAGVEAEA*
>SPBIB_v1_200030|ID:27162878| conserved exported protein of unknown function [Uncultured spirochete bib]
MQKISRFTLSLLAVAFIAFVLPLAAIAGDNALTITGTVVEVQKYGNLTVDIKPKALYDAG
FALGDVLNVTIGGNVLKIPFCTSYSDVDTGSLVVRDDQKNNLLVVAINMGNFSTKYNAKV
GDTLTFGLAEKAGYLSEYLIRQLKRTNVRSDYATDSIFANFRSIATTGIKPGLVYRSSNP
INNEIGRAAYADALAQAVGIKTVLNLSDSEADIQKYLAAPGFKSNYYKSLYEAGKVKALN
MGVDLTAPEFGAKLAEGLRFLIQNDGPYLLHCTEGKDRAGFVSAVLESLMGARLQEVVAD
YMMSYENYYGVKKGTDQYTAIANSNIVTSLTTAMSGLPKGTDISGMPLAKFAQWYLQSIG
LSSDEIAALKVKLSASAVLKAPNVTGTVTQIEKYGHAVTDITIADFNALGFKFGDMVTVV
FDNGFVLEAPYLDGYYVNNGDPLVRAYPGQTNIAVCINYGKLNVVAQVDVGSKFTIMLSR
PGAYLTQYEIRRLVRSNKREDYTSDEVFANFRNIALGGIAKGVLYRSSSPIDNQLGRAAF
ADRLIKEAGVRTIVNLSDSADNMKNYLAAKDFASPYYAALAGSNQVIFLNMNLAFASDEF
RANVIKGLVFMAEHNGPYLVHCTEGKDRTGFFAALVEALMGASKDEIVADYMQSYIDYYG
VKKGTDQYTLISQDVLGMLKVIAGTNDLDKADLAAGAKSYLLQGGMKAEQIEALKAKLST
PIAASDASPAGTLISAIFNAMRHQSREVFVS*
>SPBIB_v1_200031|ID:27162879| Oxidoreductase, 2-nitropropane dioxygenase family [Uncultured spirochete bib]
MRAINSKLLHVAVKEITLISRELKALVIGDLSARIPIIQGGMGVGISLSGLAAAVANAGG
IGVIAAAGIGLLEPDGFKDFLGANIRALRREIRKARSLTKGVLGVNIMVALSNFADMVRT
AIEEKIDIIFSGAGLPMNLPEYLHGSTTKLVPIVSSGRAATLLARRWLERYNYLPDAFVV
EGPMAGGHLGFKAEQLEDPAFALENIVPEVIQAVRPFEEKTGRKIPVIAGGGIYTGADIR
KFIEMGASGVQIATRFVATNECDASIKFKNAYIEASKEDLRIIKSPVGMPGRAIDNKFLD
DVEQGKKKPFTCPYHCIITCDVEKAPYCISLALLNAQKGNLENGFAFAGANAWRIEKIVP
VQDLMDELVEEYEASYKG*
>SPBIB_v1_200032|ID:27162880| Ketol-acid reductoisomerase [Uncultured spirochete bib]
MNTTFKSRVFAVEKVQFGPNEEYVVRGGRHLFPLLPKAFDGIRQIGVLGWGSQGPAQAQN
LRDSLEGTGIRVKVGLRQNSSSWKLAEAAGFRTENGTLGEMMDVARESDLVLLLISDAAQ
AELYPQIFKALKPGATLGLSHGFLLAVLKIHGDKFPANINVIGVCPKGMGPSVRRLYEQG
KHINGAGINTSFAVEQDIDGKATDYALAWSIAIGAPFTFQTTLEKEYLSDIFGERAVLLG
AVHGIVEFLFRHYQAQGMSPEEAFVHATESITGPISKTISRKGLIGVYNSLSEKDKEIFR
KAYGAAYRPNYEIHREIYDEVASGNEIRSVILANKRIEAMPMAKIDGTFMWKTGEKVRAQ
RNEAAIPLEPFTAGVYIACMMAQIDLLAEKGHAYSEIVNESVIEAVDSLNPFMHFKGVAY

MVDNCSITARLGARKWASRYDYILMQEAEPLWAEGKGEDEALMQKFLQHPVHGALSVAAS
MRPSVDIAVQ*
>SPBIB_v1_200033|ID:27162881| exported protein of unknown function [Uncultured spirochete bib]
MKHSSLFKHYWWAALVSLLFLAMSCASAPSAQAPSAPQSQAAVASSSGALPAPQQAAAQP
GVAAPKSDIWPKIYDDETYLTTFDSSWSEGEAVGAIKNLQNAMVEFTAGIPFSELDVDPY
GLRAKWSWTEGSFIKSANLIIPFDQVSYLLLEHYPMLNKEYKWGLLVGIANANPASIRTP
TRDAAERLGKAILVLAKARGAKVSLPNVRFGASLSPLSEAQAQAAGILQSGGIIISWVFK
ESPAEKAGFSPQDIITKADGKPVQKSDDLFSAINDAAAAGKSQIKIDGIRRSYRIDNKMY
VEIFVPVTFTLAIEQAGGAK*
>SPBIB_v1_200034|ID:27162882| exported protein of unknown function [Uncultured spirochete bib]
MNRKRIACAAALCAVFAGALFAQGADPKTVTPAWVTEKQIAQQTQTQTQAQPEAPKAKGF
NVFFIGYDYPTLSGALAPALAAWNSPFNFSLGFESANSAGSSMLSGLELEFFIAAPSKGL
RLQMNELVMIGYSLALKPVRFNIGARLGLSLLDVTDNNSASNTYTGLGGLVGPEASLYAE
LAPDFWLWVRGRYALSYFMSLISNGASPLDTGSNSLNTLSLEAGLAFRM*
>SPBIB_v1_200035|ID:27162883|aroQ| 3-dehydroquinate dehydratase [Uncultured spirochete bib]
MKNLNIAVIHGPNLNLLGTREPGIYGAETLDQINKEIEQEAKKCGATTEFYQSNIEGQLV
DYIHSCAGKIDGIVINAGAYTHYSIALRDAIAAVNIPAVEVHLSNIYKREEFRHTSVIAP
VCIGQISGFGAYSYILGLHALLHALGQAS*
>SPBIB_v1_200036|ID:27162884| Methylase involved in ubiquinone/menaquinone biosynthesis [Uncultured spirochete bib]
MNHRERHARLFNRIAIPYSWFFAGQTRSYARCFDIGRTALPSPQGKRALDIGCGTGAFTS
ALRAEGWDVEGIDVAQEMIAHAAKSGLRCSVVDILGAHSIADTSFDLVSAAYVAHGLPLE
DRLILYRECKRISKDTVLFHDYSENKRLLTSIVEYLEGGDYFNFIRTVRQELADNFSDVK
VLRVGPQSAWYICKP*
>SPBIB_v1_200037|ID:27162885| protein of unknown function [Uncultured spirochete bib]
MRPKDIDHRTAKTGFCRMRNHFLSHINAFDIPSFSPQRRRERPCAAADIERPLALRRRQG
CSSDIETSGIGSRLAGKKPAVGYGDAVEKPSMALAMIHVQTILYLVVSAKSG*
>SPBIB_v1_200038|ID:27162886| putative Membrane-bound serine protease (ClpP class) [Uncultured spirochete bib]
VHRSGKRNLLRYAGWLPFFMMLITLPAAAHAASPRDIIFGGIAWGLRQTSGPVDPGPNTF
SNSPDQVWVDEQGRAHLTLQKRDNVWTASEMMAKKDAGYGTYRFTVSSSVSSLDPNIVFG
FFTWDKAPEAFNREIDIEISRWGVKDGPNGWFTVQPYDVPGNQHAFNLPKAASYTFEMRW
DKGLVEFVLYCDGKLSEYWKYAGRVPEPGRARLRINLWLFRGKAPQGPGPYEVVISDFSY
VPSR*
>SPBIB_v1_200039|ID:27162887| protein of unknown function [Uncultured spirochete bib]
MRKRVAVMGAGISGRHSLNRNWRGKWYRWLRRSKKRMRLRMGILYYVLRSGHIPLKTKLL
GSLALGYFLSPIDLIPDFIPILGQLDDAIILPLLIAFTLRSVPKELVRDCARKAIREPVS
LSKNWRAGAVVVLIWIFILALLGCWIIKCIHA*
>SPBIB_v1_200040|ID:27162888| conserved protein of unknown function [Uncultured spirochete bib]
MDMTKDGEKMKLTARIFFIAGFYVVRYIIYGIKISGTKNIERCKSPLVFVSNHEGAFGPV
SLLSTLPVRAYPWVTHEIMSRRECAAYLQKDFTEAALKLGPRLNAIVSKIICYACVAIMQ
ALRAIPVYRNSRKILGTLQYSLNMLEQGKNILIFPEIPSTAKNTQLGDLYTGFLHLARMY
YSRHSQPLTFVPIAVNKKARRVSIGSPVIYNPDNPFWLEKERLKQEISGEIQDMFSSMEE
ARISG*
>SPBIB_v1_200041|ID:27162889| FAD-dependent pyridine nucleotide-disulphide oxidoreductase [Uncultured spirochete bib]
MKIVIIGNGIAATSAATKIREFDASCEITMLSDENTPFYSRPRLLDYLAGKVSFEQITIK
SEQWYNKLNIELVSGAKVFSITPQENKIITSKGDISYDALLVASGASAATPPFFRPDLKN
VFRLRTKEDADRIIAAAQHVRTAAIIGGGLLGIETGFALTSRGLTTTVIEIADRLLPKQL
DAESSLLLKASLEEKGLRFLTGKQAAALDADREGLCITCTDATVLRADIIVVSAGIRPNV
GFLEGSGVNVGRGIIVDEKLRTNIGNIYAAGDCAEFRSTLYGIWPAAKEQGEIAGAIIAG
QDAVYNGSIMSAKLKVVGIEVASIGDIAVGLGTRTESQREGSSFRKLFYENGRLKGAILI
GDTSDYFKLQKEIALPAPRQSA*
>SPBIB_v1_200042|ID:27162890| MATE efflux family protein [Uncultured spirochete bib]
MELAFLHQSTAQRRDRILNGPISQTLLFLAAPTLMLGIVQALMPLMDGLFINNIAGTLVA

SSVTFSEPVINMAMALAQGLSVAAMAIIGQLNGRGSFDESKRTSTQIVVMGSILGCLSAP
LLVVAAAIISARINQQISHNVFLYLSLYALVLPFSFLESIYNGLKNANGKPEAPFIRMVL
MLALKILFNFVFIYALRLGIVGCVLSSLAANILITVWMFWELFVKKGPDRLELRGFRFDM
MAVRQLFQVGAPAMLNTFILNLGFFLINTEVEKYGPVVLNGQGIANNITQVAFILPGAFS
SAVTTMVSMNIGAENPGKAKKSLVGGAVLSAITAAIVIAVIVPLSSHLTILFTRRADVLE
IANRALHIYTYSVIGFGVTITIQGAFIGLGKTRVPLVLGVLRIWFLRYLFILATEKYLKY
YAVFWGNLFSNYTASFIAIFLISRTRWESAIRQKEPKSS*
>SPBIB_v1_200043|ID:27162891| transposase (fragment) [Uncultured spirochete bib]
VTDRQGKRTKSLVNERQIVVYSAQYAQRARMERALAVEKASGQIYALSKDAKPSAYGAAK
YLKKVPFDRSTGECMPETEYLVMLDEARIEKEEQFDGYYVICTNVIGREQGEAPFSGPYR
YTPDGFFQLNRTVTDDDIIEIYKGLWRIEETFKVMKSELKARPVFLSTESHIRAHFFICF
VSLLLMRLLEYRLDWQYSAAAMQQALASACGTSVGKNLYVFDYYDAVLEAIGKNLGIDFS
KQSRTLQEIRHLLAQTKRKH*
>SPBIB_v1_200044|ID:27162892| protein of unknown function [Uncultured spirochete bib]
LLASNDIGADDIVAVELLLLFDPGFIEHHQVLSLGHAFAGAAVEGHLLEVLRRPIGAWFC
ILRERIDLSRGFLDRERALHAGTLGILRTIDDNLPFVHKALGPFPLAVGHPYLSGRIATL
VFGKERAVRIVDGSIAXX
>SPBIB_v1_210001|ID:27162893| transposase (fragment) [Uncultured spirochete bib]
MFLGTLYEKKTGRTRLKIVEAYRQDGRPKQRMIQDLGYLDELQKIYPDPIAHFKQVAQQM
TAEKKAQKESRQVQLDPYAALRPFDPAQPATISRKNIGYAALSFLYHELGIDTVLNNRQR
GTKFSANLNSIFKMLVFSRALFPDSTRGAWENRGIFFEEADYSHDEVYRALDYFLAWRPA
LVRQLHERLKTRYGRQTLLFYYDVTNYYFEIDEPDSLRAKGVSKEHRPNPIVQMGLLLDA
QGLPVSYELYRGNTNDGTTLPSMLDDAVLDLGMHHLIMVADKGMMSGDNIAKLRLGHHGY
VMSYSVRGADKAFQEYVLDEEGYTAVYDTDGTLLAKHKSRYAPREIWVTDRQGKRTKSLV
NERQIVVYSAQYAQRARMERALAVEKASGQIYALSKDAKPSAYGAAKYLKKVPXX
>SPBIB_v1_210002|ID:27162894| protein of unknown function [Uncultured spirochete bib]
MXEYSTVLNALQCITYNIMKNIVKKFLAAYAKRNYFSVPLKETAKVRRREKFI*
>SPBIB_v1_210003|ID:27162895| conserved protein of unknown function [Uncultured spirochete bib]
MPLLQVRDCPEDIYKKIVLAARRKNRTIAQQTVVLLGKSLGQEESNIERRKRLLEKIQTR
NISETTKEIDAVALLREDRDR*
>SPBIB_v1_210004|ID:27162896| PilT protein domain protein [Uncultured spirochete bib]
MTAVLDVSAAIELLLQRDKKDLFTATYEKASWIIAPDLYIIELSNVLWKHYKAQKISHAE
CMQYVEDGIDMIDDFFEVKELWKEALGEGIRNAHSIYDMYYAVLARRNDALIMTNDEQLS
SICKKLNIEVVG*
>SPBIB_v1_210005|ID:27162897| Mannitol-1-phosphate 5-dehydrogenase [Uncultured spirochete bib]
MKPMPTLVQFGAGNIGRSFIGQLFATSGYEVIFIDVAKPLVEAINARREYNIVIKRSSAA
DEVLTIRNVYALDGNDHAAVAEAVADTDYVATSVGLGALPHIFPVLARGIELRAQRYPGR
PLDIIIAENIHDGARYFRQSLEELLPPGFPLAENVGLVETSIGKMVPIMRKEDLATDPLL
LFAEEYNELIVDKHGFRGLLPQIPTLNPVENIKAYVDRKLFIHNLGHAAAAYFGFSQSPG
TRYIWQALELPGLASKVRTAMRQSAAALVREYPNDLTLASLEGHIDDLLRRFANKALGDT
IYRVGRDLYRKLARNDRLIGAILLAARHDLPYSVIAEAVRAAISFRAVDEEGRLYPRDTE
FIANEVPKGLAGILRDVCGLRESDVIDREVIARILY*
>SPBIB_v1_210006|ID:27162898| PfkB domain protein [Uncultured spirochete bib]
MLTISGTGCALMDYLYADVRLDSEAFVRYRSRQSGDGGLEPGKLVFVEDLERFAGKPFAG
ILEELTGGAKPDRANLGGPSIVALIHAAQMLEGHNAHIRFLGARGNDASADEIMNIVARM
PLDVSGYRAFEGHTPFTQVFSDPTYDQGHGERTFVNERGVADLFAPEHLPDSFFDSDIVA
FGGTALVPQIHNNLTALCKRARAGGAVVVVNTVYDFRNEAKGGRWLLGESDETYGFIDVL
VMDKEEALRLSGTESSEAALDFFRAKGTGAAIITDGVRDILFYSDGSLFSVPALSGLRFL
APGKGSLPACSLPVSAEIRRVLASPDKPKGDTTGCGDNFAGGLLASIAIQLEAGQKRGSL
DLVDACSMAIVSGGFSCFYIGGTYQEARPGEKKQEIEHYYKLYREQLSANAADRARGE*
>SPBIB_v1_210007|ID:27162899|zraR| Transcriptional regulatory protein ZraR [Uncultured spirochete bib]
MTKTATILIVDDEEGIRHGLTRLFEGEGYAVAGAEDVAQAEKIASAKKIDIAILDVRLKG
SASGLDLLARLKNEDSDLPVIIITGYGSIESAIEAMKRGASDYILKPVDNEALLALVRRN
LEVAQLKRDNRYLKKELLQKTYQRHIITKNPEILNIIARLDSVKDSTASILITGESGTGK

EVFARYIHFTSTRSAGPFVSINCAALSEELLLSELFGHEKGAFTGAIERKIGKFELADNG
TLFLDEIGDMSPSVQAKLLRVLEESSFERVGGTKRISVDIRVVAATNHDIHELIRTGKFR
SDLYYRIAIVEIKLPPLRERIEDIPYLAEFFINMYAERYKKHIDGISSEVMQRWLSYRWP
GNIRELQNVIHQAVLLCTGNRIEADTLLNCGDEAAGIGQGTPVASGAGAFRPERYPSLKE
LGEAAAAYYERQRIESALAKANGNKSSAARALGITRKTLLEKLRRYGIC*
>SPBIB_v1_210008|ID:27162900| putative Histidine kinase [Uncultured spirochete bib]
MRFFARTFNSFISVIILQAILVILLVSGSITRSQEEDSKKELKTEALNVYDNFNSWKRAL
WGTIVDLNKSGELRHMIAAQKHIAPDEPLEAYLRKAASQAGAEFLIIKNNWSTFSAVRPL
TEKPVPSPKAQDFYIDRPHPYVEMVLADNTLYFCGSVRVSADNGRYLDIFIVKRVDEALM
RQLSFNSRVRALVSLNSHFVVGTIAGTGFLEWMHGRSFSTSYTVVNELVEEGVPYAVVIQ
QSGNARLAGSQTDSPASLGNVPAKTEATLYICTFLSLSEYRARVNFINRSILLVSLLVAL
FTILLSAGLSKAVTDPVSQLRKAMIRLKSGEKPALLSGPKKGEIADLFQGFNDMSAQIAE
DSRALSAHIQEITRIKEYNDKIFNSIQEKILVINAQFAVEKANRAFLEYCGQAEDAVLGR
NIDELSLALFDEPVHTSIRAIISGGKSSDAQIRRTSSGHSFEIKFYPLLEHEAATTSIHC
IMVIEDITAKVAYEEKIRQAEKLASISMLSAGVAHEINNPLSSILTNVQNLIKAERDLER
LKDLQLVEQETKRIARIVRSLLEFSSSTRTEKPATDINAAIRNIVQLVGYSFREESSIVI
EPHLGENLPPAAIGEDECKQIILNLIKNALEAIGTSGRIRIETAFLPSESMVQCVVSDTG
KGIPNALLPRIFDPFFSTKAESGNSGLGLSVVYGLVSKFEGLIDVESEVGKGTTVRIKLP
AAPSGLRQ*
>SPBIB_v1_210009|ID:27162901| ABC transporter [Uncultured spirochete bib]
MAHLLEAKNITKRFGGLTAVDNVSFHVDEGEVVALVGDNGAGKSTLIKIISGVHKPDEGE
IFLNGQEIHIDSPIDAISHGIETIYQDLALAENMDVPSNIFLGRERTKKILGFVNVVDYE
HMSSESRKVLDRLDIRIPSLKSNIRNLSGGQRQAVAISRSIYWNAKVLIMDEPTAALGIS
EQKKVLELVSSLRKQGIAIIIISHQMYDVFSVADRIIVMRRGIKAGERLVKDTSTEEIVS
LIVGAESVEKRSQ*
>SPBIB_v1_210010|ID:27162902| putative ABC transporter permease protein y4mJ [Uncultured spirochete bib]
MQTETAEKQGTLPELLGRNWIALFIVLLVVFFSVVARSFFSFDTAQLIFFNGTEVFLLAI
AELFVIITGGIDLSVGFVMGFATVVSSKLMVACVALGFSPFWSILAASVVTLLIGLVPGL
VNGWLVACLRVPAFIATFSMLGVTHGISELLTQGIPTKNLPALAGDIGNGSFFYVAPGGA
ISFFSRPQVARGQTVLAIIPNMVIFAFLFIFLFAFILGKMKFGRHLYAIGGNIDAAIRSG
INVKRDLIKAYVISSLFASLAGLSYVMKYITGKPDAGANMLLEAIAAVVIGGASMAGGSG
TVGRTILGALVIAILETGLRIIGMQTFMTYILVGVILILAVIIDQVFPNRNR*
>SPBIB_v1_210011|ID:27162903| putative Ribose transport system permease protein RbsC [Uncultured spirochete bib]
LAAESTSNGERLKQGAPGGGGFFQTAIGRFSARNWALIFLFIMIVIFSLGSSGFFDIVNF
QNIIHLSTGAFLLAAAELLVVITGGIDLSIGYVVGLSSVLGAKVMQILYAGGAGGAGSMP
VWQVIALGCLAALSVSILPGLANGVLVTRFRVPPFIATMGMWGICNGITLFLADGFMPVM
GAPSEINAIGNSYFLYIDPSKSISFFAKPSYITQANIRSVLRLVPNSLFLSLAVLLVLGF
VLGRTRFGKHTYAIGGSMDAAIRSGINVNRHLVMIYVIASFVSGVAGLFNLFQTGIGNYT
PSGANYELMAVAAVVIGGASLTGGKGRILGTAVGVLVLAVLENGLQIIGVSAFYRYIGVG
VLLTIAVIIDRAFPDLF*
>SPBIB_v1_210012|ID:27162904| putative amino acid ABC transporter, substrate-binding protein [Uncultured spirochete bib]
MKKRYSAVFAILLILLTVAPVFGQAAKKQLTFLFMPGVQDPFYTTMEKGVRAKCKELGVN
LIVAEYPKAWGPEYQVPILQAYAQRGGFDGLLIAPTSVDALKAPLKAIYDKGVEIITVDT
FLGDGDYSKPNTDYNFPLSYIGSDNYLGGKMIAEHLAKLVGEKGKVFCEATNPDASSVAA
RVKGFRDGIAQFPNMKLVGVEWCLDVQQKAQEQTLAALQKDKDIVGIFGTNVFSAQGANQ
AVVNAGLVGAIKIASWDATITNIENLKKGVIDLVLAQKPGEMGSLGVEWLYKYLTQKVQV
PKKVIPGFEFFTKDNVNDPNMQQYIYQ*
>SPBIB_v1_210013|ID:27162905| PilT protein domain protein [Uncultured spirochete bib]
MLNSILIDTGPLIALFDKDDTYHNKVKDFIKNAKYRFITTTAVITEVSHMLDFNINAQID
FFEWIMKEGVILQEISQKDISRIIELTKEYSDRPMDFADATLVIAAEKTGIKKIISIDSD
FDIYRLPGKVKIENVFHLDHFRGSFE*
>SPBIB_v1_210014|ID:27162906| CopG-like domain-containing protein DNA-binding [Uncultured spirochete bib]
MTTVRLPIEIEQRLEILAQKKHKSKTDLIREALEKLFIQEESEKDSYELGEEYFGKYGSG

DGSLSITYKDKLKDKINAKLNSH*
>SPBIB_v1_210015|ID:27162907| protein of unknown function [Uncultured spirochete bib]
VYAEDSGWGPSRGRNFVFQVPAGVGFIEVREGQSQTGIELSDLRFVGEP*
>SPBIB_v1_210016|ID:27162908| conserved protein of unknown function [Uncultured spirochete bib]
MIYRACEPYLRKIASQFPVVLITGPRQSGKTTLARYAFPDYSYVSLENLDTADRAREDPR
GFLAQYRLKVIFDEIQRLPSLLSYLQQIVDEHPQPGSFIITGSQQFNLMAAATQSLAGRI
GRLELLPFSTGEIYSYNPSLLTDSTTSIRRGWYPPIIDRSLDADLWYENYIATYLERDVR
QIDNIRDLLTFRRFLSLCAGRTAQLVNLSELAGECGVSHNTIKAWLSVLEASYLVKLVQP
YYRNFNKRIVKTPKLYFLDTGLAARLLGIRTDDQLANHPLRGALFETYVFSELLKKRMNG
QAPWEIYFWRDHGGIEVDFILESGGTLIAVEVKSGATFHPDFTTNLSRFAGFAGSDLTHA
ALIYGGMEAFTFKDVSVVPWNQMDLL*
>SPBIB_v1_210017|ID:27162909| protein of unknown function [Uncultured spirochete bib]
VKTGEVLGRGTTPDFGVFDRTKPNAFIRPSRYEPLMRYAQPPFGYLKEDISSRMLSLISR
TGEPKGGSFVYDQEGRLIGNWFAVPDAKLHEMSWDDMLAFAPHYLDTRRIEMGFSGRLWS
AFTSASPST*
>SPBIB_v1_210018|ID:27162910| Major facilitator superfamily MFS_1 [Uncultured spirochete bib]
MSERAPAHRWFMVAMATLLMICLGTVYAWSYFQTPIMKAYGWNTTQVSLIFSFTILFLGV
AAAIGGVVLPRVGPRRLALSGSILFSLGYAVAALALSIKSLALLYIGYGFIGGTGLGLGY
VTPVTTIAKWFPDRKGLATGIVVMGFGFGALVMSKVLAPLLLSLTGSNLVTTFLLFAVLF
FAITMTSSSFLRTPPPDWKSGASPSKTDSVGNAPSAMLSQFPRTAANVPSPLEAFRAAKG
DILSARFLLLWLMFFCNISAGITVVGFQSPMFQKLLAQSNPALTSVALAAMGATLIAVTS
IFNGVGRFFWGAVSDRVGRIATFRIMLGSELVIFILLIITHQPILFAALLCWVLLCYGGG
FGIMPSAVAELFHPNKMTVIYGAALTAWAAGGVVGPQITAFIQDSVPERASTLSFIVGAC
FVALGFALSLIIQIRAKKSGRRA*
>SPBIB_v1_210019|ID:27162911| protein of unknown function [Uncultured spirochete bib]
MQYIAFDSHKRYTYAVVEDQDKGRVMEQRIAHRKGEIREFLSRHEPGSPVALETIGSWYW
IVDEIEAAGMLPRLVNARKAKMMLASSKKTDRLDAKGLNTLQRAGTLPTVWIPPGAVRDI
RELFRTRMVLTREVTRLKNRILSCFSKYGISFNDTSDAFNVKGRASLESALSLLPSHTCF
AVQNLLGQLDAVKHSIKEFEARINQVKNIAPR*
>SPBIB_v1_210020|ID:27162912| protein of unknown function [Uncultured spirochete bib]
MRHEALHFRHVVQLYERLKATKGHGVAIGAVARHLAEATYWVLTTKSAYREPNHTKCSTV
VSSRKG*
>SPBIB_v1_210021|ID:27162913| conserved protein of unknown function [Uncultured spirochete bib]
MSSKAMSLKGRINHYAKKNNIAAQVVLQNYMFERFLERLSKSEYQEKFVIKGGMLVAAIV
GLDTRSTMDLDTTLRNLPLTEEQITQAIQSICGIDLKDEVAFKVVSVASIRKDDRYGGFC
IRMDAVYDTIVTPLSIDISTGDVITPSAVLYEFSSIFDESVRIRLWGYNIETVMAEKVET
ILSRGIFSTRPRDYYDIYILGTTQKYDKALFLEALSATAEHRGSKAILSAPEAIFENISE
SRDLRQMWAKYQKKFPYAQDVTYEAIIEVLRSLLFQ*
>SPBIB_v1_210022|ID:27162914| putative abortive infection protein AbiGI [Uncultured spirochete bib]
MGINSEILQEMKKNNNVITTSQVLQLGYSKALLTKYVKAGLLERSGHGVYILPDAVNDDM
YALMLRSSKIIFSHDTALFLNGLSERTPFRHTVTIPSDSALPASIKDECTCFYIKPELHR
LGMIEKKTTFGNTVRCYNMERTICDFLRSRNRCDEETVISAVKNYAVSKGKNLNLLEDYA
KRLRVEKVLKRYMEVLL*
>SPBIB_v1_210023|ID:27162915| Phosphonate-transporting ATPase [Uncultured spirochete bib]
MISVHDILVSFSLGGGAEHTVLRNLTLEVKRGQTVSIIGSNGAGKSTLLNAIAGTVPLQA
GKILFDGQDVTPLPEWERAIHVGRVRQDPLAGTAGDMTVLDNLALAARKGPRRFRIATPP
KFAREMAERVAELGMGLENRLHENVSRLSGGQRQALTLLMAVLSRPSVLLLDEHTAALDP
ANAEIVGELTRRFIEEFQLTALIVTHDMKRALDQASRVVMMHEGSIIADLSGPEKDRMDV
PGLVRLFKQARGAEYAEDRDLLS*
>SPBIB_v1_210024|ID:27162916| ABC-type transporter, integral membrane subunit [Uncultured spirochete bib]
MIEGILVEGLIYSILALGVFVSFRVLDFPDLTVEGSFPAGAAAGAVVAQAASASPLPLVH
ALAVPAGLLAGFIAGGLAGFVTAGVHHRLKVPPLLAGIVTMTGFYSINLRILGGKPNLPL
ITNNPMLAQARSFLSSLLSPEGALLASCVLVCLVLFGLLDLFFHTEIGIAMGALGDNENA
VIQAGIQPNRLRTWGIMLANALPGLSGAMAAAYQGFADVNLGQGVVAAGLATVMLGELVV

HSQFIDVQLARVFLGSIIFRALMYAARSWGYVAGITPNDLRLLTALLIIASVAISRYGRK
KR*
>SPBIB_v1_210025|ID:27162917| ABC transporter substrate binding protein [Uncultured spirochete bib]
MRRTRMFFVALAIIGMVLLSCAPKNAKVIGVAKFVSHPALDAIEKGIVDELSKSKSDYKI
DLQNANADMNTAAQIAQRFKQEKVALAVGIATPTAQALANQIKDRPVIYSAVTDPVSAGL
VSSWDKGGANITGTSDMTPVREQLDLLRSLKDVKRVGNIYSSGEANSVAIAAIVKQYCQE
NGLEYVESTITNSSEARQAILSIANRIDGLYLGNDNTVFSALSGIAEVALEKKIPVVTAD
PSSAETIPVLAALGYDYYRMGVATGKIVVRVLNGEKTADIPALLPKDSEDMSFVLNLDTA
KKIGISVPQAVVDKAKVIISDGQVTRK*
>SPBIB_v1_210026|ID:27162918|braC| Leucine-, isoleucine-, valine-, threonine-, and alanine-binding protein [Uncultured spirochete bib]
MKRTYMLLAVFALAGLMVFSGAQNAFAAPTIKIGVAGAHTGDLASYGLPSVNAAKLVVKN
INDKGGINGQKVELVIEDDQCKPELATNAAAKLVSAKVVAVIGHICSGATKAALGIYKDS
KIVTISPSATNPPLTQSGEYPNFFRTIAPDDAQAKLAATFLAKTLKLKKIAVLHDKGDYG
KGFAELVKQYAEEYGVQVPLFEGINPGAPDYSAVVNKISNAKVDGVVWGGYHPEASKLVQ
QMKDKGMNIPFISDDGVKDNTFIEVAGKYAEGVYATGPTDTSTNALAIKAIEEHKKTFGT
EPGAFFLNAYAATQALLNAIEKAGSTDYNKIVAALKSNYVDTPLGHISFDQKGDIIGFGF
SVYQVKNGVFVELK*
>SPBIB_v1_210027|ID:27162919|livH| leucine/isoleucine/valine transporter subunit ; membrane component of ABC superfamily [Uncultured spirochete bib]
MEYFLKLFLSGTAKGSIYALIALGYTMVYGIIQLINFAHGEIYMIGGFTALILGGFFFSN
GMPVWLVLLFSVLLSIIYASAFGYTIEKIAYRPLRGKPRLSALISAIGVSIVLQNFVLLA
QTEKFMPFPSYLPEFSWLQPYKEYINSTQLIILGVTAIIMVFLTLLIKFTRIGKAMRATA
QDMHMAQLVGVDVNQVISVTFIIGSSLAAIGGVLICSYMGQINYYIGFVAGIKAFVAAVL
GGIGSIPGAVLGSFILGWTESLGTGYISSDYEDAFAFVILIVILLIKPDGILGRTQRQKV
*
>SPBIB_v1_210028|ID:27162920| ABC-type transporter, integral membrane subunit [Uncultured spirochete bib]
MKSRSLLADIGKSFLIAIWFMVLLFPLMVMKVNVSKGVAQVQFRWNMLPLVGIAAFVLSL
LWRFALEWNEKRGNDSRAGGPLAKLKALTSQYLGIAAVRKGALFILLAFALAYPFLFGMY
HTNVMITAFIYIILALGLNIVVGLGGLLNLGYAAFFGVGAYTYGLLWKYVGHSFIAAGID
PGWLFWISLPLAGIIATLFGILLSLPVLRLRGDYLAIITLAFGEIFRMVMQNSGDITGGA
TGISLIPRPWLFNIKLTPQKAATYIYFIAIVLVLITIFVVRRIEDSKVGRALEAMREDEI
ACQAMGINLVRNKLITFALGAFWAGIAGVVMAAQTTYINPDSFTLWESIIILMAVVIGGT
GSIPGAIGGAILLKLLPEYFRALAQYRMLIYGIAMILVIIFKPDGLIPRKRKQYTFKEKE
LAK*
>SPBIB_v1_210029|ID:27162921|livG| leucine/isoleucine/valine transporter subunit ; ATP-binding component of ABC superfamily [Uncultured spirochete bib]
MSTQQLQSKQSILEIENLSMAFGGLRAIDGVSMHIDEGEIAALIGPNGAGKTTIFNCITG
VYKPTEGTVSIRNKNGQIEHIHGLKPDIINHKGLARTFQNIRLFNNMTVLENVMIGRHNS
LKAGIFKAIVRDSSTKEEEQRVIEESYLILKKLKLDMYINETAGNLPYGEQRRLEIARAL
ATDPFLLLLDEPVAGMNAQETKELEETINIIRDQEHITILLIEHDMSLVMNVSERIYVLD
YGRLIAEGTPHEIKRNPDVIKAYLGE*
>SPBIB_v1_210030|ID:27162922|livF| leucine/isoleucine/valine transporter subunit ; ATP-binding component of ABC superfamily [Uncultured spirochete bib]
MALLELKNVSTFYGNIHALKSISISVEEGEIVTLIGANGAGKTTTLMSICGITPVRSGEI
LLKGQNITKVSPNKIVQMGVSQVPEGRRIFPQLTVSENLDMGAFLRRDKEGIKRDMEEVF
SIFPRLAERRNQLGGTLSGGEQQMLAISRALMANPHLLLLDEPSLGLAPLIVQSIFEIIQ
RINKERKTTILLVEQNANMALKIASKGYVLQNGVIKMADTAAHLLENEEVRKAYLGL*
>SPBIB_v1_210031|ID:27162923| putative signal transduction protein with CBS domains [Uncultured spirochete bib]
MKVGQRMTRNPITITPDVTVPEAQAIMRREKIKRLPVLDNKGKLVGIVTTLDLIHASPSP
ATSLDIYELHYLLSKLKVEKVMTRNVITVDEDLPIEEAARIMADNGISGLPVMRGNVLIG
IITETDLFKLFIELFGARHKGIRLTLLLPEKKGELAKVSNAITKAGGNIVSFATFEGEDP
TNAYCAVKVTGVEKDALIQALTPVVEKVVDARET*
>SPBIB_v1_210032|ID:27162924| Resolvase domain-containing protein (fragment) [Uncultured spirochete bib]

MRIGYARPSVLQPDAELQVKALQDAGAEKIYTENVLSNEADRPAFEEALGSLGAGDTLIV
ASLDRLARTTTELFYIIEEIIKRGAAFASLKEALDTSADDGEAGRVFLRTLGAITEFERA
ILKEWQRESIEAAKTRRGHESKSGRRRR*
>SPBIB_v1_210033|ID:27162925| putative transcriptional regulator [Uncultured spirochete bib]
MELSELFKALSDPTRLLILRLMIEEQQSLCVCEIVTILGIPQYQVSRHLSVLKQADLVQF
QKIGTWAYHYFNDSTPINQMLAELLRKAMPVEKYQTEFNRLRDRLALRFNGKCVVGFEEE
KGTAGIKKPARKS*
>SPBIB_v1_210034|ID:27162926|arsB| Arsenite resistance protein ArsB [Uncultured spirochete bib]
MEGAKKLSFVDRYLTLWIFLAIGAGIAIGYFWPGFGQFISAMSIGTTTIPIAIGLILMMY
PPLAKVKYEKMGQVFKNKKLLALALIQNWLVGPLVMFGLAVIFLRAHPEYMIGVILVGLA
RCIAMVIVWNDLAGGDRELGVGLVAFNAIAQVLFYAVYIWFFLTVLLNMFGLANGIQINI
SMWESAKTVLIYLGIPFFSGMGTRYILQKIKGSEWYETKFIPKISPLTLIALLFTIVVMF
SLKGEFIVKQPLDVLIVAIPLVLYFVLMWFVTFFIGKAMGANYRQTISLAFTAGSNDFEL
AIAVAIGIFGISSGEAFATVIGPLVEVPVMILLVNAAIKMKGLFKQKPSFEV*
>SPBIB_v1_210035|ID:27162927| putative OsmC family protein [Uncultured spirochete bib]
MNEHPQSKKYEISLSRENGKIALASAHGSTLRMSLVGTEPELGFTPPETVIAAYGACIMS
NINKVAQAESLKIDDIRIDFTAQKRNDPLGLEHIHCKIAVKSSAPKEKLQQLLTKATTDG
TATNALQEGLKADFQFEV*
>SPBIB_v1_210036|ID:27162928| protein of unknown function [Uncultured spirochete bib]
MEKIKQNSAGDASLGVLDRIQPLLLVAAIAIGLLLASIMPAFARRLEPSAT*
>SPBIB_v1_210037|ID:27162929| protein of unknown function [Uncultured spirochete bib]
MSDTIRVRVIAPPAAPNACGDTWEHAVQLIETRISARYPGTVTFEFVPLFSKAFFDMPLV
VQALGDGTAQMPVILVGDRIIQSGGKISESKIRAEIEGILHGKDQTEQRG*
>SPBIB_v1_210038|ID:27162930| protein of unknown function [Uncultured spirochete bib]
MEARLQQAADRFISTLGNNPKVSTFRNANDDFFNDSELAALRKRYNENLIIARKKQYDGT
LTQEELNTIRTLQAQVSNHPITQRFVQARAEVLDVLTECNSAISELLGFNYAQAAAPPAN
CG*
>SPBIB_v1_210039|ID:27162931| conserved protein of unknown function [Uncultured spirochete bib]
MTDIMLEAESYVQASRSLAKAIAATGNYAIFEKARDRLSTDAEARNMLKQLEAAEQKAQL
SASWGGLSDHERRKLERMQQETFKQPTILAFLEAQKSLIAELQELNRYMTEKLGIDLADM
TKPQTGCCG*
>SPBIB_v1_210040|ID:27162932|arsA| putative arsenical pump-driving ATPase [Uncultured spirochete bib]
MKAQYIFFSGKGGVGKTTMACATAVHYAESGKKTLIITTDPASNLADVFESRIGHKITPL
GIENLWGMEIDPDKATEEYRERILAPMRAVMPVSVMKVMEEQFNSPCTTEIASFDRFVDF
MVMEQSENEIPYDVIIFDTAPTGHTIRLLELSVDWSKHIEESAKGGGNTCIGPVASIQEN
KKKYDEATRLLGDPSRTDFVFVLQPEGTSLYETKRSKAELEGIGVKSVRLIVNGILPQEV
CTHPFFRSRYEMQQKYLSQIRQDFKESITYMRQRDGEIKGIESLRAIARDLFSKDRTTSS
YRLSTSFETPTQGPLVQAASLFDLINPTPGKTKAVFFTGKGGVGKTTVSTAVAYGLGQKG
YKTLLLTTDPASHIGQVLQQSVGSSISKIEGAENLWATMIDQKQAAEEYKARIIADAEKK
YSPDTLAAVREELESPCTEEMAAFDKFMGYVESDEFDVVIFDTAPTGHTLRLIELPFDYE
DQVGMMVATTAESASVKSETQHRFEKIIARMKDPEHSLFAFVVVYPESTPVVEAYRAMLDL
KDAGIETKFVVANQVLPAEICTNEFFMKRRAMQVKYLAEIETLFKKPITIMPLLDTEITG
LPMVAEASELLLAERKTESA*
>SPBIB_v1_210041|ID:27162933| Arsenical resistance operon trans-acting repressor ArsD [Uncultured spirochete bib]
MPAKIELFDPPMCCPGGLCGPAIDPVLLDLNEAILRLKKEKGIAVERYLLSQQGKKFMEN
PEVLALLQQHGTEILPATAVNGHVVKTKNFPTYEELKQWASEPAPITQGASA*
>SPBIB_v1_210042|ID:27162934| conserved protein of unknown function [Uncultured spirochete bib]
MKDKDMVDEYDFDKSIKNPYIKNLKKSVTIRLEPDTVNYFKSLSRDIGIPYQTLINLYLT
QCAKEKKKPEVVWQ*
>SPBIB_v1_210043|ID:27162935| Oxidoreductase, short chain dehydrogenase [Uncultured spirochete bib]
MLRTRPCLDPAGGLPYPVDIMSWSLSGKVVWITGASSGIGRSLAVEAARRGAWLILSGRN
VPALEETAALCERARLASENQQNAQKDEQEASPGARNHAACTLLPFDLADPEARLEAAQK
ALTIHGRIDVLMLNAGVSQRAKFVETSADVFNLIMETNFFAAVDIVRAVLPQMCSHSSGV
IACVSSVAGLMGAPWRTAYSASKHAQAGFFSSLRTELYGSGIQISIVYPGFVRTAISENA

LAGDGTRHGKLDPLQKFGQNPEVTARTIWDKLETGKLDIKVAFELKARLGVFLSRYFPAL
FVRSISRHGGL*
>SPBIB_v1_210044|ID:27162936| putative type I restriction enzyme HindVIIP M protein [Uncultured spirochete bib]
VARQKRNAQEAEVSGATIGYEAQLWKMADALRGSMDAAEYKHVVLGLIFLKYISDAFEEH
HATLQAERAQGADPEDPDEYRAVNVFWVPPEARWAHLRAQAKQPTIGQLVDDAMAAIERD
NPALKGVLPKDYARPALDKQRLGQLIDLISNIKVGDEAARSKDVLGRVYEYFLSQFASAE
GKKGGEFYTPRCIVKLLVEMLEPYRGRVYDPCCGSSGMFVQSVEFIRAHANGNGNGGKAR
ADISIYGQELNYTTWRLAEMNLAIRGIEGQIAQGDTFHNDRFPDLKADFILANPPFNVSD
WGGERLAGDKRWQYGVPPKSNANFAWVQHIVYHLSPTGVAGFVLANGSMSSNQSGEGEIR
KNLIEANLVDCMVALPGQLFYSTQIPACLWFLARDRQDGRFRDRRGEILFIDARKLGRMV
DRTHRELTDKDIKKIADTYHAWRGEKECGQYADVPGFCKSAPLDEVRTHGYVLTPGRYVG
AEAQEDDDEPFEEKMQRLVAQWREQQAEAAKLDAAIAANLKELGYGE*
>SPBIB_v1_210045|ID:27162937| conserved protein of unknown function [Uncultured spirochete bib]
MANKSRKPEQNARVPEQTEARRLDAGIAGDLPADYATLLTELKQRIREERVRVVLAANAA
MVLLYWDIGRTILQRQAQEGWGAKVIDRLSADLRREFTDMQGLSPRNLKYMRAFAAAWPD
RHIVQEVLAQITWYHNIALLDKLDAPEVRLWYARKAHEEGWSRNILVLQIERRLHERQGK
AITNFAATLPPADSDMAAQIFKDPYLFDFLGTADPRRERELEQSLIDHIQRFLLELGAGF
AFVGRQVLLEVGDRDFFVDLLFYHLKLRCYVVVELKAGPFDPAYVGQMNLYLSAVDDLLR
HPDDKPTIGLLLCKGKDRLVVEYALRDVTKPIGIAEWETRLMATLPEELKRSLPTVEELE
AELENVAHKTQEEGYGG*
>SPBIB_v1_210046|ID:27162939| protein of unknown function [Uncultured spirochete bib]
MGGEVRPFGQLFVEPTRNGLTRPKTVRGNGVKMVNMGELFAYPRLCNAPMERVPLRPSEY
ERFLLKEGDLLFARQSLVLEGAGKCSIFLGDDEAVTFESHITRVRLDPTKANPCFYFYYF
QSHHGRAAIRSIVEQGAGASGIRGSDLESLDVQWCPLPEQRTIANILGTLDDKIDLNRRM
SETLEQMARALFKSWFVDFDPVRAKMDGRWRRGESLPGLPPDLYDLFPNRLVDSELGEIP
EGWEVKPIGDLADVVGGSTPKTERSEYWEGGTHHWVTPKDLSGLSMPVLLDTERKITDAG
LAQISSGLLPEGTVLLSSRAPIGYLAIAEIPVAVNQGFIAMKPRRDMSNLFLLRWARSAH
EDIVSQANGSTFLEISKSSFRAIRTVAPAASVMDAFDRISRSMYRKVVEHERESRTLAVL
RDTLLPKLISGELRVKDAEKFLKERGL*
>SPBIB_v1_210047|ID:27162938| protein of unknown function [Uncultured spirochete bib]
VVRLKIVKVEAGVGFSRIKPDTGYV*
>SPBIB_v1_210048|ID:27162940| conserved protein of unknown function [Uncultured spirochete bib]
MSEDNANEIDRFEIGGENLLSKVSMGFFQTSNDTLPLGTETLPREELDDIRVRIGTLLKT
ETVSFLLGAGASVDCGGQLIGLVPLAVERDLHRKDTAGTTEAQVAQWLKVFYLAVSHSGG
GDTTPVSEDEIRSRQQKLEGKEVKSLLANFEQVLATLHRWRSALPSTGGRLRVDSTSTVT
LDANAEDLDECLRRATRALAIACNLPTEDKEGGISTYKAFIRKLLTRPLNLKRVNIFTLN
YDTLVEQASDADGVVLLDGFVGTYRRVFRPESYEQDLYFPAETTEGRVHRFDRVLHLYKL
HGSITWRATKPSINNPYGIESERFNLNDTQPVLIYPTPAKYGETLGMPYAELFRRFAAAV
VRPQSVLFVIGYGFGDEHVNAIIRQALAVPSFTIVIVDPAPNSDFVKALRKQNDPRVWIS
EGSRIGTFEGFVKEVLPDLREEEILKKVVATHQSLSKKENTGRGDMSDDN*
>SPBIB_v1_210049|ID:27162941| ATPase-like [Uncultured spirochete bib]
MTTDFEIGRVVAVDTAQVTIELNSDLKGMSRSTYEGPHEVGRINSYVIIPVGARRLVAMV
TRVVLVEEAEMKADRTMVTLPAARRLMKATLIGTIDGDKFRQGVSLFPVLDNPVYLAGRA
DLDAIFGPIESKQTTPPNMDEPGYCIPIGESAVVQGRPIRIDPDVFFGKHAAILGSTGSG
KSCTIASLIQSIRKQPTVKRTTFVILDTNGEYRSAFQRQKKDGTWEDIGSRKVLYIPSDP
SKAAERLVIPYWFMNAEDFVRIFQASKGVQRPVLLESLRLARNEAGSASPLATLREELIL
ELNRIWSLSGKDEKTSKDVRDLAKGLKTRIEQEDLSQGWTDASTEFSLNKDEVAKALDNV
IKTADNNIDNGTYPKVLPADARKQIRDAIDPIYQNLTGTHIGDSTNVAGRSADAPSYFDK
LKFRSRHLEQVLRREESGGARARDYTGTMLLRIDRLLADTRFDFMFGPVDGVLPNPVHAL
ASFLRDTLGIGSLQNGDLSKVEDIPTGRLPFYDRQRNNEGPVDVVILDLSLLAAEVLENV
TALIGRLILEFLQRLGEYGGEEARGSLPVILVLEEAQNYIQQPRSAEDESISRVVFERIA
REGRKYGLSLVVASQRPSELSKTVLSQCSSFIVHRLQNPEDLRYFKEIVPAIYGPMLDQI
PALAPQTALVLGECVSAPALVKIRTASPVPRSRDPKFYRYWVSDNPPEVDFESICEKWEG
THNEIDAVPSKQKRNEDNTR*

>SPBIB_v1_210050|ID:27162942| putative type I restriction enzyme HindVIIP R protein [Uncultured spirochete bib]
MNSFTESTVESAALAWLESAGWQVAYGPDIAPGMPAAERADYGEVVLPQRLRDALARLNP
GLPAEALEDAFRKLTRPEGADLIQRNRALHRLLIEGVNVEYREPDGSIRGAQAHVISFDF
PLANDLLAVNQFTLIENKHERRPDIVLFVNGLPLAVIELKNAASENATIWTAFHQLQTYK
TEIPSIFLTNAVMIVSDGLEARVGTLSAGREWFKPWRTISGEALADARLPELQVMIQGLL
APHRLLALVQNFIVFEDDGSRIIKKMAGYHQFHAVQAAVQETLRAAGLGQLEYVNDRSGG
RYMAGPNHAGKRGDRRVGVVWHTQGSGKSLTMVFYAGRIIREPAMANPTLVVLTDRNDLD
DQLFRTFSRCQDLLRQPPLQAESRAHLRKLLSVDAGGVVFTTIHKFFPEEKGDRYPVLSD
RRNIVVIADEAHRSQYDFIDGYARHMRDALPHASFIGFTGTPIEKADANTRAVFGDYISI
YDIQRAVEDGATVPIYYESRLAKLALNEAERPKIDEDFEEVTEGEEVERKEKLKTKWAQL
EAIVGAEKRLRLVAQDIVTHFEQRLEAMDGKAMVVCMSRRICVELYREIVSLRPEWASEA
DEQGAIKVVMTGSASDPIDWQCHIRNKKRREALADRFRDPADPFKLVIVRDMWLTGFDCP
SLHTMYIDKPMRGHGLMQAIARVNRVFKDKPGGLVVDYLGLAHELKAALATYTESGGTGS
TAINLDEAIAVMREKYEICCGLFHGFDRSTWTTGTPAERLGLLPSALEHLLAQENGKERF
LHAVRELSQAFALAVPHEEALAIRDDVSFFQTVQTALSKRVPGDTKPEEELDHAVRQIIS
RAIAPEGVVDIFAVAGLEKPDLSILSDEFLAEVRGMPQRNLAVELLQKLLKGELATRRRK
NLVQARSFAEMLEQTIRRYQNRAIEAAQVIEELIQLAKEMREASARGEQLGLTEDELAFY
DALETNDSAVKVLGDETLRSIARELVNIVRNNVTIDWTLRENVRAQLRVLIKRILRKYGY
PPDKQEKATQTVLEQAALLSAEWTAA*
>SPBIB_v1_210051|ID:27162943| conserved membrane protein of unknown function [Uncultured spirochete bib]
MSDILLILFTLLFPVLLLYLKERVRFMAKWSTLIVCYIAGLALGNIGILPASASALLDTL
SSVAVAISIPLLLFSVDIKKWKELSGKAILAFVLAALSVSLVSGIAYAFFRTKSAESANV
AGLLVGLYTGGTPNLAAIKTALNVDKNVYLAVHTSDIVLSAIYLLIVMSIAKPILKHILP
LKNWDGNIASPENLSFTTRFSDYFRKGLWKKIVSGFGLALAIVGVSLGVSMLVPPDFQTM
VTILLITSMALAASFVPRIRTLPMTFATGEYFLYVFAVAVGAMGNITQIFNSAGTYFIYV
AIVLFGSFVLHAALCALFNIDVDTMLIVSTSAICSPPFVGVVAVSLKARKLILPGITTGI
IGYAVGNYLGIALAQLFKAIGG*
>SPBIB_v1_210052|ID:27162944|fumI| Aminopentol aminotransferase [Uncultured spirochete bib]
MSGEMGNGPGEQSEGPSKAKQGQSRAEKRFETSIAYFERAAKVIPGGIYGSKSPGFLVPG
HFPYYLSHAKGSRIVDVDGNEFIDYLCGYGSQIVGYGNPAVDEPALAQARKGDLLNQPHP
VMVELAERLIGLVDGMDWAVFVKNGTDATTLATSIARADTGKQIIIAAEGAYHGAANWCS
TNVFPVFTLAEQRDVRYFPYNDVAALEALFRQHREKIACVILTPYHHPTYKPQQLPTPEF
IATVERLCHSEGAYFIMDDIRANFRLSMKGSHSFFGAHPDMITMGKALANGYPLSVLLGT
EGLKKTASSFFITGTYWMSAVPMIAAMATLDEMERLGGTERLAQLGRMLKEGLESLGREA
GFSARISGPPAIPYLTFDEDPDLFLNQRFCAAMADRGVFMHPHHNWFISLAHTEADIAQT
LEAAKGAFAQLRDSRKPVTLHQSFTQGSEH*
>SPBIB_v1_210053|ID:27162945| protein of unknown function [Uncultured spirochete bib]
MNTKLSQLSSEEKAVLARLKAWLALQLAAEAVRNDEGLRQACPAAGISLKLAIRGAGAGM
QLVVKNGIARVYSVGWPQLVLFFPGSASAIRVLSGSKGTAVPLPLRPGAFKALGFFRKAS
SRATELLRGAETPEDVRARLLLAATLYGLEAVAGDTYLARRMQIIPDGVVAVRAGEIEYF
VEKRGNAIHVIEAQQRPDAVLSFADYQSAIDVLSGKRQAVVALGSGKVRIEGLLPLVQGL
FVVLDRLSWYLGVAL*
>SPBIB_v1_210054|ID:27162946| protein of unknown function [Uncultured spirochete bib]
LPALPGGAQGAMLASGLTNEYKVVSIIERRKGRSGEVEGVAGIAARC*
>SPBIB_v1_210055|ID:27162947| DNA mismatch repair protein MutS domain protein [Uncultured spirochete bib]
MKVFLMYKDRDFDPQAPLAPQTDALMQDFDVDILLKAMSAGDRFLYDISKSALFQSLMDE
GTILYRQQVLDDSIRNEKIVRDIYALADEAVEAKRKNWFGVFGAYPGSILHGAVRMLEAY
LPMLERLRVIAREKQSVFRSKGFTRFFAMIQNELNDEYVRTVSQYLKDLRFDSGVMISAK
LGEGNELTGHALRKPNIKDKNWIRRILGPKTPSYAFSIDARDEAGTRALGELRDRGINFA
ANALAQSADHVESFFRILKNEVGFYIACLNLREKLASIGEAVCFPVPSAPKSLQLEFSGL
YNVCLSLFSGKKSVGNDVCANGKNLAIITGVNEGGKSTFLRSVGIAQLMMQAGMFVPATR
FRADIRSGVFTHYRRKEDRDMGSGKFDEELKRMNTIVDQIKPHSLILFNESFASTNEREG
SEIARQIVSALLESGAKVFFVTHLYTFAAQFREKANKTTILLRAERRQDGTRTFRIVEGA
PSQRSHGEDLYYKVFEEEV*

>SPBIB_v1_210056|ID:27162948| DNA mismatch repair protein MutS domain protein [Uncultured spirochete bib]
MIAVSMLFENPEDTRRASAFDMPEYFVDLNLDQILEGINGGLSEYGLQHIFYQHLSNARE
VQFRQAIMRDMEKPDFLAKIKAFSSRFRSMRRSLASLEKLYNLHHRQGWFLEAVAAYCES
VESLAGTLRDAELGSVGLRSLRDYLIAYASSESFLTLKSEMEGLMQSLGEIHYSIIIKNL
TVRVKKFENEIDYSADIDDTFSRFRQGATKSYLLKIPSSIGLNHVEAQILDCVAKLYPEI
FASLDEFCKAHASFIDETIDTFEREIQFYISYLDYIGPLRRAGLSFCHPEIAESDKNTYA
QGFFDLALAKKVSMTSTRVVPNDFSLQGKERMIVITGPNQGGKTTFVRAFGQLHHLAAIG
LPVPGTGVRLFLYDAIFTHFEREETIKSQRGKLHDEIIRIHNSLERATPSSIFILNEIFT
STTVKDSLFLSRKVLEKILALDAIAVCVTFLDELASMDDRIVSMVSCVDPQDPSIRTFKV
ERRLADGLAYARSIAEKYHLTYESLKNRFARAQGGRNPREMRGNDSMARFGEALS*
>SPBIB_v1_210057|ID:27162949| exported protein of unknown function [Uncultured spirochete bib]
MRKKKALGRLLAAFFLVMCASIAFAQPQEPTLADLPSILDHMLDNHYMPSAQVGLGSFTY
TDTQLPTPFARWFEDELRLAFAKTAKMKLFDKQVAAAMDPAIRALYGDFFGTDRADSILY
GKYAQDDRGVLVTMTLTDLSTGGLISETRYAVPASAIPSNVNVQPSVKMVQTAAALSQLF
TSSGAGGQAGSGQQGAAQDFVVTLSTDRGQGAVYRDGERLTLLVTSSKDAYLKIYHVDVN
GVAQLIWPNRFGGSGKIKAGEALKFPGPNDKFQYVLGRPYGTEYIKAVASTKPFATMEAD
FSDLQGSAVAAISRGLTVVSSDTTRAEALVVYEILP*
>SPBIB_v1_210058|ID:27162950| Major facilitator superfamily MFS_1 [Uncultured spirochete bib]
MSIKDRLPALASRDYRLWFVGQGISVIGTWLQNTGQAWLVLKLTNSPLKLGILTSVQYLP
SLLLSLFIGPILDRYPKRSILLWTQSLFAFSAALLAAIVFSGREQYWHVLAIAAFTGLVT
AVDWPARQSFVSEQVEDKDAVVNAIALNSTIFNIARVIGPAIGGALIAAIGIPWTFALNA
LSYLAVIASLWAMRSGRVAYAPKSGHYRSDIREGLAYIKKRKSIGLLLAIVGVITTFLLN
FNILIPSYAKLTLGLGADRYGILMSAMGVGAMLAGILMALGGRALQPKPGFIFGAGFILS
TAMILIGLQRNFIISAILLAACGFGMSSFATMCNTSVQIQSSSAMRGRVMAAYNLVFVGS
TPFGSLYTGQISDMLGSDAGFLISGAIGLLFLLSMRLFIAPKVFSGVKSFAQLKDSEG*
>SPBIB_v1_210059|ID:27162951| Phosphoglycerate kinase [Uncultured spirochete bib]
MQGPRLKIRPVTELELHGKTVIFRPDINSPIDPTTKRIVNTNRIEKTVPTLNLMLERGAK
VALIAHQGDTLDYQNLIPLAEHAEILSRLSGHRVSYIDDVCGPAAQAAVKALSPGEAVIL
GNLRYLAEEISTFETVVKLTAEEMTQTWLVRSLAPLADYYVNDAFAAAHRNAPSMVAFQE
ILPTAGGIQLMEEYTALKSVMENPRRPCVYVLGGAKISDAFDMMRKVLTDGSADYILTAG
VTGIVMHIARGVDFGPTITKFLADRALDTFIPEAKSLLKEFSSHYVLPVDFAYDANVGQG
APSSAQAGTKPGQSTSHNSKPVRAEAPVGSLPKDRLLPDVGRQTIELFKEYIGKAGSIFV
NGPAGMYEHEPWSDGTREIWQAIADAPGYTVIGGGDTITAATRFTDLSKYGYVCTGGGAM
VRFLAGKRLPLIEAMERAFERNLQAPLRR*
>SPBIB_v1_210060|ID:27162952|gap| Glyceraldehyde-3-phosphate dehydrogenase [Uncultured spirochete bib]
MAKVKVGVAGYGVIGQRLADGVALQGDMELVGVADVAVTLSVRALREKGMPYKFFLAAPE
KREEFDKAGIPVSGTLEDLVQQVDVMLDATSAGVGAKNRLLYEKYGKKAIFQGGEKNSVA
DVFFHGYANYEKGIGAQFLKLTSCNTTGLIRAVDCIDRKVGVEKVAITIIRRVADPGDYH
RGLTNALQIDKAPSHQALDLMTIMPHVDATGILVHTPVTHGHIITVVATPKKSVTPENVI
EFFREHPRIRVVSIAEGFLGNASLFRYARDLGNPRGDMYEIAAWEDTVVMSGKDVMFAIN
VPQEAVVIPENMDAVRACMRMQLDRIEATDTTNRYLGIGKWRNPG*
>SPBIB_v1_210061|ID:27162953| putative NADH oxidase [Uncultured spirochete bib]
MSESKYVNLFKPIKIGNFVAPNRICHVPTDISSANADGSVNQRVITYHEEIAKGGTGFII
VGASTPDKATGRPTVTCIAVDEDPLIPGLAELAEAMHRHGAKCAVQIQHPGRQSAWPRHD
MISATDMVVDLPGSAGHEVVYAESKAKGKSIRAMSVEEIYDLIEKFAEGAWRVQQAGFDG
VELHGAHGYLIAQFMSPYVNKRNDRFGGSFQSRMRFPLEIIARIQQKCGKDFPIGIRYSG
DEFIEGGRNLEESVKVAQVMEEAGVAWLDISAGIFELPGPTMDPMYYPQGWNTYAAEEIK
KHVHIPVITSHSLREPDYCEKIIAEGKADMVGLSRQLIADPYWANKARASQTEEIRKCIS
CLVGCWQESLMIKRHMRCAINPAIGDERFINFGPASKKMKLAIVGGGPGGMEAARIATLR
GHDVTIFEKGEELGGAILYCCTVPGKSKMRWYADWLRRQIANLGIKVVVYSTIPSAEQLKE
FDAVFIATGGKVEKPDIPGINSSRVFTFEDVLRCKVKGCEFWPKDGKKAPADVGETVLIW
GDHFGAADTAEKLGMEGKKIIIVTENSQFAAWMEPCHKDVFDKHLKGGQGEGLKGKVFAH
PAQVITDSTVLEIKDDGSIVLLSGKFEKSVVKADTVVLAKVVPNDSIAEEYRKAGLLVTV
IGDAKKVRNLRNAVADAADRAFVLGENAQLNANKEIIANLPTGISL*

>SPBIB_v1_210062|ID:27162954| putative Transcriptional regulator [Uncultured spirochete bib]
MGIETFRPASRLSLSEQVSKHILAKISSGELLPGDRVVEAQICKELHVSSIPVREAIREL
VANHILEYVIHRGAQVREVSMRETIDALEVKSVLEPLAANLAGLKNLRNILQKLRKYIPQ
MRKALEANDHVAFQASNQEFHRHIVEAAGNAILLRLWEQLAFDIRTKPLMDYLRIADPEQ
LIGEHQNVIDAIEEGDTKKIGYMLEIHSVHLVNHLREKMAENAEEAEKHILVINKRRKQV
*
>SPBIB_v1_210063|ID:27162955| Xylose isomerase domain-containing protein TIM barrel [Uncultured spirochete bib]
MKLGFVSAILPEYSFEQVIDFASENGFKCVEIMCWPKGKAERRYGGVTHIDMDLMDKQKA
DYIFEYISNKNVHISAVSYYPNNMDPDPQERQFYNEHLKKVIIGARMLGLHNVNTFVGRD
QNKNLSDSIADFRKIWPDIVKFAEDNDVRIGIESCPMYFTMDEWPGGKNLAGSPKIWREL
FSIIDSRYFGLNYDPSHLVWQRMNYIAPIYEFKDKIMHVHIKDAQLYQDRLDDVGALAPP
LEYHYPKIPGLGDIQWGKFISALRDIRYKGAACIEIEDYSFEDSLEDRILALRQSKAYMN
QFIL*
>SPBIB_v1_210064|ID:27162956| putative ABC transporter permease protein y4mJ [Uncultured spirochete bib]
MKAQAHVSTALDSSFFSKYKKLIVAALLIIFLFVLGEIIVSDFLSVGQILLTIKLSSFIA
LFGLCQMIVIAAGGSGLDLSVGYTATVTAVLTAKLMDGKNENIWIAILVALALGFIIGVL
NGFFVSYIKLPPLVVTLAMSQMIQGAINVYTAGKNITGKPSPILQLIAAKTTGFVPNIIF
LLILLTIIVMLILNKTRIGILLYGVGANPTAAHLSGVDIKRVRFLSYVVSGILASFIGLL
LLGNMGIAFKDMGSNYVMPSIAAAVVGGVSLAGGDGNYLGVVLGAIFLQTLTNLLVALGW
GDAGKWTGFGIVLFLLLIVYVSNRRKR*
>SPBIB_v1_210065|ID:27162957| putative Monosaccharide-transporting ATPase [Uncultured spirochete bib]
MQGKSMETKKRTLRDFTSKPEFSSLLILLVMFALTAILQKNFFEIKSIMRNINAFAPLIL
VTMGQAVVIISGGIDLSSGTALSLLTCVLTSIMKKNDPITGLYGIIVAFIVALLIGLING
FGIGYLRIPPVISTFATSFIWLGIALFLRPTPGGESVSWFGVFYNFDTLKDISGFFGTLS
GFLPPSLLLIIIGCILWYVISKTKTGRYIYAVGGNSESAYESGINTAKTQMVACMINSIF
IFLAALFFVGQTGSGDARMGDPLTLRSIAAAVVGGIALSGGRGNVYFALVGALILSFVNK
IIFFANIPYAYQTLVGGAIVIVAIAGSQAYISYSTKTKEIKIG*
>SPBIB_v1_210066|ID:27162958| ABC-type sugar transport system, ATPase component [Uncultured spirochete bib]
VNILEAKNIKKNFGGVAALSGASLTCREGRITGLLGANGSGKSTLSKIIAGVYAPDDGEI
TYLGKKIHYKNPHEAKQQGIGMVYQNLSLVPELTVWQNIVLGDERNAGYFLDDKNARERT
TDIVHKLLPTLDIEKKVFELTPSEMQVVEIAKAVSRKPKLLILDEPTAALEKVQVTSLFN
MMKELASSGVAIIFTSHRLWEVVEICHDLTIFRNGRNVGSMDFDKDEKDVKKIIFYITGS
QSETSIAKTCAETCGETRLSIKNLSLGTILRNISFDLKKGEILGVGGLAGQGQQELMLAL
AGNFPKIGCDARIDGKPIKLTKPSKAIRNGIVLVPGDRETEGLFSKHSVFSNLIFSKIGV
DKKIFIIPHRKLKKECEQIVDILALKAASLDAPVSTLSGGNQQKVVIGKWLGLEIKVLLL
SDPAKGIDIGAKADLYSYIVNQVRSTGMSVVLYASDTKELIEYCDRLLIMYEGSIVATLG
TEEITEENIIRASMHVEKE*
>SPBIB_v1_210067|ID:27162959| putative Periplasmic sugar binding protein-like protein [Uncultured spirochete bib]
MRRKGFGIVCILLVLLLGVGVSLGAAPKKAPVVGISTGSSGTSWRNIMIDALQTVGKEYK
AAGKIADYLIVNNVTNGDATEQANIIRDFISRGVDIILVNPNSPDALNGVIKEAQAAGIL
VVAFDATVTAPNVLNVTLDHYAWNLKNVEYIASTLKSGNAIQIYGLDGHPANNDRIRATA
DVLKKYPNIKLIANTTGYWDQTKAKEVAAQIIGSGKQIDAVITQDGEAYGVLSAFLDAGK
LPKVMFGDPGTAFFKEWKKLRDKKADFKACAQPNPPGIGGTAFRMALNLYNGKTFKPGVL
KDNTYFYKVGMFITDDNFEEGWALLKDKPDDYLLSEIMSQKDVDALFQ*
>SPBIB_v1_210068|ID:27162960| conserved protein of unknown function [Uncultured spirochete bib]
MKSSVQAKPVVESGFRNAVALGNGIFRVLVDAEGGMIPELSRKCGDTFINAHWLPWFRGM
APSPEAGNLQSHELFQKIPLLERIAGNFPCVPSFGDSDPVDGVEIPPHGWTANEQWQLES
AGTDENKRYAWARFQMTSPSDRMKLEFEKTDLVFADESVHYSILRIKNLSTKDIEINAAW
HNTIGFPFLTKNCKISASAKNFSTPPKGSEFDMTGRLAIGSKFTDLDRAPLRDGGTVDLA
IVPDMIGYTDFITGAVPKEAKLGWSACVNSELSLAYITWFMGPDTVQTDEIGLCFNNFWM
QYGGRPYAPWAMYEGAPDQTFCLGMENSIGAWANGLADSIAKKELLGRPTTLKLSCGEQK
TLLYATGLFDLGSEALDEPVKHIEPTSAGLRLEFNHVAISVKADGSLERLRSIARSNDCF
A*
>SPBIB_v1_210069|ID:27162961| putative dipeptidase YkvY [Uncultured spirochete bib]

MCRLPENVLMLSGHWPLCGDAVLVFPAEGNMVCIVPETQVEEVSAEIWDAQILAYAHGRH
DSPDQEREIERCLIDLANKNSWKTIGYEGTFEGIAAPGNVAEPRVPSKRFEQLLKSATSK
ATFIDAQELLDLLRKTKTAWEIEKIRLANEIANIGFEVFFEMVDAGRSAIELAAAVEQAI
MVKGTGYKKARRVRAFAQVAVGKDETLAGWRPFEVTTNRKLQKGELAILELAVVADGYWA
DRTRVRVAGSASPELQRLSDIVKQAQDAAISAIYPGVCASKIDKQARGIISKNGLENEFV
HITGHGVGFRYHEGYPILAPGSSQALLPNAIVTVEPGVYLPDFGGIRIEDDIVVTSEGPE
ILAPFSRQLS*
>SPBIB_v1_210070|ID:27162962|gdh| Glucose 1-dehydrogenase [Uncultured spirochete bib]
MSAEQYSPPNEAPKRKIYEVLKGQKALVTGASKGLGAGIAAALAEAGCDVLVNYASDRKG
AEETAAVVEKFGRKAHIFKADVSKEEEVLAMFGEMKRIFGRIDILINNSGIQVNAPFHEM
TLDQWNKVIGINLTGQFLCAREAVKAFMKQGINPEISCSMGKILHISSVHDVIPWAGHVN
YAAAKGGIMLLMKSIAQEVASLKIRVNSLSPGAIRTPMNVEKLVSPEEYKRILLDHIPVK
RIGEPEDVGRAAVWLVSDDSDYVHGATLYIDGGMTLYPEFASGG*
>SPBIB_v1_210071|ID:27162963| Uncharacterized oxidoreductase HI_0048 [Uncultured spirochete bib]
MLENLSSLFSLKERVAVITGGGGDLCGTMSRALSIAGCKVAVLDILLSKAEAVAEAIRAE
GGIAQAYACDVLDEASLKSAYEAICSSLGVPDILINGAGGNNPKGSTKAEFLDPEKLDDP
ESSNFFDLDFDGVRKTFDLNFLGTFLPTKVFSRGMVEKGKGAILNISSMGAFNPLTQVGA
YSAAKAAVANFTKWLAVHFARTGVRVNALAPGFFMTEQLRFLHIDQKTGEPTPRSRKVIS
HTPMGRYGNPEDLLGAVIYLLSDSASFVTGAVLPIDGGFSSYSI*
>SPBIB_v1_210072|ID:27162964| Xylose isomerase domain-containing protein TIM barrel [Uncultured spirochete bib]
MISLGFVSAILGDKTFEEVVDFASANKFACVEMMCWPVGKAERRYAGVTHIDVDRLDDAK
VAYIKNYLSQKKVFISGLGYYPNPLDEDETKRGVYIEHIKKVIHGAARLGVPVVNTFIGR
IPLKDPEYNFSLFKEIWPDIVKYAETQGVKIGIENCPMYFSKDEWPSGKNLAYSPAHFRK
IFEIIPSKNFGLNYDPSHFVWQQMDYLKPIKEFKNRLFHIHIKDVKVDKDRLDDVGILAT
PLEYHSPRLPGLGDVDWSKFFSVLVESGYRGPACIEVEDRNYEKFPEDVRTALLQSRNYV
SQFLVLE*
>SPBIB_v1_210073|ID:27162965| Oxidoreductase domain protein [Uncultured spirochete bib]
MKELNVGIIGLGFIGPVHLQALRKIPHLNVVAIADKAGSSIKEKVEALGVEKWYENYEDL
LARKDVDAVHICSPNHLHAEMAKKALLAGKHVVCEKPLAVSSAQAKELVELATQKNLVNA
VHFNIRYYPLIRHLRTMVQKGDIGRIFSIQGSYLQDWLFYETDYNWRLESKLSGESRAVA
DIGSHWMDLIEYVSGLKIKQVFADFATIHPIRKKPLKPVETFAGKMLKPEDYSDVPIDTE
DYATILFRFENGERGVLTVSQVSAGRKNRLYFELDGSRQSAVWDSEVPNQMWFGHRDGPN
EIIMKDPSLVYPESRKLITLPGGHQEGFNDTPFQLFSEFYEDVRNGKPSEKPSYPTFKDG
LRELVLCEHILKSNRSGSWVTVE*
>SPBIB_v1_210074|ID:27162966|xylA| D-xylose isomerase [Uncultured spirochete bib]
MAEYFVGHKEYFPGIGKIRCEGPKSDNPLAFKFYNPDRKVGQKTMREHLRFAVAYWHSFG
ADGTDPFGAATHIHPWTSDARNPMEAYEHKLDAAFEFFTKLGVDFYCFHDRDLAPEGQTP
IESERNLAHMVSLAHERQKATGVRLLWGTANLFSHPRYMNGAATNPEFGVVAHAAAQVKA
AIDATIELGGQGYVFWGGREGYMSLLNTNLKREKDHLARFLTMARDYGRAHGFKGTFYIE
PKPMEPMKHQYDFDVETVAGFLRAYGLDKDFRVNIEANHAELAGHDFQHELETAAALGIF
GSVDANRGDPRNGWDTDQFPINVYDTTLAMLAILRAGGFTTGGLNFDAKIRRNSVDPADL
FEAHIGGMDAFAAGLLAAQRIIEDGKFDAFVSQRYSSFDTGDGKRFEDGRMKFEELAALA
PAGPDVGRTSGKQERLENLIMQYVLGVE*
>SPBIB_v1_210075|ID:27162967|xylB| Xylulose kinase [Uncultured spirochete bib]
MYSVCGIDLGTQSCKIILYDPGAKRILAKAQAPLDIIAENNGTREQKAEWYEAALADCFA
AIDPGLRATIAAIGVSGQQHGFVPLDAEGKAIRPVKLWNDTSTAVECAELTSSAGGQAAL
LAETGLLMLPGYTAPKILWLKKHEPQAYARLRHVLLPHDYVNFLLTSNYVTEYGDASGTA
LFDVRVRRWSRRISDLIDPQLIDFLPSLIGPDKPAGRVSAKAAQRFGIPEGALVAAGGGD
NMMSAIGTGTVRDGFLTMSLGTSGTLYGYSDRPVVDPEGNLAAFCSSTGGWLPLLCTMNC
TVASEQVRALLGMSIEELNARAEAAPIGAEGLVVLPFFNGERIPNLPNGRASINGATAAN
FSKENLARAAMEAAIFGMRIGLESFRKLGFAAQEIRLAGGGAKSRLWRQIAANVTGLPVR
VPEEEEAAAFGAAIQALWCFEQASGKKKGIEELVDEHVALRDGSTVEPEPESVARYEEAY
ANYSKYLAALSLLYR*
>SPBIB_v1_210076|ID:27162968| Transcriptional regulator, AraC family [Uncultured spirochete bib]

MKIKDAVYVYRLISGERLAWHGRYHAHARGEYEFHYFMEGQGALLINKAKYIIDGGQIYF
VRPREFHSILPEAVEKPISYYAILFEPEAGNPVDEDIIALMGHIWRARTRALPADTKDRF
LLDDLYRLSKSPSNQSRKAAEHALLSILYRWFDHFDSPSPALGSSRAKNEHVERALSLMS
KLVREKLSSDDLAAKLGLSEEYFIRLFRHHLGMSPFQYFTRLKIEAASAVLVDNQLTISD
VAYRFGFENPFHFTKVFKKCTGLSPREYRKMFFEAGKPPSAAVAEKT*
>SPBIB_v1_210077|ID:27162969| protein of unknown function [Uncultured spirochete bib]
MNEPETTLEFAVDSLLEVLPSVWDRIRSNFRAAGTSKFGITLEQFHTLRHIYKGCCYTGD
IAEKRQVSCSAVSQAVDVLVTKGLVTRAQESGDRRQFRLELTPYARRVLDENYMENRALI
KQKIARLSREQRAIVVQSMEILKAVFISA*
>SPBIB_v1_210078|ID:27162970|yfiB| Uncharacterized ABC transporter ATP-binding protein YfiB [Uncultured spirochete bib]
MKEFKKLLHYAKPYWKLALFSLVMLVAMVGFDLAVPRLVGRIIDKGIRQKDLQVVLTTSA
VMLGLSLLSALVAVLNSISSIRVGESIARDLREAMFVKIQHFSYGNIDKFSTAKLMVRLT
SDTAAVQRLFQMSLRIGTRAPLSMIGSIVLMFVTSKTLALAMMPILVAGGIVIVYFSVRL
EPIFRTVQQRLDRLNTVLQENIAGAHLVKAFVRGPYESSRFEKANKNLADENIRVMQLMS
SMSPILTMLINTGIVLVVWLGGGQAIHGNLSLGQIVAFTNYLMATLQPLTMMTQLSNNWA
NGLASAKRINEVLDAELEVSEAPDAVSLPEPSRGEIEFEGVSFHYNGATDLAVLEEICTR
AEPGKTTAILGATGSGKSSLVNLIPRFYDASSGRVRIDELDVRRLRSDSLLEHIAVVPQD
TVLFTGTVRDNIRYGRPNATEDEIIAAAKAAQAHDFIMRMPKGYDTHIEERGVNLSGGQK
QRIAIARALVCKPSILILDDATSSVDVETETQIQNAIAKAALGTTVIMVAQRISTVLNAD
KIIVLDQGRIVAEGTHKVLLKSSPIYKEIYDSQLGAGVRHGL*
>SPBIB_v1_210079|ID:27162971|yfiC| Uncharacterized ABC transporter ATP-binding protein YfiC [Uncultured spirochete bib]
MAYETTPSVQDVRASFQRRAGRPAKFEKAADPRKAMVRLLKYLGPFKSLLILVVGFIVLY
SLLGLAGPYLMGRAIDKSIGGKDLHGLLVTALLMLAAYFLSNFFNIIANLIMARISQNAL
KNLRGDLFAHIQTLSMRFFDTHPAGGLMSRLTNDIDAINQTVSQNIISLIASILTMIGIL
ASMFILNHWLALASLVVIPIMYWFSNFIAKYTRKGFRELQKDLGELNAVAEETVSGFKVI
KAFRRNESAIETFRQKNNAVFKSAVYANSYAMLLMPLTGVLGSFFVIVLASLGGWLALKE
LVSIGMIATFINYAQNFTSPLRQLSNLYNSIQAALAGAERVFEIIDTKPETPDLPDAVPA
GRFKGEVSLRNVVFGYNPERVIIRNFSLEIPAGQTIALVGPTGAGKTTITNLLTRFYDIQ
EGQIRIDGIDIRAIRKADLRRNLALVLQDTFLFADTILENIRFGRLDATDEECFEAARMA
EADHFIRQLPRGYHTNLSERAGNLSQGQRQLLSIARAILANPSILILDEATSSVDTRTEL
RIQKALLRLMEGRTSIVIAHRLSTIRDADSIVVINNGEIVEQGNHEELLERRGFYHHLYM
SQFKGNEI*
>SPBIB_v1_210080|ID:27162972|crcB| putative fluoride ion transporter CrcB [Uncultured spirochete bib]
MAMKDLLLVFFGGGLGALSRFVFSRGISMRFFTAIPSGTLFVNVTGSLLMGFFFNLLNAS
LLPAGYRALITVGFIGAYTTFSTYALETVALLQRKEYLPAFWNFLLNNVLTFVAIVIGMV
ISSALYSVIRSEGILHR*
>SPBIB_v1_210081|ID:27162973| conserved protein of unknown function [Uncultured spirochete bib]
MHFEEDDMLLRIYIGESDTYKGKPVYEQIVLKARALKLSGATVIRGEMGFGAHSHMHSAN
VLSLSTDLPVVIEIVDKEENIEKLLPFLDEVLLEGLVTKESIHVYRYQAR*
>SPBIB_v1_210082|ID:27162974| conserved protein of unknown function [Uncultured spirochete bib]
MERRDIPIDQFHYDPAQLREHWLVLCAGDFEAGAFNTMTISWGSYGQIWNKMFFQVFVRP
TRYTFEFMEKYGTFTLNAFEPKYKPALSIIGSKSGRNGNKIAEAGLTPMVSRVVAAPCFK
EASLVIECRKMYWQDLEHAHFLDPSIEGNYPKKDYHRMYFGEIVHVSRI*
>SPBIB_v1_210083|ID:27162975|ltaA| L-allo-threonine aldolase [Uncultured spirochete bib]
MNYIDLRSDTVTWPTEAMRKAMAQALVGDDVYGDDPTVNELERLAAEMTGKEAALFVPSG
TFGNQLSLFTWCPRGSEVILGEQCHIIQHEAGAASVIAGVQTRPIFAPDGILPLETIEER
IRGNDIHYPPTSLICIENAHSLGRVIPLSYMKEVAQLAHAHGLPVHLDGARLFNAAVSMG
VSAIEISSMVDSVMFCLSKGLCAPVGSMLAGSREFIEKARRRRKIMGGGMRQVGVLAAAG
LIALKDMTRRLAEDHENAHYLASQLAWIPGIEVEKEALDINMVFFKLPAGIDGSALVRYF
KERGVLINPPEQGWCRFVTHYWIHKEDIDKVIGLMNDCIGTL*
>SPBIB_v1_210084|ID:27162976| conserved protein of unknown function [Uncultured spirochete bib]
MANKGLLMCVCQGNCPSFQQMNIFDVGNAIRREKLVDYVGIHPQLCSTDGDKFLSTLLKG

ESTDELYVAACDPTMQVKMFRDALESAGFSKEHIHGVDIRNMTTEQAVGSIKNMLGADKK
*
>SPBIB_v1_210085|ID:27162977| putative Dinitrogenase iron-molybdenum cofactor biosynthesis protein [Uncultured spirochete bib]
MNIVVMAKGAGLGAWVDDDFAHARQIVLVKDSGGFEAAANPFASSAGAENSAKLAQFILS
KFQDIGAIVAGAFDEDAKANLQEKEISMFIAAKGSVMELAEAARAGSLTRA*
>SPBIB_v1_210086|ID:27162978| conserved membrane protein of unknown function [Uncultured spirochete bib]
MSISTIYILVAAGLLVFSFIKNREKTTKALMVAGRVALMVLPVLLFVFVLMGILEVFVPR
EVFTSLLGSGHGVLGIVLGELLGVFALIEPAAVFPFAGFLHQSGASYGAVVGFVMSAILI
GIVTLPLEIQQFGARFTITRNVVALLLIFGLGLVFMVAL*
>SPBIB_v1_210087|ID:27162979| conserved membrane protein of unknown function [Uncultured spirochete bib]
MKMTHENAASEQHKKNHKGSAELVKSAIIYVALAVVVWLGTTIITPQGGVHVAGIVYKTF
SGVLAIIAAVFVLIGLIQVWLTPEQVSKVLGKEAGWKGLLLSATLPMILGGSLFVIFPLL
KTLREKGARTSSVVAFLFAWSAKAPLLPLEIKFLGLPFAALRLVSIPVLAILGGLLAEWI
IDKPLRDAGLEIPER*
>SPBIB_v1_210088|ID:27162980| conserved protein of unknown function [Uncultured spirochete bib]
MNLLEIQNLSLFKEGRPILNNLSMDIWDGHVHAIIGPNGAGKSTLANTIMGLSGYRDFEG
DILFEGESIKALSVDERARKGITLLFQEPARFEGLGVSQFILAGAKEKSRTVVEEALQKA
GLSPEKYVKRAVDKTLSGGERKRIELASIYAMKPRLVLMDEPDSGVDIDSIKYIFAVIKE
MRHMGSTVILITHSPEVLKHADHAFLICAGTLVDKGEMSRMFDYFNGKCVPCVHVGQPSP
NTLLSGSSAPAVAVS*
>SPBIB_v1_210089|ID:27162981| SufBD protein [Uncultured spirochete bib]
MSEQATADKEKLLAELLASINQHSFAPDVAHVEIHGNQVLNKNLVEGLLVESETIEDGVK
VHIRVRKGVTLKNPVNFCFGLIPDNGIQRIIIDTVIEEGAHAKFIANCTFPNAVNIQHLM
NAVITVEKGASLSYFERHVHGPKGGVTIVPLTKVIAREDSRFSTEFELIKGAAGVIELDY
EAEVEKNATVDMKTRIFGRSSDRIHIKEAAHLSGEGATGVLTSHIALKNTASANIENEIV
ADAPYARGHVDCKEIVQDEATARAIPIVQVNNHLAHVTHEAAIGSVDSKQLETLLSRGLN
EDQATDLIIQGLLGSDPFS*
>SPBIB_v1_210090|ID:27162982|dppF| dipeptide transporter ; ATP-binding component of ABC superfamily [Uncultured spirochete bib]
MADIILSIKNLKKYFEPHQSFAQSIIGGGQKKLIKAVDDISFDIRRGEIFGLIGESGSGK
TTTGKLVMKLIEPTSGQMIFNGEDVTRLDKEKLKEYRRKVQMIFQDPYASMNPRFKIKDV
LEEPLIIHKVKATIEERTKMIVKALEEVKLTPAEEYMGRWPHMLSGGQRQRVATARTLIL
NPLMLVADEPVSMIDLSTRAEILYMMKEVQKELGLTYLYITHDLSTARYFTDRIAVMYLG
RIVEIGRADDIIDNPLHPYTQALIEAVPEPESGKVNIIKELPIYGEIPSPANIPRGCRFH
TRCPYATDACKTEPEPELMLDGEEHYHACRRAYEIKAERSAKAAG*
>SPBIB_v1_210091|ID:27162983|oppD| oligopeptide transporter subunit ; ATP-binding component of ABC superfamily [Uncultured spirochete bib]
MNSLLKVENLEMYYFTSKGAVRAIDNISFELMPGETIGLVGESGCGKTSLGTSILRMPTP
PGRYVGGKIFVDGLDVIPLPEKEVRKNIRWQKISMVFQGAMNCLTPVYTIGHQMMETLNE
HADMSRADADSLIKEYLGYVGLPPEIMGRYPHELSGGMKQRAVIATALFLKPEIVILDEP
TTALDVIVQAQIINLLKKLKKQFNLSFMFITHDLALEAEVSDRICVMYAGKIVEFGTNEQ
IYGRQGPAHPYTERLLAATPRLFKKVEKLHFIPGTPPDLIAPPKGCRFSPRCNKAFGRCF
EEEPPLIEIEPGHMAACWLHHRG*
>SPBIB_v1_210092|ID:27162984| ABC-type transporter, integral membrane subunit [Uncultured spirochete bib]
MTFGDIKYRLNEFWSEFRKERSGLIGLGILIIALLIVIFEPLILPWKDVNTKWRSIDYWQ
DNSANAPPAWTNAFTKLKAPVTMRLEDAKKEETELDGGIKLVTYTFDYHYTADKAPLDVI
LHITGHGDIPFQVAIERPDGETIDLAQRFEQGLSGQDIRLSIDNDGREAAFTFVKNYESQ
EALEQFSGKSLRTADVLFNIAQPGMAKEFKPLKGTYKLIAKAMLLDPENSSVEEPYIVVT
GSVSGILGTDDSKRDLFSGLIAGLKWALLIGFLTSAISVLIGVMYGIISAYFGGAVDSVM
QFVYQIVNSMPVLPVLIVISAIFKPNIYMLILVMVLFFWTGSVMTVRSMALQIKEETYIE
AAKALGAKKSRIIFKHMVPILIPYSFASMALSVPSAIVYESSVSLLGLGDATIVTWGQIL
HDAMQGAAILKGIWWWILPPGLLIAIMGMTFAFLGFSMDKILHPKLKTR*
>SPBIB_v1_210093|ID:27162985| ABC-type transporter, integral membrane subunit [Uncultured spirochete bib]

LHAKNDEYFDILFNQKEQVAFMYKWFALKRVLKGIFTYIIIIFIMSVLFNTVNEQTMRAS
IEDQVRAESMRLKNMQPSAIQRFQQQRRDELIHLYRLDRPLLERILFRTWNTVTFNFGKS
TIIKSSRGERDVITIIGEAIPRSLILFTLAAAIEIAVGIILGLKKAQKPGGTLDRTTSLL
TMIVYGMPTFWLAMILIMFFVYQLKIFPSAGMHSVPPPQGIMYFIDLLWHMTLPLLTLLL
IGFWGVSFVVRNIVLSILQEDYIMAARARGIPENRVLFGHTLRTAAPPLITISVLSLLAS
ISGAIIFEGIFSWPGLGNLYWIAVQQNDIPVLMGDLAVTVGLYQLGLIMLDLTYGFMDPR
IKVGGKA*
>SPBIB_v1_210094|ID:27162986| ABC-type transporter, periplasmic subunit [Uncultured spirochete bib]
MKKILFVSVLAVAVLFSAAAQAKNGPIADKVIYDVRMDQTIAIKDTAEGKTDVFFTGLDG
KTFKGIQPADAEKLSAYAVPSGSWSLLLNPIPNKAPYTFVHKDGRTIFNPLAIREVRYAI
NWLIDRKKIVDEILLGAGEPALTAQTPGQPGTYKFNLVPAKLGMTVRGNEKKALADIEAA
MKEAANLPENKGKLVKSGQWWTYNGEPVTIRFIIRVDDPSGRLLEGRYIADQLEKAGLKV
ERLEYDRSKAGKLVYSGDPAAWEWSMYTEGWGAGATRAWWDVTISQMYAPYYGYMPGGMT
DGFWNYENKEIDKIAQKNINGWFLTADEYWNDNMKVQEMALKEAVRIYVCSQVQYYVANK
ARFNSRMLYGLGDGLNNWSVRSADVKPNDKGEKVLRVTQYSARGGLFMSSWDPVGVDGFS
DVYSAAIVEACTDPSTFEAPNSAKDTPLRVKYDLKKVETKLAAGKEGEPPVGLIDVDKAA
LIYNSKTKKWETGVEFKDVGDGKYDYVKNNAIKSYSKLSDLQYIYGKWHSGQPVTLADIM
YATAFAYEWANKDSDDDKYYDEAYASQYQSTLPISKGTVLNKDGTFTTYFDFNWPMDKDR
VAATGTVSPKAGNPGRQTMVSWEIYEALAKLVAEGSKSGTQYSFSSDPSVTEVDVINPKC
VADIKAKLQDFVAAKYVPDTIKQWVTPEQAVARYNAAIKFIDTYGHAYISNGPFFISKVD
YNANYIELSAFRDYPYKSDYFPKLFRTTITRIDDVKVPATAQRTADAKIDIQVSAVTYPD
DTAKAADSKAKVTVSMILADNSEKLYTAKYVSAGNFQVVIPAKDLGVLKPGAYTLVVQSV
LASESPSVQASSLVLF*
>SPBIB_v1_210095|ID:27162987| conserved protein of unknown function [Uncultured spirochete bib]
MEGNPMNIAGAENGHFVDANGRWLILRGVNLGGSSKIPKVPDGRTHLREGFYDGAHISFI
GRPFPLEEADEHFARLVRWGQHFVRLLVTWEAIEHEGPGIYDVQYLDYIEGIAEAAARHG
ISLFIDPHQDVWSRWTGGDGAPMWTLEAVGFEPRRLHASGAAMLHQEMGQQYPQMQWFSN
HLRLGCATMFTLFFAGNDFAPGVLASGEPIQDFLQRHYIEAFGALARRLTAYPNVVGFDS
LNEPGEGFIGLSDIRKNRSDFVLPGLAPTPLEAMSAGEGLTTFANEIRIKGLGLQNVGRK
PLGTPGKRAWKDGEVCIWRRAGVWDIEGGTAVAKKPQWFALKDNLAGGSPHAFFNEKYLK
PFIERYAAHIDAASASSRKTRRFMMFIESSPVGDMPTIKQGAAWNFVNAAHWYDALTLTM
KRWTGFIAYDSATQKVIIGPRAVRHYFKEALARIANHSKEHMENAPTLIGEFGLPFDING
GHAYRTGDFKIHERALSAYYDGLDANLLSATIWNYTADNTHAYGDGWNGEDLSVFCREDG
GGRALAGFVRPYAMAVAGKITLMEFDHARGTFVLEFEPNKRIKAPTEIFVPGLQFLHGAD
IVLSGGELMDLSNSSPAALQEEKKLSGLSAKAGKRYVLSGMERESFLILRIEADPDAQVC
RLSILRR*
>SPBIB_v1_210096|ID:27162988| conserved exported protein of unknown function [Uncultured spirochete bib]
MRVLCLMVAIIALVAFPLMGQEKNLVLEGDWEGILKVGSASLRIIFHIASAQGRYTATMD
SPDQGARGIPVSAVRLDGSSVVLEVASIGGSYTGSVDASFSKIEGKWKQSGMAFPLVLNK
SKKGAQAQQNTPELQDAPALQGAPAPQPQNQSVQVSQEPTPPFPYTAKDVEFINEKAGIV
LAGTLTVPNGAGPFPAAALVSGSGPQNRDEEILGHKPFLVLADYLTRRGIAVLRYDDRGV
GLSKGDFQSATTFDFADDAEAALEFLARQPGIDAHRLGVIGHSEGAIIASMLGARDPDVA
FIVMLAGPGIRGDALLLMQNTALGRASGLSEAQIAQANELNRRLYDIALEDGDAAILQKK
ILDVLENAIDTAEELTQEQKTAQKEKASLIAAQLVSPWMRSFFAIDPSEYLKQMRIPVLA
LNGSKDLQVPASENLAAIRAALESAGNRSATVVELDGLNHLFQHATTGLPSEYGEIKETF
APEALALIGDWILKMNVR*
>SPBIB_v1_210097|ID:27162989| Ribokinase [Uncultured spirochete bib]
MRILNFGSLNIDYVYRVDSFVKPGETKAAQSLSVFPGGKGLNQSLAMARAGLAVAHAGKV
GKEGGFLLETLQKGGVDTRLIETSAIATGHAIIQVDDKGGNCILLFGGANQDIDEHYIDR
ALAGFGSGDMLVLQNEISSMASIISKAKSRGMAIALNPSPYSESILSYPLELIDIFIMNE
IEAEGISGTHEPEAAASLIARSFPQARIVITLGENGSLCLYKGELVRQKAYKVQAVDTTA
AGDTFSGYFLAGLVEGLSTKNALDLAARAAAICVTRKGAADSVPWRKELE*
>SPBIB_v1_210098|ID:27162990| Aldo/keto reductase [Uncultured spirochete bib]
MIPQIPFGRTGHNSSRLLFGAAALANVSQAEADSALNLVLDSGINHLDTAASYGQAEERM

GPWLAQHRHEVFLATKTEKRTKKEALAELERSLRLLHTDYIDLWQMHVLIKDDEWDIAMG
EGGALEAFVEARQKGMVRFLGVTGHGKAAPAMHLRSLERFDFDSVLFPWNWPLAQDTAYA
AAVRTLLYVCKKRNVAVQIIKAFLRRPWCDRPHMRATWYEPLEAPADISLALGWAWGIEG
AFVNSAGDIHLLPTIIQEAARQPARPTDEAMAQMALRLGMQDLFA*
>SPBIB_v1_210099|ID:27162991| protein of unknown function [Uncultured spirochete bib]
MSRTDGAEAGKLKIGDHWNVISIIALSQNNPLKAVAEFVENSIDASARNVAIIRGKQKNE
YYLRITDDGCGIQDFRYVATHIGDSIKRKLKVQGSGDIQGEFGIGLLSFWTIGETLTLTS
AGKDGVARTMQLVKGNPSYAIRETGMLFARPGTELLIHPLLPGIRSISAEKIQNFLASEL
RDRIARSGVHIAIIDHTTRRELVVEPRKFRGRLIHQLPEARCPFGEIYVELYLAESPQEA
AVGLYKHGTRVIADISRLERFARSPWDSRCIEGIIDASFLQLTPGTRDGIVQDEVYDSFC
VALEPVEEALGALLEEQKRALEEEASRQLLNRVTRAFREAFLYLPEEDYSWLAARVREPR
RAPSAGTSSQSGAGEASANGEGSDYRESSSRTEEQNPATHASESSSDSLGDSENAALGEY
IPEPNRQQSKTVQKSFFEFAGPLHKLMISPSSSVIGTSERRKFKAIARDRAGRLIDSGID
FAWHLSEGGGALDGINSPYAEYIAPEEPCVATIELRASQGDITLTATAIITVTAELIKRA
SGTGSAQRRGLPGYTYKKAPGELWRSRYDAENAIILINNAHADFIHASRQPMTKLRYIAR
LFAKELVLANFPEASREEILERLVELTLYTEENLK*
>SPBIB_v1_210100|ID:27162992| conserved membrane protein of unknown function [Uncultured spirochete bib]
MLEIPALAKILVIFALIVVAAARRIHLGLAAAVGGIALAIWQGMPVSAIALAVAKELTNP
DLILLVILLTGIMMFSAAMKKSGAMDAFSNAIAESAPSRRIALAVAPLLIGTLPMPGGAI
ISAPLVGAMNDDDGRGPETLSAINYWFRHVLELVWPLFPAFILTVGLTNLSVMQLVLLNL
YAPFAVFGLGLIFILPKNGKTAATMPASKTAVNTTFRDTAKFGRLVRGIAPLGMILGSYV
VLDLIWELVAPGVALDAPTKSLIGRYVPVLCGLLIGSLYLRKNTKGEKIFKHSITASTIE
LIAVIVGIRLFSALMSAADLAHAASMELANAGIPAIFVIALLPFISGIVTGVGMGYVGLS
LPIVLGLLASSDIPFKVGVVIAGAFGYSGMMLSPLHVCMVVTAQHFKTTLPATIRKFALP
LGIFLVIAIAYTALLSVVLR*
>SPBIB_v1_210101|ID:27162993| conserved membrane protein of unknown function [Uncultured spirochete bib]
MLLPYSFYNSAWKFLSIALVVGLAVGGYFVPELGLGVIALILFALLTNARSSRSFCAGFC
PNGRSLSVVFEKTSKHRKLPPFLASREFRRMLCALMMFCVISLLSQSNGSLAAIGKVFWA
IYLASIGISTIAGLLWKPRAWCAFCPMGTLQDTIKGH*
>SPBIB_v1_210102|ID:27162994| HAD-superfamily hydrolase, subfamily IA, variant 3 [Uncultured spirochete bib]
MEGSWRPDDSSLKGYAMSDIIFKAVLFDMDGVLVDSERLIAESAVRMFKERYGLSVRHED
FVPFVGAGENRYIGGVAEKYGLAIDIEKAKAWTYEIYGKLAKAHAAGMRAIPGAVDYVRQ
CRAHGLKTALASAADRVKVLINLEYLGLGPDEFDVLLTGGDVAHKKPHPEMYLKAAEALG
VLPSECLVVEDAVNGTIAGVRAGARVLGITSSFPEAELRKAGASWIAADLARAPLPWELD
*
>SPBIB_v1_210103|ID:27162995| Cupin 2, conserved barrel domain protein [Uncultured spirochete bib]
MFGKHSDAGFHQPIPGNHIKTLCYGAHTLLVEVRLNKDALLPEHQHPYEQTGYLVSGRIR
MFIDGKARELDPGDSWCIPMDVLHKVEVLEDAVVVEVFSPTREEYLKYENPEDIIR*
>SPBIB_v1_210104|ID:27162996|czcD| Cadmium, cobalt and zinc/H(+)-K(+) antiporter [Uncultured spirochete bib]
MVMDAHDHHHDHSSEALEVEESGGKLLASILITSATLIAEVVGGILTGSLALLSDAAHVF
LDIFALGLGYIAVLMSKRAATGKHSFGFYRMKVLAAFINGATLLIVSFEIIREAIGRFAH
PQPIIAGPMLIVAIIGLVANLIVAFVLKGHSHDDLNARAAFLHVLGDALSSVGVIAAGVI
ILFTGWYWLDPLAGILIALVILRGAFSVLKEATHILNEGSPENADSAEVAREIAEIEGVK
GVHDAHVWALEPGYRVLSAHVVLDDQPLSATAEIMDHIKDMLHDHFEIKHTTIQFECGDC
GQCGSAIRA*
>SPBIB_v1_210105|ID:27162997| conserved membrane protein of unknown function [Uncultured spirochete bib]
MSVKEWSPNKKLLVLFGVFLAAYFIPFQAPRVSGAINEAFLMLSEYAQQHVILCLIPAMF
IAGAITVFLNQQAVMRYLGPDAKRLMAYGVASVSGAILAVCSCTVLPIFKGIYKKGAGLG
PAIAFLYSGPAINILAIVLSAKVFGLKLGIARTVTAILFSVVIGWLMAQIFKKDDEKRAA
DARLFKAPLGEEPRTLGQTSIYMASMIGILVFANWANSRGGSPVWDAIYHAKWWITGGFA
AVLAYTVIRWFNADDRMAWVVATRDFALQILPLLFGGVLVAGFLLGRPGHEALIPNSWVA
GLVGGNSIFANLFASAAGALMYFATLTEIPIVQGLLGAGMGQGPALALLLAGPSLSLPSM
LVIAGEIGWKKTLTYVGLVVALSTLAGLVFGAIA*
>SPBIB_v1_210106|ID:27162998| Redox-active disulfide protein 2 [Uncultured spirochete bib]

MKIQILGPGCPKCRLLEQHAREAVNELGIQAEFEKIEEVDDIMAFGVLMTPALAIDDEVK
VVGRVLTKDQIKALLS*
>SPBIB_v1_210107|ID:27162999| Regulatory protein ArsR [Uncultured spirochete bib]
MKQLERRYERRAEIFKALANPLRLVMLEKLADKPWCVCALADELGVDKSIVSKYLSQLKS
AGLIEDTRKGTLVEYRLVAPCILQVASCAEETIQEQKKKLLSEE*
>SPBIB_v1_210108|ID:27163000| Citrate transporter [Uncultured spirochete bib]
MSPNISAVYVLIILAFVGIASGKFPRLAMNRASIALTAAVLLVVLGGLTTQEALAVVDTE
TLALLLAMMIIVANLRVSGFFLIAGGRVLSIARSPRILLAIVVLVSGVLSALFLNDTICL
MFTPLVAEMARRSGRDGRPYLIALATSANAGSCATSIGNPQNMLIASQSGIPFGTFVLAL
GLPSLIAMLLSYWMTVLMFPSEFRANTMLRQPVGCVTADAEAGHAEMSPYLMYKSLAASA
LLLVLLLAGVRTSHAALIAASILLITRRVHPERIFAEVDFTLLVFFSGLFVLTAAVARTD
AFAMAMQWLTPHLQRPGGLFAAAIALASNLVSNVPAVMLLSPAARAMANPQPAWLMLAMA
STFAGNLTLLGSVANLIVAEQSEKSGIHIGFVDYLKVGLPVTLLSIAAGTLWLGIIL*
>SPBIB_v1_210109|ID:27163001| putative NADH dehydrogenase/NAD(P)H nitroreductase AF_2267 [Uncultured spirochete bib]
MDLNDTLKTIFSRKSVRAYAEGAVSKEQLEMLVRAGMAAPSAVDQRPWEFVVVTERATLN
ALASRLPYAKMAAHASAAIVVCGDLRRQWGGPDSVMWVIDCAAAAENVLLAAESLGLGAV
WTAVYPYPERIRSVRELLGLPDYIQPLALIPVGIPRGGEKAKDKWNPERVHWEKW*
>SPBIB_v1_210110|ID:27163002|arsC| Protein ArsC [Uncultured spirochete bib]
MVKVLFLCVHNSARSQMAEAFCKKYGEGKFVAESAGLEPGKLNPYVVRAMAEVGIDISKN
ATKSVFDFHNEGRTYNVVVTVCSKEAAERCPIFPGAPITLHWPFDDPSSFKGTDEEIMQK
VRSVRDAIEQKVMEFVSQWPRVSE*
>SPBIB_v1_210111|ID:27163003| Dihydrolipoyl dehydrogenase [Uncultured spirochete bib]
VESKQYDFVVIGAGPGGYVAAIRAAQLGLSAAVVEKDLVGGVCLNIGCIPSKSLIHSAQL
FAQGKALLEKTGAKVDLSNFDYSLVWKASRLAADRLSKGVNFLLRKNKVDLIKGKAMLSG
ASTVDVETPGGSVQLKAKAILLATGSRPRPIPGFAFDEKKIFSSTGMLMSDKLPKRMFIL
GAGAIGMEFAYVLNAFGVEVTVAELLPRVLPLEDEEASKIVEKEFRLRGVTMYTGARASG
AEVKDDHVVIHLTDAQGAPLDIEADAVLVSVGRATNTEGLGLENLGVRMERGYIITGDYH
ETSCAGIYAVGDITTYPQLAHTASKAGEIVAERVAHLLKGTPNPRERTVDRLHVASAVYC
EPEVASFGLSEASAKEKGIRHEVARFPYRGNGRAVATEAPEGQVKIVFDPDTKAILGASI
VGEGAADTIHELLLASKAELTIEDVAELVHAHPTVSEAIMEAAKAALGRAIHI*
>SPBIB_v1_210112|ID:27163004| putative Lipoate--protein ligase [Uncultured spirochete bib]
MPIVAQSYKVVSDSPLDIISFEECLVNSWAGEEALLLFYVNAPTVIIGRNQNYWREVSPS
CKVPVFRRVSGGGAVYHDEGNLNWALIVPRAIHSQDDELAAVASAIGTLGVYARPGARGG
IFVSLGDGETQGKISGTARRFGTQNVLHHGTLLVSADIAALKASLGGIKVFEDSSIASVP
GHPVNLSRFLSSLSVEELMLHLSRTLAGSAPATIELADFWNKEKTIARRVFKNPEDLERE
FDFCVECEEFEAHRKQFGSKEWIIDRSPPFSVVVSNGRAKAVVRVQEGKILQVVPLEAGD
SVSARYADELNAHFEGMAFDFRMPEIMGKEYQRGIEAI*
>SPBIB_v1_210113|ID:27163005| exported protein of unknown function [Uncultured spirochete bib]
MKLVKVIGGLLVALTLFSCAPKLAQKDLDAANAAFADAQNAKADVYAPDEFKAAQDAKAA
LDAELAAQDAKTSGKSYKQANDLIKAFADASKKAKDAAATNMDKVKGEVAQLITDIDAQY
AAVQALAQAASKDAKKAAKAKLNVKDINARVEAAGKSISDAKATNDSQDYAGARDQLNTV
KATLDELKASLEAAGFVAQ*
>SPBIB_v1_210114|ID:27163006|speE| Spermidine synthase [Uncultured spirochete bib]
MKKIIERLNPHCGFYYDITKVLVRVRSAWQTIELAESPEFGKVLLLDGITQVALKGEERY
HESLVIPAMLSHPDPQRVLVIGGGDGGVLREVLTFGTVRNAVMVELDEAVISFSKQYMPE
ISAGAFDDPRAEIVVGDGRAYVEQCPDTSFDVVIMDMTDPFGPSEKLYTREFFAQVKRIL
RGAEGVFAMHGESPIARPAAYACIEKTLRKVFSCVKSAFTYVPMYGTLWSFKFASGTTDC
ASLSAKDVGSLLNARCERMPKYCMPQTYKAIFAPEPAAIEALFHPEGRVIEDRRSHFPDA
FDPQGA*
>SPBIB_v1_210115|ID:27163007|speD| S-adenosylmethionine decarboxylase [Uncultured spirochete bib]
LVRRVKGKTLKLEGFNNLTKSISFNIYDICYARSRESQIEYLEYVDEEYNSKRLEEIVEN
VTRMINAKLVSVSTQDYDPRGASVVALINEGSPVADEHIKSDKEQTGPAPFVIGHLDKSH
IAIHTYPEYHRLTGLASFRVDIDISTCGMISPLSALNYLIESFDSEVVIIDYRVRGFTRD

HKGRKVFIDHDISSIQDYIDQDILERYITYDINILSDNNFHTKMRKKEIRLKDFLFGDEV
TSLDPKEKERIRRALLHEIQEIFECRNLNMRSGRL*
>SPBIB_v1_210116|ID:27163008|potA| Spermidine/putrescine import ATP-binding protein PotA [Uncultured spirochete bib]
MSVRLVNITKEFADPEREGGKLVAVRDFNLEIQEGEFVTLLGPSGCGKTTTLRMIAGFEM
PTTGEVWIGGKNVSNDPPNKRDTSMVFQSYAIFPHMSVEQNVGFGLELKGRPKAEITREV
RQIMEVMGIADLAHRRPDQLSGGQQQRVALARAIVNRPSVLLFDEPLSNLDAKLREQMRV
EIRRIQQTFKITSVYVTHDQAEAMTVSDRIVVMSKGRMMQVGSPFEIYSRPANRFVADFI
GRVNLLSVTVERLEQEKNIVRGTDGSRYEAASAASGVAEGGKALLMVRPESLSVESSAGA
APAAEGRMAFAQGVIDKTVYLGATVEYEIDAGYERPILAVSHDPVDAGFWRTGDRVVLRY
SPRAAHLLPPESE*
>SPBIB_v1_210117|ID:27163009| conserved membrane protein of unknown function [Uncultured spirochete bib]
MAHGISKAAAELRRAARDPVLLGVILLVAGSLIVFVLYPLISVGAASLDVGGGKTGLGVY
RDVIRSAYLRKAFYNSLMMSTLTALVGTLIGFLFAFAVTRAKVPLSKFFNILAIIPIVSP
PFIGALAIIMLFGNNGLITWNLLKIQNFPIYGFNGLFLAQVVTFFPVAYLTLKGVLESMN
PVLEDAALDLGGSRWKVFTKVTLPLAFPGIASSVLLLFVETLADFGNPLILAGSKFPILS
VQAYLQIVGMYDLPKGAAISVLLLVPSLIFFYAQRALVNRRKYTTVTGKPQQSTTQIVSP
GARIALFVFCLFVALVVGLIYFSIVVGAFAKVWGFDHHLTLDHFKFVFGVGFKAVRDTLM
IAGIATPFAGLLGMVIAFLVVRVKFPGRKFVEGVSMLSFAVPGTVIGIGYILAFNHKPLA
LTGTLAILLLNFVFRYIPVGIESGKALLASIDPSIEEAAVNLGADSRKTFSRITLPMILP
AFFSGLVFAFVRAMTAVSAAIFLVSSRWNLMTVHIMAQVESGNLGAAAAYSLLLVLIVAA
AISLIRVALKSQYSKYGGAMLHF*
>SPBIB_v1_210118|ID:27163010| conserved exported protein of unknown function [Uncultured spirochete bib]
MKARRMILYMLAIIAIAVPAFAKPGQTLVVYSSVDEVNAKKIFDAFTADTGITVQFVQLS
SGPAYARIKAEASNPQADVWFGAPSENHIIAKEEKLTVPYKGPNFDKLSKEFKDPDGYWR
SFYMNPMAFAVNKEVLARIGAPKPTSWADLLNPAYKGQIQMPSPQSSGTAYNIVASLVVM
MGEDKAFDYMKKLSPNVQTYTSSGTAPSKAVQLGQCAIGIQFTPAFFEAIDKGFPIEVIF
PKEGVWFEAPAVSILKGAKNLEAAQTLVDWLTTTKGQSVYTEKKTYFYPVLPGVPLGAGM
PAFESLKTIDVDAIWAGEQKKRLVERWVNEVLTAK*
>SPBIB_v1_210119|ID:27163011| putative Beta-lactamase [Uncultured spirochete bib]
MRSTVSSMSNEIQRLTIDAIEHQWFAGAVIRLEQEGEVLYEGAFGSAIKTAQLQAPMSAT
TQFDIASLTKLFTTTAILKLVSEGRLDLDAPLVTVADGKLAVLLGAEESGSNRRVADVLE
KVSTRALLTHSSGIHYWYPFYAARESIKASARSIAGSPAGSPVENPAASPAGEPYSAFDS
IFETVLARFPLQHTTIYSDINFMLAGFIVETVSGLPLHEAIPKLVCVPLGLERCAYLKDA
SSARNAFEAGGDSTYAATEFGNRIEMKMVADLGLHFSGWRSIDQPIVSAPNDGNCHYFFH
GVAGHAGIFTTARDLCRLGNLYAGVDNANYIADDLLEEAVTDHGNGRGLGFQFGPRYPQG
CGHTGFTGAYLFVSRNRHLSLSILTNRLHVPEPRDIDPFRKAIAELAIITI*
>SPBIB_v1_210120|ID:27163012| putative GCN5-related N-acetyltransferase [Uncultured spirochete bib]
MQIKIEDAVPGDIAQCARIACDSEIGQRYGFVEETLAARIQMRLQEGDVVLVARDMGAIE
SASSSNGAPSDIIGFAWIDTKGGFGQAPYLKLIAIDASRRSTGAGSLLLSAFEERTRGAG
RAWFLLVSDFNDRAIHFYKKHGYAVVGTLPDFAKDGITELIMYKRQEA*
>SPBIB_v1_210121|ID:27163013|oppF| oligopeptide transporter subunit ; ATP-binding component of ABC superfamily [Uncultured spirochete bib]
MNTIQPLLEVKNLKVYFPIEKGIVIRKRIGWLKAVDDVSFLVQKGEAVGLVGESGCGKST
TALAIAQLQRITNGQILFEGRNLSALPPSELYAERRNFQMVFQDPYSSLDPRMTAFDSIA
EPLRIQVRRGSLNLSEADIRKKVLDLMDKAGLSRTFAHRYPHEFSGGQRQRIGIARALAL
SPKLVIADEPVSALDVSIQSQILNLMKDLQAELGLTYLFIAHNLAVVEYFCGRIVVMYLG
TIAEIAPSEMLYHEPLHPYTKALLSAVPIPDPPRERQRSRILLKGDVPSPSEERVGCPFF
ERCPSAMDICNAQRPLLREAGAGHMVACHLYAREGDSHANKN*
>SPBIB_v1_210122|ID:27163014|oppD| oligopeptide transporter subunit ; ATP-binding component of ABC superfamily [Uncultured spirochete bib]
MIIGTEPVLSVRNLSTRFMTSSGTVKAVDGVSFDVRKGEIFGIVGESGSGKSVTNLSILR
LIPMPPGQIAEGEIMLYGKNLLAANEHELQMVRGGSISMIFQDPMTSLNPLLRVSLQIAE
ALELHHGLSRKEAEKKAVELLKAVGIPDAERRAQEYPHQFSGGMRQRVMIAMAIGCNPDL

LIADEPTTALDVTIQAQILDLIRRLSDTRTMSVILVTHDLGVVAGMCDTVAVMYAGKIVE
QASVDPLFREPKHPYTQGLLASVPRIDRAWSERLYSIPGAPPNLVDLPPGCAFAPRCEHA
MPICSQSYPPQIEMDDGSGNKRTVRCWLHADNVPGVHR*
>SPBIB_v1_210123|ID:27163015|yliD| putative peptide transporter permease subunit: membrane component of ABC superfamily [Uncultured spirochete bib]
MARLLKNPIALVGLAIIALYTLIVIISQFWLPFDPLQMDVAHILESPSFRTGHLFGTDEF
GRDILSRIMKGTGISLLIAVLATAIGATIGTFMGVWAGFLGGAWDHWIMRLADVLFSFPS
LLLAIFVMAILGENTYNVIIAISIVYIPQFARVSRGAVLAIKNNEFVRAAFSSGADRRFV
LSRHLLPNIMMPIIVQSSLSLSVAILLESALSFLGLGVQPPHPSWGNMLSSARKVMMLAP
WTAIYPGLAIMGLVLGFNLLGDGLRDILDPRLRNIK*
>SPBIB_v1_210124|ID:27163016|nikB| nickel transporter subunit ; membrane component of ABC superfamily [Uncultured spirochete bib]
MGRYIFRRLLSLIPVLLGVSIIVFFLVRLIPGSALQMYLGTQVEATPEQMEELRRIFGED
RPVPVLYVEWLGRLLRGDLGYSLRTGREVLPDILQRLPLSIELTFYALFLSLIIGIPLGI
LSALKHDPVTDVSIRITGLLGLSLPQFWLASLMVIAFSGFRGWIPMGNYVGLFKAPLDNL
RMLFFPSLAIGIGLAAVIMRYTRNSMLEVLQMDYIRTAQAKGLAKRIVIVRHALRNAILP
VITVAGFNTGYLLGGAIVIEEVFALPGMGRLALYAIYQRDYSLIQGIVLVIATLFVLVNL
VTDLIYAAVDPRIRLETREG*
>SPBIB_v1_210125|ID:27163017| ABC-type transporter, periplasmic subunit [Uncultured spirochete bib]
MKRLLAIGLALAMVAGIVPAVFAAGEEKVLTIGVDQEAIGLDPHIVTSFSSHRRVDLLYN
KLVRHDENLKIVPDLAESWEIPDNQTYIFNLRKGVKFHDGTELTAQDVEFSLNRILDPKT
ASPGRSYISSIKSIEVLGPYKVKVTLSAPLASFLEGLASNNCAIVSKAAVEKYGNLQKVA
VGTGAFMLKEWIPDNSMTLVKNPNYFEKGLPYLDKIIFRVIPEQASLLAGIKAGTLDMAQ
INDASTILLAKRDSNIVVMQKPGINVRTFGFNTTRKPFDDARVRQALSLAIDRNEIVTVA
EFGMAQPTGPIPAGATQWALPLSKLPYYTPDYAKAKALLAEAGFPNGFTFKIVTANSFEG
GLSVAQVIQNQLKKIGVNAELEVVEWGIYIDRWVKRDFDSMVELRGGSGEPDRFLYRTIH
STGGVNNFLFKDADVDRLLDKGRVLTTYAERKPVYDELQVVLAEKAPIIFLYCPFETHVL
SKQVKGFKQVGYGSLYYLIQTQK*
>SPBIB_v1_210126|ID:27163018|anmK| Anhydro-N-acetylmuramic acid kinase [Uncultured spirochete bib]
MESEKTASSLFRLSAYQSRHERALIGIMSGTSFDGIDAVLVRIITDERGHIASLAMEGHY
SLPYSQELQKRLLALCSPETARIDELVYAHAALAEWYAGAVEGLLGMAGRKAQDVYAICL
HGQTVWHAPVPRAFPGPSGQSVAARGTLQIGSAPLLHERTGIPVIYDFRSRDMAAGGEGA
PLAPFVDALLFGSLERGRIVQNIGGIGNATVIPAGAGNEAVFAFDTGPGNMVMDELAKRF
SNGALAYDKDGLLAARGRVSHELLEPLLEDPYFSRKPPKSTGREVYGAEFAARFAVRGRA
LGLSDADIIATATAFTAETIVRAYRDFVFPLVPIDDVIVGGGGALNPSLMAMIRERLPAH
ATLRTTADYGIPVLAREAIAFAVLGHEALMGHPSNLPAVTGASHEVILGSLTL*
>SPBIB_v1_210127|ID:27163019|rbpA| putative RNA-binding protein RbpA [Uncultured spirochete bib]
MGKKLYVGNLSYNTTEDSLKERFEAFGHVESVRIITDRDTGSSKGFGFIEMGTDAEAQAA
IASTNGQEVDGRMLKVNEAMDKPRDDRSRDNRRRGSYSY*
>SPBIB_v1_210128|ID:27163020| NADH-quinone oxidoreductase, E subunit (modular protein) [Uncultured spirochete bib]
MAMNGKGKAGETDDAVGNTSILPNTTAPKTQIKYRDTLLFRLHEVQNRTRGKTWITKEEL
AGIAQDYGIPLAELDGIVSFYTMFSRERRGRHVIRLCDSLSCRICGSLDIYQHLRKKLGI
SRGMTTGDGAFSLEIVNCLGACSTSPNIMIDDHLISGLSLAEIDAMLDRLAKEEV*
>SPBIB_v1_210129|ID:27163021|nuoF| NADH:ubiquinone oxidoreductase, chain F [Uncultured spirochete bib]
MSNIQYITGFATLENPLDIDSFVAKGGFSGLHSALAMTPAEVISEVKRSGLRGRGGAGFP
TGLKWESVAGDDERYLVCNADEGEPGTFKDRFVLEHAPFLVLEGMAIAAYATGAQTGYVY
VRGEYPHVVASLSSAIASAVKAGYLGEYILGSNFSFNVEVRKGGGSYVVGDETALLNSLM
GNRGYPFLKPPFPTEKGLWGKPTIVNNVETLAYVPYIFTHGAEAFASIGSPDSPGLKLFS
ISGHIAHPGVYEFPMGTTVRELAAAAGGILGNLKAVQIGGTAGPIFDTAALDYPLDFASM
RKAGGALGSGALVFMNTTVNMAHVLEVTMRFFAEESCGQCFPCRYGTRQLAFMANKIASG
QGKLEYLDLMRDTTHVMVGASFCPFGQSIAFPLESLLDRFGDEIVSFIKQQEFLKEAAV*
>SPBIB_v1_210130|ID:27163022|hndD| NADP-reducing hydrogenase subunit HndC [Uncultured spirochete bib]
MKNVTLTINGKKISVPEHTTVFSAAEKAGISIPALCRHEDLEPKGACGMCIVKIEGQPGY

KRSCVTAVEEGMEVLTSTAEIRDIRRGILELVLAAHPADCLQCIKHGKCELQTLAERFEI
RDLRYDRYTRGLPVDRSSFGIVRDMNKCIGCGRCVQVCNEVQTVASIFFHGRGSNTIVSP
AYGTTMGDSVCVNCGQCIVYCPVGALYEKEAIEEVWKAIDDPDKVVVAQIAPAVRVAIGE
EFGLEPGELSIGKLYSALKALGIDVVFDTNFSADLTIVEEATEFLERLKESGPFPLITSC
SPGWIKFGETFYPELLENVSSCKSPQQMLGALIKTHYAESRGLDRNNIVSLSIMPCTAKK
FEAGRPEMRSSGARDVDYVLTTREIARMIRQVGIDFRNLPDGTPDPLLSRYSGAATIFGA
SGGVMEAALRTAYELATGKALEKVDFVDVRGMTGVKEAEIDVDGTPIKVAVTNGLANARK
ILDRVAEDKRRGRSSYHFIEIMACAGGCVGGGGQPIPNTLARRARRIEGLYREDRSLPLR
KSHENPEIRALYAEFLGSPGSEKAHALLHTKYSPREQYQFSE*
>SPBIB_v1_210131|ID:27163023| protein of unknown function [Uncultured spirochete bib]
MFILKIDIVLVGYTLYGAVHVPFRFRVSPGIQRTVL*
>SPBIB_v1_210132|ID:27163024| PilT protein domain protein [Uncultured spirochete bib]
MTKILLDTNAYTAFMAGDQHVLSYMVDSEVVYISTIVIGELFAGFYGGDKVLQNKEELKI
FLSKDSIKIIDVTMETAEIFGELKSALARKGRMIPLNDIWIAAHAIEYGSKLITYDEHFK
NIDGVRIWEYLN*
>SPBIB_v1_210133|ID:27163025| conserved protein of unknown function [Uncultured spirochete bib]
MKSITIHSIDPDLDKKISEKSKELGLSQNRTVKAILQNALQSDNKAARKEMFSDLFGKWK
PEERAAFEKQIIDLEELNDSDWAK*
>SPBIB_v1_210134|ID:27163026| exported protein of unknown function [Uncultured spirochete bib]
MVKAKKTKEKIVKQKSFIVAALALVMVALAVGVAGAQTREPLVPQKAREK*
>SPBIB_v1_210135|ID:27163027| conserved protein of unknown function [Uncultured spirochete bib]
MMKPAIVYVTRTGHCRTLAEMASRIAGGRVWEIGDKERRNGFFGFIRSGRQSTFNMATPI
EDPNADLHDIDTLILVTPIWAGKLAPPVRTWMRSHVEEVRGKRILLIASNSGGSTVRLQQ
SFEKEFLPIDGLLSVPIQLSEEQKLEKLEAFLGKS*
>SPBIB_v1_210136|ID:27163028| ABC transporter, ATP-binding protein [Uncultured spirochete bib]
MAQLKLVDVTKKFGDVIAVNKLNLEVEKGECFSFLGPSGCGKTTTLRMIAGFEDLDEGEL
SVDGKVFSSSFKKYYLPPEHRDFGMVFQAFAVWPHLTVYDNVAFPLQIRKIPRHEIAKRV
KMALQSTGLTHREESYPGKLSGGEQQRIALARAIAINPKVMLLDEPLSNLDAKLREEMRF
EIKDLQKKFGFTIIYVTHDQAEAMALSDRMLVMDHGIVQQVGHPLEIYNKPANKFVFSFI
GLSNFIPVNIEEGKAVIDGSNETASFEAPLELGKKAFLACRPSEVDFVEEPYTGANQLPP
AIRARVARRTYLGDIVDYKVAVGNAEIRIQKPRRVQGPIEGSLCTIRINRTLWYPLE*
>SPBIB_v1_210137|ID:27163029| ABC-type Fe3+ transport system permease component [Uncultured spirochete bib]
MNTGNKLSTANIVLLFSILILVVIVAVPVILIFITAFFENGKFNIAGIAKILSDPDTYQA
LLNSLIIAAGTTLVSTTVGTFFAWLVARTDLPLKKTMKALFLVPFMLPSFIGALAWKVLL
SPRAGYINKLLMAIFGLSKAPIDILTIGGIIAIESMYLFPFVFLQVEGSLERMDPTLEES
ARISGANLFTITRKITLPLVMPSILAGALLVALYSLAHFGVPAILGTEKGIYNIPTKIYE
RIYASGGSFNAIRVGTILSTILVISAALILKLQDMLLKKGQYQIIAGKSMRPMVMKLRGL
KIPLLIISILYIGITVILPTVTIFLVGGLKTYGLSFELKNMTWNNYLKIFQWKMTKDAIW
NSLYLSLGAAFVTMLAGTMISYVIVKMKVRGKFILQFLGVLPFSLPGTVIALGVILTWSG
RFGINLYNTPWIIFVAYIARYMAFSIKSNSASLSQVSDSLEEASRACGATHWQSLKNIVI
PLIKPGMISAFFLIFLPALRELTTSVLLYGPTTRTIGVAIYALNEDGETVRAAALASIAL
VIIFAGETIIRKFLARLDQAKVKVQE*
>SPBIB_v1_210138|ID:27163030|potA| Spermidine/putrescine import ATP-binding protein PotA [Uncultured spirochete bib]
VADLQLSAVSKSYGTHRVLDKITFKVNSGECFTLLGPSGCGKTVILRLIAGFESPDEGEV
SIGGHVVATAKHSLPPEERHIGVVFQDYAVWPHKTVYENIAYPLEIAKMPHEQIKSAVSD
AIDQVNLAGLAQRYPYQLSGGQQQRVALARALVTKPDVMLLDEPLTNLDANLREEMRFEI
KELQRKTGTTIFYVTHDQEVALAISDKMAVMDDKGIIRQIGSPDEVYERPIDAFVYQFLG
IPNFIPVAIKSGQAFIDTDAVQGAAELPLQALADFPSQRFPGPKGKLACRPMDVILSRSI
HPETMVKAKIVRLSLLGPIVDYLLDIGGIAIRAQMQTEAAMAQDLLIEEGQECGVRFAEP
KWFSEDGEAIR*
>SPBIB_v1_210139|ID:27163031| ABC-type Fe3+ transport system, periplasmic component [Uncultured spirochete bib]
MRRALLVLMALLVTGIVPVIAQPKEKLMVYTSMKEVLIGEIRDAFTKKYPNIQFDYYSAG

AGKIMAKIAAERQSGKLVVDVLWTSEIPDFYSLKKEGVLQRYESPEAKYVVSPLNDPDYE
FTPARLGTLGITYNTNKIKTPPTQWSDLLGPAYKDGFAIANPALSGTSMVSVGMIANNLG
WDFIQKLRANGAKMGQGSGQVVDDTAAGDLAACIGVDYITIDKIKLGAPLGFAYPKEMLV
IPSPVAIFKGTPNLAAAQKFVDFLLSKEGQTIIANNYTLPVRKDVPIVQGVGLVAPDEAV
KRAMKIDYLKLMVEKQAVIDKFTEIMQKK*
>SPBIB_v1_210140|ID:27163032| exported protein of unknown function [Uncultured spirochete bib]
MPMALGIAVLRGLEKKPFSKRIVAGISLALISFAVMVVDIQRSIKQEDPAAYAAKILVLR
SRRIAELAASIEAISFQFFMNRLLNTTLSAYVNDAERYDISKWNTVFSQHMEGLAQTVPE
IEDAIFFAIPDPNKIPLMMTDSLTRATYQPVRSIIMQKAIAASGKSVWDVCMGASKSAAQ
NVNKLLVCARLITNIDTHVPLGVLVLLVNPDRIARAVSGSSWDEDANAAPKTDYTLLIDE
NSKILVSASLSQIGLQAQAQVPGFSEHFPLNKAASPSGKYKARALSGAKATQGLFWVIYE
RVPDRLWTLVSILPVSVDPLPLFFRIFIIAGFAASVWLILSVLRGGEATPDQSQPPAQHL
EAIPDWFKALAPREKVVMVLLLKGLSNKEIAYHLAIREQTVKNYLHGIYKKLGVQDRFSA
LLLMQNAHLNIETIKDYAEKNPSEFSVSIDILA*
>SPBIB_v1_210141|ID:27163033| protein of unknown function [Uncultured spirochete bib]
MQNQNRGFRTIQFKTIQSGACFKSYGLSRYHGNGTIFQVLQMKDFLVKFIRSPLAVGLVS
ANAVLGAAAGAAGFLPWLVVVPLFLVISGVELLLTLRSEAGAKAILAEEDRERKERDAAI
LADVAQARKRLALLRIAEPEVKAAVERLVYAAGMYLESCAKGNERDPFAEDAVLSVTKIV
DEYLRLADAERMGARLKSESLEDFVRASEFSSEIGPSSSSNPDKPDRSATTDNDSAAQEP
EPSLAQRMVRLLDKAASTISERLALPLGGMEGDHAVLDAMKAREELEE*
>SPBIB_v1_210142|ID:27163034| conserved membrane protein of unknown function [Uncultured spirochete bib]
MKRAGIVFVVLFIFGMVLQPLGALEARLVSAEIYLTLGSDGKAVVQHSLVWNVSSGTMGG
FYFQGEKAPFVWDMERCWADLPDGTRQPLEIKRAGDKWDILLARGKRTNGLSTWVLTYGT
DLLAADMAGLTQKADGEKLFFFHWAPPEWDQALDHRTVTIILPIEVSRGESGDSRSERLA
DLGFATEKQVNEKNKIDWYAADGSDGKSYLAIRFHQEKPAAYASQELQFYLSASKVEAAF
GPLFGALAQTGTGAASGGAARSTSASGSSANAGKGRSASPGASTQNFDWQRYPLLALFLC
GGVAVLGVLLYRKKVLGFPKAVAKAEGIAWAGDSWTPPRIVEGTYAVPGKVAEDLHPVEV
ALLFELPLPRVAVVMIKILEDEGTARIVQEAPLRLEILKASSADPYGDIFLKAFDSEGWV
LSGFMADFFEEALKRLQEKTWDCDIEATKAFYRKKIEEEEAAAGAESEAAADARRRRSYW
YPFMYVYSHPYYCANLGLPKEFSGSYASFMASSACFSGCFSPKNLSGTAGACYSACHNAC
HSACHQACHSACHSACHSACHSACVSGGPH*
>SPBIB_v1_210143|ID:27163035| Radical SAM additional 4Fe4S-binding domain protein [Uncultured spirochete bib]
LSLRIGRSGLVGRASASFPIPGSPEGTGTDLAWIDDFWSKAGEHIFCREDDGALILPPNR
VYKVNATAASLIAYLKRGGKVAKLKLPSAEAQRDTALFFGDLASLYRGDEPVNGTVSAET
YSFSFTRLPVLGEIALTYRCNNACRFCYAGCGLTKSLTPFLTRRPARPTADYKHIIDIFA
DEAQIPFFSFTGGEPTLRSDLEELIAYAQQKKLITNLVTNGTLIDAARAHALKEAGLGSA
QVSIEGPSADIHDNLAGRPGAFTETLAGIKALQEAKIPTQTNTTITRANLAVIHQMPAYL
ASIGIHRFAMNLFIPTILGEEADRLFVSYEEIGPYVDAVAKEAKKLGMTFFWYSPTPFCN
YNPIARGHGNKSCAAADGLLSVAPDGSVLPCSSWDEPIGNLFSQSFRDLWFSEAARFYKE
KRFAPAPCTSCESFVACQGACPLYWRYTHSEPSGFKKT*
>SPBIB_v1_210144|ID:27163036| membrane protein of unknown function [Uncultured spirochete bib]
MNPVTVQYRIRGLSTEARLILFGAASLCGILLQVFWPVAGPLVGTLLLLLPLFLLSAQPW
SNKPKDKGEEDWQPVGDAELDRIADAFKSAREIKIPLWYRPGFGIPFTVALAILTSIFGT
GLTLPGLLAMDALILFWPALNFLKIKIWVPKEFEMVMQAIQAARSVPLPDGILCTPYLRL
DRDDQGLRIPENARLMLEPRRKPEDLVGAQLQVAINKGPNGPVPYLYAVVLTHGKGQSWQ
KARSLRIPGFEVEPGGDADYGTVVIRQSTDRGGYHTKPEDCRRLVQVVVVQVLKEL*
>SPBIB_v1_210145|ID:27163037| Cell wall-associated hydrolase (Invasion-associated protein) (modular protein) [Uncultured spirochete bib]
VKGEDAGLLYPRSCVPNYLIVPKIVMKVLASLAAKSIDALRLAALGIAVCLFASSCSIFI
SYEIVPASVEIRTAAIRYAQKYAELGAVYEWGGQDPLPRKIAVDCSGLVIRCYEYACGDY
GASLLFKDTTAKGLRQYSSLIASPEKGDLIMIGDNNEISHVALFTRKEDGRLYFIDATSL
TGMVSMRSYAENDPKFIEFRRMNVINKK*
>SPBIB_v1_210146|ID:27163038| exported protein of unknown function [Uncultured spirochete bib]
MRLPSKPESLFWAILAMVMLLTACTMNNNEDDIWTWGNYPELGTGPVEFKVVPLEDGTFN

EITPMGHMNLPQHPIPTSAGGFSFLSNGYSQPVKAPADGVITAIRYTHVFNVDGTKSFPD
YAVRIYHTNTFITWYDHLSAIDDTILAKTGTLAEGWTKAYVPVKAGDVFGKTAASSEQVT
GLGMYAYDKEKTLDFINPEKYGPFGAHAVFAFDYFRNDIKTQLFAYIKRTAEPRGGKIDF
DVAGTLSGNWIVEGSNVISSPNPWNYWLSFSYDMYYPESRRISIGVELGTTIGKSNGILT
QPVEGPDYTAARTGSDPVRYKLADAMEGLSSLPATSDIKYTLLAQVVEENKIKVELFDGN
IDNPSFTENAKYYTR*
>SPBIB_v1_210147|ID:27163039| Fe-S oxidoreductase [Uncultured spirochete bib]
MDGLKKVFAKRAVNLTLRYLEGDPQENLSKAVNALMPLAKRDGIKRQLESAKQIMAEKDN
PYRNLILRVFHEIAPNVRRRFVTNFVVNASMIGPDRAQMLKYKHDCNIPWAILMDPTSAC
NLHCKGCWAAEYNKTDSMDYALLDRIIREGKELGVYFYIYSGGEPTIRKNDLIKLAQVHN
DCMFLAFTNGTLVDQDFALALGEVGNFALAFSIEGFEEATDFRRGAGTYTKVIEAMQYMK
QIGAPFGFSAVYHGKNTEQVGNEAFLDFLIEQGCMFGWYFTYIPLGKDADTALIASPSQR
EYMFHWVREMRDKKPIFLLDFWNDGQYVDGCIAGGRSYLHINSAGDVEPCAFIHYSNVNI
RNVSLLEALKSPLFKQYKELQPFNKNHLRPCPLLDNPEALSAMVKASGAHSTQPIDKEDV
DELTAKCEKAAKEWAPVADGLWAEELPEYSKAKARREEEERKTQEQIMKQLKTMRPLKTK
EDKKKIFSLRL*
>SPBIB_v1_210148|ID:27163040| protein of unknown function [Uncultured spirochete bib]
MPKIVDHDNRKREILEKALELFSLGGFHASSFAEIAESCGLSRTNMYNYFKSKEEIFFYA
VEDIFNRISVEIDRIARQKNVSIVRKLSSIYEIFTDNSAIGRYSFIITELAQKLKNENAE
FLAKLQEAAKALRQKIESLLYREGNPILAPQVPVIATLFFSLVESSISHSLFIDKGFIQN
NIASLLNLESFV*
>SPBIB_v1_210149|ID:27163041| conserved membrane protein of unknown function [Uncultured spirochete bib]
MQRRNLLFAAVFAIAMAYLEAAIVVDLRKLYGITDIMASLSSFDAQISRIEIGREAATLV
MLWALGWIAGKKLQSRIGFFIFAFGVWDIFYYIWLHIFIGWPHSLLDWDLLFLIPLPWWG
PILSPVLIALIMAIIGVQLVVSEARGIAIQPKTAEWLLLVSGILAMLYAFMADALAVRHL
NLEMLGMLRPSSFQWPIFLVGFILSVASCGKFLAAVKSDDKAGGRITT*
>SPBIB_v1_210150|ID:27163042| conserved protein of unknown function [Uncultured spirochete bib]
MMLQKETKTDDTEAFCLVLGGGGAKGVYHIGVWRALKKLGIHVDCFIGTSIGAVIAAFLA
QGSDEILEQIGHIISLDNILDLPEDFIKNGELKFNLQSLRSLPDFFISVINNRGLDTKPF
RQLIESNLDEKALRNSGKDFGVVTINVSDLKHQEVFIEDMEQGSVVDYLLASSAFPGFKN
PKIEGKSFIDGGIYDNIPYAMARKRGYRRIIVSDLSGMGRTRKPDIAGCITVYIKNSIDM
GWVLDLNRNFLDSFMLLGYLDTLRTFGHLAGYSYFIEFDTNAEKNFAPKISIPFSQSPKY
MCYDRRKLLIALECAASVLNVERIRTYTYAELRTAIAEHRYEVENKIAAATASSRKGIMN
LAKVVSEAVATRQFTECPYYYYCLVNKLFPKSTARILKNTLVRIFPELPVGAAFIEGMES
DSLNF*
>SPBIB_v1_210151|ID:27163043| putative Phospholipid/glycerol acyltransferase [Uncultured spirochete bib]
MSFDVVHTEKRIAQSAGVSGPRNAEDYDPEKELDANRYALIVQQSKVPIIALFNAAVKED
TSFLLPLMLLATIRTMRRIPRTFFIRNGPEYERYVFKTIHWWARRICRIGRIRLQVSGAE
KIRPDRAYLFVSNHRSPADIPVLFDAIPQNAAFVANGMFRMIPAFSFWMRASGTVFIEQG
NQKAELAAFKTMIRRLKQGRSLILFPEGHTHQGPGIDTFSRGGIYAAVLAGVPIVPVCLS
GTDKVIRPGSLHINRRSRVIVEFGAPIETRHLGMAAKKNIDTILHDTIAGMLDRQTSKQG
GRPRQA*
>SPBIB_v1_210152|ID:27163044| GtrA family protein [Uncultured spirochete bib]
MNAARVIAGFAKAQVSAFLGGIVDYSVMILFVQLFHIHYAIGIIAGGIVGAAVNFAINRY
WSFYSPKGRYSDPVTVQGMKFIPVCMASIAMKATGTYWLVQAVSLDYRISRLAVDAIISL
AFNYQMQKRWVFKKVRSFEKKH*
>SPBIB_v1_210153|ID:27163045| conserved membrane protein of unknown function [Uncultured spirochete bib]
MKRPIHSVTEILDAIAQGRQRTNLLRKYEQRFIAFLVRFIPSWVSSNMLTAVGFLGNVLV
FLSFVLAAKISGYWLFLGIAGFAISWFGDSLDGRIAYYRNKPRKWYGFALDIAVDWLGIV
LIGLGFILYANGYLKIVGYAFITFYGLEIIIALMRYKITGNYSIDAGLLGPTEARIAISL
FLLIEIVFKGTLQYMALVAVAVLIISSFSELKKLLDAADAQDREEKRAQDRESKNIVELK
RAEKR*
>SPBIB_v1_210154|ID:27163046| transposase [Uncultured spirochete bib]
MAITKEVLDELLKEYKGPEDLTGPEGLLKQLTKALIERAMDAEITTHLGYEKHDQSEKDT

TNRRNGRTKKTVRSDQGPLEIEIPRDREGTFEPAIVPKHQREFKGFDDKILSMYSRGMTT
REITEHLKEIYGTEISPELVSRVTDEVKELLEAWRARSLETSYPIVFLDALMINVREDGK
VVKKSIYMALAINWEGRKELLGLWIDQAEGAKFWMRVLSELKNRGLQDILIAVVDGLSGF
PEAIATIFPKTEIQLCIVHMVRNSLKFVPYKDRKAVAADLKALYASPSEEAALAALDIFA
AKWDSRFPMISRSWRMRWPEVVTYFKFPEMIRKAIYTTNAIESLNYTVRKVTRNRLSFPS
AEAAMKLVFMALQNISKKWTMPIHEWKSALGQFIIFYGDRVPL*
>SPBIB_v1_210155|ID:27163047| Phosphoglycerate dehydrogenase [Uncultured spirochete bib]
MSKRVLITSRSFGQVSEEPLNIFKENGIEVDFQNDEYNEEKFQSIIGNYDALIIGAHEFS
PKAMEKAKKLKIICKHGAGLDNINLEAAKKYNIRVTNVPATNSNAVADLAFGLMLDVARK
ISFAASRVKDGLWERVIGTDVCYKTLGVIGFGAIAKNVAKRAGGFGMKVLAYDPYVTDLP
DGFPHVSLVTFEDVIKQSDFISVHVPLNDATRNLIGREQMEMMKKGAFIINTSRGGIVNE
EALYEYLKNGHLAGAGLDVTEKEPPTGSPLLTLDNVTVVPHIGMYSKEAINAVSVICARN
VVKMLNNEQPDHIVV*
>SPBIB_v1_210156|ID:27163048| conserved protein of unknown function [Uncultured spirochete bib]
MYNIYFDSGTSNTRVYLLKDEEMVDVAQKNVGSKDSSIAGSNQVLLQGLKELYDRILDNN
GLNDAQIEGVYASGMVTSPFGIKEVPHLSTPITVEKLYSSMYTHYEGEFFKRDIHLIRGA
KTIAEGFKVDKYNISVANNMRGEEIEVFGILSDLSDEWKRGNTAIFLPGSHTHIVYVKQG
IFHDILSTFSGELFHAISTSTILSSSISSEVDQLDEEMVIQGYRNLNEYSLNRALYIAHA
MKIFNASNKIERKSYLEGVINGGVVLGFENTVKRKWKEVDRVIIASSSNIAKVYEILLKE
LERKFEILTLVASGKESFAVKGFIEILKKGGVLCQKECL*
>SPBIB_v1_210157|ID:27163049| putative 2-dehydro-3-deoxy-6-phosphogalactonate aldolase [Uncultured spirochete bib]
MASNIKDEIFKGKIVAIIRGIGSDRILDTVGALLEGGIKLLEVTFNQEDESKALDTLKCL
ELIKSKYDGQVCLGAGTVITEDQVQKAVDAGAEYIISPNTDINIIRRTKELGKISIPGAF
TPSEMVAAYNAGADIVKLFPAGILGIEYIKAVLSPLSHIPVVAVGGINVDNVNQFIKAGA
KGVGVGGNLVDKKAIYSGEYHKITLVAQTYLKKLEES*
>SPBIB_v1_210158|ID:27163050| Putative KHG/KDPG aldolase [Includes: 4-hydroxy-2-oxoglutarate aldolase ; 2-dehydro-3-deoxy-phosphogluconate aldolase] (modular protein) [Uncultured spirochete bib]
MQNLKELFFATGVIPVIKIESAERADGLAGALRAGGLRVAEITFRTKAAAGVIEKFASRH
PDIVVGAGTVTTKTEVDSALSSGAKFIVSPGFNPEICAYCIEKKIPVFPGVSNPSLIEQA
MSLGLTILKFFPAEVSGGTKALKAFESVYQAVTFIPTGGIQENNLNEYLTIKNVLACGGS
WIVPTDLIEAGKFDAITALIQSCRRSMLGFLPLTSEMKLDNASGADADRLSSPVLELKTP
SIRRTLAMLGMNEETSSSAKSAIISLGDTRIRIIE*
>SPBIB_v1_210159|ID:27163051| conserved protein of unknown function [Uncultured spirochete bib]
MDVIKELLEGIPLPKMVKVKMAFSAKKVADVEAAVRQELAKPGICDSIKPGMHIAVGVGS
RGLANLPTLVQVLVDELKKRGAVPFVVPAMGSHGGATAEGQTEVLANLGVTEASAGCPIV
SSMEVVEIGRLDNGLPVYIDKNACSADGIVFIARVKPHTAFRGPNESGLVKMVAIGLGKQ
KGAESCHSYGFKYMAEHIVAMTKIALARTPILFGLATVENAYDEIARIVAVPAADIIETD
CKLQVEAKANMPSLMFKQIDVLIVDQIGKDISGDGMDPNITGRFPTPYASGGPDVSKLVV
LDLTDKTHGNANGMGLADFGTHRLANKVNFQMTYANGLTSTVVLPTHMPTILESDYDAIR
AAIKTCNARDYSRVRVVRIKDTLHLGEIMISEAMLAEARTIAGITVVGEPQAMKFDAAGN
LVREED*
>SPBIB_v1_210160|ID:27163052| conserved membrane protein of unknown function [Uncultured spirochete bib]
MKLELVTEIGILAFGIATLVQSFHIRILKFGGSMGGDVFPKALSIVLIILSAIVFIIDLG
KYLQNKYLKKTRASATEGASVVKSAKERGYLNVLFFTVLFAIDIFSIRYIGFVMAMIPLI
FANYLLQKKEIRKTDWLIALVYAVGVTVAFWALFEKVFEVMLPQGIWF*
>SPBIB_v1_210161|ID:27163053| conserved exported protein of unknown function [Uncultured spirochete bib]
MKKIALSMVAFLVMFSAFFVAISPAAAYPDKPVTVVVPYSPGGASDITARLLAEYWKKYT
GKEMVVTNVVGAEGAVAARQVLNTKADGYTVLWYHQAILGNYYLGVADISWKDLTPACVV
MKTSRVTVTRNSSPWKNLREALDDAKKNPKKYIYGAGAGGIAYLEYGPLEMDAPGAFRVV
PNEGGDAQRIAALLGNHVDAIPVALISVVQYLKSGDIKCLALHDVESDPFIPDVPTASSQ
NIKNLVFPMTNTFFFPKNTSQNIVNDFNGIIAKIVVDPEFKKKLADMAYTVPFFKTGKDL
EKFWSDQDSVYKEVAKYVK*
>SPBIB_v1_210162|ID:27163054| conserved membrane protein of unknown function [Uncultured spirochete bib]

MDAFLQVFTFGMHTVFQPLNLIVIAIGTLWGMIFGAIPGLTATMGVAIALPFTYKMDTAS
SLALLSSIYIGAISGGFISAGLLNMPGTPSSIATTFDANPMVKQGKASLAMSLSLASSFA
GGILAVFIAIVATYGLAQVALKFGPFEYFSVGLLAFAGCVGMFGDKLFKSIISLVLGLVL
AMIGSDVLTGVNRLTFGIPNLAAGIDILPLLMGLFGMSEILFALDNKESNVVPPDARNNL
MGWKSFFSAFKLIISRSHIFNFIRSFIIGFAIGVFPGVGGATSSVVAYGVAKSTSKHPEK
FGTGIPDGIIASETANNATIAGALVPLLSLGIPGDSVTAVMIGGFIIHGLFPGPLLFREN
PQPVYIVFASQIIANLVMVLLGVFLMKFLIRTLSIKSHILLPVITAAMIIGSFALYNRVF
DIGVTLVFGFVGYIFKKIDFPTVPLITSFILGPIVEKNLRQGLASSGGSLLPLFTRPLSL
GLLIGAVILFAIGMYVSFFAAKRVESQK*
>SPBIB_v1_210163|ID:27163055| conserved protein of unknown function [Uncultured spirochete bib]
VVVFFETVGAANVAAAPKPITFKFAIIDDESSSHYRGAKKISLEMAAAIKTQINIEIANS
GALSGECDTVEIAMSCVIDITTADNFVLTTGFMCCMS*
>SPBIB_v1_210164|ID:27163056| conserved protein of unknown function [Uncultured spirochete bib]
MNKPVSLLEFAYEKIKEDIMHGKYTPGEKLNIGEIAKDFNISCTPVKEALNRLASEGFIE
AIPRHGMMLKVLSVKDIEDMLNVRKMIEMYSAKLAAANVQKHPEIIKRMKKLLPLMEALG
DHEYVEATKLEQEYHGLFVKLTENNRLIEMYEKLFGVSFAFYVVYSIGNFPLARASEASRE
HMQMYNYLLEAKGDELSELMGRHMQATIELLEDLISKDTEHRFKIN*
>SPBIB_v1_210165|ID:27163057| conserved protein of unknown function [Uncultured spirochete bib]
MSVLILETSTSSAKALLYDEIKGIVSTKIIPYNKEKNSIAMQDCDDVCRSVLEAGRQIAF
SQDVEAIALCGTWHSIVVCDRSMKPVSPSYSWAYTDSMPEIANIRKDHRLTLDLYHRTGC
MVHSTYGFYTLMHLKNSGMEFRDKLFASQAGYIFYTMTGERVESSSTMAGTSLLNVHSKQ
YDPFILSMLGLDAEQFGRLVTYRDTFPLLKNIADALGVKPGIPVVPAYPDGAMNQVGSGA
LEYGIMTMSVGTSAALRFTVDKPMIPEEPSTWCYVGVDSWVSGAAISGAGSCVDWLRRSV
FNGNMSFKTLESAAASQESTPVFLPFVFGERSPGWQDDRLGGFFDVRPQHDYGSMYRGLL
EGICFNLRQCYSLLTSIAGEPKEIRLSGGIINSPFWTQMLSDVLGRELYVSRFDQASSLG
SVALALFALGKLAKLSDFKVDSYTKVLPRESQEYKNKYERYLYWYNLTR*
>SPBIB_v1_210166|ID:27163058| Phosphoglycerate dehydrogenase [Uncultured spirochete bib]
MFNIIVTARSFGQANPEPIRLLEENGCRIEKLNIMNPLSASELLALVAEADGIIAGLDQY
DALVIENAKRLKVISRYGVGYDNVDLEAAKRKGVAVTFTPGTNENSVADLAMTLLLCASR
NIVSMDKYVKSGLGSRVIGSEMWKKTLGIIGTGRIGKGLARRAVGFSMNILAYDAYPDTA
FAQQVGLTYCSLDRLLRESDFISIHCPLTDETRSLIGAAEFAVMKPTAVLVNTARGGIVD
EEALYNALVQKKIAAAALDVTVQDPPTGSPLLGLDNCIITPHIGGYTRDAVLNMGMLAAR
NLIAVLKGEPCEFRVV*
>SPBIB_v1_210167|ID:27163059| protein of unknown function [Uncultured spirochete bib]
MRTARHGPSNMSPITHVVNIHVDLAAQNVSIQEWSGTEPRNLVIQGLKEPKHVLLVIFLG
IAEYSNGYVCANEKLG*
>SPBIB_v1_210168|ID:27163060|rhlE| ATP-dependent RNA helicase RhlE [Uncultured spirochete bib]
VNFSSFSFDGTITKAIDRAGYTDATPIQAQAIGPILEGKDVLGLAQTGTGKTAAFALPML
QRLLTGKRCTARALILSPTRELAEQTRKAFEALGKETNLYALSIYGGVSTSAQIRSLKRD
MPEILVACPGRLLDLMGQRVVSLKDIEMLVLDEADQMFDMGFLPSIRQIIEALPTGHQTL
MFSATLPSEIRSLAREFQKDPVRIEIGNSRPVETVNHIVYPVMQADKYAVLASILQENAT
GQALIFTKTKHRAQKLATQLLDAGHSAAALHGNLSQGQRDKAMLKFRTGKARIMVATDIA
ARGIDISGISHVINFDMPDTAEAYTHRIGRTGRMMHSGIALTLATPDDRSMLKIIERLIG
KPIEHKIVSLAEIDVTASIEEHQKVAWNQRGSYRLEHGERSDRRPDTHEARPGMHTNRPE
ARKSRPDSRGLKPFKQSGAQNGVSHGKTHDAAHNAVHHFRDTRNSAPNAALRSRDSRNAI
RSAAPRSGGRYPKEYASIP*
>SPBIB_v1_210169|ID:27163061| conserved membrane protein of unknown function [Uncultured spirochete bib]
MPYCPDCGVEIGNAPRCPLCGSPNPKAVPDAQNPCKDADITGKHPAANIIFEGGEGIFSK
EEKRTVLWEVLSVAFAIAIVVLGAVNLFESRRLSWSLYPVVSILLLWVEATAFLVLKNMQ
VLRIVLGAIAPPAFLLALGFVSGSPRWAFGLAIPIAVLVESLTGALVLAIGKSKRKGLNL
VAYVLVAVAVLCIGLEMFIDLFARGVVVFDWAPICAIALLPIAGFLLYLHYRVVKATNLR
RLFHL*
>SPBIB_v1_210170|ID:27163062| Elongation domain-containing protein [Uncultured spirochete bib]
MHIWARRSREQQFIHTRREDEQGEWYALDNAAIIMPAVANEITTSLFRFEAELDAPIDVD

VMGKALELTVRRMPYFNVRLRRGFFWYYFEPCHTLPHLYPDSPSPSQLWDINKRGTRMFR
IRCEANRVAGEFSHALTDGTGGMTFLKTLLANYFLLKGIAPGAELSEGEFRDILTSQSFI
ESPSIPSDEFEDAYQRYFPKGLPFPDTGPNAFHMKFKCLPKGQYRVISGELNLTEALGEA
RRHGASLTELMTAVYLDALQQIWHETVPRPKEHYIAVEVPLNLRTMFPSKTFRNFSLFVL
IAEDMRLGMRSFEELVMRTHYQMKLEYDRMSIAKHLSRNAGSARNPVVRAVPLFIKDIFA
RIFFAKFGESMLSGFISNLGQVKMPPGFTPHIQRFGFIPAPSLTTLTNASMLSWGDKLIV
DFGSLTESRELERLFFRRLRSLGLQVRVSCRLDQAEAKAQSKEEPNAILS*
>SPBIB_v1_210171|ID:27163063| conserved membrane protein of unknown function [Uncultured spirochete bib]
MIATFINAAAVIVGSLIGLLIRKGIKEEYRKVVFTAAGLTSLTIGIQMALKTSHILSFAL
ALMIGGLLGTLLDVEGGIERFGERLKQRFASKSEGAFAAGFLNASILFCAGAMAIVGSFR
AGTEGNYSLIFTKSVLDGFVSIIFAGAMGIGVAFSALSILVYQGALTLLSVYIKPYVSDL
MLAEITGIGGALVIMIGFGLLEIKSFKTGNFLPALIIIVVLVLVMPVLKFL*
>SPBIB_v1_210172|ID:27163064| conserved exported protein of unknown function [Uncultured spirochete bib]
MNRRIFRAFALITLALLVSIFASCASSPPPAPPDASAQKIIQLAQERYDAYDLNGAQYYY
QVLLERYGSDPEYMLNAKYEMAFIEYKKGNTEKAIAGFKEVIARYDAPDGQTLSQTWKVL
SEKLLAKLTGGK*
>SPBIB_v1_210173|ID:27163065| conserved protein of unknown function [Uncultured spirochete bib]
VRRFRCTRCGKSFSTQTYSTHYYLKRIVSYRDVLYRLAGGESLRGMSRALGCSLSLLTNR
IDRLARQGIALHARMLRARRGKEDICIDGFVSFDCSQYFPNEIPIAVGRTSGFVLDVSHC
TRKRSGSMRQDXKAKAQALYSTIRLERGGIXRSFREVLATSLAVQPPQPXXPFVLITDEK
PDYARLVRKLAAYWGEGXGRLVHLXISSHXPRTIHNPLFSSNYTDRELRKDLANHHRESV
CFNRNVANGMLRLWAYLMWHNYLKPYRIRWPKGRRPXTHAEASGIDATXLEXVYRSFFEE
RAFLTRSPLSPXMARSWKKEWRTPGKEKAEYIPRLALG*
>SPBIB_v1_210174|ID:27163066| ABC transporter related protein [Uncultured spirochete bib]
MAFIQLSGVALSFGARDIIRDATLNLQAGSRAALTGPNGAGKSTLMKIAAGLLKPDAGDV
IISKGARVVYLPQTGIRFEYGTVHDIAEEGFRFFRNLVEEQERIGRQLESSGLSEKETAR
LLDQHHEIGETLENAGYWRREEKIADVLRGLDFKHRDFDRPAAELSGGWQMRLALARILL
EDPDIMLLDEPTNYLDLEARTWLENFLKDYRGAVLVVSHDRYFLDVTVREVYELFSGTLT
RYAGTYSQYEARRSQELAALFEAWERQQEEIQRIEDFIRRFRYKESKAPQVQSRIKMLEK
ITPIEIPEGMKRIHFSFPPAPRSGQVVVRIRDLSKAYGNQLVIGAFSLEVERGQKLALVG
PNGAGKSTLMRLISGSDPYLTGSISLGANVQIGYFSQESAELMESSATVEEEAASVCPPD
MLPKLRNLLGAFLFRDDDIEKPISVLSGGERSRLALLKLLLKPSNLLVLDEPTNHLDLTS
KDILLEALKKYEGTVIFVSHDRQFLDELADRVLELSCGTSPRLYHGNYAYYLEKKAQEAA
GETLSQSAFSRPVENVGQNSPPVTDWENEKARKAHLRKLKRREEEISTRIGAIAAEKQQL
QEQLAHPATYMNGEKTRATLASIEALEQEAENLNEEWMEIAEMLSAEES*
>SPBIB_v1_210175|ID:27163067| protein of unknown function [Uncultured spirochete bib]
MSLNSNENMHAARFLLIGLVLALVFSTLGIGCARLEGWGVVIWSVKGTTAKAGAVVPVYL
KSNISKTYVIGLPDDSKAKIEVPLSTLEFFSSKNAAEKRAKEFAPFASLYLSAGRDGLPV
REKPSVSTRRVYRLRLGESVKVLSRVDGEAVFTGGRALPGEWYFVQATDGTRGYVFSNTM
VLYEEKENAAAPVIGTSPAPSAALLDMIYAQPWRPAYYQLMLDDDAVDPDLFALQYGLFA
DAKNSQVRIELPGYSSVFRFSSVSQGGDWLIFEGSKLRVRFENESTIVADWSGTDSVLPD
EGWGSNAQSARFIKLDTSIPVVLSGENSRRDSELKAFFQRSAMAQSNATSASTSTSTITF
LSDLAGILSIDARGTFEWSHTDQLPAGFAPEILAGSAGAAIPGAPATGAPTTAGPASNAP
ASSAATASVPIKGKIRFGLHLGTSLSSAWMGGFSLLVGDKPDREDYVYRFENGQLVIAKA
SPVTLRGTTESLDSRFSFVNYYLVVK*
>SPBIB_v1_210176|ID:27163068| Ppx/GppA phosphatase [Uncultured spirochete bib]
MSDTQPDKTARASTQTPRLHAAIEIGSTGIRLIVAELDAKGSLRILDRASKQSRLGRDVF
TMGSISRDGLRETIAILGSFSELLQGYGLKPDDIQVIGTSALREAANRDTFIDRVNLQTG
FHVRIVEDIEENHLMYLAVQKALQDERSFLSRANAMILEVGGGTTEVMLLKKGQMVSSHS
LSIGTLRMDEQIRESARQPREFIEMYLESNIKTACDVLAEDLPLDSVRTFVLIGSDARFA
AYCLSGKNFDHYAVLDRNQFVEFADSVASMSLQECMAQYHLSWSEAEGYAAGLTIERMFL
EKTGADIVIVPNVSIREGILLAQVQGMDQKIEQELRRQVVASARSLAKRFHYDEDHATHV
KNLSLELFDALKKEHGLGKHERMLLEVAAILHDIGTYLRTSGHHKHGEYIVANSEIFGLN
REDINIVSNVVRYHRKSPPLATHVNYIALPREARIVVLKLAAILRVADALDRSHSGRVRE

LSFERTEDRFLMRPSQSLDFSLEKLSLAEKGDMFEDVFGLLPVLI*
>SPBIB_v1_210177|ID:27163069|ppk| Polyphosphate kinase [Uncultured spirochete bib]
MNKLPMLNRELSWLDYNARVLAEGLKPSTPLLEKLNFMRIFASNLDEFFMVRVAAVKSAL
LNKQTSEEWAGMHPRDLLDAIHQKVQQQYDTLYAHLHEIVLPGLAANAIHLLSQDAWSSR
EWRYLERFFAEKVYPLLTPLRLEPDYFPSTGSLQVHVAFRLEGEQSIAIVQVPKNIDRFI
ILPQDLTIAAEPEAGQPQGNVLRIALLEDVVLAFGSKLFSGLKVQHGLIFKVNRDADFSV
DEDRDDDFLAAMEEVLTGRQNSTPVRMVYTGSDQILVDALMRSLALGPLDVYHVDWLLDL
GRTTALCDPEFYRGHGLVPAPSLFFSQWKPVRSFDEKGSIFDWIDEHDRFVNLPYESFDV
VQRFIEESANDPSVLGIKITLYRTSGMGSPIVHALAKAARSGKQVVVVLELKARFDEEKN
ISWATELEQAGAIVTYGVARLKVHAKAALVIRRRQDGSIARYLHLSTGNYNEKTARSYVD
FCLFTAHESLCTDVALFFNILTGYSSIQSLSFIAVAPFDLKQRIIALIDREASQSTPESP
GLIIAKLNALSDPDIIKALYNASAKGVQIMLNVRGVCTLIPGLPGISEHIEVRSVLGRYL
EHGRMLYFRNAGSEELYLSSADWLSRNMEKRIELLFPVLDAELRARCKDILMAYFKDNTH
SYRQDQLGEWEDLSQTGTGGEARLCVQERLYKLAKKALHSREEKEEILQVRRSKSATAAP
ASKNPGD*
>SPBIB_v1_210178|ID:27163070|nagD| Ribonucleotide monophosphatase NagD [Uncultured spirochete bib]
MKATEETLKNIRKKQAFIIDMDGVIYHGNVLLPGAAQFVDWLRRQKKRFLFLTNSSERSP
EELSQKMARLGIDVGPGHFYSSALATAAFLANQKPNGSAYVIGEPGLIHALYRVGYTMNN
INPDYVVVGEASSYNLDTLIKAVRLVIGGAKLIGTNPDLTGPGEGGLVPACGALVAPIEL
ATGRKAYFVGKPNPLMMRHALRTLGATREETVIIGDRMDTDIIAGIESQIETVLVLTGVT
ARQDLPKYAYAPDHVLEGIFEIPEAIEHTPDEAAAGKM*
>SPBIB_v1_210179|ID:27163071| Alpha amylase catalytic region [Uncultured spirochete bib]
MISETDAIYQIYLRNFTKEGTFRAAIPQLAHIASMGFTWVYLTPIHPIGKEARKGLLGSP
YAIYDYRAINPELGTLVDFEAFVEAAHANELKVMIDVVYNHTSPDSVLAREHPEWFLKGP
DGKPARKCEDWSDVVDFDYSASPHLWVELISTLAMWRDRGVDGFRCDVASLVPAEFWKQA
RTRVNQYDPGVRKELRPLVWLAESVHPAFLRRMRSEGYGAWSEPELHAAAFDLTYDYDGW
ERLEVKKFKKRGQTPIFKYFEYLYAQETLYPKGAKKLRFLENHDQERAASRFGRGDMLKA
WTVLYQFLPGVALTYMGQELALEHRPSLFERDPIEPSGGDPHFEQFFVSSLKATTEAKRD
APFFSWSIFDDDVVFCLRSTAPAREATPIEEAMVKSGNYLLIARAGGGGRGQTSRAARLL
APFDIHGTELLSGEPVNYPRGSDIPPSPALLVRLGN*
>SPBIB_v1_210180|ID:27163072| exported protein of unknown function [Uncultured spirochete bib]
MNKGIYDKNSGPAHNIAVASLAMAAIWLITPLRPAVSANEQKTAMLAWGGDLVVQTVYGQ
RIAEPPSRGTRKK*
>SPBIB_v1_210181|ID:27163073| conserved protein of unknown function [Uncultured spirochete bib]
MSDAKSPSFAENPRPLDTETTPFPGGPRAPETDFDDAFAAAARDYRMLLDKEYPVDASLK
LVGDRYRLSHNGRMMLYRGILSRSASQINLAKLVRASWEGAELGIDGYNVLFTLTNYLHG
HPMFIATDGFLRDVGGAHGRIADHRGFERMAEVVAEMLSQLNLSKVTFFLDAPVSSSGKQ
AAFLREAFARCAVNAEVRLENGVDHFLACWEGELIATADSAIIARTSAHVFDLARYILER
QFNAHFPPLASLIA*
>SPBIB_v1_210182|ID:27163074|plpC| Outer membrane lipoprotein 3 [Uncultured spirochete bib]
MKGSWMKGTKVSGKAAAAVKRAPSLGRTLIAFAFLALFVASGFAQTGGKLVTLKVGATPI
PHGDLLQLVKPDLEAQGIKLEIVELTDYVTPNILLAEKQLDANFFQHLPYLNDFCADRKL
QLESAGQVFVAPLGLYSRKYKKLEDIPAGSVITLPNDPTNEARALILLENKGLIKINPKA
GLKATIRDIAENPKRIVFREIEAPQLPRTLDDAAAAIINGTWAMQSGFIPARDSLILEGA
ESPYANIVAVRKGDANDPRVVALVKALQTQKVKDYLISKYNGSFVPAF*
>SPBIB_v1_210183|ID:27163075|metI| DL-methionine transporter subunit ; membrane component protein of ABC superfamily [Uncultured spirochete bib]
MNEALSLLAKPLGETLLMIAASTLIAGILGGLVGIFLFALSNDRFAGKHRGARFAKRLAD
RAINMLRSFPYIILMVLVLPLSRLIVGTSLGTLAAIVPLSVAAIPFLARIVESALSEVDS
GVIDAAISCAASKTRILFAILIPEALPSLVSGLTLTMISLLGYSAMAGVIGGGGLGDLAI
RYGYQRFRGDVMAVAVVTIIILVELIQFSGTAIARSIRAKR*
>SPBIB_v1_210184|ID:27163076| DL-methionine transporter subunit ; ATP-binding component of ABC superfamily (fragment) [Uncultured spirochete bib]
MFHAGRPEEAANQALIAHPPYQAPLAGDRTMPSGAVFQDIPVSASIFPRTALRLDAISKA

FGSHTVLDEISLNVPKGQIHGIIGKSGAGKTTLIRIAGLLDKPDRGSVFFEGINAPVHEL
RGQELLEARRKVGFIFQSFNLFASRTAGENIAFPLEAAGWPSAKIRARVQELLDLVGLAD
KADRPSSRLSGGEKQRVAIARALANHPSILLSDEATSALDPETTRSVLDLIRNLRDMLGL
TVVMVTHQMEVVRRICDSVSVIASGSIVEEGLVSEIFAHPRSAAAKAFLKEGQHE*
>SPBIB_v1_210185|ID:27163077| putative Positive regulator of sigma(E) RseC/MucC [Uncultured spirochete bib]
MKEAATVTKIDGDMVTIAVKMQEGCGVCGNNGTCKIRMSNLLAYNKNHISIKEGENVIVE
VPSVEQAKSAFWVLGLPLIMLFVGYGVGALVFRAPTEGPAVASAGAGFVLALLVGMMVQR
RKRLESFPYILAKEGEGLY*
>SPBIB_v1_210186|ID:27163078| Phosphoesterase family protein [Uncultured spirochete bib]
VAITIVFFGEIVGKTGVFTVKSTMAEIRRRFQPDFVLANADSATGGAGLGIQHAVYLRKL
GIDCITMGEAAYYKPDMTEFYPKAGWVLRPANLPEGDPGRGWKIFQKEGKKIAVVALLGQ
SGFARVHADNPFFAVDQLSHILHRETSCIVVNFHAATTAEKLSLARHADGKVSALLGTGG
KVLTADARILPKKTAAITDLGRIGSILSVGGFDPEVKVKEFLTGVPAWGKDASAQPEAQG
VCIRFDDEGVALSIESFRIQGKEIVDEGSSDSNQN*
>SPBIB_v1_210187|ID:27163079| CMP/dCMP deaminase, zinc-binding protein [Uncultured spirochete bib]
VSDYRRPTWDEYFMEVANAIAKRATCDRGRSGCVIAKDNQILATGYVGAPAGLPHCDDVG
HQMKKMLHEDGTITEHCVRTVHAEQNAICQAAKRGVAINGATLYCRMTPCRTCAMLIINC
GIVRVVAEYRYHDAAESEEMFKMAGIKLEYVHNEVLKYDRQ*
>SPBIB_v1_210188|ID:27163080| Peptidyl-prolyl cis-trans isomerase [Uncultured spirochete bib]
MKHRSVFSAIILVLSSFSIISCQAQESRPDGMYAILKTNKGNIVISLEFEKAPLTVGNFV
GLAEGKLDATKGKPFYNGLTFHRVVADFVVQGGDPIGDGTGGPGYRFPDEIVPDLKHDAP
GVVSMANAGPNTNGSQFFITLEAAPWLDGKHSVFGKVIEGMDVVKQIQQGDRIESVTIQR
FGAKAKAFDASQKAWDQRLSAAYATLNALNAQKRESDLALIAQKWPDLKPDEKGIFQKTI
RKGFGDTPKTGSKVSVIYKGMLLDGTVFDQSSLSGGPFSFTIGKGEVIDGWDSVVSTMQK
GEKRFVIIPPELAYGAQSIGDVIPPNSFLAFEIELVSIRE*
>SPBIB_v1_210189|ID:27163081| protein of unknown function [Uncultured spirochete bib]
VQHRNPYFGTIGDIEYLQPIINAIGDFGNSDSRISLIFMIISF*
>SPBIB_v1_210190|ID:27163082| exported protein of unknown function [Uncultured spirochete bib]
MKGISRIAIRSFLAIILILSLPFAASAQTPGKTSATIQLAVYVPPVLQLSLDFAPNGAAN
IVGYLGTNPGGFRNGFELKPYSIFNLGAARLVSNLTSSYSIVVQSMNGGKLKNQSSGSEI
AYDLLIGGMPAARYGDSFRMVTAMKTARDGTDLPVSIALGNIPASASYGVYSDSLLFNVM
AN*
>SPBIB_v1_210191|ID:27163083| OsmC family protein [Uncultured spirochete bib]
MTKEIQMNWLDGMAFQSELDGHTLVIDADDSVGGTNRGPQPKGLLLVSLAGCTAMDVISI
LKKMREPVSWFNLKVSGDLTEEHPKKFTSFKVIYQFKKSDGLNPDNVQKAVELSQNKYCG
VSATLRKAADLAWEIEYI*
>SPBIB_v1_210192|ID:27163084|fusA| Elongation factor G 2 [Uncultured spirochete bib]
MAFDIQMMRNIGISAHIDSGKTTLSERILFYCRKIHAIHEVKGKDGAGATMDSMDLERER
GITIQSAATNVQWKDYAINLIDTPGHVDFTIEVERSLRVLDGAILVLCSVAGVQSQSITV
DRQLKRYHVPRLAFINKCDRTGANPFKVKSQLGEKLGLNPAFVTIPIGLEDKFEGVIDLI
DMRAVYFDGPNGEELRFAEIPAHLVKDAERYREELLEAASMFSDELAEAYLEGEVTKDLL
LAAIRKGTIEEKFVPVFVGSAYKNKGVQLLLDGVVDFLPNPSERHNVALDLSNNEAEVEL
KADDKAKTVALAFKLEDGQYGQLTYVRIYQGMIRKGDELYNTRARRKFKVGRLVRMHASS
MEDINEGSCGDIVALFGIECASGDTFCHPDLNYAMTSMFVPEPVISLAINPKDTKSSDQM
SKALNRFTKEDPTFRTYVDPESNQTIIQGMGELHLEVYIERMRREYKCEVTTGMPQVAYR
ETITQRADFNYTHKKQTGGSGQYGRVAGYIEPLKDEHYEFVDMIKGGAIPAEFIPSCDKG
FRAAMEKGTLIGFPVTNVRVVINDGQSHPVDSSDIAFQLAAIGAFREAYEKAKPAILEPI
MKVAVETPNEFQGNVFASLNQRRGIIMANTEDGTFSRVEAEVPLSEMFGYSTVLRSLTQG
KAEFSMEFEKYAKVPTSISEQLRKDYLEKRKKDQK*
>SPBIB_v1_210193|ID:27163085| conserved protein of unknown function [Uncultured spirochete bib]
MLKEEFIRKSPVRIFENSIEGGLHAGDIGVLASKKGIGKTSVLVQIALDKLMQGEKVIHV
SFNAHTSYIISWYENIFSEIARRKNLENIDDIKEQLVRNRMIMNFTQEGVSVDQITRTIK
ALINEGGFNAKTIIVDGFDFTRATAERFEKVKAFLAEMQLEAWYTCTLAEAEPVLDPQGI
PVVLREIEPLISVIVVLEPKKDFIHLRVVKDHERRNPSDMNLLLDPRTLLIAEA*

>SPBIB_v1_210194|ID:27163086| conserved exported protein of unknown function [Uncultured spirochete bib]
MTYAACFLLGAAPAGADAKASRTTSGRGCWRSLALIFFIIAARSGLFAQTLQRLSFSGFE
WYVRQTTEPEGPMNNLFGGTGTSVDVLPDGALRLSITYREGDWYAAEVWTTKSLGYGTYT
FRIRTPLAQLHPDLIFGAFTYSRALGYFHREIDIEFSAWGKKVSDLRGQYVIQPFDKQGN
MHSFPAKPYAGPSSQQFTWLPDRIEFASWLGYGEKPPAGDPRLIDAWTFNDAKSIPKPSA
AIHMNLYLFDGRPPGKKDGLQLVIIDGFEFKAAPK*
>SPBIB_v1_210195|ID:27163087| putative 6-phosphogluconolactonase [Uncultured spirochete bib]
MILHVYTSDDVWLRAASEFIFSGLSEAREVRNAREAPQRSLHLCLAGGATPKPIYEALAR
DPQCAEIANARSIHLWVGDEREAEPGSGFRNSEMIAAAFKYSPTAFVLHQWPPGRRALAA
RTYEEELSTFASERVRANQPIFDLTILGMGEDGHTAGLFSLDDFQSPADSLKRATILPNS
LVMLTEAPQEPKSRMTLAPEILRSSRRILVLMQGLTKVRVLIANLVGEREDPIKHFLNET
CEVIARI*
>SPBIB_v1_210196|ID:27163088|zwf| Glucose-6-phosphate 1-dehydrogenase [Uncultured spirochete bib]
MNIATDMPYCRLDATRFSPQPALFIVFGASGDLANRKIFPALYDLYLEKQLPDELLMVGA
ARRNYSTKEFREILRASCLAHSRHQDEALHTDAWHAFSQRVFYIQNDVADPQSYEPIRRL
VGEQDRSVLEGLAPHIAIPHNILYYLAVTPEFFPVISENLGRMGCGSNASAPGWRRLVVE
KPYGKDQRSAASLTEALHKWFEERDIYRIDHYLGKEAVQNLLHFRFANTIFEPVWNRNYI
DRIEITVAEQEGIGTRGGYYDGFGAARDMLQNHLTQLLCLTVMEPPATLSPEHIRDEKVK
ILRAIPDYSPQEILARARRGQYAVGTNARGEPVPDYLNEAKVRPDSTTETFASLTLSVEN
WRFSGVPITLRTGKALAEKYSEIVLYFKRPPSALFAAQCGDLLAPNSLTVRIQPDEGIWL
SFNAKVPGEPAIRANSLRFSYREVADYFPEAYERLILDALSGDSTLFIRADESELAWQVI
DKLEAAWASADPKAAPESGGLLKYQAGISLRDLIAQVREPVLREPVLREGLV*
>SPBIB_v1_210197|ID:27163089| putative RNA methyltransferase YpsC [Uncultured spirochete bib]
MRAIAVCAIGLEKVLARDLAHLGFREVGRSAGRVVFNVSTNALAEDLTKANIGLRTADRV
LLVVGEFKAFDFDGFYQGIYSLPWEAFCAKDTRVLIERARANGCKLHAQATLQSMAQKAI
YAALMEHFHVRNMPETGRMLEVRIYGDNDWWQIVIDTSGEALSRRGYRRFTHEAPLKETI
AASLLFLAGWSRSRPLIDPFCGSGTIPIEAALYACNAAPGLKRRFAFEDFPEMDQNKVED
MRLFFRQQVRRDIRTDIRASDLDTKALELARRNATLAGISEFIKFFNADARELVPPEAAI
RSAEVRGDMAQSGIVTAGAASPGAASGPPAGTRGIMLANPPYGQRLASPDEARALYRELS
PMLKRYLDAGWECGFLSADKEFGEAIGVEPTSVRMIVSGQETIFFNWFSAGIAEAGGHAP
RSSNEHRRSRAPISPNEHRSGRPSGSPQADPQRRPSGSPQEIRQGRAPRSPSQ*
>SPBIB_v1_210198|ID:27163090| Iron-containing alcohol dehydrogenase [Uncultured spirochete bib]
MQNFEWVTPTKVFFGAGILNRLGLETSLWGKRALFLYGKGSIKTSGLYNTVVAQLRGANI
FFVEHADVQPNPRIAHATEGARKAKEHDLDCIVAVGGGSVIDEAKAIAVGALHDEPLWDF
YTRKAVFRDALPIIAVQTLPATSSEMNGASVMTNEETHEKFSIRGEMLYPKLAFLDPSLT
LSIPVQYTAYACTDILSHLMEGYFTATADFPLQDGMVEGVCRAVMGSLETILKTPRDFEA
RSTIMWAGALAWNGLLKAGVEGASIPNHMIEHPLSGMYDIAHGAGLSIVIPAWLKYMKSH
IAHRIVLFGQRIMGLEKQLDGKPAIEASDIVIEALEAWYQHIGTPIRLKEAGLQALDIDA
CTKQAMALSKLWGVPGYTEADIRAIYRLMA*
>SPBIB_v1_210199|ID:27163091| exported protein of unknown function [Uncultured spirochete bib]
MRIRRMYVSWAVMLLALVIAMMSLPDLLAAAQEISNLKPLLHVQTRWFDIYAPHELEAQA
WRLSSFADNTYSKLNGFFDTQPSRHRIPVLISDIAHSLNGYSTLLPSNRIVIFLASADPR
GQLASMADELESVFLHELVHCITLNERKGGWKALAWLGGDWVAPEGWMMPQALVEGTAVW
AESRLSAENGAAGESRAEDDVAGRENQASAKSGRLNDPAALQIVRIEREWGQNRLLWDVS
GLADFYGSGSLAYLYGGLFADYLAERFGPEILGQLWHDAAAGNIFRGFDGTALSKGILEV
KTGVRPQTLWADFLSWIDSGMEKAGEVRDAVELFSGYIGAFDVGEGVVYYFDGDKKGVYA
RAILDGQRKGVESQGKEAESRGQGTEPRRLFAADENLRNIRFNVRFHALDIDWIRPLPDG
RTIPARYQFDFETKTLSYIRDLPIPPAGAALAVAQNGSSRENIFLYDPWTDPESGTDYGL
VRLGSTILPARRTAEGKTEMIDIPGYAVRWISPGFRLDSEGSAASRGIRFALTLIPENGI
SRLGILEEEHGSWLLLVQNQAPEGGICQPVYANVTKIVYLSSKKDGLKALNVLDIGEIDK
QSTSASSLFSTIPVEWQDAVRWAQLHYLEAMQEPKQTAAIKKTDTLFPALFSSSRIPYAE
GNTAGLVLTGSDLSERAAWQVFAGWDFSNWRPSESAHIQLGAGEWHVLFSVADQSVLYAS
LGRVSSVSAVLKWERSFVPLFRSLSADLHGIFAGFQDAYPLAEVFQLSPDSTSWAIGLDA
QYQSLYRSRKPPYDQYGFALAAGADYEVASRSGFGGMSLGGSITVPGRVGLSAYGAYAPF

GGVAFSPALRLLASGSSIMPSAVQAPYPSYREYSSLISLSNWYAFGEVDVRLFSIEAGWM
LRMPLSPSWALRRIIGRAGVRGAGLEIDSTPSALCSAFARVEFDLAILAGMVATSHTMFS
VEAAWAFMAQKVGGSPLHINIGINAEL*
>SPBIB_v1_210200|ID:27163092| HAD-superfamily hydrolase, subfamily IIB [Uncultured spirochete bib]
MKNSESRLQCLRPIASLTPQEARAIRYVLMDIDDTLTTDGKLPPESYAALWALHKAGLIV
IPVTGRPAGWCDMIAREWPVDGVVGENGALVFWESEGRRLKSMTHPDAVPNSDPRLAALR
DQVLAQVPGTRLARDQFSRLYDIAIDFAEEEPILPLTAAQSIKALFEGGGAHAKISSIHV
NAWMGEYDKLSMVIRYLSARFSYDDDRNRATVVFVGDSPNDEPMFAHFQMACGVANILKY
RELISQLPHFVTRRESGAGFAEFTATLLERRAAKASP*
>SPBIB_v1_210201|ID:27163093| conserved protein of unknown function [Uncultured spirochete bib]
MAVVISASRRTDIPSFYADWFFERLMEGFVDVRNPFNPKLMRRVLLDPESVGCLVFWTRD
ARPMLSRLDELEPYAFYFHITIIGYGAALEPSGPLASEAVEMLKALASEIGQNRVIWRYD
PVLLAGCYDANWHIRNFAALAKELSGSVRHCVISIYDAYRHTDVRLKKAGFLPLCGARGG
EGASEKSSAHYQLLAKELTEIAASEGIFVSFCAEPELQQVQPSLGLGCIDSAIISELTHQ
SFSSRKDKNQRPACKCIESVDIGSYGTCPRGCLYCYADRTGKKQARNSSG*
>SPBIB_v1_210202|ID:27163094| Phospholipid/glycerol acyltransferase [Uncultured spirochete bib]
MDTIKSKYAHLIGEIIAHVPQNAHVSEENVYQQGIAALLPYIDTIIHDYIDDCSDIEGIE
NLASLHAHAKRGESCLMLMEHYSNFDLPVFHYLLRRSGQKGEEIASDIVAIAGIKLNEEN
PAVHAFARAYSRIVIYPSRSMQIIKEKYKDPKELYQEIKRSISINHAAMKALSAAKESGK
LILVFPAGTRYRPWDPSSKRGVREIASYIKSFSKFCLVSVNGNILRINPSGDMEDDILQK
DRVIYNVSPVHDSKEFLASVKYDLHFRDDKKQAIVDTIMDLLDAMHKETEDRCKG*
>SPBIB_v1_210203|ID:27163095| Rhomboid family protein [Uncultured spirochete bib]
MQTNSILRRPFRFRFFNATLYLIAANVLIFALGYVWPMVTYALALSPQAVMAGWVWQVFT
YMFAHANLTHLLVNMLGLFFFGTPVERTLGSLEFLLYYLLSGTFAGIASFAIYAFSGAWN
TMLLGASGAVFALLLAFGVLYPKAMVYLYGIIPIRAPLMVIGYTAIEIVFTLLGAASSVA
HLTHLAGFVAGAAYFPVRLGMNPFKRLRER*
>SPBIB_v1_210204|ID:27163096| conserved exported protein of unknown function [Uncultured spirochete bib]
MKKYALVALFAVAVFGMAFAVVPPTTTQSGTVSISGSVAEVFSLTVPETYTGTIANGSTA
ETWSIGNVVVTSNVKNWTISVSSANSGYLVHSVDNTEKIAYTMTLGSLVTDQSLASAWTS
AAQPRTAKVGNSYALSVKFGPSADYYQAGTYGDTLTVTISHN*
>SPBIB_v1_210205|ID:27163097| protein of unknown function [Uncultured spirochete bib]
MKTEPDSEGRKGSTFSIALSCVLCALAIFSFLMLRESAYRSESALLKSVSEKVAGQISAK
IQTILEIGEQFRQLLLTDSGKTYASLKLLADESLAKLQYIDSITVAPGAIVRYCFPEEKA
SGAIGRDLLDGPDRMQALVRAVSRKKAVLQGPTLSADGRNLAFLRVPVFNGADLWGFVSV
GFDTDRMIEYLELGAAFPGMLIAVASSEPEESGNSVFWGDQSAVQGYPARVKAGVENVSW
TVYAASNYPARQAIWWGVALVALSILSLGLFVSRIFTGRHLPERTMGVNIKAELALKEAS
FAVAPFVPQEKVEKVVWPGASEAECDELPRQAALAEDEADARIPGAAPSSEQPGPPPGSP
ISMSGAPVSPAQPGTNETIRLAPERPVSVLVVDDSEVNREILLRMLALKGYEAEAVDSGA
AALSTLKKRTFDVVLIDCIMPDMDGFALAREIRKSEASTGDKKKPVALIAMSPRHDLEEA
ERSSQAGFDSLLVKPFTMTALDQKIGERVL*
>SPBIB_v1_210206|ID:27163098| exported protein of unknown function [Uncultured spirochete bib]
MKKFFLLVLAAMFSGLPVFAADSVSTTLQATVGAALSITTTIPGTKALDPTQTSAALGSV
TITSNLTNWKIVIHSANGGKMVRSGSTDVYPYLVTFGATTGIDLATDYEIIKSAPQSAVT
TNVSVTYQTAATLGISAGTYEDVLTISLVAI*
>SPBIB_v1_210207|ID:27163099|secF| Protein-export membrane protein SecF [Uncultured spirochete bib]
MKRLIRFSKFFLPAMIISSTIIVLGLIGYFTKGFNLGVDFQAGINETIQLAYPAGEVSFT
GKGNAELTVSETQLMLVFSGAEAQKRTVALDYKSYPTLADIKAALGKETGISVKLSEGSD
ALPSSSIVPTYQGNTLLSVNPVKLYRAPINDSERFASIDIVREALKPLGQVSVQTINPVT
SQRYLVRVRDDGKNPQFTTMIPQTIRQLIEDKVGKDRVVVVKTDYVGPRYSQTLGQQSAW
LVLVTLLLILLYSTIRFKIEYALGAVLAIMHDALIMVAFIVWTRMEFNTTTIAAILTILG
YSINDTIVQFDRVREERKIHPSERFVDVLDRALTLTLGRSVITTLTTMLAVLALFLFTTG
SIKDFALALLIGMTSGVYSTIFIASAFVLMWENRRAGKERKQAKLQKDVPDKAPGKAPAG
V*
>SPBIB_v1_210208|ID:27163100|secD| Protein translocase subunit SecD [Uncultured spirochete bib]

MSKFRRLLVVIAVLAVAFMFLFPSIQWYFLTPKEDQAIAVGSREQIREYSRRMTYLIITD
LKQKATAKDTTDLSGQKMYSVAIESAKKAYSLAKKPAPKTWDAIAILSAFSGERSMFDSI
ESRYREHVLSLKNVHSNAVQLGLDLAGGMSAVIQADLKGLSEKLGHDLSSAEKEDAMKRA
VEILNSRIDKFGLTEPVIRRQGEDQIYIEIPGAADPERLNSIIMGKGNLAFYLVDSEAST
AVSSYLATNPVGIDEQTMVVDKPGLVPEGKIVRKVYKKDSYGLDEFTGEYLVLEGKPGLD
GSHIQSATVSSDPITGKPETNFVLDKEGGDLFYKLTSENVGKTMAVVLDDRVKSYARIQE
PIRESVRITGFSSDEAESLALLLRTAALPISLSVVSQQAIGASLGEDSIAQGRTAILVGI
LSIFVFMFAYYKWAGLNATIAQVLNLYLMLSVLTAFKLTLTLPSIAGFVLTTGMAVDANV
IIFERIKEEIRAGKTRAAAIDAGFHKAFWAVMDSNITTIIAALFMAQLGTGPIQGFAISL
AIGNITSLFTSLFVSRLIFDFETDVLHATHTSISWRVR*
>SPBIB_v1_210209|ID:27163101| Preprotein translocase, YajC subunit [Uncultured spirochete bib]
MNGIQNGLVLLQGTTAANPTGQLVSTIVTFGLVFVIFYFLIIRPQNKKQKDMQKMIAGVK
KGDKIVTIGGIHGVVAAVKETTVMVKVDDGTRIEFSKSAIASVSPAKAEEKASEPVEEAA
EKAEDK*
>SPBIB_v1_210210|ID:27163102|yqeA| amino acid (carbamate) kinase [Uncultured spirochete bib]
MAARKTIVVALGGNAIIEEGTEGTTEQQFENTRKSMKAIVGMIAEGHKVVLTHGNGPQAG
VHLIRNEAASAQVPPSPLNVIVADTQGSMGYMIAQSLANALRAEGIEKDIVTVVTQVEVD
PNDPSMQNPSKYVGPFYKAEQVEKLRERGWIIKEDPMRGYRRVVPSPKPLDVIEKDTIKD
LIDDGKVVIAVGGGGIPVKRDTDGMLSGVDAVIDKDRASALLASLIDADELIILTGVDKV
AINFRKPDQQVFDHLTVAECEKYMAEGQFPKGSMGPKIEAACDFIRRGGAKVIITSMENA
TAAVDGKAGTVITA*
>SPBIB_v1_210211|ID:27163103|ispH| 4-hydroxy-3-methylbut-2-enyl diphosphate reductase [Uncultured spirochete bib]
MCMGVRRADELARQAAQEAANEGARVFTYGPLIHNPQAVAELEALGVHVLDTKEVEDGSA
SIPDLNDAIVVIRAHGASLAAISRMREMGCRIIDATCPRVIKSQRLARHYEKLGWQVVLI
GDPRHGEIAGILGHTSNAIVVDGPTTALKIARKLKDKPCALIAQTTIRQEDYDAVIAIFQ
ANVPTITAEKTICPATRERQQALVELCGQVDAVLIVGGKNSENTKRLYASAIECGKPAWH
IETASELTAEMARYERIGITAGASTPDFIVDQIQEVLGAMASRRKFTLAGGQS*
>SPBIB_v1_210212|ID:27163104| Nitroreductase [Uncultured spirochete bib]
MNILHEIEDRRARRGLSEEPIDERIAAELVYAATLAPSCFNNQPWRIVMVSNEPGSAQAE
GIRAALTPGNAWAQKASWFFVLCTAAHLDCRMDEGRDYAYFDLGQAAMAIELQAQHEGLI
AHPMAGFSPSKVRAALSIPKEIVPLVVLAIGKPGPTDALSEQQKASENSERIRKPLGEVV
FLGSFGNPLQG*
>SPBIB_v1_210213|ID:27163105|udk| Uridine kinase [Uncultured spirochete bib]
MPPVKIIGISGGSGSGKTTIVRKISEMVSDFVFLPQDNYYKSAEFINNRNITAFNFDHPD
AFDNDLLIQHLTSLKNGESIDMPVYDFVHHRRTEQTIHIQTCKLVIFEGIMIFTNKQVRD
LIDLKIFVDTPDDIRFIRRLSRDIKERGRTVDSVVEQYLTVVRPGHYEFIEPTKAYADII
IPEGGFNERALDVLVTFINSVVYPAEGREKADSVPERKPPAVL*
>SPBIB_v1_210214|ID:27163106|msrA| Peptide methionine sulfoxide reductase MsrA [Uncultured spirochete bib]
METGEKTEKALLAGGCFWCLEALFKRVPGVLSVTSGYCGGAAPHPTYEEVCEGTTGHAET
VEVVFDLAVISYREILDFFWKFHDPTTLNRQGADIGEQYRSVIFYLDANQRELAELSKSA
AQAAFDRPIVTSIEPAGHFWPAEAWHQKYYERNPDAPYCRFVIAPKLAKLR*
>SPBIB_v1_210215|ID:27163107| ABC-type transporter, integral membrane subunit [Uncultured spirochete bib]
MNILTFVVVNVLSIGIPFSVALLLASTGEMFNQRAGVFNLGCEGIMSMGAFLAFMPAHLLQ
NTSLAPYANLIGIAAAIAIGALFGLLFALVTVTFRAPQGIAGIGLHMFGWGVAGTLFRYF
VGGVTGVPGLKNLPIPLLSRIPIIGPLLFNLNGLAYAAFILVPASWYILFKTPWGLKVRA
VGTYPRAADTMGVNVSRTRYQALVAGGMLAGLAGAYLSLGQAKMFADDIVAGRGFIAVAL
VYFGRWNPYLILGGSLLFSVAQSFQLAIQVLGIRFPYEFAVMLPYVLVIVVLALTPGSVT
RGPAELGKPFDREKRV*
>SPBIB_v1_210216|ID:27163108| ABC-type uncharacterized transport system, permease component [Uncultured spirochete bib]
MKSTYRALYIFAISLAAALAAILLGAVVLEILGKDPLAVYKVILTEPLKDLFGLTEISVR
MVPLILVALGIAISFRSGILNIGAEGQIQMGVIAAAAAAIYLPPLPKIVILPLVLVAGAL
AGAIWASIAGWLKAKLNVSELLSTVMLNYIAAQLYGFLLRGPMIDPAELQVGSGTPQSVR

LPKAAWIDRIIPGMRLHYGFVIAIVLAILVYVFLWRTKWGYALRAAGAGPKAARYGGIKV
ETCLIAAMALAGAFAGLAGAIEVTGLHRRAIEGISSGYGFSGIVVALFGGLHPAGIVPSA
FFFGLLLIGADMTQRIMSVPANMVLVLQGAVILSIIAAKMLIADQYLFERFARMLEKRGR
KAAGGKTKAGAGKAGAAKVDRRREQP*
>SPBIB_v1_210217|ID:27163109|yufO| Uncharacterized ABC transporter ATP-binding protein YufO [Uncultured spirochete bib]
MNNEPIHSLAVEGITKRFPGILACDSISLDVGEHEILAIVGENGAGKTTLMNILMGLYQP
DEGKILINGREVHFRTPVDAYATGIGMVHQHFMLVPNLTVAENVLLGMREFQKFLWQDME
GVRKRIREISERYNLPVQPDAYIWQLSVGEQQRVELVKTLCLGARFLILDEPTSALTPQE
TDDLIALLKRMSSDLSIIFISHKLAEVTALSDRIAILRQGKVVYRGTTSGQSVSNLASLM
TGHEVVLPCNECTGGSGETILEIRNLKVRSDRGFFALDGLSLSLQKGEILGLAGVSGNGQ
RELADALAGLRKVESGSILFRKKELANKSPRSIIEAGIGYIPEDRNEEGIVPPFNVRDNL
ILKDFRSKHFCKGPFFRFDETRQNAEELQKRFDIRCASLNVAAGSLSGGNIQKVILAREL
ARKPEVLIAVYPTRGLDMGAEEFIHERLLELRSQGTAILLISEELDELLNLSDRIAVIFK
GKILATMPTRETSKKKLGLLMAGISGEGAA*
>SPBIB_v1_210218|ID:27163110| putative ABC-type transport system, periplasmic component/surface lipoprotein [Uncultured spirochete bib]
MKRFLCVLIALCLIAGAAFAAPKTKVKIALIVESTVDDKGWCQSMHDAITAVQKKYGTAL
VEYSYSEKMKPVDAGSAARQYVAKGFDIIICHGAQYKNLVLEMADDFPKTTFAFGTSGEI
GPKNVFTYMPESEETGYINGIIAGLVTRTNKIGLVGPVDGGDAARYNRGFILGVKAVNPN
AVVQVAYTGSFGDFVKAAELAHTQIKAGADVLTGSAQQALGALRAVAEYKDKPIWWLGQD
TEQLKVPESYKVIAAASYAYAAVVEQLIDKRQSGVLGGEVIPLNFANGGFIYKFNDNVGP
VLTADIRKQAEKALADITASKLTIAWQSVK*
>SPBIB_v1_210219|ID:27163111| PP-loop domain protein [Uncultured spirochete bib]
MKHARSTLAKLTEVAIRRYNMIEEGDRILIAVSGGKDSSCLAWDLALKRQWWDVPFEIAA
CHVATDLASAGYSNPVDDSWLKAKMADWNIPFVRIDVPVAGRLKPSETMNCYWCATQRRT
ELMKYAQANGFNKLALGHHMDDILETLLMNMMRKGEFATMPPVMPYRKYPLTVIRPLALC
EERQIIACADELGLTSHTCTCSFNLDGERKKTKRLIDQITGGSSHVKRNIFASMSRIRPD
YLA*
>SPBIB_v1_210220|ID:27163112| putative 2'-5'-RNA ligase [Uncultured spirochete bib]
MRLFAALPLPAAAQHRIAEYCRTIAPSFALARPSWVPEENLHLTLHFFGELDQKNAATLQ
NLLEAEAINCPSLTLRIGNLSVLPSLRAPRVLYLHTRIEPAAPLFSLIDRLRIIAAQIGA
ETDSRPWKAHLTLARLKEPWIPELSSLPAAPEISFAIDAFELMQSRLTREGAMYSCVRRY
AFLTCSR*
>SPBIB_v1_210221|ID:27163113| putative Diacylglycerol kinase catalytic region [Uncultured spirochete bib]
MKPDDLAEGLNAIFARSPVFPEKSLIIDVIANPKAGGFSRIHHSRKRFNELKEIVRRSLQ
LPERASPYALKMHLTERCGHAAAIVQRILDRSPSNGKDSCHLIITAGGDGTSLETAERLT
KLPESEKDRFGLVRLPFGTGNDGSEGRDLLVALGRFLGSAAFERRAALRVTPSEEGGGLP
RYSFNIASIGLDAYVVADMTNRLKRHFPGDSYRFWVNIGTLFYDRIYNVGDMHLKAWGEDG
KIVMDSTGPRLLVAMGVSGNRQYGSNKKILPNDTNCVAVSQTSLFRKLILKGPIEQGRHE
NIPELVHFSAEKLMIEYGERIPLQCDGETNELAKCDFPLIMERVRNAYNVVVPVLA*
>SPBIB_v1_210222|ID:27163114| Multi-sensor signal transduction histidine kinase [Uncultured spirochete bib]
MILDASSLIPALSFCIYIPFIVFGLASKKERVNFSFLEYMGFMALWSFGSFMMHANTGLF
TPLIWNRVMLVGLLGGPITIFGTLIYFSRTEKRRYRILLYFGYIIYIFLLYLNFSGKIVT
DAGFEGNVFHYSLGPGAPIAYSLSYFYLILAILLILWELRTNPDRFLKRSLRLVVAGVVI
MLLGVAANLYAPLGRYPVDLLAATINAAIIFFSVYKYRLVHYSAVMLNIFLTLLVIIFAS
VIYMLFFVPVFALDRVIPFSELALLSVLLGIVSALLLSPLRTATQALLERIYGGKSFLYY
QELRKFSADLTSIVNLEDLAKLTVDNVISTFKLEWAFVLINDYNARNFRIIASTGLAFAE
TYGETTDQHVIVPRTDQFVQAYQLRTIQQNQAAYQRTISIKLSKGEINETVQASLVLPLK
FKERLNGFIVLGPRIEKDYYNQYDLEILQFLTDQASVAMENAISFERLRQQQKRLQETNE
ELTLNRNKLEAFFDGIATPISIQDINYNIVTANYAARRYFEKPLEELVGSKCYKVYFNRD
RPCAECLAQDCLHTKLPFSAEKQDSRTQLTFALNFYSIPTPKGSVGSFIEFFQDITKQKT
LQEELIQSEKLAGIGTLVSGIAHEINNPLGGILGTADLMLPETPESSTLREYTLDIIRYA
QSAAEVIKDLMTYSRKTRTSAEPINIITILENSLKMAMRGIDFGTIIVRKSYDSVKEILA

NPTELQQVFLNLIVNAVQAMNSDGILTLSCRQEEDDVIITVQDTGTGIDKENLDKIFNPF
FTTKEAGAGTGLGLSIAHHIVTKSGGRILLDSQEGKGTTFTVILLAASQDKDRLHLIHAK
ETRHFEDSFYLQRKVLVGEKGYQEETIRRKCDEVAFHILAYKGLQPIGTVSLHLSEEEGR
VPIEENFSIAHYLDGSLYAEIDRLAVAKEERGSLIPFSIMALAYLYARGRGAKKIFLDVF
SDEVKLIKMYEKLGFEIIGSYSKPLPCTVMMMDHVSRYEQEVSRMEHFVKPFFSRLVPKI
DFSGQDLHYVMKAMDEISAKFPKESADEQQVGEAM*
>SPBIB_v1_210223|ID:27163115| conserved membrane protein of unknown function [Uncultured spirochete bib]
MVSIIFIFCGFAVLMFAEWRYVHFSRKSQRALRGKTPGLLPGHTALQAAGYIAVAAGMLI
SLLHEGLALHIPPLVRTISLCIGLLGGALLVWTVFIEIPVGAKKHAVEPGCAYHYGSYGM
CRHPGFWWFLIFTLGIALWKSDFYAFLLFFFENALNLLLILLQDKYTFIVQFRDYQEYQK
KVPFLLPGPKQDG*
>SPBIB_v1_210224|ID:27163116| Response regulator containing a CheY-like receiver domain and an HD-GYP domain [Uncultured spirochete bib]
MTTLQKRTVLIVDDDVSMQKVLSLSMKKIGYEYAIAKNGREAIDLLESGDISPDAILLDI
RMPVMPGLAALPQIRSILPSVPVIMLTAFGDLETGLAAMKSGAFDYLVKPSSIDKIQETL
EKALSYRDIMEMKAEEERKREQYRLELEHRVESSYQELGDVYRKLKQMNIQAAYALAETI
EAKDRYTQGHCERVRWLSSLLGKVMRLPDEEIEQLEYAALLHDIGKIGIPDSILNKEGPL
DDRERDVIRMHPIIGAQILSTVEFFAKAAAAVRHHHERWDGKGYPDGAQGEQIDHLARII
SLADTLDAMATSRPYRTALDFDEVISELKSMRGTQFAPDVVDAFFAAELDKAYLKRLKEE
TGKNII*
>SPBIB_v1_220001|ID:27163117| conserved protein of unknown function [Uncultured spirochete bib]
MTEALSFAEREQRKRTIISYAVAIGLYALLFGAGLLFDFLRPPIMDLSNKTLIVNIQGPV
VNDTGKGSPVEKKDTVATERPAPPPSPAPKPQAQTAAKTAPATPSPAPQAEAVPVPVTPA
VQQPVEPWTPGERGPGSRVSSSESSVLSPGEGQVPWGTGQAIKIMKAEKGNTVETTLGGS
SETVGQSLYVPIYLGMPLPSSVSADLFNAIPDEIIPPHTVITSADARKRAFLNYYVKNGN
EYVLKNPAPLDVREKLWEMLEDAGYNAELADYKAGRSLSPVVIGFSITKDRQLRGVELLQ
SSGDPDIDAAVLYGFKRASFWNKSGDTIQGRFVYRF*
>SPBIB_v1_220002|ID:27163118| conserved protein of unknown function [Uncultured spirochete bib]
MARDHRIQPKINFDLTPLIDIVLQLVIFFMITTTFRTAPGISLQLPGSKTAQSIATPELR
VIVVSANEIYLDKTKTNLAGLELLLRKRVSGSSASQIRAVLEGQSGAEYQLIISVLDALR
MNGIENVGLITRKEKVVQ*
>SPBIB_v1_220003|ID:27163119| putative tolQ-type transport protein [Uncultured spirochete bib]
MLDFISKGGPILWVIMALSVVALAIIIERLLYLRRISIDEEKLFLRIKTSLMEEHYSEAL
AICDQNVSPFSALLKVGIENRNQPEYLQRELLKDAAAMESINLERGLPALGTISNISTLL
GLLGTVTGTMKAFGVLGKFGAVSDPSALASGVAEALITTVGGLVVAIPVIMFYNYFASKV
NYIITRLETQVNSLVDLISTYAGESSANTAEKPAKEG*
>SPBIB_v1_220004|ID:27163120|purB| Adenylosuccinate lyase [Uncultured spirochete bib]
MVFLIQFLGHNCYYVRMQNFDQFQSPFSWRYGSQAMRIIWSETYKRRLWRRVWVGLAKAQ
SRYGLVTSSQIAELEAHAADIDIEKAQEIEQSLRHDLMAELRTYASQCPIAGGILHLGAT
SMDIEDNADALRIRDSLNLLKDKLADLLSVLVDKIERYAAVPAMAFTHLQPAEPTTLGYR
YAFYAQDLADTYERFNDALRNIKGKGFKGAVGTSASYAELIGVEHLDEFETALSEELGIR
FFDVATQTYPRIQDYRVLSLLAELAAVLHKMAFDFRILQMPLIGELSEPFGSRQVGSSAM
PFKRNPIEAEKIDSLARLVASYPGVAWGDAALSGLERTLDDSANRRVILPEAFLACDELL
QSMKKLIEGQSVDETAIQRTAQAYAPFAATERVLMALGKKGADRQEAHERLRDLAMIAWN
AVREGEPNPLRQLIQSDEFFASRLSSDAIESLFDVDTYIGAAEKRARIVAARIRSLFR*
>SPBIB_v1_220005|ID:27163121| Single-stranded DNA-binding protein [Uncultured spirochete bib]
MNHLNSILVEGNLVRDPNLRGTPSGNQVCDFTVATNRSYKVADQKYENEVSYFDVEAWSR
LGAACAQNLKKGRGVRVVGRLKQDRWTDTEGKPHARVKIVAEHIEFKPMTKANKEETSKS
DETFIEEAKRSEASMAVF*
>SPBIB_v1_220006|ID:27163122| putative HTH-type transcriptional repressor AllR [Uncultured spirochete bib]
MSESAGKALDILFYLAKVGEGVSLARLSKETGMNKATALRYLTVLESKGVVERCLAGWTL
GLSLFELGSKVPVRQLVAEKVRPIIERLARETGESSNLAYLAGDTAIYLDRAEANRSLRM
RSMPGDRLPLYCTGVGKAILSQLTEERIRAILGPGPLPKITDSTLTDPEDIIHEAMRARE
LGYGVDREEFEIGLTCYAMPLRLPGSDFVGAISISGPTARMKNPEIRERFLAILRQAVQD

ATDMLSPYHYFENLPENRGD*
>SPBIB_v1_220007|ID:27163123| Peptidase U32 [Uncultured spirochete bib]
MNYNTRSWGGGAGPQPRPGPSGRIFPLHKVENTTKCLHQRAIHDYKDTVKRIELLAPAGS
PEALDAAISEGADAVYLGLRSFNARMRSANFAFNQFEAAVYACHDRLRKVYVTVNTVFEE
READRLYQLLEYLVSVGPDGIIVQDVGVAKMASEYFPSLHLHGSTQMNVASSAGCNQLSR
SGFKRAVLARELSMEEIRHIRQYTSLELEVFVHGALCVSASGLCLFSSYLGGKSANRGMC
TQACRRLYEGENSTGYFFSPDDLQLIEYLPALIDAGVNAFKIEGRMKSAEYVGTVVSAYR
YLIDNYEIDTERAVLKAKAMLQADFARSKTNFFITGAPDIYIHPDQAGGTGIHLGRIRDA
RTIDDKRWALMNTYEGLAERDSVRIHRADDSGRITAKIQAIKYGVDGMLLQLDGDWRQND
DVYLIQTAGMARRYKSILPKNLDRFRKFPSTHAAPQPVLPRVDLKKLDAALPLGTYVLAG
KVADLHAALTFRPKRAMILFDKLNAEVMRREEATLPFKRERLILWLDPYCTETDLAWLQL
ELEHWIEKGVSVIVANNQAHFSLLRGKDVCIIAGPWLYTFNQWALAYYLEQGAQAIVPPY
EISRQDLYRLAEYLPAQIFMPVLFAYPDLFRIRADLSRTYGQTHFADRDGNNFELVGRRD
YSVVIPEQPFSLVDLVPNLKKQGFSRFILDLSNAEPARGLYRDIARAAEQFKPLPNTSRF
NWKDGFWSEEKMHSADEAPKAPASRSSQPGQLARRSATKASAPARSGKKDNEKSKPPRSA
KKAARGRFPSPSKKPPRQP*
>SPBIB_v1_220008|ID:27163124|fusA| Elongation factor G 1 [Uncultured spirochete bib]
MSLEKIRNIGIMAHIDAGKTTTTERILFYSGKTYKIGEVDDGEATMDWMDQEQERGITIQ
SAATTTFWRDHQINIIDTPGHVDFTAEVERSLRVLDGAIAVFCAVGGVEPQSETVWHQAD
RYHVPRIAFVNKMDRLGADFEAVLQDMKAKLGAHPVPLNIPIGKEGGFEGVIDLIEMQEI
RWNEDGTEMTRTPIAPEREAMAHRWRENLLDAVSSHSDIVTSLYLEGASIPADIIRPEIR
KACIAQRFVPVFAGASRRNIGVQPLIDAIVDYLPAPDEVGAIKAFHTKKEEEVDIPCQTS
GYPLGLVFKVQYDREAGSLCYVRMYSGTLHNSSTVYNTSKKKRERITRLLRMHANKSEPI
DSVSAGDIAVFVGLKLAQTGDTLGTEGYPVLLEKMHFPEPVISVAIEPKTLSDRDKLKEA
LEILAREDPTFAVHEDEETGQIIIRGMGELHLDVLVTRVVREFKVQAKIGNPQVTYRESI
STKVQHTERFHKVIAGKENTAGITIEVEPLPRGSGNQYKKAIRAHEVPEEIFDAIERGIT
SAFSSGIQYGYPAVDIGVSLVSIEYSELTSTPFAFEACANMAYDAACRAASPVLLEPIMK
VDILTPKEFVGEVMSLVSQRGGFIHGSESKAVAEVIHAQAPLATMFGFTTSLRSVSQGRA
SFSMEFSHFEPKR*
>SPBIB_v1_220009|ID:27163125| protein of unknown function [Uncultured spirochete bib]
MHIYTIGFAAVLLMLSGPGAQIKENIIDAILPRQSDPQGCGYAVAAALINFVKTAICLAD
IQKGSASPTFSLEKIQIAASLMHSYGKTPPLSLADLEKLLSMEGISSTPFRVPKDESLKL
IAGAALPVVVHIGEPYPHFLLGLGGDEEHILFFDPGSGLVALTHEESIALISGYFLIPEK
TIAARPLEDYEKAFRKLKQLIWNVYWDCAQN*
>SPBIB_v1_220010|ID:27163126| conserved protein of unknown function [Uncultured spirochete bib]
MPMDFVNSPRVDTLVTESEKKVFELFETMVRTTGQERVQSAIALANLLGNPGEFSFYIDC
TEDQRIIRVFHLLRVFRENMTLLIHKTWVDGSENLQQDQLLGDLARFIQEFRDGRIVSAF
RSFVGISRQIPSLLFGSLGKANDFLEYAFRIDPKFGLFFWYIAEIDLQLRNIESIPEHRE
LFELEVLIGTFVISCF*
>SPBIB_v1_220011|ID:27163127| conserved protein of unknown function [Uncultured spirochete bib]
MAKEIRILPETKVGEGFIPPQYLGPDETEYRIRDEQLRLLGKDPSDYRSLTTHELEILVR
NNNTCNDWSHFLVKDPFCPELVRNNLFSGLIRISKLEHVSLEHHDIAMAAGIFNSQIIAC
DIGDNCIISNCAYLSHYIIDDHCILINNAEIQVSNHAKFGNGIVMESENESVRITLDVMN
ENGGRWIYPFNGMLSGDAWLWAKFRDRTRLQERFRQMTDARFDKRLGRYGIIGRNSVLKH
NRIIKDTYIGESAYIKGSNKLKNLTINSSEDAPTQIGEGVEMVNGILGFGCRVFYGCKAV
RFVMCDSSALKYGARLIHSVLGENSTVSCCEILNNLIFPAHEQHHNTSFLIASLIKGQSN
LAAGATIGSNHNSRAPDGEIEAGRGFWPGLCTSVKHSSRFASFCLLAKSDYKHELNIKFP
FCLVDSDETHGRLMLSPGWWWHSNSYALMRNEKKFKERDKRVDTSIRFVYSPFAPDTMSE
IFEGLAILEHAAGEAAMRAISSNDSDGSMFGDRIFALLAKISDAAVEPKAGTEEELKLVR
QLGALILSSSKDPLPFEVTVRGFENSNRPQVIIKPCAGWRSYREMLLWFAIQTVIPALEA
APLSSDPYEYLSSLLSASACKNIETEWENLGGFLVRRSKLEDLIERIESGNIQSWDALHE
EYRRLSAEYEHDALAFAWCILGYLSLAIEESAIWDHNEAHLPEPSRETFIQMLRDAHVLA
SEIAQRVFATRAKDWANPFRKATFRSEAEQIAVHGKVEENSAIQAVKKDMHEVQRAIDRA
LERLGTALNLG*

>SPBIB_v1_230001|ID:27163128| Urocanate hydratase [Uncultured spirochete bib]
MNSSPYTISLDFDELPPEPVFEPGIRRAPRREAELSPAQERLALKNALRYIPQKWHEKLA
PEFLKELRERGRIYGYRFRPQGRLKGLPIDQYPGKCLEGRAFRVMIDNNLDFDVALYPYE
LVTYGETGQVCQNWMQYRLICKYLEILDENSTLVVESGHPLGIFASHPYAPRVIITNGLM
VGMFDNQKDFNIAAAMGVANYGQMTAGGWMYIGPQGIVHGTYNTLLNAGRLKLGVSKDGD
LAGKLFVSSGLGGMSGAQGKAAIIAGAASIIAEVDRSRIDTRFSQGWVSRVADSLENAVR
LAIAARDKGEPIAVAYHGNIVDLLEYLDAQNIRVDLLSDQTSCHVPYDGGYCPVGITFEE
RTRLLAEDRTYFRALVDASLRRHFEVIMRLVRKGTYFFDYGNSFLKAVFDAGVKEISKNG
VDDKDGFILPSYVEDILGPELFDYGYGPFRWVCLSGKKEDLIKTDHAAMECIDPNRRGQD
RDNWIWIRDAEKNKLVVGTQARILYQDAFGRMNIAHKFNEMVRKGEIGPVMLGRDHHDTG
GTDSPFRETSNIKDGSNVMADMAVQCFAGNAARGMSLVALHNGGGVGIGKAINGGFGLVL
DGSERVDRVIRMALSWDVMGGVARRSWARNPHAMEVCDEFNRERAEFGVITMPNLVDENL
LDAVISALNLV*
>SPBIB_v1_230002|ID:27163129|trxA| Thioredoxin [Uncultured spirochete bib]
VTENLNKETFLEKVFNYEKEKEWKYNGELPAIIDFWAPWCGPCRMVGPVLEKISDEYAGK
LIVYKVNTDEEQELAAAFGIQSIPSILFIPVNGQPQMAVGALPKSTIESAIKDVLGVEAA
*
>SPBIB_v1_230003|ID:27163130|mdh| Malate dehydrogenase [Uncultured spirochete bib]
MDNDVTWWKFDEVEAFMKAGFEAVGVAPEVAAVCADVLISADKRGVDSHGVGRYKPIYLD
RIWAGILNPKTTFDVVRETPTTAVIDGHNGMGHYIAKRAMELAIEKAEKYGIGMTVCRNS
THYGAAFYYARMAVEHGMIGLTTTNARPAIAPTWGVEPMLGTNPLTWGMPSDEDFPFMLD
CATSVTQRGKIELYDRLGKELPDGWVIGQDGKYRHDTHQVLTDLTQDKAALTPLGGLGED
LGGYKGYGYAMVVELLSSALSQANFMKALADIGPDGKKKPIELGHSFLAINISAFCDLED
FKHHVGEVSRQLRASKKAPGAERIWTPGEKEHEIWLYRKDKGVPFNPPLKKAFKEVKERC
NLNIELPF*
>SPBIB_v1_240001|ID:27163131| protein of unknown function [Uncultured spirochete bib]
MARTPYREIWGIERAAEEVWNWEKVVISALNLV*
>SPBIB_v1_240002|ID:27163132| conserved membrane protein of unknown function [Uncultured spirochete bib]
MAKSRLHKLYRSILSTLSFSAELRKMRRELNAKIDQPESITPPPPFHPQGANRWFKRRRI
TIAESYLMVVRDLDSRHSSARLDALRKLADVAFHSANIDYPLNTARVQSALIKEVVKHRS
NKRRQLELLYDFSMSTRGQHQVIRKLCDELNIIELPEKGMQIGDLGYGWDGHVHDTATSG
RKNPTQLIIDAFIKGISRVTVAYGSVSDLDMMEEALEAGNILGLHVSIALEFSVMVDGAR
YHFMAELPRFNSKEELRGFFSSHASDLTSFFQGLDINRENRLDAVRRLLETFNADTLPTI
NEGFEHKPEYCLAPLSLDELLATIPNMNITPLHLAEFMYMRYRPVLQKRVWFYKVLREKI
RSSSLADPARKAEKDTIEKKYAELRKELRNLSPDTLLSEYFEDPHAISYQTVFEDIESLS
DMLHQAGCTIKFIHPLEYGVEKATRVLEQCSHCIDSAEVYNTQDCAGRSPEEVEAFARIF
NERNKRAHSKGRKVIIPVCGSDATGRNPKIPGMGFVFEDRIIGKLRQRYIKRHVALPPLV
SAMVRAEDAPVDEEHLDLRSVPCIISMGKVSAGEGYTSTSEDEVIGPVRAWRYFNPAFKN
TIRTLIGFFVATSFIGPWYALLWLGITGFRNSIADLISYRGTRLNQWKLKSINFDNVAQS
LFWTGFSVPILGFVKANFDAIWPWVHDGFVFNLVKFFFISFANGLYLASHNTLRGFDKSV
VRANIFRSILAWPLATVFAPLGNALAIPSIVQTKIWSDVVAGFIEGGNKYRKVLRQRQKT
LEEIIPTIIHSKGSTQYIAMLDILYLFSEEPRMQSIIKAVLSPYVLFTRRLRKNSSLRLT
LLVELHKTMCEERVWTELIDYIVANYDEEMADDLVDLVADALPDLQEWLGHLIAKYGESS
GLLKKFGIGRK*
>SPBIB_v1_240003|ID:27163133| protein of unknown function [Uncultured spirochete bib]
LDSVLQSRRYKAVCGRAGWMAPQETTVQPLAAVEETVDAPETADGAWLARRNGSPFRAFN
QRGPWWNSGAQHMNYAFPKKYFDSMGLVSMIDRLCLSR*
>SPBIB_v1_240004|ID:27163134| RNA-directed DNA polymerase and maturase, group II intron origin (fragment) [Uncultured spirochete bib]
VTRTEETNPETTKLIERLVERGTMREAYARVVGNKGAAGVDKMTVEALKPWLQAHWVEVK
GRLLRGTYRPEVVRGVEIPKPNGGKRQLGIPTVVDRFIQQALHQILSPIFEPEFSANSYG
FRPGRGAHDAIRKAKEYQLAGKRWVVDIDLAKFFDEVNHDLLMARIAKKVRDKKVLRLIR
RYLQAGIMKDGVVVWDRDKGTPQGGPLSPLLSNIMLDALDKELEKRGLSFCRYADDCNIYV
GSERAGMRVMESITRFIEGTLKLKVNREKSAVARPWERKYLGYSFTNERKVRIQVSASSI

ERFKEKVKALFRGAHGRNLGRFIRETINPFVRGWIQYYSLADTKQFAEELDGWLRRKLRC
NLWRQWKRPWTRLKRLMERGLPEETAVRSAPLTSAARGGTLAPST*
>SPBIB_v1_240005|ID:27163135|serC| Phosphoserine aminotransferase [Uncultured spirochete bib]
MKRVINFNAGPAAIPLEVLQKAQEEMLDWNGTGMSVMEVSHRSKEYEAMHNEAQDLFRKL
AGMGPEWKILFLTGGASSQFFMIPMNYLFGGRKATYLVTGHWGKAAIKEAKHFGPFDVLT
TENPDGTFTRVLRQDEIKIDPSSTYVHMTSNNTIFGSQWHYWPEVGKVPLVCDMSSDIFS
RPFPADKFSLIYAGAQKNLGPSGVTVVAIREDFYNLAQEQAKLPTMLSFRTHAENNSLYN
TPPCFSIYILNLTLKWLLNTVGGLDKMAKINEEKARILYQAIDNSDGFYCGPVEKDSRSQ
MNVVFRLATPEMEETFVKEAKAADIIGVKGHRSTGGIRFSTYNANLVENVKKAADFMEEF
RRKHR*
>SPBIB_v1_240006|ID:27163136| D-isomer specific 2-hydroxyacid dehydrogenase NAD-binding protein [Uncultured spirochete bib]
MVILLADAFAPDLPGRLATFGEVTSDMSRLGDAEVLIVRSKTKVDKAMIDAAPKCRYIIR
GGVGVDTIDVEYAKSKGIAVDNTPEASSLAVAELAFALMIAMPNHLVRADNSMKEGKWLK
KELERTELNGKTLGLIGIGRIGHELALRAKAFGMSVIAYDKYVTSSEVASLVSLDEVYAK
SDYISLHTPLTDETRGMINAASIAKMKKGVYLINTCRGQVVVEQDIADALNSGKMAGYAT
DVYNKEPPEGSPLLKAPNVLMTPHLGASTEENLLRLGDSIVARLKKYSGK*
>SPBIB_v1_240007|ID:27163137| conserved protein of unknown function [Uncultured spirochete bib]
MIGARERLERMGVTVPEIYLPSAGIDYRKWAVVACDQYSSEREYWEDANREIGEAPSTLR
LIFPECYLEDADKDARIANIQRTMREYLEKGILESKGYAFVLVERTTPFEAKPRLGLVVA
IDLERYQYGKDSKSLIRPTEGTIVERLPPRMAIRRGAALELPHIMILIDDPARTVIEPMY
ERRQYFERAYDFDLMKNSGHVRGWLVREENQLARVAEALERLADPKAFRAKYGKDEVLLF
AVGDGNHSLATAKAIWEETKAAYAGSPGAEAILAQHPARYALVELVNLYDEGLPFHPIHR
VLFNIDAQDFLKKLVEAGANIAYMPDAAQAFAAVDASAAKDAGSSHVFAFVSDSRAGTIT
FETPRARLAVATIQEHIDAYRASHSEVGIDYIHGTASTETLGRKKGNLGLYLPPVDKSSF
FSVVIHDGVMPRKTFSMGEAPEKRFYIEARRITND*
>SPBIB_v1_240008|ID:27163138| exported protein of unknown function [Uncultured spirochete bib]
MSKMPANKGKIVLFVLGATAANLVLMAACFALLMVFYSLALSKILPPEALIWAIAVAFLL
ALVISTLVYRKLLKFLRDRYHLDEYLGIPSK*
>SPBIB_v1_240009|ID:27163139|lspA| Lipoprotein signal peptidase [Uncultured spirochete bib]
MKKAIFWAPLVCAVLIIIADQIVKALVVSHIPEGRIYARFFGDFVWIVHARNTGAAFSLG
ASSAPLVRFLFFIALPVVVLGGVLVYYFRAAHLTLLLRWSMGLILGGGIGNLIDRIFRPE
GVVDFISLKMYGFLGMERFATFNIADSAITIGEILLIIGLLIAELQRSKVHPS*
>SPBIB_v1_240010|ID:27163140| putative GTP cyclohydrolase 1 type 2 [Uncultured spirochete bib]
MTITEFDAWARSFLEIDQLQQIDDSLNGIQVSCSSAKQIKKLAVAVDACAESIRRAHAAG
ADLLFVHHGMFWGKPEPVSGSLRERLKMLLEFDMGLYACHLPLDRHPEVGNNAQLVNLLG
VTERRPFGIYHGITIGWAGTLPNPMSLDEIILKILPDRSSPKSVIAAGPSEINTVAIVSG
GAPFESLEALAAGIDLYITGEPSHSVYHYMLEGGMNFVAAGHYATETWGVKAVAEKAHAE
LGLETIFIDLPTGL*
>SPBIB_v1_240011|ID:27163141| protein of unknown function [Uncultured spirochete bib]
MIYWNADLAAKISCSRQEMETLLPYIDHLVDVSRTLHQTGIQPSADQFSSEKDIFFRYGL
LLVSEGLAGDILEEILAVLLYVSEEKGIAFLRQCVAAEAILSIANGDDQDTLLRKLLPYC
GVDRALAALAKGKTEHAD*
>SPBIB_v1_240012|ID:27163142| protein of unknown function [Uncultured spirochete bib]
MLIERLGHMYALWGPSGKAPERNPRIDQTIQSVRRFIAAIQETSEDRKALFTQSQQSRRK
AMKLAYLLLASESDAAAIRDAFLSSGLLSSGQRASKLDALIWLAGTEAASRGIHPYTIFL
IMTAFFGPAAAETELRWLQEKAKATIPTLEEFIVPGDLTDTIEEALKEPARLQRTIRIAG
MPLSASAFAGCSLYYIEKILVLIGPIGASILAEMIHSARQRLMSDEISTAQQAFLDLFAQ
EEGETEAPENQRIKGLEAFEEEEVFADPDLIRSTTKIVMLAEAKVLKATLSSMSDNEIAS
ILRCMEAIAHERLLSLISPGRQKHVLTVIQKTGNTTNSRMLRDAQLFAQKLLASYAPKNL
KPGESLSIPEEVRALISSLLSRE*
>SPBIB_v1_240013|ID:27163143|proC| Pyrroline-5-carboxylate reductase [Uncultured spirochete bib]
MRYKLGIIGAGKIGEAILAGSLAKGLLAPSDVILSVRTEQHRQNLEEKYHAATVLHNREV
AKQSEIILISVKPRTIFEVLQEIADIIPDESVVVSTAAGVTLESMQTRLARKIALIRAMP

NLGVSVCEGMTALAPARFTEEDKVEKVKQLFESVGRAIVLDEQYMDAVTGLSGSGPAYVY
LIIEALADGGVKMGLPREVSIELAAQTVLGAAKTVLVTGEHPAKLKDQVTTPAGSTIDGL
MELEDGGLRVTLIKAVVKATERAHQLLHK*
>SPBIB_v1_240014|ID:27163144|dapD| 2,3,4,5-tetrahydropyridine-2,6-dicarboxylate N-succinyltransferase [Uncultured spirochete bib]
MNSAADELKRIFEESGSFCDAHWFVFLDALEKGEIRVVEQQADGSWKVNSWVKTAILSGF
KKGGLSAWPWPASSPAAALGSDPGSASPSGKTIKGSSGGSRRGEENGFFDRPAFPPRHFE
IGDGVRMVPGGSSVRRGAFVASGVVIMPPSYINVGAWVDEGTMVDSHVLVGSCAHIGKRV
HLSAGVQIGGVLEPPQASPVIIEDGAFIGGMCGIFEGIIVRKRAVLAPGVIITKGTKLFD
LVNGRELSGEVPENAVVVSGTRPAHGEFASEKGISLSVPCIVKYRDEKTESSVVLESALR
G*
>SPBIB_v1_240015|ID:27163145|dapA| 4-hydroxy-tetrahydrodipicolinate synthase [Uncultured spirochete bib]
MKQKIEGLGVAIATPFDDAFNVDYAGFERLLDFLCGRDFESAAGEYAEAAFRDEAARAKF
QRFWQAESGGADFIVVLGSTGEGATVEPDERRELIRRAVKRILGIPIVVGTGSNSTKAAV
RLTEEAVDLGADAVLVVVPYYNKPTPGGLNAHFEAVAKAARGKPVVVYNVPGRTGLNLVP
AVLNQLWQISGVEAVKESSGNLAQIGEICRTLPQGKTVLSGDDGLALPAIAVGAEGLVSV
AANILPRRYKALVDAARAGRRQEAKALHARLLPFTDALFLESNPIPLKAALKLTGLCGEA
VRLPLAPAVQTTRERLASVLAALGARELA*
>SPBIB_v1_240016|ID:27163146| putative 4-hydroxy-tetrahydrodipicolinate reductase [Uncultured spirochete bib]
MQIGIFGAGKLATAIAEEANALNVKAGSSADVVRFDIVWMVDMGDPMPSRHADVAIDASV
PDAVEAHLAWAIDTGTPFVIATTGWEIPDIAARVGQKTAVLVSPNFSFAVTFMRRMATQL
ATFVDWYGEGDLAVFEHHHAAKKDAPSGTAKSLAQAIISGSKRYAGWNASGWEQWDMSKV
PIASLRAGTETGVHELVFDAPHEQLSIVHRARNRRVFASGALKAAQWIQGRKGVFGMDDV
LESLLGNA*
>SPBIB_v1_240017|ID:27163147| Aspartokinase [Uncultured spirochete bib]
MTALVMKFGGTSVGNAERIREVARIVQSRQGRKRIVVVSAMAGITNLLFEMAEAARQHDL
DRAVQILSKIEQMHLEASRSLDLATPDIIGRIKEIVENLQRRIHGIDLLGELSPRTMDEV
ASSGERLSSILVAGYLGCPLLDARKVIRTDSHFGTARPKLAAIKKLAALHIVPLLESYDI
IVTQGYIGSEDAGDTTTLGRGGSDYSAGLIGAALQADEIEIWTDVEGILTADPRLVPSAR
TVEVLSYQEAAELASYGAKVLHPATVRPALDAGIPVTIRSTFKPDGMYSTISPGESSGRP
CVAIAMRRNVVIISVTQESMTDQAGFLAKLFDVFGRRGVSVDLVSTSEICVSVSLDKSAP
LEALTQDLEKLGQVSIARERAVIAVVGDLLRKTPEVLRKTFIAIDGIPVDLISMGANAIN
LSLIVQEAQADRAVQNLHAAFFEEGER*
>SPBIB_v1_240018|ID:27163148|asd| Aspartate-semialdehyde dehydrogenase [Uncultured spirochete bib]
MANIQHHSAFSPKKKIPVTILGATGVVGQRFLRRIAEHPWFYPAFLAASDRSAGKTYSEA
CQWHLPGLPYAGCGDMVVVPCSPEAAFSPIVFSALDAGPARDIEPLFAAKGAYVFSNASA
FRMDEDVPLLIPELNPEHFGLLENQKAKRGWKGAIVTNPNCTTVMLAAPLAALQSRFGID
AVMVTSMQAISGAGYPGVPALDIVGNVIPFIRNEEPKVESESNKILGTLTAGLGGLAITP
APFVVSATCTRVPVIDGHTLSISVRLAAKATIDEVAEAFTLFEPKTAPYSLPSAPEKFLQ
FLKAEDRPQARKDVEEDSGMRISVGRLRTCPILDFKFISLGHNTERGAAGASVLNAEMAL
AMGVLQGVGE*
>SPBIB_v1_240019|ID:27163149| protein of unknown function [Uncultured spirochete bib]
MSLSISNAMNSFSRALLRSDEPGIAIGEYLNELSQILRPLSYAACGRSTHQESLRCIVLG
GDEQVYGSNIAVGDEVRLGAAPCGFALEVDECVQKRIREGIVIEAPAKQNGSTAFVLSMM
FSADAAPELLLSASQALRTAAQLVEEYLSMRARLAEEIERDSGRPVGSDPVLTWCGIVGN
SDVMREVFTMVEQVAGTDASVLLLGESGTGKELVARAIHQKSSRANNPFVAVNCAALPES
VIESELFGHEKGAFTGAFEQRKGRFEQANGGTLFLDEIGELSPGLQVKLLRFLQDHRFER
VGGNTSIEANVRVIAATNRNLQESVDQGDFRPDLYYRLNVFPINIPPLRERGADILLLAD
HFVAKFNRENTKKIQRISTPALDLLMIYHWPGNVRELENCILRAAILSTDGVIHAYHLPP
SLQSAASTGTEPSTGLDAAIARLEKELIIEALKLENGNAAAAARRLGATERRVRYAMQRY
RIDARRFKTKL*
>SPBIB_v1_240020|ID:27163150|glnA| Glutamine synthetase [Uncultured spirochete bib]
MNDASSTFSLPDHFGSRCFSNDVMRERLPRSAFEAILEIQAGKRELTLDIAEIVAAAMKE
WASDLGATHYTHWFHPLSGLTAEKHESFVSPQPNGGMLMEFSGKELVKGEPDASSFASGG

LRATFEARGYTAWDVTSPAFLKRNADRYVLCIPTAFVSYAGHALDKKVPLLRSMEAINRA
GLRLLRLLGDTESERIITYAGPEQEYFLVNAELYRKRPDLLLCGRTVLGTMAAKGQELED
HYYGAIEEKAAAFMNELNSELWAMGISAKTQHNEVAPNQFEIACIYSQANIAADANQLVM
ETIRKVAEHHGMAALLHEKPFAGINGSGKHINWSIGTDKGANLLDPGEVANAPNEKDAAY
KFMRFVLFSLAVIEAVDRRAGLLRAATATAGNDRRLGGHEAPPAILSVYLGDPLHDLFDS
LETQNGAQKIANAFLELGIRSLPKLPKDFSDRNRTSPFAFTGNKFEFRMVGSSQSVATPL
TMLNAAVAEAIEHIALDIEQELSKGISILDAARLAARKSWQEHRRVVFNGNGYSAEWTRE
AEKRGLPMFRSAVDAIQELTKADNVRMLTSLGIFLAEETEARQVVYLERYSKQIKIEAGM
TIDLVRRSVIPATSEAASRYSAACTNIASLGAPSVIQETLAKRIAQLTGKAAEALDRLEK
TLNDASHIEDELERAKAFRDSVVPRTDEVREICDELERLTPKDLWPLPTYGELLFTL*
>SPBIB_v1_240021|ID:27163151| membrane protein of unknown function [Uncultured spirochete bib]
MKFLRSLVPWILVIVIILAALYLPDLGPENYKDFDSFFANERIRQGIAGYEVAIIKDGEI
AFNKAYGFDGERKTLESATPLYLGPASEILTGTLLCQLVNEKKITLDAPIAQFIPELASL
KARRASSAALDMPLTLRQLAAHRVAFPEKDLAAFDPGTTGLEAGLPDPELFLKSHFPTEK
YTRSRLSYRIVGAILEKATGKPYSDLLESMITIPLGMYLTTAKPSSIAQIAVGSGSFFGL
SFPYREKVPIDAAASDGIVTTSADIAKFLKFVVSPQRGQAIPGLSSSQTPLLYQPLYKDG
DTGFGWRILESKDGRFIFQGGSIRGYASRIVIYPERNAAIAILSSQNGILISNFILPMLV
TSAEQILFNGESRRPFPSWRAELVAGFIFFIYLLSIILQTLSSYSWARDLLKYRESSISQ
LYARFAFARTIGGLLLRVLAVVLAPFAAGYLVGRAVPYQELIAFEPGIASVLMTAMLFGV
IRNISRIILYMRLRRA*
>SPBIB_v1_240022|ID:27163152|livF| leucine/isoleucine/valine transporter subunit ; ATP-binding component of ABC superfamily [Uncultured spirochete bib]
MLEIQNLSVHYGGIHALQGIDLSVPEGKIVTLIGANGAGKSTTLRTIVGLVKPTGGKVLL
RDNVISGRQTQEIVQMGIVLVPEGRRIFPNLTVEENLLLGAYARNDKEGVLKDLDHVYDL
FPRLRERRLQKGGTLSGGEQQMLAVGRGLMSRPKLLMMDEPSLGLAPLIVKMIFEIIRQI
NAEGTTVLLVEQNAKAALEIADYGYVLETGRITLQGKGRELLSDDRVRKAYLGEAQ*
>SPBIB_v1_240023|ID:27163153|livG| leucine/isoleucine/valine transporter subunit ; ATP-binding component of ABC superfamily [Uncultured spirochete bib]
MSENTSDLMLSTAHLTMKFGGLTAVSDLNLEIRKNEIVGLIGPNGAGKTTAFNVITGMYY
PTEGKVLFKSRDITGLKPHSITALGIARTFQNIRLFKEMSVLENVLVACHMSINTGIFGA
TLHLPGYRSRESEARRFSMELLEKVGLVHLAYEKGTSLPYGQQRRLEIVRALATKPKILL
LDEPAAGMNPQESRELMDFIRKIRDEFDLTIFLIEHHMQVVMGVCEHMYVLDYGVTIAHG
DPASIQKNPKVIEAYLGVD*
>SPBIB_v1_240024|ID:27163154| ABC-type transporter, integral membrane subunit [Uncultured spirochete bib]
MKATTGKQPFALTRNKLLTIIALAAFAVLAIIANSFFDAFTLRVFNMCAIYIVLALSLNL
LNGFTGLFSLGHAGFMAVGAYVCALLTMTPELKEMNYFLVPIVPWLRNITIPFLPAILIA
GLASAFVGFLIGAPVLRLRDDYLAIATLGFGEIIRVLITNAQPITNGAQGLKGLPRYATT
FSVWVAAGLVTLFMVLLMRSSYGRAMKAVRDDELAAEAMGINVFRVKVTSFTVSSFLAGV
GGALLGHMITTIDPKMFTFMLTFNILLIVVLGGIGSISGSVISAIVVTILMEALRFLDEP
MNLIFFKTEGLPGLRMVVFSILLMVMVIYRQRGLMGNKEFSWDMLQGLFVKKGRKGGDHV
*
>SPBIB_v1_240025|ID:27163155|livH| leucine/isoleucine/valine transporter subunit ; membrane component of ABC superfamily [Uncultured spirochete bib]
MTLSLLFQHIVNALSLGSLYALIAIGYTMVYGILRLINFAHGDVFMLGAYVAFYGITLVS
LPWWAALLVSIAFTALFGIGLERVAYKPLRDSPRISIMISAIGASFLLESLAVLIFGGRP
KGVPVPDVLSKVIHFGSVSMITISLIIPLFTFALLGVLVWIVNKTKTGMAMRAVSTDIEA
ARLMAVDVNKVVSFTFGIGSLLAAFGGIMWSFKYPQLNPLMGMMPGLKCFIAAVIGGIGS
IGGAVMGGFLLGIIEIMIVAFLPTLSGYRDAFAFILLIVVLLVKPTGLLGKRQAEKV*
>SPBIB_v1_240026|ID:27163156| Extracellular ligand-binding receptor [Uncultured spirochete bib]
MKKAVGCMLLVLGIAALIVACGPAAPKTVKFGVFEPLTGANAGGGALEVEGIKLANEMYP
TVKVGDKEYKIELVIADNKSDKVEAANAAQRLVDKDKVNVVLGSWGSSLSMAAGPIVKNA
KVSAIGLSCTNPLVTKGNEYYFRVCFIDPFQGTVMANYAFKNLNAKKAVIIREVSNDYSV
GLAKFFADSFKQLTGDENAILAELNYNTGDTDFSAQLTEVKKFKPDVIFAPGNYTESALI
IKQAKELGIKTQFLGGDTWETPEFIDVGKQAVEGVVFSTFFATETPITDTSKVFLDAYRQ

KYNKEPAAVTALGFDGYLVARDAIQRAGSLDKEKVRAAIAVTKDFPGATGMITLNEDGDA
VKSAVIKTIKDGKFTYMATVQPY*
>SPBIB_v1_240027|ID:27163157| conserved protein of unknown function [Uncultured spirochete bib]
MERYDVVIVGTGPAGLGAAFELLGRKPSLRILLLDKLQVSSGGLRNDCKMNFTWPIGFPL
ECWDEATGTYYLKRVEAFLEPRIMEKRNIDVYARRAEKIGVKLIDVRQSHLGTDGGLELI
KALTARLTALGAEISLGEEMLSVDQATRTVQTDKRAVQYRYLLVAPGRGGFAFLQQLMEH
LGVEYRDNVVDIGIRVETREEHYPIVRDYYDPKFLFPKKTRTFCTNSRSAHVVQEKYGDE
KGGYWYSVNGHAWSEQRAANGLVNFAILKTVTLTQPLASGQEYAQMLGRLAALLGGGRPI
MQRIGDFRLGKRSFADEFTGDLYDFKPTLPSCTPGDISLCIPAKTMRAIWNAMKLLDTVV
PGVMHPSTIMYYPEIKLYANRPVFIDEYFQVVPGIFFAGDGAGTSRGITAAWASGLRAAD
GMLKEFDF*
>SPBIB_v1_240028|ID:27163158| conserved protein of unknown function [Uncultured spirochete bib]
MSEHFMIDKDKQQKLQNIILKLHQGASVAEVKKEFATLIQGVSAAEVAQMEQALIDNGMP
VEEIQHLCEVHVEVFKASLDKEKISSKVPGHPVQTMLDENKAARARLRALKKAVLAWRFG
LGQKEQVLAALDDMSKIIVHYTRKENQLFPYLEKKEFTGPSRVMWGKHDEIRALFKEAHS
KVEQNDKSAISSLHPLAGKISRMIFMEEHILIPEAVRRLSDEEWAHIRIGEDAIGFAWIR
PGAVYDPYLVLAGAVTAKPQPAAAIPESTAIPAVGATHHASPVPASDLVELATGKVPAGL
LSAALATMPVDISIVDAQDKVVYYSDSPDRIFPRSPAVIGRAVQNCHPQKSVATVNRILD
AFRKKEKTRARFWLEMGGRFILIEYRALYDAAGAYIGTLEFSQDLTELRTLQGQRRLLDW
D*
>SPBIB_v1_240029|ID:27163159| protein of unknown function [Uncultured spirochete bib]
MKAFDSADLAGISIGSSLDGFQKKLAGSGISFTDYVRESHIRKWTGEALAHALKIRTALV
LGLQEFLVSQNILNIDRVSMSPVTDPLCHNVEHAPVILFQNTPYRTTHSMIYSKMLACMN
PFVPGVYIDSPNIRLELPSETARHKYLIDFSQMDIELKRSRKLTPEDYFDRPEVTKAELE
QERDRALDFFEDLIIYAVKKILAFAPESLRALGVTLAVPQKPFPRFFKDEEEDHDASSLE
ERLGQKAGVQFFWVLGLLRENYDLVYPYLSRDGKRPPKASITSRQIFNYDLCAAPLYQDG
SLGRAYEVLSGGLREWVYEAIVARLLDNGILREEPQFDDHGNLLNMATLDGYGPFLTVAR
MCDEKGEGLFPETFGGGLGIERTLFALLHGPSVKDIDELTCFGKNPDKVLPFLF*
>SPBIB_v1_240030|ID:27163160| protein of unknown function [Uncultured spirochete bib]
MMNIFCNTIGTGVPGIGIAIYAPIEINAAKRHAIIAFVSLSFMLSIGRTMAKKYVFVNTA
QRGTA*
>SPBIB_v1_240031|ID:27163161|bioY| Biotin transporter BioY [Uncultured spirochete bib]
MESMNERLTNAIIACLFAALISIGAYIAIPIPGTPVPIVLQNMFIILAAFILGPWWGLAA
VVFYLVLGAVGMPVFSGGTGGLVKFAGPTGGYLVGYIPAVVAMGLLSRLGKSRWYFNMLA
GIAGMAIVYLFGVARLKAVLHVDWAKALATGLIPFIPGDIAKIAVAGILAPPILKALSQI
EQQSTNA*
>SPBIB_v1_240032|ID:27163162|cbiO| Cobalt import ATP-binding protein CbiO [Uncultured spirochete bib]
MPELFRADALRHVFPDGTAAIDNVSLSIAEGEFLIIAGRNGAGKSLLMRHFIGLSKPSSG
AVFYRGQPVPAQIPLIRREVGYVFQDTEAQIFGQTVEEDLAFGPANLGMQGQDLEDAVQH
ALKEAHLEGMENRRPNTLSGGEKRRLAIAGVLAMSPRCVILDEPFANLDFPSVQEILAVL
EVLRAEGKTLIVLTHEIEKVFALATRLVILDKGHIVFDAPPGEAQQSDFGSSGLVCPYQE
CYPWLMTH*
>SPBIB_v1_240033|ID:27163163| membrane protein of unknown function [Uncultured spirochete bib]
MADDTLNPFAFEAGKSWLAVFHPVAKLAFLIAVASAAMRSEPGLLAALFLIAFIGQFSMP
RAGKGALYSISVLILFSALVRGILPGDGRIFDVGTLSDSAIYALRLLTVYLYSRLFYATT
RVSEIGDWMTAFTRTMRRIVGVGQRDVSAQSSAAAAVNNRPTIHSNNIRQASILSDPGML
FSLVLLFLPRIFDTYQRIKEAGEVRAINLSRRNLRRSLAMLEQLIIASIVQAWRTAAME
IRAYSPARTLRLQKFAWGDWAMAGAAIALLFLAKL*
>SPBIB_v1_240034|ID:27163164| putative Adenylate/guanylate cyclase with Chase sensor [Uncultured spirochete bib]
MEHDRSSMPEFHSAMSRKARFFWALAVGLGIMLVFLWLGATEHLGMAIYDSQMRAHAAPT
KRSAMSSGGGAQSAEHGKIALVMIDQSSLDWVQKELGLGWPWPRELYGILAGYLRDAEAQ
AYDILFTEPSTFGPEDDAKCAQAMTEAGNVVLATLASKKPVLDVPNALSGHVAAIVDSDG
ICRKYQAWMSQDDRRLLSLGLAAVSKSSNPKELAASLPADGAVLLKFGSPARFERYSAAQ
VIAAAMDSVQGNAKGSATGEASPQPSPQIDFSGKVVVVGLSAPGLMDRQATPIDPALPGM

EIHATFIDNMFNHTFMQRTPIWLEMLVGALGAALMAFAPMVHKRLIAALAVIIAVGTPIA
LCLVLFGNLVFFNPISALVAAFAALVAAIGLGYQAEGRQKAYLRRAFAQYLSPEVISSLV
DQPETLQLGGELKTITTLFTDMVGFTSASEKLNPTQLAAFMNEYLGIISEEILAMGGTLD
KYVGDAVVAFWNAPLSVDDHAYRALATAVRIQARLGAASKELEQRYGIAPRTRIGVATGK
AIVGNLGSSRRFAYTAVGDSVNIASRLEAANKAIGTSILTMRDTVQHAIRNAEPSSSTEA
EIALGLPEGGAMIVRRLGLASVEGKEQPVELWSVEHSHGSVSVEPWDGVRRIPK*
>SPBIB_v1_240035|ID:27163165| putative Peptidase, M48 family [Uncultured spirochete bib]
VHVVSLRSRSLVAVLAGLVLALATLLFSSCTVSPEALQLFLNQALLQGMITPEQARLIRE
AARSFQTESRPFTYEEEYAIGRVVAAAVLSQYPVYNQPELTAYVNKIGQGLSFFSARPLL
PQGYHILILDSDEAHAFAAPGGFVLVTRGLLSLATSEDMLAALLAHEISHTALGHGLVSL
ASYRMGDLAMYYAALSTQAANTENKETAALFSAAVHDFVSLISVQGYSQAMEFEADAEAM
HILYEAGYRTSALKELVSALADTQNEHIQQYSMQHPLPADRLARIAVLEQQYEQAAELKA
RRAAARNTAGMFRLESQFSDIGYPASEGSVLEASINAFGPRPQVFGTVAPLELIRRERFE
AMKRLF*
>SPBIB_v1_240036|ID:27163166| conserved exported protein of unknown function [Uncultured spirochete bib]
MKHRFSILIVLVMVIAAVTYAQGKPAETTMLIRVEKTAVRATPSFASPVLLFAAYRTPVA
VVQIENGWVLGFVQGLSKPGYIHISALAPAKVSLSSDAASAPPPLQESEIVLAGKGFSST
LESALKEGNAFNFDAVDEMEKLTYSFAECLAFIQGIDFSPGEL*
>SPBIB_v1_240037|ID:27163167| Exonuclease I [Uncultured spirochete bib]
MPATMLWYDLETFGRDPQHDRIAQCALIRTNEALEEIADPIVLYCRLPPDYLPDPEACYI
HGITPQEAERKGISEYEFALRIMHEMAVPNTTVVGYNSIQFDDEFIRRLFYRNLLDPYVR
EWKNDNSRWDIINLMRAVHDLKHEGFEWPVNGDGNPSFRLEELAKANNIAHETAHDALFD
VRATIGLAKKVQHTYPRLYEWYYHHRRRENLAPLVNLSLREHMLVHTSQLYTRPNGCTTV
IAPLGLVEADRHALVAYDLRFKPEPFAQMSEDELRDLLFTERLEGATGPQRPPITMIRLN
SCPYLAPVKALTKDSSKRLGIDIEQCRQNLRELEAVPGLKERLIAAYRHEKKTSEELDPE
YALYGGGFYPDEDRKELERFHEVLREQGPHAAKNEFLRITYRDQRIPTLMGRLLARNFPE
TLTAEQHVSWRKHASGWVQLPLEKGATALADYAQLEAQIAQMREDDPRKPIARALLDWKA
HIEDFIRER*
>SPBIB_v1_240038|ID:27163168| conserved protein of unknown function [Uncultured spirochete bib]
MKRQELYVQLISELANYLLHGNPKKMVISLHMEADGFHIAAIDDRPRTDSEIAEIEHSLM
IQQRPELASYYGTMVGHDLLGTARLEMIGWQIKGVKLERIPEGGIMINLWIGSEGFDPKM
FTLEQGT*
>SPBIB_v1_240039|ID:27163169| conserved membrane protein of unknown function [Uncultured spirochete bib]
MVQGTKTLKVFAVTVMLVLLLAGIIDGDVGPAVQGFIRLQVTPARLVSDFVAVEGVGATL
FNVAAVGFLGYFFLLLNGLEITGAALAALFTMMGFAFFGKTLFNCIPIMAGVSLSALIVK
KKPRDYALIAMFGTAMGPLITFLAFEVGVKPVFALPGSFLIGLGIGIILPPIAIAMLRLH
QGYNLYNVGLTAGFVGLFTASLSHAAGADILPLSMWGTEREPLLIAIVPAIVLIAVVCIV
AENPGHGHSNIGQAGRDMLKIMAMSGRLPSDFSDFVSSKGALLNASIIGLLFWIFMVALG
APLNGPVLGGLFTLIGFAFFGKHPKNVFPVVLGIIAAIFVFGKSILAPGPLLAILFGTAL
APLAGEFGPVIGFVAGFLHLVIVDRTGAWHGGMDLYNNGFAAGLTATLIVSIIDWYKSSK
SD*
>SPBIB_v1_240040|ID:27163170| NAD-dependent aldehyde dehydrogenase [Uncultured spirochete bib]
MNNGIFHIPRPVNEPVFNYAPGSPERAALKQEIERLRKDRMDIPLIIGGKEVRTEKTAPI
RRPDAHNEVLGVYHIAGEEEARMAIKAALDAKAEWAALPWEERAAVFLRAAELISVKYRA
LMDAATMLCQSKTAHQAEIDAVCEVIDFLRFNPWYMEQIYNQQPDNAKGEWNRSAYRPLE
GFVYAVTPFNFTSIAANLPTAPAMMGNVVVWKPASNAVYSNYMLMKLYMEAGLPPGVINF
LPGSGGAISNVVLSSPDFAGFHFTGSTEVFRSLWKQISSNLEQYYVYPRIVGETGGKDFI
FIDPSAELANTVTAVVRGAFEYQGQKCSAASRLYIPASRSKEFIDALVAEISAIPMGEIT
DFRNFVGAVIDEAAFNTICGYIERARTSGEAKVIAGGACDKSRGWFIEPTLILTENPRSE
SMVNEIFGPVLTAYVYDDHDMEAAYRLVDSTSPYGLTGAIMATDRQRINRALLALQNAAG
NLYINDKPTGAVVGRQPFGGARASGTNDKAGSYLNLLRWVSAVAIKENFAPPSSWRYPFM
LGDD*
>SPBIB_v1_240041|ID:27163171| conserved protein of unknown function [Uncultured spirochete bib]
MKINEVVAVVTGGASGIGETVAKYFAAQGAKVVIGDVVQEQIDRVVGEIKAAGGKAVGVK

TDVTKDADVAALMDTAVTAFGAINVVVPCAGIIRDGVMINTDKETGKVKRVMETDQFRAV
IEVNLIGSFITLREAARRMVDNKWQGVLFTISSVNKVGQVGQINYSSTKAAVAIWPRILA
GEFQMKGIKGIRVMGIAPGYVGTPMVKGMNQDALNEILKDVHIGRLIEPEEIARAIAMVV
ENEAFDGTCLEITGGVTFGARAIAK*
>SPBIB_v1_240042|ID:27163172| Regulatory protein TetR [Uncultured spirochete bib]
MVEKPPLSKRKLQARETRKRILESALSLFREKGFDQVSIDEITSAAGVSKGSFYTYFQTK
SDIIIEEFRLIDDYYQKKESAIMRSPEAVSRLIAFTKYQLDYIHKNLGFRTLSILYINQM
SAFYDQKILANRERTLVRLVSKIIADGQASEHIRQGDPIELAEWMNRCMRGFFLDWAISK
GSLDIRKDGMRFFSEFVLPALIAHPARQS*
>SPBIB_v1_240043|ID:27163173| conserved protein of unknown function [Uncultured spirochete bib]
MAHYRNLVFKGGGVRGIAYLGALQYLYEHNYMQHVERVAGTSAGAITALATALNLGSFDE
LKRISDSLDFRKVPAEEDERSIRNNPPRLIALTDRYRELALFKNLQCSMRLVQEKGWYSS
DYFYKWLRALIAGQFAVEKEFYTFADFRDASLHRDGREFLNLYVTGTDITNRMARVFSFE
TTPEMEVALAVRISMSIPLFFEAIEYQYPGTDSPQLYADGGVMWNYPVNIFDEPRFGRLQ
ENGINQETLGFFIFTSPDRTHYKPVKNMIDYVSALFESLLLVQEHLTATGEKNFGRTVFI
DDCGVRPTDFDIDTRDDRYAALFESGYEATKDFFSAKTDWASFFRGLRARLGWKEIG*
>SPBIB_v1_240044|ID:27163174| putative Methyltransferase type 11 [Uncultured spirochete bib]
MREQRFAMSMLTVILLFSATFTARSQTPAMAPTASTWMPHADACKEHTMALYENMASVFD
MLFPLDRNCITALEMLVRSESGSPGSPMVLDLGAATGTLVAALNEKGWNAVGIELNPAMA
ARAKSPVIEGSMRDAVEIARRWFSAATDTRPAADAAFLDAVLCLGNTLPHLEPGERAPFF
SNIRSLLRPWAPFVIQILNYGRADIGPGFAFPEISAVNVRFSRRYAPGPAPGSLSFLTRL
RIECEIYEDTTVLYPISPSTLAALLGRAGFSHIDFYSGWSLQLFDEGKDMYCICIARL*
>SPBIB_v1_240045|ID:27163175| ATPase-like, ParA/MinD [Uncultured spirochete bib]
MSFVPEKKPNPSIKRVIGVVSGKGGVGKSMVASLLAESLASRGLRVGLLDADITGPSIPR
MLGIDSFRAESDGEHLYPIENEDGIKVLSINLFNEKEDEPVIWRGPLLAKAIDQFWSDTI
WGDLDYLIIDFPPGTSDVALTAFQTIPFSGIVVVATPQDYVSMIVRKSVNMASMLKTPVL
GVVENMRTMVCPHCGNEVALFDDGTQNGAQRMGLPLLASLPWRKELAQSRALRWSALSEA
ARKDADSLANEVELALASSTGAAATNTGASNVGTGASSSASPQT*
>SPBIB_v1_240046|ID:27163176| Dinitrogenase iron-molybdenum cofactor biosynthesis protein (modular protein) [Uncultured spirochete bib]
MDDDKLARELFAGADLVVLESDEAEALRLADLENLYQGKAAQLMGISRQTFGRILDRAHR
KVADALLNGKALKIEQARAPCLERPPFNEKFKSGGQTPFLNLEETSMKIAFVSDDGTTIS
QHFGRAAYYVVLTIENGKVIQKEKRSKLGHTQFSGLHEEHHEHGTAGAGHGFEPGAMERH
ASMAQAILDCKALIVGGMGAGAYQGIKQAGIEPIVTDLESIDEAAAAYIAGTLKNHIEYL
H*
>SPBIB_v1_240047|ID:27163177| Serine--glyoxylate aminotransferase protein [Uncultured spirochete bib]
MKGKRLLMIPGPIEFTDEVLAEMSLPTLSHVDPQFIEEFGQAIEKMRKVWLAPNGQPFAV
AGSGTLAMELAAANITEPGDNVLIVHTGYFSDRMADIFRLHGANVDIVPSEVGDLPSVEV
VRAALTIKKYKILSITHVDTSTGVRADVKNLAAAAHETGTLVVVDGVCAVAGEELRMEEW
GVDIALTASQKAVGVPPGLALLVASSRAIDAFKARKSPVRAYYCDWQYWLPVMEAYEARK
AAYFGTPPVNLVRALNVSLGQILSEGMDARFARHAKNARAFRAGLTAMGVKFVPAREEIY
ANTLTAVWYPEGIDASVLGYIAAEGAVLAGGLHPAIKTKYFRIGHMGMSDASEILATLGA
IERGFARAGYRFSPGSAVAAAQAVLAKG*
>SPBIB_v1_240048|ID:27163178| conserved protein of unknown function [Uncultured spirochete bib]
MYYEPMWNYFEPKDTRWYRWKLDGASIWMRKNGEEWRFTLVSIPFKVIQSDARGPEQADP
PADVSISLAVASGKKVALRPYPSPIPYLVSARNDVKIHPGTEAWFTIALPPIMRIELEAG
HALFEGSPFTTTSTWFGDKTSGNLCLSLPVELDPECKNEQGYAASSISRDEAIPADPLLQ
ESLQKAARYLACKSLIQCRIVVRNKSKEVLDLKRLAIFTDLMNVYEKDGMLISDTVVITG
TADGSLQTNIDDTSCKKLKKIYSAPKTGLNEVLIKRGVSFLRSITGL*
>SPBIB_v1_240049|ID:27163179| MscS Mechanosensitive ion channel [Uncultured spirochete bib]
MKSSILPDFFTRLYNEALTPAFWQKAIGIAFAAILILAFFRILQVIVSRTLRKTMPEPKA
QLIRKTIRYTGYVVAIASILQSMGINLSALLGAAGIAGIAIGFAAQTSVSNLISGLFLIS
EKSFQIGDVIQAGDITGIVMSIDLLSVKLQTFDNKFVRIPNETIIKTNVVNITRFPIRRL
DITVGVSYNSDLKKVTELLKDIAAKNMYALDNPEPLILIDKFDKSSINILLGVWFEQSKL

VDLKNSIIIDIHERFDKEGIEIPYSKMDIYIKEKP*
>SPBIB_v1_240050|ID:27163180| Glyoxalase/bleomycin resistance protein/dioxygenase [Uncultured spirochete bib]
MQFCWVTLNVNDMEKSLWFYRDIVGLPLNRTFSPAPGDQIAFLGSGETQVELIRNEKNAP
TPFSQNISLGFKVDSLEKTMELLKANGVPVHAGPFQPNPSVRFFYVLDPNGLRVQFVEDV
KQG*
>SPBIB_v1_240051|ID:27163181| Pseudouridine synthase (fragment) [Uncultured spirochete bib]
MPIPVMQHRKERRAPPVSKAPAGHLPAGRAQFLYEDSDLIVVDKPAGLPVIAPEGSRAKT
LYDIVTAHMQKTNPRGRAAVVHRLDRDTSGVMLFAKNARVKKALMDNWNKLVRRRCYTAL
VEGLMPSETGTLDSWLIENRAGQVYETKPGTRGALRAITNWKIIAAAEGAPYSLVELELE
TGRKHQIRAQLAAIGHPVAGDARYGARTDPASRLCLHAHLLEFEHPFTHKVLVFESPVPK
VFYQSENPGPHGRL*
>SPBIB_v1_240052|ID:27163182| Glucose 1-dehydrogenase [Uncultured spirochete bib]
MSTISDLFDFTGKTVLVTGASGGLGAGIARVFAAAGAAILIHFHSGRERAENLRDSLPGP
GRHECIQADGSSENEIGSCMEQVARLCGESGLSVLINNAGVYPSSALLDIDLATWHAVMD
TNLSSMHLFTREAAKIMRPGSAIVNIASIEGLKPVRAHAHYAVSKAAVIHYTMAAALELA
PLGIRVNSVSPGLVDRPGLVEDWPEGYKRYVSAAPLGRVGTPDEIGYSCLFLASKAADWI
TGVNLVVDGGASVVAPQG*
>SPBIB_v1_240053|ID:27163183| protein of unknown function [Uncultured spirochete bib]
MEERALWRHRASHGIHGMRLKSCEGFDELSAVWVAFKQFGPPYEREVLQGKRWRHGSAEQ
GPSAARRETRTLNNEGWNDGLWDFARPKI*
>SPBIB_v1_240054|ID:27163184| Purine nucleoside phosphorylase (fragment) [Uncultured spirochete bib]
MNEAVQKAINVIRPKLSSVPCLALVLGSGLGVLAEEGKHATTISYADIPYFPVSTAPSAI
LFFFMEVYFDVAGLSRRHHPYPGS*
>SPBIB_v1_240055|ID:27163185| Basic membrane lipoprotein [Uncultured spirochete bib]
MKRIFITALVLSLVFALGAGAQSAKPFVAMTTDANGLGDGSFNDGVWAGLKKAEAEGLAR
VKVVEAHAMTDYIPNLSGLAEDGAQLVFGVGFLMVEQIQEAAKMNPKTFYAGIDHFYDDP
IPPNLIGISYKEQEAGYLAGIVAGYMTNKYSKNSKLLNDKNVIGAVLGMDIPPVERYLAG
YIAGAKSVNPKVDVRYIVAGTFSDRAKGKEAALALADQGADIILQIAGLTGMGVIDAARE
RKFFALGADVDQNSAAPDYVLTSALKGTTMSAYITIKDLAAGKLKGGVNRVFGLAEGAID
IAPFHQFDSIVPAEVKNAVAKAKADIASGKVKVPASLAELGIKK*
>SPBIB_v1_240056|ID:27163186| Inner-membrane translocator [Uncultured spirochete bib]
MIDFFAALLTPQLFEATVRLATPVTFAALAAVVCERSGVTNIAMEGVMLVGAFFAAWVGW
ISGNPWLGLLGGIAASALFSLLFAWAVVSLHINHVVTGAIMNILAFGLTRYLMMVFYGRQ
GTSEAVAKTLHHYKFAIPLLSDIPYVGPAFFNQTPLVYLSIISVFAWAWIFKRTRLGLSL
IASGEHPMALETLGRSVYRVRYLAILISGVLCGFAGAFLSIENSNSFTEGMTNGRGFIAL
AANIAGGWRPVGVFIASLFFGFVDALQMRVQSLGIVKIPSEIFIVFPYVATIVAIAGLVR
KSRPPAAVGKDFNIEGDGE*
>SPBIB_v1_240057|ID:27163187| Inner-membrane translocator [Uncultured spirochete bib]
VSVSAPRRIRMVLKALSLILAVPGVLLYLASYFWGAWLLLCAIALYAADRFLAVGSAVFG
SEARKLLSRAIVPVAGIGSALLIGAVIMAITGYNPISSYGALFYGGLVRNWHISVLNATP
LIFTGLAIAFAFQGGFFNIGAEGQYYIGVIAATWMGLGSTLPGIIAIPAIFAVAAIAGAA
LNAIPIILKVKTGAHEVVTTMMFAYVVRTLSPMFIRAHGGDPALTSHPYTTDLISESVWL
PMFKDFLPGANYRLHIGVLLAIGAAFLVRFILMKTDLGYKIRAVGHNPVAAMTQGISVAR
ITAASLFISGSLAAIAGATQVLGLDHRMFQDLNAGYGWNGISIALLARNNPIGIIFTSLL
WGVLDAGGQYMARTTQTPNAIIEIVKGIILFLMLAEVIYKRAGSVFGRWAGRFKTAARKG
AGA*
>SPBIB_v1_240058|ID:27163188|yufO| Uncharacterized ABC transporter ATP-binding protein YufO [Uncultured spirochete bib]
MPKSDESPLVLETRGITKRFDHIIANNNISIKLRKGEIIAVLGENGAGKSTLMNILFGLY
KPTSGSILIDGLPVSFDSPRDAIAKGLGMVHQHFMLVPTLTVTQNIVLGKEPVRLGHVLY
KRARRSVVELSKRYGLTVNPDARLEDLSVGLQQRVEILKALYRNARVLILDEPTAVLTPG
EVEELFAVLRNLAADGTSIIIITHKLEEVKALSDRVYILRRGELVGECRTASASAAELAN
FMVGRDVVLAVERPAPRPKPEPIFSLESLFVHSSRGLPALKGVDLAVGRGEILGIAGVEG
NGQKELCEVIAGLITPDSGKILFKGEDVSRWSVRRRMDAGMGYVPQDRRVSGLVLPFSVE

ENLALGRSDRPPLSRHGFMKFAALRAQALQLMENYDIRTSGPEARVSTLSGGNQQKIILA
REFSRNPDFLLISQPTRGLDVGAIEYVYRRILELKERGVAILLISMELEELFALADRIAV
LHGGQVVFESPTATTNEAQIGEYMIRGRYERSIS*
>SPBIB_v1_240059|ID:27163189|mtnA| Methylthioribose-1-phosphate isomerase 1 [Uncultured spirochete bib]
MNDSGSISASSVPRSLRWEDEEGGELYILDQTKLPGEVVEERQDSAEQVWESIRQLKVRG
APAIGIAGAYGLVIGLKPLVDLDLREFLTQAERIAAYLNSSRPTAVNLSWALNRMLAFAR
SNAEKAGTSAVLYKLLVGEAKLIHAEDRAICRGIGESGEVLLNEGCGVLTHCNAGALATS
ELGTATAPMYLAFSHGKRFRVYADETRPLLQGARLTSWELKRAGLDVTLICDDMAAYVMS
KGLVNLCIVGCDRVAANGDTANKIGTLGVAILAKHFSIPFYVACPSSTFDFATATGADIH
IEERSAEEVTSFGARETAPKEIKVMNPAFDVTPHELISGFITEKGIVRPDYRKNLERAFR
*
>SPBIB_v1_240060|ID:27163190| Transcriptional regulator [Uncultured spirochete bib]
MLAIERRNRILEKLRNQGIITVAEMAAEFSVSEETIRRDLYKMEVTDGVQRTYGGAYIAK
AVRTDIPISIRENIYLPGKETIADLCATLVDEGDTVMLDSSTTSIHIADHIKTKRNIVVI
TNSLKIVDTFANSADVKVICAGGTLRHSQLSFVGPAAERTLEAYYADKAFVSCVGLDMEK
GATDADELEAEVRKLMLNNAREKILVADATKFGKFSLSLIYPLDGINVLVTDRQPDAVWL
RELTAKKIECLYGTGNPE*
>SPBIB_v1_240061|ID:27163191| Class II aldolase/adducin family protein (fragment) [Uncultured spirochete bib]
MLNGMSGSIKEDICEIGRRMYASGFVAANDGNISVRVSDNQFVVTPTLVSKGFMVPNMLI
TVDLDGHVVEGSFKPSSELKMHLAVYRARPDVGAVVHAHPPAATGFAVAGKALDRPYMPE
LFVNLGVVPLAPYATPSTEEVPRSIEGLIAEHNAVLLANHGVLAWGRDLAEAYSRLETIE
LYAKILISSFAVGEPQPIPDNKLRALAQIREALGVKGDISTAM*
>SPBIB_v1_240062|ID:27163192| protein of unknown function [Uncultured spirochete bib]
VWVAGLQENAVASWLSAQAAARFTLTTARSLCYFIRAPYEHVGHFGQ*
>SPBIB_v1_240063|ID:27163193| conserved protein of unknown function [Uncultured spirochete bib]
MYTVTKIVYSESAPDPEIACFLEDIYQECNRRERLELDPLAIVRRYSEPADIEVAGLVCS
TLAFGSVELILRACEKALVPLGEHPAVALAGMRPEEISQAWGSFQYRFCFPKDMIALMTA
IQRVLIDYGSLEVLFSKGDDGAGDRDGGGMSAAPSILSAASAFVGHLRQFASEAVPGGLR
QNLLPDPARGSACKRLFLFLRWMVRHDEIDSGCWKSVDPARLIVPMDTHMVKTCRERLGF
LPAASGAQVAPGGSRKGGLKTSGPTLADALRVTAAFRLYAPDDPVKYDFALTRPGIDPRP
GDERFGCL*
>SPBIB_v1_240064|ID:27163194| exported protein of unknown function [Uncultured spirochete bib]
MKFRFRFSIAIRRYFRAWIIIVVFMASGTPLITEAQQALPKIDTEQLKKISGESAEIEGL
IIRATPQSITDARARIETSPYLSAPDKAALGAIASGVGLILYGPKDSRFEIPVAAQGASQ
KYITYLVGLVDVFAGKNPQIFASEPCSTMTELISSLSYFRSAKSEVREKVNGALSRFDAL
GGVSVVPMLIRGQTALDSKEYSIAEKEFSSALLLDTKSEKSACGFARASLALGKPQAAKN
ALDPIAAGIITSSTSAEFKALYGLTLYNLNNVLDAEAWLASALNDDPGRTEVLVPLAQAA
IQRRDYSAASRYLESAAKAFSQDRTWLVLKSQYALENSRQADAERFARSAVRYFPKDPVA
LSQLIQALEKSTDEARHAEAADLAKAVLDLAMNEDTALVPLEEARRAQAKNQALQFLVSE
SYNHQDWARAAAYLQAAGSLPLDKNMVATILRKSGNVQASIQFASSWYAQEPGSEKAVEA
YLRSLAMATGGGLASVAPASDVHTGLGIALSALGVGKEGSANSALLDLVLKFIAAPYSKE
LKSFLYYMSASLQSDENKAIDQLKNALAERADNVEALVSLADIYLARYNRQFDKRDTTNR
DRAMRYLGQAKALSPTDKDLLARISQLEAQLK*
>SPBIB_v1_240065|ID:27163195| conserved exported protein of unknown function [Uncultured spirochete bib]
MKSANKGRGVVFVFTSLCVALFLCFALFLSSCSNDLLAPLGIGSNDFSISNKAQAKDLRD
ALEILHSPRLKKSGYYPVQRMLAARRISTILIEDGQQARAARFLSSLAEEHDAYEAWYLF
AAGAVYESMGSAPIARILYERIIETVPDMTIDGQSIHRTCLVKLIDSDNPPEKKTAWFKE
FIQRFPDADEIGPFHFLLAKEYEALGQWTQAIEEYRRFLPYFGVSVPGYSDAHDYARKMV
EFNDSPKDWTYADLGELVAGIRKAIAARSPQTLRKYMSKAGFFAMSWYKEGGEDSNSNVL
FNFSNFMTKGPIYVAPALDPSFNDSEAFLRTSGWTGYIPVWYLCFRKVNFPADPKVHGNW
EWAGIYFGEKMQ*
>SPBIB_v1_240066|ID:27163196| putative Gamma-D-glutamyl-meso-diaminopimelate peptidase [Uncultured spirochete bib]
MIRKCCLCMLASLCIAVPLISATEAEGAFHISAVSTEGSSSALSETPSSSSAAHTPLAPA

PTSQAAPDSASIVPPEAPEELPSSPLEESQEPIDLSQLVPEELSLEEHAKSLQYDIPMPW
GHELFEKYRQYYLSDRGRKILLSTMEKAAPFMDYIESKVAEYGAPQELAFLPVIESAFSP
FAVSRSGATGIWQFMKNSINGYGLTITEWVDERRDFMKSTDAAIKKLIDNYNDLKDWPLA
IAAYNAGLGALRRAVNAAGGDTDFWNIYDKGLVSKQALDYVPKFLAVASILRYPDLYGLA
LPPRQLKWEAIPLDRQVDLKLLASKAEIPLDTLKLGNAELHYTITPPDDSHLLKVPAEKS
DQVKAILSDPNAPLVRYDIYKVKQGDTLTAISRHYGVSISMIAKVNPGLNPDRLKIGQTL
MIPLSLQPLNPSMSSNASSGSASAQSTSHTVQKGDTLYGIARRYGTTVQKIASLNGISVN
SVLRIGQKLKVPTQP*
>SPBIB_v1_240067|ID:27163197| Acetylornithine deacetylase / succinyl-diaminopimelate desuccinylase [Uncultured spirochete bib]
MTELAQISKFLDLQEKGIIELESLLTAYPALAPESGGIGEIEKAAALEKWLKAEGIQNIQ
HYDAPDERVPSKVRPNLVATIPGKRKDSPVWIMAHLDVVPEGERSLWDSDPWKVVVKDGK
VYGRGVEDNQQGLVSSVFAALAFVKLGIVPERTIKLLFIADEEVGSKFGIQYLLEHHDLF
SKDDIIIIPDGGSPDATEIEVAEKNICWLKVTTKGKQTHAAMPDKGANAFLAGCDLALRI
HRLEKTAFTARDALFSPDRSTISPTKKEANVPNINTIPGEDVFYVDMRLLPVYPVSMALE
EISRLSHAIEAEYGVKVSIDVVQSNESRATPATAPMVEMLARAARQVYGVEAKPIGIGGG
TVGAYLRKAGYDCVVWSKMNETAHQPNENADIANIIGDAKVFATLAMME*
>SPBIB_v1_240068|ID:27163198|galE| UDP-glucose 4-epimerase [Uncultured spirochete bib]
MNVLVIGGAGYIGSHVARYFADRGLKIDVLDNLSTGTKDNLFPDYSFFEEDILDYKAVAA
IMRRGYDAVVHLAAAKAAGESMLKPEKYAVQNLTGTINILNAACEAGIKKFVFSSSAAIY
GEPKYLPIDEAHPKEPANFYGFTKLEIERMLEWYDRLKGLKFAALRYFNAAGYDPEGRVR
GLEKNPANLVPVIMEVAAGLRPHLDVFGTDYPTPDGSGVRDYIHVTDLADAHYRALLQLD
AHAESFAVNLGSERGLSVLEILEAARRITGRPIPAHITGRRPGDPATLVASSAAARKLLG
WQAKFSDMETIIATSWRAYAQHHSHSIH*
>SPBIB_v1_240069|ID:27163199| protein of unknown function [Uncultured spirochete bib]
LSLTTKALISERCAECQALSSVTNLAYEFEVKASFLASIRRGGFHASFSFLPKPGX
>SPBIB_v1_250001|ID:27163200| conserved exported protein of unknown function [Uncultured spirochete bib]
MHRFGLHCFTLKYRKTFLATFLFLLGMSAFAFTFMPMSISISPSGAQSIASFRLTNDGGQ
QIAIVIKAMTRDIDENGNEINEPADKDFTIFPTRVVVQPNSFQNIKVQYKGTPKLAKEAA
YRIIAEQVPIDFTQQQTSGVKVLFRYIAALYVTPTNVAHKLSVSKVEYAEQEGKKGFLVT
ITNSGTRHALINDPVLKIAGGTGISITLKDDAVKAIQGQNLLAGNVRRFFVPSDDAKPNT
TYTGTLSATIE*
>SPBIB_v1_250002|ID:27163201| exported protein of unknown function [Uncultured spirochete bib]
VFFRKLMSVILLFFVMGISISFAQTPPSQPAISNAPITHDTIVPDLEITIALSINGKPAG
EVNAGIWQNQAVLYKKLAAQLLIEHLRPDIYSVIFDSVFRDLTWLTADDFALVGISFEFD
SAQLTISITIPPEYAPVVDIDFVPEQVPNYKPILRAAPFSGFIQNESALQISNVTSNTLN
YSTQLLAMIDLMGTHLVGSGNISVSNAALSYSVDNAYALWSDFARKLQISLGKISIPVVG
AQTQSTLYGISIGTSDFEKYIVRQGFIDDMTEFTIHKLARVSVEVNGKPIRTMLLAPGNY
RILDLPFTSGLNEFVLRIEESDGNVQVLRRIIPRESNILQVGTSKFALSAGTGIQDWKEL
FASGYWLFGISPAFSGGFNIQADIRSAMGGLTWVSALPIGTINGSASLVGRWDGWGEMFA
PAASANYVFSMPGNSSIPSLGLSFSYRGKGFMPPATSAPDSAPPSSLLSFSASLYSAIFS
RTNASIGYSLNRILSSPTAMTHEIYASISQSFIKGGNLSLSGRLSFPSSGSPTFSAILTF
SIMPRESYARSLNFMQTTDGTTSLGILDKFSAFGQVFDFNINTSSFLPGSGESSVFNIGV
RNTSEFYDVSAAGSFAHDSSLNTDTLTGNLQFRTTLAFVGSHVAFTRQIPDSFILIATSP
SLKKESALYRLSSGAQYFAAKGQNIILPLTSYTTTVLSVDLVESPINLNPRYPFVLVSPA
FRSGILFQSDVVKRYMLYGRLVDTTGKPVEYLPGDIYDLGGSMVTSTFSDEMGHFEIYDI
LPGTYTIEWPEGYGTTQFELSESGEDTIDLGDVVVHSRGK*
>SPBIB_v1_250003|ID:27163202| exported protein of unknown function [Uncultured spirochete bib]
MSRKLLLLAVIYVGIVMAAGSQTLSLNPITAPATVTYDPDSSSDTTGPQFSITVRLSKKP
RYTFYFFVTINGSPTVNSRDLVLTDNSALSILSKFYKDSAFTQEILGGNGSGMTSTQVLY
GQFNKNTSVLTQTFTVYPWIGKNQQVPYGTYAGTFTARLYQGTVGVAGTLRDSENFTYTA
IVNQKVDVRIGPATGSYDTGSAIYNINLGEVSSGASGTFAIFLKANTAYTLKMSAASGGY
LTSTTTSDKIQYMLTIDGASKTLGPDIIIDQETAKQLYSKVLLGAISVAAGQDVEAGQYS
DTISFTISAN*

>SPBIB_v1_250004|ID:27163203|malQ| 4-alpha-glucanotransferase [Uncultured spirochete bib]
MQIPRSCGILLHPLSLGGPWPCGTMGEEARAFVDFCAHAGIGWWQVLPLGPTAYGDSPYQ
SPSAFAGNPYLIDPKALMDEGLLAPEEVEGYLTGFNGAESSSRASGVSTGSSTNYIDYAL
LFRENARLIEAAWRRFGSREGGARLAKFQQFGNAQASWLDDYALFAAIKEHEGHRPWNEW
PEAFRCREPDAIARFVSEQAERIGRIKFTQYIFWTQWQKLRQYAHERCVRIIGDIPIFAA
YDSADVWAHPELFQLDAEGMPTHVAGVPPDYFTATGQLWGNPLYDWTAHAKTGYAWWKAR
VQHTLSLVDVLRIDHFRGFEAYWAVPAGEPTAEHGRWEKGPGHALFNALKEALGELPIIA
EDLGFITTEVIELRDSLGFPGMRILQFAFESQTDNNDYPHNYPQHCIAYTGTHDNDTAMG
WIEHAAPQARKRALAYLHGGRRTFSWDLIRAAWSSPAMLAVAPMQDFLGLGSEARMNYPG
TTGGWWRWRLPEGALSPALARKIKRLSKIYFRI*
>SPBIB_v1_250005|ID:27163204| protein of unknown function [Uncultured spirochete bib]
LYSFSRSRLVGKRTCPAIARGPLAQLAEQRPFKAWVPSSILGRLISNIFYYKQLAAFARA
AYLFFGDMRIFYEKGSVRKAKKVKKRGQTPI*
>SPBIB_v1_250006|ID:27163205| exported protein of unknown function [Uncultured spirochete bib]
VEYRVNRAVAIMALAMLSSFSLAAQTVVPGLLPKDARSMGMGGSFKVFAEGYSSLWGNPA
GLAARGSLTVADSATWAYVQPTPLNIKNIIRVLGQQATHDESVATLDGLINENDFGMGES
LGFGWTGNGLGLGLTSIVDALVTGDALAGSKLAITSQTNAVLGMAWPVNLGPFGIRIGAS
ARGFYRIETLPGGWPFDSLAEAALNRQDLYSIIKVYNVQGGFGIAIDAGATFSIGPFSIG
LMMRDYGDKFAMNESTIEDIANSFMVPSGGIDTYVFKPVYTAGLALKLYQDFLFSPSFYV
EADDPLALFPLVTRDIDAMFSRLHIGVDLKFLKFISLRAGFNQGLFSFGAGLDLIILEVD
AALFSEPISAAGGRIGRTGIALQGAIRF*
>SPBIB_v1_250007|ID:27163206| protein of unknown function [Uncultured spirochete bib]
LLTDQWYNSKVERIVILEQQAAGAMGQRLVVCFSMLFFAFVQLFHPIL*
>SPBIB_v1_250008|ID:27163207| conserved exported protein of unknown function [Uncultured spirochete bib]
MKRKAMIFILLIMAVLAATAQTTLQFTSPMPKDARSMGMGGAFRVFSEGYSSFFGNPAGF
AGMSSLTLIDMSTWAYLAPTSENLGRVQSIISGSASNSDILSWAGDWLAKNNGLGAGASI
GAGWVGAKGISLGVNLVTDELAYGNSLLGAKLLSTTQANGVLGLAYPIHLGPVWFKIGVD
GRVFYRVQSKPTTGWGFNTILTDFLNDTFSASSLTLLGGYGFAADAGIILGLGPVMLGFT
ARDFGMEFKVGDFTAQDVIDKQFSALPLSGTTTAALTPSYTAGLGIRLFENGMFEPSVYA
EVDNPISLVSSTDILSDVLNSLHAGAQLRMLRFVTVRGGLNKGWYSLGAGIDLAFVEIDA
AIFTEELGLYAGDKGRSGISVQAAIRFGR*
>SPBIB_v1_250009|ID:27163208| exported protein of unknown function [Uncultured spirochete bib]
MRSKYVAYAALLAIIVLFFTGCDALITNVFKDANLGQPSAATIQNQDAATLIQQAGITSG
AVSDTFIQTVISDETTKNQVLATLQGTVDTGTPAEAQAAQALILDIKLADIGADAVMDNL
NAAVGQLATLANSNEQLNPQDIINALLPAELANNPANLASFIDNLAGLGTDVDTLAQKID
ANNGTVADGLDIATIAQTAALVKFVEIVEPASEYSTGEAIANAVEDLKNDSNADVTKYFA
AQPDIASLADNTTLQTLFDAAGLSALLDKMKSGS*
>SPBIB_v1_250010|ID:27163209|mtaB| Threonylcarbamoyladenosine tRNA methylthiotransferase MtaB [Uncultured spirochete bib]
MTLLRVAFQTLGCKLNQLETESVADRFLATGAKIVPFHEEADLYVVNTCTVTSKAEQKAR
HDIRAALAKVPHAVVVATGCYAQMEPGALAALGERVIVIPGDLKASLLALAEWLSENWQG
HGELLHAVQEWQEEIQSAAGVAAAGTPLAAGTSLGALVGAQSAPVNASSVPGTSLGALPT
DRFAFNPERFAMHSRPALKIQDGCNNRCAYCRVCLARGPSVSLPAQEALARVRKLEQAGK
AEVVLTGVNLAQYRDSALGLDFSGLLAFLIQGTERINFRISSYEPERIDDAFLAVFANTR
VRPHMHLSVQSGCTETLRRMARPYSAERVRRAVLDLRASRHDPFLAADIIAGFPGETDAE
FEQTFSLLSELDFAWIHAFPFSPRPGTRAADMKPRIPERIAGERIARLIALAHSGKQSYI
ERWIGREVEMVVEHEGFGQDDAEQFDQALTRRNADTHRGLPKVGQPEADSSSVIAALSNL
RIIGTTENYLKAEAVQKGSGRQSGIPAEAFKTDRKALEALGLRPGDTVRILIQNVLADNN
AEVSAFFVDTWTS*
>SPBIB_v1_250011|ID:27163210| putative TPR protein [Uncultured spirochete bib]
MMHKRLKQAHILVFCVLLLLGGDIGYVFSQALPSSIAAPYGMTGSDLLRMSQEGKHNEVA
LLAPEVLRKNPQDLDAYIAYAWSMNALKEYTKARDIAQGGYAKFNDARLAQALGEALYYL
GENAAALSNFQEYLAKFPEGNKVASTYYLCGELFVRMGKFNHADIAFSTALQYNPSNARW
WERLGWVREKTSRYLAALKAYEQAISLNPSLQDAQSGRMRMLAKIKD*

>SPBIB_v1_250012|ID:27163211| conserved protein of unknown function [Uncultured spirochete bib]
MSEEEVKLSVVDEEEVDTVIGSEIEFDGEIESSQSLMIKGKISGSIRCKTELYITEQAEV
NATIHAATVVVRGKVRGDVSADSLIAVLDWGHVEGDLSAPDIYLSPDCFFNGKTVFTS*
>SPBIB_v1_250013|ID:27163212|rsmA| Ribosomal RNA small subunit methyltransferase A [Uncultured spirochete bib]
MTINFDSPVSIRTFLEAQGLAVSKKFGQNFLVDRGMRERIIEALDVQPGMSVWEIGPGIG
SMTEIILQKGADLTTFEIDYGFVRVLEEMFGSDSHFSIVKGDMLKTWRSVSERPDRIFGN
LPYSAAFAIIADLLEQDCVPTRMVFTLQKEAARRMAARPGTKDYSSLSVLCASVCDVRIL
FDIGASAFWPQPRVTSSVVLLVPKKDPVAPEKRKDFSDFVRAAFSSRRKTLRNALHVWAH
THIPSMNETEFESLLAACLAKIGARPDVRAEALPPRQLWDLYDLLASASNSASTGTGACT
STSTNASGSPA*
>SPBIB_v1_250014|ID:27163213| membrane protein of unknown function [Uncultured spirochete bib]
MRVEPACIAAIGLWLGFLVGGSGLLDLVLAWVGAAFAIAMECWPRKTRGMPRTLMIAGAL
CFGLAMSAGVLRYQAQQGSMAQQISTMQQKSTAQAALLEGGLHLEAIEGVLVGDTQVTSK
ENRRFDVSVQAAEFGAPGIKVRCEWKRAVFLKRPAFSLALITGAGERMLAGTTIRAERLK
GGDFVWVDERDLKVSGTLPLLARLRAAIAARFASALFAVAGRAGPLTQALLLGVKDELDT
EFKGLFQSAGCAHMLALSGQHLSIICTLAALAAKRVLHREKLARRASFVFAWLFVWLAGP
GPSLLRAVFMLTAAEVGRMLDRPQSSFALLSLASLMLALFDPSSITSLSSIYSFAAMAGL
VLFAGRFDYALRPYMPARISQAFAASCAAVCGTAPVSILVFGTFVPAGILAATIAAPVML
VFMWLGLGAGLIGVFLPIVASVSAPLLGTLQDVLLAILSFGAWLPALQTGTGMVDRALFC
AGIVSIWVLIYAVPMLQWKKSRAQMQRMRLILPSISPWGGLSL*
>SPBIB_v1_250015|ID:27163214| membrane protein of unknown function [Uncultured spirochete bib]
MADLSLKRFSGYIIMLLAYITAWADILLAIPEPPAFSALWFISVLLKNVIRIAFVLYIAS
QLTLFAKSKWKEALVQAPSRHDARQALVVMFISLGCAGVGVLVSWLSGIANPLFIAHNRL
PLLVLFPLILLSSLSVGYAEELFFRFFMIDGLMEAGAPVNAAAVVSILIFALSHHAQGIF
GIGFAAVLAAFYTSLRFKGFGLHSLALGHALYDGIILLIVLA*
>SPBIB_v1_250016|ID:27163215| protein of unknown function [Uncultured spirochete bib]
MKPEVRAINSLLDSLSALAGRAWTEKDLSLAIDKWLPMLPRSTREALTGLAVEKLKTDPA
RMPLAIATFAGVLLKEYDGTPLTLAEWRELRDVISDCADELDMDNVTYAMSLIMDYGAL*
>SPBIB_v1_250017|ID:27163216| Methyltransferase type 11 [Uncultured spirochete bib]
VKRSVKKPVKKHEWFEDEGFWDAFAPFMFDETRWALADPEVEHILALTGIQPPAKVLDLC
CGVGRHSLAFARRGFQVTGVDITASYLEAARESAEAEKLAIEFAQADARQFVRPGEFALC
VNLGASFGYFSDAREDFGLLKRCRENLAPGGALVLETIGKETAARDFVEEEKIARDGWEA
SARYSIIGEWEKLGNFWKAQKGALVFERSFAIRLYSGTELRALLRKAGFTHIEIYGDLDG
RAYDQHATMLVAVAKLR*
>SPBIB_v1_250018|ID:27163217| exported protein of unknown function [Uncultured spirochete bib]
MKCFSAMLLAFVVCVAPFPLAALDPESSPGSSPGSSVLQVPQASETPEAPSQLPPQMLPI
LTEPADPSGFFFAAEQMRMQAGDAGLAPEQARLAEFMYTLAFRFAKGPAALEAGRSLFLL
LSTQGRLAEAASTAREWLQSFGPDWTMYRNLYDTLMAQGSFPDAFALVSELSKAMPSTAK
SRSTELSWMEYNARFGMQDYSWAANGPPFIKTRTPDSYIAKIYRLYAAVPNVPQNQAALA
LFRAAATEKNYADAIAYARPILPTFSGSDTPRAWISELGKSFYGAGLYREGIDFFLAALG
FALPAPSPAPSQSSLEAPLSAPVPERTPFMNSAPIIDRAHVSTELSWVMAFYLARMYQGL
GENQTAASVFIDLVPFAFSSEDSDSALWYWLDITMNAIAATDSTLGDLGQDDANGIQAKR
SLELSALSQAAALWKSPSYFEDIVANYMRRLLREGAWNDVVRLCVLMSQKLTASMKGPLL
YLSGRLIETGRASADLSTESDFWKWLAPTNQNQSSPNAATNAVSQNPPDSGSNSQIPSAP
SAPLALNTSPLFFQSLLRENGIEEHYRTLAAWRLGKEPPILADTPVLDKNFDIVSAITSQ
LPQSDTPPVSDEKLKEVLDFITQSLDYGLEALAAKQVAVLSPFSTDSLLWLAAKFTEHEQ
YYPALRIGSEALTRKPAAKPELAYALLYPRAWPEHFSELTAPRNIAEPLAYAIVRSESMF
NQRAVSRSGAMGLSQLLPGTAAETARGLKMSQYALFDPKDNLTIGLTYYGYMLQRFDGRP
ARAIAAYNAGPSRMAQWARDWGNLEDDILIELYPVAEPRQYTKNIIAAALNYGKMYYGIS
SKDMLDFMLAGKALPQPASVQPAQNAATSAPASAPTAQSLSEPAPAPAAPAPDTSQPTLA
PAP*
>SPBIB_v1_250019|ID:27163218|sua| Threonylcarbamoyl-AMP synthase [Uncultured spirochete bib]
MEILSTSEADLAKAARALREGKLVAIPTETVYGLGANAYDERAVARVFEAKARPTFDPLI

VHIARIEDLNEVAREVPPSARALANALWPGPLTMILPKKPEIPDIVTAGLPTVAVRFPKH
AIAQRIIALAGVPVAAPSANPFGYISPTTAKHVIAMLRDKVDFIVDGGPCDVGVESTVID
MTGTSPIILRPGGMALESIEAIIGNVSIQAQPGRSPGTTNTLDTAGVPEKGLPSPGQTLS
HYAPSTPLYLFDADSLPRSAKESEIPHPSVALVFDSIRAKELESYHIFEQVVALAPSGDM
REAAARLFSLLHEFDARGYEAIFAERVPEAGLGRAINDRLYRASKK*
>SPBIB_v1_250020|ID:27163219| B3/4 domain protein [Uncultured spirochete bib]
METKLIFAQDTGLKKLRLALIELEGLHWERAAEVAPEALFEIILEQARASGTEARVSQPQ
SPARDSRLTESRTRAVRDMLRNGSYKPAGRAKPSSEYLLAAALDGDFPRVNPFVDAVNLA
SLKYLYPMSIFDADKAGGALVCRLGAAGESYVFNSGGQIIDLEDLLCVCAKAPAVESGIS
RRAAAPEAGAGGIPIVNPVRDSMATKLFECAHNAIAIIYAPSGAEGADLEQAAADIAAWC
GRACDRTAIRLF*
>SPBIB_v1_250021|ID:27163220| protein of unknown function [Uncultured spirochete bib]
MNVAIVCEKVNGRAQMAGQARFYEKHKLGSTYGETPVCCYNIMPWKPN*
>SPBIB_v1_250022|ID:27163221| protein of unknown function [Uncultured spirochete bib]
LKACGIVESVVLAISSCHKLLIQYLKDFWTYETSPYVREKHLNGQCIGRRYVLESLHRGS
SRTPKYESVCRCVHGPLL*
>SPBIB_v1_250023|ID:27163222| exported protein of unknown function [Uncultured spirochete bib]
MRRLIAIRLAAFFSLIAVIALPLCAQQYEALDRIALVIGNSNYEGTAKLKNPVNDAQDIG
ATLSSLGFDAEVLTDADLYSMEDSVLRFRDKLAKSPNSVGFFYYAGHGVQSGGENYLIPV
DARLNSESMLRTRAVPLQFVLDSLSEAHNKLNIIVLDACRDNPFSWARSSARGLAVVGQL
PPASIVVYSTSAGRVAQDGTGRNGMFTEELLKHLPTPGLDITEVLRRTGEGVQAKTDGAQ
IPAIYSQFFGFLRLAGEGEGGGLAQGSAAQSATYGSAQGGTQEALGDASLDYLPAFFDDA
PRNTKLAIAKAEELTWDEKWLSAWRLLEKTDPNDRDPYILAEKIRTALDGNSYTDNYRGF
SFYDTPPDGDVDKARADGTIDEEYFDFDPHAAVQVLLDRGVEIPPVLALALGDFYYNVYN
YYPYYPDDWSLTSDEILADGLRWFDLANENYIVVDTTSVIHYAELLMKAGRNTDAVAILS
DQVEWEPDDRYLRQTFVDALVRAVQIDEALGQLDMLIANTGSKEEALDYYKQAAQIAFDN
QKSAQLEHYLAALEKDYPDDWFGPAMRHRLAVNTGDKLRAQKIAEDLLTHFPPSVSTDAL
ESILTNWLLDPAGPSAGLSFLDSSILAAKGKPRELGIFYLYRALYRYFTVEKDPAFKARD
AAIQRSFEDIDSAEKYLRQAGEPEDGMLESIKHIREEWGKGK*
>SPBIB_v1_250024|ID:27163223| conserved protein of unknown function [Uncultured spirochete bib]
MIRRPWWEARIRALWERKPIVWLSGVRRAGKTVLCKSLEGVQYYDCELPSVRREMQDPES
FLRAANGKFLALDEIHRLDDPALLLKIAADHFPDVHIVATGSSTLGASAKFRDTLAGRKA
ELQLTPLCEADSNFPQYSDRTRRFLHGGLPGFFLSDMRDDRDYSEWMEAFWARDILELFR
LERRSSFLKFAELLMAQSGGRFNAASFAASCGVSRPTIMNYLAILEATHLVLVVRPYHGG
AAKEIVATPCVYAFDTGFVAWAQGLHEIPAKEKGFFWEHLVLNELDAILQGSPSIMHWRD
KEGHEVDFVLAPRGAPPIAIEAKWSADAFEPEGMQAFRALHPGIVNLVVSANVDQPFERE
MKGLAVTFCPLSALANLKTGSDPDF*
>SPBIB_v1_250025|ID:27163224| conserved protein of unknown function [Uncultured spirochete bib]
MLGKSGSDPDLQIIFKKRGQTPKLKKNSKPRPAIAICKFNFQLAKAKHFYYHSYMIPRIA
EHRIRSLCAQFPAIIITGPRQSGKTTLAKALFPDRPYLNLELPDTLERISHDPRGTLYPL
RRSGAVIDEAQRFPEISSWLQGIIDEEPKAGTWILTGSDQPGLRQSVSQTLVGRAAYARL
FPFVSDELSGNPIFSSSSTDEIIQRGWYPPLFDRPFVPSDWYEQYVALYLERDLSRLINI
KDLSQFRKFFSLCAGRTGQLLNLSDLARDTGTSHTTARSWLSVLEQSFIVFELQPFYRNF
QKRIIKSPKLYFYDSGLAAWLIGIREPGQVTNHPLRGNLFETMVIADIIKRSEAIGTGER
FSFWNAPSSGEVDLVIERGTEISAIEVKSTATFRPELASGLEKWSRLASLPPERLAIAYD
GNERFQFKGINIIPWRAFGQ*
>SPBIB_v1_250026|ID:27163225| Putative oligopeptide transport ATP-binding protein YkfD (fragment) [Uncultured spirochete bib]
VVLDESPSALDVSIHAQILLLLDSFSKEKNLTYFFITHDLGVVKHFCDRILIMYLGNVCE
LAPTKKLFHKPLHPYTTSLLAAVLRPVVHKKSTQESILQGEVPSAVAPPPGCPFHTRCPK
RMEICTQAKPELAEIEPEHFVACHLYSKAERAPSRENALARQ*
>SPBIB_v1_250027|ID:27163226|ggt| Gamma-glutamyltransferase [Uncultured spirochete bib]
MNFDPTYLPYASARYPIYAQHGMVAASSPQASAAGLEMLRRGGNAIDAAVAAATTLTVVE
PTANGIGSDAFALVWIEKERRLYGLNASGWTPRDISIEKVLAAPGSATGGSEMTGGARGA

EARSTEASGAAQSGKMPTYGWVSTMVPGAPKAWAALVARFGALSLSEVMAPAIDYAENGY
PASPNLARMWQRAFEKYRKTCVGPAFEEWYRTFAPEGRAPAAGETVRLPNHARTLRAIAE
SSADAFYRGELADRIVADSREFGGFFCKEDFAEYEAGWVEPISVNYRGYDICEIPPNGQG
IVALMALNILKEFEFTTRESPETFHLQWEAMKIAFADGLAHVTDPAAMRVAAADLIQPGY
GSLRAREIEPGGPARLYTAKTPPKSGTVYLCTADSEGNMVSYIQSNYMGFGSGVVVRGTG
ISLQNRGADFSLDPAHPNCLAPRKKTYHTIIPGFILKDGRAFGPFGVMGGYMQPQGHVQT
VTNLVDFHLNPQQALDAPRWQWILGKKFMVERAFPAEIAEALTRRGHEIEVSPDSTPFGR
GQIILRLPNGTLVGGTEGRTDSNIACY*
>SPBIB_v1_250028|ID:27163227| Major facilitator superfamily MFS_1 [Uncultured spirochete bib]
MNDTIENNKNLSFRFGLNYFLLLAIYGISSPYLQLMVRRLGYSPAAVGIFLGFFELVGIT
GPIFLAQKADRFGRLKPFLFASGIMAIAGLALLAPFRIPLVTLISLTFLSLGLKTPIPLL
DTSLLRAIEKSAELGKKQPNYGLLRGIGSIGFVVVAIIVQSIPGFDASSAGTIAFAQGIL
ILLFLAGLFALPEVGHTQPRNEKIAFSFKWLDSTFMLGLAVIALGRLAMASVGSFFSMYL
TEELHWHAVGAMWALSATVEIPFIMLSWKFIQRKSPMLAVAIASGAIVVRLLIYAIFPTP
AGAITGQLLHSLCYGLFQPAAIAFVNLKTPPAERATGMAIYMGLGIGLPSFLGSALGGTI
VEALGYRWLFASYTLFAIASLVLFWRHREELTGVR*
>SPBIB_v1_250029|ID:27163228| Cupin 2 conserved barrel domain protein [Uncultured spirochete bib]
MLSIVTPMVRHESEIESVEVKGEGISGVRKQILVGPNDGWQGYMRVFALEVGGNTPAHRH
PWWHVNYVLEGEGTVFIDGVGHVVKAGSVAHIEGGKLHQFVNTGAVPLKFICLVPPEGDH
Y*
>SPBIB_v1_250030|ID:27163229| putative enzyme [Uncultured spirochete bib]
MNTVRWGVLSVSNHYRLRVHHQLAGSELSKVVAIASRDTAKAETAACELGIRRSFGSYEA
LLADPEIDAVYLPLPNHLHAEWVKKAADAGKHVLCEKPFAMNAAQAADAIRYAESKGVKV
MEAFMYRFHPQWAHAKEVVQSGEIGKVLFVHVQFTFNNKDPRNIRNILEVGGGAIMDIGC
YACSSSRFIMGKEPVRAISLVSRDPEFGTDALSSGMLDFGDARALFTVSTQAFPVQQVDI
VGTSGSISIFIPFNMYGDVPAEMRIVTGIGPRTVRLGPAGQYRLMFDAFSKSIIEGTPVP
TPPEDAIANMRALDALFRSEKSGSWEKII*
>SPBIB_v1_250031|ID:27163230| NUDIX hydrolase (modular protein) [Uncultured spirochete bib]
MKKTKEQNELQPDDSTQVSSDAYIRSLEDKMNHALERLGGIGAAASAFHFCPSCGSPKLY
SVRSRMWKCPTCGFEYFHNVATAAGIIIESRGAILMLRRHKDPMKGKFALPGGFVEPGER
AEDAALRECYEEIGWSPAHINFLATFPNVYQYQGVPYATCDVYFFSRGNIVDFDDFELDP
EETESLQFVNVTAIPWADIAFESAVRALRYFLLHESIGIPPQMPDFE*
>SPBIB_v1_250032|ID:27163231|uvrC| UvrABC system protein C [Uncultured spirochete bib]
MSMKTREGVPAVSTVGESKEVITRPVNPAAPVGRDPNAPEIPHPETDTERRARLKTLVKQ
APQNPGVYLMRDETGEIIYVGKAKHLRNRLSSYFSGKKDIKTRHLVSRIHQIEWVLAAHE
YEALLIENNFIKEHSPRYNINLKDGKTYPSIRITSEEFPRVFRTRRIINDGSEYFGPFPS
AETIDTYLDLIKRMFPLRRCVKMKKRATPCMYYHIGRCPGPCAGKISREDYLERVDEVRK
LLSGDTESLVKDLEREMRRAAGELRFEEAARLREAIKAIQSFIGHSNAVDFNNLDARDYV
AWEAEGEQVSYVVFQMREGKLRNRDSFIEHLVSDELETLDAFLARYYEEGRTPPATIFVR
RADSTDAIARYLSERFDSRIDVRVPDSERHWAVMNLAVQNAKEELAKRRRMHGDFAALAE
LKEALGLGSMPLRIEGFDIAQLGGKHTVASLVSFRNGIPDKKNYRYFKIRSVEGQIDDFA
AMREAVARRYTRLINEEAELPDLVLVDGGTGQVSAAKEILDDLGLDIDLAGLAKRDEEVW
LPGRSKPVVLAKDSPALRVLVAVRDETHRFATGLSRKLRISDATFRVLQEVPGIGDVRAK
SILREVGSLEAVAQAEPGYITSIAHVNEETARKVIDAARKTVDVARRTVDTEEEPETED*
>SPBIB_v1_250033|ID:27163232|tktB| transketolase 2, thiamin-binding [Uncultured spirochete bib]
MNIPALQSIALSVRSLTIDAIQKANSGHPGMPMGAAELGAYLYGVELRYNPGDPKWLNRD
RFVLSAGHGSMFLYSLLHMAGYDLSLDDIKAFRQVGSKCPGHPEYGVTPGVETTTGPLGQ
GISTAVGMAIAERMMAAHFNKPGQEIIDHYTYVVAGDGCLMEGVASEACSLAGHLGLGKL
IVYYDSNHISIDGSTNIAFTEDVGKRFEAYGWQVLKGSMYNFEELASLTAQAKADTSRPT
LIMLESVIGKGAPSKQGTSGVHGSPLGEEEARKAKEALGIPLDKPFWVAPEAYAYFKAHR
MALEGEYQSWQARFEAWRNANPDLAAELERWLEGKPAHELDLPAFKLGEKVATRNASGKC
LAAVASAWPNLVGGSADLTSPNVTQLPANSDGMPLVFGKENPAGRYIHFGVREHAMAAIA
NGIAVHGGLRPFVATFLVFADYLRPSLRLSALMRLPVIYILTHDSIYIGEDGPTHQPVET
LASLRAIPNLKVIRPADAEETAVAWRIAMERTDGPTALVLTRQNLPVLEKADPDWPYTMV

LGAYVVQNPARSPEVTLVASGSEVSLATAAADIVRKQRPELAIRVVSVPDRQAFYAAPAP
VREAVLAPGSLVFVAEAGVAQGWEHLAPPERILSIERFGESGPGDKVAEHLGFTAQAFAD
MILAQFSNF*
>SPBIB_v1_250034|ID:27163233| putative Histidine kinase [Uncultured spirochete bib]
MSDENISGSSPFSHIRKLRPHERAMFAGIFFVLVIVLGAVGYFSWQSILRDKKLYTELAS
RFKAEQIASWYNNHMVEAAEISASSVIFDAVADAIADPGSPKLKRIENYLTPILRSFNYA
DSAVMTPDRKILVSLTGFAQPDCDMIQKELAARNNSEPFMTSLHLVSPYAKPGLHIVIPL
VDKKTQKPIAYVVHTVFSDDFLYPMLAQWPGNEHTGETLLLKRTDGMIQVLNPLKLVRIS
AFSLQISASDPDTVEARAGRGETGFLSGKDYRGKRVLAVASRVPELDWTVLSKIDISEAF
SGWFLTLVILILFGIIAFVALVAGAYVLLSSRAISTYRARLDLLQRTERNEALLNAILER
IDSLVVIPGKNLAVQFSNQAYQARFGTEWPKDLPDPQQATPEGPKPEQARKIDIVDKNGN
HARLLIFPIKIELRGQEPLLGYVMRDITELESALDVVQQLNQELSQKVQEQTQRIIDADE
ELRAIAATISHDLAAPVRSIESFSELLYQEISERSSAEAMDYLMRIRRASASMASLTSDL
ITFLSLDTVPLSLSEFDFSLAAQEITSDIIRRNPGRRYQITIMPGLKMQCDRKLMNIALR
NVIDNAFKYCSDNTITAIEIGKIGQSGIFVKDNGIGMMPDEIKKIFTPFSERKDERYKPG
FSVGLAVTKKIIERHGGRIEIESELGKGTTVYFKF*
>SPBIB_v1_250035|ID:27163234| conserved membrane protein of unknown function [Uncultured spirochete bib]
MKQSFLSVTILLILITDPLGNIPLFISSLKHVPGARRPFVILRECAIAFLVLVGFLFFGH
VFLGALGLSDQILRISGGVILFLIALNMIFPGTGGRLVEDEVDGEPFIVPVAIPLIAGPS
AITYVMLLMKSDPSRSMEWVGAIFVAILVTMVSFFLSGKLKEWLGPRALSAIERLMGLVL
TAIAMEMLLGGLAQYLGNLPR*
>SPBIB_v1_250036|ID:27163235| conserved membrane protein of unknown function [Uncultured spirochete bib]
MRRDIKGILAMVGTAIFWSLGGLFIKLIDWHPVTIAGARSLIAALFILAVARKPKLNFSR
NQVLAAVAYSITMLLFVYANKHTTSANAILLQYGAPVYVMLLSGIMLGERPLPEQIGALV
AIVVGMGLFFADSLSLGHLVGDIAAVLAGITFAIHILFMRRQKEGSPLESLLLGHGMTAV
IALAISVFLPAPVMSGKAIAAIFGLGIVQIGFAALLFSYAIKRISAIQSSLIAIVEPALN
PVWVFLAIGEKPMGRAILGGTIILAAVVMSSVVSISEASRNGRAANAEGSA*
>SPBIB_v1_250037|ID:27163236|yjjK| fused putative transporter subunits of ABC superfamily: ATP-binding components [Uncultured spirochete bib]
MAGDERNLSIRGDLAVWNGTNCIENAQRERGRHGMSIAQSKGSVRTQTLKNQCLPLYLKL
GRSVRPLTQTSIFRYSVRMPTTDDKKIIYSMYRLSRRYGTKQVLKDISLSYFYGAKIGVI
GLNGSGKSTLLRIMAGLDTEYSGEVACAPGYTIGYLPQEPHLEPGKTVRDIVAEGVQEIM
DLHAEFERVNEAFGDPDADFDALAVKQAEIQEKIDALDAWDIDAKLEFSMDVLRCPPPDQ
VVDTLSGGERRRVALCRLLLKKPDILLLDEPTNHLDAETVAWLEQFLRSYPGTVIAVTHD
RYFLENVAGWILELDRGEGIPWKGNYSSWLEQKRQRLEEEEKGSSMRARTLDRELEWIAM
SPKGRHAKSKARIERYERLLNQDTKEKIRDVRLVIPPGPRLGDIVIEAKGLAKAFGDTVL
FEGLDFTVSPGAIVGIIGPNGAGKTTLLNMIVGKEKPDAGTLRLGETVRLAYADQMRARL
DPEKTVWETLSEGLDIIKLGGRIEVPSRAYCAWFNFSGADQQKKVGILSGGERNRLNLAL
MIKEGANVILLDEPTNDLDVNTLRALEEALDDFAGAAMVVSHDRWFLDRVCTHILAFEGD
SQAIWFDGNWSEFAEWRRAQLGIEADRPHRLVYRKLER*
>SPBIB_v1_250038|ID:27163237| Methylated-DNA--protein-cysteine methyltransferase-related protein [Uncultured spirochete bib]
MATALTLRILDAIRAVPYGKVASYGQIALVAGHPRGAGGARDVVRILASMSEKQNLPWWR
IVRKDGSIALSPGSGAELQRALLAQEKVEFTKDKKATPGAFWNGQPAK*
>SPBIB_v1_250039|ID:27163238|nagB| glucosamine-6-phosphate deaminase [Uncultured spirochete bib]
MRVIIQNSYELVARWAAYHVMQRINEHPANAPFVLGLPTGSTPLGMYHELIRLCSEGKVS
FQNVITFNMDEYVGLPAADPRSYHHFMWENFFSHIDIRKENVHILDGMAPDLDEECRRYE
EAIQHAGGIDLFVGGVGADGHIAFNEPGSSLSSRTRIKTLTRDTKLANARFFDGNPDRVP
SYALTVGVGTIMDAREVMILVSGIEKARALHNAVEMGVNHMWTLSCLQLHPKAILVCDDD
ATSEMRVGTVRYFKEIEGPHLVNWA*
>SPBIB_v1_250040|ID:27163239| N-acetylglucosamine-6-phosphate deacetylase [Uncultured spirochete bib]
MRKTVHSAFCIHNATVIAGYARMDRSAVLVEDNAIADIFSERRFAQKTFPPEVELIDAQG
AYLTPGFIDTHIHGIGGYGTEDLSADSILEMSRILPAWGVTSFTPTIYPMPEEDMIMAIR
AVVSAMGREPGAEIVGVHLEGPFISPAQLGVQRVEFVRPVDLGLMQRLWDASEGNIVSMT

VAPELKGMRELALFCIRLGIVLQAGHTDASYENMIEGMQAGIRHSTHMFNAMSRLHHRNP
NAVGAVLIQPDLSCEIIADGLHVHPDLIRLLFRDKPEGNIVLVTDSLKPTKQASDTPMFA
NDEEVYLANNLFYRASDGVIAGSSLTMVEGVRNLISFGVPLESAVKMASANPARIFGLHA
RGMISPGYRADIVLADSEFDVQMTIIGGEIKYSCAAGGTTGGAAAGA*
>SPBIB_v1_250041|ID:27163240| exported protein of unknown function [Uncultured spirochete bib]
MKRFAVFALAFIAAIALLGAQEPQTPASFYAEATQDTQEVINASRLLQSKGQWSKAWNLL
AAFDADNKNGYVLAEKIRLALEGNVANGMLISFGFVDLAEGQNLDELRANPPEKQNMADF
NPLDLAGALEKSGEAVPPVLSYEMGNYLYQVYKDFGDNWIQDSQTIQQAAVENYDRALAY
ETYTDRSLSNQAELLINLQQYDGAEKVLRKAIAAYPEELNFQVNLASTLNDLGRFDEVYP
IVDAIIAKPDTGDATYNAYVEGIKAGLNSGDEAKTDAYVEAMIARFPDEYVPMLIQHLIA
VRMGNEDKANAAADAVTAKFAVDPNVVRSLLSTWLNAQDPAAGFAYLNRAYEKYASEDKA
AGTLIFYRALMYAQTAATADDLKLALADLQEANTRFSTVYEPDNPIFQTINQLNDEWKQA
LEQPAPEAGTAPEAAPADQSQQPAQTPEVAPGAQDEPAGASSSDATSGATE*
>SPBIB_v1_250042|ID:27163241| protein of unknown function [Uncultured spirochete bib]
MLLIRHNQAFWDTTVGQQILTLDLIYRKISDAQRTFIDSASQHGIELACPSGCGKCCHGF
MPDVLPIEADYIAYYLLSNSKVFSAAEAPSLQIASSAENPSPCPFHDPDRPGENCRIYPA
RPLVCRLFGYSAIKDKNNRLSYRLCRYMPAPQGLDARILDQDTIISKVGALPPLMTDFAM
EVLSLDPSQAAERKPLIEAIRPALARIGTTLQYCPEPNAA*
>SPBIB_v1_250043|ID:27163242| Deoxynucleoside kinase [Uncultured spirochete bib]
MSRKYVVVAGNIGAGKSTLVNLLAEHLGFIAYFEPVSENPFLKDFYADMPRWAFQSQLFF
LANRADMHRRLSADPHSVVQDRSLYEDAQVFAKNLYQQGIMGEREWRVYQNLYETLAEIL
PHPSLVIYIRASVPTLQARIARRGRDFEAAIPDSYLEGLNRLYEEWISSFTLAPILTIPG
DSLDFVAESRDLQVILRTVRKRLEDNQPYLFPFEM*
>SPBIB_v1_250044|ID:27163243| conserved protein of unknown function [Uncultured spirochete bib]
MDTIVTGLFNDGYVPIMDGVTVTVRNYALWLRKKLGPTYVITPFVPNYKDTDPFPVIRFL
SIPTIVRPPYRIGLPDLDIRLQFILKNRDFDIVHAHSPFAAGNLALRVAREKHIPVIATF
HSKYRDDLMRAIAFKPIVDEQMKRIVDFFYSVDHVWVPQESVALTLREYGYKGPYDVVEN
GIDFEPPADITQCWARGAEYLKLPEEYSVGLYVGQHILEKNLEFLVRSLPAVMDALPNFR
MVFVGKGYAKETLVDLARQLGISDRVIFHDVIYDRELLKDIYARADIFLFPSLYDTSGLV
VREAAAMKTPSVLIRGSDAAEFIRDQENGFLCSEDKVDFAQTVVRALSDKDSLKKVADQA
QKTLCRTWENIAEEVAQRYSSILERWAR*
>SPBIB_v1_250045|ID:27163244| conserved exported protein of unknown function [Uncultured spirochete bib]
MAKLALSLAVVCALSTAPGFAQSWPEGKDSDRFDAGQGALEIAFLGHGSLVLLYKGSYIY
VDPVSQYANFSKYPKADLILVTHEHGDHLDAGAIAVLSKNGTRIILPEASRQKLGKGEAL
GHNHTVEAAGARVMAVPAYNVTPNRTGYHPKDRKDNGYVITAGSLRIYIAGDTEPIPEMA
NLDPIDITFLPMNQPYTMTPEQAAASARVIKPKILYPYHFGSTDTAALQKLLAANPEIEV
RIRNLQ*
>SPBIB_v1_250046|ID:27163245|mntH| manganese/divalent cation transporter [Uncultured spirochete bib]
MALTDRKTQAGAHPGTHDEANPASSHPNGHSKSDAKLIKSATDVLEGKSKKGFFARILPF
MGPAFIASVAYVDPGNFATNISGGAQFGYLLLWVIVASNLMAMLIQALSAKLGIATGKNL
AEQCRMRYPRWFVVALWLLMELVAMATDLAEFLGAAVGFNLLFGIPLWAAAILTGITAFI
ILALERYGFRSLEAVITGFVSVIAISYVIEIFLGKPDWGQVAHHAFVPEFKGPSSILLAA
GILGATVMPHAIYLHSSLMQNRVVVREPALLKKLFRYEIIDVVVAMGVASLVNGAMLIMS
ASTFHAQGLTEVGSLEEAYRTLEPLLGKAASWVFAISLLFSGLSSASVGTMAGQIIMQGF
MNFEIPVWLRRIITMVPSIVIIALGVDPTKALVISQVLLSFGLPFAIIPLISFTSNEKLM
GVLVNRKTTKALAIFVTGVILLLNVYLLYVTFAGG*
>SPBIB_v1_250047|ID:27163246| putative UspA domain-containing protein [Uncultured spirochete bib]
VYKNIMVPLDGSSTAETALPMAMYLAQAFEADITLFHAIEKNPPEQIHGERHLEHAEEAR
RYLEEIARRFQGKYAFKGRILSHVHLEESADVATSIADHMTELGPDLVVMCSHGRSNFTQ
VLIGSLATRVIGLGSAPVLLLKSDAAERSATKSGAPGIAHVVVALDNASIHDKAIDYGKE
ISEAVHARLVLLSVVPTFSSLKGKDGGWGLMSPATSSAILDIEEQKLREHLSAHQKSLAE
AGIACSISVLRGDPAHEITGVAKKLDQPLIVLGTHGRSGMKAFWKGSVAAKIVSLSSAPI
LLVPLHE*
>SPBIB_v1_250048|ID:27163247|tpx| lipid hydroperoxide peroxidase [Uncultured spirochete bib]

MATITFKGNPVQTSGTLPAKGTKAPDFRLTGSDLKDVGLADFAGLVKILNIVPSLDTGVC
AASARAFNKAAASLGNVVILTISRDLPFAQKRFCEAEGIDKVVTLSELRDREFGKTYGVE
MITGPLAGLLSRAVVVLNKDNTVVYTQQVPEIAQEPDYESALLAAKKAL*
>SPBIB_v1_250049|ID:27163248| protein of unknown function [Uncultured spirochete bib]
MIQPMELQYVVRGYDCGYGGPLKPFALANFFQEAAGAHALALGIGMEDMWAKGLTWMLAR
IDIRIERLPPEGQTVVVRTWPVGTKRLFAQRCIELVDSSGVKCAGAMYEYIVVDIKTRRA
VRPERTLPIDLRTDIPWPFPDLEPGIDGAAFAALAQALQSANFGQHEEADIPSADFIAAQ
GFMPAFSIEARARHIDHNGHVNNAHFINWLCDAVPMPPGAHLSRIKIDFVHEILKGEHVH
AWAKANDLATFESSLSSAPDGSLHNPAPQSENTHAQTWLTALSVGKELVARGMLSITTAE
*
>SPBIB_v1_250050|ID:27163249| putative glutamine-fructose-6-phosphate transaminase [isomerizing] (Glucosamine--fructose-6-phosphate aminotransferase) [Uncultured spirochete bib]
MCGIVSLVYKDENPSMGKEAEALLKRLEYRGYDSTGASFIGKDKRITLRKQVGAPSKVCP
LLNIPSFSGQRFIGQVRWATYGAVTDVNSQPHHVRCKVELVGAHNGNISNTDTLKAWLTA
RGHAVVSDNDGEIIVHLIEEQYAAAQSLASSELAHLRKAYANAGIIEGVPDGVLRMIEAI
RKAESLAEGSYAAAVADPQLPGVFAIKSGSSLYAGMGSDSHGDFIVLSSDLTSVLSKTRA
LIPLSEGEGIWFTDKQYLIFSLQGQPFFSHPRLKRSKLSIKDTQLSPEYQHYMEQEISSS
PNNINQVLRYYFRDPSIEPLAELFEERKDDCKETADKIASLSERFGSEALISGMREVFDS
SAWNEVEARVQNAGAAHFVGLDFISDEAELLKELAALDPLSRDRLLLMDKVIVWRKRRAV
LRYTGELKNAIHEASHSGGRIYLVASGTSYHAALTAAFFFNEMAHIPVYPCNPGIFRSMY
FNCLSSNDLIIGISQSGETKDLVDIFQDVKKASPSVRLVSIVNNENSRIPQELSDFYLPL
LCGPEIAVAATKSFTSQIAILYLIAASFSMPEQRIRDNLLKAKDLMTETIETRMGDIETV
AAHLFAAPSIHILGTGLIGLAREGALKIREVVLNHTEGYDAAEFKHGPNTILGRNTILSL
ADIEKLVGTEPIRGIGNELFEKLTNNYPLVFICPPEERDRRITISQIHTHKIRGADIVLI
AEPQQELALAVEGKPMGAESYWSKYIPLPPSGDPCLFVFSATIVLQYLAYRMSVRKMEWL
DSLGIQNHGVHPDAPKNVSKSITVD*
>SPBIB_v1_250051|ID:27163250| UTP--glucose-1-phosphate uridylyltransferase (modular protein) [Uncultured spirochete bib]
MGRIIKALRYTASGFWVKVRGWIEGQSAAAGAASGAARPGLVRLGIARNVRIAGARPGFR
FFSTKLHETPPQVHSGRPELQRAAPPVTIIAMKGIIVAAGYGTRFLPVTKTIPKEMLPIG
VTPSIAYIAKEFVDSGITDIIVISSRRKKALEDYFDREIELEELFMREGREDRLEKIRPF
PARISFVRQAEMKGTGHALMQVSHLLGGEPCIVAYPDDIVIGTVPLARQLMSVYEQTGKC
VLATIYEPGDVSRYGVIDPAPDGVSVRGFVEKPAAGSEPSHEVSIGRFLYTPEFFGWLAQ
GWEHHGSGEYYHVYALNKMIEKGKVAYARVDGTRLDTGELGGFFEAQLYNAMQEPALKLV
LARFVAEHGAELGLGGSRPDRK*
>SPBIB_v1_250052|ID:27163251| Major facilitator superfamily MFS_1 [Uncultured spirochete bib]
MRPIRKFLNVYSGLPSSLYVLFAATVINSVGMFVFPFLTLYLTGRVGMTQQEAGFFIMIV
NFAYIPANFIGGKIADTFGRKKLMVAAQVLSGLCYIPCGFAGIGGNVRWFLLASVFFDGL
TDPARSAMMTDLTTPETRRRAFSLTYLGHNLGFAVGMMIAGFLFEKSTSWLFWGNAIAIL
AAVSLVALKVPETKPSREQVEASFGSGATDEAHRGNIIQALLSRPYLVMFTILTGLYGFV
YAQHRFALPLQTKEFFGARGSVIYGTLMTLNATMVIVLSTPIMALTQRWKPINAVALAGI
LFALGFGLIGLAPSVLLMYLTTAIWTLGEIVNATNEGTYVANHTPISHRGRFQAVLPLIG
GLGWSVSPPVVGWFTDRFGLKTAWPYLGLIAALACIGIWYLGIIENRPGRKLGKARAM*
>SPBIB_v1_250053|ID:27163252| Phosphatidate cytidylyltransferase [Uncultured spirochete bib]
VRIDHVVTRADRIDHEQNRRIHFQSPAEEIKIELTRKSLHLLIIICIPLLDISPAVPIVA
LLIGMLVYSTSEWLRHRGIIVPIIAPITEHAARRRDGNRFVIGPITLALGALLAIIIFDP
LQAKVAILALAIGDSFSSLIGKSFGRIPMPLSGGKSFEGSVFCFLTVLVAVFVFTGKFIP
ALIIAAATASVEFWPTKDWDNILIPLAAGIIASIVL*
>SPBIB_v1_250054|ID:27163253| membrane protein of unknown function [Uncultured spirochete bib]
VTTRNFRFTYWIFSIVSVLALAGSILLAIRTGTDKRLPFFLADARLYTEYFFFGAPVGIQ
LAAALQICFLLLCSSAVLIIVLFTFQKTVSAEVYLIALWAFLVDAEALRLVMLIISQRST
SIGPLVSLTRVVYGARFTGLISLFISGLYAVGIGHERPQTGYLLAILFGAMIAMLIPISI
DRFQSSFMLAAGYRDITMIVTISLIVINFIDYVVAARLKEDSSYVFAGIGLTIAAGSWVW
LWNTRPLMGGIGIALLFAAGLFGVIKRLHKIYIWK*

>SPBIB_v1_250055|ID:27163254|proS| Proline--tRNA ligase [Uncultured spirochete bib]
MAEKITPREVDYSQWYIDIILNAKLADYSPVKGSMVIRPRGYAIWEKIRDEFDRRFKETG
HENAYFPLLIPMSFLEKEAEHVDGFAPELAVVTHGGGEKLEEPYCIRPTSETIIWAMYRN
WIQSWRDLPLLINQWANVVRWEKRTRLFLRTSEFLWQEGHTAHETEKEAREETLRMLEVY
RDVVERYLAVPVVPGLKSESEKFAGAVDTYSIEAMMQDKKALQAGTSHFLGQNFAKAFDV
KFQSRQGELEYVWATSWGVSTRLVGAVIMTHSDDKGLVLPPTLSPEDAVIIPIYRNDTKA
AVTAFAEKLQAELRAAGFKIVLDIDDSSSPGWKFAEWEMRGTPVRIEIGPRDMQAGQVVM
VRRDTGEKLFVPVGEAGARLRELQTQIQASLYERAKAFHDANTVPVSTLGELIEFFGVET
AGTSGAKGGFAEALWCGSAECEASLKEKTKATLRCIPLGKQDHIEGKCAICGAPAKHRAI
FARNY*
>SPBIB_v1_250056|ID:27163255| protein of unknown function [Uncultured spirochete bib]
MSSAEKKEIYLKDALKDAAMSSAPSFPDFADVYRKAEQRPRKVRRIAFSAAITAVAAAAS
FWIGIAWNSQTSRDIALIDSWTVSAAPVAVSVSTTQGAQNEIVLTAYQTSPVNLFVQDLW
ESSAGTGL*
>SPBIB_v1_250057|ID:27163256| putative RNA polymerase sigma-H factor [Uncultured spirochete bib]
MNSNELLVRAMSAEKKEFGRLKPDDSAATAQVSLPAGDDPVDLSLAEHAAKGDKGAFERL
MWRWWDRIRGYCATFVAFDPELAEEAAQESLIRIYKALPRWRKESSLGGYLYGICRTASL
DVIRSRARHSARSMSVEDFDSLSLESPHSTGEANVLQEEANQMLAKAMQRLDPEDRSMLY
LHEVEGKGLAELGAMYNLPVGTVKSRLFRVRDKLSVMLKEMGYELR*
>SPBIB_v1_250058|ID:27163257| exported protein of unknown function [Uncultured spirochete bib]
MKSNRAKLLVMLFLIAAVALVSAQGGPGMGMQGVMGPSAVREWFKSLNLTNDDLDKLEKT
LAARELELVKAQNEIKILQTKVANMLLEPNPDMAQIEDAVAKSLEWEKTVRMIQIERQVE
IRKILGEQRWQSVLLLVREARMSEKAGKFANSFSAKGLSPDEADRYTRLLKVLRRIM*
>SPBIB_v1_250059|ID:27163258| exported protein of unknown function [Uncultured spirochete bib]
MRTSRKAIVFLMVGAMLVAVTAGAFAQGFGRMGGPGVAVPGYGFNRATPVAPSYGYGARG
WATVPGTAVPRTWNNKSFNPSLGFMGRMGMMGSSGYGALHYFQYQKLSTEDKAKVDKLVQ
DTAEQILPLQNELRSLKLALNGYVWDTNPDKAKIDETIAKIADLQKKIQELRTNSILEIN
KIFQTAQ*
>SPBIB_v1_250060|ID:27163259| protein of unknown function [Uncultured spirochete bib]
LKKHRLGLLAGMKARMKAQQVLSPVPKTVLRACLKGIKAVELKKEKLRDRSMHLRKNRSL
LPARTRAATAKLGVALAQTADSFWLVALMK*
>SPBIB_v1_250061|ID:27163260| protein of unknown function [Uncultured spirochete bib]
MTDSAARAVYCGYENGGGMEKVTAKRIWHKKTMTISTSFFACIAMAVLMLSAPSLLFAQE
VEAWFKKPANAALYSQVREDVIAMASQMQALGLSDSILASRLEEGARKKVSPNILQASLQ
GDLQRAIAAALILKENGLFPSDKKKATAAVEQILILMRAGTSEDEFRISLKAAIAKSGKR
ESALSRAISALSVVAEAQATYQLNGTDRERLALALIASDMNEKRFDSILASMNAFAQAGY
RGSEALTKALEKASLGASGGHESADEGATGSVPGTQNGSQGMPQGNQGGGAQEGKTQGSQ
HAPTEKPQPPASTNQGSHGKTGGSPGSNSR*
>SPBIB_v1_250062|ID:27163261| protein of unknown function [Uncultured spirochete bib]
MMNCTTTHAFVDSWEKGQVPEGSVAEFKVHLEQCQECAQQFSILLPLIERDTAALQGQEY
TAGMRITSGQDQFVDNVMAAIATRNPSIEPAHRAPVRRALTRQARVLRFSPALAAAAAAI
FIVGLGLGLFFGTRNGWTVTVRFVLNAPDAQSVQLAGDFTSWSPEGYALKKIGAEGTWEI
TVPLKKGHIYVYNFVINGTTWIADPKVPAMVDDGFGGSSSLLRL*
>SPBIB_v1_250063|ID:27163262| putative RNA polymerase [Uncultured spirochete bib]
MEKEAASSVQSTMSKAKANQGSFDEKVVVEEILAGNSNAYRRIVETYSARVLAFCRSRMR
SEEDARDAAQEVFIRAYASLASFRSNENFASWLFAIAANNVRTHFRLFSSRKHKEEAFAK
EIAVAPIPDPAEEAEQNLRAEALRQAVASLPLDLRKPVELYYFAELSVEETASVLKLGQE
AVKSRLFRARKALRQAIEKNKAPSHPFAEPEPAAHQRADHVQPKQSS*
>SPBIB_v1_250064|ID:27163263| exported protein of unknown function [Uncultured spirochete bib]
MIPKQKLLVFVLVLAVGFGSLYSAAAQTAETTPATTTDSTDLQDEIAAVDEAAQIDNTAL
LTKLSAQFQVDLAVLQDLNAQGYTTGQIWLALEISLQSGKPLADAVAQAASMNTSGHGWG
VLAQALGIDPGSQEFFALKEKLQIRTRTMASEVAAEHGNRVMTQQQTQNRAQTGADQDNA
HGAGSGKSGANGAGGAIGAGGAGGGAAAGHGSANGAGASGNDPKR*
>SPBIB_v1_250065|ID:27163264| protein of unknown function [Uncultured spirochete bib]

MRGLVEDSVRTGDGNFLIDTGNDNLATLSEYLRLNIFERWCVSRTSLPFS*
>SPBIB_v1_250066|ID:27163265| Peptidase M16 inactive domain protein [Uncultured spirochete bib]
MSFILQKETAVPELDSILRLYRHEQTGARLLSVINKDENKVFGITFRTPPRRSDGVAHIL
EHSVLCGSRKYPVKEPFVELMKGSLNTFLNAMTYPDKTCYPVASTNLKDFYNLVDVYLDA
VFYPNLTEYTFLQEGWHYEVDPQTKEFSYKGVVFNEMKGAYSDPEDMHDDLCRRSLYPDT
SYGLDSGGDPLEIPTLSYQEFLDFHKKYYHPSNSFIFFYGDDDPERRLALMEEWLAPFGL
ASIDSLPQAQMRFDEPRYIEYMYQAGEQDQPKAYAAVNWALRGHGNVLHAMQASILFHIL
TGTSASPLRKALIESGLGEDLAGFGYSEELLQTAFSVGLKGVEPTNVREVEKLIFSTLEA
LAREGIEKDAIEASLNTIEFALREKNTGRYPRGLAVMLEALNEWLYNQDPIEALAFAGPL
EKTKKAYADNPRLFSELIDELLVGNTHRTTVVLLPSPDANKAREEAEKAMLDKARMAFSD
AELAQIMRATDELRRLQDTPDSPEALATIPVLSLEDIPKEAPVLPSELLLLTETPQEGGT
VAQGKQSAMDARRSETASSAFYHELATGGIVYLDLGFSFAKLESRLLPYVSLLGRLLLEM
GTDKESYVQLIQRIGINTGGIRSTSVSASRWDSREPSPWFFLRAKALPEKAESLADILID
ILTGARFDNPERLRQIVLEEKAQAEAMLVPAATRMVSLRLRSRFNPADWASERLYGIEHL
FFLRSLAQKIESDWPSVLADIEDVRRAILVRDNLIVNVTTDRATLNSCIPALRRIVKALS
PERAHVTSGTRSARNSWIHEAISACDPASVSKPAAETLELPAQVNSVGMILPLWNSKAPA
GAWLVVTKFLDTTYLWEQVRVIGGAYGGYSNLDLASGLLLLLSYRDPNIERTLDVYRNVA
AFLKTCSVPPEEIQKSIIGTIGDIDAYQLPDAKGFNALMNTLTGYTQEARQQIRDSILAA
SPDDFRHLGALIEEALGTSMTGVLGAPDRIEQALHALPAPLERLSMY*
>SPBIB_v1_250067|ID:27163266| Peptidase M16 domain protein [Uncultured spirochete bib]
MKFAICERYRRLSRLFAAVMFVFLIVASAGAQTSFIRENAPTFMKTVLSNGIPVYMKVQD
ANRVFHVSLVLQGGSLVTPPEQAGWENIALKTMARSSQNYPYEKAAAILDRTSSSIGTAV
QFEYSTFSLTTLDKYRDELLALWGDMLTAPSFSADDFGKAKDEVSLSLQSVEQNPWSTTQ
KVMNQAYFKGHPYAVNPEGTEESISPMTATDARKWYAEHFSADRIFVVAVGDFNPAELAK
KLEALLGQVSGPKLGAVSRPSPFPMGPSGSLIMQEHEQAKGTLYLRGDFAAPAPGSDDYF
AMALAARMFSDLLFSVVRDKYGAVYTPSAMIRSFLANYGSIMIYKTSAPDKIKQYIDEAA
GLLARGRVVSVDPAKTEADGYMPIGEAIDTYKQLYMNEYFEAVRSNAAIASLIIRSVLRT
GDPADWLKDQKRIDMLDADAVQRAFNTYVLGGAFLWVAVGDQTLLDKLDPRAFEAIRF*
>SPBIB_v1_250068|ID:27163267| Peptidase M16 domain protein [Uncultured spirochete bib]
MNLKKKAGRQVLALVMALISVLAIPGQQSLPQSQIGGAELSAAPGLIQNAKLENGLELFV
VENHSVPLVTVCVAFRGGALAQTPQTAGLFHLYEHMMFNGNDKYPTKDAFTAALNRMGTT
NWNGATGKEYINYYITVPSDKLADAVDFWAHAVMNPTLNYAVLENEKQVVLNEIRGYHSD
PAQIASNALESRMFPQFPWRKNIDGPESNVAQATVADLRDMQHAYYVPTNMALMVGGDTT
IDQVRALAEASFGKWTWAPEPVLGEPPQGPIPQGIRLVTSEDQFYRGIAQVQFRWRGPDV
TRQTFDTYVSDVLLFLLSSPSGPFKSAIMRKVFGLYDAEYIDFTYPTARDGGNYIFSTYM
LVQKPAAEEPVLQRVENLRQIVLSEFEQIAKDPKAYFGEGALEEAKTKLVDQNIYALESA
GSFVTDTLTFWWSTAGTDYFFDYEANCRKVTWEDISSLIRKYLIGEAGAASGTADSSPAA
SARPPAATLVRLRSSTFSADPRMDSKITELGYTRVTADNAFWWQK*
>SPBIB_v1_250069|ID:27163268| Phosphoribosyltransferase [Uncultured spirochete bib]
MNKHHYTYQEIHRLAQSIASRVNESGFNPDVIVAIGSGGFIPARILRTYLSKPILAVGVV
YYDMNDKPMEYPRKVQWIEEAEKQLAGKRILLVDEVDDSRVTLEYCTRELLAYHPAALAV
AVLHDKRKEKRGQFPPGVDQVFVGQTLDDVWIVYPWDAIDIDEHDRNARARPKEGL*
>SPBIB_v1_250070|ID:27163269|fur| Fe2+/Zn2+ uptake regulation proteins [Uncultured spirochete bib]
MNELVKFEQYLRTHGQKMTRPRRIILKAFIETEGHLTTEDILREAKKVDPGIGQATVFRT
IRLIADAGLAREALQDDGARKFEHLADHPHHDHLLCVGCGKIIEFLSPAIEKEQQKIFSQ
YAFLPRGHMMELLGLCPECQAKQKKGE*
>SPBIB_v1_250071|ID:27163270| protein of unknown function [Uncultured spirochete bib]
MLLAELGPGQKFKVLRVTFGGEIGKRLADLGFISGAEGHVVRAALFHGPMQIRLADYDLI
MRRHEAKLVEIELLPQPVPVSEPASEAHAVIDIRPNATGMRPKGMRHLNRGFHHGRPGCP
*
>SPBIB_v1_250072|ID:27163271|feoB| Ferrous iron transport protein B [Uncultured spirochete bib]
MQEKRALTIALAGNPNSGKTSIFNAITGAHHKVGNYPGVTVEKREGTLEYHGWRIHVVDL
PGTYSLTAYSLDEVVARDFVLNEKPDIIVDVLDSSNLERNLYLLLQLIELGLPVVAALNM
ADEAAAKGILIDDKTLSRTLGVPMIPTIGSRGQGVTELLDAVIETFETHKMPRMPSYGED

IEAHIAALVHLLEQDEAFAAAYSPRWIAIKLIEKDPDARRRIEQHVRAAEIHTALEAAWH
WITLHYGKDAEIILSEQRYGYIHGAVAEAVHRKRLRGISLTEQLDRFVMHPLVGLPLFFF
VIWGIFKLTFVLGEYPVAWLEQLFGSLEGLVQNSVLPGAVRDLLINGIIHGVGGVFSFVP
LIVILFFCISILEDTGYIARAAFLTDHFLHAIGLHGQSFMPLMLGFGCSVPAIMATRTLK
SPKERIATILAIPFISCGGKLPIYVLLAGAFFARNASTVVMVMYASGVVLSVLSTFFLRR
TVLKGQSMPFVMELPPYRIPTLKGVLWHVWEKTWSYARKAGTIILAVSVLMWLITSYPKA
RGIQEYEIHARQSVAEAQPGLSEAAISARAEVLVRQYQLEHSIAGQFGKLIEPVIAPIGF
DWKVGVALIPGLAAKELVVSTLGVLYGAPVDENSESLSLRQSLREAPDWSPRIALALMVF
ILVMPPCFASLATIRAEAGDKWLAFQVVVYSLVLAWVISFLVSLAGRAFGM*
>SPBIB_v1_250073|ID:27163272| conserved exported protein of unknown function [Uncultured spirochete bib]
MRKAGFAMILALLVLCVGILPAGAQPVVSAKQDIAVFALGYYGWFIPSQALGTIDLRIQQ
VFSNIGRFTVFGTAQRLSSEGVQQFIDALRQAKQANFVLPEKYQFGEAFLTKAEFERLTG
AFIVVVPVVTNFAVYWDSKAGSWNCNITTGVTFIDATGGTVISIESIDTSGSDKTNQNNA
VMSAINSIPSELEYRIRAIPAFQISTRILEAKGSRVKIQLGSNMGLKKGDEYAIVTKQTI
GGIEDSKETGLILITDVSQQLSMGRVLYRSGALDGNTQLKEIARRGTDLDLYFHMSGGES
LPGLRATVSRGFYPLRPYIGIQMPLSLAFSFLGVTIIPVNAVIGAEYMMPIGRLHIAPYA
GVGLTYAHLTAIWRSYESDYLSHIGLQAGGRVGYLLSRDMRLFVDAGLDYWLALDNLLFN
DYGGVTIGAGISFKL*
>SPBIB_v1_250074|ID:27163273| 2',3'-cyclic-nucleotide 2'-phosphodiesterase [Uncultured spirochete bib]
MKRFFVILCALLAMTMSLAAQSKTVDLTFIETSDIHGSIYPYNFITAKPAATSLAQVASL
IKEERSVPGTEVVLLENGDSLQGQPTVYYYNFEKTSGPHIWSQAVNFLGYDAVGVGNHDI
EAGHAVYDKMYEELQAPVICANAVKPDGTPYFQPYSVIERNGVKIAILGMITPKIPDWLP
PQFWTGMEFEDMVQSAKKWVPIIKEKEHPDLLIGLFHSGVDYTYGNVTRDTPNNENASQL
VAEMVPGFDLIFVGHDHAGWSGQGWDPVNKKKIDVKDPNGKVVPIYGPLNAARNVAVVKM
NLTWNDQTQSWDKIVRGGLIDMTKYQPDPDFMAKFQPGFDEIKKWVDRPIGKMDGVITTR
DSMFGDSAFVDLIHRIQLDLSNDPSLGLKPADISFAAPLSADSKIPSSPDGTLYVRDMFN
LYVYENFLYTMTLTGKQVKDFLEYSYQYWFDTMPNEGNHLINFQKDKEGKLVFDNRTNMP
MTATRYYNYDSAAGINYVVDVTQPPGNRISISCMSDGRAFNPDATYTVAINSYRGSGGGG
HLELGAGLDKETIRTMKLVNGATTKDLRYFLLKWFEKQDGPVTVMPIGNWEVVPSDLAEL
GTKTDYPLLYPSK*
>SPBIB_v1_250075|ID:27163274| Heat shock protein Hsp20 [Uncultured spirochete bib]
MNKRPYMDIGSIFDDIFEAAKDFGEKMKNFAPGFEGPGPEDNCEGPRPGPGCHGMGGGAW
FEAHGDENVDYYPNYSYPPLNIYMLPDKSLVFEFALAGFDEKNMSLSFQGDYMVFSAKME
MEQPEEGVRYFKRRLKLKDIDRQKYYVPADKFDQEKVKAVFRNGILKVTVPPKEVVDTNE
GIKIEIVKEGE*
>SPBIB_v1_250076|ID:27163275| conserved protein of unknown function [Uncultured spirochete bib]
MQELICITCPMGCHLSVDTDAQGEMKVTGNRCVRGEQYAREEIFNPRRVVTFTCTAVLPD
GSLPGPSSNLPRRVPVRTTAAFPKERIPELLDVLRGITVRLPVARGSVVLERALGMEVNV
VVSRSIGA*
>SPBIB_v1_250077|ID:27163276| FAD-dependent pyridine nucleotide-disulfide oxidoreductase [Uncultured spirochete bib]
MKQLTFDAVVVGGGAAGMSAALELDARGHSVLILEREDTLGGILMQCIHNGFGLIEFNEE
LTGPEFAQRFEEKVSARNIQASLRTTVLDIREEEGRRVVYAVSPSQGMMRIEARAVVLAM
GCRERNRGNIRIPGTRPAGIYTAGLAQRLVNIEGYIPGKDVVIIGSGDIGLIMARRMTWS
GCKVHAVIEILPYPSGLTRNIVQCLNDFGIPLYLSHLVTDIYGKDRVEGIEVTPIESGAL
MHEKAFKIACDTVLLSVGLVPENELSRNAGIEIQPTTNGPWVDSMLMTSLPGVFACGNVL
HVHDLVDYVVEEARRAGANAAAWLSGYRPSREIRIKTGSNIRYVLPARLNPERENRLYMR
SLVVKNDARLEVRLDGNVVRSRKLAHVQPSEMLSLTLGPEDVAAAGRDSVVEIALV*
>SPBIB_v1_250078|ID:27163277| FAD dependent oxidoreductase [Uncultured spirochete bib]
MEQYDVAIIGAGVCGANIARKLSQYELDVALLEKEIDVSLGTSKANSGIVHGGFHDHIST
LKARLELQGALMFDRLHEELDFPFERCGILVAALHEDEMRAIEQLYLQGVENGVIGIEMC
SRERMLELEPKLNPDVVGGLYAPSGGILEPYRFVFSLVESARKNGVQVKTEFEVAQAERD
GEFWQIQSKAGETIRARYVVNSAGLHADTISAAFGAERFTIAPRKGEYYLLDRTTKAKPS
RVIFPVPTEVSKGILVIPTVEGTVLIGPTASPAQDKEDFATTRDQLEHILHSARMMVPSI

SENDVITSFAGLRASYGNDFYIANSEKAPAFVQVAGIQSPGLTASPAIGEYVKDLLKKAG
LRLVEKPSWDPYVHKVPRARDADPYTLDTLVAQDPAYGDIVCRCERVSEAEIVKAIRAGH
TTLDGIKYYTRAQMGRCQGGFCTYKIIRILMRETGMSWDQITKHGGNSAILKGSL*
>SPBIB_v1_250079|ID:27163278|glgP| Glycogen phosphorylase [Uncultured spirochete bib]
MRIVSHTIKPALKGRLARLDTLARNLWLSWNFDAVSLFIRIDNDLWLAAGQNPVKMLGML
SQEKIDELAADQSFLSDLDEVYARFQRYLKSKPWYRGPSDSIIAYFSMEYGLDVSLPTYS
GGLGVLSGDHMKTVSDLGLPLVGVGLLYRQGYFKQYLNADGYQQETYPENDWYNMPVERC
VDQSGAPILITVDMAGRSISAGIWRVDIGRAQLYLLDTNIPENQPEDRTITATLYGGDKE
MRIKQEILLGIGGIRALKALGIKVAATHMNEGHSAFLALERIRSLMEEFHLDFHSAMQAF
IPTNIFTTHTPVPAGNERFGIDLMDKYFRAFVSGLGLDWNEFLSLGRENPFDNQESFCMT
VFALHLSAKSNGVSKLHGEVSRKMWQGLWPELELKEIPIGHVTNGVYPRTWISHDMLSLL
DRFLGPKFYFEPDNPEVWASVDRISDEELWRTHERRRERLVATCRQKLAASYVRLGMPDG
EVLRSSDVLSPYALTLSFARRFATYKRGTLLLEDPDRLIKLLSNKDMPLQLVIAGKAHPH
DQSGKDLIRELVHFSRREDVFGRIVFIEDYDMAMGRYMTSGSDVWLNTPRRPLEASGTSG
MKAGMNGVLNCSVLDGWWAEAYTPDIGWAIGSGEEYQDQDLQDKIEAQDLYDLLEHEILP
LFYSRGRDNTPRAWVAKMRASMKAIGSQFATHRMLKEYYSQYYEAALNESRALEADGYKA
SVSLAAYIDKVRRAWQRVRIVEFKDDGAPVVSRGTTITVSVLVDLAGLSPDDVAVECYKG
RLSSKGEILDGARLPMTLVGPEGHYFRYQCAMHGEITGQIGHSVRVLPSHPALDGRFIPG
LVRWAQ*
>SPBIB_v1_250080|ID:27163279| protein of unknown function [Uncultured spirochete bib]
MERFLQRIGCAEDQVIVSFARLIGDQFFAQKKDILVNDENAEYRAPLIAALAGKWLEASA
QEGMEHPRVLLLAEKGEVLAQLRGLLEQAGIESTICVSKEETESSAERLATASFVGISAD
LFMTAVLEQSFKPREFGLVIIEGADLFGELPSELQRKVWGYLLPPWERRAVLFAQRLGVR
AKNTAIDFANSPGTVTLKKARASLDAISAEFYRVLSEEKFKALLSHLAAAGAQGHHAAIV
FCNLRQTSREVEARLRLNHIPAEHVSAAAPPAKNEMLFQRFAKLQEIGDQQENREQGAEP
LESPENLESAHMQLVLVVSNDNLEALPDELALFAIHYDIPLDADIYLERVRAMRSRGASM
LGLVCERYEVGLSAIESRFGIRVECKEATDMANIKDASEGVPLDLERGHVTKRADFPQQT
GVPETRVSERRAPETRAPAARPDRPRSIQERKMHTRISERGKFERKKTDRKKGRQQPTRA
GSKNSDRNPSLYSMSTEERLAYFRNKYRGILKTPSSEASPAPQHGAQVSPPDSTNPENDG
IVKRLIGKFFSGGKAAE*
>SPBIB_v1_250081|ID:27163280| NADH dehydrogenase [Uncultured spirochete bib]
MATKKILILGGGYGGVWAGKILEKHFRKREDVEITLVDKRPFHTLMTELHEVAGWRTEPE
SVQVLFKKIFGAKRINVVLDTIEKVDFEAKQATGKVCTYDFDYIILGVGAEPEFFGTPGI
AENAFTLWSFEDAMKVRKHVEEKFYEASLETDPEKRKKLLTFVVAGAGFTGIEMIGELLD
YRDVMCRKYFLDPKEVRVLNIEALPSILPILEEPLRAKAEKYLQKRRCDVMLNSAIIGAE
PGKVLLKSGQIVETETFIWTCGVKGSSFAGQLGLPAGKRNRIDCDMELKSTKYPFVYVVG
DISGLMQEGKPLAQIVETAHFTAEAAAKNIISDIDGGERHQFKPNYHGFMISIGSHYGVS
NAGGMKTSGFIALAIKHLINVWYLLNIAGINQVWEYLKHEFLDMKNRRSIIGDFASYKIR
PYWPLLLRMWLGLMWVFEGINKIGEGWLAFASGSKSGWMFSPGVVQAGVKAAEATSGASA
WESAGQAAATAATGAAAAASAAPAVAATSAASAVASSAAAAAPAATSAASAAAAAPAATG
GTQAATQTFKAVWDLTKPIFDPNSGLVTWFRHTFMDGIFAYLPYTFFQAMIVVMEIGIGL
ALFGGCFTWLAAAASIGMCIVFTLSGMFAWNQLWFVFAAILMLGGLGRSFGIDYWLIPAI
KRWWNGTRFARKWHFYADDPTK*
>SPBIB_v1_250082|ID:27163281| Trans-hexaprenyltranstransferase [Uncultured spirochete bib]
MTQYWNDFPDIKDFLSETEALILSILENSTLPLREEVGRLVESGGKMLRPALVYVGGEFG
LNPDRKKWSKKQEKRLVNIAAAIELLHTATLIHDDILDRASHRRGIPTLHQTLGTTNAIL
AGDWLLSRCYRIIAQYVHPSRAVLLAEFVSAICKAEINQDLTKYTFALSRRRYLQTIAGK
TAALFALALHIGSAETGCSTFTTQALRRTGYNLGMAFQIIDDILDYESSLEELKKPVGND
IREGLCTLPLIYALEEKGEKIRPLLSDIRAHPDSVGSVLNLVHETDAIKRARKDAERYTR
LAHAEIARLPEGKPRRDLELIVNSLLVRTY*
>SPBIB_v1_250083|ID:27163282| Bifunctional protein: zinc-containing alcohol dehydrogenase quinone oxidoreductase (NADPH:quinone reductase) Similar to arginate lyase [Uncultured spirochete bib]
MASTMKAMVIRRTGGPDVFEEAELLIPEPKAGQVLVKIAATSVNPIDCKVRSGAVAILPP
FPAVLHGDLSGTVVEVGQDVRKFKEGDEVYGLVGWTGGEGGALAEFAVCDEQLLAHAPRS

LPLDEAAVLPVVGITAYQGLRRKIRLEKDRMLLVQGGSGGVGHIVVQMGKAFGAQVAAAA
SSDEKIAVVASAGADWTIRYDQETAAQYVERITGGKGFDVVFDTAGGANLPLSFEAARRG
GDIVTIAARAIVDLSLMHSKGLNLHVVFSLLPILYGEGRAQMGQDLEAIARLVDDGLIRP
IIYPRTFSLSSIAEAHRFLETESHYGKVLVHVQ*
>SPBIB_v1_250084|ID:27163283| Acylphosphatase (modular protein) [Uncultured spirochete bib]
MSAGRIVFLGWHFHFLGFTIIRKRDIQLEHRESVSENMQAFHAIVKGEVQGVGFRMSAII
RAEQLGLTGWVRNTPDGDVEVWAEGNTQALEHFYAWLQVGPSAARVDEVIKTDEQPRGIY
KRFSVAF*
>SPBIB_v1_250085|ID:27163284| FAD-dependent pyridine nucleotide-disulfide oxidoreductase [Uncultured spirochete bib]
MKAVDVLVVGGSATGLVAATTAKSNYPEKSVAVIRKEEKVMIPCGIPYIFNTVGTSNNNI
LPDEGLVKLGVEIIVDEVLEINGKAKTCTTKNGLEIAYDKLILGTGSLPSVPRWLKGADL
ENVFTIPKNKDYLDQLQQKLQTMKKIVVVGAGFIGVEVSDELNKAGKEVTLVEILPHALG
ATFDEEFAAEAEALLIQRGVKLRTNIGIEEILGDKKVAQVKLNNGELLDADAVILSLGYQ
PNTELAKKMGLELNAFGFIKADQYRRTSMPDVFAAGDCAEKIDFSTGKISKVMLASTACS
EARTAGLNLYELNTFATFRGTVGIYSTCIGDTAFGVAGLIEKTAAAEGFKIVVGSFTGVD
RHPGKIPDAHKETVKLIVSKHSGVVLGGEVMGGKGAGELINAIGIAVQNHMTINDLLMMQ
IGTQPLLTASPAAFPLIKAAEVAAKALKK*
>SPBIB_v1_250086|ID:27163285|copA| fragment of copper transporter (part 1) [Uncultured spirochete bib]
MPVYKTFALKGMTCAACVAANERAVGKLPGIARVSVNLATERMEVEFDPTAVDVAQIQAA
VKKAGYEAELLEKPSSAPPPIYEAYGWTRFTVSAVFAVILLYIAMGSMIGLPLPPFIDHR
TAPLAFALVQLVLLIPVLVAGKSFYIHGFKSLFHLSPNMDSLIAIGTSSAILYSLFSMWR
IASGDHSASMQLYFETAAIIITLIMLGKNLESRSKRRTSEAVRKLMKLRPAIATILEGEQ
ERKVPVDDVKPGNLLLVRAGERIPVDGKVRSGNSSVDESMLTGESMPVEKASGAPVYSGT
INLNGSLVMIAEKVGEDTALARIIRLVEEAQSSKAPIARLADQVSAVFVPVVMGIALIAA
FGWLIAGHTITFALTVFVSVLTIACPCALGLATPTAVMVGTGKGAELGILIKSGEALETV
HKIHTVLFDKTGTITKGKPALTDVVPAEGYSTEEVLGLAASVELASGHPLADAIVSQARE
KGLAISRPDHVETIPGKGIVAKISGGVAGSATLAVGNAALAESLGISIPKHASDLQEKAT
QLAGMGKTPMFVMKNSLPVGLIAVADQPRPEARAVVAALKKLGIDVAMITGDSRCTADAI
AAQVGISEVYAEVLPSEKASIIKRIQASGRKIAMVGDGINDAPALAQADVGIAVAAGTDI
AMESADIVLMHNNICDVATAIHLSRRVIKTIRQNLFWAFGYNVLGIPIAAGLLYVFGGPL
LSPVFAAAAMSLSSVSVVTNALRLKGYRKPEASCE*
>SPBIB_v1_250087|ID:27163286|copA| fragment of copper transporter (part 2) [Uncultured spirochete bib]
MKKLLKIEGMSCGHCVMHVQSALEDVPGVKSAKVNLLEREAIVDGDNLDDQALRAAVADA
GYRVVQIIP*
>SPBIB_v1_250088|ID:27163287|dnaJ| chaperone Hsp40, co-chaperone with DnaK [Uncultured spirochete bib]
VAKRDYYEVLGVPKTATKDDIKKAYRKLAIQYHPDKNPGDKNAEEKFKEATEAYEILGDE
QKRQAYDQFGFAGVEGMAGTSQQDYANVFHDFEDLFSGFGDFSSIFGSFFGEGAQQRGGS
RVNRGANLRYDIELPFEKAVFGTTIEISYSRDDTCKTCSGTGSRDGQGRKICSMCKGTGQ
IRRSSGFFAISQPCPVCHGEGTVIENPCPDCGGIGVVKKKQKIRVTIPAGVEDGKRVTVP
GQGNAGPNGGPPGDLHVFIHVRPHDIFERQDDDLYCAAYVDFATAALGGEIIIPTLEGRK
ISVQVPAGTQSGKLLRIKEEGVPISGGRRGDLYIKIFVKVPVRLSRRGRELLEEFRTVEG
ETNQPELLRLKDVPH*
>SPBIB_v1_250089|ID:27163288|dnaK| chaperone Hsp70, co-chaperone with DnaJ [Uncultured spirochete bib]
MGRIIGIDLGTTNSCVAVMEGGEPIVIANSEGQRTTPSIVGFTAKGERVVGQPAKNQMIT
NAENTIFSIKRFMGRRYAEVQQEIKMVPYKVIDNGNDVRVEAGGKLYSPQEISAFILQKM
KKTAEDYLGEPVTEAVITVPAYFNDSQRQATKDAGRIAGLEVKRIINEPTAASLAYGFNK
DQKKEKTIAVYDLGGGTFDISILELGEGVFEVKSTNGDTHLGGDDFDRRIMQWLVDEFKN
DTGIDLSQDRMALQRLREASEKAKIELSNMQQTEINLPFITADASGPKHLQKTLTRARFE
QMCMDLIERTREPCMKALADAGLTAAQIDEVILVGGSTRTPRVQQMVREIFGKEPSKGVN
PDEAVAIGAAIQGGILGGEVKDVLLLDVTPLSLGIETLGGVFTRLIPRNTTIPCRKSQIF
STAADGQTAVSIHVLQGEREMASQNRTLGRFDLVGIPPAPRGVPQIEVTFDIDANGIVHV
SAKDLGTGKEQKIRIEASSGLNDSEIDRMVKEAEAHAEEDRRERERIDARNEADSLIYTT
EKALKEVGDKIGAGEKAAVENAIKDLKSVMEGKDTELIKQKTEALKQASYKLSEELYRNA

SAQSGGAAGGSASGAAGAANESASSESNDQTKNADDVDYKVVDDDK*
>SPBIB_v1_250090|ID:27163289|grpE| Protein GrpE [Uncultured spirochete bib]
MSKHKHEHGQSSNTMKPEQTMSAESLSSLQNQQAEGQAGVPEVEIQPDAEPDMAGQPGAQ
PGAVTTPEAQLAAAKAEIELLKAQLAELNDKYLRALAEQVNFRKRMTKEKEEYQQYALST
LLSDLIPVLDDFDRSLEAAAQSQNDVSKVIEGIRLVQKRLLDTLANKYGLARYDAEGSVF
DPHMHEAMFSDQGDVTEPTVTQEFMPGYKLHDRVIRAAKVKVTVPANNGAEKAPAQMEKA
QGQAQDESSTANSSSSGQNDV*
>SPBIB_v1_250091|ID:27163290|ccmL| Carbon dioxide concentrating mechanism protein CcmL [Uncultured spirochete bib]
MRIASVIGTVVATMKQEKLSGRKLLLVREVNTAGKASGEPFVAIDTVDAGKGDIVVVTEG
SSARQTTFTDGLPIDAVIVAVIDSLEVEGKVTYRKQ*
>SPBIB_v1_250092|ID:27163291|eutA| reactivating factor for ethanolamine ammonia lyase [Uncultured spirochete bib]
MDNTRRMISVGIDIGTTTTQVVFSELSLVAIARAGQIPRLDIADRSVLFESDIVFTPLID
SRTIDADKLSSIIRAQYEKAGILPSQVETGAAIITGETARKQNADKVLAAIAGLAGDFVV
TVAGPNVEGMIAGRGSGAAAYSRQHFATVANIDIGGGSANGALFHLGEIEASAAMNFGGR
VIELDGPVVRAITNAGEAICASVGIKLKPGDRPSLGDLRKITDAMAELTILLVEGGTSPL
AEKLYQTAPLPGNANLKNIMFSGGIGYYYYNPIGLNSVEDVACHDDIGPLLAESLRKETR
FSRYTILQPKETRRATVLGASAQTLTLSGSTIWADPSILPLKNVPVTALVSGSDPSDITE
ALRRWDLNPSNDIFAIALDMQHGLDYKELSRIAENLAVFAKDLPPDKPLIVITKRDYAQS
LGQTIKGIDKNRPLLIIDQVGLEEGDYIDIGSPLMDGRVVPLVVKTLIFYH*
>SPBIB_v1_250093|ID:27163292|eutB| ethanolamine ammonia-lyase, large subunit, heavy chain [Uncultured spirochete bib]
MALRTKLFGKTWEFPDIRVLMGKANEEKSGDRLAGLAADSAEERAAARIVLADIPLSALK
ENPAVPYEQDEVTRVIQDGINETIYNEIKGWTVGELREWLLSEKSSGDDIARISTGLTSE
MIAAVAKLMSNIDLVYAASKIRVERHAVNSIGLPGMLASRLQPNHPTDSPEGIRAALYEG
LSYGSGDAVIGINPADDSYASVARLLDMSYDVVKTWEIPTQTCVLAHVSTQMRCLKSGSP
VGLVFQSICGSERGNASFGISVGMLDEAFTLAKKYCYHGGPHYMYFETGQGSALSANAHN
GWDQLTMEARNYGLARRYSPFIVNTVVGFIGPEYLYNSEQIHRAGLEDHFMGKLSGISMG
VDVCYTNHANTDQNASENLSVLLTSAGCNFFIGVPMADDAMLSYQSLSFHDIATLRELFG
LRPSPEFEAWLESIGLMRNGRLTERAGDASFFLSR*
>SPBIB_v1_250094|ID:27163293|eutC| ethanolamine ammonia-lyase, small subunit (light chain) [Uncultured spirochete bib]
MIDAKTIEALVDAVVSELESLAEKPKIAAAKPQSQQGPQSQPADGSAVAPAMQTALMSGV
KNPVDPEGLLALKQTTTARICAGRAGPRLRTEDLLAFQADLAITKDALEKQVDPLLLEEF
GLFTVRTKITGGKEEYLLRPDLGRRLSDEAKKIISERCIRNPDIQICVGDGLSARAIEAN
LAKIFPVLKAGCETAGLSMGTPFFIERCRVGVMNDIGDLLEPKVVILLIGERPGLGRADS
MSAYMGYKPHAGLTDADRDVICNIFDGGGVNPLEAGAYAIRLAERMIKAGASGIQLKLLE
GEAK*
>SPBIB_v1_250095|ID:27163294|eutL| putative carboxysome-related structural protein with putative role in ethanolamine utilization [Uncultured spirochete bib]
MSVLDPIFAHALAVRIIPKVSDEYARAIGLEPSQTSLGFITADNDDALYVSIDEATKMAD
VEVVYAHSFYAGARHSSGIYSGEIIAMLAGPDPTEVKAGLDAAIRYLETRALWYSADAHD
GVAFFPHVVSRIGSYLASVSGLPEGASVAYLVAPPLEGSFALDAALKAAAVRIAAYTAPP
SETNFMGAILTGEQSACEAAALTFQEAVLDVAQHPIMY*
>SPBIB_v1_250096|ID:27163295| Acetaldehyde dehydrogenase [Uncultured spirochete bib]
MTEIFDADLVSIQEARRLANAAKEAQKKWAGASQEEVDRVCAAMAEAGAAAAEKLARLAV
EETGFGVVEHKVLKNLLTSRDLWTAIRDVKTVGIIGRDESRGLVRIANPMGVVVALIPST
NPTSTAFFKTMIAVKARDAIVVAPHPSAVRCTFEATRVMAEAAVKAGAPEGLIACMEHVS
LQGTDALMRHKATSLILATGGTPMVRAAHSVGKPAYGVGPGNVPCYIDRSADLPQAARLL
VASKAFDHSVICATEQAAVVDKPIAGRFAELVQAEGAYFVSESEANALRRYLFKPDGAIN
PTAVGKSPQVLAKAAGFSVPDSARILIARLSKVGPEEPLSREKLTTVLGWYEVDGWREGC
NRSLELIHFGGEGHSIALHARDEAVITAFGLEKPVHRIVVNTMSSLGAVGVTTGIAPSMT
LGPGGVGGAITGDNISVQHLFTVKNIVYGLRDAPAPAMTDGTDFEHLHAPRTTVSAEEIE

QIAQRVLAELQNRHVQ*
>SPBIB_v1_250097|ID:27163296|cchA| putative carboxysome-like ethanolaminosome structural protein, ethanolamine utilization protein [Uncultured spirochete bib]
MAELSQIALGMVETKGLVGAIEAADAMVKAANVTLIGSEYVGGGYVTVMVRGDVGAVKSA
TDAGGAAAKRVGELVSVHVIPRPDPSVEMILPQKTKGSFSGRGND*
>SPBIB_v1_250098|ID:27163297| conserved protein of unknown function [Uncultured spirochete bib]
MAESFQLRVYSYLDRMQPQFAAFVGTITQGDLPTAGMAALYIEVAPGNEVFRLVDIAVKT
TEARPGAQIVEREFGMFEIHSHSQAEVLEAGHIVLDRLSLHEEERIKPFIASVQVITNVD
PYQAQLLNRFRRGSMIVPGETMLVLECAPAAYINFAANEAEKGATIKLIHISSVGRFGRM
WLSGSESEILQARDAAINALESLNGVEGR*
>SPBIB_v1_250099|ID:27163298| protein of unknown function [Uncultured spirochete bib]
MIITEAELREVWKNGHGTMPEIPPGARLTPAARDFLVTIGSSAIANPQSVETGSVSNNVS
GGSMELSASRGRLILTSTDIDDILAAHPATLIVHPEVTITDVARDRLRNAGIRIIPFARE
KPAAPGPISYNMTAQPPSAMLAAQARAGRTETAPQNSRNEQNDETFNAVKKAVMARLNGE
VDDYLVDAVLKRVLGTLK*
>SPBIB_v1_250100|ID:27163299| ABC transporter substrate binding protein [Uncultured spirochete bib]
MKKISIVLIVAMLSGSMAFAQATSTQDMNALIAAAKKEGQLTVIALPRDWVNYGEIIDTF
SKKYGIKVNELNPEGSSGEELEAIRANKGNKGPQAPDVIDVGLSFGPLAKQEGLIAPYKV
QTWDSIPNEIKDKDGYWYGDYYGVLAFEVNADIVKNLPKDWSDLLKPEYKGKFALSGDPR
TSNQAIMTIVAASLSQGGSLTNMEPGLRFFDKLNKAGNFVPLIAVPGTVASGETPITVRW
DYNALSNRDKSAGNPNIAVIIPETGVVAGVYVQAISAYAPHPNAARLWMEFLYSDEGQIL
WLKGYGHPARFNDLAKRNKIPADLAAKLPPASAYAKAVFPTIEEQNIAKKYIAEQWDNIV
RIDVQKR*
>SPBIB_v1_250101|ID:27163300| ABC transporter permease protein [Uncultured spirochete bib]
MTDSLKKERSNQKGAEQTTGASGALSLHSAAKSNWKDWLGLIPFFLFIFAFMVIPAGSLL
VGAFQNRDGSFTLINFSHLLKPNIISSYLITIRLSATTALLGGLIGFLLAYAITIGGLPE
GVRSFVSTFSGVASNFAGVPLAFAFISTLGRVGLVTVILKTVFGIDLYESGFNLYSFWGL
ALTYIYFQIPLMVLIIQPALDGMKKEWREASENMGASTFQYWFHIGIPVLLPSLLAALIL
LFGNAFGAYATAFSLTGGLINIVPILIGAQIRGDVLHDPNLGYAMALGMVLIMAITILAY
SWLQKISSRWVK*
>SPBIB_v1_250102|ID:27163301| ABC transporter permease protein [Uncultured spirochete bib]
MSNGKTGRKALSWVWLVLGLAYFFIPLISTFLFSLKGKKGVLSFVAYQHVFADPNFLKTF
TFSLQMSIYTILVGLILIVPTAIWINLKLPKVKPLIELFSLMPFVIPPVVLAFGLIKSYA
RPPLALVSSPALLVAGYVVISFPYIYRSIDTGLTSIDLKVLTEAAQSLGAGWGSILFRVI
LPNLRTALLSAIFLTFATVIGELTLAVLLAWPAFGPYMAHVGRDLAYEPAALAILSFMMT
WGSISIIQILSRGIPGSRSNVGGLH*
>SPBIB_v1_250103|ID:27163302|potA| Spermidine/putrescine import ATP-binding protein PotA [Uncultured spirochete bib]
MGFIELHGVSKSFGKNTVVRNLFLSVEKGEFVSFLGGSGCGKTTTLRMIAGFEIPSSGKI
LVDGKDVSNVPPAKRNLGMVFQNYALFPNMTVRKNIGFGLKIAGVSAHEIEKRVDEMLAL
IHMEEFANRYPHQLSGGQQQRVALARAIAVRPKALLLDEPLSALDAKIRVRLRDDIRAIQ
QKLGITTIYVTHDQEEAMSISDRIAVMRNGKIEQIGTPFEIYNRPATSYVASFIGTLNLL
EATVVDANNGIVSIGSMKLKTASSLDRMQDKPVKLTVRPEALSFANPADPVTADTNVLSG
TIETIKFLGSTVRYVMKFGDTFMYMDRFNNPQLELPKKGDPVSVIFSKEACRILPADEES
ANSLKGTEEEL*
>SPBIB_v1_250104|ID:27163303| RNA polymerase sigma-54 factor [Uncultured spirochete bib]
LQTQRPVLAQQQQLKLSPRMIQAIRLLALPLQELSEQIRQELEENPALELLTDNAELSLD
VMEDSLDTFRDSDETLREAEYSDEAFGFNNDENDRTAIIEQTIATEESLQEHLSGQLGLL
PLNDAERQVAERLIQNTDENGFHILPPQEICPDCEPGTLERILSLLQRLDPPGTCTSGYR
ESLIVQAQIAANAPRKTIEVLTSHFEDLQTRNRSAIKKSLGLNDRELDHIIDFIRTLDPF
PGRSFSRARPRYIVPDLAMWLEDGEIKVRLNDDIVPKLGINRLYTELASRASRGEAEKFA
REKVENARFFIDSIKRRNETLLKTAYAIADAQKAFFVGGPQALKPLTLKEIAQKIEVHEA
TVSRVVNGKYVQTDWGTFELRRFFTNAVNHDNGENISKEAAKAQISQIIDELASRREKIS
DRIIAEILARRGINVARRTVAKYRSELGK*

>SPBIB_v1_250105|ID:27163304|raiA| Ribosomal subunit interface protein [Uncultured spirochete bib]
MKIDVRSVHFDLSDQSRQYLDTKISRIDYAKDMIVDLLFVFTKDAGQFRLEVTANFRWGK
QAHIVETSLELNPGIDVLIDKLDQKIVKEKEKIQEKK*
>SPBIB_v1_250106|ID:27163305|hprK| HPr kinase/phosphorylase [Uncultured spirochete bib]
VKEQEPVNFTVLDMLSLELKEQNDLSLRCVAGRAGLSREITVPDLNRPGLALAGFYDSFA
WERLQIFGRGESAYINKIEADGVDEPIYAKLFSYQIPCCIFTNNIQPTAHFAAAADAAGC
PVLITTLSSVEFSIRVIRILHDIFAPKIIMHGVLVEVFGIGILLLGESGVGKSETALALI
ERGHRLVADDVVEIRCLNGTLLMGQGTNKVIGHHMEIRGLGIINITHLFGVGAIRDKKQI
QLVIQLEDWDPNKVYDRIGMDELTIDILGVKVPKLEIPVKPGRNIPIIIETAAMNERLKK
MGYHSAREFNQNILRWLESESARAMYFSRDDY*
>SPBIB_v1_250107|ID:27163306| Transcriptional regulator, LacI family [Uncultured spirochete bib]
MKNSKISIYEVARLAGVSKSTVSRVISDRGGSVSPETLERVNRAIEQLGYRKNSIAAGLR
TRKTYIVLVMIPDVANPFWSEIARAVQDKLEPESYSVVIGSTYWSEEREARYYELAQMSR
FDGVILNSVTDNIELIKKLGVPAVLIGERVAAQNIDTVGTDTMQATKVGMQYLYDLGHRR
IALATSEHGSERYLSLRARAYKDFLHEKGLPFDPSLVFSVNLNEEGGRELAKRFLAMPDW
RNRADSLFCGNDILAIAAMSEFRKFGIIPGIDISIIGMDDVPAASQTYPPLTTVRKPRER
IGQAAAELLLKRMENPAGQPEKHLFPGELVIRDSVIDRRKR*
>SPBIB_v1_250108|ID:27163307| ADP-ribosylation/Crystallin J1 [Uncultured spirochete bib]
MKTRSEVLSDRATYESRAIGAFIGLAIGDAVGDLGRSQEHRNKYGLITKLLDDGKSTDDT
EFGVLTARALLDTKGALTSDTAASAWRRYILDRGGAKKRAGRPLYGAIENLSRGMQPPLT
GRYNVMNIDDGAAMRASPHGILWAGDPERAAASAAADACVSHDADGIWAAQATAAAVAIA
IANGSVDEIVEAARRCLPAGSWIASAMNTAMDICNRHPDIEEAYEELHTKLWTPEHAAAP
EAIPQMFAIFRLCAGDPRRGLLWSANFGRDADTISGLVCSLSGAVHGAEAFPTEWTESLR
KPSGTCLDFAAEEDIVSLATELVALSCELGA*
>SPBIB_v1_250109|ID:27163308|lplB| Protein LplB [Uncultured spirochete bib]
MVALAKKKEWSLLLLLAPALLYFLIYHFIPISGMVMAFQDYRIFGKSPFVGFKHFRILFA
SPAFLQVLRNTLIISFMKIVLFFPLPIIFALMLNEVRSSILRKFIQSTTYLPHFLSWVVI
AGIWIAFLSPANGGLNQLREMFNLAPKDYMTDKGAIRWVLFASETWRSLGWDSIIYLAAL
ASISPSLYESADLDGAGALQKMRYITLPELLPTMVTVFILNMGFFLNAGFDQVFNMMNDS
VISVVDILDTYVYRIGLLNMQYSFSTAASLFKGVIGLVLILAAHQGSRKITEKGLW*
>SPBIB_v1_250110|ID:27163309|ytcP| putative ABC transporter permease protein YtcP [Uncultured spirochete bib]
MVKKVKTPAVELFWRWLFTALLLLVCVIVLVPMLNLLALSLSNPARVPEVRGLTVFPKGF
SLINYRVLAANPLFVRSFLNSVFITVVGTALNLLVTSMAAFALTRPKLPFRRFFMVLIII
VMILEPALVTEYLVIKKIGLMNSLWAVILYKTVNVYYLIILMRYFEEIPPSLIDAAQVDG
ASNMTVLRKIVMPLAKPALATLGLFYGVFHWNEYFRASIYLTDPMKYPLQLILRQFVVLD
DTASLIGSGTLFSYDEAARLSYDALQASTIIVAILPVLIVYPLILKYYARGVMEGGVKE*
>SPBIB_v1_250111|ID:27163310| Extracellular solute-binding protein family 1 [Uncultured spirochete bib]
MKKHSIVLWLMVAMLVTGVALAGAQPKVRLVLKDLDTDPNAAQFIQLIEKGMAAAGTPVK
IEIVKVPSGNYAEKISLMIMSGDIPDIIYFQGGDDKIAMQNLLEDLRPYVAKSKYIKNVL
DPHSKTRLESYPYLVWVAPPRVRAPVIRKDWFDTLPSAKAVATNPTVDTYYALFKDLVQK
KGAKFGISITGATNGVEELDSIFDGAFGITSTWMKGADGQWVYCRVTPNEKQKLEFYAKL
YKEGLLDPEYLTKKWDTKEQVFYEGKAGIIAGVAPGSIDVYANKMMKTQNTTLVPLPPPA
GKAQGLGPFVDVIKESRGFAIPVTSKVKDAAFAVLDFMWSPAGLKIAKLGIPGLHYIEEP
NRYVLTDKYAEWYNGWFGDSFNGFVPDKPLSRPIMTEAAVEAGKLAVKYMMPDKTFLIPE
QYIANWDAMTNLYKEYNADIVTGKKPISAFDEFVQKWYSAGGTELTKYAQTKLK*
>SPBIB_v1_250112|ID:27163311| ADP-ribosylglycohydrolase [Uncultured spirochete bib]
MPTIPSDYLERVYAGLLGKNIGIRLGAPVEPAIWTYERIREVYGDITSYIKPYRNFAADD
DANGPVYFVRPLLEKVERLRNKEGGWLGSLPQSAVGAGEGNPVAPLAIEPSEVARAWLDY
SREGVGMFWWGGYGRSTEHTAYLNLKHGVPAPESGSIARNGKVTAEQIGGQIFIDTWGLV
WPSNPEMAAEYAVRAASVSHDGEGLHGARFIAGCIAAAFRETDVEAVVGAGRAMLPADSA
YAAVVDAVLRFHAQHPSDWRACQEYLLAEWGYDKWPGLCHIIPNAGVCVLALLYGKGSFA
RTVEIATMCGWDTDCNAGNVGTIAGVLYGLDALPAHYRAPVNDSIVLSGLPGTLNILDIP
TFAKRVAEAGYMLAGEAVPASITEALQRGDGERAIWFDFVLPGSTHGMRVSDPTRFAARH
RSGALEFQFERLVKEESGKLFFKPFYRRDDFDDERYNPIFSPIARPGQKVRIEFMLERWE

GQSIGVEGYVRDTFTRQDIITTGTIFPQEGEPCRLDFQIPSLDGSFPDEIGLKITSFSGK
GKRDSGRLFIRHFSIEGKASYTIDMAKQSVEFGCVTPFSHDGGTWSIESGRLHLMTPVQA
ASYTGGYRVGDQRITAQVRPIAGDCHMLLVRTQGATRGYWAGFDGPGHVAIYKNDAGFTR
LANAEYSWQAGRDYVMALEAIGSSICLSIDGEKVLEIDNDRFDSGMLGCGTLRASRALYG
PFTVEEL*
>SPBIB_v1_250113|ID:27163312| putative HpcH/HpaI aldolase [Uncultured spirochete bib]
MTEYTPSTAGTLSKSDCLVVYRPSKEPVSVTVRSSVGRLFGRSIEDTAKSMLGVFGVKTG
LLEIEDDGALNLVLSARIETTLRLAGFELNISLLERDRTFSAAKIQREPSAKQLPRRARL
YIPGNQPNLMINADLFGADTLIFDLEDSVPPERKLEARILVRYMLSDTHLFRESELAVRI
NPIDSSCGREDLEEIVNSRPQAIVLPKCESARDIQVLDAELARLEAREGLPLGSILILPL
IETAAGVLAAKEIAAASPRNAALCFGYEDFISDIHSSAGERSLRSASTPANHDGEMPHSS
SQPEALLAKQMIVLAARAAGIDPLDSVISDTEDKEALRSSCEEARMLGMSGKAVIHPVQI
PAVKSAFHPSEEEIMQAKAIVEAYERSVVEGKGTAALGDTMIDVPVVERARRLLNDYRIG
D*
>SPBIB_v1_250114|ID:27163313|citF| citrate lyase, citrate-ACP transferase (alpha) subunit [Uncultured spirochete bib]
MTIGSGTDMEWKTNALGRAIPLEIEGRKLVPFAGAFADPTGAAIAASKDISTQSGKKKLR
PVPWRGKPDKLTKSWDDLFDRLDIHDGATISFHHHLRDGDAIVLEVVKRLAERGIRNLTL
APSALFSVHDALVPYIRAGVITKIEGSMNGAVGAACSRGEMPKTCVLRSHGGRQRAIQDG
DLKIDVAFIAAPCADAFGNANGLYGKSACGPLGYPKPDSEYANKVTVITDNLVSYPCLPW
AICGSNVDYVLQVESIGDSSKIVSGTTRLTRSPTQLLIADLTARFIEAAGLMHEGFSFQA
GAGGISLAATVFLAERMRRAGVKASFAHGGATKVLVDLLKEGLLSSIVDLQSFDLDAVRS
CAENPSHVASDPFISYGPRSAGCVANMLDVAILGATEIDLGFNVNVVTHSDGLLLHGIGG
HSDAAASAGCTIITAPSFRKRIPIVREEVTTVTCPGEVVDVLVTERGIAINPRREDLLER
AKKAGLPCIKISELLHDVREITGAPELPRFKDRVVAMIEWRDGSIIDVVREIESI*
>SPBIB_v1_250115|ID:27163314|ptsH| Phosphocarrier protein HPr [Uncultured spirochete bib]
MVELETTIKNRAGIHARPSALIAQTAIKFASRVYLEKSGNRINAKSIMGIITLAASFGTK
IKIITEGPDEQQAAEAIKALFESGFNEELG*
>SPBIB_v1_260001|ID:27163315|argD| Acetylornithine aminotransferase [Uncultured spirochete bib]
MKERFMNTYARTGLVLDHGVGARLFTVDGTEYIDFTAGIGVNSLGHGHPRLVEAIAAQAA
KIMHVSNYFLTEPSIELAEKLTEATGFDTVFFCNSGAEANEGMFKLARKYGSSKNPDKNV
IVTLKQSFHGRTITTVTATGQDKFHKFFGPFTPGFVYAEPEDIAGLDALLGPNVCAFAFE
PIQGEGGVRPLSRAYLQAAEKMCRERDILFCADEVQTGVGRTGAVLACERLGVRPDVTAI
AKGLAGGVPIGAVLARGKAADVFQPGDHGTTFGGGALAASAALVVLSELESPGFLDEVDQ
KGRHLMELVEGFRHPLVKDVRGMGLMIGIGVKVDPHKIVDAARAHRLLTLTAGDDTVRLL
PPLVITMEEIELGVAALKQALDDVSSAPNLVPELK*
>SPBIB_v1_260002|ID:27163316|argB| Acetylglutamate kinase [Uncultured spirochete bib]
MDEQFNADVLIEALPYLRRFSGKIIVIKYGGAAMINEGVRQSVIQDLILMQQVGMRPVLV
HGGGPEIDRMLKKLNKESLFVEGLRYTDDETMEIVQMVLAGKVNKDLVELFSRMGGKAVG
LCGSDGGLFMANRFRKNGQDLGLVGDIQSVNTELLRTLLDNGIIPVVASIALRNDEEGGF
YNVNADTAAGALAIALKAEKLILLTDVPGVMEDKSDLETLIREMDVQMAKSLIKRGVIKA
GMIPKVEGCLQALAKGVSAAHIVDGRSPHSLLVELFTDTGMGTMIR*
>SPBIB_v1_260003|ID:27163317|argJ| Arginine biosynthesis bifunctional protein ArgJ [Includes: Glutamate N-acetyltransferase ; Amino-acid acetyltransferase] [Uncultured spirochete bib]
MKQTRGGVTAPKGFLASGVHVGIRKNREKKDLALIWSEVPCAAAAVYTTNRVKGQPLIVT
QEHLADGRAQAIIANSGNANTCTGEEGIKAARRMAELVAQHLPVKAEDVVVASTGVIGQQ
LDVSVIEAGMGELVAGLSKGGSIDAREAIMTTDTMKKEIAVVEEIGGVLVTIGAIAKGSG
MIHPNMATMLGFITTDCAITSEMLDKALKFAVRKSFNRVSVDGDTSTNDMVAILANGMAG
NPLIDQEGNNFDEFRDALEFVCVELAKMIARDGEGATRLIECTVSGAVSEEQAEALSKGI
ITSSLVKAAMFGADANWGRILCAMGYTRNDFDPDKVDVAFESPRGYIEVCKAGAPLPFNE
EKAKKILNNQEVEIIVDLHAGDAEATAWGCDLSYEYVRINGDYRT*
>SPBIB_v1_260004|ID:27163318|argC| N-acetyl-gamma-glutamyl-phosphate reductase [Uncultured spirochete bib]
MIHVGILGATGYAGLELTRILLQHPRVDRLSASSVSFEGKSLEDVYPSMVNVVDGSALPP
LEKAEDVMARADIVFSCLPHGHAETAGWQCVEDGKPFIDISADFRFGTHEELFKKWYGAG

YEYPEVHAQAVYGLPELHRTAIKSARLIANPGCYPTAAELGLMPALQEGIADLSSIVITS
MSGVTGAGREPSQTTHYPEAADAASPYKVGSHRHQPEIDLLLSEMAGAEVHSVFTPILAP
MNRGILSTICFGLVHTISAADLHALYADRYAGEPFVRVLPLGSTASNRFVKYSNYCDISV
HLSADGSKAVIISAIDNMVKGAAGQAVQNMNILLGFDETEGLRFIPPAF*
>SPBIB_v1_260005|ID:27163319| protein of unknown function [Uncultured spirochete bib]
MENLLAEKTDSYRLQAIIGVVLKAGNIENAIAFCFALAAAALRRA*
>SPBIB_v1_260006|ID:27163320|hppA| putative K(+)-stimulated pyrophosphate-energized sodium pump [Uncultured spirochete bib]
MLTSGVLWAIAAALLALLYSLVTSSRIRKIPLANSDIQRISGYIEEGAYAFLTREYRTVA
YILPIVALMLLFWNVGIMKLQSISFLIGAILSASAGWFGMSIAVKANSRTAEAAQKGIQP
ALRVAFSSGSVMGMTVVGLVLLGISGVLGLLLLIPGADSVLGETIFPIVSGFSLGASMMA
LFARVGGGIYTKAADVGADLVGKVEVGIPEDDHRNPAVIADNVGDNVGDVAGMGADLFES
YAGSIIGCMVLGIAVQASPFMQIKLSILPLIAAGTGVIASLIGTLFVHINRSGNAQKALN
AGSVGAALISMLLLLPVIRLTIGTERFVSGTHEAGWLGIYFACVIGLAAGTAIGLITEYY
TGSDTPPVRKIAKSCTTGAATTIISGLGVGMSSTVFPILVIGLAIGFSNWLAGLYGIGMA
GLGMLLTLGIQLSVDAYGPIADNAGGLAEMSEMPPEVRKITDSLDAVGNTTAAIGKGFAI
GSALLTSLILIVAFISATGLQPSVMNLTNPHVLIGLFIGAMIPFFFSALAMDAIYQAAFA
MIEEVRKQFRSKPGILEGTEEPDYRSCVDISTQTALKSMILPGISAIAAPILAGLIGGPA
VLTGLLVGATVSGGLLAIFMSNAGGAWDNAKKLIESRPEGGKGSGDHRAAVVGDTVGDPF
KDTAGPSLNILIKLMAIISLVLAPLLKLAWG*
>SPBIB_v1_260007|ID:27163321| conserved protein of unknown function [Uncultured spirochete bib]
MASEKHLYMIIHPNHSLIASQLEPEHFIRHYVQGSTRYFEGRLIFVEVDPDYRHPYFDID
TAYAELKPHEDGRPKATKFIKSYRVLEHVDYNALGKLYISNSLGDYVELESKPYEPSIDT
DEFRIMLEVNPLRFIVLTKLNCHDFGQFITDPRNSKGAPKMAFTLLEFDTQEFLKEYEEN
PLIRCYVPGIHPARLVSAIEEVRTTPGKVVKGISLDCPIDRISYKYLKEGFMFAEHGKPC
KFYPYLDLDTVERKFYKFWKSM*
>SPBIB_v1_260008|ID:27163322| conserved protein of unknown function [Uncultured spirochete bib]
MELKIGLVAEKEIVVEDKHVASHLGSGGVPVYATPTMVLHMEETSRQAVDHLLGPDGATV
GAYMAVKHLAPTPKGMRVRIRAELTKIDGRMLTFKVEAWDEVEKVGEAEHVRAIISMAKF
AERIEKKKRAAGA*
>SPBIB_v1_260009|ID:27163323| Alpha/beta hydrolase fold protein [Uncultured spirochete bib]
MIQLQTLIVLPSVANHGDLGNRARILQNEQKVRYYWHMAVAVALYLVAVLLLAGIAYPLM
FRPKCRSLEFTKQYSLEHGEIDESFLGLDWKDFSFSSPNGYTLRGQYLRGKEDAPAALFV
HGITWTRYGMAKYMKPFIERGWNVAAFDLAGHGESKAPRRFHPSFGFYEKFDVKEGVKAL
RALFPKALCVGLFGESLGSASVLQYALLAKKDSSAEVDFIVADCSFSSAWDELLEQLRMV
HVPNCIAWPAAQGVRGLARLLRGFDLKDAAPAKAIMEADMPILFAHGMEDRYVPTIMSVR
MASARLSGGIGLTELVLIPGATHAKGILTDKERWLSAVNAFIDRVCDAKKKAAAK*
>SPBIB_v1_260010|ID:27163324|trpS| Tryptophan--tRNA ligase [Uncultured spirochete bib]
MERKRILTGDRPTGKLHLGHYVGSLANRVRLQDEYECFFIIADLHTLTTKPEKENIEQLS
ENVHSAVLDYLSVGIDPKKSVIYLQSAVPEVTELALFFQNLITVPRLSRVPSLKEMAQNA
HIEEMPFGLLGYPVLQAADILLPRAHLVPVGKDNEAHVEVTREIARRFNYLYGETFPIPE
AMISEFGSLAGTDGNAKMSKSLNNAIFLSDDEKTVIKRVMSMYTDPKRIRADIPGTVEGN
PVFIYHDAFNPNKAEIEDLKARYRAGKVGDIEVKEKLARALNTFLDPIRERRAYYESQKG
LVEEILYEGTMRMREEARQTLSLAKKAMGMSSVWNRISRKAEERQKKAASGQVGE*
>SPBIB_v1_260011|ID:27163325| putative Diguanylate cyclase [Uncultured spirochete bib]
MEIDSSAIRSLFIDSSLSEFFDETDLFYIDFQSSIARSCSTLRYLGYEDADLLDILGAFP
VHEADRERVELMQKRIESGETDKVIDSFRVASKKDSRFPWLRFSIKVLGREPDGKPTALI
GHVTDVDDLIASQEEIRERLVEIDAMRELIGAINKSLDFEETFKRIIEQLHRIIPFDRAT
VQAFENDSLTVIAGYGYPKSDIMGLVFPAKGIDNPAVRAIQTQRPIVCNDVSHDFPGFIA
PSKDFTSLSWLGIPMVYEGQAIGLIALDSHLPNFYTDQHVRVATAMAEHIATAFEHARSH
QLVTVQAMTDRLTGLANRYGLEMRGTELFQKAIENDQPIGVFMLDIDHFKQVNDMYGHSY
GDMVLQQIASTIRTQIRNEDYAVRYGGEEFVVLLPGLGPRESLIVAERLRIKISQTEVEK
GRKLPTVSIGIYAAVPGSLDILHEFIRKADLALYTAKEAGRNRSRVWSTSPEFYVSE*
>SPBIB_v1_260012|ID:27163326| protein of unknown function [Uncultured spirochete bib]

MVYMNFVAKRLFDAAMARHAAEWYGISKAEGLALGNKGQVQKIAENLRALQRGLTGDRAR
VASGYMDEKSALQAYLLYYWPVSFYETAAVLAELAERRTLPNIQTVLDLGSGPGPGSFAA
SLFGAERAVLVDASQAALDVASKIAEKGRQGGSSMELSALAKDLQDFSVGILSRFSSSSI
EPSAGFSAGRALLEPPFDLIIASHSINELWHDEPERIERRRNMLLSLLPALREGGLLLII
EPSAHYTSIPLLALRDSLLEAAGSFTGGNFAVSDEARATLAADPAAAHLFCVGPCPHSDP
CPMRAIGDRPCFSEWKWDAPTLVRKLAEMAGLDRTSLKASWVAFQKTGEKLGAVFARNMG
ADFGGQGIVFARQGADFAEHAANSAAPPPAAARPSGLPPAAGPAISGRIVSEPMLNKAGR
IRYIVCTENGQLSTISAPQNDPTAIHAQASTLGFFNLARGDLIEASGLEERGRSHFGIIP
ASSLRIKMKAPRF*
>SPBIB_v1_260013|ID:27163327|hndD| NADP-reducing hydrogenase subunit HndC [Uncultured spirochete bib]
MVNVKVNGIPVQVAEGSTVLEAAKKANVKIPTLCYNPDLSPWASCGICVVKIEGSNKMLR
SCCTPVSEGMSIISNDPDLVQTRKTVIELILSTHPDDCLACPRNQACELQTLAQEFGIRE
QPYKKMVRDIPQDTSTGSLILNPSKCIRCGRCVEVCQEMQGVWAVEFLGRGESIRIAPAA
DVKLGDSPCIKCGQCSAHCPVGAIYENDQTKLVWDALMKEGPEAKTCAVQIAPAVRVALA
ESFGLPPGTDLTGKIYTALRRLGFDAVFDTNFSADLTIMEEGTEFVHRLTGALKAGMQQA
TADKSMPLITSCCPAWVDYMEKYYPDMIPNFSTAKSPQQMMGAMIKTYWAAKAKVDPAKI
YSVSIMPCTAKKFENSRDESMYSSGYKDVDVTLTTRELARMIKQAGIDFLNLPESEPDSP
LGPYSGAGVIFGATGGVMEAALRTAYFLVTKEELKDVNFTAVRGLSGIKEATVHINGIEL
RVAAAHQMGNIATVLDQVRKAREEGRETPWHFIEVMACRGGCIGGGGQPYGATDEVRKLR
MRGIYDNDEKQEYRCSHDNPYIKQIYAEFLQKPASHKAHELLHTQYKERPLYLK*
>SPBIB_v1_260014|ID:27163328|hndC| NADP-reducing hydrogenase subunit HndC [Uncultured spirochete bib]
MAYKNFILVCGGTACESSHSEEIYRNLIKEAEALGVSRDVQVVKTGCFGFCEKGPIVKVL
PSESFYVEVKPEDAHEIIAEQVVKGREVTRLLYRKDKKSDTAKLEDIEFYQKQFRIVLRN
CGVINPESIDEYIARDGYKALEKALFEMAPEEIIAEVKASGLRGRGGAGFPTGLKWEAAR
KSPGDVKYIVCNADEGDPGAYMDRSTIEGDPHSILEAMVIAGRAVGANFGYIYIRAEYPL
AIERLKIAIAQAKEYGLLGKDILGSGFDFDIEIRLGAGAFVCGEETALLQSIEGKRGMPR
PKPPFPAVKGLWGKPTVINNVETLANIPVVLTRGAQWFAAIGTEKSKGTKVFALTGKINN
SGLIEVPMGTTLREIIFDIGGGIKGGRKFKGVQTGGPSGGIIVEKDLDTPISYESLTALG
SMMGSGGMIVMSEDDCVVDVSKFYMAFCVDESCGKCAPCRIGTKQMYGLLDKISKGQGEE
ADLDKLEQIGRAMTKASLCMLGGSAANPTLSTLRHFRAEYLEHIRDKKCRAGKCKDLVVY
EIDPAKCIGCGLCARRCPVPCITGEKKKPHVIDGSKCIKCGECYTACKFGAVIKR*
>SPBIB_v1_260015|ID:27163329| NAD(P)-dependent iron-only hydrogenase iron-sulfur protein [Uncultured spirochete bib]
MAKMTLEQLRAFREGKRQELEKRNVEGKDIQVIVGMGTCGIAAGAKLTFDAIVAALEKHN
MADKVVVRQTGCMGLCYVEPTVEVVMPGMPPVIYGKMTSDVAEELVVKHLVEHSLLDNHI
FDRPAVDIVAH*
>SPBIB_v1_260016|ID:27163330| Histidine kinase [Uncultured spirochete bib]
MHYAIADFVLDLVQNSVEAGAQSIEVDIDEAPDGLRVRIADDGCGMDEHEKARALDPFRS
GGAKHPGRKVGLGLPFVQQAVTLAGGWFSLKSEKGKGTEVQFFFPGKSVDSPPLGDPVAL
FTALLSMAEKSELKIRRTRARDGLSYEVKKSELAEAVGELASASSLSLVRQFLASQEEG*
>SPBIB_v1_260017|ID:27163331|hndA| NADP-reducing hydrogenase subunit HndA [Uncultured spirochete bib]
MVAGDIEFSTELQAYIDEWCKKEGGLVMMLHRIQHEFGYIPRAAAEKLSRISGIPLAKIY
GVITFYHFFKTTKPGKHRIAVCMGTACYLKGGQDLLEETRSILSIEPDEVTDDGLFSIDE
VRCLGCCGLAPVMMIDDEVHGKLAKEQLPDIIAKYRAL*
>SPBIB_v1_260018|ID:27163332| putative Tetratricopeptide TPR_1 repeat-containing protein [Uncultured spirochete bib]
MANSEKNKLIADLNNQAVSLIDAGRYQEAINLLDKALVLDPSRTGVLFNRAEAHRLAGNF
DAARSDLLSELQMEPDSPEVLHALGLLAYEQDDFAQASEFYRKALDKDPLYASAWNDLGV
VAFRSEKYDEARAAFEKATSLDPDFSDAWFNLADTYDELGLADKRAYALKKLHAARMRTG
EKPDSEDRA*
>SPBIB_v1_260019|ID:27163333| putative Protein kinase family protein [Uncultured spirochete bib]
VNPGDIIGTYVLDRRLGKGVTASTWLAHLAQDTPSAAAGNERSENGAAENQPERISATHP
ESPPNNAPTSPPVVLKIFDLAETSSWKPLDQFKREAEILRTLNHPRIPKYIESFDLAQGG
HFEFVLVMQYIEGETIESHVSSGKKFTEAHIEQMLAELADILAYIGSLRPPIVHRDINPR

NLILQPDGHVALVDFSGVQDAIRSALYPGATLVGTAGYIPVEQVSGRATHRSDLYGAAAT
AVFMLTGRNPAELPMRNLKIDLSGLLYLSPGLAYVLGNWLDPDQDRRFLSAAKAAAILRG
EEPVPTAALASSPSNARSSENTRNIESLQSSRRALISHILSEPDTGRESVDYPAELPSDS
RLEIQPLDPGLYIKLPRGKRAGSSSGFAFFPIVWLGFVFFWTTMTLRMRAPMFFPLFSIP
FWIAGVAMLKSFLKPSITDTELFITPDGLLKKSSSFISNSAQQYAISDVGTAKVQRSSVQ
LNNRYLKELAIEAGTGTLTFGLGLSDRELYYLEKRINEELEKLKKLPRE*
>SPBIB_v1_260020|ID:27163334| Transcriptional regulator [Uncultured spirochete bib]
MENPVQHTFFRYLTYSDEDEQWEMVCTDAGYTEVPPYTIYPPNKEGHPRIFQRVAVGRTL
NEYQIIYITKGEGVFETSGRHYDVKPGSIIMVFPGVRHFYKPVYEIGWMEYWVGFKGGHF
ELLRERGFLNPQEPFIEIGLQNNVLDLYNEIIAEVRDQKPLYQIVASSKILALIAEINAC
ARRRAQASHAAQVVEAAKCMMVEKIYGDIDINSIATALGISASRLNDIFKTYTSMTPYQY
YIHIKIHAAKSLLEQGDLSVKEVAYRLGFEDQYHFSRLFKKKTGIAPSQWRAFMYE*
>SPBIB_v1_260021|ID:27163335| conserved protein of unknown function [Uncultured spirochete bib]
MHMHARNIYHKCKNIHCALGERPYTRWYMKENSAHIPTVGLFGIGLDTYWAQFPGLRERL
LSYQAETAALLEGAGAMVVDAGLVDTVDKARAAGALFREKKVSLVFLYITTYALSQTVLP
VAREAGAPMVIINVQPAKNIDYEWFNSLGDRALMTGEWLANCQSCCVPEIANVFNRNGIP
YHIITGYRGEPFVEAELAQWVRAARVAEALRKTNIGILGHYYEGMLDVYTDVTRMAGVFG
SQFNLIEMDYLAQLREKVSPAQIEAKIAQFRSEFEVVSECEESELVRAARTSCALDALVE
EKSLGALAYYYEGTAGNVHQDIVTSVIAGNTLLTAHHVPVAGECEVKNALAMKIMDLLDA
GGSFSEFYLIDYEDDVVLWGHDGPAHSAIAEGKVKLVPLPLYHGKPGKGLSIQMSVRNGP
VTLLSVVEGASGKVFLLTAEGESVPGPTLQIGNTNSRYRFPLPARDFSEAWSKEGPAHHC
AIGVGHVAAEIEKTAALFGIECHRIC*
>SPBIB_v1_260022|ID:27163336|rbsA| fused D-ribose transporter subunits of ABC superfamily: ATP-binding components [Uncultured spirochete bib]
MAEYILELRHISKYFPGVQALKDVHFQLKPAEIHAIMGENGAGKSTLIKIITGVYQPDEG
EIYFNGERIHFKSPLDAQRLGIAAVYQHVACFPDLSVLENIFLGHESFDPVTRKIRWREL
RKKAEILLQSLDADFSPSTTMGNLSIAQQQIVEIAKALSVDAKILIMDEPTAPLSRRECE
DLYHIVETLRDKGVSIIFISHRIEDMYRLAERVTVLRDGNYIATWDVPGLEQSRLVQGMV
GREITQYFPSRHATIGEEIFRVEGLSRTGFFKNISFSVRRGEIVALTGLVGAGRTEICES
IYGIAPRDSGRFFLEGKEITINTPAEALMQGIGYLPEDRMKQGLVLDWDLIRNITLASLR
SFCKNGFMQPSKERDMALSLADNLKVKASNIYAKAATLSGGNQQKLIVAKLLTSKLKLII
LDEPTKGVDVGAKTEIYRIMNQLAEEGYGIIMISSEMPEVLGMSDRIVVIREGRKSAEFE
TPNASQQAILESAMTANGREAHAIAG*
>SPBIB_v1_260023|ID:27163337| Ribose transport system permease RbsC [Uncultured spirochete bib]
MQSQDKMQTGQNADGQTKAASRPDAIISSRAESTSRAETLARNTETYRELGLAIFIVILS
VIFQIRNPKFLSLSNIKDMLANTAILSILTVGMMMVIITRGIDLSIGSTMALAGMITSLT
VSANPSISPFLSLAQGMAVGLAAGLTIGVLVAYFNILPIIATLGLMNILRGMTYLISKGK
WVSAYQMSAGFKNLSTGTTFGINNLIIFAIVIYIIYAYFINQTKTGRYIYAVGSSPDTAE
LIGIKRRRIILLVYALMGLLAGLAGVLWVSKFASAQGDTAVGYELNVIAATVLGGVSVAG
GRGKVSGILLGSILFGILANALPLINISPFWQQFIQGLVILAAIISNVLLQRRNDRLALK
KRVI*
>SPBIB_v1_260024|ID:27163338|rbsC| ABC transporter periplasmic subunit [Uncultured spirochete bib]
MTEKTSTIDVNKPRGLRKYQWEIFLVVLFLAVNVVNSFLSPYYLTLDTFVSTPMNFLDKA
FLVLPMTMIIILGNIDVSVGSIVALTSVLMAVSYNAGLPMPLAMVLALVISTTCGLINGL
LQIKFRELSATIITLSTMTVYRGIAYVILEDRSAGRFPSWFSFLAWGYIGKVPFILIVFV
LAAIVFAILLHTTRFGRMVFAVGNNKTACEYSGIRTGRIILGVSVITGLMAGFTSLFLTS
RMGSTRPNVALGYELDVIAMTVLGGISTSGGKGRIAGPLISIFLIGFLNYGLGLRNVSAQ
VLLIILGFLLILSVLVQNFTDRPRQKSSSKSGKSAAPTHRSDAGSGSDIHGNSAQGG*
>SPBIB_v1_260025|ID:27163339| Sugar ABC transporter substrate-binding protein [Uncultured spirochete bib]
MKKTFTVVALVLIALALVMPASVFAAEKAKYAIVFKNTGNPYGEKQMEGFKNGIEEQGFE
AILRAPDLPTAEAQIQIIEQLIAQKVAAICIVGNDYDALVPVLKKATAQGIKVFSLDSSV
NPQARLTHVNQADSEKIGQTLIKAAYDMAGGKGEIAILSATSQASNQNIWIDFMKKELAK
PEYKNLKLVKVAYGDDLRDKSVSETEGLLRSYPNLKVIIAPTTVGIAAAGKVLTDKGLKG
KVMLTGLGLPSEMAEYIENGVCPYMFLWNPIDVGYLGAYVGTALVSGKITGKVGDKFSAG

RLGNYTITKAPDGGTEVLLGPPFKFDKSNINDWKKVY*
>SPBIB_v1_260026|ID:27163340|yiiL| L-rhamnose mutarotase [Uncultured spirochete bib]
MKRNAFAMQLKPGNEAEYKRRHDQIWPELKAELRKAGVSDYSIYLDPETMRLFAIQSLAD
DETSSTLPESPIVRKWWDSMKDLMETNPDNSPKVWPLTEMFHMD*
>SPBIB_v1_260027|ID:27163341| putative bifunctional rhamnulose-1-phosphate aldolase/alcohol dehydrogenase [Uncultured spirochete bib]
MNLETLIEISRRYGADPDYVLAGGGNTSLKDGDVIAVKASGALLGTIDENGFVQLSLSKL
RALFDMALPSDSEAREKIVLEKLLEARLPGQTRRPSVETLLHALFPYRFVIHLHPALVNG
MLCSRQALSAAETLFRNEASVLPYITPGYILADAVRRVFEHRTSSGMSPPKILFLQNHGV
FVAEDSVEEIEALYAHIFETLKAKVQPLPEEGLRGGPARGYEAAIAAVAEKAVETAATKF
NVRFFFTSNSLIGHFLASPEAFVPLSSPFTPDHIVYAGAWPLFVNISEAQSSEHLISKID
EYRIRYQEMPKIIAIQNLGIFGLGQDESAAKRACELFGDAAKIAWYARSFGSVHPMQPAD
IEFIRSWEVEKYRASIAASSNPAS*
>SPBIB_v1_260028|ID:27163342|rhaA| L-rhamnose isomerase [Uncultured spirochete bib]
MSRIEQEYKSARETYADLGVDTDQAIKTLSSIPLSIHCWQGDDVIGFDGATSLSGGILAT
GSYPGRARNAEELRADAAFAFAHIPGQKRFNLHAMYAETGGTKVERSALQPRHFDAWVAW
AKEQKIGLDFNPTFFSHPLAASGWTLAHPDENIRKYWIEHGKASRRIAQAIADSLKDVVV
NNLWVPDGSKDMPADRLGPRLRLKESLDAIYAEKLPNDRVLDAVESKLFGIGSESYVPGS
HEFYFGYASQKAIGLCYDMGHFHPNESVADKISATLLYVPYLIIHLSRGLHWDSDHIVIW
NDAITDVCREIIRMRVWDRIHLALDYFDSSVNRISAWIIGARSAQRALLYALLEPVEAMR
TEEERGDFGSRLALIEESRSLPFAAVWRHYCEMQNVPAGPEWIEDVKDYEKKVLEERG*
>SPBIB_v1_260029|ID:27163343| conserved protein of unknown function [Uncultured spirochete bib]
MAVISIRLNEEEEKMLAFLEKQFAKDKSSLIKYSLNELYENYIDNKLIEEFEAKEKETGP
KFLGAKEILADI*
>SPBIB_v1_260030|ID:27163344| conserved membrane protein of unknown function [Uncultured spirochete bib]
MTEFVNNIWRWVSDLPSALGAPLILLVGWAIAKILKLIMPKILAVLKFDVLAGKIGVNEF
LRKGHVQYAPSKLIGVFLYWVVMLLVLAMAASVLDQRAAESIWSWLFGAIPTILAAGITA
AIGVLIVNFLSNFVLTIMNNAGMGSATLVQRLIRAVGYIIVVVMVVDQLGLGQSIVSILL
LLLIGSIALGIAIAIGLGCKDMARQYVESLIRAIRERERASHGTDLEG*
>SPBIB_v1_260031|ID:27163345| putative opine dehydrogenase [Uncultured spirochete bib]
VKKTDWSGILEHLERVAAKGKDMVWCIAGAGNGGISMAGHLGLLGFPVQIYNRTDEHLNA
IRWYKGIEVEGAVSGFGPVRLATSRIGEAIAGTDVIMIVTPSTAHYGLGTLMAPFLEDGQ
IVVLNPGRTGGALEFRAALDREGCTARVVIVEAQTFIYASRMISRNKGHIFRIKNGVPVS
ALPSFLTPAVLKVIDVAFPQFIAGSNVLATSLENIGAVFHPALTLLNAAWIESTGGDFEY
YLQGISPSVARVLQAIDDERLALARALGIRTVSAREWLYLTYDSVGADLCSAIRATISYA
GIKAPKTINHRYIWEDVPMSLVPMASIAAMYGVETPAINLTIDLANLMHGVDYRANGRTV
HTLGIEGLSVEELHRLVTEG*
>SPBIB_v1_260032|ID:27163346| Cobalamin B12-binding domain protein [Uncultured spirochete bib]
MYKVSQNDVIGLIKPAIDAHTLGILAFAQILRDCGIRVEIADEDASKELDALVGRPSIER
LVRWVRAKGIDVLGFSYRLDPDRGVELFAQLVEHLKSAKMLAKDGGPIKALWFAGLPPAC
KKAKQRVPFVDAVFHGDETYQEILEILGLPAHLVPQQASAELAYDKARLEFGTEIVKNRA
YLDVAPVDRSGSPGFGTWGERVIDRVRHGIAHNLPPLMRAHVGPYAEDRSQAVALFLDWT
SRLAKSGLLDVLSIGSSQLTQSHFGEDWTGLSNGGGVPINSPEEYRRVWQAARPMLVRTY
AGTKRVPDMAKMHEQSLDICWHALSLWWFSKLDGRGDNSVLENLVEHFEALRHIASSGKP
MEPNVPHHFAFRGCDDVGYIVSGYVAAKAAKLQGIRTLILQTMLNTPKYTWGIQDLAKAR
ALRMLVRDLEGPDFAVILQPRGGLDYFSPDMEKARAQLAAVTMLMDDIEPDDPASPQIIH
VVSYSEGVRLADPDVVQESVRITRYALSEYRRLKRKGDMPEYGKHSMVEARTRHLYEEAR
RMIKFIEATIPEPYSPRGLYAMMKAGVFPLPWLSACRDEFDAAVSQPVQFMHGGVQTVDK
DGKPLAVSQRLFGIARNLERMGFAPQEEHGEKN*
>SPBIB_v1_260033|ID:27163347| putative D-alanine--D-alanine ligase [Uncultured spirochete bib]
MRLQIIMKKKTDRKIAVENEVRYARMTGIYNGAGFEAYFSYAANINDLETIIRENLPDLV
LCGIDHLPEEKDGITVSTNVHGWFESHNVLYIGSDPEVIERALSKASLKRKWEADGIRTP
PFVHVEAGEAGLKEGIQKLKVLDAFPYIIKPENLGNSKGIDENSIAWNVDELSRVLARMI
SLYSGHILVEHYLGAYPDVKEITCAMIQGQGAMQCMPAFLGLAQPKRFHLITSDDKDGHH

TVMSPLEPEQMERFVPFAQSAFESAGVRDYARGDFFFADGKFWAIEINGQPMIPDRWFEE
AAGFDGISEAQYLVGIVAAGYRRLRAQGKLTSAFPAGARALLERTRLAEDLSAETPHKGG
LQDV*
>SPBIB_v1_260034|ID:27163348| protein of unknown function [Uncultured spirochete bib]
VHHEIALVFALIAGDHCFAINYSTKSRLAFFKTKFVIIADMWYILK*
>SPBIB_v1_260035|ID:27163349|pcm| Protein-L-isoaspartate O-methyltransferase [Uncultured spirochete bib]
MITCNERKDERNFMVHTQIAARGVRDKRVLDAMRKVPRHRFVPVSCSSEAYEDFPLPIGS
GQTISQPYIVAFMAEMLRLNGSERVLEIGTGSGYGTAVLSLLAKEVFSVERIAALLERAR
EVLEALKVENVHLRLADGFFGWAEEAPFETIILSAAPFDIPNAIVNQLADGGRLVAPVGS
LGAQTLVRITRRGAHFDTEALLDVAFVPMRPGVSEL*
>SPBIB_v1_260036|ID:27163350| conserved protein of unknown function [Uncultured spirochete bib]
VWTVSATDEYIEWFCSLPGKAQEALLAKVLLLEEFGPQLGRPHADTLKGSRIKNLKELRA
RTHAQVLRVLYYFDEERQGLLLIGGDKKGRNEKDFYSRLIQMAEEIIERYRR*
>SPBIB_v1_260037|ID:27163351| conserved protein of unknown function [Uncultured spirochete bib]
MAIIKNAIEAMEAVMTPEQVSRARAAAEREMLAIRLAELRERKGIRQSDMKAFSQTAISK
IERRKDMKLSTLIDYLEGIGMGLEIRVYPKNAKGSHKSQTILKV*
>SPBIB_v1_260038|ID:27163352|mtaD| 5-methylthioadenosine/S-adenosylhomocysteine deaminase [Uncultured spirochete bib]
MALLIRHALVEGAESDILIQGERIARVVPEDSIPLDGSDEIEVIEARGMAAIPSLVNAHA
HSAMTLLRGIAEDMDLMPWLNEAIWPREARLTAEDVYWGTRLAAIEMIRAGTTLAADMYF
FPEAQAQAARDSGMRFVISFPLIDGLDEARGLAQQKACDEFFKRLPDCGPASIFALAGHS
VYATSASSWRYIARFAKEHGLFIHIHLAETETEDRECRAKTGMSPTAYLDSLGVLGPNTF
AAHCLWLDARDWDLLAQRGVTAVHNPVSNMKLASGPAFDYEAARRRGVRVLLGTDGAASN
NSLNLFSDMKMAGLLQKHHYKDPKRWPVREILAAASRNGHEFFGTGGGRIETDASADLVL
IDLARAEMTPCHDLASNLVYANAGAAVDTTICAGRVLMLHGVIEGEKEVREEAARRAQAL
K*
>SPBIB_v1_260039|ID:27163353|punA| Purine nucleoside phosphorylase 1 [Uncultured spirochete bib]
MHSFEEIQAAAALLRERLGEAPACALVLGSGLGGFADELERPVALSYKEIAGFPLSTAPG
HAGRFVAGYVEGVKVLAMQGRFHCYEGWDASQIAFPVRVLRAYGVKVLLLTNAAGGVNTD
FRPGDFMLIRDHINLSGRNPLVGANDERIGPRFPDMSKAYDPALRELAKGAARDLEIGLH
EGVYAWFLGPSFETPAEIRMARTLGADAVGMSTVPEVIAAVHCGMRVLGISCITNLAAGI
LDQPISGDEVLEISAKKRPEFSALVKAIIARIGAEDAALAGMPRGAAWRS*
>SPBIB_v1_260040|ID:27163354| conserved exported protein of unknown function [Uncultured spirochete bib]
MHNMKIVPIGTFFVVLVLLCAGCGRNPAGSGDLPQVNTNTAFDLQGFNICAWGKNWWSDA
ALVDKALKFALEEGANFLALDWPVNFNDDGTMVAFEHSLHPHWNDMQRLIEKAKQMGFYI
MLKPHTTKAESAENRNIWNTDTAIFLPGTFFSAYKGYLEELADFATQNNVDAICIGTEMN
HLDTGFRDQWSELVAAVRARFAGLVTYDAFFNRWCCSVPDIGEVCFWDLVDMIGVSLYVP
VTRNDDASVEEIRRGWFESIEPEFEISDVIAYLKGIAQDADKPLMALEGGYQSVSGGLYD
MTGPSTHKTVNYDLQSRGLDAYLRVLSENKDSWFKGVSLWQLTPSMLSSNNLQTIWHTQE
FTVYQKPAAEIVKQHFFK*
>SPBIB_v1_260041|ID:27163355| protein of unknown function [Uncultured spirochete bib]
MNSLRWLPTEKKIYAKIFINAAGVYGDVISRKAGIDAYTITARKGEYILLEPNEKYNVHH
IIFPSPTKTSKGIIVTKTITGYILLGPNAVDMPETEKSNNYTTREGLKEVLKKQKSLCQH
YRRNWRSRHLPDCVLSRIPMTLFWKTMLYQLTLSLRLAQDFGVRNQLYNFTCYADLELGV
DLGASWQNQLIQWIKGKGLGTSDILYRHSGSWQV*
>SPBIB_v1_260042|ID:27163356| N-acetylglucosamine-6-phosphate deacetylase (fragment) [Uncultured spirochete bib]
MRHLAAGGTTQHLATIVTRPHEHMVRNLKAIVRARQESALLRFAIHGAHLEGPWISLEDG
PRGAHDPRYIRKPDFEEFQKLQDAAQGLIKKAPRLRFSQRS*
>SPBIB_v1_260043|ID:27163357| protein of unknown function [Uncultured spirochete bib]
MSIRQILKSRMIVCTVPDARKQDAVYMALNAPPRRWRNNTASCNNCHSPS*
>SPBIB_v1_260044|ID:27163358|gltB| Ferredoxin-dependent glutamate synthase 1 [Uncultured spirochete bib]
MQQRKKEMHNYRIGILGLSSGAPGPGASSSIKRANIIKQQLEPSRGAYSTLSPNPSGLYL
PEHEHDACGLGIVVDLKKKSSHSVISDALTILQNLEHRGAVGGDKKTGDGAGILCQIPDR

FFRREMAINDMPYGIGMFFLPASHNAFVSAKKLVSNIAAERGFGLISWRDVPILPQVLGE
RANKTMPRISQAAFVQLNPPAIVESEDDARTRASLRSPSDDFERSLFILRKYIEQEAKRA
GFSFEEFYIPSLSSRIIVYKGMFVASQFANFYPDLRDPLFESQFAIVHQRYSTNTLPSWP
LAQPFHMIAHNGEINTLRKNINSMKARQATMASPLLGADFFKDIAVLDEAGSDSAIFDNI
FELLVHAGRSPEQVFAMMVQEPFGEGLRISHDKRAYYDFHAAMLETWDGPAAMSFTDGRV
VGAALDRNGLRPFRYSLTKSGYFIGASEAGVLDLDESDIVERGILRPGEMVLVDLENGRL
INDAEIKTRISRQKPYRRWLETNRIELRGLFSAPDVRPSSAELAHLISYFQYDNETMNIL
KPMLLQKQEAVSAMGTRKPPAILSRTPVPLYAYFRQRFAQVTNPAIDPYRESAVMSLENY
IGTQKNLLEETPEHCKQLKLQRPILSNSDMEKLKNSRVPGFSSATVSMLTPAARIRLEEE
LDRMCREAEHHIDQGANLIILSDRGLDEQHAAIPALLAAAAVHTYLVDAKKRHLAGLVVE
TGEVRDVHEIAVLLAYGASGVNPWMVFELLPSISDQLEERPSAEMLADHYIEAVNKGILK
IMSKLGIATVSSYRGSRMFEAVGLSEGLVKRYFRGTESRFGGIGLKEIADDMLSRHRAAF
GSEQESAILPDRTGHQPVNEPRQAAGGAANQATDQAADQATDQVANQTVRQTAVPWPPSL
AAKLTKAVREGDAAAWRAYADGMDSAARPPFALRDLFSFKSGSPLTSSSPLPLDQVQPAE
EIIKRFSVAAMSLGAISPEAHETLAAGANAIGSWSNSGEGGEDSERHTYKDKGLDSQNAS
KQIASGRFGVTARYLATGLELQIKIAQGAKPGEGGQLPGAKVNEYIAKLRHSKPGVTLIS
PPPHHDIYSIEDLSQLIHDLRCINPSARIAVKLAAQAGVGTVAAGVAKAGADCIIVSSGD
GGTGAAPLSSLDYAGSYWEAALPEILQVLAMNSLDLNTVIQVDGRLRTARDVVIAAILGA
REFAFGTAALIAMGCIACGRCHLGKCPVGIATQEPEFRAKFKGRPEHLIAFFKFLAEDVR
KILASLGAKTIDEVIGKYELLDFSGRASTAREKMLDFDHIKEALEIARRYPLVEGQDLAS
DTTPAIPLPEAGLRNFRPEKRQDFSISDAEAALLQKCARLLQERASTSRLEFSLPIRNSD
RSVGAALSGEIIRAGLKLEPDTVSVTFRGTAGQSFGAFLAQGVLFRLFGEANDYLGKGLS
GGRIIVRPRRESRCQPEHNVIAGNVCLYGATAGEVFLNGKVGERFCVRNSGALAVAEGTG
NHACEYMTEGIVVILGKTGINFGAGMTGGVAYVFDEDQLFDTRCNLSDVDIAAVTSAEDI
DQLRTIIERHQNLTGSPRAKLMLSEWDSYLPLFLKVVPR*
>SPBIB_v1_260045|ID:27163359| putative amidase [Uncultured spirochete bib]
MNRKRLSRRCGQGLEPGIPDTCEEYTLRDFQREFGAGRLSSAQLVDYYLGRIAVLDCPGA
GSDCEGQALRSLLYLNPEARSIAEERDRERRAGIVRGPLHGIPVVLKANINTADQMPTTA
GSQALHGFLAPEDAPLVAKLREAGAVILGKANLSEWANFRSTHSSSGWSSEGGQTKNPYV
LERNPSGSSSGSAVAVSANLCALSVGTETDGSIISPASINGIAGIKPTGGLVSAEGIIPI
SFTQDTAGPMARTLEDAIVLLEAMSGRDYSGVLGGNLAARPLEGMRLGYAEKLSRFLPQV
EDIMKKSIAALENLGAEIVPVDIEPDEEVQKAEYQVLLYEFKYGIEQYLARYVAARADSA
STPWPRTLRDIIAFNTAHADTAMPHFAQEILIEAAGKGSLEEPEYKNALALCRAFEKEKG
ISAFTAKYRLDAIVAASNSPAWKTDHVLGDHYVGGNTSLAAIAAAPHITVPAGFVGELPI
GLSVFGVPHSEEVLFRIGLAFERAVKARKAPCYKGV*
>SPBIB_v1_260046|ID:27163360| Transcriptional regulator, RpiR family [Uncultured spirochete bib]
MILGKIKETLPRMSPNFRKIASFILDNDNNVAFTSIYSLSEALDISTATLVRFAKSLGYK
GYQSFKKDLQEEIQHRLQPYDKVSLSKLGTLPEEKRLQKLIQNEYNNLRSTLNNLQLKDF
ETMIGAVQSARRIFIAGFGITRHFAQILQTTFLASQGKDVFVITGSVSDYSPQLKSFGAS
DIMFLMTFPPYSAEVKHVASVAKERGGFLCLFTDSASCPVYSKADVVIKCTTNSLLMSNS
FVGLVSVIHVFIHMLLLSSENGGKNIRNGLEMEKMGYSIIADAGEQSCS*
>SPBIB_v1_260047|ID:27163361|iadA| Isoaspartyl dipeptidase [Uncultured spirochete bib]
MLVIQNAEVFSPAPLGKLDILVGGAKILAMEPHIDPAVLPGETEVLDARGMLLVPGFIDG
HQHFTGGGGEGGFQTRVPELTISMNFSNGVTTAVGLLGTDSLTRSIENLYAKTQAFNAEG
MTAFMLTGAYWHPSPTLTGSVARDMVFLQPVIGVKLALADSRGPHIDAKDLAALASDVQV
AALVANKPGIITVHTGIRTQGLDLIFEIVEQFEIRASIFVPTHINRKGGKLTSQALALAE
KGCVVDATCSQAPLEPDSPRLTAADFACMARDNGLLGQVSFSSDAGGSMPIWNQDRSRII
GMGVGMPSSLLFELDLLVNKKGVPLEQALMPLTTTPARIYGLENSKGALRVGMDADLLTI
DPASFEIRDVVALGSVAVRNKIVEKKGYFEHGSDTSIHA*
>SPBIB_v1_260048|ID:27163362|yliA| putative peptide transport fused subunits of ABC superfamily: ATP-binding components [Uncultured spirochete bib]
MAVTHPFMHEDEILTIKDLSLSFPGYHGYTQVLHNVSFSVKKGETLAIVGESGSGKTVTM
RRVMQLLANVRTDSGRILLRKRDGTVLDITNISHKDATKIRGADLSMIFQEPMTSLNPVF
TIGDQLLEAVLIHQRISKAQAMQKVMELLELVRIPDKARRVNDYPFQLSGGMRQRVMIAM

ALACNPQVLIADEPTTALDVTIQAQILALIQSLQEKLHISVVFITHDMGVVAEIADRVVV
MYQGQVVEENDVHSVFSSPSHPYTQALLKAVPKLGSMTGRPFPATLPILEMSKELSRKPG
DAAPQEKTIDTADYTKPPLLQVRNLTTHFIADKTFFGRTTHVVRAVQDVNFDLFEGETLG
VVGESGCGKSTTGYSILKLVPAQGKVLFMGSDILTLPDSELKHYRRDIQFVFQDPYASLN
PRIRIGTAIEEPLVIHNIGTPEERKKRVEYLLDCVGIPRKHKDRYPHEFSGGQRQRIAIA
RALATQPKIIIADEAVSSLDVSIQATVLNLMLELQKEFKLSYLFISHNMAVIERVSNRVM
VMYLGHVVESGTRMQVFENPMHPYTRKLLAAIPVPDPTRRSNFGMLSGEIPSTIHRIEDA
TPRIEYIEVEPGHFVASTKEGISFGA*
>SPBIB_v1_260049|ID:27163363|dppA| DppA2 [Uncultured spirochete bib]
MKAKHLVLALMMVLIISAIPLFAAGKDIIYASDSTTKTLDPHDTSDTYSGAIERAICQGL
LGFDKNLNIIPLLAESYTYNDSATEFTFKLRKGITFHDGAPFNAQAVKVNIDRLMTGKYV
RSSLMAPVKEVKIIDDYTVKFILKEPFGAFLNALAHPGALMLSPKALQEYGDDVSKHPVG
TGPFMFSEMVSGSYVLIKKNPNYWRGTVKVDSIKFVPIPENGSRVAMLRAGQAQYIYPMP
AELLKLVENDPNIDVIKQPSIIERYLILNTKSKPLSDERVRQAINYALDKKAIINIAWGG
AATEADSIFPSALPFFKKQGPWPYDLAKAKALMKEAGYENGFKVVFLTPNASARLRATEM
VQQQLKAIGITGDIQSMDVASFYDKLAKNKLETVGETPFIAFGGWSSSTGDADWATRPLI
STEAFPPNMSNYGFFEDKTVDNLIKAGLTTADPKVRANAYAQLQDYVWSKAPWGYLFVDT
LIAAKSKNIKGIYPMADGAFTVEEAEIVQ*
>SPBIB_v1_260050|ID:27163364|yliC| putative peptide transporter permease subunit: membrane component of ABC superfamily [Uncultured spirochete bib]
MLKYFIRRCLSILPVLLVVSILVFLFVHLLPGDPARIMAGPDADEATINRVRTELGLDKS
LPEQYLNYMGRLFRGDLGKSLRSKLPVAQEIALRFPPTLYLALASMVWSILFGVLFGAYA
AMHRGKWQDYTAMLVAVSGISMPQFWLGLLLMQLFAVHLGWLPVTGFTGKFKELILPSLT
LGATVAAIIARFTLSSFLDVLQEEYINTARAKGLSERVVMWKHAFRNALIPVVTMVGLQF
GFLLGGSVVVETVFAWPGLGRFMIESVSVRDYPVLQALLLLYSFQFVVINLLIDMLYAVI
NPEIRYK*
>SPBIB_v1_260051|ID:27163365|yliD| putative peptide transporter permease subunit: membrane component of ABC superfamily [Uncultured spirochete bib]
MAEKTITPFMIFWQKFKKQKSAMVAGIFIFFLILAAIFAPFIAPYDPYAIDYGNAMMPPS
PQHWAGTDIYGRDILSRIIYGARISLTVGISSVTIGAIVGVSLGLISGFFGGFLDEIIMR
SADVLFAFPGILLAIAVVAILGPGLVNVVAAVAIFSIPTFARIVRSNTLSLKESLYVRAA
RSMGASNKRIMFVHIMPGTLSGAIVYFTMRIGTSILTASSLSFLGLGAQPPTPEWGAMLA
ESRDYIGVADHLTIFPGIAIFLTVLAFNIFGDGLRAAFDPKLR*
>SPBIB_v1_260052|ID:27163366| exported protein of unknown function [Uncultured spirochete bib]
MKRILPIAAIFLLFALIPVIAQTEAELKATYENAVKLAAAAPQDYTLNWQAARAARKYGD
YLVKNEVPGWKDTARAAAKEGMKYGEIAFKLNPTGIEGWYWYGLCVGTYSDCVSVLKALA
EGLKGKTQMGFENAYKFDKTYDNGGPILSLGRFWQVLPGIAGQDRKKAEQLFNEYIQLFG
SSPDANSDVWYFRGQLYKDTNRAALAKADLEKAAAMGNKDAQKLLGEMK*
>SPBIB_v1_260053|ID:27163367| protein of unknown function [Uncultured spirochete bib]
MKKIELVPIESMSVDFQSYGVCVSVDNRQPDYDSPEFKFWNKLGIIESQNKCSICMVESY
PQKQSLSTVFECHTRTGETLIPVENDVLLVLGLSKNSQHNEMDYDSVKAFLVRKGTAVIL
NPGTWHYAPIAQHEVVHTFVVFDHATPDSDVIKIDSQDAGVGWEIAN*
>SPBIB_v1_260054|ID:27163368| putative 2-hydroxy-3-oxopropionate reductase [Uncultured spirochete bib]
MNIAVIGTGIMGTGIGLTLLSKGHTVHCYNRTRENAQELIAAGGKYFSSPAEAAKDAEYI
IIFVWNKEALYNVLNGESGLVMGARQGQIYIDMSTQLPETAKEEVKMFKAKGADFIDAPV
HGSKAEARNGGLWIMAGADSQVFAKALPLLKEIGETVHYMGPVGSGCVAKLCGNHLVSAI
VASLGESLVMAKKSGINSEELLKLWSEVDFRSPIIDGVGHSMINHEFDVSFHLRTMVKDT
ELIRNYSESIGVPVLISNIVHELNKVGQNMGWGEQNASAIVKVFEKFAGVE*
>SPBIB_v1_260055|ID:27163369| Dak phosphatase [Uncultured spirochete bib]
MEALLREHLEPLFFSIKESFERNKEFLIDLDSKTGDGDLGLTMSKAFSAAHESVKASNSS
NLGQILAMAGIAISRAAPSTMGTLMATGFMRGGKAIENAQRIGTKEMADFWLAFSKGIAD
RGKATEGDKTILDVAAPVAKSFSASAEKNESLAVAMRYALDAAGKALEATKSMIAQHGKA
AVFREKTIGLQDAGGSALYILVKTMHDFVLA*
>SPBIB_v1_260056|ID:27163370|dhaK| PTS-dependent dihydroxyacetone kinase, dihydroxyacetone-binding subunit

DhaK [Uncultured spirochete bib]
MKKFINKPENFVDEMLKGIYASHPQQVMFVANDLRCYVRKEAVKGKVGIVTGGGSGHLPL
FLGYVGEGMLDGCAVGGVFQSPSSEQILEVTKAVEQGAGVLYLYGNYTGDILNFDMAAEM
ADIEGIKTATVVGNDDVASAIKGEENKRRGVAGIFFLYKAAGAAAAEGMSLEEVKRVAEK
AKANVRTMGVAFTPCIVPEVGKPSFTIGDDEMEIGMGIHGEPGIRRGKLLPADKIVDEMV
YPIIKDLPFEKNNEVAVLINGLGATPKEELYILARRTLHILEEEGLKVFHVYVGEFATSM
EMAGASISLCKLDEELKRLIAAPAFTPFFSQKQL*
>SPBIB_v1_260057|ID:27163371| Sugar ABC transporter, membrane spanning protein (Sugar) [Uncultured spirochete bib]
MDGKLLSGKRRYMAADRSILQLLLIAIVVFIIMALLNPGKFLTLLNIESMAYQFPELGII
SLGMMIAMLSGGIDLSIVSIANMASILMGLILTKMMPQNAANNQIVLFVAFALLISLLTG
LISGLINGFLIAHVGIPAILATLGTSQILTGIAIVITKGYAVPGFPEIFLKIGNSKLFIF
PIPFLVFLACAIVLFFLLQKSSFGIRLYLIGTNIKASVFSGIKVKSILMKSYALTGVLAS
IAGIVMTARTNSAKADYGSSYVLQAVLVAVLGGVNPAGGFGSVIGLSIAVLSLQFLSSGF
NMLRFSNFATEFTWGMFLLLIMGINYFQNKRIKSSK*
>SPBIB_v1_260058|ID:27163372| putative ABC transporter permease protein [Uncultured spirochete bib]
MKKLLQQHEFVVTIVFFLVVILIGAINPAFFSIGNIFSLLKSMIIRGILALGVLIVIISG
NVDISFTAISAFAMYTTSKIMLALFPEGGLLLAFAIGGLIGLLLGSINASFVAGMKLPAL
IVSLGTSSAIRGFMLAFIGVKIINNLPASLIAYSRSYIAKFTDASGKIVTLPSAIIMLIA
VTIIVWFILRYTMLGRGVYALGGDPVSTERAGFNIKAIQYFIYCFMGFLSGIAGVLHSVF
MRNANPFDIVGTELIVIASVVLGGASITGGKGTVYGTLLGVAFTVLIENSLIIIGVPSYW
QKVVIGLIIVVSTAASALREKFAKLEIS*
>SPBIB_v1_260059|ID:27163373| putative ribose/galactose/methyl galactoside import ATP-binding protein [Uncultured spirochete bib]
MEQVFLAVENIDKSFAGVHALNDVSLKIKKGEIHCLAGENGSGKSTLIKVIAGVEKPDSG
QIIIDNKKYHHLHPIDAIHEGIQVIYQDFSLFPNLTVAENLALNSQLEMRKKLVSWKQVK
ETAEKALQLINVDLDLNRLVENLPVADKQLVAISRALMQDAKLVIMDEPTTALTQKEVER
LFKIIKSMQEKGISTLFVSHKLREVLDISERLTILRNGQKVIEGNVEEFDETKITYYMTG
RNVSSNKFISEKKSGDEKPILSVTNLSSDGFFDDINFDLYKGEVLGITGLLGSGRTELAL
ALFGYSPADKGEIHINGQKIHIKSIQDAMRNGIAYVPEDRLNEGLFMKQSISRNIYVGIL
DSIKKALGLINVSEADSIAASLIKDLNINADDPLRPVQTLSGGNQQRVVLARWLSTKAKL
IILNGPTVGVDIGSKTDILEKLKELAKEGLGIMIISDDIPELVQTCSRILVMHKGKIIRT
LEGDEIEEKAISNILNNLD*
>SPBIB_v1_260060|ID:27163374| ABC sugar transporter, periplasmic ligand binding protein [Uncultured spirochete bib]
MKRRILLGMLLAMMVVGLVSAQTATTASKKFTIATVVKVDGIAWFDRMREGVVKFGKDTG
NTTFLLGPAKADAAEQVKIIEGLIAQKVDAICVVPFSPEALEPVLKKARDAGIIVISHEA
TNQQNIDYDIEAFVNEEYGAHMMDNLAKFMNYEGEYANFVGSLTSKSHNQWMDGAEARQK
EKYPKMKLVSRRNEEYDDQNIAYQKTKELLTKYPNLKGILGSASTTAPGAGLAVEEAGLQ
NKVSVVGTSLVSSSGQYLKSGAVKMIFFWDPADAGYVMNKMALTLLTGGKITDGMNLGVK
GYEKIKRDPQNPKVFYGQAWVDVTPANMNQYNF*
>SPBIB_v1_260061|ID:27163375| Regulatory protein LacI [Uncultured spirochete bib]
MAGTLQDIADAAGVSIATVSLVLNGKEGISAETRQKVMQAAEALNYRPKKHQSLMSANSR
NTIQFLKIAMHGHTVNRDHNVFISDYIDGMFREAQRQGYKLEIANIKGESIDYIIDSLIS
RKPEGAILLGTEFSQNDVIALKSIDFCPIVVLDTYFDYLDLNFVDMNNSDAVYKIIEYLV
SCGFRKIGFINSNVQTRNFYLRKQAFIGSMQVLGQKVDENYIVDVDSTFNGAYKDMMVHL
SRGIKLPECYFCANDIMSYGCIKAFKENGIRIPEDLSIVGFDNLPMSASMDPPLTTIDVS
KQKMGHFAVTLLDELIQSPERKNSVKILVGSDLIIRKSVAPKI*
>SPBIB_v1_260062|ID:27163376| Xylose isomerase [Uncultured spirochete bib]
MPRYSVILGNLGNTCDRFLSTGYKNQPSKEIMIRQASEIEGVEGIELVGSWDIDEHNADQ
IGDLLGKYNLSCVSIIPDLFSQKKWGTGSIASKDPDIRAQAMDYLFSICEISRKIGCSLI
NIWPGQDGYDYCLQANYLAERQIIIENIKKLAFFYPDLRFSLEYKLKEPRTHSFLARASD
TLLVAMATELPNVGVTIDTGHALFAYENMAESAVMLDMSGKKLFHLHFNDNYRYWDDDMI
VGSVHLVEYVELLYWLKKIDYNGWYSMDQYPYREDGKHAIEESVKFIQAIERKLTPSAMK

EIDNLLAEGKAVESQKWFREFIFS*
>SPBIB_v1_260063|ID:27163377| D-tagatose 3-epimerase [Uncultured spirochete bib]
MRKIGIYYAFWTREWDVDFFPFIQKVKSLGFDQLEINGGTFAEMSAAEQKRLVDEAKRCG
IVLSYGIGLTANHDVSSLDESVRQSGVRFMQQMIEAVGEAGGGMIGGTVHSAWPSTLPEG
TTNKRPYLEQSKKSMRELVKVAEDNGVILNVEVINRFEQYLLNTCEEALAYVEDIQSPAC
RILLDTFHMNIEEDSIGGAIRKAGRHLAALHLGETNRKPPGLGRMPWKEIREPLDSIHFD
GPLVMEPFITKGGQVGRDIAIWRDLIPNPDYDRLARDAAAFVRKTLCE*
>SPBIB_v1_260064|ID:27163378| putative Carbohydrate kinase, FGGY [Uncultured spirochete bib]
MSQTCAPLHIGIDLGTGSLKLSAYAGERAFSASCGYDIFSPEPGIAETDPEAWIAALKTA
WLAVCAQLQNAGLPIELASIGLSAQMHGFVPISETGAALHNAILWADLRGAAYAGLYSSL
LAGSFDRLMNAPAAGLTALILLWMKHHEPELYAKTHYILFPKDYLRFRLTGDIATDPGDA
SASLLYDFRTCRWAEDAIEALGMDHSKLPVIRDSFSPGGVVTGKASRETGLPEGVLVATG
SADKACEIYGSGFFGEYFRELALAGSPDMGIMSRQAPARGKFLNSAPLRGTGMNSALSRE
DMHSAPFRGHDSDWQSPKAAQVSIGTGIQVVIPVRGLAPYEPGLNFFESCVPSVRYRMAA
MLNGGLALEWVLSMLNADWESLYRAMDEGKTRLPQDLLFLPYLTGERSPYQNPDARGAWI
GLGLHHTRNDLLSAALLGVACTIRLGMESLGVAPDASVYCVGGSTRFKTWMNIVSEVTGR
ALFVTDQPDASVRGAAAIGRAAAETGAFDARRLPAPLETTRIEAESPAWIEHYYSRFKSS
YEALFGGHE*
>SPBIB_v1_260065|ID:27163379| NAD-dependent epimerase/dehydratase [Uncultured spirochete bib]
MKVLFIGGTGNISSACTDEALGRGYEVFHLNRGTHPEREKPGVRTLKADIRDTQAVKAAL
RGLRFDSVVQFLAFRPEHVKADIEIFDGITDQYVLISTCSAYRKPSLTPVITEDTPLENP
FWEYSRLKIACEWVLAGSGAASHTGNGEETARTEEKVRIIKEKARDEDAVFSEAEQRSFP
YTIVRPSHTYDNGWIPGCFGSASYGLAWRMLNGLEVVVPGDGQSLWTLTHASDFAVGLVG
LLGKREALGQAFHITSDEHLTWDAIHSIIGEALGVQPKIVHIPSDFIAKVLPERGAGLLG
DKAVSVLFDNSKIRRFVPEFAPRISFTEGIHRSLAWFDAHPDLKVPDKAMNADMDAILER
WQLCAQ*
>SPBIB_v1_260066|ID:27163380| putative dual use protein Tyr:Ser/Thr phosphatase [Uncultured spirochete bib]
MIFSELKIPGSSGIIGMASCPGRWTQLAAKSSMKAAALLGPQLAAQAAPSIAPQPAPPDE
QTHDYLVRDLAIIEAWGAEILVSLIEPSEYVLAGVERLPELVPIGIRHIELPIADFSVPD
NRWENAWEREGALVRSALRRGGKVCIHCMGGYGRTGMVAARLLIEFGVEPEEAIARVRSA
RPGAIEMPQQEAWVLKLRAQ*
>SPBIB_v1_260067|ID:27163381| exported protein of unknown function [Uncultured spirochete bib]
MRQKAAIRSLFLAVVCLLAVSPVLAQKVQGEAGIGFMAISPNLTDTIANFAVGDMLYGVT
VSYAVKPWLGVSTDVLYLGDSYYGPGSGMFNEGPSSWAGLQSASGDKANWKYYESFIYAP
LSINLMAPLGIVRPYLGLGPAFYFHFPSTNQDTAFTDYLKTHYGSAAVVGRIGQGFTARV
GFDVFLGDSLSIGAGYVVREDTPVTVFKDIGDLNFYKEKGYLFLVGRFYIK*
>SPBIB_v1_260068|ID:27163382| protein of unknown function [Uncultured spirochete bib]
MTENQFKESAAGWQNEFSASITIIASDFTILYMNDKSAAVNAKWGGKALIGKDVRACQQG
RNERRVH*
>SPBIB_v1_260069|ID:27163383| TRAP transporter, 4TM/12TM fusion protein [Uncultured spirochete bib]
MRKLDGKVKIAIYVYIIAVGVFHLYTSIFGNFEAYLQRALHLSMVLPLVFVLYPISKKLK
DSAVPFYDWVLAGLSFLPGAYIAANYWAISTRIVQVDPITTTQLVLGILLVVLLLEATRR
IVGMPLTIIALVFLLYMGVAAKIPGLFQGISFSVPEIVEEVFLTDEGIFSSPLGVSATYV
MIFLIFGGFLEKSGVGDFFMRFAQAFTGTQPGGPALIAVTSSCLFGSISGSAVANVYGTG
TFTIPLMKKIGYPSFFAGAVEAVASTGGQLMPPVMGAGAFLMASFLGLPYRTVMIAAIIP
ALMYYGAVFLMVRLSAHKYGLKGLSAEELPKKKDVLKDSYMVIPLVGLVYFLLAGATPMR
AAVFGIVLAWLVSFFKPKSQNQAKRRLSIIIGAVISVLITVAFVFPRFVEPIIKGNIGLW
ILVAGFVAATFLNDGMKPKDVLDAIYSGTSGIPLVAIACATAGIVLGSVALTGIGGKLVG
FVLSFAKDYRFLGLLLIMAISILLGMGLPTTGAYILASALGAPILVKMGIPPLSAHMFVF
YFAVISNITPPVALAAFAAASISGANPNKIGFQAMRLGFLAFVVPFAFCYDQGLLLQASP
IANVLAVATGIISVLAFGYFWVGYIKHPIPLWMRAILLVAGVMALAPQVLYVAISAGLVI
VCYILSASGKVVAVHQKAAVLKE*
>SPBIB_v1_260070|ID:27163384| TRAP transporter solute receptor, TAXI family [Uncultured spirochete bib]
MRRVMILLLVFVILVGASAQSKAGWPDQLKFMSGPPGGNWFALGTALSEMWSKNVIQTTS

SSGGGVSNILNVDAKKADFGFTVASLLGAAIAGEEDFEGKAVKNAVIMANLYTQYTYFIM
RKDFAEKNGIKSVDDMIAKDIPVRFATLKPGTASEFVIKALFKKGYGTDYDKLKKKKWTF
EFTSYDGGADLLADNHLDCFAFSVGKVASVVMNIESNTPVVILPVGQKALDALAAAYGTT
TFTVMPGVYKSVTTPIKTVGDYTCIVIRKDFPDNLVYELNKSMWANRDNLAMAVKDISEL
NPKEALPEGLPAHPGSVQFWKSAK*
>SPBIB_v1_260071|ID:27163385| Transcriptional regulator, IclR family [Uncultured spirochete bib]
MAEVNSVKKALAILDLLSDRNSRSLSEISKALALPKSTVHSLLETLTLARVLERDVESGS
YSLGIRLIELGYCAQTGLDLVRIAAPFLKGLNVRFDETVHLTVLDNDEVLYIDCIESQRR
LRTYSVIGVRAPLHCTSVGKAILAFLDDGEIRRIIAERGLPGFTENTITTEARLWEEIAH
IRDQGYAIDDREHEDHLRCIGAPIFNARGEVFASMSLSGPAERNTLERLESMVSALLEAT
GEISHRLGFRNREFSRARGENHITPANSY*
>SPBIB_v1_260072|ID:27163386| DctP family TRAP transporter solute receptor [Uncultured spirochete bib]
MKKAVFAVVALLMLASPAIFAQQKPVKLVFTSVSVPGDAHTQAMFVFKDEVEKLSGGQIQ
VDVYHSGQLFTQQAEQDAIRKGTVDMVYTSAQWLAEFIPYLSMFGAAYTFQSYDQMTKTF
NGPIGKKIFEEVAQKTGIRPLVAYYLGTRQLNLTAKVGPITRPEQMKGVKLRVPNSPTWI
AMGKALGANPTPMAFNEVYMGLKTGAIEGQDNPLPTDKNAKFYEVTKYIVLTNHVVDSTW
PSINEKKWQSLTKEQQGWLMQAAEKARQFCDKTNLDNEKNILDFFRQQGLTVIENPDRAA
FAAYAKNSYLTESKDISKDWDLNLYEEIQKLK*
>SPBIB_v1_260073|ID:27163387| conserved membrane protein of unknown function [Uncultured spirochete bib]
MPFWLKKTGKFLVDCIELYLPMLTFTVLFVSFMSQIVARYLFKPLIWPEELSLICFIWTA
LLGGLYAKRTGSHVAFTMLYDAAKPGLQKAMRIAGNALLLTSLILIFIPSWNYIQFMAYK
KSDALRIPMNWAYFPFIVFLADMIVRLVIDIVKDIAGKASGGGKP*
>SPBIB_v1_260074|ID:27163388| TRAP transporter, DctM subunit [Uncultured spirochete bib]
MNLALIVFFVSFTLIFLLRIPIAPGMMMASAFYFALSKNPAASLDMVAMQFLTNMNASFI
LIAVPLFVFMAEIMNSGKVTNMIFSFANALVGKRKGALGHVNVVASIIFSGMTGSALADA
SGLGSMEIKAMREQNYDDGYTCAITAASATIGPIIPPSIPMVFYSMLSGASIGYLFLGGV
LPGLLIGVGLMIYTAIIARARNYPEGQSLKPLEFWKLTLRSIPALFSVVVLLGGIYTGIV
TPTEAGALAAFYALLVSVFLYRAFGWKDFKQVLVNTVRTTGTLSLLVGSAYAFSYIVTIE
KIPNFVASLMLGLTTNKYVMLLIINVVFLALGMFIDTMAITLVFIPIVLPIINQLGIDLV
HFGVVIVLNMMIGLSTPPYGMLLYVVSGISGTPLKTIIKEIIPMIFVIVVVLFLITYIPD
IVLLIPRLSGYR*
>SPBIB_v1_260075|ID:27163389| Dihydrodipicolinate synthase [Uncultured spirochete bib]
MNKTIPDGVWPTMITPYHEDGRIDWDALEKLVEWHIERRVAGLFAVCQSSEMFHLSLRER
LELARMSIKFAAGRVPVLVSGHVADSLDDQIEEAKMMADTGADAFILVTNRLAKRHESDD
VFKRNLELFLDRFDRDILLGFYECPAPYKRLISPELMRFVVGTGRFGFLKDTSCRMGDIK
AKLEAARGSGFKLFNANAATLLASLRAGASGYSGIMTHFHSDLYVWLCRNWASRPQEAEE
LQDFLGLASVIEYQLYPVNAMYALQLEGLPITLYSRRADARQFTESMRLEVEQMMRLSHK
FSERIRSLD*
>SPBIB_v1_260076|ID:27163390| Class I peptide chain release factor [Uncultured spirochete bib]
MNKELLAASILDNAVIDFARSSGPGGQNVNKVNTKVIARVPLCTLAGLDDKEMELVRTRL
AGRINAKDEIVVSVQEERSQVQNRERALNKLIALIVAAARRMPPRIPTKPTRASKERKLT
TKRRRAAIKGYRQKPQLE*
>SPBIB_v1_260077|ID:27163391| exported protein of unknown function [Uncultured spirochete bib]
MKKKSGIALKLALIVALAVIALASCDINAPAGTVVDLNSSEKSLSADASGLSKGKPDKTP
VVEKIVGTAAIAPDCFFFEFDMVGQQKFARALVPVSPDGYIKNGKLLYNTKRYKIASFVY
DQASGYLQGTTAQEGGVSYAFAGLYSQDSGFFGTISKFEQGVEKSGYFIGSPMLGGKNIA
NYIGVATYLFPTPTPQTLVFNTILDFDAGTAVGTWSESGEGWNYSLHGPIAGTVEGDSVN
FNASVLPIFRPYMLYDMTAVGSGTFKNKGKKTVSGDFTIYYGDLVLPSLLTATKEMP*
>SPBIB_v1_260078|ID:27163392| NYC1 chlorophyll b reductase [Uncultured spirochete bib]
MNIQDSKSLVVIITGGTRGIGRGLAKCFLERGASVVISGRIGEVVRRAVEELKAEVAGPG
SALKSAEAVPVAGTECDVREFAQVQALWDYAISSFGRVDIWINNAGIGQPQSDIDELKPD
LIRDIFGTNCTGALYGCKVAMTGMRAQGDGAIYNLEGLGSNGSIVRGMAAYGASKRALAY
ITDSMAKEAEGSGVIVGALRPGMVVTDLIIGEYAGKPEEWKKVRRIFNILSDRVETVAPW
LVDQMLQNRRNGRRIVWLTGLKTMGRFLSAPFVKRSVYPEME*

>SPBIB_v1_260079|ID:27163393| conserved protein of unknown function [Uncultured spirochete bib]
MENQKTRIAIAYDFDGTLARGSLPEHGLLQDLGIESASFWNEVKERAETQDADEILAYMC
LLLEKAKERGIEITESRLGSYAANIPFFEGVVEWFGRINKYAAELGVGIDHFVISSGLRE
IIAASAIAPCFKFVFASKYLYDERGMASYPAVSINYTTKTQYLFRINKGIFNFFDNKRIN
TWIRLDRRPYPFKRMIYIGDGDTDIPAMKMTRYQGGYSIAVFDPLKWDDLSQQAKIYKLI
AEDRVQYVAPAVYSEGSLLDIAVKGIIGKIANEP*
>SPBIB_v1_260080|ID:27163394| RecG-like helicase [Uncultured spirochete bib]
MTVYKGLARDFKDDVARNVIDEKIANCFLRQFGYYPGSSERNSWTYSLRFMDTIIRIGSI
PDDCGVLIEFNIPNTNKRIDFLITGKDENGGSNFVVVELKQWQEARATPYKDVVITFVGH
GERTVAHPSYQAYSYNTFLVDMNTSIQQNRIGGHACAYLHNYQPKRNEPLLEQHYMETVL
KAPIFFKHDVRKLQDYIASKVGRGNGTEVLKHIENGEVRPSKELIDCVVGLLKGNDEFIL
LDDQKVAFEAIMSICESSRDKTVVMVKGGPGTGKSVIALNVFGKLLQGKKNVRFVAPNAA
FRNVMLETLVKNDPRQRVRVKNLFMGSASLWECRDNEFNVLVVDEAHRLKNEMAYGYKGD
NQIEDIISSSRIVIFFVDDRQRIRPEDIGSSDEIARIAKKYNADLHEFSLDAQFRCAGAK
DFIAWVEKALQIDSSARTTAWDRESFDFRIYSTPNKLFKAVKEKNDEGFKARLVAGYAWD
WTSLSENNDRAQILDVSMPEYDFAMPWNQRTKSELWAILPNGLEQIGCVHTIQGLEFDYI
GVIIGNDLRYNRATGKVEAVWNEYKDKAGKKGLKNDPVTFNALVKNIYRILLSRGMRGCY
VFCRDKNLRDYLQGLYDSKLEYADYFVEENNSALWAAEGGHGKPEDKNSYSI*
>SPBIB_v1_260081|ID:27163395| conserved protein of unknown function [Uncultured spirochete bib]
MEKKPIIVVVDGLGGGIGSQLCTRVRQAFGHDVEILALGTNASATEQMIKAGADRGASGE
NAIRVSINLGQIILGPIGIVLPDAMMGEITPRIARAVMHASSKKILLPVNQPHFILAGLP
PSSLSRCIEEAVALVASELGLAVH*
>SPBIB_v1_260082|ID:27163396| putative cation ABC transporter, periplasmic binding protein [Uncultured spirochete bib]
MGIGSSHSKRSLVWMFLALLCLSALPLAAQDKPVIASTSWVQAYAIAAGAKNIVTIAPFE
LQHPPEYEIKPSDLLAVQHASLIVYSGYEKFAKKLAETAQNSNLRILAVYTDNVPSTIIA
ESKKIAEALGTTQAQEQWAKSFTAFSDAMRERVAAALPDKRVVVQAYMKTFALWLGLDVV
GTFGPGEPSPAVVLDLIKKKPVMVLDNYHNPGGKALAESLGVPHVLLINFPGKDGTRTIE
DVFLYNEKAILSQIGK*
>SPBIB_v1_260083|ID:27163397| putative Zinc import ATP-binding protein ZnuC [Uncultured spirochete bib]
MLDDTSMPGDMPTPGDMRPANPPLLSAKNLSIGYKRSEPVVRNICIDLTARTQRRTVIGF
VGPNGAGKTTLLKTCLGLLPPLDGELRLLGVDTRTAAFAQTRKKLAYIPQNRPEGNSGQL
RISVREAVSFGRLGKLGLTGRFGRLDREAVEAAIAYCGLEDIADKAVQDLSGGQFQRVSI
ARAMAAEPALYLFDEPGSYLDEEGQKAMRTLIHSIAKSGVPLILVTHDRRLVKLCDAVIV
FRKGTAQLLNTSAFLAETRGREI*
>SPBIB_v1_260084|ID:27163398| putative ABC-3 [Uncultured spirochete bib]
MMELLHIPAFSRAIIALFASGLAFPLLGIYIFSLELIPARFGVMHLSLLGATVGLILGVD
PVLFAMLFSVAAGFAISGLSVRRSGGTSGGAMALLMTASLGIVFILFYKTNIHAIEAFNL
FWGNVLALDKTEVLLVVIVSALILAGTARFLRPISAVLFDRETAFAFGFPSTVVYTAILV
VVCAGIGLGMRITGALMVDAVTLLPALAAQSLKKNFKPTMLLASLFGVCINLAGFALAVI
FDLPTSPAIIVVGTVCVLACRLAARRNLKKGSDPVS*
>SPBIB_v1_260085|ID:27163399| Na/Pi-cotransporter II-related protein [Uncultured spirochete bib]
VTSWFQIVLQLAGSLAIFMYGMTLLSDGLQRAAGERLQRILQFITANRVVAVLTGCLITI
IIQSSSAATVMTVALANSGLLSLKQSIGVIIGANIGTTITAWIIAVVGIAKFNVLAAAIP
LVGIGYLLTLMEKRSEKLKDAGIALLGLGFLFLGLEYIATAVPKPSPELLAFLGRFSPET
QPLSLILCVAAGAVLTMIIHSSSAATAMIIPLAVKGILSFEIAAALTIGANIGTTIDAFL
ASLKGEATAKRAAWAHILFNVIGAVWAVIVFRPFLGLVRLVSGYGNGAGAIGIAIASLHT
LFNGINTLLFLPFINQYEALLKKLVREREGGEHRMVYIAPSLHPAPELSLLQARAEIRAM
AGRCRGMFDASLDLVLSNEKKDMRGRLEWFQREEQYLDDMREALVGFLLKVQTADMPESL
RARVLGKMQIVAELESASDECFSIAELMVKKSRRDLKFDEEAALSLKPYGEAVKQFFDFI
TSNLDRGLDATEFGKAGEIENQIDSFKKELKKMARKRLNAGADARTELLYIDLVRHFEKI
GDCLYAVAGELERI*
>SPBIB_v1_260086|ID:27163400| conserved protein of unknown function [Uncultured spirochete bib]
MSFRWNEEKNALLKQERHMSFERIVVAIEEGHLIGVLEHPNKEKYADQMILVVEIDEYAF

CVPCVKEKNGDYFLKTLYPSRKYTRFFKLGGKL*
>SPBIB_v1_260087|ID:27163401| conserved protein of unknown function [Uncultured spirochete bib]
MTIDEDRELIQSVENGEWQPVKDFDSVREKLVKAAKETALKDYRMNIRISKRDVELLKAK
ALEEGLPYQTFVTSILHKFITGKLKEGV*
>SPBIB_v1_260088|ID:27163402| membrane protein of unknown function [Uncultured spirochete bib]
MEAKQKTHAKASVVVARKPRTRASAAGGSAAGGRSNGGRTKPSVFKSARGIVAIAFIVLG
ASALLSMLIWTRYPSVWKPIVVPGKMLIGIFSWAAYYLPVWLFWAAALAFVPKFCPRLTY
LLGISALPFVVVTAFARLSSNPGQFFESHPSMAKVGLSSLYAALGFLFAASLVIVVAGYI
KLTEWLKTNGYIKTRAERAQAGKGQSFGEKLATFVKNISMRREERRKRKEEARLARAEML
AQQNISLHIPEVPAPDPLKSGGQTPNFNFSGPAPGAAAAQANPSPAATAALDAAGISFTS
VPPARPAQPGDEPKKAIVERILERKAAKTYHVPIDGLLNTYPDGQYWIIDDKTRASAGVL
KETLAEFGIEAEVTGIRKGPVITMFEILPAHGVKISKITNLSDNIALRLAASSVRIVAPI
PGKHAVGIEVPNERRAIVSLRELIESDHFRQTKMEIPVALGKDIAGEVQLVDLTQMPHLL
IAGATGSGKSVCVNSIILSILYKRTPEEVKLMLIDPKIVELKLYNDIPHLMTPVVTEAKK
AFQALQYCICEMERRYSMLDSLGVRDIRSYNRRVRERNLAQEKLPYIVVIIDEFADLMQT
TGKELESTLARLAAMSRAVGIHLVLATQRPSIDVITGLIKANIPSRIAFMVASKFDSRTI
IDMVGAEKLLGRGDMLFSGAQDPFPVRMQGAFVSEEEVERVVAHVKTLGEPEYIDDEIFI
DEEDLEEPTLFDEEGSDPLFEKALEIVLQQGKASVSFIQRRLKIGFNRAARLVEMMEEKG
IVGPAQGSKPRDVLRNPEVFDSPEGGGEGRSGHGGLHSGPEEEEG*
>SPBIB_v1_260089|ID:27163403|slyD| FKBP-type peptidyl-prolyl cis-trans isomerase SlyD [Uncultured spirochete bib]
MEIKKDRVVTIDYTLRDDTGRLIDSSAGSEPLVYLHGNENIIPGLEKELEGKNPGEAIEC
SIKPGDAYGDRDEALVFKVQKKDFGENVEVAPGMQFEAHGENGVQIVTVVKVDGEEVTLD
ANHPLAGETLHFDVKVVDVREATPEELEHGHVHTGHDHEEFEENEEDFEVEDSSES*
>SPBIB_v1_260090|ID:27163404| conserved protein of unknown function [Uncultured spirochete bib]
VPRASIFMKFYSKVRVRFLDLVIFGVAAALVVGTSLLVLNRQSGTRYVQMTGETGEWIAP
LNKDAEYQIPGPLGITYVHIHDGTAAVVDSPCKNKLCILAGAISEPNQWVACLPNKVFVR
ITSSGTNDSGGVDAGAF*
>SPBIB_v1_260091|ID:27163405| putative heptaprenyl diphosphate synthase component I [Uncultured spirochete bib]
MPAPSSPSCGNVRSRFSNRADVTALLAAFCFFLSAIEYMLPKPLPFMRLGIANLPILLAV
DMLPFNWFLVLAAAKVVGMSIVSGTLFSYVALFSLAGTMVAALVMRGVRRTGGHAVSLVG
VSVAGAVTSNAMQMLIARYVIFGEVAWLIAPLFLVTGLITGTLMGFFAEFFVEHSGWYSC
AMGESDDLAFGFEDEEGGSGESGASGDSAAAESKDSASARVQGASGNAADFSSQNEKKLK
PDRGARKSQRNERRFERRHRYEDMFEPWVGALLGAGIAIFFLLQRDIVAKVLYLAVFILW
TWFAGKRFSLISTLVVSAGIVAANLLIPSGRVLAKIGPLAITQFALTEGLSKALTFEGLI
YLSKAAIMPGLRFPGRFGSIIAQAFQYYDRIIEYRGKVHAKTVISDIDRLLQVVWTSARI
SGEAEASAASKGAQAGPAHRSRVHRSLILAAGIAAAVVIIPFAAKLIAS*
>SPBIB_v1_260092|ID:27163406|leuS| Leucine--tRNA ligase [Uncultured spirochete bib]
MAKYPFKEIEQKWQQRWEEDQTFRAVEDEHYPKEKRRYVLDMFPYPSGAGLHVGHPEGYT
ATDIYCRYLRMNGYNVLHPMGFDAFGLPAENYAIQTGTHPAVTTYTNINRFRTQIKSLGF
SYDWSREVTTCDPEYYKWTQWIFLQLFKKGLAYEAEMPINWCPSCKTGLANEEVKDGHCD
RCGTKVVRKRIRQWVLKITAYADRLLEDLDKLDWPEPIKLMQRNWIGKSEGATVIFKIDG
HDATLEIYTTRPDTLFGATYMVLAPEHPLVPSITTPDRLAAVNAYIDEAAAKSDLERTDL
AKDKTGVFTGAYAINPVNGARIPIWISDYVLISYGTGAIMAVPAHDSRDWDFAKKFNLPI
IQVVAKPGETGPFDREPAECFEGEGIAVNSSEFNGLPTAEFKQRITAWLEERGIGKKAIN
YKLRDWLFSRQRYWGEPIPVVHCEHCGIVPLPEESLPLTLPEVKSYAPTGTGESPLATIE
EWVQTTCPKCGGPARRETNTMPQWAGSCWYYLRYLDPHNPKAFADRSKIDYWMPVNLYVG
GAEHAVLHLLYSRFWHKVLYDLGLANTDEPFMRLVNQGMILGEDNQKMSKSRGNVINPDD
IVANYGADTMRVYEMFMGPLEVSKPWSTAGLVGVSRFLERIWALSERAIIDALMSPELEK
LLHKTIKKVTNDTATLNFNTAISQMMIYSNELAKLESIPRAAWEPFVLLIAPYAPHLGEE
LWAKLGHTESVSKAAWPTYNEALTHDEEKEIVVQINGKVRSKFTAPAGTAREQLVEMARQ
AEKISDWLAGKEIIKAIAVQDKLVNFVIKG*
>SPBIB_v1_260093|ID:27163407| protein of unknown function [Uncultured spirochete bib]
LLWILLKWPMHGKYSVMNLAYEFEVKASLYASIRRGGFHASFSFLPKPGLCVSPARTERT

LVRXX
>SPBIB_v1_270001|ID:27163408| conserved exported protein of unknown function [Uncultured spirochete bib]
MMKERQTMWRVGLVVSILAASLVLAEVFSKQVEHPAFSRMQGAADRMTRAEAAIAARRVA
LGIAIDPNLDPYRTGLIGAESSFLTTTVGDIVAKRTTTTTAFAALVVRYFYELGLRPGDR
IAVGSSGSFPGILLAVLSACAETGVEPVIITSIGASEYGANIEGLSNAEMVAACRDAGIW
PFMPAAISPGSDADQGISSLYRLEPSDELARYAHDIAARLQVPFIKGNNLEESIALHLRT
YQQAGPIKAFVNIGGADVNFGGDTDSLKLKPGLILPSVGRQSKLGGLVGYYLSRGIPVIH
LLNIKGLALQSGIPIDSNPFAPLPADVTYSRKKLEWLLILGLVLASAALA*
>SPBIB_v1_270002|ID:27163409| CapC protein [Uncultured spirochete bib]
MAGTSIIISIVLGFVFSELTGWLTGGLVVPGYLALYVDQPLRIVMTYAAAALAFLAVKLL
ARVTILFGRRRFMAFILAGICAGALLDMMTGMLPPSGSDLRAIGYVVPGLIANDTWKQGP
AKTLLASLAVTLATRAVLLLIAH*
>SPBIB_v1_270003|ID:27163410| putative Capsule biosynthesis protein CapB [Uncultured spirochete bib]
LIQAIAAAAAFGSLAVAGIIERRAVEHDLSCVLHRILVNGTRGKSTVTRLIAAGLRAGGI
TTIAKTTGSAARLVLPDGSEQPIHRKGQATIMEHAWFAHMAQKARAQAMVAECMAIRPDT
LSVVENALSQSTIGIITNVRLDHEDTMGQNLASIAGCLSLTIPRNGKLFVGIPKEPELLA
IIRKRATERGAALETVAPGPEIEGYCARFAYPMFAENLALALAACAACGVPREIALQGML
DAAPDPGVWPTLDLEWKGKTFRIINGFAANDAESTLALWHSAESHFDVAAHQIDVKVLVY
NHREDRPWRLDQVLSLVRPIGADLVCVIGAPRALTKRRIAKSGLALPFLVLDSADPEYLF
AQIESHVPQAAPQATAHVPPQATAHVPAQAAQKAARIGILLTGNIKGAGMALTEGFSAMA
ARAAAGASAARAAKEAKAEKQAARRERT*
>SPBIB_v1_270004|ID:27163411| protein of unknown function [Uncultured spirochete bib]
MQTKGLVNMPPNNIVPVISPDDVLDSFLALGKCNDLRMPSWIFETARTSALAKNDCQVLH
APSWAIRHGVAFFSSRERDLIIIRADSTESNESDGWTAKQTVRPVAGAGLEGAQDQLVLS
LPHRVEHRRWFVSYYIEDCFGAGFRARFEDRLKSPHEERCTYHSLKNTSGWIEMISGNPL
VCTPPKVQAGQRLFIVAVPYPS*
>SPBIB_v1_270005|ID:27163412| conserved exported protein of unknown function [Uncultured spirochete bib]
VNYNKRGLAVALAVAALFVLCIAQAFGQQGSQKREQPAIGLSIVLLEVRPGPGVARIFLL
SDLDSGLAGTAFDTPVFEFADEAGQADGVLHPEGANHTEGELLQPALFVCAGTHENEIAG
ILAAYWLIEKGRVHGAKLFVMPRANAAGATWSLQNPQSPRILQAIPGISRTFRYGARLSN
LAYETVSDPPLFSPPAAPAGFPALAGQEMRNLNREYPGSTDSSMTARLAFAITSLLKKEN
ISIAIDLHEASAGSNLGYSIVTRPESLDEAALAVLDLEETAGKSFRLEESKPEFAGYSHW
EWGKLGIHAFLVETFNPAQPSDDPAVDQFNNAKSPLAERVYAHLWAVQALMANAGVSLGI
SVNIEGLPASVLEVEAWLKAPLSGAAP*
>SPBIB_v1_270006|ID:27163413| conserved exported protein of unknown function [Uncultured spirochete bib]
MKRTVYFILTLLFVLGFGSTSAQTAALIPPQLAGIFEAPGFITSAGQTSDAAIVKVLVNT
RLKLNFGYDIAAKPQAVSGAKTLVLVLGASNKGLGAAGLSIEQETERVKAVLALAKSEHM
RIIAMHTGGTARRGESSNAIIALCVPHADVAIVVEGGNNDGFFTDLCAKEKVPLVMVPSI
AEAGNVLKGLMAK*
>SPBIB_v1_270007|ID:27163414|ggt| Gamma-glutamyltranspeptidase [Uncultured spirochete bib]
MMKTKMHGERESTGNQHRWLGGFSKILLAIGLLFFVLSPSVFAADTIKPEVTARNGMVAS
AQPLASAAGLEILMAGGNAVDAAVASAFALGVVEPNATGLGGEGMMVIYLADKKVTTSID
YRSMAPLADMSKVKFPPDGHVSVAVPGTVAGLCLALEKYGTMSLAQVMAPAIRYARNGFI
VSDTLASTIKDRFDPISKNDALLAILAPEGLPLEAGQIIRNPDLATTLEKIAAGGPDVFY
KGEIADAIAEDMAKNGGYITKADLAAYTAIEREPVRGTYRGYEIISAPPPVGGLSVIEML
NILENFDLASEQPLSPRNVHLMAEAMKRGFADNSAFIGDPAYTKVPVAGLLDKEYARQRV
KEIDPAKMTPAVKAGTPPEHPSTTHLSVVDKNGNMVALTQTISSFWGACVAVPGTGIILN
NEMQNWSSKGPNSYAPGKRMRTTIAPTIIAQDGRPFVTMGTPGAGRIISTMVILAVNLLD
YHMGVQEAIESPRFYARDTEKNLSIEARVPKETQDWLKSIGYSIKEYPDFDLFFGGAQAI
VVDLETGIMHGGADPRRDGAVFGF*
>SPBIB_v1_270008|ID:27163415| conserved exported protein of unknown function [Uncultured spirochete bib]
MRKRYSKALVMVVLLLVAAAGLPAQGASTILHDVRPDPAVTKIAWLSEWFSPLAGTPADT
RVFILDSGKPGATVFVAGGTHANEIAGILTAVLLVESAVPQTGRLLIIPNLNNSASTWTE
STQLPAWIAIRTPSGTRFFKYGARYTNRVHQGTPDPEQYIHPKSNERLEGQESRNLNRVY

PGKPDGTLTERLAWAVMQLLKQEHVDVAFDLHEAGPESRLANMIVANPKNLDMGAMAVLS
LELEGIKMKLEPSSESFYGLSHREWGDETQACAFLIETPNPAQSLDSADPAEDSAYPLSV
RVATHLASIQAILDAWNADAPEGKKATFTRWSEWENIARQGVGSFLK*
>SPBIB_v1_270009|ID:27163416| CoA-disulfide reductase [Uncultured spirochete bib]
MARYVIVGGVAGGASTAARLRRMDERAEIVMFERGSHISYANCGLPYYAGETIKERERLF
VMTPEKFKAWLNVDVRVRTEVEAIDRGAKSVRVRELDSGREYSLTYDALVLSPGSEPIRP
PLPGIDDPRIFTLRSVSDIDQIKSFIDEHRPERTIVVGGGFIGLEMAENLHTRGSFVTIV
EALDQVMNPLDFEMAAIVHQHLKQKNVELYLSSAVARFEKAGSRAVVVLADSTRLDADMV
VLSIGVRPETAFAKQAGIETAPNGAILVNENLQTNDPSIYALGDAIAFPHPILGMAMPIP
LAGPANKQARIVADNIVKGAGTRRWSGAIGTSIAKVFDITAAAAGVAEKLLKKNNIPCMS
IITHGSSHASYYPGAQPLTIKTVFAPDGTLLGAQVVGYDGVDKRIDLIADYIRRKAKVTE
LGEIEHAYAPPFSSAKDPVNIAGMTAENVLTRLSRHAAWSEVAVLKGNGAYILDVRTEEE
FQLGAIPGAVNIPLDNLRTRLSEVPRDRAVLVYCGVGLRGYLAERIMRQNGWTEVFNLSG
GYKTWELASEPQSHKGVYKPGLAAIQSASAKGAQELLHTTFAEGSGMPESIAAKRQAIVQ
IDACGLQCPGPIMRLKTEIDRLPEGGRVVISATDPGFVRDAASWCNVTGNLLISMEESKG
VYTAMIEKAVKQSAMAAATGMAASQGGAPIVQYSPKGASIIVFSNDFDRALASFVLANGA
AAAGKDVTMFFTFWGLSVIRKPDAPRVAKDFMGKMFGMMLPKHAGVLSLSKMNFGGLGQL
MMKARMKAKRVDMLESMIAQARQAGVRLVACQMSMDIMGVKREELMEGVEIGGVATYMDA
ASQSGVNLFI*
>SPBIB_v1_270010|ID:27163417|gpmA| phosphoglyceromutase 1 [Uncultured spirochete bib]
MHTLVLVRHGESEWNKENRFTGWTDVDLTDKGVSEAKESGKALKNAGFQFDIAYTSVLKR
SIRTLNFILDELDMQWIPVRKEWRLNERHYGALQGLNKAETAKKYGEEQVHVWRRSYDIR
PPELALDDPRFPGKDRRYAGLDPSELPRAESLKDTIARFMPLWHNEISGDVQKGARVIIV
AHGNSLRALVKYLDRISDEDIADLNIPTGIPLVYELDDMLTPARHYYLGDEEAVKAAAEA
VANQGKAK*
>SPBIB_v1_270011|ID:27163418| conserved protein of unknown function [Uncultured spirochete bib]
MNGTTKTRRLAIRGGLLVIYVLLMVLMIMTGRRHTILIDNKDAADGSYSAINGMEVAIDR
QESSEYYPGDRDKAMVQGQTHKIKVTIFDDNKTIEKEFKVPLWSDVMIISVPKLIAGVEP
WIEPFTMAEQIQEAQESGPPAGETSFQSIGPAQPMEVEQVPPTP*
>SPBIB_v1_270012|ID:27163419| ABC-type transporter, integral membrane subunit [Uncultured spirochete bib]
MSAQKKFVKFFSQYAVVIIFVMITVAAIPPSGLSIKYILQEIITRLGRNSFLVLALLLPI
YAGMGLNFGMTLGAMAGQVGLIFAVNFGISNWHGLLFALLVGLPISVFLGWICGLVMNRA
KGREMVTGYILAFFINGIYQFFVMYMLGSVIPMGNRAIVLSRGYGIRNTLNLESVRQSLD
NILVLRVGGYAVPVVTFIIIALLCIFIVWFKKTKLGQDMRAVGQDMVVAEAAGIPVEKTR
IIAIIISTVLACAGQIIFLQNMGNLATYNAHDQTGFFAVAAILVGGASVTHASIANVFIG
VTLLHSMFVVSPLAGQKLFGSAMIGEYFRQFIGYGVIALSLVLYAWRTRKAAADARLGLR
GGTNGSTKVSAVKNGANGAAGNGGQA*
>SPBIB_v1_270013|ID:27163420| ABC-type transporter, integral membrane subunit [Uncultured spirochete bib]
MRNLKTFIEDFGWPRIIIFFFLIFLFIMAPFVRVRLDASISDVLNRFGQNAIMVLAMVPM
IQSGCGLNFGLALGIVSGLLGATLSLQFQLHGILGFLGAIALAAPFALVLGWFYAKLLNK
VKGEEMTIAMYVGFSFIMFMCIMWLILPYNNPTMVWGYAGKGLRTTITVEGYWLKILNNF
LAIRIGQFFVFPTGLLLFVGLFAFLMWLFLRSKTGTAMTAVGSNPDFARASGVDVDKMRT
ISIIISTFLGAVGIIVYEQSFGFIQLYTAPSPMVFPAVAAILIGGASINKASMVNVLVGT
ILFQGLLTMTPSVINSLLQTDMSEVIRIIVSNGMILYALTRKIKVSK*
>SPBIB_v1_270014|ID:27163421| Monosaccharide-transporting ATPase [Uncultured spirochete bib]
MANGSPLLSIQHVSKDFYGNVVLHDVSFDLREGEILGLVGENGAGKTTLMRILFGMPVIA
ETGGFQGSVIIGGSEVHFSSPFDALDAGLGMVHQEFSLIPGFTTTENIVLNRESMRPSIL
NDVFGDRMSILLRDKMRERAEKAIGKLGIELDPDMLVSEMPVGHKQFTEIAREIDREQTR
ILILDEPTAVLTESEAEVLLQTLKRLASEGIAIIFISHRLQEVLAIANRIVVLRDGHVVK
DTPNEHVSMRDIASWMVGRSLGETGARSEGRKFSDIALKVHHLWVDMPGETVRDVSFDVH
KGEIFGIGGLAGQGKLGIPNGIMGLFPAGGEVILEGNTVPLNAPAKALAMGMGFVSEDRR
GVGLLLDESLEWNIAFTAMQVHRQYLKKVLGGLFSQRDERAMEDLCKEYVSLLDIKCTST
KQKAKELSGGNQQKICLAKAFAIKPSLLFVSEPTRGIDVGAKKLVLDTLHHYNRDFGTTI
VMVSSELEELRSICDRIAIVDEGRIAGILPPTADISEFGLLMSGAAMEAVNA*

>SPBIB_v1_270015|ID:27163422| conserved exported protein of unknown function [Uncultured spirochete bib]
MKKLVAVVLVLAFLVVGADVFAQAKAKFHIGVVTGTVSQSEDDLRGAEQLIKEYGSVKDG
GMIQHITYPDDFMSQQETFISSVVALADDPLMKAIVINQSIPGTAEAFKRVRAKRPDILL
LSGEPHEDPLVIQGAADLALSADFVSRGYTIIWAAKQLGAKNFVHISFPRHMSYESLGRR
RAIMEQACKDLGLNFYFETAPDPTSDVGVAGAQQFILEKVPQWIQKYGKNTAFFCTNDAH
TEPLLKQLFAYGGIFVEADLPSPLMGYPGALGLDLSKEAGDFPAILKKVEAAVIAKGGAG
RFGTWAFSYGYTLTAGLGEFAKRVIEGKAKKDSMKDVFDSLGKYTPGAKWNGAYYVDMGT
GVRAKNQILIYMDTYVFGKGFLPTTQQKVPEKYYTIKMHN*
>SPBIB_v1_270016|ID:27163423| putative Adenylate cyclase [Uncultured spirochete bib]
MAQASEHYRDFPPARMDATIEPMQFSRKRFADELYYYSNQFVLFFVMIISLTSGSWQNAI
VTSLIVLLFMLVQTGLLTVQGHIPVLRLLYSFITPAGYSILRATLSDIAFTETATMLLWG
ASVYIAVFQTLSLVLRRPFLKRLTETALSVGSALIFILFYAYLDLRISVTGAFSRGEIDS
EAMIQALRIQSFPSVFDKFIRAPQHLFALFGVISFDFMLLVARMRAITLQQRLTQLLEMP
KEDQRILAASHTEAESEGQSKEPESHTISEPVAQSAASTLSTANSPELSGLPLQSPTSLS
VTVVSSDIVGFTDLSEQLGRTAATTLLNRYYAIWTHCAGSFGGRIASLSADTVVIIFGLV
DSDRSADRALNATYAFLEELEGLREDMVVQSLPSDIKISVGIHAGFVTAALLGPPGQQKM
SFYGDTIAVAARLDSLCREFHQNLLVSHAAYRRLTLESQVTLERFSEVLLRKSTRPMPLY
AKKP*
>SPBIB_v1_270017|ID:27163424| exported protein of unknown function [Uncultured spirochete bib]
MNLRRNNLSGKSRARISENAHLQSRASHKIDALVSFAALLVVVAATATAILFVSCSGSLD
ATLKTDGSVRAAVRLDVPDALSGRVRQFVGLGSREPLFNTEAVRNQFLGRTSIMLVDAST
PSPEVLTSVVWVPNIDALIADTSLVPQGMIQFRKIPAQGSAPAMRELSVSLSRENAPYML
KLFPGVDKRIVESLSPPALEPDPVSAAEYRLNLEQVIIGKKYMAAFDACAVDIVITVPRA
IAAAAGGSFSGPIFRAKLPLFDLLTLEKPIVFSVRWPE*
>SPBIB_v1_270018|ID:27163425| PHP domain protein (modular protein) [Uncultured spirochete bib]
MILTFDFHNHSCLSPCASLENSPSEMAKRARQKGIEIFALTDHNSALNSATFAIACARQG
LIPFFGLEMNPFEEAHLLAIFPDPLAALAFSDQVYRYLPQLEIDPGQFGDQVVVDPKDEI
LAMPAAWYGNALQESFAFFADAAHNAEALVIPAHVDRAQFSVYSQLGFLPPGAYDAVEAV
GADPDPVLSGRHCVISDSDAHVLEHVGRRSSAVEIADEAIVNELRAGLAAMVERWKQWGH
SENAQHMKSADGSDDICKAGISTAIPGLAPLVSFLAEWYPRRESLALLEAMRRSFHEKKA
WSVYKRPAL*
>SPBIB_v1_270019|ID:27163426| conserved protein of unknown function [Uncultured spirochete bib]
MKHNTMTTRQIFELIDAEVIQGDFDEAPVAGVYTSDLLSDVLANGRNATVLVTIQAHQNT
IAVASIVGISLIIICNSRPIVDNMAEAAVQERIGVVRTSLSQFEVSGRLWQALHAQGEPD
DSHIRFS*
>SPBIB_v1_270020|ID:27163427| ABC transporter, ATP-binding protein (modular protein) [Uncultured spirochete bib]
MGAPIFAHNANAQKKAGKLMIELTNISKTYAKSGTKAVDSLTLRIPDGAIFGFLGPNGAG
KTTTIRIMTGAIEADSGSVLIDGIDLAKEPMEAKRRFGLVPDTPDLFSRLRAYEYLNFVA
DVYGVSASDRITVIEELAARFELSDALKSPIGSMSRGMRMKLNLIASLIHKPHNWILDEP
IVGLDPHAAFALKELMRAHAASGGTVFFSTHVMEVAERICDKLAIINKGKLVFTGDLEGL
RELRESRGRLSGQVPLVGAPNFSAREAGMVNIEGADQREESLEALFLALVEEGEQVPEEV
SKL*
>SPBIB_v1_270021|ID:27163428| putative Membrane protein [Uncultured spirochete bib]
MRSPFISLLSLSLKSVFAIHLPTQKELASPKVLLKTFGWIVLAVVLLADFGFMFAMMDIG
MHDALAPFGMQSLMLIYATVTASVLVFLFAFITSLSFFSSAPNEALFLTMPFKPSELLAA
RMATVYAIEAPIAFLVMSIAAGVYGIKNSPPFDFYVWMLLNALALPLVPLAVSYAVLMPL
VSASRWLRRKNTILYVGGFIGLALALGFNWYLQTMMARIEDPVLLRKLLIDNELNFADIA
NWWPPAWLTMTAIGNSSMPAAFAATLANLALGTALAAAAATIFGRSYTKILANFGELAAV
KGKIGRSQAQAVFAPRPVFISLVQRELHLMNREPMYFLNGPFVIVLLPVILAISLIAQGQ
QIQQALQQLRPLLDGQAGYLIPAGIGVFLASSTSIACTAFSRDAKAIYFLKSLPLEPRDI
IAAKLVHALLFAALGIVFGVVGVGLLLGIRLVDIAVALVLAVLGAVTLNICGLVIDTFWP
RLSWENPMSALKRNPNTIIVILGTMGLVAGLGALSASLPFQKYSFALLYGTVFLAAGIGF
GVLLFRKGSAFLRQMEP*

>SPBIB_v1_270022|ID:27163429| Holliday junction resolvase-like protein (modular protein) [Uncultured spirochete bib]
MRFGWLFSRAAAVYAECMNIEGLHAGLQAGPAIIAALIVFSALVIAAGVLAYRAGRQRGL
LEAELSEGERMDLARKDAIERSRAVLSGQISEQIAPWLPDFPANPSDARFIGKPVDFVAF
CGADEGVVREIVFIEVKTGRSALSPVERSVREAITQGQVRWVEYRLE*
>SPBIB_v1_270023|ID:27163430|pckG| Phosphoenolpyruvate carboxykinase [GTP] [Uncultured spirochete bib]
MQFPSYVKHARLLSWVSSMKDLCKPDFVYWCDGSEEEYNTICQSLVASGTFIPLNPEKRP
NSFLARSDPSDVARVEDRTFICSRRKVEAGPTNNWMDPKQMKSILTSLFDGCMRGRTMYV
IPFSMGPIGSPMSYIGVEITDSAYVVANMRIMTRMGRKVIEALGENGDFVPAMHSVGMPL
AVGQKDVPWPCNKENKYIVHFPETREIWSYGSGYGGNALLGKKCFALRIASVIARDQGWL
AEHMLVLGVESPDHEKTYVAAAFPSACGKTNFAMLIPPKAFEGWKVTTVGDDIAWIRPGK
DGQVRAINPEAGFFGVAPGTSAKSNPNAMATIRANTIFTNTALTPDGDVWWEGMTPNPPE
GLIDWQGKPWDPSSGKPAAHPNARFTAPLYQCPSVDPEWENPEGVPIKAFIFGGRRSTVY
PLVYQSFNWIYGVYLAATLGSETTAASTSAVGHVRRDPFAMLPFCGYNMASYFNYWLRFG
RWSCNPPRIFGVNWFRKDKEGNYLWPGFGENIRVLKWIVDRANNHGKAVESPIGWMPRYE
DICWDGLEFSEEKWAELMSVERDAWMSEILSHEELFMKLYDRLPKELIFVKELMISSLWR
APEHWEFAKVEISSYDD*
>SPBIB_v1_270024|ID:27163431| conserved exported protein of unknown function [Uncultured spirochete bib]
MRVVYIIILIAAVIIAVIFAVQNSAPITVAFFGWSATASMSLVLVLTLGAGILIGMLILV
PSIWKRMRALSVQKKKTREFQKQQKEDEKSSTPEAAQAENPSPEQNKGK*
>SPBIB_v1_270025|ID:27163432| conserved protein of unknown function [Uncultured spirochete bib]
MDFLVASPKDMERRRLVPGSFDAVIVSGDAYVDHPSFGAALIGRWLEAHGYQVAILARPD
PDDPEVFRMFGKPRLAFLVTAGALDSMVSSYTANKKPRSHDEYAPEGDRSLCLRADGTIG
KSKLGRVNARPDRAAIVYSNLCRQAYKGVPIILGGIEASLRRLAHYDYWSDTVRRSVLLD
AKADLLLYGMAERQILEVMEKLAQASKADSSTGHASGAVGTEVDLRGIRGTVWAVHASKF
EQALAAAPQPGTEVLPAFEEIQPDSDEGRRAFARSFKIQYRNTDPWSAKRLIEPCGDRFV
IQEPPEFPLSREELDKVYELPYQRAWHPMYDPFGGVPALTEVKFSLNSSRGCFGACSFCA
LAFHEGRVVTSRSIDSIVREAQSFAKMPDFKGYIHDVGGPTANFRAPACDKMAQKGACID
RRCLAPTPCANLEVDHSEYRELLKRLRSLPGIKKVFIRSGIRFDYLMLDRDRGFFRDLVE
HHISGQLKVAPEHVSDRVLDLMGKPPIETFDRFAEEYSRLNQKFGKQQYLVPYFISGHPG
ATLEDALELALYLKRFGFIPDQVQDFYPTPGTLSTAMWRTRMNPLDGTSVYIPRGTKERA
LQRALLQFSKPENHEMVREALRMLGRTDLIGRGHECLVQP*
>SPBIB_v1_270026|ID:27163433|nanA| N-acetylneuraminate lyase 1 [Uncultured spirochete bib]
MVRNRKAASLAKGIFAALPTAFDQAGNVDEKALCFLVRNLLQKGVEGFYVGGSTGECFLL
SEAERKEVLEIVLDAAEGKVPVIAHVGAMSTIVAVSLAQHAAACGASAVSATPPFYFSYN
LDKIYSYYADIAEASGLPVLVYNIPAFSGRNFGHNEIDRLLDIPGVAGLKHTSMNLYELE
RIRVKNPDKILFSGYDEVFLPALVSGADGMIGSTVNLMPELFLDIKKLFILGDIAAAQKL
QAKVNSVIEALAGGLFPRRLNMRLALPGCPAANAESHFFQFLAKRWN*
>SPBIB_v1_270027|ID:27163434| 3-oxoacyl-(Acyl-carrier-protein) reductase [Uncultured spirochete bib]
MSNERIEEDIFSVEGYIAVVTGGMGQLGRQFVKSLAERGAKVAIFARHAPDRASIKQVFA
GLSERIIAVPVDITKKETIKQGLSIVMNTWGIPHVLINNAGLDTQPSAPPEVSGPFENFP
DEVFREVVEVNLVGTFLCCQVIGAEMAKAGRGSIINIGSIYGMLSPVQDIYAYKEAKTGI
PFVKPVAYSASKSGVYNLTRYLATYWAAKGVRVNTLTPSGVWRSTQDPEFQANYCAKMPM
KRMAREDEFNGAIIFLASQASSYMTGSNLVIDGGWTAW*
>SPBIB_v1_270028|ID:27163435| conserved membrane protein of unknown function [Uncultured spirochete bib]
MVQYLFNGFANLISSGSIFALFIGVFLGIIIGALPGLTATMGIALLIPFTYGMNPAFALS
LMIGIFAGGIYGGSISAILLKTPGTPAAGATVLDGYPLAQKGQAGKALAISTIASALGGL
IGALILTFLAPQIARIAVLFGPPEYVLLGAYGLTMISYVAGKSMAKGIFMGLLGLLISTI
GIDPISGFPRFTFGQLNLLSGLSLLPVLIGLFAAAQAFEGVEKHNEIQVKQVKITKIGIS
GHEFIQILPHIIKSAFIGTFVGAVPGTGTDIAAFLSYGEAKRSSKHPEEFGTGRIEGVAA
PESGNNACVNGAMIPMFTLGIPGEAATAVMLGGLMLLGLQPGPLLFKENPQVIYTVFAST
ITSNLLILVLGLIGARFFAKVLQLPMKLITTLIFVLAIIGSYAMRNNVFDVGVTIIAGFL
GYILVKADYPVPPVLLGLILGPLVESNLGRTIIISEGNLLVFFTRPLCWLFWTLILYTII
SNILKSRKTVKASSQEAKE*

>SPBIB_v1_270029|ID:27163436| conserved membrane protein of unknown function [Uncultured spirochete bib]
MVSFKVKWLDLFLGLIGIALLIGVYILSESFPEPHEAQLGASVFPRIVSALLAVVGIFIA
LQSLKKKSNAELHIENAKMVFFTFFILLLYGLLVKKAGFLILTPVFIAVLLNIMKYSTII
INLLTSILTTAGIYLVFKIFLSVPLPEGILGF*
>SPBIB_v1_270030|ID:27163437| conserved exported protein of unknown function [Uncultured spirochete bib]
MHKRIFGIILLMSFLVAMGISAQAKFPTKPIKIIVPWNAGGGTDAVARALARSGEKYLGV
PVVVENKPGGSGAVGLAEVVQAAPDGYTLAILPVELGFMDKTGVYPFGFKDFTPIMNLNT
DPAALTVKAGRFKSVQEFIAYAKANPGKLKVGHSGTGLLWHLAAAVFAKEAGITLTYVPF
DGAAPAIAALIGNQIDAVTVSGAEVQSQVKAGELTMLACMGDKRLANFPQIPTLMELGYK
VNISTFRGIGGPKGLPADRVTILHDAFKKMMEEPEFIETLNKMGLGIDYRSTADYKKLAD
DTAAALEPVLTELGLLRKK*
>SPBIB_v1_270031|ID:27163438| Dihydrodipicolinate synthase [Uncultured spirochete bib]
MKKGKKFSGIIPPIVTPFDESGNIDRTIFRREVRHLISCGVHGLSPGGSTGEGAALTDEE
LAELIGIIREENTEGLPIVAGVIRTSTQAAVKTALAAKAAGANALMVTPVFYNVLVPDEK
GNYSFYEAIAEAADLPVIIYNVVPQNEITPDAFSRLLNIPNVIGIKQSVGGIMAFYDMVL
TNGDRGMIYSATDEMLYSTFDLGADGAISAILSLFPKLCVKMWNLAKDGDNAKARHIQDA
IYPVWKAVRGPQFPARMKAALKILGRDCGFSRSPMSEVTESQVAQMKLLLENIRDE*
>SPBIB_v1_270032|ID:27163439|dapA| 4-hydroxy-tetrahydrodipicolinate synthase [Uncultured spirochete bib]
MVKDFGRILLPLITPFDKNEEVDYDKYKQLARYALEKNYCDSIIVTGTTGEFNTLSFDER
VNLLEAALEAVDGKVPVIAGVGCASTRETVALAQKAEKLGADCLMVVAPFYCKPTQDAVY
AHFKAVHDATNISILLYNIPIFTGINMEPALVGKLAALSRIIGIKDEAGLNPIQITDYYL
ATKQIKPGFLLYNGDDLMLMPTIPQGAIGIVSGGAHLVGDRIRKVFDLYEAGKNAEATEV
YRDIFRLFRGFGVNGRVHPNPMLRAAIEIVTGIQVGPPRRPLDPITSSEREFLIALLKEV
KLI*
>SPBIB_v1_270033|ID:27163440| GntR domain protein [Uncultured spirochete bib]
LNQKIRKIERVSITDQVVKQLQALIMSGEFKIGDKLPTENDFCAQFGVGRSTVREALRVL
AAMGMIQIQAGKGAYVTKDKDNSFDAIKYWFAEKHAELSELIEVRMGIEPLAVRLAIQRA
SPESIKQIQEIHQAFKLAAIRKDHIELATLDESFHNAIIQASGNSLLIKIGKLIADSMME
FRARSFAVIENINHATIPHEKIVEAILKKDERAGTLAMIKHLEISKHDMEQVAHEIEKT*
>SPBIB_v1_270034|ID:27163441|dapA| 4-hydroxy-tetrahydrodipicolinate synthase [Uncultured spirochete bib]
MLQKEKYGKRLVPIVTPFKNDMSVDYDAIVKIGEILLKRDYADSFVLTGTTGEFFTMTSD
ERIKIFETMKEAFGARIQLIPHTGAASTQEAIALTRKAKELGFDLVMVVAPYYTKPSQKE
IINHFSLIADVVDIDIMLYNIPIFTGVNIEPESLRELSKKSNIVAIKEEAELRPKQISEY
LLATPEDFIVYCGDDTMVLEALAQGGNRIGGFISGGCHLFGDRMKKMINLFLSGNVLEAG
RIQLELLPLLRSLGQNGRTNPVCLLKGAMAMVGYPSCLPRLPLTPATDDEMIKVKSIMVR
IGLI*
>SPBIB_v1_270035|ID:27163442| protein of unknown function [Uncultured spirochete bib]
MKNAHVKLRTSREFPTISIDDAFKINRNFEKRRNDKTVAVSWWVCYAVLDLGLRQPGYCS
S*
>SPBIB_v1_270036|ID:27163443| protein of unknown function [Uncultured spirochete bib]
MRLSVGVDLHKTQFTVYWRREGDSEGRFAQYLTNEEGYCCFEEELEKAFEQGHEVAVVVE
CTGNARYFKNRVEKCGVRVVIVNTLKFKVVNESVRKTDRHDAATLAEFLEKGMLPEARLC
SQHSEELRRMLKERSSLVRTIVSVKNQIHGLLLCLGIESTRAGLQSQKEHRLIQDILAAH
GYVGGAVDPLFDILDRLDEEVKKLGKLIAELTADNPVVQLIRTIPGAGVINAATLRAYID
DISRFTTPRQLAAFASVVP*
>SPBIB_v1_270037|ID:27163444| protein of unknown function [Uncultured spirochete bib]
MVQVVLGMVRQKRWTGRYRLIVRYDRMKKAKGSGRSIIAVARALSEILWHMLTQNEPFDE
AKMIDPKIRRKAVEMQAAAFDVVA*
>SPBIB_v1_270038|ID:27163445| TRAP dicarboxylate transporter, DctP subunit [Uncultured spirochete bib]
MKNWKKLLVIVVLFLCVVGDLLAEQPIVFKLAFTDPPYLKIGDLQVMHHSYAGMVAFKSA
LEKLSSGRIVVELYPYGRLGDANENIQQIFSGTLQGATPADGAVAPFYKDIQVFSIPYAF
GSALEAYNLWDGPVGKALFDNMAKVSGLRVLAIYDNGGFRSFSNNKKPIKTAADMKGLKI
RTMTIPVHMEMVKALGASPTPIAWLELYNALQTGVVDGQENSAATILGGSLQEVQKYYTL
DEHFLGSAMIVTSEKWLKSLPPDLQTAIIQAGKIAEYAARGTSRANDSLALEALQKAGVQ

IYYPTSAEIATFKAASQQPVIDWLKKNINPSTVDTVLSALK*
>SPBIB_v1_270039|ID:27163446| putative C4-dicarboxylate transport system permease small protein [Uncultured spirochete bib]
MRTEKGIFAFIEKIITYITDILMAANIILILLSVFFRYVLGHALVWSEELAKFILVWTVF
LGASNAIRRWENLRVTFILDKLHLNASRVFDMVLKAVASGFIAFLFILALKSIPNVWSRE
MAPALGINMVIPELGIIVGLALMVLQCIGLIISFFMRSNL*
>SPBIB_v1_270040|ID:27163447| TRAP dicarboxylate transporter, DctM subunit [Uncultured spirochete bib]
MTAFWISTLGLIIFITLGMPIAFSIGMASVGFILMTNPMSLVIVPLRMFSGVNSFTLLAL
PFFVMSAEIMVRGGVSSRLFALVNMLVGRVRGGLAYVNVVQSTIFGSISGAALSDVAALG
KMEIDAMVENGYDRDFSCALTAASSIQSPLIPPSSTAILYAGIMSMSVGGVLLGGLVPGL
LIGGTQCLYILFRGRKLNLPKVTLTYDRKTKAKIWRDGLVALGMPAIILIGIVGGVFTPT
EAAAVAVFYSLIAAFLIYREAKPKDIIEALEATVKTSATLFMIVALAASYSWALGSERIP
EQIATFILGISHNRYILLLIMNVLLIIVGMWMETGAAIILFAPILGPIATMAGVSPLHFA
VIMIVNLVIGLITPPVGVVLYATCTVGQTTLERLVKALKPFYILSFSCLLLITFVPDIAL
FVPRLVGLVK*
>SPBIB_v1_270041|ID:27163448| 3-dehydroquinate synthase [Uncultured spirochete bib]
MRNGNPYLGGGRYIQIEGAVHLVGQELKRYKAKRAFIIGGHTALSIALSPIETSIKSERI
DYLVEEFQGHCTLKKVASLREKAERFDADIIVGVGGGKALDTAKLLANDMNLSVITVPTS
AATCAAFAILSVVYNDNGDVLYSVFHNREVASVLVDMEILTSHCPTRMLAAGIADALAKF
PEIAFSMEYASDWEKTVLPSAALVLSKHNWDLFFQKGKKALEDAKTGKLSPELEDVVCAG
IALTGTVSSLVSGGRQLAVAHSFYDSICKNFKQQQRKFLHGEIVSAGILLQMLVNGMESA
QIDETRNFLSSIGTPVSLRDLDIEPTSFNIDAIYTYIRTTMNLLEGWMTERLRAGLNGLL
SL*
>SPBIB_v1_270042|ID:27163449| Electron transfer flavoprotein subunit alpha (modular protein) [Uncultured spirochete bib]
VHIIVTIKQVPETDKVKMDPETGTMVRSGLESIVNPLDLYAIEEALVLKERFGARITVLS
MGPPDAERALREALSMGCDEAVLLTDRVFAGSDTWATSRVLAEAVKKIGAFEIILAGERA
TDGDTGQVGPALAAWLGIPVISYASGLELFADLGSAGAEKGGVPFAPSTVRARRLTEEGY
QILETEMPCLITVVKEIASPRLPTLRGKKFARSAQVLRWGARELGLGTEDAGLPGSPTRV
TKIFYPKVSRDGERLVARDEEGIARAVNRIFALLEEKGCLSSVPEAALATAHQERTSLYS
KASTTHENLERASLDQEAARNHSAAKLSPTEPSAAELSASSPGAAKSAVPKPEVWIIAER
RHVGLDSVSFELLARARTLADALHTSLAAIVVAPSLADEEVQALIAYGADSVIALESLNF
DDFVCETWADALFALARERKPDIVLAAATTTGRTLMPYLAAKFGTGLTADCTELAIEEGT
GLLLQTRPAIGGNIMATIKTPKHKPQMATVRPHSAQPLSPDFERKGIVLRTQALQPAESR
AGTQQIAVRTRVLSMERNTKGFENLEGARMVVSGGRGLKKAENFKLVRELADALGAVVGA
SREAVDRGWISYPHQVGLSGKTISPEIYLCAGISGAIQHLAGIRTAKTIISINSDPEAPI
HAVADLAVVGDLFEILPRLAERVRSAKTGISTEGNHAL*
>SPBIB_v1_270043|ID:27163450| FAD linked oxidase domain protein [Uncultured spirochete bib]
MLYNRVTEEIIRELAVIAGARNVLLDPAKLEAYSHDETSKEEYAHMPEVVVTPESTVAVA
AIVKLANRAHIPLTPRGAGSGLSGGAIPVFGGIVISLEKMNHVREIDYENLTMTVETGIV
TNEINNLVKDKGLFYAGYPMSLETCMLGGNIAENAGGGKAIKYGVTSRYILGLEFVTPAG
DIVWLGGKLAKDVTGYDLVHLIVGSEGTLGIATAAIIKLIGLPAAKSDLLVLFRSPQEAI
SCVPVILSKGLIPTAIEFMDRRSVETSCAYLNENLPYAQCGAMLLIEMDGRDPALVESEA
EAVGDLCMEQGAIEVYVADNRTTQERLWAIRRNIAEAFKVYCPVQSLEDIVVPPASIPAV
IPELDRIAAQFGITIPCYGHAGDGNLHATLVKDPEMSMEDWHRIEPQALEALYEAISKLG
GKISGEHGIGLKRKEYMKRFMSPVELELLKAIKRAWDPNLIMNPGKMFDLEKGIG*
>SPBIB_v1_270044|ID:27163451| exported protein of unknown function [Uncultured spirochete bib]
MKGLKMEASGVFRSSARLRALMLLAVLFMFAPSAQPVHAQDVSLSIQQWSFFADTAGERI
SGPGQHVSIGLIGGLTPRLELDFSAVTMLTPKPGDSLLGAVSLGYSLLAERWINDRVPPN
WVSMLAEIGFLGGGTELWARGFSGATPKAQVFVRFTPLVLGNSFYRRRDRLLALGFAYDL
LKQRGSFFFNFFAIDASLRR*
>SPBIB_v1_270045|ID:27163452| exported protein of unknown function [Uncultured spirochete bib]
MERLLNMKHSMRFVIFIMLMCYVAASSGMVYAAEPWHVGIVVGTEGFAAKGFDNGEYMRA
GLLFDPFPPKGIQPAWFLGALVPVNPLDFSGLMLNIGIELRYMARTAHSSFFVREWKYAP

AVSAEMLIDPAGGREVGFVVSAQLVRFWFGESYVSFLGTELVLDSKGLVSGWGASLIRLT
AFVH*
>SPBIB_v1_270046|ID:27163453| exported protein of unknown function [Uncultured spirochete bib]
MQKNYYSPMLRGMPFYRFAFGVLALLATISAMVILGGCATSQGAGSLANSIGLVNSVSLA
NSGADSTQSIEAFLESNGFSRIQTNEPIIHYSAESWKASLLNTIAQAKKSVFVLVFLANR
NEFNAEIYDALIAKAAEGVPVYFLFDATSYERIFHNPKWNDEIVAPSEVFRGTKVKWAEF
NPLRLERIAALPWLLVRDHRKIVMVDGETIYAGGYNLNIYSFAPLDQNGNEDAMVEIQSP
DAGRALAPSLAETWNQFSVESLNLEDIAPAVATGAGGSIGATAAASPEASAGSQTASMDS
TTSQDTPLACPPNANKGAVNAWLGNQVIGETRSIEALYRAIFDYAQEEVWLVQAFAMPTE
TIMQMVKQALQRGIQVNIMLSDDQISPAMDKAVHYTVLPLLEAGAHVFIYKSAGNALLHY
KLAMMDRKIVALGSPNFNYRSVRLSNEIVFVFDNERTIGVVSQNLSELQKRARPVSIEEA
QKWRGFSYFMSYLLSVPGG*
>SPBIB_v1_270047|ID:27163454| putative dTDP-4-dehydrorhamnose reductase [Uncultured spirochete bib]
MCRIASIYTSEKGRFAQGRRALAGLEAEGGAGWRFFLQNTECGIFISMKLLVTGASGLLG
RSLMKLLASYPNIETKGVAFSRAKAPLEKLDLTEERSVALFFAHFRPDVVIHLAAERRPD
IVDTDLERARAINIDATQIIARECAKRGVFLLLISTDYVFDGSAPPYFPNSPVHPLNEYG
RMKVEAEKVAETALSGIVRAFGGRSVGAVLRIPILYGPVEYLEESSVTEIALALKSTEPR
NIEHWAHRYPAHVDDVSTAIMAIVEACMQRTAACGPFPRFLLSGKEAYTKYEMAKAMAAA
LGIDASHIRPDPSPPKGAPRPRDCRMDTSLLESLGWRQEKYFSSSIGDIIKPFFS*
>SPBIB_v1_270048|ID:27163455|pdxS| Pyridoxal biosynthesis lyase PdxS [Uncultured spirochete bib]
MEQNFWSSRQSEQWRIKVGLAEMLKGGVIMDVTNAAQAKIAEKAGACAVMALERVPADIR
AQGGVARMSDPKIIKEIQDAVSIPVMAKCRIGHFVEAQILEALGVDFIDESEVLTPADDR
FHVYKHNFKVPFVCGCRDLGEALRRIGEGAAMIRTKGEAGTGDIIEAVRHMRTVRDEIAR
LTRLPDEELMTAAKELGAPFELVWEVAKTGKLPVPNFSAGGVATPADAALMMQLGAESVF
VGSGIFKSGNPERRATAIVSAVVHYKDPKILAEISEDLGEPMVGIGLDELNENQRIAGRG
W*
>SPBIB_v1_270049|ID:27163456|pdxT| Glutamine amidotransferase subunit PdxT [Uncultured spirochete bib]
VSSSRIGVLALQGDYEAHLKALARASENAVETIEVRRPADLESIAALIIPGGESTVMGML
LERFGMFELLKSRIANGMPVFATCAGIILLAKNIEASEQTRLGVLDVTVRRNAYGRQVDS
FHAPVQTTIPEIGTIDGVFIRAPKIIALGPGVEVLAVYQGDPVMVRQGNIVAATFHPELL
PGAPVHRWFIQSF*
>SPBIB_v1_270050|ID:27163457| conserved membrane protein of unknown function [Uncultured spirochete bib]
MPTEFLVLLLLILLNGFFSLSEMALVSSRKARLKSEADKGKSAYQLAFKTRESPSRYLST
IQVAITLIGILTGTVSGTTISQRLAAWLATFPALEKYAASISIAVVVLAITFLSVIIGEL
VPKSIALHNPEQIAAFTIRPMHFLSVLFFPVVRLLSAATDGLVRLMGFHRAAESAITPEE
VKILVEQGEESGVFETAEREMVEGVLNLDDRRVTMFMTPRTDVVALDTADSREAHVRAII
EHAEFGYLPVVEGDLDHAIGMLEVKRALTRLAQGRFAGIRECMQSAVMIPESFSGLQALG
ILKNAKKSAGLIVDEYGGVSGLVTIGDLLEVVANLSEIEQQEEPQIFKRSDGSYLVDGSM
PIDEFAEALELNVEQFSREDYDTVAGFVLHRTGTIPKAGDTIEWPPLSIEVVDMDGKRID
KVLVKKTE*
>SPBIB_v1_270051|ID:27163458| exported protein of unknown function [Uncultured spirochete bib]
MKPPKKSALEGLYILLLLFVLALIHPFPQTLGAQENEPARDPVTASFDQAFADGDPVRNV
IEPVFVGEAGFVQRLNIRTTRSGTQGSPTDAQASVHPPLGLVLSGGSARAYAHIGVLKEL
EKAGIYPDFIVANSMGAIIGMLYAAGFSPDDIQLLIHAIPLDSYFDIVLPTNGGFINTEA
FAAAMRMLVGNLDISETAIPIIVTAEDLPSRRQIWFAEGSFERIMTAAFSMPAVFEPQHV
GNFILIDGGIAAIVPIEPAQRFTDRLIVSTALYNRAMSFSNPLTVVNRAIDIGKTRTSMG
ALEKTSAFIIRNDVETLSYMQFSDPNLIIARGEESAARAIAALDAQERAQLENPPPPSLV
ELRARMHQKLIKTIEALRTGVMPASAPTIRAAPALRLFPPILGFAGETETIPRAGLSAVF
SAWRTKTSLSYFAAIAPETGKQWALDGSININPIEALVIGASARLWGDYSSTAILGHTPA
MWEFAGSLKSATFVGATKIGFVASADVIREIGEGSATWQSIGLVSAASVTPGFDQDAHAI
VPWYSLEAGGFAENTVSAQLSAGLEGTLMIGLHAGLFSPRVRGFAKVALNGNAFLESEFD
GFRGGAARAAAASSLITNAELAIAPRSLFFDIAETALVRNLELAPFFDTKWSAPAGGAGS
FAMKDWAAGLCFSFQASAFGLAPATISAYASYSGSHVFTFQVRAGALFPSR*
>SPBIB_v1_270052|ID:27163459| conserved membrane protein of unknown function [Uncultured spirochete bib]

MKTWKVALLAAALAIIVAGPVLAQNAVKGVTTDVHAEKGDFSVLGGIGYGWRGFGMSGGV
EYIFQKFDIPGFPLTMGVMGLAGLDFGSGFDVSAAGMATLHWGLKAYKDFPEFLRKFDWY
MGLGLGVGIIPFGFGMSSGGGVSYYVNEKLAIDLHSFYVNHFVGGASGVGGTIGIRYKL*
>SPBIB_v1_270053|ID:27163460| DNA-3-methyladenine glycosylase I [Uncultured spirochete bib]
MNRCPWCGSDPLYLRYHDEEWGVPVHNERTHFEFLLLETQQAGLSWRTILGKREAYRAAF
AYFDPEKVARFSGPEIERFLADPSLIRNRRKLEAAVANARAFLNIQERYGSFDAWIWRFV
EGRPILNTWKDICELPARTPLSDLISNEMKKAGFAFVGSVTIYAHLQAIGIVNDHLVSCF
RHEEIARLAASSAPG*
>SPBIB_v1_270054|ID:27163461| Ferroxidase [Uncultured spirochete bib]
MLSQKMIDRINLQINREMYSAYLYLAMAARMNEAGYTGIGKWLTVQYHEEMFHAMKFAKY
LHDQNATVAYTKIDTPEFKEKDVKSLFQHVLAHEQSVTASIREIMDLAIAEKDYATQALA
QWYINEQIEEEKNDTQILQNIDLLGNSAQGLFMLNIELGKRDPSVPLDFNKI*
>SPBIB_v1_270055|ID:27163462|thrC| Threonine synthase [Uncultured spirochete bib]
MVIRYTSTRNGRVNASIAEAIVQGMPQDGGLFVPVQIPALPPEALNEPGLSYAELAWLVL
APWFDWSEYELRPLIERAYIAQNAPGGEALFDVPEIVPLRAAGSLGNHPLFLLELFHGKT
CAFKDLALSLLGRLLQESLEKCDIDEPLLILTATSGDTGSAALAGLGGQPGIRIAVIYPA
EGTSEIQQLQMTSVPVEGRLVVGLRGNFDDAQRAVKRIFQIAGNPDSPFHAIRLSSANSI
NIGRLLPQIVYYVKAWRELYFSRFLRQGEPFDVVVPSGNFGDILAARYAKAMGLPIGKLV
CASNSNRILHDFFSTGVYDRRREFTKTLSPSMDILVSSNLERLLYHALEENPELVAMTMQ
KFERKGVFELSEKNRATIADFRASWADDAQTLETIRSMWESHGLLVDPHTAVACKVARDV
YTKGTEVSGETTDSAPIVVAATASPFKFPAACLEALQGKKGPTSEKGKDLQLAFELSRLT
GLPVPKPIAALVSAKINHSTIIDESDLERVLTEFAQQEAGIARIL*
>SPBIB_v1_270056|ID:27163463| putative Homoserine kinase [Uncultured spirochete bib]
MIEIEVPATSANIGPGFDCLGIALSLSNTVRVEKASETRLKGCPPQWAGPDNLFLRSFRQ
ACALLQTSVPEIEVEFITRIPPARGLGSSASLAVAGAAAALLLYAQPKAGTELDILKEQK
NQHLLLKAATAVEGHPDNAAPAIYGGFTAAAIAKEDISIIRADAPAEWRFAACIPDFDLE
TSVARKALPDVYSRADVVHAISHAVLTALAIRNADISSLGRVCEDRIHEPYRRPLVPDFE
DVENACRANGAAAVWLSGSGPTILAVFESSVPAKSLASDIAAHAKRRWQVVPLHADNMGL
RAQISP*
>SPBIB_v1_270057|ID:27163464| putative Methylated-DNA--[protein]-cysteine S-methyltransferase [Uncultured spirochete bib]
MIPIQWKETQAFSTSVPTRFGSIVVVWQGEEAPILSRIYLPRNDCALVSQAHTDFPILEQ
WNDRRLLPDLISRATEAICAAAESKPAQFPFKLLRPGLQYLNSFRQTILSLEASIPFGHV
STYLELAKASGKPSAFRAVASALSHNPFPLLIPCHRVIASNGSLAGYQGGLEMKRFLLER
EGVPFSSSGMVNLHLARLWHFS*
>SPBIB_v1_270058|ID:27163465| conserved protein of unknown function [Uncultured spirochete bib]
LKWQPACSILNNMNKALSPIVMPALFVGHGLAINAALNNDFTESLRSLRTRLPSPDAIAV
ISAHWSTPEVRVTSSPFPKQIFDSIEFQEELYAISYAPPGKPDLADTICSLLLTAGINAK
SDSTRGIDYGAWGILVHLFPEAQIPVVEISLSYHIDTKKIVNVGKALAQLRNERILLIGS
GALVQNLYKISKNIKAKPYPWALEADRKIAAIIQAGDASALSEFALRNLERSPAMPTPEH
ILPAIAILAMKEKDDRISFFHESFQNASISMRSFIIEHG*
>SPBIB_v1_270059|ID:27163466| conserved protein of unknown function [Uncultured spirochete bib]
MHHFHFCPNPACAYHQHAPKERWFVAAGFYVTKTFGKVQRFRCSFCGKYFSTQTFSLDYF
AKRXX
>SPBIB_v1_280001|ID:27163467|gidB| Ribosomal RNA small subunit methyltransferase G [Uncultured spirochete bib]
MNHEPLLARGLAALDFDISSGILDKLIQYLDEIERWNPLYGLVSAEGEDLVIKHVLDSLS
PWRVLAELLASLDSSIARNGMATVIDIGTGAGFPGIPLAIAFPERPFVLIERLEKRVRFL
ESAITLLGLSNVEIRQGTAENEQEAFQCVVFRALRPFGDKKLFRSIWKKIQPGGALFAYK
GRLMHARLELAELSEDAVLGNLAARAQIVSVWVPFLEEERCVVIARKA*
>SPBIB_v1_280002|ID:27163468|pyrG| CTP synthetase [Uncultured spirochete bib]
MRKLLFVTGGVCSSLGKGIAASSIGALMEARGFSVQMVKIDPYLNVDAGTMSPYQHGEVY
VTDDGAETDLDLGNYGRFTTAKLSRANSITTGQVYKAVIEKERAGEYLGRTVQVVPHITN
EIKQRILAVASNPEVDMTIVEIGGTVGDIESVPFLEACRQLIHEYGKQNAVSIHVTLVPA

VSGGEIKTKPTQHAVKELQEVGIQPDALILRSAKPLSDDVRRKISLFTNVEEEGVISGYD
APTIYEVPLAFYNQKLDTFLLRKMNVESRHAELGKWQAVVQAVTKPSRKVKIAVVGKYMD
LHDSYKSIWEALCHGGIAHNAGLEIEKVDSSRLEDSNADLEAIFKGIHGILVPGGFGERG
IEGMIRAARYARENKIPYLGICLGMQIMVIEYARSVIGWQDAHSTEFNKATPHPVVCLLE
EQTRIKNYGGTMRLGAYEAVTKAGSFIRAAYGTEQISERHRHRYEVNNDIRQELEAHNLT
ISATTPNGELVEACEWPEHPWSVGVQFHPEFKSRPTSPHPLFKEFIRAALSHAGA*
>SPBIB_v1_280003|ID:27163469| exported protein of unknown function [Uncultured spirochete bib]
MAGNARAWKTFARSNARAFCALCLLSLCMSVLLACSPPAEDVTQSAQAEAPDARFSDLTR
DEYRNGKHSMRITAQIATWYEPDQRLEIEHLAFTAFNTDDGSISASGEAEKATFYESSGD
IEFSGYVHLKSAEGDISFKTDHLAYKRALDIFETPLDSDVTINAKNQLFMAGRGLLFDVK
QKYYEIRESVSGSVYQ*
>SPBIB_v1_280004|ID:27163470| exported protein of unknown function [Uncultured spirochete bib]
MKFANQYPVPFINKMEYYSIMKTRAPFWLLIIVMIFTAFQAGFAQASKPAASAQPTAPAS
SQQAPAPSQPAAPSGSPQPAASQTTEGKKPITFSARSVQGVLAKGKENTILTGSVKITTG
SLVITADRVELSGEDYVNVVCSGNVVANDSEKGFSLVAEKLNYQRDSEIGIAQGKVAVND
TKNNTVINAEWVRFDQKQSIFEAAVSVLVLKEDMSIRAEYAHYNRDTEEIRLHGGAVAIT
ETGQLQGDTITASSDWSNLQISGEVSGNITSKEKTATQ*
>SPBIB_v1_280005|ID:27163471|lptB| putative lipopolysaccharide transport protein B: ATP-binding component of ABC superfamily [Uncultured spirochete bib]
MSIAILEAAHLRKSFGRKLAVADVSFSMQTGEVIGLLGPNGAGKTTVFYMIAGFLKPSAG
TVHFDRKPIDRLPMFKRAMLGISYLPQEASIFRRMTVEENILAVLEARSSLSPAGRREKA
RMLMEEFGIQHVAKQPAYTLSGGERRRTEIARALAIEPKFLLLDEPFTGIDPIAIREIKH
LIQSLSSKGIGILLTDHNVRDALAITTRAYIISKGVIVAQGEPQSIIDDPLARETYLGQD
FEM*
>SPBIB_v1_280006|ID:27163472| Hydrolase, TatD family [Uncultured spirochete bib]
MRYFDTHAHIGLIYDDPIEQLLVCQQAKLAGVSRIVSICNSLVDFKQVYENLKPAEHVYH
AVGVSPSEVNNPGKDWQRYIEEATRLPRVIAIGEIGLDYYHKFGDKRSQIELFIDQLEIA
ARLNLPVIIHNREAGHDVLDILRDRIPPAGAVLHCYSENAAYAADILDLGLDVYFSFAGN
LTYRNARNLHETVAFLPLDRILLESEAPFMVPAEFRNKRNMPEYLPSTAQFLADFLNIDV
EEVAETTYQNACRFFRLNP*
>SPBIB_v1_280007|ID:27163473|dus| putative tRNA-dihydrouridine synthase [Uncultured spirochete bib]
MSTKKMPPFLLDEKKWLAPYFLAPVAGYSDVAFRAICAEMGAALCYTEMVSAEALVRNHA
KTKELLARDPAETHYAIQLFGANPATLAKAAEIVSAFQPAVIDLNCGCPVPKIIKAGAGS
ALLKTPERIGEIVRAMRNATSVPISVKIRTGWDEKSINYLETAGMAIEAGACAITLHGRT
RAQGYSGKADWDAIKALAAQTSVPIFGSGDVFSADDAIAMQEYTGCDGVMIARGAIGNPF
IFQELCQRAAGQRIDEALSPKVVSQTALHHLELAVKYLGEQTACIEFRKHFCAYTKGFVG
GAALRAQAVRCSTFVEFQRLLELFAESAA*
>SPBIB_v1_280008|ID:27163474| protein of unknown function [Uncultured spirochete bib]
MKREGFFRGVQACVGAMALCVLVLFNSCNQSPQSALIWTDIPELVVAAQLFNRENDQFAV
DVEYKENAATALMKTNKPPSLIIAKFLLSKPVIRKLDPLDDLFSRYYVDPNDMYSALLDA
GKQGVQQILLPVSFDCMVLVERKSDSLGAGASMLDLDAIREAGDKFTRTEKGVLSAMGFS
SVWNMEFAESWLLAQDAGFSPNPNWKQTSVPKSGDAKTWPLLWNKDRLEQSVLALNSLDS
KVKKDDADAFAFSYFNKPGYQLVLENRVLFWPMKASDFFKLPYSAKSQLRYRYPAANQKL
ILTEGTRYLGVPKGAKGKKAAFAFSKWLLTPANQEKIWKEMETQRLLPDHVGPLDGFSSL
KQTNETVLSRYFPEYAQNKIVAGTLRSPVALPDYWDSFSRDFFWVWLKTMLTSGEGTESD
AQKGTIGEDFQASFEQYLGTMPDWLASSR*
>SPBIB_v1_280009|ID:27163475| Radical SAM protein [Uncultured spirochete bib]
VQANQKNSKPLINWHSDRLKERYGEHVWRLGIDAGFSCPHRSPDRLKGGCRFCAPDGNIA
AYQKAASSVPSVAQQIERALVFTRRRYHANAFFLYFQAYSCTNAPVKALAAIYDNAIDAF
WMALERITPLERDGLNFAQKGENAASTSFLTSTSPLKGLVVSTRPDCFDSEKAALLASYR
ERGMEVWVELGLQSAHEETLLFIRRGHGVKEFLDAMEIAGKAHLRRAVHLMLGLPGESHS
MMLETARIVANTGTEGVKFHDFRIAKGSAFARAFPCGEITAMHPSRLPGLLADCLEVLPP
STEIIRISADFRPEEAININIHAPLDKHHLARLVEDELRRRSSCQGQRFNTSP*
>SPBIB_v1_280010|ID:27163476| DNA polymerase III, gamma and tau subunit [Uncultured spirochete bib]

VPQSAWLYALCLLYPSPPSCYFSYMPFEVTATRKRPQNFEQLAGQNFVAATLISSLEQGR
IAHAYLFSGPRGCGKTSTARILAKALNCEHGPTPHPCGTCASCLSISSGSSLDVIEIDGA
SNTSVDNIRQIKDEVLFAPNSGRYKIYIIDEVHMLSMSAFNALLKTIEEPPPYIVFIFAT
TEPHKVPATIKSRCQQFNFRLVPAETILELLRQAAAETGVETEEEALLWIAREAAGSVRD
AYTLFDQIASFSGTKITAQNIRDTLGLVGLDRLNALFRAIVANDTKAAFTTLDEILIRGV
SPEQFLSDAVDYCRSILLIHNDIQKEGLLCAPRSTFDADVLGVLTKERAEYAIAVLLDTY
RHLKETIDPRFELELAIAKLSRISAYISQSELFDAIKAIKHTFLPQGSSQKGLPLPFQER
ASKNDSAEEAPLLRDLRSRQPESQSAQSARQTSRPSQPPETLAQKNNSQYSSPLDQEKPS
VQLSASRSGTEVEPASSVSSSPSELRKRIIAKLRAENLFLASALEKSGEWIPKHNGFIIP
VVNKVELDLISRFSALIASTGAQLMGAPCAIEAHLQKLQEVLHDEDSPKKSALMRDSETG
NAHAQISSDEVPQHDQTPETNLLKEKSTLSSFQSAEDAFNGSDEGSLPQNNPAPPSSLDE
NDIRLIELVRKMFKGKVIETKPAFEPSAESRTSAHASVPIIEPPDIPEEPEEFPDTDTEE
ESYD*
>SPBIB_v1_280011|ID:27163477| Nucleoid-associated protein Spica_0202 [Uncultured spirochete bib]
MDLSNIFDMLKNPQALKAQAEEMQKKMNAIRATGQAGGGMVKITLSGNMEMLECEISPEL
SEFKDIPLLQDLIRAAHHNAAEKIKEEIQNEFAGSLGGFGSGMPFGNLPGNFSGTEV*
>SPBIB_v1_280012|ID:27163478|recR| gap repair protein [Uncultured spirochete bib]
MKAIEDLVSLLTRLPGIGRKSALRIAYSLLKSDIAFSEALADHIRTLRSTIHFCAECGSY
AESELCQVCSDASRDRSIMCVVEQPQDVITIEASKEYKGLYHVLGGLLSPLEGVGPDKLN
IGKLIQRIHSSISTSVPIREIIIATNPTIEGDTTALYLKKLLEKEPVKVTRLATGIPVGG
DLEYADRLTLARSFHGRSAL*
>SPBIB_v1_280013|ID:27163479| conserved protein of unknown function [Uncultured spirochete bib]
MRASAIAHVLNAEVITCQELADDLEIITACGSDMMSDVIAFVKDRVALLTGLTNPQVIRT
AELMDIRLIIFVRGKKPSSEMIQMAKEQSIMLMSTQDSMFIACGKLYQAGIMGDIAYQAR
*
>SPBIB_v1_280014|ID:27163480| Anti-sigma regulator [Uncultured spirochete bib]
VLIEYIIPRMDFSAAGKASTDMKRRLTQLGLPVALIKRIAIAMYEAEMNVAIHGDGGKAE
VEILEDCISTRFTDKGPGIADIELAMQEGFSTASEEIRNMGFGAGMGLPNMKRNADELII
QSEPGRGTTVIMRFKLQTGEL*
>SPBIB_v1_280015|ID:27163481| Fe-S cluster domain protein [Uncultured spirochete bib]
VNNKTLQNQENNIRTFHSVQLDADLCVGCTTCIKFCPTKAIRVRDGKAKIFEDRCIDCGE
CIRRCPKGAKKAISDPLFILDGYDLKVALPAPSLYAQFGTRYSQSDIFAALHHMGFDEVF
DVAWGAIVATEITRSILAKPGPAPRISSACPVIVRLIQQRFPSLIPNLMPILPPSEIAAR
EARKRLSEISRKIGIFFLSPCTAKVTAVRMPLGYSQSSIDAVISFSDIFLPLKRALEERK
PLSPKKGFKSSVPQASSGHSPILPMMDNLEPGMGWARSDGELDALRIPDSVSVDGISNVI
DLFEAIENGNIESIQYIEALACPGGCVGGPMAVENPHIARSNMRQRCQKDNLAAEASSSP
MKAIKPPSPKLPEQEELPSYIWTEQVSPKPVLVLDADLSKALEMAEKIDTIHAQLPGIDC
GACGAPDCDCLAEDIVRGFASIEDCRLLEHPYSKGTKENIHSEGLST*
>SPBIB_v1_280016|ID:27163482| conserved protein of unknown function [Uncultured spirochete bib]
LNKKDFPHIHFYDQDFVDIYDRTWAWIADCWTNGGSQTKIGKIKFFYYPEKKKLDFLEQV
FSSFFLVYSNRIYSASNGLDALYALQEPDGAIRTAYDIETGQAVMPADNPLGVTLPLLAW
AEFNLYHKTANKKRVKEVMPALSKHYKWLEANFKMPNGLFATPLSSTGMDNSPRENARYF
IDFNAAIAMNALYLSALGDILNDKDASFLYKRDYFSIKTRINSKMWNADDGFYYDLDENE
NQVKTKTIGTYWILLAEIPNEERAEKLVAKLKDPHCFGTENPFPSLSADHPAFDRKGQGF
RGSVFPWLTFMVIKGLEKYYFYELARDSAIRHLYYILDSMHASDHESNRHHSRPTVWSAY
QPMNEGKAIWDEHPDWPLPNHFPSNGLSTVTLIIENIVGLYISLPRKTVDWIIPNLEVMG
IENLSLKRNMITILSSKSGRGWEIHMESEKLYYFTINVLGKKKKTLPIPSGKCSMLIDKI
*
>SPBIB_v1_280017|ID:27163483| protein of unknown function [Uncultured spirochete bib]
MSRERRDRSARERNSHDQGFADPREKALDQLTNLYVKDAISLEEYERLADEMQKASDPQS
VLNSVIVAGSGGKSAQEGSNRTPFDSTDKPLSLNQDISRSKASFEPLSPNSALNSFPRPG
DFFLCIMGERKVNGRMLLGKSASSITLMGSTVIDLRDIEVPASGMHIEVIAIMGETRIIV
SPDMLVHLSVVPIMGEAISRADVPFGSNNQGILEIGGVALMGSVSVRVVP*
>SPBIB_v1_280018|ID:27163484| conserved protein of unknown function [Uncultured spirochete bib]

MNIVISLGGSIVAPQEGPSGIFLMQFRNILLSWLNGDDRRAIIVVGGGAAARNWQKAYRE
FLSNFSEVNGDRGLRDFSCSNEALDRIGIAATRLNAQLVKEVFADCCIDPIITDPSSGFE
ANGRILVAAGWKPGFSTDYDAVLLAERFSAKKVINLSNISQIYDSDPRINPAARPLSRIT
FDSLIKMTGADWNPGANVPFDPVAAARAREIGLKVIFASGTNLQNLSDILYEKAFVGTII
E*
>SPBIB_v1_280019|ID:27163485| putative Peptidase M20 [Uncultured spirochete bib]
MKKKIEIDDTQALAIRLVRAPSVTNTSGESSFPDVLISILREQPFFSLHPELIHDVPIEG
DSKGRRNVLAFAPGKGSSCVVLAGHYDVVDTNAYGVLEPFAFDPEALSKRMLQMLSSKTN
LSSSELLLKKDLESTAFIPGRGMLDMKSGLAAGIAAMVRFLKKDDRTGNILFIAVPDEEG
SSTGMKAAKKMLSDFADEHALHYEAIINLDASVDSGDGAEGKAVFLGSVSKLLPFVVFVG
KPAHAGSPFDGFNPVIASAVFAREIECNSDSMNIRALVPGEEPPPPSILYYREMRNRYDV
TMPADVFCAVNILTFEKDPEKVFDHFKTIVQKALDNSISLIYERASAFSRKKNEHVTMYK
FSSEIIEFHELAKRAERVVPGILDRLQRFAEKSFPEDKVQQTFKIVHELLSYAQIEGPSA
IVGFAPPFYAKAQLDSERDQGFLSIIKDEVMSFNRDYGESIKLRPYFPGISDMSFLAPSV
SREAIEFVKKESAVLQSYLDKYLESPLDTPVINVGPWGREYHQNGERAHRHYAFSLLPEL
LFRLCNRLFHANN*
>SPBIB_v1_280020|ID:27163486| AMP-dependent synthetase and ligase [Uncultured spirochete bib]
MLKEAEQKYPDIPYALKRTDSGYMPTTFKEVRQKARQFAAWLLANDLKAGDRAAIIAEGS
PEWLISELGVLSAGMISVPLSIKLLPDEIPFRIQHSEAKVICTTHNQLEKLANALNLAGL
KKIDIVYLDDDIEWGQSILKKHKVRDDSFITLQKALSVGSVLLKEKKEYEERLDLIENET
KENDVVTISYTSGTTGNPKGIMLTHLNYWSNCHDAVELFDNPLYFRTLLILPVDHSFAHT
VGLYTAMLCGISLYFVDSRGGGIATLRNIPINLKESNPIFLLTVPSLSGNFMKKIIAAIE
EKGGFIEKIFKSGINAGILWHGDGFHTPPFQDRLKSFFPYFIARFLVFNKVKKALFGNSI
RFCVGGGALLDIKQQQFFAALGVPVYQGYGLTEAAPVISSNSPRIHKFGTSGAIAPSVKC
KIMDEMGTELTPGTIGHITITGENVMLGYYKNPEATAEALREGRLWTGDLGYIDEDGFLV
VVGREKALLIREDGEKYSPEEIEEAITASTEVFDQIMVWCDHKKYTIALVTLDENKVKHL
IRSNSINTAEQLLSILTDEFYRFKTDPKAKKVQPAWIPAVFQILEEPFSEQNGTINSTMK
LVRHKAAQIYQDLIAFSYTSEGSTTLNKQNLEKIRKHFSLK*
>SPBIB_v1_280021|ID:27163487| protein of unknown function [Uncultured spirochete bib]
MVIEDSFLRIKQQLLNEGRRFAVISLVNTQTSVPQRAPQNAQKLILCENGQSYGSLGGRE
FEMEAIEQAKACLALRSSKFVWIEEIPPRIDESFPDSTKRKIEVFIEVYEPQPKLIIIGA
EDISLATAIIAQNLDFKIIILDERSEIANKSKFPMAHSIICGPNLEQALSPILDQSSYIV
VNFQFNYERAVRYCLGKEWAYCGILGSKKKIVSLRKKLIEEGFSTEQLNRINAPIGLDIG
AQTQYEIAISIIAEIIAVRHNKKAVFMKSVM*
>SPBIB_v1_280022|ID:27163488| Ribose-phosphate pyrophosphokinase [Uncultured spirochete bib]
MSYSDPTHLGIIACPGAEAFANQVIRKLSGIYKHRFARKAQAIASRYHITPELVIRKINF
ANDCNTGMLFLPGNTDRYRPPQFKINVRHTFFANGEIKTEILESIRGRNIYIFQDIENHQ
PISFNDGTVHKVLSVNDHIFNLFVAVDAAMQAGAAEINLVLPIYPYSRQHKKKGREGLTA
ARIGKMLESLGVNRIITLDIHSKEIENAFDRLRMENLHASFQIIDKLMSIVDIAKEEMVV
LAPDTGAVDRNKFYANALQKPLALLYKERDYSKVSKNAADSNISEVRLLGDVTNKVVFMA
DDMIGTGGTLIKAMRYLKDLGATKVICAVSLPLFSGNAVDDFDAAYKEGLFFRIIGTNAI
YHNELLKKEWYIQSDVTGLFAQVISLLHHNRSLSSLLDNRDVVARLIKRKLESNSNTKSV
PSNPQSDQRDGASNSIDDVIPYGAADKQ*
>SPBIB_v1_280023|ID:27163489| putative hydrolase (HAD superfamily) [Uncultured spirochete bib]
MKLFLRPRLHRAGVLAFDLDGTLYHNPEYLKFQEESQIEQLADYLHIPNQAASSLVLQRK
KLRKERQLPPTSLANIFKEIGVPDDLIIEWRKRKLRPHNWLAPDPLLYESLNALKKFYQL
ALITNNPRVIAEESLDALGVASLIATIIALDDTGKSKPDPAPFQFLLQSMNQSAENCIVI
GDRYDIDIHPALNEGMSGILIDNVKEIYLLPKLLLP*
>SPBIB_v1_280024|ID:27163490| conserved protein of unknown function [Uncultured spirochete bib]
MSLVTFGEIMLRLKSPGQERLFQSPLLEATFGGSEANVAVALARLGIPARFVTLLPDNQI
GHECMRELRSHGVDVSGIKFKKGRMGIYFFENGANQRPSNVIYDRAYSTLSEMEPSDIDW
GKAFEGAKWFHVSGITPAVSQKCADATIFAVQTAKKMGLMVSFDLNYRAKLWNYGIEARA
VMSEIARSADILIGNEEDYQKSLGIKGPKEASSGELDIDEYQTMCESTLNAFPNASIAAV
TLRESHSADSNDWSGMVVARGQRFISRKYHIADIVDRVGAGDSFSAGLIFGLIKYGEPGA

ALEYAVALSCLKHSIPGDFALIEPGEVEKLLGGDTSGRVQR*
>SPBIB_v1_280025|ID:27163491| 2-dehydro-3-deoxyphosphogluconate aldolase/4-hydroxy-2-oxoglutarate aldolase [Uncultured spirochete bib]
MKDLKALFYSTGIIPVIKIESLDKADGLAQALRSSGIHVAEITFRTKTAPGVIERFASRH
SDILVGAGTVTTKEEVDSALSAGAAFVVSPGFNPVICSYCIEKGVPVFPGVNNPSLIEQA
IAMGLSVLKFFPAEVSGGVRALKAFESVYSSVSFIPTGGIQENNINEYLALKNVLACGGS
WIVPTDLIEAGKFEAIEGLIGSARRTMLGFLPLQHGDRGLNREKADAGARIGDGAAIEVK
TTSLRRSLAILQAEGFSSVLGSEVMRGKSMAAAEIEIPSLHGRIRLVE*
>SPBIB_v1_280026|ID:27163492| putative beta-D-galactosidase [Uncultured spirochete bib]
MIVTRLEDINRYRGLSTNLDTAFDWLLKEKWQNLEQGKHTIFGEAIYALVQSYKSKDHAA
CRFEAHRSFIDIQMLTSGEEIIEALPREGLEVLEPYKPDIEFYVTPKDSAACQLFMKPGL
LAIFFPEDAHRPCMIAGDGPEDIGKIVIKVAV*
>SPBIB_v1_280027|ID:27163493|kduD| 2-deoxy-D-gluconate 3-dehydrogenase [Uncultured spirochete bib]
MILDSFKLDGKVAIVTGSSTGLGQGYCIGLAEAGADIVGVDYVESPETAQIVRARGRRFL
EIKANLMSIEPIQGIIDAAVKAFGHIDILVNNAGIIRRQDAIDFSEKDWDDVMNINIKTV
FFLSQAAARQFIAQKTGGKIINIASMLSFQGGIRVPSYTSSKSAVMGLTRLLANEWAKYN
INVNAIAPGYMATNNTAPLRADPSRSEEILSRIPAGRWGTQEDVQGAAVFLASSASDYVN
GYTIAVDGGWLAR*
>SPBIB_v1_280028|ID:27163494|kduI| 5-keto 4-deoxyuronate isomerase [Uncultured spirochete bib]
MHTREAVNSEFVKALDTQRLREHFLIERVFVPGEMSMVYSHVDRMIVGGITPTSVPLALP
VSKELGTDFFLERREMGIINIGGSGAIEVEGIRHQLGPREAFYISMGTKNVVFLSENPNE
PAKFYFNSAPAHRACQTRRVTLDQAKHVEMGSDAECNKRVINQYIHPAVLETCQLVMGMT
MFYEGNVWNTMPAHTHERRMEVYLYFDMPEDRVVFHFMGKPEETRHIVVRNEQAVISPSW
SIHSGVGTGKYTFIWGMAGENQAFTDMDAVPMAVLL*
>SPBIB_v1_280029|ID:27163495| Transcriptional regulator [Uncultured spirochete bib]
MAEIKVQSLDRTFNILEILAREPNGLTLAEIARQLDLPRSTAFRLLAVLLQRDYVRKALD
TNRYRLGPGFIELSSYYLNNLELKTESAPYMRELAAALGTIVFLARRQGSMMVYIDKQDQ
FTSLRKYAIIGQQKPLYCTSLGKALMLDMDDDEIRRLHAAVKFEKFGPNTHQNIDSLIED
IHRCKVRGWAQDNEEAEPGMNCVAAPIRDYRGQIISSISTSWIPESRPDLEPGKVAIQVM
KAAREISAAMGFRGNSH*
>SPBIB_v1_280030|ID:27163496| Nitrogen-fixing NifU domain-containing protein [Uncultured spirochete bib]
MLFEEVKSAIENVRPSLMADGGDIELVSVSDDGVVKVRLTGACGACPYSIMTLKQGVEAY
LKKVVPQVTEVQQD*
>SPBIB_v1_280031|ID:27163497| Protein Soj homolog [Uncultured spirochete bib]
MARIIVFVNQKGGVGKTTSAINIGASLALQHKKTLLIDFDPQGNLTSGIGGTLTHTNIYH
VISGLAEIKNVIQPLSIPRLFLAPSSVDLSGATIELVDRPDRNDYLKRAIAPILEDYEFI
LIDCPPSLGVLTLNGLAAATEVVIPLQCEYFALEGLSLIIQTIGLVQKSINPNLKIGGIL
LTMFDARTRLSQEVVRQVTEYFKDKVFKTIIPRNVRLSEAPSHGLPAILYDPTCIGAKSY
ESVTKEIMNRG*
>SPBIB_v1_280032|ID:27163498|spo0J| Stage 0 sporulation protein J [Uncultured spirochete bib]
VAKFGLGKGLGALIPERQQFFEQSGQEASESPVRVVSIDSLSPNPDQPRKTFSQESLNEL
AESIRRHGLLQPLLVQEQAPGKYIIIAGERRYRAARLAGLLELPVILRSSEDESHLELSL
VENIQREDLDPIEEAQAYVKLMEITGATQERVAEMVGKSRVAIANAVRLLKLPEDIQSAV
KEGSITPGHARNLLSIEDENERQKLFDRIRHENLSVRQTEQEAQKLQAALQNQKGKARKS
RSGSSDDYEAALDPLLRELKEKLIERLGTKVEITGSVDAGSIKIQYFSQDDLQRIYDILD
IQ*
>SPBIB_v1_280033|ID:27163499| exported protein of unknown function [Uncultured spirochete bib]
MHCLKRGPKHASKTHKGVLPRTSTPFHMFQKWGQRLKKPVFAHFLTIILIMIPASGLFAQ
QSASSSATVSISKMTSALPKTFQATLPQGVTFQQGSPILRTAEHIGGKLMLAPSLEGVQT
IINHISSATTGTNLIIEALVFLPSPTLGGSYSASSQIDALGLLFNQFRSLQGIQYWSASR
KIMRTLYTDAFRVDNPNDKNKIKDPETIAEFRAILPQKTYIYQKDQTFSGIITEVQCAVS
QTTFLMTNTNVTPLRLIGIPVLSADGIRTGFLAAPSPEGVFLYFVTSIKSPSIGRDRVFE
SASNKALALLHWFTEAASARSIIEPVHLPWNIDDLPPEIRLKQAANNSP*
>SPBIB_v1_280034|ID:27163500|eno| enolase [Uncultured spirochete bib]

MSIIEYVEAREILDSRGNPTIEVDVVLEDGTLGRAAVPSGASTGEYEAVELRDGDKKRFL
GKGVLTAVENVNTTIAGEICGLDALEQVDIDRTLIELDGTENKAKLGANAILGVSMATAR
AAAEYLGVPLYKYLGGAHTTLLPVPMSNIINGGKHADNKIDFQEFMIMPVGAESFREAIR
MNAEVFQTLKGILKSEGQNTTVGDEGGFAPNIENEQALQYIVRAIEKAGYVPGEQIAIAL
DPAASELFEEGGGKGYRFWKSNPDKIFSSDDMIELYMRWVEKYPIVSLEDGLDQNDWDGY
VKLTKTLGKKIQVMGDDLFVTNTKRLAKGIEIGACNSILIKLNQIGTVTETIETVNMAKR
AGYTAVVSHRSGETEDTFIADFTVAMETGQIKTGSMSRTDRIAKYNQLMRIEDEMEGIAE
FWGKKVFYNVRG*
>SPBIB_v1_280035|ID:27163501| Membrane protein [Uncultured spirochete bib]
MLTRRLDEAKEAWKRRDSEAARRAHLPTKSAEERHTTGKGKYLKSIIYGGLDGTITTFAA
AAGVAGAALAPGVVLIVGLANLLADGLSMSIGDYLSSKSESEYEAAEREREAWEVENYPE
GEKLELEEIYRARGMAEADAKAVVDIISKDKKAWVDTMMVEELGIMQSDESPVANAIATF
ISFAAFGFLPISAYVVALFVPSLDAIRFPLACFLTGATLFGLGALKTIITGTKWFISGLE
MLLVGGVAASAAYLVGVLLGGLA*
>SPBIB_v1_280036|ID:27163502| DJ-1 family protein [Uncultured spirochete bib]
MKKACLLLAEGFEEVEAITPADFLRRAGIEVTITGIASRKVKGSHGIVIETDAGPEALAK
DYDVIVLPGGQPGANNLAASPAVRDLLIRHSQKGKLIAAICASPAVVLHGSCNLLQGKKF
TGYPGTEVSVKGAHFVPDRVVIDGNYITSRGPGTAGEFAIAIIAALEGRQKADEVAQHAL
QK*
>SPBIB_v1_280037|ID:27163503|yjgF| Enamine/imine deaminase [Uncultured spirochete bib]
MSKECVFASGAPAALGPYSHANKAGNFIFASGQLGLDPATGALVEGDVKAQAKQALQNLA
IVLKASGCSLSDVVKTTVFLKDIRDFAAVNEVYGSFFQKDFPARSAIQVAALPKDGLVEI
EAIAYKE*
>SPBIB_v1_280038|ID:27163504|patB| Cystathionine beta-lyase PatB [Uncultured spirochete bib]
VNYDFDSIIDRTSTHSLKWDTRRLPPGCKDALPLWVADMDFACPPEVVSAIQERAAHPIY
GYTGRSQGNYQSFIAWMQRRNGWKIQKDWIVFSPGVVPALNLAVLAYTQPGDKIILQPPV
YYPFASAVLNNGRQLVENPLVLDNGRYTMDFSDLENKIDSRTKLLILCSPHNPVGRVWRK
EELERLVDICAKHDIVIISDEIHSDIILGTEVHHCTATISEKAAAITVTLTAPNKTFNLA
GLQIANAIIPNKRLRDAFAIQTENIGLGLSNIFGMVAQEAAYEKAEPWLEALLAYLKGNF
EFLKNFLAEHIPAIKVLPLEGTYLPWLDCRALGLSDAELHDFFLKKAKLWLDDGTMFGTG
GSGFMRINIACPRAILKQALEQLEAAALQAS*
>SPBIB_v1_280039|ID:27163505|mntP| putative manganese efflux pump MntP [Uncultured spirochete bib]
MLTYILVGFALAADAFAVSVSAAACTDVLPFLIGLRAAFMFGLFQFLMPIAGWLLGSAFS
QFIQGFDHWIAFGLLGFVGGKMLFEAIRARRKQECPDPDDSPKSHGILKLNTLFLLALAT
SIDALAVGLSYNILGHPVLVPSLIIGITTFLVCLIGIEFGKRLKEVLEEWAEIAGGSILV
LIGLKILVEHLSNGR*
>SPBIB_v1_280040|ID:27163506| Flavin reductase domain protein FMN-binding protein [Uncultured spirochete bib]
MNSIYEEIPANEAYSLQNPGGVIFLCTRGSVPEGALRESRYNFAPLAWCTPYEYDPISKI
LLVCDTSHKTFRDIQENSEFAIALPSFDMRDLIEQAGSVSGFAVDKFAHFSVQYFESKSI
DVRIPSGVVGWMECTLERIVIEGTSGIVFGAVKQAFAEPDAWARRLHYVNDETWYKPGER
L*
>SPBIB_v1_280041|ID:27163507| exported protein of unknown function [Uncultured spirochete bib]
MQRRPLFLLAMLALCCTTQIFAAPGIAFTLNSIKTNSDIIAGLPLPTGADLTLELPLGST
STAFTLRAAAGYESRMILRSTATMFPIAEPPAIDGVNRFFWTNALAELGVKQYLLHKEHE
QAWLFGLVRGRYENNSPSFPTTLFPDAQAVRSASGVGGIAFDSIRWLGKNVKKGVSAELS
YEYSPDFANFSGTPADFGRANFTAKAFVPLSASNFATYLALYGVGDYALGSHIPHEVLTT
FGGITGTKGIGDMVRGAQPWGYEAPAKTYVSAELRVAGPSMFRDFAIYPVGYLFADAAAY
GSLYGSPLADNSGMIASAGAGASVSVLNFLWLGAYAGWRLPVNDPLSAIYYGSAHGFFWN
FTFVAHY*
>SPBIB_v1_280042|ID:27163508| conserved exported protein of unknown function [Uncultured spirochete bib]
MKKIIVALLVLVVASAVWAQTPGGLSAAKSTRMEADFTKIVTDMGWGTVDTTRPVLAERM
WDYYFFYPVLNKDKQITGYVWWAKDIRIASHYEDILVIINPDGTLQNWWVSANTRHADFY
TDFAKKTQGAAIQKFIGMDSKRDWNQATDAVSGSTFSAYKFFGELKAVLACFKIYVIDAG
KLIK*

>SPBIB_v1_280043|ID:27163509| putative Phosphoesterase PA-phosphatase related protein [Uncultured spirochete bib]
MAFIESMHAAELQFIQQLQSFLGLGFRIPMQFVSFFASEAFVIAVVPVLYWCIHRKKGAE
FGLLILGSAFINLWVKQLLAWPRPYEIIPSLALAKESTYGMPSGHSQLSVVFWAFIAEFL
PAGLRVPAMIIMPLLIGFSRIYLGVHFPSDVVGGYIIGVAFFGLFKAFGLKVEPMLRDSG
WRLRVILAAALSFIMNLLLPSDTMISGAFLGASIGFTFASRNVPIDQRDDVRHKLLRYLA
GLATTGIIYLALKFASSPLASISGNNQEQLIRFIRYALVGGWVSYGAPFMFLKLGLAKRE
SS*
>SPBIB_v1_280044|ID:27163510|apbE| Thiamine biosynthesis lipoprotein ApbE [Uncultured spirochete bib]
MSFSRTYSIRITRAFLALSLALLFGTISGRAIYAEDLTRTDFVLGTVCTIRLIDGGNANT
LSEAFARLRSIEDHMSANKDGTEIAQINANAGKEPVKVSEDTFYVITKALEYARLTNGAF
DPSVGPLVKLWNIGNGGEKVPPEREILAAKALVGWQQVVMDAATRKVFLKKPGMRLDLGA
IAKGYAADEVAKILIAHKVKAAVVDLGGNVLVFGSKKDKSPWRVGIQNPESARGEYLGIA
TGAQMTVVTSGVYERYFIQDGKRYHHILSTETGWPVDNGLVSVSIISKSSIDADALSTSL
FILGIEKGMALLKNFPDTYAVFIDKDKKVYLSPGASKVFTLQEKNYHLAEK*
>SPBIB_v1_280045|ID:27163511| putative enzyme [Uncultured spirochete bib]
VASTIWRLKVGPIGVNCYIAESNGSTVIIDPGAEPEKIHAFLNAHSLKPSMVVLTHGHLD
HSAAIADLFELLGTELPIAIHPDDAQYLGADGQQTNRALFEAIKAPSYFHSFWKPLPPAT
VFLSDETVVPGTTLHVIHTPGHSKGSICLYEASLDAGSYGTDEDEGRGAAGWSCIISGDT
LFRDGVGRTDAPDSDPVALERSLRKLAQYSYMTLVFPGHGPRTTIGRELSAPFHE*
>SPBIB_v1_280046|ID:27163512| protein of unknown function [Uncultured spirochete bib]
VPNDNAAPKFNEKTLEAFLEGLLDELKHPQDAKLLEEVRRAFRKKVPFHMRSYASALMIL
RAAGIYRPKATRQAPQEQLQRQPAKREQFKSEQPRSEQLRSESPRNQQAQNEQSRSEPPG
AEHTNKEMISLFVSMGKRHHLKPLELKKRIAERAGISPDSLGRVHLLENYSFIEVPASES
QRIIAAMAGAELNGRAIEIKPAKKRSESVSEGQ*
>SPBIB_v1_280047|ID:27163513| 3-dehydroquinate dehydratase [Uncultured spirochete bib]
MSKKGKPKICICLSGKTIEENLRILERYRAVVDYVELRADCLEPSERFNIRSFPEKAQLP
CILTIRRKQDGGNFEDGEGVRLVLFAKALSFPKPDAKANYAFVDLEDDFRTPVIEEACHT
FGTRIVRSHYFHDGIPENFDAAYEQIAQEDDEIPKLVVNPQSSADFIRFLEWATRVPKRE
HLLIATGAYGIVSRVLAWRFGSTWTYASPLHSGMEIAAPGHFDPFDLRNIYNFDLVNEQT
ELYSLIGQHSILASLSPYLHNKAFREMGKDALLVPTPVDSFADGLKILELLGGKGAAITV
PFKEDVLPFLAFHSTDVKRIGACNTLVRRGDAWAGYNTDADGFERSLLEFLGKQDLSGCR
ATIIGAGGAAKSIALALFRKGARCLVLNRTYSAGRDLARKYNFLYSHLDDRASDLISEYS
DIIVQATSVGMREELDPISFYEFKGSEVVFDLIYHPSKTLLLKRAEEAGCRVLNGYKMLC
YQAAGQYKLWMDEPPPDIYAQLEQPKFS*
>SPBIB_v1_280048|ID:27163514|era| GTPase Era [Uncultured spirochete bib]
VPKSAFVAIIGRPSAGKSTLLNALCGAKVSIVSSVPQTTRNAIRGIVSHPEGQLIFVDTP
GYHLSDKKFNLKLRDLVMQSLSDVDLILYVIDATREPGKEEEAIVELLLKEAPRTVIAIN
KIDHPDAKPLLVEEFILPRMPEAKRITISAAQKTNLAALLEQLFLLAPAGPLWYPEEIYT
DQEPVFRIGEIIREKAILHTRDELPHAIAVEYRESTKRADGVLFARFDILVERDSQKPIL
IGRQGSVIKRIREEAEADLKELFDYPVKLQLQVVVDPDWRRNEETLSRMIF*
>SPBIB_v1_280049|ID:27163515|pth| Peptidyl-tRNA hydrolase [Uncultured spirochete bib]
VRVTLRRIQLVRFIMIRLCAFLGNYGKEYRLHRHNVAWLFLESMRISADLHWSTKFKGTI
AQADFGYGKVSILKPHTFMNLSGESVVEAARFYRCSPEEILVVHDELEMPFGAFGYKFSG
GLGGHNGLRSLEKHLGTRDFWRLRFGIGRPDHSDIAGYVLSPFAADELDTLKTKVFPEAE
KTFYTLFTEGIEGYEERYRKVVCKQAP*
>SPBIB_v1_280050|ID:27163516| PHP domain protein [Uncultured spirochete bib]
MISNLHLHSCFSDGMSWPEDIALDAARSGLDMVALTDHDTMAGVARFCAACASRGMQAIP
ACEIDVNEPQIDYKSELLAYFPGAEPAAGAPATRAILRESLRNRRARLEFLINSARELYP
DKELSFEDLFRDKTRMEYDSALADEISWSKVDLFLYLKARRCIDPDMNYKAFKKKFFANG
LLKKYKLDKPDIASVVAAVHADNGFVVLPHFGHLWDDDAALMHKETEKLRSKLAWFRRAG
VDGVELYWYNADYRSAKINELVRTVARPQGFFFTYGSDCHGPGSGKHTITKFKGNFEGFP
VRQADGHQSEGHQGSAGHASPNTAG*
>SPBIB_v1_280051|ID:27163517| protein of unknown function [Uncultured spirochete bib]

MFQLTDAILERIVFAMEDQTNEWLIDLRTGDIVDRSEVSDEDLEAVPERYTDLPFWSSRQ
GFTILEAFAATVTSPPELKLALNAALRRGKGVFKAFRHALSSNDALYRRFQEFKLNAMRP
TIEKWMHAIQEEDALAAVKEEPEDLADIIASELEIQTVALSEAPFDVGQVISDYATETYT
TYPPALSKWALLEIHEKLTSWIREPWIAYASIDGTHPLVLGIYSLKPLEGTACCAVWGIF
ASKECATMGIEWPLLDRISSEATAAGASLIILDGPLFPSSLNEEATSHGFMQAGSSLWKM
L*
>SPBIB_v1_280052|ID:27163518|fabD| Malonyl CoA-acyl carrier protein transacylase [Uncultured spirochete bib]
MSGGSMKTCFLFPGQGAQYPGMAKDFYETSKGVRELFKTASEASSMDLKTLLFESDEETL
KQTKNTQIAIALAGAAAALSAKEHGIVPQGAAGFSVGEWPALAEAGVISHYDMFRLVSER
GRLMDEAGAHSGGSTMSAVLYLSPEKIEQVIAEAGLTQCWIANYNSPSQCVISGTEKDIG
IAEEKLRQAGAKRVIRLKVSGAFHSPLMHEAYEAFKELVEAITFSDPTINLYSNVSGKRM
ESGAEAKKYASLQIISPVRWIDEEVNIAADGFELCLETGPGTVLTGLWKASGSSISCIAA
GTLDALVSFSL*
>SPBIB_v1_280053|ID:27163519|fabG| 3-oxoacyl-[acyl-carrier-protein] reductase [Uncultured spirochete bib]
MGKLLEGEVCVITGASRGIGQAIAVRFKEEGALVYGLSRSKPEANIEWIPCDVADEVSIE
SAVNSIFQKENRIDIVVNNAGITRDGLIMRMKTEDWDAVLNANLRSAFIMSRAVSRMMLR
QRSGCILNISSVVGLHGNGGQANYAASKAGIIGLTKSLAKELASRNIRVNALAPGYIETA
MTSVLPDAAKQSLTTSIPLARPGTPAEVAEAALFLCSPRASYITGVVLNVDGGMGM*
>SPBIB_v1_280054|ID:27163520|fabF| 3-oxoacyl-[acyl-carrier-protein] synthase II [Uncultured spirochete bib]
MNRRRVVVTGLGVVSPLGNDIPTFWSNVKANKSGIAPITMIDSSELAVKIAGEVKDFNPG
LRLDPKESKKMDRFAQFAVCAALEALESAQLQKESLDPLRTGVCLGTGQGGSATIEEAGV
RLTERGPSRVSPMTVAKGLANFGAAQIALSLGVHGPNMTVVTACAAATDAIGQAMHLIRD
GHADIMFTGGSEASIVRMCMASFINIQALSSRNDAPEKASRPFDKDRDGFVMSEGAGILV
LEEYEHAKARGAKIYAEIAGFGATCDAHHLTAPDPEGMWVAKAIELALNDAGMKPEDIDY
ISAHGTSTPLNDPIETKAIKHAFGPHAYKLKVSSLKSMIGHCIGAAGAIETIAAILGMNE
NYIHPTINLDNPDPECDLDYVPNKGINMPVRAFVKESMGFGGQNAVLVVTKA*
>SPBIB_v1_280055|ID:27163521|glgX| Glycogen operon protein GlgX homolog [Uncultured spirochete bib]
MSGHFRALPKELHIFPGNPLLPGAMPHPQGVYFSVFSRHATGMILCLYESPHDDKPAASL
RLDPVRNRTGDIWHCYIPGLKPGTLYLWRAEGPFAPHEGHRFNPNKVLIDPYAKALTDGT
LDLCSALAYDESSPDKDLSFSEKPNDGCMPKCVVIEDNFDWEGDTPLNYPLKDCIIYETH
VRGLTRSSSAKVKHHGTYRGVVEMIPYFKDLGITSLEFLPVQEFDSAERFRHNPVTGEQL
SNYWGYAPLAFFAPKTSYAYPDGVDHDSYDPEFPVKEFKYMVRELHKAGIEVILDVVFNH
TAEGNELGPTLSFRGFDNSIFYMLAEDKRYYRNYSGCGNTLNCNHPVMRTFIKECLRYWV
VTMHVDGFRFDLGSILGRDSQGNLLPNPPVIESIAEDPIMRNTKIIAEAWDAGGAYQVGS
FPGGRWAEWNDRYRDDVRKFWRGDEGMAPHFATRITGSSDLYLKNGKRPFHSINFVTSHD
GFTLNDLVSYHQKHNEANGEDNQDGLNENYSSNYGIEGPTDVPFIEEIRNRQIKNFFATL
LLSIGTPMICGGDEFRRTQNGNNNAYCHDTEMNWYDWSYLKQHKDIYRFAKLLIGFRKNH
QAFRREDFFRGETDQNTEANPEAPDILWFDEHGNPPLWTSLGKVLVARINEHSKSENSSK
VPIYSYLLAFNASPEAIQIQLPDDNTEDTWKRIVDTSLPAPHDFEENEYLALGAPHTYVM
GPRSFVLLIAGH*
>SPBIB_v1_280056|ID:27163522| putative SEC-C motif domain protein [Uncultured spirochete bib]
VKTTSGTSKAAFYLRKGIYELAHHRPAKALALLRQSVEITPPSCENDLSKALYWLSIALL
RLDQRELAVKSLANAQKIRRQSYARKVYVRNINEYGMLKRPTRELDDLYAFVSIQLSSYL
LKKPQHRFGSQAEHSAVLKLILETWKQLKDSDEFKSLECGEKLLLFRKLKIDFPIFGSYS
TSPRYSERFAMNIASVGMRTCSCGSGLPFEQCCGRVQGLSEI*
>SPBIB_v1_280057|ID:27163523| RNA polymerase sigma factor [Uncultured spirochete bib]
MMERKKTKETRSSAQFDDENVLSMYLREINRIPLLSREEENEYATRAALGDELAKAILAK
SNLRFVVNVAKKYQNQGLPLADLIAEGNIGLLNAIEHYDVTKGYHFISYAVWWIRQAILK
AICEKSRMIRLPLNRANELVQIEKARNLFEGELSEDAEIREVAEFLEMEPEHVKDIISIS
RDLISLDAPVYDEKNSTTVGELIENEHHESPEQNAIQSSLREDINKVLAALTAKEREIIE
YRFGLNGKRPMSLKEIGDRMHLTKERIRQIEKAALKKVAVPETMEILQAYMA*
>SPBIB_v1_280058|ID:27163524| putative Polyprenyl synthetase [Uncultured spirochete bib]
MEHLFLEFTQQAKATISAHIASILHENQPMFSRISGTGAWLASEMEDFADSGKMLRGSLA
ILGAQLLGMQNQKNSVSLAAGLELLQAGLLVHDDIMDHDEKRRGKPTFHIRIRNKIQWPD

QALSAPLAVQLAEAQSICAGDLFFFLAWQEIAYLPQRVSVTVAQEHAKVTLAQMQDVALG
YVEGFPDISSVIEMYRHKTARYTVALPLMAGALLLDNCTSEVLKSLERLGESLGIVFQLQ
DDRLGLFGDETAIGKPVGSDLKEGKKTPYILALIPQLSESEMKRFNSIFRAQALEPSELE
WIRSLIVSRGVDAKIRSLIDNQIELARKALADLGQFEAVSEETMILLADFIDYSLSRER*
>SPBIB_v1_280059|ID:27163525| protein of unknown function [Uncultured spirochete bib]
MMRLSLYLLGHNYLKPFRIRAHKGMHPRTHAEAAGIPVHLVQHFVQALTGGIRDFLSRCT
LSETMRRTWEKRWKTPGKDKAEYLPKYALA*
>SPBIB_v1_290001|ID:27163526|gyrA| DNA gyrase subunit A [Uncultured spirochete bib]
MSESGNIIQIPIEEEVKTSYLNYAMSVIVSRALPDVRDGLKPVHRRLLFAMEELGLRNNA
PTKKSARITGDAMGKYHPHGDLSLYDALVRMAQDFSLRYPLVQGQGNFGSIDGDPPAASR
YTEAKLSKIGEEMLADLDKETVDFVPNYDESLKEPSVLPSAIPNLLINGSSGIAVGMATN
MPPHNLREVAQAIEAYIENPDISIDDLMNYVKGPDFPTGGIIYGMQGIRDAYATGRGRLT
VRGRFIIETMKSGREQIVFTEIPYALNKTTLVTRIAELVRDKQIDGISDLRDESDRDGIR
IVLELKKGAITKIVLNQLFIHTPLQSTFGVINLALVNGAPKCLNLKELITYYVQHRFEVV
TRRSQYELKKAEERAHILRGLVIALQNIDEVVAIIKASRNVDTAKTNLRERFGLSDAQAQ
AIVDMRLGRLTSLETEKLLAELKETEARIEYLRALLADSAAIHAVIKKEIHELAEKYGDE
RRTEIVPNQVEQINIEDLIKPEEMVILISNKGFIKRISVNQYRSQGRGGKGSNSTSLLED
DFLQQLFIANTHDYLLFISSWGKAYWLKALEIPEASRQARGSHIRSLLAISQDEEITAVV
DFSDFSDKQYILMGTLKGVVKKVATKEFANAKTRGIIGISLDDGDRLVSAILTGGTDEVM
LISRKGLALRMQESQVRQMGRTARGVRGLTLQEEDELAAMLRVDSEESMLIITQNGYGKR
VKYDLFTPHSRGTRGQIIYEPDESSGEVIKAITVREEDEVMVITSMGKTIKLNVSAVRQM
GKAARGVRIVNIDPPDLVIGMDKIVQQVELEGSAPNLVPELK*
>SPBIB_v1_290002|ID:27163527|gyrB| DNA gyrase subunit B [Uncultured spirochete bib]
MESTVNNYSASTIQVLKGLEAVRRRPGMYIGSTGIEGLHHLVYEVVDNSIDEALEGYCDN
VVVALEKQDIVRVEDDGRGIPVDIHPTEGISALELVMTRLHAGGKFDKKNYKVSGGLHGV
GVSVVNALSTWLEVYVHTGGKVYYQKYLRGIPEKPVVQIGETDKRGTIVRFQADPEIFEE
TVYSFDILSNRLRELAFLNKGIKISLLDERLAQPKRIEFHFEGGLREFVEYLNKNKSAIH
REVIYFSGTRDDVEIETGIQYNDGYNETMFSYVNGINTREGGTHLVGFRNALTKVINDFF
KKSKYNKKLEENLSGDDVREGLTAVLSVKVQEPQFEGQTKGKLGNSEVKGIVESFLADQL
DLYFQKNPDVINIILDKCTMAARARIAARQARELTRRKSLLEASSLPGKLADCQEKDPTK
CELFIVEGDSAGGSAKMGRNRVFQAILPLWGKMLNVEKARLDRVIGNDKLQPIIASLGTS
IGEDFDVSKLRYHKVIIMADADVDGSHIRTLLLTFFYRYMTELIEHGHVYLAMPPLYRIV
YDKEVRYAYSDEEKDHILSEINKDSSKIAVQRYKGLGEMNPEQLWETTMDPERRMIKRIT
MEDAVLAEEMFVTLMGEQVEPRRLFIEENALAVSNLDV*
>SPBIB_v1_290003|ID:27163528|dnaA| Chromosomal replication initiator protein DnaA [Uncultured spirochete bib]
MIDESNETVWQKALEIAKSQVPESEFIMWFRLGYLGFENGTILLRANNTFLRDQFMRKYG
DFMREILSSILGMQVNLSVEAQPSAIEQRLPLQKAAATSLNSSAKKAEKPNPQQAASPVE
TSISDEEISNNSSYPAYSRHTQESRTANLQPRYTFESFVVGENSNFAFNAAAAVAEIPGT
SYNPLLIYGGVGLGKTHLMHAIGNRIAERHPELRIICVTAEDFTNEFIKTIHDRTTNEFK
NKYRNIDVLLIDDIHFFQSKHGVQEELFHTFNALYDSGKQLVFTCDRPASELKDFSERLK
SRFMMGLKTDLTTPGFETRAAIIRKKLELYKRTFSEDVINLIAKSIETNVRDLDSCIHQI
IAYSDLINVEPTYDIAQNIIKQLTNAFKPAAINVSSIIKTVADYYKLSISDLKGKKRSKN
IALARQVAMYIIRETTDYSTTEIGSEFNGRDHTTVMHSCQKIEDLIKFDSIFASAMNRLI
QDCKENSST*
>SPBIB_v1_290004|ID:27163529|dnaN| DNA polymerase III subunit beta [Uncultured spirochete bib]
MKFICDRDTFSREIAIAQDIIASKNAFSIMSNVYLETTDSKLIIRATDAKVGFETEIPVS
DSEPGSLTVFCDKLMAITNSIPEGDMLFEQRESSVEIRPLSKKVRFQLKTIAGDKFPELP
RIEENQYFILQAHDLRKMISQTIFSVSNDETRFFMNGVFMEKLPDDVVAMVSTDGRRLAF
ISTPVGNIPDFASAIIPPKALSVLMKRAPDEGQILVAVNEKNIFFRFGNYQLSSFLIEGK
FPNYQKVIPHDQRNHFIVSKDDLQAALRRVSLFVEKSNKVILNVSSKGLVLESEETELGA
AREELPCEYEGEDVVILLSNRYLEDPLKVLDTDKLAIEFTDPSRAITLRPEPAANYFHII
MPMQQP*
>SPBIB_v1_290005|ID:27163530|recF| DNA replication and repair protein RecF [Uncultured spirochete bib]
MAFEQIRIARFRNIEETELELGAGNIFLLGENGQGKTNFLEALYCLSYGNSFRTHQDREL

PMHGSPEFLLRAQWREINSPAQEQISIRYSIEGRKEIRINDKLLSDRKELVAHNPAVVFC
HEDLAFAAGVPEERRFFFDQCAGMLWLDYIDLLRFYRRVLKHRNIALKTDTFSILDTLDE
QLAQYGHEIMAYRKRLSQLFIARFPQMYELVSQLGTEVSIRYSPSWPLDSSKEEIMALLA
EYRTKDIEAATSLSGPHRDRWIFTREGRNFAAFASTGQLRLASLILRIVQVHLYAELSAA
EVRCYPVLLLDDVLLELDVAKRRRFFSLLPAKDAGAQAVFTFLPEEPWREYADESTIVYR
VSNGRFSREESR*
>SPBIB_v1_290006|ID:27163531| protein of unknown function [Uncultured spirochete bib]
MDDFRVKKAGEILSRFFDEKTLRSATQFESFRASWKNIVGQRLADHSKPKSILRRTLLIS
ADHAGWIQLLQIDQERILQRIAKNFPELEITSLAFTVEEAASEAKQSPQVQARQPGSPLA
PLAESLPLQPEPDAAHARKASKSVLPAPLKEIFARLQRPRGD*
>SPBIB_v1_290007|ID:27163532|rpmH| 50S ribosomal protein L34 [Uncultured spirochete bib]
MKRTYQPSRTKRNRKFGFRARMKTKGGRLILKRRRAKGRIKLSVADEKKKY*
>SPBIB_v1_290008|ID:27163533| Ribonuclease P protein component (fragment) [Uncultured spirochete bib]
MQVLYKGEQESRAAFIAVRSFSGAVARNRAKRLARESWRLLRHSVKSGFDIVFVLYPGFA
ALSDCRSAMQYLLHKADLLR*
>SPBIB_v1_290009|ID:27163534| putative membrane protein insertion efficiency factor [Uncultured spirochete bib]
MNIVNKILSFPLLVAIRIYQLVISPLFPPSCRFYPTCSSYSYQAIRKYGPFKGLVLSFKR
VIRCHPGNPGGFDPVP*
>SPBIB_v1_290010|ID:27163535|yidC| Membrane protein insertase YidC [Uncultured spirochete bib]
MSNIFEQEPSSKEDRRRTIIAVVLSTVIVSAGFMVQNALFPPATQQSTATQTPAQGTELP
AQNTSAPAVAVPSPIPVKTASSSSIPVPAAERTYIIETDVMSATLTNAGGEIVSLTLKNH
RDKSGAVDLIVRGSSGANGLSLSFGSATAPVRELMNAQWLDESKTAIEFSRMFEAPLAGS
DQKVPFVLKRAYSFKKGEYLFALGISIEQPDGKPIALGANGIAYRLDLGPQIGPRFDQLP
KNADYRKYIAEIDGKKKSEQPKANTPTVIAPSASWLAMSGKYFTFIAVPKAPMSGYEIET
AQDPLIKQTNTLSLLRTSFSGVSTTDTYYFYFGPKTSAELGKYEYADKNGFGLASLKLED
AMEGSGMLGWLENFLKFLLNFFYKLIPNYGIAIILVTVLIKALFYPLTKKSSMSTARMAE
LQPKIQELQAKYKGNPQKLNQEMAELYKRENYNPMSGCLPLLIQFPLFIAMYNLFNNHFD
LRGAMFIGGWINDLSLPESIINFGNFRLPIVGWNDLRALPIIYLFSQLLYGKFTQSPQSA
QSNSQQASQMKLMMYGMPIMFFFILYDVPSGLLIYWITNNVLTILQQIVINDLMKKHKLA
RAEAATSTGGGPVSAGTGETSSRTAGNATKPAASKLASGKKAAVDAKARPVGRAGSKEGF
SEKVTKWLENKAGKTEKGSKRGATGKSGPNSGSGSAKGSPKKKNN*
>SPBIB_v1_290011|ID:27163536| putative RNA-binding protein [Uncultured spirochete bib]
MIYEFEGKTEREAIELAAQELGLDTSSFDVEIIENQSGGLFKKGKVRIRVHTKDAPRQAQ
QDAPEEKSEKQERHDVEPIPMDDFERKMVEWTKEVIEHMGYSSDVSVAFREPKKLGLRID
TESASILIGKKGRNIDALQLLANVYAGTLGHNDMKIVLDSENYRLRREEALVRIAYETAE
EVRRSGRSVLLEPMNPFERRIIHTTLNDIIDIETKSEGEGLMKQVRVMLKGRK*
>SPBIB_v1_290012|ID:27163537|rnfC| Electron transport complex protein RnfC [Uncultured spirochete bib]
MVIWGGFVIIFQHIASNAKKLVFSAESALIFKGDLSTMSIRTFKGGAHPPERKDRSGGKP
IERLTSVPQVVIPVNQHFGAPIQPLVKVGEFVKAGQKIADAEGRMTVPLHASVAGVVKKI
EPRMQSNNTEGLCIVIEPKDPPEGESAQDFMPPLDPFACTKEEALARIRAAGIVGMGGAG
FPAHVKLAPTKPISYVIANAVECEPYLTIDERTLIETPEKIVDGVAIVMKTVNAPEGVIA
IEENKKHAIPYVQKAIDQSPQVKAGMKIRIQELKTKYPQGGEKMLITAITGKQVPSGGLP
MDVGCVVSNVGTLCAIVDAFREGKPLIERGFTVTGGACKTPKNLVAPIGTLISDLVKASV
VETDEESLAKVIFGGPMMGVAVPSYEIPVQKNTSGVLLMSKEEAMQYEEETCIRCGRCIR
ACPTAVSPALLAIAIESGNYAEAEKIGLLDCIECGSCSFVCPAHRHLVQRFRVGKQVLRA
LKQKEAQNVR*
>SPBIB_v1_290013|ID:27163538| Electron transport complex, RnfABCDGE type, D subunit [Uncultured spirochete bib]
MSDRKLLLSSSPHIFSPVDTPRIMLSVVIALMPATAYGIYLYGLPALGVVVTSIAAAVVS
EFLFRKLIKASMTVGDFSAVVSGLLLALILPPSTPLWMVALGAIFAIVIAKEFFGGLGAN
PFNPALIGRAFLLMSFPAAITSWSMPKGLGLPADALSAATPLNLLKQGNALADVAKYFGA
SDTGAFYRQLFMGYRSGSIGESSILLVLLGGLFLLGIGVIQWIVPVSVLASTFVFSWLLG
MDPVLGLLTGGIVFGAFFMATDYATRPLTPYGQAIFGIGIGLITVLIRKFGGYPEGVTYA
ILIMNILTPFLNKLRVKKYGFVPPPKPARPSKEATK*

>SPBIB_v1_290014|ID:27163539| Electron transport complex, rnfabcdGe type, g subunit [Uncultured spirochete bib]
MKKDMVKLGITLALFASLACASLAVVYSFTKESIEKQSEMQLTASLKEVFPEASVFDQVQ
DLASPDPNVKFEAVYEVKSDKAPLGLAIKATGSSYGGNATLLVAVDLHRSIAGVRVMELK
DTPGLGMNASSPTYYVDKTKKTTFTGQFNGKYLTDAFEVKKDVIAITAATITSKSLTKII
KTAGDAAIAYMDKKAAMSTGDAGAPADANAQTQAGGN*
>SPBIB_v1_290015|ID:27163540| Electron transport complex subunit E [Uncultured spirochete bib]
MKKLYSIFKQGIIFDNPLLMLMIGLCSAIAVTTNVSNGIGMGLAMTFVLLFSEVIISLFR
KLIPSAIRIPVFIIVIAAFTTMVDLVMKAYFPDLSKSMGVFIPLIVVNCIIMGRVEAFAS
KQNLVSVIADSLGMGLGYTWVLIGLAAVRELLGNGTLAGIQIMPSSYQPILFFILPPGGF
FVFSLFIAFNIWLKKKMEASQRTKELAAKKEAA*
>SPBIB_v1_290016|ID:27163541|rsxA| putative inner membrane subunit of an electron transport system [Uncultured spirochete bib]
MDLMKIFLLALLVNNIVVMRFLALCSYIGMTSDVGQSVGMGFAVTFVTVLATAATWPIYN
YILVPLNLTFLQILIFILVIASLVQLVEFYLKKNVPGLYSAMGIYLPLITTNCAILAVTF
ENISFKYNFIESIAYSVGVSLGYLLAMVLLAGIRDRMKTSPVPKFLQGTPILFMATALIG
IAFMGFSGLIK*
>SPBIB_v1_290017|ID:27163542| Electron transport complex, RnfABCDGE type, B subunit [Uncultured spirochete bib]
MVGTILITFGFSAVLALVLGVALGFFREKFKVERDPKIDEVREALPGANCGACGYPGCDG
YSEAVATGRAPTTKCSVGGSATAEALAKIMGVEGGAAEDKVAVLLCQGAVDKTVSKGEYN
GIQTCRAAKLSTGSIKTCSWSCQGFGDCVNVCKFDALAMGEDGLPHVNYDNCTGCGLCVE
ECPQKILTLVPRDRVGSIVLCSNRSTVKASVIKTCKVGCIKCELCVKSCPEGAITMVNGI
PVTDYAKCTSCGICVEKCPTKCYKMLEVDVFGTKKLVPVMEQAQA*
>SPBIB_v1_290018|ID:27163543| putative Lipoprotein [Uncultured spirochete bib]
MQLKSRPIGRLKYFIFMQVLSDNNTMKFLRGGARGRSIGAVKSISVAHIIAARFIAARIV
AIAALWSALAVLPAYSAVTAPPLLGRASADSRQAVIKAAEAFLGAPYRLGGKTKSGIDCS
GLVYAAFLKATGIKVPRTVRDLSKWVLVIPRQDLVPGDLVFFDLEASATATSVPSQTSVP
QNAAFLSKADHVGIYIGEGIFIHAASSGSPKGVIKSSLSEAGWKHRFLFAGRALPASALS
GIAAEWGGGVGLGHLDSLLSSPFVSLRSFNGWGELSLPIANNFAAGIRLGIAWDRYLGVV
RVPAELDIGQISGFSLFAGPALTFGSSVHVIATGGIRWSPLLFSSGAQRFGIYAELRYDH
YVPLAGQPDNAVADMNACVSFSIGFRLRSVKY*
>SPBIB_v1_290019|ID:27163544|yefM| antitoxin of the YoeB-YefM toxin-antitoxin system [Uncultured spirochete bib]
MDAVSYSDLRQNLKSYMDKVYKDHDPLIITRKNNENLVLLSIDEYNSLMETTYLLSNDAN
AEHLKKSIAQYTAGNVSKRELLNDE*
>SPBIB_v1_290020|ID:27163545|yoeB| toxin of the YoeB-YefM toxin-antitoxin system [Uncultured spirochete bib]
MNKLFTDEAWKDYIYWHETDKKQLKKINDLTKEIDRMPFEGTGKPEALKQNLQGFWSRRI
DHEHRIVYKVEENQVIFISFRYHYSK*
>SPBIB_v1_290021|ID:27163546| transposase [Uncultured spirochete bib]
VKLLCSIAQVSRSGYYRYLLITQKPNKDARLAEKVREVQEAVCYSYGAKRMAHVLSEQEQ
VPINHKRIARIMQKYLLGARIRLRRHPEYWYRQRHKVRLSDRQCAPNILNRDFLAPCPLK
KLVTDVTWISCADGTLYLSAVMDLFNRQIISYTLSRRNTTATALSPILELVGSYNLQGTL
VHSDRGPAYRSEEYQALLRKHGIVTSYSRAGNCWDNALMECFFGHMKCELGFVTGSQKKQ
RVAQVSQKIHEYLVFYNKTRIQKNLGWVSPMGYKMLYTSSIESNNVST*
>SPBIB_v1_290022|ID:27163547| protein of unknown function [Uncultured spirochete bib]
MKYRKAERGSMKYPLELKQKVLKDYFEGKDGIRGLERTYGIQHQLILSWIKTCQQPSYRM
RQGKVPKEAALHLNEPPVFNDPRKELEYLRAENAYLREMLMLSGVRKNGRKKKALPPSND
SAHEATP*
>SPBIB_v1_290023|ID:27163548| Tryptophan synthase beta chain 2 (fragment) [Uncultured spirochete bib]
VSTDRGLVQSETGKFKPTLTIIACVGWGSNFAGLAFPFVKKKIEGADIEIIPVEPASCPS
LTKGKFTYDLGDVAGMTPYLAMYTLGHTFMPHPMHAGGLRYHSMSPLVSQAAAEGLLSPS
AVPQL*
>SPBIB_v1_290024|ID:27163549| protein of unknown function [Uncultured spirochete bib]
VAPETSHALAQVIREATKAKKEGKEKVILFSLSGYGLLDMQRYADYFPGKLSNLELSEAE

IEKALAGLPKLETVG*
>SPBIB_v1_290025|ID:27163550| DNA and RNA helicase [Uncultured spirochete bib]
MRIIADLHIHSHYSRATSPKLTPPYLDRWARIKGINLVGTGDCTHPGWLAELMEQLEPAE
GGFFQLKSTLRREFDAGEALAEGLPAPTGDSPVRFVLTGEISTIYSRDGRTRKIHHVVIL
PDFAAAAAFQARLERMGNVSSDGRPILGVDSRDLFAALLDADERSILVPAHIWTPWFSAL
GARSGFDSIEECYGDLAPRIGAIETGLSSNPPMNWAVSALDRFAIISNSDAHSPEKLGRE
ATVIDMENSFAGLAGALTGGGPAGRIAGTVEFFPQEGKYHYDGHRACGVVLSPAESAAYG
GLCPVCGKPLTPGVMRRVAELADRPVDETASCPPDYAGTNRRPYKSLIPLPELLAELLGT
GSGSKKVAAAYGTLVERAGSEFALLLDRNEAEIETLGTAGVPGELLAMAVGRMRSGQVSI
KPGYDGEYGVIHAFAPGERIESKAGAGLFDDETGTGEEETAPAGRARAGTLTASEPAGRK
KDATPPAALDKAPKSVPESRTAAFGLDPAQEAAVNHPGGPALIVAGPGTGKTTVLALRIA
RLVEGGLDPSSILAITFTNKAAAELRGRIEGTIGGARAARLTAATFHAFCLSVLREHTAD
ASLLSSFGVLDEEEKTAFLNLAVRPSQGRGGSGRAEASRTLADNAQTDLLQASLTKTSRP
QKVRRLASYIEERKRFLLLPGDRVPRLGPGAPDGLAELAAELGVPPLDVDLDVAYARYRD
ELKRAHALDFDDLVAGAARLLAARPEILSAYRARFRAIFVDEYQDVNFAQYALLRLLAPG
TDSAELCVIGDPNQAIYGFRGSDRRFIERFLADYPGAAIYRLVRSFRCAPGIIGAAGRLV
GAELGGSGTVVALSRCEFPTDASEAEGIAREIDRLIGGTRFFAIDSGVAGSVGAGSGAGL
ASQAHTASREGPALSSLGECAILVRAAALAQPIEKALLDHGIPYRFIGDKPWWEGEPIRS
VLSLVRAAARIDVRPDAGIKRAVEATACAIIGQHNTRQGQASPEKVLRARLPGLSPAEAV
RAAIDLLDGEAAAGASKAVAAGVPRVAFGTRPGESLERLISQASLYPDFASFLDDLALGS
PQDGYEAHAECVSLMTIHAAKGLEFDYVFVAGLEEGLLPFTLFEGHGGARAGDRKSGESE
ENSGEAGREDSARRRERIEEERRLLYVAMTRARVGLYLSWARSRHFLGRKLSLGPSRFLA
QIEDLVPKVEQTPPRKPKEPQLDLF*
>SPBIB_v1_290026|ID:27163551| putative Methylglyoxal synthase [Uncultured spirochete bib]
VSVDLSSNLSKCLDFCKRLVQINGESLDSIKRIALVAHDKEKRELAEWVQYNWKTLVKHD
LISTGTTGRLVEETMIAKAGDVRPEGLRLTLLKSGPLGGDQQVGALIAEGKIDIMIFFLG
SYVSPSPRRRCQSPFEDSRSLQHSHGL*
>SPBIB_v1_290027|ID:27163552| transposase (fragment) [Uncultured spirochete bib]
VTFLYSVMDSAYDASPIREYISGKGHVPLIEINTRRGCTRSFSPAQEARFAIRTTVEGAN
AHLKDWLLPAQLFVRGVTKVTFRLLYGVLCLAALKILQYFILPSLQTE*
>SPBIB_v1_290028|ID:27163553| protein of unknown function [Uncultured spirochete bib]
LCKKRSEKGIRVIGRSPQPRVYLLKGGNMGFYDPQHRKKRCVLSRELVFKALLQIITQVQ
KDLHSTKNFCYTLHGRGHSC*
>SPBIB_v1_290029|ID:27163554| protein of unknown function [Uncultured spirochete bib]
VTSPMKSIAEILGGMQILLDLSYDLQECFED*
>SPBIB_v1_290030|ID:27163555| protein of unknown function [Uncultured spirochete bib]
MYRKKEKVAEFEKFDMVFGGSLRRANRWARLTDLIPWDEVEACYASLFVEQNSRPAISVR
VALGSLILKENLQYCKARGIRLSGPKLGRPLSDIAKNQKQLREEQRIARQDERMRNAVEG
KRRYSLDRIMTRLQATSESTIMMVFLVMNLGRLLRKTASAFFALLVSLISRLLRAQHIVS
NHAAQAA*
>SPBIB_v1_290031|ID:27163556|metK| methionine adenosyltransferase 1 [Uncultured spirochete bib]
MTQPLLRPFTSESVSEGHPDKICDQISDAVLDACLAKDKHSRVACECFTTTGLVLVGGEI
TTHTYVDIQQVARSVVREIGYDNPEYGLDWASMAVLNSIHNQSPDIAQGVEGHGLKEYEG
KQGAGDQGMMFGFACNETPELMPTPIILAHKILQKASAVRKAKEIAWMRPDGKSQVTVEY
DGHKPVRISAVVLSQQHDEINGSGHYLEYEEIRQTLIEKVIKPVLAPTGLLDERTRYFIN
PTGRFVIGGPAGDTGLTGRKIIVDTYGGMGRHGGGAFSGKDPSKVDRSAAYMARYIAKNV
VAAGLADRCELELAYAIGVPEPISVLVETFGTAKVDEFAIEQAILKVFDLSPRGIIETLQ
LKNPIYRATAAYGHFGRDSFSWEKTDKIEALHKAIR*
>SPBIB_v1_290032|ID:27163557| putative Zn-dependent protease-like protein [Uncultured spirochete bib]
MMIATIREAIASINAHDTIKISDWRLVERRLERQERYYVASKAEQARAVDETLYSLTVYV
DTIASGAGGSGGTSSGAGTGSLPNAASGSASSAGTNNPSASPALKKFRGEATITIQPTFS
RAECEAKIRQAAFAASKSRNPWFELPEPSAPKTSVPVSGFEALSPAERIETVRHAFFSAA
NRFGGNARINSLELFITKETKRLLNSRGFDFASTIWRGYSEFVVEADSSSGTVELFDDIE
FSEPDASRLSEAIETRLAQVRDRAHAAPMPTLQGLSVILRGKEAEEVFGWFFHNSRTEMI

YSKASAFEAGKSVQESDKGEAPVEPLDVFAEPVISGLPSSSAFDADGFPLERTAVIEDGI
LKTLIGSIRHADWLGVPRKGAFGLFSVSPGTMSLDEMHAAPYLEPVMFSDFRLDPVTGDF
GAEVRLAYYFDGAKVVPVTGGSISGSLSALRAAMRRSKEVGLAAHSLCPKAVMLQGVIIT
GAC*
>SPBIB_v1_290033|ID:27163558| Peptidase U62 modulator of DNA gyrase [Uncultured spirochete bib]
MPKRTHSPFLLEAKPVLGEVIEHLKPWFDYISVLATDDRGLSYFALPGETRTAEPMWVQR
GFVFRAQREGKVAEYACAVLPGAAQESVSFASTRSRASASAIEIADAVRARLDSLLADPT
LIAFPQIADEPAAAEFLGEIEEDPFTADPEDVLSRLAVLREKLTDGKVVVMAQSRYECVD
VSRLFLSPNRSLMQSFLWSQAYLFGVARRGQMSKMSYRPASGRKGLEILRGLETSVPELA
QELADLLEAVPMEPGEYEVILDPDMAGTLAHEAFGHGVETDMFFKKRAKALEYLGKRVGS
DLVTMYDGAAGVDQTGSYLFDDEGTLATKTCVIENGILKGGISDTLSALALGIPRTGNGR
REAYSHKAYARMTNTYFAPGTSTLEEMIASVKHGWQLSQLNSGMEDPRNWGIQLICMVGR
EIKDGKYTGRVASPVVCSGYVPDVLSAVSMVSSDFELGGSGACGKGHKEYAKVSSGGPYV
KTRMRLG*
>SPBIB_v1_290034|ID:27163559| putative redox protein [Uncultured spirochete bib]
METSKREMKVLITGKSENPTKINLRAGKFELAIDEPKSLGGTDQGPSPVQVLLMALAGCL
NVTGHEVARQKGLTLHGMKVRIEGVMNPCTFLGCSFEERAGFQKIMVNITPDFEDATDAQ
IEEWMKETENRCPVTDNIRADTDIEVKVAKE*
>SPBIB_v1_290035|ID:27163560|gltL| glutamate and aspartate transporter subunit ; ATP-binding component of ABC superfamily [Uncultured spirochete bib]
VEKYIEINNAVKSFGSVKALCGVSLTVKKGEVVLVIGPSGSGKSTLLRSVNRLERLDSGE
IFIDGESVTDPNADIRHIREEVGMVFQSFNLFPHLTVIDNITLAPITVLKKTKDEAIDQA
RSLLAKVGLSDKEKAWPEQLSGGQQQRVAIARALAMNPKVMLFDEPTSALDPEMIKEVLD
VMTELAREGMTMLVVSHEMGFARAAANRIIFMDAGQIVEEAPPETFFTSPREERTRKFLE
HIL*
>SPBIB_v1_290036|ID:27163561| Polar amino acid ABC transporter, inner membrane subunit [Uncultured spirochete bib]
MMKGSPLSGRDSKNPNQTTINVTDGTLIPLKDEGSLLSAWNISFFGAVALLVILPLFSPD
PYLRILSFVPDGLVATFSVTLISIFFALLIGLFAGLGRISQKLFINRIATVYVEVIRGIP
LLVQLFYIYYALGPLLRLKGPPAAILAMSICYGAYMAEIFRAGMQAIPKGQMEAALALGM
SRSMAMRKIIIPQTIRIILPPIGNEFIALLKDSSLVSILAVSDLLRRGREYASTSFRYFE
SYTIIALIYLVMTLFFSRLVGMMEERLRHRGKVH*
>SPBIB_v1_290037|ID:27163562| ABC-type transporter, periplasmic subunit family 3 [Uncultured spirochete bib]
MKKSWPVFASVILIAIVALGVTSLSSCAKKTTKITVATDATWPPMEYVDENKNIVGFDID
LVNEVAKAAGFEVEIKNTAWDGIFAGLAAGNYQAIASSVTVTDERKATMDFSEPYVNAGQ
VLVVVRKNTTGVTTLADMIGKKVGAQIGTTGAIEVGKVQGVELKTYDEVGLAFEDLANGNI
DGVVADSPIAANFALQNASYKEKLMIVGKPFTDEWLAFAFKKGDTKTQKLFNDGLAKVKS
SGKLDELVKKWLQ*
>SPBIB_v1_290038|ID:27163563| protein of unknown function [Uncultured spirochete bib]
LAFAAAAFLRHLIQSHREVRGTIEFLGMRGFARALLRALAVSIFMLHS*
>SPBIB_v1_290039|ID:27163564| conserved protein of unknown function [Uncultured spirochete bib]
MSFNNDISLQRRPDAQPLELLSPERTMEIVLEALRELVEYELAVVMRLEAPDRLVVRKAV
GPLYTPRLEGYSISLTRRQDIARLLEHKEPKLFGEHEAHIDTYYDILDLPNGHSCLVAPL
YIESTLIGLLTLDHRACNRFSPSIVRFISTISKLISVSMVQSDASRSLMEKTRELTEERN
RLLDESADAFRDLVGVSAVWRRVLDAVRLVAVADVPVLLTGETGTGKEQVARKIHQLSHR
ASGPFIAVNCSALVSSLAESELFGHEKGAFSGAVAMRRGRFELADGGTLFLDEIGDLPLE
LQPKLLRVLQDGKFERVGGEHSVAVDVRIIAATNIDLESAIAAGKFREDLWYRLHVFPIH
LPPLRERDEDAALLAEYFVSQIRRRPGFESLALSPSAIERILATEWRGNVRELRNAIERA
AILARGGSIFAEHLVPATNAYQARQAQGAYQADSAATSASTAISEPSSMYFPPLDDALRA
HIIAALKKSGGKIYGSNGAASLLGMKPTTLQSKMKKLGIRSSSFSAPPDTKP*
>SPBIB_v1_290040|ID:27163565| putative peroxiredoxin [Uncultured spirochete bib]
MEQQNFHMPLLGDDFPTLTLQTTQGVMNLPGDLKGRWFVLFSHPADFTPVCTTEFVGFQR
LMPEFEKLGVKLIGMSVDQVFSHIKWIEWIKEKLGVEITFPIAAANDSIANALGMLHPGK
GTNTVRAVFVVDPQSKVRLVIYYPQEIGRNMEEVLRAVKALQVADKNNVAVPANWPNNEL

IKDRVIISPAKTVTEAATRLKEYDGYDWWFCHKAL*
>SPBIB_v1_290041|ID:27163566| Thiol-disulfide isomerase and thioredoxins [Uncultured spirochete bib]
VKPVQSYNEITQLIQQNEAVLFYCSAPSCGVCKSLKPKVIELVQSHFPNLPMYYVDIDAI
PEARGQLSVYSVPAVLVYFKGKELIREARNFGIMELGAKIDRYYSMIFESAV*
>SPBIB_v1_290042|ID:27163567|hipA| regulator with hipB [Uncultured spirochete bib]
MPRLSKNRSLCIWMNGLYVGRWSINRQGKHEFQYDASWLDSPEARPVSLSLPLAPADFVY
SGKIVESYFENLLPDSTEIRQRIRARFGAASLSSFDLLAEIGRDCVGALQILPEFEVPGT
SEVLEAREVNESEIESILNRITSNGPMFQAFDDAFRISIAGAQEKTAMLFHGGQWKVPLG
STPTTHILKLPLGRIGAAGIDMSTSIENEWLCSRIVSSLGLATAKCDIARFGDAKVLVVE
RFDRRFAHDGQWVLRIPQEDFCQANGYSSSQKYEADGGPGIVSIMKFLLGSQNPQRDREA
FFKAQIVNWLLAAPDGHAKNYSVFIERQGRFHLTPMYDIMSAYPVIGHGKNRIPPEHLKM
AMAFVGKNRHYEWDKIRLSHIRETARRCDYERDAEELILSLLSRVPGALSEISSTLPERF
PESVAEPILNGMLKAAQRLAHEVS*
>SPBIB_v1_290043|ID:27163568| Transcriptional regulator [Uncultured spirochete bib]
MTYLVTTTHQLAMALKIARKTLGLTQAQAGALVGLLPKTISALENRPESCSVDSLFRLLS
ALNLELSLSPKASSEPESTSMQAQEGW*
>SPBIB_v1_290044|ID:27163569| Signal transduction histidine kinase [Uncultured spirochete bib]
MKLRVRLSMYFLLVICVVAGSSLIFIQHTTENLFRSFIFSGDSEKAKIYATILADYYAEN
KGWQDLQSFLSEIPQIAFSGIDRRIHGGQGTLRISGYPEKTISALLSDRIAVADKNGVIV
ADTAAKILHTVHPASHLKYGIPIFANSEHVGTVLVGSMIDSSITGINERFLSSIVTSLIW
GVLISAGLALFLGLIFSAQVTKPIVSLTGAVHHVAEGDLSIVVNAEGNDEIADLAKSFNR
MIEELKRLENARKQIIADSAHELRTPVTLIQGTIEGMIDGVFPIDIPTLKSVHEETVRLS
RLIDMLRELEIIESGKLVLSLETINVMDILHKAEALFASSAKEKDIALSVDPPDAPPLIK
ADYLRLSEVIYNLISNSIKYTPSGGRVRLRTKNDEKRAVTRIYVEDSGPGIPAEERERIF
ERFYRIDKSRSPDRGGRGLGLAIAREIVKAHGGNIAVTDSELGGAAFIIEMKAAS*
>SPBIB_v1_290045|ID:27163570|regX| Sensory transduction protein regX3 [Uncultured spirochete bib]
VSAKILVADDEEKIAKMVATYLEASGFQPILAYDGLQALHAFREHLPDCIILDINMPGPN
GLDVAREVRKTSSVPIIFLTARADELDRIVGLELGADDYISKPFSPRELVARVRAILRRT
MQSGKDTSQTELLRHGPIEIDLKKRLVRVSGETKDLTTIQIDILSFLMREPGRVYSRMEI
LEACIGSAYEGYERTVDAHIKNIRKALGDDSDNPKFIATVRGAGYKFMEQPE*
>SPBIB_v1_290046|ID:27163571|copA| fragment of copper transporter (part 2) [Uncultured spirochete bib]
MKKLLTIEGMSCGHCVMHVKSALEEVPGVAKAEVDLIKKSAMVEGEDLNDIALKHAVTEA
GYRVSAIYGRH*
>SPBIB_v1_290047|ID:27163572|copA| copper transporter [Uncultured spirochete bib]
MIKVAQMQINGMTCAACAQASERAVKKLPGVEEAAVNFATEKLFIKFDDGKLGIDEIKAA
VAKAGYEAVELAAEKKHTQSHAAIDEHQAAKEREIRILWTKFIVSALFSAPLLYISMGAM
LGWPLPAVIDPMNYPLRYALIEIALVIPVIAAGHRFYSVGFKAIVHRSPNMDSLIAMGTS
AAILYSLFSVLNIANGNFKAVENLYFETAGVIITLILLGKSLEAVTKGRTSESIKKLMGL
QPKTATIIRNGIEVEIPIDEVEVGDIIRVRPGEKIPVDGVIVTGRTAIDESMLTGESMPV
EKEVGDKVIGASINKNGSITFRATKVGADTVLSHIIKLVEDAQSSKAPIAKMADIVSGYF
VPVVFAIAVIAAVVWLLLGHTLVFALTVFVAVLTIACPCALGLATPTAIMVGTGKGAEHS
VLIKSGIALETAHKIDTIIFDKTGTITKGKPEVTDILPVKGFSEDEILGFAASAEKGSEH
PLGEAIVHAAEEKGIALYEASDFNAVPGQGIEATVLYKHVLLGNAKLMAANNIELGEGTS
AADHLSAQGRTPMFIALDGTYAGLIAVADVVKESSAHAIAALHSMGIQVAMITGDSKKTA
AAIAAQVGIDRVLAEVLPQDKALEVKKLQAEGRKVAMVGDGINDAPALAQADIGIAIGSG
TDVAMESADIVLMRSDLMDVPTAIKLSKNVIRNIKQNLFWAFGYNVLGIPVAAGVLYAFG
GPLLNPIIAAAAMSLSSVSVLTNSLRLKRFKPFE*
>SPBIB_v1_290048|ID:27163573| conserved membrane protein of unknown function [Uncultured spirochete bib]
MGMKRAVLFVTDMICSACETRIARAVKAIDGVIAVRVTLKGGRVDIEYDDEKTTSEAIKA
AIENAGYTVGKNKNAPTMIAIGIGFILVSLYIIANASGLFNAIPAIDSSLGYGMLFIVGI
LTSVHCVAMCGGIALSQSVAQLDGCQMRGDTSRVLLADEKYKNLIPGLLYNSGRVVSYTI
VGGAVGALGSVFSFSPVAKGIIAAAAGFFMIFFGLKMLGILSALPRIPRVIPAPIQNVAN
RFSSALRNRGPFAVGLLNGLMPCGPLQTMQLYALGTGSILAGALSMFIFSIGTVPLMLVF
SLAAAFIPKKFVPVMVKASAVLVIFLGLITFSRAASLAGIALSGFPPFGSGLVQLSNGLP

EAQTVRQSENGTNGGFIKANLQGGVQTVLTDFGSSGYYLPFAVQAGIPVKWTIRVTADGL
NGCNNPITIPSYGIRKTLEPGDNLIEFTPEKEGVIVYTCWMGMVSSRITVVKDLAQANSL
PGSKGLTPSDLLANGLQQGSGGISGLSGCCTAQPPVNQP*
>SPBIB_v1_290049|ID:27163574| High-affinity iron transporter [Uncultured spirochete bib]
MGAALLITFRETLEAALVIGITLAFLAKADLKHLKPAVWVGAAVGAAASAIAAYLFIAIL
GGFEGRAEQIFEGIVMLAGAILLTTLILWLNKKDMKAAMESRVMATASAGSFWGIALLAF
VSVLREGIETVLFISSTLRSSGLAGFLGAAAGIVLAIALGVAFFRSGARMPLKNFFAVTN
ILLLLFAAGLVGQSVHELNEAGIVPPFVEQIWNLNPPSNGEAYPALHEKGAIGGFLKGLF
GYNGDPSLTETAAYLAYLISIGTILLRRNNKLKSHYSR*
>SPBIB_v1_290050|ID:27163575| exported protein of unknown function [Uncultured spirochete bib]
MVMVTLIIRVATVLVGMIVMLMPVAVMMMVITLAACLFDWNKLHAAHRALARLVIGLVSL
TFHETRVFHKSLLLMILPHNRIHP*
>SPBIB_v1_290051|ID:27163576|copA| fragment of copper transporter (part 2) [Uncultured spirochete bib]
MKYACLMKCEGNKTYDKPGKCPVCGMKLVPVEEASSQGNDHHHDSHGHEHHDHTNKHGGD
SDDQSHHDHSHHNARNLCICGTENCDGRCVTNCQCTFQERNLAMPVPLAVSGKAEAYICA
MRCEGDKTYPKPGDCPVCGMHLVKVVAFGAPSGEEEDEEMAAFKAMRGKFILSALFALPV
LFLSIGELIPGLGEFIGSLFSRKTNLLIQLVLSLPVVFWSASFIYAKGIKSVISKNLNMF
TLIALGTGAAWLFSVVSTLAPGIFPDSLKTADGFVPVYFEATVIILALTILGQMLELLAH
AKTNSAIKELLNLVPAEALVIRNGKEMKIPLAEVVVGDLIRVKPGEKIPVDGSLREGSGV
VDESMITGEPVPVEKGQGDKVTGGTINTNGSFIMVAEKVGGDTLLARIVAMVNEASRSKA
PIQRLADTVAAYFVQVVILISLATLFIWGFAFSKWDHGIVNAIAVLIIACPCALGLATPV
SIMVGTGKGAKSGILIKNAKAIEQMRKVDTVLVDKTGTLTMGKPAVTVVKGYSDFSEDEV
LRLAASVDRNSEHPLAAAIVSAADKKGLALSPTTDFTSVTGKGAKALVDGRIAAVGNAKL
VREMLGEPATESNDDYERNLQAEGHTVMYVIEGDRIAGIIGVSDPIKESTPNAVKRLKHL
GVHIVMLTGDNKTTAGAVAKSLGLDDFVAECLPEDKFETVKKMQAKGAFVAMAGDGINDA
PALAQADVGIAMGTGTDVAMESADITLVKGDLTGIARAKALSVAVMRNIKENLFFAFAYN
VLGLPIAALGLLNPIIAGLAMALSSVSVLSNALRIKGVKLD*
>SPBIB_v1_290052|ID:27163577| putative sensory transduction system regulatory protein (modular protein) [Uncultured spirochete bib]
MKDRIHIRNTVKGNILVRYSLLIAAAVAVTTIVTGVILVSVLENHYIRLHASLYAELSST
VRGEKNSSEAFIAILSRFPHVRSTAIWTASGASVMAPETGPLSGVGPLSRTAGKNAMNGA
LKGKLGIVPAPNEGLVILIPILTHEGAVEGIVGLLEDDTELRKDIQFVQLTVAATVMSVG
VLLYALLFGIFLKANQEQKKAADRLRKTFDVIVFAMSSLSGLRDQETGGHLERTQAYTEV
LLNGLRRSAKFRKTLDKDYIKDIITSAPLHDIGKVGIADSILLKKGKLEPGELESMRQHT
VLGGDLLAKAANKLPFPSSLTVAESIARHHHERWDGSGYPDGLAGESIPLAARVVSLADV
YDALRSTRPYKKAYSHEDAVRIIREGRGTQFDPDLVDVFANVESRFAEIFDTMKFD*
>SPBIB_v1_290053|ID:27163578| putative Nitroreductase [Uncultured spirochete bib]
MKGFLVSLLLAVTVFGAFAQDIELPRPAVKSGVDLLQAIKDRRVSKAFVKREVPASELST
ILWSGLGMRAADAVSSATKAGRNISFSGDNAYINVYVLTEKGAWKYIPDTYKLDFIAKGD
LRATVSKASVPDAAIMFLFTVDNALMPSFLKGNPGIFQQMANATAGFAAQNMALVANTYK
LATVVQYTLSPAGAASALRLGKDEAPLFIMQAGYTE*
>SPBIB_v1_290054|ID:27163579| Amino acid-binding ACT domain protein [Uncultured spirochete bib]
MKIKQINVVLENTAGQLFELAKLLHKGDIDIRSLFVTESREFSTVHLIVDKFDTAKRLLK
EHGYFINEVEVFALKAEDRPGGFMKILEVLKANGLNIEYVYGFGEKSDNHAVFIFRITEI
DKAIELLKSGAVGYLTADNVAGKRQGSSWELIDDF*
>SPBIB_v1_290055|ID:27163580| conserved protein of unknown function [Uncultured spirochete bib]
MMIWGYGPGIFGTFGWGGIIMGLSMMAIPILGGVFLFRYFQERREEPPAWKSALEALRKR
YARGEITEEEYQKIKSDIIKSEFENSLHSH*
>SPBIB_v1_290056|ID:27163581| Hydrogenase large subunit domain protein [Uncultured spirochete bib]
VPEVFERMRGLYTPCTDIRRKIFVGVTRFVLAGKKPEDIDYLPFDIIDMGKPTYRCCSYR
ELSIVKQRIRLAFGLPLIEERDNTPVSAGIGEAFTDRKVISAPLVNVIRAACEKCPEDKV
IVTDMCQSCMAHPCSIVCPVNAISFPNGGKAFIDQKKCVKCMKCVKACPYQSITRMVRPC
AAACGVDAIHSDPDGYATIDQDKCVNCGLCTVSCPFAAISDKTELVQILHQLMGPEDRRP
YAILAPSFVGQFGRLASPGAIVAGLRAVGFRGVREVALGADCDTLLLAKRLAEKSSPDNP

QHKTFLGTSCCPSWVKTARRHFPEFADNIAESFTPMVETAKIIKDQDPSARVVFIGPCIA
KKAEALEPEMQPYVDHVLTFEELAAIFVAKDIDLAEITPVEFPDEASALGRGYAVAGGVA
TAIAETARQKYGKENIAVARADTLRNCRAMLADIKSGKTSPELVEGMACPGGCVGGPGTL
IGLLSARNEVGKFAGLAPKNPATLTK*
>SPBIB_v1_290057|ID:27163582| conserved protein of unknown function [Uncultured spirochete bib]
MKPIKVRVCVGTNCCYAGSETLLDMLENDVDLSAFIEVEAVPCKNKACDGGRNSPVVEIE
NKVLLNATPEIVMEEIERLATARIIEETQRA*
>SPBIB_v1_290058|ID:27163583| conserved exported protein of unknown function [Uncultured spirochete bib]
MKRVGVVSLLIALIVAGALFAQGMMGGNYYGGSGMMGNWDYIPPGDAKPLSIEQAKDQAQ
KYLSLWGYDDLTVSEVMEFSNHFYVEIAEKEGKNKAFELLLNKYTGAVSPEPGPNMMWNQ
KYGRMTGSMMGGNFIAQSETDSMTISEAKAHDLAQKFLDAQNTGLKVEDGADRFYGYYTI
HVLKDGKTYGMLGVNGYTGQVWYHSWHGAFIDMKEFDGSM*
>SPBIB_v1_290059|ID:27163584| Fe-S oxidoreductase [Uncultured spirochete bib]
MEAATQEALKKLFESKLNKAMRLYLDTCAHCGLCVEACHVYQAMPETKYTPVGRAEVVRK
LFKRYFKMQGKIAPWLGEVIELDDKSVNMAYEAAFSCTGCRRCVTVCPFGIDTQQVMNIA
KLLLIGAEKQPQILAMLSDMSIAKGETWKETANDYIAALNNLAPEVEALAPTPPGRPAIP
IDVENANVLYVGLSGKHSIVPAAAILNAASENWTISHFEAVNFAAWLGDGEKQRLIAKRI
IMEAERLKVKEVAVVECGTATRVLKFMTGSHPFRIVSIVELIARYIEQGRIKVKPGTISG
TLTYHDPCQLGRNGGVMEEPRYIIGKLTDKFVELTPNKEANWCCGGGGGLISLGEHEFRM
KTGKVKVDQMRASKADTICTACENCHTQLTELNEHYGLGMKVESLTNLVAHALTGFGA*
>SPBIB_v1_290060|ID:27163585| membrane protein of unknown function [Uncultured spirochete bib]
MDPQAVSAASQAVVHTGFALFLDKLYYFIMVPLVYISVIVMIVGIVAKIVSIVRAPIPAF
SLKTYPAKKRPFLAALGDTFGMPMIRKEKPLFWFFLMMFHIGIFFLFFGHLDLFPGVNLM
PPESRHMIGAGAVGLSVTIPTFYFLFRRFKGQERHISVPSDYLLLLLLLFIFLLGDMISW
GNSWTANGFVMTKADFKNYFGILKSFSFVDPRTVLHGSHYHFVVLHVFLAELFMMILPFT
KIVHTFFSLPLNLLRRR*
>SPBIB_v1_290061|ID:27163586| putative redox protein, regulator of disulfide bond formation [Uncultured spirochete bib]
MSDYTVAKTLDCKGLACPMPIVRLSQEIAKIKVGEVIEMFTTDPGSLADVPSWAKSTGNA
VLENKQESGVIHFFVKRQK*
>SPBIB_v1_290062|ID:27163587| conserved protein of unknown function [Uncultured spirochete bib]
MDIEKELADIKAKLEAMPGKENKLSMVIFSGDLDKQIAALIIATGAAAMGMKVVLFYTFW
GTSALRDPAKKVNGKSFMGKMFGFMLPKGRNKLKLSQMHMAGMGTAMLKDLMKKKNVASL
DQLFEAAGMLGVQINICQMSMDLMDLKREEMIDYPHLNVCGVATFLNDAKESAIQLFI*
>SPBIB_v1_290063|ID:27163588| Biofilm growth-associated repressor (fragment) [Uncultured spirochete bib]
MADLKHDASRAAVIADLLKALAHPVRLRIVALLIENEATVSALEQLLGVPQSVVSQQLRI
LRMADLVQYRRVKGFATYSIKQPRLVDLIHCLEGCRTGDL*
>SPBIB_v1_290064|ID:27163589| Methylenetetrahydrofolate reductase [Uncultured spirochete bib]
MPVIKMCQRNMSKALLCFPESNIYAIVLCTMKSILQVIKEGRPVLFDGAMGTELYKRGVF
INRCFEEANLAAPELVLSVHRDYRDAGAEVLTTNSWGAGRYKLARHNLQDHVQEINKQAA
SLARQVAQDSLYVAGSVGPLGPRLEPVGALSEHDALDAFREQMEALIGGGVDFILLETFG
DAAEAAQAVRAARSIATSVPVFASMTIDMSGNPLFGLTLEEAMDRLIEAGADVIGLNCSV
GPQPMLRAAKRMHSHRSDIPLLIEPNAGMPRQIEGRMMYMSTPEYFATYTKYYLQEGVRF
VGGCCGTTPDHIRSMARTVRQYRAMCKDHDFAGEECAGKGLGDRGPGGRGPSEISGAGKI
VSFVPIGSKSGGAKPEAGKAEVERIPFAEKSQLAAKLARGQMVYSLELVPPSGTTLDTII
DKARIAKQAGIDAINIPDGPRATSRVSTLVTAIMIEQQVGLETILHYTCRDRNLISMQAD
LLGAHAIGLRNILCITGDPPKLGDYPDATGVFDIDSIGLTRMIYRLNGGFDISGKPIGKP
TGLSHGVGVNPGRANFETEMERFRKKIDAGAEWAITQPVFDRAVMEEFLDYIERNGLHIS
IIMGIWPLSSLKNALFMKNEVPGIEIPDEVIQRMEHCASAESAREEGVAIARELYESLKG
SIQGVQLSAPFGRIDLALRVIGK*
>SPBIB_v1_290065|ID:27163590|metH| homocysteine-N5-methyltetrahydrofolate transmethylase, B12-dependent [Uncultured spirochete bib]
MKRSEKYALLERLAFERILILDGAMGTMIQRRRLSEADFRGQRFADHPVNLAGDNDVLCL
TRPDVIRDIHRAYLEAGADIIETNTFNATAISQADYKLEPWVREISRAGASLARQACDEA

QASNPKKPRFVAGSIGPTAKSLSIAPDASDPGKRAVSFDEMAAAYRECALGLIEGGADVL
LIETVYDTLNAKAAIWAIHSVFRELGKELPLMISGTIADASGRLLAGQTARAFWHSIAHA
EPFSIGFNCSMGAESLLPFVEEIAEEAECAVSLHPNAGMPNELGEYDDSPENMARVLREF
ARGVEKNERHGANIIGGCCGTTPEHIRAIAEALDGIRPRFFRPKTFASGWSSHQKHSVLA
GLEPLDISPETLFVNIGERTNVAGSRAFARLIREGKLTEALEVARAQIEAGAQIIDINMD
DPLIDSANTMARFLDLVAAEPDIARVPVMLDSSNWNTLLEGLKHLQGKGIVNSISLKDGE
ETFLGRARTIRRLGAAILVMAMDEHGQAEDYERKISICERAYRLLTEQAGIPAEDIILDP
NIFAIGTGIEEHRNHAVDYIAAVAWIKRNLPGALVSGGVSNLSFAFRGNEALRSAMHAVF
LYHARAAGMDMGIVNPAQLVPYDEIPPDIRERIEDLVLNRRPDAAERLIEVAAGLSGSAS
GQPGEASEKLSPKDRVIHALVSGKSETLKDDIEELRRQYARALEIIEGPLMEGMNKVGSL
FGEGKLFLPQVVKSARVMKEAVHILQPYIEAEQAGQPSSRGKILLATVRGDVHDIGKNIV
SLILSCNGYEVIDLGVMVSSERIVEAAIEYRVDAVGLSGLISPSLEEMGRVASLMEARGL
HIPLLVGGATTNPLHTALRIAPEYSGPVIHVGDASQAPGVMDKLLSEGRRNSYLEQVRME
QENTRTRYQEGRELVRYVSLEEARRNASASNLVPPVRPHEPGPAVLAFKVADLVPMIDWS
HFFRAWGLKGRYPDILQDPQKGEEARRFYRDAMELLERAARSGLLEVKSVFGIFPAARAQ
DDILIYAGEDRTSLRARLPCLRQQRFKPEGGYLCLADFLQAAESGKPDWIGAFAVNAGGN
LEESKIALDAKADDYRALLIASLADRLAEAASERLYMLVRDAYWGFGCSGVEGIRPAPGY
PTCPDHRDKALIFSLLDVNQRIGLSLTESFMMRPASAVCGWYFASPSARYFSVGKIGRDQ
VEDYAARRGEPPEVAEAWLIGALNYDPSAKASSENIPACPSCLRPR*
>SPBIB_v1_290066|ID:27163591| conserved protein of unknown function [Uncultured spirochete bib]
MSKGAGRPHRGAPVRERFYRDTMGKRFRSWTIQYKESDLWIGVSPESWSPGMEGTAIAAL
MDARKQIEGYGRSRKSAANFFETLAPMDDDLSAPPVVRAMLQAGLKAGVGPMAAVAGAIA
EHVGRALISAFGCKEIIVENGGDLWLAFSSPLEIAVFAGNSPLSGVLGIEIQPELSPCGL
CTSSGTVGPSLSFGYADAAVILCRDAATADAWATAAGNMVQTADDIEPALASLRKASEVL
GALIVVGDRMGMQGCFRLKPLPKGHN*
>SPBIB_v1_290067|ID:27163592| NIL domain-containing protein [Uncultured spirochete bib]
MKEKYILEYTEQVVEEPIIYSLVKHFDVKVNILRAEISPGREGSMLVEIECEPDSLARAE
AFLNEHSVRMIPIAESLGFQQEACIDCGACTAVCFSGCLTIGEPDWKLHVDRSKCIACGL
CVPACPLGLFTLKFGD*
>SPBIB_v1_290068|ID:27163593| conserved protein of unknown function [Uncultured spirochete bib]
MQKTYEEINEKIARGEAVVLTAEEVARMASELSPAEIAARVDVVTTATFGAMCSSGCFIN
FGHPEPGLRMESVSLNGVPIFGGLAAVDAYIGTTEVSPSNPCYGGGHLIEALVRGERLVL
EAHGKGTDCYPRRSARTIIDKDNVNEIIMVNPRNAYQNYPAATNSSNRIMYTYMGTLLPR
FGNITYSTSGELSPLLNDPELKTIGIGTRVFLAGAQGFVSWNGTQFNTEKPRNEFGLPVS
NARTLMLTANVKKMDPAFIRAAYFERYGVSLFLGVGIPIPVLDEDIARSVSVSNQQIQTT
ICDYAAPGHPGIAKVSYAELRSGYVTLEGKKIRTAPLSSLTKAREIAEILKKQVASGLFP
LAPPVQSLPDHAVVRGLRVEGDAI*
>SPBIB_v1_290069|ID:27163594| Small GTP-binding protein [Uncultured spirochete bib]
MNPSSENPAHARATNESPLSESISIVIAGIRNAGKSSLLNTLFEKEVAIVSPVAGTTTDP
VVRKMELPGLGPVAFIDTAGIDDEGELGAMRTQKTKARLASSSIVVFASPAHRAPQPQEI
RFLEQLKSSKKPYISVLTHADCGFDAAKLQWLSKEQYIAVDNLKGYGAAEVRKALSALKD
IVHFEPSPLEGLVQEGDTILLVAPVDLAAPKGRLIQPQVETIRDGLDRDCTVIIVKEREL
LETYNRLMEKPRLVITDSQAFSKVAADIDPDQPLTSFSILFARKKGELDTFLRGISALNN
FPAGGHVAVMEACSHHRQADDIATVKIPRLFQQLVQPNARFSVARSLEVVKTMPCDLIIH
CASCMLNETAVHARIEAIAGLGIPVTNYGLFFAWANGLLPRALEPFPEYEMVYRPFFRNG
HAT*
>SPBIB_v1_290070|ID:27163595| Aspartate ammonia-lyase [Uncultured spirochete bib]
MYGEQTRHAVENFGRGQTPRDFIAALAEVKKAIFTAIQESEHRYAPEVYAAICTAIEEIR
CGLHDAAFPLPLAQGGAGTSLHMNFNEVLASLVQSRTNVPFHFLDEGARYQSTNDVIATA
VIIVACRRLREIEAGVIALQEALVAKEQAWRGIVVTGRTEWQDALPMSLAQVAGAWAGAV
ERDRWRLHKIKERLRTVPLGGTAIGTAFFVPPEYLFLAEKHLRAITGLPLSRSQNLPDAI
ANQDQLAEVASGFSLVAGTIIKLCNDLFFYTSSAVRELVHPSLQWGSTIMPFKTNPVMIE
YAKGLAMRANAYCSAVMQYAHEGNLQLNAFLPFILDGLLHASDDLGEALRALSEKLLPRL
EASVTKIVYNLHSSLALLNALRAVVPYEALKTFYESHCKEGACFPFASIEELVRTLSKES

GIAEEILYSQLEAFAPTKPGAMSAPPEKP*
>SPBIB_v1_290071|ID:27163596| Radical SAM domain protein [Uncultured spirochete bib]
MSSPAFLPQLTEQEVRALAQLPLEDLTPRAHAVSLEYHGTSVFLRGLIEATNHCAQNCLY
CGIRRNNAKVRRYRLSREEILAIVERGFAFGFRTFVIQGGEDPCYTTEFLASLCEEIKNR
TAGKAAITLSFGTKSLAAYRSLARAGADRYLMRFETADPVLHEKLRNGNSLASRLKALDC
IRESGLQVGSGFMVGLPGETEETLIQNIMLAGKINMDMGGVGPFIPHPDTPLRCAVQKPI
ELAIRATALLRLMLPHCHIPATTAAGSLDPLGREKMLAAGANVLMPNITTVEHKKDYQLY
PGKICIDESGFQCVSCQAMRIASVGLSISWDRGDALRLKERSHVW*
>SPBIB_v1_290072|ID:27163597| Biotin and thiamin synthesis associated [Uncultured spirochete bib]
MLTATQTFLDYAKLEELSGLSAPSFAQVEKILAKAKRLKGITTEEAALLLAVQDPAQLSL
LLAAAHYIKEEIYGKRLVLFAPLYTGNFCTNDCLYCGFRRSNHSLERKRLTMDEIAQQTS
ELLKQGHKRILIISGEAGNQSLEYTLEAIQTSYSVREDTARVRRINVEIAPLSVEGFRAL
KKANIGTYVCFQETYNPELYAMYHPDGPKADYRNRLYVMDRAMEGGIDDVGIGALFGLGN
YRYEVLAILEHAHHLEAAFGCGPHTVSVPRIEPAKGAPLSFNPPAPVSDIDFKKLIAVIR
ISLPYTGIILSTRESPALRKEIFAYGVSQISAGSKTDPGGYSAHKEDNGQFSVGDNRTLE
EVISDLIDDGYIPSFCTGCYRRGRVGADFMDLAKPGLIKEFCLPNGLVSFAEYLYDYASP
PTRQKGLALIEAMKTTAPAKVAHVLDKALLDTASGERDIYL*
>SPBIB_v1_290073|ID:27163598| conserved protein of unknown function [Uncultured spirochete bib]
MEKRIGVVAILIEGRDNIPAINSILSNHSDIINGRMGLPFKDRGVQIISLIVEGNPDQIN
ALTGPLGRLKGVQVKSILTRAIGDDADAHRDTNFS*
>SPBIB_v1_290074|ID:27163599| conserved membrane protein of unknown function [Uncultured spirochete bib]
MNKDKASYSFVFLVLFSIFLACLLISNIIAGKLILVFGMVLPSAVILFPITYILGDVFTE
VYGYEKTRSIIWIGFAANMLMSMIFMIAVALPYPGFFKDQSAYATVLGLTPRIVLASLIA
YWAGEFANSISLSILKKLTKGKFLWVRTIGSTVVGQSFDTVLFISISFAGNVPLSVLLQM
MAAQYLFKVAYEAVLTPFTYKVVGYIKRIEGIDTFDHGVEYNPFKLGAK*
>SPBIB_v1_290075|ID:27163600|queF| NADPH-dependent 7-cyano-7-deazaguanine reductase [Uncultured spirochete bib]
MAEYKFEILGKKVSEPMRKLETFPTPEHVKKVVLDCDEVTSLCPVTGQPDWETVRIEFEP
DKFCIESKSLKLYLWSFREEGAFCESLSARIAQDIFDVCKPRWVKVTVAQKARGGIAITA
ESRIPDLEQGAGA*
>SPBIB_v1_290076|ID:27163601|ribD| fused diaminohydroxyphosphoribosylaminopyrimidine deaminase;5-amino-6-(5-phosphoribosylamino) uracil reductase [Uncultured spirochete bib]
MHEFFMQSALDLARKGTGLVHPNPLVGAVLVRDGEIIGRGFHAFYGGPHAEVMAIDNAIL
SGNTDFSNTTLYVTLEPCCHFGKTPPCTSLILNSGIRSVVVGMEDPNPLVAGKGIRILKE
AGIDVEVGILEAECRELNRVFITYITTGKPFVLLKSGLSLDGKISTASGESRWISCEESR
KDVHRLRSEYMAIMCGIETVLSDDPALTVRMVEGRNPIRIIADSHLRIPMDSNIVTTAHK
IKTIIATTEDSDTAKAEKLKDAGVHLIYTRQTEGHVDLQKLMTILGQMEIDSILLEGGGT
LAFSALKAGIVNAVRFYMTPVLLGGQRARTVIAGEGFSALADACKIENIRIGSCGSDILI
EGTVCSPA*
>SPBIB_v1_290077|ID:27163602|ribE| Riboflavin synthase [Uncultured spirochete bib]
MFTGIIEEVGYVRSITRGAHSARITILAHTVCQGTRLGDSIAVNGVCLTVTEISSSVFVS
DIMAETLQRTTLGELQCGDAVNLERALTLATRLGGHLVSGHVDGAGTIRSILKEGIASIL
EITCSPELLKYIAVKGSIALDGISLTVCGVSDSSFSVSLIPHTQMQTNVYLKKVGDTLNI
ECDLIARYVEKLIMSKIPDEAEHPRPVLTEELLRLSGFIGGEL*
>SPBIB_v1_290078|ID:27163603|ribBA| Riboflavin biosynthesis protein RibBA [Includes: 3,4-dihydroxy-2-butanone 4-phosphate synthase ; GTP cyclohydrolase-2] [Uncultured spirochete bib]
MNSVEEAIRAFRAGKMVIITDHEDRENEGDVCIDAQFATPEVINFMAHFACGLICVPMEQ
RRLEELELQPMVHHNEDNHGTAFTVSVDHIETATGISAFERAHTIQKLIDTSCGPADFRR
PGHIFPLAGRTGGVLERQGHTEAVLDLVKLARSEMPASSNTAIAGVICEILDSDGTMARQ
KSLENFAKTHNLCMISVADLVRYRKDREGGITRETETILPTEYGNFKLVGYTENATGKEH
LALILGDISGDEPVLCRVHSECLTGDALGSLRCDCGEQYKEAMRLISRQGRGILVYLRQE
GRGIGLINKLKAYALQDTGIDTVDANIHLGFPADMRDYRIGALILRDLGVRSVRLMTNNP
QKIESMEQNGIKVFQRIPLHIQANTYNKFYLRTKQERMHHQLETLQILNRSAK*
>SPBIB_v1_290079|ID:27163604|ribH| 6,7-dimethyl-8-ribityllumazine synthase (Lumazine synthase)(riboflavin

synthase beta chain) [Uncultured spirochete bib]
MKIMEGNLIAQHIRIGIVAGRFNEFIVSKLLEGALDGLKRHGIDDTNIIMAWVPGAFEIP
LVAKKLAERTDIDVVICLGAVIRGSTPHFDYVAAEVSKGVAQVSLSSEKPVIFGVLTTEN
IEQAIERAGTKAGNKGYDAALSAIEMANLMRALSE*
>SPBIB_v1_290080|ID:27163605|thiC| Phosphomethylpyrimidine synthase [Uncultured spirochete bib]
MYSTQMDAARKGLITEQLRLAAAWEQLDEEVLRALVAQGKAVIPANKRHKNLVPRAIGRT
LKTKINVNLGTSRDCSDQEAEMEKVRTALALNADALMDLSSFGNTHSFRKRLIDESPVMI
GTVPIYDAVVYYNKPLREITAEEWLATVRMHAEDGVDFMTIHCGLNRATAERFKKNPRLT
GMVSRGGSLLFSWMEMTGKENPFYEHYDELLAICREYDVTISLGDACRPGSIADASDAVQ
IEELIVLGELTRRAHDQNVQVIIEGPGHMALDEIAANMKLEQRLCDDAPFYVLGPLVTDI
APGYDHITAAIGGAVAASAGAAFLCYVTPAEHLRLPTLEDMKEGIIASRIAAHAADIAKG
VNGAREWDNSMSQARKNLDWKEMFALAVDPEKARRYRAESKPELEDTCTMCGKMCAMRNI
NTIMRGEEVDVI*
>SPBIB_v1_290081|ID:27163606|thiD| bifunctional hydroxy-methylpyrimidine kinase and hydroxy-phosphomethylpyrimidine kinase [Uncultured spirochete bib]
MSSSSMRHVLSIAGSDTSGGAGIQADLKTMSACGVFGMSVITALTAQNTQGVQALHQVDA
DFVGAQIDAVFSDIRVDAVKIGMLGTLAVMKVIGKRLRWYRPKWIVVDPVLQAKNGYPLM
EDTAKDIFLSEIVPLADLITPNLPETAALAGFVPQTMAELESAGKKILSLGAKAVLIKGG
HRKTDADDVLVTNLGVHVFKGERLTSQHTHGTGCSLSSAITAYLARGLTILEAIERAKNY
VRVGIEHGLNIGHGIGPIHHFYNLYHGEVPQL*
>SPBIB_v1_290082|ID:27163607|thiM| hydoxyethylthiazole kinase [Uncultured spirochete bib]
MNQTNFEAMNLADVLENLRSSSPLIHHITNAVTINDCANITLALGGSPVMADSPEEASEM
VGFAGALVINMGTLHPSAILSMIEAGIRAREMGKPVIFDPVGAGATQFRRNTAEEILRKV
RPTIIKGNASEIKFLAGEASNQRGVDSLDSEADQAAIALARSSGAIVAATGAIDTVTDGK
SMYKIGGGTAMLGRITGTGCMTSSLVGCFCSVMSSHLFAAILGIMAMKLAGEKAQKALKP
GQGTGQFRINLFDAISLLTPSDFLWKERITYVTL*
>SPBIB_v1_290083|ID:27163608|thiE| Thiamine-phosphate synthase [Uncultured spirochete bib]
MLRFDSSLCLITDRSFCPKADFFPILETAIDSGVTWVQFREKSGLADREMFELGQKVREL
TARKGVPMIVNDRLDLAMALDADGIHLGQGDLPLWAAKKLWRPDKIYGVSAQTLEQAEKA
LHDGADYLGIGALFPTISKNDAIMVHHRQIELIRMLGVPLIGIGGITPERIKDIRRFGFS
GIAVISAIWGAPDPAKAVKTFVQQWKDV*
>SPBIB_v1_290084|ID:27163609| Haloacid dehalogenase domain protein hydrolase [Uncultured spirochete bib]
MLTSKAVLWDKDGTLLDTFGSWIDVERKLAEAMAAEYGFSSVKEQDYIQRVLFRLGVKPD
GTVSGQGVLASGTSEAIIGEFYSAFLEMDSFISEKKQSFFSLAKSKLAEILEQSPITPIP
AQGAQEALRFFKDLGLCQGLATSDSLANAQRDMAAAGFLDYFTFIAASDTIEQPKPHPCS
VYAFADFCGVFPSEVLVIGDTPADEAMAVAAGASFVAVLSGTGVVQDFSPKANVVHDLLD
LPGFFALPPFCIFSSSRMPRRN*
>SPBIB_v1_290085|ID:27163610| conserved exported protein of unknown function [Uncultured spirochete bib]
MKYLSTQNRKARRTLYLAAGILILFTASCSTQPAPIGQASLPPSASASSPQAKEGVMYSM
ALPQSSAGPLAFVAQTGLPGSNGQTAALGSQPEFNTEEYQRIVDNPFRNTLGNPVSTFSI
DVDTASYANVRRYLMESRKLPPKDAVRIEEMINYFTYSYAPPTGEHPVAATLTMAPAPWD
PDHWLLRVAIKAKEIETANLPPSNLVFLMDTSGSMDEDNKLPLLKQSLKIMVEKLRPQDK
VSIVAYAGTAGLVLDATSGAKKETILSAIERLEAGGSTAGGAGIKLAYEVAKRNFIQGGN
NRVILCTDGDFNVGVSSTSDLERMIEDKRKENIYLTVIGVGTGNIKDSRMESLADKGNGN
YAYIDNLLEGKKVFGKELWGNIFTVAKDVKIQIEFNPAIVSEYRLIGYENRLLAREDFND
DTKDAGEMGSGHAVTAFYELVKRGLDPQLNDLTFQQITIVPSNDVLIFKLRYKTPGDGQE
ASKLISMRFDQQALYKEFAACDDDFRFASAVVEFGMLLRDSPYKGSSSWKSAMDRARAAK
GADPDGYRAEFVKLVEMAELVAGE*
>SPBIB_v1_290086|ID:27163611| Radical SAM domain protein [Uncultured spirochete bib]
MAELMVGLRKTSLVDYPGRIAATLFLPGCNLRCPWCHNRELVEPQGFAAAQNLNINEAHF
RAATQNFSVTGAQSPAQAPENAPALIPLSQALDIIQKRKNVLGGIAITGGEPTLRPELPD
IVAAIHRLGLDVKLDTNGTLPLSLEKLLREDETRPDYVAMDLKLAPDRYRKLLPGGRLSA
HKLAAHEPQKMSDEDSHASPGQNLRRSVELLRQYGIPHEFRTLALPGSFITEKDIEELAA
LVDESPWYFAGFRGGNCLDPQWDLLKEPAPSEVEALVQVARALGKHAMVRGV*

>SPBIB_v1_290087|ID:27163612| Anaerobic ribonucleoside-triphosphate reductase [Uncultured spirochete bib]
MFNEIQSSAKSTVQKPQYVVKRSGALEEYDAAKIKSALQKAFAAVGFLVSAPETQERIDR
ITSFVEELVTQLLASRHPHSIPAIEEIQDLVETALIESKEVSVAKAYILYRARHEAIRDT
RKLLLDIDATMDGYLNQSDWRVNENANVNYSLGGLILHNSGTITARYWLKNIYSEEIAEA
HRNADFHIHDLSMFSGYCAGWSFLQLIQEGLGGVPDKIRSKPARHLSTLMQQAVNFLGCM
QNEWAGAQAFSSFDTYISPFVKEDHLSDKEIKQCLQSFIFGVNTPSRWGSQAPFTNITLD
WVVPEDMKNMAAVVGGTMMDYTYGDCQEEMDRVNRAFIELMLEGDADGRGFQYPIPTYNI
TENFNWDSDNARLLFEMTSRYGTPYFQNFVNSDLKPQDVRSMCCRLQLDKRELRKRGGGL
FGSDEFTGSIGVVTLNLPRIGYLAKDEDDFFRRLGKLMDLAKESLVTKRKVISRLLDAGF
FPYTKRYLKHLDNHFNTIGIVGMNECLLNFFSKDVTLATEQGQAFALKTLQYMRDRLVAY
QEATGELFNLEATPAESTSYRLARHDKERYPDIITAGTAEPYYTNSSQLPVMYTNDIFDA
LDLQEELQRAYTGGTVFHAFLGEAIDDWRTCRDLVKTIAYSYRIPYFTISPTYSICPTHG
YLNGEHFSCPVCGAETEVYSRIVGYYRSVRNWNKGKREEYKERKMFALKAD*
>SPBIB_v1_290088|ID:27163613| conserved protein of unknown function [Uncultured spirochete bib]
MPESLKLVLHVAQVDKWEGAISNAANFLKPAAPGEHLEIRIVANSDAVLACAPCSPKLLE
LLEMLTKHGVEIFLCKNSLKKHGIPADSLPPILKTVPAGIRTLVDLQNEGWRYYVRP*
>SPBIB_v1_290089|ID:27163614| conserved membrane protein of unknown function [Uncultured spirochete bib]
MAKKMPRIQLIRRISLGIFLVAVTVLTILHQKVQNMPSIDALDPFGGIETLFKYLAGGEF
IKKIVPANIVLLVGIIILGVVLSRFFCGWICAFGALQGVFGWLGKKIFKRRFVVPSKVDR
VLRWAKYPILVAIVYFTWKTGTLVIRPYDPLAAYGHLSAGLSAVWAEFAVGFVILVLTLV
LSMFYERVFCKYVCPLGAVNAILSRIPLFRIKRITTTCISCARCDKACPMNIDVSHADTI
SSPECIACMECVSACPTKKNSLVTTLAGKTVAVGKIAALGLAIYLGTMGVGALLGKLDFV
GPTLSQKATSGTLAIGDIKGSSTWAEVATAFGIDLERLYREAGVDASKVPPETKLKDTGA
LIGKTFEADTARFAVSKITGIPYAGENAAPASPAATPSGAAPSSAEPTSAAPSSSAAAAP
ASPTPAAAPAAVSPVPAPTSTPANAAQSSAPAASATQLTVPPDFALEGTMTVKDIAAALK
TSEVAVIKKLQLPADFPTDKPLRDLKDQYGYTMTVLKERFAK*
>SPBIB_v1_290090|ID:27163615|palH| Alpha-glucosidase [Uncultured spirochete bib]
MKLVLIGAGSAQFGFGTLGDIFSSKVLKGSHIALVDINGESLSRVQAAGQAFIEANGLDF
TLSAHTDRREALKGADFVIISIEVGNRFELWDQDWMIPLQYGIPQVYGENGGAGGLFHAL
RITPAILDICEDCASLCPDAFVFNFSNPMTAITTTVLRKFPNLRFIGLCHEIASLERYLP
LMLDVPFSELELRAAGLNHFSVLVSARYRTSGKDAYPDILAKAPAFFEKVLGYSDILEYI
QTTGNIPRTEGETGLPDIGRTESSRAWSDRGLFRHILERFHLLPITSDSHIGEYIQWAHE
VADHKGILDFYMLYRASLSMLKPTIELKVKERVVPIIEGILGDTGYEEAAVNIMNTGLIP
SLPDSVAVEVPARVWRKGLEGIAFPDYPKGFAALLRNYTGVYDLTAEAILQKSKDLAIQA
LLVNPVVTACARVPELVNVMIERQAQWLGYLK*
>SPBIB_v1_290091|ID:27163616| Transcriptional regulator, LacI family [Uncultured spirochete bib]
MVTIIDVAKAAGVSPSTVSHVLNGKRPISDATKKRVHDAIVALGYEPNPNAQALRSTSSG
IIAFYASDITEVFSTLIIQGAERICRENNYYMIFASGVEFNNDIAEAMHFLKRRRIDGLI
VSFGIRKRIENRLPKALDFPVVSINARVSDDIPSIQPDDYSGGREAARYLLQRGAKNPAI
IGGPESRLASEERIQGFVDEMAAQSIPFDRDKMIVYGDFSFDSGRTCMATLLQRDRSIDA
VFCANDYMAAGAITEAQKQNIAIPQQLRIVGYDNREFDWFWPIPITTFSLPLAEMGEKGA
STLIRMIKGEKPEPFHVVIPSSLVTRASS*
>SPBIB_v1_290092|ID:27163617| Glycoside hydrolase family 31 [Uncultured spirochete bib]
VKLEIKHLSKLSIRNSKAEIYGENGALFITPFSNFFHIEYKIGFSRDLTSEADQNLASEF
DVPTINCCNFESIKELEDRFVFVQNDARLEVLKETAVVSVYWKNNLAFGGTIGNSDTVLP
KFPLRIRYESQKAEHQGIGEEWPAQFNFRSEPDDIFFGLGEKTGKLNKAYRRFKMFNRDA
LGYEPEYSDPLYISIPFYFQESRRHSTIAGVYFPSEAVEEIDFLVESNFYTAVSMAKGPY
AYIVFVGDTYKEILAHYLTLVGTPALPPKFAFGFFGSSMSYAEPKDAQQKILEYFQKVEE
YGIPCEGMYLSSGYIKAENGRRYTFLWNTAKFPNPKEFIQSIRERGYRLCCNVKPGFLLD
HPWYEALKTKGYFIKNTLGEAAVEYYWGNYASLIDFSKEEAYNWWKEQIKKAFLELGILG
IWNDNNEFELEDDEIQMTKTLPVLMSKASYEASQEVFSGKRPWVISRSGFTGIQKFSSTW
TGDNVSDEKSMIMNIPMGFNLGLSGLFFYGHDIGGFYGPRPGKDLLLRWCQSAVFQPRFI
MHSWNPDGIPTEPWLYPEILDAIRGLIRLRYKFLPYIYSSAIRSVLESVPLEIPLWLEFF
SDDSLDVDSANHLVGDALLVVPPQSVGEHTIDCRFPQGTMWLSSDLATWYWGGSTVQMQY

PEQEPLFFFRAGSAIPFESRQSGPLEGYSDSVHVLIIPIPAELTQSTVFHVYEDDGLSEM
QDGSYNCFEFVQHKLGDTSVQLSITMKSQAHNAPKPRTLVLSLPPGYGFYDSPAGASTNS
ECTMKLFSKGQTESVRIVKLPDSWSSALI*
>SPBIB_v1_290093|ID:27163618| putative Sugar-binding periplasmic proteins/domains [Uncultured spirochete bib]
MKRRFLLASIFLLVVGIFAFGQAQKELTALWFYDDPSELEFLNKKAAEYSTSHPGVTIKV
NTVAYNDLFTRLTQLVAGNNPPDIVKLTDIRPEIEPFILDLSKYHGKDFLKPFIPGVAAA
LNREGRILGAPLDVTANGIILNKTLFDKAGVAIPSKEKAWTYNEFLQAISDVKKKTGALY
ALVWDVTPHRWSTYLFENGGSFYDSTGRKSNFNSPAAIQALRGFADMFDKGYIPKSIWIG
SENPRDMFFSGQAVAWMSGSWQVKAMLDNIKNFEWTAGPNPYVTTRSSVLGYKFVSAFST
SKYPDIAADFIKFFTSKENNSDYAKSLMTIASRTDTGTINYGNAQASSALNNLAYELAIS
PLPASTDIANPVMGYVWNTVKENVIAVLTKTKTAEQAAAAINAQIEDSLRIVYGK*
>SPBIB_v1_290094|ID:27163619| Permease component of ABC-type sugar transporter [Uncultured spirochete bib]
MSKRKIPIAPYIFIAPAILIFLLFIVVPAMEGFLQSLFTRGIIVRQDIPALRSRFVGLFN
YQKLVSDPRFLTAFGKTILFTLITVPSIMVVSLSLALLLQEKIHGVGIARAMVYWPSMIS
PIIIGIAWRWILGYDTGILNYFLTLFHIDKQPWLIDNARAFISVIVVSIWAQTGFFMVIF
IGGLNTIPDSYYEAARIDGANKWQQFSGITLPLLRPTTLLVLVLSTINAFKVYQLVTVLT
SGGPGRATAFLVQNIYEEAFSKPLSVGYAAAQSVVFFFVMLLLSIAQFKLSKEESV*
>SPBIB_v1_290095|ID:27163620|MalG| Sugar permeases [Uncultured spirochete bib]
MKSRKVLIGFLKYLIVIIVSVIFFFPMLWVVISAFKGESELFLNPPRILPVHFTLDNFKS
GLKFGNFTQYFVNSAYVTITSTIITVIISTMAGYGFAKFKFPGRDVLFIGVMATLMFSLE
IIMIPMFLTLKGYGLINTFIGIIIPPAATPTGIFLLRQHMRSIPDELLEAAKIDGANEYQ
IFTKLIVPLSVPAISTLTIFSFVWRWNDYLWPFLVINDDKKRTVPLALANFVGQYAVRWG
DLLAMTTLSIIPTLIIFLIFQKYFIKGISMTGLKG*
>SPBIB_v1_290096|ID:27163621| putative LmbE family protein [Uncultured spirochete bib]
MPSSIERIDVLVIGAHPDDAEIGAGGFLLAAKNRGYRTGIVCISDGSAGASGDVSQRKRE
AENAAFVLKIDALHFLGMEDLHIPFDKTAELIEKLLVEMRPGLILTHSPDDWHPDHRLVW
QIVDAAWALANRRSRHGEDRIERPRMLQFSTDVLRAHKPALLVDISAFAEEKQKALSCHA
SQSEIVKNVLAFNALWGASIGALYAEPFFTSEPLVLTSSLGLLEKL*
>SPBIB_v1_290097|ID:27163622| putative Glycogen debranching enzyme [Uncultured spirochete bib]
MSRLQDIGNGWLAGDAPFLLPFSGSRLQGFFNHRGSLDIALWGAGKIGSLYVRAAERAVG
RRSFVEYRVSPNVLELKSETRTVRLSAMVDEPVIVVYQESRGDPSNQNITFVPAKNYLWQ
TESGKQIHTSFIFGESARLSAIHEELFICYENSVHDSQGKKLRVSFFVIGSAQETKRALE
KLKSHIHNPSEANAFVARSIPTEQYMSKLTQHGLSEHSCTTQAIHCFHAAFSCVKSDDSG
NFAGIAAGIGYSIPARSYFRDSYWTCLALLPFTPDIVRQQILFLEKGVYENGEAPSGLIF
PTDAGMRYWEERKKADPALARDHVRPLDWWSDHFDSPLFFVNLVFDYIDFTADWSILDEG
TGQTILEKIRTIFSGYKALEDADGVPVKPFHDRDWADNVFRQGAVTYDVALYYGALAKAS
RLDGAFAARAQALRRAAAKRLWLDDKGYFAEFVQSDGYAETHLAIETITAIHFGLATEEQ
SRKILKAVKQLLFTRNNQAQPFGDWGVMSVFPGYSARTKRRGKSLFPYRYHNGADWPYWD
GLLAWILAERNDPDYQYALTRWWEYGLEQGWPEPVEYFSPPFGRGSPLQAWSSLSLRAIY
EAKKKKLL*
>SPBIB_v1_290098|ID:27163623| putative Signal transduction histidine kinase [Uncultured spirochete bib]
MSKFHRSFSFRYRLTLWYIAVFSISLLAYQTFSYFELKANLISQMDAGLLSAMDQTIQNL
DVENGNLALQNVEKTPIKPLSETQTALRIRILDADGNVIYSASGFPAIPIHQSALHALGD
HSKPIYQTIQLGTEHYRVVTAVLIIAGTQTKNFLQIANSLSNIDKELSSFIVRFLVGLPF
ILLIAAAGGLFLANTALKPIVEMKNLAEHVREDRLSDRLHYQGPSDEFGSLAATFDSMLD
RLEASFAREKRFSADAAHELKTPLTALKGKLEVALSRPRTAKEYEEALTNMKGDVERLIS
LSADLLLLSRMGNVALSSRAEKIDVGELLDSCVDQIITAFPDKHVTVKRNYEHHVEIEGV
RDYLVRLFLNILDNAVKFSNTEVMLSISIKQVQARKAAVIEISDNGHGIPKSEIPFLFQP
FYRVESDRSRLHGGAGLGLAIAHEIAEAHGGQIEISSAVGEGTRATVALPLSLKQ*
>SPBIB_v1_290099|ID:27163624|cusR| Transcriptional regulatory protein CusR [Uncultured spirochete bib]
MRILVVEDEKSIVDFLSQGLKESGYAVDIANDGQTGLDYALATQYDVILLDVMLPKLNGF
AVVRELRRASIKTPVIMLTARSTVDDRVHGLDAGADDYLVKPFAFAELLARIRALLRRPS
ISTDTAISFGDIELDTIKHEFRRRGERIELSGREFSILEYFMRNPGQVLTRTQIAEHIWN
FDAYAGSNVVDVYIGYLRKKIDEDPSHSYIKTIRGVGYRFSPEG*

>SPBIB_v1_290100|ID:27163625| exported protein of unknown function [Uncultured spirochete bib]
MKKAKIAILALMVGLIGLLGVSAQSASATPPVTAPAAATSTAPAAPAGTAQADQEGQHEF
EGEENDGAESQEESASVENDEKDEKNKETEAVAESSESQSAADTDNIEVQE*
>SPBIB_v1_290101|ID:27163626| protein of unknown function [Uncultured spirochete bib]
MLLFAGLIVSFIPAQEASPAPAAKHDSLQALGFNITIEDLVRLLDLQRSLFLDPAAKVNT
DWQDKFDELVVREIPATANRYAYAVDSDQGLQYVVIRGTHNLRNAILDLEYWKDRSPILG
INLHHGFEKAALAVFNDLEPRLKPKMPIVVAGHSLGAAEAIIVGMLLTKNGYTVEKILAS
GPPKVTDDEGWEQFESLPVIRVVSAYDPVPFLPPKSFYPESPYTQAGMLLMLLDGPYVTI
AEPTYFDNMPAAFKGVQKLDAHFDVIDHRIWIYADRAREKLAALEYVPFAGWEQYAKPRG
DAKDAPKK*
>SPBIB_v1_290102|ID:27163627|icd| isocitrate dehydrogenase, specific for NADP+; e14 prophage [Uncultured spirochete bib]
MKAFSKLTAPTEGSAIEIDKGGSLKVPDNPIIPFIEGDGIGKDISPVMRAVVDAAVQKAY
GGKRKIHWFEIYAGEKALSVYGENEWLPTETLAAIREYKVAIKGPLATPVSGGIRSINVA
LRRELDLYACVRPVRYFPGVPSPMKHPERLNVVIFRENTEDVYAGIEWPAGSPEARAIIE
TINATPHEGRKIEDIAATAIGIKPISETGSKRLIRRAIRYALAMGLPSVTLVHKGNIQKY
TEGAFRTWGYELAQKEFSEQTIQGTEAAPAPGKVAIKDVITDAMFQELLLRPEEYSVIAT
TNLNGDYLSDACAAQVGGLGMAPGANIGDNAAVFEATHGTAPKLAGQNKANPGSIILSAA
LMLEHLGWKEAADMIQKGMVGAIAKKRVTYDLASQMEEATMLSCSEFGKEIIANME*
>SPBIB_v1_290103|ID:27163628| Aconitate hydratase [Uncultured spirochete bib]
MAGMNLVQKLIKSHAVNVSLADAERGASLSSGKEVLIKIDQTLTQDTTGTMAYLQFEALG
IDHIKTERSVSYVDHNTLQVSFENADDHNYLQDVSAKYGIIFSRPGNGICHQVHLERFGV
PGKTLLGSDSHTPTAGALGMLAIGAGGLDVALAMAGFPFSFPYPKIVRVNLKGRLGPMVS
AKDIVLEILRRLTVKGGVGKIFEYSGEGVKTLTIPQRATIANMGAEMGATTSVFPSDEQT
KRFLEAQGRGQDFIPLSADPDAAYDEEIEIDLSVLEPLIALPHMPDKVVPVRSVAGTPVD
QCFIGSCTNSSYYDLAMSAKILKGRHVAEKTSLVIAPGSRQTLLMISRAGILTDLIEAGA
RILESACGPCIGIGQAPRTGGISLRTSNRNFEGRSGTKDAFVYLVSAETAAASAIMGVIT
DPRDLGNIEPPVVPERFAVDDAMIIFPKPEGRSQSIRRGPNILPLPEFKPIPNTIEGEVL
LVMGDNITTDHILPAGSKIMSLRSNLPAISEYCFSAIDPSFPKRARAASGGILVAGENYG
QGSSREHAALSPRYLGVGTVIAKSYARIHRQNLVNAGIVPLIFETPQDAEKIRQGNRLAI
LNLFEGLQSNRIEVQNLTTGAVFMTKHGLSERQIEILRAGGALNAARCEFEKHKYAKSET
SDCNSD*
>SPBIB_v1_290104|ID:27163629| Citrate synthase [Uncultured spirochete bib]
MDMQYYLSKIFENNKIEEDLYARYDVKRGLRNANGTGVVVGLTRVGDVHGYIMDEGERVP
VDGKLYYRGIDVEMLVKHAQQENRYGYEEAVYLLMFGNLPTRSELDAFTRYLGEKRALPD
NFAEDSIMKAPSPNIMNKLARSVLACYAYDPNPEDLSPGNVLRQCLELIARFPTIVAYSY
MAKKHYYDRESLFIHTPAANGSTAETLLSLIRPDQKFTRLEAEVLDLALIIHAEHGGGNN
STFAVRVITSTDTDTYSAIAAGVGSLKGNKHGGANIKVMGMMENIKASVKDWSDEEEVAA
YLARLLKGEAYDRTGLIYGQGHAVYTLSDPRATLLRSKAEELAAEKGFEKEFQLYRTIEH
LVPTVFQQVKGSTKPICTNVDFYSGFVYQMLGIPEELFTPLFAISRVAGWCAHRMEEIVA
GGRIIRPANKCVQPRLAYVPLAERG*
>SPBIB_v1_290105|ID:27163630| protein of unknown function [Uncultured spirochete bib]
MMTNGLLDPIFPIEGAKCIAEAAERAYATTTCDGLITANNKHYKYIPNLECRRFEPEHS*
>SPBIB_v1_290106|ID:27163631| putative xylanase/chitin deacetylase [Uncultured spirochete bib]
MKKNHLVKLSSLALLFAFFALTSCATAPKPSYLDPIRWWDQGQQDPIKELAIETPEQLAA
RVPKRTDPKIVLLMYHNIVFGRTGGEYNRDLYNFEHDLVFLRNRTQIIGLDELPGIQSGK
KKLDTDASIITFDDGDLSIYAVVFPLLKQYDIKATFFIITDFVGTTGYVSWDQLKQMSDY
RNAKGEKLFTIGSHSLDHKRFDEIPADQIPRELSESKLAIESKIGAPVRYFALPFGAGAG
RKEIIETAKNLGYWGIRSSTTGAMTPSTIDMFNIPAFYMTNERADILAQQIYAKLLGR*
>SPBIB_v1_290107|ID:27163632| protein of unknown function [Uncultured spirochete bib]
LPPRVVPPATPAIRNIYSILFAPKVKLDYSLASYELKQSINSAADLPPYYQDSIKISYW*
>SPBIB_v1_290108|ID:27163633| Extracellular solute-binding protein family 5 [Uncultured spirochete bib]
MNRKRKAIAGLLFLAALSVAVAANIPKDTLVVASNSEIFKSFDPGVCFEVEPSVYVKNIY
ANLVTLKMVDGAFTPVPDLAEKWEQSSDGKTWTFYLRKGAKFANGDPVKASDVEYSYRRA

ITINKSPAWLFKDTLGLTVDSIKAVNDTTVTIVTNGAPSNIVLSVLAANIGGVLNEKEVR
AHEVSGDMGQAWLTDHSAGAGAYVLKEWKRKTQIVLTANKNYFNGEPPIKTIIVRDVPEA
SDRYLLLKKGDVDVAWQITVEQAASLRDKSEDVALISTPAQSLEYVAMNASWGPFKDNRV
RQAVKYAIDYDAIINKVREGFAIKNQQFLAIGYFGYKESNPYKRDVEKAKKLLADAGYPN
GFEVEIVTSESDIRRAEAVVVQSNLADIGIKSNIVVMPAAQMYDKYRNQGLQMIVAGWGI
DYPDADALAKPFADYTVKQLAWRNAWLDDKAAAMTAAAAKEINDKKRAQMYIDLVQYWQV
NGPFAMLYQPIEYWGIRKEVKGYDKAFEGYNVHCNFTLISK*
>SPBIB_v1_290109|ID:27163634|ddpB| D-ala-D-ala transporter subunit ; membrane component of ABC superfamily [Uncultured spirochete bib]
MELSSFILKRLILMVVVIFGVLIIVFVVTRIIPADPVGAILGGNAPPAAVDAMRHQLGLD
KPIFMQLLDYLGGVLRGNFGNSLVSSRSVITDILEYLPATIELAISSIIFAVLLGTTLGL
LSAVFRNRFIDHFSRVFSILGISLPGFWLGLILILFFYYRLGWLPSGGQYDMFLSPTRVT
GLVLLDSVITGEWNVFFNAIKHLILPTFILGYSSTASIARIMRASMLDVLHQNYIRTARA
KGLPKKTVILRHALRNALIPVVTIIGLEFGGLLSGAVLTETIFSWPGLGRYIVNSLLTLD
YPAISGGTIFIALIYSVVNLVVDILYAALDPRMRA*
>SPBIB_v1_290110|ID:27163635|ddpC| D-ala-D-ala transporter subunit ; membrane component of ABC superfamily [Uncultured spirochete bib]
MRKPSSDKKISGQSAFREFLRERKPMLDDVRHTVYLWKKTPLALVGTVIICLFLLVALFA
PLLAPYSPIATDLSRKLEAPSAAHPFGLDQFGRDVLSRVIMGTRIEVSIIVIISVISIVI
GLIVGIVAGYFGGIIDEILMRITDIFLAFPRLVLAMAFAAALRPTLTNAIIAISLVEWTV
YARLARAEAMKIRSQPYIEAIRAVGASNIKIMVFHVLPMSISPIIVQLTMRMGTIILTAA
SLGFLGLGAQPPLPEWGAIVSDGRSYLMNNWWITAFPGMAIAVTVLGFNLLGDGIRDILD
PRIRR*
>SPBIB_v1_290111|ID:27163636|oppD| oligopeptide transporter subunit ; ATP-binding component of ABC superfamily [Uncultured spirochete bib]
MEQQRGDLLDISGLRVVFNTYAGQVKALNGVELWMNHGERLGVVGETGCGKSVTALSVMR
LIEEPGEITSGALWFEGRNLAQLSENDLNSIRGKDIAMIFQEPVAALNPVMKVGAQIVEN
IECQRRSADAGQIPDKRAFSEIMRDMLGRVGLDWQRTENLYPHELSGGMAQRVMIAMALS
SKPKLLIADEPTSALDVTIQAQILNLLNKLVRETQTAVLLITHAMGVAAQFCDFIAVMYA
GNVVEYGSVQSIFRNPLHPYTKGLLNAVPKIGRTDELQSIPGIVPDLIDPPSGCRFHPRC
PYRKPDCDTLEPTLRAAKTEPAADRHHVACFLYQNDA*
>SPBIB_v1_290112|ID:27163637|dppF| dipeptide transporter ; ATP-binding component of ABC superfamily [Uncultured spirochete bib]
MQSSTSDDMLLQVSHLQKFFAVRKNLYSKPLYVKAVDDCSFSIQRNTVFGVVGESGSGKT
TLGRVLLRLIEATGGSVLFDNTDILSLNTQELRLFRRRMQIISQDPYNSLHPRKVVKKLI
GEGLQIHFQLSAQEIDTRVRDILTLVGLREEHMYRYPHEFSGGQRQRIAIARALVLKPEF
IVLDEPTSALDVSVQAIILKMLKDLKKDFALTYMLITHDLAVIDYMADYVAVMYLGQIVE
SGSKDDIFKRTAHPYTSLLIDSVPPPTPSEHWSGHILNGEIPSPIHSPSGCRFHPRCPQA
KEICKTAEPEVVEVQPGHVVMCHFPLGS*
>SPBIB_v1_290113|ID:27163638| protein of unknown function [Uncultured spirochete bib]
LEARLMPSARRMMRAEHALYLARQLSADFVVSIALDIEVNPLRADPEFYRQAHPFC*
>SPBIB_v1_290114|ID:27163639| putative Beta-lactamase [Uncultured spirochete bib]
MQMTETQRAAQFIEQYIPEAMHETKTPGYSVAVMKDDELVYGGGFGWRDRERFLPATADT
LYGIGSCTKAFVATAVLLLAERGALTLEDPAGRYIPLKLRATGGTPDGAPNIGAFDGSAE
SGPITIHHLLTHSSGLPSLSTSEILLQQGLGLETGVPLASAEDFYRWVNGAQDEISALPG
ERFFYSNESYRMLGHIIQTVSGMPFYQFIAENILKPLGMERSTFVRSEYERDADKMRPYW
AKPDGTIVPAEFPYPDIGLIPDFSFIVAAGGLISSVRDMMKFLSANMPGRAAKPGRAAIS
ENVSLTGNAAPLLKPETLGRMQTKYIDRPAGYYGSNGYGYGWTITDNFLGHKMVAHGGSI
EVSTAYAAFLPEKRMSVMLAANSSGMPHATIAEGILAALLGQDPYEAIPVFDIKKRMNLL
AGTYETYRSAVSVRIFAKGGLLYLEQKNPFIDSVVPLIPEDDYLRNFRFYTLTEGVRLPI
EFRVGPKGIDLYIERDRYHKVG*
>SPBIB_v1_290115|ID:27163640| putative Branched-chain amino acid transport system carrier protein BraB [Uncultured spirochete bib]
MADTKKQALSFGAVLVVSAAVFSGHFGVGDTIFPAMLGRTTGASWFIAALGYGVVNSFMV

FLAYLAISRQNSSLFGLTSMVLGKGFAIAYTTIAVLIMGPVFILPRVSSATHEMAVAQFF
PSIPIWVTLLVYFALNYYFAYNRSKVIDRIGKYLAPALIVFMIILVIKGIFAPLSAVAST
GSSTAFSDGIINGYNTMNALGASIFGIWIINELKRRGIEDAKSRSSTIITIGIFAALALL
LTSTGLTYLGASSGGLFPDAAIGVLSVKIAEGLLGYFGKIVFAVIIALACITTSVGLTSA
AGDTFEQMSGGKIKYKFTVAASSIIGFLLGLIGLSKIVGYTVPWLMLIYPALVIIIIMSL
FTDFLKVKLATQAGVLVAILFSIGDFLAGLGIANTPFSKLNAAMPLGKQGLAWLFPSIAA
IVVFQIIAVIGRGKKAPDEAVKP*
>SPBIB_v1_290116|ID:27163641| putative Metallo-beta-lactamase domain protein [Uncultured spirochete bib]
MNTMDMLDNLSVRVVAEDSVMYESPFWGQHGISLYVTASKDGFVRHILIDVGQSHEALLH
NMRLMGIDPASIDAIVLTHCHYDHTQGLAEVLRAIGKTDIPVIAHPDLFRLNFIDKPYLR
HVGITSDDAKSNLEKIGAAFFLTADPLQLMPGLATTGYIQRQTDFEEVGIPLKTIDTQNH
LVQDPMNDDISVIGAVKGKGIVILSGCSHAGIVNITKHAIAMSGISEVTSIIGGLHLVEA
PMERIAKTVDALDSLVVGSIYAGHCTGFNAQVELRKKFGTRFMPLQTGNYFEYP*
>SPBIB_v1_290117|ID:27163642| conserved protein of unknown function [Uncultured spirochete bib]
MLIEVQFSMYPIREAHISPFIQKAVNIIESFGLPVQVGPMSSITYGESATIFKAFDRIIE
EFAGSTQFVLITTISNACPVDFSQNKSSSKSSL*
>SPBIB_v1_290118|ID:27163643| conserved protein of unknown function [Uncultured spirochete bib]
LHKTRSIVSAKESIQAYIYFWRDNIGTEIDVVFEESLKLRALEIQSGKTFSPNSPRISKH
GSVFGFVAGRLRAHLYKTQALVCYHANNHNLDMEAGSSSPVTDPKVLFDDFANEYDSWFL
TPAGRKVFAFELDLLLNSLPALSGIHLLEIGIGTGLFAIEFQKRGANVEGIEPSPKMRAI
AEKRGLEVKYGLGEAIPYPDNSFDVVLAMTSIEFSKMPERFLQEMVRVTKPSGIVAVGVL
NLWSLYGISRRVKGLFKKSLYDEAHFYSYRELKKFLSTYVNNVEVKSTVFLSPSPPNFIL
ERADAIEHFGRHHLVPFGALLVGTGKKAEQRK*
>SPBIB_v1_290119|ID:27163644| NAD-dependent protein deacetylase (modular protein) [Uncultured spirochete bib]
VLILDDTVAGEPPKLVKRVRLPSVALDEWPANSGPFCYTDNMTSQSQISRDCDSASPENL
QALYDMLLASRHCVAFTGAGVSTLSGIRDFRGKNGLYKTPDADKIFDIDIFRQDPSFYYK
MTKDFIYGLGDKKPSVVHKVLAELEKRGILKAIITQNIDLLHQKAGSRRVIEIHGSPSLH
HCPRCAWSMTFSEVAAIVRAGDIPRCKKCDAVLKPDITFFGESLPAEALAEAIEEARSAD
LLLVLGSTLLVYPAATLPEYTLEHGGRLVIVNDMATHLDAAASLRFSDLGSVFDFLEKKL
KC*
>SPBIB_v1_290120|ID:27163645| protein of unknown function [Uncultured spirochete bib]
MSNEEAMRALMSIEGFRSLNQDIETIINKSEAVKKLKREANDRQLAKEEKLKLSEEEKEY
KSKRRQIQEKLIKFATRIPAFMYLTDYRERTLKDVITQLEPGLFKKVTGLTVKDFELLIS
LGVFNSALMNDAVYKFKRYEDASLRYVGIDRHEGEDIGLYDTILRKEEYDQTFMNIAAER
*
>SPBIB_v1_290121|ID:27163646| conserved protein of unknown function [Uncultured spirochete bib]
MPMSRGTVFTDHSNLYLSGTPFRAIASGEFNEFDLNEFFRAEGIGKDARFKYENEVQKWL
DLIRGSFLETTVDNLKMGARKPPFPYSDARLLNVLTHTLWFLPSVASCYAMRNLMAQRQN
KFYHDYTVVVAAGNAAGIGVAALEPVLKAMDDPLKTKTITLSCGKLTTGVTVRPWTGIFM
LRNSSSPETYFQAAFRVQSPWTLKNPDGQSPNEELILKEECYVFDFAPDRALRQIADYAC
RLDVNEANPEKKVEEFISFLPVLAYDGSSMKRIDAAGILDMSMSGTTATLLARR*
>SPBIB_v1_290122|ID:27163647| protein of unknown function [Uncultured spirochete bib]
LSWSHYAEILKADNDLEISFYTRQCEKEHWSVRELKRQMKSMLLHRLHSVKTNLVC*
>SPBIB_v1_290123|ID:27163648| protein of unknown function [Uncultured spirochete bib]
MQKQAKSKQRVANHGEVLTGEREVNAMLDLVKPETEQIDSRFLEPACGTGNFLAEILKRK
LRVVEDRYAKSQIEYERKEITVGTEMIDKLWNSSRKAVPKPSRGLTCWIGFPRT*
>SPBIB_v1_290124|ID:27163649| GCN5-related N-acetyltransferase [Uncultured spirochete bib]
MKIKIANLTEESLQDAPEWDGYPFSCKYCLYWEFPGESTGPIEQSKDKAIRMKTAWLQRT
NEHFGNCGKIAYVDGVAEGYAHYAPPGFFPRVAEYQAGPPSQEAVLISCLFIPRHRFRKL
GLGSQLLDNVLAELKQRGIGAVETFARKGKADNPSGPAEFYFRNGFRIYRDDAEYPLLRL
VL*
>SPBIB_v1_290125|ID:27163650| Helix-turn-helix domain protein [Uncultured spirochete bib]
MPSSFGQRLKAARIMAGLSMDALVAKMGHRVSKQAISKYENNLMSPDSETLIALSEALEV
KPDYFFSKYEIAVDSINFRKKAALGKKAMETLKARIKDTIERYAELESFFPASLPFTNPL

SDYVISSLDDIRKAAMELRKAWGFGLEEPISYIINCLEEHSIRVLEINGSDSFDGLSGWV
KQSPFIILNSNAPSDRKRLTALHEFAHLSLKFSDSLSNSEREKLCHSFGGAFLLPRGVLV
KELGLKRTEISFFELDRLKKQYGISMQAIMYRAKQEEIISEYVYESFSREISARNWRKQE
PNRYPIDEHPLRFEQLLHRAISEEMISISKAAYLANTSIEDIRCQELLKNDTAHP*
>SPBIB_v1_290126|ID:27163651|adh| Alcohol dehydrogenase GroES-like protein [Uncultured spirochete bib]
MKAAVFAEFGAPLTISEVPDPKAPDGGVVLAVDATGICRSDWHGWQGHDPDIKLPHVPGH
ELAGTIVEVGKSVQNWKIGDRVTMPFVAGCGHCTPCLTGNQQVCDNQFQPGFTHWGSFAE
FVAIRYADMNLVRLPDAIDSATAASLGCRFATAFRALEAQAKVRAGEWVAIHGCGGVGLS
AIMIAAAMGARIIAIDIQSDKLAMARELGAEVVINSREVPDVLSAIRDVTGGGAHVSMDA
LGSRQTCFNSIACLAKRGHHVQVGLMLADQSHPEIPMDLVVARELEIYGSHGIQAHRYSV
LLGMIAAGKLHPERLITARLSLAQGVEFLHKMDQFPGTGINVITSF*
>SPBIB_v1_290127|ID:27163652|ymdB| conserved hypothetical protein [Uncultured spirochete bib]
MKTLYRSPDGRCIVAVGDITTLECDAIVNAANSSLLGGGGVDGAIHSAGGPQILEECRTL
RAGPLKEGLPPGRAVATTAGRLPAKRVIHTVGPIWRGGEHHEDETLESCYRESLAVAARE
GLACVAFPAISTGIYGFPKVRAARIAWKTVQEFLAGSPASMPRVIWFVFFSEADAALFMN
ENKLTENKSIV*
>SPBIB_v1_290128|ID:27163653| Response regulator receiver protein [Uncultured spirochete bib]
MGKLDKKVRTFSALEIAKLCGVVNQTAINWIRAGHLKAFTTPGGQYRVYAEDLVTFLKER
DMRIPEELHEDLTMPVDLGLALIVDDDKDLNTILKRLLERKIEELRVAQAFDGFEAGRII
AERHPAIVLLDLNLPGVDGFSLCKKIRSDESFGQPAVIAMTGIAQEEAQPKMMEAGADAF
FAKPLNFDALIAKIRELLEARKNQQGKIEE*
>SPBIB_v1_290129|ID:27163654| conserved protein of unknown function [Uncultured spirochete bib]
MEATTVLDSSSIEALTIDESGIAQRIAQELAIRLSQVMAVLSLSAEGCTIPFISRYRKER
TGNLDEVQVRDCIQKFQSYKNLEERRLEVLKGINALGKLDAFLYENIQKAKTLAELEDLW
APFKKKKKTRGMLAQEKGLGPLAELMAKAPLEEVLAAAPSFVRLDDEHPELSVPSAEEAR
AGARDILAERLSQDAEIRAYVKHVVMQNGVLSVKGIGEEEKREQSTYQMYWDYKEPLSTL
KHHRVLAVNRGEREGELDVSIEIDDVLVEELVLERVRPANAQHKEALADGLARLLLPAVR
REIRSDLTESAEMHAIDVFSTNLRNLLMQPPLRGTRVLGIDPGIRTGTKCAALDETGKFL
DYFVINQETKPDQGKKDVAAAVTKHKLSVIAVGNGTGSHEVQKLVAEAIAENHLDCRFAV
VDEDGASVYSASDLAREEFPELDLTIRGAISIGRRLQDPLAELVKIDPKSIGVGLYQHDV
NQKQLADRLDEVVSSVVNQVGVNLNTASYSLLKYVSGINGSLAKKIVKFRDASGVIRNRS
LLKEIPGLGEKTFEQCAGFLKIPEGDNPLDNTWVHPENYALAAEILPIVKSGRDPSREER
AALKEKYGVGDITLDDIITELKKPNRDPREDLPPPLLQQGVLSFEYLQPGMKVKGKVKNV
VDFGAFVDIGIKESALIHVSEMGDRFVKNPMEVLRVGDLKEFTIISIDPVRRRIGLSLRS
DAKVQAGGVQANAEKPKVVTDSRTNLAGSATTSEGRRADKRHAPREPEGDGMTYNPFADL
LKKR*
>SPBIB_v1_290130|ID:27163655| Prevent-host-death family protein [Uncultured spirochete bib]
MKNLSVGELKNQFSEVLEKIQKGESFGILYGRKKKPIAMIVPYAEKTKKSKRQIGLLEGK
VDIRFADDFKMTEEELLGQQ*
>SPBIB_v1_290131|ID:27163656| PilT protein domain protein [Uncultured spirochete bib]
MNYLLDTHVFLWALTKTEELSRKAKEAIENPGNEIYISAISFWEISIKTRLKKLDLGPLR
PDDLLQYAEKMEFQAISLTPEEAMTYYKLNENSHSDPFDRMLVWQAISRNMALISKDNEF
AKFIPYGLTLVW*
>SPBIB_v1_290132|ID:27163657| putative Acetyltransferase, GNAT family [Uncultured spirochete bib]
METHHVPGARQENRAGSPSLSGRALMIRSATLEDSPALASLAGQLGYPASPEKVRERLPR
YIGAPEARVIVAEHEGQVIGWTSIEVVDHFYLDKFAEISGFVVDERFRGQGVGHALMQEA
ERWTAAHGLSTLRLKTNVVREDAHRFYENLGFERTKTQYTYVKKLARLAADL*
>SPBIB_v1_290133|ID:27163658| conserved protein of unknown function [Uncultured spirochete bib]
MAINFERLAGNTFVARGPTNIGLYICRREGVPGESVLAEGVPIEAQSGKAASQAHGGSRA
FLIDSGGDADAGRRILRECERLGVHLAGIINTHSNADHCGGNAFLQARIGCAVCATEAEA
AFLGYPLLETSFLAGGYPQKALKNKFLMAPVSRATHILNPPCALSLDAEGQIQMRTLFKG
QVQVRALPEGVVSGEPAPRAEPESQSDPQSECRIVSLPGHYFGMVGVMTPDRVFFAADAL
AGKPILEKYHIFFFYDLAAELETLAMLETIEADWFVPSHAEPTQDIRPLVELNRRKINEI
ADVIVGLCAGGQDVRESGGHSLEDIFAGVCAHYGISLDDNQHVLVGSTIRSYLSWLSNQG

RLEYSFINGRMIFKVKQ*
>SPBIB_v1_290134|ID:27163659| exported protein of unknown function [Uncultured spirochete bib]
MSKKKANQARGCTRNFNKRVAAGFGRTAMLVALLFLIAGGSGTAFAQGHLAVDLGHPVYS
VIETAELRGVVTRLSSVKPYTSQQVAELLAQMLGHMEAFSPSEQALIQHYAKEFKAGKGV
EQALWQDAAGTARAGIRVEATTRFGVGELVDLMDGSGATALKDLWHINSRWVPYLAGEPL
PWLSMKGEAGFTFDKIEKNLYLPYEFTKEWDAGHIWLGTPRYSDGTLDYPTASYDIREDI
AAETDSGSLMVRLSRFRRDWGIGSGSFSLSGTARPFVGFETVFRPSKFFAISGLVGSLTN
WEKGENEKSTITSLIADTNGDGLINNLDTPVFSALSYQKMLGLQRMELFPFDWLTISATS
TLVGAKRFELGYFSPLLFSVMYQNQLADVDNLGVQVDGQILVPRIGKFYASFYADEMEIT
NLSELFTKVRNMFALQGGVKVPIPGLSFATVTAQYTKIEPFVYSHYATWYPDYRLRVDTS
YTQDGENLGYYLPPNSDEFLVNLEAMPAPGWRVGLKYRFVRHGDNPQADWDNHLPEIFGD
VNKYMDYNQLYSYNKNFLHDGIYDYNHIGTINVSWKPIQPPKLLGASIPFELGAGYGLSY
TWWEDGTGAGRPVSAPQWKNVLTMSVKLFL*
>SPBIB_v1_290135|ID:27163660| conserved exported protein of unknown function [Uncultured spirochete bib]
MRRIGIFAVVLLVAASGFTFAQSSSLTRVGCILGVGGLGDLSFNDLVYAGLQKAQKELGI
QFDYVEPQSISDFETYLRKMASMKAYSVIISVGYDQVDPLTKVAPQFPGQKFAIIDESLN
LPNVVSYVSKEEEGSFLVGALAGFLKKANTAKNPTIGFIAALDIPLLDKFYAGYEAGARY
VNPSVKVIANYIGGNAPFSDITTAKEIALKQFNQGANIIYHAAGGSGLGVFQAAKEKNFY
AIGVNSNQNPIDPNHIIASMLKRVDTAAYKIAAAAKQGNLETGKTVILGLADGGIDYTVE
GSKVTIDPKILKAVNDIKQSIVDGKLIVPDTHAKVEAFLKANTYK*
>SPBIB_v1_290136|ID:27163661|yufO| Uncharacterized ABC transporter ATP-binding protein YufO [Uncultured spirochete bib]
MPAILMKQISKAFDKLKALDHVDFEVDAGEVHCLLGENGAGKSTLMNILYGLYHADAGEI
YIHGKAVHIANPKIAQQYHIGMVHQHFMLIESMTVLQNIILGNEAGRFTIDYAKNRSRID
ELEKLYHFDFDLDTPISALSVGEKQRVEIFKALYRGADIVILDEPTAVLTPQEVDVLFRI
LDDMRKQGKTIIFITHKLHETMVLSDRVTVLRNGKIIGSLRTEEVTPKILATMMVGYAID
FDIVKSTFKPGKPVLELRQLKVLAASHATISLQVRQGEIYGIAGVDGNGQQELEGILAGT
AKPLSGRIIFNGEDITAMPAAKRKAKGIALIPSDRLQNAILPGMPIIDNYLLGFQHNSMF
SHFGILRFNVLKKHADNMVNQYSIKISALEQPISQLSGGNQQKIVFSREAGLNPLLLVAV
QPVRGLDIGAIDAIHRQLLQLRDRGKAILLISTELSEIMEMSDTIGVLYKGELIAQRKAG
EVTIEQLGLFMAGERPE*
>SPBIB_v1_290137|ID:27163662| conserved membrane protein of unknown function [Uncultured spirochete bib]
MTRNRKYLEDVGFSLVAILLSFLLVAVIMLILGYNPIEAYKALFEGAFGSSYSLSLTLSK
SVPLIFVGLAVGFALQGGLFNIGGEGQIYIGGFVAAITGLLLSHTIPAFITLPVAVLSGM
AGGFLWGGTIGLIKAKLNINEVIVAIMMNYIAQLMTSYFVNGPFIAAKSMTPQTEILPLN
LQLPKIIPNTQLTTSLYIALIMCVLYGFVMKRTVFGFEVKAMGNNLHSAATGGIYVARNT
VLIMALSGALAAMAGIFEVFGTYGRFIDGFSSGIGFTGIAIAFIGQSTPIGIILASLLFG
SLQAGAMQMSMMAGISANINNVIQGLVIVFIATPNIIRFAFGKRRS*
>SPBIB_v1_290138|ID:27163663| conserved membrane protein of unknown function [Uncultured spirochete bib]
MATMMFVVNMLTMTFRLAVPIALASIGVSISERAGIINLGIEGIMLLGALGGAVGSYVFS
NAWMGILLALITGIAIGVLYAFFVLKFEANQSVIGIGLNIIASGLTVVIVKFIWKKEGIS
GIVTQMSTYSVPFLRNIPVIGAFFTDQSPFILLTLLIALACSYGMWRTKIGLRLQAIGEN
PLAAATAGIPVARYRTGAIVVGSSLAALGGAYLSIVHSNLFVSNMVAGRGFIAIAANILG
GWNPMGSLLASLLFAFAQALRFQFSAVHFPDQISQIIPYVITLLVLVGVGRKTRAPAKLG
VLE*
>SPBIB_v1_290139|ID:27163664| Amidohydrolase family protein [Uncultured spirochete bib]
MSRIFLRHGIVLTIDDCDAYYPDGCIVIEHEKLIFVGEDAQHPGIHPEDSVLDMRGKLIM
PGLINTHVHSHSPLFRNFGEDVPLQVWLNDIMWPAESHMTEEHAYYAALHTCLEFISSGV
TTFADQFYFADAVARAVEKSGLRAFLCSSIFENGKSERGQTVQTAADFIAAWKGRNPLIT
PGLGPHAPYSVSAEQWRSIVALSQATHTLIHTHISETKTENRKSFAEKGESPTKWLESLG
VFSCPTLAAHCIYLSDEDIAVFRNHNVHVSYNPVSNLKLVSGIMPYLKLKTAGVQISLGT
DGAQSNNTLDLLQDLKLAVLIQKQKENDPAFFSVFDAVRLVTIEGAKALGLDSVIGTLEA
GKQADLIALDIQQPHLQPLHTDSIQMLYTMLVYCASGKDVTDAMVQGTWLMKDREILTLD
VHSVLHNTADISEQLRKKAGLTGD*

>SPBIB_v1_290140|ID:27163665| Phosphorylase [Uncultured spirochete bib]
MALDKNAITKNTKQYHIELNVGDIGEYVLLPGDPARSDRVAKYLDDAKLMANHREHRTFT
GYYKGIKVSVTSTGMGCPSTAIAAEELIHIGARHLIRIGSSAALRPDIKIGDLLITTAAM
KNEGTSKFYVPENFPAVPDFEFTHLLIDVAKEMTSGSDKRVFAGISSTDDAFYGETPEFI
KKLQDLKIQNIEMEASALFTIGHLRDVVTACICGASGNLTTGEVIYTTENYKLAEAWDLE
IRVVLEAIYRNEQRKKSDVRM*
>SPBIB_v1_290141|ID:27163666| putative Regulatory protein GntR, HTH [Uncultured spirochete bib]
MNNEESLKFRETKKPLYVEIYEKLFQFIEERNFHEGDRLPGENSLASELGVSRTTLRQAL
VLMTEDGVIHRIHGKGNFIANLAPRNLAGLENIANPVKAFTTVPVDEINIDMRFEAASEY
VMKLFGYEKSKVVMASDRWYRSGPRLIACCFAFSDLDVISSHNIDIQDAEIVKAFLEKQV
YELSSVSLNHINITNRKEFSNINIKMDSNTLILLSETLYSSENKILTHNKYYLDPLYFDI
VANGRHINRI*
>SPBIB_v1_290142|ID:27163667| putative Acyltransferase [Uncultured spirochete bib]
MRYRKGAPLTNDTPFSRLIWDILIALIALVGWPISRAFYRIKIGRGCDATSSAGMDEGSP
SRLNTPNATRTSPIASVPPKLPSPAILVSNHCMPLDPLFHGLAIFPRRTFFTLLEETCEA
PVLGSFVRLLGGIPLPRNRARLNDIEQAVAHALSTRGLIHFYPEGECFLGNQNIYPFKAG
AFYFAIKFGAPVVPIVTVLKKRAGHSTFRGQKSSRIQVTVHVLNPIQPPACGATAHETLA
RAIRFSNQVQDLMQAEIERCGGDKSLYRGPMPRIKGVND*
>SPBIB_v1_290143|ID:27163668| putative Glycosyltransferase, family 2 [Uncultured spirochete bib]
MSIALLTAYGIIVFAACIFFFFVTVSNIFWLVHTQSIEPKESGPSVAVLVPARNEALRIR
PCLDSLLKQNYRSYQIYVIDDNSTDETWEILKSYMQRYPRKFKAFRAEPLPEGWYGKPHA
LQELSGHVEEEYMLCTDADTVHKPDSIGRAIAVAERYKADLVTGYVHHVMPTFAEASVEP
SIYILTMLAMPLYLIPLVKSSKISHAIGQFMLFRRSFFEKIGGYEPVRKQATEDVKMANI
VKQQGGRIAFVDLRMSVECRMYNDYRGAIQGIAKNAYDYLGKNTLFLFLGTVAVPLVFFV
PIIVWFINIPWLGPAVPFLKASAILTLYTWILEAIDRRLPAYVPFIYPLIFVNALSALWS
GYRQVRKEGGVEWKGRKVI*
>SPBIB_v1_290144|ID:27163669| exported protein of unknown function [Uncultured spirochete bib]
MRVLHSRRQKVGQLASAMAFVLLLLNGYYTGAQQAPAQSAFLAPPQNPSSVGNFQFPFAG
ALSPKQELLPGVAFPQELSADAVKAFLGLRDDVYNNVPPETIDAMAASLLKIVAGAPLSP
ADRSLMNARIAYLAGRSWNDHKNKKQAVPWFEQAVSAARSVIDISGETPTSLIVLAEPLG
ELSILKDLGFLLANGPKIGQYASKALESDPHNIKALLLKASALAYPPPIWGGNYKKALEA
YAAILPITEPGLPRDVLFDLRVGIATAYANLKLSEHAAWWFRAALELYPENNYAKSELEK
LSQ*
>SPBIB_v1_290145|ID:27163670| conserved protein of unknown function [Uncultured spirochete bib]
MCDTVILMDNSGKPAFFGKNSDRHPEEPQALLYISERAPSTVMLREGVEYQDKGFALLLS
KPSWMAGGEMGVNSQGLSIGNEAVFSRFKPDEHGILGMDILRAALSACASAKEALDFITS
FIEKYGQGGNGAYKGKLIYNNSFLAADPQEAYIIETAGKRWAWRSAMVADAISNAYCIED
DYKRLDMQTRKEIAPVNKRAACSDESDPGRKGKRNSWKAHVEDRKYLFFTKGEQRRSSSL
GGLMRIAAERASSAQTEAQNEAQSEPKAQLEKIFSLLRSHEGATKPGPFLSRMKNLCVHP
GLFPQSATTASMVVEYLPGGALVWHTDSSYPCISLYKPVLLKDGRFYSLWKPLCAENKAE
ERYASWNSHRQWAVKSGHLQLSAKEDFALSRDAAQASIIRIAQQAFDSVSREKGSPERIS
SVYANEVAAIVGEWEKRWGN*
>SPBIB_v1_290146|ID:27163671| exported protein of unknown function [Uncultured spirochete bib]
MDRTRPSSLVILLFILSIILAVATVAVTRIIEHSRPDVCAQFTIYLEKLQPQSMLVAATT
QERYSASKEFTAKLLAIFKIKAKIRLSALADVTYVISAADPSAWSARWNPKKRILSISTP
PPDCLLPAVHTDTIEIVSENANLLTNTIFRLKEEAARMQSELSNDLMVHAKASLDEPQVL
QSIEDGVRRFALVFCESAHIGKPAAIDIQLAPRLTER*
>SPBIB_v1_290147|ID:27163672|ggt| Gamma-glutamyltranspeptidase [Uncultured spirochete bib]
MEAHMKKLAFVFALFVAALSALSAQLPVNLYGRAASGPNGVVAAAKPEASQVGIDILKKG
GNAVDAAVATGFALGVLEPNASGVGGGGFMIIKMKDMTEPVVIDFREMAPSKSTPTMYLG
ADGKVVPNSTIEGGLAVGVPGEVKGLLYALEHYGSGKLSRADIIQPAIQWALTGVPVTVN
LASIIKDNYGKLVKYENGAQIYLKDGLPYEVGDVIYNPDLAKTLAKIVKEGTDAVYKGEI
AEAIVKEVQKRGGILTLEDLANYEVKIRKPVEGSYRGYKVYSVPPASSGGTHLIEILNIL
ENFDVKKLGFQTAQSAHLWSEILKLTFADRSKYMADTEFVKVPLAGLTSKEYAKEQAARI

DLNKPLAPVTAGDPWKYESGSTTHFSVMDKEGNMVAVTKTINYFFGSGVTIPGWGFIMND
EMDDFVATPGSVNSVEPGKRPLSSMSPTLVLDPQGRSFMTIGSPGATRIFPTVAQVISNV
IDFGFPIQEAILAPRVWQGASGALNIEGRYPASALDGIKKLGHDVTIRGDWDAYFGGVHA
VVYDYDKGVLYGGADPRRDGQAAAF*
>SPBIB_v1_290148|ID:27163673| conserved exported protein of unknown function [Uncultured spirochete bib]
MIGRQRKLPASLQRAFSRLPHGAGCFSRKGFSMKRSRSVLLLVAAVIAVLAPLSLAAYNL
KAPFASQPALLTSIGQSADVEMAKAIINRLKISFTMDSLVKAQGLASSNAKTLIIVIGGS
SKGLGAAGISADAELERTKALLAEAKKRGMKIIGLHIGGEARRGELSDRFINAALPSCDY
FIVVEDGNNDGLFTKLCGTKIPLDVVQKISQVGEPLAAAFLK*
>SPBIB_v1_290149|ID:27163674| conserved membrane protein of unknown function [Uncultured spirochete bib]
VSQEFLLFILMVGVFAAGCFALKWPVSVSMLLAAVAGALAGGVQNPIRHLVEGTFGYVDT
ILTIATAMMFMTGFRDSGALEALTAAVIRKFNKLPGLLMILLTFVIMVPGMITGSSTASV
LTAGAVVAPMFASMGMSALDSGALISIAAILGMIAPPVNIPAMIIGGGIDMPYVGFEIPL
ILLTFPLAIAFALWFGWRNVRKVDRSKVVESLDFSALNQYGAWRLFSPLILIFVLMILDK
TVPRVFGLGMPLIFLLGTALTWMTGKRFEILKSAKKAIEDVLPVLGILVGVGMFIQIMTA
TGVRGFIVVNALSVPPALLYVMIAVSIPLFGAISAFGSSSVLGVPFMLALLGKDQIIAAA
SLSLIAALGDFMPPTALAAIFAAKVVGLEKYGGVLKKLVVPGLIIAVYGILFIVFSKQIR
ALY*
>SPBIB_v1_290150|ID:27163675| conserved protein of unknown function [Uncultured spirochete bib]
MFYIYLAILAVVLALTVWVLFDTKKLSLQVTAAMVIVPLLLRLLMIK*
>SPBIB_v1_290151|ID:27163676| Succinylglutamate desuccinylase/aspartoacylase family protein [Uncultured spirochete bib]
MQKHTISAIICLAGALVAAAIAGASFLAMRKPDLIVRGPGVTDVKMLSDWFPGLKGTPGD
TEVYILKGADDGASMLVLGGTHPNEPAGHIAAIMLIENAQPKSGTLYVIPRANASGFTAN
DPQEGAPQSFTIQTPNGPRVFRYGSRATNPIHQWPDPNVYIHASSGQTLSGSETRNLNRG
YPGRPDGTLTERVCYGIVQLIKKENVTITVDLHEASPEYPVINAIVAHPKAMQLASDMVL
GLEMQDIKISLEPSPVNLRGLTHRELGDFTDTLPVLMETANASQGRLRGRTDEALVLTGK
DKWYVRAEKLGRLYVPYDENGHPLEERVGRHVAAISELMNSWNLEHPEKPLEVEGLPGYE
DFLTKGIGAFLLPVPAKN*
>SPBIB_v1_290152|ID:27163677| Plasma-membrane proton-efflux P-type ATPase [Uncultured spirochete bib]
MAQGVQPSTGAFSDSTEAFKTLSLEETLAKLNASVDGLTAAEASARLARLGLNAIEEKKA
HPLAQFLSRYWGPMPWLLEIAILLAAFLGHVTESVIIFLLLSINAVIGFMHQRNASKALE
LLKKRLAVNARVLRDRNWQTIDATELVPGDIILVKLGDIVPADVKIVRGNLSVDESSLTG
ESLPAERSENSIVYSSSIARQGEATCLVLNTGGNTFFGKTASLVKIAKPVSHQEEVMLTI
VRYMMYLGIVSSFAVGIYALVLHIPFLMILSFIVTFLIGAVPVALPAVLTIVQAAAGSQM
AKKGALVTRLDAIEDAASISVVCFDKTGTITQNKLSVVGSAASPGFSEDDVVRIAALASS
ADNMDLIDSAILNAAAARGLATKGCAQLSFTPFSPATRRTEAVIECAGSRYHVLKGAVPI
ILELCSDTSESARSDINAHVALFAQKGYRTIAVARAPLGAATGNVQAAPESPPELIGILA
LADPIRSDSRAMIADLRALNVKPIMLTGDSLAIAREISVQAGIGPNIISIKELDGMDEGA
QARKVAQIDGFAEIFPEDKYRIVKLLQKSGYMVGMTGDGVNDAPALKQAEMGIAVSNAAD
VAKASASVVLTQEGVGVIVQAIRLSRRTYQRMLTWVINKVTKVVQVLGLLIAGFFLFRNM
ILSMLDMSLLVFANDFVTMSLATDNAKDAQAPNQWKVKNITLASLAIGLLLAAQGIGGIF
IGQDLLGLDFGHMQTFVLLMLIFTSQFRVLIVRERRCFWDSMPGTLLLASTGSTMAIFFL
MGAFGFIIPALGARETGLALAYSLVFTAVLDCPKRLFFKLFHVD*
>SPBIB_v1_290153|ID:27163678| NADP-dependent oxidoreductase domain protein [Uncultured spirochete bib]
MFYRRLGSAGIKVSVLSLGSWVTYGSQVDTEAATQMIKFAYDHGINFFDNAEVYAGGKSE
TIMGQAFRKLGLRRGSYLVSTKLFWGINEGPNEKNTLNRKYLLEGIEGSLERLGMKYVDL
LFCHRPDPETPIEETVWAMHDIVSSGKALYWGTSEWSAEQIREAWEIADKRNLRKPQMEQ
PQYSLLVRTKVEKEFARLYEGIGLGLTTFSPLASGLLSGKYKNGIPKDSRFALPGYEWLQ
ERWYHDEVIATIENLRPIAESLGCTMSQLALAWVARNPNVSTVITGASRLSQLEENLGAL
DVLPKLTSDVLQKIDKALEPILQKSS*
>SPBIB_v1_290154|ID:27163679| putative DNA repair exonuclease, SbcD [Uncultured spirochete bib]
MPGARLYFNVGARRIEPFMKTYYTDVRKGRFAMDERAGIFKILHISDLHLGKSLRSRDLA
EDQAHILSSIVRETARLAPDLVLIAGDIFDRSIPSESAQTMFGRFMADLRRALPFDGRIL

VIPGNHDSARRVAFAAELFEAVGIHLVSNVRPVPALVLEKGGKRAAVWALPFVTHGAFHE
FRRTWLDAADEFPETGAGRMADHMASIIANLRPHFAEYDMNVLAAHCYVRGAELSESDSA
FIGGTEAVPASLFEPFDYVALGHLHRMQALSPHMWYSGAPMAMSFGDGGSDKNSDKGFLC
VEIDAANGHLTVSPIALEPLRKMKRVRGSFSELTKETPEPADQDYIEVVLTDTDPVYNAF
SELAKRFPNLAGVQQEAFQKIALGGAGDNLGASAQPWLSLSRERNVHAAVLEDFRSFAKY
ILDEEPAEELVAAFEALIAEMAEERE*
>SPBIB_v1_290155|ID:27163680| protein of unknown function [Uncultured spirochete bib]
MRPIRLAFEHFGPYAERQDIDFSALEDFFLIYGKTGSGKTTIFDAIAYALYGEAIGGRSN
LEREFASRFSSQGSKPWVEFEFSASSAQWKVYRSVPYKKQNRKGKESEAAAEAALYRMNE
ASKSYELVADRITPVNEALLDLLRLRADEFSKIVLLPQGEFQEFLEMKTSDRAAILEKLF
DVSMYEKATEMARRKVDLLSSSLSAKNDELERLASELGSDPLARIAAMRKAFEGFRAQIE
ETKENASHLDTEIAQINERIELWQAFMSARAAFIELEHSKPSFEARAASLDAAKNLATLA
GLAQSVRSQHEDFLAVLKEAQRAGTELSKLEKARLEVEKNKERLPGLKNRQNELQRTAAL
YQRALDAWRRKEETARNLDELQKKISIALDSCAAREQELALTRKRISEFEALLAKEPEAQ
ALTQNNAVAIEALKRAEGLAKQLERLNSQRAQILREIQSTEDSLLTASRDLASAEAARSR
LEDAFRLAQAGIFAAALEEGEPCPVCGSLHHPHPASLPNYAPDEQTVRQAQDAVETQKAR
HAGLVQKKESLAQRLQDIEHEIGSLQQSISEEWQNREELASLRQEAEEISSKAIEALAQS
LKERKRILDTELEQYRLTRDALRTSRQQFEAAEEAFNQARQEREQLEKTKAELQARLDSL
AEQAGEHDPEPLLENTLRALSDLQGEIIQLEQRAQEWQMAYRATSAQLDIHLSSLELKGN
SLGPAFVRFFEESERLDVRVLLRTLEKPGIAKDAALQTSVPQKIFVPEESPHEAPARAKP
PSALDALSDLAGILLQTAKGNETADRKARVGRTASILSEAFRAAGQKEAEEEAEAEADAL
SLVAHVQQTAWPTDRLRSEEQAVSAFREAYARAKAAYDALAARARRLNEDAAKSFSDQEI
ESEEHALIDARDALVLRKKDLEDTLAELQRARAEIGAQLDQFKRSLERYNELQQQYSAAT
QEFGKMEKLSRLLSGDLIKGKKLPFKNYVLGAQFREIAARASERLYRMSSGRYIVEADPL
SGTGNQKIGLELFITDAWNGARRPVGTLSGGEKFMLSISLALGLADSIQERAGANRIESL
FIDEGFGSLDAESLSLAISVLDELRGDKTIAIISHVDELYSRIPSRIVVEKGVGGSRLRL
ERD*
>SPBIB_v1_290156|ID:27163681| Alcohol dehydrogenase [Uncultured spirochete bib]
MHEFYSPTKIIFGEGCASETAPRLREMGATKVLIVTGKSSTASSEGFRDLCKGLDAAGIQ
WVHFAEVSADPETSTVDKGAEVYRANDCNAIIGFGGGSPIDCAKGIAASIGEGRPIRDFV
GTGLAFTKPVPPLVAIPTTAGTGTEVTNAAVFTIVDEHGHRSKKGTSSQFYFPRLAIVDP
LLHMSMPPSLTAATGMDALTHAIEAFVSRFHTPVSDMYCLEAVRRIGRSLRAACRVGSDS
GTGDGAFGAGRGLKASGNSSSGNRALLEARSDMALAATLAGVGLSQAGLGMVHGFAHPVG
AMAGLAHGLANAILLPFVMEALIPDAGERLSAIGEALTGKRGTSAQDAVRDIARLGRDIG
IPENLEAAGVPQRYFEDILADALSYRRRKASPRAFTDDELRGLLARMYSGKVEA*
>SPBIB_v1_290157|ID:27163682| putative Cl-channel voltage-gated family protein [Uncultured spirochete bib]
MQEFFSIYPKSCKLQPDTYCAPIEHPDATRLYGLKNGNLYTHTRIYAYAALTGAVSGLLV
VAYRTAIVSAEHFRDALIGNAPPAKVVLLWLGIVATGAIFTALFVRRSPLIKGSGIPQVK
AFLMRRINFDWKRELPLKFAGGTFALGAGLSLGREGPSIQLGALAGTAIEDIFHIPDYRR
FLVTAGAAAGISAAFNAPLAGVLFCIEELHRNFSPVMLTVTMIASFMANVVMWIFFGTSP
IFELTILETLPLRYYFTVILGIGVLAGALGSLFNIGLLGFQKLYRRVVPREEFRVISAFL
MAGAISIVFPMIAGGGNHLVSFPLLSTMSFFAIALLLAAKFVFTLFSYASGAPGGIFLPM
LAIGSVLGGLVYSLLGLFGYQSPYLPNYILLGMAGFFVAVVRAPITGAVLITEMAGSFAH
FPAFIFVSIIATLTANLLRTKPIYDSLLAQIPPLYPEQITHAAATLHIPFMEGSSIHLVS
ELKERLPGECILTGIMRGEERLFPYPSMEILPGDEALIEVDQRAARLLKEELLKLGMPTE
DEQEIKSNGAAFT*
>SPBIB_v1_290158|ID:27163683|oppA| oligopeptide transporter subunit ; periplasmic-binding component of ABC superfamily [Uncultured spirochete bib]
MRKLLFAMIALLAVVGLSAQDFTIVNGAEPASLDPHAVEGVPEHRIYMALFEGLTVSDPR
TNRAIPGIAESWSFSKDYKTITFKLRKGAVWSDGVEITADTVVKSWLRKMDPKNAFQYAD
LPAMYIAGAMDYLEGKAGPESVKIRAVDKYTFEVQLVGPCPFFADMTTHYAFAIVPIHAI
EKYGQDWVKPGKIVSNGPFVLSEWKPQEKIVVVKNNKYWDAKNVALKKVTFIANDDINVG
YNLYKTGAADWTETVPLELMDEVKLRKDFHVAPEYGTYYYIFNVTRKPLDDARVRKALAM
AINKNDLVNKVTRGGQIPANGFVPPSAGYTPAKGYGYDPETARKLLAEAGYPDGKGFPTL

QILYNTSASHKRIAEFIQAQWKDNLGINVGLLNQEWGTYLDTRSQSHNFDMARAGWIGDY
LDPSTFLDMWVTGGTQNDGLYSNPKYDELMAKSRVTSGSERYQIMMDAEKVLIDQDMAVL
PLYYYVTQNLIDLNKWDGWYPNPLNTHPWKFVRPKK*
>SPBIB_v1_290159|ID:27163684|oppB| oligopeptide transporter subunit ; membrane component of ABC superfamily [Uncultured spirochete bib]
MARYFIRRALSLIPTLFIVITLSFFLIRLAPGGPFAREREVPEAILQNLMKRYHMDEPLF
KQYLRYMGDIVRWDFGPSYRYRDLTVNEIIDRGLPVSMSLGVISLVLAAVGGVAVGIISA
LKQNKWQDYVAVSIAVIGISVPLFVMGPVLQLIFGMKLKILPIGQWISTHGLKAVILPAL
TLSFPYFAYIARLSRASILEVLRSDYIRTARAKGLKESVVVWKHVLKGALLPVVTYLGPA
FSGIVVGSIVIESVFLVPGIGRPFVQSALNRDYTLIMAEVVVYSIILIIANLVVDLIYGL
LDPRISYK*
>SPBIB_v1_290160|ID:27163685| ABC-type transporter, integral membrane subunit [Uncultured spirochete bib]
MANKKLQQAVNEFNQVMKPVSLWADAWKRLRKNKMALISLFIVSIYILISLFAPILTSAG
VLIDYRKQVITNASLPPSLKPAGELVVKKIEARIASLEQRVKEKAEEQSAFGSFFNFGAT
DQTQPESAAEATPSDQGDTSAFTFTYQGEGFSGNTTDPVVRELISQKETLEKVKAELDTN
PDYKLVYILGTDDLGRDMFARIIYGGRISIAIGIVGTLTAALVGILIGAISGYVGGWLDN
LLMRFVDIMYGLPYMLIVIIIMAMIGEKARGSFVVLFVAIALVSWLTISRVVRGQIISLK
NSEFVEAARSMGASSWRIIFRHLLPNTLGVIIVFSTLMMPSFIMNESFLSFLGLGVSAPD
ASWGTLVSEGVRAMESYAWQLLGPGIAMTIFLFCMNFLGDGLRDALDPQSKNRT*
>SPBIB_v1_290161|ID:27163686|oppD| oligopeptide transporter subunit ; ATP-binding component of ABC superfamily [Uncultured spirochete bib]
MTDEVILEVKDLKTYFMVDEGLVKAVDGVDFQLHRGETLGIVGESGSGKSVTNLSIINLI
PVPPGKIAGGQVLFHGKDLLKMPPHEIRDIRGNKISMIFQDPMTSLNPFLRISTQMVETI
ELHQGLDKKAAKEKAIEMLKLAGIPAPEKRIDQYPHQFSGGMRQRVMIAMALSCNPEILI
ADEPTSALDVTIQAQILELMQELTRKLGTAVIMITHSLGVVAGMCDTICVMYAGRIVERG
RTEEIFESPKHPYTVGLIHSVPRLDQETKGRLYSIPGQPPNVIDLPDCCPFFPRCEKAMD
ICKKKYPPSVSFENGQSASCWLYAKEAQNA*
>SPBIB_v1_290162|ID:27163687|oppF| oligopeptide transporter subunit ; ATP-binding component of ABC superfamily [Uncultured spirochete bib]
MPDHKVLLDVRGLKMHFPIKASFFSKAKNFIYAVDGIDFQVRKGETLGLVGESGCGKSTT
ARAIAQLYKPTAGEVILNGKDLTKLPPQEMIEARKNMQMVFQDPYASLNPRMTAGDIIAE
PIRIFQKRGLIDMSTEERNDRVEHLMEKVGLSRFFKNRYPHEFSGGQRQRIGIARALALN
PELILCDEPVSALDVSIQSQILNLFKDLQDEFGLTYLFIAHDLSVIKYISNRVAVMYLGL
IVEIADAADLYKNPLHPYTQALLSAAPIPDPKIEAKRKRIILTGDVPSPDKQRPGCNFYD
RCSKHMDICKQARPMLKEASPGHEVSCFLYHKPE*
>SPBIB_v1_290163|ID:27163688| Dihydroorotate dehydrogenase [Uncultured spirochete bib]
MVSLQVSRFGLVFPNPVIAGSSGLTGNIKSIKELARNGIGGIVLKSLFEEQILMEARREA
SKGGVIYGQEEIDDYIGYYEKKHSLSEYLALIRDAKHEVSVPVIASINCVSGGEWTEFAS
EIRSAGADALQLNIFGFRNSDPGSIVRAVKDKINIPLIAKIGYYFSDVSAVVSEIERAGA
DALVLFNRPYSIDFDIEKLSLKQGAYFSSREEMSVPLRWISMLFGKVHAPIFASTGIHNG
DDVVKMLLAGAAGTEVVSSLYRNSPEVIAQMKARLESWMDAHGFETIEAFRGLLSQELSS
APETYERVQYMKYYGELQERRDGAGN*
>SPBIB_v1_290164|ID:27163689| Multisubunit Na+/H+ antiporter, MnhE subunit [Uncultured spirochete bib]
MKHGREAVILFIGLFLVWVLITWSVQPQELLTGAALVLVLTLFMRSVYPLLEGIRPGITP
LFSMVAYIFIFIWQLILANIDVAKRVLSPSLPINPGIVKVKTGLKSPLAKVMLTSSITLT
PGTLSLNVQDDEIFIHWIDVKGNDVESASKAIVAVFEQGLKGVVE*
>SPBIB_v1_290165|ID:27163690| Putative antiporter subunit mnhF2 (fragment) [Uncultured spirochete bib]
MITISLILIGLSAVLTVFRLIKGPSLFDRLVASDTLSVIGMAFLVVLAEIVQTTSYMDVA
LVYGIVGFLGTVTIARFFMRVSK*
>SPBIB_v1_290166|ID:27163691| conserved protein of unknown function [Uncultured spirochete bib]
MTVVGEILMLIGGIFLFLGSLGLVRMPDVYNRLQTGTKASTLGAMSIGLSAIFLLPGAAG
KAVLLVVFIVIANPIASNTIGRSAYLSGIKLSEKSVSDAWGVQASGEAGMVEGAAADATI
TGAAPEATDNKGA*
>SPBIB_v1_290167|ID:27163692| conserved membrane protein of unknown function [Uncultured spirochete bib]

MVAMVIVVSVLLIGLAAGAIFMRSLGSAVILLGTVSLLVSATFLLLAAPDVAITEAAIGS
ALTTLVYVLVLKRTNSVDSLEDGSNLQTGKRSESAHNGGSPAGGSHA*
>SPBIB_v1_290168|ID:27163693| Na+/H+ antiporter MnhB subunit-related protein [Uncultured spirochete bib]
MRKVIGVLLIASFAFFIFSLVMRDLWVEDPAAVRTLGQAAIEQTIPQSGAANTVTAIVVQ
FRGLDTLGEVTVLFLSALGVALLSGEFAGRGLKEVFRDDGGFILYNGARLVLPIIVLVGV
YIVAHGHLSPGGGFPGGVLVATAVYAVLMTGVKNELSTRLMAVIEGFAGLAFVGLGLLGL
LSDRFSFLANVLPKGSPGMLFSAGIIPLIYAAVGTKVAAELSTLVTSLTGSDYASAEKEA
QGGKR*
>SPBIB_v1_290169|ID:27163694| NADH-ubiquinone oxidoreductase chain 4L [Uncultured spirochete bib]
MISVSAVLYSTSMILILLGLYGVLTRKNLVRVVISLDVMDLGVNIFLVTIGYIRDGRAPI
YLSDAPLSLVDQASNMVDPLPQALVLTAIVIGFGVTAVALTLIMRLYERNGTVMVSDLRG
LKW*
>SPBIB_v1_290170|ID:27163695| putative NADH-quinone oxidoreductase subunit N [Uncultured spirochete bib]
MVSPMLLIAMPLGAAFLIVLLGSKMAARIVAIATVAFLAVLAPIWLSAAISEPFVIVLAD
VLPPLGIAFGLDAVSAGFALLIAVSGLLVLLYSFPYIPHAGEFKGKEKAEVRYYAIFLLL
LAGAFGLVMTKDIFNLFVFFEILCISSYLLVAYSQEERALEAGFKYMVLGSIGSAFMLVA
IGLSYRLSGSLAMADIAKALAAAPNSYATLVSVLFVLGFGIEAAVFPLNTWLPDAHSSAP
SSISAVLSGFVIEVALVVLVRLSLNVFSGAMLSVLAVLALAGILVGEMAALGQKELKRAL
AFSSIGQVSMMLFAFTLGSEIGKWAGLGQLLMHAGAKSALFLVSGYFIVRTGSHEIEAYR
GLGRKMPAAGAFFAIAALSLVGMPPLFGFFTKLRIVEAAAAAHSAIGYAGIVVILFATVI
ESIYLFRIVRTLYGANAEVAASGAASGDLPAFAWIAVAGFVLLVLVGGFMIPSFDRLLQP
MAAVLATI*
>SPBIB_v1_290171|ID:27163696| putative NADH dehydrogenase (Quinone) [Uncultured spirochete bib]
MLTSMLLILPLAGFLTSLAAAWIVPASSKARASGAVQKIIAGIGFAALFVFAFLAKSEIG
TGYLGIPLFGLWLNFGLTQLSWYFVAMVAGISLVTVLFSLQGLRDDGRYPFYYIWLFSKT
FGMLGVLLSADMLTFFIMWEVMSWCTYFLLQQGHEKAKRAGAGYLVYAVTAGMILLAGLL
YVYFNVGSFDFRTIQKAFSGFGAGRIIVAMLLVLVPMLIEAAAYPVHWWLPSAYANAETG
ISAYLAAISTRIGIYGMTMFLFAGFGLTALNLVTANKYVSANEVLMIIGAFTMVVPTFTA
LFQHDAKELMTWHSIGQGGYMIVGIASSTTLGIAGGLFHVFNHMTYVSLILFSIAAVEYR
TGTTNLNKLGGLIKKQPVAYLGLLFGIIGLAGIPPMNGFVSKWLIYRALILSGHPFIALA
AFIATLGTILSVYKLIHNMFLGQLPERYNDIKEVGWGMRLPIIFMMAVVWLTGAFPGTVL
AVVADIQQSLGLERVAYSVHGVAAEMGQLNMLVINIVFIGGLFVSWLIYLAGGKRKHIGQ
YDNYAAGHFLDKSIPYNYNYNFYAGMEHIFERLLHKPPVKRAEQALATFVSGTSEYVCRI
FTGNITTYSAYVVAAVVVILLVLGGGR*
>SPBIB_v1_290172|ID:27163697| Respiratory-chain NADH dehydrogenase, subunit 1 [Uncultured spirochete bib]
MSVLSIVVWIIAYPLIIFCIGVLLGGYERKFSARVERRIGPSWIQPFYDVIKLMSKKTNV
SHGYMHDVAILMLLGGTMLTLYFVPVPGFSYFSQFGDFLVISYLLLIPSLGMALGVGETA
NPNGSIGISRALQMLAGYEVPFILTFIGVAMKEQTTSMLDIIRVQQAGGFWAWGLIAHPL
LGVAALIALQGMLNEKPFEVIVAPHEIATGPMTEMGGKYLGIMFIQHLIAVPLELTLYIN
LFLGGASNWLEYLVKLFVLWTALISVNMVFGRFRVDSAVKFMWKISLPLAALGVIGIMLN
IA*
>SPBIB_v1_290173|ID:27163698| NADH ubiquinone oxidoreductase 20 kDa subunit [Uncultured spirochete bib]
MAKELTQKAAWDRVLAKLKKNSLWMLHYCTGCGAIELPPTMTARYDMERFGIMPMVTPRQ
ADILLITGYLSVKTLKRVILVYEQMQAPKYVVGFGSCTINGGMYWDSYSTIKSLDQYLPV
EMYLAGCMPRPEAIIKGFSWLIDRVGKGEANSWRDYYENYEFYRKNQEELFGGPIQSYQD
IPADAQKFGILEARQ*
>SPBIB_v1_290174|ID:27163699| NADH-ubiquinone oxidoreductase chain C (modular protein) [Uncultured spirochete bib]
MKRRVIDVTARLYKATSSAEHARSESEGAAQFVEDDVSNKMSLEEMRARIEKTAGFPGFV
VLQQKQKRIQGEIPADKLYGFAVTLKNTLGFEHLSAISCVDWIDEGRFELVYHFWNYEQG
ILVQAKISIDRENPSQPTITDLWQPAKFFERDIHEMFGVDFPGNEDLSKYILSDWQGPPP
MRKDFHTREFAHSQYHFKDYEPEWDDKVQGGYHSNHAVPDAEAQEVIMPGRKAHASSDRW
WKEAQHE*
>SPBIB_v1_290175|ID:27163700|nuoD| NADH-quinone oxidoreductase subunit D [Uncultured spirochete bib]

MSEKMNIVTVNDGRTLYPGASAPENREYNMFIGPNHPGIEGNFALKLTLEGDTIVQARTD
AGYLHRGFEKLMEQRLYMQNIAIVPRICVPDPDPNEENYARAIEAIAHIEVPRRAQFIRV
MMLELSRIASHLFAFGGFAGTLGLYTMPNWSISDRDRILELFEALTGARIYHIYIIPGGV
RRDLPEGFAQRVKDTLFYIESKLPEYDNLLFENSIIRKRSRGIGVIPPEKAMEWGVTGPN
LRAAGFAHDVRIDDPYEVYNELEFDIPIAHESDALARMLVRRKEVEESIRIIRQVLDKMP
SGPVWNKQPNPLKWKVPAGDAYVRTESSKGEYAYYMVSNGDVRPYRVHVRGASVTHGVHV
LEKLLIGTRIEDASQVMFSLDACPPEVDR*
>SPBIB_v1_290176|ID:27163701| 4Fe-4S ferredoxin iron-sulfur binding domain protein (modular protein) [Uncultured spirochete bib]
MFNPGSILKPLTALRYLGEKPDTIKVPFVERPAADRYRGFHINDLDKCIGCGTCAEICDN
DAIRMVPVEGKEAGVGRTQYRPAIDYGRCCWCALCVDVCTTGSLKMTREYIHISPDANSF
FILPNEKSIHGESAPKEGWTADKQVNFLDLDRVPMKELSAEERGDSFIEIVKGFSKEEAI
KEASRCVSCGLCTAVCPANMNIPEYIEAIWNDDIHEAGRQMYKTNPLSDVCGRICTHKCE
TACSIGNRGKPLSIRWLKRYAMDAIPQEQYLSLVDQNIVKKGGKKVAIVGSGPSGLSASY
YLALMGYEVTVFEAYPKAGGMMRYGIPSYRLPDAVLDKDIGLIESLGVTIKTGTRVGKDV
QFEDLHQQFDAVLIATGFHEGRSTKVPGTDHPMVFQAIDLLSRIRRGIEFPVEKEIVVIG
GGNVAMDIARSLARLQKQKYGEVHLIVTSLETRDIMPADKEEIEEAEEEGIVFHPGRGPE
EVLIEDSNIVGLKTSRCTRVFDEQKRFNPAFDKNDIEIYKGNMVVEAIGQGPNMSYLGDY
ATKLEYDGRRIKVTPYYQSSLGWLFVAGDIIKGPDVINGVAVGHKAAIGIDEYLAQTPDD
HVTTIDDCIKIAREYEKQEIRQLDALLKNKDLQEAEFGEGAAALGLVLQELLDQHNRNLA
QLLMLLGNENFRIHLRQELKRELRARDFSRYANLAPLPGTPGGAPFSEGDLVAFLALREQ
KGFELYNDIFYLSGHKEFEFIIESLREAQRKAVEKVEAL*
>SPBIB_v1_290177|ID:27163702| protein of unknown function [Uncultured spirochete bib]
MEIPLRLVARKDGSLFYGISLSFRLVMGGMLILLGAAIVLEEFRAGPIGWIAIFLLLLGV
IYKEDWLFDVNTKTVSGLVGFYPMLKKTQLTFEEIGYIQLAAFSKGTVPGSKEEESSNRD
AFESMRGKDSREGFKSSFDFARRKKLYIALLLITKNEEHYLLDMTPARRAIRLAQAGKAL
AALIGCSFLDNIAE*
>SPBIB_v1_290178|ID:27163703| Transcription factor/trp operon repressor (modular protein) [Uncultured spirochete bib]
MSRNPELMRESIAEMASALEGADSALIEQFFYSLFTVAEADEIAKRWALVKELAKGTPQR
KIAENLGLSLCKITRGSRELKKPGSPFRQLLDRLASLTATAADQNAQPDPSPPKE*
>SPBIB_v1_290179|ID:27163704| Phosphoglycerate kinase [Uncultured spirochete bib]
MQGPRLKIRPVTELELHGKTVIFRPDINSXIDPTTKRIVNTNRIEKTVPTLNLMLERGAK
VALIAHQGDTLDYQNLIPLAEHAEILSRLSGHRVSYIXDVCGPAAQAAVKALXPGEAVIL
GNLRYLAXEISTFETVVKLTAEEMXQXWLVRSLAPLADYYVNDAFAAAHRNAPSMVAFQE
ILPTXGGIQLMEEXTALKSVMENPRXPCVYVLGGAKISDXFDMMRKVLTDGSADYILTAG
VTGIVMHIARGVDFGPTITKFLADRALDTFIXEAKSLLXXXXSHYVLPVDFAYDAXVGQG
APXSAQAGTKPGQSTSHNSKPVRAEAPVGSLPKDRLLPDVGRQTIELFKEYIGKAGSIFV
NGPAGMYEHEPWSDGTREIWQAIADAPGYTVIGGGDTITAATRFTDLSKYGYVCTGGGAM
VRFLAGKRLPLIEAMERAFERNLQAPLRR*
>SPBIB_v1_290180|ID:27163705|gap| Glyceraldehyde-3-phosphate dehydrogenase [Uncultured spirochete bib]
MAKVKVGVAGYGVIGQRLADGVALQGDMELVGVADXAVTLSVRALREKGMPYKFFLAAPE
KREEFDKAGIPVSGTLEDLVQQVDVMLDATSAGVGAKNRLLYEKYGKKAIFQGGEKNSVA
DVFFHGYANYEKGIGAQFLKLTSCNTTGLIRAVDCIDRKVXVEKVAITIIRRVADPGDYH
RGLTNALQIDKAPSHQALDLMTIMPHVDATGILVHTPVTHGHIITVVATXKKSVTPENVI
EFFREHPRIRVVSIAEGFLGNASLFRYARDLGNPRGDMYEIAAWEDTVVMSGKDVMFAIN
VPQEAVVIPENMDAVRACMRMQLDRXEATDTTNRXLGIGKWRNPG*
>SPBIB_v1_290181|ID:27163706|iolE| Inosose dehydratase [Uncultured spirochete bib]
MEKNIRLAIAPIGWTNDDMPELGGEIPFEQCVSEMALAGFEGSEVGNKYPRDPEVLNKAL
KLRGLTICNAWFSSFLTTKPLKEVERDFVQQCDFLYAVGARVIGAAEQGNSIQGKPLPVF
DAKPRFTKDEWKRVTEGLNHLGAVAKQKGMRLVYHHHMGTGVQTTEEIDRFMEMTDPDLV
GLLYDTGHLVFSGEDHLAVLKKYLKRIWHVHLKNVRFDVVERAKREKWSFLQAVRNGAFT
VPGDGGLDFAPVFEILKNAGYKGWWVVEAEQDPALANPLEYAIKARQYIREKAGI*
>SPBIB_v1_290182|ID:27163707|iolD| 3D-(3,5/4)-trihydroxycyclohexane-1,2-dione hydrolase [Uncultured spirochete

bib]
METIRMTVGQAIVRFLDNQYIEFDGKVEKFVGGVIGIFGHGVVVGLGEALAAPGHNLPFI
QAKNEQGAGHVAIGYAKQNNRRKIMAVTSSIGPGALNMVTAASTATVNHIPVLFLPGDVF
ADRQPDPVLQQLEVPHDATISANDAFRPVSRYFDRLTRPEQIMTALIHAMSVLTDPAQGG
AVTIALPQDVQGVAYDYPVELFDQRIHHIERRPPTSAQVARIVSLIKSAEKPYVICGGGV
RYSEAGRALADFCKKFDIPLGETQAGKGSVPWDHPCNLSGIGVTGSLAANRLAKEADLII
GVGTRFGDFTTCSKWLFQNPKARFVSINVAPFDANKMNAEPIIADAKLTLLAMSRALGAA
DYKARWGDKIARAREEWAREVDSLYTQEDPMGLSQIRVIGELNDVLLPKNAIVVSGSGSI
PSDMQRTWRTRVPGTYHMEYGFSCMGYEIAASLGAKLAMPERECVAIVGDGAYTMLHTEL
LTAVQEGRKIIVVVCDNSGFQCIDNLQHSQGIAHFGNEWRKRDPETGLLAGEQVQVDFAK
NAESWGALGLRARTVEEFRKAVRRALRAKGPVVIHARVSPKSMTHGYESWWRVGTAEVSD
NPEVVRAAEEMKAELAKARKF*
>SPBIB_v1_290183|ID:27163708| Myo-inositol catabolism IolB domain protein [Uncultured spirochete bib]
MVLHHSGDYAEGYELLVLRSGDRAHMLMDFASLSLQKGTSWRSNANDERAFLLAEGMVEF
LVSTKVEWVGSSEDWIPDLKSIYIESLAEGGTRIRASRTSLLEENPIVLHLPAGTEVEIQ
ALSERAVLYVSATSNDARFAPRLYLPFECQTEFRNEGTLQETSTRIVRTVFNKTNAPWSN
LVLGEVVNQPGRWSSYPPHHHPQPEIYHYRFYPEQGFGMTALGTEAYLVRNRDTLLITDS
KDHPQVAAPGYAMWYLWVIRHLDGNPYISPEFNPDHVWVDKPGAQIWEMRRERQ*
>SPBIB_v1_290184|ID:27163709|yrbE| Uncharacterized oxidoreductase YrbE [Uncultured spirochete bib]
MSDKITVGVIGAGRIGKIHVSNIVYYMPEARVKTVADANLTPEIEAWAKSLGIPHVTKDA
EEMLRDPEISVVLICSSTDTHADYIIKAAHAGKHIFCEKPVDLTIAKVKAALAAVKEAGV
RLQIGFNRRFDHNFAHIRELAKSGALGQVQLVKITSRDPAPPPLAYVKVSGGIFLDMTIH
DFDMARFQAGSEVEEVFASGAVLVDPEIGKAGDVDTAIITLKFANGALGVIDNSRKAVYG
YDQRVEVFGSGGCAATENDTPSQVRLSDGSGVHGDKPLYFFLERYRDAFIAEMRAFFEAI
KTGSETPVTGNDGLMNLAIGLAAKKSLAEHRPVKVSEVL*
>SPBIB_v1_290185|ID:27163710| Xylose isomerase domain protein TIM barrel [Uncultured spirochete bib]
MIETRRFALNRIAAPALRLAEFFGLAAELGLSAVELRNDIRDGAVTNGLKASEVVQLARE
AGVRIITINALQQFNLPSARAKALDELDSLLALCKEIECPALVLCPNNRADDARSPEQKY
HDTVEALNTYAPLFSAAGVLGYVEPLGFSISSLASAEVALKAIHESGASCYRVLVDTFHS
YLGPDSPAMFDDPAVIQKIGLVHVSGVEASIPKSQFSDAHRVLPGPADVMNSAALMARLE
KAGYRGMYSFEPFSAEVQALGAKELASALRASIEYLSHA*
>SPBIB_v1_290186|ID:27163711| Inner-membrane translocator [Uncultured spirochete bib]
MAQQSDVGKFINRYAIFIVLVVMIVVLSFLSPSFLTAQNLINVLITESGRGILAIGVAFT
IISRGIDLSVGSIVSLTSVIAASLVQEPSYSARIFPNLPLLPPIVAVIAGLSAGTIVGIT
NGALIAYTAIPPFIATLGSMIIARGLALILTNAYPVPMLRPEFKIIGQGLLGPIPYVVIV
FALVALIAYVILSHTRFGKNVYAIGGNVNAARTSGIKVSKNLIAIYAVSGFCASIAGILI
TARAASGIATLGNNYELDAIAAATIGGTSHTGGIGTVPGIIAGILILGILNNGLLLLGIS
PYLQQVIKGVIIVSAVVFDMRKNARQK*
>SPBIB_v1_290187|ID:27163712|mglA| fused methyl-galactoside transporter subunits of ABC superfamily: ATP-binding components [Uncultured spirochete bib]
MEDYILEMKGIVRTFPGVRALNGVDLQVKTGRVHGLMGENGAGKSTLMKCLVGINPPDSG
EIWLRGKKVTIPNPYTALKLGIAMIYQELNPVLERSVMENIWLGREPMMRGAAGFLVDHK
AMYEQTRQLLSELEIDIDPKIKVKELSVAKMQMIEIAKAISYNADIVIMDEPTSALTPNE
VKHLFDMIRKLKERNVAVIYITHKMEEIFQIADEVTVLRDGNHIITEPIEKMTIDSLISA
MVGRRLTEMFPKMESKIGDVKMSVRHLEVPGLLHDISFDLRKGEILGVAGLVGAGRTELM
EALFGLRQRSAGEILIDGKKTDIRTPADAIKAGIGFLTEDRRLNGIIPVLSVKINTIVAN
LAHYTKFGVFLNHKKISKDCEEYKERLKIRTPSLEALIQNLSGGNQQKVLVARWLLTNPE
ILILDEPTRGIDVGAKAEIHTIITKLACEGKSIIMVSSEMPEILGMSDRILVVSRGQITA
VLDRKEADQETIMRYAAAKYNNNQVA*
>SPBIB_v1_290188|ID:27163713| Periplasmic binding protein/LacI transcriptional regulator [Uncultured spirochete bib]
MSIGKKLILAVIALTLVAGTAFAQKTYHIGVTLYDRDQFISSLEQAILEAAKKYPNVVVD
SQDAKQDVNQQMAQVDVFATKKYDAVIVNLINTDTVETIIQRARGIPVIFVNRRPRDAVI
DGVKTAYVGSQEYDAGRMQGEFLAKFFKGKTELRYVLFMGQLGLENTNERTRGVKETLEK

AGFKLVKVYEDTAEWDRWKAMNQMQQLLGTGKQFDVVICNNDEMALGAIEAMKALKMDLK
KIPVVGIDATPPAKEAVKKGEMAMTVFQNAKAQGAVALDFALKAAQGQKIEKFGWVPFEP
VTIENVAKY*
>SPBIB_v1_290189|ID:27163714| Oxidoreductase, NAD-binding domain protein [Uncultured spirochete bib]
MNKVKIGIVGLGRLGKRHAYNLANLVPGAELIAACSPIRAECEWAGTELGVPELYASFDE
MLEQASLDAVWIASSSTYHAQQALAALERGLHVFVEKPMGVTLEECAEIERAVQAHSNQV
FLVGFVRRFDPSYRYAKDLIEAGKIGTPFLVRSQTCDLDEYAEFQLEFVKTGGGIFLDMN
VHDIDLARWFIGSEIAEVHSVGGSFVHQGFATLGDADNTVCLATFKNGAIAVLSASRTAF
HGHDTHTEIVGTKGILKIGTTPRKNRVEIFDSAGARTDCVRDFYERFEEGFITEAREFVA
CIREGRKPEIRPQDGTAATRVGMAMTESFRKGTVVKLQI*
>SPBIB_v1_290190|ID:27163715| conserved membrane protein of unknown function [Uncultured spirochete bib]
MLVEKRFFPLYIVLFCVYTLFGISMTVIGATLPKIFADFGWSYTTAGAVIALGSVGYFLS
SYVAGILLSALGFRLTISFALLFIFIGLEFFAATPTPWLNMLLYFIVGVGQGGIELAVDW
GTLRMEGPGGGRAMSLMHGAFSIGAFVGPLIIAILISARLQWTLVYRGIGVLFFLIAIFL
QFMPLSALGRDRGHAPGSSRRDLFKHPAYWLGFFALFVYVGVELGISNWIAEYFVSVFKT
PIPTGSFMVSLFWAGLLLGRFGLPVIQRSITREKILISLSVVMAVSVIGLATVGFLGTGA
LLNVIAGMLVMLSGFGCSIIYPTVMSIIGDFFPHTQSEAVGFAAMGGGMGGFAFPYLMSG
ISAAWGIRTGFLTYALFSLVIVALNVSIVRAEHNGGIIKST*
>SPBIB_v1_290191|ID:27163716| protein of unknown function [Uncultured spirochete bib]
MKADRRRTADYRALILPTEGAFFKQSLDENLFRSYDSII*
>SPBIB_v1_290192|ID:27163717| Transcriptional regulator [Uncultured spirochete bib]
MTSIKDIARLAGVSPSTVSRVLNEREYVREEVRQRVLDIVKEQGYVQNHVARSMVLQRSF
TIGVVIPYLFNMFQRQLFASMEFFLEQHGYKTQFFFLRWSEASEQAFLRRLKSETLDGII
FIHELDNPIIYQYIQERELPVALCTFEKSEFGFPAVHVEEEASASLAVNYLLQKGHRNIA
LITGEHFSFGRQREAGYKKALAAHGLQVNPELIIRAASYNPEAGRASMLTLIKRKQPMSA
VFAVTDELAIGAMKALYEAGLRVPEDVSVIGLDDIDISAFTTPGLTTVRQPIFEMGKKSA
DLVCDWIAHGRAQGSAELFSTTIVERESVQARVKE*
>SPBIB_v1_290193|ID:27163718| protein of unknown function [Uncultured spirochete bib]
MDELMQAIKNAMKSEIDSVTIYSEAALRAEGDVAQFFALRADEEKRHYNWLLSYYKELLG
GQMPSTNLAAEIQTVQHRSPIFSQEFLTRVASDRHVSAAVSSAVLLELNATQHYQKMAEL
AKEPHLKAFFNSLSEWEMRHYEELLKIQEESRQFWFEEQQFEPF*
>SPBIB_v1_290194|ID:27163719| ABC-type transport system, periplasmic component [Uncultured spirochete bib]
MKRMLRALFFILFALGLAGASFAQAKKPALKIAVSIPPMQEWVSRIAGDRASITLVLPPG
SSPHAFEPSPRQLAELGQADIWFTISVEYEYSLRPKVSAMFPKLSIVDVTKNVKFRTLRP
GEQETGEPASVGQLDPALTNRDQHTWLGYEQAKAEITFIRDTLIAKDPAGTEVYKKNYDA
YLKEIDAVYNSLRTKLAPLAGSKVFVYHPAFGYFLDMFNITQEAVELGGKEPTQKELTAL
VELAKKERAKAIFVQAQFSQSAAQAVAKAVGAAVVPIDPLASDWLANLGRMGQALLNAGQ
RGK*
>SPBIB_v1_290195|ID:27163720| Sulfate-transporting ATPase [Uncultured spirochete bib]
MRGKGVSSMGSAVAAKVAAETAAASTAGAVAAPSAAGATECADSINAAEEHLPHLPENVA
IWCQHLAFRFKDIEVFDDVSFHVHRGEFVALTGKNGAGKSTLLKLIVGLLEPSRGKVLVF
GAPPRGRIESIGYVPQHAGFDPAFPIQVEEVVGMGRLSGLSPIAKHQKKEEIEWALRQAE
VHDLRARPFSALSGGQRRRVLVARALVGHPKLLVLDEPTANMDMDSEKKFYETLARIKGT
TTVLIATHDSDFVSALADTVLCVGQNVDHPHSVHRHAIERAVNAPAELYGGEVVRVRHDI
ELPDNACCEGKE*
>SPBIB_v1_290196|ID:27163721| ABC-type transporter, integral membrane subunit [Uncultured spirochete bib]
MSGFLQALLNPAVPFVRNALIGSVLAAFLFGVLGSLVTVRRIAGLAGAISHTVLGGIGLA
LFLASRGAPKWISPMLGALVFAILAALTISVVSLKAKQREDTVINALWAIGMSLGVVFLA
KTPGYADPMSYLFGNILLVTSGDLYLLAALNVIVIALVARYYRHIEAASFDEEFAATKGV
PVTAIYLGLLLIIAIAIVLLQTFVGIVMVIAMLTLPAGTAGYFSRNLAGMMIHATILSLV
FSVSGLAVSWTFDLPAGAVIVLLSGAVFLAFSIGNMLSKRSKRKADQQTGLEGSA*
>SPBIB_v1_290197|ID:27163722| Ferric uptake regulator, Fur family [Uncultured spirochete bib]
MTKARMHVLESISRAKEPLSARQLCERYAQEHDPATIYRALHYLEEKGSLDSFILHCSEH
GTERYYVVHAGEHRHWFHCEHCHRFTDLGLCRFDTLVDQMSKEKGLSVTSHTFYATGICK

ECRDKGF*
>SPBIB_v1_290198|ID:27163723| NQR2 and RnfD family protein [Uncultured spirochete bib]
VIKFLKQPMMRKVLIALAPIYLFSIWMYGLRVVAAAVVVFVCGIATEWLFERKRQGKVSE
AVLVSCALFALAFPPKTPLWILAVGIIFAVAMAKGVYGGFGRNIFNPAIAGRAFVYISFA
IVLSRAYTGFGNFGIGAVDVLSSATPLAQMRAGAKVPLSSLVFGLRPGALGESMTLLIVL
AGVYLILTKTASWKIIVSTLVGGAVTNLILLAVGAAKALPMESLLAGSFLFMSVFMATDP
VSAPKRQQSHYVYGALIGATAVLIRTFSAFPEGTSFAILFGNTFANLIDIAVDSLVKKAP
AQAKAPAKPEGGA*
>SPBIB_v1_290199|ID:27163724| FMN-binding domain protein [Uncultured spirochete bib]
MKLNRNSVIYAAVFTFVVCVAFVIILAIANQVTLARVNANKRLESRTAVLKAFGLADSTT
PSSEIDSKYTQLVTEKSVEKATAYTATIDGQTYVAVKLTMPGLWGPITAVLATDPAGTVV
RGFEIVDQQETPGLGGRISEPWFSAQFKGKKVLPDGTIAFEQGSGKGNFDPNNGTVDAIT
GASRTSDFVRALVNRSLALARQIGGTL*
>SPBIB_v1_290200|ID:27163725|nqrD| Na(+)-translocating NADH-quinone reductase subunit D [Uncultured spirochete bib]
VSDTGKTSAPAKVSPAGKVMRDGLWYNNPIFVQVLGICSTLAVTNNLRNTLIMVLGVSFA
TSMGSMTLSALRELIPRKVRMIVQVLVLSFYVIIIDIVLRAYQPAISKQLGPYVGLIITN
CILMGRAEAFAASNPPGLAFLDGLANGLGYGWVLLVIAIVREFFGFGSLFGFKLVGDWFT
PWTIMVMAPSAFFLVAIALWIANNYKAKVAAAAKAAAPGAAGAPAGQGGKP*
>SPBIB_v1_290201|ID:27163726|nqrE| Na(+)-translocating NADH-quinone reductase subunit E [Uncultured spirochete bib]
MIDAPSVGLLSLFLASILTSNVLLANFLGTCSFISISKDFKSSMGLGVAVTLVIGITAAV
CWAVLYFIIQPLGIEYLSFIIFIIVIAGVVQMLEMIIDRFSPALYLALGIFLPLITVNCA
VLGVILFMQIRKYSFPQAVIFGFGSGAGWWLAIMLLASIRKKLDSNNAIPAGLKGTGITL
ITIGFMAMAFVGFSGMIAVQ*
>SPBIB_v1_290202|ID:27163727| Ferredoxin--NAD(+) reductase [Uncultured spirochete bib]
MSPILVGPLTVAGISAVLALIIAVLDKALNNYGDVEISINGGERKLEVKGGSPLLSTLAA
ENIFVPSACGGRGTCGACKCKVISDVGPHLPTELPFMSPDEIAHNVRLSCQVKVRKNIEI
ELPRELFSIKKYKTHVEKIRDVTHDIKEVLFALDSGEIDFVSGQYVQLVVPPYGDIKEST
QRAYSMSSKPSDKKHVELLIRLVPGGIATTWVHKFLKEGDAVELVGPFGEFRVHDTPAAM
ICVAGGSGMAPFKSIFYNLYETSAFPEKDIWYFFGARTSKDMFYLEELRELQAKWPRFHL
VAALSEPSPEENWAGDTGLITDVLDRYLQTVIPKNDKGWEGYLCGSPGMINACINVMKKN
GITEDKIYYDKFA*
>SPBIB_v1_290203|ID:27163728| conserved protein of unknown function [Uncultured spirochete bib]
MKQSPADKQAFERMAPGIITAAGFLGFDERSPDRIIAEDERAFEELGLDFNNVADRLEEL
AHKGEAGLGEPITVGELLVQSGDARGMLPCPWEDGFFHKNAVSVSPADTPLEACVEGEDM
LIYSELSIHMLRAHHFCQGRGSPFRLEPTLLKQLLF*
>SPBIB_v1_290204|ID:27163729|pyk| Pyruvate kinase [Uncultured spirochete bib]
MEKRTKIVATIGPACESLATIKDLIREGVDVFRLNFSHKTYEEATRDVTMIHEARKALDR
PVAIMADIKGPAVRLYGYAEEIALKPGTELTIESADPAGIEKLVSSKPLHVYTNLPDIDR
ICAIGQKVILMDGYFSGKVVRKAPKAVVVAVENEGGLRPKAHLTLPGVDYPIPFLSEKDI
NDISWAVEKDIDYIALSFVRCATDVEEVRRLVQRTALSQGKTTNIKLIAKIESSRGLENI
DEIIRAADGIMVARGDMGVEMPIENVPIAQKQIIRKCYLAAKPVITATQMMESMMENPMP
TRAEVSDVANACFDSTSAVMLSGETAIGKYPVQVVRMMRTVVETVEKEFDYIDFHHDVPP
EVQNGDIPAIMSYNAVSVAYRCDANALIVLTETGHAARLLSRLRPRMPIYAFLTNERLYH
QMALNWGVEPFIHSGKGTRLDAVVAEAISICKRKGLLKSGDRVVIVAGLPLAHQGTTNMI
RVETIE*
>SPBIB_v1_290205|ID:27163730| putative tRNA/rRNA methyltransferase (SpoU) [Uncultured spirochete bib]
VKSERAAKGAMLDSGQAAVMKSGDMRPEIDLELLSKLKEKNLAALGLIVLEGRIVIEKAL
EAGVVPRLLVCTEAEEEYWQEKSRNRTVVQGSAAPQTRTPAQNSSKPQTGASSFPVRVMN
HEALCAFVDFKFHRGAIAIAEMPRIRRFEAAQNELSASQASMLSMARLPRPDDSVARTIY
ERSREAFLCLWDITDPSNLGALIRTAAGLGASGILLGPGCANPYYRKAIRASMGNAFSVP
LWSADLALLDTLRLGGAEIVGATLSEQAVSLDVWAAQQGGFLILILGNEGYGLPQEVLSR
CTSEVSIPMARGVDSLNVAVAGGILMYELFGKIRGTEKESQQKALKKVPKLGINGDRYPD

SVDLIREDREQ*
>SPBIB_v1_290206|ID:27163731| Extracellular solute-binding protein family 1 [Uncultured spirochete bib]
MKRKSIAMAFLLIVAFGFLAVQGAFAQKSRLVFMTAGDVNMLALGQNVFAPGFMAENPNV
QVITIHTGPGNAGSQTIYEKLLAEKKANKAAGDVDVALVHEIFMKWSMDQDLLQPYAQMT
NTWKYVTSPFAKTSLGINIEGYVMPLFQSQTAIAYNPDYVKNPPKSYAELKEWVKKNPGK
FGYNGIKGGASGVAFVMGWVYAETGKYKKYAVTGPFDKAEVDSWEPAYKALKEFNKNIVI
TAGNVGTLDALNRGEIWMGPVWVDMFFTFMNEGKLDPKMRLALPEPGMPGQPMFFVIPKN
APNPELSRKFIEYVTSPKIQANEIVIRFNWYPGIDGSALKGSIPQEVFDKIYRDVTPADL
QKKGLAFPLADYYNAMLEAYERWAQ*
>SPBIB_v1_290207|ID:27163732| Binding-protein-dependent transport systems inner membrane component [Uncultured spirochete bib]
MTSLQPKMWGERKLPPYVLLLFPAVTFMVLMYAWPFISSVLQSFIISGKEGGLTLANYVK
VWELYRKDIAFTFAVTIFNTFLVGVVAVIFSVYFRLRQNRVSALLSAIFKLPIFIPFVVV
SQMMNSFLAPHGLLNTALAQLGLVNLDKPLQLFNFAGLSFGFAWKQIPFAVLIIHGGFQM
IDNSYIEAARSIGAKLPSVILRVLVPMNKSSIIVALVLVYSQIIGTFTLPYMLIGGKVPT
TITVDIAHRVNYFRDFGVANALGVCSYIMVLFTAAYYLRSKIKEARQEVSQ*
>SPBIB_v1_290208|ID:27163733| Binding-protein-dependent transport systems inner membrane component [Uncultured spirochete bib]
MKRILKKSAELALLVTVFLIVIGPMVSLFLWSVAQKWYWPHPLPQAITFDYWAQALGLKT
SLAIGAVSIVSAFTLSILIALATVVIAMAIAIPAGYALAKYKIPFGTIILFLFLLPNAFP
QQPVFVNILQVFTKIGLSGTIPGVMLIHILVGLVYGVWITNATFRSIPPSMEEAARSVGA
TAFKAFYKISLPLAAPGLFSSAVFVFLTSLDEFTGTFFIGLPFINTLPLTLYSSSGYNMQ
FASVIALILLVPSVIFMLLIQKVLKAEYVGGMG*
>SPBIB_v1_290209|ID:27163734|fbpC| Fe(3+) ions import ATP-binding protein FbpC [Uncultured spirochete bib]
MAELVLKDLVIKYGKVTALNRLNLTIKDGELVTLLGPSGCGKTTTLRSVSGFLPVQEGDI
FLNGKRITDIAPENRGIGLVFQNYALWPHMTVFKNLAFGLELRKLPAEEIKKRVAAVLAS
VKMDEYADRFPRQLSGGQQQRIALARAIILEPDILLLDEPLSNLDALLREQMRFEIAAIH
NRLGITTMYVTHDQTEAMVISNRIVVMRNGKVMQEGKPEDIYMRPRNKFVAGFMGTTCFL
EGIVERFDGERCILKTNDGFELYGIGTELQIGHKAYAAVRPEYILLGKRAEASYGSSERN
CFKAIVDRSNFTGELLDYQLKLGSNFIRAKEYEAKTIYGIGQTVDVKIDEERLPIVHDTD
PEAVAV*
>SPBIB_v1_290210|ID:27163735| putative Ribonuclease Z [Uncultured spirochete bib]
MRLCFFGTSSAIPSARNGYTSFLIELANKTVLVDTGDNAIRSMLEMGRDPMMLDAIILTH
EHADHLGAFPAFIAALECMNRTKKLLVIMQPELEKRVLTLLALFDYYPEQLHFELEFAAE
WHTDGLKIDLLPGNHSKFTMMPRFVADNRTLLYTADTRYKAGQYAALGAACHTLIHEATY
SHPKLPQDTRHSSALEAGMAASEIGARNLFLCHFQDDAYKNPTEPAIEAAQSFGGEIIVP
GLMQWYTIKED*
>SPBIB_v1_290211|ID:27163736| putative HTH-type transcriptional regulator DegA [Uncultured spirochete bib]
VTNATIEDVAKACGLSRATVSRVINGEPNVKPLTIEKVKKAIAELGYHPNIHAQALSGGG
AKTVGIMLPRIWRSYYSSLLSSIEEVAAQEGYYALIMAKDYLKNAERIFDEGRVDGFIFR
NMDHVEEHERLFLRLRKRNVPFVLIGNPLRDYPAITIDNVGGGRAVAKHFAMHGFRKIVY
LGGPSDNVDSNDRYFGFRIGFEESGMNPEGILRINGDYSTKSGYNAISEIMLTQKPDAIF
AANDRMALGAILYLHKNGVCVPQDIAIAGFDDSYFAKYITPALTTVRQPFREMGTAAMKQ
LCSMLKGSSANPGKIILPAKFIVRASCSCPYSHDDSLDVLDAVENSE*
>SPBIB_v1_290212|ID:27163737| putative Major facilitator superfamily MFS_1 [Uncultured spirochete bib]
MEHTSYRVYPYRWVILAATMLVNFTIQTLWIAYSPITSAAAAYYSVSEMAVGFFAMSFMI
AFVPFSIPASWLIDRFGYVWTVGAASMAVGIFGLLRGAAGHNYALAFAVTCGIAVAQPFL
LNSWTKIPAAWFPARERATAVGLITIASILGTAAGLVLTPMLAERMSIAQVQLVYGVAAS
VAAGVFVLAAREKPRLPPDESACRERAFMIEGLKHALRTPSFVRFLVIAFIGMGIFNGVT
TWVEGIVRPRGIGSSQAGMLGAIMLVGGVAGAVIIPFFSDRSARRTPFIILGLAGAIPGL
LGLALAPGFALIAASAFVLGFFLIAVNPVGMQYAAEVARPTPEGTSNGLVTLAGQVSVLL
VYAMEAINNATGSFTTSLMAVTALLAFSVFWGTRLEEKTGKKES*
>SPBIB_v1_290213|ID:27163738|yoeB| toxin of the YoeB-YefM toxin-antitoxin system [Uncultured spirochete bib]
MKLLFSEHAWEDYLFWQNSDPTMLQRINQLILEIQRTPFSGKGKPEPLKFALKGYWSRRI

TEEHRIIYKVVEDTVFIAQLRYHY*
>SPBIB_v1_290214|ID:27163739|yefM| antitoxin of the YoeB-YefM toxin-antitoxin system [Uncultured spirochete bib]
MEAITYTKARANLAKTMDLVCDNHDPVIITRNSDQAVVLMSLEDYQSLEETSYLLKSPAN
ARRLLESIQQLESGHGIEHSIDS*
>SPBIB_v1_290215|ID:27163740|glpK| glycerol kinase [Uncultured spirochete bib]
MRYNTPMDRNSNYILALDQGTTTSRAILFNHDGIIIGVKQIPFRQIFPKPGWVEHDAEEI
WQTQLAAARGAIEAAQIESGQIAAIGITNQRETTVVWERATGKPVYNAIVWQCRRSSGIC
DELRGRGAEPEIRARTGLVLDAYFSGTKLTWLFREYPELRARAERGELMFGTIDSWLIYN
LTGAHATDPSNASRTMLFNIYERRWDEELLRLLEVPPQILPEVRPSSGIFGKAKRELFGA
EIPVAGVAGDQQAALFGHACFEPGDVKNTYGTGCFTLMNTGPFPVESKHNLLTTVAWDLG
KGYTYALEGSVFIAGAVIQWLRDQMDLLADSAESEQLARSVEDSQGVYIVPAFVGLGAPY
WDSEARGTVVGLTRGTSRAHFVRAALESIAYQSMDLFEAMKKDSGRALSCIKADGGASAN
SFLMQFQADITGTRVILPEVSETTALGAAYLAGLAIGYWSGLDDVRKNWRMRREFMPRMA
EEVRAELIDGWRKAVATARNFR*
>SPBIB_v1_290216|ID:27163741| SagB-type dehydrogenase domain protein [Uncultured spirochete bib]
MQYERFEGRWFLKSNWHLVKSGQSDQMKGVPVPLQEEPPAPEDCVISLPPPDALLAHDGG
AAGSVASNALSTALYRMLVGRKSRRKYSKEPLSLEELSYLLWAIEGVKENKGKFSFRTTP
SGGARHPLDVYVFAHKIKNLNVGLYRYLPVEHQLVLERQGDDSEALDEALNGQFWNAACI
VMWAAVPYRSEWRYGKAADKLVALDAGHSCQNLYLACEALGLGTCAIGAYDQEKLDAYLG
LDGEDMFALYAAPVGKLQGGA*
>SPBIB_v1_290217|ID:27163742| conserved protein of unknown function [Uncultured spirochete bib]
MTIDTVKLAFPEDCNIIVGQSHFIKTVEDVAEIMVSSVPGVKFGLAFNEASGPCLVRTEG
SDPALIEAAQKMALSVGAGHTFYLVIGPGAYPINVLDRIKASPEVCRIFCATANPVEVVR
SVTEQGAAILGVVDGFAPKGVEGESDKAARKAMLRKFGYKF*
>SPBIB_v1_290218|ID:27163743| Achromobactin ABC transporter, ATP-binding protein CbrD family protein (fragment) [Uncultured spirochete bib]
MSPERLPRHIPGIPETSRGLVEPQLAGSRLAGITFKQNEAPGRASLEASSLAIGWEQGRK
KHILAQNISFKVYAGHLVALVGPNGAGKSSLLRTIAGLQAPCGGLLSLLGKNIAQIPVEE
RASLLACVFNERMESGYLTVSEFVAFGRYPYTNARNRLTSEDKHKIAAALALVGMNSFAQ
RTFVSLSDGEKQKVQIARAVAQDTPVLVLDEPTAFLDAPSRIEIFRLAERLAQEAGRAVV
LCTHEVDLALKTADELWVLDREHRFTAGAPFVVARSGAIGRAFDLPTVAFDSLTGTFRPR
SAR*
>SPBIB_v1_290219|ID:27163744| Transport system permease protein [Uncultured spirochete bib]
MLVWLGLCVLILALIDISIGSVRIPLVDVWNTLAGKPPSPEWAAIIRIFRLPKLATAIVA
GASLSISGLVLQSIFRNPLAGPDSLGIGAGASIGVAVLMFAGSAVGALGAGAGGVGVAGG
ALLGNLPALGYTLLVVAASAGAGAVLLVILVISRRFEQVVTVLIMGLLVGYLTSSIVSLL
VYFGSPQKVQLYLAWTYGSFGGVRTGELPYLAGAAALGFVLIIGETKALNAFLISERFAS
TIGIRVRTSRTKLLIAASILAGSVTAFCGPIAFLGIAAPHGARMLTRSAEHRILVPSAAL
IGICFALLADILSSLPGKGAVLPINPLLALIGSPIIISMFFRREKGPIGEEAR*
>SPBIB_v1_290220|ID:27163745| Periplasmic binding protein (fragment) [Uncultured spirochete bib]
MRRTVGIFLAFLFIASTCAFAQSGGLQAGSQTGAQIGPQTSVQIAAQAGVPAGSQIRAGF
SIEQRTGYKILTVSNLWPGANATRTYVLYPRGSQPPKDIKADLFIQVPVQRVVLYSTTYI
PALEGIGELEAIVGVDNASYVYSPALRERIQAGKVIETSKNWMPDIERLIALKPDVIFNY
GLGNEWDTFPKMQEVGLPVVLLGDWNEQDPIARAQWAVFIAAFFNKEEKALERVNALAKA
YTTLKSLAAGSATRPKVLVNGPFRGIWTVSGGQSFMARIIQDAGGDYLWADSKDTGGLNL
SIEAVFERAMRADIWLNPVYGANRIADIRALDPRFAALPVLQKGQIWTNELRMSPGGSND
YFESAVMNPNLVLADLIAIFHPELLPGHKFMYYKKLNE*
>SPBIB_v1_290221|ID:27163746|proB| Glutamate 5-kinase [Uncultured spirochete bib]
MQLSELAAEAAAKAETAATEVETKATPEATAEAAAARAALAKANRIVVKIGTATITKPAS
TPARLTRAFSPDGKSFLPEESATAGGRTNIDTAYIYHVAEQFAGLVQEGKQIILVTSGAI
GMGARELGLTKRVTEVRMRQACAAIGQPILMEEYRRAFGVFGLVAAQLLVTRDEWDDRAS
YLNLRETVETLLESRVIPVFNENDSVSTAEIGNAFGDNDRLSAYVASKIDAELLIILSDV
DSLYDSDPRENPNAKPIPYVRELSEKHLAAAGGRGSEFSTGGMKTKLAAVAIARDAGCRV

VIAHGREPNVIARVAAGEPIGTLFDAAHALKNRIRWLKNSQPRGRLTIDQGALAAIRERN
SLLPRGVIAVEGDFGRGAVVLVNGVVKIISNFSSAELLAVMGKRSDEIDALLGPDAPHVV
ARPEEMAFLDE*
>SPBIB_v1_290222|ID:27163747|proA| Gamma-glutamyl phosphate reductase [Uncultured spirochete bib]
MNMQERLEHASRAAAQLLSVGIEVKNAALMRIAQKLEESREEIARANQRDLAQAQASGLP
APLLKRLVFDDKKLADVLAGIRDLLAMPDPVGRVLEARLLDDGLVLRRVSCPIGLIAMIF
ESRPDALVQMASLAVKSGNAIVLKGGSEARASNFALASIIARAGEEAGLPQYWLTLLESR
EEIGELLMYDQYVDLVVPRGSKEFVARIKATSRIPVLGHADGVCHVYVHEDADPAMAAAI
AIDSKTQYPASCNAAEVLLVNFKFGGLTPLLKFLAQAGVVLDVCPRTARMISPDIPFNLK
SDDDWSVEYLDLRMAVKIVDSLDEAIAHINRYGSGHTDTIVCASPLAARTFMQGVDSASV
YHNASTRFADGYRYGLGAEVGIATGKLHARGPVGLQGLLTYKWLLEGQGHIVADYASGAK
RFLHKDLPLDESPHAQ*
>SPBIB_v1_290223|ID:27163748|ykwC| Uncharacterized oxidoreductase YkwC [Uncultured spirochete bib]
MPQKTIGFIGLGVMGGAMAGHIRASGERLLIYTRTKSKAQMLLDAGAEWRDSPKELAHEC
DVIFTMVGYPSDVEEVYFGPEGLIENARPGAILVDATTSRPDLAVRIYEAAKARGLGALD
APVSGGDIGAKNATLTIMVGGDEEAFQSVKPLLEVMGKTVIRQGGPGAGQHTKMANQIAV
AGNLLGAVEAVTYARSAGLDPRRMLLSIANGAAQSWQLSNNVPKMLDGNFDPGFYIKHFL
KDLRIALDAAHVMKIELPMVALAERLFAKLVAEGMGDLSTHAIYLLYERGLV*
>SPBIB_v1_290224|ID:27163749| protein of unknown function [Uncultured spirochete bib]
MSDAYDQWQRIFASSAAGFVVHVDGVIADINETAIKILGGADKNDFIGKRFMEDFIHPDF
HPIIKLRREQLKSGGQVLAPVEMRIRRLDGFLVDVLSAATGILSGESLYVEVLLIDITSV
KRRERLLEAFSEMMNITTEEQNPLDIKGAMRHLQQAMQCLYPESAYAGYVSFFSMAKHLP
FASRTMQKYMQPTVYTFSPLPAGFEKWLMDTASDYSQPWDDDSALTAIIDIPSSVENEGK
AVLQPFVMDGRAFGCFFWTFKKGGIPIRLDEDKERLKAFSLAVLSSIKTFFLRQENVQRT
SDLGILHQATLQIGKIDNLQDIANAVLDILQKEKGWRPAVIRFKSRTADILETTACRPAR
AMTEEELRLYIRKINDLVRKPGQGMTGYVIEHGEPIRSLDLPSDPRYIETDPGIRFGIYA
PIPIEGKVEGAIGVESADYAFSESDLAFLSSLGELTGMAVRSVRLIEVLSERVRWLEILH
EINLQVGVEAKPEELYGILINRAMKATGAESGALLIYDAEQNVLKSHAARGWLKTITKEP
FTARDGITGTVFSTGRTRLSPHLEDDPLLLSRSRAFVPAGRANIAVPVKAGGMVIGVFHL
AMKAPVSFSQEFIELVEMFGSYAGIIIRRIQLIDAQRSAQSELRKAYDETLEGWARAIGM
RDDETLRHTSRVVKIALAIGKAMHLDAQSLEDLRRGALLHDIGKIGIPDSILRKPGSLSK
EEQRIMQTHVSFANELLKPIKYLERAIVVPYSHHERWDGTGYPQQLKGEDIPLLARIFAV
ADVYDAMTSDRPYRSAHTKDEALIYVRTQAGKHFDPKVVEAFLSVVDSIDAQMDEQ*
>SPBIB_v1_290225|ID:27163750|thyX| Thymidylate synthase ThyX [Uncultured spirochete bib]
MQDSTIKTARAGECANRTVRKLALQALGLFCYNAPMAHCIVPEAEAILDKEFPVLDKGFV
RLVDYLGGDQRIVQAARVSYGEGTKSYREDAALIDYLLRHEHTSPFEQVVLTFHIKLPIF
VARQWIRHRTARVNEISGRYSIMKDEFFLPEPEALAPQSEDNKQGRADIPFDPETANRIR
AILKEGGELSYQQYQRLLDLGLAREIARIVLPLSLYTEWYWQIDLHNLFRFLMLRLDSHA
QREIRQYAEVILDITRKVAPAATASFEKHILHGVRFSGPEMDELRRRLGLGQHDRADSAE
SAPGTNQPPQFSEPAANQPSSSSVSLLSKKELERFEEKLKSSRQL*
>SPBIB_v1_290226|ID:27163751| putative Glycoside hydrolase clan GH-D [Uncultured spirochete bib]
MNPTIEATFQKIPGDVLKILFRAREDFSCDEPLLELGEFEVRELCHALGNECRAAEEASG
QKAVALERSRSRFVWINGWQSWSFAGEIARMERPRRAFYKRVLNVFVDHPAEVALRQRAR
RFLHRHDIISHFMLGLRSGDLRLALVSDNVARYMGTSAIGAAGVGRASGTASSGSATDEK
AGDSAVSGARLVDTFGASLYLPPISFLLRGNKIRIFAYAEGGNFRRGQIVARVAVLIAPG
YFALKDKIAGLWGAHGRFEDLRWLSLEAQSAKGAGFEGGGASSGARAGQGSEEPAHPFQG
VIGGYESWYNHYTAIDERIIGADLASIGANNNIVNNYFIRRGRPTVFQIDDGWELAIGDW
EAHPEKFPSGMAALACRIRDKNLIPGLWLAPFLLMPDSKTAKAHPEWILRDRDGAPVRAG
WNPNWGGDVWCLDLSLPEVEANLASLFDMVVNEWGYRYLKLDFLYAGLMRGAFAGRKGGA
WEHYARIMARILEFSIAADGSPVAFLSCGAPIESTAPFMPLMRSGADTREHWEWPQLRLI
GHQGRPSAKLNMQDSIGRAILDKTLLLCDPDVIFCRTERTSLKDTEKFLVGMVAAMFGSQ
IMSSDDPAGFGRVPPQSGQLSEPEFTNQILDWYQRIESKKFGVERSSVRVREVYRFFSRE
KAVYGMINLSDREQFSDGAPVPKHSMLIFGA*
>SPBIB_v1_290227|ID:27163752| Inorganic diphosphatase [Uncultured spirochete bib]

MSKTVYVIGHKNPDTDSVVAAAAYAALKRAQGQPQVKAARAGAVNPQTEYIFQRFGVPLP
EFIPDLIPKAEYYIDELPPVIREHTPLWEALVLLEQSPRQVMPIVDAIGAYKGLFYYNAF
AKNILTKINPHRKAVIPTSIGHLIDTIKAQPLVPTDCEAMFNGRIVVASLSAERFQDYIH
AEPAHNKVVLVGDREEVMRIAIEAGVRALIITNGFIPSKDITKLAEAKGVAILISSYDTS
STSLLALYSTPVISVADASIKPIGPRDFMKTAKLAIAASPARAVPIVDDGGKVVGLLTEG
DLIREPNIELILVDHHEFSQAVDGIQNYHIQEVIDHHRIGTFSTPYPITFINRVVGSTST
IITSMYRESRTPLDRAIASILLCGILSDTLVFKSATTTDTDREMADYLASITDLTIEELG
RDIMGSASLAARLPIDQLLRMDRKEYEVQGKKLTVSQIELTNSQELLARQEEVLQGLAHI
RTEMGAYLAALMATDITKFESILYIDADREFYAYLSYPMQQPGIYILKDVLSRKKQLMPA
LTEMVQAALG*
>SPBIB_v1_290228|ID:27163753| TraB family protein [Uncultured spirochete bib]
MPQTIRTIQLGERQIILVGTAHISKESIEEAKETIRQEEPGRVCVEIDMGRYQAIQQDSR
WEQLDIIKVLKEGKGFLLLANLALAGFQKRLGADLGTKPGEEMMAAIETAQEMGIPWSAI
DREVQLTLKRAWAKSNLWNKSKLLASLIESAFSREKVSESDLEKLKESNELESMMNELAE
FMPSVKEVLIDERDRYLATKIFETTEQKVVAVVGAGHMNGIEQWLGKLQRGEVAADVSDI
EDMPKPGWFAKSAGWLIPLLIVALIAIGFFRSGSQASLAMIERWILLNGSLAALGSLVCM
AHPVTIIASFLLAPVATLNPVLAIGLFAAVIEAYFRKPTVQDAENLADDVTSFKGFYKNR
ITHILLVFFLSSIGGMIGNFIALPILASRAIG*
>SPBIB_v1_290229|ID:27163754| putative DNA polymerase III, delta subunit [Uncultured spirochete bib]
MAEALIPPVWILAGPEIGLKEAFVADIIGKARKLGGDEPEVHRIYAGDTLPLEAISLLQN
SSLFSSWVIVEFRNAEQLSQKADIDALASYCGAPAENTVLLLETDGYSVPKAIEKTVPPS
CRKTFFELFENELAGWVRCELSGAALTLDEEALESLLELVPHDTAALKAACLILSASFPS
GTKLAAPDVEAAILRSKPEDAFTLFDRIAQGDLAVSLEVLNSVLDSRRGDANQIIAALVW
SFRRLQRIGEALARGERFEDVCLREQARSKTIQRQIRAALQRYSPADCRRIIVALSETEG
AIRGPLGSVFDRQLLHLLITSIITEKGQGLAAAGWSEQGIYPHLVL*
>SPBIB_v1_290230|ID:27163755|lexA| LexA repressor [Uncultured spirochete bib]
MKDLTSRQKEILNFIHEYIQTNNYPPTVRETARAFSISVKGAYDHLKALEKKGHIKTSEK
RSRSIELIAPPQGDAPVIQIPLLGEIAAGRPIFADENFERTIAIPADLVANRVPHFAVRV
KGDSMIGAGILSGDIAIIEQCETAQNGDIVVALLEENVTLKRFFIENNRYRLQAENPRYA
PIYTQDLRILGRLRGIYRSY*
>SPBIB_v1_300001|ID:27163756| protein of unknown function [Uncultured spirochete bib]
MMRLSLYLLGHNYLKPFRIRAHKGMHPRTHAEAAGIPVHLVQHFVQALTGGIRAFLSRCT
LSETIRRTWEKRWKTPGKDKAEYLPKYALA*
>SPBIB_v1_300002|ID:27163757| Xaa-Pro aminopeptidase [Uncultured spirochete bib]
VKHSKYSRRQAALADELAKRGLGAVMVSDFEHGRDSSLRYLCGQPSDAIFILSAKGTSAL
IAWDINMASRLGEADHIAAYTDFGRQPQKALAAALKLLDVPKGAKVALPASTPYPDYIDF
VHALTDFDLVCEKSGVGTFIQEMRARKDTDEIALYRRIASNTDQLIDMIEKEICAGMLLT
ETDIALFLEREARRMGAEGMGFETLAAGPERSFGIHAFPTFTNEHFAAEGLSILDFGARF
EGYTSDVTMSFVSDSLRTEQKTMVGLVEEAHRIAVEACGPGVPLLSVAKLVDDFFAKAGW
TMPHSLGHGIGLDAHEAPTLSMRADPSALLEPGHIVTIEPGLYHPEFGGVRLENDVLITE
NGAEVLTHSRIVRL*
>SPBIB_v1_300003|ID:27163758|pyrC| Dihydroorotase [Uncultured spirochete bib]
MTDRFVMPMPDDFHAHLRQGEPLMQYARQHAGQFGRVLLMPNTMPPIFNVARLTEYRADV
ERALAAIPDSERFEPLFTFKILPSMLAAEIESFARAGAIAGKYYPSGSTTNAADGPRSFD
DVDEVLSTMEESGLVLCIHGENPEVPVFERERAFLPQVERLLARYPHLRIVLEHLSDEES
VRFVEQGPANLAATITAHHLLFTVDDMIRDSMNPHLYCKPVLKFARDREALRSAVLSGSK
KFFFGSDSAPHPREKKECTAAAPGVYSAPAALPALVELFDSLGSLDALIPFMAEYGAAFY
GLAAPSRTITLERAPWQVPQLIDGVVPMCAGKTLAWRIWGRN*
>SPBIB_v1_300004|ID:27163759|murA| UDP-N-acetylglucosamine 1-carboxyvinyltransferase [Uncultured spirochete bib]
MDKYIIEGGYPIKGMLTLSGNKNAALPCIAATTLADEPVILRNIPAIEDVLVMFDVLRNL
GASVEEIDAHSWRIDPRSISRYEVPEELARKVRASILFAGPLTARFGKAVLPPPGGDTIG
RRRIDTHILALQELGARIAIEATLNFSAVKLVGAPVFLDEASVTGTENAIMAAVLAEGTT
TLTNAACEPHVQDLCAMLIQMGAEIEGVGSNRLVIHGKKSLHGVEFAISSDYMELGSFIG

LAAVTGGDLRIEGVRTEDLPPLKVGFSKLGVTWDLKGNVLRPNTAAALRVVPDIGGQIPK
IDDAPWPGFPADLTSIMTVVATQAEGVALIHEKMFESRMFFVDKLISMGARIVLCDPHRA
VVYGPSKLVGNVMVSPDVRAGMAMVIAALCAEGTSTIHNVYQIERGYENLSARLSAVGAH
IRKEQED*
>SPBIB_v1_300005|ID:27163760| conserved protein of unknown function [Uncultured spirochete bib]
MGMFERLKTVISSNINSLISKAENPEKMLNQMIIDMNEQLIESKKAVAMAIADEKKLERD
LMENRAKADEWEKKAMLAVRAGRDDLAKEALLRKQEFEGYANQLAQQWEAQKQSVEKLKE
ALRQLQSKIEEANRKKNILIARAKRAEAQQRINQTMSSLSGNKSAFDTFERMERKVDEIE
ASAEAMKELEEASSGASLEKQFAQLESSPQAADAMLEELKKKMLTEDAGRTSSQP*
>SPBIB_v1_300006|ID:27163761| conserved protein of unknown function [Uncultured spirochete bib]
MSSLPKIEQYLIDLGISYQEISKNAWLIEDENKGYPKMMVSLSDPIVIISADVMPVPKEN
AETLFRMLLELNATDLLHGAYAISGNDIIVIDTLEHASLDKDEFLASIESISFALLEHYK
KLASYIHTTEE*
>SPBIB_v1_300007|ID:27163762| protein of unknown function [Uncultured spirochete bib]
MPSRLLSRIQKIPQAGSDALISSQISSDFPDCAVARFVGAVTDSNLEIVAKSFENILSGG
IQYLIVDFSAIDDISPAGIGLLLALRQKLRDRRGDLILCSMRPRMERMNRVLGLEGYFSA
AIDISAAKTGLKELTTYVYPISVRCPACDSLIDIDRPGRGRCQTCEAVITAFPDGSITLG
*
>SPBIB_v1_300008|ID:27163763| Short-chain dehydrogenase/reductase SDR [Uncultured spirochete bib]
MNGIPSGKARGEMVASSTFNGRFALVIGGSGGIGRAVSLELVRRGARLLVHGRQAEERMK
TSSPASLSTFDYAFDSPRNFIQALDARLADMRAEPDIVICAFGPFSEKPLEMVSAEEWEY
LTMANLALPGAMASHFLAGMKARGYGRFLFFGGTRTDAIRPFRKTAAYAAAKTGLGVLAK
SIAASAGDRNVAAVVVCPGPTDTEYLNAETRARHASLTATGSLLPAKLIAHASLDLIDAD
PCIASGAIVALDGGFDPESRR*
>SPBIB_v1_300009|ID:27163764| protein of unknown function [Uncultured spirochete bib]
MKKLLYFEQDSSRIRPIVVALSKHFEVSVAGDLLELAATLRQKEPRILLFGLASLPGAFS
IHILDRIKQIMLNLHEEKLASRSALSVVVMHGDDSRWQQICEPYIAALREIPFCDIYVIP
AATNPDELAVSIINIKRANRHLRTARMSATAEVPIIGNSTKMRHVIEQIRTYADKHHPVL
IVGETGTGKELAARALHFWGKRSHHHFVALNCATIPETLFESEMFGTERGAYTDASTRMG
AIEQADNGTLFLDEIGSLSLASQPRLLRVLETGEYRRLGDPHPHSAQFRLVSASCINPID
LAAKNKFRSDLVYRIADLVIEIPPLRHRAEDIPLLAAHFCSHFSKDAFSLEQNALDKLCA
YHWPGNVRELRSVIARACANNSTGRIFADDIQFLYQWKFEGNSQPEVSA*
>SPBIB_v1_300010|ID:27163765| protein of unknown function [Uncultured spirochete bib]
MESLNIPCALVEGVRFDFPSRFAISALPYIWDDEKNHQICFVSSSQWNAAKRRDEFGSMI
ASADLVLPKDRAVAGRVLAQGAEASRSWPVPFVHQMLLDAMHESAEQPELEIPSIEHIPE
MYRPQKVITTLLSAIEQRNGSVFLLGGAPVTLIKAKKNIRATFPGLAIVGSMHGLYRPQE
EAALIEAIQKSTPMLILAGAPLPSGERWIPHHMAATRSGIFLYHGAILRWFAGS*
>SPBIB_v1_300011|ID:27163766| putative Ribosomal RNA small subunit methyltransferase E [Uncultured spirochete bib]
MRQIVFPGALRGKTAYLLDAKTAHYLIDVRRMKAGDVFEAIDEEGSRFRCTIVSRENEGA
HVRLESIEPDDLAAQQDRQLQIALVQALPKGQKFDLIIRQAVELEVNLIVPVITKFCVAE
EAPERAHSKLERRRRIIREALQQSGSSALTEIVPAARLSELDKVLREHGFDRENSLRIMF
HESDAANSKPLHELLAHMQNRVAICIGPEGGFSEEDYSALAEMGFMVHHAQGPIMRVETA
AIFAISAVRVIALEREIWNR*
>SPBIB_v1_300012|ID:27163767| Carboxyl-terminal protease [Uncultured spirochete bib]
MNETETSEVRTPVERTPVEHTPAEHTPVVRKRLFSLLAFVVILALSFITVAVPESYAQQA
KTPDAKQYSQLLQNIFQFILQNYVEEPDPTKLYQGAVKGMLDSLGDPYSAFLDEDMMSDL
MSDTTGTYGGVGLYISKQTTVPTEDTPRYIEVVSPIEDTPAWKEGIRSGDLITKIDGEDT
SPLSVDKASAKIRGEAGTTVTLTFKRGNYEFEVTFTRSKIEIPAIKNAIITTASGNIGYI
RIVEWNPNTPVRMKAVLQDMHAQGIDRWIIDVRSNPGGLLSSVVDVSDLFLSSGVIVSTK
GRTISENYEYKAKPDLAIPATDRIIVMTNQGSASASEIFAGAMKDTKRALLLGQKTYGKG
SVQQIFPLDKAGFKLTMARYYTPSGVNIDKVGIEPDIKVPEPELSDKELAEVQRLYDAGD
IARFLLKNPTPSVEQRKQFAAELAQKYTVPALILEKLVRDESERSQPARIYDLEYDVQLQ
KALDLIESQDFLNQLESAKTLADLKKSTN*

>SPBIB_v1_300013|ID:27163768| membrane protein of unknown function [Uncultured spirochete bib]
MLVAGILAFIRIFALGVAAVSLGMTDFGGASTAILRVFQLYGLLFVYLLFLQYFRPSARE
ALKTPCLLISAAAPVLSVLTLLAFVKGVGKSIPVDLVKTSSSIFVVILMDLLILGLVGMD
SLKAKFPREGGQGKQAQSRGVPTVDAPDSPVPAHNSDTTNEVS*
>SPBIB_v1_300014|ID:27163769|lgt| Prolipoprotein diacylglyceryl transferase [Uncultured spirochete bib]
MVAMLEFPKWLSPEIIPGLPFRWYGLMYLIAFSVAWLLFRRESKRLRAPWTEDEAANFFI
WSIVGILLGGRLAGTLIYEPSNYYWIKPWFIFWPFDESGAFVGFQGMSYHGGFVGLIVAT
LIWCKVNRWNWLDWADLIAVSAPLGYTFGRLGNFINGELWGKVSTAPWAMLFPNAERFSA
KEPWVQEVARAVGIRLVSMNDMVNLPRHPSQLYEALLEGIVLWLVLWFFVRKRRTYRGKS
VAIYAIGYGVARFIVEYFREPDSGLGYILALGDPQAPTARFVSLFNFSMGQILSFLMIVG
AVIFMLYARKHFRIAPGEPMFVDAAKRGESGDFGAGRQKGTSGARGTDGIRENSGPRTSK
FNAPSARKLRKKIK*
>SPBIB_v1_300015|ID:27163770|nadE| putative glutamine-dependent NAD(+) synthetase [Uncultured spirochete bib]
MRVSLVQMNSTIGDFKGNVDRMLEFVGQILKAFSATERPDLIVFPEQSICGYPPMDLLDQ
ESFLFGSISALRRLQKLLPQDIAVGVGYVDKNREGAGKSLVNAYSVLYGGKVVFSQAKTL
LPTYDVFDEARYFEPARARQVFSQPFGRVGFAICEDFWWEAPDASQKYSIDPVKELMDAG
ADILIVPSASPFIAGKLQTRLRLAEKAVRAGSIPVLYCNAVGANDSLVFDGRSFAMDERT
TIRGICGWGEEVLTYDTVSGKAGRLILGAMHGEGATSPTAEIEKSSDAAHTDATEAATTQ
ATAAQAIDLQTERSEEIRRALIVGIQDYLRKSGFSRVCLGLSGGIDSALVAVLATQAIGP
ENVTCIAMPSRFSSAGSLDDAVELCRRNKLRLERIPIEVPFTAYLNALAGPFAGRPYDTT
EENLQARIRGTLLMAWSNKFNTLLLTTGNKSELATGYCTLYGDMNGSLAPIADLYKTEVY
ALAAHINRSAAAQGREEPIPDTIIAKAPSAELRHNQKDQDTLPEYPVLDGILRLYIEENR
TMDEIIDAGFDRATVRKTLEMTAKAEYKRRQAAPAIKVSKRAFGVGRRLPLARTIHEIQ*
>SPBIB_v1_300016|ID:27163771| Glycerophosphoryl diester phosphodiesterase [Uncultured spirochete bib]
MQHPSLLPDFPRPLLFAHRGLNRIFLENTIEAFRAAHDAGVPGIELDVHLTLDGALVVFH
DDTSGRIEKQVNPSAPERNLSIEGSTLAELRALSIGPRIPLLDELFETFGNRMYYDIELK
CRSASDTGLASSVAASIHRHGLEKHCVISSFNPFALRHFRKAEPDIPIGIIWNKSKELYW
FLRHGEGTLIANADFLKPEFPIAAHLLLYLRILARRPIVPWTVNDVSIARKLLKGGAEGI
ISDEADAVRAAVWH*
>SPBIB_v1_300017|ID:27163772| protein of unknown function [Uncultured spirochete bib]
MTPQELSRLDYEGASEMLLACATDAKKYEKEIASLQAQAAEWKSKARLAQDRNMEELSQA
ALQKSAELEAKAQELALELNSIQRDIEDLRTALPIIKAKQRSVDPDQLLAELSMLVGLEE
TAQTPESDTKPNQNPEEPTPGTRLGALQEGHSPVDDALAELKKKMGLL*
>SPBIB_v1_300018|ID:27163773| protein of unknown function [Uncultured spirochete bib]
MNRIAQKVTRFVGLDVHAETISVAIAETGVEVRYLGSISNRPESVSKLIKKIGTGGKWKS
LLRSRTDGVCIVLAIDKSEDRV*
>SPBIB_v1_300019|ID:27163774| protein of unknown function [Uncultured spirochete bib]
LYWQLTKARIGCEVIAPALVPMRAGDRAKTDRRDAEQLAQSYRAGELTPVWVPDEAHEAL
RDLVRAREAAVQDRLRVRHRFKEVFASVWSAAGEKDDAMDTSLPGMDQEGGDV*
>SPBIB_v1_300020|ID:27163775| transposase (fragment) [Uncultured spirochete bib]
MTPWTLRYLEWIKKEVTFEEEALQWTLEEYMNEVEHCSLRITRLDGLIDKAVENAPEAMR
EVVAALQALRGIGKVTAVTIVAEVGQLSRFPTARQSMGYIGTVPSEHSSGETIRRGGITK
TGNAHIRRIIIEVAWLQRHKQMLSQAVKLRQERLDPEIQEIALKAMHPLHDRYFRLTARG
KSKQQVVTAVGRELLGFIWAIGILVERKNAA*
>SPBIB_v1_310001|ID:27163776| conserved protein of unknown function [Uncultured spirochete bib]
MPMQSFPFCPNPHCVWHSNAPDFAWAKPKGFYTTKXFGRVQRYQCAACHRTFSSXTFSLA
YYVKRPVALPDIVARLVSGESLRAMSRNLAVSLNLLSNRIDRLTRQAIXX
>SPBIB_v1_310002|ID:27163777| protein of unknown function [Uncultured spirochete bib]
VAGGTLVALDAAKXLGGIEALGLRPSEIWCVTVPDTVRIRAEREGLHRHLPP*
>SPBIB_v1_310003|ID:27163778|mnmE| tRNA modification GTPase MnmE [Uncultured spirochete bib]
VAISYWRMPRSYFDRNTPIAALATPEGRSALAVVRTAGANAIELVARCFSNRQALLDAQG
YQAVYGWFIDPSSGEYIDEVIALVFRAPHSFTGENAVEIMSHGSPAVVERILDVLYAQGF
SPALHGEFSFRAFVQGKTDLVRAEAINELTHASCEAARHDALQRLSGALSRKLGEIRDLM
VDLLADINAKLDYPEDEGPEESSRWLEIMHHARDALSVLIQSYPGGRLRQEGFLVVIAGR

PNAGKSSLFNLFAREERAIVSPEPGTTRDWIETWISIGEFAVRLVDTAGLRSSQNMIEAE
GVRRSQSLLERADVILYLVDGVAGLNDEDREFAAKHPEALLLWNKADLSRCLPAPEGWIA
LSAKDVRSFGEFENIVIDRLRSYARTTQKAGTLERQIRIASERQAGLLKQSDLALSQALE
AYTAGAGLDLVALHIREAAEAIGEITGDIAADEVLERVFSTFCLGK*
>SPBIB_v1_310004|ID:27163779|mnmG| tRNA uridine 5-carboxymethylaminomethyl modification enzyme MnmG [Uncultured spirochete bib]
MADFEAIVIGGGHAGIEAALALARLGTKTLFITQNPDTIGRMSCNPAIGGLSKGNLVREV
DALGGQMGILADATAIQVRMLNQSRGAAVQAPRAQSDKALYASLARQTLEAQPNLTIFMD
TVTDIIVSEGSSGARAGYAVSAAGTASAPSSRHIEGVRTERGNEISAKVVVLTTGTFMEA
KLFIGLWSGSGGRLGEPAAIGLGTALRAKGFPVGRMKTGTPARIKRDSIDFSKLEAQYSD
PRKIFFSFLEQDYNRPDVPCYIVYTNQNTHEAIRAGLDRSPLFSGVITGKGPRYCPSIED
KVVRFPDRERHQVFIEPEGLSTDEMYLNGLSSSLPEDVQERFYHSIPGLEHAVIVRPAYA
VEYDYLDPAALYASLESKLVEGLFIAGQTNGTSGYEEAAAQGLMAGINARRKLDGEPPLI
LGRNEAYIGVLIDDLVTLSPKEPYRMFTSRAEYRLALRHDTADLRLTPYAIEIGLADERR
RECFEKRLQGIDEAKALLSRRKIQKEDSAHIPELEKHIGQTLADAIRDPKVGALLDDEAE
ALSHIAQLLPETAAMQASSVLTAVLNERYKGYLEKEERLAARLSKADKMLIPDKFDYSQV
KGLSKEASEKLSNQRPLTIGQASRIPGVRKSDVALLYIAISRPSP*
>SPBIB_v1_310005|ID:27163780| conserved protein of unknown function [Uncultured spirochete bib]
MAVIKSALELALERTKNLQVDESLQKASEAKLEGRKAASRWLEEPESVDFKALAGAIPPE
HRQTFLRAAFEVLSTQVQLPLNTGIDKAKLEAAGKAIIVLCGLSSRFGSEREAKLAQQQV
QSLFQQILQFLGQYSEEMKRAEQAIRNQWAPKLKEKERQLAAQLGQNVRIDPMSDPEFAE
FYRKNIDAMRKNYANALEEAKNQLAALCGFATNE*
>SPBIB_v1_310006|ID:27163781| CDP-alcohol phosphatidyltransferase [Uncultured spirochete bib]
MDEGERIASLRRSILATTSIFFVAQCALFLIFAVSAGFVTQYWSLFLPISAGFHIILLIM
LLLFQDDFVIESNGRKLDRINLANVITLSRVSTLPTLLVLVIAAKQYRIRIPLLILVALI
FLTDFFDGRISRKTNQVTRVGRMMDSASDYTLLVVLSVIFQYYLLIPGWFFVLVIVRLGI
QAFLMAILIVVRRRIEPKSTFMGKVTVASIMVLYTLEIIQLALQERFLLVFRIAEWIVAI
IIVIGIFDKIVSFFDALKSKPSAP*
>SPBIB_v1_310007|ID:27163782| CDP-diacylglycerol/glycerol-3-phosphate 3-phosphatidyltransferase [Uncultured spirochete bib]
MTAADKITLSRIVIAPLFFFVFMVQFIPRQIAIVLLWIMFGWMELSDLIDGRVARANQQV
TSFGKLFDPFADVISRVTYFLAFTSIGIMPWWVLVVVLYREFGILFLRMLLGLKGIAMGA
RAGGKLKAGVYMTAGLVSLMLYTVRVTGFLGGVASLLETLTIVVYIAAAVLSLASFADYL
VQFRKLYK*
>SPBIB_v1_310008|ID:27163783|ilvI| acetolactate synthase III, large subunit [Uncultured spirochete bib]
MEYTGARILMESLLLQSVDTVFGYPGGSVLYIYDELYRYRDRIRHILTSHEQHAAHAADG
YARSTGKTGVCIATSGPGATNLVTGIATAHMDSVPLVAITGNVATSLLGKDSFQEVDIYG
ITMPIIKHNWIVKNVEDLAEVVREAFFIARSGRPGPVLIDIPKDVTIAKTEWQAVADANN
LSVPIPGSAMLSRRARLSSRNEQTTFSDKDIDQAITMIETSERPFVYAGGGVIASNACEE
LRELIERIKAPVALSLMGISCLPPSHPLYTGLIGMHGSVASNRSVQEADLILAVGARFSD
RVVSRADSFASQAKILQLDIDPAEINKNIQTEHHIIGDLKLVLSRILGGMKSKQELTPPS
LAQKRAEWLSKVEAWKSIHPEANFKPSAFHPRFIIETVAQRLGEDAIVATDVGQHQIWTA
QFYPFAKPRTFLSSCGLGTMGSGLGFAIGAKTAHRERPVVLFTGDGSFRMNCGELATAVN
YHLPILIVILNNRTLGMVRQWQTLFYDERYAETTLDKRPPDFVRLAEAYGAAGFRADSKV
GFLAALDEAMAHIAEGRPAVIDALIDQDEQVLPMVPGGKAIDEQIM*
>SPBIB_v1_310009|ID:27163784| putative enzyme [Uncultured spirochete bib]
MAELKITPQMEILDTTLRDGAQGEGISFSLQDKIAVVKALDQLGVSFIEAGNPGSNPKDM
EFFREAPKLELEHAKLCAFGATRRKGTIPEQDEAIQSLLAAQTDTIVIFGKSWDLHVLHV
LRVSHEENLAMIAETISFFKEKGKTVIYDAEHFFDGYRANKEYAMATVQTALSAGADSIV
LCDTNGGSFPDLIEEGVRTVLGLGSGKVGIHAHNDAGMAVANSVMAVKAGARHVQGTLVG
FGERCGNAALAAVIPSIELKLQLHCLPKGKLENITETARLVAEIANVPMPDDMPYVGLRA
FAHKAGMHADGILKTRTSFEHIDPALVGNWRRFLMSEMGGRAAIAERIKKLDPSVTKEHP
VTAALAAKLKSLEAEGWQFEGADASFELLARRELGKYKPLFSIERYEVQSTHPSEEGNIC
STAWVKVKVDHSTEIAASEGNGPVNALDSALRQALKRFYPELSKVRLTDYKVRVIDSRSA

TGARVRVLIESTDGVHVWTTIGVSTDIIEASSKALADSIEFSLIQATETKNF*
>SPBIB_v1_310010|ID:27163785|ilvH| acetolactate synthase III, thiamin-dependent, small subunit [Uncultured spirochete bib]
MNRHVVSALVENRSGVLSRVSGLFSRRGFNIDGLTVGETENPSVSRMTIAVTGDEQVLDQ
IIKQLGKLVDVIAVRELDPASCIRRELMLIKVKADESVRPAVIEIASIFRSRIVDVSPST
ITIEATGDSEKLEGLLLLLRTYGIIELARTGLVAVERGSSILNITI*
>SPBIB_v1_310012|ID:27163787|trkH| Trk system potassium uptake protein TrkH [Uncultured spirochete bib]
MHLKSILRMLSAILMIVSLFILACGFFSITEKNQVRVIVSFFIPAGVGLAFFLFMLFICR
KDSKPFLTKRDGFLFVTLSWVFASAIGALPFVLSGYIPSYIDAFFETMSGFTTTGASILT
NIEVLPRALLLWRATTHWLGGMGIVVLLVAILPTLGFSAIRVIEAEAPGPSIDRIAPHIS
STAKVLWLIYLGLTGLEVLLLVIGGMTLFDAICHAFATMATGGFSPKNASVAYYNSGFVH
AVITIFMFLAGMNFTLHYRLLSGNLRSILKDTEFRVYFAIFAISSVIIGVDLLVSGMYRN
FGESLRFSSFQVSSILTTTGFATANFGQWPEVSQTILFFLMFIGGCAGSTGGGIKVIRIV
VLFKMAVTEMRYVANPRGVYAIFLGNKALRKNVIYDIAALVFLYFAFFFISVIVISFSGV
DILTSVTAVIANLGNIGPGLGKVGPAFNYAFFPWWAKLWLAFAMLVGRLEVYTVLILFSR
KFWRSF*
>SPBIB_v1_310013|ID:27163788| putative TrkA-N domain protein [Uncultured spirochete bib]
MKIAILGAGLLGSLIAHELITENRDVVIIEKNPNVAKAISNDLDCIVEEGDGERLDTLMS
AGVADADWFIACTGSDETNIVSCGIVAEAFKNVKTIARTRNPYFASFKNTGKRILGVDYI
INPEAETAEAIARIIFRGMSPEIIDVKEAGIQLRRIHCSSQARIIGKPLSDVRSLIGPEF
MVPAVMRKGELIVPAGDFVFEEDDSAYILGEPSWLDRLFGPCKSTLRKFSSLVIFGAEAL
TSLLLQELGIEQIVAHTRRSRDRGALSLLGNPRIKVIDGNREALKALSTTFPDIEPINHQ
MSEERLIDEENIGSADIVLCLTSHQSINILTALLAQEAGARRTLALVTNDLYSNILDPLN
INIVINEKTVVSGTILDRVRKAKIRRLYSFPRNEYELIEIQISKSFERLGFEIRNLNLPK
GFLVTFIIHESKTIVPTGDTRIQENDLVGLIVKKEQIGRLESIFGA*
>SPBIB_v1_310014|ID:27163789| putative Ktr system potassium uptake protein D [Uncultured spirochete bib]
VSIRIQIGKEKSLLILYFTGLIAAGTILFSMPFATKTGSMRFVDALFTATSAVCVTGLTT
VDTLSLTRVGQFVLILLIQLGGLGIIAFSTLYLFSPRKRISVITRGFTGDYTVPSVEFRT
RKIITQIIGWTAGFELLGTLIIWPVLSRSGYTLFDSLFHAISAFCNAGFSTLRSGMENFR
DEALMNVVTMILIICGGLGFIVLQDIARLIQKKKLHLTYHSSIVLRTSVGLIIIGAILFF
ILENNHAFKGMSGASKIMASFFQSVTSRTAGFDTVPQAKLTSAAQFVTIILMFIGASPGS
TGGGIKTTTFYLMLLVALRFREGGGVLVDRNRAVMPYSIFKAAAVVVRAVIIVLAVTVVL
LIAEQVHGNPLGIETAIFESVSAFGTVGLSIGITPHLSAVSKLALVSAMFMGRVGLFAMA
IPSSKYSTERYARSPRADILL*
>SPBIB_v1_310015|ID:27163790| putative TrkA N-terminal domain protein [Uncultured spirochete bib]
MRQFAFVGLGSFAMSMLERIAEITDQIIAVDDDQARIEHVKEMVSTAYTMNLLDGEAFER
VFHDPVDVAIVDVESNGAAVLLVTFRLKKLGVPEIIVKSNSEEYEELLRLVGATRVVNSD
REAATRITPLVLSSSLTNFMPISGDLVLAEVIAPDFVLGKTVIETDLRRKHHVNVVAVKH
SLQRNVNEEAEGVFNDLDIAYRFQAGDVLLVTGKESDVFVFSGVQKTAEHEKKKVNFSVL
LKSMFSRKKQDDTKK*
>SPBIB_v1_310016|ID:27163791| 8-amino-7-oxononanoate synthase [Uncultured spirochete bib]
MDIFQKCFDFDLDKQAMEKGLYPYFQPLDGLEGTEAVFHGRRLIMIGSNNYLGLTMHPKV
REAARKALEEFGPSCTGSRFLNGTLKLHEELEARLAAFVGKDAALVFSTGMQTNLGAISA
LIGRNDVVITDKDDHASIVDGCRLGIGEMKRYVHGDLDQLERILQKIPPESGAMVVVDGV
FSMGGDIVDLPKLVDVCHRYGARLYVDDAHSLGVLGGGRGTAWHFGLTDKVDLIMGTFSK
SFASLGGFVAGDKDVINYIKHTARSFIFSASLPAPNAMAAFAALEVMETEPEHVQRLWEN
AHFMMKGFRELGFNIGNTQTPIIPVIIGEDETCFRFWKELFDGGVYTNPVISPAVPEGMA
LLRTSYMATHTKEQLQRALDIFEKAGKKFGII*
>SPBIB_v1_310017|ID:27163792| DNA repair protein RadC [Uncultured spirochete bib]
MEEWQAYEKAALSIESSTSPLDIRERMVRFGSGSISNLDLVVAILGTGIPGKPVRRLARE
VLDQITQSTHSFDINKLMVIAGMGEAKSCAVAAAIELGRRIFSTRGTRITMPKDAYPLLI
HFADLRQEHFIVISLNGGHEVNAVREITKGLINKTVVHPREVFADPITDRACAVIVAHNH
PSGNLEPSEEDLDITKRLRQSGDILGIPVLDHLVFSETGYFSFVEHGLIAPTK*
>SPBIB_v1_310018|ID:27163793|rbr| Rubrerythrin [Uncultured spirochete bib]

MKSLKGTKTEVNLLTAFAGESQARNRYTYFASKAKEEGFIQIQLIFEETANQEKEHAKRL
FKFLEGGTAKVTAEFPAGVIGTTRENLAEAASGENYEWKEMYPGFAKVAREEGFDAIAAV
FEAIAVAEKQHAKRYEALMANIDAGRVFKRDKKVVWRCLNCGYLFEGTEPPKVCPACAHP
QAYFELLGENW*
>SPBIB_v1_310019|ID:27163794|dfx| Desulfoferrodoxin [Uncultured spirochete bib]
MTQKKQIYKCMKCGNIVEVLHEGEGELVCCGEPMKLFVENTTDAAKEKHVPVLEKTKDGW
WVKVGSVPHPMEEKHYIEWIELLADGNSYRAFLAPGQEPKAFFPVKAEKVQAREYCNLHG
LWKA*
>SPBIB_v1_310020|ID:27163795| conserved protein of unknown function [Uncultured spirochete bib]
MISREDFVFCIGYDGDTAIVDAKAKKEFGRLSTMELAEKGLYRAAFASALYSEKPEEMQS
FIEFFNKKAGTQYTEAGQLSRLFGVYLESVSKVKAL*
>SPBIB_v1_310021|ID:27163796| putative SAM-dependent methyltransferase [Uncultured spirochete bib]
MVFKTYYQIEILKHRLSSRSRAFFTFFTFCAFLSLLTFLSFPSLPPSARNRYCISMATII
LKSKEDIRIRRGHPWIYDNEIARVEGEPEPGGEVEVRDARGLHVGYAFFNPNSKIRARVY
SRTAKKADEEFFVRAFEAAWKWRKFCFGEPAIERRLSLRLVYGEADSVPGLIVDGFVGAT
GTEGLWLSVQFLSLGVEMRKAEILKALRSVFNPDGIAERSDAPVRALEGLPESVGVLWGS
VPERIVIEENGALFEIDLLGGQKTGWFLDQRANRAAAAKYVSRAQWADSPNKAIGARVLD
MFCNQGGFSVLAAKAGAASVLAVDSSRDALALLRKNAALNSVSDRITIVEANAFDYLRDL
EKMGQRFDLIILDPPAFAKNRAALESARRGYKELNLRAMHLLERGGVLVTFSCSHWFSPE
MFDAVLADAAFDSNRRLHILEERTQDLDHPILSGYDESRYLKCRIVQVV*
>SPBIB_v1_310022|ID:27163797| conserved protein of unknown function [Uncultured spirochete bib]
MPRPMKYRIINGRPITNIFKPAGIPQRELEEVILTLDELEALRLADLERKYQEDAAKLMG
VSRQTFGNIIASARKKVADVLVNGKALRIEGGFVDISGGSFVCLDCRQEWNMPSGSETPH
NCPHCGSSNIRSRT*
>SPBIB_v1_310023|ID:27163798| Fe-S oxidoreductase [Uncultured spirochete bib]
MYAYGPVPSRRLGRSVGVSPIPEKTCSYSCVYCQLGRTKTLTTKRHSFFPKEAVFADIEK
VVRANEGKIDYITFVGDGEPTLSLDLGALIHECKQNFPYKVAVITNGSLLWMEDVRQDLR
EADVVNITMATSDPDTFKGMHRPHGSLRFEQVWQGIQQFAAEFKGQIWAEIMLVDMVNTD
TEAMKSLKTLIDSVHPERTYVMAPTRPPAEPWVHMPRPETILEALALFGGENVTQLEEGS
FGLDEFHSAVEAISEICRRHPLRIAQARTIEAYFAQRTLDRLIASGKFKIISYQNRKYVL
PSEFVFGKQEQS*
>SPBIB_v1_310024|ID:27163799| Cobyrinic acid ac-diamide synthase (fragment) [Uncultured spirochete bib]
MCFRPNLSLGSRNNHRASERNLILTVTIMSGKGGVGKTTVAVSFATQLARRGEKVLLADL
DVEEPNAGLFLQKQRKDSVDAVVYRPKWREDLCIFCGKCQGFCKFNAIAALPKYIIVFPE
LCHSCYACSDLCPASALSMVPAKIGQIQQYSVNENLVFIEGRLDIGQEIASTLVRQTRDK
ALREAQTAGIQWLVFDAAPGTACASREAMIKSDAVILVTEPTPFGLHDMSLAYQLAKASR
KPCALVINKDSPGYAGIEKFANEEKVPIIARIPYNSRLAQSYSEGNIPVGAFPALEHALS
RIASWLLKVRAGGQP*
>SPBIB_v1_310025|ID:27163800| Cobyrinic acid ac-diamide synthase [Uncultured spirochete bib]
MIRIAILSGKGGTGKTSVAASFGYLAGKRAVLCDCDVDASNLALVAGAQNIQSREYSGGM
VAVIDPEACIGCGACARVCRFDAIEKAGPKYRIEAASCEGCGYCPRVCAFNAISMVERKS
GDIFTGQSRFDSAIVYAELSIGAENSGKLSTQVRRLADEIAEEKGAEVIIIDGPPGVSCP
AIAAATGTNYILFVSEPTRSGVSDLERAMEMARKLKIPGGVLVNRADINEVLSRKIEAIT
MQTGNDFWGAIPLSSDFVRAVRNGKTVLEETDDERITSALTQTWRSILERFQKWQ*
>SPBIB_v1_310026|ID:27163801| Dinitrogenase iron-molybdenum cofactor biosynthesis protein (modular protein) [Uncultured spirochete bib]
MALDIGTISEMAITAIMTKTTMGGKLKIIFSASENKGKDSFLDDRFGRAAGFVVYEEETD
QWSWIDNASNVNAAGGAGVQAGQAVVNSGATVYIGAQLGPKAMAVLARSTMKLFAGVPGK
TARENYELFKAGKLEKIE*
>SPBIB_v1_310027|ID:27163802|radA| DNA repair protein RadA homolog [Uncultured spirochete bib]
MKKPSIIFRCSACGHEEPKWLGRCPECGQWNTMLEAKTAGRFEKDSSAFSLPLESVNPAL
GTRVSSGISELDRVLGGGFMRGSAILIGGEPGIGKSTLLLEASAKLGAKGKALYISGEES
AAQIRLRAERIGALSKQIEIFCGNDLHACLSVMDSVHPLLTVVDSIQTIHSPEAGAVPGT
PNQIKFCTQELVEWAKSHDSIVVLVAHVTKDGMIAGPKAAEHLVDAVISFEQAENALRVL

RTSKNRFGSSDELGFFLMGGEGLAELPDPSGIFMVRREGHLPAGIAVAIVHEGSRILLAE
VQALTIPSKSGIMRVYSDRIDPLRVSRIAAVLEKQTRLDFSSQEIYVNVAGGLRLTEPAV
DLPLACALYSARTGQALPLGSAIAGELSLAGEVRPVRSMDRRAKSASQLGFERIIGPGVL
LPGEGLSKGTLSDQAARAAQHASQSFRWTRASSLKDTLRLLWNQENRT*
>SPBIB_v1_310028|ID:27163803| putative 4-alpha-glucanotransferase (Amylomaltase) [Uncultured spirochete bib]
MKCIHNNERISGLVVPLLSLRKNKSAACGVFSDLTELAKLAKQWGLSLIQLLPLNDTGNG
TSPYSALSAFALHPIYISLPEVPKTMESLGAPLAQSAIDILKKAENKLARAHAGNKRVAF
EAVLSDKLAALRSVWNESQEYCRPLAELFAAQETWAKSYACFMALKEKYGLAPWWEWPEY
RDVTPTDIETLWDSSETGEEARFRLWLQILAREQLKDAAHSVAGMSIDIMGDIPILLAKD
SADVWCDRDIFILNSQAGAPPDMYSPRGQNWGFPLYNWDALKAGDYAFWRQRLAYADQFY
TSYRIDHVLGFFRIWAISAFESEAFLGEFEPSERLARSELSALGFSDERITWLSKPHIPE
WKIQNMEARCIDAFNRAEGAPHVSQAGDTKASADRRISLVQRLDHLRQVCFSRIKNEPLF
LFSAQIRGSADINSKVAALKGDFPQAASALSDYAQSMCEWWTDRALYEVEPDRFVFTWEY
HNTTSWKSLSMQEQEALEQKAAELASKSLAEWEARGRELLSMLVASTGMQPFAEDLGAVP
PCVPKVLKELKIPGLRVLRWEREWDKPGQPYIQLETYESLSVACTSVHDSSSMRQWWEEE
ADRAQLWAMFKKMAEKDALLSSLLANCSQEAPEALEPSAAAVLVRALVLSASNVVVYPLQ
DILACHPEWREPDARDERINVPGTTLPSNWSYRVRENLRTLASDKDFAAFVSRIAVRGAS
K*
>SPBIB_v1_310029|ID:27163804| Nucleoside recognition domain protein [Uncultured spirochete bib]
VNFLKDELRRIAGKSLKRTIDTSWFLIKIMVPVSLLVTLLGWSGLLAKIAVVLRPLMRLL
GLPGEAALVYISGALLNNYSSIAVMSSMNLSLRDATILAVMCLISHNLLVETAVMKSIGS
SALKMALLRIGTAMVGGFLLNLILPASFAQIQLFSASATANSAFWPTMASWALSTLKLTA
RIIVYILALMFAQTLLEQFNLMEWLSKWLGWLMRLFGLEPSMGFMWIVINIVGYAYGAGI
IKAARDEGRMTLQEGDLFNHHAAISHSLLEDTVLYAAISIPVLWLIVPRLALAVVVVWGE
RLRRYLFRRSLKAGVV*
>SPBIB_v1_310030|ID:27163805| conserved protein of unknown function [Uncultured spirochete bib]
MNILLDTHYLLWAFLDTYRIEKRLMDIILAEDNEIFYSQASLWEISIKYNLGKLVFEGIS
PEDLYREIENSFLRCRTFTNDELISFYRLPIEHRDPFDRILVWQAITSDYFFLSSDGKAD
AYTKYGLKILH*
>SPBIB_v1_310031|ID:27163806| conserved protein of unknown function [Uncultured spirochete bib]
VGGGARAGMINVQLDHYVLIRYTTIMKTFQVAEIRAQFSSVLKEVEAGSEIGITFGKKKE
TIAVIVPIEQYKKMKERKLGTLEGKAKIRISEDWTISDEELLNS*
>SPBIB_v1_310032|ID:27163807| putative Cytochrome c biogenesis protein transmembrane region [Uncultured spirochete bib]
MNQPDLIAAFAAGLVSFISPCVLPLLPAYLSLLSGLTIKELSGQQKRAKLLTASLMFSAG
FTLAFTLLGIIFSGGMSFAGAGASRLFGQIAGIIVIVLGFNIMFDFIRILNNDARLIQKF
AGKGRGQVNSFLMGLAFAAGWSPCIGPILASILLMAARNANIAAAALLLVAYSAGFAIPF
IASALFFERLSPLLGFLKKHGNGVRIVSGLLLVAFGIVMVLGSVSRISALAAQAGIVLLS
FDASSPFFSRLIGAAVWLLFAALSFRVILNRRRTHLQEEAIAPDQTQPASPQTTANSGST
RRPTLPWLLSALFFVLAILEIAGAIGLVRIIGGWLTFTGI*
>SPBIB_v1_310033|ID:27163808| conserved exported protein of unknown function [Uncultured spirochete bib]
MAHSQNRPLSSRLIFQSSSFILLSLIIVLSSAQPAAAAETRPWYASGLEALGFYVFDQPF
EQPNFSVAAINGSLKSRLSTKGNITLLNFWATWCPPCKQEIPTIQKLHETMKGEKFEIMA
IDLGEPPASVKTFLEQNKITYPVYVDPKNSLAALYASRGIPTTYILDKNGKFIAGIIGAF
EYDNPEFVRIMKELARK*
>SPBIB_v1_310034|ID:27163809|clpP| ATP-dependent Clp protease proteolytic subunit 2 [Uncultured spirochete bib]
MKLAEPQTTETEAKRPEEPLSERFLKTRTVLLVGEVDKDLSEKVVRQLLLLDSMSEEPIT
LLIDSPGGDVYAGFSIFDVIRFIKAPVRIVGIGLVASAAALILLAVPKERRFGLPNSSYL
IHQPLSGMNGVATEIEIHARELEKTRARINEIIAEATGQQLEKITKDTDRDFWMNAPEAM
QYGLIGKVIASRNELI*
>SPBIB_v1_310035|ID:27163810| putative Endonuclease/exonuclease/phosphatase [Uncultured spirochete bib]
VRPAFLFFAAIAMLVISCRCSLGSVDQAQEIRIVSYNVHNLFDAEESGNEYPEFKPSKGK
WTKELYAKRLASVIEAVRSLGKGKDSGSAQARDPDILCVQEIENEKVLADLAEHFRKGAY
RYWAISGPKDSIIHTGVLSRFPVTAMHTHSVMDAWGFGPLRDILEVELDTSGATSDADRI

LLLICHWKSKREGEQETESARRAAAALLAQRIREIESEKPDLPIIVCGDFNESPDEYLRI
SRRYPTALMPLESGSPPAAAEGEPLWVSQSFLQPGGSSPDDASRVALYSPWAEKPDGFSI
AYRDKREQFDGFLLNAALQDGFGLEYEGFCTSESPVLFDASGKPFEWNGTSGYSDHLPVC
LVLQKNAGQARVRY*
>SPBIB_v1_310036|ID:27163811| Gluconate transporter [Uncultured spirochete bib]
MVSGVVALLLLLLLAVILIIILTGRYKMNAFLVLIGVSFVFGLLIGLKPLDVIAGIKNGFG
GTLTNIGIVIVAGTIMGTILEKTGAALSMTQAILKLVGKSRAPLAMNVAGYIVSIPVFCD
SGYVILNPLNKALAKESGISMAVMAVALSTGLYATHTMVPPTPGPIAAASALGADLGKVI
LLGLIAAIPASLAGLLWATKFAKRYEIEPEIHETYSEIVKKFGKLPGTFLSFLPIVLPIA
LILLKSVAEFPSKPFGNGGFRIFLSFIGDPVTALIIGVLVSLLLVQKGELKNAISGWMGE
GIKESAIILVITGAGGAFGQIIKASPITEFIKTNMAGMQLGIFLPFIISAALKTAQGSST
VAIVTTAGIMAPLLQTLGLDPALTTIAIGAGSMVVSHANDSYFWVVSQFSGMPVNIAYKA
YTSATAIEGAVAFIAILILSLFVH*
>SPBIB_v1_310037|ID:27163812| putative Tetratricopeptide TPR_1 repeat-containing protein [Uncultured spirochete bib]
MNKLYATLCERFGLNAYIAGDYAKAERWFRKLEQSEPNSIRVLRNLGVILLAQGRSEEAE
RYLLKEEKLYGSSFYRHSALADLAYATGKRKEAERRYRKALQEPECAEGGKAFHMRLLME
KRLAICEDEKRFAATRKAMELFKQAEDLREAGAHEDAVAMFLKSFELDETNWPALNNAAS
ILFNKQSKPEEAKPLFEKAFELSHSIQVARNLELCQQSLNRTKKRNS*
>SPBIB_v1_310038|ID:27163813|ttuD| putative hydroxypyruvate reductase [Uncultured spirochete bib]
MMNTKTMLNLRQDAEAIFRAAVERVRPERLFSYSLSLDGSKLVVGDGQAVRGYDLDKYER
IVLAAFGKASIPMAASLAALLGERVSQGLVITKAPEGGQPLAFDPETRTLFTERSIHIIE
AGHPVPDQRSMLAGKEMLALASQVREWEKRGSHTLVCVLISGGGSALLSSPAEGLTLEDK
AAVTRLLLACGATIHEINAVRKHLSAIKGGLLARALFPAETLALVLSDVMGDDLDAIASG
PTVPDTSTWQDVKAIFDRYGLFDALPESVVHIIEEGCEGERPDTPKPGDPIFRACSTILV
GTNFHAIIEAERKSKDLGYHTLVIGTRLSGEAREIAKVFSGAVQDILLHKIPVATPACII
AGGETTVTIRGHGKGGRNQEMALSFLEELAHYPSALRGQLDRIAFLSAGTDGNDGPTDAA
GAFADAAVIERAKAHALNPAHYLAENDSYTFFDKAGALFKTGPTGTNVCDIQILIVK*
>SPBIB_v1_310039|ID:27163814| conserved protein of unknown function [Uncultured spirochete bib]
MKSDSQLKLAVLIDADNTQPAIIDGLLAEIAKYGIASVKRIYGDWTNPNLRGWKERLLEY
AIQPVQQFAYTTGKNSTDSAMIIDAMDLLYTENLDGFCIVSSDSDFTRLAARLREDGKLV
LGFGQRKTPKPFVAACDKFIYTEILQENVGETDEERESHPDKDAASKRAAAQPDIKDDKK
LKALLLSAAEEAADEFGWAYLGEVGTYIANRLPEFDPRNYGFRKLGELIKATNLFEIDER
TNPMVPGKQVYLRIKHRGRS*
>SPBIB_v1_310040|ID:27163815| conserved protein of unknown function [Uncultured spirochete bib]
MTDTQFARFCDVREKIRLYISSISENAQWILEAQRTVYNARGYYEADLETPVVYNLALED
ITAKSEPRFIIVADNPGIQEQKAKNHRYLVGQSGKLAVSWFRENLGIDFRSSTLIINKTP
IHTPKTAELRLLVRAAGSRSDELADLLVDSQREMARFAFDLLEILECPLWVSGIGELRPK
GIFRPWAEELRALCLGAPFELRERVWLFRHFSMNQFAIEYANARRAMMVDPKEAQNTQKA
SRVPNPGVSDPGATYAMLAEIGRKNRTTILGF*
>SPBIB_v1_310041|ID:27163816| conserved exported protein of unknown function [Uncultured spirochete bib]
MRVFSLIATTLAAAFAFVSSSACAAQKNDTLSRLPADIRAEAQAALSAVRLGDEDRSRIA
AAINQAPSEFASLFHEVRAVMNADPNLLRRVDKTVALPASFIPGDLVSLDGAFPYAVSKK
GMMLRKPAREALARMAEAARKDGITLVVSSAYRSYEYQKTVFERNVKEMGKIEAERVSAP
PGMSQHQLGTAIDFGSITDAFAETAASKWLAAHASTFGFSLSFPKNMEEVTGYRWESWHY
RYISPAAAQMQDRFFLGVQQYLIEFLSGV*
>SPBIB_v1_310042|ID:27163817| Cof-like hydrolase [Uncultured spirochete bib]
MNRNKPAAPIKLIALDLDDTLLRADLTISEQNKQALQRAENLGIHIVLASGRNYHSMRRY
AEELFIHRRGDYLIGSNGAQLIRASSGRLIEDLKLSAAFCKEVASDLERKGYLWQLYIGG
KIYCNRMNKWAIMDQEFTGQPLEVIRDFDTILSEDQTKILVCGEPEQIEALYQGLVNRIG
DRAEIVTSKPYFLEILPKGATKGAALERLTARLGLDMESVLAIGDARNDYDMIFKAGWGC
APANAADEIRAIARFVSARTNEEDAVADILEWGLTPFLKI*
>SPBIB_v1_310043|ID:27163818|murJ| Protein MurJ homolog [Uncultured spirochete bib]
MAPQENHENSAARASTPETAASTPAPTLESDAASAKMVRSAGMLSVLTMISRVLGLIREM

TKARFLGTGIYSDAFTVSFIIPNFTRRLFAEGSITVAFIPTYKGYLHAQNDEETRTFLSA
SLTVLTICVTAAVALGIALAPWIVKLFGSEPVETALLTRIMFPFLALVSFAALLQGILNS
HEIFNPSGLAPILFNICFIVVPWLVGSRLGNPARAMAVGVVIGGLAQALVQLPAVLRLGI
RFGFMNPARAFLNPGMRKVFALIAPTILGMAAYQVNDLVSTAFASRAGTGVASSLQYSLR
LQELILGIFAVSAGTVLLPRLADAVREHAWRDYSSTLGRTMRSLLLLTVPVAVFSMAFPE
RIVTLLFKSGDFTDVSVRLTASAFFWHQTGLVFIAMNRLIAPAFYARSDTKTPTWAGIAS
FGVNVVLVLALAFRFQGPGIAFALSFSSAMNTGLLVWALIKGNIEGVRHELWDALKYAIR
MAIFSAVAVVPALFVDRAVYAKVADAHSRLISAGVPLVAGTVVFAAVGIALLVLTKDSVA
ASITNAFSRKTRGRGR*
>SPBIB_v1_310044|ID:27163819|rpmG| 50S ribosomal protein L33 [Uncultured spirochete bib]
MASKKKQNVEIIALQCTECKRRNYTTKKNRKTTQDKLELMKYCKWDRKHTLHRETKVK*
>SPBIB_v1_310045|ID:27163820|secE| Protein translocase subunit SecE [Uncultured spirochete bib]
MKKIIQFFKDSYAELAKVVWPSKEDVIASTKIVVISTVAVALVLGLIDFLLVLGIEVVFR
*
>SPBIB_v1_310046|ID:27163821|nusG| transcription termination factor [Uncultured spirochete bib]
MAKAWYILHVYSGYENKIEKTIRMLIDSGELSRDIVTDVKVPAEEVAEIKDGKKRTVSRK
ILPGYILVEMDLPENGWKATCAIIRNITGVTGFVGSASNVKPVPITMEEVKRILQRTGEL
KGERQVHFRQTFTVGEQVKIIEGPFESFSGVIDEVNAEKSKIKVSVQIFGRSTPVEVDMA
QVEKM*
>SPBIB_v1_310047|ID:27163822|rplK| 50S ribosomal protein L11 [Uncultured spirochete bib]
MAKKKITAIVKLQCPAGKATPAPPVGPALGPHGVSAPMFCQQFNDRTKNMEPGLVIPAII
TIYQDKSFTFILKTPPASVLIKKACGIEKGSALPQKDKVAKLSKAKLEEIAKLKLPDLNA
NDLEAAKRIIAGTARAMGVEVEW*
>SPBIB_v1_310048|ID:27163823|rplA| 50S ribosomal subunit protein L1 [Uncultured spirochete bib]
MKHGKKYNEVAKKVDPSQAYELGAACALVKELKTAKFDETVEVHIRLNLKKSQSVRDTVV
LPHQFRGEKRILVFCRPERVKEALEAGATYAGDAELIEKIKGGWLDFDIAVATPDMMKDV
GKLGMILGRRGLMPNPKTGTVTNDLPASLNELRKGRTEYRTDKSGIIHLPVGKVSMEPEK
IAENLNTLVEEISRKRPADAKGDFIASIYLTPTMGPSVKVVLNKSEKR*
>SPBIB_v1_310049|ID:27163824|rplJ| 50S ribosomal protein L10 [Uncultured spirochete bib]
MAIKASKLQPRKIEAIGMLKEMISSSNDFIFTEYRGLTVEQITSLRRQLREKGVELHVVK
NNFARLAFEELGFSQDVAPVLTGPTAVAFVKTDSNEVAKVLLDFAKETPALVIKGAMVGR
QFMDSHQVEALSKLPGRNQLIAMLMSAMQGPAQNLVYVINAIPTKLVRVLKAIEEKKAQE
NA*
>SPBIB_v1_310050|ID:27163825|rplL| 50S ribosomal subunit protein L7/L12 [Uncultured spirochete bib]
MAALTKEQIIDAIASMTVLEVSELVKAMEEKFGVTAAAPVAVAAAPAAGAAAAPVEEKTE
FTVVLKGNVPADKKIAIIKEVRAITGLGLKEAKDLVEAGDKPLKENVSKEEAEKVKKQIE
AAGGQVEVK*
>SPBIB_v1_310051|ID:27163826|rpoB| DNA-directed RNA polymerase subunit beta [Uncultured spirochete bib]
MAYTEQKIKRIYIGKDYRDVMDIPDLIDIQVSSYERFLQRDKLRKGEPLDLIGLEEVFRG
TFPIESPSGDLVIEYEHYSLDEKAIKYSELECKQKGLTYSVPLKAVINLVFKKTGEIRQK
EIYLGDIPLMTDRGTFIINGAERVIVSQIHRSPGVVFSHEKGVFSARIIPYRGSWLEFEI
DQKHDLIHAKIDRKRKILGTLFLRAIGIQTREDIIAAFYTTKQVELSENQTEKDALVGKI
LAKAVYIELDGEKKRLFRAGDKIHLHEIDELINLGIGSVEVINFDDEKSLHNEMILNCFE
REDVKLVKETPDQDEPTKEDAISAVYSVLRPGEPISLENAERDLHNMFFTARQYDLGSVG
RYKLNKKFDFDFKTLTLTKDDIVATMKHLINVYYGEANVDDIDHLGNRRVRAVGELMSNV
MKVAFSRMERTAKDRMTGKDLETAKPQDLITIKPIVAAIKEFFGTSQLSQFMDQVNPLAE
LTHKRRLNALGPGGLNRDRAGFEVRDVHYTHYGRMCPIETPEGPNIGLIVSLATYTTVND
YGFLETPYRKVVKGKVTDEIEYLSAIDEDKYYIAQASARIDTAGKFLDQSISCRHQGDYT
MRRPDEIQYMDVSPKQIISVSAALIPFLEHDDANRALMGSNMQRQAVPLVFTEPPRIGTG
MEWKTAYDSGVLMKAKRTGTVEFVDANRIVIRPDKMESPDDTDVYQLIKYQRTNQDTCFN
HRPIVKRGEHVLKGQVIADGPATSQGELALGRNILAGFVPWNGYNYEDAILISERVVKED
LFTSIHIKEFILEVRDTKLGPERITRDIPNTSEKMLDNLDGEGVVRIGAKVKSGDILVGK
VTPKSDTDTTPEFKLLNSIFGEKAKDVRDSSLRVPHGIEGTVIDVQRMSRANNDDLAPGV
EETVKVLVAVKRKLKEGDKMAGRHGNKGVVARVLPVEDMPYLEDGTPLDICLNPLGVPSR

MNIGQIMETELGWAASTLDEWYACHVFQSPSQEQIEEKLQQAGLPVSSKVMARDGKTGEY
FKNPITVGVVYFLKLHHLVDDKMHARSTGPYSLVTQQPLGGKAQFGGQRLGEMEVWALEA
YGAANTLQELLTIKSDDMTGRSKIYEAIVKGEPHTAAGIPEAFNVMVQELRGLALDIKVY
DSKGKQIPLTERDEDIIKKQSGAF*
>SPBIB_v1_310052|ID:27163827|rpoC| RNA polymerase, beta prime subunit [Uncultured spirochete bib]
MRDIQDFDNISIHLASPEMIRAWSYGEVKKPETINYRTLRPERDGLFCERIFGTTKEWEC
YCGKFKSIRYKGVICDRCGVEVTNSKVRRERMGHIELASPVSHIWFYRSVPSRMGLLLNL
PMATLKSVLYYEKYIVIDPGETELKKMQLLTDEEYREAMDRYGGAFTAGMGAEAIRSLLE
QIDLDKLSQELRQKMIERGAKADKRLLKRIEIVEAFRSSGNRPEWMILTVIPVIPPELRP
MVQLDGGRFATSDLNDLYRRVINRNNRLKKLLQLNAPDIIIYNEKRMLQEAVDALFDNTR
RRRAVKGAGNRPLKSLADMLKGKQGRFRQNLLGKRVDYSGRSVIVVGPELKMWQCGLPTK
MALELFKPFIMKKLVEKDVVYNIKKAKMLVESETPEVFAVLDEVVKEHPVLLNRAPTLHR
LGIQAFEPILVEGKAIKLHPLVCKAYNADFDGDQMAVHVPLTHAAQAECWTLMLSSRNLL
DPASGKTIVYPSQDMVLGINFLSRHRPGTKGEGKRYSSPEELLLACEYGACDYQALVYVQ
TPKEPVWDSYAKPATLNGSKLIKTSAGRILLNEALPKEIPYINYALTDKDIRSLIEYVVR
TNGPFITVNMLDTIKEMGFKYATFFGATIGMDDIVIPKEKTEMIEKANKEVEAIQQQYLA
GHITQDERYNRVVEVWSKTNEELTAVMMKTLEKDRDGFNNIYMMAHSGARGSKNQIRQLA
GMRGLMAKPSGDIIELPIRSNFREGLSVIEFFISTNGARKGLADTALKTADAGYLTRRLV
DIAQDVVVNDDDCGTINGAIHTAIKDGDEIIESLKERIIGRFTLDPIRHPITGEIIVGSN
EEITEEIAEQIEAAGIEEVSVRTVLTCEAKHGVCRKCYGRNLATGKTVDIGEAVGIIAAQ
SIGQPGTQLTMRTFHIGGAATKAAEENRISLRYPAYVVDIEGTYVKTDLGHFLFTRRGYI
YVNRVFGAFELSKGDVVLVEDGQRVMKDDPVIRKADGAEIKSPSISYAKVLEGKLLLIAQ
EQKVEIRNGSEVVVTKGQILLANETIATFDPFNDPIISEHDGIARYEDIVPGSTLKEEIN
AETGNVEKIITDVGSERDTKEPRILITDEAGNDIETYYLPEGAYVNVEDGEPVKAGKILA
KILKESAKAMDITGGLPRVGELFEARKPKTPAVLAMVSGTVSFGGSLKGKRVINIRDSFG
KVYKHLVPKNRRLLVRNGDKVAAGELLCDGSKNPHDILAILGEQACQRYLMDEVQQVYRA
QGVTINDKHIGVIIRQMMKKVEIVNPGDTVFIYGQQIDKHKFHDENRRVEEEGGQPAVAR
PILLGITRASLKIDSFFAAASFQETTRVLTDAAIKGEIDQLRGLKENVIIGHLIPAGTGM
KQYRQVRLFDAVNDDLNSLVEEVLEKRKQERELAPPAILQDRTTNDVSFDEPSVFDNDEG
FSEDVEPSE*
>SPBIB_v1_310053|ID:27163828|rpsL| 30S ribosomal protein S12 [Uncultured spirochete bib]
MPTINQLIRFGRKQNQWKTKSPALMGCPQRRGVCTRVMTVTPKKPNSALRKVARVRLTNG
IEVTAYIPGIGHNLQEHSAVLVRGGRVKDLPGVRYHIIRGTKDALGVEDRKQGRSKYGAK
KPKA*
>SPBIB_v1_310054|ID:27163829|rpsG| 30S ribosomal subunit protein S7 [Uncultured spirochete bib]
MGRKKKSIDRGHAPDVKYNSTTVSRFICRMMWQGKKSICTSIMYDALELLQQKSGQPALD
AFNKALENVKPSIEVKSRRVGGATYQVPIEIRESRREALAMRWLINAARARTGKAMAECL
ADELFDAFNNTGTAIKKKDDMHKMAEANKAFAHYRW*
>SPBIB_v1_310055|ID:27163830| putative Elongation factor G [Uncultured spirochete bib]
MPTESQPRGWLFLFVQQKYTDPAMNMQAAPISIRNIGILAHIDAGKTSITERVLHVAGVR
EKAGNVDEGTTATDYLTIERQHGITIKTAAVRFIHKGVRFHLLDTPGHVDFSAEVDRVLR
VLDGAVIVLCAVSGVQVRTGFIASGCRAHKIPRMYFINKMDRRGADFFAAMQEIAGELGE
SVLPLQVPTFEGHQWNGIADLVNLRWFPAMLDMSENIAQAGESPASLDSIGLPIDQAPIS
EKSQRQIEQYREKILDRASMYDDRLTEMIVNGQQISPAHIRDALRPAIQRQDIMPALCGS
SFSDISTALLLDAVIDYFPDPLERGCPPAKDLKNGSELNLPAAPDAPFSAYIFKSVLSQN
LEPLGWLRIWSGTIREGMKVMAMPARKHVQIQRLYGVNGADVEHIESAAAGEIVGARLSF
MEAGGTLCAANLQIIYESFKNPEPLVFMVIEPSSQAELPQLREALKHFSMEDVSLSVSEE
KDTGRITIAGQGELHLDIIRERLKKEYGLRVRAGNPQVPKIERLKKAARLADQFVGDFGG
ERLAVGLGVFLENEKDNNANSLHVASGIRLSANYEACILRAMETVLAVGPCEGWPIVGTK
VIVESFMPPGGAAGSTDKRSETAVEAAASSILRKAVNLAGTVVFVPIVQVSVEVPDDWFG
SALASLQARGARIEAVEDTGGVKSIVAYAPMENLFGYATSLRSITEGRGVYQAKFDHYGS
AREY*
>SPBIB_v1_310056|ID:27163831|tufA| protein chain elongation factor EF-Tu (duplicate of tufB) [Uncultured spirochete bib]

MAKEKFERTKPHMNVGTIGHIDHGKTTLSAAITMYCGRKFGDKVLKYEDIDNAPEEKARG
ITINTRHLEYQSNKRHYAHIDCPGHADYIKNMITGAAQMDGAILVVSAPDSVMPQTREHV
LLARQVGVPAILVYLNKVDLVDDPELLDIVEEEVRDLLNFYGFPGDKTPIIRGSAFKAMT
EPDNPEATKSIQELLDAMDSFFPDPERAIDKPFLMPIEDVFSISGRGTVVTGRVERGVIH
VNDPVEIVGIRPTKQTVVTGVEMFNKLLDEGQAGDNIGTLLRGIDKKEVERGQVLAKPGT
ITPHTKFRGQIYCLSKEEGGRHSPFFSGYRPQFYFRTTDITGTVKLPEGKDMVMPGDNSE
IEVELIYPIAMEKGLKFAIREGGHTVASGQVTEVIA*
>SPBIB_v1_310057|ID:27163832| protein of unknown function [Uncultured spirochete bib]
LLLLPAASGWLLDKQFSECYYSVPLKGGGTLLCEMVPRAH*
>SPBIB_v1_310058|ID:27163833|rpsJ| 30S ribosomal subunit protein S10 [Uncultured spirochete bib]
MKDRIRVRLRGFDVRLVDDSAKSIIKTVQNAGSKVSGPVPLPTRINKFTVLRSPHVHKKS
REQFEMRTHKRLIDIIDPTPEVMDALMKLELPSGIDVEIKQ*
>SPBIB_v1_310059|ID:27163834|rplC| 50S ribosomal subunit protein L3 [Uncultured spirochete bib]
MIGVIGKKVGMTQIFDETGRVVPVTVVQVVPNVVVSKKTAEKDGYNAVVVGVYEKKKSRV
TKPYAGQFPEGIAPTRILREFRDFEKEVDVGQSIDASVLDGVRFVDVIAKSKGKGFQGVV
KRWGFEGGRATHGSKFHREPGSTGNSTYPHHSFKNIKMPGHMGNERVTVQNLKVVRVDAE
KGVVLIRGALPGPRNCDVLIRKSVKKS*
>SPBIB_v1_310060|ID:27163835|rplD| 50S ribosomal protein L4 [Uncultured spirochete bib]
MEGKVISLSGKELRTIELSDAVFGLPINEDVIWYAVNNELANARVGTASTKDRGEVHGTN
RKPFAQKGGGRSRHGDLKSNIYVGGGISFGPKPRDYSYAIPRKAKQLSLKTILSLKIQDG
LVSIIEDFTVESGKTKDLLKVLSNVVDLKNPARTVLILKDDDPMIKRAARNIPWLSYLTY
NRLRAHDLFYARKVVMLEGASAKLNEFYASETKES*
>SPBIB_v1_310061|ID:27163836|rplW| 50S ribosomal protein L23 [Uncultured spirochete bib]
MEYDAILIEPVLSEKSNIMREIGQYIFKVDARATKPMIMEAVAKTFNVHPVSCKVINVTS
KPKRLRGRPGRTAEWKKAIVRLQKGETIRVFEGA*
>SPBIB_v1_310062|ID:27163837|rplB| 50S ribosomal subunit protein L2 [Uncultured spirochete bib]
MAIRTFRPVTPGLRHRVQVINEEITSNESKPVKSLTKGKRSTGGRASNGRISVRHHGGGH
KRKYRIIDFKRDKFGVPGKVSGIEYDPNRSANIALITYADGEKRYILSPKGLKVGQIILS
GPEAPAEVGNALPLDKIPVGFTVHNVELTLGRGGQLARSAGVSALIAAKEGDYVVLKLPS
GEQRMVFKKCIATVGQVGNEEHMNERIGKAGRTRWLGIRPTVRGTVMNPVDHPHGGGEGK
GKGYKQPVSPWGQPAKGYKTRDTHKPSGRFIVKRRK*
>SPBIB_v1_310063|ID:27163838|rpsS| 30S ribosomal subunit protein S19 [Uncultured spirochete bib]
VSRSVKKGPFIEKSLYKKVTEMSRAGDKKLIKTYSRTSTIIPEMVGQTISVYNGKTWIPV
YVTENLVGHKLGEFAATRVFRGHAGSDKKAEKK*
>SPBIB_v1_310064|ID:27163839|rplV| 50S ribosomal subunit protein L22 [Uncultured spirochete bib]
MANTKTGYRATSKWLIASPFKVRPVADLVRAKPYTEAMAVLENMPHKGAKLIRKVVASAA
ANALNKNRKLGEDMLYIKELRIDEGPRMKRIWFRARGRADMQLRRMCHISCVVDEIGKKT
EA*
>SPBIB_v1_310065|ID:27163840|rpsC| 30S ribosomal subunit protein S3 [Uncultured spirochete bib]
VGQKVNPIGLRIGINKDWSSHWYVEPREYAKTLHEDLAIRRRLLELPETKGADISEIEII
RHPQRITIVIHSARPGVLIGIKGANIDKIGAEIQKITQKKLQIKIKEIKKVDTNAQLVSQ
NIARQLENRQSFRRSMKSAIQNALKAGAQGCKVRLSGRLGGSDMSRTEELKEGRIPLHTL
RADIDYGFSEANTTFGKIGVKVWIYQGMVYAQDKNEDAGQLARKAKREPAGEARGERREG
SQERRDRRERGERSERGERKPSAKPDADRAEGEARS*
>SPBIB_v1_310066|ID:27163841|rplP| 50S ribosomal subunit protein L16 [Uncultured spirochete bib]
MLIPKRVKHRKQQRGRIHGEAHSGNTIAFGDYGLVCLEPHWITNRQIEAARIALNRYIKR
GGKMWIRIFPDKPYSKKPAETRMGRGKPGPEYWVAVVKPGTVLFELSGVEPKAAEEAMRL
AGSKLPVKTKFAVREEME*
>SPBIB_v1_310067|ID:27163842| protein of unknown function [Uncultured spirochete bib]
MRFVVFVVGFNTLRLVHILSVERMQLAHDNFYDNGLIHLVGNDNALQNKLALSHFLDVRI
HVPSPEELFLLRDAAALGFGDPGFDPRDLAAKISLLERILYVPDHHLESKVKVFLSKLIA
LDEHLFHLQIFE*
>SPBIB_v1_310068|ID:27163843|rpsQ| 30S ribosomal subunit protein S17 [Uncultured spirochete bib]
VDTNIQKMTEGKLVLQGIVVSDKMDKTIVVEIIMRKLHPLYRKYVNKTKRVKAHDENNEA

HVGDTVSVVECRPLSRDKRWRLVEIVERAK*
>SPBIB_v1_310069|ID:27163844|rplN| 50S ribosomal protein L14 [Uncultured spirochete bib]
MIQVESMLNVADNSGAKTVQCIKVLGGTRRRYASVGDIIVVAVKSALPNSAIKKGSVEKA
VVVRTHKEYKRPDGTYIRFDDNACVIIDANKNPKGKRIFGPVARELREKDYMKIISLAPE
VL*
>SPBIB_v1_310070|ID:27163845|rplX| 50S ribosomal subunit protein L24 [Uncultured spirochete bib]
MANAKYKLRKEDLVQVIAGKDKGKQGKILKIDHEKGRVIIAGINMVKKAMKKKSQTDRGG
IVEVEAPLHISNVMIICKKCNKPVRIGYKFEGEIKKRVCRKCGEVL*
>SPBIB_v1_310071|ID:27163846|rplE| 50S ribosomal subunit protein L5 [Uncultured spirochete bib]
MAEAKVYIPRLKKLYREKVAPELFTELGYTSKMQVPRLVKVIVSMGVGEARENKKLLDAA
VGDIEIITGQHAVKTKAKKSIANFKIRKGQEIGARVTLRGNQMWEFLDRLMNVALPRVKD
FRGVNPNGFDGHGNYTLGLTEQIIFPEIDFDKIEKVMGLSVSIVTTAETDTEAKSLLAKL
GMPFRR*
>SPBIB_v1_310072|ID:27163847|rpsH| 30S ribosomal subunit protein S8 [Uncultured spirochete bib]
MSVSDPIADMLTKIRNANAARHEKVDIPTSKLKLEIVKILKTEGYIKNFKKIQADGQNFI
RIFLKYDEENNPIIHGLSKVSKPGRRVYTGYREMPRILNGYGIMVISTSDGVITGRKAVE
KQVGGELICTVW*
>SPBIB_v1_310073|ID:27163848|rplF| 50S ribosomal protein L6 [Uncultured spirochete bib]
MSRIGMRPVAIPQGVKVTVEPTVFHVEGPKGRLSQEYRPDVAIAVKNGEAVVERKNDSKQ
AKAYHGLYRSLLNSMVTGVSQGFKRALVINGVGYRAEVQGNFLVLSIGYSTDVVAVIPDG
LKVSVEQNGQKVVIEGIDLAKVGKFASEVRSLREPEPYKGKGIRYEEEKIRRKVGKTGVK
*
>SPBIB_v1_310074|ID:27163849|rplR| 50S ribosomal protein L18 [Uncultured spirochete bib]
MSVRELSDKIRKRDRRRTHVRKTIVGTAEKPRMTVFRSNLHLYVQVIDDTLGHTLASVST
MEQQFRSLRATVQTGGVLGEEIGKRLLEKGITQVVFDRNGYKYHGIVKAIADGARKAGIK
F*
>SPBIB_v1_310075|ID:27163850|rpsE| 30S ribosomal subunit protein S5 [Uncultured spirochete bib]
VERTRRDDSGVQDGYTEKLIQLNRTAKVVKGGRRFSFSALTVVGDKQGHVGFGFGKANDV
TEAIRKSVERAKRAMVTVPVKHGTLPHEVTGLYKATTVYMKPACPGSGVIAGGPVRAILE
CAGYTDIISKCVGSRTSVNVVRAVFLGLDQLLDAQTIAKNRGKTLKDMWS*
>SPBIB_v1_310076|ID:27163851|rpmD| 50S ribosomal subunit protein L30 [Uncultured spirochete bib]
MARIQITLVRSTIGQKPNARATVRCLGLRKIGSSTIQEASPQILGMVNRVRHLVETKEVE
*
>SPBIB_v1_310077|ID:27163852|rplO| 50S ribosomal subunit protein L15 [Uncultured spirochete bib]
MTDFNLHAPEGAKKRKHIVGRGDGSGWGGTAGRGNKGQQSRSGGKTYPGFEGGQMPLYRR
LPHRGFSNYPFRKEYQIVNIRDLEGKFQAGDTVDTVSLFMKGLVRKPELPVKLLGEGQIE
IALTISVDAVSSSAKTKIEAAGGKVEVPAATGSSADA*
>SPBIB_v1_310078|ID:27163853|secY| preprotein translocase membrane subunit [Uncultured spirochete bib]
MAGTLVDIFRVKELKDRIFFTLFWLAIFRLGAFLPIPGINASALQSYFANQANTGGGITD
YLDFFAGGAFKNFSLFMMGVMPYISAQIIMQLLLVIFPKLKNIAEEEGGRRKISRWTRIG
TIGIAIIQSFSVTVWADRIPNAIAMSNRTMYTIVAIITVTTGTMFLVWIGEQITKRGIGN
GISLLIFAGIVARLPNATYELIKKIQLGEINAVYAIVVLIMFVAVVALVVLEQQGQRKIP
VNYAKRIVGRRMYGAQNTYIPFKINPSGVIPVIFASSLLTFPLQIAQSFGVQAKWLRDFS
YWLRPSGFWYNFFYVVFIIFFAYFYTQVTLNPQEISKNIRENGGSIPGIRSEKMEEYLTR
VLNRIILPGSLYLGLIALIPTLVQRLFNFPSQMAYLLGGTSLLILVGVDLDTMAQVEALL
KMHHHDGLVRKGHIRSRNL*
>SPBIB_v1_310079|ID:27163854|rpsM| 30S ribosomal subunit protein S13 [Uncultured spirochete bib]
MARIAGVDLPNKHVDIALTYIYGIGRTSAIKICEATGIDPAKRINDLSNEEINELRKVIE
NEYKVEGRLRTEIALNIKRLMDIGCYRGLRHRKGLPVRGQRTRTNARTRKGKRKTVANKK
KAV*
>SPBIB_v1_310080|ID:27163855|rpsK| 30S ribosomal subunit protein S11 [Uncultured spirochete bib]
MNVAATTTKTKKRKEKKSVYEGNVYIQATFNNTIVTVTDLNGNAISWSSSGSHEFRGAKK
STPFAAQTVTEAAAQKAIQAGLREVHVFVKGPGIGRESAIRQLGVMGLKVKSINDVTPIP
HNGCRPRKARRI*

>SPBIB_v1_310081|ID:27163856|rpsD| 30S ribosomal subunit protein S4 [Uncultured spirochete bib]
MARYIGPVCRLCRAEGRKLFLKGDRCRSDKCPINKKRLPPGKAPKTRLGKKSEYAVQLRE
KQALKRNYGLMEQQFRNTFEKALGLPGKTGDNLIILLERRLDNVVYRLRFASSRAQARQL
VNHGHIAVNGKRVNIASYLVRAGDVISVIGAAKKMESIRQALEEVQKSGVMPWLEVNADE
MTGRFVTNPQRQDVTDIADIKEQLVVELYSK*
>SPBIB_v1_310082|ID:27163857|rpoA| DNA-directed RNA polymerase subunit alpha [Uncultured spirochete bib]
MARKNLLKGFKKPKGITYEQSESTDTYGKFVAYPFEPGYGTTVGNTLRRILLSSIQGYAI
TAVRITRYDDEGVPHVITSEFETIPGVVEDTIEVLNNLKKLRVKLPNDKEELTLEYEIKG
MEELRGSFFADQSSLIVLNPELKIASLDPSVHLGLELQVNLGRGYVPSEVNEKFIDVVGT
IPMDAIFTPVKKVKYSIEPTRVGQRSDYDKLVLEIWTDGTIKPEDALGEAGKIAKDHFSI
FINFDENEAGGEETSDEIEERVRQILNMPVEELELSVRSFNCLKNANIRTIGDLTKKTEE
EITKTRNFGKKSLQEIKTKLAEWNLSLGMADYSALRNHIKLNMKKEESDEA*
>SPBIB_v1_310083|ID:27163858|rplQ| 50S ribosomal subunit protein L17 [Uncultured spirochete bib]
MKHKIGYNKLNRVAAHRKALIRNMVTVLFKQEKIVTTKAKALEARRAAEKLITRAKEDSV
HNRRIASARLYGEDAVAKLFTDIGPRFKERNGGYTRILKLGNRANDNASLVVLELVGQKA
DEKKAEREKRKAERKTKKQA*
>SPBIB_v1_310084|ID:27163859|livF| leucine/isoleucine/valine transporter subunit ; ATP-binding component of ABC superfamily [Uncultured spirochete bib]
MLKVTNLKVNYGKIEAIKDVSFEVPDGKIVTLIGANGAGKTTTLRAISGLERAAGGSIVF
EGKDITNLEAHKIVPLGISHVPEGRKIFPTLTVKENLELAGWTIKDKTEVNRRIDQVCEI
FPRIKERLSQLGGTLSGGEQQMLAVGRALVTGGKLLLLDEPSMGLAPVLVDEIFDRIVAI
NKQGTTVLLVEQNAMEALEIADFAYILEVGYTTISGEAKAIEHDQRVREAYLGV*
>SPBIB_v1_310085|ID:27163860|livG| leucine/isoleucine/valine transporter subunit ; ATP-binding component of ABC superfamily [Uncultured spirochete bib]
MTPLLKIEHLTHFFGGLRAVYDFNFEIPEGSIYGLIGPNGSGKTTIFNLITGIYDPTEGA
INFQGENIKGMKSFRIVHKGIARTFQNLRIFGNLSAIDNIRIARHYTTTSSLFASILRLP
SFKSEERAVKREALELLDLMHLSNRAMETAKNLPYGELRRLEIARALATKPKLILLDEPA
AGMNPKEVDDLMRLISRIRDEFKVTVFLIEHHMQVIMGICEWIKVIDFGETIAEGLPDKV
KNDPRVLEAYLGKRGT*
>SPBIB_v1_310086|ID:27163861| Branched-chain amino acid ABC transporter permease protein [Uncultured spirochete bib]
MNVRKLPRFLIAVVLFYIVVEVLYRTEILNGYLLHIINLSLIYVILATSLNLINGITGQF
SLGHMGFAAVGAYVSGSLTTLILKLSVTDPASMLPFILSIIAGGVVAAFVGFLIGFPSLR
LKGDYLAIVTLGFGEIIRTIFNNIDAVGGPRGLLGIPKFSNFTVILVATFLTVVIIRNLI
ESSHGRAMLSIRENELAAELVGINTTRYKVMAFTFGAFFAGIAGGLLAHLIQLAHPTQFG
FIKSVEVLIMIYAGGVGSLTGSILAAFGLTFLSEGLRLGIRALADATGLPIGGEWRMVVY
ALLLIFIMLFRNEGLMGMRESRIIKNVEVD*
>SPBIB_v1_310087|ID:27163862|livH| leucine/isoleucine/valine transporter subunit ; membrane component of ABC superfamily [Uncultured spirochete bib]
MGSIFSLQQILNGLQLGSIYALIALGYTMVYGIVRLINFAHGDFFMIGAYAAYGSFYLFN
ALVAGRSGTLAFLVLLAAMIGGASIAYLTNKFAYKPLRYKPKLSSLITAIGVSMFIEYFF
SALPAIGPSYRSFPDIIVRKEIRLGNAVISNLVIIDLAVAAILMVGLTLLVRKTTLGKAM
RAVSQDKDAARLMGIDIEKVISFTFMVGGAFAGAAGLLAGMTYPRIFPYMGILPGLKAFI
AAVLGGIGNIPGAMLGAYIMGIAETFASAYNSLIGEGIAFAILILVLLIRPRGLLGEKIA
DKI*
>SPBIB_v1_310088|ID:27163863| Branched-chain amino acid ABC transporter substrate-binding protein [Uncultured spirochete bib]
MKKVLFVMALVVLVAASYAQDIKIGGIGPVTGEAATFGISTKNGMTMAVDEWNAKGGIFG
GRKAKLIFEDDKGDPSEGAIVWNKLIQQDNVVAIVGTVMSKVSLVGAPISQAAGIPMISP
TSTNEKVTLVGDYIFRACFIDPFQGTVGANFAYNDLKARSAAAIFDLGNDYTKGLAENFK
KQFEKLGGKIVAFEGHPTGTTDFKAQLTKILQGKPDVIYIPDYYNDVGLIAKQARELGFK
GPLVGGDGWDSPELVKIGGTAVENGFFTNHYSSEDKRAIVQDFVKKYKAKFGAEPDALAA
LGYDAMYIMLDAIKRAGSTKGAAIRDALKATDINVVSGRIKFDENRNPIKSAVIIEIKNG
KQVYRTTVNP*

>SPBIB_v1_310089|ID:27163864|rarD| Protein RarD [Uncultured spirochete bib]
MAQEKAQTSMEHETRTGRFSQKQGLFAAIGAYGLWGIFPLYWKLLSSVLPLQILAHRILW
AAVFCLMLLLARKEMSSLGVALKDPRKRVLVGAAGLLVTVNWGMYIYAVNSGHVLETALG
YYINPLLSVALGAMLFREHIDRWTRIAVGIALVGIVAAGILYGQVPWISLILAITFALYG
AVKKGLGLAPITSLALETFSVAPLAFAFLAVMHAQGAGSFGNAGLRVTALLALSGVVTAV
PLLLFGVAAISISLQLIGFIQYLTPTSQLLLGLFLYKEKPNAAVVAAFVAVLVAVVIFAV
TRFKKGEPA*
>SPBIB_v1_310090|ID:27163865|hutI| Imidazolonepropionase [Uncultured spirochete bib]
MAENRQKALFVHNIGELWTAEGLRAAAGPAMGRVKKFRDTCLYIEDGRISAIGEEARLRA
AATAAPTFDAEGRACVPGFIDSHTHFIFAGWREDEFYWRAQGIPYMEMHQRGGGIQKSVD
ATRRASLEELVRLGKARLETMLSLGITTVEGKSGYGLDKETELRQLQAMDELSTVMPLTI
IPTFLGAHSIPPEYKDRPREFLAFLVREVFPLFKKRGQTPFVDIFCEEGVFGIEDSRWYL
EQARAAGFALKLHADEVKPLGGAGLAAELGATSADHLLKASDQDIQALAAAGTIACLLPL
TAFNLREPYASGRRFIDAGCAVALASDLNPGSCYSQSIPLIFALAVLYLGMSIEEALTAL
TLNGAAALGLAAQTGSLEPGKDADFLLLNAPSPGHLAYHAGMNLVHSVFKRGILVWQNGN
VLGPARI*
>SPBIB_v1_310091|ID:27163866| conserved protein of unknown function [Uncultured spirochete bib]
MVTKRDHGIHLDRTSPQERSRLISYINIKLESLGLPIYSKEGTGFVQLAADMLESFRQKE
RLLPRVLPPSDQRIQTFIDRYLADLGLARVPQIPSNTLVLDRYGMARELSLPPDGHKHIS
PTLTSYRVRNGVLHNPKSDRRTTEGVFHIVEGGLPIPPDKKAVPKLAFARILEAALNPPP
ELLELPFTAGENEKARTFVSVLLRPVVRPEIPGYCEERSMEVRFFAPGSLVANVDFVESI
FGNSGDPFIPENDASIDPVHWTGTTGCIILATHLTELTKKELGLPHWDEATERQRCDGMC
WREPTEKYNDGRAFKVCARDESGVILTIIADNYFGYSKKEIKSHISYSANLLGLVEEEHA
GGALVFPSYNLGTRFVPDTNLKSKGHSLHDVFTIMRGRIEMKQEGYAVDLTFPNIIYLPE
DAYISLEDQMAHWVSDGREESLRVLPDYIYVHPTGYRVHMERHPATGAWRLIGTAAEGLL
CHKPCTVSGGGKSEIAKSIHDAITYMPIIIADFDKDMRAVKEIIEKDYGNRFRDEAENHG
KDARPVLSPKRSLGSVIKLLSPSPAYNDEYNEWLRSIPERIKSLVFLVKRFYRPDWGDDW
LSHFSVDAVNGTTGNILKFEGRPIMGSYLRVGKNEHGMNRFFKLRQDFMPAFKLQWEDDI
SASIVVPVDQLEDLPEWAQNHLSLKFAKNCEARFFQRPDDAIIRGYDKQTEKDFARQDNF
VSNFEPLTRADASRIIENTINFYEYTEPMRKFIEETEKDPNFEYFVASNCFRLVDGAPSK
NPRYLQLDPTYVNPQAKYLAELGPRLYRRIPADKALPQPVGAVLPGRRNNPADPKAGIRP
LAVYGPIHYQDLPELFMDFVCSLTGKSPSTTGAGSEGALTKGPFNALVPTTDLNNALLAF
ILTGYAGFTTAAGHIGHKYKMDHDISLMMPELWARMGPEERDPEFLKSHGYLEKVEDFKY
KGRLIPASRLGWRITPLFAATYLGRLFDTPSVVFPEDMLRPELQSLEEFVDGIENIAAAM
EKSAKAYFEDGSVEAAIPPLKAVLSVMAYGNYEGKSIQDPEVRKLFDREYVLSSDWYRER
LERYREHEIAYIESSISYLRKFLAERAEPKSLTERRVQAELSSAHGRLEQLMAPNYLKRI
WGSIGLDPLYQGQQKA*
>SPBIB_v1_310092|ID:27163867|hutH| Histidine ammonia-lyase [Uncultured spirochete bib]
MQCYRENRRLSITLLYGILKNMNDVIIDGCSLSIPELVVVARHKIPVRLDPDAEERMRAS
REVVELAVKKKLVKYGITTGFGKFCNVTISDEDNALLQKNLIMSHACGQGEPFAPEIVRA
MMVLRANALAVGNSGVRPLLVQRILDLVNADIVPVVPEKGSLGASGDLAPLAHIALVLIG
MGEAFYKGNRMPGALALAEAHIAPLVLEAKEGLALINGTQAMTAIGALAVYDALMLYRSA
TIASALTFQALRGITEALDPRIHELRRQKGQIDAAQDLRWLLEDSLLTTKPSGTRVQDAY
ALRCTPQVHGACVAAIQHVWDIISAELNAVTDNPLIFPHVLHEGGDILSGGNFHGEPLAL
VLDYLGIAAAELANLSERRLERMVNPALSEGLPAFLTINGGLNSGFMIVQYSAASLVSEN
KVLAHPASVDSIPSSANQEDHVSMGTIAARKARQIIDHSQRVVAMEILAACQALDLRAIQ
LGLSSIDESLSPATRSIYRMLRTVVRFTDKDRIMYPDIDAVVELVSDGTLPESIDRWSWN
PGPVVPCEEYV*
>SPBIB_v1_310093|ID:27163868| conserved membrane protein of unknown function [Uncultured spirochete bib]
MKRRESAASLFGVIVAVIWGMTFLSIKVALRELGPMSLSLFRFVIASVLLAGIMAASRTS
FAIAWRDVPLLALSSFVGVTLYFFFENNGILRLTASESSLIIGVIPVVTLIGEILFLRTR
PGRLIALGIVLSFVGVALIVLRSESASASPLGYLYMVGAALSWVVYGFATKPLSGRYPML
AITFWQMVIGAIGCIPFAVAERQVWAGLSATALFNAAYLGIFGSAIGYWLYVIVLEHLGP
GRSSVFINLIPVVSVAASFVILGERLSALQIAGGIIAISGVYMATVSSQAKRHQ*

>SPBIB_v1_310094|ID:27163869| conserved membrane protein of unknown function [Uncultured spirochete bib]
VEKFFKLQERGTSVRTEVVAGITTFMTMAYILAVNPGILSATGMPAGGVFTATALSSAIA
IFFMAFLANLPIALAPGMGLNAFFAFSVVLGMGYSWQLALTAVFLEGILFVLLSFFNVRE
AIVTAIPTNVKRAVSVGIGLFIAFIGLQNAGIIVKNDATLVSLGKVTQNTGLLAIIGLFI
MGFLLAKKVKGALLIGILVTTVIGIPFGITKIPESWSPLARPAAPLLFQFQFDKVFTLDF
FVVFFTFLFVDIFDTIGTLVGVTTQAGLINKKGEIPQVKGALLADAIGTVAGACLGTSTV
TSYVESASGVAEGGRTGLTALTTGVLFLLALFFAPVFLLIPSAATAPALIIVGLFMMSPI
KEIDLSDYTEAIPAFLTIIMMPLAYSIADGLMFGIISYVVLKALAGKFKDITVVTWIVAI
LFVLKLIIG*
>SPBIB_v1_310095|ID:27163870| putative Transcriptional regulators of NagC/XylR (ROK) family, sugar kinase [Uncultured spirochete bib]
MNEQWIFGIDGGGTSTRLRIENLEGSLLFQSEGPSLNPRSAGWDGSQRTLVQLFSEMYEQ
TGLVPDSCLSGFAGVAGIDRAEDTGTMLGLIRNAARLGPDTALEVKNDSIPALAGAFGEL
RGILLIAGTGSIAVGANGAGRIIRSGGWGHILGDEGSAYWVGLRALNAAIRFHDRRGPQT
DLLGSALAFFEERDPFALIPAVYEPFDKARIAAFARIVAEERERGDEVAEQIFKEAAEEL
ALLAISIATRLGEVLAGGRIAFAGGFFSNNERLWHDTEARIIASLPRHRIYSPQADAASG
ACLLARQNIEKPRF*
>SPBIB_v1_310096|ID:27163871| putative Beta-lactamase [Uncultured spirochete bib]
MAHNSSTDLSVEKASTPDRVLAAGPGVPLDALLDSAIANKLCTGAAGAVSIDGKIVWSRC
AGKTALESDEAQLARTRATSVSEFSISEPPVLEPAVSPPAPITTQTLFDAASLTKPLATA
LLVMKAVESGALDLDAPLGKYLPEIHSQAAHIPLIALLTHTGGMPAIPALERFFPDSARL
DRQEALAQLFSIRPEFAAGRHVEYSCTGYILVGAVLERISGMLLGTLFEQEIARPLALSC
AAFAPRILDSGEPSAFKGAAQTEFCPWRKKRMDGQVHDESAYCLGGHSGNAGLFLSLEDA
LVLGNLFLQEGISRGKQLLRPETMRTMLQERTIGLEERRSIAFKLHGQDTADGPLWPAQS
FGHTGFTGTSIFFEPGRKLVAVLLTNRVYYGREATAGLIVAFRKEFHSGVWRAFC*
>SPBIB_v1_310097|ID:27163872| conserved protein of unknown function [Uncultured spirochete bib]
MHISNLPATPVRCGLDHFIADSSRRNKRYGLATNPSAITSTGIPAWKAFMQNSLGPVCLF
GPEHGFRGAAQDAVHVKDELFKGIPAYSLYGNRFKPEPWMLEGLDAVVFDMQDVGCRYYT
FLYTLAYMMEACETVGVPLIVLDRPNPIGGRKVEGSPIPKEADSFVGGYGLPPRYGLTIG
EFARYLRGEYYPDCALEIVPLSGWDARQPWAETDLPWPLPSPNLPSLSCAELYPGTCLVE
GTWLSEGRGTSRPFEIIGAPFVDGEALREALSALGLPGLVFMSLFFTPTASKYKGALCEG
VLVSVVDREAVRSLDTGIAVVHTIHRMYPHDFRWREDWENPELSFFDRLAGNTTLRCMID
SGAPLEECLAFAHEGEERFLKLRQNYLIYGAI*
>SPBIB_v1_310098|ID:27163873| Binding-protein-dependent transport systems inner membrane component [Uncultured spirochete bib]
MMFKLNGLQKAIAWIFLALLAFVNLYPIGIMILSSFKSTREIFLKPFNLPAVWRWSNYVS
AWQRADFATYFKNSILVTAASIAAILLVSSMAAYVIARFDFKFKRGIYLFLLAGLALPTR
LAIIPIFLIMRSLHLLDKLSGLVIVYAAGGIAFSVFLLVNFFKKLPRDIEDSAKIDGAGP
FRIYWQIDLPLLKPALVTVAMFNFIDVWNDFFFPLILLSSRVKKTIPLGLQAFFGEYTIE
WDVLFAALNISVLPILIIFLILSKQFIAGMTEGALK*
>SPBIB_v1_310099|ID:27163874| Permease component of ABC-type sugar transporter [Uncultured spirochete bib]
MRGRAGRSLSDRPGKAGSSEARPILYLSEVDVRKYSFLWMLFPALLLYTVFIAYPFFSSL
GLSFYDWPGIGPKKFIGLSNYKNILSGFMAPEFYRALGHNIVFFIWSLILSVVPGLFFAF
LLSADIKGTKFLKVIYFFPNTLAIVVVGFLWGLLLNPQWGLVNQLFRVLGLGFLAKPWLG
NTSTALPTIIFVTAWRGLGFYILVYLAAILGIDREMTEAARIDGATEMQIAGRIILPHLL
PIIATTSMLKFIWTFNIFDIVYAMEGTQGGPAGSTDVLGTLFYRIAFGGLGSSQVGMGLG
ATVVTLIFLLVFPVSIFYVFIVEKRVERGE*
>SPBIB_v1_310100|ID:27163875| Extracellular solute-binding protein family 1 [Uncultured spirochete bib]
MAKRFLMAMLLLCLMMPAWAQSKPATVTIWGWRAQDVDVWKSVETALKAKGEQITIKYEV
FPPTEYDSKLLVSLQGGVGPDLMYTRRLPGARTQALIDNSYLVPLDSSVDLSNFDNVSLS
FIRSNNKTWGVPFANQIVGIFYNKAMFDKYKLKEPQTWDELVQVAETLQKNGITPFFVSG
KEAWTLAMQNAMVGVSYPGDAWIGKLAEGKAKFTDPEYVNMLKDLNALKKYYQKDFMANT
TAEQDVAFAMEQVAMVFYGVWGNTNWLKTNPDLKFDYFPVPPKDKNLPAKAYIYMDGAYG
LSSASKNKDAAIKVLNYAGTKAYGELFSSTTGEITAIKGVTMPASKPILVKCYNKMVTIA

STNRYWVGSPFDAGMPSVYNILQENMQKMYLDQMTPEELAKKLQDGISTWYPAFKK*
>SPBIB_v1_310101|ID:27163876|yfeU| putative PTS component; possibly regulatory [Uncultured spirochete bib]
MDFQALDDYLERATTESRNARSANIDKLPTIEILRIINEEDKGVPFAVEKVLRDIAALVE
DIVRAFTSGGRLVYIGAGTSGRLGVLDASECPPTFGVPPGMVVGIIAGGEAALRNSIEGA
EDEPEEGMLALRNIDFCSRDVLVGITASGSAPYVLGAMQYAREQGSVVGAISCNRESKTF
ALAHHRILLEVGPEVITGSTRMKAGTAQKLVLNMLTTTSMIRIGKVYGNLMVDLTPVNRK
LVDRSKRLIRQATGCTKDEAEAAFLASGGKPKIAILMIALGISAETAQELIQQGQGRLAD
AMRIYSQSKKGR*
>SPBIB_v1_310102|ID:27163877| Transcriptional regulator, RpiR family [Uncultured spirochete bib]
MNYGALETIAQSMPRLADTERRIAEFVQASPQKVLHLNIAELARQAGTSSAAVVRFCKRI
GAEGYSDFKLWLAKDVYQGWNEKYLPDLGLESQTPASKAIHDMAEAVRRTMSALAATLSP
QAVEEAAERIRTAAMTALFGVGASGLVAADFHQKLSRIGLPSSYLFDTHAQITASCALRP
QDIAFVISYSGETDAMIEVADQAKARGCFLIAMTMAGTNRLCGRADLALSIPAVERVYRS
AAELSRLSQLAAIDILFNVIISYDIDGAIAALERSMQATHGF*
>SPBIB_v1_310103|ID:27163878| putative Diguanylate cyclase with PAS/PAC sensor [Uncultured spirochete bib]
MSIETLRTILYATSAIVGFLVIYSAARQKSHLARYFFFAALCAFLYIFGYASELGAHSLE
EIRFWLKFEYFGLSFVSSFWFLLSWKLWYSRNPRFRMVVLVFVIPAITLFLVSTQEFQGL
FYRSLRLSDIDGHHLAIIEKGPWYWVQLVYQSAFIIMSIILQWLSWRRRGGVFKSSGFWM
FVSTLSLLPWIVIYQLGKSHNGIDLAPFGIALSIFFIAIAVLRYGTLSSEEVFLYSIFAG
IDDGVVILDRDGRISDFNAAAQKIFPWLNSSSIGMPISDPADASLFNFDAPLKMEKVVEL
AGNKRYYQGRFTLIYEGRSVLGRVYFFRDITDSRRLTNRLRKLANFDALTSLYNRRRFME
RAEKVLANAQRAWKNIALLMIDVDHFKNVNDRFGHAVGDRVLAAVGRIIRRRCKQKGFAG
RYGGEEFVVLLRGAQREQALDIGEGIRRSIEGIAIEKRMIPVRVTVSIGVSSCSEKEGAY
DLDELLMSADRALYLAKNRGRNRVEG*
>SPBIB_v1_310104|ID:27163879| Isoform II [Uncultured spirochete bib]
MKKFLSMIALFAMLLPAVWSQDNHIDTIRADAPALADYGPYTIGVTTLTFVHPGQPDIVN
YKPGQPMPTYDRPLTCEIWYPATLVPGQAPSGEYLNVVTRDPSLKVTLYGRAVRNAAPDK
SAAPYPLVLISHGYPGNRFLLSHLGENLASKGYVVVSIDHTDSTYSDQGTFPSTLYNRSP
DQFFVLDSMARLNADDRGTGLAGMIDADKTTLIGYSMGAYGVVNSLGGGLSANIVKNATF
SPGNILEARAAGNPAYTASLDQRVKAGVAIAPWGMNYGFWDAEGLKGIKTPIFFMAGSKD
TVAGYSPGSHTIFDLAINADRYFLTFENGSHNSAAPMPAPKEVRAKGSLGAKVASHYLDA
VWDNVRMNNIAQHFITAFLAKYIKGDPNADSWLALIENGADGKWSVDEKGQPKPDHTYWK
GFGNNQAVGLKLEHRLPGAR*
>SPBIB_v1_320001|ID:27163880| protein of unknown function [Uncultured spirochete bib]
MMRLSLYLLGHNYLKPFRIRAHKGMHPRTHAEAAGIPVHLVQHFVQALTGGIRAFLSRCT
LSETMRRTWEKRWKTPGKDKAEYLPKYALA*
>SPBIB_v1_320002|ID:27163881| conserved exported protein of unknown function [Uncultured spirochete bib]
MKRKVAVFAVLIGLVAAFAVFAETSLPLYGNAAAKADNALTIESMLTYAIQDEYLARAEY
VAIMAKFGQIAPYSNIKQAEEQHISWLKDMFATLKLAVPADEAAKYIHAPGTLKEAAQAG
VQAEIDNIAMYERFLAQPVLKDPRYASVVDLFTLLRDASKNHLAAFQKQLQKY*
>SPBIB_v1_320003|ID:27163882| DGC domain protein [Uncultured spirochete bib]
MAENSGTNNVEFRRLQGCSTDTENMDIIFACDGAASVGQVGNHVAIDLTNAGVGARMCCT
AAVGAGSDAHVNIGKRARRVIAINGCANRCVSKIMEQRGVKVAHEYVIATMGVSKIPTLD
FNEADVERISKKIAEDLGYDIKAKN*
>SPBIB_v1_320004|ID:27163883| Two component transcriptional regulator, winged helix family [Uncultured spirochete bib]
MTIPTKILAIDDEPDVTRSIRLTIAVQEPEWTVVEASSGEQGLMLLSQEKPDLVLLDLRM
PGMSGLETLRRIRAFSNVPIIVLTVTNNELDEVNSLEEGADDYITKPFGHLELVAHIKSV
LRRSRGHEPGHEEKYYDGEFSIDFGRRIVAIGYREVPLTSTEYALLKILADNPGQTIPAE
TLLARVWGPNALDNRNYLKVYVRRLREKIEKNPAEPEYLISVRGLGYRLERHSK*
>SPBIB_v1_320005|ID:27163884| putative Histidine kinase [Uncultured spirochete bib]
MAYMARSSGKFRAVSGLRSIRKLLPLRLPFQKSTIEHKPSSIKEQTIDYFLPVARAGVVA
VSALIILAEYHQIQSVVPALWLFLELVLYNIAAGLYSRTSSMLSKKRYLAIIISDMLEAT
ILVGVTGGYDSPFFPMFLFVMAELALYFDWKIGALSIITMNGLQIIFTAIQMAGAEPQVK

FEVIENRFFRLIIVGLLFVILAESLRKEDLARKRAIHLSGLISRLNDIFAQLGNAQFDID
KVLHTILQGANALDSVAFSAVMRQVVPDKKWKIQAALGDSVCTPYIPLDSLGNLIDSMGD
SEIMATEKIKDFSPCSSAVQQFVMIRLPAYSSKENGVLLVGINTTDALEPEDKTFLHALS
IQTQLALHNAILFNETKQQYEALKSFKKTQNTFFSSAAHELKTPLTVLKVLVSTLAMTAS
SWTKDQREMLATIQSNVERLETLTTNMLATARLEAMNVALSRQPVDMKAIAERTVREMLT
LIEEKNLTITIAPEEPWPPFFADPARIRDIIANLLSNAIKFSPVGGLIGIHFEQTGAMGR
ICVSDAGPGVPPTERDKIFEKYYTSQDAGARAGTGLGLYIAHQLALLHGGDLWFDSRGSD
RTRFCFSIPLYLEGVTEDDHSDENSGNR*
>SPBIB_v1_320006|ID:27163885| conserved membrane protein of unknown function [Uncultured spirochete bib]
MKTAKTLGWLTGVVGVWEILAPFIIGYSNVGGATANAIILGILLAGFGLWVGLGKAAGTV
RTLSWINALLGLWVLLAPFILGYSGTSGGATWNDVIVGIVVIILEVWGASAAKKEA*
>SPBIB_v1_320007|ID:27163886| protein of unknown function [Uncultured spirochete bib]
MKNPQEKMKSIPGSVASPDPLQNLLNVYLKLIPSEFTKLIPSVDPRVYRGSVCFS*
>SPBIB_v1_320008|ID:27163887| protein of unknown function [Uncultured spirochete bib]
MRLLEYRLDWQYSAAAMQQALASACGTSVGKNLYVFDYYDAVLEAIGKNLGIDFSKQSRT
LQEIRHLLAQTKRKH*
>SPBIB_v1_320009|ID:27163888| IstB domain protein ATP-binding protein [Uncultured spirochete bib]
MIRTPQERERTRITIASMSRKLMLSSRVVELCESEATPRQEEFLLKVLSEEIDRRERGKK
ARLLNRAGFPVFKSFEDYDFSEIRFPPALSKEELLRADFIPEKKNLVLYGGVGTGKTHMA
IALGIAACEKGLSVRFLTVTELVLKLTEAYKAGTLERLIRDLKQLDLLILDEWGYVPVDR
EGSQLLFRIIADSYESKSLILTTNLEFSKWGGIFTDEQMAAAMIDRLVHHGHLLLFEAKS
YRMTHALMRQPGPGTAKPKTTVEAGSRLGEGGA*
>SPBIB_v1_320010|ID:27163889| protein of unknown function [Uncultured spirochete bib]
MNNPGAWKNSGIRELIPDPLKSLMDRQQRTQLHATLKTMHTLSSEYGFEIAVQALEEGVQ
RSRTSFHDAAILAARIAGYGLNMAPERGQDLHVYDEFLEGVQV*
>SPBIB_v1_330001|ID:27163890| transposase [Uncultured spirochete bib]
MFLXTLYXKKTGRTRLKIVEAYRQDGRPKQRMIQDLGYLDELQXIXPDPIAHFKXVAXQM
TAEKKAQKESRQVQLDPYAALRPFDPAQPATISRKNIGYAALSFLYHELGIDTXLNNXXR
GTKFXANLNSIXKMLVFSRALFPDSTRGAWENRGIFFEEADYSHDEXYRALDYFLAWRPA
LVRQLHERLKTRYGRQTLLFYYDVTNYYFEIDEPDSLRAXGVSKEHRPNPIVQMGLLLDA
QGLPVSYELYRGNTNDGTTLPSMLDDAVLDLGMHHLIMVADKGMMSGDNIAKLRLGHHGY
VMSYSVRGADKAFQEYVLDEEGYTAVYDTDGTLLAKHKSRVAPREIWVTDRKGKRTKSLV
NERQIVVYSAQYAQRARMERALAVEKASGQIYALSKDAKPSAYGAAKYLKKVPFDRSTGE
CMPETEYLVMLDEARIEKEEQFDGYYVICTNVIGXEXGEXXFXXPYXYTXDGFFQLNRTV
TDDDIIEIYKGLWRIEETFKVMKSELKARPVFLSTESHIRAHFFICFVSLLLMRLLEXRL
DWQYSAAAMQQALASACGTSVGKNLYVFDYYDAVLEAIGKNLGIDFSKQSRTLQEIRHLL
AQTKRKH*
>SPBIB_v1_330002|ID:27163891| protein of unknown function [Uncultured spirochete bib]
MXEYSTVLNALQCITYNIMKNIVKKFLAAYAKRNYFSVPLKETAKVRRGSI*
>SPBIB_v1_330003|ID:27163892| protein of unknown function [Uncultured spirochete bib]
MKNPQEKMKSIPGSVASPDPLQNLLNVYLKLIPSGLLQFLSKAQRNNSFSHKLLEIFLQY
FS*
>SPBIB_v1_330004|ID:27163893| protein of unknown function [Uncultured spirochete bib]
VGFSKSHDVNKMMTNIAKIIMSFSFFNFFLL*
>SPBIB_v1_330005|ID:27163894| exported protein of unknown function [Uncultured spirochete bib]
LKKLKLIMIFAMLVIILFTSCDLLKPTGTLTLSATDPWLPTITFSGQKTQLEKDQTMTVT
ATVSPAPDTYAWYLDGKAISGATTNSLTIGSTLEIGTHVITLIVGKGDYRSSEEFSMEVI
PVAAAATLAINPATITNGTINTTYTFTLTAASLPANLTQVKFNWNFGDGTTDSETVTVSN
KTASTTISHAYTADSAYGLVVTIDDGTNTLATAYASILIGTANAGTNYDLTVLDQWTAAN
SGGYGITVDTWDISTLPNGVVFDLKFDAYSMPDKFIVEYPDGTIVYNSGWRGDASYEGPK
YPGGIAGIGAGQSLAMFAKGTQQYFKITIIGGESGTAWEYSMKARQP*
>SPBIB_v1_330006|ID:27163895| Cna protein B-type domain (modular protein) [Uncultured spirochete bib]
MMQITKRISILFLILMVAGLLFGCMQPLSQSAGKGDVEVLILDGARTIQPSDVEIKIVSY
VLSGTGPNGATLANKTFTASPYTESKLAEGAWTFTVNGLNAAGSIVASGSNTATIIANST

VSVSVVLTPVKDGSGTGTFSLSGTIPSAVTLSSFTGVIAPSTGGANTTFAVTVSGTNFSY
SNSTLPVGSYILTLVASQAAGAWRGVYALRIYQGKTSTLSLNLTADDFAGATINGVAKYY
DKAANQYSGISVTITSTTSTDFAPASVTTGNAGSFNFTGLDAGTYIIEAKDPNAVYQPTS
ITITIGSNETVTTPDLVLTKAGNHVVIFRDSNTEWELAGVPATVMGDLIETEIGLTEGTG
ANQYEYKTSSDMAAYTPSLGDVVIIGGDQSQAFYDAYTTNKSKFDTFVNNGGTMYWIACD
NGWAEGDFTSSLPGGVTWRDSYEYYIDIVYFQHPITKNFPTQLYGYYASHGGFDNLDVAN
ITGLMVYVKEDAGALPTYIEYRYGLGKVLATTTPLEYYVTNGSTDMPTGFNTTYKDLFKL
MLVRSIKYIMGKTVSDTIPASDAGAIPKALTPIRMSH*
>SPBIB_v1_330007|ID:27163896| putative Subtilisin-like serine protease [Uncultured spirochete bib]
MKKIALLIAGIVLLILGCQPTITYFSLAVSVSGTGGSVVTYPNTTSIEKGSSVLLTAVPS
PGYVFDSWSGGITSNQNPLSFAMNQDYSIVAHFKVLPADTSKTVKYTLDAQWESYEKYDR
LINTVASVKSIGSKAVPNFKTADYFSLDVNASSRPKAILVKYKTGVSQKSADSLKSIGTK
DLMLDASVPFQRIVVNSDKYAEIPSMLSFLKSQPDVEWAEEDLISKALYAPDDAYYSYQW
NFDKIGMPAAWDYTMGESDVIVAVVDTGAYFPLTDLGQTRFVQGFDFVNNYLNPFDDNGH
GSHVIGTIAESTNNGIGVAGMAPGIKIMPVKVLAWDGFGYASDIAQGIYFATNNGAHIIN
LSLGGNYSAAIEDAVNYAISHGVLVVAAAGNDGTSTLNYPAALSNVLSVGATNDLNVLAP
YSNYGYGLDIVAPGGDFSRILHNNEYNEDYPAGILQQTYYGGSIGYWYFQGTSMAAPHVA
GLAALLKSKNNSLTAADITAIIENSATDLGASGYDTQYGYGLIDAAKALGIPGYIVSDQV
TSSIVLKPDEIEKWQINAAPGALIATLGFASDTGSLSFSLVNSEGTEVAVGSQANNAINL
NYIVTSANKGTYYFVIKYKP*
>SPBIB_v1_330008|ID:27163897| Hydrogenase [Uncultured spirochete bib]
MIKALRSRLHQGYKTNAFPAENPRLPERYPGAPRLETERCTVCGACKAICPTSAISVQSG
EPAGAEPRLALDLGRCIFCRKCETACPEGAIAFTGSFRMGARAKAGLVLGSVPHVSSTRT
SRLDVSNDAHKAVSTGAGNQALNSEETRLIEALDRKMLKIFGRAMKIRQVSAGGCNACEA
DVNVLETIGFDLSRFGISITASPRHADAIIVTGPVSKNMELALRKTYEAMPSPKLVIAVG
ACAISGGIYRGLPDVCGGADAVIPIDLYIPGCPPHPMTILNALLELLDRVPRKN*
>SPBIB_v1_330009|ID:27163898| NAD-dependent dehydrogenase subunit [Uncultured spirochete bib]
MKLFTNGSAMSVEDIPVVDFEFFARSIAEAVKSGASIVALFAVPDYGKGAARTAAELTAL
PTNAATEKRARLFAFLGLPTKGMIGGIAADIGSAFPSLTPKIPQAHMFEREIFENTGIVP
VGHPWLKPVRKIFDYDFYSLEGGQTHEVAVGPVHAGVIEPGHFRFQCYGEEVEHLEIALG
YQHRGIEKALAGGPDIRTIHFMETSAGDTTIGHATAYAMLIEALTSTQPDARAEAVRGIA
LELERMANHIGDLGALAGDVGYLPTKSFCGRLRGEVLNLTAEICGNRFGRGIVQPGGVGF
DIDEKMATSIAARLQKIVKDTDEAVGLLWDSPSVLARFERTGMLKKETALELGLVGPAAR
ACGIERDVRSDYPSGIYKYAAIPVATYPSGDVYARAMVRMLEYQRSASAILDVLAALPAG
NTGKPASRALPARSVALSLVEGWRGKICHCAITDNRGAFLYYKIVDPSFHNWPGLAYALR
GQQISDFPLCNKSFNLSYCGFDL*
>SPBIB_v1_330010|ID:27163899| putative Hydrogenase-4 component F [Uncultured spirochete bib]
MNMLFGGFLAVPVFGVLLILVSKSSRQALMIMLATALGGALLSGALAAQVAQAGVIEVAG
GLFRADHFSAWHMIILAIIVFLPSSVYAYTYFEGGKGIDLGYAKRFGMLWLGAFAAMSTA
LLSGNIGIMWVAIEASTLFTAFLIFRQGNAASLEAMWKYIIMCSVGVAFAFIGTLLMAAA
GQQAGLEGGDSLLLQKIYLVRGALKPSLALTAFIFILVGYGTKAGLAPMHNWLPDAHSQA
PGPVSAMFSGFLLNAALYCILRFLPLFSPSDGGASVLQGGKAGSMLVGAALASSKAQIIL
MGFALLSLVISSAFIFFQKDAKRMLAYCSVEHMGIIAFAYALGPVGTIAGLFHTLNHSFA
KTAGFFSAGSLGKKLHTNELARFSGASKRYPLYAIGFAVSLLALIGVAPFSVFMSEFMLV
SSTVAARRFVPLAIFLAATSIVFLVMLKHAIAILWKSPPDQAQAYTPGCAPGGTFSGAPA
IAPHKNANPTEFSAPSGVPPISEYLVIGLPLAFLLVLGLWMPHGLSDFLTKALDSLWSMK
*
>SPBIB_v1_330011|ID:27163900| putative NAD-dependent dehydrogenase subunit [Uncultured spirochete bib]
MTSLQNMLLIVVLITDLTMAANSRLGTCINITAIQGFALGFLPLAIGLEKGMNFELVAIA
VIGIALRGILFPYILHRSLLNSGTSRELKPIVGYFLSVAIVLVLIFASLWISIRIDFIAP
QLLLLSTVALSTIFAGTFFIVGRRLAIMQILGYILLENGIYCMGLAFVQDIPMIVELGVL
FDAIVAVFVMSVATNKIHEEFEHFDIQKLDSLKG*
>SPBIB_v1_330012|ID:27163901|hyfC| HyfC [Uncultured spirochete bib]
MNDILAKLLPLVVALVFAPLAMGIIQKVKAFFAGRKGFPVFQMYRDLYKLMHKGIVYSRS

TTWVFRLAPLLVLAVSLIAVAWLPLGGLRGLFSFQGGFIFVAYLFGLARFATLLASLDTA
SAFEGMGANREALFAILAEPATLLAFAALILTSGNSSLWGAISWGQESSFAEFGVVPLLA
LVSFFILALSENSRIPVDDPTTHLELTMIHEVMILDNSGPDLAYIEYASALKVWFYLVLM
ANIITAIFSISGWAQILAMFGFAFLLAGIIGVVESSIARLRMKKVPDFIGSAVAFALLAL
VLEGIL*
>SPBIB_v1_330013|ID:27163902| Hydrogenase-4 component B [Uncultured spirochete bib]
MMLFGIGIGVLVGGGLLTLVLKKSAGINKLGMASVVAGNVFVIVAALLGLSGRYAGEVYQ
FFLPLPVGAVLFSLSPLSSFFLLIIAVVTGLAAVYAPKYLAHYGEMPWRFKAHWLLYNLL
AASMMLVVTAQNAVFFMLAWEGMSLSSFFLVLFQNERTKVRRAGWIYLVFTHVGAACLLV
MFALLAQVSGSFDFDAMRAAAAGFSPGLKAAIFVLALVGFGMKAGFFPMYVWLPEAHPAA
PSHVSAVMSGVMIKTAIYGIVLTLGLLGGGQGTELWMGWTLVVLGLVSGIFGIAFAAVQQ
NAKRLLAYSSVENIGIIATGLGVWFIGMASSNEVLARLGLMAALLHTVNHALFKSLLFMG
AGALQLATETLDLDRMGGLLKKMPRSGLAIIAGAVAISGLPPFNGFVGEFLLYSSVFAQG
FGSSLVRAVYVIAILGGLSIIGALAVFTFTKLVGTSLLGEPRTDHAAHAHEVPASMYVPM
LVLAGLCLFIGLFPLPVMSALKLVLADSGLAVVGQLPSGIVSSLAAMQWVAFALIGLIGV
FALVRSRVLSLHKVENTSRHKVGSTTWDCGYHNPTPRMQYTASSFAQPLASFAQPLYSPH
RDGSIGPDLFPAPVARKISFPDLILDRAIKPMYSWISRAFETFSIIQHGNTHWYVLYIVI
ALLAVLFWSFVI*
>SPBIB_v1_330014|ID:27163903| DNA-binding protein, excisionase family [Uncultured spirochete bib]
MKLSVKDVSRLFNISDKTVYRWIQADNLPHYRIGGQYRFSYSELLEWAAAHGAKIRAELF
DEVAEADDCDVSIATALEAGGINYRIEGSTKEEILSSIVSLMRLPESINKDIFLQFLLAR
EKLGSTAIGNGIAIPHVRNPVVVHSETPQISLFFLEKPVDFGALDGVPVSTIFLIVSPNI
RCHLKLLAQLMFVLRDEKVLEMLSKVASRNEILAAIRNAERPVLERRQSEIEDMSRAHTA
SEQQGGEP*
>SPBIB_v1_330015|ID:27163904| membrane protein of unknown function [Uncultured spirochete bib]
LPLPGAFRAAFRDEPCYLRLSSNTDVLMNIELVAAAEFAKSEARNYLSYGIIFGILIAMA
FYGFFLFVSLKYRSYLYFILYTVSVGLWLFYVQGFAKVLFGQMPGFDQAMLWFWAGMFIT
WGTIFTISFLELQKTSRFLFYILAAALGKATQHAALVEMSLSFSCPAFQRTRHFMLQSES
ASISKPRACA*
>SPBIB_v1_330016|ID:27163905| protein of unknown function [Uncultured spirochete bib]
MRIIDGQSISATISLGIVDLQGNESAESFIERADSAMYAAKQRGKNCSVVM*
>SPBIB_v1_330017|ID:27163906|appF| Oligopeptide transport ATP-binding protein AppF [Uncultured spirochete bib]
MNNVILELKHVSKHFALSGLRNISKVVHAMDDVSFFLNKGESLALVGESGSGKTTTASVI
AGMYEKTSGEIYLDGQPVLPFKTREASLRYKKKVQMIFQDPFASLNPTHTIRSILERPFL
IHKLAGSTRELEAMLCDSLEMVGLSPAKEYLHRFPHELSGGQRQRVNIARTFAVSPEIIL
ADEPTSMLDVSIRIGIMNMMLDLKEKKHVSFLYITHDLAGARYMSTRIAVMYAGMIMEIG
PTEEVIANAVHPYVQLLKASAPAPQEGVRKAAIPAKGEIPSLVHPPSGCRFHTRCPYAKK
ICSEEVPAMRDIGNGHFVRCVLV*
>SPBIB_v1_330018|ID:27163907|oppD| Oligopeptide transport ATP-binding protein OppD [Uncultured spirochete bib]
MHADAILELRNLKAGYATNAGFVRAVDDVSLTLHRGEFLGIAGESGCGKSTLAYSIMRLL
RDNARIESGSIIFDGTDITSLDEKAMNEYRWTRMAMVFQSAMNALNPVLSVEEQLVDAVL
AHKQVTRADAKKTAMEMLELVDINAERIASYPHQLSGGMRQRVMIAMALLLKPDLLIMDE
PTTALDVVVQRSILQKIEELRHIFGFSVIFITHDLSLLVEISDSLAIMYAGKICEYGPSR
EVYASPDHPYTKGLMNAFPTLTGPISRMGGIEGQAPSLLSPPPGCRFAPRCNVAIDACTK
EVPLLQVTSPGRLCACRLCGGNHE*
>SPBIB_v1_330019|ID:27163908| ABC-type dipeptide/oligopeptide/nickel transport system, permease component [Uncultured spirochete bib]
MIRDFFKTLIKNKKSLSGLVLLGFFILVALFAPLLAPYPPKTDRVEHTTLLEENKGDPEV
LSEETEGQNREKSVYTVTITQKYEKTTEYFPTRAKPSKDHILGTNHAGNDLFSQIIMGTR
VSLFVGLATGLFVTFISLSLALLSGYLGGILDDVISFVTNVFLVIPGLPLMIVIGTYVPM
HGVLPIILILSLTSWPYPTRLLRSQVLTLKNRDFVRASKMIGERTSFLIFREILPNMISI
VMSEFFTVSLSAILGEATLEFIGVGNITVVSWGTILYWAQANGAILMGAWWWFLPPGLLI
ALTGTAFVLINFGIDEISNPRLRKR*

>SPBIB_v1_330020|ID:27163909| Binding-protein-dependent transport systems inner membrane component [Uncultured spirochete bib]
MKYLARKFGMLIFTLFVSMSINFFLPRLMPGDPAKALMDRMEGVEAEKLESVRAAFGLDT
KDSLPVQYGKYLLNTVKGDLGISLSRFPMPVSSVLASALPWTIGLMGLCTIIGFSIGTLL
GIWAAWKRQTKLASFTVGFFTFIRSFPYFWLGLFLIYIFSFKLKVFPLGGAFSVDQTRGS
AGWWLSVVQHGILPAFTITLSSIGAWLLMMRNNMINVLAEEFITLAIAKGLPMQRIRLMY
AARNAILPSVTGFAMSFGFIVGGGLITEMVFAYPGMGYMLYQAVNAKDYPLMQAIFLIIA
ASVLVANFLADIAIMLLDPRVRDGAK*
>SPBIB_v1_330021|ID:27163910| ABC-type dipeptide transport system, periplasmic component [Uncultured spirochete bib]
MKKKLMVAMVLLSLFVATTVFAQAPARGGILKMTPSKQGVLQKNFNPFSPAVLESALGCI
YETLIYFNNANGTANPWIAESWSWSNDLKEITFVIRQGIKFNDGTPLTADDVVFSAMLGK
TNKALDMSGLWSEGLQDVTASGNEVTFKFATVNVTTLEKFGNLYIVPKAIWSKVQDPLTW
TGNTNPVGSGPFMLDPASFNEQSYKLVRNPNYWQKGADGKPLPYIDGIQYVSTTNEQIAF
KLMNGEYDWAAYSLPNVDAYVAADPANNKYWFPEGNLVFLYMNNLRPPFDNVNVRKAFAM
AISQKDITRKMSPSPVPADMSAVKKSFSSIAEEGKAKYNITYDKAKARKLLESEGYKLNA
KGIYEKDGKALSFKLYVPTDWTDWIGAAETIASMLKDIGVEAIITQSAWPVPFQTSLETG
DYDMAVSFITTGTTPYYQFNRWLYSANYAGLGEKAKVFSNMRYKNADIDKNLEAYRSEPN
PEKQKVYISAVVTQFMKDTPCVPLFFNPTWFEYTTRNFVGWPSADNPYAWPSVIGMQKVP
ILLNIHKK*
>SPBIB_v1_330022|ID:27163911| putative Uncharacterized lipoprotein YbbD [Uncultured spirochete bib]
MRWEYNDFPMIEHTSWQYKKHLPLEWKIGQMLAVGFPAGADGIASLARVVKASYAGNVIL
FTRNTPDVAETRKTAASVKTLIREVTGVSPLIAIDQEGGIVARLKGGLTPLPGAMAQAAA
YLGGGIQKSDIKALGKICAEELRSLGINWNLAPVADVNSNPSNPVIGVRSYGENPKLVAD
LASAFAAGLEEGGVLATAKHFPGHGDTSVDSHLDIPLVPHGIERLEAIEFVPFRRLVDEG
IPAVMTAHIRLPSVEPDMLPATLSSRVLQGLLRKKLGFEGIICSDCMEMKAIADHFPNAF
VMAVKAGVDILFISHTAEKQLYAAHSIYEAVKKSEIAESQIDASVERILAAKEKINQTSA
FDAYKKTVAGPHAVALAKKISRTSLSVLAQGSITKPPSGSVLVDVVPGNITNAEDSLHSV
SIHAELTRQNAPIFSCAVPLNPKEEDIAAALSCAERYLSAPASAIQETEAKPALILALHA
PMLHSGQMKLVQASVRFAQDRSCPLLVILTRNPYDAPSIAKEAARANLPAPTIVCSYEYS
EASVASIVAFLTGKLEAKGICPVTVEG*
>SPBIB_v1_330023|ID:27163912| putative Acetyltransferase [Uncultured spirochete bib]
MDYAINTEDPLLVKNELLSLWNRCLGSEFPLDIRLLVQQISLDRDLHVFFTARDEASGML
IGAVLAKRATRPNLNGEIPARSYISCILVAPEFQHKGIGKNLLKTAESWCRHQHAATIAL
GSDYFHFFPGPPYNATAESDHAIAFFEASGYTKGTIEEDVIANVQSLDIPLPPASRTTKA
PGFHIACSTPELRPRIFDFFIRAFPGRWNNEIHEAFAAGMRDEDLVLVIRDSDQSVAGFA
RIYDEESPILGPGLYWRALLGPNPGALGPIGIDAAYRGLGLGMDLLRAALAELKARGIRN
TVIDWTDLGAFYAKLGFVPWKRYVMMQKKIE*
>SPBIB_v1_330024|ID:27163913| protein of unknown function [Uncultured spirochete bib]
MAHSLPVSTSRPPRARGLKPVQNTTSNSYNLVAPPAGAWIETNVVVGIVKRIASRAPAGR
NNKHFSAGTQKNKPSTLQK*
>SPBIB_v1_330025|ID:27163914| protein of unknown function [Uncultured spirochete bib]
MLLEAGLNLVVAPPAGAWIETDPLKSDITNDIVAPPAGAWIETE*
>SPBIB_v1_330026|ID:27163915| protein of unknown function [Uncultured spirochete bib]
MLLEAGLNLVVAPPAGAWIETTKETVDENGTRQSRPPRARGLKHKPWL*
>SPBIB_v1_340001|ID:27163916|cas| CRISPR-associated endoribonuclease Cas2 [Uncultured spirochete bib]
MMVLVSYDVNVTNPGGARRLRRVARTLSNHGQRVQFSVFECVVDPAQWVKLRADLENIID
KENDSLRYYYLGSNWKHRVEHIGAKPSLDLDDPLIV*
>SPBIB_v1_340002|ID:27163917|cas1| CRISPR-associated endonuclease Cas1 1 [Uncultured spirochete bib]
MRKLLNTLYVTTQGSYVHRDGETVVIEQEQKKILQVPIHMISSIVCFGNVLCSPALLGFC
AERSVSIAFLTEYGHFLASVNGPTTGNVLLRRNQYRMADSEESCRNISANIVAAKCANCR
LILHRGTRDHGEKIDTHSIKMASSSIERLIGQIDSASDTDAIRGIEGMAAAEYFAVFNQL
IVDQKDAFMFNDRNRRPPLDPVNALLSFIYTLLVHDIRSALETVGLDPAVGFLHKDRPGR
PSLALDLMEEFRPVIADRLVLSLINRRQVNPKGFSRAANGAVTMDDETRKTVLVEYQKRK

QEEVYHPYIEETVPIGLLFFIQANLMARFIRGDIDGYPPFFWR*
>SPBIB_v1_340003|ID:27163918| CRISPR-associated protein Cas4 [Uncultured spirochete bib]
LNEDADLIPISALQHLLFCERQFALIHIEGIWSENLFTAEGKVLHERVHQEHHEKRRLYR
QEFGMAVRSLKSGLIGKCDLVELYLEPSGKIAEAFPVEFKRGKNKEEEEDKVQLCAQAMC
LEEMLDIMIPEGQIYYLQDHRRTRIALDQALRKKVVQLADRARELILKGETPLAIFEKRK
CTTCSLVEQCMPSSTGLQSKSVSRFIRAQLKATESECDK*
>SPBIB_v1_340004|ID:27163919| CRISPR-associated protein Cas7/Csd2, subtype I-C/DVULG [Uncultured spirochete bib]
MSLEKRYDFVLFFDVKDGNPNGDPDAGNLPRIDAESGHGIVTDVCLKRKIRNYVGLVKNE
QPPYEIYVKEKAILNKQHERAYIALGIDLSSDEGKRKGGDKVDIARDWMCKNFFDVRTFG
AVMSTGINCGQVRGPVQLTFARSIEPVVASEHSITRMAVATEEEAKKQGGDNRTMGRKYT
IPYGLYRVHGFISAPLADQTGFSEEDLNLLWDALEHMFEHDRSAARGLMSSQRLIIFEHN
SKLGNRPAHELFARIEVVRASDSTAPARDFSDYKTMLDGNEIKAFKTVVPV*
>SPBIB_v1_340005|ID:27163920| Csd1 family CRISPR-associated protein [Uncultured spirochete bib]
MILQALKEYYDRKAADPESTIAPPGWELKEIPFVLVLDETGSLVQIEDTREGQQNRGKKF
LVPQAEKKTSGIKANLLWDTPAYVFGIQDLEGLFEKEKERKRQRLPQQRAAFLDRIRNDL
PDTAKRKALISFLENATEEHLSEYSHWPEILKTNANISFRFLDELDLYCRSQEAIQAVNA
KLHSRETNGICLVTGENDEICELHNPIKGVQGAQTTGGNIVSFNLAPFCSYNKKQGTNAP
IGYMSMFAYTTALNSLLSKESRQRLVLGSAATFVFWSSEKTDFENDFSIFFSEPPKDNPD
VGTENIRALLLSPLSGGYRNDTGNEKFYILGLSPNASRLAIQLWLPGTVSSFKDKIAQHF
EDFNVEKRESEPRYYSLWRVLTNIVPQDKTDNIPPPLVHQFMYAILTGTPYPQSLLQSAI
RRIRSDAEDRVTPVRAALIKAYLNRYIRYYSKNTEKELTMALDIEQPSIGYQLGRLFAVL
EKIQEEANPGINATIRERYYGSACSSPVTVFPTLMRLKNHHLAKLGNEGRVVYFERFIGG
IVGKIDDFPSHLDLHEQGRFAVGYYHQRQDFFTKKD*
>SPBIB_v1_340006|ID:27163921|cas| CRISPR-associated protein Cas5 [Uncultured spirochete bib]
VKGFCLEVWGDYACFTRPEMKVERVSYDVMTPSAARAIFEAILWKPAIRWHITRIEVMNP
IQWVSVRRNEVGCIFTSPTKAQLLGANGDPMGFYIEDNRQQRASYFLRDVRYRIHAWFEF
IPPESRKTNHSVNQEIWADIEEKATYERKDENEAKYAAMFERRAKKGQCFHRPYLGTREF
ACNFMFVEQPELAKPPLSLNQDLGWMLYDMDYGHPENPIPRFFRAKLENGVLLTDENIVE
VRS*
>SPBIB_v1_340007|ID:27163922| CRISPR-associated helicase Cas3 [Uncultured spirochete bib]
VLYIKLPKLISKADWVVAPAQKTGENDQEITMSEKDTSCMQAIAHVRKNPDGSWAAPHSL
QEHIKNTATRAAKFAESFNSSEWARSCGILHDAGKARDEWQLYLRRKSDYWEDEANLKNG
SNRIEHSASGAKYAEEVLGKGIGRILSYCIAGHHAGLPDLEGDNSSLLNRFEHAKTEDIQ
CWYKDSVIRELPDKKHLSPPWKLNQQEQQSILMSLWIRMLFSCLVDADYLDTESYMEPER
VSSRGKYPSLSEIKTRFDDFIKKKIRDSLAKGDTPVNQIRRHVLDQCRLAGSWEPGFFSL
TVPTGGGKTLSSMAFALEHAIIHKKERVIYVIPYTSIIEQNANEFRKALGNDAIIEHHSN
IAEDVDTGKARLAAENWEAPVIVTTTVQFFESLFAAKPGRCRKLHNIANSIVIFDEAQLV
PPEHLKPILETLDALVAKFRVSAVFCTATQPVFEKQKDFPSFPGLSNGKVREIIKDVPSI
FSAMRRVAVKIPEDLSTPLSWEKLADALKKEPQVLCIVSDRRSCRELHALMPEDTYHLSA
LMCAQHRSEVINEIKGKLKRGDQVRVISTQLIEAGVDIDFPIVYRAIAGLDSIAQSAGRC
NREGYLNASGKLGRVVLFIPPRLPPQGILRKASETTLILLKQGLSDPISHEAFKAYFSEL
YWKLNSLDLYGICDLLYPQPPALGIMFRTAADKFRIIDDSAMKTILIPYEKGASLIRELK
QIGPSSRILRKLQRYTVNVYANQFERMRARESIEEIFPRIFVLRESCMLEYSLKVGLLAD
DVPNDPQLFIG*
>SPBIB_v1_340008|ID:27163923| conserved protein of unknown function [Uncultured spirochete bib]
VSQMERIFFIDRTIQERGGVNVRTVSSTFEVSERQAKRDIEYLRYRLGAPVEWSAPKRQY
IYSAPWDGLKFASEKSLFALAFFRAILDRYSYIPVLSEELIDELKRKIGARYASIAEHVR
YELPDLQPISDDISYRICRSLLEQHNLRITYRDAHDVESERTIVPLRLINYAGKWYCAAY
DSLSGKLRVFAIARIKEAYEAEPSHFPLPGDDEIERFLSSSYGIFKGDPVGTATLRFRNG
AARAIRDQIWHPDQVLTIPDNAGDEYTVDLSLPVHDWTELLGRALRCGADCEVLGPQEFR
ERWKEEIERMREMAGKGD*
>SPBIB_v1_340009|ID:27163924| Sodium/hydrogen exchanger [Uncultured spirochete bib]
MSRKRLLIAGLALFALALPVFAQSEQSTPTEQITQFVFQLAVIIFAARLGGIAAKAISLP

SVLGELIAGILIGPFALGAIPLPGFPAGFFPVSGGEPISIMLQAFSNIAAIILLFSSGLE
TDLDMLMKYSLAGSVVGIGGVVFSFLFGAELGTFFTHQPLFAPVNLFIGIMSTATSVGIT
ARILSDRKKMDSPEGVTILAAAVFDDVLGIIFLAVVLGIVAVLEGSKAGGLSAGAILGIS
AKAFGLWLSFTALGLIFSKQISAFLKKIGGEGHFNILALGLAFFIAGIFEMEGLSMIIGA
YIVGLSLSKTELSFIIQDKTKVLYELFVPIFFVVMGMQVQISKILEPSVLIFGLLFTLVA
ILAKVLGCGLPSLFLGFNKIGALRIGVGMVPRGEVALIIAGIGLSAKILEPGFFGASILM
TILTTILAPILLNIILDKGGRGTRKEAKTTTSEEFVVELPNADLAGLLSSYLLEDLQREG
FYVQLMSISDDISHIRKGDVSLSLKVDGSKLILEAVPEDMPFVRIMVYETMVKLDANLDK
LKETYDPESMRKTLDVETIRKDPTFRKILDPQCIEVDMKATNKEEAIVELIELLDRVHHL
KDKNKVLADVLEREKIMSTGMQHGIAIPHARTEGTDKPVLAIGIHKKGIDFQTIDGTPAH
IIALIASSAGDPAPHMQVLTSLGSTLGSDIVREQLLHAKTREEAAKILGV*
>SPBIB_v1_340010|ID:27163925| Pseudouridine synthase [Uncultured spirochete bib]
MKKHWVRQMCHQGWRRTLPAHTFFLRAFFCYDEVYMLSILFEDDEILVVDKPAGLPAQPG
EGVKDDVVGVLERQLGYKPFPVHRLDRDTAGCMMLAKTKAAAAQWSQLIASKDIVKRYYA
WAAGEPQKKNGVIDAALDGRKGAQEARTFWSLKEAWRLADAQAVSLLELELKTGRMHQIR
RHLAAIGLPILGDDRHGDFALNKALRRFGIRRLMLWAYMLMLPGGTIVRASLPPHFAAFK
EMLARSGAIQQ*
>SPBIB_v1_340011|ID:27163926| protein of unknown function [Uncultured spirochete bib]
MRKPFEIAMGAWWIVPDGRTIAVPSFHESWLASHPAIASGCLHTVDFVQKSGWLSVTLYS
EGMIEIISRDISDARQREAIHQLFEMNKSLIKKIVVFQPSIEGCLTAEGPLVASWERFVA
ELDGFLARALPAAQVSSEAEPLAKGLPAAEALPAARASSEATPAAQQE*
>SPBIB_v1_340012|ID:27163927| conserved protein of unknown function [Uncultured spirochete bib]
MHIGNVHIGTCGFSYDEWKGQFYPADLPAHEMLRYYSLVFSFVELDHTWYRIPSPAQLER
MALVTPSDFRISLKVHRSLTHEVDEHWQTHAAEFCNAVSALASTGRLGCVLVQLPYRFSY
TPENRKYLANLLATLAEFPLVVEFRNATWYQERVFDALKARNVALVVVDRPDLPGLPPES
VIITSDICYYRLHGRNADLWWSGDATSRYEYRYSDQELKDRARLVRAMSRQAKTVFVAFN
NHARGNAPANAAFLQSILAEQQA*
>SPBIB_v1_340013|ID:27163928| conserved membrane protein of unknown function [Uncultured spirochete bib]
MIFTALARIVSVVISVYMLLCAVRIFFSWVPSLAQTKWGSLIARLTDPYLELFRAIPLFR
TPTVDFSPIVALAVLSVINNLFVTLSYAVRITVGFILSLLLDAGWSAISFLLGFFLVIAL
VRIIGYIAKLATLHPLWQILDGIINPLLFRVNRFIYRGRAVDYLQGLITGFVVVLLARAI
GGWAIRILTSLLMSLPF*
>SPBIB_v1_340014|ID:27163930| conserved protein of unknown function [Uncultured spirochete bib]
MLTSKQRSMLTALASKDCVMQALGKNGLTAQFVATIDGLLAHHELVKIKFLDFKDEKRGI
AEELAARTKSELVRVIGHNAIFYRQNPDPEKRRINLA*
>SPBIB_v1_340015|ID:27163929| protein of unknown function [Uncultured spirochete bib]
VRICRAAHRKPSRFLNRGLTPFLKFFDLCKVDAALFGVGVLAVENGIVADNADKLGLGSS
GQFLGYSALFVLKIEKFYLYKLVVSQKSIDRSDKLSGQAVLAERLHHAIL*
>SPBIB_v1_340016|ID:27163931| Major facilitator superfamily MFS_1 [Uncultured spirochete bib]
MSVPLSSQKLSKARSTFNIFNLFNSFSFVFVSGSIITLFALRMGAQNSIIGLLNALVYIT
FFMLPLGKRLVRRTSIIKIFGWGWVIRYIMLLPILFAPVLASKGNMGLAILLLIIGTAGF
HISRGVAMIGNNPVLGLLSSGGGDKPRSDQGRYLVNVSVVNSVAAIFANIVLAFLLGQQA
SLWTYAIAIGIGILSGLLGCVFLFKVPEPESFSSNNEQSLWNVTREALLKPEFRAFIFTF
MLLSFLSGMGRAFLPVYAKEVFAQGDDAIMVYSIVAGLGSIAMGLITRLVVDRLGSKPLF
IIYSMIGLISFLPIALIPGSGALMGAPAIIGLFLSVVHFLSSFGFAGEENAGQVYYFGLV
PKEKMLDLAVIYNLAYGIGGSLGSFLGGGLLEIFSNVGLSQAGSFRLYYALISVVLVFVI
YSMRNLPHMGAKSVGQSLSVLFSPRELRAFDLLTRLDQSASAHEEVRILQELGKSESKLT
QKELVEYLHSPRFEVRSEALLALESIPELDAETIGALIREVRTNVYSTAYVAARILGKQR
SDVAIPVLIDSLKEPDYMLQSMAMIALARLGARQAIPEIEEVIRSTENPRVRISGAYALE
LFGSIDSIPSLISCLKAEDRPANVSDELVLAISGILGIDKEFYLLYSEFIDDAKSGVAQL
TAHAHDVPLEKERLEAWMAAIQELFAAKPNPIPLARLLLNTTRITGSLLILSDALLDPKL
EYPGFNFLCAYVALHTENLRDF*
>SPBIB_v1_340017|ID:27163932| MATE efflux family protein [Uncultured spirochete bib]
MKDLTKGDEATTLITFALPMLLGNVFQQFYNMVDSFVVGRFVGTNALAAVGVAFPVIFLL

VALIMGITMGSSVLISQFFGARDRERLASTISTSYIFLFGAGIFMSVLGFFSVPFILNIL
AVPPEIYAEARSYMSIILGGMLITFGYNGVSAMLRGVGDSKTPLYLLVAASLMNVVLDLV
FVIVFHWGVAGAAWATLISQAFSFITAMVIFNRTESHMKVELKKLSWNKEIFGSMIKIGL
PTGIQQTLVSLGMMMLTRIVNEFGPATMAAYTAAARIDSFASMPAMNLSQALMTFTGQNM
GAGKTERVKKGHRAAIIMNIVISLSITLLVTLAGHWLIGIFTTDGAVIDIGARYLLIVGF
FYIIFGTMFINNGVMRGAGDVFIPMISSLLALWIVRIPCALLFTKVFGMGSDGIWWSIPA
GWFVGFVFTTWYYRTGKWKTKVLVRRPPLAEEIE*
>SPBIB_v1_340018|ID:27163933| MazG family protein [Uncultured spirochete bib]
MEIDTIPLETLAGQMSEQILPEAEQAAAAFKLFFTIVARLRAPDGCPWDLAQTPTSIRGN
IIEEAYELAEAITENDSAHIKEENGDLYLLATMVGYMSQQEGRYHVADALREAARKLIRR
HPHVFGDSTVDSPEQVVEQWNDIKERIEGRRKKDSLLDEVHRHLPPLEKAYKIQKKAAKA
GFDWQKREDIWLKLAEEMEELKTAAGNAESAGADSAGAEPAVGMESAGAASSVRKAPIAN
PDALEEEFGDLLFTVINAARLYGVDPMIALHRSNEKFSRRFRHVEARMKASHLAMTPEHM
TQMDAFWDEAKRAEDGARADNARRAEDSLRARGTQPTEHAQPASRLDIPRSRP*
>SPBIB_v1_340019|ID:27163934| membrane protein of unknown function [Uncultured spirochete bib]
MEQAYSQSIEHTAAAMRKENKHATPIDASTMNLLQKSGFFFFSVASLFLVLYSIIVILLP
DTAAIPRYPPEMLFLIGLFAIGFLLASIIAELVWLEPVLLLAFLPLHLSDHITSMYSMCA
FIIAIIELYRIDYFKTGGLAKIFLCFAYYALSIALVGTALRVFFVEIAMPIIFIFLFFLY
LLPVFKGKWQIRIARPRQTIHYKELNLTDRETDYLKECLHGAAFKEIAAHHHVSESTVRN
TFAHIYHKFSVTDKAELLSKFADFDIID*
>SPBIB_v1_340020|ID:27163935| membrane protein of unknown function [Uncultured spirochete bib]
MIDAWRAFLLQCRQVMSILKIYKWTVACGFLVALLLLLPSQIFPKTPRGMLELDKIVHGF
LFGVVTAVFCAEHRRWTKVSPPFLLSFAIIGAFSFLTEVSQLATKTRHFDLKDFGADLIG
IAAGLLLMRLIARLTQK*
>SPBIB_v1_340021|ID:27163936|ydbK| putative 2-oxoacid-flavodoxin fused oxidoreductase:conserved protein; 4Fe-4S cluster binding protein [Uncultured spirochete bib]
MTNSKNMVMIDGNAAAAHVAHACSDVIAIYPITPSSPMGELADEFSSMARKNIWGSVPQV
VEMQSEAGAAGAVHGALTTGALSTTFTASQGLLLMVPNMYKIAGEATPTVFHIAARAIAA
HALSIFGDHQDVMATRQTGWALLASNNVQEVMDMALVAHAATLKARVPFLHFFDGFRTSH
EVSKIEELSYDVIRAMIPEDKVREHRARGLNPEHPEIRGTSQNPDVYFQGREATNKLYEA
TPAIVEETMAQLAKLTGRTYNLFDYAGAPDAERVIIIMGSGADTAEETAEYLAKQGEKVG
VVKVHLYRPFSVEHFIKAIPATVKSIAVLDRTKEPGAIGEPLYEDVRTAIGEAMGKGTGP
FKSWPMVVGGRYGLGSYEFTPAMVKSVFDNLKAEQPKNHFTVGIVDDVSFTSLPYDESFH
LPSEGVVECLFYGLGSDGTVGANKNSIKIIGDETDNSAQGYFVYDSKKAGTYTISHLRFG
PKHIKKPYLVTKADFLACHKFSFLEKIDMLANLKEGGTFLLNSPYSADEVWDNLPVEVQK
EIIDKKVRFYVIDAMSIADKSGMGSRINTIMQTAFFKISGVLPEAQAVELIKKYTKKTYM
RKGADIVQKNLDAIDMALGATHEVKVGKATSTKHMLPPVPPSAPEFVRETLGEIIAQRGE
KVPVSKIPLDGTFPTGTTQYEKRNIAEKIPVWEPSICIQCGNCTMVCPHAVIRMKAYDPA
LLANAPATFKSADGKGKELAGLKVTVQVAPEDCTSCGACINICPAIDKANPGRKAINFGP
QMELRETEKANWDFFLSIPETDNKFLNLSIPKGIAMKRPLFEFSGACAGCGETPYVKLVS
QLFGDRAVMANATGCSSIYGGNLPTTPYTKRADGRGPAWSNSLFEDAAEFGYGMRLTTDK
QAEYARELLDTAKTNGVPADLIDRILANAQENEEQIEAQRANVAELRKALASKKEDWAKE
LDNVADALIRRSVWIVGGDGWAYDIGFGGLDHVIASGKNVNLLVLDTEVYSNTGGQASKA
TPIGAIARFATSGKDIFKKDLGMIAMSYGYVYVAQIALGANMNQTIKAIREAESYNGPSI
IIAYSHCINHGIDMMKGMDQQKIAVQSGVWPLYRYDPRLKAEGKNPFQLDSKEPDYSKLE
QYMYAEVRFKSLVQANPERAKMLLEKQRSLIERRYKEYRYLADRPF*
>SPBIB_v1_340022|ID:27163937| dTDP-glucose pyrophosphorylase [Uncultured spirochete bib]
MKLLVLAAGIGSRFGGIKQLAPVGPNGETLLEYSLFDALRAGFSEIVFLVRPEIEEDFRT
MVLSRLPASVQWQLAYQTSTSMLDEKSIVRARENGRIKPWGTGHALLCARAQLEQSGPFA
VINADDFYGPRAFAGVQANLAANPQEFCFAGYRLDDVVPQEGVVSRAICTIDADGLLTGI
VEHKRVWRRGQEFISEIDGKQYVLDPQTVVSMNLWGLSEAIFAWAERMWRDFLSEPANYG
SKEFFLPDIVNAMVAHGAVRVRMIPASMRSFGLTNPGDLEETRERIARMIAKGDYPAPLW
RGK*
>SPBIB_v1_340023|ID:27163938| putative UDP-N-acetylglucosamine diphosphorylase [Uncultured spirochete bib]

MSDFPRLWNIEESEFADLFAQLEAPWEALHSLDGYLASLLQKADVSGQVVLSPVPEGVHI
EGLVYIAEGCVIESGAFIRGPAWIGAGCEIRSGCYIRGGVIAGSGAVLGHASEFKHCILL
SHAQAPHFNYVGDSVLGIHAHIGAGVILSNYRLDGKPVPVRAMGAGERIETGLAKFGALI
GDYCEIGCNTVLNPGTILGAQSVVLPLSNIRGTWPPGSRLPG*
>SPBIB_v1_340024|ID:27163939| conserved protein of unknown function [Uncultured spirochete bib]
MSIPHFAGKFSSKPLFNPTDFLAYLERIGKPLSHRPAPDAVILSYQKSLFDHVVEHYPVE
RAQGYFGQFIYYLDGPRNIPQTRRQLAIAAGFGVGAPAAAVMLEELIAWGVRDFVSMGMA
GSLREDLPPGSIVLCDSAIRDEGTSYHYLPDDSDVFPDRALTEKLADELSAAGLPFARGA
TWTTDAIYRETPVEVEYFRKKGALVVEMEAAALFAVAAHRDARIASCFSVSDTLAYLEWK
PEFHSEATRETLEKLFRVASDVLLR*
>SPBIB_v1_340025|ID:27163940|sudB| Sulfide dehydrogenase subunit beta [Uncultured spirochete bib]
MHGTHRIVSKTQLSEEVFRIEVEAPLVARERKAGQFVIVMYDEEYSERIPLTIADADPVR
GTVTLIFQTVGASTHKLALKNPGDEIVLLGPLGNPTHIERFGWAVCVGGGIGLAPLYPIA
KAMKEAGNRVTVISGARTKDLLIMQKELVAIADEHIVVTDDGSFGRKALVTEPLKELCMQ
SPPPDIVVTIGPPIMMKFCAETTRPFGVKTLASLNTIMLDGTGMCGGCRVAIGDQTKFVC
VDGPEFDAHLVDWNGMMMRLGTYKEIEKEAHDRCHLEMQIAKLEAHAADKESAK*
>SPBIB_v1_340026|ID:27163941|sudA| Sulfide dehydrogenase subunit alpha [Uncultured spirochete bib]
MSHKEDFTMDNAELELAKLRAAPITPKIRLQIPPQDMPAQDPAARRSNMEEVALGYTPAQ
AMLEAERCLQCKNAPCISGCPVRIDIPGFIGKIKEGDFLGASKIIKKTNLLPSICGRVCP
QETQCQAPCTVGKALGGVEKAVQIGRLERFVADYERAQGAMEMPHVAPPTGRKVAIVGAG
PAGLTCAVDVRREGHEVTIFEAFHKPGGVMVYGIPEFRLPKVLVQSEAEVLECMGVDFEL
NFLVGRTRPLRALMSEDGYDAVFIGTGAGLPKFMNIPGENYVGVFSANEYLTRANLLKAY
MRGMAGTPIYPSRRVAVLGGGNVAMDSARMALRLGAEEVRVLYRRTREEMPARAEEVAHA
MEEGIIFDFLKSPTRVLGDDRGRVAALEVLSYELGEPDESGRRRPVPIRGSEQIIPFDTV
IVAIGNESNPLISHTTPELQVDKHGHILVDEKQKTSIERVYAGGDIVLGAATVILAMGEG
RRAAQAINEILQ*
>SPBIB_v1_340027|ID:27163942| putative Tetratricopeptide TPR_1 repeat-containing protein [Uncultured spirochete bib]
VSRGRLIPLRFFSLFLMLSSIVGGVGQLWAEDTYNAALSLFWEGRRLEMAETRQAESQQA
FQRSLAMTERLLATDAANPDLQTLKTWNLFRLRRYVDTVVYAQSVLNSRQDYRILETMAE
ALYFLGKNDEALSAFSRYFTLSPENDDRRSSAYYYVGEIYFRIKKYEHADIAFSTAVALE
KNMYYWWYRLGIVKEVLGQYRRAYEAFNTSLSLKSNFKPALDAKERVKAKSSL*
>SPBIB_v1_340028|ID:27163943| putative p-aminobenzoyl-glutamate hydrolase subunit B [Uncultured spirochete bib]
MTKNISENRACSADLAVFVARNAEVYDSIARDIWEHPEVSHEEARSSAVYRERLAARGFR
VQEFPEMPYSFCAEWGESGPVIAFMGEYDALPGMSQACATERQPVVAGAPGHACGHNLLG
MGSLVAAEGLVFLLSQTGMRARVRFYGCPAEESLGRIPLVKAGRFNDVDAAMTWHPADVN
TPHRYRTSANLSFVVSFQGKASHAGMAPHAGRSALDAVQLFNLSIEFMREHVPPGTLFHY
VISDGGAKPNIVPEHASVHLYVRAPDAQSVHEVMKRVRKAIHGAELMTETKASIEIKAGK
CDFIPNTPIQDTILEAMTAIPLPEPTPEEIEFARELQKKVAPADRQATLAPIGAPLSLLK
QPLHLEVGDFGEGKRIGGSLDTGDVSYVVPTGQMNAATWPLGVGAHTWQSCAASGSAWAM
KAARYAGASLAYAGFLLVSKPERLARAKDEHRSVPTYRSTMDY*
>SPBIB_v1_340029|ID:27163944|mtaD| 5-methylthioadenosine/S-adenosylhomocysteine deaminase [Uncultured spirochete bib]
MQPENPLDRRPVLLPDLCIRGGAVLTLDAKWRIFDPGFVAIKDSRIVAVGHLAEAEQWPA
RETLDVSGTLIMPGYVNAHTHISMSPFRGAMEDAPDRLTRFIFPLENAMVRSKLVYDAAM
FTIAEMVRSGTTCFADMYYFEEEVARAADAAGVRALAGETVVDFPAPDADRPYGGIERAE
HLAKDWKGHPRIRPCLAPHAPYTVDASHLAEIAALAEKLDIPVMMHVAEMDSEHARFSAT
HGSVLRYLDTTGILSPRLIAVHMLYLDEEDIALAAKRGISVAHCPVSNAKSGRPICPAWR
LSEAGVSVGLGTDGPLSGNTMDMQTITSMYPKLQKVREHRRDIVPARTALYAATMGGAHV
LGLSADIGSLEEGKRADIQVVALDDFNVLPVHDWYATAVYALQAHNVRDVLVDGRFVLRD
GKLQFADERELKERLLYHARQSAATIRALQPAPMHPHHTAGT*
>SPBIB_v1_340030|ID:27163945| conserved membrane protein of unknown function [Uncultured spirochete bib]
MIHFIAGVVTSANYWFIVLRSTAPILLVTLGAVITAQAGIMNMALEGSMLWAALTGVIVS
AATQNVWIGLLAGIGASVLVALVLGLFALKLHANPVLSGVALNLVATGGTVFVLFSLTGD

KGISSSLASKVLPSLSIPVVKDIPWIGKVVSGQNIVVYIAAVSVVVIDLILRRFAIGRRI
RATGENVEAARAAGINPVRIKFFTLIISGILAGFAGCFLSMGYTSNFTSGIAAGRGYVAN
AAQVVAGGTAVGSIFAAMLFGLTDAMATYLQNIGLMPELIQALPYAVTILGFVVVTFVRD
RREAERKRKLFAGTAMQKEKADAA*
>SPBIB_v1_340031|ID:27163946| ABC-type uncharacterized transport system, permease component [Uncultured spirochete bib]
MNSIKRFFTDRESLAPALVTLFAGLFSLLAGIVLIFIVSKNPVQSVRNFLLAPFLRSYNF
YYLLIVMVPITFTGLALCIVYQLRYMSLIVDASFYTGAVVATALGTLISLPSGVHPAVAM
IAAGIVGGLIGLLPAILKIRWRANELVSSLMLNYVFYFLGSFVILYYLRDRSQTILSSLP
FKKTFLLPRLVANTQLHVGFLIAAGFVVLVSIFLYRTRWGYEIRLTGANPRFAMYAGIPV
TSVILYAHFISGFLGGVGGAVEMSGLYRAFIWQMNPSYAWDGVIVAMLARRNPKFVPLSA
LFFAYLRVGADFMSRRGDVAFEFITLLQGLIILILASDRIVNYFRERQLRKQLLKNSRRE
SR*
>SPBIB_v1_340032|ID:27163947|yufO| Uncharacterized ABC transporter ATP-binding protein YufO [Uncultured spirochete bib]
VNYIVRTEKLSKIYPNGTFANKSVDFAVMEGEIHGLVGENGAGKTTLMKMLYGEEQPSQG
SILIDEKPVSLASEFDAIKAGIGMVHQHFMLVPSLTAAENLVLGKEPKRKRTRTFDLAAA
VSITEELSKHYNLPVFAKARVADLSVSMRQKLEILKALYRGARILILDEPTAVLTPQETE
ELFVELKTLKQQGKTIIFISHKLKEIFNLCDRVTVMRDGRMIATHNVADVDVHQISNEMV
GREVRFEIARPPMAPGEPALRVRELSYVDDFAIPKLKHVSFTLRRNEILGVVGVDGNGQT
ELVEIITGNEKALSGKVFAYSTEVTNRSAGFVRRCGIAHIAEDRMTVGTALPASIEQNLL
ADRYRNRQFSTRAGFLRSREMAAYAGRLVHEYDIRCNSANQPVASLSGGNMQKVVVAREF
TANASILVISEPTRGVDIGAIEYIHTKIMEMRSAGKAILLVSSDLQEVMALSDSLIVMHE
GEIVAYFPDAAAVTDKELGLYMLGIKRQSDEELRKVLHE*
>SPBIB_v1_340033|ID:27163948| putative CD4+ T-cell-stimulating antigen [Uncultured spirochete bib]
MKKLKVVVLLLIAAIFLFPSTAQTQKKANVFLLISGSLGDKGFNDSARGALDMIKQKYGA
NATVKYTELGTDVAKFAPALEDAGAEGYDVVFCSNNFNSAVQEVAARYPKTLYVMYDGWI
KEDIGNVYSIQYKNSEAGYLAGALAGLMTKYTADPKINADNVIGFVGGRDITGINDFFVG
YIAGAQKMNPSIKIIYNYVNSFTDTAKAKDQALVQYKSYNADIVYHAAGRAGLGVFDAAV
ETGKYAIGVDTDQASLFGNEPKKADLILTSTMKRVDLTLFQAFEKFMNGEKLGGKKVYVG
AAEGVIAYAPFNKAMPASIKTELEKLQADIKAGKVSIPSAFSMTADEINKMRDSVNAAK*
>SPBIB_v1_340034|ID:27163949| Peptidase M20 [Uncultured spirochete bib]
VLPTLCRMNCFGAAKYIAWTKGAGYGDVMNISELKTAVDAEILSQREETIKLAFRLASIP
EAGFEEFETMKELERQLAACGVKAEVGLARTGIRATIGPAGAPNLVLLADMDALPTRGAP
NDIMHSCGHHAQMSIMLAVFSALHALGIPEKLGFRLSLVGAPAEEYTHLDERKALRVKGE
IRYLSGKQELIRLGVFDDAACVIKYHSMADSPERMATVNGTLNGFVAKQALFVGKAAHSG
AAPDQGVNALNAAVIALMAIHSQRETFRDSDHIRVHPILKEGGTTVNTVPDRARIETYIR
GASIEAIQNAARKVDRALGAGAMAVGASVTVSTTPGYQPFRPSDALGVRLAASAARFLPE
TAIDLHDYSYASDDIGDVACLVPACQLGFSGFSGTIHSADFKASDPERAYILPGRILADL
VLDLGADGGRKAQEIRSAFKPTLDKAAYLAYLDSCFEEKTYSFKD*
>SPBIB_v1_340035|ID:27163950| conserved protein of unknown function [Uncultured spirochete bib]
MFKAVIFDFGNVLCGIDRESFVRKAAQYTSKMSADELLRALWGTQLEFEFETGRFDSHEY
FRRVQEMAGLGPSYSYEEFVEDYKRIIIPNPDGEKGLETAAKLGMRTFVLSNTSFLHASV
IFDNEILGTYPELYILSYKVGAMKPDPRIWLKLLEYARLDPADCLYIDDIEQYCQAARSL
GMSAFCYDFCIHNLSQVLNNMLQ*
>SPBIB_v1_340036|ID:27163951| Iron ABC transporter, substrate binding protein [Uncultured spirochete bib]
MKKFAVVALCLLIAVSAWAAGGKVVVYTAHEETIINALVPMFEKETGIKVEYVKMGSGDV
IKRAKAEAARPQADVIWSIGGEQLEAENQILAPYTPKEWDKINPVYKVGTNWLPYTGIMN
VFIVNTKMLTPDKYPKSWTDLMDARFKKNISSARADKSGSSYMQLCNVLSIYGDKGWDIY
KGIMKNMVISASSGAVPKFVNDGEQAVGITLEDNAFRYFAGGGPVAIVYPTDGTVAAPDG
IALLKGAPNLENAKIFIDWCLSKPVQEYLVDAMARRPVRIDTKDPKGLVPLSQVKTIPYD
FGWAAKNKDAFVKKFVDIAMELGL*
>SPBIB_v1_340037|ID:27163952|fbpC| Fe(3+) ions import ATP-binding protein FbpC [Uncultured spirochete bib]
MASIQIIDLEKHFGEVKALSGINLEIDKGEFFTLLGPSGCGKTTLLRTIAGFYHQDKGDI

YIDGELINNVPAYQRNTGMVFQNYAVFPHMTVFDNVAYGLRVRKLPKDEISRKVSAALKS
VHLEGYEQRTPEKLSGGQQQRVGLARAMAIEPRVLLFDEPLSNLDAKLRIEMREEIRAVQ
KALGITSIYVTHDQEEALVISDRIAVMNQGVIQQIGKPWEIYTNPQNMFVAYFVGKINML
TMHLEEGAEGNLRRARLGRLSLLIPAEGTKDLQEVVVAFRPEDMVEAEAAGRKNEITGLM
KTVSFMGSVARFELDVEGQLITVDRHRPREADMPQKGAPVAFAVPVESILLFDPKSGLRI
GKGAQQ*
>SPBIB_v1_340038|ID:27163953| ABC transporter, membrane spanning protein [Uncultured spirochete bib]
MSQAAETRRGTATAPGNSGLPAGRSSLQHPGRRADFWTLVIFAGFAIVVVFLIYPLFDIF
RYSFLDKTTKAFSMSNWKEFFSKMYYLRAFWHSIVIALLTTFFSIVLGIPLAFFTTRYRI
RGTNLLTTLSVLALLSPTFIGAYSWITMLGRNGFLRLMFQSIGINLPPIYGPLGIVLADT
LQYYPFISLMTAGALMTIDRSLEEASENLGARSVRTFFDITLPMIIPSVTGGALIVFMMS
LSNFGTPMIIGGNYLVLPTLAYNLYTSEISETPGMASTVSIILMLCASVVIVLQQWMSSR
RKYASMLVNRPIVKQLKGIKSVVAHFVCYLIVFLSTLPLAVIVYYSFRKTSGPVFKSGFG
LDSYRQIFFDVPKTVMNSFLYSLIAVLLIASVGTLLGFVIARKRNAAVRALDPLLMIPYM
VPGTVLGIGFIVAFNRPPIILAGTATIIILTYFIRRLPYSVRSAASILKQIDPALEEAGI
NLGSPPGRTFRTITLPLMQGGIISGAIMSWVTSMNELSASILLYVGKTMTMPIKIYLSVA
DGYFGTASAMSTILLVVTGVAMFIVNKYFNRGSETFIAA*
>SPBIB_v1_340039|ID:27163954| Fructose-2,6-bisphosphatase (modular protein) [Uncultured spirochete bib]
MYPRFESKFSLSDEETTALQRLQADGEFFSVLDHSMSVFILRHGQSEGNARNTYQGRLDF
PLSAQGEKQARAAGEWLSQFKPEIMLASPMQRARRSAEIVGEAAGISAIEFADMLIELDT
GIFSGVDPDTAARKYPEIWNEFLARSWDAVPGAESSSMLYARALCVWQKILELGQQGTSR
IVCMTHGGLVQWLLKSTMGVHSWLPLLPMSNCGISEYEIEMVKKGNPAFVQWTAINFHPP
AAPEGPKPVF*
>SPBIB_v1_340040|ID:27163955| exported protein of unknown function [Uncultured spirochete bib]
MRIRTAIAVAVCLLLGAAMQPFAFESGCEPSDPAFWAGIHGIPLDQDECAAIVGGDVRMS
LNPDRTKLIVNVIDNEYEARLGRIPPKQVLELDVHNRIVNTTNAPFMPATRDGRELAAAV
STTTKPSPFPEGYWTITAIKPRDDKYGPYFISTNAIGRVEVYENDGAGGQIPIGIFKDLG
YALHANTNPFNVSKSYGCLVLRQDDVATLARILEADKQENPKAVQKILVPPSRARMRDW*
>SPBIB_v1_340041|ID:27163956| exported protein of unknown function [Uncultured spirochete bib]
MKKNYFFFLFMAGIVVLFMLASCKQAKVTEKPLQTVPQQAASQPKTSESGAPHPEAATKM
PAIERKNESILLVYKHTGFGYVESYGFDTYVFRLLADPSGQISGGIVYQRTSDGEVEAIH
YNVTRSSDEITLAASGADKKSWSATLKLKDSSIDVSGQQKLSAKLDKALVFASTDGNYSE
SYSIDQAAPEMPSEIKKGDSTLEKGKWTFPENNKAMYVQTKPDDTENTEGFQVSLWREDN
GDLRFRTEGPTPINEVYASGLSAVLAGDHALSNAVLLDLMLGESRYLRPVYAFAISRRGA
GK*
>SPBIB_v1_340042|ID:27163957|deoD| Purine nucleoside phosphorylase DeoD-type [Uncultured spirochete bib]
MSIHIAAQKGQIADKILLPGDPLRAKFIAENYFENPICFNQVRNMLGFTGTYKGQLVSVM
GTGMGMPSLSIYVNELIREYGVRRLIRVGTCGSMREDVKVRDIVLAMSASTDSAVNQVRF
RGMDYAPTASFTLLKAAWDAATARALRVFAGPVLSSDMFYTEDPEQWKLWAKFGVLAVEM
ETAELYTLAAKYGCDALSVLTISDHLVTGEATNAQERQLSFRSMMEVALVAILA*
>SPBIB_v1_340043|ID:27163958| protein of unknown function [Uncultured spirochete bib]
MLFSVGNLVTLAIVVAMFGAYHLLTADNRSLEKLKRMGEKLKDELGAYVESKAEEIKHYG
IDLDVQQKAAKVVLEKIQEAQATIDSESESITAIADRFKEYDEILSQLMDMTKRVDQNIA
HLAEKNEFIEALARRLEVAGKKMDAIEADLPKVKDQFAQDAKRILDGFRDDILDQLNERL
TGIVNLFDQSKGDAESAIAQASALNNQIDQKTELALEKAASRAQNIEDEAFQTLRARLAA
DFKELSRQTDEQIAALSQKLDMLVQSVQAKLGETNASTEAQVKESQRRLSEAKASIAAMS
AEMVRIQKETAEASAALAAKFQAERASLEEQFAQFGQAFEAHRASFEKEFMSEISALRES
LENAKTQAETLRAAAEGNVSAALNEFEQGIYKDLDSHKAAVYEQVDKWLSSMDAKIRKIS
QDAVEQRKAEEARLAQEVNAELKRIKDSLYTQAQKIERDIEALKNV*
>SPBIB_v1_340044|ID:27163959|rpsU| 30S ribosomal protein S21 [Uncultured spirochete bib]
VRTIRQVIVEDNEPLEKALKRFKRMVEKEGIIREWKRREYFEKPSAIRNREKKALERKLM
KKTRKSQEGKY*
>SPBIB_v1_340045|ID:27163960| conserved exported protein of unknown function [Uncultured spirochete bib]
MRPRYASLLSAVAVCSLILIFFAACGQNTSSQGIDKPNPKLKTVTLTIGEATVKAEVAMT

ETERNRGLMYRTSLRDGEGMLFVFDRDQQVSFWMKNTKLPLSIAYIISDGTIVQILDLVP
YSEEPRPSARSIRYALEVPQGWFGRAGVKVGDKVAIPPL*
>SPBIB_v1_340046|ID:27163961|def| peptide deformylase [Uncultured spirochete bib]
MLNIVTYGDPVLTEKAQEISAFDHDLEKIVIDMQDAMKRDHGIGLAAPQVGISKRLFLVG
LDDERLRIFVNPRIVAMSKETNEYEEGCLSFPGLYFTVVRPSAIDIEAFDIKGKPFRISA
DGLLARVIQHEYDHLDGILFIDRVSPAKRRRALAHYSRMLNM*
>SPBIB_v1_340047|ID:27163962|fmt| Methionyl-tRNA formyltransferase [Uncultured spirochete bib]
LRVLFAGSPEIAVPALEAIAASHHEIAAVLTNPDSKVGRGLKVEQTPVASAARRIFGERV
PIFTFETLKAEARSGISACKPDILVSFAYGKIFGPKFMALFPKGGINVHPSLLPRWRGSS
PIQHAILAMDSETGISIQTIAPEMDTGDLLLVSRIPLGGRETARTLSELCARLAAPLVVE
VLDQIEHGTVKAQPQIGTPTYCSKISKEDGLIDWTRASREIDARIRAFDPWPGSYTFLRG
MRLSILEAEPVDDFSPSDVPGHVTNVQPGTIIAVVAPKGMVVQTGQGFLAVKQLQLATRK
ATGYREFANGMRDLIGTILGLL*
>SPBIB_v1_340048|ID:27163963| PASTA domain containing protein [Uncultured spirochete bib]
MAKKDITQTIKEAASKAPEKVTAAVKSAVDIASEQHKRISEWTKPQKRTFVLFAILMFVV
MIIFAVFVFFLTLKGEERTMVPDVRRMDLADALVKMQQRELYPRLTLRFTDNPLDRNLVL
DQSPPPGSIVKAGRRINLVVSRGAVLDKIEDYSGRNIDDLRLYLQGLSTTTKGLVSIREP
PLYIFDISAPGTILDQNPKPGTEISGPTILDLVVSKGPEAKDIAMPSLLGLTLREMASKA
ATTPLIFSYKMRAAHDKEAPGTVVEQSEPADKNLKQLDRVQITIAAPAARKGYTAGVFEY
TLPEYPYAVPVALDVIVPNGTRTTLYSVKHPGGSFSAPFSVQSGSVLVLMVSGAEVTRKE
VR*
>SPBIB_v1_340049|ID:27163964| conserved protein of unknown function [Uncultured spirochete bib]
MNAQGEIPVEIIHLYTYDIGRAIDLKKVASLVPAHHDMEFAKRRDTPVSLTLPKPLVLSI
STDECDSKCFDKFSAQAKIYEDGAITLIVRYKTMSRFEDLPQKANMLIRDISSANSIEQF
AESSFKMVHEALRTAVAGYKELTKNDRETYTAFCLLECPGGDPRAFIDANKDVAASLLSG
EPVGTLHMSQIEQILNKPFSYRKDDIAIFDLERCLIIDPAADYDDVIMMAEHANYRLIEL
RELDFLLDKWLGEAEQDIRRLYLSGNRRKFSDRTLRLKLAQIQGLRFDALFTLENLDNSS
KIIGDYFLGSIYHRLCEIFNTDEWKISIERRLNALQNIYELVKNDSTEQRMITLELVFIA
VCVIFPILQILQVMLLK*
>SPBIB_v1_340050|ID:27163965| putative Beta-galactosidase [Uncultured spirochete bib]
MQRAWNAGWYYRPGPAEPVFLSEEWAGPDAVAGAAKTTDRGMTTTTGAAMAAGAATQDSP
VGLPAAGWIPVHLPHDMIGAPLNSFDERTFARKGCYAKLLTMEQLARAWSDVDEASPDKK
ENLGQEAAPSEKDGAQPGAVPVPTPDKEDDFSPSSAHRSGAPAIFLRFEGVSVSCRVWVN
GRLAGSHTGPYTPFEVRIDPHLKLEPEAPPSDRPAVPGAQPSARHNVRPAWILVEVDSSE
DAGAPPFGGVVDYLVFGGIYRGVSLEVHTGAWVGTVWCAPRPLGDMLEGQWIAEVKVQIE
AAEQLPPQAKIRAQLFSGNDLIIEESAPVESPAGHAATKAKEVLLSLAVNKPRLWDIDDP
FLYRLEVALLDESGRAVDMQEIRTAFRTAEFGPSGFYLNHRRVFLRGLNRHQEYPYTGYA
MGPDAQRRDAEMLKQELGCIIVRTSHYPQSPYFLDACDELGLLVFEELPGWQHVGDASWQ
EHALRDLRDMILRDRNHPSIVLWGVRVNESQDNHDFYTRTNALARELDPFRPTAGVRYIA
HSELLEDVYTFNDFIYDGEGTPFISEPAKVLPKHHKKAPYLITEHTGHMFPTKSFDQEER
LVAHSLRHAHILDTAMGNPRIAGCIGWCAFDYHTHKDFGSGDRICYHGVADMFRIPKYAG
FLYASQVSPSERLVLEPASHFAKGERDAARMLPVYVFTNCDAIDVYRGASLVGRFSPDKT
HFANLPHPPVVIDDLIGKRIEAEGWPGRDAALFCKLASKAMAAGASRLSLMEKLQMGLFM
HRRKLSMRQIEELVIRYGMGWGSADESIRLVGILNGKEVAERTFGADSTAQGLVAEADSN
QLCAVSEEEWSSTRIVVRAVDQYGNTTPFVFEPFSIETEGPISLTGPSLRALAGGASAFW
VAAGGLKGHARIIVSSPRFESPALVELEIV*
>SPBIB_v1_340051|ID:27163966| protein of unknown function [Uncultured spirochete bib]
VNGSMSESEFDAAKAQASAGSAESPGTDKPPITPQQFFDSLLLIEDFLRYGCRSGRKVEI
SLPGDLPIETGLATRKAQLEALAAQVSECTACALSESRTQTVFGEGVSDPEVLVIGEGPG
AEEDRQGLPFVGASGKLLDKMLAAIGLSRQTNCYIGNIVKCRPPNNREPTPIERAACIGY
LRAQIAILKPRFILCVGRTAAHGLLDLTDPISRLRGRTFEFEGIPVVITFHPSALLRDAS
LKRPAWEDLKRFRALIDGSTRVEE*
>SPBIB_v1_340052|ID:27163967|priA| Primosomal protein N' [Uncultured spirochete bib]
MSASFVRVAFPIPVEETFWYRNPGFAPLEIGMRVEAPLGKRAKATGYAIEIADSVVLDPA

RIREILRVVDAEPLFDLEMLGLGKWLAAMYHCSLGEALSAMLPSARRERQDLSEEFDEVE
LSSSPLELTPGQRAALQGILAEPEGMFYLYGPTGTGKTEVFLQLAEAALAKGQGVLYLVP
EIALTRQVEQDARRRFGGSCAIIHSRLTPSRKLAEWRRIAKKEARIVIGARSAVFAPIPD
LGLIIIDEEHDTGYKSGTTPRYHARQVAMHRASLAKARLVMGSATPSPEAWHACLQGQIH
RFDLATRPAGGSFPLIHIVDMRGREGLISDELAEALREAKTAGRQAILFLNRRGFARTLI
CQTCGTEITCKHCSVPLTYHKSSGRLTCHYCGYSEPKLLACPTCGSLDLAWASYGTERIE
EELRERFPDMSLARLDTDSAGTKGKLEETLRAFRNGQIDMLVGTQMVAKGLNFPGVRVVG
ILMADQGLAMPDFRAAERVFSLIVQVAGRAGRHRPDGLVFVQTRKPDSPIIRLAAAGDVL
GFLERELEMRRHLEFPPAGRLCRLVFRSRLAHDARNAAESAAKAARHLAEEPQFADIEVL
GPSECPLAIIADNHRYQLIFRARRFQALQHFVKMLKPRIELPSSVYLEIDIDPVNMM*
>SPBIB_v1_340053|ID:27163968|ackA| Acetate kinase [Uncultured spirochete bib]
MVILTLNCGSSSVKYQVYDWDKKDVLASGIVERVTIGGSFITHKVRGRPDYEVEHECPNH
IVAIELILKTLVDPEVGVISDLSMIKAVGHRMVHGGSKFAKSSIINGDFLETFKSLNELA
PLHNPANLMGVEAAKTILPDVPHCAVMDTAWHQTMPASSYMYALPREWYEKYMVRRYGFH
GTSFLYNAKRAAVLLGKDPFETNLIIAHIGNGSSINAVKNGCSYDTSMGLTPLEGLVMGT
RSGDIDPGIIFHVMRKTGMTASEIEKKLNKESGVLGLTTKWADRRDVENAAEKGDPIAIL
AQQVEAYRIKKYIGAYYAALGHVDAIVFTAGVGEMGPEIRKLATHGLEELGIIVDEKKNA
LAKCRNAELDITGAGSKVRVFVIPTDEELVMTEDTVALINGTYDVHTNYRYYFENRDYVN
KARAEGLQRDLEKKPWLKDIVARIP*
>SPBIB_v1_340054|ID:27163969| exported protein of unknown function [Uncultured spirochete bib]
MKKLIVLLLALAMVGAVFAADAPKAVATFGAYLDSYGDFMDGLGPTLYSETYYNYSSGGV
GFSATVTGGEDIFKQVRNYSVSYQVLPELKVLAGRLRETGGARLTSYIDGNGFSTRIANV
QNGMMGQVSYQGVGLAVFSKLDTLTNTNVGLSYTAKGVAKLVGGYLGTSSEIWVGADIQA
VKGVVARVGFRNTPTKNYIYATAGSSALVKNLDLGLDADIVLATGAFNYGAQVKAQYALT
DKYSAGVKVWYDNGDAWYGNNGLAAKAYGVINFAQGDMVVGVKYNAATSTFSIPFDFEVW
F*
>SPBIB_v1_340055|ID:27163970| exported protein of unknown function [Uncultured spirochete bib]
MNITKRPSAGLPLYSVLGVAISVMVSLLVFSCATARKYDPGAPKVAIARASDAEIAKYGP
NFLVNPFKEPSIIVLGKTYDFYIVKISLNLDKQTKIKVIASSQGPSGADTPAAYTQAEFV
HFWDVVSNEGGPNDGEYERRKTTIERTVIPELSFSEGSGQNSYFLVFVGKHPVKRPITYE
VQVILDSGEAFVFSETLE*
>SPBIB_v1_340056|ID:27163971| conserved protein of unknown function [Uncultured spirochete bib]
MPLQELILQHDVWYLTDAESMQEALGALVKTVDLPEGLDREALLDALLAREDLASTAIGN
GIAIPHVRRFGSESLQHDIVVVAYLFAPVDWKALDGIPVHTLFLVLAKDEASHLQLLAEI
AHVASDESFVAFLKTMPDRAALVERIQKIEQLD*
>SPBIB_v1_340057|ID:27163972|rlmE| Ribosomal RNA large subunit methyltransferase E [Uncultured spirochete bib]
MAGYERPDYWTLKAKKEGYPARSVYKLEEIVRKFGLLKGFGGRTGAGKEDSVRSSPSSGA
SGAGAPGPAILDVGAAPGSWSLWLLRQMKGAGRLVAVDIQDLGIAPADSNFSFIKGSILV
DSVRARLREAGPFNLVVSDAAPATTGNRLVDQARSEELVEAVMGLALEVLAPGGALVMKI
FQGGEEKRLIAELKKHFAQARAFKPEACRAESFETYLVASGFARRDEQDKRL*
>SPBIB_v1_340058|ID:27163973|htpG| Chaperone protein HtpG [Uncultured spirochete bib]
MANYAFKTEVNQLLNLITHSLYSHKEVFLRELISNASDAIDKLKYLTVSDDAFKTLSFEP
RITVTLDEDAKTITVSDNGIGMDEEELADNLGTIARSGTRAFLERLGENERKASNLIGQF
GVGFYSAFMVADHIEVVSLKAGTDKAFVWKSDGKSGFEIEPAVRAGFGTDVILHINDEGT
EYLSRWSLEELLKRYSNHIAWPIHLVSKEKTYAKDPAKAESKTVDEQVNSASALWRRPKP
ELTDKDYIDFYKSLTGDEKDPLLWIHTKAEGTIEYSSLLYIPSHVPADLYIPTREQGIKL
YVKRVFITDKELQILPPYFRFVRGVIDSDDLPLNVSREMLQHNRVMMTIQQATLKKLFSE
LEQLAATNPEKFKTFISLFGVQLKEGILSDWTNREPLLKLVRFKSTLAEDWTSLADYKSR
AGEGRKTIYYLTGENEARLRASPLLEVFKEKGIEVLLGTDEIDELAFGGIGEYEGMQFKS
VQQVDAEEELGKDQSEHKDRGEKAVKILKRVLGDRVKDVRLSKRLGKSPSCVVVDKDEFT
PQYRKLMERLTNEPAPEVKPILEINAEHPLIERLVALDTRKELSGGDLATVQEIEDLSHV
LFYEAMLAEGEHFEVPQDFAERLNRLLAKSS*
>SPBIB_v1_340059|ID:27163974| putative Peptidase M23 [Uncultured spirochete bib]
VPEGLRSWKEKEKRWYSRMVQGISSGLDVVRVAVRRFFSWGRTRFVFAVIPQSEKPARKL

ELSRFSLIAIGVAALVFAGSAIASISAYGAAALKASALHTQLAKANESIDKMRETTENLL
SKTAQFQEKLGQIVTIVQSKGKPDPAQSADNILKTAGLSDLIPGRFSDDPVSRDVLRLEG
IAGTLDNSVAALDQIANVLASQKDIMTEVPNIWPIKGNLGHITMYFGQNENPFSTGQWYL
HTGIDISTNRVGDPVLATADGKVIDIHYNASLGNSITIQHSHGFTTRYGHLRGFAVKKGQ
RVSQGDVIGYLGNTGKTTGPHLHYEVYLGTSLIDPLRFLNIRKEYHE*
>SPBIB_v1_340060|ID:27163975| protein of unknown function [Uncultured spirochete bib]
MSKQGFSDCVIGQGVKAEGRIEAPGMVRIDGEFSGDIISGASVIISKDAVMHAHIRARDL
LVAGFFSGTAIVQNEVRYVETARVRADCTGKMLVVEPGALVQGKFMRNSDAQ*
>SPBIB_v1_340061|ID:27163976| Stage 0 sporulation protein YaaT (modular protein) [Uncultured spirochete bib]
MSDEYTDHNLPEEMFDILHEEEYETPKQSKAPPANAEELPQPIYRLKTLHSSETFHAYYV
PTGSETHFMKQCDLEAALEGPRPQEKPQAPSLEQCRVSLAVNTAEPSAAQAVVAAAFIIP
NRSLVIAPTKYGRDLVEVLGVVRDLSVVNADELVLIERPATVDDYRRYLENLEREKEALA
KCRERIEAHNLPMKLVSAHCLLEDGKILFFFTAESRVDFRDLVRDLVSLFHARIELRQIG
VRDEARVTGGCGVCGRILCCHGLSDKLNPVSIKMAKDQNLSLNSLKISGPCGRLLCCLSY
EHQFYRDARRELPNEGNRFTYDGTLFKVIEVNVLSNRIRIAGEDGRLLDMDAARLKYADG
HWTIIEEEQV*
>SPBIB_v1_340062|ID:27163977| Cytochrome C biogenesis protein [Uncultured spirochete bib]
MIATIAFGLLLLSLVIQIVFLFKKEGGQDGISPWLSLAAGILLLVEIVRRSIAIRFVALT
GMFESLIFLACFLALLIFALRYFKSAKGNRVIPFGATIIAVIFLALASSPLAPSDIKPPI
PALQSGWLVLHVSFTFIGEVFFAVGFVSAILQLVSKDEEKRRSYDRITYTSIAVGYPIFT
AGALIFGAIWAEKAWGVWWSWDPKETWALITWLTYTAYLHFRLVRKSTSKLVPALVIIGF
VIAMFTFFGVNFLLKGLHSYA*
>SPBIB_v1_340063|ID:27163978| membrane protein of unknown function [Uncultured spirochete bib]
MKAFFDKLYNALRSVKLAVVLLVLLALFAIAGGIIPQGKAASYYIEHFSGAGSRIILALA
LDHVFSSFLFLLVSAIFAINLTVCTFHRLTNELFKPREKRRHGPDLLHIGLIILLFGGIL
TSRSRTETFINLRKGDSARLPGGAKIVLVDLKYEQYPDGRPKSWESTVAIDKNPPPEASA
PVLTHEEEGGDTLGTTGFADPTLNNSLSSAKASSSEPATSSAAPAAPAISAETGAGPGLP
SSPAPSTPIAELPQYRIRVNTPLRLKGYSIYQQNWHTEKQVVLRDAMGMQFFLEPGMRET
TKDGFVLFMALDTSQGAGGAAAVAVAAAPAVAAAPSAIFLLEQNGKRTVVKAAPGEKIGS
FTFSGYIDQPVSGLAVVSDKGYPFVAAGFILVILGVFITYIRKLKGILA*
>SPBIB_v1_340064|ID:27163979| Split soret cytochrome c [Uncultured spirochete bib]
MAEVEKILEKDVSRRSFLKTTGIGLGVAAGALVFGTGIAGAQTQTGTVAEAPLPYVELDP
EEARLRGHAGYYMAGCMYGAFYAILSMLREKVGGPYNQIPYRMAGYGRGGAANWGTLCGA
LNGAAAAINLVAPDADTNKLVSELLGWYTITPFPSDKSNAIASAKGFKYDAKPYPTLVHV
TSVSNSPLCHASVANWIKKSGYELGTAERKERCARLTGDTAAHAVELLNAWKAGTFVPTF
KVDDQTAACIQCHEGDMEGKMACTTCHEPH*
>SPBIB_v1_340065|ID:27163980|aspS| Aspartate--tRNA ligase [Uncultured spirochete bib]
MLFEQRTNTCGELRAADIGKSVVLNGWVHRKRDHGGITFINIRDRYGVTQVVVDADSPRE
LLDIAADLKMEYCLSIRGTVRPRPDTMVNPSMPTGEIEVKAERIQILSRCAPLPFMIDES
TDAKEDMRLTYRYLDLRSFSMQKKIRLRHEAAFAVREYLVGNGFYEIETPTFIRSTPEGA
RDYLVPSRLYPGKFYALPQSPQLYKQILMVSGFDKYFQLARCYRDEDSRGDRQPEFTQID
IEMSFVTREDVLTLVEGLMGHVFKKTLGVQLPAQFVRIPYDEALDRFGTDKPDLRFGMEL
QDFAPFVDEHCSFQAFHEVVTKGGTVKALVVPGKADYSRKQIEELEAKAKVYKAHGLAWM
KVSPAGVLEGGASKFFSDHARIIQDLGAKPGDLILLVADEKKKIACTALGAVRTQLGKDI
GLCDRAVFAFAWIVDFPLFEYNEEEQRWEAAHHMFTMPQAQYHDSLEADPGAVKGDLYDL
VLNGYEIASGSIRIHDPELQKRIFRIVGMTDEEAQRKFGFLLEAFKYGPPPHGGIAPGFD
RLVMLMAGETSIKEVIAFPKNSFAVSPMDQCPNEVDERQLKELHIKIEKEE*
>SPBIB_v1_340066|ID:27163981|bcp| putative peroxiredoxin bcp [Uncultured spirochete bib]
MIKEGERAPNFALKDASGKTWTLADFRGKPFVLYFYPKDNTPGCTTEACNFRDRYDEFKK
RGIEVVGVSADSEKSHQSFAAKKNLPFILLSDPGKTTINAYGAAGEKKLYGRVFFGTIRC
TFVIDGEGVVRKVFPKVTPAGHADEVLEWLSSVLNLG*
>SPBIB_v1_340067|ID:27163982| GntR domain protein [Uncultured spirochete bib]
MEKKRGELVQQLRKMIAEPGRFPDSKLPSERELAQSLGVSRNLLREAVITLEALGYLEVR
ERQGTFITAPAADDFVASLRFASLWPEEMLCQLMEMRLVIEVPIAGLAALRRSDEEVKAM

RRCIADLEQASLRPDGGASEGAQWDSRLHMLIVNAARNPLLSRLYEGLAATMERYISMSR
VILLAIDGWPAKIVAEHEALVEAIARQDPQGAEEAQRHHLGSALKALKALTKKN*
>SPBIB_v1_340068|ID:27163983| TRAP dicarboxylate transporter, DctP subunit [Uncultured spirochete bib]
MKRVLAVAMLMAMVGVPLFAIDIKLAHVVNEQDAFHLAATKFKELTEKYTNGEVKVTIFP
NAVLGDERTLLERMKMGIVDAGVITSGPFVNFVPKFGVVDMPFLFRDAEHAYKVLDGPIG
DKLFADLEPQGWKGLAWAERGFRNLTNNKRPINKPEDVAGLKIRLMQNPIYVDSFKALGA
NAVPMAWTEALTALQQHTIDGQENPLNVIVSFKLYESQKYLAITRHAYAPAPIIMSMMTW
KKLTPAQQTAVLKAAREAAQYERDYDNQNEAGWLKELADKGMVITRPDLSAFLKAVKPVY
DTYSDKFGKDLINAILETK*
>SPBIB_v1_340069|ID:27163984| Tripartite ATP-independent periplasmic transporter DctQ component [Uncultured spirochete bib]
MAESDKAGRNASPLHLLSDGVNKVTEGALFASLLVLVGVTFLQVVFRFFFTALTWSEELS
SFLLVWVSLLGTAVAFKRGSHIAVTFVLDRLGPLARRLVQTLVALLGLAFFGIVVWYGGV
MMGSEASQVTPAMGLSMRWIYLMYPIAGVIIVLHIVDSLVAVWQKEA*
>SPBIB_v1_340070|ID:27163985| TRAP dicarboxylate transporter, DctM subunit [Uncultured spirochete bib]
MGYLLFGAFFVLMLIGVPIGLAIGLSALIVFVSMGIPLQMVPQTLLEGNNSFALVAVPFF
VLAGDILAKGGISERIVAFAEAALGRIRGGLSVVSVLASMFIAAISGSGAATTAAVGASL
LPDMKTKKYDVAESAALIAAAGTIGVVIPPSVPMVLYAVIAGESVAKLFVGGFIPGILMG
LGLIGWAAYKAKVRGYPRGERLPLKVVGKRFLSSFWGLMSPVIILGGIFTGIFTPSEAAA
VAVVYSIVIAAFVYKALDFKKFYKLVVGSGVTSALIMFIIATAKLFGWGLAFYQIPQAVA
SAMLKLTGNVPFLVYLMIGIIVLLAGMFMETASALIILTPIFIPAVVGVGGNLVHFGVVL
TIGLAIGMATPPVAIDIYVASAITGLPMGKISKPILPMILILALVFFVVMYVPDIVMVLP
RLVMGKG*
>SPBIB_v1_340071|ID:27163986|ilvD| Dihydroxy-acid dehydratase [Uncultured spirochete bib]
MISDRAKKGASRAPHRSLMKANGFADWEIERPWIGIANPYNQIIPGHVHLRRITEAVKAG
VYAAGGLPIEFPTIGVCDGIAMNHIGMKFSLPSRELIMDSIEVMVRGHAFDAIVLVTNCD
KIIPGMAMAAAKLNIPALVISGGPMLAGRYQNRDVDLSTMFEMVGKHAAGTISDEELAEI
ENVACPGCGSCAGMFTANTMNCMMEALGLALPGNGTIPAVYAERDRLAKDAGRAIMALVE
RDIKPRDILTRAAFENAIAVDLALGGSTNTALHLPAIAHSAGIDLPIDLFNEIGERVPHL
CSMSPGGPHHIQDLYAAGGVQAVMARLSERGLIKTGALTVTGQTVSSNLKRARVRDDNVI
RPLDKPYHEKGGLAVLHGSLAPLGSVVKQAAVAPEMMVHTGPARVFNSEEEAYAAIMAQK
INDGDVVVIRYEGPKGGPGMREMLSPTAALAGMGKDKTVALITDGRFSGATRGSAVGHVS
PEAAEGGPIALVHEGDLIRVDIPGKKLDILVGEAELARRRAEWKPYVQPVESEFLNRYRR
AVTSGARGAILE*
>SPBIB_v1_350001|ID:27163987| protein of unknown function [Uncultured spirochete bib]
MGGGSSAFRPADTVGLEVVVPHKVGPEAQHAVCDIAVEADTLTMVVRQVLSELAIGVVRR
KEWIVDRPXX
>SPBIB_v1_350002|ID:27163988| protein of unknown function [Uncultured spirochete bib]
MLRLWAYLMWHNYLKPYRIRWPKGRRPATHAEASGIDATALENVYRSFFEERAFLTRSPL
SPTMARSWKKEWRTPGKEEAEYLPKLALG*
>SPBIB_v1_350003|ID:27163989| 6-phosphofructokinase [Uncultured spirochete bib]
MPDFSIETLGKCSVDSPLGLAGLTGTGGPVFVRDDQFICYNIEARPGERPLLTRDQLLEV
AGPRQKIYFSPSHVHAGIVTCGGLCPGLNDVIRAIVRSLWHLYGVRRISGIRYGYKGFLP
EYGLEVIPLDPDIVDDIHKIGGTILGTSRGGGDRTSEIVDTIEQLNLNVLFAIGGDGTQK
GALAIAEEIERRKLKIAVVGIPKTVDNDIEFVEKTFGFDTAVVKASEAVAAAHMEAHSQI
NGIGLVKLMGRDSGFIAVHTVLAVHEANFVLIPEVPFDLEGPQGFLPMLEKRLARRGHAV
IVVAEGAGQHFFTQEVKFDASGNRKLGDIGLFLKEKIEEYFKAKNKEINLKYIDPSYMIR
SAIAEPVDSMYCERLGNNAVHAAMAGKTKLLIGLVNNEFVHIPIRAATSKRKQVNPEGSL
WRDAIEATQQPMSMVNPK*
>SPBIB_v1_350004|ID:27163990| protein of unknown function [Uncultured spirochete bib]
MNDEGLYVGIVTRAKNTGVARPEAWLAECGWDEWHAGRGKREETLNSAGAAQALCVRLPR
WVRGFGAAKPGATNLRQTLKAVRNAVEADVRAGRVMFLLAEFPHSFTYSVGNRLWLDRVL
GELEGLPLVVGFWSAEWYTSRLIEGLKQRNVALCLYDAPRLPGLPPAVEVLTADRVYARF
LSRNGAAWKNGQFGHVLDYRYTKKELANIVPRLLMWRRKAEAVGMVFANGRWAAESAALM

NRLLSAHVEAQLPKGGGP*
>SPBIB_v1_350005|ID:27163991| putative DNA-directed DNA polymerase [Uncultured spirochete bib]
MTSGTIIHVNVIGLMAAVEEIVDPGVRGRPFVVARPDIPRAIVLDLSPEAYREGVRRGML
VQTACARVRALKVVPPRPELYEKVDQSLLEAAFCFTPLVERAGRGHLFLDVNGTRRLFGA
PEDVARRLLGEIADQTGLVPTVALASTKTAAKVASRVFRPFGFAPLSVNDEGELVARQPV
ELLPGVGVKLLPRLISLEIGEIGTLASLSMDEAQAISARGPELVLRARCVDDSPVNPEPP
SRRMLAGETVFEPDCADPDLLAWRAREIVAELGFRMRKEGFGAHFVMAKLVYTDGAVASR
SVRAGQGASFVADIQLCEAAERAVRQAWERRVRVRKLQIELGGFEPAGPELDLFEPLPFF
KAEPEIDSTGIQTRRIAMHMRVQSALDRVHGKYGQSAIVPAAAFLYREGSLVC*
>SPBIB_v1_350006|ID:27163992| Error-prone DNA polymerase [Uncultured spirochete bib]
MLTPLVTHSAYSLLWGVLTPQQLVEAAASAGWNALALTDCNGMYGLPVFLDACKERGLRP
IVGVECTGGWGRVVTIARTKEGFSRLTRLLSARAAVLNEAGLPAYTRFVTLGRDNKQHSE
ALAKADTLLWCELAAIAAMSDPGLFLLSDSPIFLREVPCSPWLFALLSTAHASQWRALLK
TGHKPVLSPEITFLEPGQREVQRLLAAIGLGVTVREVPGLMLAPEAAAFEYARAADNAGE
IGLSLASDNSGSAAWFASAPLESRFCDLESSLAEAATANRCITEEALSEPFGDFVFPMWQ
AAGATDEVAGTCAPNDDAGAGISAPVALRRLAYEGAARRYGSLPPAVRERLEYELGIIEE
KGFCDYFLVVNEIIKKAKRICGRGSAAASIVSYALGITDVDPIAHNLFFDRFLNPGRKDP
PDIDVDFAWDERDSVLEETVRMFGAEHCARVANHNCFRFRGALRDTARAFGMPDEETSAM
ERRIEHNRDAALASADSVWKDIFLLAIRITGLPRNLGTHSGGIIIVPGNSADHVPVERTG
SGILVTAWDKDGVEDAGLVKIDLLGNRSLAVVRDAIQNLHENGIELDLERWRPEEDADTI
AMIARGDTMGVFYVESPAMRLLQKKTGVGDFAHLVIHSSIIRPAANRYIEEYIERLKGKA
WKPLHPVLAELFSETYGIMCYQEDVSKVAVALAGFSSSDADAIRKVLTKKDRGHRLEMWK
QRFYEGAAARGVAPDVIDAVWDMIMSFSGYSFVKAHSASYAMLSFQSAWLRAHYPAEFMA
AVLSNHGGFYSTLAYASEARRMGLELLPPDVNESALRCKGKDGAIRWGLGMIAGLSESTL
KALVEARGGDTTPRRSSIFRPFVSVEDFAARVPFSREEAEAFVGSGALDSIAKGMGRPAV
LMALLKACTARARHAGPGLFEDPERISRGAHISSTSHTRISSEGSPSSSKTSERRIALSQ
MQWLGTTLSCHPLSLVPGALQMPRLQAVALDRHIGEYVRLAGWLITAKEVLTTHEEPMEF
LTFEDETASFETVLFPAGYKQFRPALLEGGAFVVEGRVEESHGAITVTIAALRRLPAIPK
LAETPRAPPPSGFGYASKFGYQAVSRN*
>SPBIB_v1_350007|ID:27163993| protein of unknown function [Uncultured spirochete bib]
MDRIVAVSDTVNAGIHALALAEANGGSISAREAAERLQVSPSYLAKIMQKLAIKGLLTPT
RGLGGGYALTKPAEQISCLEVLTLLEGDLPRRECLFAKAVCRTGTCALRTFCADTEKRLR
TALETITVAAVARSF*
>SPBIB_v1_350008|ID:27163994|hcp| Hydroxylamine reductase [Uncultured spirochete bib]
MYCNQCQETMKNTACTLAKGVCGKPAETANLQDLVLHVCKGIGYWASKALPLGFYSEDDA
FYVTRMLFATITNANFFTQDFEHWIAEGLERRERIRLAYVAKGGAIDSTPPDAATWSAAN
VEAIRTAAAAGKGALTEIADEDIRALKALVLYGLKGMAAYVEHAYAIGISERGIFEFMLK
ALAQLADERVSKDALVSLVLKTGKAGVDAMALLDRANTGAYGKPRITKVRTGVRNRPGIL
ISGHDLRDLHDLLEQTKDTGIDVYTHGEMLPAHYYPFFEKYDNLYGNYGNAWWKQDVEIE
KFHGPVLFTSNCLVPPKESYKKRVFTTGIVGFEGCTHIPDREPGKMKDFSAIIEMAKTCE
PPEQLEDGTIVGGFAHDQVFALADTVVEAVKSGAIKRFIVMAGCDGRQPKRSYYTEVAEK
LPDNTVILTAGCAKYRYNKLDLGDIGGIPRVLDAGQCNDSYSLALVALKLKEIFGLDDVN
KLPLSFDIAWYEQKAVLVLLALLYLGFKNIRLGPTLPGFLSPRVAEILIENFGIKGIATA
DEDVAAMVAGR*
>SPBIB_v1_350009|ID:27163995| HAD-superfamily hydrolase, subfamily IA, variant 3 [Uncultured spirochete bib]
LIKLYIFDEGGVLIRNHTIVDKVAAALRMESEEFRRLMLQDIFALSRGEIDSAEFWRRFA
TRSGIEPGEDYLRTLFKPARDEPTFELVKELAQHARVVCGTNTINSHHQINIELGMYEPF
HSVYASHLLHYAKPDPEFWRIILKAEGVSPAEAFFTDDSPDNIEAARSLGIHAVLYTDAA
GLRRELVALGAPLRSPFIR*
>SPBIB_v1_350010|ID:27163996| conserved protein of unknown function [Uncultured spirochete bib]
MSEFHPWLYEARGQKAVAALAKNGFDALYLPSAAQAAEKVLEYVEPGAKVGFGGSMTIKS
MNIAALAEQKGAIILDHNRPGLGAEEKMEILRAQLTCDVFISSANAVTLEGFLFNVDGNG
NRVAALSFGPRKNIVVAGINKVVKDLDEAYERLKAYAAPLNNKRLSKPNPCTTAGYCADC
ALPTRICRIYHILKRKPSLSDFTVIIVGEELGF*

>SPBIB_v1_350011|ID:27163997| Response regulator receiver protein [Uncultured spirochete bib]
MDVAMPVMNGIEATRQIKKEGIDSIVIMLTAFGDEKAMEEAAEAGADDYLTKPVEMHALK
ARIELAKKARTFHVARSKLLIENKMLIGVKEEEINACIEENYSLSAELLYRLSLAAEFRD
DDTYEHTRRVGRNAAMIAQYIGKDAEYIYLLTQAAPLHDIGKIGISDTILLKPGSLDNDE
FETMKTHTIIGKKILNGSQTKILQLAESIAYSHHERWDGSGYPQGLQGESIPLEGRIVCI
ADSMDAMFSKRPYKGKMPPEKVKEELQKNRGLQFDPTMVDAVLEHWEEIADMYHAEAGG*
>SPBIB_v1_350012|ID:27163998| putative two-component system hybrid sensor and regulator [Uncultured spirochete bib]
MNTAQKQIKAYLRSAFFYSAVVAVLYFFLRNLHFGYMEHLLFEFSSIFFAMTLFVLYVIA
PRWKQSEGLIIFGIVLLFVSLLDVVHAVRFSGFPGTSDMPSSVTFWVLARGLQAFGLIAA
ATRKDILAGKKTSTTLSILLPITAGIGLFILSYVLPSGIFFIEGKGTTALKANLEILYAI
LFLLFAILMRHEPEAFIAGALFAFSEIAFITYVNVTDPRIILGHILKATGFLALGLFSLK
KYFLMPLKEMAEVQRTYSAENQGLKSTYETLTQRWNEMIKCREKIIQCNSIDAILSLERE
FFADPSKTIRLAKFEKDRLLFKNDPGLPEEKSAYLPEQFERIDMENGTTVFLDKESGFYL
NIYKPFFLFIDLAKKNLLEKNQIALLNIKLVEEEKYRIDFLRSFSHELKTPLNVIYGYLQ
LFSTEAFGAMSAQAKTALNEMIESVKKSNEIINDLLNLAKAESGAVSVKCDRIQLKSFLN
RILVEAKKNALLKNLDFVIECDGEPEISVDPKLFGLVISNLASNAVKYTDKGHVRVFARA
DDKTGIMIEVKDTGTGIPAEEIDAIFKPFKKGEHARGGFGLGLSLVKTYTQLIGGKVSVE
SQVGKGSTFRIEIPPLPKIDVSKVAKGQKADFLLIEPDKTTRDMLKILLKDSVVEEAESD
EKGIVAALSTLPDMIITDFGLQRTTGDQLIKRMRTYPELADRKFLLLTGRRSSDLTLDDI
PVIEKGNIDLQHLAKVLMAIRNDAIALVFCKGESEERLDRAKGIVRTQLAGRDSVEIDIS
EPGLSELCCLFDEAYFLTPTDSESRQKIENIIFRRKTYFSKENVVLEV*
>SPBIB_v1_350013|ID:27163999| PIN domain protein [Uncultured spirochete bib]
MLRTQNIPKFISVITYGELIYGARKSKHPEKNMATALRIAELFPVIDIHRGIIEIFGELK
AKLETSGSRIDDMDLLIASTAMYMNFSLVTNSKGHFGRIEDLPLENWNREDI*
>SPBIB_v1_350014|ID:27164000| protein of unknown function [Uncultured spirochete bib]
MSKEPSGIERSCAIREIKLAGGGLARRWQARHQPRHAGGKGGWAGGPRGSSGA*
>SPBIB_v1_350015|ID:27164001| Metallophosphoesterase [Uncultured spirochete bib]
MNQEGKKPGAMPARRTMFLAALVALVLGIIMNSYHYSLEGARGLYVWHPLLALKILLFMG
LVPISIAIVSLPLEKLAKKWPMLVMRWISMAASILVGAISIALLAFLIIVPRIGSLEPAR
LELIDPAKGIHALGTQPAEASFSAGTTNKGAAQPGIALQQPQSAALQPAIIAQARSPLDM
PLLRLSFSSDPHWGADTSNAQARTQILESIAQRKPDAFFMLGDTVETGNSATQWNFALSD
LEALIPHVPVRPLLGNHDALFGGQYLYRKAFFPKGFSSDSGSPYYWSIDAGAATIVAVDL
PWGTENFGARQRAWLEKTLAAADPHKPLIVLSHSYFYASGYDDPDFGSPWYDHYQNIPAL
VPLFEKYGVDFVISGHNHYQELLAHNGIAYAIVGSMGGIPDPQPSYRSPWSQWIAVGVHG
WLDVEVQPGKLILVFRDEQGAERHRAELRY*
>SPBIB_v1_350016|ID:27164002| putative sucrose phosphorylase (Sucroseglucosyltransferase) [Uncultured spirochete bib]
MNEYYMNKIKDLLIFIYGREQGMLTFDSLQQLIPKKTTSSKAAKLRDSLTEKDTCLITYG
DMLQPSKKEPETHSQTALEQLKEFLERWNHGSFNYLHILPFHPYSSDDGFSVIDYREIDP
ALGDWNDIAALAKEYKLVFDLVLNHGSKQSAWFQSFLENKESYRDWYITKPADFDVSSVF
RPRMHPLLTPFFRKDGSVVFVWTTFSEDQVDYNLANPQVLLEFIKIFFEYIEHGARIVRL
DAIAYIWKEDGTSCIHHPKTHAIVKLLRALIDYLELDVLILTETNVPNEENLSYFGKGDE
AHLVYNFALPPLVLHAAVSGDASPLRTWANTLPQPGEGPIFFNFLASHDGIGVLPINGLV
DDLSFKKTLNTVIERGALVSYKNAQSGPVPYEINCSYLSAVAPLSLGTSKERARAFLCCH
GVLFAFAGLPAVYFHSWVGSEQWEEGPQLFGYNRAINREKPRKDIVETALNDEHSLRASV
YTGFEKLLTFRKSERSFAPDIPQQILPAEGSVFALARGPDPSGRYVLCIQNLSNNQAKFD
LRTAPKIEILIQHGIDSEMLLSPWETRWIGYGGGKAISMLEI*
>SPBIB_v1_350017|ID:27164003| conserved protein of unknown function [Uncultured spirochete bib]
MHIQQMWIERCAGVLGKNLPEQNVLEKLHYRYTCPAREKGTYLPQWLWDSCFHAIVYRWF
DPDMAWEELQSLLMHQFEEGPDTGMVPHMSHLAENGALSAQQLFQNKNSSTITQPPLISI
AALAVHKKAPNKNILAKLYPKLLAYHDWFDRRRDDDHDGLAAIIHPWESGWDASQRWDSL
MGLHACTKEELFELEQKRKNLVSILAAHKYDAKTLAHVPEGFYAEPADFNAIRAADLMAL
AEIAGELGKIAESRELEMRAKAVQQAVHDKMISFKDGELFVHDLLGASEEKSAVDHAGKF

VLLFCQCLSASEAESISLQLFRSADCYATPFRVPSTSRFDALFNPKEYWRGNVWLPVNWL
IWRGLLSYGYKEKAHTILEDSISLVQNSGFCEFFDPITGKAGEKYGQSCPRNQSWSTIVL
DMVLDSEQIIEGKADE*
>SPBIB_v1_350018|ID:27164004|ugpC| sn-glycerol-3-phosphate import ATP-binding protein UgpC [Uncultured spirochete bib]
MVSYLKLNSLKKTYPNGVTAVKDIDLEIEKGEFVVILGPSGCGKTTTLRMLAGLEEVTNG
SIILDGRDITHLPPRQRDISMIFQSYAVWPHMTVAENIAYPLKLRKMDKHTIHSKVQEVA
RICNILDYLNRYPAQLSGGQRQRVAVARALAVDSKLSLMDEPLSNLDAKLRTSVRTFLKE
IHRNTGATTIFVTHDQAEAMALADRIVVMNEGKIEQVGSTREIYNQCGSLFTAQFMGTPP
ANIQKVSLISNQGRLIANDKNAETQFSLDLGNQSAFPGIDRYIGEEIFLAIRPENINISK
PKGINETSIEIVEPQGAYTILVTKVFGSEWKIMLEGDIDIRIGQKVSLIIDPGKIMLFDA
QSKKRVNFS*
>SPBIB_v1_350019|ID:27164005| ABC transporter permease [Uncultured spirochete bib]
MSMHPGKLWSRTVFLLILATIASTVILPILYFLTISFASNYEAYQFPAKIFPTFSYSAQL
RYNAEKGSYTLLLKKGSTYEAVKTTQDVSDFSLYCKSQLNVLLSSAAVQELFDKAKNTDH
ALPIKLKKDFFRNYIVFFILAEGTMRALFNSLKAAGWTILISLILGGVSGYIMARYKFKF
SNTFSTSLLVVRMFPAVALSLPLVVYIMKMNLYDTSFALAIVYAVPNIALTAWITSSIFK
GISVELEEAAMVFGATRLKTLMTITFPLAFPAIIASSLYAFLAAWNDSITALIMTNNNPT
LALLVYRTVGSSTIPNLPAAGAVVLLVPSLIFTFIIKNYINQLWGKVAL*
>SPBIB_v1_350020|ID:27164006| conserved membrane protein of unknown function [Uncultured spirochete bib]
MNRMRRAGFFKSPIWFLVPVIIYYAAFWIRPTISVIIESFTDKSGVFSFANYVRLFSQKS
MRTAFLNTILFTLGSAILQFILAFALALWLNKKFRFSNLVLFITLIPMAFPPAAVGILWK
TGLYRFGWINSFLCSLGLMNPANPVDWMSFRNLYAVILLIIIDTWTVLPSVMIILLAGLQ
NFNKEFEEAGWVFGANKFQTLKDIVFPIMKPTIITAMILRMIAAVQVWLIAVMIFGYNVV
PFLVERIAYNVDVITFAKYARKDAYTISVIVAAIVLISVSIYLRVSGEKQKGGEIA*
>SPBIB_v1_350021|ID:27164007| putative Extracellular solute-binding protein family 1 [Uncultured spirochete bib]
MAKSRVTFIAVFLILIGLASPFFVMAQGTQKGEKIKIVSQMFYDPAQQQYIKEQILPKFT
AETGIEVELQVVANASELYKVIQAQQQTGKWSTDILIAHDSTAVPIVQEYRAVQPYTKIP
AGTYITQFDDNFVQGGKRYFVPLQADVYLTIANRKALPYLQKLGYDINNLTWEQLAEWVR
LIKKETGLPKYVFPALAGKFATYEFNAVQLAYGDKYVPAFNTPASQAAFKLVASMKEGIL
PSSPTIDFPTASLATEEAWITVFHQAYANASFSQAPDKFIVAPVPIGGSGKRGTIIGGHG
IGIIAGSTHKAAAEKFVEFFLRDDILYAVMKNTGPWIPSKAEITSKLKDDPSDQIMKMGL
ATLTGPTLIDRVRVAEYQDWGQVKRLYEEVIGDILAGKDINKQYLDAKQKELESLKVN*
>SPBIB_v1_350022|ID:27164008| Oxidoreductase domain protein [Uncultured spirochete bib]
MNAQKGAIQSILIGAGNRGAETYGRFALAHPWLFKFTAVAEPNPVRRTNFAAQHDISEEK
STDSWEKLLEFVTPRDVVFVCSPDRFHYEQVLALIERGCSIVLEKPVVVNAQQCATLSKL
ALGKNTNIIVCHVLRYTPFFSTLKRLLDKGKIGRAITFNLQENIAYYHFAHSYVRGNWRN
TSLASPSILAKSVHDLDILYWLAGAPPETVVSMGDLSWFKKENSPENAPDRCLDGCPHAD
TCPWYAPDLYLTENTGWPTSVISDDTSLASRMQAIETGPYGRCVYRCDNDVMDHQDVLIR
FKNGINASFSMNALTYNKTRIIQISGSEGEIVGDLDEGWIEVRTFLHGTKERIQLGSSVG
GHNGGDVGLMNDIASIFKQIDDSPEPETNKTIQIKNSKSSIEESLEGHWMAFAAEESRKK
GIFINMDAYKRDGVSTRETVNI*
>SPBIB_v1_350023|ID:27164009| putative AraC family transcriptional regulator [Uncultured spirochete bib]
MLVLFCIECCTNLHDLSIDSILFDMNPVREIIYPTPGYSFCIQYFDDRRLHFNWHYHEDI
ELVLIKNGEGQAHIGDLVKHYKAPAGFLLGPSLPHGLLSIGFLQGWIIQFQEKHIKYPNA
PSEFINILNVIGESKKGLSFSSPAIVDCLPLMENLNSSNGLDKWLWLLKVLNVLSQDKNR
ELCSLLPHNQEVLTDRFEQAITQIFNEIDKTYNLEEVSRKVGMKVSSFCKTFKKRYGLSF
IEYIHSIRINNAKKLLIQTKFYIDDICYESGFNNVSFFNRKFKEVTGLTPSEYRKRYREA
*
>SPBIB_v1_350024|ID:27164010| 5'-nucleotidase/2',3'-cyclic phosphodiesterase-like hydrolase [Uncultured spirochete bib]
MLQRIGKFAVLVAAIFTLAFTGSLSAQGSLPNLPANVQITFAHTNDMHGRIVESKDVIGF
PKIYAAVQELKAKNPNTLLVDVGDTFHGLPVVNIDQGQTAVKLMNELGYAYMTTGNHDYN
YGFARLLELAKMANFKILAANVYKDGQRVFPAYDIRDMGGVRVAFFGLATPETAYKTDPK

GIQGVTFSDPIVEAKLVVAELAGKYDLLVCLSHLGVDDSSDPTSITLAKFVPQIDIILDG
HSHTSLADIQKKNTTNTLITSTGAYGTGLGVVDVVVGSDRKVISKTARTITPQNSPDLKG
DPRIATMLSDLVKAQDAVLSQVVGQTSVPLEGKREIVRTQQSNLGTLIANAMLYVTGADF
ALMNGGGIRDSIPAGNITLKQIYTVQPFGNYIQTGKVLGKEIDAILENGVGKLPAPDGRF
PHLANLTYALDASKPAGDRVSDIKIGGVPVDPDKEYTIATLNFLFNGGDDYRMLVGKAKN
DFPSDAEVFIAYLKHLGTVTNENMVYQK*
>SPBIB_v1_350025|ID:27164011| putative PfkB domain protein [Uncultured spirochete bib]
MTFTQPSEHQFDIAFLGQYTKDTIITKSETRIVDGGAYFYGSAAAAALGLKVAAITRLAA
SDFASFRFLLERGVTVKAIPTEKSTCLQLLYPSDNPDERVLSVTSVAEPFEPADVASITA
LVWSIGASIRGEVSLDVLKAIKAKGARIGLDAQGFVRVVRDGTLAYDREWPEKAEVLSLV
DVFKADVVEAEILTGTRDLRQAARELAGFGVAELVLTHADGVVVYAGGSFHEAPFKPKSL
VGRSGRGDTCLASYLAARITMPPNEATFWSAALTSLKMEALGPFAGTQADIEAALHERYG
LHKVENT*
>SPBIB_v1_350026|ID:27164012|fbpC| Fe(3+) ions import ATP-binding protein FbpC [Uncultured spirochete bib]
VSVRDFSARSSNVTLIHLTKKFRSLDGSGEIVAVNDVNLEIQAGELVTILGPSGCGKTTT
LRMIAGFEYPTSGSILIGGRDVAMIPPNKRGLSMVFQSYALFPHLSIYENVAYGLRVQKL
PAQEIRERTERALELMQLTTMARRFPSQVSGGQQQRIALARAIVIEPSVLLFDEPLSNLD
AKLREYMRDELRKLQKRLGITSLYVTHDQSEAMAISDRIVIMKDGCIQQVGTPREIYAYP
HSKFVADFMGKANFINVNVLGMEGETVGLGGREGAGSSEISAAAERAARIEIEGRQFVVP
RPGAVAPKAGKALLVVRPETLKLVPLGESASEADLSAGSARGSPGISALKGRIDRFTYFG
NIARYEVSTENAPLLIESYNPGASQILEEGAAVGIVIDFESARLLPADEQEHRA*
>SPBIB_v1_350027|ID:27164013| ABC transporter permease protein [Uncultured spirochete bib]
MRKNTRFSIAHLVRDPVLLFLVLLVFTGLILFILFPLYKIFVYSVTNNEGRLSFQAAIDM
FTSKAYARPLGNSMLLGITTGVLATLIGYIFAYALTRTDIPLKGFFRTIATIPIISPPFI
LSLSMIFLFGRNGLITRKLLGIEDANVYGMHSLVVVQTISFFPIAYLTLTGTLSKLNPAV
EDAALNLGASKARIFRTVTLPLSVPGILSSLLLVFIQSMEDFSNPAVISGSFSTLSVEAY
RTITGMYDMRGGSLMALMLLAPTLIAFVLQKYWLAGKSFVTVTGKPTTARAQSRDPKLVW
PLFAFCMLVAAIIILFYGTVLVGAFVKIWGINFSFTWSHFKYVMTLGWQPLRNSVILALA
STPIAGLLGMIIAFLVVRKEFPGKRAMEFVSMLTFAVPGTVVGIGYILAFNDKPFMWTGS
AFLLIMAFTFRNMPVGIESGTSTLIQIDRSIEEASTILGATGAVTFRRISLPMLKQAFFS
GLVYSFVRAMTAVSAVIFLISPRWNLATISIFSLFEASKYSDAAAYIVVMIVIIVVAIGG
LNLLVGLLGNSGTVASGGNSERT*
>SPBIB_v1_350028|ID:27164014| Extracellular solute-binding protein family 1 [Uncultured spirochete bib]
MRKIFLSMFALMFCVIPLLSVGAQGTAEYKLLTVYTALPESELPTYFSQFEKDTGIKIQY
VRLSAGELLARVRAEKNNPQASVWFGGSYDNFVPASKEGLLEAYQSPELKNIPKVYWDPE
GFANPFYVGAIGFACNTEWFKKKGLPYPTSWDDLLKPEFKSQISMAHPGTSGTSYTILAT
IVQMRGEEGAWKYFTALNQNIRQYTKSGVTPPMDVGLGEAAIGITFSHDGLKPAFEGYPV
AMSFPKDGTGYEIGCMALIKNGPAKETANGKRFIDWMLSKRGQDLFETSKSFRIPVNTLA
TPPKGAISVDSLKVINYDAVWAGQNRNRLVEQFTKVVAAASNLK*
>SPBIB_v1_350029|ID:27164015| PfkB domain protein [Uncultured spirochete bib]
VKYDIIMLGHISKDIIIDEKGQENRLYGGALLYSSISAARSGARVLAITKAAKGDFGALD
VLKQQEGIELVVLESPASTSIRNVFLSSDHERRQTTLLSRAEPFSAADIPEEAEAEIIDL
AGLFVGELPDSLIEELAQREPGKRASGERKSGKRAKIAVDAQGLLREAQPDGRMIFRDWK
NKHRYMPYVSYFKADAAEAEILTGLTDREKAARVIASWGGSAGGSPWAGREPEVMITHNT
EVIVLAGGQIYRAPFTPSNRSGRTGRGDTTFAAYLAWRLSHGPEESVRFAAALCSIKMET
PGPFSGTIEDVFKRMR*
>SPBIB_v1_350030|ID:27164016|plsY| Glycerol-3-phosphate acyltransferase [Uncultured spirochete bib]
MLAMILGIILAYLAGSFPTGLVVGKLFFGKDPRQQGSKATGATNVFRVFGAKAAIPVALV
DVGKGALAVFLAAWVGRNAALPREALQVAGAVAAMVGHVFPVFAGFRGGKGVATGAGALI
MMAPVAAIFCVLGFLLVVGLTGIISASSITAALILPIAIALGAQGSPPNPWLLGLGIAIA
IFIVFTHRANIGRILRGEEKSFEQFRFLRRKKHD*
>SPBIB_v1_350031|ID:27164017| putative DegV family protein [Uncultured spirochete bib]
MRKLGRRSHARSTIASNEKQAGALICPALPFDIECNIVNLMKISYLDGPRLSRALTAGSQ
TLIQNSASLDAINVFPVPDGDTGTNMASTVRAITSSLAAFRPKNAGSVLKRAAQSALAGA

RGNSGAILAQFFSALAEELQHDARIGAKRLANAAVSAAEKTRRALSIPKEGTILTVLHDW
AHAMHEKAQQSDDILHVFIAAYESAKASLARTRNMLPEMKRAGVVDAGAKGFVHMLEGIV
QLIRSGSLKEAARADKSLQASTGAMLDFQAPNDVDVLLASSDSQFRYCTEALVHGEGLDL
DAIRTQLSQYGDSVVVAGTESLAKIHVHTDAPYQVFDFLDSQGLVDSHKVDDMELQKLLA
HRAQLAGQNQRAAATEKPQTCAIVTDTGCDLPEAFLFEHGVIKVPALITIDGKTRPDGPA
LDIRAMHRLMREHPDFSMSTSQPTDAAFSRAFAIAAGHSNEILYIGLTAALSGTFQAGVR
AATSLFRGSAAPAGQTGQDGRTERAGHTEQTGQTERSPQTPPHFVAFDSRTVTAAQGILT
SRAVEMAEHGLSAGEIARALETLRDRMVFFVAVRNLSSLIRSGRLHGVKSVILRKFGLRP
LLTTNKEGKAETAGIYAGEKNTVSALLSRIKKAFSAGSRAELHISHVDAPEEAQKLADLC
AAYLHPESKIVISEMGPVLASLAWLGAISVAGLPESVPKLV*
>SPBIB_v1_350032|ID:27164018| putative CoA-binding domain-containing protein [Uncultured spirochete bib]
MAEKYKRGLTPVRLDAKARALRDEAIRIAARAKAEGRSALTELEGMALLSAMGIRTPRYW
LVSSADEFLGRLGEKTSSNVSIMPGPFPGAKAVVKVISPEILHKTEVQGVEIVDNTTASI
VDALRRMEGRFAGARLDGFTVNEFIAFEPKLGHEMIFGYRFAPDFGPVVSFGPGGIYTEY
LATKFRQGAANLILSPRVSNPKALEALLRENVIYGLLCAGLRNTKPELAPEALQRAIQHF
LDAADALAAAGIGEFEVNPMVLSHAGELVALDCLVTLKDFSSMGLASDKDGLPVNMAQQT
RPVQEIGRILKPASAAIIGVSEKGMNNGRIILRNLIENGFDTSHLYVVKAGIESIDGCRC
VPDVASLPEKVDLFVLVIPAASTPATLAQIATYDKAWSVIVIPGGLEEKAGSESIVAEMR
RALSEARGQGKGPLINGGNCLGIRSVPGKYNTLFIPEHKLPMPKGKVEPLAVLSQSGAFA
ICRISKHPAINPKYTITCGNQMDLTIGDYLDYLAQDPDLHVIAVYVEGFKPLDGEKTLEA
CRRITESGRSVIFYRAGRTQAGAGAAASHTASIAGDYPVTRQLFAQAGAIVCDSLDEFDD
AITLFTLLDGRKARGNRLAAVSNAGFECVAIADNLGGMVLSSFGEATKAELEEIFRAARI
TEIVDIHNPLDLTPMAGDDAYEGSFRAALLDPSADLGIVGIVPLTVMMNTLAADPAVHSE
DVTREDSIAARYGKLMRETDKPFVTVVDTGPLYDPLCRELEKYGVPVFRTADRALKMLEL
WRNSPAAR*
>SPBIB_v1_350033|ID:27164019| Indolepyruvate ferredoxin oxidoreductase [Uncultured spirochete bib]
MTCDIILCGVGGQGGLSVSVVIARAAMASGFLVKQSEIHGMSQRGGQVLANLRISDKEIF
SPTIPKGKADIIFAFEPLEALRYLSWLSVERGTVVAATTPIRNIPSYPDIESVLAEIRKL
PRARLIDADSLAKQAGNVRSANLVLVGAAADLLPVPPEAIEKEIVALFSRKGEAVVQANL
KAFEYGRSIQHG*
>SPBIB_v1_350034|ID:27164020| Indolepyruvate ferredoxin oxidoreductase [Uncultured spirochete bib]
MAEMVLSGDEAVAQAALDAGLSGGFAYPGTPSTEIMEYLQEHLGDKRAGAGDADGSAETG
SVLEVRTAPRVAQWCANEKTAYESALGVSFAGKRAMVSMKHVGLNVAMDPFTNSALVRIH
GGLVVVVADDPGMHSSQDEQDSRLLADFARVPCFEPSDQQEAYDMTRLAFDYSEEHEVPV
LVRLTTRLAHARALVTMAPARDVPARGKCTDPYSWILMPEMARRRWLALLDKQPIFRADA
EAASRLVLGSKELGVITCGLARAYYKENEDEWARAHGGERPSHLHIGRYPVGAEKIRALV
AHVKRVLVIEEGYPYIERDLRGIFGTPVPVAGKMTGEIPLAGELSADSVRLALGLQAREG
SAVAGIEVPGRPPQFCQGCPHADSITALKKALEGEAEFFTASDIGCYTLSALPPWNAVES
CVDMGASVGMARGAASVGQKHAIGVIGDSTFYHSGMTNLIDAVRYRTPMTLLILDNGTTG
MTGAQPTISGTSQLPKLLEGLGVEKEHIRVLEAHKRALDTNVAAIREEIAYDGVSVIVMV
RECIEWLKKARKS*
>SPBIB_v1_350035|ID:27164021| Glutamine synthetase, type I [Uncultured spirochete bib]
MKLSEWNGDFAGIDYVSFIVIDIDGRMRAVSLPSSYASEAVLSKGIGFDASNFGYAKVHA
SDMVAVPDMESAFVEQKDNFNILHVFCNVQTMDGQFFAQYPRSVIRAAQHALQASGIGDD
AKMLVELEFYVFEDVRYSTTAHHSYYYVESSEGIGEEYSDTPRLGMSQGYHRMAPEDRYQ
LLRNRAVKTMIDVGIPVKYHHHEVGAAQLEIELDFISMVKAADAVSLAKWILRNEAEGLG
LHVTFMPKPMYGVAGSGMHVHQFILKDGRSIFPGEGLYGLSDKGLAYTAGLLSHALTGSL
LAFSNPSTNSYRRLVPGYEAPVSATFAQGSRAAAVRIPGYLGKGEARIEFRTGDATANVY
YFLAAMLLAGLDGIQKGLDPVALGYAKEKPSEKHIFPMGLFHVLSGLKKDRSYLEPAFPS
ELIETWIARKEKEASYVYNAPVPQEYELYFD*
>SPBIB_v1_350036|ID:27164022| protein of unknown function [Uncultured spirochete bib]
MLSVCVSFFDTIVSYLVGLGKPGYNILAHELKCWGRSRGPSFKARVCELNRQGVLLRARL
GADMQAKTAAATDDAVRSSERGLP*
>SPBIB_v1_350037|ID:27164023| protein of unknown function [Uncultured spirochete bib]

MACASGKGACALHMPAQRQRSRPRYASARAQSSKLCAHERYAQMVELADTLDSGSSAKAW
GFKSPSGHFQALFGEPFLLP*
>SPBIB_v1_350038|ID:27164024| putative Site-specific recombinase, phage integrase family [Uncultured spirochete bib]
MGAKIRLRGKQKYFYLDICLNGKRRFEALHFALPADRQGQREAWALAEEIRRKRELQIAA
GRYQLVDPVGSKMTLIKYAEKIAAEYDKKMHLPKSLKYLRQYAGDTALVDVDERFIDAYR
AFLLKQGELGAKTAQHYLAALKAVLARAERERLIERNPAKGVKPIRAPEHEKPYLTVEEI
QRLYNTPVEGELADEVRRGFLLSCFTGLRLGDIRSLLWGDIRREPEPMIKKRQNKTGDIV
SIPLAPIAWELIDDKRLHRQDELVFPRLTATKGVNPHQPLIAWRKRAGIEKAFGWHAGRH
SFAMMTLEASGDIYAVSRLLGHSDIKITEVYLRMTDQRKKEIIASLPQVKKDSERIIKLA
AEKK*
>SPBIB_v1_350039|ID:27164025| protein of unknown function [Uncultured spirochete bib]
MPKLKTNIPIETFTKKVKSLAHEDAGTILTPSGIDRGILPQDAGTLPVRILQKYLRGTAG
LAYIQRKSLRAFRQEEHLSRTDLAKLFNVKVSQVARWLAPKKNDNDLIPILVSDLMKAKI
YLIPKDLERVFSSITKVADDFKG*
>SPBIB_v1_350040|ID:27164026| protein of unknown function [Uncultured spirochete bib]
MNAAGYKKSPRGWGGHGAKGNSRTIINIDKNRENVKLPDWMRRKIAKIAARKDDFALFEE
QAVILLAAECSTFPKQAARIPESLREKLTYQRPDMLDPLERASIAIIAAQVEDLADIFVQ
HPYEDFFDLKARALAITLEEGGRV*
>SPBIB_v1_350041|ID:27164027| conserved protein of unknown function [Uncultured spirochete bib]
MSSDPYLDTVDELGAEERAKKERLAKLEIVSGAALLTTHYEKPKYLWQGVLPDAGLAICA
ASKASGKTLLLLQLADAISRGRDFLGIPTTESKVLYLQLELSQRRTAQRLLKMGIVPCAN
FDFAFRWPTGAEGLQALADSIEAQGYRLVIVDVLQLLWPIEADANSYQDVYAVLAPLRQL
ANDLGVMIALVTHRRKMETADYLDGVMGSVAMQANADVLLTLIRSRGEENAVLFLDGNDI
EAQKLALDFCTDPLGYRLSTASPEELRQTPERRRLIEYLREHGGHGRTSEIATALGIDDS
TVSRLLRRLADNGLIVRTQYGEYTLLQKGIQTVQSVQT*
>SPBIB_v1_350042|ID:27164028| conserved protein of unknown function [Uncultured spirochete bib]
MEKAQNPCTALERDKMYQRPDKVLECGEKLESIGDILHTHACKCYHYVCMEQNKIKRLFL
ANGGYLRMSQAIAAGMSRHAFYALRDKGVVIQESRGLYKLSGQPDHPYPDLCVMSLRYPK
AILCLVSALSFHNATTQIPKEVNIALPRGSRTPAMRYPPLHAYFFSKASYETGIEEHIIE
GVGVKVYDLEKTIIDCFKFRNRIGMDVFLEAIKLYKAKAKVQPSRLAEYAKICSIEKAIT
PYLEAIL*
>SPBIB_v1_350043|ID:27164029| transposase [Uncultured spirochete bib]
MAITKEVLDELLKEYKGPEDLTGPEGLLKQLTKALIERAMDAEMTTHLGYEKHDQSEKDT
TNRRNGRTKKTVRSDQGPLEIEIPRDREGTFEPAIVPKHQREFKGFDDKILSMYSRGMTT
REITEHLKEIYGTEISPELVSRVTDEVKELLEAWRARSLETSYPIVFLDALMINVREDGK
VVKKSIYMALAINWEGRKELLGLWIDQAEGAKFWMRVLSELKNRGLQDILIAVVDGLSGF
PEAIATIFPKTEIQLCIVHMVRNSLKFVPYKDRKAVAADLKALYASPSEEAALAALDIFA
AKWDSRFPMISRSWRMRWPEVVTYFKFPEIIRKAIYTTNAIESLNYTVRKVTRNRLSFPS
AEAAMKLVFMALQNISKKWTMPIHEWKSALGQFIIFYGDRVPL*
>SPBIB_v1_350044|ID:27164030| conserved protein of unknown function [Uncultured spirochete bib]
MNNAVYTKFVIGSSRLIKRDISASIRQRLFDLSKRENRPFGEVLQYYAIERFLFRLSKSK
YADRFILKGALLLSVWHSEVLRSTMDIDLLGRLENSEETITAAIQAILEQNVIDDGIVYH
ASTITTEAISEDARYRGIRVSFEATLAVAKIRLKIDIGFGDSMYPAPQSQEFPVLLDQEA
PHLLCYSRENAIAEKFEAMVKLGNLNSRMKDFFDIWLLSRYFSFDQAILAKALKLTFTQR
ETKMDTSVVFSPEFSSLKQVQWEAFRKRQKLSYSPAQFSEVVNSLSRFLLPCVSSEMADV
GEAMIWKPRGPWERKVKE*
>SPBIB_v1_350045|ID:27164031| transposase (fragment) [Uncultured spirochete bib]
MRKVTNAKASAILQGMQDRELYEKLLGLKEPWSVENVTLDLPSATVTVAISHPKGAKFPC
PVCGTERPIYDHQKRRWRHLDTCGFTTILEAEVPRIQCPEHGVKQVNVPWGEPGSRFTAL
FEAIAISLLKVASFSDVARHLRISWDAASGIMERAVRRGLARREAQPLRRXXXDETSFQK
RXEYVTVVFDQERSCVVDVLDGRKKETLKTWLAANQNALGTLESVSMDMXDAYIXAVRXX
>SPBIB_v1_350046|ID:27164032| protein of unknown function [Uncultured spirochete bib]
MAFRAQELCCRLLDPPGARCLQVASSKRTECRIRGTFAQPGMFGPRQWAFLSPLFEFPVF

LGAHLVDRLIEVLRHMKPVKTDFLXAIGXX
>SPBIB_v1_350047|ID:27164033| protein of unknown function [Uncultured spirochete bib]
MGGDFVREELLDSVQFKTMLQHACDMYCEIMCYAPPDTKSYGNSEIAQALSDGRVAMAVS
WGGQAAPIVKAGRNNGIEFSITPLATSWNATWGIGIISAIDSARAAQVLCMLLELMDKHL
DRLVAEYAGSPVRISTYASAEINEKCPWLKAQLEMIQNARHLPSDSSLVESVNKIGERIA
KAVHGAEE*
>SPBIB_v1_350048|ID:27164034| Helicase domain protein [Uncultured spirochete bib]
MIHRYVFQRSRSMPGKRFLEERLTGATGYDRIAGYFDTSLFELAGEALEQVQGSIRIICN
SDIRARDVEAAAAAAREQAQRLSFFKHDPEDLAKGGSDRIARLARLLSGQGTAKLEVRVL
PDDVFGLIHGKAGVMRYADGRRTSFLGSANETFAGWALNYELVWEDDSDEACDWVQQEFD
RLWSHPLAMPLSKAVVQEVERLSRRTEVRLEDWKKNPDAGGVAVESPVYREQFGLWPHQK
YFVSQAWKAHQAHGARFVLADQVGLGKTVQLGMVAQLIALSSEKPVLALLPKTLMEQWQV
ELWDLLQVPSARWNGTAWVDEESHEYQPRGRDPILACPRKIGLISQGLIVHSPESLQCLL
EREWSCVIVDEAHHARRRKLPAPDERGPAIRNPDTECNRLYAFLFKLASKTESMLLATAT
PVQLHPIEAWDLLYLLSQNNPHVLGDVGSFWLDPEKALPVVLGEAELPQEAAEIWPWLKN
PFPPSWESPHAKRLRLDAHMSERTAVCGLSFHELRPAARTWASSLCSVFFDQHMPFVRNI
IRRTRSYLESTINPATREPYLTLIGIELFGEDEPIPVTGYLANAYQEAEAFCLTLSQRIR
AAGFLKTLLLRRIGSSIEAGRRTVAAMLARSQAPLTEDDEANEPENNGQEVQTETLSALY
PLTEKETEILQRCASLLEASEEDPKWQVILHFLKDEGWAEEGCILFSQYYDTAYWVARLL
STAFPDLPIGLYAGADKSMLIHQGRELRKERTELKRMVKEHELSILVGTDAASEGLNLQT
MGTLINIDLPWNPTKLEQRKGRIQRIGQKRESVKILNLRYKDSVEDRVHQVLAGRLQDIY
RLFGQIPDVLEDVWIDIALGEVEKAKQELDRLPHRNPFDERYGRIDSVADWERCAEVVNR
IEKIEVLKQGW*
>SPBIB_v1_350049|ID:27164035| ATPase AAA [Uncultured spirochete bib]
MREERIRSILTGGEHETVEFKQALTQVPESLYETICAFLNHKGGDILLGVDDDGQVIGIP
EDKADAFCLDIANATNNNTLFSPPYLLYPQKLLFEGKWIIAVQVPESSQVHRLRNMVYSR
GASGDYRVTVPEHIAKIVNRKRTFYSEAKVYPYLHVSDLDEKTLQKTRERIRNIRPNHPW
LDLSTEDFLRKAGLYCRDLETGTEGLCLAAALLFGKEETILNILPFYKIDALLKRSNVDR
YDDRCYVQSNIIEAYLQLMEFIERHLPDPFYLEGDVRISLRDKIFRELVANLLVHREYTK
ADQTRILIYQDRVEFSNPCIPHWRGRIDPSSVVPFQKNPLLSKMFLQLGWVEEIGSGLMN
VMKYLPLYTRSGHAEILEYEDFRVIVYLEAAPQATPQATPQATPQATPQAPSDRIATILE
FCKIPRSREEIQDMLGLKDREHFRKEILEPLIITGKLELTIPDKPRSPHQKYVTKGYTP*
>SPBIB_v1_350050|ID:27164036| conserved protein of unknown function [Uncultured spirochete bib]
MPHTPFIEIQFPLARLSAESYKERKANLGQTLTGLGKWWGRKPLILVRASLLGLLMPASD
DPEKDRQIYYKILTMDEDGLWRRKTKTMSAADIAQRLEPREYEGLIERKGSTASWVRGVA
KADKERLERIAFARLPYEDKLAYCCRPEEIDGPSPEAWKDINAHLGTSAHSLPELFEQLS
QKAFGHRARVGDCFCGGGSVPFEAARLGLEAYGSDLNPVAVLLTWGAINLIGGGKDVQEE
VRRAQEAVWQKVDEQITAWGIEHDGRGNRADAYLYCVEAKCPATGLWVPLAPSWVISEKY
RVVAVLRCNDARQGYDIDIISGASDDQMRRAKQGTVKDSEMIDPEDPSRRYSIASLRGDR
RGPNGETIYGLRLWENENFVPQPDDIFQERLYCVRWVTPQGERIYMAVTEEDLARERKVL
ELLKERFAEWQEKGYIPSKKITEGVETSRLFRERGWTYWHHLFNPRQLLVHGLFHYFSDN
INKTRYNIFSMGRIADWNSRLSGWMPHEGNAKGNNTFYNQSLNTLFNYSTRPVLKLDTNW
FINFDKAVYFAKRKTITLMPLDARDVTYTADLWITDPPYADAVNYHELADFFLAWYEKHL
PRLFPDWYADSRAALAVRGADEDFKKSMVEIYANLARHMPDDGLQLVMFTHQDASVWADL
GMILWAAGLRVTAAWTIGTETSSGLKQGNYVQGTVCLVLRKRLEQRRAWIDELYPQVEDE
VKRQLDAMQAIDDTLQPQFGDTDYQLAAYAAALRVLTSYDSIEGRDITHELFRTRAKNEA
SEFERIIDRAVSIATNYRIPRGISSFSWRNLEAIERLYLAGLELERHGELRQGAYQELAR
GFGVAEYKFLLGDAGANQARFKTPSELKRLYLSASQNSNDKGTGAFAASLLRHLLFAIHE
AVRTENPREGLNYLKAERLDYWSRREDLINLLDYLAAARNLSALTYWEKDAESAGLLAGL
VRNDYVGSR*
>SPBIB_v1_350051|ID:27164037| SMC domain protein [Uncultured spirochete bib]
MISRIQLSNFKTFEALDIEVGPVTLLIGPNNTGKTTVLQALTLWDIALRKWWEQKALSSA
RTRTGVTINRQDLFAIPIPEARLLWYNLHTRSGMTSNANVFMTMRLEGITENKQWHAALE
FYYANPESLYARPVKGEDDPENLKLALKESFSYLPPMSGLSSSEERLEEGSIKRRIGEGR

TAEVLRNLLWKIYNKEETKWKDLTSAIKKHFGTEVLPPVYNPATSLISCRIRESGKPEMD
LTASGRGFQQALLLFSFLHSQPYTVLLLDEPDAHLEILRQKSLYMALASEVKAKNAQMLI
ATHSEAILDLASQNDTIIAFIGRPHQSKKSSELKKALIEIPYSEYLSAEQTGTILYLEGT
TDLEFLRAFAEVLQHPVREKLERPCVKYLNGNDMDLARSNFNGLKEALPDLRGLILTDNL
QKIHEMPPGLVHMQWERNEIECYLPLPHVLERYFAGATPDSPSLFETQTMGTLKRIIEDQ
TPPAALRDMQDDFWKETKISDKWLGTVLKKYYAELGLPVQINKGDYFLFARHARPEELDK
EIIKKLDILNDFLA*
>SPBIB_v1_350052|ID:27164038| conserved protein of unknown function [Uncultured spirochete bib]
VKAKLPGFGFDPEKHAHFFAVIFPSASKGESTIKVIEHFEWPGEVPDDTTISFDNRDLKV
FIKREFFNEVADAIKAEFNRRLTAHGLPTGRWPARGGTALLSASFGKELLLLLWAIEDAS
TSDIQNAVHNWLGLSPEERWWLYTMTNAATGQALAGRNRGWRKAVRFALCENPVSGVIIR
KRLALEPSLFEEVEE*
>SPBIB_v1_350053|ID:27164039| conserved protein of unknown function [Uncultured spirochete bib]
MTIFDNCKPRQSVFDRTRRDTVLNLSDFLEGKLDEQAAMQFFTENFVTAGMKMLIQKSFE
RLSGTHDQPSAYLLSQAMGGGKTHSMIVLGLLARFPYLRAKLGHDFHLGSRPYKVIGFDG
RESDYPYGLWGALAEQMGKRELFNPLYSPLQAPGVTSWVNLLKGEPVIILLDELPPYFNN
ARSIQIGASNLAEVTATALSNLLVAANKEELSNVVIVISDLSSTAYEAGTGFINQALENF
RQETRRNVVPIEPVATQGDEIFHILRTRIFESLPDTSVRDEVARHYAQAVENAKQMELTA
ESPASFAALLRESYPFHYSLRDLYGRFKANAGFQQTRGLLRLMRAVVANLWESERAKQLE
LIHPYDIDLNDQEIFSEFSTINPNLNEAVRVDIANHGTSHAEELDTKLGGGTQAQDAAKL
LYVASLSTAQNPELGLRDSELIAWLCRPGHDISRMRTDVLEQLPASAWYLHVSNDGRFYF
KNVRNLAATLHGYVESYTQETRIKELREYLKSLFKPNRSDVYQDCLALPTWEEVQPAQDR
TILVITEPYPIHVSDQIPLHPDLIRLYENLEYKNRIIFLTGDRDTMNEVLKNAALLKAIR
TILAEQETEKLSERDPQRIEAKKNEDRILINLRSSIQQTYSVVVYPSSDKLRKEIIRFNF
DSNNYDGEAQIRAALLNARKFSEDTGNLDSWIAKVEERLFDNQNPVRWKDVKARAAGKTN
WQLHLPRLLDDIKAHAIRTGKWREEGEYVRKGPFAKEPTSVNVQVKSRNDDTGKVTLDIR
PVGGTKVLYEIGDSRPTTASEAVPNYSAFETGELKLTFLCVDENDPAREAKPVVWKNRIS
LKSRIFEQAGERFIELIAAPNAPIFYTTNGSDPRSKGASYAGAFPIPKGCRLVQAIARKD
DIESELLRRDVTQAEQVSVDPLKPLVWKTKRFQNLTTADAWQLITRLTEYGAQADGITIY
FELPDTGGEEINYTSPEGIQKNGEAIRAILDLVGNFAGNVNISMTVQRIHFSRGQDFLDW
ANKDRIPYDINSEVRQ*
>SPBIB_v1_350054|ID:27164040| conserved protein of unknown function [Uncultured spirochete bib]
MRFYLAMTDNDWFRYLRSLQPVPEDINFWQPSGKKPANLAKGEPFLFKLHAPFNRIAGLG
FFSAFATFPLGLVWEAFGERNGCDTLQALSRKIAHYQGIRGEYDPRRLVVGCNILTDPVF
FNDSEMIDAPLDWSSNIVRGKYYDTEEAVGARLWDQVMQRLEARRFLEREAVGEIGTASV
VVPEPQWREVIAKVRVGQGAFRFMVTEAYRRACAVTADHTLPVLEAAHIKPFSEHGPHLV
SNGLLLLRADLHKLFDEGYMTVTPDYHIEISKALKEDFNNGKIYYAFHGQQLANLPENPAD
RPSREFLQWHNEVAFKVG*
>SPBIB_v1_350055|ID:27164041| protein of unknown function [Uncultured spirochete bib]
MHTECRSTRVLSGYSRPAILVLAVLLSGCALFQPYRNVTAYPPDSKPQWQDINSAAQICR
VYLDYPPLSIHAVKVDLQAQNLNIAVYPLAASEAGEGTSLSKKVSTFAKENHCFVAVNAN
PFAPSSATEGDLRTITGICVANGNTVILIVIDGRTSESVGATEEEVALWMRYFGADDAIT
LDGGGSSAMAILDGTIVLENVPVHGNVPGVERAVATCIGFALRE*
>SPBIB_v1_350056|ID:27164042| conserved protein of unknown function [Uncultured spirochete bib]
MSAQDVRWIQRFNHFSKAFAQLQEAVELSKQRKLTKLEEQGLIQTFEYTHEMAWNTLKDF
LEERGTKKLYGSKDATREAFKTGLIENGEIWMNMIESRNLTSHTYNEETATEVISAILHS
YFAEFSDFHARMQELKEERE*
>SPBIB_v1_350057|ID:27164043| DNA polymerase, beta domain protein region [Uncultured spirochete bib]
MTARFGLKETTIKKIHSVFEKYPQVEQAILYGSRAKGNYRNGSDIDLTLVGGDELTMNVL
YQIMDELDDLLLPYTFDISIYRDISDSDVRDHILRVGAVFYQRAAEPTHF*
>SPBIB_v1_350058|ID:27164044| conserved protein of unknown function [Uncultured spirochete bib]
MVPTKDMLDQIAKEEAELVLDQFTEEDAWELGCLMVEEAKKRQARIAIDIRRPGQIMFHA
ALAGATPDNDEWIRRKSNVVFRFGKASLAVGVSLALAETTIEQKSFVSPLEFSPHGGAFP
IRVRGCGLVACATVSGLPQEEDHALVVACIRKFKERKTPR*

>SPBIB_v1_350059|ID:27164045| Glycoside hydrolase family 13 domain protein [Uncultured spirochete bib]
MNTQEIRKQKTRLCIIVGIVALILTACAFQPKIEEPSPLANYQTVEPGTIENMGNPKMEF
IVPAHDALKPAADDIVIYYYRNDGNYEPWGFWLWAIPGGDGALVWEKTKNLEVIGNVGYL
RFKKDGSTFGVNVIGSSGMFGLIPRKDSAWEKDGDQDRIIDSRAGNEWVVFQGDQKTYHY
GPYVPSIEAARLISPNEIVLDLSGRYGLSLEPGPSGFSVRYADGSGEIAVVDAVNNADPA
NRKNNYARRVKLTLGEEVRLDRPIEVVHPSFLAPTTVNTSGLAATMADNIVPPEGFKLGA
IYDAVHKSVEFRLWSPFASRVTARLYRTSLASTADYSLDLAKDPNTGVWQGSFDSVDPDG
FFYEYSVFFGNKENIVLDPYALSMDAFTGQGPGRGAIVDPSKAQPEGGWQGYTDYQLEKR
EDAIIYEISVRDFTIAPDAGTKARPGSFLAFVEKLPYLKELGVTHIQLMPVLNFYYNNEL
ETAYEATGRANNNNYNWGYDPHNYFTPEGWFSSNPRDPYARMRELKTLIKEIHKAGMGVI
LDVVYNHTATPSILDDIVPGYYYRRDAKGDLTNNSGCGNDVATEREMASRLICDSLYYLA
DEYKVDGFRFDLMGLIDVNTLLKARAAISAIPGKEDILFEGEGWKMYHGPALTVMNQDYM
TQTNEVSVFNDEFRDILKGGGLNDRTKGLVTGRPVNTSLVFNNLAGRPMLYYKADDPGDS
MNYVSAHDNLTLADNIAFNVGLSPKYPEERAEIAARAKLANFFVLTGQPIAFLHGGCERG
RSKPKLNSTSEVIGEYVHNSYDASDDINQFPWTVPSEYAAMAEWVKGLIAIRKAEPGLHI
GDAATISSAMKQIPHADQLSMGWTVNYGGTTLAMLVNANFDTSIDFDVGIDLSKAVVLVD
ADEASPQGVSKPSGIQVSGTKATVAPLTAVMLKLLTP*
>SPBIB_v1_350060|ID:27164046| Pullulanase, type I (fragment) [Uncultured spirochete bib]
MNQNWKEFLTIDIAPGENRRIAWADKVGGFLDLMSASPCAGEGYVHGQESRLKDVLEVSG
RTIRSRNLCHVQVLPGEVAFSKEKAGGTKNILRLALLMEADAFWIELDTSQASERADGPT
SADAFAIESDGNRGTVEGGGRSVCLVLPEAQAGEADWPQPHEYRYTAFSLWASAGIEIAI
ASRQPFAIDQSPLESEVQIPHGFGTIRVEFSVPRDAYGADVPGRLAGHNHTLYIAWGKNA
EEALTRALQLAAEDAIQHHRAATSDILSSMDIATGNDEFDKALRWAAFSGWSLVTREYGL
GIWAGLPWFRDNWGRDTFIALPGILLVTGHFAEAREIIRTFANRQNTDPTSPDFGRIPNR
WRSPDDVIFNTADGTLWFIREVWEYVQYTGDTAFALEMKPFLDRALTADITRSDEHGFLQ
HGDADTWMDARIRGKEAWSPRGDRAVEIQALFYTALLCGARIAELAGDFGHAAHYREAAS
KLRSSFLHYFWRPEANRLADRLYSHSEEADLRVRPNGCIALAASAILPEEQALLPIEIEA
VVLADCVSRLVYPYGVASLWQEDPLFHGRHDGSPLYHKDAAYHNGTIWGWNAGPVIQELL
KFGQISLAQSLAAELARQILHDGAAGTMSENLDAMPGPDGKPVPSGAYSQAWSVSEFVRT
AFQSFLGFMPRLADGELIAAPYPGFSGTVKFGTDQQLYIAASRGNSIFEYQIEWLGEIGA
DSSLELHLHIATNKGIIKAAIPIRQGQRLHLRYDEASGKMHSVSSYPPLKQAHSVIETRL
VIPIQPAFKNLHFASPHLPPWNKPAREPQYLERIILGSADTANPAVYLIKRC*
>SPBIB_v1_350061|ID:27164047| Alpha amylase catalytic region [Uncultured spirochete bib]
MKRLGAFLLVAIALLSPLAGQESGQLWWKDALGPAYQVLIYSFADSDGDGYGDIKGLTNA
LNYLNDGNPFGGNDLGISAIWLSPINSASSYHGYDVKDYKAIDPRLGTMEDFEHFVADAH
SRGIKIILDMVFNHTSREHPWFLDAMRSASSPYVAYYRTKQPGTQYGSGGMGRFYRYTRP
DGSVFEYFSAFWEGMPDLNLDNTDVVNELKDILAFWIGKGVDGFRFDAAKHAFDPNEMPA
GTPTLALNKSFWNDLRRYCRRIKPDVMFIGEVLTESSAEIAAYASVFDGLFDFPAARLTI
DAVNRMGPGAFPNSYLNNYRQYQRIPSFQPLPLLTNHDQDRAMSTLLSGLGLNATAGAEP
ESGDNQATLAAKALALTKAKLAAAISQTLPGLPFVYYGEELGMTGIRYQNDDISRRDGFL
WTSHPGIPNTDWAKKSGKAVPGQNRLTSPLDIQLQNPESLANFYGSLSLLRAASPAIRRG
GYVPVSWPGFDNASILAWLRGDDAQTVLVIHNLGTTPFHAATPEGIQLRLLWASDEELSR
SAGLKTSQAALDVTVPAESSAVFEVLKP*
>SPBIB_v1_350062|ID:27164048| ABC-type transporter, integral membrane subunit [Uncultured spirochete bib]
MNASITIVSGKLSRRAKARRTGLKIFQYTWLVLTCLVVLVPILWMIVASFTRGKLLSGVP
LLPNFKNFSLEHYTYLFTYKSQSGARFSDFVASFLRSLSIAVVNTAAVVLLSSMTGFVFA
RFRFRGKKSLLVSFLLLQMFPSFMGMIAIFMIFRTFGWLNKPLYLVFIYAAGAIPYNTYL
IRGYMRSISLSIDEAAMIDGASKTQVFFKIILPLSGSIIGFVAVNAFMAPWMDYMLPNQL
LDMQNQTVAIFLYRLTDPFITMYYNPLNFMAGALLLATPITLVQFYMQRFIVYGMTAGAE
KG*
>SPBIB_v1_350063|ID:27164049| ABC-type transporter, integral membrane subunit [Uncultured spirochete bib]
MANAPIATNKPKSWKAKPLAVGIASLLVPGLGQWLNRQRLKALIWFFIPLFILAIELGSS
QWGRYLELKQGKVPPEALATAAYQNASATQEETSSQSDYMSALFGDSGQEGQTAGSGDPF
ADSTNAQENNTPAGSGDPFAQAAVSEENNKPGSTSEAKGADAEQGNYFNATYVVPNYRPG

EPHYPIRDFGGFFTRGIWGLVTLGRLVIDQPYAGGYIELYNKISPWLSADNSIVLLGNGL
ICLAVLLILGLLWVLGAIDAYSTRKRILSSGQVERFSAFWKRTWDSLYVYIVSAPAFVLI
IMFTVIPIFFTFLLAFTNYTYKIKLGAKLIEWVGFGTFSYLAVDPGWLSIFGQIFLWTIF
WAFMSSFTVYALGFVNAMIVESPLVRYKKFWRTIMILPWAIPSLVSLMMFRNAFDKDGLV
NQFLFATGLMEPVSKLLFNIGLAGQADQPIFWFQPIYNGKLARFVVILVNLWLGAPYHMM
MIIGVLSTIPKELYEAAAIDGASGSQRFRYITLPMVLSATVPALIMTFSFNFNNFGAVYF
LTGGGPVWDPAKVPDSMRIVGSAMPGQTDILISWIYKLSFTKGFEQYNVAAVYSIIIFMI
VGGFSVFNLLRSKSFREEAGE*
>SPBIB_v1_350064|ID:27164050| conserved exported protein of unknown function [Uncultured spirochete bib]
MKRRMAFVMVLILIGTSVFAQSKAVWDAKNRQFQIEKGAKIRLGVDNDKVGAAIVQLWDQ
LHPEAKGIVEYMNLGAAGSADQITQLQGDAPDVVLAIDGEVSRNAQSLLPLHKVIADAAK
SFAVEPFFSGANSGMAVKFIPVAYDGMTFAWNKTMMDALKLDTKDANKDGLPDAFDTWEE
IFALAKKWQTSRPTYKGKPVTIVYPMCLDEVWSGYSNLTAGGWEIFPKADPANPGFDQDS
FRAGLEFIKAAADAMISVEVNGARTPAASMVWRWDDALNNETAPFFLVGTWMDVNGAETK
GGYDIKFGPMPTWNGKRLTPFVKTKGWIINGFTKYPSAAHELYRILYTRDGLQAMVNNSS
YIPALKPNATNTPSYRDDPNKAEMAKAFAFNYPEPSITLPKNPAKKAMDGYYGIGVNLEL
RDTWDGKKTPAEAQANLINLYNKWYEENNK*
>SPBIB_v1_350065|ID:27164051|nplT| Neopullulanase [Uncultured spirochete bib]
MNVAAIYHRSTDAFCYSPDGRSLVIRIQTASDDIESVEVWVGDPYEWRINQESIQAVWQC
VRIKANRIGSNGLSDYWEARWVPPYKRARYAFRLYGRDGSICDLGEKGLVRIDPADRDKP
IDYWNSFLFPYIHQTDTFRAPAWVASTVWYQIFPERFRNGNPNNDPPGTKPWSRGPVQNR
EFYGGDIEGIIQGLDHIEALGCNGIYLTPIFASPSAHKYDTTDYFCIDPSFGTEEDLKRL
VDVCKAKGIRIILDAVFNHAGKEFGPWKDLVERGPDSVYRDWFRIRDFPLFPSGQDSGDS
RHTNFETFGFTTRMPKLNTNNPETREYLLEVAVKYVRDFGIDGWRLDVANEIDHEFWREF
RKRVKAVNPDAYIVGEIWHDAMPWLRGDQHDAVMNYPFGSAITDFVLGNAWALTGKDFLH
RINAISFSYPDTVLQSTFNLLDSHDTDRIVTRFQDRELAKIALCLLFALPGSPCLYYGTE
YALEGSRDPDCRRCMIWDPQPDEKEFERFVASLVNLRRTWWKTFAYGTRESSFNDSFPGF
VALIIHDDAMRLALLVNRDTSAVPEAAWRDALGLHSSAPVEVILASQPFAGFLGGRSAVY
LHM*
>SPBIB_v1_350066|ID:27164052| conserved exported protein of unknown function [Uncultured spirochete bib]
MRKILSFIAAFLILGGIVFADVSVKEVGDGKVEVTFFFGSTKANQVVIAGDWTDWQNGAI
PMTKVENGWEYKIVVPANTVMKYKFIADGTWIFDIKAPDKIDDGFGGFNGLVDVAELLAA
KKGGTTTAAAAPASGGSKLRFQTWSMVGTQAKFDTTNNMEVQSAGVGVKSYFKISGNALP
SMPIYAEIALAENDGFENLYKKGSLAFSDGLTNLLVDTIFDPIYYIDGQRKAATYLGHLK
LGFDSDYVNFVTGYKYAKLPGHTNVSWITVDSEWEAGWNETGGFAVFSLGPALRKIGDWT
VNATIGPNKTADRAGNQYGLFSYATLQNANHYFDFQYNGAFGTTYKTIFDEIYEADFIGG
YAGKFDPVTIKANALYNLWGANVINDSYITAYNPSTSDVSGAKQGLGFLPNFAANTQVAY
AGDYFGTTLGYRLRGFQANMMYVEQGADGHTHLVDQLGALNSQRVWLDVFAYPLDTLSVG
LYGYVDLVLDKTAAKLPYNDKDNIQLYAKPSFELKLDRVFDIKTTLNGYVKLTYNTATAD
QFTRGTNTSQFLFGEGGLKLSMADVSKTVKGIDLYYGFDNNNANYLFNTLIGAVKLPSDL
TLQGGAGLRTPNAGVAASSSPFGFFVGVAQKFKALQKPTAYAQFVYNMDPYNDFGSGPSS
FNLNDFVTYNGVNNYVGTAAVRIGLHWDL*
>SPBIB_v1_350067|ID:27164053| conserved exported protein of unknown function [Uncultured spirochete bib]
MKRIMFTLAVLMAVIALTGCPIQHTQVYPAYSLPIKQNVSTTTNTAAIQVTATETANSYS
KYTVTIKNLVADVGKKFVVAGASIGSTSSNIGDNWNVTASSVTDAGLVGTVDNTGTFSIV
FYGKAPSWGNAADGAQFKICIYDDPSWNLVLGDAGGNNFYVAGSTGNDIELTIDPNKL*
>SPBIB_v1_350068|ID:27164054| putative Lipolytic protein G-D-S-L family [Uncultured spirochete bib]
MYANEVCLFGDSIARGVILDACGSYKPIRESFAFLAASELGFSLINKARFGCTIAKGREI
IERFLGRNAAVTTCSASLADPAGISRAPSVPQTATNIMPENASPNVAIHIRTDNSHAADS
SRPRLAFLEFGGNDCDFRWDEISADPHREHLPATTPERFFHMYGEVLQMLKQNGFKPVLM
TLPPLNAERYFAWFTRAGLDQAAILSWLGDVQFIYRWHESYSSAIWEIGEAHNVPVVNIR
KAFLEQRNYSRFLCKDGIHPNTEGHRLITSEIIKFAQEHMSV*
>SPBIB_v1_350069|ID:27164055| conserved protein of unknown function [Uncultured spirochete bib]
MDEKAYEAVKAYLDALLAGKPPAWEQLPDISLYMDQVVSYLERQLGGLFAAEDERAITPS

MINNYVKAKIVPRAESKKYNQEHIALLLSIFTLKRALSVQDLGALLDGKPEAGSYREFYE
LFRKALIECAASTAEEIFAGLGIKKSERALSGEAEEKANIANIDESKLRELALKLAVEAS
LRSLAAERLLAIARSRELSGTQKPSSSRALSAAQEPSGRREAKKG*
>SPBIB_v1_350070|ID:27164056| conserved membrane protein of unknown function [Uncultured spirochete bib]
MRTKIIAILLALCTLFLLSSCLPGDGKNTVDRPANFLTGIWHGWIAPISLIVSIFNGKIR
IYEVNNTGWWYDAGFYMAVISGFGGLAVSRRKRRREE*
>SPBIB_v1_360001|ID:27164057|leuA| 2-isopropylmalate synthase [Uncultured spirochete bib]
MARKIYIFDTTLRDGEQAPRCSMNLAEKLEVARQLEKLGVDIMEAGFPAASPGDAAAVRE
IARTVKNVRVAALARALEKDIDTAWEAIRKAESPRIHTFIATSPIHMEYKLRMAPEQVIE
AAAAAVRYAKHYSSDVEFSAEDASRSEPDFLCRIFSAAIDAGATVINIPDTVGYAIPEEF
ARLVAYIKANTPNIDRAMISVHCHNDLGLAVANSLAALRAGADQVECTVNGLGERAGNAA
LEEIVMALRTRRDFFDLATGLDTAQIFSTSRLVSKVTGVSVQINKAIVGENAFAHEAGIH
QHGVLANRQTYEIMTPESIGLPQNSLVLGKHSGRHAFEERLKYLGFALDSARIDELFAQF
KILADKKKTVTDRDIEALARGASGTIPETYKLERFAVQSGTQLAATCTIRLARKDGSVYE
KAAVGDGPVDAAFKAIDEIVGRTLELEDFRLSSVTSGEDAQGEATVKVKFEGRTWNGHGV
STDILEASIKAYLAAINTMEWEMAVYE*
>SPBIB_v1_360002|ID:27164058|leuC| 3-isopropylmalate dehydratase large subunit [Uncultured spirochete bib]
MNDTAYTEKAMTMTQKILATKAGLKAVEAGQLIEAKLDLVLGNDVTAPPAIGEFEKLSCT
RVFDPEKVALVPDHFTPNKDIKTAEQTLKMRQFARAQNVAHYFEVGQMGIEHALLPEQGL
VTAGDCIIGADSHTCTYGAVGAFATGVGSTDLACAMATGTTWFKVPSAIRVELSGTPRGW
VSGKDVVLHLIGRIGVDGARYKSLEFSGAGVGALHMDERFTIANMAIEAGAKNGMFPVDE
HTRTYLAYVGAKEPVVYAADPGAFYDDIVRIDLGAVPLTVSLPHLPSNTRAIEEVEGMPI
DQVVIGSCTNGRISDLRVAAQILKGRKVAKGLRCIIFPATQRIYLEALREGLIETFIEAG
AVVSTPTCGPCLGGHMGILAKGERAVSTTNRNFVGRMGHTQSEVILAGPAVAAASAVAGK
LASPEKVSDAVPDRAWLYRM*
>SPBIB_v1_360003|ID:27164059| 3-isopropylmalate dehydratase small subunit 1 (modular protein) [Uncultured spirochete bib]
MQQGQKGSEHMSEYGNEQRKGGQSTFSAKTGAQILRGTVHAYGDNVDTDVIIPARYLTTS
DPAELAAHCMEDIDREFLKKVKPGDIMVAGENFGCGSSREHAPLAIKAAGISCVIAASFA
RIFYRNAINIGLAILESPEAARALSDGDEVAVNFESGEIRSVKDGAIWKAQPFPPFIREI
IEAGGLVEATKRRLAVRQRDYGAEAYQPATRTKDAL*
>SPBIB_v1_360004|ID:27164060|leuB| 3-isopropylmalate dehydrogenase [Uncultured spirochete bib]
MNYRIGIVRGDGIGPEVVGAALGVLEAVGARFGHAFAKEELLAGGCAIDASGEPLPEETV
ARAKACDAVLLGAVGGPKWDTLPSHLRPEKALLGLRSALRVYANLRPAVLLAPLREACPL
KDEIVGSGFDILIVRELTGGIYFGKSGRTEDGQAAFDTEFYTRKEIERILRVGYEAARKR
RLRLCLVDKANILESSRLWREVNARISAEYPDVETSAMYVDNCAMQLVRAPGQFDVIVTS
NMFGDILSDEASMLTGSIGMLPSASIGEPGAPGKPTMGLYEPIHGSAPDIAGKDIANPLG
TILSVAMMLRYSFNLNKEAEAVEQAVARVLEAGWRTADIALHKVESTTRIVGTKEMGRLV
AKEVQRSRI*
>SPBIB_v1_360005|ID:27164061| AzlC family protein [Uncultured spirochete bib]
MESSRSGKRRVVLAAAFKASIPVLLGYTTLGLAFGFTLVAAGLPWWMSPVMAIFIYAGAA
QFMGIGLITGGAGLFEIALLTLLLNGRHAVYGLSMLEQFKSAGKWKPYLIFGLTDETYGL
LTTVKPPAHVEQARFYAAVTALNQSYWVLGCTVGSLLGSALAFDARGMDFALTALFIVLL
VEQVRAVQRFEPYVAAFAACILALFIAAPRDFLLVALLFATGLLSLLRPRLDGRKPAPAS
TQGPQGGGEAQ*
>SPBIB_v1_360006|ID:27164062| Branched-chain amino acid transport [Uncultured spirochete bib]
MTRLFAIVVIMTLVTFFTRAVPFLFFSRTKPPAFLDYLQRYIPPVVMTILVFASYKDIEF
TQTPFGIPAAAAGVLTALLHIWKRNVLVSIAGGTAVYMVLIRVL*
>SPBIB_v1_360007|ID:27164063| putative acetyltransferase [Uncultured spirochete bib]
MEIRKGSIEDVPAWFELVYAVKSENLPTLFEMTTPITLESSYRYMEKILSVDGSFVLICF
DGSKAVGSLDCLRKERKEENHVASIGMCVAKASRGQGIGRRLLLSLFEICKQEKKIEKIE
LDVFSNNPGAIALYKSLGFELEGVKKGSIRKDGEAIDLICMGKFLY*
>SPBIB_v1_360008|ID:27164064| putative alkylmercury lyase [Uncultured spirochete bib]
MGTMNITFLYFNGCPNSEPTLENLKTALAELNFDADLSIIEVKDPQQAASLGFLGSPSIL

VEGRDLETGRPPAGSAFSCRIYEIEGRRTGRLPKDYIKARLQSLAQTLG*
>SPBIB_v1_360009|ID:27164065| putative reductase [Uncultured spirochete bib]
MLKIAVIIGSTRPGRNGEAVGRWVYEQAKKRSDAQFELVDVKDFNLPLLDEPVPTSMHQY
TQEHTKKWSEKISGFDGFVFVTPEYNHATTGALKNALDYLYTEWNNKAAGFVGYGSMGGA
RAIENLRLIMGELQVADVRAAVYLSLFTDFENFSVFKPLPNREAELNTMLDQLVAWAEAL
KAIRT*
>SPBIB_v1_360010|ID:27164066|ppiB| peptidyl-prolyl cis-trans isomerase B (rotamase B) [Uncultured spirochete bib]
MANPIVTFKTSKGDISIEVFEDNVPITAANFLEYVKLGFYDGTIFHRVIPGFVIQGGGFA
PGMEQKKTRAPIVNEASKAPPNARGTLSMARTSVPDSATSQFFINLVDNKSLDYRGPGAG
AGYCVFGRVTEGLDVVDAIAQVKTGRRPPHADVPVEDVVLISAKVV*
>SPBIB_v1_360011|ID:27164067| conserved exported protein of unknown function [Uncultured spirochete bib]
MKPNQSFGSIVVCMGSSCYARGNIYTVEHIQSWLARHGLSASVELSGSLCGGRCKEGPVI
IINGKVYTNVMPETIEDILVHEFEKAGDPELVQPDSTCSNTSAGDSWRVDP*
>SPBIB_v1_360012|ID:27164068| conserved protein of unknown function [Uncultured spirochete bib]
MNRQVIYTETMRCQDCYKCVRECPVKAIQILDGHARVVEDICILCGHCVMVCPQSAKKVR
SDVERVRRLLELKPVAVASLAPSFAAEFSGCTAGQLIASIRKLGFAAVSETALGADLVSS
AMRNELGQLADNGRNFLIGAACPAVVRYVSFYRPDLVPFLSENGSPMIAHARYLRSRFGE
NALVVFIGPCIAKKKEADESGGLVNAVLTFAELRQLFEEENIDPSAEKPTSNDTFFPQRP
SKGELYPIEGGMIASIKQHGSTDVPCMSFSGMGHIQAALRDLPDGIPETGIFLELLACEG
GCINGPCSDASRGTVSKRMQILLYEKQGESEPFNTEIACQVKHSAAKPVQRQVREEEIRA
ALALVGKKEPSDELNCSGCGYDSCREFAKAIVIGHAEPTMCLSYMRNLAQKKANALLRAM
PSAAVVVNADLKVIECNEPFVRILEKDAQFVAEAKPTLEGADLRKLLPFWERFNDVLHHS
PGDAFIESDFRCGERILHGTIFSIEKQMIAGGLFQDITAPWIQKDRVIKEAKKVIRQNVR
TVQKIAYLLGENAAESEAALRSIIESFGGEVGQ*
>SPBIB_v1_360013|ID:27164069| Stage II sporulation protein E [Uncultured spirochete bib]
MIESENKPMHADFFVEIGHWQVAKYGETSEGDVFISRKTEDGRIIAVLSDGLGSGIKAGV
LAALTATIATGCSAARIPIKRTARIIMRSLPVCSERKISYATFTLVDIDTSNRIKVVEYD
NPAYMLVRQGVEVEPLRSMVSVTKGGGRIAGHPKPATLRSSRFKAEFGDRVIFFSDGVTQ
AGMGSRNLPLGWGLEGVRRFVSQMIAENAEISARDLARRIVTWALLLDGDQAKDDITCAV
VYFRAARRLAVFTGPPLHPAHDNELARLFVSSPGRTIIAGGTTATIIGRELGRKITMDLK
HRDPELPPVASMEGADLVTEGILTLSKTADYLEQGAAPEELRQNAATAALRLMLDSDIIE
FFVGTKINEAHQDPNMPVELDIRRNVVKRLAHLLEGKYLKQTTIRYI*
>SPBIB_v1_360014|ID:27164070| conserved protein of unknown function [Uncultured spirochete bib]
MKKVHVTICAGTACYVLGGAELLGAIDSWIAKWGEQVEFEGTPCLGFCKSAGEAKAPYVL
VNGLVVEHTTAEKLEEMVNSALLGTEEEASSADAAARPGNAITRKDGGYASGR*
>SPBIB_v1_360015|ID:27164071| Hydrogenase large subunit domain protein [Uncultured spirochete bib]
MPREDNTTRIRRELLVRLGRLALEGTLADSIDDVPFDMTAEGWETMRCCVHHDRAILRLR
SYALLGGDVRGMDDVRKPLSVLAEGFQNSRCQDGAELGESGRFPLQVLGEACNACAKSRY
VVTDACQACVARPCKVNCPKGAVTVNGRSHIDPDKCVNCGLCEKSCPFHAIVKVPVPCEE
VCPTGAISKGEDGIARIDEAKCILCGKCLRACPFGAPIEQTQYLEAASWLREQARPLIAL
VAPATMAQFPVSSGKFMAGLYRLGFAAVAEVAEAACATAEREAAELRERMHRSEGFLATS
CCPSWVLASKALGDVAGHVLHTPSPMAIAAKWARKRYPEARIVFIGPCLAKRAEARGFPG
EDGRKLVDAVLSAEEIGALFTAKDIQLQALEEARAVQLAEQGSSLDLCYGRGFAQSGGVA
ASVQSVLENENSRIEVGSSWTIRTLVIQGLSKQVLALAALWNKQAPDADLVEVMACEGGC
IGGPLAIAQAKSAAVFLQRYMSEPPPEAKVASSSEAVEKAV*
>SPBIB_v1_360016|ID:27164072|carB| carbamoyl-phosphate synthase large subunit [Uncultured spirochete bib]
MNAQNTLRKPQKVLVLGSGGLKIGQAGEFDYSGSQALKALREEGVTTILINPNIATNQTS
EGMADATYFLPVTPDFVRQVIEKERPDGILLSFGGQTALNCGLALSREGILYKYAVDVLG
TPVEAIELTEDRKLFAEHLRTLGLKTPESKAAVSVEEAIACADEIGYPVMARAGFALGGA
GSGICRNRRQLVARCEKAFALSTQVLIEEWLGGWKEIEYEVVRDSADNCIAVCNMENIDP
LGIHTGESIVVAPSQTLTDEEYHSLRSIALKLIRSLGIVGECNIQYALDPNSGDYRIIEV
NARLSRSSALASKATGYPLAYVAAKLALGYSLTELRNKVTGVTTACFEPALDYCVVKMPR
WDLSKFKAVDTQLGSEMKSVGEVMAIGRNFEEALQKAIRMTGISELGLRADGKLLGKSFT
NLATALKKPTDRRLFAIYRALAEGWTVKKINSLTGIDSWFISKIATIQKCEAELAATVRT

ASRGGTSAASPSVCPPELLRKAKRLGFSDAGIARILGCSEQEVRSWRESIGLRPAVKQID
TLAAEYPAQTNYLYTTYCAAQNDIAPSHTSILVTGSGPYRIGSSVEFDWCAVSAAQTCHQ
LDKKTIMVNCNPETVSTDYDICDRLYFEELSFERLMDIYEFEQPSGVLLSMGGQIANNLA
LPLFEAGALILGTSPQSIDRAEDRHKFSRLLDELGIDQPAWKELTTIEEARAFASTAGYP
VLVRPSYVLSGAAMNVAWDDESLATFLGLAADVSSEHPVVISKFIENAKEIEIDAVARHG
KLIYHAITEHIENAGVHSGDATVVFPPQRLYIETVRKILRITEQIAAALNITGPFNIQFV
ARQNHVMVIECNLRASRSFPFCSKVSRVNMIDLAVRAMLGEPVTAPPSLMYDQPWVGVKA
AQFSFSRLHGADPVLGVEMASTGEVGCIGSEFNDAFMKAMVSVGYALRGKKILLSTGPLE
DKLDFIDSARTLAEMGCELYASRGTALFLKHFGIPVTQVHWPLEKAEPNILTMMRAREFD
LVINIPKNNRRRELKNDFLIRRMAIDLNIPLFTNIKTARQYIESLAYTRRNGIEIRAWEE
YR*
>SPBIB_v1_360017|ID:27164073|carA| Carbamoyl-phosphate synthase small chain [Uncultured spirochete bib]
MFRKEKAAMLVLEDGTVFEGYSVGADVSSEGEVVFSTGLVGYPQSLTDPSYCGQILAFTY
PLIGNYGVPALKRNSHSIPLNFESDRIQVSGVVVAEASFEPSHHSTNLSFPEWLKQEGVP
GIAGIDTRALTRLLREHGVMKGKILVEGAGLAHTNLEKADSPVKVVSVKEPIRYPAGPGA
PKLVLVDCGVKANILRILIDSGVDVTRVPWDYPFETLEYDGLFLSNGPGDPKACTRTIAH
LRHALTQKRPIFGICLGTQLMALAAGADTYKLKYGHRGQNQPAIERTSGRCYITSQNHGY
AVQEASLPPGWESWFTNGNDGTVEGIRAAGAPFKAVQFHPEGCPGPRDTEFLIHQFLDEV
QNRRSAK*
>SPBIB_v1_360018|ID:27164074| Auxin Efflux Carrier [Uncultured spirochete bib]
MLSALQSVFSVILMIGLGFFLAKRRWFEGSSSALISRLVVNVALPAYMISNLMGGYDRAK
LLDMLPGLPVPFLVMIVSYVAAMGLARLIRVQKDRRGAFQSMFALSNSVFIGLPVNVLLF
GDASLPYALLYYIANTTTFWTIGVYGIAVDGSIRKNGPRPSLVSLSGLRRILSPPLLGFF
AAVIMIMLGIRLPKSIMDTCRYLGSMTTPLSMLFIGIVIARVEWKALRFERDFLVILAGR
FLVTPALMFFLVRGLDFPLLMKQVFLMQAAMPAMTQNPILAEAYGADAEYTAIGTSLTTV
LSMLSIPIYMTLVGVLF*
>SPBIB_v1_360019|ID:27164075| conserved protein of unknown function [Uncultured spirochete bib]
MTDFDTLFLKRKSVRAYEDRPIQPRVRAQVLAATMRAPTAGDLMLYSVLEIEEQALKEKL
AETCDHQQFIAKAPLVLVFLADYSRMMAYFEHHGVPEWCARTGRPAIKPRESDLLLACCD
ALIAAQTAAIAAESLGLGSCYIGDIMENWEIHRELLALPKYTFPITMLCIGYPTQQQKDR
LQPPRLPEQLIVMKNRYRPAAPEELASMYQGKGYGKFALHGDAENAAQALYDRKFAADFS
QEMRRSVAAMLKDWE*
>SPBIB_v1_360020|ID:27164076| conserved exported protein of unknown function [Uncultured spirochete bib]
MKGALIAFGLGTLPTGSLYVEIQFMGLAFAAWRFVLTLGALTITGLIMQRIISTKKKKA*
>SPBIB_v1_360021|ID:27164077| Sel1 repeat protein (fragment) [Uncultured spirochete bib]
MKRLFVFLLVFFIAFAFASAQTSASDIASLRAAAENGDPKAQNSLGIKYFNGDGVAQDLA
EAARWFEKAADQGNATAQNNLGFLYEEGKGVPKDYAAALGWYQKAADQGYTAAQCNVAWL
YENGLGVAKDYEKAFHWYEESAAQGYAYGQYSVGWFYDKGLGVQQDFGEAVKWYTLAAEQ
GLPDAQYMLALKYENGEGVDADYNEALYWYNESAYGENTDAMLALGFYYEFGFAVDDDPV
RALAWYMIANDYGSEEAAGYVTDLSKELTEEQIAKARLIADDF*
>SPBIB_v1_360022|ID:27164078| conserved protein of unknown function [Uncultured spirochete bib]
MSTSEKSDTSASNIPPSTQLARKIIEQLSTVIDPELQSDIMSLQLVENLFVGEDGSVSYT
FRPTSFACPYGVNLAMEIKKAIASVKGVTRQEIHIEGFVAAKELEELLN*
>SPBIB_v1_360023|ID:27164079| conserved protein of unknown function [Uncultured spirochete bib]
MPYRDGTGPMGRGPMTGRGFGYCGAGAGFGAGAGFGPGYGMGYGRGFGRGMGFGAGFGWR
AAAYGAVPYAVPAVPTIEAQKSALEYAQKALELRLEAVKAELDALKKSESEAPKA*
>SPBIB_v1_360024|ID:27164080| Transcriptional regulator, PadR-like family [Uncultured spirochete bib]
MPGGPHGRFGAGRGMGRGPAGPMFLQACLLVLLHKGKSYGYSLAQDLKQFGFDPARIDIS
IIYRALRGLEMQGLVETAWDENSLGPQRRMYEITPQGEAVLSAWMQNLHQRKLEIEALES
VYDEVTNRHP*
>SPBIB_v1_360025|ID:27164081|trpE| Anthranilate synthase component 1 [Uncultured spirochete bib]
MNGRRGDEIVFSMPGERFTPFTLAKKLGSKAILESASFKQGRERYSLLLVDEAFRVRQER
DGVSFVVNGISRPWTPKSELAERRKGKYDILDALSYIASQNAGVAPHLPVPASGIGFLGY
DFAARCDTVKITAKPDAIGVPEAEFIVGHLYLVFDHFTDTIHGIALNYTEHEVDLDARIR

DLKKRLSDLDFSHLAPPEEAVSFNVISDEEADKRVYMDGVEKIRERIIAGDIVQAVLSRR
LVVESSLDALEGYRRIRSASPSPYLFYIDFGTYALVGASPESLVRLRNGVASIRPIAGTR
RRGSTPDKDIELEKELLADPKERAEHLMLVDLARNDLGRACTPGSVRMTRNFEPERFSHV
IHIVSEVEGIPAKDHTNLDVLRSAFPAGTVSGAPKIKAMEIISDLEPMDRSFYAGAVGYL
DAAGGFDTCITIRSALVKDGRWYLQSGAGIVYGSKPEREWEETNEKLAALRAALSGGK*
>SPBIB_v1_360026|ID:27164082|trpGD| Bifunctional protein TrpGD [Includes: Anthranilate synthase component 2 ; Anthranilate phosphoribosyltransferase] [Uncultured spirochete bib]
MTILIDNYDSFTYNLYQLLARLSNEPVIVIRNDEINIEGIEALKPKRLVISPGPGRPENA
GISVAAIQHFAGKIPILGVCLGHQAICYAFGGNIIQAKHIKHGIAEEISLDGRGLFRTIG
NKSVFTRYHSLVIDEATLPSCLEITARSADGDIMGVRHTFFNIEGVQFHPESIASEAGEL
LLKAFLTYRREPFAFKFTLEKLLTRQDLDRETAESFMEELTDGALDTPKTAALLTAFAAK
GPAAVEIAGCAAVLRRKKTPFTTKVPVTDTCGTGGDEMGTFNISSMAALAAAACGLPVAK
HGNRAVSSKSGSADFYEALGVPVNIPVAAAQTLLEKTNFVFLFAPFYHGAMKHAAPARKA
LGIKTIMNLVGPLSNPADASYQIIGVYDKALLRPVAEAARLLGVKRVMTMRSRDGMDEIS
PCALTDIVEISEDGIVKEYVFDPKAEGFGTYDHNELKGGDANDNAKLALELADGKGYPAL
EAATAINAGAALYISGRTKSIVEGAKQAAAALASGAVAAKIEELKMAARIFAAADGGAYH
G*
>SPBIB_v1_360027|ID:27164083|trpCF| Indole-3-glycerol phosphate synthase/N-(5'-phosphoribosyl)anthranilate isomerase [Uncultured spirochete bib]
MADILEKIVADRRRDLAKFGPSFGSRIPTRRLRPLVPFLAEPAAILEIKRASPSRGDIVQ
NLDPRKLVAAYAAAGARNISVLTERNYFKGSLEDLVDVAIARPELAYLRKDFLLQEEEIE
LSYRAGADAVLLIARILDVDLLRRMAALCRSFGMTPFVEVREYEDYAKLTAAASDGVVLS
GANARDLATFAIDPLIPAAARSRLPGRAVYESGIDSAGAAAYARRLGFDGILVGEAAIKD
PGGISAIINGFQSAKEDASGIFWRRIAERRETRVNNLKRSLVKICGLARTEDALLAADLG
ADLLGFVFAESPRAASKQVVREVYQYLYKQQRDLSDKDDPKPLLVGVITEIHSTKAETAL
SLAREGILDAIQWHGESTPAALAALDVVLEGKAGRYAAARIGNAEDLAVIDLLRRSGEPR
ILADARAENVAGGTGMAIAAELAKFMADRGFLWLAGGLGVGTVGHALKSYAPELIDASSK
LEVEKGKKDHGLMAAFFKEIDEYAN*
>SPBIB_v1_360028|ID:27164084|trpB| tryptophan synthase, beta subunit [Uncultured spirochete bib]
MPTSDKIMARRSDKGYFGPYGGRYVPEVLRSAIEEIEEAFFEEIADPSFNAELERIRSEF
IGRPTPLLYASNASKLLGGADLYIKMEGLAHTGAHKINNAVGQALLAKRMGKRRIIAETG
AGQHGVATAAACAKLGIECVVYMGAIDVDRQQPNVATMELFGAKVVPVYSGSKTLKDAIN
EAFRDWAESFSNTFYLIGSALGPSPYPDMVRVFQSVIGRETKAQLAERGVTPEVLVACVG
GGSNAIGFFEPFLDDETPRLVGVEAGGIGQGKGEHASRMTGNGARTGIVHGYKSRFLLDD
DGQVAETHSISAGLDYPGIGPELASLGRSGRIEFRRATDTEALEALTFLAKTEGVVFALE
SAHAASAALSLARSLQPEKAVVVNMSGRGDKDLFITAPLLRPKEWKDFLERELVAMNVSQ
GAKK*
>SPBIB_v1_360029|ID:27164085|trpA| Tryptophan synthase alpha chain [Uncultured spirochete bib]
MSVERIPLMAHLVAGYPDASGCRAAARGLVEGGATYLEVQIPFSDPSADGPTIRDACSSA
LEKGSSVKESLALVADLHATYPEIPVFVMTYASLVFAPGIVTFVDAAAKAGAAGLIVPDL
PFDADEGLAQACVAASEKIPGAICSIPVAAPSMKPIRLAAMIALGRPYIYAALRAGITGA
ATEINADTKMFLSAVNKGGSKIFGGFGIRSGAQARAVAPYVHAVVAGSVFVETVSAVVLA
QSPESKTASARSLQIRNEAIRRAVYKKAKEIIAG*
>SPBIB_v1_360030|ID:27164086| Radical SAM domain protein [Uncultured spirochete bib]
VESIVKPPEKITLALSHTDSIAFNKLIHVSPQTIDKLIDAPALRKAIAKVFEWSTYRSSL
SFKEYPRQVQLDKFYITRAFIKAIDAALASAQKSPVFRHSLVNSVLPAMFSAIDKTAERE
EAFKRRYGFGPPRFLVLSPTKFCNLHCTGCYANSDSASREKLSFETVDRIIREKTEVWGS
VFTVISGGEPLLYESEGKTILDLARAHQDNYFLMYTNGTTIDKEMARKLAEVGNITPAIS
VEGYENETDARRGKGIYKKVLSAMENLRAEGVPFGVSLTATKENAHLIPSKELVEYYTRL
GALYFWIFQLMPIGRASMDLVVTAEQRLDMYNRMFDLIKNDRYFIADFWNGGTLSNGCIS
AGRMRGGGYLYIDWAGHVTPCVFNPYAAGNVNEIYTRGGELSEVLVSPYFESLQEWQKDY
AMNDEKYEGNWVLPCPMRDHYADMLKILEKTNPEPTDEAAAEALHDPTYHAAMIQYDKEL
EQTIGPLWKKKYLKH*
>SPBIB_v1_360031|ID:27164087| putative Transcriptional regulator, TetR family [Uncultured spirochete bib]

MENQVRERIVDQSKELFFNRGVRNVTMDELASACGISKKTLYVNFPSKEELLHYIMMQLR
DELVERVRCILSDTSQPVFSRLRDVINAVSLHSMQFAPIFLEDVKRFQPGTWRELQTYKK
NGIADAIRTLIHDGQAQGYIRKLPENFIIHVCFALIDDILTPETMLELSMPFHDLFEHLM
SLFFEGFLTEDGKKAIYYIESS*
>SPBIB_v1_360032|ID:27164088| membrane protein of unknown function [Uncultured spirochete bib]
MAKFVYKFRFFIIAAFVLLTLVAALFIPNLRFDGDIQALAPTNVSSVEEYKSVSQTFGGA
DSLAFSAQSEHLFTPEALSEAAAIGKELQSLPDVSDVVSVFTYVEPVNTTDGLSFIPYVD
PDALPQDQASADALRARLSGIKTVSGKLLSADGDTLLYIIQAAPNVSRIRLIEGIQGIMK
THPGFSFQMAGTSFIDYQMVSYMKHDVVVLVAFGLVAVLLVLLIGFRSLRGIFLPLFTIG
CSLIITFGFMGLLHAKLTIVALIIPVMLIAICNNYAIHFLTRFYEDVFVNGMTDKVEIID
SSFKSLSKPVWLAFLTTVASFISFVTSPIPRIAEMGLFIAFGITYAFAMTMFFIPALLSV
LPPPKKHRRYHANEALISNIAQKAGRRITKRRLAILAVTLVIAIAGIIFIPRIVVDSELA
NYFNPKDPVRQGIDFINKKVAGSQIIKVSLPLDPRTAEGLAKARQFQDYAESLGSVGSVF
SIVDSIQTLNRVFHDNDPAFDTVPDNDEAAAQLLLLVETSLSPERLVQLVSEDYQNLCFS
IQLKAVDSATLQKTADMLRAKAHEIVGPQGVAIAGVSLISNQLNHLVIISMLQSFAIAFV
LIFAFVAFGFRNIPAGLASAGIMIAIVLVVFGFMGFAKIELSTATALVASITLGVGIDYL
IHFSARWFSERTSGKSLEEATSRTLSLTGRGIIINAMAIIFAVIPPFFSQFRPIMYFGIL
CFMSILLSLIGGLAVMPAMLSAVPERFFKRIRVG*
>SPBIB_v1_360033|ID:27164089| exported protein of unknown function [Uncultured spirochete bib]
METLKISRIHAPRRARAPRGRHVVMVLALLSVGIAGPAWAQASLSGEISAFSGVFFTDSG
SFKAGDYFQPSVSLKLAPSYASGPLTMKGELTGSISLADTGYAASMKLGETYATVDVAEG
LSFTAGSKIISWGTALVANPEGFINPVDSLSQLVSENRSDWLLPVPLASAKYIKGPFSFE
AVALPFFRPSTIPAATSRWYPAQLAVLDAKNGSVIPASLPSFPGATFSVSTTPADPALNI
ETMQGAGRTSLALGAVDVGLSGWYGFTKTPAFDVTTTVGMPINVDITASYKRQGAVGMDM
SATVLDSSVVWLESALYLPEYYIGTENSGLPVALEKNTLKSAFGMDRTFSIGSIGDLYCA
LEGNLSWILDYDSRLASAIKETGLGATLVTEYRSPSQDFTVRLVVMEPDFLSVDTTKQYL
VRLSMKAKLADGYSLSAGATLFEGTSGTIGQYANNDFAYVAVTASF*
>SPBIB_v1_360034|ID:27164090| conserved protein of unknown function [Uncultured spirochete bib]
MNNCAQIAGARASRAPGKPAHCGATTIFAAKSLFVLLFGLVLMITAGAQTTSQSGYDIMK
KANDKPTGNDMTANVLMKITSKTGSVKTREFVMYELKQKDGSSSILIKFLQPADVKGTSF
LTLESGNGEKSQYIYLPSLKKVTRIAASDKNKPFMGSDLTYDDFGSRKLDDYSFNLLGEE
ILEGRACWKIESVSKDTSQSTSKIISLVDKESYIVLKVDFYDKTGALYKQMVVQKTDKIS
GFWTMLELEMKNLSTGSSTSLKFDVVKYNTGLSPSMFSKDGLGK*
>SPBIB_v1_360035|ID:27164091|uppP| Undecaprenyl-diphosphatase [Uncultured spirochete bib]
MLVFQSIILGIVQGLAEFIPISSSAHLVIIPWLFGWNNPTLTSLTFDVALHLGTLLAVLV
FFASDWARLIGAWFKSIFQFKIGDDPDRRMAWYLVLACIPGGISGVLLESKIGQAFHSDP
IPKGSMLFMAGAIALLALLLWLADKLATHRRAFGKIKARDALYIGLAQAFAVIPGVSRSG
STITAGLAVGLEREAAARFSFLLSAPIIAGAGLKSLYDLLKQVKAGAIAGTELAIFPIGF
VAAAISGFLCIKFLLAYLRKHSTAVFVWYRFALAALVLIVALARG*
>SPBIB_v1_360036|ID:27164092| transposase [Uncultured spirochete bib]
MFVQTLFEKKTGRTLLFYYTARRVKGKIVKTKVKRIGYLDEFLDAYPDPLTHFRQEAKRL
TQEAQLKTLTVTFSMDEHFSFGAGFAATEDAAVEKADRTFHYGVLPLLQLYRELKIDAFL
RIKAQYTKVDFNHNHLFQMLVFGRILFPESKLATWRDRTRILQHSDFSDDAVYRALPFFA
QIKDALVQHLHEQVQRQYHRDTTLLYYDVTNYYWEVDREDELRKRGVSKEHRPEPIVQLG
LCMDNSGLPVTYGLFPGNTNDVATMRPMMQHLAESLGTKHLIYVADKGMMGGMNIAQIIL
EHNGYVISSSVRKADAELRRYILDHEGYTELAGGSFKYKSRLVPCTLYVDTPDGRKKQIR
INERQVVFWSEDYWKKARHDRDMAIAKAMARAGYGENTVLNNHAGNRFIKKEIFDPDTRK
EVDHPEFSFALDQELLDSEEELDGYYLIRSNVVGVREGDAPFNQPYRWHAKDNLFELNRP
VVDLDIIDMYRGLWRIEESFKITKSQLKARPAFVHRQDSIEAHFLSCFVALLLLRLLEKR
TGEKIPVATIVESLRKAQLVQLEDETYVNACCDNVIEAIGQALELDLTKKYYTKGELKAL
RGKTAKSR*
>SPBIB_v1_370001|ID:27164093|fruA| PTS system fructose-specific EIIABC component [Uncultured spirochete bib]
MILQRAFKPASIKIGLESEDKDELLEELVDVLAKTYPKDKEFPREQVLDALWAREEKMST
GIYKGVAVPHATVEGIDSLHGVLGISKKGIEYESLDGSPVYLVFMLISSPGEAELHLDAL

RKIALLIQDPVLLDNLMKAARPEQVFALIREFEASAVL*
>SPBIB_v1_370002|ID:27164094| protein of unknown function [Uncultured spirochete bib]
MDSVDSIDKLIEIERQAAAIIKEAEDKASRILLDAKNKSESLLKDKLAEARMKFESDLAA
SRDALRTQTQKEIEDYKASLKNIPLNQTALSAALEDLLKQEA*
>SPBIB_v1_370003|ID:27164095| protein of unknown function [Uncultured spirochete bib]
MADSFEIAYLYARVCGAFSKMQLGEAGRELMRNPGGIPALWKLYFNEEPPTIPESKLIFE
AERKIIQRSISSFLRLAKPLSDSDAFIHALISKFEITAIKSMLFRLRSGEPRPEEISYSS
PVIEQALSAWPRLADMFQDTPYAWLDNSWLDNIALAENKLDRQYYLDLWATASRIPKPKL
GAILDLVRWEIVYQNVIWALRIRRYYNMSRQDATSMLVDIKGIDTTSLALQTFDFDIDRL
DSFDSWPLKKLLSNQTSAHLDVPVLEIRAQEELFTMVRRALHLHPFSYTPVYCYFKLLEY
ETSLLLAVLESARLGVTAEQKSKYLWIPGEETA*
>SPBIB_v1_370004|ID:27164096| V-type ATPase 116 kDa subunit [Uncultured spirochete bib]
MSSVRMRRVELLLLKSDIDAVLKYLGSQQCFQIIYPDEIERIAHEKLAGIVESDEEKALA
SRLDNAKSKLDFIGTFFGLPVPQNIIEDAHLPDEEMLSKLDILYERCADLKTRIAEQEAK
VDQLAESLHEAKAFSGLSQPFDELEKFSYVSIQIGHIAKEKLDALEKALGARAVIIPLDN
EGTILAAASRKGRFALETELSKAGFEKKSPPKEASGVPAEAMSALEHAYQAEKLRLENLL
QEKKSLSEQYGSLWQVMVSSVRLKQALILVETKLEQTEWTYRLSGWVPVDRIDRMAKDII
GMLGERVSIRIFNPDETKSSGEKDTSEEVPVLLKHNVFVSAFQGIVLSYGTPLYGDIDPT
PLVAFFFTLLFAIMFGDLGQGLVIAGLGLAMLKMKKGFLASYKRYALAFIAAGVGSMVMG
LLVGSFFADDKVLVPLERILTRLILGQPKDRFLQIMPQGNINSMFYFFGFTVGIGVIINS
TGLVINMINLMRRKEYGEALFTKTGLAGSLAFWWAIGMGVRVILGAKLGWIDIVGLGIPL
LAMFFAEPLKALVDKAQGKKPEQVSLMDSLVGGAVELIETLSYYASNTMSFLRVGAFALA
HAVLSFVVFTMAELVRGKSSSGMAFQLIVYIIGNVIIIGLEGLIVTIQVIRLQYYEFFSK
FFARTGKTFKPVSFSE*
>SPBIB_v1_370005|ID:27164097| conserved membrane protein of unknown function [Uncultured spirochete bib]
MKKKLLAGVMTFASSIAAFAQTQTAAVPASAQTAKYIGAAIAVSTAVLAGGFAVAKIGAA
AMGAMAEKPELSGKALPYVGLAEGICLWGFLVALLIILL*
>SPBIB_v1_370006|ID:27164098| Vacuolar H+transporting two-sector ATPase F subunit [Uncultured spirochete bib]
VAEIFVLAEEEILIAFGMIGVKGKAVTSRDDAIATFRSIVQNKTCTIDRGVVDLSDCKML
ILSEDISDMIGKELADWQLSGEFPLIVEIPPLSGTSAQHTRLVDAVRQAIGIKIQ*
>SPBIB_v1_370007|ID:27164099| putative ATP synthase (E/31 kDa) subunit [Uncultured spirochete bib]
MEEIRGTEALEREILDDARKRAERIVRKAEDEAKALQAQTEQKIKAAIDALTQEYQTKQQ
TAEKEVRSRLPLEKMRLEIQYRDEVLRNAVAEVLAKIDPRLFGAWCLRGLKRQVELIRGS
KATISVKGLDSAYIEEIKTLFQDAGSVSIKESEAMKARGIIVEPDDNSYRISITENELVS
WLLDEERGELASALFGQTDSSATNTAGSPTGAGTSALKGARS*
>SPBIB_v1_370008|ID:27164100|atpA| V-type ATP synthase alpha chain 2 [Uncultured spirochete bib]
MSAGYITRISGPVIYAQGMDDAGLYDVVKVGKAGLIGEIIKLKGDVATIQIYEDNTLMRV
GEPVECMHRPLSVALGPGLIGSIYDGIQRPLPVLKNVSGAFLAPGLAGEPLDTSKKWHFV
PLLKAGDPIAPGTAFGIIQETASIQHRLVFDASVPASTADWIAPEGDYTCTATLVKSREG
KEYTAVSWWPVRAARKFTEKQITHEPLVTGLRVIDVLFPIAKGGSAAIPGGFGTGKTMTQ
HAIAKWCDADIIVYIGCGERGNEMTEVLTEFPSLIDPRTGRSLMERTILIANTSNMPVAA
REVSIYTGITIAEYYRDMGYQVAVMADSTSRWAEALRELSGRLEEMPAEEGFPAYLPTRL
AEFYERGGMVKTLEGKKGSVTIIGAVSPPGGDFSEPVTQHTKRFIRCFWALDRDLANARH
YPAISWNDSYSEYTEDLIQYWDSIDPAWDALRTKTMDILKKERKLSEIVRLVGPDALPDE
QRLILLTADMIKNGFLQQSSFDTVDMYCAPPKQTFLLTCILTFHELAENAIKNGAPLLKI
SALPIKEKIIRLKSTLENNKVQEGQAVIQEIQQAFTQLGVGAQGGLSI*
>SPBIB_v1_370009|ID:27164101|atpB| V-type ATP synthase beta chain 2 [Uncultured spirochete bib]
MRGLEYHGILRADGPIVIAAHRENVGFSELARVRDRTGELRLGRIVDISEQAIAVQLFSE
NTGISIDESWIEYLEKPLTFRVGDGIVGRIFNGLGDPIDGYPPITSSDLRDINGRPINPS
ARVYPRDFIQTGISAIDGMNTLIRGQKLPIFSGNGLPHNRLAAQIVRQARVLSGESQFAI
VFAGMGIKYDVARFFIDSFEKSGVLSRVVMFLSLADSPSIERLVTPRTALTAAEYLAYEK
NMHVLVVMTDMTNYCEALREVSAARNEVPSRKGYPGYLYSDLASLYERAGKIENCEGSIT
QLPILSMPNDDISHPIPDLTGYITEGQIVLERDLSQRGIYPPIAGLPSLSRLMKDGIGEG
MTREDHRDLAAQLFSAYAYVRSVRDLAAIIGEEELSDRDKIYLNFGDRFEREFLAQGEFE

NRSIEQTLDLGWKMLSILPEAELVRIPPKLIEKYLPRTGNGDPAKE*
>SPBIB_v1_370010|ID:27164102|atpD| V-type ATP synthase subunit D 2 [Uncultured spirochete bib]
MAEPFPPPTKTNLIKEKRSLQLALQGYDLLEKKREILVIELMNRMDALELLEQEIAKMTE
KAYATLRQMLLSVGRERALSISSLPVRDITLTASHVNVSGMTLPTLDVHASAPVLHYSFM
NSFAVCDETVIEFTELLQKLSTAAGMRSIVWRLAREVRKTQRRVNALDKMVIPRSREIVK
FIDASLDERERESLFAVKMLKQRLSEQDQT*
>SPBIB_v1_370011|ID:27164103| UspA domain-containing protein [Uncultured spirochete bib]
MKNMLETVLVAINGSDASISAFKYALAIKKSLGSRIIACYVVDTATIRQLALSRIFVPEE
SEEYERNLENSGRRYLNFCIELARQKQLSIETAIRKGSVSGEIVKASMELGADAIILGGN
PADTLYRDAIADAYREILKNARCPVLFVKPPIGDELFKAL*
>SPBIB_v1_370012|ID:27164104| protein of unknown function [Uncultured spirochete bib]
MLLCKWHLILSLAPYFWYYFEMMRSKYSLYPLAAGIVIIVLGLSSCVSPLSWSSYSENLN
LLDAAAWKSDMSTLESELLGHPKLKSDTALQAQLSTAISQAMSDIDGAPDGESRRDAAIA
GISKACAIVGDGHTRINASPTARYPVALRFFPVSQSMASTEYELRVFAASDENASLLGGK
IARIGSKTVEEVLDILAPALSIESALGKPGLEELKNAAIRAESLNAFMNPILMRGLGLAD
EQGLHLSFDTEASPTGCPAVYTVLESSDAFTWNYAIDPNAPPALSRQKPNENIWYTIPEA
HPETLYLSFQSCESDAGPIFDEVIQLLKSSPAPNRLIIDMRTNSGGNSMPGTRFAQQLAD
TEVAKRKGGVVILVGPYTFSSALMNAADILKACGARGDPGSGNAVLAGEPLIEPMDHYGE
VVRFSLPNSGLVIGRSSRLWEYSKTSGITPDNGFLGPTPDNLRAPTFEEYRRGQDPVLEM
VL*
>SPBIB_v1_370013|ID:27164105| putative signal transduction protein with CBS domains [Uncultured spirochete bib]
MKKTAGDILEVKGHEVAAVSPDDTVLHALEVMAEKNIGAVLVMDEQGMVLGVFSERDLAR
KLIIKSHSCENEKVGNIMTKKVIFVQLDTSIEQCMNMMTQNRIRHLPVLDNNRVVGIISI
GDVVKALIEVKDRIISEQAFELGQHERASTPGAV*
>SPBIB_v1_370014|ID:27164106| AMP-dependent synthetase and ligase [Uncultured spirochete bib]
MITLKKFTLAEVPELSAKQFASRPALSMVGGSVFTYQDFERESRSIAESLLHAGVKKGDR
VALLAENSPHWVMTYFGIARAGGIVVPILTDFIPAQICNIIDHSGARIVFVSEKLRAKLS
DLPKDIEVRNVKTGRPFEEPGASPESASARTLFPDVEADDLAMIVYTSGTTGLSKGVMLT
HRNILSNATACKSIIVLRRTDRLLSILPLAHAYEFTIGTVIALLSGSHIHYLDRPPAASV
LIPALQAVRPTIMLSVPLVIEKIYRSSIKPTLDGMKLYKSPILRPLIIRFAGMKLMKTFG
GKLRFFGVGGAPLAADVEEFLKKAHFPYAIGYGLTETAPLLAGCGPRTTHLRSTGPALKG
VQLRIADPRPDTGEGEIQAKGPNIFKGYWKDEARTREVFTDDGWFRTGDLGYIDEKGRLF
IRGRSKTMILSASGENIYPEEIESIINQSPEVAESLVVEDEGGLTALVYLKSEVLENLEA
RLQDGLDAAGALSARVSQAISSAEKSVAGTVSHAVVDVEKAVEHLLENIRKEANSKLAAF
SRIQNVKIHREPFEKTPTQKIKRFLYGKKKEGSREQGAAGDNHASSGIHQAGKS*
>SPBIB_v1_370015|ID:27164107| Response regulator receiver modulated diguanylate cyclase [Uncultured spirochete bib]
MSSIRILLVEDSITQAMRLRYVLEAEGFDVDVAANGADALTFLENNAPDLIISDVMMPKM
NGYELCRKIRENPRYKETPIMLVTTLSDPTDVIRALEAGADNFTTKPYNEQALISRIRYI
LANAEIRKQRGSEIGIEVFFSGKKYFINSTRIQMIEFLLSTYESAIQKNQELFISNNKLK
EALDNIITLQRNYRQLLETSQDAILVYDKDKMIRYANPAAHALFSKEQKGLIGARLPIEE
DISTQKEIEIKDPYGNTIFLDARSVSTDWDNETMTLSVLRDITESTQLRKELEQISLTDD
LTGLYNRRGFKILSERMIKLARRLQANMFILFGDMDGLKTINDTLGHLEGDNAIRTMASI
FKSAFRESDLVARMGGDEFAVIGLINENFVPNRLVERMNELIGSFNAKGEAKFKLAVSIG
IERIAYDSQVPIEEMLSVADTKMYKEKEGKR*
>SPBIB_v1_370016|ID:27164108|cheB| Chemotaxis response regulator protein-glutamate methylesterase 2 [Uncultured spirochete bib]
MIKVLIVDDSIVSRQLLKYIFYLSGDIDVVAEAKDGAEAIRIIDDRKAPRPDIITMDIEM
PGIDGYETTKHILERHPVPIVIISNAMQYYSAEKAFRAMQAGAIAAVGKPPGLGSPDFYQ
KARELIDLIKRISGIKVRPRLSTAELEGHQKDSVHKSQVVEQKPLSRDQLQTWAQQNSPG
IVAIGASTGGPPAIQAILARLPADFPLPIVIVQHIASGLAKNFAEWLDKTCPLHCKLSEE
SERMRPNTVYIAPNDIHLKVAYPDTLQFQMPDANELIVPAAEVLFHSVAEVYGSRAIGIL
LTGMGRDGSRGLLELKKAGALTIAQDRESSVVYGMPGEAEKLGAACCYLPPESIAKILLE
IYNAARKEKEQTR*

>SPBIB_v1_370017|ID:27164109| putative Chemotaxis protein [Uncultured spirochete bib]
MLNQEEFLKQLREAFAIESKEHIQTIASALLALEQTHGEPDQELIETTYRAAHSLKGAAR
AVDLKDIEYICHSMESVFQALKRDEIRPESQLIDTLHEALHLIELELSNSAVPATEIGAI
CERLDQQAKKGNAILSTSLAPPSPTQASVAPPSPVPPSASTYKPETRPAIPSTIRVQAER
LDTLLRESENLIAIKQLHAQIQAQLKSVLSLLSEASSGNQARKDSIHALSAISNRLASES
VHISTIIDTLIEEAQSVLMLPCSVLFDSFPLAAYDIAKSLGKEVSLDISGQTIELEKRIL
EKLKDPLMHILRNAIDHGIEPPGEREAHGKPRRGHISISVSHLDARHIEIIISDDGQGID
PESIRSTAVARRMFSESEAQKKTAGELYGLLFESGFSTSQIITDLSGRGLGLAIVKNAMD
ELEGSVRIKSEPGRGTSFHLVIPYTKASFKGNFVRVSEQTFVIPSSALEQGLRISQDAIT
AAGSANVIEYQDETIGIVHLGDILGISRPMETETPDHVIAVVCRSREKACAFIVDEIMGE
QDVLIKPLGSLLQKVRGIYGATTGANDEVIPILNMKEILELAFSSLFGPPGRVQPEKASV
ASASTEKTNAPKRILVVEDSITSRTLITSILSAAGFQVQAATDGIEGYTLLKSEPFDLVV
SDIEMPRLDGFGLTERIRNDATLANIPVILVTALESKEHKEKGIAVGANAYITKSNFAQS
NLLETVRRFI*
>SPBIB_v1_370018|ID:27164110| conserved exported protein of unknown function [Uncultured spirochete bib]
MLKNMKMRAKLILGFGLVIIVMISLAWYSFTEQAAINRHLDDIINTHFATLTLVDEINIA
RVQLSQNVSDFVAASTASEIEKLYQSVQNDIQTIQTNITKLQTINTETETLKPLADLNAS
FARWLIIYNEEVGLKNAKKQKELVQSFNTKGKEAFALVAEKVANIAESTKKRAADAYQNA
LAMQKSATTITIILNILGIAFAVLFAFVIGENIVRPIKQLVANLEEVANGNLQKHGIDDK
RKDEIGMLIKAFESFLDRFREQISEMNKMVLHLAASANEMSATSAQLGSSASETAASVME
TATTTEEIRQVAQTTKDLAGTVTASSDQSYALSKESMTAILQMGENVQKIKNQMDNISQS
LLNLSEQSQMIAEITAAVDEIAEQSNLLAVNAAIEAAKAGEQGKGFAVVAQEVKSLADQS
KQSTRQVRKILADIQKATSGALMAVEQGNKEMQQGAEKMNQVDAAMRAIADSLSKSVQLV
QQIAYAVEQQFTGIDQINSAMNNVKTASAQNADAARNLEQVAASISDSGNKLKSFIEVYK
L*
>SPBIB_v1_370019|ID:27164111| protein of unknown function [Uncultured spirochete bib]
MQNVSKNSLLAANNSYPSASPVPESPEHSAHTTRTAEDILLARAKELAKGQRHTTKKTDI
RCIEFALSGMSFAFEMHSIREIMRNSMPITPLPFLPKSFLGLINVRGEVVPVIDLAHYLG
LSRPKEAKEVLKSLIILQKNEVAVAFPCERIVRIREFSKNELQPTIAAQSQVIASLAKGC
GRGGITVLDADAFMLQLQKSLNPE*
>SPBIB_v1_370020|ID:27164112| putative MCP methyltransferase, CheR-type [Uncultured spirochete bib]
MNKEQRTLHAAFDEHALKTLLEQNRNQIQRSLEANFGLKPGAVHYYSLKKAAHGLQEAEK
KPLPEIIRLLCEIPMREDVVRIIVPHITVYETYFFRHPEHFAWLRDSFLPGLEIRKRAKG
TLNITCWSAGCSTGEEAYSLAMTLFDYFKGQQGWELKVVATDINHESLDIARHGCYGEWS
FRDLPRETKERFFYECNDALELVPGLPRLKRYHIDTRIQQMVTFAELNLSAPQWDVSELS
ARTFDLIVCRNVLIYLHQDAAKALISRFAEYLEPNGALIVGPSEPWFLNDSPFVAQQIRN
GTIFIKKENSAQSEQHTKQLPAQRLENPNSIKTVAESLTPQKTAPAESLSPPQPTLKTTF
PVTMHEAPLRAKEEREEVIANARHLADSGEAARAIELLSELIQKETTDPELYYLRGLAYI
HTRDLSSAESDLKKSLFLEPDNVVAYIALASIAKERGNESEMKKHYSNALHFLSKIEETA
VVPGSNGIPAKAMRDMISSLMAR*
>SPBIB_v1_370021|ID:27164113| protein of unknown function [Uncultured spirochete bib]
MPDSGTITPNISKQDLPANAALNVCIFRVGQRLYCLPAASVEYVYPYCEITPVPHEAESI
EGVINLRGSIIPVIDMGMKCEQRPLERDPAHKLLVARVGSHRIALHVEDVMDVIDVEPSG
WEETDAILPGIQVLTGIIKYQDGLALLYDPEHFFDTELAEREHAQKK*
>SPBIB_v1_370022|ID:27164114| conserved protein of unknown function [Uncultured spirochete bib]
MDPEMRKKIDETFAKVREPQSDAPIVDLGLVEKVTYSEKEKTLLVRLAIGTPRWQCPACS
AINGVVKEGIVRRAKEAFEAAFPELKILVE*
>SPBIB_v1_370023|ID:27164115| conserved protein of unknown function [Uncultured spirochete bib]
MNPFLFTGILILGVGATVNYFLGVKKNRWLGKRLSVQSEEVLAPTETNYVNIGGAIGYNF
RYQLKGLWNEAKGTFTFVPRHSLLYMPVSFLIRGGDHYYLNIYTDKRLPGEGHIILASHL
RKAKIDGLEAMSRKDIELGGRKFVLLWRESRILDKLEKTALGFPEPQTLVHFCCFADNKT
LFLHLSPKNGVIQNNLSYFLKNAQVFLEK*
>SPBIB_v1_370024|ID:27164116| Arsenite-transporting ATPase [Uncultured spirochete bib]
MSPHISHKTAFFLGKGGVGKTTLSASFALALARAGYKVLAASLDPAHNLGDALGLKLKSE

PQHVEPNLDALEIDLDAWVNTYLEESRSQLKSTYSYNVTLNLDSFFKILKYSPGTEEYAM
LWAIEDIHCNLAPLYDVVVLDTPPTALSLRFLSLPTISGLWVKELAKLRETILSKRNTIV
RLNPGSPVKESCLDKDDDKVYGKLSSIRQRLDALEHLFQKESFLNVIVNPDDLSVSEALR
IKDELDRLEIPISTICLNKRGMVNGQWHIHESLASIPLFSFDFESNGIRTRDQLTELGIQ
PLIDIFLKKGSLSA*
>SPBIB_v1_370025|ID:27164117| protein of unknown function [Uncultured spirochete bib]
MTEKMKLLHLVRRGAKTFIQVQREKATGMLDFELKELENIFALLLLGGFAGIPSPPAPIA
IELLPYLEREIVVLLARSDLSTDPIGALMGMLEID*
>SPBIB_v1_370026|ID:27164118| Carbon starvation protein CstA [Uncultured spirochete bib]
MSTLIALLALVIYFGFYFLYGRTIRDKVLKSQEAPQAPSKRLSDGVDYVPTSKYVLFGHH
FASIAGAGPITGPAMAVAWGWLPGLLWIWLGNIFIGSIHDYLSLTASVRYDGRSMQFVAQ
DLIGKKAGKAFSWFILFLCILVVAAFGDIVAGQFAADGRVFSAFFFFCVAAIITGYFMYH
TKLGIGGGSIIGIVLIILAFWLGEILPINASKDTWFIVIFVYIVIASALPVNFLLQPRDY
LNSFLLYFGLLVGGLAAIIAVKGFDSLPATTSFSAVVIGGKPTPFWPAVPLVIACGALSG
FHALVASGTSSKQLRDEKDALFVGYGAMLTEGFLSTIVVISIAAFGIAAMGEGNVLKTAA
LNRFVLSYGTMVSTALPFLTPSFMKLFAAVWVSSFALTTLDTTNRLGRYLVQEMVLPIKE
KSPGVYNTFNNKWVASLIIAFFGVGLAWTGNYTVLWPAFSGANQLIASIVMLTVAVWVKK
KLNPAYTLVVLIPAIFLWVTVTAGIIWYEIVIVPSFFATVANAAAQAKNYTTGVVVGAIN
IFMLVLNFIMIVAFFRNWNTKQAS*
>SPBIB_v1_370027|ID:27164119| protein of unknown function [Uncultured spirochete bib]
MKNVHGPVDLGGGYFRIGSSQLVGSLQSNIYLLIDGDEAVLFGPGSAIDLAELVRNIESV
APKDPIRTVIVHEQDPSSSSALSLLEKEGLRFDVVTHWRTWNSLRFYGLSSEPYIIDEHS
WVLRLASGRTLQFLPTPYLYQPGAFATYDRATRTLLSGALFSSYSSEFSLYGEGADYLEQ
LKNFHRATMPSKDFLAPVVHSLASWDIGRILPAYGPIWRKNIRGILNQLADLECGELARS
KETQALQQQIVKENGSQEEVAHLKAELEKLRKVNEELNHSISVSRDRALRDPVTGLYSEF
FYKSFIEEEVAVRISDMGPQDNVLGVFGIDENIAQIEYKYGSREVEALLHGVAAIITENL
PQTSMAFRLHGATIAVWMPAVLFDDAVALFDKIRYQVENSKAFVEPVTVSAGVATLSEAA
NLQPDLERLGADLTDLGIRRLRLARRRGGNMVYFASAEENESESKARILIVDDDEVNVDV
LRTFLSNEGFSVLSASDGQEALSILGKEIVDVVITELMVPKIDAYLLKESMLSKSATKDI
PVILISHLKTESTIRRAYRLGIVFYLQKPIILEELLGIVLNLTEAGSRA*
>SPBIB_v1_370028|ID:27164120| Glycosyl transferase [Uncultured spirochete bib]
MTIPSEYLLGAAVLFLWLTVFVVLRTLLNKARLAIKQETLKSQLRQLPQIFAPVYEQSKH
MMRMLLDHILYLTQNVNISAQQKQRLFNAETVSYFERTQMRRLASHNRFMRMNAARLLAS
FGTDNARKAIEKALLREKHFPTRLYLANALSDIKDARSLHALIQSLKGAHYWYRNKVNML
IASYGDALKDFMRGYYWSADIEIRELLVDLAGVIPCVELKEYLADILHRGFGEIGKIEKQ
TANLPSKCCYYCVHGQIEAGKDHRQCPYRGKVANNFHCWRYRTLVTSLSPAVSYHRLMVR
AAETLERFYPDELDCEYYLEHVDKDIQSIAVRSLGHSARERNVRTLLFYLERDETAQAAR
AGLRLLLNSHPQYMPQMIEAFRNSDGVLHNRLAEVLSSRIEYVLTKLLGYEHDFAAGVVN
ELITMGRVSELIEFLKRNRNKELEAKLVDIIRSRLSDSEVLARECGLFLPDHLLQELGIS
RISPLAKKREEKLDRKMVRRLYVMLSFAVVFFPLLYVLKYYDRLGAMPFVQHLKYFVLNF
NINFAYYAMAVNAIYLGLLFLSKLNIRRASRLWELKGMTMLFKPRMLPSVSIIAPAYNEE
MTIIESANSLLNLKYPDYELVVVNDGSKDNTLRTLIEHFDLKKTDFSYQERLKHYPIRGI
YTNPAIPRLIVVDKENGGKADTLNAGINVASKEYFCGIDADSLLESDALLKIASLTLDYG
VETPALGGNVFPINGCKVDKGKIEKIGVPRNWLARLQMVEYLRAFMCGRLGWDYLNSLLI
ISGAFGLFRKERVVAAGGYLTSSGQYQKDTVGEDMELVVRISRLMREKKLKYRISYAFNA
NCWTEVPEEMSSLKKQRNRWQRGLIDIMNFHRKLIFNPSYGVMGLVAMPYYLIFEMIGPL
FEIQGYLMVAIAAIFGMLSVRLALLLFFASILMGIFISISSVRIAEHLNVYFSYRDTLKL
LKTAILENFGPRQLFSFWRVLGFLNAMRKPQGWDKFARKGFAGSGQAGGKAA*
>SPBIB_v1_370029|ID:27164121| conserved exported protein of unknown function [Uncultured spirochete bib]
MKVKTKEYFKTSLLWIIGILVFALFTALIAMPFVLWIAKPYRQLDIWVVDKTVPYSDFRE
HAGLFWLLNNEKIAKPATRVLYDKRSDYYGFYPTGKNEWRSLKLPQTGKKPDLIYIADTY
GVYRDDYMQSKMTAALPSLIYGGLNSEDYRVIQASLGGGNTIIAEFNTAASPTNSRDRAL
FERLIGLAWRGWIGNYYESLAQGGGVPDWVVANWETAKKEKWNYSGRGIVLLDENGNVEV
LTEAEDLGPKGMKVQFDGLLGQKLGIKKPVSYRYWFEWVAADPLVEQVAHYSLDLTAAGK

KKLEALGLPQKFPAVLKFENSQYTSWYFAGDFADLQFSGTPYRMEGIRAIKRILADDTVD
NNSYFFWKVYAPLMHYILASAEKHRAEVRSEAALAGPTALRVNVRAVKDGIQLRTKNHEW
ETLFLRGVNMGLAEPGKYFTEFPYDTDTYVRWFNYIGALGANTIRVYTLLPPEFYRAFRI
YNEAHPDAPLLLLQEIWPEEHPPGNDYLQPAYQEAFLKEIDYGIDAIYGRANIPERKGRA
WGIYTTDVSPWLIGWLVGRELESAEVLQTDANHKGATYKGTYVSAGAKASPTEVWLAESL
DAVAATEAERYGQLHPVGIVSWPTLDPIEHDTEWDPNTGKKNRWNDRASIAVEHLDITDK
MTAGLFGAYHIYPNYPDFIVNEPAYNNYKDEYGVLRYGGYLQEFIKTHSRYPAIVAEFGI
ANGAGVAHLSPDGLNHGGIDETTAGRDILRMQAAIQKEGYAGAIIFEFMDEWAKKTWITE
PFKIPFDRRIFWHDVVDPEQNYGLIANESIPPEKPEQTISGSNALSSLEVAHDASYLYLT
IHLASGKKPQDCEVLLGIDTYNRTLGQMSWPRSLGRTASGMEFLLDIRNEEARILVNPSY
NIASARYATSLRQDGVFENMNMLVNGKVVTKDGREIPEKRFDISLMRQGPFDESGNLWNV
EGNTINVRIPWGRLNVTDPSSLRVLNDTRTNLGDPGIDTLKASITDGFVFDGLVLDKHSG
SVIGRLNANVTSPYIWNGWEETPPYRERPKKSYSIVQDAWKVASEKDKPVRADFVGAHSK
*
>SPBIB_v1_370030|ID:27164122| Peptidase M29 aminopeptidase II [Uncultured spirochete bib]
MQDQRLKKLAHLLVNYSTKVQPGDKVLIENVNPEPDFVRLLIEEVHAAGGLAFVSYRDKR
LERTLFMNAPEEQFALQAEFERARMDKMDVYIGFTNMQNSFAWQDLPQDKIELYNRHIWK
KVHIERRISNTRWVVLRYPSAAMAQNAGMSEDAFEQFYFDVCTMDYAKMSKAMDPLVALL
ERSDRVRVKGPGTDLEFSIKGMPAIKCDGTMNIPDGEVYTAPIRDSVNGVLSYNTPSEKD
GFKFENIVFEFKNGKIVRAEANDSERINKILDIDEGARYLGEFSFGVNPYITKPMLETLF
DEKIAGSLHITPGNSYDDCFNGNRSALHWDIVLMQDAASGGGEVWIDGVLVRKDGLFVQP
ELVALNPEALRT*
>SPBIB_v1_370031|ID:27164123| protein of unknown function [Uncultured spirochete bib]
MLRLWAYLMWHNYLKPYRIHWPKGRRPATHAEASGIDATALENVYRSFFEERAFLTRSPL
SPTMARSWKKEWRTPGKEKAEYLPKLALG*
>SPBIB_v1_380001|ID:27164124| conserved protein of unknown function [Uncultured spirochete bib]
MADKTSRLAGNAAGKWYVDSSCIGCGLCSSTAPDIFTLGDEGQALVIRQPQTASEIELAT
QALNDCPVQSIGNDGE*
>SPBIB_v1_380002|ID:27164125| Spore protein SP21 family protein [Uncultured spirochete bib]
MNSLVLYDNNPFDLIEKFFDDDWLAPRFRTPAIDVYEEGDKYMLEAELPGLSDKDIKLEV
RNGQLTLSTAKSEKTEEKSKKGRWIRKERREFRFARSFTLPEDVDASAIEAHFKNGVLQV
IMPKKPEAAPKAIEVKID*
>SPBIB_v1_380003|ID:27164126| Extracellular solute-binding protein family 1 [Uncultured spirochete bib]
MKRVIIVAFVLLLGVTMVFGQQKVGAYTTLEEPLAKELFDEFQKETGIVVNWQRLSGGEV
ESRLEAEKSNPQASIWVGGVGLNHISAKLKGLTTPYKSKMLENTPLQFRDPENYWVGLYI
GPLCFVTNNKVAKAQGLVPPTSWADLLKPEYKGKIRMPNPTTSGTAYNTLTTLRYVFGGD
ENKVFDYLSKLDKNIDQYTKSGSAPGKSVAIGEIPIAIGYAHDNVKLRVEGADITITFPS
EGTGFEIASMSLVKGGPDPVNAKKLYDWVLSPKAQEIIAKWYVIPLSKLAKKNPNAFSIS
EIKTVNQDVIWDAANKERLLERWVKEIGSKR*
>SPBIB_v1_380004|ID:27164127| ABC-type Fe3+ transport system, permease component [Uncultured spirochete bib]
VFTKKKLIVFAIVCAIFAAGEYWIFSTIQASFEKNAAKEITALARAIAASAPENNESLAS
WTEQLPSTYHGYRFFAFKGVPGEEGSEPAGGDSALSELWKLPSNADIAAKAAESVSYLEV
FRWPSRVTFAGVPYTLIIAPVPDAEGSSARAALLIAADASGYVAFGRLVQTLAVVTFVLF
ALGFGIATFSRDPISGYAILVLAAVVVAFVAYPLFEAVRLTLIKQGRFSFDVWKTILSNR
QYLQALWGSIQLGIVTATISTFIGFMYAFAVARTSMKGKKLVTALATMPVISPPFSLTLS
IILLFGKNGIITKQWLGLQNFNIYGLGGLALVQTISMFPIAFLTMQGVLQAIDSTLEDAS
LDLNASRWHTFSHVTLPLAAPGLLSSWLLVFTNSLADFANPLLLSGSYRVLSVESYIEVT
GMSRLNNGAALSLLLLLPTLTAFLVQRRWVSRKSFVVVTGKPSTRLSDLAAPGARRALTG
FVVVVSGLIVALYLTIVAGCFVKNWGIDYTFTLVNIHEALTRGKQALISTITLAGIATPI
AGVLSMVAAVILVRKKFPGKRILESLIMTPFAIPGTLVGISFILAFNKPPLLLVGTGAIL
VINYVIRELPVGLEGGVAALRQIDPSIEEAAADLGADQATIFRTIILPLIRPAFISSMSY
TFVRSMTAVSAIIFLISARWYHITIQIYNFSENLRFGLASVLATTLIIIVLAVFGLMRLL
IRQSEFLEKTVTAQ*
>SPBIB_v1_380005|ID:27164128|fbpC| Fe(3+) ions import ATP-binding protein FbpC [Uncultured spirochete bib]

MATKSVPVTLSHVTKIFKDPKSKAEVHAVEDANFDVRSGELVTLLGPSGCGKTTTLRMIG
GFELPTKGKILIGDTDVTYLPPNGRQTATVFQSYGLFPHMDVFDNVAYGLKIRKLSHTEI
ENRVRTVLELVGLAELAHRAPGRLSGGQQQRVALARSLVVEPQVLLLDEPLSNLDALLRE
QMRVEIRRIQKSLGITAIYVTHDRIEAMSLSDRVIVMRNGVIQQIGTPGDIYERPVSMFV
AGFVGKVAFFPAQLEGIEAGGRAICTVGGKRVRAGSAAEGLGTGSPAVIMARPESLRLVE
KEDAISSGTVTARVYLGSSVEIYVKTETGEILVQIDDPSGKAIPSEGEAVWIGLNEALVR
VLPAE*
>SPBIB_v1_380006|ID:27164129| taurine transporter subunit ; ATP-binding component of ABC superfamily (modular protein) [Uncultured spirochete bib]
MVSLEDISFFYTVHGIPKPVFEHFSAQFEHGRITAVIGPSGCGKTTLLRLIAGLNTPQSG
RIVVEGRAEEKLQSRKVPAAGPRIAFIFQDFGLLPWLTVERNAGLGLEALGIPPAERHAR
VRGILEELGLSEWRKAYPVRLSGGMQQRVAVARALAINADLILMDEPFSSLDALTRESVQ
DMLRSIQRAHNTTIIIVTHSIEEAVYLADSVVVLDGSMPANYCQVVDNPHVWEQESAARA
PHVMQAPVPQQPPASPPEASLNPRETSGYLAAVGALRHTFNQAVAQKAEAEMAAAAFAVP
AAAVTPLAAAVPAVPAVSAAPDVPAAPAALPAHKKLAPSVTRFLARSAQIFAAAVFLVAA
WWAAAALVQRPFLPSPSLAFAQFAVNLQKGVFQIHVLASARRVFFALLAAGPLAWVLGLL
AGRIPFFDNIFAPLVYFLHPLPKVAFLPILMLFLGLGDASKIALMGLVIFGQLFVTGRDA
AKGIAPALLDSVRTLGFSRLSIIRLAIVPSTTPSLMSALRVSLGTAIAVLFLSETFASID
GLGWYIMDAWSRIDYPDMYAAILALSLFGLFVYFAIDAIEAVLLRWRERT*
>SPBIB_v1_380007|ID:27164130| ABC-type nitrate/sulfonate/bicarbonate transport system, periplasmic component [Uncultured spirochete bib]
MKISAILIFALAVLIQPFTQASAQQVQAAPLSIGIFADADSLPLIVADNEGLFQKEGISV
KLVRFSSAIERDSALQAGKIDGAVTDVLAVVLANQGGFPLKITSLTNGRYGVAVSPKSNI
QSMKELEGKNIAVSLNTIIHYFADWSAREAGLKADSLNLVPVPKMPVRLEMLLSDQIPAA
VMPEPFLTTAKLRGAKILVSSDDSGLEAGVLAFPPSVIAKNADTLKRFYRAYWNAAQRIN
AQPDQYRNMLIESLSFPDEAARVFVFPRYVKPRLPVQTSIETAAQWLLSKALIKALPKTA
DLVDASMVTGW*
>SPBIB_v1_380008|ID:27164131| putative aromatic acid decarboxylase [Uncultured spirochete bib]
MAMRYLVCITGASGALYSLRLMSALASRGVILHVVASQWGARVLLQETGRPLGYWLGKLR
LQGGPDGGPALITMHDSSDFSAPIASGSFRLNGTVIAPCSMGTLGSLASGVCSNLIHRAG
AVALKEGWPLIVVPRETPLSLVSIRAMATLKEAGAVILSASPSFYSKPESIESLVDTVVY
RILDHLGIIDKNIYRWSREEP*
>SPBIB_v1_380009|ID:27164132| putative 4-hydroxybenzoate polyprenyltransferase [Uncultured spirochete bib]
MALSSTEPGILKRIGMIGDSVMITHTLFSLPFVVAAILLETEGRPPAAKLLWIIVAAFGA
RNAANALNRIIDRDIDAKNPRTAGRHLPSGKLAPRELWLFTFAMLALLVLGAAMLNPLCL
ALLPVAGLLLLGYSYTKRFTWLSHYWLGIACSAATMGAFLGISGTFHLRYFPLTAGVALW
VAGFDIIYALQDIEFDRKEHLYSIPARFGETGARIIAAASHLGAFAGFVSIYLFWDSPGL
FAAIGLALCFVLLVAEHLIAAQKTQNRIKLAAYTLNQIIPIIYLCGVALDIYLR*
>SPBIB_v1_380010|ID:27164133| conserved protein of unknown function [Uncultured spirochete bib]
MAYSGLQEFISFLELKGELVRISAEVDPILEITEIADRVMRRGGPALLFEHVRGSRWPLL
INAYGSEKRMAWSLGAESLDEKAGEIDSLIQWAWSQLRDFSLLSALPEALSRLPIARSLM
PKKVGRAPAREVKDEGGFDTLPIIQCWPQDGGRFLTLPVVCTIDPETKSQNWGMYRMQIY
DDRTAGMHWHIHKDGAHFFQKYRAQGRRMPVVVALGGDPAVTYASTAPLPEGIWEAMFAG
FLRGKPAEVVEIGDTGILAPADAEFILEGYVDPDEERLEGPFGDHTGFYSLPDDYPVFHL
ERMTRRKTPVYPATIVGIPPKEDCFLAKATERLFLPLLKQLCPEITDINMPLEGVFHNCV
LVSIRKRYPGQARKVMHFLWGMGQLMYTKLIVVVDDDIPLKDLSMVAWRVFNNIDAKRDF
EFSDGPLDALDHSSPKPRFGTRLGIDATRKWPEEGHDREWPDPLAMRLDIVELVNSRWKE
YGIELD*
>SPBIB_v1_380011|ID:27164134| ABC-type antimicrobial peptide transport system, ATPase component [Uncultured spirochete bib]
MSDIIISLDKVKKVYFLDAVEVEALKDISLDIHAGDFVSIAGPSGSGKTTILNLIGCVDK
PTSGTVVVGGKRTSDLDDDSLTELRHKSIGFIFQTFNLIPVLNIRENVELPLLLDNQSAG
SQSKIESKADRKEWVDFLIESVGLKDRMLHKPAELSGGQRQRVAIARALVMKPAIVLADE
PTANLDSATGESILRLMRHMNETFGTTFIFSTHDPDIVEEADHIIRLKDGRIIEDRRRNG

ASAALLAANLETAEATGTEGGQL*
>SPBIB_v1_380012|ID:27164135| putative ABC-type transport system, involved in lipoprotein release, permease component [Uncultured spirochete bib]
MIAARLAWGNLGLHRRKSLLIGMIMGMGILILFIGNSFIDTALSGLQRMFVQGYTGDLMV
TGPTEFPTTVLGETAGSEEVLPHIAQIKKYEDFLAGRNDVAATLPLLSGQAAFGIGETEL
DSGYCFGVNADDYRKFFPDNVTLVSGEWPKAGDGPWVLLSEPTLAILNKSATSPVQPGTH
IIMTALANSAGTVIREVTVKGVIKFNQSNKQLEGLTLVDADTMRDLLGFASLRDGAPVLT
GQEASFVSNFDPNALFENLFASDTTGAAPAAAAPASTGREGSDPSISSSATSTVPSAQAT
VKAEVVPAWQFLLIRVKPGASSSRLLSDLSAFSKQIADGDRVQDWIAGAGKVARTAVTIR
LAFDLLVAVVAVVVVMIMMNVLIISVNERLYEIGTLRAIGAKRRVVRNMILYETAFLAAI
AGAAGLVIGFVVLLLLGKFGIRAPNLFFEALFGGQVLKPMVSAGAAIRAFIWILAMGLGA
SWYPTIVALRIEPVIAMRGD*
>SPBIB_v1_380013|ID:27164136| membrane protein of unknown function [Uncultured spirochete bib]
MRLIHLKLAARNVRRQPHRTIALGGAVAFSALVMTLISGFVNGMDSAIQDNVTLYSGGHV
LVSGYTVSWSGRLQSRFADEAVANIVQSAYPEIRTASPISQTRATVVFGSREVQLSLRGV
DWGKDELYHSSLILSKGDWKSLAAPRTMLMSAQTAARFGLSTGDAVLVRLSTASGQQNVT
DYTVAAIYDDGAAGGMSTAFVAFSDLTADLNMKEHEQQAIAIFLKNSTSAENAAQTITKA
LSEKGYTVSRGAAPTSGALSASSESFVSSVVGSGAAGTAATGTTTGTAAGSSGAGASRRA
AAGTVTYYVSTVQELAGEISSALGSIRWIGYAVFALMLIVSATGISNSYRMVLLERTKEI
GMLRCIGYKKKDVFSSFIAEGILLAGGAALVGVILGLPVGFGIGLVPFNPHGDFGTALVQ
GHLKFVPSIGQLALILVFVTIVAVAAVFLPAKKAADVVPVEALRKTA*
>SPBIB_v1_380014|ID:27164137| conserved exported protein of unknown function [Uncultured spirochete bib]
MVNMQRRMTSKLSLVLFGLVLIFSAKAQGAPPAGGGQGGGAPMGGIGLPLGFLEQNKDYQ
FPDDITKYSATDFLMLVRRTDIRSSFYDSDMTATITTVSVSPDKGTFVRKEQIFRRDKDD
AFLMLTLEPESRKGQGMLRVDNNMWRYDPTSRKFTHTTLKDTYENSTVRNNDFRRWQRSI
DYSVEKITTGTLGNYTVIIGELKANNDEVPFPYIRMYIEKDRKIVLKVEEYSLSMKLLRT
AYYTQYVQIGNSFVPTVQIFQDGLIPEKRSQVTYTDISTKPVPDYYFTKEYLERVSQ*
>SPBIB_v1_380015|ID:27164138| exported protein of unknown function [Uncultured spirochete bib]
MKARNRPVSIALAAAALLVLSAGGLWAQTGTAPASQTPPAAIDENALFGGPKDIVTVIDP
NVAATSVVQLVQDTKTYPVFLISGTAGAGLFGNYTPVGAASTDPQKLYGAVNLSGLEFDY
LPTKSLHFSVSADLLAVPSEILSASVGAYADLRASELVRLYAAGSFDYDPNAYLSTKTYA
TPTFSLDELFVDTELGKKVFFRLGKQRISWGVGYWYKPSDVLSLSAIDPDNPTAAREGPF
AFKIDMPMKLNHAMLYVVPPINGDAGSFSAASKYDLVVNGWELSFAGFGRTDMLARPRGI
FTFTGAIGAFDVYGENVILYGSDRTYVQESSTAPGTYETYRVENIPFVQSTLGIKYSLSK
SNGLSFSAHVQGYYNGTGYADSTILQKSAARTAVEHSATYQPTDLTQAGMWYLAGSTSIS
GRFGEGRNLTVVTASAYALTNFSDRSVRFNPQLSVQVGDQGGRATLTLSDLTAIGALYSE
YAPNGNKTTPALTLTVFDKLSLQASVPITLDTDFSIKKVQTAFSVFWNAVSY*
>SPBIB_v1_380016|ID:27164139| Two component transcriptional regulator, winged helix family [Uncultured spirochete bib]
MHGRILIVEDTKELAELYQLYLQNEGFECRLAFDAESALPLASNEEWDLIVLDLNLPGMD
GFEFLERLRKIKSTSVMILTAREADEDVIFGLGVGADDFVTKPCPPKVLAARVRAHIRRV
VALQTRQNGAIVRFGPFELDIEGMYLTKNSEPVSLSAREFNILKELVMHPGKPFTPEDLY
AHVWGQEFGDISAVAVYIQRLRKKLEDDPANPYYIQTQYGMGYRFNPEAFAEARP*
>SPBIB_v1_380017|ID:27164140| putative Integral membrane sensor signal transduction histidine kinase [Uncultured spirochete bib]
MKLKLRTQFAILTIAVTLFPIAFGLWFFTGQQAKRDPRQPTQLFLSEVAQLWSKDKNLTF
ADIRDAGERAELPIIDAALIDADGTVLVSSFKGLPAGSKLQVADLARPPLMPQGKPGPEL
RFLPVDSNVTKSPLLLFDIRPFWTREDVRNRNFMLISSFALGAFLVAGLMSLLILRSTNT
AIKNLIDDTTTVASGKLDHEVKGSGAEELRSLADSINHMRITLRDMISRRMNMLIGVSHD
LKTPIALIQGYTDALADNMASNEATRQHYLEIIREKSAQLEDLVSDLIEFMKIDDLNMPM
ETVDLGSFVLALGKRFEEDARLLGLEFVYGFGSAFSKEPPDGALADAALRGVPHGAAPSS
ARQSSAALHIKMNRMLVERAIENLVSNAFRYTGTGGRVEFSLQFDSGQPVLVVADNGPGI
PEEELPYVFDAFYRGTHSRQEAGHGLGLTIVKSIVDLHGWEIEAHNRTVDGAAIGVAAGS
AASHGLLIAIRMKAAL*

>SPBIB_v1_380018|ID:27164141| conserved protein of unknown function [Uncultured spirochete bib]
MHISEIQLHRSISDSELEALVDKTIEASGALLAKCILIIPPDITRFHSRAGQITDALVAK
LGPRVAAILPALGTHSAMTEEERAHMYPGSPSQLFKVHDWRHDVVELGRLPEERVSEISN
GKVRYSYPVQANRLLTSGDIDFIFSVGQVVPHEVAGMANHAKNIFVGTGGKEAIDRSHYL
GAVCGMEGIMGRADTPVRALFDEGLSLAAGKLPPITWILTVIGSAEDSSLALRGYFSSQD
RRCFEEAAALSARVNIQLLEKPIQKAVVWLDPEEYRSTWLGNKSIYRLRMAMADGGELLI
LAPGLRSFGEDPNIDTVIRRYGYRPSTEIQTLVAREPALADNLSAAAHLIHGSSEGRFRI
TYAPGPLVSKEEIESVGYQWADLGQALQRYTPQGRLTGSHSTRDGEPFFFVQNPALGLWS
TAARMQNTRSLRPSMRLG*
>SPBIB_v1_380019|ID:27164142| conserved protein of unknown function [Uncultured spirochete bib]
MKLSRFSIGTGDRFGMEGEAQIGAFQQLREHGGEADIVWNKSNREHVIIGSTPADQAKAA
AEAVKHAGWNGNWFVDADHISLKTVDWFLPHCNFFTIDVAETIGKQAPADARAAYLLRAS
FLLKEGAAPIPVAKADLESVADKFLAAVLEAGTTYRYIAARKPAGSFVVELSMDETDAPQ
TPAQLAAILVAVAAEKIPLATIAPRFPGKFLKGIDYVGDPAEFLAAFEAETRVVLWASSA
LGLPEGLKLSVHTGSDKFSLYGGIHEIVTKLGAGLHLKTAGTTWLEEIIGLAEAGGEGLA
IAKRIYATAYARIDELTAPYANVVEIDKARLPSPQAVASWDSDTFIRALRHDPGSTAMQA
DLRQLMHVAFKVAAEMGSQYTNAVRAHHASVARNVQHNLYARHLAPIVLGSTRRL*
>SPBIB_v1_380020|ID:27164143| putative Integral membrane sensor signal transduction histidine kinase [Uncultured spirochete bib]
MQKPHAESLKSSKRFPRLGIRGKILACFSIVLAFFLGLAVVMQNESIQLSREYGVNLSSY
HLVHRFRLNLANFHGLTDRYLREPLSANPELIYNGIASLNAQYAELLPLEDYSIHAGFEV
RATGYGLDVYLPLVSRAVGLRAGGSPDYYQAFVKATRIQGYIDVYLNRLLSELMQSGEET
YAKLSRRSEILNKTILLSMIIASILAVIVIVLVTEAITAPLRKLAKEAEKLASGDLEAGI
VEAHTNDEVETLARSFASMASNIRAMVEGLKEKAELERLLHEEELALVSMGKALREAQFM
NLQEQMKPHFLFNALNTIARSALLERAERTESLALGLARLLRATIKDSGALSSLEEEITL
AQAYIEFQHARFGDRLKWKIDVPSALQSLKVPRLMIQPLVENAVRHGLEPKVEGGTVYIK
ARRRNTSLILWVLDDGVGISKEKLEEIREHIVFSLELKNRKPEELPVPTDAELPNSDPGE
SENELLNGTGIGLANLATRLAILYSGQCRFDIQADSDKGTLVRIMIPLGATTS*
>SPBIB_v1_380021|ID:27164144| protein of unknown function [Uncultured spirochete bib]
MIRILIADDEALEREAISHILRHIHLEEECVVDQAVNGYEALEAAEKYEPDIAFLDIRMP
GMDGLEVAERLSRLGNPPFIIMVTAYDSFAYARMALRYGVLDYLLKPASSEEIEAAVRKA
IYQIRRRREEAARRAEMHSIAADMEQIARMNIVDQLEAGSVADSDVTRLVKLRFGTEMWS
CAAIVAGARDGSSSASLSLALLEQHRFLSGIAERHLASDLGIPENLPSLFFAGSGERITS
MLLVAPFLDEKRSAESEPHAAFHAFQHIRSRLAQFRYRCIDAGAGDLRFGACVSDAGLAS
AALAAAKAAFNLTAAECPILVLSPMPLAHGREAAAAGSLAGRTISWLQDHFMESIGLADA
AEYLHVSPSHLSRMLKKETGIGFGEMLVRTRIAHAKNLLANGIPAKEASFLVGFRDQSYF
TKVFIKVAGVSPSRYLDLGR*
>SPBIB_v1_380022|ID:27164145| TRAP dicarboxylate transporter, DctP subunit [Uncultured spirochete bib]
MKRMHKAALILMLILVPAMLFAQAKPIVLRLAETHPKGYPTELGDEEFARLVKERSGGRI
VIEVYPGSQLGEEKAVIEQVQFGAIDLTRVSISPLASFVPKLNAFQMPYLYRDSDHMWKV
LKGDIGKELLASLEPFGFIGLGWFDGGARSFYNSKKPVRTPADLKGLKIRVQESELMMGL
VRAFGAVPTPMPYGEVYSGLQTGVIDGAENNPPSYFSASHYEVAKYYTLDEHTMVPEIII
GSKISLGRLSKADQDLIKQAAFDAIDYQRAQWAAYVKLSMDKVIAAGCTIIPVPDKTEWI
KTVQPMYKTQSKEIQDLIARIQAVK*
>SPBIB_v1_380023|ID:27164146| conserved membrane protein of unknown function [Uncultured spirochete bib]
MKRTLITIVTLIHILLINLSKLMIVAMLLLVFANVVLRYVFNSGIYWSEEIALVLEVWFI
FLSLGLGVKHRLHISINLVKRESMPAWLNKALDLLADIVFIIVGAVMVIYGIRLTQFTMR
SIMPATKWPAGILYLVLPLSGFVVIVEALFHILGYNMFDEKIDAYLSGKGGKLGDIFRSE
S*
>SPBIB_v1_380024|ID:27164147| conserved membrane protein of unknown function [Uncultured spirochete bib]
MTDPTIATVILMGGFALLMVLRVPITFSLALASIATATYLNIPLMAIVQQMVQGVKSFSL
LAIPFFIIAGEMMGRGGIALKLINLANVLVGRIRGGLAMVNVVESMFFGGISGSAVADVA
STGTIMIPLMEKSGYDDDFSVAITVTSATQGIIIPPSHNMIIYSVAAGGVSVGRLFLAGF
VPGVTLGIALMIISYIISIKRNYPKEKKYSFKEAVKIFGDAFLGLMTAVIIMGGVVSGVF

TATESAAVAAIYAFIITFFVYKEIPLKEFKGILYSTLKTLTMVMALIASASAFSWLMAYL
RIPAIATEFLISITNNKILLLMLINVLLLLLGMIMDMAPLILIVTPILYPVVVGKLGLSP
IHFGIILMINLAIGLCTPPVGSALFVGCAVGKISIEKAAKAMIPFYFVMVIVLLLITYVP
QITMFVPNLIMPGG*
>SPBIB_v1_380025|ID:27164148| 3-oxoacyl-(Acyl-carrier-protein) reductase [Uncultured spirochete bib]
MSFVESLFGLQGKVVVVTGGGGALPSSMAIAFAKAGAKVALWGRGTHHPMSEAADEVKKH
AGLPAESRQVFGITVDTASTDACENAFDETVHSLGMPDILLNGVGGNMGRSSFVDFDEKV
FEEVLRLNLMAGLMVPSRVFARRWINAGIKGSMINMTSMASYRGLSAVWAYDAAKAGVLN
LTQALAKELAPYGIRVNAVAPGFFVGNQNRALLFDEKTGELTPRGKAIISRTPFGRFGEF
EELWGAVLYLASEKSAGFVTGVSIPVDGGYLSDNI*
>SPBIB_v1_380026|ID:27164149|uxaC| Uronate isomerase [Uncultured spirochete bib]
MAHFIDENFLLKNETAKRFFFGTAKDLPIIDYHCHLSPKAIAEDKPFDDIARMWLEGDHY
KWRIMRANGTPESLCAGDGPWEEKFVAYASALTKAAGNPLQQWSHLELRRIFGISEVLTP
HNALKIREEANRVLATKKISPRSLLEQFNVKVICTTDDPIDDLHWHKAIADEQRAGSAFQ
TKVLPAFRPDKAMNTADIAAWNTYVDALGKSAGIEIGSYAALAEALARRHAYFHAMGCRL
SDHALLVPVFMPASESELDAIVQRARNGTVLSASEQAKLMTAVLSHLARLNAQKNWTMQL
HIGALRNVNHQLFKAFGPDVGGDCTSDEPIIAPLAAFLGALDSERKLPKTILYSLDPAKN
NPLVALAVSFTGASPFESGAKGSSRASGTMQVQVPGKVQVGAAWWFNDQKDGMERHLKEY
ASVGLLGAWVGMLTDSRSFMSFPRHEYFRRLLCNTIGEWVEAGELPDDPLYTESVVRAIC
WENAASYFGML*
>SPBIB_v1_380027|ID:27164150|ugpE| glycerol-3-phosphate transporter subunit ; membrane component of ABC superfamily [Uncultured spirochete bib]
MAELTHNSNKAAHRAKLIRRTFPHIMLWIGIAIIVFPIYFIFSASSHTSAEILAAPMPLN
IGPHLIENYRQALFEGTKNMGTPVLTMMKNSLIMALGISIGKIVVSLMAAFAIVYFKFPL
RNFCFWTIFITLMLPVEVRIMPTYKVISDLGMLNTYAGLILPMIVSATAVFLFRQFFMSI
PREIAEASQIDGATPMEFFSHILVPMTRTPVAAMFVIQFIYGWNQYLWPLLITTKPEFYT
LLIGINRMMSGADVQIEWQIAMATTLLAMLPPVLVVVIMQKQFIAGMTETEK*
>SPBIB_v1_380028|ID:27164151|ugpA| glycerol-3-phosphate transporter subunit ; membrane component of ABC superfamily [Uncultured spirochete bib]
MSKQYEFKAPILPYILVLPQMVIVFIFFFWPAAQAILQSFYLQDPFGGRMIFVGFQNYSR
LLADPDYWQSFTVSTAFAVCITVGAMTISLFLAIQANKKIRFASFYKTMLIWPYAVSTLV
AGAIWLFMFNPNVGVIAYMLKHTFHVDWQYLLNFNQAFLLLTLAATWKQLAYNFVFFLAG
LQSVPATLIEAAEIDGASPSTRFWKIIFPMLSPTTFYLLVMNLVYGFFETFPIIHQITQG
APGKSTGILVYKVWRDGVINLDLGSSAAQSVILMIMVILLTVFQFRFIERRVTY*
>SPBIB_v1_380029|ID:27164152|ugpB| glycerol-3-phosphate transporter subunit ; periplasmic-binding component of ABC superfamily [Uncultured spirochete bib]
MKRVILLMAVLLMAVGAYAQTTVEFWHAMTGHNGEMVQVIADKFNASQKDYKVVPVYKGS
YVDTMNAGIAAFRAGNPPAIIQVYEVGTATMMAAKNAIKPVYQLMAENNMKWDPNMYIPT
IKSYYSTSDGRMLSMPFNSSTAVMYYNKDAFRKAGLDPEKPPVTWPEFFEVAKKLKASGM
EGGFTTNWISWIQLENFSAWHNLPFGTRSNGFDGLDTQLVFNSPIHVRHFQNMYDLSKTG
VFIYGGRENKANPLFTSGRVGLHFESIGGYGNMKANCKFEFGVARLPYYPDVPGAPQNSI
IGGASLWVFNGRPQAEYKGVAEFFNFISQPEIQSLWHKETGYLPITNAAYELTKSEGYYK
TNPGLEVAIKQLLNKAPTANSMGIRFGNFQIIREIEDQVWEDILAGKISVKDGLDKMVRD
GNKTLREFEKLYK*
>SPBIB_v1_380030|ID:27164153| Glycerophosphoryl diester phosphodiesterase [Uncultured spirochete bib]
LPEKARSSTIKLMKHCIACILAFLLSFASVSFAQPDRPMQANLSMEKIIMQTSRPLVIAH
RGFRGIAPENTLVSAQKGFDAGADMWELDVAASSEGELVVIHDNTLVRTTNAASIFPKRS
PWTVYDFSLAELKSLDAGSWYALTDPFKQIKSGRVAAKELQAFAGLQIPTLREALELTKR
LGWRVNIEIKDATGFACDAWIVEKTAALIRELDMVPSVLVSSFNHEYLRRMKKTAPEIAV
AALIDKPIEDPVSVLKEIGAVALNPNAKYLDEATVRTVRAAGFGVLPWTVNDPADMKRLI
QWGVTGIITDFPDEGLKQVAGHL*
>SPBIB_v1_380031|ID:27164154| MutT/NUDIX family protein [Uncultured spirochete bib]
MIAGDGNGARKWAMLSSRKVYECNVFSVFERESRGPDGRVGRFAVMEAKDWAVVVPYVRT
ENGTSFLMVRQYRHGADEVSLEFPGGVVEPGEEPAHAAARELAEETGWVSARILHAGTVF

PNPAIQDNHFHVFVALDPEPKVTRNLDSNEIIDAHLMPADEIRQKMGTGELRHALMVTAL
FLADKCVAALDEKPARQAES*
>SPBIB_v1_380032|ID:27164155| Fumarate hydratase class I, aerobic [Uncultured spirochete bib]
MPHVSIEQFISFGPESEFEPLALPRPLQANGRIILEPESIRECAKLAFSRIAYKMPRSQL
AAFSAVLESPESSVNEKFVAASLLENARIAAKGTYPICQDTGTALVYGWRGDQVDIVHNE
EIEDLLAGGTAEAYAQESLRNSQLGPISMIEERNTRDNLPAGIQIRSVRGDSLALVFVAK
GGGSTSRTSLTMESPAILRQDRLEETLRKRIQSLGPSGCPPYTIAMVLGGTSPSETLYAA
ELAALGLLDNLPQSAGDDGTPLRDRFWEEKMLEIAKESGIGAQWGGVHLAAATRAIRLSR
HAANLPLAVAVSCAANRHIAVIAGPKGWAIQKLAHYAKDSYPSFETLRKDVFDAAPHIEL
SKSSQDWLAQLRSFDAGTIVTLSGPVIVARDAAHARLLKILEKEKHLPDWFLGLPVFYAG
PTEAKPGHATGAFGPTTASRMDSYLEPFMNAGGSVVTIAKGSRGAEAKKAIIAHQGVYCA
AIGGAAALNAAHHIRSLSIIQFEDLGMEAVRAATLETLPVIIAIDARGNDIYSKE*
>SPBIB_v1_380033|ID:27164156| conserved protein of unknown function [Uncultured spirochete bib]
MDATHADVALLRCEDYDRTKLDAIIAQAAEIAGFPDVRGATILVKPNVLNASPAAKAVTT
HPEFVGAVIRFALSQGAKEVLVGDSPGWQPGALAAKTSGIYDAVKQNGGKWVDFREASPH
AVNHGKRLRNIPLTTWLEHVDIVINLPKLKNHRLMTYTGAMKNLFGLIPGTAKSAMHMQY
PGVMEFGEMLVDLALSIPRCFTFMDGIVAMQGEGPGSGTPYSLGIVLASQSIAKLDWIAA
RCVGYDPALIPYLADGLRRTLQSEAIIEPAISPLSVQDVSHEGFELLPYHSELGRKLGAI
PNAMRAFAGSLIRLRPVFHTDKCIGCGACVQICPANALALDSKNKTHIVRIDDKLCITCF
CCHEVCPAKAISIGRVPMRLWHRAKRS*
>SPBIB_v1_380034|ID:27164157| protein of unknown function [Uncultured spirochete bib]
MRYILVERVPRRMAIRLDRNNWVYYIGRGSNLRILLFSIGCMLFLLGSSFRPAPGPLSAR
GGSDATPFAASEDIVMPEPEDKLVKYTVYEVKRGDTLSGIAAQFDVSLDTLISINAFTSA
KALKPGQLLKVPNQSGIVHIPTKDATVQEIAAAYNISADRIIDANGLLTENIHAGRPLFL
PDVRLPAAQLREIAGTLFRWPVSGRITSWFGWRKDPFTGRRSFHNAIDIAAPYGSPIKAP
MDGRVIETGYSPILGKYVMMSHSGGWKTLYGHMSEIVVQEGQYVSQGRTLGRIGTTGYST
GPHVHFEVIKNGSLVNPLNYLP*
>SPBIB_v1_380035|ID:27164158|ptsI| Phosphoenolpyruvate-protein phosphotransferase [Uncultured spirochete bib]
MRITKGIAASPGVSIAPAFLFIDDSADIPQYHITTADIPAELDRFHEASLLAKREIEALR
DRAKLEAGEEQASIFDAHLMMLEDPEVLDQIERSLNENLFNVESAILSFETEMVDKLSSS
PDPLIQERVSDVHDVIRRILGHLLKKERISLADLETEVILVAKDLLPSDMVGMSRSMIKG
IVTESGGRTSHAAILARAFEIPAVLGVGPNFVEQVKPGQSIYVDGDKGVVAIDPDEIFIQ
REKAARVARIQREKENRELRSIPAQTKDGTRISLVANIEMPDEVANVIEYGAEGIGLFRS
EFLFLGGHVPGEEEQYKAYSKVVQAMEGKPVTIRTLDIGGDKVLPELGAMGEKNPLLGWR
AIRFCLEKTELFKTQLRAILRASIHGKIKIMFPMISTIDELIRAQGLLAEAKWECKAHGY
EIDPNIETGIMIEVPSAAICADVLARSCDFFSIGTNDLSQYTLAVDRGNQKVAYLNEPYS
LAVLRLIRMTIGYGEHAHIGVSLCGEMAADPASAVLLVGMGLRSLSMSASAIPAVKRAIM
SVTIEQATALAHQALSMNNSAQVSALLASKLSL*
>SPBIB_v1_380036|ID:27164159|der| GTPase Der [Uncultured spirochete bib]
MNKTSQEDRQLKKPKSIKRKASADSSAPKRKQTSAPAASQTNEEAALPAFRYENLPLVVL
AGRPNVGKSTLFNRLLGKRRAITDPTPGVTRDPIEEECELRGTDKKVIVVDTGGFKIERE
GLDELVVARTLAYIERANLVLFMVDAMEITPEDEEFAAFLRPYAKKLILVVNKADSPERD
ALVWTHAKWGFDPVLFISAEHNRNIDELSEAIAARLDFTSVRAVEIEKADIRIAIMGKPN
TGKSTLLNALLGQERSIVSSEPGTTRDIVEGRFTWKGRGITVLDTAGIRRKKKVTDNVEY
YSVTRSIAVVNRADIVILLIDAEEGLSDQDKKIAAFAVEAGRPVVFALSKWDKMPKMKNA
FEAARDRLRFFFGQMAFAPVVAISAKEGEGIDKLMSTIISLYSKANRAIETAKLNQAVAR
WIEETPPPAGPRTRFKLRYAVQASMNPQRFIFFVTRPEAVAESYRSFLKNKLRLEFGLDG
IPIQLELRASHKDRMRS*
>SPBIB_v1_380037|ID:27164160| protein of unknown function [Uncultured spirochete bib]
MPYLSIGQLERLISVPASTLRFWEKTVPFLTPMRSKSGRRTYSLSEAALIARLKHLALDR
KFGLRQAQQLLEFELLYGDQDMRAQINALRDEILLLVLDCEKWREEFEEIRAAITSEKEF
FNGPENQALDGIH*
>SPBIB_v1_380038|ID:27164161|apeB| M18 family aminopeptidase [Uncultured spirochete bib]
MDQKIKRLMEFIDASPTPYHAVDNLIGMLDAAGAQRLDERQVWQLEPGAMYYVSRSGNSL

IAFRMGLRFSGDTGFLLAGAHTDSPSLKLRPEKQLKSKGYLRLALESYGSPILSGWLDRP
LGVAGNVAVREGSDIHSRLFVSDKPFAVIPNLAIHLNREINKGFEYNLHQQMPAVFGLAR
AENQEESQAGGSHAEREISEILERIGEQMAVDPANIVSADLRLFDYGPSQIIGEELINAP
RLDDLAGCLAVMEAFSASEAQEATQLACFFDAEEIGSMTPGGAQSAYLRDILARIVLASR
NSGQDFYRALANSACISVDASQAWHPGYPEKFDEFYTPVLGKGIAVKSNANMNYATDFTS
LQIAETAAAKAHLQIQRYMARADIQPGKTIGPITASRTGISTVDVGHPMLAMHAIRETIA
ASDHLDMIALLRAFYS*
>SPBIB_v1_380039|ID:27164162| exported protein of unknown function [Uncultured spirochete bib]
MKFLRRFLPLLVVVALIFASCATNTGQVPTGTETPPTVTQGGQPQPQTPVPQEPSAEKYE
LQNLFAQANTLKTEATDFALGSVLPDKFLEANREHDAVSAEYKDLVETPATYDGVKAYPL
KEKLEHLVSTWDSLIQEGMPLRAGVEKDSADEMRFAAMNVDAPIQAPTQYDAGLQYYSQA
AMFADASNYAQAISTYQFAAAAFGNSAAAAQVNSLRSDIEKNDYGKYESIQMYFTMGEEK
YKEAQDLWAKGTLDDIAASTAALKEAQTYYSFVDTKGAELRAFEGKDKAASAQQEALSVK
ADINAPDEYQQALDILAEAEKNMADSSYVSAYSWYLDAASAFGTAHDAATARGPEVAEAI
AKAESNLADAKDRASELGLEENIYLTAAAEHLGKAKALKEELQYPDAIFETNEVANYIGL
SDNLVQQEAQIREKARIEQLKKDKAAADQAIADAKTRIAWADQVKLKDDYPKEYASASSS
MAAAEKAYEIEKYVPAKTLAEQVSSTLSDEFQQQVLASREAAKAKAAQAEEEARLKAANK
QAADVAISDAEARMAWANENSVRTDYPDEYKSASVAMVGAYVAYGNEDYVVSKQKAEEVS
SILSNDFQAQVAADRAAKAQLARDKAEADQVLPQARARMAWANQANIKADYPTEYNSASA
AMTAAEKAYQAEKYAPATTLAKEVLSTLSPDFEAKVTSERAAKQMQDEIAKAGKAADALG
KAHSRMAWANQVNLVADYPDEYRDASTSMQAADTAYSENKYDATTSLANDVIRILSDDFI
AQVNAEQAAKAQLARDKAEAEAVMAQAQARMAWADQANIKTDYPTEYNSASAAMTASEKA
YEVERYAPATTLAKEVLSTLSADFEARVAVERAAKENQAKIAQNQKDAEAALGQAKDRYD
WATSKNAKNNYPDLYKQGGEALADANTAYNAADYVRAKNLANQAYWTLMQIDEFAPLPAT
YKVRLIPERRDCLWRIAEYPFVYNNPYKWPVLYEANKKTFKDPSNPNLIFPGQILKIPSI
KGEVRQGEWDAKKTYQPLAK*
>SPBIB_v1_380040|ID:27164163| protein of unknown function [Uncultured spirochete bib]
MRANAICRPHISGRKHIRAAFIGAAALAWLMLWDGCSYSHSFPAGFVRFAPQLDAWENRS
EGFCLVLVSSSDRAQGFLEWNSQGIYGRIVQAQKNGNTVAITLGDGGMNGLQLSIFLKRK
SIIAEYFRKDKSALQPFRIEAWHVSSEEGMRVLGATFVEKLPNEGALSLRLQYVSAQDAA
RSRILDEILRRGMSPYNMAELRLAQQKEAAVAVQNRAPPPQEYEEIEYPVFISDQYLSIA
TQHYLFDGGAHGAASTTFDIIDRAEGKRLSAQDIFAGDWKAGLKLLLAAELMRQSAFWGN
SGQEADLKALGLFESELYPSDDIFVCAGGVGFQYDRYQIAPWYMGEFIIILPWNELTSYL
SPAFKIPQ*
>SPBIB_v1_380041|ID:27164164|ileS| Isoleucine--tRNA ligase [Uncultured spirochete bib]
VYKPVDPKVNFPQQEESVVQFWEERNVFKKSISQREGAPEFVFYDGPPFATGLPHFGHFV
PGTIKDIVPRYKTMRGYRVERRFGWDCHGLPVENLIEKELGLNSKTDIERYGVAEFNEAC
HSSVLRYVKEWRQIVTRLGRWVDFDNDYKTMDPDYMESIWWVIKSLWDKGLIYEGYYILP
YCPRCSTVLSNHELSLGGYKDVHDPAISVRFKIVGVLASNKDPALADLANGSTYFVAWTT
TPWTLPSNLALALGPDIDYVCVIDGKERYILAESRLSAYYKHPESLQIAWKKKGHELAGI
QYEPLFPYFADLSAKGAFVTVLGEHVTTEDGSGIVHTAPGFGEDDYEVLKGTGIPTVCPV
DAECKFTDEVPDYKGIFVKDSDKAIMERLKQEGKLIKRDQILHAYPHCWRCGSPLIYRAV
SSWFVKIEPIKEAMLRANKSIYWLPAHIRDGRFGKWLENARDWAISRNRYWGNPLPIWKC
DTCGKTICVGSRKELAELGGDVPEDLHKHHIDKVTLPCSCGGTMHRIPEVLDCWFESGAM
PYAQNHYPFENKEKFDEHFPADFICEGLDQTRGWFYTLTILAAALFNKPAFKACVVNGLV
LAEDGKKMSKSMRNYTDPSEVMDKFGADALRLFLMNSAVTKAEDLCYSDDGVKEVLKSVI
IPLWNAYSFFVTYANIDGFTADGFDCTDVENPLDKWILSVCEKLVADVTAGMEAYDMQAA
IGPMVEFVDSLNNWYIRRSRRRFWKSENDADKKSAYRTLYRVLRRLTLVMAPVIPFVTET
IYQNLKLENEPESIHLANWPTEEPQYRNLELERDMRIVRQAVSMGRALRVANDLKIRQPL
ASVQLITRQPEEQEVLARMEDILREELNVKSVFVHEKEEDLVEYHAKANFRVLGKELGAE
MKEAAAKIAALDTSAIKRILVGEQVPLTLASGRTVSLDASKVEIVREEKPGLKVLNEGTL
TVALDTTINPELLYEGYVRDLIRGIQNARKEAGLEVTDRIKLALFGDDELHTALERFSQL
VAEETLAVGIEWTKPKDGITVEAGEKSWTVSLQKA*
>SPBIB_v1_380042|ID:27164165| exported protein of unknown function [Uncultured spirochete bib]

VKCQGVRRMLLCQPTPNLFSCPAPSLVLCLVVGLLAAANPMAANAQSASLVFTTATGALA
GIAAPGSIAQGGFDEEIALKGRMNTGPVRASALISASLSSGYAALARWYGSSSTQTMRFS
WPGWTKGALAPDVLAAIEIQELALAVSAGHLRFEAGKFPLKWGVGKASRPSDIFRTMDYT
SLIPVSKNLPAVRFSIFPSSMSRIEALASIDGDSTITAGARYFTSISMGGSANGGLTGGL
SGGASSPFFVSEVGSFALSAGWRRKQGSADEFAGSFELQVDAGPFAPYTEFSARTTDSTA
YALAMLGTSLAISDLSLTMEAQTALGQSSAGTQLFFLGTWKASDLTSISFPVFWLADAHI
LAASSIAQFQGILGGRLDFSATASWIFAGSPASFLWKIGAYWTRSLSTY*
>SPBIB_v1_380043|ID:27164166| exported protein of unknown function [Uncultured spirochete bib]
MKKASNYFVIISRFAKVFLFAAGALLLMAQAVMAQGTPGQDGGAAAAGSTAGSSREADAL
LEAIDSAQRYSSISYIGLMEITQGSRVLKKQFIANARGSDKVILEFTNPEDKGTRMLRLG
STIWMYFPSEGETVRISGSLLRQGLMGSNLSYEEVAEGESLREAYSAQIIKEEMLDGRLC
KLLKLVSSRTDISYPVRILWVDASRKIPLKIELYARSGILMKTMYVKEIEAIAGRQLPTK
VEIVDALKKNSKTVFSIQSVKIDVAMPDSMFSMEALTK*
>SPBIB_v1_380044|ID:27164167| membrane protein of unknown function [Uncultured spirochete bib]
MNLISYAWRNLLRNTRRSLLAVLSTFLATLLVVAGNGLTEGFLDSMVRNYAKNETGHVNI
TTSSYRERARFLPIDDYIADADQLVSEIEQMDGADAGAASAGSGITAAPRIRFSVLLSSG
LFTKPAVAIVGDPEKEKSLLMLNRNILPGGSYCDEPGTAIIGSGLAKDLGLKPGDKLKII
ATRADGGIGFKSLKVSGIFQTGTNALDGSIFQMRLDDAQNMLGMPGGAQQILVMLPDYRL
ADAWQKKIGAVLAAHGQGQLSVLPWSAIGDYPKLILMTSSIYMAMWFFIALLGAFIIANI
MTMVVLERRRETGILMAMGMPPRRIILSFLLEGTMMGFGGSVLGTAAGIAFNAAFSRKGF
DLSSALAGFSWPIDNIIKPTISVSGVLAGILFCTVAAAIMSLLPSWRASRMEVVDALKAV
Q*
>SPBIB_v1_380045|ID:27164168| membrane protein of unknown function [Uncultured spirochete bib]
MKASSSVLFRIAARNVLRHRRRTLITAIVLTVGIAVFIMFQSLLAGMDRVTIDTAIDYDS
GSISLRSPGSDPLFKNIAIKEPKAIKDRLPALLPKGSTWTQRTRFYVQVSNWIDETPALA
FAVEPEKDKEVFKTSSTVQSGAWVSPAKAVIGLDLAKDLGVRIGDLIAVTAILPDGSLNA
IELEVGGIADLPLFSLSQQAIYLSAQDAEALVGAPLPVTEIDIRIPPASRLDTLVAQSDA
AASALEKAFSGIEAMSIGEAMRDYLAMRNMKSKFAYIVVVIVLLISAVGIFNTVLMSMYS
RIREIGVLAAYGLDPGQIKRLFSLEGMLIGIIGSAGGVLLGAVFVWWLTSRGIAFGGMFG
NLDLGNLPKNLLIKGEWNPLAFIQSFSFGVLVSWLASLMPARKASHIEVTEALRFV*
>SPBIB_v1_380046|ID:27164169| conserved protein of unknown function [Uncultured spirochete bib]
MAEIILSLSDVTKIYGKGETEVIALKDIDLVFEQGEFAAVVGPSGSGKTTLLNIIGCLDS
PTRGELSWRGAPMKSMNRTQLAAYRRMHVSFVFQSYNLIPVLTVAENVEMPLLIEGMHDR
TAIRERALSIVEMVGLSDKANRYPTELSGGQEQRVAIARALVKKPSIVLADEPTANLDSH
TAGEIIKLMREINEKTQTTFLFSTHDPRIMELAQRVITLRDGQIERDERGAP*
>SPBIB_v1_380047|ID:27164170| Transcriptional regulator, ArsR family [Uncultured spirochete bib]
MNLPDLDKIIHERARLRILASLSSQPARTCTFTELRDNLDMTAGNLSSQLSVLESSGYIL
IEKKFKGKKPLTEIKLTELGNEAFRNYLETLDSMIAALKPHT*
>SPBIB_v1_380048|ID:27164171| membrane protein of unknown function [Uncultured spirochete bib]
MNISDQKLKEFEAALREIREAASHEAGIFHDKDLRKTFIWLSLGFSIVIVAFCIAGHFLI
SESGTSGTSSAGIIFWTFAIMLAVGAAIKITLLSRIMSRKNKSIASLLRVIYGKGPASVI
IAALLAVVVICVFLVTEGLGPLAVSMSAIFAAFAVFALDIRIQLPEFSALGWTLLALGLV
SLFFIRGSPWLWGGIVWGGSFVSLGIAGIVASRAS*
>SPBIB_v1_380049|ID:27164172| protein of unknown function [Uncultured spirochete bib]
MRTEGVDIDFRFLEDVQSTENIRRIVIRVSEGDVSVAVSNDSDLRYSIELEGIPGEIDAW
SCSVRRAESIAILDAMANDFVRIAKCNIHVPSYISDIEVHSSRGSIEVRDISANILVISE
RGSITVSGAKFVEASSVNGSIHIENSEGCSARSIDGNLVCHRISGSVQAEMQNGSVTIDQ
VAKNVAVVSDQGKISVRRVGGRVRLISNKGDVELEVSGPFGGGEIQTYSGDISIQLEHSS
VEFRAETLSGRIDASHPVNSAGMGPQRCAFRTGDGARRLYVKSVLGDIEVN*
>SPBIB_v1_380050|ID:27164173| Long-chain-fatty-acid--CoA ligase [Uncultured spirochete bib]
MADTIPQMFIEVTSKQPDVNVQLSKDSQGKFQPTTYQTLLREASICALGLRNLGIQRGDR
IGFISDNRKEWLIADLAILGLGAADVPRGCDATAQEIAYILSWSECAFAILENDRQLQKI
LDIKASRLPFGSQSLTEAPYAHLKTIILFDPPTSAISEEAARNGLQVLLFSDIMDTGRSL
YATDPDFYTKEAKAGARGDLATIIYTSGTTGDPKGVMLSHGNFLHQTEYLPSIIGVKSGE

IFLSVLPVWHSFERVVQYIILQAGATIAYSKPVGSILLADMQAVQPHWFTSVPRIWESIK
DSVYKSVRQSSAVKRAMFSVFIAIGEAHAYFRNMLLDRLPKFSPRSRAVDIGVAVIPFVL
LSPLRALGDVLVFKKIKEKLGKRFIAGVSGGGALPPSVDKFFDALGILILEGYGLTETAP
VLGVRLKNHPVSGTVGPIHRGTEIRIVDEHGNALPPGHKGVIQVRGPQVMLGYYKRPDLT
AKILSQDGWLDTGDLGMLTVHGEIKIVGRAKDTIVLRGGENIEPVPIEQRLGESAFIQQA
VVLGQDQKYLAALIVPRQEALIAWAEENEIPFNDYPSLLDQPEVKELIDSEINSFVSMKN
GFKPFERIFRFALLPDPFEPGRELSAKQEMKRFTINELYKKQIRELFAE*
>SPBIB_v1_380051|ID:27164174| putative tRNA-dihydrouridine synthase [Uncultured spirochete bib]
MVPFPRGSLFLAPMVGITNRAFRTLVYELGAPDWFVTEMASAEAFLSGGRNESIYLDPSP
RSLQTSVQFAARSPETLAKACNAVSQLPAEYRPAGIDINMGCSAPHIKGYGRGAALLDNP
SLAREMVAAAREQWVGMLSAKIRISTSLGEDGTIALAQSLASAGLDCITVHARLDTQKFR
RKADHRFAVLLAEVLPIPVIANGDITNREECAALLDSTHIHSLMIGRAAVQKPWLFASLK
SGAGAGEGERGTPGSSEPGAEPQYGTRHMPEIDLLAIALRFIDLAETLLPPEWQRETCRR
VFSYYAANVSFAHHLRFSLVNAPSLGAMRQILRDYFNQVPQDRIFAQDQILA*
>SPBIB_v1_380052|ID:27164175|rpsP| 30S ribosomal subunit protein S16 [Uncultured spirochete bib]
VSVRIRLKRFGAKARPFYRIVVMDSRSARDSKTIDELGYYHPIETEDKQLLINETKVRDW
LSKGALPSDTVKSLLNKKGIQLG*
>SPBIB_v1_380053|ID:27164176| conserved protein of unknown function [Uncultured spirochete bib]
MERDLVEYIVKSIVDNPDAVNIKVIEGEKSTILELRVAESDIGKVIGKNGRIARAIRTIL
SAAASSQSKRVILEILD*
>SPBIB_v1_380054|ID:27164177| putative Ribosome maturation factor RimM [Uncultured spirochete bib]
MDKKQKAEYLAVARLGAVRGLEGEIKLHSYSGEFSHIEKAGEIMLGGEAGLEDARPIRIV
QFLQGGWGASVIFEGFDTPEKARTLVGRELFLPREKACPLRPHEYYVADLVGMTATVNGL
AIGVIAAVLDGGADALLEVKRDSGGSALVPFRKEFVGEIDEEKGELEILSSWILE*
>SPBIB_v1_380055|ID:27164178|trmD| tRNA (guanine-1-)-methyltransferase [Uncultured spirochete bib]
MNIKVLSLFPEILEAYFSASIMKRAVGKGLVSFELVNIRDYAYDKHRKCDDEVFGGGAGM
LLKPEPLDRALAAAGSPEVRTVYVTPSGRLFNQAMAADFAKEENLVILCGRYEGIDQRII
DTRVTDVVSIGDYVLSSGEVAAMVVIDAVYRLVPGVISGESLSEESFSAGLLEYPQYTRP
EEYGNMHVPEVLVSGNHAAIARWRLRASLIKTLAYRPELLQTIDLTGEIGRLLKEVIDGG
GENEPRAANSGGTGKN*
>SPBIB_v1_380056|ID:27164179|rplS| 50S ribosomal subunit protein L19 [Uncultured spirochete bib]
MNLVQQIQAEQARTDLPAFRVGDTVRVSFKIVEGRIERIQAYEGLVIAIKNSGASKTFTV
RKNSYGVGVERVFPLNSPRVDKVEIVKTGKVRRAKLYYIRTKVGKKSKVKTLVGGRKSDK
PEA*
>SPBIB_v1_380057|ID:27164180| conserved protein of unknown function [Uncultured spirochete bib]
MFALQKCAVQKGRDGEDEAVRFLLKSGWEVIYRNFRSRRGEIDIIALRDGILAFMEVKIA
NRFTQEDLQHVIRAKKRRSIIETSKLFLAMNRKYSQYHIRYDVILIAENACARHMEGAFA
ENDEAE*
>SPBIB_v1_380058|ID:27164181| protein of unknown function [Uncultured spirochete bib]
MKQNKVSRDLPGWAGSTPDDFLLADYDIAEEMKASSWKRLYELKHKLIDDHYLDGAIASI
AEMLSNRVKGKSR*
>SPBIB_v1_380059|ID:27164182| conserved protein of unknown function [Uncultured spirochete bib]
MNNEPNASPSKRPSGRFRSHSFRKKRPAKPSSPSLEPTESQPDPQAERVKKQELKICKVC
GKPIFDLAGAIASRDDGEPIHFDCAIEILSKEETLASGEKIFYIGSGSFAVCYQAPGGKL
EIRRKIRWETAGSTQPWRKPMVSSPRLP*
>SPBIB_v1_380060|ID:27164183|serS| Serine--tRNA ligase [Uncultured spirochete bib]
MLDIRFIKENLEAVRQNIRDRYMHADADLVVSLYDRRNALLRELEDARRLRNENAQAMKS
PMSAEDRAARIEEGKRLKETIARLETELQEAEASFAVEMAKIPNMAHPDAPRGKEDKDNL
EVKRVGSPTQFDFPPKDHVELGQSLDIIDFDTATRVSGTKFYYLKNEAVILELALVRYAL
DILSRHGFTLMATPDIAKTEILEGIGFNPRGAESNVYTLEGEDTCLIGTAEITLGGYHSG
TILDKSQLPIRYAGISHCFRREAGAAGQFSKGLYRVHQFTKVEMFVYCLPEDSGRIHEEL
RAIEEEIFSSLGVPFRVVDTCTGDLGAPAYRKWDLEAWMPGRNNGEWGEITSTSNCTDYQ
ARRLNVRYRDDDGKNRFVHMLNGTAIACSRAIISILENFQQADGSVRIPDALVPYCGFDV
IRPRKK*

>SPBIB_v1_380061|ID:27164184|prfA| peptide chain release factor RF-1 [Uncultured spirochete bib]
VTERIDQMRARIAEIDELISRADMMRDQQKYRELMKERARLAEVVATFDETQSLAVSLHD
TEALLEGETDPELRQLIEEELGQLRERFIRIEEKLKTLVMPRDPFADKAVIMEIRAGTGG
EEAALFAADLYRMYSRYADSRRWTLEVMSMSETELGGIKEIIFSISGEAAYERLRYESGV
HRVQRVPATESSGRIHTSAVTVAVLPEMEETEIEIKPDDLRIDVMRASGPGGQSVNTTDS
AVRITHLPTGIVVHCQDEKSQIKNKAKALRILRARLYELEDQKRQAARSAERRSQVGTGD
RSERIRTYNFPQNRLTDHRIDLTLYKLDLVMEGDIDELLDALVAWGKQQAFESEGHALA*
>SPBIB_v1_380062|ID:27164185| putative Release factor glutamine methyltransferase [Uncultured spirochete bib]
MTVRELLLEAARTFSGSDTPFLDAVILLAHSLDVPKEKILAMLPEEACDVPASFFEMADR
RRRGESIAHIRGFREFYGRTFLVNDDVLSPRQDTEILVEAVLEAGDMCASMPNASRPLKV
LDLCTGTGAVAISIAAERPAWHITASDISLKALEAAQQNAHRLLALPSAVEFVASDLFSG
ILGQFDIIVSNPPYVPRAQAEQLISDGWKDPLVALDGGIDGMDFVRAIIAQASEFLYKNG
VVLLEMDPSQVSEAVALFKLAGFCEIRIWKDLAGRERVAGGRYG*
>SPBIB_v1_380063|ID:27164186| (P)ppGpp synthetase I, SpoT/RelA [Uncultured spirochete bib]
MDELAQNFLSKAEIRFTAEKRDILSSAVMYCQHSHGDKKRASGEPAFYHDLRVAEILLDL
GMDLESIIAGLLHDTVAERVSGYAPGQAAQAASRRKVPGASEPARTERSKSEKETGTLNS
LLAAPNPQIEEMYGTDTALIVAGVNRLSSVRAKNKSVHAAETMRKMLFALTSDIRVILVK
LADKLDSMRTLKYLPEDRQKEIATECIDIYAPLADRLGISWLKDELEDLSLKSLNREIFD
QIKDIVNARKEERQEFLKKVTEKILSSAAKENIEVEVSARAKHFYSIYQKMRKRGKSAEE
LYDLLGIRLICRSVNDCYSLLGLVHRLWKPIDGRFKDYIAMPKSNGYQSLHTTVIAYGGQ
LLEIQIRTREMHLVAEYGVASHWLYKKGSTAELPRLEDLPIVNKLKQWNQFIIEGESYLE
EIKRELLRDSIFVFTPKGDVIELPAGATPIDFAFAIHTDVGAHCLGAKVNGSIASLDSEL
KNTQIVEVITSPNAKPNINWLKIAHTSKARSKIRQLLVQTGQAVAIDKHIVAARKGEKTL
PETGQARPHAHERPSEAPHAKSEEFPSVVEFHSMQPGLVLRSEKAGVSVGGIRNMMIRIA
GCCRPVTGDRIVGYVSRGRGIIVHREDCRSLASIADFEERRIDVKWEDEAMNATARYRVT
TKKTFDIFSEIESTVRKCSGRLKEGKLGERGDGTLSGFFILEVDARDDLKRVAKALRTLP
SILSIEEEAL*
>SPBIB_v1_390001|ID:27164187| exported protein of unknown function [Uncultured spirochete bib]
VRQNQIYSIAIFLLISILAILPVAAEPQLKTLGNGMTVLLNPIESEENVAILLAYHAGAD
AQTAQTAGLFKLLELALFNGPAASPGTTEPAAAIDVLEPNRIAGGASLDRFEFGFSVSKE
KLVPALDTILYLFSQERREFLLAQEDGVENARQSARALIKDSTLDSNAIHDMAIAKKLFS
RAPFRLDVLGADYVLEKADAATLRTFAATWLVPNNAFIAIAGNFDTDQIMNLLDERFGQL
PKASNPWSAPLAAFPKPGVTRPLFLVFPDSTMPSGMMQVELRYRGPDPKETSSFAALRML
EELANTPSSRFFTAIRKGMPKGAEPKEVRIAYSPNRNASWLSIIASIPLPSGKKPTDIVF
SFKELARGTELYMIKSNPAYFSAAEYQSARQALLEKTMAIQGDPLDAAAHAAALWSWGIP
SFVFQESDAIMKTGQKELSQAVDTYVQKNLEVVTVRLNPDLYEANKKIFSSNGFETVTAQ
NAFWWR*
>SPBIB_v1_390002|ID:27164188| protein of unknown function [Uncultured spirochete bib]
MDYVRYCLASNLRLRRAILQMSQEELAQRADLSPGFIANIETGRNFPSSKAILKISAALK
IEPWKLFLDPQKQDLFFTRDEIFQWFEDSRKRLFGYPQNPEEEQKGKNRPEDSTSPED*
>SPBIB_v1_390003|ID:27164189| Peptidase M24 [Uncultured spirochete bib]
MFTSETYIKRRTAFRQSLYDRGILNGTLLLLANSECPRNYPSNCYPFRQDSTWKYLVGLD
VPGIAFTLDIDSGHSCIYADDPGIDSIIWTGGTPTRSQLASQAACDEAASFSTLSALLAR
ERAAKKPVFIFPPYRAEQVSALVALLGLPDASPADFLKEHINTSVIAALVSLREIKEPEE
IAEIERAVTITKAIHEDILHNFRAGWTEKQAANRVLSLASEYGCELAFATIATCKGAVLH
NAPTAYAATSNDTFLLDAGVEMPSGYAGDLTTTFPVGERFDVQSREIYEVLSRVFNEAIS
VLGPGVRFIDVHKRASVALAQGLTSLGIMKGDPEKAVQAGAHALFFPHGLGHQIGLDVHD
MEGLGEDIVGYGDELGRSDQFGLHALRLAKTLKPGMVHSVEPGIYFISELIERWRAQGIC
KDYIEYDNINKWMHVGGMRIEEDWCITESGARRLGPEFDKKVAAIEAARSGK*
>SPBIB_v1_390004|ID:27164190| protein of unknown function [Uncultured spirochete bib]
MKEVFACAFLEDAIVVKSMLESAGIQAELLSDQMLDINPFYSIEPKGAKIVVSDSDEADA
KVIVDSFREHTRSDN*
>SPBIB_v1_390005|ID:27164191| putative Metal dependent phosphohydrolase [Uncultured spirochete bib]
MKKKLLIPSIIVLLTAFAVAGVFLLMQLYIADKSFDTLDRALKSLIDLQASAAYNGYFAW

TKMYDLVMQGNLTEANALADEIPKSFPLVASAEIMQGVPPEENFVVGADGELLRIVYSIR
DDDEFRTAPNAVAVTIIQASKLLDIVSPNGFKIDMTGGRKTTYGIHVSANLMHISLWNII
AMVLLTILFASLLIQRYVKKTAFFLDTRGLESIIYLFEQTEKMSASHSRNVAVLALFLGK
KLGLRGKKLRNLYVAALLHDIGKIGVPTNVIAKAGRLDSKEYMQIKQHPVISARILKSFK
EFERLSPIVLYHHEREDGSGYPEGLTGKDIPLESKIIAVVDVFEALVGERPYRNPIHPFS
AFEKLKKMPLDQRIVKVLIDNYSELNSFQPPRWVLSYSPWLLGV*
>SPBIB_v1_390006|ID:27164192| conserved protein of unknown function [Uncultured spirochete bib]
MREIYRQLADAGILFFEYGVTALDAYYDEKPRPVRFVVAECSVIDLARCFSELQYPNLPY
ADAAFDTCEASLALRTSFAESGGAPAGAIQESSMQESSVRFLCTEDALHCEVGGSWANFR
RNPQSDIFYDQGNIYDDLKAKAIPSCNTDTDNALFESAALATFHPEGYRLAKEPIPLPAC
TSSYFQKDLLITVLTGPYPAAGLELLKKSGFIAREWPEIALLDDVDHAKDYHPEGNGWNH
TLQTFSYRKERDLVLSLALLLHDIGKPESLSREGKRFDQHAELGARRARKFLARLGFSTT
TQEDVAFLIRYHMMPAALSVLPIQKTEPVITDPRFPALLELFRCDEFSSYKDPEGYYEAC
NAYQTIRRNMKNPYRNADGSLKSRSKRTIAHY*
>SPBIB_v1_390007|ID:27164193| protein of unknown function [Uncultured spirochete bib]
MMQVDVEAFWANFEKEIGEKIISRTMGQHFSTRKSQGEWGLLVLSETALRFRPTPGENWF
DSLFRMAAPRVPKEPLADILVPLESIASIELPKKHFFDFLFSPPFTVFTLRYRIGNTEHD
LLLGADSKCELFRWLLSNLPVAKGT*
>SPBIB_v1_390008|ID:27164194| conserved membrane protein of unknown function [Uncultured spirochete bib]
MSRTLEREAGKCYSPAMSRARGIAAGIGYSTIFGFSFLVTKETLAVLSPVELLASRFFIA
ALLMSGLALAGAIRIDFRQKPLGLLLLMCLFQPVAYFIFETYGVANAATSVAGIILGALP
AGVAIVGAIVLKERLSALQYSGLAMSIIGVGLVALLGQGQNAETKPIGVVFLTASMLSAV
IFNIASRKASERFSPAERTFAMMWSGAISFGIPAALQNIAGKGNIAALFNTAGSRFVPSW
AGILYLGVLSSVLAFFLINYSLTYLKASQSAVFTNMVTVITVLAGVLIRHEAFSWNQGLS
ALLIVIGIAVANSAGKREIQERRS*
>SPBIB_v1_390009|ID:27164195| Amidohydrolase 2 [Uncultured spirochete bib]
MKNAAFYDVHCHLMNLSHPAFVSIIESLRHRPREVIYSQIASFEYLASSMLKRGGEKVRN
LLAVMDNDCADILFLMEDDLAGQFEDAKNAKFKEPAPLREGSLHIGELSFDSLVLTPLVM
DFSTPSTLTPDTYYKRPPRKPIEAQIVDIMYAIRRYRADRPQGFLRVYPFLGLNTKNYRA
EGLLAYLEKWFAGYKKDESEFERRFMSMSANAPDCTPESYGLFAGIKLYPPLGFDPWPDD
PAEREKVEILYSFAESKGIPITTHCDDQGYRIIPLEESLLFTAPARYKPALEKYPNLVLN
FAHFGKRYIRMIGGRAQEDWRAQIVDLVLAYPHVYTDFSFTAGEPSFYGELANLLDSLSQ
NEAEKLSHRILFGSDFMVNLFKVRSYRDYYELFSQSGLAEEMKLRFCSENPRVFLFGSGE
*
>SPBIB_v1_390010|ID:27164196| Aminotransferase class I and II [Uncultured spirochete bib]
MFEPFGLERFFAEHEFNARYLLCTSDCESMPISTLLELSDNPGHALESLTKTWLGYTESR
GSPILREKIAKLYKGLEAKSILVHSGAEEAILNLYMATIQPGDTVIVNWPCYQSLHEIPK
SLGAHVIKWEIQRSPARWFFDPVELEQLIESAEGPDRRHGPGASFGFGSGPSPSLNARTN
SGSARKFSPGPNPTTHVKMVVLNMPHNPTGALMTQEEFSRVVEICAKRGITLLVDEVYRL
LELDGREPLPAACEVYENAISLSVLSKAWGLAGLRIGWLATHRSDILDRVAAVKDYNSIC
ASAPSETLACIALDNSEKILARNKAICQSNLAHYRAFFDQHVELFSWIPPQGGSVAFPRL
SATAENAPWNEADKLAHELVNDTGALILPGSLYGEEYAAHFRIGLGRAAIPQGLDVFSKW
LENRRKI*
>SPBIB_v1_390011|ID:27164197| putative tRNA threonylcarbamoyladenosine dehydratase [Uncultured spirochete bib]
MSAMAMESPARFTDRTRIIVGENGLDALAKARVAVYGLGGVGAACALDLVRAGIGYLHVI
DFDIVEISNLNRLAIGFSRFVGMPKTEAFMQAAREINTDIEILAEKTFFTSETATKVIAR
ECEIHADCVDLLSPKVSLLAAMRTQNLVFISSMGTAGRLMPERLKIGLMSEVKGCPLARA
VRQRLAKLQIPLDFPVVWSDEPAIKPMPREGQRGIQGSAPFVPQAAGHFMASWIVRKILE
DTHV*
>SPBIB_v1_390012|ID:27164198| putative Hydrolase, TatD family [Uncultured spirochete bib]
MYCDAHIHLVDLDSREPGFARRPLPFPWRAAVVSHDPDEFAASEKMQALLSPTIAGFGVH
PQNPDMRNADFLAALCREKKIRFIGEAGFDFFGDGPLRSRTPEAMKLQTEAFVFQARLAA
FEGLPLVIHLRKAADVLMAHGRILADIPSVIFHCWPGRLEEANMLLKKGINAYFSFGTPL

LRDSSHAIESLIGLPLSRILSETDAPWQPPHGSAWTRLEDIATVVRKMSMVLKIEQSALE
SILEANFASAYLLNE*
>SPBIB_v1_390013|ID:27164199| protein of unknown function [Uncultured spirochete bib]
METILMDVRRFMRSILDLAHARSDVQVGSGRKADTHSKSASYRSIDTGSSVDVYGCRGRI
DIYSGKEAFEFYKKMSFDKMPPL*
>SPBIB_v1_390014|ID:27164200| Transcriptional regulator, PadR-like family [Uncultured spirochete bib]
MSAAEELYEKWKSQFRKGFLELCVLELLSKSGRSYGLAIMEQLNNAGVDVTEGTLYPLLM
RMTKDESIEAQWETPDVGHPRKYYVISSTGSALLGQMREEYERSAAARRTIMTGGHAE*
>SPBIB_v1_390015|ID:27164201| protein of unknown function [Uncultured spirochete bib]
MTRKEYIDILTQRLGGLDEASCADIILEIEDHIDGLVREHPEKSPEEIIEGLEPPESLAD
SLREAAGLGPYEPPHAEQPKSGQKAKKNVHITIDDIELEEAIRKAFDIARIFRRNRDGTF
KESQESQTSEDVHGSYEFGDSDLASVHEVSVRCRSSDIRVLVADTSLAVRTPGEEHPRLE
LRYDSTRGTFGISTAHGKSEPDLIELRIPSTVDSLLVSTISGDVQVLDRIGSLSIKTASG
DVDVAACAGDIEVRTASGDVSLAQCQENITVTTASGSVSIEADELCNAIEVSSASGDVLL
YYPELWDARVAVSTISGDIEHNGNATGRGTIVFGSGLAPVRITTVSGDISVRQMR*
>SPBIB_v1_390016|ID:27164202|cat| 4-hydroxybutyrate coenzyme A transferase [Uncultured spirochete bib]
MNLQDEYRTKLISIPEAVSKIKSDDNVVVAMCASEPQGCMGQFHTVADRVENVRVFSCLT
LQPYDFYMKPEMKGHFELASWFHAPGSRAALKNNTGTVTYVPNMLHRSATDFIFARKPHI
FYGTCTPPDKHGYVSLSLGITYEKDILEAADLVILEVNPLLPRTFGDTHLHISEVDFFVE
HEQIVPELPSPQPSETDLTIGRYIGELVEDGSTIQLGIGGIPNAAALALRDKKDLGVHTE
MIVDSMMELYEMGVITNRKKSFHKGKLVATFAMGSRKLYDWLDDNVAVEFMRGRWVNNPA
VVSQNSKMVSINTCLMVDLTGQVASESLGPVQYSGTGGQSDTAEGAVEGIDGKGKSIIAC
YSTAKGGKVSTIVPVLPEGTAVTLHRSLVDNVVTEFGIARLRGKTVRERARELIAVAHPD
FRAELTEKAKALGYL*
>SPBIB_v1_390017|ID:27164203| 3-oxoacyl-[acyl-carrier-protein] synthase, KASIII [Uncultured spirochete bib]
MAKAAIIGTGLYAPGQPIDNETLKKLTGVEFDSQRHEEKLGIKSRHIARLSGLKETTADF
AEHAARDALQAAKVDPKDVGLFIVATDTPEYISPATSILLQGRLQGGETGAKSFDVGASC
ASFIEALDAAARHLITDSSMRYALVVGVYNMPAYIRDGDAFGWSIFADGAGAVVLERQEQ
GQSGYIDGAFVTDGTQWNFVGVYAGGTKLPVTRERLDSGEYGLQLLQKLPGDRNVKLWPP
LVQRLLEKAKIPQTAVSHYLFTQINKSVIEQVMAVLGEPLHKTTMVMDRYGYTGSGCIPM
ALHEAVMAGRIKKGDLVVMVASGAGLAVGASLLKF*
>SPBIB_v1_390018|ID:27164204| putative Transcriptional regulator [Uncultured spirochete bib]
MGSDTRKALIESAASLFSARWYGIVSVAEICRSANLSNGAFYRHFKNKEEIFCAILDYVV
GQIESALAPLAQIEPNDRLRQFVRIIYDFSQNNTPLVRVFREGQYRLFEYERKLKDVYEK
AFAVVFDRKPSTAEYLFALGGLRFASIRRAFHQIPVQPEAIVSILHSGILKEQPINADRV
FSTSITPLPIELLPDSREQLLAEGRKLFGEKGYFETNIHEVASNAGLAIGSFYRYFESKE
AFYKEIIQSVGRDVRHFITLNLGNGLNRLERELRGLWLFILFLSMDRYCYNIVREAEFVL
PEEVRSYYDAFHRGYLKREDSTITCDTTTCIEFMLGVAHYLGIEVIFDKSPDNARQVIEE
IGYLYTHGLSGDERLHNARN*
>SPBIB_v1_390019|ID:27164205| Beta-ketoacyl-acyl-carrier-protein synthase III [Uncultured spirochete bib]
MHVGIAGIGLYIPEHQMTASDLAKATSVPEDVIALKFGIKTKPIAGQEEPTSYMGFMAAQ
AALEDAAIPAEDVDLVIWCGAQHKDYPCWLAGLNVANRLGAIRAWSFDMEAMCGSMMAAI
DVAKSLMLARDDLNTVLLVSGYRNNDLIDLSYPPTRFMMDIGSAGSACVLRKNLGRNVVL
ASAFKGDGSLSEMCVVPVLGSKAWPPKPEDAQHASFIVPDEQVFKQKLGEVTMPNFYAVI
RESLKRSGLSERDIDYLAILHFKRSAHLAVLQELGLRENQSTYLEDYGHLGQNDQLLSIK
LGLETRKIRDGSRIVMVGAGLGFVWASTVVVWGPVAADHA*
>SPBIB_v1_390020|ID:27164206| Leucine-, isoleucine-, valine-, threonine-, and alanine-binding protein [Uncultured spirochete bib]
MKRMMLVLAVLVLFAGSAFAQGTTKLEGTIKLGGIWTLADITGKQGSAAAQLAVDEINAA
GGVLGKKLELIVVDDEGKADKAAAAVEKLATVDKVDVFVGGMASGAELGKIPAFKKYGKV
VMSTGAAGSATVEKALGPSPESDFYFHLHPWDYNQGASYAEGWDAIQKKYPQIQIKKIFL
AYEEGAFGKSSWDATKVLFGSDKRYTIDGASFKSALLGGGDYTAVLEAAKAFKPDLFIWA
GYDADALPMLEQAKAMKFSPGIYLGAPPGWPIGFGSSKLARNVMLYGMWSPAINDINPVS
AKFYKNYIAKYREEPATYFAPLAYSAVYIVAEGIKAAGTTETGPLVKALEQTKYVSPLGE

VITFKPSNIIKHQGITRQKILQWQNGIQEVIWPFETATAEPVYPFPAWK*
>SPBIB_v1_390021|ID:27164207| High-affinity branched-chain amino acid transport system permease protein LivH [Uncultured spirochete bib]
MGPSLRRHNLNIQAVKMAIKQRKAIIFAISIATAVVLILWKPTVLIYGLQAAGLYAAVAI
PMGLVLGIVHIVNLSHGEFMMLASYATYTVCRALGVDPLIALIPAALVTALFGWVVFQLT
IRRALKAPELNQLILTFGLAIAFSQIINLIFTSQTYKLSLEYSSMSLDIGDLSFGVWSFV
FVAVAVIYAVGLQMFLTKTRTGKAALAVGQNPKGAAIVGIDVYRTYGLVFALAIGLVGAM
GALFLTKSAIFPGVGSPFTMKSFSLVAMAGIGNIPGILGASVLLGLAENFLRSFRGTRDW
AEIVYFVLIIAVILSRSLKGKKS*
>SPBIB_v1_390022|ID:27164208| Inner-membrane translocator [Uncultured spirochete bib]
MNRTLVRILLILIAVAAVALPAFAGDYPLQVARNIMLYMALAISWDMLLRSGQISFGIAG
LFGIGAYAAILGVVRAGMPTWLSIPFAAVFAALVAFLIGFVILRLRAMYFSIVTLALGEI
FRIIVHNLHDFTGGPEGVVMQQGVMFGGNASKLYWLVLAGLLVAIAASYWFEKSRIHFAL
TAIRNNEISAKSSGVDIFRWLLLAFVVTSAIQGMLGGIFVMSYGFAAPDVVFSADFTLLP
LAMALLGGVYSTRGPIFGAILLGLVAEWLKLKIPYGHLVVYGIMIIIVILFMPQGLKNLL
KPAAKRAEGRSA*
>SPBIB_v1_390023|ID:27164209| Branched-chain amino acid transport ATP-binding protein LivG [Uncultured spirochete bib]
MIKLRTTNLTKRFMGLVAVNNVSFSMEEGEILGIIGPNGAGKTTFINLISGIIMPSEGRI
EYKGQDITWMPAHERARIGIARTYQLIHPLENLTLIENVMVGSIFARGHSLKEGRRRAEN
LCHELGLTDLERDTSRLTILEVKKMEIARALANEPEVLFLDEVMAGLNSDETKELIAMVQ
KIAREKNLAVGVVEHVMGVIRELTHRVIVLEAGELIAEGKYEEVSRNPRVIEAYLGGGAA
*
>SPBIB_v1_390024|ID:27164210|livF| leucine/isoleucine/valine transporter subunit ; ATP-binding component of ABC superfamily [Uncultured spirochete bib]
MLKIQGLECGYGRMKVLEGINFEVGAESVGLFGPNGAGKTTLISAVMGMIKPWKGSIELE
GVRIGGAETFTIARMGVALVPQERELFPGMSVEDNLMLGAAYIPHAKDDIPNQLEKVFEL
FPILKERRTQYAGTMSGGQQRMLAIGRALMSKPRLLILDEPSLGLQPSIVAEVFEVLKSL
KSSISILVTEQNVRESLKAIDRGYVLENGRIVLEDSAEGLKTNPHVVKSYLGL*
>SPBIB_v1_390025|ID:27164211| putative hydrolase [Uncultured spirochete bib]
MASGFVAVNGIKLAYELSGTRGVGSADAPLVLLNGIAMSISHWKPLIAALPEGTRCLCHD
FRGQTLSEKPAGPYSLVMHADDLAALMDALHIERGHIVGTSYGSEVAMEFAIRYPERCAS
LVVIDGVSELDPVLDAAVVSWMETARTDARLFYKTMLPWTYSSGYIAAQKAILAAREEAV
AKLPPEWFKAFVELCKAFLDIDITPRLGAIACPTTVLVGEKDILKHKGFSEIIARNIPGA
MLHVIPDAGHAVVIEQPAVVAEEIWQAVS*
>SPBIB_v1_390026|ID:27164212| Putative hydrolase or acyltransferase of alpha/beta superfamily (fragment) [Uncultured spirochete bib]
MASGFVVVNAIAMFFVEAGVATTAGAVSMAGAAAMAGAPSGAGADPWVGVPVVYVHGNTG
SSIWFSLVMNVPGCHVVAFDMPNFGQSSPLEGDISIQRYAAYVAGFMDAKGLKGAVVVGH
SLGGCVVQALALERPDLVKAMVLVDSGAPNGLITPKDRHPAIELMRTNRAVLEQALRAVV
PTLKNDELFKAIVDDAQKMAQPAWIGNAEALSHFDITARCAEYKGPVLVIRGALDPIITA
DMAETTAKAYPGARLVTLEGVGHSVIVEQPDTFVRLLGDFVGEHVTAR*
>SPBIB_v1_390027|ID:27164213| 3-ketoacyl-CoA thiolase [Uncultured spirochete bib]
MSKVSIVGAYNTQFGSFVKKNKETGEVTDLKSIYDLMLEAGRGAIADAAIEAKDIDGVWV
GSFAPGLFANQEHLAAFATEIAPDALRFKPMYRCEDACASGSAAIYNALYALEAGRANIA
LVLGVEKMSLLDTKGTTKALAAASYWAEEGAKGYTFPGLFAEYAKGYAAHYSLDPATFSK
MLATVAALCYKNGIDNPLAHFGKGGPADRLGLTTAQAILDLPPEKNPMIAEPLRLHDCSL
ITDGAAAIVLVRDDTSLAKSDRAVKLAGFGHVNERIPISVRPNLYQLMAGKEAVRQAFAE
AGITARDVDLAEVHDCFTITQLLITEALGLSKDGQAGYDYMAGRFTRDDDQCAVNLSGGL
KAKGHPVGATGVSMHALVYKQLIGEPIGAILTKKRPTVGVTFNVGGSGVTNSVSVLSR*
>SPBIB_v1_390028|ID:27164214| conserved membrane protein of unknown function [Uncultured spirochete bib]
MNIGDFLKRNKTMVAMVVMVILVGLGEKMAERFLPLYILAIGGSTWVVGGLNAMDNLLSA
LYSLPGGYAADKIGFKRSLFLFTALAMGGYLIVILFRSWQAVLVGAVFFISWTAVSLPAV
MSLVSKAVPKERRALGVSIHSFFRRIPMALGPIIGGLLIGAYGTKNGVLVAFGAAFIMAG

FSLFLIQRYMDNDAGEGKDRGSQKIPFKDMFNPALRSLLASDILIRFAEQIPYAFVVVWA
VNSNGLTALQFGVLTTIEMVTAMLVYIPVANLSDKYGKKRFVLITFGFFTAFPVVLLFSH
SFPMFVVAFIIRGLKEFGEPTRKALIMDLAPEAAKARTFGLYYLIRDVIVSIAALSSAAL
WNIAPAVNFLVAAGFGVAGIIVFAIFGKDLTMVSAQKAPASK*
>SPBIB_v1_390029|ID:27164215| protein of unknown function [Uncultured spirochete bib]
VQLVQHFVQALTGGIRAFLSRCTLSETMRRTWEKRWKTPGKTKAEYIPKYALA*
>SPBIB_v1_400001|ID:27164216| Long-chain-fatty-acid--CoA ligase [Uncultured spirochete bib]
MFLGHTPSGAPPISSFPPHSRLLNAGWPNGYGRAAHSSSKEKCHKNIAKPLLQERGKDDI
SRVQALCLFSMELMCSIIYDMRETEPWKFLDAYRGRVFTGKWPTLPQLFDIVTERFPDRP
CFTVYSPERVSLTYKEAQAVTVKVAAYLHSIGIAKGSHVAVTGKNAPEWAVAYLAVLYAG
AVVVPIDYQLPTREILALIKAGDVSAAFIDEEKFEEMQKELSTMPCFSLAKGTRNYIYDL
APPSGTKFEPPVVPGQSLAAILFTSGTTGIPKGVMLSHENFVSDCFLAQSNLNISETDVF
YALLPIHHSYTMLAVFIESLSVGAETVFGKKMVVKQILSDLKQAKVTMFLGVPMLFNKLL
SGIMKGIKEKGAVAYALIRTLMAISGFIKKAFGVNPGKKIFHSVLEKASLSSIRICISGG
GPLDPQVFRQYNQLGIDFVQGYGLTETSPILTLNPVEHYKETSVGKVIPGAEIRIDNPNE
EGIGEIVVRGPMVMQGYYKMPEETAEVLSEDGWLRTGDLGKLDDENYLYLAGRIKNLIVT
EGGKNVYPEEIENEFQLYDEIEQILVRGYIPEGKSVAEEIEALVYPNQDWAAQNKLTSEQ
IEAQIQEIIAEVNQRLRPYQKITRIQLLNEPLEMTTTKKLKRQTSGSASNLVPELK*
>SPBIB_v1_400002|ID:27164217|valS| Valine--tRNA ligase [Uncultured spirochete bib]
MKSVELPKAYDPKTFERRVYEMWKTEGHFQPKVDKRKRPFTIVIPPPNVTGVLHMGHALN
NSLQDIQIRYRRMKGIPTLWLPGTDHAGIATQNVVERKLRKEGRERTQIGREAFIEETWK
VAKEHRAFINNQLERIGASVDWSRERFTLDEGLSKAVREVFVTLYERNLVYRGEYLVNWC
PSCGTALSDDEVEHEDEPGSLWHIWYELVDGPTPEWPSGRIEIATSRPETLLGDTAVAAH
PNDQRYTALIGKMVRLPLTDRIIPIIADSYVDPEFGTGLVKITPAHDPNDFLVGQRHKLG
STNILNPDATLSSAVPEKYRGMKVLEARKAVLADLESQGLLKSEEKIVHAVGHCYRCHTA
IEPYLSRQWFVRMKPLAEKALKAWQDGDVVFFPKKWEHTYEHWLENIRDWCISRQLWWGH
RIPVFYCKDCGATMVSREDPVVCTQCGSSNIYQDEDVLDTWFSSWLWPFSTLGWPKDTAD
LRYFYPTSALVTAYDIIFFWVARMIMAGMEFTGQSPFKEVYIHGLIRDKQGRKMSKSLGN
GIDPLEIVDEYGSDALKFTLAYNCAGGQDILLDRDSFKMGSKFANKIWNASRYILMNLEG
RTYLPESEIVYNDTDRWILHRLNLAAKNVSQALESWRFNDAAQTAYSYFWDDFCDWYIEA
SKLSTKSSDEHEKDRATTVLLNMLDASLRLLHPFLPFVTEEIYSMLPNANGKLISARYPE
WTAERDAPETEAHFVALKEIVTLVRTLRSEFQIPPEAAIPLNLVFDANFLHAKFITENIA
LISLLAGASSVSIGAPPPSGSISLAGNGVTAQVQVRGLLDISKLIERLSKEKEKEKAYIS
KLEAKLSNPAFLSSAPADIIEKEKEKLAASASKAAKLEMYIRELS*
>SPBIB_v1_400003|ID:27164218| conserved protein of unknown function [Uncultured spirochete bib]
MNSGENGPQDTGPNGSKSPAAESSQDAESPQDAKSPLSRQGSILRSDRLTMDKAAMLRLK
RMHLAPYIQLATCLIGKLRRSGGNMFRHQLDTMAILMDYGYIDSVLLKASLIHDLLEDAP
ETDPDTILSIDDESVDVYKLVLEVTRRPVENKAEFLNRIRDFGSLRAKVLKSADRISNMI
SLGYVTDDVFVRRYTDETEQFVFPIAERADERMLSELKELVATRREYLRNRIEI*
>SPBIB_v1_400004|ID:27164219|deoC| Deoxyribose-phosphate aldolase [Uncultured spirochete bib]
MNRKWTKAEIAAAIDHTLLKATATSEQIRTLCAEAKEYTFKSVCVNPCWVPLCASELAGS
GVLIATVVGFPLGANSTAIKVEEARRAVAEGAREIDMVINIGKAKAGDWNAVRDEIAAVV
HASKPAIVKTIIETCYLTQDEKIAACRAAVAAGAAFVKTSTGFGTGGATIEDVRLMKETV
GDAAQVKASGGIKTYEDAVAMLEAGATRIGASAGVTIMKEAEA*
>SPBIB_v1_400005|ID:27164220| Uncharacterized ABC transporter permease protein YufQ (fragment) [Uncultured spirochete bib]
MFFKEIYPTFYIAIAMVFITWFVVYKTPLGLRMRACGEHPQAAASMGINVAKMRYFGVLV
SGALGGLGGAVMVLTADIQYTLVSIHGTGFISLASLVFGKWNPFGVLGAGLFFGFSTALS
FYAKDIKFLAGLPGEFFYILPYLLTIIALLFFAGKSVGPKAAGEIYDSGKR*
>SPBIB_v1_400006|ID:27164221|ams| Amylosucrase [Uncultured spirochete bib]
MQQGFDTFKKEFSARLSARGLPRYSGWPEFSARLEDELERLTGLLFELYGDRPDFAYWVE
NIVLGAFEAYRKRPTWLKARDREYPPESGWYLREDVIGAVCYVDRWAGTFRGIEERLPYL
KELGIKYLHLMPFFKSPERENDGGYAVSSYRETNPALGTIDELSALARKLARHSIALIAD
FVFNHTSDEHEWALAAKAGDEFYRDFYLTFPNWKEPEEYSKTLREIFPEARRGSFTWNEE

MQRWVWTTFHSYQWDLNYRNPAVFEAMAGEMLALANQGVAGLRLDAVAFIWKEKGTPCEN
LPQAHTIIRAFQCVARLACPSLVFKSEAIVHPDQIVQYIDLRECQLSYNPLLMAELWEAA
ATKEVRLLAYSIKKRHNLPEGCAWVNYIRCHDDIGWTFADEDAAALGIKGFDHRQFLNRF
YLGEFPGSFARGLSFQYNPVTHDMRICGTAASLAGIERDMRRDPGKNRETALRRFLLLYG
IAFSAGGIPLIYLGDELGMENDPDWDKDPAHTKDSRWVHRPVWQEVLFRERHNPATVTGQ
VFASIKKMIEQRSKHSVFAVQGIEVLESGHPSVLLFRKKSKSETLVVVGNFSECEATVPR
EVCARAFEGRLAVDLLASGAAAGEGASVGPLTLKSCELKWLLIEG*
>SPBIB_v1_400007|ID:27164222| GGDEF domain protein [Uncultured spirochete bib]
MIRFSRRIFTDLAIYMMGFGFVIGVVFPFFMTLLGVPASYTMQLWFFIVCIIAGLIVGAM
NILLARSVVGSRLHLIANKMKQIGGYISSKTHEEIVTTCSPQECMVEKDSDDELGECAES
YNQLINALYASFQQETMVKSFSQLLSSRLELDDLAASVLPSMLEHTGAEAGALLVEENDE
LRPAASFRINGVETLKDREFIKRVAQEKKRITIELPENIAVEGALLTFRPASIILEPIVY
HDVVLAIMLLAFIREPGEEAQRTIDLISPNLSVAIRNSLSYDQLQRLAANDSLTGLFNRR
FGMARLQEEFGRAVRNSAPIGVCIFDLDHFKLVNDTYGHQVGDKVLVFLSKIIRSALREG
DIAMRYGGEEFMTILPGASMTDAYQIAERIRRMVEESEFQYGPQRIRLTISGGATSWPDF
DASSADALVKRADESLYAAKEAGRNRVTVL*
>SPBIB_v1_400008|ID:27164223| Cyclic nucleotide-binding protein [Uncultured spirochete bib]
MIDVNALQKYSLFGGVSPEQIEKIKPLLGTAHFEAGECPQIEGQPSDKIYFILSGEVEIV
KQGLVIARLKEGETFGEMELLDIMPSIATVRAATPLEVVTISNRALYEISKLDLKTFSMM
IMNLARDLSRRLRRMDELACLDSNDILKK*
>SPBIB_v1_400009|ID:27164224| Extracellular solute-binding protein family 1 [Uncultured spirochete bib]
MLKRNNVLVVLLFAILAFAVSAQTVTITYWQYYYESKVKLMDELIQKFQAANPGIKVEQV
TFPYESYNQKVAASIPAGEGPDIINLFYGWLPTYVKAGYLAELPASDFNKAYFDKNFYPF
VAESVQFGGKYYSVPTAVRTLSLFWNKKLFKDAGLDPNTPPKTLEELEAYSKKLSKYDAQ
GNLIQAGLAMQPTGQGHNWIREILFRQFGNTPYSSDYRKVTYADANGVAAFKWYTDRITK
DKVGYPNFATDDVTAFKSGKAAMNIDGSFRIATLKAVKDLEWGVAELPAYKGIKSNYASF
WTHGIVAGTSGKKLDAAIKFLKYITSPEVQELWLEKVGELPANPGLSEKYKNDPVISPFL
KGLAYAHATAFVDEAGQRTVLVDAVDEVVLKKVDPAVALKNAAAKEQALIDDFWKQ*
>SPBIB_v1_400010|ID:27164225| Binding-protein-dependent transport systems inner membrane component (fragment) [Uncultured spirochete bib]
MTLATKRALTAYLFLALPLVFFLCVRLGPMVYMLVMSFTNWGLLRKTVKFIGFENYIILF
NDPVFLQALGNTFRYAVFGAPIVIILSLLIALLLDSIPKGKGLFRLIYVLPYITPVVAVS
WVWRWMYQPPPLGIINGILGILGLPAGEFLNSPTQALPSILAVNV*
>SPBIB_v1_400011|ID:27164226| putative ABC transporter permease protein YesP (fragment) [Uncultured spirochete bib]
MGYCTTIFLAGLQTIPKEMSEAARIDGANDQQVLRRITLPLLMPITFFLMVMEGIQFLRI
FTQVYNMSMQAMGGPLNSTKSAALYIYQKAFTNFEMARAASASVVLFLIIMVVTVLQVRF
FDKRVNY*
>SPBIB_v1_400012|ID:27164227| Binding-protein-dependent transport systems inner membrane component (fragment) [Uncultured spirochete bib]
MKHRNQMQRWGKVLPAYFILLFLSIIMLFPFVWMLSSSLKDVTQIYRLKLFPERPTLENY
RYILFSASTKFPQWFLNSVIVAICTTASVLFFDSLVGYTLAKFRFPGKKIVFFLIISTLM
IPTEMLVIPWYAMARALKWVNTYWGIMFPGMISAFGVFLMRQFMSTIPDDLIDAA*
>SPBIB_v1_400013|ID:27164228| transposase [Uncultured spirochete bib]
METALSELPDDVESLKKLVVEKARRAHELEATSKQLKEENAALQLKLLEVTDKYETLQQK
FFGSSSEKRKKEDDNPKQALLFNEAETYAEAPPKPEKSIPVKSHERKVRGRKPLPANLER
REFVYELSEAERTCPSCGAVRPEIGQEVREELEFIPARFVVNAHILKKYGPCQCASCSHP
IVQAEGPAKLIPGSSFSNTTIAFFLTSKFVDSQPFYRMEGILSRWGIDTSRASLCKVAVS
AGRAIGELLDELRKDLAASPVLQMDETVVQVLHEKNRSAQAKSYMWVARGYAEGKPVVFF
HYHPSRAKEIVQKFLHGYHGFVQTDGYAGYNEVGFSPGITHVGCLAHVRRKFFEAEQQGS
TEASAFLERIAELYHAEKLVRKKFEEGVLTTEEFLAARTKEQGPRLSEMKAWLIAKQGQS
PPSLSFGKAVHYALGQWDRIEKYLQHELLTPDNNAVENAIRPFVIGRKNWLFSNTPLGAH
ASAGIYSMIETAKANGHEPYKYLCYLFNALPKARSLEEKRALLPYKLAPNSY*
>SPBIB_v1_400014|ID:27164229| conserved protein of unknown function [Uncultured spirochete bib]

MYFKKDRAIHLVAGVTDMRKQINGLAQIANEKKSDRVFSGDYFIFLGKTHKVMKVLYWDR
TGFCLWVKRLEEETFPWTRKQKGIITLKREKLKLLLKGIDIFREHTEKRYVSVL*
>SPBIB_v1_400015|ID:27164230| Amidohydrolase 2 [Uncultured spirochete bib]
MRVIDSHVHFPENWIIDNGDPFEVSAPGKGTGYTSGRTKSSPSDAAKPAANKHAQWMENE
KARWESAWQFPAPEEISLEEGEKRWSNECSRYDFLKAVVFVTAGSNQFASELVARHPNQF
RAYAHHDPMLSDAAERLEKALTEQHLCGYKILGPKVDTPLSDKRFDPIWEVAQSHEIPVL
IHFGIMGAAGGIASHVNINPLAIHDVAKRFPDMPIIVPHFGTGYLFETLNLCWACPNVYI
DTSGSNQWMRWMPFDVTLESLFRKFHETIGASRIIFGTDSSWFPRGFTIDYLKAQNRAMS
DVGYSEDEKDMVLYRNIVYLLKMEHL*
>SPBIB_v1_400016|ID:27164231| Fumarate lyase [Uncultured spirochete bib]
MADKKFSQVYVENVLDPAYRNWKANFYAASLKVHKAHLCMLYETGIISKDKAKEIAGGIA
YLEHEFSAPGHIPEGVEDLYFLFEKALGQQIGDENAAWLHTARSRNDMDTTIFRLVLREE
LAVLIERLQVFSKALLARCLQGENELTVLFTHGQPANPSTTAHYLSSFLMETIGDMKWIV
QALADVNQSTMGACAITGSGFPVDRQYVSDLLGFENYIPNTYQAVSTSHWLVNAASTIRN
LMIDIGRFAADLLHKASSEVGLYRFPDDLVQVSSIMPQKRNPVILEHIRIQAEMVAGACD
AVAHSFLNVPYQDVNENADMIISKLIETIHESTSVIDLLQESVLKMKSDPARAREICRQF
GVTTTELADSLVRIYGIGFRKAHHICAEYVASGYDIQTLRDSFFKETMQALDLSNSRIEE
ILDLGTFIQVRKTPGGPSHEGIKAVYARAEEMLGDMEKNIISVKSKWHRADDALEEAFKA
LIAG*
>SPBIB_v1_400017|ID:27164232| conserved protein of unknown function [Uncultured spirochete bib]
MIEHDTPDPSRLRFIAMDMDGTILDDGYQLSAHVAQTLRALQNKGKKLIIATGRIYASAH
EFLQGAFEPDGFVCTNGADIYGPNGVRIASHHIPAEAMPALIDASRRHSALVCCYIGDNW
IYERYSDMVAFYEQRSGMKGIQRSFDSLSGEDILKFLAIGPHEELLAIREKIAQKASRLL
ETVFSHSTMLEIMAGGVSKRRGLEECLEYFGGTMEETIAFGDAENDLEMLKAVGVGVAMG
NAHASIQAEVPHVTESVDEDGVARFLERLFGI*
>SPBIB_v1_400018|ID:27164233| Carbohydrate ABC transporter substrate-binding protein, CUT1 family [Uncultured spirochete bib]
MKKALIVLVFALAFASMVSAQTINVLFWDDAYPRTLMERIPEFEKATGIKVNIEILQPPQ
VFTKTSVSVSKDRTDYDLVCVDEGNIPLFASLMLPYDQWAPGKVFKKVDPNTVTPAMLDV
AQWDGKLIGLPINGNLYVWMTRKDLIENPKYKADFKAKYGYDLGVPQTFQQMLDEGTYFY
ENGIVSGGFGPFNGGPAGVFGEAIFMWESYGTHFIEWVKGKPTLVVDKAKAVQGMEFYKK
LMKISPKGAETMAHVERQAAFAADPKGVFTQFIWPAQIASYEDPDKSLVAGKIVYSAPPA
GPAGRFAIRGTWAVNIPLASKNKDAAAEFVYWWASKDIATWLVEKNTVPARTDALTDPRF
AKTKPWLAAIADSMKYAAARPRFNEVAQVQDIVKKYWIMGITGQMPCDDAMQKIIDETKD
VLKKAGY*
>SPBIB_v1_400019|ID:27164234| Carbohydrate ABC transporter membrane protein 1, CUT1 family [Uncultured spirochete bib]
MFSRKPATELPRAQGQAGKARGRAPFLFLAPAIIVVFAVLIFPIIFSLFVSTYNWPLSQG
AGVRHFVGLGNYAALTQDPEFWNSLKLQLGFIFIAIPIELLFGFAAALLLNREFFGARVV
RSLLLLPVFFLPILSGMTWRFMLQPRYGPLNALLLKLGAPEITWLGNPATAYAAIIVQDI
WRMWPFMFMLLYAALTSIPQELMEAAEIDGAGFWLKLRSLTIPLLTPTILTAVLLRIIDA
LRVFSEVYVMTEGGPGSATMLFSLYTQRQAFGYQKVGMASAMAIFLLVISIVFALTLVRK
NMSLDVLEEKAGA*
>SPBIB_v1_400020|ID:27164235| Carbohydrate ABC transporter membrane protein 2, CUT1 family [Uncultured spirochete bib]
MMKSLLKKRTRKIILNTIIAIVAFLVLAIELYPIVITVINGFRRDINILSGQPFQLKQLT
LRSYELVLKNAGFRLGMQNSIIVGLLSTAISVLIGAMASYGIARFHFKGRNGLAYSFLVF
RMLPQISLVISLYLMFSAVGLRDTISGITLAHTSFNVPYVIWLLLPFFTAVDKAYEEAAM
VDGCTRQGVFFKIFLPIVAPGLVVAAVFAFLNSWNEFMYALILTGVKAKTAPIAINGLLG
GETLTWGQACAAGTIMLVPVFIFTLGMQKFLIRGITTGGVKG*
>SPBIB_v1_400021|ID:27164236|yihQ| alpha-glucosidase [Uncultured spirochete bib]
MPIERISFVKRSDGFSLYADERLVLSHSESAPFLFMGRGEARIAMHHGNFDIEDEIRELY
PLAQWDLDADGELVRLWSEGGRWAVTIRPEIVEGRLVVHISRAGIVARDSLFEGEVRVRL
SLPARPAEHVYGCGEQFSYFDLRGRKFPIWTSEQGVGRNKSTRVTLEADLADHAGGDYWW

TFFPQPTFVSSDRYWVHMDSSAYSVFDFRRGTAHEVLCWELPAALVFGQAESMPAVLEDL
TEYFGRQLLLPEWVYKGVILGIQGGTEVCQKKLDKARAAGVPVAGIWAQDWEGINITSFG
QRLRWNWVWNPERYPSLDAAIKAWRTEGVRFLVYANCYVGKGWSLCEEAAAKGYLVKNAQ
GEDYYVDFGEFDAGIVDLTNPEAFYWYAQALARHIIEFGASGWMADFGEYLPTDAVLFDG
TPALLAHNLWPALWARCNNEAVRLAGAEGEVLYFMRAGFTGSQRWCPMMWAGDQNVDWSN
DDGLPSAVRAALGLAMSGHGLHHSDIGGYTTLFGMRRTKELFMRWAEQAAFSPLMRTHEG
NRPAENWQFDSDEETLAHLARMGRVHVLLADYLKACVAENAARGMPVMRPLFLHYPEDEQ
AWTIDNEYLFGADLLVAPVIVEGARERTVHFPGDVWLDFWTGEPIWESRLAGDLIVKAPM
GKPPVFVRADSPWKDLLRSVSNLVP*
>SPBIB_v1_400022|ID:27164237| putative N-acetylglucosamine-6-phosphate deacetylase [Uncultured spirochete bib]
MADSHTHETYIIEGADLVLPDCILPHAALAVREGKIAHIQASGRSADIAKKDSAFSGAPV
LHMPDSYVMPALVEMHIHGCGEWGFEKLAGPTDLVSAAAFLEQKGVGCFVPTLLWDEETV
ANLALAIERCGLPEYTLPGLYLEGPFVNKARRGGIHPERIHAPEPDFVRKIVEAAGGHLK
IVTLAPELDGIEAVYRIFQEAGALVSLGHSDARLPAAKLPAHPYSITHLFNAMSGVDHKE
GGLANLAFSGDPDFVELNGDGIHVNATCLRLAARAVSADSLVLISDAVISAGMVHGSYRY
YGHEVISSERGVRYADTDVLMGSNRLGIDIVRNFVEQAQVPLWQAVRAMSLTPRKALGQA
EEYGSLEVGKFADIFLWDKDLEIPVRPETLLARTDLRAE*
>SPBIB_v1_400023|ID:27164238|alr| Alanine racemase [Uncultured spirochete bib]
MDHRGTTAWIDLAAIRSNYRQLVKLAGGSAVCPVIKADAYGHGALAVAHALEREGAPYLA
VALAKEALELRESGIQTPLLIFGVPEHSWSSIAIRLGCAQTAYTEEHVRILASAAKSLGK
RAKAHIKIDTGMNRQGLSWTKARAFAGLLSDYPEIAVEGIYSHFADADNPDPSFTRLQLG
RFNEALAAFAEAGIVPKYRHIANSAALLFHPETRLDMVRPGILLYGLSPAPGIALPEGFA
PAMTLTTSIVHVREVPAGESISYGRTFSTERRSRIGVLPIGYADGYPRALSNRASVLVRG
MCSPVVGRVCMDMTMIDLTGIPGARAGDEVIVFGGRDLPVSEISGLLGTIDYEVACMVSK
RVPRVHFDEPAQDS*
>SPBIB_v1_400024|ID:27164239|dapF| Diaminopimelate epimerase 1 [Uncultured spirochete bib]
MVDRSIPEGFHTHKYNALGNDYLVIDPAEFDVSLNEEAIRALCDRNRGVGSDGILYGPLA
SFEGGIWRVDGETLAEADNESRVARTSPPFFLRIFNPDGSEAEKSGNGLRIFSLYLHESG
RVGFAPFEVQTKGGKVACTVLPGEGPERRITVAMGAPEILSNRLTFLIDGIEFRAVSVSM
GNPHCVLLGSRPSPELARRIGPIIEHHPDFPNRTNVQFLEVLDRENIRIEIWERGAGYTL
ASGSSSCAAAAAAKHLGLVDDQVAVHMPGGVLEIDMREENIRMTGPAVRTFDALLAPALY
SAAYARH*
>SPBIB_v1_400025|ID:27164240| Amidohydrolase [Uncultured spirochete bib]
MENLLKEALAMQDWLIDTRRTLHRIPEPGNEEIKTSQAICAQLDALGIPYTRIGTGVVGL
LEGARSGRCIALRADMDALPIEEPQDRPYASQHPGYMHACGHDAHMTIALGAAKLLARRR
HEFSGSVKFLFQPAEETTGGAKPMIEAGCLENPHVDFVLGLHVIPELPAGQIEVKPGPFY
GASDNLEIRIKGKSSHAAYPEQGIDAIVAASAVVQMLQTIVSRSISALDSAVITIGKIQG
GTRSNIIASEVVLTGTIRTLSEQVRKFIGASVEEVCAHTAAAYGASSEVHISPSYPVLVN
DEAATEFVRQTAIQVLGAPNVHLRQKPTLGVEDFAYYLRERPGAFWHLGCGKHPPLAADS
VGSISAGATAALHSPDFDIDESCLPIGVALQASTAMRWLLDI*
>SPBIB_v1_400026|ID:27164241| protein of unknown function [Uncultured spirochete bib]
LGSLSTNFETLQDIWISNAQSATLKSGLELPLIIILRSKDPNLTQGVYSDLLTICINQF*
>SPBIB_v1_400027|ID:27164242| Peptidase C26 [Uncultured spirochete bib]
MAQPLIGITSFADRARQPAPYISLKESYVRAIEAAGGVPVILPVADAVQACAIIPRLDGI
LFSGGGDIAPWYFGQEPLPGLGSYDTHRDEWEIELCNAAWDAKLPMLGICRGCQLMNVAR
GGTLIQDIERTKPDALQHNPAVPHDELCHDIAIEKESALAKLFGTGRLRVNSFHHQAVER
AACDFAVTARSSDGIVEAMEAKDGRFALALQFHPEGLFMRYPDFLAPFEALTRAAAARLS
SLRS*
>SPBIB_v1_400028|ID:27164243|lysA| Diaminopimelate decarboxylase [Uncultured spirochete bib]
MQRILHAKGQRMVTTHFPEGIVADDARWLFDGADLAALAKEFDTPLYVMSEALIRQRCAE
LRSAFLARYPGTRAYYASKAFQTLDMLRIIKDEGLGVDVVSGGELYAALKAGIPASEIIF
HGNAKTDEEISYAVQSGVGRLVVDNLEEIYRIEREAAHAGIAQTILFRVTPGVDSHTHRF
ISTGSLDSKFGIPLEASETARYVAALRECPHVEFAGLHFHVGSQLMENSSHLAALRIVLD
FAARLVREFGLAVKELDMGGGFGIRYLPEDNAPALSDFVDPMIALVREWSEREKLPLPAC

AIEPGRWIVGEAGLTLYRIVAVKEIPGVRAYVAVDGGMGDNIRPALYEAKYHAAIVGRAD
AGLGAASNQLRRVTIAGRYCESGDILIRDIDLPDPRPGDILAVFSTGAYCYAMASNYNRA
PRPALVMVKKGKARLSVRRETYDDVIHREK*
>SPBIB_v1_400029|ID:27164244| transposase [Uncultured spirochete bib]
MFVQTLFEKKTGRTLLFYYTARRVKGKIVKTKVKRIGYLDEFLDAYPDPLTHFRQEAKRL
TQEAQLKTLTVTFSMDEHFSFGAGFAATEDAAVEKADRTFHYGVLPLLQLYRELKIDAFL
RIKAQYTKVDFNHNHLFQMLVFGRILFPESKLATWRDRTRILQHSDFSDDAVYRALPFFA
QIKDALVQHLHEQVQRQYHRDTTLLYYDVTNYYWEVDREDELRKRGVSKEHRPEPIVQLG
LCMDNSGLPVTYGLFPGNTNDVATMRPMMQHLAESLGTKHLIYVADKGMMGGMNIAQIIL
EHNGYVISSSVRKADAELRRYILDHEGYTELAGGSFKYKSRLVPCTLYVDTPDGRKKQIR
INERQVVFWSEDYWKKARHDRDMAIAKAMARAGYGENTVLNNHAGNRFIKKEIFDPDTRK
EVDHPEFSFALDQELLDSEEELDGYYLIRSNVVGVREGDAPFNQPYRWHAKDNLFELNRP
VVDLDIIDMYRGLWRIEESFKITKSQLKARPAFVHRQDSIEAHFLSCFVALLLLRLLEKR
TGEKIPVATIVESLRKAQLVQLEDETYVNACCDNVIEAIGQALELDLTKKYYTKGELKAL
RGKTAKSR*
>SPBIB_v1_400030|ID:27164245| exported protein of unknown function [Uncultured spirochete bib]
MKKFVVFLVIGLVAGSLAWSATIGITVNAAQKLEVTVDAATWTIALDNTGTAIDVPNQGQ
LTVKSSKASYTVTFTSTNGGTLNNGSYTMPYKVKVDTSSWVAGVATNNLSSYTQLTSAKP
IIFQLKTPTAGKTFPIGFNIEAYTDYYVDGAYTDTITISIAQS*
>SPBIB_v1_400031|ID:27164246| exported protein of unknown function [Uncultured spirochete bib]
MQAKNTAECNMKKYLTLLLLLLLASIAVYSQSIGVTVIAAPVGANVTMNSYVWNITLAND
GVTSYLNDYQGIMTVKSGSSVNYRVDFSSQNLGYVKQGSSYQIPYYVYVTQLTTGHIGII
GTPAITLGYVQLTSTQSIQFNKKTPTGGIQFYVGFKITATAGEFFESGAYTDNMSITFTA
L*
>SPBIB_v1_400032|ID:27164247| exported protein of unknown function [Uncultured spirochete bib]
MDKIRPYLLAGLCGLLLGAGIVGGCWIYRAGNDAATNRAVISAYQSAMADWQRRATELAA
TLDDVRRDARQSVELAGRINAVVTGYFSDIAKARTENELAVEQLRLAIDIYNVLRGFYDK
GFNATPKTGATAKP*
>SPBIB_v1_400033|ID:27164248| exported protein of unknown function [Uncultured spirochete bib]
MSGKKFKAWLIMTTVLVGIYMLTLFVAADILTSGIGATIVGAIAFGAVGYGGVQVADSWQ
RAKYYRPELDRTEEEK*
>SPBIB_v1_400034|ID:27164249| protein of unknown function [Uncultured spirochete bib]
MIIKDGIITAGFDELRPLSQPMNRRDHFHGALDIARGDGIVLSPVDGEAQGFVIFRGVEP
NVQVRSWTQGEKPDILALPWREYWQDIYGAIIVIIERGTKRLHILCHFWPSRVLNHDPEF
DGPFHSVYYLEERQKTRWPSHILMTDEVYVKQGQRLAPVGNAGFSTGPHVHWEVHHQADR
LDEYAKRVNPAKEYL*
>SPBIB_v1_400035|ID:27164250| protein of unknown function [Uncultured spirochete bib]
VDLGEKLSALELSHHELASVVWGDDKKRDNGLRARVTKMAEVEIPAIQSSITSLNSCVDD
QGKRLEAHIKEHSENKKDDATIRAEKVRAYGVIVAALLGNAATIITVLLTRGK*
>SPBIB_v1_400036|ID:27164251| protein of unknown function [Uncultured spirochete bib]
MDYEQIQSLEAEIENLKKDLALTDWYVVRFAETGKPIPEEVLAERQEKRQRINDLQEQIR
SMMAKN*
>SPBIB_v1_400037|ID:27164252| protein of unknown function [Uncultured spirochete bib]
MGVVSTGQLTLYDVNDPTSSLFTEFWDGDWASRWVNYVGSGEMSVAVDSTLPVGALALTV
GNNSGNDMVWLIAKQNIPFDANKLYKLTFYVKRTAGTGTTYLGVAGVAPDGVTLVNNTGV
NSFSNQHYIAASGVSPGSSWTKYVGYIKGTAATGTTSACPTPESPGKLHTNCRYFRPMIL
VNYTAAAGTNVVGAVTVEVMPELADIPNGNDTRDKAVASDAQLTAMASDSIITPQEKLFA
LGRWCEWYNDTAATSALPTAPTTDGRYKRIVDSANSVSGWTPTTAGTASKAFYDALEALR
AYLFSSPGVVLAGTWSTNITITKSTWLTLWVTAEATAQALETEIAGKQGLTANLSNDSHL
VPTDADGNNGNFAGCATTMSIYKMGVDDSANWTVAARPSSGVTGSLSGKTYTVTALSVDA
GYVDLTASRTGYPSITLRFTIVKAKGGAAFWLVSSADAIQKSQAGVYTPSTITFTGKRSA
SAGVVVDYAGRFIIAETTDGSTYTDKYTSSANETSKTYTPSAGIKALRCRLYLAGGTSTL
LDEEIVPIVVDGPTGPTGPTGPQGPTGPAGQDAPRYLGLYAYANRGSITGMIAGDLVVLY
SATQAERGIYAYVSSTWTKQSTPTQDQIMRCMVGVLDAVRQGYGVSADYIGAGATSFETL

LVNFIYAQYALISGSIRAGTRYDQAGNETNPTKEGVWIGANGKIKGAINDVEPDQVTPSF
TRRQLFTSSGTWAVPAHVKWVRVTAMGGGGGGGGSGGVTASAEVGTAYDGNAGQNGGASS
FGSYVVANGGAGGTGTGGGGSGGTGQNGGSSDFIGGYNGANGGAGTAKGTTNSAATGTKT
SGAGGGRGASSGLYDANVELSNASGGLTKTPTISAGRSKTNSASASVGIVVSNGGAGDAG
LYGAGGGGATGGCYAYKSPDASFSAWGASGGGGGAGALRQRVFRINEVYGQSITVTVGAG
GTGGTAGGGSTATGAAGGNGGKGWVLVEW*
>SPBIB_v1_400038|ID:27164253| conserved protein of unknown function [Uncultured spirochete bib]
MGIVSTGQITLYDYNDAAPITAFISASKGTAQVFTKDESITSYNPDYSSAANVLTAYVYV
NGVNVVNNLTNRKWGTTLGGSDLGTNVSSITKSTNIDPANPVYNVYFEGDYTDPVTMLVT
HVNAMITLHCVKSGTNAVFVQVNGQLVIEGNPTGGTKNTASVTADLMRAAGIDNTGVTYQ
WFKSPYAAADQLDANHVDVVAGKYSFKNTAGSAASAPADGTWADVKTLVVREDAVNDIGL
YLVKAKDADGNIYQAYFQIYDVSDPYDVKVVATNGNVFQNGNGTKNLTPEVWYGNTKIDI
TNYTFTWKLYDKNGKKSGFIDTARTSAAKTISSHTTGSSAVFTISSALSSAPVAGDVIRV
ISADGLKIESFEVASATTTAITIRSPQNGFSNNYPASTSDYANGKLWLYVGNGATAGQKT
TTGSTALSVTGDDIDGLGTVFCDAINPNV*
>SPBIB_v1_400039|ID:27164254| protein of unknown function [Uncultured spirochete bib]
MIRKIFVELAFPGDSAWTDISALVLDKSWTIDRQAFNDEKKSTLDKFTCSLKFDQTILSK
IRAAEERIGIRVKDLDGNPLFFGAIEPAVSHETSDHIGDIAIEAADNSWRLDDKIKTTRQ
MPANVTDAGFYIWNPADQQHSICHILLTEAGYTEAEIDGSLSDLRQIEMVSVEAEASTYR
EVLDTLLMEHELVLHAKPDGTLTLLSWRPAAITATIDKNSLSTVKPFRFENRFDKRDGAK
ITWAQPEIIPDVLVYRENLPVDENGVFTGEAIAAGDYFPKDSDIEDIYQSYVENWLDRPY
LARETRIKNKDLTLIATSEPRLFYDADDGITPDIAEFEYKRAHIRFINNASETKKIYNFE
IKANVLIRKKIGITKASPLGTETDLLEYTSQYIFDSTAADALAQALAFDALAEEQYSFGL
NEIIEIGSRVRIIEQRNGTDVEAIIQKTRLSGESPVNEYEAVQFLYEGNLQLNHDYSLGN
QIWPPYDYQQVVQRLEDVSRVFYDQPVGPYKRGDLWISNGILYQSKETRGTGEFFDSDWE
WCIRSNLTTIIESTNGDVFKPGENTTTTLIARCFRNGIEITDTLPDSAFRWTRVSRFPQQ
PPNDDETWNYNHASGYRTVEVTTDSIYARATYKVEILE*
>SPBIB_v1_400040|ID:27164255| protein of unknown function [Uncultured spirochete bib]
LIEDEKKKWEDYYKYLKSQAKETAKEVLASGLLDQFNSIGAAMASGASSADAAEAAMHKF
FQSALQQTSMLALNAGLKLLVEGGLPMLPVALGLFALAGISGIAAGAIGAAGGVRQTDYD
QYIVNPIVDAETDLAKKRVDIIKQQLEDEKKLRDENLKKIEESFNTEYEILKDQWQRGLI
STEQYKQQSASLRSQEESAKAEANKPVEEAEALLAQIEAARDQKLSYLATEAKKRQDELN
SMSGWDKFWSGRDEELVAELDVLDARIKKVKEAQSLPEISAAKYGADFVTQGPKLMLVGD
NPGGRERVRVEPIGTPNRYGPQPEQITIQISGDIYGIDDLYQKLEAAGRKLLKAGRVRTG
VFA*
>SPBIB_v1_410001|ID:27164256| protein of unknown function [Uncultured spirochete bib]
LRYSSRNSKRSKLQRWRKCAMADVEDLKIILRAEVDKAIADLKRASREGKNAEKDWQSIA
DTFQKNIQHSLSLKNAFSQLTMQIAGGLAIYDLAAKGIRAIGQFAADSIREYKEAAEAHA
RLAAQIKATGGAAGFTAAQLEDMASRLQGSTRIEAEEINKAQSALLKFTSITGEQFAKAT
ELSLDLAAVLGGDVSGAAQTLGKALEDPISGMSSLRRAGVVLSEGQEQLARSFAESGNTA
KAQEIILKALQDRIGGVAAAVGKEDPAGMKRLQLAIKDVKEEIGELLAKNMKPFLDALAT
DIENFRKRLANSKTDAAAVVQAVKFGDLIDVGDVSGIRQAVQNLSGKVDKEIVKAWIEEA
KKRNPLATQAQAAAIRAAEEELAKMPALQVAGASGSANNDADIKAQEHIKNVTEELNKNI
QAIKLRASALGQEASSQELLTQYLNAYVKLIQNSDGLVTENNPAAKLLKIQIQALVNGQK
ELSQAEALATYQIEKAGEIEAXX
>SPBIB_v1_410002|ID:27164257| protein of unknown function [Uncultured spirochete bib]
LAFADCAADDPDWENFAPDEYEILPSSIDESGKMLVKRRDVPNLLTDPLFLSGWKEWTRY
RRFGLPHGGGWRNEKPLVIRVIEVFEQEFEAKQAAEMEKMRHG*
>SPBIB_v1_410003|ID:27164258| protein of unknown function [Uncultured spirochete bib]
VTTYEPDLSWNIWKEQPKNNAPGIPMPDDEKVKVEITWPDIETFERLIGNDSTFATFITL
TKHCATKITGFSLHGEPIETGAELAAVRAGRTSKARELAINIGSYIFKESLLDEEEKN*
>SPBIB_v1_410004|ID:27164259| protein of unknown function [Uncultured spirochete bib]
MAVLKIRAGANPADPADVQLWFGEEAIENAKVIEAYNDHGKKRVIAEIIWEEKLGRKEVE
KSDKRKGDNLRAGSVVEHMEGTAQK*

>SPBIB_v1_410005|ID:27164260| conserved protein of unknown function [Uncultured spirochete bib]
MANRFSGTKNYLYLGSLGTEVTTGSLSGEKFFKITAKGASSAFPADSVANDVVYNKPAIT
LASGDKAKPIELTKLGFVTNVPQSASKEKYENTVQTDVAKSYEEGDKPEISGTIDGYFTD
DANADLILKRFFRLIDDNGAGQKTYQPIETGVLHFFLGRRETTTVGQVEVMEYMPAIIDS
LTVDKPMNGPQTFNFAYTVIGNEMPSITRRTITA*
>SPBIB_v1_410006|ID:27164261| protein of unknown function [Uncultured spirochete bib]
MKIWNIYRTVKGYLEQNLNSKIEALATDANIACAAAKAFWVGWRDPFNLKDYNSVFVVPD
TLKRNDDAVTDDVSIAIIAALKSPTPDSLSDQMGIYADAIATVIEDDPTLGGVAFEANVI
DFDFSLPAPGSPLIGALTAIIMVRMDRI*
>SPBIB_v1_410007|ID:27164262| protein of unknown function [Uncultured spirochete bib]
MISMTVQTKKYGDLGKFADNELLKMSARITSLWGESLANYIRENQLSGQVLQVVTGETRA
SMGFYKLKKDKKATMVVRPGKGVKGHLNYLAGMQRGMLAGRGRKVLIRPKPFMKPGFRAW
RATGEPRRIKEEVFQAYLKHSFASGGSA*
>SPBIB_v1_410008|ID:27164263| protein of unknown function [Uncultured spirochete bib]
MMLSTDALTSWETAKTMLGFADDQQSVVEFLINAVSATANRISGRRLKARDYDLRLNGTD
KNSIVLPEYPIVSLSKVYIDGNREFPPESEIDPDMVSIDPDGGIIRLHDMIFPAGIGNVR
VIAKLGYDPVPQDLELAVLEAISYNRRRLESGTTGMRQVSVDGTVTSQYELGLPLSIREV
FEGYRSNL*
>SPBIB_v1_410009|ID:27164264| Phage major capsid protein, HK97 family [Uncultured spirochete bib]
MDSDVKEMLDNLGKEWKAFRDVNDQRLAAIEAKQGHAELDAKLAAIEKELTESKAQINRV
MLGARLGGAEEKSELYRAFTDWMRDPGRSQQFKAAVQVQTGGDGGYLVLPELEKTLQRVV
SDSVAMRQLANVKTIGGKSYLKNVNKGGITGGWASEGETRSGNNTTPGLAQIEIIPRELY
TLPSASQEALDDLDFDVAAWLAEEAGIAFSSLEDAAFIAGDGNGKPKGFLAETMVANSSW
AWEKIGYILSGAAGAFPTTHPGDVLIDLIYALKAGYRNGAAWIMNDLTQSVVRKFKDGQG
NYLWQPSFQLGKPDTLCGYPVMISDSMPDIAADAYSIAFGNFKQGYQIVDRKGVRVLADP
YTTKGAVTFYTYKRVGGAVADYNAIKVIKFAAA*
>SPBIB_v1_410010|ID:27164265| putative Endopeptidase Clp [Uncultured spirochete bib]
MIRSKWYAIDIAPDYAEISVFDEIGGFGVSVTDFKEQFDSIKNAKQIRLLLNSPGGAVTE
GMAFYNLLASVRDKLTVEVIGLAASMASVVALAGSKLVMDEGTYLMIHNPWTITWGDADQ
LRKDADVLDKMRSELISIYAAHSKLSPKEIGQMMDDETWLTAQEAFDSGFADEVRETVQA
AALYDVSKIGFKKIPMALKHLDFRSIKTIRDFEAFLRDAGATRAEAAAIASGGWKALQRD
AEQPKSEDDGEIKEALSGLISILKGESNGL*
>SPBIB_v1_410011|ID:27164266| Phage portal protein, hk97 family [Uncultured spirochete bib]
MKFGERLRLAARALIFGSDDWVRALKGYYETASGQIVTADTAMRIATVNACVRILSETVA
SLPLHVYQRLDNGGKERASDHPLYELLHSRPNPWQTSFEFREQMMSHLLLRGNFYAVKLY
HGDLIIDDLIPLNPDNVTVLQLPDYSLQYQILTGTGSESIILGQKDILHIRGLSKNGILG
ESVISQARDVFGSALATQEYAGKFWRNDATPAGIIKVAKKLEKGEADRIREIWNDDHAGS
GNAHKLHVLGDGASFEKIEMTAEDSQLIETRRFQRSEIASLFKVPLMLLQADTQTTTYAS
SEQFMLAFTMHSIRPWLVRIEQALQMQLFTAPQKYFPEFNLDGLLRGDLKSRYEAYKIAR
DAGWMSKNDIREKENMNPIENGDDYRSLAELQNAKNLTGGA*
>SPBIB_v1_410012|ID:27164267| Terminase [Uncultured spirochete bib]
MRNKWLFLAVAIMTASLRPEILYAEKVLNGEIVACKLVKLACQRHLDDLKRQNTEKFPYT
FDAARADHAIAFIEQLRHVEGPSASTIGGRDNRIKLELWQKFFVGNLFGWRRADGTRRFR
HVYFEVARKNAKTTLGAGIANYIFWADRPADPGCQIYFAATKQEQAALAWRIARLQIERH
PVLKNLGKTYESKQYIVKTMKDAKGRPISDWSSRMRPLGQDSKTEDGLNPSLAIIDEYHA
HPTSEILDVLESGMMARLQPLTLILTTAGSNFDGPCYQVERPLAVGILEKTLQPIPEDVF
ALIYTLDEGDDFADPKVWIKANPNLGVSVMPQLLESRVAMALAAPARARDVKTKNFNIWQ
QSINRWITDDIWMACAEPVDEETLAGRHCTLGLDLSTNTDLTAICAAFPPAEPGEKWKTI
WRLFMPMDNLLERERQDKVPYTEWARLGLIIPTDGNTVDYDFIEQEIRIFGDKYLIDEIA
YDPFKAGEVVAHLSSEFTMVAVPQRYNPMAIYSDIFERLVRKGELAHGGHPILRWMMSCT
EVKADRQGNIMPMKPRRETSGKRIDGIVAAIMAIGRASITNGGETGFAKADEVVG*
>SPBIB_v1_410013|ID:27164268| Phage terminase, small subunit, , P27 family [Uncultured spirochete bib]
MPKPRVPTQIKVIRGTFRRNEAPANEPQPDLLKAAPKPPAHLNKWAKRMWKDIAGKLLAL
GMLTDIDLYTLEVLCEQYGIYRELKDAITHRDTPAGREKISIAQYLAGQNSQTIPEYAAM

RAAFERYTALLKEFGLSPASRSRMDIPREPPKAVDPMEELLNAK*
>SPBIB_v1_410014|ID:27164269| protein of unknown function [Uncultured spirochete bib]
MDVAARHVYMAAKGLWMPTKPARMCKYPYCPNLTYDPSGYCDIHAAMRQAGRMPDKRPSS
PRRGYGRDWQKVRAEVLTKAGIPRDLWPLYDVDHNPPYNPAIEPDHRKYTLIPRLHGEHS
SKTNREDGGFGHRRGESISLQSFAVNRMRCSMSHTTDSRGKGVRHA*
>SPBIB_v1_410015|ID:27164270| protein of unknown function [Uncultured spirochete bib]
MKTEQMAENLRQLYVAGKIEIGKTYHVLPSDCSGNGMDIALKNRLDESADYDLGNGVVAK
CRCSVQVGSTRWGDGIPTLYSIEVYCPDNQSKIDLSKLSNESLRKLNALARKAGYKSCTE
AYYDKIDEPVIKHEPWGFETKSGKFFPGIRTTKWWKSGNYRPAKTIIYLPESFNL*
>SPBIB_v1_410016|ID:27164271| conserved protein of unknown function [Uncultured spirochete bib]
MSRRTTVYLNKNLEALLKKYAERLNTDEGEYGQSATLTEILGRYDELVRAERRRLRDLFE
ENEINLLLNNALSTIYSYQTIIGAVLADTEDEDPSQFEFFGVDRAALIEKLRNLTPGQQF
ALVDWLEEMRSAS*
>SPBIB_v1_410017|ID:27164272| protein of unknown function [Uncultured spirochete bib]
MRNLELEMEAYRKANSYLYSKHIRNAEEIIRVALLQCKNPAIALSGGKDSVAMAGLVCKF
CSPKIIWNDSGLELPESKSIIYEIADRFGLEIIVAKGCAIEEAEAIGRGELSRYDKKVIN
SIDTIINPVRKALIEHGIDLEFVGLRKKESIKRRMLLSQYGPIHYSKKWGIKIAWPMMNW
DGRDCLAYICENNLPIHPAYLMASDPAMARVSWAFDTAREAPAETEVVRRNYPEIYRKLR
EAGLCQ*
>SPBIB_v1_410018|ID:27164273| protein of unknown function [Uncultured spirochete bib]
MHAINLIAQSVRTLAPALPCDPIEATCAVTGQVCPCLPRREVLGDSFTNIDCLRAPESEF
VGVDVYIAWFFGYKTTEDKKRLKCPERMASWICDGNEFRELSRIEIREMVLNGIPYKQWA
AYVTTSYKKHGSLWARVNIGPRGIWRFEMIDADCRDENKTREYWNRMNAAVNAGIGRGII
ETLDMPPYLTMKVGMKTWIEYEKWAKPIYQSGLYQFLAYLLPSAEERINAKSGIGNGGIS
KSKQLSLF*
>SPBIB_v1_410019|ID:27164274| protein of unknown function [Uncultured spirochete bib]
MKDILLTIFAVLRFIYNHSKITGKRNGSEFFADRIVQAATEKTLLAFAERLSKLVDADIG
SIWESRGVEFLRISGTQDAAKVYQWIRLYPRIVAMISALPRMEQVEEAIESIEIESVKED
GCAIPQGKYEIPIEITTLSPLAHGADIKAGNATLFRRMQVLSDTGATLSLPFYSGNALRG
QMRDLLADDFLRAIDIKPSRTNPPIALWFFHTIYAGGALEENSDAAKAFGKLLGTNGAIK
AEGIYQFRDTLPMISALGCSIGNRIIEGRANFGDFRPCCYEWSNGNIKVSELMTWEFLTR
REDFEGHEDGDNKSMIATIECIKPGVVLQGGIDIRGHASELERSVIGKGLSLLADRGFIG
AYSRQGFGKVRIEIENAPDGKLYENYLAENKNKIIDYLESLGAICMPSI*
>SPBIB_v1_410020|ID:27164275| protein of unknown function [Uncultured spirochete bib]
MSIGKDLDEIAAELMRLDYGELHIVVRGGEIVSYSIIRSKLVLKRNEKEHKQLQEKQY*
>SPBIB_v1_410021|ID:27164276| protein of unknown function [Uncultured spirochete bib]
MSAPWRQVHKTFWSDPKVVDYFTPEDKFFYLYLITNEHTTQCGVYKISLKQIGFETGYSI
ETVKNLVERFQNGLKRIRYNPETNEIAILNWAKYNYPTTIKDNRFLCIQEEITAVKDKSL
IADVLQNAVPEIRDALLMKGDVSTSDAPYKGLGSPSEAPYKPLGEEKEEEQEQKQEQKKE
KEREPRAREASFPQEKPENEKPASVRLAEDWFRRFNSMTGAKTKPDSKSLELSRRLLEFL
GNDLDMALLAVDYYFSHWRELWFACERWSRSGPEDLRKWEFRFASFADPENCQEILSRIA
RQQETKSHDSFSAARPGDDEPPIDPEENAKAWQRINEFLRQKIAAHEAAMTA*
>SPBIB_v1_410022|ID:27164277| protein of unknown function [Uncultured spirochete bib]
MRIGSFWVKKNDDGSKTMSGEIGSDVGLSVPAGQRLYAKLVRNDKKKDAKSPDYFLEAWM
PREKERKDYKMQFDPQRNDDAVF*
>SPBIB_v1_410023|ID:27164278| protein of unknown function [Uncultured spirochete bib]
MEYLPLDEQTKMPGLVLKLPQLEVIEQLLHAALDGPGQRWYTLEQAHYRKHGSLPGGVSL
KTIRNTLALQPRGGIPDGWVSGRKAWRAETIEEWCQIDDSNLEAYLKMYAPHLRVPKRIQ
EANARHAIEYTAFADKETEHEEEDPAARS*
>SPBIB_v1_410024|ID:27164279| protein of unknown function [Uncultured spirochete bib]
MANGKNEYKSDNEYVKFDRRSFASFKIFIEKLSGAAINPKHHAAMAILPKGWRNILAFSL
WKNREEFEANLDEAAARLGAKILNDWWASADGILISVFVPSSAFTFAQSIKDFHMLDEPA
VDKASQKQILKGTRK*
>SPBIB_v1_410025|ID:27164280| protein of unknown function [Uncultured spirochete bib]

MITEVVEYWVDGRKVTTLDALLRIVGAKSNKEIDDVRACLAQSKPYKVHRIKQVYGIPAI
RTGALLHDPCIHRLG*
>SPBIB_v1_410026|ID:27164281| protein of unknown function [Uncultured spirochete bib]
MIRLFIPGEPRGEPRTRATRTMSGKVLVYHDASADRWKQQVQLAFALKRTAMLTGPVAVS
MEFISKPKDPHGYYSTKKPDADNLAKAALDALTKARAWKDDAQVACLIVTKRFAQPKTGE
QAGAFIEIEELPQTMDPAFPASLAGYMGRGFLRVCQ*
>SPBIB_v1_410027|ID:27164282| exported protein of unknown function [Uncultured spirochete bib]
MINRKICIFSLFFFLLLTLNAIAQSDSNYIVNYFVDDFGDPTEHKYLFTIVTGKFSNSAT
KDSILKVGVIIADSEISFALYEYGSNRVTGSDYTDYVIKMKFPNGHIIEKTTKIDKYSGR
LEISSIFDSSIDFDTMFSGNAPIKIYIYEKDRPYANYSFSVTFPSKEFISNNLGSDFFWI
Q*
>SPBIB_v1_410028|ID:27164283| Exonuclease (modular protein) [Uncultured spirochete bib]
MIIFTLLIVGLIIYLIIRSKKPARKDIREPIYPRAMPPQKVHPHRDMVVREIKEWLEGIE
LTDVIVFDTETNGFYPDESSLLSIGAIRYSWDPYEKLKEKARFERYYYPKEPFNERAIMV
NGLTKEKIAELRGDATYPEHFLDDYLAFKEFCEGVRLIAGHNVSFDVGFVPFLKNRRKLD
TMKSNADVVCVEWMDSKNDWKWPSLEETARFYGVPFNEDDAHGALYDAMIAGEILKRMLD
RAEVKVQII*
>SPBIB_v1_410029|ID:27164284| conserved protein of unknown function [Uncultured spirochete bib]
MGKTSGLKPGQKAPASGQYQTVGPKGGKGKEVTAVKGEPLPPTDKPGSTYNLVDGTKNKS
GKA*
>SPBIB_v1_410030|ID:27164285|rflA| Phage P1-related protein in restrction modification operon RflA [Uncultured spirochete bib]
MDDYINIYCDESCHLEHDHQKAMTLGAIWCEKKKIKEITRRLIEFKGKYGLRHESEIKWS
KISSNHYQPYLDIIDYFFDDDDLHFRGLIIPDKSILNHQKFNQTHDEWYYKMFFRLLTPI
IDPNYKYNIFLDYKDIQGAERITKLHDVLCNNMYDFNHSIIESIQLVKSHHVELIQLTDI
IVGALSYNARDLNTNAGKNKIIERIKQRSGYSLTRSTLYRESKFNIFIWDPENEDHNA*
>SPBIB_v1_410031|ID:27164286| protein of unknown function [Uncultured spirochete bib]
MLNFLPPLLTLEDVDYDPNRLIMEAYRIFRKDFIDNKPYFHATPIKLAQGIGNDGKENTF
WHIISGTDKKITISKILILTA*
>SPBIB_v1_410032|ID:27164287| putative Integrase family protein [Uncultured spirochete bib]
MGTKYHLFRRNLINSKGKKYTVWYYWYKENGKQIRKPCGRGCRLKRDAEEFIAKLEAQES
TSAPSIHVLKSKNSNFASATYRTFQDLAAEMFLPDSLHLKRQTLADGIAIKETTRLAHRG
RLENYLIPKWGRSPFARFEDEGFAYDFQDWLVGLRKAGKDEPISNSLRNNILETMSICLR
EAKRKRLIKTVPDMGTIRFIRHSRRQNTLSLEEIYRLFPEDPAALDAVWRRKDSRDYPGI
GILFGTMFCLGLSAGLRSGELRAVSRDQFVIHSLQDGSYLYGLIVDRAYDSTGQISSALK
KGNDENLRHRAVILSDKTMRIVNLYLDASAVVDGPIFLFHGHPVSKELLNARWKVGLANA
QIDMSGNRRMTVHALRYTYNTRMKALVSEQTLHEFIGHNSSEMTDHYDRPHMEERLLQLA
DQRGAVERFWG*
>SPBIB_v1_410033|ID:27164288| protein of unknown function [Uncultured spirochete bib]
VRGANAFARANFAIRCVAVDPFRAVAQGLARLVRDQDVGGSNPLSPTTESHSIERLYLFM
*
>SPBIB_v1_410034|ID:27164289| conserved protein of unknown function [Uncultured spirochete bib]
MSEHSLLYSFRISADAASALEKLPACIDEANPVPERKRASLRYSIRDLWRSLTMERALRN
IDYLNNPAFFSAYLRYFLPWNLVRLIALLTELPLELKNGATIVDMGSGPLTFPLALYCAK
PELRKVPLTIICADRAPRIMEAGKLILELLAAKHGGELPPWNIELRHLRFGEPIREKADL
FCAVNVLNEFFWHHEGMLADDAAEVLRKIEHYCTAGGSMLIVEPGEPRSGGLMSAIRAAA
ILSGNVVEAPCPHANACPMPGIFKSGQEYLTGRASQFAHKEAKDEGQKERAIKDNRLLEP
VQMPSPRTKYPWCHFSVPAEFAPRWLRKLSDESGLTKEKLSFSFLYIRKAEGNTSRGRVE
QARESLCRIVSDPILLPRDRAGKYACSAAGYTLVTAASGMELPASGSLVRMHKGKIERPG
TPEIDRKSGAIIVSY*
>SPBIB_v1_410035|ID:27164290|tdh| threonine 3-dehydrogenase, NAD(P)-binding [Uncultured spirochete bib]
MKALVKRKNEPGLWMEEAPVPKIGDNDLLIKVKKTAICGTDVHIWKWDTWAQRTIRLGQI
VGHEFVGEIVEMGRSVQGYKIGERVSAEGHIWCGVCRACRAGRRHLCPNTVGIGVNRDGC
FAEYISVPAQNAWHVHDAIPDEIAAIFDPLGNATHTALSFDLVGEDVLITGAGPIGCMAA

AISRHVGARNVVVTDVNPWRLELAKKLGATRIVNVSKEDLKEVQKELEMVGGFDIGMEMS
GSPQALEQMIENMYNGGRMALLGLLPDGAGIDWSRVIFKGLFIKGVYGREIFETWYKMQT
MLLSGLDISPVITHRFSFDDFEKGFAAMLSGEAGKVVLSFE*
>SPBIB_v1_410036|ID:27164291| conserved membrane protein of unknown function [Uncultured spirochete bib]
MEKLALLAAFGTAVFWAFSAIFFEDASKKVGALAVNFWKVTFAFVFLAISGTVVRGVPFP
YDAPLRTWIFLGLSGLVGFVISDYFLFNAYVLIGSRITVIFQSLTPVFTALFAYIFIAER
MQAHRFVGMAVTIAGILIVVLTRSKQSRQNNEGALSAKGLLFAFLSSVFQAGGMVLTKAG
LGDYSPISGTQIRAFIAIFGFAINALLIGQGAHVFLKVPKIREAFSSTIKGSVFGPFLGV
ALSLFSLQNTQAGATSTLMALTPVVIILPSVFILKQKIHALEVVGAAVAVAGAALFFLL*
>SPBIB_v1_410037|ID:27164292| Transport-associated protein [Uncultured spirochete bib]
MAVICISRELASYGEETAQELAKLNGYRIVDKEHIEAALTAIGIDANKQARYDEKNPGFW
ASLSQQRDEYLHFLTQAIYETALENNCIILGRGAHAILKGIPNLIGIRIGASKAVRVERV
RKAQNIDSRHALQIIESSDHDRAGFHKYFFSVDWYNPSEYDMTLTTDRCNPVHAAAAIDA
FRKAFVGEAQEKEAVAKLQDLLLGSKIVTEITYNRKIPIHFLEAAVERGVVVLHGVANTH
AAVDSAVAAANLVPGVKQVESAIQLVQEFTVVP*
>SPBIB_v1_410038|ID:27164293| RNA methyltransferase, TrmH family, group 3 [Uncultured spirochete bib]
MQAALNLQKVVLVNYISSFHSIIETLRASPQGCMLYVAEGGRHAGPRIRQIIEEAEKRGI
AVRHLPEMDLSRMCPEHRGLVLAAPLEARGMLSIEALCEHSSSKDAGLVLVLDHIEDPHN
LGAIVRSADAFGVDAVVIPTRRASPLTDAAERSAAGATAWLPVIQVKNLRAAVDQLKDAG
FWIYAADMSGSALGAKPLGKKVAILLGNEGKGVSRILKDAADECISIPMFGHVESLNVSV
SAAILMYEYRRAHALL*
>SPBIB_v1_410039|ID:27164294|pyrBI| Protein PyrBI [Includes: Aspartate carbamoyltransferase ; Aspartate carbamoyltransferase regulatory region] [Uncultured spirochete bib]
MTRTGPFKGRTISVVNDFSLDEQRYLYRKTRELKEAALSGSDVSKFRINDLDYQVYLIFM
ENSTRTRESFRNAGKFLGARVNVFDAATSSFNKNESITDAVKMLFGYSGESCFVLRTKLE
GVCTWLDEAFTEYAQITGKPKPSFINAGDGKHEHPTQEFLDEFSFLEQLGWNEDHIHIAM
VGDLYHGRTVHSKADGLRIFRNVEVDLIAPELLSMPSYYVEKMKANGFQVRIFDSIEEYL
AQARVAPIWYFTRLQLERMGEAVLERTPYLRQAVTFRKDFLGKLPDGCRFYHPLPRDRNF
PTIPFFLDELPLNGWDGQSINGYWTRITEIAMLAGRIGEDFEGEHARRPKFVDDFVHEAQ
AQEKRKPEYKVGIKPVEEGIVIDHIATGKPVGEIWDTIDAVRKILKLDVRSSHGVYHSNR
GPDIFKGIISLPDIISFGEKDLKKLAAIAPGCTLNLIRHAHVAKKYRLSMPPRIYGFDEI
SCKNENCISFPANNEGVLTEFIRKGETTFVCKYCEREHKFRDIWDV*
>SPBIB_v1_410040|ID:27164295| putative Uncharacterized RNA methyltransferase TDE_2619 [Uncultured spirochete bib]
MTAEMLTLRVEKLSSHGEGIAFSEGKAVFIPYTIPGETVLCEITESHASFLRAQLLEIKT
ASPHRVDPPCPLFGICGGCALQHIDYTHQIRLKQETAQETFRRIGGFDPGELEIVTGEPY
HYRNRTQVHACKDSGLGFTKAGSRETVRTPHCPTLVPVLDRWFASENRKARPFHELSALI
GDRPRFTAFAQDERIYIEGRDAYAHATVLGKEFRFPVAHFFQSNISVLEKLIARYIEPLE
GASALDLYSGAGLFSLFLADRFESTECVESDSVAVEAARGNLSNARSAVRFSDIPAERWI
KTPHARQSFECIVVDPPRAGLTAEVRAWLAGTDARILTYVSCDHASLARDLRDLHSASWQ
TLSLTLFDFYPQTGRLEAVARLSRGV*
>SPBIB_v1_410041|ID:27164296| Beta-lactamase domain protein [Uncultured spirochete bib]
MSITIRSLGAAEEVTGSKHLLDVDSTKILIDCGAFQGKRAKADAKNRALLGDDVDPASID
ALLLTHAHYDHCGLVPYLVKKGFKGSIYSTSATRDLANLIMTDSAHIQERDADYLSRQAE
KKNETFDWKPLYDEFDVIQAMDQFVTVGYHRPILIADGIQAEFLDAGHILGSAFIRVIAK
DKEGHTAVIGFSGDLGRKNKPIIKDPECLDGIDFLLLESTYGDRLHESTDDAVARLEKLV
NRTAETRGKLIIPAFAVERTQELVFYLHLLHDKGRIPEMPIWVDSPMALNATSIFAIHPE
CYDKETYDLFTTHAKNPFGFASLNFSRSVEESKMLNQAKGPMIIISADGMCEFGRIQHHL
MHGLGDPANTVLIVGYMAEGTLGRRLKDGAKEVRIHGDWYQVRADIQEIDAFSAHADWQE
AVDWLGCIDKKRLRKTYLVHGEGEALTAMQKHVLDAGVQAAEIMKPGEIYTIA*
>SPBIB_v1_410042|ID:27164297|rpiB| Ribose-5-phosphate isomerase B [Uncultured spirochete bib]
MKIVIANDHGAVALKQHIVAWLKAQGHEVVNLGVDVEERVDYPDQAELAVQEYQKGSYDF
GIVCCGTGIGISIAANKHKGIRCALIHDSYTARMAKEHNNANFVAFGGRVEYAEPVEQML
SIYMNAQFEGGRHTQRVAKLDALC*

>SPBIB_v1_410043|ID:27164298| conserved protein of unknown function [Uncultured spirochete bib]
VIRVGKKNAAEECRMGLEPGDTFECTYETPAEFCPTSFIKIFPAMEVVRCNGDLRALGGN
GPYETTFICPDGVVLFKLTAERKAK*
>SPBIB_v1_410044|ID:27164299| transposase [Uncultured spirochete bib]
MFMRITNSGKNGAYHYAQLAESRRDPATGKVKTSVIYNFGLVEQLDIPGMERLIASIRSI
LPASSAMAQGAPQDYEFLGSREIGPVWLLDQLWKKLGIDTALASLVKEHQYRTPVERMLF
AMVAQRIVAPGSKRSIERWLEKDVLIQGLGEVDVHRLYAAMDLLIESNEAVQHALFTHVT
KKLDLDVDILFLDTTSTYFEIKGEDDHEGGLRKRGHSKDNHPELAQVVAFAVTKGGIPIR
AWVWPGNTSDHEIAQQVKHDLSQWNLGHLVMVQDAGFNSARNRQILLQGGGDYIIGEKLR
AGSKGEAVEALHRAGRYKTIEREGKVLLCKDVLIDEGSATQRRYVIVQNPEAAQRDKETR
SQIVEEARRRLAALAQLSGEPHRKAACRLRSHGIYGRYIGQRKDGTLFIDEEKMKRESLL
DGKFLISTSLMHCSTEEIVYGYKQLWEIERVFRDVKHVVDIRPVYHHLDDRIRAHVLICF
IAMVLVRVAERTLSMSWRDIAYTLTDIRVGHTKGPDGELWLMSPLSEMQRNLFSTLKVKH
PPKIWDF*
>SPBIB_v1_410045|ID:27164300| transposase (fragment) [Uncultured spirochete bib]
MTERLVIDAFLQGIGKEQPGSGLIVHTDQGSQSTGANFMAVGKKKGAIPSHSRKGNPYDN
ALMESFYRILKRELVDDAGFRTRDQLVLNSSC*
>SPBIB_v1_410046|ID:27164301| conserved protein of unknown function [Uncultured spirochete bib]
MSRRKFDKEFKVAAVRLVVDEELPVCQVARELAVHQNSLYRWVSEYEKYGESAFPGNGSK
IYNYQAEINRLEKRNRELEEEVELLKKFRVFL*
>SPBIB_v1_410047|ID:27164302| Sulfatase (fragment) [Uncultured spirochete bib]
MIVRWPGIIPAGTVCHTPWWYADFRPTIDEIIGNSMPTDCDGISILPLLYGNVEQLDSRY
YERIFYWEGYTYGFEQAVRWKNWKAVRKISAESGETSFELYDLASDLAEKNNLATNYSKL
VEKLRHQMISNRTDSPFWPIQFH*
>SPBIB_v1_410048|ID:27164303| IstB domain protein ATP-binding protein [Uncultured spirochete bib]
MRRPRTNRTLLREEISQLLKKMAFSQTAVQLCEEEGTAPMEQFLLNVLQSEMASREQARR
ARFLREAAFPVYKTLEGFDFTSVSLPPALSRDELLSMQFVAEKKNLVMYGPVGTGKTHLA
TALGVEACNRSMRTKFFTAAELVVRLSEAHKEGMLDKLLKAVLRADLLIIDEWGYVPVDH
QGAQLLFRVIADSYEQRSLILTTNLEFSKWGSIFTDDQMAAAMIDRLAHHGHILLFEGES
YRMKHALMRQKDQIKLPMA*
>SPBIB_v1_410049|ID:27164304| Integrase catalytic region [Uncultured spirochete bib]
MLTMNQIDQIKELQRQGYGPGEIASRLRIDRKTVRTYMKREDFNESLEAHTTWPSKLDRW
KPLIDEWLAEDQRMRFKQRHTAKRIHQRLCGEHAGEYDCSYPLVQRYVKAKKVAQRQMDG
FLELVWAPGEAQADFGEAEVMEAGVRKTIKYLTLSFPYSNAGFTQVFEGETAECVAQGLQ
DIFGYLGGVPRRIVFDNATGVGRKIQQHVALNQLFLRFKCHYGFSVSFCNPNAGHEKGNV
ENKIGYTRRNFFVPLPVVDRLVDLNMQLFKKALQDHARKHYKKGQTIAALFAEEQTALSP
LPARPFNVERYERLRTDGYGKFCLDGKHWYSSAPEYASGEVTVGIKAREIVVYGPDGEVR
GEHRRIYGEERSDSIDWHTSIAALMHKPSAWQNSHFRAATSPSVRQALDALPRDRLRDVL
KGLVQSSERFGFEVALASLEEAVSVARLDSYSVHAVAARHVYDGLYGIPAAGPDLGVYDR
AFIGEKEHTP*
>SPBIB_v1_410050|ID:27164305| N-acetylgalactosamine-6-sulfatase (fragment) [Uncultured spirochete bib]
MKKEIKYPNVIFILCDDFGYGDAGCYGGTSIPTPNIDKLAEQSMRFTQCYAGAPVCAPSR
CVLMTGLHTGHCTVRDNFAWKGGLPPEGRVSLRRDDSIIAEDLKHVGYTTGIFGKWGIGE
EGTEGIPNLKGFDEWFGYLNQRHAHSYFPTYLWKNQKRIDFSPAKDGQQGPYSHDLIVNY
AIDFITRNADKKFFLYLPWCLPHEPYEIPAQYAWNEAKATWKDEEKAYASMVRKIDTDLG
RIMELLEKLRITNNTLLFCVM*
>SPBIB_v1_410051|ID:27164306| protein of unknown function [Uncultured spirochete bib]
MHDTDKLPNMIHISMIHIRIIICSITNLGGIQHEERDKVSQCDFYFMR*
>SPBIB_v1_410052|ID:27164307| Arylsulfatase regulator (Fe-S oxidoreductase) [Uncultured spirochete bib]
MKKAFFLIKPVSGNCNLACHYCFYRDLLEHQIIHNFGFMDSSTVDILIERAFELDADILT
FVFQGGEPTLAGLPYFEHFVKKTQTCTKSNLRHQHPQINFSIQTNAIALNTEWARFFKRE
SFLVGISIDGARIIHDTYRTHYDGSGTWAEVMHGIELLRKEGVLLNALCVVTDFAADNAE
LIYNRLRNLGFEWIQFIPWLPPLKEESVLSSELLTPGAYAHFLKAIFDLYYQDWLHGSVV
HVQWFDNLVAIAAGMPPESCGMLGCCPVNFTVEANGSVFPCDFYVSDEWCLGNIHDLSFQ

SMKEGIIAHHFIERSSYIDPSCRICFAFFLCRGGCRRYREPFFDGKLKINRYCTAFKEFF
QYAGEHIMNMAAFYRN*
>SPBIB_v1_410053|ID:27164308| putative Ribose operon repressor [Uncultured spirochete bib]
MASIHDVARLAGVSAATVSRVLNSSGSVTDATRAKVRAAMKRLGYIPNPSARTLRNNRTG
LLALITPEIINPYFAAVASGVQDMCRKSNYQLILCNTGGDESIEISYLNLLSNKQVDGII
IAPPGTHTNPKSDVRIRNLIDSNYPIVMIGKRFDSDYVNCDIVTTNTAIGTREAMQHILD
GGHSRIAYLGGPNPSVAKTRLATFRSSLSAHGLIIDENLIFQTNLTLEDGYNICKKLLDI
TNRPTAIFAVNDMVAIGAMIALQEAGIAIPSEMIVVGFDDIPLASIFRPSLSTVAQPKYD
LGYIAAERLIARIDRLIENFETISLPTHLIIRESSMSNKTPKMMINFG*
>SPBIB_v1_410054|ID:27164309|dppF| dipeptide transporter ; ATP-binding component of ABC superfamily [Uncultured spirochete bib]
MNDKEDQILLEVENLRKYFAVKHGVLRRKKGWLKAVDGVSFELFRGETLGLVGESGCGKS
TTGRIILRLIKPEPNSQIFFHDNDKVIDIAKATPQELKYLRTRMQIIFQDPYSSLNPWMK
IGEIIAEPLLIHKIMVGKELTNKVHSLLEMVGLDASYAMRYPHEFSGGQRQRIGIARALA
LNPTLVVCDEPVSSLDVSVQAQVINLLVDLQERLHLSYIFIAHDLGVVRYISRRVAVMYL
GRIVEIGSTEEIFHNPAHPYTEALLSSMLSTDPDSSKERLQLEGDVPSPINVPSGCPFHP
RCRYYQQNKWPECVSDIPTPTSIYSDTHKVSCHFAPNLNLCGVETEGTVKIGVKNGFNT*
>SPBIB_v1_410055|ID:27164310|dppD| dipeptide transporter ; ATP-binding component of ABC superfamily [Uncultured spirochete bib]
MEHLLEIDQLKTYYFTDCGIVKAVDGVSFSVEKGQCLGIVGESGCGKSQTALSIMGLVSK
PQGRIVSGKILYRDKKGHITDLATLSPNGYEYRSIRGKEIAMIFQEPISSLSPVYTIGDQ
IVEALIEHTTLSKKEATHRAVELLKTVGIPAAEERIHDYPHQLSGGMCQRAMIAMALSCE
PRLLIADEPTTALDVTIQLQILRLLKELQERLSMGLMMITHDLGVIAEIADYVIVMYLGK
VVEKGPVRKILREPKHPYTQGLLQSRPSGTAGKVRLNPIRGVVPSAIDPPAGCPFSTRCP
KVLDKCSAFPPFALWEEGHAAACWLYENFM*
>SPBIB_v1_410056|ID:27164311| putative Extracellular solute-binding protein family 5 [Uncultured spirochete bib]
MKSSRVVIAFLAMSAFAVVTTAAAPSGTYKEAPQLAAMVQSGKLPPVELRLPPNPVIVKP
LDSVGKYGGTIRKVYTGVNDWWNLALFGVRAEPLLAADLEGNIVPNLLESYEYISGGNIL
RLYIRKGIKWSDGVPFTVDDIIYDLQTRGNKNMPLESAGLASKVVADKIVKIDDYTVDLP
LKEKYPLEFTIAYDPTVSPKHYLIKFDPRYDSTKTWQDLATAWSPSRNSAALVNLPVLSA
WKVVEYVENVRIVAERNPYYWKVDTNGQQLPYIDRVVFNYVASTDTIPAMIMAGQVDFQA
RHLSLADFPFYKQNESMGGYRTAVLPNTNLGPAIHLNYADKDPDLRKLFRTKEFRIALSY
GIDRLAISNTLFFGQAKPWGFSPLEGSPANPGNPYSTMYTQYDPSAANKLLDELGLKDTN
GDGFRELNGKPLSIIIDMDKGGGAGPVQVVELIASQWQKIGIRAIANTIDRSLILARWKE
NSHDAFAWNVNGGVDPLQFTFAWSTTAAPDFMWGNVGVPLNEWQTSGGKTGVEPPAFVKE
MNGLVLEAQQELNAKKRNELARHITKIASENLYKIPTTTLVSVGVVSTKLANVPNSWTDG
ISVLSPRNIQPWQFFYK*
>SPBIB_v1_410057|ID:27164312| ABC-type dipeptide/oligopeptide/nickel transport system, permease component [Uncultured spirochete bib]
MNFVLETGIRSPERCSCIRLKRDGMTGYILRRIGIAFVTLFFSTILIFAIIKLPPGDFVS
YAISQSGGEGGLSSIADRMRQFYGLDKPAIEQYSRWIGGLVRGDLGYSFLYRQPVLKIVW
NQMGWTVLITGLSMIFSWSIGSAIGIFSALKKYSFWDYLFTAFGFLGLSIPSFFLALIII
YMCISAGSVVTGGLFSPEYAVAPWSWARFLDLLKHIWIPIVAIGTAQMAQVIRIMRGNLL
DVMNQPFIKTARAKGLSERKVIFKHAVRIAMNPLISLAGLSAPALVSGIVVTAVVLNLPV
IGPTFIVALKSQDMYLAGGYLLLMVVLLLIGNILADILLAWSDPRIRFD*
>SPBIB_v1_410058|ID:27164313| Binding-protein-dependent transport systems inner membrane component [Uncultured spirochete bib]
MWKRFKKHKIALTGLVLLFLLIIPVVFAEFFAPYGLNETSSNSYAAPTRIRFTDEKGKFS
LKPFVYDAQPSFDLNTGQTFWKEDTTSPHYLRLFIHGSAYSILGLKLDIHLFGAEGGHVF
IMGTDALGRDVFSRLLYGGRVSLAIAFITALFALIAGSLAGIVSGFFGGVADMIIQRVIE
LFITIPNIPLGLALAAFLPPNLNPILLIAAISLVIALVMWANIARQVRGKTLPLRDAVHV
QAAVALGASTPRVLLKHIFPAIYSHLIVVVTLAIPQAMLAEAGLSFLGFGVRPPLTSWGA
LLQDAQNFRTISLYPWVAVPGLAIAVTVLLLNFVGDGLRDATDPYLN*
>SPBIB_v1_410059|ID:27164314| conserved protein of unknown function [Uncultured spirochete bib]

MNNYSIRGLEVHDWSHIWNFKTMRRYMKFMVENDMNTLVLHHVGVLDLITFPAKFLGGGT
PTNSIFEVYNQIDQNIYRYALRENLNLYRRDFLKQLIKEARQCNIDVYIEDKELWFSDFI
LNYKPDLIKNGVLCPSEPFWWEEFLPAKYEELFVALPDLAGVVISFGTGESRLAIANMHS
CNCDLCKKMTPAQWHTNMILGTYGPFKNRGKRLVVRDFIYTKEEQEQFAEALKVIPQEVV
LSLKNTPHDFYPTFPDNPLIGRVGERPQWIEYDVNGQYFGWGVVPSIMFDDIEHRLAYGL
DHKVSGFFMRTDWEGVQDLSCFDGPNLLNLYAAAILGKNPAADRREIVLRWLEGESMLDT
ACTPMQINETLDWLLKILEPTWSIMRGAVYVNGTVFSDNSLFHVSFGQPQWVAETHHSLK
NWFAEARDALGLTTKNVKFILEEKDRASKIMDEVLDVLALGRHALTETAYKDLLDRFELM
KAYVQGFCLETRLWVFGRLWVENRLDMLPGFDRPVKSFLEEAFVKIKEYIASVKGMPQLT
SYPACVLLNIERMECFIRDAEKKIMAMRPLE*
>SPBIB_v1_410060|ID:27164315| ADP-ribosylation/Crystallin J1 [Uncultured spirochete bib]
MQNYKERNSSRKRLSELVFRDKVLGCWLGKNAGGTLGEPWERKFGVDEMFDVHWYSHIPE
GGIPNDDLELQLIWFQALKERGPGITAWDLAEYWLDCVMYNFDEYGLSKANLKRGLVPPI
SGWHNNWFKNCMGSPIRSEIWACIAPGEPRIAAKYAFEDALCDHAGGESVFGEVFNAVLE
SCAFFESDKYRLIELGLASIPESCLTSRSIRDAWDMHKKGIDWREARNILKDRYSIPLAQ
YSPVNMGFQIIGLLYGEDFGDAICKAVDCGWDTDCTAATVGAILGIIEGASRLPEKWIKP
LGYNISTNLRTGGIRNLRAPTDINELTDQVCAEAKRVLKFWDAEVAIEDASEETRDESSC
FFRKYEFKIDRIAPYEPNVATWNLGTVSVSLRYLDSASIFSDRSTPLELEIHNPHPEAIL
IEISVLLPEKWSVEPIRQQTFTIEAYDTAKVAYNISVSDGIIEDSNRGYFIISAKNRMAL
CAVPLVLLGGSKWLVSPLFEGKTLEDDCGIDESLVPSTMLEEWSIYWRSGNDLSPESFYR
GKPGSVFFFNSIWSPEETEVILGVSNTGRMKIWLNGQLCHTTVQPTCLRPNLGNGGGDGS
NYCNIILHSGWNDMLIKLERGEKLLEAHVTLGKPNREYPKNLGEPCLGLRRAYFSWERGE
WL*
>SPBIB_v1_410061|ID:27164316| Lipolytic protein G-D-S-L family [Uncultured spirochete bib]
MVIDSNILAIKEKYLKYIWNPDDGSVSFFPALKDGTVIVFQGDSITDANRDRSVLAPNDM
FGLGGGYVFLAAATLLSHLPDRRLRIYNRGISGDKITTIAKRWEQDTLALNPDFITVLVG
VNDYWHISKHGYKGSVELYKETYKNLIARTMKVLPQARLIICEPFAIRCGYVDESWFPEF
SKYQAVAFDIAESSGAIFLPFQSLFEVALRVQEATYWCPDGVHPSAAGAYLMASAWLNCI
AMLDDR*
>SPBIB_v1_410062|ID:27164317| protein of unknown function [Uncultured spirochete bib]
LRKRLLLMATYSKLRTSPEMIQKTGYLSQDKSLVIESVEIWVNTLLFASKMGLTYES*
>SPBIB_v1_410063|ID:27164318| protein of unknown function [Uncultured spirochete bib]
MGLSMKERQRIIAETATRYREASKKEKGRILNELTALTGYNRLYAMHLLTW*
>SPBIB_v1_410064|ID:27164319| Integrase domain protein (fragment) [Uncultured spirochete bib]
MCGKRLKVSIRENLAFLALHEEYAITDAVRAELAAISPATIDRLLAKEKQTXWXXKRHST
TSEAANHYKTKIPIRTYYGSEEQRPGYLEIDTVFHSGVTVHDEFCCTLDATDTMTGWVEL
RALPNRGQRWVKEALVDVRSTLPFRLIAIDSDNGSEFLNRQVYDWCTREQISFTRSRSYH
KNDNPFVEQKNSQYVRQFVGYARYDTTEEFEALREVYQVLCPLLNLFYPSTKLIAKHREN
ATIHKTYDTPQTPFSRVLASPFVSLRAKEQLAVRKTGYDPVVLRYRLDTARQTTPCA*
>SPBIB_v1_410065|ID:27164320| Transcriptional regulator, LacI family [Uncultured spirochete bib]
MRTSLKELAKKSGYSVNTVSRALRDADDVKASTKSLIKQLAEEMGYIPNVVAKNLRLGKT
NTIGVVSADSSNPYFAEVILAIEDAARSHDYHILLVNTEESPEGEREAIDTLIERQVDGL
LIIPVFGEDANLERLKNLSVPFLLVGRWLPGLEDHSILTDEYDKAKQVTSLFLANGHTHV
LHLAGPSFVSSSFDRIKGYRDAHSEAGIPVNEDLIVETDGHIEDGHRHINALIRKELPFT
ALFAFNDLVAIGAMRALKEAGYLIPQDVEVVGFDDLDLSRYLYNSLSSVRIPKQELGRIA
FESLFEHMTDPKKLYRRQTIESRLMLRETTTFENIGDSQIQGGIK*
>SPBIB_v1_410066|ID:27164321| Extracellular solute-binding protein family 1 [Uncultured spirochete bib]
MKKNRLVLALGLILIALMTVGAQKPIEISFWSLFTGGDGEFFDAMVKAFNESQNEIVMKT
DTVKFTNYYTKLTAALSAKNAPDVVVMHQDRLVNYVPNGVLMQLDEYVQKANIDLSTFSP
APLNSCKFNGKLYAIPLDVHPLVMYYNKDLMAKAGVTKVPETLAELISAAKAIQDKTGAI
GIAADNTTATYKAYTLTRLFMSLLMEQNVTILDASNKRANFNNAAGVKAYQALSDMVNKD
KLTPKQLDYDSSVSSFKLGKAGFHFNGVWMVGGFESQSGLNFGVVQFPALLGKNAGWTGS
HTLAVPVQKTTDPDRIMAVMKFIDWMTQHGEMWAKAGHIPTRTTVYSKPEFKNLPHRAEY
ADAVKNTFAPPATHKWPELYDAISDSLEESIALNKDAKAALDQLEKKVNDILK*

>SPBIB_v1_410067|ID:27164322| Binding-protein-dependent transport systems inner membrane component [Uncultured spirochete bib]
MHLAGHTKRDIILFSAPYLLVYLVFLAFPVVYGIYLSFFDWNILSGKAFVGLRNFVQAFS
DEKFKSSFFHTLQFVGLSTPILIVTGFLMALISVRPTKLGKAAESIFFLPYMFSTTVVGT
LWAWLLQKNFGLVNQLLQAIGLKGVGWLTDPNVAMLSIVWATLWWTAGFNMILFSAGMKQ
IPDEIYESARLDGAGKFVTLTRITIPLLKDTTLLVVILQIIASFKVFGQVYVMTGGGPYG
TTRVLVQYVYETGFNYFKLGYASAMSMVLFVVILVISAVQLLASKDDVQ*
>SPBIB_v1_410068|ID:27164323| Binding-protein-dependent transport systems inner membrane component [Uncultured spirochete bib]
MKKILLNMVVAVLIALWLVPLLWLIITSLKMENDVVTDHLTLFSAAPTFKNYIKALTTTP
IKQWLLNSFFVASVTTVLTLFLDSTMAFVLSRMRFRGRKALYLFVLAGMMAPFESMIIQL
YLEFNAFKLLNTLAAVIIPRLALPVGVFILTQFFNGIPTALEEAAYIDGASRFKVFTSII
LPLGKSAMFAVAILSFIGSWNDFLWPLIAISNSAKYTVTVGIANFQGTHGTEYALIMAGA
VIASIPQILIFLFFRKNIVQGIAMTGIKG*
>SPBIB_v1_410069|ID:27164324| putative Glycoside hydrolase family 2 sugar binding [Uncultured spirochete bib]
MMKEASGNRFVEDSHIFAGHPNPSWARRTWMSLDGIWTITHRATKKEIRVPFPIGSKESG
VDFKDSGLFVYEREFILEPFLLAKRYLLHIGACDYMTTVFVNGHLVGSHVGGYASFSFDI
TSVVKPGKNRIEIRVLDSHSPFQVRGKQTFMRNAFFVWYEGIAGIWQSVWLEEVGLRYLE
RAETSFDFFGQKLAVSVFLKPLDNKSEHIPDSSLRLRIDVKSSDGRGKEATRLLEADNGV
FKATFGFDEFNAALWSIETPNLHPMALTLLCGNTVFDTVETYFGLREISAGKDGFKINGK
PVFLKMVLNQGYYPGGVYTPLDYSRMAEDIRTIKALGYNGARIHEKVEAPYFHYLCDRLG
LLTSFEMPSFYLPSKKGFRRYESELKELILRDSMHPSCIMRMLFNETWGIWGVCRKSSST
RSFVEKMYRLAKHMDPTRPVIENSGWEHFMTDIVDFHHYLRNASLARNLYSKIRNGDEYT
LEGFSLRRVLEFYIKNQVPFATRSVFLEKPSNADVLPLFLSEYGGFGWYDTEKKNAVEES
IEEYTRDIVHSGLFCGYCYTQLYDVGSEVNGLMTFERNPKVDIECVRKANNWEKQ*
>SPBIB_v1_410070|ID:27164325| protein of unknown function [Uncultured spirochete bib]
VRAESEQLGEAIIGQQNSIPMLGNKSGYTLTIQRTRFFLGNLTGYLASLECLIMQMSQLV
IISRFYFRLN*
>SPBIB_v1_410071|ID:27164326| conserved protein of unknown function [Uncultured spirochete bib]
MSRRKFDKEFKVAAVRLVVDEELPVCQVARELAVHQNSLYRWVSEYEKYGESAFPGNGSK
IYNYQAEINRLEKRNRELEEEVELLKKFRVFLQKKKHVRFRYLAECVM*
>SPBIB_v1_410072|ID:27164327| protein of unknown function [Uncultured spirochete bib]
MLKMNQIDQIKELQRQGLGPGDIASRLGLDRKTIRKYLHKEDFNQPQRVPVVHPSKLDRW
KPLIDAWLAEDQRMRFKQRHTAKRIHQRLCEEHAGEYDCSYPLVQRYVKARKAAQRQPGG
FLELVWAPGEAQADFGEAEVVEAGVRKTVKYLTLTFPYSNAGFTQVFGGETAECVAQGLQ
DIFRYIGGVPSRIVFDNATGVGRRIQQHIALNQLFLRFKCHYGFSVTFCNPNAGHEKGNV
ENKIGYTRRNFFVPLPVVDRLADWNDGLGQTARSCGTKSRSF*
>SPBIB_v1_410073|ID:27164328| IstB domain protein ATP-binding protein [Uncultured spirochete bib]
MAFSQTAVQLCEEEGTAPMEQFLLKVLRNEMASREQARRARFLREAVFSVYKTLEGFDFA
SVSLPPALTRQELTSLQFVAAKTNLVMYGPVGTGKTHLATALGVEACNRSMRTKFFTAAE
LVVRLSEALKEGMLDKLLASVLRADLLIIDEWGYVPVDRQGAQLLFRVIADSYEQRSLIL
TTNLEFSKWGTIFTDDQMAAAMIDRLAHHGHILLFEGESYRMKHALMRQKDQVKSPWPDG
EICTIFLGILA*
>SPBIB_v1_410074|ID:27164329| conserved protein of unknown function [Uncultured spirochete bib]
MNSSIRFGTCSWNYPSWVGLVYAETQRRAAAYLREYSQKYDTVEVDSWFYKIPDREEVAD
YLAQVLPSFRFTCKVPQELTLTHLRGNAGASTGVPNPSFLSPDLFARFLEAVEPMIPRLD
AIMFEFEYLGKDKMPSLEAFLEKLDDFLTAVRERARGLPLAIESRNRNYLSTAYFSFLRD
RRLIPVFSEKRYLPHVYEVYWQYRNLVDTDIVIRLLGCDRAEIEKKTNEQWNTIHEAKPD
KQLILQMAMDIASQGHKVTINVNNHYEGSAPLTIEVMQKIQQKLGAEKKSSGS*
>SPBIB_v1_410075|ID:27164330| exported protein of unknown function [Uncultured spirochete bib]
MKRHLALFILLLIIIFSVAADQVELYYNGNYIKNFYLKDATGYSVLNLSTKSANAALTIT
IDETYGKHNQYFLNRLYRITSISKMKDGTIFTYESLLSLSQESTSFVSYAWIMPSAQYDG
YFSLRNSDGEILAVYFDFNLNITRIE*
>SPBIB_v1_410076|ID:27164331| transposase [Uncultured spirochete bib]

MFMRITNSGKHGAYHYAQLAESRRDPATGKVKTSVIYNFGLVEQLDIPGLERLIASIRSI
LPASSAMAQGAPQDYEFLGSREIGPVWLLDQLWKKLGIDTALASLVKEHQYRTPVERMLF
AMVAQRIVAPGSKRSIERWLEKDVLIQGLGEVDVHRLYAAMDLLIESNEAVQHALFTHVT
KKLDLDVDILFLDTTSTYFEIEGEDDHLGGLRKRGHSKDNHPELAQVVVTFAVTKGGIPI
RAWAWPGNTSDHEITQQLKHDLSQWNLGHLVMVQDAGFNSERNRQILLQGGGDFIIGEKL
RSGSKGEAVEALHRAGRYRTIEREGKTLLCKDVLIDEGAATQRRYVIVQNPEAAQRDKET
RSQIVEEARRRLAALAQLSGEPHRKAACRLRSHGIYGRYIGQRKDGTLFIDEEKMKRESL
LDGKFLISTSLMHCSTEEIVYGYKQLWEIERVFRDVKHVVDIRPVYHHLDDRIRAHVLIC
FIAMVLVRVAERTLSMSWRDIAYTLTDIRVGHTKGPDGELWLTSPLSEMQRTLFTMLKVK
HPPKVWDFKKSKKAPIGV*
>SPBIB_v1_410077|ID:27164332| transposase [Uncultured spirochete bib]
MGKVTKAEVSAILQGMQDRELYEKLLGLKEPWSVENVTLDLPSATVTVAISHPKGAKFPC
PVCGTERPIYDHQKRRWRHLDTCGFTTILEAEVPRIQCPEHGVKQVNVPWGEPGSRFTAL
FEAIAISLLKVASFSDVARHLRISWDAASGIMERAVRRGLARREAQPLRRIGIDETSFQK
RHEYVTVVFDQERSCVVDVLDGRKKETLKTWLAANQNALTALESVSMDMWDAYIGAVREA
HPDGEQKICFDRFHVAQYFNKAVDKVRAEEHREFKTRGEQSPLTRTKHAWLRKGAPDAAF
SSLARSNLKTARAWRIKEAAAELLRTKSHEEAARDWRKLLSWMMRSRLGPVVKVAAMIRQ
YLWGILNAARLGATNAKNEAVNATIQKLKVRACGFRNRSRFKMVILSHLGGLSLLPEVMT
*
>SPBIB_v1_410078|ID:27164333|chpA| toxin of the ChpA-ChpR toxin-antitoxin system, endoribonuclease [Uncultured spirochete bib]
MVKPKKYVPERGDIVWLDFNPQLGHEQKGRRPALVISFKEYNEKIGLALFCPITSKVKGY
PFETEVKGKIINGCVLSDQVKNLDWTVRNVEFIEKIEDEKLNDIIDNILLLIEK*
>SPBIB_v1_410079|ID:27164334| Transcriptional regulator/antitoxin, MazE [Uncultured spirochete bib]
MQTVVQKWGNSLGIRIPSVYAKEFNLKHGNSVEIVKEGGKIIIIPPKKTLEEMLSKVTKD
NIHAPIETGSSLGNEEW*
>SPBIB_v1_410080|ID:27164335| PilT protein domain protein [Uncultured spirochete bib]
MLNSILIDTGPLIALFDKDDTYHNKVKDFIKNAKYRFITTTAVITEVSHMLDFNINAQID
FFEWIMKEGVILQEISQKDISRIIELTKEYSDRPMDFADATLVIAAEKTGIKKIISIDSD
FDIYRLPGKVKIENVFHK*
>SPBIB_v1_410081|ID:27164336| CopG-like domain-containing protein DNA-binding [Uncultured spirochete bib]
MTTVRLPIEIEQRLEILARKKHKSKTDLIREALEKLFIQEESEKDSYELGEEYFGKYGSG
DGTLSVTYKDKLKDKINAKLNSH*
>SPBIB_v1_410082|ID:27164337| Phospholipiddiacylglycerol acyltransferase protein [Uncultured spirochete bib]
MPISKSTNWGRKEKMITYTESLRGVSHYSLDGFCEGWQQPLTPLQLRSILKHSTYRILAM
DTEHTRIVGIITALSDKIHWAFIPYLEVIPAYQKQGIGKRLMELMIEKTKGIVCIDLTCD
TEMQAFYEQFGMVASHGMILRRYMDEK*
>SPBIB_v1_410083|ID:27164338| conserved protein of unknown function [Uncultured spirochete bib]
LANFKIAETETFEKKIQSSQYKFLYKKIQHYIYPLLRENPYFGPNIKKLKGEYKEIYRFR
IGDYRLFYTISEETVIIFILDIENRKDAYK*
>SPBIB_v1_410084|ID:27164339| conserved protein of unknown function [Uncultured spirochete bib]
MAKTITVRVEDTTYDIFKKAAEGQKRTISNYLEFAALNYTVNETVVDDSEMKEILAFEND
IKKGLADISAGRYKVIG*
>SPBIB_v1_410085|ID:27164340| Ribbon-helix-helix protein, CopG family [Uncultured spirochete bib]
MKKTNTITLRVPEELKERIENMATLQGVSMNQFALYAFTKELSELENSQYFKKYLKGKKK
EEIIKGFDDVFSKVKPRNVDQWDTL*
>SPBIB_v1_410086|ID:27164341| Toxin-antitoxin system toxin component, PIN family [Uncultured spirochete bib]
MKVVLDTSVLFQALYSSTGASHAILKLIREGALQLPISIPVFEEYREVLLRQSSLDLFEL
STNDVQKIIDFIALIGVKTDIRFLLRPNLRDENDNIFIELAFASGAHYVITKNVNDFKYD
ADLRFNEITIATPAEFMKIWRNTYEKD*
>SPBIB_v1_410087|ID:27164342| Plasmid stabilization system protein [Uncultured spirochete bib]
MSAKYTIVFSRYAEDDLSEIIKYYAEKNSQYALKLLDTLETRVQELRELPARGRIVPELA
QQNILEYRELIEGNYRIIYVIQDSMVVIHAILDSRRNLEELLMQKLMRFYS*
>SPBIB_v1_410088|ID:27164343| Prevent-host-death family protein [Uncultured spirochete bib]

MNVNLKDTIKPISYIKTNAADMMKFINDNKSSIIITQNGEAKAVLVDIDSYQEMRDAFSL
LKIIKLSEKDIAAGNYKESDAVFSNLRTSLSK*
>SPBIB_v1_410089|ID:27164344| conserved protein of unknown function [Uncultured spirochete bib]
MNKKVFIDSDVILDLLSKRQPFYPAAAEIFTFGDYGQLDLYTSSLVFSNVFYILRKSVGN
EKAKELLRKLRILIRIIPIVEKNIDMALNSTFTDLEDAIQYYSSCSFGIDVIVTRNTSDY
KNSEELLIQSPEEFINIIKSIEKE*
>SPBIB_v1_410090|ID:27164345| conserved protein of unknown function [Uncultured spirochete bib]
METKLTLKLDQAIIQRAKQYAEEHNRSVSKLVEDYFRNLIVNRKDKTHYSPLVEELSGVI
TPDDINNSDYTSYLEAKYE*
