# Supplementary material for: Fermentative Spirochaetes mediate necromass recycling in anoxic hydrocarbon-contaminated habitats
Source: ISME J. 2018 May 30;12(8):2039–50. doi: 10.1038/s41396-018-0148-3 (PMC6052044; doi:10.1038/s41396-018-0148-3)
Supplement: Supplementary file 9 — Supplementary Database 2 [file 41396_2018_148_MOESM9_ESM.pdf]

>SPSA8_v1_10001|ID:41144770| putative TPR domain protein [Spirochaetes Bin 1 SA-8]
MSIFFKRRTSRTSSRHSKAKSVAILAVSVMAAAGVIVLLVLLFSGKASFSTLISGRRKSE
VLASWNAGDRVSTLQLCRQELEKNPLDPFYLSFNGFSAYYLAMEKQEGEERLSLLDEAVT
SLRKSLAVDEKSPVLGQEEYVLGKAYYQKGLPWYDLSLKYLEKAKLDNYSAPDLDQYLGL
LYAASGDHGTAIKHFETALKNNPSDVLMISAALSYKESGNTAKAVEILNSAISSSSDDLV
VLKCRMMIADIEFDTGNLKKAEELYNALVKADPSLADAWFRLGVIYEAWKDPIRARAAWR
KAIAQDPNHVEARKKLSERL*
>SPSA8_v1_10002|ID:41144771|mreB| cell wall structural complex MreBCD, actin-like component MreB [Spirochaetes Bin 1 SA-8]
MAIKNIFESFSQDIGIDLGTCNTLIYVRGKGIVLNEPSVVAVEKGTNRLVAVGTEAKGML
SRTPRDIIAKRPLRDGVIADPDMTEKMIKFFIQKVVPRNWFFKPRIVIGVPSCITKVEEN
AVIDSAYKAGARSVKVIPESLAAAIGAGIPIVEPAGHMICDIGGGTSEISVISLKGMVVT
RAIRVGGDEFDEAIIRHIKNVHNLIIGDQTAERLKIEIGNASPERTIEKVEIKGNDAITG
LPRRFEVDSVEIREALIASVNQIVDEVKKTLSETPPELAADIVERGIVLSGGGALLKGLP
KLIAKETGVPVILAEKPLECVAIGAGKYFETMNSHEGSESVYESLNL*
>SPSA8_v1_10003|ID:41144772| Cell shape-determining protein MreC [Spirochaetes Bin 1 SA-8]
MPRKQKKARVPFLHTTAIIMVLSSLLMVSISTKTLNDFPARAFRAVGSSVQEIFYKVGSF
FSNTILSIQKLKNLQNQYNSLAEKLESYYQMERDFADTKAENERLKEQLGFYQTINTVKT
SAQIVASEPGNLYETYTINKGSSDGVQRNMAVVAFQNGMEGLVGKIISVNASTSVVLPLF
DKRFYAAARFSRTRSAGLVNGRGSFLDDLVMEYVPRPNASEIQIGDLVVTSGLDSIYPAD
LAIGRVKSIETDVIGTAVTIHLDPALDFSRLEYVFVIETQKIVDNSNGEVIKSEAKP*
>SPSA8_v1_10004|ID:41144773| putative Rod shape-determining protein MreD [Spirochaetes Bin 1 SA-8]
MIKSVSLASMLAVLILFIQTTWFKNGILASATPDFTLLLILWMSFSNKNIEGLLAAFVIG
IAHDFLSSSPLGFHAFLYVVPAYVALLIRASIFIDRILMPFAAALLATILKALLSIFLAR
LFGKGAIIAYSFGNIRFWMECLMNILLAPLVFWVLSKTGKAFITKSHTE*
>SPSA8_v1_10005|ID:41144774|mrdA| transpeptidase involved in peptidoglycan synthesis (penicillin-binding protein 2) [Spirochaetes Bin 1 SA-8]
MNNKKSIPEKTKYFKILVAVIGVVYSIYLFSLQVIQAQNFRTEALRYASQSMKLPAIRGE
ILDRTRNVSFASNEDSFSLAVIPAEIEKGLHESLFLKIAEATGISLDSIMKKIPPKYYHL
YQAIIIASSVDSSAIIKIAENREQFPGVYWNPQPKRKYPAIGSMAHVIGYVGEITRDEYK
LLYNKGYSANDVIGKAGLEKVYDEVLKGKDGYVLKNVDVKGKDLSAYESKVEKPVNGKNL
VLTIDAEIQKTAERALGQRMGSVVVLKPATGEILALVSYPWFDPNLFTSNKAGDEYARLL
QDEKKPLLNRAVQSSYPPASTFKTVLTAADLEEKAFPIDGKIVCKGVIDYGGREWNCWIH
RPGHGPLDLKGALAQSCDVYYWTLGRDYLGIDTIVSYAKEFGYGAPTGIDLPGEASGFVP
TPQWKEKTYNERWTPGDTMNLSIGQGYMLASPLQVANMMAMVANSGKIMKPHLVREVRDS
GTGALLSSVEPELLATSNISKESFLHLQEDLRGVIVNGTARTPISTKVVQIAGKTGTAEV
GLKDRWHSWFASYGPYDAPVDQQIVVVTMIEATNPWEWWAPYAANVIYQAIFGGQDVESA
AKTVGVNIDRMNIRGRTE*
>SPSA8_v1_10006|ID:41144775|mrdB| Rod shape-determining protein RodA [Spirochaetes Bin 1 SA-8]
MKTLQVPKFLSDMDYLIFTSVFFLIGIGILSIASAGVDADGFRFSNEYLKQIIWAVSGFI
LMLLAISLDISRLKDYFIFAYLFILLILIYTRFAGKVVNGARAWIGVGDYGIQPSEFAKI
ITILFLARYLDAAEMESSFKRLVISSLIIGIPVLLILSQPDFGSALVFFPILIAMLFMAD
VDKRYVYFILGTLLTTFLLLIMPLAGSYFFPKGNPVELLYQRRIILNVITMFSIAVFALS
AYGWIKFKKRYYFWISYFFSIFNVSIIISFIAQKILKEYQIMRLMVFLNPRVDPQGAGWN
IIQSITAIGSGGFSGKGYLQGTQSHARFIPQQSTDFIFSIIAEEWGFVGSLVLVGLFCFF
FYRIVLLIESTKDRYSALVASGIFGMLVFHFIINVGMAIGIMPITGIPLYFISYGGSSLW
AAMTAVGILLGISARRYRM*
>SPSA8_v1_10007|ID:41144776| Radical SAM domain protein [Spirochaetes Bin 1 SA-8]
VKKLSLINPVEAFGSQLFSIIKPARYIGGEVGSNKPVAETEQRFIFALSFPDLYEIGMSN
NAVRILYNDLKTMSDSVVCERVFAPAPDFENLLKQKNVPLYTLESGIPLKQCDLIGFSIG
YELLATNILTILDTGKIPLRVSDRGDEDPIVIAGGPAATNPLPFSSFLDAVYIGEAEAGF
YNLLKKLAEIKKSGGSRSAMLQAIADENSFWLSPESPVARDPDGNMRLRKAVRAVQAEFG
RQAKDTAYPIPVLNTIQNHGVVEIMRGCPNGCRFCHAGFFYRPQRIKPPQQIQEEVRNLI

SKGGYREITLASLSSGDYPGIIELVHSLNDTWKSKKVSFQLPSLKVESFTLPLLSELSET
RKSGLTFAVETPVDSWQKSVNKTVEFEKITAILHEAKNYGFRSAKFYFMIGLPVPGRGMG
EARAIVDFLLKISHVEKIAINVNIGTFVPKPHTPFQRSGQLPEAEALAAIHYIKDSLRPY
KFISVSYHSPFTSMLEGIISRGDERVGELLLNAYRKGARLDAWEEHFDRNRWKEILLESS
LQLGFDLEKEYLQPIPENHEPPWEREINLFVSKTYLEDEAKKSIESEMTSVCDDECDHPC
GSCNSRFSIVNKFIQSEVRDRASMPQPEDFGYKITKTQDSILEDQRFGIVFKKTGKGIFY
PLHSISNIFARALSILEFPVRFSEGFNPLPKMEFTQPLGLGIGSEYEVLAVWLKDIIQIT
DSDLFIKSFNCQMPQGIEVVSVRFGKKRSAGKNSIGTLYSGSSFQISLKEKMDINSLLIS
DKFNLLKSELEKRTEVESYDEVQKNVSVYVNDSHGGENNIQHVLKDILQIENIHDECRIV
KIQSWAQIQENDGNRRIPLMEAL*
>SPSA8_v1_10008|ID:41144777| protein of unknown function [Spirochaetes Bin 1 SA-8]
LGIEYPSVRQVPGFFYSKRGIAAMRTRTMIKYPTLFGFILLCLVSISAGSCGLPKILYLY
PPLSFYASGSSTVILVHDTKNYDALEGANQSFKGYEIFYRIYDTESKAISDISLINNKID
IAPDSPDIIMRYAADTLKFLRMKNSSTSSQPLITIGNPENSGQFDLTLNQDSEWTISGET
LGISIDVVRTITDSTRLSFSQKSNYLSSDSDYSGTTDSPDSLYAVFFAVAYAHDPETIGQ
IVYSIPVVLSVPIQF*
>SPSA8_v1_10009|ID:41144778| putative Tetratricopeptide TPR_2 repeat-containing protein [Spirochaetes Bin 1 SA-8]
VDKKSTAAKPANAAPVEKKSLTDKINEFFTKNRTAIIAAVGAILAVIVIIGAYTLISNSV
AENSSRAMELARTKLEQWGQESDETKKTELESAITADLDAIIKKWPRTFAAQHALFTKAG
IATANKKWEDAEKFALEAANKLPKTYLAPIALEAAAVAAEEQGKADKALEYYQKYVKTYV
SDTPGLAHAYFSLGRLAEASSNWKDAIANYEKLTADFAASDWAKLAKDRLIYLKAQGLDK
*
>SPSA8_v1_10010|ID:41144779| Transcriptional regulator, NifA subfamily, Fis Family [Spirochaetes Bin 1 SA-8]
MKVMTESIDPAKFALLIETSALINSNYADAKTLLTQIIEFSARLVDAEAASLVLYQADKN
KLRFEVAVGPRQHELTGQSIDAEKGIIGWVIRHGKSLIVNDPESDVRFSPDISRSLDFPT
YSILAVPMKVKGILVGVIEVINKAGKRYFLEEDLKWLEYFALQAAIAIENARFLEKARNE
IAYLSEKAQELEGVHEIVCASRIMLEKVELADRIAPSDASVLILGESGVGKELFAERIHR
KSKRHDKPFIKVNCAALPETLLESELFGHVKGAFTDAVQDRVGRFEAADGGTIFLDEIAE
MSLKLQAKLLRVLQQKSFERVGSSQTKIVDIRIIAATNRNLEAMVEQNLFRPDLYYRLNV
LPLWIPPLRERKEDVPVLADLFIKRYARETNKCLQGFSDTAMELMLSYSWPGNVRELENA
VERAVVMSQDSTISPADLMLGTKTADIDEFRGKNLKDAVNIFKTHFIRKALELHGWNQTE
TAKSLKIQRTYLSRLVKELSIAQPKE*
>SPSA8_v1_10011|ID:41144780|rpsA| 30S ribosomal subunit protein S1 [Spirochaetes Bin 1 SA-8]
MDNKEVEMDAAAPARDEFQTQLQEQYLKSFEGLEEGDLIDGKVVQINGDFVFVDVGYKSE
GKIPIMEFGDDLPNVGDDVKVILIKKETHSGEVIVSKKRADEKVFWKSIAVAFKEHKPVD
GLIEKEVKGGYEVDLGHGLKGFLPSSKADIQRLEKTDILVGQTAKFYLERLYSEKKINII
LNRRKWMEEDIELRRNEFFQNVQIGDTVKGIVKSFTSFGAFIDLGGFDGLLHINDMSWGH
VTRPKDFVRKGQEIELKVIRLEPEEKRINLSLKHFTQDPWFTFEDKYHVNEIVKGKVTKL
TDYGAFIELEEGIEGLAHISEFSWIKKVHKPEDMLKPGDVVDCMILGYDLQAGKVSLGLK
QVQENPWDTINEKYPAGMRLTRKIVKVTNAGAFIELEEGIDGFLHIDDYSWTARIKNPSS
VLHEGDEIEVIVLESNPEDRNIRLGVKQLSDDPWRSFARAFRVGSIVEGTVSNVTDFGVF
VKVQGDIDGLVKKQDLTSDRDESYEDALKKYTPGMQVKAVVIDLNPDRQKLGLSIKDLVQ
RQQREELSKFIQDEESDAGYTLGDLIKERDNSRKNS*
>SPSA8_v1_10012|ID:41144781|cmk| cytidylate kinase [Spirochaetes Bin 1 SA-8]
MIVAIDGPAGCGKSTIARMLSQNLGFLYVNSGNIYRAISLWAVEHGIPPEEGSKIIEEAR
KLDLRYQSDGSLTMNGRKLDRELRSAEVDAVVSQISAIPEIREIVNMIVHESAKGRDAVA
EGRDITTVVFPNAEVKFYLDASVEERARRRLNEHVSNQNEDEIIRNIQMRDQIDKTKPVG
ALKIAEDAEYLDTSGLTIEQVYEKVYSKILYTREAHGQ*
>SPSA8_v1_10013|ID:41144782| Pseudouridine synthase [Spirochaetes Bin 1 SA-8]
MAEEKKRLQAYLAAAGLGSRRACEKLISSGRVKVNGKPAQLGQSVFPGDEILLDGKPVIH
QEIKHYILLYKPRGYLSSMKDPEGRALAIDLLKDACPERVYNVGRLDQWSEGLLLFTNDG
NLALKMVHPSNNLEKEYEVETDKDLPSDFKLRFENGVDIEGVRYTAKSVSVTGAKNARIT
LIEGKNREIRRVLEFFGLRALSLKRVRIGSLVIGNMQPGEYRKLSDAEIQSLQKSFKPNR

GGSFNDSSN*
>SPSA8_v1_10014|ID:41144783|scpB| Segregation and condensation protein B [Spirochaetes Bin 1 SA-8]
MEIEETTALVGAVLFLESEPIDEKGISRITGLDEAVVAEALQQLCVEYENEIHGFAPMRS
AGGWILAPKVTLWEKLKDHYGKKNETKLSKAAMETLAIIAYSQPVTRAEIEAIRGVSADG
MMRFLLARGLIQEVGKKDIPGKPMQYGTTTEFLKYFKLASIADLPRLDELENSRFSLDES
EKG*
>SPSA8_v1_10015|ID:41144784| Segregation and condensation protein A [Spirochaetes Bin 1 SA-8]
MNTSTTYQTESGMTVPSAQFHEHQENRRNFRLKDFEGPLDLLLFLIKRNDMSIYDIHIAS
ITEQYLECLNSEEDISLDELSEFYNLAATLLLIKSRMLVPSSEIEDDDFEDPRQGLIEKL
IEYQKFKKLSTLMEQREMEVEWTLERSCMQRTLPFASAEAEDVWMDIDAWDLLRTFSSLV
QNFSSERIIDMYEEISINEKVALILEKLDKNQSLSFTELITRPRSALDLACAFLAILESV
KNKLITIKQHKLFGDIRIFKRNSEVMHGN*
>SPSA8_v1_10016|ID:41144785| 2-amino-4-hydroxy-6-hydroxymethyldihydropteridin epyrophosphokinase [Spirochaetes Bin 1 SA-8]
LESVRVFLGFGSNIGDGIANIQNAACEIENRTGSVVRLSGFWRSKARYYLEQPDFINAVG
EIRCCLGAEELLALCNAVEAEMGRDRSLVAEKGPRVIDIDILLYGKEIIASDRLIVPHPG
IRERKFVLLPLMQLDSTLQDPVTGTLFQDILSRIPRQGIYPILSQRYDAPYPA*
>SPSA8_v1_10017|ID:41144786|recA| DNA strand exchange and recombination protein with protease and nuclease activity [Spirochaetes Bin 1 SA-8]
MARKSAAAASLDNDTGIPELSSQKGNASADNSEKLKALEVARLHIEKQFGQGSIMKLGAH
ESSQGIEAVPSGSILLDEALGIGGYPRGRIIEIYGPESSGKTTLALHAIAEAQKLGGIAA
FIDAEHALDPVYARNLGVDIDELWVSQPDNGEQALDIAESLIRSGAVDIIVVDSVAALTP
QAEIEGEMGDSHVGLQARLMSQALRKLAGTLAKSKTILIFINQIRMKIGIMFGNPETTTG
GNALKFYASVRLEVRKVETIEKGDEDAIGNKVRVKVVKNKVAPPFRRAELEIMFGKGISR
SGSLLDAAVKYNIIEKKGSWYTWGEEKIGQGRENAKLFIEQNADYAEKIEKLVREKIFEK
PASASSSDSTSKTGQIAAEPRPELNPKSENQDVITADDLF*
>SPSA8_v1_10018|ID:41144787| conserved membrane protein of unknown function [Spirochaetes Bin 1 SA-8]
MKHQPELFVAYRILFNKDIDSGKVTRKRSRLSRGSFGGAVLGIGISLIPLIIVLLVSDGM
IEGITNRYMETKTYHLQIALPLEAGKDAREIMSALQDAFPGDRFFEERSGPAVAVAGSEN
HSVYLRAVDQSFCSDPGTIRYVRIEDGEFGFSASKSIVLGKPLAEALGISVGDTLTVLTP
RKAEPGDSEHLESIANYQPKLSFYRVSGIISSGYHELDAQLAFIPLKEGEKILNFAGASS
FIGIKTDKPYGKELEIARNKIAAIVSQIGLSWFEPVFLRSWIQIERSLFRSFEVTRSTLL
FIMAIAIAVAAINLSSALLTFVADHTTEIAIFRSYGASSRSIGAIFLWAGTLTGAVGTFV
GMVLGITISIFINPIIRGMEWLVNSLNVLFALLAGQPALPVKLLDPSYYLEKIPVTVNII
EIGIISTASLLVCIMASLIPARNASKISVLELFRKT*
>SPSA8_v1_10019|ID:41144788|lolD| outer membrane-specific lipoprotein transporter subunit ; ATP-binding component of ABC superfamily [Spirochaetes Bin 1 SA-8]
MNEIFCSNVCKSFVSKAERLDVLRSLDLSLPASCTCAIMGASGSGKTTLLSLIGGLEGFD
SGEIQVGVHKLHTMDEKKLAIYRSETIGFVFQFHYLLKDFTALENVALPAYLKGAEKKKA
WEKAEALLEKVGLADRKNHFPSELSGGERQRTAIARALVNDPILILADEPTGNLDRHNAE
SVAELMFRLPELSGATVILATHDEALARRAQFVFRLSLGSLEAV*
>SPSA8_v1_10020|ID:41144789| conserved membrane protein of unknown function [Spirochaetes Bin 1 SA-8]
VKPGPLLYVARRWGYSSRKKASARRSMLASAGIAAGVAALIVVVSIMGGLQKGYIDAILE
ISSFHVRVVLSPDTDPISASEKIKNVGGIVSVLPFQETTMLAAGPGRKTATMLIRALPDD
AAERDPSMMQALNFKAIETSLSDTRIFMGSEAAFLLGIGEGDSLELLNIVSDDQSGILPF
STAMQAGPMFRSGYYEFDSGMAFISSKSFLAASMKGKPWVLGIKLNQKYRDFQAKNQIAA
ILGSDIVSIQTWREYNRSFFGALRTEKTVMMLLVGVIFLVVGMNIFHSMRRTIASKMQDI
AVMKSFGVLGGELNSIFIMEGLEIGILGALAGFAAGMILSSNINEIIRIFLNLAANLAAV
FQSLGLKISQYTYQRLTTSYFYLDKIPVSITSFEIFFIIASAIASSVFAAFLASGKTALA
RPSEVFRNE*
>SPSA8_v1_10021|ID:41144790|ftsY| Signal recognition particle receptor FtsY [Spirochaetes Bin 1 SA-8]
MGFAEKLKNFFKRSTLDDTVYDDLADLLVEGDIGASFAFDLTDRLRMQCKKQKVEDVETA
RKILKDLLRQYAVKTDFLLDSSNLNIVLLLGVNGVGKTTSCAKLASYVMKKNPGSKVILA
AGDTFRAAAVNQLKIHGERLGVRVVAQHQGADSAAVLWDSIEAAQSDKASLVIADTAGRM

HTRQDLLKELEKMDRIIEQRTNSANYRRYLVLDATTGQNGIRQAETFSGAVKLDGVILTK
YDSTAKGGMILSLARQFGLPTVFIGTGETYKDFAPFDPEQFLNDFIGI*
>SPSA8_v1_10022|ID:41144791| protein of unknown function [Spirochaetes Bin 1 SA-8]
MAVLLQLMRKTIQLNSKRYGKLIILIYLVFEVFFSYAQSPAKKIGFASYFVSNSSLLDSL
EKSASMAEFCQISAAMEGSAVQKTNPLAMTLPALMRDALRGMQRGNSRLAVSPFEARFLD
GNFFSVNELLKEAEKLQNLDGIIGGAFLVKDSKVDIISFYQEVKTSQFFIHRAVFDVFSI
EEFPPTVLASFSKLLWSQVYSREELKSALRSSSGTDAEAVQLIQVSQDILAWQFKEAHSI
AQKKLTASMSRTIVSIPVTLLLFGFYQGYREAAFRNSDFTAASNFFLGTALAGSAVSLGF
LVDTFINLRQLLRTSF*
>SPSA8_v1_10023|ID:41144792|prfB| Peptide chain release factor 2 [Spirochaetes Bin 1 SA-8]
LTYGGVFDAASLEQQVAEKEKLTESPDFWNDAAKAGKLYAQIKQIKDRIEIWRSLCTDIV
SLEEYLELALAENQQDFEAEIQETLNALRERFDQAVVLELLSGEADSTDAYIAIHAGSGG
TEACDWASMLLRMYTRWAERRNFKTEVVDILEAEGGIKSATLQISGSYAYGYLKGESGVH
RLVRISPFDANARRHTSFASVYVLPVLDDSITIDIRPEDIRIDTYRSGGAGGQKVNKTDS
AVRITHLATGIVVTCQNERSQYKNKDVAMSVLKSRLYEYYRQEKEKENSKFQSDKKEIAW
GSQIRSYVFQPYTLVKDHRTKFSVGNVQSVMDGDIDPFIESFLRWKWNGGTVAADEEDDS
IE*
>SPSA8_v1_10024|ID:41144793| Aldose 1-epimerase [Spirochaetes Bin 1 SA-8]
MSVEKKYFGTLADGSDVSLYILKAGRFQAAFSDYGATWVSFLMPDRNGVIEDVILGFSTL
SPYAAKHPFFGSTVGRFANRIADARFKLDGKEYQLFANNGTNHLHGGRRGFDKVLWSASI
FESENAVRFSRKSPDGEEGYPGNLTCQVFVSLSNDGRLKLRYTATADARTPVNLTNHAYF
NLEGEGKGTILNHILTLNASRYLPVDSRQIPLPGAPLTVEGSVFDFRKPKRVGADMGSEL
SGYDHCYVIDRGETTSIMECAAISAPISGRRLKVSTTLPGVQFYTGNNISSLTGKQGSLY
DKYSGFCLETQYFPDSPNRDDFPSCILSPGQTWEHETEYCFSIS*
>SPSA8_v1_10025|ID:41144794| conserved protein of unknown function [Spirochaetes Bin 1 SA-8]
MKQDGKVDQIPIALVTGATSGIGKAVAIELTERGYKVYGTGRMPPQPGKTDEHGIVFLAM
DVLSETSVTEGIGRILEKEHQLDVLIACAGMGIAGSVEDTSMSDADLQMRTNFLGTVLVV
KTCLPIMRQQKHGKIVIIGSLAGRIGMPYQAFYSSSKFALEGFTESLRHEVKPFGIDVCI
VEPGDFNTGFTAARKKFGISGSPYSKAFLRTISIQEHDETHGAAPSIAARAVAGILEKKN
MPLRLVVGPVFQKFAAGLKRIIPAKLFELLYKIYYRL*
>SPSA8_v1_10026|ID:41144795|cmk| Cytidylate kinase [Spirochaetes Bin 1 SA-8]
MEPIIIAISGKSGCGNTTVSRLVAEALGLKFINYTFRALAAEKGLALADILEKADKDPAW
DRLLDARQIELARQGNCVIGSRLAMWLLPEATLKVYLKASQSARVDRIHQREGGDKEALA
RFTAERDARDQKRYKEIYNLDTDDISPAHLVIDTERWTPKQISHIIVSAVGEIDMIL*
>SPSA8_v1_10027|ID:41144796| conserved protein of unknown function [Spirochaetes Bin 1 SA-8]
MFTSMTGFARREFKTDIISGVMQVKSYNNRYLDVAISLPPQLSMFEPLFQQALVENIRRG
KIEFNLRVKSFSYPISVQPDTSAAKAIYSALAEIGKSLELTEKPDLALIAGFDGVLQYDR
EVDVDIVWQSLRPILDELIAEFNEHRTREGEATQTNIFSELERFASMLAIIRQHASSMDE
MIKNQLRERFAELLPNGYDEQRLLQEVAVQLVRLTINEEIARLEAHIAAFKIVSAEQMPA
KKLDFLCQEMNREVNTIGSKNILIPVSHAVIEMKDALENIREQLKNIE*
>SPSA8_v1_10028|ID:41144797|murC| UDP-N-acetylmuramate--L-alanine ligase [Spirochaetes Bin 1 SA-8]
MIQQFPENLNSARFHFVGIKGTGMTALAEILVRAGAQVSGSDVPDVFYTDKILSSLGIPV
YENFSEKNVPADVTTIIYSAAYNPATNPELLEAKRRCLSLFSYPEALGALSRTMISAGIA
GVHGKTTTTAMAGSIMAQASFPATILAGSAVASFGDRCTMSLGNRYFIAETCEYRRHFLN
FSPAVIVLTSVESDHQDYYPTYDSILEAFVEYGQRLPKNGTFIYCNDDPGARKAAECLRE
DRKDLNFIPYGFSASGDFSVQSSRQEEGRTAFRLGLFSEEFTLHVPGKHLVLDAAGAIAV
CTTLYKIENPQTTAQELQSFLLSAQTALAAFQGSRRRSEVIGEAGGILFLDDYGHHPTAI
KATLEGIKAFWPSRRLVVDFMSHTYSRTRALLDSFAESLTPADAIVLHKIYPSARETPDP
ELNGRTLLKKVEELPMPGKWSFLLYTEEPMDALDKLETLLKPGDLFVTMGAGDNWKLGRA
LYEKLAERTQ*
>SPSA8_v1_10029|ID:41144798| putative 4-alpha-glucanotransferase [Spirochaetes Bin 1 SA-8]
MQGTKVIFGTYNSMPHGISSRIFERTYQNSWRPFLSGLYKFPTIHAVLYYSGTVLQWIEE
NHPEFMLLLEEMLGKKQIELLGGPYFDPLYPVIQPGDRLGQIEMLTTYLRKAFGKRPSGT
WIPEYSWDASLPSVFRNSGLSYSFLPLKLLDTDNGDSDTVFKPRITEDQHKLFYIFPVVD

IEEDLETIASFEDIVQNQFSSNPNLPLISVMLRGSAIPRMWEVSGLASPDVLFEKTFAWF
QKNCLIFETITAQSYMKQVRSGSLFYLSPCASSRMINKIASPIKQNSYCNANPVRKLIMQ
NPYSKRLFDKMYYMHAIMNLLRGDKTRKKVAMEELWKGQNGNAYWEGDFGGIKRPEIRAR
AYHALIHAEKATRAHGSFSPGLVFDDIDCDGESEAAYQANDYNCYVSNKGAAVFELDSLK
SKHNYCCTYSSNMLIPPECLRDKFYALGSFLDEQLDMSKLQYSLIEADKNINRVVFCKEI
SLRQGDSIHPLFLRKNYLFQKHVISVDYEIINRSTTAIQFRFGPEIRFQLALDMRDLEFF
RYESHDRIPIPAEAESLEFQSIAFLSYNNIFKENLELRSPKNTWFSISHSIEELPPSDEY
NTFAVMPYPYTEAQSDFQAVHERIYQGTVIKTGWDAILPPDSASQFSLSIHLHT*
>SPSA8_v1_10030|ID:41144799| Integral membrane protein [Spirochaetes Bin 1 SA-8]
MNTISKPPFSALIANPIFLSALISLFIAQLVKAIIVLLKVRSRKELEEEXKETXIIFLWR
TGGMPSSHAAVAVAITTSIAIEEGFSNLFILAFFITLIVIRDALGVRRSSGLQAKTLNRL
GQELASRLGIAYSALKEVHGHTWPEVIAGAMLGLGIAICMAHIIA*
>SPSA8_v1_10031|ID:41144800| Pseudouridine synthase, RluA family [Spirochaetes Bin 1 SA-8]
MESTIFQATPNDHGRRLDRILRKLLKNQSLSFIYKSLREKKILVNGKSAVPDYRCQSGDF
IEIFLSIHASLPVSVQPEDQQKETADRLKNMILLNTADLLILNKPRGILAHDGKDSLDAL
VQAFYQSSHPEKSLAFKPGPLHRLDRNTSGIITFSQSIEGARQFSAGMREGKLKKTYLAI
LDGCLSEPESWTDTLERDEKQKTTVLAEPLSNNSLKSARTDVWPLACHEGFTFAAIRLFS
GRTHQIRAHSSIHGYPLSGDRKYGSRTNLQYYFLHAWMLDLSFLKLPMLPEQIQAPVPPE
FLFFLSKRLCLSEKEVYSLLQSYAKNF*
>SPSA8_v1_10032|ID:41144801|hflK| Protein HflK [Spirochaetes Bin 1 SA-8]
MPGTVFKPKIPKINLKGSTIIIGLIIIGVIAFLSTSFIIVDQTENAVITTLGRYTKTVGA
GLHYKLPLGIQKAYLVQTKIIQTESFGFRTIKPGVVTQYSNKKYPEESTMLTGDLNIVDV
EWIIQYRISDPKAWLFNVSDRNKTIRDISQSVMNRLVGDRSILGVIGPDRQTIQDSAVIL
MNEILHQYGLGIDIVQVQLQNIVPPQGVQDAFEDVNKAIQDMNRLINEGKEAYNAEIPKA
RGQADQTLEIAQGYATERVNKARGDVARFISVLDEYRKSPDVTRKRLYYEMIDEVFTGAE
NVELIDKSLNNFLPIKNLGQNSSGVTK*
>SPSA8_v1_10033|ID:41144802|hflC| Protein HflC [Spirochaetes Bin 1 SA-8]
MKKSILILIILAIVIGGVVIAKPFFILNEGEQAVIVRFGRIVKVHTEAGLKFKSPFVDEI
HKFPKRLMSWDGEPQRIPTKENQFIWIDTTARWRIVDPTKFYESVSTLENAYGRLDDVID
SAVRTVISSNYLREAVRNSDEILKSGKAETFQTGDTEGSTSLEQLTISQTQYEKIEKGRQ
VLSSEMKTLIAGIAPSFGIEVLDIIPRQIKYSEELTDSVYQRMIKERNQIAQAFRSYGEG
KKAEWLGKLDNEKRSILSSAYAKAETTKGKADAEATKIYAEAYGRDPSFFEFWRATESYK
RTMPNFQKTMSTSMDYFKYLYSPTGR*
>SPSA8_v1_10034|ID:41144803|ybiT| putative transporter fused subunits of ABC superfamily: ATP-binding components [Spirochaetes Bin 1 SA-8]
VITVTDLSMGFGERSLFKDVNLKFVPGNCYGIIGANGAGKTTFLKILAGEIPYDRGTITI
SAHSRLATLKQDHFAFEEYRVLDTVIMGYEKLFKVMQERDTLYAKEDFSEEDGIKASELE
AEFAELGGWEAESQAGILLSGLGVDERNHEKLMRDVDESVKVRVLLAQALFGNPDILLLD
EPTNGLDLESIAWLEEFLIEFPNIVIVVSHDRHFLNSVCTNICDIDFGHIRMYAGNYDFW
YRMNKILVQQQKDEKKRREEKSRDLKDFIQRFASNASKSRQATSRKKILEKLEIDNLVPS
SRRFPYVSFKPAREIGNNVLRVVNLSIPGEARPLIEDFSLTVNRNDKIAFVGHEHLAKST
LFSVLAGETAPLRGEIYWGQTVQKGYFPKDSSQLFDSDLTIVEWLQQYTDSDDETYIRSF
LGRMLFSGDDALKPVRVLSGGERVRCILSRLMLISANCLILDEPTNHLDLEAITALNDGL
IDFPGVLLFASHDHEFVDSIANRIIEICPGGIIDRYMSFDDYMGDSQVKELRDQYYHGHV
IVEI*
>SPSA8_v1_10035|ID:41144804| Basic membrane lipoprotein [Spirochaetes Bin 1 SA-8]
MKRTLIVVLALLSILVVSCGNNQAKAKAPAMPTTRLLTDATGIDDKSFNAAAWRGILSFY
GDSWENQAQKGKLYDVVTAQTQDMYIPNLKQASDEKYDLVIVTGFTWADALGEVAPKYEK
QKYLIVDVDWVGKPNVMEATFSEHEGSYLVGVAAALKAQADGIKNPKFGFIGGVPGPVIT
KFEMGYVQGIKSVLPNATIVDYYANDWGKPELAKAQAKNWYDSGVYAIFSAAGGTGNGTI
AQAKEYRSQGKNVWAIGVDSDQYEDGIYAEGKSAVLTSMLKRVESATKYALEQVKANTFK
GEVVVFDTKADGVGFSDKNSELGADIVAKVNEAKAKIISGEIKIIPTYAEAKQAGVVPGN
LQASDNQ*
>SPSA8_v1_10036|ID:41144805|yufO| Uncharacterized ABC transporter ATP-binding protein YufO [Spirochaetes Bin

1 SA-8]
MEDFAIEMLGITKAFPGVVANDDVTLRVKDNEVHAILGENGAGKSTLMSILFGLYQADSG
TIKIRGKEVRITDPNHATRLGIGMVHQHFKLVHNYTVTENIILGSEPRNRFGNTDVKSAE
KRVSELSRLYGLDVDPRSTIDSISVGMQQRVEILKILYRNADILIFDEPTAVLTPQEIDE
LMKIFARLKQEGKTIVLITHKLREIKQAASRCTVLRRGKAIGTVDVAEASESEMASMMVG
RDVKFSVDKAACNPGKVILEIKDLTVRNNRGLPAVKNLSLNVRSGEILGIAGVDGNGQSE
LVEALTGLRPAEKGTILVNGKDITKTSIRERISAGIGHIPEDRHKHGLVLDFRLDENLIL
KTYQSKNYAKPFGVLDPKPVRENAERLIAMFDIRAGQGPATKAKSMSGGNQQKAIIAREI
DLSPDLLVISQPTRGLDVGAIENIHKRIVEERDKGRAILLVSFELDEIMALCDRIAAISK
GEITGIIDQVEAEEHEIGAMMAGIHKTASPKAASV*
>SPSA8_v1_10037|ID:41144806| ABC-type transporter, integral membrane subunit [Spirochaetes Bin 1 SA-8]
MKKNTTSFIYKALASDAYVSIFVVILGFLLGTILVVIVGKNPAGMYQAMLQTLTGLDITR
GVWNSRYIGEWLTASVPLILCGLSMGFAARTGLFNIGAEGQYIMGLTVAQFVALYFPAVP
FLHWLVALLAAGLAGALWGGIVGWMKAKFSVSEVVATIMLNYIAFFFSRWASMQIPGANT
YKTPNFPETARLSSPLLEMLTNGSRLNRGLWVALFCVLIYWFVIEKTKLGFELRATGLNK
EAARYAGIKVTRSVTTSMLFAGMFAGLAGAAVALGSFNYGRVLAGYDNYGFNGIAVALVG
NSTAGGTLASGLLFGMLASAQPLMQSRQIPKEITWIISGLVVVLISLRAGVKMFLDWRAR
KLAKELKEANHE*
>SPSA8_v1_10038|ID:41144807|yufQ| Uncharacterized ABC transporter permease protein YufQ [Spirochaetes Bin 1 SA-8]
MNSLIAMIPSVLMIVSPILITAIGGMISERAGVVNIALEGLMGIGAFTAATVHYFLEPST
QLSVPIALSAGLAAGLVFSLIHALASVTLNADQVISGTGINLLSTGATAFLAQIIFKMER
TAPFRLGMMPGLWGIYPTAWIALVIVLISWFILYKRPFGMRLRACGEHPQAAASAGINVR
KMRYIAVMISGALAGLAGGCLVLTQTIQYTIYTINGAGFIALAAVSFGRWLPKGVAGASL
LFGTAVALAIYMVNIQSLRFLPSEFFSVMPYIITLITLILFSGKEYAPRAAGQPYERSGS
*
>SPSA8_v1_10039|ID:41144808|deoD| purine-nucleoside phosphorylase [Spirochaetes Bin 1 SA-8]
MDNSFFHQIEERGLAHFSNGTPHNTAKIGDIAKLVLMPGDPLRARFIAETRLSDVRQINA
VRNMFGYTGRYKGKDVTVMGSGMGGPSMGIYSFELFAFYGVEQIVRIGTCGGMTDAIDVG
DLVIAMTASTDSNYAHQYNLHGSFSPCADYGLLEKAVTSARKRGIKFWTGGIFSSDVFSL
YSALPPEEGWKKWADMGCAATDMECYALYCNAAYLKKRALTLLTCSDSNISRKEMTPIER
QTALNGMFDVALDLL*
>SPSA8_v1_10040|ID:41144809| Rieske (2Fe-2S) domain protein [Spirochaetes Bin 1 SA-8]
VVLGSWQLKDKPLGVTRLGGKLVFYRTRKGKPVCLADKCAHRGVALSIGKLCEHDTIQCP
FHGLEYDPAGKCTIIPANGKNTPVPPNFKVRSYHTFEDHGFIWIWWGDSAPSPEKPEFFD
DIPEEAKYANSIDHWNAHYSRVIENQLDCVHLPFVHYNTIGRGNRTLVNGPGIEWVHKNK
FFMYVYNEVDRGQTPKKNSEVPIPSPSRYKIEFLFPNLWENRIDDKVRVVAAFVPVDEEN
TLLYLRFYQAFARVPGIGTLIAKSAMPFNLFVAHQDRRVVITQRPKASALASGEDLFQGD
YPIIQYRKRRNELKKLAGLEE*
>SPSA8_v1_10041|ID:41144810|tyrS| tyrosyl-tRNA synthetase [Spirochaetes Bin 1 SA-8]
MNKALETLKARGFIQQCTDIEGLSKVMDEGPVTFYVGCDPTGPSLHIGHMVPFFALRHLR
DAGHIGIALLGGGTARIGDPSGKSEMRKMISYEDIDRNADLFKTQLDRFIGFDGKHSFTA
NNKDWLANLNYIEFLRDIGKHFSVNRMLSFEAYKIRLETGLSFIEFNYQLLQSYDFLQLY
KRHGCRLQIGGDDQWGNIVAGIELIRRVEQAECFGLTFPLVMTSDGKKMGKTEKGALFLD
PAMTSPYDFFQYWRNIQDADVERFLLMFTFLPVDECKKLAAGKDAEINKAKERLAWEVTA
LIHGKDEADKALSAARAAFGDGGNADELPSVTLPAKRLETGIGVLDLFVESGLACSKSDA
RRLVQQGGASVNGVKISNETATISTEDLDEGILFLRAGKKRVCRVIVQ*
>SPSA8_v1_10042|ID:41144811|hupA| HU, DNA-binding transcriptional regulator, alpha subunit [Spirochaetes Bin 1 SA-8]
MADKATAMTKNELVSALAEKTGLTKKDAKAALEVLAQIAYKEVKKNKKFTLPGFGILKVS
KRKARMGRNPATGESIKIPARNVLKFSVSKACKDAVL*
>SPSA8_v1_10043|ID:41144812| Selenium-dependent molybdenum hydroxylase system protein, YqeB family (fragment) [Spirochaetes Bin 1 SA-8]
MDSVNVFAHAARLCSAGVNFAITSIVQARGSTPRASARMLVEENGTCIGTIGGGLLESMV

VKDALECIKHGTSAVKTYVLTSCSSTNESPIQEAGPYSDTARLDMNCGGTVDIAIDVVAG
RRQLVIIGAGHVGLALANLADMTGFSVVVVDERPALASKERFPMAAAIYSNADLVKAIHQ
VPEQEGQIVVIATHSEDERALRTMVSRKWAYLGMLGSRRKVKLLLEKLAGEGVPAEILAK
VHAPIGLDIGSETPEEIAVSIIAEIMKTASGTSGLSLSEKETV*
>SPSA8_v1_10044|ID:41144813|glyQS| Glycine--tRNA ligase [Spirochaetes Bin 1 SA-8]
MGDTQVTMEKIVSLAKRRGFVFQSSEIYGGQAGAWDYGPYGIELKNRIARFWWKEMTQLH
DNIVGLDAAILMHPRVWEASGHVENFTDPLVDCKKCKMRFRADQIPEENLASKTCPECGG
ELTDTRKFNLMFKTNIGPVDDGSGLIYLRPETAQGIYVNYKNIVQSNRMKIPFGIAQIGK
AFRNEIVTKNFIFRTCEFEQMEMQFFVKPGSDVEWFNYWRDQRWNYYKKLGVNMEKLRWH
QHGPGELAHYAKDAYDIEYEFPMGFKELEGVHNRSDYDLSRHQQYSGKDLQYIDQDNNNE
RYVPYIIETSAGLTRQLLMLLCDAYEEQKVADKGNDDDYRTVMHFHPIVAPVTVAVLPLM
KKDGLAELAQEIRGDLKEDYATDYDQAGAIGRRYRRQDEIGTPFCVTIDYQTKEDGTVTV
RDRDSMEQVRVPCPNLSDFIKTKIKEYKRQA*
>SPSA8_v1_10045|ID:41144814|glnS| glutamyl-tRNA synthetase [Spirochaetes Bin 1 SA-8]
MSESQKEESGYSDFIREAVAKDLKEGRYKEVHTRFPPEPNGWIHIGHAKAIFVDFEVAAS
FGGKCNLRFDDTNPEKEDMSYVEAIKRDVKWLGYDWEDREFYASDYYETLYELARKLIRK
GLAYVDDLDEEQIKEYRGTNVPDKNNITYTPPGKNSPYRDRSVEENLDLFARMRAGEFPD
GSKTLRAKIDMAHPNLLMRDPVMYRIRREPHYRTGTQWCIYPMYDFQHPLSDAIEGITHS
LCSLEYEIHRPLYEWFVSNCEVFPSRQIEFARLNLTHTVLSKRWLIQLVREGKVTGWDDP
RMPTIAGMRRRGYSPGAIRDFLGRIGIAKTDXMVDIQLLEHCIREDLNKKASRYMAVLNP
VKLIIDNWPEDKVELLDAVNNPESPEAGSRKIPFSNVIYIESEDFREVPPPKYYRLFPGN
QVRLRYGYVITCTGFEKDPETGRIIAVHSEYDPDTRGGDAKDNRKVKGTIHWVSAKSAVP
FEARLYDHLFEPERPMDVAPGHSFMENLSEHSLEVIPKAFGEPAIAAIPVGETVQFERIG
YFCKDPDSTRDSAVFNRTVGLRDTWAKIEKKNS*
>SPSA8_v1_10046|ID:41144815|gltX| Glutamate--tRNA ligase [Spirochaetes Bin 1 SA-8]
MEVRVRYAPSPTGNQHIGGVRTALINYLFAHSTGGKFILRLEDTDRTRYSEEYVRNLYDT
FKWLGFYWDEGPDVGGPAGPYVQSERFDLYRKYAEQLVQMDKAYYCFCDEDRLQKLREEQ
QASKTGEQGYDRHCRSLSPEEVKEKLEQKTGYVIRLKIPLEGTTVFHDSLLGRIEWKNSD
ISPDPVLLKSDGFPTYHLANVVDDHLMGITHVMRAQEWIPSAPLHKIMYDAFGWEQPELC
HLPMVLGQDGHKLSKRHGATSLNEFRKAGYLPEALINYVAQLGCSYEDGRDLYTLDELEH
LFKIEKLNKAPAVFDYQKLEWFNGQYIRQKQDAELSQLVRPFLIDAGLRKESDPGADALE
LKAMVLVKERLKLLSEAPAMMAYLYKKPQLPEAKEFLPKKLDLAATIEMLKRDLLLVDTI
DFSDILKAEEAFKEYSVREGVKLGDLLMPLRVAVTGARVSPPLFESIGILGVEEIKERIQ
VAIEWLSKALEQSGTAEA*
>SPSA8_v1_10047|ID:41144816| membrane protein of unknown function [Spirochaetes Bin 1 SA-8]
MENPVENPNLQDSNLEDSNLEENKNKPPSVLPGEQIADKTGTKIVKARKIFSFIVIFSAL
VSVAVLILFNTGSESSLPFWTLQTRTQALLAAVVFFVLMTAGKFFIGLYYRIFKLEKTSW
MNLAGLLVLVAISLVFFLLASIVITLAKGYVF*
>SPSA8_v1_10048|ID:41144817| Histidine triad (HIT) protein [Spirochaetes Bin 1 SA-8]
MEYFFNFNKLGYLKGKRPAGCILCLVGTGSDEVENLIVYERGTMLVSLNLYPYNPGHLLV
FPKRHCIDVRQLTDEEKSDLFNTQDKCLGVLDSLYRPAGYNIGFNMGLAAGASIEHLHMH
IIPRYSNEIGIAELLGGKKVLVQNPLETRELLLKAFGS*
>SPSA8_v1_10049|ID:41144818| putative Thiamine pyrophosphokinase [Spirochaetes Bin 1 SA-8]
MNCLVITGGDAPKAETLRLLSARCQLVIAADSGIDTAVSASIIPDVAVGDFDSTAYSIAS
IASQGVSVVRYPEDKDDTDTEIALRLAREKGADYIILAGAGGGRLDHLLAVFTIFSRRFH
PDEWQTKEDSIFCVPIGMTKHFRAAEQSFVSIFPLGESSAGMRSKNLKWALDGLIWDRGS
FGISNRCVGPEFSVTAGTCPLLVITPSGTESF*
>SPSA8_v1_10050|ID:41144819| tRNA adenylyltransferase/tRNA cytidylyltransferase [Spirochaetes Bin 1 SA-8]
MRLRKFRIPHEILFLIEKIEKSGKKAYIVGGAVRDYLLGRSIANDFDIATDASPQEIIRL
FNRVVPTGIKHGTVTVLIGEHSVEVTTFRVDKGYSDSRRPDTVEFTPNLIEDLSRRDFTM
NSIAYDASEANLIDPFYGQSDIARKIIRTVGNPLERFSEDGLRPLRAVRFASQLGFSIHE
DTLAAIPVCMEAFKKVSWERIRDELIKILLSPVPSVGLRLLESTRLMEHALPELLPGRNC
LQKGMHVYDVLDHSFLTVDSCKPDLELRLAALFHDAGKPFVKSSDEYGIPTFYNHDKKST
ELAVQALRRLKFPNDCIDTVCHLIALHMFHYEDNWTDAAVRRFIAKAGEQYLERLFLLRL

ADGSATTGIPADPRSLVPFRKRIDAILAEQSALQLKDLKISGNDLIDIGIPKGPVIGRIL
NELLETVLDDPAQNNRETLVKIAMGLKSKYL*
>SPSA8_v1_10051|ID:41144820|rnc| Ribonuclease 3 [Spirochaetes Bin 1 SA-8]
MDKQNEIPRPELLHRFQNSLSVSFININLLESALTHRSCRDAEMPHNERLEFLGDSVLGL
AVATIVYTELEGRPEGELASLKSRVVSEQVLSRIAAQIGISELIRLGKGEELSGGRQKKA
LLADALEALIGAVYLDQGFEAAFQLVKRLLEQEISDSLKHSSKDWKTILQEFAQKEYKSL
PTYTLGKTEGPDHARLFHVTCEIAGKIHGPYAGKTIKEAERLAAEHVCRTLARSSKRAAK
LLDEIAGLQR*
>SPSA8_v1_10052|ID:41144821|acpP| acyl carrier protein (ACP) [Spirochaetes Bin 1 SA-8]
MDELFEKIKKIIADKLEVEESKITLDASFRQDLGADSLDTYELVYGIEEELGIQIPDEKA
NEFETVRDAYEFIKTQVK*
>SPSA8_v1_10053|ID:41144822|coaD| pantetheine-phosphate adenylyltransferase [Spirochaetes Bin 1 SA-8]
MKAIFAGSFDPPTCGHEDIILRAARLFDTLYVVVAENGVKMPFFSMEERIRLVTDIAKSH
QLSNVVVARAEGLLVSFAREHGCRVLVRSIRNSRDLLYEQNMASMNRRLDPEIDTVFLPA
RHELADISSSAVRELIALGNLPDGIVPLIVRKAIENRHGLLT*
>SPSA8_v1_10054|ID:41144823|lnt| Apolipoprotein N-acyltransferase [Spirochaetes Bin 1 SA-8]
MVRTLALSLFSAVLCAISLPNEIFPYGVWLLGFIALAPLYFALLKAENHRQAFSVGALFG
AAHHALTSYWLFFYKDFAFWTLGSTTIAYAVVYGVCAMYGAFLVRSTSGWRRPLIFAIAW
TVFEYQKSIGFLGYPWGLIPYSLTSLPILLQIADITGVYGLSFILAYSSAVISDISNSAQ
NKKFLLAQGIALILAILGLLVYGGLAFSANYEKKAEIQAVLVQQNTDPWIAGEMAALEAN
IRNARKAMDEVQDSSGRTADLVIFSETSLRRPYTDFKPWFSTNPSDLPLTDFLRHYKVHL
LTGAPVVLDWNTYEASNSVILIDSEGVQHGSYAKIHPVPFAEAIPLWEYEWFRKFMREKV
GIEGGWVMGTRISIFTLPLQTGGRVQFATPICFEDAFSDLCRQYILDGAELFINLTNDSW
SRRESAQIQHWAIARFRAVENRRTLVRSTNSGVSCVIDPTGRSILELPQFTGTAALASIP
VYISHSYTFYTRYGDLFARSMVAFLCLWASALLIKAKGKWPRLIFIHVSV*
>SPSA8_v1_10055|ID:41144824| conserved protein of unknown function [Spirochaetes Bin 1 SA-8]
MSITEYLESPPFFEIERYHDHSAMDAVSFIGTLRKHPYDPEKCLLLTDRQDSLKWIEAGV
IIEFRITDVLAADELPSLVDEMGTARQLVRIWVRRGAIALRYEPFEVNEKSSGPRDSEHL
RERFNAFFHAKTGGK*
>SPSA8_v1_10056|ID:41144825| Radical SAM domain protein [Spirochaetes Bin 1 SA-8]
MKPDLTEEPRQAQAAFFQVLDDSRVQCVLCHHACVLKPGQWGICKVRTNNAGTMSIPLYG
HISSLAIDPVEKKPLLYFMHGSYTFSAGFWHCNMKCPFCQNWSISHPNPGIAKRTAVLPP
EALVQKTLDSGCPSVSLTYSEPTIHIEYALETFKIAKAANLATILVTNGSICAEPAQSLL
KLTDAVNVDIKTASEERYRSILGGDLQTVKNFIQIAYQYSHLEVTSLLVPGILDQPDDIR
IIAEFLASISPQIPFHITPYHPDFNYMQAPLTYRQMQEIAKPAYELLECVHLYRPWQDR*
>SPSA8_v1_10057|ID:41144826| protein of unknown function [Spirochaetes Bin 1 SA-8]
MCGNSSVVERHLAKVDVAGPTPVSRLNNVAIRNLAESPFFLRRKDIY*
>SPSA8_v1_10058|ID:41144827|tig| Trigger factor [Spirochaetes Bin 1 SA-8]
MVITDTKFEKLEKSRVRLTLTVPAAEVRKEYDSMMKEYINSVKIDGFRKGHVPASVLERK
FGESLRLDAMGRVLEKAVEEGLKTSEEKPLMYDSPELDGKPEFAVDKDFTFSVLYDVYPK
VEAPAIDGIEITLPKVEISEADIARELEQIRQRNAIVVEKTGPAAKGDIATVNFFELDED
EAPISGSAREDFTFEVGSGMNLYKFDEDVEGMAAGDEKTITKTYPADSEYPELAGRTVKL
NLKVTKVKQKDLPELDDELAQDVSEKFKTLDDLKQDLKKQLQTSLDERLRNLKEQAIVEQ
LLARTKIEVPETMIAAELSMRWDALKRDMGIDSDEKMEKIAAYSGKTRESLYQDWRPAVD
KAIAGRLLLDALIEKSGFTVSDEDVQAEYARIAEGTALSAEEVKAEYEKQNYVEHLKNDL
LERKFFDSVLANAVIKDGETVPFVDFMSKNQ*
>SPSA8_v1_10059|ID:41144828|clpP| proteolytic subunit of ClpA-ClpP and ClpX-ClpP ATP-dependent serine proteases [Spirochaetes Bin 1 SA-8]
MNEQMNNLVPIVIEQTGIGERSYDIYSRLLKDRIVFIDGEIQDLTADLIVAQLLFLESQD
PSRDISIYINSPGGSVTAGLAIYDTMQYLKPDIQTICLGQASSMAALLLAAGTSGKRFAL
PSSRVLIHQPWGNASGQASDISLQAREIVRLKKLTIEYFARHTGKTLEQVSKDMERDFFM
SAQEAVEYGLADKIMEPRKNVQTQK*
>SPSA8_v1_10060|ID:41144829|clpX| ATPase and specificity subunit of ClpX-ClpP ATP-dependent serine protease [Spirochaetes Bin 1 SA-8]

MSKPRSNPPGKEEFCSFCGKSASFAKQMIAGPGVNICDECVRVCEQILDQEEQHVSNAFL
GEVPKPKEIKAYLDEYIIGQDYAKKVLSVAVYNHYKRIHHRHSADSDVEIEKANVLLLGP
TGSGKTLLAKTLAKKLKVPFAIADATTLTEAGYVGEDVENILLKLIQAAGGNIEAAERGI
IYIDEIDKIARKSENPSITRDVSGEGVQQALLKIIEGTIANVPPQGGRKHPNQEYLRIDT
TNILFICGGAFVGLDRIIESRVSSHPLGFGADIKTRAEKNLRELFNQLHPDDLIKFGLIP
EFIGRLPIHVALDDLTRDDLKRIITEPKNAVIKQYQASLKXX
>SPSA8_v1_20001|ID:41144830| Glycoside/pentoside/hexuronide transporter [Spirochaetes Bin 1 SA-8]
MAQSPERKIKFKSLVSYGIGDLYGGGSFLIISTLFLFFLTDVVGLRPALAGLVVFGGKFW
DAVSDPLMGCISDRTKSKYGRRRLYFLIGIVPVFISFFLLWISFKTGSEFVSFLYYFFAY
ALFCTVYTMVMVPYSSLPAEMSLDYKERTRLSGARMIFSQLSSIISGVVPGMLVNTIYKD
NPRMGFLLVGLIFGLFYAMPWFFVFKGTWELPEAEQNMPSSSLGKAFSDMRTLFRVRSFR
IHIGMYILGYSALDVLSALFVYYLTHHIGARGYYTACLGTMLISQLIVLPLYLFIANQLG
KGKAYTIGAVIVVVIGSLFYGIPAGAPLSTLMLLSAGLGFGLSAVVVMPWAMLPESADAD
ELVNGLQRAGSVAGSFTLIRKLVQALVLWLX
>SPSA8_v1_20002|ID:41144831| exported protein of unknown function [Spirochaetes Bin 1 SA-8]
MKKSTHRDHFGIVLTLFLSFCSFVSCASSDPTKTDVVSAPSMYHRAESDIRTEIQPIMNA
GKVLVVYYSQGTAGRQVAEDIAVLTGADLEEIKEVHERKTGFFGFMITGAASTFKFSSKI
EPPQRDTAQYDTVYVITPIWSWSLSPPVRSWLKLSAGKIRQAAFITISGDTKPDKVVKDM
EKTGKVVPFAFVGYAERDFLPENRAVYVEKLKTILMGLSESKP*
>SPSA8_v1_20003|ID:41144832| MATE efflux family protein [Spirochaetes Bin 1 SA-8]
MKKLPVQKLPLFGPASFYRDALALAIPVMLQSLITGLVSLVDNFMVAGLGDARMAGVNIA
NQINFVFLIVVNVACIAGGIFLSQFRGAGDKKGMAQAFRFKIVAAGLIAAVYFLLCQMFP
EQLLSLMVAGNREGAEIVEQGVRYMRAVSWSFVPLAISAAFGSSFRDIGVPKIPLIVSTT
AALVNTFGNYLLIYGNLGAPRLEVTGAAIATVIARIFELAAFFIMLKRMKPVFVFRPSAL
FALERKFLREVLSRSAMMFFSETAWVVSETIITALYNGRGGAETVAGMAAGWSVANLIFL
VFPAIHTVANVVVGSTLGAGRLDEARQKARWVLSGSVVMGIAGGLAAALSTLGIPLVFGN
LTDSARVVTRGLIFVIGIYLPMWCLLNAQFAISRAGGDTLMGLWVDVGVTYLIFIPAAFL
IARLTDWGPVLLFGIAKISDIPKALVAAWWLNKERWVKNLTSL*
>SPSA8_v1_20004|ID:41144833| putative Diguanylate cyclase [Spirochaetes Bin 1 SA-8]
MDGTTILILLALGSCIQLLFGALARMDGNKNAFRACLAGVFLLTIGLVAAGSQDVLLPWV
SKGTGPLTIIAGAFLISLAASSLSDSREKKFQTRLGIGAGVAYSATVIFSALLPGWIWIA
TAFASAPFFLIAGIFLFEAEDRTLLKIVSGIMFSSIAALLMLAAGLKTAGFSVGILRQSN
VLLALFVMEQIVWAVFLLLFSEANGFRLAQASTHDELTGLYNLKGFSEETSKTLSMCTRH
NIAYSFFMFDIDHFRKINDELGHLAGNQILMDFAHRLSSRIRVYDILCRTHGDEFMLFLQ
SVDHEKIEPVMDRLCGGFSFQTEEGIRYSVSASVATVDHPAGRAVRFEELLAAGIQGLLA
AKQQGGARLEQVPF*
>SPSA8_v1_20005|ID:41144834| Transcriptional regulator [Spirochaetes Bin 1 SA-8]
MAHAGTRWPGAKAMEESSLEERDWKIIDVLYAEKSISRAAKTLYISQPALTSRIHLIEQD
LGTTLAIRTSKGVKFTPQGEFLAEKARTMLREYAAIRELLQAMSDDISGTLRIAASQFMM
KYYLPDLLKEFKNQYPKVEFNLTSAWSKDVYGIVRCRDAHLGFVLDTLEWEEERFHLFQD
PMCIASAFPIDLVDLPNLPRIEFHTNPSNKAVLDRWWQSNFDTPPRISMVVDILDTCHEM
VRHGLGYGILPKMIVQSDPLLHLIELKDLEGKPLTRDGWMIYSARSRELGIAERFIEFVK
G*
>SPSA8_v1_20006|ID:41144835| conserved exported protein of unknown function [Spirochaetes Bin 1 SA-8]
MSFMKKAFLILGLLVVAGGFAFAQKAPANYPTKAMEFIAPSGAGGGWDTTIRMVAKALQD
EKIVSVPMPVTNRTGGGGGINLAYMQTKKGADDLISVYSPPLLFIKLNGTSKFGYKDTTP
LARLIADYGAFYVAKNSKYKTMKDVLDAVNANPSSVKIGGGSAAGSMDHIVFLYVAKAYG
VKDLKKIQYIAFQDSTGPTQLMGGFIDVLSSDIASLRGLVESGDIRCLGVTSATRIGTGI
VATYPTMKEQGIDVEFANWRGLFGAPGMPDYAVSYWRDAIGKMVKTATWKKIMDEQAWTP
YYMDQPDFMKFLDKVNAEYTEIFDEIGILVK*
>SPSA8_v1_20007|ID:41144836| putative tricarboxylate transport protein TctB [Spirochaetes Bin 1 SA-8]
MVLTIQVISDLFYLVLALAYGIGALSLPEAMFGDPWAPRIYPLIIACGMAILSVVLLVGE
LKKQRSGKADAAVRFSIDSDGMIVAFVTVASLFYTFLFNRLGFILSTFLFMESIMLFISK
AKKMLWPTVIAILFAVGVYFGFVNLLGVTLPAGILEF*

>SPSA8_v1_20008|ID:41144837| Uncharacterized 52.8 kDa protein in TAR-I ttuC' 3'region [Spirochaetes Bin 1 SA-8]
MESIFQNLGIGLAVAIQPINLLWVTVGGILGTIIGMLPGIGPASGVALLLPLTFKMGPVG
ALITMAGVYYGAMYGGSRASILINTPGDGAAIASCWDGYPLATKKKRPEAALAISAIASF
IGGMIATVFMIFLSAPLAKLAIKFGPAQYLMLYLFALSATASMSGKGKVIKGFVAMFLGL
MISTIGIDSLSGVHRFTFGLMELQGGIDFLIAIIGIYALGEVFESLKSIGKGKTKIQKKF
GRIWITKDDWKRIWPVILRSTPLGFLVGVLPGTGATMASIMAYNQEKNLSKHKEEFGEGA
VEGIAAPEAANNAASVGALIPMMTLGVPGSGTTAIMLGALMMLGLQPGPLLFTRTPEIAW
GVIASMLIGNIVLAIINLPMAGMLVRILSVPERILYPIILGLAFLGTYSISNTTFDFLIV
IGFGLLGLFMKKSGFPTSPMVLGAIIGGAIEEKFRQALTISNGSYRVFVSDPITIVLLVL
TLGSFLLPLFKKKGIKA*
>SPSA8_v1_20009|ID:41144838| putative Protein CitXG [Includes: Apo-citrate lyase phosphoribosyl-dephospho-CoA transferase ; 2-(5''-triphosphoribosyl)-3'-dephosphocoenzyme-A synthase] [Spirochaetes Bin 1 SA-8]
MEIMDVKGKAFSSDIDKLKLVRIAILDARESRAEAQKELYRRYAVPIVSMTLVSPGIKKN
TRTRRAALHAAWSILLQQLSSAGFTILQSRCRITAAGPECLLAVEGSASEIKELCLQLEE
CLPFGRILDIDVLSDSGKGLASLHREAFGRSPRRCLVCDSDAFLCIAEKRHTAAQLEKAC
FQLYRMTADAMEEDIAEMAVASLIAEASLPDKPGLVSPGNQGAHRDMDFSHMVSSAHALR
PYFFEVAKRSFSYAGNLEMLLEILRPLGLEAEQRMYAATGGINTHKGAIFALGFFTAAAG
YLAGHTELFSEYASYAEALCDIIRKIANNVASSDYEKHERLVPTNGIRWYREYSVTGARG
QAERGYPLVFNTIFPVLAQGLSHGTHETSMVLLNALIRSIAELDDTCLLSRGGRAGLDEA
RRRASELLIKLGKSENSAEAHNVILEFGAWMREHALSPGGSADMLAAGIFLYKLNERYGG
FSGWQP*
>SPSA8_v1_20010|ID:41144839|citD| citrate lyase, acyl carrier (gamma) subunit [Spirochaetes Bin 1 SA-8]
MATVKQISQAGTLESSDILVQLSPAEPGTGIKLALESPVRKQFGTRILQIISAELHRAGI
NDVLVSANDRGALDYTIEARIQAAIARASGGEEART*
>SPSA8_v1_20011|ID:41144840|citE| citrate lyase, citryl-ACP lyase (beta) subunit [Spirochaetes Bin 1 SA-8]
MKPSLRRAMLFAPGNNPGLLQNAGIYGADSLIFDLEDAVSIYEKDSARLLVRNAIRTIQY
PCEVGVRINHITTPWGLRDLEEVLSAKPDFIRLPKCEDAEELKLIDDIISKAEKKHGFAE
GSIQLMAAIESPKGLRNAYQIATAVPRMMALAIGGEDFATSLKTTKTKDGTELLVAKCMI
VFAAREAGISPIDSVFSDINDEENFIKETELAKQLGFDGKSCVHPRQIELVQRVFQPTQK
EVASARRIIKAYNEAMERKLGVVAVDGKMIDAPMITRANRVLANARASGLIEEENLV*
>SPSA8_v1_20012|ID:41144841|citF| citrate lyase, citrate-ACP transferase (alpha) subunit [Spirochaetes Bin 1 SA-8]
MKNKAGREIPDTIQGYGTVIPYQGPFALKPDGYRAGSALKMAKPGQEKVLRSIEDAIAVA
GLKDGMTISFHHALRNGDRVMNLVISAIARMGFRDIRLAPSSFLDANDELIPYFEQGVIT
GAQNSGARGKLGAYLTHNRLPKMTVIRPHGGRARAVIAGELHIDVAFLAAPACDRLGNIT
GVLGPAACGSLGYAMIDARYADTVVAITDNLVDGALCPVSIPHHLVDFIVPVESIGDPRG
INSGSLRLTTNPRDLLLAKMTALVIESAGFYREGFSMQLGAGAASVAAGRFLRESMMRDK
ITASFAIGGIPGPFCDLLDEGLIHTLFDAQSFDSRAIQSLRDNPRHQEYDIDQYANPWNK
GAMVNFLDYTVLGATEVDVDFNVNVITDSNGVIMGALGGHPDASAGARCTIITTPLLRGR
LPIITDSVQTIATPGETVDIVVTERGIAVNPLRPEIAENLKKAKLPVMDIRELRDLAYSF
SGIPEPVRVTDEVVAVIQYRDGSVIDVVRKPAD*
>SPSA8_v1_20013|ID:41144842| Citrate lyase ligase [Spirochaetes Bin 1 SA-8]
MYGTPHLEIADLTDSGVVAEVQEFLSRHALGFDVAVDHTVILRLSGRMIGTGSFKGEVLR
NIAVDEEVQGEGYTATILNELVQEQGRRGIMHRLIFTKPQAASRFQNLGFRLIADAKPWA
VLLEAGLGGLDDYLARVKKICDDLLPRRTAVVVNCNPFTRGHLGLLETAARESEAVIVFV
VSEDRSLFPFKDRIELIRRGTVHIQNLRVVETGNYMVSSATFPTYFTREEHLADAQAHLD
IALFAQKIAPALAIVARYVGDEPYCAVTEAYNRAMRDILPKAGVSLKILPRYTGPDGKPI
SASTVRDLIRSGDWEKIRPLVPDVTWDYLRSPEHLDIIEKIKASHSRH*
>SPSA8_v1_20014|ID:41144843| Class V aminotransferase [Spirochaetes Bin 1 SA-8]
MKGKRLLMIPGPIEFTEEVLAETGKPTLSHVDPQFIKEFGQALSMMRKVWLAPSGQPFIV
AGSGTLAMELAVANITEPGDSVLIVNTGYFSDRMAEVFKVHGAVVDIAPSELCDVPQTEI
IRSMLKAKRYKIVSITHVDTSTSVKTDVKAIAEAAREAGALSLVDGVCAVGGEELRQEAW
GVDIALTASQKAIGVPPGLALLVAGDRALEAFKTRKMPVRSYYLDWKYWLPVMEAYEAGK
PAYFGTPAVNLVRALNVSLGQILAEGMDARFLRHRRNAQAFRAAMSAIGLSFLPVRENLL
ASTLTAVWYPEGIDASVLPLIAEEGAMLAGGLHPAIKTKYFRVGHMGMSDASEILATIGA

IERGFSRAGYRFDHGTGLAAAEKILLA*
>SPSA8_v1_20015|ID:41144844|rhaA| L-rhamnose isomerase [Spirochaetes Bin 1 SA-8]
MSTKLYREAQERYGEIGVDTDKAIRTLSQIPLSIHCWQGDDVIGFDGAARLSGGILATGA
YPGRARNAEELRRDIEKAFSLIPGTKRLNLHAMYAETNGAKVDRDQLEPKHFDGWLAWAK
GFGIGLDFNPSFFSHPLAESGFTLAHADPSIRNFWIRHGIASRQIAQFFADELKDTVVNN
LWVPDGYKDTPADRLGPRIRLQDSLDQIYHDSLPNDRVIDSVESKLFGIGSESYVAGSHE
FYLGYAAKHALGLCLDMGHFHPTEQIADKISSVLLFIPKLVLHLSRGVHWDSDHIVVWND
DISNVAHELVRIQAWDRVYLALDYFDASINRIGAWVIGARSVQKALLSALLEPSEILRDE
ERHGNYFKRLAYLEESKSMPFSAVWNYYCETENVPENATWIDSISEYERLVLSKREG*
>SPSA8_v1_20016|ID:41144845| protein of unknown function [Spirochaetes Bin 1 SA-8]
MLQNHGIFIAADSPVEIDKLYETVISAILKKISIQPDMSPITVNTQDMDKIISTIKKYFP
DQAIHIANTKACSSFLETNETFKPLAEPFTPDHIVYAGAKPLYVKKDTAMDPEKLMLAIN
QFIETEEFSPKIIAVEKTGVFALSDTRRKAELAMALFLDTVKIASLAERFGGSHHMNKAD
IDFIKNWEVEHYRAKVTAEKKRINL*
>SPSA8_v1_20017|ID:41144846| protein of unknown function [Spirochaetes Bin 1 SA-8]
MNTIKSLIEISRKYGSNPEFVLGGGGNTSCKDNGILTVKASGFALGTIDENGFVKLDLKQ
VLAILENTFSTVPAQREKQILDALQDARLQGKHFGQV*
>SPSA8_v1_20018|ID:41144847|yiiL| L-rhamnose mutarotase [Spirochaetes Bin 1 SA-8]
MKRNAFIMYLKPGNEAEYKRRHDEIWPELEEILRSAGISNYSIYLDRNTNTLFAFQMLTD
DATDNELPVNSVVRKWWDSMKDLMETNPDNSPVCNPLIEVFHMD*
>SPSA8_v1_20019|ID:41144848| putative sugar ABC transporter, substrate-binding protein [Spirochaetes Bin 1 SA-8]
MKKIVIAVAMIIAIMGMVLPASVFAADAKPKYAIVFKNTGNPYGEKQLEGFKTGIEEQGF
EAILRSPDLPTAEAQIQIIEQLIAQKVVSICIVGNDYDALQPVLKKATAQGIKVFSLDSS
VNPASRLTHVNQADSEKIGQTLIKAAYDMAGGSGEIAILSATSQASNQNIWIDFMKKELA
KPEYKNLKLVKVAYGDDLRDKSVSETEGLLRSYPNLKVIIAPTTVGIAAAGKVLTDKGLK
GKVALTGLGLPSEMAEYIENGVCGYMFLWNPIDVGYLGAYTATALVTGKIKGAVGDKFSA
GRLGNYTITKAPDGGTEVLLGPPFKFDKSNIAMWKKVY*
>SPSA8_v1_20020|ID:41144849| Branched-chain amino acid ABC transporter permease [Spirochaetes Bin 1 SA-8]
MSDRTIEIQKPRTWKTFIYQWEWFLLLLFILICSINSSLSPYFLSFDTFLNAPMNFLDKA
FLVLPMMMIIVLGNIDVSVGSIVALSSVIMAVSYNAGLPMPMALILALVVSTLCGFINGM
LITRFKELSATIVTLSTMIIFRGIAYIILEDRASGKFPEWFSFLAWGSIGILPFIFIAFI
FAAVIFALVLHRSRFGREIFAMGNNSTACRYSGIKTDDVLMKVSLLTGFMAGITALFLTS
RMGSTRPNVAQGYELDVIAMVVLGGVSTAGGKGRIGGPILAIFIIGFLNYGLGLVNISAQ
VLLIILGLLLILSVMIQNARFLKRNQKSARPLTIKS*
>SPSA8_v1_20021|ID:41144850|rbsC| Ribose transport system permease protein RbsC [Spirochaetes Bin 1 SA-8]
MSDKTALKSGERFRELGLLIFIIVLSVIFQLRNPSFLTLANIKDLLANTAILGILSVGMM
MVLLTRGIDLSIGATMALSGMVCSLTGIAYPDMSPLVSILQGMAIGLVVGSIIGILVAYF
NILPIIATLGLMNIIRGLTYLVSKGKWVSAYQMSAGFKNISKGTFLGINNLVALAIIIFI
IFYYFINHTRTGRFIYAVGSNPDTADVVGIPRKRIICMVYIFMGALAGLAGVLWVSKFAS
AQGDTAVGYELNVIAAVVLGGVSVSGGRGKVSGIILGTVLFGILANALPLIHISPFWQQA
IQGIVILAAVIANVLMKRSNDKSALKKRAI*
>SPSA8_v1_20022|ID:41144851|rbsA| fused D-ribose transporter subunits of ABC superfamily: ATP-binding components [Spirochaetes Bin 1 SA-8]
MADFILEMEHISKAFPGVQALDDVHFKLERGSIHALMGENGAGKSTFIKIITGVYQPDSG
IIKIDGKPVQIHSPLDSQHLGIAAIYQHVTCFPDLSVTENIFIGHETINPLTRKINWKEL
HENAKNLLDQLDADFSPQTVMGILSVAQQQIVEIAKALSSNARIIIMDEPTAPLSKRESE
DLYRITESLKAKGVSIIFISHRLEDMYRIASTVTVLRDGHYINTWPLDAVTEENLVKAMV
GREITQYFPQRDSAPGDVIFSAQNLSRTGFFKNVGFTVRKGEILALTGLVGAGRTEVCEA
IYGITKLDSGTLMLNGEKLKISSPMDALKAGIGFLPEDRLKQGLVLDWEIFRNISLSALK
RFSRHGFMHDSDERKTAKELAEKLEVKATSIYDKAATLSGGNQQKLIVAKLLTGDMKILI
LDEPTKGVDVGAKTAIYRIMNSLAEAGYGIIMISSEMPEVIGMSDRIVVMREGRVTAIME
TKTANQQAILEASMSIAKNHDSEENQ*
>SPSA8_v1_20023|ID:41144852| conserved protein of unknown function [Spirochaetes Bin 1 SA-8]
VACNVFYITWEEKSIAALSEGLILRIMTKAGLFCIGLDTYWKQFEGLRERLSGYQQRVAD

RIGKAGVQVVDAGMVDTTDKARKAARLFKKEGVDIVFLYISTYALSQTVLPIALENPKTP
LVILNLQPSAALDYESFNALGDRGKMTGEWLANCQACSVPEIAGVFNNASINYDIVSGWL
DDETSWKQIKEWLEAASIRKVIRDSNFGILGHYYEGMLDVYTDVTRLASVFGCQFTLLEM
DYLKTLRDSVSAQEVAAKISQFSNEFSVSPECELPELERAARTSCSLDKMVEAKRLGGLA
YYYEGVSGNDHENIITSIIPGNTLLTSHNIPVAGECEVKNLIAMKILDAAGYGGSFSEFY
LSDFTDDVILLGHDGPAHASISDGKVRLVPLPVYHGKPGKGLSIQMSVRKGPVTLLSVVQ
GPGGSTKLLIAEADAVPGPILLIGNTNSRYRFPISARTFINNWSKAGPAHHCAIGTGHLA
GILKKLAALLGIESIVICESGETSNG*
>SPSA8_v1_20024|ID:41144853| putative transcriptional regulator [Spirochaetes Bin 1 SA-8]
MGSNESNRTKTYFQYLTCSEEDEKWQLVCTDAGFTEVPSYTFYPPNKSGHPRAFQYVATG
RTLNEFQIVYITKGKGVFETLGKKFDIIPGSIMIVFPGIKHFYKPIYEVGWYEHWVGFKG
PYADSLVKNGFLSLENPCFIIGLQNQIIELFSEIVDEVREQKPLYQIKAASKIIALIAEI
LATERRQAQPSQSEKIVEQAKFLMEESIYDDIDLNSIASQVGVSTSKLNEIFKTYTSMTP
YQYYIHIKLHAAKALLEQGDLSIKEVAFRLGFEDQYHFSRLFKSKTGIAPSLWKDFVYQ*
>SPSA8_v1_20025|ID:41144854| putative Beta-lactamase [Spirochaetes Bin 1 SA-8]
VAITQKTALDSLIEAALSDKAFAGIACGVWKDGVPVYKRYAGFAVPDTSPVCADTMFDLA
SLTKPLATAPLVMMARDEGMLELEKTVGNYLPHIHQDTAPIPLISLLLHLSGLPAIPALQ
TEFPDASNFSKEKACTKLFRLKPDPALFGKVLYSCTNYILLGLILEKIYGMTIGQLFKCK
IADRLGLEHCGFAPGMLKNGEPQVLEKAAATEYCPWRKRRICGQVHDESSFCLAGQAGNA
GLFANLDDTASIGEQFLRHGGKPLLSRASHMLMTSVQTVNLAERRSCGFRFHDDTTADGP
LWPHDSFGHTGFTGTSIFFAPRQDLMTVILTNRVYHGRDTTLDKIVQFRKDFHSLVWGLW
GEEAL*
>SPSA8_v1_20026|ID:41144855| conserved protein of unknown function [Spirochaetes Bin 1 SA-8]
MRIHYTRSGLDSFLADVSRRGKRYGLATHPAAVTFKGEPSWKAFMNAHLNLACLFGPEHG
FRGASQDAVSVEDEIFLGIPAYSLYGKRLVPEPWMLEHLDALVFDMQDVGCRYYTYLYTL
AYIMKACSDARLPLIVLDRPNPAGGIKVEGSPIKPEDESFVGGYGLPPRYGMTIGEFARY
LQGEYYPSCELEVVRLSGWSRDMQWLETGLPWPLTSPNLPSLSCAEVYAGTCLVEGTWLS
EGRGTSRPFEIIGAPFIDGETLRTALAKLDLPGVVFLSLFFVPTASKYMNEQCEGILVTV
VDVESYRSLDTGIAIIHTIKSLYPDQFRWREDWENPALSFFDKLAGGTTLRHMIDSGRSL
EECLAFAHQGEAAFLAGRQKYLLY*
>SPSA8_v1_20027|ID:41144856| ABC-type sugar transport system, permease component [Spirochaetes Bin 1 SA-8]
MMLKFTRFQKMVAIFFLALLAFINLYPIGIMILSSFKSTREIFMKPFNLPSVWRWQNYVS
AWQRADFAVYFKNSILVTAISIAAILLVSSMAAYVIARFDFKGKRYIYLYLLAGLALPTR
LAIIPIFLLMRSMHLLDKLSGLVIVYTAGGIAFSVFLLVNFFKKLPRDIEDSAMIDGAGP
FRIYWKIDLPLLKPALVTVAMFNFIDVWNDFFFPLILLSSKAKKTIPLGLQAFFGEYTIE
WDVLFAALNISVLPXLIVFLILSKQFIAGMTEGALK*
>SPSA8_v1_20028|ID:41144857| Permease component of ABC-type sugar transporter [Spirochaetes Bin 1 SA-8]
VRKKAYLLMLLPALLLYSLFVAYPFFSSLGLSFYDWPGIGPKKFIGFGNYRNILTGFMAP
EFFRAAWHNVIFFIWSLILSVVPGLFFAFLLAAGIKGTKFLKVIYFFPNTLAIVIVGFLW
GLLLNPQWGLVNQLFKAIGLGFLAKPWLGDTKLALPTIIVVTAWRGLGFYILVYLAAIIG
IDREMTEAARIDGATEMQIAGRIILPHLMPIIATTSMLKFIWTFNIFDIVYAMEGTQGGP
AGSTDVLGTLFYRIAFGGLGSSQVGMGLGATVVTLIFLIVFPVSVFYVFIIEKRVERGE*
>SPSA8_v1_20029|ID:41144858| Extracellular solute-binding protein family 1 [Spirochaetes Bin 1 SA-8]
MTRKLAFLALMVCLIFPLWAQSKPATVTIWGWRAQDVDVWKSVETALKGKGEQITIKYEV
FPPTEYDSKLLVSLQGGVGPDLMYTRRLPGARTQALIDNGYITALDGLLNLSAFDPVSLS
FIQANKKTWGVPFANQVVGIFYNKAIFDKFGLKEPQTWDEMVAVAETLSKNKVTPFFVAG
KEAWTLAMQNAMIGVSYPGDEWIGKLAKGEAKFTDPEYVAMLKDLNALKKYFQKDFMANT
TAEQDVSFAMEQVAMIFYGVWGNTNWLKTNPDLKFDFFPVPPKTKNVPAKIYTYMDGSYG
LNSATKNKDAALKVLQFTASKTYGELFSKTTGEITAVKDVTMPASKPILVKCYDKMNTIA
STNRYWVGSPFDAGMPSVYNILQEHMQSMYLDLITPEELAKKLQDGISTWFPAFKK*
>SPSA8_v1_20030|ID:41144859| putative Glycerol-3-phosphate-binding periplasmic protein [Spirochaetes Bin 1 SA-8]
MKRGLAILAILLSAAIAMAAPVKVTLWYAQTGVYAKSLLEIVDSFNKQNQDKIFVEAVYT
GNYQDTMQKLLAAMVAGDVPTLAQIEQSRIGQFVDAEAFQDLNAYIKSDQAFAATLSDFW

PRFINANTFSGRLLGLPLNCSTPLLYINRDLFRKAGLDPDKPPKTWTELYAAAKKISALG
PDIFGYRFGIDDWLIESKTWQFGGEIISEDGRKMLIYSPETVAGWKYFQKGVKDKAFIFG
VTGGNELDISGRIGMVVRSTGSIQYMKDNAKFDLGATVMPMEKRKIVAIGGANIFMFASR
PKPEKAAAWEFLKFLTNTENSRKWAMSTGYMASRISAFESKEMQAVLKADPRFGLTYEQL
KESAVRRPWFGPYPEVHAMMTSAWEQVMTNTDADVDAILKKLQVEAQKVLDTYYK*
>SPSA8_v1_20031|ID:41144860| Binding-protein-dependent transport systems inner membrane component [Spirochaetes Bin 1 SA-8]
MIRKYPLSLKIAVTLFLFFLAILINLPFFWMLVTSFKTEDQAFSIPPSFLPTIFDLRNFV
KAASFIPIGRYILNTLYVSFAVVALHFFTNSLAAYGIARIKFKGASVVFAILLGTMMVPP
EVTMVPLYVIVKKFNMMNKYSGLILPFASSAFGIFMLRQFFMGIPKELEDAAIIDGAGRM
RIFFSIILPLSKPALFTMSIYSFLAVWNEYLWPLVIINDSARQMIQVGISQFVSGWETQW
TMRMAASTIAVLPVIILFFFVQKQYVEGISVSGMKE*
>SPSA8_v1_20032|ID:41144861| ABC-type sugar transport system, permease component [Spirochaetes Bin 1 SA-8]
MVRSRKDELLGWLTVTPALVSQILFIYLPLATAISISFNNWNLIRPMRWVGLDNYKTMFT
NADFWNSLKITAIYVVGTVIPSVVLGLVLALFLNIKWLKGKGMLRTLFYIPVITSMAAAA
VIWGWLFESNYGLVNVMLGWFGIGKIAWLSDPNYSLVALMIIGIWKRVGYNMVLFLAGLQ
AIPSVYYEAADIDGASSYQKIMSITLPLLSPTTLFVFIVQFIASFRVFVSVSVMTRGGPA
KSTQVITYYLYENAFRYLKFGYASAVAVFMFLLMVFFTVFQFRASKKKVFYS*
>SPSA8_v1_20033|ID:41144862| conserved membrane protein of unknown function [Spirochaetes Bin 1 SA-8]
MKSGFSPFEEYRSLPRSIWALFVVRLVIAAGNFVFPFLTIILTTKLDWTAEKTGTFLSLI
QIAALPGVLFAGRLSDRTGRRLIIMLCQAAAAILFAISLATGFTDFLPYLVGAASILLAM
TWPVSGALVADLVPPERRKTAYALLYWGNNIGFSIGPIAAGFLLHRAPSLIFLGNMTALI
ISINILYRYIPAKIPAQPTAEASERSGVNAMEANYEGGLARVLKSRPTILVFAFIVALLN
LVYSQHSFSLPLYLSEKMGSAGAEVFGAAMTVNGLTVVIFTLILSKITQRLPVLIIMVIA
AMLYAAGFGLLSFHPSLLLVLGSTVIWTWGEILGATNINVYIASRSPQSHRGRISSITSL
VSNLGSLSGPILAGQLIKRFSSAFIWKAAPAIAITAALFLLLLWSHDRTKGLQ*
>SPSA8_v1_20034|ID:41144863| Thymidine kinase [Spirochaetes Bin 1 SA-8]
MRGYDTSDTGQLLKNLGFPLLKVHASYSHHDFITPGRRILVIGPMGSGKTEFTARVWRDS
KVALRKNGKAVNLTTTNGVDRREVFVVRASLDKARFPDYPEDALAFRGGYERCGDHIGSA
RDSFALEVLLAENPSIGTWIVDETAFFDERIAYLMTEESESRSVTFICPTLVLNFRREIF
NQTARLLLENATDVFPLTAYCEHEDCLLDSLYTYRYYLIDGKECPALYFDPLIIIGGDRT
KTDGREPNYCTRCDTHHYLPGKEYTFFTLKPLGEQAARGNIAPLLNELNALAGNIEASRL
YASMEKRYIACDEPCPVPMNALLVPCIAERALVYLYAEQNLLSTEQVIFIVNKLGLDREY
LAQRLADNRRPLEF*
>SPSA8_v1_20035|ID:41144864| Bacterial extracellular solute-binding protein, family 5 [Spirochaetes Bin 1 SA-8]
MKTRYFASAILLLALMFLLPAFVFGQSENPDEFVTVYGTSLPEFDPHKAIFSSEAQLFTA
LYEGLFSYDPATLEPVNAAVATWSKSKDGLTYIFVIKNNAFWSDGSPLLARDFRDSWMRM
LKTNADYATFFDIIDGAEEFRTGRDTNPEHVNIEAVSMKTLVVRLKRPAAYFTRLLCHHA
FAPIHPSMLAHDNFSDGIPFPVNGPYRFEKFSDDEIILAKNEFYWDKASVSIERMKMLFS
EDDAIASTMFNNGNAHWLFGIGDYDSILMEEAIQVNPVFSTNYWFFNCSVKPWSDPKVRR
ALALLLPWNNIRSKEVYSVPATTLVLPLPGYSKAKGIETSNKTEAMQLLAEAGYPEGKGL
PAIQIFYADSKENRRIVSLFKSAWEALPGLIVQGLSLHPAEYYAAIAEMKNMPEMTLAHT
TWIGDFADPEAFLQMWTENSPLNDAGYASAEFSQSLAKSYGAEGTERMNLLAEAETILLQ
QAAVLPIYHGFGVSVIDIDYIEGWYQNALDLHPYKYFKFGTPSIQPNIAQSGNPSQSKDI
*
>SPSA8_v1_20036|ID:41144865| conserved protein of unknown function [Spirochaetes Bin 1 SA-8]
MGLAKEFDKEILVMGVLVSDSIIESQAMEALQANFGSIDVMTEHEIFTWTDYYNLEMGQP
ISRFFLCFQNRVAPDRLAEIKLLTNDIELNYAVDGKRKINLDPGLLAPGRFCLATTKDRA
HRIPLKDGIYAELTLIFQKKEFHPLPWTYPDWASEPVRNMLADWRKKLFL*
>SPSA8_v1_20037|ID:41144866| exported protein of unknown function [Spirochaetes Bin 1 SA-8]
MACFLACTVLLFAFACSSCSAMQRWYVHRLEIAMPQIPAVWDHIAPPVYELAWTDEEGQA
RTAYVPSGQSAVVTVARGVKQAVLAFPIVFQKRLKPAGGLYPDYLKYPGRPFPSDEPDEL
TIDFEGGYIASVASIIAKAGFNPWILRLSCLKQDWISRHADPWGVDPALAASRLIDGTYS
SAVFSQKAESVTVSLPPPAGWLPASPFARLREEDGKILAEVNEGFHLFYRSDQIAMVSAS

EGRVVLQVSRISSGQADGENRALPDDAFY*
>SPSA8_v1_20038|ID:41144867| putative PAS:ATP-binding region, ATPase-like:Histidine kinase, HAMP region:Histidine kinase A-like [Spirochaetes Bin 1 SA-8]
MKPSMFRKTVLLVNVAILTLTLLLMTAGALLADRAYLASSSRYLLESAYLASALITSDSS
QAFNLHQLSNSGSIRLTLIAADGTVLSDSSADPASMENHALRPEVAAALQGKSSWAVRIS
ATTGMRTLYAAVPLALYKSSAGSILRLAMPLPSFFARLSGLAWIPPIFLVLVALFSVAAS
LLIQQSVTIPILKLTEKAERYSDSESFSEIKLKSIPRELLPINAALDTMAKKILSRSLEN
RRMRSRYEAILESASEGIIASDESLRIQEVNKAAAGLFGINETERTQCIGQSVLKGLRNS
LINEIFETCSHEKRLITREIPFFSENGERHYAVHASPFEDEETHGIVAIVSDVTEIRRLE
RIRKDFVTNVSHEIRTPVQVIKGYAEIMSDTLAHAEPTSADGDLFSQLREQSGIIHHSAL
RMEEIIKDLLLLSRLERDPGTWIIKEPCRIRPILEVVKTALANQAREKNMPILIACPEEL
EAQVNSGLLEQALTNLVGNALQYSFPGTAVEVSASTKDNTLIISVRDHGAGIPAKDLDHI
FERFYRVDKSRNRSTGGTGLGLAIVKHIVTAHGGTASVESVVGQGTVFTISLPAGNTRNL
*
>SPSA8_v1_20039|ID:41144868|regX| Sensory transduction protein regX3 [Spirochaetes Bin 1 SA-8]
MPDILIVEDDEDIGKLVSLSCRNEGWHPVHVKTAEEALIRIRSDRFDICVLDLMLPGMDG
LSFLKVIRSMSILSFMHIIIASAKDDDTDIVAGLELGADDYVVKPFSPRVLVARIRAALR
RNADSTTANSQKPTVFEHRGIAINKERHSVTVSGKPVDFSATEFGILELLVREPGRVFTR
EQIINHVKGPDYPVTDRAIDVHILSIRRKLNELADLVETIRGIGYRLKDS*
>SPSA8_v1_20040|ID:41144869| Phosphate-specific transport system accessory protein PhoU [Spirochaetes Bin 1 SA-8]
MQSMSLKIKIDHLNELFLSMSIKVKEALEKSRFALRQGDKEIVKLIKEEDAEIDRLQQAI
DDEVAQTMATQQPVASDLRLLVSLLKIASDFERAGDYAVHLAKAGKHFVNMPVWKQFDIL
DAMAQQCGVMIDGTARAFIERDSIHARSIAKMDDDVDHAHKAVVKDTLLLIHQNPDLAEQ
AAKIITLSGYLERLGDHMTNACEAIVFMVEGIHTELND*
>SPSA8_v1_20041|ID:41144870|pstB| phosphate transporter subunit ; ATP-binding component of ABC superfamily [Spirochaetes Bin 1 SA-8]
VKSMEYGNTSGVATVLPQFVSDHVALEVKNLVVEYRDGVQAVKNISFSVPRQAVTAIIGP
SGCGKSTALRALNRMNELIPGTKTTGEVLFHSKNIYDPNVDPVTVRRHIGMVFQKPNPFP
KSIYENIAWAARIHGLSDNLDDLVESSLRQAALWDEVKDKLKKSALSLSGGQQQRLCIAR
SIALKPEIILMDEPTSALDPISTASIENLIHDLKQRYTIIIVTHNMQQAGRISEMTAFML
TGDLVEYAPTNRLFVSPEDERTERYITGRFG*
>SPSA8_v1_20042|ID:41144871| conserved membrane protein of unknown function [Spirochaetes Bin 1 SA-8]
LIELSAQTITFLMLGGLFILILTGYPIAFVIGSVALGTGLLVFGPNTTFHILYTRFYDLS
LNYPYLAVPLFTFMGVILQHSGATKELYDNLYETLGSLKGGLAIVTIIFGTILATCLGVI
AASVTILTMMALKPMVTKGYDKALASGSVIAAGTLGILIPPSIMLVVYAPQAGLSIGQMM
IGAFFPGLLLSALYLLYVGIRCYRNPQLGPSIPKEEMSPFTAKRLWGLVKSLLPTIVLIF
AVLGTIFAGLAPPTEAAAIGCLASIILAIIYRKFSLELLKKAAIETLRVSAFVVMIAAMS
YAFVGIFMSAGAGDVVAKLILSVPGGKWVSFAVIMLIVFFLGMFIEWIGIVFIIVPVFSP
IFVKLGFDPLWAGLMVCVNLQMAFMTPPMAMSIFVLKGSAPPELGITMKDIIRGVLPFVG
LVMIALVLLTIFPEIITWLXQKMLGK*
>SPSA8_v1_20043|ID:41144872| conserved membrane protein of unknown function [Spirochaetes Bin 1 SA-8]
MGALKKIVKTIDTISEKSGQIGKWAAVALVVIGAYDTITRHFFNAPSDWAYDMLCMSGGA
LYLLGASYDHAHEAHTRVDIFFNMLKPRTRSLVNVIAALLFFFPLIGMLLYMGINWAIKA
VRVNEVMFTSFWYPPAYPYRILFVIGIFLLFIQGIANFAKDLYFLIRGEKID*
>SPSA8_v1_20044|ID:41144873| conserved exported protein of unknown function [Spirochaetes Bin 1 SA-8]
MKTRKAVVLLVLLLAVLPMAMAQTKIVWKSSGHGPASDPSQIFHDEVCKAITKATGGRLE
IKPFVGGSIVPAYKELDAVNENVLQMAYTCPMYNLDKWPAAGLISSRPGALANEVLRSWF
DYGGGADXMNKMMAGYNVMTFPGALSPLPEEVFLHSKVKITKVSDIKGLRIRAMGDGGEV
MKKLGASVVIINGGDVYESMQRNVIDAFEYSTLASNWNMHFNEVAKYVILSPIRAPSDPQ
VFFVNKTAFEKLPEDIQVIVQEVISSYTQKQHEYLIAESIKALQKFKDAGCEVYRLPKEV
EDAVAKAADEFYTEKSKKEKPIFAEIYNSMKNYGIAYDSMK*
>SPSA8_v1_20045|ID:41144874| conserved protein of unknown function [Spirochaetes Bin 1 SA-8]
MRTIITTLIENSPGEHLSLKSEHGISFLIEKNGEKILFDTGQSEAFIHNAEKLGKDLSDV

SQVVISHGHYDHSGGLRSLLAINSSFTFVAGNGFFRKKYATDGKSFEFLGNDFDEQFLKD
NCIPIRIISSPLEKILPGIFALTAFERKYPDETINPRFVLDTGTTFIPDDFSDEISLAVE
TDKGFIILVGCSHPGIKNMLDAAASRLPGPIYGVLGGTHLVEASDRSLAKSIQYFSDSNF
GALGVSHCTGKKGTQELERSAASFFRNTTGHAIIL*
>SPSA8_v1_20046|ID:41144875| GntR domain protein (fragment) [Spirochaetes Bin 1 SA-8]
MLEPKAAYYAGIKALDSDISGILKVCAEFAEKAPSNNIPELIQIDEQFHNEIAKSTKNRT
LSILMKTMAKSLPVGWISSLNVPGRIEKTVHEHLSIAQAIQEHSPQKAEQHMTIHLQNAQ
HDIISFMESR*
>SPSA8_v1_20047|ID:41144876|pyrD| Dihydroorotate dehydrogenase B (NAD(+)), catalytic subunit [Spirochaetes Bin 1 SA-8]
MSADLSVRIGNLLLKNPVGVASGTFGYGLEYENLVAIDELGALYTKAVTLEPRAGNPPPR
LVETPQGLINSIGLANPGVEAFITEKLPSLRTRSCAIVVNVAGSTEDDYCAVIEAIEKAA
PLSAKGRKTGVDAYEINISCPNVKHGGMAFGTNPQQVQSLTGRLRLLTRRPLIIKLSPNV
TDIAAIAMAAEAGGADAVSCINTLVGMAIDTETCKPFIAMGTGGLSGPAIRPVGVAAVWK
VAKAVSIPVIGLGGIASASDALEYLLAGACAVQVGTALFSDPQAPHKILEGIQTWMQRKG
IERVTDIRKMLKG*
>SPSA8_v1_20048|ID:41144877| putative Dihydroorotate dehydrogenase B (NAD(+)), electron transfer subunit [Spirochaetes Bin 1 SA-8]
MNVSRQFTAAITSIHEIAPSWYLAGFSWPRDLALPQPGQFFTYNPSLLPGAGKSLLRKPL
AFAGFEDQRAYTVFQARGEGTKALAQLEANEKLDLIAPLGNSFPQCDETEKAFLVGGGIG
LGPMLYLASEFLSNHFDFEVVLGFRTMAQVPDFSACINTAPIAATLQAIMKAASIATDDG
SSGFKGTAIDLLKEKAFGLSNTADFQSNDEKKLTKNHVHLFGCGPFPMLKALHNLAGQYQ
APAHVSTEQWMACGVGACHGCVLPASGGGYVRVCADGPVFCSDAIDWGKCP*
>SPSA8_v1_20049|ID:41144878| conserved membrane protein of unknown function [Spirochaetes Bin 1 SA-8]
MSAILDIAVLLAFLGGGAVAAKSKFTPSGKVVDSIIRWVLWLLLFSMGLRIGNNPVLYAN
MKTMGAVALATAILTVLGSVAAVFLASMVIPGMKKEPAKGKSASSVMFLAHLRAPLFLLA
IVIAGFSAGILLPAVRFDIGIITGWTLNALLFFIGMQFALSGIDLKDVLATPAALAVPLA
TIIGTFGGSLVLTVLFPISAGKALALGGGFGWYSLSGVLISNLGDPVLGSTSFLANMFRE
SLALILIPFLGRSRVPSLAVSVGGATAMDVTLPLIEQSAGPWIVPLSFMSGAILSLIVPF
LVPLFFRLG*
>SPSA8_v1_20050|ID:41144879| conserved protein of unknown function [Spirochaetes Bin 1 SA-8]
MNQEAVYRILLETEPTPSPFTLVFSGRENRKVNGLYKPDSAEIVIHNRNFDSDNQLIYTA
LHEYAHHLHHCRNAGLSQARPHTQEFWAIFHDLVSKAEGKGLYKNVFETEPEFQELTRQI
KERCMRANGEILLEFGRLLSQAAALCRTHKMRFEDYIERVVGIPRATATAAIAAEQIGLD
PGLGWDSLMFVSKIKNPDDRAQVIESLSAGASPTAVKGMLKSEQLPDNSEERLLKEKHRI
ERSIENLTRKLEDIEEALSAIQA*
>SPSA8_v1_20051|ID:41144880| Uncharacterized glutaredoxin-like 8.6 kDa protein in rubredoxin operon [Spirochaetes Bin 1 SA-8]
MSVVIYTTPSCSYCRLAKDYFRQNGITFTEYNVAADMRKADEMVRKSGQMGVPVIDINGR
IIVGFNKPEIERALHR*
>SPSA8_v1_20052|ID:41144881| exported protein of unknown function [Spirochaetes Bin 1 SA-8]
MGMRAGLKMLIFWLCAFSVGAAESSAYSQTTEGRLVLEPENDTTCVSLLYRFSELKEHFE
LTARMIGRVSALSELWSPHDAGGAFQLRWRSLSAFLGEGAPGTSRAFRSSSGSSLLGTGM
LWSFSNQSESGESPKKILMGFDSPYLRVFRFLEYSAEYPSTNVLVEPEYATSGVEFSLPI
SHGLVSISGSMLHPAIQPSGAGLKYPSYWASLYSLSNFKAADVGIWAGWSRGYITPAGIA
AALEMRFGTMKTGSQKPPPFWYFMQSHIFAANEWYTTYRSAFPGQDFYFRQYVSASFGEC
RLYSSFSAWSIFSGSSLRRIFDPSVSVFTKYAWLWALDGADEKLAVSIPGWFLQSRFSWD
RNGLRRIEPTLRRERPFLGGRLTFLLSLKADKTTDDPESGGSDDSLDFEPSVEGDFENSI
LDVAGTSPHLPLAIQSALFQIRYQQQIYASGDSRLTGSFSGTIKLPSLNEGTLSPEIQIA
GEMILKAQKNATLAIKGKAAMKIEAMESLSFTKASVSVSYTWKSSS*
>SPSA8_v1_20053|ID:41144882|pncA| Pyrazinamidase/nicotinamidase [Spirochaetes Bin 1 SA-8]
MKRALIAVDIQNDFCPGGALAVPGGSDIIPVVNKLLEIIPVSVLTQDWHPAGHISFASTH
GKPDYSIDDSEKNLGVLWPVHCVAGTHGADFHPALESFRGKLILRKGSTFDLDSYSAFFE
NDHCTSTGLAGWLREREIQQVIIAGLATDYCVLYTAMDSVRLGFATSIVIDAVRGVDASP

GDAERALRTMEAAGCTLLLSNRIDK*
>SPSA8_v1_20054|ID:41144883|pncB| Nicotinate phosphoribosyltransferase [Spirochaetes Bin 1 SA-8]
MKKHTTSALFTDFYELTMAQGYWKRGLYEHRVVFDYFFRRQPFGGGYSVFAGLGSLVEAL
QTFSFSEHDLEYLESLSIFDTGFLDYLKNFKFEGTLYAVREGEVIFPQEPVLRIEGNLIQ
SQIIEGVILNTLNFQSLVATKSARVWLAANKGKIMEFGLRRAQGSDGAISATRAAFIGGA
AGTSNTLAGKLFNIPVMGTMAHSWVMSFPSEIEAFRSYASIYPDATTFLIDTYNTLESGI
INAITIGKELASKGHSFGVRLDSGDIDYLSRQVRQKLDEAGFQNAKIVVSNELDETIIEH
LVAARAPIDVWGVGTNLVTGGNEAAFTGVYKLAAMIQDDGTAKPVMKVSDNPEKSTNPGI
KNIYRLYNDCDSARVDLITLGDEAPLPDHEITVHHPAGDYRQVKIAPSRVEPLLKKIMEN
GILMEPLPDLKASRNYFSERISNFDSTYLRFLNPHIYKVSISTHLKELKLSLIKKYMRKH
*
>SPSA8_v1_20055|ID:41144884| Transporter [Spirochaetes Bin 1 SA-8]
MSDVKRDSWGSKLGLILAMAGNAIGLGNFWRFPYQAAKNGGGAFMLPYFIALIFMGIPLM
FVEWNLGKTGGRYGHGTLGPMVYLQAREKLRPKSALWFGVLAGGLAFSVVVLVNSYYNQI
IGWTLGYSWLSLTGGYADKAKSTVDVFLDYIGSPVNMIFWVISLGALAFAVMRGIQKGIE
AWAKIMMPLIYVFGIVLIVRTLTLGSPVNPDWSPIKGLEFIWTPRWADLNWGAALAAAGQ
IFFTLSLGMGIIANYASYLKRDDDIVLSGFTTVALNEFAEVILGGTIAIPIAFAFLGPDG
VGQSVGLSFIALPNVFRTMSGGSFFGAFWFLLLFFAGFTSSIAMFNYLVALLEEDMGIDR
KKGSWIVFALYIAVGLPVGIEPMLIRGSTAYLDSVDFWVGNVYLIVLGLIEVAVLGWMYG
KKGLEQINNGSYWKMPKWFYALFIQFLTPVLIIVFLVGFISQLKFDSLVASPWAVAGQIV
VVAVLIIGIFQTYKAITRTYGKEISANKVLVVKE*
>SPSA8_v1_30001|ID:41144885| protein of unknown function [Spirochaetes Bin 1 SA-8]
VKQSWLXISRRSEMEETSFDXIGLTGKQKALRDSFASLLQKPRSENRDSLTETEGIAMLK
AMDIALPAHRFVSGSAEYHGIAESSGSEVLFPGAKAVVKVISPQILHKTEMGGVDIVENN
PEMILASIRAMEERFASFQVEGYTVNEFVQFEPKLGHEIIFGYRFAPDFGPVVSFGPGGI
FAEYLAENFKTGSANVFFSPSLATRDLVEQTLAHNVVYGLLCAGLRNTKPVITGSELVDA
LMKFIEAAPALAASGISEFEVNPMVISKRSGKAELVALDCLVKLKDFSAMGLAQDADGVP
VNPAQKTRPTANIDHILKPQSAAIIGVSEKGMNNGRIILRNLLENGFDARRLYVIKAGVP
DIDGCRCVPDIGSLPEKVDLFVLVIPASGTPETLAQIAELDKAWSVIVIPGGLEEKSGSD
AIVARMKKSLEDARAQGKGPLINGGNCLGIRSVPGKYNTLFIPEHKLPMPKGRLAPMAVI
SQSGAFAICRISKHPDINPKYTITCGNQMDVTIGDYLEFLAQDQEIRIFAVYVEGFKMLD
GLKX
>SPSA8_v1_30002|ID:41144886| putative indolepyruvate ferredoxin oxidoreductase beta subunit [Spirochaetes Bin 1 SA-8]
MNYNIILAGVGGQGGLSVSVVIARAAMASGLQAKQSEVHGMSQRGGEVLAHLRIADTEIQ
SPTIPRGDADLILAFEPLEALRYLPWLSADHGVVVTASSPILNIPNYXDLELVXNEIRAL
PRHRIIDADTIAKAAGSXRAANIVLVGXASDLLPIAPEAIEREISQLXARKGEAVVAXNI
KAFRDGRNKF*
>SPSA8_v1_30003|ID:41144887| Indolepyruvate ferredoxin oxidoreductase [Spirochaetes Bin 1 SA-8]
MHELVLSGDEAIAQGAIDAGLSGAYAYPGTPSTEIMEYLQDNLDRYKARIAESERAAVVA
QWCSNEKTAYESALGASFAGRRAMVSMKHVGLNVAADPFINSALVRIKGGLVVVVADDPG
MHSSQDEQDSRVLADFARMPCFEPSDQQEAYDMVREAFDYSEKHEVPVLLRITTRLAHAR
AAVKTAAVRQPNPLSKSDDTMSWILMPAMARNRWKILLEKNRQFEKDSEAAAPFSLRSKK
LGVITCGLGLRYYLENEADWSATNDGEMPSHLHIGRYPAGKEKIRQLAASVEKLLVIEEG
YPFIEREIRGVFSAPVPVLGKLSGEIPLDGELSSDSVRRALGLAQKASVQLPGISIPARP
PQFCQGCPHSDSVTALKEALKDEAEFFTASDIGCYTLSALPPYSAVETCVDMGASVGMAR
GASTVGQKKAVAMIGDSTFYHSGMTNLLDAVAHKTPMTVLILDNSTTGMTGAQPTISPGS
RLPELLAGLGVEKEHIRELEAHRRSHEANVKVMKEELDYDGVSVLIMKRECIEYLKKARK
A*
>SPSA8_v1_30004|ID:41144888| Glutamine synthetase, type I [Spirochaetes Bin 1 SA-8]
MKLSAWNGEFADIDYLSLLVIDIDGHLRAVSLPKSYVNEKVLKKGVGFDASNFGFAEVHS
SDMVAVPDLSTAFIEEKDGFRILHAFCTVNLTNGEPFAQYPRTVIARAFEKIKASGIGDD
AMALVELEFYVFDDVRYSTTFDHSYYFVENSEGIGEESEDQPRFGLSKWYHRVSPQDSYQ
LLRNRAVKTMTDAGIPVKYHHHEVGSSQMEIELDFISISKVADMVTLAQWILRNEAEELG

LKVTFMPKPMYKIAGSGMHVHQFILKNGKSIFPGEKMYGLSDEGLSYIAGVLSHALAGSL
LAFSNPSTNSYRRLVPGFEAPVSATFAQGSRAAAVRIPGYLGKGEARIEFRTGDATANVY
YFIAAMMLAGIDGIVKKLDPIALGYATDKVTAKNTFPMGLYHVLNGLKKDNSYLEGVFPK
ELISEWIRRKEKEAEYVYNAPVPQEYELYF*
>SPSA8_v1_30005|ID:41144889| protein of unknown function [Spirochaetes Bin 1 SA-8]
MDPQIKKRIDSALAKVKEPQSDVPIVDLGLVEKVTYSEKEKTIILTLVSGIVPYECPACS
AINGVVKEGIIRRAQEAFATEFPELTVIVQ*
>SPSA8_v1_30006|ID:41144890| conserved protein of unknown function [Spirochaetes Bin 1 SA-8]
MNMNPFLLTAIILLGVGATINYFLGVKKNRWLGKAMSTQAENVLNPKETEYVNIGGAIGY
NIKYKLRDPWREAKGTFTFFPRHSLLYMPFSLAIGGSDRFFLNLFTDRKLAGEGHIIEKK
YLKKAKIDGFQEMEKAEMSRDGREFVLAWRHGDIKDTLTRTLMAMPDPQSLVHFTCFDGN
KTFFLFLKPKKGNIEKNLRVFVAQCSEFFR*
>SPSA8_v1_30007|ID:41144891| Arsenite-transporting ATPase [Spirochaetes Bin 1 SA-8]
MKNTAFFLGKGGVGKTTLSSAFALELARSGKRVLIASLDPAHNLGDTLYTKLNGQPRAVE
KNLDALEVDLSEWVEKYLEESRQQLKENYSYNMTLNLDSFFSILKYSPGTEEYAVLWAIE
DVHCRLAPNYDIVVFDTPPTALSVRFLSLPFISGLWVAELSKLRERILEKRQTITRLNPE
SPVAASCVDKDDDKVYGKLTNIRQRLAALATLFSKHSFMSVIVNPDDLSVYEATRIKDEL
QRLELPLAALCINKQGVSDAEWKLSAELSSLPRFTFDFTRDGIHSREDLAKIGLKPLVQL
FFKEGK*
>SPSA8_v1_30008|ID:41144892| conserved protein of unknown function [Spirochaetes Bin 1 SA-8]
MERKLHHILKAAFHTAIQVNREKATAIIEFEVKELENIFSLLVLGGFAGLPSPPTPIAVE
LLPYMERELTILLSRTDLSQDPLATLMDMLQID*
>SPSA8_v1_30009|ID:41144893| Carbon starvation protein CstA [Spirochaetes Bin 1 SA-8]
MSTIIALIALAIYLTFYFVYGRIVRDKLLKSNEAPEAPSKRLSDGVDYVPTSKYVLFGHH
FASIAGAGPITGPAMAVAWGWLPGLLWIWLGNIFLGAIHDYLALAASVRYDGRSVQFVAQ
DMMGKKAGKTFGWFTLFLCILVVAAFGDIVAGQFAADGRVFWAFVFFCVAAVISGYFMYK
TKLGLSWGSVIGVILVIAAFWLGNQFPVKWSKDVYFLIIFVYIVLASTLPVNLLLQPRDY
LSSFFLYFGLLVGGISALISFAPFTSIPAVTSFSAKLIGPAGNLQPSPFWPTVPLIIACG
ALSGFHALVASGTSSKQLKDEKDALFVGYGAMLTEGFLSTLVVTSIAAFGAAALGEKLMT
TPALGRFVQSFAKMVSTNLPFLPMSFMTLFAAVWVSTFALTTLDTTNRLGRYLVQEMALP
LKDKNPGLFKFFENKWVASIAIAFVGIFLARSGGYTVLWPAFSGANQLLASVVMLTVAVW
VKRKLNPAYVASVLIPGIFLWVTVTAALIWYEVAIIPVFFVDMSKSMNVITGAVVGAITL
FMLILNFIMVGSFLKNWKTQPAA*
>SPSA8_v1_30010|ID:41144894| Diguanylate cyclase (GGDEF) domain-containing protein [Spirochaetes Bin 1 SA-8]
MSQRKSAEELGNGYYWVGSETFAYNFQSNPYLFLNGDDAVLFDPGSTIDIKEVVKNIESV
TSLDKIRYVVLHHSDPDIASAVPYLESLGMKFTIVTHWRTWTLVRYYGVASPVYLVDEHG
YSLTLGNGRVLQFIPTPYLHFPGAIVTYDRQTGYLLSSDLFGAFSNAWSLYADENYVEGM
KTFHEHYMPSNDILRPVMDLLGRLEISMILPQHGSIIKKNIPEYIEILRNLDCGNLTRLA
HKDLSSDGGYTAPANAVIERAKGLFGAETVRILGEKLGLTLDDQASRILDFPMSGEELWN
RISEELYLLHGVKALIVLHPLILKLSQEYGIIVPKIFNSALGESQLEKDKLAREIARIEE
INRQLMQSAGKSQDSMMKDTVTGLLNEAFFRNFIDEEAAIALGMEGSEDDVLAVIGVDEG
LARIEYQYGPREVEEILKGVSRVIVEQKRENQVAFRLHGATFTLWMPRILFHEANDICDK
IRKMVEDSKAFIEPITISIGLAAAVEIKSMTEEKMENAGSALTELGLRRLRLARKRGGNI
ICSSSEVGNDIDTKARILIVDSDPVDADVIKTFLENADYFVAEASDGDEALRKISEEGFD
LIISELMIPKVDGFMLKEALSHRSGTKDIPFILLSHLKDEKSVIRAYNLGINYYLRKPFL
LAELLGIVQNMPLSGEGK*
>SPSA8_v1_30011|ID:41144895| Glycosyl transferase [Spirochaetes Bin 1 SA-8]
MNLQIEQILVAAAFLLWLDIFIVIRTLYNKTHLQLNANRXAQXLAELAHLFEANTDWDKK
KVRRLFTNYLQLKQSIDLPEKERMRILNLAGADRIEPQLNRQIRSPSRYKRMEAALKLAL
ISNDSARLTLQKALLREKDYPVRLFFANALADIKDPRCIAVLAESLLGAHRWYRDXTNML
IXSFGMASSDYLRGYLWRTESEIQEMLIDVAGQCVCENLKQYLDDFLHRGQSEIGKLTLS
VKDCSEHSCAYCAYGRKLTEDLRRHCRYHGIVESNFKCSHYRFMVTTKDPANNHHRLMVK
AAETLARYYLDSLNNQYYLEFPDREIRNLAVKSLSNSNKGENVYKLIHYLSDDEVSQSAR
QGLSLILTAHPRLIPLVVDEFFKAGDKRLKQHLAEVLAGRIEYFIYKLGGKDRERYELII

QEVISLGKFSQIIEFLKINKDIEIENAVLKIIRDDITQNEALARECGLYLPDRILEKLGI
ARQVPPVVKRQEKMDWKMVRGLYVILFFALAFFPFLYIVRYSDRIWSIPFISQLKTFVVD
FNYDFAWYSLTVNAIYLVLLALSAMQVTIATRLWNLKTMSMLFKPRMLPSVSIVAPAYNE
EKTIIESANSLLNLKYPDYELVIVNDGSRDNTLNTLITYYDLKRTEYHFQQRLKTKMVRA
IYTNPLLPRLIVVDKENGGKADSLNAGINIASKEYFCGIDADSLLEPDSLLKVAALSLDH
GIETPAMGGNVFPINGCEVDRGKLMSIGLAKNNLAKLQTIEYLRAFMCGRLGWAQINSLL
IISGAFGLFRRERIISIGGYLTSSERYQKDTVGEDMELVVRISRLMREKKLKYRIGYAYN
ANCWTEVPEDMKTLKRQRDRWHRGLIDIIFFHRKVIFNPSYGKMGLLGMPYYLIFEMIGP
LFEIQGYIMVFLAAIFGLISTKLALLLFVSSILMGILISMGSVLIAERQLYYFNYRDTLK
MLLIAFIENFGPRQMFSLWRVLGFLNAMKKPTGWGKMERKGFGPAAGAAEKPAQPPAEPK
KG*
>SPSA8_v1_30012|ID:41144896| conserved protein of unknown function [Spirochaetes Bin 1 SA-8]
MLIKNVREHSRLMILSILLIAVTIAAVLLFLAPFVIWQIQPYRPMNIWVVDKTVPYPDYR
EHQGLFWVLKNEKIAKPGSRKLYDEKSDYFGFYPYGKNEWRESPLPPSGPRPDLIYLADT
YGVYKDDYMQRRLSGEISPKIYGGLNANDNEVIKRNLGANNTFIAEFNTAASPTNAFDRR
NLGRLLGVKWSGWIGKYFENLTRGQEVPRWVVANWEEQNKQEWNYYGRGFVLISDSDEVQ
IFTMEDDVGPKGLKFKYRDEWAELLKSRKEISYRYWFEWVQPDLSIETVADYHFDVTARG
KERLEKMGLSAVFPAVVRYKNPQYTGWYFAGDFADLNFAKTSHSVWGMQWIKKLLVDDSV
DSNVYFYWNAYVPLMHKILADIQAAKIARLSMAEENKEPEVTVRAFGKGFQVLGKDGAWH
DLFIRGVNMGLAEPGKFFTEFPEDMATYLRWFDQIADMNANSIRIYTLPPPEMYKALYAH
NTQKPDKAIFFFQEIWPEEHPPNEDYLAREYRESFLKEIDYGIDAVYGRANVPERKGRAF
GIYTADVSPWLLGWLVGRELESSEVMATDARNKGVSYQGTYVVYSGKNATPTEVWLAESLD
EVAAIEANRYGKLHPVAIVSWPTLDPISHDSEWDPVTGKKNKGNDRASVSIDHFEITPAM
KAGLFGAYHIYPNYPDFINNELSYGLYKDEHGVLRYGGYLREFMQGHKRYPALVAEFGMA
NGFGIAHFAPDGLHHGGQTEEEAGRDILRMLAAIQREGYAGGLIFEWMDEWVKKTWTTEY
LMIPYDRHVFWHNAMDPEQNYGLLANDVVPKEKPEMVLPGQGKISSISIAADASYLNLEI
QLAKALDFKSEELIIGLDTLGRQLGQMRWPAGNLQTKSGMEFVVRLSAQDKADLLVIPSY
NSAESRFATIRLWDGSYERINMLVNGKVTTKDGREIPEKRFDASALRKGIFDESGNLWYT
EGNKIFIRLPWTLINVTDPSSLKVLQDNRTTFFNPERDALKVQTTDGFVFDAIVWDKGKG
STTGSLSSNVDKPYVWEGWESTPPYRERLKKSYTIIQKDWANYLHELSALR*
>SPSA8_v1_30013|ID:41144897| protein of unknown function [Spirochaetes Bin 1 SA-8]
VFAQYIQSMTEKNILSSEAFANVSIEFPIQFDLKIIYLTAEAQDLESGLERLLAELSIPC
SLIQGISVPGKKYGKLGARVTVSSRETMDSLYAGVAKLPGVKFAL*
>SPSA8_v1_30014|ID:41144898| Peptidase M29 aminopeptidase II [Spirochaetes Bin 1 SA-8]
MKDPRVAKLADILVNYSIKAKPGDKVLIQDTNEEPEFVTALIRAVHKAGAHAFVTLRNKT
IERELYHEGQEEQFALQAEFEAARMKRMDAFIGFTSLRNAYAWKDIPSEKMDLYNRYIWK
KVHIEQRIPHTRWVVLRYPSAAMAQNAGMSEEAFEDFFFNVCTMDYARMSKAMDALVEKL
KATDTVRILGPGTDLKLSIKGLPPIKCDGTMNIPDGEVYTAPVRDSVNGVLSYNTPSEKD
GFKFENVRFEFKNGKIVDAKANDTARINRILDIDEGARYLGEFAFGINPFIRAPLLDTLF
DEKIAGSVHITPGNSYDDCFNGNRSSLHWDLVLMQDPESGGGEIYFDGVLARKDGRFVLP
ELEGLNPENLM*
>SPSA8_v1_30015|ID:41144899| putative ferredoxin [Spirochaetes Bin 1 SA-8]
MADKSAKNPGSAAGPWYVDSSCIGCSLCAGTAPDTFAMSDDGSVAVVIKQPEGADELELA
AQAMTDCPVSAIGNDA*
>SPSA8_v1_30016|ID:41144900| conserved protein of unknown function [Spirochaetes Bin 1 SA-8]
MRTIIRIDQELCNGCGACETGCPEGALKIIDGKARLVGESLCDGLGACIGHCPQGAIQTI
QTEAEDYDEYAVMAKVVPQGESVILAHLAHLEHFGQDVYIKQALDYLEKHALPVPEGYSA
SKPVLEPKKISFSKPCSPDVRPMKAIGTSGTIRDKAASSTVFPEKPSAPSALSHWPVQLH
LINPRTSSFNNADVVIAADCTAFALGSFHTDILKDAPLVIACPKLDSGREIYLSKLASII
SQAKSVRVVVMEVPCCSGLLRLVREARTISGRSIPIHSLVIGIDGGFVARNSD*
>SPSA8_v1_30017|ID:41144901| Heat shock protein Hsp20 [Spirochaetes Bin 1 SA-8]
MNNLTLYGTNPFDLMDRFFSNDDFLYPKFRAPAIDVREESDKYVIEAELPGLSEKDIKLE
LKDGILSMSTAKKEAKEEKNDKLRWIRKERREFRFSNSFALPEDADAEKIEAKFKDGVLS
IILPRKPESAPRIVPVKVA*

>SPSA8_v1_30018|ID:41144902| Extracellular solute-binding protein family 1 [Spirochaetes Bin 1 SA-8]
MKRIVALFAVLLLAAGFVGAQQKVGVYTTLEEPLAKELFDLFQKETGIQVNWQRLSGGEV
ETRLEAEKANPQASIWVGGVGLNHMSAKMKGLTTPYKSKLLENTPPEYRDTENYWVGLYL
GPLAFITNNKVAKEQGLVPPKSWADLLKPEYKGKIRMANPTTSGTAYNVITTLRYVFNND
EEKVFDYLKKLDANIDQYTKSGSAPGKSVAIGEIPVAIGYAHDQVKLKVEGADVTITIPA
EGTGFEIASMSLVKGGPDVVNAKKLYDWILQPKAQEIIAKWYVVPLSKLAKKNPVAFSMD
EVKTVKQDIVWDSNNKERLLGRWVKEIGSKR*
>SPSA8_v1_30019|ID:41144903| Binding-protein-dependent transport systems inner membrane component [Spirochaetes Bin 1 SA-8]
VFTKKRIIIFVTACLIFLAADYWMFSTVKASFVKSMTQNAKMLASAVARSVPEDPDQSKE
WLASLSQTYPGYEFFYTRGLPWEDGYEEVGGNADIAQFWAQNKESPAIAQGLDSVSYLET
YIVPGSFHIGPTNSTMIMTPVPNANADSAAGLIIIAMDASRIGGFVSLINALIVIAFALF
ALGFGVATFSRDPISGYAILVLFTIVLAFVAFPLFEAVRLTFMKNGRFTLEVWKAILGNV
QYLKALWGSVQLGIFTASFSTVIGFLFAFAVSRTSMKGKKLVTALATMPVISPPFSLTLS
IILLFGNNGLITKQFLGLSKFSIYGLGGLTLVQTISMFPIAFLTMQGVLQAIDSTLEDAS
LDLNASRWHTFTHVTLPLAAPGILSSWLLVFTNSLADFANPLLLSGSYRVLSVESFIEVT
GMNRISNGAALSILLLLPTLTAFFAQRAWVSKKSFVVVTGKPSMRLSDLASPGTRKALSA
FVVVVSAFIVALYGTIVAGCFVKNWGIDYTFTLANIGEALTRGKQALVSTMTLAGIATPI
AGLLAMISAVLVVRKKFPGKRLLEGLIMTPFAIPGTLVGISYILAFNKPPLLLVGTGAIL
VINYVIRELPVGLEGGVAALRQIDPSIEEAASDLGADQATIFRTVILPLLRPAFISSMSY
TFVRSMTAVSAIIFLISARWYHITIQIYNFSENIRFGLASVLSTTLIIIVLAVFGLMRLL
IRQSEHLEKTVTA*
>SPSA8_v1_30020|ID:41144904|fbpC| Fe(3+) ions import ATP-binding protein FbpC 1 [Spirochaetes Bin 1 SA-8]
MAVQSVPVKLEMVSKTFRDPKTKAEVHAVRNADFEIQPGELVTLLGPSGCGKTTTLRMIG
GFELPTEGRILIGGKDVTYLPPNGRETATVFQSYGLFPHLNVEENVAYGLKIRKLDDATI
QARVKQALALVGLEALAKRSPGRLSGGQQQRVALARSLVVEPQVLLLDEPLSNLDALLRE
QMRVEIRKIQKSLGITAIYVTHDRIEAMSLSDRVIVMKDGVIQQIGSPNDIYERPNSVFV
AGFVGKVAFFPVILESVEPNGSCRCLMEGKLVKAGNKAEDARAGEKAVIMARPESLRLLP
PEEGLVRGTVTARVYLGSSVEVFVKTAYGEILVQVDDPSGKKIAAEGETVSIDFNENLVR
VLPEAKIEQQE*
>SPSA8_v1_30021|ID:41144905| Taurine import ATP-binding protein TauB (modular protein) [Spirochaetes Bin 1 SA-8]
MVKLEHVSFSYSLKGKALPVLRDINFQVEKGSITAIVGQSGCGKTTMLRLIAGLNRQDEG
IITRGDASGSTSRPNISIIFQNYGLLPWKTVKANAELGLLARGVKNRTRSAAVAPILEEL
GLSGFSRLFPLRLSGGMQQRVAIARALTTSSDILLLDEPFSSLDANTRESMQDMLLATQK
KHGTTVILVTHSIEEAAYVADTVYIASGKNPSTIIERMETSRKSKVLPAEPSRSLSISYQ
DTSDTSALPYRESAVYFQDVARLRQRFSEIAGSLQDTSQLAETEGILRAGKAGSTGKPRV
LNPGLRKFGNKLLHLVFAMLFAGLLWQLAAASVQKAFLPPPLSALRRLFELIMSGSLNTH
TLQSLKRIFLSLGIAGPLAWILGLLSGRHTTIDAVTSPIVYLLHPLPKVAFLPVLMLFFG
LGDASKVALMGLVIFSQLFVGARDASRAIPQSMIDTVRSLGGSTSFVTRHVILPSTFPAL
ISSLRISLGTASAVLFLSETFASMDGLGWYIMDAWSRVDYFDMYAAILTLSLVILILFLL
LDLVETITLRWRELD*
>SPSA8_v1_30022|ID:41144906| Nlpa lipoprotein [Spirochaetes Bin 1 SA-8]
MKKTILIMILLVFLGLTADTRIFADSPSAPAPLKLGIFTDADSFPFMICEAERIFQEEQV
IVELVRFQSAIERDSAFHAGSVDGIISDLCAAVLARQGGFKVRITSLTDGRFGIVAGPGV
KAKSLADLAKKPIGISSNTIIHYMVDSYMKNAGIRQSDISLMPIPKLPVRLELLLSGQIA
AAGMTEPFITMALVKGSTLLASTDEYPLGAGVLVFHERSIEEKTEAIKRLYKAYWKAAQR
INEKPDSYRSLLAQKAGFSDEAARAFKFITYQKPKLPNQEDIRRAEAWLLSKNLLKTEIA
PSELVDGRFTAGW*
>SPSA8_v1_30023|ID:41144907| Phenylacrylic acid decarboxylase (PAD) [Spirochaetes Bin 1 SA-8]
MARYLVCITGASGSIYPLRLIAALAAKGAIVHTVCSPWGARVVLEETGRPIGYWLGKVRA
SGGPSCRPAIVTYHSSEDFSAPVSSGSFRLDGTVIAPCSMGTAGSLASGATANLIHRAGA
VALKEGWPLIVVPRETPLSLIHIRSLQTLKEAGAIILPAAPSFYSKPQTIEQLVDSVVYR
IMDHLGVNNIHAYRWS*

>SPSA8_v1_30024|ID:41144908| putative 4-hydroxybenzoate polyprenyltransferase [Spirochaetes Bin 1 SA-8]
MASESSSPKASFSVFSRLGTLGDAVMIRHTVFSLPFAVAAVLLETSGRPPLHKVFWILVA
AFGARNAANALNRIIDRRIDAKNPRTASRPLPQGRISELELWLFTAAMLIMLIVGAAMLN
RLCLALLPLAGILVFGYSYTKRWTWLCHYWLGIACSAATMGAFLGISGAFHLRYFPITAG
VALWVAGFDILYALQDIEFDRKEGLHSVPARFGAKGARAIAALGHAGTILGLASIFFFWP
SPGPASAGAIALCATLLAGEHLVSRGGTEKHIRIAAYNINEILPFVFLLGVAIDMYLV*
>SPSA8_v1_30025|ID:41144909| conserved protein of unknown function [Spirochaetes Bin 1 SA-8]
MAYSGLQDFISYIESRGELKRISALVDPILEITEIADRIMKNQGPALLFEHVKGSKWPVL
INAMGSERRMAWAFGAESLDQKADEISGLINWAWSQVRDFSLFSAIPGALPKLPIAKSLL
PRKTTRPPCRQVIDDTAGFDSLPVLQCWPEDGGRFFTLPVVCTMDPETGAQNWGMYRMQV
YDDRTAGMHWHLHKDGAHFFEKYRALKKRMPVAVSLGSDPAVTYSSTAPLPEGVWEAIFA
GFLRGKSTQVAKASLSDIMVPADAEFILEGYVDPDETRLEGPFGDHTGFYSLPDQYPVFH
LERITRRENPVFPATIVGIPPKEDCYMAKATERLFLPFLKQLAPEIQDLVMPLEGVFHNC
VIVSIKKRFPGHARKVMNFLWGMGQMMYTKLIIVLDDDINLKDLSTVAWKVFNNIDAGRD
LVFSEGPLDALDHSSPVPRFGTRLGIDATKKWPEEGHTRPWPEPLAMTDAIKELVNRRWS
EYGF*
>SPSA8_v1_30026|ID:41144910|ybbA| putative transporter subunit: ATP-binding component of ABC superfamily [Spirochaetes Bin 1 SA-8]
VENSGSFVVTVENVHKNYALGKTEVKALNGISFTITKGDFISIAGPSGSGKTTVLNLIGC
VDTASSGTVTVLGQRTDSLDDDKLTELRHRGVGFIFQTFNLIPVLNIRENVELPALLDPS
ESGMAKKELDAWVDHLIEGVGLADRMKHKPAELSGGQRQRIAIARALVMRPPIVLADEPT
ANLDSATGESILELMKRMNREFGTTFIFSTHDPGIVSIADHVIRLKDGLIVENYRTSGQN
GNGRAEVHAGRVLA*
>SPSA8_v1_30027|ID:41144911| putative ABC-type transport system, involved in lipoprotein release, permease component [Spirochaetes Bin 1 SA-8]
MILARMAYGNLFLHKAKSLLLGLIICFGIAILFVGNSLIDTTISGLQSMFVSGFTGDLMV
TGPTSFPTTIFGETAGGEEVIPHIAKYQDYVEFLKADKRVAGYLPMLSGQVAMGLGEQII
GRGAAFGVNIDEYRKFFSGNIELVAGEWPASGAPPWILISETSAAMLARSGGKKLGPGDK
IVLSALGETAGTVIREVTISGIVKFNQSNQQLARISLVDADTLRDLLGFASLRDKAVSLT
AEQMEFVTSFNPDALFDDMPVEQEKASETQFQDSQASVSEIVPTPAWQFLLVKTVKGANT
GAVMKSLQKFAISLERNDHVQDWVAGAGTIARTAVTVRLVFNLLIVVIAVIVIMITMNVL
VVSISERISEIGTLRALGAKKSFIRQMILLETTFLTVLAGAVGLLLGLSILLILKSSGIQ
APNLFFEALFAGKVLRPVISSGAALKAFAWIFGMSVLSSLYPVAIALRIKPVVAMQGD*
>SPSA8_v1_30028|ID:41144912| membrane protein of unknown function [Spirochaetes Bin 1 SA-8]
MIQQIMAVRNSVRQPRRTILLGGAIAFGVIIICLVSGFISGMEKAVQDNVTLFSAGHVLI
NGITISESGRAQNRISDATLRERVKALVPEAVSVSQTAQAQATVVFGSREQQLRIRGVDW
ESDRLFSGSLVLTEGSWNEARKERMIVLGAQSAKRFGLGLGDTVMVRLSTASGQQNVTEY
AVAAFYDDASSGGMNTALVPVSDLLSDLNMKKGEYQALAVFLRDALLADETAQAVEKGLT
AAGYRFIASGQGGAGGVGNAAGGAMSGGAMGGSAMNGGAAMPSGSALFGGTQISRNMPAG
TTMYRAVTVTQMSGQTGAVLATIKWIGITVFLVMLVLVAAGITNTYRMVLLERTKEIGML
RCIGFKRKDVFAVFMYEALMIALAGSIAGILLSLPLGILVQLIPFDPNGSLGSVLTRGHL
VFAPNLGSLALVVAAVLAASCLAVLSPARRASRLKPVEALRTVA*
>SPSA8_v1_30029|ID:41144913| conserved exported protein of unknown function [Spirochaetes Bin 1 SA-8]
MINRLSDTRKALVLSIFLLAVLLGASAQGAPGGATGGAPSGGLPPPGMYGNPEDTTTATK
PGSIQEYSAKDFLALVRQTDIRSSFYQSDMSATITMVDVSPERGTRTFKQMIYRRDKDDA
FLMITIEPESRKGQGMLRVDNNMWRYDPTSRKFTHTTLKDQYENSTAKNSDFRRSQRSWD
YSVQTVESGKLGKYDVWIAELKANNDEVPFPFIKMWIEKDRTIVLKVEEYSLSKKLLRTS
YYTNYVQIGTSFIPTTQIYQDGLIPEKRTQVTYTNISTKPVPDDYFTKNYLERISQ*
>SPSA8_v1_30030|ID:41144914| protein of unknown function [Spirochaetes Bin 1 SA-8]
MKNLKPGWGIGTLGLLFLVLACPLEFANAQTSLPALDDDALFGGSNDIVTVIDTNVAATS
VVELVKDTKTYPVFLVEGDLGAGLTSSLVPYGASGSDKNLLFGAVHLDSLAFTYSPVKDV
DFNITTSATLLPNSATDVSVSAYANLRASEYTRFYTSGKYNYNVSSSSTVVTADGLALDE
IFLDTAINRKLFFRLGKQRVSWGVGYWFKPADVLSLAQIDPDNPTASREGPFAFKADMPF
GLNHATLYAVPPIDGSFGTFSVAQRTDIVAGGFELSFAGFVRTDMGARPRLMFMFTGAIG

PVDVYGENVALWGSDRNYVREKDGGGYELYRVENQLLFQSTLGLKYSWQNSNGLSASFHV
QGYYNGTGYEDSSILQTTAARNLVKASSAYTSNDLTQAGMYYLGANVSFGARFGEGKKLT
NTTLGSTALFNFSDGSMRFKPSWSLTVGSGGSALNLAVSALTSLGKLYSEYAPQGNMVTP
ALTATINKTVQVSASAPLKLDTDYSLKKASLALSLYWNVISY*
>SPSA8_v1_30031|ID:41144915| Two component transcriptional regulator, winged helix family [Spirochaetes Bin 1 SA-8]
MKGTILIVEDTRELADLYALYLEREGFSCTIALDAETALPLAKSQSWNLVVLDINLPGMD
GFQFLEEFRKFSAVPVMILTAREADEDIIHGLGVGADDFVTKPCPPRVLAARVRASIRRT
LAKTDDQEANLFHFGPFELDAEALYLTREGHPINLSPREFGILVFLIKARGKSFSPEEIY
QQVWGQSFGDISAVGVYIQRIRRKLGDDPAAPRYIQTQYGLGYRFNPEMLQ*
>SPSA8_v1_30032|ID:41144916| putative Integral membrane sensor signal transduction histidine kinase [Spirochaetes Bin 1 SA-8]
VRLKTQFFILTGAVTLVPILFGLFLFSFQQAPRDPREPSRIFMASISEKWRLGETLNAQV
LTEEAEKAGMPLRGVAILDASGTILVSSFSHLQEGSQATLKDFTPRPRATPDEPKAELKL
MPLDSKNPSSPYILFDIQPFWSKQDIRNRNILLIGSFVLSLFILTAILSFFILRSISKAI
GTIEADTAIVATGNLDHEVAGMGNHEILMLAKSINLMRLNLKDMLARRSKMLMGVSHDLK
TPIALIQGYADALSDDVAKDEAEKKQYIEIIRNKAKQLEDLTEELIEFMKIGGDGAVSVE
EVAVDELFISLARRFEADARLLSRNFEWGFGERLEPYPDFPAPKLPMNRLLVERAIENLV
SNALRYTEADGNIQFRLTSNEQGSSIAIIDNGPGIAAADEPYIFDAFYRSSNSRADGGYG
FGLTIVKAVADLHGWSVRVGRRSDGQRGTEAVLAIIEPGKAKG*
>SPSA8_v1_30033|ID:41144917| conserved protein of unknown function [Spirochaetes Bin 1 SA-8]
VVGRADDGRLVLRGFFSGTDNDCFYKTAKLSAEVNITKLDKPIAKAVVWLDPSEFRXTWL
GNKAVYRTRMAMADGGELIILAPGLAHFGEDAGIDMLIRKYGYRTSAEIRKAVHDNADLA
GNLSAAAHLIHGSSENRFSIRYAPGPSVSKTEIESVGYGWADLERMKTEFAIDKAVAGWN
IAPAGEPYFFVPNPALGLWTTKERFSLSPSPAR*
>SPSA8_v1_30034|ID:41144918| conserved protein of unknown function [Spirochaetes Bin 1 SA-8]
MTIIEYGSQNTEISSEQLESFVADSIAALGNVRSMIALPPDITRFHSRAGLITDMIARIA
GPRLKAVMPALGTHVPMTAEEIHAMYPGTPKGLFRNHDWRNDVTELARLDADFISEVSEN
TVAYDYPVQANKLLVSSGHDAIISIGQVVPHEVVGMANHAKNIFVGTGGKEAIDKSQKPL
PWRGLWHGADDGESRHTGSSCVQQSARIRQPHPAPHSLDIDRGRPR*
>SPSA8_v1_30035|ID:41144919| conserved protein of unknown function [Spirochaetes Bin 1 SA-8]
MKVLSRFSVGTGDRFGREGIAQIEAFRMLAEKGVDAAIVWNKSNREHIIIGTSPADQRRS
ADEAIAAAGFKGMYAVDADHIGLATVDRFLPYCDFFTIDVADYIGKPTEPEKMEAFAAKY
RRLFDDVRCPVSITEADVAAGSRKYLYAIEEAAKVYRRILAGKPDGNFHIEVSMDETDVP
QSPAELALILAGLADEKVPVQTIAPKFSGRFNKGVDYVGDAAAFEREFEQDVRVTQWAAQ
ALGLPTSLKLSVHSGSDKFSIYPAIRRIIQKTGCGLHLKTAGTTWLEELVGLAEAGGEGL
EMAKLVYRKAYARYEELAAPYATVIDISMSRLPVPKIVDGWSSEEYTAALRHVPSNKAFN
PDFRQLLHVGYKXAAELGQDYLRALEAHRESISRNVTENLYDRHLRALFLE*
>SPSA8_v1_30036|ID:41144920| putative Integral membrane sensor signal transduction histidine kinase [Spirochaetes Bin 1 SA-8]
MAEQTEVRHQRRILHHIGIRGKLLAFLAIAVVFIITLEIVAQNATYRAAEEYEQDLAHYH
LVHRLRATLDSFRLNSDRFIKDPNSIPIENLFETIATLNSLSSSLAPVEELSIEAGFEVR
AIGYGFDAYLPILSRSLSSRASGRTDYYADFARADRIAGYIDTYLSRLLSILLRDGEARF
KIISSRMKEINKIILIGIAIAGLLSLGYVFLIANSITKPIRKLASASEKLARGELEIDPL
PRKSRDEVGVLAESFFIMSQNIRLSIENLREKAELEQKLHEEELTLLSMGKALREAQFMN
LQDQMRPHFLFNALNSIARTALLEKAKATEKLAISLSKLLRATIKEGGPYIPISEEVDIV
QEYLAFQKARFGDRFEWHIIFDPELNACRIPRFLLQPLVENAIRHGVEPKEDKTEILVSI
RRRGERLRIAVADTGTGMAAEKLAELNLRLRQPFDSVSAKAGSDNLVSANGIGLVNVANR
LTILYGQEALMRIYSVPGKGTIVRLSLPLKGIARWPESL*
>SPSA8_v1_30037|ID:41144921| protein of unknown function [Spirochaetes Bin 1 SA-8]
MARILIADDEELERAALRLIMTEMAPGSSHEIYEARNGHEVLQLAAAQPFDVIFLDIRMP
GLDGLETAETLRSRGINTPIVIISAFDTFAYAQKAIKLGVYEYLLKPASAEEVLSALGKS
LESTSGGMDNPARTLQEAREQLRRQLIDQLQRGIPDSALFTEYEKLASLEKKPRTAFAAR
LEPNTHFGRIASAILSSALYAAQNILASQRTSFLASVNESALRILVYAESQAELETYVSN

LVPEMKRRLLSEFSANLLIGVAGPGRAESAQLLERADEALMLAKPSNPLIKLSEPASIEP
EQLVHLSGQRNPRSLGLRALEYIRTHYNQDISLSSAADALGVNPFHLSHAITRELGIGFS
ELLARMRTNKAKELLLAGSSVKEAGYLVGFSDQAYFSKVFKRFQGISPGQFASKTAKKYK
KE*
>SPSA8_v1_30038|ID:41144922| TRAP dicarboxylate transporter, DctP subunit [Spirochaetes Bin 1 SA-8]
MKKATFIIIALLIAAVLMPMQVFAQKTIVLRLAETHPQDYPTTRGDYEFARLVKERSGGR
IVIEVYPGSQLGQEKAVIEQVQFGAIDLTRVSISPVAAFVPRLNAFQMPYLYRDETHMWK
VLKGDIGKELLASLEPFGFIGLGWFESGARNFYNSKRPIRTPADLKGLKIRVQESELMMG
LVSAFGAVPTPMAFGEVYSALQTGVVDGAENNWPSYFSTSHYEVAKYITLDEHTRVPEII
IGSKISLGRLSPADQELIRQAAFDAIDFQRAEWAKYEKISIDKTVAAGSQVVEIADKSAW
QALMAPLYAKQSKEIQALVERIKAVK*
>SPSA8_v1_30039|ID:41144923| Tripartite ATP-independent periplasmic transporter DctQ component [Spirochaetes Bin 1 SA-8]
MILRQLAFLFIFCKESTMKDLILRTINFFHIVLVELAKLMIIGMVMIVFINVILRYVFNS
GIIWSEEVALLLCVWFIFISFGLGIKQRLHITINLLRKDKISPKLDAILDLMAELIVVFI
GMIMIIYGARLVQFTMRSIMPATQWPAGILYLVVPFSGLTMVLEAILHLLRWDTYDQKID
DYLSGKGGKLKDIFGGNHG*
>SPSA8_v1_30040|ID:41144924| conserved membrane protein of unknown function [Spirochaetes Bin 1 SA-8]
MADPVIASTILLGGFILLLILRIPITFALASVSILTAMYLNIPLMAIVQQMVQGVRSFSL
LAIPFFILAGEMMGQGGIARKLIDFANILVGRVRGGLAMVNVVESMFFGGISGSAVADVA
STGTIMIPMMKMQGYDDDFSVAITVTSATQGIIIPPSHNMIIYSLAAGGVSVGRLFLGGF
VPGVTLGLSLMIMSYFISIKRNYPKGEKISFKQALKTTKDAALGLLTAVIIMGGVISGVF
TATESAAVAAVYAFFITFFVYKEIPLKAMKGILYSTLKTLAMVMALIASASAFGWLMAYL
RIPAKATQLLLSITSSKVLLLLLLINALLLLLGMIMDMAPLILIVTPILYPVVVTTLGMSP
VHFGIMLMLNLAIGLCTPPVGSALFVGCAVGKITMERATKAMLPFYLTMFIVLMLVTFIP
EITMFIPNLIMPGN*
>SPSA8_v1_30041|ID:41144925| 3-oxoacyl-(Acyl-carrier-protein) reductase [Spirochaetes Bin 1 SA-8]
MSFVESMFSLDGSVAVITGGAGGLPRSLAVAFAKAGARVCLWGRGTNHPIEDAVVSVKAE
SSSPDVFGVTVDTADSQACEHAYDVTVKQWGVHPAILLNGVGGNRGKSSFVDVDETVFEE
VLKLNLLSGLVTPTRVFARRWIEHGIKGNVINMTSMTSYKALSGVWAYDAAKAGVLNLTE
ALAKELAPHHIRVNAIAPGFFVGNQNRSLLYRDDGELTERGKAIIARTPFGRFGEHSELW
GAALFLASDQASGFVTGVSIPVDGGFLADNI*
>SPSA8_v1_30042|ID:41144926|uxaC| Uronate isomerase [Spirochaetes Bin 1 SA-8]
MKPFMDKNFLLDTEAARDLYFGQAAPLPIFDYHCHLSPKEIAENKPFSDIAEIWLKGDHY
KWRIMRSNGADESLCSGQASWEDKFLAYAQALEKSAGNPLLHWSHLELQRAYGIDDILTQ
GNALKIRQWANDIIRERKDSPRSMLTRFNVAVVCTTDDPADDLGWHRQIAADPEFGTEAA
RAGKKTTRVLPTFRPDRIMNVQDAAAWKNYAGTLGNAADIEIKDFASLQEALARRHAYFH
ANGCRLSDHALLTPAFALASEAELNTIIGSLLEGKAADTEAAEKIATAVLIHCARLNAKA
GWTMQLHIGALRNTNTRLFRLYGPDGGGDSISDSNIVLSLARFLDYLDAQNSLPKTILYS
LDATRNASLAVLAQSFAMHGSPGKIQYGSAWWFNDHKDGMERHLVETASVSLIGRWVGML
TDSRSFLSFPRHEYFRRILCRIVGRWIEDGELPSGEAYADSLVRNISWYNARDFFGMEIP
RWAKN*
>SPSA8_v1_30043|ID:41144927|ugpE| glycerol-3-phosphate transporter subunit ; membrane component of ABC superfamily [Spirochaetes Bin 1 SA-8]
MREHSRIQRILPHIYLWIGIIIIIFPIYFIFVGSSHTSAEILAAPMPLKLGPHLFENYRR
ALFEGTSNMGTPVLVMVKNSLIMALGISTGKIIVSLLAAFAIVFFDFPGRKFCFWAIFIT
LMLPVEVRIMPTYKVISDLGMLNTYAGLILPMIVSATAVFLFRQFYMSIPREIAEASQID
GATPMQFFRSILVPMTRTPVAAMFVIQFIYGWNQYLWPLLITTKTNYYTLLIGINRMLSG
ADVQIEWQIVMATTLLAMLPPVIVVMIMQKQFISGMTETEK*
>SPSA8_v1_30044|ID:41144928|ugpA| glycerol-3-phosphate transporter subunit ; membrane component of ABC superfamily [Spirochaetes Bin 1 SA-8]
MTKQYEFKAKGLAYLLVAPQMIIVFVFFFWPAAQAILQSFFLQDPFGGKMIFVGLENYTQ
LFTDKSNGYGQSFVVSVFFAVGITVLAMSVSLFLAVQTNKRIRFETFYKTMLIWPYAVST
MVAGVIWLFMFNPNVGIIAWVLKHKFNVEWSYLLNFNQAFILITIAASWKQLAYNFVFFL

AGLQSVPETLIEAAAIDGAGHGLRFRKIIFPLLSPTTFYLLVMNLVYGFFETFPIIHQMT
AGGPGKSTAILVYKVWRDGVVNLDLGSSAAQSVILMLMVVALTIVQFRFIERRVNY*
>SPSA8_v1_30045|ID:41144929|ugpB| glycerol-3-phosphate transporter subunit ; periplasmic-binding component of ABC superfamily [Spirochaetes Bin 1 SA-8]
MKKIALLLFAVLLVTGTYAQTTIEFWHAMTGKNGDMVNAIAEKFNNSQKDYKVVPVYKGS
YSDTMNAGIAAYRAGQAPAIIQVYEVGTATMMAAKGAIKPVYQLMAENNEKFDPKVYIPT
ITSYYSTSDGKMLSMPFNSSTAVMYYNKDAFRKAGLDPEKPPVTWPEFFEVAKKLKASGM
EGGFTTNWISWIQLENFSAWHNLPFGTRSNGFDGLDTQLVFNNPVVQRHFETIYSLSKDK
VFIYGGRENKANPLFTSGQVGMHFESIGGYGNMKANCKFEFGVARLPYYPDVPGAPQNTI
IGGASLWVFNGKSKAEYKATADFFSFLSLPEIQALWHQQTGYLPITQAAYELTKSQGFYT
TNPGPEVAIKQLLNKLPTQNSMGIRFGNFNIIREIEDQVWEDILAGKISVKDGLNKMVTD
GNATLRKFEALNK*
>SPSA8_v1_30046|ID:41144930| Glycerophosphoryl diester phosphodiesterase [Spirochaetes Bin 1 SA-8]
MGHKNISRITIVMLVAMVLSMAFGQTEPAQPSVVPGQGSAEASKGISMNTTSNVLVIAHR
GFRSVAPENTLLAARKAFEIGADMWELDVAATTDGELVIMHDDTLLRTTNVKEIFPARDP
WSVYDFSLAELKSLDAGSWYKKADPFKQVLNGNISREEAASFAGEKIPTLREALELTKAR
AWSVNIEIKDATNRTCDAWIVEKTVDLVQELGMEAVVVISSFNHTYLERAKKAAPEIKVA
ALIDRPVKDPVTILKRLGAVALNPNLKYLDEKTVITVRAAGFGVLVWTVNESADMEKLIR
WGVTGIITDYPDRGLAVVNSKR*
>SPSA8_v1_30047|ID:41144931| putative Peptidase C39 related protein [Spirochaetes Bin 1 SA-8]
MAERITSMYHYLNTYDVGRSIWVFSKSTGFAEVLSYSRHESCPGKAQGLENAHEGLVLTS
GARRGTLITDEIEIPDADWLTPWWNADIAGSGGLSVYLQAEVSGQWSSWYPMGSWKHNPA
SRTFSDETGGVLVDTLKLAKPARKFRLKLELSAGTSPALPGSVLLRRVGVITRSSAGQHS
ASKPYLLKESALLVPRRTQMAEDESVKNRICSPTCLAMALDYFGINLPTSFVAASCFDTG
AQIFGNWSFNVAALWSLGLRARLEYFLNFEFASAELYTGKILIASIRYGKGELAGAPVQQ
TDGHLVAVTGLKKDKTKGYMVLVNDPAAPDVQTVQRAYPLEQFEKAWTGYAYVIEGKR*
>SPSA8_v1_30048|ID:41144932| exported protein of unknown function [Spirochaetes Bin 1 SA-8]
MAIFLLSAVFLILNTAAGYPALARDIQKARNLTIGQPEQRKLALQLTETWAAAKNLGIYD
NMLVFDAYRIRARVLEREGKTAEAQALYNTLKTWYAHTRPAESLP*
>SPSA8_v1_40001|ID:41144933| protein of unknown function [Spirochaetes Bin 1 SA-8]
LIKFADEATIIVASGKGGDGCVAFRREKYVPLGGPAGGDGX
>SPSA8_v1_40002|ID:41144934|rpmA| 50S ribosomal subunit protein L27 [Spirochaetes Bin 1 SA-8]
MAHKGVNGRDSNPQHLGVKVYANQAVSAGSIIVRQRGTKINPGTNVGIGKDDTLYALVDG
MVSYKEFRGKKYAQVEPFPAE*
>SPSA8_v1_40003|ID:41144935|rplU| 50S ribosomal protein L21 [Spirochaetes Bin 1 SA-8]
MYAIVEINGKQYRAEQGKKLTVDRIEADQGSSLSLDKVLLVSRDEAVTVGTPYVAGVTVK
AVVEEEVKGDKVIVFKYKPKKDYRRTQGHRQPYTVLSVQEIAGV*
>SPSA8_v1_40004|ID:41144936|argS| Arginine--tRNA ligase [Spirochaetes Bin 1 SA-8]
MTELKAAWKQRVYAALRKFAQSGNLAIDTLSEDMIVAEFPPKPELGDIGFPMFSYAKIFR
MSPANLASQVAAALEPAKGDDGADLGAAKAVGPYLNVYFNREALSKYIFQNGTSFARSPL
AGRKVMIEFSCPNTNKPLHLGHLRNNVLGESLSRIIKTAGAEVRKVNLINDRGIHICKSM
LAYQVYGGGRTPEDEGLKSDHFVGKYYVLFNTLKSEDPSAEEKAQELLRKWEAEDPDVMN
LWKKMNTWAVDGIKATYARQGISFDQYYFESQTYMKGKEEILAGLERNIFYREEDGSIWA
NLEDIGLDRKVLLRKDGTSLYITQDIGTAIYRHVDWPFDQLIYVVASEQQYHFRVLFEIL
KRLGYAWAKDLYHLSYGLVNLPSGRMKTREGTVVDADDLIDELASLAQKEIAERERESAI
DDAAKVAEKIALGALHYYLLQTSPVKDMLYNPEQSLSFTGNTGPYIQYMGARACSILRKY
HKGEGNAGKGTVTPAKLSSDADWMLVRRLLALQEAVELAARSKEPSVVAAYAHDLASDFS
SWYRDNPVLASEDPDLSATRVELVKAVKSALQNVCWMLCIPFLEEM*
>SPSA8_v1_40005|ID:41144937| protein of unknown function [Spirochaetes Bin 1 SA-8]
MNDLSYMVKQARVFFPVTAIPKASQDKVDGIRNEALLIRIQAAPEDGKANTAIVNVLSKA
LKLPKSEIRIEHGHANRHKIVSIPQNSLXSLQKLCLINLSGKEPSDSSP*
>SPSA8_v1_40006|ID:41144938|ligA| DNA ligase [Spirochaetes Bin 1 SA-8]
MEQDQTAARVRELEILIKKHQDLYYNAEPEISDEAFDALWDELARLDPNNNLLKKVGEDR
LDGWPKARHLIPMGSQSKAADPESFSSWAMKNPSNLYLVQYKLDGASIELQYKNGRLEKA

VTRGDGIIGDDITPNALRMIGVTAELPIPFTGGVRGEVLMSHEIHRTKYADKANCRNAAN
GLMKRKDGTGSEDLQVICYDAAGMAAEQETAAPDIRVHLDFKTEMDKLGWXEKMGFXTVP
SVXLKSPEEIIEYRGKIMAIRKDLPFDIDGLVIKLPEIDPEDAAKPRPEKQIAFKFSLEE
AVTTLVAIEWSESGVNYTPIGIVEPVRLAGTTVQRANLCNPDMIRTMRLKIGSRVVIVKR
GEIIPKIETLVENPPDAEEIGQPEHCSRCGTALVDEGTRLYCPNPACPKRALHRIEKWIS
TLDIRELGDVLIQKLFDAGKLRSIADLYRLTAEDLERLERMGPVLARKIIRSIEAVREIP
LEVFLAGLDIEGVGPLVARNLVVNGFNTLEKLLSAREEDFLTLDGIGLIMAKSIVGGLKE
CKNEIEEILMTSKIRIQTHVADSAEAVLAGKSFCFTGELVSMKRQEAEKLVARFGGTTKS
SITKDLSYLVTNEPGSGSEKNKKARQYGIPVINETEFLALIDNAKEQVAKNE*
>SPSA8_v1_40007|ID:41144939| putative Xanthine dehydrogenase [Spirochaetes Bin 1 SA-8]
MSSMKPGEQLPILTLLEVRDMLFASTIRSTVAYAKEFSVQRSQLPFGYRMILPEDMVKDG
MSLEFSDGIPLFATDTIRYRGEPLGLIVGPDPVLCDNLSLNVKVTYRESEPNFSWTTFPS
SRIAARKRIRIGKIEVSTPKGISDQQWETTTHWTGSFDHEYSEPSSALAVWEYDKLVIYC
ASQWPNHVRSSISEVLKVPQKDILIRPTDLGQSIEGRIWFPSLIACHAACAARLCNKPVK
ILYTRGEDYCFAPKQARSCISISSLSDERGRLASLDIRLVINIGAYNPLAEELLDHAVLS
LLGIYACPVVSIDAFAVQSNVIPLGALGNLGSIHGFFALDAHLNHLARKYQKSPAEIKAI
NMIAKGMTVHGDVQVSSVIPFSKIQQKLEIMADFQRKHASYEVIRKRDPASRKGIIRGIA
GSFGYLTGDAFLNIPEKNRYSVEVQLEKDLTIVIKTEAAISSENARLMWKKTVSEILSLP
ERSIHVIQESVHTNSRCGPMVVSRGVFAINKLIERSCRALQKKRFRETLPLTVKSQMQCN
PSIQWTEDGLSGKAFDAASWCGTIIELEVDAITGQVKPLTIWMAVDAGRIINKPVAESSL
RAQAVNALRLCLLDDVVPETIDSSSYLAKKRLPFNEIPDIVIEFMDSDRTSALGIGELPF
ATIPSAYYGALSQALGLNLPSIPASPHDVMRIWEAT*
>SPSA8_v1_40008|ID:41144940| Isoquinoline 1-oxidoreductase subunit alpha [Spirochaetes Bin 1 SA-8]
MTITFQLNGITKNVKIRSGDRLSTILTQEHPPIKSLIPDCLSGHCGKCLVIMDKRLVYSC
LVPAFRARDAEITTIEGLSDSDDIRDIQKGFQEAHCHPCNFCKNAKTLVIWDLLNRIALP
NESQIEESLSVVSCSCTDPVSLKKAVFLAADLKNRRKYHRADK*
>SPSA8_v1_40009|ID:41144941| protein of unknown function [Spirochaetes Bin 1 SA-8]
VLISEIHYPASVTDALALLDEQPETILLAGGTEITGAQVSRFLQFPQSIACIAKIPELKK
TTRTEQFMEFGSCTSLTGLLSLGPGILPHPLAQAIQSIGNPAIRNIATLGGNLASRRQFM
DLWPFLVCMDAQVEIRNRTNSHWANLFYLADSSSKPFFPEKSILTKVRIPLVEYSNVFYR
KIGGQGFPHHESAVFVCMAEIQQNKIESFRLVFSGTKVFRLQEVELSMFAKKITNNSREL
AFARQEYIREFSRSGFLKPAIFSSLVTDAFETLFGASM*
>SPSA8_v1_40010|ID:41144942| putative Uroporphyrin-III C/tetrapyrrole (Corrin/Porphyrin) methyltransferase [Spirochaetes Bin 1 SA-8]
VSAKGKLLLLPAPLQPYSRDAWTLANLESSLPARALFEYRRLSTFITESSKTAYRLLSAI
RPNEDLASLSINILDEHSKEEDIFNFIRLXENGEDAGFFSEAGLPCIADPGAALTAAAHK
KNITVVPISGPSSIILALIASGLDAQRFSFLGYLPQESVQRKHVIRRIGQDTAKDRITRL
FIETPYRNRALIQDLLRFLPGDMHFCFAANLDTSEQLIISQSVDQWKTFPVTMPAKVPAV
FVIGYPATIKPVNTV*
>SPSA8_v1_40011|ID:41144943|mfd| Transcription-repair-coupling factor [Spirochaetes Bin 1 SA-8]
MNKKEPQVLLDFLRHYEPYTALRRSLENGEKTIDIKEAELSFLAMMAAALHRFEKKRLCV
VVPTDQDAENFIRDTGFFGAKADVLPWWKTGAYRSVSVRSRIFGERVRTLADLLHGKASL
VIASARAYMTPVPPRDYLREKLLVFNRLSRFDPERIADRLSQYGYLRVPRVSLPGEYALR
GEVLDIFMPGDNTAFRIQFEFDMIEKITAFDPITQTASGKLDSILLRPIKEVMWEPETLK
SVQEKGSGYPQCRERMNILADSLQRQEDGKAEEYWFGSAFDSPHSVAEYFDSDTIILFAN
IERILAQQESARKEYAGMYRQALRYGAVPPPERCLVEFGTDFDGIHTIVRSFALKDETQM
ERLSLGSEPGHSYFGNIAYLKEDISNFEKAGYRQYVAAGTDIQAQRLASLISNENCAFLI
GDLSAGFTIPGLKLRVIQEQEIFGRKKRTPKSLAASQSSAIENFVELAPGDFIVHVNYGI
GKFLSIERMKVLGNERDYIKIAYADAETVFVPIEQANLVQRYIGNEGEAPRLDSLGSKSW
ENRKKKVRKSVEELAERLIRIYARRKAARGYAFPRDNEWQTAFEASFPYNETIDQLRCID
EIKKDMESPRPMDRLVCGDVGYGKTEIALRACFKAIAAGKQVAFLAPTTILAEQHYENFK
ERIEQYPVTTAMLSRLVAPKVQKKVLKGLADGSIDMVVGTHRILQKDVVFKDLGLLVIDE
EQRFGVKDKERLKEMKAGIDCLTLTATPIPRTLHMSLLKIRDMSILQTPPAERQPIETFV
EEYKPEVVARAIRNEIERGGQVFYLHNRIESLQEVEAFLRTIVPEVLTESAHGQMEPREL

EDIMHRFIHGAFHVLVSTTIIENGIDIPNVNTIIIDRADMYGVSQLYQLRGRVGRSDRQA
YAYLLYPDKRALTEIAMKRLQIISDFTELGSGFKIAMKDLEVRGAGNLLGREQSGDIYSV
GFDMYLKLLDDAVNALSGFEPEEEPYLELEYSGFIPEDYINQPMLKMEIYKKIASVSRQH
ELDALQEEMENRFGPMPEEVQSLLSLAEIRVICRKLSVASLREKKGMVHVEFSKVSKISV
ERLVRLIKESNGRIRLDPEKPNAIILMTKAIGLREKSEFIRERLASLL*
>SPSA8_v1_40012|ID:41144944| protein of unknown function [Spirochaetes Bin 1 SA-8]
MIAVLVADDDAFSRQLSHRLRLQGYDLIRYRDPVKLSDNIAELAPDLVIVNGRDFPLHWQ
VLSAEISMLGMTGTTVILASSSCPQNFRQFKELKMSWLEYTGSIDKNMFRACLDQASQAK
HISVSDTE*
>SPSA8_v1_40013|ID:41144945| conserved protein of unknown function [Spirochaetes Bin 1 SA-8]
MSSDLNSPCDESYLEPYASKFKAIGHPVRLKILCLIAHQDVPCVGDIWRCLNQPQPVVSQ
HLAILKKTGIVASEVVKTRRVYSIKDPFIKSLVESIICNIEDSATGHEDERQTPTSPV*
>SPSA8_v1_40014|ID:41144946|ispG| 4-hydroxy-3-methylbut-2-en-1-yl diphosphate synthase [Spirochaetes Bin 1 SA-8]
METETVKVGSVMMGGSWPVSIQTMWKEPLPAFSGTSDPRLAETAARIKQLHEIGCDILRF
AIPDERSVESLGLLAAMSPIPLVADIHFDWRLALKCLDFPIAKIRINPGNIGARWKVEEV
VRKAKDKDVPIRIGVNAGSLPEDLRSNEDTAHACILAAEREIEVLEELDYHTIVVSMKLN
EPAEVLKANREFASRFSYPLHLGVTEAGPLISGIVRNTAALVPLLQEKIGATIRVSLSDS
MEAEILAGREILACAGARNAGARIVSCPRCGRASFDTHAFIDRWSERLYRIQKPITIAVM
GCVVNGPGEAKNADIGITGAGDYVLIFRKDREPLKIKPAEADSAFSQALEEIINE*
>SPSA8_v1_40015|ID:41144947| conserved membrane protein of unknown function [Spirochaetes Bin 1 SA-8]
MNIAMIGAACVLGFSAVGSAIGAGLAGQAAIGSWKRSYMSNKQASFLLVAFAGAPLTQTI
YGFILMSRMIASTKDPLLVLAAGMAGGLAIGMSAVAQGQAAAASCDAFGETKKGFSNYMI
VIGLCETVALFVMAFTFGAI*
>SPSA8_v1_40016|ID:41144948| V-type ATPase 116 kDa subunit [Spirochaetes Bin 1 SA-8]
MIAPMKRFTIVVTSHDIDSAPHYLRKLGIAHIEPLAGAGQHFQNLEQRKKDAELAKIILA
SYRNKKQKTETVSQLSTKELISRAIEANALINSSRDTIADAEKELERIRSWGEFSPDLFQ
RLKTDRIHIRLFEGLEKNLSELSSSVEYLRLAAPKGKVRIAVFSNEDVPASFMEFHVPEE
NAATLQNRIAQEKEKSAKAEADLRELSKSIARVEQEIRIIDAEIKIESLRSGMPREEQLA
YFSGYVPARESEKLKKEAKKRKWALLVDDPSDEETPPTKIENPPAVKIIQPVFDFLGTVP
NYREYDISAYFLVFFSLFFAMIFGDGGYGMLLLGLALFMLLRTRAQKAPVGDSLKLLFVL
SAVTIAWGFATASWFAIPYETLPSLLQKLSIPAISSNNPNAGTNVKIFCFILGTLQLSIA
HIKNIKRDFPNPKFLAQLGSLLMVVGMFNAVLNLVIDSRRFPLQDWALVLIGGGFFLVFL
FGNWNGSLVGSLIEGLKGIIPTFLGTVSVFADITSYIRLWAVGLAGLAISQTVNGMVVGM
FGPSGRIISFIIGAVMGLVLLFVGHALNIVMTVLSVVVHGIRLNVLEFSSHLGMEWSGYK
YEPLGEIAENINEQEKEL*
>SPSA8_v1_40017|ID:41144949|atpD| V-type ATP synthase subunit D [Spirochaetes Bin 1 SA-8]
MAKIKLTKNELKKKKDALKMYRRYLPTLQLKKQQLQMEIRGVEARLEQLLAQKAAFEKAF
EAWIAVFSEPQYTHDQNGMPLLNIKKTIVSEGNIAGVTIPVFESVEFDLSEYDLFLTPLW
VDKALELMKNVLSVDVEYSVVEKQKDLLMSELRTTTQRVNLFEKVKIPETLADIKRISIY
LGDQQTAQVVRGKIAKNKVGGRE*
>SPSA8_v1_40018|ID:41144950|atpB| V-type ATP synthase beta chain [Spirochaetes Bin 1 SA-8]
MNKIYSRIESITGNVISVRAENIAYGELAEVSTRYGTSLAETIRLDKDLVFLQVFAGGRG
VSTGDEVRFLGHPMQVSFSDNLLGRIFDGAGEPRDSGPALKDHLITIAGPSVNPYKRIIP
REMIRTGIPMIDLFNTLVKSQKLPIFSVSGEPYNQLLARIAMQAEVDLIILGGMGLKFDD
YLFFRDTLEEGGALSKTIMFVHTAADPTVECLMVPDISLAVAERFALKGKNVLVLLTDMT
NFADSMKEIAITQEQVPSNRGYPGDLYSQLASRYEKAVDFSDAGSITILAVTTMPGDDVT
HPIPDNTGYITEGQFYLRGGHIEPFGSLSRLKQMVNGKTREDHRAIMDGMIKLFASYRDT
LEKKSMGFQMTSWDKKLLKYGELFESRMMDLSVNVPLEKALDEGWEILAACFEPRETGLR
SELIQKFWPAREGALKSQAQ*
>SPSA8_v1_40019|ID:41144951|atpA| V-type ATP synthase alpha chain [Spirochaetes Bin 1 SA-8]
MIQTNGHVIAVNGNMVSVHVEGVVSMNEVAYILSGGRQLKSEVIRIRGSEVQVQVFEITK
GIRIGDPVSFSGEMLSVELGPGLLGQIYDGLQNPLPEIAEKTGYFLEPGIYLKALNRSIK
WRFTPAAKPGDVVRRGYTLGTVPERNFIHSIMVPFNMFGDYTVASIAPEGDYTIDEEIAI

VEDSRGNRKSLTMTFFWPVKRAVDCYAERLKPTQPMVTKTRIIDTFFPVAKGGTYCIPGP
FGAGKTVLQQVTSRNADVDIVIIAACGERAGEVVETLQEFPEIIDPRTGRSLMERTIIIC
NTSSMPVAAREASVYTAVTLAEYYRQMGLDVLLLADSTSRWAQAMREMSGRLEEIPGEEA
FPAYLESVIAAFYERAGIVKLYNGKKGSVTIGGTVSPAGGNFEEPVTQATLKVVGAFHGL
SRERSDARKYPAIHPLDSWSKYPGILPEKAVSYAHHYLERSSEVGAMMKVVGEEGTSLDD
YVIYLKGEFLDAVYLQQNSFDPVDAAVSPERQKTMFTLIIRILGSRLEFSNKEEARSWFY
ELRQLFIDLNGTAQDSDLYQQFLDKIEKALESKKPVLQHGAKAVLSLLE*
>SPSA8_v1_40020|ID:41144952| conserved protein of unknown function [Spirochaetes Bin 1 SA-8]
VSRYWYFPATLPGLLFGSASPMTDEQFMEMARQALSPSDFAELETCLDISGMGAKEKTYN
SRLLADYRAWERAFRNELARLRSRKAGIDEEQYLRQGPCKDEAAAAAQSCFTCQDPLEAE
LAAEHERWLAVERYSALSSFDLDSIIAYRIKLGIVNRLKNLEKERGILEYSRLYKEIMEK
AASRDAYSPGDEE*
>SPSA8_v1_40021|ID:41144953| putative V-type ATP synthase subunit E [Spirochaetes Bin 1 SA-8]
MELQVQELLERIKSEGIDSAKAEAGRIIAEAEAKAAALLEDAEKAASGSEEAARLRIQNM
ENASRVALAQASRDTLLALKESVQTFFDNLIVASIGEAFDANFVQKILPEILKEMAKNLN
GDIIVSVPEKVQASLDQALSVRMSKELSRGVEFKVFPGIDAGFRVSGKDSTIQYDFSAEA
VAAIISARLNARLSEIVNESVKGSRNL*
>SPSA8_v1_40022|ID:41144954| Oxidoreductase domain protein [Spirochaetes Bin 1 SA-8]
MKETTIGIIMNGVTGRMGTNQHLERSIMAIRKQGGIRLADGNYLFPEPILCGRDEGKLKK
LADTYGISKYSTQLDTALSDPYYKVYFDAQVTNLRPEALQKAMKAGKAVYCEKPTAGSTR
EALQLASMAEKLGLKNGVVQDKLWLPGLRKLKYLADTGFFGRILSVRGEFGYWVFDGFSQ
PAQRPSWNYRKEDGGGIIMDMFCHWQYVIENLFGKIDALSAIGAIHIDKRVDENGRQYAA
TAEDAAYGTFMLDNGIVCQFNSSWDVRVRRDDLLVIQVDGTRGSAVAGLRECFIQDEAMT
PKPVWNPDIPSSIDYFENWARMPDRQHYDNAFKVQWEKFLLHVAQDEPFPWTLREGAKGV
QLAELGMQSWKEKRWLSVPPLASEE*
>SPSA8_v1_40023|ID:41144955| Xylose isomerase domain protein TIM barrel [Spirochaetes Bin 1 SA-8]
MRDLADMAIHTITTKPWKLDTALEKYAQAGITGVSVWKESLEGFTLPEARSLLETSGLKV
VSLVRGGFFPASSLAQRMKKVEENKQLIDTATAIGAPLIVLVCGADPGIPLQEARKQIEE
GIAQILPYAEDKHIKLAIEPLHPMYADARSAIVTIRQAHELCIKFDXKSLGIAVDVYHVW
WDPDLEYQIEKAGXSNSIFAFHVCDWKTPTLDMLNDRGLMGEGCIPIRTIRQWVQKAGFS
GFNEVEIFSNRYWSMDQDQYLSMIVKAYEEHV*
>SPSA8_v1_40024|ID:41144956| Glycosyl hydrolase, family 88 [Spirochaetes Bin 1 SA-8]
MISISSNHDFLHLKEKIRRFFEMAGVKTLNLAGRWEGIQGAPVYTVNGIYTQRGWTDWTE
GFQYGNAILVYESTHDPRLLMYARRKTREKMAIHVSHTGVHDHGFNIMSTYGNLLRLSRT
GLIDADAWEKEYYLLAIKLSGAVQASRWTSLHEKLGYIYSFNGPHSLFADTIRSLRVLGA
SHLAGHYLKAEQDETINLLERLLAHAETTARYIVFEGKGRDRWDVRGRVAHEAIFNPVNG
SYRCPSSQQGYSAFTTWTRALAWIILGFAEELEFVSMLHEDAIQSLELPFVKTKTEIIQR
FTEVTRACADYFIENTAPNGIPYWDTGAPNLHKLGDYLIKNPDPYNPWEPVDSSAAAIAA
QGLLRLGLFLKERQLADAEKYLGCAITISQTLFSDEYLSLDEQHEGLLLHAVYHVPNNWD
NPSSKSSLPAGESCMWGDYHILELARCIQQMNSGINPSRFFDGILEAD*
>SPSA8_v1_40025|ID:41144957| Short-chain dehydrogenase/reductase SDR [Spirochaetes Bin 1 SA-8]
MLDVMDGMNTMTISPRIALVTGSSRGLGRGIALELSRNGYSVAIHYSENKDAAFETLRLC
GISKINSLQKFEIFKADISSKKDRHRLIDAVLEAMGSIDVLVNNAGRAPDKRADILIASE
ESFEKLLSVNLEGPYFLTQYLAQRWMEKASDFEATQSEKNCKIIIFVTSISAETASTGRG
EYCISKAGLAMAAKLWAARLAESGVQVFEIRPGIMDTDMTRAVHDTYELRISQGLVPQKR
WGIPEDVGKAVAALASGNFGFSTGSVIHVDGGFHISRL*
>SPSA8_v1_40026|ID:41144958| conserved exported protein of unknown function [Spirochaetes Bin 1 SA-8]
MRRTAGSLLIAILALCIITVGVSAQATWKPNKPITIIVPWGAGGSTDQITRITAAELETA
LGQKIVIVNQPGASGSVGTKNTLDAKKDGYTWTAGAAADLATYKVQGMLDTDIRTDWHIF
LSVANVSIVGVNANTPYKTIDELLAAFKAKPGQIAVATAGQSSAGHIGIEMIRKFTGIEY
KHVTYDGGNPAVIATVSGEAQVTTQLAVEQADMIRGKKIRPLAVLSDKPLYLEGYGEIAP
ITNWIKDFKSGPNYFGIFIPKGVPQEVIDTVSALWEKIIFNSQKIKEYARSRGALFAPSY
GQTAQDRAFSYYQPVAWLYYEAGKAKVSPDTVGIPKP*
>SPSA8_v1_40027|ID:41144959| conserved membrane protein of unknown function [Spirochaetes Bin 1 SA-8]

MQEKTMVKSDFFASIILIVFGILIVAISLSMPTMAEKEATIYSAPGLVPAFIGAVLAVLS
FFMLIRSVARIKKTGLPLLPTKSSFSNFFRDVATRRIIITLALCLGYALLLGKIWFPALT
VIYIFLFIVAFEFDWQKSFQSQLKKIFIAFIIAVVATAVIAGTFRYVFLVNLP*
>SPSA8_v1_40028|ID:41144960| conserved membrane protein of unknown function [Spirochaetes Bin 1 SA-8]
MLQGLQMFSNALLGFLNPRSLFDALWATQLGIIIGMLPGLTATMGVALLTTLTFSMAPNN
AILILICMYIGAIYGGSRSAILLNIPGTPANAATTVDGYPLAKQGRAGEAIGIATTGSFL
GSVIGMLALAIFTPLIGSVALSFQSFEFVWFSIFGIIICGNLTAPKDPLKGWISGFLGLF
IAMIGMEGIHAYVRFSFGNTDLSGGISLIPAMVGAFGFAEIISVMRVEHYEVVKTKISRV
LPRPRDVLKYWKTILRSGIIGTFIGAIPGVGEDIAAWVSYDIAKRSAKKEDQEQFGKGSI
EGLLAAETGNNACVPGAIIPVLTLAIPGSAPAAVLLGAMLIHGVRPGPLIMIEFPTFIYE
VIAMVLLATIAMFVLGLSLVRPLVKVLMVPRQKLMPVIFLLCVIGSFALQARLFDVLVML
VFGILGFILREMDYPMAPLVLGIILGDILDKNLRRALILSNGSIAPFFTRPICLILFVLT
LLIVVTRAKWYKTALAKLKNSLKRAA*
>SPSA8_v1_40029|ID:41144961| Dihydrodipicolinate synthetase [Spirochaetes Bin 1 SA-8]
MDTAAQAKIAYDRFAAGTVIPAIPLALDEHRHFDERRQRTLIRYYLDSGAGGIAAGVHTT
QFQIRDPEYNLFEPVLRSISGFIDDHVKKTGKAVIKIGGICGKTDQAVREAKFLSAAGYH
AGLVSLAAMKHETISSMLEHCESIAQEIPVIGFYLQPLAGGVVLPYEFWKKFCCIENVIG
IKIAPFNRYKTLDVVRAAAESGRQGEIALYTGNDDAIVYDLVSTFRIPVAGVEHVLEIKG
GLLGQWSVWTSKAVEIFETIRTIKKNNESIPNWLFQYAQGLTDANSAIFDAAHDFAGCIP
GIHEILRRQGLLEGTWCLRKEEILSPGQKENIDRICAAYPELQDNTFIQEHLSSWLA*
>SPSA8_v1_40030|ID:41144962| conserved protein of unknown function [Spirochaetes Bin 1 SA-8]
MAVYPLHFDSEDQLEDFMATPSQEALETLSQLTGDIAILGIAGKIGVTLGMMIQKSLVAL
GSARKIYGVSRFSDSEARKKLDVAGIITIPCDLLNAKDVANLPDAEHVVFMAGRKFGTAG
NEELTWAVNTIAPRNAAERYKTSNMVVYSTGCVYRLLSPETGGATEDSLPDPIGDYAQSA
LGRERIFQYFSKIYGTRAAILRLNYAIDLRYGVLRDLADKIMSETIIDNPDGAFNCIWQG
DVLNQTICSLGQTSSPAAIINITGPETVSIRWAAEELGKRLGKRPIFRIDSAMPDRTYLS
NAAKAAQLFGYPEIPLLAMIDMTADWVLHGGRSLRKPTHFETTDGRF*
>SPSA8_v1_40031|ID:41144963| N-acetylglucosamine-6-phosphate deacetylase [Spirochaetes Bin 1 SA-8]
MEACKDLRLTNCKILTPFEMLEEGSITITEGKIAEVNRGISEESGSLKTLDMRGALVIPG
FVDLHCHGALGEDFMSASEEAVQKAAWFHLQHGTTSLLPTSLSAPLPAILSMLKTMQSAM
QNGTVPNLAGVHIEGPYLSELQCGAQDTRYLKNPDPEEYYKILEFSSIIRRWTIACELPG
ALELGRILARLGILPSIGHSNAQYSEVVEAVSSGFRLVTHLYSGTSLVTRKRGIRYPGVV
ESTLLLDTLAAEIIVDGMHLPEALVQLVFKIKXADNVIMVTDAMAXAGMGDGIYRLGDAE
SGQDVSVHDGVAWTSSGEAFAGSIATADHLLRKAVLWAGISLKDAVTSLSATPAKLAGIY
KRTGSIEPGKNADILILDDNLEILMVIAKGRIVGSGGSYGSISSSF*
>SPSA8_v1_40032|ID:41144964| Xylose isomerase domain protein TIM barrel [Spirochaetes Bin 1 SA-8]
MILSGFADEAGDSLDEQIAVTRELGWRYVELRSIDRKNVLDLDDRAFEQCADKLTKAGIK
VNCLGSNIANWGTSINDDFAATEKTVDRAIKRMKLLGTEMIRIMSYAILFGSNGKPLADQ
RAEERIRRLKIICSKFADAGITCVHENCLNYGGMSFSATQKLLDEIPQLMLLFDTGNPCL
TPDFSKPEPWPNQNPFEFWKLFSPRIRHVHIKDGWRNPTTGKEEYFMPGEGPCKLEQIIE
TILKAGYQGAFTIEPHIAVVYHNPSVQSSSKQRKTMYIEFARKFETMMSKLEYREEHGSL
*
>SPSA8_v1_40033|ID:41144965| Dehydrogenase-like protein [Spirochaetes Bin 1 SA-8]
VKFGIIGTGLIAEFHARALSEIKGAELEACMDVNSERAAGFGAKYHCTSYNSIEHFLKHP
GLEIVTVCTPSGLHRDMAVAAAHAGKHVIVEKPLEISTERCQQIIDACQKNNVVLSGIFP
SRFFSAASTVKKAVDAGRFGKLTMGTASVKWWREQSYYSTGGWKGTRSLDGGGALINQSI
HAIDLLLWYMGDAEEVYACMDTRAHSGIEVEDNAVVAVRFKNGAIGTIHGSTSVWPGFLK
RLEISGTAGSAILEEENLTFWKFKEENPDDEHMRKQFAGATHTGGGASDPAAISHHGHKL
QFLDIIDAIQNNREPLIDGEEAMKAVRLIEAAYRSAELDKPVSLGEM*
>SPSA8_v1_40034|ID:41144966| putative Xylose isomerase domain protein TIM barrel [Spirochaetes Bin 1 SA-8]
MDRFKLAVCNELFGSMDARTYIPVVKRSGYEGIEFAPYTIFPDFNVHQARKRAVEIHELL
KGEGLAFSGFHWLLAYPDGMHLLSDNSDARRKAWDHLKLLFELSFEFGGGTLVLGSPRQR
TIPDNTERNSAIRRFIDEMVFFIESIEGSKSMLLIEALSPDQTNFINTLQEAAELVQQID
STYFQTMFDFHNARDEKQPWDKLIEEHFKCIKHIHINEIDGGAPGTGKSEYRNAFSAIKK

LRYEGWISMEVFTTPADPERYISAALQFQKKIFHLVEE*
>SPSA8_v1_40035|ID:41144967| Malate dehydrogenase [Spirochaetes Bin 1 SA-8]
MKAIERNKLIRFSARIFMACGVPEQQAFEQSMVLVAADARGIPSHGIARLPRYVAGLQQG
SMIPNIQPEAIKETLVSLLVDARGGLGAPVSIATMKKVIEKAELAGMSFAAVRNSNHFGI
TGYYAMMALPHDMIGMAMTNTAALGVPTHARQVMFGTNPIAFAAPAFEEKAFVLDMSTTA
VTRGKIEVFARLGKPLPRGWAVDRDGAPAGNANALLDDMLHRSGGGLVPLGGDDEIHAGY
KGYGLGVMVDILTGVLSGGLFGQQVADTETTSARVSHCFAAMKIDMFQKPEEFKKSMDSM
LKALRTAEPARGKARVYYAGQKEYEAEEEASKFGIKIDDTTFRILTDLGKKFDINPEDLL
FH*
>SPSA8_v1_40036|ID:41144968| conserved protein of unknown function [Spirochaetes Bin 1 SA-8]
MQIKADGQTYAPRGMVSPVCEPGTFKIGVIGLDHGHIYGMCNGLVEAGAEIACVYDNERM
KMDAFCKVFPDAKKAESEHEILENPDIALVASAIIPDRRTELGIRVMKAGKDYFCDKPPC
ITKQDVSQARRAVEETQKKFAVYYSERLHVEASVCAEKLIKDGAIGRVIHVAGWGPHRIS
IPQRPSWFFDKARYGGILVDIGCHQIEQLLFFCGASDAVISSSRAGNYKYKQYPRFEDFG
DAALLMNNGTTGYFSVNWFTPDGLGAWGDGRTFIQGTEGYIELRKYIDVAADPEGDHVIL
VNNKGEQHIHAAGTCGFPFFGRLIKDCLERTSTAYDQELTFRAIELAIEAETQATAVQ*
>SPSA8_v1_40037|ID:41144969| putative signal transduction protein with CBS domains [Spirochaetes Bin 1 SA-8]
MNKVKDILAKKPKAVFTVQSDATLYQALEIMADKNIGAILVTDKNGKLAGIFSERDFARK
IIIKGRDGEKTTVGEIMTTQVLYVDPETTINDCMTLMTEKKIRHLPVMDKGELVGIISIG
DVVKAYCANQDSIIAEQEFQLGQLERYINQSP*
>SPSA8_v1_40038|ID:41144970|fbpC| ferric transporter subunit ; ATP-binding component of ABC transporter; CP4-6 prophage [Spirochaetes Bin 1 SA-8]
MSVKLIHISKEFPDLEREDRKMLAVRDMNLEINEGEFVTLLGPSGCGKTTTLRMVAGFET
PTTGEIWIGGRCVNDDPPXKXXTTMVFQSYAIFPHLSVEQNVGFGLELKGRPKNEIKDEV
RKVMEVMGISDYAKRRPDQLSGGQQQRVALARAIVNKPGVLLFDEPLSNLDAKLRETMRV
EIRRIQQTFGITSIYVTHDQAEAMTVSDRIVVMEKGKVMQVGTPFEIYSRPENRFVADFI
GRVNLFPVTIKKLGKEDSGGTVLNNKGKEIRAASIASGLCEGDAAFMMVRPESFVLASKE
SETSFGSGVVTKAVYLGSVVEYELDIGEDKPILAVTHDPINNGFYSNNERVWFSFTEQAC
HVLPQ*
>SPSA8_v1_40039|ID:41144971| conserved membrane protein of unknown function [Spirochaetes Bin 1 SA-8]
VKTSSLTRASAEIRRIARDPLLFAIIVITMLSLLVFVIYPLVSVGLQSLKTDKGLGFSIY
GDVLKSAYLRKAFFNSLYMSVLTAAAGTLIGFLFAFAVTRADVPWKKLFNAIAIIPIVSP
PFIGALAIIMLFGNNGLITWNLLKINNFPIYGFNGLFLAQVVTFFPVAFLTLKGVLESMN
PVLEDAALDLGGSRFKVFTKVTLPLAIPGIAGAILILFVETLADFGNPLILAGSKFPILS
VQAYLQIVGMYDLPKGAAISMLLLIPSVTAFFVQRKIVGRRKYTTVTGKPQSSSGKIVSP
GARIFLFIFCLFITIIVGLIYFSILIGAFAKVWGFDHRLTLDHFKFVFGVGFKAVRDTLM
IAGISTPIAGLFGMLIAFLVVRVKFPGKKLIETTSILSFAVPGTVIGIGYILAFNHKPLA
LTGTLAILLLNFVFRYIPVGIESGTAILAQIDPSIEEAAIDLGADARKTFSRITLPMIVP
AFFSGLVFAFVRAMTAVSAAIFLVSSRWNLMTVHIMSQVESGNLGAAAAYSVLLVGIVGL
AIVFIRMVLKAQYGKLGGTMLHF*
>SPSA8_v1_40040|ID:41144972| conserved exported protein of unknown function [Spirochaetes Bin 1 SA-8]
MKASRGFLIICMALCLIVPVFAKPGATLVVYSSVDEQNAKKIFDAFTADTGIQVQFVQLS
SGPAYARIKAEASNPQADVWFGAPSENHIVAKQEKLTIPYKGPGFDKLGKEFKDPDGYWR
SFYMNPMAFAVNKEVLARINAPKPTSWADLLNPIYKGQIQMPSPQSSGTAYNVVASLVVM
MGEDKAFDYLKKLAPNVQTYTSSGTAPSKAVQVGQCAIGIQFTPAFFEAIAKGFPIEVVF
PKEGVWFEAPAISILKGTKNLEGAQLLVDWLSSTKGQNVFTEKETYFYPVISGAKLGKGM
PEFDSLKTIDVDPIWAGENKKRLVERWVQEVLTAK*
>SPSA8_v1_40041|ID:41144973|atoC| Acetoacetate metabolism regulatory protein AtoC [Spirochaetes Bin 1 SA-8]
MKILLIDDEKQLGLSLKSYMLDDGIELDVAFNGKSGIQKLEDESYEAVVTDLRMPGLDGM
QVLSWIRDERPGMPVIMISAHGEIQDAVTAMKLGAQDYLVKPFDPDELVLRLKKAVESRR
VMQKLQAGMAAGHEEAGMIGESPKMKEIVKLLEKASPSAATILITGESGTGKEVAARFIH
ARSGRTGPFLPVNMGAFPENLLESELFGFEKGAFTGADTRKQGLFESAQGGTLFLDEIAE
LPLHLQVKLLRAIQERKIQRLGSVKSIPIDVRILAATNRDLEKDVKEGKFREDLYYRINV
IRVRLPPLRERKEDIPLLVGRFIRRFSQASARKISSISKESLDLLLQYDFPGNIRELENA

IERACILAESDVLGAEDFDFLRPARSILGEENIETLDVHSGNRAGEAESRNGPKTIEEME
KQLIEAALKRNAFHREKTAMELGITRRTLLNKMKEYGLEP*
>SPSA8_v1_40042|ID:41144974| protein of unknown function [Spirochaetes Bin 1 SA-8]
MDNANLFSSLNQRKKIRAPTVSGIFYPQSPEALTEQIRILLESAAIELSERFSAIICPHG
SLEYSGDIAALAWKSVSRMNIETIVIITPSHHCFENGIFLPESEYFTIPGCELRVDKKSI
KALLHCNSAYVKDDLPHLEEHGIEAILPFATHLFPDSSLIPIIAACSDVPSLITLFNCLS
DILEDRLSRTLFVLSSNMAVSETDEQTVQLTEKFRSALLSRDITTMEAFSHGEPSFCGAR
IIQAYLQSSCSTGKAPVFLGSRTSKLMTEADEPVVGYASFGFPA*
>SPSA8_v1_40043|ID:41144975| conserved protein of unknown function [Spirochaetes Bin 1 SA-8]
MDIELSKQDRDILKTIARETLAARLEHRSPLYPEHSRALEKRYGIFVTIKVHGQLRGCIG
RMSSNQPLIETVKDMALAAGFEDPRFPPLSHNELSLLEIEITILGPLEPLSPPDNFTLGI
HGLFIASHGRSGVLLPQVAVEYGWNKTEFLQHVCLKAGLPPETWKSPDVVLYRFEGTVF*
>SPSA8_v1_40044|ID:41144976|ppdK| Pyruvate, phosphate dikinase [Spirochaetes Bin 1 SA-8]
MAKKKYVVYFFGNGVAEGDSSMKELLGGKGAGLAEMTKIGLPVPAGFTITTEVCDLYYKNG
KQWPEGLDKEVDKHLKLLEKTVGKKLGDPKDPLLVSVRSGAAISMPGMMETILNLGLTDA
SVEGLAAKTGNRRFALDAYRRFIMMYGSTAMGIDREEFDHAFNEVKEKFTRPRLKIAAGK
KVLDTDVNAEELEKLVAEFKAIYRKYIKAEFPQDPREQLKGAIDAVFNSWMADKAVTYRK
VEKIQGVKGTAVNIVQMVFGNMGDTSGTGVCFTRDPNTGENTFYGDYLVNAQGEDVVAGI
RTPIKLIELEKRDPKAYKQLLDVRKKLEANYKDMQDLEFTVEEGKLYMLQCRTGKRSPIA
AFKIAVDMVKEKLITKEQAIMRIKTSDIEGIFYPMIDKTQAAALKDAFLVQGIDAVPGAA
AGKVVFNAKDAEAWAEKGEKVILVRKETSPEDVGGMHAAQGILTATGGKTSHAAVVARGW
GKCCIVGCEKLNIDYTKGTFSVGEKTVKQGDFITLDGSSGNVYVGQLKLVKPEPPEAYTT
LMGWVDKIRTLKVRTNADTPYDAENARRLGAEGIGLCRTEHMFFDSEERILAIREMIIAD
DEDTRKKALAKLLPFQTKDFEGIFKAMDGYPVTIRLIDPPLHEFVPHDEAGQRALAEAAK
VSFEKVVQRVNQLHEANPMLGHRGCRLTITYPEILDMQVTAIITAACNMTKKGISVLPEI
MIPLVIDAKELKILETRARQVADAIIAKSGVKLSYMVGTMIEVPRAALLADQIAEVAEFF
SFGTNDLTQMTLGMSRDDAGKFLPDYVDEKKAGIFKADPFQSLDQVGVGLLVKMGIEKGR
STRAKLKVGICGEHGGDLTSVKFCHRVGMNYVSSSPFRVPVSRLAAAQAVVEEQAAMATA
APAKKTAAKAKKAPAKAAASKGTASKGTASRGTASRGSAVRGRKAAAEKVAAKAKPAATK
KAAAAKKTSVKSAAKTVTKKAPAKTSAKTIAKKAASKTAAAAKRPVGRPKGTSKKAK*
>SPSA8_v1_40045|ID:41144977|dinB| DNA polymerase IV [Spirochaetes Bin 1 SA-8]
MERIFFHVDMDAFFASVEQADNPALAGRPVIVGAAPGRRGVVSTCSYEARAFGVHSAMPI
AEAARLCPHGVFLPVRMKRYSEVSAIIMSILFTYTPDVLQLSIDEAMLDMTGTGKLWGTP
EECAFKMKKTIRDRTGLTISIGASANRYVAKIASGMSKPDGFLLIKPGDERAFMRSLPLE
KLWGAGEKTRERLKARGLTTMAAIQDAPVSLLMSIFGNAGGRFLAMACQGQDPGVFTRDD
ENKSVSTERTFEADISDRTILIDELRMLADELSFRLWDNKMHTGCVGIKLRFADFTSITR
QRSLAVPTASADCIFQEAMQLFDANWTCGKALRLIGIFLSSLVPESHIENSLFQEEPPKS
ALAEQVVHELQKSGKGNLIRGTRLNRRG*
>SPSA8_v1_40046|ID:41144978| exported protein of unknown function [Spirochaetes Bin 1 SA-8]
MKKIFTAILLTTLGAILFSSCMGMETAITINSGNSGTVTAEYRLSEELVNFGAIEANKAL
LPIPITKADIENSLASAKGLKLVSWSSKKSGTDTVIKTVISFDSLEALVFYLDPQGQMAR
YSVSDAEKKIIFSLGDTMPPMDEQMKSLAKEAFAPYAFKFTVTTPSKIIRAGSSLSIISA
ATDGKKAVFEGKMQDIVTSGEAPVIEFTW*
>SPSA8_v1_40047|ID:41144979| conserved protein of unknown function [Spirochaetes Bin 1 SA-8]
MSLLFMRDTIIPQGRGCQESPSVIVLKQMVVSMLLLVLELPETTSLKDKRKVVSGIKHRL
HNKFRLSCSEVDLQDSLGFAEIGAALVSNSPVFGEKVLNEAVRFVEENFPVNIYESQIHS
EVYG*
>SPSA8_v1_40048|ID:41144980| Methionine--tRNA ligase (fragment) [Spirochaetes Bin 1 SA-8]
VSRMKRRLITSALPYVNNVPHLGNLIQVLSADVFARFCRSRGYETLYICGTDEYGTATET
KALEERISPKELCDWFHAIHRDIYSWFGISFDKFGRTSTPEHTEITQSIFLDLEKNGYIV
SQTVEQLFCDHCGRFLADRYVRGTCPVCGYENARGDQCENCGKLLDPTELKNPKCSSCGA
TPHPKATTHLYIDLPKIKEKLEPWIQSASEKGFWANNAIQMTQAWLRDGLKPRAITRDLK
WGIPVPMSGFEDKVFYVWFDAPIGYISMTASLAREKGFDWKSWWQNPEEVDLFQFIGKDN
IPFHTVIFPSSLLGSGKNWTLLHHMXX

>SPSA8_v1_50001|ID:41144981| ATP phosphoribosyltransferase (fragment) [Spirochaetes Bin 1 SA-8]
MTSSTRLYASVSAWRNPGKRSAAESLVLILRSVLEARKRVMLEVNVSAENLEELVKILPC
LRSPTLSPLEGGKDFAVRVAAPRSILADLIPEIKRRGGTDIVVTELCQLVP*
>SPSA8_v1_50002|ID:41144982| putative Histidinol-phosphate aminotransferase [Spirochaetes Bin 1 SA-8]
MEKKPGRLLKLDANEGRCVLTQTELASLLQPEVAQRYPDARPLENRLASWLGLRAGQVIA
TAGADDAIDRIFRAYGGPGKTIITTTPGFVEFLDTCLRTKSTFKSIYREPGEAFPLEAFL
AALNREKPAIAILASPDNPAGTLIDEQAVFAIAETCKKTGTLFVFDITYIDFAEDYPLLE
KSLAVLPWIVLTGSFSKSRGLAGFRAGWAACSSENTTIIDTLRDFGPPYSLSSPAIQAAL
MAIEAGDRFQAFVERVQTERRILTAAMARLGAKTWQSQANFVTVAIENSKEFAECLLQEG
IRVRTWPESPESRKLVRITCPGNADEFEYLLSALEAVQAGGKS*
>SPSA8_v1_50003|ID:41144983| putative Imidazoleglycerol-phosphate dehydratase [Spirochaetes Bin 1 SA-8]
MSEVEKSAESPVWVRAKRESKETNIEIELALEPGTIEIDSGIGFFDHMLKALAYHAGWAL
KLRCKGDLEVDDHHSIEDCGIVLGSAFGQALQLMPAIQRFGSAFAPLDEALARAVVDISD
RPWASVDLGLVREKIGEAASENLRHFLETFAGNARITLHVDVLKGSNDHHRAEAAFKALA
LALKMALQPRRQPRQEEGLQTAAQTGAADNPPSTKGSPRLVIETAGGPTVPKGI*
>SPSA8_v1_50004|ID:41144984| putative Imidazole glycerol phosphate synthase subunit HisH [Spirochaetes Bin 1 SA-8]
MEERRKTLAIAATGTANLASVLALCARTGVKALITTDPAIVADAEYALLPGVGAFGPAMA
NLRACGMGMALRERWLKGKPLMGICLGMQMMCRESEEAPGVPGLGFIPAAVQKFRGSLPL
PQLGWNSIVPGNGSILESGWAYFANSFRVAAMPATAPAEFSAAKPGRFVGSQAQYGETFA
ASIEFFPEGSALPTLLLCQFHPELSGSWGEALFKRWMGLDYSPAQQRQEAAR*
>SPSA8_v1_50005|ID:41144985|hisF| imidazole glycerol phosphate synthase, catalytic subunit with HisH [Spirochaetes Bin 1 SA-8]
MSGLAKRIIPCLDILGCRVVKGVNFKNLADAGDPAELARRYEAEGADEIALLDISATLED
RKTALETVRRVRLAVSIPLSVGGGIRTVDDGERLLNAGADRVSVNSAAVRNPEILSLLAE
RFGVQCVIAAIDAKSVTAPQLTGGKGGRAWEVMIDAGKTATGLDAVAWATACANRGAGEI
LLTSIDRDGTGTGYDYALLEAVAVAAGIPVIASGGASRPEHFLEGLTAGAQAVLAAGVFH
RHERTIEEIKEYLKKNGMEVRL*
>SPSA8_v1_50006|ID:41144986| conserved protein of unknown function [Spirochaetes Bin 1 SA-8]
MIIPSIDIQNGQAVQLRGGRHPVLVVGDPEKLAEKLSRLGEIAIVDLDAALGKGSNKDLI
LKLVSKYPCRVGGGIRSRELALEYLDAGARQIVIGTRAARDFLEALPRERLIAALDMDKG
TIMVEGWTKATSQSIKERITELAPLVGGFLITTIEREGELAGLDMDLARDLIAVSKGTRI
TFAGGAMGGEKGVQQIAELDRLGADVQVGTALATGALKLSEAFAAQLVSDRSDGLWPTAV
CDEGGRFLGLVYSDLESLEMALNTGKGIYKSRSRGLWIKGETSGNTQRLIRADLNCERDS
IRFTVAQEGQGFCHLQRRNCFDDGFGIEKLSRTIADRFKQAPEGSYTRRLFSDRGLLAAK
LREEADELAKADGARDTAFEAADVIYFALVKATREGASLYDIEQELERRSFKVQRRPGNS
KPGYDSSDGGASWTSIH*
>SPSA8_v1_50007|ID:41144987|hisD| Histidinol dehydrogenase [Spirochaetes Bin 1 SA-8]
MDQYPLRRISLNQIPEPQARLFDLEVDAVAAKAIEAVRLGGEPALRMWAEKLGEIAEGDT
IFYNRGDLEAAFQAISEAEQRLLQRVQGRVAAFAEAQRQALGDVDIAVPGGRAGHRFIPV
ARAGCYVPGGRYPLPSSAIMTVTPAKVAGVREVVCAGPKPQTITLAAAFVAGADCFLACG
GAHAIAALAFGVVSPRCDVIVGPGGRYVASAKRQLFGSVGTEAPAGPSELLVIADDSADA
EIAAADLLAQAEHDPSSVPMLACRSEAFAAAVETALARQLAALPEENRNTAMQSLKNGWA
FVSPRMEELCKAAETCAPEHLELMVAQTDAYAQRIQSAGAVFVGNNSAEVFGDYGAGPNH
TLPTGGAARFAGGLSVLNFLKARTWLALSEAEPLIADTAQLAKLERLEAHARAALKRASR
*
>SPSA8_v1_50008|ID:41144988|ypwA| putative metalloprotease YpwA [Spirochaetes Bin 1 SA-8]
MRQEIAKLVELDRQRTLLTHIGAVLGWDQETYLPEKGIEERSEQLSLIEGLAHEKAVDPQ
IGELLAATEAAKDLSPEEAAYVKLTRREYDKETKLPSEFVMEYAKQASLSQAAWSQAKSA
NDFSLFAPHLETMIRMNKERAAYLDSGRKPYDVLLDLFEYGSTEASVAAVFSVMKKDLVA
LLDKIAAQPPVDDSFLHAPVSAEIQAKMSRYLMDAVGYDQKRGRLDTTAHPFTTTLGRDD
VRITTRYVENYFPSSIFSTIHESGHALYEMNIDPHPDFRGTRLAEAVSMAVHESQSRMLE
NFVGRSRAFWEKHYPAIQAMAGEPLANVDVDRFVKAINKVERSLIRTEADEVTYGLHVIV
RFELESAIISGNLAVKDIPQAWNAKIKELLGLDVPNDRLGCLQDVHWSMGAFGYFPSYAL

GNLYAAQFWEVMQEQIPNLEQKISRGELGDMQQWLKVNVHSKGSLYLPGELLQKVTGKPL
DPSCFARYLNKKFS*
>SPSA8_v1_50009|ID:41144989|recG| ATP-dependent DNA helicase RecG [Spirochaetes Bin 1 SA-8]
MNVPTFVSLKKAQDRKHYRRTRKSCYTGFVFLFELNQPVSALRGAGSAVAAKLARLGIVT
ISDLLSHYPRDYEDRTRYVPLSQFAKYPSVHTIAQVVRHDYIGYGRMKTLKIKIQDETSQ
AELICFNRNFLESRLPVGSHILVHGKFQFRYGELQSSTFEAEPVREGQQPSQGILPVYSL
TEGISQGIMRKLVRQALAMYGLKIENELPADLIQKEALCDKALAIKQIHAPESMDALNRA
RASLAFEELFYFQLKIAMRIQSRHQQKLPRKKIQGLLRSRLLERLSFQLTADQKKVLEEI
TADQEAEHPMARLLQGEVGSGKTIIALLAALNEVERGGQAAIMAPTELLARQHAATAAKL
LEPLGIRLAFLTGNINDQSRPNLLAALKEGAIDIVIGTHALFSDDVEYRNLQLVVIDEQH
RFGVLQRIALFKKGRIPDTLMMTATPIPRSLALTFFGDLAVSSIRTMPPGRKPVQTHLAK
LGHETKVYEFVRNQLMQGHQAYFIYPLIDESDKSDLRNATEMAKKLAEDIYPEFSVALLH
SRLKEDEKNRIMDDFVAGRCRILTATTVVEVGVDVHNATVMVIEHAERFGLSALHQLRGR
VGRSDQQSYCFLIYSDSITDDAKKRLKAIYESTDGFKLAEEDLNIRGPGEMLGTAQAGSL
RLAIADPVRDLEMLKKARFEAFAVIKQDPALLNPEHLIIRKVLDSQEKIADSL*
>SPSA8_v1_50010|ID:41144990| conserved protein of unknown function [Spirochaetes Bin 1 SA-8]
MKKYEKIILDSRTPDEYVDNSLKSKLTPAEKAALARVWMQQTGFTKEDILHARNRHPYWK
KKKMEGADERTQRRLAMHDYTKGKTVVWDQRKLQEFLELNKKDASGRYVHKDWELAKRFG
TTIPSIQYMRRKYKKVRDLLGPKARKEKIIEYMGYSEIVLSNGGPAKKR*
>SPSA8_v1_50011|ID:41144991| protein of unknown function [Spirochaetes Bin 1 SA-8]
MIDLSALVNKTVDFAYRGSRLALDLSHALFSSYTIDAGTRLLLKEVAHDEAIVHADNLLD
AGCGIGVIGIAMAASSPAMQVVMRDRDLLAVAFAERNCWRNGLPATRLDLDGQPAPAIEK
KSPKHKCRTERTAPIIIAPGLLGEDDVFGPYDAILSNLPAKAGPHILSYFIAACEKKLLK
PGGRFGFVIVNTLADMAEGWCREAGLSVYSKAATKSHTAFLTEKKAPVHSASPETARTLT
VIPEEGILGSLKKRASMIEFYTRNRLDTQLEHARLQTEGFWGLPEFDTLSYQTQLACEAF
SQHAGSLLVRNALVFEPGIGIAALWIVKMFGPQSLHAVSRDLLSHFAVEHNLSKSAKTTI
AHHSALELEQVEQASLDAVFWFPDETPMYDFYTPAWETLARTMKTGALAVITAQTGVIAR
FEKSKPKELRKAGEKKKKGFSALIVQRV*
>SPSA8_v1_50012|ID:41144992|efp| Elongation factor P [Spirochaetes Bin 1 SA-8]
MVRGGEINIGTCLLINGNPHIVVEREFVSPGKGSAFARVKAKNLKSGNVITQTIKTADFV
EDAQVDLVDCQYQYSDGENFMFMNNENYEQFGVPISLHEERKPYLKEGETYTLVMWEGEP
IDIKIPYKMVFTVVESENYVRGDTVSGATKPVVTETGLVVRVPLFIKQGEKILVNTETND
YVERVNE*
>SPSA8_v1_50013|ID:41144993| Lysine--tRNA ligase [Spirochaetes Bin 1 SA-8]
MQAFWYTVLMDIRMMEIRSRLLTITREFFLSRGYLETDTPALAPSLIPESCLEVFATEYV
HPIEGRTPLYLIPSPEIWMKKIIAETGRSVFQLCKSWRNAESRSRIHSPEFTMLEYYGLG
LSSRDNIRLTEELFSALKTELSPSQALPPFRIMTMKEAWAEFARADLDALQSAEAMREAC
RQRDLLVSPATSWEDAFNIIFLSLVEPNLPQDRPLVLNEYPAGIECLAKDIPGTVYKERW
ELYVSGIEIANCFTEMANPSAVRAYFTSQSMKKKDALVPHNVDAEYADIFEHFPPCSGVA
VGFDRLLMVLAGKKDIADVQYFPFAGMGNADSRRKSLQNLNFPQ*
>SPSA8_v1_50014|ID:41144994| protein of unknown function [Spirochaetes Bin 1 SA-8]
LIYLDEEAGRWYADKILQAAASRHFFSYNLLRKENWEPVVRSWESATHADLWQCYQLDNL
GALLVAAFRYAPDVLPESSESRAERGLPALPSVSESYMRACSIGRFARANWYHEVLARLS
DCARLVTEEAKTQGKALFPPRQWHRFVNSKFPEKALAIIAGLGTIGRNGLVIARRTDFTG
LPGDDDCDTAVVEAGWSSAVVLGLMFLPFDFSAETDLPPRIMLEPLSLCGSCARCINACP
SKALQFKEVPAYHRESCIQNYTSSAAPIPESVQKAWDRQLYGCDICLEACPWFKPDSAAI
SVFGCIGGFFDADAISEMPDAQLRLMLKGSALDQKWIEPEALRRNARLV*
>SPSA8_v1_50015|ID:41144995|glyA| serine hydroxymethyltransferase [Spirochaetes Bin 1 SA-8]
MSFIESFDPELWAAMQSEAARQRDKLELIASENYASKAVMEAVGSVLTNKYAEGYPGKRY
YGGCEFVDMAENLARDRAKQLFGSDHANVQPHSGSQANMGVLFAALNPGDTILGMDLAHG
GHLTHGAPVSFSGKMYKVVRYGVRRDTETIDYDQVAQLAREHKPKMIIAGASAYSRIIDF
DAFRKIADEVGAYLMVDMAHIAGLVATGRHPNPIKIADFTTTTTHKTLRGPRGGMIFVGN
DRENNRGIMTPKGDALKKWSEVIDGTIMPGIQGGPLMHVIAGKAVAMKEALSPAFAEYID
RVIANASTLAGSLSASGARIVSGGTDNHLMLVDLSPLGITGKEAEKWLDEANITVNKNGI

PFDQKSPFVTSGIRVGSPAVTTRGMGPAEMEKIAAFIMDVLKSKGDSAVIAKVKGEVLAL
TKLFPLP*
>SPSA8_v1_50016|ID:41144996| protein of unknown function [Spirochaetes Bin 1 SA-8]
MRKTSFGIIAALLLIAAIGGAYWYFRPVIEEIEPKVFEPGQELSVQGRNFSILNSKPEIS
FDGVPLPRYDFSVISDKVITVKVPVTADSGILRVRSNLGWSNPRIVIAKEALPQQAAQHS
AQSIGPEISGIKPDEARIGDLIEISGLNFGTSTAFSAVSFPDSAPELLAGISSSGLRDAV
SGLAANQRLAELWDDKTIMIRIPDFAGSGSLVVHTPQGNSNPVFLRIRQGTGSRTLQNPA
IYAVRQKVKIQSRNAKAGDTVMVYIPTPALSALQRTSVVQESDTGVQGAVRKASESDGSY
SIFEIYPAVSAEVTAEQRFLVAVYAVESDLVGYRDGFGAGGVPEYLQEYLKPDGLVPAGV
TNLVDTAKKIIGKEKNLQKSAVLLWNWVQKNIKYVPEKEKPMWPLANSAASAFAAKKAGT
RSFVLIVSALFRAAGIPALPVSGFLVRQDNVTIPHFWLEYYLPAVGWIPFDPVLAMNKAP
AGFIPEFRDTGRYFGSLDNRHIAISRGLPEAVSADPAERNVQNKALWSFQNIYETSRTSS
YESFWFDLEIVGRY*
>SPSA8_v1_50017|ID:41144997| protein of unknown function [Spirochaetes Bin 1 SA-8]
VTMRDVMAHERRTALSAFLLFFMLSAELWAQSGTQPKPGTSSGTGTDSTLAGLIFQIRPA
FTLSMEMVNALQAQEGSVSGSSKAQANPEILQRIDWGQTITQVTQGAAPLDVKILGTNIV
VLMQIFPVETSATKVSFLVQGQVWAKMPDNSLSYKTTVQTLSLAYGSRLYYYPLGIDAKA
GAPLAVEIQVKKQSSH*
>SPSA8_v1_50018|ID:41144998| protein of unknown function [Spirochaetes Bin 1 SA-8]
MCPDDELLSAYCDGEVPSPWKERIEAHAAICSKCSERIRTYAELHALLNSLDTEEEREAF
AAARQRVFTAIQSKDFDFSRKSAGTIGRRLSEFWARPIVLPMPVAAAALVVVAFLGGLSL
GVLNPRAKDARVLASAVQTLPASNASFDSILQFAESQTSPQTVTIRVQKDALIIQQGTPV
IVTLPSNSIQEVSTTTVGGL*
>SPSA8_v1_50019|ID:41144999| RNA polymerase, sigma-24 subunit, ECF subfamily [Spirochaetes Bin 1 SA-8]
LLVSGRHGGGTDFRSLYEAHFDMLMKVACRITNSMEAAEDIVHDAFSKIVERNMDFPTSN
DAKFWLIRVVKNNAINYSKRKGREARAYEKWWRSEASPIENIEAGSVTIKETGTEAGVQP
SLEEDVVRDEASRELRKALSALSPKLKEVLVLKEFSGMSYKEIGAVLGVSEGNVKVRAFR
ARAALLTLLGKEDRHVSR*
>SPSA8_v1_50020|ID:41145000|cysS| Cysteine--tRNA ligase [Spirochaetes Bin 1 SA-8]
MRVMRLFNSMGRELQDFIPEQAGKVGFYGCGPTVYNYAHIGNLRTYIFEDLLVKTLKRFG
YEVTHVMNITDVGHLSGDDDTGEDKMVKSAQEKGESVLKIAQFYTDAFFRXTQALGIARP
TIVCKATEHVGDMXNLIKRIEANGYTYMAGGNLYFDVTKLPDYGKLANLRIEDLKAGARI
EVDENKRNPHDFVLWFTKSKFENQALVWDSPWGRGYPGWHIECSAMSMKYLGETFDIHAG
GIDHIPVHHTNEIAQSEAATGRKPWVKYWLHAEFLVLDKGKMSKSKGGFLTLQSLIDAGY
DPLDYRYFCLGGHYRSQLSFSWEGMEQAKAGRKALLERVLALKERVGASDLPALDQVHPA
VRERLEQFDGFLAEDLAVPRALAELWQLLKDPALPPADVLAGAYSMDQVLGLGLEQVKKS
ETQVDESLVREIEGLIQERTAAKKAKDFAKADSIRSALKERGILLEDSPAGTTWKKNG*
>SPSA8_v1_50021|ID:41145001|murB| UDP-N-acetylenolpyruvoylglucosamine reductase [Spirochaetes Bin 1 SA-8]
MVMETLWQISQKINIDPRRILSNEPMSRHTTFQIGGPADIFLXXASLNELSSALSFFXGR
GIPCFVLGGGSNLLVGDRGIRGXVLDLSGMRGCXRXGTVIKAQAGCSVEALCEEALASGL
GGLEDFYGLPGTAGGAIFMNARCYERDFSAPISTITAMSPEGIIRTIPTAELRWAYKKSL
FQPGEAMDGWIILSADFQLAPADAGTVASIMRMRKTDRMMKGHYRYPSAGSLFKNNRAFG
KPTGAILESLGLKGYRIGDAGIATFHANIFINYGKASARDMLTLIQTARDKAWQKLGIEL
EPEVLMVGEF*
>SPSA8_v1_50022|ID:41145002| membrane protein of unknown function [Spirochaetes Bin 1 SA-8]
MDFFNEPVGIFSVILTVALVAPFLADKLGIPIIASMTLAGIMLGPELLGVLDATMFLQFM
ASLGTAYVFFISGTESRVQLSPASGGKSLLFGGITFTLPMLAGFAFGRFAFGMDLASSLL
LGAFFASSGHFRLPLLFRKDVLAHPSARVGKEGAGIXRILAMLILLFFSXVIPGQPLRFG
IQHLVFALVYLALIAAAVPXIARAVFRKIKXDSSLGTTFILLVMFAASFGALLAGAPTYF
GAFAAGLALAPVFDSSRGASSKLDFIGETLFLPFLLIFLGISADFSAAAPISAIIILIAG
SVLFGMGSKIAAALTAGKILRYNRTDTILLLSYSASFASFSLVFASVATNSGLFSKPLLS
GAIILVMVSSSLASLMARHSGSLILSEDHQEDAGTSKAQPERILVALSKPATANRLVELA
CLAAQSSHRLIFPLAVITDTSGNIEARNAAETMLSAAVMRADASRTVVLPMTKTSVNPAE
GILSAAEEANADTIVIGWNKXPRLSNAFFGSVIEQTVNASSQMVVIARAMDAFAAPHLVL

IAPPLCEHHPGFPQAISFIAAIAEKSRAKVHLATLSGNASTISSVLNSKGLKIEHPFLEI
SAWKDFSQLMKKLPAGNKQFALLSARPSEPSWHPAMEKLPHIVGESFPDSDLLMLYFSNS
GITPLNAPQKESAELETQETAAGLSVPNMASEALTLLRNAINQGRVRVNMEHTAISDSMF
ELVASAFPYDRAFTGKMSSRLVEILQRQPIEMSPGVLLVHERVPSITESIVCMGSSRNGL
RISSLDQPIQIIILIFVPEEQIPEKHLALLGKIAYLFNEKNLAEYLIEAATPEDVFAKLQ
*
>SPSA8_v1_50023|ID:41145003| conserved membrane protein of unknown function [Spirochaetes Bin 1 SA-8]
MIDFLTATGISFLVNILFFLIAVRRKSDIVTDLSYGLSFFLTALVLVYLHGIAGAVSLVP
FILVMLWSLRIAGFLFLRILTFKVDHRFDGRREDPVKFAKFWGLQAISTSIIMLPVLATI
SNPSSSFSFLQIIGSLIALTGIVLEAIADHQKFKFKKAGKTGFISTGLWSWSRHPNYFGE
MLVWWGLYLYAVPMLAGWWHLAILGPVYITSLLLFVSGIPLLEKSADQKFGQDPEYAAYK
ARTSILIPRPPRLPGKTGKRSTMGIPAVHSLDMEKFKGRWYELARIPLPVARDWVQTSDV
YEEGKDGFWNVRYEGKAGGASGPAKVLKQKLRIPDPAKPGEMEVSFIPGVWMKYRLIYFS
EDGKAMLVTSKKMKYLWIMSRLPQIPEEAYASLVAKASEFGFDVALLEKVPQ*
>SPSA8_v1_50024|ID:41145004| Ketoisovalerate oxidoreductase subunit VorA (VOR) (2-oxoisovalerate oxidoreductase alpha chain) (2-oxoisovalerateferredoxin reductase subunit alpha) [Spirochaetes Bin 1 SA-8]
MKEKTFMAGFGGQGVISLGQMWVYCAMKEGLQVTFFPFYGAEKRGGIARASVIVSDAEIA
SPLVTKADSVVVMNQDSVPIAEELCKEGGVILVNSSLVKKDPNRPDARIVHVPCNEIAEK
IGDVRIANMVMMGALSKVTGAVKLDTFEAVLKSFFPPAKHKLIPMNIQAVQEGKQAV*
>SPSA8_v1_50025|ID:41145005| 2-oxoglutarate synthase [Spirochaetes Bin 1 SA-8]
MELVYGHPKSLKPIQTKYCPGCGHGVIHRIISEVIDEMGLQTKAVITNPVGCSIWADLYF
DFDSVQPAHGRTPAAATGVKRMLPDHLVICYQGDGDLAAIGTAEIIHAANRGEKFTTIFV
NNAIYGMTGGQMAPTTLVGQYATTAPLGRDPGDAGMGYPIRVCELLATLQGTKYLARGAV
NNPINVKKTKKMIRTAFEAQMRGEGFTMVEILSQCPTNWKMDPVDSIDWLEKNMITYYPL
GEIKNTLAPAGDAASAQN*
>SPSA8_v1_50026|ID:41145006|vorB| Ketoisovalerate oxidoreductase subunit VorB [Spirochaetes Bin 1 SA-8]
MSEEIRLMKGNEAIGEAAIRAGCTAYFGYPITPQNELTAYMAANMIPKGRTFIQAESEVS
AINMVYGAAATGVRTMTSSSSPGISLKQEGISYLCGADLPAVIVNVARSGPGLGGISPSQ
GDYFQSTRGGGHGEYYTIVLAPKGVQDAADLTYEAFDISDRYRIPVLVLADGLIGQMMEG
VVLPEPKDPESLPVKPWKVGHQAEMKRDINHVSSMNLVPIELETANIARFKRYEQVKKEL
CRYEEIDVEDADIILVAYGTSARISLGALQAARKEGLKVGLFRPITLWPFPYDRLNQLAS
MGKRFLSVEMSMGQMVEDVRLAVNGKAPVDFYGRCGGIIPSEEEVLAEVKRILAR*
>SPSA8_v1_50027|ID:41145007|ptb| Phosphate butyryltransferase [Spirochaetes Bin 1 SA-8]
MNHITDIIEKAVSLGRKKIAVAAAQEASVLEAVADAWKAGITEPILVGDPELIEKARQEA
NGGKGLDLSAFEIIPVKDLYASAVKAVELVRNGEAAFLMKGIIDTSLLLKAALNKETGIN
AGRLASHVAVMEVPTYHKLFVVTDAALNIAPDLPALIDIIASAVKVSNSLGVATPKVAML
AAVEKVNPDKMPCTVAASILTQMNRRGQIKGCIIDGPLALDNAISAESARIKKIVSDVAG
DADILVAPDIEAGNILYKCLLDLAQAKGAGIVMGAAKPIVLTSRADTAETKLASIALAAL
ASGAGK*
>SPSA8_v1_50028|ID:41145008|buk| Butyrate kinase [Spirochaetes Bin 1 SA-8]
MPSCILVINPGSTSTKIAVFEENTRLFDKSVSHSSDEIKKYPSIAAQYGFRHEAIMDALK
AQGFDMKRLVAVVGRGGLLHPIPGGVYRVNEAMKQDLESARYGEHASNLGALIADNIARE
LGIPAFIADPVVVDELSDVARVSGNKLFTRKSIFHALNQKAVARRFARERGKRYEDVTVI
VAHLGGGVSVGLHQEGKVVDVNQALNGEGPFSPERSGTLPAGDLAKLCFSGKYTEKEIMK
LITGAGGMVSFLGTNDMRDVEKLYHEGDPAGTLYYNAFIYQVGKAIGALAAAACGKVDGI
IVTGGIAYGKEIVEGLEKMCSFIAPVIAYPGEGELEALAQAGFGALAGEIVIREYTA*
>SPSA8_v1_50029|ID:41145009| conserved protein of unknown function [Spirochaetes Bin 1 SA-8]
VKHGFTKHDYEIIVKDTMIRVLNSIFWFLRGIKVYALVGSTGTGKSFRAKLVAQKYGIEM
IIDDGILIKGDQLIAGRSAKKEAMYLGAVKTALFHDKAHRDEVVKALQHEKFRKILIIGT
SEKMVQRICERLAIPPPFRIIKIEDIATQDEIQKALQSRKIEGKHVIPVPALEIKRNYPS
IFYDSVRVFLKRSFGASASMPKIYEKSVVRPEFTKRGRVAISEAALSQMVVHCVDEYDAS
LRVRRMAIREDTQGYRITIFLDVPYGTRLASNAHALQDYIVDNIERFTGILVAEVNIVID
RMRVKSGKNSRQLN*
>SPSA8_v1_50030|ID:41145010| Phosphoglucomutase/phosphomannomutase alpha/beta/alpha domain I [Spirochaetes

Bin 1 SA-8]
MSMIFHPETGFDIGDPDIAPLALERLPSRETVLENASSLILSASGWRKIFALHTHDAEMA
PWSLECQAEDSLSTQISDSDKVIAALMAKTFGEFILAAAGTAKPTVLLGIDTRPTGPTIA
DIFVRVLTSMGIGVHYCFIISAPEIMAYAAYAGSLPSDSEQHADGFAYISASHNPPGHNG
IKFGLAKGGVLSAAEIAPLITRLKALLSGENPSAEAYSLIQAAIPAVVADCFSKTQHWKR
QALSAYILFSHQVITGEAELEDQRLLLDMIAEQCRIRPLGIVAELNGSARSLSMDSSFLE
SMNLKSKFYNAHPRNFVHRIVPEGDSLNLCIKKLDEMHTADSSFQLGYVPDCDGDRGNLV
FYDRHLAQSRLLEAQEVFALSCLAELAFLHAQGKASQCAIVVNDATSMRIEAIASRFGAS
VFRAETGEANVVDLADSLARKGWLVRVMGEGSNGGTIISPSRVRDPLSTIGALLKLLRLP
ALPGKPDLFKLWLELSGQEAFYRPDYDLTDIVYSLPGWATTSVFEKRAALKVATEDKTVL
KRIYQRIFLAELERKQGELARRYGIVSWKAMASIGIGEKDASTDFSASGSGGLRIVFSDA
GGRPKAFIWMRGSGTEPVFRIMADVDGGTSEDEAYFLDWQTSMVRAADSEASSA*
>SPSA8_v1_50031|ID:41145011| conserved protein of unknown function [Spirochaetes Bin 1 SA-8]
MGIRGELYSTRFVCNERTYFFNVKQNRTGDVFLSIVESKPGEGESFDRRSIVVFGEYMDG
FLKAFQSALKFIDKTGIKVEPDQTLYRERDGEAHQDEGREGRERKAQPRQSRFRDTDTAR
RPDSRGERSPAPFSARRTSPYPQSSSTDRHHDHAPRNAAPRSPGGRSSAPRGASSREEGS
ASKPVRKIVVRKTRKSDSN*
>SPSA8_v1_50032|ID:41145012| protein of unknown function [Spirochaetes Bin 1 SA-8]
VDSPWIFHQTAAIAAAKPINFTLPDKKKRLAGATFALQMSFSQLLSLFLVFLTTIFRTGF
EAEPSSREEAPRGAELRPPGLRGAAFLGAWS*
>SPSA8_v1_50033|ID:41145013|rnhB| ribonuclease HII, degrades RNA of DNA-RNA hybrids [Spirochaetes Bin 1 SA-8]
MAQKEFAFLTESSLCKRSPVLDEIALRGILHSVRFVCGVDEAGRGPLAGPVCAAAVILPA
DFPGECLGDSKAMSAGKREKAYTIIVEQSLDWAIGWATHDEIGTMNILQASLAAMGRAVA
LLELCPDIVLIDGNKVPALKYPAAAVIKGDSRVPAIMAASILAKVARDRVMDRLDAIEPE
YGFSKHRGYPTLEHRKAIARYGPSLWTRPGFSIKLPQ*
>SPSA8_v1_50034|ID:41145014|trxB| Thioredoxin reductase [Spirochaetes Bin 1 SA-8]
MAEKHELIIIGAGAAGLAAAQYGARANLDTLVLEAMAPGGQALLIDQLENYPGILEPVSG
YDISETMRVQAERFGAKFASATVKSVKKTGSFFTIETFDGVYEAEAVIVATGSQHRHLGI
PGEEEFIGKGVSYCATCDGPFFKGKRMLVVGGGDAACDEAMYLAKLSDKIRMIHRRDRFR
AQKALAERVLANPNISVRFNTVAKMLKGTKKLEAVVLQDVVTGETWEEAIDACFVFVGSD
PQTAFIDDAAKDETGYLITNDTMETSIPGLFAAGDVRASPFRQIVTACADGAIAAHAAAQ
HIDELKGSAYK*
>SPSA8_v1_50035|ID:41145015| protein of unknown function [Spirochaetes Bin 1 SA-8]
LTGSLLTCQLSARLNGNADVPISLTIRNLYPTMNVMEEQQKVFLIAHESLMSELPAFLRR
FDGGRTNWQIEAVIAPDSKLLNRITFEKAILAADAALIPEQLKAMLLEDSLPLVLLVSAG
SDAQPFLNTRHDTFIRLVLCEHGVCASQIAESLISIRRNFEREEHLAARQDEIGSRFQNL
LKALPDIVYILDEKGSFVFLNDAVRELGYEPHELIGRHFSEILHEEDRTRVSREAVLAHI
RAQEAFPEIPPKLFDERRSGLRMTRDLEVRIPNKTTGQILYGLVNAYGESSQALIDGMND
VYHGPVTMGIIHDITAVKLYQKSLEENLAAKELLLREIHHRVKNNLQVIASLTHLRQMEV
DDEKTRDVLAGLIAQIKSIAMVHEALYQTENLQGVSAKEYFERFALFMEQTYSYIGSPIS
LEVNAQDCLVDVEQLSYLAMIASELVSNAYKHAFPGNRSGFIRISFFLIGEEYIMSVEDN
GVGVRAEAESGNGLGLEIVQALARQLGAHLEQKFDGGINITLRMSGRRLD*
>SPSA8_v1_50036|ID:41145016| putative Histidinol phosphate phosphatase, HisJ [Spirochaetes Bin 1 SA-8]
MLFSYHTHTEFCDGRASAASMAHHAHQAGYAYLGFSSHAPLPFDTNWNMRWERLGDYAAT
IRALGNTYGPKEMTILLGLEIDYLENLVHPSDSAYDRIKPDFRLGSVHYITPEEGEAFTV
DENATDFARHVKLSCLGDHSMLWKQYYRNLTAMIEKGGFDIIAHFDIVRKNNQNGMYFDE
DAPAYLDAAFEAIDAAAQSDIAVEINTGGIARGKTLTPYPAMILLKRMHDKNVRITIGDD
AHGPAHLGAYQQTARLWAKEAGYSSLFYLKQNGEWAETGID*
>SPSA8_v1_50037|ID:41145017| C4 zinc finger domain protein, DksA/TraR family [Spirochaetes Bin 1 SA-8]
MEKTFTEQMRELLVSQKKEILDALATSNEEFRTIVAEMDPKDVADVASDDIDRKMLEALS
SQDIKRLKAIDAALTRISQGRYGLCMVCGKKIPQERLTAIPYAVMCIDCQKSEERRNR*
>SPSA8_v1_50038|ID:41145018|infA| translation initiation factor IF-1 [Spirochaetes Bin 1 SA-8]
VAKEEAIEVEGIVREALPNTMFRVELQNGHIILAHLSGKMRKHYIRIVPGDKVRVGLSPY

DLSRGRIIYREK*
>SPSA8_v1_50039|ID:41145019| putative Smr domain protein [Spirochaetes Bin 1 SA-8]
MDMDKLLKQWDELKKQEARKPKNTIEKAPGKVPGEPRRPEEKKERSIHDQLGNWITIHGV
EDKDSEYLDDEGQRSAAEAARRFRLLEPQAVLDLHGYSAAEAEPLLERFLQTSARKGLEK
VLVIHGKGNHSTTEHVMTDLTRRVLERNKNAGSFGPADRRHGGRGATWVKIRN*
>SPSA8_v1_50040|ID:41145020| putative 1-acyl-sn-glycerol-3-phosphate acyltransferase [Spirochaetes Bin 1 SA-8]
MKSAQRYIPTNSVFFTRLVQNTYGVWLKRAYNIHAEGLEIFSKIKPPFLVVGNHATLLDP
FIVNAFIPHPVHWVASDGNMRNPIMRFLLIKLVGSIPKSKVIPDIETVNWIVNIIRKQKG
VVGFYPEGQSSWNGSSLPSVGSSAKLIKLLKVPVIAANSRGAYMTKPRWAYTRRPGGVIV
HFSELFSPAQCKALSVEELDTALDKSIYHDDIAWCQQTGRSYDHPQRAECLELALFACPS
CGALNTLSSKGTAIRCHQCGFSADYQPDGSFSSIQQGQKNPAAIKHDFFRSLKNWDLWQH
EHLARLIEEKFSKAPEKIIFQDADAILLEGKRMDTMRRIGRGTLTLTADKLELVSSGNIL
ASFRCQDIEGPGVLKWNFFEFYVGKNVYRVRFRNRNTSGRKYASALELLLKTPR*
>SPSA8_v1_50041|ID:41145021| Na+/glutamate symporter-like protein [Spirochaetes Bin 1 SA-8]
MDFSWKVIIDAGLISAALLLATLIRSKVRFFQKYLIPNALTAGFLLLPLYNYVLPPIGYS
KNGLGDLVYHLLNISFIAMSLRSSPPKVKGHKGGGVLGMSSGILMGYATQALLGLFLTLL
FIPTVHHAFGLHLPLGFALGPGQAYAIGKGWEGMGFEGGSSVGLAFAAIGYLWACFGGVI
LINKGIRKGWVSADVLSMMKNTDLLRGTIAKDEEKPIGARLSTDSEAIDSFSFHVAAVIV
VYFLSFLFLKGLTFLLSFAGKAGMDLAANFWGINFIFSAIIAIVVRKLIDRFKISHVFDN
ETLTRISGFSVDYMVAGALAAISLVFIGKYWLPLLIMSTICGIMVFITVPWMSSRMFHDY
RFERMLMLYGVSTGTLSTGLALLRVMDPEFKTKVSSDYMLSAGLTFLLAIPFILSINLPA
KAFMTGSMSYYWLMIGIAGLYLLYVLVTYVILARKKSFAKPLQIWYED*
>SPSA8_v1_50042|ID:41145022| putative Transcriptional regulator, Crp/Fnr family [Spirochaetes Bin 1 SA-8]
MDVRDLALLAGCSLFSGIEPTQLAANLAERKVSFLHFNKNASILLAGCKYSSLFILIQGE
AWAEMISDEGKIVRVESFKAPEAFAAAILFSPDQILPVSVFAKTDCRVAVFGKDSLIALA
MLHQPVMEALLAETGAKLQFLTEKLRSAQFGTLREKLADWLIRRFELSGSKTIITEANRE
RLAELFGVARPSLSRELGNLAKKNIIEFEGRQITILDLKALQRLRSGHD*
>SPSA8_v1_50043|ID:41145023| NADH pyrophosphatase (modular protein) [Spirochaetes Bin 1 SA-8]
MMLIDPSEYAVFFNGLDVLVPLRKAEEDFEAALFSDTEPFSRLAWLFPDEDTSGDASGKA
FSMLGTSQIPTARFLHGSFRYVSLSLDDAAAEQLSRVLPFERIPVRQMLSLVSQEAARFL
LKAHAHAHWSEVSRYCGACGSPLATGHDHGDPGGKYCSACKRFFFPKISPAVIVLIHNGD
SILLAHNRKFTAGRFGLIAGFVEMGETLEETVHREVMEEAGIEINNLRYVKSQPWPFPDS
LMVAFEADYKSGTARPDGLEIDQLGWFTRQTLPEIPPKGSVARSLIERYLQAGSAGKQ*
>SPSA8_v1_50044|ID:41145024|upp| putative uracil phosphoribosyltransferase [Spirochaetes Bin 1 SA-8]
VSKIILKAQDLDGLLTDKDNEYLARMDSLYRQAMVSFNILSTTRSEREQKKEESSLIELY
NTMGSLMHEICATEPRIQVYSFATPQENHGEASRLIAKLRDIKTGRQEFVYYIQRAFELL
FNLAYGGAGSVNKNYLIVKTPVDIPVQNFAVHKIPNVDDQLENTVMCVMLRGALLPSMII
SKEIQEYSSTGYLTPFALFKIKRDDTKSENTMEYILDLDRSYFNLAELDGKDLLFADPMN
ATGGSLVTVVKYLLDQGIKPRSVKFFNVISALKGSLRIIRALDNITMYTLWMDPVLNERA
YIMPGLGDAGDRINGKDEDEHPRDMIRLVADYGTNITGLYRSQLRIIEETVLHH*
>SPSA8_v1_50045|ID:41145025|pduL| Phosphate propanoyltransferase [Spirochaetes Bin 1 SA-8]
MPETKVLVNLSNRHIHLSREDVEALFGKGYQLTKTKDLMQPGQFACAETVTIKGPKGAFE
GVRILGPERKETQCEILASDQFKLGVPGCPVRESGQLDGSAPFEIIGPAGSVKKDKGLIV
AIRHIHFDPESAHRFGVVDKQKVSLKVGGERGAVFLNVVCRVNPAYALECHLDFDEGNAV
GIASGAIGEIVAL*
>SPSA8_v1_50046|ID:41145026| putative Pentulose/hexulose kinase [Spirochaetes Bin 1 SA-8]
MIYSIDIGTASMKGGFFTRSGSCVAFAKAPIRAEKGNIPPIHEISAFAWLDAFKELTRML
AGQSAAGGLGLHQPIEAIVVSGNGPTIVPVDKSGQPLMNAITWLDRRASAEAAEASEALE
YKLDPAYNLPKILWIRNHAPDIYEKTRWFVSCPEYLCAQLSGNYVSFLPAEGFKKIIWDE
QALARLFLDAEKFPPFLPPASAVGVLTQPARAEFGLENLAEPGIPIIGSGPDFLAALVGT
ATIEPGRACDRAGTSEGINLCAAEAELRDSRLLYMPHIIEPYWNISGVISTSGKAVSWIR
GTLGLSDLSYDEFFAMAETAQAGARGLLFLPYLSGERAPIWDPAARGAFIGLGLHHGKAE
MARAVVESTGFAIRDVVELMEAAGARVDDIRVTGHPASGRVWNQIKADILQKPVRAPEFY
QAELVGNTCMALAAMGEFGDMAEASRAIVKTGLVYEPSAGKKALYDELFGLYRESYQALK

GIFPRLAGESA*
>SPSA8_v1_50047|ID:41145027| putative HTH-type transcriptional regulator CueR [Spirochaetes Bin 1 SA-8]
MKKSDNGLKIGDLASMFGLTARTIRYYEELGLLKSNDRTEGIHRRYPEKNIVYLNRIKYL
KDYGLSLGEIKEFFDLAEKDRSGAACKELLLSKYQEKIAEEENNLEKIRIRLASLREQVA
ALQSLEHFFSCPGDSCETCEFSMFCDEPEDEAP*
>SPSA8_v1_50048|ID:41145028|tpn| Membrane lipoprotein TpN38(b) [Spirochaetes Bin 1 SA-8]
MKRTLTIFTILLASLALSAQVFAAPLKIGVFIPGIREGSPTYDGLAKGAERFAAENPGSS
VKVFEAGFNQAEWEEKLISLTAEGKYDVILTSNPSMPDLANKVSALFPKQKFICLDGQFA
GNPNMYTVLYNQIEQGFITGYLAGLVSTSSMPGANSAKKAGMLIGQHYPVMDKLIIPGFE
KGLKAVDPAFQLDVRVLGNWYDATKALELTKSMYDSGSDVVLPIAGSASQGSIKAAQDSG
KYLVFFDADEYSRAPKNIVGCTALNQERLAYTVLKDAAAGTLPFGKADVVGIKEGYVEFL
DRSPVYLALLPAAIRSKMDMKIQEIKSGKLTFAIPQL*
>SPSA8_v1_50049|ID:41145029|yufO| Uncharacterized ABC transporter ATP-binding protein YufO [Spirochaetes Bin 1 SA-8]
MSYLVMNNIRKTFPSSGTVANDGANLSVEAGEIHAIVGENGAGKTTLMKILAGLLEPDAG
TITIAGKPAVFRSPQDTKAFHIGMVHQHFIMVPEFTVAENIVFGQEPRKAGFLFDTKIAE
KEAARMIERFGFSLEPDALAADLSVSERQQLEILRQMYRETQLLILDEPTAVLTEQEIQH
FFELLKTLKGQGITVLIITHKIREVKEIADRVTVMRDGRTIATLNTGDISEYDLSCLMMG
SASCLDFSARRRNPAGARTILDVKNLSITRKHHSMPLLHQVSFSVASGEILGLCGISGNG
LKEIEEVLAGFLQPSSGTLLFQGKALPKSRTAPWLPNGIAYVPSDRMKRGVVLQKTWAEN
YIALDRKRFFPRGIADRKSARETALRRIQDFSIKAQPDSRMDELSGGNIQKSILARELAD
EVPALCVLCEPTWGLDSLSASAMHEKILSLRDSGAAVLLISSDIDEIMALSDRIAVLRRG
TITAQVHNSSDVTREYLGALMLGIHNEH*
>SPSA8_v1_50050|ID:41145030| protein of unknown function [Spirochaetes Bin 1 SA-8]
MSPGNIWAHSCWGFTMSIDKQGMNTREGFKIIIFTLTVSVLLTLVLLGAFASSFPDAVTA
FFVRPLTNRYFFGNLLAAAIPLMIAGLGIAFAFSSSNFNLGXX
>SPSA8_v1_60001|ID:41145031|nth| Endonuclease III [Spirochaetes Bin 1 SA-8]
VYLPPELWYGIASVRLGQVWASLEVKVEMEMNAIENSRLSEKLAKMHEILAPLWPDAVPL
LHYRTCFELLCSVVLSAQCTDEQVNAVTPVLFSRFPDAESMAEADIAQIESIIRPTGFYH
AKARHLSESAKLIMSRFNGQVPQTIEELVTLPGVGRKTANLVVSACFGTPGVIVDTHVMR
TAVRMGICDKPDPELIEMTIRKNLPESQLTAFSHALNRHGKHVCSARKPLCLTDMQRCPV
AGX
>SPSA8_v1_60002|ID:41145032| putative Ornithine carbamoyltransferase [Spirochaetes Bin 1 SA-8]
MNNSAMRGRSLLSWSGFSKEEIRLILETAGKLFQEKSKNKLGHRMKNKVLFQLDNRSGAG
MQTAGNRFSETALALAFQKESGILIPHFPLAASEQLLAPAIEDMARIAGQLANAVSCGGN
QEYLDALAGFSSRPVFNVGTNQENPLLALAVILLLQNEVGDLRGKKLALTGEGDNPTIRS
LLILAGKLGMNAALASPQALWPDKDFFNLCKKEAESSSAAVVVSSELTEVLLDADCIFTD
SWLKSEPDPALCVNVENLARTGKSRVILLHGMSAGRGPEMSLDVFESPDSRVFSQFDALT
AAARALCILFVK*
>SPSA8_v1_60003|ID:41145033| Phosphoesterase RecJ domain protein [Spirochaetes Bin 1 SA-8]
MKKVPNGIQQFIEQHDCFYVIGHREPDGDCIGSELALASFLRSMGKRTHVLSSGPFTKSE
ILQFEHKFKNSAPPENDYERAAIVLDCSSLSRIGSVGETMPDVPIAFIDHHASGEVPGE
NDYLDETAPAVTAMVYLLMKAFGHKPSKEEAELLFFGLATDTGFFRHLDEKSSDTFEVAA
RLVESGVSPKRIYSDINGGKTLFSRKMLGEILMRLEPHYDGRLLVSYVTLDDQQKYGMAS
RDSDILYQLIMSVGGCEACFVVKQEAENSCTVGFRSRERVNVAEIAAKFGGGGHRLASGL
VMQGTIDSVMDTLIEAFRPLFAEEEL*
>SPSA8_v1_60004|ID:41145034| protein of unknown function [Spirochaetes Bin 1 SA-8]
MESRASAPELGTKTKFLDEFDEKTSAAFFLLVRDVFRQPHYYGFISMEEASEVLQAYRSR
IRSILERGLECNARQPAYLAICMRYLAKTYRREQQRQELREAVLESSWYSGFQFHDTAVK
SMPTAIAENFPQEEFYQTIPPAVFFNPLKTEYRRILFLLLKCACELDDAMILEASVRMGV
PYAWLLYLVHRARLIMEPQRLLKNQLQEKLNSTWVDLQILESRWMADEKSGKDVTRLSVR
LEAVRKRYYRLLERRASMKLLLPNKEIAKILQVPKGSVDSGLYYFKIRGTIKTRLSTGKR
ENLAQA*
>SPSA8_v1_60005|ID:41145035| Non-canonical purine NTP pyrophosphatase [Spirochaetes Bin 1 SA-8]

MEIVIATWNAHKADELRPLFPGHRLLLPSEVGLADCDIDETGSTYLENAFIKAETVFKLC
GRPTLADDSGLSVAALNGAPGIKSARYGSADGTTKLDASARNALLLEHMKGQADRRCAFI
CCLVLMYGDNRFISVQETCPGLLTEVPRGFGGFGYDPVVYLPELGKTVAELTPEEKNRIS
HRGRAASRMNRMLSILEPNK*
>SPSA8_v1_60006|ID:41145036|rpmE| 50S ribosomal subunit protein L31 [Spirochaetes Bin 1 SA-8]
MKQGIHPKYEKATITCACGNVIETRSTVKNIQVEICSACHPFFTGKQKLVDTAGRIERFN
RKYGIKSTETK*
>SPSA8_v1_60007|ID:41145037|rho| Transcription termination factor Rho [Spirochaetes Bin 1 SA-8]
MASIRKSRMKDASAHNDIQEQEETTMNSENSSQDSQSLFESHREDPSRSESPENRAPDES
AAQSRGNEASSAGRPQRRLKVRRKNTQPAAAPSMAAPAAQSGGFMEGAAAPEEPQEKLAE
PETQTVEELQNGGLIRATFEPGSAIQEEKHNRGSHARKQEQPERISSDRSHDAAKLDHNE
TRARLSINDLSKMGIKELRELGAKYGINHEDLIVLRKQELIFYILKSHTEHGGIIYAYGS
LEILPDGYGFLRSPQNSYLPGTDDIYISPSQIRLFNLKTGDTVYGQIRSPKEGERFFAML
RIEQVNFDEPAVAQNRIPFENLTPLYPNQKLNLETVTEETSTRIINLFCPIGKGQRALIV
SPPKTGKTILLQKIANAITTNHKEVYLIVLLIDERPEEVTDMERSVRAEVISSTFDEQAT
RHVQVAEMVLEKAKRLVEHGRDVVILLDSITRLARAYNQTVPTSGKILSGGVDSNALHKP
KRFFGAARNIEQGGSLTIIATALIDTGSRMDEVIFEEFKGTGNMEINLDRRLSDKRLFPA
INIKKSGTRKEELLLTEEELQKIWVLRKVINPMDDSDIIELLIDRMLKTKNNEAFLKSMS
SPVYNGND*
>SPSA8_v1_60008|ID:41145038| protein of unknown function [Spirochaetes Bin 1 SA-8]
MAKSGTTRKPLLFSTVLSKNIKATGNLSSSSSILIEGRFEGSLRTSSHVGIAKTADASFS
ILSCGSAEIAGKASGHIAAKEFIEALPKASIAADLEAPIVRIPDSCKFEGSIHTSMIDRL
*
>SPSA8_v1_60009|ID:41145039|hup| DNA-binding protein HU [Spirochaetes Bin 1 SA-8]
MAEKLTKAELIDALYANLPSSSKTTRKDIHELIDGLFAEIKTAILSGKTVELRGFGTFEV
KLRKGRSKARNPKTGEIVSVQDHGVAGFRPGRELKKAAWDMEALKAPK*
>SPSA8_v1_60010|ID:41145040|rpsT| 30S ribosomal protein S20 [Spirochaetes Bin 1 SA-8]
LSTKNLSAEKRMRQDEKRRIRNKAAKSAIRTAAKKVVTASEKKDAETAKAALLDMIKKID
TAARKGIVKKNTAARKKSRMQKLVNKLGA*
>SPSA8_v1_60011|ID:41145041| putative 1-acylglycerol-3-phosphate O-acyltransferase [Spirochaetes Bin 1 SA-8]
MVNLFIVVVILLCFSFWEIELLLAYLILPRSRSMRLAKRFESRAIRLIFSMFSTYRHFRI
RPINKISGPLPERFLVVANHQSLLDIVVLMKILPEGLNARFVAKHELAWGIPLISLLLRT
TGHCLVKRKNDALNAMRAVAAMAKRSKRDKTIPVIFPEGTRSRTGDLGTFHSAGYRKLLE
TDSLPILVIAVEGGYKVAKLKDFIRDFGTVPYKVSLLGVLPAPGGKKEALASLETARTMI
ENELIQMRKSP*
>SPSA8_v1_60012|ID:41145042| protein of unknown function [Spirochaetes Bin 1 SA-8]
MKKFLKLLVTFGIMQYFYICGTTAKLLFMRLLLDVAAGNSSFSGGLFLPHPIKLETSQSG
KVDTTAKACIS*
>SPSA8_v1_60013|ID:41145043| conserved protein of unknown function [Spirochaetes Bin 1 SA-8]
MNSTYNQIAEQDFYKARTRSLLTRIYSIINRNDTRLLPFEEAKNLIRPESETYSGLATVP
LEKIIGSEGRYRDFDRHFLPQKDMLKERWINIDKTHYEDVPLPPVRLYEMGGVYFVRDGN
HRVSVARTRGQEYIDAEVTSLKSRIALTEDMSIETLKQRVIEYEKRQFYEKTGYLAIVRR
DDLNFSEPGRYDTIYEHIIVHKYYLNQKSPQEIPVSDALYSWHENVYMPIIHAIYEETLL
DLFPGRTDADLYLFLVRHWDELKHASKRDVGVGEAARHFRQTLKASKAEAGRLQGSALFR
ALAEFFRKLQKK*
>SPSA8_v1_60014|ID:41145044| Metallophosphoesterase [Spirochaetes Bin 1 SA-8]
MKTVNILCVADEIDLLVYSSNIKERFGDVDLVLAAGDLPDEYLGFITAMLNKPLLSVAGN
HDPSDVNEKHAFSQSQAMLFMHGRERTSLGRLSFKIKKEAGVSVLGLPGSMFYNGGPNQY
SDFAMWMRVIFLLPELLIRRLFLGRGVDIILAHAPPKGIHDAEDRCHRGFSAFNWLIHFA
RPRYFLHGHIHVYDTQQLREQKAGDTAVVNVYGHRLISINLEKQP*
>SPSA8_v1_60015|ID:41145045| putative DNA mismatch repair protein MutL [Spirochaetes Bin 1 SA-8]
MGIIHVLSRETSRLIAAGEVIDRPASALRELLDNALDAGASDISVLTEKGGIDLLRVIDN
GSGMDRDDLALSIQEHATSKILKADDLLSVRTMGFRGEALASIAAVARLEITSCEKNSEI
GWKLQSEPGKPPVLTPVAARQGTVVTVQGIFEQYPARRQFLKKPSAEALLCRQVFTERAL

AHPHIQFSWSSGAETSRYPAGSLLQRMSLLYPELPAHLLSEVEINFETAAFHLILAEPSF
HRNDRRYLQVFVNRRKVPEWGLMSVVEYAYSEYLPGGMKPCAFLFADIEPSLADFNIHPA
KREVRIKTIESLKSLLYSSLKEQLHTLYGSGSMNMAEYSPAAGLAFETVPPSQKPDQSFW
ETVRTLNETPIKESDSSAFGTTGYAGERPTFRYIGRAFGPYLLFSIDDTLFILDQHAAHE
RILYDSLLERPVSSQALLVPYIFEPESDDMKNRLASILPEMKNFGFEIEKQHDAFIIHAV
PARLGEKAIPAFLDFVQKPPENSRVSGIVATMACRAAIKDGDVLDDFSARELIAKSLQLP
FPRCPHGRPIWIRLDRDYLDKMSGRNPG*
>SPSA8_v1_60016|ID:41145046| conserved exported protein of unknown function [Spirochaetes Bin 1 SA-8]
MKKIVALSLLLALLIPSAAFAQEGIGAGILGWARENLAMEMGSVTIDGVGYSKIVLAPEV
RMGRLKLGLYLPVIYKDDLFNPSTWYAPGGNNEWDFGAAYWGTNTMKALLDVASDLVLKI
KYLEYGQPLEDKFFFKVGNLHDLTIGHGLIMRNYRNDTDFPSIRRTGVNVGYDFGAVGFE
ALANDLPIPEIVGTRLYVRPIKGFKLAFGISGVADLNAGKDLAGTAWETAGENLVFIGSG
VDLDLPIIPANPILGVRAFAGAAVTMPYVKANFTSPNNASTVTQGFRTDLIWDNAGPHNY
GAAAGFLGNVLFIDWRLEYRYFTGIFRPSFFDSTYERARSLFVQEYVGYLDGTKGINSAP
TVMGVYGEAGVSLFKEKLSFSAGYMWPWSFDAGFTLGDADDELHAGIVLKKGLIPKINVA
GAVYYDKWGLVNQLASGTFQFFDAKSALSGEIEIPVPSTPNLAVGVIFKTVVARDAAGNV
LYVDDDPSKGVKIAPAITIETRFHF*
>SPSA8_v1_60017|ID:41145047| conserved exported protein of unknown function [Spirochaetes Bin 1 SA-8]
MRKFALVLAILIAVFPLVAQETGTPSPGFSAGLNLGSDLLPTGAGGALESWSKLGFQPDL
SIGKFGVGLDFTIRFKLYPNGATPIEIYSPDWIPAGGQTIFDVYLPKIMYVRYGLRGVDP
IYVKLGSISDFSLANGLVISDYANTRFLPDTRIFGLQAGLDGAAFKFPFLGLEALTGNIS
KLDVVGGRVYIRPLAFFGNGIPGRIQIGATAVADQNPLLYIPTGDLGSYPASKLIYVLGA
DVTVPVLTGDLLSLTMFAEGAREMNGAMGAIGGLRGKLIGIVKYGAQVRYLQEGFIPTYF
DANYDLYRADRFKYIEDTVPGSFTPGWLANIGFTLFNNLFVFDATLDGPFAAIPSVATSN
SGEYPHLKGSLLLKDGLIGGLSFEGGYEKYFIGRAKPFLQDIIDPTDAVIGLAVHYKTGA
TVLTLNYAYRWDPSIDDWDVSSSLSASVRF*
>SPSA8_v1_60018|ID:41145048| conserved protein of unknown function [Spirochaetes Bin 1 SA-8]
MDFFAKLESRVQSLNTNLCVGLDPSFSQDEISRLGSEACMRSALERNLTIIEKTAPFTAC
YKPNIAFYEAFGSLGLEVLKKTIDAIPKEIPVLLDAKRGDIGSTAKAYAQAVFGYLGADA
VTLNPYMGKDTVEPFLEWDDKGVFILCRTSNPGAPLIEELPVDGKPLYLKVAQEFASWSP
RVGLVVAGNDPASLKAVRDVAPSVWILAPGIGAQGGKADEAMRAGSRADGSGILVVAARS
VAEASDPGEAARVLRDTMRQTVSIRDPVADPLKQELIKYLIETGCFKLGEFTLKSGIKSP
FYIDLRKLISNPAAMRAAGKAYARLASQLVYARIAGIPSAGLPLATAASLSTDKPMIWPR
MPVKEHGTGNRIEGSFERGEIVLLLDDLITTGLSKLEAIDILRAEGLEVMDLVVLIERGR
EGRADMEKAGVQLHAFLQVGELFDYCEQNGIIDSAKKHELEDFAGLKA*
>SPSA8_v1_60019|ID:41145049| Transcriptional regulator, BadM/Rrf2 family [Spirochaetes Bin 1 SA-8]
MRTTTRGLYALKSLLALAGNASSEKPVPLHRLAENEGISPEFLQQIFFKMRKAGLVAAYR
GPGGGFYLAKAPEEITIQEILLAAGETLSISPCSPLGLSSDCNNQQCKKLDDCKASRFWL
GLQQEISSFAGSRRLSDFL*
>SPSA8_v1_60020|ID:41145050| conserved protein of unknown function [Spirochaetes Bin 1 SA-8]
VTPESGFNDKIEEQAAYLVNRLLKNEKSLRRWARKNDFHALRLYDRDIPEIPLAIDRYET
DRGIKLVAALYERPYEKSDKEESAWLDAMVGAVCTALSLDSGSVFIKVRKRMRGLSQYDR
NSDSHAEAIVREAGLSFLVNLSDYLDTGLFLDHRLTRGMAASQSKGKSVLNLFSYTGSFS
VYAAAGGCRLVTSVDLSNTYLAWCRKNFLLNNVPDSIHEEVRMDVMLFLREARKAGRKWD
FIIADPPTFSNSKKSPEDFDINLDWPELLERCASVLEPHGQILFSTNSRQLKWDGSKCPL
EWQDITDATIPPDFRNKKIHRCWLLKAADR*
>SPSA8_v1_60021|ID:41145051|rex| Redox-sensing transcriptional repressor Rex [Spirochaetes Bin 1 SA-8]
MPNHKKISFAPSIRRLPSYLHIIRSAQARSEVYISGTVIANELNLEPIQVRKDLAITGIV
GKPKKGYPVAELISSIEHYLGWDVNHRAIIVGAGNLGSALSGYQEFRNHGLNILAAFDID
PVKIGKNIHGVPVYSLDELESETVKLNPEIAIITVPSPFAQSTCDTIVASGIRAIWNFTN
VKLKVPDNVAVWREDLSSGYAMLSVMMQQR*
>SPSA8_v1_60022|ID:41145052| protein of unknown function [Spirochaetes Bin 1 SA-8]
MILPYLLHADWEEPPLSMEMELPYGRLLQRIIETLTFLRQSNPQTSEASVSMEQVNAAIQ
LYCMAPGIVNVMLNYKICVEHDLPLHPTVYYELKEARKYRINHEIAEIEKANRLYRRSIH

LARDVVMMTAGIEETLDQFRHGIPRELLGFVYSGIRDKYTWRGSDPALLEKLADMVRASY
KPDILIAAAHGSIMPSLLLAELLDIPLYFVRLSMFKRNDEEPIIAISDEAWLFSYRESRA
LLYDEDVAGGRTLSLFQERLKPLFAETRTACSIRHAGAKIKPDYAARTWWD*
>SPSA8_v1_60023|ID:41145053| putative Formate dehydrogenase family accessory protein FdhD [Spirochaetes Bin 1 SA-8]
VQASENNPENVAVAVLAGGSSSRCETGDKRELVIDGTALGRRAVLNALSLSDRVFVIGSP
HPSYQDLPISCFQDEIPGFGPLSGLHAALDRAETGWCYLLACDMPLVSPAWYALLLEKAR
TAPEWGAFIARTSNGYLEPFHGLYSSRLKKPLEVFLQKHQTGGRRQSISSFLATVDFCAV
EAEEVLACISDWQLFSSINCQREYFDFIKNYRY*
>SPSA8_v1_60024|ID:41145054|tatA| Sec-independent protein translocase protein TatA [Spirochaetes Bin 1 SA-8]
MFGRIGPMELILILAIALVIFGPKKLPEIGKAIGEAIKAFRKGSEKVTEEIDKATSPDAD
EKPKVTDAKNGKDDSTSATPKA*
>SPSA8_v1_60025|ID:41145055|tatC| Sec-independent protein translocase protein TatC [Spirochaetes Bin 1 SA-8]
MSTAIGEEKDGPSMSFFGHLAELRKRLIAALAVFLLASIASFNWSEKLADFMIAPAQDMA
FVFLSPPDLFMLYIKIALSMGLVISLPVIIFELWMFISPALLPREKRSIFFSLLAGGLLF
ISGAAFSYFVIIPFTLRFFMSYQSAYVKPMISINEYFGFVISLALSFGAAFELPVVASLL
GAFGVLKAELLIRIRRIAVLLIFIAAAILTPPDIVSQVLLALPMLALYELSVMILKRQEK
RRAKKEAQVQISI*
>SPSA8_v1_60026|ID:41145056| DEAD/DEAH box helicase domain protein [Spirochaetes Bin 1 SA-8]
MTFESLGLSPRMLETLAKKGFEEPTPIQSLAIPRLMAPGNAIAAKARTGTGKTAAFGIPL
IERLSEARSNTQTRPVSAIILVPTRELAIQVTEEISSLKYAENPRLACVYGGASMRDQLR
KLAQGVDIVVGTPGRVKDHIERGTLDLSTVSFFILDEADEMLDMGFIEDIEFILDSAAKD
ARILLFSATLARPVLNIVRKRAGDIDIIEDTTETLPTELAEQVWIEVHERDKLEALSRIV
DSEEEFYGIVFVSTKVEADKLAKSLGERGYDCEALHGDLSQEARERVLGKFRDKRISILV
ATDVAARGIDIEKLTHVINWSLPHDPESYLHRVGRTGRAGNSGTAITFVTPQEYRLLFRI
RKISGTALKKGKVPDVEGVIAIKKERIAARILARQEAEEADDGIWLALAKDLMEKLPPEK
ALAYALAEGFAGQLDPGRYRELTEVSVDEAGVSRLFISAGKRDQMTPRSLVTIIKKLSNL
PDRLINSIEIYDEFSFVSVPYKDAEKIITEARKKGGFPQFSLAAPRGGARGRRDGPPQSP
RRASSQQGTKARRASSPRFSHSDERPGTGKRKPRF*
>SPSA8_v1_60027|ID:41145057| Glutamate formiminotransferase [Spirochaetes Bin 1 SA-8]
MHRDAVVECVPNFSEGRDTKKIEAIAGAIRAVNNVKLLDVDPGADTNRTVYTFVGSPDAV
VDAALAAARAAWEQIDMASHRGAHPRIGALDVCPFIPVSGITMDECAELARDFGKRLAEE
LNVPVYLYEKSASRPERTSLADIRTGEYEGLSSKLQDPAWQPDFGPARFVPRWGATVAGA
REFLIAYNVNLNTKDKKLANEIALTIREGGRSAKNPDGSIARDAQGNAIKVPGRLKAVRA
IGWYIDEYRCAQVSINLLDYHLTPLYEVFEVVKEEAEKLGLLVTGSELVGLVPLAAIEEC
GRHFAAKAGKSAGLPEKELVEIAIQSLGLRSVSAFEPEHKIIEWNFRKPAPLARSRILDF
LDELSTDSPAPGGGSAAALAGSLGAALAAMVGNLTVGKKGYEAGFDELGAMAQKAQHVKA
KLAAGIDRDTEAFNAILEAGRMPKATEEQKKAREQAMEKASREAVLVPLENAKTCLEALV
CTLTAAKLGNRNSVTDAGTGALLAKAGLESALLNVEINIKSMTDSQFTGQIAAETRTLRE
QSGQILSEVLTQVEKCLSGAEK*
>SPSA8_v1_60028|ID:41145058|potA| polyamine transporter subunit ; ATP-binding component of ABC superfamily [Spirochaetes Bin 1 SA-8]
VKGSDVAVSNVSKSFGSFAALKEVSVSINKGEFYSLLGPSGCGKTTLLRLIAGFEDPDQG
QITIDGADVLGLPPDKRHCNTVFQSYALFPHLSVFENVAFPLRIRKVPSRLIKELVFKHL
ELVQLEAHADKKPSQLSGGQKQRVAIARALINEPSVLLLDEPLSALDAKLRQHMLMELDA
IHDKVGITFIYVTHDQQEALSVSDRIAVMNAGKVLQIGSPQQIYENPASEFVARFIGEAN
LFTGVVTRREGSLNLVDTDGFGPLLVDDETEVAVGDRIVATIRPEKIRISTDRLGGPSIN
VLHGIVHEPVYSGFQTKYIIRADSGALLTVFRQHANWSEGIPDIEWKDEVYLSFSARDMV
IVERNS*
>SPSA8_v1_60029|ID:41145059| ABC-type transporter, integral membrane subunit [Spirochaetes Bin 1 SA-8]
MKRNYGFAYAAPQALWLTVFFAAPILIIIVYSFLKKGLYGGVEPQFSLEAYSALANPTLL
TVAFRTLWVSAAATFLTILIALPCGYSIARAKNQALRLFLVIVPFWTNFLVRIYAWIAIL
GNEGFLNHALRFLGFRAEGYQFLYNQGAVILVLVYMYLPYAILPLFSTIDKFDFSLLEAA
RDLGASRMQSYLRVMLPNIKPGIFTAVLFTFIPIFGAYAVPLLIGGKDSYMLGNVIADQV

TKTRNWPLASSISMTVTLITAILVFLFALRKPKEQKLVRDQEALSPSASGGAA*
>SPSA8_v1_60030|ID:41145060| ABC-type transporter, integral membrane subunit [Spirochaetes Bin 1 SA-8]
MSSLRHHGLKPVRIAMLFHARRALRKSAKAASKLLPRKFAGSDMIFWIVILFLFMPLIVL
FVYSFNASRSGEWKGFSLIWYQKLFTESPDLWKAFENSALIALTSAMMATMLGTLAAVGT
ARYSFALKSYVATMSFIPMILPEIVVGVSLLVFFAGVGLKLGLISVWIAHTTFNIPFVYL
LVSARLAESDPTIVEAAKDLGATEIQTLFKIILPMALPGIASAFLTAITLSLEDFVITFF
VSGPGATTLPLYIYSMIRFGVSPVVNALSAVMVAGTVLLVYPLRNFLKVFAAR*
>SPSA8_v1_60031|ID:41145061| Extracellular solute-binding protein family 1 [Spirochaetes Bin 1 SA-8]
MKHILRRIRLDVSALVLIVAALVFSGCQPKTAATPKKLFIFNWTYYTPDSVIEKFEKEYG
VDVIYDSFASNEEMFAKLKAGGSSYDICFPSGDYVSIMIKENMLQKIDHKALKNFANVAP
EVLAKCDFDPGNEYSIPYYMGAAGVAVNKTKVQNYEHSWSIFSRKDLAGKMIMLDDMREV
MGDALKFLGYSVNTTNEAEIVKARDLINNVWKPNLLKFDAEAFAKNFAAGEVWVAQGYAE
SIYAELDKSRWNEVDFFIPKEGGPSYIDSMVILKNSKNVDLAMKFIDFIHRPEIYAEFCD
YFGFPATANTPARQLKKGDTWYSAESLDPCELKKDVGADLDKYNAAWQEIKVGK*
>SPSA8_v1_60032|ID:41145062| Rhodanese-like protein [Spirochaetes Bin 1 SA-8]
MAFMNAVEQKIKAGARVVDVRTWDEFEDEHFPGAVCVPVDEIMAKAEELGPKNTPIVLYC
ASGARSAYAARILKSMGFADVTNAGGLYDMPGF*
>SPSA8_v1_60033|ID:41145063| CoA-disulfide reductase [Spirochaetes Bin 1 SA-8]
MKLVIIGGVAAGATAAARARRLDENAEITILEKGPYVSFANCGLPYRLSGDINKRSALIL
QTAEGFFSRYRVKVMLNTEAIRLDRAGKKVIARGKDGELEIPYDKLILAQGGIPFVPQVE
GSDAPNVFRLWTIPDMDKINNYINETKPTSAVVVGGGFIGLETAEAFVKRGLSTSIVELT
SQLMPAADPEFGKQIQLAFEEAGASVYTEKSIRKINDKEKYVELNDGTRVKADIVLMSAG
VRPNLELAQKAGLVIGQAGGLVVDEFLKTDDDNIYAAGDMIEVVRRVDGAKVRIPLAGPA
NRQGRIAATNALGGSMRYRGAFGTSVFKAVEYTFAQTGLSEKAAKAAGFDVRAVHIHKAH
HATYYPGYEDLSIKLVYTADGRLLGAQAFGKEGVEKRIDVLATALAGRMTLEDLAELDLS
YAPPYSSANDPFQMATFAAQNDLSGFSPFISPAEALKLFNDKGGTVFIDVRNYGDYLKGH
LEGSVHLPIDELRDRLDELDAQKPIILISKAGFEGHLGVRILKQHGFKDVRYVTGGIMSM
RLFPGYQETTEE*
>SPSA8_v1_60034|ID:41145064| Aminotransferase [Spirochaetes Bin 1 SA-8]
MKFSDRTAFAIQQNELEKARQARLLQGLPVLNASESNPTKTGLLHEADELANALDNPQNR
VYIPDPKGMLSARRAVAEHLQKKGHDGQPDRLFLCASTSEAYAYLFKLLCDFGDAVLVPR
PGYPLFDHLSMLEGVEATGYRLEYHHPGGWRVDLDSLETTLLQDTERRIKAIVVINPNNP
TGSYIHREERAAIERLCARHGLAIIADEVFFDFSLENTSEFQSFYNACSILTFVLDGFSK
RLCLPQMKLGWIQVNGPEAEAAQAIERLEIISDSFLSAGTPIQNAAAALLEEEERFQRLV
RERIKAVMTVYRTMFEYPGSPYRLLTCEGGWTALLQCPRYESEKNLSLGLLQDKGIFLFP
GYFFDMEYEAFFAFSLILEPEKAAEAGRALLEYFRTLESA*
>SPSA8_v1_60035|ID:41145065|folD| Bifunctional protein FolD [Includes: Methylenetetrahydrofolate dehydrogenase ; Methenyltetrahydrofolate cyclohydrolase] [Spirochaetes Bin 1 SA-8]
MSAKLIDGKAVAEQIREEIKSRVAQLRKKGVIPGLGVILVGEDPASVSYVTAKEKACEEA
GIASFEHRLPAEVSLEGLLGYVRAFNEDPRVHGILVQLPLPRHIDEKAVIAAIAPEKDVD
GFTPVNVGRMLLEEPCFLPCTPNGVLELIKRTGVKTDGAHVVIVGRSNIVGKPLANLFIR
KSINSTVTLCHTGTHDLASYCKKADILVACAGKPRLITADMIKEGACVIDVGVNRVADPA
AKKGYRLVGDVDFAGASEIAGFITPVPGGVGPMTITMLLQNTVESASRT*
>SPSA8_v1_60036|ID:41145066|folE| GTP cyclohydrolase FolE2 [Spirochaetes Bin 1 SA-8]
MVDVQSMKDERQIPIQKVGVKGVRYPILLKDKTEQVQHTTAVVNLYADLPHDFKGTHMSR
FIEVFELYRHDLSMPNFLAMLRTIKKELDAETAYADVHFPYFITKTAPVSGQRSIMSYDC
SYEARVSPEGHRFIVGVTVPVQTVCPCSKAISKQGAHNQRGLVTLKVNLGPFFWIEDMVA
LVEESASSDLFTLLKREDEKFVTEKAYDNPRFVEDVVREVYLRVDALGKFPWFSVEAENF
ESIHNHSAYAFVEKHR*
>SPSA8_v1_60037|ID:41145067| membrane protein of unknown function [Spirochaetes Bin 1 SA-8]
MADNYPVEQKDLSEKGTKGIMSAAGGVGLLAVNALLGIPVLGWVLSAGLLGLGAVGLFGK
TKTDKVSGSLLSAAGVAGLMTVFMPGLARTILFTGGTGLLIYGLVNIGSFVMGLKKKN*
>SPSA8_v1_60038|ID:41145068|miaB| (Dimethylallyl)adenosine tRNA methylthiotransferase MiaB [Spirochaetes Bin 1 SA-8]

MTYFFETYGCQMNKAESSAMEHILSESGWMTATESDADLIIINTCTVRATAENRAWSRIR
KYAADKKKRKLVLMVVGCMAEQYKDEMKKKVPAIDYVLGTFQKQTFGLILEQIQQGTTYD
VHDETPSYVFSKSHHEPGSFRAFVPIMHGCNNFCTYCIVPYVRGREVSRNPLDILAEIDS
LDRAGVREITLLGQNVNSYAWKGDEGILDFPGLLQRISGHLKARQTGIGWIRFLTSHPKD
LSDRLIQVIADEPLMCRHVHLCIQSGSNRVLSLMNRKYTKEYFLELVDKMKTSIPGLSLS
TDILVGFPSETEEDLQDTLDVMRKVEFTYSFMYHFNPREGTPAVKLEGKIPEAVKKERLS
RVIELQKAITSKLMQERIGQCDLVLIEDQSKKSKNEVLGRTARDEMVVFKADASRIGTFA
TVRLIGISGNTFKAEEVNA*
>SPSA8_v1_60039|ID:41145069| exported protein of unknown function [Spirochaetes Bin 1 SA-8]
MRKAAVVFLFLSAVFWQAFCQTSTTIKQQPIQAPPKIEFGKITIEQARKELAKVQGTSNM
SAYLEAYALALPLDKALVLYREFFPKLPAAQQEAAARTVISLALIAGRLDDAIFFLPSIS
TAQARMLELRLCLATGKLQEAQMVLQRIRNPEQSKKTALSLQEDAEIKLLEAWLLYFEGF
IEQSFSAAKPLVTTENEKSIRNEAFLLLWYIANSDAFAQIKSAKKGFDEKSIKAAIGKES
PYSIEHGIIAGALKIAPSAWILESTRLSGLALSAKPAVAALSGQSAQQDKAGGASDSSAG
FSRLQVGWFSVRENAVSFQATLVKKGFKTVVEEQKNKDGELRWAVIVDAEGDWSQMQARL
KDAGFESYIVQ*
>SPSA8_v1_60040|ID:41145070| protein of unknown function [Spirochaetes Bin 1 SA-8]
MSDKTKKIVWLSVGISFFLLVVAGAAFLLFLPSPTNSATPFSLTGKSEPKSKTPQDYVAN
LPVSTMETTTTTKSGDIIIVYGNGDSTTTTIQAPVPSSKPNTTSTQPAPVTTTIVVAPPA
PKSIVTTTTTVVKQQQASPPTSTTAKTTATTATTAAKPVSSDYWIQAGSFSSKATADKLK
EAFQERGMVAAITVKDINGKSYYQVKVGPYPSRDEAKKWIQTVKSVPGASAEAFVTQ*
>SPSA8_v1_60041|ID:41145071|coaE| Dephospho-CoA kinase [Spirochaetes Bin 1 SA-8]
MKTIVGLTGGYCSGKNQVAVILAAYGYYCIDVDALGHRALELSKKELAGTFGSCIFDEYG
SIDRKALGALVFSSKSLLEKHESIVHPVMLSLLDAELERSERVCINAALLYKFPQVLKCN
YIIEVTAPLELRLERAKTRDGADRETALARIAHQEYLWKLRPRVHPPVFFISNAGCIDDL
ESSLARIPGLLPQDI*
>SPSA8_v1_60042|ID:41145072|polA| DNA polymerase I [Spirochaetes Bin 1 SA-8]
VKPSLYLLDVYAIIYRSYFAFMNRPLRNSSGENVSAVYGFFKFLFALFDQRKPEAFAAVF
DSKGKTIRHHMYEAYKATRQKTPEDLTAQVPLVEEILQALGVPMLRKEGYEADDVIATIA
NRCREEDRACFIISGDKDLLQLVGGSVKALRPAENFSFKLLDHDDVIGEWGVSPVQILDY
LSLTGDASDNVPGVKGIGDKTAVKLIAQFGSLDAIYANLENITPESLKKKLIEGRDSAYL
SRKLITLETSLDLGLESLDALHLGALNRAQATPLFLALGMKSFAAPEPAPSRASAHSNGF
LFDDGTPPEVSGGATSADTAGASAAATQETVAQASDANAGTPKEGALSGPGVYAAITDEA
GLSKLVDDCISKGCFAFDTETDSLDIMEAAPVGFSLCHEPKHSFYIPLVSPDSPVIARES
AVRQLERLFSSRATLVAHNAKFDIHVMRRFGIRIENPIFDTMIAAWILDAESNSFSLGSL
SERILGTSGLLYDDVVPKGKTFSSVPIQEAVRYAAEDADFTMRLHQYLDGELKRSDLAKI
FYDIEMPLIPILTDMEERGISVDANALRQFGDELEIELARIEQEVWQLVGHEFNLASTKQ
LQDVLFVERKLPVQKKTKTGYSTDTTVLEELAPLDPVPRLILRNRMLSKLKSTYVDTLAE
LAEKRGKIHTSYVQTGAATGRLSSRDPNLQNIPIRDEEGRKIRSAFTASPGNLLISADYS
QIELAVMAHLSGDSNLIQAFRDGVDIHRRTASFIFGIPEAEVTPEHRRVAKTINFGVIYG
MSAFRLARDLGIPNARAQAFINSYFSTYSGVAAFIRDVITQTEKTGFASTLFGRRRKIPA
INSRNKTEQMAAQRVAVNTPIQGTAADIVKIAMIQVQRELDNYLPDVRMLLQVHDELVFE
CPAGIVSEAEKLVKDVMEHAVTLRVPLRVSVETAFSWGDMH*
>SPSA8_v1_60043|ID:41145073| putative Heat shock protein DnaJ domain protein [Spirochaetes Bin 1 SA-8]
VDQLFDRLERLVKSWINAGADASFSPSGFSSSRRTSSGDPDLDAAMAELDDFLDTSKTET
ERREAEAKRREEAMRAEAERREWSARQEQNRFYRSGPNSGSGVSQEETSKAIREAYAYLG
LPAYAPFPEVKSAYKKLLFKYHPDRNSSSPEALKKATETSTRINAAYQIIEAYEEARKGK
QGAFR*
>SPSA8_v1_60044|ID:41145074| putative Pseudouridine kinase [Spirochaetes Bin 1 SA-8]
MKHASKGFVALAGGINLDIQGISNSAFRSGDSNPGRIISSPGGVCRNIAENLSRLEIPVT
LISAFGKDPEGESLKRDCLNKGIDISLSLTAAGNTARYLCLASPDASLIGAVADMEIMEA
LTPDFFESRKAALAGAAAIVVDTNLPEASIGWFARQFGRTGRVWRAGGKGPVLILDTVSA
AKAPKARGVLAEFDLAKPNLEEAKVLAGLECSAEVEASQILEALHSSRALPAELYISLGE
RGILYADSELSETGLVPLPPHNVRPEAKNRSGAGDAACAGLVYASLHGLPMNEKARYALA

MAVIAAAALQTVHPEVDLLMLKREKERFYESIS*
>SPSA8_v1_60045|ID:41145075|yeiN| conserved hypothetical protein [Spirochaetes Bin 1 SA-8]
MNPYLEISDEVASALREGKAVVALESTIISHGMPWPKNLDTALEVEDVVRAHGAVPATIA
IMNGKMKAGLGKRELEALAKSGHEVAKASRRDFPRLLAERATGATTVAGTMIVASFAGIP
VFATGGVGGVHRGAEASWDVSADLEELAKTSVLVVCAGAKSILDLPKTLEYLETRGVPVI
GMGTKELPAFYTSHSGIPLEEQADSPAEIASRMRAKWACGLSGGIVVANPIPAEFAMDPT
TINLAIDQAVREAEAAAVKGKELTPFLLKRIVEITGGDSLESNIALVKNNAALAAEIAVA
YAL*
>SPSA8_v1_60046|ID:41145076|glcD| Glycolate oxidase subunit GlcD [Spirochaetes Bin 1 SA-8]
MTSTYGRLNSAIAAKLKEICGAQQVLYEDKGAMERYSHDETAEKQYAGMPEAVVFPQSTA
QVSAIMKLASEQHIPVTPRGAGSGLSGGCVPVFGGIVISFEKMNKIKEIDLENLMIVAEP
GVVTNSINEALKKDGLFFAGYPMSLETCFLGGNVAENAGGGKAVKYGVTSRYITGLEVVL
ANGSVMRLGGKLRKDVTGYDLIHLMAGSEGTLGIVTEITLSLLPAPRYRAILLASFSSVE
SAISAVPRIMTGSRIIPTSIEYMDRASILASCSYLNETIPYGEAHAMLLIEVDGTAKEEV
ARSYMSIGDVCESCGANPVYVADNRTTMDRIWNVRRNIAEALKVQSPHQSLEDIVVPTAA
IPGFMRELAELSRKEQVQIPCYGHAGDGNLHATIVKPDSWSMETWLARLDGILGELYRIT
RKFGGTISGEHGIGNKRKKFISITLDPVALEMMKQIKQAFDPDGILNPGKIFP*
>SPSA8_v1_60047|ID:41145077| Electron transfer flavoprotein subunit alpha (modular protein) [Spirochaetes Bin 1 SA-8]
MTIIVLVKQVPETDSLVMDEATGTVVRKEDSSIINPLDLYALEAALRLKDANPDTKIIAV
SMGPGHAAAALAEAVSMGCDDAILVSGREFAGSDTWATAKALAAAIKPAGIPDLILCGER
ATDGDTGQVGPEVAAFLGLPVMTYASSLSMEAGSLLIERILETDIEKIRMPMPCVISVTK
AVGEPRLPTLSGKKRSYGRSIPVLGIKELMLNPETVGSKGSPTRVVRISSPKIARHSAFL
DAADPEGKKRAIAAVIAALEDRGLLPETLTLSASAVASAQASAYAGTQRQDDSPAETQTA
ARGSSGTKRSVWILAEARHGKVLPISFELLQWARSLGPEENILTTAVLCGPASEPEILFS
YGADRIIHCPDSRLAPHEIALTARYLDSLLASESPDVFLAGATTYGRSVMPYLAALSSFG
LTADCTSLSLDIASGELLQTRPAAGGNIMATIRTIGKRPQMATVRPHSQKPHLMQSAASC
VFEKRDLDGPFSGNSVELLEEKPLKLDETLDSKVRIVAFGRGLKKKDNIHLIRDLAQVLD
AGIGASREAVDRGWADYPRQVGLSGHTVSPDLYLACGISGAIQHLAGMQTSGFIVAINTD
PDAPIFSVSDIAVRADVFDFLPSLTEALKNHLSAREAQR*
>SPSA8_v1_60048|ID:41145078| conserved protein of unknown function [Spirochaetes Bin 1 SA-8]
MKILRYEKGGLEFYGFLDGQAIVRAEGDLSIGLTPTKHQDSLAEVRLLAPCVPTKGVCIG
LNYRDHAMELGLALPTAPVVFLKPSTSLIGPGDWIDYPELSHRVDYEAELVAVIGKKAKN
VLPEQALEYVLGYTCGNDVTARDLQPKDGQWTAAKSFDTFMALGPWIETDLDPSNLKIRA
ILNGAQKQSSTTANLIFDVPHLVSWLSRIMTLLPGDVIMTGTPSGIGPMQKGDEIAIEIE
GIGTLINRVR*
>SPSA8_v1_60049|ID:41145079| Malate permease [Spirochaetes Bin 1 SA-8]
MVVFSRVLSIFILVVVGFAARKSKILDGSMVKGLAGLILNVAIPFTIIASFDRSIPSSAM
PDLLRMALWAAAIHAFGILFSAALYRRMPAAERKVLTYITVFSNCGFMGFPVTESVFGKV
GVMYASIFVIIFQIFIWTYGIYLFSGQSGKDQLMRAILNPGNISVAIGLVIWLLPFSLPD
ALTTAISNLSNLTTPLSMIVVGATLAEVPIKGILKGGELWFGTTIRLIAMPLAVFGIMRL
FGFHDLPAKVAAFLTAMPAAAQTVIFAERYQADVPLASRIVFLTTVFSAITIPIFALFLV
*
>SPSA8_v1_60050|ID:41145080| exported protein of unknown function [Spirochaetes Bin 1 SA-8]
MKKAFFSILVLALAISLSGCASMLKSMGGVTKAELAAQEDRLTSKIESTNAALAKTNAAI
AEIDSIKARLEDLSKQVEKATLSALEMEAIKAQLAQITTDLEKISDTTLLNLAKLINDAL
SQTGATESK*
>SPSA8_v1_60051|ID:41145081| ABC transporter substrate binding protein (fragment) [Spirochaetes Bin 1 SA-8]
LAVFLLVETWLIRYFPGFPAKSVFQYNTVEEKMKRLMIRLCGTFLFLALLVSCGKPDTKL
IGIAKFVAHPALDAVEKGIIDEIAKSKPEYKLDLQNSNADMSTAAQIAQRFKQEKVSVAV
GIATPTAQALANQIKNIPVVFSAVTDPVSAGLVPSLEKGGANVTGTSDMTPVRQQLDLLR
ALQGNLKRLGHIYASGEANSAAIAKIVQAYCEENXX
>SPSA8_v1_70001|ID:41145082| protein of unknown function [Spirochaetes Bin 1 SA-8]
MVLDRQIDVGFIGRKVQYAPRTHLNEYLVKKDNLVAIVSSTHPLAGKKSISLDELSRIPL

ILPHRDVLTRRSVEERFHQLGYSVDLAFEVSNTEAIKRMVACNLGASVLCQSSVQKEVEA
GWLSALLVDELDLSRFIYIITRKTEKIPPGLDDFIQFAIASFSGNL*
>SPSA8_v1_70002|ID:41145083|mdh| Malate dehydrogenase [Spirochaetes Bin 1 SA-8]
MSEDITWWSFDEVEAFMKAGFEAVGVPSEEAAVCANVLIAADKRGVDSHGVGRYKPIYLD
RIWAGILNTKTNFEVVRETPTTAVIDGHNGMGHYIAKRAMDMAIEKAEKFGIGMTVCRNS
THYGAAFYYARMAVEHGMIGLTTTNARPAIAPTWGVEPMLGTNPMTWGMPTDEDFPFMLD
CATSVTQRGKVELYDRLDKDLPDGWVIGQDGKYRHDTKQVLVDLTRDLAALTPLGGLGED
MGGYKGYGYAMVVELLSSALSQANFMKALSDLGPDGKKKPIELGHSFLAININAFCDLED
FKHHVGEVCRQLRASKKAPGAERIYTPGEKEHDVWLYRKDKGVPFNAPLKKSFKEVKERC
KLDIELPF*
>SPSA8_v1_70003|ID:41145084| conserved membrane protein of unknown function [Spirochaetes Bin 1 SA-8]
MELINNLLTGFGSLIGIAPILAITAGVIFGIWVGAMPGLSPSMGVALMLPFTFRMPSNIA
LIMLTAVYLASNYGGSITAVTINTPGTPSAVATAFDGYPLAKMGKAGWGLGISLVASVAG
GFIGIIILILFSKPLAAVAVKMHPAEYFMLAVFGLTTVASLGGKNGIKAFAAALLGLLIN
TIGLDPISGVKRFTFGQVNLFDGFDFIPALIGLFALSEVFSRLEEGFSKEKIDYKLEKKK
EQWPTLKDYWKLRRVTVQSSVVGTLIGIFPGAGSAIASFVSYDLAKRTSKTPETFGKGNP
EGVAAAEASNSASVGGAMVPLLTLGIPGSAATAVLISALMIHDLVPGPMLFANQPVLVYG
LFASMLVANIIMLIIGALGSQLWIKVTRIPQQLLIPLIISIAVVGSFAVKNSMFDVFACI
GFGVLGWILKRHEYPMAPIVLGMVLGKLAEANFRRAVIMGGYSIFFTRPASVVLLVLSIL
SMALPLIQTYRDKKKAA*
>SPSA8_v1_70004|ID:41145085| conserved membrane protein of unknown function [Spirochaetes Bin 1 SA-8]
MNAIGRLGSFLNSKTGLLIVAGVFLAYLVIGYIAAKPLAKKHYRGQFVVILALIFISILF
LIITYSFRKSGVVHSGVIPRLWIFGILACCVYLLITIFTGKESPDPEGGSLQRPIIFILV
TLIYIVLMPYLGFFIASFIFLAAAIIMLEYRRWGVILAVSAGWIAFNYFLFYKLLFVPFP
QGIILKLLQG*
>SPSA8_v1_70005|ID:41145086| conserved exported protein of unknown function [Spirochaetes Bin 1 SA-8]
MKRFVFAVLAVMMLLSAFMAGAQAKFPSKPIRLVVYTAPGGLIDITARKMVQIAAKYTDA
TFVVENKAGAGGLIAWEYVLSQPADGYNLFAVTRSNIANLISTGSDMDPFTLDWMALLVS
DPEAIIISTKSKLQTLPEIIADAKARPGKQIWIAPPGIDEYVVTYKFWEKTGITGAFVPFD
SGAQAMAAVIGGQGQVYVGNAADLAGKPDLKMIAIAAEKRLAQFPNVPTLKELGIQGMDM
ESIWRGFTVKKGTPADIVKWYDDLFEKITNDPEWKAFFEKDGMELVHFRKDRFEALIRSD
MVEMKKFLKP*
>SPSA8_v1_70006|ID:41145087| conserved protein of unknown function [Spirochaetes Bin 1 SA-8]
MADVFAKNVATSAEEAAKIVPRAEFRVFGQGVIDIVTEKIWNGKTVLFQKRKMPKETYFL
SIHTNEANVKVRDGLLDIKTKVGETPEGYEIFQPRGKFQFPVKKEDLATILSHLKVSMNL
TQDSYTIEEFIEMARKHPDLTPVAVEKLRFGFTIDGIICEYAQVWFNGAMIESACVESEN
YAGMKLVVESLGLSGMPNTNYLKAAKRVVGMEA*
>SPSA8_v1_70007|ID:41145088| Inorganic pyrophosphatase/exopolyphosphatase [Spirochaetes Bin 1 SA-8]
MSARTNAGGLSGFLQEVRINKENAREVWVVMGNEAADLDSMASAVCLAWQLARKNRQAGT
VFLPFIPILREDFRLRTEAAFLFSEAGLSETDLFFADDVNLEALGKNGTLRLVLVDHNKP
GKAYAAYLPNVAGVVDHHQDEGLFPELKARNIEPVGSACTLVAEKILKNGMSDISSDVAV
LLAGTILLDTVNLDPAAGRVTPKDEAMVSALLPIARKSQQVLFDRLQFEKFNVASLSTFD
LLRKDYKEYTAGAVRYGMSSVLLSLESWKKKDSKLPEELKRYAESRKLSLLFVMNAYTEP
KFTRELVVYSPDSALFKKIDGFLKATDLGLSTVPAPAGSTPDCSGWFAQANESYSRKKLQ
PIIQQFLES*
>SPSA8_v1_70008|ID:41145089| protein of unknown function [Spirochaetes Bin 1 SA-8]
MTAYNIFDLIGGLAIFLFGMIMMNDNLTAFAGTKLRSIMILLTKNKFRGYLTGLGVTVLN
QSSSATTVLEAVLVGAGLMTFQQSLAVTLGAELGSTVLDSCLPSQR*
>SPSA8_v1_70009|ID:41145090| putative Na/Pi-cotransporter family protein/PhoU family protein [Spirochaetes Bin 1 SA-8]
VAIGFFTFILAKNKKQKSLANTILGFGLLFLGMDFMSRAMEPLRHSAVFMSLMSRIEEPI
LGVLVGLLFTMIIQSSGATSGLVIAMAISGAITLEQAVPINLGASIGTCITAILGSLSLN
REAKRSAYIHVVFQTIGVSIAFILLMIPFRNERFYIYLAKEFSALFPGGRNNLARQIAMA
HTLMPMINHVFIFPMLPVITKIFNKLVPPEPPKEVFGAQYLSETMLSEPAIALIQVKKEL

LRMAGIIESMLDKARIMLVDRSLDTGKEIKKADKMVDTLRTEIIVYLTKIAKNPLSEQES
RMQTAYLFLASELENLADVIERNVLDRIKKLVNKDLYLSEEGFSDIMKMSEIVIGHFKLF
VKSLAEEDAGKALEVLKDVENSRELLNELRRKHFQRLNNGVSISLDTTEIHMDLLSHLHR
INKHTNHLAQTLVEMSAS*
>SPSA8_v1_70010|ID:41145091|trxA| Thioredoxin [Spirochaetes Bin 1 SA-8]
VVEHLTKETFLSKVFDYENKKEWEYQGELPAIIDFWAPWCGPCKMVGPVLEALDKEYEGK
IQVYKVNTDEEQELAGAFGIQSIPSLLFVPKEGQPQMAVGALPKSTFQSAIKDVLGVAL*
>SPSA8_v1_70011|ID:41145092|hutU| Urocanate hydratase [Spirochaetes Bin 1 SA-8]
MNLVDFEEFPPDPVFEPGIRRAPRRDAELSPSQTLLALKNSLRYIPPKWHEKLIPEFLQE
LREHGRIYGYRFRPAGRIQGKSINEYPGACIEGKAFRVMIDNNLDFDVALYPYELVTYGE
TGQVCQNWMQYRLICKYLEVLDHNSTLVLESGHPLGMFKSHELSPRVIITNGLMVGMFDN
QKDFNVAAALGVANYGQMTAGGWMYIGPQGIVHGTYNTILNAGRLKLGIPHDKDLAGRLF
VSSGLGGMSGAQGKAAVIAGAASIIAEVDASRIQTRYSQGWVSSVAKTCEDAFAQAIASA
EKKEAIAVAYHGNIVDLLEYAETHGIHIDLLSDQTSCHVPYDGGYCPAGISFEERTQLLA
EDRERFRQLVDKSLRRHFEVIKKLSSKGTYFFDYGNSFLKAVFDAGCKEVSKNGLDEKDG
FIFPSYVEDILGPELFDYGYGPFRWVCLSGNPEDLRKTDKAAMDCINPERRGQDRDNWIW
IRDADKNKLVVGTQARILYQDALGRMNIALRFNQMVRDGEIGPVMLGRDHHDTGGTDSPF
RETSNIRDGSNVMADMATQCFAGNAARGMSLIALHNGGGVGIGKSINGGFGLVLDGSERV
DSIIRMAMPWDVMGGVARRAWARNPHAIEVCGEYNDRYADLGNITLPEPVDEDLVKKLAQ
DEGVI*
>SPSA8_v1_70012|ID:41145093| conserved protein of unknown function [Spirochaetes Bin 1 SA-8]
MAKNRALGQGGKNQNFESLFLLEMKPRIPDNWLMRNDIKIIRELGLGTNFIAAPYLQKGE
SEYSIRNQQLAALGKSPNSYRKLKPFEIESLVRNHNTCSDWSLFLVTDPFVPELINNNVF
SGLIRIGKLERVALEHHDLHLFAGISNSKIIACDIGDNCVISNSAYIAHYIIGDNCILLE
NAEIHASNHAKFGNGIVMEGEDESVRITIDVMNEQGGRWVYPFEDMNCADAYLWARFRDR
PKLMEQFIRMTDASVSHKRGRYGVIGHGSVLKSNRVIKDVMIGESAYIKGSNKLKNLTIK
SSEEARTQIGEGVELVNGIVGYGCRIFYGCKAVRFIMRDNSALKYGARLIHSILGENSTI
SCCEMLNNLIYPAHEQHHNTSFLISSLVKGQSNIAAGATIGSNHNSRAPDGEIEAGRGFW
PGLCTSLKHSSKFASFCLLSKGDYRYELNIPFPFSLIDNDYRHDRLVINPAYWWTSNLYA
LMRNESKFKSRDKRMDKRLNFEYSPFAPDTAQECLAAMALLEELTGESASKQAASGTIAD
PTLEKLIRSEIQQRTADEKPRDAAGQKMIYRKIGRAIILNASDDLPFAVRASSMEHSDRE
CIIYKPVRAWHAYREILLWFAMRTISDYALARGIGSNAYEALAIELAADTWICADTTWEN
IGGILVSDARLNLLLEKTEQGMYPTWAEFHQEYKSLSEIYPLDKARYAWAILQILNQPSQ
DSEETAEKPDSVPRKVLIKALLEAEKLCSSIEAGVTSSRAKDYSNPFRLATYRSKAEMDA
VLGRLEENPFITHTKIEMAELKKAFSYLRELLSHEM*
>SPSA8_v1_70013|ID:41145094| protein of unknown function [Spirochaetes Bin 1 SA-8]
MRMDYTETSMMDNLLSDSDKHALELFYSLETAKGSMRESYAIRLADVLGNPGEFSFYLQC
SEDQRLIRTFHLLRVFRENVELLIHKTWVNESDEKPKQMLLEELKIFIEDYREGRIVSAF
RRFVSIAKAVPALLFGPTGRAPDFLEYAFRIDPKFGLFFWYVNELEKQIRNAQTLEDRES
LYRIETLLGAYILASM*
>SPSA8_v1_70014|ID:41145095| exported protein of unknown function [Spirochaetes Bin 1 SA-8]
MSSALQLAFLLASLFSLTAGTDQQSLKYSIVPRQQEVNTCGFAVAAGILRAATDDVPSVV
PDEAALTAIFAQHDSAGKPETATMLDLLRIFSQHGIAAIPFRGNIEDAVGMLSQGIPLVT
HLKSPTEHFILAARLSPFGMAAADPDVGFQIMPMDDFSARASGFFIALPDLRNNASFLDR
VNKANALELRQMGFFSHLIGGGILQKPAPPGKGVQSGKVQRESRESSLGFSVALFGSTTA
SAAPGLLLPAEAVEVSARLDGRLSSALSYGAVLALIPGLWSRFSASLAFRLDNGHESPRL
KKALLLKAGSALEVRLAEVPGSGYRLFPEPFLGLSSSSVSDASMVSAGFVAGTSLAAGVG
VLPFFQAEAGTILALSADLALQAGLTARLKMEADSGRRFVALYSTCEFGIHIPADRLCER
LPGSLYAGVRISLSPAQEKTNAVIVSMQL*
>SPSA8_v1_70015|ID:41145096|fusA| Elongation factor G 1 [Spirochaetes Bin 1 SA-8]
MELREIRNIGIMAHIDAGKTTTTERILYYSGKTYKIGEVDDGEATMDWMDQEQERGITIQ
SAATTTFWRNHQINIIDTPGHVDFTAEVERSLRVLDGAVAVFCAVGGVEPQSETVWHQAD
RYNVPRIAYVNKMDRLGADFEAVLEDMRLKLGAAPIPVNYPIGRENSFEGVVDLLTMEEI
RWNEDGHEMTRSPIAETRRHEARVYRETLVDALSAHSDVITELYLNGEEIPAQIIKAELR

KACIARALIPVLCGASRRNMGVQPLLDAVIDYLPSPDEVEPAKAFHLKKEENVEVACNTS
GYPLGLVFKVQWDREAGPLCYVRMYSGKLNNASVVYNVTKKKRERITRLLRMHANKSEPL
DSVSAGDIAVLVGLKNAQTGDTIGTEGWPVVLEKMHFPETVISVAIEPKTMGDRDKLKET
LDILSREDPTFMTRENEETGQLIISGMGELHLDVLVTRVIRDFKVQAKVGNPQVTYRETI
TATVSHTEEFKKVFAGKENTAKLTIEAAPLPRGTGNIYRKAVRAPTVPEEIFEAIERGIQ
SAFASGIQFGYPSVDIGITLTDIEYSELTSTPFAFEACANMAYDAACRAAGPVLLEPIMK
VDILSPKDFVGEVMSLVSQRGGLIHGSDSKAGSEVIHAEAPLSTMFGFTTSLRSVSQGRA
SFSMEFSHFQPKR*
>SPSA8_v1_70016|ID:41145097| Peptidase U32 [Spirochaetes Bin 1 SA-8]
MTIYTKRETFFNSNGRKFLPNDEICRLAILKPEAKTFYKDSVKRVELLAPAGSPEALDAA
LGEGADAVYLGLRSFNARMRSSNFAFNQFEAAVQVCHDKLKKIYVTVNTVFEEREADRMY
QLLEYLDRIKPDGIIVQDLGVVKMVRDHFPGLALHASTQMNVASSRGTNFLSRNGFKRAV
LARELTLEEIRTIRQNTSLELEVFVHGALCVSASGLCLFSSYLGGKSANRGTCTQACRRL
YESETGKGYFFSPDDLQLIDYIPDLIEAGIESFKIEGRMKSAEYVGTVVSAYRHLIDSWQ
IDKERALVKAKALLQSDFARAKTSYFMPGGNPDYIHPDQAGGTGIHLGRIRDIRVIDDER
WALMASFDGLSEGDSIRIHRSDDSGRITAKIKGVKPQINGMLLKIEGDWRQNDDVYLIQT
ASMARRYKPVLPNNLDRFRSFPSRHLAPKPQFVMPVSSKKNSLPDGFYTLIGKVADLHAV
LAVRPQKAMILFDRLNAESMRKNEQTLPYKRERLVLWLDPYLPESDASWLEPELDYWISR
GVKQIVVNNLGHFSMLRGKDVELIAGPWLYAFNTWSSAFLFDQGVKYLIPPMEISKQDLQ
KLVDAFHPGVVLPIVFSYPSLFRIRGDLTARYDSKRFMDRDGLTYTLSGRRDYNVLVPDK
PFSILDRTPFLRKEGFTKFILDLSNADSTRGLFRDIAKAADEAKILPNASRFNWKDGFYN
EENASKA*
>SPSA8_v1_70017|ID:41145098| putative HTH-type transcriptional repressor AllR [Spirochaetes Bin 1 SA-8]
MSESAGKALDILFYMAKAGDEVSLARMSKETGINKATALRYLSVLESRGVVERRPGGWSL
GLSLFELGSRVPVRELVVEKVRPILDRLARESGESSNLACLAGDSAIYLDRAEANRSLRM
RSAPGDRLPLYCTGVGKAILSQLPEERIRAILGPGPLPKINDSTLTEPEDVIREAQRARE
LGYGVDREEYEIGLTCYAMPLQLPGSDFAGAISISGPSARMRNPEAKERFLNLLKEAKEE
ALKAISPYDYDQNTTAPSVSSISSII*
>SPSA8_v1_70018|ID:41145099| Single-stranded DNA-binding protein [Spirochaetes Bin 1 SA-8]
MNPLNSILVEGNLVRDPITKTLASGNQFCGFTLATNRSYKLNGETRETEVSYFDVETWSR
LATACSQYLKKGRGVRVVGRLKQDRWVDQEGKPRTKIKIVAEHVEFKSDKQKDKEPVKPG
SLSDQIMDEIEETEGAVVF*
>SPSA8_v1_70019|ID:41145100|purB| Adenylosuccinate lyase [Spirochaetes Bin 1 SA-8]
MNDFSGYLSPYSWRYGTPQMRRIWSEEGKRRLWRSIWVSLARVESEFGLVDGKLADELEA
HAGDIDLARSFEIEEEIRHDLMAEVKTFAEQCPNAGGIIHLGATSMDIEDNADVLRMRES
LRLVIEKLKGLLGIFADTIDRTAGLPIMAFTHIQPAEPTTFGYRCAMWAQDLLTNLEDLE
SVLASLRGKGFKGAVGTAASYSELIGPERVEQFEERLAEELCIQFFPIATQTYTRLQDYR
VVAALAGLAASLHKMAFDLRILQSPPVGEVSEPFGSRQVGSSAMPFKRNPIDAEKIDSLA
RLLSSMPQVAWSNASLSLLERTLDDSANRRTILPESFLIADELLKTAAKIVSGMVLNTEA
MERTFAVYAPFACTERIMMALAKKGGDRQVLHEKLREHALSAWAAIKSGQDNPLFGMILE
DAFFSELAPRSELEXLMDVRTYVGNAPERARKFAQKIKDXVRDT*
>SPSA8_v1_70020|ID:41145101|purL| Phosphoribosylformylglycinamidine synthase 2 [Spirochaetes Bin 1 SA-8]
MLRNAQENSLKKSRXLFGILDEDADWAVIMPMEIGQNIFRFTIKELQPGGKSSAVIVRNA
QRIGLDKVCAVHESDMYFVRGNIAPEELDLLGRFLFSDPVLTAFSVEQLPCKAEPDVQYI
EVGYHVGVTDSVAEEIIRAARELGVTGVDAAATGKRYTVSCRNGTSLKQEELLRLAEKVL
VNPVVQRYSIGPITPAFPDESAPRIPAELFNIHEMNDEELSALNLRQRSALSMEELHAVR
DYFRAEKRPCTDAEYEMIAQTWSEHCIHKTFKAKIRVEGGDADGLPPVVDNILLTYIKSA
TDAISAPWVVSAFKDNAGILAFDEEHEISFKVETHNHPSAIEPFGGANTGVGGVIRDILG
VSAKPIAVTDVLCFGLDDRKDFFLANQSLQTLHIQDGVIAGVQDYGNKMGIPTVNGGIHN
HEHYTSNPLVYCGCAGIAPKGSHPSAQKIGDRIFVIGGKTGRDGIRGATFSSMTMNNSTF
EHAGSSVQIGAPLVEKKVTEIILAARDKKLYSAITDCGAGGLSSAVGEMTSDLGGEIEIA
KVPLKYAGLAPWEIWLSEAQERMVLAIPAENTTEFIELCESNDVEYCDIGYFSGTGRLVV
RYYGDVIIDLDCSFLHKGIPQRHLIARPAIRTAVNDEAAILKKALLEDAYRSEGNIFFEN
LLISVLKHHAVHSKEHAIRLYDHEVQGATVIGPFAGYSQTGPSDAAVLAPAEVKSGKGIA

VSNGFNPRYGEADSYNAAFSAFDEAFRNAVACGADPERIAVIDNFCWGDPKNPENMYTLL
RSAKACRDAALLYQSPFVSGKDSFNNEFVDEKGNRRAIPPSLLISAIGLVPDVHKAVSAD
LKETGNSIYLVGEFRPELSASVAAQLLDANFSEQTADGKTHVDFSAGTGPSPSRKAAQVY
RALHRAMQNSLIASCHDISDGGLLTAIAEVCIAGERGASISLEKLKLSGSAWDIALTLFG
ETNGSLVVEVRREKEEDFLKEMGENECVKIGTVGKLNQNLDLRITSQALLSFSLDAMKTA
FHVQSIAGGVR*
>SPSA8_v1_70021|ID:41145102|purQ| Phosphoribosylformylglycinamidine synthase 1 [Spirochaetes Bin 1 SA-8]
MKPKALILHVAGINRDNDVCQALSLAGADPHIVHVNQLKTEKKLFANFQMLIIPGGFSYG
DALGAGKLFALDIASYFKEEASAFIASGKPVMGICNGFQTLVKSGLLPGGESGTQKSCTL
THNEHNRFECRWVSLSLPKSSCIWTGGITRIVRCPVAHGEGRFLTADNAVLQNLRNRGQV
ALTYVQSNGAPAEGKYPDNPNGSIFDIAGICNEAGNILGLMPHPENNIFEGRNGSDGSGL
LIFENGVRYAAQI*
>SPSA8_v1_70022|ID:41145103|purC| Phosphoribosylaminoimidazole-succinocarboxamide synthase [Spirochaetes Bin 1 SA-8]
MVNREVVETALHQPFKGLMSSKSEVLSGKVRDWYSLPGKQRLLVTTDRLSAFDRNLGFIP
WKGQVLNQLSAWWNEKTQDIVPNHLISMPDPNASLVHEVAPIPVEVIVRGYITGVTETAL
WYRYSLGERHIYGYDFPEGLVKNQKLPKPIVTPTTKGGATGHDERLTCSEIVEKGIVEPG
LWKQIEDTAFALFERGRELAEKAGLILVDTKYEFGLDDEGRLLLIDEIHTPDSSRFWKKS
SYEDRLSKDLEPESFDKEFIRLEYAKMGYRGEGEIPQMPDELWVTASMFYQSAFEMLTGN
TFLPGEYPVEPRLMKNLQKAGIIV*
>SPSA8_v1_70023|ID:41145104|purE| N5-carboxyaminoimidazole ribonucleotide mutase [Spirochaetes Bin 1 SA-8]
MKSCKAIILMGSRSDMPHAEKIAEKLDRFGVPYVFRIGSAHKVPLYVLDIIHEAENESQD
IVFITIAGLSNALSGMVDWATRFPVIACPPPPAAFAGSDIFSSLQMPPGVAPAVILDPLN
AALFTAKILSFSSPDIALAIRSYREEQQAKLLNDDAETRQKRNGSN*
>SPSA8_v1_70024|ID:41145105|purF| Amidophosphoribosyltransferase [Spirochaetes Bin 1 SA-8]
MTMLRPDRNAMGPIDFYTGPREECGIIAVYDPQSEAARTAFYGLFALQHRGQEAAGIASY
NERMVHLKKDIGLVSQVFSEEDIRKLPGRLVIGHNRYSTTGIPSAKNAQPFVVTGKYGPI
GIAHNGNIVNAQELRRELLDKGIGLTSTSDTELMLMSLGLASGKTWVQRIRNAIKKWIGA
FSFVLLTEEGFYAVRDSWGFRPLSYGKTADSAFVAASETCALSTLGCSEYFEIPAGAILF
CSNSGEFEVIDSGLKKKPSACLFEYIYFSRPDSIWNGLSIHLVRRKFGEELAKVAPVDAD
MVIPVPDSSISAAIGYAHESRIPYGEAFVKNRYIGRTFIEPTTTLRQKGVRMKFNVLKDS
VEGKRVIVIDDSIVRGNTIKPLVTLLRNAGAREVHVRVASPPVKYPCRMGVDMGESEDLV
ANRLDAQELARWSGAVSLAYLPLESIRNVLGIYSACCTACFSGDYPFKVGTNNSKNQFEP
*
>SPSA8_v1_70025|ID:41145106| phosphoribosylglycinamide synthetase phosphoribosylamine-glycine ligase (modular protein) [Spirochaetes Bin 1 SA-8]
MKILLLGSGAREHALAWKIKISPKCDMLWCAPGNAGTAEIGTNIALDIANPAEVAEFVEK
EQINLVVVGPELPLAAGVADTLSALPADSRPLVFGPVKAAALMETSKVYAKKFMRAHGIP
TAEFRVFSESDKALSFIESAPWPFVIKASGLASGKGVFLPENTEEAKHIIKNLLDDKALG
DAGSEIVIEERLFGEEVSLLGFCDGYTVLPMPLAQDHKRLLEGDCGPNTGGMGAIALGTQ
DSLRQAQEWAGKFLLPVIAGLQEDSVHYHGVLYAGLILTSQGPKVLEYNCRFGDPETQAI
LPLLDSDLIELLLTACAEGRLAEAKPKWKDEASASVVLASSDYAQDTKAFCDSIIRDFGGT
EKNAVIFYAGARREGDVIRAHQGRILCASGWDVSLSLALEKAYARISRIEIAQGRYRKDI
GFNRQFLHNLEKKEGAAASSLAPSSEAYRSAGVDIDAGDRAVALMSAAVKATYTPHVLAG
IGAFGGVIDAEAFSAVENPVLVASTDGVGTKVKLAAMRGSYTTIGQDIVNHCIDDILVQG
AKPLFFLDYFAASSLKPEMVAQAVSGMAEACKASGCVLIGGETAEMPGVYEKGEFDIAGT
IVGVADKGKLLPAGNLKAGDMLIGLPSSGLHTNGFSLARKIFSEQELFAPFADTGELLID
ILLKPHRSYLRYLEKILFAAENPIKALAHITGGGFEGNVARILPDSLSAVINTSRWPIPP
VFAAIAEHGNIAAEEMYRVFNMGVGMVAVISPEQFEAVRASIDLDCWNIGHLQPGNGKVI
MQYGKAGRR*
>SPSA8_v1_70026|ID:41145107| Phosphoribosylglycinamide formyltransferase [Spirochaetes Bin 1 SA-8]
MKLVVLVSGNGSNLQAVLDAIKDGRLASTVELVISNKQDAFALERARQNRIPFLAFPSRS
SLPRETYDSILARTALAFEPDYILLLGWMKILTSVFIDNFPGRIINLHPALPGTFPGTHA
IERAWEAYQAGTIHVSGVMVHYVPDEGVDSGPVIGKKEIAMTGIKTFEEFERITHETEHE

LIVATLQKLEKNWNKLCGKEIQA*
>SPSA8_v1_70027|ID:41145108|purH| fused IMP cyclohydrolase ; phosphoribosylaminoimidazolecarboxamide formyltransferase [Spirochaetes Bin 1 SA-8]
MKYALISVYEKTGLEKLAKTLTGHGWNILASGGTAKLLSAAGIRVREVSDYTKSPEILAG
RVKTLHPAIHGGILARGTEEDRLQLENLGFEMIDLVVVNLYPFEKTVAIPDVSETEAIEQ
IDIGGVALLRAAAKNHDRVTVVSDPADYQVIIDIVEAGKEIPAELKKKFALKAFRQTASY
DTAISDWFAKQVGGESSRTFTLKGYHAKTLRYGENPHQHAEYYSAEPGAGIFGGTVLQGK
ELSYNNILDLDAAWQAVNRFERPAVVIVKHLSPCGIAQAPLSAAGNAAENAPDDASLVSA
LAQAIACDPVSAFGGVIAVNREFSGECARNLGSLFVECIIAPAFSEAAIEILGKKKNLRL
VVPGACKPQNEIRTVLGGFLRQDIDDGDPENATWTVVTKRTPTAEETAALGFAWKACISV
KSNAIVLANTNATVGIGGGQPNRVDSVRIAVTRAGERARGSVLASDAFFPFPDSVEEAAK
AGITAIIQPGGSIRDQEAIAGADSAGIAMVVTGVRHFRH*
>SPSA8_v1_70028|ID:41145109|guaB| IMP dehydrogenase [Spirochaetes Bin 1 SA-8]
MNLQSEKLFESGMALTFDDVLVVPSYSELLPSQVDIRTRLAKDIVLNIPLISAAMDTVTE
SSLAIALARMGGMGIIHRNMSPEKQATEVDRVKRSESGMILDPVCLEKNASLAEARELMQ
KFKISGIPIVQGEEKKLVGILTNRDIRFCKDDDFKKPVSEFMTSDKLITARVGTTLDEAR
DILQRYRIEKLPLTDEAGCLKGLITVKDISKKADYPNAALDKLGRLLVGAAVGVGADLEK
RIQLLSSRNVDAVVIDTAHGHSRGVIEAIRRIKAVVPGLPVIAGNVVTAEGVRALAQAGA
DAAKIGVGAGSICTTRIISGAGMPQLSAIYECAKEAAKFDIPVIADGGIKYSGDMVKALV
AGADCIMLGSLLAGLEESPGELVLYNGRQYKTYRGMGSLGALQGYGKDRYATGLGGGEKL
VPEGVEGMVPYKGKLSDFVFQLIGGLRSGCGYAGARTLQDLKQKTGLVRISNAGLLESHP
HSITITREAPNYQRGDF*
>SPSA8_v1_70029|ID:41145110| putative tolQ-type transport protein [Spirochaetes Bin 1 SA-8]
MIDFIIQGGAVLWVIMALSIVACAIIIERLLYLRRISVDEDKLFLRIKASLLEGHYNEAL
AICDQNLSPFSTLLKVGIENRHQPEQTQRDILKDAAALEAPKLERGLSALGTISHISPLL
GLLGTVTGTMKAFGVLGKFGAVSDPAALASGVSEALITTVGGIVVAVPVVIFYNYFVTRV
NLILTKLENQVNTLVLLINAANNGNGGKGTSEEA*
>SPSA8_v1_70030|ID:41145111| conserved protein of unknown function [Spirochaetes Bin 1 SA-8]
MNSRRLSPKISIDMTPLIDVILQLIIFFMITTTFRAAPGISLDLPGSSTAQSVSSSALQV
VVLSEDEIYVDKTRTDLKNLSATLRQRVAGSDGTHIRAVLEGRAGASYQLIISVLDAFRF
NGIENVGLITRKEKMVQ*
>SPSA8_v1_70031|ID:41145112| conserved protein of unknown function [Spirochaetes Bin 1 SA-8]
MKDLAKPSASFLEREDRKRRLTAYGLTILLYALVFGFGIFIGILSPKEQAFSNSTVVINL
AGPEKPEIGLGSVAPSEKGEEKKLEENPSPPKPAVESPKAETPKTKTETAKSVEKTVQPP
KQPANPSTSMVSDSTEPLSTAKTPGPSSSTVQSETAPAEPWVPGQRGPGSRVSSSNSAVN
VPGQGEVPWSQGNSVQISKKEKGNSSETSLGASQGTVGHNIYVPVFYSLPLPRTVPAYIY
ESIPDLVQPPNTILYTAQARKKAFSNFYVREGDLFRLKNDVPLDYREQLWQILEDAGYDV
MNADYKQDKNLMPVVIGFTVTKDNQMKGVEILQSSGDPEIDRSVVYGFKRAAFWNKTGET
VPGRFTYRF*
>SPSA8_v1_70032|ID:41145113| Response regulator containing a CheY-like receiver domain and an HD-GYP domain [Spirochaetes Bin 1 SA-8]
MKAPALRSILIVDDDPAIQKVLSIGLGRLGYHIHVAQNGREALNYLESGDYDPDCILLDI
RMPVMTGREALPKIREIKPITPVIMLTAFNDLATGLDAMKNGAFDYLVKPSRLEHISETI
EKALKYRDVVAEKAEQDRKNEAYRLSLEKKMQSKAIELNDTYMKLKAANMQTVRALAETI
EAKDQYTQGHCERVRKLAVKIAAALKLPAEKIEPLEIAALLHDIGKIGIPERILNKQGAL
DPDEVDIIKMHPIIGAQILSTVEFFLPAINAVRHHHERWDGNGYPDGVAGEDIDQLARII
TLADTFDAMAMSQPYRKALPLDGVLREIQEESGKQFDPEAVRVFFSERLYQDYEDAAPRQ
AE*
>SPSA8_v1_70033|ID:41145114| putative Phospholipid methyltransferase protein [Spirochaetes Bin 1 SA-8]
MVLIFIILAGFLLLIAADQLQIRKKSILSRNVRRAGYLLVLASFVLIALSSTLDTRPSLL
FLGTAIFSGILLLWSVFFEFDIERKKHDIPENGLVTTGSYRFCRHPGFWWFALFSLSLGF
LRGISAYFFTILLMNLMNLLLIFIQDRYIFPKVFQGYDEYRKQVPFLVPRIPKAMRKP*
>SPSA8_v1_70034|ID:41145115| putative Multi-sensor signal transduction histidine kinase [Spirochaetes Bin 1 SA-8]
MILDASTVIPALTFCIYVPFIIFGLSSKKEKINFSFLIYMGFMALWSFGSFMMHANTKLL

TPLFWNKVMLVGLLGGPIAILGLMIYLSETNKRRYRIFMYVGYGIYLFLLYLNLKGTIVT
DAGFDAEGFFYKLGNGAVIAYSLSYFYLIISIALIAREIAVNENKFKRKTLRLVAGGAFI
MLAGVAVNLYGPLGKYPIDLAAATINAAIIFFAVYKYRLVHYSSVVLKILLTIFVSLLSG
LIFLFIFIPIFHLDRRVPFQQLTLISLILGIFSSVVLSPLRTTTLEFLERLYGGKTFAYY
QNLRNFSASLTAIVELEKLGTLTVERIMATFNLEWAVMFVNDYGARNFKLNAAQNLPFGN
TIISGYDESIILQRGDELIRKYLQHSGGNKKTADYITVPQDTITINLERNGSRESVNASL
ILPLRFKERLNGFIVLGPRTDKDYYNQFDLEILQLLGDQCSVALENAITFERLRQQQKRL
QHINNELVISRNKLEAFFDGITTPISIQDINYNIVAANYAARRYFEKSAEEIIGSKCYKV
FFDRDRPCLECMAQDCLHTKLPFTAERQDAKGIITFSLNFYPIPVPKDSQLIFLEFFQDI
TKQKTLQEELIQSEKLAGIGTLVSGIAHEINNPLGGILGTADLILSEAEEGTAIREYAQD
IIRYAQNAADVIRDLMTYSRKNRSSSEMINIVNVLENSLKMAMRGIDFRSVTVRKNYNQT
DMIQANAIEIQQVLLNLIVNAVQAMNGDGVLSLDVKQEDSDIIIVVQDTGKGIEKENIDK
VFNPFFTTKEPGVGTGLGLSIAYHIISKAGGRILLDSEAGKGTTFTIILPAANQERNRIR
FIHAKETRQLEDSFFIQRKVLVGEKGYQEETIRRKEDESAFHVVAYKGLQPVGTCILHMS
TDEGGIPIEANFDLSGFKDEYPYAEIDRLAVVREERGSIIPFSIMTLAYLYGRARGAKKI
FLDVFSDENKLIKMYEKLGFKIIGSYNKPLSCTVMMMDHTSQYETEASRMEHFVKPFFSR
LVPKIDFTGEDRDHVMRVINAIKAKFPKDKDQSEES*
>SPSA8_v1_70035|ID:41145116| Oxidoreductase domain protein [Spirochaetes Bin 1 SA-8]
MEKIRFGLYGYGKVAQLHATALASLDNAQLVAVCGRNLQKAHDFACRWNIAARSSCEEMV
QLDKVDAVLITTPHPLHAEHAMEALRAGCHVIIEKPMALRVQDCDAMIELAKDRGLTLSV
ISQRRLYPATQRIKKAIDDGLLGTPMLGQITMLGWRDKAYYESDPWRGSWAMEGGGVLVN
QAPHQLDLLSWYLGPVAEVAGFWANVNHPYIEVDDSAVAVVRFKSGALASIFVSNSQEPG
IYAKVHIHGSSGASAGVQTDGGAMFIAGRSGVLEPPVNDLWTIPGQQHILEQWKQEDSAF
FSTIDATWYFFARQIEDFSSAIMHGKKPMVSAEESREAVKILEGIYRSHASGSIIRF*
>SPSA8_v1_70036|ID:41145117| Alcohol dehydrogenase GroES domain protein [Spirochaetes Bin 1 SA-8]
MKALVLTAPSTFEYVDYPDPEIRNPDDVLIRIRAAICGSDVHGMDGSTGRRKPPVIMGH
EAAGEIAAVGSGVTGFKPGDRVTFDSTEYCGECEFCRRGEVNLCSNRKVLGVSCDEYRRD
GAFAEFLVVPQRILYHIPESLDFTGAALAEPSAVAAHAAAITPLKMGDTIAVVGSGLIGL
LLIQILRAFSPGLIVAFDPDRERLRAAEKAGATAGFDPSNPAELEAALRLTGIRSFDRVF
EAVGATAPIQTALAAVRKGGSITLIGNVSPMIQLPLQSVVTRQISLLGSCATAGEYPLVL
DLLASGKIDYASVVSAAAPLSEGAGWFRRLHAREKGLLKVVLKP*
>SPSA8_v1_70037|ID:41145118| PfkB domain protein [Spirochaetes Bin 1 SA-8]
MKTLNISESPGARDFAALGALVTRLDPGLIPFSFADTYQVHVSGGEFNVAANLARCFRKN
TAVVSAMVDYPIGEKVESEVRKMGVDGIYRRFKHDGVRGPNIAQVWSDQGKGVRPPVVFY
NRANEAAAQLKPGDFDWDSIFSRGIRWFHSGGIFSALSSTTPELVIEGMKAARKAGAIVS
FDLNYRAKLWAANGSSTAAHEVLGRIVDEVDVLLGNEEDLQMGLGLKGPDIHSASKLDPE
AFYRTIEEVSQRWPNIKAVATTLRDVKSTNRHLWSAVLWLGGDRYLAPTMELDVLDRVGG
GDGFAAGLIYGFLEGMSPQEALNIGWAHGALLTTFPGDTTMASLEQVIALSKGGSARIQR
*
>SPSA8_v1_80001|ID:41145119| protein of unknown function [Spirochaetes Bin 1 SA-8]
LRRKQTMVDSFVTIAGIAMFYVVAGEGVPIVYIHGNTGSSRWFSRVMX
>SPSA8_v1_80002|ID:41145120| putative hydrolase [Spirochaetes Bin 1 SA-8]
MPTAEIQGVSFYYELSGQGNETIVFLNGIAMSVAHWAPYVQAFQGEYRCLCHDMRGQTLS
EKPEGEYSLDLHAQDLAELMDMLGIARAHLVGTSYGAEVAMAFAVRFPEKCLSLTLIDGV
SETDPLLEAIVDSWIQAAEKDARLFYRTILPWNYSPGYIGANRELLAKREASVTNLPREW
FEGFNRLCAAFLKINLTESLSAIQCPTLIMVGEKDILKPYKFSKIIEQRIKGSQLIILPG
AGHAAVLEEPALVQLHLRNFLAAR*
>SPSA8_v1_80003|ID:41145121|braG| High-affinity branched-chain amino acid transport ATP-binding protein BraG [Spirochaetes Bin 1 SA-8]
MLKIQGLECGYGRMKVIDGVSLEIGNESVGLFGPNGAGKTTLINAIMGMNKPWKGSIELD
GTNIVGLETHVMARRGVALVPQERELFPGMSVEDNLRLGAVYIPHAKDAIQEQMESMFSL
FPILKERRKQLAGTMSGGQQRMVAIARALMSKPKLLILDEPSLGLQPSIVSEVFDVLKEL
KKTVSILVTEQNVRESLKSIDRGYVLENGKIVLEDTAKGLETNPHVLKSYLGL*
>SPSA8_v1_80004|ID:41145122| Branched-chain amino acid transport ATP-binding protein LivG [Spirochaetes Bin 1

SA-8]
MTKLETANLTRRFMGLVAVNNVSFKLEQGEILGIIGPNGAGKTTFINLVSGIIMPSEGTI
TFNGRDITYMPAHERARLGIARTYQLIHPLENLNLVENVMIGSIFAKGSSLKEARRKAER
LCEEMGLTNLERDTAKLTILEIKKMEIARALANEPEVLFLDEVMAGLNSDETKDLIATVK
KIAAERKLAVGVVEHVMGVIRELTNRVIVLESGELIAEGPYEEVSKNPRVIEAYLGGAA*
>SPSA8_v1_80005|ID:41145123| Branched-chain amino acid transport system permease protein LivM [Spirochaetes Bin 1 SA-8]
VNRNLSRIIALSAGAIAIXLPLFAGDYPLQVARNVMMYMXLAIXWXMLLRSGQISFGIXG
LFGLGAYAGILGMIRGGMPAWLSIPFAAFVAGFAAFLIGFMILRLRAMYFSIVTLALGEI
FRIIIHNLHDFTGGPEGIVIQQGVIFGGSSSKLYWLTLGGLAVAIAASYWFEHSKIHFAL
TAIRNNETSAKSSGIDIFKWLLVAFVVTSAIQGLLGGIFVQSYGFATPETVFSADFTLLP
LAMALLGGVYSTVGPIFGAILLGLAAEWLKLKIPYGHLVVYGIIIILVILFMPNGLLGRI
RQTTGKRAGRTA*
>SPSA8_v1_80006|ID:41145124| High-affinity branched-chain amino acid transport system permease protein LivH [Spirochaetes Bin 1 SA-8]
MMAPKQRTAILAGLAVIGTIALLAWKPTVVIYGLQAAGLYASIAIPMGLILGIVHIVNLA
HGEIMMVAAYATYFVCKALGMDPLLAIIPMALVMAAFGWVLFQLTIKRALKAPELNQLIL
TFGIAIALSQSVNLLFTSQTYKLQLDYVSSSIDIGELSFGTWSFVFVAVAILFAYGLKYF
LTKTTTGKAALAVGQNPRGAAIVGIDVYRIYALVFGLAIAMVGGMGALFLTKSAIFPSVG
GPFTMKSFCLIAMAGVGNIPGILGASLLLGISENILRAFRGTRAWADIVFFVLIIVVIMS
RSLKGRKS*
>SPSA8_v1_80007|ID:41145125| Leucine-, isoleucine-, valine-, threonine-, and alanine-binding protein [Spirochaetes Bin 1 SA-8]
MKRLMTVLAVLLLLGGSVFAQNTIKIGGIWTLADITGKQGSAAAQLAVEEINKAGGINGK
MLELIVVDDEGKADKAAAAVEKLATVDKVDVFVGGMGSGAELGKIPAFKKYGKVVMSTGA
AGSATVEKALGPSPESDFYFHLHPWDYNQGASYAEGWDAIQKQYPXIQIKKIFLAYEEGA
FGKSSWDATKVLFGSDKRYTIDGAPFKSALFGGGDYTAVLESAKAFKPDLFLWAGYDADA
LPLLEQSKAMKFTPGIYLGAPPGWPIGFGSSKLSRNVMLYGMWSPAMNENNAASAKFYKA
YVAKFKEEPATYFAPLAYSAIYILADGIKAAGTTETAPLVKALEATKYASPLGELITFKP
SNIIKHQGITRQKILQWQNGFQEVIWPFEAATAEPVYPFPAWK*
>SPSA8_v1_80008|ID:41145126|fabG| 3-oxoacyl-[acyl-carrier-protein] reductase FabG [Spirochaetes Bin 1 SA-8]
MERMKDKICVVTGGARGIGRTIVDRFSDEGAAMVFALDVNAAGFADLEASGRKNVRPTVV
NVTDTAAIQAFVDAVMAEFGRIDVLVNNAGITRDALIQKMSDEDWDAVINVNLTGVFKMT
RAIAPHMMNQGVGAIVSISSIVGLDGNIGQSNYEATKGAVVTMTKGWAKEFARKGAKVRV
NSVSPGYTRTPMIETVPEKVLEPIIAKTPLGRLAMPIDIANAVLFLASEEAAFITGQVLR
VDGGLVL*
>SPSA8_v1_80009|ID:41145127| 3-oxoacyl-(Acyl-carrier-protein) synthase III [Spirochaetes Bin 1 SA-8]
MHVGIAGIGIYIPPQRMTAGDLAVMTGVPEDIIAQKFGVKSKPVAGPEDSTADMGYKAAL
KAVEEAKIQAEDIDLVIWYGAQHKDYPCWLAGLNVANRLGAVKAWSFDMEAMCGSMMAAL
DLAKSLMLTRDDLETVLLVSGYRNNDLIDLTNPQTRFMMDIGSGGSACVLRKNLGKNVVL
ASAFRGDGSLSEMCVVPVLGSKKWPPSAEDATHPYFVVPDEEAFKKKLGEVTMPNFYAVI
RESMKKSGFEEKDIGYLAILHFKKSAHDAVLAELGLKPEQSTYLDEYGHIGQNDQLLSIT
LGLAADKIHDGSVVVLVGAGLGFVWAATTVRWGPWNE*
>SPSA8_v1_80010|ID:41145128| putative Transcriptional regulator [Spirochaetes Bin 1 SA-8]
MENSSSKERLILSSIDLFSRKWFGTVSVAEICRNAGLSNGLFYKYFKDKEAIFKEILDLV
IAKISSAVAQVAGESALERLTSFIDIIYSFSQENTALVKVFREGQYRYFEYEKRLKTVYE
RALFFIFKAEPDIADYLFGLGGLRFASIRAAFHQIPVDLKRLLAILKNGIFHGLPIDEKK
VFSTSITPLPLQIMPDGRERLLIEGKTLFGEKGYFDTNIHEITDKAGLSTGAFYTYFDSK
ESFYSELIHRVGREVRHFITINLPPGLNSLERELRGLWLFIVYLSIDRTCYGIVREAEFV
LPAEVKIYYDAFAEGYRKHQDPGIAALDPTGIEFLLGVAHYLGIEVIFDESPANARNVIL
QLGSYYQTGFSGKFEL*
>SPSA8_v1_80011|ID:41145129| 3-oxoacyl-[acyl-carrier-protein] synthase, KASIII [Spirochaetes Bin 1 SA-8]
MASVRIAGTGLYAPGPAISNEKLKALTGIEFDSAKHEEKLGIKRRHIARLSGLDETTADF
AEKAARQALANAQIDANDVGMFIVATDTPEYISPATAILLQGRLQGAERESRAFDVGASC

ASFVTALDICSRTMLSDASMRYAVVVGVYNMPAFVRPNDPFGWSIFADGAGAVVLERVAG
QEPGFIESAFRADGTQWNYVGVYAGGTRRPVTKELLDAGTYGLELLQRLPGDRNIKLWPP
LVRRLLEKAGVEQKAVSHFIFTQINQSVIAEVMSLLGEPMEKTTTIMDEYGYTGSGCVPM
ALHTALQKGTVKKGDLVVLVASGAGLAVGAVLIRM*
>SPSA8_v1_80012|ID:41145130|cat| 4-hydroxybutyrate coenzyme A transferase [Spirochaetes Bin 1 SA-8]
MNWLDLYKSKFISIPEAVNKIESNQDVIVAMCASEPQGCMSQFHTVADRVKDVRVFSCLT
LKPYDFYMKPEMRGHFELASWFHAPGSRAAIKAGTGTVTFVPNMLHRSASDRIHAHQPDI
FYGTCTPPDKHGFVSLSLGITYEKDILEAARLVILEVNPRLPRTLGDTQLHISEVDFFID
NEQEVPELPAPQPNATDLAIGQHIADLVEDGSTIQLGIGGIPNAAALALREKKELGVHTE
MFVDSMMELYEMGVITNRMKTLKKGKFVATFAMGSRKLYDWLDDNVAVEFQRGKWVNDPA
VIAENSKMVSINTCLMVDLTGQVASESLGTVQYSGTGGQSDTAQGAVAGKDGKGKSIIAC
YSTAKNGTVSTIVPVLPEGTAVTLHRSFVDHVVTEHGIARLRGKTVRERVKELVNIASPE
FREGLLAKARELGYI*
>SPSA8_v1_80013|ID:41145131|pckG| Phosphoenolpyruvate carboxykinase [GTP] [Spirochaetes Bin 1 SA-8]
MQFPLNLRNRNVKDWLEKMVALCKPDFVYWCDGSEEEYATICQALVKSGTFLPLNPEKRP
NSFLARSDPSDVARVEDRTFICSKNKVDAGPTNNWMDPKEMKAKLRGLFDGCMRGRTMYV
IPFSMGPIGSPMSHIGIEITDSAYVVANMKIMTRMGSAVLDALGENGEFVPCAHSVGMPL
EPGQTDVPWPCNKEEKYIVHFPEERAIWSFGSGYGGNALLGKKCFALRIASVIARDQGWL
AEHMLILGVESPEHEKTYVAAAFPSACGKTNFAMLIPPEEFKGWKVTTIGDDIAWIRPAK
DGQVRAINPEMGFFGVAPGTSAKSNPNALASIRANTIFTNTALTPDGDVWWEGMTEEPPE
GLIDWRGNPWDPASGKPAAHPNARFTAPLTQCPSLDPEWNNPLGVPIKAFIFGGRRSTVY
PLVYQSFNWAYGVYLAATLGSETTAAAAGEVGKVRRDPFAMLPFCGYNMAAYFNYWLRFG
RNIRNPPRIFSVNWFRKDANGKFLWPGFSQNIRVLKWIVDRANNHGKAVESPIGWMPRYE
DMCWDGLDYPKEKFSELMAVDRQLWMNEILSHDELFMKLYDRLPKEMIFIKELMLSSLWR
SPEHWEFLQCDFEPTED*
>SPSA8_v1_80014|ID:41145132| protein of unknown function [Spirochaetes Bin 1 SA-8]
MTRQEYIQRLEAGLSGFDAETRRDLLLEIEDHIDTLSASRPEASESEILACLENPENLAR
SLCLEAGIKPEETSRTSTEDKKNKGPRITIDGQDLDEVIRRALDIARMFGTKRKTEAQFD
VQSAVAGKARKVSAAFRSADVQVKLSVDGIKVMSDKDGPGISIINHDNQQVEIRSASPKE
DPDLVELWIPADVDELKIVTSSGDIRILDRVGDLLLQTASGNLTIDCCSGNVTARTASGD
IEAARCSENTILETASGDIQLEIDEQANSVKIATASGDVSLRYPEDFDAVITYTTVSGEI
DHDCMQTGNRTLKIGQGFVPIKIQTVSGDMKIRRA*
>SPSA8_v1_80015|ID:41145133| conserved protein of unknown function [Spirochaetes Bin 1 SA-8]
MDNTDELFEKWKSQFRKGFLELCILELLAEEGRSYGLQIMESLRVAGIDVSEGTLYPLLM
RMTKDGSIISSWETPDIGHPRKYYSITTGGQNLLSNMLEEYERSEIARSRLSKKGKSI*
>SPSA8_v1_80016|ID:41145134| protein of unknown function [Spirochaetes Bin 1 SA-8]
MEAILVDVKRVMRSLLGMVRAVSGAFRNTSVDLDADGHKGRAAGSAGVTLADVYGRRGRA
DIYTSKETFELYRKMSFDKNPPL*
>SPSA8_v1_80017|ID:41145135| putative Hydrolase, TatD family [Spirochaetes Bin 1 SA-8]
MLADAHLHLADLAERDPGFPARIPHDSWRGAAVSHDPEEFVRTEELLRQLPPTLHGFGIH
PQNPREDTADFLAELCASQKIDFIGEAGFDFFSASPEGVVAERVRNPENLGRQQLAFEFQ
LGLAVRYRLPLVIHVRKAMDILLAYGRELSSAPSVIFHCWPGNPLEAEAILKKNVNAYFS
FGTPVLRGSKHGLASLRAIPRERILAETDAPWQPPHHAIYTCWENIEDVTIAIARQLDLA
KTAACTLLYDNFQIAYRTGI*
>SPSA8_v1_80018|ID:41145136| putative tRNA threonylcarbamoyladenosine dehydratase [Spirochaetes Bin 1 SA-8]
MAFNERLAIITGSNGLEKLKNARVAVYGLGGVGAVCAMDLVRSGIGTLYVIDFDRVEESN
LNRLYFGYLSTVGKTKIEAFEQFARDVNPAIKIIARRIFFSGETAASIVDTESDFHADCV
DSLNAKVQLIAALVRGKHRFIASMGTAGRLAPEKLKLGKIQDTRGCPLARSVRTRLKHLN
IGANFPVVWSDEVPAKPIEPEVSAEEETQTAESNPDQQAGSSAPRGRKRLTQGSSPFVPQ
TAGHIMASWIVRQILAESEYPSRQGD*
>SPSA8_v1_80019|ID:41145137| Aminotransferase class I and II [Spirochaetes Bin 1 SA-8]
MFQPFRLERYFAQHEFSARHLLCTSDCESMTIDELLTIGAPAGSMSGPAAEPESAAALAM
AVPVTAKGNSYACLGKTWLGYTESRGAPALRAAIAALYRDLDMEEVLVHSGAEEAILNVF
LAMVGKDSLVIVNTPCYQSLMEIPAGLGAKVIPWQIRCEGEAWRLDIDALDGLLDSARRG

QDSSAPLVVLNMPHNPTGALISAEEFDSIIQVCRRHGAILFMDEVYRLLEYDEKDRLPAV
CEAYENGISLSVLSKAWGLAGLRIGWLATKRRDLLDKVAAIKDYNSICASAPSEQLALLA
VGNTATIVGRNRAICAENRVLFSQFFKAHEELFSWIPPKAGSIAFPSLKAELKKTGIPSG
NSAAISADLSDALAEQDAELFARVLLAEIGVLLLPGVHYAFDKAFFRIGLGRRSVPVSLS
IFDAWLREKGY*
>SPSA8_v1_80020|ID:41145138| putative Amidohydrolase 2 [Spirochaetes Bin 1 SA-8]
MKNRMFFDIHCHAHTLAHPSFLALIQTIRNRRLEAVYSQLTAPEYLVSSFFHRGGEKLRN
MLSVMENEPGDIFMMMEDDLAGLYLPDFGQEKARAASKDRLLPLISQNKLKLGGKTYDRL
AICPLLIDFNTSGEYKPDTYYNRYPRKSMKAQIVDVLMGIREYRKQRPDGLLEVYPFLGV
NPSRYSLPELERFLAEWFPKPMLKAPAEPEAQTMTNPGLRQSVCMTQRRSQFADMIDFQS
EGNFGQDNYFAGIKLYPPLGFDPWPSEPSEREKAEALYQFCETNQIPITTHCDDGGFRVI
QLEKAWTYTAPERYTEVLLRFPNLRINFAHFGYLHNQIKRINDQHEWRNRILKFMLEYPN
VYSDFSFDGVYPQFYQELLEMLANLSESDRKVVEQRVMFGSDFMVNLLKVNSYLEYFTIF
EESPLDSALKHKFGSENPARFLFGV*
>SPSA8_v1_80021|ID:41145139| Permease of the drug/metabolite transporter (DMT) superfamily [Spirochaetes Bin 1 SA-8]
MSKSLSLLAGTGYAAIFGFSFLVTKGALAVLDPMELIFVRFLVAAILMTILAATGIIKIR
YSGKKIGILAGMVLFQPVLYFIFETYGVRESATSTAGIILGALPAIVALLGLVFLKERIT
KIQSLGLGLSVLGVVIVVALGAQSQTGAEGTLRGAILLIGSMLSAAFFNIFSRKASRTFT
DYERTFAMMWSGALAFGIIVLVRYMANGGSLPPPSAGAQNLVLSSLPARALSVWFAIFYL
GVLSSVIAFFLINFSLTRLKASQSAVFANLITVITVVAGVAVRGEKFDIVQGVGAVVIIL
GIFLANSGKNEGKSAD*
>SPSA8_v1_80022|ID:41145140| protein of unknown function [Spirochaetes Bin 1 SA-8]
MTDTSIEAFWADFERQTGEPVLCRTMGQMFSTPKDRGDWGLMVLSPTSFRFRKTPGENWF
ASIFKASAPVVPRQQEEDMIIPYTAMLEIKPPKKTILDILFGSPFISFSLIYTKGDETMQ
VHFAVDPKSDFLARLKELSKL*
>SPSA8_v1_80023|ID:41145141| HAD-superfamily hydrolase, subfamily IA, variant 3 [Spirochaetes Bin 1 SA-8]
VLNNDSKAFEAVLFDMDGVLVDSEELIALAAQMMFKAKYSVEMPRDAFLPYVGAGEDRYL
GGPAAERGIEIDVPSAKALTYEYYGQLASTHIRVLAGAREYLSACKKSGMKIALASAADK
TKVLINLKVLGVEEGFFDAFVTGSEVEHKKPSPDIYLLAARKTGVQVDRCLVVEDAVNGI
IAGVRAGCRCLGITSSFSESELKAAGALWCAKDLASAPKPWELTTSIAL*
>SPSA8_v1_80024|ID:41145142| conserved protein of unknown function [Spirochaetes Bin 1 SA-8]
MQALSQTFSLPFFTEKAILNLMTDFYALINASSLPYFEYGVSALDRYKGLKPQEPIRFFA
VHGSLVDVARLFDSVEYPGLPYADAMIGRASIGFEARIKCIEDPGQAEKHASPFMEFRWN
PRTGHFLDPASMYQNVKQDELVLSPADSENALFETAILLSRFPCQEDQAAGNKPASQGLA
TCKMGKTNLPVSVPEVFQKDLLELILQGKYASQALDFLERSGFVKKFWPLLAALTDVDHA
KDCHPEGGGWSHTMEALSQRKTFDRAISLAILLHDIGKPLSTQSEGRKFDRHAEIGADLA
ARFLRSLHFEEKLIEDVRFMIRWHMMVAALPRLPLASVRSIVFDKRFPDLLELFRCDEFS
TFKGPDVYYASCAAYRQIMKNRKNPYRSDTGEKLRVGAPLGTKRQL*
>SPSA8_v1_80025|ID:41145143| putative Metal dependent phosphohydrolase [Spirochaetes Bin 1 SA-8]
MKRALSKMAFFLIILFGLSFLVLVPIRHKEQELKLDSIRTAMSSVISMQATALEDGYFSW
SELQNLIKGRQMAAAVQLLKPIYEAYPFVQNITVQPGVPPSESFEIEGLNQSIYLAYSIK
DDFGYEALPGWKAVVILDAQKIINAIHPAGDLVIDPLHGTVLVYGIRVRFSTPLFSLIDY
LVAAFASIALGYPLWMYLKKRTVYFYETKGLESIIFLFEQTEKSSANHSRRVAALALFIG
KKLGFRGAKLRNLYTAALLHDIGKISIPSDILTKNGPLTIEEIQYIKTHPLISSRILKNF
KELSHLEKTVLYHHERMDGSGYPEGLKGNEIPLDSRIIAVLDVFEALIGDRPYREPVDVL
SAFETLRSMSLDQEIVEVLARHYHEFTNFQSPKWAVAYDPISVV*
>SPSA8_v1_80026|ID:41145144| protein of unknown function [Spirochaetes Bin 1 SA-8]
LKTVYRAMFQDDALVLASMLKSAGIEAEVFADRMLDVNPLFSTDINGVSIVVPDEQEQDA
MAIVNDYKKNTSFSGMKA*
>SPSA8_v1_80027|ID:41145145| Peptidase M24 [Spirochaetes Bin 1 SA-8]
MFHQSTYRTRRAKLLAQLKDRNVETGFIVLLANGESPRNYPDNCYEFRQDSTWLYFIGLR
EPGMAALXNIAXGETTLFGTELTVHDMVWTGPRPSLEELAALGGISQTASIDKLAEHIGQ
AARYGRIHYLKPYRVDTAQSFSRFLGMPLEQVGIGASLELTKAVIALREVKEDEEIGELD

AASAVSAEMHQAVLSSLHPGWRAWEAASLARYVAESRGFSLSFPSIATTHGEILHNHDYG
AVCEDGQLFLLDAGAESQNGYAGDLTTTFPVGRQFTARQKEIYTILLAMMEASIKKTGPG
VRFLDVHLAAAQALAQGLVDLGLYEGEPDEIVALGAHALLFPHGVGHMIGLDVHDMENLG
EDLVGYDGQPRSSQFGLRSLRLAKTLKPGMAHTIEPGIYFIPGLFELWRSEKKFKHLIRY
DRLAAWIGAGGMRIEEDWLVTDTGARRLGPLLDKGIGAIEAQRRVMH*
>SPSA8_v1_80028|ID:41145146| protein of unknown function [Spirochaetes Bin 1 SA-8]
MDYVRECFVLNLKIRRKIMRMSQENLAEAAEVSSGYIANIETGRNFPSTQVILKLAKALN
VDHWNLFVDPRKEDIGFSKDEVFSIIDNFKNYLMGELPKRYPSKVDSDGQKS*
>SPSA8_v1_80029|ID:41145147| exported protein of unknown function [Spirochaetes Bin 1 SA-8]
MPFFLLAAVFYISIIFPTQAQTGQAAQAPVAVPVALESGIPYTETLPNGLDIILLPQNGA
EKAGFSLIFRGGTDAQTTKTAGLFKLLEHLIFRGVAASPGEPEPAGVLESLSAQDLHGGV
QQDRFFFSFTTRPEQATQALDTLLYLFSGLRLESAFADPKALSDAKDMSLLFINQEFSDP
SAIYENSIARKIFVSAPWRFDIGGPDYIIDSVTEDILKDHAKTWLLPNNACLIVAGNFDP
EAIRKEIPKRFSTWAKGPNPLAKPLPVFPKPGITRPTFMVLPDPSISEGKALIEMRYRGP
DASSTRYQSALLWSVMADNPEGRLAKAIKKGLPASSNPQNLSTRYIAAPFASWLSVSAEI
AIAAKSNLPDTILTFKELVRSTEMYAMKTNSAYFSVSEYEKAKSEVKEQRMAAFSDPAEA
LLELGGYWILGGLSFIRDMPNRIEKVTSKDIMAFADEYFMKNLEVLAVRLSPEDYAKQKK
SFDSYGFEQMTAQKAFWWR*
>SPSA8_v1_80030|ID:41145148| (P)ppGpp synthetase I, SpoT/RelA [Spirochaetes Bin 1 SA-8]
MHSEERVRKFLQSFQNVFGSAELAKAEQALAFAQTRHGQEHRASGEAAWEHNLRVAALLI
EMGLDEDSVLAGLVHDTIDKSEDAQKKTLAVTPDQELTERFGLETATIVAGVSRLSSVRA
KVKTVQGAESMRKMLFALTGDIXVILVKLADKLDSMRTLKWLPEERQKLIAAECLDIFAP
LADRLGISWMKDELEDLSLKALNREAFDQIKEIVSAKKDEREKFLARVSSAIRSLAEAEG
IQIEISSRAKHFYSIYQKMRKKAKEADELYDLLGIRILCATETECYTLLGIVHKLWKPIE
GRFKDYIAMPKANGYQSLHTTVMSYEGQLLEIQIRTRLMHAVAEYGVASHWLYKRGTTNE
KPVLEDLPIVNRLKQWNEFLSQGAGYLEDIKRELLKDSIFVFTPQGDVIELPAGSTAIDF
AFAIHTDVGAHCLGARADGSIIPLDAELKNTQVVEILTSPLAKPNLNWLKLAHTSKARSK
IRALLVQSGQVLAIEKSIVVSKHPEKEKSEDRSKEQAAEEAKLHGSRQQEAPAFEPGKTE
LEFRSYRTGQELRSDKAGLSIGGARNMMIRIAGCCRPVTGDAIVGYVSRGRGITIHRADC
RSIAAVADFEERKIEVRWEDGSSAIARFRVTTKKVPGIFSEIESAVRKYSGRLREGKLGE
RGDGLLSGFFTMEADNRESLKKVSKSLRGLPSIISLEEEFDTK*
>SPSA8_v1_80031|ID:41145149|prmC| Release factor glutamine methyltransferase [Spirochaetes Bin 1 SA-8]
VNIMSTYRDILSAVSATFTDSETPFLDACLLLAHNLGLSRESLLTRLSESAPPLPASFYE
AVRRRSEGESIAYILGKKEFFGREFIVDSRVLVPRPDTEILVESALECGDVIEQEHPGRL
SVHDACTGSGAVAVSIASERPGWTVSASDISEAALEVARANAVKLLGKPLLLTVSNLLGA
VRGTFGIITANPPYVPSCETDALLRQGWKEPRLALDGGLDGLVLIPELISQAFRLLEPGG
FLLVETDSLQVPDVCAMFEKMGFVAIFVKRDLAGRDRVTGGRKPLA*
>SPSA8_v1_80032|ID:41145150|prfA| peptide chain release factor RF-1 [Spirochaetes Bin 1 SA-8]
VTDRIAQLQERLAELDAQIASPEIFKDQARYREVMKERSKVSAILSIDEKYQQIATELSD
AEAMARHETDSELKAMAEEEISVLKEKAEACRQELENLLAPKDEMADKSVIMEIRAGTGG
EEAALFAADLFRMYSRFADIKGWKVEVMSESATELGGFKEIVCSIDGEGVYEQLKWESGV
HRVQRVPATENSGRIHTSAVTVAVLPEMEETEIEIRQEDLRIDVMRSGGPGGQSVNTTDS
AVRITHLPSGIVVHCQDEKSQIKNKAKAMRILRARLYEIEEAKMQAERSQERKSQVGSGD
RSERIRTYNFPQNRLTDHRIDLTLYKLDLIMEGDLEELVSALVKHFRQLALAGSLQ*
>SPSA8_v1_80033|ID:41145151| putative tRNA-dihydrouridine synthase [Spirochaetes Bin 1 SA-8]
MLTIQRGSLLLAPMVGITNRAFRTLIAEIGAPDYFFTEMASAEAFCARAQYEEVYTDPLP
EPSRTSVQFYARSSESMAQACRRIKERPEHLRPAGIDINLACSAPHIRRQGGGSAWSTDA
EQTSELVAAARANWAGMLSAKIRTGPDDNYDRLLRFAAALASAGLDFLTLHPRTDSQKFK
GTPNWDLIGRLSCDLSIPVIGNGDIKDAISLRSLRTVQNPYAFMIGRQAVREPWIFRTLK
ILENKDSPDTRDIQVSFDRLAIACRFIDLVQNLLPEKWKLETLRRFFGYFTESFSFAHHA
NVLLANSDNPMRSKEILERYFEQVPQDRYVVIE*
>SPSA8_v1_80034|ID:41145152|rpsP| 30S ribosomal subunit protein S16 [Spirochaetes Bin 1 SA-8]
VSVKIRLKKFGAKARPYYRIVIMDARSARDSKTIEEVGLYHPIETEDKQIRIDEERVKYW
LSKGAQPTDTVRSLLNKKNIRLG*

>SPSA8_v1_80035|ID:41145153| conserved protein of unknown function [Spirochaetes Bin 1 SA-8]
VERDLVEYIVKSLVDEPDAVDIKVIEGEKSTILELKVAEADIGKVIGKNGRIARAIRTIL
SACASAQSKRVILEILD*
>SPSA8_v1_80036|ID:41145154|rimM| Ribosome maturation factor RimM [Spirochaetes Bin 1 SA-8]
MVANPEERTLAVARLGSPRGLHGYLKVHSFSGEYGHILKLKEALLYPPQSASQQSSKPRK
LKVRGFEEGDWGIAIAFIGFESPETARELVGMEIVVPREQACPLRDNEWYIGDLVGMDLV
YQGTVMATVASVLDGGPDPLLEAQLPEDGRTVLVPFRNEFIGKVDTSAGSMELLVDWILS
*
>SPSA8_v1_80037|ID:41145155|trmD| tRNA (guanine-1-)-methyltransferase [Spirochaetes Bin 1 SA-8]
MKFTVLSLFPEILEGYFSASIMKRAVGKGIIQYSLVNIRDFAHDKHHKCDDEVFGGGAGM
LMKPEPLDKALESAGSPGVRTIYVTPSGRLLNQAMAQDLAREEQLVILCGRYEGVDQRII
DTRVTDEISIGDYVLSSGEVAAMVLIDAVYRLHPGVISGESLTEESFSGGLLEYPQYTRP
AVYATMQVPDVLLSGNHAQIARWRLKASLAKTIAYRPELLARTDLPEAVGRLLKELIDEG
GLNERTAVHTG*
>SPSA8_v1_80038|ID:41145156|rplS| 50S ribosomal subunit protein L19 [Spirochaetes Bin 1 SA-8]
MNALQSIQAEQIKSELPVFNVGDTVRVSFKIVEGKTERIQAYEGLVIAIKNAGIGKTFTV
RKNSYGVGVERVFPLNSPRVENVEVLRRGKVRRAKLYYIRTKIGKKSKVKELIVSKKENA
N*
>SPSA8_v1_80039|ID:41145157| conserved protein of unknown function [Spirochaetes Bin 1 SA-8]
MASRFLTTLYGRQGEAVFADRLARDGWQILARNFRRRTGEIDIIAERNGIVAFIEVKTWR
YLDCADLSDVINAIKIHRIIETSKIFLKMNRQYNWKRIRYDVVLLAPDGTAQHFEGAFDE
TQ*
>SPSA8_v1_80040|ID:41145158| protein of unknown function [Spirochaetes Bin 1 SA-8]
MKHGKTVSNDPLSWAGSDKTDVQRYHDLMVDNSGQDVYKILNLKHKLEDNTYVEDALSRI
ANFLANEANKKGKA*
>SPSA8_v1_80041|ID:41145159| conserved protein of unknown function [Spirochaetes Bin 1 SA-8]
MNPNRSAHDNAGQGKNYPHKKPSPESGRERGKQAQQNRRPVAPGLTEKPKMSPPKTCAIC
QKPIFDLAGAIADKESGEPVHFDCAIERISKAESLGPNEKVVYIGAGNFAVVEYKNGADG
AFTVKRRFHWEKEGEKQIWRKEISSYISRI*
>SPSA8_v1_80042|ID:41145160|yfeU| putative PTS component; possibly regulatory [Spirochaetes Bin 1 SA-8]
MDISRIDDALEHAVTESRNIKSMNIDSLSEAEILRLINDEDKTVPFAVEKALDSIALAVQ
DIVAAFRDGGRLVYIGAGTSGRLGVLDASECPPTFGVPYGMVIGIIAGGDDALRKSIEGA
EDNPEEGRKALEAIHFSSNDVLIGITASGSAPYVLGALQYAREIGAKTGAISCNRESKTF
LLADHKILVEVGPEVITGSTRMKAGTAQKLVLNMLTTASMIRFGKVYGNLMVDLTPVNRK
LVERSKRLIRQATGCSRDEAEQAFLASGKKPKVAILMILLEIGADHAQSLLARGSGRISD
AMRLFNAEKAEKAAQGG*
>SPSA8_v1_80043|ID:41145161| Transcriptional regulator, RpiR family [Spirochaetes Bin 1 SA-8]
MSESEVKQGAMDKIRSMLPEMSEAERKIALFVLSEPTKSLHFNVVELSRHSASSSAAVVR
FCKRIGVDGFNEFKLWLAKDVFQDREEKFLPDLDLESKTPAERAIHEVIDYARKSLQDLS
RTLDPAQLDRAAQKIRSASATMLFGIGASGIVAADFQQKLLRIGLTTSFTYDSHAQITTS
CSLKPTDTALIVSYSGETELMIEIAKQVKQRKASLITLTMEGANSLRQYADIALLVPASE
RIYRRGAETSRLSQLTVLDILYRIIVSYDVEAAIEAIERSMEATHRKRKXTGXFPNIXXQ
RFLLISENPQKGTRTSGILMEPSACWKFSIMAMIAREQAMAVPLSM*
>SPSA8_v1_80044|ID:41145162|serS| Serine--tRNA ligase [Spirochaetes Bin 1 SA-8]
LQESSAPVIILPMLDYKFIKENLEAVKQNIAERYMQADADRVVSLYDERNTLLRELEEQR
KNRNENALSMKGKLDPETRTAKIEEGRQLKEKIAALEARLAEVETQLQAEGMRIPNIAHP
EAPRGKEDKDNLEIKRVGAPTVFNFAPKDHVQLGQELDLIDFDTATRVSGTKFYYLKNEA
VLLEMALVRFALEKLRSKGFALFETPDIARTEILEGIGFNPRGSESNVYTIEGEDTCLIG
TAEITLGGYHSGSILEKSALPLKLAGVSHCFRREAGAAGQFSKGLYRVHQFTKVEMFVYT
LPEDSDAMHEQLRLIEEEIFSDMGIPFRVVDTCTGDLGAPAYRKWDLEAWMPGRNNGEWG
EVTSTSNCTDFQARRLNVKFKDDDGKNKYVHMLNGTAIACSRAIIAIMENFQQADGSIKI
PDVLVPFCGFSEIRRKR*
>SPSA8_v1_80045|ID:41145163| Long-chain-fatty-acid--CoA ligase [Spirochaetes Bin 1 SA-8]
MANTIPQMFIEKAESQPDVNVQYSKNEKGDFIPTTYRELLNNICSFAAGLNAVGLKRGDN

IGLISDNRKEWLTADLAILGLGAADVPRGCDATLQEISYILSWSECRLAILENEKQLEKV
LEVQKSLPALKTVILFDPPSGALKEKAESSGLTVLEYSAVFEKGAALEKANPGLYRAEAR
KGKRDDLATLIYTSGTTGDPKGVMLSHGNFLHQTEYLPSIIGVRSGELFLSVLPVWHSFE
RVVQYIILQAGAGIAYSKPIGSILLADMQAVQPHWFTSVPRIWESVKDGVYKSIRQSGTV
KQILFKVFVGIGESYAYFRNHLLGRIPAFAPRSRVLEIAASIIPFLLLFPLWKLGDILVF
KKVKAKLGKRFIAGVSGGGALPASVDRFFDALGVLILEGYGLTETAPVLGVRLKNHPVLG
TVGPIHKGTEVRIVGEHGEILGPGKKGVIQVRGPQVMLGYYKRPDLTEKIMTKDGWLDTG
DLGMLTVHGEIRITGRAKDTIVLRGGENIEPVPIEQKLCESEYISQAVVLGQDEKYLAAL
IVPTQERIIEYAEENNIPFADYESLILQPEIHELLDFEISELVSSKNGFKPFERIFKFDV
LAEPFQPGRELSAKQELKRHAINTLYHQRIKALFKN*
>SPSA8_v1_80046|ID:41145164| protein of unknown function [Spirochaetes Bin 1 SA-8]
MRSEGTDLEIRYFQENVDPEIAERFIIRLPEGAIAISNSADDMIHIDCELHGTTSTIPSW
KPIVRRHESILVLGSEASADVYTASIEIKVPVRIKDLEVHSLKGEIDIRDCPVNVLAMTE
LGGIHIHGAHSVEASTVQGQITLINCGSISVRTIDGSIRCSKINGIINAETQGGDIQISR
VKGNVVALTQTGDISVLRPEGRIRLITRTGDVELELGPVFGGGEVNTYDGDVNLMLEQAN
IEFRAETLSGHISSPDETIAPGSEPRRCAYKIGQGTKRLHVKSVLGDIELE*
>SPSA8_v1_80047|ID:41145165| membrane protein of unknown function [Spirochaetes Bin 1 SA-8]
MKDEKIQQKDLQEIADCLRDLRTAIKTNSPLLHIVASSRLYPILGLVFGVLASGYSIMMH
ILTTASDTAEPGFQPASLTWIFLVFLLVCGGVVKVFYTNRLIRNLKNASFGALLKTIYGG
RISGFLLSAALTITAAIVFLVRMNHPWYIVPVAAIFTSFVVQVMNFLIELAEYSIFGFSV
LIFGLVSLFVIDTWPWLCVALTFGGSFLVFGIAGLIRASGITGKK*
>SPSA8_v1_80048|ID:41145166| DNA-binding helix-turn-helix protein (fragment) [Spirochaetes Bin 1 SA-8]
MKSRTGTTASLPDPDRIIHERARLRILVYLASSLSAKVGFTELKDSLGMTAGNLSTQLX
>SPSA8_v1_90001|ID:41145167| protein of unknown function [Spirochaetes Bin 1 SA-8]
LDSVELVFDDDAIDAIAEKAISQKTGARGLRSIVEKLMTDIMYEVPGAKGAIKVRVTRDA
VEGREPCRIEPNKKTA*
>SPSA8_v1_90002|ID:41145168|tbpA| thiamin transporter subunit ; periplasmic-binding component of ABC superfamily [Spirochaetes Bin 1 SA-8]
MCKVLFLQGGRKKPFSFTACQLFLMTVMLAVPIMLTISCQAQAKTKSLTVWTYDSFVSEW
GPGGSLAKLYKEKTGTDVTFVSKGDGGALLAALLAEGNASQADAAIGLDNNLADKALSSG
LFSPLNLKNLASVDKNLLIDDKNRLIPYDYGTFAIIWDSKTSITPPKSLEDLTRPEYRKS
LILMDPRTSTPGLGFLAWTEAVYKDAWQDYWTRLAPSVLAMTPGWDTGYGLFTKGEAPLV
ISYSTSPAYHKAYENTERYKALAFSDGHPMQVEFAGILTSSKQKKEAEKFLDFLLSPEAQ
AILPETQWMYPANMTVNLPDSFSVIPQGITTIKAEVKNLEKDPEIAAAILSGQK*
>SPSA8_v1_90003|ID:41145169| putative ABC-type transporter, integral membrane subunit [Spirochaetes Bin 1 SA-8]
MDEKNRQSASSFLTAFVMLLTLIPVFSFIVFALSKSVKSGTAGFSVFITANQILTKANLR
ALTFSLQQAALSTISAVAIGLPGAYFVARYRFRTRRLFFSLAAVPFCMPPLLVILSFILY
FGKSGFLAAAFSRAGLPFLKSSSFLYSMTGLVFIHAFYNFPIVIQTVGALWSQLPDSREE
AARTLGAGRAKAFLVGTLPYLLPSLFQAASLIFLFCFFSFTIVLVFGGLSGSTLEVEIYR
ATRFSGDYSRALFLGFIQTAMAMLVVSIFVHFDNKTTQIAKGFGAGRTRLEPRGQAQIAL
FLYQLMIAMFFIGPLVALVLEGFTVRPSLAGNTHFGITNFTKLFRDGGKPLLPALLNSLI
IGSVVAAIATLIGLGISASDHYGRRKTSIWTALPLALSPAIVALGWATLLQSSSIAAIIA
GQSVMAWPFVSRNLGASFKSIDRNKYEAARVLGARRIQAFWRVDMPMLLPSIASAGAFAF
SIALGDANIPIILGXGKYETLPLLLYRLVSAYRFSEACAVGIVLAIVAGIAFYLKEKTSE
LL*
>SPSA8_v1_90004|ID:41145170| putative Polyamine ABC transporter, ATP-binding protein [Spirochaetes Bin 1 SA-8]
VSYCNIESLRFVRRGFAVNVSFDLEKGKTAVILGPSGSGKTTLLRCIAGLEHPASGGIFL
NGKNIISLPPEKRNIGFVFQDFALFDHLTGRQNIAFGLKLRSIPAGEIERIIDRLARKFQ
IAHLMDRKPHTMSGGEKQRLAFARSLAIQPDLLLLDEPLSSLDAPLRKELRSFIREKLSS
EGLTAIHVTHDVQEAFELADIVFLMDKGVIRHAGTPAALHETPPDAWTARFLGLGNLIPA
VPLEIRDATADVMTAIGTVRQVNKPQGRSPSDSHHSEDLFLFIPDSAFIIHDRGVSAAAR
HDLQLHGKVSKNILEGSSMRAEVMIETDSKEKAIKLELPVSSGSLAAGDIVEIGINPDRC
IVLPEQSIDYE*
>SPSA8_v1_90005|ID:41145171|aroK| Shikimate kinase [Spirochaetes Bin 1 SA-8]

MSPIISLIGMMGSGKSMTGRSLACILECPYVDLDTEIEKTAHCDIPRIFAQHGEAGFRDI
ESAALADILANNRQRLVLSCGGGIILRKENRTMLRARSFIVWLDVPIHELQRRLTAERSN
RPLIAEGDLKTRIKKLSAQRGSLYEETAHFRLIWNEGMNAEDCAKLIHNQWIAQAEQCSD
RD*
>SPSA8_v1_90006|ID:41145172| Membrane dipeptidase [Spirochaetes Bin 1 SA-8]
MHETQLTDSCAVIDAHCDTLLEIAGIGYESERGTPPRDFFRNNEHAHLDLPKLLAGHVRC
QFMALFCDDAYLDSAYEHTLALIDIFKKLCSGSGGRFFPVLSAKDLDCAVPGDKVGTLLS
IEGAEALGPNAEHLPELYSRGIRAVGLTWNRSNAFARGVRGEGEGGLTEAGHRLVEQLEA
MQLIIDASHLSDEAFSDLAQQARRPFIASHSNARAVYNHPRNLSDTQIRAIADSGGAVGV
VFVPNFISGESGVSYFDRLIDHINHIVKVGGIACAALGSDFDGFKDEPEKRVLSDASEYP
KLLEALLQRGYTTSDVEKIAGGNWERVIREILQ*
>SPSA8_v1_90007|ID:41145173|uvrB| excinulease of nucleotide excision repair, DNA damage recognition component [Spirochaetes Bin 1 SA-8]
MKPFKVVAPFEPAGDQGTAIAALVEAARSGKRYMTLKGVTGSGKTFTMAKVIEALQQPTL
VISHNKTLSAQLYREFKGFFPENAVEYFVSYYDYYQPEAYVPSKDLYIEKDSSINDEIDR
MRLAATAALMERRDVIIVATVSCIYGLGSPDLYKEMRIYIDRGKVIDLDTVKRNLINLQY
ERNDMVLDRGRFRVRGDILEIYPAYAEDAYRIELEFDTVRRIRKIDPLTGKAGDDLDEAI
IYPAKHFVMPEDMVQRAVQRIKDELEMQYAYFLDNNKPLEAERIKSRTEYDIEMLEEMGY
CSGIENYSAPLSGRKPGERPGVLLDYFPKDFITFIDESHVTLPQIGAMYAGDRSRKTSLV
DYGFRLPCALDNRPLTIDEFETIVDKVIYVSATPSPKELERSAVIAEQLIRPTGLIDPEI
QVMPTEGQMEDIYQRVRERSQKGERSLIITLTKKMAEELTEYLTGLGLKVRYIHSEVETI
ERVEILKQLREGLFDVLVGINLLREGIDLPEVSFIAILDADKIGFLRSATSLIQIIGRAA
RNAAGLVVMYADRESEAMKTAIEETNRRRAIQLAYNREHGITPVTIKKTVQDILERHHEE
KVDITLGEIGLLKKSHSLLVPEQKKALLKALEARMLEHAKNLEFEEAALIRDEIQRIKDG
SDA*
>SPSA8_v1_90008|ID:41145174| Peptidase S1 and S6 chymotrypsin/Hap [Spirochaetes Bin 1 SA-8]
MKLYSKKAVLSIAIVSFLSAAALIAVSSLIIVRSGIWSSRSIDTGPSPTTSITQPVSGQI
TETALSDSGYTLDEMENISIYERYNESVVNITTEVMALNWFLDPVPQSGGSGSGSIIDEK
GYVLTNNHVVKDAYKLYVNLADGSRHEATVVGTDPQNDLAVIKFSPLAGTRLKPIPFGTS
KNLKVGQKVLAIGNPFGLERTLTKGIISGLGRPIQKDSSTILQNMIQTDASINPGNSGGP
LLNARGEMIGINTMIYSPSGGSVGIGFAIPVDTAVRIVPELIKEGKVRRGWIDMDAFILV
PDLVEYMRKAGYPVPVEKGLLVSQVRSGSNAEKAGLRGGTKAVRYGSSVFNIGGDIITSV
DNHEIASVADLYTALEDNKPGEKVWVEFYRGNQKNSVQITLTERTQTK*
>SPSA8_v1_90009|ID:41145175| protein of unknown function [Spirochaetes Bin 1 SA-8]
MLVVGLGPVSIDLLLQIPLRTIINDDTAMSAAAERKDTIAMLRTAFFE*
>SPSA8_v1_90010|ID:41145176| Cob(I)yrinic acid a,c-diamide adenosyltransferase [Spirochaetes Bin 1 SA-8]
MSIITRTGDEGETGLWSGERISKDDIRVEAYGTIDELSSALGMARHLCFQDEVLYAIDEI
QRLLFRAAGELASVGIPFDRPITEKDEERIAEKTLSIEERVVLTGFVIPGMTAGSAALDL
ARTIARRAERRVITLARREAVSPDLTRFLNRLSDYIFMLARLEEASAGKLTFA*
>SPSA8_v1_90011|ID:41145177| Putative arginyl-tRNA--protein transferase (modular protein) [Spirochaetes Bin 1 SA-8]
MLIFNKILSKPLLLQAGTGKIFHDVHSLDILICMDRKISRLTTACPYLPDRKACMLFMET
AWDEADYFVYENLLVQGWRRSGHLLYCYQCPGCNRCLPIRIPINRFVPSKSLSRIIRKNR
DISVRLQNASYSDERFRLYEKYVTARHFESAARDADLEHRLAFLDMTAGLKAQIIEYRDQ
AGILLAEGFADILPEGISSVYFAFAPDSGWRSLGRFSVHAEIQIARQLQKSFYYLGFWVP
GSGKMDYKADFAPFELAVDDQKSQTADFSRDPLMNLHEKAWRTFSSKEEALSWLEAAGYL
DR*
>SPSA8_v1_90012|ID:41145178| putative PpiC-type peptidyl-prolyl cis-trans isomerase [Spirochaetes Bin 1 SA-8]
MASQINKSETQETKKTEEKKAPKRGVKNPFVYGGTIVILIITIIAFVFIPSISSGFSSGS
APSFGSWKGKPITYEADSYFANQVAQINDYLRQQGMSDQNFQYYAYQVWQMAFQSTAVRT
ALIDIVKQSGFRVTEKGLDEAVATNAAYQQDGKFSVEKFNKTPLATKLSIRNTTKEDLFV
RRYYEDIYTIAPSTAEIAFVASMAKPQRSIEYTAISLADYPDEEAAAWGAKNADLFKTVG
LSRITITTSEADAKKVLAQVKENKLSFEDAAKSHSKDSYADKGGDAGAVLFYVFENDFTN
KDDAKKIASLPKGEVSDVYKLGDKVWAFFKINSELVPADFTKKAVLDEVKAYLYEKERGT

LESWAIAKANSSLDLTNAAAFAASAKKAKLALKKAGPFIINIGSPTFYAYGQQIPLLQGP
YANNDPALDGAEQDEAFMTELFAAPKGKVSKPVVLGDSVVVFSVTDDTPASDDETALVKF
AFPYFHQQVVDNQTRSTILKSKNFKDEFNKMFFKIYSTQASAK*
>SPSA8_v1_90013|ID:41145179|lepA| GTP-binding membrane protein [Spirochaetes Bin 1 SA-8]
MHEKELIRNFCIIAHIDHGKSTLADRFIEKAHIIDQRKMQNQMLDNMDIERERGITIKSQ
AVTLPYTARDGKTYTLNLVDTPGHVDFSYEVSRAISSCEGAILVVDATQGVEAQTLSNMY
MALEHNLEILPVINKIDMPAADVEGVRAQIDKDLGLDGSSALAVSAKMGIGIDELFEAIV
ERIPSPEGSPDEPLQALIFDSHYDAYRGVVIHVRVFAGKLEKGMKIRFMSSQAVHEVEDV
GLFKIIPTSTDMLSAGEVGFCIAGIKTVRDVRVGDTVTGDDNPCLAPLPGFKEIKPVVFS
SIYPIDSADYDELKDSLEKLTLNDASLVFEKDNSLALGFGFRCGFLGLLHLEVVQERLER
EFGQAIIMTSPSVRYKLHLHNGETIFIDNPSEYPDPSRIESAEEPYIKAEIITPVPYIGN
LITLCVNKRGEQKSFIHLDEKRVELVFEMPLAEVLFDFYDKLKSTSRGYASFDYEVIGYR
PTELAKLDFLINGEPVDALSMLVYKPGAYERARKVCERLQRNIPRQQFKIAIQGAIGATI
IARETVNPVRKDVLAKCYGGDITRKRKLLEKQKEGKKRMKMVGNVELGQDAFLAVLRTDD
DEER*
>SPSA8_v1_90014|ID:41145180| putative Major facilitator superfamily protein [Spirochaetes Bin 1 SA-8]
LFLATLEGVTNLVYSTCTMIALKHLPRNASASIKLEPLWALFGPLVTYFMPLYQKELGLS
EVQMGIINSIAIATGFVFYALASPITNKLGRRYTSIIFDTVAWSIAMLVWAFSRSFAWFL
IAQILNSVVRIVVVSWNLLITEDATDQERAAIYGWIYVIGAFGGLSTFLGGIIIQKFGLV
PSMRIIFILGSISMSAMFVLRFIWTRETAPGQYLKEKLKKEPFVKLVLQQIPSARDALKD
GNFLLMTGIYILASAVLSIDFFRILYLAEKKLIPSWTISMIPATGAISCMAVFFIVLPRR
KHIPHIKSLKSGFLLSFLFQTAFLLIPVRSAALAILIVSGLQTSYFLIQTFRDTVFMNTV
RSDQKSELFSLVQALMLFFTIPMGWLAGWLFEFSNNLPFILAIILYGLGFLLSTNLEKQN
SAQETGSSV*
>SPSA8_v1_90015|ID:41145181| Glutaredoxin [Spirochaetes Bin 1 SA-8]
MNLIDLPYITKEGFRNDHSIVVYALSTCGFCKRALAFLDQHGFSYKYIHVDQIPLETKTE
VKKILKERFNENVAFPFAVIDEDKHLVGFIEPDWVLTLGVTEGE*
>SPSA8_v1_90016|ID:41145182| Ferredoxin thioredoxin reductase beta chain [Spirochaetes Bin 1 SA-8]
MNQKTLDQTRLFTRMVAEKQGWTLNPDTEFYESLVEGLTANYNRYGYYLCPCRDSDGSRE
VDKDAICPCVWSRKDIPEFGHCYCALYLSKEFSESRAPCRAIADRRYEA*
>SPSA8_v1_90017|ID:41145183| conserved protein of unknown function [Spirochaetes Bin 1 SA-8]
MIYLDWAATSKPDEGILSESLAETLQIFGNPSSQHELGRKARELLESSRLRMLAAINNVQ
GKASTKSQPGYPKLIFTASGTEADQIPLLSLLNKKNISKLAEEKANRPHLVISAIEHAAI
HSQTLQLQNLGFEVSFIRPDPEGFIQPKAVTKAIRPSTVLIAVMLVNNETGAVQDIPGIG
RAIEESSIALRMKAPWFHVDCVQALGKIPFSLPQNVSSAAFSAHKIGGPKGIGALLLFKE
LIPLAQGGGQESGYRSGTENLAGIDAFSRCVQKSLAGLNKNLEHVRQLEQLLLSGLSAIP
GCQIIPASRRPGDSRFSPWIISASFPGLSGEVFQRALSEQGICVSTGSACASSHGAKAKG
RRILDAMGMTPEASFCSIRISLGSDSAEDEILTFIETAHRLYSSLKT*
>SPSA8_v1_90018|ID:41145184|thiI| putative tRNA sulfurtransferase [Spirochaetes Bin 1 SA-8]
MSAIFLIKTGEVLLKLGNQREFVHQMKSQIRNRLGAIPHKIEEYPGRFFLEVSDENAPVA
EFVLSHCPGVNGYAPVTVCAKTTEAILAASVVAANHEAEISGKRTFKVETRRSDKSFPLD
SYSMSSLAGETILSRVQGSQVDVHSPDYVITIEIRERAYVYGPTLPGPRGLPSGVSGKGI
LLLSGGIDSPVAGYMMARRGLALEAAYFHTYPYTSIEAQKKVEALAVKIAAFCGGIRLWV
VPFTDVQMKIKKLANPEATTIMLRSAMMEAAHALALKVGASCIITGESLGQVASQTAENM
RVTQAPTTFPVLRPLIGTDKEDTIAIARKIGTFDISILPYEDCCVLFSPEHPILKPNFRE
AVNAYKEIDLSAEIAASVKNAEALRFYYSDVLKEQFGN*
>SPSA8_v1_90019|ID:41145185| RelA/SpoT domain protein [Spirochaetes Bin 1 SA-8]
MTREKSVLVIPDKSKLAEIYAASVQDYEKALSVFVEDLSRLLNQAGLRPTIKSRVKQFES
YYSKRLRLLKNAWMESGIASPVNDMLAVRVVCPFLGDLAVAENAIKEAFTIEEIERKGAE
RSFREFGYESIHVLVKIPEGLEPLCKHLDRAVIEVQLRTILQEAWAEVEHELVYKAEFTP
FDEPMKRKLAALNANLTLSDIIFQEILEYQKRLNTELAKRRQAFYHKIEEVSEPELAGGE
KKTAQKELEEETQRANQSSESKFELENFKSFGMDGLLLAALEAHNNADFDKAEQIYSEII
LKKPEKEILAVVYKHRGMAYFAQSRYKEALRDFSACLILDPECYKALYYRGVVKSILEDH
SSAIDDFSSALKIHPYHFFSRYRRALSYWKLGDSAQALADCEIALRIEPDNALARHLLNQ

IKDRISQTDF*
>SPSA8_v1_90020|ID:41145186| protein of unknown function [Spirochaetes Bin 1 SA-8]
MPMQTKQHAHGALIILPILLAAGVIAYGLTLSSSSCTLYFPDQHATNLVRERRVLSPIGN
PEERSRRVLDELALGPMNHKLQPLLPNGAQIRLVMQRSGTLYVDIELANLADSKIRFPLI
KEAIEKSLKTSVPGSGSIKLFINGIETSK*
>SPSA8_v1_90021|ID:41145187| Cell wall hydrolase/autolysin [Spirochaetes Bin 1 SA-8]
MFLRNRINLLALVIVLFISLPFVFGQQADAAASAQDLQTSLSVQLFYDPLLKTGYFVREG
KYLSFATDQPYLALDFKQIAKVSAPYNKEGVLMFPPDTKRVIENYYSAMQKETDGKYSVA
AILIDPGHGGKDTGAIAEFGSTRLLEKNLTLEVSKRVVELLSERYPNRKILTTREGDSYP
TLDQRVEMANSIPLKPSQAVIYVSIHANASFNKNAKGFEVWYLNPEYRRTVVDAVTVQEK
GKDIAPILNAMLEEEFTTESIILARKVYDRLGSMIGDASPGRGIRAEEWFVVRNAKMPSI
LIEMGFITHPEEGLLLADSGYLRKIADAIYNGIVDFIDHFER*
>SPSA8_v1_90022|ID:41145188|nrdJ| Vitamin B12-dependent ribonucleotide reductase [Spirochaetes Bin 1 SA-8]
MKFERRFTKKGKGPYAGITWEKRISEIRNPNGKLVFHMDSVIVPSFWSQIATDIIAQKYF
RKAGIPADKARAWEAFIPESQRQLAGDSPIPGSEHDARQVFHRLAYTWCAWGRKAGYFDS
EEDEKAFYDELLYMLAHQMAAPNSPQWFNTGLHSVYDIEGPAQGHFYVDPETNEVVKSQS
AYERPQPHACFILDIQDDLVNEGGIMDLVTREARLFKYGSGTGSNFSKVRARNENLSGGG
VSSGLLSFLKIADRSASAIKSGGTTRRAAKMVILDADHPDIEQYINWKTEEEYKVACLAA
GSAALNRYAKDMKLAIDSFGPDRESAFSLAENKPLRAAVRGAIKAGIPAAWIHQVLTLYY
QSGELITTPQYDTGWESEAYNTVSGQSSNNSVRVTNAFMNAVLSDGDWDLIARTTGKKMS
SVKARKIWDEIAQAAWRCADPGIQFHTTVNEWHTCLNDGEIRASNPCSEYMFLDDTACNL
ASLNLLSFYDQKEETFDDEAYRHAIRIWTTVLEISVVMAQFPSKEIARKSYDYRTLGLGY
ANIGSLLMVMGLAYDSDEGRAVAAALSAILSGEAYAQSARMAKEWGTFSRYPANADVMLR
VVRNHRRAAYDAKSEEYEQLSIIPQGLKEWACPGNLIAAARESWDDALSLGQQHGFRNAQ
VTAMAPTGTIGLLMDCDTTGIEPDFALVKFKKLAGGGYFKIINSSVPPALKALGYSEEES
QKILEYALGTETLEGAPAISIEILKEKGFDDASIAKIETSLKSAFSLESCFSVHTLGPDL
MSRLGISETTYSSPDFSLLEHLGFSDSEIEAAELYACGTMGLEGAPILKPEDLPVFDTAT
PSGKRGTRSIDWRAHIAMMAAVQPFITGAISKTINMPNTATLDDVKNAYMLAWKSMLKSI
ALYRDGSKLSQPLSALAPGSDLIADSIVALQSGEMEDEAFNEQPVSKETGVSETVRAPEL
PGMPVSPRGVRRSLPNRRTGYTQKAKIAGHSVFLRTGEYEDGTLGEIFLDMHKEGAAFRS
ILNSFAIAVSLGLQYGVPLEEYIDAFTFTRFEPNGVVQGHDYIKMGTSVLDYIFRDLAIS
YLNRHDLGQIKPEDLVATSTQSKDGNRTGERRTAFENGMSSHPSRPVQTNSNTVSQLPNV
GAEKGVPTKKGNPTLDSNTKFIRAARQKGYEGDPCPVCGNMTLVRNGTCLKCETCGSTTG
CS*
>SPSA8_v1_90023|ID:41145189| putative Arginine deiminase [Spirochaetes Bin 1 SA-8]
MERLSITSEIGVLKRVMLHTPGAEIEAMTPREAEQDLYNDIIPLQAVLQEYTSLKEFLSQ
VARVYELSDILAESLEDTGDKFEFLTDLSRCFPIRSVMETLMAMPARELSQAIIQGIPSP
KLSLSSRLSEHSFISRPLPNTYFMRDSAAVIGNCAISAATAYDVRMVEAIITRFILTHHP
DFLARTLLFDGPSERNRYLTAEGGDIMVLSPTVLAIGMSERTTSFAVERIAQNAARASET
DVTIFAVDLPKSRATIHLDMTFTMIDREMALVFEPVILGPQRSRVFRIDAKKGGKLSYHE
EHSLLSGLEKVGIQLKPVLCGNGHRIYQEREQWWSGANSFAFAPGKILMYASNLYTLEAL
SNAGFSVVPSKDFIAGKDNPANHERLAVTFEGIELARGGGGARCMTFPIEREDL*
>SPSA8_v1_90024|ID:41145190| conserved protein of unknown function [Spirochaetes Bin 1 SA-8]
MAKRRIVIIGAAGRDFHNFNTRFRNDPSCEVVAFTAAQIPDIAGRKYPAELAGPLYPHGI
PILLESELPNIIKSMNVDECVFAYSDVPYNHVMSLSALVNAAGASFTLLGPKDTQIKSTK
PVISVCAVRTGSGKSQTSRKIVQMLMKRGLKVVAIRHPMPYGDLVKQKVQRFAKVEDLAY
HKCTIEEMEEYEPHIVRGNVIYAGVDYEAILREAEKEADVILWDGGNNDFPFYVSDLLIT
VADPHRAGNEVSFYPGEASLRMANVIVINKMDTASPENIQTVRENITRVNPNAMVIDAAS
PITVDKPELIKGKRVLCVEDGPTLTHGMMKIGAGVVAARKFQAAEIIDPRPYVTGKLAET
FRIYPNIGTLLPAMGYGDEQIRDLEETINRTDCDSVVIATPIDLNRVVKIKKPTVKVGYD
LQEIGKPDLEEVLDDFCTAHKL*
>SPSA8_v1_90025|ID:41145191| conserved protein of unknown function [Spirochaetes Bin 1 SA-8]
MTSDLCKEFNFSETGSNRGTPPRAIMTTLLAGDMKFRIEEENPKRQRFFNLMGIQPESVR
SLELLHTREVLVTSRAESLGDLNARAFASGGADGLVTQDPGCILGLTVADCMPIYLYDAE

TQAFGLLHSGWKGTGILRQALSVMGKAFSTRPENVHVILGPAIGSCCYRVQADRAFQFQK
EFGPGSVIVKQHGSGEPEYSLDLVAANKKLARELSIASVSVVGECTCCSSDFGSFRRQNT
QVSGEGAFSRNLALIGYFP*
>SPSA8_v1_90026|ID:41145192| putative Ribosomal large subunit pseudouridine synthase D [Spirochaetes Bin 1 SA-8]
MKIVPFSETAQPFVVQESADYVFLYKPPFFHSVSLRSADEPSLAAWYRENCLEWATAFQQ
SCLEYSEKNRISDALKTRLFSEFGMLSRLDYQTSGLVAFAKNPEGILRFLRIAAGSALEN
GIARGFGLLSKEYLLVCTPGGNGVRGSKPLVIPGFPFDELQKTKISGYFRSWGEKGCRVA
CIAPEFVSSVQAGKKLSRELYETQFEMLEKEWRDRYAGAKPERTILAKAVITRGFRHQIR
AHSAWTGHPIMGDAEYGGQPSRRLFLESFAMTIPSEEGKPQRIELYPEHELI*
>SPSA8_v1_90027|ID:41145193| ABC-type multidrug/protein/lipid transport system, ATPase component [Spirochaetes Bin 1 SA-8]
MADFFETDDVTKGYDSQITRRILTYIRPYRLIVAITLLALALSTAGELLSPVIIRRAIDD
ALMKTWYGFLPETRLTEANRELKLSDKDPFIGGKIYVRTSRLSGLSTAEKKDLEAKGLFD
TREMYVFTVDPASEAQAALRAARPELFAAMDGPYGVIPMEKLRALAAADAAALRQADSAQ
IASYVLLLLGILATVLVSTFLMVYFSNLLGLKVMKDLRMQIYSHVLTRSMAYLSRQPVGR
LVTRMTSDVETINQFFTDVLSAFIKDATIMLGALAVLFFFDVRLALVVTATLPFVLTVSN
IARKKARDAFRNQRKWTSKVNSYIAENLSGIEVVKLFVQEDKTRKTFMGIDEALKKANLG
EMYVFATFRPAVDFFATMTSAVVIAVGAGFYLAHAISLGTLIAFINLINMFYSPIKDMAE
KYILLQSAMAGGERIFALLDADDTIPDMPKLPMPSRVRGHIEFDSVWFAYKEEEWVLKDL
SFNVEPGQMVAIVGYTGAGKTTIANLITRFWDIQKGEIRLDGSPIRDLPLHGLRRAIQPV
PQDVFIFSGTIEDNIRLGTQITEDRMKLACKAVHADEFIEALPNGYTTILSEGGSNLSQG
QRQLLSFARVLAHDPSIIILDEATSSVDTETEQIIQRGIEGLLSGRTSIVIAHRLSTIRR
ADRIIVLAQGKIAETGTHDELIANKGLYYNLYLLQNSGRDV*
>SPSA8_v1_90028|ID:41145194| ABC transporter related protein [Spirochaetes Bin 1 SA-8]
MIMLKEFKTIFPYLSKYKFRYIAGILFLMLVDAAQVLIPRFVKQAIDIIALGTFSIQEIW
KPVLSMLAVALVISIGRFLWRLFIIGASRQIETEMRDALFSHLLDMSASFYQKNKTGDLM
ARATNDMNAVRQALGMGFVAFVDGVFMSSMILIAMISDNPAVALYTIIPLPLITVLIFFF
GRLVGGQFKKVQEVYSKLSDIAQETLAGMRVVKAFVKEKHFSDQFSSENDHYRDAVMDLV
KTFGFFFPLISFLSGISTVILLSVGGSAVIKNRMSPGGIIAMISYLEMLVWPLMGAGFTV
NIIQRGAASLKRLNEIMNEKPEIASASNTVAQPEGTGIEIRNLNFSYPDSSRKALSGISL
RLEDGRTLGILGRVGSGKSTLLKTLPRLVDTEKGSVFIGGIDICEFELKSLRSMFGYVPQ
ESFLFSDSVRANICYGKPDITEERFHELARIASLERDVKLFPAGWDTVVGEKGLTLSGGQ
KQRIAIARALALDPPILIMDDALSAVDAETEEKILANLLVERQGKTNIIVSHRVSTLRNA
DLILVLDGGHVVQLGTHEALMAETEGFYAEIARLQELEAAAVSGSKEV*
>SPSA8_v1_90029|ID:41145195| conserved exported protein of unknown function [Spirochaetes Bin 1 SA-8]
MKFCVKKLLAFSLVISFSLAPAFLFAQNTAFSVSLEAETGTVKVFSHTYRVGPAPANTNF
DFVTMGGQEILFPFSRLSAELLIANRHSVRFLYQPLEIATQSVARTAFTIDSITFAKDSP
IDIIYSFPFYRTTYLYDLLKGENFLGVGAAIQLRNASIRFSGFDGSRNEVRTVSQNLGIV
PALAVAGKFSPPGNFYAGFEATGIYASSALFNGATFQFEGSILDASVRVGKSFSNGVEAF
LNARFLGGSAAGVSQYPDLYWTESQDDETANYLATGSLTLGARLTL*
>SPSA8_v1_90030|ID:41145196|rnhA| Ribonuclease H [Spirochaetes Bin 1 SA-8]
MDEIIIYTDGGCSGNPGPGAWAYVMRYRERLREDSGFSPETTNNRMELEAVIQALSFVRQ
RRHAIAAQSARHPSWLLSPIKIHTDSQYVKNGITAWIAGWKARGWKTSARQPVKNQELWM
RLDELVTELQPEFLWVEGHAGIPDNERCDFLVRETMEKHSQSSP*
>SPSA8_v1_90031|ID:41145197| protein of unknown function [Spirochaetes Bin 1 SA-8]
LKITVEYLGFLEIDSIKSGSAMELPDGTTAGEVFSRLGLKGSYLKYVLPIINGTRAGHDQ
PLKDGDKLFIYLPVGGG*
>SPSA8_v1_90032|ID:41145198|fusA| Elongation factor G [Spirochaetes Bin 1 SA-8]
MPFSTEQIRNLAIAGHGGTGKTTLLEHILFQGGVISKPETVESGKTVSDYGEDEIARKIS
VRSSLTHCTYKDTKINFLDTPGSSDFVGEVILSFRACESALLLVDARTGVQIETIKLWRN
LDSRTKPRMVFIAKMDEDRASFQNALDDIKAKFRMAPVPVSIPMGEGLDFNGVIDVINAK
AYVKPASHDQKEVASEIPSEYKDAFEAARAQLFEAAAEGDDDLMEKYLNEGELSHEEMLT
GLKKALALAKIVPAFAGAGSRNSGITPLLDFIVASGPSPKDLAPEEVVDADGNITQVSVD

AAKPASAMVIKTQIDQFSGRLSFVKVITGALVLELDVVNVRDLRKERISKLYTAQGKKLE
EVSSLPAGDIGIIAKSASLKTNDTISAWDKPFAYKPLKLPTPVHMLAISALNKKEEDKLN
ELLLKAAEEDLTFQVNYNTETKETVIAGMGELQINMILDKIKAQSKIAAETRIPRVAYRE
TITKKANAEYTHKKQSGGHGQYGRVVFDVEPLERGKGYEFENKVFGGAVSKGFMPGIEKG
IHQAMEAGVVAGYPVVDVKTSIIDGKEHPVDSSEMAFKLAARGAFREAMRQANPVLLEPI
MNLTVYVEEKNLGDVMSDLSGRRGKISGQNPIGGGIVQVDAQVPQAELLRYAIDLRSMTS
GTGSYEVEFSHYSPITGKIADDVIKAAQAFKTAEAEEE*
>SPSA8_v1_90033|ID:41145199|nrdR| transcriptional repressor of nrd genes [Spirochaetes Bin 1 SA-8]
MRCPHCGSLDDKVIDSRSLANGEAIRRRRECLSCGLRFTSYERIEEKQLLVIKRDGRREP
FERSKIERGLIRALEKRPVSQMSIENLINEIEDESAQAAKATNEVSSELIGNLVLSKLYR
LDKVAYIRFASVYRKYETPDEFMREIELLEKNKQDQ*
>SPSA8_v1_90034|ID:41145200| SPFH domain, Band 7 family protein [Spirochaetes Bin 1 SA-8]
MLWFILGFSLCIAWWLSFALIAFVRKQKPVYIFLLAAGLVWGAAELILAFAYESLAVFPL
FLVPVVIMLPMMRIINEYQRGVLFRLGRMMAVVGPGFNLILPFGLDVMRTVDMRTFTIDV
SKQEVITRDNVPVIVDAVVYFNVFDPTLAVNKVADYTKSTSLLAQTILRSVLGQHELDEM
LAKRAELGQILKELLDEATDPWGIKVTAVEIKAVELAETMKRAMARQAEAERERRAKVIA
AEGELQASEKLAQAATVMSAAPSSLQLRYLQTLSEIAVEKNSTIIFPLPMEILKYFSAEK
KEP*
>SPSA8_v1_90035|ID:41145201| conserved protein of unknown function [Spirochaetes Bin 1 SA-8]
MDFSEKVKSFIEKSIDSSKDFIAKAGSQAQVWSAMGKLKFEIIQLRSQAQHLMTQLGTET
YRLLVEKNEPMIGSSTQEISPLLTQLQKIEREIAEKEEAFKAAGGKDADLDGDGKPD*
>SPSA8_v1_90036|ID:41145202| putative phosphoglycolate phosphatase, bacterial [Spirochaetes Bin 1 SA-8]
VRKNEVRAFLFDLDGTLADTLDDIAFSVNQVLKRHGFPIYPASAYRMMVGDGFARLMERV
LPAEAQANMSFFESLKEEAAASYKDNVMRSTKVFPGIHAVLQEFTQKGVPFAVLTNKPDH
LAKLLIGELFPDFKFLAIAGDLPGKLRKPDPTRALEISGISGIPASDWAMVGDSGVDMEM
AVRAGMLPCGCLWGYRSESELRKSGARHLLSKPWDLLTLV*
>SPSA8_v1_90037|ID:41145203| Phosphomannomutase (fragment) [Spirochaetes Bin 1 SA-8]
MHTHHGKGVFMEKAQLLARAQEYMHQESDRAFAQEVEKLIASDDFKELEDRFYRDLEFGT
GGLRGIIGGGTNRMNTLVVTKATQGLCDYLKEQYPTKSLSACIAYDSRRRSSDFALATAL
VFAANGIKAYMFTSLRPTPELSFAIRX
>SPSA8_v1_100001|ID:41145204| 2-isopropylmalate synthase (fragment) [Spirochaetes Bin 1 SA-8]
VEAGASQIECTINGIGERAGNAALEEIVMALATRHSIFNAATRIDTTQLYKSSRLVSTVT
GAKVQINKAIVGENAFAHEAGIHQHGVLANRQTYEIMTPESVGIPKNSMILGKHSGKHAF
EARLHELGYSLRPAEIELVFAKFKELADRKKTVSDRDIEALVRGASAIIPEKFKLASFVV
NSGTQITATATIRLAIQGNGTIEEVAAGDGPVDAAYKAIDSIVKRKLELADYRLNSVTSG
EDAQGEAMVKVRCEGKTWNGIGVSTDVLEASIKAYISAINAMEWELESFQQGGEQ*
>SPSA8_v1_100002|ID:41145205|leuC| 3-isopropylmalate dehydratase large subunit [Spirochaetes Bin 1 SA-8]
MKQSTAMTMTQKILAHKAGKKEVRTGELIEAAIDRALGNDVTAPPAIDEFEKLGKPAVWD
PEKVVLVPDHFTPNKDIKTAELTKKMRLFSRKMNISHYFEVGKMGIEHALLPEQGLVTAG
DCIIGADSHTCTYGALGAFATGVGSTDLACAMALGRLWFKVPPAIRFKLSGSLSGWVSGK
DLVLHIIGKIGVDGALYKSMEFTGEGVGSLYMDERFTITNMAIEAGAKNGIFPVDSRTTA
YLEYVRAQKPQAFSTDPDAQYEKTIEIDLAKVPLTVSFPHLPSNTKTIQEAAGLPIHQVV
IGSCTNGRISDLRIAASIMKGRKIDDSIRCIIFPATQRIYLQALREGLIETFIEAGAVVS
TPTCGPCLGGHMGILAAGERAVSTTNRNFVGRMGHKDSEVYLASPAVAAASAITGRLTDP
EEICPVPLSRDVLYSMEF*
>SPSA8_v1_100003|ID:41145206|leuD| 3-isopropylmalate dehydratase small subunit 1 [Spirochaetes Bin 1 SA-8]
MAYQKTASGRVHKYGDNVDTDVIIPARYLNAFKPEELAIHCMEDIDAEFSGRVQAGDIIV
AGYNFGSGSSREHAPLAIKASGISCVIAASFARIFYRNAINIGLPILESPSAVSALQNGE
RVEVDFSDGTIRSLDSGRLWVTVPFPKFIQEIIEAGGLVEYTRRSLDAEQERTI*
>SPSA8_v1_100004|ID:41145207|leuB| 3-isopropylmalate dehydrogenase [Spirochaetes Bin 1 SA-8]
MRAKIGVIRGDGIGPEVVGSALEVLRAVSRKFHHNFEEVELLAGGCSIDTFGEPLTAQTL
EQAMQCDAVLLGAVGGPKWDSLPGAMRPERALLGLRSGLGVYANLRPVKLIPQLKEASPL
KLEILQRGIDLLIVRELTGGMYFGNRGRFTDQAGNAAAFDTETYSAPEIQRILEVGYEAA
MSRSKRLCVVDKANVLESSRLWREVAGSVAGRYPEVTTSYLYVDNCAMQLILNPSQFDVI

VTSNMFGDILSDEASVLTGSIGMLPSASLGDMRGGKAESGTVRRGLYEPIHGSAPDIAGT
GKANPLGTILSAAMMLRYSFNLETEAQAIEKAVETVLDSGFRTADIAGHGEAKSEKLTIP
ATTEDMTREVVRAV*
>SPSA8_v1_100005|ID:41145208| conserved protein of unknown function [Spirochaetes Bin 1 SA-8]
MRIITFGEIMLRLKSPGQERLFQSPLFEASFGGSEANVAVALARFGLEAAFVTLLPDNQI
GKECARTLRYHGVDTSFLRYKKGRLGIYFFENGAAQRPSNVIYDREGAVITLIDEKDLNW
KNIFSDASWFHVSGITPALSKQAADATLLAVQEARNAGLTVSFDLNFRAKLWNYGEPAQK
RMRQLAEYTDILIGNEEDYQKSLGIAGPQSADSGIIDSQAYLRMCRQALEEFPHVRYAAV
TLRESFSADHNAWSALLVSRSGFWKSRRYDIADIVDRVGAGDSFSAGLICGLIEHSNDPQ
QALEFATAASCLKHSVPGDFAVLEKAEVEKLVAGNASGRVQR*
>SPSA8_v1_100006|ID:41145209| Putative KHG/KDPG aldolase [Includes: 4-hydroxy-2-oxoglutarate aldolase ; 2-dehydro-3-deoxy-phosphogluconate aldolase] (modular protein) [Spirochaetes Bin 1 SA-8]
MQNMKELFYSSGIIPVIKIDDAEKADSLAGALEAGGLQVAEITFRTQAAAKSIELFATHH
PSIVVGAGTVTTKIEVDTAINAGAQFIVSPGFNPEISAYCIEKKISVFPGVNNPSLIEQA
MSLGLTELKFFPAEVSGGTKALKAFESVYSAVSFIPTGGIQENNLNDYLALKNVLACGGS
WIVPPELIQAGNFDMITALVKSCRRTMIGFLPFVPTTESHNAQDAALEIKTPSLKRTLAM
LGLSGTYNSKEKSKYLALGDTIIRIAE*
>SPSA8_v1_100007|ID:41145210| conserved protein of unknown function [Spirochaetes Bin 1 SA-8]
MILTSLQHIERFGAISSNLAAAASWLKENDFQTLPEGRYSIDGDKVFALVQQYQTKSPEL
CFPEAHRVYIDIQLLVSGREIVEVCGAEGLAIKEPYKPDIEFFQPAADAQKILLKPGFAL
ILFPEDVHRPCMSESSSPEPVKKIVIKVAL*
>SPSA8_v1_100008|ID:41145211|kduD| 2-deoxy-D-gluconate 3-dehydrogenase [Spirochaetes Bin 1 SA-8]
MILDEFKLDGKVAIVTGSSTGLGQGICLGLAEAGASVVGVDYVPSTETEAQVTAMGKKFL
SITANLLSIEPIPGIIKQAVDTFGRIDILVNNAGIIRRADALDFSEKDWDDVMNINIKTV
FFFSQAAARQFREQKSGGKIINIASMLSFQGGIRVPSYTASKSAVMGVTRLMANEWARYD
INVNAIAPGYMATNNTAALRADAERSEEILKRIPAGRWGTPKDVQGAAVFLASGASSYVN
GYTIAVDGGWLAR*
>SPSA8_v1_100009|ID:41145212|kduI| 5-keto 4-deoxyuronate isomerase [Spirochaetes Bin 1 SA-8]
MHTREAVNSEFAKQLDTQGLRDSFLIADLFQPGKLSMTYCHTDRMIAGCAFPLDTPIQLP
ATKELGTDFFLARREMGIINIGGAGIVSADGREFELDNQDALYLGMGTQDINFKSKSPDA
PAKFYFNSCPAHRACQTRRITRAEAKHVEMGSPAECNQRVIHQYIHPAVLESCQLVMGLT
SFYEGSVWNTMPVHTHERRMEVYLYFDLPKDGIVIHLMGKPEETRHLIVRNEEAVISPSW
SIHSGVGTGKYTFIWGMAGENQTFTDMDAVPMSALK*
>SPSA8_v1_100010|ID:41145213| TRAP transporter, DctM subunit [Spirochaetes Bin 1 SA-8]
MNLAAMFVMLFLLLFLGVPIAYSIGGASILYFLLKAPVFLLAMPQRIWSGTNNFIIIAMP
LFMLAGEIMNKGGLTKRLINFSMLLVKPIRGGLGEVNVVASMIFGGISGSSVADTSALGS
ILIPAMVEKGYPLGFSAGITVASSTMGMIIPPSVPMLMYAMASGASVGKLFLAGFVPGAL
VGITQFVLTYIISKKNHYHPIHERSSFRTNFKIAKDGTLAILMPLLIIVSISAGIATASE
SAAIAVLYALILGFFIYRELSVKHVLAALKKTAIMSSSIMIIGGFTMVFTWILAVEQVPA
MIANFIISSGISTTMVFIFLDILILFVGTFLDVTPCILLLAPILLPVMKQFGMNELQFGA
ILIVGLAIGLVTPPVGMCLNVANKICGLSIVRIFKEALPFIICNVIVLILITFIPELSLW
LPGLIK*
>SPSA8_v1_100011|ID:41145214| TRAP-type C4-dicarboxylate transport system, small permease component [Spirochaetes Bin 1 SA-8]
MSVPQHIAAPIIRNKSAHGQGKRMGKKSGGSWLNTLLHSLDKTLAILLGALVFILATGVM
ISVFLRYVFGVSFAWAEELLTMTFIGTTFFGSALGIREEEHIALGIFKPKNPILRRLFIG
INSILLILILSFVFYYSLQWIAKVGSVPSPSMGIKYLYFYIMVPVSFALSIFYAFCNILG
LFMHLDPPSTKSIFEDVTAEGGMQ*
>SPSA8_v1_100012|ID:41145215| Tripartite ATP-independent periplasmic transporter solute receptor, DctP family [Spirochaetes Bin 1 SA-8]
MKKAGALLIMCFLVFSMVFAQSTAKVYKMKLGHASSTESTRHKALLVFKNYVEEKSGGRL
LVEIYPAATLGNESDMIESMKLGTQEAFVGGVFDAQTPKLNLILMPFFFNSQADLMKVAK
SDIGKMIMKDAEKYGIKMLAFGDGGSRHFTNNIRPIKSPADMKGLKXRTPPIESIMKCMQ
ALGXNPVSIPYGDTYMALKTGVADGQENPXANIGDMKFFEVQKYLTLIDYQFHPEPFDVN

LAFYNSLPADLQKILEEGAWVYTEXQNENRRNMNDYYFNMISNXGVQIYKPTDAERQLFI
KACEPVYDYFVGKGLFTKADLDEMRKIIASK*
>SPSA8_v1_100013|ID:41145216| Transcriptional regulator [Spirochaetes Bin 1 SA-8]
MPEIKVQSLDRTFRILEILARAPNGLSLVEIAAVVDLPKSTAFRLLAVLIQHEYVRKTDD
ALYKLGPGFIELASHYLNSLELKTESAPLMRELAANLGTIVFLARREGAMMVYIDKQDQF
TNLRKYSIIGQQKPLYCTSLGKSLLLDMTDAEIRLLLADTDFEAFGPNTHKNIDSLLADL
HRCKARGWTHDDQEAEPDVNCVAAPIRDYRGQIISAISTSWVLATRPDLEPEKVSVHVMK
AAAAISAAMGYSGKNSEE*
>SPSA8_v1_100014|ID:41145217| protein of unknown function [Spirochaetes Bin 1 SA-8]
MRVSQRFFKTERNRKTNFNQLSPKYEIASVGQIFEHPLQNSTQASGLETFGALSPFVYTP
CVQKSAHALQFTHFDSSTIGYHVAAIEFPPLDFSIDMFHNIATLIDCQQ*
>SPSA8_v1_100015|ID:41145218| conserved protein of unknown function [Spirochaetes Bin 1 SA-8]
MAKKGLLMCVCQGNCPSFQQMNIFEVGNAIRREKLVDYIAIHPQLCATDGDSFLTTLLQG
DSTEQLYVAACDPTMQVKMYRDAMEAAGFSKENIHGVDIRNMSTEQAVDAIKKMLSV*
>SPSA8_v1_100016|ID:41145219| putative Dinitrogenase iron-molybdenum cofactor biosynthesis protein [Spirochaetes Bin 1 SA-8]
MNIIVMAKGAGLGAWVDSDFAHAHQIVYVPARGGFDALGNPFLPDSSEIEEVSEALAEFV
LSKFPDAEAIIAGSFNREICEYFESHNVAVFTEPFGSVLELVEKIRAGVQA*
>SPSA8_v1_100017|ID:41145220| putative membrane protein [Spirochaetes Bin 1 SA-8]
MDVGNIYLIIAAGLLVFSVFKSKEKTKKALIVAGKSALTVVPVLVVVFLLMGLISAFLSK
ELISQVLGDKSGLPGILISEVLGSIALIVPAAVFPFTGMLHEKGASYPIIFAFIMTAILI
GISTIPAEIKFFGARFTIVRNILTFVFIFALSILFGMLIA*
>SPSA8_v1_100018|ID:41145221| membrane protein of unknown function [Spirochaetes Bin 1 SA-8]
MKKILKEAVIYLVIAAVIVLAVALFIPAKTENVLSVAWNALLGAIKIMISVFIFIGLLQV
WVSTEKLSALLGKEAGWKRFVLSSIIPIFIGGSLFTIFPMLKTLKDKGASYAAIFAFITA
WSGKGPLIPLEIHFLGLKFSILRILIIIPFAVVMGLLGEFIMEAIDKRDASS*
>SPSA8_v1_100019|ID:41145222| conserved protein of unknown function [Spirochaetes Bin 1 SA-8]
MNLLETKNLTLMKDNRPLLNNLSLEIWEGHIHAIIGPNGAGKSTLANTIMGLSGYRDFSG
DILFMGESIKGLSVDERARKGITLLFQEPARFEGLGVSQFILAGAAKKSISLAEEALHKA
GLAPERYLKRAVDKTLSGGERKRIELASIYAMKPRLVLMDEPDSGVDIDSVKYIFSVIKE
LRKEGSTVLLITHSPEVLKHADHAFLICAGTLVDKGEMTRMFDYFNGKCVPCVHVGAPES
EIQLKSLAAV*
>SPSA8_v1_100020|ID:41145223| SufBD protein [Spirochaetes Bin 1 SA-8]
MSEMTSVDKEALISELLASINEHKYGPDIAHIEVHGNEVLGKNLVEGLEVESVSIENGVE
VSIRVKKGYTIKNPVHFCFGIIPDNGVQRIITTTIIEEGAHAQFMAHCTFPNAVNVQHIM
QASIRLEKGSSLSYFERHVHGPEGGVTIKPVTRVTIAEDARFSTEFELIKGAAGTIELDY
EAECGKNATVDMLTRIFGRFGDRIHIREAAHLKGDGAVGVLTSHIALKNSASAVIENEIV
ADGSYSRGHVDCKEIVQDQAVARAIPIVQVNNHLAHVTHEAAIGSVDSKQLETLLSRGLN
EDEATELIIQGLLS*
>SPSA8_v1_100021|ID:41145224|argD| Acetylornithine aminotransferase [Spirochaetes Bin 1 SA-8]
MKMEALDLKRNYLVPCSYHLYQRPPEIVRASMQYLFDANGTQYLDFFAGVSVMNCGHCNP
QINEAAKAQIDKLQHTTTLYLTRPIGELAEQLARILPGSIKRSFFCNSGTEANEGAFLAA
RLFTGRRKIAALTRSLHGRTNLAMSATGIPMWRTDPFLDTAHTVILPGPYDETAGMVTDA
AADASLSTISATLACRDFAALIIEPIQGNGGIIPLPDKYIQQLRQLCTETGTLLIADEVQ
TGFARTGTMFAIEHSGVVPDIMTVAKALGNGFPIAAYCTTDTIAQAFTKPSASTLGGNPV
SSSAALAVIRYIEQENLVHRAKELGEKLMTGLLELKNRHPMMYDVRGRGLMVGAELQKAD
GSPNPEGTDIVLEFTKDHGVLIGKNGLYRNVLAFQPPLIITEENISKMLSVLDDALTHLS
KTSAS*
>SPSA8_v1_100022|ID:41145225| Peptidase U32 [Spirochaetes Bin 1 SA-8]
MRCYFGKQVELLAPAGTYQILESLIDAGADAFYLGGKRLNMRLHRSEYNFSEAELIKARE
LTRRTGKNLYITVNNLLSEKELSEARQWLEFLAEEVQPDAVIVQDISLLSIAREFGLQIH
ASVMMNAHNIPGIRELQACGVTRVVLSREASFEQARKIHEATGIEIEYFAHGDLCSAHGS
QCYYSGMLFGMSSNRGLCMKPCRWPYSASYVVDGKEQSSTRFFPLAVRDMFLYPHLQDAI
EAGIMSFKIEGRMRTAEYLKPIISAYAKFLDALCGSKAFDPLQEYEQLYQNRMRDFTCAY

AFGKPGSAILNERWEGTGKFYSTGKVFSVAKEEPEAGEAQIQRVRSRIESRNTGDRAAPA
GIGKKPELSVRVHSRDQAEKALQNGADILIVPLDSLLPGFQPHAHDLIELKRAFPEARLA
LSLPRMMNDDEFEEIRQVLSRLNTRYDELYISHLGQERYFREAAPALCADFSTNIYNSRA
LKFWKSRGISTAGLSLELKALELLDLLEAVSAEQAANADNTAHPELLAYGRPTVMYMELD
PFQAVREKSGLGRGQPMSVTKLVDEKGYQHPVVGDTRGRFHVLPVKPVNLYRLIPELAKA
GVRRFRIEGATMEPEQLGIVVRAFADLFQTGIIEPDRIPRDQAGEWLGALGMLGPEGKEE
Q*
>SPSA8_v1_100023|ID:41145226| conserved protein of unknown function [Spirochaetes Bin 1 SA-8]
MTEVQSWSNLTRAERTKNALIKHGFDALVVSTKEEALAEVMKFIKPAAKVGFGGSMTVKA
VGVQEKATAAGAIILDHNKPGLSAEEKMEILRAQLTCDVFISSVNAVSMEGELFNIDGNG
NRVAALTFGPRKTVVVVGANKIVENEEAAWARIRAWASPMNNKRLNKENPCVKTGQCMDC
DNAGRICRVYQVLRRKPSVSDFTVILVGEELGF*
>SPSA8_v1_100024|ID:41145227| Metallophosphoesterase [Spirochaetes Bin 1 SA-8]
MKISWMDERLTAPQKRVILWSLGALLLCLAMNAYHYSLEGAKGLYLWNRTGLFRGLILMA
FIPGSLALLSFPLEKLKQKSASKIMRGISLASSVVVALLSAGILAFLIIGPRSGSVEPPR
LKLINPVQGIQPSSPSLAAGGIFSNSAAPLTAQPAGQSVVPASLAPPLLRLSFSSDPHWG
ADTSNTQARSDILAHIAERKPDAFFMLGDTVETGSKALMWNFALADLEAIIPSVPLRVLL
GNHDALFGGQYLYKKAFMQKDFKSDSGSPYYYSIDAGAAEIIVLDLPWGTEQFNARQKKW
LTKTLTESNRTKPVIVLSHSFFYASGYDDPELDKPWYDHFQNIPAITPLFEQHGVDLVIS
GHNHYQEYLEKNGVRYAVIGAMGGIPDPKPKYVSPASKWIAVNQHGWLDVDIYEGEMVLS
FRDQNNEVLHQEQIRY*
>SPSA8_v1_100025|ID:41145228| conserved protein of unknown function [Spirochaetes Bin 1 SA-8]
MDMVRLQFEKGRRYRCCFSFLFLAITFYSAVLTTTLGAQDLPGVKEIPDIFSNSDLLAGT
NTGLIRIRKDGEKTTLWQEGRVTAIAKTAGGWFFLSSKGVLFSADLRKFENRSAGLPKKT
IKLVVSEDPLSTQLVKETVNLTAIAVDPNQENRLLVSTGSEVFFSETFGRTWISLGSPSV
SGGIKAAAFGPPSQNSKNSSVWVSTAIKGVFTRDLDAKNSWASASGGLPPVLGSNYEEVS
GFAYSTSLPSRGLYAATTFTGQIFQWDSSAMKFVPRFSAGKDFSSIEAFSISHKSGAYFL
SDGIITRLDSQNDGKLSAVQLKPATNILAARLKNALSDGNPAINCLALLLEEQKPNEEPL
IFNECWQLPSFAAAKPESSAALSKSEQRKIAANGKRGLYLQTGFLIDSASRSKYFSFMQE
KNLDTIVLDLKDDSGKLRFAPRDPLLLAMASPGALLDLDAFAAEAKARGIYLIARIVVFK
DEALYRWKDGALAVRNTQDGTPWQGVKKDGQLVQEYWVDPYSPLVWQYNTAIAREVVSRG
IDEIQFDYIRFPTDGENLKFIRFPYQESEMNQESALESFLRYARARLDAPLSIDIYGANG
WYRSGSRTGQNAELLAEYVDVICPMFYPSHFEQEFQAQAPAELRPYRIYKLGTLRNYHIA
RGKALIRPWVQAFFLNVSYDRQYYNETYVANEIRGAQEGTNQGFTFWNNSGRYDDIPRLE
PGVMNLAYGF*
>SPSA8_v1_100026|ID:41145229|dppF| dipeptide transporter ; ATP-binding component of ABC superfamily [Spirochaetes Bin 1 SA-8]
MSDIILSVQHLKKYFEPHQTFTQSITGGGQKKVIKAVDDISFNIERGEIFGLIGESGSGK
TTTGKLVMKLIEATSGKIVFNGEDVTVLNKEKLQEYRKKVQMIFQDPYASMNPRFKIKDV
LEEPLIIHKVKSSIEERTKMIVKALEEVKMTPPEEYMGRWPHMLSGGQRQRVATARTLIL
NPLMLVADEPVSMIDLSTRAEILAMMKEVQKIMGLSYLYITHDLSTARYFTDRIAVMYLG
RIVEMGKAEDIIDNPLHPYTQALIEAVPEPEPGKVCIIKELPIFGEIPSPANIPPGCRFH
TRCPYAVDDCKTLPEPELMLDGDGHLHACRRFKEVAQIRGRKIVH*
>SPSA8_v1_100027|ID:41145230|oppD| oligopeptide transporter subunit ; ATP-binding component of ABC superfamily [Spirochaetes Bin 1 SA-8]
MNSLLRVENLQMHYFTSKGAVRAIDSINFDVMPGETLGLVGESGCGKTSLGTSILRMPTP
PGRYVGGKILLEGTDIIPLPEKEVRKDIRWARISMVFQGAMNCLTPVYTIGKQMLETLNE
HSEMPRNDAENLMKEYLGYVGLPPEIMGRYPHELSGGMKQRAVIATALFLKPSLVILDEP
TTALDVIVQSQIINLLKKLKAQFNLSFIFITHDLALEAEVADRICVMYAGKIVELGTNEQ
IYGSSGPAHPYTERLLAATPRLFKKVEKLHFIPGTPPDLIHPPKGCRFHPRCHKAFERCR
EEEPPLVAIEPGHFSACWLNHKG*
>SPSA8_v1_100028|ID:41145231| ABC-type transporter, integral membrane subunit [Spirochaetes Bin 1 SA-8]
MNLNDTKYRLLEFWSEFRKERSGLIGLAILITALLVVIFEPVIIPWKEANSKWRSIDYWQ
DNSPSAPPAWTNAFSKLKAPVTVKLEKGTKEETELDGGVRLVTYTFTYDYKADKAPLDLI

FHTSGTGDIPFSVSIERPDGQVIDLAQRFEQGLNGQDIRISADNDGREASFAFIKQIESE
TAIEQFSGKSLRSTDLLFNEARAGMSTEFKPLKGTYKLIAKAMILDPENFKIEDPYVVVT
GSVSGILGTDDSKRDLFSGLIAGLKWALLIGFLTSAISVLIGVMYGIISAYFGGKVDSLM
QFFYQIVNSMPVLPVLIVISAIFKPSIFMLIGVMVFFFWTGSVMTVRSMALQIKEETYIE
AAKALGAKKSRIIFKHMVPILIPYSFASMALSVPSAIVYESSVSLLGLGDATIVTWGQIL
HDAMQGAAILKGVWWWVLPPGLLIAIMGMTFAFLGFSMDKILHPKLKTR*
>SPSA8_v1_100029|ID:41145232| ABC-type transporter, integral membrane subunit [Spirochaetes Bin 1 SA-8]
MYKWFALKRVLKGIFTYIIIIFILSALFNTVNEQTMRANIEEQVRAESVKLKNMQPSAIQ
KFQQQRKDELIRLYKLDRPIMERIIFRTWNIITFNFGKSTIIKSSRGERDVMTIIGEAIP
RSLILFTLAAAIEIFVGIILGLKKAQKPGGTLDRSTSFITMIVYGMPTFWLAMILIMFFV
YQLKIFPSAGMHSVPPPSGFMYFLDLLWHMALPLMTLLLIGFWGIAFVVRNIVLSILQED
YIMAARARGIPEKSVLFGHTLRTAAPPLLTISILSLLGSISGAIIFEGIFSWPGLGNLYW
IAVQQNDIPVLMGDLAITVGLYQLGLIMLDLTYGFMDPRIKVGGKA*
>SPSA8_v1_100030|ID:41145233| ABC-type transporter, periplasmic subunit [Spirochaetes Bin 1 SA-8]
MKKILTVSLVALALLMSVNAQVKNGPIADKVIYDVRMDQTIAIKDTAEGKTDVFFTGLDA
KTYKGIQKADLDKLSTYAVPSGSWSLLLNPIPNKAPYTFQHKDGRVIFNPLAIREVRYAI
NWLIDRKKIVDEILLGAGEPAITAQTPGQPGTYKFNLVPAKLGMTVRGNEKKAIADIDTA
MKAAADLPENKGKLAKTGQFWTYNGEPVTIRFIIRVDDPSGRLLSGRYIADQLEKAGLKV
ERLEYDRSKAGKLVYSGDPAAWEWSMYTEGWGAGATRSWWDITISQMYSPYYGYMPGGAT
EGFWNYENAEIDAIAQKNINGWFLTADEYWEGNMKVQEMALKEAVRIYVCSQTQYYVANK
ARFNARMLYGLGDGLNSWSVRSADVKPNDKGEKVLRVTQYSARGGLFMSSWDPVGVDGFS
DVYSAAIVEACSDMSTFESPNSAKDTPLRVKYDLKNVETKVVAGKDGKPEGTIAVDPAAM
IFNSKTKKWETGVEYKDVGDGNYDYVKNAALKSYSKLSGLEYIYGKWHDGQPVTLADIMY
ATAFAYEWANKDSDDDKYYDEAYASQYQSSLPISKGVVLNKNGTFTTYFDFNWPMDKDRV
ASSGTVSPKAGNPGRQTVVSFEIYEALAKLVAEGSKSGTQYSFSGDPSMTEVDVINPKCV
ADIKAKLQDFVAAKYVPDAIKQWITPEQAVARYNAALAFIDKYGHAYISNGPFFISKVDY
NANYIELSAFRDYPYKADYFPKLFATTLTRIDDVKVPATVQKTSDAKIDIAVSALTYPED
TAKAADNKAKVTVTMILADNTEKVYTAKFVSAGNFQAVIPAKDLSGLKSGAYTLVVQSVF
GTEAPSVQPAALVLF*
>SPSA8_v1_100031|ID:41145234| 5'-nucleotidase/2',3'-cyclic phosphodiesterase-like hydrolase [Spirochaetes Bin 1 SA-8]
MVKKLRAVVLILAVLLSLSFVQNVVAQEVKITLLHTNDTHARIVESKTELGYSRIAAAFA
DAKAKNPNTLLIDLGDTFHGLPIANIDQGASVVKLMNELGYSFMTTGNHDYNYGFDRLME
LDRMAGFQILAANVYKDGKRVFTPYSIQNIDGVRVAFFGLATPETAYKTDPKGIEGVSFT
DPIVEARMVLSEIAGKYDVLVLLSHIGVDKSSDPTAIQLAQALPQIDVILDGHSHTSLAD
IQKENTTGVLITSTGAYGAGLGVVDLVIGADRKVASKTARTITVANSPELKGDARIAQMI
TDLGKAQDAVLMQVVGKTAVALEGKREIVRTSQTNLGTLIANAMLYVTGADVALMNGGGI
RDSIPAGDITKKQIFTVQPFGNYIQTGKVKGSEFKAILENGVGKLPAPDGRFPHLANLTY
TLDVSKPAGDRVSNIMIGGKPVDPDKEYVLATLNFIFNGGDDYRMLVGKAQNDFPSDAEV
FIKYAEYLGTITNENMVYKK*
>SPSA8_v1_100032|ID:41145235| putative DegV family protein [Spirochaetes Bin 1 SA-8]
MKLTYIEGPDLGKALVSGSFQVMKNVESLNEINVFPVPDGDTGTNMASTVQTISAAFAEG
IPDNVSQVLDVAAASALEGARGNSGAIFAQFFHGLAKELRNEARVSMRRFSEAAINAAEH
AQRALSHPKDGTIISVIRDWALQLQTHAEKSSDVTEVMDSALAKARLSLEETRNILPEMR
SAQVVDAGAKGFVHFLEGVLDYMKTGVLKSFKAQPSLRLAGQALHTAHDSIEHGRYCTEA
LLQGDRIDAEELKTFLEEHGDSVVVAGSETRARIHVHTDHPSVIFDFLETKGELSSHKID
DMVLQVRLSGMKKRKCAIVVDSACDITDEFRLEHGILRVPLTLIANGRPRPDGDGYSDAA
LRNRMRTDPTLVMTTSQPSDAVFKRAFEIALSSAEKALYIGLASALSGTFEAGLRAAREF
SGRVLCVDSRELTAGYGLLVTRAVRAVAAGKSAEETARLVEQWRRNLALFVAVPDLTSLE
RTGRLGGVKGLVLRKFGLRPVITPGPDGKAESGGLYLGIRKGPEKLFRLMRKKFGKHERL
EIQIVHVDNPTAARELADMVEQHFTPSRPIVISQMGPLLSSLAWLGSVGIAALPEGEELL
ASSAEGGKA*
>SPSA8_v1_100033|ID:41145236|plsY| Glycerol-3-phosphate acyltransferase [Spirochaetes Bin 1 SA-8]
MSWLVAGLAAAYLLGSIPTGVWIGKWLFKKDPREGGSGAMGATNMFRQFGAKAGIPVILV

DVAKGSAAVLFAVWCWNMGAVRNAVGATGTAAMLYQLSAALLAVAGHVFPIFAGFRGGKG
VATGAGALIIIAPLAALWSALGFMIVLGFTGIVSASSMVAALILPVAVALGAQSRPPSIP
LLAFSTVLALFVLFTHRANIKRIFQGEEKRFEKLRFLARLVNKKNTR*
>SPSA8_v1_100034|ID:41145237| putative DegV family protein [Spirochaetes Bin 1 SA-8]
MKIRYLDGTRLRRAILAGTATLIANTENLNAINVFPVPDGDTGTNMAHTVKTIARRLASM
KPVRKEKAGAVLEQTAVSALQGARGNSGAILAQFFCGLADELKADARVNARRFAEAVRNA
VQKTKTALSTPKEGTILTVLSDWALALQEKASATEDIVHVFMAGYEKAKESLARTKTMLP
EMKKAGVVDAGAKGFVHLLEGIADYIRSGSIRHFARKQAETQMSAPSDSSIEFHDALQHG
FEPMLETGCRYCTEALLSSSSNSLDAIRLSLNGLGDSIVVAGNGRMAKIHIHTDTPSSVF
DALDALGTIETHKVDDMMLQQSLAVRAQAKGKANICAIVVDTGCDLPPEFMLEHGIIKVP
ARIDINGLVRPDGPALDAGRVVSLMRHEPGFSMSTSLPDESSYTRAFSLALAHAGDVLYI
GLSSALSGTFEAGVRAAKKLSPSIVCLDSRSVTSGTGALVARAVQLAEVGMRAEKIKKEL
AALSSQIAFFVAVRDLSSLIRSGRLHGLKSVILRKFGLRLILSTNSEGKAETAGVFFGRA
DTARVIFKKLASRLPKGENCAIFISHVSAEHEARELQTLCARHFGPQTEIHIAEMGPLLS
SIAWLGALSAAGFAKAPAS*
>SPSA8_v1_100035|ID:41145238| protein of unknown function [Spirochaetes Bin 1 SA-8]
MTLFTLLDGKKARGNRLGAVSNAGFECVAIADNLGGMKLAAFSESAARELQDIFVQTKIA
DIVDVHNPLDLTPMAGDDAYEMSFRTALLNEGTDLGIVGIVPLTVMMNTLAADPSKHTED
VTREDSIAFRYGRLARETDKPFVAVVDTGPLYDPLCRQLEAQGVPVFRTADRALKMLELW
RIRQR*
>SPSA8_v1_110001|ID:41145239| protein of unknown function [Spirochaetes Bin 1 SA-8]
MEKEMSKIMKTTSLTSLLERIAGEYQAKDSIFEIPAAVFSNVFSMEAESTGMQVMSGKAS
IPIGPAAGPHTQIAPNIVAAYLAGSRVFELKTVQENXX
>SPSA8_v1_110002|ID:41145240| putative Allantoinase [Spirochaetes Bin 1 SA-8]
MIFKNLLAALPGENEFRKVDIQVTGSILRAISPAGTLKQSFPEEEYFDGEGLMAFPGAID
PHVHFDEPGFTHREDFFHGTCEAARGGVTTVIDMPCTSLPPVTSTEALKNKLEIIQTKAV
VDYALYGGLHGAMSKRQIEDIVQSLKDYVVGFKTYFLSGMDTFPTISHDLFREAIKACSA
VSRPLLLHAEDDKVVAQFWKHQEMESAGAEPTWKDYYATRPMEAEIAACRHALELAAENA
ATLHVVHVGTDEAAHMLKKGGASCETCTHYLAFDETDFERLGASLKTAPPVKEPTQKALL
WKSLADGTISFITSDHAGAPDYEKFTGNPLTAYGGIPGTGTIFPYLLSEGLFAGRLSLKR
FLEATSSAAARRYGLDDRKGSLEPGKDADLILVDPDATTHFLPENMFSKSKITPFAGMRF
AGRIVGTFVRGELVYGTSRLLGGNIPHSIRKHQLSASGTIVAAQGYGNNLQWRKK*
>SPSA8_v1_110003|ID:41145241| Xanthine dehydrogenase/oxidase [Spirochaetes Bin 1 SA-8]
MKNIAESALNVRGQTQYIDDLPEPAGCLQAAVRLSDSAHGRIVAIKTDKALALDPSVQVI
TAKDVPGVNQIGFNKPDEPLLPEHEWEYWGQPVALVLAKNREIARAAAKLIEIEGEELPV
VTDPREAARNSDFILPSRTIQCGDTEEAFKHCTYVIEGRVESGGQEHVYLETQGAMAEVR
EGGKVFVISSTQGPSGVQRAIAQSLGLPMNMVEVEARRLGGAFGGKEDQAATWASLAALG
AWVSKKPVKLYLNRHEDMRATGKRHPYSSDFKIGAAQDGTILAFEADYYQNSGSTCDLSP
AILARTVLHATGAYKVPNVRVTGYMCKTNLPSFTAFRGFGAPQAFFVIESALDALSQASG
IPVAEIQKKNLYREGDSTYFGMELERVRTHEAFDRLLEKTQWHNLQSEISEFNRKSRTVK
KGAAIIPVCFGISFTKLPMNQAGALVHVYIDGSVLVSTGAIEMGQQVSRKIATVVSETLG
VPLENIRVERTTTLTVANTVPTAASTGADLNGMAARIACLEIKKRLAGKAGELLGANPDA
IRFENGMIFKNGKKTELSWKDLIVKANEARIDLSAHGFYATPGLFYDMKAERGKPFAYHV
YGAAAVVATIDTIRGTYDLESVSIVHDIGHSLDISVDRGQIEGALAQGLGWSLLEDLRFG
PDGKPMSDTLSTYKVPDISFMPRSMDIEFLPEIENPTTPFNSKAVGEPPLQYGIAGYFAI
LNALRAANPEGKTFYSIPLTPEKAAGFLDGEEL*
>SPSA8_v1_110004|ID:41145242| xanthine dehydrogenase, Fe-S binding subunit (modular protein) [Spirochaetes Bin 1 SA-8]
MHNMTNQKIMVNSQWRVPALSFGSPLLDFIRNELGLTGTKEGCREGDCGACAVLMGEKLS
DGTIRYRAIPSCISALGDFEGKHLITIEGLTANSKEGLTPVMRAFLDENASQCGFCTPGF
IIALTAWLAEPRTLDIAGAITAVDGNLCRCTGYGSIRRAAEKLCRDFADLPLDPKKRLAV
LVEREVLPKSVLAFMDDPGKRQNSPVSASAKPAEPAVLIAGGTDYYVRNPYPAQQFSPVL
LRTIGHLSEISEVQDQFGHHVSIGSAVTIHDFFTSKLVQLTVPGIETYETQFASTLIRNI
ATIGGNIANASPVGDITAMLMGLGALLCIGIPDDTSKQETRIMPIEKFFLGYKKIDLKPQ

EAILAVRLPLDNKPANPLMFSFEKIAKRKKLDIAAVNTAISFKRENGRFTSVRISAGGVA
PTPILLEKTAQLLEGKLCNRGDAKGLAELALQVSAAAESEVKPISDVRGSADYRKRMTGR
LVLAHFARLFAKDGITEELFP*
>SPSA8_v1_110005|ID:41145243|ade| Adenine deaminase [Spirochaetes Bin 1 SA-8]
MGKEIIDAAIGKTICDSNLIHCKMLDLFSGRILDDATISIRKGLIVGINDKLEAKETIDL
KGMYLAPGLIDAHVHIESSLLTPAEYAKVVLPHGTTTIVADPHEIVNVLGYDGMRFMINA
SRDVPMDIYFMVPSCVPATDFDSAGASLYASDMYPFLQDSRVLGLGEVMNYPGVLAQDPR
LMDKIALFKNAGKAIDGHAPGLSGAALSAYVAAGIGSDHECTTPEEAIEKAAKGMYIMMR
EGSTAKDLLKLLPAVRPETASRFMICSDDRHSNDLRDEGHMDYSLKLLLSNGIAPMDAFR
IACFNPSQWFKIPGTGAIRPGYKADFIAFSSFDDFRVEKVFKNGSIVAENGTLKRNFESV
FPPLRDSVNIKWLTADDFKIPDKGRKVRVIEANEQSIITGAAAVYPKVEAGYCVSDTKRD
IVKIFVIERHTGSGNIGKGFIRGLGLRRGALGGTISHDSHNMILAGVDDASIFKAARHLN
KIKGGLVFTVGDEVILDLPLPVAGLMSDKNAEFVIQRLREFEQLFKEEGLSNTSPLMTLS
FMALPVIPSLKITDKGLIDVDKFEPVSLYCD*
>SPSA8_v1_110006|ID:41145244| conserved membrane protein of unknown function [Spirochaetes Bin 1 SA-8]
MEKLFKLKEHGTTVRTEIVAGITTFMTMAYILAVNPGILSAAGLPAGGVFTATALSSFIA
IVLMALLANLPIALAPGMGLNAFFAFSVVLGMGYSWQLALTAVFLEGIVFILMSFFNIRE
AIVASIPKNIKNAVSVGIGLFIAFIGLQNAGIIVKNDATLVSLGHVTSGPALLAIIGLLF
TGILLAYRVKGALLIGIIATTILGIPFGVTKVPENFSPVSSPAAPLLFQFQFDKVFTLDF
FVVFFTFLFVDIFDTIGTLVGVTTQAGLIKKDGTIPKVKQALLADAVGTVAGACLGTSTV
TSYVESASGVAEGGRTGLTALTTGVLFLLSLFFAPIFLLVPEAATAPALIIVGLFMMAPI
KEIELGDYTEAIPAYLTILMMPLSYSIAEGLVFGMISYVVIKAFAGKLKDIPVVTWIVAL
FFVLKHILG*
>SPSA8_v1_110007|ID:41145245| Inner-membrane translocator [Spirochaetes Bin 1 SA-8]
MDLITSILTITIRAGTSLVFATIGEILTERAGILNLGIEGIMLMGAVTSFAASFYSHNLV
FALLVACGVGAFMALIHAFLTVTMRANQVVSGLSLTLFGTGFASFLGQKLGPQSNNFRLV
GLKGAKFTPINVEALSQIPVLGALFKQDILTYAVYLIIPLAWYFLYKTRPGLWLRSVGED
PQTADAMGIDVIKTKYFYTILGGILIALGGAHLSLSYTPGWSENLTGGRGWIVIALVIFS
MWNPARSIWGAMLFGGINAVQFRLQASGTNIPASFLNMLPYLGTITVLVVMTWWEALSKK
IGAPAALGAAYMREDK*
>SPSA8_v1_110008|ID:41145246| Inner-membrane translocator [Spirochaetes Bin 1 SA-8]
MKILFEKRKSTSVPALFLVPLFSFMVSLMLTGLVLIIFGTNPFSTFSAMAKGAFGSSNGF
SETLVKAIPLMLTGLGVAIAFRLKFWNIGAEGQLTLGGVAAAWVALFLSPSIPSALLLPA
VLLVGCLAGALWAGIPAFLKTRLKVDETLVTLMLNYVAVLFSEYLYYGPWRDPKGYGFPG
SAPFPDEAWLPRIMGRAHIGIYFAVVAALILWVVFKRTRWGFELSIIGASQKAARYQGIA
VERNIFLAILLSGALSGLAGASEVTGISHRLQQGLSLGYGYTAIIVAWMSQLNPLAIPFV
SLLLAGILTGGDQVQLMMGLPSAMGIVMQGLILFPMLAGSLFTEYRIKFIRKQPKAACVE
GEA*
>SPSA8_v1_110009|ID:41145247|yufO| Uncharacterized ABC transporter ATP-binding protein YufO [Spirochaetes Bin 1 SA-8]
MEKDKRVPVVEMRSVTKTFPGVVANECVDLALYAGEVLALLGENGAGKSTLMNVLCGLYR
PDEGEIFIRGRKAEINSPRDSQRLGIGMVHQNFKLVDSMTVLENIILGMKDQGFIPDMKK
VRAEVTSLSEKYNLSVNPDASIWQLSVGEQQRVEILKLLYRKAEILILDEPTAVLTPQES
QELSKVIHLMRTEGKSAIFITHKMEEVMTFSDRVMVLRKGKVAAELSTSSTSPRDLAKLM
VGREVLFELEKKPFQPGEAVLRLEKVCALDDRGLPALKDVSFEIRKGEIVGIAGVAGNGQ
RELAETITGLRRLTAGSVRIQQKDVTNAKPLTIIKAGVAHIPQDRSSVGAVGDMSVASNL
AMKDYRKKPLSVGIFLIPQRILSMAKKLIDTFRIATPSPETHVKFLSGGNIQKTILAREI
GSCKNLLVAVYPSRGLDIGATESVRKQLVEQRDKGLGVLFISEDIDELIQVADTILVMFE
GKIMGNVPAASADPEKIGLMMAGVSQTENEGIDISQGVMTS*
>SPSA8_v1_110010|ID:41145248| Purine-binding protein BAB2_0673 [Spirochaetes Bin 1 SA-8]
MKKLILGLLVVAMVFSLAMPLAAQAKKPFKIAFVYIGPPGDLGWTYEQDRGRLALEKKFG
DRIETKYIENVPEGPDAERVIRQYAAAGFDMIITTSFGYMDPTLAVAKEFPKVIFEHCSG
YKTAPNMATYFGRIEEARYLTGIIAGRMTKTNVIGYVAAFPIPEVVRGINGFTLGVRSVN
PKAQVKVVWTNTWYDPVKEREAAVALLDSGADIIAQHQDTTEPQKAAQERGKLSIGYDSD

MGKFVGDTVLASAIWNWETYFIDTVQKALDGKWQTHQYWGGLKDGVTKLSAISKRVPDSV
KKEVEAAQAKILGGWSIFTGPIKDQSGKEVYPAKAVIPDDKQLSMDWFVEGVVGKVQ*
>SPSA8_v1_110011|ID:41145249| protein of unknown function [Spirochaetes Bin 1 SA-8]
MKRVIEVAVFSIFVLAFSAFVYFFAQQRKESLNASIPVAGRFVELSSQSLVQNTASAGNT
AIKQAKINPDWDETFLSVLEINLDTDEDLEQIITIKSTTKPQSPIAIAIADFQPLTGNYI
RIFKGETKAVRTNEFVVQPKPLLSQDSTEILCFGIDNNDLQCLTVFSRAENQLSKYTEIF
SEAGRVILIDDSQSQFPVPLTVVSKEQTRSVFSYNAKIKKYEAGKSEVLSGTPAENIVKE
PAVYLDAQSFEKHLAGTWYRTVSGRNSNEPDPILLEFSPEQKKIKMIANNSMTEWNWMST
LVSKNSIYISIENSALQEVRRLLTITTQTEDTIAVTAIAQQTVRFTQKEPWDGSFSRMSA
RSAPLPDGAAASSETAASPRLKDQNNFSFLYTFLPGWYRNETGDEIIFQNGSMTMTVNGR
SAKGFFILQQIDGAAILDFQEVNNAGTPQSRSSFLLSATRSKTGDIGTLSLEAVRISEKG
VELLYKPPLKFLKIEKII*
>SPSA8_v1_110012|ID:41145250| putative Integral membrane sensor signal transduction histidine kinase [Spirochaetes Bin 1 SA-8]
MKKKSSLLLYILALTVMVATVGLLWVVIIRESERRNLMLEYEAFKASSVIIDEYRRDSSF
SPKNDPRIKGVGFYSVDGTAVQKYGTAPARMTIPSRLKLPGSGESGAVPASNLLSVISAD
RNSIELFRYLGPQTAGRGMGMGMGRGRQSFLVNPPAAPYPPNSLPPGSADPGLLSNLNTP
LYVWIEYSADSYFRARNSLYLAAFFISAVLLILFGFLTILFQHNESLKEKEMANRELIQL
GEAARTLAHEIKNPLGIMRIQTARIRRAAAEKEDSARASLEKNEAVIFASAENIDNEILR
LSSLTDRIREFLKAEPIDFHDINLVTFLRSYKERYRHLENEGFVFSLELPDVAEAVVQAD
QNRLESVLDNVLANAKEACTAIGLEDKRITIRLYIQGKYWCVAVMDNGPGIPKELESRIF
TPFFTTKEKGSGIGLAFAKKIMQSFRGDLIYEGSQESGAVFILKFPAAVKQ*
>SPSA8_v1_110013|ID:41145251|rocD| Ornithine aminotransferase [Spirochaetes Bin 1 SA-8]
MKSQDYIALDNTYGAHNYHPLPVVISQAKGCKVKDPEGKEYFDFLSAYSAVNQGHLHSHI
VNAVKEQLERVTLTSRAFHNDKMGLFLKKLCDFTGYETALPMNTGAEAVETALKAARRWG
AEVKKVPNGKQQIICAEGNFHGRTIAVISMSTDPESLVNYGPYVPGFVKVPYADANAIEN
AITPETVAVIIEPIQGEAGVIVPPAGYLEQVRNICTKHNVLFILDEIQTGFCRTGKRFAW
QHENARPDIMCLGKALGGGIMPISAIVADHSVMNVFTPGTHGSTFGGNPLAGAVGIAAIE
VLENEKLEEHAERLGQLFRKEISAIPCPKMKLVRGKGLLNAVVFEEGFEAWNVCMALKDA
GLLAKQTHGNIIRFAPPLVITEKELMEAVAIIKNVFMNIQ*
>SPSA8_v1_110014|ID:41145252|ddl| D-alanine--D-alanine ligase [Spirochaetes Bin 1 SA-8]
MKAVAVLYGGKSGEHEVSLISAASIVKNLSTEKYSVTLIGIAKDGKWYLQEMPAFSSNES
SMAGMPAIQKGKRVMVLPGAGLWYESSPSRYKKLPCDIAFPVLHGTFGEDGTVQGLLECA
DIPYVGAGVLGSSIGMDKAITKALWAEKNLPITDYLVVRSEEMKQPDINAFLTRKIEARF
GWPCFVKPACAGSSVGASKVPAGESLLEALHRAFLYDNKVLIEQFIPAREIECAVLGNDN
PVVFAPGEIIPTHEFYDYEAKYKDPDGARLEVPAKLLPSQEETIKKLALEAYTALTLHGM
ARVDFFMDKRTGNIMLNEANTIPGFTAISMYPRMCQAGGLSYSELLDRLVELGEEEYTKK
QKIAYSFAN*
>SPSA8_v1_110015|ID:41145253|murE| UDP-N-acetylmuramyl-tripeptide synthetase [Spirochaetes Bin 1 SA-8]
MAKPLDALTVSIPVLSRKGSVNPTIEGLAYDSRLISKNYLFFAFQGLHTDGHAYIDAAIQ
KGAVAIIHDKELATQNPEISYIKVPDARLAMSQVADDFFDHPSDSLYVIGVTGTEGKSTT
VYLIYQLLKLAGFKAGFFSTVMSDSGSGEAPNPEHQTTPEAIAVHQYLASMRDAGCSFAV
VESSSHGLSARTGRLANVHFDCGIMMNVTHEHLEFHGTWEQYRSDKANLFRNLDTHCHSK
QVAGLETPLPSFGIVNADDPSASYFTACTQKPVYSFSLNTNSATVHASNLVPDERGCSFI
LNDQEGKHHPARINLPGTFNVMNAMAALLAVSKTTGLACRDLIPLLSQLKPVRGRMMRIS
RGQPFEVVIDYAHTPSSFETVLAPLRKQYAGRIICLFGSAGERDTAKRPLQGSIAEKYCD
ILVLTDEDPRGEDSMRILEEIAEGCKHKIGNTDLFLIPDRPAAIRHAFSLAKPGDLVLLL
GKGHENSIIYHDRVMPYDEEKEALQALSELGFN*
>SPSA8_v1_110016|ID:41145254| conserved protein of unknown function [Spirochaetes Bin 1 SA-8]
MKKTLIVLFLILALVPSAFGQFRFEIGANAPVAVGFITGDGSLYGDFLEVIENVGLIPIP
NLSLMLQANLGGILRIGAGIKAQSILVASLAYPAALVELSLGALSINAGIGGLYFGYYAL
GNIYGLEEMDVLLPELSVWLGFGKKRSFRIGGGAIGILSSSLDISEVPFAAYAGFKVVLE
*
>SPSA8_v1_110017|ID:41145255| 6-carboxy-5,6,7,8-tetrahydropterin synthase (modular protein) [Spirochaetes Bin 1

SA-8]
MCRSGTDRAFSALFCAIGPVAERAPYQYYMFMYHTRIESTFSAAHHLTHYNGKCERQHGH
NYKVYLWARGGKLDEGGMLVDFGRMKTALKEVLAILDHQDLNELGFFSADPSAERIAEFI
FNQVRENNPELPICAVDVFETDTSMARFSLD*
>SPSA8_v1_110018|ID:41145256| putative Radical SAM domain protein [Spirochaetes Bin 1 SA-8]
MPGHEILPAPSQAEIKNPSWDKASARILIVRLSPFFDVDVSTSHHVLYAELAAAENSYFI
DFSFFPGKDQRALYQKSGNPWFTSIIAGRSPKDFDLIMISNAYALELINLPYLFSTAGIP
LSARKRMESEDSPLVILGGSNAAAAGAVVAANGKYPASDALVDGIFFGEAEGAVTKLVYF
LCDTAKTKRRRLAAAASIPGFWPCLLSEGTRRQVSNLRPASVLKPLLFNGPNAKTVRLAI
TAGCPGYCSFCLEGWDRRPYREKPLAILLEEAQVLKKNTGADTLEIYSFNFNTHQDIFNL
IFSLNRIFLKVSFMSQRLDILAATPRLFKAELAGGKRSFTLGIEGISGRMRSFYRKGFTD
KDLSTCIDLVAAQGVKELKLFYILSGFENEADLTEFDGFLKELSEKRRNKAPGLRILASA
GYLVRLPFTPLQYAPLVLDRDRLEKISSRLETSCLANHIEFRLASNFSEYYIDQLMTLGG
HLLFGWLNNLPASGIVFDGTADKKAYHALREFIARNTIAFEPLMAEKDASFTPDFGFMEP
LSHFATLFRHYREASEFKNRPLCLGSTCSGCEVCDSKEEIRFMTSHTMTEPKEAEYAQRL
AKLMEAKARFKPVFIDVTMSRDFAFSSSEYREASLLKYLFAAFSGSDRLLFTAKELIFSK
NEAFESILKPWYGRFGMSRFALYGPDSVRSEAMLAEILKKPSADSGLVSLLTAQARAPAQ
ALKASILCPPMNSAVLARLFGTYLTNAGLSPIMRKSSGKIFFDLTAKSIQKKMIISAVLA
EGENPEISIACGHKTDLSALFSMIEDEAGRPPLLQIVEIVNALD*
>SPSA8_v1_110019|ID:41145257| Helicase [Spirochaetes Bin 1 SA-8]
MSMNDFLSLGLESSVCEALSKLGYSTPTHPQKLAIPEILAGKNIFLQSETGTGKTIAYAA
PILTKIAGKSASAGPLAIILTPTQELAVQVERKIDELAQLSGKPISIFTLLGGSPISRQE
AALKKKPHIVVGTPGRTADLVTMRALSLKNLAFFVLDEADRLFSQEYRDEVETLLARAPE
ESIKILASATIDKKTRAAAREFIKHPVSLDLLEEGVLSADIEHWAFYVEHRKRIDFLRKL
ETALRPSRCLVFASSSERVIKAGERMQEMGLPAAYLISRQDKEEKRVAIERFTTGSLRYL
VTTDLGARGLDIPNISHIISLDVPEDASIYIHRAGRTARAGKKGVSIILADQIDLSKASR
IAVMRGFVFRTKMLENAQVLEPTTEEFFAYVEEAEKERQEYRKNRGLKG*
>SPSA8_v1_110020|ID:41145258|rnz| Ribonuclease Z [Spirochaetes Bin 1 SA-8]
MNLEAFILGCGGMMPLPYRHLTSVLLRREGDLFLFDAGEATQISIRRLNLRWKKITAIFI
SHMHADHVTGLPGILMLNSQVDRDEPLYIFGPPKIAEYVEQSRRSLDMYINYEIIVREIA
EPAVVWEEKEFRIRSFGLRHTKMCFGYTMEEFQRPGMFFPEKATALGVPRGPLWSRLQNG
QSVTIPGDITILPEQVMGPARSGRKFSFVTDSLYFPEISNEVADSDLLICEGMFEDALLT
TAMEKKHMTARQAAQIAANAGMTKQLGLIHYSPRYADKELKLLLDEAREVFPATILTRDR
MNLPIEFVD*
>SPSA8_v1_110021|ID:41145259| membrane protein of unknown function [Spirochaetes Bin 1 SA-8]
MRKKLRVAIGILSLVAAAFTIISFIFRLSEIKASGELASGAEYSILRNAVVSVTTKEDFQ
DRFIRDRLLALYDGSTRLLAAQVLDENGLSVWKIPAESKYFALPNQKGGRAGFSSPQWST
VVFTTPLSGNMRLAALYASVFTSEISNAARLPSFVIGGWFLIVLFLSFFLTKDKEEGLLP
EPIDTKAAAERQGSEPIREMVQTQEGLIEMPPLEKTSGSGTEPVSVQEKESLEPEISKPE
TSQMLEQQREAELSPELLAAYGLSLDTLEKSGMTSDGNAQTTFESKQGSIQGPLSKSGNF
EESLQKFEEEIVLWTSRQKPAEPAQPALFESQALKHPAVQPEIASKTLPSTLETHTEEAS
ASHSEESETQELPEGLDQEIEETLEELETEDSKPLNHDEPEIRPYKENDAAIPTSAEKES
QEQEKRDISALPMPLSISEPNLENLLDNELAHGMERETSLMLIHCELSGPQDPASNALFA
TIRDYFVAKELMFELYKGGFAVVMPGMDLGSSLKMSEDLADVLAATMNLYKDLEGEPPVF
IGISSTGGRAIDAQKLYREASTALHKAYSGSPWHILAFRPKIASQM*
>SPSA8_v1_110022|ID:41145260| conserved protein of unknown function [Spirochaetes Bin 1 SA-8]
VKNNVRPSMLPSGWYPPDKASMQAFLRDALRPHVQKPEELALGSEMTDRPSSAQPAGACA
CIAPHAGWYFSGNLAARAIASLAPAETIVVVGGHLHRSSPVCYAPEEGFETPAGVLTADT
ALLNALLSELKEEGLPPVVPDMQPDNSVEVLLPMVKLLHPGSMVLWLRSPPRFEAKLLGQ
ALARCAGTLERSVACVGSTDLTHYGPNYGFMPAGRGKEAVAWVKNKNDKPFLSALLAMDC
EAALNLAQKNNSACSAGAAVTALAFALGRGAEQARLIEYGNSYDSMPSDSFVGYAALSFR
*
>SPSA8_v1_110023|ID:41145261|murD| UDP-N-acetylmuramoylalanine--D-glutamate ligase [Spirochaetes Bin 1 SA-8]

MDNFTFSLSDIPGMKVTIMGLGLHGGGLESARFFCDRGALVTVTDLRDETVLKPSIEALG
SRRIRYVLGRHEANDFSSADIVIKNPAVRKDSPYLAAARQIESDISVFLRFSRSPFVAVT
GSKGKSSTASAIYFGLKFANLKALLGGNITVSPLSFIEETAPERPVVLELSSWQLGDLKG
KGVLKPKVSILTSIMPDHMNYYRSMEEYVADKRVIYSEQDETDFTICDFDSEWGRSFATE
TRANVLWFSGKAASESAFRRGAWLSADTDGKIFGYCRFEQTKESVIEEILPPNLRVKGEH
MRKNLLAAGLALRAMGLDSAITAKAMGEFPGVEHRLEFFAEARGIQWYNDSAATIPDAAE
AAVSSFSEPVVLITGGTDKNINFAPIQKAFEKAAVIVLLKGSGTDKMIPILKATGKLFEG
PFDNLNDAIAAAANKAKPGMAVVLSPGCASFGMFLHEFDRGEKFKAAVRNYLKDSAAYPT
NESEGILS*
>SPSA8_v1_110024|ID:41145262|livF| leucine/isoleucine/valine transporter subunit ; ATP-binding component of ABC superfamily [Spirochaetes Bin 1 SA-8]
MANLLEVSNVSLHYGAIKALDNVSISVEENEVVTVLGANGAGKTSLLRAISGLGKLSGGT
IRYKGKNLTDNLPYTLAGMGIAHVPEGRHVFATLTVRENLLLGMYGVKRFRPGSSQKSKE
DRVYELFPILKERKNQLAGTLSGGEQQMLALGRALVSSPSLLLLDEPSLGLAPIIVQEIF
DLIRKIHIEEKVAILLVEQNARKALKAASKGYILELGKIVMEGEAKKLAEDERVRHAYLG
GQASSC*
>SPSA8_v1_110025|ID:41145263| ABC-type branched-chain amino acid transport systems, ATPase component [Spirochaetes Bin 1 SA-8]
MSITRSLLDRIKLGSGSGLPPSLGKKLSYAAIVLILVFPFIPFIDNYWIDIGFYFGIYAL
LGLSLNIVLGEVGLFDLGHTGFYAIGAYTTAILNTHFHIPILILLPVSAIIAGLFAWLVT
SPVIHLKGDYLCIVTIGIGEIVRLTMINNPLGLTGGPNGINGIDTPVFFFPIISSRQFYY
LIWIVMGFVTFGLLRLQRSRIGRAWNFIREDETAAEALGVDVRHYKLGAFILGAALAGIA
GNIYASKQMSVSPESFTFMESSLLFCIVLLGGLGSIPGTMLGALVITVFPEVFRPFAKYR
LMFFGLALLFMMIFRPAGILPRKRDSLETLLKAISGKRKGRKEQAAEAGAEKSLTHPSVQ
PVPTANKRGGMLLSVRDAHLSFGGVMAVAGIDVEVERGKITALIGPNGAGKTTLFNLITG
IYKPQKGSISFNGEEISGQIPHRIVQKGIARTFQNIRIFPTLTCLENVLCGQHCHGKAGY
AASIFHLPYQQEEEQAMFDSAKRCLERVGLGDSLDLLASSLPYGKRRYLEIARALAVKPD
LIVLDEPSSGLNDAETDELADLLIDLVRDGYSILLIEHDMHLVDKVSDHVIVMQSGKKIA
EGEMCVVRENPKVIEAYLGTDED*
>SPSA8_v1_110026|ID:41145264|livH| leucine/isoleucine/valine transporter subunit ; membrane component of ABC superfamily [Spirochaetes Bin 1 SA-8]
VKTFIEQLVNGLTVGSFYALVALGYSMVYGVMKLINFAHGDLFALGAFLGYSLLTGAAGK
ISETIGLWGGIAFAAFVVSASIALAGILVERIAYRPVYPSGRLSLVVSALGMAIFIQNGI
MAIWGARPQAFPASVVPSARIAVMGVPMTVLQLTILGISFLLMLIIWFIIEKTPFGAAIR
AAAQDRETATLVGIDVRKVIIFVFALGPGLGGMAGLMNGLYYRAISFNMGWNYGLKAFTA
TILGGIGNIPGAMLGGLLLGIVESLLSGYVSGAWKDVFVFIMLIAVLIFKPTGILGEKVA
EKV*
>SPSA8_v1_110027|ID:41145265| Amino acid/amide ABC transporter substrate-binding protein, HAAT family [Spirochaetes Bin 1 SA-8]
MIKERGMRKHAVFFSAVFGLMIFAVLAGCGTAQSGKTIKIALQAPITGDYAYEGQMAKQS
VTVAAELINKAGGVLGKQVEIVIVDDASNPKDSALAAQKAVSQKVVAVIGSYGSSVTEPA
ADIYEKNKLVSVGYGCTAVRLTMDKERKFFFRTCGRDDAQGLFFGKYAVETMGAKRIAIM
HDNSTFAKGVADEAKKALDPYIAEGKAEIVYFDAITPKEKDFSAAVTKLRETKPDVWYFT
GYYPEAGLLIRQARDAGLTCPFIGGNAAINDDFVKIAGIDVAKGALMTQEPLVTDVSTPI
AEQFKALYAEKYKELPSSPWPVYAADALYGIVGAIAKAGKADSTAIADVMHSKMDGVEGV
TGPVLFTERGDRKDVPYKMYIVDNEGKLQVYSK*
>SPSA8_v1_110028|ID:41145266| SoxA1 (fragment) [Spirochaetes Bin 1 SA-8]
LMPRIESHPILAVEKTKKDVGFLFNGEAVRGLEGEPVSSALIALGFFEFSRHPKDSAPQG
LFCANGQCSQCTVLIDGIPRKSCVTPLEEGMDVRTLKGLPVLPETDMPLKQAERRTIHTD
IVVIGAGPSGLSAAYELGKMGFQVLLADDKEKLGGKLVLQTHKFFGSEEDCYAGVRGIDI
AAILEEKLSRMPNVTILRNSPVVAIFKDRKAGIYKGYSSYLLVEFTALVVAAGAREKNIL
FPGNDLPGVYGAGAFQTLVNRDLVKPARRILIVGSGNVGLIAAYHALQAGIQVAGIFEIM
GRINGYKVHADKIRRMGVPIHLNTTILRAEGEGKVSKAITAKVDDQFKPIPGTSREYEVD
TILIAAGLASCDEFLRQAREYGILAVAAGDAEEIAEASSAMFGGRIAAFTLAKMLGKRAE

INPEWLPKREILKSRPGDTFSRSPVVPGPQWRPVFFCSEEIXX
>SPSA8_v1_120001|ID:41145267| conserved protein of unknown function [Spirochaetes Bin 1 SA-8]
VVREDAVVDIGLYLVKAKDAEGNIYQAYFQVYDISDPYDVRINAANGNVFQNGNGTKNLT
PEVWYGNTKIDITNYTFTWKLYDKNGKKSGFIDTARTSAAKTISSHTTGSSAVFKISVAL
SSAPSAGDVIRVISADGLKIESFEVASATTTAITIRAPVNGFSNNYPASTSDYAGGKLWL
YTGNGATAGQKTTSGAAALAVTGDDIDGLGTVFCDAINPNV*
>SPSA8_v1_120002|ID:41145268| protein of unknown function [Spirochaetes Bin 1 SA-8]
MGVVSTGQLTLYDVNDPVSSQFVEIWDGDWASRWVNYAGSGEMSVATDSTLPSGALALTV
GNNSGNDQTWMIAKQKIPFDANKLYRLTFYVKRTAGTGTTYLGVAGVASNGVSLVNASGS
NSYSSQHYIAASGVSPGSSWTKYVGYLKGTASSGTTSDCPTPESPGKLHTNSRYFRPLVL
VNYDSVAGTTVVGAVTVEIMPELSDIPNGNDTRNKAVATDSQLAAMANDSIITPQEKLLA
LGRWCEWYNDTAATSALPTAPTTDGRYKRIVDSANAVSGWTPTTAGTASKAFYDALEALR
AYLFSSPGVVLAGTWNTNINITKATWLTLWVTAESTAQALETEIAGKQGLTANLSNDSHL
VPTDADGNNGNFAGCATTMSIYKMGVDDSANWTVAASPSTGVTGSLSGKTYTVTAMSVDS
GYVDLTASRTGYPSLTRRFTIAKAKGGAAFWLVSSADAIQKSQAGVYTPSTITFTGKRSA
SAGVIGDYSGRFIIAETTDGSAYTDKYTSASNETSKTYTPSAGIKALRCRLYLAGGTSTL
LDEEIIPIVVDGPTGPQGPTGPQGPTGPQGQAGQDAPRYLGLYSYANRGSITGMIAGDLV
VLYSATQSERGIYAYVASTWTKQTTPTQDQIMRCMVGVLDAVRQGYGQSADYIGTGATSF
ETLLVNFIYAQYAMITGSIRAGTRYDQNGTEVNPSQEGVWIGANGKIKGAINDVEPDQIT
PSFTRRQLFALSGTWAVPAHVKWVRVTAIGGGGGGGGSGGVDGSGDAITVYDGNNGGAGG
ASSFGSYVVANGGAGGLGTGATGGTGGSGQNGASSDYIGGYNGANGGTGNTKGTGVTSGT
SGAGGARGSSAGLYDASVQLSNASGALTKIPAISAGASKSNSVTVPTSGTKTSNGATGKA
GAYGGGGGGASGGCYACKLTDSLTTGRGAFGAGGGAGALRQRTFKITEVYGQSITVTVGA
GGAGGSAGGGTTALGAAGGKGGDGWVLVEW*
>SPSA8_v1_120003|ID:41145269| protein of unknown function [Spirochaetes Bin 1 SA-8]
MRENSMRTKTNNMGGPVSFNEKISELELAHHELATVVWGDDKKRDNGLRSRVNKMAEVEI
PEIKTTISILQGCLDDQREKLETHIVAHKETRKDESTIRAERIKTYGAIVVSLIGNAATI
ITVLLSRGK*
>SPSA8_v1_120004|ID:41145270| protein of unknown function [Spirochaetes Bin 1 SA-8]
MEVIKGGKITAGFTDKRPLSVPAEKRNHIHGAIDIAGGDGLVRSPVDGIAQGFAIFRAPG
GGWDHEKEKRLILDMPWREYFADIFGAIITIEERRTGRLHILAHFWPEVVIDGSSKATVQ
FQYDNYLETAKEDRFVSHILMTKRIEVEKGDILAPVGNAGFSTGAHVHWEIHHSTKRLDV
FEKRVNPEDYL*
>SPSA8_v1_120005|ID:41145271| protein of unknown function [Spirochaetes Bin 1 SA-8]
MKGKKWQAWASMTFVLVSIYILTLVVAPDVLTSGVGSTIVGAIAFGAVGYGGVQVADSWQ
RAKYYRPELDKEEK*
>SPSA8_v1_120006|ID:41145272| protein of unknown function [Spirochaetes Bin 1 SA-8]
MNEKVKLCIVACLIGIILGASTATWIVGRNSARERAGYESTLADWERRNNELNNQLREAR
ALDTERRKLDEERRVMDAEIERTLREGLGAISQARSDYERGLIQLRFAKDIFEVLRKRYD
PAYHGAAPAVP*
>SPSA8_v1_120007|ID:41145273| protein of unknown function [Spirochaetes Bin 1 SA-8]
VSVPRDYQIIKRNGIYYFRFRDPVTGKVLSGKSSRLRNRDAAIAWAVHEYEKIKAKFGSS
TNPYRDWAAKFFTSECPHISRVLNEGKTYADSTRDDNRKYLEILLEDPIADIPLNEITRP
DVVELQDRLVRKYGRTRKTQMLYSTFRITINEAAYRGKIPISPCAGLKQISYKKKPRQAL
GKEDMEKFLSKENWDNETHWKMTMTARYTGMRAGEIRGLCWEDIDKAKKLIYVRHNLPQN
VGIEGLTDPKWGKRRIYPYTKELSQILESDRKKEGLVFNTDWKPIDYWAWLDSVKDARKK
SGAKGGIHALRHTINTELASNGVSRDIRKALFGWSNDATADGYTHAEMFEISSYAPLIEA
TVSPDTPKPRRKK*
>SPSA8_v1_120008|ID:41145274| putative Transporter [Spirochaetes Bin 1 SA-8]
MKLWFKYLLGIIFGLALYAVVPRPMLESGGLFAVLAETAARIGFYCVGAYLFSSLVVAIP
KLFEEKRFWRLFLKGALFYLGSLLAASGLGILAAMVFLPLRIPFSIETPATNIPTPSNLQ
FSIFPMNLVSLAANAHEYLLPLMLFAFALGLAIAHDPMAARPVFLFFDSLSRIFHNINTF
IIELLPVLLIPMTARALFDIEKPLSLTTFGSFVTVLALCTGFFLFIVIPAAFYFLSGKQN
PLKSGYAILGAALASLISGNMRFSLGTTIYHVRENFGVKRRYNALFFTEGVFLGRAGTAF

VSAVSFAAILSSYSRLGIPAETALWLLIFIPIATIIASAGLQNGPIMVLAILCSYYGKGF
ENGFIIMIPAMAMLAMVANLLDTVWLSFSTICVARREIENNVKPLNHII*
>SPSA8_v1_120009|ID:41145275| putative PTS IIA-like nitrogen-regulatory protein PtsN [Spirochaetes Bin 1 SA-8]
MDLMTVLNPEMIALGLKGTTKLEIIDELIDVAVKSGKVSDPTAAKASVLERENRMSTGMK
HGIAIPHGKTSAVQELVACIGVSEKPVDFDALDHQGCRIFIMTLSPPEKTGPHLQFLAEV
SMLFRSEEKRQAILKAKTPEEIINILKA*
>SPSA8_v1_120010|ID:41145276|groS| 10 kDa chaperonin [Spirochaetes Bin 1 SA-8]
MNIKPLGDRILVKMKESETKTASGIIIPQTAQEKTQTGVVVAVGTDADVIKVKVGDEIMY
DKYAGTQVKIDGADHLIVKMSDILAVIE*
>SPSA8_v1_120011|ID:41145277|ftsH| ATP-dependent zinc metalloprotease FtsH [Spirochaetes Bin 1 SA-8]
MAGQKKDWKFKPPTTPNGKNGKDPQPTGPFGGFQFKFSIGYILTMLIAVSLFNYLIFRTD
NTLVPYSTFKEKISSGEIKRVEIDLNYYTGYPDKKNNEPSSPLPATAKTGAVFKTVPIDD
PGFTALLDEKGVVYSASPREGSAVLSFILNWVLPFGIMFLVWRTVMKRFSGGNSNVLAFG
QNKATIVAEGDIKTRFGDVAGVDEAKAELVEVVDFLKNPGKYTDIGGKIPKGVLLVGPPG
TGKTLLARAVAGEANVPFFKMSGADFVEMFVGVGAARVRDLFKQAREKAPCIVFIDELDA
LGKSRVSGVMGGNDEREQTLNQLLVEMDGFDATSGLIILAATNRPDVLDPALLRPGRFDR
QVLVDRPDMTGREAILKIHARNIKMDDTVDLAKIARSTPGFVGADLANLVNEAALLAVRA
GRKKVNQDDFNAAIEKVIAGLEKKTKVINPKEKQVVAYHETGHALTAALTKGADPVRKVS
IIPRGFGALGYTLQIPLEDRYVVSEEELLSQIDVLLGGRAAEEIIFGSISTGAANDITRA
TDIARKMITEYGMSERFRHVALTKRGLGFAGQSAADPFIAREYSEETQKYVDDEIAKTIA
ARYDHVLALLQNKRNLLEKIAATLLEKEVIEEAEFVALTKDEQTEQNPVDSLTAV*
>SPSA8_v1_120012|ID:41145278| putative Na+/solute symporter [Spirochaetes Bin 1 SA-8]
MSLFLIVYTLALLLLAFLSFRKKDSFQKYILADRNQPKFLIIASMLASTIGGGLTIGTVN
KAFLIGFPAFWFVASGSIAHLTQGLFLSEKVRKTEALTLPDLAAKLSSPQVGRLAGIIIV
LTWTGIAGAQFLALSKVITTITGLGHAEAIIAAAAFLVIYTIMGGQRSILKSDFLQFGLL
AISLIIAVVWLFTARPVSFPSLGISLFSPKFTPSDLLYYLVVVAGSYLICPMMFGRILSS
DSAKSARTASFVSAAGMLLFAFVITAFGLWARASGFNPGSSDPLNAMISGVFPPILGFLM
TFGILAAILSTADTVLLTAASVLEHDVIQGDSLYRTQTWVGVVAFFAAFVALFQTDIVDL
LLKTYQGYTSGIVPALFIAIALYGRRRVRPIFLFAAILSGYLLGFTGSFLPSPALQKGFA
FAGILVSTLISLLGIERKPSPSQTN*
>SPSA8_v1_120013|ID:41145279|fabH| 3-oxoacyl-[acyl-carrier-protein] synthase 3 [Spirochaetes Bin 1 SA-8]
MSVVIRSIASYIPEIRIDNNELAARVDTTDEWIRSHTGIGARHIAPDGVLTSDMAALAAK
SALEKAKISAREIDILIVATATPDYFGFPGTSCIVQDKIEAYGCTAFDIVAGCTGFIYAL
DVASAMLEARQARNALVIGAESLSRVVDWSDRSTAVLFGDGAGAAVLSRIDEPGRGCLSF
ILGAEGSGAKDLYLVQAGRPNAFSRETPQTPVIFMNGKKVYDFAVKSITVLIERIMHATA
YRLDDFAWIVPHQANARIVQAAGKRFKIPEEKFYLNMEEYANTSAASIPLALAEMDSKGL
LKKNDLIMLLGFGAGLTYGAAVIRW*
>SPSA8_v1_120014|ID:41145280|fabF| 3-oxoacyl-[acyl-carrier-protein] synthase II [Spirochaetes Bin 1 SA-8]
MKKMIAVTGLGVISPVGNDIQSFWDSLINGKSGIGPVTRFDASRLDAKIAAEVKSFDPSV
WMDKKEARKMGLFSLYAVSAAVQAWNDAGLPDTQDSMALLPYDRSRIATCIGNGIGGIEI
FQDSFTKLLQSGPDRMPPMTVPLMIANEAAANIAMRFGINGPAYTQVTACSSGSDAIGQA
IDLIRSGRVDAVVTGGTEGAITEFAMGGFCRLKALSTRYNDNPSLASRPFDKDRDGFVIG
EGSGILILEDYEKAKARGARIYALAAGYGGSCDAYHLTAPHPEGLQGARAIQLAMEDAGL
SPEDVGYYNAHGTSTEMNDPIETAMVKRAFGAAVKNLKVSSTKSMTGHCIAAAGAIEAIV
CIKAIETGILPPTINLDNPDVEAGCDLDYIPNKAIRHPIKAALSASLGFGGHNGVLAFTR
A*
>SPSA8_v1_120015|ID:41145281|fabZ| 3-hydroxyacyl-[acyl-carrier-protein] dehydratase FabZ [Spirochaetes Bin 1 SA-8]
MQNIEAYLPHRKPFLFVDDVSIDGELIRAKRQFHPDEWFFQGHFPAYPVVPGVLLVEMMA
QAGGVGAKLLGIKPQSMFVFAKIRSAVFKRQVRPGDLVEMEITNIKSGSLILHQKGIGRV
AGEVAVEAEWIAMASGVPE*
>SPSA8_v1_120016|ID:41145282| putative reductase TDE_0597 [Spirochaetes Bin 1 SA-8]
MKLIPMVRNNICLNAHPEGCAQEVRLQINRVAGLASRVKTLGSAITRKPKNVLVIGCSTG
YGLASRIVAAFGYEAATIGVSYEKEPSPAKAGSPGWYANQTFDTEAQKRGLTAKSFNMDA

FSNEAKAKVIETARALGIQFDLVIYSLASPVRTDPDTGVMYRSVIKPIGREYTGKTADIF
TGKISEAKVQPADEEEIRQTVKVMGGEDWARWISELSSAGLLTESAITLAYSYIGPELSW
PIYRDGTIGRAKEDLEKTARKLRADFEKQNLKAFVSINKAVVTRASAVIPIIPLYVSTLF
KVMKERNIHEDCLDQMIRLYAERLYLNGLNSIPLDLEGRIRLDELEMGQAVQAEVASRLA
SINESNLSSLADIEGFRMDFLRAHGFEVPGVDYDKEVESFQ*
>SPSA8_v1_120017|ID:41145283| Ribosomal RNA small subunit methyltransferase E [Spirochaetes Bin 1 SA-8]
MNMLFIASESDSTSGDITIPRADRRYEHILKVLKKAPGNEISAGSADGFLGKARILSVTK
AEIRLSFQPEKPAEPLHPLRLILGFPRPIQANRILKDISSLGTAQIWLTVTELTEKSYLK
SDIFANREFSPALIEGAEQAGNPRLPEVKTYWSLKRALDALDSLDTRNAADKPGSAELSQ
PSGCGTRIALHPDPHAVPISAVSLLETPVTLAIGSERGWTEQEAALLQHHGFTICSMGTR
ILKTETATVAAVSIILSKLNLL*
>SPSA8_v1_120018|ID:41145284|yjbG| Oligoendopeptidase F homolog [Spirochaetes Bin 1 SA-8]
MAATIPSRHDVPEQFRWNLSALFPTDAAWETGLADLTAKIGTIEQLKNQFGADKAKFKEI
LAFYSDYMMLEERLGYYAHLRMTEDEGNSESRGRFARYMGTATQGQGAWAWFIPAIHSLD
SGFISDCIQDKQFADYAVFLQKLLRFKPHVLSEKEERLLALQSEANQTAQETFGVLTNVD
LDFGTVDTEEGPKPLTQSTFXXFMRNXNXDIRQKAYFQFYXAYEKHKNTLASLYAGSVKL
DKYQAQVRNYASAREQALFPDNVPEAVYDNLVQTIHDNLPALHRYYELRQKILKVDELRH
YDVYVPLVEEKKSRHTYEEAVDIVTEALAPLGDEYVSTLRKGLLGGWVDRYENKGKRSGA
FSAGSFCGEPYILLNYKEDVLRDVFTMAHEGGHSMHSWYSARSNPFLCYSYTIFEAEVAS
TFNEQLVFKYLYERSNSEAERASLLANRIDDTLATLFRQTMFAEFESLTHAMAERGEPLT
VDSLRSVYRKLLQQYFGPSMKLEDVSDLEGLRIPHFYNAFYVYKYSTGISASIALSERVL
HGGKAELDDYFAFLKSGGSRFPIEALKVAGVDMATPQPIEMACQRFAKDVETLGKLLGV*
>SPSA8_v1_120019|ID:41145285| Transcriptional regulator, LacI family [Spirochaetes Bin 1 SA-8]
MRATIKDIALRAGVSKTTVSFALNNPSRISKETYERIMAIVRELGYYPNPLARTLTTKRL
GALGLLLPQPIAEVMGNPHLCEIISGIGKECDSHRISLTMLPPVQGKVIEAARRSFVDAI
VTIGVGPEHEVVNFFHKHNIPFVTIDGQETEDTINVGIDDESAAFALMNHILELGHKHIA
ILCVRRDFKSISDINASIVLEKRLKGFSRALEAYSLGLSHEGITVLETEGSLEGGKRAGL
AVLKSAERPTAIVAMADIIALGTYAAAQDLGIVIPEQLSVAGFDDIPQGSYAHPTLTTIH
QPSTEKGQVAASMALQLLDGKKVEHRRFSFRLEVKGSTARVP*
>SPSA8_v1_120020|ID:41145286| AAA ATPase [Spirochaetes Bin 1 SA-8]
MRQISISYDSGETVSFPVGVKASEAIAEIGKLPWPLAAVLVNNELKCLDTELESDCALKP
VLVNSSLGAAVYRRSLCFLLALASREVFPKRRLIAGMAIGTGFYHYYFDERPLNENEIQQ
LSDKMKDLVERDILINQEKWPWEKACRYFEKTGQSDTLALLENLNDPFIPLNECAGFRDL
HVAPLVSSTAVLATWELLPYHGGMLLRYPHKETPNELEPFEDVPVLYRIAEEYKERGRVL
GVGSVGKLNKLTNIGQIHEYVMVAEALQNKKIAALAESVARNADKVKVVLIAGPSSSGKT
TTAKKLAIELKVLGFEPIPIELDNYFVDRTRTPLDREGKPDFECLEALDVEYLNQQLLAL
FSGDEIELPLYDFKAGMRRSSGKMLRLMGNEILILEGIHGLNEELTPRIPRENKFKIYVS
ALTQLNLDDHNRISTTDYRLLRRMVRDHNFRGHSAQATLAMWPSVQRGEQRFIFPFQNSA
DAAFNSALDYELGVLKVFAEPLLRTIKSTSSEFAEARRIQSFLDNFIPIPAQYVPGNSIL
REFIGESDFHY*
>SPSA8_v1_120021|ID:41145287|oppF| oligopeptide transporter subunit ; ATP-binding component of ABC superfamily [Spirochaetes Bin 1 SA-8]
MNNVILSVKNLEVXFKNGHESFTAVRDVSFDXYEGESFALVGESGSGKTTIGRTXIXINP
ATKGEITFAGKRISGVIDKALDSYVIRNIQMIFQDPMASLNERATIDYIVSEGLYNFNLF
KDENERREKVFKALNDVGLLEEHAERYPHEFSGGQRQRICIARVLAMEPKFVIADEPISA
LDVSIRAQVLNLLRDLKQERGLSYLFIAHDLTVVRYFADRIAVIHQGELKELASTDELFA
RPLHPYTRSLLSAVPLPDPDVEKQKKLIVYDPGMHDYAKDKPTWREVFPGHFVQANEKEA
AEYAAMYQ*
>SPSA8_v1_120022|ID:41145288|oppD| oligopeptide transporter subunit ; ATP-binding component of ABC superfamily [Spirochaetes Bin 1 SA-8]
MNYDIATPILKVRDLVVKFRVRNKELTAIRKISLDLFDGETLAIVGESGSGKTVLTKTFT
GMLESNGRIDAGSIEFEGRELVKIKSDRDWETIRGVKIATVFQDPMTSLNPLKKIGVQIT
EVIEKHQKLPKEEAYRIAVELMTKVGIKNAEKRFNDYPFQYSGGMRQRIVIAIALACKPK
ILICDEPTTALDVTIQAQILDLIRALQKDYGFTTIYITHDLGVVANVADRVAVMYAGQII

EYGTVREIFYDPRHPYTWALISSLPTLASRDEPLYAIPGTPPTLFNEIKGDAFAPRNRYA
LNIDFEEEPPFFXVSPTHFAXTWLLDPRAPFADPPLAVKLMRKKGNIYKEPAAEGSK*
>SPSA8_v1_120023|ID:41145289|amiD| Oligopeptide transport system permease protein AmiD [Spirochaetes Bin 1 SA-8]
MAKTAERFAFATFDVLQSERIAGPRYSYWKSVFQTFFKKKSSIFWLLLLLTLTMMSFIQP
IVSGYDPAVSPNINKPSTWYLSPSGEHWFGTDDRGNDLWDVVWAGTRMSLSIAFIASAIN
IGLGILVGAVWGYSKRLDPILLALYNVISNVPAILRSMMLMYIFGRGFWQMILAMTITGW
LGIAFFIRTQVMIIRDREYNLASRCLGTPIKRMVTRNILPYMVSVIATLVYQEIPGIINT
ETVLSYLGIGLPSTYPSLGRMIDVYWSFVDTYPHMIVFPGLIMGLITISFYVVGQLFADA
SDPRTHR*
>SPSA8_v1_120024|ID:41145290| ABC transporter, permease protein [Spirochaetes Bin 1 SA-8]
MKGYVLKRILQSILSIFIVSTLTVFLVYSCIPRQLVFKGDQQLPKLANKPDEYHDYKFRT
WENLGYIDYVTMKEYAQTVFGKDSPEVSDAIMQGSTFYEKFRADFQAKGYKVDIQPVTGY
LYATKDIPTMTRVLKWYANLIQVDTPYRIKDPNNPNLKRGYYFTKDWSGMPALAASGTLY
KYQIWLGGSFPFIHXNIIKLNMGRSYPTYDGMPVLEVIGSGQGRTLRREVTTKVNGEEVT
FYTSIDEHSLKYKPELDRLDRNKFDDNYADGNSIYNDPSMLGTSFTIGIYALLISLFFGL
TVGVVAAMRKDRIFDKITMGYIVFISSIPTLLYIALFARFGMKVLGLPDKFPFLGSGNML
SYILPTLSLSLGGIAGEALWIRRYMVDQMNADYVKFSRSKGLSQREVFFKHIIRNALIPV
VHSIPMAVIGTLAGALITESFYAVPGMGKMFPSSISDYNNAMIIALTFIFTVISILAIFL
GDILVTFVDPRISLAAKKEAR*
>SPSA8_v1_120025|ID:41145291|aliB| Oligopeptide-binding protein AliB [Spirochaetes Bin 1 SA-8]
MLKKIVLAVTLAAVLFAGTAGAATPKSKDYVYVFSTDPRSFNYLNDQRATNTQHITNFVD
GLIEHDRYGILRPALAESWTVNDDFTVWTFNIRKGVKWVTGDLEVYADVKAQDWVDAMKY
MLDNKSQLTYLIDGFVKNAGLYLKGKVTDFSQVGIKAKSDYVLEYTMEKPTPYFDTMLTY
NAYWPVNGEFLKAKGKDFGKVDKNGILFNGAYVLSNFTSKSVIEYDPNPTYWDKAHVYIS
HVKYVVYYDGKDPDSLFNNFDAGVYVSAPVYTDNEALFARAQAKYKDYIFRARQDSTSFVY
AFNYDRGAFASPADPTKGKTPKSEKAIEDTKKAILNRNFRKAIFFGIDRPTILAQRNGEV
NKLAAIRNTYTAPELSSDKAGKDYVKYVEDALKARNPADFPASFKVDDAQDPYYNPTKAK
AYMAKAKAELTAKGVKFPIELDVAADVSYTKGMKMDQSLKAGLEALFGTDTLKVNIIEMD
SDNYDASTYYAETGSQSNYDIGNTTGWGPDYGDPYTFLQTLEPVVGALLTPIGLDPVDEG
TDKAAATAVGLYDYAKKVEAANNEYKDISKRFKLFAEAEAQLLDDAIILPYMSFGGAFSI
SRVVPYTAPRSAYGADEYKFKGVIIGDKVVSLAEREKAKATWEKERAAEFKKLNAK*
>SPSA8_v1_120026|ID:41145292| membrane protein of unknown function [Spirochaetes Bin 1 SA-8]
MNRLSSKSPGTMGSIPLFIVLICGGVAAGLLFAAFQQESSFRLAFSNAVLLVSFLLFGVS
WILYLKKDGVRFFQPRRKNPLSQSASWLDRTGAPGEVPQAPSPIPDASGPDSEAYQKLSE
AEQKLRMRMLGANSQENEPNQAVKTSASLKHSLIAGFFLLLAGLALQYLLKF*
>SPSA8_v1_120027|ID:41145293| membrane protein of unknown function [Spirochaetes Bin 1 SA-8]
MEDQISFNSHAKALLLLFLACAFFAVFATVDTSEHYRAWRTARKWTDEQKSAAPAQDANS
MYSLILAALAVETSIIAISFIGALGLGIATSTESAKTYALARAFGWLSAGLGLAYTILMI
YYQMRVGPRVVLKGPIFDYDLSMQIPILLAVIGYPATMAVYALFLYKKRVTRKD*
>SPSA8_v1_120028|ID:41145294| NAD-dependent malic enzyme [Spirochaetes Bin 1 SA-8]
MKLDMSLSNLSHAFPEDLSQDQKARAQTLFLKKLSEKAHRFYGGKMQTVPKAGLYGFNWF
NVWYTPGVSKISTTIRDNNDDSFALSNRGNLVAVVSDSTRVLGDGDCTPPGGLGVMEGKA
FLMKYLGGVDSIALCVDSRDENGKHDPDKIIQFVKMLQPSVGAVNLEDISQPNCFKVLDE
LRESCDIPVWHDDAQGTASVTLAGLLNALHLAGKKLQDAKIVLLGAGASNTTIARLILTD
GGKGENMVLFDTKGALHRERKDIESDTRYYRKWELCLATNPGCVKTEAEAIKGADVLIAL
STPGPDTIKREWIRSMAPKAIVFACANPVPEIWPYAAKEEGAFIVATGRGDFPNQVNNSV
CFPGILKGALAVRARKITDGMAIRCAHSIADFARKRGITPDNIIVTMEETEVFAQEAADV
AMQAIKEGVARITDTWENVYNKALADIKESRKVAQDLMELGHIAPPPREMLDEALEEAIA
EVRK*
>SPSA8_v1_120029|ID:41145295| putative dihydropyrimidine dehydrogenase [NADP+], similar to dihydroorotate dehydrogenase [Spirochaetes Bin 1 SA-8]
MAELSTWFMGLKLKNPLVVAASGLTSTPEGVDKAVQAGAGAVVLKSLFEEQILAELGSEA
QGLDLDAYPDAEAFVSRTAWEEGTERYLDLVRKAKEKAQDIPVFASINCVGTGKWANFAR

EIEKAGADAIELNIAFMPFSTAASAKEIEEHILSTIKEVRLSTRLPIEVKLGQQYSSLPN
LAKSIAREQANALVLFNRFYKFDIDITGMKLTSAPVTSSPDEYHESLRWISLLFQRAGIE
LSAATGIHSAQTALKFLLAGASTFQVCSGIYKNGWKFVSGMLDDLSALMDEQGFASVDAV
RGRLSAHNAQKPEEYLRLQYIKALTGIN*
>SPSA8_v1_120030|ID:41145296|thrS| Threonine--tRNA ligase [Spirochaetes Bin 1 SA-8]
MAVDVERMRHSSAHVLAEAVLRLFPGTKVGIGPAIDDGFYYDFLFATPITNEDLPAIEKE
MRRIIAGNHPFVRREVSKDEARKLFADQPFKLELIEGLEEGTISVYEQDGFLDLCRGPHV
ETTRDIRPDAFKLRTIAGAYWRGDEKRPMLTRIYAYAFASKAEXEAHLKMLEEAERRDNR
KLGKEXDLFSTHEEAGPGLIYWHPKGGRFRVELEKWWRDEHFKNGYEILFTPHIGKSWLW
ETSGHLGFYKGNMYSPMTIDEEDYYIKPMNCPFHIMIYKNSTHSYRDLPLRWAELGTVYR
YERSGVLHGLMRVRGFTQDDAHIICTPEQIEDEIAEVLRFSLAMWKTLGFKQIKAYLATR
PQDSVGEPARWDQALESLRKAVEKEGLAYEVDEGGGAFYGPKIDLKIKDAIGREWQMTTI
QFDFNLPERFDMTYTDRDGQLKRPYMVHRALLGSIERFFGVFIEHYAGAFPLWLCPEQVA
VIPVAPTFSDYAKKIADELKKRDIRAKAMLSDERMNAKIRDAQNQKIPYMLVVGQKEMEE
NQVSIRYRDGRQENGVSFERFVGQVLDKIATKALDL*
>SPSA8_v1_120031|ID:41145297|clpS| regulatory protein for ClpA substrate specificity [Spirochaetes Bin 1 SA-8]
MSSMNGQTDSDTTIVTQTEDHVEPAEPDEYRVILLNDDFTTMEFVISVLMTIFHKSLPEA
SKIMLDVHRKGKGVVGEYTYDIAATKINQVHLLARQMGFPLKCVMEKV*
>SPSA8_v1_120032|ID:41145298| ATPase and specificity subunit of ClpA-ClpP ATP-dependent serine protease, chaperone activity (fragment) [Spirochaetes Bin 1 SA-8]
MKVSQEVQAIFNAAYNEAKLRSHEYLTPEHLLYAGLSFEKVRLVFENCDADIDQLRRGLE
AYFEQKVPVVRNNAEPVQTSAFQAVIERAVMQSQASGKSEVEVSDLIVSLYDEERTYAGY
YLRKLGIKRLQLLEVLSHGIMDMPDEEEMIEGIDEAEGRQESEGKEKRSMRPGPLERFAT
DLTALAAQGKLEPVIGRETEIERTIQVLCRRLKNNPIHVGDAGVGKTAITEGLAQRIVAG
KVPALLKNYTIYSLDMGALLAGTKFRGDFEERVKKVVDVLLKKEKAILFIDEIHTIVGAG
AVTGGSMDASNLLKPALTSGKLRCIGSTTYDEYNKIFEKDRAXX
>SPSA8_v1_130001|ID:41145299| 50S ribosomal subunit protein L5 (fragment) [Spirochaetes Bin 1 SA-8]
VRGGSLMAEAKTSLPRLKKLYREKVAPELFKEMGYESFMQVPRLVKVVVSMGVGEAKENK
KLLDAAVTDLGIITGQHAVKTKARKSIATFKIRQGQEIGARVTLRGDYMWEFLDRLMNVA
LPRVKDFRGVXX
>SPSA8_v1_130002|ID:41145300|rplX| 50S ribosomal protein L24 [Spirochaetes Bin 1 SA-8]
MTDSKFKLRREDLVQVIAGKDKGKQGKILKIDREKGRVIVAGVNMIKKAMKKKSQTDRGG
IVEIEAPLHISNVMIVCKKCGPVKIGYKFDGDTKKRVCRKCGEVL*
>SPSA8_v1_130003|ID:41145301|rplN| 50S ribosomal protein L14 [Spirochaetes Bin 1 SA-8]
MIQVESMLAVADNSGAKTVQCIKVLGGTRRRYASVGDIIVVAVKTALPNSSVKKGTVERA
VIVRTHKEYKRPDGTYIRFDDNACVIIDANKNPKGKRIFGPVARELREKDYMKIISLAPE
VL*
>SPSA8_v1_130004|ID:41145302|rpsQ| 30S ribosomal subunit protein S17 [Spirochaetes Bin 1 SA-8]
VDTDVQNKKKGKLVLQGVVTSDKMDKTIVVEVMMRKLHPLYKKYVTRSKKVKAHDEANSA
HKGDVVRVEECRPLSRDKRWRLIEIVEKAK*
>SPSA8_v1_130005|ID:41145303|rpmC| 50S ribosomal protein L29 [Spirochaetes Bin 1 SA-8]
MKNSFKELKYEELLAKREDLRKKYFDLRFQMVVGHVENPLEKRNLRRQIARVETLITQAK
KKLAGEQA*
>SPSA8_v1_130006|ID:41145304|rplP| 50S ribosomal subunit protein L16 [Spirochaetes Bin 1 SA-8]
MLIPKRVKHRKQQRGRIHGDAHCGNTIAFGEYGLICLEPEWLTNRQIEAARIALNRYIKR
GGKMWIRIFPDKPYSKKPLETRMGKGKPGPEYWVAVVRPGTVLFELAGVDAKVAEEAMRL
AGSKLPVKTKFATREETE*
>SPSA8_v1_130007|ID:41145305|rpsC| 30S ribosomal subunit protein S3 [Spirochaetes Bin 1 SA-8]
VGQKVNPIGLRIGINKDWSSRWYVEPRDYAKTLHEDFAIRKRILELPEVKGADVAEIEII
RHPQRVTVIIHTARPGVLIGVKGANIDKISLEIQKVASKKVQIKIKEIKKTDTNAQIVAQ
NIARQLEGRGSFRKTMKSAISNAIKGGAQGCKVRLSGRLGGADMSRTEELKEGRIPLHTL
RANIDYGFYEAQTTFGKIGVKVWIYQGMVYASDKNEDAGMLAKKARREPAAQGAERREGG
ERRPRRERQDTSRADDRARS*
>SPSA8_v1_130008|ID:41145306|rplV| 50S ribosomal subunit protein L22 [Spirochaetes Bin 1 SA-8]

MVDNKTGYRATAKWLIASPFKVRPVADLVRGKPYTEALAILDNMPHKGARLIKKVVVSAA
SNGLSKNRKLDEDMLYIKELRIDEGPRMRRVWFRARGRADMLIKRMCHISCVVDEIGKKS
EA*
>SPSA8_v1_130009|ID:41145307|rpsS| 30S ribosomal subunit protein S19 [Spirochaetes Bin 1 SA-8]
VSRSVKKGPFIEKSLYKKVIDASKSGDKKMVKTYSRTSTIIPEMVGQTISVYNGKTWVPV
YVTENLVGHKLGEFAPTRIFRGHAGSDKKAAK*
>SPSA8_v1_130010|ID:41145308|rplB| 50S ribosomal subunit protein L2 [Spirochaetes Bin 1 SA-8]
MGIRTFRPITPGLRHRVQVVNDEITTNKSNPEKSLTKGKPSTGGRASNGRISVRHHGGGH
KRLYRQIDFKRDKFGIPGKVTSIEYDPNRSANIALITYADGEKRYIISPKDLKVGQTIMS
GPAASLEVGNALPLENIPAGFTVHNVELTLGKGGQLARSAGASALIAGYEGDYVVLKLPS
GELRMVFKKCIATIGAVGNEEHMNERIGKAGRSRWLGIRPTVRGTVMNPVDHPHGGGEGR
GKGYKQPVSPWGQPAKGYKTRDPHKPSGRFITKRRK*
>SPSA8_v1_130011|ID:41145309|rplW| 50S ribosomal protein L23 [Spirochaetes Bin 1 SA-8]
MEYNSILIEPVLSEKSNLMRESGQYVFKVDSRADKLMIMEAVAKMFNVHPVSCNIINVAS
KPKRLRGRPGRTAEWKKAIVKLQKGETIRVFEGA*
>SPSA8_v1_130012|ID:41145310|rplD| 50S ribosomal protein L4 [Spirochaetes Bin 1 SA-8]
MESKVISVEGKELRNLELSEAVFGLPVNEDVIWYAINNELANARVGTACTKDRSEVHGTN
RRPYSQKGTGRARHGDTKSNVYVGGGISFGPKPRDYSYMMPRKAKQLAMKTILSMKAQDG
TLAVVEDFTVETGKTKDLLKKLSAVLNVKEPERTVLILKDDDPMLKRAARNLHWLTYLSY
NRLRAHDLFYGRKVVVLESAAKLLNEMYAG*
>SPSA8_v1_130013|ID:41145311|rplC| 50S ribosomal subunit protein L3 [Spirochaetes Bin 1 SA-8]
MIGLIGKKVGMTQVFDASGKLIPVTVVQVVPNIVVGKKVADKDGYNAIVVGAYEKKKSRV
LKPYAGQFPEGIAPARILREMRDFEKEVQVGEALDVSLLEGVRFVDVTATSKGKGFQGVV
KRWGFGGGRSTHGSKFHREPGSTGQSTYPHKTFKNVKLPGRMGRERVTVLNLKVVRVDAE
KGVVLIRGALPGPRNCDVLVRKAIKKN*
>SPSA8_v1_130014|ID:41145312|rpsJ| 30S ribosomal protein S10 [Spirochaetes Bin 1 SA-8]
MKDRIRVRLRGFDVRLVDDSASSIVKTVQSAGAKVSGPIPLPTRINKFTVLRSPHVHKKS
REQFEMRTHKRLIDILEPTPEVMDALMRLELPAGVDVEIKQ*
>SPSA8_v1_130015|ID:41145313| protein of unknown function [Spirochaetes Bin 1 SA-8]
VPWSLRAPSCHAQGMTDSLRIPRKKQGIIAEKIKAV*
>SPSA8_v1_130016|ID:41145314|tufB| protein chain elongation factor EF-Tu, possible GTP-binding factor (duplicate of tufA) [Spirochaetes Bin 1 SA-8]
MAKEKFERTKPHMNVGTIGHIDHGKTTLSAAITMYCGRKFGDKVMKYDEIDNAPEEKARG
VTINTRHLEYQSVKRHYAHIDCPGHADYIKNMITGAAQMDGAILVVSAPDSVMPQTREHV
LLARQVGVPAVMVYLNKVDLVDDPELIELVEEEVRDLLNFYGFPGDKTPIIKGSAFKAMT
EPDNPEATKSIQELLDAMDSFFPDPERLTDKPFLMPIEDVFSISGRGTVVTGRVERGIVH
VNDTVEIVGIKPTRSTVVTGVEMFNKLLDEGQAGDNIGTLLRGVDKKEVERGQVLAKPGT
INPHTKFKGQIYCLSKEEGGRHSPFFSGYRPQFYFRTTDITGTVNLPEGKEMVMPGDNTE
INVELIYPIAMEKGLKFAIREGGRTVASGQVIEVIA*
>SPSA8_v1_130017|ID:41145315| putative Elongation factor G [Spirochaetes Bin 1 SA-8]
MAESQPVRNIGVLAHVDAGKTSITERILSLSGVKADAGSVDDGTTSTDYLSVERRHGITV
KSAAVQFSWNGAPVNLIDTPGHVDFSNEVHRVLQILDGAIIALCAVSGVQARTEVISDSC
KERSLPRIYFINKMDRAGADFSGVLQDLRSSIEPAAVAIQLPVFSGRNWTGIVDLVTMRR
VDCETGRSADLAGGNPAEKEILAQAVAARQKLAEAIAEHDETILALFAEDKDIPNELLAE
SLKELTWKSVLVPVVCGSAFNTASVALLLDASLRYLPDYRHARVPDCHSPDSNQPVRLKP
ALEETLAAFVFKTTADAEQKTFSWVRLWAGALQPGASALNSQTGKIVHLKKLYSIQASEV
LEVSRAQAGDIIAIDAGLASPGATLCAKHRPLFFEELRIPPPVVVQILEPMDIHETPGIR
KALHHLAREDASLIVRDEADTGRFEIAGQGELHLQIIVERLYRDFGFKVRTGNPRVTCKE
RPKKAVSVSEGFDRDFGGARIRTSISLNLEPCVDLAGNTMVFAPELRLPAPYREAVLRGL
EAALSVGPNEGWPLVNAAITITHLIPPDQNSGRNGETAVEAASAMACRKALLNSDSVVLE
PVMETAIECPEDCFGQVLALISNRQGQIDSVEDGISRKHITTRITMRMLFGFSSDLSSAS
QGHAQFQARFIGYEPRR*
>SPSA8_v1_130018|ID:41145316|rpsG| 30S ribosomal subunit protein S7 [Spirochaetes Bin 1 SA-8]
MGRKKKTIDRGHQPDSRYNSVAVTKFICRMMWQGKKSICTRIVYDAFEVMQAKSGTPALD

VFNKALENAKPVVEVKSRRVGGATYQVPVEIRETRREALAMRWLINASRSRSGKAMAEKL
ADELLDAYNNTGTAIKKKDDMHKMAEANKAFAHYRW*
>SPSA8_v1_130019|ID:41145317|rpsL| 30S ribosomal protein S12 [Spirochaetes Bin 1 SA-8]
MPTINQLIRLGRKPNVWRTKAPALMECPQRRGVCTRVMTVTPKKPNSALRKVARVRLTNG
IEVTAYIPGIGHNLQEHSVVLVRGGRVKDLPGVRYHIIRGAKDTLGVEDRKQGRSKYGAK
KPKA*
>SPSA8_v1_130020|ID:41145318|rpoC| RNA polymerase, beta prime subunit [Spirochaetes Bin 1 SA-8]
MRDIQDFDSISIRLASPEMIRAWSYGEVKKPETINYRTLRPERDGLFCERIFGTTKEWEC
YCGKFKSIRYKGVICDRCGVEVTHFRVRRERMGHIELACPVSHIWFYRSVPSRMGLLLDL
SLASLRSILYYEKYIVIDPGETELKKMQILTEEEYIAAQDRYGGAFTAGMGAEAIRSLLQ
RIDLDKLAAELREKMVAKGPKSDRRLLKRIEIVESFRNSQNKPEWMILSVIPVIPPELRP
MVQLDGGRFATSDLNDLYRRVINRNNRLKRLQNLNAPEIIIRNEKRMLQEAVDALFDNTK
KKRVVKGASNRPLKSLSDMLKGKQGRFRQNLLGKRVDYSGRSVIVVGPELKMWQCGLPTK
MALELFKPFIMKKLVEKDVVYNIKKAKMLVESETPEVYAVLDEVVKEHPVLLNRAPTLHR
LGIQAFEPILVEGKAIKLHPLVCKAFNADFDGDQMAVHVPLTNAAQAECWTLMLSSRNLL
DPANGKTIVYPSQDMVLGINFLSRPKKGVKGEGKRYSSSEELLLACESDACDYQAEVKIP
APKNLVWNKLNQVSTLPPNRVISTTAGRVLLNEALPAEIPYINYSLTDKDIRALIEVVYK
FHGPFITVNMLDAIKDMGFRYATFFGATISMEDIVIPAEKAQMIEDANKQVESIQKQYIA
GHITQDERYNRVVEVWSKTNEELTAVMMKTLEKDKDGFNNIYMMAYSGARGSRNQIRQLA
GMRGLMAKPSGDIIELPIRSNFREGLSVIEFFISTNGARKGLADTALKTADAGYLTRRLV
DIAQDMVVNIEDCGTINGAVHTALKDGEEIIEPLRERIIGRFTLDPIKHPITGDLIIDSN
QEITEEVANAIDAAGIEEVTIRTVLTCEARYGVCQKCYGRNLATGRTVEIGEAVGIIAAQ
SIGQPGTQLTMRTFHIGGAATKASEENRIYLKYPVLVNRIEGSYVPTKDDTYLFTRKGYV
YVSRIFFKTEFSKGDTLLVQDGARILKGSAIIRRKDGTEVVATDISYAKILGSLLLLIAQ
EQKIEVRNGSELLPKEGQIVAANETLAVFDPFADPIISEFDGYVRYEDIIPGSTLKEEIN
EETGNIEKKITEFASERESKQPRIIIADEAGNEIFAYLLPGGAYLNVEDGEVIKAGKTLA
KTLKESAKAMDITGGLPRVSELFEARKPRSIAVLAMVSGKVTIKGNVKNKRIVVITDKFG
KEYKHLVPMGRRLLVRDNDQVEAGELLCDGNKNPHDILNILGEQACQRFLMDEIQQVYRL
QGVTINDKHIGVIVRQMMKKVEIVSPGDTRFIFGQQVDKYKFHEENERVVKEGGQPAVAR
PMLLGITRAALKIDSFFSAASFQETTKVLTDASIAGAVDSLRGLKENVIIGHLIPAGTGM
RDYKGIKMSDAEHEDLDAFVEDILEKRKREKEMAPFPALEDRADASSSFEEESVFENDAG
FEQVSEDEE*
>SPSA8_v1_130021|ID:41145319|rpoB| DNA-directed RNA polymerase subunit beta [Spirochaetes Bin 1 SA-8]
MAYTKNKIQRRYIGKDYQEAMDLPDLIDIQLSSFERFLQREKLRNGEPLDSVGLQEVFEG
TFPIESQSGDMVIEFERYNLDESAIKYSELDCKQKGLTYSIPLKATVNLVFQKTGEIRQK
EIYLGDIPLMTDRGTFIINGAERVVVSQIHRSPGVVFSHEKGVFSSRLIPYRGSWLEFEI
DQKKELIYTKIDRKKKFLGTLFLRAIGFTTREEIIEAFYKTKTIELSDLEAAKEQLVGKV
LAKAVYIEFDNEKKKLFRAGEKLHLHEVDELGNLGIPSVEIIDFDHPDSMHNEMILHCFA
REDVKLEKETSDQDEPSKADALSAVYATLQPGEPITVDGAERDLMSMFFSSRKYDLGKVG
RYKLNKKFNYAVPVQEYTLTKADIVATMKHLMDVYYGEAYVDDIDHLGNRRVRSVGELLA
NVMKSAFARMERIAKERMALKETETVRPQDLISIKPIVAAIKEFFGSSQLSQFMDQVNPL
AELTHKRRLNALGPGGLSRDRAGFEVRDVHYTHYGRMCPIETPEGPNIGLIVSLATYTTV
NDYGFLETPYRKVKDGIVTNEIEYLSAIDEDRYYIAQASARIDTAGNFLDSTISCRHQGD
YVMQTPKDIQYMDVSPKQIISVSASLIPFLEHDDANRALMGSNMQRQAVPLVFPEAPRVG
TGMEWKTAYDSGVLVKAKRSGVVKFVDASKIIIKPDTMDPLDEDTYILVKYQRTNQDTCY
NQRPIVQFGEKIMKGQVIADGPATNTGELALGRNILAGFVPWNGYNYEDAILISQRVVKE
DMFTSIHIKEFQTEVRETKLGPERITRDIPNISEKILDNLDGDGIIRVGAKVKSGDILVG
KVTPKSDTDTTPEFKLLNSIFGEKAKDVRDSSLRVPHGIEGTIIDIQRMKRSANDDLSPG
VEEVVKVLIATKRKLKEGDKMAGRHGNKGIVARVLPVEDMPYLEDGTPLDICLNPLGVPS
RMNIGQIMETELGWAGSTLNEWYSCHVFQSPSQEQIAEKLVEAGLPGSAKVKVRDGRTGE
TFKNDVTVGVVYFMKLHHLVDDKMHARSTGPYSLVTQQPLGGKAQFGGQRLGEMEVWALE
AYGAANTLQELLTIKSDDMTGRAKVYEAIVKGEPHTAAGIPEAFNVMVQELRGLALDIRV
YDAKGKQLALTERDEDIINKNTGAF*
>SPSA8_v1_130022|ID:41145320|rplL| 50S ribosomal subunit protein L7/L12 [Spirochaetes Bin 1 SA-8]

MAALTKDQIIDAIASMTVLEISELVKAMEEKFGVTAAAPVAVAAAGPAAAAAPVEEKTEF
TVILKGGVPADKKIAIIKEVRAITGLGLKEAKDLVEAGDKPLKENVSKEEAEKVKKQIVD
AGGAVEIK*
>SPSA8_v1_130023|ID:41145321|rplJ| 50S ribosomal protein L10 [Spirochaetes Bin 1 SA-8]
MAMRATKLQPSKVEAIQMLKEMIQGSNDYVFAEYRGLTVEQITNLRKQLREKGAELHVVK
NNFARIAFEELGYTKEVAPLLAGPTAVTFVKSDSNDVAKVLLDFAKEVPALTIKGALVDK
GFMDKAQVEAFSKLPGRGQLIAMLMSAMNAPAQNLVYVLNAIPTKLVRVLKAIEEKKAQE
A*
>SPSA8_v1_130024|ID:41145322|rplA| 50S ribosomal subunit protein L1 [Spirochaetes Bin 1 SA-8]
MKHGKKYIEASKKVDRNVELEPVAACALLKDIKFAKFDETVEAHVRLNLKKSQTVRDTVV
LPNQFRGEKRVLVFCKPERVKEALEFGAAYAGDAELIEKIKGGWLDFDVAVATPDMMKDV
GKLGMVLGRKGLMPNPKTGTVTNDLKSALNELRKGRTEFRSDKTNIVHLAIGKASMEAEK
IAENLMALLEEINRKRPSDVKGDFITSIFLASTMGPGVRVAVAKNEKR*
>SPSA8_v1_130025|ID:41145323|rplK| 50S ribosomal protein L11 [Spirochaetes Bin 1 SA-8]
MAKKKITAIVKLQCPAGKATPAPPVGPALGPHGVSAPMFCQQFNERTKGMEPGLVIPAII
TVYQDKSFTFILKTPPASVLIKKACGVEKGSPVPHKQKVAKLPNDKLEAIAKQKLPDLNA
NDIQAAMRIIAGTARSMGIETELKK*
>SPSA8_v1_130026|ID:41145324|nusG| transcription termination factor [Spirochaetes Bin 1 SA-8]
MGKSWYILHVYSGYENKIEKTIRMLLDTGEISKDIVTDIKVPTEEVTDVKDGKKKTVTRK
ILPGYLLVEMDLPDNGWKAPCSAIRKITGVTGFVGSNLSSKPQPIPSEEVRRILQRTGEL
KGDTQIRMKQSYAPGEQVKIIEGPFESFSGVIEEVNAEKNKLKVMVGIFGRSTPVEVEMT
QVEKV*
>SPSA8_v1_130027|ID:41145325|secE| Protein translocase subunit SecE [Spirochaetes Bin 1 SA-8]
MKKLIQFFKDSYAELAKVVWPSKEDVISSTKVVVISTVFVALLLGLIDFLLVMGIEVVFR
*
>SPSA8_v1_130028|ID:41145326| 50S ribosomal protein L33 (modular protein) [Spirochaetes Bin 1 SA-8]
MRGGLLVGYRERTENMASKKQTVEIIALQCTECKRRNYTTKKNRKTMQEKLELMKYCKWD
RKHTLHKETKVK*
>SPSA8_v1_130029|ID:41145327| Protein MurJ homolog (fragment) [Spirochaetes Bin 1 SA-8]
MFPFLALVSFAALLQGILNSYGIFAPSGFAPILFNLCFIIVPWMVSSLTANPARAMAIGV
VAGGFAQALCQLPAVLKTGSKFGFIAPRKAFKNAGMRKVFMXIAPXILGMXXYQINDXVS
XAFASRXGLGTASSLQYSLRLQELILGVFAVSAGTVLLPSLADAVKESNWLAYSSRLGRT
LQSIFLATIPVTVFSMIANTEIVTLLFKWGSFSEESVRLTASAFFWHQTGLSFIAANRII
APAFYARSDTKTPTFAGIASFAVNXALVMLLSYPFKGPGIALALSLSSLVNTILLVTMLI
RTKTAGIAEEFAKSARYALKLLVFSIASGAVILALRVPLLSLFAGRGRLVSAGLPLIIMT
LLYAGFGIAMLALSKDPLALSIVKAVRKRSFKNEKQRS*
>SPSA8_v1_130030|ID:41145328| protein of unknown function [Spirochaetes Bin 1 SA-8]
MPAGEPDKQSLNLKESLQNPERPGLDNHEKKMIRDAGVLSVLTMISRVLGLVREMTKARF
LGTGMLGDAFTVAFIIPNFMRRLFAEGSVSVAFIPTFKGYLHEDDRKATEEFLSASLTVL
TPDCPDHSGKQHCRAGHCLRSLDRKSFWF*
>SPSA8_v1_130031|ID:41145329|ltaA| L-allo-threonine aldolase [Spirochaetes Bin 1 SA-8]
MNKIDLRSDTVTWPTEEMRTAMASAPVGDDVYGDDPTINKLEELAARMTGKEKSLFVPSG
TFGNQLALFTWSPRGSEIVCGEQCHIIQHEAGAASVIAGVQTRMMPAPDGIMPVAAIEAR
IRGNDIHYPPTSLICLENAHSSGRVISLSYMREVSALARSHGLPVHLDGARLFNAAEALH
VEAAEIAAEVDSVMFCLSKGLCAPVGSMLAGPAHFIEKARKKRKIMGGGMRQAGILAAAG
IIALEKMTRRLNEDHRNARYLAQKLAAISGISVELDALDINMVFFKLPDGIDGQNFASFL
ASRGVLINPPEAGYCRFVTHYWIKREQIDTVVELISNFIAEKR*
>SPSA8_v1_130032|ID:41145330| Cof-like hydrolase [Spirochaetes Bin 1 SA-8]
MKIRLIALDLDDTLLRSDLTISAGNRKALVNAENAGIEIVLASGRNYISMREYARQLGLD
RPGNFLICSNGAETLAAESGEIIEQLRLSXEFCHAASSEIESRGFPWQVYQDGKIFCSXI
NPWALRDKHLTSQPVEKVEDKQALFRNGQIKFVIPGEPEKIAELYAEFRMLFSDQAEVVT
SKPYFLEILPKGADKGQALGRLAEKLKIPMKAVMAIGDAMNDLGMIEAAGFGCAPANALA
VVKASARHISQRTNEEDAVADLIYSVAL*
>SPSA8_v1_130033|ID:41145331| Peptidase M15B and M15C DD-carboxypeptidase VanY/endolysin [Spirochaetes

Bin 1 SA-8]
MNRGAIFTIICIMALMSCGSGSAQTQQKQNASSQAINAALTALEQSEIQPEDKKNILAAI
NKSQPRFTEILAAITETVKQDSALLLRVDKKKGLSETWEPSDLKLLDKTGISIARAGLSL
RAPALIALQEMNKKALAEGVTLLVSSAYRSYNYQREVFARNVKELGEAEASRVSARPGSS
QHQLGTAIDFGSITNDFARTKAGAWLAANAGRFGFSLSYPQGLEQLTGYQWESWHYRYIG
KDAVALQNEFFSGIQHNAMLFLDAFFAIMPPSK*
>SPSA8_v1_130034|ID:41145332| conserved protein of unknown function [Spirochaetes Bin 1 SA-8]
MTEGQFTEFCKIRDEFRAYVDELSARASSWLLPLQEELRQLQGYSDYSVETPVVYNRALD
DVQAGDDIRFIIVADNPGKNEQLGKNQRYLVGQSGKLARSWFKAELGLDFERQAIIINKT
PIHTPKTAELRLLRKLADQRPTEFDVLLAGSQKRMADFAFRLHVCLDCYLWISGYGELAG
RGLFKPWAEALSGYYQKKSDDIKRKVLVFRHFSMNQFAIEYKSNQAGLPVIGKLEAIGQA
NRRRILGW*
>SPSA8_v1_130035|ID:41145333| putative Acetyltransferase, GNAT family [Spirochaetes Bin 1 SA-8]
MTMEIAAFNLDQEGRIRLATPKDSHAIAQLRMNFLEQIKWFSEEERQETFASQKTMFEEG
IRNGSMQVMLFEQNQTVAATSALRKLSLKTGSGAQAELLAVYTRHEYRKKGIASRLVEAT
IDEARRSGFKTLVLQPTEDSFELYRKFGFIGTARHMSLAMQNQGGPRNGTPA*
>SPSA8_v1_130036|ID:41145334| conserved protein of unknown function [Spirochaetes Bin 1 SA-8]
MKNDNNQLKLAVLIDADNTQATIIEGLLAEIAKYGVASVKRIYGDWTNTNLRSWKDKLLE
YAIQPVQQFSYTSGKNSTDSAMIIDAMDLLYTENLDGFCIVSSDSDFTRLAARLREDGKL
VLGFGQRKTPKPFVAACDKFIYTEILRESDEEKESKSEKDAQVKSQNDIKTDHRIKALLV
SAVEDAADEFGWAYLGAVGTYIANRQPEFDPRNYGFRKLGDLIKASALFEIDERANPMDP
GKQVYLRLKAKTR*
>SPSA8_v1_130037|ID:41145335| protein of unknown function [Spirochaetes Bin 1 SA-8]
LLKIAELRSAAQIWSFEITRESAKKAFAEGYAASGILDELANLAQGSVPEMLAMTLESWE
REHVSLRIMKGYVVSFDDEMAGILEAGKAFDNLRVTRLAKNLVFLDNHQAKLIPEILGKI
GLPIPPVMTSSGSDHGKPLLPALEARASALSNTVRNYAGSSEFAIIDFDRQCEQEKLPLP
KDSLSLRQLLEELSAMETSDRIRSVLEERIRRKLIISAEQLQMLKADMEHDERLGRAMNG
GHSAEGLNFQGKLHIIKGALKNKKARLTVQWISEGRELKTEMRPASLEKTDKDYLLTGED
LASHSPLTLRVGTIRYLALSEGIIS*
>SPSA8_v1_130038|ID:41145336| protein of unknown function [Spirochaetes Bin 1 SA-8]
METLKPENSQWRSAMLKMEKRNLKALVRNYLQPGPDEASPEKLVSQLAEYLQKTETQERI
IALLDDFDLVLIGLVLLKGSLSRQDAILILRGEVSEYELDFRLDSLVERLVLYLEKDAIY
NLTPFFKQPLLFRWTIQDLVFGTHHSGNGPAESRYSLQDLFISAAFIVKFEPSLFRKNGQ
LTARAVTKLQQYFKTDEQAPEIIKTIIADFEQKSFLSRKDHSAQLNEDVFARFLLDSAAN
NPLSLVLTPYPEISTALLPFLSHAFRFSGKGLMRFLRILAIANSIKIDPSEIVEKLTRFG
LLLKDNDDYICIAQRARMRGIKKKKKKRRGA*
>SPSA8_v1_130039|ID:41145337| Helicase domain protein [Spirochaetes Bin 1 SA-8]
MQSSGPLIVQSDMTILLDVHAPDAEPAREALGAFAALEKSPEHLHQYRISPLSLWNASAA
GLRPDEVIESLEKFTRYEIPRDVPFIIRDTMSRFGALVLREASEKDGRGSDFLELHCRDE
ETRTRIISDQKLAKWLVPQGKKFLLRLVDRGTVKVELLKRGYPVKDEAPLLEGSVLEIAL
KFEKPDGTRWVLRPYQDEAVRAFLGGNKPGYNYGVIVLPCGSGKTIVGMAAMVAIRKKTL
IITTNIVAVHQWRNELLDKTNLEREQIGEYTGAAKEIKPVTIATYQILSWRPDKESDFPH
FDLLRKQDWGLVICDEVHLLPAPVFRVVAEMQAVRRLGLTATLVREDGREGEVFSLIGPK
RYDVPWKILEKKGFIAEAFCKEIRVPMDDATRLSYAVAERGRKLRIAAENPLKDDVVRFL
LSHHRGESILIIGQYISQVSRYAALLDAPLITGTTPDSRREELYDAFRRGEIKVLVTSKV
ANFAIDLPDASVAIQISGTFGSRQEEAQRLGRILRPKEQNSFFYSLVSRYSVEEEYADNR
RKFLVEQGYRYSIEAWE*
>SPSA8_v1_130040|ID:41145338| Glycerate kinase [Spirochaetes Bin 1 SA-8]
MINMFEKSHYDARNDASEIFRAGVDRVNPLKLLDVTLALTGKVLTVKHAKGEERYSLDAY
RHIVAAGFGKASAVLAEGLERKLAGMVEEGIVVTKSAETAQCATIKILEAGHPVPDERSV
SAAHKILSLAAKVREWERKGEHCLVIMLVSGGGSALLCAPLPGISLQEKALATKLLLGSG
ATIQEMNAVRKHLSAVKGGKLAEAFAPAEILSLVLSDVIGDDLDAIASGPTVPDSSTWQT
VLSVLIARNVMDQMPPSVRDMVLKGVAGKIPETPKLHSLDSEPSFFNNTKTVLIGSNMLA
LQAARCKAEALGYKTFLLTSRLTGEARETARLFPALASDIAEYGIPVTKPACLIAGGETT

VTLRGNGLGGRNQEMALAVLTLLDDNKPGHRNAVFLSAGTDGNDGPTDAAGAFASMELKM
AAEKQGLSALEYLQENDSYHYFEQIGGLLKTGPSGTNVCDMQILIVP*
>SPSA8_v1_130041|ID:41145339| putative Tetratricopeptide TPR_1 repeat-containing protein [Spirochaetes Bin 1 SA-8]
MQSFISTLWERFGLNAYIRGDYEKAERWFRKLEQREPDAISVLRNLGVILLARGDAEGAE
SYLLREEKLYGKSFHRHAGLADIAYARGKRKEAERRYALALQEPECQPGGKAEASRSLLE
KRLSICSSEDAFARSRESMKIFREAEELSHEKKYEEAVLKFKESAALDETNWPALNNAGS
ICLNQLGRNREAQEFFEKAFEISKNIQVARNLDLVMRKNSKELK*
>SPSA8_v1_140001|ID:41145340| Glucosamine-6-phosphate deaminase (fragment) [Spirochaetes Bin 1 SA-8]
VPRLKAFHAIAGDAADLSAEVKRLGDVISKATIDVAFVGIGENGHLAFNDPPADFENEQP
YLIVKLDQACRQQQVNEGWFPSVEATPDRAISMSVRQILKSRCIINTVPDERKAKAVAAC
LGGEVSPLHPASILQLHPECYTFLDTASASLLFGRTSRGGGQ*
>SPSA8_v1_140002|ID:41145341|gpo| Glutathione peroxidase [Spirochaetes Bin 1 SA-8]
VDQNKLYEIEFKKNDGTVMNLAGYRDKTLLIVNTASKCGFTPQYKGLEALYKKYAARGLV
VLGFPCDQFAHQEPGSDADILQFCQINFGVTFPLMAKIEVNGPGAHPLFAELRKRTGGLL
GNSIKWNFTKFLISPGATVIKRYAPSVEPEKLEKDIEELLASTPAGSSK*
>SPSA8_v1_140003|ID:41145342|ziaR| Transcriptional repressor SmtB homolog [Spirochaetes Bin 1 SA-8]
MNTVHDVVELCTCTEVHPEAVAQARKGELPVPQLLSMSELFKILGDPTRLRIVNALAKDA
ELCVCDLSRALDKTQSAISHQLSLLRRARLVQYRKEGKVVYYRLDDDHVERLVSIAAQHV
AEQRSDVL*
>SPSA8_v1_140004|ID:41145343|ziaA| Zinc-transporting ATPase [Spirochaetes Bin 1 SA-8]
MKDTCCSSGTCAVDVATIKPVGYTLSQSQKKELVLILTALLFAVTGILLWEWTGRDPQGL
APRGLGIAKAAGVFFLVLSYILAGAEVLKGALKNILKFQPFDELFLMSIASLGAFAIGAM
EEAVGVMVLYRFGEFLQDVAVTRSRQSIKALLDLKVEKARVQQDGQWQEIESSQVKVGDL
VQVRPGERVPVDGEVVEGSASFDTAAMTGESLPRLAEAGSEALAGFIVSGGAVVIRTLRQ
AKDSAASRIIRLVEDATRSKAKPELFIHRFALIYTPIVVFSALAVAVIPPLGFPGQSFSL
WLYRALTMLVISCPCAFVLSIPLSYFAGLGGAARKGILIKGAAVLDTLANARSVVFDKTG
TLTKGVFTVKNLYPAEGFSADDLVYHAAAAGSASNHPLSQAIQRFYNSQKKNHSGFPKDA
RFTEIPGHGSVALLDGQEILAGNDRLLHLKNIPHQCEAADETVIHVAVAGTYAGKIELGD
SIKDDARPALQELQQLGIKDLAVLTGDSEGPAKRTAFELGDIAVHHSLLPEDKLAKVDEL
LKAYENRGSVIYVGDGINDAPVLARSDAGIAMGGIGTDAAIESADVVLMTDEISRIPQLI
SHARKTKRIVLQNILFALGFKVAFLFLGALGIATMWEAVIADVGVALLAVLNATRALR*
>SPSA8_v1_140005|ID:41145344| putative enzyme [Spirochaetes Bin 1 SA-8]
MAESCIRVDSLSFRYENGGQVLSNISFSLSRGSAVALVGANGCGKTTLLWLIAGLFEAES
GSIYIEEILLNRRNTRTLQKKLGLAFQNPDDQLFMATVQQDVEFGPRNFLLPEQEIQHRA
REAMHRTNCLHLADRPPYRLSGGEKRMVSLATILASDAEILLLDEPTNALDPRARRTTIN
LLRSLPHTKLIATHDLDMVLDLCDEVILLNHGTIAAKGPSREILSNGPLLEAAGLELPLS
LQG*
>SPSA8_v1_140006|ID:41145345| putative Cobalt ABC transporter, inner membrane subunit CbiQ [Spirochaetes Bin 1 SA-8]
VTTSTKQGYRFRRFEEFACISSRIHQFHPAVILLAAFSYITLLTSFPATAVIELAPYFLF
PILLAQFARIPLKPLFASTLKVLPMILLIGFLHPFLDKELAYHGSMELNRGWFIFISLML
KGFLTILMNFLMISILGIGGLQKAMKSMKIPPLFFMLFTLIYRYIMLLLEDLYRTLRAYE
LRSGRKPRLTRNEWGSLPGGILIRTYEQGLRVQHAMELRGFNPAHPYGSVQKLSIPDIVF
IMLWLSLVVLFRIYSLPEVLGSVLVAFCGEGAGLGVLHG*
>SPSA8_v1_140007|ID:41145346| Cobalamin (Vitamin B12) biosynthesis CbiM protein [Spirochaetes Bin 1 SA-8]
MHMADALISPQVGAALWIATAATGAYCIKKVQHDECETPTALAGILGAFVFAAQMINFSI
PGTGSSGHIGGGLLLSILLGPHLGFLAMSAILLIQALFFADGGLLSYGCNVFNLAFYTCF
IAYPLIYKPLAKKEKRSKAAIVLASVAGLQLGAFSVVLETLLSGKTELPFGTFILLMQPI
HLAIGLVEGLLTAVIVGYVQKTAPQLIRHTERIPENDKAATVRQKIMALFILAALITGGL
ISWFASENPDGLEWSIAKVTGSTELSSASSTHALFSAIQEKIAILPDYSFKASETSSGQS
ASRFGTTFSGITGALLTFVLLLLTNLGIKTFSWRKKPVPGSTQRDNLD*
>SPSA8_v1_140008|ID:41145347| Hydrogenase expression/synthesis HypA [Spirochaetes Bin 1 SA-8]
VHELGIMMHIVETVEAFARDQGVEHIKTLVLQVGELSPVIPQYLRACYPAAVDGTSMQDT

ELEIEIVPGNGVCSECGKVYNLVTYNKHCPVCDSTNAEIISGREFLIKEIVAS*
>SPSA8_v1_140009|ID:41145348| Formate dehydrogenase-O, major subunit [Spirochaetes Bin 1 SA-8]
MARDKILDLANKISKTKRGSKNEIKPEYPEYQILDPIVTDEMADVALQVEFRKPQSAEEI
AARCGKTVEETTKLLWDLAYAGVTIVNKIDGVDKFWHEVWVPGHMEMIVNNKENVRKHPE
VARAFDAYGKRRGPVAAGNFPVGAGAMRVIPIQRAIDGETRHASFEEVEKYINDAHLISV
SDCSCRTSREIMGEGCGHLKEDMCIQLDHAAEFYIRTGRARQITKEEALEIMHRAEDNGL
MHSIPNLDGPGHTHAICNCCGCGCFSNRIANMYNNPDMVRSNFVAQVDAEKCVACGECVE
NCPSNAAKLGQKICTTEAERKKVTKVLPYDTEWGPEHWNPDYRTNKKVVEDTGSSPCKAG
CPAHIAVQGYIKLASQGRYSEALELIKKENPFPAVCGRICPRKCESQCTRGDLDDPVAVD
EIKKFIAEKDLYADTRFIPEKRHNYGNRIAVIGAGPAGLSCAYYLALDGYQVTVFEKEQK
LGGMLTFGIPAYRLEKDVIDAEIDILRVLGVEFKTGVEVGKDVTIPELRKQGFEGFYLAI
GAQAGRKLGLEGENAEGVFAGVDFLRMVNLGQKTDIQGKVIVIGGGNVAIDVARTARRSG
GEQVAMYCLESRREMPALPEEIEEAMDEGITIENSWGPKRIIVKNGKVAGVEFKQCTRVF
DETGRFNPAFDETATITVEADRVLLSVGQAIDWGRLLEGTTIELNPNKTIKVDPVTFQTA
EPDVFAGGDVVTGPKFAIDAIALGKEGAISLHRYVHKGQSLVLGRLKRDYKPIDKENLNL
EGYDAIPRQRPIVEVKEDDKLSFRDTRGVLTEEQIKKETERCLSCGATVVDEFMCLGCGQ
CVIRCKFDAIKLVRRYNAEQLPYEKIKPTVMKYILKRKVRIAGKKVLTNVGSMFGKKD*
>SPSA8_v1_140010|ID:41145349| [NiFe] hydrogenase nickel incorporation-associated protein HypB [Spirochaetes Bin 1 SA-8]
MNTYRVLEIKKTVFSGNDERAKILRNNLEQQGVFLLNVMSSPGSGKTTTLVRTIQNMKNQ
FKIGVMEADIDSDVDASTIAKTGVKVIQLHTGGMCHLDAEMTREGLDNLQLQDLDLVFLE
NVGNLICTAEYDTGAAKSMMILSVPEGDDKPLKYPVMFQKVDAILVNKIDALPLFDFNLE
RFKEYIARLNPNAAIFPISAKTGEGMDVWISWLNKEVTSWKAIKTNQ*
>SPSA8_v1_140011|ID:41145350| Regulatory protein GntR HTH [Spirochaetes Bin 1 SA-8]
MELPKLQAPSLKELFIKEMEAQILSGKLPIGSKLPSEREISEAMGVSRAVVNAGLLEMAR
KGFLEVKPRSGAFVADYRRRGTSETLLSIMNFNGGMLGRQEIKSILELRLVLETLALELA
ITRITEEDLAIIRKHLDTLATAIDPQTAAEAAFCFHYEIGFMSGNTLLPLIFYSFKAPVT
MLWQRYITLHGKDMLYQNTKALYEAIEKRSTAEAIHLFRESINQTISGGVSIYYE*
>SPSA8_v1_140012|ID:41145351| Transcriptional regulator, AraC family [Spirochaetes Bin 1 SA-8]
MKIKDAVYVYRLMSGERLAWHGRYHAHARGEYEFHYFMEGQGALLINKAKYIIDGGQIYF
IKPREFHSILPEAVEKPISYYAVLFEPEAGNPADDDIVTLLGHVWHARSRAFPVDSKDRF
LLDDLYRLSKSPSEQSRKAAEHALLSILYRWFDHIDSPTPALSAPRTKNEHVERALSYMS
KHIREKLSSDDLAARLGLSEEYFIRLFRHHLGMSPFQYFTRLKIEAASAVLVDNQLTISD
VADRFGFENPFHFTKVFKKCTGLSPREYRKIFFEAGKPPSARSEE*
>SPSA8_v1_140013|ID:41145352| Xylulokinase (fragment) [Spirochaetes Bin 1 SA-8]
MGVVCGIDLGTQSCKIILYNPHTRHILAKSQAALDIIAKNDGTREQKAEWYETALSDCFA
AIPPDLRADIDAIGVSGQQHGFVPLDADGHAIRPVKLWNDTSTAVECEELTGKAGGNAAL
LAETGLLMLPGYTAPKILWLKKHEPDAYARLRYVLLPHDYVNFLLTGAYVTEYGDASGTA
LLDVRARTWSRRASAASSIQISSIFFLI*
>SPSA8_v1_140014|ID:41145353| Xylulose kinase (fragment) [Spirochaetes Bin 1 SA-8]
VSAEAAKRFGIPEGAVVSAGGGDNMMSAIGTGTVRDGFLTMSLGTSGTLYGYSKTPVVDP
EGNLAAFCSSTGGWLPLLCTMNCTVASEQIRALLGMSVEELNLRAESAPIGAEGLAVLPF
FNGERIPNLPNGRASINGATAANFSRENLARAALEAAIFGMRIGLESFKKLGFTAREIRL
VGGGAKSRLWRQIAANVMGLPVRVPEEEEAAAFGAAIQALWCLEKAGGRARNIEELVDAH
VAIRGDSAVEPEPEAVACYEEAYENYSKYLAVLSPLYR*
>SPSA8_v1_140015|ID:41145354|xylA| D-xylose isomerase [Spirochaetes Bin 1 SA-8]
MAEYFVGHKEYFPGIGKIRYEGPKSDNPLSFKFYDPDRVIGGKAMREHLRFSVAYWHSFG
ADGTDPFGSATQFHPWASDARNPLEAHEHKLDAAFEFFTKLGADFYCFHDRDIAPEGATI
AESEKNLVHMVSLARERQKATGVGLLWGTANLFSHPRYMNGAATNPEFGVVAHAAAQVKA
ALDATIELGGQGYVFWGGREGYMSLLNTNLKREKDHLARFLTMARDYGRARGFQGAFYIE
PKPMEPMKHQYDFDVETVAAFLRTYGLEKDFRVNIEANHAELAGHDFQHELETAAALGIF
GSVDANRGDPRNGWDTDQFPTNVYDTTLAMLAILRGGGFTTGGLNFDAKIRRNSVDPSDL
FEAHIGGMDAFAAGLLAANRILEDGKLDAFVRQRYSSFDAGNGKRFEEGQLSLAELASLA
PADPDVGRTSGKQERLENLIMGYVSRI*

>SPSA8_v1_140016|ID:41145355| protein of unknown function [Spirochaetes Bin 1 SA-8]
MIAVSVLFENPEDPRRASAFDMPDYFIDLNLDQILDGINGGLSEYGLQPMFFRHLANAQE
VRSSTRQSTALSGKFNSTSPIWTISGR*
>SPSA8_v1_140017|ID:41145356| DNA mismatch repair protein MutS domain protein (fragment) [Spirochaetes Bin 1 SA-8]
MTSARVVPNDFSLQGKERMIVVTGPNQGGKTTFARAFGQLHHLAAIGLPVPGSAARLFLH
DGIFTHFEREETIKSQRGKLHDEILRIHGSLERATPSSIFILNEIFTSTTVKDSLFLSRK
ILEKILALDAITLCVTFLDELASMDDRIVSMVSCVDPQDPSIRIFKVERRSADGLAYARS
IAEKYHLTYESLTSRLAGAHDKRGERKSEGKNSVPDASEVKA*
>SPSA8_v1_140018|ID:41145357| DNA mismatch repair protein MutS domain protein [Spirochaetes Bin 1 SA-8]
MKVFLMYKDRDFDRQAPLPAWAEALMQDFDLDILLKAMSADDRFLYDISKSALFQSVMDE
GNILYRQKVLDDSIQNEKTVRDIYTLADEAVEAKRKNWFGVFGAYPGSILYSAVRMLEAY
LPMLEKLRAIAREKQSAFKSEGFTRFFAMIQDELSDEYVRTVSQYLKDLKFESGIMISAM
LGEGNELTGHALRKPKSKDKNWIRRILGPKTPAYSFSIDPRDEPGTHALGELRDRGINFA
ADALAQSADHVESFFRILKSEIGFYIACLNLRRKLISMGETLCFPVPFAQGALTFEFSGL
YNVCLSLFSGRKSVGNNVRANGKNLAIITGVNEGGKSTFLRSVGIAQLMMQAGMFVPAEQ
FSADIRSGIFTHYRRKEDRDMSSGKFDEELKRMNTIVDRIQPHSLIFFNESFASTNEREG
SEIARQIVSALLESGAKVFFVTHLYTFAAQFSGKANTSTIMLRAERRQDGTRTFRIVEGA
PSQRSHGEDLFYKVFGAEKEPGAE*
>SPSA8_v1_140019|ID:41145358| protein of unknown function [Spirochaetes Bin 1 SA-8]
MLMKLSQLTKEQKAILARLKAWLALQLAAEAFRSDAGLQKRCPVGGVSLKLKIMQNGAGR
GGSVHSSSGRDMAINPAASASSADESAATATKPAATLQLFVKKGNAIVGARNWPQLVLVF
PGSEAAIKVLSGGKGAALPLPLSPGAFKALGFFKKASSRATELLREAQTPDMVRASLLLA
ATLHGLEAVSGDSYLERRMLIVPDGTVAVRVGQIEFFAAKRGRDIRVMESAQHPDAVLSF
SDYQSAIKVLSGKRQAVVALGSGEVKIEGLLPLVQGLFAVLDRLSWYLGVSV*
>SPSA8_v1_140020|ID:41145359| Aminotransferase class-III [Spirochaetes Bin 1 SA-8]
MSSGKKFARSREYFERAAKVIPGGIYGSKSPGFLVPGHFPYYLEGAKGSCIVDVDGNQYI
DYLCGYGSQIVGYGNPAVDGPALDQARRGDLLNQPHPVMVELAERLVDLVDGMDWAVFVK
NGTDATTLATSIARADTGKQIVVAAEGAYHGAANWCSTNVFPVFTSAEQRDVRFFSYNNI
AQLEKLFIDNKNNIACVILTPYHHPTYKPQQLPTPEFLKAVEVLCQREGAYFIMDDIRAN
FRLSMAGSHSYFGAHPDMVTMGKALANGYPISVLLGAEALKKTASSFFITGTYWMSAVPM
IAAMATLNEMERLGGPHRLAQLGTLLKNGLESLGEKAGFKARVSGPPAIPFLTFDEDPNL
FLNQRFCAAMADRGVFMHPHHNWFISLAHSEEDIERTLSAAREAFEELRGGFSALFMNKI
HEQ*
>SPSA8_v1_140021|ID:41145360| putative integral membrane protein [Spirochaetes Bin 1 SA-8]
MMHVLLIGFVVLFPIVLLFLKERIPFMAKWSTLIVCYLAGLALGNIGLVGESATGLLDTL
SSVAVAVSIPLLLFSVDMKKWKELSSKAVLAFVLAALSVSVVSGLAYALFRMRSNEAYKV
SGLLVGLYTGGTPNLAAIKTALSVDRDTYLAVHTSDMVLSALYLLFVMSGAKSVLRKFLP
TRDWDANVASSEIVSFTSRFSDYFGKGLWKKIGAGLGLAIIIVGISLGLSGLVPADYQTM
VTILLITSLALAASLVPSIRALPMTFATGEYFLYVFAVAVGAMGNISQIFSGAGIWFFFV
LLVLFGSFLLHAILCAIFKIDVDTMLIVSVSAICSPPFVGLAAVSLKARKLILPGITTGI
IGYAVGNYLGIALAEFLKAIGG*
>SPSA8_v1_140022|ID:41145361| Short-chain dehydrogenase/reductase sdr [Spirochaetes Bin 1 SA-8]
MALEAAQHGARLIISGRNVEALADVAAHCSKSCAIVPFDVADAGARERALRIVLEKYGCP
DVLILNAGVSQRARFEETDEDVFNSILETNFFSAVAMTRMILPEMRRKNSGVIACVSSIA
GLMGAPWRTAYAASKHAQSGFFQSLRAELYGSGIQISMVYPGFVRTAISQNALAGNGARH
GELDPLQKLGQDPAETAKIVWRKLEQGSLEIRVAFDGKAHLGVFLSRYFPALFAKSISRH
GGL*
>SPSA8_v1_140023|ID:41145362| Transcriptional regulator, LacI family [Spirochaetes Bin 1 SA-8]
MATMHDVAQRAGVSVATVSRFFNGGYTSPASRASIEKAVEELQYSPNSIAKALSAHSSRL
VGLVVPSVTNPFFPELARAVEEETARKGYQLILCNSEGSIEKEQRFIEAMTSHFADGIIT
STGNCGEVYKASGMPVVSVDRELGTGAPHVTSDNYRGGMLAFKCLAEKACKKFAFIGAPQ
ESTSQRHRREGFFHEAERQGYAAPEIFLADEADEYRALEDEGEHLSRYDGIFAWNDHAAV
QAIRALHQVGRSVPRDVMVIGFDDVHIARLYIPSLTTIAQPIYEMGRAAAELLMRQMNGE

KLDNVTYLLGIELISRETTAVSANLRVFQHADAF*
>SPSA8_v1_140024|ID:41145363|rbsD| putative cytoplasmic sugar-binding protein [Spirochaetes Bin 1 SA-8]
MKKYGILNAPIAEVLAAMGHTDSLAIADCGLPIPNDVRRIDLAVKKGLPSFLEVLSAISD
DMVIEKIVLAAEIQEKNPAVFNAILQMLHGIPIEFVQHTEFKALTKSCKAIVRTGEATPY
ANIILYSGVNF*
>SPSA8_v1_140025|ID:41145364|rbsA| fused D-ribose transporter subunits of ABC superfamily: ATP-binding components [Spirochaetes Bin 1 SA-8]
MDGIRLEMRGIKKSFGPVVVLKDMKLDIEPAEIHALVGENGAGKSTLMKILGGVIQKDAG
EIRINGQPVEIRRVRDAERYGIAVIHQELSILPYLSIAENVFLGKELKTRLGLVDARRMH
IEVAEKLKELGLAVNPDMPAGSLSVGQMQLVEIAKALIHRAKLIVMDEPTSALTDHEIEK
LFGIIRSLKTKGVSFIYISHRLEELFSICDRLTVLRDGEYIGTANVRDLSFNQVVSMMVG
REIGDRFPRKDTAPGETILQVNNLGRGKAFRNVSFSVRKGEIFGIAGLMGAGRTELVRAL
FGAEPAETGTIQLKGKNVSITSPLDAMNLGMGLVTEDRKAEGLFLDFSIEFNIAAANFDH
LAKAGFVHPATIRDFAEQFTQSLRIKTPSVDVPVSNLSGGNQQKAVVAKWLGRSPEILVL
DEPTRGVDVGAKREIYEIMTKLAAQGVAIIMVSSELPEVVGMSDRVLVMRQGEQAGILEE
NITQERIMALATGVVYT*
>SPSA8_v1_140026|ID:41145365|rbsC| D-ribose transporter subunit ; membrane component of ABC superfamily [Spirochaetes Bin 1 SA-8]
MRKLSFNRSKYGALAGLIGLGALLSILSPSFLTLSNIMNVLNQVSLNGLVAIGMTFVILS
GGIDLSVGSILALSGAVLASMLKGGMPQIIAILIALVMSAVFGLLNGVFIATFNLQPFIV
TMAFMTIFRGSTFVFTQGRPITGLGKQGIFVAAGRGDIAGVPWSGILLLACLIITAFLLA
RTVYGKTIYAIGGNPEAARLSGLPVKTARASVYAVSGFFAGLAGMVLTSRLDSAQPLAGQ
SYELDAIAAVVLGGTSLSGGRGFMVGTLIGAFIIGILNNGLNLLEVSSFYQQIVKGAVIL
LAVLLDRRK*
>SPSA8_v1_140027|ID:41145366|rbsB| D-ribose transporter subunit ; periplasmic-binding compoent of ABC superfamily [Spirochaetes Bin 1 SA-8]
MKKLLVFVLVLVSVAALVTGCAGGGGSSKKIGLAVSTLNNPFFVTLKEGAEAKAKELGYE
LIVTDAQDDPAKQASQVDDLIQKKVGVILLNPCNSDAAATMVEKATKAKIPVISVDRGVN
GAEVLSHIASDNVAGGKMAGEELIALVGEGAKVVELQGIPGASAAIDRGAGFHQAVDGKL
NVVASQPADFNRDKGFTVMQNIIQANKGIKGVFAHNDEMALGAVKALEAAKLTDVVVIGF
DATDDAVAAVKAGQMKATVAQKPALIGSMAVETAVKKLKGETVEKSIPVPLELVK*
>SPSA8_v1_140028|ID:41145367| Ribokinase [Spirochaetes Bin 1 SA-8]
MRVLNFGSLNIDYVYQVDHFVQPGETEAASSHQIFAGGKGLNQSLAMARAGLDVTHAGMI
GPEGDFLVRTLAESGVKTDRIGRVSMPTGHAIIQVDRAGRNCILIFGGANREIDAAMIDA
ALSGFGDGDLLVLQNEIAGIGGIMQKAHQRGMKIVFNPAPFTAEIAAYPLHLVDIFVVNE
VEAAALAASNVSASQRSEASLAQLDPVPSGASTALSADLRSVIKTLANRFPSALLIVTLG
EKGALAVQNGKEIFQPAFKVNAVDTTAAGDTFLGYFIAGLSENRGLKASLELAARASALC
VSRKGASDSVPFRKELL*
>SPSA8_v1_140029|ID:41145368| conserved exported protein of unknown function [Spirochaetes Bin 1 SA-8]
MKKGFMLVLAVLFIGLPVFAGTTISTTLQAVVGADLNITTTIPGTKAVDPTLTSAALGSV
TISSNVASWTITVHSANGGKMVRAGGGDYPYTFTWGATTGIDLSTDYVQTKNAPQAATTY
NLAIAYQTAASLGLSAGTYEDTLTITLATL*
>SPSA8_v1_140030|ID:41145369| FAD dependent oxidoreductase [Spirochaetes Bin 1 SA-8]
MTEQSTVIVVGGGIAGLTAAAYVAKKGVDVLLIEKNSTCGGLINTFARDGFVFDGGVRAL
ESAGIIKPMLADLQIDLPSIKSNVSVGIAGDVINVESEANLADYEKLLLRFYPDSADDIG
RLMDAIKEVMRNMKILYAVDNPLFKNFKEDGAYFVSKYLPWMFKFLLALRKIDAMKGPVE
EFLAGIIKNQSLMDIVDQHFFAATPTFFAMSYFYLYTDYFYPKGGVGTLPAKVQEKLLAF
GGRIKNDTKIVTLDPAARLLTDSEGNTYQYEQLVWAADLKHLYSIADTNGLPQEKASKIK
AEKDLLLSRKGAESIFTLFLAVDMPPEDFKKISHGHFFYTPSPKGLGETHRSELQGLLKQ
WGSLDKQTVLAWLDRFCELNTYEISIPVLKDSEAAPPGKTGIIASLLFEYELVRRVKEDG
WYEEFKSHLEAKMIDVLSSSIYPGLKDHVLFQFSASPLSIEALVGSSGGAIIGWSFKEPV
PVTSSMLGVNDSIKTSIPGVFKAGQWSYSPTGVPTSIMTGRLAADAAVKAIKAIKARKG*
>SPSA8_v1_140031|ID:41145370| Short-chain dehydrogenase/reductase SDR [Spirochaetes Bin 1 SA-8]
MAEKKVRQSRVRKYWPQYKWSNIAAMLKNNRIPPKLCDLDFNGKLVVITGATSGIGYKTA

RRYAEGGADLIVINRNREKSRALCEEIQRDFKVRCDEIIADLSQLDDMHRAADQLAALER
PIDVLIHNAGVYLKKRSVTRDGLETTFAVHFLSSFIINWKLLDKLKRENRARIIFVGSEG
YRFAVWGLNLDDLQFERGGYSGLKAYGSAKTAQLLAMHRFARLFEGTDVCINAMHPGMVA
TNTGRENHAFYRWYKKNILDRNSLPPEVSAQALFYLGVSPDLRGVTDSFFNLTTKEELAP
PALDLEVADELWQKSLELGRLA*
>SPSA8_v1_140032|ID:41145371| conserved protein of unknown function [Spirochaetes Bin 1 SA-8]
MERLMDTDAFVITDIEQFTALWSKTYNREGKPDWSHIIPYYDESIHFKDCIQEVRGIEEF
KALTERLARRSNELSMALKNAVMLGRLIYMEWEMTILYRNTRSSVIYGASRIMLSEQGKI
IDQRDYYDLWGDIFDNIPKFNRRYRSFMKKVFG*
>SPSA8_v1_140033|ID:41145372| exported protein of unknown function [Spirochaetes Bin 1 SA-8]
MKRIFTFLFLCLFLASGLFAQNLAEKFSSPDQTIEYHFMLEIKNLNEKDISTAFQSAIVE
PLKEIVSVGALGKFKPGIYIDSRSYGLEKQNLIVRVRTGQITIKARASSPDALLDLEKGT
SKKYEMDYFGTPDYSISTDIKFPEAEFDIQPSAWTPAKLWDFIEKKSPAIMKQIQPAIQA
TPDIEIPGVAHMYSANIEIKHPLAQKLKEMDGLVESGLAVWFFPPTDSYLVELAYTAKVK
AQPVAEQLYYDLIEKLKSSGLYASNQISKTQQYFAAYFGPKK*
>SPSA8_v1_150001|ID:41145373| Uncharacterized ABC transporter ATP-binding protein HI_1470 [Spirochaetes Bin 1 SA-8]
MKAVSLENISFSRLGRGRILEDISFSIEAGKKVAILGANGAGKTTLLSLISGHLKAESGK
IELNGQPLRSLSPLERARMIALLPQIERLPFNYSVLDFVLMGRAPHIQALSQPSEQDYAM
ARSALAELHLEHLEKRSAAEISGGEFQLIRIARCLAQEADTLVLDEPTSLLDPANSQRVA
RELALLAEKNRTIIFSTHDLALVQSVSDLVLLLYNKKIMMFDIPX
>SPSA8_v1_150002|ID:41145374| Transport system permease protein [Spirochaetes Bin 1 SA-8]
VNNPDNGRNLGRMILLVVLLLGVFILYLFIGRYPRLGFTSIRDLLHDTTTQRVFFSLRLP
RALGAFLLGTVLGGAGAMLQTIFGNPLVDAGFLGVSQGSAFGAAIALVLGSGPSLAKNLG
DKGLAVFVMSLSFVMALGALALSLFLAKKFKFGGQVLRLVLAGLAVSAFFSSLLAAVKYV
ADPLKTLPDIVFWTMGSLSAMTWQKLAGIAPLAAASVIVMFVLRWRSSLLSLDDQVSRSL
GLHPEAERAIAAVFAALGVAAMTASCGIVAWVGLVVPQGARIIFGPDGRDALPASMLGGA
VFVLLADGIAKNLFSGELPLGIVTALMGALLFVFVLTSRKTEMAR*
>SPSA8_v1_150003|ID:41145375| putative Periplasmic binding protein [Spirochaetes Bin 1 SA-8]
MKIKFFPVLIFIALVCGNLPAQERIVLGGSKAVIMVVDAVYLFPEAGSKVVASAKGDQGL
GIFLAAIDPSYAKRPIFETSAGAETYASFKPDLVIMKSANKKSLGPALDALGIQQLYLNL
ENPDDYYSEITLLGKLFKNEKRAAEVVAWYKSEVKKVTDKTAFIKQTEKRKVLVLQPSAG
AEGVWEVPPASWIQTMMVELSGGIPVWTSANPGSGWAKVNAEQIAAWNPDVIFMINYKDN
PEKSAENLLKDGRLAGVSAVKSSSVYGFVKDFYSWDQPDTRWILGLRWMAATLNPALFPD
FSAISLAKNFFNFAYGMDEKLFNQTIMPRLSGTVEGKAGK*
>SPSA8_v1_150004|ID:41145376| putative ABC transporter, transmembrane region:ABC transporter related [Spirochaetes Bin 1 SA-8]
MNKIHHIRWIWYVWKEKKGLIVLLLFLTLLSSAVAVAFPLLTRTLFDLFQNILANDTRYA
EPLKEIRKIALYFVALGFAGFIAGFFPGIRGALNVVFDYIVRNKYFKAVTEKDYRFFSKY
SSGDVVTRLTSDISDFPKLSWFLCSGIFRAVESGSKVIFCAIAMFLLDWRLALFSIISLP
FMLYIFSKTQDTIYDKVNRNEQAISHINEQLEMSFSGARIIKAFGSEKKYERFFSDALSR
RFETEMGVIKLETVLNLIYQNIDYVAQIALIFAGGLMVVRNSITIGTFYAFYNYLNLLIY
PILDIPQLFIFGKRAFVNIDRLEEMKDFPAWRDFAEAQSGQGVLEHFESLKAHNVSFSYE
GRLLPAVRHVSFEMKKGERILVIGPVGSGKTTLIKMIAGILPAQEGEISINGKNLQEIQP
ASWARLFGYVPQEPLLFSGTIHENVAFGTKEEEPNLTHDDFWRYIETAQIGEEIRTFPLK
ECTVLGQRGVSVSGGQKQRLAIARALAREPELLLFDDMTASLDTKNEGMLWKALEGKDIT
IVAVSHRLSSVQYVDKVLFMKEGEMIAYGKHEELLYSCDEYREFFAGNISLVDVK*
>SPSA8_v1_150005|ID:41145377| Xenobiotic-transporting ATPase [Spirochaetes Bin 1 SA-8]
MKLKQTYNLQLLKYVADYRYLAVPGLLVMLVITGGRIVQPLILRAIIDQAVPHNDAGLLL
KFAFLYLGIVILTGGLNYLGNIMVSKLGLSIVTRIKQDIFSHFLRLPASYFDAHPVGELM
SRTENDTEKVRDLFSNLGVTFIVSVLMMLGMFGVTLTIAPALALIMIAVTLVFLVILLLF
FSRLMKLYDASRSLHARVTAKVTEFVQGIEILRAFARTDWARKSLDKTGKEKMRNDVRVS
LFEYSAMSALDSLVGPLFIVALILLYAPRVLTGAMTLGTLLLFFEYGASLLRPVVEIAES
IRRIQQARTSLKRITTIMALPEEAGRKNEIAPIFNHEIVFENVWFAYKDEDWVLKDVSFS

IEKGKMTAVVGASGSGKSTTVSLLCGFYHPQKGRITIDGTPFSSLNLVEWRKKIGLVLQD
VYLFPGSVFENVRIYNDEIPESEVRSALARVHAEDSVQKLPSGIATNLWERGGNLSAGEK
QLLAFARAIVVNPELVILDEATSNIDMETEQKIKASLEILLKGRTSLIVAHRLSSILNAD
QILFFSEGKILASGTHQELYENLPAYRLLVEQQFLKQGNDNE*
>SPSA8_v1_150006|ID:41145378| protein of unknown function [Spirochaetes Bin 1 SA-8]
LVIGRNPGWLADSSGLRLNQKSFEEYTVAILIMKHLHHELMIFSFVHKKPYYHHFPKTQV
GK*
>SPSA8_v1_150007|ID:41145379| putative Cys-tRNA(Pro)/Cys-tRNA(Cys) deacylase EbsC [Spirochaetes Bin 1 SA-8]
MSHEQPASRQKTNAIRFLESKAIDFDILEYEVDEDVSAIHVAEKLSMDPDQIFKTIVLKG
ERAGYFVCIIPGSCEIDLRKAAKAVGDKSCDLIPLKDLEPLTGYIRGACSPFGMKKKFPT
FLDETANMFDKISVSAGKRGMQIFIAPELLAQLAEAEMTDLIQ*
>SPSA8_v1_150008|ID:41145380| putative 4-diphosphocytidyl-2C-methyl-D-erythritolsynthas e [Spirochaetes Bin 1 SA-8]
LPLSFALDILLLMKPSLLNPEPVQQLPCILLAAGASSRMNGTNKMLLPLAEKPVLRYLAE
TILSATRPLIVVTGSVQAEFRRILEGLPEVIFAHNADWQRGMVSSAQTGIRTMLEAGDFS
GFFLHHGDIPFVTEKVFSALASEFHARQRQGLKETALVSAFHGRNGHPVLFPASYASAIL
SLGDGERLKSILLELGSVSVETGCEGVLEDMDTKEDYEALRKKFLPEMYRGVRE*
>SPSA8_v1_150009|ID:41145381| conserved exported protein of unknown function [Spirochaetes Bin 1 SA-8]
VKNRIISAVFFLLSFTLLQPGAQQIHPLEGDWTGILSPGGMNLRIVFHIVQKNGKLTATM
DSPDQGVKGIPVSRVSLVSDILELEVGSVKGMYSGKISANGMKIEGVWKQSGAEFVLNLE
RQEPNASHAQSNQQSTKNSPTQRPQEPKPPFPYTSHEIVFKNTKAGISLSGTLTIPAGKG
PFPGVVLVSGSGPQNRNEEILGHKPFLVIADYLARHGVAVLRYDDRGVGASRGKFAGATT
FDFADDAEAAVEFLASRDEILKDGIGVIGHSEGGLIAPIVASNNSKVNFIILLAGPGIRG
DQLLALQNAAIGRASGMTEASISIAWDVNTKLYAIAIQNEEAAALERKIVNEVVKSQDPD
GKADAEQRKTLEAQGRQVANQLLDPWMRSLLALDPAPYLEKTRVPVLALNGSMDLQVPAD
ENLAAIQKALIIGGNQHVTTTKLDGLNHLFQHSKTGLPSEYGIIEETFAPEALNIMLDWI
VTLFSVQQ*
>SPSA8_v1_150010|ID:41145382|argH| Argininosuccinate lyase [Spirochaetes Bin 1 SA-8]
MAKLWDAGTASEVLDKDVELFLSSLAVDSRLLREDLECSIAHSRMLGKTGILPAEESAAI
TAALESMLKELAEGKLTVDTDSEDVHSFIEAELVRRLGEAGKAVHAGRSRNDQVAAAFKL
HMKRACKNTAFDVLDAIEAILSAAKNHTETLMPGYTHLQRAQPVTLAHHLLAWCAALERD
YSRFTDSMNRLDESPLGSGALAGSGLPTDREYTADLLGFARPSRNTMDAVADRDACIDYA
SAGASLMVHLSRFCEDIALWVSTEYSFMKLTGRASTGSSIMPQKRNPDPAELIRGKAGRV
FGNLQALLVMQKGLPYAYNRDLQEDKALFFEIEDILHGVLKAFAVLVTSLVPDAAKMEDA
LKDGYLEATDVAEFLVIQGIPFRTAYKAAKELVQICVRKGKRLRQIDQSDLAEAHQVFAE
CAVAPDVLADYLAPSASVERRKQTGGPAPEKTRSEIARIESFIAAQRKA*
>SPSA8_v1_150011|ID:41145383|argG| Argininosuccinate synthase [Spirochaetes Bin 1 SA-8]
MKKKIVLAYSGGLDTTVIIPWLKENYDCEVVAVCGDVGQEADWEAMGKRAVSSGASKFIR
LDQKEEFVKEYLWALVKAGSPYEKKYLLGTSAARPLLAKGLAEIALQEKADAVAHGCTGK
GNDQVRFELGVKAFAPEMEVIAPWRIWDIQSREEEIEYLEKRNIPVPMKKSDSYSRDDNL
WHISHEGLDLEDPANEPKFEGMLKMTVAPEKAPDKAEYVTLAFENGVPVSVNGEKLDPVS
LVKKLNQIGGKHGVGIVDMVENRVVGMKSRGVYETPGGAIIMAAHDKLEELCLDKKTLSF
KMTVAQRFAEILYEGEWFSPLCGALNAFIDWTQKTVTGRVTLKLYKGNILSAGASSPYSL
YDASLASFTTGPLFSHHDSTGFINLFGLPTKVRARLNARIAAHGTAAGPDVVKPGSSGYT
APAGD*
>SPSA8_v1_150012|ID:41145384| Sodium:dicarboxylate symporter [Spirochaetes Bin 1 SA-8]
MAEKKESKGLLSWYFNTNLLARILIGLVLGAIVGIILGFFPSSVKPFVDNSKFFGDLFIR
LLKMIVVPVILFSLISGAASIAPSRLGKVGIKIIVFYLLTSAFAVAIGLIFANILQPGAG
FNVVGDAAIKGKEAAAPTIIQILLNIVPTNPIDSLAKGDVLPIIFFAVVFGIGVSYVKDS
KNKSVANAGTVILDAANGAAEAMYKVVAGIMQYAPIGVFVLIAQVFAQQGPKAIGPLLVV
TLAVYLGLLVHAVGVYGGLLSIYKLGFLKFLKGANEAMITAFVTRSSSGTLPITLRCSEE
KLGIPRAISSFTLPLGATINMDGTAIYLGVCAMFIGYAIGQPLTFNQQLTVVITATLASI
GTAGVPGAGAIMLLMVLESVGLKVTEGSNVAAAYAMILGIDALLDMGRTCINVTGDMAGS

AIVAKTEGELDLSKWK*
>SPSA8_v1_150013|ID:41145385| Aspartate racemase [Spirochaetes Bin 1 SA-8]
MRKLTLGVLGGMGPEATVYFFSKLVAFDKVEKDQDHLHIIIENNPSIPDRTRHLLFNEEN
PIPAMLSSLRLLEQAQADVAAIPCMTAHAFLSTLRTQAKIPILSAFEIMAEKIPILFPGL
RKLGILATTGSRELRLYESHLPAFECLWPDQEIQEEMVMEAVYGQEGIKAGNSGSYPRNL
LVTASRNLAAKGAEAIIAGCTEVPLVLRQSDLNMPLIDPMTLMAEHMVEYARNHGSAT*
>SPSA8_v1_150014|ID:41145386| membrane protein of unknown function [Spirochaetes Bin 1 SA-8]
MYLSEIISAMTDFFASMPPILGAFLSFIAGWIIAKLFQIILPKLLSFLKFDRLSEKVGIT
NFLKKGNVEYAPSKLAGMLVYWFLMVIVLSNTLAILDTNAAASLSDWLRSALPRTIAALL
IVIIGVVIVSFLSNFFITIAKNAAIHNPILIGKIIRYVGFIILGTMVLEQLGLGQTIVST
LLLVLFAAIAFGMALAIGLGCKDMARKFVEDFVRNIREKERLKHGSDLEG*
>SPSA8_v1_150015|ID:41145387| NAD/NADP octopine/nopaline dehydrogenase [Spirochaetes Bin 1 SA-8]
MDDQKSWSEIENKLRGKALLGKKAVWCVAGAGNGGLAMAGHLGLLGFRVHLYNRTDEHLN
AVRWYGGVDIEGAVEGFGPIALATSSIQEALQGVDVVMIVTPSTAHYSFAAKMAPFLQDG
QIIVLNPGRTGGALEFRQVLKSEKCSARPVIVEAQTFIYASRMLTRHRAHIFRVKNGVPV
SALPSFLTPGALAVLNNAFPEFTAGTNVLATSLENIGAVFHPALTLLNAGWIESTGGDFE
YYIQGISPSVAKVLQKIDDERLALARALGIRTVSAREWLYLSYDSSGDTLCEAIRATSSY
IGIKAPSTINHRYIWEDVPMSLVPMSSIGAMLEIPTPTIDLIIKLAEDMLEADYRSSGRT
VETLGIKGMNIEDIHRLVTEG*
>SPSA8_v1_150016|ID:41145388| Cobalamin B12-binding domain protein [Spirochaetes Bin 1 SA-8]
MIKIDKSDVVGLIHPHIDVHNLGITSFSRILEDCGIRTELCDETVSLAIENLNEHNARII
AAWIKNKHITISGFSYRLDPDDAVRLFDIWIDFLKSSNLLASRGGPIRGLFFAGLPDACK
KVRVRFPFVDACFSGDETIHETIEKAGIPRSNMPSALSSSLTYDDDRLALGEDVIHTGNY
FSVSAVNRHGYDGYGKRGDSVVSRIRHGDEHHLPPLIRVHAGPYLADRKEAVKLFLEWSK
QLAKGGLLDVLSIGTSQLTQSNFGENWEGMHNGGGVPINSPDEYRQIWEASRPMLVRTYA
GTKDVLWLAQMHEQTIDIAWHALSFWWFCKIDGRGPNTVFQNLQEHFDAMHYIASTNKPL
EPNVPHHFAFRGTDDLGYVLSGAIAARAAKASGIKTLILQIMLNTPKYTWGIQDLAKARA
MLRLTKELEGPDFTVFLQPRGGLDYFSPDSFKAKTQLAAVTALMDDIEPLNERSPQIIHV
VSYSEADRLADPAVIEESIKITRYSLDRYRALKKSGDIPDMTNNPEVAEREKRLYTEAKQ
MLAFLESAIEKPYTPTGLYRMLKAGVFPLPWLTSCREEFPKAVAVQTRFHNGGVIAIDQA
GGMLPFEVRQEMVRAALLEQKP*
>SPSA8_v1_150017|ID:41145389| conserved protein of unknown function [Spirochaetes Bin 1 SA-8]
MIYKPELYHGFAKKPPFFEGWYYKQISADGINTLILIPGVFFGKSADDTHAFIQVYDGKS
GTSWYCRFPADSFIASRNEFSIHVGGSRFSRESVHLDIHQDGVVLVGDLNLKNHHPWPVT
PLSPGAMGPYGFLPFMECNHAVLSFTHSIEGSVSLNGADIDFSQGKGYMEKDWGSAFPST
YIWFQTNHFKATDTSLFGSIADIPWRNSSFSGFILGFMLKGKLYRYATYSGAKIRKLQVS
EREIELIAGDRKTELEVNVERHHGAALYAPYSGEMRTKIYESLSAKTMVRFYSIAAGLRH
LEWEGSGEHTGLECVGSLNFKF*
>SPSA8_v1_150018|ID:41145390| conserved protein of unknown function [Spirochaetes Bin 1 SA-8]
MLFYDRPDGKKIKQPHAMNALMPYMMRGRNESAVYYEKDIDMENALRYIRQKNSALAPSS
QSLKAEYRYSLFGLVLAAAVRTVALRPELNRFIHTRGLYQRNHIAISFIVKQKMTEEAPE
ANAKVFFEPADTLDSVTEKINAAIRHAREYGEGGDGEKIAKIAHSIPGGKALIMALYRFL
DRFNIAPWALIKTDPLYSTAYFANLGSIGLDTPYHHLYEWGNASIFVVLGKLFQKETRQG
SSYAHHHYINFKVTLDERISDGLYFARSAALFARFFSHPELLELPLDELRELLKKKQEA*
>SPSA8_v1_150019|ID:41145391|kbl| glycine C-acetyltransferase [Spirochaetes Bin 1 SA-8]
MYGAVKEELARKLSQIQSEGLYKAERVIITPQGAAIRVRDGREVLNFCANNYLGLSNDVR
IREAGIKAMEAWGYGLSSVRFICGTQAPHKELEKQVADFLGMEDAILFSSCFDANGAVFE
PLLDEDCAIITDSLNHASIIDGVRLCKAKRWVFKHADMRDVEEIDSDTGKQAKGLERCLK
ESQGARIRMIATDGVFSMDGDIANLQAVCDLAERYNALVMVDDSHATGFVGKTGRGTWEH
CGVAGRVDIITTTFGKALGGASGGVIAARKEIVEYMRQKARPYLFSNTLAPSIVGGTLKA
LELLTATTELRDRLAANTERFRTRMTEAGFDIRKGVHPICPVMLYDEKLAHAMADALLEE
GIYVIGFSFPVVPRGKARIRVQISAAHTEDQIDAAVTAFVKVGKKLGVV*
>SPSA8_v1_150020|ID:41145392| Rhomboid family protein [Spirochaetes Bin 1 SA-8]
VRIKYNAPVTLTFTFISAIVLALSLTILPSLTSLWFSTPVPFRSKVFGDYLKLITYIFGH

ASVQHYMGNFTMILLLGPLLEAAYGSGFILLSIFITALTTGLLNIFLFPNTVLLGASGIV
FMMIILASITNFNKGEIPLTFILVMVVYLGAQVWDALSKQDNISQFAHIAGGLAGSFLGF
FRTKLH*
>SPSA8_v1_150021|ID:41145393|exoA| Exodeoxyribonuclease [Spirochaetes Bin 1 SA-8]
MKKFLSWNVNGIRACAQKGFLDWLSSENADFVCLQETKAHQEQLPPELRNFVDEAGHPYK
IYWSQAQRKGYSGTAILAKQEAEKIETLGIPEFDDEGRTVIADFGAFILVSAYFPNSQEA
GARLDYKLRFCDAVLAFCENQRKAGRHVIVAGDYNIAHTPIDLARPDQNEGNPGYLPEER
AWMGKYLSAGYIDTFRHLCPEPGHYTWWTYRVPNARTNNIGWRLDYHCLDREFLPALLDA
GIQSEVRGSDHCPVSLTLDI*
>SPSA8_v1_150022|ID:41145394| ROK family protein [Spirochaetes Bin 1 SA-8]
MTNEDSGPVRSTTIRERNELTVLRLIFRTGSLSQSKAVLKTGLKAPTVFRIFAQLEKRGL
IHEIAAESKPDTDKRPRKGRRPVEFAVNPDAAYVVGLDFWARSASAIVQDFSGQVIASRS
VCFPMAPNAESAVQVLSTLIHDMLREVRIPEEKLLGIGVGAPGSVSLATGVVHFYSRIPG
MVEFPLGERLEKEFHAPVSVTNNASIVALAEERYGLAKGSGSLFAFLIRAGVGGAYLQNG
RLISDRGRTAFEVGHLSMDRNGPECSCGNRGCLELYLSEDSILAALNSAFPGIDMDRIDE
ILTTECEKAIPLLAPLFDIASHAVRDVRRLLAPEAVLIVTRSQKLSQCIAEAAKTDFARS
DQRFGPPGAKIIAAEYDSLLACKAACDLVYESYFSKGSKASAEQRNRTAARKGIQE*
>SPSA8_v1_150023|ID:41145395| protein of unknown function [Spirochaetes Bin 1 SA-8]
MFRKQHYFSSRWKESRHVADFEISADTQAESFMKALQELSHEDEELPLPSELGPVVFRDG
IFQIDMEPSGKTASLDPALKGLIDSILGS*
>SPSA8_v1_150024|ID:41145396| exported protein of unknown function [Spirochaetes Bin 1 SA-8]
VKFRNTVICLLFFLQAVSLASAQAVRNLDTLGRFSTSRFEIYYPQSLSDEAVRLAGFADR
TLAELESFFGAAAERRKIPVLLSDTRMDLNGYSTTFPSNRIVIFCAEPDIASGLASLDDP
LYLVFLHELTHEVTLNIRSPLWKFLKSVLGDFVTPSGWIMPAALVEGQAVYIESGFSGRA
GVSEAQKAGRLNDPAALELVFDEKRAQLARSLWDVSGAMDFPGSGSLPYLYGALFVRYVG
ERFGQAALVQLWREAGNGNILIGFENSLLAEGIVKKVTGVNADQLWVDFQDWLGGVASGA
GSHGDRISENTPPAGGSNQLLRSGLVPISSRRIGAFSAGSNAIFYLDKEKQAVFRQDFSG
ESFSHPRKLFNADGYVRQMALSDDEKTLLVDWRAYDSNQNLRPAQYRFDLEKGRLIQDRA
PSDAQALADIASRAQLQYLCPVSVNGQIIIYGLLRVGTVVYPARVFADGRIEIWKGMPGA
VRSISIWRSRKPAAEKNSESIGIVLGLVGDDMISRPMLLEEDEGNWSAREVVCALPSGGA
HSPALLTEDRLLFSSPGLNGINILYWSEFSRTRNVPGRWEPYEKETAAAEQDLGNTMQAP
AQTRRFPEVFRTSRLPYADAESVGISFQAMDLTERLSWSANLGFNISASAPEASLVIALS
TLKGRMILAVQDGASAGPGSSVTRISSILFDYNRHRGFMPVWKSLDFSIDARYAGLQNNY
AADSFFSLNPDYMAVSFGIKTQGSTIRQSSFAPFNRHGVLMMAALDLEGTSVSTASLSFA
LQGSAALSSAGIQADFYLSGSITSDELRFAPSGRYYAFSGNNWISGRPAEYPIFQEYAAI
DGKRFYAFSELSARLFSAGFFNKEGQSSVIKMPLSPSLLARRAGMVAGSRFAFLFDNLAS
QFLSSLFIRAELDFSVLAGLAAQNRLVAGCEFALPLAPSLAGRSFLVSFSLKNGW*
>SPSA8_v1_150025|ID:41145397| DinG-like protein [Spirochaetes Bin 1 SA-8]
MKNKALHESGKKFLPEAISSLREAIAEISGNEVFAIGACDKSGMISEVEIVARGSVSSVP
ALESWFEKGSVLIHNHPSGNLLPSEADVEVAAQAGSYGVGSYIVDNDVAYAFVIAELVQQ
KPERRLDCEDMGAVLEYGGKLSRVMPSFEPRESQVLLARDVAAILNDRGILAAEAGTGVG
KSFAYLVPSLAWAIANDTRVVISTATINLQDQLFTKDIPIIASMFKKPVKSVLVKGRSNY
LCKQRLGEAIAEEGIFLKEDDSLKKILDWDNSGGSGDRADIPFSVEDSLWSRICSESESC
LALHCGFREKCHVLQVRKTAASAQVIVVNHHLLFADIASRNRSSGGEQTAILPSYSALIL
DEAHSLESSATSLFTEEFTKFSIFRHVSRLKKSSKTRTSGLLPRLAKLPEIDRAALEKAE
NALKPLQNTMAELESEALRMLSSVSSRRLRPADSQAWRGLTERCEQLQKGLLAVSSALAS
SLANVEATLSNETVIAEAKLLMRNMNDTAVLASRFKDYPQEEDMIFWIQKEHAQRKDPYI
SFNATPLDVAPLLEKTVFKKIPAIICTSATLAIGGSFQHWMRRSGIDPSRPDISAQIYRS
PFPFSSNALLAIDTESPSPQKSPQAFKEYVIRAIPRLVMASRGRALVLFTSYDLLNAAYA
VAEPLLIQAGIPCLRQGMDSRSRLLSMFKEDIPSVLFATDSFWEGVDAPGETLSQVIITK
LPFRVPNDPVPEARAEAIEKSGKNAFMEISLPEAIIKFKQGFGRLIRHSEDRGAVVVLDS
RLVHASYGQLFINSLPESILVVDSLTAIEKKVENFLA*
>SPSA8_v1_150026|ID:41145398|manA| Mannose-6-phosphate isomerase (Phosphomannose isomerase) (Phosphohexomutase) (PMI) [Spirochaetes Bin 1 SA-8]

MPLLEKHCFIMKNTTRHNAWGSYDGFQKYAHIQQALDKPSAEYWMGDHPNDPSYLMFSDG
TSLPLDKLIRTNPKAFLGEEVFRQFDDLPFLFKVLSASMPLSIQVHPDKAKAKAGFEREE
ARGIGLTAPERNYKDKNHKPELAVALSDFRALCGFRPAQETARLLGPELTAFFSFSADAF
QDSLIRLLKKALSLHDHEKYQLEAMALKQAETLRQSSNRADRLAGDAVQECYRHYPHDGG
AVSPLFLNLFTLEPGQGLYVPAGVMHAYLSGTILEIMATSDNVIRGGLTQKHIDVDDLIA
VIDFAAQPELIQPVTKNAWKIWNTQADEFTLMEFSAVQQGRTSWSVTGPEILFCNQGSFQ
IECPAGSASLVEGTSVFIAGSCKNCTLAGTGVAYRAMVPLQKDQP*
>SPSA8_v1_150027|ID:41145399| conserved protein of unknown function [Spirochaetes Bin 1 SA-8]
MTVWVDGDSVPRDVRRILLQRKELTEDIQNQSQSQVLRFITTVRLPDVPLPYITKVSPGP
DAADTFIEEHAKAGDVVITRDIPFMDRLAEKGIWALNDRGELITKENSAERRSLRDASLT
LRNLGVLPDSPKGSQRTAADTKRFADSLDRLLARILK*
>SPSA8_v1_150028|ID:41145400| protein of unknown function [Spirochaetes Bin 1 SA-8]
VEDGKFYSHHGLDFEAFKRAKEINDKLGKPLYGGSTLTMQVARTLFLVPEKSYVRKYFEI
ITALELELILSKDRILELYFGYAEWGRGIFGIEAAARHWYGKGLASLTKDQAARLIALLS
SPIKYSPETLNKSGILRERYDYLVRRYIENQSAPPDLALTVTPPPVSPEPGMDEGDSETT
EQPAMPGEAGTQEVSAQLSDRVPTQPSQEQTSDPTDTAKQAPTE*
>SPSA8_v1_160001|ID:41145401| conserved protein of unknown function [Spirochaetes Bin 1 SA-8]
MTTMEMLERLKKYQELLSERVALEEEIATLPKTLETQNEMLSRIKKSFMEKDEEFKRVEE
KVHELRQALQEAEMTREHAEQQMDGITTQREYEAINKEIRDATDKEQALRKDIQQEERVF
SEIEEELRKNAGTIEQLEVEIAEKTKVIDSQKSEKLQAIEQLRLQEAEVSQDISKEILFK
LERIIRNKKGVGVVPVHGVVCSGCHMILPANFVNEIRDSESNTVHFCPYCSRILFYEEVK
DESGFLGDIESGALADLAFDTEEEEEEAEDEDVAESEELEKPKASKKKASKKKAVIADDF
LMDQED*
>SPSA8_v1_160002|ID:41145402|rpoD| RNA polymerase sigma factor RpoD [Spirochaetes Bin 1 SA-8]
MSDMDLDPAISKLLEYAKEKKSISFDELSDFLPESVLNSEKIDGILALLESNNIRLEEEE
IQIEDEQTLKKELESKRLVNNSKDSGTDDPIRLYLFEIGKEHLLTAEQEVELSKLMEEGE
EIIKAILRKSGMLIPEFYQLTVKALKKELKDTSGQRKENTEKIAERRRLNQFYRDVIKDT
LQELREYVDLKKKIIAKGGDIFADPDLVERREKLLEKLQSTEIHPEEISSFSEKFIIAAK
RIKKYRHEQDRIQRILQINEMRDIRVLGRNLTIKEKREEIEDRLGLKSDEIKEKIRQLQI
TEKKLKELEASFESNCDQIITMAREINRGRKMMKNAKDRLIKANLRLVVSIAKKYTNRGL
HFFDLVQEGNIGLIKAVEKFEYRKGYKFSTYATWWIRQAITRSISDQARTIRVPVHMIEQ
INKVVRESRQLMQKLGREPNDEEIAEQLGWPVSRVKSVKNVAREPISLETPIGEDEDSLL
GDFIEDKEIENPSNQTDYKLLQEQIKMVLSTLPPREQEVLRMRFGLDDGYSLTLEEVGLY
FNVTRERIRQIEAKALKKLRHFKRSQKLRDYMDH*
>SPSA8_v1_160003|ID:41145403| DNA primase [Spirochaetes Bin 1 SA-8]
MAKIPEATIQEIMDRTNFLAVYQEKVRLIKKGNKWWGLCPFHTERTPSFSIDAERGLFYC
FGCHKGGSIIDFLMETEKLSFFEAVSDLAEKANVPLKLEQNGDTREESERFQLYEMYEKL
ARAFHWLLINHDSGREALQILEKRGLSDKEIQGFLLGYAPADKNWLYGFLKSKGYSEEFL
ARTGLFSQRNRTFPLFADRIIFPISDPKGRIIAFGGRLIHGEGPKYINSPDTAIFKKQEN
LFAFDKSMEAIKETGIAVICEGYMDAISFHAAGVRNAIAPLGTAFTPRQAQLVRRRAEKV
ILTFDADSAGKKATERAISIAANAGLEVMVASFRDFKDASEILEKQGPQALKKSLEYCIN
AGVFLIQQARETFDLTSMEGKARAVAFFYPYLDALDSEVKQNSFIEEVSRSFGINSAAIQ
ADYLRAKSKIPTARNLQKIDGRPILGNANGSARTADLVFMAAVALKPQSFSRLSEMVKLE
DLDDIRARDIYTALDQMYRDNPDSFSTARIVELIENESAKRFILEVAASGELDENIQKVI
DDGALGVRKRSLEKKRYRLTIELERAMSDASGEEKKDGIINEFLKEKMLLDAELSKLKGE
MNE*
>SPSA8_v1_160004|ID:41145404| Aminodeoxychorismate lyase [Spirochaetes Bin 1 SA-8]
MKKRHLILAAAGLIIFITIAALLMVLNAPPHVSTDLSDAAGSEPVSVVFAKGQAVTFLAE
ELAQKKLIRSSEFFKLYVKVSGNDSKLKAGSYRISRGLSTVQIARLFVDGKVHTIRITIP
EGYTSRQIADMLEREGITGAREFLEAANSPELLKEIGIPSQSAEGFLFPDTYMLSENTPA
QEIVRLLSANFFEKLKKVNGNAGIDGKDLLDKIILASIVEREYRKAEDAGKIARVFLNRL
KIGMPLQSCATIVYILTERMGKPHPEKIFFSDLEIKDPYNTYRNRGLPPGPISNPGLTSL
NAVFNPPENDYLYFRLSEDGSGSHVFSRSFDEHIQNGISVKGY*
>SPSA8_v1_160005|ID:41145405|surE| 5'-nucleotidase SurE [Spirochaetes Bin 1 SA-8]

MRILVTNDDGFDADGLAVLVSYLERKNSLPGNSDVWEITVLAPESERSGVSHAMTLKKPT
KVRKLSEKRYTCSGTPADCLIVAGLGVVSEKPDVVISGINRGPNLGTDIIYSGTCGAARQ
AALEGIPAIAVSCAEYKGDLHYEACAAFVADNLSALISAWQPDTFININGPSSIEENLEA
VWAVPGRNRYIDQLKCFDGADGYMYCFLTEGRHERTPDPVSDHHAVMSGKIAISLIAVHP
VSAHDNRLNGKPFKVWA*
>SPSA8_v1_160006|ID:41145406| Galactokinase [Spirochaetes Bin 1 SA-8]
MKDIFALHLAEYESAPEVIAVAPAVMKLLGEHTASHEGLVIAAPISYEMRVAISRRKDTS
MRFFAADLNERKRSSAGNLKFKREDRWANHVKSVYDYFIRNFNIEARGINVTINGTVPPG
LGLGISAAINMSAALALKTLYGLSLKNEELAEIACKAQSVFFEKPVSLVDYLAGTAPGLR
SLSIIDLRSSRRRAVQFLDESWKIILTDSKVPRASVEAELKQRHDDCRKCLQHIPGRSAK
TLRDIKLSELDELMGVFPESMRRRCIHVVEEIARAQEAEDALIRQDAPAFGRVINKSQAS
LRNHFEISCPEIDWLAKRSLEIPGVLCSRMTSNGFGGCVYSIMKEDALDAYRKRLEEYER
IFGFKAPVYEISIKGNLRATLYNGNEP*
>SPSA8_v1_160007|ID:41145407| conserved protein of unknown function [Spirochaetes Bin 1 SA-8]
MNFSAINGMLQLEHVQLAKKVERLKNECGQRPKGSLVLKRRGSQQYIYLVKRVSGKVVTE
YIGKQDSWKAKGLMAKIAERRKYEDDLKLAEAELARLEKMMKASGVFFVEPNP*
>SPSA8_v1_160008|ID:41145408| Maf-like protein TDE_2348 [Spirochaetes Bin 1 SA-8]
MDSLILASSSPRRRDILTALGIPFSIIEPDIDETAHDHLPPEERVIRISEEKGYKARKLY
EDSASLSSEMPCHSWLILSADTLVAVEDWDHNTWTTIGKPSSRDEAREMLEILSGRTHRV
FTGMTLLDCRTGFPYSKTVAASVTFAPMTDQEISWYLDSDEWKGVAGAYRIQGKGSLFIE
SIEGSWSCVMGLPIRELYGMLRDVRFSF*
>SPSA8_v1_160009|ID:41145409|rpsB| 30S ribosomal protein S2 [Spirochaetes Bin 1 SA-8]
MAVVTMKNLLESGVHFGHQVKRWDPRMKKFIFAERNGIHIIDLQKTIQAIKDAYDAVQKV
VGSGKSVLFVGTKKQAQTAIQKEAERCEQFYVNNRWLGGMLTNFSTIKKSIQRLKKIERM
EVDGTFESLTKKEIASLQKEKTKLEKNLGGIKEMKDLPGILFVVDTRKEAIAVAEAQRLG
IPIVAVVDTNCNPEGITYPIPGNDDAIRSISLFTQIIANAVIDAGASEGLKIIENLPSDE
AEDIAADAAKDDDVEIDINAYTAVAEDASSAAAAEPADAADELPLVDEDKLYETK*
>SPSA8_v1_160010|ID:41145410|tsf| Elongation factor Ts [Spirochaetes Bin 1 SA-8]
MEIKASDIKNLREKTGAGMMDCKKALTESNGDFAAAEKLLREWGMAGVEKRAGRATNEGR
IFIAEKDGEIAMVEIACETDFVARNADFIASGNKIAEKALAAKANGPTPELENMVKDIAS
VIKENIALKRVFFLKAGAGEYLHSYTHGEGRISVVVKAASSSSETFANDKVKAFIHDVAL
HIAAFNPMFLDQSKPDASWVKEQQEIFAKQVELDEKMKGKPANVIEGILKGKLKKLMAEV
CLMDQGFVKDEKMSVAQALEAVKKETGLSFTIAGYTYVRVGENA*
>SPSA8_v1_160011|ID:41145411|frr| ribosome recycling factor [Spirochaetes Bin 1 SA-8]
MDSTRTACEERMKKTIASLKDEFNAIRTGRASPALLDKIRVDYYGQKTPLSQVATVSVPE
ARLIVIQPWDRSLFSDIEKAILKSELGLNPSNDGKVIRIAIPPLTEQRRKELVKSAKAIA
EQSRVAIRNIRRDGLEELKKLLAAGGVPEDQIKKEEDELQKLTDSFVAQVAKVLEEKEKE
IMEV*
>SPSA8_v1_160012|ID:41145412|ispU| undecaprenyl pyrophosphate synthase [Spirochaetes Bin 1 SA-8]
MISKEHLPSHVAIIMDGNGRWATRRNKARTEGHRAGLETAKKIVEYASLTGIKYLSLYTF
STENWKRAQSEVSFLMNLIKNHLRKELDFYRDHQIRVVHTGNRDTLPADILEEIDSVVSD
TASFKGMTVNLAINHGGRDEIIRAANKAIASGTRKLTEEDIRNNLDSPLVPDPDLVIRTG
GELRLSNFLLWQTAYAELYFSEKLWPDFQIDDFMLALEDFSRRERRFGGLK*
>SPSA8_v1_160013|ID:41145413| putative Phosphatidate cytidylyltransferase [Spirochaetes Bin 1 SA-8]
MTGNTKQRLLFFFIGIPTFILAVLFLPFWNNALFFLIVAALQVMAIYEMRGIFRAKAITI
DAVRIAIFSFATSGAVFASSFSAKESRTQFSSLEILLIMSAVSLLLIAGQYAFIKKDDFE
SALAEISASVFSFFYIGILGAFIVYTATCFPQKKEPVFLFTLMTFGNDSLAWLFGVTIGK
RRDLFDVSPNKSIAGFIGGFLGSIFAGILGYFLFPEAGFSSISPLILMGALMGCAVIVGD
LLESALKRSANVKDSGLVVPGRGGILDSSDSLLFSAPVFVFLSLLFGFFK*
>SPSA8_v1_160014|ID:41145414|dxr| 1-deoxy-D-xylulose 5-phosphate reductoisomerase [Spirochaetes Bin 1 SA-8]
MEKKAKKIILLGATGTIGIQTLEYVEQANREAPGSFEIVGMCAHSNEKKLLEYARFGTAA
KLALAEKSETGRIHFSGKDAIRFLLENTEADIVVNGIAGSAGLEASWHALRSGKHLALAN
KESVVMGYRLLAQLANANDRMIIPVDSEHSALFQLIARIGRSDIASIGITASGGPFLNKS
LRELETITPDEAVRHPVWNMGRKISIDSATLANKGLELIEAVRLFGFRQDNVRVLIHPQS

LVHAFIQTRDSVLWPHVSFPDMRLPIAIALTWPEKTDLSYGNVDLAGKTLSFENPDDSRF
PMLKLARNALDIGEGGTIAYNAADEVAVHAFEAGLIRFTHISVVTERVLSASWPRLVDNF
EHIREIDSHARETAKQFIQEIEC*
>SPSA8_v1_160015|ID:41145415| Membrane-associated zinc metalloprotease [Spirochaetes Bin 1 SA-8]
MLTILAGLIGLSIVVIFHELGHFFAARSVGVEVEAFSIGWGPKLVGKKWGKTEWRISALP
LGGFCKMKGEDDFKKALDLKLDTIPGSSDSFYGASPWKRIIILFSGPLMNVVFAIIMFSF
VSLVGITIITAPNRIVLASEFNKTTEAHASSNPADLAGLQSGDIILALDGKTVRDYSDIQ
ELIGFNPGKTMDIEILRDGKKIATTITPMLDKNSGQGLIGIYAWVDPVIEKIESGTSAEI
AGLLPGDKVLSVNGQSVRNTIDIMKILESKPEVIELNVERSAQRITKKLVVVYSDANSLN
LGIQFKSFVRHDKAAGVGDAFINGVSEFSKTFSLTIRGIGLLFSGVNVLKAISGPARLTY
IIGNAATESIKQSGLAGIVPILNFLAFLSVGLFIMNLLPIPALDGGQILLSLSEMIKGKP
ISTKSIYRFQTIGMVMILALFLFATFSDVLFFIFRAGQG*
>SPSA8_v1_160016|ID:41145416|dnaB| Replicative DNA helicase [Spirochaetes Bin 1 SA-8]
MIQNTQLKDKIPPSNLEAEQACLGALLIDPDSLTSVLRYIRSDDFYSLANSEIFSAILAL
HEKGQKADLITLTDELKLAGTLDKAGGPAYIASLTSMTPSAANVEYYAKIVQEMSTRRSL
IKLSSTVMSLAYDDSIETGPILDQIQSRIFEITQNRKTASYHSIKDLIPEIISIIEELSQ
NPNAFTGIPSGLADLDAMTSGFQNSEFIVIGARPSVGKTALALTIASHIAIDLKIPAAFF
SLEMSEHALMNRIIASEARVPXDRIRTGRLRTSDLNDIMEAAGRIYEAPLYIVDMPNMKL
LDLRTMARRLVLEKGVRIIFIDYITLITHENSDLPRWEQISEISRSLKALAREMKVPVVA
LSQLRREAEGKQPNLSDVRESGSIEQDADLILFLHREREINKSADQRSEQIETDLIVAKQ
RNGPIGKVTVLFKPAYAKFVSLDRREK*
>SPSA8_v1_160017|ID:41145417|rplI| 50S ribosomal protein L9 [Spirochaetes Bin 1 SA-8]
MKVILNQDVQNLGEIGDVKEVADGYARNYLLPRGYAVAHNSKNVSLFEKRKAEIEAIKEQ
KKKSSASLKEKLEAEELALLMPAGANGKLYGAVTNATIADELLKKGIEVDRKKIEVPGKA
IKSTGNYKIIVHLYDKEEAIVKVAVAGHEVKAETKESSEDQPRKQRVKRTRAEHAEPATE
EEQAAAFEAAINAGRMN*
>SPSA8_v1_160018|ID:41145418| membrane protein of unknown function [Spirochaetes Bin 1 SA-8]
MENRNRTIIQNQILIDSVPGLVSGVLYLSYILSFAFLVPIQYSFASKGRKPGLITVATAF
AIIVFGQLGRMISSGIASFGLVASAVLPPALFLVSMIYINGEYQAKKQSRRLLLAAIILS
LISAPFVYLAVTNHEVLESLTSMAVATLGGGSMSEELSTNARLAVQAATATISSAFSAFI
LWILAVSYWLGNRYASRIRMRHEVSLEAAKSQTTLALADIRVPVTLLWPTLIVWASLFAI
IVTKTGGIISAVITNASLFLGSLYAIQGIAILSYLANHSRVFFFIKVLLPLLFLFSFLNP
TVGTIILIALPVLGITEVWFPYRNLKGA*
>SPSA8_v1_160019|ID:41145419|rpsR| 30S ribosomal protein S18 [Spirochaetes Bin 1 SA-8]
MSDSRDMMEARAGRGDSDQGDGRKDQDDRNGGVRKGRGFFKKKVCKFCTQKSKIDYKDAD
TLRRFTTERGKILPRRITGTCSKHQRSLAVNIKRARALALLPYVLK*
>SPSA8_v1_160020|ID:41145420|ssb| Single-stranded DNA-binding protein [Spirochaetes Bin 1 SA-8]
MRDINVVVLIGNLTRDAELKYVKNGTAIVHFSIAVTRTRKQNDEWVDEPNFFDIDYFGKG
AESISKYLLKGRMVGVEGELRQDRWEQDGQARSKVSIVASNVKLLGSNPGSAGGGYAKPR
TENSSSPSKAPAQSEQPDTAGFDDDIPF*
>SPSA8_v1_160021|ID:41145421|rpsF| 30S ribosomal protein S6 [Spirochaetes Bin 1 SA-8]
MRQYELVVILPSEEEPFRKAKESVTAEIAQFGAAEVKEEDMGDRPLAYPIRKKERGHYML
YKFNLEPNKVIPMERAFKLNTNILKYLLVKVES*
>SPSA8_v1_160022|ID:41145422|hisS| Histidine--tRNA ligase [Spirochaetes Bin 1 SA-8]
MPAYIEPRVLKGFRDYLPTAEIQRKKLIQKLETAFSHFGFVPIDTPVLEYAEILLGKGGG
ETDKQVYRFKDHGDRDVAMRFDLTVPFARFMAQHVEELYLPFRRYHIAKVWRGENTQRGR
XREFMQCDFDIVGTDSASADADIVLTIITALKSLDIGGFMVRINHRGLFNRFLENKGMSE
KSSSILRTVDKLEKIGVQATRELLVAESDEKTADEILYFIHPEVEFQETLNKMRALCAAD
DPLADEAYQRINAVYAIVKKAGFADCIQLDPSITRGLDYYTGIVFETSLGKMPEIGSVCS
GGRYNELANLYTDRKLPGVGASVGLDRLIAALETIEAQKSQQPCPAVLIGALDEALFADY
HALAMELRIHGISVEVYPEAKKLPVQFAFAEKKKIPWMVLLDCKALDANEIPLRILANRE
TRSFKSREGLVSFLKEQLASLNRESGQDNG*
>SPSA8_v1_160023|ID:41145423|trmB| tRNA (guanine-N(7)-)-methyltransferase [Spirochaetes Bin 1 SA-8]
MADTHPIRTFVVRAGRMTDAQKRALELHGPSFIIPYSNQKTDFERFFNEEKPLVAEIGFG

MGQATWQIAIQHPEFNYLGIEVHTPGVGRLVMEIANHEIKNLKIIQHDAVEVFENMIPDA
SLAGIHLFYPDPWPKKRHHKRRIVRPELVSLFASKLAPGGYFYFVTDVEEYALASLSVLS
ENSQLRNPFEGFAPRQAWRPETKFEKRAKTAGIEKAAWELYFLRTGN*
>SPSA8_v1_160024|ID:41145424| Helicase-associated domain protein [Spirochaetes Bin 1 SA-8]
MARKAKFRYYSNMIPHELPVYQQKEKILSALEGNQVIVVESPTGSGKTTQLPIILHEAGY
GDHGIIGVTQPRRIAVLSVSDFISRQVACKVGELVGYKMRFEDKTGIKTKIKIMTDGIXL
QELKLDPYLSKYQIIMVDEAHERSLNIXFILGLLKKIIEARHDFKXIVSSATINAEVFSE
YFGECPIVKIETQTYPVTVIYDPIVDRAEQNKEAESNVLRYSDEALYEKIHAVLLRTLEN
RDEGERGDILIFLPGEKTIKGCMVRLATSPIYPRLHLVPLYARLGKDEQERVFDPAPEDK
IKVILATNIAETSVTIDGVTVVIDSGLAKLNYYNPRTFTSSLIEAPIAKASSNQRKGRAG
RTAPGVCYRLYARKDFESRPLFTTEEIYRTDLSEVVLRMAELGITDFENFDFISKPPKSG
IAGAIDVLNLLDSLNPDRTLSSTGQMMCAFPLLPRLSRMIVEAIMKYPEVLHEVLVAAAF
LSTMSPYVLPPGQELEARSAHHAFRDPMGDFITYLRLFEAFSAAPNKTKFCEKNYLDERT
MFEIVRIREQLEIIVSDMGVPILRGGSKFDYLCAVSRGLIQFVCARQERGLFRSLTAEKI
QIHPGSAMYRENPEFIVAGEIVRTTRMYAMSVSPLPREILQKISPVLASRLLAIAPSGRK
EERTGTKKPAKRSAEIMRGIPAKALADTSASRDFTNQIKIGSQVFDIKPAAGKRKKVILD
WAKFKLVIPELDRDAGDGFKGLLGCVYFNGYSLLDNEKLATIIKIGSWLSLDADFGKAWP
RGGNFETVNQDGSVNAEGFQNLANHLHHVFQVVPAKKNEKSLGFVTLFTDGNGNFWFKIS
RGFHTALNESLSSLESLADIASKDSVSAAEREKVGALYRRLSAFFDE*
>SPSA8_v1_160025|ID:41145425| Citrate transporter [Spirochaetes Bin 1 SA-8]
MSLYIPAVYAILILAFTGIASGKIPRLAMNRASIALTAAILLIVFRGITAEEAFAAIDIE
TIALLLAMMIIVANVRISGFFTLAGSKILSIAQAPRSFLAMVVFMSGFLSAIFLNDTICI
MLTPLVAEIALLSKRDGRPYLIALAVSANAGSCATIIGNPQNMLIASQSGISFLAFFLYL
APPAILAMFLAYFVTIRAFPEEFSKQNQIKLHYGQTRVSQGQSENEHIDKALIAKSLFAS
ALLLFLLAFGVRTSVAALISASTLLVTRRIHPEKVFLEVDFTLLVFFSGLFILTSTVART
PVFKNFLIDVLPMLHRPGGFFAAVIAVLSNIVSNVPAVMLLAPVAKSFSDPMTAWLMLAM
ASTFAGNLTLLGSVANLIVAEQAKKSSIRISFMDYFKVGFPVTIISIALGAVWLGFVR*
>SPSA8_v1_160026|ID:41145426|xseA| Exodeoxyribonuclease 7 large subunit [Spirochaetes Bin 1 SA-8]
MMNPYRPGKELESYSDDENPVMKEHGIFTVSSLTQMIRNNLESSFSNIQVDGEVSNAKLS
SAGHLYFSLKDREALIQAVMFRFQMNSLDFQVSDGMKIRATGSVSVYAQRGQYQLIVSTL
RKQGVGDILAMLEERKQRLAREGLFDSEKKKPLPRLPKKIVVITSQQGAALHDILTVLKR
RNSGIDVVILPAPVQGEDAPPVLVKRLHQANIFNLGDVIIIGRGGGSLEDLLAFSDESLV
RAISKSRIPVVSAVGHEVDWSLADFAADVRAPTPSAAAEIVSENKEAIASQIEQFQDELI
TSIKSRLNYAKLAIKSFSPQDIETRFSRILMPLARRLDDSIDSLRQSIKDRTINMLHTLA
LLESNLESANPLAILNKGYAVVYRKDPKSGHLSKEPVSAAQDLQHEDVLAITFAKGKADA
TVKEIYTDEKL*
>SPSA8_v1_160027|ID:41145427| Exodeoxyribonuclease 7 small subunit (fragment) [Spirochaetes Bin 1 SA-8]
MKSFEERLEKLEQLAEQIKDPKLPLEKAVSVFEEGINLSRSLKKELEAIQGKIEILLNDP
EDAENPKTAVLDIEDQ*
>SPSA8_v1_160028|ID:41145428| ADP-ribose pyrophosphatase [Spirochaetes Bin 1 SA-8]
VGEHRSVAGIAVRDGKILVGKRKAGGSIGLLWEFPGGKVESGETDEQALIREFLEEFNLM
IMPIRKLGESSFLNRHGRSELAAWEVALPKTVSDDLSLIPLHEHTHLRWVSMEELAALQM
PESDRSLIETLSKQFSELFY*
>SPSA8_v1_160029|ID:41145429| putative YjeF-related protein [Spirochaetes Bin 1 SA-8]
MKRIVSAAEARALDKRTREALAIEDVLLMEKASLRLWSLLQKIILKEQPRFFVFLCGKGD
NGGDGMAVARHAWSSGIEKEKLQVIVAKEAGGEASQLQLKSLKAIGITVTAWEKGCLENI
ASKNSGKIAVIDSVLGTGASGQARGTVREMIDSINNLSGNLGRERVVVAAIDVPSGLGDG
WMEGFPIVRADYTLTLDPAKQALYHPAARLFAGEISIVEDIFPHTIVQTMSEGCLMETSD
IPLNRVHLSNNAYKISRGRVTVFAGTVGTLGAALLCAKATQAAGAGYVQFFTQDEAYTLL
VSAMPAIMAIPESAFYSGKLKADTIVAGPGWGKSAKHAEILKNLLELDLPLVLDADALRI
LAADSSTDFLNTIQKRTAPLVLTPHPGEFESLVQRYIAAPENLAFAELLARVAETLNAII
VFKSHVTWICSPKGKAENRFSIWDGQCPGLGTAGSGDVLAGFLAGDLAIRSAANNKETYE
LWELALESARQAVVCHGYAGRLLHGKQGWFSASELLPFLKKLACEVAENKNERLDGSKAS
G*

>SPSA8_v1_160030|ID:41145430| exported protein of unknown function [Spirochaetes Bin 1 SA-8]
MIIMRIIKAVCSILVVAALSSCAASAPATELKYTELFKIGYGYSDSQITRNSFENRNSDK
SAHSADNISLEARNGLYHILSGNNGKIMRFSSYGDLLLVIQDARYIKNLMNADTEKTEIS
ENEEDKSRLSPRINPEIGFQQLSLLAVDSAGTMYVSDRLPPSVKTFYDEKSQAFSDAIVR
RFSKDGTELGFLGQEGVGGSPFPVMQGLRVTDDDKVAVISASSYSYHIFFFNADGSLLSS
LKISRSTIPLPDSESSADSALASSLASIETLLPLPDTSPKPLVIAKVDYYQQNLDAVKNL
QSWLFTIDASRGEIMQSIRIPANGKETEAPELIGVRGKRYYLMQKADAAGSGTASANNRF
TLSILLENGVISKRYLVDLPKDVTEATNFAIGKDGLLYCLGFSLDYVSCLSWAIP*
>SPSA8_v1_160031|ID:41145431| protein of unknown function [Spirochaetes Bin 1 SA-8]
LNVSRATSIFTAMALNLLTAFVGSKKDRDIKTLLPLLHSVNERESWAMSLSDSEFPQKTE
AFKKRIKDGESLDILLPEAFALAREAARRVLGERPFDVQILGGIX
>SPSA8_v1_170001|ID:41145432| polynucleotide phosphorylase/polyadenylase (fragment) [Spirochaetes Bin 1 SA-8]
MVQTVRCQVGNETMVFETGRMAKQAAGSVFATYGGSAVIATACCSDKVTEGLDFVPLTVE
YNEKYYAAGKIPGGFIKREARPKDKEILVSRIIDRPMRPLFDKAFGREIQVVPTCISTDQ
INPPDILAVNAASAAVHISSIPFAGPIAAVRVCLLDGEYIVNPTFDQIAKAKLEIVVAGT
AVGITMVEGGSHEVSEEEMIAAIECAKEPISRLCAAIEELRALAGKEKLPLAPLTITLNR
ADEIRAYAHEKLSKALFTKVKQERGRAVSQAIKETETAFADALTDDTQKKLFSTLMDDLQ
YEILRSSILDKGLRVDGRGLEEIRPITCEVGVLPRTHGSALFTRGETQALAITTLGTVFD
EQILDDIEGDRRERFMLHYNFPPFSVGEVGRLGTGRREIGHGDLARRSVEAMLPXX
>SPSA8_v1_170002|ID:41145433|rpsO| 30S ribosomal subunit protein S15 [Spirochaetes Bin 1 SA-8]
MALTKEEKSQIILEYGKDEKNTGSIETQVALLTGRITYLTEHFKTHKKDTNSRRGLLKLV
GQRRKLLKYLQRTDLEGYRALVQKLQIRK*
>SPSA8_v1_170003|ID:41145434| putative riboflavin biosynthesis protein RibF [Spirochaetes Bin 1 SA-8]
MNILSWEEMTAQKGAVPRAVTVGVFDGVHLGHQRLLAKVADQDNSLCRTVVTFKENPKKL
LHPATYKGSLFSLDQKISSFEDAGMEACVLIDFSQNFGKLTGVEFLSGLAAGGAKYLCVG
PNFRCGYKMDTDAQALVTICASRGVKAEILEPVMHNGHPVSSSRIRTAILDGRLNEARAM
LGREYEIPVMAIDNGSEAHLIFKTPEDAVLPPEGLYFVKCRDGKIEYEAEAEIAARRVCI
HSTANRRWEKVAIVAMLSKE*
>SPSA8_v1_170004|ID:41145435| tRNA pseudouridine synthase B (fragment) [Spirochaetes Bin 1 SA-8]
MISGGFLLLDKPSGITSFSSLVGVKKVFPGLRIGHTGTLDKFASGLMIVLAGDYSHLSPW
FTGLDKVYEAEIEFGIETDTLDPEGEITGRSPPPDRASLESAIKHYTGPQKQRPPEYSAI
HVNGKRSYELARSGKQVALTARDIEIYSISLQDYKENLAKMTVHCSSGTYIRALARDIAH
ACGSLAHVKNLRRTRVGVFELQKAIPSEHLPNHEDFCSLTPQIAESLGLNVGTIPLEAEK
AFLNGNPKALSQLTLAAELRKSGDIGGGCDAAIFSENSAFLGIVSISRGKLAYRKVMPIP
GVGVQ*
>SPSA8_v1_170005|ID:41145436|rbfA| Ribosome-binding factor A [Spirochaetes Bin 1 SA-8]
MDQIRLRRLEELLREEISNLINRGEIKDPRVSSLXSVTRVEASRXGSHAKVWVSCLDSEN
LSLDEAVQGLEHAAGYIQSQIARSVRLRLTPILHFIADAGIRDGFEMTEKIKGLVKE*
>SPSA8_v1_170006|ID:41145437|infB| Translation initiation factor IF-2 [Spirochaetes Bin 1 SA-8]
MTDSTDNSEKKVHLIKQPKPVTPEKPAPSSSAAPAADEESSQEKKKVVVVKKKVVVKKPQ
AKVLAHHDKEQSQPIETPGQTGAQEKPQQEKQVQHASLLKAQVEKAGEINQTAPAQTSTA
SSESVHSVTDTAGNAAKADASSQPASPGSKTQSQQAPAEKFQSQGYQGQRSSSASSSNRG
YQGSNYQDQSASGYNRNQQPQGYGGRPQGSGYQGSSYQGGSRTGSSSGYQGSSRSGQPGG
YQGSGYQGSNYQGQRPSSGYRGQPGSQPSGSYPSGGYPGSRPSGYQGSPGSYPQGGSPQG
GYPQRQVRPGSTGPVGRVGGYPAPGSQPRSGGYPSSSPGGRPGGYRPQGAPGAGGSRPGA
YRPGAPGTGRPGGYAGRAPAGSPPPMESSKTAQRKTATKRKTTFVRREDELEKELQLKKK
EESRAIAIPKSIDIMENITVSDLAKKMNIKPAEIIARLMKLGVMATINQKIDADTATILA
SEYGCEVKIVSLYDETVIEKAAEKPEDLMHRPPIVTVMGHVDHGKTKLLDAIRKTDVVSK
EYGGITQHIGAYQVETPHGKITFLDTPGHAAFTKMRARGSQVTDIVILVVSAVEGVMPQT
KEAIDHAKAAGVPIIVAINKIDLPEANPDRVKTQLSEMGLIPEEWGGTTQFCEISALQKK
GIPELLDAILLQAEVLELTASYKQLAEGKIIESRIDQGRGIVATIIVEKGTLRIGDPFVA
GIYPGKVRAMFSDKGARVDEATPSTPVEVLGFEGMPMAGDPFEVVEEEKYARAISLKRQE
LKKYEEGRNVKKVTLDNLYETISAGEVQELKVIIKGDVQGSVEALKGMLEKLSTREIRLN
VIRAAAGAISEDDVMMAAASDAIIIGFNVRPTPASKALADREKVDIRKYNIIYRVQEDIQ

KAMEGMLSPELKEQDIGKAEVREVFKVPKVGIVAGCYILEGIVRRNSQVRIIRESIEIFQ
GKINSLKRFKDDAREVSAGYECGIGIENCNDLQVGDILEIFETVEVARTLGQAVDNGSDQ
TQKA*
>SPSA8_v1_170007|ID:41145438| protein of unknown function [Spirochaetes Bin 1 SA-8]
MRQKPMLPHSLRPRVLKHKASKRQRKNSKAKDIRGSAHQALRPRTEAIREATIRINQHPG
IIEISSRRAMAADRKVQDIRVRVIREEAAQGQALAIRVQAAQANQADTRDRDIRDPIIKV
SGLLPVIADSLVLSHREAIHQAVIRGRAHLVIRVRRAVIRREALLRAVILSAR*
>SPSA8_v1_170008|ID:41145439|nusA| Transcription termination/antitermination protein NusA [Spirochaetes Bin 1 SA-8]
MASEMAEAIRQLINEKGISEDLIINTLQEALLAAYKKKFGSNENAIIRIKDNYEGVEVYA
KKVIVDFDEFEDEVTQIPLEEARKFAEDAEVGDILELPIDLKEFDLQSVMVAKQTTRQNF
RDISRDTLYAEYKSKQGELIIGYYQRERNGVIYVDLGKIEGIFPKKYQSPREVYHVGDRI
KALIWEVEKGKSGLQIVLSRSHAEFVKKLLELEIPEIYDKTIEIFKIVREPGYRTKVAVY
SRREDIDPVGACVGPKGMRSQLISQELEGEKIDFLKYDVDPRQFIKNALSPAEVKSVYIL
DESKHMALAIVQEKELSIAIGKMGQNVKLANRLCDWNIDVKTEEQYLQDSRNIELKKAAD
SLFAVEGQEGEETTEEYVRLSDLPGMKQDWISAIQKANLDMVEDFLEKEDDELLAELGLK
QAELDSIKAIIEENFEIVQEEEEVVIPEEEEYTCPDCGSIVTPDMTHCPSCGVELSFEF
GDDQQA*
>SPSA8_v1_170009|ID:41145440|rimP| Ribosome maturation factor RimP [Spirochaetes Bin 1 SA-8]
MSVENEKNIEAEIAELLSGAGLQLLEFSLGRQKGNVKVKAVIYSPKGTGTDECTKAYRLI
LPRIQLATNNQNPDIEVASPGIDRLIKTKREWQAFIGNAVKVLEKDQTEWISGILKSADE
TSVVLENRGGRKTYEYTAIVKARLDSAYKGE*
>SPSA8_v1_170010|ID:41145441| Phosphoribosyltransferase [Spirochaetes Bin 1 SA-8]
MMRFAQQLQKLLFPHYCVLCGSLLNDKEDKGYPLCAVCESSLPVPGNNRCCRCGRPLISE
IDTCMLCRQHAFAFDRLIPLYAYQDEKAGALVRAYKKGKRYSLARFWAAEIERVLKIEFG
AAIIVPVPPRPEKLRSGEQDQVETMASMLEQRGFAVARILARGESIQQKKLNRSMRQESA
RKAYHILETCKNAVPQKLVLIDDVFTTGATLDTCAKILKANGAEWVGAIVLAYD*
>SPSA8_v1_170011|ID:41145442|mutS| DNA mismatch repair protein MutS [Spirochaetes Bin 1 SA-8]
MPERVTMLDQYRKIKARYRDVILFFRLGDFYEMFYEDAIEASALLDLTLTKRQEQPMCGI
PYHAAKPYIARLLKAGKKVAICEQLTAPGSKGIIERDVIEVVTPGTSLEEDFLDHGSNNY
IVSVCTLQKQCCIAYLDVSTGEFLAYRHDNAHEDASQFLRSEFYRLNPREIIIQQSMYEN
PSILSLLREFESMLIEKRPDWSFDIEKASQYLKDRFKVASLKGFGFDDHAPELAAAGAVL
EYIEENSKTRCPHIRSILPYQKDDYLVVDEATKKNLEIVRNLQDSGKRDTLLFVLDRTKT
AGGSRLLKQWLLQPLRQKNRIEERLDAVEHLYRDQLLLNEARRRLAAILDTERLTARLAM
NKAHAKDLLALADSVHASLELTAFMSQTLGSNQEKIPKLLAIRLSPEQLEDCRKLVHSIR
TAIKDEPSILLTEGNLIRNGWNETLDHLRNLKKNSTSVLEGYLEQERAESGIQNLRIRYN
KIIGYYLEVTKGKLDSVPPHFIRRQSLVGAERYTTDKLADLESEINGATEKIIELEKSIF
LEVRENLKQYIPSMEQLARQISEIDCLVSFAQAATEGGYCRPQLLEAPILDISQGRHPVV
ETCIPQGSFVPNSITLSTQGTYFALITGPNMAGKSTVLRQTALITLMAHLGSFVPADEAR
IGLTDKIFCRVGAQDNLARGESTFLVEMHETAYILNTATENSLVIMDEVGRGTGTLDGVS
IAWAVSMYLLERIRCRTLFATHYHELTSMEHPGLKNMSMAVLENEGEIVFLKRLKEGASA
GSYGLHVASLAGIPAAVLENARVLQEKLSRFEHSLSLTDTVFKQAEEKQAEQPASQKAKA
GELFSAEDLALTRLRGLDPDSLTPLQALTILHELKSML*
>SPSA8_v1_170012|ID:41145443| putative Anti-sigma factor antagonist [Spirochaetes Bin 1 SA-8]
MENEQIEIGISDRRLVVRPIGHVTAKLCQVLKTRLFSQMDAAGVKQIEFDMSACKYMDST
FLGLLLLIEKSARAHGFEPITMHQTNDVCRSLLSTMGMERKFIFADIPCGACKRLEILSA
PRDVSAQFLLETHRELSQLSPENEEKFRLLTTLLEKSEGSDRRP*
>SPSA8_v1_170013|ID:41145444| Outer membrane protein [Spirochaetes Bin 1 SA-8]
MKRNPLRIYIIALVMIFGSASQTFSQQITRVAVLDIGRVLSYYSKEAAALANFEKKKAEV
QAEVDKRSAEIKLLQSKKAEAQSRNDQELASSLDLEIVRKTADLKEFLAARQNELDLLAK
ALSSSSSFLQRLNSTIAKVAEAEGYSLVLNMKPQDQNANLVLWNSPSIDITDKVIQALAV
K*
>SPSA8_v1_170014|ID:41145445| Outer membrane protein assembly complex, YaeT protein [Spirochaetes Bin 1 SA-8]

MNMKRAFATLIVLLAVLSAAFAQETESDWFWNKPISSVQWEGIRRANRNELDALMKSYIG
QQFTEDLWLTIQARLYELDWFETIEPQAVPANDEKSRMILKFIVTEKPAIQSVRVAGNSA
VRTSEILNVISTKGGEIFNSTKANQDDTAILKLYIEKGYPDARVSHQIQPVQNDPSQLVL
TFQVTEGSQVALRSINFTGNASVSEKSLKAAVTLKEAALFQPGTFQESKLEESRKLIVDY
YQSKGFVDAKVLDVFREIEKDPDTGKNWLNLTFSISEGKQWKFGGMEFSGNEVFDSEKLK
SLVSQKPGNMLNYKKLLQDKQKIDDLYYESGYIFNQISMKEKRDEASQSIVYQISIVERS
RAHIESLKFSGNEKTKEYVLAREMPLEEGDIFSKSKIIEGLRNLYNLQYFSAVEPQISQG
SAENLMDLVINVEEMSTADIQFGITLSGLGQTGNFPLSGFVKWNDRNLGGTGQNLAVNAT
VSPTEQSLDTSFGESWLFGKRISRSFGLNITHNSQTTGQDILAPIFSNEDIPDPFTSLEG
ANSWSGSISSIPAEYLMPYDNYSMSLSFSAGYTMRTAVGDLGISGGISSGLGMVSFDENK
YRPYEKEMRETNNTWLLTNKLFARFNINNLDFWYNPSRGYFASQRLTLAGFLGFERQHYI
KSETKLDAYLKLFTIPLSETWKFSWVLAGHTGYQTLFAQPWTALSVTKDWVSLDGTFNAR
GWKELYSTKGTDVWENSLELRMPLVDQMLWMDLFVDAGAMKTQGGWLDMTQTTPVPSAAA
FGWKNFAFSTGFGFRFIVPQFPFRFYLVKRFTFDGSAFAFKTAGANFDFVLSITQPLY*
>SPSA8_v1_170015|ID:41145446| protein of unknown function [Spirochaetes Bin 1 SA-8]
VVKFSGSHGLFGEVALEKGVVKGKVQAEGLPVVLGEGVGRFGFDAEFRYGGAGDWEVQGK
GLKVGYEGLQAVPEVMIEGYKADGKGFELAKIAIQSGTDIYDGTLRGSYKNNSMDELLQI
EASFKNRSRVAERASLSYKNENNQLEALLKLENIDLDKYLPSLKIKGLAYGKIEAKGFFS
LAALTQAIRDKQDLWAILPPATFEFDMKNGELQNAPVNLAGNGTFEGGNLLLNIPVLEIM
RSKITKANLYLQIPTGNFALDSHLETNLASSLLETDLGVSGQAIFSTAEGFALQSIKMAG
NLKNMQYHETSILPWAFSGSYQNGNLNFQGGKGAVSLSMLADGTFDMQLEKDLPILGRLR
GILSPDGTVNANIENLQADLKSLGKYLSTKTFDIRQGIAKGNLTIKGSLEDPEIFGELTM
TDAAVYSRELVQGLIGPFFAKVVINGKNIETMPTEIPLEQGKVTVAATGLFDRWSITDLR
FNVSTKPDSIIALSGKIAGITVIDAKAKVDLTLSLNDELVIAEGKLYLEKGQILIDPQGF
LPENAPPVDENALAFRVKLAMTFGKQLEVFIPDNKIPLVKGFTNPSSFLSLQYDSNSQDF
SLDGKVDLRTGYVLYYFRNFFLKSGVIEFAENSTKFNPLITAIAELRESTLTGPVKITLS
AEKSPLENLNPRLTSVPFMTETQLIALMSGGVLALDTSKPLDIREAAIASSEFLPQFNIF
KTFEQTVRKALGLDIVYIRSSFIQRWLLDLTKPATEPATEDPLARYLDKSELYMGKYLTD
SAFLYAALKFTENPLVSSSRLRLDSEFGIELDAPFGILDWSITPSLSEGSLVTGQKLSLS
WQFQY*
>SPSA8_v1_170016|ID:41145447| protein of unknown function [Spirochaetes Bin 1 SA-8]
MTKKRGRLSTVLILALISAVLFAVLILPPIHREANYQIEKAVNQAVNLVYEATSLRLGYE
YVMFTGIDHVSLHGLELFQEETSQYAVSRRKVASIKQLDFIFNPFAALFGRTSGILKEIV
AEGSVIELFLEKDISIYNRLSAYLAKQAEQPSPRLSVLLKDTDIVVHAGLVDVFHVNSSA
VQISTKAGAPEISSPLIKADIRLSSIGLDTVFVDIFSISSQLAADFSSSHYQATADIAYR
GISVRNLAIEADQGTGGVHCKVSPSDSLIIAGNYDFKDLVGFSIQMANYIPSADVAGTEG
LLKELLGLRYEGNIRGGYHAGSAGKGLWYSGEISARGAKGQKIDGVDVSDALAKIAGKGD
GTGFSGEAIARQNGYEARYNGKIAYRELELGGKITLKGKDLLAEGLINGKSGNYSLKAGG
EYSSGKEKLSFSADAVLKNAESYELKELKLGYGKEEVTGRGRGKLGGGLGFEGVIGYKGQ
EYEVSVGYGQGVVKFSGSHGLFGEVALEKGVVKGKVQAEGLPVVLGEGVGRFGFDAEFRY
GGAGDWEVQGKGLKVGYEGLQAVPEVMIEGYKADGKGFTVKRAAVSGKGYEVIGLVSGVY
GQGGVTVQGSLTGKGQVMGNGARYEVRVSYGEGFGKVRVGVLGYEAAEGLVVNGYVEAQG
GLDLAKVMSGEYGELLTWRGEGQAGLVGKGYEVREQKFGIEGFKEGGREGVAIQLKGGGY
SGTLAAGFDGQMRGEITLREFVPGTVVTAEAGVLKELGVLRYTGVVRAGMSGGESGVKTG
TSGMWYEGDVTVQGAAGDLVFGVDVSETEVKLAGKGDSAGVRGEAVVRRGEAEARYEGVV
RYEALELGGALRVKGKGFEGAFDIAGKSGKYTAQGSVQAKAGAAWGIKAQVTEQADRYDI
GLEAGFGKGRLEAAGSYGKEKGWYEAQLKAEGVGIEEIRKLFELAGVGDAGLLAGVDGGT
VSVEGSLQGQGKKVSWVIGKAEASGSMKGTSVRISGKGIVGDEQSYELKELKLGYGKEEV
TGRGRGKLGGGLGFEGVIGYKGQEYEVSVGYGQGGVKFSGSQGW*
>SPSA8_v1_170017|ID:41145448|tmk| Thymidylate kinase [Spirochaetes Bin 1 SA-8]
MAGKDTNFSVNNFIVFEGIDGSGTTTQMAYLASVFSRRKTPVFFTAEPTERPEGKLIRRI
LKGELDADPGTVAHLFASDRYQHLYGKNGIIEHLQHGEIVICDRYVLSSLAYQGISCGND
LPRYLNARFPPPGLTVFFRIEAGKAMQRVQRRQELEIFEKLPFQLAVEAAYEAEIASAMQ
SGWKIEILNAEESLENVSRQLVEIIDRHLSESA*

>SPSA8_v1_170018|ID:41145449|queA| S-adenosylmethionine:tRNA ribosyltransferase-isomerase [Spirochaetes Bin 1 SA-8]
VKTDDFNFDLPERLVAQYPVKERGSSRLFVLNRSTGQYEHSMAAELARFIEPGTVMVFND
TKVRRARLYGSAIETGAMVEFLLLEPVADSTGKADASLILENKGSQLWRTMTSKVKRQRK
GRRYSFPEGLQGIIVEELGEERVVEFSEPISEAYLERNGHIPLPPYIKRNDEAEDAQRYQ
TIYARNTGSAACPTAGLHFTPHIMDSLLERGIELRWVTLHVGLGTFLPVRSETVENHVMH
EEWYHVPHETADAVNKAKAEGRKVLSVGTTSLRTLESASQSGELKAGAGSTSIFIYPGYQ
FRIVDQLFTNFHTPKSTLLMLVSAFAGRERILSAYEEAIRLEYRFFSYGDAMLIR*
>SPSA8_v1_170019|ID:41145450|ruvB| ATP-dependent DNA helicase, component of RuvABC resolvasome [Spirochaetes Bin 1 SA-8]
MKNENSKMDPQFDPLEDEKEATLRPQILSEFQGQARLKENLSIFIRAAVERKEALDHLFL
MGPPGLGKTTLALVVASEMGVDFKPTSAPALEKPKDLAGILTTLRPGSVFFIDEIHRLKP
AIEEMLYIAMEDFELDWIIGQGPSARTVRVPVPPFTLIGATTKAGMVSAPLSSRFGISLR
FDYYSPEELELVLRRSSRILAIDITDDATQLLASTSRGTPRIANRLLRRMRDFAQVAGSD
SITRAIVADGLNRLEVDDLGLERIDREILKAIILKYAGGPVGAETLAITVGEAVDSLEDY
YEPFLIQAGLIARTPRGRIATPLAYKHLGLETSRENGRFQF*
>SPSA8_v1_170020|ID:41145451|ruvA| Holliday junction ATP-dependent DNA helicase RuvA [Spirochaetes Bin 1 SA-8]
MFNRIVGILTGCTDESLFVMTAGVEWDIAVPARAIPLFGPVGEETEVFTWLHHFEDGMRL
YGFPSASERRLFLDLVKVEGIGPRQALKILSGIAPQELALALEQSDLKALQKISGVGPKL
AQKMVLALKGQLVELAEGTAAGAALHNASPWSDVITALVDMGYDRKAVEAAVRKNADSVA
ASDDKEKELFRLVLLDLSFGGSRQ*
>SPSA8_v1_170021|ID:41145452|ruvC| component of RuvABC resolvasome, endonuclease [Spirochaetes Bin 1 SA-8]
MSVWQAPGAEEPGRIILGIDPGLASVGFGVIREVSGRLLHVDHGTIQTSSGQPAQERLFA
IYAGILTLIDSYRPSAGGIEGLFFFRNVSSALPVAEACGVIKLAFRQRAIDLSEFSPNDI
KKAVSGTARAEKKQVQEMVKMLLGLAEIPKPDHAADALAAAICRAHFEGPSGLSIR*
>SPSA8_v1_170022|ID:41145453|yebC| conserved hypothetical protein [Spirochaetes Bin 1 SA-8]
MSGHSKWATIKHKKGALDAKRGQMFTKLIKEISIAARMGGGDPDSNARLRTAILKAKAAN
MPKDNVERAIKKGTGELEGSTYEELTYEAYAPGGGAMLIEVLTDNKNRAAAEIRNILTRS
GANLGTSGSVSYLFKRKGVLTYDGEKYTEDQIMEAGLDAGADDIVNEEGSIIVYTDPANF
ESVLNAMNAKGFETLGAEISMVPDTYVAADGDTAGKVQRLIDKLEENEDVQNVYHNIELP
EVEE*
>SPSA8_v1_170023|ID:41145454|mutS| Endonuclease MutS2 [Spirochaetes Bin 1 SA-8]
MMNWNDGIWMRMDEHTASLLDYMTVLNKTADYSVSPEGRELCLEMPPLKEPASLLQLKHE
CELFVKALEEYPAPECGFPDIEIVLKKLAVEAMVLELEELHALSVWIRDYEKLLNFARKA
LVFQSTEKQAKNGTENYTIESDMQYQDRLVPLSLLIQRAPSLNVVYKKIQEILTPDGQLR
DLPAIKSIQTAIARKNKEILALSGSYSRNPELRDALQSAEPTQRDGRTLLAVRANFKGRI
KGIVHEVSASGQTVFMEPFDIMEKNNELVELDAQLKAEIRKILRTATEEIRPHRLFIKEG
RRITAWIDMRLARAIQAKRENLIFAQECSEGLTLWKARHPLLGKKAVPIDVLLPENIRTL
IITGPNTGGKTVTLKTIGLHVLMNQACLGLPAVVGTKLAIFDNVYADIGDEQSIDQSLST
FSGHIKVISAIARNATKKSMVLLDELGAGTDPEEGCAMAMSLLDYFIRKGALTISTTHHG
ILKNYGYTKPGCLNTSMEFNSASLSPTYRIAMGIPGESRALDIASGIGLGQEIVEQARSY
LSKERTDIGELIRGLSEKHREIELLEREQRKKLKAAVEDQRKADLALLRVRQKELELKRN
GLKDMKKLLDASRKTLENLVRELREHGTAVEKTREVKQFLTDLAETVEEKWTELNQEETD
LEKEAEEIDVPEADKTAPASAGELREGMLVFFGSYKKQARLIRKEDDNTWLVEVGSLRMR
ASESDLYPLKIKQEQKPLLQIELAADSAGAPSVAVFQLDVRGMRLQDALRMVEKQLDAAS
LQGLKLFSIVHGTGEGILGRGIHEFLKQNPAVADYYFARPEEGGFGKTIVRLGSD*
>SPSA8_v1_170024|ID:41145455|rsgA| putative ribosome biogenesis GTPase RsgA [Spirochaetes Bin 1 SA-8]
LNGTIIATSNNLSRVLCEDGTVRICTIKGKRIKTLIGSYNSLAVGDEVTIILQDGDRGQI
EALQDRRTVFGRYNEKGRAEQAIAANIDMVCCVTSPSLPPFRPRFIDRVAVLAELAEVPL
HIICNKCDLGLPPDVTDRLDGYRYLGYQVHLVSAETQEGIDGLKKALAGKLSVFAGQSGV
GKSSILNALIPGLERKIQSVSVKYERGRHTTTMAEMIFSDSMRIIDTPGIRRLALRSLPL
EALPACFPEIRALSADCALGARCSHVDEDGCAVKRAAEKGLIHPDRYESYLRIRSELSVP
ETWKKEGVRDPGRKERAVNPGGTRRKGRFLRNFSAEHDDDELE*

>SPSA8_v1_170025|ID:41145456|murI| Glutamate racemase [Spirochaetes Bin 1 SA-8]
MYSVAFLDSGVGGLPYLQAAKEIIPGMSVQYLADNAGFPYGTKTASSLKEILADRVRRLR
SRFLPDALVIACNTASQIGLTSLRKAHPDFPIIGTVPAIKPAAQETKTGRIGVMATESTV
KDIYLQNLIAEHASDVEVIKLPAQALVSFIEKKYLFSTPQERESKIKPYIDFLLEKKVDR
IVLACTHFLHVEKDIEQYLAKAAANSVKVVESQWGVARRLAQILASCEAPAERAEQENRF
LLTSDPPFDPMYALWAERFGLSAPDKL*
>SPSA8_v1_170026|ID:41145457| conserved protein of unknown function [Spirochaetes Bin 1 SA-8]
MQIGFSAICPDCGKDLHVCLMCRFYAPGAHWDCRETIDALVQDKEKRNFCEWFAVNEKYF
TKAGQVPAGIGKAESAKKKFDSLFKS*
>SPSA8_v1_170027|ID:41145458| protein of unknown function [Spirochaetes Bin 1 SA-8]
MFRTNQCAFPGCSRPAVSGSGFCSQHDSDQENSIKELVRHIQREKSAVNVDFSNLRLLGT
DFSRIRFIGCRFSKAILSHVMFTGGSFRLX
>SPSA8_v1_180001|ID:41145459| Tryptophan--tRNA ligase (fragment) [Spirochaetes Bin 1 SA-8]
MERKRILTGDRPTGKLHLGHYVGSLANRVRLQDDYECFFIIADLHTLTTKPEKEHIEELA
ANVRSAVLDYLSCGIDPAKSVIYLQSAVPEVIELSLFFQNLITVPRLSRVPSLKEMAQSA
HIEEMPFGLLGYPVLQAADILLPRAHLVPVGKDNVAHVEVTREIARRFNYLYGETFPIPE
ALVSEYGXX
>SPSA8_v1_180002|ID:41145460|msrB| Peptide methionine sulfoxide reductase MsrB [Spirochaetes Bin 1 SA-8]
MSGDFCKTTAVAAMVLYHYIFITMLVINSEKSARLILVIAAVAISAVFLHACSSPRSAAK
VNPAQENTAVQTPVPAQPQGGQLVILSEKEAADVCYVQPTEELKKRLSPEEYAVLVQAAT
EPPFQNPYWDNHAAGIYVDRIDNTPLFASSTKFDSGTGWPSFWQPIDQNALVLVEDRSYG
MNRIEVRAKKSGGHLGHLFNDGPNPTGLRYCINSASLRFIPKEKMAEEGFASLLPLVESR
*
>SPSA8_v1_180003|ID:41145461| conserved protein of unknown function [Spirochaetes Bin 1 SA-8]
MELKPGIVAEKTIIVEERHVAAHLGSGGVPVYATPSMILHMEETSRDAVDHLLGPRGATV
GASISARHLAPTPVGMKVRVRSELVRVDGRLLTFKVEAWDEVEKIGEADHVRAIIDLDRF
GVKIAQKEALVGKTGKGS*
>SPSA8_v1_180004|ID:41145462| conserved protein of unknown function [Spirochaetes Bin 1 SA-8]
MTLEELSKLDYQGAMELMVAYSTDIKMQEKELEPLKKEAELWASRVSLAESKALADLAQA
ARERHGQIQEKIIALENSILELKRDVARLKEALPGLKAKERSIDPDQLLAELSMMTGEAM
DPEKAKFDREFAALEGSGAGTSAPQAEPQSSPKPASPVDDTLAALKRKLGL*
>SPSA8_v1_180005|ID:41145463|nadE| putative glutamine-dependent NAD(+) synthetase [Spirochaetes Bin 1 SA-8]
MIISIVQMNSVIGDFEGNANKIVSYVNKACSRKSALPPPELVIFPEMALCGYPPMDLLDY
DSFAEGSLAGLRKLQKELPSSVAVGVGYVDRNRSGSGRALINAFSVIADGKIVFTQLKTL
LPTYDVFDEARYFEPAKVRKVFTPQQARIGFAICEDFWWEAPPSPDFRYPIDPVKELLDS
SIDILIVPSASPFIAGKLKTRLRLARNTAREGHIPVLYCNSTGANDSLVFDGRSFAVDSK
GDMIGMCGWEDEILTIDTTSMTAENLVLAQTARVQHLETILAREQPSGEPVPATVQNPVN
QNEIEEIHQALIVGIKDYLDKTGFSKVCLGLSGGIDSALVAVLAAEAVGADNVVCIAMPS
RFSSEGSMNDAIELCRRNRIHLEKLPIEVPFTAFLSQLAVPFAGRPFDSAEENLQARIRG
CYLMAWSNKFNSILLTTGNKSELATGYCTLYGDMCGALAPIADLYKTEVYALSRHINALA
RQAGKMDPIPEAIILKAPSAELRLNQKDQDSLPEYEILDKILVHYIENNKAFDEIVELGF
DAALVRKVLLMTARAEFKRRQAAPAIKVSRRAFGIGRRLPLARAIYEADSI*
>SPSA8_v1_180006|ID:41145464|lgt| Prolipoprotein diacylglyceryl transferase [Spirochaetes Bin 1 SA-8]
MLLTLEFPAWLKPEIIPGLPFRWYGLMYVIAFGLTWILFNRESKRIKAPWNEDQAANFFL
WAIIGVLLGGRLAGTLIYEPTDYYWRHPWFIFWPFDEGGQFTGFQGMSFHGGLAGVIVAT
LVWCKVHKQRWIEWADILAVSVPLGYTFGRLGNFINGELWGKVTTRPWGMIFPYAERFSA
KETWVQQIAEKTGISLNSLNDMVNLPRHPSQLYEAIFEGIVLWLVLWFFIRKKRLFPGLA
AGSYVIGYGIARFFIEYFREPDAGLGYIIKLGSPDAPTYLFQTPLNFSMGQILCLLMILG
GIIFLQVSARKAKLASLETSQNSARPTARKLRKKIK*
>SPSA8_v1_180007|ID:41145465| membrane protein of unknown function [Spirochaetes Bin 1 SA-8]
VFSILGHGALKHLDPIPSRLLDSRIMKRNPIHAIAAALTLARPYLAIDLMVRLGFANLDG
ASGALFRYLVLPMAILPVTLAFFYYNEEKYRTFGSLALLMTGSSLVLLALAGFAISTNIQ
AATLFSGSVTNLVHGGFDFLFLLILDLSVSLTLGISLFRKEKKAPALESSPALRAELDTE
PRKE*

>SPSA8_v1_180008|ID:41145466| Carboxyl-terminal protease [Spirochaetes Bin 1 SA-8]
MAGFRNEKTRTIGLWLIACVLVLFLSFTSVAIPQASAQAASGSQDDAKRYSQLLQSIYQF
ILQNYVDQVDPAVLYEGAMKGMFNSLGDPHSVFLDADMLSDLMRETDGKYAGVGLYISKQ
PGAAGENETKYVEVVSPIEDTPAWKAGILPGDLILKIDGESTADLTVDQASNKIRGPAGT
KVILTFRRGASYEFDVEFTRATIEIPSIKTGSFEQNGAKYGYIRIIEWIPQTPEKIQEAL
SRLVKEGISYLIVDVRSNPGGLLSSVIDVSDLFIDSGVIVSTKGRNKQENFEYKAKPGVA
VPSSVKMIVMVNKGSASASEIFAGAMKDTRRALLLGEKTYGKGSVQQIFPLDKTGFKLTM
ARYYTPSGVNIDKTGIEPDVSVPDLVLSEKQMGLYEKLYTSGLIQKFAESNPNAGPAERK
AYAEHLIAVEGFDIPQAFIEKMLRDEAEKTKPAQVFDLEFDVQLQEAIRILASSEFDSML
QNSKTVEQLLSAKPDTGAVTQRQ*
>SPSA8_v1_180009|ID:41145467| putative Ribosomal RNA small subunit methyltransferase E [Spirochaetes Bin 1 SA-8]
MRQLILPGDRTRDMCCTLDDKTSRYLVRTLRQKVGDTFSALDETGTRFDCEISSIEKGRV
VVSLSARDSKDSNEERGGHAGDEPSQFESAGMPELILVQAVPKGQKFDLIIRQAVETGVE
LIIPLLTRYCVVEERDARDKLAKLERRKKIVREALQQSGSTTLTEIAESVPIRNLAEQLA
QRGIRPENSLFLLFHEKPLARQSLHEYCSRSVNSIVLVIGAEGGFSDEETALLEQSGFNL
VHFSGPIMRTETAALFAIAAVKAIFLEKTIWKLSR*
>SPSA8_v1_180010|ID:41145468| protein of unknown function [Spirochaetes Bin 1 SA-8]
METLAIKSLLLDDVRLDFPTERQLGALISVWDDGKNHQIAFLDSAGHRQAQKVNEYASMI
ASSDLVLPVSGNLVERASALQTSSDAGACIHERLFPIPRQHIEYVDYHFLSQDEPAAFRP
YNALKVLNSLLSALEMKSGTVFLLGGENAILQKAEMNLRATFPGLRIVGRAPGNFSAANE
AAIILAMQKSTPSMIIAGSHLETGELWIPRHMCFVKSGIFFYKQSIMETLA*
>SPSA8_v1_180011|ID:41145469| protein of unknown function [Spirochaetes Bin 1 SA-8]
MLTVIYWDCVACRIRKVTDILQKHFSVLSVPTFADITKHQPIQSVCAVVVGCNDSCLEKV
SVSIEKLKNSIPLPIFMISESIMPDTKKTPDGVFFCNSSSVDQLGEKIQDIVLGNPAAEI
ARSVMPSFLGKSAAIRNVIRQVEQYAPFDNPVLILGETGTGKELVAHALHENSMRKGKPF
IPLNCSAIPETLIESELFGTARGAFTDAVYRSGAFSRASTGSLFLDELGSMSLMVQPRLL
RVLESGEFMRVGGEKIEKSDFRLISASCRNPLDLSDAGLFRHDLMFRISDLIIMIPPLRE
RREDIIELAHHFCAELSMNRCGLSENALEKLYCHNWPGNVRELRTVISRACVHTREGAIT
EREIMFHSEFKNILKDCPLG*
>SPSA8_v1_180012|ID:41145470| RNA methyltransferase, TrmH family, group 1 [Spirochaetes Bin 1 SA-8]
MTAGHNISIVLCRVKESGNVGSVCRAMKTMGFSKLVLADCPDYEEEKVRMMAVHAFDVFE
HAQHVSSLDLALSSSALSAGFSRRKGEHRKSFFMSLRPFIRSRLLSLPSDTPVSFVFGNE
RDGLSDSELALCSLGVSIPTSESFPSLNIAQAVQIACWEVFSAFQDSESSEFRAAEQKPT
VPAKTIFLSRAQVDERTAAISTSLAQIGFFKKSSDEYARRFFRDMIERAQLSKLEADYLA
QIFSKAAILASHEGKPRRKIPHDRNESTSNFTA*
>SPSA8_v1_180013|ID:41145471| Short-chain dehydrogenase/reductase SDR [Spirochaetes Bin 1 SA-8]
MSIQEQEEQHKTPSEESRHSVFAGKRALVVGGSGGIGRWLSKILAERGASVFIQGAHESK
VAGLVEEIKRAGGTAEGCAHDIESASEFCAFLEGYGDFDIVISAYGPFLQKPLTQYSPQD
WEHLVGHNLALPGALASLFLPGMLHRNFGRFLFFGGTRTDALLTYKTNAAYASAKAGLNV
LVKSLAAEGRDRNVASLLVCPGLVDSEYLDTAAREALCAKAPGGKLIPARTVAETALNLL
DADPCAASGAIVSLDSGFSPS*
>SPSA8_v1_180014|ID:41145472| protein of unknown function [Spirochaetes Bin 1 SA-8]
MGSRLLAKARNSIVPENGADKAADASPEILTAHAAEHSAKVNKASSLRGKASQLREIRES
RSQTHNGYFTIETLGSHPDVLIVHPLKPLQIDTVQSMHAELLKTIDSFRSILVWDFSTVE
NIDAAGVGMLIAVQKKLREQSGDQALSGLRPRLMRFIAMLGYADYFCVEPDIPHVLDRIE
EETSGIFPLMATCPACGTGLQISEPGRGRCRACGAVLSIFSNGSVELG*
>SPSA8_v1_180015|ID:41145473| conserved protein of unknown function [Spirochaetes Bin 1 SA-8]
MIVQSRVEEYLIDLGISYQEIAKDAWLIEDEEKGLPKMVVSVTDPIVIIRANVMPIPEKN
REALFELLLSLNARDFLHGAYAIDGNDIIALDTLEYQNMDRNEFSASLEAMAFALSQHYP
KLSVFA*
>SPSA8_v1_180016|ID:41145474| conserved protein of unknown function [Spirochaetes Bin 1 SA-8]
MGIFDRIKTVISSNINDLVTKAENPEKMLNQMIIDMNEQLIESKKAVAMAIADEKRLERE
VVENKAKADEWEKKAVLAVRANRDDLAKEALLRKQEHESYVGQLATQLQAQKESVEKLKD

SLRQLQTKIEEASRKKNILIARAKRAEAQEKINKTMSSLSGNKSAFDTFERMAKKIDEIE
ARTEAQKELEDTTSGASLEKQFAALESSPTAADAMLEDLKRKLLTEDAGGSAQK*
>SPSA8_v1_180017|ID:41145475|murA| UDP-N-acetylglucosamine 1-carboxyvinyltransferase [Spirochaetes Bin 1 SA-8]
MYKYVIEGGYPVSGKVRASGNKNAALPCLAATVLANSPVTLRNIPEIEDVIVMTEVLRYL
GAGIQHPEANVWIVDPRSINRFDVPVSQARKVRASILFAGPLVAKFGSALLPPPGGDTIG
RRRIDTHVLALEELGAKVSVKEFLEFSAKSLHGTALFLDEASVTGTENAIMAAVVAKGKT
VIRNAASEPHVQDLCNMLNAMGAHIDGIGSNVLTIFGAEKLEGTDFTIGPDYMEIGSFIG
LAAVTRGELTIEGVNPDDLPPLKVGFSKLGISWTLSGTSLFVPSSQSLKVVNDISGQIPK
IDDSPWPGFPPDLTSIMTVIATQTHGTALIHEKMFESRMFFVDKLIGMGAQIVLCDPHRA
VVSGPARLSASVLVSPDVRAGMAMVIAALCADGESTIHNVYQIERGYENITERLRNIGAH
IVRREDS*
>SPSA8_v1_180018|ID:41145476| putative Phosphoserine phosphatase [Spirochaetes Bin 1 SA-8]
MMSNIPAGQAEPISVSESFYALDTMAAELLAYFELNVESKKRELMRRAAMLARDLYSDTP
VPAKRSVFSLFRIISLLNRLHASGGSDFGDSISQLLAKTVCSARTAVQVIHAGLHAEEKT
HFFGHGEHLPFEREIESYKGQILLALDPMLADAIQPVLYRLEHVPRAVQSGDELLTWLGL
VKNEDADTGQEYRGPEPERVSGAKQISIAREGKLETYSLRDGFQLPDVIITDLYSAGFAG
FEIQELLKSFSRLADIPVIILSPLLDSRIIARAIQLGADDVLRYSVEPSVFFARIESSIE
RNKMREKHRVNVMALAKTRARLEAELKSGAEYVRCLLPGKIKSQLLSTDWIFIPSASLGG
DLFGYHRLMDGRISVFMIDVSGHGVQSALYSVTIFDTLRLEGLRNTDFGDPASVLRSLNH
AYRMEERNNLLFTVWYGVWDERSRTLVHASAGSPPAVLILPGGGAVELKSPGLVAGADPD
ADYENMSIQIPRNSRLFLFSDGIYEFPAQDGEIFGLEAFVQLLEKMAVSASETMMSVGEI
IARVKEQGRVVRFEDDVSLLEFSFG*
>SPSA8_v1_180019|ID:41145477|pyrC| Dihydroorotase [Spirochaetes Bin 1 SA-8]
MKLKIRKPDDFHVHLRQGSRLKDYVVREARYFNRALVMPNTLPPITTPEMIAAYRSEISK
IIADSDPDCRMTDFTPLMTFKLLPGMKGELVQSCARAGAIAGKYYPQGSTTNASDGPSKP
GDIQEALDAMEEAGLVLSIHGEDPSSPVLEREQRFLPTVEYILERWPDLKVVLEHLSTKE
AVEFVKNGPDRLAGTITAHHLLFTIDDMIGESMKPGLFCKPVLKTGADREALRHAVCSGS
LKFFFGSDSAPHPIDTKRREASPAGIYSAPTAVGMLVEVFRNLGSFAELENFWSKNGALF
YGLPIREDYIELTQEDWVVPEEIDGAIPLGSGTKLSWK*
>SPSA8_v1_180020|ID:41145478| Xaa-Pro aminopeptidase [Spirochaetes Bin 1 SA-8]
VTTLHSIYQTRRKKVAQVLKQRGIHAVRFMDFESMRNPAIRYLCGHPGDALLIISSDEKA
ILVPWDVNMAEKMAFADLILPYTKFGRSAIAATRSVLQELAIPGGSKIEVSEATAYPDYV
DLVSELEAWDFICEKGGIDAEILKMRAAKDEGELEIYERASALTNYLIDAIEQKVKSGEI
TTELDAALFIEKECRTHGAEKTGFDTIAAGPSRSFGIHAFPSYGSGPFATKGLSILDFGI
VVDGYTSDVTMTFAKGPLNKEQEKMLTLVQQAYDLAVAACKPGIRSRDIAMLVDDFFKEA
KMTMPHSLGHGIGLEAHEAPGVNIRDDNDAVLVPGHIITIEPGLYHPEFGGVRLENDVLI
TKDGGKVLTSSRIVRL*
>SPSA8_v1_180021|ID:41145479|argD| Acetylornithine aminotransferase [Spirochaetes Bin 1 SA-8]
MSAQTIKTDRFMNTYKRTGLVFEKGKGAKLYTADGSEYIDFTAGIGVNALGHGHEKLVAA
IAEQAAKVIHVSNYFLTEPSIKLAEELTSLTGYDKVFFCNSGAEANEGLIKIARKYGSSR
KPDKNVIVTLKGSFHGRTVTTVTATGQDKFHQFFGPFTPGFVYVEPDNLSALETALTDTV
CAFIFEPIQGEGGVRPLSTQYLQAAEKLCHERDILFVADEVQSGVGRTGAFLACEKLGVR
PDMTAVAKGLAGGVPVGAILARGACAETLQPGDHGTTFGGNPLAAAAARAVLKELTQPGF
LAEVERKGEKLKAAVRSWQNPLIKEVRGMGLMVGIAVTCPPSDVVAQCRTNHLLALTAGE
DTVRLLPPLVITDEELDAGIAILKSSLDELAQRA*
>SPSA8_v1_180022|ID:41145480|argB| Acetylglutamate kinase [Spirochaetes Bin 1 SA-8]
MITDTMRADVLLQAMPYFKAFSGKTVVVKYGGAAMINEGVRVAVIEDMILMQQVGIRPVL
VHGGGPEIDKMLKKLGKERIFIDGLRYTDDETMEIVQMVLSGKVNKDLVALIENAGGKAC
GLCGGDGGLFRATRLLKDEKDLGLVGEITSVDGAAVSSLLNAGYIPVVSTIGLSDDAEAL
YYNINADTAAARLAAALGAEKLILLTDVPGILTDTSDAATLLSKIRKEEVANLIQQGVVS
GGMIPKVEACVEALHSGVTSTHILDGRSPHALLIELFSDQGMGTMIV*
>SPSA8_v1_180023|ID:41145481|argJ| Arginine biosynthesis bifunctional protein ArgJ [Includes: Glutamate N-acetyltransferase ; Amino-acid acetyltransferase] [Spirochaetes Bin 1 SA-8]

MKQVRGGVTAAKGFLASGMHVGIRKNKEKKDLALVWSERMCAAAAVYTTNKVKGQPLIVT
REHLSDGKAQAIIVNSGNANTCTGEAGIAAAKRMAALVAERLPVKPEDVVVASTGVIGQL
LDVSVIEAGMDSLVAGLSKAGYLDAREAIMTTDTVKKELAVQFELKGKTVTIGAMAKGSG
MIHPNMATMLSFITTDCAIDQGLLQKALSASVRRSYNRVSVDGDTSTNDMVVILANGMAE
NPIITSADDDYQEFYEALDWLNIQMARAIARDGEGATKLIECVVEGASDEEKAEQLAKGV
ICSSLVKTAFFGADANWGRILCALGYTRNDFDPGKVDVSFESASGYISVCKNGAPLPFNE
EKAKKILSYQEVTVYVDLHEGDAQATAWGCDLSYEYVRINGDYRT*
>SPSA8_v1_180024|ID:41145482|argC| N-acetyl-gamma-glutamyl-phosphate reductase [Spirochaetes Bin 1 SA-8]
MFENQISAGILGATGYAGAELTRILLGHSKIQTLFLSSISFQGQNMENVYPSLLNCGNKK
SPIFGTPLVLESADSVIEKSDVIFSCLPHGHAEKAGEACINQGKLFIDISADFRFQDDEA
TFSAWYGKVYANKELHNLAVYGLPEMNREAIKKAKLIANPGCYPTSAELGLMPALRHKIA
DTSSIIIDSASGVTGAGREPSQSTHFPEANDAISPYKIGAHRHQPEIDRYLSIMAGTPVE
TIFTPHLAPMSRGIVSTMYFKLSVPLSIDELHEIYASFYKDEPFVRILPVGSFASNRTVK
FSNFCDISLHLSSNKKTAIIVSAIDNMVKGAAGQAIQNMNIALGFDETEGLLTVPPAF*
>SPSA8_v1_180025|ID:41145483|hppA| putative K(+)-stimulated pyrophosphate-energized sodium pump [Spirochaetes Bin 1 SA-8]
MGMGTGIVIALFAALFSLLYAIRKARAIIDLPVENEDLVRIGSYIEEGAHAFLSREYYVL
AFFIPIVAIFLAILQGGSIRLQGAAFIVGALCSGLAGWFGMGVATKANSRTAWAAKTGIG
EALAVAFSGGSVMGMTVVGLALLGIGSVLGVLLLILGQSAMVLSESVFPILTGFSFGAST
VALFARVGGGIYTKAADVGADLVGKVEIGIPEDDHRNPAVIADNVGDNVGDVAGMGADLF
ESYAGSLIGCMVLGINMGSSAEMKIRLTILPLIIAAVGVLASLIGTLFVRMKPGKSPQKA
LNAGSLSAACIAIVLLFPVIRIVIGTESFGDDTIRGFGYLGVALSAVIGLAAGAAIGIIT
EFFTGTDTAPVKRIAKACITGXAXTIIXGLGVGMLSTMPPILIISAAIAGSYFAGGLYGV
GIAGLGMLLTLGIQLSVDAYGPIADNAGGLAVMAEMPPSVREITDSLDAVGNTTAAIGKG
FAIGSAALTALILFSAFIAASGVTEHLISIMNPAVLIGMFIGAMIPFLFSSLAMDAIGVS
AFKMIEEVRRQFRAKPGILRGEEIPDYKSCVDISTRSALKSMIIPGLIAIITPIVAGFAG
GTALLAGLLAGATVSGVLLAIFMSNAGGAWDNAKKLIESRAEEGKGSVSHRAAVVGDTVG
DPFKDTAGPSLNILIKLMAIVALIIAPILRTMGR*
>SPSA8_v1_180026|ID:41145484| conserved protein of unknown function [Spirochaetes Bin 1 SA-8]
MAEEKHLYLIMHPNHSLIASALEPEHFIRHYVQGSTRYFEGRLIFVEVDPTFRNPYFDID
TAYSELKPHEDGRPKATKFIKSYRVLENMDFSALGKLYLCNSLGDYVALDYADYNPSLDT
EEFRIMLEINPVRFIVLTKYNFKQFGAFITDPKNTKGAPKMFFTQLEFRTEEFLRDFEEN
PLIRCYVPGIHPARLRNAIMEVRNTPGKFVKGLSLDCPIDKISYKLLRDGFMFASQDGSY
RYYPLLSLDEVEHRFYKYWKSM*
>SPSA8_v1_180027|ID:41145485|mnmE| tRNA modification GTPase MnmE [Spirochaetes Bin 1 SA-8]
MPRTYFDTKSPIIALATPPGRSALAVIRLSGENTISLFSKCFSTPDKILNASGYTLHYGY
FLEPESGEKIDEIMVAVFRKPHSFTGDNSIELFCHGSPAVVNRILSILELQGCKPALPGE
FSFRAFINGKRDLVETEAINELAGARSETARSDALLRLTGILSQKFSELRKQMLDLLAEI
EARLDYAEDEGPDQEIQWLERLHQYSTELQLVSKSYIGGRLRQEGALIVLAGRPNAGKSS
LFNLLVREERAIVSPEPGTTRDWIETWLEIAGFPVRLVDTAGLRPTEAIIEAEGVRRSIE
LIQRSDILLYIVDGEIGLLEEDLQFLQQFPSALKLWNKTDSPRCKQIPESWIGITAKALS
TAPILEKAIFNLLDRMSNPSLSEISETQNIISGDQSQKQQKPRENIVAIAEERQKKLVDS
CLKSIEHAILDLQKGSSFDIIALDIREAANYLGEITGELVNEEIFDRIFSKFCLGK*
>SPSA8_v1_180028|ID:41145486| glucose-inhibited cell-division protein (fragment) [Spirochaetes Bin 1 SA-8]
MKDYPAIVIGGGHAGIEAALALARLGTKTLLITQNPDTIGKMSCNPAIGGLAKGNLVREI
DALGGQMGILSDMTAIQVRMLNQSRGPAVQAPRAQADKALYSAMARKALEIQPNLTIFMD
TVVDIITDSSGKTIEGVITERGKKISADTVVLTTGTFMEAMLFIGPWQGSGGRLGEPAAV
GLGTALRKRGFPAGRMKTGTPARIKASSIDFSKLTVQHGDSKKIYFSFLEKNYERPNIPC
HILYTSESTHKAIRSXX
>SPSA8_v1_190001|ID:41145487| protein of unknown function [Spirochaetes Bin 1 SA-8]
MAVEGKPMGDDSYWSKYIEIPHTGDPYLFVFGATIVLQYLAYRMSVRKMEWLDELGVDEH
GVHPDAPKNVSKSITVD*
>SPSA8_v1_190002|ID:41145488| conserved protein of unknown function [Spirochaetes Bin 1 SA-8]
MRGFYGYGMGYSFPWWNIVSWVIGIGVIAAIIFFAVRSSKQKKPEVPADNRDALQILADR

LARGEITKEEYLETKELLER*
>SPSA8_v1_190003|ID:41145489| Fumble domain protein [Spirochaetes Bin 1 SA-8]
MTIGIDIGGTTTKIVGLSQCNEIIYTTVKANDPVASAAGAIGKFISVAGIKIQDIKRLAV
TGVGSTFIKDDVLGIKAEHVEEFQAIGLGGLKLSGAERALVVSVGTGTAIVHAEGRNARH
LGGSGVGGGTILGLGKALVGISDFDNLAQSASRGKVEGVDLTIGDISSRAVGSLPQNATA
SNFGKMADRPSPDDISAGIVNMVFQTVGMLAILAARAENDPAIVVVGNTMQIPMARQVLD
GLYPIYGVRFIVPDLAQYATALGAALYIC*
>SPSA8_v1_190004|ID:41145490| UTP--glucose-1-phosphate uridylyltransferase [Spirochaetes Bin 1 SA-8]
MKGIIVAAGYGTRFLPATKTVPKELLPVGLKPSIAYIVDEFVASGITDIIIISSRRKKAL
EDYFDREIELESIFLKEGKLDKLALIQPPEVSMSFVRQTEMKGTGHALLQVKNLVGSEPC
VVAYPDDLHLGTVPLARQLISIYEQTGKCVLATIHEPGDVSRYGVVDPKPDGVGVRGFIE
KPAVGKEPSHEVSIGRYLYTTEFFDKLEEGWAQHKSGEYYHSYALDRMIEADKVAYCRIE
GTRLDTGDPAGYLEAILHNAAQDPQLKPVLMKFFRSMQ*
>SPSA8_v1_190005|ID:41145491| conserved protein of unknown function [Spirochaetes Bin 1 SA-8]
MSTVVFEKLPALDYENACVALPKEYGTPAWEALSEVAAIRKALFSLLEKLDAHEEIVSKI
RGKPVVIKPNLVIVYSNIGTVKPEFPETTDPRVIDALVLWLKQYTDTITIAESSGRGSPT
RASFAISGLDRLSKKRGCELVVLEERPCELYRLPKARVSRTLYIPDTFGKVARGEAFYIS
VPKLKTNLYTGVTLGFKNSMGIIPYNERQHAHHYDINRKLVEMMYLVKPDLTLIDGIIGG
NGPCPAPVYPVSSNMLIAGSNPVETDRAAVRFMGFDPKGIPLLTIADELGFNDDTVRIIG
EAAPANGFSSPDYSLLSDRIIRHFPNIRVLYGIDKHGLGNPENSDVDVRAIEARCRGGCV
ATTRFAFEMLIAEGKSSARQGTVIIGSGIDGYWLDASGKKYSVQDIAALKGYKLCIGSCT
KSISGICDQYVAGCMPLANAPHAALHKFTRTRCAILGFGNRHFPLFIKALLETRHARRKL
LKAGATLDMDFPVIDSAAIAVEPGAETDASPPWQALSVPARTDPKTLKRQLRFEDDSLFA
SLTGTFIPNRIPKALYRVQAVLTTLITISPLCAGFLALFGLLPGIPPSLLFSIFIGILIL
HSFEVPFAIVAEAKRRRRKKLSQISTSSETSGRDAVKVMIKTMLIGYPAWLPEIQGIHDE
DEAFGCRH*
>SPSA8_v1_190006|ID:41145492|aor| Aor1 [Spirochaetes Bin 1 SA-8]
VINNMGKILFVDLASGSFEERALSDSLYREYLGGIGLAVRILLDEIPAGADPLGPDNILA
FMAGALTGTGSLVTGRWMVACKSPLTGGWGDSNCGGNLSPAIKQCGYDGIFFKGIAPAPV
IFICDAEGPRLEPAAELWGRDAIETELAIKAQWKVQKKPAVAAIGYAAEKLSLISGISND
GGRYAARSGVGAVMGSKRLKALVLAGNKKIGCSDPEKMHELSSRFSERVKKSNVPKFVSY
RQLDLLGKMLGLPIVFRLNGILSTAFFKKWGTIYNNTGGIVNGDTPIKNWAGSVRDYPKE
KYEKINAHWVTDRETKKYFCYSCVIGCGGICDVSGIVENQGTADSSASHAHKPEYETHAA
FGPMCLNDDENSIFICNDICNRSGLDTISAGSTIAFAIECCERGILTEQQLEGLDLRWGN
SEAIVQLLKKMANREGVGAIFADGTRKAAERLSELTGGDVSAFAIHAGGQEPGMHDSRMD
PLMGVAFSADPTPGRHTVAADVYYNVMRLWKKVSWAPAVPHIYLKKREYEATTEEARKAV
ASTLYKQIADMAGGCLFAMIAGVQNWDLFDMLNAATGLKCSPDEYMEIGRRAQTLRQLFN
LKHGVDPTANFIPLRLQGKPPLPAGPLRGKQVPVPEMVSLYWREMGWDERNGVPLKQTLE
RLGL*
>SPSA8_v1_190007|ID:41145493| putative pectin degradation protein [Spirochaetes Bin 1 SA-8]
MEKIFFPDKEIKTDIIELGNIFRKIRAHDSKMMMVEVFFETGAKAPEHKHFHEQITYCIE
GEFDFCVEGVSHRMKSGDSIYIQSNKLHYVVCHAKGRLLDAFTPQREDFLS*
>SPSA8_v1_190008|ID:41145494| Permease of the major facilitator superfamily [Spirochaetes Bin 1 SA-8]
MRLARKATIIMSEGQDTTISHALKGLFKVYGGLPRPLYALFGATIVNSMGIFVFPFLTLY
LTARLGMSQSEAGKFMFLISLVYIPGNFIGGKLADKFGRKKLMVITQIISAALYIPCGFQ
VFSRYVPWLILASVLFDGITDPARSAMMTDLTKPDNRRAAFSMTYLGHNVGFAIGSLIAG
FLFENASSWLFWGNAIAIFAATAIVALTVPETKPTQEQIDATIGSGSTEEAHEGNILSAL
LSRPFLILFTMITTWFGFVYAQHRFALPLQTKATFGLSGAAIFGSLMTLNATQVIFLSTP
IMALTKKWKPINAVALSGILFAAGFAMIGVAGNIWLLYVSTFIWTLGEIVNATNEGTYVA
NHTPISHRGRFQAILPLIGGIGWTISPPVIGSLIEKRSLAAVWPLLGIIAFASSVLLWLL
GRIEDAQIAKKVKQLEFSDK*
>SPSA8_v1_190009|ID:41145495|cdsA| Phosphatidate cytidylyltransferase [Spirochaetes Bin 1 SA-8]
MRQETGTLLLREKPKESEEADHRRHHDEKLEPSIKTSSIRSRATPEEISIEVTRKSIHLL
IALTPSLLAFSRTFTIALLAAGTLAYSIFEALRLRGIWIPLVSWLTSKAARLRDNGNFVT

GPVTLGLGALVSVLIFPPMQASIAIYILAFGDGFSSLVGKAFGRITLPLTDGKSLEGSLT
CFLTSFLSSYLVCGQLFASIGIALAATAVEAAPTKDWDNIILPLTAGVMAVILL*
>SPSA8_v1_190010|ID:41145496| membrane protein of unknown function [Spirochaetes Bin 1 SA-8]
MLSCVTLLVRRRTYWAVFLVSAFMLALVIAASIFLLIRKDVPVFLIEHGAYKVHPFLGIR
IPSFILSIFDIAVSVLFSFSISIFILKVFKKTVSAEIFFFCLWVGTVAFEALRLVGLAAA
ILDLSYIFISFIAKLYMGAKFLGLVMIFISGLYAAGMRSEKHFALVAGGIGVSVFFASIL
PVNTGIFGMNLLYRIGYSRMFEGFSFAVILITVMNYLIAARLRSDRAYFMIALGIAGITV
SYFLLSLDMAPAVSLISILTMASGSLLYVIRLHSFYLWQ*
>SPSA8_v1_190011|ID:41145497|proS| Proline--tRNA ligase [Spirochaetes Bin 1 SA-8]
MPIPKLEQGFGKAGSIARQRYKMAEKITPRETDYSQWYIDIVLNAQLADYSPVKGSMVIR
PRGYAIWERIRDEFDRRFKETGHSNAYFPLLIPMSFLQKEAEHVEGFAPELAVVTHGGGE
ELEEPYCIRPTSETIIWSMYKNWIQSWRDLPLLINQWANVLRWEKRTRLFLRTSEFLWQE
GHTAHETEVEAVEETLKMLEVYRDVVERYLCIPVIPGVKSETEKFAGAVRTYTIEAMMQD
KKALQAGTSHFLGQNFAKAFDVKYQTRGGTLDYVWATSWGVSTRLVGAVIMTHSDDKGLV
LPPSIAPEEAVIVPIYKNDSKAAVLEYAGKIYSQLKALGLRVVLDDDDSSSPGWKFAEWE
LRGTPVRLEIGPRDMQNGKAVMVRRDNGEKTVIDAAQAGEKVKETLALIQASLYEKAKAF
RIANTKPIGNREELLAFFGAEGMGTADAQGGFAEALWCGSAECEAELKAQTKATLRCMPL
DRQDNISGTCALCGAPARHRAIFARNY*
>SPSA8_v1_190012|ID:41145498| putative Diguanylate cyclase containing PAS/PAC sensor [Spirochaetes Bin 1 SA-8]
MPLERMILIIVEFLYFFVFVFSLTRRRNYLAQLFSLAVLAGMVYVGGYFFELGSQSVEEL
QFWLKVEYFGLSFMPTLVLVFAWKFNRKQAMPSNLTMVIFAIPFVTLLLSSTNEYHHLYY
RQLSFVRIDGMSIAHIAKGPWYYVFTVYMNCIVLLSLAIFFQVFRKNKSVIRAKAFWFFA
GTLIVVGMELLYAFGLTPYGIDIVPFSFFFAVLCFAIAIFRYEFLKSNDLFKEIIFTKLS
EGALLLDQNGRIADYNDAAARMFSWLSPEAIGYRIQDLPVAKELLSQIEAGKTVNINCNG
REFYYELKITNLKEQGAEMGKVYLFKDITRLRRIMRTLYRLANFDSLTGVFVRRRFFEEA
EKELARSIRYKKQFAIFMIDVDNLKEINDTFGHQAGDAVLKALAKALKARLRKSDIFGRY
GGDEFSMVLVEIDYLNSMLMAESFISMVRNLEISFHDRILKTTVSIGVMFFKGVESDMTI
EHLLSEADKALYQAKKEGRNRFVAAKPL*
>SPSA8_v1_190013|ID:41145499| protein of unknown function [Spirochaetes Bin 1 SA-8]
MADDYSVFRHPDLLKDAALRESPVFPDFESVYKADVKQHTIRNRILLAVFLCLTMAISAA
GGFLLGSLSNFDGPLFAGKPLVTSWSLQPEMPAAQTREGEVGIYLAGLWDAASSTAVYQD
R*
>SPSA8_v1_190014|ID:41145500| RNA polymerase sigma factor [Spirochaetes Bin 1 SA-8]
MEDKPRYFTLSQHEPSDETFDAELAAHAANGDRGAFERLVWRWWDRIRGFCSAIVGFDPD
MAEEASQETLIRLYRSLKNLRKKSSVGAFIYAICRNAASDVMRRSARVASRQVSLEDLPG
LELSASGETPEQGILRQDLQLQLIRALRELDPTDRALIYLHEAEDASIQELARYFSLPEG
TVKSRLYRARIRVARLLTEAGYGR*
>SPSA8_v1_190015|ID:41145501| exported protein of unknown function [Spirochaetes Bin 1 SA-8]
MRKSTRKILTAAVVFVLFFASAALVSAQMRPMGKDAVRGWFQNLRLTDQELQEIAKIIEA
DEAELARAQAEIKIIQSKIARLMLESAPDLDAIKEEIGKSAEYEKTIKFIQIKRQLEVKK
VLGEERWQTVLMLVREARVSEKFGKFADSFSSKGMNPKDADRWGRMMLMLKRFM*
>SPSA8_v1_190016|ID:41145502| conserved protein of unknown function [Spirochaetes Bin 1 SA-8]
MDEVRPRLFMSRCLGFEHCRWNGEVINDEFVRQLEPFVDIIHECPEMAIGLGCPRDPVRV
VRDGDTAELLQPATGRRFGPVMRSWAEEYCGNLEAPDGFILKSRSPSCGFKDVKVYASFA
PESGSKPGTGIFGAEVVRAFEGIPIEDEGRLRNFTIREHFLGAVWTLARFRAVKKKAAMG
ALVDFHASHKYFLLLHNQKEMRLLGSIVANLDKKPLSEVFSLYEIGLKKALASPPKYTSL
INVVQHTFGGFSESLSKEERALFVNLIEQYRDERIPASVLLQLVRSWAVRFGEEYLLKQV
LFQPYPLSLVEITDSGKGRSY*
>SPSA8_v1_190017|ID:41145503| protein of unknown function [Spirochaetes Bin 1 SA-8]
MHTEELLLFAADTARLMLESGGETYRAEECAISIITSQGGAEAECFATATGLMLSFVSDD
GKTHTVVRRIKKRIMNLEKVYRIDRMVRDLKTDQIGFEEAESELDRIERLPERSGKTRIA
AGAFGAAFFALLFRADVREAFAAFFIGAFVALVVRLSARLRMPDFIANLAGGRSQLSAPC
CWFKSDSCIGRM*

>SPSA8_v1_190018|ID:41145504| conserved protein of unknown function [Spirochaetes Bin 1 SA-8]
VTIIGVIMLLVPGVAITNAIRDTIAGDFVAGLARGAEAFLAAAAISAGTGGAFALWQILQ
PFFGKGA*
>SPSA8_v1_190019|ID:41145505| putative Membrane spanning protein [Spirochaetes Bin 1 SA-8]
MLAPLYAAIATSAFSILFGLGWIDASIASCGAALGWAVYQWFPALHAEAFSVFFAALAAG
LFSEIASYFRRKPATVYMIASIIPLVPGGGMYYTMLSTLEGNTAKSVDTGIATLMTAFSI
AIGLAGANVIGRIAFGNRYRPLRSQK*
>SPSA8_v1_190020|ID:41145506| Pre-sequence protease [Spirochaetes Bin 1 SA-8]
MSFTLIEERQVQEIASIVRLYTHDQTGARLLSVINADENKCFGISFRTPPEHSDGVAHIL
EHSVLCGSRKYPVKEPFVELMKGSLNTFLNAMTFPDKTVYPVASTNLKDFRNLVDVYLDA
VFYPRITEDTFRQEGWHYEVDESTGELSIKGVVYNEMKGAYSDPEDLHADLCRRSLFPDT
AYGLDSGGDPAVIPTLTYEQFKKFYQTYYHPSNSFIFFYGDDDPKERLALLDEWLAPYSA
QKIESLPGIQKPFSKPISLTVPFSSEENKAYTAINWALREHGDQEFNLAVGILSHILTGT
PASPLRKALIESGLGEDLAGFGLEDGLRVSAYSVGLKGVAPERVGDVEKLIRTTLENLVA
QGIDPDTIEASVNTIEFALREKNTGRFPKGLAVMLEALGEWLYDHNPIDALIFEPSLSAI
KARLARKERYFENLIQTLFIDNKHASVVTLLPDSEIEARRSAKEAEKMAEIRAQLSELDI
ANIKQEAEHLKKLQQTPDSPEALSTIPSLALSDIPTQAPRIPLEIFENGGQTTLFHDLPT
SGILYLDLAFPFTHIESRLLPYLSIFGRALLEMGTDTEDYVTLSQEIGKHTGGIGASAFA
ATRYGSAEAISALTVRGKTLLENSEKFFSLLEKILLKSNFSNRERFRQIVLEEKAQAEAS
VIPSGHRLIGLRLKSKLTRADWISERINGLENIFFLRKLAERAEHEWEAVEQDLRALRDN
IITRNGLIVNVTVHHENYSSLRTSIEGLISHLPQRGAQNTEGIASVAAQAELEVSARQPG
RAEALTAPTQVNFVGKAFPLGTAGARLPASFQVVKKYLDTTFLWEKVRVQGGAYGGFSIL
DMNAGIMLFLSYRDPNLVDTLAIYDQAAEFLSSLEISQEELTKSIIGTIGDVDSYLLPDA
KGFTSMMHYLTGYSQDIKQKTRDEILATGTHDFVKLADALRSALPQALEAMLTSKSTLDT
LDSDQKAQLEISPLF*
>SPSA8_v1_190021|ID:41145507| Mpv17/PMP22 [Spirochaetes Bin 1 SA-8]
MKRGDFIWGGVFAAITAFLVIPETRHLFEAATRSHPYIMGFIKFAIMASMGELLTVRMLQ
KHWKKPAGFAAKAIVWGIIGMVIVAMFALYSDGVAGLLRRGLLYAGQGKIAAFLTALFTS
AIMNGTFGILLMLSHKISDTYIDLRTDREKPNFQMTLKAVDWPAFFSFVIGKTIPLFWIP
AHTITFLLPAEYRVLAAAYLSVALGMILAYAKLRNQEKK*
>SPSA8_v1_190022|ID:41145508| ABC-type transporter, integral membrane subunit [Spirochaetes Bin 1 SA-8]
VTTNDIKKMVRSNLRDYGMYIALALIMIIFTVLTKGLFISSRNIANLLNQSGYIVVLAVG
MTLVIVIRHIDLSVGFLAGFIGAVTAIALVSWKLPLILVLPGVLILGVLAGLLTAYPVAK
LGIPSFVTSLAGWLIYRGALLLVTGQTGTIIIDNPTFNALGNGYIPDIIKSETFLPGVHK
LTLILGALAIVWFIAGQIRNRKSKMEYGFDVLPTDMFVMKLIFMAAILGFITWILAGYNG
MSWTVVIMLIVVFAYDFITQQTVLGRHIYAVGGNPEAASLSGINVQKITMMVFGSMGFLT
ALSGILFTSRLQSATPQAGTLFELDAIAAAYIGGVSAAGGVGKVTGSLIGALVYMSLMNG
MNLLGTDISLQYIIRGIVLVTAVVFDVSTRKTKV*
>SPSA8_v1_190023|ID:41145509|xylG| fused D-xylose transporter subunits of ABC superfamily: ATP-binding components [Spirochaetes Bin 1 SA-8]
MSPSFLVVRNNVCGARNNEAGMETAILEMKNISKSFPGVKALSNVNFSVQRGEIHCLVGE
NGAGKSTLMKVLSGVYPYGEYEGQILFNGTEQKFKAIHDSEEAGIAIIYQELALVPEMTV
YENILLGNEIRKGIAIDWNKTIQRATELLEKVRLKVNPATKIKDLGVGKQQLVEIAKALS
HNVKLLILDEPTAALNENDCDNLLDLIVGLKEQGVTCIMITHKLKEVIRIADKVTVLRDG
QTICTLDAHKGEVNENVLIKHMVGREIQNIYPAQETRELGSIILQTKQWTAFHPVTGRKV
LDNVDFYIRKGEIVGLAGLIGSGRTEFARSLFGNPDNYQLSGDLILKGKKAAFNHPEKAI
KAGLAYVSEDRKGNGLILIQDIKNNITLANLKDIMKSWNVVNKNLEVKVAEEYRAKLNIK
TPNVEQKVVNLSGGNQQKVSVAKWLFSKPDVLIFDEPTRGIDVGAKYEIYCIMRDLAAQG
MSIIMISSELPEVLGMSDRIYVVSTGRITGELPAAEATQEKIMHMATN*
>SPSA8_v1_190024|ID:41145510| Periplasmic sugar-binding protein [Spirochaetes Bin 1 SA-8]
MKRAAMLIASLLFLVVLGNAAFAQVKVGIVLPTKDEPRWIQDQTRFLDALKTAGISAEVL
FSQGDSAKERANVESLVSKGIKVLIICPHDGTAAAAAADVANKAGVKVISYDRLIRGTAS
VDYYVTFDSVQVGMAWGDYLVSKASGKGNNLYLYAGAASDNNAFLFFEGAWKVLQPKIAD
GTFIVRNSDKAVALSKKATLTRDEMASIIGQVTTNWNFADAKSKAEANLTAAKKEMKGTV

YIAAPNDGTARAIADAFAADKDVTKYYITGQDAEIASIQYIIDGKQSMTVLKDVRILVKD
AIAAAVDFLNGKVPPKTTTYNNGKVDVPAKPTMITTVTKENVKAAIIDSGYWPASNFKGL
*
>SPSA8_v1_190025|ID:41145511|xylF| D-xylose transporter subunit ; periplasmic-binding component of ABC superfamily [Spirochaetes Bin 1 SA-8]
MKIKIKKPLLILCAILAAGTFIVSCGGSAKKSQDKKLVIGFSLDSLVVERWKRDLEIFEK
SVREMGADFIYEVADQDADRQAEQVRSMVGRGIDVLVIIPNDADKLTASVKYAKSKRIPV
LSYDRLVRNAGVDLYVSFDNEKVGSLMAESLVQVVPRGNYVIINGARTDNNAIMLNTGMH
KVLDPFIERGDIHILEEIWPETWDNEAARKGLEQVLSRTRAIDAVIAGNDMLAEAAINVL
SENRLLSTTRVVGQDAELAACQRIAEGSQYATIYKPIDRLALRAAGFAVMLAKKEKITTD
ATINDGLKKGGVPYVRLEPILVTKPLLDETVIKDGFHSRADVYRNIRSSY*
>SPSA8_v1_190026|ID:41145512| putative Two component transcriptional regulator, AraC family [Spirochaetes Bin 1 SA-8]
VYRILVVDDELPVASGIAHIIKRDFSGVYELAGIANSGREAVEKATRTAPDIILMDVKMP
GFSGLEAIREIKVKGVNPSFIMITAYERFEIAREALELGVVDYLLKPVAAEALALSLKRA
EKYLENRDKLEKTSLILHELKEECKTLAAQNLIYSMVLGTLSEQKARLLQSMLDMNTEAG
IACAIYAAPADLDEILNDIQYKTSILAEKISPAILAAFIPIKNTEAAEASKEAFGELDEI
LKRMQGALRGFGSLCQTAESPASWEAAIGNLNNTAQKKSGNSWKPLLAALENELADSLQT
GKTEPVLPLLEEIRICARASGLPSESEIGTFIALVGRVARELRERSCLSEEEAEDIMRVK
SLCLGSSADMFAAEAERKIGLLLKHLEKAPGKYSRIVADAMSIVHNNYAKPLSLESVADA
IGISPGRLSRLFAEETGQGFSRYLVMFRIDQAKKMLERPGASVKEVSAACGYPDQNYFAR
LFKKLIGTTPTAYMSEFARKTGGAHENKD*
>SPSA8_v1_190027|ID:41145513| Signal transduction histidine kinase, LytS [Spirochaetes Bin 1 SA-8]
MMKNMKSKRKPDTVRSRMIRHSALILLIMMSATLYTGLASFQLADSVSVLFRNNLLMKDL
RESLARTETSLTNYLTSKNSDALKEYIKNSTHLSDTARKLNQEIRNDESLLLQRDLARSI
EQYIIKTETSVSGKRGRDVQTYSTAYYESEKIADVIRFLIEKNEKVFIADSIAAFSNYKS
VISPAVTTNAILVVAATLLGLMLLISYSYTLTEPLAVLAEAANALGTGNYEAELPKPERM
DEISVMATAFSTMRDNIKQSFNSMQEKAEMEKSLMQERMHVLDMEHKLKDAELLALQSQI
NPHFLYNTLSAGMGIAWEENADRTSNFLEDLAGFIRYALKPTFRTVLVSEELECAQRYIR
LLQARFGARYQFEIHAEESALSAKTPALLLQPLIENAITHGLAKKEEGGTIKVAIALDGS
DVVMQVEDDGEGMSEHEIARLYQELDTNHEPARKDGSGIGLRNVMRRITLSTNGAGVVEI
HSRLGGGTVVTIRIPRKGD*
>SPSA8_v1_190028|ID:41145514| protein of unknown function [Spirochaetes Bin 1 SA-8]
MSGEESAASMLDSREPVNALICSSARDTIGAVQVVVDRNAVGKVLIVGTDETPEISRYIQ
NGVVAASIVRDSSAIGRSALRAFSDMLSGIKNPARYESGFLVLDQQSLKAALR*
>SPSA8_v1_200001|ID:41145515| exported protein of unknown function [Spirochaetes Bin 1 SA-8]
MKKTMPLVFILLAIILSSAYAQMYGYEQEIMQKKLRTDLGIDDFKFLGMRGIKISYLGDL
QLYEIRFFSQKLKYNISILPTAEHLGYFILNNGKFVYLLHNDTEIAAIMKTYFFGKKLSE
QETIEFLLFIYSQTANVWNENVYIIRSFGDLENPEFKKYYSNSNENYYTLKNSIINKYHD
YQFPMVIDENDLSCTFYIISGWMHKIDTLQKITISVKNDTIEYNNELLEKDIYDNIPLIA
Y*
>SPSA8_v1_200002|ID:41145516| conserved membrane protein of unknown function [Spirochaetes Bin 1 SA-8]
MNPELSKNKIEHATPAFIFLSVLYGALLIISNITVVKLVKVGPFLLTAAFFTYPLVYVLS
DIMTEIYGYRLSIKAIWANFAAQALMSVVFAFTLSLNGTDPDIQNAMATLFSTTWRIVLG
SLTAYFIGDWLNSYLMSRMKVAQKGKNFFLRAMASSLPAHFIDTALFNLIAFGGVWTSGD
ILRNTLSESSLASVYEFLVFPLTFIVVRYWKKLEKIDVIDEGIKYSLF*
>SPSA8_v1_200003|ID:41145517| Chloride channel protein EriC [Spirochaetes Bin 1 SA-8]
MSSHESRTPANARKQRTRIEIYSLSLLTGILAGLVVVAYRFAISAAEESRSSFLQLHRTS
LPHLAAFFLLLAGAGAVTFLMTRARPLIKGSGIPQVKAFLLRRVNFDWKRELPFKFSGGV
LALGAGLSLGREGPSIQLGSLTGKAVEEIAHKPEYQRYLITAGAAAGISAAFNAPLAAVL
FCVEELHRNISPVMLTSALIASFSANLIMWIFAGNTPIFGIELINVLPINLYVPAILAIG
ISTGILGALFNHGLIRTGEWFKKLFHQHLPGMMLAFLVGGLVCILLPEITGGGNSLINQL
NLNSFTFLTLIILLAAKFVFTLFSYASGAPGGIFLPMLALGALIGSIANRAFVFFNLPDG
YLPNYLLLGMAGFFVAVVRAPITGAVLITEMAGSFSHFPAFIFVSVLASLTASILKTPPI

YDMLLAKIPASSQNKANIHSAEEAEPVVLHIPLQEGCLYTDCAGLSAHLPAPALLSGILR
GENELFPEPGLEIYAGDIALILVPRYRAHQLKESLLELGKSAAVCPDSNEET*
>SPSA8_v1_200004|ID:41145518| putative PAS/PAC sensor protein [Spirochaetes Bin 1 SA-8]
MTQNQHNGIIKELKILIIDDNEDDAALMEHFLRKQWPEALVTVIDSREGFLDLLDSNFQF
DLVISDWSLPQFSGMDALKMLRGSDIQAPFILVSGKIGEESAVQAIREGAYDYVLKNNLT
RLPIAANRALQDRAREQAEQKMQEQLHLQATGLQAIPIAIVIISASGIIESVNPAFEVLT
GYEPEEVIGHSVAEFCDSGLCMQDFQDWKGNHIKTSRVTEKRKNGSHYFEEWTICPVYND
KDQLTHFIVAKTDVTSEEQRKRHNELELLYSNLSAQANSSSILIKRTINFIAEQFPASHP
GFHIHATSHESAVDSWIGTPHEKSLNEFTYEIHFFDQPFGQLAMNYSSTSVLNEFELYAV
IAKAFESALLKLAAKERAESQIKKISFLSYITKSINSVFDADLMLGNILKQSAKLLNADT
MALYLYNKETKDYVCRAHYGLRSINLTGVHIAPGQPYVGTAASDQQFLSCYTFDNLNGQD
QFIALIQTEKFLSQHCAPIVVGGMTLGVLEVFQRKEFTLTDEWVALFEAIAMQTGVALEY
NTVFLELQSVYRDLENSYEATIEGWSSAMDLRDQETEGHSKRVTSLTVAFATRLGFSQDD
IVRAKRGALLHDIGKIGIPDSILRKNGPLTEEEWAIMKEHPSKAYGMLYRIPYLRNCLDI
PLYHHEKWDGTGYPQGLSGKKIPLAARMFALIDVYDALTSDRPYRKAWTKEKTVEYIREQ
AGRHFDPELTEAFIALIKD*
>SPSA8_v1_200005|ID:41145519| Response regulator receiver protein [Spirochaetes Bin 1 SA-8]
VNGIGEFQTILLVEDNPDDEELTLLAFKNNHIANSIIVVHDGAEALDFLFRRKAYANRDV
RSTPRLVLLDLKLPKLSGLDVLIELRNNEMTRFLPVVVLTTSNQDEDIINSYKYGANSFI
RKPVEFSKFNEAIKSLGLYWLVLNEVPLSTKTALAKG*
>SPSA8_v1_200006|ID:41145520| putative Histidine kinase [Spirochaetes Bin 1 SA-8]
MVLSIIQNISIIIAAEVLYHFLSQSFSGKAYLIAILNGILFGLTAVLAMMTPFRFETGII
YDGRTIVIGIATLFGGPLTGSIASAIAAGYRIFIVGGTGYLAGVLSILEAFVIGAIFNRI
RKHAKLAHEWPIIVAASMLIHVFMLLAQLFLPNQRWRLVLPVISPVVLTIYPIGFILIST
LFIEAEKKAQAHKELEASEERYRQIFNNRHTVMLIVDPQTGAIIDANPAAEVYYGYSHEI
LCRMKVADLNTLSPEEVRQEMARAKSFQKNAFQFRHRLASGEIRDVEVFSGPIIFSGKQY
LYSIVHDISEKMEAKRKIVEMNENLEKIVSRRTEALESANLELESFAYSVSHDLRAPLRA
IEGFFGFLMQEIAEINPSLSDNARHYQERIRFNITKMNLLIDDLLNLSRIGTQELSLQSV
DMSELAKEVVNELQSENPGRRAAIIIDPAMTCVADKALLKIALTNLFSNALKYTSTVIQP
AIRFSSMDKDNERVYFIKDNGIGFDMQYAGKLFTPFQKLHSDKRYPGTGIGLSIVRRVIS
KHGGKIWAEAVPDKGATFYFTLGG*
>SPSA8_v1_200007|ID:41145521| Methyltransferase-related protein [Spirochaetes Bin 1 SA-8]
MDFQALIPLRCPLCAQETERLNFPIRGRDRWYHRCGSCGFMYLAPAHHLSAEAEKARYLQ
HQNSNDNGYRDYLETFIKAAVLPHLKPGAEILDFGSGPVPALSNMLNERGFQCYPFDPYF
SPDSAWQSRSYDAVMLHEVAEHLADPAAVFTAAGACIRNGGFFIIRTRFLESCADFASWW
YRMDSTHISFYTEIGMRKIMASQGFLPILFQIPDIAVFRKNTR*
>SPSA8_v1_200008|ID:41145522| Biotin/lipoate A/B protein ligase [Spirochaetes Bin 1 SA-8]
MAYEFRLLKTGFHDAFFNMGLDEAILTAVAEGRVLPTIRFYGWEPHAISIGYFQGVYEEV
NLEACREKGVDVVRRITGGGAVFHAAEVTYSIVIPESHPLSRPSILDSYRLLCSGIIEGL
SRLGIEAEFAPINDIIAGGKKISGNAQTRKKNCILQHGTILISVDVDTMFSLLKVPQEKA
RGKLIQEVKARVTSIENLLGKAIGFDETALALEQGFSSALDLALSEAEPSEEETIAATGI
SAEKFSSPAWTFKR*
>SPSA8_v1_200009|ID:41145523| conserved protein of unknown function [Spirochaetes Bin 1 SA-8]
MKISVIGKPEGCKLLRIFLEVTEPLNRESIIISLNIHGDFFAIPEERFEAVEMELAGTRL
GNLAENFDAAIASHHIQVEGICGRCLMEILRSVVHGI*
>SPSA8_v1_200010|ID:41145524|lipA| putative lipoyl synthase [Spirochaetes Bin 1 SA-8]
VIETGKKLPKPEWIRVRLPSGNEWQHVDAILSNYGLHTVCDEARCPNKGECWGCGTATFM
ILGDTCTRGCRFCAVKTAKTGLPVRQNEPEALAAAVKEMKLTYAVITSVDRDDLPDRGSG
HFQRCIEAIKTMSPDVKVEVLIPDYYGEELRTVLAANPDVLAHNIETVPRLQSVRDARAS
YEKSMRTLREAKRMGARLTKTSILLGMGETREELLATFQDLRKIDVDILVMGQYLQPTPQ
ELPVFEYIQPEIFPELAKLAKAQGFKSVVSSPFARTSYHALEASESLQ*
>SPSA8_v1_200011|ID:41145525| conserved protein of unknown function [Spirochaetes Bin 1 SA-8]
MGLDDWQERKREEVYASVLRGLERRRQADPTFTIDDARSALEHLYVLDGNDWLGRGELGD
IISAATLAAYEYFIHEWMKEKEQA*

>SPSA8_v1_200012|ID:41145526| conserved protein of unknown function [Spirochaetes Bin 1 SA-8]
MSVEIRKVTTSAELKAFITFPERLYGDNPNWVPAPVFDDLNTLRKDKNPAFEFCEAEYWT
AWKDGTMAGRIAGIINHRYIEKWGNKYARFGWIDFIEDFEVASALVKTVEAWAREKGMDG
VHGPLGFTDLDREGMLVEGFNEQATFATIYNFPYYPEFMERLGYKKDIDWIEFQVQTPEA
IPEKVLRVNEILSKRSGVRIYEWKNKKELVAKFADDIFELIDEAYADLYGTTPLSRKQVE
VYIKQYLGFVDPRFTKVLVDQNEKLVGFGITVPNLSDALYRCKGRLFPFGWFWLLRALKK
PTKIDMLLVAVKKEYLARGVVAILMTALNKSAIENGIKVSETNPELETNYQVHGLWKDYP
KRQHKRRRVYLRLLKD*
>SPSA8_v1_200013|ID:41145527| putative Peptidase, M22 family [Spirochaetes Bin 1 SA-8]
MNILVFDTAAEKLEVAVSAAAENRFAFRSVSGFRHSETLLPTISSVLDEAKLAMNSIALI
ACTQGPGSFTGLRIGMATAKGLSMALGIPWVAVPTLDCLARPFGELAGPVVPVMDARKNR
LYAAMYFHGRRQGDYLDISFQDLVEKLDSYPDILFTGPDAELFRELAGERPGFVIDEGST
EARMRGLVSLALDKLNTEGPEPDTSGPLYLREPEIG*
>SPSA8_v1_200014|ID:41145528|tsaE| tRNA threonylcarbamoyladenosine biosynthesis protein TsaE [Spirochaetes Bin 1 SA-8]
VRFILKLIRTTTPAQTMELGSIIGKNAPEGKVIAFSGDLGAGKTTMTKGIARGLGIQEEV
TSPTYTIQSDYQGRLILHHIDAYRLSGADDFLETGIMELLGTPGSLSIIEWSERIAPLLT
SGIALEDLARIRISVAEDGARTIELEGAWLEELFS*
>SPSA8_v1_200015|ID:41145529| putative Hydrolase, alpha/beta fold family [Spirochaetes Bin 1 SA-8]
MTESGLLFTASDGKNIAYQKVLPAGKPKGIILIGHGMNDYGSRFMHLALPLGEAGFAVFL
PDLRGHGDTDKDANRGYLADENGFERVLEDIMELGDLAVKECGQIPLFYFGHSFGALLGM
ALAGIYGKYFEGIVLSAPPQKPDPLLDFAGGIVVSIGLKSKGPHAPAKLPRQMTFGAYAK
TVPNPKTDCDWISRDQDVVDAYVADPKCNFICSYSFYQDLMHGVRKVYSDGFFNSIPTSL
PVLLFAGSNDPVVGMRAGFQKIEKLFKSLGLTDFESKCYEGNRHESINELNKDEVIADVC
DWFTRHIA*
>SPSA8_v1_200016|ID:41145530| Poly(A) polymerase [Spirochaetes Bin 1 SA-8]
VFIRYHRDKNGKLVQQAVVYTEKEHGIARDAIDADAIKVIERLKEAGFKAFIVGGAVRDL
LRGKNPKDFDLVTDAIPARIKKIFRNSRIIGRRFRLVHIYFGDKIFEVSTFRSIVNGSVG
NEFGTIDEDVLRRDFTINALYFDPIENIIVDYVGGFEDVREGILKPIIPLGDIFAEDPVR
MVRGIKYAIAANCVIPPNVQKQIRKDSQMLAGVSASRMTEEFFKILASGKSAEIIQSLCN
FRLFEYFVPHIWARMEASPEYREALLADLAALDAQNVSLARTLLQDESAEALNQEKKSVS
VLLSYLLKRYVSEKLASLKSDGTGELPPSEVFRASLLDARAFVMPMNPPRVELEAAMMMI
FKNLGISPLRKPERKRRSWKPVSKQGERLLADKEQR*
>SPSA8_v1_200017|ID:41145531| conserved protein of unknown function [Spirochaetes Bin 1 SA-8]
VNPEKLSLLADEELILLADRMGIFIPEGLDRRFIIEEIITALDDDDHERVFSHDGTGHVE
EKKFSGSRSLPAVGFEKSGFPERYNETMIRALPRDPAWIYTYWDLSEQKRESLSGDDENF
ELFLRVVELQPESGHKKDFFDISISHDDWKWYINIPSPGETYRIDLCVRKNGAAKVLARS
NEVRMPIFFVRHAQRIPKMTKSLLLLSGIEDLHLFEQKEENPSRILFDNGE*
>SPSA8_v1_200018|ID:41145532| conserved protein of unknown function [Spirochaetes Bin 1 SA-8]
VHNQRPMLALVLNAHLPFVRKPEFPEFFEERWLFEALSETYLPLLRLFKKLESDSIPFKL
SLAISPTLLSMLSDKLLADRFVKYLQKQIELGEREKERLAGDPVFAPLAALYHSLYLRNL
ADFETLYGREILPAFDYYYKKGKIEILTSGATHAFLPLYQDVPEALLAQIETAIVAHRSI
FGKNPQGFWLPQLGWYPALGKILREYNIKYTIVTTRGALLGNPTPLFGSFSPVTTSDGLN
VFIRDAGATKAVWSETEGYPADPVYRDFYRDIGYDLDSSFVKPYLGDSVEHGFTGFKYWA
ITGRTDQKRPYEPGAAAAKVLEHAHAFLEERKTTARAAGFLMKDRKPLIVCPYDAELFGH
WWFEGPLFLEALFRTAARKHGPEELEFVTLAEYLRHNPENPHSDPEFSSWGEGGYAEVWL
DGSNDWVHRHSHKILERMVELAERFPNESGLRERILNQGAREALLAMTSDWPLLLRSGKS
SGFAQQQIVEAVKNFYRIYEMLCANTVDTEWLTTLEKRDNLFPAINYRIFAPKR*
>SPSA8_v1_200019|ID:41145533|purA| Adenylosuccinate synthetase [Spirochaetes Bin 1 SA-8]
MNVVIIGAQWGDEGKGKIVDYLARDAGIVVRFSGGANAGHTIVLSGTKYALHLVPSGILY
SDKIVILGSGMVIDPEALFTELDGLKAKGIDWEGRVLISDRAHLVLPRYKDVDREMESNR
KKPIGTTGRGIGVAYAFKASRDGIRIADIDDEDRLQNLSEEDKAFINKWRDKLKPMATDL
AAYISRHRNSWTLFEGAQGALLDIDTGTYPFVSSGMSCAAGAAAQGGIGPRRIDRILGVF
KAYTTRVGNGPFPTEFKDDSPDSLSNYIRTTGNEYGVTTGRPRRCGYLDLVALRYACMVN

SIDRLVLTHLDVYDALAEFEACVAYRIDGKVVEDFPASIRDLERAVPVLKKFDGWKMPIS
ACQSWEELPTKAREYVEFIETFTETPISIVSVGSDRQQTIVRESPWTRF*
>SPSA8_v1_200020|ID:41145534|guaA| GMP synthase [glutamine-hydrolyzing] [Spirochaetes Bin 1 SA-8]
VDKILILDFGSQYTQLIGRRVRELGVYSEIIPGDSPLNETHLKDCTGIILSGSPFSAYEP
DSPIIHSSVYDCGLPILGVCYGIQRMTLDARGRVAKLEEREYGKKAVAVRTGIDDPLLKG
IPASFISWMSHGDSIVSPGEGWEVIGDSDGNIPAILRHEKLARWGVQFHPEVSHCEFGTV
LLSNFVFGICKTKAGWSMQAYLDQEAEKIRTTIQDRPALLLISGGVDSTVAGAFLLRVLG
PEQVYLLYIDTGLMRKAESEEVMANLARLGAKHLYKADAEARFLTALAGVADPEKKRRII
GDMFITVQEEEVQRLALKDAFLVQGTLYTDLIESGKGVGKKAQVIKSHHNVRSPLVEEKR
RKGLVIEPLDRLYKDEVRALGRLLGLPESVVGRHPFPGPGLGVRILGAVDREKCDILRNA
DAVFIEKLRELGLYDKIWQAFCVLLPVRSVGVAGDERKYGYVAALRAVTSEDGMTAQVYP
FDAADLIAISSAITNRVPEIGRVVYDVSSKPPATIEWE*
>SPSA8_v1_200021|ID:41145535| putative L-lysine 2,3-aminomutase [Spirochaetes Bin 1 SA-8]
MQKPWQHDMRQRITELPDHEHSALIAELIQKNALPFAATAYWLSLADEDYRTCERDARGV
PRDPILAQALPSPEELEISALESLDPLNETRHSPYPRIVHQYPSRVLLRTTGECALFCRH
CFRRSLLPQERGFISESAEHRLIDYLKAHQEVREVLISGGDPLTASDARLAKLFSGIREA
GANILIRLCTRMPVTLPSRVTREFVGMLKRFKPLQVVLHINHPRELSDVFVEKARLFIET
GLPVHSQTVLLRGINDNLETLVSLFSRLTRIGIDPYYLFQGDLAAGTRHFRVPLSKGLAL
YAELRKQLSGLELPRYAVDAPDGAGKLYLPESVVGREGDFWILRAPDGSLHQYPEETD*
>SPSA8_v1_200022|ID:41145536| Phospholipase, patatin family [Spirochaetes Bin 1 SA-8]
MAAYRNLVFKGGGVRGIAYLGALQFLYEKGLMRSIERVAGTSAGAITATVVAMNFSSFRE
IHEISDSLEYRKVPSEGTQTEEHVRFLKANPLAIRNQFRAVFKNVQCSMRFIQDRGWYSS
DYFYTWLKDVIARQFTVKKESYTFSDFRNASIHAGGREFLDLYITGTDISNRMSRVFSYE
TTPDMEVALAVRISMSIPLYFESIQYQYPGTSEPQSYVDGGIMWNYPISLFDDQRYGRKI
VNGVNTETLGMFIFTSPNETRYKKVDSLVDYLGALFESLLLVQEHLVAASERNKGRTIFI
DDKGVPITFFDVDTGDETYRKLFDSGYESAREFFENRTNWDMFFHGIQQKLGWIR*
>SPSA8_v1_200023|ID:41145537|argR| Arginine repressor [Spirochaetes Bin 1 SA-8]
MKERLERLKVIRNLIKTHRIDSQDTLLQLLVEEGYTVTQATLSRDLKYLKVGKVADGHSG
YYYTIPSDEERKESERHLVQDFLRGYVSVDWNESLVVVRTFSGHSDSVALAIDNMGLDEV
LGTISGRDNVVFIALKQGVKGEDFIRALKEKIPDFDPE*
>SPSA8_v1_200024|ID:41145538| conserved protein of unknown function [Spirochaetes Bin 1 SA-8]
MEDFKAVILHGFSNEEALTIMRAVKSLGISASQTAFATTTPTSLQWKVEYLLEHLHEEHA
MMQTRLAEQKKAGH*
>SPSA8_v1_200025|ID:41145539|clpB| protein disaggregation chaperone [Spirochaetes Bin 1 SA-8]
MNYEKFTLKAQEAVRDAASLAQNHDNPSVEPEHILLALLDQEEGVIPSLCERIGVDIASL
SSELGARVEKLPKAYGSASQVYLSPAASKAIAKAEAEAAAIKDEFVAAEHLLLGILAVEC
PAAELLKQLGITKDRILATLKSIRGNRRVTDQEAEEKYRVLERFTRDLTALARAGKLDPV
IGRDEEIRRVMQVLSRRTKNNPVLIGEPGVGKTAIVEGLARRIMEGDVPDSIRDKTVLAL
DLGALVAGSKFRGEFEERLKAVIEEIQKSDGKIILFIDELHTLVGAGAAEGAMDASNLLK
PALARGELRAIGATTLDEYRKHIEKDAALERRFQPVYCAPPSVEATIAILRGLKERYEVH
HGVRIRDDAIIAAAVLSDRYITSRFLPDKAIDLVDEAASRLKMEIESQPTDLDVLERKLI
QLKMEAQALEREADAASAERLAKMRKEIADLTAQRDSMKMKWEAEKQKISEIRNLKQQIE
ELKLEADRKLREGDLAKASEIKYGRLVEAQKKLEALTSELKSRAEGQTLLREEVSEDDIA
RVVSNWTGIPVSKMLSSELQKYVELEQKLAMRVIGQEEALRAVSDAIRRNRAGLSDPRKP
LGSFLFLGPTGVGKTELAKALAEFLFNDEKSLTRIDMSEYMEKHSVSRLIGAPPGYVGYD
EGGQLTEAVRRRPYSVVLFDEIEKAHPEVFNVLLQTLDDGRLTDGQGRVVDFRNTIIIMT
SNLGSDLILEAKGREEIRQGIDALLKTAFKPEFLNRIDEIVIFDRLDAPMIRRIADLRLA
ELADRMSQRSIMLNIDDEARDFIANEGYDPLYGARPLKRAIQALLENPLARKVLSGDIRE
GDTVQVAKTQDGLDFIVIRTAEGRNA*
>SPSA8_v1_200026|ID:41145540| protein of unknown function [Spirochaetes Bin 1 SA-8]
LNRLEADLLYTDTHAHLSLCEQRIGRQKLFSLLDEYDQLWQGSADTPKPFIVDVGTVAGD
LASRLGVFGRYAWLKFSAGIWPSQTALEHPDESLFRLKDDCGSGRVAAVGECGLDYFHMA
GSRNAQIRLFEQQIELAXX
>SPSA8_v1_210001|ID:41145541| protein of unknown function [Spirochaetes Bin 1 SA-8]

MERISLRPDSDRSKGQNCADCHQWTHRRRNPDLGASLRGRHPHAGSRIYLYAWNAEIPDP
RHNRRRGKRLIKHKVAMKVPTMTVETKGAGFRLLFDDRAVLVHMPDSPFIYAGKGSQSIT
MNCGNFSIEDEIEELLPLIDWNLEESGPGFAKVRFSAFSRYSLDAQFTINDGRLVVNLKS
GDTQESGNQQANRYRIVLPADSEEYIYGCGEQFSYFDLRGRKFPLWTSEQGVGRNKKTRI
TLEADLQGNAGGDYWWTFFPQPSFVSSAGYWCHADTSAYAVFDFSKPDRHSLYFWDLPQK
LLLSRQFNKENMAGLLX
>SPSA8_v1_210002|ID:41145542| Carbohydrate ABC transporter membrane protein 2, CUT1 family [Spirochaetes Bin 1 SA-8]
MKKRTRTILTSTIIAIVAFLVLIIELYPIGITVVNGFRRDIIILSGQPFKLSQLTTRSYE
LVLKNPGFQLGMKNSIIIGLLSTGISVLIGAMASYGIARFRFGWRNSLAYSFLVFRMLPQ
ISLVIALYLMFTNLGIRDTVGGITLAHTSFNVPYVIWLLLPFFTAVDKAYEEAAMVDGCS
RMGVFFQIFLPIVAPGLVVAAVFAFLNSWNEFLYALILTGVKAKTAPIAINGLIGGETLT
WGQVCAAGTLMLVPVFIFTLGMQKFLIRGITAGGVKG*
>SPSA8_v1_210003|ID:41145543| Carbohydrate ABC transporter membrane protein 1, CUT1 family [Spirochaetes Bin 1 SA-8]
MSSKKLVTDSRPARNKEGKAREHAPFLFLLPALLVVFAVLIFPIIFSLIVSTFNWPLSEG
AGVRRFVGLGNYIGLTQDPEFWNSLKLQLGFIFIAIPLELLLGFAAALLLNREFFGARLV
RSLLLLPVFFLPILSGMTWRFMLQPRYGPLNSLLLSLGAPEITWLGNPVAAYIAIIVQDI
WRMWPFMFMLLYAGLTAIPQEMIEAAEIDGAGFWKKLRSVIIPLLTPTILTAILLRIIDA
LRVFSEVYVMTEGGPGTSTMLFSLYTHRQAFGYQKVGMASAMAIFLLILSIVFALTLVRK
NMSLDALEEKAGG*
>SPSA8_v1_210004|ID:41145544| Carbohydrate ABC transporter substrate-binding protein, CUT1 family [Spirochaetes Bin 1 SA-8]
MKKALFILLLAVAMVGMVSAQTINVLFWDDAYPRTLMERIPEFEKATGIKVNFEILQPPQ
VFTKTSVAVGKDKTDYDLVCVDEGNIPLFASLMLPYDQWGPGKIFKKVDPSTVTPAMLDV
AQWDGKLIGLPINGNLYVWMTRKDLIENPKYKAEFKAQYGYDLGVPQTLQQMLDMGTYFY
KNGIVSGGFGPFNGGPAGVFGEAIFMWESFGTHFIEYVNGKPKLVVDKAKAVQGMEFYKK
LMAISPKGAETMAHVERQAAFCADPKGVFTQFIWPAQIASYEDPDKSLAAGKIVYSAPPS
GPAGRFAVRGTWAVNIPLASKNKDAAAEFVYWWASKDIATWLVEKNTVPARTDALTNPKF
AASKPWLAAIADSMKYAAARPRFNEVAQVQDIVKKYWIMGITGQMPTADAMQKIMDETQD
VLKKAGY*
>SPSA8_v1_210005|ID:41145545| putative Sugar phosphatase YidA [Spirochaetes Bin 1 SA-8]
MEKNSIKYIALDMDGTILDKDYTISPAVIDALQRQKLSGRKILLATGRVRASALKHAHKF
GGADGYICSNGADVYGADGRRIAQRHIEEPVSRLLVELARQTTSHFHAFIEDDWYYEEEK
PYTEFYHRRSGLEGRPTNFDYFSALTFTKFMFLDDHEKLEPIKTKIELDFPSSLQAMYSA
PFMLEVVARGVDKAAGLKAFLDFAGASLSEVCAFGDAENDLDMLKAAGIGVAMGNAPEPV
RQAVRYHALSVDDDGVAVFLNTFLS*
>SPSA8_v1_210006|ID:41145546| Fumarate lyase [Spirochaetes Bin 1 SA-8]
MSKKRFSEIYWRNVLDPSYQNWLRLFKTASFQVHRAHLLMLFRQGVVSRDIVDSIKKALD
KLERTFEFPAVLPNGVEDLYFILEQALGEEVGQENAAWLHTARSRNDMDTTIFRMVLKNE
FRNLIQKLTAVCRAMELKAQHSEGLLTVLYTHGQPANPSTFAHYVMAIAQELADTAGYCL
DALDDIDKSTMGAAAITGSGFPIDRAMVAQFLGFSAYAVNTYQAISTGHWLERPAQAVQL
LMVDIGRCIADILHKASCEVGLIIFPDDLVQVSSIMPQKRNPVILEHVRIQASSIIDSCS
AMLQHFRNVPFQDVNENADAPIAQFLDTLRKSVALLDLFEEAVLKMEPDTERAREISRQF
GVTTTELADDMVRRTGVGFRVAHELCSIFVSSGSSFDVLRKEFNKKTGNTLPYDDDELRR
ILEPETFIRVRSVPGGPAQDGMKENYIELEKKVFALQRSVAESLERESAGIKSMMDGLSE
I*
>SPSA8_v1_210007|ID:41145547| Amidohydrolase 2 [Spirochaetes Bin 1 SA-8]
MRVIDSHVHFPEDKIIDDGLEHDEKASSDGIGYSSGNTEKSAEALKPHQAWLRAEKNRWE
SAWRFPEPVAVSKEHGEALWLSEFEKYPYLEKVVFVTAGSNQFASELSERNPGRFIGYAH
HNPMLKDAPDRLEKAVKVQKLRGYKLLGPKVEKPLSDPFFDPLWETAQDLQIPVLIHFGI
MGAAGGIASHINMNPMAIHDAAKRFPDIPFIIPHFGTGYLFEVLNLCWACPNVYIDTSGS
NQWMRWMPYEVSLEILFRKFRETIGPSRLIFGTDSSWFPRGFSEKYLDEQNRAMSYVGFS
DDEKDMVLYRNIAALLKIQGN*

>SPSA8_v1_210008|ID:41145548| Binding-protein-dependent transport systems inner membrane component [Spirochaetes Bin 1 SA-8]
LNRSIMKTGGHKKISINILFSYFFLTLFSIVMIFPFIWMILSSLKDVSQIFTLKLFPSKP
TLDNYRYILFSGSTKFPQWFLNSLIVAACTTVSVLFFDSLLGYTLAKFRFPGKKIVFLLI
ISTLMIPTEMLVIPWYAMSRALKWGNTYWGIMFPGMISAFGVFLMRQFMSTIPDDLIDAA
RVDGQGELRIFLTVILPLVTPALATLAIFNFIGNWNAFLWPLIVSSRPRMFTLPVGLANF
SGEAGSDWHYIMTGAAVAMAPLVVVFLLFQKQIVRGIAMTGLKG*
>SPSA8_v1_210009|ID:41145549| Binding-protein-dependent transport systems inner membrane component [Spirochaetes Bin 1 SA-8]
MTLAGKRALTAYTFLFIPLIFFICVRFGPMLYMLVMSFTDWGLLRKTVKFVGFGNYRAIF
SDPVFLKSLSNTFVYAFGGAPIVIIISLGLALLLNLIPKGKGFFRLIYVLPYITPVVAVS
WVWRWMYQPPPLGLINGILGALRLPAGDFLNSIHQSLPSILAVNVWVELGYCTTIFLAGL
QTIPHELSEAARIDGANDRQVLTRITLPLLLPITMFLTIMEGIQFLRIFTQVYNMSMQAM
GGPLNSTKSAALYIYQKAFTNFEMAQAASASLVLFAIIMTITLIQLKFFDRRINY*
>SPSA8_v1_210010|ID:41145550| Extracellular solute-binding protein family 1 [Spirochaetes Bin 1 SA-8]
MKKKTLMIMVLLAIAVAGTFAQTVTITYWQYFYESKVKLMDELIQKFQAANPGIKVEQVT
FPYESYNQKVAASIPAGEGPDVINLFYGWLPMYVKAGYLQELPATDFNKAFFEKNFYPFV
AESVDFGGKYYSVPTAVRTLALIWNKKLFKEAGLDPNVPPKTLEELEAFARKLSKYDGQG
NLIQAGLAMQPTGQGHNWLREVLFRQFGNTPYSSDYRKVTYADANGVAAFKWYMDRIVKD
KVGYPNFATDDVTAFKAGKAAMNIDGSFRIATLNAVKDLEWGVSELPSYKGIKSNYASFW
THAIVAGTSGKKLEASIKFLKYITSPEVQVLWLQRVGELPATPLLSESYKNDPVIAPFLK
GLAYAHASQFVDEAGQRTVLVDAVDEVYLKKVDPAVALKNAADKEQALINSFWAK*
>SPSA8_v1_210011|ID:41145551| Cyclic nucleotide-binding protein [Spirochaetes Bin 1 SA-8]
MVDTSQLQKYSLFGGVLPEQIEKIKTLFGYEQFNTGETPMKEGAPNDKIYFIISGRVKVS
KKGIPIAELKEGETFGEMELIDVMPSIATITALEPLEVVTISNRALYEISKLDPKAFSMM
VMNLARDVSRRLRRMDELACK*
>SPSA8_v1_210012|ID:41145552| GGDEF domain protein [Spirochaetes Bin 1 SA-8]
MIRLSKSVFNDLAIFMMGFGFLVGIVFPFFLILLGVPASITLQPWFIFVCIMAGLVVGLV
NIILARSVVGARLQKLVKRMQYISNHLINDSHEDIVSNCKTSDCLLEVDSSDVLGESAKS
YNVLVNSLFASLKSELMVRQFTSMLSTRLELEDLGVTVLPALLSFTGTEAGTIVMERDGE
MEVIASYRIRDNAGVVKSDVVTRAIVSKQRQLLQMPADIAIESSVLDFSPRTVLVEPLVY
HDVALGVIVLASVSAISQETLAMLELVNASLAVAFRNALTYDQLQKLAANDSLTGMFNRR
FGMARLQEEFGRSIRSGSPVGVCIFDLDHFKNVNDTYGHPMGDKVLVHVSRLLKSALREG
DVALRYGGEEFMAVLPGASLTDAFQIADRVRRLVEETVFQHGSQNLKLTLSGGAASWPDF
DASSSDALVRRADEALYQAKEGGRNRIVAL*
>SPSA8_v1_210013|ID:41145553|ams| Amylosucrase [Spirochaetes Bin 1 SA-8]
MEGFKQSLYRRLERRSITKSVWWHEFFIRLENELDRLVSLLYELYGERPDFAFWVEDIVI
HAFESFRTRPAWLKERDRQFPPESGWFRSEKQMGAVCYVDRWAGNFQGLRSRIPYLEELG
ITYLHLMPFFKSPEKENDGGYAVSSYRETNPALGTMKELALLAQTFSEHGIALVADFVFN
HTSDEHAWALQAKKGESWYRNFYLTFSDRSEPDEYEKTLREIFPESRRGSFTFNEEMQRW
VWTTFHSYQWDLNYRNPAVFDAMCCEMLSLANHGISILRLDAVAFIWKQKGTSCENLPQA
HTLIKAFQSMARLACPSLLFKSEAIVHPDDIAKYIDLRECQLSYNPLLMAELWEAAATKE
VRLLSHSLEKRHKIPAGCAWVNYVRCHDDIGWTFADEDAWALGINPFDHRQFLNRFYLGD
FPGSFAKGLSFQYNPITQDRRICGSAASLAGVDRADAVRDPEKLERGMARLILLYGIALA
AGGIPLLYLGDELATENDPAWDKDPAHKADSRWVHRPLWNETLANERHDSSTVTGKVFST
IKSLIAQRAAHPVFAASDLAVRPSGHKSIIVIHKEVPGSQLVIVGNFSESSVPISQTRLT
EILGGEPYTDLVTDSIWNARSAEALAPCQLLWLFR*
>SPSA8_v1_210014|ID:41145554|yufQ| Uncharacterized ABC transporter permease protein YufQ [Spirochaetes Bin 1 SA-8]
MKATLILEMFPIALMFASPIIMAALGGLFSERSGIVNIALEGIMMVGGFAAASVTVLLES
STPLAPWIGLLVGIAAGMLMSLLHAFASINLKANQVISGTALNILAGGVTIYLSQIIFHQ
QRTRSFSNGIQKMTVPGLSRIPLLGKMFFIENYPTFYIAIALVLITWFLVYKTPFGLRMR
SCGEHPQAAASMGINVAGMRYIGVLASGALAGLAGGVMVLTADIQYTVMSIHGTGFIALA
SLVFGRWNPFGVLAAGLFFGFSTALSFYAKDITFLAKLPSEFFYMLPYIFTIVALIFFAG

KSVGPKAAGEIYDSGKR*
>SPSA8_v1_210015|ID:41145555|yufP| Uncharacterized ABC transporter permease protein YufP [Spirochaetes Bin 1 SA-8]
MTKRTEKVLLPLIAVFLGFLLGSIIVISTGRSPVAMFSAMIRAVSGIDLVNGMSFNPRYI
GEFIIQSMPIILTGLAFAFASRTGLFSIGAEGQLMVGSLAATAVSLLVVAPAAVHVPLVL
LAALLAGALWGGIPGVLKARFNVHEVVVTIMMNYIAFHLNNFTILNVFGSVDRVKTAFFP
QSALLKDPMLESLTNGSRLNWGFVPVILALLAFSFIINKTTFGYSLRAVGYNKEAARYAG
MKVNRNIILSMMIAGAFAGLAGAVITNGTFNFGRALPAAEGYGFDGIAVALVGANEASGI
LFAGLLFGMLKAAQPLMQSQGIPKEIVGIIQASIVLFVAMQYGIKLILEKLARKKNAPAE
GGQE*
>SPSA8_v1_210016|ID:41145556|yufO| Uncharacterized ABC transporter ATP-binding protein YufO [Spirochaetes Bin 1 SA-8]
VAYIIEMLNITKDFPGIRANDNITLQVEEHSIHALLGENGAGKSTLMSILFGLYQPDAGK
IRIKGKDVRVTDPNVATRLGIGMVHQHFKLVHNFTVTENIILGMEPRKGPVIDIRAAEEK
VAAISKLYGLAVDPRAKIEDISVGMQQRVEILKMLYRDAEMLIFDEPTAVLTPQEIHELM
HIMRRLVEEGKTILLITHKLKEIKEVADICTVLRRGKVVGTVQVADASEEQMAEMMVGRE
VSFHVAKGPAQPKEVMLRIENLNVKNNKGVLGVKNLSLEVRKGEVLGLCGIDGNGQTELV
QALTGLTRVESGKIYVGDTDITALSIKARTDLGLGHIPEDRQKHGLVLDFTLSENFVIHN
YYEKPYAHWGILNPKAIRENADRLIREFDVRSGQGGDTPARSMSGGNQQKAIVAREIDRS
PKVLVVAQPTRGLDVGAIEYIHQRIIAERDNDKAILMVSLELDEILDVSDRIAVIFNGEI
VGIVNAKETDENELGLMMAGSLRKNVPPARQSEPSASVQGRNA*
>SPSA8_v1_210017|ID:41145557|tmpC| Membrane lipoprotein TmpC [Spirochaetes Bin 1 SA-8]
MKKAVLVLLVAALVAAPVFAQFKVGLVTDVGGIDDKSFNQGTWEGIVRFAQDYKLPKANY
KYLQSSAEADYVPNLTTFADEKLNLIVAPGFLFNNAMAEVALKNPTQKFLIIDSVVQDAK
GKNIPNVANAVFAEHEGSFLVGVAAGLKAKADGKNVVGFLGGMQFPLIEKFQAGFEQGVK
AVFPECKILVDYAGDFAAPDKGQAIAQKQFNAGAYIIFHAAGGTGNGMIKEAKERSQKGD
IRWGIGVDKDQYADGLYGNKSAVLTSMMKRVDVAAYDVCKMTMEGKFPGGQVLTFTLANK
GVGIPEKNPNLSADIVAKVREYEALIASGKLKVSEVPGK*
>SPSA8_v1_210018|ID:41145558|deoC| Deoxyribose-phosphate aldolase [Spirochaetes Bin 1 SA-8]
MEKKWTKADIAAAIDHTLLKATGTESQIRELCAEAKKYRFASVCVNPSWVPLCSQELQGS
GVMVCTVIGFPLGANASEIKAAEARLAVGQGAHEVDMVINIGKAKAGDWSAVESDIRAVV
EAAGNATVKVIIETCYLTNEEKVKACQAAMKAGAHFVKTSTGFGTGGATAEDIALMRKTV
GSTMKVKASGGIRTLQDALAMLKAGADRIGASAGVSIVSEIEG*
>SPSA8_v1_210019|ID:41145559| conserved protein of unknown function [Spirochaetes Bin 1 SA-8]
MNPEGRPGPQEIKDQYSGVPQSSLPLRSEAGFMDAESMIRLKNMHLAPYIQLATALIGMR
RRNGGNMFRHQIDTMGILMDYGYIDSILLKASVIHDLIEDVPGISGDSILAIDEESAEVY
KLVLEVTKRPIENKLDFLARIRDYGSLRAKVLKSADRISNMISLGYVTDIKFVQRYTEET
ENHVFPIARLADERMLHELEELVATRKEFLARRFEI*
>SPSA8_v1_210020|ID:41145560|valS| Valine--tRNA ligase [Spirochaetes Bin 1 SA-8]
MKAIELAKAYDPASFEDAIYAMWKSEGHFMPKAEKGKKPFTIVIPPPNVTGVLHMGHALN
NSLQDIQIRFRRMTGRPTLWVPGTDHAGIATQNVVERKLRKEGKDRRQMGREAFIEETWK
VAKEHRAFIDNQLAKIGASVDWSRERFTLDEGLSKAVREVFVTLYERGLIYKGEYLVNWC
PSCGTALSDDEVEHEDEAGSMWHIWYELADGPCPECPKGKIEIATTRPETLLGDTAVAAH
PDDERYKQLIGKMLKLPLTDKTIPIIADAYVDREFGTGLVKITPAHDPNDFEVGNRHNLP
RVNILNPDGTLADTVPEKYRGLKVLEARKLVLADLETSGLLKSEVKLTHAVGHCYRCHTS
IEPYLSEQWFVKMKPLAEKALKAWQDGEVVFFPRKWEHTYKHWLENIRDWCISRQLWWGH
RVPVFYCRHCGAVLVEREDPTYCKKCGSSDIYQDEDVLDTWFSSWLWPFSTLGWPRETAD
LRKFYPTSALVTAYDIIFFWVARMIMAGMEFTNQSPFQEVYIHGLIRDKQGRKMSKSLGN
GIDPLEIVKENGADALKFTLAYNCAGGQDILLDRDSFKMGSKFANKVWNASRYILMNLEG
REMLDLNSIQLNDTERWIAGRFQEAVQRVSQALESWRFNDAAQAVYSYFWDDFCDWYIEA
TKLSTKFGDDKEKDRATTVLLGMLEKSLRLLHPFLPFVTEEIYRMLPNAKGRLISQPWPL
DKESARDGQLDASFESLRQLVSMIRTLRSEFQIAPEARIPVDIRLDADSATSDFIRRNAS
LIALLVNSPEPAFLAPADARPEKTVALAGKGFEVFVHVRDLIDLDKLLATLRKDLEKERG
FASKVEAKLSNQAFISGAPEEIVAKEKEKLVESEIRMRKLSRYLEELA*

>SPSA8_v1_210021|ID:41145561| Long-chain-fatty-acid--CoA ligase [Spirochaetes Bin 1 SA-8]
MSDTTPWAFLDGFRGSFFKGEWPTLPELFRISAQRFPERPCFTVYNPDRQSLNYTQALRK
IEEIAWYLKAKGIQKGDRVAVSGKNSPEWAIAYLGILEAGAVVVPIDYQLSSEEIAHLVS
ASEARILFIDEEKHTELEALCTGLEETIALYESHPSYLYRIAASAWGKKNEKSEADSDKA
EEEDLAAILYTSGTTGNPKGVMLTHRNLVSDCFLAQANLHIYYTDVFYALLPIHHSYTML
AVFIESFSVGAETVFGKKMVVKQILKDLKEAHVTMFLGVPLLFNKLLNGILKGIREKGIA
VYGLISAMMWLSGFIKKVFKVNPGKKLFHSVLDKASLASIRICISGGGPLAPSVFRKYNQ
LGIDFVQGYGLTETSPIITLNPVEHYKETSVGKILPGMEMKILSPNADGIGEIAVKGPMV
MKGYYKMPEETAEVFTPDGWFKTGDLGYIDEENYVYLTGRAKNLIVTEGGKNVYPEEIEY
RFQLFDQIDQVLVRGYIENXDSKSEKIEALLYPSPDWVAKLTEEAGSQAKAWEQAELKLK
SFVDEVNLKLLPYQKITRVIILREPMEMTTTKKVKRFTSKIES*
>SPSA8_v1_210022|ID:41145562| Anaerobic glycerol-3-phosphate dehydrogenase subunit A (fragment) [Spirochaetes Bin 1 SA-8]
MEALYDNLNAAPLPLPACSMKKNGHHVCIIGGGGTGGALAYDLALRGFAVTLLEKGELTS
GTTGRHHGQLHCGARYAYGDRNIARECFEESVILSRIAGSCIEYNGGFFVALTDEEADFE
KAFIEGCLESGIPARKVKKEKILAIEPRLGAVETGVFVPDGTFDAFRLPMMFFSAAKMLG
ARIMPWHEVVGFDITNGRMQAVIAQNLLTRESGASVSGKELRIEADYVINASGAWAGKIG
ELAGADIPVTPAPGAMLAVKGRLVDRVISRX
>SPSA8_v1_220001|ID:41145563|ispF| 2-C-methyl-D-erythritol 2,4-cyclodiphosphate synthase [Spirochaetes Bin 1 SA-8]
VHYIPGERDNKKITFKEDIPAMSSSTCEKLRIGEGWDIHPLVPGRPLLLGGVRIENPLGE
DGHSDADVLWHAIIDAVLGAAGLGDIGTHFPPSDRTWKDADSSELAKMAAKLVKEAGFAI
QNIDTTVVLESPRLGPHKEAIRTKIASVLEIPVDCVSVKAKTAEKFGAVGAGIAIEARAI
ALLASKRN*
>SPSA8_v1_220002|ID:41145564| protein of unknown function [Spirochaetes Bin 1 SA-8]
VFLFEQWQDGFVLTVEGRRILHHRKRHPALFVLKPRDTRTGADNPETPLSWKALRSFSIV
NENSESRLLRFDETILIRFFYANKVLRCQFRVLDPAVKGLRLVLDAESEEMLFGLGGYCL
EAQAQGNLKGLKIKTECAQSASLPDEPVVFSSRNWWIQTERKACPEFDFQLDKTIVTMSP
VPDELLAGFGAKPASALVLLTSHKLKKWDDGVIPVKKRFPLPGWVFEGAIMQQAACLNPE
MMKPLRTAAVVTDQADEAGAVRAVNQAAWNHLLTVSAVRVLEAQSFLGKKADMMVRLLLA
NSFSGVGYQSLTIALPPVSNFAGQDSVVPFLARILDMAVFSPLLTVNLGGVGEHVEQGSP
VQHFLKNLMRASELHAKLAEYHEYCSYLWQSKGLPVFCHPAIFYPEEKVLWNFDDAYLYG
SDVLIAPSLPNDGRTRQLYLPDDEWVHLWTSRNFSGGVAVVDAPLGRPAVFYRKNSAFAA
LFDEVRKIAVRLS*
>SPSA8_v1_220003|ID:41145565| L-fucose isomerase [Spirochaetes Bin 1 SA-8]
MKQHIRLGAVCLSRTTFDFKAAKELWADIRSQLAALPNVTLTAPENLVFEVDEAKAAASE
LARAEVDGVMIISGTFHLGHLALEIAKSVKAPILLWGLPELPYNGGKIRLNSVCGVNLNA
SNLYKAGIRNFRAVVRPDVDTDWVDALRVLAVLKNSHIGIIGYRAHGFFNLSFADLSLYR
ETGILLDHYELSDMWNFPITDEEAAERKKDYLKVFDCSGISEAQLDKVARLAVKMKKFLD
KNKLDALAIRCWPEFAAEYGISPCAAMSLLQSEDRILACEGDVEGAVSMLMHRAVGAETP
FLFDFSQVDFEKNHALFWHCGVAPCNLWDGKCNRSLDTYFAGGKGVTADFVLKSGEVSVL
RLDSADGKYRVFIQKATAVPMEKELKGTYLKAVFDSPVQEVLAKVVNNGIAHHASVVYGT
YLEPFRIAAKIAGWEVIE*
>SPSA8_v1_220004|ID:41145566|bcsP| 31 kDa immunogenic protein [Spirochaetes Bin 1 SA-8]
MKKLLVIALCLLAVTGAFAQQRFISIGTGGTAGTYYPLGGAMAEIWNKNIAKMNATAQST
GASIANVNLLRDGKVDVIILQNDVAFYALHGVELLQDKAYKDIRGMACLYNETVQLVALE
SSGIKSVYDLKGKKVSVGAAGSGVEANARQILEAAGLTYNDIKVQYLSFGESASNLKDGN
IDAAFTTGGFPIAAIQDLAVSRKTVLVPMDKALVDKLMAKWAFYTPTVIPAGTYKGIDVA
TPSVAVKSMLAVSAKLDANLVYEMLKTMYENGPRLVAAHAQGANIKLETALDGMSIPLHP
GAEKYFKEFKK*
>SPSA8_v1_220005|ID:41145567| conserved protein of unknown function [Spirochaetes Bin 1 SA-8]
MRPFKFFRGNLEKRKAAVFFVAFLLVCGFAALFLLQRIPSLSIYDEHGRLLKKMPLPDGE
FVHHYIHSIHKTPVDEVFIVKNGELILTQVTYDSYGVGMPTGEEEGFTLQDGRFIVNLRR
TFKQIDIRVSPVPDHGIILGDILYRFTDWAGVEDLLVLKPSVSYSIHFGRNSLP*

>SPSA8_v1_220006|ID:41145568| TRAP transporter, 4TM/12TM fusion protein [Spirochaetes Bin 1 SA-8]
MSEEIKTEDQAIDSQEILKKFDKEADYRTYTGFFAKAIAALAITFSVFQLYTAIFGVLDA
MIQRSIHLSFGLCLIFLLYPTSKKWSRSKLHPVDAILAVLGVLAPMYIVMNYQSLVGRAG
QTTTFDLIAGIIGIILVLEAARRVVGIPIIVISLIFIIYAFLGPYIPGKMAHRGARVDTL
VQHLYFTTEGIFGIPLGVSSTFIFLFILFGAYLEKTGLGQLFIDLANAVAGWAAGGPAKV
AVXSSALMGTVSGSSVANVVGTGSFTIPMMKKLGYKPEFAGAVEATASTGGQLMPPIMGA
AAFLMAEFTNVPYARIIGAAAIPALLYYFGVWSGVHFEAKKLGLRGLKREDLPKFKVIML
ERGHLMIPLIAIIYLLVTGFTPMKAALWAIVLSILASMIRKSTRIKPIDIIKGLEAGARS
ALGVIAATACAGIIIGVVTLTGLGLKLGSTLVEIAQGNLIITLFFTMITSILLGMGVPTT
ANYVITSTIAAPAIVMILAQKAGLDPNVAAPAAIVLPAHMFAFYFGIIADVTPPVALAAF
AGAGIAKANPMKTGFNASKLAIAAFLVPYIFVLNPQMLLFNVTAIDMIWMLITSLVGIVG
VAASVNGWFLTNMAWWERLMGAAGGLLLIVPGLVTDAAGLGLVGLVLILQRIKKAKKA*
>SPSA8_v1_220007|ID:41145569| Aspartate kinase [Spirochaetes Bin 1 SA-8]
MKVLKFGGTSVGSVQSIEKLIGIVRQSGERIIVVSAFSGVTDMLIRAVNQAASSGDFRDT
LESLETRHFDAARSLISSAANRPDSLHEAERHIASMLGEIENLLTGISLLHEKTPRTMDL
AMSYGERLSAYIIAKAFTARGMPAEFVDAREIVRTDDQFGSAHYLEQESRELIRARLVDN
ASIAVVTGFIGSTDSGITTTLGRGGSDLTAGILGAALDAEEIQIWTDVDGILTADPRLVR
DAFVIPEISYAEALEMSHFGAKVLHPPTILPAMVRGIPIRIKNTFNADAPGTLIAKKAAS
SGFPVRGLASISEIALIRVQGPGMPGVTGIAARMFGALAGAQVNVILITQASSELSICCA
INPLDSQKAVKAISGEFELEIKAGLIQKPSIEEGLSVVAVVGERMKRKTGISGKVFSALG
RNGINVVAIAQGSSELNISIVVDRRDRGKALQTIHDAFFLAGIRTVNLFLVGTGLIGSTL
LAQIARQQGDLRSKHSIAINVAGICNSRYMLLKEDGIGLETWRDVVASAEKADLDAFIDG
MITMNLPSACFCDCTASDEPGKRYEKILKSSIAVVTPNKRANAGEKAYYNRLINLSREKG
NIYRYETTVGAGLPVIGPLQDLVASGDTIHRIEAVLSGTISYLFNTLEEGRKFSEIVKEA
KAKGYTEPDPRDDLSALDAARKTLILIREAGFNLDYKDIRIEPLLPQSCLAAPTIAEFLE
ILPSVDEAYETKRQEAAKHGNVLRYVSEITPVKASIMLKEFGPRSPFFNLAGTDNMVVFT
TDRYTENPLIVRGPGAGADVTAGGVFADILKLAE*
>SPSA8_v1_220008|ID:41145570|gcdB| Glutaconyl-CoA decarboxylase subunit beta [Spirochaetes Bin 1 SA-8]
MSEFLTEFVQSTGFVNISAGQILMLLISFILLYLGIYRKYEPLLMVPIAFGMLLANLPIT
GIVDGPVGQMPGGLIYYLYQGVKLGIYPPLIFLGIGAMTDFGPLIANPMSLLLGAAAQLG
IFIPFIIATYIGFDLKAAASIGIIGGADGPTAIFVTSKLKPELLGPIAIAAYSYMALVPL
IQPPIMRALTTPKERMVKMAQLRQVSKTEKIVFPIAVTVLVGLLLPAAVPLVGSLMIGNL
FRETGSTDRLSDTAQHALINILTIFLGVSVGSTATAERFLNPETLKIIALGLFAFACGTA
GGVLFGKLMYVLTKGKVNPLIGSAGVSAVPMAARVSQVVGAEADPTNFLLMHAMGPNVAG
VIGSAVAAGVLISILG*
>SPSA8_v1_220009|ID:41145571|gcdC| Glutaconyl-CoA decarboxylase subunit gamma [Spirochaetes Bin 1 SA-8]
MKQYRITVNGQSYDVLVEEVSAGSAATASAPSAAAPRPAAPAAAVPAATTSAASAGAISV
KAPMPGTVMSFKVTKGQAVKRGDVLLILEAMKMENEIVAPQDGTVVAFRVPVSASVNTGD
PLVDLA*
>SPSA8_v1_220010|ID:41145572| protein of unknown function [Spirochaetes Bin 1 SA-8]
MLNKWLIMIAGVGTVFVTLILLAVMLSFFPMIFGGRKEKKHPNPAPIPEMSGPSDHQPAQ
NQVSKAPVAAENDHSLIAVLTAAIAAASGTSASSFRIASIMPASRQEGSFNTPIWGRVER
YTRE*
>SPSA8_v1_220011|ID:41145573| Methylmalonyl-CoA carboxyltransferase 12S subunit [Spirochaetes Bin 1 SA-8]
MDEKLKALEAKRAKVLAGGGSKRVAAQHSKGKKTARERIEALLDPGSFIELDAFVEHRCT
DLGMDGVEAPGEGVVIGYGTVNGRLVYVFAQDFTVIGGSLGEMHAAKICKVMDMAVKVGC
PCIGINDSGGARIQEGVDALKGYGDIFYRNTLASGVVPQISVIMGPCAGGAVYSPALTDF
VFMTEGSSNMFITGPQVIKAVTGEDVSADDLGGAHIHTEVSGVAHFRYPDEDSTIAGIKK
LLSYLPQNNIEDAPILDAGDDPTRADASLADIMPEAPNKPYDVREVIRRVVDAGDFFEVQ
PDYARNIVTGFARLAGRSIGIIANQPAVLAGVLDINASDKASRFIRFCDSFNIPLVTFTD
TAGYLPGVGQEHGGVIRHGAKLLYAYSEATVPKMTVILRKAYGGAYIAMCSRHLGADQVF
AWPTAEIAVMGPEGAANIIFKKEIDESSDPASTRKAKIEEYKTNFANPYKAATRGYVDDV
IDPAQTRARLCAALSMLLGKRQTLPSRKHGNIPV*
>SPSA8_v1_220012|ID:41145574| conserved protein of unknown function [Spirochaetes Bin 1 SA-8]

MKLEKLDHIGIAVEKLDDSLAIWEGTLGLVLHGTEEVVEQKVRTAFLPLGESEIELLEST
DPEGPIAKFIAAKGQGIQHLAFRVDNIEAALEELKAKGVRLIDEKPRYGAGGARIAFLHP
KSTGGVLIELCERK*
>SPSA8_v1_220013|ID:41145575| putative enzyme [Spirochaetes Bin 1 SA-8]
MQTNHEDLAQRCLSGDIRAIARLISLLEDGDRQAYQIFGTLKNSLGRAQVIGITGPPGSG
KSTLTDKMITGLRKRGKRVGVIAVDPSSPFSGGAILGDRLRMQGHATDPGVFIRSLASRG
TLGGLSKATASAVHILDAAGYDCIIIETVGVGQSEVDIVKVADTVILVSVPGLGDDIQVI
KAGIMEIGDIFAVNKADRDGADRVVREIRAMLETQAMLKFGKAVPAGPDTLAARNAEAVH
HGRLMETPQKTETEQDPHAALELPPVLKTVAETGEGVDVLLETALKQFETARLSGELEKR
RMRAIRAQLQDLVSWQVMEYFLSHEGVRHLDRLSKAVFSHSIDIYSAAETLFEEFFHGGI
K*
>SPSA8_v1_220014|ID:41145576|yliK| fragment of methylmalonyl-CoA mutase (part 2) [Spirochaetes Bin 1 SA-8]
MERKIRVLVAKPGLDGHDRGAKVIARALRDAGMEVIYTGLRQTPEQIVQAALQEDVDVVA
LSILSGAHNHLFPRVVELLKENKAEDILVFGGGVIPEDDIPFLKSKGIAEVFGPGTPTSV
TVDFIKANIKRK*
>SPSA8_v1_220015|ID:41145577|yliK| fragment of methylmalonyl-CoA mutase (part 1) [Spirochaetes Bin 1 SA-8]
MSDFEKLEQELNGWNAKVDKSIAKFPERKAKFVTGSNAPVERLYTPLNLKNQDYLQDVGL
PGEFPYTRGVQPTMYRGRFWTMRQYAGFATAEESNARYKYLLEQGQTGLSVAFDLPTQIG
YDSDHALAAGEVGKVGVAIDSLEDMETLFGGIPLDKVSTSMTINAPASVLLAMYIAVAEK
QGVSPDKLNGTIQNDILKEYVARGTYIFPPAPSMRLITDIFEFCSKQVPNWNTISISGYH
IREAGATAIQEVAFTLADGIAYVEAAIKAGLDVDDFAPRLSFFFNAHNDLFEEVAKFRAA
RRVWAHIMRDRFGAKNPKSWMLRFHTQTGGSTLTAQQPENNIIRVAIQALAAVLGGTQSL
HTNSKDEALALPTEDSVRVALRTQQILAYESGVAETPDPLAGSYYVESLTNSIEEGVLAY
IKRIDDLGGAVKAIEQGYVQQEIQDSAYAWQMDVEKGERIVVGLNKFQIKEMPPKGLLRV
DPSVGERQVAKLKALREKRDNAAVQKALDNLKLAAQGNDNLMPPILAAVKTYATLGEICD
VLRSVFGEYRPSVMF*
>SPSA8_v1_220016|ID:41145578|korC| 2-oxoglutarate synthase subunit KorC [Spirochaetes Bin 1 SA-8]
MRVEFRLSGSGGQGLLLAGIVLAESAILDGKNAVQTQSYGPEARGGSSKAEVVISDTDID
YPKATDPDYLLALTNDAYKTYGKLMTKGTIIIDDSVTRDPEITAKTISLPILETAAKKVG
KTVVANIVALGVLGGISSIASVDSMKAAVKARVPKGTDELNLKALQAGLDLAAESVSSR*
>SPSA8_v1_220017|ID:41145579|korB| 2-oxoglutarate synthase subunit KorB [Spirochaetes Bin 1 SA-8]
MAEILDYFRKERLPSIWCPGCGHGTVTGALIRAIDKLGLDKNNIVMVSGIGCSSRAVGYL
DFDTLHTAHGRAIAFATGVKLAKPNLKVIVMTGDGDCAAIGGNHFIHAARRNIDITTIVM
NNNIYGMTSGQYSPTTPHGMLGTTAPYGNVEYAFDLCELAKAAGATYVARATSYHVTLLE
DLIEKALQNKGFSVVEAMTQCPTYFGRKNKIGGAVELLNWIKDGTINVKAAAALPPEKRE
GKILIGELHRAERPEFTASYLEMTSKVQRTYNDYKGGNRGCELNSD*
>SPSA8_v1_220018|ID:41145580|korA| 2-oxoglutarate synthase subunit KorA [Spirochaetes Bin 1 SA-8]
MPEIVKLMQGNEACTMGALAAGLDFYAGYPITPSTEIAEMCSEELPKVGGRFIQMEDEIG
SIAACIGASLTGKKSMTATSGPGFSLMQESIGYASLCEIPVVIIDVQRMGPSTGMPTSPA
QQDLMQARWGTHGDHAVIALAPGNVKECFDLTFKAFVLSEKYRVPVIVLTDEVIGHMREK
VIMPDPRTYKIPVRAMPATPAGYKPYEADPDGGVPLMAPFGKGYRWHCTGLFHDEKGAPS
GKASVAETLLYRLKRKIEKNEAELVDYFAESMEDCDVAVVSFGSSAMAALSAVRKARAQG
LKAGMLRIKTVWPFPDAAVRELTKTAHTIIVPEMNQGQMALEVERVTRCNAKVVKLGKVN
GDLFRPEEVFEVLKEAF*
>SPSA8_v1_220019|ID:41145581| Ferredoxin-2 (modular protein) [Spirochaetes Bin 1 SA-8]
MAEQKKGYFHIIREKYCKGCGICIAFCPKQLLVLKNGKVFPERPEICIGCHMCEYRCPDF
AIEVKPIVEETNHSADVEGLDYSLPPEAHHA*
>SPSA8_v1_220020|ID:41145582| conserved protein of unknown function [Spirochaetes Bin 1 SA-8]
MNHELKIRDLTLRDGQQSQFATRMNQAQIDTVLDEYRNAHFYAMEVWGGAVPDSVMRYLN
ENPWDRLEKIKAGIGNVSKLTALSRGRNLFGYNPYPDSVIEGFCRNAISSGIDIMRIFDA
LNDIENMKSSIRFVKEAGGMADCAVCYTMDPKFTIGEHIHAFFSGKALPINLFTTNYYVE
KAKELEKLGADMITIKDMAGLIEPAAAAKLIKALKNEVSVPIDLHTHCTPGYGVASLLAA
MVNGVDIVDTAILSFSGGPAAPAYEIIRIFADKLGLDTGVDNAAVAKIDAHLREARKQLA
AYDQYKKLPPVLDLSKTAISQDLNLLFDDALEATVGKKYSKALELCRKIEAAFNYPEPDK

IVQHAQIPGGMYTNMMSQLKEAKLEQHLEEVLKAVPIVRLDCGVPPLVTPTSQIVGVQAV
NYVVSKTQGKDPYSNISKNFAELVKGSYGKTPWPVDPEFRFRVCGTREEIPYDTSAYKKQ
ANPLIPEAGNMLLAMNEKEELLLELFPSVADRFLRGKRISEWKAAHPGEEPAPISASDGA
QAISSAAGTKGSVMAPASFAVPAPPAFSSNAASLPDDYAIRDGELSPEFWDFAIQNAE*
>SPSA8_v1_220021|ID:41145583| Phosphate ABC transporter, inner membrane subunit PstA [Spirochaetes Bin 1 SA-8]
MEFENQFEKSANRTRRHRKDKFFALSLILPGIIVLAILVLILIMLVAKGVKAFNPDFFLS
VQKPFGVEGGGIVNGILGSLLILAMASLISIPLSLIFSVTLIEKKDSRIARLLELCLSSF
QGVPSIVFGVIMYSWLVVPLKSYSALSAAIAVSFIMIPTVSATIKEVLLLVPSSYKEAAI
SLGVPRWKAMTGVIVPSAMPGIKGAVALGLARIAGETAPLLFTAFGNPFLSLDPAKPVSA
MPLIIYDYIKSPYADWHQKAWGTALLLVLMVFALTLSISARQKNAHPH*
>SPSA8_v1_220022|ID:41145584| Phosphate ABC transporter, inner membrane subunit PstC [Spirochaetes Bin 1 SA-8]
MNDRRYDSILTGAAVAGILLFMVFLATLVLQSIPAIQANGLGFVTGKEWNPVTVKFGALP
FIAGTLLTSLLALALSLPFSLAIGIVLGEYASESKISTVANTIVNLLATIPSVVYGLWGL
AVVVPLVRNIELALGVPPYGVGIASASLVLAIMIIPYLSSITREVLLNVPSSVKQGALAL
GSTRSEMITGIALPYVRSGILAGVLLSLGRALGETMAVTMLIGNATRMPTSIFSIGNTLA
SIIASQFNEAESDVHRSGMIGLALILFMITFIMNYLGRRVIQRTGVHHGI*
>SPSA8_v1_220023|ID:41145585|pstS| phosphate transporter subunit ; periplasmic-binding component of ABC superfamily [Spirochaetes Bin 1 SA-8]
MNKTTRVSVLLSMLVIFTLGMGLPVFAQKSGSNITLLGSGATFPAPFYSKIFDVYASTKG
VKVNYQAIGSSGGLKNIMDKVVDFGGSDSFVKDADYAKYSAPIVHIPTVIGAVVLTYNLP
GVTSLRMTGEVIADIYLGKISRWDDARIAELNPGAKLPKMAIMPVYRSDGSGTTFNFTAY
LAAISKEWKEKVGNANSVSWQAGQGAAQNAGVAAVVQQTPGSVGYVELAYANQNKMPVVS
IKNAAGNWIEPNLNSISKAAALALPADTRILLANTQAPEGYPICALTWIIVYKEQNYSGR
TIQQARELANLLSWIVRDGQKYANDLDYAALPEQAVKTAEAIIKSITYDGKPIL*
>SPSA8_v1_220024|ID:41145586| putative acetyl-hydrolase LipR [Spirochaetes Bin 1 SA-8]
LIFRHIETPSIRSMLFERMLWASGLKSSQEETQDFKRRDYSKSISSPNPPEYLKLRINIR
RETVEGFPVFYLSSRVRATDIQPKTILYLHGGSYLFTFTRQHWGFLTRLVEATGCTIIAP
DYPLAPKHFYAHAYLLLTALYEFIIADIDPGNLLFMGDSAGGGLALGFSMFLRDEGFPLP
EKLIMLSPWLDITLTNPAIQEVDNQDPFLNVSALKKAGLAWAHGANPRKCLISPIYGRFD
GLPELYLFIGTKDIMIADCRRLLGYCLASNVHLNYFEYENMLHVWMLLTFKEAKEAMEQI
AEIVESAGKPPVLPD*
>SPSA8_v1_230001|ID:41145587|topA| DNA topoisomerase 1 [Spirochaetes Bin 1 SA-8]
MYMTDIKKSKAVPKSGRKPAEKSKTAAKTRSKILVIVESPAKAKTIEKYLGSRYKVLASM
GHLIDLPKSRLGVDVENNFEPEYLTIRGRASVLKDLVTEAKKASAVMMASDPDREGEAIA
YLIGKYIEDKVPGVPVRRITFNEITQSAIKAAVAEPRDIELPKVDAQKARRVLDRLVGYN
LSPLLWKKVKNGLSAGRVQSVALKLICDREREVESFIPEEYWTIEALLKLHRTSLKTELA
LFEGEKPALKNEKQTQELIHRLEREKAIVSDIRSADKVIRPKPPFTTSKLQQTAANRLGF
TSKKTMQIAQHLYEGVNIGSHRQGLITYMRTDSTRISEGALQEARDWLGENFPAQLPESA
QRYSSGKQAQDAHEAIRPTSVSLTPETAAQYLSKDELKLYSLIWERFVASQMIPAVTRVT
TADISIGDGIFRTSATSFIEEGFYKVIRIAASKEERTSHVLSLAVGQELQVEKIHGIQHF
TQGPSRYTDASIIKALEELGIGRPSTYAPIISTLLERYYVTREARQLMPTTLGRMITEIL
EQFFPDILDTGFTARMEGLLDEVEEGKSDWAAQMKEFYTPFKQRVDAVMSTLPVKKGAMD
EVTDAVCEKCGKPMVKKLGKFGFFLACSGFPACKNTKSIPLAVCPKCGGDIIARKTGKGK
RKEFYGCSNYPECDFVTYDKPTDSACPKCGWFLVEKQDKVSGXX
>SPSA8_v1_230002|ID:41145588| DNA protecting protein DprA [Spirochaetes Bin 1 SA-8]
MKKSRPQTLQTSLFPAFSRKEVQPLPESRQYDILFLLAVNRMHLLRPHEKVLLVQSIFGK
EDLVSLLPLELETIILRKIDWTQWLPLRFWEQAVRDFEYIARYSIRFVSIFDAKYPPLLR
ETYRPPFGIFVRGVLPDPDKPSVAVVGTRTPTALGVNCAFEISKSLSDSGSCVVSGLARG
IDTSAHRGALRGQGRTLAVMPGGIDMIYPVSSKPLAAAILDSGGALVTEHPPGTSIQKYR
FPERNRIIAGLARSCIVVEAPEHSGALITAEFAADEGRDIFVHKKCLGSVRNAGARKLAS
EGAQSIETVQDIYADWAGNFELGERVRARQYGKVKACAGNDNHEEGCI*
>SPSA8_v1_230003|ID:41145589|xerD| Tyrosine recombinase XerD [Spirochaetes Bin 1 SA-8]

VSQLGSSYKAHLLMVRKRSSLTVEVYLRELSRLEQFLEHSGKTPLTASSEDILAYLVKRQ
SEGLSRTTMARVVASVHSLFKYLQLEGLREDDPSLLIRTPKQSRTLPEVLRPEEVDRFLA
SINPDSPNGLRDRAIFELIYSCGLRISEAASLTFDRLYLKERLLRVTGKRKKERLVPFGD
DALYWLEKYLEEGRPHLAKGKHSNLVFLNQEGNGISRKGIWKRFSGVRAAAGVHAKVHTF
RHSFATHLLAGGADLRTVQELLGHSDISTTQIYTHIEEDSLREYHKEFFTRN*
>SPSA8_v1_230004|ID:41145590|ftsZ| GTP-binding tubulin-like cell division protein [Spirochaetes Bin 1 SA-8]
MAIEIIDEANAANPTVIKVIGTGGGGSNAVNRMIASGLKNVQFIAVNTDLQDLGRSKAEI
KLGIGTKVTKGLGAGGKPELGEKAAMEDRNMIEQALRGADMVFVTSGLGGGTGTGSAPVI
AQIARDLGALTVAVVTRPFGFEGKVVNKIAAEGLERLRQAVDTVIVIPNQNLLKIVDKKT
PIKQAFLLADDVLRQGVQGISDLITLAGDINIDFADVRTVMEGQGDAIMGIGIGQGENRA
LDAASKAVNNPLLEDAHIEGAKNVLVNVTGGEDFSLIEYEQIVDLITQKASEDAHIITGF
VTDAKIGDEVRVTVIATGFGTSKPSSSSMGTSQSSLKTKSGIRMPNSDYVSPAEYEMMTQ
NSPAVTKGYSFDPANGEDELDVPTILREMKFGTEKQGARA*
>SPSA8_v1_230005|ID:41145591|ftsA| Cell division protein FtsA [Spirochaetes Bin 1 SA-8]
LCIESRRHKLADNYIASLDIGTTSTRVVVGEFNEEDNLEITGVGVAPSTGLRRGVVLNIE
ATLNAVNASVDAAELMSGCEIHEVVLGLSGANVDSMNSRGVVAVAGKGREITSEDIARVH
EAARAVSIPMDREILHVIPRTYMVDTQDNIHDPLHMIGVRLEADMHIITGAVTTTQNLEK
CVTRAGYRVEKRVLNCYAASRAVLTQEEKDLGCLLMDIGGGTTDLMVFSNGAPVYTCAIP
AGGAQVTNDISIMMSILLDNAEKLKREAACCWMEAVDPEEFIVVPGIGGRDPSEIPRRKL
CSIVQPRMEEIFLMAKEKLEKSGYLKEIRAGITLTGGTAQMEGVCELAEFIFNVPVHIGL
PPVIGGLESEYRNPAFATAVGLVMLAADEMAASALSRGKGLKQGIRPRGPAKQGSPGKSS
AGKIWNWLKDKFI*
>SPSA8_v1_230006|ID:41145592| protein of unknown function [Spirochaetes Bin 1 SA-8]
MIELKEKIQPNTAERAKPQKIEHDSASNLLERSTARRESSTPGISSRAQSESFENRVRAI
PLKPFPTKREKLYDRNAEKLAASAQRHARELERKAEQKKMNARLGIALAIILCLSLALFF
AIPEVTRITKVNVRGMQTVSETDVVSALGLSSEIHLFNANIVEMEKRISADPRIASVRIQ
RVLPDKLVVNLAERVPVAVILHTDEFGTQSLVVDREGVVFGKLDGAAQSSLLPVLSGIRF
EQFTPGQRLPSMLLPLLADLDRLEKTDASLLNAFSEIKIMKVSEDDAELVLYPVTHAIPI
RTQMKLDKAMLGEALLVLDILASRKGTEKIQEIDFRTGTIVYRVKEAQAG*
>SPSA8_v1_230007|ID:41145593| Cell division protein FtsW [Spirochaetes Bin 1 SA-8]
MKRTGFKEKSFSPSDPRTVTGAFALLLLVGFAAMWSSSMGYSLRIGKSASYFALRQAFFI
LPSVLVFVFFSLVKLETLRKYVGILTLMSLISLLLPLLPGIGVTINGGRRWVNLGFTNFQ
PSELWKPMSILYAAHMLDKKNELIKASSGEAFFPFFVISAGIVFIFFQNDFSTAILALIS
VAAVFWAAGVPFAFFIGVFMLGVISIVLMVGTSEYRMMRIVGYLIPDYDAHGINYQVQNS
IRAIMSGGFWGKGLGLGTRKLSSIPEIQSDFVFAGFVEETGLLGVSVVLACWIFIGYRVL
KTASNIAGFKAFLAVGLFSLLSFQVLFNLGVISGFLPATGIALPFFSQGGSSLIATAITA
GLIVNTIRDDGFAPLNATGGYHD*
>SPSA8_v1_230008|ID:41145594|mraY| phospho-N-acetylmuramoyl-pentapeptide transferase [Spirochaetes Bin 1 SA-8]
MLRELLLPLVKYFSPFNIFKYLTFRSAYAAVTALLLVLLFGPLVIEKLRVLKFGQSVRTE
GPQSHLAKTGTPTMGGVLIIASVVVSVILWQDVRNPLTWISLFGLVGFCFIGMADDLLKI
KKRNSDGLSPGKKLFFQFLVSGVVAVLLYYQGGQGMTKLYLPFIKQHVVDLGILWIPLAM
VYVTTWSNAVNITDGLDGLASGLMIMAIIAFSILTYVSGRADWAQYLNIPYIKGAGELTI
FNLALLGACTGFLWFNAHPAEVFMGDAGSLSLGGILGILSLIVKKEILLLLIGGVFVMEI
GSVMLQVGYFKLSKGKRLFRMAPIHHHFELSGQKETKVVVRFWILGGIFALLALSTLKIQ
*
>SPSA8_v1_230009|ID:41145595|murF| UDP-N-acetylmuramoyl-tripeptide--D-alanyl-D-alanine ligase [Spirochaetes Bin 1 SA-8]
MTDMLKQGLFSCGEAAAILGASCKGNLDSIITAVQVDSRAVVPGSAFFALMGERVDGHDF
IGKALDAGAVCIIAAENQKAKAFDAMQQCCPGWPHIPASVKDSRANPPVVPCLILVPSVL
NALQKLAREYRARMKSLVRIGVTGSSGKTTTKECIGAILAANFGTEAVAMNKGNLNSDIG
LALSMFTLKPEHRVAVFEMGMNRQGEIGELAWILEPDYAVITNIGTAHIGILGSRQAIAE
EKKKVFSRFTGKQVGFIWEDDDFRDFLREGVNGRVRSFGFSSFGNEVSIAPQGLSGWEIR
WKNQIIQFPLPGKHNCLDALAALAVADELELDPAATARGLSTVKPLFGRSQIFQGSIDLL

FDCYNANPDSMSQAIEFCDSVPARGRKVLVLGSMLELGEMEEEEHRLLGEKAYYSSAEAV
FFFGPETKASLERFSEFMEGTSAVATKAGNATRTVFHTDSMDELAGAVLSFLKPGDLVLL
KASRGRALERLADHLQSAGLVKLDSEKEDRHAS*
>SPSA8_v1_230010|ID:41145596| protein of unknown function [Spirochaetes Bin 1 SA-8]
MNRNRFWVSIFVSLVFVFGLLVQVSRFTMLSRQASRLEMEQEAIAAENRKLEAEIAVLTN
RTRTDVLAGRSNFVTIQPEEKLTIIIEPKKTDTSTDPVNTAKLPQGARHD*
>SPSA8_v1_230011|ID:41145597|mraW| S-adenosyl-dependent methyltransferase activity on membrane-located substrates [Spirochaetes Bin 1 SA-8]
MTQATETANDHESQHYHYPVMLPEILELVRSLEKSNPAILDCTIGAGGHAEAILAALPHA
HYTGIDADPEAIERAGARLSAFADRLTLINGYFDEAVARLAQDPDFRPDFILFDLGVSTH
HYTESRRGFSFQSDEPLDMRLSPEAELSAADIVNRFREDEIADILYLYGEERHSRRIARA
VVEARKIAPIKTSARLAEIIAKSVPGDYRHGRIHPATRSFQALRIRVNNELERDSAAIAG
ALGLLASGGILAVISFHSLEDRIVKQLFKQWSTEHGYTLIWKKPCSPREEEILRNPPSRS
AKLRAIQALASEGRRA*
>SPSA8_v1_230012|ID:41145598|mraZ| Protein MraZ [Spirochaetes Bin 1 SA-8]
MNLLTGEFRNTLDEKGRLSLPARLRSELPGTSLVLTQGVDKCLWLFPPLQWQELSDKLMA
STSLFQARSRLVQRRILAPAQEVEIDRLGRISIPQSLREWAGLKGECIVLGITKYIEIWN
AEEYQKYLDANESEFLQATEGIEGLTL*
>SPSA8_v1_230013|ID:41145599| conserved protein of unknown function [Spirochaetes Bin 1 SA-8]
MSESALYSSVSSQFDHVIRDPVWGDIPMPESIFELTRAQPFLIADNIRQLGPVSMVYPGA
THSRKAHSIGVYSLARRMLLALIERGQISFVSREGARSFLVAALCHDLGHYPYAHSLKEL
PLTSHEALAGDIMLEEPMRSLAGSCGADPEQVAAIIDLDRDEPHDRETAMFRRFLSGVMD
PDKIDYLTRDAFYCGVPYGIQDADFIMRRLFIVDDRLAVDERSEISVEAVLFSKYQMYRA
VYWHPLVRSATAMVKKSIVLALLGNEIKMQDLYGLDDEGFRSLVLGKDSDSFLLARRVFS
GKLLHPVAELSYDPANIVHTDLDDLEKRLGAESALAMAADLPENEIVIDIPEPINFEVEL
PVMTAQGLEPFIAHSSIFSESAVRDIRNSLRKIRIYAPKKNRDARIQELARQLLS*
>SPSA8_v1_230014|ID:41145600| Methyltransferase [Spirochaetes Bin 1 SA-8]
VNIQFLHGKSFHAARGFLNHLLEEIPGFTRIDNELISCISIPGKETAMPELPESVYWTRN
VWEEPFLLEFDSISEAARALKAIQRNWAPYPVHCSRRTMLISDALPPLPTKPKQFPFTLP
AAPMGAFTLLDEHQLLGSARCSSPFPNGEFSFIEDKEGPPSRAYQKLWEALVLAGRMPAP
GERCIDAGACPGGWTWALASLGASVTAIDRAPLDEKVAAMERVTFLKHDAFTLKPSELGP
ADWVFSDVICYPEALYRWISQWLESGLAKNFICTIKMQGPSYDRATTRLFAGIPGSRVVH
LWHNKHELTWMLLSGA*
>SPSA8_v1_230015|ID:41145601|pcrA| ATP-dependent DNA helicase PcrA [Spirochaetes Bin 1 SA-8]
MNFSKELDPEQYAAVSRTEGPVLIIAGAGSGKTRVITYRIARLLLDGVSEKSILALTFTN
KAAKEMSDRAQELVRKPLKELMVSTFHAFGAWILRHEIHRLGWKENFTIYDEQDRMSSVK
ECAREIGLAMDSIDVQDIVRFISQCKTALLPGRAASEDCYESLEVFRQAGEVYQRLFREY
RKALYIYNAVDFDDLIAMPIELFARFPDITARLRSRFSHIMIDEFQDTSLQQYEFIMAFA
GNNICAVGDDDQSIYSWRGANFGNIEKFERDHPGYFEIKLERNYRSTSTILKAANSVIAH
NVKRKKKNLWSPSDLQGVPIVCSEEKDEAEEAERIVEKIRLLRISENLSWQDFGILVRTN
SQARIIEETLMEAGVPYRTAGGPSFYQRKEVKDMIAYLRAAANPDDDVSVMRIINVPRRG
IGKVGLEKMISYARSHNQSLHDALEALARSNHPLCQQHATRDAIEFFEYLANARSAMLSR
KIPVSAQLREMVKEIGYWRYLLEEHKSDDKVAGWKYRNIELLIASIERWERDPDNFDTSL
FAYLARISLITRDDPEDTEGKVSLLTIHSAKGLEYEIVFLPGCEEGIMPHARSVEEGDGD
IEEERRLFYVALTRARKRLYLSRCLERKHYSQILTPAPSPFLEELPKELVKDEDGSEKDG
RTEEEIQKELFARFKAKFASRAG*
>SPSA8_v1_230016|ID:41145602| Small multi-drug export protein [Spirochaetes Bin 1 SA-8]
MTTTTLLLTALLALAPISELRGAIPFAVLRGISLPAAVLISIGFNALVPLVVFIFLSTLH
KFLYRWNLYHSFFDRVVEKTRIKVHPKVEKYGYLGLLLFVAIPLPVTGAWTGALGAWILG
MQKRKAVPAIIAGVCIAGIIVGILVFVFGAGTRTIFFKQF*
>SPSA8_v1_230017|ID:41145603|miaA| tRNA dimethylallyltransferase 1 [Spirochaetes Bin 1 SA-8]
LIRLLNTNSLPTDIIIVTGPTASGKTELLSQLFAKGAPDFFQSLSGKITAPYDHAEIISA
DSMQAYRGMDIGTAKPDAALISALPHHLIDIKDPDEQYTAGEFVRLADSLIGGMRSRILP
IISGGTGFYLKNFIIGPPPAPPSDPAVRKQVQEDLRRFGISTLRDELLNADPESYARIAY

NDVYRLTRALEIIRASGKPLSAFAPSESPRHGYRFLVLGVERPREELRERIERRVEAMFA
AGLPAEVARLRKMGYNADCPGMQAIGYREFFELEDSGMDAIMAAITLHTRQYAKRQMTFF
KSLPGIQWIQPEASQLAGKIRDFLMFRL*
>SPSA8_v1_230018|ID:41145604|rpoZ| DNA-directed RNA polymerase subunit omega [Spirochaetes Bin 1 SA-8]
MIMPLEELINYNKNAYELTVAINRRAFHLAVMKTPDVEKNNGKVVSLATRHIFDKTVEYK
FITD*
>SPSA8_v1_230019|ID:41145605| protein of unknown function [Spirochaetes Bin 1 SA-8]
MSSTSSDGSKSESAMIESTGTPRIVSIEILGVSFSIKTDDDPEYIQGLVSELKKRLSDIS
RQMKIVDPLKLAIITNLLTLDEMHQSKEQKQTVSRDYEASSLLQDIDKRLGELGL*
>SPSA8_v1_230020|ID:41145606| protein of unknown function [Spirochaetes Bin 1 SA-8]
MVSLEQIRALEARVEKAVVLIDKLRRENAELEQKLIDATRAEEQVRNAYSELERKTALSV
RLASESSSRIEELTSKAREAEERAKEAELRAAESAERAAAFERKANAAEAEVASYRDRAL
AAEHRAAELESHAEELRKEQARIEDGLVHALEKLDAFEDLVMGISVQKSTDLEESVQSRD
EKVEAVDESQSTLVSQDATFHGADNELDIF*
>SPSA8_v1_230021|ID:41145607|rplT| 50S ribosomal subunit protein L20 [Spirochaetes Bin 1 SA-8]
MPRAVDGTRRKDHRKKILKIAKGYWGRRHSNYKVAKDAVAKALSDAYKDRRDKKGVFRTL
WIARINAACRAQDITYSRFIEGLGKAGITLDRKLLADLAVRDPAAFTAVVDKAKSALQA*
>SPSA8_v1_230022|ID:41145608|rpmI| 50S ribosomal subunit protein L35 [Spirochaetes Bin 1 SA-8]
MPKMKTKKAASKRFTLTASGKVKYKKMNLRHILTKKTTKRKRNLRKPGFVAAGPVHQIKK
KLLPNG*
>SPSA8_v1_230023|ID:41145609|infC| protein chain initiation factor IF-3 [Spirochaetes Bin 1 SA-8]
LADKDLRINEQIRVREVRLIDEKGEQRGIVSTLEALRIAREVGLDLVEVAPQSVPPVCKL
LNYGKFKFEQEKKIKDARKHTKPTEIKEIRMQPKIAGHDLDFKSEHIRDFLEQGYKVKVT
IRFRGRELAHTEIGEEILKKILAKLEGYYVMEKLPLMEGRFMSMVLQPKASIIKKQQAEE
SPASGVTAE*
>SPSA8_v1_230024|ID:41145610|fba| Fructose-bisphosphate aldolase [Spirochaetes Bin 1 SA-8]
MTSYKELGLVNTVELFKKAVEGGYAIPAYNFNNMEQLQAIIQACVETKSPVILQVSAGAR
KYANATLLRNMAKGAVEYAKELGYPIPIVLHLDHGDSFELCKDCIESGFSSVMIDGSHLP
YEENVALTRKVCEYAHSQPDYVSVEGELGVLAGVEDDVSAEHSHYTRPEEVEDFVKKTGV
DSLAISIGTSHGRAKFKPEQCTRNADGVLIPPPLRFDILEEIEKRIPGFPIVLHGSSSVP
MQYVRMIEMYGGKLSDSVGIPEEQLRKAAKSAVCKINIDSDGRLAMTALIRKVLAEKPDE
FDPRKYLGPARDELKKLYMHKNLEVLGSAGKA*
>SPSA8_v1_230025|ID:41145611| protein of unknown function [Spirochaetes Bin 1 SA-8]
MKGVLADIKSKRLLACLLLFLAIMALVSCSSEKTNDNGRLHFLFDDAFSQLFPDLTARIE
KAVQAGSAGQSGPERGRVSPIPSLKGSISNLISQWTQKGEPATIIASPLAQKLILDDGRL
LGSAVLVVPFGDVVYDYESSYAKVAENLAGRLNSIQKKEKRQAVCGVLFQENFMRQASAL
ARLEGVLKQKAEGASLIIKKAALDSDTVDTDSRFTAQFEAILKEKPDVLLIAIDHPELVQ
RWSRDSQQDKGKLFFAADASSWGGFSFDTRLFNLTILGNEAVMADRVLQKARSIVSGQQV
SESPELVALPIRTTWFRK*
>SPSA8_v1_230026|ID:41145612| Hydrolase, TatD family (fragment) [Spirochaetes Bin 1 SA-8]
VHSRDAFEDTLSVLKTSAASLSVIIHCFSYSPAEAEQLLRQGFYLSFAGNLSYRNASGLR
EALRIVPDSQLLLETDAPYLNPEPFRGKHASSADIVRTYEIAASLRNSTSEKLAGIVQQN
AETIFGTMLF*
>SPSA8_v1_240001|ID:41145613| conserved protein of unknown function [Spirochaetes Bin 1 SA-8]
MSVKRDFGLRLNRLSQQERNKLISYINIKLASMGLSVYSKEGTGFVELASDMLESFRQKD
RILASYLPPVDQRIQDFLDAYFSDLGLASMPRIPSKTLVLDRYGMARELSLPPDGHKHVS
PTLTSYRIRNGVLHNPTNDKRTTEGVFHIAEGGLPVPADKKAVPKLVFARLFEAAFNPPA
EMLDIPFTAGEGEKARAFLSVLLRPIVRPEVQGYCEERSMEVRFFVPGSLAASLDFVESI
FGNSGDPFIPENDAALDPLRWTGTTGLIVLAPHLTLLTKKELGLPHYDQATARQRRDGMC
WKEPTERYNDGKPFKICARDTRGIIVSVIADNYFGYSKKEIKSHISYSANLLGLAEEEHA
GGALVFPSYNHGVRFIPDTNLNSRGHSIHDVFELLRGRIEVKPEGYAVDLTYPNIIYLPE
NAVLSLEDQKAHWVWEGREESLRILPGEVYVHPTGYRIHMERHPVSGAWRLIGTAAEALL
CHKPCTVSGGGKSEIAKPITDAISYSPITIADFFEDFAAVKKIIEKDYGNRFRDDSENHG
KDARPILSPKRSLGSVIKLLSPSPLFTDEYNEWLKSIPERIKSLVFLVKRFYTPDWGDDW

LSHFSVDAVNGTTGNILKFQGRPILGSYLRVGRAQNGMQRTFKLRQDFMPAEKRQWEDDI
TASVVVPSKNLKNLPDWANRHPSLKFCKNXX
>SPSA8_v1_240002|ID:41145614|hutH| Histidine ammonia-lyase [Spirochaetes Bin 1 SA-8]
METVVLDGCSLTLDALVRIAREKAFVALDETALQRMASSRSTVESCVEERLVRYGITTGF
GKFCNVIISKEDNAILQKNLIMSHACGQGEPLPEDVVRAIMALRANALAVGHSGIRPRTV
QHLVDVLNAGIVPVIPEKGSLGASGDLAPLAHMTLVLLGMGEAFYRGERMAGSKALALAG
LKPVTLEAKEGLALINGTQVMTAIGALAVYDARNLVQTANLVSSMTIQALRGITDAFDPR
IHELRRQSGQIQCAADLRRLLENSRLTSRQGELRVQDPYTLRCIPQVHGASIDAIDYVQR
CIENELNAVTDNPLIFPNPESDNQQKGSSKHKASGHSSEHVHGQDPGDILSGGNFHGQPI
ALAMDFLGIACAELANISERRLERLVNPALSEGLPAFLTTSGGINSGFMIVQYSAAALVS
ENKVLAHPASVDSIPSSANQEDHVSMGTIAARKARNIIWNAQRVLGMELCAACQALDLRA
IQIGTAETASLLSPRTAEAYTTVRSVVSMVDSDRIMYPDLDAVASLVADGSFSSGMSEAV
P*
>SPSA8_v1_240003|ID:41145615| conserved membrane protein of unknown function [Spirochaetes Bin 1 SA-8]
MQDRMNKGKGSALPGFGIAVIWGLTFLSIKVAIQEFQPMTLALMRFIMATALVPGIALLT
RTSLKVARRDLPILAASGFVGISLYFFFENNGILRLSASESSLIVGTIPVLTLLAEIFLY
REKPRAKVVLGILFSFLGVALIVLRSESARSSSFDGYLYMAGAAISWVVYSFLTKPVSNK
YPLLSITFWQIFFGTLGCLPFALLENKSILHVSGAAWLNVVFLGVFGSAIGYWLYVIMLD
NLGASRSSVFINLIPVVSVIASFFVLGERLAPLQLLGGAAALAGVYLATARQ*
>SPSA8_v1_240004|ID:41145616| Sodium/hydrogen exchanger [Spirochaetes Bin 1 SA-8]
VKIRKILILLIFLVTASALFAEGTEAPSLTEQMTTIVLQLAIVIFAVRAGGALARKINLP
SVIGELLSGVIIGPYALGAIPLPGFPGGLFQASEGFAVSPVLYAFATVASIILLFSSGLE
TDIDMFLHYSVAGGIVGLGGVAASYTLGAGIGALLTHQSFFAPGSMFLGIMSTATSVGIT
ARILSDKKKMDSPEGVTILAAAVFDDVIGIVLLAIVMGVVAVLTGHKSGGLSPWGITSIA
LKAFGIWLGFTALGLIFGKKIAELLKKMGGEAMYPVLALGLALLLAGFFEMQGLAMIIGA
YIVGISLSKTDIAYLIQDKLKPLYDFFVPIFFAAMGMLVDVRQMLNPAVLVFGAIYTLMA
VLAKVLGCGIPALFLGFNMRGAVRIGTGMVPRGEVALIIAGIGLSAGILEPSIFGVAILM
TMVTSIIAPPLLSMVLTAGGPGTIKPVKGSVTETISAEFPTRELAELVSTTFLRELEREG
FFVQLMSIRDDISHIRKGDIAISLITGKNFITIETAPEDASFVRATLHETLVKLDANFDR
LKDSFDPEKLRSELHVDSGRKDTTFRKILDPACVTTELKGSTKNEVIEELVGLLDKAGKV
TSREQLLKDILEREARMSTGMEHGIALPHARTTGIKSQTLAVGIHKNGVDFQTIDGTMCH
IIALIATPADDEAPHMQVLASLGAILGDDAARMALKNALSPEEVYSILKKA*
>SPSA8_v1_240005|ID:41145617| Pseudouridine synthase [Spirochaetes Bin 1 SA-8]
MMLTVLFENSDILIIDKPAGLASQPGERVGASVVTLAESQFGFKPYPVHRLDKETAGCMM
LAKSSRAASEWSERLSERKLRKVYWAVSFSTPSRDMGVFSDSLDYQGKSQKAETFYRVLR
KFSTENMPKNAAGSAGSEALRMSLLELELGSGRMHQIRRHLALHGLPILGDDKYGDFKLN
KRIKSLFGVKNLLLWARQLEIPGIGMAVSAQPAHFSRFFAVWDDQEPGQTADSASSDVWH
*
>SPSA8_v1_240006|ID:41145618| protein of unknown function [Spirochaetes Bin 1 SA-8]
MAIEPFKLSHGALWLIPDGRVIKIQGFHDSWLRAHGALASGAKNTVEFVQKSGWISAVLH
DQGFCELIIRSRNEDRLRQAVWNILSVNQGILNKVVIMVVGLEGCLMLGKAEIESREAFE
RALDSDVFS*
>SPSA8_v1_240007|ID:41145619| conserved protein of unknown function [Spirochaetes Bin 1 SA-8]
MEGAGLFIGTCGYSYEEWRDAFYPKDLPKQGFLRFYSLIFPFVELDFSWYKMPNPRTLEM
LANQTTPGFLFSIKAHRSLTHEPSDEWQRDAETFLEAVYPLHNRRKLAGILIQLPYHFSY
TAENRKYLAELCKILESVPLVVEFRNSQWYLPRVYEELSSRSITLAMIDRPDLEGLPPET
SIMTTRTVYYRLHGRNSQQWWNGDVTSRYDYEYSEKELKEKARIIHALKSKADRIFVAFN
NHARGNAPSNAKLLMNLLKKLEI*
>SPSA8_v1_240008|ID:41145620| conserved membrane protein of unknown function [Spirochaetes Bin 1 SA-8]
MLISILARILSVITSLYMILCAIRVFMSWAPGLQLGRLDYYLARIVDPYLSIFSRMTLFR
TRQFDFSPIIALAVLSVANNLFSTLALTGRLTLGFAASLILGAAWSAVSFVLSFLTACVL
IRIVVYLLKFNSVHPVWMVVDSIINPVLFQINKLIYRGRVVDYLQGLITGFLVLLLFRAG
LGALIRIVSGLLLALPF*
>SPSA8_v1_240009|ID:41145621| conserved protein of unknown function [Spirochaetes Bin 1 SA-8]

MLTPKTRSFLSGLAAKSPVLMHLGKGGASEAVTAQLETLLAHHELVKLKFIDFKGEKKEI
AESLASSTHAELVRVIGNTAVFFRQNPDPDKRKVIVP*
>SPSA8_v1_240010|ID:41145622| Major facilitator superfamily MFS_1 [Spirochaetes Bin 1 SA-8]
MPNAISPGKLQKARSKFYYFNFLNSFSFVFVSGSFLTLFAIRLGASKAIVGLLNAVAYLT
FFLMPAGKKLVQKHPIVKVFGWGWVSRYIALLPVLFAPLLSAKGHNGAALGLLVAGTTGF
AISRGVALIGNNPVVGFLASGGGEKPRSDRGQFIVNTSIINSLASMVSGLLIAIFLGEQA
SPWSYALGVGIGIVTGLAGCILLLQTPEPTNYKPETKNTLLSTTLESIKDPAFKRFILIF
MVLSFASGMGRSFLPVYAKDAFHQADDAVMVYSLLASLGAVAMGLLSRLVVDRLGSKPLF
IIFSALGLASFIPMAIIPSGRSVLASGTVAALFLSFIHFLSAFGFSGEENAGQTYYFSLV
PRNKTLDLSVVYYIAYGMGGALGSGFGGVLLDLFDSFGLSQNNSFRLFYALLSLMLIAAL
SAMRKLKRLGSASVSESIGVMFSLRDLKAFDLLARLDRSETPADEIRLIHELGQSPTRLS
QKELIEYLHSPRFEVRMEALLALEAMPMLGHAATQALVRELEANTYTTAYVAARILGRHR
IQAAIPVLSKAAMAEDYMLQGSAMVALAKIGDINSIPLIETILQNSRNPRVKISAAYALE
LLNARSSLPVLASTLRHDDPPAFVSDEITLAMASILGVMKEFYPLYSAFIEDESDGIAQL
KSTAQDIIVDVPTLQRWNAAVDALFSKTAPDGKQLSALILDASIDSQIDIILAEALLDPR
LCYRGLRFLAASYPLFVKRI*
>SPSA8_v1_240011|ID:41145623| MATE efflux family protein [Spirochaetes Bin 1 SA-8]
MKDLTKGNETTQLILFALPMLLGNIFQQFYNMVDSFVVGRFVGTTALAAVGTSFPIIFFM
LSLIMGVTMGSTVLISQFFGARDHEKLKKVISTSYIFLFLAGTVMSFIGVFSSGFILRAM
SVPADVMPEAAAYLRIIFAGMLATFGYNGVAAMLRGLGDSKTPLYLLIASTLMNIVLDLT
FVLVFHWGVSGVAWATIISQAVSFIGAIVLLARRKEYVAPDFRHLEWNKEIFSKMIKIGL
PTGIQQTMVSLGMMVLSRIVNEFGPATMAAYTAAARIDSIASLPAMNLSQAITTFTGQNI
GAGKAERVKRGHLSAIAVNTAISLVITIAVLLFSKNLMSLFTTNTEVIAIGSEYLVIVGI
FYVLFGIMFINNGVMRGAGDVFIPMINTLLALWLVRLPCALLFTRVFKMGTSGIWWSIPA
GWGIGFVFSTWYYLTGKWKTKAVVKAKREVESIVEP*
>SPSA8_v1_240012|ID:41145624| MazG family protein [Spirochaetes Bin 1 SA-8]
MNAIKSDLARLAGSSHETILVSAEKAKEAFERLYTIVARLRAEDGCPWDREQTPASIRNN
IIEEAYELVEAISDHDPPHVKEEVGDLFMLASMVSWMYEQNSVFTASEALEEVSQKLIRR
HPHVFGKSDADTPDKVVEQWNLIKEKVEGKRKKDSMLDDVPRHLPPMERAYKLQKKAAKA
GFDWQKTQGKTENPPDGLWEKIQEEIQETREALKTGSEEQVEEEIGDLLFSLVNLARHHR
VEPSLALQKANEKFTRRFIHVEKAMKKAQMPMDAEHLQHMDLFWEEAKSQEQPTRKRENQ
*
>SPSA8_v1_240013|ID:41145625| membrane protein of unknown function [Spirochaetes Bin 1 SA-8]
VVQEDSLHGQERAELFRKTGLLFAGLLSLYVLIIYIGQIFKLYEKQPLNFDMFLLLIMAA
VIIAFIASALHEHLNWLQPVLLLFITPLPMFRFGESMFSLGAFSAATILLFRLGFFEKKR
LFKFLIIMSYYYFCQLLVGITIGAPLLSLVMPLIFMTIFIMFLLLVFREKWIIYIQNEKP
ELSLSQLGITKTEAEYLRETLTGKTFKEIAIDMQVKESTVRNTLARVYKKMGVVDKASLA
TKCEKYLIID*
>SPSA8_v1_240014|ID:41145626| conserved membrane protein of unknown function [Spirochaetes Bin 1 SA-8]
LRVKISCFKFTILTACLILVALLVPGTTYRSLPSFFGIDKLAHLGLFFLFSLSYSLEYKR
AYTRLPAVSHAFGLFLVFVLISELLQLLTSSRHFELTDMVFDIIGASAAYAAVALIGSGQ
KR*
>SPSA8_v1_240015|ID:41145627|ydbK| putative 2-oxoacid-flavodoxin fused oxidoreductase:conserved protein; 4Fe-4S cluster binding protein [Spirochaetes Bin 1 SA-8]
MADSKKMVMVDGNAAAAYVAHACSEVIAIYPITPSSPMGELSDEFSAMGKKNLWGTVPQV
VEMQSEAGAAGAVHGALTTGALSTTFTASQGLLLMIPNMFKIAGEATSTVFHIAARAVAA
QALSIFGDHQDVMAARQTGWALLASNNIQEVMDMALVAHSATLKTRVPFLHFFDGFRTSH
EVQKIEEIPFETMKKMIPEDLVRAHRSRGLSPEHPEIRGTSQNPDVYFAGREAVNKFYAA
TPGIVEETMNQFAQLTGRAYKLFEYVGAPDAERIIIVMGSGADTVDETVTYLASKGEKVG
AIKVRLFRPFSVEHFINAIPSTVKSIAVLDRTKEPGSIGEPLYEDVRTAIGEAMGSGKSH
FKGWPTVVGGRYGLGSFEFTPAMVKGVLDNLAQAEPKNHFTVGIIDDVTFTNLSYDENFH
LPAEGVVECLFYGLGSDGTVGANKNSIKIIGDETSNSAQGYFVYDSKKAGTYTISHLRFG
PKLIKKPYLITKADFIACHKFSFLEKLDMLVNAKEGGTFLLNSPFPADKVWDELPLEVQN
HIINKKLKFYVIDAMTIAEKAGMGTRINTIMQTAFFKISGVLPADEAIRLIKKYTEKTYH

RKGADIVEKNLAAIDLALNATEEVKIGKADSKLRIQPPVPASAPAFVRETLGEIIAARGD
KIPVSKLPEDGTFPTGTTQYEKRNIAEKIPVWNQEICIQCGNCTMVCPHAVIRLKAYEPK
YLEKAPKTFKSADGKGKELAGLKATLQVAPEDCTGCGACVNICPVNDKAVPGRKAINLEH
QLPLRESEKENWEYFLSIPDTPAKYLNLNLPKGIGMKRPLFEFSGACAGCGETPYVKLMS
QLFGDRAVIANATGCSSIYGGNLPTTPYTKGDDGRGPAWSNSLFEDAAEFGMGMRLTADK
QTEYAREILTSVKGRGVDTALAERILNNPMESDEDIEVQRTAVAELKKALAANNEEWAKE
LVNVSESLVKRSVWILGGDGWAYDIGFGGLDHVIASGKNVNLLVLDTEVYSNTGGQASKS
TPIGAIAKFATSGKETMKKDLGMIAMSYGYVYVAQIAMGANMSQTIKAFREAESYDGPSV
IIAYSHCINQGIDMMKGMNQQKIAVESGVWPLYRYDPRLKAQGKNPFQLDSKEPDYSKLD
TYMYAEVRFKSLRDANPERAKMLLEKQRVLIERRYKEYRYIADRPF*
>SPSA8_v1_240016|ID:41145628| conserved protein of unknown function [Spirochaetes Bin 1 SA-8]
MKLLVLAAGMGSRFGGIKQMAPVGPAGETLLEFNLYNALKAGFSQAVFLIKREMEADFRK
LIAERLPLNLEYEFAYQDVLASVPESVKSKLIPYLASQGRIKPWGTGHALLCAREFLASS
PFATINADDFYGAEAFKVTADFLQRGAKTAFCLPVYQLGRVLSRNGSVSRAVCDLDSSSM
LKEIVEHTKVLQKNGQILSVDADGKERVLAPDSPVSMNLWGLTPAIFSYAEALFADFIAN
ESNWAKNEFFLPYIIRSMLERGTAAVKALPVEDACFGLTNPDDLSEVQSNLAALSRNGSF
PTPLWKQGGIGA*
>SPSA8_v1_240017|ID:41145629|glmU| Bifunctional protein glmU [Spirochaetes Bin 1 SA-8]
VRDGLYRYTGLPELFQQDDFAHLKLFKSPGYPWDALKNLPDYLAGLFETLAGGSNPGNSR
IWTQEHVVIEPGAFLEGPIFLGEGTIVQAGAYIRGPAWIGRGCEIRQGAYLRGMILAGDG
CVLGHASEFKYCILFDGVQAPHFNYVGDSILGNHAHIGAGVILSNVRLDKKPIRVQLIGE
GYQGARIDTGLEKLGGILGDACEVGCNAVLNPGTILGAACRVAPVSSIRGTWKEGSILPI
L*
>SPSA8_v1_240018|ID:41145630| putative Purine phosphorylase family 1 [Spirochaetes Bin 1 SA-8]
MPVPRFREKFTSKPFFNPSDFLGYMKKIGALSDRPAPRGVILGYQNSLFKYVVENYKTSA
AEGYFGSYLRYIDEPFAGSGQVAIAANFGIGAPAAAFIIEELIAWGVQEFISMGMAGSLR
KDLPPGSLVLCTSAFRDEGTSHHYVESDELAYPDPELTLRLEKALRTRNLDFSKGPTWTT
DAIYRETPAEVVGYRDLGALVVEMEASALFTIARFRNVPIASCFSISDTLAELAWRPEFH
SDTTREGLEKLFHAAVDALMA*
>SPSA8_v1_240019|ID:41145631|sudB| Sulfide dehydrogenase subunit beta [Spirochaetes Bin 1 SA-8]
MKTEHLIVSKRQLSEEVYRIEVLAPLVARERKAGQFVVVMYDEDYSERIPLTIADADPEK
GTVTLIFQTVGASTHKLALKEPGDSIVLLGPLGNPTHIEKFGWAVCVGGGIGLAPLYPIV
RALKEAGNRVTVISGARTADLLIMQDEMNAIADENIIVTDDGTAGRKALVTVPLAELCAA
TPPPDIVVAIGPPIMMKFCAETTRPLGIKTLASLNTIMIDGTGMCGGCRVSVGGATKFVC
VDGPEFDAHQVDWGQMMSRLGAYKDIEKEAHEKCHLEMSIKQLEKERS*
>SPSA8_v1_240020|ID:41145632| Glutamate synthase (NADPH), homotetrameric (fragment) [Spirochaetes Bin 1 SA-8]
VPSGNVHQAVGERTIMSHMETSFYQQAAQAELEKIRSTPLTPRARMAIPVQDMPCQDPEV
RRHNMHEVAAGYTREQAILEAERCLQCKNAPCIAGCPVKIDIPGFIRKMKGGDFEGAGQV
IKQTNLLPSICGRVCPQEKQCQLPCTVGKALGGVDKAVQIGRLERFVADYEREHGLQTVP
HAGPASGRRVAIVGAXX
>SPSA8_v1_250001|ID:41145633| membrane protein of unknown function [Spirochaetes Bin 1 SA-8]
MNHPWYIVPVAAIFTSFVVQVMNFLIELAEYSIFGFSVLIFGLVSLFVIDTWPWLCVALT
FGGSFLVFGIAGLIRASGITGKK*
>SPSA8_v1_250002|ID:41145634| Transcriptional regulators, marR/emrR family (modular protein) [Spirochaetes Bin 1 SA-8]
MKSRTGAKDSLPDPDKIIHERSRLRILVFLASSLSSAVGFTELKEGLGMTAGNLSTQIST
LEEAGYIRVEKRFLGKKPNTSIMLTTEGKTALETYIADIEILMTAARKQEGVLKEAQH*
>SPSA8_v1_250003|ID:41145635| Uncharacterized ABC transporter ATP-binding protein TM_0352 [Spirochaetes Bin 1 SA-8]
MESILSLVNVKKIYRKGDNEVAALAGINIEFERQEFCAIVGPSGSGKTTLLNIIGCLDRP
TEGSVTYLSKPLASLDEKSLSAYRREHISFIFQSFNLIPVLTVVENVELPLEIEGKTAKT
DVRKKALAMLEKVGLSDKANRYPRELSGGQEQRVAIARALVKDPVVVLADEPTANLDSHN
AEEIVRLMKKINSDLHTTFIFSTHDRMIMQEAGRIIELHDGAVASDTRRS*

>SPSA8_v1_250004|ID:41145636| conserved membrane protein of unknown function [Spirochaetes Bin 1 SA-8]
MNRTLLSIASRNVVRYGKRTVITAIVLMFGLACFIFFDSLLAGMDRMTIDAMTDYTASSI
IIRTAEYQKNHRGLPLDYGIDAPEKLIEDLKQTAPEASGITPRALFAGNASNRIESIPIL
GTVVDPFRDATVFRIRDAVKQGSWLSPGIEEKTAVLGITVAQDLGLSVGDYFLLSARTTD
EATNADEFKIVGIVDVPAPEINQGGVFISFDNAREFLGSNLPVTEIDIALPRQPSIDAGM
KKAGEAALKIAQRHPDLATISIADTAKDYMAMRAMKSKFSSIVILVVLLIAGVGIVNTIL
MSIYSRIKEIGVLRAYGMEARHIRRLFSLEGIMIGTVGSLGGALLGALLVWWASTSGIPL
DMFLGKISVGSIPLAGTLYGEWHIGTFIFSIAFGILASWFSARIPAKKAASLEVTEALRF
I*
>SPSA8_v1_250005|ID:41145637| conserved membrane protein of unknown function [Spirochaetes Bin 1 SA-8]
MKVFSMAIRNLQRNRRRSLLAASSVFLSIFLVIVLQGFMDGFLDSLIRNYTKNETGHINI
TTAEYRERARFMPVDTFLEDYAALSDAIRTELGSIGNTAVIAPRIRFGVLLSSGNLTRQA
LALAGDPEIEKKLLMLDKRLLPGGQYCDSPGTVIIGEKLANDLGLSTGDAIKVVAQKADG
GIGFKKLKISGLFRTNVNSLDESVFQMSLNDAQSLLAMGNGTQQLSIMLPASENSASRAA
SLSRQLESKGFAGLSVLPWTAIGEYPKIVAMAGAVYFSMYIVIALLGAFIIANIMTMVVL
ERKREIGILMSMGMKKSNLLSLFLLEGSMLGFIGAVGGSIFGLAVTGIFSKIGFDLTSSM
AGFSWPLDNIIHTRFSIGAGIAGITLGTAVAAIVSWLPSWRAANLPPVEAIRSI*
>SPSA8_v1_250006|ID:41145638| putative Negative regulator of sigma E activity [Spirochaetes Bin 1 SA-8]
MKRESFVMLACFVFFMMTGMTYGQSAQSIIEKVEANQTYQSISYEGTMEIISGSRIMTKT
MKAWTVGSDKAFIEFTNPEDRGVRMLKLGKNLWMYFPSEKDTIKITGALLQQGMMGSNIS
YEDMMEPDILKSKYEASISGEETINDRSCVILTLKALSSAVAYSKRVIWVDKEHFFAMKT
ELYSRGGQLLKTISTLSTEKIGQRYFPIKIEVIDGLKKNSKTVFSMRSVSIDIKIDESIF
SLKALSK*
>SPSA8_v1_250007|ID:41145639| protein of unknown function [Spirochaetes Bin 1 SA-8]
MKKAFLTAAIALAAIANSFSQEISFRLKLEQAASADFSMSYFTSIDFGGSSSASLALKMT
QGAFSMEGSGSGTLLYGTNAENLLAAKALGKNQFLEIIIPDLSVITLDLRLSTFFASFAS
GNLKLEAGLSQINWGTGKVFSPADIFARYRRVGLSTEREADILLRAFWYSGPTSIAEAVF
VPYLPYLHEKSGLAFGARFYTALFDTIGAGLQAAWFSAHGLEPAAAVMAMEAQTDLWFFT
PSLEAKLSIPFDAAQHPVWQMMAGLTIPFGSVSAFAEYLYDAAGVFHHSVYASLSLKADE
WITLSLPILLYPENGFFQGGVSTAGVQLLGAQMTCSALISHFAPGAFNIQFSAYLTKKF*
>SPSA8_v1_250008|ID:41145640|ileS| Isoleucine--tRNA ligase [Spirochaetes Bin 1 SA-8]
VYKNVDPKVSFPKLEEDIVRFWEENKTFQKSVSEREGQPDFVFYDGPPFATGLPHFGHFV
PGTIKDIIPRYKTMKGFHVERRFGWDCHGLPVENLIEKELGLNSKTDIETFGVANFNEAC
RSSVLRYVNEWRAIVTRLGRWVDFDNDYKTMDPDYMESIWWVVKALWDKKLIYEGHYILP
YCPRCSTVLSNHELSLGGYKDDHDPAITIRFKIKKAPAGLEDLADGSTYLVAWTTTPWTL
PSNLALALGPDIDYVCVIDGNERYILAESRLGAYYKDSASLTLAWKKKGSELAGLTYEPL
FPYFADLEKEGAFVTVTGAHVTTEDGTGIVHTAPGFGEDDYQVLKGTGIPTVCPIDAECK
FTDEVPDYKGIFVKDSDKAIIDRLKKEGRLVKREQILHAYPHCWRCDSPLIYRAISSWFV
KIDPIKQKMLQANKKVYWVPAHIRDGRFGKWLENARDWAISRNRYWGNPLPIWKCDTCGE
TVCIGSRKELEEKSGVYPQDLHKHFIDGITFPCSCGGTMKRIPEVLDCWFESGSMPYAQS
HYPYENKERFEQDFPADFICEGLDQTRGWFYTLTILAAALFDSPAFKACVVNGLVLAEDG
KKMSKSLRNYTDPMEVMNKFGADALRIFLMNSAVIKAEDLCYSDEGVKEVLKSVIIPIWN
AYGFFVTYANIDQVQPSDPLPGSLANTLDAWILSVCEKMVLEVTEGLEAYDMQAAIAPIL
EFIDSLNNWFIRRSRRRFWKSENDADKAEAYNTLHRVLKRLVLVSAPIMPFITEAVYQNL
KTAGEPESIHLCAWPEYDERFRNPALERDMASVRHAVSMGRALRVANDLKIRQPLASAQL
VTKDETERSVLARMEEILRDELNVKSILLKDNEEDLVEYSAKANFRVLGKELGKDMKAAA
AEIEKLSAQQIIQLINGQTIQLAVGERTVSLTKDGVDIRRTEKAGLKVLNEGTLTVALDT
TITKDLLNEGYVRDLIRGIQNLRKESGLEVTDRIHLAVSGDEDLAEAFRQFHALVAGETL
ASAAEWKPDLGSEAVSIEAGEKTWNAAIKKA*
>SPSA8_v1_250009|ID:41145641| conserved membrane protein of unknown function [Spirochaetes Bin 1 SA-8]
MFQSRNEQLNNSRDFKLFILASVFMGLGACVNSSXFNNYLHDXYALDMVRRTFLEIPREM
PGFLVSLFVGXLAALGDIRIAGLAQITASLGMLALGWIPPNYVLMLGMLFIYSSGTHVYM
PLGNSIGMSFSETGNEGRTLGKIQASTTLALVLGAGALLLAFKYLNLGYRLAFTLGSLCY
AVSGLFLFSLTPRPKAKNPTRFVFSPAYSRFYLLSILYGARKQLFLTFGPWMLVDLFKQP

VATMSLLFFVVSTGNVIMKPAVGRWTDRFGPARILLAEALFTIVICLLYAFSPLLLPWKI
ALVVVCLCYIADQMADSVSMTRAIYVKNIVKKPEDLSPTLSLGISIDHIISMVLPVAGSM
LWLKSGSRGYTWIFLGGAVVALINVAVSLGIAKKGKSR*
>SPSA8_v1_250010|ID:41145642| exported protein of unknown function [Spirochaetes Bin 1 SA-8]
MHKGRNLFVVLMTIAALLLASCASNPETPKPENPPEPTTTTTVAAPTPEQTPVTPVTTVA
AAPLEAPKPSVSEVELRNLFGQANALRSDAQKLQLDELVPEEFASAETAFTKAKTAYDKA
LDAALFDGVAAYPVKGLLEESITQWEAVIAKGLPLRSDAEQEKARDMRFMAMQVDAPALA
EERFQGAEQLLGQADAFARVEDFELAIPAYKQAAAAFDVSAEMAKANAYKQKIEANDYAR
FNPNYFEMAENKYKMEADYWDSGSLEDLVNGADVLREANYYYDYVIKSGAEYRSFEGKDA
ALAAQAKALGIKADVNAPDEYSSANDILNEGLANQQAGNYESAYLWFRDAVPAFDAAHDA
SLALQAQNELVIAEAAERIEAAKAKSDEAEIDNNPYLAAADESLAKARALYSEKHFDDSI
MDANEAVNFAGMSDTFVDKTILEREEEAARLLAEAKAAADPAMADARNRIVWANNNNIKA
DYPNVHKQASASMTAAEMAYDNERYVPAKALAEEVSSVLSDDFQAKVLADRKAREAEKAR
IDEIEKAKEVASVALKNAEARMAWANENKIKADYPDEYNAASSAMIAAYTAFGNENFNLA
TQKAHEVSDILSDDFKASVQAARDAEKARLEQLAKDRAAADPALADARNRIVWANNNNIK
ADYPAEYKQASSAMTAAEMAYDNERYVPAKALAEEVSGILSDDFQAQVLAERQAKEDEKA
RLAQLAIDKAAADPAMADARNRIVWANNNSIKADYPAEYKQASSAMTAAEMAYDNERYVP
AKALAEEVSGILSDDFMAKALAKKNEPEQTPPTPPTAPAQPSQPAQPSEPVKPVVDQTAQ
LKAAAEQNIAKANDKLAWAVSKNAKNNYPELLAKGSGELDSAKAAFASNNYQEASDKAVA
AFNTLSAIAEFAPLPAKYTVRLIPERRDCLWRIAEYPFIYNNPLKWPVLYEANKKTFKDP
SNPNLIFPGQVLQIPSIKGEKRDGMWDPKKTYQPLAK*
>SPSA8_v1_250011|ID:41145643| putative M18 family aminopeptidase 2 [Spirochaetes Bin 1 SA-8]
MKESTNALCQFLDASPTSFHAADNIRSYLLAHGAQELDEAASWQIEPGVPYFVCRSGTSI
VAFRAGLKSMGDAGLVIAGAHTDSPGLKVRPGTHKQNQNMVRIGVDVYGGAILSTWLDRP
LAVAGRLFLRDSESPASLKSVLYNSGKPVGIIPNLAIHLNRDVNRGFEYSPAQHLPVLVS
SLREKSDTQSANLSWLHEYIGEDTNLDIEPGDIVASDLFFYDTQKSAIFGTRHQSALPDD
QERDILVNAPHLDDLAGCHAILEAFSSAVPSEFGQVACFLDSEEIGSMTMQGADSSFVRD
VLARLAIVTGAGAEDFYRMSSRSLFVSLDAAQGWNPAYPEKYDEKLSPLLGAGPAVKINA
NQRYATESHVESLIAIIANQEKIPLQRYMSRADMQPGTTIGPMSAARLGIRTIDIGHPLL
SMHSIRETINSFDHAMMIRLLAAVYKNPPIFS*
>SPSA8_v1_250012|ID:41145644| Uncharacterized HTH-type transcriptional regulator in himA 3'region (modular protein) [Spirochaetes Bin 1 SA-8]
MSGYTTGEVEELLDLPASTLRFWEKEVPFLTPRKDVFGRRVYSPLDLCILSRLKFLALKR
GLGLKKACAMLEKELFLADPTLKSEIXQAKINLLSLKAESRXIREKXSVLHPDPYAAEES
AXNIVSPASKLNSPSLFEQELS*
>SPSA8_v1_250013|ID:41145645|der| GTPase Der [Spirochaetes Bin 1 SA-8]
MKEIETGKSHSAHSSGGRVRKTAAIQKKPEKSDNDTIQDIAEGLPEKLVRYENLPQVVLV
GRPNVGKSTLFNRLLHKRRAITDPTPGVTRDPIEEECVLPGTEHRVRLVDTGGFKLEREG
LDDLVVARTLSILSRADLILFMVDAVSITPEDEEFAELLRPMSDRLLLVINKADXPERDY
QAWAHMKWGFPATLFISAEHNRNIEDLVEAIASRLDFSAVKTVDLERADIRIAIMGKPNT
GKSTLLNVLLGESRSIVSDIPGTTRDIVEGRFTWKGRGIVVLDTAGIRRKKKVNDNIEYY
SVTRSLSAVTQADVVILMIDAQEGLTDQDKKIAAFAVDEGRGVIFALNKWDLMPDIKNSF
EAARDKLRYFFGQMAWAPVVALSAKDGQGIDKLMNTVISTYSKLCRKVETSRLNKAVAQW
METTPPPVAPRTKFKLRYALQTSMNPQKFTFFVTKPEAVADSYVSFLKNRIREEFDLGTI
PVMLELKASRKDWRER*
>SPSA8_v1_250014|ID:41145646|ptsI| Phosphoenolpyruvate-protein phosphotransferase [Spirochaetes Bin 1 SA-8]
MREIHGIAASPGMASGPAFLFYDEVTDNVPTYPIASETVEAELERFSSAVSAAKKEVELL
RDRAFKEAGESQAAIFDAHLLMLDDPEVAERVKNDLYSTLMNVESIVFALAREMVDQLSA
SNDPYMQERVSDVHDVTRRLLGHLLKRERINLADLQDDVIIVAHDLLPSDMVGMSRHHVK
GIATEAGGRTSHAAILARAFEIPAALGLGSFMSDLKMGTKVIVDGDKGILVVDPDESALK
KVQASMLLRLQKVKEFENIKALPAATKDGTRILLKANIEVPEEVESVVEHNADGIGLFRS
EFLFLGGHIPGEEEQFRAYKAVVEAMNGKPVTIRTLDIGGDKVLPELGAQNEKNPLLGWR
AIRFCLSKTEIFKTQLRAILRASVFGNVHIMFPMISTTEEIIKARGLLGESKYECRAQGY
KVPDHIKAGIMIEVPSAAICSDILARSSDFFSIGTNDLTQYTMAVDRGNEKVSALHEPFN

PAVLRLIKTTINNAQHACIDVSLCGEMAADPYAAVVLVGLGMRELSMSSASIPAVKKQLL
SITLEDAENIASAVMKMTSANQVLTYINNRFAL*
>SPSA8_v1_250015|ID:41145647| LysM domain/M23/M37 peptidase domain protein [Spirochaetes Bin 1 SA-8]
VRRNRSHWHNEIILLFASGSTLKRTLFALVCLITLLGSSFRPQPSPEFAKGSIDMEGSGL
ASISSEDESAGEGLFYTVYQVQKGDTISGIAESNNLTADTIISINGIQSAKSLKPGQLLK
LPNMSGIIYTAKAGETVAAIAKKFEISEDRLIVANSLMKTDFSAEKVLFLPDARLPKAQL
REISGDLFKWPVRGVITSWYGWRRDPFSGRNSLHNGIDIGVPTGTPVGAAMEGTVIETGY
SPIMGKYVILSHAGGWKTLYAHTSSILVQEGQYVSKGSRIALSGNTGYSTGPHLHFTVYK
NGKPVNPANVLQ*
>SPSA8_v1_250016|ID:41145648| protein of unknown function [Spirochaetes Bin 1 SA-8]
MSFKKKQHLRPLILVILLFLTASICPALEIKTDNYTITTEAVTSPGFALVSITSNASGTL
IQIDNQTVGSSPWTGRLAAGNHIINMSAPDHYDLRFLLTCQENTKYSISCRLDPHTGFLD
VTIRPEDAMLYLDGVKMVNQFAEVPVGYHTIAARKFGFEEKSLKILILKGKISRVSMMLE
PSSFSVRDFRLTRKVFNPDNHGLFGKTTLAFSVTAPGYGRIEIRDSTGSLIFQHTLLPFT
TWAQSFSWNGRTASGSAKDGSYTARLLLWPEKKQETPAVSAAPVPGDFTRTDSAEQASIV
QEISFRIDRSQIIAPAGYENARTGLAFVSNPSMQELPVFGLDIGGVIAYDSTASASAGVG
LSGGIRLGSSAALGFAGSFFGNGDAGLSASLNGAVLKGPQLDAAISARYEIADFSNSGGG
ISNDIELNFPLAFKTGAVRIGLEPGIRLNLDDTSIAPQTGVGLWYQSPALFAGFSAHQVF
APGGIACDANPLSLALDAKYMVNGVPLTMGVRFSAEMTPEFEKLQAWFGIGLAF*
>SPSA8_v1_250017|ID:41145649| Iron-sulfur cluster-binding protein [Spirochaetes Bin 1 SA-8]
MSIAAFDVSIERCSAYQKDSVERVIESAAEKAGFPSILGKTILVKPNILNASTPEKAVTT
HPVFLAAVLQFLRKNGAGRIIVGDSPGFQPGSFAGKAAGLHQVVLDAKAEWDEFKQGSLH
QAPEGKLVKSFVLADVLGTCDLVVNLPKLKTHRLMNYTGAMKNLFGLIPGLAKSAMHMRY
PDKEQFGTMLVDLALSVVPTFTFMDAIVAMEGEGPGNGTPYPLGLVLASASMPLIDWTAA
SIIGYKPEKIPYLADAMRRIGRNPEKPEYSYGPESVTAVRAENFKLLPYETAHPSALKAL
PGFLQKLAVSATMRRPIFHEKKCIGCSACVKICPGKALVLDRKASGINKILIDDAACITC
FCCHEVCPVNAISAGKVFRRSRRDRKLP*
>SPSA8_v1_250018|ID:41145650| protein of unknown function [Spirochaetes Bin 1 SA-8]
MSQFNLHEFLDFGSPDDYERLDFPAPQKHDGRLLADPVILRELANRAFTEIAYRYPRGHL
EELSKILKDNTAKEAEKFVAASLLRNAAIAAEGFYPMCQDTGTALIYGWRGNGLGGGTAD
EAAWLAEGASEX
>SPSA8_v1_260001|ID:41145651| Beta-lactamase domain protein (fragment) [Spirochaetes Bin 1 SA-8]
MKIHPITDKIFALHADIKSDDLFEGIWPIPYGVSLNSYLVKSEKIALIDLVRDWVGAPGE
LAGQLASIGVGLDDIDYLILNHLEPDHTGWLNEFRAIXX
>SPSA8_v1_260002|ID:41145652| conserved exported protein of unknown function [Spirochaetes Bin 1 SA-8]
MNSKVSSRFGRFFMLTVGALLLLSGIAFAQEAGSDVNLDELFGGDVVTVEQDSPAPSQNA
VTSSLKSDKVRIGGSFSGSLGAAATWNNPWDGSFDLFNADKTEPSAQAKATVFFDARPAE
DFRVYGSLNTSWPYAVADSSSDKSIIPNVSVFELFSDFSWNDQVFFRFGKSTVKWGAGYF
WSPADVINLEPINIFDPTSQRAGPLNLKIHVPVLGTQNNFYFYTILDEKNLAFSTTALAG
KAEFLLGKYELGIGGYYRCDVAERGMLTLTGPLGNFDIFSEAMISRGSAKTFVTDIAVLP
PFTVSTSKVEDHRSSFYASASAGFMYSNQNSNVSAIGQYYYNGEGYSDSTRTDLIGKATT
VIAALGGADTVPGKAMAQALSGLLFGSGRHYAALSLSKSKFLSDDFSVSILALANLSDFS
GIIRPQASWKIIDRLSVTASPMFIFGGENTEYASLFGGDILAFSVSVSIGSGSF*
>SPSA8_v1_260003|ID:41145653| conserved protein of unknown function [Spirochaetes Bin 1 SA-8]
MLPEANKGQGYLREDDNVWFYDPSSRKFSHSSVKENLQNTEAKNSDFTAGSLADDYEITA
ITEGTIGKYPVWIFDLKAKTNEVAYDRVLLSIRKDRIMVLKREDYSLNGRLMRTTLYPKY
VEVAGKLLPSQTLIIDEINKGEKSQITMAEQSVEKLPDKVFTKAFLEQVNR*
>SPSA8_v1_260004|ID:41145654| exported protein of unknown function [Spirochaetes Bin 1 SA-8]
MKKKSISPVLILVIMLAVLAPAALSAQTLSMTEAKKILAQLDDLGNFPGKDFTALFTIVS
EKPGEKQSVTQVRVFR*
>SPSA8_v1_260005|ID:41145655| putative ABC transporter, permease protein [Spirochaetes Bin 1 SA-8]
MNRIVPKIALRNLSRQKKRSILLGGAIAFGIMIVTLINGFAGAFITNVSENFAYLMAGHV
FVQGQEKTASGKRISVIRDDALIFNAVENAGIKYKTATKASEVMATIVFEGKSIRQNLTG
LEMEKSAFLRDRLMLKQGSWEDTMKPDALIISEKIAKKLNVLPGDRITAQFQTVTGQNNV

ADFTVAGISADVSIIGSVMAYVNLAYLNAAIGIGPSEYMSLGFMLDNLKDAASFSDRLFT
SMKGMGLQLFDRTKQAENGSSTPFMAMLRSQNQETWSGTKYRVYTIDDQLAQAKQIVVAL
DSASLVILIVLFAIVMIGITNTFRMVMYERIREIGTMRAVGVQRSEIRSLFLYEAFFLAL
GGALSGILAAFIVMNLLSLINFGMNSPAFLILKNGHLSFFLPPLRALANITIIAVLTLFA
AYFPARAAAKMEPAVALRTLK*
>SPSA8_v1_260006|ID:41145656| conserved membrane protein of unknown function [Spirochaetes Bin 1 SA-8]
MPVTLRMALRNIFEHKAKSLIIGILLALGAFILVLGNSFIDASQAGIKSTFTDNYTGDVF
ISGLSEDGSVSLFGVTSPGGLSPTPVIPDYDKVLAIVKASPLVDKATGMATGFALVTKGE
EADIRSETPEDTANTDPGARFLFLFGVDARDYWSVFNSVDLTAGSLLEPGQTGVVLYDKH
MDKIAKWLGHPLKIGDSIVIQGISSSGFKIRSVPIVGTYHQKGEGAAPEQMAFIDIDTLR
VMAGMTVGAGDDQNLTASSTAMLSIEDTDSLFGDDLLDAAPSESGFNEKKLIAQLADTTA
RERANTADSGSWQFIXIKTKRASDAKVLTANLNSAFQKEGMKVEAGDWQKAAGPYGQSVD
VVRIVFVIAIVILSIVAVIIIMNTFVISVIERTGEIGTMRAIGAEKSFIRKLFAVEAAVL
SLVFSSLGALLALGAVQILRAMKIVAGNPFFEVLFGGKYLSPFVTLPNFLTGIVAMLAVG
YLAHLYPVSVALKIQPVRAMQNE*
>SPSA8_v1_260007|ID:41145657| conserved protein of unknown function [Spirochaetes Bin 1 SA-8]
MGIIEVQDVKKIYPLGKVEVPAVKGVTFSIEKGDFISIAGPSGSGKTTILNMIGLIDKPT
SGEVIIDGKKTSGLSDKELTRFRHEVLGFIFQSFNLIPVLNVWENIEFPLLLGQTRITKA
EKNDWIDWLIAEVGLTEWKTHKPNELSGGQRQRVAIARALVTKPQIILADEPTANLDSAT
GEQIIELMKKINRELLTTFIFSTHDAKIVGIADHIIRLRDGLVTENRRRGEDINGSSQIV
EE*
>SPSA8_v1_260008|ID:41145658| putative enzyme [Spirochaetes Bin 1 SA-8]
MADFSPEFSKNSMNMKKSVIRELLKLTQQPEIISFAGGLPAPETFPVEDLRVAADAVFKK
HAAMALQYGTTEGDKSLKAALIKYEEANGVKIGENNLLITSASQQALDMIPKIFLDPGDY
VLAGRPTYIGAIQAIQSYQGKVIGIPFSMDNDGFDMVELEKRYRRAVDTGSKIKYIYVIP
DFQNPSGICWSLEKRKALLEFAYEKKLFIVEDAPYREIRFMGEPIPSIYQLDQQGAKAGN
VINLKTFSKILAPGTRIGWIMAREDIIQKFVVAKQAMDLCTSPFTQLWLAEYLSSGKLNS
VIKHTCDIYRAKRNHMVEMLEKYMPKRFDLRWTKPEGGLFLWISLPHYIDTDKMIFKAVE
KKVAYVVGSAFYFDEPEHNAMRINFSYSSLEQIEEGVKRLAEVIKEEIEAHESGPRGQTA
PEI*
>SPSA8_v1_260009|ID:41145659|ychF| putative GTP-binding protein [Spirochaetes Bin 1 SA-8]
MALNCGIVGLPNVGKSTIFSALTSAKAEAANYPFCTIDPNFGIVAVPDPRLDKLVEMFKP
KRRVPAVVEFVDIAGLVKGASQGEGLGNQFLSHIREVGVIAHVVRCFDDPDIIHVANRIS
PKDDIEVIHTELALADLETLDKRLEKLQKQLKVQDPAVKKETEREIAVLTKIRPALESGK
PARTVPLDDDEKAAVQSSFLITMKKEIFVCNVXXSGLAGNXYVEEXKQXAAAIGSEAVSI
CGKFEAELADLENEAEREEFLQGIGLEESGLSSLIHAAYRLLGLRTFFTAGEDECRAWTI
KAGFKAPQAAGVIHTDFERGFIKAEVYHYDDLIKLGSEHAVRQAGKLRQEGKEYIVQDGD
VMFFKFNV*
>SPSA8_v1_260010|ID:41145660| conserved protein of unknown function [Spirochaetes Bin 1 SA-8]
MSCSSNSMSEHTDSDEAMLTGLPPFPDYAEGISPLFLILGSFPSRASLAMKTYYAHPRNH
FWRIVGEIFNMDLEKASLKEKSTFLRMHRIIIWDSIAACRRPGSLDNAIQDAVPNDIAGL
LARFPSIRAVGLNGLQSARVFFTRILPEPAKVPAIGEILTLKITKNRPSSVLVMRLPSTS
PVPTRAYRDGASKIPHWKQFFTIHL*
>SPSA8_v1_260011|ID:41145661|uvrA| ATPase and DNA damage recognition protein of nucleotide excision repair excinuclease UvrABC [Spirochaetes Bin 1 SA-8]
MDKLIIKGAREHNLKNIDLELPRNKLIVISGLSGSGKSSLAFDTIFAEGQRRYVESLSAY
ARQFLGRLDKPDVDYIEGLSPAISIEQKTTHKNPRSTVGTVTEIYDYFRLLFARIGIPHC
PKCGRVIKEQSVDQILDTILSWPDDTRIQLLAPVVRAMKGEHKKILEDALKQGFTRARID
GEMVDLEEAPALEKQKKHTIEIIIDRIKLNRDARRRIAEAVETSLHIAESIVIVLRDEGD
GTIKEEFFSQKGACPECGISLPEMEPRLFSFNAPQGACPVCTGLGVKLEFDPDLVIPNKS
LSFNEGGCIPYNPQSAWNRSRFEALAKHFKFSLDTPFEDLPEKAMAAILHGTDEEIHVRY
DNRENTGHFEYQTRFPGILEDLKRRYMETTSDGIKQWLEHFMTEKPCSSCDGKRLRSEAL
AVTIGGKNIHELSSFSVEEALSFFDSVRLTDVEQQIAKQILKEIRARLSFMKNVGLEYLT
LERKASTLSGGEAQRIRLATQIGSSLVGVLYILDEPSIGLHQRDNQRLIDTLTFLRDIGN

TLIVVEHDEQTLRTADYIVDLGPGAGEHGGKVIAAGPPEQIMLSEGSITGRYLAGKLSID
VPAVRRIGSSNRLVVRGAREHNLKNIDVAIPLGMFVCVTGVSGSGKSTLLSDVLYPAISN
RVMRTSMTEGLYDSIEXIEFIDKIINIDQSPIGRTPRSNPATYXGVFTAIRELFASLPES
KARGWKAGRYSFNVKGGRCENCQGDGTLKIEMNFLPDVYITCDVCHGKRFNQDTLDIRYK
GKNIADVLDMTIEEAAEFFEHIPPIRHKLITLLSVGLGYIKVGQSALTLSGGEAQRVKLA
FELARRATGKTLYFMDEPTTGLHFADVKQLMDVIHRLVDTGNTVVMIEHNLDVIKQADWI
IDLGPEGGGRGGSIVAQGRPEDVVQSSSSYTGAYLKPYLSV*
>SPSA8_v1_260012|ID:41145662| protein of unknown function [Spirochaetes Bin 1 SA-8]
MLLAMFAARKRNTALVLLLFVVSLSGILPSRLLWAQESDKGSEYLDSLSALERKTLALDI
AVSSYYELMDLAQRFNLPTEGTSQDLRQRLYGFFKLENPVKPSATALTIESASSFSTFSA
EQKTSNLIQLSGPVILKISTSDGFLHRISADRILYDSEKNIAEAWGNIEYTREGNGRTDR
FAGSHLIVDLAQYSGVFLDGSFNLEPSSAQQRTLLLHFDRLVKRSENVSALESATLTACN
ELPLHYYIKAAKVWLFDSGDWALSNAVLYIGVVPVLWLPFFYYPSQEIVFHPVVGARTRA
GAYFQSTTYLIGEKPVSTSAMSVFTALNQGAAMGKTEMSGVFLKRTGSTEQSSATAAGTQ
GSSSIQSSKSLKLMADLYSALGVYAGIAGYFPIAKSSGKLEFAAGIGVSRSLFPQSTGYY
SPFDYAGNYVSVWNQSSFFGAQLPFRYGMTFSYKNTVKNGPFSATFSADIPFFSDPYFEQ
DFLDRNESSNVFSMFGAVQKTISRRSTLTDTLQGSLAWTFASSSRAPLLKALSFSRLQSQ
LISRTRSQPTSGLTAQQKRLLSVDPQRDFYYPDEFRVLDASMAASGTLFSFKKSKPSGTG
QGTVTASQGQQPSLPPAQADSEAAVLQNASSPAAGTQKRDFSQFASRLDWSISASGMANN
KFRSNQWNTPVDIDASSQYFLLGYRGNLRFALASSFAENLMSQQLTLNFNAQDQYRPYLY
DERSSPTSPHPYRLTDYAYRSNSADFANELSFQPLPAGTAFSSTSLQYSLKGTLFSTKYQ
GLSGSGVDAAPIYATDWFSWTSAYIQSHTLSINIGYAKTGKPSQRLSFSASLPPLDEKYA
TQYSLQAKQYAFVAQGSLTRNSGTSQLEPSSLNAQLKLGGGTLPSLNSELVWDFVTSAPL
SLAASASWKTFSGRFSARKAKGYDFSSGTWIPDGTEYFRPYDYSLAFAPIWQTKSKAAIA
SAPAAVSAISTDEVEDEAEAEAPASETQAPSPQPSAVIGQSVYFSLKPKLSYTQNIIRFT
ESTLAFSLDASIATEKGTKLTFSSSSINKSAWRYWPGMFPITTGLDPNAYARNPITDIYN
SLAIWDSARLKTTLFKLQTLSLSLSQDLHDWTINAGLGMSPVLITPDSGRPYYQLDFSFT
FAVTWKDVPEIKTQLSYKEGSLANE*
>SPSA8_v1_260013|ID:41145663| PhoH-like protein [Spirochaetes Bin 1 SA-8]
LNSESIVLENTDLLSEICGVNDYNLSIMGDLLGARVLSRGNELFIDSDDTDIQSLFSSLV
EALAKSIAGGLPATPDLIIALHAELTPLGNEHSSALGGAASSPTKNASCDDFLDLCIQIP
GSFTKVYPRSRGQGLYLKGLATHDLIFSIGPAGTGKTYLAMAWALKEVLTKAKRKLILTR
PIVEAGESLGFLPGDLEQKINPYLRPLYDAMEMILPYETIRKLEESRSIEVAPLAYMRGR
SLTNCVVILDEAQNTTKEQMKMFLTRLGENTKAIVTGDITQIDLPRKTESGLVHAMQVLE
NIDEIMISKLDGRDVVRSQLVRKIIEAYEHE*
>SPSA8_v1_260014|ID:41145664| putative 7TM receptor with intracellular metal dependent phosphohydrolase [Spirochaetes Bin 1 SA-8]
MNKLIKDSFIFWNILACILLASILLALLGPGANAVRRNFEGIEAGKIADRDIVAGKDVFY
IDTEATRLRIEAEERLVLPVFQMDEQASLRITRQFQDFRNAFKDLVSDDVSAESSILMLQ
SKFPGIFSRSLLASLLSTPLKSQALVYTEDVLQSLLSTGIVLLPSEGLSTYNQDYYELRR
LINNKIESEQRAFSSMITKQDVPEQINREISEKHLSRQLGGIVSGLVQTFAQENVFFDET
ASKARIKAVRNKVEPVKRAIGRNEILIRKGEMVTPETFARVQAIRSAASRADFGLVFGGL
GLLVVAAFISVILLGQNVQPGGNGDRKIVLMALYSATLFFILILVAARLTTGQLPLESSY
IIPISLFVGIGTAISGQTFGYTYSLILGLVSAPASNLNPHLLVFILLSGIFSSFMISISK
TRLALVRASFFQALFQGLLTLVLLLPYAGKFQLVAGLSFMQALNGFLSGALILAILPVIE
QGLNLPTRFRLLELSDVNAPALKELMTQAPGTYSHSLNVANLAEAGAEAIGANALLARVG
AYYHDIGKIEQPEYFVENQKGVNKHDDINPRLSATVIRSHVKFGVEKAKALGLPKEVVEI
VAEHHGDSVITWFYEKAKAEDETVRREDFSYPGEPPRSKEAGIVMIADTVEAATRTLKKP
TVPRLEQQIRQLILDKVQAGQLDNCSLTIKDLESIRHAFTRILAGQFHSRIEYPRQKENG
*
>SPSA8_v1_260015|ID:41145665|ybeY| Endoribonuclease YbeY [Spirochaetes Bin 1 SA-8]
MNQVLVDFQDIEEPSCIVPVSEFMQKILEFKKISDWEVSILFCSDAFIQNLNKEYRAIDA
PTDVLSFSMGEWYENEKQKIYRAGDIVISMPALHRNAEEFKVDPDEELKRLLIHGILHLE
GMDHETNDPSEPMLIRQEAILREFAGSKLL*

>SPSA8_v1_260016|ID:41145666| Hemolysin C [Spirochaetes Bin 1 SA-8]
MNGIFRIFNRKHHPVTEENQQVLAEQEEMKEGIEHLAETTVKEVMVPRTDTVFISHDASR
QEILGTLVGSGHSRIPVYRETMDQVVGIIYAKDVLASLVSNGKIETSALMRKPFFVPETK
KIDSLLKEFKRKHVHIAVVVDEYGGTSGIVCLEDIIEEIVGEIQDEFDNEKEPIIQTAPN
TFICDARTRLDDISKELSINLPAEEFESLAGYVFDLFGRIPAPQERAETDDAEFTVMEMD
AHRILSVKIQLKNRNNDTTSI*
>SPSA8_v1_260017|ID:41145667|gdhA| glutamate dehydrogenase, NADP-specific [Spirochaetes Bin 1 SA-8]
MNAYIKQVLDDLRARYPWESEFLQAAEEVLESIAPLIEAEPKYKAQKILERIVEPERTVM
FRVPWIDDKGEYHVNRGYRVQFNSAIGPYKGGIRFHPKVTLSTLKFLGFEQIFKNSLTGL
PMGGGKGGSDFDPNGKSDNEIMRFCQSFMTELFRHIGPDTDVPAGDIGVGGREVGYMFGQ
YKRLANEFTGVLTGKGLTFGGSLVRPEATGFGAVYFAKEMLKLKGQTLEGKTVSVSGFGN
VAWGACIKASQLGAKVVTISGPDGYVFDPDGVGTKEKWDYLEEMLVIDRNKVENYVKKFP
KAKFFAGKKPWEQKVDIAMPCAIQNELNGEDAKVLIANGVQTIVEVSNMGCTPEAWKLFI
EKKIPFAPGKAANAGGVATSGLEMSQNSMRLAWSAEEVDRRLHEIMTNIHKSCVETAAKY
GFEGNYVIGANIAGFKKVADAMIAQGLV*
>SPSA8_v1_260018|ID:41145668| 3-phosphoshikimate 1-carboxyvinyltransferase (fragment) [Spirochaetes Bin 1 SA-8]
VHTAGFFYAHFMVYSATIMNTTCLYTSVSGIISAPPSKSSMQRAVACAMLADGISLIRNP
AFCDDARAAMGIASAFGAGAELSNDSVQISGSPLFKNRTTMSENAGSLEVSCGESGLSMR
MFAPISALLSRPVRLVAHGSLTRRSMVMVQEALNAMGVSCDSADGLPPLTVKGPLLPGIY
HIDAGASSQFLTGLLMALPLLAGDSVLRVSNPVSTGYLDLTVATMGLFSVKVEHINNYSR
FEIPGGQRYVPATMSVEGDWSGAAFLLAAAALGAKERPLRIENX
>SPSA8_v1_270001|ID:41145669| Fe-S cluster domain protein (fragment) [Spirochaetes Bin 1 SA-8]
MFFISPCTAKVTAIRMPLGYERSLVDAVFSFQDIFPALKKALSRPSETPSRFAPLQERIA
RMRRGHGMDWARSDGEIDGLGIEQAVSVDGIQNVIALLEEIDNGKFSSVPYIEALACPGG
CVGGPMAVANPHVARAAMKHIAAAEKTAAAQSGQSEQPAQAGFGWERELQPKPVFVLDRD
MLKALQLAEQMELITSQLPGLDCGACGSPDCRALAEDIVKGKAVIEDCLVMMRKKTAFNL
TNE*
>SPSA8_v1_270002|ID:41145670| conserved protein of unknown function [Spirochaetes Bin 1 SA-8]
LNKKDFPRVHFYDQDFVEIYDRTWAWVSDCWTQGGPDSGIDKLKFFIYPQKPVLDIMEQV
FSSFFMVYSNRIFTASNGLDALYALQEPDGAIRAAYSVETGKPILQDDNPEGVMLPLFAW
AEFNLYHKTANKKRVKDVMPALKKHYKWLESKFKSANGLYATPVASTGMKNSPRPNAMYL
VDFNAAMAMNALYISALGDILNDKEISFQFKRDYFSLKTRINSKMWNPEEGFYYDLDAQE
QQVKTKTIGTYWLLLAEIPNEERAEKLIHKLKDPAYFGTENPFPTLSADHPLYDRKGNGY
CGSVVPAFTFMVIKGLEKYNFYEFARDCAIRHLYFVLDTMHQNSEHREGGHQRDHKPIVW
SAYQPQSEGKAHWDAVPDWPYPHYLPFNGLTTVTLMIENIVGLYISLPRKTVDWIIPNLE
IMGIENLSLKRNMITILSSKSGRGWEIHMESEKLYYFTINVINKKKKTLPIPSGKCSMLI
DKI*
>SPSA8_v1_270003|ID:41145671| protein of unknown function [Spirochaetes Bin 1 SA-8]
MDAFEAERQRALDNLTSAYALGKITLEEYEVRADRIQKALSIDGITSQVSDLPVPASETS
SRSTAPLSSRRERMPESLSQPGSYLVERRQGSPEFLPCIMGDRKMVGDWLNSDTATSITL
MGSTTLDLTETAIPPGRLKIEAIAVMGEIKILVPRGLPVRMSAFPFMGEAHIQKGVEQRV
DRSLPYVEISGLALMGSIVVKSV*
>SPSA8_v1_270004|ID:41145672| conserved protein of unknown function [Spirochaetes Bin 1 SA-8]
MLYAISLGGSIVAPPEGPDTGFIAAFAERIAAWLAQHPEHRLIMVIGGGGPARTYQNAYK
KFLEITAATLPTLQPGASLDTALDWIGIAATRLNAQLVKSVFGPLCADAVVTDPSADIAF
SGRILVAAGWKPGFSTDFDAVWLAERFGASMVLNLSNIAKVYTADPRKDPAAAPIDAISW
PEYRAMVGSTWTPGANLPFDPIASEKAEKAGIKVVCASGKDLDNLFKILDGKAFIGTMIG
*
>SPSA8_v1_270005|ID:41145673| putative HAD hydrolase, IA, variant 1 family protein [Spirochaetes Bin 1 SA-8]
MKCGLAASEEPLDTTAPEISTVIFDCGRVITMDQDSACAQAMADFFSPSADLAEFIDTYT
RERPAYDQGLIDAFAYWNNVGRHYGKTVSAEDVSKLIRWDMRSWFTINPETVHIIQELKR
RDFRLMILSNMNEEGKAEMFGASRYCGETDWISLFDDILLSCDLFQLKPQEEIYRTCVAR
AGTEAEACLFIDDSPANIQAARECGLHGIVFNHASQLRSTLRDSYRLLN*

>SPSA8_v1_270006|ID:41145674| protein of unknown function [Spirochaetes Bin 1 SA-8]
MPIQTNTMVNAIRRNRAGLRYNTGLRFALILFLAVFLVFTTSCTTPLQWGTIPPSADDLA
VQAWLSDISLLKNEMLKHPKLVSDQTARQTFIHEIEALQQTLETADNLIGSPADTAIPGI
QRALAAVGDGHTRINASPSDLYPVAMRFFLDSRHADEGADAPMEHWDLRIAAINQPQTEY
LGLIVTRIGPYATQEAIQQIAPYLSIESALGKEANPYLYEHAVRSEIMESFMNPYLMRRL
GMANQNGLTLTVLREGVEEELSVPLTSRQDNHWVRVLDSIPSIPFTRSHPEARWWYDYIP
GHPSSIYLRYDDCDQAAWPLLQEVLALLPDKGSQQTSPDHLIVDLRFNSGGNSMPGYRFA
QALAKKQISAEQGGVILLISGTTFSSAMQNAADIIKACGGKNKNTGTAILIGEPLTEPLR
HYGEVKRFSLPASGLIIGRSSRLWTYDTSTGIFPPRGVLEPSAGHLVWPDFDDYRNGIDT
VFEHALELIP*
>SPSA8_v1_270007|ID:41145675| Peptidase M20 [Spirochaetes Bin 1 SA-8]
MSKTIDFNDVRSLSISLTRNASITDTSGEKAFPAFLLDIISQIPYFRDHPEDLRLHEIPG
DPKERSNLTALVRGKGKDCLILTGHYDVVQTSMYGSLEPWAFDPETLSVKILEQLADVSS
KDNPLYQLKEDLQSGEFIPGRGILDMKSGLAAGISVLARFAAPYERIGNILFLAVADEEG
KSHGMKAARAMLPEFLKQNGLKPAAIFNLDSAVDKENGEAGRAVFTGSVGKTLPFVYFLG
QCTHAGAPFDGINAVLAASEFAREIECNPDAMQERQAAPGEEAPPPTILYFRESRAHYDV
TTPSSVFCAINLLSHQRSPEDIMENIRRITQSAMNKAISLLRERASTLSRRISEHFKLPA
AQPAVIEFSEFSQRAERTSPGIVQTIRNLAEKEHPDDQVLQTIFILNHLLPYTAIEGPAA
VIGFAPPYYARAELDSERYQNFVSLLRETLSAYSLETGTAVRVRPYFPGISDMSFLAPAD
TREQRSYLASQSPVIDSTLKEGEHGTIGCPVINIGPWGRDYHQMGERVHAVYAFKQLPEI
IWRLAQGILD*
>SPSA8_v1_270008|ID:41145676| AMP-dependent synthetase and ligase [Spirochaetes Bin 1 SA-8]
MKRTVLRMLAEASRNWADDPYALRKTDKGYEATTFAQAQVKARQFGAWLVKEGFGKGTAF
AILGEGSPEWICGEYGIMSAGCVSIPLSIKLLSEELPFRLNHSESKGILTTHNQLEKVLT
ALDAVENKALRIIYLDSDVEWAASMLEKHGVSKNLLTGFAQALEAGEKALADKSSEIAEK
LDRIFEEAEEDDVVTISYTSGTTGNPKGIMLTHLNYWANCHDAVDLFDNPIHFRTLLVLP
VDHSFAHTVGLYTALICGIALYFVDSRGGGIATLRNIPINLKESNPIFLLTVPALSGNFM
KKIISGIEEKGGLIEKIFKSGIAAGIAWNGNGCNTPPFMTRAKAFFPYFLAKALIFNKIK
KMVFGDSIRFCVGGGALLDIKQQEFFAAFGVPVFQGYGLTEAAPIISSNVPKKYKFGTSG
VVAPSVSCKIMRQDGTEAAVGETGEITIVGENVMKGYYKNEEATAQALRGGRLWTGDLGY
MDKDGFLVVVGREKALLIAEDGEKYSPEEIEEAVTMSTDVIDQIMVWCDHKKYSCALVSL
DTAKVERLMKAKGISDATKLCAALQEEFYRFKTDPKAKKVQNAWIPAVFQIVPAAFNEKD
GTINSTMKLVRYRVAQVYKDLLDYSYTKEGSVTTNARNIQTLKELFKLK*
>SPSA8_v1_270009|ID:41145677| conserved protein of unknown function [Spirochaetes Bin 1 SA-8]
MTKNMGSIDRIIRLSAAIIIALLIGTQVLTGTAAIILGILAVVFVLTSAVSFCPLYVPFK
FSTKKKN*
>SPSA8_v1_270010|ID:41145678| exported protein of unknown function [Spirochaetes Bin 1 SA-8]
MKIRNAIAAAAILLLSLVNFSAQNLYAQGNPAAVSGLVFVEGGTFRMGVENSKDPGLAAH
DVTVSSFYICDHEITVGEFRKYMQEFGIGTIADFDNSGQVVYRNGKWVTDENANWENPGFP
QTDDHPVTMVSWYDTLFYCNYLSIMEGLEPVYDILKTGEVQANLNKNGYRLPTEAEWEYA
ARGGVRSKGNKYIGSINPEAVAWLEHNSGNTTHPVKKKMPNELGLYDMGGNVYEWCHDWF
GPYKPEPVKDPVGPESGENKVLRGGSWASKPAETLPTSRIEGGYYFAGSDVGFRVVRPSG
PGTSKPIIAKPSTTIVSSTTTTTTVAKKLSSLKGMVQMTLVEGGTFIMGTESGESNEKPAH
TVTVSTFLIGTYEVTVAQFAKFIEVTGYVTEAEKGKGGWIRENGQWIQKSDANWFNPYIK
QTAQHPVVHICVEDAMQFCNALSEAEGLQPVYRITASTISVDLSNNGYRLPTEAEWEFAA
RGGLKSKGCQYAGASDLSEVGWHSANSGGVTHPVGSKKPNELGIYDLSGNVWEWCQDRYS
PYTADSQRDPLGWSEENKKVLRGGAWTTGPAYCRVYKRLSEKPDVTDFTFGFRVARRP*
>SPSA8_v1_270011|ID:41145679| Polygalacturonase [Spirochaetes Bin 1 SA-8]
MSTKISLDDFGALSDGSADCSQAFQAALRELDKLGGGTLQVPRGTYTTGPLELKSNTHLY
LSSGAVIRFIPDPSRYPPVETRWEGLVCNAMHPLIYARDSSNISLSGQGTIDGSGLWWWK
TYREKRARNQRSPELAIELELANLNGSTELQPSGGGGREMQFLRPPLIQFYRCSKILMEG
ITFRNSPFWTIHPVFSDNIEIRGITIINPSDAPNTDGIDIDSCSDVTILHSTIDVGDDCL
ALKAGSGHQGLAENKPTRNVIIQGCTFLNGHGGVVIGSETAGGVENVEVLNCQFDGTDRG
IRIKSRRGRGGIVQNLDFRNLLMRETLCPLTINLYYNCGASAEERPLLFSSFPEPLSSLT

PRIRNVKISNLTALDCRSSAGFIAGLPESPIENLLIENFHASLSQEKLKKVSNSEMYEGL
PEIESRGVRIRNASCIIHGLQVEGTQDHEKIIEEQGSRISGLGN*
>SPSA8_v1_270012|ID:41145680| protein of unknown function [Spirochaetes Bin 1 SA-8]
MEDHEKDGKAIRQTGREGVANERIHRDKCRRVAFMLASIHTGAAKKIWAELFKVSHRDRL
ILYIFPGGRLKAPDSHESLRNGIFNFISQNNVDGAVSWASTLSGFVSEKEVEQFHYSVIN
VPLVTFGLKFENAPCVQIDAYSGMIKLISHLAEKHNKKKIAFIGGPNVHSSAEARYHAYR
DALKNLGLTFDPRLTVLNNSWDEGQDAAIELLDNRKLIPGKDFDALCAASDLLLFSAVQV
FHGRGFAIPKDIAIGGFNDSEESYLLSPTFTTVNMPFARQASHAFKSIKQILSGHIMGKD
VLLGTKLIIRQSCGCHIESVRLIERAARKTRISKAASMKAATAFFHDELAHRLAAIFSSS
MHVDTGKAEYMAKELIEASAALFYKEKNDEFLAVLDRMLNEAILEGKEISIFQDILTMFH
SYFRKSMEYRGVLDEMPERVIHQARVLVSDAEKRMSNYQAWKERQIEHVLNSFNHDLLCA
VDVASLVQSVRRWLNPIGIHACHLVMYYSEKESCYCGGYDKDRALIPDSESGEYLSRYHF
PANRILPDHLAPSLPGVHVVLPLVDRSASIGYAVMETEYIDPSLLEEIRAQLSSALRGVL
LFREATSLRIRAEKAEKIKTELLARVSSELQKPAASIAEISMELMSQSEVGHESITAQES
LRKIHALALAQQQRVYTLLELSRAQAEDLPVHASFFSLKDLFSDFIESCSMEFSQAALDY
APLSCRMPVLFGDRQRVLQAARILVPALTVNFNAEKIGISCSILPYGVSIDIIASVSSVP
NENKIAQIAAAVDREKWEKISAFSRSASGLELELAQRLMYFNHGKIEFFNHKSEIGFHLK
LPYPAAMGRYFETEHEFTPQEIILVNGDPVFFSKSGFEDTCPQSRLEMEKLVSPIWKQNI
PRLCYAELSSLTAGQAQSLLMLLESEPPGAWRFYFSCGENSNHDSLNGFINGQYKDLHQF
LEMKLRQGTALLMVARDTALEDLNILKQALSDSGYAVHVCHDPETAPPLYEKCSPDLLIS
VGQNSSFLYELNTVFRAVSGVPTLCLAPLFTEQQFEKAIISRPNTCVLNLGDLFEPAIRA
KIKELASNKNALGAAAGRLVMKSIFYINHNFRNHLSRWKLCGEVNASEDYLSRIFHQHIG
IGLWDYLNRLRISYAITQLNTTNDTIYEIAERCGYQDQAYFCRVFRKLSGMAPTAIRKSA
VSNVSKVQ*
>SPSA8_v1_270013|ID:41145681|lplB| Protein LplB [Spirochaetes Bin 1 SA-8]
MTATIKGLSDKGSGMILRRVPAQGRWFRRFAPVYLLASVSLAYFFIFKYIPIWNAQIAFR
DFQALEGVWKSPWTGLANFREFVSSYYFTELLRNTVFYSIGKMLFSIPAAIFLALAIYEC
RILVLRKSVQTLAYLPHFLSWVIMYAILLSLLSPSNGVVNDVIKLLGGQPVSFMTDTNAF
PWIVILSDTWKEMGWSAIIFLAALMGIDPTLFEAAMVEGASAWQRVRFITLPSISPVIVI
VFLLRIGTILDAGFNQIFMLYSLPVYSVADIIDTWIYRQGILEFRFGLATAVGLFKGVFG
MILLLISNRLIKKRTGSGLY*
>SPSA8_v1_270014|ID:41145682|lplC| Protein LplC [Spirochaetes Bin 1 SA-8]
MAGTVRLSKSDKGFYLFINISLVCTLCILIIPIWSTITLSFRPNDFIGTALEGMFLPPWQ
WSTAAYRSLLGHRSFLLSAVNSFKIFFEGVAAALFLTVPLAYLLSVKNLPGRGFLYIFVI
IPYLFNPGLVPNYLLVTRLGLVDKLAAVFLPGAVSVYNTLIMKSFFESLPEDLKESARID
GASELAILIKIILPLSKPILMTIGLYYGVHFWNDFFNAMLYINSSKLQPLPILLRNILIA
SGMNEYVEASAFGEAPIQAIKAASVFLAAIPMLVAYPFIQKYFVKGTMLGSMKG*
>SPSA8_v1_270015|ID:41145683| Extracellular solute-binding protein family 1 [Spirochaetes Bin 1 SA-8]
MKKTLSAMLMIGMLVMAVGMVQAQATKPVTIELWYGAAITEAGPPPDDWVVYKIVRDKLN
IDLKITALPSSESDQTTKILAAAAANQLPDIFMVQRTPWLKLIQQGLLADVDDMYPLMPH
RAKNYFNADSQEFTTVKGKSFGLAFPGALQKNEGLVIRKDWLDRLGLKMPGTIDELYDVC
YAFTFKDPDGNGKNDTYGYGAFVELFDQEQYLGRRFDPIMGAFGVAGTWNMTKDNFGLNV
KKPEYFSALSFVRKMVENKVIDPDWPVYKKDDFRAAWKQGKFGIMREQFAALAAAANYAP
FDKNFPNGEWVPLAPPKGPNGKNATGIVDANYRIYAVSAKAAKAGKKEAIARLLDWMSTE
EGYRLIGWGVEGVNYSLDENGNITDKNVPADTKFSSPKGQTVTQLRNMVFYNSDMELISR
YPYFTTINGRKLGPLMYLPKFQSYPWVNVTGAGTILPPSNAGDLLRYINQGVQEFALGKK
PLTKENWDAFIAQMDKLGAGAWEKSAKAQMEEDGYLK*
>SPSA8_v1_270016|ID:41145684| Candidate d-4,5-unsaturated beta-glycuronidase Glycoside hydrolase family 105 [Spirochaetes Bin 1 SA-8]
MEKEGLLHLLEGVLNTAANSIELQLREQKLRWNYETGFLLEAMYSAAPIAENEKIVQLVR
QSIDSLVDAHGYIAGYRREEFNLDQINPGKLLFRIWKDSKDSKHEIALRALAAQLEYHPR
THCGSFWHKKIYPWQIWLDGLYMFGPWYARYSIEFGKPELLEDLHIQIVNIREHLKDQNT
SLYYHGWDESRQQSWANPATGCSSCFWGRALGWLAMALVDIWEIAHRMIAWENELTQMIC
DLAASIFAYQDSSGLWYQVVDKKGLDGNYFEASASAMFAYFFAKGIRLGLLPNAPYCAAS

GKAMDGLAGKLTHLDKEGGVHLSGICMVAGLGGNPYRDGSFKYYCAEPVVVDDFKGLAAL
LFAIAESVKIIQ*
>SPSA8_v1_270017|ID:41145685| conserved protein of unknown function [Spirochaetes Bin 1 SA-8]
MADSLASAGFSLEVAGRAFSLMDSYIYGFELQRRSMTSGGEEDQRKNAETFSSNLATGCY
PCLSRMVELSMETGYDEKADFEFGLNLILDGLERLLLSEKKSDA*
>SPSA8_v1_270018|ID:41145686| protein of unknown function [Spirochaetes Bin 1 SA-8]
MSEKARPATSSEKPAEARESAIRRRKEEKHEMKTFQKVGGISAIGAAATYLFAMGMAATV
LNPMMVSTLSIQDYMSFLMSNRILVFXX
>SPSA8_v1_280001|ID:41145687| protein of unknown function [Spirochaetes Bin 1 SA-8]
LKSPRAIQKVPELIHFQKRGISGMGPMAEFSKGRKNSDIPHGFNRKSILRISAAEFILVT
CAILVILTGLGIYASSQYAQLAAQTEKLLASIASQKSDKIESFFKLFLVGAQKNSQNTEF
SSFGVNIARGDNNKEPLERAAAYLNAVEESYHYSASXX
>SPSA8_v1_280002|ID:41145688|tkt| Transketolase [Spirochaetes Bin 1 SA-8]
MDIHSLEKIALSVRSLTIDAIQKANSGHPGLPMGAAELAAYLYGVEMKYDPADPSWLNRD
RFVLSAGHGSMLLYSILHMAGFPVSLDDIKSFRQIGSKCAGHPEYGVTPGVETTTGPLGQ
GIANAVGMAIAERMLAAHFNTPDAEIIDNYTYTLVGDGCLMEGVASEACSLAGHLGLGKL
IAYYDSNSISIDGSTSISFTEDVSSRFEAYGWQVLHGDMYDFEGLARLTAMAKAEKHKPS
LIVLKSIIGKGSPGKQGTSGVHGAPLGEEEAAKTKAALGIPQDVPFYVAPEAYEYFAHHR
KELSARKADWQKIYEAWGKAHPELSEELSAWFFNRPMSEPDLPSFAQGEKIATRNASGKC
ITAVAKAWPNFVGGSADLTSPNVTQLPPAPDGSSQVFSKTNPKGRYIHFGVREHGMAAIA
NGMALYGGLRPFAATFLVFADYLRPALRLAALMKLPVIYVLTHDSIYIGEDGPTHQPVET
LASLRAIPNVSVVRPADAEETAVAWRMAMEKTDGPTVLVLTRQNLPVFAKDDPEWPYTMA
LGAYVAKNTATPPDATILATGSEVQLAWQACAFVAEKRPELQIRIVSVTDMRAFANAPAP
VKEAILSPGATVFAVEAGVAQGWERFARPDRIFSIERFGESGPGDKVAEHLGFTAKKFAQ
LLLAHL*
>SPSA8_v1_280003|ID:41145689|uvrC| UvrABC system protein C [Spirochaetes Bin 1 SA-8]
MKDDERKAPRQLSPLAVKNAEFRALARQAPEAPGVYIMRDENNTIIYVGKAKILRNRLLS
YFSGKKDIKTRHLVERVAHIEWVLAGSEYDALIMENNFIKEHNPRYNINLKDGKTYPSIR
ITAEEFPRVFRTRRIINDGSEYFGPFPSAEVIDTYLELIKRLFPLRRCTVMKKRESPCMY
YHIGRCPGPCAGRISHEAYMERVREVRKLLMGETDSLLADLRRKMEEAASTFKFEEAAQA
RDAIKAVEQFAGRTSVQDFDPEARDYLAWHSDGDLISLVVFQMREGRLRGRDSFIAPLYS
SEEEAIETFMMSYYSQERKPPARLYLKSKIPVRPLKRYFKAQYHVTPEFLIPDSSLHSAS
MQMAVQNAKEELIRRRREIGDMAALAELKSILDLETLPVRIEGFDIAHLAGKFTVASLVS
FHNGIPDKKNYRYFKIKSLGGAIDDFASIREAVARRYTRLINEEAELPDLILIDGGAGQV
SAAREILDELGLDCGLAGLAKKNEEVYLPDRIGPIVLPKDSPALRVLVAVRDETHRFATG
LSKKLRTENLKFTVLEAVPGIGEKKARKLLKIFGSLKEIAMADVERIAGEAGISRETALM
VKEKAGGSYGL*
>SPSA8_v1_280004|ID:41145690| MutT/NUDIX family protein (modular protein) [Spirochaetes Bin 1 SA-8]
MGSSTKKNRNSDNASILQEQNERAAKTLLHLGGIREAAAAFRFCPACASPKLYSVRDRMW
VCPDCGFEYFHNVATAAGIIIEVNGSIVVLKRAKEPRKGSYALPGGFVESGERAEDAALR
ECREEIGWAPEKIEFLASYPNIYKYHNIPYATCDLYFSAKVPSFNSAELDIDPEETSEVL
LLSADTIPWDMIAFESAQRALRKYLLSRSIGLPPRSMEWEALE*
>SPSA8_v1_280005|ID:41145691|pyk| Pyruvate kinase [Spirochaetes Bin 1 SA-8]
MDKRTKIVATTGPVSENPETIQALVEAGVDVFRLNFSHKSHDEADRAIAMIHDVRKKLRK
PVAIMADIKGPALRLYGYSEKIQIARGQELSIQSHDPIGIESSKSDEPGEVFTNLPNIDR
ICAIGQKIILMDGYFTGHVVGRGKDSVRVKIENEGELRPKAHLTIPGADYPIPFLTEKDI
ADINWAVSRNIEFIALSFVRCATDIEEVRRLVQRTAVSMSKHTHIKLIAKIESAKGLANI
DEIVQAADGIMVARGDMGVEMPIENVPIAQKSIIRKGYLAAKPVITATQMLESMIENPMP
TRAEVSDVANACFDSTSAVMLSGETAMGKYPVQVVSVMRSIINTVEKQFDYLDFHHDVPP
EVQNGDIPAIMSYNAVSVAYRCNAKALIVLTETGHAARLLSRLRPRMPIYAFLTNEGLYH
QMALNWGVTPFLHSGKGTRLDAVVDEAITICKKGNLLAAGDKVVIVAGLPLAQQGSTNMI
RVETIE*
>SPSA8_v1_280006|ID:41145692|ribA| GTP cyclohydrolase II [Spirochaetes Bin 1 SA-8]
MIIPSVEQVIREDVEFLRHCPHADNCAACSEEVCVMVASVADFPTEYGHFTIIGFVNNKD

GKDHIIILKGEIGDGEDMLLRIHSACLTGDALGSMRCDCGPQLHHALEIIEKAGRGAVLY
HQAEGRGIGLVNKLRAYALQDAGYDTYDANLALGFKADERDYAIPAAMLKKIGIKSVRLM
TNNPEKVAELEKHGIVVKERVQHELPAHEHDRAYLETKKERFGHFLDLDH*
>SPSA8_v1_280007|ID:41145693| conserved protein of unknown function [Spirochaetes Bin 1 SA-8]
MNFLPACLTDLTKAGLKPGDLDVIFVTGDAYVDHASFGVALLARFLESRGYKAGILARPD
PDDVEAFRQLGKPRLAFLVTAGAVDSMVSNYTANRKLRSEDEYAPGKARELCQRADGTIG
PGIKNRSNARPDRATIVYTGMCRQAFKGVPVVIGGLEASLRRLAHYDFWSDSVRKSILLD
SKADILVYGMGELQLEEILKRLTEGSALDGIPGTVRAVHVGAMPEHELKQAYPEALILPG
YSAISGADPVSRQHFAESFAAQYRNTDPFTAKPLLERYGDRLVIQEPPIRLLERGELDAL
YALPFTRQWHPMYQVFGGVPALAEVKFSLLSSRGCYGACSFCALTMHQGRLVSSRSAEAI
VREAQELAALPDFKGNIHDVGGPTANFRKPACEKMAKKGACVDRLCLAPKPCKNLSADHR
EYVEILRKLRALPGVKKVFVRSGVRFDYAMLDPSDEFLKELVEHHISGQLKVAPEHVSEK
VLQLMGKPPLSSFEAFKKKYEALNQSLGKKQYLIPYFISAHPGSGLAEALELALYLKKTG
FVPDQVQDFYPTPGTLSTAMWWCGMNPLDGEPVYVAKGAHERSLQRALLQFNKPQNRELV
KEALQMLGRTDLIGNRPGCLIR*
>SPSA8_v1_280008|ID:41145694| conserved protein of unknown function [Spirochaetes Bin 1 SA-8]
MSISLIVVLSISALILVMAGVAVGVKLGKVIASSKAESRLDLERSDAVKRSRAVIGGQVL
EQVAPYLPDFPCHPQDVRFLGKPVDFVAFSGSNENDIDEIVFIEVKSGDSALSAAERSIR
QAIIDGRVRWVEYRIP*
>SPSA8_v1_280009|ID:41145695| conserved membrane protein of unknown function [Spirochaetes Bin 1 SA-8]
MNSRLLKLISLYIKSTFAISLPAKAEWKKPKVLFKWIGIAALTILVIADFSFIFIMMNLS
LYDGLKPAGMQSMMLLNAATTSAVLVFVFAFLMALSMFSMAGIESSFLVLPFSARQLFAA
KIALVYVSEVILGAFMLLVAMVIYGIKESPPFMFYVNGLITAFALPLLPSAIAYAILVPL
MNTSKKFRNKNFILYVGGFMGMGFALLFNLYLQRSMANVANPAQLALMTGPDSFISKFGQ
AWIPSWLAWISMRRSDSLTGFLAVLGNLAIGSAALAAVVFLFGKAYVRSIQDFSETTIRK
RRLSRKEHDSLFRASPRLLSLIKREFRLMNREPMYLLNGPFVILLMPAILAIVFIAQKDA
LREISGLLAPMLQGPAGYLVPAGFGAFLGSSTSIACTSVSRDAKTIPFIRSLPITPGSYF
LAKFVHAETFSVLGALFGCLSGLYLLKNGLSDTLMAFALALLFTTAVNIYGLWLDTANPR
LRWDNPIAALKQNPNSIIVILLVMGLVGXLGYLSSILKLSRAGYFLVYGGILLALDIAGA
VFFGRYAARRMEYLE*
>SPSA8_v1_280010|ID:41145696| conserved protein of unknown function [Spirochaetes Bin 1 SA-8]
MIELKNISKTYGKSTVKAVDSLSLTIPNGVIFGFLGPNGAGKTTTIKALSGALRLDEGSV
AIDGISMADAPLEAKQHIGLVNDNPELFNRLKAHEFLNFIGDVFSVPSDIRKERIDDLAR
RFSLSDVLTTSIGSMSRGMKQKLSIIASLIHDPENWILDEPMVGLDPQASFELKEIMRER
TKAGKSVFFSTHVMEVAEKVCDRLAIINKGKIIFAGNLDDLRLLKSGQQNSLEELFLSLV
DSTNGVS*
>SPSA8_v1_280011|ID:41145697| CBS domain protein [Spirochaetes Bin 1 SA-8]
MAAMATVPINTDSGPSVVLELLYQLKVRDAMSGSVVTGSADMTLREIQLLMKEHAITGVP
IVEQGRLLGLVSIGDIIQALDDGHINQLVREGMNTSVISLDEDMPLSFAVTYFERYNYGR
FPVLGPGGSLVGIITASDIVRRLLVAMNEEISKLERRISGQNIDRDSDAPTVMEMSFAIA
PLDFAKAGLASTKFKKALIDRGCPPELARRAAIISYELELNQVIHSVGGVMRLKVMDSII
EITAQDEGPGIPDLEKAMQEGYSTANEWIRSLGFGAGMGLPNAKRVADRFTITSKVGKGT
TAAAILICSTGRN*
>SPSA8_v1_280012|ID:41145698| conserved protein of unknown function [Spirochaetes Bin 1 SA-8]
MRIRELIQLLGAEVVQGVYEDVEIAGGYTSDLLSDVMGHAKAKEALITIQAHRNTIAVAG
LVGMPAIVICNSRPIPEEMISAAREENIAILRTDLSQYEVSGRLWSVLSAGKSA*
>SPSA8_v1_280013|ID:41145699| PHP domain protein (modular protein) [Spirochaetes Bin 1 SA-8]
MTLKADLHNHSCFSPCGDLSMSPSVISRTARKRGLKLVALTDHNGALNTLAFAVTSARDG
ILPLFGLELETLEEVHLLAVFATPREALEFGQLMFRYLPKMPLDSVRFGDEAVVDADEQV
LGLHEIWFGASLDLSLDEAAMMAKQGGALVIPAHVDRPVNSISSQLGFLPDGPFDAVEAI
RPVGAALSGGLPVITGSDAHFPEHIGRRAFSMELPDSLTSALNLSLEAFYVKSWRFEPEL
ETLDYKRFIESSVCREYPEEEAHAVFEAVRREGLPGAAAFPFS*
>SPSA8_v1_280014|ID:41145700| protein of unknown function [Spirochaetes Bin 1 SA-8]
MKHAVSKSSPSARALDFSPWTKRFLFPALLLMLAFSSCSGKIEMAVRSDYSARIAVKMDI

PEALSARIRQIGNIGASASLFDVAKIRNEFSARKTLLLVDASSASKDAMTAVLWVPNLED
LARDMSLTPPGMIRFETVSGTQAQGVLRELSITIDKSNAARAFALFPGIDSRLVDSLSPP
ALEEQSITANEYRMNLENVIIGKKAMPAFDACALEVNLTAPKTIVSATGGTVAGQTFKAK
ISLFDLLVLEKPIVLAVRWIN*
>SPSA8_v1_280015|ID:41145701| putative Adenylate cyclase [Spirochaetes Bin 1 SA-8]
MGAMKTRLRRFSEEAYYYSSQLVVLFVVIIFLTSGSLSMGITSSLIIVFFMIAQIMLLVS
QGHLPLLRFLFSLITPLGYSILRAATSNYAFTETTSLLLWAAAIYIGFFQAVSLSFPQGF
IKRIAETALSLGSAVIFFAFYYYLDLRISLQKAYEMGEISLEVFRTSLQIGSFAKGLQTF
ITSPQHLFALLGIISFDTMLLASRMRSISLHARLDAMLAQKAPGPEQVPAEESTFGQDIR
RDSGPVRLIVSAVSSDIISFSSLSEMLGAQKAADFLNRYYALWTHAASPLGGRIISTGSD
SVIVLFGLVDETNQADRALQAVYDFMDKFSGLKEDLAELALPNEVRISIGVHTGSVVAAT
LGPPGEQKKTVFGDTIAVAARLDSLCREMHQEILVSHSTYRRLSLEQQATLDRIGEVLLR
QSSRPVPVYTKK*
>SPSA8_v1_280016|ID:41145702| conserved exported protein of unknown function [Spirochaetes Bin 1 SA-8]
MKKLAKLTAILLALTFILAGADVFAQTKAKFHIGVVTGTVSQSEDDLRGAERLIKDYGSV
KTGGMIQHITYPDDFMSQQETFISSVVALADDPLMKAIVINQAIPGTTEAFKRVKAKRPD
ILLLAGEPHEDPLVIQSSADLALSADFVSRGYTIIWAAKQLGAKTFVHISFARHMSYESL
GRRRAIMEEACKDLGLKWAFETAPDPTSDVGVPGAQQFILEKVPQWIEKYGKDTAFFCTN
DAHTEXLLKQLFQYGXLFVEADLPSPLMGYPGALGLDLSKEAGDFPAILKKVEAAVVAKG
GAGRFGTWAFSYGYSLTAGLGEFAKNVIEGKAKKDSLKDVFTALGKFTPGAKWNGAYYVD
MGTGVRAKNQVLIYMDTYVFGKGFLPTTKQKVPEKYYSIKAKK*
>SPSA8_v1_280017|ID:41145703| Monosaccharide-transporting ATPase [Spirochaetes Bin 1 SA-8]
MGHSGPLLSLKNVSKEFYGNVVLQNVSFDLHEGEILGLVGENGAGKTTLMRILFGMPVIA
ETGGFKGSISIDGSEVHFSSPFDALDAGIGMVHQEFSLIPGFTTTENIVLNRESMHPSLL
NNVFGERMSILDRPIMKTRAEKAISKLGVQLDPDMLVSEMPVGHKQFTEIAREISRDKSR
LLVLDEPTAVLTETEAEILLQSMRKLAKEGVAIIFISHRLHEVLTVSDRVVVLRDGLVVK
DVPSKGLAVKDIASWMVGRSVSGETDRSNEERHFGEAILSVKNLWVDMPGETVKNVNFTV
RKGEIFGIGGLAGQGKLGIPNGIMGLYPAGGEVNFNGKPVVLNDPAGALKAGMAFVSEDR
RGVGLLLDESLDWNIAFTAMQVHGQYLKPVLGKLFSLRDDRGMEDLCKEYISLLDIKCTS
HKQKAKELSGGNQQKICLAKAFALKPNLLFVSEPTRGIDVGAKKLVLDTLHRYNREYGTT
IVMVSSELEELRSICDRIAIIDEGRIAGILPATEDVAEFGILMAGETVSSEETANA*
>SPSA8_v1_280018|ID:41145704| ABC-type transporter, integral membrane subunit [Spirochaetes Bin 1 SA-8]
MRDLKTFIQDFGWPRIIIFFFLVSLFILAPFVGVRLDASVSDVLNRFGQYAILVLALVPM
IQSGAGLNFGLALGIIAGLLGSTLSLQFELRGFIGFFGAILLAAPFSILFGYLYGKLLNK
VKGEEMTIAMYVGFASVMLMCIMWLLLPYNNPTMVWGYAGKGLRTTITVEGYWLKILNNF
LSIPIGQFFVFPTGLLLFVGFCAFCMWIFLKTRTGTAMTAVGSNPDFARASGVSADKMRV
ISVIISTFLGAVGIIVYQQSFGFIQLYTAPSPMVFPAVAAILIGGASINKANIINVLVGT
FLFQGLLTMTPSVINSILQTDMSEVIRMIVSNGMILYALTRKTKVTK*
>SPSA8_v1_280019|ID:41145705| ABC-type transporter, integral membrane subunit [Spirochaetes Bin 1 SA-8]
MSAHKKSFTKFLSEYAVVLIFVVITLAAIAPSGLSIKYIVQEVITRLGRNSFLVIALLLP
IYAGMGLNFAMTLGAMAGQIGLIFAVDWGVAGWQGLVFALLVGLPISILLGWITGKVMNK
AKGREMVTGYILAFFINGIYQFFVLYMMGSVIPMRNPAIVLSRGYGVRNTLNLESVRQSL
DSILMLRLGGFAVPIVTFVIIGLLCVFIVWFKKTKLGQDMRAVGQDMAVADAAGIPVEKT
RIIAILISTALACAGQIIFLQNMGNMATYNAHDQTGFFAVAAILVGGASVTHANIPNVFI
GVILLHAMFIVTPMAGQRLFGSAMIGEYFRQFIGYGVIALSLVLYAWKNRKAIEDARSGL
RQGQPVQKEQNDKGGKA*
>SPSA8_v1_280020|ID:41145706| protein of unknown function [Spirochaetes Bin 1 SA-8]
MNLAQTLKSKKFIIRALLVAAYILLGIXX
>SPSA8_v1_290001|ID:41145707|zwf| Glucose-6-phosphate 1-dehydrogenase [Spirochaetes Bin 1 SA-8]
MNVINQDYPVCNLEAHRFAPEPAFFIIFGATGDLAHRKIFPALFDLHQEGALPEGMVIIG
FARRSLQHAEFRKQVLEDVRAHARHSTFADSAFDAFSSRIWYLQADLEQAEGYQTLAKII
AGENPGFFAADDGNPRSLSLPRNALFYLAVGPEHFGGIAENLGTAGLGTIAADSGKPDSP
ASGNSWRRLVVEKPYGRDMESARALTNLLHRHFREMDIYRIDHYLGKETVQNLLYLRFAN
SVFEPIWNCHHISAIEISVFEQDGIGQRGGYYDTAGAARDMLQNHLTQLMCLVTMEPPAT

LDPESIRDEKVKVLQSIRRYSASELVRHARRGQYIEGTGPNGAVIPAYRKENKVSPDSAT
ETYAQLTLELDNWRFSGVPITLRTGKAFAEKFSQIVIRFKRPPSALFAAQCGEKLVSNAL
TIRIQPDEGVWLTFNAKLPGVPAIKASHLRFSWREVSNYLPEAYERLIADALAGDSTLFI
RADESEHAWAVIDELEKAWRETDPEATPEAGGLWRYPAFSPIPPSQA*
>SPSA8_v1_290002|ID:41145708| putative 6-phosphogluconolactonase eukaryotic type [Spirochaetes Bin 1 SA-8]
MMIETFASREAWLSRTSQAVIMVLKHAAVSEKENFHICLAGGSTPKELYRIIAGSSECRA
LSAGIQIHLWQGDERCVAEGSPFLNSLMIIDAFRQTSEGAERWPNPPIFHRWTCDIQNLT
PEASAVRYAAELESQLAASGTNAFDLCILGIGADGHTASLFSLQDAIDSSKPLAFPTMSP
QEPRLRVTLRGNLLKSSSEVFVIAAGKGKKAIIEAVSKRKMMYPVEAVLPEQAHVFYLET
*
>SPSA8_v1_290003|ID:41145709| conserved exported protein of unknown function [Spirochaetes Bin 1 SA-8]
MRHHQRTIGTFFTACAFLLSASIAFSQPRPPVEKISFSHFTWTVRRTEEPQGPGSNYFGG
RDLSVFSNPDGSVTLKLGYKEGIWYAAELTMDKRLGNGTYVFQIDTPLAQLDSNLVLGLF
TYSRQNKFNHAEIDLEFSAWGIKKAPVLGQFVVQPYTIDGNYKTFPLPREEGPTSYAFTW
LPDRIEFAAWLGHGPLPSTASPSLIAQWSYTNTQYIPKSGQEYLHMNLYLAEGCSSPSGN
GRTSVMIRNFEFMPAK*
>SPSA8_v1_290004|ID:41145710| conserved protein of unknown function [Spirochaetes Bin 1 SA-8]
MLKEEFIKKSPVRILERSIEGGLKPGNIGIIASKKGIGKTSVLVQMALDKLMQGEKVIHV
SFNAHTSYVISWYENIFTEIAKRKNLENISDLKDQLVRNRVIMNFTQEGVSVDQIIRSLR
ALIVDGAFEAKTIIVDGFDFSRATAERFEKVKQFIQEMNLEVWYSCTLGGEEPIFDKHGT
PIVLREIMPVISVLIVLEPLHDYIHFKVVKDHERVNPQDLSLKLDSRSLLIAEN*
>SPSA8_v1_290005|ID:41145711|fusA| Elongation factor G 2 [Spirochaetes Bin 1 SA-8]
MSTDIHKMRNIGISAHIDSGKTTLSERILFYCRKIHSIHEVRGKDGAGATMDSMDLERER
GITIQSAATNVTWKDYTINLIDTPGHVDFTIEVERSLRVLDGAILVLCAVAGVQSQSITV
DRQLKRYHVPRLAFVNKCDRTGANPFKVKMQLQEKLGLNPAFVTIPIGLEDKLEGVIDLV
EMRAVYFDGPNGEDLRFAEIPAHLQADAEKYREELVESASMFSDELAEAYFEGNVSKDLL
VGAIRAGCIAEKFVPVFVGSAYKNKGVQLLLDGVASYLPDPTEVRNVALDLNDSEKEVEL
AADPNAKTVALGFKLEDGQYGQLTYVRIYQGELKKGDELLNTRARKRFKVGRLVRMHANS
MEDISEGSCGDIVALFGVECASGDTFCHPDLNYAMTSMYVPEPVISLAIEPKDKKSSDQM
GKALNRFTKEDPTFRSFVDPESNQTIIQGMGELHLEVYIERMRREYKCEVEVGRPQVAYR
ETITQRADFNYTHKKQTGGSGQYGRVAGYIEPCEEQVYEFVDQVKGGAIPSEYIPSCDKG
FKASMQKGSLIGFPIVNVRCLVNDGQSHPVDSSDIAFQLAAIGAFREAYEKAKPTILEPI
MKVNVEGPTEFQGNIFASLNQRRGIITASTEDGTFCRVEAEVPLAEMFGYSTVLRSLTQG
KAEFTMEFEKYGKVPTQIAEQLRKDYLEKRKKEQQK*
>SPSA8_v1_290006|ID:41145712| OsmC family protein [Spirochaetes Bin 1 SA-8]
MNRVWKAPQLAMTAKVAIFYLLHENAEIKGVRMMKAEIVSRWKGDMAFEADVNGHPVMMD
ADEAVGGHDSGPRPKALFLASLTGCSGMDVISILKKMREPCTWFEMRVEGTTAEEHPKKY
TQIKLVYRFKKSDNLNPDNVRKACQLSQDKYCGVSANIKDSADLTWDVEFV*
>SPSA8_v1_290007|ID:41145713| Major facilitator superfamily MFS_1 [Spirochaetes Bin 1 SA-8]
MQNWKKSFNALMVAELFAIMGFSTSNPMIPLFLRELGVHDATGLNWWTGAINGLSSLALA
VFAPIWGALADHYGRKLMLLRAMIGGAVIMGLLALANAPWQVLMLKILQGCVTGTVAAAT
VLTASLVPAAEAGYYLGLMQMSVFIGNSIGPLFGGVITDLAGSRVNFLATSALLALSAFL
VIRFVAEDFITDKKTGSLLKNAVPDFSAVRGNASLKALLILIFTIQFANAVVSPIIPLVV
LHMTEAFRGTGSLSGLIIGIASIAGALGALITGKVSNRIGYGKALLICIAGSFLFYFPQG
FAVTPYQLLVLRFFTGFFLGGTMPSANALIAQQTSKEHQGAVFGISSSISAGGNALGPAF
GALAANLVGYPAIFFIATLMLGVLAFFMSKRILVSSQR*
>SPSA8_v1_290008|ID:41145714| exported protein of unknown function [Spirochaetes Bin 1 SA-8]
MESQTKRIVLAVLMIIVCASVFAQNSPRSTSATIQIVAVVPSILKLNLDFANSATATLLG
YLRSDGTTNPMPAGKGNQFEIKPGATVELGSAHLFSNVPGSYTIIAYSANGGALRNSYSM
TNDAIPYSLILGSMQAVPQGGAFRFTASGKSTKDGTELKVALALGAVPANAPSGFYADQL
MFSVSAN*
>SPSA8_v1_290009|ID:41145715| protein of unknown function [Spirochaetes Bin 1 SA-8]
MIISTASTIRFVWLSIAYLLFLIYFKYTFEKTPGRGNIGASIRNIMKNNRIF*
>SPSA8_v1_290010|ID:41145716| Phospholipid/glycerol acyltransferase [Spirochaetes Bin 1 SA-8]

MDSIRDRYKPLITEMMTHMGSQVSVSEKDVLQEGNKELLPYIDTIIADHLLPGSGIVGFE
NLAALQQLSDKGEPCLILAEHYSNFDLPAFHYMLRQHGVDGSKIADKIVSIAGIKLSESN
PVVNAFAKAYSRIIIYPSRSLEIIKKNYKDPNKLYHEVRRSFAINRAAMKALNSVKERGR
IVLVFPAGTRFRPWEPETKRGVREIASYIKSFSKFCLVAINGNILRINPSGEMEEDLLHP
DKVVLTVSPVFDSKEFLAGIKLDHHFRDDKKQEIVDHLMGILEKMHDDAERDRLS*
>SPSA8_v1_290011|ID:41145717| Rhomboid family protein [Spirochaetes Bin 1 SA-8]
MKPDNLLRRPFRYSYFNATLYIIAANIVLFALGFMFPYLRAYMALNPAAVLSGYVWQFVT
YMFAHASLSHLFVNMLGLFFFGPPIERKMGSKEFILYYFLTGTLAGIASFAIFALTGAWY
TMLVGASGALFAVLLAFAVIEPDAMIYLYGIIPLKAPVMVLGYAGIEIVSQLLNFRSSVA
HLTHLAGFVFGWLYFLVRFRVNPLKRLITRR*
>SPSA8_v1_290012|ID:41145718| protein of unknown function [Spirochaetes Bin 1 SA-8]
MSKAQKQLLILSLPMILFVISCLAFWGAGASVVRTKGTIMETLLTRLKFDIAESVDSIVG
KGDQLAALLAGNASVDEPGITALAERILREIPCVKGIAIAPDAIVKSYYPASGNENLIGH
DLLTNPERRESLALAVESRKPIVSGPFSSVDGTVFFIRYPVFQHEKLWGFISLTISFDEF
KNAIGLEQRYPGLQISISRSNRPFSAQSSQLQLHGEAWDMGIQFGAASTLFSIALVLLFA
ISLSASVFLFILLRQNNKIAQGKPAPRAGQKAAAVSTEHTQASSPALKAENLTQHNFRNT
DNAKPEAVSRTKREKVEFQGPDVPGQLFMPEMLVEGNPAHVFAAYAGKKTEPALEAESLP
RLEKPASTVISFIKAETKPESPKIAVNHEEKAVQPEPLELDFGKEQGMAREQTPVSIHDI
SILVVDDSEANRDIMGRMLALRKYNAEFASSGSEALALCASTQFDVIFMDCFMPGMDGYK
TARALRDQKLAERSVIIGMSAKVGQQELDLCMQSGMDDLLPKPFTISELEFRIRKHLS*
>SPSA8_v1_290013|ID:41145719|secF| Protein-export membrane protein SecF [Spirochaetes Bin 1 SA-8]
MKRIIPFTRYFLPAVLVSSALILFGLFGFFTKGFNLGVDFQAGVNQTVQLAYPVASISYE
GKGNAEMTVSETAITLVFSGAETDSKTVVLNYKDNPTVADLAKALEALPAVKVQLEPGQE
NSASTLLVPTYQGNTLINLKPALVHRLPASDTERFASIDKVREAVKDMGRVSVQTINPTS
QQRFMIRVQDEGKDPRFTETARAGIISGLENAFGKNRVVEVKTDFVGARFSQGLTQQSIF
LVLATMVLILLYSTIRFKFEFALGAVLAIMHDALIMIAFVVWSRMEFNTTTIAAILTILG
YSINDTIVQFDRVREERKLRPTDKFADILNSALTHTLGRTVITTATTMLAVLSLFFFTTG
NIKDFALALLVGMTSGAYSTIFIASAFVLFWNQRKEKKGHASSIPAASAAGKAQPAK*
>SPSA8_v1_290014|ID:41145720|secD| Protein translocase subunit SecD [Spirochaetes Bin 1 SA-8]
MSKTRRLITVIVVLAIAFAFLFPTIEWYVLTPKEDQAIAVGSREQIRDFSRRMAYSVLID
IKNKALAGDSTDLSNQSKDLALVVSVAKKAYSDAKRPQPKKYDAASILAAFSSERAMFDA
VEARFRERTLALKNLHGNALQLGLDLAGGMSVVIQADMKALAEKLGHDLSPAEREDAMKR
GVEVLNSRIDKFGLTEPVIRRQGEDQIYVEIPGTPDPERINSIIMGKGNLAFYIVDSEAT
AALSSYLATNPLGVDERTMTVAQAGLIPEGKIIRKVYKKDSYGLDEFTGEYLVLEGKPGL
DGSHIQSATVSSDQITGKPETNFVLDKEGGDLFYDLTSKNVGKTMAVVLDDRVKNYARIQ
EPIRESVRITGFSADEAQALALLLRTAALPISLSVVTQQAIGASLGEDAIAQGKTAIIIG
LLAVLVFMFAYYKGAGVNATVAQAVNLFIMISVLTAFKLTLTLPSIAGFVLTVGMAVDAN
VIIFERIKEELRSGKTRKAAIDAGFHKAFWAVMDSNITTIIAALFLAQLGSGPIQGFAIS
LAIGNMTSLFTSLFLSKLIFDFETDVLKKQNVSISWRAE*
>SPSA8_v1_290015|ID:41145721| Preprotein translocase, YajC subunit [Spirochaetes Bin 1 SA-8]
MNAISHGLVLLQAASANPTGQMVSTLVTFGLVFVIFYFLIIRPQNKKQKDMQKMIAGVKK
GDKIVTIGGIHGTVYSVKEGTVVVKVDDDCKIEFSKSAIASVSVAKAEEKAEEPASDKTE
EKK*
>SPSA8_v1_290016|ID:41145722|cpkA| Carbamate kinase [Spirochaetes Bin 1 SA-8]
MTGRKTIVVALGGNAIIEEGTEGTIEQQFANTRKSLDAIVGMIQEGHKVVLTHGNGPQAG
VHLIRNEAASSQVPPSPLNVIVADTQGSMGYMIAQCLSNALLKAGVKKDVVTVITQVEVD
PADPSMQNPTKYVGPFYKAEQVEKMRERGWVIKEDPGRGFRRVVPSPIPLDVIEKETIKD
LIDDGKIVIAVGGGGVPVKREADGTLSGVDAVIDKDRASALLANLIGADELVILTGVDRV
AINFKKPDQKFFDRMTVAECEQYLKEGQFPKGSMGPKIEAACDFIKRGGDKVVITTMENA
TLAVDGRAGTVITA*
>SPSA8_v1_290017|ID:41145723|ispH| 4-hydroxy-3-methylbut-2-enyl diphosphate reductase [Spirochaetes Bin 1 SA-8]
MIIHRAEVLGMCMGVRRAEALAREAGAAGAASGKQVFTYGPLIHNPQAIESLRKYGVQVL
DPEQFELGLLDDQVKNSIVVIRAHGAPLSTFNRLKALSAAIIDATCPRVLASQKKAVQLL

HDGYVVIIAGDASHGEVAGILGHAPGAMVAENPESARRIANSIKAGKAALIAQTTIKESE
YKAIENALKEKFPGLLVIQTICPATXDRQEALRLLADSVDALVIAGGKNSANTKRLFMAA
LESGKPAWHIEKPEELPDEVFDKEVVGLTAGASTPDFIVDEVEKYLMQGANRHGIAE*
>SPSA8_v1_290018|ID:41145724| Bacterioferritin comigratory protein/NADH dehydrogenase [Spirochaetes Bin 1 SA-8]
MELLNEIEKRRARRALSSQPLSRECLTRLVKAATFAPSCFNNQPWRLIVLEKATASPEYE
AVAAALTPGNAWALAAPVLFLFATGNHLDCRLDDGRDYAYFDLGQAAMALQLQAVHEGLY
AHPMAGFAPAKVRAAVHLPPELVPVCVIAVGAPGDMALLNEGQQKTEVSARSRKNISEVA
FMNFYGNSADLG*
>SPSA8_v1_290019|ID:41145725|udk| Uridine kinase [Spirochaetes Bin 1 SA-8]
MNPVKVIGICGGSGSGKTTIVRKISEVVSDFVFLPQDNYYKSAEFISNSNITAFNFDHPD
AFDNDLLIEHLTQLRSGSPIAMPVYDFVHHRRTEQTIQVTPRKLVIFEGIMVFTNKSVRD
LIDLKIYVDTPDDIRFIRRLNRDIKERGRTVDSVVEQYLNVVRPGHYEFIEPTKSYADII
IPEGGANERALNVLVTFINSVLNGAP*
>SPSA8_v1_290020|ID:41145726| protein of unknown function [Spirochaetes Bin 1 SA-8]
MSKKPLSLTIVPMAVIIVSVFVIGIAGANLLGVWKTTNTKEPVKIKTGEFAGLPNPADIR
GSYSWADIGKAFNIDVQHLLAAFMATDPAVKVNTLEAIYPKEILPAGMEIGTSSVRLFVS
LYTGIPLAAEEGTILPLSAIAVLKEFGKADPGLIAAAAAKAYDPSAKQPAPSPVPQPEKP
VKPTETASAVPAASSTTVTTIKAALASETAAQTTTAKTTSTTAAGTAETHEVKTGTVTGK
TTFKDLKDWGLTEEKIKSATGGKIGSDNTVIKDWAEPNGLTFSELRTKLQALLDGK*
>SPSA8_v1_290021|ID:41145727| conserved protein of unknown function [Spirochaetes Bin 1 SA-8]
VLAWVSYMTIITGKLIFQDYDPYYTLFNFWTGEVAITGYIALALVIILSLFVERPFCKYA
CPYGAFLGIFNLFRIFGIRRNAPTCISCKICDKACPMNIEVSTSKVVRDHQCISCLECTS
ETACPVPATVQMLALPGTVQDSFKKKSETSEAAK*
>SPSA8_v1_300001|ID:41145728| protein of unknown function [Spirochaetes Bin 1 SA-8]
MRGSMKSIGKNFCTHRMLEEYHENYYQPAMAEGERLKAEHYESSRSLAAYLQKAYSQWPQ
ISVLQVSDDAGPVIARGTRITVNAIVNLAGLKPEEVRVECYRGKLNSQGEIKDPERTEMK
PTAQEGSRCTYQAVVNGSSTGQVGYSIRILPEHPALNERFIPGLVTWA*
>SPSA8_v1_300002|ID:41145729| conserved protein of unknown function [Spirochaetes Bin 1 SA-8]
MELSLFTMDDYDAVYALWQNTPGMGLNSIDDSRESIARYLQRNPATCFTAKEGKTLAGVI
LSGHDGRRGFIYHLAVAASYRRQSVGRQLVERAAEALKAEGIAKVALVVMAKNELGNVFW
ESIGFHQRNDLVYRDKVITDKTMRRMDT*
>SPSA8_v1_300003|ID:41145730| HAD-superfamily hydrolase, subfamily IIB [Spirochaetes Bin 1 SA-8]
MQPIDELTAETARKIRFVLMDIDDTLTTDGKLLAESYAALWQLKKAGLVIVPITGRPAGW
CDLIAREWPVDGVVGENGAFAFWESEGQRLKALTHPQATPNTSPLLQEIRDAALQQFPGL
RIARDQFSRLYDIALDFAEEEPRLPLATAAEVKKLCEQYGAHAKVSSIHVNAWLGEYDKL
SMAIRFLGERFGYDDAAMRGQVVFVGDSPNDEPMFAHFPNACGVANVIRYETMIKTLPAF
AADQEGGLGFAQIAAMLIARRTC*
>SPSA8_v1_300004|ID:41145731| FAD dependent oxidoreductase [Spirochaetes Bin 1 SA-8]
MVESYDVAIIGAGVCGANIARRLSQYELRVALLEKEIDVSLGTSKANSGIIHGGFHDNIK
YLKAKLELQGAMMFERLHNELDFPFERCGILVVALHEDEMRAIEQLYLQGVENGVIGIEM
CSRERMLELEPKLSSDTVGGLYAPTGGIVEPYRFVFSLVESARKNGVELITQFNVAEAVS
ENGFWTIKAEDGRLIRARYVVNSAGLHADEVSKAFGGEEFTIKPRKGEYYLLDKTTKSRP
GKVLFPVPTAVSKGMLVVPTVEGTVLLGPTATTVEDKEDFSTTRAELDGILKSARSMVPS
ISETDVITSFAGLRPVYGDDFFIDISKKAVNLIQVAAIQSPGLTASPAIGEYVKDLLKKL
GLRLVEKADWDPYVRKVPRARDLSPFELDELVKKEPEYGEIVCRCERVSEAEIVKAIRDG
HTTLDGIKYVTRAQMGRCQGGFCTYKIIKILMRETGMSYEEVTKRGGDSVVLKGEL*
>SPSA8_v1_300005|ID:41145732| FAD-dependent pyridine nucleotide-disulfide oxidoreductase [Spirochaetes Bin 1 SA-8]
MKNFNFDAVVIGGGAAGMASALELKKRGSSVVIIEREEELGGILMQCIHNGFGLIEFNEE
LSGPEFAQRVHEQVEAQTIPSFCGTTVLDIRPEGDRKIVYCVSSREGVMQIQARAIILAM
GCRERNRGNIRIPGSRPSGVFTAGLAQRLVNIEGYIPGKDVVIIGSGDIGLIMARRLSWV
GCKVHAVVEILPYPSGLTRNIVQCLNDFNIPLYLSHLTTEIYGKDRVEGVEITPIQNGAL
VYEKSFRIPCDTVLLSVGLVPENELSRNAGVEINPATNGPWVDSTLMTSVPGIFACGNVL

HVHDLVDYVVEEARRAGSFAADWLAGKHPVRELRVKTGANVRYINPSRVDPSRQNKLYLR
SLIVKNDSVLEVKVDNRVVKQLKKAHIQPSEMLTVSLGPDELAQSGMDAQIEVSLL*
>SPSA8_v1_300006|ID:41145733| conserved protein of unknown function [Spirochaetes Bin 1 SA-8]
MQEMICITCPIGCHLSIEKISDEEISVTGNKCQRGAQYAREELLSPKRVVTATCRLKQLS
NQTATVGQAAAGSLYKPRRVPVRTAKAFPKERIPELLTLLYQHEVSLPVERGQVIIPDAL
GTGIDVIAERTMA*
>SPSA8_v1_300007|ID:41145734| protein of unknown function [Spirochaetes Bin 1 SA-8]
VTIGVTGAFGFLGASCIAELLADPLAPEQRLVAFGSSRFSSPLFDGSAVEVRSLDILDSN
SLASAFADIDLLVHSAGRVGFAAKEKRSVWDANVLGALYVFNAARKAGVSKIINIASVSA
LGPAPEFPAPEISAPEPGISLCGNPAAFCKPNYLSWQDKNRWYRPLTEQDRPYDDPGSPW
SFKXRDEALAAVRMSLAGDYAFLRRVSCIYLDSKLANLELAHELYQDKGLPIVNILPGTA
IGRGEAHTGIGDLVRALASGRLKAVLPGACSFMDSMDFAQGVKKALLFGRSGEDYILAGA
PENNLSFSQLASLVLESCEPGMPSVPARRPVHSGEILELPCFPARLAGYLAERLLPGLAL
SEGTVLSGCVKAPCSSEKAIRELGYAPFSPLKRAISEILK*
>SPSA8_v1_300008|ID:41145735| Heat shock protein Hsp20 [Spirochaetes Bin 1 SA-8]
MKGNRMYVDIGSILDDVFEAAKDFGEKMKNFGPGFEGMDPGCNPFKGEGGDHGSPWFESQ
ADENADYYPNFSYPPMNIYLTPDRNIIFEFALAGFEEKNISLSFQGDYMIFSAKIDLEQP
MDGVRFFKRRLKLKDIEKQKYYVPADKFDQEKVKAVFKNGILKVTVPPKEETESTEGIKI
EIIKEEN*
>SPSA8_v1_300009|ID:41145736| 2',3'-cyclic-nucleotide 2'-phosphodiesterase [Spirochaetes Bin 1 SA-8]
MKRIALVLCILMVSFSAFAQMEPAQKSIALTFIETSDIHGAIYPYDFVNAKPLATSLAQV
ASLIAEERAANPNVVLLENGDSLQGQPTVYYYNFEKTAGPHIWSQAVNFLGYDVVSVGNH
DIEAGHNVYDKLYEEIQAPVVCANAVTPDGSTYFKPYTIIEKAGVKIAVLGLTSPKIPDW
LPPQFWTGMEFEDMVESAKKWVKVIQETEKPDILVGLFHAGVDYTYGNVTADTKFNENAS
QLVAERVPGFDLVFVGHDHSGWDGMGWDPVNKKKVEVKDPNGKTVYIYGALNAARKVPVV
NMFLDWNDTTKSWEKRVRGGLVDISQYKSDPDFMAKFQDGFDGIKAWVDRPIGKMDGVIT
TRDSMFGDSAFVDLIHRIQLDLSRDPSLGLKPADISFAAPLSADAKIPTSADGTLYVRDM
FNLYVYENFLYTMTMTGKQVKDFLEYSYKFWFDTMPNEGNHIINFQKDKEGKLVLDNRTN
MPLTATRYYNYDSAAGVNYYVDISKPAGERVVITSMSDGRVFNPDATYTVAINSYRGSGG
GGHLTAGARIDPTVIRTMKLVNGATTKDLRFFLLKWFEAQTETVTVGPIGNWEVLPADLA
AVGAANDYPLLYPPKK*
>SPSA8_v1_300010|ID:41145737| protein of unknown function [Spirochaetes Bin 1 SA-8]
VNCCISRNRCAKPSAAWMATSTEAEPNYLAATIRITRLRNLALPFKTTI*
>SPSA8_v1_300011|ID:41145738| putative Protein kinase family protein [Spirochaetes Bin 1 SA-8]
VKASDRIGNYILIKELGRGISASTWLAEQIRAQDAAADAAAESDEPSPLAVLKILDLSEA
GSWNIVESFRREADALKTLSYPGIPRYFEYFEAAEENRIRLGIAMEYIEGRNLESIIQSG
QKFTETEVEHILAQLCDILAYLGSLRPPIIHRDVNPRNILMKPDGTVALVDFSGVQDAVR
TALFPGATLVGTAGYIPLEQVAGKASHRSDLYGAAATAVFLLTGRNPSELPSAGLRINLD
GIVELSPRLKAVLGSWLEPDVAKRSLSAADAASILKGKRDAPLVRENEVYDDIQQARTND
RDIRISGLARKIREAIQEQESQGLYSQRSSPPVEYPEKLPSDSKVTVTGDETGLVIKIPR
MGMKGQSGAGIFFPIFWLGFVGFWTMMTIGMRAPIFFPFFSLPFWGVGIFMAKTMLGPAL
TSKELVITSDGLLIRTGLPGFEKATTWPLSDIGKVRVVNSATQVNNVRPKELLIEAGSRH
LRIGTGLSERELLYLEKSMRETLSRMDGHQH*
>SPSA8_v1_300012|ID:41145739| putative Tetratricopeptide TPR_1 repeat-containing protein [Spirochaetes Bin 1 SA-8]
MNTADSPGSVLANLNKKGLNLIDAGKYESAIAIFSEALGHALSLGPTDAAGILYNRAEAF
RLSGNLTSAKADILKALEFSPEDADIMHAMGLVCYEEDTFEEAAAWYRKAIDIAPRHEKA
WNDLGVIHFRSGEYESARQSFEKAVSINPDFADAWFNLADTYDELGLKAEYRRALEALNT
ARMHVAEKDIL*
>SPSA8_v1_300013|ID:41145740|hndA| NADP-reducing hydrogenase subunit HndA [Spirochaetes Bin 1 SA-8]
MPLAQIEFSKELTEYVEEWGKKPGGLVMMLHRIQHEFGYIPREAAEKLSLMVGLPLAKIY
GVITFYHFFKTSRPGKNRIAICMGTACYLKGGQDLVEETQSILGIKGDEVTDDGLFSIDE
VRCLGCCGLAPVLMVGDEVFGKVTKDQLPDIIAKYRNK*
>SPSA8_v1_300014|ID:41145741| ATP-binding region ATPase domain protein [Spirochaetes Bin 1 SA-8]

MHFAVTDFLLDIVQNSCEAGASLIQLSIEETETSIAIKVADNGKGMDEAELKKALDPFYT
DGVKHPSRKVGLGLPFLKQATEQSNGRFEIRSEKGKGTEVFFQFFKHEIDAPPVGDIPGA
LVSVLCMPGGHEMLISREKREAGIRYELSRKELAEILGSFERMDSLILLRDFVRSQEEQD
*
>SPSA8_v1_300015|ID:41145742| NAD(P)-dependent iron-only hydrogenase iron-sulfur protein [Spirochaetes Bin 1 SA-8]
MAKMTLEELRALRDSKRQDLARRDVEGKEIQIIVGMGTCGIAAGAKLTFDAIVEAVQRHG
LQDKVLIRQTGCMGLCYVEPTVEVVMPGMPSVIYGKMTQDIADKFVRKHLVEHTLLDNHI
FDRPAADIVKK*
>SPSA8_v1_300016|ID:41145743|hndC| NADP-reducing hydrogenase subunit HndC [Spirochaetes Bin 1 SA-8]
MAYKNFILVCGGTGCESSRSDEIYRNLLAEAEAQGVKSEVQIVKTGCFGFCEKGPIVKVL
PSESFYVEVKPEDAKEIIAEQVIKGREVKRLLYRKDQKTADNVKVEDIEFYQKQFRIVLR
NCGVINPESIEEYIARDGYSALEKVLFEMTPEQVVAEIKASGLRGRGGAGFPTGIKWETA
MKVDSDIKYIVCNADEGDPGAYMDRSTIEGDPHSVLEAMIIAGKAIGANMGYVYIRAEYP
LAIERLKTAIAQAKEMGLMGQNILNSGFDFDVEIRLGAGAFVCGEETALLKSIEGNRGMP
IPKPPFPAIKGLWGKPTVINNVETLANIPVILTKGAAWFASIGTEKSKGTKVFALTGKIN
NSGLIEVPMGTTLREIIFDIGGGIKGGKKFKGVQTGGPSGGIIVEKDLDTPISYESLVAL
GSMMGSGGMIVMDEDDCVVDVSKFYMAFCVDESCGKCSPCRIGTKQMYNLLDKISKGNGE
MKDLENLEKIGTAMTKASLCMLGGSAANPTLSTIRHFRDEYIEHIVDHKCRAGKCKDLVI
YSIDAEKCIGCGLCARKCPVPCITGEKKKPHVIDASRCIKCGECFKACKFGAVVKS*
>SPSA8_v1_300017|ID:41145744| protein of unknown function [Spirochaetes Bin 1 SA-8]
LQLIMIVFPPFSALDYRAEFAGLEALAALDAAGSVNDMRLFLFPGDAGNRALSCAQTAAD
ALFSIDRVDNQVFAFARAALMINNMLDILVAEMTDRGKRRVGC*
>SPSA8_v1_300018|ID:41145745|hndD| NADP-reducing hydrogenase subunit HndC [Spirochaetes Bin 1 SA-8]
MINCKVNGIPVQVAEGATILEASKKANVKIPTLCYNPDLAPWAACGICVAKIEGSNKMLR
ACCTPVAEGMNIITHDPDIVETRKTVIEMILSTHPDDCLACPRNQNCELQTLAQEFGIRE
QAFPKMLHDLPIDDTTGSIVLNPEKCVRCGRCVTVCQQMQNVWAIEFLGRGETIRIAPAA
DAKLGESPCIKCGQCSAHCPVGAIYENDQTKIVWDALRKTGDDAKTCVVQIAPAVRVALG
EAFGLQPGTDLTGKIYTALKRLGFDVVFDTNFAADLTIMEEGTEFVKRLTSALQNGLGTA
TREKSMPLITSCCPAWVDYMEKYFPDMIPNFSTAKSPQQMMGAMIKTYWAEKANVRPDKI
FSVSIMPCTAKKFETHRDETMCSSGYQDVDVSITTRELARMIKQAGIDLLNLPESEPDSP
LGPYTGAGAIFGATGGVMEAALRTAYYLVTGNELKDVNFTAVRGISGIKEASVHVNGVVL
RVAVAHQMGNIEQVLNEVRKARDEGRDTPWHFIEVMACRGGCIGGGGQPYGATDEVRKLR
IRGIYDHDEGKEYRCSHQNPYIKKVYNEFLEKPGSHKAHELLHTHYTERPLFLK*
>SPSA8_v1_300019|ID:41145746| protein of unknown function [Spirochaetes Bin 1 SA-8]
MHSFTLTLFKEKRALGVVRMQKLVRLVASRFLEEFVVHFLDVRVLV*
>SPSA8_v1_300020|ID:41145747| protein of unknown function [Spirochaetes Bin 1 SA-8]
MNQEKQSGKAASGQNYAVSITCDGREIPLVPYIETLFGATIGAMLGTLKGAEDAKSITIS
LTKRTPPSQS*
>SPSA8_v1_300021|ID:41145748| putative Molybdopterin-guanine dinucleotide biosynthesis protein B [Spirochaetes Bin 1 SA-8]
MKQCVISVLGWSGTGKTTFIEQAIRECKKRGIKTAAIKKSRHEPAYPAVGKDSNRYLKAG
ARQSLFISEQAYIHETGTLSSGIQPLPPGDAVTIERWALSQIHGADIIFCEGLRPQHSLA
ILACAGARSESELKYPLRDVDILITDSPELAQTAEECAKKAFLPDAAASFIEYILGIED*
>SPSA8_v1_300022|ID:41145749| putative Methyltransferase type 12 [Spirochaetes Bin 1 SA-8]
MSAINSVLEHYYPYLAAIAKNSKNTKESLNRSDVMRYGNSLRELQRGLTGNRELPGSGYL
EHSDQLAVYTLYYWPISFVQTFIALSEIAQRGRLPRIETLFDLGSGPGPNSFAALEFGAK
KCSLLDSSERALNTALTLAERIISNTSNFSISYVNLETMPDDALPGAAETCDLIIASHSI
NELWKNEPDAASRRERLLRNAWEQLAPDGLLLVIEPSAMVTSRPALLLRDRLLTSLPDAE
CVAPCPGSNPCPMLQKGEARTCHSTWLWEPPQPVAELARAAGLDRDAVKATWYALKKSKT
RQTASADPCSAFGDTASALEGRIVSEPMLNKAGRIRYLVCTETGLKTVSAARTDSHAETL
GFFGLKRGDLVRFSTLQAREGVFNYGLSEKTSLHFIMKAPDVDSCQKPPRKP*
>SPSA8_v1_300023|ID:41145750| putative Diguanylate cyclase [Spirochaetes Bin 1 SA-8]
MEGMPPFSSVNQMFEVPRLRNILATYGFSLLDYRTCKGVLSDTLTARGLSPETFFANSFE

AHLSSEDAGKMRQAIQSLPASPTGKTSNQFRFINQETGNESWILMDFELFLAGEDGKPAL
VLIHDQDVSELIKAQEEIRERLVEIESLKDLVLSINKSLDFNETSRKIIEHLHRILPFDR
ATVQVLEGTNLHIIDSYGYPEDAVMGMTFPARNIDNPARRAITTRRPIICNDVEHDFEGF
VQIPGVAPTKSWLGIPLVYEGKTIGMFALDSSELHFYKDHHIRIASGVADQIAMAVAHSL
QHSKVKEAALKDKLTGAANRYGLETVGQTMFAAVQKEEQSLGILMLDIDHFKEVNDTWGH
AYGDLVLKSLASEVNKDLRVDDYLVRYGGEEFLVLLPKATAREALVVAERLRQRIPQIEI
IPGRSFPTVSIGIFAGIPGPLDQLHEFIQKADLGLYEAKESGRNRCRVWRANPEFYDKSN
KNLVL*
>SPSA8_v1_300024|ID:41145751| Tryptophan--tRNA ligase (fragment) [Spirochaetes Bin 1 SA-8]
MSKSLNNAIFLSDDEKTVYKRVMSMYTDPKRVSADIPGTVEGNPVFQYHDAFNPDKAEVE
DLKARYRAGKVGDVEVKEKLAKALNEFLDPFRERRAHYESKSGLADEVVYDGTMKMREEA
RKTLSIAKKAMGISAVWNRISRKAEDVKKKQAERL*
>SPSA8_v1_310001|ID:41145752| Aminomethyltransferase (fragment) [Spirochaetes Bin 1 SA-8]
VLHDWHAAHGAKMAPFAGYEMPISYPTGAVEEHAITRRSVGLFDIDHMGQFEVSGPGADA
YVARMVSAKTSDMEAPMARYSLLLDERGLVLDDLFIYKLESSWWIVVNASNREQDLAWFM
EHKTPGVTITDHSDATYMIAVQGPNAIALLDKVGAPAVSALERFTWGRIRIDSIDILFGR
TGYTGEDGGELFFPAEHAERLWNYLLEKGGELGIETKPIGLAXX
>SPSA8_v1_310002|ID:41145753| putative Lipoprotein LpqB, GerMN domain protein [Spirochaetes Bin 1 SA-8]
MAVKKRKKQQSSGTGCLLFTILFSVLLVLFIIKFPQIKATLEKTGLLQKLSTTVTTVPPV
PPEKTQIPSSATSVPQAEAPASPGTTTIQPREGNQVPQISTSTAETTRTVSLYFVSIADD
GVISSHEVKRAIKATDTPLSDAVKALLEGPSENEIRSKLISLIPRGTKLKGVSMRGSTAI
IDFNEAFMYNRFGTEGYTAQLKQIVYTVTAFPSVQDVQILIEGKLVDYLGGEGVFTGKPL
SRNSF*
>SPSA8_v1_310003|ID:41145754| conserved protein of unknown function [Spirochaetes Bin 1 SA-8]
MQGRVELAQAFIGETMERFQQSSSLPDKTLLDSLLHEAVAALTAENPEKRPYSVSAREKL
PGGFVRLSAPHVILIPDLHARFDFLSAVLTASIQAEGLWDESRTLLDSMDSGLLEIVCLG
DILHGESPESARNWVLAAEKFAKDGRDESLFSSEMDAEMGTSIAALMLVLKIKALFPESF
HCLKGNHDNMSNSSCDGDFAFYKYAREGAMGAAWFRLRYGDDLLKQMRTYERFLPLVAAG
RWFCASHAEPRRAISLEDLLNYRERPDVVTDLIWTGNGSAEEGSVRESLANLMSPGDVAG
FEGLWFSGHRQVNGHYALRQDGALVQIHSHAREQVVLLPNSRISGPWGVQNGLKAPRAVF
LELDRQYLVPSAFLR*
>SPSA8_v1_310004|ID:41145755| putative Tetratricopeptide TPR_2 repeat-containing protein [Spirochaetes Bin 1 SA-8]
MNNSERIRDRAEKAMSTIVFIQARQSILEQLGYQGLDESIPLPVQLPGPVARFDASMITE
ESIITGILRFLAWKPDAEHAEYYRTLVKALRPSLLEELSNAGIAKAEAHEWEVAEEIFLA
LAGLYPEKPEILLDLAMLHEEHAKALEQEQDEECAEAEDDAAYDYYKMLLGREPPYAPAY
YYAALFFLRKKTFDRASSLLTTFISLSDDERRVQKAKDILAKLKKLGYLDTTFKEAFDFI
RMGEDEKGLAKAQEFIERYPDVWNGWFLAGWAHRKLRNWEKGKEAFQKAIDLGSEEADTF
NEMALCQLETGDFTGARTSLERALRFEPENVKIIANLGALEYRMGRTAEARGFFEAAVEM
DPDDTVSKTWLEKLDKEAGGE*
>SPSA8_v1_310005|ID:41145756| conserved membrane protein of unknown function [Spirochaetes Bin 1 SA-8]
MISFFIKKAFFDGWDNLFSLAALNAVHLILAFVSIVLPMSMGADGLTAFACITVGIFLLS
LWQSICAFAMCEVANYRSLGIKATLAFFPKALKPGLLTGLFSVLIWFAVSVGIPFYIMQK
GIVGVFLAGLLFWTCLVGLLVWQYYLPLEARLGGGFKKNIRKAFILFLDNPGFSLFLFGY
SILTIAVSALPAFLAPGLAGLALAQADAVKLRMLKYEWLESTPNADKHAVPWNELLAEEK
EMVGVRTLKGMIFPWKEGK*
>SPSA8_v1_310006|ID:41145757| Ornithine decarboxylase [Spirochaetes Bin 1 SA-8]
MKEQYHFPLENYMSRERFERIKSFAKDKETPCLIIDLDVIKKNYEDLRKNLPFAKIYYAV
KANPDDAVVSLLRDLGSNFDVATRYELDQLLRLGVTPDRMSFGNTIKKEKDIAYFYEKGV
RLYVTDSITDLDKISRAAPGSKVFFRLLTEGLGADWPLSKKFGSHPDLTRQLIKTAVRFG
LEPYGISFHPGSQQRDVGQWSSALTTVGQLFAWARDDLKVELKMINMGGGFPASYIEPTD
TLPQYAEDIKRFLDNSFKNQWPEEIIIEPGRSMAGDAGVIVSEIINIAKKSVHERYPWVF
LDVGKFGGLIETLDEAIKYPIYFESEGPAVEVILAGPTCDSMDILYEKTTYMMPENTRIG
DRVYIFTTGAYTQSYSSVYFNGFPPLKSYILPKS*

>SPSA8_v1_310007|ID:41145758| protein of unknown function [Spirochaetes Bin 1 SA-8]
MKAEEQKTRKKSRPKSGRLPAVMSVLTLAAVLGLFVIAIGQKSGSLRISAQETVLSPPKT
FPAETPAITASARPIRIANWNIHDCAFYNAKTKEREPLHQYVAKALHEAKVDIVVFEEIQ
GDTKKGGDIALLSVSLAKEGWSMPYVALSTSKSEDDIAVFSRYKIVESGQVLVPGSSDLW
PRPGIYARISLGSQDLDIYGFHFKAMDDEKSHEARLAQAKALADLLISRYGAELSQKPII
LAGDFNTVSSADFLENHSVLSFLSLKEDTDSGNDFLPVNYSFYPAEPTFVDKNYRSLLDH
ILISASLAKNASEKNTFVMQPPSTEAGIPVSDHRMIITDITMPVSP*
>SPSA8_v1_310008|ID:41145759| Aminotransferase class I and II [Spirochaetes Bin 1 SA-8]
MNQLAEELNSILANTAAGRLLSEMGRRMYFPKGILTQSAEATEKAYKYNATVGMAYEHGE
PMMLDALKEALPGLTPKEAVAYAPTGGIMPLRKRWKESLYRKNPSLIGKDFSMPVVVPGL
TAAVSYLTDLFVEPGDTVVVPDLHWPNYRLIIEERKTAAGLTFPIFSNGGYNLEALAARL
REAGARRGKAICILNFPNNPTGYSLTAQEADKLIAMLVDIANEGTDLLVIADDAYFGLQY
EKNLLEESIFARLVDAHQNILAVKADGPTKEDYVWGFRVGFVTFGGKGLTDVHQEALVKK
LLGITRSSVSSSSGLAQHLLLKALEFKGYEDQKARYRAILEGRYKALKAAISKAELPDAF
IPLPFNSGYFMSFECRGFSAERLRIRLLNNHGIGTISMQDKYLRVAFSSVEEEDIPDLLD
KILLVVREIVENFKG*
>SPSA8_v1_310009|ID:41145760| conserved exported protein of unknown function [Spirochaetes Bin 1 SA-8]
MKRFFSVLLILAAVTGMAFAQVDLSKVKNGVYFAQDEAFGKTGWKEQVIIEVNNGKIVRA
VWNGVSNIAGAVDKKSYAASGKYGMIKASSIKAEWDAQAKAVEEYLVKTQDINFSKIKAD
GKTDAITGATFTVSEFFTLAKKALASAPVAKGAYKDGWYYAEQPNFDKSGWKDYVVVTVV
NGSIVDVVWSGIPKDPAKKSKLVEAQLGTYKMNAKQGEWNVQSERLCQAIVKAGDPAKIT
LKADGKTDAVSGVSITANAVTLAIEALKAAK*
>SPSA8_v1_310010|ID:41145761| conserved protein of unknown function [Spirochaetes Bin 1 SA-8]
MQKKTAFIAPSASVTGDVLMKEDSSIWHNATVRGDIAPIEIGKRSNIQDGAVLHVAHNHP
CIIGDDVTVGHGAIVHACTVHDCCLIGMGAIILDGAEIGTESIVGAGALVTGNKHFPPRS
MILGNPAKVVRQVSDEEARKIRESAEHYIVLARKASTDSMTAES*
>SPSA8_v1_310011|ID:41145762| Carboxymethylenebutenolidase-related protein [Spirochaetes Bin 1 SA-8]
MKEQSAEKKKFRPGWSVIAAAILVLLFIVGSGFVAGLPDLSDPQLKLSSNTVSVEKIGKD
LYFLPKAEKKPLGFIFYPGAKVPEGAYSYLARALAEKGYPAVLLKMPLGYAIFDTKAAAR
AMQQLQETKAWVVAGHSLGGVAAAMFARDNPGTVKGVVFLASYPAGGSSLAQTDLQALSI
SASNDMLATAEKIEKAKPLMPPKAEYQVIQGGNHAQFGTYGDQKGDGVAEIPASLQLSAV
IESVLAFLAKTM*
>SPSA8_v1_310012|ID:41145763| GHMP kinase N-terminal domain protein [Spirochaetes Bin 1 SA-8]
MIPSAVPILHSLKELYSEAEYPAALARYRKLEDRVRAWTRSDSHSNAPQTEAVTWFSCPG
RTELGGNHTDHNHGRVLAGSINLDMLATVLPRQDSLVNVWSEGYKPFSVDIADISIRANE
KGSSAALTRGLAAFLSKKANSPKLRGFDACMDSTVLPGSGLSSSAAFEVLIGSIIAAVNS
IEASPTELAIGGQYAENEYFGKPCGLMDQIACALGNICAIDFTKPSRPGIEQLAFDFSAH
GYELIVVNSGSSHADLTDDYAGIPAEMKAVANLFGKQTLNEVSEQEILAQAPEIRKKIGD
RAFLRALHFAAETRRAASMKEALERGDLSVYFELVKESGLSSWRLLQNIVPAGAVRDQNI
AVALALTEKFLGKDGACRVHGGGFAGTIQVYLPSGRIAEYIDYMKKVFGEAQVFPLQIRS
HGVVCLDRI*
>SPSA8_v1_310013|ID:41145764| Major facilitator superfamily MFS_1 [Spirochaetes Bin 1 SA-8]
MNEKKAGFKPYLKLTFLIGFGFFTMGLMDPLYDTYVPIFLGKYVSSMTLVGFIMTLDNIF
ALFLIPLASVVSDRTDTRIGRRMPYIITLLPLTALFFGLLPYAAGISLGALIALIFCLNL
VKQSARGPVVALMPDIIPAEFRSEANGVINTMGGIASIVGTIGLARLMDIDLVLPILGHT
KDRLPFPLAGLFVIIAVLFLFFFIKEKKQPGKADNEERVPILQSFKSVASQQDKSALYIL
VSLFLWFLGYQGVLPFIGKYSVDVLKTSSGSAALAAGMVGIAYAIFAIPSGYVAHRVGRK
KTIRTSLIVIAIITGILFLHDPLTSGLSPSIRLFSFWLIMFVFGMFWVSVVTNSFPMLWQ
MASWGTMGVYTGLYYTASQAAAILAPVLTGAIIDLAGYRGIFLFCSVCMVAAFLVMGLAT
SGEPDSKTR*
>SPSA8_v1_310014|ID:41145765| Amidohydrolase family protein [Spirochaetes Bin 1 SA-8]
VYLCNPAFQTAQAVAVKDGRIVGIGSRDELEIRYSARRFLDLSGQYVVPGFIDPHCHFLS
YGYVLQRADLFSTASWDEVLERLVSYKAKKPAFWILGRGWNQNDWVTKEFPDRTRLDELF
PDNPVLLTRIDGHAAIANARALRLAGITADTRVEGGAVSAENGSPTGLLLDNAIDLVRAV

IPAPSREEMTDALLRAQQNCFAAGLTSVSNAGTELDEAILMYRLQAEGRMKMRIYVMLAA
GARETAFALGKAPDGCPLPGSIFQNESLSIRSFKMFADGALGSRGAFLLEPYADDPDNRG
LLTCPQQDLERQCSIAADCGYQMNVHAIGDAAARMVLDSYARFLKPGNDLRWRVEHAQLI
HADDLPKFGRYGIIPSVQTTHATSDMKWATARLGKHRMPYAHIYRELLAQNGWLANGSDF
PIEKIEPLRGFRSAVFRTNDEGEPKGGFQPENALTREEALKAMTIWAAKANFEDHLKGSI
EAGKLADFTVLPADLMEVSEERLRSIKPSMTIVGGEIVF*
>SPSA8_v1_310015|ID:41145766|ycjV| putative sugar transporter subunit: ATP-binding component of ABC superfamily transporter [Spirochaetes Bin 1 SA-8]
MAKVELKKIGKVYDGNVRAVDNANITINDKEFVVFVGPSGCGKTTTLRMVAGLEDITEGE
LYIDDKMVNDVPPKDRDIAMVFQNYALYPHMTVYDNMAFGLKIRKLPKEEIDQRVKEAAR
ILDIEKLLDRKPKQLSGGQRQRVAVGRAIVRKPKVFLFDEPLSNLDAKLRVQMRAELIEL
HDRLQATMIYVTHDQVEAMTMGDKIVVMKDGKVQQIGSPLYLYNNPINKFVAGFIGSPPM
NFLNVKVSEENGVIWIDEGSFKIKPAEAHVPLLKAYVGKEVFFGIRPEDLPIPEEHHDNT
FRAKVTLVEPLGAEIHLQAATATQTMTARIPPHHLYKHGDEIEFAPVMTKAIYFDKETEK
SILPVKWNEQE*
>SPSA8_v1_310016|ID:41145767| RNA methyltransferase, TrmH family [Spirochaetes Bin 1 SA-8]
MMNIARLKNLHGIARLRKIALMLSQIEHEIACSEAQPEILNLLHQAGLIADFLEASGEST
PELLLAARQFKASAGNAQTQNFDLCFRALNNFRHTAMRGAGQAPADWDFWDYRGTSAEQV
RDERFFYPGLKVYLEDIRSPFNVGTIFRTAEAFGFEEVILSPGCADPRHPRALRSSMGTI
DMIPWRRASLDEASPGASGIFALELGGTPLGSFTFPRSGLLILGSEELGVSQNALSLARV
HAGILSIPMKGIKASINVAVAFGIAASAWASSPTADFNFEKSSLTEAENQSKLNLIT*
>SPSA8_v1_310017|ID:41145768|rsmI| Ribosomal RNA small subunit methyltransferase I [Spirochaetes Bin 1 SA-8]
MGELYIVGTPIGNLEDITLRALETLKKVDYIACEDTRQTLKLLSHYNIHKPLVSCHANDE
LKASRKIVSLLAGGASVAYCSDAGTPGLSDPGAVLAREVRREGYRVVPIPGPSAFAALIS
VAGFGGRSVLFDGFPSPKASRRKTRLAELLAREEAFVLYESPYRIAKLMRELALLAPERE
VCIGREMTKLHEDIRTGTACELAALFEAESSGTSLQETPIPARGEFAILVSGRENQAGLP
EDECSQGRD*
>SPSA8_v1_310018|ID:41145769| conserved membrane protein of unknown function [Spirochaetes Bin 1 SA-8]
MFSRYPVTMNKTDTKKQVFLIFFLALFLLVARLFYPFLTVIIWSGLLYAFLEPLFEKLAS
GMGQKNSSARKRPLAKTITAGLFAFLGVFILVVPFIYLFISLLRQVVDLAGNLIRFAESH
PDIVSLSATSPVGGFLFRVSGGTIDLSSINVVHELKVFLTNSSSRIIGLSGALLKNSAGL
LITLAFMVFTLYFLLMDGKALFDIVVSAIPIEKSYTAMFMKKLRESGKHLVLGFFLVALY
QAFAMFILALAFKFKSPLVLAFLTAISSFIPMVGTGLVWGPLVLYLGLTGSIGKAVLFLL
CAGFFISFVDNFLRPMVLGNRLSIHPLLIFFAIVGGLSLFGFNGLILGPLILIIFFSAVE
LYEMQDQLADDVQEKSGKSS*
>SPSA8_v1_310019|ID:41145770| PHP domain protein [Spirochaetes Bin 1 SA-8]
VDGITEAALAAAEAGITFIPGIEIEIEFNPGEFHLLGYGLDIKAPALTAALQELKKAREE
RNATIASLFTSSGIGIDIEKIKTERKSSYIGRPQIAEALVAQKIVKTKQEAFDRFLGKGK
PYYLPKECLSFERALEVIRQAGGVAVVAHPYSLFVSKSKLASLMDEWKERGVQGIEAYHP
AAKPGPCRTLELMARERGFAVTAGSDYHNPGKPLCGIGRTSGNVPISDDFYQELLPLFSW
TSSAN*
>SPSA8_v1_320001|ID:41145771| putative Carboxymuconolactone decarboxylase [Spirochaetes Bin 1 SA-8]
MTIQERFEARQRNGAEIQKPMQLFGSLDEKSTHAHLDAKAQAFAPGALSVKMKALVALGA
AVALDAPSCIMNNVKLAKNNGAAKEEIMEAIAVAKFSKSATVLANAAPALEWLMTQEAAS
AENKA*
>SPSA8_v1_320002|ID:41145772| conserved membrane protein of unknown function [Spirochaetes Bin 1 SA-8]
MISAAVLFKGLIAALIVGFVTSSIRSWIDRVFLVIMLITIVGLPINEAIQINLLVLAFSA
LFHILRNGKQLKNDLPAGSSEWLIIVISASIGGVAGRLANSAAAPAVLMGTLGVYAILVG
LRILYIKPIPEKEIKAHPVLLSPVSLAGGFLTGLISAGGKPFIVPAYNNAMGHHPKRAYA
LASLGVVAGAFASILTQAVLPGSFSADQLLLALYEFTLVTLVALLVNKFWTEKLAKIVNL
TIAPILMLVGIKFLSSILK*
>SPSA8_v1_320003|ID:41145773|arsC| Protein ArsC [Spirochaetes Bin 1 SA-8]
MVKVLFLCVHNSARSQMAEAYFKALAGDRVVVESAGLEPGVLNPYVVEAMAEDGIDISSN
PTKSVFDFYKQGRLFDYVVTVCSKEAAERCPIFPGNAVRLHWPFADPLQFKGSHDEIMAQ

VRDVRDAIKQKVIEFYQSLNI*
>SPSA8_v1_320004|ID:41145774| putative PTS IIA-like nitrogen-regulatory protein PtsN [Spirochaetes Bin 1 SA-8]
MDTILDALQEGRLFELPENDKTHALQFLAHIIEAFPQIPTGTDIVGNVMARENLTNTALG
KGWACPHARVDFEEDLMCVVGWSPTGIDYGAPDGKPVSIITMYLVPSNQRNHYLREISVM
AKVLKSAQDTEKLLKLADLNEARNYLLDLVSASKDTIGPDARARMIRLQSKTAMATQPIG
DLSNLVIEPVSIITGTGPKPLALTQNQELMIFIESLPGLADKLENEGSYQNGTWRIIRRH
TVAYQGGRMVYDCIALANVKANSSAVQQAKQNA*
>SPSA8_v1_320005|ID:41145775|mgtA| Magnesium-transporting ATPase, P-type 1 [Spirochaetes Bin 1 SA-8]
MAKQMFPVKKESTDALNVQRQADEARLRDLCICPIFEVFQNLKTNANGLSDKEAEDRLSR
YGPNELSKAESKGFFRDMAERLQSPLVIQLLVIAAVSGIIGQFTSSIIVSLMIVLSVGLS
YILDARSNREVEALGKRVQSRTWVLRDGKEVEIKMAEVVPGDIVLLQAGAIIPADVRLIS
AKDFFVSESALTGESMPVEKSANLSGNSSSAVLELPNACFLGTSVTSGSARAVVINTGAQ
TLFGEVSRKLGEKRIETDFDKGIRAFTWLMIRFMLVMVSVVFLIVGLTKGNWLEALLFAL
SVAVGLTPEMLPMIVTVNLAKGALAMAKKKVIVKKLPSIQNLGAINILCTDKTGTLTQDR
VVLEHHVDIIGNKSEEVLNYAYLNSYFQTGLKNLLDRAVIEHVDLDVDECKLVDELPFDF
QRRRMSVVVDYEGDNVLICKGAVEEIYSCCTHYQIDEEIYPLIDMIRADLFEEVEKLNRE
GFRVLGIAYREFPKYKNVFTVEDESQLILLGYIAFMDPPKESATEAVKLLDRAGVKVKVL
TGDNGLVTEKVCRDVGIAFANSMTGAALAQLSDAEFAEAVVKNDVFVKLTPSQKEKIVLE
LRKQGNVVGYMGDGINDAIALKAADVGISVDSGVDVAKEAADIVLLEKSLLVLEEGIMEG
RRIFANIIKYIRMGASSNFGNMFSVLGASYLLPFLPMRPLQILTNNLLYDFSQTGIPMDR
VDPEQIAKPVRWDIQNIKRFMIWIGPISSIFDYATFALMWFFFKASAYLNPALSLVQKDG
LERLFQSGWFVESLLTQTLIVHIIRTRRIPFFQSRASAPMLMTTLAVMAIGAWLPYSPFA
GFLGLVPLPAIYWLWIVGFLVTYSILTHKVKRIFLKRFEGA*
>SPSA8_v1_320006|ID:41145776| Transcriptional regulator, ArsR family [Spirochaetes Bin 1 SA-8]
MSITTLDKVVKYCNIYPMMTEEEKRRCELRANMFKALAHPLRIYMLEKLKERPWCVCELA
AELGVDKSIASKYLTILKDAGLVGMQKRGILVEYTLIAPCVLNLAACAESDVFSNRKKAL
LG*
>SPSA8_v1_320007|ID:41145777| Redox-active disulfide protein 2 [Spirochaetes Bin 1 SA-8]
MKVQILGTGCSKCRLLEDHVRQALHDYNITAEIEKVTNIDDIMAMGVMMTPALAIDGVVK
SVGRVLTKEQLLPFLKGEK*
>SPSA8_v1_320008|ID:41145778| conserved membrane protein of unknown function [Spirochaetes Bin 1 SA-8]
MAGKTEWTPNKKLLLVFGVFLAAYFIPFQAPRVISATNEAFLMLSEYARQHVILCLIPAM
FIAGAITIFLNQQAVMKYLGPDANRLTAYGVASVSGAILAVCSCTVLPIFKGIYKKGAGL
GPAIAFLYSGPAINILAIVLSAKVFGLKLGIARTVGAIIFSILIGFIMSVIFKKDDQQRT
KDSRLFNAGMEEGGRTLGQMAFYMASMIGILVFANWANSRGGSPVWDAIFSAKWYLTAAF
GIMLIFSLRKWFSKDDQLAWVVATRDFALQILPLLFGGVLVAGFLLGRPGHDALIPSSWI
ASLLSGNSLFANLFASVAGALMYFATLTEIPIVQGLLGAGMGQGPALALLLAGPSLSLPS
ILVISGEIGWKKTLAYVALVVVFSTLAGIAFGAIA*
>SPSA8_v1_320009|ID:41145779|czcD| Cadmium, cobalt and zinc/H(+)-K(+) antiporter [Spirochaetes Bin 1 SA-8]
MEHNHSHEEDADEFASGKKFILSIAITTITLVAEVVGGILTHSLALLSDAAHVFLDIFAL
ALSYGAVRLAMKAPNSQHSFGFKRMKVIAAFINGSTLLIMAFEIFLEAIKRFSNPVEVLA
GPMLIIAAIGLATNLLVALVLKGHDHEDLNARAAFLHVMGDALSSVGVIAAGLLIMLTGQ
TWLDPLASILIVAMLLFQSIKVLKSAIHILNEGNPDGAASDEVQTKLEALPAVKDAHDIH
VWTIEPGYRVLSAHIVVDDMAVSSTALIARDIKAMLHDHFNIEHATLQFECVHCGQDCQD
PGCDGSHN*
>SPSA8_v1_320010|ID:41145780| exported protein of unknown function [Spirochaetes Bin 1 SA-8]
MKYKSILLIVSVLLFVPFFSAFSAESSVDLVINAEVRPLLSIVMTSPPVFQLIDADGNAI
VSRNLGTAIVKSNYRNWTVQISSSNKLNPATGRLKKIDDNVYIPYTFELRTGDSVLVSQF
DTPSAAQGITASAGRQLSLVFNFVDPGETAMWEKGIYQDTITIVVVAT*
>SPSA8_v1_320011|ID:41145781| exported protein of unknown function [Spirochaetes Bin 1 SA-8]
MKKLVALAVVAFVAIGASFAADLVINGSVTAALSVAITNSPITVTLDGTGTAGTVDRTAT
LNAKSNKKNWTVSFDSANDGKLKSALTGVEIPYLLQASTPAWGSAGVTNGLATAVNPEGK
TIVVTDGKTPRDGVNFTLTVTVPAQDGAAELFEAATDYTDTITISIAAS*
>SPSA8_v1_320012|ID:41145782| exported protein of unknown function [Spirochaetes Bin 1 SA-8]

MKKFALVALFALVVFGSAFAGTSGTVGLSGTVAPQFELTLPGNYTAGTMNDGASEVNTWT
IGNVAVVSNYKNWTISVSSANSGYLVHSTDNTEKVQYTMTLGSLATNQSLGSLWTSAAQA
RTPKTGSTYALSIKFTASATDFWQTGTYTDTLSVSITHP*
>SPSA8_v1_320013|ID:41145783| exported protein of unknown function [Spirochaetes Bin 1 SA-8]
MKKRALVTLLVILASLPVFAATAGGDGTLSIQGTLNAHVELSLPPSFEGEIIDTAGYLNS
WDLGTLNVNTNFKNWKFYLSSTNAGSLVLFGDPTETIPYTIKLVRVSDEEKVFDNVALDS
PLISDVQPRTPKNGLDYELFLEFTADETSQWENGVYVDTIYISIATN*
>SPSA8_v1_320014|ID:41145784| ATPase-like, ParA/MinD [Spirochaetes Bin 1 SA-8]
MSLVPEKKSNPKIRHVIGVVSGKGGVGKSTVAVLLAQSLASQGRAVGLLDADITGPSIPR
LLGVDTMKGESDGEHLFPIENEEGIRVLSINLFNDNEDAPVIWRGPLLAKAIEQFWQDTD
WGDLDYLIIDFPPGTSDIALTALQQIPFSGIVVVTTPQDYVSMIVRKQINMATMLKAPVI
GFVENMRTLVCPKCGEVIKLFDDGSDAQKALGLPILASLPWRQEVAQACSIRWAMLPEAV
KKEALAIAQQVEMSAASAK*
>SPSA8_v1_320015|ID:41145785|lysA| Diaminopimelate decarboxylase [Spirochaetes Bin 1 SA-8]
MTNAVSIKNGRLFFDGVDMTELAAIYGTPLYVVSESIIRERIERIKIEFLNKYPGTIAAY
ASKAFQTLEMCRLVASEGLGFDVVSGGEIFATLKAGADPADLIFHGNAKTPDELRYAVQH
GVGRIIVDNLNEIAQLERLGAVFGKKIPVLYRITPGVDSHTHRFISTGSLDSKFGIPLNQ
EVREMYMHAVLRSPHIDFRGFHFHVGSQLHDNFSHLAALDILLDLVRQVKAEFGFAAREI
NLGGGFGIQYLPTDPDPGLSYFIEPMMKKIEEGFSSAGIPQPTVIIEPGRWIIGEAGLTL
YTIGAKKEIPGIRTYLGIDGGMTDNIRPALYEARYHALIANKAERTGDTLVTIAGKCCES
GDILIRDIKLPDPQPGDILAVFSTGAYNHAMASNYNRIPRPAVVMVKNGSHHLSVRRETW
EDLISREL*
>SPSA8_v1_320016|ID:41145786| Amidohydrolase [Spirochaetes Bin 1 SA-8]
MPETTLEMIRRYQPWLVEIRRQLHQIPEPGDAEFKTADSLCRILETLKIPYTRKRTSIVA
LIEGNKPGKTVAIRADMDALPVDEPENRPYASCHKGYMHACGHDAHMTCALGAARYFAEH
RAEFSGAVKLLFQPAEETTGGAENMIKDGCLEDPHVDAVIGLHVQPSISVGKIEVKYDAL
NGSSDYLKIIIQGRGAHAAYPDTGIDAIMIAANVINALQTIVSRTVSPLEEAVLTIGTIE
GGKRNNIIADEVTMTGTIRTTNPEVRADIARKVTALVQTIPQAFGGSGFVEIIPSYMALI
NHNWIVDIVAEEVQRLLGKDSLVWKSKPSLGVEDFSFFLKERPGAFYHLGCGNPEKGITA
PLHARDFDIDEACLPIGSALHAAVAQRLLAEENHK*
>SPSA8_v1_320017|ID:41145787|dapF| Diaminopimelate epimerase 1 [Spirochaetes Bin 1 SA-8]
MKQHFFKYHALGNDYIVIDPAVFNFTPTPEAVRLICDRNRGAGSDGILYGPLPGSVPAVP
LLEIYNPDGSEAEKSGNGLRIFSLYLAEHGYRGLESFQIQTKGGLVTAQVKSLAPPLISV
DMGAPSFDAQKAGLATDQAEFINQRLDAGSHPLKAVFVSMGNPHCVIFGEQPSPSLAKTL
GPLIESHKLFPQRTNVQFVEILSRHDIRIEIWERGAGYTLASGSSSCAAAAASRKLGLVE
SPVTVHMPGGVLHLELADTITMTGPAVSVYDGVFSSAMLAELARLSAVPAKAGASIAAAS
LAESGFLGANMHEEPSHA*
>SPSA8_v1_320018|ID:41145788|alr| Alanine racemase [Spirochaetes Bin 1 SA-8]
MIPRKVVAYINIDHLEHNIALIRSRIGKARLCAVAKADAYGHGALPIARALESAHVDSIA
VAYLEEALELREAGIRSPILILGITDPRNSELLIEHDIAQTIYTADSLLALDAAAARHGK
KGRIHLKIDTGMHRQGIELENLPAFLEKLTSCRYLEIEGVYSHFAEADSPQRDFTLSQIE
KFNKGLDMLAQRGIKPPLRHIANSAGILNYPEAYYDMVRPGILLYGMSPDGSLKAPDGFK
PVMRLAASIANIKTIEAGESVSYGRIFTASRRTRVGLLPIGYADGYARSLSNKAMVLIRG
KRIPLIGRVCMDQFMVDLTDAPEVIVGDEATLFGTEDLPAGFLTRLMGTLDYELTCGISK
RVPRIVCRGGECQ*
>SPSA8_v1_320019|ID:41145789| putative N-acetylglucosamine-6-phosphate deacetylase [Spirochaetes Bin 1 SA-8]
MDSFILKNGTLVLPDYQIDNAVLIAQQGIIIYAGAGPLEAVIRHFPEAASLPIEDCRGGY
ILPPLVELHIHGAFSVGFEQGPSAEDLVRLSVRLDAEGIGAFVPTMLWEEHAAAELVHAI
KVSGLSPGKIPGIYLEGPFVNPEKRGGIPLQNIARPSKQLLQKILEVCDGFLAIMTLAPE
LEGVEELYPLLESAGVVIALGHSNTSGIGMKLPASRFSVTHLFNAMSGLDHRSGPGLAGY
VLGGGKQRWAELNADGIHVNRFAMNVASRCLDPSQLILTSDAIAAAGMPEGMFSYFGQDA
LSDKTGVRYKETGTLIGSNRLGIELVKSFMAASGWPLHACVRAMSTNPWNLLNRAQPGLV
PGAPAELFIWDRDLNHCWRSTT*
>SPSA8_v1_320020|ID:41145790| alpha-glucosidase (fragment) [Spirochaetes Bin 1 SA-8]

MAGLLEDMSAYFGRQPVLPDWTHDGVILGIQGGTDVCHQKLEKARAKGVPVAGIWAQDWE
GINITSFGQRLRWDWIWNRERYPGLDSQIKAWRSQGIRFLCYANCYVGAGWTMFKEASLR
GYLVKNQAGEDYLVDFGEFYAGIVDLTNPAAFSWFADQLVANIIDLGISGWMADFGEYLP
SDAVLYDGTPALLAHNRWPSLWAQCNYEAVTRAGAWQEVTFFMRAGFTGSQKYCPLLWAG
DQNVDWSNDDGLPSALRSALGAAMSGHGLHHSDIGGYTTLYGMKRTKELFMRWAEFAAFT
PMMRTHEGNRPKDNWQFDSDDETLEHLARMTKIHCALKPYLQTCEAENAARGIPVMRPLF
LGYPEDPAAWIIDSQYLLGQDLMVAPVLEEGAVSRSVHFPAGTWLDFWTGDVVYSAAAPA
NITISAPLGTVPVFYQQGNETAEHLAEAVKANLERRWHRAD*
>SPSA8_v1_330001|ID:41145791| protein of unknown function [Spirochaetes Bin 1 SA-8]
MSIFGNAGGRFLAMACQGQDPGVFTRDDENKSVSTERTFEADISDRTILIDELRMLADEL
SFRLWDNKMHTGCVGIKLRFADFTSITRQRSLAVPTASADCIFQEAMQLFDANWTCGKAL
RLIGIFLSSLVPESHIENSLFQEEPPKSALAEQVVHELQKSGKGNLIRGTRLNRRG*
>SPSA8_v1_330002|ID:41145792| exported protein of unknown function [Spirochaetes Bin 1 SA-8]
MKKIFTAILLTTLGAILFSSCMGMETAITINSGNSGTVTAEYRLSEELVNFGAIEANKAL
LPIPITKADIENSLASAKGLKLVSWSSKKSGTDTVIKTVISFDSLEALVFYLDPQGQMAR
YSVSDAEKKIIFSLGDTMPPMDEQMKSLAKEAFAPYAFKFTVTTPSKIIRAGSSLSIISA
ATDGKKAVFEGKMQDIVTSGEAPVIEFTW*
>SPSA8_v1_330003|ID:41145793| conserved protein of unknown function [Spirochaetes Bin 1 SA-8]
MLLLVLELPETTSLKDKRKVVSGIKHRLHNKFRLSCSEVDLQDSLGFAEIGAALVSNSPV
FGEKVLNEAVRFVEENFPVNIYESQIHSEVYG*
>SPSA8_v1_330004|ID:41145794|metG| Methionine--tRNA ligase [Spirochaetes Bin 1 SA-8]
MKRRLITSALPYVNNVPHLGNLIQVLSADVFARFCRSRGYETLYICGTDEYGTATETKAL
EERISPKELCDWFHAIHRDIYSWFGISFDKFGRTSTPEHTEITQSIFLDLEKNGYIVSQT
VEQLFCDHCGRFLADRYVRGTCPVCGYENARGDQCENCGKLLDPTELKNPKCSSCGATPH
PKATTHLYIDLPKIKEKLEPWIQSASEKGFWANNAIQMTQAWLRDGLKPRAITRDLKWGI
PVPMSGFEDKVFYVWFDAPIGYISMTASLAREKGFDWKSWWQNPEEVDLFQFIGKDNIPF
HTVIFPSSLLGSGKNWTLLHHMSSTEYLNYEAGKFSKSAGIGVFGTDVMATGIPADIWRF
YIFWNRPETSDYTFTWADFKEKVNGELIGNLGNLVNRTLTFIVRYFDGTIPAGKPDELFW
SKARELESKATECLEHADLRDAFRAIFELSNLANKRFQDGEPWKTRTSNPEAAASLLSDL
CYILKDIAIMIEPYMPAAAKKIGDFLGMKIGQDGLSWKDLGKLEGLSLVHSPEVLFAKLE
DAHIEELRERYSGSQKDRKEKQEAVEAHSIQKAEPSSAPKASSEPAAAKEKALPLADLPQ
EERFAKLVDLRAATIVKIERHPKADKLYIETLDDGSGAERVIVSGLVPFYKEEELLGKTI
ILVNNLKPAKLRGIESKGMLLAASRHGEEGKEAVEVLTAPWAKPGTRIHLETTLGETLFD
QIDADTFFSIPLEAKDGFAFAGKEKLIIDGKPIMLEKVRDGEIG*
>SPSA8_v1_330005|ID:41145795| conserved protein of unknown function [Spirochaetes Bin 1 SA-8]
MIGESDTPVPQNLNQAADIRIDAFLAVNAENFSECGEDETSSFLQSAFWSVFKTKTGWRA
YYCRFRLGDSHTSFPLFILCRNLALGFSLAYIPHGPPVCPESIEPGIFLSNLAKALVDKL
PENPLFFRFDLAWDAETAQSQMLSSAEYLKLHHLSRGTAVQVPDTVLLDLRQSEEEILAG
MKPKWRYNIRLAEKKGVSVSWSGKDQLPVFMQMYRETAQRDGIAIHSSGYYEKIFEIAMQ
FNKTLHENRADVRLWTASHDGDVLAGIITLFYRGHAIYLYGASSNAKRNLMPAHALQWAA
ICAAKEAGCYDYDFFGIPPNDDPNHPMAGLYKFKTGFGGRIVHRAGCYDTPIAILPYRAF
MFMEKARLFWHKNVKKHFARKARQKTPSE*
>SPSA8_v1_330006|ID:41145796|dtd| D-tyrosyl-tRNA(Tyr) deacylase [Spirochaetes Bin 1 SA-8]
MRAVVQRVSDASVSIQDGDSSIITGSIPNGLLVYLGVGKHDTEKDAEYLADKIANLRIFM
DVDEKMNLSLLDMGMPALVVSQFTLFADARKGRRPSWSNAADNDTAFTLYQRFCDELGKK
GVPVQTGKFREIMKVRYTNEGPITILLDSEKTF*
>SPSA8_v1_330007|ID:41145797| exported protein of unknown function [Spirochaetes Bin 1 SA-8]
MKKALLALFFVGSVFSLSALDIRDGLVRIRIDEINGRVLLYRMTNVSGSQYEPIFFDSDP
RTSTLMVSIDGRKARLGDSSEYKFNVRRTATGAEVEYSSVVATITEKISFIKSLDSRVSN
GFKVEYVIKNLSSREYKVMLRRIWDTRLGEPKGRHFSSNLSDKIEDEQVFTAESPESYVM
TPGESASMAVLLAKVPRPDAVVIANWKRLSDAAWMYSTYMKGFSMAPYSLNDSAIGFFWD
ERILKPGASVVFTSYLVTGGPWTEFRDALISGGYGINEDLPGVQPTETGIARKNPGLDIE
AIRQLLALLDSAIDNIDAISESDITMLMNQVDDLLNPYEAIK*
>SPSA8_v1_330008|ID:41145798| protein of unknown function [Spirochaetes Bin 1 SA-8]

MGIKKSIIVLIAVLFCSVLPTYSQKLDPEIAFAKAFDLFRQGNFREAALAYGWLFSEISS
SDSSLAEQSAYMQLISTFNAGQYDETKILAEAYLARFPESERFVDIQYQEARIAFAEKRY
DDAIQLFTDFITQYESIKDLQEFVSQALFWRSESFYQLGRLTEAFSGFSALADNYPNTSK
KNEALQRIEIIGAESRERFQRRVIDFNLKESLQMQSRMEQAEKYAEQLLERYALLVRHLR
SIYGLGQPGRLPLYANALAMPPTPAPPPPQIVIVEQSQPVNAISNLEIARNNRLKKLLSA
KQRVLSLLADRLLNYAGEVLK*
>SPSA8_v1_330009|ID:41145799|rpe| Ribulose-phosphate 3-epimerase [Spirochaetes Bin 1 SA-8]
MNRPVIAPSILSADFSDIASAIKLIEEAGAEWIHADVMDGQFVPPISFGSKMIADIRKRT
RLPIDVHLMTVQPENHIDDFILAGADYITFHAEACVHAHRIIQRIREAGRHPGISIVPST
SVDSIRESLPFVDLVLVMTVNPGYGGQAMLPFCLDKVSKLKEIRSMLGLRYLVSIDGGVS
LETAPLIASHAPDILVIGSAFFSAADKSDLAESMRNHCGTNPVC*
>SPSA8_v1_330010|ID:41145800| Tetratricopeptide TPR_2 repeat-containing protein [Spirochaetes Bin 1 SA-8]
MAKDDSVNDLLSNGYECLKNADLATAGRFFDEALGKDYDNEEVLFAMKCNLFWIEQLEEA
SGVEPLLERGDFMIEQWRGFQVFLARITGNSDRALYAFKRMVFSVALDFFLALPDESKKS
LGTALDFRIGRCRKALGDYETAIKHFERAAKFKKDDARFLAELADCYAISGDQRLSKMMF
RDAFLFEPDKVDLEFLEAGMIRKLEELAREAGHSGLAVNEWIPVYGELSGILDIKRDLSQ
AEVARLNTSILHMENERKTYPARTEPLKPRLLNQYFRYIDYLKAAQSDSSRLEKVLLKIR
LLDEDVYKKYVS*
>SPSA8_v1_330011|ID:41145801| Transcription elongation factor [Spirochaetes Bin 1 SA-8]
MSDNQLVDKVQHMLNEEKWTRTTLSNYSISSLKELDALIEEAESLNCKTELLDLCLEHLA
NTRNSVIALYIAGIANMSKQPVDESYMVQLMDLFAESKRTNIVEYLCERILDFGENRIAL
ARLAECKEGEEKADEKFALWERLIKIDYEEADIVKLIAERKDKQGDRQQAIEYYKKALIR
YIGKVLPTNVREIWQKLVEYCPQDLDYFYHIQKKIASQISPDKAAMLLMDLYNHYKNLKD
WDTALDILKLILEYDEKSPAVRREFIECYSGKYAGHSRLQDYLRLSNINQSYRSLHEAIA
EFEKHISFDIGNFVFHRTWNIGRIANIQGDEVIIDFAKSRGHKMSLKMAIDSLMPLSKDH
XWVLKAIWKKEKLYAKVKNDPEWALKTIIKSFDNQADLKRIKAELVPSVLSLSEWNSWSA
KAKDILKTNSMFGNSADDISVFIVRDRPQSFEEKIYNQFKAEKDFFARVSYFREYIEENR
FDSDYFTEMLGFFTAYLKTVQTADEYVVSSYFIIKELVSIDGRYAQYLTVNFTELFALIE
DPLSVYLKIKDTELRNSFVQSIKTFIPGWPSIFIKLLPYARSMKMLEWLESAGHEDLLKK
MAMDIIENYRDHREAFIWLVKDLNEKPWFADLGIVYEKILLTLLHIVDITFKEIENHKDT
TENKKINRIVQQLLFKDRLLEKFLLAPDTTVQTIERVYALMMDIREIDPAVKMDIKRKVA
EKFPSFKFGAEEEKLTVSRGLMVTAKQYEEKQKLLAHIMENDVPQNQREIAFALSLGDLR
ENAEYKAAKEKQDELNAKVGKLKTELEKAQIFDKTTVTTSKISFGTRVVLKNELSGENEV
YTILGPWESDPSNNVISYLSPLGKKLFNHKPGEIISFSINDRKFQYRVEKIEAADF*
>SPSA8_v1_330012|ID:41145802| exported protein of unknown function [Spirochaetes Bin 1 SA-8]
VNRILFRHIIFLALAAAFLTAAVSQTFAFTLIRSSDSVDQDXASFLPPIRGIPFDELPHW
LPEGPPIDPGEENEELLVLIAQSLTWLGTPYRAGGFSRNGIDCSGFMHTILTEVLPGKGP
FPRQSAEFAKIGKKADNIRPGDILLFAINGEIYHVGLSLSSSTFIHAASEGARTGVIISS
LYEGNWRARLFAIRRIWN*
>SPSA8_v1_330013|ID:41145803|tgt| Queuine tRNA-ribosyltransferase [Spirochaetes Bin 1 SA-8]
MSEKIFFEGHYDKNSRARTGEIHLPHGIIQTPAFMPVGTNATVKAVPPEDLQAMGFRIIL
ANTYHLYLRPGHELIRKAGGLHGFSGWRGNFLTDSGGFQVFSLAPFRKIVDEGVKFRSHI
DGSSHFLTPEKIVDIQTAFNSDIQMQLDVCAPWGEADKKAWKAMMLTFEWGRKAKAQWEK
AKHEGYQGKLFGIVQGNFYKELRKESAERTIEQDFPGIAIGGLSVGEPTEVFSEFLEYTA
ELLPNDKPRYLMGIGTPDYILEAVRNGIDIFDCVYPTRTARNGLLFTSSGQITIKKALYE
DDFSPIDPECSCPVCLNHSRAYLRHLYRNGEILYSILATRHNLHFLGDLVRKIRLAILQD
RFEEFRKDFLNRYFAQP*
>SPSA8_v1_330014|ID:41145804| conserved membrane protein of unknown function [Spirochaetes Bin 1 SA-8]
MKLLMKPLRLQKLLIGSVLRRLAGGELFLVMIFLLAELFSSLWRFLASGAEVGKIFLWVA
AGIPRHSIEVFPVAFLFALTMSLSELQANGELMVIYSSGVSLRKLNIPVFMLAVASAFAI
FGMNEYLAIPATVQRDNLYQNMLGAKTTGFNPQDITILAQKGQVVYRCASFNPLTATMRD
IDILERDESGAPSRRILADSAIWTENRWLFSGARVFSRTQSNFWTELPGGNFSDNAFSEP
PESFAMITKNPAMMRLMPLKNYISFLINAGLPSVEAEIEYNKRFSTLLTIPIVFGLSLVF
SGLFRKNILLMSLLFSLGTATVYYVAQMLGSLAAKSSLVSPFFGVWSITILFSLLAGLCF

YKAKT*
>SPSA8_v1_330015|ID:41145805| putative Permease YjgP/YjgQ family protein [Spirochaetes Bin 1 SA-8]
MHEKNFAPFRQNKIFLYISREFMLSLFISFCFFFVIFFINQILLMAEEILSKNVPLHKTI
LLLFYSLPSIVTISFPFSALAGGLMASARLNSDNEFLAMAASGISIRTMYLPFIIIGLLS
SFISFQSNDYFLPRGTKAFQREYGRLVAKSASVELGAYSVKNYAQMTIVTGEKKDDAIGA
VLLYPQSGTPGVSSKTLIAAGKARFSISPDQYNALVTMDSVWQQEIDTKKPNHFSISRAE
QVQYRIKISEPIAGFSSAGPSEMSSAQIKVKITDKTANLEKRISDSRIQKSRTRVRLISA
YGTELYSAPEEAIPVKTVKXKNNPAEAPLAALAKSTNEYRASVTALPQDRTLQIYKLEYH
KKFSIPAAGFFFSLLAFPLGLGTGKAGRTAGFGLALLLSVVYWALLFAGQTLGLRSALEP
GLAMWAPDLLVLAAAALMLVLRHFSSRRFL*
>SPSA8_v1_330016|ID:41145806|dut| Deoxyuridine 5'-triphosphate nucleotidohydrolase [Spirochaetes Bin 1 SA-8]
MQKLNISIPVLLDEGAALPEYKTIGSAGADLRANLAETITIKPMERVAVPTGIRIELPEG
HEAQIRPRSGLALEKGVTCLNTPGTIDSDYRGEIKVILVNLGTEAAEIHPGDRIAQMVIA
QYTKAVFLPVEAIGETSRQEGGFGSTGR*
>SPSA8_v1_330017|ID:41145807| putative Peptidase M16 domain protein [Spirochaetes Bin 1 SA-8]
MLTHVSLHDGPTLLVDHRQAANSFFCSIWFPFGSRHEASCERGFLHFIEHMLFKGNIRYS
AQEIWSLAEGSGGFLNGFTERDTLCIHAHVPKSEWQTALNLLFHMAFTSTFPICEFEKER
NIIAAEILQTQDDIEERLLDDFFIQFWQGQPASFPIAGSLRDIKALTRENVIEFYQHVFR
PQNAIIAVSGPVPASKITRFLERILAEISAPFDSGRVTKLCTPDLAPKPYAGRIYKKTES
SQQFHIEAFQLDRPFPGFDYYSLALVSNLIGESSSSRLFRKIREEEGLAYTISSSLYHSR
TEALLLIVSAFEEENTARLLSLIDDELKNLFSEPLREKELEDHRRRLAGSFQIMLEDPEF
RARRMARNFIDSGQVLDENEELERYLSVDMQQVKTVLMMVNQAPCMQFLYGPIGKNKAEE
LGFLNAKSRRESPCKS*
>SPSA8_v1_330018|ID:41145808| polynucleotide phosphorylase/polyadenylase (fragment) [Spirochaetes Bin 1 SA-8]
MLPPKEKFPYTIRVVSEVLESNGSSSMATVCSSSLSLMHAGVPMKKPVAGIAMGLITDSE
RYAVLSDILGDEDHLGDMDFKVAGTKDGITGFQMDIKISSVSTEILTKALAQAKNGRLHI
LGIMEATMAEPASDISEYAPKVVVAKVDQDKIGAVIGPSGKNIKAMCEKFDVQINIDEDG
NVTIYGKNQKSAYEARDAVLGMVEEPEIGKVXKGTVKRIMDFGXFIEILPGKEGLAHISK
LSKGRVEKVTDVVKEGDSVEVKLLEIDHMGRLNLALVGALDENGNAPASAPRPDSRPRDP
RRFSDDRRDSQHRR*
>SPSA8_v1_340001|ID:41145809| Chromate transporter (fragment) [Spirochaetes Bin 1 SA-8]
VPGLVAAMVYKSAKKRSWNVLRAAETMILAILLALFPTLSLPILLGGIAFLYGIETVCTR
H*
>SPSA8_v1_340002|ID:41145810| Chromate transporter [Spirochaetes Bin 1 SA-8]
MHETLTLVWTFFKVGIIAFGGGWSTVGIIKNAVVPTWINESDFRALIAIAQSTPGPIALN
AATMIGWSHGGFISAILSTLSVVAFPLMAITATTLLTGKIKLNQGALDLSLKTGSLAMML
MTLWVLKPVSLDPLLLVLMLGAFYISAFTKINALWAILGSGAINAIAGPWIRGLFGG*
>SPSA8_v1_340003|ID:41145811| conserved protein of unknown function [Spirochaetes Bin 1 SA-8]
MIYPNQPNAVREPCTAVRVDRLTTQEHENALSFRYSLAALCTSGNDTACVTLLAGAGTRW
VASLKTSGMDFSFPADMPRGLYPVENFIGGNPHKVPLAAYSMDAVRNLGEQVIVIRGWQR
EIEQQVLEPLGIDSRTVAFVTQLPGPNGKVAGHGDAIWQAQEAWMNSRYVITNFGGDANS
PITVRAGLAMIKMLDEAGIEIGLLLPVALQKNPAYPVTLDESGLPVSFGHNKLGGGPSAM
VTANPQHADGSAYTNVGIRIYRKDWLLRAISEIRSNFWSPDASYEIPGNDPAGHEFALDN
VDAWLASKHMARIAAIANPEELTPAKSFSDIGKFEAAVREVRKEWDMFLSLQKAL*
>SPSA8_v1_340004|ID:41145812| putative ABC transporter permease [Spirochaetes Bin 1 SA-8]
MNIPHYPEFAPIDLSMKDAMHPAFNLIKDGISEFTFANLYLFRHVYHYKVSMLPDKGIAI
SGIKEGKSFFYLPCCFAPPSIFDDLMDKHDYMKNLSESQAQAHRINLEGRGYLVFEDRDN
FDYLYYRKDLAELSGREYHKKRNLVNGFVSNYVCEQRPLKKDNVQDALAVLEEWRESKGI
EGDYRAAREGLELFHELGMRGAVFYIENAPVGWCLGEAVAKGKMFAIHFEKACDRFKGIY
QFINQAFAQSLPEYFKLINREQDLGEEGLRQAKMTYRPCDFVRKYRVIHPDKKEFKPHDI
SEPAECGPGTMHSCEG*
>SPSA8_v1_340005|ID:41145813|pepT| Peptidase T [Spirochaetes Bin 1 SA-8]
MIMKNHNQFLSKPYVDGMVERFIRYVKVDTQSDRHIEDIPSTKGQWDLAWMLESELKALG
LADVSLDNHCYLIARVPASPGMENKPSIGLMAHMDTASDVSGANVKPTLVKDYQGNAIEL

SAGYMLDPKNYPDLTEHIGDTIITTDGTTLLGADDKAGVAEIMTAVEYMIKHPEIKHGPI
EIFFTPDEETGKGMDKFPLTKAKSTACYTFDGGKAAEIESECFTAYSVHAEFSGQVIHIG
AARGKLANAVAMAASFVNLLPRSESPEATDEWFGYYCPIEINGSLEHAELDIYLRDFTSA
GMERRIEAIKAFAKTVEAQYPLGKVSLTIKKQYLNMKEELDKHPDVLSNLVKAIEMTGAE
HGMRPIRGGTDGSRLTEMGIPTPNVFTGGYNYHSRYEWASVSEMSLAVQTILNLVQCWI*
>SPSA8_v1_340006|ID:41145814|atoA| acetyl-CoA:acetoacetyl-CoA transferase, beta subunit [Spirochaetes Bin 1 SA-8]
MEYVENKEIIARRIARFFKTGNVVNLGIGLPTLVANFVPAGETVILQSENGLIGLGPAPD
ADHVDKDLINAGAQPVTLQVGGCYFDSATSFGIIRGGHVDFTVLGVLEVDQEGNLANYKI
PGKLVPGMGGAMDLVAGSKKVIAATTHFEKSGSSKLKRRCALPLTAQKEVDYVVTDLGVF
EIKNNTFVLTEYFAPYTPEYIVEKTDADVVVSEHCKLSDI*
>SPSA8_v1_340007|ID:41145815|atoD| acetyl-CoA:acetoacetyl-CoA transferase, alpha subunit [Spirochaetes Bin 1 SA-8]
MIAKPMISPQKAAELVQDGMVVHVGGFLGCGSPERIIQALLERGAKNLTLVCNDTAVYDP
KTGRKNGVAPLVLAKAFSRVIVSHIGTNPETQRQMNAGEMIVDLVPQGTLAERIRAAGCG
LGGFLTPTGVGTEVENGKQKFEMNGRTYLLELPLKGNIALLKARKADKAGNLVYSKTARN
FNPLMAMACDIVIVEAEEIVEIGSLDPDQIHTPSIFVDYIVQA*
>SPSA8_v1_340008|ID:41145816|kamE| D-lysine 5,6-aminomutase beta subunit [Spirochaetes Bin 1 SA-8]
MAHIVRPYGDTLDDGKVQLSFTLPVPHGLKAQEAARIMVLGWGFSDCEIVHSARISDNFT
FFVAYAKTERGVDYDAVEADRNEEEKSMSMDEVNEFIRTHIGRKVVVVGACTGFDAHTVG
IDAIMNMKGYNHHYGLERYSMMEAHNLGAQVPNERLLEYAVKVKADAILVSQVVTQKDTH
IHNLTNFIELLEAEGLRSKFICIVGGPRISNKLAVQLGYDAGFGRGTYADNVATFVVKKL
AERMEL*
>SPSA8_v1_340009|ID:41145817|kamD| D-lysine 5,6-aminomutase alpha subunit [Spirochaetes Bin 1 SA-8]
MKEKLGLSKDKIAEARERAIAICAPVLDFIHEHTSVTVERATLRLLGADGANADGVPVPN
LVVDSLRQNLSEGAALRYANALVVKGMTPQELNSAIASGLDISKIPLGDKAFARVKAEQL
AQAHLDRIRNNRGQREKKLAEFAGRNNSPLLYLIVATGNIFEDVKQAQAAVLNGADVIAV
IRTTAQSLLDYVPYGATTEGFGGTYATQENFRIMRQALDEAGEREKRYIYLTNYASGLCM
PEIAAMGALERLDMMLNDSMYGILFRDINMYRTFVDQKFSRMINAYAGIIINTGEDNYLT
TSDAYEKAYTVLASQFLNERFAYAAGLQAWQMGLGHAFEMDPSIPNGFLYEFAQAQMARE
IFPEHPLKYMPPTKHMSGDIFKGIVMDNMFNFVSKATGQGIHLLGMLTEAIHTPFMMDRY
LAVDTARYVMNNIASFGDDIEFKKDGIMVRHAHEVLDKTLEFLREVEKTGLMDAIAQGLF
AEVKRPKDGGKGLEGLLAKSPDYFNPVESLLEEELGIL*
>SPSA8_v1_340010|ID:41145818| protein of unknown function [Spirochaetes Bin 1 SA-8]
MQQSEKTALPAHEATSAWTFADMDVVWHEFKPLTPYGKDYVYARTVHTDPLLLGEIYDDT
EQMAKFHESRTKTSSEQEKLAWHLSRIPRLPAALDDSLDIFLIKKFLSNYRALGESLDEE
SRKRFGFVFYSHALLEELNKGGSDSETFFIASSYDSELEAVRIGIREISEKLAQNRKESE
ERITRTWGVDFKNREFLTVPAELAVKMSAQAGQAGCPAIAVESFDDSYCLIRPLPDEAAL
ELQRQHAMLREKEAIRERAVLQKLFEMVGAELDNLSTYAKVVERLDLARARYLLKKKYGL
QKPDFDAQVMDIREGRFIPLEKECIAASRRYTPLSMRSEKPVAVLFGSNMGGKTIVLRTV
LFLQLMAQTGLGVPAEVFRTRIYTSIRYVGDNGQDLQKGLSGFGMEIHALADIIETAKRG
ACLCAFDEFAHSTSSDEAEALLSAVVAWFSRNPENRMLFATHFRRIARTSGADYYAMAGL
DVMRAVQVIRDSLGADSAGKEKNLIERIRMINEMMRYVVCPEKSIGKSADAPLQETESGN
GSDALAVASLLGLETEIIHLATKILKEKHA*
>SPSA8_v1_340011|ID:41145819| protein of unknown function [Spirochaetes Bin 1 SA-8]
MLPGAWLFIDSVNLVTGPGAASGKTTFARNAIAALHAQNHSVAVLAVGLEGDRTARIAAG
ASAGLPAGAGWGKRALAAASQFSFQPGDVFLTTSAYLEAADCQPEILDVLPGSSALGRLA
LARARRGGSAALVGPEHNEYLALAVSRILDNQWASTIFIDGALNRITQAASVSNAQLFYS
ARVSRSECKSLIDKMRYLLDLLSLPVYQPDTDIHADKAVFIDGALTDSAAERLRNSDEII
VVRDFSSIFLDPQAFRRFGGSARICVRNKVHFGGFSIILRSIQKDEFEQLAGPELTEKIV
SWNTWEGGENQNAAE*
>SPSA8_v1_340012|ID:41145820|kamA| L-lysine 2,3-aminomutase [Spirochaetes Bin 1 SA-8]
MHEYPSYDYRRIPLFKNVSPEEWNDWHWQMRHDIRDVETLAQVIPLTEKDKSDISEVLKI
FRMAITPYYASLIDPSNPVCPVRLQAVPRLPETRIDASDQDDPLHEDVDSPVPGLTHRYP

DRVLLLVTNLCSMNCRHCTRRRLVGFEDVHMSDENIDAAIEYISKTEIVRDVLISGGDPF
TLSTERLEKIISRLRAIPHVEIIRIGTRTPVVMPMRIDDQLVNMLKKYHPIYVNTHFNHP
KEITAEAKAACEKLANAGIPLGNQSVLLRDVNDCPVLMKKLVQKLLTIRVKPYYIYQCDM
SRGISHFRTTVSKGIEIIENLRGHTTGMAVPTFVIDAPGGGGKIPVMPDYLITSNEKRVI
LRNYEGVITTYDEPHFYEEDCGQCRICFDEPWLKPTCGVARLLDGQEKVLEPKNLARVAR
SHHHDDV*
>SPSA8_v1_340013|ID:41145821|kce| 3-keto-5-aminohexanoate cleavage enzyme [Spirochaetes Bin 1 SA-8]
MAKMVITCACTGAETTKAQNPALPVTPEEIAEAAYEAWRAGAAILHLHVRDENGRPTQNP
AVFKKAIDRIRSKCDIIVEITTGGAVGDTAEERLRPIMELEPEMASLDCGTVNFGNDYIV
NTLPVMRKFAEEFKRHRVQPTLECFDLGHVYASHILINEGLLEPPYHYGLVLNVPGAVPY
SADNLEVFKRALPEGANFTVMGIGRASLPAQYGAVATGGWIRVGFEDNVYYSKGVLAKSN
AELVERAVRIAREAQFEIASSDDVRKMFNLKG*
>SPSA8_v1_340014|ID:41145822|kat| 3-aminobutyryl-CoA aminotransferase [Spirochaetes Bin 1 SA-8]
MPAQEYPKLKLDESMRLFEQAKEVCPGGVMGIRRPYNFIQGEYPIFIDRGYKGHIVDVDG
NDYIDMLCAYGPVILGYDEREINDAVKAQMDEKGFCFSLVQKVQNDLEERLTHIIPCAEQ
VILVKTGSDATSLAVRIARGYTDRKYVLRCGYHGWHDWCVEVHGGVPEEITNLTVEFEYG
SLESLESALQAHEGEVACVIVTPVGHPLAKPVMAPPAGYLEGVRKLADRYGAVLIFDEIR
TGFRAALGGAQERYGVTPDLGTFGKALANGYPISAVAGKREIMKVAEQKVFVSSTFFPNS
LEMVAAMKCLDILEREKVPDSIWQRGTVFLEKLRKIAENSGLPLTVSGIPPMPFLTFDKS
EKDGEKTYKARREYFYTQTIRRGLFIQPYHHWYIAHRHTDRDLDYALGAISEALELTAKK
FPIGR*
>SPSA8_v1_340015|ID:41145823|kdd| L-erythro-3,5-diaminohexanoate dehydrogenase [Spirochaetes Bin 1 SA-8]
MPMGDKYGTHRVIEPAGAMPQTAQKLDNDMSKLYDNEILVDVIALNVDSASFTQIEEEAG
GDEAKIGEIIMNIVKTRGKQQNPVTGSGGMFIGKVAKIGSALKDRDLKVGDKIASLVSLS
LTPLKINRILAVHKEIDRVEVDAQAILFESGIYAKLPEDMDEGLALAALDVAGAPAQAAK
LVKPGDTVLVLGAGGKSGLLCCYEAMKRVGPTGRVVANIHSARSRGILEELHLAHEIVQA
DASKPVEFLNKVLPANKGREFDISINCVNIQGCEMASILPVRDGGIVYFFSMATSFTRAA
LGAEGIGKDVTMIIGNGYTKDHAEITLWELRENPALRKFFETQYVK*
>SPSA8_v1_340016|ID:41145824| conserved exported protein of unknown function [Spirochaetes Bin 1 SA-8]
MKNRTVFLLCVFLIAAGSSAWAQLAFGVTGSANGSTNASIDDFKDRFERGEGIFYGGFVE
LGMRNLCLGTSWGKSSYSVYFGGSDLTPMQDLMTYTYAQGHLFSYKAFLDPFFEVGFGKF
KADYSNSNDDGDKSNPLKASRFINAGGGVGLNFGHIGIFLKVLYAMPFGQPVRDSTNSYN
LEAYSIEPLEVFIGTKIIL*
>SPSA8_v1_340017|ID:41145825| exported protein of unknown function [Spirochaetes Bin 1 SA-8]
MRKRILTMLLVFLAGTSMVFAQSKTSLNIQSNQVGAKVYLNDNLAGYTTPNFSALVTPGL
YYIKVTKDGFPDFKTTVVVGSTPITITANFGPSQPPSPPAPPPAPVQHQLSVDSNVRNAH
VYIDKAFVGTTPFVGFYKPGYYTITIRVPGYEDYSQKVNLTSSFKLYATLNLLPLPVYFD
VNNVRGASVYRDGQFMGTTPYSGSWLPGSYSLRVSAPGFADYNERVALSGPLVMQISLAP
LLVDYEIRVPDYFMTIPGSPPMYRNLQLYLDGRRVDQLYGKALPGRHVLTVFINDLKLEA
EFELIGGKPAVIEPFFGIRVQ*
>SPSA8_v1_340018|ID:41145826| protein of unknown function [Spirochaetes Bin 1 SA-8]
MSDFISLQEFERSASIAGIFKGVEIDLLHETLLSWKSSPSKPYTVLELRDGKTLAAYAII
CKITGRDSTYDIRYFVVDRDYRHTEGSKHLLALIDQELLKAYPYAVIRAEFSTRKLIGLG
EKTLEEAGYRLIGHIKDYHGEGDDYYYFIHVAYRNKPVFEKPELNIDSN*
>SPSA8_v1_340019|ID:41145827|gmuE| putative fructokinase [Spirochaetes Bin 1 SA-8]
MRIGAIEAGGTKFICAIAETEFQGDGWSKPLIKESISIPTAKPEVTLQACLGFFSSAEPI
ERLGIASFGPVELDPLSPQYGFITTTPKPGWKQTDIVGFFKKTLQIPVFFDTDVNGAVLS
EHRWGNGRGLSDFVYITIGTGIGGXIMANGRLIHGAMHPELGHFLPQKHAQDNFAGVCPF
HGACLEGLASGPAIAARWGIPGEKLADNHPAWELEAYYLAQVSAMLAMTLSTRRILLGGS
VGMRPEIVGKTRKILSTVLAGYLSWLESPEAVESFLMNPLLGSQAGILGAAALTLD*
>SPSA8_v1_350001|ID:41145828| protein of unknown function [Spirochaetes Bin 1 SA-8]
MTLLGRQPSEPNLALLFDLQRTLTLYPDLDRGVAVCVQKIAEAFPRLNIMAGLEESDSSR
IGKIFGEPXX
>SPSA8_v1_350002|ID:41145829|asd| Aspartate-semialdehyde dehydrogenase [Spirochaetes Bin 1 SA-8]

MGLYRTPANSPRNFHISFAPSTDLEGSANSIALLEKNPLVKPYLQQGKKIPVTILGATGV
VGQRFLRRLANHPWFYPAFLAASEKSAGRRYDEACAWHLDGEPWAGMGAVTVTACTPEEA
YAPIVFSALDAEPARQIEPLFAQAGAYVFSNASAFRMEDDVPLLIPELNPDHLAMLGYQR
KKRSWKGAIVTNPNCTTVMIASALAPLEKAFGIEAVIVTSLQAISGAGYPGVSAMDITGN
VIPFIKNEEPKVEIESNKILGRVAALGEKMEFVNAPFAMSATCTRVPVLEGHTISISVRL
KGKPTLDEVRSALQNFVPATAGFELPSAPEKFLELNPLENRPQPRRDAEKDGGMRIIVGR
VRECPILGFKLVSMGHNTERGAAGASVLNAEIALAMGVXQ*
>SPSA8_v1_350003|ID:41145830| Aspartokinase [Spirochaetes Bin 1 SA-8]
MNTAMERTERPEADRPLVMKFGGTSVGSAQRMKDVAVLARVNAQNGVLVVVSAMSGVTDS
LFRAARLAFEGNLEAANAVCEDLRAKHYETHRELSPRTETADEFAYIDEIIDALKERLHG
VALLRELSPRSMDAIASSGELLSGQLVARAIGCRYIDARPLMKTDSRFGEARPDMEAIKA
AVNEHVAPLMRPWEIVVTQGYIGSDQENATTTLGRGGSDFSASIFGAALGAREIQIWTDV
EGILTCDPRIVPNAKTVEVLGYFEAAELAAFGAKVLHPATVKPALDAGIPVTVRNTMKPE
GKYSTIKPGKSSGRPVVALAMRKNVTIISIKQEDMTDQYGFLAKLFKVFGDAEVSVDLIS
TSEISVSISLDSSSPLPKLKKELQKLGAVDVLSGRVVIAVVGDLLKRTPAVLNKVFTVLE
QIEVDLVSLGANSVNLSFIVKQEDAEFAMKKLHEVFF*
>SPSA8_v1_350004|ID:41145831| putative 4-hydroxy-tetrahydrodipicolinate reductase [Spirochaetes Bin 1 SA-8]
MKIGIFGAGKLSGAISEEVRLTKSVSGSKPEIVWVVDQGDSVPPVAVDVAIDASTGSAVM
EHIQWAIDTRIPLVVAATGWSIPNLPELVSDKTGLLVAPNMSIGVAFMKRMSSIMALLAD
LDSSGELSIFEHHHSQKKDAPSGTAISLANACVQASSRYKGWTAGPAEPSSISVVSLRSG
AEPGYHQILFDSPLETLTLSHRARDRRVFAKGALLACSWILGKKGVFSMDDIVESLIREG
GVSNA*
>SPSA8_v1_350005|ID:41145832|dapA| 4-hydroxy-tetrahydrodipicolinate synthase [Spirochaetes Bin 1 SA-8]
MSMEAIMDTAKALNGLGVAIATPFFPDGTVDYQAFSRLMRFLAGKAPDKQGSPSAKPDGS
SRGVFAGQWDSFWTNESGGANFLVVLGSTGEGATIEPDERRKLIQTAVEEKTGLPIVVGT
GSNSTAASVRLTAEAVSLGADAVLVVVPYYNKPTPEGLQAHYKAVAEAAGGRPVIVYNVP
GRTGLNLTPAVLQKLWQIPNVTAVKESSGNLGQIGEIARTLPPGKILLSGDDYLALPSIS
LGAEGLVSVAGNVLPRRFSALVEAARAGRRDAAMKLQHQLLPLMDSLFLESNPIPLKAAL
AMLGLASDFVRLPLFPAQAGTRQKLEGALTAILETGQK*
>SPSA8_v1_350006|ID:41145833|dapD| 2,3,4,5-tetrahydropyridine-2,6-dicarboxylate N-succinyltransferase
[Spirochaetes Bin 1 SA-8]
MDIEAIQQKLAAEAPVSEDVWEAFLAGLESGEISTVTRDESGTWHTNVWVKSAILKGFRA
GGMKPFFWPPSLGLGTFENPDFFDRPAFPPRAITQEDGIRMVPGGSSIRRGAYVARGVVI
MPPAYINVGAHVCEGTMVDSHALVGSCAWIGANVXLSAGVQIGGVLEPPQAQPVIIEDGA
FIGGMCGIFEGILVKKNAVLASGVIISRSTKIMDLVNEKEYYGVVPENAVVVPGSRPASG
PYAQRLGIGIQTPCIIKYRDEGTSAAVVLETALR*
>SPSA8_v1_350007|ID:41145834|proC| Pyrroline-5-carboxylate reductase [Spirochaetes Bin 1 SA-8]
MKFKLGIIGAGKIGEAIVAGVLAKGLYQPQDIVLSVRTENHKEDLEKRLGIQSTLDNVFV
AQNSDIIILSVKPKAILEVVNQIQSVLKPGNLLISTAAGVPLELIESRVPEFVSVIRTMP
NLAVSIGEGMTVLSCGKNVSPGNKTMAEQIFASVGNALFLEEQHMDAVTGLSGSGPAYIY
IIIESLADGGVKLGLPRDVATQLAAQTVLGAAKTVLLSGEHPAKLKDLVTTPAGVTIDGI
LELEEGGLRVTLIKAVVKATERARQLLHKA*
>SPSA8_v1_350008|ID:41145835| protein of unknown function [Spirochaetes Bin 1 SA-8]
MVHLFIVQGPRQSGKTTFLCGLYNSARLSGISVYAVIEENERDSQGIPISLVFHTLETGA
IRLLAERESSRPYPPFQFRQDVFDAVLDELRQASRTRDLVIIDEVGPYEILEQKGLWPFF
RDLPTDRRLTLAIGIRPDLVDSLLLALADWNVPYKLEAILPMAQNETPTKQFADTITACC
QSISLDL*
>SPSA8_v1_350009|ID:41145836| protein of unknown function [Spirochaetes Bin 1 SA-8]
MIYWNRDLAARLKCPEKEKQQLPTFIKKLMVYIEKIHESGVSATFPNVERPLFPYFYQGL
SLIMDNLSTESIEEIMAVYLSVSQESGLQFLKQCIETEAVLSLASGDTPEFTFRKIVPYA
GIEQADWLIQSAANKGTL*
>SPSA8_v1_350010|ID:41145837| protein of unknown function [Spirochaetes Bin 1 SA-8]
MLMQRLYRLQSLFNLIQKTESGAGAETEGPSSISFPPVQKSEADQLMAXAKVFXGYIATL
REKGKEAFGKAIADEKRRKLKFCLRILLESQPTVNLQQSFLSGGMLXARDAHEALETAMY

FQGTSSALKNSHPAVVLKVMSAFLGVEASAQADEWIAERYKHRSALEEELILPGDMPDII
LSNLGNTARLERTIRLAGPQLAGTALSGCPREILSQLEHRIFSTCGTILLHHDIEVARAD
YSSDEIADAQNAFMELLETMKISEMEETSGMIEPEKQKMPQDPLLVADLSNLIMETEEKL
LKDILIKLSPQAIAVIVQGVEPAAHDRIFSTLPAGKARRVLDALEASTGIDTLTLTRNAQ
IFAQQILSEISQHHKITAKQLPITGRLRQLLSAILSRE*
>SPSA8_v1_350011|ID:41145838| putative GTP cyclohydrolase 1 type 2 [Spirochaetes Bin 1 SA-8]
MVSLLPWKALLLYSIFMDIREFDQWCRSFLKIDDLVGIDDSLNGIQIGRSLAPVRKVAFA
VDACQESIQRAVQAGADVLFVHHGLFWGKPERIEGSLLKRLKTALDADLALYACHLPLDM
HPEVGNNAVLSSMLNLQNRIPFGIYHGIPIGFSGSLPSPLSLEEIRKKLLPDNSSPRLVI
PGSRKLIETAAVISGGAPFESFQAISKNIDLYITGEPSHSIYHQVVENGLAFMALGHYXS
EVWGVKAVAXKLTQEKSIETFFIDLPTGL*
>SPSA8_v1_350012|ID:41145839|lspA| Lipoprotein signal peptidase [Spirochaetes Bin 1 SA-8]
MNDSTITLQRRSLPFLVSFIIILADQISKAFIVAKIPVNTIGSRLLGDFLWIVHAKNLGI
AFSLGDSFSQLIRVALFIILPLGFIGFGIAYCLKTEKLSTFQRYAIAVIIGGGLGNLIDR
IFRADGVVDFISLKFYGILGMNRFPTFNVADSCITIGALLLFASGVLWSPAEETER*
>SPSA8_v1_350013|ID:41145840| conserved protein of unknown function [Spirochaetes Bin 1 SA-8]
MKVEKKTALALFVLGASAFNIIITAGIFLALLALYSLTFAKILSPEFLMWAVVGAFVISL
VLSVLIYKKVLNWARKKYDLDARLGLK*
>SPSA8_v1_350014|ID:41145841| conserved protein of unknown function [Spirochaetes Bin 1 SA-8]
MNNMSRRFMKVGVTLPRILIPAESIDRRSWAVVACDQFSSEREYWEKVARTVGDKPSTLN
LIFPECYLEDSDKNERIAAIRASMKTYLERDYFTELPEGFMLVERHTPYEEKPRVGLMIA
VDLEQYRYGKDSKSLIRPTEGTIVERLPPRMDIRRGAALELPHIMLLVDDPEKSVIEPLY
ASRQYLPKVYDFDLMENSGHLRGWHVSDTRYLEKILSAMEKLADPSSYKNKYQQDEVLLF
AVGDGNHSLATAKAIWEETKKNLQAGLPDHPENAQLLENHPARFALVELVNIYDEGLPFH
PIHRVLFKANEAELIGIMEKAGAVVRRTTSYEQAFKLADAPQEGKTQTIAFVSAADSGVL
VFEQPKSKLAAGTIQEILDAYLKAHPETSIDYIHGEGSLVALAQKQGNFGLYLPPVDKSS
FFGTVIADGVMPRKTFSMGEAPEKRFYMEARKIQP*
>SPSA8_v1_350015|ID:41145842| D-isomer specific 2-hydroxyacid dehydrogenase NAD-binding protein
[Spirochaetes Bin 1 SA-8]
MTILLADAFAPDLPGRLAPFGNVTADLKQLPEAEVLIVRSKTKVDKAMIDAAPKLKYIIR
GGVGVDTIDVEYAKTKNILVDNTPEASSLAVAELAFALMLAMPNHLVQADNSTKEGKWLK
KELERTELNGKVLGLVGIGRIAREVASRAKAFGMRVVAYDKYVPSSEAAEMMSLDEVYTQ
ADYISLHTPLTEETRGMINNESIAKMKQGVRIINTCRGQVVVEEDLVAALVSGKVAGYAA
DVFYKEPPADSPLLKAPNVLLTPHIGASTEENLLRLGDSIVARLEKYTASK*
>SPSA8_v1_350016|ID:41145843|serC| Phosphoserine aminotransferase [Spirochaetes Bin 1 SA-8]
MNRVINFNAGPAAIPLDVLKQAQEEMLDWNGTGMSVMEVSHRSKEYEAMHNEAQDLFRKL
AGMGPEWKILFLTGGASTQFFMIPMNFLHSGRKATYLVTGNWSKGALKEAKRFGTCDAYT
TENPDGTFTRILKPTEFSIDPNSTYAHMTSNNTLFGSQWHYWPETGSVPLVCDMSSDIFS
RPFPADKFSLIYAGAQKNLGPSGVTVVAVKDSFLQLAEEQAKLPTMLSYRTHDDNNSLYN
TPPCFSIYILSLTLKWLDKNGGLEAMQKINEEKGRIIYNAIDASNGFYRGPVEKDSRSLM
NVVFRLDTVEKEELFVKEAKAHGIVGVKGHRSTGGIRFSMYNANLVDNVAKAAQFMEDFR
KKYS*
>SPSA8_v1_350017|ID:41145844| putative Transcriptional regulator, LysR family [Spirochaetes Bin 1 SA-8]
MDIVKLRAFLTVAKLKSISEAARELNYTQPAVSAQIRDLESILGISLFEKSGRKLRLSEP
GRIVLPYVERLLRDYDLINAAIPQALDSSRAYLRIGASSLPGVHLVPQLLADFGRSFPDI
CISLSIEKANRIERMVLDRQIDVGFIGRKVQYAPRTHLNEYLVKKDNLVAIVSSTHPLAG
KKSISLDELSRIPLILPHRDVLTRRSVEERFHQLGYSVDLAFEVSNTEAIKRMVACNLGA
SVLCQSSVQKEVEAGWLSALLVDELDLSRFIYIITRKTEKIPPGLDDFIQFAIASFSGNL
*
>SPSA8_v1_350018|ID:41145845|mdh| Malate dehydrogenase [Spirochaetes Bin 1 SA-8]
MSEDITWWSFDEVEAFMKAGFEAVGVPSEEAAVCANVLIAADKRGVDSHGVGRYKPIYLD
RIWAGILNTKTNFEVVRETPTTAVIDGHNGMGHYIAKRAMDMAIEKAEKFGIGMTVCRNS
THYGAAFYYARMAVEHGMIGLTTTNARPAIAPTWGVEPMLGTNPMTWGMPTDEDFPFMLD
CATSVTQRGKVELYDRLDKDLPDGWVIGQDGKYRHDTKQVLVDLTRDLAALTPLGGLGED

MGGYKGYGYAMVVELLSSALSQANFMKALSDLGPDGKKKPIELGHSFLAININAFCDLED
FKHHVGEVCRQLRASKKAPGAERIYTPGEKEHDVWLYRKDKGVPFNAPLKKSFKEVKERC
KLDIELPF*
>SPSA8_v1_350019|ID:41145846| conserved membrane protein of unknown function [Spirochaetes Bin 1 SA-8]
MGKAGWGLGISLVASVAGGFIGIIILILFSKPLAAVAVKMHPAEYFMLAVFGLTTVASLG
GKNGIKAFAAALLGLLINTIGLDPISGVKRFTFGQVNLFDGFDFIPALIGLFALSEVFSR
LEEGFSKEKIDYKLEKKKEQWPTLKDYWKLRRVTVQSSVVGTLIGIFPGAGSAIASFVSY
DLAKRTSKTPETFGKGNPEGVAAAEASNSASVGGAMVPLLTLGIPGSAATAVLISALMIH
DLVPGPMLFANQPVLVYGLFASMLVANIIMLIIGALGSQLWIKVTRIPQQLLIPLIISIA
VVGSFAVKNSMFDVFACIGFGVLGWILKRHEYPMAPIVLGMVLGKLAEANFRRAVIMGGY
SIFFTRPASVVLLVLSILSMALPLIQTYRDKKKAA*
>SPSA8_v1_360001|ID:41145847| protein of unknown function [Spirochaetes Bin 1 SA-8]
MDSCAHGVDALRNAYHRHRETACTGSILSTASNSRHKSMGYLCRTDRRRYKAVKEEALRF
ALRTADPTGRLNMLREYIQAQALRSLHESRAFEALSFVGGTALRFLFDLPRYSEDLDFSL
EIPENYFPDAWMAKLKRDLEFQNFNVEMSWNSTKPVHTGWIRIASVMKDAGLSSRPEQKL
SIKIEIDTNPPAGAATMVRLVNXX
>SPSA8_v1_360002|ID:41145848| conserved protein of unknown function [Spirochaetes Bin 1 SA-8]
MKLERLLQAFAGQPYFDYPAVRLHFQSESDHTSRTALSRFAKRGILRELKRGIYAFSEPY
RSQPLNPLQIAQALYSPSYVSELWALSWYGVIPEKVTLITSVTTRATRIFKNALGEYRYR
TIDGRFFHSWQTVEILSAEVRIATPEKALLDLWYLEKGEWTAARMESMRFETRIIDTGKL
HVLAQSCPPRLIRAIKAWAIYAGQTEGGTKL*
>SPSA8_v1_360003|ID:41145849|bglA| Beta-glucosidase A [Spirochaetes Bin 1 SA-8]
MKRTDGFPDDFIWGCATSSYQIEGAAQEDGRGVSIWDTFCHTPGKVAEGDTGDIACDSYH
RYPEDIALLRELNANAYRFSTAWPRIQPDGSGKPNPKGVAYYDKLVDSLLEAGIEPWMQL
YHWDLPQALEDRGGWANRETAFRFQEFAEIMFVHFGDRITHYSSLNEPWCSAYLGYREGA
QAPGKRDKVLANRAVHHLLLAHGLAQKSYRATGLSGKFGIVINPGRQRPATARPEDYAAA
EIASIESTRIWLDPLYARGYPALFTQYFGADLPIVDGDMEIIGSLPDFLGVNYYFEHVVK
AVSADERNPFGVALAGTWHEKTAMGWDIVPEGMLRLLRYIAGNWPISNLYVTENGAAFDD
VPDADGVVHDKERIEYLRAHLQACRDAIAEGIPLKGYFLWSLLDNFEWAYGYRKRFGIVR
LHPETLNRIPKKSFYYYRDVIAGFGL*
>SPSA8_v1_360004|ID:41145850| Auxin Efflux Carrier [Spirochaetes Bin 1 SA-8]
MFTALQSVLSVILMIGLGFVLARKKWFEQNGQTILSRLVVNIALPTYMISNLMGGYDRVK
LLSMVPGLPIPFGIMIASYLIAMLLAVLFKVKKTRRGAFQSMFALSNAVFIGLPINILLF
GEQSLPFALLYYIANTTLFWTIGVYGIARDGALRTGRPAPSLVSISGLKRIFSPPLLGFL
IAIVLILLEIKLPKFVMDTCKYLGNLTTPLSMLFIGIVIARVDWKKLKFERDYLVILAGR
FLLTPLMMFFLVRTMDLPLLMKQVFIMQASMPAMTQNPIVAEAYQADAEYAAIGTSLTTV
ISMMTIPVYMSAILVLI*
>SPSA8_v1_360005|ID:41145851| Trp repressor [Spirochaetes Bin 1 SA-8]
MSRNPELMKHNIAEIAKALEGADANLIESFLYSLLTPAEADEMAKRWALVKELAQGTAQR
KIAEELGLSLCKITRGSRELKKENSAFRILLNKLDEASAQDPKKQG*
>SPSA8_v1_360006|ID:41145852| protein of unknown function [Spirochaetes Bin 1 SA-8]
MELPLRLKKLKDGSLIYGISPWFRVMNGVFVALIAAAFLMNDIPMSVLAWILEVGLALGV
LYEENWKADAAGKRLVHKGGVYPFIKSTPVDFSEIEHFLLEALARGTVPGSSEERDDKAR
AQTMMNGGSDERNAPGGMFARNLRRKPFVNLILQTRSGEAYLIDSLPARKAKRLEIAGTA
LADSCGCQLEKDF*
>SPSA8_v1_360007|ID:41145853| AzlC family protein [Spirochaetes Bin 1 SA-8]
VPFEASGKARFGSIFTAAFKASVPVLLGYTTIGFAFGLVLTGAGLPWWLSPVMALSVYAG
AAQFMGVGLMAAHAGIAEAALLTLLMNARHAVYGLSLLDEFSKAGKAKPYLIFGLTDETY
GLLTTVKPPTPADQTAFHTIITGLNQFYWFLGCTAGGLIGSSLPFDTKGLDFALTALFVV
LLVEQMKTVKTAAPYLLAIASAILALIFVSGRNFLLVSLALASCLLLLFRKRIHQ*
>SPSA8_v1_360008|ID:41145854|azlD| Branched-chain amino acid transport protein AzlD [Spirochaetes Bin 1 SA-8]
MIRYYLLVIAVMTIVTVFTRAVPFLFFSGRKPPQWLDYLQKFMPPAVMTLLVFNALKDVS
FAEAPYGLPALGAALVTAILHLWKRNVLLSIAGGTAVYMLAIRLPL*
>SPSA8_v1_360009|ID:41145855| conserved membrane protein of unknown function [Spirochaetes Bin 1 SA-8]

MKFQIVTKLYKNLISTLSFSSELRKMRRELNAKIDMPDTISPGMHAHALGVNRWFKKRRV
SIAESYLMVVSNLDSKYAEKRLDALRVLADVAFHSKNLDYPLNTARVQMALIKGVVKNRT
NKRRQLELLHDFTISSRGQHQVLARLCDELNIIELPEQGLRLEDFSYGWDSHVHDTATTG
RKNATQLIIDAFIKGISELTCAYGSADDIATMEEVLEAGSILGIRIHLGIEFSMEVMGKR
GHFMALLPDSIKTPADVRAFFDTNEQTLKEFFDGLATNQLNRMASVRQLFREFNQTYLVE
LNEGFPDSPLFRVPELTLESLQEFIPTVSINRLHIAEYLYNIYKPILQNRLLHLKVLRAG
MKQGHGRKKPDSPEFRKIDKLYSALKKELKNLTPDILMERYFSGPRIIDYTSVFKDIEKL
KSQLTQAGCRLKMLHPLEFGLDDAEKILIRYRGIIDEVELFSTQDCIGRKPEEIEALAVM
INRLNALDGPPLVSVCGSDSTGRNPHVPGMGFIFEDKIIGARKNSYVRRHMALPPIVSAM
VNAKGRHVSEEDILRAPVIISMGKISTGKERDGQNEFINPWRAFRYLNPLVKNIILVSIG
FLVADSFIGWGYALLWLAITGFRNSIADLISTRGTKLNEWKIKSINFDNVVQSLFWTGFS
VPILGFVKSSFDTVWQGVHDGLVFNLVKFFFISFANGLYLATHNTLRGFDRKVVRLNFFR
SVLAWPFATIFAPVGNLIGIPSIVQTKIWSDFIAGFIEGGNKYLKVLRLRRQNLEEIIPL
ISDAEESTRYAAMLDLLYLFREEPRTKSSLRAIFDPAYKPLIVLKGYVKPKPQGYEAFHR
AIFAPDLAEKLSLFILSQYEAERAEDLANLAADTLPVLRSWLESKLNAVIAAV*
>SPSA8_v1_360010|ID:41145856|rbsA| fused D-ribose transporter subunits of ABC superfamily: ATP-binding components [Spirochaetes Bin 1 SA-8]
VAEPLISVRNLNKSFPGVHALKDVHFDLYPGEVHTLVGENGAGKSTLMKVLAGVYQKDSG
EILVNGKPVEIPNPRAAQRLGISIIHQELNLMNHLTAAQNIFIGREPKKYLGLFLDEKQL
NRKTQDLFGLLKMDIDPRVKIGELTIAKQQMTEIVKALSYNAKVLIMDEPTAALNEIEIE
ELFGIIRHLRSQGVGIIYISHRMDELFRISDRITVMRDGQYIDTLKVSDTNLDNVISLMV
GRKIYESAPQIDLAVNRPVVLKVSGLNQGKTLKNVSFEARKGEILGFAGLMGAGRTETAR
AVFGADPIDSGEILVNGNKVTIRHPSDAVRHGIGYLSEDRKRYGLAVGMDIETNIALASM
QSFTGFGGFINIKATRKAGSDMIQKLSIRTPSLSQKAKYLSGGNQQKVVVAKWLIRNCDI
LFFDEPTRGIDVGAKQEIYKLLNELAEAGKTVIMISSELPEILRMSHRIVVMCEGRVTGE
LLASEATQEKIMKLATQRESMVKTAQ*
>SPSA8_v1_360011|ID:41145857|rbsC| D-ribose transporter subunit ; membrane component of ABC superfamily [Spirochaetes Bin 1 SA-8]
MPEKIIADKAAKDSFSSSSAKQKILAFSSLIILLIVFSFASPNFFQSNNLIAIMLATAVN
GVLAIGVTFIIITGGIDLSVGTMMTFTAVMAAQFVANWHMPVMVGVLVAIMTGMVCGFLS
GVMVAKLKIPPFIATLGMMMLTKGLSLIIAQSKPIYFTESPTFALIAMGSIFSFIESFKI
PNAVLIFFALAIVASIILNKTVFGRYTFAIGSNEEAVRLSGVNTDRWKIGVYAFSGAICG
IAGIIIASRLNSAQPALGPGYELDAIAAVVIGGTSLSGGEGTILGTVIGAFIMSVLTNGL
RILSVPQEWQIVVTGVIIIFAVYVDMLRRKRSK*
>SPSA8_v1_360012|ID:41145858| Periplasmic binding protein/LacI transcriptional regulator [Spirochaetes Bin 1 SA-8]
MKNRRIMTVLIVAMMLVAVTTVAVAQTVYIPLISKGFQHQFWQAVKEGAMQAAKDYKVTI
TFEGPESEAMVDKQIDMLSAALAKKPAALGFAALDSKAAIPYLKQAQAAKIPVIAFDSGV
DSDIPLTTCATDNIAAAALAADMMAKLIGGEGEVAVIVHDQTSRTGIDRRDGFVNRIKEK
YPKIKIVSVQYGGGDHLKSTDLAKAIIQANPNLKGFFGANEGSAIGVLNAVKEMGKIGKI
TVIGYDSGAQQIAAIRSGEMAGAITQNPVGIGYKTVESAVKALKGEKLPKFTDTGFYFYD
KSNIDDPKIKAVLYQ*
>SPSA8_v1_360013|ID:41145859| Fructokinase [Spirochaetes Bin 1 SA-8]
MLPCPVWMRQPGAQAPEFRHNKRRICMIACIGENLIDMVPLTAVENAPYGAFKAAPGGCP
YNSAIAAARLGSTVFYIGKISNDFLGDRLVNRLAGNGVRLDYVARAENPVTLAFVEKSAS
GEARYAFYASNAADRSLEAGDLPAMLHPDIHFLLLGSISLLLEPGAGSILNFTERVQERV
LVSYDPNVRASMIPDRAKFRSSFEAMCARCAIVKASDADLAWIYDSPFDREIVAHILDCG
AKLVLVTQGSQGSLAATGHAFASAEAVPVRVVDTIGAGDTFHAAVLSFLDSNKWITAKDL
ENLSAAQLEKILRFASAAAAMDCMREGAEPPTLEALLSRFPDLALQ*
>SPSA8_v1_360014|ID:41145860|fucU| L-fucose mutarotase [Spirochaetes Bin 1 SA-8]
MLIGIKPCISPDLLTVLYKMGHGDELVLADAFFTGDALNARVVRADGLRIPDLLDGILRL
INLDDFVDNPVIMMQPVPGDTLDYEVERRYREVIDRHWPQTPPIVRMERFAFYERSRKAF
AVVMTGETVKYGNIIIKKGVPPVGEFA*
>SPSA8_v1_360015|ID:41145861| Transcriptional regulator, LacI family [Spirochaetes Bin 1 SA-8]

VGKQSITIKEVAEYAKVSTATVSRVINNDSRVIDDTKERVLKAMDELGYKVNIVARSLKT
NQTKTIGVMAPDLSGDFFMYIAESMDKELAANGYSLVVCASHDSLEEEAKRLRHLAERLV
DGVVVIPATDRGSHFRFLEEFGIPIVFVDRTVTDFAVDAVLTENEEGAYEATKALISDGY
SRIGFIGGKPEISTSKERFSGYSRALREAGLPIVDEFVRFGPPTLPFGYHAMEEMLKNDR
SPDAWFLVNAFTHIGATNYIMSDESTRRRNIVIAAFDEMPYAPLLRFCRYAVQQPIAELG
RTAARLILKKIMEPDPTRTQIVRLKTRLIKHNYREVSYGRI*
>SPSA8_v1_360016|ID:41145862|fucI| L-fucose isomerase [Spirochaetes Bin 1 SA-8]
MEEFEMQEPQLRLRGGLPKIGIRPTIDGRLGGVRESLEAVTMGMARAAAKLISENLKHPS
GHAVECVIADTTIGGVAEAVAAQEKFAREGVAVTLTVTPCWCYGSETMDMDPNTIKAVWG
FNGTDRPGAVYLAAVLAAHNQKGLPAFSIYGHDVQDLQDTDKIPSDVAEKILKFARAGLA
VAWMRGKSYLSVGSVSMGIAGSIVDADFFQSYLGMRNEYVDMSEITRRIEEKLYSEAEFE
RAMRWVRTYCAEGPDNNPPEKQHSRSKKDKTWETSVKMAIIMRDLMVGNPGLRRKGLGEE
ALGHNALVAGFQGQRHWTDHSPNGDFMETVLNSSFDWNGIRQPYIVATENDSLNGVAMLF
GHLLTGSAQIFSDVRTYWSPEAVKRVTGWQPEGIAANGFIHLINSGATTMDGTGKQRKDG
KPVMKPWWEVTPEDAKACLSATSWRYASLGYFRGGGYSSDFLTEGGMPVTMSRINLIKGL
GPMLQIAEGYTATLPEHVHDSLDLRTDPTWPTTWFVPRLTGTGPFKDVYSVMAAWGANHG
ALSYGHIGSDLITLASMLRIPVSMHNVEPSTIFRPSYWSAFGMDPEGADYRACSALGPLY
*
>SPSA8_v1_360017|ID:41145863| protein of unknown function [Spirochaetes Bin 1 SA-8]
MKSVKNVLIGLLLLTAVATVFGQYAGGTFQTDFGKMMLQQNGSQLSGTYDYAGGRIEGTI
MGNTATGWWYQTNGKGRFIFVFNQDFSAFTGKWGYDSSEPSATWNGTRIGAAPMQIPPPA
SYGTMGQATGVYLSDFGDMTLEQNGSSVTGRYTHADGRIEGTLNGNVLTGWWYQSNGKGR
FSFVFNSAFTAFAGKWGYDSNEMTGTWNGTRKESSKPSTNQQTVIQVEGTEVFSNWNKAG
VANLPPSHTYFYIAKSMTITRIVNYHWNDGRGQYPGYISLRSSDGKTYGPWQAVGTSGTG
GAQNVNWIVYPNVLIAMGLYQIVDSDPGSWSHNGMSFGSGFSSVTAK*
>SPSA8_v1_370001|ID:41145864| Radical SAM protein (fragment) [Spirochaetes Bin 1 SA-8]
VLNLDEQIARAVAFTRYRYKARLFFLYFQAYTNTYLPVGQLRALYDHAIAVMEAIAPGTL
RGLVISTRPDCIDREKVELIASYQRQGLEVWLELGLQSAHDQTLKRINRGHTVSDFVAAS
KLAHDAGIRRAVHVILGLPGENRLMMLETMRFLQKNGLEGIKFHDLRLAKGSALERSYRA
GEFAPLHPDRLPPLLADCLELLPASVEVLRLSSDFSRDSVLDLFPHPDKNNLYMAVEAEL
ARRNSFQGRLVE*
>SPSA8_v1_370002|ID:41145865|acyP| Acylphosphatase [Spirochaetes Bin 1 SA-8]
MKAFRAVIRGDVQGVGFRASAAWEARRIGVTGWVRNTDEGSVEVWAEGNEPELEEFFHWL
EHGPSTAWVREVQRVWEPPTGRYSSFGVFLGD*
>SPSA8_v1_370003|ID:41145866| Short-chain dehydrogenase/reductase SDR [Spirochaetes Bin 1 SA-8]
MKTGLSGKIVFLSAATDGLGFAIARQALVEGARVFIGSRSEEKVRIALEKLAPLGDASGI
PLDMASGESIKDWVKAGLALFGGCDALLVNAGGPPPGNFESFGDDSAWQKAFELTLLSAV
RLIRESLPALKASGHGSILTITSSAVKEPWPELILSGVMRSGVSSLVKSLSRELGHYGIR
VNNIAPGKIMTSRLEKIIRSEAEARDIPYAAQLENTERDIPLGRIGTPDEFAQVAVFLLS
DAASYVSGQTVLVDGGITKFLY*
>SPSA8_v1_370004|ID:41145867| conserved protein of unknown function [Spirochaetes Bin 1 SA-8]
MPFNNKISVQQRAVPQPLELLNPERTMEVVLEALRDLVEYELAVVLRLETQDKLVVRKAV
GPLYTPRLEGHSISLAKRPDIARLLEHKEARLFGEHEAHIDTYYDILDLPDGHSCLVAPL
YIESTPIGLLTLDHRACNRFTPSIVRFISTISRLISISIVQSDASSSLLETTRQLTEERN
RLLDESSDAFRDLVGVSAAWRRVLDAIRLVAVAEVPVLLTGETGTGKEQVARKIHQLSHR
ASAPFVAVNCSALVSSLAESELFGHEKGSFSGAVAMRRGRFELADGGTLFLDEIGDLPIE
LQPKLLRVLQDGKFERVGGERSISVDVKIIAATNADLELAIAAGRFREDLWYRLNVFPIH
LPSLRERDEDAALLAEYFASQIRRRPGFEALALTPPAIREILNTKWRGNVRELRNAIERA
AILARGENILAEHLMPAVNAYRGNQAQGPQKRLTPVASSATGVSEPDSPYFPNINEALRT
HIMTALKKSGGKIYGSDGAAALLGMKPTTLQSKMKKLGIDRNNQEL*
>SPSA8_v1_370005|ID:41145868| putative peroxiredoxin [Spirochaetes Bin 1 SA-8]
MEQQTFHMPLLGDDFPSLTLQTTQGVMNLPGDLKGQWFVLFSHPADFTPVCTTEFVGFQR
LMPEFEKLGVKLIGMSVDQVFSHIKWVEWIKEKLGIEITFPIAAANDSIANALGMLHPGK
GTNTVRAVFVVDPQGKVRLVIYYPQEIGRNMEEVLRAVKALQVADTNKVAIPANWPNNEL

IKDHVIIPPAKTIAEAAARLKEYDGYDWWFCHKAL*
>SPSA8_v1_370006|ID:41145869|hprA| Glycerate dehydrogenase [Spirochaetes Bin 1 SA-8]
MFVTWCKTDTMKIVVLDGYTLNPGDLDWNALAALGDVAVYDRTEASSIVERIGDAELILT
NKTPISAETMQACKNLRYIGVLATGYNIVDTAAARAAGIVVTNIPTYGTDSVAQFTFALL
LEICHHVGHHAQTVRDGEWTKSPDFCYWHYPLIELAGKTMGIIGFGRIGRRTGEIARAFG
MNVLAYDEYPNTVSEKEGISCTTLDRLLAESDVISLHCPLTDRNKGMINTDTIAKMKDGA
ILINTSRGPLVDETALAAALNSGKLAAAAVDVVSTEPIRPDNPLLKARNILITPHIAWAP
KEARSRLLNIAVENIRAFIDGSPINVVF*
>SPSA8_v1_370007|ID:41145870| putative type I restriction enzyme HindVIIP R protein [Spirochaetes Bin 1 SA-8]
MPHLTESEIESATLSWLSDLGYQTLFGPDIAPDMPAAEREHYGQAVLEGRLRNALARLNP
HAPAGAREEAFRTLTRPDSPSLIGNNHAFHKMLIEGIPVEVQRKDGSYGHDQILAIDFAH
PDNNEFLAVNQFTVVENHIERRPDVVVFINGLPLAVMELKNAADENATIWSAFNQLQTYK
QQIPALFAYNEALVISDGVEARIGTLTADRERFMPWRTIEGEELADARLPQLEVVLKGVF
DQKRFLDMIRHFIVFEDDGGGVLIKKMAGYHQYHAVNVAVKETIRACRCPENTREDSGTY
FAKGQKDARPGDRRVGVVWHTQGSGKSLTMAFYAGRIILEPAMQNPTIVVITDRNDLDDQ
LFATFARCHELLRQQPVQAESRSHLRELLKTDSGGVVFTTVHKFFPADDEDRHPLLSDRR
NIVVIADEAHRSQYDFIDGYARYLREALPNASFIGFTGTPIELSDKNTRAVFGDYISIYD
IERAVKDGATVPIYYESRLAMLELNEEERPKLDAEFEEATEGEEIEHKERLKTKWAQLEA
LVGTEKRLKLIAEDLVKHFEARLAAMDGKAMIVSMSRRICVELYKAIVSIRPDWHHEDDD
KGMIKIVMTGSASDPPEWQSHIRNKLRREELAQRFKDPRDPFKIVIVRDMWLTGFDAPCL
HTMYVDKPMRGHGLMQAIARVNRVFRDKPGGLIVDYLGLADQLKYALASYTESGGKGETA
IDQEKAVALMLQKYEICCDLFHGFSWSAWKTGGAQERLSLLPAAQEHVLAQEDGRNRLAK
AVAELSKAFALAVPHESALEIRDDVAFFQAVKSVLTKSTPDDRRSPDEIEHTIRQIISKA
VSSHEVIDIFTAAGLKKPDISILSDEFLEEVRGMPHKNLAVDMLRRLLEGEIKARGRKNI
VQARSFAELLENAIRKYQNRAIETAQVIEELIALAKDLREADKRGEKLGLTEDEIAFYDA
LEVNDSAVAILGDATLRLIAQELVKAMRSSITIDWTVRENVRAQMRVIIKRILRKYNYPP
DKQAHATELVLEQAEVLCRDWAE*
>SPSA8_v1_370008|ID:41145871| Peptide-methionine (S)-S-oxide reductase [Spirochaetes Bin 1 SA-8]
MSNQMGFSVRIFIPSGNPDGLRIIEKSNWTGQGLVFPRALYAEIRQRNEISRTGVYILWG
PQKSGNLLMIYIGEGDSVLSRLDQHAKQKDFWTHAVIFTSKDQNLNKAHVQYIEARLVQL
AAEAKRAELDNGNVPQPPNLSEADKADAETFLDNILLCLPIIGLNVFEKAKVLGEKSHDL
ILKSKGIEARGQDTAEGFVVRTGSLAIKDEAPSIHGFLSGLRRTLLDQGIFANEGTHFRL
TQDYTFNSPSTAAGVLLGRSANGRTEWKDEQGRTLKKIQEDESSGP*
>SPSA8_v1_370009|ID:41145872| Reverse transcriptase [Spirochaetes Bin 1 SA-8]
MGTQAKGQSKSQTLKNKDFDVLAMSCKKARAFFLKPESYCNIDLPSYFNFKKILSSVAKI
LDKRELSSMSRKPYEHEGVNYSLLSNKDGRYAWRPFQLIHPALYVSLVNYITEQKNWEFI
RARFKKFHVKNCRCLSIPIKSKSKRKDKAAQILRWWQEIEQKSIELSIEYEYIFHADITD
CYGSIYTHSIAWALHNKEIAKINRGNQIIGNIIDRHIQNMRHGQTNGIPQGSVLMDFIAE
MVLGYADLQLNNKLDQAGIGEYQILRYRDDYSVFVNNPQTGEAVLKALTEVLIGLGLKLN
TSKTTGAQSVISNSLKADKLAWLRARQEDENLEKHLLIIHAHGLDYPNAGSLLQPLQDYM
KRLSHVEHIQNPLALISIAVDIAFNSPRTFPVVTAIVSRLLLFLKSDEECAEVIKKVHAK
LSRFPSTGLMEVVWLQRIGHFYGIGNYEEKLCHIVTGKEHVLWNNDWITLKEMKEAINPAD
IFDKVKFKAMKPIVQPFEIDLFNEYY*
>SPSA8_v1_370010|ID:41145873| Restriction modification system DNA specificity domain containing protein [Spirochaetes Bin 1 SA-8]
MGGEWRKVRVEDIAEKIASGPFGSNLREKEYTDGGVPVIRGNNLTGPRFNNRDYVYVSEQ
KADQLRASLAYPFDIVFAARGTVGAVGIVTKCRFSRFLLSSNLVKVTVDQAKADPHFVFY
YFRSADGQNEILAHVNTTGVPKIERALESLRRFRINLPPLPEQRAIAHILGTLDDKIELN
RRMSQTLEEMARAIFKSWFVDFDPVRAKAAVRREHPDWTNAQVSRAACPNLKPEIAELFP
DRLVDSELGEIPEGWKIGCIGNEFNITMGQSPPGNTYNEVGEGLPFYQGRTDFGFRFPMR
RVYCTAPTRLAKKGDTLISVRAPVGDINIAAEDCAIGRGVAAARHKTGSRSYTYQFMHAK
ADVFACFEAEGTVFGSIGKKDFNMIRCLVPPKIVVTAFDRLIVQLDDRISINEHQSYNLA
ALRDELLPKLISGEIRVKDVERLLERTT*
>SPSA8_v1_370011|ID:41145874| conserved protein of unknown function [Spirochaetes Bin 1 SA-8]

MGNEPAPVPPEGQLLIYHDGALRVQVRIDGKTVWLTQRLMAELYQVTTANINQHLKAIYK
EGELSPEATIKQFLIVQTEGSRQVSRMVDHYNLDAILAVGYRVRSARGTAFRQWATARLS
ELLVKGFTIDDERLKQGRTLGDDYFEELLERIRDIRASERMFYQKITDIYATSIDYDPNA
EITKTFFATVQNKLHWAIHGHTAAEIIFQRADASQPNMGLTTWKNAPYGPIRKADVIVAK
NYLSEEEIRELNRIVSMYLDYAEDQARRRKPMYMADWVKKLDAFLAFNERDILMHAGKVS
QALAEEHAHVEFEKYEAERRRVEASTPSSDFDRFVEQTKQMIENEESLPKIEELAQRNRE
SKR*
>SPSA8_v1_370012|ID:41145875| putative type I restriction enzyme HindVIIP M protein [Spirochaetes Bin 1 SA-8]
MAKSSNNGANLGFESELWRAADALRSNMDAAEYKHVVLGLIFLKYISDAFEEQHAKLEAE
RAQGADPEDPDEYRAVNIFWVPREARWSRLKAEAKQPTIGKTIDDAMLAIERDNPSLKGV
LPKDYAHPRLDKQRLGQLIDLIGNIGLGDRENRSKDILGRVYEYFLSQFASAEGKKGGQF
YTPRCVVRVLVEMLAPYKGRVYDPCCGSGGMFVQSEKFIEEHGGKIGDISIYGQESNHTT
WRLAKMNLAIRGIEGNLGKEHADTFHRDLHPDLKADYVLANPPFNDSDWGGERLKEDKRW
KYGIPPAGNANFAWVQHFIFHLAPTGYAGFVLANGSMSSNQSGEGEIRKNIIEADLVDCM
VALPGQLFYSTQIPVCLWFIARDKKNGSFRDRRGETLFIDARKLGTMIDRVHRELTNEDI
KKIADTYHAWRGDKDCKASYQDIPGFCKSATLDDIRSNNYILTPGRYVGSAPQEEDDEPF
EEKMQRLVAQLREQQAEAAKLDAAIAKNLEELGYGK*
>SPSA8_v1_370013|ID:41145876| Dual specificity protein phosphatase [Spirochaetes Bin 1 SA-8]
MTFTSLNDPIHIDSVKISGRNGEIGMTFCPGRRGAGSLGYYERDLVADLRAVREWGAEVL
VSLIEEHEYRMAGIEDFDMKLPEGLKHLRMPIPDGGTPDETWERQWKADGCVVREVLERG
GKVCLHCMGGLGRTGMIAARLLVEFGLAPDEAIAQVRAARPGTIETAGQAEYIRQCARNS
TVA*
>SPSA8_v1_370014|ID:41145877| DNA polymerase III/DNA elongation factor III, tau and gamma subunits (fragment) [Spirochaetes Bin 1 SA-8]
MPYEVTASRKRPQIFEQLAGQEFVAATLEKSIETGRIAHAYLFSGPRGCGKTSTARILAK
ALNCESGPTPHPCGTCDSCRSITAGSSLDVIEIDGASNTSVDNIRQIKDEVLFPPNSSRY
KIYIIDEVHMLSTSAFNALLKTIEEPPPYVVFMFATTEPQKVPATIKSRCQQFTFRLVSP
ETVKNLLAAAAGDLGVTAEEEAXX
>SPSA8_v1_380001|ID:41145878| protein of unknown function [Spirochaetes Bin 1 SA-8]
MAEPGKIIHVPIEDEVKTDYLTYAMSVIVARALPDVRDGLKPVHRRLLFAMEELGLRNNA
STKKSARITGDAMGKYHPHGDLSLYDALVRMAQDFSLRYPLVYGQGNFGSIDGDPPAASR
YTEAKLSRFGEEMLADLXX
>SPSA8_v1_380002|ID:41145879|gyrB| DNA gyrase subunit B [Spirochaetes Bin 1 SA-8]
MDSNYSANTIQVLKGLEAVRKRPGMYIGSTGIDGLHHLVYEVVDNSIDEALAGYCTDVLV
VLERNDIVRVEDNGRGIPVDVHPTEGISALELVMTRLHAGGKFDKKNYKVSGGLHGVGVS
VVNALSSWMEVQVHLDGKLYFQKYQEGIPVDPVAEIGITDRRGTVVRFKADPTIFEETVY
SYDVLSNRLRELAFLNKGVKIILVDERLSTPKSIEFKFEGGLRQFIEHLNKNKNVLHKDV
VYFHGTRDDVEIEVGLQYNDSFNEIVFSYVNGINTREGGTHLTGFRAALSRTVNEFFKKS
KYVKKLDETLSIDDVSEGLTAVLSVKVMEPQFEGQTKGKLGNSEVKGIVDGFCSDQLDLY
FQKYPEIVNAILEKCVLAAKARIAARQARELTRRKNLLDSASLPGKLADCQEKDPTKCEI
FIVEGDSAGGSAKMGRNRVYQAILPLWGKMLNVEKTRIDKVIGNDKLQPIIASLGAGIGN
EFDVSKLRYHKVIIMADADVDGSHIRTLLLTFFFRYMTPLIEKGHVYLAMPPLYKISWDK
EIRYAYDDEEKDRILSGVEKDPSKIAVQRYKGLGEMNPEQLWETTMDPERRNIIQISMDD
AVLADQMFTTLMGEQVEPRRQFIEENALAVSNLDI*
>SPSA8_v1_380003|ID:41145880|dnaA| Chromosomal replication initiator protein DnaA [Spirochaetes Bin 1 SA-8]
MDTFDGSAIWEEALNQAKKDISEQEFLMWFRLSYEKLENGEMTVRAPNTFLRDQFSRKYI
PYMLNVLKSLTELDLKLVVRAFKPDIAGGQDTAERDPAPSSGTDVAEKNGQLNKSDPAPS
KAIFFDSPSHSREKSSTPASIASTEPRKHTSLRPNYRFENFIVGENNRFTYNASEAVAKN
PARTYNPLLIYGGVGLGKTHLMHAIGNSIYENFPEKKIICITAEEFTNEFIQMIHDRSSQ
EFKNKYRSADVLLIDDIHFFQNKPGVQEELFYTFNALYDSERQMVFTLDRHVKELKDFSD
RLKSRFDKGLVVDVQPPMYETRVAILKQKMLQGNRTVAVAEDVIDLIASNVSSNVRDLEG
SLTKLIAYAELVHKDLTVDIARNLLKELFNTKKHSAITVDSIIRMVADFYKLSLSDLKGK
KRSKNIALARQVAMYVIREVTEYSTTEIGVEFGGRDHTTVMHSCQKIEQMAKFDPSFDAS
LQRLLRDAREHESS*

>SPSA8_v1_380004|ID:41145881|dnaN| DNA polymerase III subunit beta [Spirochaetes Bin 1 SA-8]
MKFTCDKTSLLREISFAQEVIASKNALSIMSNVYLEAVESRLYIKATDVKVSFQTSIPVV
DVVPGSLTVFCDKLAGILASLPDGDIVVEQDENRVTLKPTSKKIRFQLRTLSGEKFPEIP
RAEDERFFEIQAREFRKMIGQTLFSVSTDETRYFMNGVFMEKSAEGDLIMVATDGRRLAF
IKNSIADEVPDFKPVIIPTKALSLINKRMSDEGLISIAITEKSIFFRFNAYEVSSVLIEG
QFPNYQKVIPQNQKLVFKVAKSDLLEALKRVSIFVEQKSNRTFFTLSNGSLVLSSEETDI
GAAREEIACDYDGDDVTIALNYKYIEEPLRVLDEGNLAVEFNDPSRAITLRPEPAGDYFH
IVMPMQID*
>SPSA8_v1_380005|ID:41145882|recF| DNA replication and repair protein RecF [Spirochaetes Bin 1 SA-8]
MSFSRIRLVSYRNLEDAEINTAADRVFLIGENGQGKTNLLEALYYLSFGGSFRGQVDSQI
PANGAKGFGLKGYWNNNSILEDEIEVSVQNSVKDIHLNGKPVRDRKELVGHNPSVVFCHE
DFSFAAGEPELRRFFFDQCAGMISLDYIDILRAYRRVLKQRNAALKESAYDLLEVLDPQF
VRYGRELMAIREIIFSRFQQQFPQLYEHVSQLGKPVELEYKPNWSQGAGFEEILQFVESK
RDIEIIQGTTRSGPHRDRWNFVCGGESFTDTASTGQLRLVSLVLRVVEAIVYAETAAAQT
GKRQWPVLLLDDVLLELDVGKRRRFLELLPGEHEGAQAFFTFLPGEPWTEYASGDTLVYR
VNHGRFEG*
>SPSA8_v1_380006|ID:41145883| conserved protein of unknown function [Spirochaetes Bin 1 SA-8]
MDDSRAKSVSQLLSAFFDEKTLQRGEQYAQFSRSWRSIVGDRLADHSRPSDIRHGVLLIE
TDHSGWIQLLQLKQERILGEIMKRYPELDIHGLAFRIGYGEGDKRTAVVDAPKAGHSPNL
PASSGNSEQAEPESQAAQMQHEAEPLHETAQPERSEGESAGKSEEKSKLPGFLLKKFDHI
RKNIEKR*
>SPSA8_v1_380007|ID:41145884|rpmH| 50S ribosomal protein L34 [Spirochaetes Bin 1 SA-8]
MKRTYQPSKTKRNRKFGFRARMSTKGGRLVLKRRRAKGRLKLSVADENKKY*
>SPSA8_v1_380008|ID:41145885|yidC| Membrane protein insertase YidC [Spirochaetes Bin 1 SA-8]
MSIFTPGTESKEESRRTIIAVVLSTLIVGVGFMVQNALFPPVQQTQPVQTTQTTESQPVA
APQSSVQPAAVPAISAQTAEPAPVAQATYKIETNLLKVELTNAGGDIVSLKLKKHKDNKD
FVDLIVPGASGAKGLSLSFGGADAPVISDFMKAEWLDAERRTIAFTRTFMAKSADSDELV
PFTYKKVYSFRDDEYMFGLAVTLENSTNKAVPLNANGVAYTLQMGPQIGPRFDQLPKNAD
YRKYIMEVNGKKKIEMPKPNTMQVIKEQPSWAAMAGKYFAFIGIPQAPFATFSFYQGQDP
DINQTNTMFISRPAITASVQTDTYYFYFGPKTSTELAKYEYSDKNSFGLASMKLEDATER
SNILGWLEAILKFMLNIFYKIIPNYGIAIILVTIVIKIIFFPLTKKGSISTAKMQELQPK
VAELQAKYKSNPQKLNQEMAEFYKRENYNPMSGCLPMLIQFPLFIAMYSLFNNHFDLRGA
SFIPGWISDLSLPESILNFGSFRLPLLGWSDLRALPIIYLASQLLYGKFTQTPQSGQNAS
QMKLMMYGMPIMFFFILYDVPSGLLIYWIVSNVLTIVQQVVINDMLKKHKLALAEAGNGS
TGTAAAGNAKSGNGQSMKNAGKTTVRNGPQEGFSQKVTKWLEDKAKEAGADTSKPGKPGD
KPKSGKKQ*
>SPSA8_v1_380009|ID:41145886| Spoiiij-associated protein [Spirochaetes Bin 1 SA-8]
MIYEFEGKTEREAIEMAAAELGLETDEFDVEIVESQAGGLFKKGKVKIRVHVRDSAGSPG
TMAGEPDSAAGLDGEAESEVPDQPARREKAVSDKPLLPNDEFERKMIEWTSEVVTRMGYP
AKVTVLFREEKKLGLKIETENTAMVIGKKGKNIDALQLLANVYAGNLGRDDLKIILDSEN
YRVRREESLVRIAYESAEQVRRTGRSILLEPMNPFERRIIHTTLNDIVDIETKSEGEGLY
KQVRVTFRGRK*
>SPSA8_v1_380010|ID:41145887|rnfC| Electron transport complex protein RnfC [Spirochaetes Bin 1 SA-8]
MTAKTFRGGAHPPEKKDRSSGLAVERLRDVKQVVIPVNQHFGAPLQPLVKVGDYVKRGQK
IADADGRMTVPVHASLAGVVKKIEPRTQSNNLDGPCIIIEAKQPEPVDGVVESSDDFMPP
LDPFTCSKEEALARIRAAGIVGMGGAGFPTHVKLAPSKPISYVIANGAECEPYLTIDERT
MIERPEDVVEGVAITMKIVNAPEGIIAVEENKMHAIPLLEKAIASSPRTRAGMKIRVQLL
KTKYPQGGEKMLITALTGKEVPSGGLPMDVGCVVQNIGTLCAMVEAFRDGKPLIERGLTI
TGGACTKPANVIAPIGTPVADIIAAGLTAVDDEALRRIIYGGPMMGVAVPNYSIPVQKNT
SGVLFMNAEEAVHYEEETCIRCGRCMRACPIRISPALLAVSVETGDYKEAEKIGLLDCIE
CGSCSFVCPAHRHLVQKFRVGKQILRTIKQKEAQNAR*
>SPSA8_v1_380011|ID:41145888| Electron transport complex, RnfABCDGE type, D subunit [Spirochaetes Bin 1 SA-8]
MPAKGMLLSSSPHSFTPINTPRMMLSVIIALIPASVYGVYLYGFQALALILVTVLSAVAS

EYLFRKLIKAKTTIGDFSAIVTGLLLALILPPKTPLWMGAMGAFFAIVVAKEFFGGLGAN
PFNPALTGRAFLLMSFPAAITTWTMPHGLGLADATSTATPLALVRQGAALTDVATYFGAS
DIGSLYRQLFMGYRSGSLGESSILLVLIGGLFLLALGVIQWIVPVSVIGSAFLFSWLLGM
DPIFNILTGGVVFGAFFMATDYATRPLTPAGQAVFGVGIGLLTVIIRKFGGFPEGVTYAI
LIMNLATPFLNKMRVKKYGFVPPVKPAKPTKEAVK*
>SPSA8_v1_380012|ID:41145889| FMN-binding domain protein [Spirochaetes Bin 1 SA-8]
MKGIAKLGLVLAIFAAAACASLAVVYAITKPQIEAQDQIALTASLKEIFPEAVNFEDVTD
KLVSKDANIKFLNAVLVKGENAPIGVAVKAAGPSYGGQATLLVGITLQRSIAGVRVLELK
DTPGLGANANNPGYFVKKAEKVTFPGQFTGKYLTDAFAVKEDVIAITASTITSKSLTKII
KTAGDSAAEWLETVTADTSAETTGGK*
>SPSA8_v1_380013|ID:41145890| Electron transport complex subunit E [Spirochaetes Bin 1 SA-8]
MKSLWKTFKQGIIYENPLLMLMIGLCSALAVTSSVSNGIGMGLAMTFVLLFSEIVISLFR
KVIPNSARIPIFIIIIAAFTTMVDLVMKAYFPELSKSMGVFIPLIVVNCIIMGRVEAFAS
KQSFLPVVFDSLGMGIGYTWVLVAIAAFREILGNGTFAGIQVMPGNFQPILFFILPPGGF
LVFGLLTSFNIWLKNRMAKSEKAKERA*
>SPSA8_v1_380014|ID:41145891|rsxA| putative inner membrane subunit of an electron transport system [Spirochaetes Bin 1 SA-8]
MDLIKIFLVAVLVNNVVLMRFLALCSYIGMTSDVGQSVGMGFAVTFVTVLATAATWPIYH
FILVPLGLTFLQILIFILVIASLVQLVEFYLKKNIPGLYAAMGIYLPLITTNCAILAVTF
ENISFKYSFIESIVYAVGVSIGYLLAMVLLAGLRDRMKSSPVPKFLQGTPILFIATALIG
VAFMGFSGLIK*
>SPSA8_v1_380015|ID:41145892| Electron transport complex, RnfABCDGE type, B subunit [Spirochaetes Bin 1 SA-8]
MVTIVLITIAFSAILAFVLGTALGFFREKFKVERDPKIDEVREALPGANCGGCGYPGCDG
YAEAVATGRAPTTKCTAGGSATAEAVSKIMGVDAAREDVVSVLLCQGTKDVAVSKGEYVG
VQTCRAAKLSTGSVKACSWGCQGFGDCVNVCKFDALVMGEDGLPHVNYDNCTGCGMCAEE
CPQKLFVMVPREKKGSIVLCSNRNVIKANVLKTCKTGCIKCEICVKSCPEKCITMVNGIP
VTDYSKCTSCGVCVQKCPTHAYKLLEVDVMGEKKKVPVLEA*
>SPSA8_v1_380016|ID:41145893| protein of unknown function [Spirochaetes Bin 1 SA-8]
MEANRRLLLAVMLAVSPAFLFAYSEFPPLSAANASRAREMVVAAAYGYIGVPYLYGGESA
KGMDCSGLVYAVFSRALNSGVITAEATAAGSASSQIPRTVSQQSQWALKIPRSQLKPGDL
LFFVLEPAAALTSAKPDHVAIYIGESRFIHAASIGTSRGVTINSLNEPRWSARFLFAGRV
IPASAALSVAIEWGAEAVFDALPEPFEGVRGAGLWSGIELPLGNNFSAGLRAHASYDSRL
AMLRVPMEATIGQTSGFSLFAGAAFTWDLNSGELFMPAGLISTIGIRWSPLIFSSSGSRL
GTSFELRYNHLLAEKNQPWNSRADQYASFTLSAGFRVRTLY*
>SPSA8_v1_390001|ID:41145894| putative capsule biosynthesis protein [Spirochaetes Bin 1 SA-8]
MIKISFVGDIMCQKWLLKAAKKKDGSFDFTSVFQNMKEIFAASDYVVGNLETVCAGKKNR
YTNDMYSYNSPDSLLEAIAASGIDMVTCANNHSLGRGSEGLYRTLSLLDAAGMQRTGAYL
PTEDWNEPFTIELHEKKITFIAATSSTNKSTLECGLAPDGHPALKLLQYQHATRSKKSIT
RKCYDWAKVMVPLELRLFINRMLGRGTRRAAADNGGMDNFSGKLRVDLERVIMRARETAD
YVIACPHMGGQFNLEPGTFSKYMMDFFRTQKVDCVVANHAHVVQRCEWKEDMLACYCLGN
FCVFPLNSDMIRGSLTEYSIMLHLYLDDSGPGLTTSGVGFTVLKNVKNNEGLLTVYPVKN
LYDSLDGENERKKLRQENSTIIKRFCPTMDKDMDVRLEYAMERRTMV*
>SPSA8_v1_390002|ID:41145895| protein of unknown function [Spirochaetes Bin 1 SA-8]
MVNLLSLFQKTPRVQKFALKLAMKMIVQPRLALLGVPIIAITGTNGKSTITRLLTRIYRN
AGYNVGACSTYGVTYNEKLVSRTDDASGLGAWRAARCSNVDLLVLEVARGGLARWGLGFR
KCTVGIVTNVYEDHLGFEGIHTMEQMAELKSAVPRATEKNGTIVLNADDPLVKGMAQKSK
AKSIYFTLAKDCKEFDNIFYVNGSHIWKKNREIEERIIDVREIPITLNGERTFNVVNVMA
VLAAVEGMKKYIRVDSASVTKTLGEFGTDPSDNLGTFHRISIQGEHVILCNAKNPASFLF
ETEIVKTIKQKEGFDYIAGTLSSPGNRSIRYYNEMSEIAASTCDLVLMTPPKDHYLRGRE
PKEIVALLSSKIPQDKILDDNNFTLSQWISFSKSKLNGRILFVIFGSITSPKINIFDESA
GIHYLAMGS*
>SPSA8_v1_390003|ID:41145896| Pseudouridine synthase [Spirochaetes Bin 1 SA-8]
MRQKTLFPNHPLPTVNGIGPSCLRIPAGPWKNILEYLQERFRTIKPETWISRMEKGAVVD

EYGAPLVPDSPCRKGALVFYYRELEHEIHIPFEESILYQDDHILVADKPHFLPVIPSGRF
LRETLLVRLKRKLKRDTLVPLHRIDRETAGVVMFSLNPETRSAYASLFTGHQVRKVYEAI
AQTLPGCHFPVTRRSRLVKGVPFFRMKETDGIPNSETVIEMMENKGTISLYRLTPVTGRQ
HQLRVHCAALGIPIMNDSFYPEVRRREADDFSSPLQLLARSIAFRDPLTGHERCFESTAK
LETLMAEQY*
>SPSA8_v1_390004|ID:41145897|rnr| Ribonuclease R [Spirochaetes Bin 1 SA-8]
MWYDSDRTMRKKKQRQELTKDAVLRIFKQEKKPVAQSELVAILSLNKKQGKTLKQIIREL
VRDGSILQLKNKRYGLPQEMNLVAGTLWCTRSGNGFVVPDNLEGKDIFIPARFIKNAFHG
DKVIVRVEHTFRGRKEGTVVKITQRKMKNIVGFVRRDRNATYIIPEDERIPHHFVVASSA
KSLRLNDNDLVAARITRFPEGGVDPICTVLKVFQGLTDSRSIIQFVQYKNALPFRFKKRT
QHEAQSLGTSMDITDRLDLRETIHVTIDGEFAKDFDDAVCIEKTDRGYVLLVSIADVSHY
VLPETGLDREAYERGTSVYFPGAVIPMLPEILSNGICSLNPDEDRLAMTVKLTFNTNGDL
TDSSFHKSIIRSVLRLTYTEVEDALIRKDRTVRKEVQRILPALEWMGELAALLSEKRARR
GSLDFDLPEPDVILDIEGGIQNILRAERLFSHRIIEEFMIAANEAVANFLTENKVNTMYR
VHEPPDTEKLKDLERLLQALSIGYKRDARNIRSLQSVLRNVEGTHYEFLVNRVLLRSMKQ
ARYSSQNKGHFGLASHCYLHFTSPIRRYPDLICHRVLKNLISDEGKRYGEKELETMANYL
SERERLAMETEREIEDRIRVLFMRDRIGKEYDGIISHITSFGLFVELINVFVEGLVLVNT
LCDDYYHFEEGKFRLIGRRTRKIYRLGDAVRIKVILADVEKNLLHFSLVNNHSKF*
>SPSA8_v1_390005|ID:41145898| protein of unknown function [Spirochaetes Bin 1 SA-8]
MTREEKNLVTAHMDQVFHGQTVRQNLPVCECGKYYDEKDLTEAPAVFFREINVFGKTFTL
IEPLCPVCKQRIHASFNILN*
>SPSA8_v1_390006|ID:41145899| Nitroreductase [Spirochaetes Bin 1 SA-8]
MELFDAIKNRRSCRNYLPEAIDDTVIESILEAGTWAPSPLNTQPWEFIVITSGDVKEKLF
SEADRCRKWALEKSSWKWLDSYQVDFLKQAPVIIAVVGDPKKTGVDMFMDEGMVGYQAAC
AAAIQNIHLAAHSFGFGTLWFTLFDKKAVRGILGMGDDKTPVALILLGKPGASPTPVPRK
NVKDKTRYIR*
>SPSA8_v1_390007|ID:41145900| Uncharacterized membrane protein HP_1331 [Spirochaetes Bin 1 SA-8]
MFGYIPLGIVFGFLLTQAGYLWYYAVLMSVAIYSGATQFLAINFFVNHAALIEVAVTTLL
LNLRHSFFGLSLIRKFSGTSPVKPYLIFALTDETYALLTSMDEPEQKSKTRYYFFIALLN
HLYWITGTLMGALLGQAFTTNLKGMEFALTALFVVLAIEQYKKIRSLKPFMIGAFVGITC
LVFVAPQYMLLASILSGTTMLLLLRKKE*
>SPSA8_v1_390008|ID:41145901|azlD| Branched-chain amino acid transport protein [Spirochaetes Bin 1 SA-8]
MSYPHTIVSIFLMAFIILVTRIVPFLFFRKKELPEMVNYLEHTIPPLMMLLLVLYCLKDV
RWASAPFGFTEFGAISVVVIVHLWKSNALLSIVSGTAVYMFMLNAFLST*
>SPSA8_v1_390009|ID:41145902| Phosphoenolpyruvate carboxykinase (ATP) [Spirochaetes Bin 1 SA-8]
MASIQGIPKSPWRAIIESAFFASSVRKTSMAELYDLAKKQPEVLITSEPMYRPEQFGLPQ
DAKILVSNDGGIFGRTARARRLVRNFTKPEKDKFQSMLAEATYQLNKRPGLWLEGMIGLH
PDFMIKAHLLSPETDAKNMLDWGFNFIPFMPPWSDLYTESRALGEPDILVLADPDWSNPE
FKDGLVIIDEDQNCIAILGLRYFGERKKGTLTIAWTVGVRHNMVACHGGIKKIGDKPPVA
VFGLSGSGKSSITNSGDHEGTLTEEEKVTVIHDDAFLIDLDHNLSIVLEPSLFDKTDAMQ
FGDPIVKYFYSAQNMAVTRMADGSQRLVGMDIRNDNGRCLKSRDMFNHAHFCEKPSTVIW
LQKDTSLPPICKITHSAMAVAMGASLSTMRAKGVENVDPKELERLVIEPFANPFRVHPLV
EDCRQFLKLFKSGCDCYIMNTHAVGMPGHLTDIPKSISLSIVTALVRGKIEWKEWKAFSG
LQIPKNGNELFSPDYDKKYSPPKRHPGYLRFLRDRMQDRITFLSNKRDLENDMESIFIDP
LVASRTVLDRILEPL*
>SPSA8_v1_390010|ID:41145903|rlpA| RlpA [Spirochaetes Bin 1 SA-8]
VKSLFSFAPFQAFLLFVLAGCWVITTPYKIVKGTVKGTYYGVKGAYEITAGTTKVVYHIG
KFSYKIVKAPLEWAFVNEDIEAIDGLSPKEAIRQGRVKNAPYVVKGKTYYPMSIEKARTY
EETGIASWYGYETLRTKGGRMTANGEAFDPRQLTAAHKYLPLPTHVKVTNLENNQWVIVR
VNDRGPFPSDSNPSSGERIIDVSEGAAKKLGFHTKGLARVKVEAIELKKEG*
>SPSA8_v1_390011|ID:41145904| Chaperone protein dnaJ [Spirochaetes Bin 1 SA-8]
VKQTDYYKILGIEKDATHQQIKETYRRLAFEFHPDRNKGDVSAVEKMKELNEAYAVLSDP
EKRKRYDSMNQQYGYEAYDHFRQGYSDQDIFRGSDINQIFEEMTRQFGFRNFNDLFREFY
GPGYQTFEFRRPGVFGRGFIFTGFPFGHQRNTQGVFSSVKTGLVTGLLGRLAGYLAKKAF

KGWDEGRQATSDRYDVITLNPKDATHGGKVSYIDPTTSRQLIVTVPAGVQEGQTIRLKGA
AGLSGDRSQTGDLYLKVQFRKGVLQMAKELYNKILSK*
>SPSA8_v1_390012|ID:41145905|trkH| fragment of potassium transporter (part 2) [Spirochaetes Bin 1 SA-8]
MRYFKNPELRLFISIIIGATIFITAIRMLSGTKLSEETVRDSLFQTVSIITTTGFITKDY
ELWPAATQIMLFLLMFVGGCASSTGGAIKNVRILVLFKYIGAEVKKLFYPRGVFPVKLEG
KSVPENLVSNIMAFVILYVLFFIFGTVAVSCLGMDMISAAGAVAATLGNVGPGLGTVGPI
DNFADVPAVAKWILSFLMLAGRLEIFTVVVLFTGRFWK*
>SPSA8_v1_390013|ID:41145906|trkH| fragment of potassium transporter (part 1) [Spirochaetes Bin 1 SA-8]
LNTTNILYVLSRLWMIVSTFFILPLCFSFYYTDGIHYHFIFTIGGILLLSCLLWPFGSKP
KELTTKEGFLLVNLCWISLTFFGCLPFLTTGYVKSFTDAFFESMSGFTTTGATILSDIEM
LPKSVLLWRNMTQWLGGMGVIALAVAIFPFTGMGGAHLFKAEVPGPSKDKISSRFSDTAK
LLWGVYLIFTILETVMLMLGGVNFFDSLCITFGTMATGGFAPPQCEHCSFFFSLCSLHHH
FFYDIGRH*
>SPSA8_v1_390014|ID:41145907| TrkA-N domain protein [Spirochaetes Bin 1 SA-8]
MRIVIIGAGDVGFNLAKRLGHEGHNLTLIEEDPEKCERAGEILDVSVIQGSGTDQEVLKA
ASLSSADMLIAASGVDEVNILACLFASKLGVKKKIARVRTSAYYKPDSILKAHDLGIDLF
IHPEEEVSEEIIRLLMRSTASEIIEFEGGKILMLGMKVAPGFPYLNKQLKDMGTAEQRRH
FRIIAVLKGDKTIIPTGDDYINKNDQVFVVTEREYLPELLKLAGMADEKLEKIMILGGGK
IGRKVAQSLEKRHIDVIVVESNKAKSVKLAGELEKSMVMHADGTEIDSLVREGILGMDAL
VAVTSDDENNMIACLLAKHLGVRKTIALVNKVNYLPLMPVIGIDSTVNTRISTVNAILRL
IRRGGIVSVATFRGIDAEAIEVEIKANDRMTGKPLKNLKLPDDALVAAIVRGSEVFVPYG
DTVINPGDKIVVFALPHAIKAIESRLS*
>SPSA8_v1_390015|ID:41145908|fabH| 3-oxoacyl-[acyl-carrier-protein] synthase 3 [Spirochaetes Bin 1 SA-8]
MKRVAIVGTGSYLPETIMTNFDIQKFLDTSDEWIYTRTGIKTRRIAEKDAAVSDLSKIAC
ERAMNMAGLKSDDIDLIILATITPDTHCPAGSNWLEAKLGCTNAVSFDITAACSGFIFAL
HVADKMIKSGANKTALVIGAEIMTRVVNWQERESCILWGDGAGAAVVTASDSGAHVLSTH
IHTDGKNGDTLLMPGGGSKTTPISHESVDKGLHFLKMIEANKSFKVAVNRFAEAVEEAVE
VNGKKVDDVDVIIPHQANLRIIQGMAKRLKVDISKVYMTIEKYGNISSATVPIALDEAVR
DGTIKKDSLICLTAFGGGLTWGSSLIKW*
>SPSA8_v1_390016|ID:41145909| putative membrane protein [Spirochaetes Bin 1 SA-8]
MTKDRIDLQGFFIILFMTMLWGLNYPAVKVSNAGLSPVFTTFLRSVIATCCGVIYCLVVR
QPLFHRGILLFHGAMTGILFGVEFVFLYYGLLYTNASRAAILLYLSPFVVAVGAHFFLKE
RLTVLKGIGLILAFLGIFLVFKGKPTAYNRLMLIGDLCEIMAAIFWGATTIYIKKYLAQK
VHPINTFLYQLAFSIPIMLIGTFLLEDTWVKGVTTPVVLSLFYQSVIVAFASYLTWFKLI
HIYPVAQLSVFTFLTPIFGVFXX
>SPSA8_v1_400001|ID:41145910|rplE| 50S ribosomal subunit protein L5 [Spirochaetes Bin 1 SA-8]
MQVPRLVKVVVSMGVGEAKENKKLLDAAVTDLGIITGQHAVKTKARKSIATFKIRQGQEI
GARVTLRGDYMWEFLDRLMNVALPRVKDFRGVNPNAFDGHGNFSLGLNEQIIFPEIDFDK
IEKVMGLNIAIVTTAKTDKEAKSLLGMLGMPFRK*
>SPSA8_v1_400002|ID:41145911|rpsH| 30S ribosomal subunit protein S8 [Spirochaetes Bin 1 SA-8]
MSVTDPVADMLTKIRNANAARHEKVDIMASKLKLEIVKILKTEGYIKNFKKIQAEGVNII
RVFLKYDEANNPVLHGLNKVSKPGKRVYSGYKEIPRVFNGYGTLIISTSDGVITGKKAVE
KQVGGELICTVW*
>SPSA8_v1_400003|ID:41145912|rplF| 50S ribosomal protein L6 [Spirochaetes Bin 1 SA-8]
MSRIGIRPVQIPQGVKVAVSPTSVTVEGPKGKLVQEYIPEVEIAVEGSTVVVNRKNDSKQ
AKAFHGLYRNLINNMVIGVSQGFKKTLVLNGVGYRAELQGNLLVMSLGYSTDFAVVVPQG
LAVTVEANGQKVVVSGIDRALLGKFASEVRSLREPEPYKGKGIRYEDEKIRRKVGKTGVK
*
>SPSA8_v1_400004|ID:41145913|rplR| 50S ribosomal protein L18 [Spirochaetes Bin 1 SA-8]
MSVKELSDKIRKRAKRKVSVRKTISGTAEKPRMTVFKSNVHLYVQVIDDTAGHTLASAST
LEKSLKDIGHTVEGGGKLGEEIGKRLLEKGITTVVFDRNGYRYHGIVKAIADGARKAGLK
F*
>SPSA8_v1_400005|ID:41145914|rpsE| 30S ribosomal subunit protein S5 [Spirochaetes Bin 1 SA-8]
VERNRREDAQPRDNFTEKLVQLNRTAKVVKGGRRFSFSALTVVGDHQGNVGFGFGKANDV

TEAIRKSVEKAKRAVVHVPVKNGTLPHEVTGVYKATNVLIKPAFPGSGVIAGGPVRAILE
VAGYTDVISKCIGSRNSMNIVRAVFNGMDKLMDAKAVAKNRGKTLKDLWG*
>SPSA8_v1_400006|ID:41145915|rplO| 50S ribosomal subunit protein L15 [Spirochaetes Bin 1 SA-8]
MSDYQLHAPEGANRKKRIVGRGQGSGLGTTAGRGNKGQQSRSGGKTYPGFEGGQMPLYRR
LPMRGFSNQPFRKEWQIVNIGDLETRFQAGDTVDAASLLMKGLIRKPENLIKVLGKGKLT
IALNISVDAVSAGXKAKIEAAGGKVETPTAVGSASDA*
>SPSA8_v1_400007|ID:41145916|secY| preprotein translocase membrane subunit [Spirochaetes Bin 1 SA-8]
MAGNPLSDIFRIKELKDRILFTLFWLVVFRLGAFLPIPGINASALQSYFASQAGQSGGIT
DYLDFFAGGAFKNFSLFMMGVMPYISMQIIMQLLLIVFPKLKSIAEEDGGRKKISRWTRM
GTIVICIIQGFSVTVWADRIPNAIAMSNRTMYAIIAILTVTTGSMFLIWIGEQITKRGIG
NGISLLIFAGIVARLPNATYELIKKIQAGEINAVYALVVLLMFVGVVALVVLEQQGQRKI
PVNYAKRVVGRKMYGAQNTYIPFKINPSGVIPVIFASSLLTFPLQIAQSFGVQAKWLRDF
SYWLRPSGFWYNLFYVLFIVFFAYFYTQVSLNPQEISKNIRENGGSIPGIRSEKMEEYLS
RILNRIILPGALYLALIALIPSLVQRLFNFPSQLAYLLGGTSLLILVGVDLDTMSQIEAM
LKMHHHDGLVRKGHIRSRNL*
>SPSA8_v1_400008|ID:41145917|rpsM| 30S ribosomal subunit protein S13 [Spirochaetes Bin 1 SA-8]
MARIAGVDLPNKHVEIALTYIYGLGRTSAKKICEATGVDPNKKINELSNEELNELRKVIE
NDYKVEGRLRTEIALNIKRLMDIGCYRGLRHRKGLPVNGQRTRTNARTRKGKRKTVANKK
KAV*
>SPSA8_v1_400009|ID:41145918|rpsK| 30S ribosomal subunit protein S11 [Spirochaetes Bin 1 SA-8]
VAVTTKKRKEKKSVYEGNVYIQATFNNTVITITDLNGNAIAWASSGTHQFRGAKKSTPFA
AQTVAETVVQKAMNSGLREVHVYVKGPGIGRESAIRQLGALGLKVKSINDVTPIPHNGCR
PRKARRI*
>SPSA8_v1_400010|ID:41145919|rpsD| 30S ribosomal subunit protein S4 [Spirochaetes Bin 1 SA-8]
MARYIGPVCRLCRTEQKKLFLKGDRCKSDKCPMNKKRLPPGKEPKARMGKKSEYAVQLRE
KQTLKRAYGLLEKQFRNSFDKALAMPGKTGENLFILLERRLDNVVYRLHFATSRAQARQL
VNHGHIAVNGKRVDIASYLVKAGDQISVIGQAKKLDMIKNALQEVAKSGTMPWVEVNPDE
MVGRLAAYPHRQDITDMADIREQLVVELYSK*
>SPSA8_v1_400011|ID:41145920|rpoA| DNA-directed RNA polymerase subunit alpha [Spirochaetes Bin 1 SA-8]
MARKNLLKGFKRPKGITYEQSESGDSYGKFVAYPFEPGYGTTVGNTLRRILLSSIQGYAI
TAVRIVRYDDEGAQHIISSEFETIPGVVEDTIEFLNNLKQLRVRLPNDMEQAVFLYEIKG
ISELKGSFFSNDRQLEVLNPDLTIAALNPDVHLDLEIQVDLGRGYVPAEVNEKYIEIVGT
IPMDAIFTPVTKVKYSVEPTRVGQRSDYDKLVLEIWTDGTTRPEDALGEAAKIAKDHFAI
FINFDENEAGGDELNDEIEERVKQILNTPVEDLELSVRASNCLKNANIRTIGELARKTED
EITKTRNFGKKSLAEIKTKLSEQNLSLGMTDFTPLRNYLKLTMKKEQTNEA*
>SPSA8_v1_400012|ID:41145921|rplQ| 50S ribosomal subunit protein L17 [Spirochaetes Bin 1 SA-8]
MKHKIGFNKLNRVSAHRKALIRNMMTVLFKHERIETTHAKAVETRRAAEKMITRAKVDSV
HNRRIAARKIQDEAILNKLFTEIGPRFKDRAGGYTRILRIGNRAQDAADMVILELVDQKV
EEKKAERNKRKAEKKAKEAKKAE*
>SPSA8_v1_400013|ID:41145922| conserved protein of unknown function [Spirochaetes Bin 1 SA-8]
MSKAHRGKGVRELPSSGRGTCPVCKRDGVKVLYEQEVEGKKTKICKVCKATLANSK*
>SPSA8_v1_400014|ID:41145923|livF| leucine/isoleucine/valine transporter subunit ; ATP-binding component of ABC superfamily [Spirochaetes Bin 1 SA-8]
MLVVKNLKVSYGKIEAIKDVSFEVPDGKIVTLIGANGAGKTTTLRAISGLEKCAAGSIEF
DGKNVTNMEAHKLVPLGISHVPEGRKIFPTLTVRENLELAGWTLKDKKLIKERMEDVFEV
FPRIKERANQLGGTLSGGEQQMLAVGRALVTGGKILLLDEPSMGLAPVLVDEIFHQIVEI
NKRGMTVLLVEQNAAEALDIADFAYVLEVGYTTISGTAKAIAEDPRVREAYLGV*
>SPSA8_v1_400015|ID:41145924|livG| leucine/isoleucine/valine transporter subunit ; ATP-binding component of ABC superfamily [Spirochaetes Bin 1 SA-8]
MSALLSIKNMTHYFGGLRAVSNFSYDIPQGTIYGLIGPNGSGKTTTFNLITGIYTPTEGQ
VFFQDKEITGMKPYKIVQTGAARTFQNLRIFSNLTALDNIRIALHFNTRSGLVSSVLRLP
SFMNEERQVKKESRELLELMHLSSRANELAKNLPYGELRRLEIARALATKPKLLLLDEPA
AGMNPKEVDELMSLIVRIRNEFKVTVFLIEHHMQVVMGICEYLKVIDFGETIAEGLPEAV
GKDPKVLEAYLGKRGA*

>SPSA8_v1_400016|ID:41145925| Branched-chain amino acid ABC transporter permease protein [Spirochaetes Bin 1 SA-8]
MTKVKLPKFLIASVVFYAIIEALFRLEVLNGYILHIINQSLIFVILAASLNLINGITGQF
SLGHMGFAGVGAYVSGTLTTIILKLNPTVTSNMGIFVLCLLAGGVAAAFIGFLIGFPSLR
LKGDYLAIVTLGFGEIIRTVFNNIDYVGGPRGLLGIPKFSNFTLIFIAAFLSIVVMRNII
QSSHGRAMLSIRENELAADLVGINTTQYKVMAFTIGAFFAGIAGGLLAHLIQLAHPTQFG
FIKSVEVLIMIYAGGVGSMTGSIIAAFGLTFLSEGLRIGIRALADATNLPIGGEWRMVIY
SLLLIFIMLFRTEGLMGTKESRIIKIEEAD*
>SPSA8_v1_400017|ID:41145926|livH| leucine/isoleucine/valine transporter subunit ; membrane component of ABC superfamily [Spirochaetes Bin 1 SA-8]
MGSIFNAQQIVNGLQVGSIYALIALGYTMVYGIVRLINFAHGDFFMIGAYAAYGSFFIFN
SMLAGKHGSIAFLVLIVSMLAGGLIAMLANQLAYKPLRYKPKLSSLITAIGVSMFLEYFF
SALPVIGPSYRSFPDIIPKVQISLGSATISNYVVIDIVVAATLMIGLTFLVRKTALGKAM
RAVSQDKDAARLMGIDIEKVIGFTFLIGGSFAGAAGLLAGMTYPRIFPYMGIQPGLKAFI
AAVLGGIGNIPGAVLGAYIMGLAETFASAYNSLLGEGIAFAILIIVLLVRPRGLLGEKVA
DKI*
>SPSA8_v1_400018|ID:41145927| Branched-chain amino acid ABC transporter substrate-binding protein [Spirochaetes Bin 1 SA-8]
MKKFTLVLLLAVLAASLVSAQDIKIGGVGPVTGEAATFGVSTKNGMAMAVEEWNAMGGVF
GGRKVKLIFEDDKGDPAEGATVYTKLIQQDGVVAIVGTVMSKVTLAGAPIAQANGIPMIS
PTSTNEKVTLVGDYIFRACFIDPFQGTVGASFAYKDLKAKKAAAIFDLGNDYTKGLAENF
KATFEKLAGKGSVVAYEGHPSGATDFKAQLTKILSAKPDVIYIPDYYNDVGLIAKQVREL
GFKGPLVGGDGWDSPDLVKIGGTAVENGFFTNHYSAEDTRPIVQDFVKKYKAKFGAEPDA
LAALGYDAMYIMLDAIKRAGSTKGTAIRDALAKTDLNVVSGRVTFDANRNPVKSAVIIEI
KNGKQVYKTTVNP*
>SPSA8_v1_400019|ID:41145928|yojE| Uncharacterized transporter YojE [Spirochaetes Bin 1 SA-8]
MKNQTSKISGTLAAFLAYGMWGLFPLYWKMLKSVEPLQILSHRIFWAAVFCLILLGALKQ
LSRIGELFKSPRKVLRVAACAIVVTANWGIYIWAVNSGRITESALGYYINPLLSVAFGVL
FFRERADVWTKAAVSVAALAIFAAAVIYGSVPWVSLLLAVTFAGYGALKKQLALDPLLGL
TIETLMVAPLAFGYLAIRQFQGTGAIFNASLLVTALLILAGAVTAIPLLFFAKAANSISL
QKMGFIQYVSPTGQLFLGLVVFHEKPTSALLVAFAGVIAAVLMYALTRKRVAEKAQFAAN
*
>SPSA8_v1_400020|ID:41145929|hutI| Imidazolonepropionase [Spirochaetes Bin 1 SA-8]
MRGNGKGFCAIINIGSLASPQGKRQAAGTAMNSLYRCKNACIVAKNGIIIYAGPASAEEG
RLALKKAQAEGAVLIDAENRACVPGFIDSHTHFIFAGYRADEFFWRAGGMPYMEIHRRGG
GIRKSMEATRSATLHDLIEAGKKRLDTMLALGVTTVEGKSGYGMDLETELRQLTAMRELS
SLSPVEILPTFLGPHSIPPEFESRPSDYIDFVIREVLPKVAADKLAVFADIFCEKGVFEI
EDSRRYLRAAQNAGFKLKLHADEIVRIGGAGLAAELGAVSADHLLKASPEDLQAMAQKAV
IATCLPLTAFSLREPYADARGMIDRGLAVALASDLNPGSCYSQSIPLIFAIAVLYMKLTF
EETLTALTLNGAAALGLAASHGSLETGKTADIVILDAPEPEHLSYHIGMDLVHTVVKRGE
LVYQQSTKA*
>SPSA8_v1_400021|ID:41145930| conserved protein of unknown function [Spirochaetes Bin 1 SA-8]
MRFFIEATEADPHFEYFAAPDKPRIVNGAPTKNPRYLQLDPNYVNPQSRYLAEIGPRLYR
RVPADNALLHPVGAVLPGRRNNPADPAAGIRPLAVYGPIHYQDLPELFMDFVCSLTGKSP
STTGAGSEGALTKGPFNALIATTDLNNALLSFILTGYSGFTTAAGYIGHKYKIDHDISIM
MPELWARLSPEERDPEFLKEHGYLEKVDDFEYEGRLIPASRLGWRITPLFAATYLGRLFD
TPTAVFPEDMLRPELQSMPEYVDGIENIASAMKKSAEAYFEDGSVEAAIPPLKAVLHVMA
KGHYEGKSISDPEVRMLFTRDYVLASDWYHERLERYRNSEIEYLEKSIVYLRSFLAEKAE
PQSLSAKRAQAELLRARERLERLMDKNYLSIIDGSLGKDILYEKA*
>SPSA8_v1_400022|ID:41145931| protein of unknown function [Spirochaetes Bin 1 SA-8]
MDRTVDSEGADSCRRIGRVIPASRQDGSYRMQQRVVRRYSAVEPRSDFRQISALRIDIIW
VKLEVSWIFCRGAVDNSRFIRCCKIFKMGISFRCFNEKAHCVGIFRELYGFFDDLCSMAA
GEGFEIADEIVLAGKILFSLLVIAADDGVIGALEEACFGVFAEFEAGMAVCPIREVFQXX
>SPSA8_v1_410001|ID:41145932|pgcA| Phosphoglucomutase [Spirochaetes Bin 1 SA-8]

MFTSLRPTPELSFAIRELGADTGVVVTASHNPPQYNGYKAYWNDGSQVVPPHDVGIIEKV
MAVKTPKTIDKESAIKQGLLVYLDDSIDNRYVAMVKSKLLRPVLIKQEASKVKVVYTPLH
GTGAMLFERIMGELGLNVMTVPEQRNPDGEFPTVSYPNPEEPAALKLAIGLGEKVHADVV
MATDPDADRLGVAVPDKKGIFQLISGNQLGSLHLDYLLHSLSELGKMPPKPACIKSIVTT
ELQAYIAAKYQVECLDCLTGFKWIADVMRQLEKEGKTFIYGTEESYGHLIETEVRDKDGI
SAAALTAEMTLYWRSKGKSLLDRLDEIYLEHGFFQEKGINKYFEGPRGMTIMSGIMETYR
SNQPIALGGIPVVHIRDIKTGLAWDVGSSVTKKLDLPASDVIQWKLRDGTMVTVRPSGTE
PKIKYYILCRTEASIGLEEAKKQSALKIAAIEADIRKVIDSAK*
>SPSA8_v1_410002|ID:41145933| putative DNA-directed DNA polymerase [Spirochaetes Bin 1 SA-8]
MGDSSYGEPIWAWLMKPGPSDLDTLSRQAWVSAEPVQPAVEGCPLPEDWDRLSYAAFDVE
TTGLYPGKDRILEVGIVLFSFDQEGAITEEQSYASLVNPGIPVPASSSAIHGITDLDVAS
APLFHEIAAEVARLLAGRVFVAHNAGFDFGFLKQEFNRIITQPQEATLWTGDEQTPEILS
FTQSLTTMTLADTLSLLKLANPALLSYNLGKAAFVLGIETGTSHRALDDARTCMNIFAYA
ARKLTGKCP*
>SPSA8_v1_410003|ID:41145934| protein of unknown function [Spirochaetes Bin 1 SA-8]
MNRAFRQGFLLLAILPLAMLLFGFISSWQKLAVEDGLILFSAAFDARSLSSDDIESFSSR
EASLYLQPSILPMLSERSIRLLAVQLTEKALKSGKAPRQAVASILPEPAKIIAMLARTRD
IDDFEDFTTIEGSAGKYYAAVTSAAPVMKPLRKSYEIIFEAAFAEMKKLGQVRNAIQDYA
LTAEYRSGITLTGLSSRPLKSQYDLAHTFALDIFLQDVEVNPVNGLQKGPMIFSLSDGLV
IAAEKNWRGGETLEEYGSGGISPKAGNGVIIYDPSQRKYFSYFHLHDVLVEPGDAIQKGQ
PLGYGGNTGTNARKKGHGEHVHVEIFDAVLGKNLRNSEIAAVIFSR*
>SPSA8_v1_410004|ID:41145935| putative Glucokinase [Spirochaetes Bin 1 SA-8]
MKPNTDSYVFLAGDIGGTNSNLALVRYKQGAFTLEYFQRYATQQENSLLDPLARFLDSAK
KAGFNAPLSACCVSGAGPVVNGEIQLTNAPWTIRQSEIQNLLGIPTFLINDFTAVSYAAV
LLNTQDKQAVTVMPHSDGSQPQPGIGIALVVGAGTGLGTGFIQKNVDGSYMAFASEGGHS
ELPCYDDLSRAFHSWMTDKLGTAPGVELAVSGQGIGNIFSFICSSAFSKSLIGERYSLAY
QALNMELSQTVSSILAKPEAERPALIAANKTNDWRCALTMEVFVNFYARKVSGLASIFLP
SGGIYLAGGISSKNEAFLLENHRFMRIFEENYAPHIRSFLKGASVMLVRDYSISLLGAAN
AALQLSRN*
>SPSA8_v1_410005|ID:41145936| conserved protein of unknown function [Spirochaetes Bin 1 SA-8]
MTPISEALRQDMAAKGIDIDLTLSILNDFNNGLYDSLKPVKAVSVPKIDGSTVIDTRLFA
AGKASVYRTDRDTASRALADAGIPMPKTAISEKSVDGKDILAFPTEALLDIGYSLLPKTA
FGVLNGGSATSYADTKKNKALGDQVFSILEAKFHELAPSCKDVPKGITPAYTNPDGSPGY
SFLELKMRARLALAKRCIKRMGVGTVCKRQGLEFLPLFQMSSSLNDEQLKAHYDAVQSSP
LLAGLAKETGLEPAQWHTGIQPMIAAYSHSSCGRPKTIFDTAWGKPDSTLPLPGGHGQFF
RILAPVLKAFHAQGIRFACLGNVDNIGYMPDPVELAILLLSGKSAAFDFSFRTELDVKGG
ILVETDTGSRTIADIGPAISVEEVARIEQAGYSILFNCASGIFDLDYLVPRIDHLARELP
VRFSDQDKDAGKYSQAEQVTWEITSLLPSFLAFAVEKNERFLAAKLLVENLLTSGIGLGD
PGMPPAIRAASEKLHAGLERLLTEQYGLILKNGRYLPDLPV*
>SPSA8_v1_410006|ID:41145937| putative Molybdopterin oxidoreductase [Spirochaetes Bin 1 SA-8]
MEMVPVFCGRDCGGDACPLIAVVENGGVIRIQHNPEGRIMKACPKGFLLPHQHYSSERLL
EPLVRTGSRGSGQFRAVSWKEALSLVKEKLENTIERYGPQSILSLASAGSTGALHNTETL
TKRFLNCLGGHTSLSGNYSSNAANYALGKLFGTSYGYSGFPPESLLGAETIILLGANILE
ARLGAAYAEYLEEAAKAGKKIIAIDPRKTKTASLEGAEWVPILPGTDSALLYAILQRLEA
DSRIDHAWLAERAEGAEEIFSYIDGKTDGIKKDADWAEQQTGIPAMTIRKIAQIYISNHP
VLLFPGYSIQRTQFGEEAMRLAVLLQLATRNFGVFGGSTGSLNNKLPGLNVGKMDDLGRG
NNQSVPILRWPDAVLEGQPRYPDIHVLYSAGGNFLNQGADIRKNIRAFMKADFVVSHEMF
MTPTARCSDVILPVKGPLQKEDIGIPWAGNYLLYKPQILKVRGKEKSDYEIFSMLASMMR
VEAEFSKGRSEHEWIAQFLQASEVSDAESFMKKGIWFGDRYDISYMEKFLFNPQGNPLGT
RSGKLEFTGDRARFDAPKHSEFSLVTPKRHNFVHSQGGDFAELLKNNPLQVNSNDARRLG
IKEGDCVKVSSETGWLLAHVHISDEIRESVVSLHEGTWFEEKTDTQDCCDAVSPSPNLLT
STAGTAESCSCVMHGVPVSVSRVN*
>SPSA8_v1_410007|ID:41145938|lon| Lon protease [Spirochaetes Bin 1 SA-8]
MAENEIIPIDQVLPNKLPIITLIGRPIFPGIFTPIMIANQPDILVVEQAMSADGMVGLVL

AQDENAPLDSANLYQVGTVAKIVKRINLPDGGINIFISTLKRFKIKKFLSKEVPIVAAVA
YLDDYNDDSDEIKALTRALLGEMKQISENNPLFSEEMRLNMINIDHPGKIADFIASILNI
DKKEQQKVLETLDVHERMERVLIFIKKEQELLRIQKKVQAEINEKIEKSQREYFLKEELK
AIKQELGLPSDAKSSDVQKFRAKIDSFQFEGETKELVEQELEKFSLMDPSSSEYMVTRNW
LELVCSLPWNSPLSNDFDMKAAQEILEADHYGLKDVKERIIEYLAVRKLKKDSKGSILCL
VGPPGVGKTSVGRSIARAMGKEFFRFTVGGMRDEAEIKGHRRTYVGALPGKIIQGLKIVK
KRDPVFMIDEIDKMGVSYQGDPSSALLEALDPEQNFSFRDHYLDLPFDISNIFFIVTANT
LDTIPRPLLDRMEIIQIPGYVDSEKLEIARHYLIPKSLEKNGLKKADVRYTRDALVFIAE
SYAREAGVRNFEKYLDKIHRKIAKSIVLEETKEKKAVIDKAAVQKYLGKPIFRDDDIKKA
TRPGMAVGLAWTSMGGDTLIIEAVANPGKEGFKLTGQMGSVMQESASIAYTFIRSIAAQK
FGIGADYFESRQIHLHIPEGATPKDGPSAGITMATALLSLVTKKKIKDRLAMTGELSLTG
QVLPIGGLKEKTVAARRNKIKDIIIPAANQNDLDEIPEHIKKGITFHPVQRMEEVIELAF
NL*
>SPSA8_v1_410008|ID:41145939| putative Adenosine kinase [Spirochaetes Bin 1 SA-8]
LEKQKGGIVTLGHLLIDHITLVQTKFIENLGLERPVYHTNSAELRKILTALQNAPAEMLK
SHTKSLGGGVAICAKTLASLGIRTTLLGSVGADEDAEFLRHALDQYGILYELGTSLKATG
VFCSMQTESNAKWLVVSPEAARDVRGMVIDEKLWSNNEILYIDGLLIDDVQWLAAIADSA
VRHGLILAMDISTVDNARYNRNIIISFAEKYCRYVFANEREYSVLKLTSQQLASSKVYWI
VKRGPKGATAIFQNRVEHASSYSVTKIVDETCAGDAFAGGFLYGLMMGLPVRHCLSLGNS
AGKLAVMSCGSLFDQTAMRKTLMTALERLLF*
>SPSA8_v1_410009|ID:41145940| putative Adenylyl cyclase CyaB [Spirochaetes Bin 1 SA-8]
MIEIELKARVSDPAAVEARINSFLSFVGTVDKHDEYWSIPVLSSMVLTTGFRFRIRHEAE
NTLITFKEKTYTNTIEVNREVEFGVQNEAAFRKFIEKMSARHLYSKQKKGKQWKGEGNLL
AELVAVEGLGNFLEVEMLFEDGSKVKPESVKKNLIEIIERCGLSASDIEPRPYSQLLGMP
KY*
>SPSA8_v1_410010|ID:41145941|gpmI| 2,3-bisphosphoglycerate-independent phosphoglycerate mutase [Spirochaetes Bin 1 SA-8]
MVSALKKNPGWKGRKGPLVLVIMDGVGYGQYKEGDAVANADMRHFRSLEASCPTTKLKAH
GTAVGLPSDEDMGNSEVGHNAIGCGRVFAQGAKLVSASIETGAMFQGPVWKDIIAQVKNR
DSSLHFLGLFSDGNVHSHIDHLKAMLKRARDEGVKKARIHILLDGRDVGSQSALDYIHPF
EEFLKGLSTDGFDYQIASGGGRQYITMDRYGANWEMVKRGWDCHVCGIGRKFSSAEEAVL
TYRKENPQVIDQDIREFVIHNDNGPVGTINDGDALIFFNFRGDRALEITRAFEEENFTFF
DRGKKPDIYYAGMMQYDGDLGIPAKYLVSPPAIDRTVGEFLANSGVKSLAISETQKFGHV
TYFFNGNRTDKFSEELEDYIEIKSDRVPFEERPWMKCAEITDKVISAIESGTYQFIRLNF
PNGDMVGHTGVYQAVICGMEAMDLQIGRLAKAVEKAGGILILTADHGNSDDMFEHDKKTK
AVLFDKDGQPKAKTSHSLNPVPCVIYDPGFQGEYDTRLVSGLGISSLGATCINLLGFVPP
EDYDTSIVRMIHLSKDNQA*
>SPSA8_v1_410011|ID:41145942|apt| Adenine phosphoribosyltransferase [Spirochaetes Bin 1 SA-8]
MTQEIEATEGFNLDDAIRKVQDFPKPGILFYDITSVLANPPAFNYCIESMLKLYRHENID
AVAAIESRGFVFAAPFCQKLGLPLLLVRKKGKLPGKTISQSYDLEYGSAELEMHIDDIKP
GMRFLIVDDLIATGGTINATCSMIKRCGAVPVRAFSVIGLPFLNYQQTIQDVSVDTLIEY
FGE*
>SPSA8_v1_410012|ID:41145943| conserved exported protein of unknown function [Spirochaetes Bin 1 SA-8]
MKLKCWYRGLFLLCVLFILPLGSAFAQQGRAAGTDSILMITQPAGTPIPLTRLSDTSGQT
VNQSPKLSKGSALGRFITISFGAFPFMFFYTNLAFDVTEFAASGFDLQYAPWPVQNRFSS
LPISEQLLRIGIASSVSLAIAVLDAVFPAPQRK*
>SPSA8_v1_410013|ID:41145944| YggS family pyridoxal phosphate enzyme [Spirochaetes Bin 1 SA-8]
MRYHADMTENYGFVQDNVRSVLENIDAALRRAGREPSSVTLLAVTKFHPQEAVLAAYAAG
IRFFGENRVQEAMLKFDESFKTIIPDAKLHLIGTLQSNKINKAIGLFHCIQSVDSEELLM
SLTRKATERDKMLEVMLELHTGEASKAGFPDIDAMLRAVESSLKMDAASVNIRGLMTMAP
FTADSQNIRVSFRKLASALKTLREHFNLPDCTELSMGMSNDYKIAIEEGATIVRIGTALF
GERT*
>SPSA8_v1_410014|ID:41145945| protein of unknown function [Spirochaetes Bin 1 SA-8]
MDGVTVLEKKLDTIWVSADGYAPFNNKPPSKNSLDAHGRLIIGELDLVRGIHVLDIMAAD

FSGNAASVQLRLTVE*
>SPSA8_v1_420001|ID:41145946| protein of unknown function [Spirochaetes Bin 1 SA-8]
LPGKSEEKLKSVLSFAFQTSGKNVEFPAFPRQYASFFERAGEYLESPITGVLKAGMSAKF
VYISNAKKVSLIHDGNFIPLDENPASPGRFELELKVPKTELLKLGVSEDGVNFSIMLSWS
VR*
>SPSA8_v1_420002|ID:41145947| exported protein of unknown function [Spirochaetes Bin 1 SA-8]
MKKFVLVVMALLAATMSFAAGKLTLMQKKPEIDAQLKAYAQAWGQKMA*
>SPSA8_v1_420003|ID:41145948| Extracellular solute-binding protein family 1 [Spirochaetes Bin 1 SA-8]
VTVVVKSIGGTSGGMGPQLKADYAAGDMPDIFAFDGLEAYKEWEGVILDLSNEPWVKNTS
VAFKYNSKIFGFPVAVEGWGMAYNADMLAKAGIDPKTLVNYDGYKKAFEKLDSMKAQLGI
NSVVSMAAAVEMGWVTAHHNFNSLLSNGLPYGDLSVVNALLAGNVDMQRLQEYADWVELL
FKYADKSVLLTGNYDAQVGAFATGKAVFLHQGNWTDPNIKGANATFKMAFAPHGSMKKAT
DGIFVAAPSFYAINKESKNLALAKKFLVDLAGTPEGAQYMVKEAGMIPAFSTIKLNPDGQ
LSKSVQEWAAAGKVYSWNQYYFTGDFRDKVLTPIYNQFAAGKITKAQFIDLMAKAFKDNA
KK*
>SPSA8_v1_420004|ID:41145949|amyD| putative starch degradation products transport system permease protein AmyD [Spirochaetes Bin 1 SA-8]
MTKKTESRLVFWIFLAPVLIAFVMVMIIPFFLGTYYAFTNWTSSARADSTLKFVGLSNFI
SSFKDPAFFYSFGITFLYTVLNMIAINVAAFLLAMLVTGQLKLKNVYRVGFFVPNLIGGL
ILGYIWQFIFNNAIPSLGKAIPFLNFMADPNNLMLSKNTTALLAMIIVGTWQYAGYIMMI
YVAAIENIPQELIEASKIDGATPWIRLRAITLPLCAQAFTVTMFLTLVNSFKQFDVNVSL
TSGGPSTMLFGQPILGTELLALNIYNTAFISNNLSIAQARAVVFFLVLAIISLIQVYVNK
KKEIEL*
>SPSA8_v1_420005|ID:41145950| ABC-type transporter, integral membrane subunit [Spirochaetes Bin 1 SA-8]
MIQEKTRKPGVVAAEIFTALLFILFLFPFLLVLINSAKTAFEVTQFPLAWPEKWSNIFNN
VVRIWTNEAVRYPSSLLASTIITVVSLVLINLFSAQAAWVLVRTKTKVSSAIFFVFVAAM
VIPFQIVMFPLLSWFRTVTSITGIRLLRTYQGIILSYIGFGAPLSIFMFHGFIKSIPLEL
EEAATIDGCKKHEIFYKIIFPILTPIQATVMVLNGIWIWNDYLLPLLVLGKGNDIMTIPL
AVSNFAGAFVKQWDLILTAILMAMIPVVIFFLFAQKYIVKGMVAGAIK*
>SPSA8_v1_420006|ID:41145951| conserved protein of unknown function [Spirochaetes Bin 1 SA-8]
MKKAWLLVIALAMVALMGSCDLFGTIKAADILGEWEFLQVTDIKGTEVFNVHLSVMADES
GKIVMLDLGWDIHDGEDLLIRWHGLNGTFSGKKFTGTYKAFYLGDTEYNIEVTFSYKNGE
IGIACKGEGPLNGVTLEHGTKAEI*
>SPSA8_v1_420007|ID:41145952| Dihydrodipicolinate synthetase [Spirochaetes Bin 1 SA-8]
MPTARETLITTLFPTGIPSLWCPPLTHFSEDGLIDAERIKAHLAHLRPSVPCLLVPGSTS
EGWELDEAEEFELLDIMLACAREMDFSIMIGMLRTQSGEARKGIIAMLNHVFKSESPSIQ
MLIERNICGFAVTAPKGKNLPQSTIHEHLQEILNLSLPTALYQLPQITENEISPETVAAL
VSNYQNLYLMKDTSGKDRVVTSGLNFDGLFFVRGAEGDYAKWYKPAGGRYDGFLLSSANC
FSRQLKSVLQYIEAGRLEDAEMVSNRVSEVMTRLLQAASALPFGNAFTNANKAIDHFFAY
GPHAELSAKIRTRSGAFLPRALLEETRTVLRNFQLMPSKGYLE*
>SPSA8_v1_420008|ID:41145953| putative Membrane-bound serine protease (ClpP class) [Spirochaetes Bin 1 SA-8]
MKFNPSLKSAFLFCTTFLFSILNLIPAAAQPRDISFGGMTWGLRQTAGPVDPGPNTFSNL
QDQVWVDAQGSVHLTLQKRGTIWTASEMMAKKDTGYGTYRFTVSASLADLDPNIVFGFFT
WDKAPEAFNREIDIEISRWGIPDGPDGWFTVQPYDRAGNQHSFYLPRANSYTFELQWAPG
VVRFSLLTDKNNKEIWTYSGAGVPEPGRARLRINLWLFRGKEPAQLWKHYEVVISDFSFS
PLI*
>SPSA8_v1_420009|ID:41145954| FAD dependent oxidoreductase [Spirochaetes Bin 1 SA-8]
MAEGMMGIIKREHNLLYKHGDMMKKKVVIIGAGLSGLATATLLAKRGFAVSVYEHAVQPG
GSSGAFKRGNAIFDVGSAMMFGFGPTGFNPHSLLFNEIEEPIAVIEHSSMYRIHFAGQDV
VFHADVESFLHALERLFPDDIADIRRFYAYLQHLYFDVVMKDPSLISPSEIPLMEMQTRF
FKDPVTQMKIIPLLFSNAASILRRFTQSVPVMQFFDKLTSTYCYTTMEETPAIMAVTMFV
ENHRSGSYYIHGSTQVYVGKLEKAIEKYGGSLVYGAEIVALEVEGGKVRAARLADGTNIE
ADYFVVYSGTVWNLYQKLLPSDAVPPVLLKKVSETVPTPSSIVLYATVKKEAFPEDIGPIE
MLVQDTDALTESEITLYIPTIDDPSLNEPGCHSILAIGPSFRTWPSPETLAKGDPKVRAA

YENAKQEEAERIIDYLASYYPAFKANLVDWEIGSPVTIERYSLKNKGSVAGPKQMVGQDL
MHRQHAGTRWKNLFCCGESTTMGTGTPAVVISGISAADVILRREGLPEYRYFSDKGFVQY
LKSPAPAYAQEGLGKIGNLCLWCEHDACRQVCPQDMDIRGIFRRLYSXNLEGARKLAKFS
EGSAGELVCLCCEKTPCVKACRRRTILGKSVPIPVSLAALFSRAQHRESS*
>SPSA8_v1_420010|ID:41145955| Zinc/iron permease [Spirochaetes Bin 1 SA-8]
MCMLEYLKTLSPVMQTLAATLFTWGVTALGAGMVFFFKSIDKRVLDSMLGFAAGVMIAAS
FWSLLAPAIELSESMGLIPWIPPAIGFLSGGIFLRLVDRLLPHLHIEYKMEEAEGIKTNW
SRSILLVLAITLHNIPEGLAVGVGFGAVGASIGSASLAGAMALALGIGIQNFPEGAAVSI
PLRRDGMSRSKSFFYGQASGIVEPIAGVLGAALVSTMRPILPYALAFAAGAMIYVVAEEV
LPEANRDGNGHLATIGTMVGFAVMMILDVALG*
>SPSA8_v1_420011|ID:41145956| conserved membrane protein of unknown function [Spirochaetes Bin 1 SA-8]
MLTTHDRVLAIFVSCSIALFMLMAILSNGMVPAVTGFWQLQISPARLISDFTLMANPGAA
LLNVSMVGILGLLVLRLNRIQLSGPSLAAVFTMMGFAFFGKTLLNCIPIIIGVSISALIV
RKKPREYVLIALFGTSLGPIVSFIAFELGFPPGIALPLSFIIGIIAGIVLPPLAIAMLHL
HQGYNLYNVGLTAGFIGLFAASIPNAGGVDISTFATWNTGRNIWLFLLIPIVAVLALALS
IALGAKKSLKEFLSIQKMSGRLPSDFTEMAGKGGALANAGLLTLLYWFFMLIIGAPFNGP
VLGGLLTILGFSLFGKNILNNVPIALGILFAVILFGKSISTPWIVLAFLFGTALSPVAGE
FGFSAGLLAGFLHLVLVERTGAWHAGINLYNNGFSAGLVATLIVSVIEWYKTNIEQ*
>SPSA8_v1_420012|ID:41145957| conserved protein of unknown function [Spirochaetes Bin 1 SA-8]
MKRREFLVHLISEVSSFLLQGNPRKMVVSLHQEADGIHLAVLDDRPRSDAEIAEIEHSLN
TARRPELAGYYGQMAGQDLLGSARLNLVGWQIKHADVLKTPEGGVMLNLWIGGEGFEFDK
FNLSKGTQE*
>SPSA8_v1_420013|ID:41145958|sbcB| Exodeoxyribonuclease I [Spirochaetes Bin 1 SA-8]
MASSILWYDLETFGINPSYDRIAQFACIRTNEELEEIADPIKLYCKPALDYLPSPQACLV
HGITPQAAHELGETEYDFAKKIHFEFSQPGTTVAGFNSINFDDEFIRFLFFRNLFDPYER
EYANENSRWDIINLVRATHDLRPEGIVWPKDNNGMPVFKLEALTRANGLDHFSAHDALSD
IRATIAVAKLIRTRQKKLYDWYYSHRKRESLKPVIDLPSRKILLHTSSEYTTERGCTTLL
APIGMDQANKNQLIAIDLRYDPERLLDLSAEEIRKLVFTKAAELDEPRVPLVRIKLNHCP
FLAPERVLSAEAAQRLGINRDECSRRADLIRNNPNLIQKLQAVFEIPMEFPSIEDPEASL
YSGNFIPKPDRSKLKRLHELIADSGGAAAGRDFVRNSKFSDERIPRLLLRFFARNFPETL
TAAERLKWRNYCAQKIQLPPAEGSAELADYGKLLQSFLDNPELPAPKRAVVHSLLDWKFF
LEKELLAYDEAVSKLNSNP*
>SPSA8_v1_420014|ID:41145959| protein of unknown function [Spirochaetes Bin 1 SA-8]
MKRILFVVFAAFLLWWGGGACTKAFAEEQPGAIFMTVQVESAGIREKPAVLSSIIATTRY
GIVVKVFEIREGWAKVEIPGQTRIGFIFATSLRKVTIPEGQMAAPLQGVTAPQVVLAGKG
FAPINANPATGMESRENSTQKQWLDSMESLTIDPWEALAFVLGKAD*
>SPSA8_v1_420015|ID:41145960| protein of unknown function [Spirochaetes Bin 1 SA-8]
MPKRYWWLTVSLIAALLFSSCLTLSDSISMLVSKAVVQGLLDETQAAILKSAATEAVFAS
RELSPEEEYYLGRAISGAILEVYRPYDCPELTRYINLLGQGLALFSPRPELYKGYHFLVL
DSDEPNAFAMPGGQILITRGLLSLTENEDELAAILAHEISHTALRHGVLSLQSYALAELA
REAAIKAGQAGSAEAVSFTNKFGTAISEFAAVLLVSGYSQNFEYEADRTAQSILAQLGYA
PSSVAAVIMKLPDKEGEAANSFVITHPEKEARIGSLESSVSNLVLFSLSAVQRWREYGLL
LREVEPDEILSVAPLGVVRADRYARYSLFFRKDAGK*
>SPSA8_v1_420016|ID:41145961| putative Adenylate/guanylate cyclase with Chase sensor [Spirochaetes Bin 1 SA-8]
MKGSCIERRIEVLAISRAARILLSVVGGLLLLYICNFSGIETKLQFALHDTAVRIEAQRF
ENSMNSKSNRPDIRLIFVDQYSLTWVQENLGFSWPWPRELYGLIADFCSQAQVQAVDVLF
SETSIYGPDDDRRLAQALEQAGNVVLVATSQSGKLSSIPVERSKVEFGTARALVDSDGVI
RRYDARSSVTGNGVLPALGAAVLKKIGLKNSFPAAVYLKFLGASPGFEAYNAAEILSAEL
HRFDENKNIDPYSGTLSPEAFRNAIVFIGFSAPGLLDQQAVPVDSAMPGVEIHATFVENT
LSGRLAYQMAPFSSWLFILLFGMLGAILPSVMRKTSLLVTGFFLLAPLVIISNALFFSAG
LVAPGGAALTLAFGSGLTGILLAYLAEGKQRAFLRRSFSFYLAPEVIEQLLNHPEMLKLG
GETRTITVLFSDMEGFTNFSEKLSPDVLAGFMNEYMTKVSEIILKYGGTIDKYIGDAVVA
FWNAPLEQKDHAERAVLAAVEIIVALEESRGSVSNLVLPRTRIGIHTGRAVVGNMGSAKR
FNYTALGDTMNIASRLEEANKGLKTSILISADTARYCMKSEVGKNNKYKLKAMGNIKVPG

KEQEIEVWQVCPFKSVSNLVLDGRQRDSTESPS*
>SPSA8_v1_430001|ID:41145962| Branched-chain amino acid ABC transporter, amino acid-binding protein (TC 3.A.1.4.1) [Spirochaetes Bin 1 SA-8]
VGLYYSGFITHAQGGCMKRALFVLVLVLSIALVGCAGTAKSDTIKVGWFGALTGDQAVWG
ENEFNTVKMLFEEYNNAGGIEVGGKKYKLEVIGYDNKGDAQEAVNVVKRLTGQDKVVAIL
GPNASGNAIPIAPVLEQAKVPDIATVATNPKVTVLDGKVKPYNFRVCFIDPYQGAVAAGY
AYDKLNARKAAILYDVSDDYSQGLREFFKANFEKKGGMIVADESFNGGDVDFRPQLSKIK
AANPDIIFMPYFFKEVALSANQARELGIKTTLMGGDGWPSDQLISMGGKAVEGSYFVNHL
DYADPAVQDYKARYKAKYGKETELNGYLVHDAVLLLVAGLQKAGKVNGEALAX
>SPSA8_v1_430002|ID:41145963|glmS| Glutamine--fructose-6-phosphate aminotransferase [isomerizing] [Spirochaetes Bin 1 SA-8]
MCGIIGYTGPRNTSKILLEGLRRLEYRGYDSAGIVVGKESEKSGELTIIKSVGKIAALKS
IVPHNLEGSWGIGHTRWATHGGVTESNAHPHTDMSGKIAVVHNGIIENHTAIKSMLQAKG
CVFKSETDTEVIPHLIAQYYEGDLLKALLKALQHLSGTYAIACIHADEPGRIIGARNGGP
LVVGIGSGEMFLASDITAMVAYTDKVVYLNDGEIVDINAGAFTITNRFEQHVDKAVDTIS
WELGSIEKGEFPCYMEKEIFEQPESIARAISGRLDAENATAKLGGLNLGRKMLSDISRVK
VIAAGTSWHAGLVGSFLLEQVARVPAQAELASELRYRNPVVEKDSLWFTVSQSGETADSL
YAMREIQRKGGTVLGICNVVGSTIARESDGGIYVHSGPEIAVASTKAFTSQLAAFYLFTL
MVARMRDMSREEGSKFLTALQEVPAKVAETLKQRDHVQRIAKKYCHAKDFLFLGRGILYP
IALEGALKLKEISYIHAEGCSAGEMKHGTIALVSPELPSVFLVSDDFLREKTISNMREIK
ARGGPVIAVGVEGDLETEKIADEFIPVPRMDPRFYPFTMILPLQLLAYFSALELGRDVDQ
PRNLAKSVTVE*
>SPSA8_v1_430003|ID:41145964| putative Biotin/acetyl-CoA-carboxylase ligase [Spirochaetes Bin 1 SA-8]
MELPFFRFKSVSSTMDTARSLIQQGLSSPAAVVARYQTNGRGRISGRTWEAKPGSSLLTT
IMLPVSLVRPEALSLKTGLALSIALEQLTSAHELAGSSFLLKWPNDILASGDHADPDSFR
KLAGILIEQSAGWFVIGMGINLQKGAYPEVLVNSAVSLEEILGIQPLSDQGEYLIRRTVE
NILAFSANDEWRTAYISRMWKLGMNIRFAEGHPEQDKFSQGILEGIDSSGQILIRLTDGS
LRSYSSGEISLVRSSAQ*
>SPSA8_v1_430004|ID:41145965| 2-dehydropantoate 2-reductase [Spirochaetes Bin 1 SA-8]
MPKINKVLIVGAGAIGAAIASRIFDVNPDAVALAASGERKQRYLRDGFIVNGKRYDFRLA
DSAGATPYDLIILAVKNYDLESAIEEIRPFVGENTTILSLLNGITSEQILRAEFGKDAVP
LAFIIGIDALREGNRIDFPRPGEIRFGDEKNDSDNLRPAIAEIADFLKSHNVPYSIPNDM
VRAMWYKFMMNVGLNQWSAVLRAPYGVFQKNAIARDLVRETMEEVVALGIQRGVAISSAD
IDSLFTILDTLGPEGKTSMLQDVEAQRKTEVDAFAKVVVDEASCSGIPVPANAMLFRMIS
CIEQSYADLAR*
>SPSA8_v1_430005|ID:41145966|hflX| GTPase HflX [Spirochaetes Bin 1 SA-8]
VTELTDPRPKAERSLLVGIEKDTIRKAEAQSLLDELKGLAKTLGIDVADSILVKLREKTA
NLLVGSGKAQEIANAAEALNADSIIFDHILTPVQQRNWETLTKKKVYDRAELIIRIFSAR
ALTKEAGLQVELAQLQYALPRLAHSYEELSRQRGGRYGTKGAGEQKIELDRRSIERRIHE
IKEELKEVQMSREIQRKRRERILLPRAALVGYTNAGKSSLLNALTNAQVLAEDKLFATLD
PTTRRLTLSSGGTLLLTDTVGFVRNLPHGLVEAFKATLEEAALADLLIHVADASDPEVEV
HMQTTEKVLSEIGAGMKPRILALNKTDIADPEKIDQWTRIHGAGIPVSAKTGEGLELLAK
AIEQALTAEMGAYEFEFPHNEYALVALIHREGSILSERTTDSGTRIVCRIPERILHRVEK
YCVTNEAQDGGYDAENQ*
>SPSA8_v1_430006|ID:41145967|argI| ornithine carbamoyltransferase 1 [Spirochaetes Bin 1 SA-8]
MPVNLKGRSLLTLKDFSTEEIRYLLDLSHDLKAKKRAGVKGNLLNRKNIVLIFDKASTRT
RCAFETACWDEGGNATFLTNSQMGKKESIEDTAKVLGRMYDGIQYRGFSQELVEDLAKYS
GVPVWNGLTDDDHPTQILADLLTIEEHIAKPLSKVKMVYCGDARNNMGNALMIGSAKMGM
HFVALAPRELWPNEKLTAEMKAVANETGGKIEFFSIEDADKALAGADVLYTDVWVSMGEE
DKFEERIKQLEQYRVTMDFVKKTGNPDVIFLHCLPAFHDTETAVGKDIQKKFGISEMEVA
DAVFRSRHSKVFDQAENRLHTIKAIMVATIGDL*
>SPSA8_v1_430007|ID:41145968| Aminotransferase class I and II [Spirochaetes Bin 1 SA-8]
MALSISEIGKAVLDTHYAVRGPIVARAQELEKAGKEIIYCNIGNPQALKQKPITYIRQVL
ALTEYPQMIERADSMFPQDAIETAKTILKESKHGLGAYSDSKGMLFVRKAIAEFIEKRDS

TTALKVESNPDHIYLTDGASKGVQTALRILIAGEQDGIMIPIPQYPLYSATITLYGGVQV
GYYLDESSGWNLSYKMLEEAYLEAHAHGVKIKAIVVINPGNPTGSVLSRENIEMVIQFAK
THGLSILADEVYQENIYRPEDSFISFARVMTEIREREVSLFSFHSTSKGFLGECGHRGGY
MEVRNVSQDVLAQITKLQSVSLCSNLPGQVVTYLMVKPPAEGSPSYSKYNEEKNSILSEL
AKRAKILADGLNEIPGISCQPITGAMYAFPSIMLPQGRTDEEYXMALLEQEGVXLVPGTG
FRQVXGTAHFRTTILPPTEQILSVVEKIXRFHLAWK*
>SPSA8_v1_430008|ID:41145969| PpiC-type peptidyl-prolyl cis-trans isomerase [Spirochaetes Bin 1 SA-8]
MEWRASHILVKDRNLAQELLKRVKQGANFEGLAREFSTCPSKSSGGDLGWFGPGKMVAPF
EYACKNLGVGSVSDVVQTQFGYHIIKLTGKR*
>SPSA8_v1_430009|ID:41145970| RluA family pseudouridine synthase [Spirochaetes Bin 1 SA-8]
VKKTIELLHEDAAFLIADKPSGWLSINDRYDPDAPVVLNALEKEFGKLFVVHRIDKDTSG
VLLFARNAQAHKILNDQFATHEVQKKYIALVRGVPDEAEWRCELPLRADADKMHRTIIDA
RRGKDAVTRFKLLETLGQYSVVEAYPETGRTHQIRVHLAATGYPIACDPLYGDGKPILLS
LLKRRWKGDIYEEKPLISRTALHAEYLSFSHPVTNALLEISSPLPRDMKAVFKQLRSL*
>SPSA8_v1_430010|ID:41145971| protein of unknown function [Spirochaetes Bin 1 SA-8]
VKIGKGIAENSEKQSKNEENKGNSG*
>SPSA8_v1_430011|ID:41145972|groL| Cpn60 chaperonin GroEL, large subunit of GroESL [Spirochaetes Bin 1 SA-8]
MAKQLLFSEEARRKLLVGVETISRAVKVTLGPKGRNVLLDKKFGAPTVTKDGVSVAKEIE
LEDPYENMGAQLLKEVATKTNDIAGDGTTTATVLAYSIVKEGLKAVAAGMDPMGLKRGID
FAVSLAVDEIKKNAKEIKEKEEIADVASVSANNDKEIGMQIADAMEKVGKDGVITVEESK
TMDTTIDFVEGMQFDRGYTSPYFVTNRDSMTTVFENPLILIHDKKISNMKDLLPVLEKTA
QTGKPLLIIAEDIEGEALATLIVNHLRGTINVCAVKAPGFGDRRKAMLEDIAILTGGEVI
SEELGLKLENTALSQLGTAKTVKVDKDNTTIINGGGKQKDIQDRIAQIKKQIEETTSDYD
REKLQERLAKLAGGVAVINVGAATEVEMKEKKHRVEDALSATRAAIEEGIVPGGGIALIQ
AAIALEKANTAKLTDDEKVGYKIVKRALEEPIRQIAENGGVDGSIIADKAKSSKKGIGFD
AARMEWVDMVKAGIIDPAKVTRSALQNAASVAALLLTTECAITDLPEKEKPAMPAGGGMG
GMGGMDY*
>SPSA8_v1_430012|ID:41145973| conserved membrane protein of unknown function [Spirochaetes Bin 1 SA-8]
MEQLHFGILSIIPPLLTIILAVTTKDVVISLFLGALSGTLIAVGGNPYFGLINLADRIAD
TLADGWNIRVLLFTIILGLLVGMLAKSGAAYSLGSWAGKKIKSRTGALIFTWLFGIIIFF
DDYFNSLTIGTCMKPLCDAKKISRAKLAYILDSTAAPVCIIAPISGWVAFVIGTYKAMPE
WQGLGVGELVFFLKTIPFNLYAIFAIFMVLFITITYKDFGPMARSEARALKGIGLFDEEK
YGTVVSQVESKVHTNNAKPFDFLVPIFAVIFIALFFFPMTTWMGAVDGENIKTIGEAMAA
IPLGQAFNDTDSSKALMYALIFATVFGYIYFILRKLLNLKAAGEAMLDGFRAMVPAAMIL
TLAWTLGGVIKSSPGDGGVGLGSYLSEVVVNGNFPIQFFPLVVFILSCIISFASGTSWGT
MGIMVPVSLPIIVQLAKMANMTPDQTVNAVALTIGVVLGGSVFGDHCSPISDTTILSSTG
ASVPHLEHVATQLPYAVFVAACAMVGTVVGGFTWSALASIIATAIPFVAGTFLLPKWFGP
KHYGLE*
>SPSA8_v1_430013|ID:41145974| Alanine racemase domain-containing protein (fragment) [Spirochaetes Bin 1 SA-8]
MGAPYLEIRLDIIASNAQAIVDFCGSFGVEVWGVTKGCCGMPEVARAMLSGGVFGIGESR
LANISRLREAGIDVPFLMLRLPHISEARSIVEAFDMSLNSEIDTIRALAAEARAMRKRHG
VILMVDTGDLREGLWPDRVSETARKIADLDGVELVGLGTNLSCYGGVIPSEANLGLLVQL
ARQIEQEFGVKLKYISGGNTSTLPLMASGKLPAGVNMLRIGEAILQGTESIHRTPWPGTR
QDAFVLHAEVIEIADKPSVPIGERGQDGFGGYPVFEDRGLIRRAIVNIGRQDVLVENLTC
LDKGVTILGASSDHMILDATHALQPLNIGDTVSFNLGYGAMLQAMTSPYIEKKPAP*
>SPSA8_v1_430014|ID:41145975| conserved protein of unknown function [Spirochaetes Bin 1 SA-8]
MGGYLAVKKLVSLGDIQAFIQYVRSFTQPISQIANISNVLQQTAASAERVFEFLEEAEEV
PEQENALVPSGIQGHVEFRNVHFGYSPEKIIINDFSADIKPGQRVAIVGPTGAGKTTMVK
LLMRFYDVNSGAILIDGHDIREYRRAGLRKLFGMVLQDTWLFNGTILDNIRYGRLEAAEE
EVFEAAKAAHADHFIRAFPDGYKTELNEETTNISQGQKQLLTIARAILANPSMLILDEAT
SSVDTHTEVLIQKAMNNLMKGRTSFIIAHRLSTIRDADWILVMNHGDIVEQGTHESLLEK
GGFYAELYNSQFEKSEAAEAV*
>SPSA8_v1_440001|ID:41145976| conserved membrane protein of unknown function [Spirochaetes Bin 1 SA-8]
MSIFEIIMLLCFGAAWPFSIAKSLKTGKTGGKSFAFLVIVIVGYISGILNKILYKPDGVV

WLYALNASMVSIDAVLWLRNRKREKSAERAESAKGNA*
>SPSA8_v1_440002|ID:41145977|uppP| Undecaprenyl-diphosphatase [Spirochaetes Bin 1 SA-8]
MTAVKAIILGLIQGLAEFLPISSSGHLALFKNVLGLEQVPLVFDVILHMATLLAVVIVFW
KRIVGMLKAFWLFVSKGRAGLQIGDGQDQGTQENLAVIPPLLAATVITAVLGYAIQKYFS
DENIKFVAVRMLITAAILGLTFFVKPGARKLGSIGIGRSALIGVAQGAGVFSGISRSGIT
ISAGIFSGLDREAAGEFSFLLSIPAILGALVLTLKDAGSMMAEVSAAHLALAFVTAFFSG
YLALKFFMKMVKNGKLYWFAPYLLLAGAAGLLFL*
>SPSA8_v1_440003|ID:41145978| Cell division protein FtsK/SpoIIIE [Spirochaetes Bin 1 SA-8]
MARSDDESHGNAGKASVVVASPPSRQNRGNGKSAKSKRETSLWSSPSKVIALLTALGAAY
LVLSLFLALKWPQGWVFLNGLGKQILIRFSWAGFFLPLWLACAAVLIVLPGFYPRLTYFL
GAAALPFLVLTIFARLSTEPGAFFENHPVFAAVGLSSLYAGVGFLFALSLVLVSLGYFKL
NEWLNLKEHAKAKGATAAKSDVLARKAGSLKKKEPFFLRAKMRIQQWYADWRGKRTKAEK
QLRDAEALAANSFDFDLPALKPVGDMKSLNQQNGNQKTIEEGSPGFSAKAASEPAGPVQT
KLDANGKEIEFTSVPPQTVSDGHSNAAGTDDSRKQDIIKKILERKALKPYTVPVDGILNT
YPDGQYWIIDEKTRRNAEVLRETLAEFNIEAEVTGIRKGPVITMFEILPAHGVKISKITN
LSDNIALRLAASSVRIVAPIPGKHAVGIEVPNDRRAIVSLREIIEHEQFKSAKMEIPVAL
GKDIAGEVQFIDLTQTPHLLIAGATGSGKSVCVNAIILSVLYRRSPEEVKLLLIDPKIVE
LKLYNDIPHLLTPVVTESKRAFQALQYCICEMERRYSMLDKAGVRDIRSFNRKIKDKGLA
QEKLPYIVVIIDEFADLMAQTGKELESTLARLAAMSRAVGIHLVLATQRPSIDVITGLIK
ANIPSRIAFMVASKFDSRIIIDMVGAEKLLGRGDMLFSGAQDPFPVRMQGAFVSEEEVER
AVEHVKTLGEPEYIDDEIFYDEEEENAEPGLFDDGDGDPLFEKALEIVLQQGKASASFIQ
RRLKIGYNRAARLVELMEERGIVGPAQGSKPRDVMRNPGVTGSRPPLLDPMDSDDSAGNA
*
>SPSA8_v1_440004|ID:41145979| putative Diguanylate cyclase [Spirochaetes Bin 1 SA-8]
MEPLKFNIVSGLWLLFAIPVIINARKGLSRSEVRGSRAFGFLNLSIAWYLVFAALELMSR
TIEQVLFFTHLEYLALCAFPVEILAFTFSFLDIEFKLKKLVLFAAGVFSSAVLLLQWTTA
RHNLFYANPRLIPYQDLLVIKFEKGPIYLLWVVALLMVITSAALLFIQRTFHARGLVKRQ
LFIISVAMCLPLAVSLLYVFQVLPIPFDFNLFSYILISILTSYALFKAKMLEFAPVSDDK
VFEFLREGCMILDEHHTIININNAAIDLLPNIGEKLIGKTIQQALPTCENLIASLVNDVP
ATIECDGSVFGGPEHLTVKTSPIKAKANQVLGWLITMENTNERFQLLEELQHFATHDDLT
GLCNRRFFFEQVRKLLGTDTPQVLTCAFIMLDIDDFKTINDSMGHQTGDGVLQAVSVCIS
QTITPDALACRFGGDEFLLFLPGLDLKTAIEEAIQLRQAISEMDSNRNSVANITVSIGVA
AAALYTAEDIDMLISAADKALYKAKRAGKNQVASQETLVTQTLKPRN*
>SPSA8_v1_440005|ID:41145980| Pseudouridine synthase [Spirochaetes Bin 1 SA-8]
MNDRKHRRTPNHPHENPPAPSHQGNTPRPRRQNSRFFEILYEDSDLIVVEKPEGLPVIAP
EGSRGKSLYDLVTRHIQKRNPHGRAALVHRLDRDTSGVMMFAKNPQTKKILMDNWNALIK
KRTYTALIEGQLPAEEGILDSWLMENRAGQVYEVPAGTHGALRAITRWKVLKNLPKFSLV
ELDLETGRKHQIRAQLSAVGHPVAGDSRYGASSNPFGRLCLHASALEFEHPRTHRVMRFE
NQLPDYKF*
>SPSA8_v1_440006|ID:41145981| protein of unknown function [Spirochaetes Bin 1 SA-8]
LSFGGGVGEYSLDAMVQVGFHADGSEFSDASGHSWFEYILEFDIRERSG*
>SPSA8_v1_440007|ID:41145982| Cupin 2, conserved barrel [Spirochaetes Bin 1 SA-8]
MVLERKDMDVEARPQMRGGKGVCNVTNLGAKPLQQHLRVLAEILIPAGASIGEHEHLHET
EYYLITKGSGTVNDDGTLKKVKPGDVVITTGGAKHSIEADSGEELSLIAVIVTDQ*
>SPSA8_v1_440008|ID:41145983| putative enzyme [Spirochaetes Bin 1 SA-8]
MDFNASLFSDLKDKDFSAQGLMVLEGRITIEKALEKGIKLIGLASIPERAEEWRTKIGSE
IPLVEMQQAELSGIVGFPFHRGSLALAIRPEITEFSAISSCQAASAQRPWFVCLWDIADP
SNVGAIIRSAAALNASGIILGPGTADPLYRKAIRASMGNVFSLPLYSLQEKTLEDLGSAG
VYRIAGALSAKAVPLAEACQLSRGRFPAILLVGNEGYGLPEKVRSACDLEVFIPMIRGVD
SLNVAAASAILMYELFV*
>SPSA8_v1_440009|ID:41145984| conserved protein of unknown function [Spirochaetes Bin 1 SA-8]
MKTKPEDRIAYEKMLPGVLTAQGFLGNDIRPLAEIIMEDEQEFIRLGLDFEEVADRLDAL
KQAGEKGLGEPITVGELLVQTGDARGMLPCPWGDGLYHKNAISIQPADKPQEACVEGEDM
LXFSDLSIHLLRVHHFCQGLGSPFRLDPVFLKHLIY*

>SPSA8_v1_440010|ID:41145985| Ferredoxin--NAD(+) reductase [Spirochaetes Bin 1 SA-8]
MTQILIGPLAVAIISTVLAFLIAIIDKVLNNYGQVKISINGGKKVLEVKGGSPLLGTLAA
EGIFVPSACGGRGSCGACKCKVTTDVGPHLPTELPYMSPAEIQKNIRLSCQIKVRKDLEI
ELPEEFFSVKKFQTKVESIKNLTYDIKEVLLALQDGQTVEFEAGQYVQLVVPPYGEITES
VQRAYSMSSRPLDKTHIELLIRLVPGGIATTWVHKFLKEGDRVEVVGPFGEFHIHDTPAT
MVCVAGGSGMAPFKSMLYHMYETGAFQEKEIWYFFGARTTKDMFYLDELAELAARWPRFH
FVPALSEPKPEENWKGDVGLITDVLDRYVQSTIDKSKGLEGYLCGSPGMINACINVMKKN
GMTEDKIYFDKFA*
>SPSA8_v1_440011|ID:41145986|nqrE| Na(+)-translocating NADH-quinone reductase subunit E [Spirochaetes Bin 1 SA-8]
MINAPDVGLLSLFLASILTSNVLLANFLGTCSFISISKDFKSSMGLGVAVTMVIGICSAI
CWAVLYFIIKPLGIEYLSFIIFIIVIAAVVQMLEMIIDRFSPGLYLALGIFLPLITVNCA
VLGVILFMQIRNYNFMQAVIFGFGSGAGWWLAIMLLASIRKKLDNNNAVPAGLKGTGITL
ITIGFMAMAFIGFSGMIAVQ*
>SPSA8_v1_440012|ID:41145987|nqrD| Na(+)-translocating NADH-quinone reductase subunit D [Spirochaetes Bin 1 SA-8]
MSPAKKVMKDGLWSNNPIFVQVLGICSTLAVTNNLRNTLIMVLGVTFATSMGSMTLSFLK
DLIPRKVRMIVQVLVLSFYVIIIDILLRAYQPAISKQLGPYVGLIITNCILMGRAEAFAA
ANKPGLSFIDGIANGLGYGWVLISIAFIREFLGFGSLFGIKLVGDQFTPWTIMVMAPSAF
FLVAIALWIANNYKAAAEAKKKAKPAVQPASQSK*
>SPSA8_v1_440013|ID:41145988| FMN-binding domain protein [Spirochaetes Bin 1 SA-8]
MKFKKESIGYVVIFTFIVCVAFVILLSIANQLTLDRVKANKSYESHFAVLSAFGLADGNT
PKDQVESIYAASIKELAAADPGVSAYSADIDGTNYLAVKLTNPGLWGPITGILAADIKAE
RIRGFAILDQQETPGLGGRISEPWFIAQFKNEKVAGDGTIAVIQGSGKGDPDSENGQVDA
ITGASRTSDFVKTLVNKSLDQIRKIGGSL*
>SPSA8_v1_440014|ID:41145989| NQR2 and RnfD family protein [Spirochaetes Bin 1 SA-8]
VVKFLKQPMMRKVLYSLIPLYLYALWMYGYRVILSAAVVFLLGVGTEWLFERKKSGKVSE
AVLVTCALFALAFPPKTPLWILGVGIIFAVSMAKGVYGGFGRNIFNPAIAGRAFVYISFA
VVLSASYTSFGNFGIGAADVVASATPLGVMRQGGQVPILNLLLGQRSGAMGEGMVLLVIA
AAIYLIATKTASWRIILSSVLAGAVLNAILYYSGVKRALPMESLLAGSFLYVSVFMATDP
VSAPKKQLSHYFYGALIGATIIIIRTFSAFPEGTSFALLFGNTFANLIDIAVDSAKKPGP
AKQGGTK*
>SPSA8_v1_440015|ID:41145990| B3/4 domain protein [Spirochaetes Bin 1 SA-8]
MLTQFSFDNDRSLANIIAAVVEAQGVSWEPYSFEQVFQDELQRCLASGESLVSPSRKKAV
RDMLRHGSYKPAGRAKPSSEYLLQAMLEGDFPRVNYFVDAANLVSLAAGYPISIIDLDKA
GSDLLLRRGKTGDAFIFNAGGQEINVTDLLCIYRKAGADWLPTANPVRDSMATKIFEGAR
NLLAVIYAPEGSEGSDLPIAAEKLAGFLKARARQVAAEILKLE*
>SPSA8_v1_440016|ID:41145991| exported protein of unknown function [Spirochaetes Bin 1 SA-8]
MKKRCIALIVAVLIAAPLFAIDFGVNYTKEQAGFQRDFFGATIRSVGEGFLGFDLMVITP
SLTSYMDPVENAKFLIAHYQDIEYAQILPFLLVNLRMKPLTLYGGLAPMIDLTYLPDDLG
NKQFDIQLYSPYIYQAKVGLQLNLLILGAYAEAGTIVDLTFKSAFENFHLTFGAVLNF*
>SPSA8_v1_440017|ID:41145992| Cna protein B-type domain [Spirochaetes Bin 1 SA-8]
MKKTIIILLAVLVLGTVLSSCDQLMQWQGNAIIKGLAKYFDKGANQNGGIRITLLSATII
IKPNQVPLDVAPLVKVTSSNGRFSFNGIAPGSYIVVAEDPSGEYQPANLYVTVAANGEVT
TEDLVLTKAIQHVVIFRDSSGEWNETTAIGDILADEVGMTEGSGANQFEYKSSAEMAGYT
PNLGDLIIIGGDQTTSFYNTYTVNKAVFDNFVDGGGTMFWIACDGGWQAGDFTSTLPGGV
TWRDRYENYNDIVYFEHPITRNFPEQLYGNYASHGGFDNLDSLPLTNLMVYVKEASGLLP
TYIEYRTGSGKVLASTAPLEYYVANGPTDKPDGFSTSYRDLFKLMLARSIKYIMGLPVSE
DVPPSAEAMGLKALPAQRMSH*
>SPSA8_v1_440018|ID:41145993| Threonylcarbamoyl-AMP synthase (fragment) [Spirochaetes Bin 1 SA-8]
LSFTDNPARIPPMMTLDISEENLKIAGRALAEGKLVAIPTETVYGLGADAFNAAAVARVF
EAKARPSFDPLIVHIARIEDLEKIASSIPPKAKLLADHLWPGPLTMILPKRSEVPDIVTS
GLPTAAVRFPQHPVARKIIEFSGTAIAAPSANPFGYISPTRAEHVARLLGEKIDYLVDGG
ACEVGVESTVIDMXX

>SPSA8_v1_450001|ID:41145994| Beta-glucosidase homolog [Spirochaetes Bin 1 SA-8]
MAQAAAYLSGNIGKSDIKALGRQCGEELRSLGINWNLAPVADINSNPSNPIIGVRSYGEN
PEQVADLASAFAAGLKEGGVIPTAKHFPGHGDTSVDSHLDIPLVPHDIERLEAIEFVPFR
RLIHEDIPAVMTAHIRLPSVEPDMVPATLSSRVLQGLLRKKLGFEGIICSDCMEMKAIAD
HFPNAFVMAVKAGVDILFISHSAEKQLFAARSIYEAVKKGEIAESQIDASVERILAIKGK
IYQTNSFDAYKKTIASPHAGNLAQKISRASLSVLVHGDTSTPPSGSVLIDVVPGNITNAE
DSQHTVSIHAELTRQNVPIFSCAVPLNPEGKDIASALSCAEGSLSSPASAIQKAEAKPAL
ILALHAPMLHSGQMKLVQASVHFAQARSCPLLVILTRNPYDAPAIVKEAALANLPAPFIV
CSYEYSEVSVASIVAFLTGRLEAHGICPVTVDG*
>SPSA8_v1_450002|ID:41145995| ABC-type dipeptide transport system, periplasmic component [Spirochaetes Bin 1 SA-8]
MKKKLIVAMVLLSLFVATTVFAQAPARGGILKMTPSKQGVLQNNFNPFSPAVLESALGCI
YETLIYFNNANGTANPWIAESWSWSNDLKDITFVIRQGIKFNDGTPLTAEDVVFSAMLGK
TNKALDISGLWSEGLQDVTASGNQVTFKFATVNVTTLEKFGNLYIVPKAIWSKVQDPLSW
TGNTNPVGSGPFVLDPASFNEQSYKLVRNPNYWQKGADGKPLPYIDGIQYVSTTNEQIAF
KLMNGEYDWAAYSLPNVDAYVAADPVNNKYWFPEGNLVFLYINNLRTPFDNVNVRKAFAM
AISQKDITRKMSPSPVPADMSAVKKSFSIIAQEGKAKYNITYDKAKARKLLESEGYKLNA
KGIYEKDGKALSFKLYVPTDWTDWIGAAETIASMLKDIGVEAIITQSAWPVPFQTSLETG
DYDMAVSFITTGTTPYYQFNRWLYSANYAGLGEKAKVFSNMRYKNADIDKNLEAYXSEPN
PEKQKVYXSAVVTQFMKDTPCVPLFFNPTWXEYTTKNFVXWPSADNPYAWPSVIGMQKVP
ILLSIHKK*
>SPSA8_v1_450003|ID:41145996| Binding-protein-dependent transport systems inner membrane component [Spirochaetes Bin 1 SA-8]
MKYLARKFGMLIFTLFVSMSINFFLPRLMPGDPAKALMDRMEGVEAEKLESVRAAFGLDT
KDSLPVQYGKYLLNTVKGDLGISLSRFPMPVSSVLASALPWTIGLMGLCTIIGFSIGTLL
GIWAAWRRQTKLASFTVGFFTFIRSFPYFWLGLFLIYIFSFKLKVFPLGGAFSIDQTRGS
AGWWLSVLQHGILPAFTITLSSIGAWLLMMRNNMINVLAEEFITLAIAKGLPMQRIRLMY
AARNAILPSVTGFAMSFGFIVGGGLITEMVFAYPGMGYMLYQAVNAKDYPLMQAIFLIIA
ASVLIANFLADIAIMLLDPRVRDGAK*
>SPSA8_v1_450004|ID:41145997| ABC-type dipeptide/oligopeptide/nickel transport system, permease component [Spirochaetes Bin 1 SA-8]
MIRDFFKTLVKNKKSLSGLILLGFFVLVALFAPLLAPYPPKTDKIERTILLEENKSDPEV
LTEETEGKNREKSVYTVTITQKYEKTTDYFPTRAKPSKDHLLGTNHAGNDLLSQIIMGTR
ISLFVGLATGLFVTFISLSLALLSGYLGGVLDDVLSFITNVFLVIPGLPLMIVIGTYVPM
HGVLPIILILSLTSWPYPTRLLRSQVLTLKNRDFVRASKMIGERTSFVIFREILPNMISI
VMSEFFTVSLSAILGEATLEFIGVGNITVVSWGTILYWAQANGAILMGAWWWFLPPGLLI
ALTGTAFVLINFGIDEISNPRLRKR*
>SPSA8_v1_450005|ID:41145998|oppD| oligopeptide transporter subunit ; ATP-binding component of ABC superfamily [Spirochaetes Bin 1 SA-8]
MDADAILELRSLKAGYATNAGFVRAVDDVSLTLKRGEFLGIAGESGCGKSTLAYSIMRLL
RDNARIESGSILFDGTDITSLNEKAMNEYRWTKMSMVFQSAMNALNPVLSIEEQLIDAVL
AHKQVSRAEAKKTAIEMLELVDINPERISSYPHQLSGGMRQRVMIAMALLLKPSLLIMDE
PTTALDVVVQRSILQKIEELRYIFGFSVIFITHDLSLLVEISDSLAIMYAGKICEYGPSR
EIYASPNHPYTKGLMNAFPTLTGPMSRMGGIEGQAPSLLAPPPGCRFAPRCASAVEACTT
AVPMLRTTSPGRLCACRLCGGNHE*
>SPSA8_v1_450006|ID:41145999|appF| Oligopeptide transport ATP-binding protein AppF [Spirochaetes Bin 1 SA-8]
MNNVILELKGVSKHFTLSGIRNIGKVVHAMDDVSFSLNKGESLALVGESGSGKTTTASVI
AGMYEKTSGEIYLDEQPVLPFKTREASLRYKKKVQMIFQDPFASLNPTHTIRSILERPFL
IHKLAGSAQELEAMLSDSLEMVGLSPAQEYLHRFPHELSGGQRQRVNIARTFAVSPEVIL
ADEPTSMLDVSIRIGIMNMMLDLKEKKNVSFLYITHDLAGARYMSTKIAVMYAGMIMEIG
PTEEVIANAVHPYVQLLKASAPAPQEGVRKSAIPAKGEIPSLVNPPLGCRFHTRCPYAKK
ICSEAVPAMRDIGNGHLVRCVLV*
>SPSA8_v1_450007|ID:41146000| protein of unknown function [Spirochaetes Bin 1 SA-8]
MCIVSIMMVQCSHEEQKMAHTCALSDAEAYLLKTLGLKIRTAPLLGVSLLN*

>SPSA8_v1_450008|ID:41146001| protein of unknown function [Spirochaetes Bin 1 SA-8]
MYAYDKEKTLNFINPEKYGPFGAHAVFAFDYFSDDIKTKLFTYIKRTAEPRGGKIDFDIA
GTLSGNWIVEGSDVIKSADPWNYWLAFVYDMYYPESRRISIGVELGKTINRLNGLLTQPV
EGPEYTDVTPASSSPAVYKLADAVEGMNSFPTQADIKYTLLAQVVEEDKIKVQLFEGNVE
NPSFTDSALYYTR*
>SPSA8_v1_450009|ID:41146002| Oxidoreductase domain-containing protein [Spirochaetes Bin 1 SA-8]
MNIGIMSAAHVHADAYVGCLRSIPGVHIAGIADHDHERGAAFARHYGLDYFASYEALLGQ
KLDGVIITSENAFHRELALKAAERSVHILCEKPLATKVKDAEDIVRAAREAGVLLMTAFP
MRFSVPVRTVREELLAGRLGTPLCFNASNQGQLPPGDRAWFTDPVLAGGGAIADHVVHLA
DIMRWYLGTEVVEVYAAANRIFHADEVQVETGGMVSIRFENGVFATIDCSWSRPVSWPSW
GGLSFDLITDKGAVRVDAFKQNLTLYPAPKASKVSAGASAVPAGGTGTAPVAAADHDWTT
AHSHGDGGLGPRWLSWGSDANQAMIEEFIAAIREGSAPAVTGADGLAAVRIIAAAYKSIQ
EGKAVRV*
>SPSA8_v1_450010|ID:41146003| putative dehydrogenase [Spirochaetes Bin 1 SA-8]
MKIGIIGTGFMGSTHASAWKKTGADLVGFLADPPSEARQIAQHYEARAFSSLEELIERVD
VVDICSPTHLHSEMAIAAARAGRHIVCEKPLARTVEQGEAMLEACRMAGVRLFAAHVVRY
FPEYALAAGRVQAGDIGKVGTAQYRRLSYRPKKPVGNWFLDEEKSGGILLDLMIHDFDIA
RWIAGDVVSVHARNVTSYNKDSPADYGMVILVHASGALSHVAGAWAYPPPVFRTGFEICG
DGGIIQHDSDSESPIEALIRQETSEAPDVGLPASPLAESPYDLEIADFYRCLTEGGEPSV
SAKDGLAALKIACAAIESAKTGQAVELEPRGERGEKVPKAGPGLADEHIASKGGRR*
>SPSA8_v1_450011|ID:41146004| Carbohydrate ABC transporter membrane protein 2, CUT1 family [Spirochaetes Bin 1 SA-8]
MKQLASKASKDAMRNRFRGFLKHVILILFSMIALFPILLILMNSFKLKKYIFAAPFALPN
AKTFSLIGYNTVFQRSNLIVNFGNSIIVTVVSLFFILLFGSMAAFALSGYKFKGNAFLGF
YLSIGIMIPIRLGTVSILKLMASLGLVNSLIGLILIYIAQGLPLTIFILTSFMSQIPDEL
KDAGRIDGASEYRIYSLILPLVRPALGAIGIFTMIPIWNDLWFPLIVAPSAKTATITLGV
QQFLGQYVSDWNAVLSALVLAMVPVLLLYLLFSRQMIRSVTAGAIKT*
>SPSA8_v1_450012|ID:41146005| Sugar ABC transporter, permease protein [Spirochaetes Bin 1 SA-8]
VLDIRGSRKFPFFLVVFLGPAAICYTLFMIYPLVESLILSFFSVQPDNSSVFAGFANFVK
LFTDSDTAPRFWGALKNNVLFFIIHMVVQNPIGLLLASLLVTKGKARGFYRTVLFMPTVL
SVVIIGFIWQLILNPLWGVAESMMRAVGLGKLFQPWLGLAEYALPAVALISVWQFIGIPM
ILFYTALIGIPDELIEAAVVDGCTEAQAFWHIKFPLILPTIGIVSILTFVGNFNAFDLIY
TMKGAFAGPAYSTDIMGTLFYRTFFGQQLQLGNPTMGATVATMMFLIILTGVLIYTFLFQ
RRIKTYQL*
>SPSA8_v1_450013|ID:41146006| Extracellular solute-binding protein [Spirochaetes Bin 1 SA-8]
MKRLLLAVMITLVALSAFAQQKVTLTIESWRNDDIQIWQDQIIPAFEKKYPSIHVVFAPT
PPAEYNGVLNSKLEGGTAGDLITARPFDASLALYNKGYLADLTNLPGMNNFSSVAKSAWI
TDDGKHVFAVPMASVIHGFFYNKDIFKKLNLKVPQTKAEFMQVLEAIKKDGRYIPLAMGT
ADMWEAATMGFQNIGPNNWKGEEGRQALLAGKQKYTDEPYIKTWKELAAWAPYLPKGYQS
VKYPDAQQLFTLGKAAIYPTGSWEIWLFEKDAAFELGIFPPPVEKKGDTVYISDHTDIAL
GMNAKTKYPEEAKKFLEWMTTPEFASLYSNALPGFFTLSNHKITLKDPLANEFLSWRTTC
KSTIRNSYQILSRGDQYGAPNLENELWQVSASVINGTMTPEEAGKRVQAGLDKWYKPKK*
>SPSA8_v1_450014|ID:41146007| protein of unknown function [Spirochaetes Bin 1 SA-8]
LLEVEKMGKKTLLLMFLILLVGAGCSLVTEVPSGSIKVGIDLSKPRSLGYSINKITLELK
HEKSGETIEREFSIVNTSANFQIRGLRVGQWQIKVKLYEDTQEVGAGMTTVSIEKDAIAS
ASITITLYTGSLNLDVDWENQGDPPEGFTLVNPSTFTMGRTKWSGQYGIAYDNELPTHLV
ELTKPYIIAKYEVSYAKWKEVYDWAIVHGYDFSNAGWKGGQSFLETQSPLTDDTHPVTHI
NWYDAVKXX
>SPSA8_v1_460001|ID:41146008| protein of unknown function [Spirochaetes Bin 1 SA-8]
LKYKTGAGTNTDYLDAQTDLMRTEADYYRALFDEEIARASLRKATGETAY*
>SPSA8_v1_460002|ID:41146009| Periplasmic component of efflux system [Spirochaetes Bin 1 SA-8]
MKKKRLIIVGIIIVLIIAVLLINHFRTKKDNSVLKLSGNIEVTETNVGFKLPGRVVELAV
DEGQQVKDGQRIARLDNAELTSVVMQYKASLQEAMTRLAELRAGSRAQEIERAQANVNAL
AADLDKAKKDYERADTLYKNGAISASQFEAAQNAYNTRAAQHKSAQETLSLIKEGPRKED

IQAAEYRVQQAKAMLNTSEERLKDTTLFSPISGVVLRKNVELGEIVSSGIPVFTIGDLEG
PWVKVYVKEDRLGQVKLGQKADVSVDSFKNKTYEGTVTFISSEAEFTPKNVQTEEERVKL
VFGVKIKVKNENGELKPGMPADVRITLK*
>SPSA8_v1_460003|ID:41146010|ybhF| fragment of putative transporter fused subunits of ABC superfamily: ATP-binding components (part 1) [Spirochaetes Bin 1 SA-8]
MNSEIAIKTQNLTKAFSGNVAVDNLNLEVKKGELFGLVGPDGAGKTTIMRLLAAIMDPTS
GNAWVAGYSVLTDGESIKDEIGYMSQKFGLYEDLTVFENITFYADIYGVSQKERPRRIER
LLGFSNLAPFKDRLAGQLSGGMKQKLGLACALIHTPKVLLLDEPTNGVDPVSRRDFWKIL
YDLLKEDITVFVSTAYLDEAERCTKIGLIHKGAILKVDEPSAIRESLEMSMIEVSSPHAR
VAREIISTVDGVNSISTYGERLHIGIKERPIADKVLAALTVNNVEVEGYREIVPSLEDVF
MELVKEEEGKRP*
>SPSA8_v1_460004|ID:41146011|ybhF| fragment of putative transporter fused subunits of ABC superfamily: ATP-binding components (part 2) [Spirochaetes Bin 1 SA-8]
MNENNTAVQVTDLRRTFGDFVAVDNISLSVTKGEIFGFLGPNGAGKSTTIRILCGLLMPT
GGTGTVGGFDINKESELIKQVIGYMSQKFSLYDSLTVEENIKFFSGVYNVPKVKKKERME
WVLEMAGLMEKRNTLTRNLATGFKQRLALGCAILHEPRIVFLDEPTSGVDPISRRKFWDL
IGEMSQAGTTIFVTTHYMDEADYCDRLALMYRGKIIAEGTPNELRKKYMSRDVLEVNVDK
VVEAFNVLSLHRIEAAIFGSTLHVMVDDRETSRAHISSVLGDSGITVKGIEKIPPSLEDV
FVTLIEVS*
>SPSA8_v1_460005|ID:41146012|ybhS| ABC transporter, membrane protein [Spirochaetes Bin 1 SA-8]
MKLLRVKAIMKKEFIQIWRDPLSLAMAFIMPVILLFIYGYAITFDIDKIPTVVFDMDKSS
LSREFVNQFHESGYFRVNSYVDNYKAIDPYLDAGRARVAVVIPEDFSKNVHKGCNVKIEV
LIDGGDSNTATIAQGYAIAIAEGFSQRFRGSRVTPFIDSRSRVWYNNELKSRNFIIPGLI
AIIMAVIIALLIALTIAREWDRGTMEQLISTPVKTPELIIGKLIPYFLIGFADTILTILM
STLLFNVPLRGSVILLLGLSGIFLFGGLSFGMLMSIVGKTQLAASQAALLTSFLPAFLLS
GFIFSISNMPKPLQIVTYFIPARYFVTILKGIFLKGSTLKILWLEALLLTLFAVLVFIAA
NRKFRKRIE*
>SPSA8_v1_460006|ID:41146013|ybhR| ABC transporter, membrane protein [Spirochaetes Bin 1 SA-8]
VFERIKNLVIKEFIYYFREKRTIFFLLATPLIQIILFGYVATMDVNNISTALYDLDRSSE
SRELVRRLESSGYFTITRMPASPEEIRDLIDKGDVLCAIQINRGFGKDIQRGTPTEVQVI
VDGTDSNTALIAMSYINTIIGKYSMDMTGGVVNANIWRLDLRNRVWYNPDLKSRNYMVPG
VIALIVMLTCLLLTSMSVVREREVGTMEQLMVTPIRPLELILGKTIPAAIIGYFDMALVT
FFGVFWFSVPIHGAVMLLVLCTGAYLLSVLGFGLLISTISKTQQQAMTATFFFFQPVILL
SGFATPIETMPEVFQYITYLSPLRYFLVIVRGIFLKGVGIDILWPQILALLALGTTILTA
SVFRFKKRLA*
>SPSA8_v1_460007|ID:41146014| putative heat shock protein related to DnaJ [Spirochaetes Bin 1 SA-8]
MLVNQLDEVALKNVRIACATLFSEELAHDDSFIENLSMDTVEKAYWQKAKRCYHHLTPLS
SRSEAQEALNDVRNSLETLASFLQSGKQAVDEAPRKGRIIAIGGAKGGIGKSIVVANLSV
LLASKGFKVTAVDLDLGGANLHLCLGNKVLLQHNINDFLRKRVDSLQDIAIKSEYGPYLI
GGDSAQLGAANIDFGRKLKLLKAVENLNSDFIILDLGGDTSYNIIDFFLKADYGIVLTTR
DSASYIGAYHFLKAAMYRRFNRLFGPESKFRMYKNHQLENLIHDILNPASGEWPKSIHDL
LERVRQEQPDHLPFMNEVVENFRSYLIVNKVPNRLLGGFDVNPIITRIQQVTKTWLAKEV
TYLGSISFQPEVENSMIDLVPVVVKYPKGKMAEELSAIVTRLFDQEM*
>SPSA8_v1_460008|ID:41146015| protein of unknown function [Spirochaetes Bin 1 SA-8]
MAVSTLRARALTKIRIEKIKNFTKYLNGIEYVYLSAS*
>SPSA8_v1_460009|ID:41146016|pcm| Protein-L-isoaspartate O-methyltransferase 3 [Spirochaetes Bin 1 SA-8]
VKFLIFSILIFVSALALSVDTAMSADAFEKRRQEMVEKDIKTRGIKDERVLSAMLKVQRH
LFVDEKQRSKAYNDHPLPIGEGQTISQPFVVALMTEAVGLKGTERVLEIGTGSGYQAAIL
SEIVKEVFSIEIKQGLYEMAKERLQKLGYRNITVKHGDGYYGWEANAPFDVIMVTASANH
IPPPLLAQLKEGGRLIIPLGSTVFYQNLTLVTKEKGKHTIKELGAVAFVPMTGQAQKK*
>SPSA8_v1_460010|ID:41146017| conserved membrane protein of unknown function [Spirochaetes Bin 1 SA-8]
MRPPYLLAFFCSLITLSFEILLIRIFSVRLSYHYASLIISLSMAGLVIGGVIVFFQHATS
RPPGFSSHQRLQRFAAALAVSCPAVFMLLSLIPLDHVRMLWENVQVVYLVVFIFLCTIPF
FLYGVFISSALSAWRDTANRVYATDLVGGATGILLVVMLMDALKMEYILVIVTAVTGWII

LSALRKTPSKIVMTAILFGLCLCIVLDASSLKMSPYKGLSQALKDDGAKHVATIYSSHSR
LDLFENPQMKFAPGLSLTFTEPVPKGLGMALDGEIAGVVMERKEQAIYKFLSYIPSALPY
LIHTPDNVVIIGARNSIDLLLPKYYGVVRASIAEHDASVVKVFSCRHGSIIHPFPVFYGS
GRTLLKKLDNNLGLIFLSKTGFFPSGSFGLQEDYDLTVEAIGTYMQRLNNNGILFIQMFL
VPPPRSEVRLAKNIQTALEKIGIHELANHLLIYRSWDTINFLIKKDGLSETEFRNVRQFL
ANRQFDVLYPDIAEQEQFIAGLDYKHLFYRILTDRASSEFSSSYAFEIRETTDDRPFFHY
FLKTSKLIDIYKLSGRKWAYFLHEGMSLPFILVCTILFTICIFVIVLILTKKKLKFSLKS
QKSPSLSCSLIYFALIGFAFMFIEVFFIHRLILPFGSPVKAFSVTLVTILVSAGMGSIGT
GWFTGKKTMVTISLAPLLIIANYFFFDLADESVLSAIFIIPIGIVLGFFFPVGLRFLVTN
KTGQVPLAYTANGAASIIAPSLVSLVAVSYGCKVLLLLAAFLYILAIAIILPVIFRVLTA
SKYS*
>SPSA8_v1_460011|ID:41146018| UBA/THIF-type NAD/FAD binding protein [Spirochaetes Bin 1 SA-8]
MKKLIEFLKTNAQGDLLPWEIHKRAMNEFGLTCRDVEEAALALEILPARYQRNRKTITTL
LQRKLFQSKVAVIGCGGLGGYIIEELARLGVGTIKAIDPDVFEEHNLNRQILCKVSDLGK
PKAKIAKARVKSVNPAVKIISVVKPFSRENGLGLLKGMDVVVDALDSVPTRIDLAECCAA
LDIPLVHGTIGGWYGQITTQLPGDSTVQMIYRNCKDSKGVERMLGNPSFTPAVIASLEVA
EVCKLLTNQGTQLRKRMLFINMLDMEVDQIEIGETTSC*
>SPSA8_v1_460012|ID:41146019| ThiamineS protein [Spirochaetes Bin 1 SA-8]
MVFLIRNDVMRITIKLFATLRQGRFDIDTLDLPPGTKIIDVVQRLGIPEKEVTLIFVNGL
HRNLISELHDGDTLAMFPPVGGG*
>SPSA8_v1_460013|ID:41146020| conserved protein of unknown function [Spirochaetes Bin 1 SA-8]
MPEFCNPFAGMRSDRKLSKDELVRAIRFMIAAEYEAVQLYQQTAESTDNRLAKEVLLDIA
NEEKEHAGEFLRLLRELDPEEEEFYREGSKEVEEMIKKVKK*
>SPSA8_v1_460014|ID:41146021|yjgR| putative ATPase [Spirochaetes Bin 1 SA-8]
MNQILIGKGEKPVYLLAKYGNRHGLIAGATGTGKTISLMVLAEGFSRMGVPVFMADVKGD
VAGLALAGVTNEKIQQRVNQIGIRDYINEANPVLFWDLYGKNGHPVRTTISEMGPSLLSR
VLEINDTQAGTIEVAFKLADDEGLLLLDLDDLKALLSFVAENRKEISTQYGLVSTQSIAA
IQRALLTLERAGGEALFGEPALELNDLMRTDLSGRGIISILSADQLILKPKLYSSFLLWL
LSELFENLPEVGDLEKPKLVFFFDEAHLLFDDAPPALRQRVEQVVRIIRSKGVGVYFCSQ
FPDDVPNDILGQLGNRIQHALRAYTPRDQKAVQTAAETFVANPTLDVAEVISQLAVGEAL
VSTLQDKGVPMPVERTIICPPRCRMGAVTPEERSIVRARSPIGGKYDSPVNRESAYEILS
QRTKEQEPNQPAPRQKGAEKPSEGGGGLGDLLWGSKHREGMVQALAKQAARTVGSQIGRQ
IIRGVLGGILGGFGRK*
>SPSA8_v1_460015|ID:41146022| putative Fructose-1-phosphate phosphatase YqaB [Spirochaetes Bin 1 SA-8]
LISTLIFDLDGLLADTEKLHCRAYQDALARHGFLLTEQDYAEHWILNGGSIREFIEARGL
SIDPDMVRRAKAKRYGELVRSGVEPMPGALSLLSKVSGWKRLALATSSYEDDAHAVLQAL
GIVSYFSCIATRSNVAKVKPYPDLFLSVAHRLGETPNNCLVFEDSEKGVKAAGAAGMKCI
AIPNIHTAKHDFGMATMVVSSLYDITKDLIDNM*
>SPSA8_v1_460016|ID:41146023| protein of unknown function [Spirochaetes Bin 1 SA-8]
MPLPLFALRETYMKDQHTPDMTADSVLHICLTGDFTLAKEFYVVLQAGFSIECQIGTSIE
HLFYDQLKFNHHLVEKRIHTIFLNGKPVDDIATSMVYDGSTLALSSAMPGLVGATLRRKS
PLASFRQSISTLQESRDTEKTSGYIKIKLFNILLEELGPFFLKMGIFIQRTEFAAFTEQL
SDSFYDRITQVLMNEKPVQLKSSAPWPSLLERYDCIHVKVLSE*
>SPSA8_v1_470001|ID:41146024| conserved protein of unknown function [Spirochaetes Bin 1 SA-8]
MKIFIVGTGRMGAWLTEELCWNHEVWVHDKDPLRMKYFIKVHRILDLNELDAVKPDLFIN
CVALGNTVEAYREVLPHLPESCIIADIASVKNGLADYYKALNRPFVSSHPMFGPTLANIR
DLHDESAVIISESCEAGKRFFREFYQRLGIKIFDYSFEEHDRMVAYSLSTPFASSMVFAA
CMKKQEAPGTNFKRHMAIAKGLLAEDDRLLTEIMFNQYTIRQLELINSQLAYLTHIIRQR
DYEEMKKFLDTLRSNIAD*
>SPSA8_v1_470002|ID:41146025| Prephenate dehydratase [Spirochaetes Bin 1 SA-8]
MTLDDLRQDIDKIDARILALLNERMEKAILTKKFKTGIEDSSREKAIFEKVERSSHCLVE
PKFAVDIYSFIISENKRLQKMNFKTIGFQGEHGAYSEVAARLLYPSFATMPCHEFQDVFE
NVEAGNFDFGIVPVENTLGGLVGPVNSILIYTNLKIVAAIDMPVRHCLVCPPGTDHRELR
TVWSHSQALAQCRNFLTRNHLDPVSFYDTAGAAKAIAETRPKGVAAIASRFAADLYGLDI

IKEDIQDAEHNRTRFFVLSTGALQNKGSKCSAVFTAGDKAGSLFSILRVFADAAINLTRI
ESVPDKPGKYAIFIDFEGSLEEERVAAAIAAVSREAQDFRILGCYDETRVE*
>SPSA8_v1_470003|ID:41146026|aroC| Chorismate synthase [Spirochaetes Bin 1 SA-8]
MNRFGKNFGVELFGESHGKQVGVVIDGCPPGIPLAENDLEPDLARRRAGLDGTTPRREHD
IPSILSGVYQGYTTGSPILIAFPNNDTRSVDYRQFTSIPRPGHADFCALKKYAGFADPRG
SGHFSGRITVGLVVAGTIAKKILSFASFETRILEAGGSPDINRVVSQAQDSGDSVGALVQ
VTVRRIPAGLGEPFFDACESVISHAVFAVPGIRGIEFGDGFAAAKMYGHEHNDPILDASG
RTGKNGAGGINGGISNSNPIVFRAAVKPTSSIAMPQETFDFSSQNLTRLEIEGRHDACIA
LRSAVVIESAAAIALADLYLTARAVSVLA*
>SPSA8_v1_470004|ID:41146027| putative 3-dehydroquinate synthase [Spirochaetes Bin 1 SA-8]
MQKKNAXLNEEHDNPGSEIEFCSLESLKTRLSKQTTAIIADSRVYALYGKFFKDFPLIQV
PAGEPAKTYSELLKLYTALAEMEADRSWTLLALGGGSTSDLAGFAAHTWMRGIRLLVAPT
TLLAMTDASLGGKNGIDLDGYKNLVGSFHVPEKIYCDIDTLYSLDAGQFSSGMAEVIKHA
ILQGESTFAFLESLAEKSRGPFPANFPASIDRADMLRMVRESQAVKLNIVARDPKEKNER
RLLNLGHTFGHAIEKMTGALHGHSIAVGLAAACEYAVRSALMDADTAERIKKLLTAFSLP
TSLETLGYQGGRKVLSDAIRLDKKRGGSRMNFVVPRKIGLVEIHSVDIEILTNFFEKEVS
*
>SPSA8_v1_470005|ID:41146028|aroF| 3-deoxy-7-phosphoheptulonate synthase [Spirochaetes Bin 1 SA-8]
MKNLKLAAARPDLLRSIIDVNGVKVGQEFVVIAGPCSVETEKQTVETAIAVKKAGAKMLR
GGAFKPRTSPYAFQGLGLKGLKILDKARRETGLPIVTEVVDTRDVSWVGEYADVLQIGAR
NMQNFSLLREVGKSGKPILLKRGMYSTLEEWLNCAEYILAEGNPNVILCERGIRTFETYT
RNTLDLSMVPAVHGESHLPIFIDPSHGTGILSMIEPMSLAALAAGADGLEIEVHIDPPSA
LSDKDQQLTVPQFELLMKKLAQAREFFGQLHAGFTGETERPGAEQRTDAQSDLPTAFADA
VD*
>SPSA8_v1_470006|ID:41146029| conserved protein of unknown function [Spirochaetes Bin 1 SA-8]
MMLLSAGKTFPKNALIQSNKDDNQLKSPGQPSCHLFFDKIMRYNPSMYFNELVEAYNKQR
HDRNIYHDLMRFRVREVLLVGSLYDSFVVESDGVLTEQIYGEYFKLNLNTIPRVVCAYTE
ETALDLFRSGKFDLVILMAGLDFDIPLAIAKAMKHIWPDIPILLMATNNSSLEYLDFDSP
EMASIDRVFVWNGYSKLFVGMIKYIEDVRNVHADTQTGQVRVILLIEDSVRYYSRYLPVL
YKVVLKQTQNLIEEERITETYKLLSIRARPKILLASSYEEAEKLFETYQNNLLTVITDLR
FPHKGKCDEEAGFDFIKMAKGKIHDLPILIQSSEPHIRSKAYEIGASFVDKNSESLELEL
ASFLQMNLGFGDFRFCMPDGKEIGRARNMREFIAKMHELPAESLVYHAEHNHFSAWLMAR
GEIQMSKVLKPYQIGDFPDIHAMRDFILKILDSVRKEKSKGIVPNFDESMLHEENYISRL
VDGSVGGKGRGIMFIHSLLENLDFSRYIPEIEIKIPRTLFIGIDEFENFLESHNLWSEAF
YKPYSNQLRQTFLSTPLSPTLIARLRAFLARTNRPLAVRSSGLFEDMLMVPFSGIYDTYI
IPNSHPDLEVRLNQVCDAIKLIYASLFSVESRSYFEAAHYNLEEERMAVLIQELVGSRHG
DYYFPHVSGIAQSYNYYPVAYVKPEDGLCVSALGLGTYVVGGGVAHRFCPKYPKLDVLSP
DHALESTQRTFHALDMSISTPDLSAGEMASLKELDVSAANDDRHFAMIASTWDAEDRRFV
PGASGRGPKIIDLANILKYEALPFAQAISMILEIGSRSMGTPVEIEYAFNLSEPSGKPAL
YILQLKPLIQADDKIELNLGALAPEDCFIVSDKTMGNGRDLSVQDIVWVNPEKFDKAKTL
EMTAEIEEIDRELKKAGRRYVLIGPGRWGTRDRWLGIPVSFAQISRAKVIVEVDLDNFVV
DSSLGSHFFHNLTSMNIGYIKIPLRSKEAFIDWDWLKSQRRAFETDHCVWSTLKAPLDIL
MDGKQGKAGLLKAGNRERPSEENDEIEMENAEYN*
>SPSA8_v1_470007|ID:41146030|pheS| Phenylalanine--tRNA ligase alpha subunit [Spirochaetes Bin 1 SA-8]
MDAISIAKTLHPLEVKIILTYDTSAIFDARKVREELGYREGQENQAFSWLSGKGLITEVS
RAQTGFFEITALGRDLAEKGLVEERILALLAAEGAKTLSAITEKLGLEQKHIGSAFGLLS
KEGIVALDDTKQACLVSEARLPQKIKILKSLLEKALGKNGILEENELSAEEKAVMSTVAK
KRGAQDAPFRSADREQVFYKLSPEADEVRRALRALGVTGEEIGALTPEILIKGTWKNAAF
RPYNIQITPARIIPGRKNPYVEFLESVKDKLVSLGFEEFDGNMVETEFWNSDALFMPQFH
SARDIHDVYYIEEPRKAKFIEEPYLSKVAAAHEHGGDTGSRGWNYSFDREFTKRLILRSQ
GTVLSARKLPTAKVPGKYFGIARCFRYDKVDATHLSDFYQTEGIVLGEDVNLRTLLGFLE
MFAVEVAGAKEVKYVPGYFPFTEPSVEVHIKHPVLGWFELGGSGIFRPEVTKSLGINVPV
LAWGIGIDRMALMALGLNDLRELFSPDLENIRLRRALLAN*
>SPSA8_v1_470008|ID:41146031|pheT| Phenylalanine--tRNA ligase beta subunit [Spirochaetes Bin 1 SA-8]

MPKIEVNEKLFFDLLGERYTSEQLEEIFPTAKAELDGWDTDGVSAEDRTIKIELNDTNRP
DLWSTAGLARQLKTHRLGKIAEYPFFVPLGQQPSVRYTVEVHESVQKVRPYLAGFVARGV
AITDAMLKDFIQTQEKLTWNFGRKRKTVSIGLYRPAIITWPVHYKGADPETTSFVPLQET
RTMTLRQILAEHPKGKEYSFILANEPIHPLLTDNQGRVLSYPPIINSADLGAVLVGDSDL
FIEVTGSDYPSVALTASIMACDLHDMGFTIEPVRVVYPYDTPFGTSVVFPSYFQETVAVE
LSEAEKLLGMSMSGEKALDALRHMGVSARIEGSKITVTPPCYRNDFLHPVDVIEDILIGI
GMQHFSPQRPKDFTIGRLSPIETLSRKVKLSMVGMGFQEMIYNYLGSGKDYIEKMGIDPA
AVVKISNPMSENFEYVRNSPLPSLLGTESVSAKALYPHRTFEIGKVAIKDDEANYGVLTR
QYLGFLNSHSSADYNESASQVAAILYYLGAAYEVRESLDPRFIPGRQAEIVSQGRKIGVF
GELHPAVLEAFDINVPCAGGEIDLDYFI*
>SPSA8_v1_470009|ID:41146032| conserved protein of unknown function [Spirochaetes Bin 1 SA-8]
MEIYAFEPFGFSGFLVRVEVDIRRGIPATDLVGLAAGAVRESRERVRAAIRNSGFEFPQD
RILINLSPADIPKEGSSYDLPIALKILALSGKVPECGNRILVLGELNLDGSVLPVRGILP
ALTGAASQGIRRCLVPAGNFTEASQQNLSEVYPINHLSLLPDILWMIQGNQSFTNMPKTE
QQRFLAWNQGYSQTKAGNEQESRVQVDYSDYLGNEPLLRALQIAAAGRHNALLFGPPGTG
KTMAALRFPSLLPDLSEQQAMEALSIRSLYGYSIESLPEMKRPPLRAPHHSASLEGIIGG
GRPVRPGEISLAHNGVLFLDETTEFRHDVLQALREPIEHGAVILTRAGRTLSYPSDFQLI
LAANPCPCGNLGNPQKTCVCSPLEIQRYWKKLGGPLMDRIDLRIPVAVPRIENIMNNTKA
NQAALRSSVLQAIERQKARNIAGSRYLPNAKLPAGVISKCCRLETRAEMLFSQKIEALGI
SVRSCHAILKIARTIADLDGKEVITEDCMQEALHFRQCGENEVYLP*
>SPSA8_v1_470010|ID:41146033| protein of unknown function [Spirochaetes Bin 1 SA-8]
MLSYKPSFRALDELIKLAPRLSSALSDFFKISQILIKLSASK*
>SPSA8_v1_470012|ID:41146035| conserved protein of unknown function [Spirochaetes Bin 1 SA-8]
VHRLGSMLARVKLKGIDGGPHKRWSMWFNSMVXX
>SPSA8_v1_480001|ID:41146036| protein of unknown function [Spirochaetes Bin 1 SA-8]
MTAAGIKKARWQGGQKAWGNGTYTLSIDKKPDNVKLPDWILKRLAVIAARQKDFAEYEHS
ALVVLAAECKAYPETASRLPARFLTAIAHCKPGILDPIERAAVLLXX
>SPSA8_v1_480002|ID:41146037| conserved protein of unknown function [Spirochaetes Bin 1 SA-8]
MSNATFISSDEAARLLGISKGYLYKLTHERKLPCYKPRGGVLLFDTEELDRYIRAGRVKP
RQELAALAENYLNNGGRA*
>SPSA8_v1_480003|ID:41146038| protein of unknown function [Spirochaetes Bin 1 SA-8]
VRVKIKLCGAKERTEKLIERAQKKQQANPCGTAAKALADLKLASTNLEVLPIRGDFGALV
ENITRGDLRFLYKERYLSHAELAKLMGIPKAKIERWLKPGKSNDCILPYKRLDFYDVEAR
APQRGERRKKLIAHALASMGKNK*
>SPSA8_v1_480004|ID:41146039| putative Site-specific recombinase, phage integrase family [Spirochaetes Bin 1 SA-8]
MGVKIRLRGKSKYFYLDICINGKRRFEALHFALPTDRQGQREAWALAEEIRRKRELQIAA
GRYRLVDPVGSKMTLIEYAEKIAAEYDKKMHLPKSLRYLRPYAGDTALVDIDESFVDGYR
AFLLKSSELGGKTAGHYLAAFKAVLARAERERLIERNPAKGVKSIRAPEYEKPYLTIEEI
QRLYNTPAEGKLSEEVRRGFLLSCFTGLRLGDIKSLAWGDIRREPEPMIKKRQNKTGDYV
SIPLAPIAWELIDDKRLHKPDELVFPRLTATEGVNPHQPLIALRKRAGIEKPFGWHAGRH
SFAMMTLEASGDIYAVSRLLGHSDIKITEVYLRMTDQRKKEIIASLPQVTKEGEVKIKLA
GEKK*
>SPSA8_v1_480005|ID:41146040| protein of unknown function [Spirochaetes Bin 1 SA-8]
MTADTISVLIFLVGDKSFCLEIDVIDHVISACAISPVPDTKSSIEGIINVRGRIMPVVNM
GSKCFSKPIPLALSDKFIIITIEKRYLALHVTDTLEILTLKKTAITQPDTILPGLSVISG
IIQYAQELSLLYNPRKFFRNEFMEASEEEPQEANHGTALEIH*
>SPSA8_v1_480006|ID:41146041| putative Chemotaxis protein methyltransferase [Spirochaetes Bin 1 SA-8]
MFSDQPAGQCNAENQEDLTSLIPRLTETIESALSIKKNSLHHYTVRKAVQALCKTLKISL
DEAISILTSPPPYGTQIVNILAEHITIGETYFFRHSEQFEFLEKIWLPSLVSERIRTGEK
KIALWSAGCSTGEEPYSLAILLNKVLPDFASWDITILATDINKNSLAFAQQAVYSEWSFR
GVSPAVIEKYFVVQKSPLLERTGFLSGSRYKLKDSIRDMVSFRQFDLAASPWAMTPDFPA
TFDIVFCRNVLMYFTRDTARQIIGRFHTMLREDGLLAVSPSEGWFARGAGFSLYPETSLS
IFKKKPINGTRLPPIGDSTSQNKTVIPAASQMPPKTSIAKSVKKAPSAGKPGPVSSTAVT

SNIKEKAQALADTGKLSEALALIEAAISXDTTHAXSHYLKALILMNLNRLPEAESSXKKA
IFLDAEMVVAYLALASIAQVKXNHDVMIRHYRTAIKLLAKLDENQIVPYSDGMPAKALAT
MISKVTEQHEHEEKS*
>SPSA8_v1_480007|ID:41146042| protein of unknown function [Spirochaetes Bin 1 SA-8]
MKDNPDTTTINAILRKRAEQLSRAEKIQEEKPSYTMLKFLISGLEYGLEIPFIREVIFNS
SGYTPIPGVPDYILGIKDIRGEIIPIVDILRILGIGQNDAEHRDFCIVAESEDIVFGIMA
DSIKNMDRIEKESVNADAASVSENVARFCYGLARDGSLLLNGNLLFTDTLIRGIER*
>SPSA8_v1_480008|ID:41146043| putative Methyl-accepting chemotaxis sensory transducer [Spirochaetes Bin 1 SA-8]
MFSNMKIRIRMILGFGLVLLLILAMGIFAYYQLNTMAGLTKQLYARSLPVSVAVNKLKTS
FTSIELLMSQAIGSRTKVNTQELNKKLSDEYFALELQLDVLREKYLGDQKEIAEIAASLE
KLKLVFRSISEILSTNLSSTADRIFVEQAIPIIEQTSKALDAIEVQVKSRASNFYSDTET
ARKSIITLLVIISASVILLSILIAFLLTESILRPLGDILTIIHKARNGDLTVDTEAKLRK
DEMGKLSSAFIELISRFRIQIQELVAAISQLATSSSEISATASQLSASASETAASISETA
ATVEEVKQTAQLSMDRSKDIYEVTQQSFTASKAGLDSLSSLMNKIHEIKNQIDRIAQSIL
NLNEQSQAISDIISAVDDIAEQSNLLAVNASIEAAKAGEQGKGFAVVAQEIKALADQSRQ
STKQVKKILGDIQKATDVAIMASEMGTKAAENGVSSSLQVESXLKXIINGXEEXTHMTQQ
ISAASQQQYSGIDQVTLAMENINXASKQNAEGSKNLEQSAHDIMQLSTRLKALIERYKI*
>SPSA8_v1_480009|ID:41146044| conserved protein of unknown function [Spirochaetes Bin 1 SA-8]
MQDQEEFLKKLREAFNLEAAEHLAVIASSILALEGMTPPYPKDLVETAYRAAHSLKGASR
AVDLRDIETICHAIEGVFSKISKGELAPDKSLLELLLAANDSMEGILKGNTILTAAQIGA
LARRIDEAAIPKPKIAVMKLSEIAAQTASPPAPPATPQEQRPLAAESKPETDHPHSVQKS
SFANTLRIPAEKLELVLRDAENIISAKLALRQIMEYLSSLSSECEELLSSKEIETTKLRQ
MVSETSKTLKKISKYAATEEYRVSTTIDKLIDDARTMLMLPCSLLLEGFPRMVHDLAQSM
GKKIEIAIEGKNIELEKHILEKLKDPLTHLVRNSIDHGIEIPADRVKAGKPETALIKIKI
ANLDSKHIEISVSDDGKGLDYDNIKSAAIAKGLITKEEAGNIDNERLGMLIFESGFSTSP
IVTDLSGRGLGMSIVQQAVDELDGFIKTESNPGEGTTFRLVVPFSRATFRATLIKTGDRK
FALPTSSIIKTMRMKVDSIFLAGGREMMYFDSRPIGILRANRLLNLPQSTMESTNRLASI
IITQSGESIAALMVDEILYEQELLLKPLGQLLKNIALISGASIAGDGEIIPVLNVKELMR
SVTARELENALIKNGALPAKERKTRKKKSVLVVEDSITSRMLLKTILESEGYAVTVAVDG
QDGYTALKSNPVDCVITDIEMPRMNGFELTAKIRADKKLGDLPVILVTSLESREDKERGV
EVGASAYLQKSNFSQTTLLDTIKRFI*
>SPSA8_v1_480010|ID:41146045|cheB| Chemotaxis response regulator protein-glutamate methylesterase 4 [Spirochaetes Bin 1 SA-8]
MVRVLIVEDSYVSRELLKYIFIKDGGIEVIGMVGRGEDAIALLESMKPDKPDVITMDIEM
PGLDGYTTTRRIMETRPVPIVIISVGFQYPSAEKTFKAMQAGAVAAVGKPPAPTHPEFPA
KTRELIGLVKAMAKVPVVRRYANIPKPQTALTLDTAAIKADKNYKLVVIGASTGGPPVLQ
TILSRLPKPFPLPIVIVQHIASGFASSLAEWLNNTCGLKVVLAEDNTIATPGVAYIAPHG
SHTTISAGGVFNLQKPAENEILVPAVNPLFKSAAAALGSSCIGILLTGMGKDGAEGLLDL
KNAGALTIIQDKITSIVYGMPREAEKLGAAQLILPPEEIASVLEEIGKKAAMGDK*
>SPSA8_v1_480011|ID:41146046| Response regulator receiver modulated diguanylate cyclase [Spirochaetes Bin 1 SA-8]
MDNNHILIVEDSLTQAMRLRYILEKEDLMVDVATNGREALDILRNIPINIVISDVMMPEM
NGYELCKAMRADPKLSSIPVMLVTTLSDPTDVIRALEAGADNFLIKPYDESTLISRIKYI
IANMEIRKTQGAEIGLEVYFNGRKYFLNSSRIQMIDLLLSTYESAIQKNEELNTTNQRLE
EALDNIITLQKNYLQLLETNVDAIVVYDNKNLVRYANPSANAIFVSKKDSLLNKKVPFEL
DTSSDSHEIEVTDPDGQVLTLDGRTMTTDWDGEMMTLAVFRDMTEATRLRKELEQMSLED
DLTGLYNRRGFNLLADRMVRQSKRFNMQLFVLFADLDGLKHINDTLGHPEGDLAISTVAS
IMKKTFRETDILARMGGDEFAIMGMINELFVPDKLIKRFNQLVEEWNEKEKRQFKLSVSM
GIETIDPSAAEPIETLLKRADEKMYANKLARKANRI*
>SPSA8_v1_480012|ID:41146047| TldD/PmbA family protein [Spirochaetes Bin 1 SA-8]
MPESHHSAFLRAARPLLKEVVGILSRQFDYISVLCTDDNGISYAATPGETRTAEPMWVQR
GFVFRAQKQGKVAEYAVSNLEVADGAQQLAEGIAMALHQLLASPGLIAYPAIPDQAMTRE
FSLQPAEDPFLAEPEQVLSRLTSLRETLTDRKVIVMAQSRYEYVRVSRMFLSPHRDLIQT

FPWSQAYLFGISRRDQLSKMSYRSASGRKGLELLQDLETTVPDLTQELHDILDATPIEPG
EYEVILDPDMAGTLAHEAFGHGVETDMFFKKRAKATEYLGKRVGSELVTMYDGAADAEQT
GSFLFDDEGTLAHKTKIIDHGILLGGISDMLSSLALNIPQTGNGRREAFSHKAYARMTNT
YFEPGTATLEEMVASVKHGWQLSRLNAGMEDPRNWGIQLLCMVGREIVDGAYTGRVASPI
VCSGYVPDVLSAISMVSKDFTLSGSGACGKGKKEYVKVSSGGPYVKTRMRLG*
>SPSA8_v1_480013|ID:41146048| conserved protein of unknown function [Spirochaetes Bin 1 SA-8]
MMNMIDTILEGVKASKIAISDWRLVRKQGTRQEQYFVGSEAEQARLVQETRYSLTVYVDT
IDGDKKFRGEATISIQPTLTADEVSAKIAQAAYAASKSRNQWFELPGPAEPKVAIPVSAF
SVLPESQQLEEVRKALFQAVPESAASGPRINSLELFLTHEENFMLNSKGFSHRSEAWKGY
SEFVVDSASPSGPVELFDDIEFSEPDRERLAETIGARLAEVRDRAAAVPLPNLKDIPVIL
RGKEAEQFFAWFFHNARTEMVFSKASSFSVGMNVQKNTDTDSVQEPLTIWAEPFLPGLPA
STSFDSDGFPLERTLVVENGVLKTLIGSVRHADWLSVPRKGAFPLFSVEPGTMPLSEMHA
SPYLEPVMFSDFRLDPVTGSFGAEIRLAYYYDGIKRVPVTGGSISGAVPMLRNTMRCSTE
RGLATMSLCPIAVKLQGVSITGMTE*
>SPSA8_v1_480014|ID:41146049| protein of unknown function [Spirochaetes Bin 1 SA-8]
VDETAIARAITKVFDLTPHGIIETLQLKNPIYRATAAYGHFGRDNFSWEKTDKVEALIRA
IR*
>SPSA8_v1_490001|ID:41146050| ABC transporter related protein [Spirochaetes Bin 1 SA-8]
MSQYKIRNISKSFGEIHVLDDISQDFETASVTAVLGPSGCGKTTLFNILSGIVKADSGNI
GQFAAARFSYAFQDPRLLPWLSVEDNMRFALSAIQDEALKKSRIHQFLADSGLEKFRTRK
PAALSGGMKRRLSLARAFACPSDVLLLDESFSSVDLKLKIELMDLFSRLWEEEKRTTVFI
THNIKEALYLADTIILLSSRPARILKIFFLDKPRNRRLFNATEYHDLEDEILEMILH*
>SPSA8_v1_490002|ID:41146051| putative Multi-sensor hybrid histidine kinase [Spirochaetes Bin 1 SA-8]
MAVIGIVEDEALIALDLKKCLDRAGYGIAGPYSNAEAFLDGVRSGSMDLVLMDITIQGRI
DGIDALRMAKEECGLSGIFITALADDATLSKIKAASPLGILVKPFSERELLSTIDLALFR
DGMERKLKASEQRYRSLFSDSISSRCILDEQGFILEANNAFNLLFQGAEKDSFPIFLKKP
ESWAFIKERLQSQPLVRAIELEMMDKAGRPLAILGSFSKIDIVRQSEAGIACELVDMTES
KRLREDLYQAQKMEAMGQLAGGIAHDFNNILTAVVGHAEMLKIDIHQDNPSYEDVLGIIG
TVGRATQVTRQLLAFSRKQPFSPKKISLSSLVMDSQKLLKRLAGESILFSLYISDEELPV
YIDPVQLDQALINLVVNARDALEGKADGRISIVVGKRVLPERLMIRGKPLEAGAYAAIEV
TDNGSGIPPEAADRIFDPFFTTKTSGKGTGLGLAIVASITTLAKGAVDVQSLYGQGTTFT
LFFPLVRENENEGVDNSASDQDTSQNPSKTLPVFSGNLQILIVDDDENLLGFLARIVSKA
SATVFSARNAGEALLILETKKIDLLVSDIFLPGMDGIKLYERVRQKQNIAAVFMTGRLDH
QIEIPSGTVLLEKPFTPQELLQSIEHQCKIISSISSSRS*
>SPSA8_v1_490003|ID:41146052| protein of unknown function [Spirochaetes Bin 1 SA-8]
MTMKKRDTSTGILCLEVLALLLTLFSLMFSSCAHGMAVITKPYTPTEMNIQNLAALAADK
PLEFFQAFTAMAMEPEKAPLLEENQIAQTLSSQAAIKLAESYKQSKAEHDYKNALKYFYS
LKAIAGMTDKRLNTAASLASALITSSELFELLSAQVQSFRNAKQYAPAKSIMIRAFNLFQ
EHETEFNQSAKKNAIDLFQEWSEYANANSDKNTLALLSKTDMVGDFKDPASPFSKLVSSV
VTVYYVDRGIKFQQGAGRPDRVLGSAFQIDPAGYYLTNYHVISSEVDPAYNGYSKLSIRPS
DNPDERIPAKVIGWNKEMDLALLKSVVAAPFTLHLTEKAETEKGQQVFAIGSPIGLENTI
TSGIISAAGRRLLTRGDVIQIDAPVNPGNSGGPLVDRSGKVLGIVFAGMKDYQGINFALP
LSWINIYIPGLFSGGSLENPVLGVLLARNLDGSMDIVYTLPQNRMFKAGDRLIMVNGEKP
QDISSAQYKIAGIPAGSLVRVDIMRDGKNWQVLKIAESSKTIPLQDAARQDDVENFMAGC
LGIKLEHVSGPRGPGSLYKVLRTWPGLAADEAGIREGDTIKFIKMSIDQRELLAGFVISV
VSRSTGYLERSFQLTVSLESTSLL*
>SPSA8_v1_490004|ID:41146053|ftsH| protease, ATP-dependent zinc-metallo [Spirochaetes Bin 1 SA-8]
MFDNKNEKEPGKPDLPQQLPPRANKAAFAVFLSLAVLFGALFLFNDKSSSKEIPYSAFIS
YLELGEVDSVQIIDQKDIEGTLKGSSGVPVQFKTTIPYVDPELLSRMREKNIKISGTVRG
ASPLQIFFELTPWFFGFFLIWIMMRQMQGNNKAFNFGKSKAKRYIESDKKITFADVAGQK
EAKYELMEVVDYLKNPQKFAKMGAKIPKGVLLVGMPGTGKTLIAKATAGEADVPFFHMSG
SDFVEMFVGVGASRVRDLFEQGRKNAPCILFIDELDAVGRTRGAGYGGGHDEREQTLNQM
LVEMDGFDTKDGVIILAATNRPDVLDPALLRPGRFDRQVVVAMPDIQEREAILKIHMKKI
PVAQDVDVMRLAKATPGTSGADLANLVNEAALFAIRKNKNQVEMDDFEDARDKVLMGVAR

TSLVIAEEERRATAYHEAGHALLHYFLPNADPFHKVTIVPRGRALGLTLSLPEKDTYSRS
RGWLFDRIVITYGGYAAEXLVFGETTTGTAQDLRQATDLARRMVCEWGMAETMGPIAYGQ
EDEPIFLGKEIARHKDYSEETARKIDEAIRSILDQGMNKAMEILKREREKLEALAKELVL
KETLEDAEIRQLLSLSAGDNSISSEA*
>SPSA8_v1_490005|ID:41146054| protein of unknown function [Spirochaetes Bin 1 SA-8]
MQSSGKISGQTAGQSSIEDSIAGLLTALVPEKEPVFVAFSGGLDSRVLLEVLVQLGKNPL
RVIHVNHNIRPREELEKERELVIATCKKLKVSLTIAKIRPGLITGYARKKKCGVEAAARH
FRYLAFKKTCKRFGFRYIATAHTADDQMETLIYRIFTGSNPESLGGIPKKREMGKEIFVV
RPMLTLQRKDIELYARERQLEYSEDSTNSGTAYVRNRIRHFAIPALDKALPDWRSGLLRS
AAQILKMVKLQNRQVRSVLQTSHRDLEERRISISYGLFSSQPEAVRGFVLRDCLQWVACK
RNISVRACASAVDALEKGAEQIVCCGVVISRSKDTIIFHPEKQYQKMDGYCFLFHEPKII
RYGEALVGFSWEAVQEKFDHESADDMYLYEGSFSFPLIIRSRKAGDVIKTEKGHTAIDDI
LKSWHIQKGMRDIVPVIEDRDGIVAVLPALLGNTACSQKRFRPFSINGNPGKLHIILKGV
SYIHV*
>SPSA8_v1_490006|ID:41146055|rplY| 50S ribosomal protein L25 [Spirochaetes Bin 1 SA-8]
MDHFILQAFNRELTTKGDIKTARKEGRIPAVVYGGGISPKNIFIPASEYIKVQKNLTEST
IITLDIEGTKIDAFVKEHQKSAVAKDLLHIDFLAVQAGKKIHAHVPLHLVGTPKSVLEGG
ILEHLAHEIEIECDPSILPEKIDIDVAGLEINHPIHIRDIPVEKGMRVLTNPETVVAIVK
YAKHEEAAPAAAAPEAVAAAAEGAPAAKAE*
>SPSA8_v1_490007|ID:41146056|spoVG| putative septation protein SpoVG [Spirochaetes Bin 1 SA-8]
MEITDIRIRKVPTEGKLKAYVTVTFDECFVVHNVKIIEGKNGVFIAMPSRRTKTGEYKDI
AHPICPDFRTKLQERILTAYSTDAGSDDSVPEFD*
>SPSA8_v1_490008|ID:41146057| putative 4-diphosphocytidyl-2-C-methyl-D-erythritol kinase [Spirochaetes Bin 1 SA-8]
MFNEIRIIANAKINIGLAVGEPLSDGYHPIVSIFQAVSLYDELGIKLGSKAGDVTIAGAF
DCLPENTTVYKAARLFLDELGENRSVGIEVKKRIPARAGMGGGSSDAAAVLAGLNRLHGN
PLDEAQLARIGKKIGADVSYFILGKGTALVSGIGDIVEPIPTRTDFGIVIVLPDMGISTP
WAYKRLDQMRHYSDIGIHPGHLLEAKFTMRKMYYEALTDWDFVNDFHEIALADFPNLMET
AEALKAQGAILVSLTGSGSGIFGIFENDVIADQAAASLKIPQTQGIMPLKPLETTLELG*
>SPSA8_v1_490009|ID:41146058| protein of unknown function [Spirochaetes Bin 1 SA-8]
MNSDLPARPLAIVTSSNSDSDLVDALEMGVIVYSSLQETETEIYATMRRNQEIVNTNLVV
LQRKSKELWRFCQL*
>SPSA8_v1_490010|ID:41146059| protein of unknown function [Spirochaetes Bin 1 SA-8]
MTPISSASTRSESEFELVTIANGLAGKSEFILRSPVHFDESYDDDFDEFEDEDDDDLDDD
DFDDEDDFDDDFDDEDDDLDDEDFDEDEDDIDYDDDDIDYEDLDE*
>SPSA8_v1_490011|ID:41146060|rsfS| Ribosomal silencing factor RsfS [Spirochaetes Bin 1 SA-8]
VFDSKSLAFAAGALLAEHKAEDVIILDLRGIAGWTDFFVIASCNSSTHMKGLARFIEDFM
SAEKIQPLNKPVFKEDESWLLEDMGDVIVHIMSKEARSFYELEKLWFSAPSFKIEAGAKQ
D*
>SPSA8_v1_490012|ID:41146061| conserved protein of unknown function [Spirochaetes Bin 1 SA-8]
MMKRIFDKSAILLWLIIVLFGGLLLGGFLYIRSESLAAQIRTDKPISILLVFEMDKKPIS
SQLLMFFPAKSKAALVDIPTNMAVILKSIKRMDRIDYVYKPQAPKTYIQEVSAYLDTAIQ
GYLIFNEKNLVKQVDLLGGIQIFIPQMIIQEGKDGIRLPGGYVTLDGDKALQYLMYRDPE
EAEADFLARRQKFSLMLLKRISQNAELLASKSNADSIVAAMNSNFSNPARKLLLSEMAKI
DTDALLIQKITGMYRTVEGQQLFFPFYDGELVRDMLKQTMTAMVSSENSISTKKSYTVEI
LNGTQEKGLASRTSDIFESFGYDVVAIGNAKTMDYEKTVIIDRYGDKDALVAIGSVIKCS
SYADSSNYKGSIKADFTIILGKDFNGRYCVR*
>SPSA8_v1_490013|ID:41146062| putative Hydrolase, HD family [Spirochaetes Bin 1 SA-8]
MDFQHLETNILQXLDLNVKESRKQHXLRTADMAVRLCERFGEDQKSGYIAGIAHDMMKDM
PLDLQWQYAQRFSLIPHSDGFRFPVPARSASMNFYDKIIHAPAASVYIFETYGVTSPRIL
EAIACHASAAETMSRLAKIIYIADKLEPGRAHVSEELRSKMDSLTMDELFFLSLESVVSW
LSSQNAAIAQTTLDLYNSLTMKAGI*
>SPSA8_v1_490014|ID:41146063|nadD| putative nicotinate-nucleotide adenylyltransferase [Spirochaetes Bin 1 SA-8]
MKTAILGGSFNPVHIGHLILAEEVLSQCGYDQILFIPANIPPHKSIDDPGPHIRLEMLEA

AIKGYPSFAVSDCEILRKSVSYTIDTIRQLKTDAGLKGQMDGKPGLIIGDDLLAGFFEWK
EPHSLVKEAEIIVAHRQFPYRLSLSYPHRYVENTLIPISSSEVRARISAGKAWHSLVPQM
VQHIIEQKGLYGFSAS*
>SPSA8_v1_490015|ID:41146064|obg| GTPase Obg [Spirochaetes Bin 1 SA-8]
VAFRREKYVPLGGPAGGDGGKGGDVIFVVKRNLRTLSYLRFKQTFRAQNGIPGMGRNMHG
KDGEDMIIAVPPGTIIKDYDTGEILKDFGMSISGTGAEREGERWVFLKGGKGGWGNTHFK
NSVNQAPKHAQPGQPGQERRLKIELRLIADIGLVGFPNAGKSSLLDAFTNARPKIAPYPF
TTKIPNLGVLTLHDRDIIVADIPGIIEGAHEGAGLGIRFLKHISRTACLAFLIDLSDDNW
KEAYGILKAELTSFSRELAEKPHMIIATKLDLPEARERFEEFRQMIAEPIFGISVFSGEG
LEDLKEAFFNQVVHQEASDEPQTSTMGDLFGLEDELLEEGQQE*
>SPSA8_v1_500001|ID:41146065| Aminomethyltransferase (fragment) [Spirochaetes Bin 1 SA-8]
MPLHGHEISQSINAIEAGFKWACDFEKDFIGKQALLDIISAGPSRKLVGIEVTGGVPREG
YEVAGADGAIIGRCVAGMYCPTVKKYAANAFVEPAFAKSGTAVKVIIRGQPKDAVVIKRP
LYIPAYRR*
>SPSA8_v1_500002|ID:41146066|gcvH| glycine cleavage complex lipoylprotein [Spirochaetes Bin 1 SA-8]
MSIDANARYAESHEYARIDGAAVVCGISDHAQAELGDIVFIELPAVGKSLKKGENFGTVE
SVKAASDLYMPISGTIAEVNDALHADPALVNREPYGAGWLIKITPADLSEMDKLMDAKTY
GKTIGEE*
>SPSA8_v1_500003|ID:41146067|gcvPA| putative glycine dehydrogenase (decarboxylating) subunit 1 [Spirochaetes Bin 1 SA-8]
VPFIPNSNEDRESMLKEIGKNTIEELFLDIPEKLRFPVLNLPEGISEMEMLSEIEALSAK
NITARKAAWFLGAGAYNHYIPSLVPALASRGEFLTAYTPYQPEVSQGTLQAIFEYQSMAA
ELFGVSVVNASHYDGATALAEAVLMAWKQNLERNKILLPKDLHPEYREVLATYLSSFEVE
VKEYEGAPENAAPDARTACVVAGYPDFSGQIHSLAKGAELAHASGALFIVHADPLLCALL
ESPGAQGADIVTAEGQCLGNPMNFGGPFLGMMGATEKLMRKMPGRIVGEARDHDGKRGFV
LTLTAREQHIRREKAVSNICSNQGLVMLQACIYMAAMGHHGMKTVARLCYDKAHYAASRI
AGLPGYKVGNTAFFKEFLVETPVPAERIFEKLAKKSVVPGLPLSRYDSSRPKELLVCVTE
MNTKAEIDRLVELLEEVSA*
>SPSA8_v1_500004|ID:41146068|gcvPB| putative glycine dehydrogenase (decarboxylating) subunit 2 [Spirochaetes Bin 1 SA-8]
MNLAPEKLIFELSSKGRVGCALPKSDVPSYDLPSGLERKELNLPEVDELTVVRHFTRLSQ
KNYSIDTQYYPLGSCTMKYNPKANEVAASFAGWKFAHPLAGDVSSQGAIELIYKLQDSLA
EIGGFEAASLQPAAGAHGEFSGVLMIRNYLRDKGETKRVKMLIPDSAHGTNPASCTMAGF
ITQTIPSDKKGNIDMKALKAALDDTVAGIMITNPNTLGLFETNIEKIARLVHRAGGLVYG
DGANLNALTGVFKPGLAGIDVMHFNLHKTFSTPHGGGGPGAGMVAAGKALAPYLPGPVAV
RKRGRYRLSMPEKSIGRVKLFYGNFGVLVRAYAYIRMMGRDGLRRIAENAVLNANYLRKK
LEGTYPLKYTRGCMHEFVAMGDIAPGIHTLDIAKRLIDYGFHPPTIYFPLIVPEALMIEP
TETENRETLDAFAEAMLAIAEEARTRPELLHAAPVTTPVGRLDEVAAARCPVLCYRGD*
>SPSA8_v1_500005|ID:41146069| putative undecaprenyl-phosphate N-acetylglucosaminyl 1-phosphate transferase [Spirochaetes Bin 1 SA-8]
LPLIPFLLAWALSATAIPAIMFYSHKKGLFDKVNDRKIHNGNIPRLGGVGIAFSFTATIV
LLIVWRYGGIDSLNDHFSLLPIVLSGSVIFLLGLIDDLKDIRALYKFLVQLAVGIFLIAY
GFRFKIIMVPWGDGTFELGVLSYPITLLWIIGITNAINLIDGIDGLAGGLSVIAASVFGI
FFWVNGSLVSAQICFTLAGAILGFLIFNLPPASIFMGDCGSLFIGFLLSLLPLLGQHTTS
VEIGLVSASTILAIPIFDTLMAIYRRRKAKVSFFTADKGHLHHILMNYNNSNWKTLASIY
GITISLSVIALSSLYSKPITSFSLKILALIAVFIFFLVVNREKLFMHKAEGEQ*
>SPSA8_v1_500006|ID:41146070| AMP-dependent synthetase and ligase [Spirochaetes Bin 1 SA-8]
MYELKQMTLVEMIQKSTALYGDRPALCMAGGEQFSYREIGTRTKTIAARLLALGIVRQDR
VAIAAESSPYWGIAYLGAVRAGAIVVPILPDFTPDQIKNILTHSEAKAVFCSDKLFNRLK
DIELPVTLLDIKQGEIRKSPLVLDPAALSKESEAILAIFEDAAAEPDDTLMIVYTSGTTG
LSKGVMLSHRNILSNALGCRSIIILHRNDRLLSILPLAHTYEFTIGFIIPFLAGSQIFYL
DRPPAATVLLPALQLVRPTIMLSVPLVIEKVYRSSVKPTMEAIHLYKNRFFKPLIIKIAG
MKLKKTFGGQLRFFGIGGAPLAADVEEFLKKAGFPYAIGYGLTETSPLLAGCNPQHTHLR
STGPALTGVAIRIANPRPDTHEGEIQAKGPNIFKGYFKDELRTKEAFTEDGWFKTGDLGY

VDEKGRLFVRGRIKTMILGASGENIYPEEIEAVINQSPYVQESLVLEDEKGLTALICLKS
EVLENLQSRLQDEADEVGALASRVGNALSNAEKSVADTMSQAFSSAEKAAEKLLETIKKE
ANDKLAAFSRIRSVKLHAEPFEKTPTQKIKRFIYTKDAKAEDKKSNQEG*
>SPSA8_v1_500007|ID:41146071| exported protein of unknown function [Spirochaetes Bin 1 SA-8]
MKRYHTVIVLAFVFLALTSCSWMQAGGTGIIQLNFLFNPGFALKKGVIEQDGAKSIVPSD
SWAPARYEIQGIGPGGCTFSLSSTNSSVKAKITPGAWTVTVTAYAQNGKELGTGQATCSL
YPGKTTNQSVTIYPKEGKGSLSLAILKNLAVPEGSLLKGRLEPYGLPGREFNPDAVPINF
QLAASENSAIIPEIDAGHYLLTIRLFNPDGSLAGGMADSVVILAGFETSGTCTITMGNPE
LSLVSEFFPSDPLASPIMSVSHTVSDNASFVPLAIPGFPEVLIPHADLTIRWFVNGEEGW
NGAQITGNYGLLPQNTYAYLPFSRPFELSLLRADCVASSDVTNQSGSGSVFVERKMANEN
NDAAWYGSYDYRTVSAPALFPSASQYMTGTGIRADVAAVAASPSGLIIVAGLDKSSAIHA
FIAGYQAEARYDDTQAPFILPPSASWIRAWRDEVKINTSWRSPDRLAISSDSRYVAAASS
ASNWLRLYTLDQNGNIIRNFDVTGSETGSMQNFSNIKGIAFSADSSVMYVLTNSKEAVYA
FNISGAAPLLMSCYQFSRINENTSLSMQDIAITTEGSIIATASEASRIYVLKDVQGNLML
DQTIEKPANGPGPVKPQSLQALINHDGFACISNGNLVQFYRKLASDQSYHIDSTFTLPAE
AANAKSLAMTQGHLAVLGAQRVSFLNLDTDELPIGIKYLVPDAIDTAGILQARSIVSVRG
ACLLGCGSLGMVSVISLK*
>SPSA8_v1_500008|ID:41146072|glgA| Glycogen synthase [Spirochaetes Bin 1 SA-8]
MGEFPLKILMVSSEAIPFAKSGGLGDAVSALAKALFRLGHDVRILIPRYYSIDKRQLEQL
EGPMGVPIGKLELWTSVYRTNLPETDLPVYFLDYEAYFGRSGIYGSDSQHEYMDNPERFA
LLSKAAFQLCRKLSWIPDIMHVHDWPSSLIPVYLKTTERNSEFKDTASVLTIHNLGYQGI
YSKEHFSFFGLDWAEFHGSGFEYHDALNLLKAGITSADSLSTVSPTYAREIQTPGFGFGL
DGLLRHRSRELVGILNGIDTDTWNPETDPYIPAHFSADNPEGKALCKEALQKTFGLPADP
SVPVIGMVVRLTEQKGIAEMFGPGYGAVPEVCRTMHAQFAIVGSGDAWCEKELKTLSHLY
PHFKAKIGYDEKLAHLVEAGSDFYMMPSRYEPCGLNQMYSLRYGSLPIVHRTGGLADTVE
NYNQETGDGTGFMFDDPTPRALYNTIGWAVWAWYNRKEHITLMRLKAMQSAFSWEKSAGE
YIALYSHALALHASKR*
>SPSA8_v1_500009|ID:41146073| conserved membrane protein of unknown function [Spirochaetes Bin 1 SA-8]
VEKGKERKVYDYNQEKVQESLLRIFRSSKREATVADLAGLTGLPLQQIQAEMPALSDEYR
GRLKVTESGEILYSFPDGLKSRYRGFGPAIKKIWKAVKKGVIEISKALFKAWIVVMLVGY
FVLFIALALFAMIASIAMQQGGSGRSSRSDSRGGGGLGGLWLTGRLFDSIIRIWFYSELF
KSPETRFRQAQSRAQRRPLHKAVFSHVFGDGDPNQGWAETEKKAVVAFLQTHKGVMTMPE
FIAITGRTPQDAETAISSYLLEFEGTPEVTENGALYFSFPKLLTRIGTTPDIYGSSAPLK
KLKSFSSNTKKADNTFRLINLANVLFGGYFLANAISVGNMFYINTARGLALRGGFPFLYS
ATGYLFEMLGSANPVNLIFWGLGITPLFFSILFFAVPILRSIRLSRENEQIKAENLRKIM
ANRILVQPLNFVPAVIEPMVPEAAPASPRTAELVSKELAAWAQAEPIEGTYRFDRIYEVQ
KEIESIRSRIDIDSLKPGKTIFDSHA*
>SPSA8_v1_500010|ID:41146074| conserved membrane protein of unknown function [Spirochaetes Bin 1 SA-8]
MNTADSDKKTAALRTFSLSPAGVFLSIISALCFSTLGIFATALYSMGFSVQQALAWRFTF
SSILLWVFLAARVAARNKQGEKTAKPDTARLAPLLLLAFFGFAPQAGLYFITVKMLAPGI
TSLLLYLYPSFVLLISAVFLGRKPSRTQLFALLLSFSGCALTFFQPGNYPVLGLVLGVAV
AVAYSAYLVWGELVLHRFDSIFATAVIMSVATLVYWGWVLLSGDSVRVPDSPMAWLSVIG
IALFATVLPITTLFSAMRKIGAANVSLISTIEPVSTVLLSLLVFHETLTQGRIVGGALIL
AAVIVLQEKSGSPRN*
>SPSA8_v1_500011|ID:41146075| protein of unknown function [Spirochaetes Bin 1 SA-8]
MNELTAQIXAIRRARGWRLYTENGRRLLDFYQDQGRGVMGWKADGAARLAKALLDKALTA
PYPSVWQKRLERAVLAWKPAFASVLVFDNETEAEQVFRNLSGLLGVVSNDTGPFTALEMP
FAEFLPNNGQDSGLLKNSKKLAIARLPLARSLAPGILLLSKDASQELQACGKLPPQAPVA
AFKLGAAVKSLQSLAAFHEKYQEALWTKIDPFIENLFSRKGPWLFPVYPALEHRSVFLNC
LASGILISPAYDFPSLIPGEFDLGEIKVLADIARPGQ*
>SPSA8_v1_510001|ID:41146076| Pentapeptide repeat protein (fragment) [Spirochaetes Bin 1 SA-8]
MFTGGSFRLCFFDQAMLDSCDFSGVDMDFCSCGDADIIDSTFENSELIHVNFTGSRIRES
TFSSSNLYDSRFILSLISHTTFENCDFKRVYFIPEEGSEAYSVNNSNFMEAIRDVEHLYL
*

>SPSA8_v1_510002|ID:41146077| conserved protein of unknown function [Spirochaetes Bin 1 SA-8]
MKIYHHYSIYGFSNTYILGNEDTKSALIVDPAEISPLMIDQIENNEYRLSAILVTHNHIH
HIRGLKTLMRIYEPTIYASNARLFGIVCRKVRDQDMLEEAGFSILAIAVPGHSQDSIAYK
VGNVIFSGDALHAGLIGKTTSSFNTQALAERLNSRILCYDDDVMIFPGHGPPTTVGTEKR
FNLGLRPGFAKLLHPNYDFFV*
>SPSA8_v1_510003|ID:41146078| putative L-fucose isomerase-like protein [Spirochaetes Bin 1 SA-8]
MQRFTFTVASSVLHDSKSVDAIFSRYQPLLEAAGGRRIGAIADAPENEPVFFFVLTGGTE
SVVLPQIQEAQLLAKRLPVVLLAHPHQNSLPASLEILAKIQQDGGEGTIVLVKSDQDEQA
MLEIEKLASIFKALIRVRSAAIGAIGEASDWLVASRHSPETVKKSWGAAIVPIDFHELQK
KIDSIRKEGGADASYGAAAAKFLANASFIRESKPNDIYKSDTIYRAIREIVAERKLDAIT
LRCFDLVLLDQSTGCFALSQLADEGIDAGCEGDIPAILALVWMRALTGKPAWMANPADIQ
PTMETSAGRGTMLLAHCTVPRTLLTGYGVRSHFESGLGVAVAGTMPQGPVTIVRIGGTVL
DSVFLAEGTIIASPHEEGLCRTQAVIEMDGSALRELLEKPLGNHLVVVSGHWADAGRKYL
KFAGLAKKS*
>SPSA8_v1_510004|ID:41146079|pdxY| Pyridoxamine kinase [Spirochaetes Bin 1 SA-8]
VAVLSIQSHVVFGHVGNRAAVFPLERLGLEVLPLNTVQFSSHTGYEGWTGMIFPAQHLRD
VLEGLRKSGMLFSCEALLSGYLGDPENADTVVNIVKELRGLFPGFLYCCDPVLGDDEHGI
YVKPEVLQFMHTEVLEQASILKPNRFEAELLSGIAVETEQDIKEACRILHEKGPAIIIIS
SLESRHKKKDTISMALSFGNAGYRITTPRLNFSRHPHGAGDLASALFLGNFLKTKNPVTA
FERMTNAVFAVFQATHQAESRELRVIEAQEAFAAPPELFHAEQIW*
>SPSA8_v1_510005|ID:41146080| putative Oxygen-independent coproporphyrinogen-III oxidase 1 [Spirochaetes Bin 1 SA-8]
MNLSQVLHEKASVLDKPQGLYINIPFCTARCAYCDFHSFVCSAVSERSRADYIEALFLQL
DELEKILGTFQFRTIYIGGGTPTSLEDVLFLRLLGELRTRFGRFAEEWTVEANPETLSDA
KLNMLGENGCTRISLGVQSLDEAELHALGRKASAEASRTALERALKSGLKVSADVISGIP
FSLQEGSARKPALLSNVEYLIKAGVQHISMYDLIVEQGTRIEQQLTRGLLILPDEDAMYE
ERKAAEAILAQNGYYRYEISNFARPHEESLHNQIYWSMESWIGLGSGAVSSLNASGSDNQ
SCATVRFEGDRNLDRFLTQVSFLNVEPELVTPSDAELEMIMMGLRTRQGLDAMHFQSRFG
RDPRALIANTLEKWAEYYIPDSRFVRLSDRGMDLLNRILVDCMDEIQN*
>SPSA8_v1_510006|ID:41146081| protein of unknown function [Spirochaetes Bin 1 SA-8]
MFGRHLTYTERVLLRRRRARIILLSLLVLAGISLVHAFFLASYTVAYDARSIGFDKAERI
LATPLPFGPETILGKLPAFRNPKLGEAVVVSAGQSPFRPSSERLWLHLVRFFSLQALGEP
ENISIGIVQAVPGQGISQGNGSYTLDQDEIFLKFAASANGDGGTGTAIVRLSEIRASVVL
VYWPLKHIRVP*
>SPSA8_v1_510007|ID:41146082| Signal peptidase I [Spirochaetes Bin 1 SA-8]
MPWSLSTPAEDASVNSMKHDEDFLTRLQRFTENLLTKRKIRRLKEKVKQQKRNPILDWVY
AIVWAACVVLVINQYFFQNYQIPSGSMEKTLLIGDMIFVDKLSFGPELLPGVAKMKGLAK
PSRGQVIIFENPSYLSRGPIFTIAQQMIYMLTLTLVDIDRDQNGEQKVHYLIKRAVGTSG
DTVKVVRGEVFIKPKAGSSFIDEPSLMKGEGLPWKAARMVSKAEYADVEHVSVASAYQEA
GLPLPASIGSPSVSNAYKDAFSYDMKRVAILRDINPAEPRYAMTAQRYSQGWYIPEGRIF
PMGDNRDNSRDARYFGPVSTSRVLGRALFIYWPFSRAGAIK*
>SPSA8_v1_510008|ID:41146083| putative PEGA domain protein [Spirochaetes Bin 1 SA-8]
MKESKLPTLEEIEKTRVVLKPVFGISPRTYLPVIYGILTIVFLFFLLVYPGIVNNGSVLV
FRGEPRLAAVYIDGAYRTDTTAPIFVKSGNHKIRFEYSGFSPHEQQAAVKGRLLGSLFFP
RKQTIIYQMQADHPGRLVEESFAAFSSWSLTGKPSALYQLPPAISEPISNLASAGMLKDS
SKFLQPSAIAGMTREAAFVRNLASSAVSAEAGRDALRAGTLVVSHGQPGPLGIIATIRTA
VSIAGADKGSAVFLYELLGKSAGKYLEKLSTLKNEAAAFSASATRPSPSGRLQAAGVSFV
MFSKTSFQMTGETPSGNKFPYTVSVPEFGLAATEITKNQWKLFMAENPFWNLKNKETLIQ
QRLVDEDYLSTWEETTDSAPIRSISWHAASAYCSWLSGKTGSSYSVLLPSEAMWESAAVA
NNGYDFGAQAVWAGADLTGPLPAASRGYGRAGLADLGGNVWEWTSDSYYAYPALSAGTYA
ASEKAVRGGSWGNAEGVIKPTSRGVMPAERCSAFLGFRPAIVKK*
>SPSA8_v1_510009|ID:41146084|smpB| SsrA-binding protein [Spirochaetes Bin 1 SA-8]
MSIPENVKVVAVNRRAHYDYFVDETFECGIELKGTEVKSIKDGKVSFPDGFAEVRSGEIW
LKNFTIAEYSFSSVFSHDPERPKKLLLHAQEIKRIARKVQEKGYTLIPLSVYLKHGLVKI

ELGLCKGKKAFDKRAVIKERDVEREIRREFRSRNF*
>SPSA8_v1_510010|ID:41146085| RsmD family RNA methyltransferase [Spirochaetes Bin 1 SA-8]
MRITGGKYCGRTVELPDSQLEIRPAMDRMRESVFAVLGDLTGLSFLDLFSGSGIIALEAA
SRGAYPVACVEKDRAKFPILLKNVSISEQRIECHAMPVERFLLRNRQSFDILFFDPPFPY
AYKEELLSLAQSGRSLKQGSLVLLHFPRENRIPTTLGALSVEDERAYGRSIVRFYRMK*
>SPSA8_v1_510011|ID:41146086| Tetratricopeptide TPR_2 repeat-containing protein [Spirochaetes Bin 1 SA-8]
MNDTIPPPFTDESFDIPETLETEISEISDASKKGYQLLKENRFDEAVESFAQILEKDREN
NYALVGLGDAFRKRGSFKEAADYYRRCLVFHPGNSYALFGLADCYKALNQYHKAIEIWEQ
YLLHDNTNITVLTRVADAYRKVHDFRKSKAIYQRVLEMEADNHYALIGLGHLHYDFKEYK
EALSYWQRMVDLQGNEVDIRVLTSIGNCYRKLKQFDKGIPYFDKALEREPENFYALFGIA
DCYRGIGKQNQSLVYWNKILEKDPKNKVILTRAGDAYRNMGDLEKATQYYENALNIEFDI
YAVLGLAVIARMQGKHEDSITSLKMLIQNDPKNYRIYLELAQAYIAAGKPQQALDVLNEF
QKLGIKNLFIQEAIAKLQSRSQA*
>SPSA8_v1_510012|ID:41146087|rlmN| putative dual-specificity RNA methyltransferase RlmN [Spirochaetes Bin 1 SA-8]
METAGKIFPSGLFPAEIAQDAGFSDAFRARQMYSWIVKGVSDFASMTNLPAAERERLASL
YGSLRSSSVSSELEDSDGSLKLQIRLRDGSAVECVLLEDIEGRKTACLSSQVGCPMGCTF
CRTGQLGFLRNLGPDEILEQYYFLKERRGFISNVVFMGMGEPLLNLPAVRKAIALLSDHD
GIGMSMRKITISTSGVVPGILDLAKNGPKVRLAVSLTTADNELRSEMMPVNKSWDLAALK
EALLTYQKITRERITLEVALTGNRNASPADARKLVQWVKPLHAQVNLIPWNSIPDLPFTE
PDSNQLRTFSDIIEKAGLITVQRMHRGRGVMGACGQLGDTLILKE*
>SPSA8_v1_510013|ID:41146088| protein of unknown function [Spirochaetes Bin 1 SA-8]
MASLKARLERLKSLGLGLEPASELGKHRKEMQNRSDDSSEDGFARKKPESETKVCTRDAD
RTQESSAQGFLPGWEKLAPHVFMKITETSLDANPCVLSPRNFEPLRFAKEAARDVAHWDV
PLEDISFFDLETTGLSGGSGTIAFLAGIGWFAGSDFITAQIFIDDFPGEGAALGQIIAML
AERSWICSYNGKAFDMPLLRTRCIMNGIRFSEKAGHVDALAASRRLWKRTLGSCALTDLE
ESVLGHSREADVPGSEIPRLWLDYSNMRSSGEVSAGMMAMMELICSHNRIDILSLARLVL
LIQKLYENPGRYLALNCSGVSVDTNRLARHLIALGRIEEGCALLEESAARGDDEALFLLA
RHYKSSRDRLNQARVLSALGADSYRACVEKAKFAEHVEKDYEKALFLAARAARIAETDSL
NPIHRENCEKRIRRIRKKMGTL*
>SPSA8_v1_510014|ID:41146089| Uncharacterized ATP-dependent helicase YprA (fragment) [Spirochaetes Bin 1 SA-8]
MAGYPGSTASFWQQAGRAGRRGAASVAVMVATSSPLDQFYALHPEYFFSQKPERGRVDLN
NLYIYMDHAKCAAFEXPFQDSADSNFGISDKEDLEEALGMLEAEGVVRKTEGRWFWASEG
YPGESVSLRSARSDNVVIVDTTNGAHTVIGEMDRPSAKELIFDNAVYIHNGTQYMVEKLD
IGNRVCRVVKKDVEYWTDSIVKTDLEVLTEDDDIAFEASSLECVVGDVLIRDQVQKFKKL
RFHTHENIGYGEVFLPPEEMQTRAAMFIVKPQSHAGLFLDSLSPAQAQDVLSGAGKLLKS
LVPAFLLCDPVDIGMIARVKDPHFQRPALIIYDKYPGGTGLSEALPDRAKDLFMAAGERV
FSCGCKDGCPSCVGADVPGIKLISRQFLVLCFAADSLLSFNEHHRPRT*
>SPSA8_v1_520001|ID:41146090| Arabinose efflux permease family protein [Spirochaetes Bin 1 SA-8]
MTLRERLPALASKDYRLWFFGQGISVIGTWLQNTGQAWLVLKLTNSPFMLGLISTIQYLP
SLLLSLFIGPLLDRYPKRSILIVTQILFSLAALILAALAFSPHVQYWHIMAIAAFTGVLT
AVDWPARQSFVAEQVGERSLVVNAVALNSTIFNIARVLGPAIGGVLIAAIGIPWTFLFNA
GSYIAVITSLCFMKAGRNPRAKGGNFKGELRAGLSYIRKEKRIMLILIMIGFFSLFLMNL
NILIPSFAKLTLGLSADRYGLLMSAMGFGSMIAGIIMSLSGEKLVPSPRLILIAGFVLAF
AMVLTGLQKNFILSAILLAACGFGMSSYATLCNTSVQILSTDEMRGRVMSAYNLLFVGST
PIGSLYCGKVSESFGVNAGFWISGALGLVFLIIVSLFVFPRVFARVSNLVLDGTQRDSTE
SPS*
>SPSA8_v1_520002|ID:41146091| conserved protein of unknown function [Spirochaetes Bin 1 SA-8]
MITFPFCPNKXCSLHQQXPADASWFSPKGFYYTKTFGXXRRXQCKVCHTTFSTQTFSQAY
YLKKLIPLPDLVSRLASGESLRAMGRNLTISPKTVQNRLERLSRQSLAFHAKALSHLKKH
DAVCIDGLVSFDTSQYFPSEIPIAITANSQFIIDFSHASRKRSGTMTKAQKEKAAALYRQ
VSLERGAVSRSFKDTLVSAFQYQPPARFRPFILITDEKPEYQRVVFRLNEFRFQTDTTWF
IHQRIWSKLPRTIHNPLFSSNYIEREVRKDMAHHHRETECFNRNVSNGMLRMGLYLMWHN

YAKPFRIRAKKGKRPLTHAEAAGIPEWIITQFLNTLTEGVRAFFSRIKLTAAMERTWVKG
WMTPGKQAKDAIPAYVLG*
>SPSA8_v1_520003|ID:41146092| membrane protein of unknown function [Spirochaetes Bin 1 SA-8]
MNPFSLSETXNXLSKLSAXSKLISLTLLSMASMNAQLPXLIALLXASXLFHFIYRIKLXD
SKKMLLFILYISIFTSIIGIIAPGDGRLFSIELLPRFLTYSLRLYTVFFFSHLFYSTTKV
SDLGNYMTRLAQKARKDRSQSELINSKYSDSMDPGMAISLTLLFLPRSFSTYETIREAAE
IRGYGLKKHKIADFLKILETFLIFSLRYALKTSGAMKIRGYSPLRTVRXQKFKLGDYFLV
IGSLILVFPILC*
>SPSA8_v1_520004|ID:41146093| Polyamine-transporting ATPase [Spirochaetes Bin 1 SA-8]
MAELFKTEGLTHIFKSGRAGLRNISLHIDSGSIIVLAGRNGSGKTLLVRHFVGLSTPTAG
KVLFHAEPVAQSIKKIRTSVGYVFQDADLQIFSQTVAEDVAFGPENLMLPAEVVEQRVER
ALSATHLEAMKNRRPETLSGGEKRRLAIAGILAMEPECLILDEPFANLDLESIRDVVEIL
VELHKSGKTLIVVTHELEKILPFADKLIILDNGELVFSGHPXDTTPELYKNHGLVCPLEG
HFPWXYQDFSSFETKHKIGSTKKSNPLRRLLR*
>SPSA8_v1_520005|ID:41146094|bioY| Biotin transporter BioY [Spirochaetes Bin 1 SA-8]
MNPVNDKLTNSILACLFAALISIGAYIAFPLPGTPVPIVLQNMFIMIAALVLGPWWGLAS
VFIYLLLGAGGMPVFSGGTGGFMKFVGPTGGYLFGYIPAAVAIGFIALKGKGTFLANILA
CTAGMAIVYLFGVVRLKFVLASSWGKAITAGFLPFIIGDIIKITLAAILSPRLIKGLEHL
SERESDG*
>SPSA8_v1_520006|ID:41146095| putative Radical SAM domain protein [Spirochaetes Bin 1 SA-8]
MLNLYFNMKRTFIRLSLVAIHPESNTAFIPLGVASIAAYLEQFIKNAGGQQPKLEITILE
NRGEELTAGWILDRKPDIVGFSLYAWNTKRVFQLAREIRNSESAPFLVAGGPDAEYCAAL
FRDNSMSSEKTAEIPFDAVFLGEAEKSFAHWFLDEFLGGTAALQQSSPEFIPSIPCEPEE
LVSPWLSGYLVPSADRDVAWEMTRGCPYHCTYCYEGRGSSRVRALPKKRLEAELELFEKR
AVPKVFVLDPTFNLNPARALSMIEMLAKKAPSIYWYLEIRAELVTPALAEAFARLPCSLQ
IGLQSADSAVLKTVGRSIDRKLFAEKIRFLNQSQAVFGLDLMYGLPGDSLESFFESLDFA
LSLQPNHLDIFPLAVLPGTILYDQRVEFDLEAEPEPPYLLRQHPGFSRYDMEKAGKVAKA
CEIFYSRGRAVPWFLAVLKPLKLKPSRFFMEGLECLQAQDWKHQSMTHRQIEELQIIALR
SIYEKMGKEKLLPAVQDLVRYFGALSRAYAEQERSRITLNYPLADIESGFLMRHIERYCE
RHQPSPAKIEISPSKKGPNVKIL*
>SPSA8_v1_520007|ID:41146096| protein of unknown function [Spirochaetes Bin 1 SA-8]
MDSLIRKSETSIAEALASWQAQLTQKGSSLEEEVAERYVSRWTSPNLSHVMKIRTALVLG
LEEYLASEGLVNIDRVTISPVTDPLCHGVEHVPVISYQNVPYRTTHSMIYSKFLACMSPH
IPGVFIDSPNIRLELPSSDGSMRKKYLIDFSQMDIERRRHTPIASDGYFRDPDTVTRILQ
EDRDRALDFFEDLIIHAVKKVRSSCSESLKALGIALDIPQKPFPRFFKDEWLEKGESEKG
LEKKLGKIAGTQFFWVLGLLRENYDLVYPFLQEDGSIPEPGDITSRQIFNYDLCAAAKYL
DGSLGDAWEVLSGGLREWFFPAIIARLLANNVLREAPVFDGKGNITNLDKLDGYGPFLLA
ASMKDSGGEPIFPQTFGGGLGIERSLFALLQGPKIRQIEEITYFGKNPDIPGPFLF*
>SPSA8_v1_520008|ID:41146097| conserved protein of unknown function [Spirochaetes Bin 1 SA-8]
MSEYLTVDPERQLKLKDIIGKLHQGVPVHEVKREFGRLIQGVSAAEVAAMEQALIDGGMP
VEEVQRLCEVHVEVFEQSLAQNGNPKKTPGHPVYTLIEENRRAMTALKELKNTARAFRWG
MSSRDTAAAALENASKIIIHYTRKENQLFPYLEKHGFTGPSRVMWGKHDEIRELFKEARL
ALDQGGKAFAPKAAAIAQKMKKMIFMEEHILIPEAVRRLSEREWAQIRLGEDAIGFAWVK
PEALYDAALVLATEQKKDQLMTEKVETPKAPAAGMLNLDTGTISAELLNLALKTLPVDIS
VVDEDDHVLYYSDSPHXLFPRSPAVXGRSXQNCHPQKSVETVNKILSXFKKSEKDSARFW
IEMGGRFILIEYYALRDKDGKYRGTLEMSQDLTELRALQGQRRLLDWD*
>SPSA8_v1_520009|ID:41146098| Cation diffusion facilitator family transporter [Spirochaetes Bin 1 SA-8]
MGFSANEADLKSETEDMEDRIRLIRTASWIAIGGNFMLAAMKIIVGFIAGSLAVLSDGID
SSTDVLIALMTLLAAGISAKPSDKEHPYGHGRIETVASAVIAFIVFFAGGQVFVSALRAI
FSSEPASMPSSLALWVTGVSIAGKLALTYSQFYLGKKSNSAMLIANAKNMRGDVITSAAV
LIGLGLTFLTGIPVIDKVLAMLVALWIIKNAIGIFLEANTELMDGTTDHGPYRDLFKAVS
DVPLAGNPHRVRLRRIGSMFVADMDIEVDPDMTVREAHEISQQVETSIKQALPEIYDVIV
HIEPHGNVEEEKFGLTSKDTH*
>SPSA8_v1_520010|ID:41146099| FAD-dependent pyridine nucleotide-disulfide oxidoreductase [Spirochaetes Bin 1

SA-8]
MQHYDTVIVGTGPAGLGAAFELQARRPQDSILILDKNEMSSGGLRNDCKMNFSWPIGFPE
TCWDEVTASYYLKRVEDFLQPAIMERKNIRTYVHRAEKIGVKLLEIRQSHLGTDGGIELI
KQLTARLQAAGVSIALGETMKTMDASAKVITTDKREIHYTDLILAPGRGGFAFLQDMMNL
CSIPYRDNIVDIGVRIETREERYPIVKDYYDPKFMFPKKTRTFCTNSRSAHVVQERYGDP
ETGYWYSVNGHAWSSSRPANGLVNFAMLKTVTLTAPLASGQAYARMLGMQASLLGGGKPI
MQRIGDFRLGKRSFAESFTGDLYDFEPTLPSCTPGDIALSAPAKVLNAIWNGIKLLDSIV
PGLLHPSTILYYPEIKLYANKPVFADQHFKVAEGLYLIGDGAGTSRGITAAWASGLRAAD
GILSERS*
>SPSA8_v1_520011|ID:41146100| conserved exported protein of unknown function [Spirochaetes Bin 1 SA-8]
LMKRIGFFALGLAVIALLFACGPAAPKTVKFGVFEPLTGANAGGGALEVEGIKLANELYP
TVTVGGKEYKIELVIADNKSDKVEAANAAQRLVDKDKVHVVLGSWGSSLSMAAGPIVKQA
KTPAVGLSCTNPLVTKGNEYYFRVCFLDPFQGTVMATYAAKELGAKKAVIIREVSNDYSV
GLAKYFVDSFKQLTGDDASILAELNYNTGDTDFAAQLTEIKKFKPDVIFAPGNFTESALI
IKQARELGIKTPFLGGDTWETPEXX
>SPSA8_v1_530001|ID:41146101| ATPase and specificity subunit of ClpA-ClpP ATP-dependent serine protease, chaperone activity (fragment) [Spirochaetes Bin 1 SA-8]
VFGQDQAIDAVVRAVKRSRAGFRAPDKPVANFLFVGPTGVGKTELARQLAKHLGITLHRF
DMSEYQEKHTVSRLIGSPPGYVGYEEGGLLTDAIRKTPHAVVLLDEIEKAHTDIYNILLQ
IMDYATLTDNQGRKADFRNVILIMTSNAGARDVGKALIGFGDRSVSNSALDEAVERTFTP
EFRNRLDAVVHFNNLPMPMIEKIVLKAISEFNTQLAEKHVELIVPDHVVHFLAEKGYSRE
FGARNIGRLIEDRIKSFFVDEVLFGRLEHGGKAIADLEGESIVFHVEAAASAAVPPTEAV
PAG*
>SPSA8_v1_530002|ID:41146102|aat| leucyl/phenylalanyl-tRNA-protein transferase [Spirochaetes Bin 1 SA-8]
MLPYLQTSRFFDESHDFVFPPVSRATREGIVCLGGNLSPGMLLSAYRRGIFPWYSDDEPI
LWWSPDPRFCVLPSTLHVPGSAKKLLKKNRFSITVDTQFRAVITECALISRPGQPGTWIT
PEMQEAYIRLHELGYAHSVEVWENGTLAGGLYGVSLGKAFFGESMFSRVSGASRAGFLTF
AAVLFEKGYELIDSQVHTDYVASMGGVDIPRARYLALLDTALKHETHKGLWTDFFTSGRW
*
>SPSA8_v1_530003|ID:41146103| protein of unknown function [Spirochaetes Bin 1 SA-8]
MKIIFVQLPVQEPNWEDAAANVTLAAGYLTAYAESRGLLNRSEWSILDAATSTYGSDAAI
VDRIQRQEPDIIAFSLYAWNLERSLFIAERLRSMLPRTRLIAGGPEVVEDMPIAHHSPFH
SLVAGEGEIPFAQILQDIRLHRPLDRIYRADTLVDLATLPNPYLAGTLPIVKDEPVHIET
VRGCPFHCAYCYYGKNFPTVRRYPEEQALELIQRACKAGSSEINIMDPSFQFHPDLDKRL
ADFAQANTARLPVHTEMRLEGVTAATAELMKKAGVQSIEAGLQSINPKALEAINRKFDME
KFVHGLEQLQNQDILIKTGLILGLPFDGYEQIVETFDFLGMHGLGQDAELYPLSFLPGTE
ARARADEWGMARMELPPYWVTSNDWISSNDMIDVIAAFEESFEREWASPPAPHFTANSGG
LTGFVDARKVENIDWMRLNPEKLANSMTLLADADDPESLSRLVRAARDLRRDNPFTLYQI
VLTSNTRIPSERLIEKIRDAFVFEDHYYELSHYFSPDLQESYQTRMFFATKNFALAYRAM
EEAQDLETMVIISGKAGYNADRLSELLPFVIFDRETIPFDRLYELLSIYTEYPHMLIEAP
EQLFRT*
>SPSA8_v1_530004|ID:41146104| D-isomer specific 2-hydroxyacid dehydrogenase NAD-binding protein [Spirochaetes Bin 1 SA-8]
MRIGSFMPVHSVWGKHLDQLKKEFPEHEFLSGLTDSSTEAATLDIILAGRLEHATYLASP
ALSAVFQPFTGINHLPLAEFAQRGVRVFNVHANAFDVAERALAMTLAFYGRLIEFHNDLR
QDLWHGFWVRAGAEDNWDSLYGRTCAILGTGAIGVELAKLLLKAFSCTVYGWRRNITKGAP
EGFDMVLSDLQETISKAEIIFIALPATPLTEGLLSHEILLSMKDKFLVNVGRGSIVDEAG
LYEALEKKILKGAAIDTWYTYPPVGKVGAPSRFPINKLPQVILSPHVGGSTNQATLKAVD
VTIANIRDYLRTGTCRNEADLSAMY*
>SPSA8_v1_530005|ID:41146105| putative ankyrin [Spirochaetes Bin 1 SA-8]
MVQKKRNRRLSILSLLSPMLLALIMLTLMLFASCQTTPAQKEASLTELVISGDLNAIKKY
YSNQEQLNMKDADGLYPLHHAVKRGDPQIAEILIVLGAKTDVLDPAGKTPLRYAIDRKQT
AMARMLVDRGASLFIADAAGKTSAEVALSAGPDMISAVFSAKNINESGADGRTALHMASD
RLMANEVSLLLDMGASVQTKDKAERTALDLALQHPEDKRAAIIAEKLIIKGANPSLPAFS

DFTWFATTVRSSDYRTVRLSNGNTPLHEAVRLFQLGFVDFLLSREVDPNKKNLSGDAPLH
LAVRTGWYDGAELLLKNLADPDIRDAQERTPLHLQSPAAVRLQMTKLLLKYKADPNLKDS
MGNTPLHLAVKYSYEPSVIEEFLKANAPVNAANLDGDTPLMLCVRAGTYQYAAALIQYGA
DIFLKNNSNESSLSMAVSRGTGAVDKIVLPSNVLQRDNSGNSVILTAVALKGSPEVVSLI
LQKGGDPNIKNNFGDNALHVAVRNNLADQGGILINAKTDIFAINAASETPVFLALSAKDG
PYDWFFTPSVITARDVNGDTVAHQAARKNLDKGLAYLKGRGSDMAAVNTTKETLLHVAAK
SNAADALRYLINDKADMEARDNNGDTPLQASVLADSLSCMQLLIASGADLNARNFSGESV
LHQAVRKHNLAITQFLLGKGSGLENRDNRGQTALSVAAREAQADIARVLLTAGAIVDARD
YSGSTPLYYGVETSNLALIGVLVKANADVLAKNASNDSPLLAAMKKGPSVMRELLSGPTK
DRADSEGKTPLRILVEARASLDTLNLALSGGADPNSKDKLAGTPLHAALRQKDFQTAGLL
LAAKADPFAPDSSGQTPISLVLTDSVALKDFVMAAGVNAADIKKEGFLFYAVRAGSREAV
QILLDLSIDKTQRNISGETAFDIAVAKGLSEIAAMLQ*
>SPSA8_v1_530006|ID:41146106| RNA polymerase sigma factor [Spirochaetes Bin 1 SA-8]
MAKRSDQDRKNFPLNQTSDSKNHDSAQPQRHKTPKRHAIDKDPLSLYLYQISKYPLLNAE
GEQRVGKQVHDCQQELKALKAQREIEQQSGAQNTAAPNPAEDTSIDEAIRLTEENLKAAK
QEMITSNLRLVVSIAKAYQHRGLNLLDLIDEGNIGLLEAVERYDYSRGYRFSTYATWWIR
QAIIKSLADQGRIIRIPVHMLXTIRKCFYASKQLTQELGRAPEISELAERVEMTEEKVLK
VIEFSQNTASLDSAIDDDNKSSLSDVISDEEGLDPYSEAFSVTMKELLDCVLDGLSTRET
TVLRLRFGLSGEDPHTLEETGKVLGITRERVRQIQERAIEKLRQRKELSECE*
>SPSA8_v1_530007|ID:41146107| putative LysM domain protein [Spirochaetes Bin 1 SA-8]
MMNVKRSILLSILLCIVTAGMAFAAGPVTHTMQKGETLYSIAKKYNIPYESLAAANGISD
PTKLKIGTVLVIPSVHLVSKGETLYGIARLYSVSLDELLTANKLAASYVLKPGDILVIPG
GKPDTGTTVTTAAAPSTTVAPSTTTTNGIATTVPPTTVAPSTTTTKSIGTTAPATSSTVP
APVPIPEPVKTQDKVVDPKAGWPAPGKVMYLDGKLEGVMIRVKPGEIAKAIASGTVVSAG
PSRGFNQVVFVQAKSGYVYVYGGNETLLVKTGDTVKAGTELGKIGIDSKDNSPIAYFFVF
RNGQPIDPALAPRE*
>SPSA8_v1_530008|ID:41146108| Diphosphate/fructose-6-phosphate1-phosphotransferase [Spirochaetes Bin 1 SA-8]
MNERSPLHNVRFAYIPKMPEIFNLPTASIKAIPTEVIEPKVDRDTLKGIFSHSYGKPAVK
FAQGENIFAGKTMAVGVLLSGGPAAGGHNVIAGLFDGLKKNSPGSKLIGFKGGASGLLDD
KSIEITAELMDSYRNTGGFDIIGSGRTKIETPEQFEKALATATNHKLDALVVVGGDDSNT
NAALLAEFFASRKAPIAVVGVPKTIDGDLKNEWVETSFGFDTATKLYSELIGNICRDAAS
GRKYWHFIKLMGRSASHIALECAFQTRPNIALISEEVEARKMSLAQIVDSIAVSVTARAA
KGENFGVVLIPEGLIEFIPEFKVLISSINDLLAHEDEAFKQLPSVAEQRKFVAKKLDTEA
SALFTSLPEDIQAQLCWDRDPHGNVQVSRIETEKLLVMMVKERLATLSKEGSYSGKFDYQ
THFMGYEGRCAFPSNFDADYCYSLGNAAFLLALNGYSGYIASIRNLTAQASEWIPGGIPL
VSMMNIERRHGKDKPVIKKALVELDGKPFKHFAEQRAKWALSSDYRIPGSIQFFGPPAVT
DATTMTLQLERS*
>SPSA8_v1_530009|ID:41146109| conserved exported protein of unknown function [Spirochaetes Bin 1 SA-8]
MKKRIILASLAFLLAAFGLFAQEIVTAEQFFNKISDKYATINDYEASIQISSGTQPMNGK
LSFRAPALLRIDFSQPAEQVIVFDGQRLVVYIPQYRAVLQQETGANSVGAGAAALASRDG
LAMMKRTYSLAWESSPQPVPLDSGSQEMVQRLMLTRKSAAEGFKTIRLSISPATLLIRRI
EGWTVANEKITFDFTSIKLNQGIPATRFLYDAPASANVYNNFLFQ*
>SPSA8_v1_530010|ID:41146110| protein of unknown function [Spirochaetes Bin 1 SA-8]
MARIGSIIQETRLKKGINLDKVADETNISVRFLKKIETDDFTGFPGEPYVVGFIRNYAEY
LGLDPEAIVTRYRADEASLAERKQEEPAAPSMAGSSETETHDSELPEEEKPHSKGAGAAS
QQAFPAGEKPAAKKTKXX
>SPSA8_v1_540001|ID:41146111|lpd| Dihydrolipoyl dehydrogenase [Spirochaetes Bin 1 SA-8]
MSDYNVIVIGGGPGGYLAAERLAHHGKKVLLAEKEFLGGTCLNIGCIPTKTLLNSAKHYH
NALDGDRFGIHVESVRCDWQKMQAWKQEVVEKLRNGIAFAEKKLGVEVVHGAAKLAASGR
AVEVDGRILSADDIIIATGSVPVMPPIPGSQGNPQVLDSTGLLGIGAVPERLCVIGGGVI
GIEFASLFSALGSQVEVVEMLDEIVPFMDRETAPVLRKALKSVRFNLGCTVEKIEGGTVF
YRKKDGELATSVSDYVLMAVGRKPMIEGWGAREAGLDVSQKGVAVDDRMRTNLPHVWAVG
DVTGRSLLAHSAYRMAEIAAANIADGEQAVRRGERMRWDCVPWAVYSIPEAAGIGLTSQE
AERRGLHALSVSVPLKTAGX

>SPSA8_v1_540002|ID:41146112| 2-oxo acid dehydrogenase acyltransferase [Spirochaetes Bin 1 SA-8]
MAHSVVMPKLGNTVESSIIVKWHFEEGQRIAQDDVLCEIETDKATMDVPAGASGVLLKRL
HAEGDDVPVLAPIAVIGEPGELFEAAAGAAASAGDSVKAAHNQEPEAQNRAAASPTGSAA
ANNPAGMVAASPRARKLAEKGMISLDGIAGSGPHGRILERDVSAVLQNAPGFSSAARGAA
GRGNPEIGAALSGESGSGLGGRLLASDVARLAEKMRESDAGAAGQADYTDEPIKGIRKII
SDRMLHSIQSSAQLTLNSSANADQLLALRARFKQSAPSLGLSGLTIGDMVAFALSRVLPR
HPALNAHALPGIVRKFRHVHLGLAVDTPRGLMVPVIRNADSLSLKEISAQSKQLAKACVE
GTISPDLLSGSTFTLTNLGAFGIESFTPILNEPETAILGLCSIVPRPKAEGNGSEMRIGL
SLTIDHRIVDGADGARFLKDLSAVLAEFDLYLLAEGLKD*
>SPSA8_v1_540003|ID:41146113| Transcriptional regulator, DeoR family [Spirochaetes Bin 1 SA-8]
MEAALNLELTERESLILDMLAREGTLAVSALSKTLGVSEVTIRSDLKSLEEKGFIYRTRG
GAYPSFHKDILERQRLNMEEKNRIARAAAEAVQDGDRIMIEAGTTTALIVRYLTGRRGVQ
IVTNSTLVFSYARLNPALNIILTGGIFRKETESLVGPVALRAIADFNVRLAFVGTDGFSL
ERGMTTQLVEGAEIVKAMRNQADETWLVADSSKYGKTGFVSVLPLSEVDGIITDERLDPA
ARQRMSEQSIRFRLA*
>SPSA8_v1_540004|ID:41146114| putative Diguanylate cyclase [Spirochaetes Bin 1 SA-8]
MTKKTLIVWGAVLSLCLIIFFGIHYAYRLPEGAAQVQTGQVVTMPSGKTIDVSGEWYIGG
IKTPDFQPGVYSVRMNVPLSEPHVGAESREDYILVFPSVDGNALRVFFNGHYIGSQGDIA
QGNSNIWYAAKFFTVPAAIVSAENTIAAEIRGVYEAGISRLPYLIQKSKSPYSLSWLSFS
TRGLVVILAGAIIILGFILIFMGFGMYPQNNSRLILGIACFLTALFLTDFMNLEYLPVSL
LAFKKMVVIARHLAAITFFIGFLKLLEHPSDIFIRIFVVIQIACSAILLYPSEVHLLKQF
YTVTYLTILPLPLYLLYFLFHKRLQKKEYSILLAGVIFAMLTAIRDVAIPLINPGALYFS
HFGFMILIISAAWFIVNDDIQHFRLLIQEKTRSEQYRHESLHDPLTGAYNRSMLEQAQRD
LPSDFSLILIDIDNLKEINDSYGHLAGDYALRNLVSRTQNLIRHDDLIIRYGGDEFLVIL
PHCPSERLASIVENFDAEFSRSEVALQDGQIFTYSVSLGSMCVMSSNRSCSDSFEDALVR
ADEKMYENKRLKKSNSRIARDQA*
>SPSA8_v1_540005|ID:41146115| Dihydrodipicolinate synthase [Spirochaetes Bin 1 SA-8]
MKNIQNGVWPTMITPYNADGTIAYETLEKLIDWYIEKGVAGLFAVCQSSEMFHLSLRERL
ELAKACIRFADGRVPVIASGHVSDTLADQIDEAKMMADTGADAVILVTNRFARKHESDDV
FKKNLDIFLKNFDRDVLLGFYECPAPYKRLLNADLISHLVATGRFGFLKDTSCRMADIRA
KVAAAAGSGFKLFNANAATLLDSLRAGAAGYSGIMTNFHADLYVWLCKNWEADPELAEKL
QNFIGLASVIEYQLYPVNAMYALQMEGIPFNLISRRADAGKFTESMKLEVRQLMEVGSQY
RKFIKQELYHA*
>SPSA8_v1_540006|ID:41146116| TRAP transporter, DctM subunit [Spirochaetes Bin 1 SA-8]
MNVALIVFFLTFIAVFLLRMPIGIGMMVSSVFYFAISKHPAASLDMVATQFLTNMNSSFI
LIAVPLFVFMAEIMNSGKVTSMIFSFANALVGKKKGALGHVNVIASIIFSGMTGSALADA
SGLGSMEIKAMKENGYDDGYTCAITAASATIGPIIPPSIPMIFYAMLSGASIGSLFLGGI
IPGLLIGAALMLYTAVIARKRNYPEGNKLPFREFIVLTLKSIPALFSVVVLLGGIYSGIV
TPTEAGALAAFYALLVSIFFYRAFKWNDFKNVLINTVKTTGTLSLLVGCAYAFSYIVTIE
KIPNLVASLLLGMTNNKYIMLLLINVLFLILGMFIDTMAITLVFIPIVIPLINALGINLV
HFGVVIVLNMMIGLSTPPYGMLLFVVAGISGTPLKKVIKEILPMIAVIVVVLFLITYIPD
FVLFIPRLAGFK*
>SPSA8_v1_540007|ID:41146117| putative Tripartite ATP-independent periplasmic transporter DctQ component [Spirochaetes Bin 1 SA-8]
MREQSSLKKAGMFFVNLIELYIPIATFVVLFVSFMTQIVARYFFKPLIWPEELSLMCFVW
TALLGGLYAKRTNSHVAFSMVYDSAKPETQRWMRLAGNLLMAAALIIVYVPSWEYVQFMA
YKKSDALRIPMNWAYFPFMIFLTDMIVRVVIDIVKDLKKPAGGGEQ*
>SPSA8_v1_540008|ID:41146118| DctP family TRAP transporter solute receptor [Spirochaetes Bin 1 SA-8]
MKKFFLLVLAVLMIATPAVFAQNKVNLVFTSVSVPGDAHTQAMFVFKDEVEKLSGGQIHV
DVYHSGQLFTQQAEQDAIRKGTVDMVYTSAQWLAEFIPYLSMFGAAYTFQSYDQMTKTFN
GPIGQKIFEEVAAKTGIRPLTAYYLGTRQLNLTAKVGPVTKPEQMKNVKLRVPNSPTWIA
MGKALGANPTPMAFNEVYMGLKTGAVDGQDNPLPTDKNAKFYEVTKYIVLTNHVVDSTWP
SMNEKKWKSLTKEQQGWVLAAANKAREFCDKTNLDNEKNILDFFRSEGIVVIENPDRAAF
AAYAKNSYLTESKDISKAWDLDLYDEIQKIK*

>SPSA8_v1_540009|ID:41146119| Transcriptional regulator, IclR family [Spirochaetes Bin 1 SA-8]
MVNTVAKALQILDALSESQPQGVSDLARNLGLPKSSVYSILETLVSEKIVEKNMQTGKYS
LGVKLIELGNCAQTGLDICRIAAPFLQGLNVKFDETVHLTVLDNYEVLYIDCIESRRRLR
TYSVIGVRAPLHCTSVGKAILAFLPDDEIRKIVAEKGLPVFTAATIASPERLAAEVDTIR
KQGFAIDNMEHEEHLRCVGAPIFNSQGSVFAALSLSGPAERNTMERILEMALEVVAAGLE
ISRRLGWRGEPQSMRR*
>SPSA8_v1_540010|ID:41146120|oppF| oligopeptide transporter subunit ; ATP-binding component of ABC superfamily [Spirochaetes Bin 1 SA-8]
MSEHDTLLEVRGLKMHFPIGKSLLAKNRGFIYAVDGVDFSIKRGETLGLVGESGCGKSTT
VRAVAQLYKPTAGEVILKGKDLTKMAPHDLLAARKNMQMVFQDPYASLNPRMTAGDIIAE
PMRIFQKRGLLDISNDERNDRVEHLMEKVGLSRFFKNRYPHEFSGGQRQRIGIARALALN
PELMLCDEPVSALDVSIQSQILNLFKDLQQEFGLTYLFIAHDLAVIQYISDRVAVMYLGV
IVEISGSKDLYKNPLHPYTQALLSAAPIPDPKIEARRQRIILTGDVPSPDKQRPGCYFYD
RCQKRMDVCKTCRPQLKTTEEGHQVACFLYHKPE*
>SPSA8_v1_540011|ID:41146121|oppD| oligopeptide transporter subunit ; ATP-binding component of ABC superfamily [Spirochaetes Bin 1 SA-8]
MTDEVILQVRDLKTYFTVDEGVVKAVDGVSFDLHKGETLGIVGESGSGKSVTNLSIINMI
PNPPGKIVGGQVLFSGKDLLKISASELRQIRGNKISMIFQDPMTSLNPFLKISTQMIETI
VLHQGLDKQAAKEKAIDMLKLAGIPAPEKRIDQYPHQFSGGMRQRVMIAMGLSCNPEILI
ADEPTSALDVTIQAQILELMQELTKRLGTAVILITHSLGVVAGMCDTLCVMYAGRIVERG
KTDDIFEDPKHPYTQGLIRSVPRLDKENADRLYSIPGQPPNVIDLPDCCPFYPRCEKAKD
ICRKKYPPVTGFENGRAVSCWLYAEETK*
>SPSA8_v1_540012|ID:41146122| oligopeptide transporter subunit ; membrane component of ABC superfamily (fragment) [Spirochaetes Bin 1 SA-8]
LVRELGYQKEALEKVKTEMNTNPGYQKVYILGTDDLGRDMLARVIYGGRISILIGIVGTL
TAALVGILIGSVSGYVGGWLDNLLMRFVDIMYGLPYMLIVIIIMAMIGEKARGSFVILFV
AIALVSWLTIARVVRGQIISLKNSEFVEAARSMGASTPRIIFRHLLPNTLGVIVVFSTLL
MPSFIMNESFLSYLGLGVSAPNASWGTLVSEGVRAMEFYTWQLLAPAVAMTLFLFCMNFL
GDGLRDALDPQSKNRT*
>SPSA8_v1_550001|ID:41146123| protein of unknown function [Spirochaetes Bin 1 SA-8]
MLKVIVGSTDIEKADLAKGARNYLIQGGMKAEQIEALKTRLASPVSLLERLSSKPFAYTI
FVTPECLFSQEFAS*
>SPSA8_v1_550002|ID:41146124| conserved membrane protein of unknown function [Spirochaetes Bin 1 SA-8]
MNEQKSNQVKTWFLWSLAAAAGFTALIAVLSPALQRFVKPEDQGPSWYYWQLAEPNFWTH
FSAWALYILHQVFTWVVAARMLKEGNHPDRITNLNKTALWGNLGFMALHLIQTHVLYDGL
AQDVPVWSSQWSVIFMLIIMIYLLIPRRGIAFGLNVPLKRRVYGWIRKYHGFYISWALVY
TFWFHPMEGDYGLLTGFFYMFLLFIQLSFAGTKLHTALGWLTVLELTVGLHGPAIALQKY
LAKYAGASIFADIWPMFLFGFVGVFVVTGQYGFKLGKNARIAVYATYAALAILVYSFRGF
GRLFEIVFIPIALIGGALLMAAAGNILSGKDGLVPLRRVSSEKK*
>SPSA8_v1_550003|ID:41146125| Ketol-acid reductoisomerase (fragment) [Spirochaetes Bin 1 SA-8]
MRNSFESKVFKTETVWFGPHEEMVVRGGRNLFPLLPKAFDGIRQIGVLGWGSQGPAQAQN
LRESLEGTNIKVKVGLRSGSASWKAAEAAGFTEANGTLGEMMAVAGDSDLVLLLIADAAQ
AELYPELFKVMKKGATLGLSHGFLLGAMKINGDRFPAHLNVIGVCPKGMGPSVRRLYVQG
KSVNGAGINASFAVEQDVDGKATDYALAWAVAIGAPFIFKTTLEYEYKSDIFGERGILLG
AVHGIVEFLYRYYQAQGMSPEEAFRHSAESVTGPISKTISHKGIMGVYESLSEADKKVFR
KAYGAAYAPAFETHYENYQEVASGNEIKSVLLAAERMKRIPMGKIDESSMWKVGEPRFRS
IPLPQGSISPA*
>SPSA8_v1_550004|ID:41146126| Ketol-acid reductoisomerase (fragment) [Spirochaetes Bin 1 SA-8]
MMAQIDLLAEKGHAYSEIVNESVIEAVDSLNPFMHYKGVSFMVDNCSTTARLGSRKWAPR
YDYLLMQQAEPIWSRGAGEDSALWEKFMHHPVHAALATAASMRPSVDIAVVG*
>SPSA8_v1_550005|ID:41146127| putative Glc operon transcriptional activator [Spirochaetes Bin 1 SA-8]
MTPALMQNSETTSADESKVFSYIQEQIVSGRLKAGVKLPSERQLCEILGVSRGYVRKALS
RLEHYGLIKTLPQRGTIVAEMGSKAISGLIANIGSFEETFTPGDLFEIRELLETYAAKRA
ARLANQDSLEEILKWHIEFKQKAENGERALDEDHLLHIAIAKASGNPVCLSLISYITPQI

IALNTDFSEKDPARFRNTFEEHDRIVKAILAKEENAAEIAMYHHMREARKRRFST*
>SPSA8_v1_550006|ID:41146128| ABC transporter, solute-binding protein [Spirochaetes Bin 1 SA-8]
MKRKIMFLFMIVASFALVMAPWAQTKPVTVTHWYWADNPKYSAEMQKIAADFNATNGKGI
TVIAQEYPWDGGAYSETLFRAVIGGGGPDTSSFKLTSTPLFSANGLLVNLDSYLASWKDK
DKIEPSLFDVMRKASGTKSVYVMPWNTQVLYVYYRPSLMKKAGITVPKTYAEFLEACKKL
TMDTNGDGKIDVYGFGMRGAKGGHEPWGSFIWARGGNFDDFTTPQAIAGMQDFIDLFKNG
YVPPTAPMDGFNEIIANFKSGKTAMTIHHIGSSIGMMDTFGDDVDAFAFPGGAGRWTSMG
DTENVMLKSAKNRDAAFEWLAYLATGKGQEAWCSATGNVPVSKDVQKLPFFQNNRFMKVS
IEGAPYAGIYPILDTTTEWISTIWPNTIAAALTGKMSAADAMKQLQKGLWGK*
>SPSA8_v1_550007|ID:41146129| conserved membrane protein of unknown function [Spirochaetes Bin 1 SA-8]
MRKKTTVWPYLLLAPALATILAVVFIPVINAVLMSFQNYDLRRPSQIRFIGLGNYIAVFK
DPLFWQALLKTVIWVSAGVGFQFVFGFMLALLLNRHFFGRGFFRSISLIPWVTPGVLIGL
MWRWIFDGNYGVLNDLLSKLGLIENNIPFLAQQSTAFPAVILTIIWQGIPFFALMLLAGL
QGIPEELYDAADVDGASLIQRFFMVTVPSLKNTIFITTMLRIIWVANSVDVIFNLTEGGP
AYSTQTLSVYVFNKGNALNLGYASTMAILLALALSSVAIPYLKKLFKSQEEAL*
>SPSA8_v1_550008|ID:41146130| ABC transporter, permease protein [Spirochaetes Bin 1 SA-8]
MRNERMLRLQRVLRRLLGNILLAALLVFLLFPFYWTFVTSIKPEKELYASVVTYWPKAPT
FTSYTRLFTEYNFLKPMLNSLLVAFFTTVISLTVSLLAAYAFSRYRFRGRKFFMTLFLTN
NMFPTVLLLIPLFAIMRKAGILYTPTALILSYTTFTIPFSIWLLNGYLDDLPRSLEEAAM
VDGANRAQAFLKIIFPMLVPCIIATGAYVFMTAWNEYTFAVMFTNERNRTIPVALKNFIG
QLGVEWGMLTAGGIITIIPVCILFFFAQKRLVEGLTAGAVKG*
>SPSA8_v1_550009|ID:41146131| putative transketolase N-terminal section [Spirochaetes Bin 1 SA-8]
MNNGFLIEKADSLRRDIIEMLYRCQSGHPGGSLSCVEILMALYYKTARVDPINPSWANRD
RIVLSKGHGCPALYAILADKGFFPREDLWHLRQIDSHLQGHPDMKKTPGVDVSTGSLGQG
VSVAAGLAMAAKYRKASYRVFAILGDGELQEGMVWEAAMSAAHYKLDNLIFILDHNGLQI
DGSNDTVMALGDIEXKFRAFGFNVASVNGHDIYSIANAIEQKQLDGKPLLIIAETIKGKG
VDFMENQAGWHGRPMNETEYLAALNVLKGKYHG*
>SPSA8_v1_550010|ID:41146132| putative transketolase C-terminal section [Spirochaetes Bin 1 SA-8]
MAEKKSLRVAYGEALAELGAINKNIVVLDADLSHATMTSIFAEAYPDRFFNFGLAEANMM
AAAAGFAHSGLIPFASTFALFGTGRAFEQIRNSICYPEENVKLGLSHSGLSVGEDGGSHQ
SIEDIALMRVLPGMTILVPCDPLETKKAVYAAAEIKGPVYIRVARPAVETLTDVSSPFIP
GKANILRKGDDVCIIAAGLMVQDALLAADRLAREGISACVVNMHTIKPIDEALILEISAS
IPAIVTVEEHSIIGGLGSAVAEVLAGRSHAKFARIGIMDKFGKSGKPADLFREYGLTADD
ITKKCVDLLA*
>SPSA8_v1_550011|ID:41146133| conserved protein of unknown function [Spirochaetes Bin 1 SA-8]
MGIQSYEYRDAGLKRVYENEHWMIGIKNWKPANDVEGIDCLERHLMSDELFTLLEGECIL
LYCETSEKEPSASDLRTLKMEKGKVYNIPKGLWHNTITFKNTKLLLVESPDTGMSNSEII
PLSSIHRKRVHAIATEMGKLESMQ*
>SPSA8_v1_550012|ID:41146134| putative Oxidoreductase [Spirochaetes Bin 1 SA-8]
MAKLRAGIIGLGKTGIKQLHALSLMHDVEIAAITTSDPEKWKKLSERYEISSCYSDYRDM
LRTADLDVVHDCTPNYLHYEIDKAIINEGKHILSEKPLALFPSEAKELVSLARQKNVKTA
VNFIYRHFPVIEQIRKYILDGKLGRIFSIHGAYLQDWLLENTDYDWRVESKFSGQSRALA
DIGSHWFDIAQFLIMSQITEIAADFATFIPERIEQKHGLSVAVDTEDYAGILFRMENGIH
GTVTVCQTCAGKHEGLSFEINGSEASMRWSYSDPFHAVLMKTGRPDTVITAKQDQYTGND
APGQFAQNWLIADFYQSILGNTNHIYADFSDGYATTCIIEAALKSMQSRAWTTVLR*
>SPSA8_v1_550013|ID:41146135| protein of unknown function [Spirochaetes Bin 1 SA-8]
MLGGTEQEYLGKIFIDTIVHPASRELILARRAKITSQHGMLEPIEAVLQKNNGSAMDVLI
AAAWQEIDTDIIINSTFFDISPIKARERLLKAVSTIASSASSQTSLDQLTNILLDAIQKA
FPYAECAGYVHVTKSLDESDTIFGKMMATCELEPELEQFIWKAVQSDACKQNSAYLLTRP
AQSGGDTYVFQPVRAENQLLGAIFLQYQRNKAPGADEQANLYTLAEAIVNAVQKVFLIQQ
NESRYKNLAVLYRASIRLGELRSSTEIARETLRILEQEKGWTHSVIRIRDEASKRLVTLS
YCAPGVHSCEEEKVIEWLNTKISKPGDGLTGWVIDHDESVLCKDLPHDPRYLETDIGMLY
GIYAPIHSEGVTIGSIGVESDRIDFSQADLELLCSLGDLIGMALNAIRTMDLLGYRVRWL
EQLYQAGTAVTLEASEDMIFHQLLEGAMDALSAEAGALMLYNAEKNLLIPVAKRGWLKDI

VDPIAPDKSLSGLVFSSGRSHISWELSQEPVVLSHIREKIPDDKRGVSVPILAESTVIGV
FHLGFSTDIHINKDIVAVAELFASFAGTVIKRVELSLELRNANNALRDAYDATLEGWSRA
IGLRDDETASHGERVTSLAVAIGKQLDLSPEMMEALRRGALLHDIGKIGIPDRILLKQGS
LSAEERATMQTHPALAFQLLKPITFLKEAVTIPYCHHERWDGSGYPQGLSGEAIPLLARI
FAIADVYDAMTSDRPYRKALSHDEAVVYIMSNSGKHFDPKVVRAFLALSRSLR*
>SPSA8_v1_550014|ID:41146136| protein of unknown function [Spirochaetes Bin 1 SA-8]
VLACDFCSPRQNQFSARGMNNSIYENFAEILLLGSTKHGQCXX
>SPSA8_v1_560001|ID:41146137| protein of unknown function [Spirochaetes Bin 1 SA-8]
MASGSKGTFRPVLVVFMAIALLTGAALGIGLGSTLNIIRNENFSEFTVALPSKLYDIKGR
LITEFYAEEKREPVPLKDLPPALIGAFLTREDQSFYSHHGFTIKGLTRAFFGKIIGKNLG
GGSTITQQLAGTLYADRRDISLRRKIVELWWALQLX
>SPSA8_v1_560002|ID:41146138|gapA| glyceraldehyde-3-phosphate dehydrogenase A [Spirochaetes Bin 1 SA-8]
MKVAINGFGRIGRLVFQSIVDQGLLGKDKIDVVAVVDVSTDAQYFAYQLKYDSVQGKMKA
EISSRKSDPSKAEDDVLVVNGHEIACVMAEKELKNLPWAKLGVEYVIESTGLFTDEKAFA
HLEAGAKKVIITAPAKSKAEDKKIPTFLVGVNHEKYDPAKYDVVSNASCTTNCLAPLVYV
LLKEGVGIETGLMTTIHSYTATQKVVDGVSKKDWRGGRAAAINIIPSTTGAAKAVGEVLP
EIKGKLTGMSFRVPTPTVSVVDLTFRANRDTSIEEIDSLLKKASETYLKNILGVSTEELV
STDFIHDPRSSIYDSLATLQNNLPGEKRFFKVVSWYDNEWGYSNRVIDLLRYMDSKKQ*
>SPSA8_v1_560003|ID:41146139|pgk| phosphoglycerate kinase [Spirochaetes Bin 1 SA-8]
MIKTXKDIELKGKRVIMRVDFNVPMKDGKVQDDTRIMAALPTIKYVLDAGVRSLVLMSHL
GDPDKDAAKAKEKAEKEGKAFDLEKYLNGKHRMQPVAEYLSSKLGKPVIFLPSCKGQKEA
IDALPDGAVAMLENTRFHKEETAKDIAVQEVLAKELASYGDVYVNDAFGTAHRAHASTAT
ISKFSSVSVGGFLMEKEVAHLEPLLTSPPKPMVAVIGGAKVSSKIAVLESLLKTADALII
GGGMAYTFLKVQGIQVGKSMVENDFVDVASNLLAAAGKANVKIILPVDHVCAETFAPDAD
PVSIDSANIPENLMGLDVGPKTLELYRAALATARSVVWNGPVGVFEFDAFAKGTEEIARM
VAAATGRGALTVVGGGDSVSAVNKFDLASQMSHVSTGGGASLEYLEGKILPGIACLEQK*
>SPSA8_v1_560004|ID:41146140|cysK| cysteine synthase A, O-acetylserine sulfhydrolase A subunit [Spirochaetes Bin 1 SA-8]
MKVAKRVSDLIGNTPLVELSRLNSEGHARVLVKLEYFNPGHSIKDRIALSMLEAAEQQGF
LVPGMTIVEPTSGNTGIALAMIAASRGYRCILTMPETMSRERRLLLQAYGAELVLTDGKL
GMAGSIAKAREIAESDPSRYFIPMQFENTANPQAHFSGTGPEIYRDTDGLVDIFVTGVGT
GGTITGTGRFLKAQKPSVSIVAVEPDASPVLSGGQKGPHGIQGIGAGFVPEILDTSIYNE
IIRVKDEDALETARRAAREEGLLVGISSGATLWAGLELSRRPENRGKVIVLIAASYGERY
LSTQLFAQLAAGTLNSPENGRGENRA*
>SPSA8_v1_560005|ID:41146141|cysE| Serine acetyltransferase [Spirochaetes Bin 1 SA-8]
VRNAHDYGFLLTEKKKSAMAEDIAAVLQRDPAARSALQVALVYSGLHALWMYRMAHRLWR
ANARFSARLLSQAARFLTGVEIHPGASIGRRLFIDHGMGVVIGETAVIGDDVTIYHGVTL
GGTSLEKGKRHPTIGNRVTIGAGAKILGNINIGDDSRIGANAVVVKDTPPNSVVVGIPGQ
IVRRTRMHHEGDRPDLNHVELPDVVGKTLRELLNRVEELEEKVAGFHHVEKHAKIQHFDR
EGAWIDGDFVI*
>SPSA8_v1_560006|ID:41146142|tpiA| Triosephosphate isomerase [Spirochaetes Bin 1 SA-8]
MRSYFIAGNWKMHKTAAESVALASELREKLAGCKEKVMIAPAFTALSEVSKVLAGSSILL
GAQNMGPEEQGAHTGEVSVLMLKDLGVKVVILGHSERRHTYLETDELINKKVLLALKHEL
EVILCVGETLEEREAGRLEEVVGTQIEKGLASVPAEKLPMVTIAYEPVWAIGTGKTATPE
DADAVHAYCRKVLAGMYGQEAARSMVIQYGGSVKPDNAATLMGMPNIDGALVGGASLKAE
TFVPIACFR*
>SPSA8_v1_560007|ID:41146143| Preprotein translocase, SecG subunit [Spirochaetes Bin 1 SA-8]
MGFFAILLLVIFVIVSLLLIFLVIIQDEDSDSLGGIFAGGSGSAFGSRASNVVIKITYVL
GALFFISAFAMALINKTPTGSIPQTVTQQSTESGNAEWWKTQPAQTPETTTETTAK*
>SPSA8_v1_560008|ID:41146144| putative PTS IIA-like nitrogen-regulatory protein PtsN [Spirochaetes Bin 1 SA-8]
MILQKAFRPSTIRIGLESEDKDELFEELVDILARSYPQDKAFPREAVLQGIQQREKKMST
GIAKGVAVPHATISGLDGLHGVLGISRKGIDYEALDGEPVYLVFLLVSPPGDAELHLQAL
RQIALILQDHDLMTALLNSQSSEKAYEILTTCDILQFE*
>SPSA8_v1_560009|ID:41146145| protein of unknown function [Spirochaetes Bin 1 SA-8]

MMGPHLALLYGKVKGATMDSLDSLERLLETEKKASRIIEEAETAAARIRLEAQEKAAALE
QEKLLEARRFFEAEAARIKKDIENEVQTELDTFKLHLRTIKIDKDRLAPLVWNIVRQDN*
>SPSA8_v1_560010|ID:41146146| protein of unknown function [Spirochaetes Bin 1 SA-8]
MAGFADFSYIYARICGAYSHMRLYSKSSSLESVKSPSQLWKIWFDEEPPALPEYVLVSLA
EKKILKDSISKFRNLAEILPETDALVRSLLLKFEISTLKNLLLRIQKNEARPEVIDYQSP
VISSLLEFWPNLKAGLRGTDYAWIDETALSDIGKTENLLDRYYYLKLWAAVQALPGGKTG
AIQDLLTREMLYQNAIWALRTYHYYGLSREKTQTMLISIPQKDITSDALKIFDCTMENLT
SFADWPLKALLKNQSGKGLDIPLLEITAQRELFLAVRRALHFFPETYTPLYCYFKLLDGE
VNLFSVAMESVRLGVPLSESSQFIWALGGEPA*
>SPSA8_v1_560011|ID:41146147| putative V-type ATPase 116 kDa subunit [Spirochaetes Bin 1 SA-8]
MSQVSMQRIEMILLKSDIDNLLHYLGKQACFQIIYPDASEREKYSGVSKSDEEQTEAYLV
EHLEEAQRKLEEMGRMLGYVMPMQIKPDTHLPDEAMIGVIDERYAAVTHLEAEIQKQTSV
IAELEETVHEAKASTNLPLANENLERLSFITIKTGTIQPDKMARLQETLGARAIIVPLDE
KGRIVAASSRKGRFALETELERAGFVKLDLPTGSQPVSSEAVDALEKTLKLRKAELSALA
VEKEKTAASFQDTWELFVLSIQLKRALLKVENRLEGTRWAYRLEGWVPANRTKGLIREVT
AMFKNVAIRLVAPDEKGKDETDIESIPVLLKQNAFTRAFQSIVLSYGTPLYGDIDPTPVV
AFFFTLLFAIMFGDLGQGLVIFLTGILFVRTKKASFEKYKRFGPAFIAAGTGSMLMGLLV
GSFFADDAILVPFEHMVTRVIFNRSSDRILDIMPQGNLDAMFRFFGFTLGVGVFINSTGL
VINMINLCRRKEYGEAIFSKTGLAGAFVFWWAIGIAVRIAAGGSLAWFDVPGLGIPLLAL
FLAESLKALLPSRDGHREKISLVDAAVGGMVEIIETISYYASNTMSFLRVAAFALAHAVL
SFVVFTMAELIRTRAPGGSYAQIFVYIIGNIIIIGLEGLIVTIQVIRLQYYEFFSKFFTR
SGKSFTPMSFEKVD*
>SPSA8_v1_560012|ID:41146148| ATP synthase subunit C [Spirochaetes Bin 1 SA-8]
MKRKWMSGIFAFAASASAFAQQTAATAAPSAGASIAKYIGAALAVSVAAVAGGMAVAKIG
SAAMGAMAEKPELSGKALPYVGLAEGICLWGFLVALLILLL*
>SPSA8_v1_560013|ID:41146149| Vacuolar H+transporting two-sector ATPase F subunit [Spirochaetes Bin 1 SA-8]
VIEIFVLAEEEIVLAFRMIAIEGLSVANKDEACRIFTAITHDKAAPGPKGGTFDLSQCKM
LIISEDVADMLGNGLTDWQLEGQFPLIVEIPPLSGHVEGHARLVDMVRQAIGIKIQ*
>SPSA8_v1_560014|ID:41146150| protein of unknown function [Spirochaetes Bin 1 SA-8]
MEQIQGTEALEREILEDAARKADKIKKKAEGDASRILAEAQSGIQKKIAVLQSEHEARIA
SLQNELHSRVPTEKSRLQSKFKESVLEAALEEFFEHEGSDLLGQWCLSQLEKSQKLIYAS
SADVFWKGLNAADQKKLEEFCGKSKSSLALHEDAGLPYRGVRVKPKDQSYECRFTERELR
DWLLDNKRGMLADALFGSNLNENPQENERAGKGIGADSMPQDRA*
>SPSA8_v1_560015|ID:41146151| V-type ATP synthase alpha chain 2 (fragment) [Spirochaetes Bin 1 SA-8]
MKKSGIITRISGPVVLAQDLENAGLYDVVKVGGAGLIGEIIKLKGDTATIQIYEDNTMMK
VGEPVVCLEYPLSVSLGPGLIGSIYDGIQRPLPVLKTLSGPMLVPGVAGESLDTEKRWHF
RPAIRPGEAIAPGKLFGTIQETPSIQHRLIFDSNAKSENAAWVAEEGDYTVTELLLKGES
GAQYSAKSQWPVRKPRQFLAKKVPQEPLVTGLRVIDVLFPIAKGGCAAVPGGFGTGKTMT
QHAIAKWCDADIIVYIGCGERGNEMTDVLTEFPHLVDPRTGRSLMERTILIANTSNMPVA
AREVSIYTGITIAEYYRDMGYHVAVMADSTSRWAEALRELSGRLEEMPAEEGFPAYLPSR
LAEFX
>SPSA8_v1_570001|ID:41146152| phosphate transporter subunit ; membrane component of ABC superfamily (fragment) [Spirochaetes Bin 1 SA-8]
MAVTMLIGNSNFIPTSIFSPANTMASVIANEFAEATGITAASLIYVGLVLFLVTTIVNVI
GTYVIKKVALEASKEVT*
>SPSA8_v1_570002|ID:41146153|pstA| phosphate transporter subunit ; membrane component of ABC superfamily [Spirochaetes Bin 1 SA-8]
MNDHTTGRMIKDKAFRIIVILLACFSILPLLLILYFITRNGIAVINWEFLTQLPRPIGEA
GGGIFNAIMGTGILIVLSSILSIPFGITAGIYLSEKTEGKIANIARLCVVVLQGTPSIVI
GIVAYIWVVAPFQSFSALSGGVALSIMMLPVIIKATEETLKLMPYHLKEASLALGVPYYK
TILKVILPTGLSGILTGILLSVARITGETAPLLFTAFGNQFMNWNLFKPIDSLPYRIFYY
AMSPYPEWHAFAWGASFILVVLVLAFNLIARGVAVKWKVQF*
>SPSA8_v1_570003|ID:41146154|pstB| phosphate transporter subunit ; ATP-binding component of ABC superfamily [Spirochaetes Bin 1 SA-8]

MESTVLKVHDFSAYFGDNQILKNINLETGSNLVTALMGPSGCGKTTLIRCVNRMHELIPG
AKVAGKMYLKDNDIYAMEPIVLRRKIGMVFQKPNPFPMMSIYDNVIAGYKLNGIRLKKDE
FDSIVERSLKGAALWDEVKDSLHRKGTFLSGGQQQRLCIARALAMNPEILLFDEPTSALD
PKSTAAIEELIVELKKTVTMLLVTHNIAQAARVSDFTAFIYLGELVEFGPTEKMFTVPKD
KRTEEYLTGKFG*
>SPSA8_v1_570004|ID:41146155|phoU| Phosphate-specific transport system accessory protein PhoU homolog [Spirochaetes Bin 1 SA-8]
MLEEKMIGLRRELIECATHVEGMIDKSIAGLVRKESSLLREVIEKDEPKANETEILIEDL
CTTMIAQYEPKAKDLRTILMTMKMNNDLERAGDHAVNIAESSLYIIERPAIKPLLDIPRM
AEEAVGMLKDAIEAFIKEDALLAAMVCERDTIVDNLGQQILRELITFMSADPTTIERAMR
LLNIARNLERIGDLSTNISEDVIFMVEGRVIKHHKDQEGV*
>SPSA8_v1_570005|ID:41146156|glgP| fragment of glycogen phosphorylase (part 2) [Spirochaetes Bin 1 SA-8]
MEIIFEINRRFLDEARLVYPGDDERLRRLSLIDETDARYVRMANLATVGSHSVNGVAALH
TKLLQKTVMKDFYELFPEKFHNITNGVTPRRWILLSNPRMSQIITDAIGDGWICNPQDTI
RRIEKFVDDVEFKNAWRKAKFYNKQELAGLIKKRIGINVNPESMFDIQVKRIHEYKRQHL
NVLHVITQYNALKNNPKLDVPDRTVIFGGKAAPGYFMAKLIIKLINSVADVVNNDPDIAG
RLKVIFFPDFNVKNGQRIYPAADLSEQISTAGKEASGTGNMKFSMNGALTIGTLDGANVE
IREEVQPENFFLFGLTVDEVLELKTRRYNPMEYYENNSKLREAIDLISSGFFSRGDKNLF
KPLVDSLLYQDEYCVLADFQAYIECQQKVNEIFLDQDQWNRMSILNVARMGKFSSDRSIS
EYAQKIWKVNAVRAIKERE*
>SPSA8_v1_570006|ID:41146157|glgP| fragment of glycogen phosphorylase (part 1) [Spirochaetes Bin 1 SA-8]
MREKSPKKELNVNETCGNVRTGLSAESIKRAIIDNLNFIQGRIPYLATPNDWYMALAYTV
RDRMLERWIRSILVFRQSPRAVCYLSAEFLMGPHLANAMVNLGIYEQVREAVAQLNLNLE
DIIAQEGEPGLGNGGLGRLAACFLDSLASLEIPAIGYGIRYEFGIFDQEIRDGWQCEVTD
KWLAFGNPWEIARPEIAYYVNLGGHTEHSYDEQGKYRVRWVPQRVVKGMAYDTPIPGYHV
DTVNILRLWKSEACESFDFQAFNVGDYYGAVEEKIASETISKVLYPNDEPVKGKQLRLAQ
QYFFVSCSLQDMMRIHRLTGDAPDTINEKFAAQLNDTHPSIAVAELMRLLVDENQVDWDR
AWAITQKTFAYTNHTLLPEALEKWPLPLFW*
>SPSA8_v1_570007|ID:41146158| Efflux transporter, RND family, MFP subunit (fragment) [Spirochaetes Bin 1 SA-8]
VEPRDTPVTFEYVAQTQSSQLVNIFSRVSGFLDKRVYTEGAIVRAGEVLFLMDKKPFQVQ
VDGYTAALARQKASFETARLNLERTKPLAEKKALSQKDLDDAIGKYETAAAAVEEAKAQL
DSAKLNLSYCTITSPVTGVTSSAMQQEGTYINAPNSQLTTVAVLSPIWVNFSISENDMSK
LREEVRRKRLIPPRGGEAGSRSHSC*
>SPSA8_v1_570008|ID:41146159| protein of unknown function [Spirochaetes Bin 1 SA-8]
MIVPQRSVQQGAKGHFVWVVTKEGTADYRPVTVGEWQENNWFISEGLSAGDQVVVDGSLT
LRPGAQVKIKPLSAQPPVSVNQAGTKTSQTSSPKKDK*
>SPSA8_v1_570009|ID:41146160| fragment of putative multidrug resistance protein mexB (Multidrug-efflux transporter mexB) (part 1) [Spirochaetes Bin 1 SA-8]
MFSKFFIERSIFATVISLIIVIAGLVAIKSLPVAMYPTITPVQIQVTATYPGADAKTVGD
SVAAPIEAQINGVDNMLYMTSTSSNTGQMTVTVYFSLDTDPDIAQVQV*
>SPSA8_v1_570010|ID:41146161| fragment of putative multidrug resistance protein mexB (Multidrug-efflux transporter mexB) (part 2) [Spirochaetes Bin 1 SA-8]
VNLATPQLPDAVTKYGVSVQKKSSSVLMILAIFNKDGRYSPDYVNNYANVYVLDAIKRVE
GAGQAQIFGMTDQAMRIWMNPDRMASLGITTSDVQSAVAKQNALFGAGQIGQQPNDQGVQ
LTFPVVTQPPFVTPAQYEDIILRASQDGSAIVHLKDVARAEVGRRAYVDDNRLNGIPATP
IAIYQQPGANGIEVSKAVRKTLEELKRTMPDGLEFKIALDTTDFVKLSINEVYHTLFEAI
LLVVLVVYLFLQSFRSTVICVVAIFVSLIGTFAGMLALGFSINMLTLFGIVLAIGMVVDD
AIVVVENVERNMTKVGLHPKQATIKAMEEIGTSLIAVVLAMASVFIPAAFLPGTTGQLYK
QFAITIVVSVALSGFVALTLTPAMCAIMLKHSTPPMRGFFGWFFRFFAWFNRGFDRFTIA
FGDVVVLMIKRMVIAFAVLAILVWALVRLFRSIPTSFVPNEDQGYLMGMVIMPDAASLNR
TVDTAKRIDELFAKDPAVDSRTMINGYSLIDGQFKPNVSTFFIALKDFEERYASTERARK
ENAGAVLRSVHAESRNIETGLFIPIAPPAIPGIGTTGGFEFWLQDKGSGDPVRLFEVTQQ
FLAKARARPELSGLSSTFRSSSQQLRAEVDRSKAVLLGVPVDDVYSALQAQFGSITVSQF
NQYSRVWNVILQSDAQFRRAPTDITRLYTRSNKGQMVPLSALVTTRYVTGPDLIPHFNGF

PAAQVTGSAAPGFSSGEAIRIMEEVAREALPQGYSYAWSGMAYEEKKSGGTSAAAFIFGL
IIVFLILAAQFESWTLPGSVMTAVPFGILGALVFNWIRGLDNDVYFQIGLLVLIGLGAKN
AVLRVTFATELRKQGLSIMDATIKAGEERLRPIIMTSLAFILGVLPLAIATGAGANARHS
IGTGIMGGMIGEATLAMLYVPLFFYLFDRLSERSARKKEKSISAETLSEPTLNPDTPGGI
SPNAEGGH*
>SPSA8_v1_570011|ID:41146162| Type I secretion outer membrane protein [Spirochaetes Bin 1 SA-8]
MRKLIILTFVTLILAACAIGPDYRRPEISTPKAWRIEEKEAVDVANTAWWKQFDDPALNE
LIAVSLRENKDLKIAAARVEEYMGRYWVGRSQLFPQIYGSGSGGRQRSTERGPSPVPSYV
DNPYTIYQGVFSGSWEIDVWGRLRRLNEAARADLLSTEEGKRSVILTLVASVANSYISLR
DLDKQLMVARQTAKSREDSYDLFKVRFEGGVISELELSQARSEYELAKSKIPSIEKFIVF
QENALSLLLGRNPGPILRGKTIDELVLPSVPAGLPSELLERRPDIRQAELDLIAANARMG
AAKALFFPTISLTGIFGWSSTDLSMLFSGHSQTWNWAGTFTAPIFTGGSILGNYKATQAF
REQALLRYEKAIQNAFREVDDALVDQKNTREQLLSQQRQVEALREYASIARLRYENGYTS
YIEVLDAERSLFNAELSYTETQGALFQALVNLYKAMGGGWILAADIAEHINK*
>SPSA8_v1_570012|ID:41146163| putative Ion transport 2 domain protein [Spirochaetes Bin 1 SA-8]
MKRLLPYLRRMWATDVALTMLLVFLLIFIFLLFPMGQIGSVKFLTNLVFSFILITGAIAA
AKNRILKRLVLLWSILAFVFIWVRHLFPHQAFVFATTSLSLAFLLLLAFLILGQAFREGP
TTSHRIMGAVAVYLLLGLIWSLAYQLTALCVPNAFIMQTPFAAGDTQSLESHLFYFSFIS
LTSIGFGDIVAVHPMARMLVILEGVTGQLFPVILIARLVSLHVETKHKK*
>SPSA8_v1_570013|ID:41146164| conserved protein of unknown function [Spirochaetes Bin 1 SA-8]
LFLKEKINKGEVKMKKIITLLVGMIFLSISVAAFAKGDFLGEYSKTLAPGPKGGAKLRWV
KPGVDHTKYKKFIVDYVVFAFADDSESKIINGDEMKKLGDACTLAIIDALKDKYPIMSEP
GPDVIRLKFAIVDLKQSRPGVSAITSVIPVGLGLSIIKKGATXX
>SPSA8_v1_580001|ID:41146165| membrane protein of unknown function [Spirochaetes Bin 1 SA-8]
MDHKEIRLAVILSMDVPGLHFSGREEEEDACLIERCGTSVRDCATEFKGEVLQSIGDSFL
LAFSNSLDAVRCARSIQRKFRSIDSENPAVALLPRIGIHLGEVFFVEHDAAGDTVTKASS
LQAVAKPGFTCVSKEVWSLVESKLEPEPGFSCKPLSPSRAKSLPKSQQSPMQVFEIEFSV
DDATETNSQNSGLNEKSDSSTRLETPIRSPRNISGFENASAGTAQNTSFNAGNQSSNLND
SSPENIVSSIKAALLDDIRVSGRRLTVDEALTKYGYYGIEAKEAIAQLADSGILIKNTRT
EGQPTFNSTSDLGKSIEQAVHAIVHEIERSVREGDFKNQIKNSSVKKAVKQEIKNSFKMT
DEQDRKRHRERRERKREVGTTNFETYKNELAKKQEKLAKSIPGGIVSFVIVNSALFFFAL
KNLNGFPWPVFVALFWLFGIIDNVFSYFRVRKQNFEVQALPDLDETETREIREIHKERDK
IGKSFINLLTIPTILMLINLATRGRDPWFLIPSGIIAVSFLAQVGRYFSTIPTRTRKFFE
KIGISGGKRGLKNASAQRMEESGIDLGEDYDALYRSAKDAAADIERSLRTHDAKEADEMK
PQLNSYLNQVLMLAKTTNEIDTIISEIPMENLARDKANLVAKLSDAEPTMRPEYEANIRE
IEKQESSFKALNEQKEIMDLRLKASVNQLQQLKMELARTRAMDAETSALEASSAIHSLRS
KSVELANYLEDLKGGMIEATQDPFDSLGSVSNLVLDGRQRDSTESPS*
>SPSA8_v1_580002|ID:41146166| conserved protein of unknown function [Spirochaetes Bin 1 SA-8]
MITIGSLVMYKSQLAIVTDSSDGRITIETETREQKKVREKDVLLLHKGPLKQLPPSLTDQ
TDFETAYEMLSADMGSEDRIPVDWAELAELVFGTANAETIAQCARFARQSPLFELKDEGL
YIISPESVAKIREKASKKQREAELRAKFIAALGKAIKSHNAQEISEQAEFVPFITELEAF
AYGISSQSQIAREAGIAADQLAVHKALLDSGIWSAERNPWPQRAGCSLSPPRLAFPETEH
PTMKLPREDLTSLESFAIDNAWSQDPDDAISIDGKTLWIHIADPAAFVLPDSDLDKEALS
RGATLYLPEKIVPMFPEKAMPILGLGLAPESPALSFKLEINEAGNILNTDIIPSLVRVHR
LSYEKADELINSGHPALNALLQKSEIRHQRRLANGAVDIDFPEVSMKAGAEEIRFLKIPQ
TKSSSMVREMMLLAGEGIGFWAKAQNIPFVYASQDAPNISESMLKSNSNPSALSIQYQRR
RGMRASITGTEALSHRGLGLPLYSQVTSPLRRYQDLLAHYQIRAFLAAKAGLPDADAMML
REDEVARRCMLAAQASGQTRQAERDSRMHWVCVWLMKNPGWKGLATVMDAGNRDALVFLH
DFGIELQMRTRRQLELDSTFFVRATRISIPNHDFTLEEAEPT*
>SPSA8_v1_580003|ID:41146167| conserved protein of unknown function [Spirochaetes Bin 1 SA-8]
MTALLVGIGLIVVAVLLVLPGIAGWWPDVLTFLKGSIPVLAIMIGAVAILIGVADIKDKR
EAEKEEQEEKAKEQAEEKK*
>SPSA8_v1_580004|ID:41146168|rplM| 50S ribosomal subunit protein L13 [Spirochaetes Bin 1 SA-8]
MKTMFVTSATAERKWYLIDAEGKPLGRVAAKAASMLRGKNKPTYTPSQETGDFIVIINAA

KVAVTGRKRQEKMYYKHTGYVGGLKDYTFRELIERDPVAPLELALKGMLPKGPLGRKLYK
NAKIYAGADHPHAAQMPITIDL*
>SPSA8_v1_580005|ID:41146169|rpsI| 30S ribosomal subunit protein S9 [Spirochaetes Bin 1 SA-8]
MVRNLGIGTGRRKCSVARVFLREGTGKYLVNDIEVSKYFKIPELVTMATRPLAVTASENK
FDILITVTGGGVNGQAGACSHGLARALAQIDPANQVSLRANGLLTRDPRMVERKKYGQRG
ARRRFQFSKR*
>SPSA8_v1_580006|ID:41146170| protein of unknown function [Spirochaetes Bin 1 SA-8]
VRIESVHFETSGMAAIAMSGGFLFQIPLVRVHELALYGFLLSDSDSSAMGQEQAREALRK
LPEIAGEFHEEDELFITISRIDEETRAFSKVSELCGIAEQNSQGLYRKLQAKGFSRSAIQ
KAIEKAQAEGLADNARFARIWARKRAEKTLCGPKTLLAELASRGISAEDARLALLDIPFD
MILEGAYMKTIRKLSKKYRGPELVQHAYRQLRLEGFDSESLKNLIDSEKNSL*
>SPSA8_v1_580007|ID:41146171| putative PTS IIA-like nitrogen-regulatory protein PtsN [Spirochaetes Bin 1 SA-8]
MDDDILTIEEVARYLRVSERTVYDWAQKGEIPSGKIGTVWRFKKSDIERWVNERLSSNRP
AGLIGSIQLQNIISPDRILFLNYSKKRDALIALADNLSSAPQIKNKQELIAEILRREELM
STAIGRGIAIPHVRLSSVTDLVVSIGISSCDITDFQSLDDVPVRLLFMIAAAYNQHAYYL
QTLSFFSARLKANELRDSLLSCKTPQEVYDLLIKQDE*
>SPSA8_v1_580008|ID:41146172|smc| Chromosome partition protein Smc [Spirochaetes Bin 1 SA-8]
VFLKSLEIYGFKSFADRTRIEFSDGISALLGPNGCGKSNVVDAIKWVVGEQSAKSLRADS
MEDIIFNGTETRKPLSVAEVTITISNEFGVLPLDAPEVAIKRRLYRSGESEYYINGKLAK
LKEVRELFWDTGIGKSAYSVMEQGKIDQILSSKPEERRYLFEEAAGITKHKVRVKEAEAK
LERTEENMRQVEGILAEVKRSHDSLKAQADKTIVFRDLKEKIFAAELDAYLLRLRQFVRD
KDRKEADFTQKKAERDRLHEETEALTRSMSESLDLVNEMESRLVEMQKTLYGLAVEKNGK
EKHKSMVADRLREVNAKIDQLEMRAKAVKEKIASLREEEEEKEAELADYRLRLKEAEKRI
EEFESGIKAAALALKANDEDAAKCRISISALEEAVRHDRTKLDEITEDIVEQLDIRLKET
GYSSQAHKALEEEIAALADTLKARMAGKAVLVEDIRRARNFDAPNTEALISRLLSSLEDL
SESAKLADLIASKFAEYRATTPAFLDEFLSPEGIITKKRSIDTSIARNLEEIARLNARIA
ELENDNRSLGEKIEAYRKTLEDARLAKARVQTQMTGAQDALAVFRREIAGQEAFLREQEA
EIFSETRRKTEIGEELEQLQDELDEIEAKGKKLAGDMHQLEQTIETKNSDLVQRKKDASR
LADRMQAIQNEMESIHLEIAQSETEIKNIRENFKELYSRDLSEFEDRMFEIRVPLSELRE
RLAFFRNQLKDLGSVNLMAPEEYREVKERYDFLSAQMADLQKARNDLKLITDQIREESAE
LFLQTYNKIKKNFHNMFRRLMGGGRAELRLSDPDHILESGIEILAQPPGKKLESISLLSG
GEKSLTAISLLFATYMVKPSPFCFLDEIDAALDEANVVSLVNMLREFSRTSQFIVITHNK
KTVSGADTLLGVTMEESGVTKAIAVRLERHQAGEASVVIAQDDGFMEEDVVFETGRQLPP
SAR*
>SPSA8_v1_580009|ID:41146173| putative Threonyl/alanyl tRNA synthetase, SAD [Spirochaetes Bin 1 SA-8]
MLQTEKKFYSHPELFADAATVLNVQKSAAGLEVILDKTIFYPEGGGQPCDKGSIQGCPVV
MVREAGKAIVHVLAAQSADFAAGDVVALQVEEARRCDHMQQHSGQHLLSGILEKDMGIHT
ISFHLGTEYCTIDVSTPSLSASQITEIERRSDFFIEREVPMLIHYCPPENPADFQLRKRP
PAEALNGEEVLRIVEIQNYDWSPCCGTHVSHLGQVRLLRILAAEKYKGNTRIYFAAGNRA
VQHLRRQHETLKSLAGRLGTSVDEADERVAALLRKSSALESAKKLLVSQRARLEVSMAIQ
EAAERGEQLKLLFLRSDDRDADEVLETVKAAQNASLDVIVFSVPAKTVIAARSPDASWDV
ASCAKPLLGEFEGKGGGSASLFRAVFQDGAKAEAYAAVLRECAIGTEIQRISFNPSNSSR
NSEFLL*
>SPSA8_v1_580010|ID:41146174| alpha-glucosidase (fragment) [Spirochaetes Bin 1 SA-8]
MPQVHSDVGGYTTLPKMARSKELFLRWLEMSAFSPVLRTHEGNRPGANWQFDSDEETIVA
VSRFSRIHANLKPYLFATAQEAAARGMPFMRPLFYHYDGDFGDDEDKAYLLGRDLAVYPV
LKPNAKSRKLILPKDDWMYLWDGTGYGGGGAEVSAPLGTIPVFYRRNSEFRELFEGLKDI
R*
>SPSA8_v1_590001|ID:41146175|hyfB| HyfB [Spirochaetes Bin 1 SA-8]
MKFFQQSGMRKRALRKIVGLLEFNLLPEGAAMAFYNAGILILLSGVVLVLFMKTSEGVSR
LGRIMTLAGDAAVGIAAAAGMAGRYAGQSYVFFWRLPLGQALFSLRPLSSFFLLMLATVT
ALAAVFAPSYLEHYHYSAGRMKYHWAFYLVLTVSMMLVVTAGNSVFFMLAWEAMSLSSFF
LVMFEHQKPEARKAGLVYFVFMHIGAVCLLVMFAILGKAAGSYDFAAMRSLGTNIAPGMK
TAVFILSVFGFGMKAGIFPFYAWMPEAYRAAPADVSAVMSGVMKKTALWGLISILSLAGC

FEAWMGWTLLVSGLITGLLGIAFAAVQNNAKRLLAYSSGENXGIISMAAGIWILGASWGN
SLLEALGFTAMLVHILNHALFKSLLFLGAGAMQSAVDTVDLDKMGGLLKKMPKTGLAVIA
GGIAICGLPPFNGFISEFLMYVSAFYAGTKSSLGNVIFIVAILGGVSMIGALAVFTFTKF
IGTSLLGEPRTEYAARAQEPAPGIYVPLYVLAGLTLFIGLFPQAVLLLLTPVFAEVGISG
FNTMADSIFPMLGIIQKVVLSLVSIMILLFIVRNRLLAKRTKAAAETWGCGFDRPSARMQ
YTASSFAEPLTSFAGPLFSPEISEGVQTSLFPAPVVKKITVRDLLLDRLIIPAYRWLAGI
FEKFSVIQHGNTHLYXX
>SPSA8_v1_590002|ID:41146176| DNA-binding protein, excisionase family [Spirochaetes Bin 1 SA-8]
MKLSVKEVARLFNISEKTVYRWIQTDNLPHYRIGGQYRFSYSELLEWAAIHGTTVQSDLF
AENGEQADSPVSIANAIELGGIHYRIEGSSKEDILRSVVSLVKLPDGVNRDIFLQFILAR
ERLGTTAIGEGIAIPHVRNPVVFHSEHPQVSLFFLEKPVDFGALDGIPVSILFLIISPNI
AGHLKLLSHLMFVLKNSEVLAVLKGSGTRNEILSAIRNAEASFAQNCRTAGIQPAS*
>SPSA8_v1_590003|ID:41146177| exported protein of unknown function [Spirochaetes Bin 1 SA-8]
MKYLKYVALALAMVLAVSCSPKLPQKDVDAANAVLADAQNAKADVYAPAEFQAANDAKAA
LDAEIAAQEAKTSGKSYKQTTELAKALSDAAKKAKDAAAANMETAKNDVATMLTDSQALL
AKVKADAELAAKDAKKAAKAKLNVKTIAEQVASYEAALAEAQAANDAQDYAGAKDKLTAL
KDQMTATQASLEAAGFAAAN*
>SPSA8_v1_590004|ID:41146178| GDSL-like protein [Spirochaetes Bin 1 SA-8]
MYAREVFLFGDSIARGVMLDTNGSYKPFADCFAVRTADELGYQLINKARFGCTISKGLEI
IKRSILGLGKALQDSDSKRLAFLEFGGNDCDFRWDEVAAHPQAEHLPNTPPELFRQLYRE
SIDILRESGYTPVLLTLPPLNAERYFDWFTRTGLNRAAILGWLGDVQFIYRWHESYSNSI
WQIGEHERCVVLDIRSAFLKRRAYDKLLCLDGIHPNTAGHELIKHEILDYAKEV*
>SPSA8_v1_590005|ID:41146179| conserved protein of unknown function [Spirochaetes Bin 1 SA-8]
MEDSHEQAIAEYLDVLESMEAPDWDKLPDIGLYMDQVISYLTRLIKPFYALHEDHKITPS
MINNYAKANVIPRAEGKKYSQEHLALLIMIMTLKKSLSVQDIRTLLDEQAGEATHRDQYG
RYQAVLDVAGKSVAAEIRTELAGLESANPADRIRASEKLRELALKLSLEGSIRSLAAERI
IDLLR*
>SPSA8_v1_590006|ID:41146180| exported protein of unknown function [Spirochaetes Bin 1 SA-8]
MKKAIFFIAVLLLATMAVSAAPSESGSMPITVTVDGFLSISVPTFTTRNINAAEDTVLEP
DGKLKIISNLNNWYILVHSVNGGLVMHSAGMGPAYSLPYTIAISGLIDETKLDSDVESAS
QSATALAGNEYNIKLRFGPSATFITAGDYSDTIVITLSVR*
>SPSA8_v1_590007|ID:41146181| conserved membrane protein of unknown function [Spirochaetes Bin 1 SA-8]
VTIPVVAKILVVFFLIVFATSRKIHLGLAAFAGGFFLVLWCGIPVSAAWNGFIAEMLNPD
LWMLVALLAAIMMFSAAMKKAGGMEAFSSSVIALMPSRRLALAIAPMLIGTLPMPGGAIL
SAPLVGAMNEDGARSPETLSAANYWFRHTLELAWPLYPAFILTVSITGIRSSQLMLLNLY
AVPTLFLLGLMFILPHEQSKSGMTRKSSRSGEGHFASLSGLIPLAIVLAVYGLVSIFSDA
LFSSFGLPAAVLALLNRYLPVFLGLAASSVYLLSKYRGKRIFSGSLTASTVRLIGVVIGI
RIFSALLNSANLASAAASELASAGIPAILVIAVLPFIAGLVTGVGFGYVGLSLXIVIGLI
PTVAGLPYLAGIALAGGFGYAGMMLSPLHVCMVVTAEHFRIGLPATIRKFAAPLLVFTGV
LLAYVWILTLVLQ*
>SPSA8_v1_590008|ID:41146182| conserved protein of unknown function [Spirochaetes Bin 1 SA-8]
MKSTQCLFTGTAFLLGAAVAEKEAVVIFVGDVVGPSGLRAVFAHLPAFAKKKKADIVIAN
GENAQRGFGIGKEEIEALISSGVDVITSGNHVWERKDAEELIGSTSVLLRPANYPAPLPG
RGYGVFKGRNHTWAVVNLQGREDLYPIDCPFKTADEIIARVKREQPDAFIVIDFHAEAAE
EKEALAWHVDGRACVLAGTHTHVQTADERILPLGTGYLTDLGMSGPLDSVIGVKTDICIQ
RSLTQIPIKMETSENEASVCGAVFTIDTATRKCMKIERFELK*
>SPSA8_v1_590009|ID:41146183|rny| Ribonuclease Y [Spirochaetes Bin 1 SA-8]
MSLKVWIFALPVAGLILGWMIRWLYARFQLTAAENEAERIRQDAVKEAEAQKREILVEAK
DQLIRERNQQEREMRERRIELQKLERRVIQKEENLDGKIEALERMEAQLKVVEKEKAIADRE
AALLDQEERYREELERISGLTSEEAKRVIIQNMENEARHDAQLLINKIDQEAQLTAEKKT
RDILVTTMQRIASDVTAETTVSAVSLPSDEMKGRIIGREGRNIRTLETLTGVDVIIDDTP
EAVVISCFDPVRREIARVSLERLIADGRIHPARIEEMVQKVTREISQKIYDEGEKVVFDL
GLHNMAPDLIRALGRLHYRTSYGQNVLMHSKEVAVIAGLLAAELGLNREIAKRGALLHDI
GKGIETDGDQNHAEMGMDLVRKMGEDPRVINAIGSHHNDIEPSCPESILVQIADAISAAR

PGARRETLDNYIKRLENLETIAESFSGVDKAFAIQAGRELRILVNYENVTDDQARELCKN
IAKKIETDLRYPGRIKVTIIRETRIVEYAR*
>SPSA8_v1_590010|ID:41146184|rbpB| putative RNA-binding protein RbpB [Spirochaetes Bin 1 SA-8]
MSKKVYVGNMNYSTAENQLRDLFAQYGEVASVNIIVDRYTGKAKGFGFVEMNNDDEAQKA
IDALNGFEFMGRQLRVNEAEDKPRRDGGNFRPRRY*
>SPSA8_v1_590011|ID:41146185| Ribosomal large subunit pseudouridine synthase D (fragment) [Spirochaetes Bin 1 SA-8]
VHRLDKDTSGVIIVARNPEVHAFLAGQFRERTTRKEYFALTHGVPKELDGRIENYLARDK
KNRKKFAAQKEGGKHALTIYRTVAVWEAEKGSKYALVQLFPKTGRTHQLRVHLAGMGCPI
VGDPLYGVKESFIPDATLMLHARRLKIRLPGEEAARVFKAPFPAHFLKAVRFLDARAKK*
>SPSA8_v1_600001|ID:41146186| conserved membrane protein of unknown function [Spirochaetes Bin 1 SA-8]
VLSKTDPNAVALDTSRWNLKPGAEGGIFGTDGLGRDIWARTWYGTRTSLLLGFVIALFDV
GIGMIAGALWGYVKRIDRIMTELYNVLNNIPQTVFLVLLAYIMRPGFWTIVVALCSTGWI
SIARFVRNKVMSIRDAEYNIASRCLATPLPRIIQKNVLPYLVSILIMEAALTIPYSIGAE
VFLGFIGLGLPVEAVSLGNLVNQGKNTFMLYPYQLTWPTVVLAAITISFYIVGNRFADAS
DPRNHV*
>SPSA8_v1_600002|ID:41146187| fragment of putative dipeptide transporter (ABC superfamily, ATP_bind) (part 1) [Spirochaetes Bin 1 SA-8]
MSGALLLSAEDLVVQFSLRGRKLTALRGASLELFEGETLAVVGESGSGKSVLAKTFLGML
DKNGTITAGSIQYRGKDLARLSRERDWLEIRGKKIAMIFQDPMTALNPLKTIGEQIREVI
ELHRGMDRRQSKTETLKTLEAVGIQDPERRYRQYPHEFSGGMRQRVVIASAVACKPEILI
CDEPTTALDVTVQAQILDLIKALKAEYAMSVLFITHDLGVVANVADRVAVMYAGQVVEQG
TVDEIFTDPRAPYTWALLASLPQLGVKGEDLFAIKGTPPSLFKEIKGDAFAPRNPLALEV
DFVAEPPIFDVSPTHWAKTWLLDPRAPAIRPPDSVLRLREKLLAERKLS*
>SPSA8_v1_600003|ID:41146188| fragment of putative dipeptide transporter (ABC superfamily, ATP_bind) (part 2) [Spirochaetes Bin 1 SA-8]
MKQSPLLSVKNISVDFGSGRKKFRAVSNVSFDVYEGETLSLVGESGSGKTTIGRAIMRVH
PISEGEITYRGERISGDLTPKAERLVHQKIQMIFQDPMASLNERAKIDYIVSEGLYSFHL
FKDEAERVAKVEQALRDVGLLPEFATRFPHEFSGGQRQRIGIARALVMEPELLVADEPIS
ALDVSIRAQVINLLNKLKRNKSLTYLFIAHDLSVVRFISDRIAVIYRGHLVELAQSEDLF
RKPLHPYTRALLTAVPTPDPQIEKLKKIIVYKPAERSPDEPAPSWIEAESGHFVLGTEKE
VNSWLKL*
>SPSA8_v1_600004|ID:41146189| putative xylanase/chitin deacetylase [Spirochaetes Bin 1 SA-8]
MKKFSMPLFALLLVLLISGCATLHSGSSAQNQPLPDTANTAPSASSYLDPIRWWDVGVQD
PTRELAEETPDQLVLRVPKRADPKIVVLMYHNIVFGRTGGEYNRDLYNFEHDLVFLRNRT
QIIGLDELPYIQSGQKKLDTDASIITFDDGDLSIYAIAYPLLKKYNIKATFFIITDYVGT
TGYVSWAQLKEMADYRNEKGEKLFNIGSHSLDHKRFDEIPAEQIPKELSESKFAIEKNIF
SPVWYFALPFGAGSGRKEIIETAKNFGYRGIRTSATGVMTPAAIDMYNIPAFYMTNERAD
ILSQQIYRLLGR*
>SPSA8_v1_600005|ID:41146190| conserved protein of unknown function [Spirochaetes Bin 1 SA-8]
MPRPFCKRTIGLNPDDNVFMPIGIPGGLLEEITLSLDEAEALRLADLEGLYQEAAARSMN
VSRQTFGRIIESARKKTADAIINKKMLKIAGGIVQLKEQGEDTMKIAVPTRGDQIDNHFG
HCEKYSIFEIEGNTIKAQSSLDAAEGCGCKSNIASTLAGMGVRLMIAGGIGEGAIRVLAS
NGIKTIRGASGSVQKAVEDYLAGQLMDSGETCHTHGADHECSHTH*
>SPSA8_v1_600006|ID:41146191| putative Auxin efflux carrier [Spirochaetes Bin 1 SA-8]
MARVILMITLIPCGIFSGWAIKRFLTAPGRLAHEQRRDRDPIAVIPGILQKIALLGINPL
AFASSIWALKLNASGAASLPFIGLGTLAAGMLAGKIVSRLLRLSRGRDTVFSISSSLTNI
GSIGSLIVFYVLGEQAFALVPFYKFFEEMWSFGVCFPMAARTGLALKSQDLPLQRTGTAQ
SLLKIFRDPFILAAFVSIGAGLGLNASGLPRPEFLRQLNAAIVPLGSFLLLVSIGMKIPE
QQSRLSKPVLLSYLSIRLLVLPAASFLLCALFGIASSPDQTALKVALTMAVMPAAFTSLI
PPVLYDLDFPFSFSLWIASNLTLAGILPALWLLFH*
>SPSA8_v1_600007|ID:41146192| protein of unknown function [Spirochaetes Bin 1 SA-8]
LAIRFYCGESARIAAVSLAYTRRLLAAFFSFCIQDRIELYGTRYSDDYPDTLRYLLRLGD
KAVSDGARQTCS*

>SPSA8_v1_600008|ID:41146193| exported protein of unknown function [Spirochaetes Bin 1 SA-8]
MKKKNGFLLKLALVSALVLAMLVSCDVAGLSGTQNVSEDTAEARGLVKPEPKLDAAGNLL
PQKIVGTAGIGADAFFFEFDVSGQAKFARAFVPVSESGFLVNGKIHYRKENYRLTTLDIL
PLPDNSGATLMMGTTATVAGTSYSFQGVYSKETGFFGNIKKTVNGVVTGSEYDFFGGTPI
FPRSDVENYIGYATFHFDSDTTALIFNAAIDNKTKNIVGTWCETKPGMGNIHGTILGVRA
GKAVSFNGEVLDMYKSIPGLYLDTMTADGAGEFTNPSEKTITGSFTIHWIGYDWPSDFIA
VKVQE*
>SPSA8_v1_600009|ID:41146194| Glutamate racemase [Spirochaetes Bin 1 SA-8]
MLKNDNPEIGILCWEAGQSPRGLVQLEALKGNSTNPDSYNFPIRFYRVKGANIHTILENP
DKTVLARMIEAGKEMVQEGVKAVTTSCGFNAIFQKELSEGIGVPVHTSSLLQVPLVQLML
GSHRDVCIITAKKAALKPEHLAAASISRTENLHIYGLEHCPQWSRIFETPDEDLDVEAVR
QEVVGVAMDALCEHPHIGAFVLECTDLPPFAEEIRAKSRLPVFDFITLVQYVYSALPR*
>SPSA8_v1_600010|ID:41146195| Saccharopine dehydrogenase [Spirochaetes Bin 1 SA-8]
MSGRRVVLLGYGMQGKACLIDLLNSAFFDEIVVADNVPDIAQTMSALRDARVRGVQVDAS
DNGALSAVMKDASVVIELLPGIFALPVARLAAKLGVNLVSAMYLANPGEQDPEKRRAQRE
ELAAIDATMKEKGCTILEEFGMDPGIDLVLGRKALDDLDEVLAFHSYGAGFPEKGSDGNP
IHYKFTWSIVGVMRSYLRPSVVIKDGKSVAIPADAMFIPSSTHILDIPEMGGPLECFPNG
DSSHFAEVFGIRNIVKNMGRYICRWPGHAAFWSVMAQCGFLSDEPIQVGNAKIAPSVFCA
ALLGSQSKFFYDEHEKDVALVRTDARGMKKGMPRRVIYQIIDKRDLATGLTAMQRTVGFP
ASIGARMILDGRITKRGIVSPSEVDFNSFMEELGKRGIFMTRQEMEWDGNLEP*
>SPSA8_v1_600011|ID:41146196|ald| Alanine dehydrogenase [Spirochaetes Bin 1 SA-8]
MVIGIPREIKNNEYRCAAIPETVSELVQRGHSVLIERGAGTGSSISDTDFAQAGAVLTDC
QTVWESSELIYKVKEILPPEYRFLRKDLTILTYLHSNAHHEMTDELLKSGCIGIAYEDIT
DAAGNFPLLRPMSELAGKGGFFMACQFMQSINEGGGLMLTRISGVKTPEILIIGAGNAGM
GAAEMAASLGNRVTILDVSIERLESARNRLPSNVQLLYSNHDNLSSCLSQADVVFNCILW
PKTRKDHLITRTMLKVMKPSAMIVDVSCDEGGAVETCRATSHDNPIYREEEILHYCVDNI
PSAFSRTATYSLANATLPYAREIADKGPVRALRENPHLRKGLTCFHGILTLEETGLKQSR
PYQRPEDIIELAR*
>SPSA8_v1_600012|ID:41146197| Transporter, NhaC family (TC 2.A.35) (fragment) [Spirochaetes Bin 1 SA-8]
MLAYELDSGSAPVCTIIPLSAWAVYIAGLLKGYGPIVNAEIGMQTFIRSIPFNFYGIFAV
ALAGLIAYQVIPNFGPMKKAEERALKEGKVIRDGATPLTGIEMDEITPAEGKKPNLFIYL
FIPVLIVIGIALGTFLILHSTKTLEAFFAAVMYQAVALTIGGYFKNIKDFMDTATRGIKA
VIPAILILALAYCINDISKALGAQKFIFEMSKSWMTSSMLPLAAFIAGAMISFFTGTSWG
TYAILTPFVLPLAMQLSGNTVNTLVLASVGAIVSGGCFGDHCSPVSDTTCLSSFGAGSDH
MDHVSTQIPYALTAGVVAAILFGVIGILAV*
>SPSA8_v1_610001|ID:41146198| putative Periplasmic/7TM domain sensor diguanylate cyclase [Spirochaetes Bin 1 SA-8]
MQKIHAWKFTNAVFFDIIDSRMNKKTYFKSAWAVLIVALAAVLPIGAESSQSVSVIDLEK
TGLMYFNADPSLGIEDVQKAEIQDRFAPIDGRPVWVGKRAPVAWIQFSLPERASAEAEGS
PDRVQDTAALPVRWLLRVRPSFSIILDSVELYIPRKDGGFSRYVTGALNPESALELDSRY
FLFSLPEDAYGRDCYLRFSSNTDVLINLELLNPKAMTAKELTTFVVYGLIFGILFAMAAY
SVFMLVSLKDRSYLFYILFTISIGLWLFYVQGFSKALFGKVPFMDQAMLWLWAGMFITWG
TVFSISFLKLKRGSFLLSVMLVLAALGAVVSAAGLLGWNQIAFTLSHYLGLVAPVVIILA
ATVRLSRGFRPALSFLIAWLFLALGGLVFSLMGLQILPVNFFTINALSIGAALESIMLGM
ALAERFGQLESEKTELEKAQKKYRELSYTDALTGFYNKLFLANYFENELAGHQQDSKDLA
GILMDVDDLKAINDSFGHFAGDSMLLALAESVRTCVRGNDLVCRLNSDELVILLPATTKE
NAFRVAERIRIRFETDSLNVVNDKAIHCTVSIGVIQLQSGEDMGSFLARADKAMYEAKHR
GKNCAVMM*
>SPSA8_v1_610002|ID:41146199| putative AMP-dependent synthetase and ligase [Spirochaetes Bin 1 SA-8]
MQSALLGVPFKTAQLFPDRTAYKWRAKNSEETRSYAQLARSIRLFAAGFKAYGIQRGDHV
GFFVNNRFEWIVSDFALMALGAVSIPRGSDTTAKEVAFIFHHSDSRHLIVETARQLAQLV
ETFEPADWERCATIFVVEEGDESLIPEFLSAKTRFYSELLQKGEEEYAKDPELLEKLDRE
IKDSDLLTIVYTSGTTGNPKGVMLSHANFIQNLVANTPRLQIDINRAETTVVMLPSWHVY
ERAFEYCGLASGLTFVYSNAGRFASDLLTEKPHLLISVPRVWESVYQKIIKAIAEMPGAK

RRLVLGLIKNNQAWMTSNHYLKGCYITLHERKLPEKAIAAVWHVLRWIALFPGHLLAGVL
FKPFKEKVGGRLRLATCGAGSLPKYLDEFFNAIGITLVNAYGMTECAPGILSRRVGYNTF
GSTGIPFDNTEVEIRKEDGTRAAVGEKGVLFVRGPQVMSGYYKNPEATKAVLSDDGWLNT
GDLAVQSENGEFIIVGRAKDTIVLMGGENVEPEPIEDKLKESVYIDHAVVLGQDQKQISA
IVAVNEEELMKLAAELKLSMNEIVISGSNSIENDAIYNILLEEVNSRISKEHGFRPFERI
TKIFPILNDFSVGKELTQTLKVKRKYVEERFKHLVDKVLKGEEKGKK*
>SPSA8_v1_610003|ID:41146200| Uncharacterized AIPM/Hcit synthase family transferase aq_356 (fragment) [Spirochaetes Bin 1 SA-8]
MPYVGLRAFAHKAGMHADGILKTTSSFEHVEPKSVGNDRRFLMSEMGGRAAIAESIRKFE
PLATKDHPAVKALSARLKELEAEGWQYEGADASFEILARREVGLYQPFFRLERYEVQSRY
PEMKESSCASAWVKVSVNGTEEIAASEGNGPVNALDSALRKALQRFYPELARIRLTDYKV
RVIDTMSATSAKVRVLIETTDGERVWTTIGVSTNIIEASSLALADSIEFSLIQNSI*
>SPSA8_v1_610004|ID:41146201| Uncharacterized AIPM/Hcit synthase family transferase TM_0552 (fragment) [Spirochaetes Bin 1 SA-8]
MIEIFDTTLRDGAQGEGISFSLQDKLVIARTLDQLGVAYIEAGNPGSNPKDMEFFREASK
LELKHARLCAFGATRRKGMSPGRDEGVKNLANAATSTVVLFGKSWDLHVQQVLQVSHEEN
LAMIAETVAFFKDLGKEVIYDAEHFFDGYRLSREYALASIAAAVEAGADCVVLCDTNGGS
FPDLIMAGIQNVRQTHSIRVGIHAHNDAGMAVANSLMAVKSGAVQVQGTLVGFGERCGKT
WHDLSSGTESLQSDRNRSENRGNRQCTPPR*
>SPSA8_v1_610005|ID:41146202| putative Branched-chain-amino-acid aminotransferase [Spirochaetes Bin 1 SA-8]
MSFESMVIPGSLEPAQLASLDWGKIGFSLTVTPYMGGSVAGPDGAFEPCAVVKTGVLKVP
PQACALNYGQAMFEGMKARIGVDGKIRLFRPEANAARMASGAARFMMPAPSTELFIETVT
KVVKANRDYIPPYGKGSLYIRPLLFGIGKTLQPAPSDTTMFVVYTQPVGLYFKGMACITI
KADDTYQRAAARGTGFVKAAGNYAPCFYPAAEAKHEGFSDVLYLDHDGEHVEEVGSANFA
MVKNGTLYVADSPSILRGITRDSVMRIAREILGIEVVFAPLELDRVLGLGAYAGQGPAEE
AFCTGTAAAISPIGAMRWKDADYSFSGGAIGPITQKLFDTIDGIQTGRIPDPFGWTVVVD
*
>SPSA8_v1_610006|ID:41146203| ABC transporter related protein [Spirochaetes Bin 1 SA-8]
MNLLSLKDISLSLKSGPLFESVTIDIDEADKIGLIGLNGSGKSSFLKLITGAAAPDHGEI
SLAKRFTYSFLPQKSEFSENTTLGDFLYLGDATEILHPAEEEPRVSLENRYSALCKELNL
PSPDTPMKFFSGGELKKAELARALAPSARLLILDEPTNHLDVDTIEWLEKKLQTSRQAII
LVTHDRWFLDAVVTRIAEIDRHTITMYPGSYSRHLERKAVILAGLERAENKRLANLKIEL
AWLNRGARARATKSERRKQEIRAMSESLLEKPPSAYSFSSSETRLGKKVCVMTDVSFSYG
TKQILKDFSFEFQPGEKLAIVGPNGSGKTSLLKLISGMLTPDSGTIDLGATVQLSYFRQT
NESLDGEKSILAFIQEHADHFKLADGSVLDAELLLERFGYDRDFRNQKIKTLSGGEVRRL
MLVRILAESPNMLLLDEPTNDLDIETIERLEEYLEDFGGTVILVSHDRLLVDKLAQNLLI
FKGSGVMERYSGSYLEWSEHKERETSALLSAEKPKSKKPGAADDRRDTQKTSVGRDKLSF
KEKREFENLLNEIDALETEKRTLEESFANPKAFGPDMQSAHARYEEIEKLLEQKMARWEH
LAEREG*
>SPSA8_v1_610007|ID:41146204| Citrate transporter [Spirochaetes Bin 1 SA-8]
LQKKRRDCTAESVWSRNFFAWSIRMTLLVPVIAAVMYLGIILVPQKKSWFSLGAAFILIV
TGIAPLREALLHYINWNILLIYLGSLVLAELFIYSRVPAYLAEKIIDKSSTLGAAIVIIL
LMTGFISAFVENVATVLVMAPIMLELSRKLKKSPAPFMIGLAVMANLQGTATLVGDPPSM
IFANYAGYSFNDFFFTQGKLSIFFFVQAGALAGALYFYAIFRNLDRERVSVPEERIQSWI
PTLLLLFMIVGLGVISGFSAGGIHLASGLLCAVLAIVGLVWLRCSMHESRDSVKKMLIGL
DWDTMAFLAGIFIVIGSISATGALDEVARWLAGVIGGRIALGYVVIIAFSVVVSGFVDNV
PYIVAMLPVAAGLAKTMQVKPELFMFGLLIGSCMGGNLTPFGASANVVATGLSKKAGSKV
SFISWLKLAGPFTLITTVVSALVTYAVWXX
>SPSA8_v1_620001|ID:41146205| protein of unknown function [Spirochaetes Bin 1 SA-8]
MKRCSLAVLVIGILFGSVIAIGAQEAVSGATKEWTIDSISAASKAFYYAKSSLTGEDLWK
AIEARAGAVVVATANEDGTPNAAVIIPGISKDRTALIFGIAPNQTLENLKARHVAVVTIY
LYTASATDKFERNKGARIIAELITDQTEIARLAAENKDRGASAGSTFLKIVKILPLG*
>SPSA8_v1_620002|ID:41146206| conserved membrane protein of unknown function [Spirochaetes Bin 1 SA-8]
MKKSVLVVLLLAGVLSFATAEGIAGDPHASVGDLMVSAGANIGWGSFGVGGGAEYIFARW

DIPGFAPLTFGGAAKAGVYFGSSIHIDLAALGTIHFGLKTFESLPDFLKNLDWYYGLGIG
FGIGTSGGLGFSTGSGISYYLNDKLALNADYFYTDYFGSGAGSSSTLGVKLEL*
>SPSA8_v1_620003|ID:41146207| putative Patatin [Spirochaetes Bin 1 SA-8]
MIFFSALFLLFAQTGNPGNFDTVFPAFDAVGSILLPFEYGVDGFAARIEHVRDMGREPIG
LVLTGGSARAYAHIGVLRVLEQAGIVPDFIVANSMGAVVGMLYAAGFSPDLIQKTIEQIP
PERFMSLVLPGHGGFLNAGGFIATVSKIVDGIDLKDTPIPILVVAEDLKTRRQVRFAEGD
FARIMAASFAMPAVFEPALVNGHALVDGGSTNLVPVAIARDFSSRLIVSTTFYDRSMSYT
NPLSILNRTFDIGKTRAGLQQIQDVNPFIIRNDVEHFSFMEFSDPHAIIGAGEMSASAAL
ADVRTYLGIGLNSGLNGLDEALQVRRDYYAGRVPGMIRELVFGALPTFEPTSRYKLRFKL
SDAFGYSVMSLDDQSYFGLFLASAVGKIRFNAGMMAGLAGVAGHQWGAIAEFIANPADTF
KFTSAARLWGDFASYPTFFLQPKSAEIAGSLSWTSQTNLSVVPFVEGKAAVSLADGQVSW
LARVGSKLSLISAKHLEFSAGLDFFIDNDDGSSLRYGPEGMLTLGYAEPLLGAVRLHGAM
RQDFSANGLSFSAGDAFRGTTPSGTAPTLAAAGLDLVWFAKSLEFAAGEVILVRNIEIGP
YLDLAWKGFSASDGYAPDAFAVGLNANLSVSLVGLAPFDVACYAAVSSSGTLMVGLRSER
LHVRPAAIR*
>SPSA8_v1_620004|ID:41146208| conserved membrane protein of unknown function [Spirochaetes Bin 1 SA-8]
MLSELALIFLLILLNGFFSLAEMALMASRKARLRSEAEKGDKAYKMALETAENPGRFLST
IQVAITLIGILTGAIGGATISQSLTAWLASIPPLANAASALAVAIVVAATTTASVILGEL
VPKNLALSKPEVISAKLIRPMAIFSAIFYPLVKFLTVITDAIVRLLGLKANREPEVTEEE
VKVLIAQGTESGIFESSEKGMVEGVLELDDRRITSYMTPRTEVAALDLADPEISPLQFIM
DHAQYAFLPAMEEDLDKIAGMIPVRPALAAFLRDRNTDIHSILVPPVFIPETISALRALA
ILRESKISTALIVDEYGGVSGLVTRGDLLGSVLSGIDEGASAEIPGVVKRSDGSFLVDGA
LPIADFSDSLDLDETLFDTDLYDTVAGLVLACMGSIPKAGESCDWQNLHIEIMDMDGNRI
DKVLVEKRDTEQSIDQPS*
>SPSA8_v1_620005|ID:41146209| conserved protein of unknown function [Spirochaetes Bin 1 SA-8]
MDYLVKEIERFYKVIKLKPFRRTEGVAFDIFPMQYLPRIDGIDRVIHKSSAVSPGAVGDI
ARPWYLHPHQEDNLLVLHGVRYVEIYTLEHRRVENFTVEPERILHNGTVIFDGPALVVWP
VNVFHRIVSGEQGSASLNFAVRHPGFDIATNFSIYDLNTETGEYRVIREGRLDQFAE*
>SPSA8_v1_620006|ID:41146210|patB| Cystathionine beta-lyase PatB [Spirochaetes Bin 1 SA-8]
MREDFDNVPDRVPTSSLKWSYRKRFCGDDSVLPMWVXXMDFACPRAIVEAVRARADHPIY
GYPVRTDGYYSSLINWMKKRNGWDIKKEWICYSPGVVPAVNFCVLAYSQPGDKVIVQTPV
YYPFSSAVLNNGRQLVENQLRLDGDHYVMDFEDLEQKIDARTRMIILCSPHNPVGRVWKQ
EELERLVEICRRKGILIISDEIHSDLIIGPTRHTCIAMLPGAADITVTLTAPNKTFNIAG
LTTANAIISDKRLRDAFMTVAGNLGLGISNVFGIVAQEAAYTYGEPWLEELLEYLRENYR
TLTDFVAARLPELSVLPLEGTYLAWLDCRKLGMNDAELKEFFLKKTKLWLDEGTMFGNGG
SGFMRINIACPKQLLTEALTRIENAFIENRR*
>SPSA8_v1_620007|ID:41146211| membrane protein of unknown function [Spirochaetes Bin 1 SA-8]
MLWFIFEGIPKVDVSRMQGFMANGFSNFLTALIMYMFALDGASVVINMGGEVKDPRKSIP
VAIIWGTVIAAVIYALLAFIATTAVDYKQLIPPVTKRAMPLGEFAKKFMSPAGFVFFISG
GAFLAILTTLNAGIMVQSRIFWAASRDGIFPRWFAQLNKSGQPSRVTITVALIIALPIIF
KFDLSFTTLITLVPGFIFSMMMPISVLFIEKKFPNLYKKSLLPLPKIWLYLIVAFSLGIS
LLLSWNGLLKIGVKNLYYFAGFFGLGTVYYFIMTARMKKEGRIYRDIKHDYDKYWIEEEA
RLAQQNK*
>SPSA8_v1_620008|ID:41146212| membrane protein of unknown function [Spirochaetes Bin 1 SA-8]
MAGTEGQDFKREITTAQLVWICVGAIIGAGVFSLTGIGIAYVGSAAFLVYLVAAAIAIVS
TIPTMIAASACPTTGGYYKYISRFLDPTLGFYYMWNRIIGIFFFFFFFKPCQSGHLLFLP
YFSLRFSTCSM*
>SPSA8_v1_620009|ID:41146213|yjgF| Enamine/imine deaminase [Spirochaetes Bin 1 SA-8]
MSKHCFNASNAPLAIGPYSHAVIAGDFVYLSGQAGLDPSSNQLVSGGIVPETRQVLTNLK
AILTEMNLSLADVVKTSVFLKDMADFAAFNAVYAEFFPADCPARTTIQAAALPKNANVEI
DLVAYKGK*
>SPSA8_v1_620010|ID:41146214| conserved protein of unknown function [Spirochaetes Bin 1 SA-8]
MLNTDVAEITGSHRFSDTDRQLLARYELVAESVARIFGSGCEVVLHSLEDMAHSVIRIFN
GQVTGRAPGSPITDLGLDVLKKSFETRNDIIGPYFSKTKAGKPLKSVTTLIRNDEGTPIG

FLCINFDLSMPLSHFLQEFSPSSEAPQTGENFAPDVTDLVGHAVADELDAISRVTGVSPT
EKNRRVVFNLEQRGVFEIKGSVELVAGELGVTKHTVYKYLREFRNQN*
>SPSA8_v1_620011|ID:41146215|pdxT| Glutamine amidotransferase subunit PdxT [Spirochaetes Bin 1 SA-8]
MKIGVLALQGDYEAHIKAIEAAAERAVPGRTASDGIVLFEVRSLQDLNKADAMIIPGGES
TVMGSLLIRFGFLDAFRDRILSGMPVFGTCAGLILLSRQIEKFSQPGLGVLDVTIRRNAY
GTQVDSFRAPLEVRFAEDGAERVETMEGVFIRAPKIVQTGPNVSVLASLAGDPVLVRQGN
ILGATFHPELVAGARIHDYFLGIVKKSV*
>SPSA8_v1_620012|ID:41146216| Pyridoxal biosynthesis lyase PdxS (fragment) [Spirochaetes Bin 1 SA-8]
MSDPKMIKEIQDAVSIPVMAKCRIGHFVEARILEALGIDFIDESEVLTPADDKFHVFKHD
FKSPFVCGCRDLGEALRRIGEGAAMIRTKGEPGTGDVAEAVRHMRAVRDGIARLTRLPEE
ELMTAAKELGAPFELVYEVAKTGKLPVPNFSAGGIATPADAALMMQLGAEAVFVGSGIFK
SGDPARRASAIVSAVVHYKDAKILAEISEDLGEAMVGIGLSELGENQKMAVRGW*
>SPSA8_v1_630001|ID:41146217| exported protein of unknown function [Spirochaetes Bin 1 SA-8]
MKKLIVLLLAFAMVGAVSAQVTTSIGLYGEVTVIDQAGQGVFTRYGNGYDTWTFKASDKD
GKYGISLTDQNILDDGGFTVRDWNFWWKGQYTKVILGNLRNADFRMTVPYWAGATIFGGT
DRITGYGVLIETLPKNGLTFGVNLPIGTAAANTVDILQNADLGVKYDVKGLGSFIALFNA
DFVAPAANVLNLGAKITSVKNLTAVGIAQLKFDADTYKAALGFAYSGIENLSANLEAAYV
YTAGVSTFSVWGQGAYSVTDKLSATVGGSIANGGAYDVYANLGYDYGNGLSSEAGVGFDG
AFYGALKVYYSVAF*
>SPSA8_v1_630002|ID:41146218| exported protein of unknown function [Spirochaetes Bin 1 SA-8]
MKTCKSPILITIIFATAMLISACATNSKFNPNQPQVTMSQAVQAELRQFGPNSDTNPYIE
PRTLIRGKLNEFYIVRLALNLSSESQISILAEAKGPDGTETAKAMDMYAFEAYWDSVTYR
EPDNDAKIQTRIANIKRSCIPSFSFTASAGQGIYYIPFIGKNPMTRPSRIYVQVTVGNSE
PIIYTADL*
>SPSA8_v1_630003|ID:41146219| putative phosphoenolpyruvate-dependent sugar phosphotransferase system, eiia 2 [Spirochaetes Bin 1 SA-8]
MNCQLLIKYLDSGDTYYGVRGKNPQDAIESLFSALIVPEGLDRNALKEAILDREAIMPTA
IGDGFALPHPRKPLEDSGLMPIIALAYCDSPVDWAALDDIRVSNFFLVVSTNQEEHLALL
GELAELLRDKNFVKFLGEKPTKKELLDYLVSENPLATR*
>SPSA8_v1_630004|ID:41146220|rlmE| Ribosomal RNA large subunit methyltransferase E [Spirochaetes Bin 1 SA-8]
MAGYDRPDFWTLKAKKEGYPARSVYKLEEIIRKFGLLKLSGGASGPAVLDIGAAPGSWSL
WILRQLKGVGSLTAVDIQDLGISPSDANFQFFKGSILDETMRKNLRDAGPYNLVVSDAAP
STTGNRLVDQSRSEELVEAVMGLALEVLAPGGALIMKIFQGGEEKRLIASLKQNFAQARG
FKPEACRAESFETYLVASGFSLTR*
>SPSA8_v1_630005|ID:41146221|htpG| Chaperone protein HtpG [Spirochaetes Bin 1 SA-8]
MSKYAFKTEVSQLLSLITHSLYSHKEIFLRELVSNASDAIDKLKYLTISDESFKSIQFEP
RITVFLDRDAKTLTVSDNGIGMNEEELADNLGTIARSGTKAFLEQLSDTDRRNSNLIGQF
GVGFYSIFMVAEKAEVTSRKAGTSEAFMWSSSGQGEFSIEPSTRESFGTDVKLYLNEEGS
EYLYRWSIESLLKKYSNHIAYPIYLVSNEPKYDKDGKEQGKKLQEEQVNAASALWRRPKS
ELQDKDYIEFYKSLTGDDKDPLLWIHTKAEGTIEYTSLLYIPSRVPADLYIPSHEQGIKL
YVKRVFITDRELQILPPYFRFVKGVIDSDDLPLNVSREMLQHNRVMMTIQQAALKKLFSE
LEQLAAANPEKFKQFISLFNVQIKEGILSDWTNREPLLKLVRFKSTLADGWTSLADYKNR
AGDSRKTVYYISGDNEARLRASPLLEAFKEKNIEVLLGTDEIDELTFGGIGEYEGMQFKS
VQQLDAEEELGKKDESSPKDSEKAVAALKRVLGDRVKDVRLSRRLGKSPSCVVVDKDEFT
PQYRKLMERLANEAIPETKPILEINASHPLVNRLSQLETRKELSSGDTEIVQELEDLAFV
LYYEALLAEGEHFEVPHDFSERLNRLLAKTE*
>SPSA8_v1_630006|ID:41146222| putative Peptidase M23 [Spirochaetes Bin 1 SA-8]
MKSMPYNILNFSRRMKAPGRGLTFFGSGWYSRKLERTVPEGLREYKKQEKLFYLRFIAAL
KALWGKTAGLFVSLYSKGRQRLSLAVIPHSEKPARSVTLSRFALVFLLLLTGIVLVFSIA
SAGRYALTAAQLAAVARERDDLRDTVDRLRDGVQTLASSALKFEREMTTLQDISKTNSAG
TGSKPSGVTASSQSDALRTAGLSGLLSIPDNPGLAGREIAQLEKISSFLDASVPDVERIS
VLLAGQQDILTEIPNIWPVQGGIGHISMYFGQNENPFSAGVWYLHNGIDISTFRTGDVIL
ASADGKVIDASYDPSLGNSITIQHSHGFLTRYGHLKSIKVKKGQIVSQGQAIGTLGNTGI
TTGPHLHYEVHLGTSVIDPLRFLNIRKAQAVRPAP*

>SPSA8_v1_630007|ID:41146223| protein of unknown function [Spirochaetes Bin 1 SA-8]
MARDSTPHLTECLLGAGSSVTGTVSGQGVVRVEGDFEGSIQARNAVILAKGAKVKAGISA
KDVFIAGEFSGTIEAAGTVRIAGGAVVAAEIQAKALELAAGASFDGHFRRMTT*
>SPSA8_v1_630008|ID:41146224| Stage 0 sporulation protein YaaT (modular protein) [Spirochaetes Bin 1 SA-8]
MTDSLDGVPIQEDEIDSIELMEPEAFTSRTGGGGKPMAEDLPEPLYRIKLLHSNETFFAY
FVPTGTGFPHFHSLEALERLMDDPTANKPDTAHKIAADAGEELVSEAEKEESARPGEMYI
PDKSLAVAPTKYGKDLVEVQGRIMDTSLVNPDDLVMIERPASLDDIRKLRENLTKETYAY
EQCRDRIWARNLPMKLVSAHYLLEESKILFYFTAETRVDFRELVKDLVSLFKIRIELRQI
GVRDEARVCGGCGVCGRVLCCNGISDKLNPVSIKMAKDQNLSLNSLKISGPCGRLLCCLS
YEYQFYKDARRELPQEGIKFIFENTQFRVVEVNALTSTVRMAGEDGRVLDMKAERFRYSE
GRWKIIEA*
>SPSA8_v1_630009|ID:41146225| Cytochrome C biogenesis protein [Spirochaetes Bin 1 SA-8]
MIATIAFGLLIIAQILQSVSLFRTTAKDRISPWLALGSSILLLITVIQRSIAIKFFALTN
MYESLLFLAMLASLMAFAITRWKPTSESKALPYSLNILAIIFLALASSPIAPSTIRPPVP
ALQSGWLVLHVSFTFIGEIFFAVGLVTAILQLTAKTEDNRKKFDRLTYTSIAIGYPIFTA
GALVFGAIWAENAWGRWWGWDPKETWALVTWLTYTAYLHFRLVRKNTSKLMPALVILGFA
IAMFTFFGVNFLLAGLHSYA*
>SPSA8_v1_630010|ID:41146226| membrane protein of unknown function [Spirochaetes Bin 1 SA-8]
MDSITDRIYSFLKSIKLAAFLLVLIGIFAIIGGIIPQGKSLEFYLSHYPKIAKTIQGLGF
DHIFTSLPFLALVALFTVNLTVCTVHRFGTELAKPREKRKHGPDLLHIGLIVIIFGSILS
ARTRTEEMFYLGTGQHLHLPDKMVLQIVALSEEKYDTGRVRSWTTEAIIRQPSPEPTHTD
EPYSGGDTLGEEGFADQKLASSLPAMPAAASQTPAETQTAVSVPDGEKVTIKVNAPLRRK
GYTVVYQFNWKKTQVPVLVDKEGIIKELEPGDRIPTPDGFILFMAFEEAPAPQGAPVSPKA
VFLVDSAGKRDLVKVQPGGNIHGYTFQRYDEHPISGLKVISDRWFPLILAGLVLALFGTA
LTYIKKLKGLNV*
>SPSA8_v1_630011|ID:41146227| Tat pathway signal sequence domain protein [Spirochaetes Bin 1 SA-8]
MADLEKALEKQVSRRSFLKTTGIGLGVAAGALVLGTGVVGAQTTQAAGTVPEAALPYVEL
DPEDARLRGHAAYWKAGCSFGGFYAIVSMLREKVGGPYNQIPLRMLGYGRGGVANWGTIC
GALNGAAAAINLVAPDADTNKLVSELVGWYTGFAFPSKEANAIGVAGGFKYDEKPYPKLE
LVTSVSDSPLCHASNAKWIAKSGYLLPTNERKERCARLTGDVAAKTVELLNAWKAGKFVA
TYKVDDQTASCLSCHAEDQEGKMACVSCHEPH*
>SPSA8_v1_630012|ID:41146228| protein of unknown function [Spirochaetes Bin 1 SA-8]
MAGETSIKEVIAFPKNSFAVSPMDQCPNEVDEKQLKEIHIRIVPKE*
>SPSA8_v1_640001|ID:41146229| protein of unknown function [Spirochaetes Bin 1 SA-8]
VTWFALGRTSQAFQMRGILIHGTHAICQMRANHQLNPIETKILGQALLGSGLSAALQKEP
GTVMLRIDSTGCAEGMSAEGMKLDNSTIHARSRIFHDHLPEAVYALDSTDKIFLPGSMTL
TRIEKNGRPVSGSVSLKTGNIAKDLAYYYLESEQIRTAVDIGLHFTPEGLAFGAGALLLQ
AMPGSDDRFIEQVETMLSGLPPLGLWFSQGGTRDNLLMSIFKEAGFVRTAEGAFAFDCPC
SWEKFLEHIISLDTATVDDIIANGPWPLKTTCHYCSSQYEYSKEAVESARKKIRSRS*
>SPSA8_v1_640002|ID:41146230| PP-loop domain protein [Spirochaetes Bin 1 SA-8]
MKNAETTLAKLIEIALRRYEMIKPGDRILVGVSGGKDSSCLVRDLAIKRSWWDVPFELAA
CYIASDLHTEGFGPPPDDSWLKKQLETWDVPYIRIDVPVIGRLKPGESLNCYWCATQRRT
ELSRYARANGYNKLALGHHMEDILETLLMNMTRKGEFATMPPVMPYVNYPLTVIRPLALC
EERQIIACAEELGFLSHTCTCGFNLTGARKTMRKQLEALTGGSSHAKRNLFKSMSAIKKD
YLA*
>SPSA8_v1_640003|ID:41146231| putative 2'-5' RNA ligase [Spirochaetes Bin 1 SA-8]
MDRLFCALCLPENLCDRISTHIGQIKPALQHARPVWTGDATYHITLHFFGEVDEAAREFI
RLQLAKEEFRHSRPRLRSSGHLLFPNRVSPRVLGLSFRITPDDCLVPLVQQIRIIARNAG
AENEQRTWKPHLTIARLKNPVFINPQSLPRPPLFEFEPETFNLVKSDLTPQGPRYTIIQR
YRFF*
>SPSA8_v1_640004|ID:41146232| protein of unknown function [Spirochaetes Bin 1 SA-8]
MADKKLDEPKKAEKENKNDSDLDPCTTSHSPESTRPTEEEDACDEGVH*
>SPSA8_v1_640005|ID:41146233|trpB| Tryptophan synthase beta chain 2 [Spirochaetes Bin 1 SA-8]
MLTKAILSESDMPRQWYNLAAEFPGKMSPPLGPDGKPVTPDQLAAVFPMNLIEQEMSADR

WIDIPQEVQAILRTWRPTPLVRAFNLERALKTPARIYYKYEGASPAGSHKPNTAVPQAYY
NKIAGIKKLTTETGAGQWGSALSFACAQFGLECKVFMVKSSYNQKPYRKFMMQTWGGTCI
PSPSDQTESGRQILREHPDSPGSLGIAISEAVEAAVTDTSGQTRYALGSVLNHVMLHQTV
IGLEVRKQLAMFGEKNPDVIVACVGGGSNFAGLAFPFVKEKIEGADIDIVPVEPASCPSL
TRGEYLYDLGDIAGMTPYLKMHTLGHTFIPSPMHAGGLRYHGMAPLVSLAAKEGLIRPVA
VPQLECFKAAVTFARAEGIIVAPETSHAVAQVISEAEKAREEGREKTIVFGLSGHGLLDL
QGYADYFAGKLVNFELVDDELKESIQSIKTLLPQNA*
>SPSA8_v1_640006|ID:41146234| putative Diacylglycerol kinase catalytic region [Spirochaetes Bin 1 SA-8]
MRPENLASGIKTILEHSPAFPKAKLIVDIIANPKAGGFKRRRFADKRKKELAALVAESLQ
LPQRKSPVSVHLHLTERGGHAAAIVQRLLDRSESNGTDLLHLIITAGGDGTSLETAERLT
HLPEKEKNRFGILRLPLGTGNDGSEGRDLRTALGRFFKPVRFERRPAVRVVPALEGGKPP
LYAFNIASVGLDAYVADMTNRLKIMFPEDSYKFWLNVATLLYDKAYPLAPMKMRVWDMES
RLVEDETSEKLFVALGSSGYRQYGSNKKILPGPENAIAVYQTPLLRRLFIKGAIEHGQHE
NLKELKHFSASRIELEYHEPILLQCDGETHSLANCDFPLTLELIPDLYNVVVPEI*
>SPSA8_v1_640007|ID:41146235| Binding-protein-dependent transport systems inner membrane component [Spirochaetes Bin 1 SA-8]
MRVSPARKIIFAIALVGIAVPVLFPFLWMLASSFKTQVDIVSWPPKLLFSPIIGNYIKVF
AEQNFLKYFINSSIIGIAAVSFSLLLGLPAAYSIARFKQQKLSVFILIARLMPGISFLMP
WYIIFSRLHLMDSYVALVLSHMLIALPIVVWIMTSYFETVPVEMEESAMVDGATRQKAFM
AIILPVSGPGIVTSITLSFIFSWNNFMFSQVLSMEKTKTLPIAVYNFVSYAEVDWGAVMA
AAVAIMTPAIILTMVFQKYVVKGLTMGAVKG*
>SPSA8_v1_640008|ID:41146236| Permease component of ABC-type sugar transporter [Spirochaetes Bin 1 SA-8]
MSKGGFVERNLKILFPLPAVTFVVVMMLFPVLYTLFLSFTNWNLTSGMPLNFVWFRSYLR
VLKEPRFLSAIGRTFSFTAIALSAESLLGLALALLLNREFKGKPLMKLILLLPLVATPVA
IGIVWNLFFDPTIGLANFVLTQLGLPKSSWVSSSAAVIPSLALVDIWQWTPMITLIVLAG
LAALSQEPYESARVDGASNFQIFSHITLPMIAPTLLTAMILRAIDALKTFDIIYAMTGGG
PGYASETMNVMAFKYSFEYFRMGQASVILVFLFLLVLLLSIVIMQVRKHFEL*
>SPSA8_v1_640009|ID:41146237| Extracellular solute-binding protein family 1 [Spirochaetes Bin 1 SA-8]
MKNKLTALVLLLAVVLGAGAQTKEIRVLLANHPYGDLLKTMIPEYEKATGVKVNVESLQE
SQLTTKLTTEFATNSSTADVFMTRPLQEGKMFYKNGWYEPLSSYDFSDFPANAMDVAKFG
SKPYLVPLVTEWQVMYYRKDLFKKAGLAVPTTFEELEKTAKALHSADVAGFASRGKGAAA
VTQISSYIYNYGGLFLDKGKAVFDSKQALDAFRFYGKMLANYGPAGVTSMSWENIMPLFQ
AGKVAMWTDASVFYGQIVDPTKSQVPAENVGIANLPAGPNGSHPFIVVSWGMAIARQSKN
KALALDFIKWATSKELAKKALISNITMARNSAWADPEVRAKVNPDLITTQAFAAKNGFPY
DRPYMSAVGNARDLIGEVIIESINTAGASAKLEQLAKQKVQAVNELLEDTGEYGVY*
>SPSA8_v1_640010|ID:41146238| Transcriptional regulator, RpiR family [Spirochaetes Bin 1 SA-8]
MSENSCLYLIHSLMDSFSDKEQKVARYILNNPADSVHPSIDELAGSIGVSISTMVRFVRK
LGYEGYQQFRIALATEALAPNLRFYEAPITENEDAIKIVFGAARTALDMTEKMLDRDAVK
KIAKKAVQARSCYIFGLGGSNVVARDAFHKIIRSGIRCQTAEDYHLQLMLASQAGDKDVA
LIISHTGANKDTLGIAEILKKQGCTIGVITTYPRSPLARSADYLLLAGAPGAAIISEAFS
ARIAHLAIIDVLYVEVMELLKNKGIESVQKMRSAIAGRRI*
>SPSA8_v1_640011|ID:41146239|gnd| gluconate-6-phosphate dehydrogenase, decarboxylating [Spirochaetes Bin 1 SA-8]
MKADIGLIGLAVMGENLVLNMESKGFAVAVYNRTTSKVDDFVTGRGKDKKILGAHSLSEL
VSMLEKPRKVMMMVKAGTAVDQTIEQLVPLLEAGDIIIDGGNSNYQDTIRRTHDLEAKGL
LFVGSGVSGGEEGALHGPSLMPGGSEQAWPHIKPIFQAIAAKVEDGSPCCDWVGSDGAGH
FVKMVHNGIEYGDMQLIAETFHIMRYRLGMSYDEMAAVFEDWNSGDLGSYLVEITRDILR
YKDEDGSPLVEKILDTAGQKGTGKWAGIASLEFGVPLTLIAEAVYARLLSAAKDQRLAAA
DILGEPDSAAFSGDKKLFVEKLGKALYAAKIISYTQGFLLLKEAASNFGWNLNYGAIALM
WRGGCIIRSIFLGKIKEAFDANPNLENLMFAPFFAEKLRASSDALRAAAAECALSGIATP
ALFSALAYYDGMRTRRLPANLLQAQRDYFGAHTYERTDRXX
>SPSA8_v1_650001|ID:41146240| Oxidoreductase, 2-nitropropane dioxygenase family (fragment) [Spirochaetes Bin 1 SA-8]
MVALSNFADMVRTAIEEKIDIIFSGAGLPVNLPEFLQAVPGAKTKLVPIVSSGRAATLLA

KRWLDKYNYLPDAFVVEGPKAGGHLGFKKEQIDDPAYALEKIVPEVIAAVAPFGEKHGRK
IPVIAGGGIYTGADIRKFIELGADGVQMATRFVATEECDASDAFKNTYITAHEDDLQIIK
SPVGMPGRAIGNQFLADVEAGKKKPFACPYHCIVTCDVEHAPYCISLALLNAQKGNLDKG
FAFAGANAWRCDRIVKVQELMDELQKEYEESADGVQD*
>SPSA8_v1_650002|ID:41146241| Heavy metal transport/detoxification protein [Spirochaetes Bin 1 SA-8]
MRKLLKIEGMSCGHCVMHVQSALEDVPGVKSAKVDLLERSAMVEGENLDDQALRAAVADA
GYKVVSIMP*
>SPSA8_v1_650003|ID:41146242|copA| copper transporter [Spirochaetes Bin 1 SA-8]
MITTKRFAIRGMTCSACVAANERATRKLPGVQLSQVNYATETLTIVYDDSIVTAEAIQAA
VKKAGYSAEEKQTLSHTSQTDSHQQEKEQEIRQQWRKFGISALFAVPLFYLAMGHMLKLP
LPAFLHPMKHPYLFAFIQLFLVIPVIIAGRQFYTVGFRSLVNRSPNMDSLIAMGTSAALV
YSLYSMAMLLGGDLEAVNGLYFETVGMIITLILLGKTLEAVTKGRTSESIKKLMQLQPST
ATVVYGETHVEIPIEQVEPGDQILVRPGERIPVDGVILQGESAVDESMLTGESLPVDKKP
GDAVSGGTMNKNGALLFRAERVGAETTLARIIALVEEAQGSKAPVARLADQISGVFVPVV
FVIALLTSLAWLISGQSIVFSLTIFVAILTIACPCALGLATPTAIMVGTGKGAEYGLLIK
SGEALETAHKVTTVVFDKTGTLTEGKPSVTDIIPVQGTSADLLLSLAAGAEKASEHPLGL
AIVSAAAQKQLSIPLPETFSAIPGKGIIANFPDYEFCIGNESFMAEKGIGMGESFSEKAR
ILSDNGKTPMFAAVNGSFAGILAVADTLKPEAASTIQTLRKMGIVVAMITGDNQRTANAI
ARQAGIDTVLAEVLPQGKAGEVKKLQAQGQIVAMVGDGINDAPALAQADIGIAIGSGTDV
AMESSDIVLMSGSIRDIPNAIHLSKKAIRNVRQNLFWAFGYNVLGIPIAAGALYIFGGPL
LSPVIAAAAMSMSSVSVLTNALRLKRFKPLLANSNA*
>SPSA8_v1_650004|ID:41146243| putative membrane protein [Spirochaetes Bin 1 SA-8]
MAETANGQAPSAIKIGKKAFLFSAGIILALMIVSGILTLVLPAGEYQRVVQDGKTLVVDG
TYQQIPRPAYPFWRWFTAPIEILFAPGNVALITIIVFIVCVGGSISILEGAGVMEELVKI
LVRRFRTQKYRLIALVIFFFMAVSSFIGVYEGMVPMIIFLVPLAISLGWDSLTGLGISLL
PLAFGFASAVTNPFTIAVAQKIADLPLFSGAWLRIIFFAVVYGLVTFFVIQHAKKVEKNP
RLSPTFADDEILRQSLLKDESTHAKAETDPIQEKQAALAKGKALIWFAVCVGIAMAIVLF
TARIPGLSDLAFPVMALLFLVGGVGGGRIAGFSGKQIAKTFARGAGNLAPGIVLILMAYS
VKHIILSGKIMDTILFGAASMIRQSSSIIAAFLIYLTTLVMNFFIGSASAKAFLMMPLLT
PLADLVGITRQTAVLAFDFGDGFSNMIFPTNALLLIALSFTCVSYPRWMAWTWKLQVAIL
VITAAFLAFAVAIGFGPF*
>SPSA8_v1_650005|ID:41146244| MscS Mechanosensitive ion channel [Spirochaetes Bin 1 SA-8]
MNGIQLPDSLQTFFQSIGTTEFWWKAISTILTVAVILGVFRFLQMAVVRTASKKFPEAKV
FLARKIVKYAGYSIAAAILLKAVGINITALLGAAGIAGIAIGFAAQTSVSNLISGIFLIS
EKPFEMGDVIQTGDITGTVLSIDLLSIKIQTFDNRFVRIPNETIIKTNVINLTKFPIRRM
DIRFQVSYNADIEKVIDTCAKIAQRNRYALDNPEPLILVDSFDNSGINILFGIWFERSQL
VSLKNSIIIDIQRRFAEEQIEIPYPKMDVYIKENGGMENG*
>SPSA8_v1_650006|ID:41146245| conserved protein of unknown function [Spirochaetes Bin 1 SA-8]
MWNYFSPEDLQWYSWELDGARAWLRKNGEEWRLALEGIDFRAIQPGAKGPIPAVPSLELP
IFFSVGKGTKIALRPQLAHRPYVIAVRNDVRLLPGAEAQFDIALPPLLRFELEGGKVLLE
AEPFTVPNTWFGDKTSGNLCHSLPMSLDPRCAGEKQAPADSAAPGTHNAERYLACRSLVQ
CRLVIRNQSKESIDLDRLAIFTDLINIYQKGEFMVTDSVVIVANQDGELQTRIEKEKSRG
LTVLQAARSSGISEILVRRGVSFLKRIAGLEPV*
>SPSA8_v1_650007|ID:41146246| Ferredoxin-dependent glutamate synthase 1 (fragment) [Spirochaetes Bin 1 SA-8]
MLTAKQEAVSAMGTKRPPAILSAGPIHLFAYFRQLFAQVTNPPIDPYRETLVMSLENYIG
KQRNLLEETPEHSRQLRLVRPILSNEDIKKLQAANLEEFKVETVSLCFAATAANTDKADF
ETMLNDALQNICREAESKIDAGANLILLSDRDISETMTAIPSLLGVSAVHTHLVDAKKRH
LAGLIIETGEAREIHDIAVLLAYGASGVNPWMVFDLLPTFSSLPEFSAVSAETMADNYIE
AVNKGILKIMSKLGISTVSSYRGSRLFEATGLSANLVQRYFRGTESRIGGIGLSEIAFDR
IQAHRDAVDLSGRMPCTEILTRSPAGRDVPWPFSLAAHLTKAVRTEDASAWRAYADGMDS
PERQPFALRDLFSFKPAQPVPRASVEPAETIIRRFSVAAMSIGAISPEAHEALSRGANSA
GSWSNSGEGGEDFNRNDAALTASRQIASGRFGVTARYAATCRELQIKIAQGAKPGEGGQL
PGTKVTAYIAELRHAIPGKTLISPPPHHDIYSIEDLSQLIYDLRCVNPSARIAVKLGAQA
GIGTVAAGVAKAGADCVVVSSGDGGTGAAPLSSLDHAGNYWETALPEIRQVLAMNGFDLS

VVVQVDGRLRTARDIVIAAILGAREFAFGTAALIAMGCIACGKCNLGKCPVGIATQDPAL
RARFKGSPEHLVRFFRFIAEDVRSLLAELGVHTLDELAGRYELLDFHGRAGTPYARLTDF
STIISSLEIARRYPLADEAEMEGFGPPALPLPEAGLRNFKPEKRKEAPVSDVESSFLSRA
IEACKHGQPFIAQVSIRNSDRSVGAALSGELARQGLGLSPDSIKVDFYGSAGQSFGAFLA
SGITFMLEGEANDYLGKSLSGGTIIVRPERNAKFEPEEQIIAGNVCLYGATAGSVFINGR
AGERFCVRNSGALAVVEGTGNHACEYMTDGVVVVLGKTGVNFGAGMTGGIAYVYDEDQLF
DTRCNLSDVDLSIIGNRDDQDRLHTILESHHALSGSPKAARLLASWERVVPLFLKVTPAA
PV*
>SPSA8_v1_660001|ID:41146247| ABC transporter substrate binding protein (fragment) [Spirochaetes Bin 1 SA-8]
LLSIAPRIDGLYLGNDNTVFSALNAVAETALEKNLPVVTADPSSAESIPVLAAIGYDYYK
VGTATGKIVVRILNGEKTADIPVYLPTESSDLLMVLNLDAAKKLKLTVPAEIVEKAAVVI
KDGSLVRK*
>SPSA8_v1_660002|ID:41146248| ABC-type transporter, integral membrane subunit [Spirochaetes Bin 1 SA-8]
MIEGILVEGLIYGILALGVFISFRILDFPDLTVEGSFPAGAAGGAMTALMLGPKFGAGGS
LFLVVCILCAGLIAGGLAGFATAEIYHRLKVHPLLAGIITMTGFYSINLRILGGKPNLPL
IAGNPLLDSARSLLNGMLSPEFSLLAASMLAVLVLFGILDFFFHTEVGIAMGALGDNENS
VIQAGINPVRLRTLGIILANALTGLAGAIAASYQGFADVNFGQGVVASGLATVMLGELVI
RSQYIGVQLARVFIGSILFKALMYAARSWGYLAGITPNDLRLITALLIIGSIALSKMGRK
SS*
>SPSA8_v1_660003|ID:41146249| Phosphonate-transporting ATPase [Spirochaetes Bin 1 SA-8]
MIEIESARVSFSLGAGEEHVVLKNLSLSVKAGQTVSIIGSNGAGKSTFLNAIAGTVPLRS
GVIRIDGVDCTRQAEWERARYVGRVRQNPLAGTAGDMTILDNLALASRKGPRRLKIATPP
AIARRMAEEVAQLEMGLESRLHENVSRLSGGQRQALTLLMAVLSKPSVLLLDEHTAALDP
ANAEKVSALTKHFIQEFGLTALIVTHDMSRALSEADRIVMMHDGEIIADLSGKEKASMTV
AGLVDLFRKARGRDYAEDRDLLR*
>SPSA8_v1_660004|ID:41146250| conserved exported protein of unknown function [Spirochaetes Bin 1 SA-8]
MKKSILALLLVLLAVSGPSAQTSAADIAALLASAGSGNASAQNSLGVKYYNGDGVKQDYK
EAFNWFYKSALQGNMVAQSNLAYLYDSGKGVTKDFNSAAYWYRKSAEQGYATSQKNLALM
YENGEGVPQSYEDAAYWYQKAADQGNATAINNLGVLYENGRGVTKNVDKAFELYAKAANL
GNSYAQNNLGWFYESGMGTPKDILKAFEWYKKSASQQNPYGQYNLGWLYEHGDTGIPVDK
DSAFIWYKRAAEQGHDQAQYQLAGMYERGEGTQKDYDEAFYWFQESAYNGYADAMVAVGE
YYEFGLAGDIDYVQALAWYLIAAQYGHDDAGYYAELVKETYELTDEEIAEAERLASEF*
>SPSA8_v1_660005|ID:41146251| Peptidase U32 [Spirochaetes Bin 1 SA-8]
MELLSPAGSIEKLRYAYRYGADAAYIGLPLFSLRAKAENIDDTDEGAPEKIARVKSFFSL
ERQRKLYCAVNIVFHDDDLRKLESALPRIAEYPFDGFIVSDTGAYDILREAFPDKEFHLS
TQADCTNWRAARHYLRMGFSRIVPGRELSLDEIKTIKDHVPELEIEAFVHGAMCMAYSGR
CFISSWLTGRSANQGDCAHSCRWHYKVYIEEEERKGRLIPVETGETAHGGYTLIMSSRDL
NMVDHLDDLRRAGVDSIKIEGRMKSLYYVALTTRAYRFALDHPSIDNPFKQDLEAVSHRE
FDTGFYYSRNGMDTSTSLSYQQSHLFIGSVEPFTSSFLINSLGEEILVAELFARFSSHEW
TAPVFMDLKNTVSLAAPLELLGPEGEFFSLAPSDYRFFDIDGNIPEKANHGKIWFFQARR
AACPADIQLQWLLRKQASRQ*
>SPSA8_v1_660006|ID:41146252| conserved membrane protein of unknown function [Spirochaetes Bin 1 SA-8]
MNIIPSAFYNSGWKIVSLIFVLAIALGGYFIPILGLAVPLLIAIALIMNARKTRSFCSGF
CPNGRALSFTMKTVSRNKVLPPYLYSTEFRRMLCGFMMFCIISLLARSDGSVQQIGRVFW
AIYIASIGISTISGLLFKPRSWCAYCPMGTLQDTMKNPPHRIKSSSRPSMPQ*
>SPSA8_v1_660007|ID:41146253| putative Polyamine-transporting ATPase [Spirochaetes Bin 1 SA-8]
MHLIELHSVNFQYENSAQPALQNISLSVDEGEYLALVGNNGSGKSTLVRLFNGLRLASSG
TVLAAGKNPAERANLQCIRRTITIVFQSPTDQIVSSCVEEDVAFGPSNLGLSQAEIDVRV
QMALQAVGLLEHAKRPTRFLSGGQQQKLAIAGALAMQPRCIIFDEATSMLDPKTRASVLS
LMKELSEQGIAIIHVTHDMDEATQAGRIIALDKGRLVYDGKPEGFFTAGERPQSLSLAES
LGLELPSVLKLARSWGLTLPLNASYQDMAKQLVAAAGQGKTGISVSSANGGSSLSQKSPQ
PRNDVLFFTQIEAIRYNNVRFSYLSGTTNEVMALKDVSFLIPKGTMIAFVGMTGSGKSTA
LQLANALLKPVSGDVLIFGRNTREKSVDLKALRMNAPFSIQRPESALFEAHAADDVAFGP
RNQGLKGPALVRRVETWMNRAGLPFIEFRDRLIKTLSGGEKRKLALAGVFALESDTLLLD

EPGAALDPGSRASVFSLLEQLHEQGKTILFATHSMDEAIRADLVAVFKDGCLMAFGPPRD
VFYRDYEESWGIDRPRIVQFAMALEAEGANLPYYPVSAEEALSLFEAGRLP*
>SPSA8_v1_660008|ID:41146254| putative Energy-coupling factor transporter transmembrane protein EcfT [Spirochaetes Bin 1 SA-8]
MKNLEFFRNISIGQYIDSHSLFHGLRPATKYLLLLGLMLLAIAAPTLAGAGFAFFAALVL
AQFSKVPLSFLLRSLKPIFPVIILSFMLQFLFRWPGDSSITLISLGIFSLTVRELWVVAM
ILIRAAAMMTIVGWFTSITTEAEAAKGIEDIARPISSKTLPIHRLALAVAAAIRFIPIIA
GELEEIVKAQASRGADFGQDTKGLLAKARAYLPLFVPVTIRALERAEMLAEAMEARCYTG
EGQTVAPKTPMKRSEVLIRFSVPIIVIGVLVVDVYFISTWIRPY*
>SPSA8_v1_660009|ID:41146255| conserved membrane protein of unknown function [Spirochaetes Bin 1 SA-8]
MDTQKKASSAASNSWMRKTMVTGALSAVSIALFLTPFGYIPWLAGASLTVMHVPAILGGV
LEGPIVGAIVGGIFGVTSLIKAATAPQGPIDVFFTNPLVSVVPRMLVGFFAWLFYKLLAK
WNIHAASIAAGIGGSIVNSVLVLGALVLFGAIPLAVAGSVFVANSLVEAVLAAVLTLGVI
TAWKGIGNRSGKAKLADEE*
>SPSA8_v1_660010|ID:41146256| putative Type III pantothenate kinase [Spirochaetes Bin 1 SA-8]
MIVVVDIRNKVVTVGIIQQDDVTAAMSEAAPEPLKWRSIRRYGAFHERTSDEFSQLISQM
VAELVPDSAEAHGFVSQESGSKAPEPVVWISSVVPALTGVLAASTEAVFKVTPHVVGPGT
RTGIKIRTDFPSELGTDLVCAAAGARRLTSRPCLIVDFGVALTISAVNARGEFLGAAITA
GPRIAAESLRQETAQLVEANLEFPASAIGKSTRHSICSGIMIGYTGLVRQLLLEMGRELR
ADEIKSGLLEMPGTDTDSLSEIEX
>SPSA8_v1_670001|ID:41146257| protein of unknown function [Spirochaetes Bin 1 SA-8]
VNDHLILCPFVVLIRGRSIDASIVSVPAHPVNLVDYIPSLDAETAAQSISRFLTGQAPEE
FMTSSLDDRAREMFESEKIRLNAPEWILGATPDFSFSISTAMSAAEFRVSAGKIAAITMR
QADSNPYFHRIIESELYKFFGRQFSFGLLDEMKACMDLYQKK*
>SPSA8_v1_670002|ID:41146258| Pyridoxamine 5'-phosphate oxidase-related FMN-binding protein [Spirochaetes Bin 1 SA-8]
MAIHELLEQLKTLVSEVGIGILTTIDEEGRPYSRWMTPVFIPRLPGSLYAVTSRSFKKAD
HIEKNPNVSWIFQSRSLDRIGTIQGKAEIIRDPSLSAEVIEAIGPHLTVFWKYSGDPSKL
VVVETRLERVSMFTPLGKGVETAEVADE*
>SPSA8_v1_670003|ID:41146259|pdhA| Pyruvate dehydrogenase E1 component subunit alpha [Spirochaetes Bin 1 SA-8]
MSKIALNDYPKDAWLDMLKTMQLIRTFEEKAGQMYGLRKIGGFCHLYTGQEAVATGMISA
LDLEKDYVLTAYRDHGHALACGIDPKAAMAELFGKVTGVSRGKGGSMHFFDAKRHFLGGN
GIVGAQIPVATGVAYAQRYQKTGGVTLCFFGDGAFHQGALHESFNLARIWNLPIVYVVEN
NQWGMGTNWKKVSANPDFAETAKAYSMKGYVCDGLDILDVYHVAREAVAGARNGEPAFIE
ASTYRYKGHSMSDPQKYRTREEVDAYKQRDAILLLKSRLEAANMLNQEEWDTIVALCETQ
VDESVEFAEKSPEPAPGELFTDIFA*
>SPSA8_v1_670004|ID:41146260|pdhB| Pyruvate dehydrogenase E1 component subunit beta [Spirochaetes Bin 1 SA-8]
MSELTYREALNRAMDEEMARDGNVFIIGEEVGEYDGAYKVSKGLLAKYGPERVRDTPIAE
LGFTGLGVGAAIAGLRPVVEWMTHNFALLALDQVVNNAAKMRQMSGGQLKVPVVFRGPNG
PAEYLAAQHSQSFAAYWAHVPGLKVVAPATPADAYGLLKSAIRDDNPVVVLEAEMMYSWK
GEVPDHEYLVPIGKANVVREGSDLTLITFSKPLRVTLESAEMLAQKGIQAEVIDLRSIRP
LDEETLYASVRKTGRCIVIDEAWPMASVGSHVAWLISKNCFDTLDAQVELVSGEDVPMPY
NHNLELLAQPSARKIIDAAKKVLYREDL*
>SPSA8_v1_670005|ID:41146261| Pyruvate/2-oxoglutarate dehydrogenase complex, dihydrolipoamide acyltransferase component [Spirochaetes Bin 1 SA-8]
MAEIIYMTALSPTMTEGTIARWKKSEGDSFASGDLLLEVETDKASMDYEAPKSASLLKIL
LPAGGKAKVGDPIGIIGKKGEDTAGLMASLAEKSQPSPVPDTPSTQPPSNQVIQNVSARL
DAAIESHNAKEKLPHDAKDMFTPVSPVTVPPSSPLARKLAKELGIDLRAVKGSGPQGRVV
KRDVEAFAVSPRTQTVQPDVLVPQPQSAPAKIPVSGKRAVIARRLSESFFTAPHYYLKKK
VSAEKLLALRQFSAELAQSTEKASLNALLMKLAAEALKRHPEINVHFASDYIEQFAATDI
ALAVALDDGLITPVVRDCADKSVRQIDTEMKALIEKARTKGLAPSEYEGGTFTITNLGSF
GIDEFTAIINPPGSAILAVGAIKKEAVPQEDGSIGVISMMTLTLGCDHRSIDGAVGARFL

KDLALFIEEPASVLL*
>SPSA8_v1_670006|ID:41146262|lpdA| Dihydrolipoyl dehydrogenase [Spirochaetes Bin 1 SA-8]
MIEKSFDVVILGAGPGGYVASIRASQLGLTAAVIEKDNPGGVCLNIGCIPSKALIHQASL
FSGGKALLEKTGAAIDLSGFDYTPVYKASRLAADRLSKGVAYLLKKNKVELIKGFGRLSD
KNSVLVTSESEEILVRGKAIILATGSRPRSLPGFEIDEEKILSSTGMLMMEKLPKSMIIL
GAGAIGMEFAYVLRSFGVEVTVLELMSQVLPLEDEESAKVVEKAFLARGIKIHTSAKAER
AEPSETGVKVSLTLKDGTAAVLEAEKLLVSVGRAPNTENIGIESLGVRSQRGYLETGEYY
ETSASGIYAIGDITTQPQLAHVASKAGEIAVEHIAHLLKGSPEPAEKYINPYTIPSAVYC
EPEVASFGLSEKKAKDLAVRYEIARFPFRGIGKAVATEVPEGQVKIVFTPDTGAILGASI
AGAGATDLIHELLLASNAELSLEELADMIHAHPTFSEGIMEAAKAGLGRAIHI*
>SPSA8_v1_670007|ID:41146263| putative Methionine synthase [Spirochaetes Bin 1 SA-8]
MSAYISASGIADVIHDPLPFARQALSCVEKKLPESAATWSESDRELVLLDLAHHISFIAT
AGMYQSTELLNDYIDWLKVLSSTLGFSPSSILQSFSCLTETGKTFLPEAVSALLADWFGK
AGARYSAHSENSSSYMKIAIENNKNASLFLTALLEGKRPFAMSIIENLVNNGATLIDIYE
NIILPSQYEVGRLWLSGRISVAQEHYVTAATLYMISTLYPDFFLTARPVKPLVIAACPQT
ELHELGIRMIADTFQYHGWDTVFLGASVPTADLLKEIQRLNPAVVNLSATIATHIPWIEE
IIRQVRNRHGSSIKILVGGRAFFTAPDLWKAVGADGTAVHCNEAVALAENLLAS*
>SPSA8_v1_670008|ID:41146264| putative Diguanylate cyclase [Spirochaetes Bin 1 SA-8]
MNDETVLNEYSQLVNDLVNTKRELSRINAALIEKERFLSRILEISPSILYVINLKTRKIE
YTNRFMPALLGYTEEDFLGTIAIPDSLIHKDDLDVLDRHYAALREAQDTDILEFEFRAAH
KSGQTSWMRCRESIFARDELNMPQKAVGVIEDITSRKMREHKLIEESTKDVLTDLLNRRG
FFLFSASLMSRCAAMSQSCALFYIDIDNFKMINDKHGHGEGDEALKALAEIMRNTFRTSD
LVARLGGDEFVVLMPDVDENTIDVIQKRFQKKLDEFALISSRPWSLSVSVGVAFQMSGQI
LPVSELLKIADENMYRNKNSMQNTLNQS*
>SPSA8_v1_670009|ID:41146265| putative Polysaccharide deacetylase [Spirochaetes Bin 1 SA-8]
MKNPSVAFYKRLILISVALLILLPVSASVYLLIENRSLKARQIFAVSQPDDVVTVGTTQI
EPNNVMKDKPVLSYQILYPDFRSDFFGFNNDLVSNKTVFITFDDGPSPGTSKILDILRKE
SVPATFFVNGKSNPFLTAQLDRILSEGHGLGMHSYTHRYNVIYASMENFLDDFNRNFLYI
KNETGVSPQILRFPGGSINIFNIGTYQSLIAEMLRRGFIYYDWNVSAGDAIPGATAQSII
DNVVHGVHLCWGPAFVLLHDNGSPLLNEALPVILETLKREGYEFRKIDNSVKPPMFIYPD
*
>SPSA8_v1_670010|ID:41146266| protein of unknown function [Spirochaetes Bin 1 SA-8]
MKKPGNFSKAMEELISGKTAASESEETAKAEEPEIYMTPDYAPKARGEAVITADMVIKGS
INSEANISISGSVVGDVSSEGDITIQGKVEGNIKAQSLMVQGGTITGDILASAHVVVADS
AAVNGNIKADRIEINGKIVGNLEASSRIVLNQKASIEGNITAGLLSVLEGAELKGNVSIK
KA*
>SPSA8_v1_670011|ID:41146267| Molybdopterin dehydrogenase FAD-binding protein (fragment) [Spirochaetes Bin 1 SA-8]
MEIKEYVRPASLDEAYSFVVEKKGFPLGGGAWVHMNVRSVDLAVDLSALKLDYIVDKGDA
VEIGAMATARQIETSALLNQAFSGAFARATXX
>SPSA8_v1_680001|ID:41146268| Dihydropteroate synthase (fragment) [Spirochaetes Bin 1 SA-8]
VKQSLLLANDRRLDIGKKPIIMGIVNITPDSFFPESRKSDPERAVEHAVRLLGEGADILD
FGAESSRPGSKPVTLEEERERLIPVLKAFRKQSNAPVSVDTRHFDIAXX
>SPSA8_v1_680002|ID:41146269|dxs| 1-deoxy-D-xylulose-5-phosphate synthase [Spirochaetes Bin 1 SA-8]
MSLLEHIHDPQDLKNLRERELRILAEEIRAKILKTVKKNGGHLSSNLGVIELTIALHRVF
DSPKDAIVWDVGHQSYAHKLLTGRAKAFRSIRKKGGLSGFPKRSESIHDIFDTGHSSTSL
SSAMGLLEARNRLGQEGYVIAVIGDGALTAGMAYEALGNISQLGLPLIIILNDNKMSISR
NVGAVSRYLSRLSASVRYQSFRSTIDSLLLRVPHVGPRLLGLVVRGKRAVKAIFFKENLF
SDFGLEYVGPIDGHNIPVLLQVLEQVKRIERPVVVHVVTTKGKGDEKAEDDPEQFHGIAP
ACPDEPAKNASFTEVFGSEMAALAAKEPRLAAVTAAMAKGTGLSVMKKLYPARVYDVGIA
EQHAVTFAAGLARGGLKPVVAIYSTFLQRAVDQVFQDVALADMPVIFAVDRAGAVGDDGE
THQGLYDIAIFKSMPNLILLAPSSAGELKLALQYAVLQPHPVMIRFPKDMAACEDAALTV
DFISGRGVFLRKKNFSRVLVCAAGPLAMTAAAVSDRLEQEGISVDVFNLRFLKPLDTEFL
SIIFKKYERIVSIEDGVVRGGVGESLQSVLASAGISLPLTTMGFSERPLPQASRAELLAS

ACLDEAGLHAALKKQANLGKGVLPSETITAACK*
>SPSA8_v1_680003|ID:41146270| protein of unknown function [Spirochaetes Bin 1 SA-8]
MRSITLYTLHLHKALEYQTFLGAGKTSRSPQDYTSVPGEEIIWLFDDAKLVINDPDNGPR
IQTPWPAAHFAGFKTEPAQTGDQASRITVKAGDYLFTQWRTKDFADPSEGFEEFIRQIWW
EQEKTEGPWILRIVREDNDIAFQGLRKKIGLKSA*
>SPSA8_v1_680004|ID:41146271|alaS| Alanine--tRNA ligase [Spirochaetes Bin 1 SA-8]
MITIHELRKKYIEFFKSKGHAEISGKSLIPENDPTVLFTTAGMHPLVPYLLGEPHPAGNR
LTDYQKCIRTGDIEAVGDPSHLTFFEMLGNWSLRDYFKKEAISWSFEFLTSPEYLGIPVE
KLSVTVFAGEDGIPRDTDSAEIWKSLGIPEERIYFLPREDNWWGPAGETGPCGPDTEMFV
DTGKPACGPDCKPGCHCGKYFEVWNDVFMQYNKNAEGKYVPLAAPCVDTGMGIERTVAML
QGKKSVYETEAFMPILAVLEEISGKKYGAEGNDPEIDRSFRIIADHVRTSTFILGDPKAV
LPSNVGAGYVLRRIIRRAVRHGRKLGIEGPFLSKPALVVIENYKAPYPELVENRQRIVDE
LKAEEEKFLETLQKGEHEFEKMLPNLLKNPQKIMSGRLAFKLYDTYGFPIEITEELAAEN
GLAINRQEFDEAYKKHQELSRAGSEQVFKGGLADHSEIATRYHTATHLLHKALRMVLGDH
VAQKGSNITAERLRFDFSHPAPMTREELEKVQAIVNEQIQRDLPVSMAMMGLDEAKASGA
IALFGEKYEQVVKVYAIGDFSKEVCGGPHVQHTGELGRFRIQKEQSSSAGVRRIYAVLE*
>SPSA8_v1_680005|ID:41146272| conserved protein of unknown function [Spirochaetes Bin 1 SA-8]
MKILVTGGFGNVGRSTVNACVRAGHETVIFESPSALKKAKAGLQSLIKTRWRACRLMTGD
VRSFQDIERALLAFEGGPDAIIHLAALIPPASERDEAKTRSINIGGLQNILAVCTKNNLH
PRIVFASSIATYGDRLKDFWIRKDDPLHPSDVYSQTKAACEQLLSQSGFEYVILRLSYVA
WAQWLPFDPLLFAMPPETRIEIIHTEDAGRAFTNAAVLPGIGGQIFNIGGGAACRTSFRA
YLDRMFRYFGLGNSDFLSDELFAKDSFHCGWYADSDEADSILQFRRKTLEDYYEEVRWEK
RLLAPFSSLAAPLVKAWLVKMSPNFKKKPKERGAGIRGRAQLKRRGI*
>SPSA8_v1_680006|ID:41146273| protein of unknown function [Spirochaetes Bin 1 SA-8]
MPRKKSELRRTVIIETAKRLFSAEGFERTSMNLLARSMQVPVGSVYTYFPSKDALLSAIV
EEGWSQFIDTLESGFSQVNEHVPDDHERPLQKLSFLVLVLLPQLFRDADLIAILLSESGK
PHHLDEKLSYLSNLIAGILDEFKAAEPGRVDLDSASIRTGLSVMLLGSLETVRLNHQTGL
AIDQNEIIRFLCASVEHVLGCRLPVPSVSF*
>SPSA8_v1_680007|ID:41146274| Asparaginase/glutaminase [Spirochaetes Bin 1 SA-8]
MQLMDTTAMRVIVTGGTFDKHYDEIKGELTFKDSHVPEILKRARVTVPVQVELNQLIDSL
NMQDQNRLSVLEACKKAEETKIIITHGTDTMTMTATLLGQAYLDKVIVLTGAMVPYRVLD
SDALFNFGAAFSAVQLLPNGVYIIMNGRIFSWNDVRKNKTKGIFQTLSEE*
>SPSA8_v1_680009|ID:41146276| protein of unknown function [Spirochaetes Bin 1 SA-8]
MKSFAGFSSILIGNFFVSETKDNISLSRLKLVLAGLLQDALSASKAAHKNTAPLARNGLY
GTILNKNSL*
>SPSA8_v1_680008|ID:41146275| Transglycosylase SLT domain protein [Spirochaetes Bin 1 SA-8]
MVPYRPLRAKGAVFLCAALLALSASCSRPASTNLSLDSEILSLVSDTKKLPISIEEKPAN
DFIAKFYKNSQTKNSVLDFFASLTNDRAVAAAILDNAVKHEVPASLAFAIAYEESRFNPK
ALNINSGSKDRGLFQLNSNTFPELKESDAFNPEINAKEGIKYFKHVLDLSGNEISALAMY
NAGRTRVTQKGAPVTTLDYISRILTYEKNIASLFTAKVVASSSLINRIKFGLLTTDQEKA
E*
>SPSA8_v1_680010|ID:41146277| Transcriptional regulator, TetR family [Spirochaetes Bin 1 SA-8]
VSISVEHDKRKEEILDKALDVFVVEGYKDTTFQKIAERCGITRTILYIYFNNKKEIFASC
IKRFMNKLEHEILLIAEAQDRTSAEKLVAIGEMVVQICEKESRLLSVVLDYLLKLKTAGG
DPDERVRRRTIRMRHILSYIIIEGKKKGEFSRDISVKATSELFYALIEAAVFRVTVRGKA
TMDLAEPLKLLASTLSDKKS*
>SPSA8_v1_680011|ID:41146278| Alpha amylase catalytic region (fragment) [Spirochaetes Bin 1 SA-8]
LGSSNRRINLAPKPREDYSTGMKQGKQKILACSLSAFGLSGIRTGDRVMEFHIHAGIRSR
LAVSSSLFASTGNLILPDFYSSRVMAEKIRSLILTEGRDAKDISAGKLNAMALIDEILHI
IFLLYRESVAPDVMKKFESSIQDAIGKEEFDTLLNEFCVQFPPRDVYQGLTAPEDYLAGS
TSRDQTGIVPPSGNQQKKVPNRELALEELILLKLANENPAFKTFKFLYDDGLRDTGTVSG
TLSAKTKYSTVFKVLEKVQLELPGFGPTGTMMPILELLRLPARMAPDSVEKQLLWIRDNW
GATFREIKDKVLKTLDLIQEEEMPRFPPGPGPIKPYQYALQRHEYEKFSSDQDWMPSLVM
IAKNALVWLHQLSVQYGMLITRLDEIPDQELXX

>SPSA8_v1_690001|ID:41146279|whiG| RNA polymerase sigma factor WhiG [Spirochaetes Bin 1 SA-8]
VHQYAPLVKYVAGKVISNLPPSVEYDDLVGYGVFGLFDAIEKFDPEKHVKFKTYAVTRIR
GAMYDHLREIDWVPRSVRQKAKEIEQAIMLLESKLGRPASDSEIAACLGLTEDEFSKLMS
KIASTAVLSLSDVWSSGTDGEHGSLGESIESPAAMNPDVNMEKEEIRRVVIQCIKELPEK
EKKVLVLYYYENLTLREIGQILEVTESRISQLHTKAILRLKGKLTSYRKGLF*
>SPSA8_v1_690002|ID:41146280|atoC| fused response regulator of ato opeon, in two-component system with AtoS: response regulator ; sigma54 interaction protein [Spirochaetes Bin 1 SA-8]
MQCTILVVDDEKNIREGLGEAFRMEGYEVLTAADGLEAKNLLDEKYIDLVISDLRMPKLD
GKELLQYIKRQWENIPVIIITAHGDITEAVQAMQLGALEFITKPLDLEHLLKLTKKALEF
RELSIKNQELREEVEAQHRISSIIGKSPAMRKIFDLVRKVAPTKASVLITGESGVGKELI
ADAIHNLSPRRDKPFVKVHCAALAESLLESELFGHEKGAFTGAQARKRGRFELADSGSLF
LDEIGEINQNVQIKILRVLQEKKFERVGGESTIEVDVRLIAATNRDLKAEIQEGRFREDL
YYRLNVVNIHVPPLRERREDIPLLATTFLYEFAHENGKDIQGFDPKARQALFSYSWPGNV
RELRNCIESAVVMASGKVITLDDLPPGPRSTQDRTEIRIPAFCTLAEAEKIIIAETLAAL
GGNKTKTAEVLAIGRKTLYQKIQEYGIEPADIEKASLES*
>SPSA8_v1_690003|ID:41146281| Chemotaxis regulator-transmits chemoreceptor signals to flagelllar motor components CheY [Spirochaetes Bin 1 SA-8]
MKPAFLEKALSMAGKLDFNDLVKLLNLVAMDDMRLEAAIQSMFDGIIVCNREHVPVIINK
SAERILKIPSDFGNKPLWQGITDEDLKQFFHEGLVSEENIMGNEFAIDCPGGTRIVAISL
STLVFSEKISGTIIHIEDITEKRKRETQLRRAESLAALTTLAAGVAHEINNPLGSLSIHI
QLMERLLKNPQKLDDRALLHHLGIVKEEIDKLEHIVKDFLFAVRPMDIQLLNQDLKPILE
EIADFVRPEAEKYNIKLKVSVQKDLPKILLDKRHLKQALLNLIQNAIAAMSDGGTLKLEA
KKADEEIRILVSDTGIGIPEELLTKIFEPYFTTKKNGTGLGLTITFKIIKEHSGDISLES
KPGKGTTFTIHLPIPQAERKSLPIYDEDAVSAGSHVEA*
>SPSA8_v1_690004|ID:41146282| putative DNA polymerase III domain-containing protein [Spirochaetes Bin 1 SA-8]
MYENLLAQDSIRQELIRMLISSEIPPAMLFSGPPASGKLTAALETARVLSCTKAGHWNCV
CEDCRRHRNLIHTDLLLFGKRSFPIEIAAARDALIEKPGKAAAYLFTRSVRKLLSRFNPV
LWQGEEQKADRASPIIQEIEEALDTIDPETITESALNEKTIKTVDEIKLACANLEPFVPE
TVSVAMVRNMDSWAHLAPSGQRKTVIIENADRMQDAARNAMLKTLEEPPESVRFILLTSR
RASMIATVLSRSRIFSFSQRDAKATRMILERVFKSNSDADSLSSFFESKMPFSPSEANRY
AELIAGLLLLDCAKTGLPVPEGYCSGIARQAAASSKTMRDVLEELSESTKSFGSKDKAFA
NSFLQVMKFLLTVFSRMLAESSGNPELMLLIDRWSNHVREAAVQYGSLNRSPELLLTVLI
STFGDRP*
>SPSA8_v1_690005|ID:41146283| putative Colicin V production protein [Spirochaetes Bin 1 SA-8]
MSTIDWIFAGIVILAGIRCFVKGFVAEVLSVAAYAAGLLAAVLFSNKVTVFLAENLKLGN
LNSTILYIIAFIICFILGFIIMKILEKLLREGLEAANLEIFDRIIGLALGLVEGLAFVSF
ILIILEMQTFFPIDKTLATSIFAKTLLPIVGPAITQNLQPALDSVGGQLKLDEIFRKK*
>SPSA8_v1_690006|ID:41146284|murG| UDP-N-acetylglucosamine--N-acetylmuramyl-(pentapeptide) pyrophosphoryl-undecaprenol N-acetylglucosamine transferase [Spirochaetes Bin 1 SA-8]
MKSGEKAGISADKALMADKGLPRDAKTIAFTGGGTGGHIYPGLAVIEALRQRGFTGRIFW
IGSQKPLEREIVEKQGVEFYSIPSGKLRRSFSLENFIDLFRIIAGYFAARALLAKTKPDV
LFSKGGYVSVPPCRAAVSLGIPYVTHESDASPGLATRLNAGKAVRILVSWPGTAQYFDPE
TKKVIVCGNPVRPSLLASSPEKGRKLLGAPDGLPIVAFIGGSQGSRQINELVREALPLLE
GKAFVVHQTGSAHFDPKAHAAIPGRYFAKPYFLEEMGNVLASARVAVGRSGAGMVWECAS
LGIPMVLIPLAGSGTRGDQVENAQLAAEAQAARVLSGDAVSGQAVASAILGYLEDEKAWK
QAHNAALALSGIDREDGSRTTSADYIAETLLNMIRQS*
>SPSA8_v1_690007|ID:41146285|pgi| Glucose-6-phosphate isomerase [Spirochaetes Bin 1 SA-8]
MTYKNLDSCASFQALKELPRVSIAESLTPERIESYTIPAAEGFLFNYAAAPVNEKIIDAL
QKLAEEQELVEKYRALIEGEIMNTGEKRMVLHHLARGQLGKAVVFQGQNLRDFYENERRK
IAEFAQSVHAGTLVGATGKQFRTVVQIGIGGSDLGPRAVYLALDRWAEAARRKRMKAAFI
SNVDPDDADHTLASLDLETSLFILVSKSGTTQETLANETLVRARLESAGLDSSRHMIAVT
SKTSPLAQSPDYLASFFIDDFIGGRYSTSSAVGGAVLSLAFGPETFLEFLEGAHAGDSAA
LEPDIRKNACLLDALIGLYERNICGYPCTAVLPYAEPLVRFPAHLQQLDMESNGKSVNRS
GEVLAYKTGPVVFGEPGTNGQHSFYQLLHQGTDVVPLQFIGFAENQNSSDVISMGTTSRQ

KLMANFIAQIVAFARGKKDQNPNKCFTGGRPATLLMARQLGPRQLGALIAHFENKVMFQG
FAWNINSFDQEGVQLGKVLANKVLSIMQGEKTDDTALETLARLTSKL*
>SPSA8_v1_690008|ID:41146286| RecQ family ATP-dependent DNA helicase (fragment) [Spirochaetes Bin 1 SA-8]
MKEDTEFFEDGVNEDPVANLACERFHIPYLMPLQRMVIANILDSLDYPDNAEQICQMALF
PTGFGKSICFQLPALFLPGLTVIVYPLLALMTDQQKSLERLGIPCSLFRGGLAKEEEQAV
FSALKSGASNMLITNPESLSRPDLLSLLQNRIISHLAIDEAHCVSEWGETFRPAYLALGK
IAAMLKPKVFSAFTATASPLVEAAIAKHLFAGSGYIRITADIDKPNIHYHVMRSLCPRHS
LERLLLECKKPAIVFCQSREGTRILAEMVERRLPFESRFYHAGLEREEKIAIETWFMESE
TGILISTCAYGMGVNKKNIRTVIHYGSPPSTEAYIQEAGRAGRDGEPAEAILLKLPAEEN
FAASDPDKTADSADSGVEAAREARRRAFLAYGMDGRCLREQLLNLMGANLDSPCSGCSVC
DGTWSQEPEGQRELVQFFSANPFRFSLYQSIGFLNMRADVAGKNARKPRCRSAGVLQDWQ
RRDSMLAIREGLRRGIIVEKARGMRKGRIGIEV*
>SPSA8_v1_690009|ID:41146287| protein of unknown function [Spirochaetes Bin 1 SA-8]
MKSVALSKKPWTVKSKALLPRHADSFAQPRHRTALHVGQAQRLSRTADMLAMAXX
>SPSA8_v1_700001|ID:41146288| protein of unknown function [Spirochaetes Bin 1 SA-8]
VEGLGNGWRFSAGRRSVNIGAGTYSLTVSGANPWYDHVLAELEAPLSAGSLRYDFLAIST
QRRSGNDGKSLFLHQIGFDFPVWSASLAEFNTVAGVPLDLQDIGPFLIYHHLFAGGSNVM
FKLDGQLKPSDRLRAWGCILMDDFQLSSESTASNPNAFGFEAGLEAVLIPGRSSERARFF
RKDYAVSLGPEQLDGRLKASLQWYWASTYLYRRVASAPNEAFYTRYFLQNQNFGWAVVEP
WFSYPLGPDRMLFRADLRYSKERLSLKAAGTFAILGSESDEYTYNSPYASNWLGPQPPLS
YSLKAELATEMTLGRQSLVSGSLYMIWNTGEATEVGLQLGFIHRFSLPR*
>SPSA8_v1_700002|ID:41146289| protein of unknown function [Spirochaetes Bin 1 SA-8]
MDTSENWKPPHLVLLIMPCILAKVKFVKITINLQEEEFPEERLSIAEILKKKNYSFPHII
TRLNGKLVERGQRDSTYAQDGDDVELYHLISGG*
>SPSA8_v1_700003|ID:41146290| putative tungsten-containing aldehyde ferredoxin oxidoreductase, 4Fe-4S polyferredoxin subunit, beta chain (AorB) [Spirochaetes Bin 1 SA-8]
VIKYLKHTDEKCVGCNTCVSVCSRLYFKEENPAKSSIVIQNTGKNQFQITVCDQECRLCV
DECPVQAISISKAGVVLVDKKKCVGCLACVAICPIGAMRYYPGQSTVFKCIACGACAKEC
PTGALEIATKEEKPKTPEAQA*
>SPSA8_v1_700004|ID:41146291|aorA| Tungsten-containing aldehyde ferredoxin oxidoreductase, alpha chain [Spirochaetes Bin 1 SA-8]
MSIQKVEFKLVTEYSYEKKPVLRGYNMRTLAIDVGKLSGGKPGAILPPESPAIAEKPVSE
EMKKRFVGGKGFGMRLLWDAIKPETKWDDPENDIVIAMGPLCGNTNYPGSGKSLVVTFSP
LTGIPIDSNVGGYFGPYLKFSGFDALELSGKSDRDLIIYIDGDEGKIQVFEAPAELESDT
HLLAEQLTHAFAANEEDRQHVAVVSTGRAAEHTWLGLLNFSLYDKRRKGIRVKQAGRGGI
GTVFRDKKIKALVCKYSHVTQNENHAADPAALQRIGLKLHREIMKHDDEQSKMRKVGTAH
LMEIMNDYDLLPVKNHRYGQDKDAISLASWVWQKYFTQGMPDGCWYGCTLACAHAVDHFE
LKTGPYKGKKVVVDGPEYETAAGVGSNLAIWDPEGVIEINFYCDTYGIDTISFGTIVGFL
MECWEYGILNEARTGGLKLEWGDWKAACELLHQMAEGKGFGVLAGRGTRYLAQYFAANFG
ADPQLMKDIALNGKGLEQSQYLSKESLAQQGGYYLTNKGPQHDEAWLIFMDMVNNQLPTF
EKKAEALHYFPMFRTWFGLQGLCKLPWNDIEPADNAKWPEPNKVPEHVDNYRQLYQAVTG
EPLTWDELIRQSERIYNFQRIFNLRMGYGKRNEDYPPYRAVGPVTEAEYLSRQERYDKQL
REKMGLNPESMTLQEKMAATRKWREQQYESLLDAVYKRRGWNNNGIPTPEHLKAIGMDLP
ELLEVVQKHL*
>SPSA8_v1_700005|ID:41146292| conserved protein of unknown function [Spirochaetes Bin 1 SA-8]
MYRKLTEAECRDAMKNGEFSQAIVSSKPSVAIVLTQSWCPQWGWMRSYLPALAEAPEMDI
YWLEYDHEPFYDEFLEFKEDRFGNREIPYVRYYRNGKLVKESNYIDKAGFLRMLKN*
>SPSA8_v1_700006|ID:41146293| conserved protein of unknown function [Spirochaetes Bin 1 SA-8]
MKHGLKRLLFSLGGRALTVIPQKVPVLFSGLGAVRKIPGELATLKVGKPLIITDAGVERA
GILAKLTEILKSASIPYAVFSGITPDPTFEVCEKALEALRVNSCDSVIALGGGSVMDAGK
LVRMGATHKKPLARFAGLLRCRNRGLPFICIPTTAGTGSEATAAAVITDSVKHRKVTVLD
PRLPPDSTILDPALTAGLPPALTAATGIDALTHAIEAYCNTLHYRDVDVQALEAARLIFA
NLPRAYADGSDLEARENMLKASHIAGRAFTRGFVGYVHAVAHRFGEKYHVPHGLANAIAL
PWFLDFYVDVCPERLADLADMVLREDTRREYTRREAYSGEAEVAAAPETHQNSEPRNPEF

NLRSMNKAERRAWYQKKARAFCDAVKALELEIGIPGKGDFIRAEDIDSIAEGAIKEAHGT
PYPVPAVLDADDLKTLIRKMMK*
>SPSA8_v1_700007|ID:41146294| Peptidase M16 domain protein [Spirochaetes Bin 1 SA-8]
MRKKSCTRKAVCFALLAFLLLPLQILSAQSAQNHVGHMVLPNGLEVFVVENHSVPLVTVC
VAFRGGASAQTPETAGLFHLYEHMMFAGNSKFPTKESFNAALNSMGTTAWNGATGTEYIN
YYITVPSEKTDEAVEFWAQAVRNPLFEPAVLENEKNVVLNEIKGYHADPARIAANALESR
MFKDYPWRKNIDGPEYNVQSTTVQVLKQMQSRYYVPGNMALMVGGDCTMEEIKALAEQYF
GDWKGQGAPSFDVPPHGKIPAGINLVSVEDQFYRGIGNIQFRWRGPDVLAQAQDTYTSDV
LLFLLSSPIGRFKDSIMKKVEGLYDAEYIDFTYPTARDGGNYIFSTYMLIQKPASEGAVL
DRVMNLKNAVLDEFKEIARDPSGYFGSNELQKAKTKLIDQNIFAMESAESFVTDTLTFWW
STATADYFFGYEENCSKVSWDDIRALIQHYITGSEASPAPEIATMLRIRTSTFGSDSRMA
AKMQEYGFEKVNADNAFWWQR*
>SPSA8_v1_700008|ID:41146295| Peptidase M16 domain protein [Spirochaetes Bin 1 SA-8]
MKNMLWKKCAVLAVAMMMLFTIGSGVSAQAAQKVKVDFVEMNRTTFRQFTLSNGIPVYIK
ENKANKVRNISFVLRGGSLTIPQEKAGWPLLAFKTAARASVNYPYAVVTDLLDETSSSIS
ASATFEYTALSLNVLDKYFAKLLPVWADMVTQPAFAKSDFDQAKSEVELNVQSKDQNPWA
KTNLLTNQKFFEGHPYAANPDGTEGSIGKARLDSIRAWYTENLSADRAFIVAVGDFSNEA
LETELEKTFASIPNLRLGPVPKATAFAKTAQGRLYTEEDEQSRGVVYMRGDFTAPSPDSA
DYFPTSLAMKLFSDLLFTIVRDQYGAVYTPGSYIRSFGANYGSITIYKTSATEKIKSYID
EAAEVMAEGRCVSVDPSRPGEESKFMIISDALEAYKQMFKNEYFNAVRTNSAMAGMMIRS
VVNRGDPADWIMDVQRIQAVTPGQISEAFSRYVLDGGFIWVALGDPGLLRKLNPADFTRL
GK*
>SPSA8_v1_700009|ID:41146296| protein of unknown function [Spirochaetes Bin 1 SA-8]
MKNAARLFAILFGLVLIAATVINADLIVKSQKALADGIFRSSSSAKPARYHLVAVFPDTD
DSYFRGLINGLNEEASKIEAVVQIFRYPAESNTEAERYFNLAMAARVDGLIMFSKRGDPL
AERKALADRSNVRFIPVFMDSPLDEDTFFVGSDLKKQGFASASAILPRLGNSARIGVILP
QTGSENILNEAFYQGVLEAMKNFPGARVVAAIRSRPGAMSGEESAASMLDSREPVNXX
>SPSA8_v1_710001|ID:41146297| conserved protein of unknown function [Spirochaetes Bin 1 SA-8]
VNDHLIRCPFVVLIRGRSINLILDIPDLELSSENFFLVAREYGICSGYIDLLIITEHADI
VVVETKLFRNPESSRKVLAQVIDYIKTLAQIDIDEFIAQIDNIKEKRIDINRKSDDRFLA
VLKRNIERGNILAVILGDDINTNLLEMVSSIHSAPHLAFSIILLSLETKKKDNLLILNPT
ILSKTKEIERSVINISIDMIDKNVKISAQAPEKKGKGSKPIISWEEFLTNFDDKERIRIL
DKFHTKWLSIDSEGFNMGTAGFSAGIIKNRIRIPIQYAYSANLELFTEKKCKSLGLKDSI
YELYKNHLKSQQNLYDKYVIANKTYIEYSKISNTELNAILEASIKVAEKIKNEEV*
>SPSA8_v1_710002|ID:41146298| Phosphate transporter [Spirochaetes Bin 1 SA-8]
MKIPIDWLIEGEPWIEYRARRDLLGQSENDPQVKSVRKSMLANAKVQDLVAELSGWPGTV
ISSHKSASQPFHKLTFIADLGLTATDPGVDTIVEHILKHQSAEGPFQLPMNIPMHYGGKG
EDQWAWALCDAPLIVYALVKLGVENEPVVQAAIKYLVGLVCDNGWPCVVSKELGTFRGPG
RKDDPCPFATLAMLKALSEIEALRNSSACHTGADTILTLWNESTTRHPYMFYMGIDFRKL
KVPFVWYDLIHMLDVLSRFPWLKKDARLLNMLEILKSKADRQGRFTPGSIWAAWKDWEFG
QKKEPSRWLTLLAWRVIGRIETDSM*
>SPSA8_v1_710003|ID:41146299| conserved protein of unknown function [Spirochaetes Bin 1 SA-8]
MKKFALFVFNGDPMCFIHVLLNALDMKAKGHEAKIILEGASVKLIPELVQPGNPLHGLWK
MNLAAHLVEGVCKGCSTKLGTIEAAKEQGLALLDDMSGHPSIAAYRDKGYEIITF*
>SPSA8_v1_710004|ID:41146300| protein of unknown function [Spirochaetes Bin 1 SA-8]
LQRQKKAKVKFSSFGISIYTVIYSSLTVNTSKTVRPYFYCKNHVQRTH*
>SPSA8_v1_710005|ID:41146301| conserved membrane protein of unknown function [Spirochaetes Bin 1 SA-8]
MITMPDKFIRRLIMTLTGVFICSFSVGIFNLAAFGVDPFQCFAQGSFLLFAKHFQYGTYY
LTISLVLLIIILIWDKHYVGIATFINMFLTGYIVDYSFTFLVHLLPDVDLLQRMLLLLVA
VLIMCFGSALYYTADLGVSVYDAISLILAKKKIAVKYYIVPFKWIRVFNDLICVTLGTLF
GKMPGVGTLITAFFMGPLISLFNKKFAEPFLQMDFMNHE*
>SPSA8_v1_710006|ID:41146302| Major facilitator superfamily MFS_1 [Spirochaetes Bin 1 SA-8]
VPFINRLLHWFIVGIGTTVMSLMLLSKGSTVDTLGFITAIYSVFIVLFEFPSGVLADILG
QKKIYLVSLALSILGYTIVLFSNNLVWLFIGFSFYGIARACSSGSVEALFISQYIQKNGK

ENLHRLMSALSSGEVIGLASGALLGGVIPMIWAQYFPGQNKYNGNLIVQIAALFILLVFT
WFGVQETRDTEKHKQLMMVHIKESLSIVLRNRNILLLILGTMTWGFCFNAIELYWQPQVK
GILGTDAQTWIFGVINSGYFFASLLGVGLINLLLKKRSIAQRLVLVFSRILAGLLIVVLS
FQSTIVAFTSVYLFLFLMNGMMNIPEGTIMNSLIPDEKRSSLLSLSSLMMQVGGIAGSVI
YSMLVKSIRISGVWILSGIVFGVSSMLYLSLKTDFKKPGV*
>SPSA8_v1_710007|ID:41146303| Pyruvate/2-oxoglutarate dehydrogenase complex, dihydrolipoamide acyltransferase component [Spirochaetes Bin 1 SA-8]
MKTRKVSEFRKLSIYGFQVTENSHNFYALLEFDITEVRKYLRERRVSGKSGSLFAFLLKA
IGRCLTIFPDFNSMIDLKRISTFEEVDISIPVEIFKNDEIFNKQLVVRDIDKKSIADIDN
EINQSKKADKNEKAYVPSPFFQKLITMMPNFIIKPLFNLLIRNHTMVKELSGTAFVTSVS
MFSNIPGYIIPYVGKPKSCSFALGSVYKKPVVINEQITMREIINITAIFNHDMIDGAPAA
RFINQLRKFIEMEYIDLID*
>SPSA8_v1_710008|ID:41146304| protein of unknown function [Spirochaetes Bin 1 SA-8]
MFDLLKIVNNTRWIDYGLNIINIFIIDELEKLQNRSRRFFNFIHSEWAHKFSFCESFKT*
>SPSA8_v1_710009|ID:41146305| putative 1-deoxy-D-xylulose-5-phosphate reductoisomerase protein [Spirochaetes Bin 1 SA-8]
MSSHRKTVIITGANRGLGKAAAKEIARAGCRVIMACRNTDAGEIARSEIIADTNNSDIHV
LKLDLASRASIHEFIDGFSRQFGSLNVLINNAGIAKQEFSKTEDGFEINIGTNFFGTYVL
TTGLLPFFDELHEKRIINLTSNIYRIGWFKTEHINRYRWFRAYAVSKRMILLYTKWLAED
KELCDFRVNAVHPGIVDTSIMYTGQWYDAVIKVLCKPFFITAEEGARTCVSLALDMAAVN
GKYFAKGEQREIRLSRRMIHERDDLLAFCRMLNSG*
>SPSA8_v1_710010|ID:41146306| protein of unknown function [Spirochaetes Bin 1 SA-8]
MSKETILTNGTIYSLEREGERFDTMLIGSDGRKKGLYRKGEQPAVDVGIAILIAVGSAVS
IR*
>SPSA8_v1_710011|ID:41146307| conserved protein of unknown function [Spirochaetes Bin 1 SA-8]
MYEKLIETLTKRFMDNSKRHQGLDWDSIAERLQKNAAALLILEAMEATGGEPDVIAYEKA
GNSFVFCDCSAESPSGRRSLCYDDHALELRKANKPAGSAVSMARKLGVSLLTEEEYRHLQ
TLGEFDLRTSSWIMTPPEIRNLGGALFCDRRYNRVFTYHNGADSYYSSRGFRAILKVH*
>SPSA8_v1_710012|ID:41146308| conserved membrane protein of unknown function [Spirochaetes Bin 1 SA-8]
MSTTLYQKGGSTMGLEQTAKGKPAVSQDESLGKLRRFNVIMGFMHLIQGIFMWVVSNDKT
YPIFTNYLSFDRATFTLKPNPQLLYQLRFGPAVALFLLASAMAHFYLSSIGYQKYRENLK
KGMNPIRFYEYAVSSSLMIVLIGMLVGVWDLGTLIALFGINAMMNLFGIMMEYHNQXX
>SPSA8_v1_720001|ID:41146309| protein of unknown function [Spirochaetes Bin 1 SA-8]
MLITRLDEIPDQELDLLAERGINGLWLIGIWQRSPASEKIKQLCGNPDAAASAYSLFDYE
IAPELGGWEALDVFRGRCMWRGIRLAADMVPNHTGIDSAWVRNRPDLFIGTDRCPFPSYT
FNGPDLSSDPAVGIWLEDHYYTRSDAAVVFKRLDRRTGKVRYLYHGNDGTGMAWNDTAQI
DFLNPEARAAVKERIIHVAQHFSIIRFDAAMVLARQHIRRLWYPAPGTGGAIPSRAEHSM
SDEAFDKAHPNEFWREVVDECAVRVPDTLLLAEAFWMMEGYFVRTLGMHRVYNSAFMNML
KTESNSMYRLTIKNTQEFDKQILKRFVNFMSNPDEETAIAQFGSGDKYFGVCTLMATMPG
LPMIGHGQIEGFSEKYGMEYKRSYKDEKPDPVLIGRHEREIFPLFRMRKLFAEVDNFYLF
DFVKDHHKVDENVFTYTNGRGEERALVFYNNYWERTSGTISISCPYAEKDGSGKRKRIVA
LSEALALDPAPGNFVITREMRSNLYYIFKTADIHSHGWHVNLEGYQSLVFTDFTRVHDYN
GSYQRLWENLHGQGVTDFEDALEEASHPELYHSLEKTIAAIIDFCLLTSEKISNAAQEKE
TGRIAEISEIYFARLAQIIVEEGGPAPLLDAVGSCVKTLKTGLKLIGSLVSDGNGALEIE
QHNDLLEEFSRTVKTLKGRMALSYAVFILAISRLQREGNRTEELRFLLEKFLLNKKMLEA
AENQAVSHIERPLGNEISALSEFDLGLLVQAFMTRPLPVLTAPQPNKSGKSKPLFSTPLE
RAFELLQWAGNDPIARQALGVNEWQGIEYFSKEKMEALLFLGPSLTLLEEVLGTKAAEKT
DLFEKLTALQPHVFSAIKTRDLILKAVVQSGYQLRSLVKLLTARGMDMNE*
>SPSA8_v1_720002|ID:41146310| protein of unknown function [Spirochaetes Bin 1 SA-8]
MNNEIPCRDVLDSPLAEAIVKHISDRRYYFVFPSQIXADSWAQAVFRHPEIEALEPGRFL
SWDRFLALIREKPRNETIRPADHYTRYLWACGALKENAENPFLRALVKPDLAPPSRYPAW
LASIANRLNFLEDIARREYASESHNAEILDFIILSEKYRIFLERNNLFEESSLPLRFPEK
SKFILFGASAYKDILWYRSMLEKSGCTEFFDVIPAEPGRSQKLHRFENFRTEARWIFANI
RSMLNNGKTPGDFAISVPALQPDMKAYLSVFAHEYDISITFRVGEKLSSSPFGLLLNLIA

AAIDDTLSQQSVEALARQTLFVWKQPGAFAKLMEFSRMFSIPRKNAGKKLMNRVWSMTFS
DAQFEEKEALHDFYLLLQKKLSAIAAAASFQSLREALFDFRLTFIEDSGISEQSEKLLAR
IFEELISLERAAQSLTGVSLTGNTFQHFRAFLDTIQYAPDSSDMAVSVYPYHSGILNAAP
VHFVLDASQDAVDSAQKFPSLPESIKKLLPDDTSYESYILRSFDCVNAIYCFADTALSGF
CVPHPWFSAEHIQTVFVNEEKQLNALSFHAREKKAWIENRPENLQPLTFGQQNAALGIFD
SELNGQPLPPPLFDQRKTDTRQTIAADNVVDCIAPALHGSSIKINPAALGDYSACPFRWF
LAYIVRLQNKIPSEPLMIGSILHDCIKNIVLRISSKQPVLRKEAIQAYSDLVIDAIQQGI
DYQTRKSGHGARPFLEAHFRRMTSRLLTYLDLEAELQEAGWTVGEFEKSFEKHFEQLSVI
FNGRTDRLMFRREGSEMACLLIDYKKKYIPQKKHLLLDEKTGQLAELQIPGYILLLTEAG
FLVMSAFYYSIENAKKQVVLGDESRAAAPDAQSYFKELEALRAMLEKASEGIWSGRITAA
SPFTKTCSNCEYRPICRANYSSERH*
>SPSA8_v1_720003|ID:41146311| protein of unknown function [Spirochaetes Bin 1 SA-8]
MTELDRWQKKAVTADGNCVVTAGAGAGKTTVLAERYLHLVLAQRIPVTDILALTFTRKAA
AEMYERIYRKLAESEDPFAQSQLALFSQAKITTLDSFCSSIVRRAAPDYGYSPEFSVDDV
SATEIAEKTAFQFVMANRDKPAINEFLQSHQMDAVVNDFFASLGTALVSPVNLVKPEFSP
MEPAMRKYISDYAETQIEKMSSLCASIRSAGLGIKAPHADCASAIDAARRFPKESTAVIR
ENLQLLEPFISAAGQLGMKSYGKDAGESCIKEYAKELRQCAKDFPQVKDLVESFPLYSDM
LKYLDDFAHQLAEQKRLADIMDFKDLGYCAVDTLIRRSDIRQELKTSIQSIMIDEFQDNN
ELQKSLLYLLAEKQSLTLDRIPTASEIEASKLFFVGDEKQSIYRFRGADVSVFKKLSREL
ARSEAPTPDAESEATSLVLGANYRSSASLIDFFNDFFSSIMNSDTAGEGKDFYARYETMS
VGNEERQKRPFPSLLKFYELAENSENESSQESAGDDDTSAGNDSIAESRPDSEFLSADES
EALAVARFIRENIGVLDVSDSDGTSRKASPADFAILLRKTSPQHLIERYLRHFSIPFRID
QPRDLFKESIANDLYNILLLLLEPANQPAFAVVLRSPLCRISDAGFALIMTRARGFEEIP
STLKLDEYDSRMIARGKAFFAKLSQNARNTSITRTXXFIWNYSGLRLDFLSRPDSRVFME
HYDFIFSLAASVEAQHGTLSDFVDELGLYIENSDRKFEPGDIPRESAPGVRIMTIHKAKG
LEFPIVIVPFVHHQNGGNKARIWGKTEFGIALGLKNPEDPESKTNNLLMSLANAQEKEEA
GAEVIRLLYVACTRAEDHLVFFGRKPSRNAPDSFYNYLNSYFNQSRKEKFSIAHRSEILE
KRIHSEKSDTGAFLKAYNAVYSQAKSEETSKQHRKERYTVSELNEISLELPVQTALSFQR
HSGSLVVSVPAINIKADIFGTLCHEILAWAVSRNGSLKDFIPSLPTREALTADQLQKAIS
HGSTLAEAFFTTDVWKNSLDNAAIESEKPFLLKSGDFIIEGRMDLVIESNDAVIILDFKT
GQEEKPQVYAIQLALYQAAMQKIAADKTIKAGIYWLESGAFSWLEGRMENKDIIMLASVL
QEKSENVPAAENQNGSN*
>SPSA8_v1_720004|ID:41146312| membrane protein of unknown function [Spirochaetes Bin 1 SA-8]
MPSSLPCIRRQCRKSLQTKQSKLGYTGLSRGPFLGLKAAWKTRTLSCWLQCYKKNLKMCP
LRRIRMDRTNQPPTPLMLFFMFAKITSVTLGGGYVIVPVIGSALEKKGWMNEDDFYTIFA
KAQAFPGPLAMSTAVLVSLRLCGFWGAFAAFWGILLPPFLALILVSDLIRTYGXX
>SPSA8_v1_730001|ID:41146313| ABC-type transporter, integral membrane subunit (fragment) [Spirochaetes Bin 1 SA-8]
MSEKKEKQAVNEFNQVMKPVSLWADAWKRLRKNKMALISLYIVSFYIIVTLFAPLLTKAG
IIYDYREQTISNASLPPSFKPAGQLVIKKLEARIADLERTLEEQKAEEAANGPSGSYGFD
FGAVPGTETETADQSAEVDPNLNFSLDFGGHGTDTESSSNPLVRELGYQKEALEKVKTEM
NTNRGYQKVYILGTDDLGRDMLARVIYGGRISILIGIVGTLTAALVGILIGSVSGYVGGW
LDNLLMRFVDXX
>SPSA8_v1_730002|ID:41146314|oppB| oligopeptide transporter subunit ; membrane component of ABC superfamily [Spirochaetes Bin 1 SA-8]
MARYFIRRALSLIPTLFVIVTLSFFLIRLAPGGPFAREKDVPEFILQNLLKRYHMDEPLG
KQYLRYLGDILRWDLGPSFRYRDLSVNEIIDKGLPVSMTLGVTSLVLAVVSGIAVGIISA
LRQNKWPDYVAVSIAVIGISVPLFVMGPILQLFVGMRWKILPMGQWISVHGIKAIILPAL
TLSFPYFAYIARLSRASILEVLRSDYIRTARAKGLKESMVVWKHVLKGALLPVVTYLGPA
FSGIVVGSVVVETTFLIPGIGRPFVQSALNRDYTLIMGEVIVYSVILIFANLVVDLIYGL
LDPRISYK*
>SPSA8_v1_730003|ID:41146315| ABC-type transporter, periplasmic subunit [Spirochaetes Bin 1 SA-8]
MKRSLFVLFALLSVVALGAQDFTIVNGAEPVSLDPHAVEGVPEHRIYMALFEGLTIADPK
TNRALPGIAESWSFSKDFKTVTFKLRKATWSDGVEITADTVVKSWLRKMDPKNAFQYADL

PASYIAGGMDYLSGKAGPEAVKIRAVDKYTFEVQLVGPTPFFPDMVVHYAFAIVPMHAIE
KFGNDWVKPQNWVSNGPFVLKEWKSQERVVVVKNPKYWDAKNVALQKVTFLANDDVNVGY
NLYKSGGADWIDTVPSELMNEIKLRKDFHVAPEYGTYYYIFNVTRKPLDDARVRKALAMS
FDKKALVENVTKGGQVPANSFVPPSTGYTPAKGFDFNPEMGRKLLAEAGFPGGKGFPTLS
ILYNTSANHKRIAEFIQSQWKENLGITVNLVNQEWGTYLDTRSQSHNFDIARAGWIADYL
DPSTFSDMWVTGGTQNDGLYSNKRYDELIEKSRTLLGAERMKVLGDAEKVLIDEDMAIMP
IYFYVTQNMIDLTKWDGWYPNPMNTHPWKFIRPKK*
>SPSA8_v1_730004|ID:41146316| Alcohol dehydrogenase [Spirochaetes Bin 1 SA-8]
MFVFNSPTKLVFGEGTAASAGSELKALGVHKVLLVTGPGRTASQETLAGILASIREAGLE
FVHYAGAGADPATTMVDEGAALYKKEGCDGILGFGGGSPIDCAKGIGASIGENRPIRDFI
GTGLAFTRPVPPLVAIPTTAGTGTEVTNAAVFTLVDGPKKAKKGTAGTSLFPRVALVDPT
YHLSMPPELTAATGMDALTHAVEAYVSRNHNPISDFFCLEAIKLIGLNLKRAWKNGDDME
ARSGMALASTLAGVGLSQAGLGMVHGFAHAAGALGGLAHGMANAIILPFVLREYISDANM
RLAAIGKALTGRQASTALDAVSAIAELGSDIGIPRNLKNAGIPESLLPELFNDAKTYKRR
PQSPRLFTDEELERLLYKAWSDSMID*
>SPSA8_v1_730005|ID:41146317| putative Nuclease sbcCD subunit C [Spirochaetes Bin 1 SA-8]
MRPIRLELKNFGPYRGTVSVDFSELGEFFLICGKTGSGKSTIFDAITYALFGEAPGARKG
HERELVSDYAEIGEEPSVRFEFSLSNARYLIQRTPPYRRLKKNGGFTEVPPEASIACIDA
AGREISVLASGVRDTNLKVGTLINLTAEEFSKIILLPQGEFQEFLQMDSAERSEILEKLF
PVAMYDTIAELAAEKYKKLKNELSAMEDEKSRIENQLGSDYESRYAALLLDQTQLETEVQ
SATGMVADRKSRLDSVRDRVERAQKAMQAVQTLQALRARKPEEDIRAERLDRARRAEQVA
HQIKAFAGANARTQEIVRKTRELESKFAELVQDQNKASEAQGKAAQLSAELQSLAREELQ
LERACKAWANKMDLEKQALSLIQQKAEFVQEIQQKETALQKRAEELSAVEPAEEEETQAA
ERWERLQTQLKEREELVRTLHELAERRREISSVTAKIEQTEAARREAELLDTLITQEMAG
LEAGLLHSEAEHLALQLEAGKPCPVCGSLDHPAPAATQTADTGDSEQTIEDLKLKLASKR
KEADASRSKLAALCATISNLTEDKAGKEKKFSVLAAHAVSACAKLAMPPALLEDLFSEEK
ISAHIDLLDDDIERIKSELADAQKCSVSLQDRRKKAKQLRDARETIRAELDLLQKKLAEL
EKAVAANTARLSEIAKEAGQENPEPSLLAVREKIAGYTREKEEAERYFQSWNQAKKQTYD
RHAELKTELAAVFEQFNLELSELSGALVSAGFAAPAGGPRTEHAQFSTPHFAWLSEPSEY
SVLQNALSWRLAEKLEIEAHNAVSALGPIHELEFSAALEAVHAAELGKAERNTEEEKSRM
YREALAKAETLAQALAHDLPQEGESLPDIAKLEQEFTEAREALDNAQTRLLENSTYLQTM
KNSMARIAELEVLRAGKRSDVSTAEELSALLKGELSGKRLPFKFFILAKYFRQVVSRASL
HLSEMSDGRYYLIPEETLSQDVSRSSAVSRAGRGRIGLGLKVRDDWTGADRSTGTLSGGE
KFITAVSLALGLSDMIRERSGGVSLEAIFIDEGFGSLDEESLDTAMNVLEKIRGGRTIGI
ISHVTSLRNRIPNQLEVVKTAQGSTLRIR*
>SPSA8_v1_730006|ID:41146318| protein of unknown function [Spirochaetes Bin 1 SA-8]
VTALGHLHFFQSPAPRIWYSGSPMPYGVPDAGRKTGLCRITLKSGDPLAHVECIPLKPLR
GFRKLRGFFGDILRYQPTAEEREDYVEITLCDTKPVVNPLHELKKLYPRIYFLKSEADEA
AAGSRPSAMSETEASLLDPEKAQSDDTIMQAFAEFHQEMKGKVPSKESMDQFELLLKEAR
NAANQA*
>SPSA8_v1_740001|ID:41146319| rRNA (Guanine-N(2)-)-methyltransferase (fragment) [Spirochaetes Bin 1 SA-8]
MPFKRSSQLSYTPDTTRRYYRKKQNHVKNCQLEYLAYSVRFHHTVSMKQTLRGIALCAVG
LEKIASKELERLGLRENERKPGRVYFDIEEKNLTQALSMTNIGLRTAERVLIEPGRFKAV
DFDAFFDGIFEMPWEMFCYKDTKIHIERVRSHESKLFAQTSLQAMAQKAIYEKLMQNYRM
RTMPETGNELXVRVYLDNDECSXGIDASGDALHKRGYRAMAGQAPLKETIAASLLFLSGW
NRKFALLDPFCGSGTIAIEAALYALNFAPGLNRRFAFESMPAVNPQGVSEARSMLESHIK
TDAEFSILASDIDPSVLNIARRNAEEAXX
>SPSA8_v1_740002|ID:41146320| putative D-alanine--D-alanine ligase [Spirochaetes Bin 1 SA-8]
MPHIILIKSYTDKPWRSPETFHLIEQALRKRWDVEPIEAENLQQLDGFISVMKKQHGEDL
FIFNISEYIDERTMQGFLPEYLEKSGIRYLGSGSMAVSTGLSKTETKKVLARNNVPTPHF
LLIPKDDDTITLQDSALRYPLFVKPDGTGGHIGIDNDSIVKNEAELRRAVRRVHEIFGQN
ALVEEFITGEGMREFSVGILDGAKRVFLPVEIDYSLMNVPVRILSHDLAMQDQEKVIPVT
DGKLFARLADLADRTFDALGAHDYSRVDIRMNEHDCYVLEINVMPGIGPASFLPEAAKFY
LDMKYEDLVRKLVEVSLERQTSTAV*

>SPSA8_v1_740003|ID:41146321| Peptidyl-prolyl cis-trans isomerase [Spirochaetes Bin 1 SA-8]
MIKHGRAEVKHACFHHANFSGGGGLLLASLASCQNTNQLPDGLYATMDTSKGTIVIQLEF
EKAPMTVGNFVGLAEGSLDVAKGKPFYNGLVFHRVEPGFVVQGGDPKGNGTGGPGYKFPD
EFSPELKHSGPGILSMANAGPNTNGSQFFITLAETPWLDGKHSVFGKVVEGMDVVNKIAV
GDKINKITIQRIGAAAKAFDSSQKAWDERLSAGYAALKAAAQAKRASDEALISQKWPDAV
KDENGIFQKVIRKGFGEVPQKGSTVSVIYKGMLLDGTIFDQSSLSGAPLSFRVGTGEIIE
GWDKVVATMKKGEKRLVVIPPELAYGSSGIAGVIPADAYLVFEMELTSIKP*
>SPSA8_v1_740004|ID:41146322| CMP/dCMP deaminase, zinc-binding protein [Spirochaetes Bin 1 SA-8]
MSDAVYRRPSWDEYFMEVANAIAKRATCDRGRSGCVIARDNQILATGYVGAPSGLPHCDE
VGHQLKKLIHEDGSITQHCVRTVHAEQNAICQAARRGVSIEGATLYCRMTPCRTCAMMII
NCGIIRVVCERRYHDGAESETMFRQVGIKIEYVFDEVQKYSNQ*
>SPSA8_v1_740005|ID:41146323| conserved protein of unknown function [Spirochaetes Bin 1 SA-8]
MNIVFLGEIAGKSGVFAVKSTLREIKARYAPDFIIANTDSTTGGAGLGVQHAVYLKKLGL
DCLTIGESAYFKLDMTGFYPKAPWVLRPANYPYDNPGRGYRVFQTERGKIAVIVLLGQAG
FSRVHLENPFHTIDRILERVSRDTSSIIVDFHAATTAEKLTFAAYLDGKVSAVIGSHCKA
LTADARILQKGTAAITDSGRCGSILSVGGMDPAAKIREYMTSVRTYMGDGTEGLESQGCA
LTIDENGRATTMESFRIPCKEQIHD*
>SPSA8_v1_740006|ID:41146324| putative Positive regulator of sigma E, RseC/MucC [Spirochaetes Bin 1 SA-8]
MTELALVKKIDGKMVTVSIEMQEGCAACSNGACKANRSALQAYNRNDVNIAEGDEVEIEI
PGAEQAKSAFWVLGLPLIALFAGYGLGRLLFPTGGEGPAVGVSGALFLVVMAIGLFIQKK
KKYETLPVIVRKFGEYGV*
>SPSA8_v1_740007|ID:41146325|ppk| Polyphosphate kinase [Spirochaetes Bin 1 SA-8]
MAEYPFFNRELSWIEFNNRVLQEALKPHIPLLERLKFLSIVSSNFDEFFMVRVAAVKSAI
RRNELSFDVTGITPTALLDQISERVKKIHSVQYTYLMKEIWPSLVKAGLEITSPADWSPH
ETRFLESYFSENIFVLLTPLRIEEDSFPSVGNLQIHCAFELEAEDGAQRFAITQVPRNTT
RFIRLPAEEQTPAANENGIQKIRIALLEDVILRFAARLFPGNRVRSSLVFKVTRDADSGV
DEDRQEDFLAAMEEVLAERHNSTPVRLTISGKSPILSAILKKGLELGEPDTYHLPGPIDL
GGFHELAAEISSLITQEPMISQLLDKPWPPIQLHEASETSIWEEIESKDILLHVPYESFD
TVSRFIEEAATDPDVLSIKMTLYRTSGDSPIIKSLIKAARNRKQVAVVVELKARFDEERN
ITWASSLEQAGAIVTYGVARLKVHAKAALVVRKGKDGSIRKYLHLSTGNYNDRTAKLYSD
FSLFTVNEDLCREASFFFNMLTGYSSVQTLSVMAIAPFDMKKRLLALIDREIQRSTAEAP
GLIFAKLNALADPEIIEALYTASQAGVRIQLNVRGICALIPGLPGLSETIEVRSILGRYL
EHGRMIYFANGGTEEIYLSSADWLPRNMDRRIELMFPIFDENIRKTCKEILWTYFKDTSH
AYRLLPSGAWEAIKPEGHEKPAGAQEMLYHRVRKLARAIQTPQEQLTVRRRFRSAR*
>SPSA8_v1_740008|ID:41146326| Ppx/GppA phosphatase [Spirochaetes Bin 1 SA-8]
VAAIDKNGSLKVLDRAAKPSRIGRDTFTMGRISRDAIRETIAILNVFKELLAGYGITPEA
ATTIGTSALREAANRDTFTDRVTLQTGFKIRIIEDIEENHLMYLAVQQALQDERKFLSRS
NSMILEVGGGTTEIMLLQRGKMVSTHSLRIGTLRLDEQIREAGSSRNYLEEYLESNIKTA
CDLMADELKLDTIKSYIVIGSDARFAAYCLSGTTYPGYSVLSREQFIEFARTISAMSLEE
CISAYKISYTEAEGLASGLMIEALFLEKTSAENVIIPNVSIREGAILAEVSGLDKSIRKE
LTAQVLASARSLGKRYRYDDIHARNVAAISLEIFDFLSREHGLRDHERLLLETAAVLHDI
GTYIRASGHHKHGEYIVSNSEIFGLNKNDIAIVSNVVRYHRHMPPSPMHLNYISLPREDR
VIVMKLAAILRVADALDRNHTGRALDSSFEKSGERFLIKPSQALDYSMERLSLSAKGDLF
EDVFGLEPVIV*
>SPSA8_v1_750001|ID:41146327| membrane protein of unknown function [Spirochaetes Bin 1 SA-8]
MTDKEISLTRAEKISKSLNQVTGIACAFFFGTMTVIVFLGVFFRYVLSAPLNWVEETSRY
LMIWGASLAISIGISQGDHVGLTIILDALKPGTGKKALILAINTLVFAFVVFMFVYSWFA
AVESKTQMTQALGISMFIPKLAVPVAMLLSAIQVVLX
>SPSA8_v1_750002|ID:41146328| TRAP dicarboxylate transporter, DctP subunit [Spirochaetes Bin 1 SA-8]
MKRTLILVLAVLMFLPGAVFGQTVLKLAHLNPQQPFDVASAAMAAVFKNEVEANSNGQIK
VEIYPNGVLGKEAETMVQVKSGVVQSFISSSGGMAQFYPLIDVTNMPFAFSSYNVGYKVY
DGPFGKELAADIEKKAGFKVLGFGESGGFFAITNAKKPIKSPADMKGIKLRTMALPLHQA
IVKALGASPTTVAWAEVYTSLQTGVVDGQMNPISIIAMAKFQEVQKYITLTNHLYAPYVW
VMNPKFYSGLTPELQKVVDDAARTAILAGRGLSRIIDASEKGLPALAEKMQVYVPTKAEM

KQFRDAAIPAAKEFMASQYKKDGEMWVDKFFDAIDKAEQELGL*
>SPSA8_v1_750003|ID:41146329| 8-oxoguanine deaminase [Spirochaetes Bin 1 SA-8]
VILLKDCYTVVTGSREAVRGSPAASAKASATGTLSGIDILIDGSRIVKIGASLSAPEGTQ
VIDASRHVVMPGLVNTHHHFYQTLTRNLPAVQNAKLFDWLVYLYDIWKFLDPEAVYWSSM
LALAELAKTGCSLTTDHHYLYPAGFPDDIPSLQFKAAADIGLRFAPTRGSMSRSKKDGGL
PPDSTVQDEDSILIHSEETIKRFHDPAPDAMRKVALAPCSPFSVSERLMAQSAALARKYG
AHLHTHLAETSDEDDYCVQVYKRRPLQVMQDCDFIGPDVWYAHGIFFNDEELDILAKTGT
GVAHCPSSNMRLGSGICRVREMLDRGIKVGLAVDGSASNDSSDMLGEARQALLLQRIRYG
SAGIKADEVIRVATKGGASILGYEEAGEIREGALADIALFDVIKLEYAGALSDPVAALLF
SGYNHGVDHLIVNGKIVVRNARLTAIDEDLIRANAEQAAQRMYRKAGIL*
>SPSA8_v1_750004|ID:41146330| Pyridoxal-5'-phosphate-dependent protein beta subunit [Spirochaetes Bin 1 SA-8]
MIDLKVIESQRKQNIQRCKEKGILLPTFAQMRDPSKIPESIKKELTGIGLWDVHPRNLFR
VNWHNEPKEFGGTYGGVNYFEIPRAITGTKARIIGLVGKWFPTGAHKVGAAYSCLVPELV
TGRFDPTTKKAVWPSTGNYCRGGAYISRLLACPSVAILPAGMSKERFEWLKTMAEEVIAT
PGCESNVKEIFDKCVELEKTRPDAVIFNQFDQLPNHLWHYAVSGPAMEEVFNTVAGPNDH
VAGVVLSSGSAGTLGSGSYMKEKFPGTRIGVAEALQCPTILENGYGDHRIEGIGDKHIPW
IHNMRETDAAIGVDDELPIRFIRLFNEEAGKKILIEAGADPAEVERLPLLGISSVGNLIG
SIKFAKYYELGEDDVVFTMFTDSMAMYQSRLSELNTERGIYTQRDADRDYDRLQGLSTDN
LLELSQVDKRRIHNLKYFTWIEQLGKSLPELRAQWNDYRNYWGSLHQQAAELDKLIQEFN
AEVLG*
>SPSA8_v1_750005|ID:41146331| putative threonine synthase [Spirochaetes Bin 1 SA-8]
MKWQYRCPQCGAAYPIEPGRYLCDICSKSNKPGMPLQGVLECVWEGTPVLHDDDIPLPVE
REFFPPVPVGNTPLWAPTRLREDLGRNKLWLKDDTCNPSGSYKDRASWLVSAFARKFGIH
EIALASTGNAASSMACIGAAAGQKITVFLPKSAPVAKRIQVLQYGAELREVDGTYDVAFD
QSLAYSDATGVLSRNTAYNPLTIEGKKTGSFEIVRDLAIQGGGEPDHVFVPTGDGVIIAG
VIKGFEDLVKLGRISKMPKVWAAQAEGSSAIARALAAGSFEFLPSNTIADSISVDVPRNG
SFALEKLKMHHGGAVAVSDKEILDAQVYLSRASGLFAEPSSACAFAGFLKVQHEIPIDDT
VVIMLTGSGLKDIKSAAAAVGAGL*
>SPSA8_v1_750006|ID:41146332|ygeY| putative peptidase [Spirochaetes Bin 1 SA-8]
MDKKLILEKAKQYRDYTAKNLSEIIKIPGFSTTEKERVALLKKMCEEAGMEDVHLDGLGS
LLARVGSGKKKLVFDAHIDTVGVGDPSQWKTPPHSGLIKDGLVYGRGASDQLGGAASMIT
AGRILKEMAYNGDYQVWFSFTVLEEDCDGLCWKYLIEEEKFIPDFAVSTEPTSCRLYRGH
RGRMEIQIDIKGVSCHGSAPERGDSAAYKAARAALALEKLNAELQPDDDGFLGKGTIVVS
QIKVHGPSQCAVPDQAMLYCDRRLTWGEDDKLAIAQVEDALKKAGVDNFTVHMPEYSQPA
YTGKLYKQELYFPTWKIPADHVLVKSGVEAYSELFGKTPVVDKWTFSTNGVAICGRHKIP
VIGFGPGDEAQAHAPNEITRIDDLEIAAAFYAALPYMLEKQA*
>SPSA8_v1_750007|ID:41146333|ssnA| putative chlorohydrolase/aminohydrolase [Spirochaetes Bin 1 SA-8]
MILFENVRLLSFNPPAVSEEMDLAAYGPDEGGEKAGTIAAIGKNLAAVYPEATVAGKGGY
LSPGLVCSHTHLYSALARGMEVAIKPSKDFAQQLKHLWWRLDRAIDTPILEASALAGCAD
ALSAGVTSLVDHHAGPECIEGSLSIIREAYEKVGIRGLLCYETTDRNGMEQARLGVQENI
RFAKEIDAMRKAGKRPLVDAAIGGHAGFTVGDATLEALAEAVQGTGRGIHIHLAEDKYDA
VDSRHRFGLDLIERMDRAGVLSPKSIIGHGVWLTPSEVEIMNERDVFLAHNARSNMNNAV
GYNALLTSHKNVVLGTDGMGADMLEEFKFAVFRHRESQGPWWPDNFLVCMDRGNRILERY
FGSDFSNSTAEGFAFGKLAVGAPADLVLWDYDPPTPISANNIAGHLAFGMSSRSVRTVAV
AGQLRIQDRTPLFDAEKIQAYAREQARRLWKNMEGR*
>SPSA8_v1_760001|ID:41146334| ABC-type transporter, integral membrane subunit (fragment) [Spirochaetes Bin 1 SA-8]
MVFPAVAAILIGGASINKANIINVLVGTFLFQGLLTMTPSVINSILQTDMSEVIRMIVSN
GMILYALTRKTKVTK*
>SPSA8_v1_760002|ID:41146335| ABC-type transporter, integral membrane subunit [Spirochaetes Bin 1 SA-8]
MSAHKKSFTKFLSEYAVVLIFVVITLAAIAPSGLSIKYIVQEVITRLGRNSFLVIALLLP
IYAGMGLNFAMTLGAMAGQIGLIFAVDWGVAGWQGLVFALLVGLPISILLGWITGKVMNK
AKGREMVTGYILAFFINGIYQFFVLYMMGSVIPMRNPAIVLSRGYGVRNTLNLESVRQSL
DSILMLRLGGFAVPIVTFVIIGLLCVFIVWFKKTKLGQDMRAVGQDMAVADAAGIPVEKT

RIIAILISTALACAGQIIFLQNMGNMATYNAHDQTGFFAVAAILVGGASVTHANIPNVFI
GVILLHAMFIVTPMAGQRLFGSAMIGEYFRQFIGYGVIALSLVLYAWKNRKAIEDARSGL
RQGQPVQKEQNDKGGKA*
>SPSA8_v1_760003|ID:41146336| conserved protein of unknown function [Spirochaetes Bin 1 SA-8]
MNLAQTLKSKKFIIRALLVAAYILLGILMVYSGKRHTILIDNKDAEDGSYQAIDGMSVQV
DNLEAAEYYAGDRDKAVVKGQKHVIRIELFNEEKKIEKSFTVPFGNEMLILSVPKLVNNI
EPFIEPFTALMEQNSQVEAQTPDQQHFGSDMSGEQIDMTAENAQQ*
>SPSA8_v1_760004|ID:41146337| CoA-disulfide reductase [Spirochaetes Bin 1 SA-8]
MARYLVVGGVAGGASTAARLRRMNEQAEIILFERGSHISYANCGLPYYAGQTIQERDRLF
VMTPEKFHAWLNVDVRVRTEVTAIDRAAKSVLVKALDSGREYRESYDFLVLSPGADPIKP
PIPGISQEGIFTLRSVTDIDSIKEYIDTKRPERAIVVGGGFIGLEMAENLHARGAFVTIV
EALDQVMNPIDFEMAALVHQHLKSKNVELYLGSAVQKFEKRGSRIVVFLSDGTELDADMI
VLSIGVRPEGTLAKNAGLATSPNGAILVNESLQTADPTIFALGDAVAFPHPILGMAMPVP
LAGPANKQARVVADNIVKGAGTRVWKGAIGTSIAKVFDITAAAAGVPEKLLKKNNIPVRS
IITHGGSHAGYYPGSQPLTIKTIFSSENGQILGAQVVGYDGVDKRIDLLAEQIRRKAHIS
ELAEIEHAYAPPFSSAKDPVNIAGMVAENLLAGMTRLIAWHEVEAFQKQGAFFLDVRTQD
EFSLGTIPGAVNIPLDNLRSSLTEIPKDRFVVAFCGVGLRGYLAERILRQNGWTEVANLS
GGYKTWEIATEPQSHKGVYHRGLANVQMNVAGRKEDLVHTTFAEGSGMPEQLANRKDTVV
RVDACGLQCPGPIMRLKAEIDRLPEGGRIIISASDPGFARDVASWCRVTHNLLVSLEESK
GIYTAVVEKSAMQAKMLSAMPGASAQGAPMVQYSPEGATIIVFSDDLDKALASFVLANGA
AAAGKKVTMFFTFWGLSVLKRPNPPHIVKDFMGRMFSFMLPKNPASLNLSKMNFAGAGRI
MMKSRMKAKNVDMVEQMIESARKAGVRLIACQMSMDIMGVMKEELLDGVEIGGVATYMEA
ASESGVNLFI*
>SPSA8_v1_760005|ID:41146338| putative Mur ligase middle domain protein [Spirochaetes Bin 1 SA-8]
MLFLLIELFFCRPTDGLNCYPFRPGVKSMTMSALPLIPCLTLIMLLLGVSESALHRKRLA
KIPIRILVNGTRGKSTVTRLIAAGLRGARQHGSSDGQSRTIRTIAKTTGTEARQILPDGS
EEPVARKGPARISEQIAFVKNAAEAKADAIVVECMAVTPENQRVFEQHLNRSTIGVLTNI
RLDHTDVMGQDEFEIAQTLALSIPERAVYIANPGEFEDFFRAECKKRGTRFISVNPEVIP
DHYTAKFSYPMFKENLAAALAVLKHCGIDEETAIERMSQARADPGVVPVFTMVLDGETMV
AVNAFAANDAESTLAFWREFGQPLIKAPQNRTDDIQESGGMQNADVVVAILNHRSDRPFR
IRELWDVVSRLPVQKVLFCGDLQSEALRFARQHPAASETDSGTKRQTHPLPELVCCGCAA
PRDILKLAVKDRQPETTTLIFMAGNMKGAGMKLTQFICDAAEAKQERKIP*
>SPSA8_v1_760006|ID:41146339|capC| Capsule biosynthesis protein CapC [Spirochaetes Bin 1 SA-8]
MIPESIALSVVFGFFLTELTGFLPGGLVVPGYLALFLDHPSRIAATLLAAGLTLACVRLL
SRYLVLFGRRRFMAYVLTGMSLLWLFDLLAPFVLPSIALPQGLDVRAIGLIVPGLIANDA
ARQGLPRTLTGLAIVTLTVRLALFLLQ*
>SPSA8_v1_760007|ID:41146340| conserved exported protein of unknown function [Spirochaetes Bin 1 SA-8]
MKNQKFSIFIAASLLTLLASCLLVTSATVMAPHPRQFEMKKAAELMIRYTAAIAEQRGGI
DLALDPNKTGLIGLENSPLTTTAGDLTSKRTSLNPDFAALVVRQFNELGIAKGDRVAIGA
SGSFPGILLAVLAACSTLDVEPVMILSIGASQFGANIPGFSTVEIIKILKAKGLPVAEPV
AVSPGGEHDSGLGGLYVEGPVDDLVRLAHGAGYPVIETPSLAANIRTRFDLYTERGPVKA
FINIGGADANFGTISETLTLGNGIVKNHRVKTSSPERGLVLEFLDRGVPVINFLNIKGLA
QKSGIPLDPVPFPPPGSSEVYFVTAPSKPIAALGLAAAAVFLVLAKLTLPGKSREKTLPA
KG*
>SPSA8_v1_760008|ID:41146341| leucyl-tRNA synthetase (fragment) [Spirochaetes Bin 1 SA-8]
MAKYPFESIEKKWQARWEKDKTFKAHEDPSFPKEKRQYVLDMFPYPSGAGLHVGHPEGYT
ATDIYCRYLRMQGYNVLHPMGFDAFGLPAENYAIQTGTHPEITTRANIERFRSQIKALGF
SYDWDREVSTCTPDYYKWTQWIFLQLFKRGLAYEAEMPINWCPSCKTGLANEEVKDGLCD
RCHTKVTRKRIRQWVLRITKYADRLLEGLDQLDWPEXX
>SPSA8_v1_770001|ID:41146342| AAA ATPase (fragment) [Spirochaetes Bin 1 SA-8]
MYLDYYQLTKEPFYITPDPEFLFLSESHKQAMANTVYGIGMKKGLILIIGDIGVGKTMVA
QSLLAKSDTEYLKTVYIDTPDVTFTELLHTVCQRLGLLTSGNTSEMVNTLHX
>SPSA8_v1_770002|ID:41146343| protein of unknown function [Spirochaetes Bin 1 SA-8]
MPHVKKITRDYGRFWPDLSFFCFLSLFLLFLNIPVIAGTIDLPKTGQSTCSNATGNPIGC

DNTGQDGDYRAGIAWPVPRFDIDISGACITDNLTGLMWSRYAWNPPITLPTSWASSVLNP
PTAFYACGFSDWRLPNINELQSLINFEKPNNVQWLASQGFLFLNMFPIKYWSSTSATDNP
KSNAWYINMVDGSIAKDSKLATYFIWPVRGTSNPEENAPVGQIAETGQASIQASYDDAYY
AITPGLNVGVPWPEPRFTITYCDATGQCPDQDADCDAESSNNIVTDNLTGLVWTADANLQ
GLTTWGNALSFVQGVTTCGYGDWRLPNTRELFSLIDRSQCEPALPADNPFIHIQYGADDL
YWASTSYANYPDRAWAIDMRDGALGPWAKTNTAFVWPVRGGKTKPYILTIKKLGNGQGTL
TADGLSCSGKTCKGEYQSYEVVSVTATPQTSSVFMGWDGDACSGSLDTTCRISVTANMKV
TATFRSKVKISVAPQSLNFKNLKQNIESSPLSIVITNKGVENLTISTLEITEDTESIFKI
MSDPADCAVIASDASCTVTITATSPDYDTKTAKLKIISNDPKNQTTVVRLKAKVKPPKIT
RKPSIVSFGKVAVGTPVNKTVTLTNKGITDLVIGAITIAGDHPADFSPLATDTCSGATLV
TGGTCTVTVTFTPSTAEKRRAILQIPSNDPNPKRSLLSITVKGAGV*
>SPSA8_v1_770003|ID:41146344| protein of unknown function [Spirochaetes Bin 1 SA-8]
VIGNTERVKTQLIALSSQLKPNAELNIGFSFCSDHTAISYEQYYMLIRGM*
>SPSA8_v1_770004|ID:41146345| protein of unknown function [Spirochaetes Bin 1 SA-8]
MKKIAVDIDNILWDFSPVFWERLRKINPAIIPPTTWNRWDFWHTYVTTKQLYGAIKEIHL
EQEKFKPFDDAPAFLLALREMGFYIIIASHREQGTFEPTKRWLSIHDLVYDEIHLSNDKS
VLFSDLCAVVDDSPSVLDKAKAAGIIRAGLRKPWNEDTGHPLFDTLPEVLTYLESACNIA
HSL*
>SPSA8_v1_770005|ID:41146346| putative Outer membrane efflux protein [Spirochaetes Bin 1 SA-8]
MTHFLRVFLISLIALPVIFFPSIPVSADRLTLSDGIRIVTENNMLVKIKKQDELIAESDT
LVARSGLLPRVYSTYSQNYTDKQQGTQIRGQSVYTQQQDFYTFSLRAQQLLWDFKGTLSL
YEASKKIQETRQLEYKRTQNFMALSFALGYFDLLESEKMITVAEKETEMLDAHRAMAENL
YNEGVITKNDLLQAEVRLSDARQKLLTAKNIRKITASRLNTMLSRPLSSSLDAEEVARPV
VVPTVDLEKAMEKAEKDRYEIRMVDTALDAIASEATAKKSEYYPRFFVEGGYNYLRNKYA
LYDGSWSVMGGMSINLFSGGATQAGLDKIKRERDKLIIERKKLIEDIRYEVERYYLELIN
AREKVKVTKDASSQAEENLRINKVKYAEGEGKATDVIDAITLLTGAETNHYRSQYEMFRA
EANLMYAMGRDLVEVYK*
>SPSA8_v1_770006|ID:41146347| Secretion protein HlyD family protein [Spirochaetes Bin 1 SA-8]
MQWAVTLWRCINNRMEESLNAVRNEKKKKPLIMGTIVGCILIILIVTGYLYYRATRVSTD
DAFVEGRVHTIASKIAGTVTAVLVTDNQAVKKGDILVEIDEVDYDIRVKEATTALDTEKT
RLIEAESRRDAAIKQVEEVRAAINAARATEELQEAHARQARIDMKRAENLFRNEAISRER
FEKAKTALDVAEAQLRASREQVKRLEASLGAQVSMVKQAEAVVRTQSAAIKQRKTLVEAA
DLNKSYTKITAPADGYVTKKSVEVGNQVHTGQPLLAVVALDDLWVVANYKETQLEKIKPG
QKVKIVVDAYPGKKFGGEVESIMAGTGATFSLFPPENATGNYVKVVQRIPVKIRLDNEAK
KENILRIGMSVVPTVYVR*
>SPSA8_v1_770007|ID:41146348| Drug resistance transporter, EmrB/QacA subfamily [Spirochaetes Bin 1 SA-8]
MNRWLVAVTVMLPTLIEIIDMSVVNVALDHIRGSLSAGIDEATWAITMYLVSNAIIIPIT
GWLSRYFGRKVYLNFSIALFTISSLLCGLAWNIQSLIIFRVFQGLGGGALQPISQAILLE
TFPPRLHGVAMAVFGIGIMFGPIVGPVLGGVITDYWSWHWIFFINIPFGIISIFMTMLVI
KDPPYMKKTRMKLDYPGLLLVALGLGCLQVILDQGQREDWFESSMISWLTIISVVSLTLF
LIVESRTQEPVIDLKVFKNISFSTGNVVMFFAFFNLFGSIVLLPIYLQTLMGYSATLAGL
VLGPGGFSSIIALAIAGRLVTKVNPKWLLVFGISTAALSTYMMSLFNLNADFITVMWPRI
ILGLGMGFIFIPLTNLTLSIISREGMGNATALFNLIRNLGGSFGIAFVTTVLARRAQFHQ
SHLVSHVTPFDLTYQIAVEKLKAFFHMLGFTESMSKMAADGSIYRTLIKQATMIAFNDAF
WLMSIMLIATLPLIFLFKRPRHGEHIETIH*
>SPSA8_v1_770008|ID:41146349| protein of unknown function [Spirochaetes Bin 1 SA-8]
MVTGIGSFPFTDVDEALDIIFATCPEIPFWPQLPKRAPYENMYIPFLEAVPCVVIHENEG
TAFMNTESVEGIEEFYEDVQNGTIEAFRISEKVAPGFSRFLERLDERRREVKFIKSQITG
PFSMGLGLKDENGKPIIYNYGYFDIIKKAIHMKAQWMVATIKAHFPEKGIIIFFDEPYMV
SFGSAYVSISKEEAIRLFDEVLGGIPAXX
>SPSA8_v1_780001|ID:41146350| Ribose-5-phosphate isomerase B (fragment) [Spirochaetes Bin 1 SA-8]
MDSKIRLAIASDLSGFPLKKEIVKHLQANHPEIEVLDFGIESEDAPQPYFMQAPKVAKAI
QEGRADKGIAICGTGQGMAIVCNKHKXX
>SPSA8_v1_780002|ID:41146351| putative transketolase C-terminal section [Spirochaetes Bin 1 SA-8]

MAEQKEMRAVYAETLTALADQGLEIVVVEADLMRANGTGAFAKKYPARTVNVGVAEANMV
GIASGLSAAGKIPFAATFACFASRRAYDQFFLSANYAGLNVKLVGSDPGITAGFNGGTHM
PFEDLALMRAVPKLAITEPSDPASLAAILKLAAETYGCFYIRLQRKPANMLYSAEEKFEL
GKAKVLKKGKDITLIALGAVMVQEAMRAAANLAAQGIEAAVIDALWLAPLDEATILEHCA
FGKVITCENHRVTGGLGSAVAELIAEKAPGTRLARIGVGKDSFGEVGTPDWLADHFALTA
PHIEKAALSLLGR*
>SPSA8_v1_780003|ID:41146352| putative transketolase N-terminal section [Spirochaetes Bin 1 SA-8]
MNKEELKELQETAKRIRSLTIEEIGHLGVGHIGGAMSIVDILTLLYFRHMNVDPANPRKA
DRDILVLSKGHAGPALYATLAEKGFFPTSWLLTLNQGGTLLPSHCDRQKTPGIDMTTGSL
GQGISAAVGYATGLQMDHTPATVYCIIGDGESNEGQVWEAAMFAAHRKLGNLIAFADWNG
MQIDGLTKDVMNMEDIVAKWKAFGWEAEMVDGHDFEAMDAAISRAKARRTQEGARPTMIV
LKTIKGKGCSFCEGLVTNHNMNYGIEQTKEALAALGTKARLIPALLNE*
>SPSA8_v1_780004|ID:41146353| conserved exported protein of unknown function [Spirochaetes Bin 1 SA-8]
MHKKAKKPALIAAWFGFAVLAGCSAPGNDQVFLPIGDARPPVLVTVNQVDSGLLELDFSE
DVAMVADSFVFQPKGIQVNPTVDGKRIQVAFSPRLDPGTACTLSGEAEDRAGNRLKFLIS
FSGYNEHPARLRLNEVQPGKNSSASNPHRDYIEFLVLENGNLGGATVEWASSVKSYRYQF
PAAEVRAGECVILHCAPEGIPAERDEIGSDCGLSGGIDASLSGRDFWTSVGGLPDSTGVI
AVRMREKDPACDGLFYADTDKAGALEIKAFGGLPKMLADANIWPSAGGAPAWEDAFIWKS
STSRPLHRTEGAANGKSAWYVGDAGSQSPGEEAPREAVSKSAAKKVNNKKTSP*
>SPSA8_v1_780005|ID:41146354|trxA| Thioredoxin-1 [Spirochaetes Bin 1 SA-8]
MSAEVTLTKENFNTEVIESPIPVLVDFWAEWCMPCRMIAPSVEQLAETYKGKIKVGKVNV
DDESEIASQFGIISIPTLIVFKEGQAVKQKVGAIPKHEIENLFKGLL*
>SPSA8_v1_780006|ID:41146355| conserved membrane protein of unknown function [Spirochaetes Bin 1 SA-8]
MEKAGHTWTFARIGGIDQVVLKKGDDIEHLAELDPKLWAILSAPTRQAKYAETYALLDTD
HDGKVRIPDILDAIAWLKNIMVSLDVLFDKKDGLTEKEIADETLIKAFKEACEILKAQGK
LAQNGQSLILNSGLAAAAIALQKNQKFNGDGVVIKNSTDESAAASFIDSLVKLGYAAQDA
SGEMGLDKANLDAFLADAEAMLAWRAKISENPELLXCGDKTDAAVELFYAIKPAVDDFFK
RCQIFALSTSDTALRDIDTAISMVLAGNQEEQDKMPLPLALPNREIVLHLDKPMLPAFMK
TVTEFFAILPSSLAAGKEIDFASWQEISRRISAYAEWKSANPASRLNDVEDALLVEYLKP
MYQNAVIALLEQDLARXEYAQSFADLRKLIILKRDFLGVLQNFVNLDAFYRDKNAVFQSG
RLYIDGRELELCMDVVNPGAHGAMAVMSSMYLLYCDLVSKEGKKKSIVAALTGGDADNIF
AGRNGVFIDNESKDWDAVITKVVVQPISIREAFFSPYKWVVRTLEEAAARRAANAESANL
AKLKTGAEQTVATVGKVDAKPDPANLAKKIDVGTVAAIGVALGSIGAMITGLLGIFVGMG
IWMPLGIIGVFILISGPSMILAYMKLRRRNIGPLLNAEGWAVNGKLKINVPFGGSLTHLG
AIPLGSARLLMDPFEKKKKPWLLYLLLVLAIVLVAAYFLGWLDGILVALSIRK*
>SPSA8_v1_780007|ID:41146356| Major facilitator superfamily MFS_1 [Spirochaetes Bin 1 SA-8]
MKTKKASLPLLFSVVFMDMVGFGFIIPLIPDYIAKFGGSPALAGILQASYAIGQFFAAPI
VGRLSDKYGRKPLFLFSIGGTFFSLLLLGFAPSLTFLFLSRILDGLTGGNITVAQSYIVD
ITDNKNRAKGLGLIGMAFGLGFIIGPLFGGLLSQFGIAVPAFVAAGIAFTNLLLITFVLP
ESLTDERKEEIAKNPRRAFSAKLLMETLKRRGTGPVLHMTIIYSFAFVLFESMFAIFAST
ALGLGTSTRGLLLAYVGLLVAFVQGGLVGFLAKKFDERKLALVGISMAAFGLLFYALSPS
LVVLVIALLPLSIGSGIGSTMLRTLLTKSVPRESSGGTLGIASSVESLTRIAGPLAGGAL
LGLLGPRAPGLVASLLAAWTSWYGFRFLIKEDCLAEGAVSCSEPIS*
>SPSA8_v1_780008|ID:41146357| protein of unknown function [Spirochaetes Bin 1 SA-8]
MSQTPDVLDLLQEWMQLVMKHSMHGFIRYSKDTGLSMSQFGALFQIHRSKICGVTDIGTD
LGVSSAAASQMLDRLVQQGLVERIEXX
>SPSA8_v1_790001|ID:41146358| protein of unknown function [Spirochaetes Bin 1 SA-8]
MDTTAQLIILRLENSLRARKYSPATRRRYIGFCQRLFQRYPTMEPAELGTVQLEAFLADL
ERQGRSASCINQAISAFSYLWVNILALPFPITARPRADQVLPRVLSPGQVESLIRGAKNP
RDRLAFALAYSAGLRVSEVAKLRIHDIDRQRMTMLVRQGKGRKDRILPLSRRVSAMLESW
LDXX
>SPSA8_v1_790002|ID:41146359| protein of unknown function [Spirochaetes Bin 1 SA-8]
MMTNNRKSLSYFPVDTMFSIPMRKLTMHYGPEGIGIVILILQMIYHEGYAITWDDETCCL
FCRENNIDKKTVQEILRFCLKQGIFDRTLYREKKILSSAEIQKQWLKICQQSKRKGMSIE

HGVSLLDQETDRFAKSSSGKTPKDSGNLPENSGRLPENSAQRKEKKIKEKKIKENKNKKN
KSEEDRSEEKRTRAEAQAPELWPALSVLPKASDPGLDDMKARFQRIIREKERQEHLGNIQ
QIAAGQPPGPKPDLEAMKARFHKLIREKNLQEYSGKTPQKAAGQPP*
>SPSA8_v1_790003|ID:41146360| protein of unknown function [Spirochaetes Bin 1 SA-8]
MTTTTAGVEMNTRETFLKFHYAHPELYLALRKEGYRAARSGKTHIEINELAQLAALQGHW
ELLDLHETCLKHYKALLAADPLLWKLLRREN*
>SPSA8_v1_790004|ID:41146361| protein of unknown function [Spirochaetes Bin 1 SA-8]
MKLLHAADLHARREGANDFNTAIKHMESIAKTQNADLIALSGDIWDGPTQNSAGTLFPDF
VEAIRSLADCAPVAMIYGTPSHDVEGSLEIFETLASKHGIRILRPGISYILKDGTIQELS
GDNHENAQLLLTGIPEPSRKWLLAENRTDSELHAQEILKTLCLALGCMRERYPGLPFVVL
AHGQTEGSLTAAGXRLDXDRNLHFSKESLKTLHADYIALGDIHEPQHIEGTRAWYAGSVY
PLTYGETHTPGCNLVTIEDAGKPVAVERISFPIPIRKHIISHAACALEIPQYRGERIWYE
IRGSRSETALIDTQEVLSRLIGHGADPASRVTIDLTPETAVRSGEIRKKNGIIEKLKVWA
ECSGEKLSASLLDKANQLARETSLKNQAPADASYRVDRLILRGAIGIWMVSHKDEIDLDL
ASCGPSVLALIGKNGAGKTTILENMHPWPRLLTRSGALKTHFRLADSFRDLYMTETLSGW
KYRFLITIRADIPTGAMECWIFRDTGKGWEPLPGINGRLEPYEAWISRLFGSLSLYQRTA
FTSQKPTLTCPDLANATKGEKKTLFSELCGISWLETYRLEAKAKTEAAAREIERLEPRHS
MLKDAWEQEASIKRELCETAERLNELTQTEEALKKDLENAQKEIRNQRELLAEREKLVRQ
RERAFNAIMSLKARQREHEQVIDVHRASLRKKNDALAILARADSLTREKEQLIHTIAQLK
EEERNRYREYSRKLEQYAAERESILGELARIRESTLTDAESLSQKKDTLSRLQDSSCPTC
GQQLPSDILATQEKTRGRLTGEIAELETRLAGSAARKHDLETTLKNLSAPGYPEVREILE
EHDLAKVKEELAQLNTQAAQETIRIADVAEGTITQARQELLRIENEIGSQSDEADRCTEK
LAQLPKKETLQQAENQLEQLAGKLSDTTMEKVRTQTRREQLEQSLAATMRQAEEYQRLTQ
TLADLTRDLCEWAQLERATGKDGIQALELDALAPSIAQTATRLLIASGNQGSIAIQTQKL
SGSGSRLHAMEDFSILYIAQDGTEQDISTLSGGEAVWIRKAIYDAFEAIRSNGSGFRMET
VIIDEADGSLDPESRLRYMRMIEASHREAGRYQTIIVTHSLELQSMATRCIHVAELKASS
PPKPAAQLGLTA*
>SPSA8_v1_790005|ID:41146362| protein of unknown function [Spirochaetes Bin 1 SA-8]
MTRQQALEFISRYPNALSYVKASHLEELSPLLEVSIEAISCRKEEFHCLPGNTYMPRKET
IDKFGQAAEISFNPLAESTRREGDCYIGKSQAMVLGPDGKQIFGDICEYEYDVAVRHEIE
IIEDLHAKFPRFHSQGKLNEDKARLAYLALRKTARQRANTGARTRAILSILGMQTGFKDL
FRPEDKPDATVTFLFSRIIINAMNEMVLNRMLVNLTAPAAMLFGPRKQQTLLPHDDLPEP
EPQEAIDAHLILEEPQQPLAMPPMDPPDTISKEKEIQLLRRNINVHLQTGQLDEKIKTAC
SQALERHGDDEAYLRAILERLKMLGKPQRDGREP*
>SPSA8_v1_790006|ID:41146363| conserved protein of unknown function [Spirochaetes Bin 1 SA-8]
MKTLENHEEKMNPGNRNEPMLLDVQNAAKLLNLSRSYLYRETRARRIPHIKIGSRILFRT
TDLQAWLEKQYIDSDSNCA*
>SPSA8_v1_790007|ID:41146364| Endonuclease/exonuclease/phosphatase family protein (fragment) [Spirochaetes Bin 1 SA-8]
LPLLLAQISTPDNPYQAVISPRLGRTASKEQYAFIYRTDLFEPLGEASVLPDPQDLFERE
PFMAMFKVKEGNLDLILVDIHTKPEDAAREISLLPKVMQYAAEKYQEPDILCLGDYNADG
SYFNETAYQEIFPPQNYLQLIPNSADTTVGSASLTYDRMTATSAMASDMTGTWGIYDFSS
LPCVMTNGINPKTISDHYPIWIEAYTNRDTD*
>SPSA8_v1_800001|ID:41146365| Fumarate hydratase class I anaerobic (fragment) [Spirochaetes Bin 1 SA-8]
MLEEKNTGDNLPAGVDIRSVPGNEYRFLFAAKGGGSTSRLSLSMESPAILKEERLKSVVA
ERIKKLGAAGCPPYTIGMVLGGTSPSQTMYALELAVYGLLDKLPTVEMERTATIFADLLP
DGKTPGKTSASAQHSFLPGTGVRSIEWEEILYELGVQTGIGAQWGGRHLALKTRAIRFSR
HAANLPLAIGISCSAHRKARAVMNEQGWFLEKLESDPARFLPASMDVLPGAVEIDLDTAQ
DQWLEKLRSLSSGTPVLLSGTVTIARDAAHARLETMAKSGVPIPDYFIEHPVFYAGPTEP
LPGRPSGSFGPTTASRMDSYLEFFMEKGASLVTIAKGNRGEEAQHAIRKARGVYLAAIGG
AAALTAREHVLESRIVDFADLRMEAVRLVRLARLPVMVVIDASGRSLYA*
>SPSA8_v1_800002|ID:41146366| Major facilitator superfamily MFS_1 [Spirochaetes Bin 1 SA-8]
MIKTAFRETQTSIRFGISYVMLFAIYGISSPYLQLMIRRLGYAPSAVGIFLGLFELIGIT
GPIVLARNADSRGAYKPYLIASGLMILAGLAILVPFRIPVITIISLSLLSLGLKTPIPVL

DAALFKAIDITAPQESRGLNYGLLRSLGSAGFVIVTLIVQSIRGFDTASSQTMALYMGIL
TAIYLGGLLFLPETGIIQQKAHKERLSFAWIDSVFIIGILVIALGRLAMAAVGSFFSLYL
TEELGWHAVGAMWAISATSEIPFIALSWKFIKKNSPMTAIAIASSAIVVRLMIYALFPSP
AGAIAGQLLHSLCYGLFQPAAIVFVSLKTPPEKRATGMAIFMGFGVGLPAFVGSALGGRI
VELLGYRGLFGGYTLFAVASLLVFYKNRKLLTAVK*
>SPSA8_v1_800003|ID:41146367| NUDIX hydrolase [Spirochaetes Bin 1 SA-8]
MVAGDGSGSILWQTVAKREAYRGHIFTVFEKTSRGPDGRQGTFSVLESRDWAVVVPLVRS
EKGDSFLMVRQYRHGADAISLEFPGGVIEPDEAPILAAQRELAEETGWVSNDVMQVADVF
PNPAIQSNHFHIFLALNPAPAVDRNLDEHEIVDAVLVPVSEVWNMMGEGEYRHALMATAL
FMAEKALLKLGIKIAS*
>SPSA8_v1_800004|ID:41146368| conserved protein of unknown function [Spirochaetes Bin 1 SA-8]
MEFEIPDFFKTLESSITVCDKNYILIYMNDKAVASYRDAGGEALLGQDIRICHRQASLDK
MKAIMETGIPNAYTISKNGIRKLIYQAPWRNGGSIAGIVEFSIPIPDEMPHYVRS*
>SPSA8_v1_800005|ID:41146369|ykcC| Uncharacterized glycosyltransferase YkcC [Spirochaetes Bin 1 SA-8]
MLISVVIPVLNEKENISALTDRLTEILSRYDAFEIIFIDDGSTDGSIELLRSLSVERKRV
GYLSLSRNFGHQNALRAGITAAQGDCVIMMDGDFQHPPEAIPLFIDKYLEGFDIVSGVRD
DHSTSSETPGFSKQISSRLFYRIINSLSDVRIQPGAADFRLISRQACSILLGMKEQNLFL
RGILPWTGLPQTEIRYQTGKRKHGTTKYSFSKMLNLAMDGITSFSIKPLRLTSFLGAVIS
ILGFVYAVYALCMRLFTTKTIEGWTSLLISVLLIGGIQLLSIGILGEYVGKTFMETKGRP
HYIIRESRLPLQNAEPELKAGQRLDKEEEQ*
>SPSA8_v1_800006|ID:41146370| putative Small multidrug resistance protein [Spirochaetes Bin 1 SA-8]
VNICLPLFGIAASALAQIFLKYAARFEVRETQWFLCMGASLVSYGASFFLYAAMLRTRDL
SRISPIMASAVAILVVLAGSALFGEQISIRRALGIAFGLAALYLLSS*
>SPSA8_v1_800007|ID:41146371| membrane protein of unknown function [Spirochaetes Bin 1 SA-8]
VVSLHGSEPGFLRRLVLSVCRHAENAGSQQNQPDHGQRGRHTGCACRICALRRTDIDQAG
AGNRLRACRALSVIKLNRTKSMLETESKRQTAGAAIFTVLSLFLVYAFMLSRFNLSMLFS
DTTLTGGDSASWYQVLHTLKNEFLPHGRLFSYSNSNFFGYLEGQHYFPLPFLAAAVAGFF
MPLTVALKLATVAGGFGLPLTSFAAAYAIQKNSKRSKKTALFSGILSACGSLVFLFNESY
TIFGGNWLSTFAGEFCYSWAIALLPLFAASVILDHLKKRSGILSGILLGILGLSHLFVFM
PAFFLPFFPVFRLFFELIRGIKKENPEAQQEKNALLMRVLTTYGIAFLLMAFWMLPMIAT
RKWAQPISMIWHFASFREFARQTLAIVWLPAIMLFLAASFGRKKIAAKRRASEAREMEAA
PETSKTDQAYLPRFFSYAVAACAFLFLAAPGLGMPDIRFVPTAVIFSLFGISLLTANPVL
RVAERLVWRFSSAERSASAPSRSGTSGIPALLIAEIAVVLASCGIALTTGRNAPSWFVWN
YSGYEAKQEWQFINKLRDTYKGSLYNGRFLWEKQDQRDNRDFGSERAFENLYLFTGHPSS
EGIHYGSSFMARAATYLQSSYSLHPVDPEAERIYSVIDPDSWTVRFAMLNVHYIIVHSDE
MKKLFSEHPSFALDMEAGKFAVYRFKAYQGHYVQALPADALSIVKSGPGGFKTDYYRFFR
DYELIRFPFISNEFADSQLVNSLKGGAREYSDYDSYRAALLPQALIYGYEPKTVMGISAE
HIDNFSISFHTEKPGQPHLISLSYAPGWKSLAGEKIYPVSPGFMLIVPTTQEVRISYERT
MWEWLGIFLTLSAIPLALRIRTRSGKSRNXX
>SPSA8_v1_810001|ID:41146372| protein of unknown function [Spirochaetes Bin 1 SA-8]
LAQGPYFAIKVTPAIHHTMGGVKIDSSAQVLREDGTAIAGLFAAGEVTGGVHGGNRLGGN
ALADIVTFGRIAGENAAARAKAK*
>SPSA8_v1_810002|ID:41146373| conserved protein of unknown function [Spirochaetes Bin 1 SA-8]
MAKENNAVRLKRRLICEVTSLVLEGKLADEIDSIPYAMTNENWETIQCCIHHDRANLRLR
LMSLLGYSTEGMLDVDKPLRTYAQEALSGKKPDAFPLSLMSAACNGCTKSRYIVTNVCQG
CLARPCKVNCPKGAVSIINNQSHIDPELCVNCGLCEKSCPFHAIIKIPVPCEEVCPVGAI
QKGEDGIAKIDESKCILCGKCLSACPFGAPVEKSEIVHVLSALIHGKKLIAMVAPAAMVQ
FPFPPGKFIAALEHFGFSKVMEVAEAADRVAEAEAREFAERLHENNKVLATSCCPSWVRA
AGSMGLEQILSDTPSPMLVAAKTAKAQDPDAFTVFIGPCLAKRWEAHRASQNSPLVYAVL
TSEEVGAMLMAAGIQVNEENEEDLATAGNASVYGRGFAATQGVTAAVKHALSPSAVEVNS
CIVSGITKDTASQLKKVQESDKPLLIEVMACDGGCINGPCQLTNPKVAGAFLERYKAAAE
HTTALKSA*
>SPSA8_v1_810003|ID:41146374| conserved protein of unknown function [Spirochaetes Bin 1 SA-8]
MNQVKVTICVGTACYVLGGAELLGMVDELTARFGHHVMCEGSPCLGFCKLSRNSKAPFAL

VNEKLLEAATPESLLKAVEEALSGVQNG*
>SPSA8_v1_810004|ID:41146375| Stage II sporulation protein E [Spirochaetes Bin 1 SA-8]
VKPHFIEIGSYQLPKAGQNAAGDVFMSRKIQETDRIISVLSDGLGSGIKAGVLATLTGTL
AAGCIAANMSINKTAKLIMRSLPVCSERKISYATFTIVDVDKGHDVRIIEYDNPPYLLIR
QGCEVEPVKTEIQVAKGKKIGSHPKPAILRGSSFKPQLGDRILFFSDGVSQAGMGTKVLP
IGWGYNGVKEYALNLVRQNPDISARELAHAVVIRAAYHDLGIPKDDITCGVVYFREARRL
LIVSGPPIHQEDDQELASKFKNFPGKKIICGGTTANIIARELKKTIQVSLKVRDLTIPPP
ACMDSADLITEGIVTLGKVVEYLEQDTKIEQLPENSATMILKHLLDSDIIEFLVGTKINE
AHQDPTMPVELEIRRNVIKRLKALLEEKLLKQVSIQFI*
>SPSA8_v1_810005|ID:41146376| Fe-S cluster domain protein [Spirochaetes Bin 1 SA-8]
MDSAQVIYTELTECQDCYKCLRECPVKAIQIVRGHARVLEDRCIHCGNCVEICPQKAKKV
RSDLERAKILIRLRQKTVLSLAPSFIAEFSGIPMQKLIAGCKKLGFSHVSETSVGADAVS
ESVSKTIAEQQPPLMLSSACPAVVQYVDKYLPEMSGFISTACSPMVAHARIIKQTLGPGT
AVVFAGPCIAKKREADASEGAVDVALTFQELRQWFCEEGIDPEDAAVRDEDSFFLNQAQD
GILYPIEGGMVASIKHLKHSDAHCMTYSGIHQIREAVRGIVDQYLENENNPGNQKTLFLE
MLACEGGCVNGPMVQQSGGTVAKRLRVLSEKEKRTERGRVLSEPVSVQDIAADLTMPQSI
RQKPVAAVAVSEEDIKASLASIGKYSRKDELNCSGCGYDSCQAFAAAMFLGKAEKTMCLS
YTRKLAQKKANALLKAMPSAAVVVDASMHIVECNKPFAELLGTDVMELYALKPSLEGADL
RKLLPFWEAFEQVLMPESQDIVASDFQCNGKIVHGSVFSIEKGLLAGGLFQDITAPWIQK
DRVISQARKVMSQNLRTVQKIAYLLGENAAEAEAALTSIIESFQSERSGDSYKSNKE*
>SPSA8_v1_810006|ID:41146377| conserved protein of unknown function [Spirochaetes Bin 1 SA-8]
MNQSGKTITICMGSSCYARGNVYNIEIIQSWLRQQGLEAEVEVVGTLCEGQCRQGPIVKI
GATVYQGVTASSISEILAHEFLEVHHG*
>SPSA8_v1_810007|ID:41146378| conserved exported protein of unknown function [Spirochaetes Bin 1 SA-8]
MTFFLQKLRNSILIAIIAASLTACAGNTTARITQNTLIAAGPRIELKLTPGPFYEYEMHT
LFAHYTVRPQVAVWIETLDGTYIGTIYATEAAVTGKFKMAPKKGRPEALPIWSGRKTGNI
DAISAPTRAGEKTIYESAAISGLQPGRYVIMLETNRSYDWNKTYTKKNSGVNGQPSVIYR
AEIDIGSGAGTASLEPIGTGSVDGSDSKIRWGLHGIDTALQLFSSLQVIYRD*
>SPSA8_v1_810008|ID:41146379| Transcriptional regulator, TetR family (fragment) [Spirochaetes Bin 1 SA-8]
MEERRLSVCLKIMKYYSRGNEMSGIKSEKNSYHHGNLRAKLIELGLEVLEAEGAEALSLR
ELAKMAGVSKTAPYRHFKNKEIFLASLADEGFRLLYADLLQARGNDPGPVERMGRSYMAF
AVKHPALYRLMNSSX
>SPSA8_v1_820001|ID:41146380| conserved protein of unknown function [Spirochaetes Bin 1 SA-8]
MPSQIIHILHGVATLQRTIALVGEAGGSTLSREGGILFSVFPKLCEPELFPWFCLGCQGP
DLFYHNQRTRPVALEYGSLLHRHSYGDFSLSLLENALKYIPGDNQATPEVAFAIGFLLHP
FPDRLLHPYIIYKSGAVSNIGAEIVKNAQNHIFLERILDMLFLEWSLGSVPEIVLWSVQK
DYPAVCSFPQSKLLSRPAREVSVSFRHIIIEAILNTFPKRANEDRNLELRIQNMFQDASH
FYELTDPGFFKLNSLKNNRLINSILVKNEKQALAIAALLYPFGVTMNADFLNMCQTKWYS
PCENGFGLSRSISIFRF*
>SPSA8_v1_820002|ID:41146381| putative Alpha/beta hydrolase fold protein [Spirochaetes Bin 1 SA-8]
MIIVYLAIIYFLAVFIFSVVAFYLMFYPSCKSLEFTRANGLEKAEFSQEFLDNPWIDFNI
PSPHGYSISGNYLVSRTGAPSAPTALFVHGITWTRHGMYKYMQPFFKNGWNVASIDLAGH
GNTWAPRRYFPSFGYYEKFDVKTAVDFLKTKFPGSALFGVVGESLGAGSVLQYSAISNGA
IDFVIADCPFASARKELLHHVRKLGFPPFFAQHVSLIVAILVRMTKGFSFSDASPEKAVM
ETATPILLVHGLEDRYVPTWMSLHLYNIRKKAGFDATSLLLVPGARHAKSFMTDPALWEK
TAFSFISEVKPVEKPVAG*
>SPSA8_v1_820003|ID:41146382| DNA topoisomerase 4 subunit A (modular protein) [Spirochaetes Bin 1 SA-8]
MAYVKKLFNENFLYYASYVIKDRAIPDLEDGLKPVQRRIMHSLFEMDDGKFHKVANVVGH
CMKYHPHGDASIGSALVVLANKELFIDKQGNFGNILTGDEASAPRYIECKASSFAKEILY
NPKTTVYCESYDGRNQEPVHFPAKLPVILLIGAEGIAVGMSTKILPYNPXEVIKAEIACL
QGKAFSLLPDFQQGGLMDVSDYQDGNGKVRVRAKFDTSDPKRIVITEIPFGSTTESLIAS
IEAASRSGQLKIASITDFTTDKIEIEVRLQRGVYTQEVIDALYAFTECEQSISTNCLVIK
DYKPVVMTVSEIVRYHAGKLLSILRQELEIERGELLDEIHARTLERIFIEERIYKKIETR
RTQETVLKAVKDGLAPFDREIGREVTREDIERLLKIPIRRISLYDIEKAKNELKQVQARL

AEVESHLAHLVDYAIDFLKAQAKRLEAAWPRKTQILSFSKVDYKEAARRDIAVRYDSATG
YIGTSVSSGEKVLEVSSFDRILIVRRTGLYSIVPVPERLFVDQGLLWVTLAEKETVAATT
LTVIYKMLDSGSPYIKRCVIESWIVGKDYSLIPENAQLLLFSTEADFEFTLSYAIKKRMR
KASETFRARNFPVKGLKATGVRLAAREVLAVESLGKRVRPSGVIEPELGLDTKVAPEEQG
QELSEAGSEDKPVAPESPMKLPKPATAVSGKAKKSRKKPEPKAGAAGKQLKNDASGGAQA
GKAPSNNGTQAASKTADKTPKKNAKTAKSADGSGSKPSGTKKKGLKADTAKIDTKQEAKA
DKEKETSQEGRGKGLLATLARKKAELAGGADSESNKRREK*
>SPSA8_v1_820004|ID:41146383|parE| DNA topoisomerase 4 subunit B [Spirochaetes Bin 1 SA-8]
VAENRTKSSVIYDESKIKTLSSLEHIRLRTGMYIGRLGDGSNPDDGIYILIKEVIDNSVD
EFIMGAGDRILVSVEKNRVRIRDYGRGIPLGKVIDCVSTINTGAKYNDDVFQFSVGLNGV
GTKAVNALSRVFRVVSYRDGRLFEAVFERGVLKNQREDSCGREKNGLLVEFEPDPDIFPD
FSFNLEFLEKRMWNYACLNSGLTLVLNGREFKAEHGLLDLLASEVGESALYDLGWYKGER
IEFAFTHTNNYGEEHFSFVNGQYTSDGGTHLSAFREGFLKAINEFYQASWKGEDIREGVA
AAVAIKLKNPLFESQTKNKLGNAEIRPWILQEVKRGVDDFLRRNPQEAKKLQEKILNSEK
LRTELNIVKKEAKEAAKKIELKIPNLKDCKYHLGDGEKGEVSTIFITEGQSAAGSMVSSR
DVYTQAIFSLRGKPENMYGKRQTAIYKNEELYNLMMALGIENDIENLRYARIVIATDADY
DGFHIRNLLLTFFLTFFEELVTQGRIYILETPLFRVRNKKETRYCYSARERDEATKAMGA
QAEVTRFKGLGEISPSEFGQFIGKDIRLVKVDIQTLSAVPELLTFYMGKNTPERRDFIMQ
NLIADL*
>SPSA8_v1_830001|ID:41146384| Protein PyrBI [Includes: Aspartate carbamoyltransferase ; Aspartate carbamoyltransferase regulatory region] (fragment) [Spirochaetes Bin 1 SA-8]
MKRNGPFKGRTIAVVNDLSIDEQRYLYQKTRELKHLVSTGGDYRHFKLNDPDYQVYLIFM
EDSTRTRESFRNASKFLGARTNIFDAASSSFNKNESITDAIKMLYGYSSDSCFIMRTKLE
GTCRWLEEALGRYAETSGGPLPAFINAGDGKHEHPTQEFLDEFTFLEKLNWNDDHIHIAL
AGDLYHGRTVHSKADGLNVFRNVEVDLIAPELLSMPSYYVEKMKANGFQVRIFESIDEYL
ANGKVASIWYFTRLQLERMGEAILERTPFLRNSVTFRKDMLGLLPENTKFYHPLPRNRAA
PTIPTFLDDLPLNGWX
>SPSA8_v1_830002|ID:41146385| putative Uncharacterized RNA methyltransferase TDE_2619 [Spirochaetes Bin 1 SA-8]
MADTTFLVKVEKLAAGGEGIAFADRKAIFIPFSIPGETIRIRPIKETRDYIRAEIAEILT
VSPDRTAPECPLYGICGGCSLQHIEYTAQLRLKAEAARESFVRIGKFDPGPLPVHSDKPY
HYRNRAQFHLTRDGGMGFMKADSDEAVRADSGCPILKPALEAWLKSENRKARPGKDMKLY
IDQQTRFIAFADDTKIYIEGKDRTAHIVLAGRSYRFPVKHFFQSNLAMAERLIHDIESRL
PEPGERAVDLYAGAGLFSKLLAERFSSLFCIENDTVSLEAARGNCQGPGIRFFPMDVETW
ASRELARTGKSAPSAEASGSAGPAAQAPASAFDCVIADPPRQGLPPILKTWLASTAIKKF
FYVSCDHATLARDIGELIKKGWELESLELYDFYPQTGHLESLAILRPAGYRAGIGA*
>SPSA8_v1_830003|ID:41146386| Beta-lactamase domain protein [Spirochaetes Bin 1 SA-8]
MSISIRSLGAAGEVTGSKHLVEVDGVRVLVDCGAFQGKRDAADAKNRDFLSGLPADTIDA
VVLTHAHYDHCGLLPMLPKHGFAGNIFSTSATRDLANLVMMDSAHIQARDADYLSRQAEK
KNEKFSFKPLYDDMDVVRTLERFITINYHQKLTVADGVSIEFFDAGHILGSALVRMTVKE
KNGRVTVIGFSGDLGRNGKPIIRDPEFLTDIDYLVLESTYGDRLHEETKDAMEHLANIVS
KTAARGGKIVIPAFAVERTQELIFNLHLLLDDGRIPDLPIWVDSPMAVDATAIFKIHPEC
YDKETNDAFTKHSDNPFGFAGLHFSRTVEDSKLLNASKEPMIIISADGMCEFGRIQHHLM
HTIENKNNTVLIVGYMAEGTLGRKLKDGASEVRIHGDFYRVNASIEEIDAFSAHADYAET
LAWLEHTDKARLRKTFLVHGESKALEAMQEKVLAAGVRSAQIVKKGEIYEL*
>SPSA8_v1_830004|ID:41146387|gyaR| Glyoxylate reductase [Spirochaetes Bin 1 SA-8]
MSKFKVYITRQIPEAGLALLRERFDVSINPDDRPLTRSELLTNIAEADGVLCLLTDKIDA
EVFDTAKKARGFANYAVGYDNMDVAEATKRKIPLSNTPDVLTNATAEMAWALLFSAARRV
VESDRVMRSGAWKGWGPLQFIGQDVTGATLGIIGAGRIGRAFAMKSRGFDMNVVYTDEYP
CTELEEKLGARKVSLEELLSTSDFISIHVPLMPSTRHLIDEKALRMMKPTAVLINTSRGP
VIDENALVKALKEGIIAAAGLDVYENEPKAAEGLADLPNVVMTPHTASATFDSRNGMAVK
AATNLIAMVEGRRPPDCINPQIYSS*
>SPSA8_v1_830005|ID:41146388| Transketolase, C-terminal section [Spirochaetes Bin 1 SA-8]
MTTLKKTEIPTRKAFSMRMAERGAESEFAVFESDIGYSTYSYLFGDKFPERYFNFGIAEA

STMAAAAGMASTGRTTIVSGYGVFLTMRAVEMVRSFVCYPNLNVKILSSHGGITAAIDGV
THQATEDIAFMTTLPNMKVLCPCDPASAKKAFDLALATPGPVFVRLMRDPLFDLYSEDDL
KIPAKFRMGGSNVIKEGTDITIASYGDILFQAIEAADILAKEGLTAEVLDLYSLKPLDFD
GITASLKKTAALLVAENHQARNGAGTAIGDYLARLGMPAIFDHLGLDDTFAESGDYQGVV
DTYGLGAKNIAETARRLILRKS*
>SPSA8_v1_830006|ID:41146389| putative transketolase N-terminal section [Spirochaetes Bin 1 SA-8]
VTALYFEIMNIDPSNPKWPLRDRFVLSKGHACPVQYAALAMRGFFPVEELGTLRKLHSRL
QGHPVAGKLPGIEVTSGSLGVGFCEAVGLALEARLEKSSWHVWALLGDGELNEGAIWEAA
QAASKFSLDNLTAIVDRNRLQNDGFCDDVMPMDSISDKFRAFGWETLEIDGHDMAQILEA
LKKAKAMQGKPCCIIAKTIKGKGVSFMENKRDWHGKPPNDEQYSQAVRELQGETL*
>SPSA8_v1_840001|ID:41146390| Pseudouridine synthase [Spirochaetes Bin 1 SA-8]
LISYEILFEDNDVIAVNKLAPMPVLRDKTKDLDLQTLLQREQASCKKFLEAVHRIDRRTS
GIVVFAKTPRALKTLDEAFKNRTIHKTYLACLEKEPAPAAGILKHGIVFDKKRNISVAIP
YESLDAAQKRLPANTQVLDAELSYTMVFQSDRYFFVTAAPRTGRHHQIRAQFAAMGWPIK
GDLKYGARRSTQSGLLMLHAWKIEFPHPVSHEMVSLTASFPPAEKLWTIFSQNMADSQ*
>SPSA8_v1_840002|ID:41146391|deoD| purine-nucleoside phosphorylase [Spirochaetes Bin 1 SA-8]
LGQLQKQFLQVQRLLGNLHFKRKEVMSIHIAAKPGQIADRILLPGDPLRAKFIAETFLEG
AERFNEVRNMFGYTGTYKGVKVSVMGTGMGMPSLSIYVNELIRDYGVKKLIRVGTCGGMH
KDIKIRDMILAMSASTDSAVNKIRFNGMDYAPTANFNLLMNCWDAAKANGFNPFVGPVIS
SDSFYTEDPEQWKLWARFGVLGVEMETAELYTLAAKYGVEALSILTVSDHLVTGEATSAE
ERQLTFKAMLETALAGIIK*
>SPSA8_v1_840003|ID:41146392| protein of unknown function [Spirochaetes Bin 1 SA-8]
MKETENRVEEDFASFGQAFEEHRARFEADVLKETTSITKEIESLQNQIVALKEQAYMNAR
GKLEGFEDAMLADLENRKAQTYQKLDAWLTEMGKTLSSVQEEAKAQRLAEEGRYAEEFRA
HLVKVRDDMYAQLEKMRENLLAVKESIQEENRAEQA*
>SPSA8_v1_840004|ID:41146393| protein of unknown function [Spirochaetes Bin 1 SA-8]
MLFSAGNAITLGIVLVFFFVYHKLTSNNRSLEKVKKFSEKLQIELGDYVSSRADELKHYG
IELDVHQKAARLALEKLKEAEAAVAEKAESIGEIAERFKQYDEVLSGLMKMTERVDQNLA
RIHDDEAFAESVNKKLDLAKKSLSALEREMPLLREAFAQDAKKTVDAFRDDILAELHDEL
ASTSQELSAVKKEAMEAYSKAVSARALVDAELEKALAVARERASSIEDEAFSMLTANLNS
RLEELRNSVEGRMEELARGVASEAKSLRESVANFKLKWEQESAALLEDFAHKAEAVQASF
EEKSAATIADLNENRQGTGSRRRTVQRGGQRC*
>SPSA8_v1_840005|ID:41146394|rpsU| 30S ribosomal protein S21 [Spirochaetes Bin 1 SA-8]
VITIRQIQVEDGEPLEKAIKRFKRMVEKEGIIREWKKREYFEKPSAIKNREKKALARKLM
KKTRKTHDSKGGY*
>SPSA8_v1_840006|ID:41146395| conserved protein of unknown function [Spirochaetes Bin 1 SA-8]
MKNLLNVQIFILFIALILFSACSPAKISSQDLNKPNPTLKTAMLRVNGIEITAEVARTEL
ERNRGLMFRKSLPDGKGMLFVFDYDQKVAFWMKNTSLPLSVAYIASDGTITQILDLVPFS
EEPRPSTRSVRYALEVPQGWFSKMNIKPGDVISIPTLK*
>SPSA8_v1_840007|ID:41146396|def| peptide deformylase [Spirochaetes Bin 1 SA-8]
MLEVITYGNEILEEKAEPVTAFDDQLAETVAQMFEAMKRDHGIGLAAPQVALKKRLFITD
VEGDKKRVFINPEIIMTSPEVSEYEEGCLSFPGLYFVVKRPASVTIQAYNEKGKPFTLDA
DGLLARVILHEYDHLEGKLFIDRIPPMRRERALLHYRRLVKM*
>SPSA8_v1_840008|ID:41146397|fmt| Methionyl-tRNA formyltransferase [Spirochaetes Bin 1 SA-8]
MRVLFAGSPGIAIPAMMEIAAHHELAAVLTNPPSEQGRGGTGKNRGPLPTEVAQAAKEAF
GASVPVIEPEKLGAAVRERIASFQPDILVSFAYGKIFGPKFLALFPRGGLNIHPSLLPRH
RGSSPIQQTILDRDTETGVCVQALAPEMDTGDLYLVERIPLSQRETAANLSEICASLGAR
MIVDVLASIEAGTAQPKPQIGDASYCRKISKEDGLLDWSLDCLTLDARIRAFDPWPGTYT
FLAGQRLNILEAIPIPDIRLPNSVPGTIIGLDKAKGIIVAAGQGXX
>SPSA8_v1_850001|ID:41146398| Transketolase (fragment) [Spirochaetes Bin 1 SA-8]
METPTPERTKALQEIARKMRFHIVDMVYKAQSGHCGGSLSAADIVTALYFEIMNIDPSNP
KWPXX
>SPSA8_v1_850002|ID:41146399| putative Transcriptional regulator, GntR family [Spirochaetes Bin 1 SA-8]
MKEMQKIDYPDLSEKVYRELRIMILEGELLPGEKLKQEVLAARLGISRTPLVAAFSKLEK

EMLIELLPRRGARVRALNSKELLDLYDIRIMLEPMGAGGMAHHATAEDIQKLRELLESYK
QLVDSSDHRAIRRADYEFHLAIAQMSRNEPLYRIISAFNIVFICNQGGLLKPARNSYIEH
AALVEAIAEHDEMKADNLMRDHLTDSRHRLLYKLEEKM*
>SPSA8_v1_850003|ID:41146400| putative Transcriptional regulator [Spirochaetes Bin 1 SA-8]
VRKIESTVVADEVYRQIKRMIIDGTLEPGSRVDRQTLAEGLGVSMTPVNEAVARLVGERF
LERRIGSARGNDGFFVPVSQRDALVHLFEIRAGIEGIAARLCVERMIDDDSVPEVQKLCS
FFSGFDFGSRPANSEETKAYTIEDMNFHEAVINFSGNTILADIDRNLGCIHVSKVKGLVR
PPEETLSEHLAIIEAFRAKDALLAQSLMTQHHLRSRDVLLETLKKQQRVS*
>SPSA8_v1_850004|ID:41146401| conserved protein of unknown function [Spirochaetes Bin 1 SA-8]
MQKRRKIIIAVAPVAHVEKTIPSGLHNPVEPEKIAQDVIDCAAAGASLVHLHVRDKAGNQ
VADLSWFKETIDRIRAESAIIIQGSTGGKSNLSLEDRSASVNEPRVQMASLNMGSTNFGE
SVYINTWPDIRYWAEKMKKARVVPELEIFDLSMIESIVALAEEGVLAPPLHFNFSLGFKG
ALSASADNVFRLKQALPPGSSWSLIHEGMRDFSLLAVAIGAGASGIRVGYEDGFELEPGK
YATNAELVRKAADLVKMLGCEPATIEEAYRMLDIMKPAAVS*
>SPSA8_v1_850005|ID:41146402| protein of unknown function [Spirochaetes Bin 1 SA-8]
MFGVSPAYFFSRYSTDFSVRDYIDGLEWLKAHGFHGFQLEVFHREKLAEWENQAFSLADA
SIQLGMQATQFVAHFLLFATRTPEALLSDAGMEELKRLPEIAAHFPGCTTLTLPLSPFVV
GASYPPLNSAEYHRFWEAFCSKLLVMDELARAGGMRLALEIVPGSLMGGTEGFLRFIHET
GNQTIGYNFDTGHAHASKECLELIPAKLAGRIYGTHLKDNAGMENLAMPPGKGTIDWTRL
LAGLEKNGYRGSFDLEIASRAPELVFEEYLEGKACIETSLNQLDSSSSKS*
>SPSA8_v1_850006|ID:41146403| conserved protein of unknown function [Spirochaetes Bin 1 SA-8]
MIKYAVISSFLGKTKDRFHEYNQALDLEQKFKIASEIPGMAGLECVYPYEVSNAEEVKSL
MKKYSLGISAVNVNVKAEPEFRNGGLTSPDKNVRNKAVRFIKEAKDFAVAIGADKVTCCP
LGDGWEFSFQTDYSKNWTYLKETFGEAAAYKPEITLYIEYKPSETRGTCFINTAAKTILL
IREIGLPGLGVTIDVGHSIYGGANPAEELALLHDSGLPYYVHINDNDAKWDWDYFAGSKH
LLAYAEFLYYLRKYGYDDYVTSDTSPTRWDVKGMFETNNRITARLLSRLEEMERKGFDKV
LESGDYMAVWKFIEEELFGLK*
>SPSA8_v1_850007|ID:41146404| putative transaldolase [Spirochaetes Bin 1 SA-8]
MSEGYFQRVQKMTATRFWINNVTRNEADRSIAAGAVGCTQNPSYVWKMLTNEEERGFAEA
QLRKILAATADDNEALILLQQELVSGIAKKFLPLYENSCGKEGYVSIQGDPFDESYDTIM
RLARMNRACGPNIMIKIPVTEHGLSAIHQCILEGMPVNATEVMSLSQALDVCDAYDDATR
GMKNPPIVYLSHIAGIFDEYLSGSDCSKTPEASRDVLFQAGKAVAKKIREYMNERKTNVG
FINGGARGLHHFTEWVGADIAVTINWKGTAEELLNIDPPVVSRFDCPIPASVIDALCTIP
EFRKAYYPEGLAPSEYESFGPVVLFCSSFRTAWQNALSRIREIRTL*
>SPSA8_v1_850008|ID:41146405| D-lyxose ketol-isomerase [Spirochaetes Bin 1 SA-8]
MIYRSKVKELQAKAAIMLKNSGFPLTDSEIDSIAVADFGLGKPECEGAQILTLFATDRIS
AKLIVLFNGQILPEHWHPPVGNDPGKEEIIRGYCGTVYYFDDFSGAPATPDVPAGKSSVY
TCRTRLDLAPGMQVIVPPGRKHWLQGGPEGGCVISFSTCVRDILDQFTDKAIIRTTEILE
D*
>SPSA8_v1_860001|ID:41146406| putative aliphatic sulfonates transport permease protein SsuC [Spirochaetes Bin 1 SA-8]
MKKAKKLALGFAGATALILIWVIASEVVNASLILPRLADVMQAIGKLSASSALAKAISGT
FLRVLEAFTLSLVIGSLTGILSGLHESIEFFLSPFLTAIRATPVLALILVLMFWMPSSQV
PVISAFLMAYPVMHTSLHTGIRLTDKDLLEMAQLFRVPKAPMFFLLRLPSALGHFLSGAK
NSLGLCWKVVVAGEVISQPQFALGTKLQEARLSLETPEVFAWVFITVLLCGMSEYLLSYA
SQSFSAGGWSX
>SPSA8_v1_860002|ID:41146407| conserved exported protein of unknown function [Spirochaetes Bin 1 SA-8]
MIRKSLFKSLAAKILIGVLLVASSGLASAQANSQGXQTVRISILKGPSGVSSAWFVSSPP
ETGSAKIEFLIAGSADIVTAKLVSGEIDAGVLPVNVAAKLYSAGIPLRAAAVVGNGMVKF
LTNDPAIKSLSDLRGKSVAIAGQKATPDYLFRFLTSKDGLEAGKDYTAVYNLSYPEIAAA
LASGKIQYAVLPEPFATQALLLGKELLSPISLDSLWQKHTGMQSYPMSIFVVSKKLADGN
RQLLTKILQSLEASVKKTVEDPKGTALLVEKLDLGLKAAVAEAAIPKSNYVYEDAASDKK
SIEALLSLFLSFEPASIGGKLPDQDFYLN*
>SPSA8_v1_860003|ID:41146408| Fibronectin-binding A domain protein [Spirochaetes Bin 1 SA-8]

MSLNYKEINRILEELDLSGSKIQKIFQPSYDTIILELYRRGERVLFLVSVASGACRIHSI
SSLSMKNERPLRFMQCLRSRIRNGIITEAVQIGNDRIVRLSIQSSGEEGEERLSLFLKLW
SGAGNILLVDSNSIIIDALYRRPQKGESSGVSFSLEKFAPSRPSQAGTKPSAAKEFTLRE
LPGEGSFNEKINRFYSEQAGQLSREALLAEAEIRYEKRKRFLEQKLAELEKRRLEYENAE
RYRQIGDILMAENSAQPSQNSETVLSKTPKGKTCFLHATDFFSNSPISIQIDSSLSLLQN
AQSYYEKFKKAKSGLGAVEEELLKLREAITALEAWKEKVFGTEDPFLLAKMLSKGGTVRE
KQKNRYPCIAIDNDGWTFLIGRSAKENDEILRRFVRGSDMWLHARDYAGSYVFIKVPRGK
SVPLDVLIDAGMLALYYSKGRKNIDGDIYYTPVKHVRRAKDGPKGLVIPMLEKNLHVRIT
PERLKALLAHANEQQSGELS*
>SPSA8_v1_860004|ID:41146409|secA| Protein translocase subunit SecA [Spirochaetes Bin 1 SA-8]
VLGERPFDVQILGGIVLHNGKIVEMKTGEGKTLSSVSAAYLNALTGKGVHIVTVNDYLAE
RDSQWMGKVYKFLGLSVGCILSSMDPAARRISYGCDITYGTNNEFGFDYLRDNMTWDPET
RVQRGHAYCIVDEIDSILIDEARTPLIISGPADDDSAKITEVSRLSQSLTEVKKNPETGE
YPDEANGESAEGDYRIDEKSKRIMFTSEGLNHIEELLQKRNLIKGSLFDESNFEYVHYFT
QAVRAHRMFSKDVDYVIQDGQVQIVDEFTGRILHGRRYSDGLHEAIEAKERIKIARRNRT
LATITFQNYFRMYEKISGMTGTADTEAPEFNKIYNLDVVVIPSNRPVIREDQDDLVFLNE
DEKFNAICDEIQKANTKGQPVLVGTVSIEKSEKLSALLLRRGVRHEVLNAKNHAREALII
AEAGSKGAVTIATNMAGRGTDIKLGGNPEFRARHKAGSSASDEDIRKIMEAEYQAWNRDY
QEVRSLGGLYVIGTERHESRRIDNQLRGRSGRQGDPGKSVFFLSLDDDLMRLFGGENFKG
MMSKVGMKPGEPIYHPLLNRTIESAQKKVEERNFEIRKHLLEYDDVLNRQRNYIYEQRDE
ILHDTKLIERIQASASEMLSEMISDFLEAMKSGSKSAQTDFAISLRDTFGIELSAAQLSS
MAHDRQQLTAFIQSCLDKDIEIKKALAGEENLNLFIKYQYLQEIDNKWLDHLETMEALRE
AVYLRSYAQKNPLLEYKIEGSDIFEQLVDSIRKSIASRVYRVRISAEEERRPAAKPGIPA
QVIHESASSFSPGQQNRAESSFAAPRQSAAMANASGVEGATVVRTMPKVGRNDPCPCGSG
KKYKHCHGR*
>SPSA8_v1_870001|ID:41146410| Ferrous iron transport protein B (fragment) [Spirochaetes Bin 1 SA-8]
MSCGAKLPVYVLLAGAFFTKNPGNVVMAIYLAGVILSMVSTVFLRKTILKGQTTPFVMEL
PPYRMPTVRGIFWHVWDKTWQYVKKAGTVILAASVLIWVITTFPQVGNESELAGRFADEA
RIAAPGATQEQLDSIVASRLSEYRLEHSIAGSIGKFLAPVIQPLGFNWKIGVALIPGFTA
KELVVSTLGILYGAQIDAGDGASLQDALRNDPGMNPLVALTLMVFILVMPPCFASLATIR
AEAGNRWMLFQVGYSLTLAWVLAFLVRVAGSLLTGM*
>SPSA8_v1_870002|ID:41146411| ABC-type transport system, permease component [Spirochaetes Bin 1 SA-8]
MNFISALLNPTMPFVRNAVLAGALASILFGVLGSIVTVKRIAGLAGAISHAVLGGIGLAI
FLAAKNIVPGLPPIVGALVFAVIAAALIGVVSLKAKQREDTVINALWAIGMSIGILFIAK
TPGYADPMSYLFGNILLVSTRDIILLAILDVIVIVLAWRFYPQIEATAFDEEFSRTRGVP
ADTVFLVLLSITAIAVVLLQTFVGIVMVIAMLTLPAGTAGYFSKNLSGMMVSATVFSLVF
SLTGLLVSWQMDLPSGAVVVLVAGAVFLLVSALRIRKLA*
>SPSA8_v1_870003|ID:41146412| Sulfate-transporting ATPase [Spirochaetes Bin 1 SA-8]
MEHKVAIRFDRVSFSHREADVLRNTSFHVHEKEFVALVGPNGAGKTTLLRLVMGLAVPAS
GKVEVFGASPRSVQASIGYVPQYMNFDSSFPISVEEVVKMGRLVGLGRGCMEKRCADVDT
ALELAEVADLRKRPYAALSGGQRRRVLVARALASNPRLLILDEPTANMDVESEKRLYTVL
GNLKKTATILIATHDTGFVSALTDVVLCVTEDGKGSHHVVRHASVPAEDVPVDLYGSHVV
RILHDTDLPDDICCGTERQGAAR*
>SPSA8_v1_870004|ID:41146413| ABC-type metal ion transport system, periplasmic component/surface adhesin [Spirochaetes Bin 1 SA-8]
MKRTHHGLHDKPMLGRGNISIIAGFAAVLFLVLPLAAISAQASSSSGQKPVIAVSILPQA
EFVQKIAGGSVSVVTLVGPGASPHNYEPSPRQMTELGKASIWFTIGVEFENALMPKIKSL
YPKLKIIDTTAGIVYRNLEAHHHEGEAAQAEEESGKDPHVWLGHDAVKAQLAVMLDALTA
LNPAGKAQYKKNHDSYVQAIDAAFVQLKKDLAPMKGQTVFVYHPSFGYFFDEFGIIQEAV
EAGGKEPSQKSLAVLIKQAQEDKVKVIFVQKQFSTNAAKTVAKAIGGSVVEIDPLASNWL
ENIMVMGNALKKAAR*
>SPSA8_v1_870005|ID:41146414| conserved protein of unknown function [Spirochaetes Bin 1 SA-8]
MTHISKALIKNRRAIQAIVATGVYIAASALGLNLIWILVAGSVLGVLFGKVYCRWACPIG
FFMELAMSFSKDGKFRQMYQYHKVGCPIAWISGTLNKYSLFRIRRDDKSCTKCGACDKVC

YISSLEPSQFSLFKRGKDNPATSFSCSKCMECVAACPTKSLIFTSK*
>SPSA8_v1_870006|ID:41146415| putative 26 kDa periplasmic immunogenic protein [Spirochaetes Bin 1 SA-8]
MRFGQKRGTALLSCIAILAMIAVGGAAAGAQPAQTIRVVGEASMSVDADMAVLVLGVVQQ
GKTPTLAQQAVSMASDRILASITGLGIDRKKMRTGNFSMYAVYDDKPGKQNDIIGYRAET
SITVTIEDLKQVALVMERALFAGANQIQNLQFQKKNEEALRLQLLKEAAENAAKKAQAIA
NGLGLSILKPVSVEEQSYSMRAPNRDMYLTKAAAGSGQDPFAAGSIELTASVSALFEVAA
EKP*
>SPSA8_v1_870007|ID:41146416| conserved protein of unknown function [Spirochaetes Bin 1 SA-8]
MVRLVNRHALFAVRHHDLPSLLAGKLNALITRPFTKGRDWYDAVWYLSKTPAIEPNADLL
GSALAQFGIPYKGDWRPLLREKLQQIDFRAARNDVYPFLEKPQEADYLTREYLEQLLER*
>SPSA8_v1_880001|ID:41146417| protein of unknown function [Spirochaetes Bin 1 SA-8]
LKTRQGLKRNCENELWQMHHSVAADLKTRQGLKRELDRSPQNVLRPLQRT*
>SPSA8_v1_880002|ID:41146418| protein of unknown function [Spirochaetes Bin 1 SA-8]
LKTRQGLKHIDSAKTKLFEIVAADLKTRQGLKPRNIILMAMIRGVAADLKTRQGLKQCII
DGGSDVLKDVAADLKTRQGLKRR*
>SPSA8_v1_880003|ID:41146419| protein of unknown function [Spirochaetes Bin 1 SA-8]
MKIEEVAADLKTRQGLKLTKSMMVDNILCKVAADLKTRQGLKLRS*
>SPSA8_v1_880004|ID:41146420| protein of unknown function [Spirochaetes Bin 1 SA-8]
MVGCSGLENPTGIETGDYKTHAATEPTVAADLKTRQGLKHTPLWEATEKVVQLQRT*
>SPSA8_v1_880005|ID:41146421| protein of unknown function [Spirochaetes Bin 1 SA-8]
LKTRQGLKRYDSFTYPQKGFVAADLKTRQGLKQREKTMIISSTDWLQRT*
>SPSA8_v1_880006|ID:41146422| protein of unknown function [Spirochaetes Bin 1 SA-8]
LKTRQGLKHITIYSPIDSADRRSCSGLENPTGIETRSSVQDDISGVTSCSGLENPTGIET
SNPHFAEMENASVAADLKTRQGLKLIQHLIYEVKEGKVAADLKTRQGLKPRD*
>SPSA8_v1_880007|ID:41146423| protein of unknown function [Spirochaetes Bin 1 SA-8]
MNRCIAYSDNSCSGLENPTGIETNPALTPSDTASSVAADLKTRQGLKRTEYDTILSFEPF
VAADLKTRQGLKRAKKNPCHRAGPIVAADLKTRQGLKLVIGLQGNAGGVWVAADLKTRQG
LKPNDYKAYAMP*
>SPSA8_v1_890001|ID:41146424| protein of unknown function [Spirochaetes Bin 1 SA-8]
MIRVKNQKQIEGIRASCALLSRLLFEVRKAIAPGVSGLELDELAEDFIRKXX
>SPSA8_v1_890002|ID:41146425|nusB| N utilization substance protein B homolog [Spirochaetes Bin 1 SA-8]
MASRRKARILAFQAIYAWEASGTPLEELLKFSWLEQDKVSNIEDEIAAFSRLIITGTLEK
CAEVDAAIKRHLAHWPFERLKKVDLAILRMGTYCLLYQKDLPAQITIDEAIEIAKEYGSD
ESYKFINGVLDGINKESR*
>SPSA8_v1_890003|ID:41146426| putative PpiC-type peptidyl-prolyl cis-trans isomerase [Spirochaetes Bin 1 SA-8]
MKRLFLFFFACVAFASTGAQTTIDKPAATIKLTKQEVISVRQIKADVEKLEKATGAKFTA
DQIKQVLDARINSMLFIQFCDREKITVSEADVANALNQMKASLGSKATDADLEASLRASG
VFVEPKVYVRQRLLFETYVQTKRADELKKALTPPTADEILKAYDLAKATLVRPDTMRISI
LYVDIRGKSDADIKKAKETMQSMASSLKLNSTKFDEYVLRSGDAAGYKAIQSMYLEKTTQ
NKTIFGQELFDAVFKLKPGEISPLVESPTGFRIVRANEFLPQKQLGLSDTVPGNQNMTVQ
DFLIYQLAGEKEQKFMDTLEADLIQKLRNDATIKIYDENLKW*
>SPSA8_v1_890004|ID:41146427| putative Heat shock protein DnaJ domain protein [Spirochaetes Bin 1 SA-8]
MENYYDVLGVSPDSNQSTIKSAFRKKAKKFHPDLAASDTIEKAEELQAEKKTANPSGKTR
NNSVASVRESAMRLILEAYRILSDAEKRRAYDRILRRQKKEEGGFNYREFLKARNKDPES
QAKLIVYDLLHNLESEALEIYERSKSFPDFRLERYLDRGDAMDAEYCLAEEYEKRGRLLK
AYSIYKKLILMEKEKAWFRYYFDVVTLRFRTLMLQKMPGRIDEEDYLDRLDEVVGLDIGL
RDSAQFLRKKAEVLIRRKQYLEARLALQEAERRMPRLPGLSAVRHRLEEVGF*
>SPSA8_v1_890005|ID:41146428| conserved protein of unknown function [Spirochaetes Bin 1 SA-8]
MRIAVLTINDNDGSNTLDRLAKVAENEFSRGGNHVEIVKDASLQLQYFDFIILLTRPVGF
SGKPSMKIRQVLKNSGNLIGKRSMVVLFKKGLFLQSMLRNAMSELEKEGLLVTTGERITN
DEQLIAVLRTAPLTRR*
>SPSA8_v1_890006|ID:41146429| UspA domain-containing protein [Spirochaetes Bin 1 SA-8]
MVKWVESVLVAINGSEASISAMKCAIALGKLHDTRVAACYVVDTATIRQLALSRIFIQEE
SEEYERSLEMSGKRYLGLCAELAKQKGVEIQLLLKKGSVPGEIIKAAMELNADMIFLGGN

PQEPQYRDVIAEANREIVKNARCSVLVVKPHTGDEIYKKLP*
>SPSA8_v1_890007|ID:41146430|atpD| V-type ATP synthase subunit D 2 [Spirochaetes Bin 1 SA-8]
MAQPMPAPTKTNLIKEKRTLALALQGYDLLEKKREILVIELLKRIDELELLEDEIRKTTE
KAYGTLKKMLLSAGRERSQAISALPARQMILKASRVNVSGMNLPVIDAQAGELKLHYSFL
NSFAVCDETMIEFTELVSKLAVAASLRSIVWRLAREVKKTQRRVNALDKLVIPRSKEIVS
FIEASLDERDRESLFAIKMLKQHMSEEV*
>SPSA8_v1_890008|ID:41146431| protein of unknown function [Spirochaetes Bin 1 SA-8]
LLRIPESLLNKYLPAGETPDGGERS*
>SPSA8_v1_900001|ID:41146432| Homoserine kinase (fragment) [Spirochaetes Bin 1 SA-8]
MIRISVPATSANIGAGFDTLGIALALPASFAVEPAETTSISGCPVEFQTEDNLFLRSMRK
ANEILGYENRPIRLHIESAIPVARGLGSSAALTVGGVLAAMRHGGARSAAPLSADEKALA
LQIATLVEGHXX
>SPSA8_v1_900002|ID:41146433|thrC| Threonine synthase [Spirochaetes Bin 1 SA-8]
VRGFFYASVLVILEPMQEILFRSTVGGADAPVPVETAIAEGMPADGGLYVPVSFPRLPDP
DRVFSPEPLSYTELAFLVMKPYFSGWDEAILKEILRKAYDQNAGAGREPYFDTGEVVPLV
PLEGIAQSPGARVFVLELFHGRTCAFKDMALSVLGGLIHHSLERLGRREPLAILTATSGD
TGSAALEGLAGVPGLKTAVVYPHAGTSEIQRLQMVTTSPENRLVLGLEGNFDDAQSAVKA
IFASAAISADFAGIRLSSANSINIGRLLPQIVYYVKAWRDLSVRGALGRDGQMNVAVPTG
NFGDILAAKYAKEMGLPIGTLVCASNANKVLADFFATGVYDRCRPLVKTNSPSMDILVSS
NLERLVFLAAGQNPERVKTLMSNLKTKGIFKLNRKETAYLADFEAGWCSDYSAGSAISKA
WSVSHVLLDPHTATALDVVLQKEHILKDGTPLVIAGTASPYKFPRACSEALGFTHSDSSA
SSDLDCALQLEAKTGVAMPRQLKDLSAKTVHHRKVVTKEALAAELAQFFRNKSE*
>SPSA8_v1_900003|ID:41146434| Ferroxidase [Spirochaetes Bin 1 SA-8]
MISTKMADRINLQINREMFSAYLYMAMSARMTEMGYTGIGKWLMIQYHEEMFHAMKFAKY
LQDQGASVKLDKLDAPEFPEKTVKELFQHVLKHEQWVTASIREMVELARAEKDYATENLL
NWYIDEQVEEEKNATEILQAIDLIGSSAQGLFMLNIELGKRDNHAPLDFTKA*
>SPSA8_v1_900004|ID:41146435| putative Nitroreductase [Spirochaetes Bin 1 SA-8]
MLNLVSAMKTRVSTRTFDGKPLSEEEQAALEKLLQDVERAEKPDFLLPVRLTLHAGPGAK
MGTFGLIASAAGFLVPAVRNAPFAMESVGYSLERAILGAASLGIGSCWIGGVFSRAKANE
AARAGKDEMVPVIAALGHPASQRSLADRIVTGAAQSRIRKPISEIFFSLAAQPRDGIDTS
EEAASAGKLSAPWDTILELVRIAPSASNKQPWRIVQVSEIPYFVFFMEENYRYNNSLGDV
HLQNIDMGIAMCHFDAAAEAYGLAGTWRPLVEHKDTSSSAEDEKVGRALAFGRSRGWKPI
AVWQQKR*
>SPSA8_v1_910001|ID:41146436| ABC transporter related protein (fragment) [Spirochaetes Bin 1 SA-8]
MSAQKDTQKDTGMPAGGMAPGNAMRPGGMRPGAAFGRGGPQAMMKGEKARNFKGTMKKLI
AYLGPYKLSILVVLIFAVGSTVFSIVGPKILGKATTKLFEGVMQTIAGSGTGIDFSYIGG
ILLTVLILYGTSALFGYIQGWIMSGVSVKITYRFREEILSKINRMPLRYFDGTNHGEVLS
RMTNDVDTVNQTLSQSLSQMITSVVTVLGVLVMMLSISWQMTLVAMLILPLSLFIIRFIV
SRSQKYFKEQQVYLGHVNGHIEEMFGSHAVVKAFNGEEKNIRKFDELNGTLYNTAWKSQF
LSSIMMPMMNFVGNLGYVGVTIMGGYLAVKKLVSLGDIQAFIQYVRSFTQPISQIANISN
VLQQTAASAERVFEFLEEAEELPEQEX
>SPSA8_v1_910002|ID:41146437| ABC-type multidrug transport system, ATPase and permease component [Spirochaetes Bin 1 SA-8]
MIRLFRYLKPYIASIILVIALLFIQANADLALPDYMSKIVNIGIQQSGISDAVPEVIRQS
EMEKIQLFLSDEEKTAVLEAYSLADSTSSVYSSLQAKYPGAKGEPLFALKTVSTETRAKL
NTVLSVPLSLVSTIEQAAADPEKAKTMTQGMPFDMSKLPPGTDLFGVLKMLPESQKASVL
ASIRGKFEALDPMLREQMGIRAVRAEYEALSANIGALQNSYILKTGALMILLTLVSVTAT
ILVGFLGARTAAGLARDLRGDVFRKVENFSFSELDRFSTASLITRSTNDITQIQMVVIMG
LRMIFYAPIIGIGGVIRATAKASTMWWIIALAVGVLLVLIGTVFKIAIPKFKSIQKLIDR
LNLIVRENLSGMMVIRAFTMQGYEEKRFEKANRDLTGTMLFVTRVMVVLMPLMMLIMNLV
SLLIIWTGAHEVASSQIRIGDMMAFMQYSMQIFFAFIMLSMMFIILPRASVSADRIADVL
AEEPQIKDPEKPLHFPEPFTGTISFKNVTFRYPGSSEDVLHQISFDARPGQTTAILGTTG
AGKSTLVSLIPRFYDVSEGSIEVDGIDVRXVSQKELRDKIGFVPQKGSLFSGTIESNLLY
AKEDATPEEIQTALAIAQASEFVNAKPEGGQAEISQGGINVSGGQRQRLTIARALVKNAP

IYIFDDSFSALDFKTDMKLRQALKSGVKDSTVLLVTQRVATIKHADQIIVLDEGRMVGIG
THRQLMETCEVYRDIALSQLKQEELA*
>SPSA8_v1_910003|ID:41146438| protein of unknown function [Spirochaetes Bin 1 SA-8]
MLDRLVQQGLVERIEDPEDRRGKRLSLTDKGMKMVQEGIYARQEWMKTLSASLSDSEKEI
ISAALALLVEKAKNINSAPRGANPHETGAIRCL*
>SPSA8_v1_920001|ID:41146439| protein of unknown function [Spirochaetes Bin 1 SA-8]
MKKLIASDGVSGDEFGYSLAAFGNRMVTGAHYANSKEGAAYVIPLKP*
>SPSA8_v1_920002|ID:41146440| protein of unknown function [Spirochaetes Bin 1 SA-8]
MKKIIVDIDNTLWDLAPVFFEYLKRYNPEIPVEDLKRGETRLKGYIPREDLYGVLKEIHM
RQDQFKPYPEARDFLSTLKEMGLYVIIASIRDEEARDATERWLMKYDLPYDELHLSNDKT
VLFNDAWAIVDDSIWTLDKAAQAGIVRTGLRNVWNESRGHLLFDTLPEIIDYLKKQCPCR
*
>SPSA8_v1_920003|ID:41146441| protein of unknown function [Spirochaetes Bin 1 SA-8]
MQLKPQRKNDDELLVGGIFSLTGYLSWSGHYKKKGAELKIEMINEAGGINGKCLRLIAYD
DRSSPEQAAKIAEILVLKHRVIAMIGTGSLPISGAVARVANKYKIPAFLNSGYAIDPLSD
LFVFNTSHKTEFAIACSFGHFVERGIGRIALLMPKGPLGDLGSWLGRQVGSHLGIKIVGE
ERFDVSVPEMTSQLDRLKSLKPQALFSFVTGEPAARLVMKMTHLGINIPLLVSHGNANPA
FLKLVSRASVPIIVPSGKTMVIDSIPELDPGKNKMKDFSTMHVKRFGEPANYCSAESADA
IDLLAEGLRSSGRPDGRILRDAVESIRQFHGMQGVYDLSPIDHYGTQVEHMVLLEVQDGA
WHLTKRFSSVGVFESIHSDNKRRLVFRLARAFNEPSTEALTLACETQGCAEKLAAQVGLS
CTNLKADPCFAVKLSSREKQELMQAIREEDYAKAKESLCRSLTISLLHYYKAFEPLKLAV
SELFHALFDAAASEEGVDLEKLIELKSKYVLEWANLKDQEALCFWTVRVFRETMELLCDR
RRERGSGLLKNILRFIEMHFAEDLTVERIAREVCLSPSRLIHRMKSQYGLTLSDCIARVR
MDKAKALLRDTDMTICEIAHEVGYADQGYFTRVFKKCLNKTPGNYRESCRIRFLS*
>SPSA8_v1_920004|ID:41146442| protein of unknown function [Spirochaetes Bin 1 SA-8]
MKKLSVLITLCFVFVMFTGVGPVIAQTKAPYKIGGIFPLTGYLSWLGEYKKKGSELKVEL
INKAGGVNGRPLELVVYDDQSSPETGSRVAQRLVSKXX
>SPSA8_v1_930001|ID:41146443| exported protein of unknown function [Spirochaetes Bin 1 SA-8]
MKKNKFLPAVMSFFIFAMIAPPSVAHAQETAISLGSWSFFSGSPGVFTSLGITQGLTPRL
EAGLSLIPRLTPEPFEDFYAEAHLGYSLAANRFQGLNQPASYINILGDIGIIAGISSIGA
PGQTLKKSIFIRLTPITLGNAFYGRRDRIYSAGALFKLDSPSLTFFLNFIISDFFIAKRS
N*
>SPSA8_v1_930002|ID:41146444| exported protein of unknown function [Spirochaetes Bin 1 SA-8]
MSHNARFSLLIMLLIFICSGAAFSQQDSSQKPAISYSAGMAGFSDSAFSAAETLRFFISL
ESERANPFAPSLFAAIQVDFNPLSLQTAYWGLGFNLTLFSLQHHFFSFASMRKTSWAPAF
SAAFYFPFDSAKNSYFEAAAEPIRLFTGSSYVSLLSPGALFSKNFKITGYSLSPFKFTYH
FY*
>SPSA8_v1_930003|ID:41146445| exported protein of unknown function [Spirochaetes Bin 1 SA-8]
MPRLFSIIFSLFLVSSVSAQAAPSTAESADPIGQYLSARSIPSYESSLPRYYFSGEAWNE
RAFELVANAKSYILIDSFLVNAHPVNQKIIDALREKAEAGVRVYIIFDSSSYFTYMPDFV
SYLPTPVVTFRGTRVNVAEYNSINGTKLFALPQLLNRDHRKFWIVDGHTVALGGMNLNYY
SMKESSSLGNIDTFVEVQSPPAIRQLISSFVETWNLCSPMPLNANSFTTTQPPEPNYQPE
TRLWLFDQDLHGRSEVNALFDSFFNFAQRELWMIQGYTFATPELLKKIKKATSRGVTVNI
LLSTNSFREVYELASNYCVLDLIRAGANVFMFDDPGKAFLHYKLFLADRTAAAFGSPNFN
FRSQYLSREIAILFNDERVGRAAYENLETLLARSRPVTKDEAERYRGLKYFIAYMEMLFG
G*
>SPSA8_v1_930004|ID:41146446| dTDP-4-dehydrorhamnose reductase [Spirochaetes Bin 1 SA-8]
MKILLTGATGLLGRALRKQLLLIPNFELTAAAYSRAVPPLVKADLTDSGHVETLFETARP
DIVIHAAAERRPDIVDSQPEKARTLNVAATETIASACARHSASLLYISTDYVFDGSNPPY
FPDSPVNPLNEYGRLKLEGERIIKNTCPKGAILRIPLLYGPVEFLEECSVTELAKKLLVT
APVKVEHWARRYPLHVDDVSRAIIAITSAMRDFSEDFPIFLLSGPVSWTKYEMLLEIGRV
LGKSTSHVIPDTAPPSGAPRPKDCRMDTSRLESLGYTPQIAFGEGIKSALLPFFNIGV*
>SPSA8_v1_930005|ID:41146447| protein of unknown function [Spirochaetes Bin 1 SA-8]
MDTIFWSARQNDAWRTKVGLAEMLKGGVIMDVTNAEQAKIAEKAGAAAVMALEXX

>SPSA8_v1_940001|ID:41146448| protein of unknown function [Spirochaetes Bin 1 SA-8]
VSHDRWFLDRVCTHILAFEGDSQAIWFDGNWTEFAEWRRATLGIDADRPHRIVYRKLER*
>SPSA8_v1_940002|ID:41146449| conserved membrane protein of unknown function [Spirochaetes Bin 1 SA-8]
VDTSAVSKESAQIAASAERKRAGLLAMAGCAVLWSMGGLFIKLIDWQPFAIAGARSAIAA
LFLFIWIRKPKFTFSLLQTSSALAYAATMLLFVFANKHTTAANAILLQYGAPVYVALLGS
YMLKEVPKLEEWLALLAICAGMVLFFMDSLAAGHLLGDTVAVISGFAFALNIVLMRKQKD
ADPLSSMLLGHIFAAIIAGIISLFLAPLVISPRSLAAISALGVVQIGLAAVLFSYGIKRV
TALQGVLTAVIEPIFNPVWVFLVTGEIPGSKSILGGLVIISAVLISSVISIRKAGKGAS*
>SPSA8_v1_940003|ID:41146450| conserved membrane protein of unknown function [Spirochaetes Bin 1 SA-8]
MKQSFISATILLVLITDPLGNIPLFLASLKHVEPSRRRIVILRECAIAFFTLVIFLFFGS
AFLDALNLSDETLRIAGGVILFLIALNMVFPGSGGKLVEEESGSEPFIVPVAIPLIAGPS
AMTTVMLLAKSFPDRKLEWVGSIFIAIMITTIVFFSSTRLQAWLGSRFLSAIEKLMGLVL
TAIAIEMLLGGITNYVRLIQG*
>SPSA8_v1_940004|ID:41146451| putative Histidine kinase [Spirochaetes Bin 1 SA-8]
MKWSTIDNIFVDEAMLSALRQSGAESKPVVSRIFNIPPLGKPGFAVVYGLYANTPDRKNP
SAYVLHLLEAETDFFPMISSWPIPSETAESYLIRRSQDEILYLSPLATMPGAALTFSHPV
SRGNSAEAQALSGKTGLLKGRNYHGKQVFAYALPIPDSDMTLFVSMHQNEVMRPWIASLI
SHILAAAALLACGIIAVHMISAARMTREMRKELTLAKQLQEEHKKFDAFMKYMPSMIMMK
DKTNRILYTNSRFDSHFPEHEWIGKTPTEIFTPEQAKITLAMDRKALEEDYCEYQEIRTD
KSGNELILLTQKFRIDIEGKDPLIGQIITDITDKEQAFKEIIKLNENLEQRVKERTEQLE
ASNAELRSFSNSIAHDLRSPLRAFDGYLQLLSERGRPIFDDEGKRYLAKLSAASERMKTI
IDDLLLLSKISSTEMILENVDLGALVNRVASDYIVRENSKSISLSIAPKAKALCDARLAN
ILIECLLDNSFKFSRDIQDIKIEFGTAKNKMFYLRDNGLGFDMEYAGKLFQPFQRMHPAD
EYPGNGIGLAIAKRIVDRHGGTIRIESSPEKGTTVYFSLESV*
>SPSA8_v1_950001|ID:41146452| Cyclase family protein (fragment) [Spirochaetes Bin 1 SA-8]
VGGDIDLLLNQRAVIGCYPWRFVDGESSIARIVAHVDDDRYEELMKKKAKAELTRFGDVA
GAKNSWLHEEAKKLHRK*
>SPSA8_v1_950002|ID:41146453| Transcriptional regulator, GntR family [Spirochaetes Bin 1 SA-8]
MAGRNLSGEIYETLADRIKSWRYLPGHRFTEEDLCREFSVSRSPVREALNMLVKANLIQK
EERKGYRVRKMDLRELIELYNTRLILELAVIDMLCDKGIPAQTIQELKARWERLQSALPS
MAQEAALEDERFHEALAEAADNRVIQRLLKEIDQDIRFVRLSDITDQERLKKTCSDHLRI
LTALETGDRELAKRTLSENIMWGRDKVASALKDALIHACDVA*
>SPSA8_v1_950003|ID:41146454| protein of unknown function [Spirochaetes Bin 1 SA-8]
MPAMLRKPAGLTAILRGSRFPKEPFPFVLHSIFPSAINISRENDELLASLVLKKSAMHPR
AVFVLSDGQEPVDFSRLNLAPGMEGHADADGFHIQSELLIPYARDVTTLPSSRISIEFSN
LHHDWLRAFEYCTAQLSALQKQKNSDLDIETLIVGESEGDLPACTSFDARQRALLIKSIQ
NLGKAMWQADAKSALRHACRLIGLGQGLTPAGDDFLTGFSYALMCRQQIAEDKQSLAASE
FLNGLKNRLRNEPGQTNDISRTFLLLSLSGEASEALKRLASAFEDGFSRQQFEFALQVLA
GIGHSSGLDAACGFIYGVFQNDRAFSKSLRTFANSQQCRL*
>SPSA8_v1_950004|ID:41146455| Carbon-monoxide dehydrogenase (Acceptor) [Spirochaetes Bin 1 SA-8]
MLHEFTYKAPVTREDLFKLLDDHGSEAKILAGGTDLLVNIRNGVMKPAVVVDVKKIPRYK
DISYSEQEGLLIRPGVTINDILRNHEIQENFPLLQECGHDLASYQIRNRATAIGNVVNAS
PCSDMAPALLCLGAQAVIASSNGTRTLPFSRFFTGVKKTVLQTGEILESIAIPASARGAK
GAYKKLKRINGHDLGIVGVAVSIKDDILRIAVSSAAPTPVVTPDLPADIPAEDAVAATMN
IISPISDVRCSKEYREFMVGVFVRRLLAEVRK*
>SPSA8_v1_950005|ID:41146456|xdhC| xanthine dehydrogenase, Fe-S binding subunit [Spirochaetes Bin 1 SA-8]
MRVKLTVNGELYERDVKDNKTLLRFLREDLGFLGPKEGCGAGECGACTVFMNGKTVNSCM
VLAAEADGAEIETIEGESKDGMLSKLQQAFDRNHAVQCGFCTSGMIMSARELIRIHPKPT
VEEIKEGLEGNFCRCTGYKQIIEAVLDATGQLDEKEGLKYAGK*
>SPSA8_v1_960001|ID:41146457| protein of unknown function [Spirochaetes Bin 1 SA-8]
MTAELIKTGFLQQSSFDAVDMYCKPARQVALLQCIVMFHEEAAAAVNAGVPLPKIAALPQ
REKLARLKSTMTNEDLSPAHVLQDEIKAMFADLRSHYEKAAHL*
>SPSA8_v1_960002|ID:41146458|atpB| V-type ATP synthase beta chain 2 [Spirochaetes Bin 1 SA-8]
MRGVEYRGLLKADGPIVIAARRENAGFSELVQVRDRNGEIKLGRVVDLSEQAVAIQLFSD

TSGITIHDAWVEYLEKPLQFRVGDDIIGRIFNGLGEPIDGYPAIFSSDLRDINGQPINPS
ARVYPRDFIQTGISAIDGMNTLIRGQKLPIFSGNGLPHNKLAAQIVRQAQVRSQDSQFAI
VFAGMGIKYDVTRYFISEFENSGVLSRVVMFLSLADSPSIERLVTPRTALTAAEYLAYEK
NMQVLVVLTDMTNYCEALREVSAARNEVPSRKGYPGYLYSDLASIYERAGKIEGCSGSIT
QLPILSMPNDDISHPIPDLTGYITEGQIVLERDMSQRGIYPPIAGLPSLSRLMKDGIGEG
MTREDHRDLAAQLFSAYAYVKSVRDLAAIIGEEEVSERDKLFLAFGERFEREFLAQGERE
NRTIEQTLDIGWKVLSVLPREELLRIPESLLNKYLPXGETPDGGERS*
>SPSA8_v1_960003|ID:41146459|atpD| V-type ATP synthase subunit D 2 [Spirochaetes Bin 1 SA-8]
MAQPMPAPTKTNLIKEKRTLALALQGYDLLEKKREILVIELLKRIDELELLEDEIRKTTE
KAYGTLKKMLLSAGRERSQAISALPARQMILKASRVNVSGMNLPVIDAQAGELKLHYSFL
NSFAVCDETMIEFTELVSKLAVAASLRSIVWRLAREVKKTQRRVNALDKLVIPRSKEIVS
FIEASLDERDRESLFAIKMLKQHMSEEV*
>SPSA8_v1_960004|ID:41146460| UspA domain-containing protein [Spirochaetes Bin 1 SA-8]
MVKWVESVLVAINGSEASISAMKCAIALGKLHDTRVAACYVVDTATIRQLALSRIFIQEE
SEEYERSLEMSGKRYLGLCAELAKQKGVEIQLLLKKGSVPGEIIKAAMELNADMIFLGGN
PQEPQYRDVIAEANREIVKNARCSVLVVKPHTGDEIYKKLP*
>SPSA8_v1_960005|ID:41146461| protein of unknown function [Spirochaetes Bin 1 SA-8]
MRIAVLTINDNDGSNTLDRLAKVXX
>SPSA8_v1_970001|ID:41146462| Ribonuclease PH (fragment) [Spirochaetes Bin 1 SA-8]
VIQKIPLRDSVAAISVGLVGGEILLDLSYEEDSKAEVDMNFVMTGKGQLIEIQGTAEKTP
FSKEQLDVMYQYAYKGIGEITRHQKIALGPMFPA*
>SPSA8_v1_970002|ID:41146463| Non-canonical purine NTP pyrophosphatase [Spirochaetes Bin 1 SA-8]
MKNIIIATENMGKFREIKALLEHEFDNFYSLRDFQEKVPVEEDSELYIENALKKARKVGD
RFGMHTLADDSGLEVKALGGRPGVRSSRYGMSDDERIFRLLSELEGIPWDQREAVFKAYL
AFYMPERERCYVFYGELRGIIGLEKHGRNGFGFDPVFYVPERNKYLAEIPMEEKNRLSHR
GKAIQSFINFIQ*
>SPSA8_v1_970003|ID:41146464| ATPase involved in chromosome partitioning-like protein (fragment) [Spirochaetes Bin 1 SA-8]
MHTMETILITWWDRTKNFIKGFFVRTSDNTLSHEQEYIRKEPPVPRLRHNESGWISPPYL
TSKHVRLDGKVVAENRCVGALARPCEIDSYKILRAQVTQLTKQEGKNTIMVTSAREGEGK
TLTAINFALSLAMEFDQTVLLVDCDLKKQSVHKVLGVESMHGLSDYLISGADLSELILWP
GIEKFTFVSGGRPLHDSSELLGSPRMKELMAEMKNRYPERYIVFDVPAVLTGADALAFAP
LVDGILLVVEAGKTSIHDVNEAISLLPKEKILGLVLNKSGLGGNV*
>SPSA8_v1_970004|ID:41146465| Chain length determinant protein, Wzz-like protein (fragment) [Spirochaetes Bin 1 SA-8]
VATVLSSLYLQENLSQREQQTAGVSKFLQDETQVLQAQLVELNSKIATFKGKNVGALPEL
LQANLQGLDRSDRDLIQLKDQLRTLRDKESYLVIQLSNIPTTGTANADKLLLKELRSKLA
QLQAKYSDQYPDVIKTKREIAELEGRVGKTGTEGTEKAKSDFIPAEQSDNEAYVTLASQL
ASTRSDIQSVQRQLVETEKKRNEYQRRIEASPRVDETYKALLLERNAIQAKYDDLTRKAM
EAKVAQGLEKGQMGERFTLIDPARVPGKPIKPNRLAIILIGFILGIGAGVGMMALQEAID
HSVRDSRTLTALTGVPVLGTIPNIVTIKDVTERKKRNRTIVISVTTVLVLAILLVHFLFM
DFTILWARLSRRF*
>SPSA8_v1_980001|ID:41146466| conserved protein of unknown function [Spirochaetes Bin 1 SA-8]
VFELKTVQENDHLDIEKPCIDALDEGHNVEWSTELTLDEARMEYLNAWIAVNLLAALFSK
KPQDFFFNMSVGYTLAGIQGKKVDGFIEGMRRPEATEYWEKAIAELQAFVAGKTFAAAFG
DANLEKAKTLAAHMPVRPVHSVTLSTMHGCPPDEIEKIGRYLIEEKGFDTYIKLNPTLLG
YDEARKILDQLGWTEISLKRESFEHDLQFDQAIKLIQTLTTVAREHSCRFGIKLSNTLAN
ANVDERLPGAERYMSGRALFPITISLAAKLAKALPNFDSRFSYCGGVSALNAADLIKAGM
GPLTVATDILKPGGYLRLSQIASEVTGSVPFAPGCVDPEALTQLSQAVLARPEYRKEWKI
GTVSIRKPLPLFDCFAAPCIEACPVNQKAPAYIKAQGEGKPEQALATILSDNPLAHTTGV
LCDHVCQEHCSRLDYEGSVRIRDVKLTSARTSPIPAAKLPEHAAKPSGKTAVIGAGPAGL
ACAYHLALSGYPVTVFDADNTLGGVPTNIIPSFRISREELKADLDRIQALGVEFVPNTYI
DSLKKLSEKGYTSFFIGIGAHKARELPLKGSGVRVISALEFLEECSKQGTQAFAGIQHVV
VAGGGNTACDAVRAATRIPGIASVRLSYRRTKKEMPADLEELENALQEASAINLADAEAP

AVLHELSLPESARPGKLTLRVMKLGEKDSSGRRSPVPTEETFEVPCDLVIAAVGEEPDRA
FFEKLGIECGKNSLPTVNPATLETSAANVYAGGDARRGPASIIAAEADGRKAAFAILAKE
GFSTPQPDYTAPALDKNKLARRGELLPSLPFDHPDFAKREAERCLACDSACLRCVEVCPN
XANIYIEMGLPFKQDAQIVHVDKLCNECGNCGFFCPYEGEPFSGKPTLFDTIEDMEHSRN
AGFCFIFSRKLPALAVRSEIGGSISRFDYASWNXX
>SPSA8_v1_990001|ID:41146467| protein of unknown function [Spirochaetes Bin 1 SA-8]
VAVASDPKIEGYGNNPSAEIFQDFVVASDPKIEGYGNLNNLPLLLWVTGVASDPKIEGYG
NLLGIISSPFLQVASDPKIEGYGNYVK*
>SPSA8_v1_990002|ID:41146468| protein of unknown function [Spirochaetes Bin 1 SA-8]
MALIVASDPKIEGYGNKTTFRAVSGLIPVASDPKIEGYGNVVSYHCFAGGSMLQVTRK*
>SPSA8_v1_1000001|ID:41146469| protein of unknown function [Spirochaetes Bin 1 SA-8]
MFICEICGEEFEDEDESDWPX
>SPSA8_v1_1010001|ID:41146470| protein of unknown function [Spirochaetes Bin 1 SA-8]
MRKRFDEQFKATVALEAVKVTANFTPSTNF*
>SPSA8_v1_1010002|ID:41146471| transposase [Spirochaetes Bin 1 SA-8]
MYFKKDRAIHLVVGMTDMRKQINGLAQLANEHKPDCVFAGDYFVFLGKTRKVMKVLYWDR
TGFCLWTKRLEEATFPWSRKRKGSITLERDKFKLLLHGIDIFREHTEQHNYSVL*
>SPSA8_v1_1010003|ID:41146472| transposase [Spirochaetes Bin 1 SA-8]
METVLPELPENVESLKKLVIEQARRAYELEKTSKKLQEELAALKLQLQVTEEQFALLQRK
FFGATSEKRSKEHDNPKQALLFNEAETYAEEPAKPVKKPSEASHERKAVGRKPLPGNLER
REIVHALSEAERTCPACGAIRPEIGQEVREELAFIPARFVVNSHILKKYGPCQCAQCQHP
IIQAPGPAKLIPGSSFSNHTVAFFLTSKYVDAQPFYRMEGILSRWGIETSRASLCKLAIA
AGRAIGDLVEVMKTDLKASPVLQMDETVVQVLHEKNRSAQSNSYMWVARGYADRKPVIFF
HYHPSRAKAIAQGFLTGYHGFVQTDGYPGYNDIGQSDAMTHVGCLAHVRRKFFDAEQQGS
TEARHMLDLIAELYHAEKILRDRYESGEYTAEAFVRIRKQEQEQRLAAMRDWLVQKQGQS
PPSLAFGKAVTYALGQWETIGHYLEHELLTPDNNFIENAIRPFVIGRKNWLFSNTPLGAH
ASAGIYSLIETAKANGHEPYHYLCYLFDELPKAKSMEDKRALLPYRLAPGAY*
>SPSA8_v1_1020001|ID:41146473| Extracellular solute-binding protein family 1 (fragment) [Spirochaetes Bin 1 SA-8]
MKKFVLVVMALLAATMSFAAGKLTLMQNKPEIDAQLKAYAQAWGQKNGVTVVVKSIGGTS
GGMGPQLKADYAAGDMPDIFAFDGLEAYKEWEGVILDLSNEPWVKSTSVAFKYNSKIFGF
PVAVEGWGMAYNADMLAKAGIDPKTLVNYDGYKKAFEKLDSMKAQLGINSVVSMAAAVEM
GWVTAHHNFNSLLSNGLPYGDLSVVNALLAGNVDMQRLQEYADWVELLFKYADKSVLLTG
NYDAQVGAFATGKAVFLHQGNWTDPNIKGANATFKMAFAPHGSMKKATDGIFVXX
>SPSA8_v1_1020002|ID:41146474| protein of unknown function [Spirochaetes Bin 1 SA-8]
MKIKLIHDWITLSVNYDVAMLERGVVTGQDIRTVLSARKAVCSGYSKLFETMASAAGFEA
KTIHGWARGLSGNFEFTPRNSHAWNMVNISGQWLFVDTTFDAGAFTDGRYKQRYSTDYLF
PGPEQLQYTHFPEDPSDQLMSRPIDRSRFINQALFLPEFFKLGLSVPAVSANPGQFMPAL
VKAADRYNLDFEAPPDVMLDASVFSSDGGEIGGSVMLSLRAAGKWRISFAFPKAGTYKAL
IFAGKTSTREASGSTALPGKSEEKLKSVLSFAFQTSGKNVEFPAFPRQYASFFERAGEYL
ESPITGVLKAGMSAKFVYISNAKKVSLIHDGNFIPLDENPASPGRFELELKVPKTELLKL
GVSEDGVNFSIMLSWSVR*
>SPSA8_v1_1030001|ID:41146475| protein of unknown function [Spirochaetes Bin 1 SA-8]
MRGDLVYAEPAPSLLSFNPRPCMRGDVTSSHRAYFDTKFQSTPLHEGRLFQSTPLHEGRP
KVPDELKGTIEFQSTPLHEGRRCFIAVSM*
>SPSA8_v1_1040001|ID:41146476| conserved protein of unknown function [Spirochaetes Bin 1 SA-8]
LHLRRCSGLENPTGIETELLLKSLRFIDDRCSGLENPTGIETWSFSSAKANYI*
>SPSA8_v1_1040002|ID:41146477| protein of unknown function [Spirochaetes Bin 1 SA-8]
LECVQCVGHVAADLKTRQGLKPSRAASHNDRDGVAADLKTRQGLKHIILDIDLRPRNQVA
ADLKTRQGLKL*
>SPSA8_v1_1050001|ID:41146478| Uncharacterized ATP-dependent helicase YprA (fragment) [Spirochaetes Bin 1 SA-8]
MRTIGAQLPSLSPLPQGLHEKIASALLQRGISSLYTHQARAYQKARARCNFVVVTPTASG
KTLCYNMPVMQSLLENPEARALYLFPTKALSQDQQAELNEISLGGAMPVRAYTYDGDTPA
SLRSIARTQGRIVITNPDMLHAGILPNHTKWIAFFSNLNYVVIDEMHTYRGVFGSHVGNV

IRRLKRIAAFYGSKPCFILSSATIANPQELASMLIEDEVELIDENGAGTSEKTIIYYNPP
ILDSLQGIRRSSALESENITLKLLSMGIKTILFARSRLKVELIASYLTEHLANRFNDNSH
IRISPYRSGLLPSERRAIERGLRDGSIQGVVSTNALELGIDIGGLDAAVMAXX
>SPSA8_v1_1060001|ID:41146479| protein of unknown function [Spirochaetes Bin 1 SA-8]
MQLQKKKTGIPRDIAEFTMYTVIMKRIKAAPSRLAGLFLIFILASCASSAETTGAAKNPV
VIDASAPLRLYRTDKPDSRVTVLSQSMASSFQKSADPERALREFTAQLVKGESDPYMKIK
LIHDWITLXX
>SPSA8_v1_1060002|ID:41146480| protein of unknown function [Spirochaetes Bin 1 SA-8]
VVSADDAQDARIKIRNNPASLLGAAFILFMITVYIVNSAMSRGMPVFFFCNCIRLL*
>SPSA8_v1_1060003|ID:41146481| protein of unknown function [Spirochaetes Bin 1 SA-8]
MNNKELAKLENGFSLIARYSDILCSADSIEKAFSEICTDIGILTGSNRAVIYENINDEQS
FSTRCVMRGSWTAESSSPLEPEFPFSWMRASSEDLAVAARIRTFFKDRKAVQLTAGEFPE
SLNFSVNAYDMHNACLMPIFVDNSIWGEIVLTSDIPGKTWDEIDFAILKSLAEILPSGVD
RFKNELTNQEARIEQEILFENRIAGNXX
>SPSA8_v1_1070001|ID:41146482| membrane protein of unknown function [Spirochaetes Bin 1 SA-8]
MHGFYFAYYVVAFLPAILMFLSRNHRAFEMVFTATLMYYVHYLFFMFFPADGPIPEREHL
FQNGYVFIPIMNAIYHYSGQQGGGAFPSTHVSLAVIIGIYTYRYFKPIRTLIVVLCVGII
VATVYCSYHYXX
