# Supplementary material for: Fermentative Spirochaetes mediate necromass recycling in anoxic hydrocarbon-contaminated habitats
Source: ISME J. 2018 May 30;12(8):2039–50. doi: 10.1038/s41396-018-0148-3 (PMC6052044; doi:10.1038/s41396-018-0148-3)
Supplement: Supplementary file 10 — Supplementary Database 3 [file 41396_2018_148_MOESM10_ESM.pdf]

>SPBDM4_v1_10001|ID:27157205| conserved protein of unknown function [Uncultured spirochete bdmA 4]
MVYRSESWKKYIPDDIKAKYEIYDYKHAAAILYSEFPEHFKEVCSVLRQFKITKDDITSR
GGNESNIPKKFSDILRPLGWEEKQLHAKVIVDDHEISHDTHWVDYLKGRVAFDLEWNSKD
QTYDRDLYAFKSFFDYDKISVAILVTRSTDLNHYFSELGSYFANDGTERKYMAKYGASTT
QMGKLLPRLEAGRNGGCPILVFGITRKSIIEEP*
>SPBDM4_v1_10002|ID:27157206| protein of unknown function [Uncultured spirochete bdmA 4]
LGCISLFVRFDALPFFSRLIKEEDEMRRRKEINAFQETTATAFRRTCA*
>SPBDM4_v1_10003|ID:27157207| Addiction module toxin, RelE/StbE family [Uncultured spirochete bdmA 4]
MSSEKFTVLIYPTAEKDLFEIKEYFETTLKTSAIPLFEKFYASIDILETNPYIHPLLKDA
YLQQLGYRMIPIDNFLIFYIVKDGVVQIHWFLYGKRDYSHIL*
>SPBDM4_v1_10004|ID:27157208| Prevent-host-death family protein [Uncultured spirochete bdmA 4]
MPIIRPISDLRNNFASISEAVHAENEPVFLTKNGSGDMVVMSLEYYEQQLARIELYQKLN
EAREEIRNGATGKDARTVLQGFMHS*
>SPBDM4_v1_10005|ID:27157209| protein of unknown function [Uncultured spirochete bdmA 4]
VRVTCERALRQQVGAIVASYAAAPLFSLPLSYLGAVFLFYPEAPQKACQERESGSRGCSA
QKPIFEFLYIIRINHRNTGAFLTFKLGFSYLFSFQRRR*
>SPBDM4_v1_10006|ID:27157210| SMC domain protein [Uncultured spirochete bdmA 4]
MIESLRIIGYKNLTDFAISFKPGINIIIGDNDAGKTTILEALNLVLTGTLLGRSIFTEIS
PYIFNNEIVKDFVNKIGTPECIPPQIIIEAVFQDTEETTRYRGINNIERNDVPGISIIID
LDKEYAEEYVQYIKNKEEVHSVPTEFYGVSWSFFNGNPVGGRSNPIRSTYIDATSIRFSS
GSDRFINKVINDALTEKEKAQLSIEYRKLRETFSKIEGVKTINTSLSEKGREITKKDFLV
SIDISAKSGWDSVLIPYLDDIPFQFIGMGEQSKLKIGFALNATLEKTSVFLIEEPENHLS
FTNMANLIDQISEMCNERQVIISTHNAFVLNKLGLDNLKLLHNKNVVSLAELNPSTVTYF
KRLPGYDTLRMVLAKSVILVEGPTEELLIQRYFLDKYGTLPVNNGVDIISVRGLSFKRFL
EISKHLKIETKVITDNDGDIEAVKNKYADFDGIDSIRIIYPNDNAISTIEIAFCNCNTID
TLNTILEKKFKNIEEAKEYMLKNKTEWALKVFESSENIIYPEYIQNAI*
>SPBDM4_v1_10007|ID:27157211| conserved protein of unknown function [Uncultured spirochete bdmA 4]
MPSKNIITIAAAGSGKSTGIVREAISNTSKRILITTFTVEGTEELKRIFIEQNYAIPKNV
DIMSWMSFLLQECIRPYQLSTFNRTVSRPIFVQGRSTKYFKKTDARYFFGTEDCIFSDKL
ADFAILCNIKTSGKVIKRLESMYDLIFIDEFQDFAGYDFDLVIELLKTNISIRIVPDPRQ
GTFSTNESARNAKYKKAGVIKLIEKLEQNGLCEIVLNNWSFRCNQELCNFSDSIYPKYEK
TISKNFVKTDHDGVYVVRGNQLNEYIMKYNPVVLRYNKKSKWTDDKIKNFGAVKGRTYDR
ILILPNKPIINYINGQPFISPAKYYVAFTRAKYSVAILYDGELKHPMIKEWKT*
>SPBDM4_v1_10008|ID:27157212| CopG-like domain-containing protein DNA-binding [Uncultured spirochete bdmA 4]
MTSVRLPTDLERKLEMIARKKRTSKTNVIREALETLFTQEETEKDSYELGKEYFGKYGSG
DGSLSTTYKNKLKEKLNVKYHSH*
>SPBDM4_v1_10009|ID:27157213| conserved protein of unknown function [Uncultured spirochete bdmA 4]
MLNTILIDAGPLIALFDKDDKYHAQVKEFIKQGKYRFITTTAVITEVTHMLDFNVDVQID
FFEWMVKDGVVLQEIEQKDISRIIALTKKYRDRPMDFADATLVIAAEKTGIKNIISIDAD
FDIYRLPGKVKIENVFKKQ*
>SPBDM4_v1_10010|ID:27157214| protein of unknown function [Uncultured spirochete bdmA 4]
MSKQYTGLFADFYDLIQNTCYELPAYIEFMKTYGNTILELGSGTGRLTIPLAHAGARITG
LDINDDMIALCQRKINDAIKNNIKIIHGDMTNFHLSDSFDLVVAPCNAINHLLSLDELTK
MLLCVRDHLRPNGRLIIDNSLPRVDELLKSNGELEINDFVNKEKNTVIRDYYVANYDFVN
GLEYDHIKLEEYANNSLMRVEEIDETTAFYYPRELRYVLMSNDYTIEREIGSLKNGTKLT
KESKEMVFICSKN*
>SPBDM4_v1_10011|ID:27157215| Histidinol phosphate phosphatase HisJ family [Uncultured spirochete bdmA 4]
VIDCHVHIERGPYTREWINEFINQAKSREITDLYLLEHTHRFREFQECYEEVAHYSEYQN
DWYKRRSGVLLQSYINLVKNYKEKPHHGINIKWGLEVCYLPGIEKIIEQLKKNNTFDFYT
GSIHWLNGWGFDHKKEFWKGEIVNDVYKQYFDTEVRLIQSELFDYIAHPDSIKCFGHYPT
YELDEEYEKVIHVAKSTTIQFEQSCGLYNNYGHNKIGLEENFLKKLKENGIQIITASDAH
RPEDVGRNIEKALALINEA*

>SPBDM4_v1_10012|ID:27157216| protein of unknown function [Uncultured spirochete bdmA 4]
VYQLFIKWLSRMSMTNITCA*
>SPBDM4_v1_10013|ID:27157217| Adenosine monophosphate-protein transferase SoFic [Uncultured spirochete bdmA 4]
MYQFYKYRYQLLTYVLICRIIGFMSSFNPFQPYNTLPLLPPDLAKTEIIAILKQETNAAA
ALAELKGLANIIPNQAILINAIVLQEAKDSSEVENIITTRDELYKALSTTIKKYDPATKE
VMYYREALNEGFQKVSQRHLISLSDILHLQQTIVKNDAGIRKLPGTALVNDTTEEVIYTP
PDGEAILQTLMANFMEYLNNDERSLTKLAILHYQFESIHPFYDGNGRTGRIINILYLLLK
EYLDIPILYLSSYIIKNKSMYYKLLHDVTSEGAWEPWILFMLKGIEETSHETLLKIKHIK
ELLELTIEKVREQASKIYSKELVETLFVNPYCKVEFITKSVGVERKAASRYLHQLADIGV
LAVYKVGKENIFINSTLIDILKQ*
>SPBDM4_v1_10014|ID:27157218| transposase [Uncultured spirochete bdmA 4]
MPWRMESMQLQKKKFITLWETGRFTKTYLCEEFGISRPTGDAIIKRYQEVGWDALEEQPR
GHKSHPFTTTKTIEDAIINERKAHSNWGGRKIRVLLLRTYEEAEVPSETTVNNILKKHGL
TVPRKPPRRKLLKSEPKFDPQLPNQIMSADFKGKFRMGNGQYCNPLTIADSCSRFLFAIV
GLERPDTESSKPIFERVFREYGLPYQLHTDNGPPFGNAASLRRMTMLSVWIMELGITPVY
SDPASPQQNGRHERMHRDLKAEATRPPGSSMVAQQRKFNHFREEYNTIRPHEALGMKTPA
EVHTWSSREYPRRIRDWDYEKDITPKMVTVNGAIRWKDKGFAMISTALGGKYVGLHPVDD
GLWLVYYRHVALGYFCEQTMKVYELNDFDF*
>SPBDM4_v1_20001|ID:27157219| Methyltransferase type 11 (fragment) [Uncultured spirochete bdmA 4]
MTLAQHTKGTITGLDIFPDFIEKFNRNAEKLGLQNRVKGIVGSMDNLPFQNEKFDVIWSE
GAIANIGFQKGLNYWKGFLKKDGYIAVTYESWFTDERPTEIEKFWVDAVPEIDTIGHNIS
IMQKAGYSLVAAFTLPETCWTDTYFNPQKAIQKPFLEENTGNKAAEAFVGYMNHEAELYS
KYKPYYGYVFYIGKKV*
>SPBDM4_v1_20002|ID:27157220| protein of unknown function [Uncultured spirochete bdmA 4]
MTKYFKYYVFSILISILLVLGCKNITQTNPTSDTSTDNNTIFNITSMRRDTTTNVIEVRI
DTFPSTWGNWQMYLNGKEVSMEGNAGEIVVRPNAALDSAPTGLFIGTLPWLTGLDGIDFP
MEGALQFYIPGKGYSNKFYYNLKDQTFDDDSSADTTHNWINHYGDLVIENNETRSIENEK
YFQQGNIYVNDNSKLIIKNSQLMMGRGDVPTIHVYIIVAPNASVEIENSKIFPDSGLVCV
LNKGNVTITDSPTSIHYFDMSDGAHLTMINSEMVFTIGGLLQVTGGETTVVNSTIGALGL
KVPANAHLNVSNLSSGVYFDSWDVHSMIPEADYTLTLTKTQILKDDFTGDLEHGPYERGW
IFFLDPDAHVRIANSELRKVFIDVIDDNATFENLKVGIPSSLKYRDIELTNVTVMGQWPF
YIKDSKVTINNSNYLFLQPTGTSTVSVNDSHICEFIPREFSGTMEFRNGLWTTAGEIIGD
VAYHSMSNDFSIKGSLKIGDGIQDNLQWKDATVTREYDIHVVKKDGTPLSGVLVQMDGQN
VSTDSDGKAKISIQFDEYNFKQNMILKISLPNNTTTETEISFFSETPIKVIIE*
>SPBDM4_v1_20003|ID:27157221| protein of unknown function [Uncultured spirochete bdmA 4]
MNMDFWGFGFQYGVSAIFSLPFYIKVIREEDRMQRKNEIGAYNEALPKTSRITCA*
>SPBDM4_v1_20004|ID:27157222| putative toxin-antitoxin system, antitoxin component, ribbon-helix-helix domain protein [Uncultured spirochete bdmA 4]
METKLTLKLDKTVIQSAKQYAHHRNRSLSKLVEDYFRNLVASDTQQKSHFSPLVEELSGV
ISEKDLNNLSYTDYLEAKYE*
>SPBDM4_v1_20005|ID:27157223| conserved protein of unknown function [Uncultured spirochete bdmA 4]
MNNTLFIDADVILDLLCKREPFYVFAAEVFTLGDKGKLHLATTSLVYANVFYILRKVLGI
EKAKELLRKLRLIVHVIPIDEKMVDLALNSSFSDFEDGLQYFTARENDIGIILTRNIKDY
KEKELLIQTPEEFLKTLPIKNKA*
>SPBDM4_v1_20006|ID:27157224| Histidine phosphatase super family protein [Uncultured spirochete bdmA 4]
VTEIYFVRHAESDFLVTDEQRRPLTPKGVSDLEKLVKLFRTYTIATIYSSPYTRAIQTVK
PIAEVKGLMVIIKEDFKERISKSNWIDNTGDLTTFVEKMWEDHRKSIDGGESIIEVQERN
IKELRNVIKENENRKVIIGTHGTALASIMNYYDEKFTYTDFMHFIGKMPYVIKMVMDGDV
FISLEEIAI*
>SPBDM4_v1_20007|ID:27157225| Histidinol phosphate phosphatase HisJ family [Uncultured spirochete bdmA 4]
VIDCHVHIERGPYTREWINEFINQAKSREITDLYLLEHTHRFREFQECYEEVAHYSEYQN
DWYKRRSGVLLQSYINLVKNYKEKPHHGINIKWGLEVCYLPGIEKIIEQLKKNNTFDFYT
GSIHWLNGWGFDHKKEFWKGEIVNDVYKQYFDTEVRLIQSELFDYIAHPDSIKCFGHYPT

YELDEEYEKVIHVAKSTTIQFEQSCGLYNNYGHNKIGLEENFLKKLKENGIQIITASDAH
RPEDVGRNIEKALALINEA*
>SPBDM4_v1_20008|ID:27157226| protein of unknown function [Uncultured spirochete bdmA 4]
VYQLFIKWLSRMSMTNITCA*
>SPBDM4_v1_20009|ID:27157227| Adenosine monophosphate-protein transferase SoFic [Uncultured spirochete bdmA 4]
MYQFYKYRYQLLTYVLICRIIGFMSSFNPFQPYNTLPLLPPDLAKTEKIAILKQETNAAA
ALAELKGLANIIPNQAILINAIVLQEAKDSSEVENIITTRDELYKALSTTLKKYDPATKE
VMYYREALNEGFQKVSQRHLISLSDILHLQQTIVKNDAGIRKLPGTALVNDTTDEVIYTP
PSGEDIILTLMTNFVEYLNNDERSLTKLAILHYQFESIHPFYDGNGRTGRIINILYLLLK
EYLDIPILYLSSYIIKNKSMYYKLLHDVTSEGAWEPWILFMLKGIEETSHETILKIKHIK
ELLELTIEKVREQASKIYSKELVETLFVNPYCKVEFITKSVGVERKAASRYLHQLADIGV
LAVYKVGKENIFINSTLIDILKQ*
>SPBDM4_v1_20010|ID:27157228| transposase [Uncultured spirochete bdmA 4]
MPWRMESMQLQKKKFITLWETGRFTKTYLCEEFGISRPTGDAIIKRYQEVGWDALEEQPR
GHKSHPFTTTKTIEDAIINERKAHSNWGGRKIRVLLLRTYEEAEVPSETTVNNILKKHGL
TVPRKPPRRKLLKSEPKFDPQLPNQIMSADFKGKFRMGNGQYCNPLTIADSCSRFLFAIV
GLERPDTESSKPIFERVFREYGLPYQLHTDNGPPFGNAASLRRMTMLSVWIMELGITPVY
SDPASPQQNGRHERMHRDLKAEATRPPGSSMVAQQRKFNHFREEYNTIRPHEALGMKTPA
EVHTWSSREYPRRIRDWDYEKDITPKMVTVNGAIRWKDKGFAMISTALGGKYVGLHPVDD
GLWLVYYRHVALGYFCEQTMKVYELNDFDF*
>SPBDM4_v1_20011|ID:27157229| putative GCN5-related N-acetyltransferase [Uncultured spirochete bdmA 4]
MKIDLVDVRFEDIKKDIILNITSYSSLIDSYYEDHVIESKHYKISVDNEMYGYLSIFDEK
MLTQYRLLERYLPLANKVFEELINKNIFSEIYVSTSDKNLLTVALDYYKTIDVQDYVFQE
SQINQCDINFVLKKALKEDKELIVENSNNFFKFVDKNIDCGELYIGRYKEELVSFGIIEN
SKLYKSVASIGIFTIEKERGKNYGAMTIIRLVEECHRIKIEPIAGCFSKNKYSRNAAFKA
GMYSNTRLLKIIL*
>SPBDM4_v1_20012|ID:27157230| Bifunctional deaminase-reductase domain protein [Uncultured spirochete bdmA 4]
MANFVVYIACSLDGFIAKPDGNLDWLNTIPNDNNDDYGYSQFIERIDGIIMGKNTFEAVVG
FPEWPYTKPVFVVSNSLKSIPEEVKGKAEIVHGDIRKIIEDLKRRGIKNIYVDGGKTIQF
FLKEDLIDEMIITTVSKIIGDGIPLLGRIGIEKTFKVKKIERLNEYLVKTYYRREEI*
>SPBDM4_v1_20013|ID:27157231| Toxin-antitoxin system toxin component, PIN family [Uncultured spirochete bdmA 4]
MKVVIDTSVLYQALYSSSGASHAILQLVRTDDIKLALSIPVFEEYCDVLLRDTSLRAFGL
SHDDVQKFLDFIALIGVKTDIRYLLRPNLRDENDNIFLELAFASGSHCVITKNTRDFMYR
PELRFEEIDILTPAVFMENWRNQYA*
>SPBDM4_v1_20014|ID:27157232| Ribbon-helix-helix protein, CopG family [Uncultured spirochete bdmA 4]
MPKTNIMTIRVPEELKEQIENTALLQGVSINQFAVYAFTKELSELENSHYFKKYLHGKKK
TDILKGFDEVMQKVKKREVESWDRLEESNA*
>SPBDM4_v1_20015|ID:27157233| protein of unknown function [Uncultured spirochete bdmA 4]
VYQLFIKWLSRMSMTNITCA*
>SPBDM4_v1_20016|ID:27157234| Adenosine monophosphate-protein transferase SoFic [Uncultured spirochete bdmA 4]
MYQFYKYRYQLLTYVLICRIIGFMSSFNPFQPYNTLPLLPPDLAKTEIIAILKQETNAAA
ALAELKGLANIIPNQAILINAIVLQEAKDSSEVENIITTRDELYKALSTTIKKYDPATKE
VMYYREALNEGFQKVSQRHLISLSDILHLQQTIVKNDAGIRKLPGTALVNDTTEEVIYTP
PDGEAILQTLMANFMEYLNNDERSLTKLAILHYQFESIHPFYDGNGRTGRIINILYLLLK
EYLDIPILYLSSYIIKNKSMYYKLLHDVTSEGAWEPWILFMLKGIEETSHETLLKIKHIK
ELLELTIEKVREQASKIYSKELVETLFVNPYCKVEFITKSVGVERKAASRYLHQLADIGV
LAVYKVGKENIFINSTLIDILKQ*
>SPBDM4_v1_20017|ID:27157235| transposase [Uncultured spirochete bdmA 4]
MPWRMESMQLQKKKFITLWETGRFTKTYLCEEFGISRPTGDAIIKRYQEVGWDALEEQPR
GHKSHPFTTTKTIEDAIINERKAHSNWGGRKIRVLLLRTYEEAEVPSETTVNNILKKHGL
TVPRKPPRRKLLKSEPKFDPQLPNQIMSADFKGKFRMGNGQYCNPLTIADSCSRFLFAIV

GLERPDTESSKPIFERVFREYGLPYQLHTDNGPPFGNAASLRRMTMLSVWIMELGITPVY
SDPASPQQNGRHERMHRDLKAEATRPPGSSMVAQQRKFNHFREEYNTIRPHEALGMKTPA
EVHTWSSREYPRRIRDWDYEKDITPKMVTVNGAIRWKDKGFAMISTALGGKYVGLHPVDD
GLWLVYYRHVALGYFCEQTMKVYELNDFDF*
>SPBDM4_v1_30001|ID:27157236| CopG-like domain-containing protein DNA-binding [Uncultured spirochete bdmA 4]
MTSVRLPTDLERKLEMVARKKRTSKTNVIREALENLFTQEETEKDSYELGEEYFGKYGSG
DGSLSTTYKNKLKEKLHGKYHSH*
>SPBDM4_v1_30002|ID:27157237| conserved protein of unknown function [Uncultured spirochete bdmA 4]
MVNTILIDAGPLIALFDKDDKYHAQIKAFIKQGKYRFITTTAVITEVTHMLDFNVDVQID
FFEWMVKDGVVLHEIAQKDISRIIALTKKYRDRPMDFADATLVIAAENTGIKNIISIDAD
FDIYRLPGKVRIENVFKQQ*
>SPBDM4_v1_30003|ID:27157238| protein of unknown function [Uncultured spirochete bdmA 4]
MIDHFMEELKELYIKEDKEEETYKNEESTLSLVLENLANELKEDYTLTPLLVQYIWSEQR
GEHYTTACQVDLVVPSPS*
>SPBDM4_v1_30004|ID:27157239|aldA| putative aldehyde dehydrogenase AldA [Uncultured spirochete bdmA 4]
MRTFEEVKKFIDGSYKLFIGGKWVDAIDGGTFKVTCPFNGEVLATCAEGNAKDVDAAVKA
AWKAWPAWSKTSQAERAAILNKIADRIEANAEKIAWADSLEVGKTNNPFGWGNYFRYYAG
AALTYEGIANSTIDHHVNLVLNEPIGVVGEISAWNAPFDFACMKIPPALAAGNCIVYRPS
SHTPIGTLMLAQLAADLLPPGVLNVVTGPSPTCGQAILDHPGIHKVSFTGSTETGINVAT
AAAKKLIPATLELGGKSANIFFADCDFEKAVAGMSMGIFALAGEVCVAGSRIFVQEDFYD
KFVNAAVAAAKSIKVGPAWDPTTQMGAIIYEQQMQKILNYVEIGKKEGAKVLCGGHRLTG
GIYDKGFYMEPTLLEGTNDMRVAKEEILGPVGVIIKFKTEEEVIAMANDSTYGMGGGVWT
KDVKRAFRVAKDVRTGNMWVNTYLQIIPGYAFGGYKKSGIGREMHKSTLDHFSQKKSIVF
NTSDTELL*
>SPBDM4_v1_30005|ID:27157240| NADH oxidase [Uncultured spirochete bdmA 4]
MALQNLFSEGRIGTLTLKNRVVFPPMGTNFPEDEKVSKSLIDYHVARAKGGCGLNIVEIA
AVHPTSRGTRTLGIYDDKFIPGLKTLADAIHEAGGKAAVQIWHAGRQTNSAVTGLPIVSA
SPIPCPLCQEMPRELTLKEIKELVEAYGDAALRAKKAGFDAIELHGAHGYLIAQFMSPYS
NHRGDNYGGSLENRARFALEIIADVRKKVGNDYPVLYRLSSEERVKNGLTFEDTKKIAKL
IEKAGVDAIHVSVGVYETLHYTVPPIDLPVGFNVPGAAAVKSAVHIPVIAVDRINDPVLA
DQILRDGNADFIAMGRGQIADPELCNKAQRGDFDSIVKCIGCNQGCVDRLLMQGLSVSCL
RNPSTGRESEYAIKPADKRKKVLVIGGGAAGLEAATTLSRRGHEVILCEKNDSLGGQFFL
AGVAPGKKEMSDAALQMGRTAEKTGVAIRLGTELTEELLGEINPDEVVVATGSVPFIPEI
PGNDKPHVVTAIDVLKGKKTTGNSVAVIGGGLVGIEVAEYLREKGKKVTIVEMLDEVAKE
LGMLRRPFAFQYIKEHAIEVYTSATCTEIKDNSLVIEQNGKRKEIGGIDTVVMATGVKPY
NAVVDILKKAGCTYHTIGDASASGKALDAIWAGASIGRAI*
>SPBDM4_v1_30006|ID:27157241| conserved protein of unknown function [Uncultured spirochete bdmA 4]
MENLEEDSLYPTLLNALITGGLEPIAQAAFDYLKHPFILVDAEYNRLVQIPNEPIGDMIW
DSIWENDGVPLNLLQLFSDEHLLQKNYNSNKSFFANWGSLEKCPRILSNISVKNSIVGYT
ATLYLHDACTEHDLAAIDLISQVFAIEFQRQNLHVTEVHTMWALFLTNLFEGKIKNHKDL
AQWELYTKHSLNRDFCVVAAQPYKGTSEQSSLPYIRKSIESFYPLLHPVISDEYLYFLLS
DISSNHSYESELKSLVANLADYNLHIGVSYRFSDVLDIGIYKDQATLAVANGSSSADPSH
VYFYQDCALGEFFLGASEHMNKQNYLHPAIHELRDYDKEKNTDYLHTLSVYILSMCNASE
TLKQLHIHRNTLPYRLRAIEKIANLDLNDARTCAHLLCSFYLLQYAE*
>SPBDM4_v1_30007|ID:27157242| Predicted DNA-binding protein with the Helix-hairpin-helix motif [Uncultured spirochete bdmA 4]
MTTADKIGILADAAKYDASCSTSGSARKNMRGGVGTGAVGGICHAWAADGRCISLLKVLL
SNTCAYDCVYCVNRRSNDIRRTAFEPEELVRLIMDFYRRNYIEGAFLSSGVIGCPDGTME
RLIHIARTLRTREHFNGYIHIKIIPGTSERLVLEAARWADRASVNIELPSSASLVQIAPD
KKPEAIFGPMRALARASGFESRLLAEHPQAENRKLPDMTPRLASGQVGHIAGAPTPPTEI
ARSNYPNPPSPPGRDRSPLSLSVMEARKKRLRNPEADIIPAGQTTQLVIGASPESDATIL
ALAENLYLAFDVRRVYYSAFIPTGNDPRLPVVGKPPLAREHRLYQADWLFRFYGFKANEI
LDPAHPFLDMSLDPKSSWALRNPGIFPVEINTADYNVLLRVPGIGPKSASRIIAARRCGT

LRIASLAALGVVMRRARWFITVSGKLAAKEFDEISMRGWHLLEHPELLRRVLLDPAFRDE
PSSQLEFQWETD*
>SPBDM4_v1_30008|ID:27157243| conserved protein of unknown function [Uncultured spirochete bdmA 4]
MGYAEQHTVIRYDGSFAGLLCAAGEASTISLKQQEGLYFKNQIEEEELFEESFFIRTDLG
HARKLWKKVAAKGYATSLRTCFEAYCSDGPNKDNHTGRVLCAILREADSSERGPHGRAPA
PGNLATLDNLNDPDILSVATVAARCRNQAQKITGLIRFSELTDGLWYAAISPDCDVLPLI
APHFALRFAPCSFMIHDLQRSIAIVHEPGMVWHIIGGVSLPNSMNVSDLPCTEREFLTRE
NWVRYFSSVAIEARRNPRLQASFMPKKYWAGLPEMCHDKVSDSSRLPSKKEVP*
>SPBDM4_v1_30009|ID:27157244| conserved protein of unknown function [Uncultured spirochete bdmA 4]
MNSINAEMVFRSTHTLLSELVDGPAPGQGTWILDRDPSAGLAGTLDSLTAAQASRHIIPG
ESSIVGHANHLLFSLTLMNRWAAGENPFEGADWQGSWKTQTVDDVQWEDLRRKLREQGHA
WLKALDTARDWDEEDFTGALASIAHLAYHLGAMRNMLDTAKQTPKF*
>SPBDM4_v1_30010|ID:27157245| protein of unknown function [Uncultured spirochete bdmA 4]
MGKGYRQSCGEGKYLSKNSWILHDETVSLFPALQNEGTLALKPDFIMILVARTVESLPWE
RLIIGEPFAVRSSGYVDEGDVYPGKGGLRT*
>SPBDM4_v1_30011|ID:27157246| ADP-ribosylation/Crystallin J1 [Uncultured spirochete bdmA 4]
MQNSKERNTFRKRLDESVFRDKVLGCWLGKNAGGTLGEPWEKKFGVDEMLDVNWYSNIPE
GGIPNDDLELQLIWFQALKERGPGITARDLAEYWLDCVMYNFDEYGLSKANLKRGLVPPL
SGWHNNWFRDCMGSPIRSEIWACVAPGEPRIAARYAFEDALCDHAGGESVFGEVFNAVLE
SCAFFESDKYHLIELGLASIPEGCLTSRSIRDAWEMHKKGVDWRDARDVLKDRYSIPLAQ
YSPVNMGFQIIGLLYGEDFGDAICRAVDCGWDTDCTAATVGAILGIIEGASCLPEKWMKP
LGYTISTNLRTGGIRNLRAPTDINELTDQVCAEAKRVLKFWGAEVIIENSSGETGDVASF
SGMSEFKIDRVAPYEPNVATWDLKTVTVSLRYLDSAAILGDRPSPLELEIHNPHPEAISV
EVGISLPDAWCVDSPARQGFLVPAYGNVKVAYNINASSKDIADSNRGYFTLSVKDRLALC
AVPLVLLGGSKWLVSPHFEGKTLEDDCGVRESVVPSAPPEGWSTYWRSGNDLSPEPFYDG
RAGSVFFFNSIWSPEETEVILGVSNTGRMRLWVNGELRHTTVQVTDLRPNLGNGGGDGSN
YCNVTLRSGWNDILIKLERGEKPLEAHVTLGKPDKKHPKNIGQTCLGLRRACFSWENGER
L*
>SPBDM4_v1_30012|ID:27157247| conserved protein of unknown function [Uncultured spirochete bdmA 4]
MKNYSVRGLEVHDWSHVWNFKTMRRYMKFMVENGMNTLVLHHVGVLDLITFPAKFLGGGA
PTDSIFEVYNQIDQNIYRYALRENLNLYRRDFLKQLIKEARQCGIDVYIEDKELWFSDFI
LNYKPELMKNGVLCPSDPFWWEEFLPAKYEELFVALPDLAGVVISFGTGESRLAIANMHS
CGCDLCQKMTPAQWHTNMILGTYGPFKKRGKRLVVRDFIYTKEEQEQFAEALQVIPQEVV
LSLKNTPHDFYPTFPDNPLIGRVGERPQWIEYDVNGQYFGWGVVPSIMFDDIEHRLAYGL
EHRVSGFFMRTDWEGVQDLSCFDGPNLLNLYAAAILGKNPTADRREIVLRWLKGESMLDA
VSTPMQINATVDWLLSVLEPTWPIMCGAVYVNGTVFSDNSLFHVSFGQPQWVAETHHSLK
NWFADAEDALGLTAENVQSILGEKESASRSMDEILNVLATGRHALTEAAYNDLLSRFELM
KAYVEGFQLETKLWVFGRLWAENRLDDALAGLDRPVKVCLEEALVKIKEYAASVKDMPQL
ASYPACVLLNVERMECFIRDTEKKIMATRPLV*
>SPBDM4_v1_30013|ID:27157248| Binding-protein-dependent transport systems inner membrane component [Uncultured spirochete bdmA 4]
MWKRFKKHKVALVGLILLFLLLLLVVFAEFFAPYGLNETSSYSYASPTQIRFTDAKGKFS
LRPFVYDAQPSFDLSTGQTSWKVDTTSPHYLRLFIRGSSYSILGLKLDIHLFGAAGGHVF
IMGTDALGRDVFSRLLYGGRVSLAIAFITALFALIAGSLAGIVSGFFGGVADMVIQRVIE
LFMTIPNIPLGLALAAFLPPDLNPALLIAAISMVIALVMWANIARQVRGKTLPLRDAVHV
QAAIALGASTPRILLKHIFPAIYSHLVVVVTLAVPQAMLAEAGLSFLGFGVRPPLTSWGA
LLQDAQNFRTISLYPWVAVPGLAIAITVLLLNFVGDGLRDATDPYLN*
>SPBDM4_v1_30014|ID:27157249| ABC-type dipeptide/oligopeptide/nickel transport system, permease component [Uncultured spirochete bdmA 4]
MTGYIIRRIGIAFVTLFFSTILIFAIIKLPPGDFVSYAISQSGGEGGLNSIADRMRQSYG
LDRPVIEQYGRWIGGLVRGDLGYSFLYQQPVLKIVWEQMGWTVLITGLSMIFSWSIGSAI
GIFSALKKYSFWDYFFTAFGFLGLSIPSFFLALIIIYVCISAGSGVTGGLFSPEYVVAPW
SWARFWDLLRHIWIPVVAIGTAQMAQVIRIMRGNLLDVMNQPFIKTARAKGLSERKVIFK
HAVRIAMNPLISLAGLSAPALISGIVVTAVVLNLPVIGPTFIVALKSQDMYLAGGYLLLM

VVLLLIGNILADILLAWSDPRIRFD*
>SPBDM4_v1_30015|ID:27157250| putative Extracellular solute-binding protein family 5 [Uncultured spirochete bdmA 4]
MKFRRVVIALLVMSVLAVMTAAAAPSGTYKEAPQLAAMVQSGKLPPVESRLPASPVVVKP
LDSVGKYGGTIRKVYTGVNDWWNLALFGVRAEPLLAADLEGNIAPNLLESYEYLSGGNVL
RLHVRKGIKWSDGVPFTVDDIVYDLQTRGNKNMPLESAGLADKVVADKIVKIDDYTADLP
LKEKYPLEFTIAYDPTVSPKHYLIKFDPRYDSTKIWQDLAMAWSPSRNSAALVNLPVLSA
WKVIEYVENLRIVAERNPYYWKVDTSGQQLPYVDRVVFNYVASTDTIPAMIKAGQLDFQA
RHLSLADFPFYKQNESMGGYRTAVLPNTNFGPAIHLNYADKNLDLRNLFRTKEFRMALSY
GIDRQAISNTLFFGQAKPWGFSPLEGSPANPGNPYSTMYTQYDPSAAAKLLDDLGLRDTN
GDGFRELNGKPLSIIVDMDKGGGAGPVQVVELIASQWQKIGIRAIANTIDRSLILARWNE
NSHDAFAWNVNGGIDPLQFTFAWSTTAAPDFMWGNVGVPLNEWQTSGGKSGIEPPAFIKD
MNQLVLEAQQELNANKRNELARQITKIASENLYKIPTTTLVSVGIVSTKLANVPSTWTDG
ISVLSPRNTQPWQFYYK*
>SPBDM4_v1_30016|ID:27157251| protein of unknown function [Uncultured spirochete bdmA 4]
MGIISRKWYRLQISEKWYNAYLNLSSFIFGFTSSPQRDSKYRNHEILNIYNYKIIKYCIK
L*
>SPBDM4_v1_30017|ID:27157252|dppD| dipeptide transporter ; ATP-binding component of ABC superfamily [Uncultured spirochete bdmA 4]
MERLLEVDQLKTYYFTDRGIVKAVDGFSFSVERGQCLGIVGESGCGKSQTALSIMGLISK
PQGRIVGGKILYRDKNARTTDLTTLAPTGDEYRAIRGKEIAMIFQEPMSSLSPVYTIGDQ
IVEALTEHTALPKKDAARRAVELLKTVGIPAAEERVHDYPHQLSGGMCQRAMIAMALSCE
PRLLIADEPTTALDVTIQLQILRLLKELQKRLSMGLVMITHDLGVIAEIADDVIVMYLGK
VVEKGPVRKILREPKHPYTQGLLQSRPSGAAGKVRLNPIKGVVPSAIDPPFGCPFSTRCP
KAFDQCGLPPPFAVWGEDHEAACWLYENLM*
>SPBDM4_v1_30018|ID:27157253|oppF| oligopeptide transporter subunit ; ATP-binding component of ABC superfamily [Uncultured spirochete bdmA 4]
MDNKEDKLLLEVENLRKYFPVKHGVFRKTEGWLKAVDGVSFKLYEGETLGLVGESGCGKS
TTGRLILRLTKPEPDSRILFRDNNELIDITKVAPQDLKHLRTRMQIIFQDPYSSLNPWMK
VGEIVAEPLVIHKIMAGRELSMKVDALLEMVGLDASYTNRYPHEFSGGQRQRIGIARALA
LDPTLVVCDEPVSSLDVSVQAQVINLLVDLQERLHLSYIFIAHDLGVVRYISTRVAVMYL
GRIVETGPTEEVFHSPAHPYTEALLSSMLSTDPDSSQKRLQLEGDVPSPINMPSGCPFHP
RCRYYQQNKWPECVRDIPASTSVDSDVHRARCHFTTRLNLHGVGDEDSVKIGVKNGLNT*
>SPBDM4_v1_30019|ID:27157254| putative HTH-type transcriptional regulator DegA [Uncultured spirochete bdmA 4]
MASIHDVAHLAGVSVATVSRVLNSSGSVTDATRAKVRSAMQRLGYTPNPSARTLRNNRTG
LLALITPEIINPYFAAVASGVQDMCRKSKYQLILCNTGGDESIEISYLDLLSNKQVDGII
IAPPGTHTNPKSDTRIQNLLSKNYPIVMIGKRFDSEDISCDIVTTNTAIGTREAMRHILD
GGHSRIAYLGGPNPSIAKTRLATFRSSLSANGLIIDENLIFQTNLTLEDGYNICKKLLDA
AHRPTALFAVNDMVAIGAMIALQESGVEIPTEMIVVGFDDIPLASMFRPSLSTVVQPKYD
LGHIAAERLIARIEGLVDKFETISLPTHLVIRESSMSQQKGMMPKTVL*
>SPBDM4_v1_30020|ID:27157255| Anaerobic sulfatase-maturating enzyme [Uncultured spirochete bdmA 4]
MNTAFFLIKPVSSLCNLACRYCFYHDLAEHRLTRDYGLMDSRTSDLLIERAFDLGVGALT
FIFQGGEPTLAGLEYFEHFVEKCRACAESDSHHRPQINFSIQTNGVSLNAEWARFFKREG
FLVGASVDGPRIIHDTYRTYRDGSGTWAQVMHGITVLREEGVPVNALCVVNNLTADNAGL
VYHFLRRRGFDWIQFIPCLAPLAEEPAAPYEALNPEAYAHFLKEIFDLRYGEWLRGSGQS
VRWFDNLVEMAAGLPPESCGMLGCCPMNFTVEADGSVYPCDFYAADEWCLGNIHELSFQV
MQEGITAHDFVESSRLIDPVCKTCSAFFLCRGGCRRDREPFSDGKPRLNRYCPAFKEFFQ
YAGARIMRMAAALKETI*
>SPBDM4_v1_30021|ID:27157256| N-acetylgalactosamine 6-sulfate sulfatase (GALNS) [Uncultured spirochete bdmA 4]
MSSSSDGEGKSTPQPNVIFILCDDLGYGDPGCYGGASIPTPNIDRLAEQSMMFTQCYAGA
PVCAPSRCVLMTGKHTGHCTVRDNFAWAEGLPPEGRVSLRRDDITVAEDLKRAGYATGMT
GKWGIGGAGTEGIPNLKGFDEWFGYLNQRHAHSYFPTYLWKNRERVEFSMSGEEQRGPYS

HDLIVNYALDFMTRNAGSKFFLYLPWCLPHEPYEIPTRYAYPGNDTWSGEEKAYASMVRK
IDADLGRIMDLLEELHIADNTLLFFGSDHGAARRWEGVFDSCGALRGQKAYLYEGGVRTP
LIVRWPGTIPAGTVCHTPWWYADVRPTIAEVTGSGTPTGCDGLSILPLLRGNTDSLDSRY
RERLFYWEGYTHGFEQAVRWKNWKALRKTSADGVETSFELYDLASDLAETTNLVEASPEL
AAELRHQMILSRENTRFWPISFK*
>SPBDM4_v1_30022|ID:27157257| membrane protein of unknown function [Uncultured spirochete bdmA 4]
MDEKDALKDIADIKALMRGMTTHVNRNAGWFFIVWGIIWIIGFMTTQVIGNDARFVWLAL
NILGIGLSFYLLRRFFGKRGGQMIPGLGRRIFLIFAGTAIFGILVGVLFGISSAEDITLL
VVLLSGLCYFMAGIVGPAKNLYLGLLLWAASLVGRLAFPSYLPLIVAIVGGATFLGLGIS
FLLRREL*
>SPBDM4_v1_30023|ID:27157258| Transcriptional regulator [Uncultured spirochete bdmA 4]
MNDDSLLIHQPTRLRIMAALSTLEEGTKVDFTFLSDELEVTEGNLSSHIRKLEEAGLLNV
EKVFVDRKPKTWLALTERGRKAFAEYVRELERIVRSDARTEP*
>SPBDM4_v1_30024|ID:27157259| ABC transporter related protein [Uncultured spirochete bdmA 4]
MESVEKVIELAGVKKKFGERQALNGISLSVGRGNIFGYLGPNGAGKTTTIRILLDLLRAD
TGVVEVLGSPADHLETRRRIGFLLDADGLYDQLSAIENLEFYAALYGCKPRRQSIMKLLE
SVGLADRAEDRAGGYSKGMRRRLALARALVHDPELLILDEPMSGIDPSGQMELRAIIKNL
VKERGKTILFSSHDLDEVERLCNRIALIDKGEIRVAGELGQLLGTGNQGRIIISTSVPVE
PTLLSEMERRLWLKVNGSGPEGLELSLPADVVSFLAARGVGIEGVRAKHTSLEELYAGIL
KEREA*
>SPBDM4_v1_30025|ID:27157260| membrane protein of unknown function [Uncultured spirochete bdmA 4]
MSARNALTKMALSELVRSRSYRVAFAVIALFLVGILHFIGVRLLLNPLVERETLDRGLLL
AAASGLVYCTVLICAGLFLNISSTQPLVRAKASGSIESLLAAPVDARDLWRALSISSILP
GIVAGWAGGLVSAIVYELLYLAPRGMTLASPWIILNSFILLPAMYAAIAFLVHAIGLQGR
ATSGAVIAQIFFPVYTSLVMNLGGRDILAVPRADLAAAQLGLSLIAVGAIALSRRGLTKE
RIVLSCRQ*
>SPBDM4_v1_30026|ID:27157261| membrane protein of unknown function [Uncultured spirochete bdmA 4]
MQAVSPWWRIAAVYGRDLGEIRKSPASKAILIVFAALSVAVAIGAKLLLDQVLAAGAGAD
ASVADEIVRKVVGAFLLENSFYGITMLPFSLLVWVFAGAIVMREKLTGNLETLLATPLSL
PEIWLGKTLALSAAATAIGWLSGILEVIAAHCIVAATLSRFVFLLSTPAIVAALLLNPLF
FSGMCALILIIALAKDAEASLLPSFLIGMGAMIGLPVAIGTGAIKIGSWIFCLYQTGASM
VLWGAVLAILFTTKKEEVVLSARK*
>SPBDM4_v1_30027|ID:27157262| ABC-type sulfate/molybdate transport systems, ATPase component [Uncultured spirochete bdmA 4]
VSLEASLYLRVASPDFHKPFVLETRYSVAQGEILGLLGPSGSGKSMSLKCIAGIVRPDRG
HVRLNERILFDSEREIDIPSRERRVGYLFQSYALFPHMNVIKNVAIGIGRRGEESYPEWK
RRAGRSARQYMEMLHIEHLAFRYPRQISGGQQQRVALARLFASQPEAILLDEPFSALDTE
LKDAIGDELKKTLLSYGCPVIFVSHNDEEVGRFCSRTIRIRDGRLESPCAEPQKDPQKGL
AVTQ*
>SPBDM4_v1_30028|ID:27157263| protein of unknown function [Uncultured spirochete bdmA 4]
LRDSKPLLRILLGLRARRFESAIPYTDRPRTEAPHFLVVVRDEDDGAAIGEQRLLQFVAD
GILELSIQGGKRFIQQDGLRLGREKAGQGNALLLAARNLPGISKSQMFYVEHLHVLPGRS
AGAALPLGVRFLTAATDTDGHILDYVHVRKEGVRLKEIPNPSFAGRDIYLSLAVE*
>SPBDM4_v1_30029|ID:27157264|modB| molybdate transporter subunit ; membrane component of ABC superfamily [Uncultured spirochete bdmA 4]
MFDLSPLYISLRAAALSSAIVFVLGTVVARLCYTLRGRKAAIVDTILTLPLVLPPTVLGF
FLLVLFGRNGPFGVLLARMGQKAVIFSWKATVIAAAVVSFPLMYRSARGAFEQIDQELLW
AGRTLGMAEWKLFLHIMVPEAWPGLMAGLALSFSRSLGEFGATLMIAGNIPGKTQTIPMA
IYFATAGGDMQTAWIWVGIIVAISCVSLTLTTHFDSRRRG*
>SPBDM4_v1_30030|ID:27157265|yvgL| putative ABC transporter substrate-binding lipoprotein YvgL [Uncultured spirochete bdmA 4]
MKKRFFVCLFVLLSLVAGFAQGASKQKTELLISAAASLTDCMNELKAVYMAKNPSIIIRC
NYGSSGALQQQIEQGAPADLFFSAGLKQMKALADKGLMDTSTVRNILENHVVLVVPKNGI
KLSSFDDLRKPSVTKIGIGDPKSVPAGQYADQVFKNLGLSDAISGKLVLAKDVREVLFWV

ETGNVDAGVVYSTDAQISDKVIVSAIAPENSHEKIVYPVGVVKDSAHPKEAKAFEDFLSS
DEAVKIFAKYGFAVI*
>SPBDM4_v1_30031|ID:27157266| Molybdenum cofactor synthesis domain protein [Uncultured spirochete bdmA 4]
VQKGTIKAICASGGKGTAKHPVEEATLKANWGIEGDAHAGTWHRQISLLSASTVDAFNRQ
GADVADGDFGENILAYGLDFPAFPVGTVLVCGEVVLRMTQIGKVCHSGCDIQKRMGKCIM
PTEGTFARVLHGGILRPGMPIEAYTAQRVFILCASDKGYAGERNDESTPALQHLVTGQGY
EVVGTALLPDDREQLSALMARVCDSYAADLLLTTGGTGLSLRDVTPEATIDIAERMVPGL
AELMRLRSLSVTARASLSRAVCATRAQTLIVNLPGSPKAAVENLQTILEPLPHGLAILQG
TQGECAVDSHI*
>SPBDM4_v1_30032|ID:27157267| Molybdenum cofactor biosynthesis enzyme [Uncultured spirochete bdmA 4]
MTDGEAGIVGIRDSFGRTHTYLRLSVTEACNYRCQYCGPGVVHRGLSDDHVVLLCSLFRS
MGIRTLRLTGGEPTVRPGLIPLIVRLSSLGFERLALTTNGTSLVRDARALRKAGIQSVNV
SLDAVDADLYASLTGGFPVRPVLEGIGAALSEGLAVKLNAVLLADTYRSQVRQLMDFAGK
FGIPLRFIELMPFGDGARCEGVSTATLVAFLGEEYGEEASVCPPQGLGANSVSKGDEPWG
SGPAEYRRFGGVDVGLIGALTSCFCSRCQRLRLTNEGWLKTCLYHSDHLNLSELLDRGCS
QDEMRGRIADFVLTKRLRHAFQSGPVEWPLSSVGG*
>SPBDM4_v1_30033|ID:27157268|moaC| molybdopterin biosynthesis, protein C [Uncultured spirochete bdmA 4]
MNDAMTHIDENGRAIMVDVSAKEDTKRRAIASGRIFMNRQAFGAIAGGTAPKGDVLAAAR
IAGIMAAKQTPSLIPLCHTLLLTSVQADFRLLPDECAVEAVCTVTSTGKTGVEMEALTGA
TVALLTIYDFCKAIDRSMHVEDVHLQFKDGGKSGRYDRR*
>SPBDM4_v1_30034|ID:27157269| Molybdopterin biosynthesis enzyme [Uncultured spirochete bdmA 4]
VKLIRTQDAVGHVLCHDMTQIIVGVTKDARFRKGHIVREEDIPILLSMGKENLYVWEKQE
GMLHENEAAEILCNATKNDHMERSDVKEGKIELRATCEGVLQIDLERLLRINSLGEVMIA
TRHNFSGVRKGDKLAGMRVIPLVIDAAKMDRVTAIAGTSNPLLSIRPYVIRRCTVLVTGC
EVKKGIVEDTFSPVVEQKLAEVGVSVSAKILTGDDEVYISSCIKQAVADGEEMVLCTGGM
SVDPDDRTPKAIRDSGARIVSYGAPVLPGAMFLVSYLGDVPVLGLPGCVMYAERTIFDIL
LPRFVARIPVTAYDIASLGNGGLCLQCRPCTFPNCGFGNGGI*
>SPBDM4_v1_30035|ID:27157270| Molybdenum-binding protein [Uncultured spirochete bdmA 4]
MAFSFYVRIPFTSDGAFFGPGVNELLHNIDVANSLSAAANMMGMSYSKGWKIIHVAEKKL
GYPLTVKNIGGVDGGGSSLTKEGHSFMERYDAFVAESRAAVQVCFERYFPEAQQ*
>SPBDM4_v1_30036|ID:27157271|sodA| superoxide dismutase, Mn [Uncultured spirochete bdmA 4]
MSFTVPELRYPFDALEPYIDAETMRIHHDKHHGAYVANLNKAVDGTEYASWSIEAIARDW
GKLPDTIRTAVRNNGGGHYNHSLFWELIGPGAGGKPTGALASAIDRDLGGFDAFRSEFTK
TALARFGSGWGWLSLGPDKKLIVESTPNQDNPLAEGHIPLLGVDVWEHAYYLKYQNRRAD
YLEAFYNVIDWKAVAARFEKAMS*
>SPBDM4_v1_30037|ID:27157272| protein of unknown function [Uncultured spirochete bdmA 4]
MILYHMNIGFPFLSEKSKICIDSKEVVSRNKKFEDEAEKWQELLAPTEGYEERVFYHRVR
PDSAGKACIELRNDENPHCGYGLRIRYSTEVLDHFVECLAFCAFSMAQEVCTSDWLLLIF
L*
>SPBDM4_v1_30038|ID:27157273|vapC| Ribonuclease VapC [Uncultured spirochete bdmA 4]
MIIADTSVWIDYVRGINAPHTDLLDNELLHDRVATGDIIIVEFLQGFREEKDIKVAKQIM
DRLEYYDFLGKGMALITAANYRKLREKGVTVRKTIDVIIGTFCIEKGFELLHNDRDFDPM
EKYLGLKVKR*
>SPBDM4_v1_30039|ID:27157274| conserved protein of unknown function [Uncultured spirochete bdmA 4]
VRTNIVIDDNLMNKAKKLSGYKTKKETIEEALKLLVAQREQSEIRKFRGKLNWEGNLEEM
RLDK*
>SPBDM4_v1_30040|ID:27157275| protein of unknown function [Uncultured spirochete bdmA 4]
VDNADTRLEFGNQFAILYGDRVPL*
>SPBDM4_v1_30041|ID:27157276| Dual specificity protein phosphatase (fragment) [Uncultured spirochete bdmA 4]
MCACPGASGRNLREDLAAIESWGAGILISLIEEYEYRFAGVERMPELVPKYIRHLRLPIA
DASIPTMDWERTWLREGPSVREALRRGGRICIHCMGGFGRTGLLAARILVEFGEEPDLAI
RAVRIARPGAIETSEQEEYVRNIASQIL*
>SPBDM4_v1_30042|ID:27157277|guaB| IMP dehydrogenase [Uncultured spirochete bdmA 4]
MGFETLIAEGTGLTFDDVLIVPGYSEILPSEVDISAELVPGMRLKSPILSAAMDTVTDSQ

LGIALARIGGIGIIHRNMSPEAQAAEVYKVKRSESGMISDPVWLPETATLADAEHLMETY
RISGIPIVDPQNGRLVGIITNRDRRFCGPEDMQKPVSEFMTSKNLVTAPEGTTVEGAKAI
LRRHKIEKLPLVDRDGRLKGLITIKDIVKKEEYPSAAVDGRGRLLSGAAIGVGPDLEERV
NLLLERGVDVLVIDTAHGHTKKVIEAIHRVKAVAPDTPVIAGNVVTAEGTRALIEAGASA
IKVGVGAGSICTTRIISGAGMPQLSAIYECAKAARSYGVPVIADGGIRYSGDIVKAIAVG
AETVMLGGLLAGLEEAPGELVLYEGRQFKSYRGMGSVGALQGYGRDRYGSGQSNGKLVPE
GVEGMVPYRGKLSDYLAQMLGGLRSGMGYAGARTLAELRSARMVRITAAAYAESHPHSIV
ITKEAPNYQKRD*
>SPBDM4_v1_30043|ID:27157278|purH| Bifunctional purine biosynthesis protein PurH [Includes: Phosphoribosylaminoimidazolecarboxamide formyltransferase ; IMP cyclohydrolase] [Uncultured spirochete bdmA 4]
MPLALISVYDKTGLVPFAQRLARKGWRFLASGGTAAALRGAGIESVDVAAYTGSPELLGG
RVKTLHPAFHAGILARPEAADMEELRAHGFEPIDLVVVNLYPFAETVRKMKERAGAAAAG
AVTGNASVPDGAVFSGRGAAAVGGNLRAGPASLDAGSEAEIIEQIDIGGVALIRAAAKNY
ARVCVLCEPGDYERVAAEAEEGAVQPATRRALAAKAFARTAAYDAAIAAWFGQQDAAEAA
VSDGEALRGASSFEAALDDVAGSPAPAGATPGAPQNSTSTGVSEGVSLFLAGPPGTKFSF
GGRIERSLRYGENPHQKAFFVLPEAEGGPLGGRVLGGKELSYNNLLDLDAAWRAVLSFEK
PAAVIVKHLSPCGAAEAETLRAAYDAALACDPVSAFGSIVALNRPLDVDTAIALKELFLE
CVAAPAIGTEVRDILKAKKNLRLVEADLAVFGRQAREFRTAAGGLLVQEPDCGDPPPAEW
RAVSARQPSAAEMEALRFAWKLVQHVKSNAIVLAVGCAAVGIGGGQTNRVDAVRQACERA
GERAVGAVMASDAYFPFADGIEAAAKAGVTAVVQPGGSVRDAEVLAAADRLGLAVVYTGV
RHFRH*
>SPBDM4_v1_30044|ID:27157279| Phosphoribosylglycinamide formyltransferase [Uncultured spirochete bdmA 4]
MSSPLRIIVLASGEGSNFQALIDAIETHDIESLRIEALICDVPGARCIDRAVQHAIPAYI
LPAPAGARRGSPERITYDERLAAIAKSFGAELILLLGWMRLLSQAFLGHFPGKVINLHPA
LPGAFPGTHAIERAFEAFQRGNIERTGVMLHLVPDEGVDSGPVLRIAEVPIYKEDTIESL
EKRVHDTEHREVVALVRDLALNRGIFKEKESNYASRVDIGV*
>SPBDM4_v1_30045|ID:27157280|purD| Phosphoribosylamine--glycine ligase/phosphoribosylformylglycinamidine cyclo-ligase [Uncultured spirochete bdmA 4]
MTVLLVGSGAREHALAMAIGASSECERLVIVPGNAGMAEVGDIAEVALDDIDGLCALAER
ISADLVVIGPEAPLAAGLADRLRGREMLCFGPSAAAARIESSKAFSKSFMQRHGIPTAAG
RSFTDAGSAKKWAREFGRPVVVKASALAAGKGVIVPESAHQTEEAIETLLVPGGEIVLEE
RLEGEELSLIALCDGKNYAVLPSARDHKRLLEGDRGPNTGGMGAYAPACSMEEAEALARL
VIAPALGGLAEEGSPFVGALYAGLMLTEGGPRVLEYNARFGDPETQAILPLFASDLLTTL
AACARGDIGQALPRFYRQSVACVVLASEGYPVHPKTGRAIRIGALPEKAFCLHAGTRLES
GQIVSAGGRVLSVVGMGATRAEALGTAYAAVANIHFEGMQYRRDIGARAARVAEFVRTSV
AAVSAPAGAGAPGTAAPQLAPSAYARAGVDIDAGNKAVELMKKAVRSTYGSEVIAGIGAF
GGQYDASSFKNLGSPVLVASTDGVGTKTSLALKFGRLKGLGQDMVNHSIDDILVQGARPL
FFMDYIAADTLDPEKVAEIVDGMAEACRDANCALLGGETAEMPGTYRHGEMDIAGTIVGL
ADRQSLLPRPDIAEGDILIGLPSSGLHTNGYSLARAITAEMDLERVQPELGEALADALLR
PHRSYLPILRNALDATPGPVKALAHITGGGLIENISRVLPPNLDARIHAESWKWPPLFSL
LQRWGNISTEEMRRVFNLGIGMVAIVSAGERARFLSMLAEPASVIGELVPGCGKVRFV*
>SPBDM4_v1_30046|ID:27157281|purF| Amidophosphoribosyltransferase [Uncultured spirochete bdmA 4]
MTGKLHEACGVVGVVAEEAARLAFFGLFALQHRGQESAGIATLDTGSIHLHKDVGLVSQV
FRGENFSTLAGNIAIGHTRYSTTGKSSARNAQPFLIDTQFGPLALGHNGNIANAPALRKQ
LLGRGLGLMTGSDSELLAMMLAGTPGKTWTERIAQAMKFWIGAYSLVLLTREGVFAVRDP
WGYRPLAWGRIDSGGWAVASETSALRVMGCSDFEEIPPGTILHFNERDPVECTRVEIDAP
HAACSFEYVYFSRPDSVWNGKNIHAVRRRLGELLAEEAPASADLVIPVPDSSIAAAIGYA
QRSGIPFGEGLVKNRYIGRTFIEPTKALRRQGVALKFSPLKETLEGARIVLVDDSIVRGT
TTAPIVALCRNAGAREVHLRIASPRILHPCYMGVDMGTEHDLIAVGREPEEIAKMVGADS
LAYLSIEGFSRAIGIDGVCRACFDGNYHIPVDENFTKNCFEGFGKGTV*
>SPBDM4_v1_30047|ID:27157282|purE| N5-carboxyaminoimidazole ribonucleotide mutase [Uncultured spirochete bdmA 4]
MSRGRVVIFAGSPADKTHVEAIARALGEFGIPSTVRIASAHKTPKRLLEIIEGYDAEPIR
TVYVTVAGRSNALSGLVDAATQFPVVVCPPPSESWAAYDIWSSLRMPSGVAPAVVLDPAN

AALFAAKLLALGEPDLRERIRQFQERNANRLVEEDEKIAGAARQAENSVTRELRT*
>SPBDM4_v1_30048|ID:27157283| protein of unknown function [Uncultured spirochete bdmA 4]
VPASYPAGPRVLKALEKARARGAGL*
>SPBDM4_v1_30049|ID:27157284| Phosphoribosylaminoimidazole-succinocarboxamide synthase (fragment) [Uncultured spirochete bdmA 4]
MRFIVTTDRLSAFDRVLSAVPFKGQVLNELSAFWFRATADIVAHHLVSVPDPNCAIVKEA
SPLPIEVIVRGYITGVTSTALWRRYELGERTIYGQHFPEGMRKNERLPHPIMTPTTKGGP
TGHDERLEPREVVEKGYLGAAIWNRVQDAAFALFARGTERAAQAGLILVDTKYEFGLAAD
GSLLLIDEVHTPDSSRFWLASSYGERFEAGLEPESRDKEFVRLFYAEKGYRGDGEPPELS
DSVWA*
>SPBDM4_v1_30050|ID:27157285|purQ| Phosphoribosylformylglycinamidine synthase 1 [Uncultured spirochete bdmA 4]
MKALILKAPGTNRDFDVRGALERAGGAAEILPLSELRSAPGSMRGYGMLVIPGGFSYGDA
LGAGKLFALDLSQFFADEVRHFVETGKPVLGICNGFQALVKAGILPGKKFEAHKFTLARN
AGGNFECRWVRLVAPHSRSIWTQGIESVFCPVAHGEGRFAADFSDAGALLQTEGCIALQY
ADAQGAPAQGNYPENPNGSIADIAGICNPEGNVLGLMPHPENAVFEWQYASLPGRSGDGA
LRLFENGLRYAAS*
>SPBDM4_v1_30051|ID:27157286|purL| Phosphoribosylformylglycinamidine synthase 2 [Uncultured spirochete bdmA 4]
MAGRNILYRFEVWNRGAGGPRIDALRADVRALGIGILRSARKIALYFVEGELAPEELSLL
GRFLFSDPVEEVFEWREVGRGGRRGQPDAALHEGEGLAVDREAASERIVEICRKPGVTDP
VAIEVVRAAHELGISGIKRAATGVRWELEADWLDDAALSKLTRVLLANAVIERWEFGEIE
PIFPEGREQGLAVEHYDLAAMNDGELLGLSAKRRLALDLEEMRAIQAWFGARGRPATDVE
LEMLAQTWSEHCVHKTFKADVEVRNSKGRPYPPVVHGIFNIYIRKTTEEIGAPWVRSAFV
DNAGIVSFDGDWDISFKVETHNHPSAVEPFGGANTGVGGVIRDVMGVSARPIAATDFLCF
GPPDMPEDQLPQGTLHPRLIARGVVAGVEDYGNKMGIPTVNGAVHFHPGYAANPLVYCGC
VGLAPAGVHRNAPRAGDRVIVLGGRTGRDGIRGATFSSMKMDGSTGDVAGASVQIGDPIV
QKRALDVLLAARDAGLYDAVTDCGAGGLSSAVGEMASALGAEIELSRIGTKYPGLSPWEL
WLSEAQERMVLAVPPRHLEAFAALCSRFESEFWDIGAFRSDGELVLRMNGEIVLGLSMEF
VHKGLPRKHLVAERSAGGTHGGHGNPDSVKYSGDVALPADAKARFLSLLSDMNIASREQV
VRRYDHEVQGGTAVKPFAGPYGDAPSDAAVLKPQGTDGTWGIALSNALRPDYGELDPYRG
AWAAVDEAIRNAVAVGADPERIAILDNFCMGDPGDSAVMWALVESARGLREAALAFGTPI
ISGKDSFYNEYLGPDGRRHAVPPSLLVSALGFVPDVKRALTSFLKKPGDSLWLVGDFRPV
HRQESKVPEVSLRACEVYKAFFEAVQEAEVVAAHDLSDGGLAVALAEMCVGGRLGARVAL
PDSAMVDASVPVLTLFGETTGCLLVEVGAGHEGSFSARFGPGLALRLGEVKAGPAMEIHG
GGGNAGCGAGGPLLSVSIDEMLCAWKGDAWKGDAWKGKAREVLP*
>SPBDM4_v1_30052|ID:27157287| putative arsenical pump membrane protein [Uncultured spirochete bdmA 4]
LNTFVLVVLVLMYVGIILFPARKAWISMGAAAVLVAGGAVTPGHALGSLVNWNILCIYLG
SLVLAELFIYSRVPAFIAERIVERSPSIGVAITIILLLTGLVSAFVENVATVLVVAPVMI
ELAKRGKKNLAPLMIGLAVMANLQGTATLVGDPPSMIFANYAGYSFNDFFFKFGKPSIFF
AIQVGAVVGAVYFFFYFRSMQSDVHTLPPERIVSWAPSLFLLAMIGGLALFSALSKEGGI
HPASGIFVLGLAVLGLLWLAFIKKEPGRARDLVRHIDWGTLAFLAGIFIVVGTLSESGAL
EVLAGKLAVLVGDDLFFGFALIVSISVLISGFVDNVPYIAAMLPVASGLAKTMQVAPELM
LFGLLVGSCLGGNLTPFGASANIVAVSLSEKQGTNVNFWYWLKIAGPFTILTTIAASGFF
WIIWR*
>SPBDM4_v1_30053|ID:27157288| putative Branched-chain-amino-acid aminotransferase [Uncultured spirochete bdmA 4]
MSRFKTMVLSGTLTPTPLSAIKWDSIGFGGIVTPFVGGSVALSDGVFEPMAVVPNGNIAI
PPLACSLNYGQELFEGMKAQRGPDGNIRLFRVDANARRMAKGAARFMLAPPSEELYREAV
IATVKANADYVPPYGKGSLYVRPILAGVGMTLSPAPSDTTLFLVTCLPVGLHYKGRAMVN
IKVETEFQRAAAKGTGWVKAAGNYAPCFLPSNEAKHEGYDDLLFLNQTGNNVEEVGTANF
AIVKNGVLYAADSPSILPGITRDSVMRIAREILHMEVVFASLELDRVLGLGAYAKEGPAD
EAFFTGTAAVISPISNIYHAGKDYHFGDQQGPFATKLRDQLVGIQTGRLPDPFGWVTVVD
*

>SPBDM4_v1_30054|ID:27157289| putative Diguanylate cyclase [Uncultured spirochete bdmA 4]
LKLAHLTKIKTFESSKRFLIKKPNEGDLIILQNQWNQLKILLHEYDAKKGPSVEFNNLSE
EIWVQANIVVTGIENSFHSYIKYYTVIAIVLFSSIIMLALISFAIKYFIQQRVEVQASHD
YLTGLFNRNYFDILLKRQLHESNRNNQNVAIMLCDIDHFKNVNDTYGHDNGDKVLKAIAE
TFMMTSRASDTIIRFGGEEFLIIASYQNIDDLMRYAERIRMNIENTKILDLNITISIGIA
ICDHSGEVNKVIKKADTALYTAKENGRNQVILYDNIKK*
>SPBDM4_v1_30055|ID:27157290| protein of unknown function [Uncultured spirochete bdmA 4]
MIIIGMVGVLGLTLALSLYITRFIGQYSVLINDSGKVRGGIQRTVKLALMGEDYASVSDE
IDGYLRFFQKSDEF*
>SPBDM4_v1_30056|ID:27157291| membrane protein of unknown function [Uncultured spirochete bdmA 4]
MSNLISFRHTFTHLREHARPILVLLILLAAILLTRLPSYLLFHILAELTSIVIGVCVFTI
AWNSTSLLESGFLLVLGIGSLYVSGIDVLHALAFKGMGI*
>SPBDM4_v1_30057|ID:27157292| protein of unknown function [Uncultured spirochete bdmA 4]
MLASIFVWNIFPSAYIEGEGLTPFKVGSEYAIIAVLLAAMALFWRGRTALALSVFYGIVG
SLAASIAAELAFTLYFGVTDTANLLGHLFKITAFYLLYRALVEVAFTRPYDLLLRQLKQS
EQTERAGRNFAEVELHACEESYLRLMNNALVGVYQTSLDGIMLFANSEFAHLLSFSSAEE
MIGGNVAERYRDLQDRARLVEILKREQYVREFETDLWTRNGQSRHVLMSASLKDRTISGM
VLDITERKRAEEKIKIQVEQLAALRAIDIAIISSAELYLTLKIVLEQVIQHLHVDAASVL
LLNPHQYTLEYAEGIGFRKEIPALPPLRLGEGFAGRAALERRIVSAVNIAESEIEFRQKQ
LIEGEDFVSVYTVPLIIKGQVRGVLDIFNRSELHPDQAWLDFLETLAKQTAIAIENATLS
EGLQKSNMDLIRQADRRRVGGNAQAYTICV*
>SPBDM4_v1_30058|ID:27157293| Cyclic di-GMP phosphodiesterase response regulator RpfG (fragment) [Uncultured spirochete bdmA 4]
MRKHTQYAYEMLAPIAYLKPALDIPYCHHEKWDGTGYPRGLKGEQIPLAARLFAVVDVWD
ALRSDRPYRKGWSDDKVYEYIKSLAGTHFDPRAVELFFKVLSENNQGAG*
>SPBDM4_v1_30059|ID:27157294| protein of unknown function [Uncultured spirochete bdmA 4]
VFSNLLMSSAAMDQLVHHAVKFVIEGKSYRVESFSAR*
>SPBDM4_v1_30060|ID:27157295| protein of unknown function [Uncultured spirochete bdmA 4]
MFATLNPEAPDLIPDTIAYQKRIHKNAISMSVGLTNISPC*
>SPBDM4_v1_30061|ID:27157296| transposase [Uncultured spirochete bdmA 4]
MQDRELYEKLLGLKAPWIVTDVNLQVAESKVTVAVGHAPMTEFACPECGVLCKVHDHRRR
QWRHLDSCSFVTMIDADVPRVKCPTHGVKQVMVPWAEPGSGFTALFEAMAISWLRAASID
AVAKRMGISWEEAWGIMERAVQRGLARREAAPIEELAIDETSYQKRHEYVTVIVDRKTGC
VIDVLDDRKKNTLKTWLECNKDRLSAVRTISMDMWEPFITAVQETIDAAGSKICFDRFHV
ASHFGKALDKVRAAEHKALGSVSPLTRTKHDWLRTKANGGYHDKRAFLELTRMHLKTARA
WMIKETATGLWSYSYRGVAERNWRSLLSWISRCRLEPVIKVGQMVRKYLWGIINAILLKA
TNAIAESINATIQKVKARACGFRNRSRFRIAILFHKGGLSLLPSGL*
>SPBDM4_v1_30062|ID:27157297| transposase [Uncultured spirochete bdmA 4]
MKSISDILGGLQILLEVSFDIQAGFEEYLTKDQRTFLAILRVIEEHLRVPYEAESRYGRP
AYSINPFIRAFWAKSYFRLLTMDDLRKRLLSDPNLRMICGFTKVPSLATFSRRMSLLSES
SLMEKSFETMVSEYYAGSIVGDVARDSTAIAAREKPCNKKSDVALPKPKKYRRGRPRKGE
ERPRKAPPVVAQHTTMSLEEALHTLDTRCSWGCKKNSQGNVSYWKGYKVHLDVTDAGIPI
SVVVTGARVHDSQVAIPLERMTEARVTHLYSLMDAAYDSDTIRSFIQERGRVPLIDHNKR
KADTRPPFDPASQRRYAIRTTVERTNSHLKDWFLSSPYFVKGIKKVSFQIMCGVLCLTAL
KILQYFIAPAMR*
>SPBDM4_v1_30063|ID:27157298| protein of unknown function [Uncultured spirochete bdmA 4]
MNDFEYILSNTIRKDLAKYIVNSILPVPEKLFRYTGLKEYVLSDLTNNQLKPVDPSYFND
TYDSSLIIDNVNGRPDDYTFIRRIYENANDMGNYRKIVEKRKKLDFAIKDHFKNHFRIMC
FSEICNDIKMWGLYADYNKGICIEALSKVMR*
>SPBDM4_v1_30064|ID:27157299| mRNA interferase [Uncultured spirochete bdmA 4]
MGMVINQYEVYLVDLDPTQGHEIQKTRPCVVLSPDEMNHNISTIIVAPMTTKSHPYPTRI
EVNFNNTDGWIVLDQIRTIDKTRLVKKMGKIDRKIIHNVKKVIKEMLVD*
>SPBDM4_v1_30065|ID:27157300| SpoVT/AbrB domain-containing protein [Uncultured spirochete bdmA 4]
MLISVVPIGNSKGIRLPKAILDQLHVSDTLELEVEDHQIILKPVAKKPRHHWNEAFIKMH

QDQQDTMLLPETNVTEAFEWEW*
>SPBDM4_v1_30066|ID:27157301| protein of unknown function [Uncultured spirochete bdmA 4]
MRSNPPFTCIENKDNEESITEFDEYTFILKLEDYERAQLTDPESKFFKLGTETVEMMRLA
GPKKQ*
>SPBDM4_v1_30067|ID:27157302| transposase [Uncultured spirochete bdmA 4]
MGNAKRTYTEEFKRNALEMLKKPGKTGADVARDLGIGSGEINRWRRAVEQEHRGEEKAFP
GKGQPRDEELARLQRENRDLKEANEILKKAVAIFSRGEK*
>SPBDM4_v1_30068|ID:27157303| transposase [Uncultured spirochete bdmA 4]
MPWRMESMQLQKKKFITLWETGRFTKTYLCEEFGISRPTGDAIIKRYQEVGWDALEEQPR
GHKSHPFTTTKTIEDAIINERKAHSNWGGRKIRVLLLRTYEEAEVPSETTVNNILKKHGL
TVPRKPPRRKLLKSEPKFDPQLPNQIMSADFKGKFRMGNGQYCNPLTIADSCSRFLFAIV
GLERPDTESSKPIFERVFREYGLPYQLHTDNGPPFGNAASLRRMTMLSVWIMELGITPVY
SDPASPQQNGRHERMHRDLKAEATRPPGSSMVAQQRKFNHFREEYNTIRPHEALGMKTPA
EVHTWSSREYPRRIRDWDYEKDITPKMVTVNGAIRWKDKGFAMISTALGGKYVGLHPVDD
GLWLVYYRHVALGYFCEQTMKVYELNDFDF*
>SPBDM4_v1_30069|ID:27157304| exported protein of unknown function [Uncultured spirochete bdmA 4]
MKKIFACVLIVLLVVSVPAFARPKQREVANLGVDTIIGASISRIKPIGGTLGIIFGVANV
VTYAITGKTIGGYAIDGFERSSEIIYSKDNPSRGWMSLGMQ*
>SPBDM4_v1_30070|ID:27157305| protein of unknown function [Uncultured spirochete bdmA 4]
MKSIIISHNKLFTICLHNILDYLTSNNKNSISNFVLIVIDATSIDSNIILQKLLFKYSDS
IYRILLIHDHVDDLTEKIVYNSRVVAVYNPRTGNSTKFGHLLQHVLDGGVMLSKEKLFEL
RQTELIHTEIVFTKREEEINQLLHNGDSIKVISSELHISQNTVKVYISRIYKKLGIHSRA
EYYINYDX
>SPBDM4_v1_40001|ID:27157306| transposase (fragment) [Uncultured spirochete bdmA 4]
MQDRELYEKLLGLKAPWIVTDVNLQVAESKVTVAVGHAPMTEFACPECGVLCKVHDHRRR
QWRHLDSCSFVTMIDADVPRVKCPTHGVKQVMVPWAEPGSGFTALFEAMAISWLRAASID
AVAKRMGISWEEAWGIMERAVQRGLARREAAPIEELAIDETSYQKRHEYVTVIVDRKTGC
VIDVLDDRKKNTLKTWLECNKDXX
>SPBDM4_v1_40002|ID:27157307| Metallophosphoesterase (fragment) [Uncultured spirochete bdmA 4]
MLGDTVETGSRSLMWNLALSDLEALIPDVPLRPLMGNHDAFFGGQYLFRKAFFPKNFSSD
SGSPYYYSIDAGTAKIIVLDLPWGTEQFNRAQKEWLQKTLEKADPKLPIIVLSHSFFYAS
GYNDPTSDAPWYDNFQNIPAIAPLLERYGVDLVVSGHNHYEEYLEHNGVHYAVIGAMGGK
SDPPPTYISPASKWMAVGTFGWLDVDITSSELVLSYRNENGKLLHEERIPYTGT*
>SPBDM4_v1_40003|ID:27157308| conserved protein of unknown function [Uncultured spirochete bdmA 4]
MFDADKWSYTVRAGRARLALEKNGFDAVVAPAAADAAALVMEFIKPGATVGFGGSMTVRA
LGIQEKAAAAGATLLDHNAPGLSAEEKYGILRSELTCDVFISSVNAVTLKGEMLNVDGNG
NRVAALTFGPRKTIVVVGANKIVADEAEGWARIEAKASPMNNKRLEKDNPCVKTGQCMDC
DSPGRICRVYQILRRKPSLSDFTVILVAESLGY*
>SPBDM4_v1_40004|ID:27157309|hcp| Hydroxylamine reductase [Uncultured spirochete bdmA 4]
MFCHQCQETMRNKGCDMARGVCGKTAEVANLQDLLLYVCKGIGFWSTKAHERGIYNEEAA
FFVDRMLFATITNANFSADDFERWILEGLKIREEVRTQFEKVDGLVSGTIPEAATWNVAD
IESLRSAAAAGKGGWAEIRDDDIRALKSLVLYGLKGIAAYVEHAYVIGKKSEEIFLFMMR
ALKDLGEEDLSADQLTALVLETGKYGVAAMALLDSANTGVYGNPRMTKVKTGVRNRPGIL
ISGHDLVDLEDLLEQTRVEHVDVYTHGEMLPAHYYPFFAKYDNLYGNYGGSWWSQGADIE
AFNGPVLFTSNCLVPPKDSYKNRVFTTGVVGFDGCVHIPDRKPGKRKDFSAIVALAKTCP
PPRELENGEIVGGFAHEQVFALADKVVEAVKSGAIKRFVVMAGCDGRQPVRSYYAHVAEG
LPGDTVILTAGCAKYRYNKLPLGDINGIPRVLDAGQCNDSYSLALIALKLKEIFGLEDVN
ELPLSFDIAWYEQKAVLVLLSLLYLGFKNIRLGPTLPGFLSPNVAHVLVERFGIKGITTP
DADVAAMLRGA*
>SPBDM4_v1_40005|ID:27157310| putative Transcriptional regulator, BadM/Rrf2 family [Uncultured spirochete bdmA 4]
MERVLGITDGTTAELHALALAAINGGSIAANVAAGRLGVSPTYLAKVLQAMASKGIIVST
RGSGGGFSLKKPASELSCLEAIEALEGPLPSRYCLFEHAVCTKKTCAFRELCIDMEKQIR
STLSTTTIEDLARSFQDKE*

>SPBDM4_v1_40006|ID:27157311| protein of unknown function [Uncultured spirochete bdmA 4]
MDAGAIAMSVFSNAIVAFSRSSGPGGQNVNKVNTKVTASIALEKIEGLSQAERIAVKARL
SGRIRGDGVLQVRVQETRNQGRNREIALELIGTVISGFLWFCVKIRPFTPDYDCFPNPNN
TTNQNFTATPISESINPTCFCCQVLMKPVFSIFRIIPLPGRRISNDFEVLSCSA*
>SPBDM4_v1_40007|ID:27157312| protein of unknown function [Uncultured spirochete bdmA 4]
MLSEIGVAVKFWLVVLFGLGKQS*
>SPBDM4_v1_40008|ID:27157313| transposase [Uncultured spirochete bdmA 4]
METYIPHREGIIQTIVSHSLTQYQAYASPSETILDRLEQFTACADWTKGVARIRCEDCGH
SYFRPFSCKVFHLCPSCDQKRTLLYAEYLAEDLLLDLPHRQFVFTIPKILRPYFKSDKRL
FGEVSKLIFSLLSDFFSLAAGQGLLCACVVSYQSFGEFARFHPHWHVLVLEGGFTTHDRF
VYLPIGADEGMLKVWQAAILSLFLRKKLIDQARVNMLKDWKHSGFSIESETRLFSKADRE
ALGQYVVRGATCAEKIHYDPASDTVTWTAAPKGFFKGRAETFRGFEFVDQIVAHLPPRRV
QLVRRYGVYAGKVRKQWQERPSIYRLAPESWQKGHPYQSQSAQGKPSEDIASAEVPDAWG
KLRKKSWARLLRKVYELNPFVCPKCQGPMSVVAIIEDPKELGKIIEWAQQQAREPHLTAC
ARSPPELALATV*
>SPBDM4_v1_40009|ID:27157314| conserved protein of unknown function [Uncultured spirochete bdmA 4]
MARYLEGKRLYLRTFRKADVEELDELMDDWPTQVMTGSVYPNTEKELEEAIDRWQRTDSR
IWFAVIDKETNKIIGETGFLRIFMPWRTTDITIEIWDKKYWGKGYGKEIAGLMFEYGFNY
LNIHRMAIGVVEKNEQAMKIGASDFCVAGVQNSREYANLRSCRIESFTRSCLG*
>SPBDM4_v1_40010|ID:27157315| transposase [Uncultured spirochete bdmA 4]
MQDRELYEKLLGLKAPWIVTDVNLQVAESKVTVAVGHAPMTEFACPECGVLCKVHDHRRR
QWRHLDSCSFVTMIDADVPRVKCPTHGVKQVMVPWAEPGSGFTALFEAMAISWLRAASID
AVAKRMGISWEEAWGIMERAVQRGLARREAAPIEELAIDETSYQKRHEYVTVIVDRKTGC
VIDVLDDRKKNTLKTWLECNKDRLSAVRTISMDMWEPFITAVQETIDAAGSKICFDRFHV
ASHFGKALDKVRAAEHKALGSVSPLTRTKHDWLRTKANGGYHDKRAFLELTRMHLKTARA
WMIKETATGLWSYSYRGVAERNWRSLLSWISRCRLEPVIKVGQMVRKYLWGIINAILLKA
TNAIAESINATIQKVKARACGFRNRSRFRIAILFHKGGLSLLPSGL*
>SPBDM4_v1_40011|ID:27157316| protein of unknown function [Uncultured spirochete bdmA 4]
LTFGDIYKSEFEKEGNLISIDGNIEEAKSISDSIKTSGGKRFKYTIRRDGQPR*
>SPBDM4_v1_40012|ID:27157317| protein of unknown function [Uncultured spirochete bdmA 4]
MIKQMSISETRKKITSLADELSYFQNELEIIGIDKRFNMDRQKPTTTEFRA*
>SPBDM4_v1_40013|ID:27157318| conserved membrane protein of unknown function [Uncultured spirochete bdmA 4]
MNLLTKFKETSISVVPILLIVLVLELTVAPIGTTLLVQFFLGGVLIIIGLSVFLLGIDIG
VLPVGQKVGATLVYKRNLGLMLAVGFVVGFFITVAEPDVRVLANQAATVDPSISKTLLVV
MISLGVGLFVAVGFARTTLGIPYRYLLIVFYIGVFILASLTDPAYLGVGFDAGGATTGPM
TVPFIMALGVGVAAVRKGKEAEDDSFGLVGLASIGPIAAVLIMGIINKGRSAPASAAAAE
SVETQGIAASFIHLIPETLADVALALGPLALMVFMFQITLLHMPRHQMMRTIKGLIYTFL
GLVFFFIGVNGGFLPVGSTIGGIIGAMEHNAILIPIGFVLGAVVVLAEPAIWVLNNQVEE
VSGGYIKKRVMLVSLSIGVAVAVALAMLRVVTGMSIWWLLVPGYTLALALTFWSPPLFTA
IAFDSGGVASGPMSSTFILSFTLGASAAAGGNPITDAFGVIAMIAMTPLIAIQILGLIFK
YKEKKAAVPHSSEAAEETVE*
>SPBDM4_v1_40014|ID:27157319| conserved protein of unknown function [Uncultured spirochete bdmA 4]
MTVTKQTIYTHGQLLLSIVERHTGERLVAATKRAGAHGGTIILGRGTAESSLLESLGIGD
SEKDVVLTLATDEQTGPIMAALRSSEMREKHIRGVAMLIDVTDILRHVRSDEADSVSSAE
RRSPMKTQDDHVLITFIVNRGYADDAMLAARKAGAKGGTVLNARGTGSEEDVKFFGISLV
PEKEILLILVEAAQSDAILQAVRKVPCLAEPGSGIAFCTAVEDFITLGTKANDQR*
>SPBDM4_v1_40015|ID:27157320| protein of unknown function [Uncultured spirochete bdmA 4]
LGKFIENETIGACIGGDEFILIVPLANRGKIADIAAGISGELAKYEDPAIPAEKRLSMAI
GYGTAYSVKEDFFKAYEDAEKMMRYVKLLDKNSDSRSIMKTHPEIGYRIAMSWSNFEELA
PYIPLAP*
>SPBDM4_v1_40016|ID:27157321| conserved protein of unknown function [Uncultured spirochete bdmA 4]
LLLISLSHHERWDGTGYPRGLRGKEIPLLARILAVADTYDAMTSARVYRKPIGKQEAMEE
IRRNSSTQFDPAIVNIFLEYLEKNEP*

>SPBDM4_v1_40017|ID:27157322| protein of unknown function [Uncultured spirochete bdmA 4]
MSHSLNIPCTAQEFRCDKKSPLNTYGDHLTKVSLFLLIILFPMTVFFFSNTLNRVNAFEN
ERIITIARMAATLIDGAKLASLRGDESDLSRPEYVYLKADLVRLHQSDTLISSAFLMRRT
EEGKYILLVDSESPDSAHYSPPGKEFARVTEEMASPFVDGTSLVSRPTTDSENMWRTALS
PVLDRETGNVVAALGIEYSDDVWNILPLKRPSYTLIGFSILGGLIILAVLFGIQNIRIRS
LNRTSQEERRRLRIFLDQLPGMAFRCLSDGRGTMTYVSPGCKALTGYSPEMLIDNRGISF
GEIIVPEYRESLHALFDRTAATGKDVHAEFEIVTKDGKRKWVLVYGEIIHGDNNTAEAIE
GIILDISEKKRAEAENEYLSNHDAPTGLYNRHYFFETLLNLESEGSIPYPALSGTSTDSA
LLTMRLATRRATGFWSK*
>SPBDM4_v1_40018|ID:27157323| protein of unknown function [Uncultured spirochete bdmA 4]
MEEGSVIAAHTGGDEFALILLSADEEKVQKTMDSLSRDFQQCRDLNMPAINRLSMDFGQR
TATSRDSDVFQAFDNAQKMMRQAKLLNKASASHGILNAMLATLYAKSGETEEHSARLAYI
STKIGVDTCPKKS*
>SPBDM4_v1_40019|ID:27157324| Cyclic di-GMP phosphodiesterase response regulator RpfG (fragment) [Uncultured spirochete bdmA 4]
MLHDIGKISIDDRILKKEESLTEDEWKIIKTHPQIGYRIAMASSEFREIASYIIVHHERW
DGKGYPRGLKGEEIPLLARILTVADAYDAMTSKRVYKDAWKREAALEELKKNSGTQFDPK
IIDIFLSLAEAEDFGCPDV*
>SPBDM4_v1_40020|ID:27157325| transposase [Uncultured spirochete bdmA 4]
MKSISDILGGLQILLEVSFDIQAGFEEYLTKDQRTFLAILRVIEEHLRVPYEAESRYGRP
AYSINPFIRAFWAKSYFRLLTMDDLRKRLLSDPNLRMICGFTKVPSLATFSRRMSLLSES
SLMEKSFETMVSEYYAGSIVGDVARDSTAIAAREKPCNKKSDVALPKPKKYRRGRPRKGE
ERPRKAPPVVAQHTTMSLEEALHTLDTRCSWGCKKNSQGNVSYWKGYKVHLDVTDAGIPI
SVVVTGARVHDSQVAIPLERMTEARVTHLYSLMDAAYDSDTIRSFIQERGRVPLIDHNKR
KADTRPPFDPASQRRYAIRTTVERTNSHLKDWFLSSPYFVKGIKKVSFQIMCGVLCLTAL
KILQYFIAPAMR*
>SPBDM4_v1_40021|ID:27157326| YhcH/YjgK/YiaL family protein [Uncultured spirochete bdmA 4]
MIVCSFKDLEKYQSVLNYLPEALRKVEELKKAGLIPGRYDYHGFFINVQRGTTKPLNEGR
FETHMRYVDIQYEIEGSELMAYAPKDALRDDLPYDEKNDIAFFNADKKDTTIAAIQAGMC
YIVFPEDGHMPCRHTDRPTNYTKLVIKLPVKK*
>SPBDM4_v1_40022|ID:27157327| conserved protein of unknown function [Uncultured spirochete bdmA 4]
MTNEELVENVKIEGIGVASNLLAERLRRLILSRELPPNFVFPNETTFCEQLGVGRSTLRE
AYKVLEAEKYITRTKRGTYVNDVDQTLSSVSFETTIKMSDFNDLLEFRTMIESELAGLAA
QRADAENIEKMKEYLDMMRRHRDNLAVLTSDDVNFHLEIAKASRNKLLINTMELAYETFL
QGTFNAFQIDTKANIDQALFYHEKVLSSIEHHDPVMAKEYMRNHIQSIKNRGVK*
>SPBDM4_v1_40023|ID:27157328|dhaK| PTS-dependent dihydroxyacetone kinase, dihydroxyacetone-binding subunit DhaK [Uncultured spirochete bdmA 4]
MKKIINEPEDFVDDMLAGVLSAHGDQLTNVENNNRNIVRANWKDKVALVSGGGSGHLPLF
MGYVGKGLLDGCAVGGVFQSPSVNQIYDITKKVDSGKGVVYIYGNYTGDTMNFEMSSEMA
DIDGIKTAHVVGNDDVASAPKGEEQKRRGVAGIFFIYKTAGAAAEAGASFDEVVRIANKA
KANIRTIGVALSPCIIPEVGKPSFTIADDEMEIGMGIHGEPGVKKTKIQKANILVAHIMN
TILTDMELEEGCEASVLVNGLGATPKEELYIVWREIAQILAVKKVKVFHVYVGEFATSME
MAGFSISVFKLDDELKMLLRAEARTPFFEQIAY*
>SPBDM4_v1_40024|ID:27157329| Dak phosphatase [Uncultured spirochete bdmA 4]
VKTINSEELVKMLVTIADSMTQQRKHLIELDSTIGDGDLGITMERGFQAAAAFAKNNADL
PPGFLLVKSGMEIVKLAPSTMGTLMGSGFIQGGKVLDQKMELTASDLTIFFEGFLNGVLL
RGKASPGEKTIVDILIPVVEQMQAYQGDDIVEVLACAEKGAQQGLENEKGMVSQHGKAAV
FREKTLDLIDPGSEAVAIMINACKRSVVKESL*
>SPBDM4_v1_40025|ID:27157330| conserved membrane protein of unknown function [Uncultured spirochete bdmA 4]
MKKIDTLNPWKTELKALSRYPWIWAVLGSIILFILLSVFAGKFSMESFITLATSATFLTI
VATGQMLVITTGEGAIDLSVSTVITLSAFYTTGIANGSNGNLWLAIPAVLLTGVVIGLVN
SFLILELKIPAIIATLGMNYIISTLILLYNKNFRVFDVAPFLTSVVRYKIFDFIPLMIII
TLVIIIGYDFLLRKTRFGKSLLAVGQNREAAALAGIQVKITQMIAYVMCSVLAAISGIFI

SARVGGAFYGLGDSYQMQSIASVVVGGTLISGGKAVPIGTYFGALFLIMLTTAMQVAGLK
FGMQNVITGLFIVAVLFLASKQSQQ*
>SPBDM4_v1_40026|ID:27157331| conserved membrane protein of unknown function [Uncultured spirochete bdmA 4]
MDNNRCLLQLRDISKTYLMTQALKSVSLDINKGEILGLIGANGAGKSTLMRVISGVTGPD
AGGEIVFGGTALERGYSARQANKAGIHVVYQELSLCTNLNVVENFYIDQHEAYKKDFHWR
KRLARHAAEAITDVFPGSNIDVNTKIGNLSLSHRQMVEIARATSMAGLRLLILDEPTSSL
SAKETQQLIDYLKKLKEKDISIIFISHRLSEVVKLADQIMVMRNGEKIWEGQNKDIAEQY
LVEKMTESTASPDAQDKREAEAARTAINEEIFVKTKNLTMNKLGGIDVSLYGGELIGIAG
LDGNGQGDFLKAIFYATKNNKQLQKAGKICYVAGDRKKEGIFPLSNIADNMTINEIGKFP
YFKFLDLNYLTERVNTWFRNLQIKAMSEKQMITSLSGGNQQKVLVARAFLSDADIIILDD
PTKGVDVGTKKQMDLLFKEVAAQGKLVIWYSTEDDEFEYCSRILVFRYGKIVRELKDGEI
KKETIIASSFEGDSLQDKSLLNKEQKFKHRSPILVPIIALIAVFTVSGLYQKNVFTQFGI
DLLLTGALPLVFAALAQMFVIGLSQIDLGVGALMGFINVICATIMYKNTSLGFLMLAGVV
VLYGIIGVLIYIRQIPAVIVTLGMSYVWTGLAYTIQERPGGNSPELVNKLFNSSFLIPSS
VLYIIIGIVVSVLIYRSKYGIVLRGFGNNATAVSNSGWSPAKAFAIGYMLCCVFSIFGGL
AITASTGGCDANSTTSYTLLTVASVVIGGSELVGGIVFPSGTVIGAIMLSIISALLGFMR
LDASWVTAMQGLILIVILASRLLRKNKI*
>SPBDM4_v1_40027|ID:27157332| conserved exported protein of unknown function [Uncultured spirochete bdmA 4]
MKKVLTAALIVLVLSTVCVFADAKGVQKGYVIALSNSFYGNSWRKQMVDCFTKSAMQAKA
AGRISDFIIVNGDGTQNTQISQLNSLILSDVDAICINAASPTALNGVIEKAIKKGIKVIT
FDSIVTQPDAYKMDFDFYGQGKTIAKYCVDRFKGKANVLFVRGIRGSDPEIQLYNGAMSV
LKANPGMKVVAEADGEADAAISQSAVSNILPSLNNIDVAITTGGEYGVVQAFEAAGRKVP
VIIGSNRSEFIKWWIDEKAKNGYETVSISSEPSIGSVVVWLAIHILEGDKVPNSIKLNSI
LVTNKTVDQYKDLLPGTVIAQDYDEQFVIDMLKNLNNR*
>SPBDM4_v1_40028|ID:27157333| putative HpcH/HpaI aldolase [Uncultured spirochete bdmA 4]
MALLLKKKLKAGKSVLGTMLCMIDNPNIVWLLKGIGFDFVFIDCEHGTYTVQAVSNIVTV
ARAVGLGTIVRIPQPDRPSIQKYADMGIDGIMLPMVETTKQIELANQFSRYKPLGSRGIS
LGATVDWKGGVDVGETIRELNDDFIILAQIESRAGVENIDQIMSVPGVDGCFFGVYDMSI
SYDKPGEIYDPIFRQNIKIVLDSAKRHNKILGHHFFGYQDLEWGMEQGVKFITWHTDTSA
LQKAYADDVKKIKSMPLFVR*
>SPBDM4_v1_40029|ID:27157334| putative transaldolase [Uncultured spirochete bdmA 4]
MGDYFKRVMSETATRFWINNATNTEAKLAIEAGAIGCTQNPSYVWKMLDGSEDSELAKAK
LKEIIKSEPNDSEALIKVQRELVANIAKHFYPMYEKSNGKLGYVSIQGSPIQEDAESIIR
FARYNCSAAPNIMAKIPVTEEGLKAIGVLVKEGIPINATECMAVKQVLDVSEIYAAASGH
MKNPAPMYFSLITGIFDEYLHSYVEKNHVDVNPDFLWQAGICVARKVHAMVKNRCYTIGF
ISGGARGLHHFTEMVGADATVTINWKGTADVLLKQDLPVVSRFHMQTPFEVEDALVEKLP
DFRKAYFVNAIKPSEYEDFGPVVLFRSGFVSNWEKALEFVASLR*
>SPBDM4_v1_40030|ID:27157335| putative Xylose isomerase domain-containing protein TIM barrel [Uncultured spirochete bdmA 4]
MSVKKSIVLTALLPAAETEQRIFEMGASTMKAYDLSVIEFYAPWDTAKRRGDFLKSHGLC
GVYLAATYQKRNHSHLCSLGNKERKSAVEDAKYCIDAASDAQVKSILFTSGRRPNDPAQY
QSAYSVLVDSFSELMEYAPKTMDVVLEPGDRDVDAMQLVGPTELAIRFANDIRKEYPNFF
LTMDTSHIAQLGENALTALRKASCVCHHVHLANCILQRANPLYGDKHPLFSCMNGVYDDE
ELHAVYDNLVVMFRQQRKDLTIAVEVISRNGGEFGWMDEVIKGSPWFFSKIIDEE*
>SPBDM4_v1_40031|ID:27157336| D-isomer specific 2-hydroxyacid dehydrogenase NAD-binding protein [Uncultured spirochete bdmA 4]
MKRILINIPNFQVLNPDAYKYLQDHGYDIILNPMKESVATYEEIFPYIDTIDAVIVGNDI
WDDRLFTHAKKLKVIAKFGVGVDAIDLAEAKKFGIKVVNAPGKNADAVAEFAVGLALDAI
KNISHQQALLKNNHWSVDWLGQEIKGRTVGLYGFGNIASKAAVIFKSFGATVIACDAIPN
YELAKKIGVELVSADELLTRSDILSIHVPNTKENYHLINRDSIAKMKDGVYFINTARGGL
VDTDALCDALDSGKIRWAALDVYEKEPAEPCDRIVKNEHVTLTPHIAGQTHESWHAISMS
IVNDIERVLNNEEPHAWVNRNC*
>SPBDM4_v1_40032|ID:27157337| conserved protein of unknown function [Uncultured spirochete bdmA 4]

MLNKNYVSPKLSFPEGPVLMDNGDFFLVEMGEDAGTISHIDGKTFTCTRVVKTGRPNGLA
IDKDGTIWCAESKHPSLLKVTQDGKVEMVLEKCEGEDFRFPNDLAFGPDGMLYMTDSGIR
FYDSETNGKLNPDYMTYPYRGKVYCINPKTLEIYKIDDYIKFTNGIALGPDDRLYVNETR
TGNIYSYGKVNGRYSNKDKKLFANVLHEGENLSPSRGPDGMKFAASGNLYCVVYGQWDVV
VLDRNGKQIKYIPTEGQNPTNLVFGPQGSGDIYVTEVAEGHMEKHHVGEDGMVLYR*
>SPBDM4_v1_40033|ID:27157338| Anaerobic ribonucleoside-triphosphate reductase activating protein [Uncultured spirochete bdmA 4]
VLIGGLQRCSLVDYPGHLCAVIFTQGCNFRCPYCHNPQLVDPARFEAPLDEEKVFNFLRS
RIGRLRGVVVSGGEPLLNDDLPEFLDRIGQMGYLVKLDTNGSFPDRLSAIVSAGLVDFIA
MDVKAPFGRYGELAGVPVDVGAIQESIRIIRNSGTEYLFRTTLAKPLLDEKDTRLIRSFL
GPGAHYRVQPINPNAEWLRPEARLG*
>SPBDM4_v1_40034|ID:27157339| Oxygen-sensitive ribonucleoside-triphosphate reductase [Uncultured spirochete bdmA 4]
MFQSIIKRNGSEVRFDAEKIGSAISKAGAATGEFDAPIAKRLALRALMMAQQAFGEKIPT
VEAIQDIVEEVLLASPYRKTAKAYILYRDQHARIREITARSNGELVDQYLNQRDWRVNEN
SNMTFSLQGLNNYISSEISSGYWLNQIYPSEIRDAHLDGDFHIHDLNVLSVYCVGWDLKA
LLVEGFRGVTGKIESRPARHFRSALGQIVNFFYTLQGEAAGAQAFSSFDTLLAPFIRYDR
LTYPEVKQALQEFLFNINVPTRVGFQTPFTNVTLDLVVPKTYADEAVIIGGEPQPATYRE
FQPEMNLFNRAFLEVMYEGDAAGRVFTFPIPTYNITPDFDWDNHELDMLWKVTARYGVPY
FSNFVNSDMKPEDARSMCCRLRLDNRELRKRGGGLFGSNPLTGSIGVVTINLPRIGYLAH
DEDEFLRLLDARLLLARESLEIKRKVLERFTSNNLYPYSKYYLRDVEKHFGCFWKNHFST
IGVIGMNEACRNLFGEGIASAVGQKFALRVMDYLRHRLIEFQESTGNNYNLEASPAEGAS
YRLAKLDKNKYPDIICANTSNDLEGGEREPFYTNSTQLPVNYTDDIFEYLDLQDEIQGKY
TGGTVVHVFSGERVEDYEAVKALVRTICSNYKLPYFTFTPTFSICPDHAYIPGEVAVCPK
CGKPTEIYSRVTGYLRPIAQWNKGKQAEFELRKTFSVKKEELCS*
>SPBDM4_v1_40035|ID:27157340| protein of unknown function [Uncultured spirochete bdmA 4]
MANDTDHLSPSLAIAYCDYTYRGSPNTDLLLLAASIRRYGGICGSAPVWVMCQDAGKIQA
QVFEKAAALHVELLPYRDVQDLTDFPFAQKALAAAEAERRAEGKASELLWFDRDSLVLND
LAPLLPCAEKKVSFRPVNVQNIGQASAGPEDVHNEFWRRACVLGGVDYRSMGTTTSYIDR
KVLRFYIAAGLVGIRPELGIFREWERLLRIFAGDRFMRAYCQEHVLYKIFMHQAALSIAV
AGKTKVAERQELPPSAMYPMNFWNSDSNAYKPAVLDEVISLRYDTVLDDDGWGNFPMSTG
LRNWINDNIR*
>SPBDM4_v1_40036|ID:27157341|ygiD [H| Extradiol ring-cleavage dioxygenase, class III enzyme, subunit B [Uncultured spirochete bdmA 4]
MAEIEKNDGAAQIVYFSHGGGPLPILGDPGHRSMVRFMEALPAKLRKPDAILVISAHWEA
HEVTLTGGESPPLIYDYYGFPEEAYDITYPAPGQPGLVELIAGLLQKSGIPSRVDKNRGF
DHGMFIPLKLMYPDALIPTVQMSLLHSLDPSSHLKLGRALRPLLEKNILVIGSGFSFHNL
GAFSWRGEAATDTKNDAFQNWLIDLCTGDLSESDREHSLEYWEEAPSARYCHPREEHLLP
LHVCAGMANRPARVIFDDFILGKRAVAFQW*
>SPBDM4_v1_40037|ID:27157342| conserved protein of unknown function [Uncultured spirochete bdmA 4]
MIVNVVFHVDEIEKWELALANVHNLLAGIEVERSQVEVVANGKGVEIFASPDEKSLHTMT
ILADRGVRFCACQNSLKSHNINEEEVPEFFVVVPIGVLELAEKQQKGFAYIKP*
>SPBDM4_v1_40038|ID:27157343|ywlF| putative sugar phosphate isomerase YwlF [Uncultured spirochete bdmA 4]
MKIVIANDHGAVALKFRLVKWLESQGHQVVNLGVDVEDRIDYPDQAELAVREYFKGGYDF
GIVCCGTGIGVSIAANRHKGIRCALIHDSFTARMAKEHNNANFVAFGGRVEYTEPVEQIL
GVYMNAQFEGGRHATRVAKLDTIC*
>SPBDM4_v1_40039|ID:27157344| Metallo-beta-lactamase family protein [Uncultured spirochete bdmA 4]
MHMSITIRSLGAAEEVTGSKHLLEVDSQKILIDCGAFQGKRAESDNKNRALLGDDVDPAS
IDALVLTHAHYDHCGLVPYLVKKGFKGSIYATSATRDLANLIMTDSAHIQARDADYLSRQ
AQKKNEKFEWKPLFDEFDVIQAMDQFVTVGYHRPIFVGDGIQVEFFDAGHILGSAFARVT
AKDKEGNTAVIGFSGDLGRKNKPIIKDPECLKDIDILLLESTYGDRLHESTDDAVSRLEK
LVNRTAATRGKLIIPAFAVERTQELIFYLHLLHDQGRIPEMPIWVDSPMALNATSIFAIH
PECYDKETYDLFTTHAKNPFGFSDLNFSRSVEDSKALNQAKGPMIVISADGMCEFGRIQH
HLMHGLGDPANTVLIVGYMANGTLGRRLKDGAKDVRIHGDWYQVRADIQEIDAFSAHADW

KEAVEWLGCVDKVRLRKIYLVHGEGEALTAMRGHVLDAGAKDAEIVKAGEIYTIA*
>SPBDM4_v1_40040|ID:27157345| putative Uncharacterized RNA methyltransferase TDE_2619 [Uncultured spirochete bdmA 4]
MTDDLLTLRVEKLSSHGEGITFSEGKAVFIPYSIPGELVFCRIVEDHASFSRAELIGTKE
PSPHRSEPLCPLFGVCGGCALQHIEYAYQTKLKQAAARETFQRIGGFDPGELDIVTGEPY
HYRNRTQIHACGDGGLGFTKAGSPEAIRIPKCPILITTLEHWLVAENRKAHPYRALSALI
GERPRFSVFGQDERIYIEGRETYAAASVRGKNFQFPVAHFFQSNIIVLEKLVERFVEPLK
GVLALDLYSGAGLFSLFLADNFDSIECVESDAASMEAARLNLSRARAKVGFSDILVERWI
QTPHANRAFDCIVADPPRAGLSPELRAWLAGAQADALLYVSCDHASLARDLRDLTQKGWK
TEDITLFDFYPQTGRLEAVARLARAAR*
>SPBDM4_v1_40041|ID:27157346|pyrBI| Protein PyrBI [Includes: Aspartate carbamoyltransferase ; Aspartate carbamoyltransferase regulatory region] [Uncultured spirochete bdmA 4]
MARNGPFKGRSISVVNDLSLDEQRYLYRKARELKEAAISGGDVSEFRINDLDYQVYLIFM
ENSTRTRESFRNAGKFLGARVNVFDAATSSFNKNESITDAIKMLFGYSGESCFILRTKLE
GACTWLDQEFSDYSHITGKPKPSFINAGDGKHEHPTQEFLDEFSFLEQLRWNDGHIHIAM
AGDLYHGRTVHSKADGLKVFRNVEVDLIAPELLSMPPYYVEKMKANGFSVRVFESIEEYL
AQAKVAPIWYFTRLQLERMGEAVLERTPYLRQAVTFKKDFLGQLPDGCHFYHPLPRDRNS
PTIPFFLDELPLNGWDGQSINGYWTRITEIAMLSGRIGEDFEGEHAQKPEFVDDFVHEVE
AREKHKPEYKVGIKPVEEGIVIDHIATGEPVGEIWDTIDAARKILKLDVRSSHGVYHSNR
GPETFKGIISLPDIISFGEKDLKKLAAIAPGCTLNLIRHAHVAKKYRLSMPPRIYGFDEI
SCKNENCISYPANNEGVPPEFIRKGETTFVCKYCEREHKFRDIWDV*
>SPBDM4_v1_40042|ID:27157347| putative TrmH family tRNA/rRNA methyltransferase [Uncultured spirochete bdmA 4]
VKHVSSFHAILETLRAAPGSGTLFVTEDERRRGPRIRQILEEAERQGLAVRAVPASKLAE
MNPEHRGVVLAVESELQRTLSLEELCERVSQVDASLVFVLDHIEDPHNVGAILRSADAFG
VDAVIIPGRRASPLTDAAARSSAGAIAWMPVIQVSNLRAAVDELKSAGYWVYAADMAGAP
VGEHPMSKKALVVLGNEGRGASRILKEAADESISIPMHGHVDSLNVSVSAAILMYEYRRA
CRG*
>SPBDM4_v1_40043|ID:27157348| Transport-associated protein [Uncultured spirochete bdmA 4]
MAVICISRELASYGEETAQELAKLNGYKIVDKEHIEAALSAIGIDIEKQERYDEKNPGFW
ASLSQQRDEYLHFLTQVIYETALENNCIILGRGAHAILRGIPNLIGIRIGASKSVRVERV
RKAQNIDSRHALQIIESSDHDRAGFHKYFFSVDWYNPSEYDMTLTTDRFDPIHAAAAIDA
FRKSFISDTQEMNATAKMQDLLLGAKIVTEITYNRKIPIHFLEAAVERGVVVLHGVANTH
AAVDSAVAAANLIPGVKQVESAIQLVQEFTVVP*
>SPBDM4_v1_40044|ID:27157349| conserved membrane protein of unknown function [Uncultured spirochete bdmA 4]
MEKLALLAAFGTAVFWSFSAIFFENASKKVGALAVNFWKVVFAFFFLAISGTIVRGVPFP
YDAPLRTWIFLSLSGIVGFVISDFFLFNAYVMIGSRITVIFQSLTPVFTALFAYIFIAER
MQPHRLIGMAVTVIGILIVVLTRSRQNRKNNEGRLSVKGLGYAFLSALFQAGGMVLTKAG
LGDYSPISGTQIRALVAIIGFGINALLIGQGTHVFFKVPKIREAFTSTLKGSVFGPFLGV
VLSLFSLQNTQVGATSTLMALTPVVIIVPSVLILKQKIYLMEIVGAAIAVGGAALFFLL*
>SPBDM4_v1_40045|ID:27157350|tdh| threonine 3-dehydrogenase, NAD(P)-binding [Uncultured spirochete bdmA 4]
MKALVKRKNEPGLWMEDAPIPRIGDNDLLIKVKKTAICGTDVHIWKWDTWAQRTIRLGQT
VGHEFVGEIVDMGRSVQGYKVGERVSAEGHIWCGVCRACRAGRHHLCPNTVGIGVNRDGC
FAEYLSVPAQNAWHVHEAIPDEIAAIFDPFGNATHTALSFDMVGEDVLITGAGPIGCMAG
AIARHVGARNIVVTDVNPWRLELAKKLGATRVVNVAKEDLKEVQKELGMVGGFDIGMEMS
GSPQALEQMIENMYNGGRMALLGLLPDGAGIDWSRVIFKGLFIKGVYGREIFETWYKMQT
MLLSGLDISPVITHRFSFDDFEKGFAAMLSGEAGKVILSLD*
>SPBDM4_v1_40046|ID:27157351| putative Small ribosomal subunit Rsm22 family [Uncultured spirochete bdmA 4]
MGEHSLLYSFQISEDAASALQKLPACIDEVNPVPERRRTELRYNIRDLWRSLTMERAHRT
IDYLNNPAFFSAYLRYFMPWNLVRLVALLTELPLELSADATIIDMGSGPLTFPLALYCAK
PEFRKAPLTFICADRTPRIMEAGRLILELLAAKHGGELPPWKIELRHARFGEPLHEKASL
FCAVNVFNEFFWHHEGILSEDASELYHRTAQYCAPQGKMLIVEPGEPRSGGLVSAMRAAA
IIGGNEILAPCPHAYACPMPGIFKSGQEYLIGHHRTLEPVRMPAPRAKYPWCHFSVPAGF

APRWLTSLSEEAGLSKEKLSFSFLFVKKQSVQKKPSGGHLPIHDSSHMQSATVAERAAGG
LTCRIISDSIALPGGRTGKYACSSLGYTLISADQNKTQPESGVLLRFRLTHPRGMEPRGS
QQEALEFDRKTGAVLISY*
>SPBDM4_v1_40047|ID:27157352| protein of unknown function [Uncultured spirochete bdmA 4]
VRFRRNAELNPSAARRAFLCVSTGFGEIRMLAYYFHGILRAVAQGLARLVRDQDVGGSNP
LSPIDKKAAISFASPGGLFIPIDGH*
>SPBDM4_v1_40048|ID:27157353| exported protein of unknown function [Uncultured spirochete bdmA 4]
MKKYVALLSLLILTGVAVCAQQSINIKAIVAPVGANITMDSYLWNIALANDGTISYQNDQ
QGVMTVKSGSTWLFHIDFSSEHLGYVKQGAYQIPYYVQVTEIPSSYSGFILPSSIMDGYV
QLTSTQSIWFLGRTPKAGIRFYVGIKIDATSGQFFESGTYTDTLTINFVAL*
>SPBDM4_v1_40049|ID:27157354| conserved exported protein of unknown function [Uncultured spirochete bdmA 4]
MKKFFVFLIIGLVAGSAAWGATIGITVNAATKLEASVDQSTWTIALDNSGTAIDLPTQGQ
LTVKASKSAYTVTFTSANSGTLNNGSNTIPYKVKVDTSAWTTGVATNNLSSYTQLTASAV
KTIVFQSRTPTVGKTFAIGFNIAAYTDYYVDGAYTDTITIAIAQN*
>SPBDM4_v1_40050|ID:27157355|lysA| Diaminopimelate decarboxylase [Uncultured spirochete bdmA 4]
MESTSCPEGIEIENSNHYFDGVDLVALALDYGTPLYVMSETIVRRHCEALRESFLRRYPG
TRAYYASKAFQTLDMLRIIKDEGLGVDVVSGGELYAALKAGVVPSDIIFHGNAKTDHELR
LAVEAGVGRIAVDNLEEIYRIEREAARTGKTQTILFRVTPGVDSHTHRFISTGSLDSKFG
IPLEPVETTRYIEALAACPHVDFAGLHFHVGSQLLENTSHLAALRVALGFAARLARDFGL
TVRELNMGGGFGIRYLPEDRAPVLSEFVDPLMAAVREWSAREKLPLPACAIEPGRWIVGE
AGLTLYQIIAVKEIPGVRTYVAVDGGMGDNIRPALYDAKYYAAIVGRTRDQGASVGRGEA
AGHGQAQNRSSVVESNVATASERPFRTVTIAGRYCESGDILVRDASLPEPRPGDILSLFS
TGAYCFAMASNYNRVPRPALVMVKNGKARLSVRRETYEDLLSRDI*
>SPBDM4_v1_40051|ID:27157356| Peptidase C26 [Uncultured spirochete bdmA 4]
MTKPLIGITSFEDRSRQPAPYISLKDSYVRAIGAAGGIPVVLPVTNKEQAHLLIPRLDGI
IFSGGNDVAPWFFGEEPLPGLGSWDTWRDEWEIELCNRAWDAKLPMLGICRGCQLMNVAR
GGTVIQDIERSDSKALLHNPAIPHDELCHHIVIEKGSSLFALFATEKLLVNSLHHQAIEE
PAQDFKVTARSPDGIIEVLEAKDGRFALALQFHPEGLFVRYPAFLAPFKALVEAASKNSG
*
>SPBDM4_v1_40052|ID:27157357| exported protein of unknown function [Uncultured spirochete bdmA 4]
MKLRYIALVIIFSLGVSAFAATNGTVAISGIVPESVILSVSSSHVNLTANRGSESMISVT
TLTVWSSGHWVIAVESKNGGYLLNSDDLQEKIHYLFTLGILTQSPEELKSPWTSEVQSPT
SKVGLALPLAILLQTEGQMIGEGTYEDLLTINIKQF*
>SPBDM4_v1_40053|ID:27157358| Peptidase M20 [Uncultured spirochete bdmA 4]
MSDVLNTIFPEARALEHWLVDIRRALHRIPEPGNEEHETSSAVRAKLDELHIPYTQIGTA
VVGLLEGERPGRCVALRADMDALPIEEPADRPYASLHHGYMHACGHDAHMTVALGVAKLL
SAHRHGFAGSVKFLFQPAEETTGGARPMIDAGCLENPHVDFVLGLHVAPELPVGHIEVKS
GPFYGASDNLGIIIKGKSSHAAYPEKGIDAIVASASVIQALQTVVSRSISALDSAVITIG
KIQGGTRNNIIADEVTLTGTIRTLSEEVRHSICSKVIDVCTQTASAFGASCEVHIRPSYP
VLVNEEKATELVRDTAVQLLGESNVHLRQKPTLGVEDFAYYLQERPGTFWHLGCRRQENR
SPSPLHSPDFDIDEACLPVGVAVQAASALRYLQAEP*
>SPBDM4_v1_40054|ID:27157359|dapF| Diaminopimelate epimerase 1 [Uncultured spirochete bdmA 4]
MAIRCIPEGFHAHKYHALGNDYLVIDPEELQIFLSQDAISALCDRNRGIGSDGILYGPLA
FFEEGARALPDDSPFTGSRNTFALRIFNPDGSEAEKSGNGLRIFSLYLYESGRVDETPFQ
VRTKGGLVECQILPGEGEERFITVAMGEPQFLPGSTTLSLEGVEYRAVRVSMGNPHCVLM
GPPPSPELAKRIGPLVEHHQDFPNRTNVQFLEVLDRKNIRIEIWERGAGYTLASGSSSCA
AAAAAKRLGLVDDTLTVHMPGGALEIDMQKENIKMTGPAVRAFDALFSPYTLSLAQKR*
>SPBDM4_v1_40055|ID:27157360|alr| Alanine racemase [Uncultured spirochete bdmA 4]
MDHRGTTAWINLSAIRSNYRRLAELARGSAVCPVIKADAYGHGALAIAHALEREGAPYCA
VALAKEALELRESGIQMPLLVFGVPDHSWSSIAIRLGCAQTIYTEEHVRILASAAKSVGK
RAKAHIKIDTGMNRQGLPPTKVKAFAGLLADYPEIAVEGIYSHFADADSPDPAFTNLQME
RFTAALAGFSQAGIVPKYRHIANSAGLLFHPETRLDMVRPGILLYGLSPAPGLGLPPGFE
PALTLTTSIAHIKEVPAGESISYGRTFTTKRRSRIGLLPIGYADGYPRALSNRATVLVRG
MRAPVVGRVCMDMTMIDLTGIPGARAGDEVVVFGGRELPISEIADLLSTIDYEVACMISK

RVPRVHIDESPQED*
>SPBDM4_v1_40056|ID:27157361| putative N-acetylglucosamine-6-phosphate deacetylase [Uncultured spirochete bdmA 4]
MTHNNSSATYIIEGADLIFPDAIMPHATLAVFEGRIAHILADHELSSPLDPAKLDPSFEG
APVLHVSDAFVVPSLVEMHVHGCGSWGFERVSGPEELLGAARFLEERGVGCFVPTILWEE
RALSSLVAAIEASGLPRERLPGIYLEGPFVNPAKRGGIQPGNIRPIDPSLAKKILDASQG
LLKISTLAPELDGIESVYRIFQGAGVLVSLGHSDAKLQSLKLPGHPYSITHLFNAMSGID
HREGGLANLAFSQSPDYVEVNGDGIHVNATSLKLSARAISKDSLILISDAVIGAGMPHGR
FRYYEHDVVSTERGVRYADTDVLMGSNRLGMDIVRNFTAQAGVPLWRAVRAMSLVPRRAL
GMAKDYGSIEIGKVADIFIWDKEMEIATRPEALLASGESFSRDSRSQFGAKL*
>SPBDM4_v1_40057|ID:27157362| putative Cof-like hydrolase [Uncultured spirochete bdmA 4]
MLDPKTLNPSELRFIAMDMDGTILDEGYRLSPHVTQTLNHLQKQGKKLIIATGRIYAAAY
GFLKGAFEPDAFVCTNGADIYGPHGARIATHHIPRQALHALAATGRHHASHEVLFCCYVG
EEWIYEHPSAMVDFYEKRSGIKGLQRNFDSLAGEDILKFLAIGPHEKLLPIREEIANSVP
GLLETVFSHETMLEIMASGVSKRQGLEECLRYFGGTLEEVVAFGDAENDLEMLKAVKAGV
AMGNSHASIQEEVAFVTESVDEDGVAHFLERLFGL*
>SPBDM4_v1_40058|ID:27157363| putative transcriptional regulator, Crp/Fnr family [Uncultured spirochete bdmA 4]
MIDVNALQKYSLFGGVTSEQMERIKPLLGSEHFEAGECPQIEGQPNDKIYFILSGEVDIF
KREVVIARLKEGETFGEMELLDIMPSIATVQAATSLEVVTISNRALYDISKLDLKTFSMV
VMNLARDLSRRLRRMDELACGEAKIT*
>SPBDM4_v1_40059|ID:27157364| Amylosucrase (fragment) [Uncultured spirochete bdmA 4]
VVRLSCPSLLFKSEAIVHPDSIARYIDMHECQLSYNPLLMAELWEAAATKEVRLLAYSLK
KRHHLPSGCVWVNYLRCHDDIGWTFADEDAAALGIKGFDHRQFLNRFYLGEFPGSFAQGL
KFQYNPATQDMRICGTAASLAGIERDLRRDPGKNREIALRRFLLLYGIVFSAGGLPLIYL
GDELGMENDPDWDKDPAHAGDSRWVHRPVFREALFEERHDPATVTGSVFAQFKKMIRARA
AHRIFAVQDIQMIESGHPSVLIFRKVSETETLVVVGNFSEHCAGVSMDVWHSLFEGITSQ
DEIPEAFDLLSDRHFVPEMPPELLPCELVWLYMPNGGRAQ*
>SPBDM4_v1_40060|ID:27157365| Amylosucrase (fragment) [Uncultured spirochete bdmA 4]
MLRGFDSFEHELIERLKARGTGRFSCWPEFERRLDAELGQLTGLLFELYGDRPDFAYWVE
NLVYGAFEAFKARPRWLKARDHALPPESGWYLAQDVVGAVCYVDRWAGNFRGIAERLPYL
KELGIKYLHLMPFFKSPDSENDGGYAVSSYRETNPALGSMAELSALARLLAGEDISLVAD
FVFNHTSDEHDWALAAKSGSAAHRDFYLTFPDFREPEEYSRTLRDIFPESRRGSFTWNEE
MKRWVWTTFHSYQWDLNYRNPAVFNAMAGEMLALANHGVAALRLDAVAFIWKEKGTPCEN
LP*
>SPBDM4_v1_40061|ID:27157366|yufQ| Uncharacterized ABC transporter permease protein YufQ [Uncultured spirochete bdmA 4]
MTFELFLQMFPIALMFSAPIILTALGGLFSERSGIVNIALEGIMMVGAFTGATVTVLLEN
STPAAAWYGLLAGLLAGVVFSSLHAVASINLKADQVISGTALNILAGGLTVYLCQIIFHQ
QSTRSFSNGIQKFSVPGLSNIPVLGRMFFDQVYPTFYIAIAMVLITWFVVYKTPLGLRMR
SCGEHPQAAASMGINVARMRYFGVLVSGALGGLGGAVMVLTADIQYTLVSIHGTGFISLA
SLVFGKWNPFGVLGAGLFFGFSTALSFYAKDIPFLANLPGEFFYILPYLLTIIALLLFAG
KSVGPKASGEIYDSGKR*
>SPBDM4_v1_40062|ID:27157367|yufP| Uncharacterized ABC transporter permease protein YufP [Uncultured spirochete bdmA 4]
MTARKEKIVLPLIAVLLGFVLGSLIVKLTGRSPLSMYAAIIKGFSGIDILNGQPINTRYI
GEFVIQAMPIILTGLSFAFASRTGLFSIGAEGQLMIGSISATAVALLLEAPKGIHLPLVL
LAGILGGALWGAIPGLLKARYNVHEVVVTIMMNYIALHFNNFVILHLFGSADRVKTAPFP
TTALLKSPFLESITRNSRLNWGFIPVILALLIFSFIINKTTFGYSLRAVGYNKEAARYAG
MKVSRNIVASMAIAGAFAGLAGAVITVGTFNFGRVIQSAEGYGFDGIAVALVGGNEATGI
LLAGMLFGGLKAAQPLMQSQGIPREIAGIIQASIVLFVAMKYGIKMVIDKFERKQAPSTE
GDRV*
>SPBDM4_v1_40063|ID:27157368|yufO| Uncharacterized ABC transporter ATP-binding protein YufO [Uncultured spirochete bdmA 4]
VSYIVEMFHITKDFPGIRANDDITLQIEERSIHALLGENGAGKSTLMSILFGLYQPDGGS

IRVRGKEVKISDPNVATSLGIGMVHQHFKLVHNFTVTENIILGMEPHKGPVIDIRTAEQK
VEEISRLYGLAVDPRAKIEDISVSMQQRVEILKMLYRDAEILIFDEPTAVLTPQEIHELM
QIMRRLVEEGKTILLITHKLKEIKEVADVCTVLRRGKFVGTVPVADTDEEKMAEMMVGRQ
VSFHVEKMPAKPGDELLRIEQLNVKNLKGVLGVKNLSLTVRAGEVLGLCGIDGNGQTELV
QAVTGLCKIESGKIYLKDKDITHLSIKERTDRGLGHIPEDRQKHGLVLDFTMGENFIIHN
YYEKPFANFGILNKKAIFENAERLIQEFDVRSGQGAMTPARAMSGGNQQKAIVAREIDRS
PDVFVVVQPTRGLDVGAIEYIHKRIIEERDKNKAIFLVSLELDEIMDVSDRIAVIYNGEI
VGIVDAAATNENELGLMMSGALRKTRSTPHPAHEAVHA*
>SPBDM4_v1_40064|ID:27157369|pstA| Phosphate transport system permease protein [Uncultured spirochete bdmA 4]
MENNVFAGQYRGRKRKDIFFRIAVMVPAGIVIALLFLILAQVVVNGAKSLNFDFFFQQQK
PFGEIGGGIVQAIVGTLMLLATAIVIALPFSLLAALYLYEHKGSKRANGLLSVVNTLQGI
PSIVIGVVIYSWVVVPMKTFSALSGGIALSIVMLPLMISMIKESLEMVPASYVEAALALG
APRWRVIVEIVLPAAGSGILEATGLGIARAAGETAPLLFTAFGNPFLSLNFKGPISALPL
VIYEYIKSPYDDWHQKAWGAALLLVVFVLSLNLFIGTRKKR*
>SPBDM4_v1_40065|ID:27157370|pstC| phosphate transporter subunit ; membrane component of ABC superfamily [Uncultured spirochete bdmA 4]
MNDRRFNALLGFAALVIVFIFAGFLFTLVVQSLPAVKQEGAAFLYGSEWNPVTTHFGALP
FIIGTLMTSAIALVIAIPLSLSAGIVMGEYVNSSWIRSILNTLINVLASIPSVVFGLWGI
EVVIPLIRKLAMLVGAPPYGAGILSASLVLAIMIIPYMSSMTREALMNVPAGLREGAFAL
GLTKQEVVTGIALPFAKNSIFAGILLSLGRALGETMAVTMLIGNATRIPTSLFSLGNTLA
SVIASQFNEAASDVHRSAMMGLALILLVITMLAGWFGRSLMSKGMGAYGK*
>SPBDM4_v1_40066|ID:27157371|pstS| phosphate transporter subunit ; periplasmic-binding component of ABC superfamily [Uncultured spirochete bdmA 4]
MEENAMKRNAVSRKLVLLALFVVGLTGVLGAQSLLGSGASFPAPAYTKMFDEYYKATGVK
VNYQSIGSSGGLKNIQDRVVDFGGSDSFIKDADFSKYPAQVVHIPTVIGAVVVTYNLPGN
PKLRLTGDVVADIYLGKITKWNDKAVAELNPNVKLPNLAIMPVYRSDGSGTTFNFTAYLS
AVSAEWKSKVGNANSVSWPAGQGAAQNAGVAGVVKQTQGSVGYVELAYANQNSMAVAAIK
NSSGMWVDPSLESISAAAAGAFPADTRILLVNTDAKEGYPISALTWIIVYREQNYGNRSR
EQADQLAKLLWWMIHDGQKYVTSLDYATLPAPALKAAEAIIKSITYDGQPLVK*
>SPBDM4_v1_40067|ID:27157372|tmpC| Membrane lipoprotein TmpC [Uncultured spirochete bdmA 4]
MKKALLVLVVAALIAAPVFAQFRVGLVTDVGGIDDKSFNQGTWEGIVRFAQDFKLAKGNY
KYLQSSAEADYVPNLSTFADEGLSLIIAPGFLFEQAMTQVATNYPTRKFLIIDTVVNLPN
VASAGFAEHEGSFLVGVAAGLKAKADGKNIVGFIGGMQFPLIEKFQAGFEQGVKAVYPDC
KILVDYAGDFAAPDKGQALAQKQYNAGAYIIFHAAGGTGNGLIKEAKERSQKGDIRWAIG
VDKDQYADGIYSGSKSAVLTSMMKRVDVAAYDVCKMTMDGKFPGGQSLVFTVKNKGVGIP
DKNPNFSADILAKVKEYEAMIASGKLVVSEVPAK*
>SPBDM4_v1_40068|ID:27157373|deoC| Deoxyribose-phosphate aldolase [Uncultured spirochete bdmA 4]
MNWTGKLIAATIDHTLLKATATEDQIKALCSEARQHKFKAVCVNPYWVPVCARELAGSEV
LIATVVGFPLGANGTAVKAEEARRAVAEGAREVDMVINIGRAKAGDWTSVREDIAAVVKA
SKHAIVKTIIETCYLTREEKVDACRAAVDAGAVFVQTSTGFGTGGATAEDVRFMKESIGD
AAFVKASGGIKTYEDAVAMLEAGASRIGASAGIAIMRDAEASV*
>SPBDM4_v1_40069|ID:27157374| conserved protein of unknown function [Uncultured spirochete bdmA 4]
MNSGEDGPQNGEISLPKQNSVLRSDRLTMDKAAMLRLKRMHLAPYIQLATCLIGKPRRSG
GNMFRHQLDTMAILMDYGYIDSVLLKASLIHDLLEDAPETDPDSILSIDDEAIDVYKLVL
EVTRRPVENKAEFLTRIRDYGSLRARVLKSADRISNMISLGYVTDDVFVRRYTDETEQFV
FPIAERADERMLAELRELVATRREYLRNRIEI*
>SPBDM4_v1_40070|ID:27157375|valS| Valine--tRNA ligase [Uncultured spirochete bdmA 4]
MKSVELSKAYDPKSFEQRIYEMWKSEGHFQPKVDRHKRPFTIVIPPPNVTGVLHLGHALD
NSLQDIQIRYRRMRGIPTLWLPGTDHAGIATQNVVERKLRKEGRSRESLGREAFIEETWK
VAREHRAFIDNQLARIGASVDWSRERFTLDEGLSRAVREVFVTLYERNLIYRGEYLVNWC
PSCGTALSDDEVEHEDEPGALWHIWYELVDGPCPEWPSGRIEIATSRPETLLGDTAVAAH
PNDPRYVAIIGKKVRLPLTDRVIPVIEDSYVDPEFGTGLVKITPAHDPNDFLVGQRHGLE
RINILNPDATLSDAVPEKYRGMKVLDARVAVLADLETLGLLKGEEKTTHAVGHCYRCHTV

IEPYLSKQWFVRMKPLADKALKAWQDGDVVFFPKKWEHTYRHWLENIRDWCISRQLWWGH
RIPVFYCKDCGAMIVSREDPTVCTQCGSGNIYQDEDVLDTWFSSWLWPFSTLGWPKDTAD
LRYFYPTSALVTGYDIIFFWVARMIMAGMEFTGQSPFREVCIHGLIRDKQGRKMSKSLGN
GIDPLEIVEEYGADALKFTLAYNCAGGQDILLDRDSFKMGSKFANKVWNASRYILMNLEG
RAFLSERELTYNDTDRWILHRLNEAAKTVGQALESWRFNDAAQTAYSYFWDDFCDWYIEA
SKLSTKSDDDKEKDRATTVLLKVLDESLRLLHPFLPFVTEEIYGMLPNAKGRLISASYPE
WTKERESPDIQTHFEALKEIVTLVRTLRSEFQISPEVSIPLDLAFDGDFAHAAFIRGHAA
LISLLTGASSVSIGGASREGSVSLAGNGATIHVQVRGLLDIAKLVDHLSKEVEKEKTYIA
KLEFKLANDAFLSSAPPDIVEKEKDKLAVSTAKAAKLDLYIRELS*
>SPBDM4_v1_40071|ID:27157376| Long-chain-fatty-acid--CoA ligase [Uncultured spirochete bdmA 4]
MRETEPWKFLDAYRGRVFSDKWPTLPQLFDITAERFPDSPCFTVYSPQRVSLTYREARTL
IVNVAAHLGSVGIKPGTHVAVTGKNSPEWAIAFIAVLYTGAVVVPIDYQLSTQEIITLIK
AGDVMAAFIDEEKFEEIQKEFHDMPCFALTKKAGNYIYDMTPPQGFKFEQPPVKMQSTAA
ILFTSGTTGVPKGVMLSHENFVSDCFFAQSNLNILHEDVFYALLPIHHSYTMLAVFIESL
SVGAELVFGKRMVVKQILSDLKAAKVTMFLGVPMLFNKLLAGIMKGVKEKGAFTYALIRT
MMAISGFIKKAFGVNPGRSLFHSVLEKASLSSIRICISGGGPLDPKVFRQYNELGIDFVQ
GYGLTETSPILTLNPVEHYKETSVGKILPGVEIRIENPDEDGIGEIVVRGSMVMQGYYKM
PEETAQVLSEDGWLSTGDLGKLDKENYLYLAGRIKNLIVTEGGKNVYPEEIENEFQLYDE
IDQILVRGYNPEGKSVAEEIEALIYPNQDWISQNRLMPNQIEARIQDIITEVNQRLRPYQ
KISRTHILKEPLEMTTTKKLKRQSAKI*
>SPBDM4_v1_40072|ID:27157377| 3-ketoacyl-CoA thiolase [Uncultured spirochete bdmA 4]
MAKVSIIGAYNTQFGNFVKKNKETGEVTDLKSIYDLMLEAGRGAISDAGIDPKDIDGVWV
GSCAPGIFANQEHLAAFAPEIAPDALRFKPTYRCEDACSSGSVAVYSALYALESGRAKVA
LVLGVEKMNLLDTKGVTHALATCSYWPEEGAKGYTFPGLFAEYAKGYAAQYGFDRPMLAE
MLATVSALNYRNGIDNPLAHFGKGGVADRLGLMTAEAILALPDEKNPMIAEPLRLHDCSL
VTDGAAAIVLVRDDLSLAKSEKAVKVAGLAHVNERLPISVRPNMYELMAGKRAVQQAFAE
AGITIADVDLAEVHDCFTINQLLSTEALGLSADGRAGYDYMEGRFTRDDGHCAINLSGGL
KAKGHPVGATGVSMHALVYKQLVGQPIGAVPAKKQPTVGVTFNVGGSSVTNCVTVLRKE*
>SPBDM4_v1_40073|ID:27157378| putative hydrolase or acyltransferase of alpha/beta superfamily [Uncultured spirochete bdmA 4]
MASGFAAVIGVSIFFVTAGNGVPVVYVHGNTGSSVWFRPVMDVPGCRAVAIDMPNFGQSS
PLDGDISIQRYAAYVAGFMDLKGIKEAVVVGHSLGGCVVQALALERPGLVKAMVLVDSGA
PNGLVTPKDRHPVIELMRTNRAVLEQALKATVPTLKDDALFKTIVDDAQKMAPPAWIGNA
EALSHFDITSRCAEYEGQVLVMRGTLDPIITDEMARATAGAYRHAKLVTLEGVGHSVTVE
NPVLFLQIFRDFLAEQGICKK*
>SPBDM4_v1_40074|ID:27157379| putative hydrolase [Uncultured spirochete bdmA 4]
MAEMMVNGVRIVYELSGECDVGVLKAPIVLLNGIAMSISHWKPLIAALPAGTCCLSHDFR
GQTLSGKPAGPYSLAMHAKDLVDLMDALHIERGHIVGTSYGSEVAMEFAMLYPERCASLV
VIDGVSELDPVLEAAVVSWMEAARKDARLFYKTMLPWTYSSNYIAAHRDMLTAREDGMAA
LPAEWFAAFVELCKAFLKINQTQRLKQISCPATVLVGEKDILKHRGFSEIIARNIPGAVM
HVIPDAGHAVVIERPALCAEEIWQAVSLR*
>SPBDM4_v1_40075|ID:27157380|livF| leucine/isoleucine/valine transporter subunit ; ATP-binding component of ABC superfamily [Uncultured spirochete bdmA 4]
MLTIQNLECGYGRMKVIDGVNFAVGAESVGLFGPNGAGKSTLIATVMGMIKPWKGSIELD
GKRIEGAETHTIARMGVALVPQERELFPGMSVEDNLVLGAAYIPHAKDDIPNQLEKVFAL
FPILKERRTQYAGTMSGGQQRMLAIGRALMSKPGLLILDEPSLGLQPSIVAEVFEVLKGL
KSSVSILVAEQNVRESLKAIDRGCVLENGRIVLEDSAEGLRTNPFVIKSYLGL*
>SPBDM4_v1_40076|ID:27157381| Branched-chain amino acid transport ATP-binding protein LivG [Uncultured spirochete bdmA 4]
MSKLKTENLTRRFLGLVAVNNVSFSMEEGEILGIIGPNGAGKTTFINLISGIIMPSEGLI
EYKGQDITYIPAHERARMGIARTYQLIHPLENLTLIENVMVGSIFAKGNGLKEARRRAEA
LCQELGLTGLDRDTSRLTILEIKKMEIARALANEPEVLFLDEVMAGLNADETKDIIAMVQ
KIAQERHLAVGVVEHVMGVIRELTHRVIVLEAGELIAEGKYEDVSKNPRVIEAYLGGGAA
*

>SPBDM4_v1_40077|ID:27157382| Branched-chain amino acid transport system permease protein LivM [Uncultured spirochete bdmA 4]
MNRNVSRIFAALFGILAIALPLFAGDYPLQVARNIMLYMALAITWDMLLRSGQISFGIAG
LFGVGAYAAILGVIRAGMPAWFSIIFAAIFSGVVAFAIGFLILRLRAMYFSIVTLALGEI
FRIIIHNLHDFTGGPEGVVMQQGVIFGGSAGKLYWLTLAGLAVAIAASYWFEKSKIHFAL
TAIRNNEISAKSSGVNIFRWLLLAFVVTSAIQGLLGGIFVQSYGFATPDTVFSADFTLLP
LAMALLGGVYSTRGPIFGAILLGLVSEWLKLKIPYGHLVVYGIIIIIVILFMPQGLKQLV
RQPANKTDRRSA*
>SPBDM4_v1_40078|ID:27157383| High-affinity branched-chain amino acid transport system permease protein LivH [Uncultured spirochete bdmA 4]
MVVKRRTAIIAAMSIAATIALILWKPTVLIYGLQAAGLYAAVAIPMGLVLGIVHIVNLSH
GEFMMVAAYAAYSACKALGMDPLLAIIPTAIVTAIFGWVIFQLTIRRALKGPELNQLILT
FGLAIAFTQTINLIFTSQTYKIPLDYVSASMDIGDITFGTWSFVFVAVAIVYTYGLQFFL
TKTTTGKAALAVGQNPRGAAIVGIDVYRTYGLVFALAMALVGAMGALFLTKSAIFPGVGT
PFTMKSFCLIAMAGVGNIPGILGASVLLGVAENFLRAFRPTRDWAEIVFFVLIIVVIVGR
SLKGRKS*
>SPBDM4_v1_40079|ID:27157384| Leucine-, isoleucine-, valine-, threonine-, and alanine-binding protein [Uncultured spirochete bdmA 4]
MKRMMLALAVFALIAGSAYAQGTIKIGGIWTLADITGKQGSAAAQLAVDEINAAGGVLGK
KLDLIVVDDEGKADKAAAAVEKLATVDKVDVFMGGMGSGAELGKIPAFKKYGKVVMSTGA
AGSVTVEKALGPSPESDFYFHIHPWDYNQGASYAEGWDAIQKQYPNIKIRKIFLAYEEGA
FGKSSWDATKILFGGDNRYIVDGASFKSALFGGGDYTSVLEAAKAFKPDLFLWAGYDADA
LPLLEQSKAMKFSPGIYLGAPPGWPIGFGSSKLSRNVMLYGMWSPAMNDNNPASAKFYKN
YVAKYKEEPATYFAPLSYSAVYILADGIKAAGTTETAPLIKALEATKYASPLGETITFKP
SNIIKHQGITRQKILQWQGGFQEVIWPFEAATAEPVYPFPAWK*
>SPBDM4_v1_40080|ID:27157385| Beta-ketoacyl-acyl-carrier-protein synthase III [Uncultured spirochete bdmA 4]
MHVGVAGIGLYIPKHYMTAADLAKATTVPEDVIALKFGIKRKPIAGPEETTSYMGMMAAK
AALDDADIKAEDIDLLIWCGAQHKDYPCWLAGLYVANQLGATRAWSFDMEAMCGSMMAAL
DVAKSLMMVRDDLNTVLLVSGYRNNDLIDPSYPPTRFMMDIGSAGSACVLKKNLGRNVVL
SSAFKGDGSLSEMCVVPVFGSKAWPPKPEDALHASFIVPDEQAFKQKLGEVTMPNFYAVI
RESMRKSGFVEQGIDYLAILHFKRSAHLAALQELGLREDQSTYLENYGHLGQNDQLLSIK
LGLETGKIREGSRIVMVGAGLGFVWACTVVQWGAWQESVKS*
>SPBDM4_v1_40081|ID:27157386| putative Transcriptional regulator [Uncultured spirochete bdmA 4]
MNSDTRQALIDSAVTLFSTKWYGTVSVAEICRSAGLSNGAFYRHFKNKEEIFCAILDHVV
QQIEKALKPLSSMELSQRLPNFVSIIYNFSQDYTPLVRVFREGQYRLFEYERKLKDVYEH
AFQVVFGRTPSITEYLFALAGLRFASIRAALHQIPVQTDALVTILSKGLFKTQPINADRI
FATSITPLPIELWPDSRDLLLAEGRKLFGEKGYFETNIHDVTSNAGLAIGSFYRYFESKE
SFYKEVIRSVGRDVRHFISLNLGNGLNRVEREMRGLWLFILFLSMDRYCYNIVREAEFVL
PNEVRDYYDAFHRGYLKREDASLTCDSTTCIEFMLGVAHYLGIEVIFDKSPEIARQTIEE
MGQLYIHGLGEMQRPDESASVR*
>SPBDM4_v1_40082|ID:27157387| 3-oxoacyl-[acyl-carrier-protein] synthase, KASIII [Uncultured spirochete bdmA 4]
MPKAVIIGTGLYAPGEPIDNVTLKKLTGVEFNAERQEAKIGIKKRHIARLSGLQESTADF
AEKAARHALEAAGVDPKEVGLFIVATDTPEYISPATSIVLQGRLQGGETDAKSFDVGASC
ASFVEALDAAARHIMTDSSLRYAVVVGVYNMPAYIRDGDAFGWSIFADGAGAVVLERQED
SSSGYIDGAFVTDGTQWNFVGVYAGGTRLPVTRERLDAGEYGLQLLQRLPGDRNVKLWPP
IVLRLLEKAGMSQSAVNHYLFTQINKSVIEQVMEVLGEPLEKTTMVMDRYGYTGSGCIPM
ALHEAIQAGRIKKGDLVVLVASGAGLAVGASLLRI*
>SPBDM4_v1_40083|ID:27157388|cat| 4-hydroxybutyrate coenzyme A transferase [Uncultured spirochete bdmA 4]
MNLQDEYKRKLISIPDAVSKIKSNENIVVAMCASEPQGCMDQFHTVADGVENVRVFSCLT
LKAYDFYMIPEMKGHFELASWFHAPGSRAALKKHTGTVTYYVPNMLHRSATDFIFARKPHI
FFGTCTPPDKHGFVSLSLGITYEKDILENADLVILEVNPLLPRTFGDTHLHVSHVDYFVE
YEQLVPELPSPQPSETDLTIGRYIGELVEDGSTIQLGIGGIPNAAALALRDKKNLGVHTE
MVVDSMMELYEMGVVTNTAKAFHKGKFVATFAMGSRKLYDWLDDNVAVEFMRGKWVNNPS
VVSQNSKMVSVNTCLMADLTGQVASESLGPVQYSGTGGQSDTAEGAVEGFDGKGKSIIAC

YSTAKNGTVSTIVPMLTEGTAVTLHRSLVDYVVTEFGIASLRGKTVRERARELISVAQPN
FRAELTEKAKTIGYL*
>SPBDM4_v1_40084|ID:27157389| protein of unknown function [Uncultured spirochete bdmA 4]
MTRKEYIDILAQRLEGLDEASLNDIVLEIEDHLDGLAREHPEKSAEQIIDELEPPESLAD
SLRDAAGLGPYGERESGAPHHTDQTGQPEGQAEDSSSPSAKKTVRIVIDDEDLEEAIRKA
FDIAKIFKRNRDEKKEGSLKDVYNLSFGIKGTELSDLRHVSVRARSADIRVLFSNGELEV
DTPEEEEPTLLMQHAGKDVLRIATLVGSSEPDRINLRIPASVDELDISTASGDVHVVDRV
GSLSIVTASGDVVVETCEGDVEVKTASGDIKLAQCQENLKVSTASGDISIQADDMCNAIE
ISSASGDIMLVYPDGWGASLSISTVSGEIEHDGLSVGRRRVRIGNGLVPVRISTVSGDVR
IQKEE*
>SPBDM4_v1_40085|ID:27157390| Transcriptional regulator, PadR-like family [Uncultured spirochete bdmA 4]
MGAAEGLYEKWKSQFRKGFLELCVLELLSNSGRSYGLAIMERLNNAGVDVTEGTLYPLLM
RMTKDESIEAQWETPEVGHPRKYYVISNIGRALLVRMREEYEKSAAAQRNLMSGGQGL*
>SPBDM4_v1_40086|ID:27157391| protein of unknown function [Uncultured spirochete bdmA 4]
MEIIHMDARRVWRSVLDLAHARRDALTLSGRMAGKQGRAPRRNIDTSTSRDVYGRENRAD
MYAREEAIELYKRLSFDKRPPL*
>SPBDM4_v1_40087|ID:27157392| putative Hydrolase, TatD family [Uncultured spirochete bdmA 4]
MYCDAHIHLVDLDGREPGFARKALQFSWRGAVVSHDPAEFLQSEALRAKMPPTIAGFGIH
PQNPDMQNATFLSSLCDEKKIRFIGETGFDFFGDGPLRSRNPDAMRQQAEAFLFQARLAA
RAQLPLVVHLRKATDVFMAYGRELDGVPSVIFHCWPGRLEEAKMILKKGINAYFSFGTPL
LRDSKHAIESLLGLPASRILSETDAPWQPPHGSAWTRLEHIVDITDKMAHVLELDLKALA
PLLEKNFTAAYLLDN*
>SPBDM4_v1_40088|ID:27157393| putative tRNA threonylcarbamoyladenosine dehydratase [Uncultured spirochete bdmA 4]
MTNLQNVPSEKSSGSFFDRTRIIVGESGLCALANAHVAVYGLGGVGAACALDLVRAGVGY
LHVVDFDTVETSNLNRLAFGFQRYVGQAKSDAFIDVAKEINPAIEIVGEKLFFSSDTAAG
IIARECSVHADCVDSLGPKASLIAALRQGDFIFISSMGTAGRLMPERLKLGPMASVKGCP
LARAVRQKLSKMQIPLDFPAVWSDEPAVRPLPREGRRGIQGSAPFVPQTAGHFMAAWIVR
RLLEDAHV*
>SPBDM4_v1_40089|ID:27157394| Aminotransferase class I and II [Uncultured spirochete bdmA 4]
MHMFEPFGLERFFARHEFSARYLLCTSDCESMSIDELLALTGQSSEALDALQRTWLGYTE
SKGSPLLREKIARFYRGLDPDSILVHSGAEEAILNLYLATVRPGDTVIVNWPCYQSLIEI
PKGLGAETLKWKVREHEGRWFFDPDELEQLICGAESSSGRQKVKMLVLNMPHNPTGALMT
PGEFNRVVDICRAREILLLVDEVYRMLELGATQRLPAVCEIYENAISLSVLSKAWGLAGL
RIGWLATRRKDILDKVAAVKDYNSICASAPSETLACIALDHSDKILARNKAICEANVVHF
KHFFERHRELFTWIPARAGSVAFPALLSAGHQHWHDAASLADQLLQETGALILPGALYGQ
EFGLHFRIGLGRRAVPEGLSVFSRWLEARGEH*
>SPBDM4_v1_40090|ID:27157395| protein of unknown function [Uncultured spirochete bdmA 4]
LGRYAAEKVSRRILYGSDFMVNLFKVRSYRD*
>SPBDM4_v1_40091|ID:27157396| protein of unknown function [Uncultured spirochete bdmA 4]
MMQADVEAFWANFEQEIGETILSKTMGQHFSSRKSQGEWGLLVLTGSAIRFRPTPGENWF
QSLFRMAAPKVPQEPLSDIVLPLAFITAIELPKKRFFDFLFSPPFTVFTLRYRLGEEEKS
LLLGADPKSEFFDKLLAELPVLNGT*
>SPBDM4_v1_40092|ID:27157397| conserved protein of unknown function [Uncultured spirochete bdmA 4]
MSDIYQQLEDTNILFFEYGITAIDAYYDEKPRPIRFLVVQGSVIELARCFGELQYPALPY
ADASFQEEARFLCTENALEDDLGGPWMNFRKNPLNGIFYDKDNIYDELRARTLPSRYAKS
ERSLFDAAIFATLRETLMPESEELVIPASTSALFQKDLLISILSGPYPASGFELLKRSGF
IAREWPELALLDEVDHSKDFHPEGNGWNHTMQTFSYRKDRDLTLSLALLLHDIGKPESQS
REGRRFDQHAELGARRARKFLSRLGFPKGIQEDVGFLIRYHMMPAALAVLPIQKTEALIT
DARFPALLELFRCDEFSSFKDPERYYEACNAYQTIRRNMKNPYRNADGSKQRANKRPPSH
Y*
>SPBDM4_v1_40093|ID:27157398| putative metal dependent phosphohydrolase [Uncultured spirochete bdmA 4]
MKRKLFVPIIITLVTILVVGVAFYGVQRYVADRNFDTIDHALKSVIDLQASAVYNGYFAW
TGLYDLIMEGKLAEANELAQDAKESFPLIDSLEIGQGIPPEENFVITLDGELLRIEYPIR

NDDETKTAPNTFAVTVIQAQNLLDIVSPHEFRIDTVHGRKTIYGIPVSPSVPFFSLWNIA
FMALLTSLIVWFILQHYFRKSDFFLDTRGLESIIYLFEQTEKVSASHSRNVAIFALFVGK
KLGFKGKRLKNLYVAALLHDIGKIGVSTSIVTKTGKLDSKEFAQMKQHPVISARILRSFK
EFEHLSNIVLHHHEREDGSGYPDGLKGKEIPLESKIIAVVDVFEALVGERPYRNPIHPSS
AFEKMKQMPLDQRIVNVLIEHYGELGTFKPPRWVLSYSPWMFAS*
>SPBDM4_v1_40094|ID:27157399| protein of unknown function [Uncultured spirochete bdmA 4]
MKTVFVSAFLEDAIVVKTMLESAGIPAELLSDQMLDINPFYSIEPKGAKVIVPDDAETDA
EAIVANFKEHKKIEK*
>SPBDM4_v1_40095|ID:27157400| Peptidase M24 [Uncultured spirochete bdmA 4]
MQNRILAFSNLLQIDHYLGNSYNIRMFTREIYIKRKEAFIRALCERGITRGTFILLANSE
SPRNYPSNCYPFRQDSSWLYFVGTGFPDFGYSLDIETGRTCLYADEPSLDDIIWTGELPS
SKELAYKAGIDYSSPFSALHSLVADRLAKQEPLFILPSYRSEQADKLAELFSQGDSSRRD
LLRQFVDPRVILALVGVREIKGPEEIAEIERAVAITKAIHEDLLHSLEPGWTEKAAADYV
LSRASAQGCELSFATIATCRGAVLHNSPTGYAATAQDTFLLDAGVEMPSGYAGDLTTTFP
VGPRLGTQARAIYEVLYQAFETTARALAPGVRFIDIHKTASLAIAKGLISLGLMRGDPHE
AVEAGAHALFFPHGLGHQIGLDVHDMEGLGEDYVGYGEELRRSDQFGLRSLRLAKTLKPG
MVHSVEPGIYFIPQLVQKWRAENICKDFLKYDIIEKWMSVGGMRIEEDWCITESGARRLG
PAFDKKAEALEKARSGI*
>SPBDM4_v1_40096|ID:27157401| protein of unknown function [Uncultured spirochete bdmA 4]
MDYVRYCLASNLRLRRSILQMSQEELAHLANLSPGFIANLETGRNFPSSKAILKISSALK
IEPWKLFLDPQKQDMFFTRDEVFQWLEDSRERLLGLSPKQDEQNDESQSSDDASPQD*
>SPBDM4_v1_40097|ID:27157402| exported protein of unknown function [Uncultured spirochete bdmA 4]
VSDCWGFLQNKTNRTTNPSQATTLHPKIDNVCFAFYYERVKHKYACLLIITLLVSFAAMP
PAFAEPEIKTLANGLTVFLNPIEGEENVAVVLAFHAGADAQTAPVAGLFKFLEYALFNGP
ATKPGISEPALAIDVLEPNSIEGGSSIDRFEFGFSSKKENLVPALDTLLYLFSQERREFI
FAQTDGIEYARQATRALIQNELSDSDMLSNMAITKKLFAKEPYRLDTLGADYVIEKVDAS
QLKSFASLWLVPNNACIAIAGGFDKEQVLALVDQRFGQLSKARNPWPAGLATFPKPGVSR
PVFLVFPDSGIPTGQMEIEIRYRGPDPKDTHAYTSALMLQELANAPSSRFQTAVRKGMPK
DSDPENLRIVYTPTRNSSWLAIESMLSVPAGKRPADLVFTFKELVRSTELYMIKANASYF
PAAEYQKARESLLEKKMATQGDPLQSAARMVTLWSWGIPSFLFQESDAIMKSGQKDISQF
VDTYVQKNLEVVMVRIDPNLYEANKKNFSSYGFETISNQNALWWR*
>SPBDM4_v1_40098|ID:27157403| (P)ppGpp synthetase I, SpoT/RelA [Uncultured spirochete bdmA 4]
MDEILQNFLSKAEHRFNAEEKNFLSSAAEYTQSTHGDRKRASGEPAFYHDVRVAEILLEL
GMDLESILAGLLHDTTVDRVSGFSPVPNSAQESSNKPDSGGQPQSSTAQNPNKVPSPQGK
SGRTRKEKDAGALNSLLAAPDPHIEELYGKETALIVAGVNRLSSVRAKNKSVHAAETMRK
MLFALTSDIRVILVKLADKLDSMRTLKYLPEERQKEIATECIDIYAPLADRLGISWLKDE
LEDLSLKSLNREVFDQIKDIVNVRKDARQEFLKKVTERILSSAAKEEIEVEVSARAKHFY
SIYQKMRKRGKSPEELYDLLGIRIICKSINECYSLLGLVHRLWKPIDGRFKDYIAMPKSN
GYQSLHTTVLAYGGQLLEIQIRTREMHLVAEYGVASHWLYKKGTTSELPQLEDLPIINKL
KQWNQFIAEGDSYLEEIKRELLRDSIFVFTPKGDVIELPAGSTPIDFAFAIHSDVGGHCL
GAKVNGTIASLDSELQNTQIVDIITSPNAKPNLNWLKIAHTSKARSKIRQLLVQTGQAVA
IDKHIVAARKSEKAPAESPQARPHAQDRAQAEAQRGRDEGFPSVVEFHSLKPGLVLRSEK
AGVSVGGMRNMMIRIAGCCKPVTGDRIVGYVSRGRGIIVHREDCRSLMSIADFDERRIDV
KWEDEAINTTARYRVTTKKTVDIFSEIESTVRKCSGRLKEGKLGERGDGTLSGFFILEVD
SHEDLRRVAKALRTLPSILSIEEETL*
>SPBDM4_v1_40099|ID:27157404| putative Release factor glutamine methyltransferase [Uncultured spirochete bdmA 4]
MTYRDMLLEAAHSFSSSETPFLDAVLLLCCSLDLPKEKVLAMLPDRVENVPTSFHEMVER
RRRGESVAHIRGFREFFGREFLVNDNVLSPRQDTEVLVDAALESGDALSKAFNTGEGASH
LSVLDMCTGTGAVAVSIAAERPQWRVSASDVSPSALDVARENARRLLPSPSISFIESDLF
FSIEGRFDLIAANPPYVSRAQAERLVGAGWKDPLLALDGGIDGMKFVRAIIMEGHKFLSQ
NGVLLLEMDPFQIPEATALFVKEGFCDIRTWKDLAGRTRVAGAHHG*
>SPBDM4_v1_40100|ID:27157405|prfA| peptide chain release factor RF-1 [Uncultured spirochete bdmA 4]
MRSRIAEIEELISKADILRDQQKYRTLMKERARLSEVIETFDETQRIAFSIHETEALVED

GQDDELKELAEQELLQLRSRSLVLEERLKSLLMPRDPFADKAVIMEIRAGTGGEEAALFA
ADLYRMYTRYADGHGWNLEVMNMNETELGGIKEIIFSISGGAAYERLRYESGVHRVQRVP
ATENSGRIHTSAVTVAVLPEMEETEIEIKPEDLRIDVMRSSGPGGQSVNTTDSAVRITHL
PTGIVVHCQDEKSQIKNKAKAMRILRARLFEIEDEKRQAERSAERRSQVGTGDRSERIRT
YNFPQNRLTDHRIDLTLYKLDLIMDGELDELLEALVAWGKQQTFESGGREMA*
>SPBDM4_v1_40101|ID:27157406|serS| Serine--tRNA ligase [Uncultured spirochete bdmA 4]
MLDIRFIKDNLDAVRQNIRDRYMNADADLVVSLYDKRNGTLRELEDARRLRNENAQAMKS
PLSAEDRARRIEEGKRLKETIARLESELQEVETSFAAEMAKIPNMAHPMAPRGKEDKDNL
EVKRVGVPTSFDFPPKDHVELGQSLDIIDFDTATKVSGTKFYYLKNEGVILELALVRYAL
DILSKHGFTLMTTPDVAKTEILEGIGFNPRGAESNVYTLEGEGTCLIGTAEITLGGYHSG
AVLENEQLPIRFAGVSHCFRREAGAAGQFSKGLYRVHQFTKVEMFVYCLPEDSGQIHEEL
RAIEEEIFSSLDIPFRVVDTCTGDLGAPAYRKWDLEAWMPGRNSGEWGEITSTSNCTDYQ
ARRLNVRYRDEEGKNRFVHMLNGTAIACSRAIIAILENFQQEDGSVRIPKALIPYCGFDV
IQPRRGI*
>SPBDM4_v1_40102|ID:27157407| protein of unknown function [Uncultured spirochete bdmA 4]
MSNEPNNNPSKHGNGKFRNHNFRRRKPARSAIQQEANASSQKPQAERATQELKICAVCGK
PIFDLTGAIASREDGEPIHFDCALELLSKEEKLTPDEKLMYIGSGLFGVCAQSAAGKLEI
RRKIRWEAAGTIQPWRKPMMSSPSLP*
>SPBDM4_v1_40103|ID:27157408| protein of unknown function [Uncultured spirochete bdmA 4]
MKQSNISRDMPGWAGSTPDDFLLSKYDITEEMKATNWKRLYELKHKLIDDRYLEGAIASI
AEMLSNTVKGKSR*
>SPBDM4_v1_40104|ID:27157409| conserved protein of unknown function [Uncultured spirochete bdmA 4]
MYVARGKLKKCTAQRGREGEDKALQYLAGAGWSILSRNFRARRGEIDIIAARGGILAFIE
VKTANRYTREDLQHVIRAKKRRSIIETSKLFLTMNRKYNQYRIRYDVMLVQGDACVRHME
GTFAEHDEAE*
>SPBDM4_v1_40105|ID:27157410|rplS| 50S ribosomal subunit protein L19 [Uncultured spirochete bdmA 4]
MNLVQQIQAEQARTDLPAFRVGDTVRVSFKIVEGKTERIQVYEGLVITTKNAGAGKTFTV
RKNSYGVGVERVFPYNSPRVEKVEIVKTGKVRHAKLYYIRTKVGKKSKVKTLVGGRKSDQ
RQA*
>SPBDM4_v1_40106|ID:27157411|trmD| tRNA (guanine-1-)-methyltransferase [Uncultured spirochete bdmA 4]
MNIKLLSLFPEILEAYFSASIMKRAVGKGLVSFELVNIRDYAYDRHRKCDDEVFGGGAGM
LMKPEPLDRALEAAGSPELRTIYVTPSGRLFNQAMAADLAREENLVILCGRYEGIDQRVI
DTRVTDVVSIGDYVLSSGEVAAMVLVDAIYRLVPGVISGESLSEESFSAGLLEYPQYTRP
EEYATMHVPEVLVSGNHATIARWRLKASLIKTLAYRPELLHTIDLSGEVGRLLKEVIDEG
GENEPRTANSGGTGKN*
>SPBDM4_v1_40107|ID:27157412| putative Ribosome maturation factor RimM [Uncultured spirochete bdmA 4]
MNDTRKPNYLAVARLGSVRGLEGELRLVSYSGEFSHIARAGEILVGGPLGLEDARPMKIL
HVLQGGWGASIVFEGYGTPEEARQLAGREVFLPREQACPKKPGEYYVADLVGMIVTVGGL
RVGVIAAVIGGGADDLLEVRLDRGGAALVPFRKEFVGNVDEEKGELEIVSSWILE*
>SPBDM4_v1_40108|ID:27157413| conserved protein of unknown function [Uncultured spirochete bdmA 4]
MERDLVEYIVKSLVDNPDAVNIKVIEGEKSTILELRVAENDIGKVIGKNGRIARAIRTIL
SASASSQSKRVILEILD*
>SPBDM4_v1_40109|ID:27157414|rpsP| 30S ribosomal protein S16 [Uncultured spirochete bdmA 4]
VSVHIRLKRFGAKARPYYRIVVMDSRSARDSKTIDELGYYHPIETEDKQLNINETKAREW
LSKGALPSDTVRSLFNKKGIQLG*
>SPBDM4_v1_40110|ID:27157415| putative tRNA-dihydrouridine synthase [Uncultured spirochete bdmA 4]
MIPFPRNSLLLAPMVGITNRAFRTFVHEMGDADWYTTEMASAEAFLSGGRNESVYLDPAP
CPPRTSVQFTARSPDSLAAACRAMSKVPAERRPAGIDINMGCSAPHIKSSGRGAALLDNV
PLAKEMVAAARESWAGPLSAKIRLSVTKGEEGTIFLAKSLAEAGLDFLVVHARFDTQKFR
RKADHRFAVSLAKELLIPVIANGDISTAEECRSLLASGNIHSLMIGRAAVREPWLFLRLH
EELAGISVASAQKHDLIAIGLRYIDLAETLLPPEWQKETCRRSFSYYADNVTFGHHLKFS
LINAPSLDEMRIILKKYFDEVPQDRFL*
>SPBDM4_v1_40111|ID:27157416| Long-chain-fatty-acid--CoA ligase [Uncultured spirochete bdmA 4]
MANTIPQMYIEITQKQPKANAQLSKDAQGKFQPTDYGTLLQEVGICASGLHKFGIKRGEN

IGFISDNRKEWLIADLAMLGLGAADVPRGCDATAQEIAYILSWSECAIVILENDRQLQKI
LDIKAGRIPFTSQSLREAPYAHLKTVILFDPPPQSPQSAAEEARNAGLSVLFFSDIMKEG
QALHEKEPEFFQNEAAKGTRNDIATIIYTSGTTGDPKGVMLSHGNFLHQTEYLPVLLGVK
SGDVFLSVLPVWHSFERAVQYIILQAGATVAYSKPVGSILLADMQAVQPHWFTSVPRIWE
SIKDSVYKSVRQSSAVKRAMFSVFIAIGEAHAYFRNMLLDRLPKFSSRSRVLDIGMAIIP
FVLLTPLRALGDVLVFKKIKVKLGTRFIAGISGGGALPPTVDKFFDALGILILEGYGLTE
TAPVLGVRLKTHPVTGTVGPIHRGTEIRIVDEHGNVLPAGQKGVIQVRGPQVMLGYYKRP
DLTTKILSDDGWLDTGDLGMLTIHGEIKIVGRVKDTIVLRGGENIEPVPIEQRLCESTYI
QQAVVLGQDQKYLAALIVPQQETLIQWAEENEIPFNDYTSLLDQPEVKELIDSEINAFVS
MKNGFKPFERIFRFALLPTPFEAGRELSAKQELKRHAINELYKNQIRLLFTE*
>SPBDM4_v1_40112|ID:27157417| protein of unknown function [Uncultured spirochete bdmA 4]
MRTEGVDIDFRFIEDVQSIENIRRIVLRVSEGDITVIKSHDVYLRYYIELEGVSGEIDAW
SCKVRRAESIAILNVMGNDFVRISKCSVFIPSYISDIEVHSSRGSIQIRDMPSNVLAITE
RGNIGVTGAKFVEASSVSGSINIEGSEGCSVRSIDGVLRCSRISGSAQVEIQNGSAFIDQ
VEKNVAAVSEQGKINVRRVGGRIRLISSKGDIEFEVSGLFGGGEIQTYSGDISVQLEHSS
VEFRAETLSGRIDTSHSVNTAGMGPQRCAFRTGDGARRLYVKSVLGDIEVN*
>SPBDM4_v1_40113|ID:27157418| membrane protein of unknown function [Uncultured spirochete bdmA 4]
MNISDQKLQEFESTLKEIRDAAISDSKLFSDHDFRRAIIWLSFGFSIVITAFCVAGHALI
GNGDAATTHAAILMFWIFIILLAIGGTIKITLISKIMARKNKTLFSVLRIVYGRGPLAVI
IAAIVSIVVTSAFFMTAGPGSLAIPLSAMFISFGVFALDLRIRLPEFRALGWSLLILGST
ALFFVQESPWLWGGLVWGGSFFALGVAGLFAMRAS*
>SPBDM4_v1_40114|ID:27157419| Transcriptional regulator, ArsR family [Uncultured spirochete bdmA 4]
MNLPDLDKVIHERARLRILACLTSQPRHICSFTQLKETLDMTAGNLSSQLSVLERSEYIV
IEKKFRGKKPLTEVVLTEKGSEAFGNYIDTLDQMIAALKSGK*
>SPBDM4_v1_40115|ID:27157420| conserved protein of unknown function [Uncultured spirochete bdmA 4]
MAEVVLTLKNVTKIYGKGEAEVTALHHISLAFGQGEFAAVVGPSGSGKTTLLNIIGCLDS
PTEGELAWKGARMKSMNKTELATYRRTHVSFVFQSYNLIPVLTVTENVELPLLIEGNHDK
AQIHERARAIITMVGLADKANRYPTELSGGQEQRVAIARALVKKPSIVLADEPTANLDSH
AAGEIIELMGEINKKTNTTFLFSTHDPRIMELAHRIITLCDGQVESDERRAP*
>SPBDM4_v1_40116|ID:27157421| putative ABC-type transport system, involved in lipoprotein release, permease component [Uncultured spirochete bdmA 4]
MKADNRMLFQIALRNVARHSRRTAITAVVLAVGIAVFITFQSLLAGMDRVTIDTAIDYDN
GSISVRSKDYEAHATSQPLEYGIQDPEGVMGGLAALLPVGSTWTPRTRFYVQVSNWADET
PALAFVVRPDTDRKVFKTAQKVVGGGWGVTTKAVVGTDLARDLNVKEGDSIVVTAVLPNG
SLNAIELVVGGIADLPLYSLSQQAIYMTTQDAEALVDAPLPVTEIDIRLPPASRLDVLVA
QADSAASRIEKNNNDLAAVSIGEAMRDYLAMRNMKSKFAYIIIVIVLLISSVGIFNTILM
SMYSRIREIGVLAAYGLEPKQIKRLFSLEGTLVGVIGSTGGVLLGAVFVWWLTSRGISFG
GMFGNLDLGNLPKDLFIRGEWNPATFAQGFIFGVLVSWLASLMPARKASSIEVTEALRFV
*
>SPBDM4_v1_40117|ID:27157422| conserved membrane protein of unknown function [Uncultured spirochete bdmA 4]
MNLISFAWRNLLRNTRRSLLAVLSTFLATLMVVAGNGLTEGFLDSMVRNYAKNETGHIHI
TTAAFRERSRFMPVDEYIPDADALAEKIEGLSPGSSQAEHLLAAPRIRFGVLLSSGPYTK
PAVVIAGDPEKEKFLLMLNRNILPGGSYCDEPGTAIIGSGLAADLELKVGDTLKTVATRS
DGGIGFKSLRVSGIFQTGTNALDGSMFQMRLDDAQTMLAMPGGAQQILVMLPDYRQANAW
QKKISGALSTEESANLSVLPWSAIGEYPKVIQLAEAIYIGMWFFIALLGAFIIANIMTMV
VLERRRETGILMAMGMPPAKIVLSFLIEGTMLGLGGSVLGTAAGIAFNATFSRKGFDFSS
ALAGFSWPIDNTIRPTISLSGVLSGILFCTVAAALMSFLPSWRASRMKVVDALKAVQ*
>SPBDM4_v1_40118|ID:27157423| putative Negative regulator of sigma E activity [Uncultured spirochete bdmA 4]
MRVQKSRSKIRFPAIVAILCVAGVISVSAQAAAGGGFSREAQAILDAIDAAQQYSSISYV
GMMEITQGSRVLKKQFAAYARGKDRVFLEFSNPEDRGVRMLRLGSSIWMYFPSERDTVRI
SGSLMRQGLMGSNLSYEEVAEGESLKDFYSAEILREETLDGQPCKVLKLSSSRTDISYPV
RILWVDNTREIPLKIELYARSGILMKTMYIKNVEPISGRLLPTTVEIVDALKKNSKTLFS
MDSVRLDIMLTDSMFSMEALTK*

>SPBDM4_v1_40119|ID:27157424| protein of unknown function [Uncultured spirochete bdmA 4]
VKVLEQRRKVPLRASGGRKDQSSHGACREDTGRARSPRALSFMRIFGAAALGAWVMLCPY
RSSAQSASLVFTSTTGVATGVAALSATPSAGFDEEIVLNGRLQTGPLRVTALTSVSFSTG
LASLGRWSVFSSGQTLRWSWPAWSSRAASPDTLASIEIEELAAALSGTLAHTGFRIELGK
FPLKWGVGKAFRPSDIFKTMDYASLIPASKGLPAARLALFPSPLSRFEAVASIDGDSTVT
AGARYLTALGDTAAIAASGGRRRQTGAADEYSGSFEGQIDAGIFTPYTELSVRTDGSSAY
AITMLGSSLAAGDVTLWLEGQASFGPATMNAQLFLLGTWKVSELTSVSMPVFWFDDARIL
SASALAQFQGILDGRLDITATGRWLSATTPPGFLWKLSAYWTRSLSTY*
>SPBDM4_v1_40120|ID:27157425|ileS| Isoleucine--tRNA ligase [Uncultured spirochete bdmA 4]
VYKPVDPKVNFPQQEESVVQFWEERKIFQKSISQRESAPEFVFYDGPPFATGLPHFGHFV
PGTIKDIFPRYKTMRGYRVERRFGWDCHGLPVENLIEQELGLNSKTDIERYGIAEFNEAC
RSSVLRYVKEWRQIVTRLGRWVDFDHDYKTMDPDYMESIWWVIKSLWDKGLIYEGHYILP
YCPRCSTVLSNHELSLGGYKDVHDPAITVRFKITSLPASNTDPAFAGLADGSTYFVAWTT
TPWTLPSNLALALGPDIDYVCVVDGDERYILAESRLSAYYKHPESLQLAWKKKGRELAGI
RYEPLFPYFASLSEKGAFVTVLGDHVTTEDGSGIVHTAPGFGEDDYEVLKGTGIPTVCPV
DAECKFTDEVPDYKGIFVKDSDKAIMERLKQEGKLVKRDQILHAYPHCWRCGSPLIYRAI
SSWFVKIEPVKGSMLRANKSVYWVPSHIRDGRFGKWLENARDWAISRNRYWGNPLPIWKC
DTCGKTVCVGSRKELADLGGDVPEDLHKHYIDKTTLPCSCGGTMHRIPEVLDCWFESGAM
PYAQNHYPFENKEKFEAKFPADFICEGLDQTRGWFYTLTVLAAALFDKPAFKACVVNGLV
LAEDGKKMSKSLRNYTDPSKVMDKFGADALRLFLMNSAVTKAEDLCYSDEGVKEVLKSVI
IPLWNAYSFFVTYANIDGFVAEGFDCSDVENPLDTWILSVCEKLVADVTAGLEAYDLQAA
IGPMVEFVDSLNNWYIRRSRRRFWKSENDADKKSAYKTLYRVLRRLTFVMAPVIPFVTET
IYQNLKLEDEPESIHLAIWPAEEVRYRNLDLERDMRIVRQAVSMGRALRVANDLKIRQPL
SSVQLITRQPEEQEVLTRMEDILREELNVKSVIIHEKEEDLVEYHAKANFRVLGKELGPD
MKEAATQIANMSGAEIVHILNGATAPVTLRSGRTISLDASRVEIVREEKQGLKVLNEGTL
TVALDTTITEELLHEGYVRDLIRGIQNARKEAGFEVTDRIVLTVSGDEELHAALERFSQL
VAEETLAVKIEWSDQAKGISVEAGEKTWTVSLAKA*
>SPBDM4_v1_40121|ID:27157426| protein of unknown function [Uncultured spirochete bdmA 4]
MSGESAFEKRHRKMVVPRGIALFGALLFIAFFVADRELISPSFPRDFVRISSRLDAWENR
QEGFRLVLVSNDDRAQGFLEWGALGLYGRVVEAKADGSARTFFLGDGGMNGIELTTAMRH
GSIEVHYRPPVHPDTKGSGGLQEATVAGTRLKAADGLRILAAQYKKTQKDGRGISLRLQY
LGAGQASQAHTFDEVLRRGESPYQMAAFRRAQQEEASLNVYQAPAELSQSQRTVQPEQSQ
RSVPPLEYEEIEYPVFISDDFVSVATQRYLFNGGAHGAASTDFDVIDRKTGVRLGLSDIF
AGDDWKAGLVPLLKAELVRQHGFQNGQNEAGANTRDSGDPARAELKNQGQDDLRTLGLFE
PDIWPSEDVFVCGSGIGFEYDRYQIAPWYMGEFILVLPWSEVKPYLSPALLSAGLFQ*
>SPBDM4_v1_40122|ID:27157427| exported protein of unknown function [Uncultured spirochete bdmA 4]
MKFVKRFLPVLVVVALIFASCATNTGQVPQGTETPPAVTQGGQQEAQPQAQPQPPSVEKY
ELQNLFQQANTLKTEATDFDLKSVDPDGFLAAGNQHDAVAAAYNNLVETPAQYDGVKAYP
LKAELENLVSTWDALIQKDMPIRADAAAKHADEMQAAADNADARTQAPMQYDAGLRYYSQ
ASAFKEASNYAQTITSYQLAAVAFDAAAAAAQVNVLRADIEANGYSKYESIQMYFTMGED
KYKEAQDLWQKDTLDDMSASAASFKEAQNYYSFVVTKGAELKALEGKDKASGAQAEALQV
KADVNAPDEYQQALDILAEADQSMQEGSYVKAYSSYWDAASAFETARDAAGAKQQEVTDA
IAKAEANLTDAKDRASELGLEDNIYLTAATEHLDKAKALKEEVLYNDALFETNEVSNYIG
LSDNFVQQEAQIREKARLEQLAKDKAAAEAAIADAKARMAWADQVDIQSDYPKEYKDAST
SMAGAEIAYNNEKYVPAKTLADEVSSTLSDEFQQQVLAAREAAKAKAAQAEEEARQKAAN
KQAADVAIADAETRMAWANENQVRNDYPDEYKGASVAMVGAYVAYGNEDYMLSKQKAEEV
SGILSNDFQTQVAADRAAKEQLAQDKAAADEVMPKARDRMAWADQNNIKADYPTEYNDAF
TSMSAAEIAYNNEKYAPATELANEVLSTLSSDFETKVASDRAAKAMQVEIEKAGKAANAL
GAAHSRMAWADQVKLASDYSDEYRDASTSMQAADSAYSQKEYDATVILANDVVRILSDDF
MAKVNGERAAAEAAVAQLAQDKAAADEVMPKARDRMAWADQNNIKAEYPNEYKDASTSMS
AAEISYSNEKYVPATTLANEVLSILSPDFEAKVAADRAEKARQAAQAAVAQNMKYAQTAI
ADAQSRYDWAASKNAANNYPELFKQGGEALADAKTAYNALDYTRAKDLAAQSYWTLMQIN
EFAPLPSTYKVRLIPQRRDCLWRIAEYTFVYNNPYKWPVLYQANKATFKDPSNPDLIFPG
QILKIPSIKGEAREGAWDPDKTYQPLAK*

>SPBDM4_v1_40123|ID:27157428| putative M18 family aminopeptidase 2 [Uncultured spirochete bdmA 4]
MEQKIKRLMDFIDASPTPFQAVDSLISMLGAMGAQGLDERQVWQLEPGVVYYVSRGGSSL
VAFRMGLKAPAEAGFLLAGAHTDSPALKIRPEKELQSEGYMRLALEPYGSPILSGWLDRP
LGVAGNVAVRENDGVLLRFYISHEPFAVIPNLAIHLNRDINKGFEYNPHQHMPALFGLAS
SLAPEKSADTPPGTSEIIARVAAELGVEPAAIVSTDLRLFDIEPSRLIDGSLINAPRLDD
LAGCAAVMEAFVGVEAQAATQVACFFDAEEIGSMTLGGAQSSYLRDILARITLASRNSGQ
DFYRALSCSSSVSVDASQAWHPGYPDKFDEFYTPVLGGGVAVKSNANMNYATDFTSMQIA
EIAAAKANLNIQRYMARADIQPGKTIGPITASRTGIPTVDIGHPLLAMHAIRETIAASDH
LDMIAFLRAFYS*
>SPBDM4_v1_40124|ID:27157429| protein of unknown function [Uncultured spirochete bdmA 4]
MPYLSIGQLEKLLSVPASTLRFWEKSVPFLTPLRTKSGRRTYSLSEVALIARLKHLALDR
KFGLRRAQRLLEFEMLYEDQDYRANIKALRDGILLLMMESGQWQQAYRDFTVNAKKSGKE
LFDGAEDQAFDGFH*
>SPBDM4_v1_40125|ID:27157430|der| GTPase Der [Uncultured spirochete bdmA 4]
MNKKEVARKISPPGSGAERQADIDISPAVRYENLPLVVLAGRPNVGKSTLFNRLVGKRRA
ITDPTPGVTRDPIEEECELWGTGRRVLMADTGGFRLEREGLDELVVARTLAYIERASLIL
FMVDVTEITPEDEEFAAFLRPYAKKLVLVVNKADSPERDALVWAHARWGFDPILFVSAEH
NRNIDELEDAIVARLDFSRVREVEIDKADIRIAIMGKPNTGKSTLLNALVGHERSIVSSE
PGTTRDVVEGRFLYKGRGITVLDTAGIRRKKKVTDNVEYYSVTRSIAVVSRADVVILLID
AEEGLSDQDKKIAAFAVEAGRPVVLALSKWDTMPTMKNAFEAARDRLRFFFGQMAYAPVI
AISAKGGQGIDKLMSTVVSLYGKANKVIETSRLNQAVAAWVEESPPPAGPHTRFKLRYAV
QASVNPQRFIFFVTRPEAVAESYRSFLKNKIRLELGLDGIPVQLELRASRKDRLRS*
>SPBDM4_v1_40126|ID:27157431|ptsI| Phosphoenolpyruvate-protein phosphotransferase [Uncultured spirochete bdmA 4]
MRITKGIAVSPGVSIAPAFIFIDDSAEIPQYHITVADVQSELDRFREASLLAKREIEALR
DHAKKEAGEEQAAIFDAHLMMLDDPELLDQIEFSLNDNLFNVESAILSFETEMVDKLSSS
PDPLIQERVSDVHDVFRRILGHLSKKERISLSDIETEIILVAKDLLPSDMVSMSRSMVRG
IVTEAGGRTSHAAILARAFEIPAVLGVGPDFVDQVKPGQPVYVDGEKGIVAVDPDDVFIQ
REKAARLARVQREKENKGLREVFAQTKDGTRISLVANIEMPDEVTNAIEYGAEGIGLFRS
EFLFLGGHVPGEEEQCKEYTRVVQTMQGRPVTIRTLDIGGDKVLPELGAMGEKNPLLGWR
AIRFCLEKTELFKTQLRAILRASIHGKVKIMFPMISTIDELIRAQGLLAEAKWECKAHGH
SVEPNIETGIMIEVPSAAICADVLSRSCDFFSIGTNDLSQYTLAVDRGNQKVAYLNEPYN
IAVLRLIRMTIGYGEHAHIDVSLCGEMAADPASAVLLVGMGLRTLSMSASAIPAIKRVLM
SITIEEASALAHQALSMHTSSQISALLTSKLNL*
>SPBDM4_v1_40127|ID:27157432| protein of unknown function [Uncultured spirochete bdmA 4]
MGIRLDRNNWVYYIGRGSNLRALLLSIGCMLVLLGSSFRPSPGPMSARGGSDINDPAVAE
EIAMPEPEDKLVKYSVYEVKKGDTLSEIADEFDVSLDTLISINGFTSAKSLRPKQLLKVP
NQSGIIYVASKASTVQEIADAYNISADRIVDANGLMSENISSGRPLFLPDVRLPAAKLRE
IAGTLFQWPVRGRLTSWFGWRKDPFTGRRSFHNAIDIAAPYGSAIAAPMDGRVIETGYSP
ILGKYVMMSHSGGWKTLFGHMSEILVREGQYVSQGRTLGRIGTTGYSTGPHVHFEVIKNG
ILVNPLNYLP*
>SPBDM4_v1_40128|ID:27157433| transposase (fragment) [Uncultured spirochete bdmA 4]
MVGKLPIGQKELIWSRVKVMVGEGQLSLKEASITLNVSYRQAKRIVSRYREQGEAGLLHG
FQGRRSNHCLGSVLCERVFEAYRTTDTAMQLL*
>SPBDM4_v1_40129|ID:27157434| Iron-sulfur cluster-binding protein [Uncultured spirochete bdmA 4]
MSDAKRADVALLRCKSYESRELDAIIAQAAGIAGFPDVEGATILIKPNILNASPAAKAVT
TNPAFVGAVIRFVKSRGAKSVLVGDSPGWQPSVLAAKTSGIYDAIQHNGGTWTEFREVSP
HPIANGKKLRNIPLTTMIEQADIIINLPKLKNHRLMTYTGAMKNLFGLIPGTAKSALHLQ
YPAVVDFGEMLVDLALSVPNCFTFMDGIIAMQGEGPGSGTPYSLGIVLASQSVAMLDWVA
AQCAGYDPARIPYLVDGMQRTVGSATIAEPATYPLSEAEIGHEGFEQLPYSSELGHRLST
LPNTLRSFAGSLIRLRPIFHTKKCIGCRACVEICPAKALRLDKKNRSNIIRIDDRLCITC
FCCHEVCPAKAITIGRAPLRIAHRPRNQPTAS*
>SPBDM4_v1_40130|ID:27157435| Fumarate hydratase class I aerobic [Uncultured spirochete bdmA 4]
MAHISLDNLIHWGSESDFEPINLSRPREEEGHIILRAEALRDCARIAFSKIAYTMPNAQY

ATFTEIIEDPASSPRERFVAASLLENALIAARGTFPLCQDTGTALVYGWRGDRIDIESDA
DPEELLAQGASEAYAQEKLRNSQMGPLNMTEERNTRDNLPAGIQIRSVRGDALKLVFVAK
GGGSTSRTSLTMESPAILRKDRLEAILDARIRALGPSGCPPYAIAVVLGGTNPSQTLYAA
ELAALGLLDALPESAPGDGTPVRDRLWEQKILAIARESGLGAQWGGSHLAISARAIRLSR
HAANLPLAVAVSCAANRHLAVYVNRGGWHIQKLVPPEQKLEETVKGLIRQVSSTAPSVEI
SHTDTAWLEELRSFAAGSMVLLSGPVLVARDAAHARLLKILETEKKLPDWFIGLPVFYAG
PTEAKEGYATGAFGPTTASRMDNYLEPFMGAGASLVTIAKGSRGVEARKTIARHRGVYLA
AIGGAAALNAERHIRSLSTIQFEELGMEAVRVAVLERMPLIVAIDSKGNDIYRA*
>SPBDM4_v1_40131|ID:27157436| MutT/NUDIX family protein [Uncultured spirochete bdmA 4]
MIAGDGNGARKWITLSSRKVYECNVFSVFEQQSQGPDGRIGHFSVMEAKDWAVIVPYVQT
GQDTSFLMVRQYRHGADVVSLEFPGGVIEPGEEPAHAAARELAEETGWVSERILHAGTVF
PNPAVQDNHFHIFVALGPKPQVDRNLDANEIIDAHLVPADEVRRRMGEGELQHALMVAAL
HLADRLVASLGPQP*
>SPBDM4_v1_40132|ID:27157437| Glycerophosphoryl diester phosphodiesterase [Uncultured spirochete bdmA 4]
MMRSRIHHRARKTSRRRTRHKPFFCVLFIFSVGLALCGQNRPENLSMERLIMQPSRPLVI
AHRGFRGIAPENTLVAAQKGYDAGADMWELDVAASSDGELVVLHDNTLVRTTDAKARFPA
RSPWNVYDFTLAELKSLDAGSWYSQTDPFMQIKAGRVKSEELQSFAGIKIPTLREALELT
KRLGWRVNIEIKDATGFACDSWIVERTVALVRELDMVSSVLISSFNHVYLRRVKKAAPEI
AVAALIDKPIENPVQTLKDIGAIALNPNAQYLDEATVKAVRAAGFGVLSWTVNDPSRMTD
LITWGVTGIITDFPDLGLAKTKALH*
>SPBDM4_v1_40133|ID:27157438|ugpB| glycerol-3-phosphate transporter subunit ; periplasmic-binding component of ABC superfamily [Uncultured spirochete bdmA 4]
MKRVILLMAVLLMAVGAYAQTTVEFWHAMTGHNGEMVQAICDQFNASQKDYKVVPVYKGS
YSDTMNAGIAAYRAGQPPAIIQVYEVGTATMMAAKNAVKPVYQLMAENNMKWDSSIYIPT
IKSYYSTSDGRMLSMPFNSSTAVMYYNKDAFRKAGLDPEKPPVTWPEFFEVAKKLKASGM
EGGFTTNWISWIQLENFSAWHNLPFGTRSNGFDGLDTQLVFNSPIHVRHFQNIYDLSKTG
VFIYGGRENKANPLFTSGRVGMHFESIGGYGNMKATCKFDFGVARLPYYPDVPGAPQNSI
IGGASLWVFNGKSKAEYKTVAEFFNFISQPETQALWHKETGYLPITQAAYELTKSQGYYE
KNPGLEVAIKQLLNKAPTANSMGIRFGNFNIIREIEDQTWEEILAGKISVKDGLDKMVRE
GNKTLREFEKLYK*
>SPBDM4_v1_40134|ID:27157439|ugpA| glycerol-3-phosphate transporter subunit ; membrane component of ABC superfamily [Uncultured spirochete bdmA 4]
MSKQYEFKASILPYLLVLPQMVIVFVFFFWPAGQAVLQSFYLQDPFGGKMIFVGFQNYSK
LLADPDYWQSFSVSTAFAVCITLGAMAISLFLAIQANKKIRFASFYKTMLIWPYAVSTLV
AGVIWLFMFNPNVGVIAYVLKHTFHVDWQYLLNFNQAFLLLTLAATWKQLAYNFVFFLAG
LQSVPATLIEAAEIDGAKSGTRFWKIIFPLLSPTTFYLLVMNLVYGFFETFPIVHSITQG
APGKSTAILVYKVWRDGVINLDLGSSAAQSVILMLLVIMLTVFQFRFIERRVTY*
>SPBDM4_v1_40135|ID:27157440|ugpE| glycerol-3-phosphate transporter subunit ; membrane component of ABC superfamily [Uncultured spirochete bdmA 4]
MAELTHNSNKAAHRAKLIRRTFPHIMLWIGIVIILFPIYFIFSASSHTSAEIQAAPMPLN
IGPHLIENYRQALFKGTQNMGTPVLTMLKNSLIMALGISVGKIVISLMAAFAIVFFKFPL
RNFCFWSIFITLMLPVEVRIMPTYKVVADLGLLNTYAGLILPMIVSATAVFLFRQFFMSI
PREIAEASQIDGATPMQFFRYILAPMTRTPIAAMFVIQFIYGWNQYLWPLLITTKPEFYT
LLIGINRMMSGADVQIEWQIAMATTLLAMLPPVIVVVLMQKQFIAGMTETEK*
>SPBDM4_v1_40136|ID:27157441|uxaC| Uronate isomerase [Uncultured spirochete bdmA 4]
MAQFLDENFLLKNETAKRLFFHVAKDLPIFDYHCHLSPQAIAEDKPFDDIARMWLEGDHY
KWRVMRANGEPESLCGGKGPWDEKFVAYARSLSKAAGNPLLQWSHLELRRIFGISEVLTP
QNALEIRGKANHVLASERISPRSLLAKFNVKVLCTTDDPVDDLHWHKAIAEEQKAGSGFQ
TKVLPAFRPDKALNAGDIAAWNKYVDELGKAAGTEIGSYEALLDALAKRHNYFHAMGCRL
SDHALLVPPYAPASETELDIIVKKGRRGAELSPVEQEKLATALLLHFARLNAQKGWTMQL
HIAALRNVNPRLFKAFGPDVGGDSTSDAPIIAPLASLLGALENEGHLPKTILYSLDPSKH
NPLAVLAASFAGTSPFDNASQVPGKVQLGAAWWFNDQKDGMERHLKEYAGVGLIGVWVGM
LTDSRSFMSFPRHEYFRRLLCNAVGEWVGAGELPDDPLYTDQLVRGICWENAAHYFGML*
>SPBDM4_v1_40137|ID:27157442| 3-oxoacyl-(Acyl-carrier-protein) reductase [Uncultured spirochete bdmA 4]

MSFVESLFNVQESVVVVTGGGGALPSSIAVAFAKAGAKVALWGRGTHHPMTRAADQVKKQ
AGLPAEDPNVIGVTVDTVSSAACEAAFDETVKLLGMPDILLNGVGGNMGRSSFVDFDEKV
FEEVLRLNLMAGLMVPSRVFAKRWIDARVKGSMINMTSMASYKGLSAVWAYDAAKAGVLN
LTQALAKELAPHGIRVNAVAPGFFVGNQNRALLFDEKTGNLTTRGEAIIARTPFGRFGEY
EELWGAVLYLASPKAAGFVTGVSIPVDGGYLSDNI*
>SPBDM4_v1_40138|ID:27157443| conserved membrane protein of unknown function [Uncultured spirochete bdmA 4]
MTDPTIATIILLGSFVLLMILRVPITFSLACASIATAAYLHIPLMAIVQQMVQGVKSFSL
LAIPFFIIAGEMMGRGGIALKLINLANVLVGRIRGGLAMVNVVESMFFGGISGSAVADVA
STGTIMIPLMKQKGYDDDFSVAITVTSATQGIIIPPSHNMIIYSVAAGGVSVGRLFLGGF
VPGVLLGISLMIIAYIISVKRNYPKERKYTFKEGVKIFGDAFLGLLTAVIIMGGVISGIF
TATESAAVAAIYAFIITFFVYRQIPLKEFKGILYSTLKTLTMVMTLIASASAFSWLMAYL
RIPAKVTEFLISITDNKILLLMLINVLLLVLGMIMDMAPLILIVTPILYPVVVGKLGLSP
IHFGIILMINLAIGLCTPPVGSALFVGCAVGKVSIEKATKAMVPFYIVMVIVLLLITYVP
QITMFVPNLVMPGG*
>SPBDM4_v1_40139|ID:27157444| Tripartite ATP-independent periplasmic transporter DctQ component [Uncultured spirochete bdmA 4]
MKKFLVSAVTLIHILLINLSMIMIVAMLLLVFANVVLRYVFNSGIYWSEEVALVLEVWFI
FLSLGLGVKHRLHISINILKRESVSAWLNKALDLLSDIIFLIVGFVMIVYGGKLVQFTMR
SIMPATKWPAGILYLVLPIAGFVVVVEALFHILGYNMFDEKIDKYLSGKGGKIRDIFRSE
S*
>SPBDM4_v1_40140|ID:27157445| TRAP dicarboxylate transporter, DctP subunit [Uncultured spirochete bdmA 4]
MKKMHRAVLVLLLILAPAMLFAQAKPIVLRLAETHPQDYPTTRGDYEFARLVKERSNGRI
IIEVYPGSQLGEEKAVIEQVQFGAIDITRVSISPVASFVPKLNAFQMPYLYRDADHMWKV
LKGDIGKELLASLEPFGFIGLGWFDGGARSFYNSKKPIYKPSDLKGMKIRVQESELMMGL
VQSFGAVPTPMPYGEVYSGLQTGVIDGAENNPPSYYSASHYEVAKYYTLDEHTMVPEIII
GSKISLGRLSQADQDLVKQAAFDAIDFQRAEWAAYVQLSMDKVKAAGCTIISIPDKTEWM
KAVDPMYKKQPKEIQDLVARIRAVK*
>SPBDM4_v1_40141|ID:27157446| protein of unknown function [Uncultured spirochete bdmA 4]
LNSAHEIQQRRGSSSNSLSAIAIAWLQDHFMESIGIWDVARELRVSPSHLSRVLKKEIGI
GFGETLVRIRIARAKNFLANGISAKEASSLVGFRDQSYFTKVFMKIEGVSPSRYIEQIQS
PQ*
>SPBDM4_v1_40142|ID:27157447| protein of unknown function [Uncultured spirochete bdmA 4]
MIHILVADDEALEREAMLHILTGAKLGEPIQVEEAANGLEALKAAQARKPDIAFLDIRMP
GVDGLGVAEKLSLLPDPPIIIMVTAYDYFAYARTALRFGVLDYLLKPASTEDVYAALRRA
LREITKKKEETARRLAAQTMAADLEAVINADICNSLRVGTVDDGDIQRLVTFRTGAATWD
CIAMVAGTARPLADDASSIAAREFSRFFYALAERFLVSDLGLGKANPMLFIASQEQDAAK
TEAVQTNSPIMNLLLVIPHAVRVPHADASQSFLDIRHVREQVGG*
>SPBDM4_v1_40143|ID:27157448| putative Integral membrane sensor signal transduction histidine kinase [Uncultured spirochete bdmA 4]
MHIPRPGIRGKILICFAVILAFFLGLTLVMQNESIKLSREYGENLSSYHLVHRFRLNLGS
FHALADRYLREPLSVDVEDVYTGIASLNAQYAVLIPLEDVSIPVEFEVRATGYGLDAYLP
LVSRAVGLRAGGSPDYYQAFVKATRIEGYIDVYLNRMLSALMQNGEETYAKLSRKSEILN
RTVFISMILVSILAVVIIVLVAEAITAPLRRLAKEAEKLAGGDLDAGIVEAHTKDEVETL
THSFATMATSIKEMVEGLKEKAELEKLLHEEELALVSMGKALREAQFMNLQEQMKPHFLF
NALNTIARSALLEHAPKTESLSLGLATLLRATIKDSGALSGLDEELTVAQAYLEFQHARF
GDRLQWKIDVPSTLLSARVPRLMVQPLVENAVRHALEPKLEGGSVYIKARKRKGKLILWV
LDNGIGMSRERLNEIRSNIAASLTSKTYGQGESFAGMNAEIPAIKQELGNENALLEGTGI
GLANLATRFAILYGDTCRFEIQSKLDRGTLVRVIIPLGAAIE*
>SPBDM4_v1_40144|ID:27157449| conserved protein of unknown function [Uncultured spirochete bdmA 4]
MRLSRFSIGTGDRFGLEGEAQIAAFKELRAHGGEADIVWNKSNREHVIIGSSPADQANAA
AASVTNTGWDGKWFVDADHISLKTIDWFLPHCNFFTIDVAETIGRQASAGARAAYLAQAQ
FLLEKGAAPVPVTQGDIETVADKFLAAVQEAGNTYRYIAARKPKDSFVVELSMDETDAPQ
TPAQVAAILVAVAAEKIPLSTFAPRFPGKFLKGIDYVGDVAEFLSAFEAETKIVLWAADR

LGLPKGFKLSVHTGSDKFKLYKGIHEIVTRLGAGVHLKTAGTTWLEEIVGLAEADGEGLA
LAKRVYAAAYARIDELTAPYANVVEIDRARLPRPDEVAAWDSKTFVNALRHEPGSQTMQP
DMRQLMHVGFKIAAEMGEEYTDALVEYRESVARNVQSNLYARHLLPIILG*
>SPBDM4_v1_40145|ID:27157450| conserved protein of unknown function [Uncultured spirochete bdmA 4]
MYIYEKNPDRSIHQSELELLVERTIEGSGALTSKRILIIPPDITRFHSRAGRITDLLVAK
LGSRVAAIMPAIGTHAPMTGEEIAHMYPGSPASLFRIHDWRRDVAELGRIPEARVSEISG
GRVRYSYPVQANKLLSSGEIDFIFSVGQVVPHEVAGMANHAKNIFVGTGGKEAIDRSHYL
GAVCGMEGIMGRADTPVRALFDEALGVAAGKIPPIAWILTVIGSAEDGSLAPRGYFASLD
RRCFEEAAALSAQVNIQLLEKPVRKAVVWLDPEEYRSTWLGNKSVYRMRMAMAEGGELLI
FAPGLRSFGEDPNIDAVIRRYGYRPGAEIQSLVAREPALADNLSAAAHLIHGSSEGRFRI
SYAPGPSISREEIESVGYRWTDLDHALKRYAPHGRLAGFHTTADGEELFFVPNPALGLWS
TATRMQGA*
>SPBDM4_v1_40146|ID:27157451| putative Integral membrane sensor signal transduction histidine kinase [Uncultured spirochete bdmA 4]
MKVKLRTQFAILTVAVTLFPIVFGLLFFTGQQAKRDPREPTRIFLNEVAERWGRNRNLTI
EEIRMAGEQSGLPIIDAALIAPDGTVQVSSFRELPAGIKLEVEDLVKPPLLPMGKPRPEL
RFLPIDTNIEESPLLLFDIQPFWSREDIRNRNFMLITSFALGAFLVAGLMSFLILRSTNR
AIQNLIDDTATVASGKLDHEVKGSGAEELRSLAESINRMRITLRDMISRRMNMLIGVSHD
LKTPIALIQGYTDALSDNMANDEATRIHYLEIIREKSAQLEDLVSDLIEFMKIDDFNTPQ
ETVDMAPLMISLGKRFEEDARLLGLELTYGFGRLGSPLSKEAPPNIPKIRMNRMLFERAI
ENLVSNAFRYTGPGGKVEFLMHVEEGHPVLIVADNGPGIPDDELPYIFDAFYRGTHSRKE
AGHGLGLTIVKSMVDLHGWSIEARNRRLSTASAGGVSAGGPRTGAVEHGLEITIRMKASA
*
>SPBDM4_v1_40147|ID:27157452| Two component transcriptional regulator, winged helix family [Uncultured spirochete bdmA 4]
MHGRILIVEDTKELAELYMLYLQNEGFECRLAFDAESALPLARNEDWDLIVLDLNLPGMD
GFQFLEQLRKEKSTPVMILTAREADEDVIFGLGVGADDFVTKPCPPRVLASRVRAHIRRT
ITLQTRQNGSIVKFGPFELDIEGMYLTKDGEPVGISAREFNILKELVTNPGKPFTPEDLY
AHVWGQEYGDISAVGVYIQRLRKKLEDDPANPFYIQTQYGMGYRFNPEAFATGANP*
>SPBDM4_v1_40148|ID:27157453| exported protein of unknown function [Uncultured spirochete bdmA 4]
MRTRNMRGLGPFAAIALFALCAGSLWAQTSTASASETPPAVIDENLLFGGPTDIVTVIDP
DTASAGTVQLVKDTKTYPVFLISGSAGAGLYGNYTPVGATSSDSQVLYGAVNLSGLEFDY
LPTKNLHFSVSNDLLAVPNDILAASVTAYADLRQSDLVRLYAAGSFNYDPSNYLSTKEYD
SGTFSLDELFVDTQINEKVFFRLGKQRISWGVGYWYKPSDVLSLSAIDPDDPTAARNGPF
AFKIDVPMKLNHLMVYMVPPVSGLADSSSMAAKYDLVVNGWELSFAGYGRADMLARPRAI
FSFTGAVGAFDVYGENVLLYGSDRTYVRESSTTLGTYETYRQENSPFFQSTLGIKYSASY
SSGLSYAFHVQGYYNGTGYADATILQVPDARTAVKNSTTNTAKQTNDLTQTGQWYLAGSA
SISGRFGEGRSLTQVTGSAYALANFSDGSMRFNPQFEVAIGDQGGRATLTLSDLTAIGAA
GSEYAPAGNRTTPALTVGILNDAFELQTSVPITFNTDFSVKKVQTAFSVFWNAVQY*
>SPBDM4_v1_40149|ID:27157454| conserved exported protein of unknown function [Uncultured spirochete bdmA 4]
MADVRGMKMRFLNAKLTSKLSIVLFGLVLIFSAKAQGGPPPGGGGGGPMGGIGLPLNFLQ
ADKDYKFPDDITKYTANDFLMLVRRTDIRSSFYNSDMSATISMVTVSPDRGTKTFKQQIF
RRDKDDAFLMLMVEPESRKGQGMLRVDNNMWRYDPTSRKFTHTTLKDTYENSTVTNNDFR
RWQRSIDYSVEKLSTGTLGNYKVIIGELKANNDEVPFPYLKMYIETDRKIVLKIEEYSLS
MKLLRTAYYTQYVQIGDSFVPTVQIFQDGLIPEKRSQVTYTDISTKLLPDYYFTKEYLER
VSQ*
>SPBDM4_v1_40150|ID:27157455| membrane protein of unknown function [Uncultured spirochete bdmA 4]
MRLIHLKLAARNVRRQPHRTIALGGAVAFSALVMTLILGFVNGMDLAIQDNVTLYSGGHV
LISGYTASWSGRLQNRFADDNVAKTVGSTNSDIRTVSPIAQSRATVVFGTREIQLTLRGV
DWAKDQLYHDSLILAKGDWKGLENSRTMLMSAQTADRFGLSVGDQVVVRLNTASGQQNVT
DYKVAAIYDDGAAGGMNMAFVAFGDLTADLNMKSNEQQQIAVFLKNSTDAEKAAQTITGE
LSSKGFTVSRGSASNTGSSNAAPSPDSSTNADSFVSSITGTGQDASRHLSPGTTLYYVST
IQELAGEIGSALGSIRWIGYAVFILMLIVSATGISNSYRMVLLERTKEIGMLRCIGYKKK
DVFSSFIWEGILLAGGAALVGVVLALPIGFGIGFIPFNPHGDFGAALVQGHLRFAPTLSQ

LLLILVFVTIVAIAAVFLPARKAAEVVPVEALRKTA*
>SPBDM4_v1_40151|ID:27157456| putative ABC-type transport system, involved in lipoprotein release, permease component [Uncultured spirochete bdmA 4]
MIAARLAWANLGLHRRKSLLIGMIMGLGILILFLGNSLIDTALSGLQRMFVQGYTGDLMV
TGPTEFSTTVLGETAGSEEVLPHIAQIKKYEDFLASRKDVASTLPLLSGQASFGIGEAEL
GSGYCFGVNVDDYRDFFPDNVTLMNGEWPAENDGPWVLLSEPTLALLNKSASSPVYPGTK
IIMTALANSAGTVIREVTVKGVIKFNQSNQQLSGITLVDADTMRDLLGFASLRDGAPVLS
GQEAAFVTSFDPDSLFNDLFAGSGGTAANAAATGSASAAEASGQGAAASSAQTRVQAEVV
PAWQFLLVRVKPGASSSRLLSDLSAFSKQIDDGDRVQDWIAGAGKVARTAVTIRLAFDLL
VAVVAVVVFMIMMNVLVISVNERLYEIGTLRAIGAKWRVVRNMILYETTFLALIAGAAGL
LIGLVLLLVLGKVGIRAPNLFFEALFGGQVLKPMVSGGAAIRAFLWILAMGLGASWYPTI
VALRIEPVVAMRGD*
>SPBDM4_v1_40152|ID:27157457|yknY| Uncharacterized ABC transporter ATP-binding protein YknY [Uncultured spirochete bdmA 4]
MSDIIISLDKVKKVYFLDTVEVEAFKDISLDIHAGDFVSIAGPSGSGKTTILNLIGCVDK
PTSGTVIVHGKRTSDLNDDSLTELRHKSIGFIFQTFNLIPVLNIRENVELPLLLDSQSGS
GRKADRKEWVDFLIENVGLKDRMLHKPAELSGGQRQRVAIARALVMKPAIVLADEPTANL
DSATGESILRLMRHMNETFGTTFIFSTHDPDIVEEADHIIRLRDGRIVEDRRRNGTSVDF
AAAPGADEPVGATGTEGGQS*
>SPBDM4_v1_40153|ID:27157458| conserved protein of unknown function [Uncultured spirochete bdmA 4]
MAYSGLQEFISFLESRGELTRISTEVDPILEITEIADRVMKEGGPALLFEHVRGSQWPLL
INAYGSEKRMAWSLGAESLDNKANEIESLIGWAWSQVRDFSLFSALPEALPKLPVARSFM
PKKVRRAPCREVADEGGFDSLPVIQCWPQDGGRFLTLPVVCTVEPETKAQNWGMYRMQVY
DDRSAGMHWHIHKDGAHFFQKYRVRGHRMPVVVALGGDPAITYASTAPLPEGIWEAMFAG
FLRGKPAEVIEIGDTGILAPADAEFILEGYVDPEELRLEGPFGDHTGFYSLPDDYPVFHL
ERMTRRKNPVYPATIVGIPPKEDCFLAKATERLFLPLLKQLCPEITDINMPLEGVFHNCI
LVSIRKRYPGQARKVMHFLWGMGQLMYTKMIVVVDDDISVQDLSKTAWRVFNNIDAARDL
VFSEGPLDALDHSSPTPRFGTRLGVDATRKWPEEGHEREWPDPLSMRLDIVELVNSRWGE
YGIGRP*
>SPBDM4_v1_40154|ID:27157459| putative 4-hydroxybenzoate polyprenyltransferase [Uncultured spirochete bdmA 4]
MASAAPESDSRQDLKAGTHRKPSPGKHVRDLFRRLGTIGDSVMISHTLFSLPFVISAILL
ETNGHPPFWKLFWIVVAAFGARNAANALNRIIDKDIDAKNPRTAGRHLPSGRLSPRDLWL
FTVAMLVLLVLGAAMLNPLCVMLLPVAGLLLFGYSYTKRFTWLSHYWLGIACSAATMGAF
LGISGTFQLRYFPLTAGVALWVAGFDIIYALQDIEFDRKEHLHSVPARFGETGGRIFAAL
SHAGAFLGFISIYYFWETPDIFTGIALALCFLLLVAEHLISARKSESRIKLAAYTLNQII
PIIYLCGVALDIYVR*
>SPBDM4_v1_40155|ID:27157460| putative aromatic acid decarboxylase [Uncultured spirochete bdmA 4]
MPIRYLVCITGASGGVYAVRLMSALAARGVILHVVASEWGARVLLEETGRPLGYWLGKLR
TQGGPDGGPALITLHDNSDFSAPVASGSFRLCGTVIAPCSMGTLGAISSGICSNLIHRAG
AVALKEGWPFIVVPRETPLSLVSIRAMSQLKEAGAVILPASPSFYGKPKDIESLVDTVVY
RILDHLGIVDKNVFRWSREES*
>SPBDM4_v1_40156|ID:27157461| Nlpa lipoprotein [Uncultured spirochete bdmA 4]
MKIFSVLLIAIAALVQPYAQSATQQALAAPLSIGIFADADSLPLIVAANEGLFQREGVAI
NLVRFSSAVERDSALQAGKIDGAVTDVLAVVLANQGGFPLKITSLTNGRYGVAVSPKSNI
QSMKELEGKNVAVSLNTIIHYFADWSATQAGLGAGALNLVPVPKMPVRLEMLLSDQIAAA
VMPEPFLTTAKLRGAKILVSSDDSGLEAGVLAFPPAVIAKNAGAIQRFYRAYWEAAQRIN
ARPDQYRSMLVDSLSFPEEAARVFLFPKYVAPRLPSQASIDNAVQWLLSKGLIKASPKHA
DLVDASMVSGW*
>SPBDM4_v1_40157|ID:27157462| taurine transporter subunit ; ATP-binding component of ABC superfamily (modular protein) [Uncultured spirochete bdmA 4]
MVTLEDISFSYTAHGVPVPVFEHFSATFEEGRITAVIGPSGCGKTTLLRLIAGLLSPQSG
NILMQRPRIGFIFQDFGLLPWLTVERNAGLGLEALGIPADERRRRVSDILEELGLSKWRK
AYPVRLSGGMQQRVAVARALAIDAGLILMDEPFSSLDALTRESVQDMLRAIQRTHQTTII

LVTHSIEEAVYLADTILILDGSMPVKDFMPVQNPHRWEHTPNHPGEQAATFSSQGQRSSE
LSSSSNPRENAAYLSAVGRLRHAFDEAVAQKAGVATAGQSPAGPSATNPAGRSPASVATL
EKAPKGVSGIISTLSPSVSKFLIKTAQIVAAAIFVCVVWWAAAALLKRPFLPSPWLAFAK
FSENLHKGIFQIHVLASARRVFLALLVAGPLAWMLGLLAGRVRFFDNFFAPLIYFFHPLP
KVAFLPILMLFLGLGDASKVALMALVIFGQLFVTGRDAAKSIAPALLDSVKILGFSRFSI
IRLAIVPSTTPSLMSALRVSLGTAIAVLFLSETFASIDGLGWYIMDAWSRVDYPDMYAAI
LALSLFGLILYLIIDAIEAYLLRWRQNN*
>SPBDM4_v1_40158|ID:27157463|fbpC| Fe(3+) ions import ATP-binding protein FbpC [Uncultured spirochete bdmA 4]
MKGNDMAAKSVPVMLSDVTKIFRDPKTKAEVHAVENANFDVKPGELVTLLGPSGCGKTTT
LRMIGGFELPTRGKILIGDSDVTFLPPNVRQTATVFQSYGLFPHMDVFDNVAYGLKIRKL
SQDEIKNRVNAVLDLVGLSELAHRAPGRLSGGQQQRVALARSLVVEPQVLLLDEPLSNLD
ALLREQMRVEIRRIQKSLGITAIYVTHDRIEAMSLSDRVIVMRDGRIQQVGTPGDIYEYP
VSMFVAGFVGKVAFFPARLEGIEDDGRTALCTVGGRKVRAGSVAGNLGAGARAVVMARPE
SLRLVGREDAIATGMVTARVYLGSSVEIYVKTEAGEILVQIDDPSGKILPSEGETVWIGF
DEKLVRALPAD*
>SPBDM4_v1_40159|ID:27157464| ABC-type Fe3+ transport system, permease component [Uncultured spirochete bdmA 4]
VFTKKKLIMFAVVCAIFAVGEFWIFSTLQNTFEKNSIKEMTSLIKAMTSSAPQNDEDLAH
WSEELSAIYPDNRFFVIKGVPGEEGSENIGGDSALFDIWKLPANATIAENAAESVSYLEV
FRWPAKVSFLGVPYTLMVAPVPDEEDSSARATLLVAIDASGYVAFGKLVQSLAVVAFLLF
SLGFGIATFSRDPISGYAILVLAAIVIAFVAYPLIEAVRLTLVKEGRFSFDVWKTILSNR
QYLQALWGSIQLGIVTATLSTFIGFMYAFAVARTSMRGKRLVTGLATMPVISPPFSLTLS
IILLFGKNGIITKQLLGLQNFNIYGLGGLALVQTISMFPIAFLTMQGVLQAIDSTLEDAS
LDLNASRWHTFAHVTLPLAAPGLLSSWLLVFTNSLADFANPLLLSGSYRVLSVESYIEVT
GMGRLNNGAALSLLLLLPTLTAFLVQRLWVSKKSFVVVTGKPSTRLSDLASPGARRALTG
FVAVVSGFIVALYATIVAGCFVKNWGIDYTFTLANIGEALARGKQALLSTITLAGIATPI
AGILSMVAAFILVRKKFLGKRILESLIMTPFAIPGTLLGISFILAFNKPPLLLVGTGAIL
VINYVIRELPVGVEGGVAALRQIDPSIEEAASDLGADQATIFRTIILPLIRPAFISSMSY
TFVRSMTAVSAIIFLISARWYHITIQIYNFSENLRFGLASVLSTMLIIIVLAVFGLMRLL
VRQSEFLEKTVTTQ*
>SPBDM4_v1_40160|ID:27157465| Extracellular solute-binding protein family 1 [Uncultured spirochete bdmA 4]
MKRVLIVGFVLLLGVTMAFGQQKVGAYTTLEEPLAKELFDEFQKETGIVVNWQRLSGGEV
ESRLEAEKANPQASIWVGGVGLNHISAKLKGLTTPYKSKMLANTPLQFRDPENYWVGLYI
GPLCFVTNNKVAKEQGLVPPTSWADLLKPVYKGKIRMANPTTSGTAYNTLTTLRYVFGGD
ENKVFDYLDKLDKNIDQYTKSGSAPGKSVGIGEIPVAIGYAHDNVKLKVEGADITITFPS
EGTGYEIASMSMVKGGPDPVNGKKLYDWILSPKAQEIIAKWYVIPLSKLAKKNPSAFSIS
EIKTVDQDVMWDAANKERLLERWVKEIGSKR*
>SPBDM4_v1_40161|ID:27157466| protein of unknown function [Uncultured spirochete bdmA 4]
VLSIHFNKINYKFYQNFFNFGKLLASYILSAEKGSGRVSWSCEDPNLPHFTTCGGSL*
>SPBDM4_v1_40162|ID:27157467| Heat shock protein Hsp20 [Uncultured spirochete bdmA 4]
MNSLVMYENNPFDLMERFFNDDEWLMPGFRTPEIDVYEEGDKYMLEADLPGLNEKDIKLE
VRNGQLTFSTLKNEKSEEKSKKDRWIRKERREFQFVRSFTLPEDVDANGIEAHFKNGVLQ
VVMPKKPEAAPKTIEVKIA*
>SPBDM4_v1_40163|ID:27157468| putative ferredoxin [Uncultured spirochete bdmA 4]
VADKSARLTGNVKGRWYYVDSSCIGCGLCSSTAPDTFALGDDGQALVIHQPETADETELAN
QALNDCPVQSIGNDGE*
>SPBDM4_v1_40164|ID:27157469| Peptidase M29 aminopeptidase II [Uncultured spirochete bdmA 4]
MEDPRLGKLAHLLVNYSTKVKPGDKVLIENVNSEPDFLRLLIEEVHAAGGLAFLSLRDRR
LERTLFMNAPEEQFELQAEFERARMDKMNVYIGFTSMRNSFAWQDLPQEKTELYNRHIWK
KVHIERRITNTRWVVLRYPTAAMAQNAGMSEDAFEEFYFDVCTMDYAKMSRAMDALVTFL
EKSDKVRVKAPGTDLEFSIAGMPAIKCDGTMNIPDGEVYTAPLRESVNGVLSYNTPSEKD
GFKFENIVFEFEKGKIVDAKANDTSRINKILDIDGGARYLGEFSFGLNPYITKPMLETLF
DEKIAGSLHITPGNSYDDCDNGNHSALHWDIVLMQDADSGGGEVWIDGKLIRKDGIFVAP

ELLALNPDALKD*
>SPBDM4_v1_40165|ID:27157470| conserved exported protein of unknown function [Uncultured spirochete bdmA 4]
MKNRSKEYVKATFLWVFGIFVFVFFAILVASPFILWIAQPYRQLDVWVVDKTVPYPDFRE
HAGLFWLLNNEKISKPGTKVLYDVRTDYYGFYPTSKEEWSTIPMPHTAKKPDLIYIADTY
GVYRDDYMQKKLPNAFSSLIYGGLNNDDYSTIQQAMGGGNTLIAEFNTAASPTNDRDRAL
FGRLIGLKWQGWIGNYYESLAKDGGVPDWVVGNWESERKQTWEYSGRGIVLLDEEGSVEV
LTEKDDIGKNGMKMIFRQEWAKTLGIKKPTSYRYWFEWVIADPLVEEIAHYDLDVTPSGK
KKLDALGLPNTFPAIVRFKNDQFTSWYFAGDFADLQFSGTTYRMKGIQTIKRILADDTVD
NNSYFFWKVYAPLMHHILADIEQRRVETRATTKPAETQVRVRAVKEGIQLKSSTGEWRTV
FLRSVNMGPAEPGKFFTEFPYDNDTYVRWLNYIGSLGANTVRVYTLLPPEFYRALKIYNQ
THPDAPLFLLQEIWPEENPPGGDYLRPAYQEEYLKEINYVIDAVYGRANVPQRKGRAWGV
YSVDVSKWLIGWLVGRELESVEVMQTDADHTGATYTGEYVSAGKGASPTEVWLAESLDAA
AMAEAERYGVLHPVGIVSWPTLDPIEHDTEWDPKTGKSNRWNDRASVAIEHLDVTEKMTA
GLFGAYHIYPNYPDFIVNEPSYGDYRDAVGILRYGGYLEEFIKTHSRYPAIVAEFGMANG
AGVAHISSDGLNHGGVDEKTAGRDIMRMYAAIQKTEYSGASIFEFMDEWAKKTWITEPFM
IPFDRRVLWHNIVDPEQNYGLIANETVPPSAPEQKISGGGVLSGLEVSHDASYLYLTLQL
TGPKTPQEYEILLGIDTYDRTLGQMHWPGNLEKTASGLEFLLDIQQNEARLLVIPSYNIA
SARYATTLRRDGAFEEIQFLVNGKVTTKTGSDIPERRFNASLMRQGPFDESGNLWNIEGN
LIHVRIPWNRLNVTDPSSRNVLNDARNIGDPGTDVLKTIITDGFVFDARVLNAKTGTEAG
RLNANTDSPYTWPGWEEAPPYRERIKKSYGIVRDTWKREAEKEKAIIADFFGIQSVR*
>SPBDM4_v1_40166|ID:27157471| Glycosyl transferase [Uncultured spirochete bdmA 4]
MTIPSEYLLGAVILFLWLTIYVVVSTLLNKARVTINLENLKSDLRELPQIFAPTYEQNPR
KMRTLLVHILYLTQNIKMSLVQKQRIFNVTAVQYFERTQIHRVASRNRFVRMSAARYLSS
INTDNARKAIEKALLKEKHFPTKLYLANALSDIKDPRSLYVLIQSLYGSHYWYRNKVNML
IASYGDIVRESIQGYFWRTDIEIRELLVDLAGSIPAEELKGYLVDVLQLGLGEVGKLKRL
TANLPAKCCYYCVHGRMEADDTHRQCPYKGKVANNFRCRKYHTLVTSLSTALNYHRLMVR
AAETLEKYYPEELASDYYLEHVDKDIQSIAVRSLGQTLRTGNLRKLIDYLERDETAAAAR
EGLRNLLNHHPKFILTLVEAFQNSEGALHNRLAEVLSNRIEYIITKLLGHEHDVAAGIIN
ELISMERVSEIIEFLKRNKNVELEDRLVEVIQPRLQDCELLAKECGLFLPDSILEKLGLK
RLFPPARKREEKVDKKLVRRLYVVLAFALGFFPLLYVLRYYDRLDTMPFIQHLKYFVLNF
NVNFSYYTIAVNVVYLILLFFSRLNIKRAAQLWELKSMTMLFKPRMLPSVSIIAPAYNEE
TTIIESANSLLSLKYPDYELIIVNDGSKDNTLRTLIDHFDLMKTDFSYKERLKHYPIRGI
YTSPSIPGLIVVDKENGGKADTLNAGINVASKEYFCGIDADSLLEPDALLKIASLTLDYG
VETPALGGNVFPINGCKVDKGKIEKIGVPKHWLSRLQMVEYIRAFMCGRLGWDYINSLLI
ISGAFGLFRKERVVAVGGYLTSSGKYQKDTVGEDMELVVRIARHMHESKHSYRISYAFNA
NCWTEVPETMSSLQKQRNRWQRGLIDIMYFHRKLIFNPWYKLMGMVSMPYYLIFEMIGPL
FEVQGYLMVAIAALLGMLSTRLALLLFFASVLMGILISVTSVKIAEKLNVYFNYKDTLKL
VGTAIIENFGPRQLFSTWRVIGFVRAMQKPQGWQKFERKGFADNRQERGDAPRGAHR*
>SPBDM4_v1_40167|ID:27157472| protein of unknown function [Uncultured spirochete bdmA 4]
MENIQDPIDLGGGFFRIGASRLLGELQSNIYLLLDDGEAILFGPGAAVNLPELKENIQKC
ASLRPISAIVVHQQEPSSSSALTYLEKEGSRAAITTHWRTWGQLRFYGLSSQPYIIDEHA
WSLRLSSGRVLQFLPTPYLYQPGAFATYDRVTKTLLTGALLSSYSENLSIYADDGQFLDR
LKAFHRANMPSREFLAPVMQILAKWDIERVMPSYGPIIQSGVKRIFSELAELECGELARS
EGVEALYAGMEQFGATGAEVARLKQEIETLRRLNEELNHSITVSKDRAIRDAVTGLYSEI
FYKSFVDEEIAIRITEAGPEDHVLAVFGIDENIAQIEYKYGSREVEALLHGVARVIEENL
PKTAMAFRLHGATMAAWIPAILFDRAIELFDKIRYQIETSKAFVEPVTVSVGVATLAEAA
STQPALEKIAADMTDLGIRRLRLARRLGGNTLYFGSTEENEGEVKARILIVDDDEVNVDV
LKTYLINEGYSILTAADGQEALSILGKEIIDLVITELMVPKIDAYILKESMLSKSATKDI
PVVIISHLKTELTIRRAYRLDIVHYLQKPIILEELLGIVQNLTHVGSGA*
>SPBDM4_v1_40168|ID:27157473| Carbon starvation protein CstA [Uncultured spirochete bdmA 4]
MSTLIVLIALVIYFGSYFLYGRTIRDKVLKSKEAPEAPSKRLSDGVDYVPTSKYVLFGHH
FASIAGAGPITGPAMAVAWGWLPGLLWIWLGNIFIGSIHDYLALTASVRYDGRSVQFVAQ
DLIGKRAGKAFSWFILFLCILVVAAFGDIVAGQFAADGRVFSTFFFFCVAAIITGYFMYH
TKLGIGGGSIIGIVLLLLAFWIGDLLPIAASKDVWFLVIFVYIVIASALPVNFLLQPRDY

LNSFLLYFGLIVGGLAAIIAVKGFDSLPATTSFSAVVIGGKPTPFWPAVPLVIACGALSG
FHALVASGTSSKQLREEKDALFVGYGAMLTEGFLSTIVVVSIAAFGIAAMGEGNVLKTPA
LNRFVLSYGTMVNTAMPFFSQSFMKLFAAVWVSSFALTTLDTTNRLGRYLVQEMITPVKE
KKPGLYKTFNNRWVASLIIAFFGVGLAWTGNYTVLWPAFSGANQLIASIVMLTVAVWVKK
KLNPSYTMVVLIPAILLWVTVTAGIIWYEIAIVPTFFAAAANAAAQTKNIITGVVVGAIN
IFMLVLNFIMISAFMKNWKTKQAD*
>SPBDM4_v1_40169|ID:27157474| conserved protein of unknown function [Uncultured spirochete bdmA 4]
MNEKMKLLHLLRRGAKTLVQVQREKATSMLDFELKELENIFALLLLGGFVGIPSPPSPIA
IELLPYLERELVVMFARTDMSVDPIGSLVGMLEID*
>SPBDM4_v1_40170|ID:27157475| Arsenite-transporting ATPase [Uncultured spirochete bdmA 4]
MVSPVSHKTAFFLGKGGVGKTTLSASFALTLARSGQKVLVASLDPAHNLGDALGSALKNE
PLTVEPNLDAMEIDLETWINTYLEESRSQLKSTYSYNVTLNLDSLFKILKYSPGTEEYAV
LWAIEDIHCRLSPLYDIVVLDTPPTALSLRFLSMPTISGLWVKELSKLRETILEKRSTLV
RLNPESPVKESCLDKDDDKVYGKLSTIRQRLSMLEHLFQKESFINVIVNPDELSVTEALR
IKDELDRLEIPISTICLNKKGVLNNEWHIHQSLAAIPMFSFDFESSGIRTRDQLIQLGTQ
HLVDTFLDKRSIPA*
>SPBDM4_v1_40171|ID:27157476| conserved protein of unknown function [Uncultured spirochete bdmA 4]
MNMNPFLFTGLVALGVGATITYYLGVKKNRWLGKHLSAQTEEILAPTDTNYVNIGGAIGY
NFSYKLKGDWKEAKGTFTFIPRHSLLYMPISFLIRGGDHFYLNLFTDRRLPGEGHLILAS
HLRKAKIDDIEAMSRKEVELGGKKFVLLWRENRILDKLERIALSFPDPQSLMHFCCYADN
KTLFLHLAPKKGIIGDNLSYFVKNAQVFLEK*
>SPBDM4_v1_40172|ID:27157477| conserved protein of unknown function [Uncultured spirochete bdmA 4]
MDPEMQKKIDETFVQVKEPQSDVPIVDLGLVEKVTYSAKEKILLIHLAIGTPRWQCPACS
AINGVVKEGIVRRAKEAFEKAFPELSIIVE*
>SPBDM4_v1_40173|ID:27157478| putative Response regulator receiver modulated diguanylate cyclase [Uncultured spirochete bdmA 4]
MSNIRILMVEDSISQAMHLRYVLEAEGFGIDVASNGIDALSFLEHNTPDLIISDTMMPTM
NGYELCKKIRENPRLKEIPLMLVTALSDPTDLIRALEAGADNLITKPYNEQALISRIRYI
LVNAEIRSQSGSETGIEVFFAGKKYSINSTRIQMIDFLLSMYESATQKNQELLLSNNKLK
EALDNIITLQRNYRQLLETDQDAIFVYDKDKVIRYANPAAHSLFSREGKGLIGAHLPIDE
DISSQKEIEMKDPYGNTAFLDVRSVSSDWDGEMMTLSVFRNITEATQLRKELEQMSLTDD
LTGIYNRRGFKLLSERMVKLARRLQSQMFILFADMDGLKSINDTLGYLEGDRAIHTMAIF
FKNAFRESDLVARMGGDEFAVMGLINENFIPSRLIDRMNELVQGFNAKGESRFKLSLSMG
IENIPYDSQAPIEEFLNSADAKMREEKQKQKKKFFNNS*
>SPBDM4_v1_40174|ID:27157479| conserved protein of unknown function [Uncultured spirochete bdmA 4]
MKRFVLFGFLAVLLVAGCELFPNITWEDIKGEWDFPDTTFNDSTIKSIHLSLMDPGEGES
EYRIDLSWENGDNYLLHYGEGSMDKNVFSGTYNLGGDYTDSTTYSVTVTFTLNNDQLKAV
FTGTGPLNGLVLEHGTLNAG*
>SPBDM4_v1_40175|ID:27157480| AMP-dependent synthetase and ligase [Uncultured spirochete bdmA 4]
MITLKDYTLAEVPQLSGARFGLRPALSMVGGAVYTYRDFERISRGVAESLIADGVKKGDR
IALLAENSPHWVMTYFGILRAGAIVVPILTDFIPVQICNIIQHADARIVFTSEKLRAKLS
ELPKETAIREIKQSYVPSPSPAALPAVAADDLAMIVYTSGTTGLSKGVMLTHRNILSNAT
ACKSIITLYRTDRLLSILPLAHTYEFTIGTVIALLSGSHIHYLDRPPSATVLIPALQAVR
PTIMLSVPLVIEKIYRSSIKPTLDGMKLYKNKFFRPLILLFAGMKLKKTFGGRIRFFGVG
GAPLAADVEEFLKKAHFPYAIGYGLTETAPLLAGCSPRSTFLRSTGPALKGVDLRIAEPR
PDTGEGEIQAKGPNIFKGYWRDEARTREAFSSDGWFRTGDLGYLDKKSRLFIRGRLKTMI
LGASGENIYPEEIESIINQAPEVAESLVVEDEEGLTALVYLKSEELENLEARLQDSIDAA
GDLGSRVGQAIVNVEKSMAGTVNHAVVDAEKALERLLENIRKEANSKLAAFSRIQHVKIH
REPFEKTPTQKIKRFLYGKKKAEGAR*
>SPBDM4_v1_40176|ID:27157481| putative signal transduction protein with CBS domains [Uncultured spirochete bdmA 4]
MKTVGDILKTKGHEAASVAPQEKVLKALEIMAEKNIGAVLVMDERGMVHGIFSERDFARK
IIVKGHSCENENVETVMTRKVIFVQPETSLEECMNLMTVHRIRHLPVLENNKVAGIVSIG
DVVKALLEVKDRIISEQAFELGQTERARQPGAV*

>SPBDM4_v1_40177|ID:27157482| putative universal stress protein family [Uncultured spirochete bdmA 4]
MKKILETVLVAINGSDASICAFKYALALKKAFGSRIVACYVVDTATIRQLALSRIFVPEE
SEEYERNLENSGQRYLNFCTDLARQKQLAIETCVRKGSVSGEIVKYSIELNADAIILGGA
PADALYRDAIADSNHEVLKNARCPVLFVKPPLGEEFYKSM*
>SPBDM4_v1_40178|ID:27157483|atpD| V-type ATP synthase subunit D 2 [Uncultured spirochete bdmA 4]
MAEPMPAPTKTNLIKEKRNLQLALQGYDLLEKKREILVIELMKRMDALELLEQEIAKMTD
DAYAALRKMLLSVGRERALSISSLPVRDITLSASYINVSGMHLPTLDVRASAPVLHYSFM
NSFAVCDETVIEFTELLDKLSTAAGMRSIVWRLAREVRKTQRRVNALDKMVIPRSREIVK
FIDSSLDERERESLFAVKMLKQRLSEQD*
>SPBDM4_v1_40179|ID:27157484|atpB| V-type ATP synthase beta chain 2 [Uncultured spirochete bdmA 4]
MRGLEYHGILRADGPIVIAEHRENVGFSELVSLRDRTGEDRLGKVVDISEQAIAVQLFSE
NTGISIDGSWIEYLEKPLTFRVGDGIVGRIFNGLGEPIDGYPPIMSSDLRDINGSPINPS
ARVYPRDFIQTGISAIDGMNTLIRGQKLPIFSGNGLPHNRLAAQIVRQAKVLSGESQFAI
VFAGMGIKYDVARFFIDSFEKSGVLARVVMFLSLADSPSIERLVTPRTALTAAEYLAYEK
NMHVLVVMTDMTNYCEALREVSAARNEVPSRKGYPGYLYSDLASLYERAGKIEGCEGSIT
QLPILSMPNDDISHPIPDLTGYITEGQIVLERDLSQRGIYPPIAGLPSLSRLMKDGIGEG
MTREDHRDLAAQLFSAYAYVRSVRDLAAIIGEEELSDRDKVFLGFGDRFEREFLAQGEFE
NRSIEQTLDLGWKMLSILPESELVRIPPKLIEKYLPRAAEPEAKE*
>SPBDM4_v1_40180|ID:27157485|atpA| V-type ATP synthase alpha chain 2 [Uncultured spirochete bdmA 4]
MSAGSITRISGPVIYAQGLEDAGLYDVVKVGKAGLIGEIIKLKGDLATIQIYEDNTLMRV
SEPVECLHRPLSVALGPGLIGSIYDGIQRPLPVLKSLSGAFLAPGLAGEPLDTQKKWHFV
PAIKEGDPIAPGTAFGIIKETDSIVHRLIFDSLVPACTAATVAPEGDYTISATLVKSSDG
KEYTAMSYWPVRTPRTFKEKQVTHEPLVTGLRVIDVLFPIAKGGSAAVPGGFGTGKTMTQ
HAIAKWCDADIIVYIGCGERGNEMTEVLTEFPTLIDPRTGRSLMERTVLIANTSNMPVAA
REVSIYTGITIAEYYRDMGYHVAVMADSTSRWAEALRELSGRLEEMPAEEGFPAYLPTRL
AEFYERGGMVTTLESKKGSVTIIGAVSPPGGDFSEPVTQHTKRFVRCFWALDRDLANARH
YPAISWNDSYSEYTEDLIAYWDAIDPAWDSLRSKTMEILKKERRLADIVRLIGPDALPDE
QRLILLTADMIKAGFLQQSSFDKVDMYCAPPKQTLLLTCILTFHELAENAIKSGSPLLKI
SALPIREKLIRLKSSLENEKVQEGQAVIQEIRTAFARLGAAQGAVSA*
>SPBDM4_v1_40181|ID:27157486| conserved protein of unknown function [Uncultured spirochete bdmA 4]
MEEIRGTEALEQEILDDARKRADRIIRKADDDAKIMQAQTDQKIKEAVDALTQEYQARQE
TAEREARSRLPLEKMRLEIEYRDAMLRKALQEALAAMDPRFLGAWCVRQLRRGAELIRSS
QAKIMVHGLDTSTMQEIRELFSGASSVSIEEVPSMKVRGLSVEPADNSYHISITEHELVA
WLLDEKRGELGAALFGSNAHVEKSATPDRAEPPAQEGSTL*
>SPBDM4_v1_40182|ID:27157487| putative Vacuolar H+transporting two-sector ATPase F subunit [Uncultured spirochete bdmA 4]
VAEIFALAEEEILIGFGMIGIKGKAVTGREDAIATFRSIVQNKTCTIDGGTVELSDCKML
ILSEDVSDMIGEELSEWQLSGAFPLVVEIPPLSGSSAQHTRIVDAVRQAIGIKIQ*
>SPBDM4_v1_40183|ID:27157488| ATP synthase subunit C [Uncultured spirochete bdmA 4]
MKKKLLVGIMTFVSSAAAFAQTQSAVATVSASGQVAKYLGAAIAVGAAALGGGYAVAKIG
AAAVGAMAEKPELSGKALPYVGLAEGICLWGFLVAVLILFR*
>SPBDM4_v1_40184|ID:27157489| V-type ATPase 116 kDa subunit [Uncultured spirochete bdmA 4]
MSTVSMRRVELLLFKSDIDAVLKYLGSQQCFQIIYPDELERIAHEKLAGVAEPDQEKALV
GQLDEAQRKLDFIGTFFGMAAPQNIVEDVHLPDDEMLSKIDILYERCATEKKRFEEQEAK
VDQLAESIHEAKAFAGLSRPFDEFEKFSYVSIQVGKVSKEKIDPLVKALGTRAVIVPLDD
EGTILAASSRKGRFALETELSKVGFEKKSPPAGVQGVPAEAISALERAYNAEKLRLGALL
NEKKGLAEQYGELWQKMTSSVRLKQSLVLVEHKLESTKWVYRLSGWVPADRIGRMNKDLL
SMLGNRISIRIFDPQEQINKKGGKVSEEVPVLLKHNAFVSAFQGIVLSYGTPLYGDTDPT
PFVAFFFTLLFSIMFGDLGQGLVIVALGFAILKAKKGLLARYTKYAIAFISAGLGSMVMG
LLVGSFFTNDSILIPLERTLTGFFLGTPKDRFLQIMPQDNITAMFYFFGFTLAVGVIINS
TGLVINMINLVRRKEYGEAIFSKTGLAGSLLFWWAIGIVLRLILGGKLGLIDVPGLGIPL
LALFLAEPLKAKIDRAQGKVPEQELSLSDALIGGAVGLLEVVIYFASNSLSFLRVGAFAL
AHAVLSFVIFTMGGVVRGSSASGLVFEILIYLIGNAIIIGLEGLIVTIQVIRLQYYEFFS
KFFTRTGKTFKPVSFPG*

>SPBDM4_v1_40185|ID:27157490| protein of unknown function [Uncultured spirochete bdmA 4]
MAESLEFAYLYARVCGAFSKMQLGETARGLMQNSGGIMALWRQLFDEEPPSLPEAKLLFE
AERKIIERSIDAFLRLAKPFSESSDLIHVLISKYEISAIKSMLFRLRSGESKPEEVSYTS
PVIEQALANWPRIADMFYRTPYSWLDSHWLDNIAIAENRLDQQYYLDLWNAANKIPARKI
GAMLDLIRWEIIYQNVVWALRVRRYYGMTEQDAVGMFVEVKGIDTVSLAIQTFHFDIDKL
ETFDRWPLKKLLSNQTGPGLDVPVMEIRTQEDLFSMVRRNLHLHPFSYTPIYCYFKLLEY
ETSLLLAVIESVRLGVSPDEKAKYMWIPGGEPA*
>SPBDM4_v1_40186|ID:27157491| protein of unknown function [Uncultured spirochete bdmA 4]
MDSVDSIDRLVEIERQAADIIHDAEEKASRAQLDAKTKAETLQNQKIAEERKKLDADYTV
FLNTLQKQSQKEIGDYKKSLTTIPLNHSALAETLKGFLNTEA*
>SPBDM4_v1_40187|ID:27157492|fruA| PTS system fructose-specific EIIABC component [Uncultured spirochete bdmA 4]
MILQNAFKPATIKIGLESEDKDELLEELIDVLAKNYPGGMVFPRESVLKALWAREEKMST
GIYKGVAVPHATVDGIDSLRGVLGISKKGIEYESLDGNPVYLVFLLVSPPSEAEIHLDAL
KQIALLIQDSLLLENLMKAATPEKVYSLIRGFEESIGAM*
>SPBDM4_v1_40188|ID:27157493|uppP| Undecaprenyl-diphosphatase [Uncultured spirochete bdmA 4]
MLIIQSVILGLVQGLAEFIPISSSAHLVIIPWLFGWNNPALTSLTFDVALHLGTLLAVIV
FFASDWGRLISAWCKSIVQFKIGDDPDRRMAWFIVLACIPGGISGLLLESKISEAFHSDP
IPQSSMLIMAGAIAALALLLWVADKFARHQRNFGQIKARDALYIGVAQAFAVIPGVSRSG
ATITAGLALGLERETAARFSFLLSAPIIAGAGLKSLYDLWKQINAGTVAGTELTIFPIGF
IVAAVSGFLCIKYLLAYLKKHSTAVFVWYRFALAALVLIVALVRG*
>SPBDM4_v1_40189|ID:27157494| conserved exported protein of unknown function [Uncultured spirochete bdmA 4]
MKFCTHNQRKAFSFMLSGLFFIGAVAAQTGNPGYEIMKRANDRPAGNDMTAKVLMKIVSK
SGTIKTREFMMYQLHQKDGASSTLIQFLQPADVKGTSFLTAESAKGEKSQYIYLPSLKKA
TRISASDKNKPFMGSDLTYDDFGSRDIEDYTFAMLGEESMAGRACWKVESVPKDKSGGTS
KIVSLVDKESLLIVKADFYDKSGALYKQMTASQSERISGIWTIVDLEMKNLTTGGATSLR
FDAVKYDTGLSPSLFSKEALGK*
>SPBDM4_v1_40190|ID:27157495| exported protein of unknown function [Uncultured spirochete bdmA 4]
MNDKLKNSQTIRNGKSFFSIALMFFIVGAVPLSAQASLSGEVTAFSGIFFQASGTAKAGD
YFQPSTSLKLAPSYTSGPLTIRAELTGAISLADSGGYDVAMKLGETYATLDIADGLSVTA
GSKVISWGTALASNPEGFINPIDSLSQLVSENRSDWLLPVMLISGKYIRGPFSIEAVALP
FFRPNTIPAKSSRWYPASLAALDALNGAFISPALFAVDTTPSDLSLNRENMQGAGRMSLS
QGAVDFGLSGWYGFTKTPAFDVETTPGIITGVDITASYKRQGAIGADVSATVLDSSVAWM
EAALYLPEYYLGEETTGLPVALEKNTLVSAFGLDRTFGIGSAGDLYCAMEGNLSWILAYD
SRLASSANETEIGATLITEYRPSAQDFTIRLVVMEPDFLTVDTTGQYLARLSMKAKLADS
YSLTLGTVLFGGTSGTIGQYADNDFAYASITASF*
>SPBDM4_v1_40191|ID:27157496| membrane protein of unknown function [Uncultured spirochete bdmA 4]
MAAFVYRFRYLIVAVFCILTLCAAFFIPNLRFEGDIQALAPNNVSSVGEFRRVNQLFGGA
DSLAVSAQSKSLFTPEALAEAAAINKELQSLPDVSDVVSVFTYVEPANVEDGLSFIPYID
PDALPSDQASSEALKSRLQGIKTVSGKLLSSDGDTLLYIIQTSPNVSQIRLIDNIKSIIA
AHPDFAFEMAGTPFIDYQMVNYMRHDVVILIVFGLLAVVLVLLVGFRSLRGVLLPLFTIG
CSLIMTFGFMGLLHAKITIVALIIPVMLIAICNNYAIHFLTRFYEDVFINGMTDKVEIID
ASFRSLSKPVWLAFLTTVASFISFVTSPIPRIAEMGLFIAFGITYAFAMTMFFIPALLSI
LPLPRKHRRYHANEALITSMSKKAGGFITRHKTAILVVTLVIAAVGAFFIPHIIVDSEMA
NYFNPNDPVRVGIDFINKKVSGSQLIKVSLPVDPRTSEGLKKANEFQEYAESLGQVGSVF
SIVDSIKSLNRVFHDDDPAFDVVPDNDETAAQLLLLVETSLSPERLTQLVSEDYQNLCFN
IQLKAGDSATLQKTADLLRTKANEIVGTGGVSMAGVSLIANQLNGLVIISMLKSFVIAFT
LVFVFVAFGFKNIPAGLACAGIMIAIVTVVFGTMGLAKIELSTATTLVASITLGVGIDYL
IHFSARWFSERNNGYDKKEATSRALGLTGRGIIINAMAIIFAVIPPFFSQFRPIMYFGIL
CFVSILLSLVCGLVTMPALLSIIPDKYFKKIKI*
>SPBDM4_v1_40192|ID:27157497| putative Transcriptional regulator, TetR family [Uncultured spirochete bdmA 4]
MHSKASGDGSASMGNEVRTRIVDLSKDLFFTRGFTRVTMDDLASACGISKKTLYVNFPSK
DALIHQIICQTQEDLIDQVSRILNDSNIPVVKRLKDVISAVSLHSLQFAPLFLEDVKRFQ
PKGWKDLQDYKRTSIADAIHLVIHDGQEQGYIRKPLPEDFIVYVCFTLIDNVLTPATMFE

LSMSFHDTFENVMSLLFEGFLTDAGRKAIYAIE*
>SPBDM4_v1_40193|ID:27157498| Radical SAM domain protein [Uncultured spirochete bdmA 4]
MESVMKPPEKDELAVKRADSIAFNKLIHVSPKTIDKLFDKPALRKTVAKIFEWSTYRSSL
SFKEYPKQVQLDKFYITRAFIKSIDAALANARQSPVFRKSFVNNVAPAMFGAIEKTNERR
EAFKQHYGFGPPAFLVVSPTKFCNLHCTGCYANSDAASHEKLSFEVVDRIIQEKTEKWGS
IFTVISGGEPLLYQSESKTIFDLARRHQDNYFLMYTNGTLIDAAMARKLAEVGNITPAIS
VEGYEAETDARRGKGVYKKILSAMEHLRAEGVPFGVSLTATKENAHLIPSRELVEFYTKL
GALYFWIFQLMPIGRGSMELVPTAEQRFEMYNRMFKLIKDDSYFIADFWNGGTLSNGCIS
AGRMRGGGYLYIDWSGHVTPCVFNPYAAGNINEIYARDGELSEVLVSPYFESLQEWQKDY
SMSGDAYTGNWVLPCPMRDHYADILSILEKTHPEPTDEVAAEALSDPKYHEAMVQYDEEL
ETIFGPMWKEKYLKQK*
>SPBDM4_v1_40194|ID:27157499|palH| Alpha-glucosidase [Uncultured spirochete bdmA 4]
MKLVLIGAGSAQFGFGTLGDIFSSAALRGSHIALVDINGESLGRVLAAAKTFIGAHGLDF
SLSAHTDRREALKGADFVIISIEVGNRFELWDQDWMIPLQYGIPQVYGENGGAGGLFHAL
RIIPAILDICVDCTELCPDATIFNFSNPMTAITTTVLRKFPQLKFIGLCHEIASLERYLP
LMLGLPFEQLTLRAAGLNHFSVLVSAQYRDRGTDAYPDILAKAPAFFEKVLGYSDILEYI
QTTGNIPRTEGETGMPDIGRTISSRQWSDRRLFRQILERFHLLPITSDSHIGEYIPWARE
VADHKGILDFYTLYRASLSMLEPTIELKVKERVIPIIEGMLENSGYEEAAVNIMNAGLIP
SLPESVAVEVPARVFRRGVEGIAFPDYPKGFAALLRNYAGVYDLTAEAVLHKSKDLVVQA
LLVNPVVPVCERVPELVDVMIGRQAQWLGYLR*
>SPBDM4_v1_40195|ID:27157500| Sel1 repeat protein (fragment) [Uncultured spirochete bdmA 4]
MKRLFIFLSMLIVALPVVSAQTGQTDLALLTATAESGDAKAQNGLAIRYYNGDGVVQSYE
QAAIWFEKSARQGNATAQNNLGFMYEEGKGVPQDYARALQWYTMAANQGHAAAQANVAWF
YENGFGTAQNYDEAFKWYQESAAQGYAYGQYSVGWFYDKGLGVAEDNTQAVQWYTLAAEQ
GLPEAQYMLGLMYENGDGVAVDFSQAFHWYNEAAEGESPEAMLALGFFYEFGISVDEDIV
QALAWYMIASDYGNKDAASYVEDLSKDLSEQEIDAARQMADDF*
>SPBDM4_v1_40196|ID:27157501| putative Membrane protein containing DUF6 [Uncultured spirochete bdmA 4]
VKESPVPRLGWACGMPILAVLVAAALGASSGLYIKGLAFSSLAMTSLRMTVPLLLVLPSA
AKHGLLLGKRGMRWQLSLASALNAIRLYLYVLAYKLTSMGNAVVLLYLWPVFALVIDSIR
LKKSLSVVRLGILLLSIAGVLVMNLNKRFSLSETDLLGSACMIASALIFAVTNIIFKQVL
TVISEIDTLYFQNAIGGIIFLPFLIAEIPHAPLSHLSIGIFYGVAVGLVGFGLFFVGMKR
LPLFQYSALSYSEVPFAVLLGIAFRGEKMTALQGLGMALIVLGSFLAQRSHSSSE*
>SPBDM4_v1_40197|ID:27157502|norM| Na+-driven multidrug efflux pump [Uncultured spirochete bdmA 4]
LKDAQHKTVYTSSSHDTTRLEIFRNASIPSAVANFAIPTVISQLVTMVYNLADTFFVGQM
GDPRKVAAVSLVLPAYTMLTAIANLFGVGGSSVISRFLGADQPDKARQTSTFCIFSAIAA
TLIFSMLIFIFKGPFLLLLGANQDTSEFASNYIVWILVIGGVPTVLGMLFGHLVRSEGGA
KQASIGMSFGGLLNIILDPIFIMVLGLGVRGAAIATMISNCAVVCYFLVYLYRKRGKTII
SFVPRGFDQELHIAGSVAAVGLPASLQTLLSLTSNTVLNNLASGYGSTALAAVGIVKKID
MLPMNVTLGISQGILPFIGYNYAAKRYNRVRQVNRFTRIIAVCFSLLCVFVFEIFAERIV
GLFINDAETIRLGATFLRILCLATPMMAISYLITTMFQATGQGKRALAISVFRKTTVDVP
LMFLMNRLVPLYGLLMVQPIVDTLSIGLAFALYRDFSKKLKAKSDTEG*
>SPBDM4_v1_40198|ID:27157503| Auxin Efflux Carrier [Uncultured spirochete bdmA 4]
MFTALQSVFSVILMIGLGFGLAKARWFEGGASNLISRLVVNVALPAYMISNLMGGYDRPK
LLSMLPGLPVPFAVMIASYLVAIGVAAVLRVRKDRRGTFQSMFSLSNSVFIGLPVNLLLF
GDASLPFALLYYIANTTLFWTIGVYGIAVDGAVRNGTQKPSLVSASGLRRILSPPLLGFL
TAVILILLGIKLPKSIMDTCKYLGNMTTPLSMLFVGIVIARVEWKKLRFEKDYIAILAGR
FIVTPVLMVLAVRWLDLPVLMKQVFLMQATMPAMTQTPILAEAYNSDTEYAAIGTSLTTV
ASMITIPLYMTLINVLIQ*
>SPBDM4_v1_40199|ID:27157504|carA| Carbamoyl-phosphate synthase small chain [Uncultured spirochete bdmA 4]
MSRKNKSARLVLEDGSVFEGLSIGAEVSVEGEVVFSTGMVGYNQSLTDPSYCGQILVFAY
PLIGNYGVPALHTNAQGVPLNFESKNIQVSGVIVSEASMEPSHYTAKQSFSEWLTVGQVP
GISGIDTRKLIRLLRENGVMKGRILVDGSEEPVRDELKAHAPVKLVSPKDVVRYKGHPDA
PKIALIDCGVKANILRILLNAGAEVIRLPWDYPLESIEYDGLFLSNGPGDPKACTKTIAH
LRHALSKPHPIFGICLGTQLMALAAGADTFKLKYGHRGQNQPAIETESGRCYITSQNHGY

AVHEDSLPPGWEPWFLNGNDGTVEGIRAAGAPFKAVQFHPEGCPGPRDTQFLIDEFLQEA
RAAKDSI*
>SPBDM4_v1_40200|ID:27157505|carB| carbamoyl-phosphate synthase large subunit [Uncultured spirochete bdmA 4]
MKSFAAKDPRPTLVHSKPRNIAVKKVLMLGSGGLKIGQAGEFDYSGSQALKALREEGIRT
VLINPNIATNQTSEGMADATYFLPLTPEFVTQVIEKERPDGILLSFGGQTALNCGLALHK
AGILYKYAVEVLGTPVEAIELTEDRQLFATHLRSLGLKTPASKAALTLEEAISHAESIGY
PVMLRAGFALGGAGSGICRNERQLRARAEKAFAVSPQVLIEEWLGGWKEVEYEVVRDSAD
NCIAVCNMENIDPLGIHTGESIVVAPSQTLTDEEYHSLRSIALRLIRSLGIVGECNIQYA
LDPHSEEYRIIEVNARLSRSSALASKATGYPLAYVAAKLALGFSLTDLRNRITGVTTACF
EPALDYCVIKMPRWDLSKFKNVDTRLGSEMKSVGEVMAIGRSFEEALQKAIRMTGISEFG
LQTDGKREKILPNDLRSALSKPTDKRLFAVYQALAEGWTVQKIGKLTGIDPWFISKIANI
QNCEAELTGTKPGASGPVRSAKNISLLYKAKRLGFSDAGIAHVLGRSEPEIRSWRESVGL
RPAIKQIDTLAAEYPAQTNYLYATYGYAMEHGAHGSPASDVRPSHGSILVIGSGPYRIGS
SVEFDWCAVSAVETCRKLGRSTIMVNCNPETVSTDYDVCDRLYFEELSFERILDIYDFEE
PAGILLSMGGQIANNLALKLFDAGVLVLGTSPVNIDRAEDRHKFSSLLDELGIDQPAWEE
LTTPQEAYAFASKVGYPVLVRPSYVLSGAAMNVAWDSQSLGSFLGLAADISSEYPVVISK
FIENAKEIEIDAVAKHGKLLYHAMTEHIENAGVHSGDATVVFPPQRLYIETVRKILRITE
KIAAALDITGPFNIQFVAQQNHVMVIECNLRASRSFPFCSKVSRVNMIDLATRAILGETV
QISSNAIYDQPWVGVKAAQFSFSRLHGADPVLGVEMASTGEVGCIGSELNDAFMKAMVSV
GYAPRIKKLLLSTGPLEDKLEFVDSARALVEMGCTLYASRGTAQFLKKYGIEVSQLHWPL
QKAEPNILTMMHRREFDLVINIPKNNRKRELQNNYLIRRLAVDLNIPLFTNIKTARQYIE
SLTYAKKNGLEIKAWEEYRKMDV*
>SPBDM4_v1_40201|ID:27157506| Branched-chain amino acid transport [Uncultured spirochete bdmA 4]
MSYFLLVVVVMTAVTFFTRAVPFIFFAKRQPPSLLVFLQKYMPPVVMAILVFASYKDIDY
GAAPFGVPALIAGAVTALLHFWKRNVLLSIVGGTALYMLLIRL*
>SPBDM4_v1_40202|ID:27157507| Uncharacterized membrane protein jhp_1251 [Uncultured spirochete bdmA 4]
MQDNHSRREWRLVLPYAFKASIPVLLGYTTLGIAFGLTLVAAGLPWWLSPIMALCIFAGA
AQFMAIGLLQSGAGLFEIALLTLLMNGRHAVYGLSLLGPFRNSGKKWKPYLIFGLTDETY
GLLTTVKPPAHVDETKFFAAITALNQSYWVIGCTVGSIVGSALALDTTGLDFALTALFIV
LLVEQIRAVRQYEPYLAAAFACVVALFVSPRNFLLVALAIATGVLALLRGKIEQSTKAHQ
DDGGGDT*
>SPBDM4_v1_40203|ID:27157508| Thiol-disulfide isomerase and thioredoxins [Uncultured spirochete bdmA 4]
MLYRFLYIFKDMKELGSYDELIRLMQQEKAALFYFSTPDCGVCKSIKPKVISLVQEHFPL
LKLYYINTEAVPEARGQFSVYSVPVVLVFFQGREYIREARNFGIMELGAKIDRYYSMMFE
KATQ*
>SPBDM4_v1_40204|ID:27157509| protein of unknown function [Uncultured spirochete bdmA 4]
MTDPFRSWNRGVFNLYESGGSTTSVGPDWWAMQIRKGRTMASQYIMRHKM*
>SPBDM4_v1_40205|ID:27157510| protein of unknown function [Uncultured spirochete bdmA 4]
MTAEWEGDWSKVDISNTVLSEFSGSYMMFNGIARLTAGKVRADGGYRFTNFDTAGFSTRI
ANAETGVLLTIQPLRGISFGTFLPVPVEAQAASTTYQRMNLGASWDIPEVAIFKASYRME
PYILYGITPKGKELAVGGQLRAIENFLFTLGYRWFDVADEHDFFIDTSYRFPSTLLSAYA
YLSVQSPLLYKGFKANIEQNIGKTALVAGTSVSWGEGPDVWWLDGWDVNPYLRYDFGGSS
VQLGVDCTYTTSFAYKVQLTYTIGF*
>SPBDM4_v1_40206|ID:27157511| conserved membrane protein of unknown function [Uncultured spirochete bdmA 4]
MKSKIIVIMLALCMLVVLSSCLPGDGKNTPERQANFFTGIWHGWIAPISLIIGIFDHNIR
IYETHNTGWWYDFGFYMAVISGFGGLALTRRRRRD*
>SPBDM4_v1_40207|ID:27157512| conserved protein of unknown function [Uncultured spirochete bdmA 4]
MNEKAYEAVDLYLESLLAGKPPEWEQLPDIGLYMDQVTSYLERQLGPFSPSEKERAITSS
MINNYVKAKIIPRAKSKKYSQEHIALLLSVFMLKKALSIQDMGVLLADASSEGSYREFYD
RFRKALTECAISTAQEIRTGLEPGSAESTARFEASAIDDAKLRTLALKLAVEASLRSLAA
ERLLALLQTEEPLKAR*
>SPBDM4_v1_40208|ID:27157513| G-D-S-L family lipolytic protein [Uncultured spirochete bdmA 4]
MYGQEVCLFGDSIARGIILDAQGSYKPIKECFASLAASELGFGLINKARFGCTITKGREI

IEHFLSRVDSETRRLAFLEFGGNDCDFRWDQISADPHAEHLPATTPERFFRMYSEVIQEL
KNKGFRPVLMTLPPLDAERYFAWFTRTGLDQKAILGWLGDVQFIYRWHESYSSAIWEIGE
THHVPVADIRKAILEERNYSRFLCKDGIHPNAEGHQLIKSEIIKFAKEHQADKA*
>SPBDM4_v1_40209|ID:27157514| conserved membrane protein of unknown function [Uncultured spirochete bdmA 4]
MRGEQTSYSPVFLGLTSLFITCLLISNIIASKLMQVGGIVLPSAVILFPVTYILADVFTE
VYGFRKTRMVIWIGFAANAFMSMIFLIAIALPHPSFFANQAAYATVLGSTPRILLASLAG
YWAGEFSNSIVLSVLKKATKGRHLWTRTIGSTIVGEGLDTVLFIGIAFAGTVPAGTLGGM
MLAQYLFKVSYEVIFTPLTYLVVGFIKRKEHIDTFDSEVVYNPFKLGE*
>SPBDM4_v1_40210|ID:27157515|queF| NADPH-dependent 7-cyano-7-deazaguanine reductase [Uncultured spirochete bdmA 4]
MAEYEFKSLGKTTRAPSRELETFPTPPSVTKVVLESDEVTSLCPVTGQPDWETVRIEFEP
GNLCIESKSLKLYLWSFREEGVFCEALAARIAKDVFDAIKPKSVAVSITQKPRGGITISA
EARLPKIV*
>SPBDM4_v1_40211|ID:27157516| conserved membrane protein of unknown function [Uncultured spirochete bdmA 4]
MLNIILIGLVSFFADMSTEMVYPMIPLYLTSVLGATPALVGVIEGIAESLASILRVFSGY
VTDKYRKKKPIAFLGYSTVLIYKVALLLAHSWAGILFARVIDRFGKGIRTAPRDVLVSES
SDPKTLGHSFGIHKALDMGGSAVGILAAFFIMKTWTGVTVYRNLFLISTIPAVVALVMFF
FIKETNKPRESKAPLNVFKGLKTLNPDLKLYLTVVFLFNLGNSSNSFLLLRAGNIGFDAA
SVVLLYFLYNLVSSLLSIPAGKLSDRIGRKRVLVTGYIVYAIVYAGFAWAPGKGVLIGAF
ALYGLYTAMIAGVERAFIAEIAPKELKGTMLGLHSTIVGIALLPASLIAGELWSAFGPAA
TFSTGSGLALVAALMLAIGLNKKPERNMLQ*
>SPBDM4_v1_40212|ID:27157517|rluD| Pseudouridine synthase [Uncultured spirochete bdmA 4]
MAREHRNGRASPSGGHWGKIDYLYEDRDIIVVSKPVGLAVIAPEGSRAKTLYDIVTDHMR
KSNPRARPAVVHRLDRDTSGVMMFAKSAMGKKILMSRWNELVRERIYVALVEGTFQGESG
LFDSWLIENRAGTVYVAEPGVRGALRAITRWRVLKKGNRYCLLELSLETGRKHQIRAQLS
AAGHPVAGDTRYKARTDPLGRLCLHATALTIEHPFTKQIMRFENPFPDEFLSFCRN*
>SPBDM4_v1_40213|ID:27157518| Basic membrane lipoprotein [Uncultured spirochete bdmA 4]
MRDISKLFLLLTIALVAVSGAFAQSTPPAAGPNLTIGLVMVGPYNDHGWSQANYDGLQYV
LAKVPGTKLVYIDKANSADRPGTTVSQLGESLVAQGAKLVIFSSDDMTDEAIKFAQDHPD
IFVILTSGSQQWKEGKNYLPLPNMINVMGRMEYMKMVAGVAAAMTTKTGKIGFLGPLIND
ETRRLASSAYLGARYAWVNYLKKDPAKLTFKVTWIGFWFNIPGVTSDPTQVADDFFNSGF
DVVISGIDTTEAVAEAAKYTAQGKQVWAIPYDYSKAIDEGKGVSLGVPYFNWGPAYVNAV
KSAMNGTWKNHFEWNGPDWKNINNPDTSAVGFNKGTLSKDASAAVDKFVGELGKGLNLWV
GPLNYQDGTVFLKNKEKATDQQIWYLPQLLQGMEGQSVSK*
>SPBDM4_v1_40214|ID:27157519| conserved protein of unknown function [Uncultured spirochete bdmA 4]
MQVELRGITKCFGALRANDNISFTVPPSTIEGILGENGAGKSTLMKILSGFYSPDSGDIL
LDGEKVKIDTPADAIRQGIGMLHQDPLDFPPLKVIDDFILGEKGGLLPNRKRASKEFKEL
SARFDFSIDPESYVDTLTVGERQQLEILRLLWLGARALILDEPTTGISLPQKEKLFATLK
KLASEGMTILFVSHKLEDVEALCSRATVLRQGKLIGTMELPFATKALVEMMFGKEMPQES
KTQLKMGMNVLNAKHIDLDGVRIQIKGVDLQVHAGEVIGLAGMEGSGQTLFLSACAGLSQ
PVGGSFCLDEEEKDMTGQTYHEFRKKGVAYLPAARLEQALIPGLSLTEHFILSEGMRGFF
IDRKKAEDLAKSRIALYNIKGRPDSTVESLSGGNQQRALLALMRDPLKLILVEHPTRGLD
VESTIYIWGKLKERCANGTAIVFISSDLDEILKYSDRVLVFFAGRVTPPLNASSLNVERL
GRLIGGKDWDMLETEAAHA*
>SPBDM4_v1_40215|ID:27157520| ABC-type uncharacterized transport system, permease component [Uncultured spirochete bdmA 4]
MRDTRTNRILFQIGAILFALIVTTLVLSLAGAQPFAAYKYIMLGAVGSWDVVSNVLVSWV
PLLLATSGLLVTFTVGLWNIGIEGQITLGAIFTTWAMRLLQDSGWNPSLILVIAVLAGML
GGTLWALLAGALKTFGGVNEIFGGLGLNFVATALNIWLIFGPWKRPGVASMSGTLPFDRS
LWMPIAAGSSRLTPATLVIAIVGIIAVYATIEGTYFGLKLKAVGRNPKSAYILGIPTWQH
MMASFAICGVFAGLAGAVQVTSVYHRLIPSISSGYGYLGLMVAMLVGYKASLAAPVALFF
AALNVGSIQLPIILKLDSSLAGVLQGTLVLFVILGQGVRTKLLKKTKVSS*

>SPBDM4_v1_40216|ID:27157521| Inner-membrane translocator [Uncultured spirochete bdmA 4]
MNNSALVTSIAGVLAAAAPILFAVIGETISERAGVLNLSMNGMILLSAMGSFAVALATGS
VVLGFLAGAAIGALVSLIVAFASLTLLQSQVAVGFVLALLCRDLSYFLGNPIMGKVGPRI
SQVPIPGFERIPVLGTIFFRQDVMTYLSFILIVLAWFFIFRTRPGLILRGVGERPAAAYI
RGANVNRLRYLYAVIGGAIAGLAGPIYSLSVKAGWKGTISGLDGIGWIVLSITIFGGWKP
FRAAFGAYLFAFLQWLGLVLQPSLPSVPSQVLQVAPFPLMILSLLLVNIGNAEWVDRSLS
RLPENVRRDIAKVLRAMNATPPAALGVPFEKE*
>SPBDM4_v1_40217|ID:27157522| putative unsaturated glucuronyl hydrolase [Uncultured spirochete bdmA 4]
MNSYRTAVYLQVLASAAAAALADCTSRPIKWNYETGVLLSALGELSRRRFAGLFDAPMQS
CVDELVLEDGTIRGYSVDEFNLDQINPGKELFSLWKATGRKAYEKAIRTLAGQLAHQPRT
ASGSYWHKKIYPDQVWLDGLYMFGPFQARYGIEFGQPELIDDVCRQFLAVQVSMKHAASG
LYYHAKDEKGVSKWADPQTGCSPHVWARGVGWLSMALVDTLDWIPPEHGQRAQIMGMLAE
LLDAVVRAQQESGLWLQVADVPSGSGNYGETSASAMFAYALYKSVRAGYSRNETQAGQWL
RAASSALEGILTFIRWECPEGAIPRLHLGGICKVAGLGGNPYRDGSFEYYIHEPVVSDDF
KGTGPFMLALNEAIHFSRR*
>SPBDM4_v1_40218|ID:27157523| Extracellular solute-binding protein family 1 [Uncultured spirochete bdmA 4]
MRKIVKAAVVLLLMMAAVSSAFGQGAKKPVTVELWYGAAITEAGPPPDDWVFYKIAREQL
GIELKLMALPSNDNDQDVKILAAAAANQLPDIFMVRREPWQVLIKQGLVADVDDMYPLMP
NRAENWFDQDSRTYTTINGKSYGLAFPGSLQKNEGLVIRKDWLDKLGLKMPTTLDELFDV
CYAFTYKDPDGNGKNDTYGFGAFVEHFDQEQYMGRRFDPIMGAFGVAGTWNMTKQNFGLN
VKKPEFYEALQFVKKMIDAKVIDPDWPSLKKDDFRAAWKQGKFGVMREQFAALSASANYA
PFDKNFPNGEWAALPPPKGPTGKSSVGIYDTNYRIYAISTKAAKAGKKEAIAKLLDYMAT
DEGYKLIGWGVEGVNYSIDANGNISDKGVPDETKFSSPKGQTVTQLRNMVFYNSNMELIS
RYPYFKTANGRTMGPLLYLKVFQNFPWTNVTGSGTIKPHPNNADLKRYINQGVQEFVLGK
RPLTKENFAAFVAQMDKLGAAEWEKTARQLMEESGYLQ*
>SPBDM4_v1_40219|ID:27157524| ABC-type transporter, integral membrane subunit [Uncultured spirochete bdmA 4]
MAGTIRLTKSDKFFHFFIDAGLVLILAVLIVPIWSTLTLSFRPNDFIGSALDGMFLAPWK
WSTAAYRALLGHRSFLSAAGNSFRIFFEGVATALFFTVPLAYALSIKTLPGRKLLYGIVL
VPYLFNPGLVPNYLLVTKLGLSNTLAAVFLPGAVSVYNTLIMKSFFEELPEELKESARID
GASELTVLLRIILPLSKPILLTIGLYYGVHFWNDFFNAMLYINDAKLQPLPILLRNILLS
SGMNEYVEANAFGEAPIYAIKAASVFLAAIPMLVAYPFIQKYFVKGTMLGSIKG*
>SPBDM4_v1_40220|ID:27157525| ABC-type transporter, integral membrane subunit [Uncultured spirochete bdmA 4]
MMEGQSVLLRNPASHALWRRIIRHRSVYLVASISFIYFIIFKYIPIWNAQIAFRDFQALK
GVTGSPWVGLKNFSDFVNSYYFWELIRNTVFYSVGKMAFSIPAAVFLAVALYECRIYGLR
KTVQTMAYLPHFLSWVIMYAILLAILSPSNGILNDIIKAFGGEPVSFMTDTKYFPWIVIL
SDTWKETGWSAIIFLAALMGIDPSLFEAASVEGASAWQRVRYITLPAIKPVIVVVLLLRL
GTVLDAGFNQIFMLYSLPVYSVADIIDTWIYRQGILEFRFGLATAVGLFKGVFGLLLIVV
SNKLIKRYTGNGLY*
>SPBDM4_v1_40221|ID:27157526| protein of unknown function [Uncultured spirochete bdmA 4]
MQAHFPRIAFLLASLHTGSARGLWPELIAAAQSHRCILFVLPGGRLNAVPGFEHMRNGVY
RYARSPNIDSALCWASTLSGYATEATVEQFLLDTVNVPLVTFGLKIGEHPFVGIDAYSGM
KALIKHFIRAHRKRRIAFIAGPRQHSSAEERYRAYCDALNEAHIAYDSSLVCLDIPWTEG
RRAALQLLDERGLVPGKDFDALCAASDLLAFEAGLVLRERGMEIPFDIPIGGFNDSEESR
IFSPPLTTVHMPFSHQALCALRLLSEMRKGTPPADMSLKTKLIIRSSCGCMLNSVRKAGR
SRTWADLSPDLIEAFKASLSVRQDRSFLVLLARMLDANIGVRGEVRGLQNTLSALRVEFL
KRAGSSGEAERTETLIHQGMVAVCLAEERKYEYRLWKEKQDEQKLNLFNQELLCAKDMES
IISAAAAHLPELGIASAYLMTRDKQGALMFRGGFRQEKEHFPVEIVRPRRSCEIVPQGCF
LPDRMMPAEPGAYFVLPLFFESTDLGHLVVQAIDIDPSLYEEIRSILSSALRGIMLFEEG
DEARKRAERAEKSKSDIVAVISRELRNSIDTFNASAESDTSGRVVSGLEVSGLQVSRLKE
LVRYLLDLSQAKSDVVLAKAALFDPYAFCVAWARGQCADSTYGLKIECAEPPSMYPALYG
DTEQLARILSILARLFAEMYQTAELKLSIAVLTRGCALTLSLPSVLSSRGDIPDLPKIEK
DIRLELARQISILHGGQIHFAKEKDGVSVTLCLPYPSMNGAIRNISDDSKVVCFIGPDQS
VIESAFSLSADESFQVLPYNAVFNQEISKVQRMFLYLDPASIDHESSAALAFILDQDVFQ
RAECYIALHASSEGAYPMLRQSADAAGFLRSVLLSDSPSAILMIGSETALHPGMVHIQHL

TQEAGIRLVACASPAAAEAALNRWHPVLFLALEPKLETQGYGPLLRRFPTVPLIAASRSF
EAKEEWTSVLERPHTIFCNTGEVFSGLLTNLVQRSMEGQNFLPSPTACIVAKCIFFINKT
YAEQLSRWKLASLLNTSEDYLSRIFHKQMGVSLWEYLARLRVVNAIDMLRSTGASLAEIA
DRTGFKDEAYFCRVFKKMTGATPGSFRIESFAEVRKVQESD*
>SPBDM4_v1_40222|ID:27157527| Polygalacturonase [Uncultured spirochete bdmA 4]
MIADIAEFGGTGDGQTDNSKAFAAAIASLKRAGGGTLKVTRGTYLSGPIELFSNITLEIG
ASAVLQFLPDMQLYSPVLTRWEGIVCFGMHPLVFTRDAHDISLKGEGTIDGNGNPWWAAL
RHKKSVHQSCPVEPIEIALAEYNKDLIDQPSGGGGREMQFLRPPLVQFFRCSNITIEGLT
FRNSPFWTIHPVFSDHVTIRDVFVQNPPDAPNTDGIDIDSCTNVRVENTTVDVGDDCIAI
KAGAGERGLREGKASKQITIRNCHFKQGHGGVVIGSETAGGIHDVSVNDCTFEGTDRGIR
LKTRRGRGGAVSGLRFRNLKMERVLCPVAINMYYRCGAKPEDKQALFSLKAEPLDSLTPS
ISDIEISGLEAQGCRASAGFIAGLPESPIGGLKMEQCHIGLAGHDMVPVSESEMFEGIPE
TSARGIRIRHASCEFRDVRVMGLGNQEKPFVNEEGAKIKIDMPAP*
>SPBDM4_v1_40223|ID:27157528| conserved protein of unknown function [Uncultured spirochete bdmA 4]
MTPSKEMLDQIVHEEGELILERFTEEDAWELGCLLVEEARKREARVAVDIRRPAQILFHA
ALEGATPDNDEWIRRKSNVVFRFGKASFAVGISLALTDTTIEKKSFVSPLDYSPHGGAFP
IRVTGCGLIACATVSGLPQEEDHALVTACIRKFKESKTAKKEHTAR*
>SPBDM4_v1_40224|ID:27157529|corA| Magnesium transport protein CorA [Uncultured spirochete bdmA 4]
MKWRWKKKGADAAESMDKPSQSANGHKKTRRVSNLPFSGVSVDTAGLPPGSPRYIGDVEP
TLATYSIYSYDEKSAVMFIPKDVSELLALIDLNVVNWININGLSGGMVEKFCAMMGIHPL
VIEDILNTEHRPKLENFDDYLFLITKMLTMHEDGTIEYDQVSFILKGKILVSFQESPGDS
FKGVRERILAGVGKLRKLGADYLLYSLVDNIVDNYFIVLESLGDNLEGFEDASATEDDGA
FLHGIQELNLELARMRRVIWPVRDSIGALARSDSIQMDYSLSPYLRDLYENTVQAIEALE
SYREHTASIIELHLSEVNMNMSRVMKILTILSAIFIPITFIAGVYGMNFAYMPELELPWG
YPAALGLMAIVVIAELIYFKVKKWL*
>SPBDM4_v1_40225|ID:27157530|fucU| L-fucose mutarotase [Uncultured spirochete bdmA 4]
MLKGIPEIISPELLKILHEMGHGDDLVIGDSNFPAASNAKRLVRADGHGVPEMLDAILSL
FPLDTFVAEPVSLMQVVKGDSTVPEVHERIREVVRRHDPRGDSAVTSVERFEFYEKARQS
YAILATTEPAAYGCIIIKKGVIH*
>SPBDM4_v1_40226|ID:27157531| Branched-chain amino acid ABC transporter, permease protein [Uncultured spirochete bdmA 4]
MTTTKSADSVRRGRSGRDPNLLRLFVMLVFVFVLMTVLRPNQFATMAGFNSMMRQFPEYG
IMAIGISLTMITGGIDLGVVGTANLSAIIAAKFLIGTVPRGAASAQVVVPYLIIAVLLSLS
VGLACGAFAGVLISRFNIPAILATLGTQQLFTGIAIGITSGRPQSGLPLLYSRIGNMEFF
RFIPISFVIFFVVAVSVGIMLSRTRFGTYMYMMGTNPRASRYAGLNNTSITLRTYMISGF
LSSVAGLIMMARANSAKADYGAPYTLQCVLIAVLGGINPNGGFGTISGVTMAILILQFLS
SGLNMFNNISNFYRDVIWGGVLILVLVFNKLLSWREERRAVRKSMN*
>SPBDM4_v1_40227|ID:27157532| Branched-chain amino acid ABC transporter, permease protein [Uncultured spirochete bdmA 4]
VISLRRITKRSEPYVLAAIILLSLLIHVRSGQFFSGNNLVDLARSLIVPALFSVGCMMVI
VSGGIDVSFPAIASLAMYVTDKLLLASNYSGNVILAYAISAGLGLLMGALNGFLIGFLRL
PTLVVTLGTSSLYTGLMFGAFSASASDVPPSIAKHGKEILFTATNPTMGLTSNMPSQILI
LIAVLILAFFLLRYTMLGRGIYAIGGDETAAERAGFNVRGIKMFLYCFVGVLAGITGIVR
ISMMSYADPSTMLGMEMTVIAAVVLGGTRVTGGMGTLTGTILGVALMTILSNSLILIGIP
TYWTRVFTGAVIIIGTGVTAHQTISTRRRRAQSQKAAA*
>SPBDM4_v1_40228|ID:27157533| putative ribose/galactose/methyl galactoside import ATP-binding protein [Uncultured spirochete bdmA 4]
MAASILRVEGITKSFGGVQALKGVSLEIGQGEIHCLAGENGSGKSTLIKVISGVHEPDAG
KIEINGRQFSHLTPKEAISLGIQVIYQDFSVFPNLTVMENLALNNELMDKRKLMSYKRAR
AIATHAVSQIEFDVDLDERVENLSVANRQLIAICRALLQEAKLIIMDEPTSALTKKEVRA
LFAVIKQLQSRGLSILFVSHKLDEVFEISERYTILRSGENVASGKTVDLDRKTFVYHMTG
RQFEDGFYRPSAAKREPILSVQGLSLEGAYSDISFDLSPGEILGITGLLGSGRTELVETL
FGISQPDAGKIFIRGKEVRIKSVKSAIGKGIGYVPSDRLTEGLFLPQSVERNLVITALDR
LSDHFGFLRRSAIADMTRKWIGELSIAAKSPTLPVWTLSGGNQQKVVLARWLANDLSILM

LNGPTMGVDIGSKYDIHVLIRELAARGLAIIIVSDDLPEVLACASRILVMRDGAFVQELD
PSATTESKLSELSTGIA*
>SPBDM4_v1_40229|ID:27157534| conserved exported protein of unknown function [Uncultured spirochete bdmA 4]
MKRKLLLAVLLVMVFALAVPLFGQSYDPMSFKSKKPMSQWKIALVPKDATNAWFVRMEVG
VKQFAKDTGINAFQKGPAKTDAAMQAQVIEDLIAQGVDAICVVPVDPAALEPVLGLALSR
GIVVIDHEGASQQNCLYDIEAFSNEGLGQTIMMELAKMMNYQGVYTTMVGNVTNASHNEW
ADAGIAYAKAHYPNMTLLAAEPRVEIMDNLDTAYERAKELFKKYPNLKGIFGTSSLSSPG
VARAIEELGLKGKAFVTSVGLPNENKAYLKNGTLQTVLLWDPKDAGYAQLALAVKILKGE
KIENGCNLIAPGWTSMQFAPGSKKVLQGKGWITITKDNVDSFGF*
>SPBDM4_v1_40230|ID:27157535| Regulatory protein LacI [Uncultured spirochete bdmA 4]
MPYHMERAIKDWCHTMSIRLKDIAEAVGVSPTTVSLVLNNKPGAREELRRKILKTAKDLG
YTAARNEKPGSLCLLHIARHGHTVNRDHDVFIADYIEGLSQGSKLAGYSLEIVTFKLAPM
EQVLDTALSKTTNGFIILGTELSREDAELFQAIRKPIVFIDTYHEFLNFDFVDMNNEDSV
FMLMSHLYERGHRRFGFVKGSIETRNIRLREMGFSKGLQKLGLEADTSFIFSVDQTYHGA
YSDMKSILAGHPRLPTALICSNDIIASGCLKAFAEAGVRVPKDISVVGFDNLPLSAISDP
PLTTIQVSKAQIGRMAIQLLVSRIQGEADMPAVKVLIGGKLVERSSVKRIDIA*
>SPBDM4_v1_40231|ID:27157536| Xylose isomerase [Uncultured spirochete bdmA 4]
MKYSIILGNLGNTCDRFLSSGYKDQPSKETMIRQASEIEGVTGVELVGTWDITPQNADEI
GELLDKYGLVCASVLPDHFSQKRWGRGAFVSLDSAIRAQALDETLIAAELARKLRCSLVN
IWPGQDGYDYPLQADYLKARQWFVDAIRSAAKAFPDIRFSLEYKPKEPRTHSFLARAADT
LLLAQEIGLPNVGVTIDTGHSFVAGENVAEAATLLSRSGNKLFHMHFNDNYRFWDDDMIV
GSVHFVEFVELLFWLKEIGYSGWYSMDQYPYREDAQGALRNSVEFLQGIEAMLDAKSMEE
IRSVIAEGDAVKSTAWLKSKIFPSK*
>SPBDM4_v1_40232|ID:27157537| protein of unknown function [Uncultured spirochete bdmA 4]
LLCAGGTTRLIGEERARQDIPKGLSRETFWMKDWKYSHPTGISRLHVAQTASGCFGVRTR
SSH*
>SPBDM4_v1_40233|ID:27157538| Filamentation induced by cAMP protein [Uncultured spirochete bdmA 4]
MMPVRYHEGRFPPTNLDWQKIIPLIGPAYASLSRYDGMLAAVSNPELLLSPLTTQEAVLS
SRIEGTQATMGEVLEFEAGQAPTSDERREDIHEILNYRAAMRRAEELLKSLPFSQRVLRE
AHKVLLDGVRGQNKSPGEYRRIPNWIGPPGCTLENAHYIPVSAEKLPVFMSEWERFAHSE
FQDVMTQLAILHAEFESLHPFLDGNGRLGRMIIPLYLWQKGLIRSPMFYISTFFEAHRDE
YYDGLLAVSRDDDWTGWCRFFLKAVQVQAEDNLKKAQGILSLYDQMKLEVPEMTRSQYSI
RAIDWIFGQPIFNHSVFIESSGIPKPTAKRILSILEEEGILSVVVPGRGRRSAVVACSRL
INIAEGKELF*
>SPBDM4_v1_40234|ID:27157539| exported protein of unknown function [Uncultured spirochete bdmA 4]
MTVFWARRFSIGVIASLAHAEEIQVIRLEVTNFQMRQFPQMILVDIEMLGILF*
>SPBDM4_v1_40235|ID:27157540| protein of unknown function [Uncultured spirochete bdmA 4]
MSSSSLYLSRASPFHFSIVPAVWSDPSIASSIRWLLSLIFSLRLLTQGALGCRTPQSELS
FNFFSRVRSWQEIALALLKKYTEHFYIYQNHLWELPHLEIRYLEPDDLNFLGVGEASDDS
Y*
>SPBDM4_v1_40236|ID:27157541| protein of unknown function [Uncultured spirochete bdmA 4]
MKQSKDLTKQLELLKSERDSLLKDIESLRTSLDLLKTKSTELEKMRIELVITLMISEAS*
>SPBDM4_v1_40237|ID:27157542| protein of unknown function [Uncultured spirochete bdmA 4]
MRNYNRISSKNREEIMVYSANGNSQETIAKKTGFSQSTISRELKKGEDRSVYNPFLAQRK
ITLFVKHRYRTLKKNDSTWRVIQNHLAIRWSPYEIMDLLHKSANDATIVPVSEKTIYNYL
NFLYEGGDEKAGAARTSPKRQNAQHEGNKKRGKPANITLIDERPEEVNARTVPGHWEGDL
IIGKDHKSALSVIVEQQTR*
>SPBDM4_v1_40238|ID:27157543| protein of unknown function [Uncultured spirochete bdmA 4]
LIDWFENYIALEVRKSIEKRLKTLEPQLVKSITCDQGKEMTEHERLASSIKIKV*
>SPBDM4_v1_40239|ID:27157544|yugJ| putative NADH-dependent butanol dehydrogenase 1 [Uncultured spirochete bdmA 4]
MNNFTYQLPTKIIFGRGTENTVGSEVANYSKKILLHFGGKSAEKSGLLGRVRASLQIAGI
EWIELGGVQPNPVLSLVRKGIERCRRDNVGFVLAVGGGSVIDSSKAIAAGVFYEGDVWDF
YSGDAAPPTKALPVGAILTIPASGSESSPGSVITKEEGALKRAVNADCLYPRFSILNPEL

AFTLPADQIANGVADIMAHLMERYFTNTPHVEFGDRLIEAALRTVIASAPLLFRGTHDYD
AWAEVMWAGDMAHNNLLDRGRVGDWGSHGIERELSGIYDVAHGAGLAVIFPAWMKYVYRH
DVARFAQFAARVWGVEPDYLDVERTAIEGIARLEAFWKSIGLPLRLSELGIGTNRIDELA
LRCTENGSKKIGHLVELDTADIAAIYRAAE*
>SPBDM4_v1_40240|ID:27157545| protein of unknown function [Uncultured spirochete bdmA 4]
MTKVQYGVSPAFFISLFGDKFTPDDVCEAMPIVRRLGFGCFQAELVNFSSVDLWMKGGAE
KVKNHATEMGLLMSQFVAHFMIEAFADENALFSDFGIEEMRKVFDIAGTLRKEAPITVAI
GPYRSKAYDQKARVRAYARLIEKLSKIAQDALRLGLKLALEIQPGTLLEPGANGILKAIR
DIGSGNVGYNLDTGHAWAAGNNVALYPEMLDGHIEGTHLCDNFGRENLSLRPGAGNIDFG
YLIPALVRSKYDSSLDLEIFVPRDKVEKEYSAGLAFVKKLVEA*
>SPBDM4_v1_40241|ID:27157546| conserved membrane protein of unknown function [Uncultured spirochete bdmA 4]
MEIIWILALMLFFIFIRVPIAFAIGLTGVISIIIKGLPLELVTQRMFTSLDSFTLLAVPL
FIFVGEAMNTGGVTERLFGFCRVLLRHVKGSLGHVNVLASVIFAGMSGSAVADAAGLGIV
EIKAMREIGFDDDFSAAVTAASSTIGPIIPPSIPMVIYAVLAEQSVGRLFLGGIVPGLLM
AIALMILVYFISKKRNYPVEPRAKFGEIVRSFLKAFPSLLTPAIILGGILLGIISTTEAA
ALSAVYALFLGFVIHKELTLKSLYEILKRTVISSVTVLIVISSANILTWIIISSGSPDAI
AALVQNITTNKLLVLLLINLVLLFMGCFLESISILTIMVPILLPIVNALGINLVAFGVII
VLNTMIGLITPPFGLSLFVVSKLTNLSVSKLTKPVLLFAIPLVVVVLLLITIFPEVITFLP
DLIMGKA*
>SPBDM4_v1_40242|ID:27157547| putative TRAP dicarboxylate transporter DctQ subunit [Uncultured spirochete bdmA 4]
MAEINAPNGKKKKNSEFFRVLDKVLEVTSVVLLANIIVLMSFQIVMRYVFDSPPTWTEEI
MKYSLVWLTFIGSVLAVRDKAHVLIDAFTNKLPAKLQRVVLFLSNCLSILFLSILVIWGF
RFARMNLSNYSLITKIPRGMVFSVIPVSAFLMILYFIRSMKEEK*
>SPBDM4_v1_40243|ID:27157548| putative AP endonuclease family 2 [Uncultured spirochete bdmA 4]
MHKLFEPLALGTNLFKGYSLEEALSYIASCGFEYVEIASIANMCEHVAPKDMSGEKAAEV
KKLIEKVGLKTYAFAGHVDLTVESDLKDFLKKIEFAAAIGAKIINTNSGPLSRLAEFREN
MKKIITQAERFNMKIGLESHGDIIGTAKESVKYLKEFNHPLVRFNYDTGNTYFYSKGHVD
VAEDIKYCFEYLEHIHLKDISIKGNVVRYCKIGEGDINFAAVAESLKSLGKPLPAGLEIP
VFMHGTIDLLSSADAPLPIELAKSAARDSKKYLENLQERR*
>SPBDM4_v1_40244|ID:27157549| conserved protein of unknown function [Uncultured spirochete bdmA 4]
MIIAAVQKTAAILGGAAIIGRYRSEDTFTIDVKERIFVMKVAKAWEEEGVTIPAPYNRTI
KVLFAPDKKGVNEITFSHALIHANSSTDSHTHDRPELIIIMSGRGIAVCNGVQMPVEPDV
VLWIEAGEEHQMINTGYETLKLATVFIPAYTAEFNYNRCLEAAKKARENA*
>SPBDM4_v1_40245|ID:27157550| TRAP dicarboxylate transporter, DctP subunit [Uncultured spirochete bdmA 4]
MSMLKKSAIVLCLMVLMVSTMPMTVFAAPTQQFTLKLGSNTAPSHPENQFARKLAELVSA
KTNGNVTIKVYDSATLGDHLERQEGLRLGTIDMTLTSIGYLGGYNPIFNIFEMPYLFKDE
KHQNKVYQGEVGQKIAKEVENYGFVMIAFLEMGARHITNSVRPIVTPDDLKGLKIRVPET
KSSIDALIAMGATPTPMAFSELYMGLQQKQVDGQENPLSNIYASKFYEVQKYLSLTGHQR
FEQICLFSKLNWKKLPKEYQDAIMQSAAEANIFVQGVVAKEEGSLLGELKNKGMLINDVD
QSAFIKKVLPLREQYAKLYGDQAKVYFDMIDAAGK*
>SPBDM4_v1_40246|ID:27157551| putative Cupin 2 conserved barrel domain protein [Uncultured spirochete bdmA 4]
LYVIDCAKIEGVLIPAPYERLVKVIVAPDTQDTVKDVSITMGIIAPHSRNDLHMHADGIE
LLYIVTGYGKAIVGDTTYELKSNYLIIAPKGVMHQQINESAETMRMLAIWTPAVKGADVI
SRAIEAAKV*
>SPBDM4_v1_40247|ID:27157552| putative Transcriptional regulator, GntR family [Uncultured spirochete bdmA 4]
MKQTEEKPKVSNASLAYDAILKAVVNHDLPPGNPLLENNLCAWLGMSRTPIREALNRLRA
EGIVEYVRSKGFYVCSLSIEKIRQSYEAIEALESMVAYLVAVDHSHSDFPLVEKAVLRME
KDVESKDWDDWVKADEQFHAMMYKCCRNEYIVRYLTLQLIPAKQVRYLITKTYIDKSTST
KAHRATYDAIIRNDPERARALAQEHFAWIRREAMNFLSNFNFLPKEIPSGFR*
>SPBDM4_v1_40248|ID:27157553| conserved protein of unknown function [Uncultured spirochete bdmA 4]
MGLAELKATEGAMGRKKLTIEPVCKHTHFTWGERLTLQYYYTGTNKYQKIRSPTLLGRVL
GKHERTIRRELQRGMVLHERGDVPFEQWDYNAEYAQNDAERKSGGKGPDLKLGKDWAFVR

QVSRLIKERHYSPYAIIQHFHTTGWPSTMRICEKTLYNYIAAGDITDVSERDLLLQGRRR
TPRERPARHSRAANAARSIDKRPAGINDRSQIGHWEMDTVYSGKECSPGCLLTLTERKSR
VEITRKLPDRTAASMNAAFDAMERQIGHRRFCRLFKSITADNGGEFSDVQTLERSALCSQ
PRTRLYFAHPYCSSERGTNENHNGILRRFIPKASDIGLINKKAVRETQDWMNNYPRKILK
GLVPLVELVNEMGEGFIIPPFLELTE*
>SPBDM4_v1_40249|ID:27157554| conserved protein of unknown function [Uncultured spirochete bdmA 4]
VILFVLTNGFPKKTEKIPKEEIEVCKRRTQDYLARGSKA*
>SPBDM4_v1_40250|ID:27157555| ABC transporter related protein [Uncultured spirochete bdmA 4]
MSILSVKDAGFSRKAGPLFEHAYLEIEAHDRIGLIGRNGAGKSSLLGLLIRELVPDSGEI
AWARDFSYSYLSQNIEVPDGVTLRDFLYLGNATEISAAVEKKERGADPADNRQSNVPTIS
LENRYRALCRELGLSDMDASLQYFSGGERKKAALARTLAPYSSLLVLDEPTNHLDVQTIE
WFESKLAAKDQAFILVTHDRWFLDSVVNSIVEIDRHTLHSYPGSYSKFLERKAVDIASLD
HMENKRLANLKIELEWLNRGARARATKSERRKKEIEKMRKSLLEEARSKVIFASNVARLG
KKVCVMRGLGLSYGGRRLFGDFSWELGPGTKVGIVGPNGSGKTSLLKIIAGQLEPTEGSV
EIGQTVRMSMFAQTNEAVDGELSVLDFIREHAEHFALPGTSDLDAESLLERFDFPRTFQK
QKLRSLSGGEMRRLMLVRVLVESPNFLLLDEPTNDLDIETIESLETYCAEFSGSVLMVSH
DRLLVDRLADELLIFDGHGQIERFHGSYFDWKLADAERAEAASRAGAAAAPRSNATSSNI
GKRTMPAQASDTAPGSPIQASKSNKLSYKEKQELAGLLKEIDALESEKRNLDTFFQDPSG
AISGGAPFPAPIDFVEAGRHYEEIDRLIAEKLARWEELASRE*
>SPBDM4_v1_40251|ID:27157556| Xanthine dehydrogenase FAD-binding subunit [Uncultured spirochete bdmA 4]
MHDFEYIRPKTIDEAISFLDVHGPDARILAGGTDLLIQLRNNDIQPSFVLDLKNIPELQT
GIQLNNGFVSIGATVTMSEIIMNPLIDRHFPALIDAAKVVGSVQIRNRATLVGNICNASP
AADSAPPLLVYGADVIAVGSKGTRRIPLNEFFVSSGVTTLQKGEFVASIELPLPNKKMGA
AHVRRTRRRGHDLASVTLCCGVEETGTVRLCYGSVGPRPILMVDESGVLADEKASEEAKA
AIFESMFAQASPSKKSMRASPAYRLAMLRVLGRQALELAISRLHEGGK*
>SPBDM4_v1_40252|ID:27157557|xdhC| xanthine dehydrogenase, Fe-S binding subunit [Uncultured spirochete bdmA 4]
MEKLIPVEMNINYQHYQLNVSPTKRLLDVLREDLGLIGTKECCGIGECGACTVLVNGRAI
NSCLMLAVEADGTDILTVEGLSHNGRLDSLQEAFLEKGAVQCGFCIPGQLIAAKYLLTQN
PHPTRQDVEEGMAGNLCRCGGYERIIDAIMSVAENGEAQE*
>SPBDM4_v1_40253|ID:27157558| Xanthine dehydrogenase, molybdenum binding subunit apoprotein [Uncultured spirochete bdmA 4]
MNLQTVGASPYRGGGFERVTGQQKYVADIKLPGMLESKLVTIDSAHAKILSIDTTDAKKV
PGVRLVMTSADLPQPVPRFGPQIADRPVLADGEVKFHGEPVALVVAETIEAAEKAVRLVH
VQYEELPVVFTVSTALDPAAPLVMDASLRPDDPLAKTNIFKEHHFGWGEIDDSKADLIVE
NTYSFPMVTQFAIEPHAFIAAPDKDGIAVWSTIQHPYWLQKILADLLKLPLSKVRVYAPD
PGGGFGGKQHTKYEPALVFAAMKLGCPVRLVLTLEETFQAVRRTSCDTHIRTGFTRDGTI
VFQDITANYLLGAYVDIADRVVAKGSYAGAGPYNVPAVRIVSRSILSHTPPATAFRGFGN
PEVNWAVESNIDEAARLLGIDRLEIRLRNIAKKGDKFIPYDKPSDGDWRQTILKAAELIE
WQKPLPPSHHRGIAVGLKLGPTTGLSYSLVRLLADGSVIISCGTSDMGQGARTVFAQIAA
EELGVPYEKVNVIMGDTATVLYDQQTSASRSTVLMGNSVLKACYSIHAQLKTRLAKVYQI
DESEITVEHGKVKLPDREAPIVEFIKPALGKLGGEIIGIGEERKEGEKDHPLGGSACFYE
FNCTAIEAEVDKNTGDITITRYVTVSDVGKSINPMQVHMQDEGAAVQGLGHTLMEHYIFD
EKGRIRNLGAADYRILTTMDLPKEFYSETVENADGPGPYGAKGMSEGALLCVSAAVACAV
GDAIGTTIKDLPLTPERVWTAIQTQKSISSPQ*
>SPBDM4_v1_40254|ID:27157559|iolA| Methylmalonate semialdehyde dehydrogenase [acylating] 3 [Uncultured spirochete bdmA 4]
MSEIKQLQYWVDGGWKTSTTTKYMDCYNPSTGEVLARAPQCTVEEVESTIASAVKAYPAW
SNTPPNKRVQVLFRMKALLDKHLDELTLMVARENGKVLDEAMGDVLKVTEVVEFACGITH
LLKGPALMNCTVGYDTTQYMESLGVFAGIAPWNFPAMIPMGWMAPLCIATGNTMIIKAAS
FCPQTAMRIMELWAEAGLPPGVLNLVTCSRHEAEIFLRHPDVKGISFVGSTSVGRHIYET
AAANGKRVQCLTEAKNHALVLKDAALERTARGIINSAYGCAGERCMALPVVVVEEEIADK
LVALLKEQLEKLKIGPAYEPTSKMGPLVNAEHKAFVSNWIEKGIEEGAELLLDGRKLVVN
GYENGFYLGPTLFDHVAPDMSIGKKEVFGPVLCVKRVKNFEEGIELMNSSEFANGSVIYT

QSGYYAREFAKRTHGGQVGINVGIPVPLGVFGFTGHKNSFFGDLHAMGTDGIRFYTELKS
VTSHWFSEDEAREGKVLNSWDGMISFPDKK*
>SPBDM4_v1_40255|ID:27157560| conserved protein of unknown function [Uncultured spirochete bdmA 4]
MAIDLNAYRAMFDLKGTNALVAGGAGGIGSAVSAGLAALGCRVFLTARHEQKAEEVAQAI
REEGGEAIGLSLDVKSMENLERFSEDLHKKVPKIDVFINCVGTHIEAPAEEYSEKDWDHI
LSVNLKTAFFLSQEIAKHQIGAGGGKHIHVTSVRGQLGISRGYISYCVSRGGMNMMIKQL
ATEWAKYGITVNGIAPTFTKTSLVAQYLEDPAFYKPLVARIPLGRVCEPSDIAGLAMYLA
SHASDFITGQIMYVDGGLTATQ*
>SPBDM4_v1_40256|ID:27157561| Beta-lactamase domain protein [Uncultured spirochete bdmA 4]
LEQLTKNVFAETKLRGCNPGYVVTSDGVVVIDTPQLPTYAIRMREECEAKGHIRYIINTE
HHVDHIFGNYFFKSAEHVISHEETQKLFMVVYPQINPYEYAKEAMPTDDPEGIPLFPDEK
TYFADPKRPDITITQDATIIVGDRTFNLLHTPGHTPGQLAVHIPEERIAFVGDTIFNHCQ
TWLYTSNLEQWFRSLDRLLDLDVDLLVPGHGPVCTKQEIYVQRAFLLEWITAVATAVAKG
WSKEECIANISFLDRFPCDIGQEYMGPEVTRNNVAALYDQLTVRMPVTVEKR*
>SPBDM4_v1_40257|ID:27157562| conserved protein of unknown function [Uncultured spirochete bdmA 4]
MKVFSEEMLSGDGMLRIDVVGGEGREHVLEEMTFLNYRLADVKLRSEVRKQKPDIVWDVE
ASKKAIRYDGVRLTLDGTWEQGELNKIVVSMLALEMEKVGLHPFHSSSVRYRDKTILFLG
GESNHGKSMSQIEGCRRGGLLVSTETTVTDDRGWAVMGSKNVYLRMRAKGTERADLPNQD
EGVSKFFSENPPYINYSDPTNIDLVIMPGIDGHFDTKVVEMNRFEREYQTFHSLMNYFGL
NQLLSPPGIVMPIIDTDALRAKRGDFCSRFAADRPYFMIRAKTPQLLFDEIEKLM*
>SPBDM4_v1_40258|ID:27157563| putative phosphoenolpyruvate synthase (fragment) [Uncultured spirochete bdmA 4]
MMFIRTFKELKKDMCEECGGKASHLGELTSLGLNVPAGYTVLGSAYYHHLEANDLTEKIR
AIVATINFDTYEDPEKKSQLIRDLIIAAPMPPEIEKEIVEYYSLLPGEKDPFVAVRSSVA
MKDSSISSFPGLMDTYHYIKGATQVVEKVKECWASVWSTRAAVTRYNKKLDHEKAVIAPT
IQLMVNSEVAGVAFTMNPITKAKDEIMIESNWGLGETVVSGKATSDLYIIDKQTSEVKNQ
TIANKEDSYVQAEGGGGKWIKVESFKAKAPTLTPEMVKELCRVAHIIEDHYGYPQDIEWA
FEKGQLYILQARKARASGE*
>SPBDM4_v1_40259|ID:27157564| putative transketolase C-terminal section [Uncultured spirochete bdmA 4]
MELGEEIAQRTAFGQALVELGAEKPNMLVLDADVGPSTQTHLFGRAYPDRFIEVGIAEQN
MVGIAAGLSTLGFVPWISTFAVFMTKRAGDQIRNSIAHPKANVKINGAYGGLPTGRAGAT
HSAIEDLAVMRAMPNMIIMDPADPREAAVCARLAMEIEGPVYLRTVRCPVPVIFPEHHKV
EIGKAVKLHEGKDVAIISSGMMTPRALKAAEILEGQGVSARLLHMPTIKPIDIEAICDAA
ESIGAIVTVENHSIIGGLGGAVCEVVAEYAPCRVKRLGFPDVFLESGDDEVLFSRYSLDI
AGIVSVAKALAEGGKKNK*
>SPBDM4_v1_40260|ID:27157565| putative transketolase N-terminal section [Uncultured spirochete bdmA 4]
MAHEHDVGFLESVAHQIRTLIIDTAHTAGAGHTGGSLSEVEILTALYFSFLNVDPECPDW
NERDRFILSKGHASLGYYVTLALKGYFPTDNLKTFDREDSTLQAHPDMHKCKGVDYSTGS
LGQGLSIGVGIALGGLKKNQQFKTVVLLGDGECQEGQVWEAAMFAGSKHIPKLIAIVDYN
KVQLSNKVDENLSLEPFAIKWEAFGWNVLSADGHNIADLLSVLSRASALSARGPVVIIAN
TIKGKGVSFMEGKYEWHGKAPNDQEYTAAKKELGSK*
>SPBDM4_v1_40261|ID:27157566| Alcohol dehydrogenase, zinc-binding domain protein [Uncultured spirochete bdmA 4]
VVEKTMKAIRLNAPNDFEYCDIPVPEPGSHDILARVETVAICGTDPHIFAGEFPGFWPKS
FPLIPGHEWAGTIVQVGEVAKNLGWKEGERVCGISHVGCGYCSMCMQGRYTLCLNYGHEE
KGHRQYGHYSQGAYAQYIAASVKSIARIPDTMGFDVAACMDPFSIALHAVMRSGMEPGDS
VLVNGSGAQGLMSIMIVRAMGAGAILTSGSGFRLQVAERLGAITIDYRKENVVARTRELT
GGLGAKRVIECSGTPEGVRQACEAVAKGGTISMVSLPSKDVEIPIRRLVLDEIDLRGNRA
NPNTLEPAIAMVQENNIDLSSLITHAFPLSKFAEAFDIFVHRKDNSLKVVVKPND*
>SPBDM4_v1_40262|ID:27157567| SMP-30/Gluconolaconase/LRE domain protein [Uncultured spirochete bdmA 4]
MKENPIRNFHIDKTKINFIGKDLQRPECILAEPDGGLWAADAHGGVMHIRPDGSQELITQ
KRSSDFTQSASDVDRFTKGTLPNGLAFAENGDILISNFGTDCLEVMKRTGETKVLMDTVD
GKPIGKVNFVLRDSKNRLWITVTTRVKNWIDALVPDLDDGYIIRLDELGPHIVADGFHFT
NEVRFDKDEEFLYVVETTGGRILRFRVDEKGELRNREIYGPSHLGAGAYPDGIAFDAFGN

LWGTMVYSDKLFVITPDGEFILLLDEGDPEKVRRLDEAFYRKGVTSDILFETGQGIAPWM
ASVTFGGPDLSTVYIGSLKETRIPYFKSPVPGLPMVHWKHQK*
>SPBDM4_v1_40263|ID:27157568| Cupin 2, conserved barrel domain protein [Uncultured spirochete bdmA 4]
MKIVHESQVPEIQVPGRYLRWIADAKSLQPEFLTSCVMRVSAGETVKPAHAHPDGEELIY
VISGEGKVYVDGEVQVLKPGTAVLFQKNSIHMVRNSGTEEMKVICFFAPPASLDTYEFHP
EVSFPEGI*
>SPBDM4_v1_40264|ID:27157569| putative C_GCAxxG_C_C family protein [Uncultured spirochete bdmA 4]
MVARRYFLRDDNRYGCAESTYIALKTIFGLPSPEDSSPAMVLNGGIAYSGGMCGAITGAA
LAVGELAGHLPRDQQDAKKLARATIQKLLEEFQKQFSSDQCGVLIPYQISIPAEHEKFIA
SGIWKTTCMRQIEFVVARLALLKDKNVWFGLD*
>SPBDM4_v1_40265|ID:27157570| Transaldolase [Uncultured spirochete bdmA 4]
MPLQSYLQWLSESTVTNWWCDSGTPSEIKLALQHGAVGITTNPPLSATAISAKPGEWQTQ
IEEIIKSATDPSAKAQELTRLVATQGAQVLSPLFARTSGKLGYICAQVDPAKAGSREDML
AMGRRFSAWAPNISVKLPATSAGLDVLEELAAEGIAVTGTVSFTVAQMLAVAEAHARGVQ
RALANGKKSAPCNAVLMIGRIDDYLTEVSADNQSKASPDDIRKAGLAIAKRAYALFKERK
YDAKILVAALRGTYHATELAGADIILSIHPKYQKPLMENEAPKEEKYPVPIESQVLERLY
TIKEFVRAYEPDGLSPEEFIGFGVTQRTLSQFTETGWRRLETVR*
>SPBDM4_v1_40266|ID:27157571| protein of unknown function [Uncultured spirochete bdmA 4]
MDNDKAYRILNDLLTEPRLGRLYSAMKTMYSELKISHNWEHIMRVTLNAVVLGTEENANL
RLVIPAALLHDIGFVTNPSEPKKHNEYGASACHSYLTEWSEAERKKISDIILKHKGKYPG
YTAYEPSTPEEKVLCDADQIDKFGWVGLLQVIKVYAEYGSIGYKNFDTLRGLAEGIEEAS
HIELYTDAARKMAEALKEPSYHEVSKSVLAELAFYEGWKD*
>SPBDM4_v1_40267|ID:27157572| conserved membrane protein of unknown function [Uncultured spirochete bdmA 4]
MAEVKTKETIPFWLAVGITAIVFLPLTMFLGKFNIPLWVSFIVWAEFFNFGGTVKKAGKY
ILLAFPAGAAICALGFIVSFSLSAGIPGLAGPNGSGMWGMWVGFGVAVCVLVYIMKFSKI
FADGSLAYFNGMTMMIAVLFTNSYPQSANAAIQVNTIIACLWTILSGYFGLFLGWFNMAI
TFPKKVSE*
>SPBDM4_v1_40268|ID:27157573| PEP-utilising enzyme, mobile region [Uncultured spirochete bdmA 4]
MDRNQVLARFFGDKEFPVEWVDEEEKKLFWWFDDNHCPNPVSPMYFSLDGWWGPTCEYMY
RRFDMPMGVKWPAKRINGYVVYTAIVPRSEEEEAATGSYYSWVMPTYAKNFLSWWKDRYVP
EVKNNFAYIDNFDAEHATLPELMVHLEEMIDIQERHFRLHWILNYAQFQVSTDFGALVKE
LFGDVPSDLLGKVNISRADRNWDGLRELWKLKEKVKSSEELSEAFKSGTNGAEIRVLLQK
TKAGSEFLKDVQAYANEFGYKSIYTHEYRFKLWVEDNTPVIEQIKNYFDTNYDYNKAYNS
CIKEQDDAIAELRKRLASKSQADKDRFEQALDLNLRMMPLTPDHHFYFDQSTYARMRIVL
LRVARKMVKEGILDDPEDIMYLEYEQLRRYIANPKTYDGRGLIKKAKAEMEKASKIAPRP
WVGTVTNWGMYMEPYHMLWGYPERFEKGSGQGIKGEVKGLAASPGIAEGVAHVVHSPAEF
DSVKKGEILVCVMTNPAWVVVFSKIAGIVTDAGGVLSHSAITAREFMIPGVVGTSNATKE
IKTGDKVRVDGSTGVVEIL*
>SPBDM4_v1_40269|ID:27157574| putative Transcriptional Regulator, GntR family [Uncultured spirochete bdmA 4]
MAESYGKKKLTRTVLSEQIKEQLMEDIFHRKYKAGDRIVESALARELGVSQASVREALRS
LIAMGFLENEPFKGITVRSFTAQDLMEVITVRIALETLAGQLAIKCITDEEIAELENITS
EMIAASKEGDTQRRIHLNIAFHEKFVQTSGNKLLVQLYNYMRFGNWSLLTGNLSEMDPIQ
ISTRHKILIDALKSRDPDKVAKALREHIESTSKPLSDMLIKEGAGVDKS*
>SPBDM4_v1_40270|ID:27157575| Glutamine synthetase, type I [Uncultured spirochete bdmA 4]
MKLSEWNGDFAGIDYVSFIVIDIDGRMRAVSLPSSYASEAVLKKGIGFDASNFGYAKVHA
SDMVAVPDMETGFVEQKDGFNILHVFCDVRTTAGQFFEQYPRTVIRAAHQTLRDSGVGED
VRMLVELEFYVFEDVRYSTTSHHSYYFVESSEGIGDEYSDTPRVGMSQGYHRMAPEDRYQ
LLRNRAVKTMIDVGILVKYHHHEVGAAQLEIELDFVSMTKAADSVSLAKWILRNEADELG
LKVTFMPKPMYGVAGSGMHVHQFIVKDGASIFPGDGLHGLSKKGLAYTAGLLSHALTGSL
LAFSNPSTNSYRRLVPGYEAPVSATFAQGSRAAAVRIPGYLGVKEARIEFRTGEATANVY
YFLAAMLLAGLDGIKNELDPVALGFAKEIHHKKHIFPVGLLHVLGGLKKDNDYLAPVFSA
ELIETWTERKGKEASYVVYNAPVPQEYELYFD*
>SPBDM4_v1_40271|ID:27157576| Indolepyruvate ferredoxin oxidoreductase [Uncultured spirochete bdmA 4]

MAQMVFSGDEAVAQAALDAGVAGAFAYPGTPSTEIMEYLQEHIGEAKDAGSSVARAPDGI
TRVAQWCSNEKTAYESALGVSFVGKRTMVSMKHVGLNVAMDPFVNSALVRIHGGLVAVVA
DDPSMHSSQNEQDSRLLADFARMPCFEPSNQQEAYDMTRFAFDYSEAHEVPVLVRITTRL
AHARASVTTAPAKPATDLGKTTDAYAWTLMPAMARKQWAGLLAKQAAFRADADAASRLEL
GSKEFGVITCGLGRAYYMENEEDWAQAHGGERPSRLHIGRYPVGMGKIQALVSHVKRVLV
IEEGYPYVERDLRGMFGPPVPVAGKMTGEIPLAGELSADSVRAALGLPRREGVAKVAIEV
PGRPPQFCQGCPHADSFTALKKALEGEAEFFSASDIGCYALAAQPPWSAIESCVDMGASV
GMARGAASVGQKRAIGVIGDSTFYHSGMTNLLDAVRHRSPMTLLILDNGTTGMTGAQPTI
SATSQLPILLEGLGVEKEHIRVLEAHRRAIDADVAAIREELAYDGVSVIVMVRECIEWLK
KARKS*
>SPBDM4_v1_40272|ID:27157577| Indolepyruvate ferredoxin oxidoreductase [Uncultured spirochete bdmA 4]
MTCDIIFCGVGGQGGLSVSVVIARAAMASGFLVKQSEIHGMSQRGGEVLAHLRISDKEIF
SPTIPKGKADIIFAFEPLEALRHLSWLSVERGTVVAAMTPILNIPNYPDIESVLGEIRKI
PRSRLIDADTLAKQAGNVRSANLVLVGAAADLLPVPPEAIEKEVVALFSRKGEAMVQANL
KAFELGRNIQHA*
>SPBDM4_v1_40273|ID:27157578| putative CoA-binding domain-containing protein [Uncultured spirochete bdmA 4]
MHNSQLHGNKLSADGKVLRDEALRIVARAKSEGRSALTELEGMALLSAMGIRTPKYWLVQ
SADEFLEKVRKSAQEIPKTKKGADGDTFSDASGGAFRVSGGGSARSPFPGAKAVVKIISP
QILHKTEVQGVEIVENAAEAIAAALRRMESRFGGVPRDGFTVNEFVAFEPKLGHEMIFGY
RFAPDFGPIVSFGPGGIYTEYLASKFKAGAANLMLSPRVSDQKTLETLLKENVVYGLLCA
GLRNTKRELEPGALQEAIQHFLDAADALAAAGIGEFEVNPMVLSRAGELVALDCLVTLKD
FSSMGLASDKDGLPVNMAQQTRPVQEIGRILKPASAAVIGVSEKNMNNGRIILRNLIENG
FDTSRLYVVKPETESIDGCRCVPDIASLPEKVDLFVLVIPAAATPSTLADIARQDKAWTI
IVIPGGLEEKSGSGAIVAEMHRALADARAQGKGPLINGGNCLGIRSVPGKYNTLFIPEYK
LPMPKGKVEPLAMISQSGAFAVTRLSKHQNINPKYALTLGKQMDLTIGDYLDYLAQETEL
HVLAVYVEGFKPLDGEKTLEACRRISESGRSVILYRAGRTQAGAGAAASHTASIAGDYPV
TKQLFAQAGAIVCDSLDEFDDAITLFTLLDGRKARGNRLAAVSNAGFECVAIADNLGGMK
LSSFGETTRSALEEIFKKARIAEIVDIHNPVDLTPMAGDDAYDGAFRMALFDPDSDLGIV
GIVPLTGMMNSLPANPSAHNEDITREDSLAVRYDKLVGETDKPFVAVVDAGPLYDPLCLE
LEKRGIVVFRTADRALKMLELWRKSPAGRSE*
>SPBDM4_v1_40274|ID:27157579| putative Dihydroxyacetone kinase family protein [Uncultured spirochete bdmA 4]
MKISYLDGPRLRMALTAGSLSLMQNIGSLNAINVFPVPDGDTGTNMASTARAISASLATF
CPKNAGSVLKRAAQSALAAAKGNSGAILAQFFSVLAEELGHEARISAKQLASAAVLAAEE
TRRALSIPKEGTILTVLHDWAHALHEKAQQSDDILHVFIAAYESAKTSLAKTKDMLPEMR
RAGVVDAGAKGFVHMLDGITQFIKSGSLKEMLRADKWRSASGEIAHDVLAEPLFDIQAHS
EFEILADADNATFRYCTEALVHGDKLDLDAMRAELATIGDSVVVAGSKSLAKIHVHANSP
SLVFDMLDLLGFVEAHKVDDMKLQTKLAHRARGVQTCTIVTDTGCDLPEAFLLEHGVLKV
PALITIDGKARPDGPALAIQKVYRLMKERPDFAMSTSQPTDAAFSRAFALASAHSKEILY
VGLTSALSGTFQAGVRATRALGGKIVAVDSKTVTSAQGLLVRRAVEMAEGGRDAQEIARR
LEGLRGQLVFFVAVRNLSSLIRSGRLHGLKSLILRKFGLRPLLSTDQAGKAKTAGIYMGE
RNTVTTLLSKVKKAFPSGSRVELHVSHVDAAADAQHLADLCAAHLHRDSKIMISEMGPVL
ASLAWRGAISVAGLPLEG*
>SPBDM4_v1_40275|ID:27157580|plsY| Glycerol-3-phosphate acyltransferase [Uncultured spirochete bdmA 4]
MGAFFGVLLAYLAGSFPTGLIVGKLLFGKDPRERGSKATGATNIFRVFGAKAAIPVVLFD
VGKGALAVFLASWMGRNAGLPREALQVMGAVAAMAGHVFPVFAGFRGGKGVATGAGALIV
MVPTAAIFCALGFLLVLGLTGIVSAASMTAAIVLPLAIALGAQGRPANPWYLGFGVAVAL
FIVFTHRANIKRILSGQEQAFEKFRFLRRKKKSGNA*
>SPBDM4_v1_40276|ID:27157581| PfkB domain protein [Uncultured spirochete bdmA 4]
MRFDIIMLGHISKDIIIDEKGRETRLFGGALLYSSISAARAGAHVLAITKAAPEDFYALD
TAKLQKGIELVTLESPSTTSIRNVFLSSDHEGRETSLLSRAEPFSAADIPGDAEAKIIDL
AGLFVGELPDSLIEELSGRAAIAVDAQGLLREAQPDGRMLFRDWKNKYRYMPFVRYFKAD
AAEAEMLTGFSDREKAARTIAAWGRNAAGHEPEVMITHNTEAIVLVNGTMYRAPFTPSNL
SGRTGRGDTTFAAYLAWRLMHNPQESLRFAAALCSIKMEKPGPFTGTLEEVFARMKRDAS
R*

>SPBDM4_v1_40277|ID:27157582| Extracellular solute-binding protein family 1 [Uncultured spirochete bdmA 4]
MRKIFLSMMVLFCLLPLFSVGAQGTPEYKFLTVYTALPESELPTYFSEFEKDTGIKIQYV
RLSAGELLARIRAEKGNPQASVWFGGSYDNFVPAAKDGLLEAYQSPELANIPKVYQDAMG
YANPFYVGAIGFACNTDWFKKRGLSYPTSWDDLLKPEFKSQISMAHPSTSGTSYTILATI
VQMRGEEGAWKYFTALNQNIRQYTKSGTTPPMDVGLGEAAIGITFSHDGLKPAFEGYPVA
MSFPKDGTGYEIGCMALIKNGPAKEMANGKLFIDWMLSKRGQNLFETSKSFRIPVNTQAT
PPKGAISVDSLKVINYDPVWAGNNRTRLVEQFNKVVAAANNLK*
>SPBDM4_v1_40278|ID:27157583| ABC transporter permease protein [Uncultured spirochete bdmA 4]
MRENTRLSVAHLVRDPVLLFLVILVFTGLFLFILLPLYKIFSYSITTNEGKLSFQAAWDI
LRSRTYLQPLKNSMLLGLTTGILATIIGYVFAYALTRVDMPLKGFFKTIATIPIISPPFI
LSLSMIFLFGRNGLITNKLLGIEDSNVYGMHSLVVVQTISFFPIAYLTLTGTLAKLNPAV
EDAALNLGASRGRIFRTVTLPLSVPGILSSFLLVFIQSMEDFSNPAVISGNFSTLAVEAY
RTITGMYDMRGGSLMALMLLAPTLIAYVLQKYWLSGKSFVTVTGKPTTSRRQSRDPKLMW
PLFAFCMLVSLTIILFYGTVLVGAFVKIWGINFSFTWAHFKYVMTLGWTPLRNSVVLALA
STPIAGLLGMIIAFMVVRKEFPGKRAMEFISMLTFAVPGTVVGIGYILAFNGKPFMWTGS
AFLLIMAFTFRNMPVGIESGTSTLIQIDRSIEEASTILGATGAVTFRRISLPMLKQAFFS
GLVYSFVRAMTAVSAVIFLISPRWNLATISIFSLFESSKYSDAAAYIVVMIVIIIVVIGG
LNLLVGRLGNSGTVSSGGSGERA*
>SPBDM4_v1_40279|ID:27157584|fbpC| ferric transporter subunit ; ATP-binding component of ABC transporter; CP4-6 prophage [Uncultured spirochete bdmA 4]
VSERDFSARSSNVSLLHLTKRFRSLDGSGEVTAVNDINLEIRAGELVTILGPSGCGKTTT
LRMIAGFEYPTSGSIFIGGRDVAMIPPNKRGLSMVFQSYALFPHLSIYENVAYGLRVQKL
PAQEIRERTERALELMQLTSMAKRFPSQVSGGQQQRIALARAIVIEPSVLLFDEPLSNLD
AKLREYMRDELRKLQKRLGITSLYVTHDQSEAMAISDRVVIMKDGWIQQVGTPREIYAFP
QSKFVADFMGKANFIKVQVLGIEGEAARIEIEGMRFTVPRAGKAAPRAGSALLVVRPEAL
RLLPLSSAQGSAALKGRIDRFTYFGNIARYEVSTENAPLLIETYNPEASFVFEEGAEVGI
IIDFESARLLPAEEQEQIS*
>SPBDM4_v1_40280|ID:27157585| conserved protein of unknown function [Uncultured spirochete bdmA 4]
MSDAQQYDIAFLGQYTKDTIVTKDGSRVVNGGAYFYGASAATSMGLKVAALTRLAPEDFS
SFRFLLEHDVLVKAISTQQSTCLQLLYPSGNPDDRILSVTSVAEPFEPSDVESISAPVWS
IGASIRGEVSLEVLKAIKAKGARIGLDAQGFVRVVRDGQLAYDTQWPEKAEVLQLVDVFK
ADVVEAEILTGTRDLYKAAKILAKYGVPEFVLTHGDGVVVYADGSFFEAPFKPESLVGRS
GRGDTCLASYLAARISMNPEKATFWSAALTSLKMETLGPFAGTRQDVESALRERYGK*
>SPBDM4_v1_40281|ID:27157586| 5'-nucleotidase/2',3'-cyclic phosphodiesterase-like hydrolase [Uncultured spirochete bdmA 4]
MLKRVGKFAVLVAAIFTLAFTGTLSAQANLPGDVTLTFAHTNDMHGRIVESKDVIGFPRI
YTAVQDLKAKNPNTILVDVGDTFHGLPVVNIDKGETAVKLMNELGYSYMTTGNHDYNYGF
DRLMELSKMAKFEILAANVYKDGQRVFPAYDIRDMGGVRVAFFGLATPETAYKTDPKGIQ
GVTFSDPIVEAKLVVAELAGKYDLLVCLSHLGVDDSSDPTSITLAKFVPQIDIILDGHSH
STLADIQKKNTTDTLITSTGSYGSGLGVVDVVVGPDRKIISKTARTITPQNSPDLKGDPK
IAAMLDSLTKAQDAVLAQVVGKTAVALEGKREIVRTQQSNLGTLIANGMLYVTGADIALM
NGGGIRDSIPAGDITMKQIYTVMPFGNYIQTGKLKGSELKAVLENGVGKLPAPDGRYPHL
AGWTFTLDVTKPAGERVSNIMVNGQPVDPDKDYVFTTLNFEFNGGDDYRMLIGKAQNDFP
SDAEVFIAYLKHIGTVTNDNMVYQK*
>SPBDM4_v1_40282|ID:27157587|livG| leucine/isoleucine/valine transporter subunit ; ATP-binding component of ABC superfamily [Uncultured spirochete bdmA 4]
MPVDMSQSSHVLLEVKNLSKSFRGLMALQNYNVQLTEGEILGIIGPNGAGKSTVFNLLTG
HLNLTKGSIVFRNIDITHAAPEEIAGLGIGRTFQNIRLFGSMSCIGNVIAAMQMKERQSL
LGAILNLSHFREQEKRFAMQARTLLALFNLQEHADSPALSLSYGDQRRLEIVRAVALKPR
LLLLDEPTAGMNPHESAEVINLIKRIHDEYHLTIIIIEHNMPLVMKVCSRLQVLNYGEII
AEGKPENVRNDPRVIESYLGRVG*
>SPBDM4_v1_40283|ID:27157588|livF| leucine/isoleucine/valine transporter subunit ; ATP-binding component of ABC superfamily [Uncultured spirochete bdmA 4]
MLQLLQVHTKYGQVEALGGISLEVQEGSLVALLGANGAGKTTTLMSISGILKPSSGKILF

RDTDLTQLDTREIVGLGIIQCPEGRRIFGNLSVKENLRMGAIRRKDKRHLDQAFARVFEI
FPLLKERMAQAGATLSGGEQQMLAMGRALMAEPHLLLLDEPSLGLAPIIVDKIFEVIQRL
KQEGVTILLVEQNAHLALEIADYAYVLEKGRIVLEGSGVSLRANSDLERSYLGEVPEENL
*
>SPBDM4_v1_40284|ID:27157589| Inner-membrane translocator [Uncultured spirochete bdmA 4]
MSAGEYISQQIINAISIGSLYALMAIGLSMVFGILRLINFAHGDLIMVGSYTTVLLLSGG
IPFVWASLAGIAASTIMGLLMERLAYRPIRGAPDVAMLLTSFAVTIFLENLAIILFKATS
RAFPVPPVLNTVVSFGNLQIASLNIISILLTIILLILLTLFVTKTETGISMRAASQDLVA
ARLMGINVNRVIAAAFAIGSFMAGVVGILWSAKVGKIDPLMGFNPVLKAFVASVIGGLGS
IPSAVLGGYVLGALEILLQGLLPANVVPFRDALVFLILILLLLFRPNGILGSTEKEKI*
>SPBDM4_v1_40285|ID:27157590| conserved membrane protein of unknown function [Uncultured spirochete bdmA 4]
MRRDRSTAIVSLAAIGITFLVLWLLHSFGGYYIERIVIGIGINLILVISLNLANGFTGLF
SLGHIGFMAIGAYASAILTLPLSLKSTNLPDLPVFLSKIQMPFLPAVLVGGLIAMVVALL
IGLSLMRLTGPYISVATMGFLVIVQVVLTNWDSMTRGARTFAGVPEYTNVWNVWIWAILV
LYIVWRIKFSSFGRKMMAARDNEIAARSLGINVMASRLLAFCISAFFTGVAGALWAHFIT
AFSPKSFYFSQTFSVVTMLVVGGLGSLSGSVVGVMLITILSEILRNAERGFSIGPLVIPE
VYGASQVIMAVLFVLFIVFRPRGLFGDAELDVRRFFKKEKEVSG*
>SPBDM4_v1_40286|ID:27157591| conserved exported protein of unknown function [Uncultured spirochete bdmA 4]
MKKTVLFLAAILVIFLVSCGGSGSKNIKLGMLYNMTGSQASLDGPSSNGAKLAAKEINAA
GGVLGKQLELVSYDGKSDAETIGKAAAQLVEKDKVVAMFGFSDSDMVMAAAPIAAKAKIV
FITSGATSPKLPSQVPDYLFLSCFGDNVQAAAAAEYSWNTLNAKSAYLLIDKGMEYTLLL
GKYFQERFTELGGSVVLEDTYQTGDKDFSAQITKLKALKPEPDMLFISSGPDDIGTIVKQ
VRDAGITSPIFGGDGYDTPLLMEIAGKGANNVYYTTHSLMDKNAGSPEVQKFIAAYEAEY
NTAPENAFAGLGYDTVYLLADAIKRANSADPKKIREALQTTKDLPGVTGSITFEPGSPIP
QKGVAIIEIQDDALKLAAEVVPEKVPAP*
>SPBDM4_v1_40287|ID:27157592| Creatininase [Uncultured spirochete bdmA 4]
MKKNPLEIQHLTTSDYKAAPFDKVILPLGSLESHGGHLPFGTDAITAHMIALDIAAKVPG
TAALPAMPFGVSEHYKEFPFCISLQFETEIALIRDVLESIYREGIRKVFIMNGHDGNISP
IEVASRSAKVAHPDFKIVSLDAWWVLVGSLVPEGFFEVWNGLGHGGEGETSICLELFPEL
CEMQYAKGVTPKLPPYVDIKWRFSELTNTGASGDPTKGSAEKGETMRKVLVDTAVSILKE
LEKKGWDYRSDEVK*
>SPBDM4_v1_40288|ID:27157593| Metallophosphoesterase [Uncultured spirochete bdmA 4]
MTQQIKRTTRGLSRRTVLISALVALVLSIIMNGYFYSLEGARGLYVWHPLLALKVLLFMG
LGPISLAIVALPLEKLAKKWAMRMVRWCSFIGSILVSVASIALLAFLIIVPRVGPLEPAR
LDLINPSQGIQSQEVQMEAGFSAGGVKDSATQSPEAEDAPGALSPLDRPLLRLSFSSDPH
WGADTANATARTQVLEGIAASKPDAFFMLGDTVETGNSATQWNLALSDLEALIPNVPLRP
ILGNHDALFGGQYLYRKAFYPKDFSSDSGSPYYWSINTGSATIVAVDLPWGTEMFGARQK
AWLEKTLAAADPRRPLIVVSHSYFYASGYDDPDFGTPWYDHYQNIPALVPLFEKYGVDLV
ISGHNHYQELLSHNGVTYAIVGSMGGLPDPEPSHHSPYSKWIDVGGHGWLDVSVLPGRLA
LTFRSETGEARQRATVGY*
>SPBDM4_v1_40289|ID:27157594| conserved protein of unknown function [Uncultured spirochete bdmA 4]
MSEFHAKLYEARGQRTVAALKKNGFDALYIPTAALAAEKVLEFVHAGAKVGFGGSFTVKS
MNIQELAASRGAVILDHNKPGLGAEEKTEILRAQLTCDLFISSANAVTMEGFLFNVDGNG
NRVAALSFGPKKNVVVAGINKVVANLDEAYERLKAYAAPINNIRLQKPNPCVTSGYCADC
ALPTRICRIYHILKRKPSLSDFTVIIVGEELGF*
>SPBDM4_v1_40290|ID:27157595| 6-phosphofructokinase [Uncultured spirochete bdmA 4]
MPDFTIETLGKCAVDSPLGLASEPGERKPVFVSEGRYICYNIERDLGASTEERPADQRPL
TRDQLLEVAGPRQKIYFSPSHVHAGIVTCGGLCPGLNDVIRSIVRSLWHLYGVRRISGIR
YGYKGFLPDYGLEVVPLDPDVVDDIHKIGGTILGTSRGGGDRTPEIVDTIEQLNLNVLFA
IGGDGTQKGALAIAEEIDRRKLKIAVVGIPKTVDNDIEFVEKTFGFDTAVVKASEAVAAA
HMEAHSQINGIGLVKLMGRDSGFIAVQTVLAVHEANFVLIPEVPFDLEGPQGFLTMLERR
LARRGHAVIVVAEGAGQHFFNQTVAYDASGNRKLGDIGLYLKEQIQVYFDAKHMDINLKY
IDPSYMIRSAIAEPIDSMYCERLGNNAVHAAMAGKTKMLIGLVNNEFVHIPIQAATSKRK

QVNPEGSLWRDAIEATQQPAQMVNSK*
>SPBDM4_v1_40291|ID:27157596| Class I peptide chain release factor [Uncultured spirochete bdmA 4]
MDNELLRASVTANAETDFSRSSGPGGQNVNKVNTKVTTRIRIAAIEGLSPSELELMRTRL
ANRITTEGELVVQVQEERSQSMNREKAIEKLISLVARAAKRAPPRIPTKPTKASKERKLA
HKKIRSSIKKNRQSPPVEG*
>SPBDM4_v1_40292|ID:27157597|bcp| putative peroxiredoxin bcp [Uncultured spirochete bdmA 4]
MIQEGERAPDFALKDADGKTWTLSDFRGKTFVLYFYPKDNTPGCTTEACSFRDRYAEFKQ
RGVEVVGVSADSEKSHKSFAEKKNLPFILLSDPEKKTIEAYGALGEKKLYGRTFLGIIRS
TFIIDGDGIVKKVFPKVDPADHAREILDIL*
>SPBDM4_v1_40293|ID:27157598|aspS| Aspartate--tRNA ligase [Uncultured spirochete bdmA 4]
MLFEQRTVTCGELRAADIGKSVVLNGWVHRKRDHGGITFINIRDRYGVTQVVVDADSARE
LLDTAAELKMEYCLSVRGTVRPRPDTMVNPSMPTGEIEVKAEKIQILSRCAPLPFMIDEQ
TDAKEDMRLTYRYLDLRSFSMQKKIRLRHEAAFAVREYLVDKGFYEIETPTFIRSTPEGA
RDYLVPSRLYPGKFYALPQSPQLYKQLLMVSGFDKYFQLARCYRDEDSRGDRQPEFTQID
IEMSFVTREDVLTLVEGMMGHLFRKTLGVGLPAQFIRIPYDDAMDRFGTDKPDLRFGMEL
LDFAPYVDNACSFQAFHDAVAEGGAVKALVVPGKADYSRRQIEELEAKTKVYKAHGLAWM
KVNQSGALEGGASKFFSEPARIVQGLGAKPGDLILIVADKKKKIACTALGAVRSQIGKDL
GLCDPAVFAFAWIVDFPLFEFNEEENRWEAAHHMFTMPQTQYHDSLEADPGAVKGDLYDL
VLNGYEIASGSIRIHDPELQKRIFRIVGMTDEEAQRKFGFLLEAFKYGPPPHGGIAPGFD
RLVMLMAGDTSIKEVIAFPKNSFAVSPMDQCPNDVDERQLKELHISIVQDK*
>SPBDM4_v1_40294|ID:27157599| putative redox-active protein (C_GCAxxG_C_C) [Uncultured spirochete bdmA 4]
MAEVEEILEKDVSRRSFLKTTGIGLGVAAGALMFGTGIAGAQTQGGTVAETPLPYADLDP
EEARIRAHAAYFRAGCAYATFYAILSMLRDKVGGPYNQIPLRMLGYGRGGAASWGTLCGT
LNGAGAAINLVASDADTNKLLGDLIGWYTITPFPSQESDNVAAVKGYTYDAKEYVTAALP
TSVSNSPLCHASVTSWINKSGFAASAPERAERCARLCGDVAAKAVMMLNEWKAGAFVATF
KVSDETAGCLSCHSSDQIGKMACTSCHEPH*
>SPBDM4_v1_40295|ID:27157600| membrane protein of unknown function [Uncultured spirochete bdmA 4]
MKSSFDKVYDALRSIKLAAVLLVLLALLSIAGGIIPQGKTTGFYQLHFPGAGSRIILALG
LDHVFSSLLFLLVAAVFAVNLTVCTFHRLTGELSKPRGKRRHGPDILHLGLIILMFGGIL
TARTRTETLINLRRGESADLPDGAKIVLVDLNYEQYPDGRPKSWESTVVIHRVSAPAPSA
ANTPAPSTPGGLPQYLIKVNTPLRLKGYSIYQQSWHTEKQVVLKDAMGMSFSLEPGMRGA
TKDGFVLFMALDNPSLGAAVPASSTPATSSAPAASSDPLPQSAIFLLEEHGKRTVSKAAP
GQGIGPFTFDGYIDQSVSGLAVVSDRGYPFVAAGFILVILGVFMTYIRKLKGMLA*
>SPBDM4_v1_40296|ID:27157601| Cytochrome C biogenesis protein [Uncultured spirochete bdmA 4]
MIATIAFGLLILSLIIQIVYLFKKEGGQDVISPWLSLAAGILLLVEIVRRSIAIRFVALT
GMFESLIFLACFLALLIFALRYFKTTKGNRVIPFGATIIAVIFLALASSPLAPSEIKPPV
PALQSGWLVLHVSFTFIGEVFFTVGFVSAILQLTTKNEEKRQSYDRLTYTSVAVGYPIFT
AGALIFGAIWAEKAWGVWWSWDPKETWALITWLTYTAYLHFRLVRKSTSNLVPALVIVGF
AIAMFTFFGVNFLLRGLHSYA*
>SPBDM4_v1_40297|ID:27157602| Stage 0 sporulation protein YaaT (modular protein) [Uncultured spirochete bdmA 4]
MSDEYNDQALPEDMLDILHEEEFEAPHAAKPRGTSAEELPQPIYRLKTLHSSETFHAYYA
PSDESAHFMKQCDLEGALAGPRPALDAASSSAEPTDQVSSEASAAAGDFVIPARSLAIAP
TKYGRDLVEVLGVVRELSAVNADELVLIERPATVDDYRRYLDNLEREKEAFIKCRERIEA
HSLPMKLVSAHCLLDDSKILFFFTAESRVDFRELVKDLVSVFHTRIELRQIGVRDEARVT
GGCGVCGRILCCHGLSDKLNPVSIKMAKDQNLSLNSLKISGPCGRLLCCLSYEHQFYRDA
RRELPNEGTRFTYDGTLFKVIEVNVLSSRIRIAGEDGRILDMDAARLKYSDGRWTVIEEE
S*
>SPBDM4_v1_40298|ID:27157603| protein of unknown function [Uncultured spirochete bdmA 4]
MNKRDFSDCVIGEGVVAEGKIDAPGMVRIDGAFSGDIISGASVIISKDAVVHAHIRARDL
VVAGVFGGTAVVRHEVRYTGTARVQADCTGDFLVMESGALVKGRFSRNNRVQ*
>SPBDM4_v1_40299|ID:27157604| putative Peptidase M23 [Uncultured spirochete bdmA 4]
VPEGLRSLKETEKRWYARLVQGLTGGADVVRVAIRRFFSWGRARFVIALIPQSEKPARRF
EISRFSFIAIGIALVLFVGLAVFSLSAYGATALRAASLDAQLTKARQNIDQMRESTENLL

SETAKFQEKLGQVVDIAQSKGKPDVQQNTSDTLKTAGLADLISGQLSDDPVSRDVLRLNG
IAGALDKSLPALDDIANVLASQKDVMTEVPNIWPVKGDLGHITMYFGQNENPFATGQWYL
HTGIDISTNRVGDSILATADGKVIDIHYDASLGNSVTIQHSHGFTTRYGHLRSFAVKKGQ
RVSQGDVIGYLGNTGKTTGPHLHYEVYLGTSLIDPLRFLSIRKEYHE*
>SPBDM4_v1_40300|ID:27157605| conserved protein of unknown function [Uncultured spirochete bdmA 4]
MAIIEIHDVKKNYPLGKVEVQAVKGVSFLIEKGDFISIAGPSGSGKTTILNMIGLIDKPT
AGEVIIDGKATSGLSDKELTRFRHEVLGFIFQSFNLIPVLNVWENIEFPLLLGKTRITKE
EKNSWIDWLMAEVGLTEWKTHKPNELSGGQRQRVAIARALVTKPQIVLADEPTANLDSAT
GEQIIELMKKINRELETTFIFSTHDAKIVEIADHIIRLRDGTVTENRRRGEDVAASATTV
EE*
>SPBDM4_v1_40301|ID:27157606| conserved membrane protein of unknown function [Uncultured spirochete bdmA 4]
MPVTLRMAFRNIFEHKSKSLIIGTLLAVGALILVLGNSFIDTSEAGIKRTFTDNYTGDVF
ISGISEDGDVSLFGVTSPGGLSSTPTIPDYEKVLSMVRESPLTSKATGMATGFALVSKEE
NNEAPNQDSQSDTSNMAILFLFGIDATEYWNVFNSTEITAGKLLEPGQTGLVVSDAHLAT
ISKKLGKEVKIGDKILIQGISSAGFRLRELPIVGTYHPKSESAMPEQMAFVDIDTLRVMA
GMTVGANEAIDLKASDTAMLNTENTDALFGDDLLEAAPQKSGFDEAKIKAQLADTTARVR
ANTADTGAWQFIVVRTKKASDASALIKYLNDSFQKEGIHAVAGNWQKAAGPYSQSVDVVR
TVFTIALIILAIVAILIIMNTFVISVIERTGEIGTMRAIGADKGFIRKLFNAEAAMLSLG
FSTIGAVLATLVAAVLRSLKIAAGNTFFEILFGGKYLSLIVTPANFITAIVIMLVVGYLA
NLYPVSVALKIQPVKAMQNE*
>SPBDM4_v1_40302|ID:27157607| putative ABC transporter, permease protein [Uncultured spirochete bdmA 4]
MNKVIPRIALRNLSRQKKRNILLGGAIAFSIMIVTLVNGFTGAFIENVSENFAYLMAGHV
FIQGSERTASGKKISAIRDDSIIFDAIANSGIKYKTATRSSEASATLVFEGKSTSQNLTG
FDLENSGFLRERLVLKQGTWDAVKAPDALIISEKVAKKLNVLPGDRVTARLQTVTGQNNI
ADFTIAAISVDSSIIGSAMTYVNLSYLNEAIGLKPDEYTSLNLMLDDLKDSAPFADRLYT
SMKGMGLQLFDRNQKNESGSTTPFQAMLQSQNKETWEGVKYRVYTIDDVLSQAKQIVNAL
DTASTVVLIVLFTIIMIGIANTFRMVMYERIKEIGTMRALGVQRGEIRSLFLYEALFLAL
GGALAGIILALVGMNILQLINFGMDSPAFLIMKNGHLSFSLPPLRAIGNIAIIAVLTLVS
AFFPARAAAKMEPAEALRTLK*
>SPBDM4_v1_40303|ID:27157608| conserved protein of unknown function [Uncultured spirochete bdmA 4]
MKSLFHAGLAGVSSPAVIFQKKGDYMHPPIRSYGNSHKSRYTVLLLLLTLAFAIPLSGFA
QTLSIPAAQKLVKELDDQSNFPGKDYTALFTIVSEKPGEKQTVTQVRTFRRDTKKQFTFL
VMLPEVNKGQGLLREGDNIWFYDPTSRKFTHSSIKENLQDTEAKNSDFTASSLVDDYTVN
AISEGTVGKYPVWVLDLKAKTNEVSYDRVKLYVRKDKAMMLKREDYSVNGRLMRTTLFPK
YVELEGKLIASQILIMDEINKGEKSQITLTEQSVEKLPDKVFTKALIEQANS*
>SPBDM4_v1_40304|ID:27157609| conserved exported protein of unknown function [Uncultured spirochete bdmA 4]
MPHGKGVKNMNVRSTRMSVFVSLFIVLAAFSVQAQDSGSDVNLDELFGGDMVENPENVPA
PTQNPVTAALATEKVRIGGSFYGSLEAAATWQDLWGGGSDLFSADSTSLDTVLKSTIFFD
ARPNEDFRVYGSTKVAWSTLTGDSIAVPSIKVFELFSDFSLNDQVFFRFGKSTVKWGVGY
FWSPADVINLEPIDVLDATAQREGPINFRVHIPVLGTQNNFYLYAILDEDNVDFSTTALA
AKAEFLLGNYELGTGIYYRNETAERAMLTLTGPVGNFDIFGEAMLSRGSAKNFVTDIATT
TPYTITTSTKTDHRNSIYFSASTGFMYSSPKDNFTAIGQYYYNGEGYSEADRKGLIADAN
TVIAALGATPTAAQYQQALAGLIYGSGRHYAALSVTQDEFLTKDTSISLLVLANLSDGSG
FVRPSIAWQVVDNFKFSLSPTFIFGGADTEYSILAGGDTVSVSLGAIVSGSF*
>SPBDM4_v1_40305|ID:27157610|htpG| Chaperone protein HtpG [Uncultured spirochete bdmA 4]
MPNYSFKTEVNQLLDLITHSLYSHKEVFLRELISNASDAIDKLKYLTVSDDAFKTLNFDP
RITVTLDEDAKTITVSDNGIGMDEQELSENLGTIARSGTKAFLEQLGENERKASNLIGQF
GVGFYSAFMVADQIEVVSLKAGTDKAFVWKSDGKSGFDIEPGVRAGFGTDVILHMNDEGM
EYLSRWSIEELLKRYSNHIAWPIHLVSKEKTYAKDPAKAESKTVDEQVNSASALWRRPKS
ELTDADYIGFYKSLTGDEKDPLLWIHTKAEGTIEYSSLLYIPSQLPSDLYIPTREQGIKL
YVKRVFITDRELQILPAYFRFVRGVIDSEDLPLNVSREMLQHNRVMMTIQQATLKKLLGE
LEQLATTNAEKFKTFISLFNVQIKEGLLSDWTNREPLLKLVRFKSTVAEGWTSLADYKAR
AGEERKTIYYLTGENETRLRASPLLEVFKEKGIEVLLGTDEIDELVFSGIGEFEKMQFKS

VQQLDAEDELGKSAGEHQDRGEKAVKIIKSVLGGRVKDVRLSKRLGKSPSCVVVDKDEPT
PAYKKLMERLTNEPAPESKPILEINAEHALVERLVALDTRKELSGGDIVTVQELEDIVYV
LFYEALLAEGEHFEVPQDFSERLNRLLAKAN*
>SPBDM4_v1_40306|ID:27157611|rlmE| Ribosomal RNA large subunit methyltransferase E [Uncultured spirochete bdmA 4]
MPGYDRPDYWTLRAKKEGYPARSVYKLEEIMRKFGLLKVLDGRTTEGGPDQIARPTRPRP
SILDVGAAPGSWSLWLLRQMKGAGQLVAVDVQDLGIAPRDANFSFLKGNILDDAVRAALR
DAGPFQLIVSDAAPATSGNRLVDQARSEELVEAVMGLAPEVLAPGSALIMKIFQGGEERR
LLSELRAHFAQARTFKPEACRSESFETYLVATGFARRD*
>SPBDM4_v1_40307|ID:27157612| protein of unknown function [Uncultured spirochete bdmA 4]
LILISGYLMKRVSFEYPEIDSLQLQQSLKDYAFPRDRVTRLLRMCRWNTRCGTTTSSRKP
SVW*
>SPBDM4_v1_40308|ID:27157613| conserved protein of unknown function [Uncultured spirochete bdmA 4]
VSLEYALRHYNLIPEAVRVVTSITTGRRKRYRTPVGDFDYVHMPQRFLPLEF*
>SPBDM4_v1_40309|ID:27157614| putative Nitrogen regulatory protein [Uncultured spirochete bdmA 4]
MQMLLDELIAEYDPWYLVDPESMPKALGELLRALPLPKGLDRDALLDALLGREEVMSTAI
GNGIAIPHVRQFGSESLQKDIVVVAYLFEPLDWKAPDGQLVHTLFLVLAADETRHLQILA
EIARLASDEDFVAFLKTMPDRAALVERMRQME*
>SPBDM4_v1_40310|ID:27157615| exported protein of unknown function [Uncultured spirochete bdmA 4]
MILSKGKAKRLALFTVFGGVLFGLLLGSCATTQGYDASIPSVSMSHVQESDLSKYGASFA
ENPFKEPNIVFIGKLYEFYIVKISLNLEEKTRIRVEADSRAAPDVSAPVAYNQEEFIHFW
DIVSNQGGPNPAQYERRKRTIEDTVIPDFAFTENPGQSSYFLVFIGKRPIKRPISYEVRV
VLENGESFLFSETVE*
>SPBDM4_v1_40311|ID:27157616| exported protein of unknown function [Uncultured spirochete bdmA 4]
MKKLIVLLLALAMVGAVFADDQKAVATFGAYLDSYGDFMDGLGPTLYSETYYNYSSGGVG
FSATVVGAEDIFKQVRNYSVSYQVLPELKVLAGRLRETGGARLTSYIDGNGFSTRIANVQ
NGMMGQVSYMGVGLGVFSTLDKFENTNVGLSYTVKDVAKVVGGYLGTSDEFWVGADVLAV
KGVTARLGFRNTPANSYIYATAASSSLVDKLTLALDADAVLSPFAVGAQGNAEYAVTDKY
SAGAKVWFDNGDAWYGNNGFAAEGYGVINFAQGAMTVGLKYDVASTTFSIPFDFEVWF*
>SPBDM4_v1_40312|ID:27157617|ackA| Acetate kinase [Uncultured spirochete bdmA 4]
MVILTLNCGSSSVKYQVYDWDKKDVLANGIVERVTIGGSFITHKVRGRPDYEVEHECPNH
VVAIELILKTLTDPEVGVIPDLSMIKAVGHRMVHGGSKFAKSTIINGEFLDTFKSLNDLA
PLHNPANLMGVEAATSILPNVPHCAIMDTAWHQTMPASSYMYALPREWYEKYMVRRYGFH
GTSFLYNAKRAAVLLGKDPFETNLIIAHIGNGSSINAVKNGCSFDTSMGLTPLEGLVMGT
RSGDMDAGIVFHVMRKTGMTAAEIEKKLNKESGVLGLTTKWADRRDVENAAEKGDPVALL
TQQVEGYRIKKYIGAYYAALGHVDALVFTAGVGEMGPEIRKLATEGLEELGIVVDEKKNA
LAKCRNAELDITGPGSKVKVFVIPTDEELVMTEDTVALINGTYDVHTNYHYSFENRDYSN
KARAEGLQRDLEKKPWLRDIVARIP*
>SPBDM4_v1_40313|ID:27157618|priA| Primosomal protein N' [Uncultured spirochete bdmA 4]
MPASFVRVAYPIPLEETFWYRNPKSARVEIGMRVEAPLGRRPKAAGYVIEVAEAPSTENK
PVSALPLDLTCLAPDKIRDILRVVDTEPLFDPGTLELGKWLAAMYHCSLGEALSAMLPSA
HREPKELAEEFDEVEMSRTPLVLTPGQNAALEGILAEPDGMFYLYGPTGTGKTEVFLQLA
EATLAQGRGVLYLVPEIALTRQVEQDVRARFGARCAIIHSRLTPSRKLAEWRRIAKSEAR
IVVGARSAVFAPISGLGLIIIDEEHDSGYKSGTTPRYHARQVAMHRARLARARIVMGSAT
PSPEAWQACADGRMRRFSLTNRPAGGSFPGIHIIDMRGRQGLISEELAQALRDAKSAGRQ
SILFLNRRGFARTLICQTCGAEVLCRHCSVPLTYHKTSGRLVCHYCGYSESKPGACPECG
SLDLVWASYGTERVEEELHEHFPDISHARLDTDTASTKGKLEDTLRAFRRGEIDLLVGTQ
MVAKGLNFPGVRVVGILMADQGLSMPDFRAAERVFSLIVQVAGRAGRHRPDGLVFVQTRK
PESPIIRMAAAGDVPGFLERELEARRQLEFPPATRLCRIVFRSKSHRDARETAAKCAAVA
HRLAREALFADVEVLGPSECPLAIVADNHRYQLIFRAHSFSHLQRFVKVFRRSISPISSV
YLEIDIDPVQMM*
>SPBDM4_v1_40314|ID:27157619| Uracil-DNA glycosylase, family 4 [Uncultured spirochete bdmA 4]
MRMNEEVSRGPGVVAKEEATQESVAKTESAPGAASQPRQFLEDLLLIEDFLRYGFRSGRQ
IEAEEGNSAFGDSSSAEDETGFANEDRQARLKDIEARVRQCTACVLSQSRSRTVFGEGVT

NPQVLVIGEGPGAEEDRQGLPFVGASGHLLDKMLAAIGLSRKTNCYIGNIVKCRPPNNRE
PTPTERDACIGYLRAQIEILRPMFILCVGRTAAHSLLEVNDAISRLRGRTFAFEGIPVRI
TFHPSALLRDASLKRPAWEDLKQFRALMDGTADGPARTSKGGE*
>SPBDM4_v1_40315|ID:27157620|lacZ| Beta-galactosidase [Uncultured spirochete bdmA 4]
MERAWNAGWWFSPGPVADAFLSEAWGGPARETADGPSAGTPAAGEGRASWIPVHLPHDMI
GVPLNAFDERTFARRGCYAKALRKEDVGALAGTVPDAQTSADQAETHKAAPTDVALFLRF
EGVSVSCRVWVNGRFAGSHKGPYTPFEVRIDPFLQPQVEACAAVSVPSAWILVEVDSAED
AGIPPFGAVVDYLAFGGIYRGVSLCARTGAWLDAVWGAPRPRGDSLDGAWHLEISVEAKS
FTGRIIEHGSLRARLLRSGGLVAEGDVLLPEAPLFEIQSAQARNDAISLSANFSLSVDKP
RLWDIDNPFLYDLEISLCGQTGRKLDTRTIRIGFRTAEFGPSGFYLNHRRVFLRGLNRHQ
EYPYAGYAMGPDAQRRDAVMLKHELGCVIVRTSHYPQSPHFLDACDELGLLVFEELPGWQ
HVGGATWQEQAMTDLRDMILRDRSHPGIVLWGVRINESQDNHEFYARTNGLSRKLDSYRQ
TAGVRYIAHSELLEDVYTFNDFTHDGEGTLYITEPAKVLPKHNKRAPYLITEHTGHMFPT
KRFDQEERLVAHALRHAHILDAAMANARVAGCIGWCAFDYHTHKDFGSGDRVCYHGVADM
FRIPKYAAYVYGSQVAPSERIVLEPASRFAKGERNAGYMLPVHVFTNCDAVDVYRGGAYI
ARFFPDRAHFANLPHPPVVIDDLIGERIAAEGWSRRDAKLFRRLAGRGMAVGAARLNIWE
RLGMGFFMLRHKLSTHKLEELVMRYGWGSADEKIRLVGILDGKEVAERGFGADGAAERLV
AEADANRLDARDEEEWASTRVVVKALDQYDNTVPFVFEPYTIEVEGPARLIGPPQRALVG
GASAFWVAGGAEKGRVRISVSSPRFEQPAVLELKVE*
>SPBDM4_v1_40316|ID:27157621| conserved protein of unknown function [Uncultured spirochete bdmA 4]
MMNGQGEIPVEIVHLYTYDIGRAIDLKKVASLVPAHHEIEFAKRRDTPVSLTLPKPMVLS
ISTDECDSKCFEKISAQAKIYEDAAVTLIVRYRTMSRFEDLSQKANMPIRDISNSISIEQ
FAESSFRMIHEALRSAVSGYKELSENDREAYTAFCLLECPGGDPQAFIEDHKDIAASLLS
GEPVGTLHLSQIEQVLCKPFSYRKDDIAIFDLERCLIIDPAADYDDVIMMAEHANYRLIE
LRELDFLLDKWLGEAEQDIRRLYLSGTRRRSGDRALRLKLAQIQGLRFDALFTLENLDNS
SKIIGDYFLGSIYHRLCEIFNTDEWKFSIERRLGALQNIYDLLNTDTTEQRMMTLELVFI
AVCIIFPILQILQVMLVK*
>SPBDM4_v1_40317|ID:27157622| PASTA domain containing protein [Uncultured spirochete bdmA 4]
MAKKDIKDFTQTIKESTSKAPEKVSATLKTVAETASDQHKRISEWTKPQKRTFVLFALLM
FLVMVLVAVLVFFATLKGEERTMVPDVRRMDLADALMKMQQRELYPRLTLRFTDNPLDRN
LVLDQSPPAGSIVKAGRRINLVVSRGAVLDKVEDYSGRNIDDLKLYLQGLSTTTKVLVSI
REPPLYIFDNSAPGIILDQTPKPGTEISGPTVLDLVVSKGPEAKDIPMPSLIGLSLREMA
SKAATTPLVFKYKMRAAHDKENPGTVVEQSVEADTTLKQLDTVQITVASPTARKGYTSGI
FEYTLPEYPYVVPVEIDVIAPDGTRKSIYSVRHPGGAFNAPFSVQSGSVLVLMVSGAEVT
RKEVK*
>SPBDM4_v1_40318|ID:27157623|fmt| Methionyl-tRNA formyltransferase [Uncultured spirochete bdmA 4]
VRVLFAGSPDIAVPAMEALAASSHTVTAVLTNPASKVGRGLKMSGTPVAQAALRLWGDGV
PIFTFETLKTTAREAVAACNADILVSFAYGHIFGPKFMALFPKGGINVHPSLLPRWRGSS
PIQHTILAMDEETGISIQTIAPEMDTGDLLLVQRIPLTGREKAGTLSGLCAHLAAPMVVE
ALDAIDQGKARPRPQTGDPTYCGKISKEDGRVDWGRPSRELDARIRAFDPWPGSYTILRY
MRLSILEAEPFDEFVPSIADEHVTNSVPGTIIAVAATKGIILKTGQGYLAVKHLQLASRK
SVCFREFANGVRDLVGTVLF*
>SPBDM4_v1_40319|ID:27157624|def| peptide deformylase [Uncultured spirochete bdmA 4]
MLDIVLYGEPILIQKAQEIKEFDHDLEKLAGDMHDAMKRDHGIGLAAPQVGASKRLFVVG
LDNEPLRIFINPRIVAMSEKTSEYEEGCLSFPGLYFTVVRPSAVDIEALDIRGKPFRLSA
DGLLARVIQHEYDHLDGILFIDRVSPAKRRRALAHYSRMLNM*
>SPBDM4_v1_40320|ID:27157625| conserved exported protein of unknown function [Uncultured spirochete bdmA 4]
MRKRYVSILQAIITSLLFLVFFAACGQKGTVQGIDTPNPKLKTVDLAIGTATVKAEVALT
EIERNRGLMYRTTLREGEGMLFAFDRDQQVSFWMKNTKLPLSLAYILSDGTIVQILDLVP
FSEEPRPSTRSIRYALEVPQGWFGRTGIKVGDKVQIPPLK*
>SPBDM4_v1_40321|ID:27157626|rpsU| 30S ribosomal protein S21 [Uncultured spirochete bdmA 4]
VRIIHQITVEDNEPLEKALKRFKRMVEKEGIIREWKSREYFEKPSAIRNREKKALERKLM
KKTRKAQESKY*
>SPBDM4_v1_40322|ID:27157627| protein of unknown function [Uncultured spirochete bdmA 4]

MLFSAGNLVTLVIVAAMFGVYHLLTADNRSLEKLKRLGDKLKDELGSYVESKSEEIKHYG
IDLDVQQKAAKVVLEKIQDAQATIDEKSDSITEIANRFKEYDNILAQLMDMTKRVDQNIA
HLAEKNNFIEELARRVENADKKMNSIETEMPKVEEQFAHDAKQILDGFRDDILDQLHEQL
TGIVNMFEKSKTDAQSAIAQASSLREQIDRMTETALEAAASRAHNIEDSAYSALRTHIAA
NFDELSRQTEERVAALSQNLDTLSQTAETKLSSLAAATSSQVSDTDHRLSEAKAALNAMT
TDMARIQKETAETSTALSKKFKAERDSLEEQFAQFGQAFEAHRASFEKEFLDEISTLRAR
LEEAQARTEDLKTTAEGNISTALGTFEEDVAQKLESSKAAMDEQIDSWLSSMDAKMRKIS
QDALEQRKTEEARLAQEVNTELKRLKDSLYTQAQKIERDIEAFKDV*
>SPBDM4_v1_40323|ID:27157628|deoD| Purine nucleoside phosphorylase DeoD-type [Uncultured spirochete bdmA 4]
MSLHIAAQKSQIADKILLPGDPLRAKFVAENYLDNPVCFNEVRNMFGYTGTYKGKPVSVM
GTGMGMPSLSIYVNELIREYGVKRLIRIGTCGGMHEYVKLRDIVLAMSASTDSAVNRVRF
NGMDYAPTASFTLLKAAWDSATARELRVFAGPVLSSDMFYTEDPDQWKLWAKFGVVAVEM
ETAELYTLAAKYGCEALSVLTISDNLITGEKTTSEERQLSFRAMMEVALDAIFA*
>SPBDM4_v1_40324|ID:27157629| exported protein of unknown function [Uncultured spirochete bdmA 4]
MRKNCSGFGFVALLLGIVTIFMLASCKQAKVVEKPIETVPSQTNAEQKTLPAPGPHPEAA
VGVPEVSKQNENILLVYRHTGSGYAEAYGFDTYVFRLLTDGSGQITGGIAYQRTIGGEIE
AVRYNTSLSGDEISLTASSPGEKSWSDTLKLKENRVDVSGAHKMVATLDKALVFSSSDGN
YRESYSTDPAQKDLPVEIRKGGSLMEKGAWSYPETGKAVYAQTRPEDAQNAEGFQISLWY
QDKGDFRFMTEGPEPINEVYATGFASVLASDHVLVNAVLLDLMLGDSRYLRPVYAFGISR
RGSGK*
>SPBDM4_v1_40325|ID:27157630| exported protein of unknown function [Uncultured spirochete bdmA 4]
MRTKTAIIVAVCLLVGAALQSFALEQEGEAFDPALWSFIPGEPLSQDACAAAVGGDVRMS
LNQNRTKLTVNVIDNEYEARLGRIPPEQVLEFDVHNRVVNTTQAPFMPAKAAGRTLAASA
SVTTKPSQFPEGYWTVTAVKPRSDKYGPYMLSTNAVGNVEVYVSDGTGGQLSIGTYSDLG
YALHSNTNAFNVSKSYGCLILKQEDVAMLAGILRQDRAENAKAVQKILVRSPGRIFDR*
>SPBDM4_v1_40326|ID:27157631| putative Phosphoglycerate mutase [Uncultured spirochete bdmA 4]
MYARYEPKNVPDPRLLEALQREQADGPFFSSLGHDMSVFILRHGQSEGNARNTYQGRLDF
PLSAHGEEQARAAGEWLCQFSPDYVIASPMQRARRSAEIVADLVHIDHIEFNDVLIEVDT
GIFSGIDPDTAARRYPELWDEFYIKSWDAIPAAEPSQSMYGRAILAWKRILELGLQGASR
IVCMTHGGLVQWLMKSSLGVHRWLPLLPMSNCGISQYEIEMVKKNNPAFVQWTKINFHPP
LAPEGPAPVF*
>SPBDM4_v1_40327|ID:27157632| ABC transporter, membrane spanning protein [Uncultured spirochete bdmA 4]
MSSGVRTSRLHGHRPDIWSLVIFAGFAIVAIFLIYPLFDVFRYSFLDKVTKTLSLSNWKT
FFSKAYYLRAFWHSILIALLTTFFSMVLGIPLAFFTTRYRIRGTNLLNTLSVLALLSPTF
IGAYSWITMLGRNGFLRLFFQSIGINLPPIYGAFGIVLADSLQYYPFISLMTAGALMTID
RSLEEASENLGARSVRTFFDITLPMIIPSVTGGALIVFMMSLSNFGTPMIIGGNYLVLPT
LAYNLYTSEISQTPGMASTVSIVLMLCASVVIVLQQWLSSRRKYASMLVNRPVVKRLKGI
KSLLAHLLCYFIVFFSTLPLAVIVYYSFRKTSGPVFKPGFSLDSYRQIFFDVPKTVMNSF
LYSLIAVLLIASVGTLLGFVIARKRNAAVRVLDPLLMIPYMVPGTVLGIGFIVAFNRPPI
ILSGTATIIVLTYFIRRLPYSVRSAASILKQIDPALEEAGINLGSPPGRTFRTITLPLMQ
GGIISGAIMSWVTSMNELSASILLYVGKTMTMPIKIYLSVADGYFGTASAMSTILLVVTG
LSMFIVNKYFNRGSDTFIAA*
>SPBDM4_v1_40328|ID:27157633|fbpC| Fe(3+) ions import ATP-binding protein FbpC [Uncultured spirochete bdmA 4]
MASIQIVDMEKHFGEVQALSGVNLEIEKGEFFTLLGPSGCGKTTLLRTIAGFYHQDKGDI
YIDGELINNVPAYQRNTGMVFQNYAVFPHMTVFDNVAYGLRVRKLPKEEISRKVSAALKS
VHLEGYERRTPDKLSGGQQQRVGLARAMVIQPRVLLFDEPLSNLDAKLRIEMREEIRAVQ
KSLGITSIYVTHDQEEALVISDRIAVMQQGVIQQIGKPWEIYRNPQNTFVAYFVGKINML
TMRLDEGAEQGLRRATLGNLSFLVPSEGTEGMHEVIAAFRPEDLIEAAKEGSQNEITGIL
KTVSFMGSLARFELNVEGQEITIDRHRPREADMPQKGAAVSFAVPTQSMLLFDPKTGRRI
GKGGRR*
>SPBDM4_v1_40329|ID:27157634| Iron ABC transporter, substrate binding protein [Uncultured spirochete bdmA 4]
VIACLLIAVSAWAAGGKVVVYTAHEETIINALVPMFEKETGIKVEYVKMASGDVINRAKA

EASRPQADVIWSIGGEQLEAENAILAPYTPKEWDKINPVYKVGTNWLPYTGIMNVFIVNT
KMLTPDKYPKTWTDLMDTRFKKLISSARADKSGSSYMQLCNVISIYGDKGWDVYKGIMKN
MVISASSGAVPKFVNDGEQAVGITLEDNAFRYVSGGGPVAIVYPADGTVAAPDGIALLKG
APNLENAKIFIDWCLSKPVQEYLVDAMARRPVRTDCKDPKGLASLSTVKTIPYDFGWAAK
NKAAFAAKYADIAMDLGL*
>SPBDM4_v1_40330|ID:27157635| putative HAD-superfamily hydrolase, subfamily IA, variant 3 [Uncultured spirochete bdmA 4]
MYKAVIFDFGNVLCKVERENFVRVAARHSRTMGAEDLLNALWGTELEYEFETGKFDSHEY
FKRLQAIADFDPVYSYERFVEDYKRIIVPNPDGEWGLKTAAGLGARTFVLSNTSFLHASV
IFDNEVLGTYPELYILSYKVGVMKPDPRIWKKLLEYTRLEPEDCLYIDDIEQYCEAARSL
GMSAFRYDFRTQYLSQELKNMLQ*
>SPBDM4_v1_40331|ID:27157636| putative TPR protein [Uncultured spirochete bdmA 4]
VSRGRRRPLLVKAFVVFFVLGWTVASLPSTWAEDSYSTALSLFWDGRRLEMVETRQTESQ
LAFQRSLAMTERLLTADPANSDLQTLKTWNLFRLHRYIDTVVYAQSVLNTKQDYRILETM
AEALFFLGKNEEALAAFSRYFTLSPENDDRRSSAYYYVGEVYFRIKKYEHADIAFSTAVA
LEKNMYYWWYRLGLVKEILGQYRRAYEAFDASLALRSDFEPARSAKERVKAKSSL*
>SPBDM4_v1_40332|ID:27157637|sudA| Sulfide dehydrogenase subunit alpha [Uncultured spirochete bdmA 4]
MSHKEDFTMDNAELELAKLRAAPITPKIRLQIPPQDMPAQDPSERRHNMDEVALGYTPAQ
AMLEAERCLQCKNAPCIAGCPVRIDIPGFIGKVKGGDFLGAGKIIKKTNLLPSICGRVCP
QETQCQAPCTVGKALHSVGKAVQIGRLERFVADYERTHGAVEVPNVASPTGRKVAIVGAG
PAGLTCAADVRREGHDVTIFEAFHKSGGVMVYGIPEFRLPKVLVQSEAEVLESMGVDFEL
NFLVGRTRPLLSLLREDGYDAVFIGTGAGLPKFMDIPGENLVGVFSANEYLTRANLLKAY
MKGKAGTPIYPSHRVAVLGGGNVAMDSARMALRLGAEEVRVLYRRTREEMPARAEEVEHA
IEEGIIFDFLKSPTRVLGDDKGRVAALEVLSYELGEPDDSGRRRPVPIRGSEEIMPFDTV
IVAIGNESNPLISQTTPQLQVDRHGHIIVDERQKTSVERVYAGGDIVLGSATVILAMGEG
RQAAQAINEILQ*
>SPBDM4_v1_40333|ID:27157638|sudB| Sulfide dehydrogenase subunit beta [Uncultured spirochete bdmA 4]
MHGTHKIVSKSQLSEEVFRIEVQAPLVAQERKAGQFVIVMYDEDFSERIPLTIADADPVR
GTVTLIFQTVGASTHKLALKNPGDSIVLLGPLGNPTHIERFGWAVCVGGGIGLAPLHPIA
KALKEAGNRVTVISGARTKDLLIMQDELAAIADEHIVVTDDGSFGRKALVTEPLKELCMQ
SPPPDIVVTIGPPIMMKFCAETTRPYGIKTLASLNTIMLDGTGMCGGCRVSIGGTTKFVC
VDGPEFDAHLVDWDGMLMRLGTYKEIEKEAHDRCHLEMRIEELEAQGDGARAPEKEPAK*
>SPBDM4_v1_40334|ID:27157639| conserved membrane protein of unknown function [Uncultured spirochete bdmA 4]
MAQRMPKIQVFRRVVLGTLLVGLTVFTFLHQKLQGIPAIDALDPFGGLETLFKWLAGGDF
IKKIEPGNIVLLGAIVALGIVLSRFFCGWLCAFGALQGVFGWIGKKLFKRRFEVPKKLDR
VLRWMKYVILVAIIYFTWKTGELVIRPYDPLAAYGHLPAGLTAVWTEFKVGFILLVLFMV
LSMFYERAFCKYACPLGAVNAILSRVPLFRIKRDKPTCISCSKCDRVCPMNIDVSHAESV
KSPECISCFECVTACPTKKDTLVATIGNKKTKLWTVVIIGFAIYAGAALIGQVTGMLRFT
APSLSELSDKGTLNVADIKGSSTYEELAGAFGVDLDQLYRELGIDKTKVPTTSMIKDTGK
LAGIEDFETDQARVAVAKLLGIPYEGEGTTPTETATPEETTAEPGAAAPAVSSESSLMIP
EGFALEGTMTIQDVAKALNATPQQVIGKLGLPQDIPLDKPLREMGSLYGYTMPQLKEKIA
Q*
>SPBDM4_v1_40335|ID:27157640| conserved protein of unknown function [Uncultured spirochete bdmA 4]
MSIPNVPKKIDSEALFRPADFLAYLGRIGVAVSHRPAPDAVILSYQKSLFEHVLETYPCE
RAKGYFGRFIHYVDLPGSTKSIAIAADFGVGAPAAAVALEELIAWGVKEFISMGLAGSLS
EDLPPGSLVVCDGAFRDEGTSYHYIEEDVPACPDAALTGRLEAALRDAGLSYAKGPTWTT
DAIYRETPLEVRRFRERGALVVEMEAAALFAVAQYRGAPLASCFSVSDTLAYLEWRPEFH
AKPTVQGLETLFSCAVRALQ*
>SPBDM4_v1_40336|ID:27157641| putative UDP-N-acetylglucosamine diphosphorylase [Uncultured spirochete bdmA 4]
MNPGERKNVSADSLKGQSDRFPALWDLKHSEFFGLFESAEFPWDVLKLLDAYLQSKLESV
SGPKIRSFIPHGVHIEGDMFIDEGCTIEEGVYIRGPAWIGKNCEVRSGCYIRGGVLAGEG
AVLGHSSEFKHCVLLGLAQAPHFNYVGDSVMGWHAHIGAGVILSNFRLDGKVVPARALGS

KERIDSGLPKFGALIGDGCEIGCNTVLNPGTILGERSVVMPLSLVSGTWPRESRIENQAM
RLPN*
>SPBDM4_v1_40337|ID:27157642| conserved protein of unknown function [Uncultured spirochete bdmA 4]
MRLLVLAAGIGARFGGVKQVVGVGPRGETLLEYSLFDARRAGFDHIVFLIRPEIEADFKA
NILSRLPASVSYSLAYQKSVSLLDEAARARFEKSGRAKPWGTGHALLCARKHLEGKGPFA
VINADDYYGLPGFEKVGAHLSSSPGEFCFAGYRLDDVVPKGGSVSRAICLIDSQSYLSEI
VEHRKVWRAGSLFVSQREEGTVELSPSTAVSMNLWGFNPSIFDYAERLWKNFLSEPANFE
SREFFLPDIVQDMMREKAVRVRMVPASAQSFGITNPEDLQETRQRISRLVWEGVYPSPLW
EGA*
>SPBDM4_v1_40338|ID:27157643| protein of unknown function [Uncultured spirochete bdmA 4]
MTFAAVSVRDSENIGIRPLGPDSEMLRRAISAFATSLIVSISIAIIISILLLTLGLTGSS
VF*
>SPBDM4_v1_40339|ID:27157644|leuA| 2-isopropylmalate synthase [Uncultured spirochete bdmA 4]
MNTPRTIRIFDTTLRDGEQAPGCSMNQVEKLEVAGALASLGVDVIEAGFAIASPGDSASI
HEIARTVRGPVIASLSRAVELDIDASWDALRGAERPRIHVFIATSPIHMKYKLRMKPDEV
VAQAAAMVKYARKRCPDVEFSAEDASRSDLEFLCRILTAVIEAGASTVNVPDTVGYAMPG
EFGSLIRSIRAGTPNIDGAVVSVHCHNDLGFAVANSLAAIEAGADQVECTINGIGERAGN
AALEEIVMAMRTRADYLRSACGTGLRCNVDTTRIYPVSRLVSTVTGSRVQPNKAIVGENA
FAHEAGIHQHGVLTNRETYEIMTPESIGLPRNRMVLGKHSGRHAFDERLKELGFRLEAER
AARLFEAFKILADKKKVITNRDIEALVRGAVPDRPAAWRLERFIINSGSTITSTSAVCLV
SAGGQTCEQVAIGDGPIDASFNAIDKIVNAKPVLEDFSLDSVTDGKDAQGEARVRIRLAD
RTYNGHGVSTDIVEASVRAYVDAINAVLGAADDASRPEDTANKRDGTKAFAETERSNQ*
>SPBDM4_v1_40340|ID:27157645|leuC| 3-isopropylmalate dehydratase large subunit [Uncultured spirochete bdmA 4]
MTMTQKILADHAGLPSVEAGQLIEARVDLVLGNDVTAPVAIKEFEKIGVAEVFDRRRIAL
VPDHFAPNKDIKSAEQCRIMRDFARKHDIEHYFEIGRMGIEHALLPEQGLVLPGELVVGA
DSHTCTYGAVGAFSTGVGSTDMAAAMAAGTAWFKVPGAIRFVLNGALEGWATGKDLILSI
IGAIGVDGALYRSMEFDGPGVATLSMDERFTVANMAIEAGAKNGIFAVDERTRAWAVAVS
PRSFKEYAADPDAEYERTIAIDLASVEPQVAFPHLPSNARPAAEAGETPIDQVVIGSCTN
GRIEDLRAAAFLLKGRSVAPGVRCIVIPATQRVYQQAVREGLVEAFIEAGAVVSTPTCGP
CLGGHMGILAAGERAVATTNRNFVGRMGHPGSEV*
>SPBDM4_v1_40341|ID:27157646|leuD| 3-isopropylmalate dehydratase small subunit 1 [Uncultured spirochete bdmA 4]
MEQGCRVWKYGDNVDTDVIVPARYLTTGDPVELAAHCMEDIDPRFAAGVLRGDIIVAGEN
FGCGSSREHAPVAIKASGVSCVIAKSFARIFYRNAINIGLAILECPEAAEDTRSGDRLTV
DFESGEIRNLSRGARYRAVPFPPFVRNIMTAGGLIPYIRGKLLKPGAHLGVGQGGD*
>SPBDM4_v1_40342|ID:27157647|leuB| 3-isopropylmalate dehydrogenase [Uncultured spirochete bdmA 4]
MKYTIATIPGDGIGPDVVAEAVKVARTVAGVSGFTVEFMETLMGGAAIDATGSPLPDSTL
RACRSADAILLGAVGGPRWDSLPGNLRPEAGLLGLRAAMGAYANLRPARIFPELAAACPL
KPEIVAGGMDLLIVRELTGGIYFGERGKSPDGEAAWDTERYTKAEIERLLEVAFRAARSR
KRKLCVVDKANVLESSRLWRQVAQAAAARYPDVETTFMYVDNCAMQLVRDPRQYDVIATS
NMFGDILSDEASMITGSIGMLPSASIGDSGPGIFEPIHGSAPDLAGKDQANPLATILSAA
MLLSYGLGRHDEAALIEGAVSSVLAAGCRCADIATGAAEETVLGTRAMGEAVARAIEEAA
R*
>SPBDM4_v1_40343|ID:27157648|ilvD| Dihydroxy-acid dehydratase [Uncultured spirochete bdmA 4]
MRSDKAKLGPERAPHRSLLKAAGYTDWEIERPWVGVANAYNQIIPGHVHLKRITEAVKAG
VYAAGGLPIEFPTIGVCDGIAMNHLGMKFSLPSRELIMDSIEVMAQGHAFDALVLVTNCD
KIVPGMAMAAARLNIPSVVISGGPMLTGTLGGKEIDLNTVFETVGKHIAGKASDADLLEV
ENAACPGCGSCSGMFTANTMNCMMEALGLGLPGNGTIPAVYSERDRLAKEAGRAVMQLLE
KGIRPRDILTREAFENAVAVDLALGGSTNTTLHLPAIAWAAGLDFDIELFNELGGKVPHI
CSMSPGGSYHIQDLYRAGGVQAVMTRLARGGLLHENCLTVTGATQGENIAGARVVDDEVI
RPLDRPYHATGGLAILRGSLAPDGAVVKQSAVAPEMLVHTGPARVFDSEEEAFAAIQARK
ILDGDVLVIRYEGPKGGPGMREMLTPTSALAGMGMDKTVALITDGRFSGASRGASIGHIS
PEAATGGPLALVREGDLIHIDIPGKKIDLLVDEAELARRRAEWKPYVQPVDSLFLNRYRR
VVTSGMKGAVLE*

>SPBDM4_v1_40344|ID:27157649|ydbK| putative 2-oxoacid-flavodoxin fused oxidoreductase:conserved protein; 4Fe-4S cluster binding protein [Uncultured spirochete bdmA 4]
MTNTKKMVMIDGNAAAAHVAHACSDVIAIYPITPSSPMGELADEFSSMGRKNIWGTIPQV
VEMQSEAGAAGAVHGALTTGALSTTFTASQGLLLMIPNMYKIAGEATPTVFHIAARAVAA
HALSIFGDHQDVMATRQTGWALLSSNNVQEVMDLALIAHTATLKARVPFLHFFDGFRTSH
EVSKVEELPYDVMREMIPEDKVREHRARGLNPEHPTIRGTSQNPDVYFQGREATNKLYEA
VPGIVEETMKRFAQLTGRSYKLFDYAGAPDADRIIIIMGSGADTAEETAEYLAKQGEKVG
VIKVRLFRPFSVQHFIEAIPTTVKSIAVLDRTKEPGAIGEPLYEDVRTAIGETMGNGTAP
FKGWPTVVGGRYGLGSYEFTPAMVKAVFDNLKAAQPKNHFTVGIVDDVSFTSLPYDESFH
LPSEGVVECLFYGLGSDGTVGANKNSIKIIGDETDNSAQGYFVYDSKKAGTYTISHLRFG
PKLIKKPYLIGKADFLACHKFSFLEKIDMLSNLKDGGTFLLNSPFSAEEVWDNLPLEVQK
QIIDKKLRFFVIDAMSIADKAGMGSRINTIMQTAFFKISGVLPEGEAVGLIKKYTKKTYM
RKGADVVQKNLTAIDMALNATQEVKVGQATSAKHMLAPVPETAPAFVRETLGELIAQRGE
KVPVSKIPLDGTFPTATTQYEKRNIAEKIPVWEPSICIQCGNCTMVCPHAVIRMKAYDPA
LLTKAPATFKSADGKGKELAGLKVTVQVAPEDCTGCGACINICPALDKANPGKKAINFGP
QIELRETEKENWDFFLSIPETDSKVLNLSIPKGIAMKRPLFEFSGACAGCGETPYVKLVS
QLFGDRAVMANATGCSSIYGGNLPTTPYAKRADGRGPAWSNSLFEDAAEFGYGMRLTADK
QNEYARELLGAAKGKGVAADLADRLLTNVQENDEQIEAQRADVAALRKALGGKNEEWAKE
LDNVADALIRRSVWILGGDGWAYDIGFGGLDHVIASGKNVNLLVLDTEVYSNTGGQASKA
TPIGAIARFATSGKDIFKKDLGMIAMSYGYVYVAQIALGANMSQAIKAIREAESYDGPSI
IIAYSHCINHGIDMMKGMDQQKIAVQSGVWPLYRYDPRLKAEGKNPFQLDSKEPDYSKLE
QYMYAEVRFKSLVQANAERAKMLLEKQRGLLERRYKEYRYIADRPF*
>SPBDM4_v1_40345|ID:27157650| conserved membrane protein of unknown function [Uncultured spirochete bdmA 4]
MNVVKSYKWTIASAVSVLVLLLLPSQLFPKTPRGTIEIDKIVHAFLFGAVTAIFCAEHRQ
WKKMNPPFFFSFIVIGAFAFLTETSQLATKTRHFDMKDFSADIIGIAAALLIMRLVATMG
ASLHKGKDK*
>SPBDM4_v1_40346|ID:27157651| membrane protein of unknown function [Uncultured spirochete bdmA 4]
MKDFENQISSSTKKETTSSTPAPDIDPSALPLLQKSSFFFFLIVSFFIVLNIVLVTLKPE
RPPLRHYSPEFLIIEGLFSLGFLIASISRSLSWLGPIMLLAFLPFHLSSHIASMYSLGAF
LIAIIELYRIGFFKRAGLFKITTLFIYYALTVLLVGIARSVFVVEIIMPIIFLFLFFLYL
LPLFQGKWQINILRPRQKIRYKDLNLSERETDFLKQCLDGATFKEIASRNHVSESTVRNT
FAHIYHKFEVSGRRGLLSKFADFDIID*
>SPBDM4_v1_40347|ID:27157652| MazG family protein [Uncultured spirochete bdmA 4]
MDLDSNSLQAIAGQTFEQILPDAEKTAEAFKLFYTVVARLRAPDGCPWDLEQTPSSIRGN
IIEEAYELAEAITENDNPHIEEETGDLYLLATMVAYMGQQEGRYTVADSLCEGARKLIRR
HPHVFGESDVHSPDQVVRQWNEIKERVEGRRKKDSLLDEVHRHLPPLEKAYKLQKKAAKA
GFDWQNSHDIWNKLEEELAELKQARAASEENAPDSGDALEEEFGDLLFTVINAARLYGID
PSIALHRANEKFSRRFRHVETRMKQNHLAMSPEYFEQMDAFWNEAKREEDAGETSSKVTK
RQP*
>SPBDM4_v1_40348|ID:27157653| Major facilitator superfamily MFS_1 [Uncultured spirochete bdmA 4]
MAVPLSAQKLASSRKTFNIYNLFNSFSFVFVSGSIITLFALRMGAQNSIIGLLNALVYIT
FFMLPLGKRMVRHTSIIKVFGWGWVIRYIALIPILFVPLFASKGQMGLAILVLILGTAGF
HISRGVAMIGNNPVLGLLSTGGGDKSRSDQGRYLVNVSVVNSVAAILANIVLAFLLGEQA
SLWTYAIAIGIGIITGLIGCIFLFKMPEPDAFSSKDEQSLWDVTKEALLKPEFRAFIFTF
MLLSFLSGMGRSFLPVYAKQVFAQGDDAIMVYSIVAGLGSIAMGLITRLVVDRLGSKPLF
IIYSAIGFISFLPIVLIPGSGAMMSAPAMIGLFLSVVHFLSSFGFAGEENAAQVYYFGLV
PKEKMLDLAVIYNLAYGIGGSLGSFLGGGMLEIFTNVGLSTDVSFRVYYAAICIVLAVVI
YSMRNLPHMGAKSVSQSLSILFSPRELRAFDLLTRLDQSASAHEEVHILQELGKSESNLT
QKELVEYLHSPRFEVRSEALLALESIPELDTETIGALIREVKTNVYSTAYVAARILGKQR
SDTAIPALVDSLDGPDYMLQSTSMVALARLGYRPAIAKIEEVIRSIENPRVRISGAYALE
LFGSIDSIPTLISCLKAEDRPANVSDELVLTISGILSINKEFYLLYSEFIEDNRSGVAQL
TAHAHDLPLGKEPLQAWLSAVEALFAPTPDTVPLARFLLSSSKITSSILILSDALLDPKL
DYPGFNYLCAYVALHIETLKDY*

>SPBDM4_v1_40349|ID:27157654| conserved protein of unknown function [Uncultured spirochete bdmA 4]
MLTSKQRSTLTGLASQDCVVQALGKNGLTEAFISQIEVLLARHELIKIKFLDFKDEKRRI
SEEIAARTHAELVRVIGNNAILYKQNPDSEKRRVVLQ*
>SPBDM4_v1_40350|ID:27157655| conserved membrane protein of unknown function [Uncultured spirochete bdmA 4]
MILTSLARIMSGVVSIYMLLCIVRIFFSWMPSLIDTKWGRLIARLTDPYLDLFRTITLFR
TSAVDFTPIVALTVLSVINNLFMTLSYAVRITFGFILSLLLDAGWSAVSFLLGFFLVIVL
IRIIGFIARIATLHPLWQILDSMINPLLFKVNNVIYRGRAVDYLQGLITGFVVVLLVRSL
GGWIVRMLTSLLMSLPF*
>SPBDM4_v1_40351|ID:27157656| conserved protein of unknown function [Uncultured spirochete bdmA 4]
MHIGNVHIGTCGFSYDEWKGQFYPADLPAHEMLRYYSLVFSFVELDHTWYRIPNPGQLER
MALLTPSDFRFSLKVHRSLTHEIDEHWQTHAAEFRTAVSALASSGRLGCVLVQLPYRFSY
TLENRKYLANLLSTLSDFPLVVEFRNATWYQERVFDTLRERKVGLVTIDRPDLPGLPPES
VITTSDICYYRLHGRNADLWWSGDATSRYEYCYSEPELKDRARLVRAMSNQAKTIFVAFN
NHACGNAPKNAALLQSILAEQQA*
>SPBDM4_v1_40352|ID:27157657| protein of unknown function [Uncultured spirochete bdmA 4]
MRKPFEISTGAWWLVPDGRTIAVSSFHESWLASHPAIAGGALHTVDFVQKSGWLSVTQYS
DGMLEVISRDILDPRQREALRKLLEVNGGAIKKLVVFVPVIDGCLTAEAPFTADWERLSR
NLDAFAGKGT*
>SPBDM4_v1_40353|ID:27157658| Pseudouridine synthase (modular protein) [Uncultured spirochete bdmA 4]
MSHFFGDIAAFAGTIFYKYNLINGGSFAPYPQFCYDTASMIPILLENDEIIVVDKPSGLA
AQPGEGVHDDVVAVLERQLGYRPFPVHRLDKGTAGCMMLAKSRQAAGKWSRFITDRDVAK
RYYAWVSGTPSEEKGVIDAVLDGNKGAQEARTLWNLKETWNLTLTGEGSSAGGRLLVAGS
ELISVSLLELELKTGRMHQIRRHLAGIGLPILGDDRHGDFSVNKALRRAGVKRLMLWARE
LVLPAQASLPGGASILASEPPHFAAFREFLMRSGTLRQPNLSQPTPRPRRSGRLGRAGPI
ESTK*
>SPBDM4_v1_40354|ID:27157659| Sodium/hydrogen exchanger [Uncultured spirochete bdmA 4]
MSPKKWLITSTIFLVCALPVFAQAETIDQTTQLTQFIFQLAEIIFAARLGGMAASAISLP
SVLGELVVGIIIGPYALGAIRLPGFPSGFFPMNGGEPISIILQAFSNIAAIILLFSSGLE
TDLDMLMRYSVAGSIVGLGGVIFSFGFGLVLGSFFTHQALFAPVNLFIGIISTATSVGIT
ARILSDRKKMDSPEGVTILAAAVFDDVLGIIFLAIVLGIVAVIEGKKSGSLSAGAILGIS
AKAFGLWLSFTAVGLVFSKQISAFLKKIGGENHFNVLALGLAFFIAGIFEMQGLSMIIGA
YIVGLSLSKTEISFVIQDKTKVFYELFVPVFFVVMGMQVQISKIFEPSVLMFGLAFTVVA
ILAKVLGCGLPSLFLGFNKIGALRIGVGMIPRGEVALIIAGIGLSSKILSSEIFSASILM
TILTTICAPILLNFIIDRGGRGTVKEAKSATSEEFVVDLPNNDLASLLSAYLLEDLQREG
FYVQLMSISDNISHIRKGDISLSLRVEGTKLVLETAPEDMPFVRTLVYETMVKLDANLDK
LKETYDPAKMREEVTAIEGSRKDTSFLKILAPECIKIDMKASTKEEAIDELVGLLDSVHK
LKDKDKVLADILERESIMSTGMQHGIAIPHARTTGVDKPVLAIGIHKQGIDFQTIDGTPA
HIIALIASSEGDPAPHMQVLTSLGSTLGSDIVREELLHAKTREEAAKVLGL*
>SPBDM4_v1_40355|ID:27157660| putative Periplasmic/7TM domain sensor diguanylate cyclase [Uncultured spirochete bdmA 4]
MEIIEKSNIHRWMFFVYTILQMVRKSKGFIFLFFMWIVFICPVGISAQEQIPPIINLEQI
SDYYFDADPALGIGEVSSPEFSSHFSPVGGRPIWTGKHAPASWLRFTFPLDRLGPGYEVG
SDIEDRAIQWLLLVKPSFSIILDNIELYVPRPDGGFDRFVSGAQVKSNPAEPHSRYYFFS
LPADAFKEKPCYLRISSSTDVLMSVELVNASLFAKNQAQSYLGYGLIFGIIIAMIIYSLF
LLLSFHYSSYIYFIFYNISIGLWLFYVQGFAKAVFGQMPGFDQAMLWVWVGQFITWGTIF
TISFLELQKRSRVLFYILAIAAVLGGLVSLAGIAGLNEIAFTMSHYLGIIVPVLVIFAAV
LQVKRGYRVSRYFLIAWCSLASGGLMFSLMGLKVLPVNVLTRNAMSIGLAFESIFLAMAL
ADQFKNLEVEKKKLEAAQDHFRELSLTDTLTGLRNRRYLMMELGQAMKEAFQNREPLSLI
MLGIDDFKLVNDRFGHEVGDDILVSLAHSVRSCTRETDSACHYGGDALVIFMPKVAKQDA
LRVAERIRTHFETESLRVINGETLGATISLGIVELAGEDTIDTFLSRADAAMYKAKSRGK
NCSVVM*
>SPBDM4_v1_40356|ID:27157661| conserved exported protein of unknown function [Uncultured spirochete bdmA 4]
VKKKLLVSAIIVGFVAVMFIPAQGTLPLYGNSAAKADNALTIESMLTYAIQDEYLARAEY

VAIMAKFGQMPPYSNIKQAEEQHIAWLKDIFASLGLAIPADEAAKYVHVPSTLKEAAQTG
VQAEIDNISMYDRFLAQPVLRDPRYAGIADLFTRLRNASSNHLAAFQKQLQKY*
>SPBDM4_v1_40357|ID:27157662| putative GGDEF domain protein [Uncultured spirochete bdmA 4]
MSIDALRMILYVASAAIGLLVIYSVIRQKSHLARYFFFVALCSFLYIFGYASELGSRSLA
NIRFWLTFEYFGLAFISSFWFLLSWKLWYNHNPRFRMIVVVFVIPAITLFLVATQEYHGF
FYKSRSLAEVEGHYLVVIEKGPWYWVQIIYLFSFILMSFILQWRAWRREGGAYATDSFWI
LLGTLNLVPWILVYQLGFSPYNIDLGPFGIAVATFFIAIAVFRFGALGSEEVLLHSIFAS
IDDAVMVLDQEGKISEFNTAAQRIFPWLDAACVGKPVSNPNDASLFNVAQNQKIEKMVEL
SDSRRYYQGKFTQIYEGRSKLGRIYVFRDTTESRLLMKRLRKFANSDVLTSLYNRRRFME
RAEKALYRAQKTQENIAMLMIDVDHFKNVNDRFGHVVGDKVLSAVGRVIRIRSRELGFAG
RYGGEEFVVLLQNVQRQKAMEVAEGIRKSIEGIAIEKRMISVKVTVSIGVSRCKAGESTY
NIDELLMSADRALYQAKSRGRNRVEE*
>SPBDM4_v1_40358|ID:27157663| Transcriptional regulator, RpiR family [Uncultured spirochete bdmA 4]
MSQGALDTIVAAMPTLAEKERKIAAFIQASPQKALHLNISELARQAGTSPAAVVRFCKHI
GAGKYTDFKIWLAKDVYQGWGEKYLPDLELESQTPGARALHDMTEAVRRTMSALSQTLDP
RAVEEAAERIRAAGMIAIFGVGASGLVASDLHQKLTRIGIPSSYLFDTHAQIAAACSLHP
HDIAFIVSYSGETDEMIEVAAQAKARGSFLIAMTMTGPNRLRESSDLILEIPSVERIYRS
AAELSRISQLAAIDILFNVLISHDLDSAIAALERSMQATHRF*
>SPBDM4_v1_40359|ID:27157664|yfeU| putative PTS component; possibly regulatory [Uncultured spirochete bdmA 4]
MDIHAIDDYLEHATTESRNARSANIDALPVIEILRIINEEDKGVPFAIEKALPDIAALVE
DIVRAFNDGGRLVYIGAGTSGRLGVLDASECPPTFGVQPGMVVGLIAGGDAALRNSIEGA
EDDPEGGVRALRDINFSARDVLVGITASGSAPYVLGAMQYAKDLGAAVGAISCNRESKTF
DIVRHKILLEVGPEVITGSTRMKAGTAQKLVLNMLTTTSMIRIGKVYGNLMVDLMPVNRK
LVERSKRLIRQATGCTKDEAEAVFLASGGKPKIAILMIALGIGAAQANALIQRGQGRLSE
AMRMYSTEKKSK*
>SPBDM4_v1_40360|ID:27157665| Extracellular solute-binding protein family 1 [Uncultured spirochete bdmA 4]
MMKKLAIALLLVAMLSPLCAQQKTVSIWGWRAQDVDVWKGVEAALKAKGDTITIKYEVFP
PTEYDSKLLVSLQGGVGPDIMYTRRLPGARTQALLDNGYLTALNGLVDLSNFDAVSLSFI
SANNKVWGVPFANQIIGIFYNKAIFDKYKLKEPQTWDELVQIAETLTKNGVTPFFVSGKE
AWTLAMQNAMVGVSYPGDAWIGKLAAGKAKFTDPEYVNMLKDLNALKKYYQKDFMANTTA
EQDVAFAMEQVAMIFYGVWGNTNWLKTNPDFKFDYFPIPPKDKNIPAKAYTYMDGAYGLN
NTSKNKDTALKVLNYTGTKIYGELFSSTTGEITALKGVTMPASKPILVKCYDKMVNIAAT
NRYWVGSPFDAGTPSVYNILQEDMQSMYLDQMTPEALAKRLQDGISTWYPAFKK*
>SPBDM4_v1_40361|ID:27157666| Permease component of ABC-type sugar transporter [Uncultured spirochete bdmA 4]
VNMSAPRPILSSHRGALISHESEVVVRKYSFLWMLFPALLLYTLFIAYPFFSSLGLSFYD
WPGIGPKKFIGLDNYKNILSGFMASEFYRALWHNIVFFIWSLILSVVPGLFFAFLLAANI
KGTKWLKIIFFFPNTLAIVVVGFLWGLLLNPQWGLVNQLFRVIGLGFLAKPWLGDTKTAL
PTIIFVTAWRGLGFYILVYLAAIIGVDREMTEAARIDGATELQIAGRIILPHLYPIIATT
SMLKFIWTFNIFDIVYAMEGTQGGPAGSTDVLGTLFYRIAFGGLGSSQVGMGLGATVVTL
IFFIVLPVSIFYVFIVEKRVERGE*
>SPBDM4_v1_40362|ID:27157667| ABC-type sugar transport system, permease component [Uncultured spirochete bdmA 4]
MMFRLSRFQKVIVWTFLLLLAFVNLYPIGIMILSSFKSTREIFLKPFSLPAVWRWQNYVS
AWQRADFATYFKNSILVTAVSIASIILVSSMAAYVIARFDFKFKRGIYLYLLAGLALPTR
LAIIPIFLIMRDLHLLDKLSGLIIVYTAGGIAFSVFLLVNFFKKLPRDIEDSAKIDGAGA
FRIYWQIDLPLLKPALVTVAMFNFIDVWNDFFFPLILLSSRAKKTIPLGLQAFFGEYTIE
WDVLFAALNISVLPVLIIFLILSKQFIAGMTEGALK*
>SPBDM4_v1_40363|ID:27157668| conserved protein of unknown function [Uncultured spirochete bdmA 4]
MYAGYCYSCLPYAQMIAYIMSQQNQQQNSVVIGLEHFLDDPKRQNKRYGLATNPAAITSR
GIPSWKAFIEHSLGPACFFGPEHGFRGAAQDAVQLDDENFKGIPAYSLYGKRLKPERWML
EPLDAVVFDMQDVGCRYYTFLYTLAYIMEVCEKVGTPVIVLDRPNPIDGVKVEGSPISSH
IESFVGGYGLPPRYGMTIGEFALYLKGEYYPGVPLEVVRLSGWDSRQAWAETGLPWPLPS

PNLPTLSCAELYPGTCLAEGTWLSEGRGTSRPFEIIGAPFIDGEDLREQLSALNLSGVVF
MSLFFTPNASKHQGELCEGVLLSVIDRHAVRSLDTGIAVIHTIHRMYPHEFRWREDWDDP
ALTFFDKLAGGTELRYMIGKGASLDECLAFEHQGEDKFLALRGKYLLYGEGKR*
>SPBDM4_v1_40364|ID:27157669| putative Beta-lactamase [Uncultured spirochete bdmA 4]
MPSLTPLEALLEEAISQNLCTGAAGAVVVEGKTLWSYCSGKTGEEETPLQEGEGIEVLAR
TSPAPITFQTRFDAASLTKPLSTALLTLKALQAGALNLDDTIKKFLPALNQRTGEIPLIA
LLTHTGGMPAIPALQRYFPDPAHLDRDVAFSRLFSIEPESPAGRHVMYSCTGYMLLGAVL
ERISGMLIGRLFQQEIAGPLNLPRATFAQGTLASGEPVPLENTVETEFCPWRKKRMRGQV
HDESAYCLGGHSGNAGLFLSLEDALILGNLILQEGAHNGKQIFKPEIMQLLQREHTRGLE
ERRTIGFRLHDNDTADGPLWPAQAFGHTGFTGTSIFFEPKRRLLAILFTNRVYYGRDATA
DAIVAFRRAFHTKVWHVFS*
>SPBDM4_v1_40365|ID:27157670| conserved protein of unknown function [Uncultured spirochete bdmA 4]
MNQQWIFGIDGGGTSTRLRVEGLARELLYQAEGPSLNPRSVGWNGSRKTLEGLFSAMYEK
TGLDTDNCMSGFAGVAGIDRSEDTGTMRSIIRDAAELGPDTNVEVRNDSIPALAGAFGEL
RGILLIAGTGSIAVGADGAGRIIRSGGWGHILGDEGSAYWVGLRALNAATRFHDRRSPQT
DLLGRALAYFGEKEPFSLIPAVYEPFDKSKIAAFARIVAEERDRGDEVAEQIFKDASEEL
ALLVISVATRLGEAQAGGRIAFAGGFLANNERLWHDTEARILAVLPRHRIVLPEADAANG
ACLLALINAGQKSRN*
>SPBDM4_v1_40366|ID:27157671| conserved membrane protein of unknown function [Uncultured spirochete bdmA 4]
MKQRQPAATLFGVIVAVIWGMTFLSIKVALREFGPMSLSLFRFIIASVLIALVMLITRVD
FRVAWRDVPLLSLSSLVGVTLYFYFENNGILRLTASESSIIVGAIPVVTLLGEIVLYRVR
PHRLVILGIVLSFLGVALIVVRSESATGSPLGYLFMGGAVLSWAAYGFITKPLSGRYPML
TITFWQMVIGAIGCIPFAIVERQVWTGLSATALLNAAYLGIFGSAIGYWLYVIVLEHLGP
GRSSVFINLIPVVSVAASYVVLGERLNPLQIIGAIIAITGVYLASSGI*
>SPBDM4_v1_40367|ID:27157672|hutH| Histidine ammonia-lyase [Uncultured spirochete bdmA 4]
MNEVVIDGCSLSIAELVAVARRRILVRLDPDAEERMLASRKVVELAVKKKQIKYGITTGF
GKFCNVIISEEDNALLQKNLIMSHACGQGEPFAAEIVRAMMVLRANALAVGNSGVRPLVV
QHILRLVNADVVPVVPEKGSLGASGDLAPLAHIALVLIGMGEAVYKGNRMAGALALAEAH
IPPLVLEPKEGLALINGTQAMTAIGALAVYDALMLYRSATIASAMTFQALRGITEALDPR
IHELRRQKAQIEAAKDLRRLLEDSLLTTKPSGTRVQDAYALRCTPQVHGACLAAIQHVWD
VIAAELNAVTDNPLIFPHVLREGGDILSGGNFHGEPLALALDYLGIAASELADISERRLE
RMVNPALSEGLPAFLTINGGLNSGFMIVQYSAASLVSENKVLSHPASVDSIPSSANQEDH
VSMGTIAARKARQIIDNAGRVAAMETLAACQALDLRAIQLGLPTIENSLSPATIPIYRTV
RAAVRFTDQDRTMYPDIDAVVSLLNDKTLAQGMDRWSWNPEPVAPCED*
>SPBDM4_v1_40368|ID:27157673| conserved protein of unknown function [Uncultured spirochete bdmA 4]
MVTKRDHGIHLDRTSQQERSRLISYINIKLQSLGLPVYSKEGTGFVQLAADMLESFRQKD
RLLPRILPPADQRIQNFIDRYLADLGLARVPQMPANTLVLDRYGMARELSLPPDGHKHVS
PTLTSYRVRNGVLHNPKNDRRTTEGVFHIVEGGLPIPPDKKVVPKLTFAKILEVALNPPA
ELLEVPFTAGENDKAHTFVSVLLRPIVRPEIPGYCEERSMEVRFFAPGSLVANVDFVESI
FGNSGDPFIPENDASIDPVHWTGTTGCIILATHLTEITKKEVGLPHWDQATERQRRDGMC
WREPTEKYNDGRAFKLTARDESGVIITVIADNYFGYSKKEIKSHISYSANLLGLGEEEHA
GGALVFPSYNLGTRFVPDTNLKSKGHSLHDVYTIMRGRIEMKDEGYAVDLTFPNIIYLPD
DAYISLEDQKAHWVSNGREESLRTLPGNIYVHPTGYRVHMERHPASGAWRLIGASAEGLL
CHKPCTVSGGGKSEIAKSIHDAITYMPIIIADFEKDMKAVKEIIENDYGNRFRDESENHG
KDARPVLSPKRSLGSVIKLLSPSPAYNDEYNEWLRSIPERIKSLVFLVKRFYQPDWGDTW
ASHFSVDAVNGTTGNILQFEGRPIMGSYLRVGKSEKGMNRFFKLRQDFMPAFKLQWEDDI
SASIIVPVDQLEDLPEWASNHLSLKFAKNCEARFFQRPDDAIIRGYDKQTEKDLSRQDNY
ISNFEPLTRSDAAHIIENTVHFYEYTDPMRKFIEETEKDPNYEYFVASNCFRLVDGAPSK
NPRYLQLDPTYVNPEARYLAELGPRLYRRIPADKPLPQPIGAVLPGRRNNPPDPKAGIRS
LAVYGPIHYQELPELFMDFVCSLTGKSPSTTGAGSEGALTKGPFNALVPTSDLNNALLGF
ILTGYSGFTTAAGHIGHKYKMDHDISLMMPELWARLGPEERDPEFLKSHGYLEKVDDFKY
KGKLIPASRLGWRITPLFAATYLGRLFDTPSVVFPDDMLRPELQSLEEFVDGIENIAAAM
EKSAKAYFEDGSYEAAIPPLKAVLSVMAYGNYEGKSIQDPEVRKFFDREYVLASDWYKAR

LEHYREHEIAYIESSISYLRKFLAERAEPKSLTERRVQAELSSAHARLEQLMAPNYLKRI
WGSIGLDPLYKGQAKV*
>SPBDM4_v1_40369|ID:27157674| Dopamine beta hydroxylase-related protein, containing doMON domain (fragment) [Uncultured spirochete bdmA 4]
MKMKAFSTALVLLVLASVPAVFAVNVDGTIASGEYSKQAVFDKGNFKLLWEFEGDKVFMA
IVAKTPGWVAIGFNPSTVMAKSDMVLGLVKDQSDVQAVDEWSSGIFGPHAPDVDKGGKSD
ILSYAGTRTDDTVVFEFSRLLNTGDQLDKVIPTSGKFKVIWAYGPSLDVTAKHKKAGSAT
IQMEGQK*
>SPBDM4_v1_40370|ID:27157675| membrane protein of unknown function [Uncultured spirochete bdmA 4]
VSLLPFHVVSMGLAFLSMISAIVIARYFKTKKWWLKAHRVLNIGAVIFAVAGLVFALLMV
SSTGGPHIRVPHAVIGAVTLVLLVVMPFLGSAIFKTKDKKAIQNLKKTHRHMGRITALML
TVTVAAGLTLAGIL*
>SPBDM4_v1_40371|ID:27157676|hutI| Imidazolonepropionase [Uncultured spirochete bdmA 4]
MQEKNTTSYFIHNINELWTPEGQRAAAGAAMQKIKKVHGACIYVKDGRIAWVGEEARLRA
SDAVIAREALSATFYNAEGRSCVPGFVDSHTHFIFAGWREDEFFWRAQGLPYMELHRRGG
GIQNSVQATRGASLEDLILLGRKRLATMLSLGITTVEGKSGYGLDVETEIRQLLAMRALS
RAGAPDIIPTFLGAHSVPPEFKGRPRAYLSFLAEEVLPLVKSEKLAHFVDIFCEEGVFGI
EDSRWYLERARAAGFALKLHADEVDPLGGTALAAELGAISADHLLKASNDHIAALAASGT
IACLLPLTAFNLREPCASGRRFIDAGCAVALASDLNPGSCYSQSIPLIFALAVLYMDMTI
EEALTALTLNGAAALELAPRTGTLEPGKDADFLLLDAPSPAHLAYHAGMNLVHTVFKRGA
SVWQNGNMLEMEKMQSAGLSLS*
>SPBDM4_v1_40372|ID:27157677|rplQ| 50S ribosomal subunit protein L17 [Uncultured spirochete bdmA 4]
MKHKIGYNKLNRVAAHRKALIRNMVTVLLKEEKIVTTKAKAIEARRAAEKLITRAKVDSV
HNRRIASARLYGEDAVAKLFTEIGPRFKERGGGYTRILKLGDRANDAASLVVLELVDQKA
DEKRAEREKRKAQRKAKKQA*
>SPBDM4_v1_40373|ID:27157678|rpoA| DNA-directed RNA polymerase subunit alpha [Uncultured spirochete bdmA 4]
MARKSLLKGFKRPKGITYEQSESTDTYGKFVAYPFEPGYGTTVGNTLRRILLSSIQGYAI
TAVRITRYDDEGVPHVITSEFETIPGVVEDTIEVLNNLKKLRLRLPNDKEELTLDYEIRG
IEELRGSFFAEQGSLVVLNPDLKIASFEPSVHLGLELQVNLGRGYVPSEVNEKYIDVVGT
IPMDAIFTPVTRVKYSIEPTRVGQRSDYDKLILEISTDGTIRPEDALGEAGKIAKDHFSI
FVNFDENEAGGEETSDEIEERVRQILNMPVEELELSVRSFNCLKNANIRTIGDLTKKTEE
EITKTRNFGKNSLQEIKTKLAEWNLTLGMADYSALRNHIKLNMKKEESDEA*
>SPBDM4_v1_40374|ID:27157679|rpsD| 30S ribosomal subunit protein S4 [Uncultured spirochete bdmA 4]
MARYTGPVCRLCRAEGRKLFLKGERCRSDKCPINKKRLPPGKAPKTRIGKKSEYAVQLRE
KQTLKRNYGLMERQFRNTFEKALGLPGKTGDNLIILLERRLDNVVYRLHFATSRAQARQL
VNHGHVAVNGKRVNIASFLVKAGDVVSVIGAAKKMDSIRQALEEVQKSGVMPWLEVNADE
MTGRYVTNPQRQDVTDTLDIKEQLVVELYSK*
>SPBDM4_v1_40375|ID:27157680|rpsK| 30S ribosomal subunit protein S11 [Uncultured spirochete bdmA 4]
VAASTTKTKKRKEKKSVYEGNVYIQATFNNTIVTVTDLNGNAISWSSSGSHEFRGAKKST
PFAAQTVTEAAVQKAMQAGLREVHVYVKGPGIGRESAIRQLGVMGLKVKSINDVTPIPHN
GCRPRKARRI*
>SPBDM4_v1_40376|ID:27157681|rpsM| 30S ribosomal subunit protein S13 [Uncultured spirochete bdmA 4]
MARIAGVDLPNKHVDIALTYIYGIGRSSALQICETTGIDPDKRMNDLTNEEINELRKVIE
SDYKVEGRLRTEIALNIKRLMDIGCYRGLRHRRGLPVRGQRTRTNARTRKGKRKTVANKK
KAV*
>SPBDM4_v1_40377|ID:27157682|secY| preprotein translocase membrane subunit [Uncultured spirochete bdmA 4]
MAGTLVDIFRVKELKDRIFFTLFWLAVFRLGTFLPIPGINASALQSYFANQSTANTGITD
YLDFFAGGAFKNFSLFMMGVMPYISAQIIMQLLIVIFPKLKSVAEEEGGRRKISRWTRIG
TIVIAVIQSFSVTVWADRIPNAIAMSNRTLYTFVAILTVTTGTMFLVWIGEQITKRGIGN
GVSLLIFAGIVARLPNATFELIKKIQIGEINAVYAIVVLIMFVAVVALVVLEQQGQRKIP
VNYAKRIVGRRMYGAQNTYIPFKINPSGVIPVIFASSILTFPLQIAQSFGVQAKWLRDFS
YWLRPNGFWYNFLYVVFIIFFAYFYTQVTLNPQEISKNIRENGGSIPGIRSDKMEEYLTK
VLNRIILPGALYLGLIALIPTLVQRLFNFPSQMAYLLGGTSLLILVGVDLDTMAQVEAIL

KMHHHDGLVRKGHIRSRNL*
>SPBDM4_v1_40378|ID:27157683|rplO| 50S ribosomal subunit protein L15 [Uncultured spirochete bdmA 4]
MADFNLHAPEGANRKKHIVGRGDGSGSGGTAGRGNKGQQSRSGGKTYPGFEGGQMPLYRR
LPSRGFSNYPFRKEYQVVNLRDIEGKFQAGDTVDTVSLFMKGLVRKPELPVKVLGEGQVS
VALTVSVDAVSSSAKAKIEAAGGKVEVPAATGSSADA*
>SPBDM4_v1_40379|ID:27157684|rpmD| 50S ribosomal subunit protein L30 [Uncultured spirochete bdmA 4]
MARIQITLVRSTIGQKPKARATVRCLGLRKIGSSTIQEASPQILGMVNKVRHLVEAKEVE
*
>SPBDM4_v1_40380|ID:27157685|rpsE| 30S ribosomal subunit protein S5 [Uncultured spirochete bdmA 4]
VERTRRDDSGVQDGYTEKLIQLNRTAKVVKGGRRFSFSALTVVGDKQGHVGFGFGKANDV
TEAIRKSVERAKQAMVTVPVKKGTLPHEVTGKYKATTVYIKPACPGSGVIAGGPVRAILE
CAGYVDIISKCVGSRTSVNVVRAVFSGLGQLLDAQEVAKNRGKALKDMWS*
>SPBDM4_v1_40381|ID:27157686|rplR| 50S ribosomal protein L18 [Uncultured spirochete bdmA 4]
MSVRELSDKIRKREKRRTHIRKTITGAAEKPRMSVFRSNLHLYVQVIDDMSGHTLASVST
MEQQFRSLKPTIQTGSMIGEELGKRLLEKGITQVVFDRNGYKYHGIVKAIADGVRKAGIK
F*
>SPBDM4_v1_40382|ID:27157687|rplF| 50S ribosomal protein L6 [Uncultured spirochete bdmA 4]
MSRIGTRPVVIPQGVKVTVEPTVFHVEGPKGRLSQEYRPEVQIAVKDGIAVVERKNDSKQ
AKAYHGLYRSLLDNMVTGVSQGFKRALVVSGVGYRVEVQGNLLVLSIGYSTDVVAVIPEG
LKVSVEQNGQKIVIEGIDYAKVGKFASEVRSLREPEPYKGKGIRYEEEKIRRKVGKTGVK
*
>SPBDM4_v1_40383|ID:27157688|rpsH| 30S ribosomal subunit protein S8 [Uncultured spirochete bdmA 4]
MSVSDPIADMLTKIRNANVARHEKVDIPTSKLKLEIVKILKTEGYIKNFKKIQTDGQNFI
RIFLKYDEENSPIIHGLSKVSKPGRRVYTGYREMPRIFNGYGIMIISTSDGVITGRKAVE
KQVGGELICTVW*
>SPBDM4_v1_40384|ID:27157689|rplE| 50S ribosomal subunit protein L5 [Uncultured spirochete bdmA 4]
MAQTKVYIPRLKKLYREKVSPELFSELGYASKMQVPRLVKVIVSMGVGEARENKKMLDAA
ITDIETITGQHAVKTKAKKSIANFKIRKDQEIGARVTLRGDQMWEFLDRLMNVALPRVKD
FRGVNPNGFDGHGNYTLGLTEQIIFPEIDFDKIEKVMGLSVSIVTTAETDMEAKSLLAKL
GMPFRK*
>SPBDM4_v1_40385|ID:27157690|rplX| 50S ribosomal protein L24 [Uncultured spirochete bdmA 4]
MANAKYKLRKEDLVQVVAGKDRGKQGKILRIDHEKSRVIVSGINMVKKAMKKKSQTDRGG
IVEIEAPVHISNLMIVCKKCNKPVRAGYKIIDGQKKRVCRKCGEVL*
>SPBDM4_v1_40386|ID:27157691|rplN| 50S ribosomal protein L14 [Uncultured spirochete bdmA 4]
MIQVESMLNVADNSGAKTVQCIKVLGGTRRRYASVGDTIVVAVKTALPNSAIRKGSVEKA
VVVRTHKEYKRPDGSYIRFDDNACVIIDANKNPKGKRIFGPVARELREKDYMKIISLAPE
VL*
>SPBDM4_v1_40387|ID:27157692| protein of unknown function [Uncultured spirochete bdmA 4]
MCFVVLVVGFYTLRPIHILSVKGMELAHDNFDNDGFIILVRNYDALKNKLALSHFLDIRI
HLPSPEELCLLRGAAALGLGDPRFDPRDLAAKISLLERILDVPDHHLESKVKIFLSKLIA
LDEHLVQLYIFK*
>SPBDM4_v1_40388|ID:27157693|rplP| 50S ribosomal subunit protein L16 [Uncultured spirochete bdmA 4]
MLLPKRVKHRKQQRGRIHGEAHSGNAIAFGDYGLVSLEPHWITNRQIEAARIALNRYIKR
GGKMWIRIFPDKPYSKKPAETRMGRGKPGPEYWVAVVKPGTVLFELSGVEPKVAEEAMRL
AGSKLPVKTKFAVREEME*
>SPBDM4_v1_40389|ID:27157694|rpsC| 30S ribosomal subunit protein S3 [Uncultured spirochete bdmA 4]
VGQKVNPIGLRIGINKDWSSHWYVEPREYAKTLHEDLAIRRRLLDLPETKGADISEIEII
RHPQRVTIVIHSARPGVLIGTKGVNIDKIGAEIQKITQRKLQIKIKEIKKIDTNSQIVAQ
NIARQLENRQSFRRSMKSAIQNALKAGAQGCKVRLSGRLGGADMSRTEELKEGRIPLHTL
RADIDYGFAEADTTFGKIGVKVWIYQGMVYAQDKNEDAGQLAKRARREPAGEARGEHREG
GQERHERRERGERGERRATPRPEADRAEGEARS*
>SPBDM4_v1_40390|ID:27157695|rplV| 50S ribosomal subunit protein L22 [Uncultured spirochete bdmA 4]
MANTKTGFRATSKWLIASPFKVRPVADLVRAKPYTEAMAILENMPHKGAKLIRKVVSSAA
ANALNKNRKLGEDMLYIKELRIDEGPRLKRIWFRARGRADMQLRRMCHISCIVDEIGKKT

EA*
>SPBDM4_v1_40391|ID:27157696|rpsS| 30S ribosomal subunit protein S19 [Uncultured spirochete bdmA 4]
VSRSVKKGPFIEKSLYKKVTEMSRAGDKKLIKTYSRTSTIIPEMVGQTISVYNGKTWVPV
YVTENLVGHKLGEFAVTRIFRGHAGSDKKAEKK*
>SPBDM4_v1_40392|ID:27157697|rplB| 50S ribosomal subunit protein L2 [Uncultured spirochete bdmA 4]
MAIKTFRPVTPGLRHRVQVISGELTSNESQPVKSLTVGKRSTGGRASNGRISVRHHGGGH
KRKYRIIDFKRDKFGVPGKVSGIEYDPNRSANIALITYVDGEKRYILSPKGLRVGQTILS
GPEAAPEVGNALPLEKIPVGFTVHNVELTLGRGGQLARSAGVSALIAAKEGDYVVLKLPS
GEQRMVFKKCMATVGQVGNDEHMNERIGKAGRTRWLGIRPTVRGTVMNPVDHPHGGGEGK
GKGYKQPVSPWGQPAKGYKTRDTHKPSGRFIVKRRK*
>SPBDM4_v1_40393|ID:27157698|rplW| 50S ribosomal protein L23 [Uncultured spirochete bdmA 4]
MEYDAILIEPVLSEKSNIMREFGQYIFKVDSRATKPMIMEAVAKTFNVHPVSCKVINVTS
KPKRLRGRPGRTAEWKKAIVRLQKGETIRVFEGA*
>SPBDM4_v1_40394|ID:27157699|rplD| 50S ribosomal protein L4 [Uncultured spirochete bdmA 4]
MEGKVISLSGKEPRKIELSDAVFGLPINEDVIWYAVNNELANARVGTASTKDRGEVHGTN
RKPFAQKGGGRSRHGDLKSNIYVGGGVSFGPKPRDYSYSIPRKEKQLSLKTILSLKVQDG
LVSIIEDFTVESGKTKDLLKVLANVVDLKNPERTVLILKDDDPMVKRAARNIPWLGYLTY
NRLRAHDLFYARRVVMLEGASAKLNEFYAGVTKES*
>SPBDM4_v1_40395|ID:27157700|rplC| 50S ribosomal subunit protein L3 [Uncultured spirochete bdmA 4]
MIGVIGKKVGMTQIFDETGRVVPVTVVQVVPNVVVSKKTAEKDGYNAVVVGVYEKKKSRV
TKPYAGQFPEGLAPTKIFREFRDFEKEVEVGQAIDASVLDGVRFVDVVARSKGKGFQGVV
KRWGFEGGRATHGSKFHREPGSTGNSTYPHHTFKNVKMPGHMGNERVTVQNLKVIRVDAE
KGVVLIRGALPGPRNCDVVIRKSVKKS*
>SPBDM4_v1_40396|ID:27157701|rpsJ| 30S ribosomal subunit protein S10 [Uncultured spirochete bdmA 4]
MKDRIRVRLRGFDVRLVDDSAKSIIKTVQNAGSKVSGPVPLPTRINKFTVLRSPHVHKKS
REQFEMRTHKRLIDIIEPTPEVMDALMKLELPSGIDVEIKQ*
>SPBDM4_v1_40397|ID:27157702|tufA| protein chain elongation factor EF-Tu (duplicate of tufB) [Uncultured spirochete bdmA 4]
MAKEKFERTKPHMNVGTIGHIDHGKTTLSAAITMYCGRKFGDKVLKYEDIDNAPEEKARG
ITINTRHLEYQSAKRHYAHIDCPGHADYIKNMITGAAQMDGAILVVSAPDSVMPQTREHV
LLARQVGVPAILVYLNKVDLVDDPELLDIVEEEVRDLLNFYGFPGDKTPIIRGSAFKAMT
EPDNPEATKSIQELLDAMDSFFPDPERAIDKPFLMPIEDVFSISGRGTVVTGRVDRGVIH
VNDQVEIVGIKPTKTTVVTGVEMFNKLLDEGQAGDNIGTLLRGIDKKEVERGQVLAKPGT
ITPHTQFKGQIYCLSKEEGGRHSPFFSGYRPQFYFRTTDITGTVKLPEGKDMVMPGDNSE
IEVELIYPIAMEKGLKFAIREGGHTVASGQVIEVIA*
>SPBDM4_v1_40398|ID:27157703| protein of unknown function [Uncultured spirochete bdmA 4]
MNKLQRPSGNINATGCYAINYLFGLSDYKQRIKIAQ*
>SPBDM4_v1_40399|ID:27157704| putative Elongation factor G 2 [Uncultured spirochete bdmA 4]
LARRKYTDIVMDSAHASGSGPSFPIRNIGILAHIDAGKTSITERVLHVAGVREQAGNVDE
GTTATDYLSVERQHGITIKTAAVRFLHKGVRFHLLDTPGHVDFSAEVDRVLRVLDGAVIV
LCAVSGVQVRTGFIASGCRAHKIPRMYFINKMDRRGADFFGALQEIAGELGEPVLPLQIP
TFEDYLWNGIADLVEMCWYPSSLDRTEARSAAESDIADAPETRALPIEKAPLTEESRRLI
AEYREKLLDRASMYDDNLMEKIVDGKAVSSGEIRAALRAPIHRQELAPALCGSSFSDISS
ALLLDATIDYLPDPLVRGCPPAKDVKSAKEVRLKAESGAPFSAYIFKTVLSADLNALAWI
RVWSGTIREGMKVMAMPAKKLTHIQHLYGINGAEIEPVQDAAAGEIVGARLSYMEAGGTL
CEPGLQIVYESFKNPEPLVSIAVEPASQQELSKLRDALKRFSLEDVSLLIDEEKETGRIT
ISGQGELHLDIVLERLQKEYGLRVRAGNPQVPKVERLKKSVRFGDEFVGDFGGEKLSVAL
RVSLENQKDKDENSLLLASGIRMSLNYETSIRRAMETVLAVGPTGGWPVIGVKAVIESFV
PPWSTDKRAETAVEAAASSILRRAVSLAGSFVLVPIVQISVEVPDTWFGAALATLQARGA
RIESVEDAGGIKSIIAYAPMENLFGYATSLRSLTEGRGIYQAKFDHYGSALEY*
>SPBDM4_v1_40400|ID:27157705|rpsG| 30S ribosomal subunit protein S7 [Uncultured spirochete bdmA 4]
MGRKKKSIDRGHAPDVKYNSTTISRFICRMMWQGKKSVCTGIMYDALELLQQRSGQPALD
AFSKALENVKPTIEVKSRRVGGATYQVPIEIRESRREALAMRWIIGAARARTGKPMSECL
ADELFDAFNSTGTAIKKKDDMHKMAEANKAFAHYRW*

>SPBDM4_v1_40401|ID:27157706|rpoC| RNA polymerase, beta prime subunit [Uncultured spirochete bdmA 4]
MRDIQDFDNISIHLASPEMIRAWSYGEVKKPETINYRTLRPERDGLFCERIFGTTKEWEC
YCGKFKSIRYKGVICDRCGVEVTNSKVRRERMGHIELASPVSHIWFYRSVPSRMGLLLNL
PMATLKSVLYYEKYIVIDPGETDLKKMQLLTDEEYREAMDRYGGAFTAGMGAEAIRSLLE
QIDLDRLSQELRQKMIERGAKSDKRLLKRIELVESFRSSGNRPEWMILTVIPVIPPELRP
MVQLDGGRFATSDLNDLYRRVINRNNRLKKLLQLNAPDIIIFNEKRMLQEAVDALFDNTR
RRRAVKGAGNRPLKSLADMLKGKQGRFRQNLLGKRVDYSGRSVIVVGPELKMWQCGLPTK
MALELFKPFIMKKLVEKDVVYNIKKAKMLVESETPEVFAVLDEVVKEHPVLLNRAPTLHR
LGIQAFEPLLVEGKAIKLHPLVCKAYNADFDGDQMAVHVPLTHAAQAECWTLMLSSRNLL
DPASGKTIVYPSQDMVLGINFLSRHRPGVRGEGKRYSTSDELLLACEYGSCDYQALVYVP
VPKEPVWDGYHKLPVLNDSGLIKTTAGRILLNEAMPKEVPYINYALTDKDIRLLIEHVYR
ANGPFITVNMLDTIKEMGFKYATFFGATIGMDDIVIPKEKTEMIEKANKEVEAIQQQYLA
GHITQDERYNRVVEVWSKTNEELTAVMMKTLEKDRDGFNNIYMMAHSGARGSKNQIRQLA
GMRGLMAKPSGDIIELPIRSNFREGLSVIEFFISTNGARKGLADTALKTADAGYLTRRLV
DIAQDVVVNDDDCGTINGAVHTAIKDGDEIIESLRERIIGRFTLDPIRHPITGQIIVGSN
EEITEEVAEQIETSGIEEVSVRTVLTCEAEHGVCRKCYGRNLATGKTVEIGEAVGIIAAQ
SIGQPGTQLTMRTFHIGGAATKAAEENRISLHYSVYIAEIEGSYVKTDPGHLLFTRRGYI
YVNRVFGAFDLAQGDVLLVEDGQRVMKDDPVIRITDGTEVKSPSISYAKVLEGKVLLIAQ
EQKVEIRNGSDMVVTRGQILLANETIATFDPFNDPIISEHDGIVRYEDIVPGSTLKEEIN
AETGNVEKIITDVGSERDTKEPRIIVTDEAGNDIETYYLPEGAYVNVEDGENVHAGKILA
KILKESAKAMDITGGLPRVGELFEARKPKTPAVLAMVSGTVSFGGSLKGKRVVNIRDSFG
KTYKHLVAKNRRLLVRNGDKVVAGELLCDGSKNPHDILAILGEQACQRYLMDEVQQVYRA
QGVTINDKHIGVIIRQMMKKVEIVNPGDTVFIYGQQVDKHRFHEENRRVMKEGGQAAVAR
PILLGITRASLKIDSFFAAASFQETTRVLTDAAIKGEIDQLRGLKENVIIGHLIPAGTGM
KQYRQVKLFDAENDDLNALVDEVIEKRKQESELAPPAILQDRTSSEVSFDEPSVFDNDEG
FSEDAGPSEE*
>SPBDM4_v1_40402|ID:27157707|rpoB| DNA-directed RNA polymerase subunit beta [Uncultured spirochete bdmA 4]
MAYTEQKIKRTYIGKDYRDVMDIPDLIDIQLSSYERFLQRDKLRKGESLDQNGLEEVFRT
TFPIESPSGDLVIEYERYSLDEKAIKYSELECKQKGLTYSVPLKAVINLVFKKTGEIRQK
EIYLGDIPLMTDRGTFIINGAERVIVSQIHRSPGVVFSHEKGVFSSRIIPYRGSWLEFEI
DQKHDLIHAKIDRKRKILGTMFLRAIGFQSREEIIAAFYTTKEVILSENQAEKDALVGKI
LAKAVYIELDGEQKRLFRAGDKIHLHEIDELINLGIDVVEVIDFEDEKSLHNEMILNCFD
REDVKLVKETPDQDEPTKEDAISAVYSVLRPGEPISMENAERDLHNMFFTARQYDLGAVG
RYKLNKKFDFDSKTNTLTKDDIVATMKHLINVYYGEANIDDIDHLGNRRVRAVGELLSNV
MKVAFSRMERTAKDRMTGKDLETAKPQDLITIKPIVAAIKEFFGTSQLSQFMDQVNPLAE
LTHKRRLNALGPGGLNRDRAGFEVRDVHYTHYGRMCPIETPEGPNIGLIVSLATYTTVND
YGFLETPYRKVKNGMVTDDIEYLSAIDEDKYYVAQASARIDKQGKFLDQSISCRHQGDYT
MRTPGEIQYMDVSPKQIISVSAALIPFLEHDDANRALMGSNMQRQAVPLVFTEPPRIGTG
MEWKTAYDSGVLVKAKRAGSVEYVDATRIVIRPEKPESPDDADVYQLIKYQRTNQDTCFN
HRPIVKRGEEVLKGQVIADGPATSQGELALGRNILAGFVPWNGYNYEDAILISERVVKED
LFTSIHIKEFILEVRDTKLGPERITRDIPNTSEKMLDNLDGEGVVRIGAKVKSGDILVGK
VTPKSDSDTTPEFKLLNSIFGEKAKDVRDSSLRVPHGIEGTVIDVQRMSRANNDDLAPGV
EETVKVLVAVKRKLKEGDKMAGRHGNKGVVARVLPVEDMPYMEDGTPLDICLNPLGVPSR
MNIGQIMETELGWAASTLNEWYACHVFQSPSQEQIEEKLRQAGLPVSSKVMARDGKSGEF
FKNPITVGVVYFLKLHHLVDDKMHARSTGPYSLVTQQPLGGKAQFGGQRLGEMEVWALEA
YGAANTLQELLTIKSDDMTGRSKVYEAIVKGEPHTAAGIPEAFNVMVQELRGLALDIKVY
DSKGRQLALTERDEDIIKKQSGAF*
>SPBDM4_v1_40403|ID:27157708| protein of unknown function [Uncultured spirochete bdmA 4]
VVPPFSGCIQRGFSRECLLMTKMLEALRPIALSARTFDPRVQAHMEG*
>SPBDM4_v1_40404|ID:27157709|rplL| 50S ribosomal subunit protein L7/L12 [Uncultured spirochete bdmA 4]
MAALTKEQIIDAIASMTVLEISDLVKAMEEKFGVTAAAPVAVAAAPASGAAAAPAEEKTE
FTVVLKGNVPADKKIAIIKEVRVITGLGLKEAKDLVEAGDKPIKENVSKEEAEKVKKQIE
AAGGSVEVK*

>SPBDM4_v1_40405|ID:27157710|rplJ| 50S ribosomal protein L10 [Uncultured spirochete bdmA 4]
MAIKASKIQPKKVEAIGMLKEMISGSNDFIFTEYRGLTVERITALRHQLREKGVELHVIK
NNFARLAFEELGYSDAVEPVLRGPTAVAFVKTDSNEVAKILLDFAKETPSLVVKGAMVDR
QFMDSKQIEAFSKLPGRSQLIAMLMSAMQAPAQNLVYVINAIPTKLVRVLKAIEEKKAQS
GAN*
>SPBDM4_v1_40406|ID:27157711|rplA| 50S ribosomal subunit protein L1 [Uncultured spirochete bdmA 4]
MKHGKKYNEVAKKVDPSQAYELGAACALVKELKTAKFDETVEVHIRLNLKKSQSVRDTVV
LPNQFRAEKRILVFCKPERVKEALDAGAAYAGSDEYIEKIKGGWLDFDIAVATPDMMKDV
GKLGMVLGRRGLMPNPKTGTVTSDLSAALGELRKGRTEYRTDKSGIIHLPVGKVSMDAEK
IAENLGILMEEINRKRPADAKGDFVASVYLTPTMGPSVKVALNKSEKR*
>SPBDM4_v1_40407|ID:27157712|rplK| 50S ribosomal protein L11 [Uncultured spirochete bdmA 4]
MAKKKITAIVKLQCPAGKATPAPPVGPALGPHGVSAPMFCQQFNERTKSMEPGLVIPAII
TIYQDKTFTFILKTPPASVLIKKACGIEKGSSLPQKDKVAKLSKTKLEEIAKQKLPDLNA
NDLEAAKRIIAGTARSMGVEVEW*
>SPBDM4_v1_40408|ID:27157713|nusG| transcription termination factor [Uncultured spirochete bdmA 4]
MAKSWYILHVYSGYENKIEKTIRMLIDSNELSRDIVTDVKVPAEEVSEVKEGKKRTVSRK
ILPGYILVEMDLPENGWKATCAVIRNITGVTGFVGSAANVRPQPITMEEVKRILQRTGEL
KGERPVHFRQTFAVGEQVKITQGPFESFSGVIDEVNAEKNRLKVSVQIFGRSTPVEVDMA
QVEKI*
>SPBDM4_v1_40409|ID:27157714|secE| Protein translocase subunit SecE [Uncultured spirochete bdmA 4]
MKKIIQFFKDCYAELSKVVWPSKDDVIASTKIVVISTVAVALVLGLIDFLIVLGIEVVFR
*
>SPBDM4_v1_40410|ID:27157715|rpmG| 50S ribosomal protein L33 [Uncultured spirochete bdmA 4]
MGAMASKKKQNVEIIALQCTECKRRNYTTKKNRKTTQDKLELMKYCKWDRKHTLHKETKV
K*
>SPBDM4_v1_40411|ID:27157716|murJ| Protein MurJ homolog [Uncultured spirochete bdmA 4]
MASHNPEDPIEPVELSGEPVYPADEPVELMEESSKVAADIAAEKAARSSAKMMRSAGVLS
FLTLISRVLGLVREMTKARFLGTGIYSDAFTVSFIIPNFMRRLFAEGSVTVAFIPTYKGY
LHAQNDKETHDFLSASLTVLTICVVAVVALGIAFAPTIVKLFGSDPVETTILTRIMFPFL
ALVSFAALLQGILNSHEIFGPSGFAPILFNICFIVVPWLVGNRLGNPARAMAVGVVIGGL
AQALVQLPAVIRLGTRLGFMNPARAFRNPGMRKVFALIAPTILGMAAYQVNDLVSTAFAS
RAGTGVASSLQYSLRLQELILGIFAVSAGTVLLPRLADAVREGAWREYSSTLGNTMRSLL
LLTVPVAVFSMAFPERIVALLFKSGDFSDLSVHLTASAFFWHQTGLVFIAMNRLIAPAFY
ARSDTKTPTWAGIASFGVNVILVLVLAFRFQGPGIAFALSFSSAVNTGLLVRALIKGNIE
GIRNELWMSLKYMLKMLLFSIIAVVPSILVDRAVVAKVGAAHSRLVASGVPLVAGTAVFA
AVGIGLLVLTKDPVAASITNAFSRKTHGRGPSRS*
>SPBDM4_v1_40412|ID:27157717| Cof-like hydrolase [Uncultured spirochete bdmA 4]
MRTMNQPASDAHIKLIALDLDDTLLRADLSISEENKRALQRAENMGIHIVLASGRNYFSM
HHYAEELFIHRRGDYLVGSNGAQLIQASTGNLLEDLKLSADFCKSITQEFEREGYFWQLY
IDGNIYCNHMNEWAIMDQRLSGLSLEVISDMDAVLSQDQTKILVCGEPARIEALYKELKN
RIGNNVEIVTSKPYFLEILPKGANKGAALERLAKRLRIGMESVLAIGDARNDFDMIQKAG
WGCAPANAAEEIRAIARFVSPKTNEEDAVANILEHVVFD*
>SPBDM4_v1_40413|ID:27157718| putative carboxypeptidase YodJ [Uncultured spirochete bdmA 4]
MFSLILVVSAASSCAAQKNMALSALPSDIRTQVEAALSAAHIGDTDRSRIVEAIGNDASA
FALLFHEVRDVMKKDPELLRRVDKSVALPANFVPSDLVKLDGVFPYVVSKKGMQLRAPAR
DALVRMTEAARKEGETLVVSSTYRSYVVYQKTVFERNVKEMGKTEAERVSAPPGMSQHQLG
TAIDFGSITDEFADTAAGRWLSANAGKFGFSLSFPKDMEAITGYRWESWHYRYISPAAAE
MQERFFLGVQQYLIEFLAELPH*
>SPBDM4_v1_40414|ID:27157719| conserved protein of unknown function [Uncultured spirochete bdmA 4]
MKTDPQLKLAVLVDADNTQPAIIDGLLAEIAKYGIASVKRIYGDWTNPNLRGWKERLLEY
AIQPVQQFAYTTGKNSTDSAMIIDAMDLLYTENLDGFCIVSSDSDFTRLAARLREDGKLV
LGFGQRKTPKPFVAACDKFIYTEILQANVSESDEERENPPDKESGKGATPPTDIKSDKKL
KTLLLSAAEEAADEFGWAYLGEVGTYIANRLPEFDPRNYGFRKLGELIKAINLFEIDERT
NPMVPGKQVYLRIKHRGRN*

>SPBDM4_v1_40415|ID:27157720| putative glycerate kinase [Uncultured spirochete bdmA 4]
MREKTKLALREDAETIFQAAVDRVMPERLFSYCLSLEGSALGASDGQMTRKYDLSQFEHI
VIAAFGKASLPMAASLVSLLGERVSQGLVVTKASEDGRPLQLAPQVEKVFAAQSVRVIEA
GHPVPDQRSVMAGKEMLTLASQVRDWEMHGSKTLVCVLISGGGSALLSAPVKGLSPEDTA
DVTRLLLACGATIHEINTVRKHLSAIKGGFLARAFSPAQTLSLVLSDVMGDDLDVIASGP
TVPDMSTWQDVKAIFDRFGLFDALPEAVVRATHEGLSGLRPETPKPGDTIFDSCSTMLVG
TNYHAILEAERTSRALGYNTLVIGTRISGEAREIGKIFSGIAQDIMLHELPVAVPACIIA
GGETTVTLRGKGRGGRNQEMALSVLREFSHFPRALCSRLKGVVFLSAGTDGNDGPTDAAG
AFADAEAIGRAQALDLNPSCFLAENDSYAFFEKADGFFKTGPTGTNVCDIQILIVR*
>SPBDM4_v1_40416|ID:27157721| putative Tetratricopeptide TPR_1 repeat-containing protein [Uncultured spirochete bdmA 4]
MNKFYATLCERFGLNAYIAGDYQKAERWFRKLEQSEPTSIRVLRNLGVILLAEGRSEEAE
RYLLREEKLYGASFYRHSALADLTYATGKRKEAARRYRLALREPECAEGGKMYHMRGFME
KRLAMCEDEKRYTGTRKAMEFFRQAEESREAGAHQEAIQKFLQSFALDETNWPALNNAAS
IYMNTLKQPAEAEPLFSQAFELSHSIQVARNVELCRQAINRGQKKK*
>SPBDM4_v1_40417|ID:27157722| Gluconate transporter [Uncultured spirochete bdmA 4]
MVSGVVALLLLLLSVIVIIILTGRFKMNAFLVLIGVSFLFGLAIGLPALDVIAGIKNGFG
GTLANIGIVIVAGTIMGTILEKTGAALSMTQVILKLVGKSRAPLAMNIAGYVVSIPVFCD
SGYVILNPLNKALAKESGISMAVMAVALSTGLYATHTMVPPTPGPIAAASALGADLGKVI
LLGLITSIPASLSGLLWATKFARRYEIEPESHESYSEIVKKFGKLPGSFLSFLPIVLPIV
LILLKSVAEFPSKPFGNGNFRIFLSFIGDPVSALIIGVLCSLLLVQKGELKNAISGWMGE
GIKESAIILVITGAGGAFGQIIKASPITEFIKTNMAGMQLGIFLPFIISAALKTAQGSST
VAIVTTAGIMAPLLPSLGLDPALTTIAIGAGSMVVSHANDSYFWVVSQFSGMPVNIAYKA
FTSATAVEGAVAFAAVLILSLFVH*
>SPBDM4_v1_40418|ID:27157723| putative Endonuclease/exonuclease/phosphatase [Uncultured spirochete bdmA 4]
VRPAFFILSAIAMAAVGCRCSLAPAGTSQEIHIVSYNVHNLFDAEESGREYPEFKPSKGK
WTRELYAKRLANVVEAVLSVSETEGAGSRRAAAPDILCVQEIENEKVLADLAEHFGRGVY
RYWAISGPDESIIHTGILSRFPIVAMRTHSVMDAWGFGPLRDILEVELELGSAERIVMFV
CHWKSKLEGEKETESGRRAAAALLAQRIRGLQTEKPSLPIVVCGDFNESPDEYIRVSRRY
PTAIMPSDAGTPPSAAEGEPICVSGSWAGLANTGEDQPFSNENAPKLILYNPWNEISDGF
SIAFQEKREQFDCFFLNANLHDGEGVEYESFGVANAPMLFDSNGTPFEWRGSEGYSDHLP
VELILSMREGR*
>SPBDM4_v1_40419|ID:27157724| proteolytic subunit of ClpA-ClpP and ClpX-ClpP ATP-dependent serine proteases (fragment) [Uncultured spirochete bdmA 4]
MNGVATEIEIHARELEKTRVRINELIAEATGQPLEKITKDTDRDFWMNASEASQYGLIGK
VILSKNELA*
>SPBDM4_v1_40420|ID:27157725| ATP-dependent Clp protease proteolytic subunit 2 (fragment) [Uncultured spirochete bdmA 4]
MKLAEPQTAETEAKRPEEPLSERFLKTRTVLLVGEVDKDLSEKVVRQLLLLDSISDEPIM
LLIDSPGGDVYAGFSIFDVIRFIKAPVRIVGIGLVASAAALILIAVP*
>SPBDM4_v1_40421|ID:27157726| Antioxidant, AhpC/TSA family (fragment) [Uncultured spirochete bdmA 4]
MEYRHSAFHRFTLHASALMLLSILIFFASAGSVFGAQSRPWYATRLEALGFYVFDQPFEQ
PNFNVTSVDGQMKSRLSTKGNITLLNFWATWCPPCKQEIPTIQKLHETMKGEKFEIMAID
LGEPPASVKTFLEQNKITYPVYVDPKNSLAALYASRGIPTTYILDKNGKFIAGFIGPFEY
DNPEFVSIMKELARK*
>SPBDM4_v1_40422|ID:27157727| putative Cytochrome c biogenesis protein transmembrane region [Uncultured spirochete bdmA 4]
MNQPDLIAAFGAGLVSFISPCVLPLLPAYLSLLSGLTVKELSDQQQKAKLLGASLTFSAG
FTLAFTVLGVIFSGGMSFIGGGGTSQLFGKIAGIIVIVLGLNVMFDFIQILNNDARLIRK
FAGKGRGQVNAFLMGLAFAAGWSPCIGPILASILLMAARNANVGAAVLLLVTYSAGFAIP
FVASALFFERLSPLLNFLKRHGKGVRIASGILLIAFGIAMALGSVSRISAIAAQTGGALL
GFEASSPLPSHIIGTAFWLLFAWLSSRRILRRRKALKAVPGAGGATPRAQKSPVIAYIFS
ATFIVLAVLELAGIISLLHLIGGWLTFGGL*
>SPBDM4_v1_40423|ID:27157728| conserved protein of unknown function [Uncultured spirochete bdmA 4]

MKYVGIIDCGTTNSRFSIVDNRGKIIASVAAKIGVKDTASQRTKEPLKKGLRKLFEEALS
ASGIAASDLNLVMSSGMITSEIGLMELPHQPAPIGLDELAKGLVLLSGSSELGIGVDIYL
VRGIKNRPDPPRGAPLSEAKNLDFMRGEETQMMGLISLYGGGVPTTAVNLSSHTKYIAID
AKDRILGSITTLSGQVYESLATDSVIAKSIVPAPRDDVVADSDLLRDSLEKASVFAAAAV
HDHGLLRSFLMPRFMDTLMWTDWRVRKRFLEACIVADDLKVLDAFPEFGFKVGDRVFLIG
LEERCRLFEAQFRHTAQGKNAKITVISNEEEIRKLAIHGALSIASKAGLL*
>SPBDM4_v1_40424|ID:27157729| Dihydrodipicolinate synthetase family [Uncultured spirochete bdmA 4]
MKLYGVIPPMVTPFKESGDVDYNALEVLVRFLATNVDGLFVCGSYGSGPLMNMDERKEVA
TKSIEFAAGKVPIIVHTGSASSREAAQLSAHAVSIGALAVSAVAPYYFHHSPKGIIEYYR
SILAAVPKGYPVYVYHNPRFSGYEIDMETMVKLADMGIAGVKDATFDIAKFSAYRRDLSP
RGFDVVLGTESMWLSARVLGAEAFIPGLANAFPELCTELHRQGMQNDYEKACKTQFVVNK
IRDLMYLAGSTQQAVYAMLSIRGIVKSYPRSPFLAANQSAQKELQELLAELGVL*
>SPBDM4_v1_40425|ID:27157730| Transcriptional regulator, RpiR family [Uncultured spirochete bdmA 4]
MTASARSIPVDILAEIKLSLNTLPPLQQQIASFIIEHPQDVVRMSISHLAMQTGAKSEAS
IVKFYRSLGFSGYHDFKVTLATQIAGSSFHRPDMTTEIQVHDDVSSIRKKIFISSMHVLE
ENNNTIDDEVLEKTVDILEQAKRVIILGYGTSAVAAYDLFVKLSRLGIDCHFSLDAHVNA
LTLAEPREGDVVFAISYSGESRDVVLQAEQVRGTAKIIALTGEVDSPLAEIANVCIAVKS
FETAYRIDAMVSRMVQITIIDILFTALAIRGGDAALARLTRSRQGISFLKF*
>SPBDM4_v1_40426|ID:27157731| Dihydrodipicolinate synthetase [Uncultured spirochete bdmA 4]
MSTQIGGIIPPVITCFDKNGKIDENAQKELVRYLSNHVQGFYPCGTYGSGPLMTSEERKR
VAEIVIKEKGSALAIIHVGAPSTAEAVDLAKHAESAGADAIGAIPPYYYAYSQEQLIDHY
RALIKAVKIPVFLYNNPELSRNPVLSESLRILAEDGLAGVKDSSFDLVNFYNYILAVKQP
GFTFIVGTEAIAAAALDAGASGVIAGLANVFPEFLEDFYTTWKQGDPQRTAKKQLDVIRA
RNALKLGPTLTMTYAGLRMRGFNPGYPRAPYTEITKEVYDKALREFKAMGLIG*
>SPBDM4_v1_40427|ID:27157732| conserved exported protein of unknown function [Uncultured spirochete bdmA 4]
MRKFILLAVILSLVAGIPAFAKDPAYPTKPITIICPWSAGGGTDNTARFIAGMLSKELGV
PVIVLNKTGGSGAVGHMEGALAAPDGYTITNITFELNTLKYLGYANLTPAGIMPIFQFNE
DADALSVQADSPYKNVKDLLDAIKAKPDGTFLFSGSTIACAWDLPRIQMLMAAGINPKRV
KFIPTQGGAAPAITELLGGHVDAVVCSYPEIAPQVEAGKVRTLAVFSDQRNPLYPNIPTL
KEQGIDVTGGTWRGFAVPLKTPDYVVKTLTDALKKITASKEFVDFMNKNQFGIKIRDQKE
FTDFINDQFKTLEGVMEAAGYLSK*
>SPBDM4_v1_40428|ID:27157733| conserved membrane protein of unknown function [Uncultured spirochete bdmA 4]
LRINREHIVGFICIAIAIAILCITPTFPKGQAGVNLTGPAFFPEVLAIAYLVLGVIQILI
GFKAQKQIKDGTPSDTQEVQKKKGRVWPTVEFIALLIAFIVLFEPLGFIISTIAFLFLLM
LLLGLKWWKSLLYAVIYTAVIYLLFGRLFTIGLPSGILSALGM*
>SPBDM4_v1_40429|ID:27157734| conserved membrane protein of unknown function [Uncultured spirochete bdmA 4]
MIQAFIAVFSPNVLIPWVLAMALGIFIGAMPGLTATMGVALIVPLTYHMQPIAGLAMILG
LSFTSIFAGDIPATFLRVPGTPASGAAVLDGFEMSKQGKGSLAIMIDLFCSGLGGLIGVV
LLITISPTLASFALQFTHFEYFWLGLLGLSMSAVITKGSNTKGLIAVVGGLLISTIGIDI
STGYPRFTFGNINLISGINFIPVMIGLFGLSEVIKDIAMHTQDQLSSAAVVERGHTTVKD
VLKVMGRNKRVIVQSSIIGTLIGALPGAGADIAAWVSYGFAKKTSKHPELMGTGTEEGVV
APTTANNAALGGTWIPALVFGIPGDTITAIVLGAMLMYGLKPGPLVFTNNKELINQIFAV
ALTSQVFVVILGYIGIKAFSFLFKFPRNVVLAGITIFSLIGAYAIRSNVFDVFLVILFGI
LGYFMEKAEIPLSPFILGLVLGPMIEDNLRIGLIKTDNRFLPFLTRPISAVFAAILLITF
LYEPVKTLLTKRRKN*
>SPBDM4_v1_40430|ID:27157735| PfkB domain protein [Uncultured spirochete bdmA 4]
MKDFLSFGEPLVGLYPPQAASVADDVPLIKTWGGDTSNFAIGVSRLGHSVSYLTAVGDDP
FGRGFLALWNKNGVDTSLITLDPKRRTGLYFVSFENKKHSFTYYREGSATSAISIENIAP
NIADEFKILHLSGISLGISGSALETGIKLMDIFQRSNRKISFDINFRPALWPSAKQASHI
LSAAISGGVDYLEITDDEMLALGWGNTLEGLTSRFPAVGSILLKHGPKGASVYGEGNYFS
VPAFEVEVKDTVGAGDSFDAGYLSATLDGAAPPEAARFAVATAALTCTGTGPLERMPYKK
DVVLFLGASEK*

>SPBDM4_v1_40431|ID:27157736| Nucleoside recognition domain protein [Uncultured spirochete bdmA 4]
VTFSGQNLKLIARTWFKRTFDTSWFLLKIMVPVSLVVALLGWSGSLEKIARALHPLMRIL
GLPGEAALVYISGALLNNYSAIAVMSSMNLTLHDATILAVMCLISHNLIVETAVMKSLGS
SAAKMALLRIGVAILAGGILNLVLPPSFAEVKLFASGAVAQSDFWPMLGAWAISTLKLTG
RIIVYILVLMLAQTLLEQFNMMEWLSRWLGWLMRIFGLEPSMSFLWIVINIVGYAYGAGI
IKAARDEGRMSLQEGDLFNHHAAISHSLLEDTVLYGAISLPVLWLMVPRLVLSLIVVWAE
RLRRALFRRSPKTDVV*
>SPBDM4_v1_40432|ID:27157737| putative 4-alpha-glucanotransferase (Amylomaltase)(Disproportionating enzyme)(D-enzyme) [Uncultured spirochete bdmA 4]
MKFIHEKNQFAGLAVPLLALKKNKNAACGVFSDLVNLGKLAQQWGLSLIQLLPLNDTGDG
SSPYAALSAFALHPVYISLSETATTMASLGVPLEDETAKELARAARALDRKYGENLRVPF
EDVLHAKLAALRAAWSVSQDFCGPLAEAFAAQERWAKPYACFIALKEKFGRAPWWKWPEW
TTVSAADIDALWFNLEIAEEARFRLWLQVLAREQLQRAAQSVREMGVDIMGDIPILLAKD
SADVWANRAIFILDKQAGAPPDMYSPRGQNWGFPLYDWDALRVQDYAFWRQRLSYADKFY
TAYRIDHVLGFFRIWAIGAFESDAYLGAFEPSERMHRSELSALGFSDERITWLSKPHVPE
LKIRNAEKECIDEFAAVTERAGGVQPYADRKAAFVRRLEDVRRACFARIHNEPLFLFNPE
IRGTGEINDLIWHVRQDFPEADHGLNDYARGMSDWWIDRALYEVEPDKYVFQWSYHDTTS
WKSLSPQEQGALEAKAAALTTASLDIWEARGRDLLSMLVSAAKMQPFAEDLGAVPTCVPK
VLREMEIPGLRVLRWEREWEKPGQPYIPLEMYEPLSVACTSVHDSTNIRQWWEEEADRPQ
LWAMFTKMAEHDALLRSLFESRAREKREAGQSPQSAVEPPKVLDTAAAAGIIRAMALSSS
IAAVYPLQDLLACHSEWRESDPRDERINVPGTTLATNWTYRMPVDLYTLAKDRDFPALIS
KLAAREAPA*
>SPBDM4_v1_40433|ID:27157738|radA| DNA repair protein RadA homolog [Uncultured spirochete bdmA 4]
MKKPSLIYRCSACGHEEPKWLGRCPECGQWNTMVEAKTTGRFEKDKTAFSVPLESVDPAL
GARVSSGISELDRVLGGGFMRGSAILIGGEPGIGKSTLLLEASANLGAKGKALYVSGEES
AAQIRLRAERIGALSKQIEVFCSNDVHACLSVMDSVHPLLTVVDSIQTVHSPEAGAVPGT
PNQIKFCTQEFVEWAKSHDSIVVLVAHVTKDGMIAGPKAAEHLVDAVISFEQAENALRVL
RTSKNRFGSSDELGFFLMGEGGLAELADPSGIFMVRREGQLPAGIAVAIVHEGSRILLAE
VQALTIPSKSGIMRVYSDRIDPLRVSRIAAVLEKQTRLDFSSQEIYVNVAGGLRLTEPAV
DLPLACALYSARSGQALPEGSAIAGELSLAGEIRPVRSMERRAKAARQLGFGRIIGPRAA
LPGDELSLSRAGKVGAATGSSQSGPHWLSVSSLKEALRILWNQESRT*
>SPBDM4_v1_40434|ID:27157739| Methyltransferase type 11 [Uncultured spirochete bdmA 4]
MTAITLKPKEDIRIRHGHPWIYDNEIARVEGEPAPGEEVEVRDARGLHVGYAFFNPNSKI
RARIYSRTAKSVDEAFFTRAFESAKEWRQFCFGRDALERGMSLRLVYGEADSVPGLIVDG
FVGTPLAKDEELSDAQERARSGQGLWLSVQFLSLGVEVRKALIVKALCKVFAPDGIIERS
DAPVRALEGLPESTGVLWGSMPERIIMEEKGALFEIDLVGGQKTGWFLDQRANRHAAARH
AAGRRILEMFCNQGGFSILAAKAEAASVLAVDSSAEALELLRRNAARNQVADRVSVVEAN
AFDYLRDLEKMGQRFDLIILDPPAFAKNRASLEGARRGYKELNLRAMHLLERGGLLVTFS
CSHWFSPEMFDAMLADAAFDSNRRLRVLEERSQDLDHPIMIGYDESRYLKCRIVQVL*
>SPBDM4_v1_40435|ID:27157740| conserved protein of unknown function [Uncultured spirochete bdmA 4]
MISHDDFIFCIGYDGDTAIVDGMANKEFSRLSSAELAEKGLYRAAFASVLYSKNPEEMKA
FIDLFNKKAGTQYTESSQLSRLFGVYLDEISKVKVL*
>SPBDM4_v1_40436|ID:27157741|dfx| Desulfoferrodoxin [Uncultured spirochete bdmA 4]
MTQKKQIYKCMKCGNIVEVLHEGDGELVCCGEPMKLFVENTTDAAKEKHVPVLEKTADGW
WVKVGAVPHPMEEKHYIEWIELIADGNSYRAFLSPGKEPKAFFPVKAEKVGAREYCNIHG
LWKA*
>SPBDM4_v1_40437|ID:27157742|rbr| Rubrerythrin [Uncultured spirochete bdmA 4]
MKSVKGTKTEVNLLTGFAGESQARNRYTYFASKAKDEGFIQMQLVFEETANQEKEHAKRL
FKFLEGGSAKISAEFPAGVIGTTRENLAEAADGENYEWKEMYPGFAKVAREEGFDAIAAV
FESIAVAEKQHAKRYEAFMANIDAGRVFKRDKPVVWRCINCGYLFEGTEAPKACPACAHP
QSYFELLGENW*
>SPBDM4_v1_40438|ID:27157743| conserved protein of unknown function [Uncultured spirochete bdmA 4]
MAEWQAYEKATLQIGRSGSSLDIRERMLRSGSGSISNLDLVVAILGTGVPGKPVRRLAKE
VLDQITESTHTFDIDKLMVIAGMGEAKSCAVAAAIELGRRIFSTRGTRIVMPKDAFPLLI

HFADIRQEHFIVMSLNGGHEVNAVREITKGLINKTVVHPREVFADPITDRACAVIVAHNH
PSGNLEPSDEDLDITKRLRQSGDILGIPVLDHLIFSETGYFSFVEHGLIAASKGG*
>SPBDM4_v1_40439|ID:27157744| 8-amino-7-oxononanoate synthase [Uncultured spirochete bdmA 4]
MDIFQKCFQFDLDKQAMEKGIYPYFQPLDGLEGTEAIFHGRKLIMIGSNNYLGLTMHPKV
REAARKGLEEFGPSCTGSRFLNGTLKLHLELEERLAVFVGKDAALVFSTGMQTNLGAISS
LIGRNDVVITDKEDHASIVDGCRLGMGEMKRYVHADLDQLERILQKIPQESGTMVVVDGI
FSMGGDIVDLPKLVDICKRYGARLYVDDAHSLGVLGGGHGTAWHFGLTDKVDIIMGTFSK
SFASLGGFVAGDKDVINYIKHTARSFIFSASLPAPNAMAALAALDIMETEPEHVERLWDN
AHFMIKGFRELGFNIGNTQTPIIPVIIGEDETCFRFWRELFDNGLYTNPVVSPAVPEGMA
LLRTSYMATHTKEQLQRALDIFEKAGKKFGII*
>SPBDM4_v1_40440|ID:27157745| putative TrkA N-terminal domain protein [Uncultured spirochete bdmA 4]
MRQFAFVGLGSFAMSMLERIAEVTDQIIAVDDDPTRIEHIKEMVSTAFTMDLLDEDAFER
VFHDPVDVAVVDIESNGAAILLVTFRLKKLGVPEIIVKSNSEEYEELLRLVGATRVVNSD
KEAALRVTPLVLSSSLTNFMPISGDLVLAEVIAPDFVVGKTVVEVDMRRTHHVNVVAVKY
ASERAANEASEGVFHDLDTTYRFKTGDVLLVTGKEDDVFVFSGVQKTAEHEKKKTNFTAL
LKNMLGRKKQGSAKA*
>SPBDM4_v1_40441|ID:27157746| H(+)-transporting two-sector ATPase [Uncultured spirochete bdmA 4]
VSTRIRIGNETLSLLLYFTGLIFAGSILFSLPFATQHGSMRYIDALFTATSAVCVTGLTV
VDTLSLTRAGQTVLLILIQLGGLGIIAFSTLYLFTPRRKISIVTRGLAGDYTVPSFEFRT
RKIIAQIVTWTFSFEFIGALIIWPVLSEHGYSFFDSVFHAISSFCNAGFSTLQSGMESFR
DNVLMNGVTMTLIVAGGLGFIVLQDIARFIQKKKLHLTYHSSIVLRTSASLILIGAVLFF
VLEYNHALKGMSGGSKIMASLFQSVTSRTAGFDTIPQAKLTSTAQFVIIVLMFIGASPGS
TGGGVKTTTFYLMLLVALRFREGGGVLVDRNRTIMPHSIFKAAAVVVRALLIVLVVAAIL
LIAEQAQGRPLGIETAVFESVSAFGTVGLSIGITPSLSVISKLALIGAMFMGRVGLFAMA
IPHSRLSPERYAMSPKADILL*
>SPBDM4_v1_40442|ID:27157747| putative K+ transport (Trk) system, NAD-binding component [Uncultured spirochete bdmA 4]
MKIAILGAGLLGSLIARELIAENRDVVIIEKNPNIAKSIANDLDCIVQDGDGERIDTLVA
AGVGDADWFIACTGSDETNIVSCGIVAETFKKVKTIARTRNPYFASFKKSEKRILGVDHI
INPEAETAEAIARIIFRGMSPEIIDVKEAGIQLRRLLCKKEPRFVGKSLAEVRSGIGLDF
MVPAVLREGELIVPTGNFVFAEDDAAYILGEPSTLDRLFGPCKDALRKFNSLVIFGAETL
TSLLLQELGIEQITAQDRHPRGKGPLSLLGNPRIKVIDGDRDLLKSLSAMFPDIEPINHQ
LRDEHFIEEENIGNADIVLSLTSDQSINILTALLAKNAGARRTLALVVNDLYSPILYSLN
IDIIINEKTVMSGTILDQVRKAKIRRLYNFPRNEYELIEIQISNSFEYLGTEIRNLNLPK
GLLITFVIHEGKTSVPTGNTKILENDLVGLIIKKEQIGRIEAIFGA*
>SPBDM4_v1_40443|ID:27157748|trkH| Trk system potassium uptake protein TrkH [Uncultured spirochete bdmA 4]
MHIKAILRMLSTILLIVSFFIFGCGFFSLIEKNAAQVSLSLLIPAALGIAFFLVMFFVSR
KDEKPFLTNRDGFLFVTLSWVFASALGALPFTLSGYIPSYIDSFFETMSGFTTTGASILT
NIEALPRALLLWRATTHWLGGMGIVVLLVAVLPALGFSAIRVIEAEAPGPSVDRITPHIS
STAKTLWLIYIGLTIIEILLLVIGGMPLFDAICHAFATMATGGFSTKNASLAHYDSGFIH
WVITIFMFLAGTNFTLHYRLLSGNVRSILKDTEFKVYFMLFAVSSIIIAGDLFHSGVYQS
FGESVRFSSFQVSSILTTTGFATSNFGEWPAVSQTVLFVLMFIGGCAGSTGGGIKVIRVV
VLFKMAMTEMRYVANPRGVYTILLGNKALRKNVIYDIAALVFLYFAFFFVSVIVISFTGV
DILTNITAVIANLGNIGPGLGKVGPVFNYAFFPWWAKLWLSFAMLVGRLEVYTVLVLFSR
KFWRSF*
>SPBDM4_v1_40444|ID:27157749| Beta-phosphoglucomutase [Uncultured spirochete bdmA 4]
MFEHEEWRIAEPGYDIHQIEKYETLFALGNGNLGLRGDFEEKIPAFHRGTYINGFFEKEP
IAYGETAYGYAENHETILNLPDAKIITISVEGEEFLLDSAEIQSYERYLDLRNGVLIRSV
RAMLPNRALIEVISSRLVSLERESIGAIRYEARVIDTPDGRPVAVRLESRVEGMATNRWA
ESDPRVGSKISRAAYSITRLECNESESRLLIATKNSGLAATAQVTHAFYGPETMALSATA
EASSAVQRWEGAARPGERIVLIKYMAYRMASAGEEALLWQSAHEELINASRDGFEELQRE
QREFLADFWNCADVIVEGGPDIQQALRFNLFHVLQSSGRNGKTSIAAKGLTGEGYEGHYF
WDTEIYVCPFFTYTKPEFARRLLEYRYSLLDKARLRAETMTEKGALFPWRTIDGEETSAY
YPAGTAQYHINADIVYAMEKYCEVTGDAEFLYGPVAEVAVETARLWVSLGHYGDDGRFRI

DEVTGPDEYTALVNNNAYTNLMARNNLYFAVRILRAMQGERPDSFESLRRKTALEDDEIE
GWRNAAGSVYVPYDPKTGIYAQDDSFMQKERWPFSETPPEKYPLLLHFHPLVIYRHQVLK
QPDLVLAQFLLPEQFTLAEKKRNFRFYEPLTTGDSSLSHCIQSIMASETGNSEAALDYFM
KTVRMDLDDMHGNSRDGIHTAAMAGSWLSVVYGFAGFRDRASAEGEIRYSFNPRLPAGWR
RLAFGLQLGPVKLSIDIGTSEVKYSLEEASRSGGSASAETGGGLRNSGALTFLHRKQKVI
LKSGESAVLSLEPALRAALLDLDGVVTDTARYHYLAWKRLADDNGWHVDEKLGEGLKGVG
RREALEVILGHNGVNLPEEKKHELAERKNAYYREFLRKITPDDILPGMKSLLLGLGQRGV
KRILASASRNAPAIIDCLGLGPIPGRFFDGIADPNAVTCGKPDPELFLLAADIAGASPED
CVGIEDSLAGIKAIKDAGMKALGVGNGLEGADLSVSSTSDITVDTLEQLIRS*
>SPBDM4_v1_40445|ID:27157750| Transcriptional regulator, LacI family [Uncultured spirochete bdmA 4]
MKKQSISASDVAKMAGVSRTTVSFILNNIQGKHISEATRQRVLQAVEALKYVPDMNAISL
AKKTHYSIGFFISYSSSVFSDAYLFRLIEGMALVLNKRHCRLDFQPLRKEKVNYLDLVRS
AGLDGIILMNTHTDDPGIQELEVARVPLVVIGSIEGLSVPQVDIDNIAAAREMVKYLVSL
GHRKIAMIAHAPLTFYAAAHRLEAYTQILYEKGIPQRPEYVEVADFSEESGYGAMQKLLG
LSDRPTAVFAGNDMVAYGAMQAILDAGLSIPEDISIAGFDDDYLSRYLNPALTTVAQPSV
GLGEAAARLLIQEITSPHESRSRGKERIILPTILAKRESCRRI*
>SPBDM4_v1_40446|ID:27157751| ABC-type transporter, integral membrane subunit [Uncultured spirochete bdmA 4]
MANKSGSVRTPLRRALGRIPLHVILILICVAWITPSAGLLVSSFRPKNMIATNGWWEAFK
TPFSFTLENYAYVLTRGNMGRSFVNSLLIAVPSTLMPIFIGACAAYAFAWMRFPFRKTLY
LMIIALLVVPLQMTMIPILRLFNKVGLTGSFVGIWLAHTAYGLPFAIYLLRNFIAQLPRS
LIESAQIDGASHPTIFLKLVLPTSIPAIASLAIFQFMWVWNDLLIALIYLGGTPDVAPMT
VTISNMVNSYGGGWEYLTAAAFISMVLPLIVFFALQKYFVKGVLAGSIKE*
>SPBDM4_v1_40447|ID:27157752|aglF| Alpha-glucoside transport system permease protein AglF [Uncultured spirochete bdmA 4]
LAWGKYKARQKNMHTNYIMVAIAALAVIPIFLWAYLMITEQIATHTSRKTAGVLRPWFWI
LPAVSLLIIFLVYPVINTIVLSFMDARSEHFVGLRNYKYIFSDSGMLVVLRNNLLWLVFF
TAACLVLGLLIAVLSDRVKYESVAKTCIFLPMAISFVAAGVIWRFMYEFRPAGTPQIGTV
NAILTAIFPNFEPKAWLFNRSTNNAALIVVGIWMWTGFAMVILSAGLKGISEDILEAARI
DGASEIAIFFKIILPLMMPTITVVATTLIINVLKVFDIVYVMTNGNLGTEVIANRMYKEM
FNFRNYGRASAIATLLLVAIIPVMIMNLKRFNTEESNG*
>SPBDM4_v1_40448|ID:27157753| Extracellular solute-binding protein family 1 [Uncultured spirochete bdmA 4]
MKRAVLIALLAVAAVGFVFAAPSGQKTINFLGVWGGQEADVFLAMVKPFEEKTGIKVELE
STRDLDAVITTRVEAGNPPDIAALPGPGKLVELAQSGKLVELSKILDMKEFDKNYSSGWK
SLGTVNGKLYGVFMKAATKGLVWYDPATFKEKGLTVPTNDWTWDQMMALSKQAISNGIAP
WAIGVESGSASGWVGTDWLENIFLRVNGPDKYREWYEGKLPWTSPEVKKVWEIWGQIVAD
PKMIYGGSSYVNSTNFGNAAAPLFTQPPQALLHQQASFIQGFITSQFPTLKPVQDFDFVA
FPSIDSRYAKAVEAAADVIGVFRDTPEIREFVNYLASAEAQAFWAAGTGALATNRNVSLV
FYSDPLIRRAADILNKSEIVVFDASDMMKPEMNAAFWSAVVSYIDNPKKLDSILAGLEKV
RQEVYQ*
>SPBDM4_v1_40449|ID:27157754| Uncharacterized AIPM/Hcit synthase family transferase aq_356 [Uncultured spirochete bdmA 4]
VLKLEMFDTTLRDGAQTEGVSFSVSDKLAVVKTLDAFGVRYIEAGNPGSNPKDMEFFAAA
SDLNLQHAKLVAFGSTKRKNIPVEDDGNVRSLLQANTSCVAIFGKSWGLHVTEVLSTTFE
ENLRCVLETVRFFREKGKEVIFDAEHFFDGCRSNRDYALDVLGAAVEGGADVLVLCDTNG
GTMPLQVYEIVELVCGRFPGMRIGIHCHNDMGCAVANSMLAVKAGAVHVQGTFTGIGERC
GNADLGIIIPNLQLKGGYECISGNLEKLCETEIKIAEIANLPVPHNKPYVGESAFAHKGG
MHIDGVDKVSHSFEHIDPTFVGNRRRFLLSEVSGKKAVLLKIRDVAPELTKDSPETAKIL
DKLKELEHEGYQFEAADASFELLVKKVIGKFRPHFDLNMYRASSESPAPDGEMNSNAMIK
IKVDGSAETTAAVGNGPVNALDLALRKALCVFYPELSNVHLIDYKVRVLETGRATGSRVR
VLIESTDGHRKWITVGVSTDIIEASFVALVDSLEYKLCMEENE*
>SPBDM4_v1_40450|ID:27157755|ilvC| Ketol-acid reductoisomerase [Uncultured spirochete bdmA 4]
MANMYYEKDCDFSVLKGKTVAVIGYGSQGHAHALNLKDSGANVVVGLYEGSKSMEVAREA
GLRVMVTADAVKASDVVMILVNDEKQASLYRKDIEPNLKSGATLAFAHGFNIHYGQIQPP
ADADVIMVAPKGPGHTVRSQYLEGKGVPCLVAVYQDSSGKARDIALAYAYGIGGARGGIL

ETSFREETETDLFGEQCVLCGGVSELIKAGFDTLVEAGYQPENAYFECLHEMKLIIDLVV
KGGLSYMRYSISDTAEFGDYTVGRRIITEETRKEMRKVLSEIQEGSFARSWILENQANRP
YFNARRRQEQNLLVEKVGAKLRAQMSWQKDGK*
>SPBDM4_v1_40451|ID:27157756|ilvH| acetolactate synthase III, thiamin-dependent, small subunit [Uncultured spirochete bdmA 4]
MIRGNRMLNHQFIVSLLVSNHAGVLTRVSSLFARRSFNIDSLTVGVTEDPTLSRMTIVSW
GDDYMKEQIVKQLQKLMDVKKVQLMQSDKIVVRELMIVKIRIKKGELSEAMEAINAYRSN
VVDLSPDSIAVEITGEPLKLNAFLNYIKRYEIVEMCRTGPTAMGRSTYCLENDTY*
>SPBDM4_v1_40452|ID:27157757|ilvI| acetolactate synthase III, large subunit [Uncultured spirochete bdmA 4]
MLLTGAQIVIKTLIEQGVDTIFGYPGGTVLHIYDELYKNSDRIRHYTTCHEQGAAHAADG
YARVTGKTGVVIATSGPGATNLVTGIANAYLDSSPIVAVTGNVATSMLGRDSFQEVDISG
ITMPITKHNYLVKDITVLADTMREAFFVASSGRPGPVLIDITKDVQTGMAEYEPALAAQK
YEPAPQISESRLEEAARLIREAKRPYIYCGGGVVISNASEEVIAFADKIDAPIGLSIMGL
SAVPYSHPHFLGMVGMHGRYGATKALYESDLLIAVGTRFSDRAIGNKLEFSRDRKILQID
IDPAEIGKNIGVTMGIVGDVKIVLKALTAKLSRREHKAWGERVKYIKGHPDNNLEMDSGR
LTPQSVIRGICRRMGDNVVATDVGQHQMWTTQYYQFKKPRTFITSGGLGAMGFGMGAAIG
ACVGSGLKRTALFTSDGSFHMNMNEMATAVSNDLPLIIVLLNNHTLGMVRQWQTLFFDGR
YSNTSLERKTDYVKLAEAFGAKGFKAETNEEYEKCLDEAFQYGTPVLIDCTIDSDERVLP
MIPPGGSIDDIILR*
>SPBDM4_v1_40456|ID:27157761| CDP-diacylglycerol/glycerol-3-phosphate 3-phosphatidyltransferase [Uncultured spirochete bdmA 4]
MTAADKVTLSRIIVAPFFFFVLKAEFIPRQPAIVALWIMFCWMELSDLIDGRVARAHNQI
TPFGKLFDPFADVISRVTYFLAFTSIGIMPLWVLLIVLYREFSILFLRMLLGLKGIAMGA
RMGGKLKAGIYMVAGLLSLALYTAQVYGSPGFSGPVPYSDMSGLTNILSTLTLIAYILAA
VATILSFIDYLVQFRKLYR*
>SPBDM4_v1_40457|ID:27157762| conserved membrane protein of unknown function [Uncultured spirochete bdmA 4]
MEEERIASLRRSILITTSVFFIVQCTLFLIFSVSAGFDSKYWSMFLPISGGFHIVILIML
LLFQDDFTIESTGEKLDHVNFANIITLSRVSTMPTLLVLVMAAKQYSIKIPLLILVALIF
LTDFFDGRISRKTNQVTRVGRMMDSTSDYTLLVVLSVIFQYYSLIPRWFFTLVLLRLGIQ
ALLMAILAIVKKHIEPNSTFMGKVTVASIMVLYTLEIIQLAVQAPPLLVFRIAEWIVAII
IVIGIFDKIASFFDALKSKPSTP*
>SPBDM4_v1_40458|ID:27157763| conserved protein of unknown function [Uncultured spirochete bdmA 4]
MAVIKTALELALERTKNLQIDESVQKANEAKLEGRKAAGRFLEAPDSVDFKAIFDTIDAA
QRQTFLSAAFEVLTAPIQLPVNTAVETAKLESSGKAIVALCGLSSKFTSEREVKLAQQQA
RSLFQQIIQFLGKYSEEMKRVEQAIRNQWAPKLREKERQLAAQLGQNVRIDPMSDPEFAE
FYRKNIDAMRKNYSDALEEAKTQLSQLCGFEKH*
>SPBDM4_v1_40459|ID:27157764|mnmG| tRNA uridine 5-carboxymethylaminomethyl modification enzyme MnmG [Uncultured spirochete bdmA 4]
MADFEAIVVGGGHAGIEAALALARLGTKTLLVTQNPDTIAKMSCNPAIGGLSKGNLVREV
DALGGQMGILADATSIQVRMLNQSRGAAVQAPRAQSDKALYSAMARQALEAQPNLTIFMD
TVTDILVSGGEQRRIAGVRTERGNSISARTVVLTTGTFMEGKLFIGQWSGPGGRLGEPAA
IGLGTALRARGFPVGRMKTGTPARIKRGSIDFSKLEAQHSDPHRIFFSFLERDYGRPDLP
CYIVYTNQATHEAIRAGLDRSPLFSGVIVGKGPRYCPSIEDKVVRFPDRERHQVFIEPEG
LYTDEVYLNGLSSSLPEDVQTRFYHSIPGLEHAEIVRPAYAVEYDYIDSSALYASLESKI
VGGLFIAGQTNGTSGYEEAAAQGLMAGINARRTLDGEPPLILGRNEAYIGVLIDDLVTLS
PKEPYRMFTSRAEYRLALRHDTSDLRLTPYAIEIGLADEQRRECFERRLRTIEEVKELLD
GRKVQREDCVQIPELGNHVGQALAEALRDPKIGALLDDEADALARIARLLPQTATMQASG
VLTALLNERYKGYLEKEERLANRLSKADKLFIPAEFDYSYLKGLSREAQEKLTAQRPLTL
GQASRIPGVRKSDVALLYIALAKRGG*
>SPBDM4_v1_40460|ID:27157765|mnmE| tRNA modification GTPase MnmE [Uncultured spirochete bdmA 4]
MSRTYFDRAEPIVALATPEGRSALAVIRTSGENAVDIVARCFSQPQALLGAEGYHAVYGW
IFDPVSQERLDEVVALVFRAPHSFTGENAVEIMCHGSTAVAQRILEVLYTQGFSPALPGE
FSFRAFAGGKTDLVRAEAINELTHASCEAARHDALQRLSGVLSRKLEEIRNLMIDVLADI

NARLDYPEDEGPEETDRWVQLMQRAQDELSDLIRSYPGGRLRQEGLLVVIAGRPNAGKSS
LFNLLVREERAIVSPEPGTTRDWLEAWISIGDFAVRIVDTAGLRNTGNSIEAEGVRRSHA
LLARADVILYLVDGTVGLDDEDRSFVARYPRALLLWNKADLSRCMPAPSAWAAVSAKDIH
SFGSFESMLKSALHSSIQEALTSGQYEAQVRIASERQARLLRQAHEAIAQAMEAQTAGAG
LDLVALHIRESAECIGEITGDIAPDEVLERVFSTFCLGK*
>SPBDM4_v1_40461|ID:27157766| protein of unknown function [Uncultured spirochete bdmA 4]
MTPQELSSLDYEGASELLLAYATDAKKYEKEIASLKSQAEEWKSKAMLAKSRAMPELSES
ASQKASELESKAQELSLELTGLRRDIEQLREALPIIKAKQRRVDPDQLLAELSMIVGDLG
DEKKPVGPGSGATAGGTVAGEAASEAAAGTSAGPGTEGPEAPPEPPTNPPSQPIDDALTE
LKKKMGLL*
>SPBDM4_v1_40462|ID:27157767| Glycerophosphoryl diester phosphodiesterase [Uncultured spirochete bdmA 4]
MRHPSLLPKFPRPLLFAHRGLNRKFLENTIESFQAALETGIPGIELDVHLTSDGKLVVFH
DDDSGRIEEIAHPDEPVRNLSLENSTLSELRSLSIGAQIPLLDELLEHFGSRVYYDIELK
SRSSADTGLTSKVAGAIRSHGLGKNCVISSFNPFALRHFRKADPAIPLGIIWSTSHELYW
FLRHGEGALIANVDFLKPESSLAAHLPLYLRLSGKPFIPWTVNDPQIARSLLARGACGIV
SDEADVIKAALDG*
>SPBDM4_v1_40463|ID:27157768|nadE| fragment of NAD synthetase, NH3/glutamine-dependent (part 2) [Uncultured spirochete bdmA 4]
MNKAPSAELRHNQKDQDTLPEYAVLDGILRLYIEENRTMDEIVDAGFDRATVRRTLEMTA
KAEYKRRQGAPAIKVSKRAFGVGRRLPLARTIHEVA*
>SPBDM4_v1_40464|ID:27157769|nadE| fragment of NAD synthetase, NH3/glutamine-dependent (part 1) [Uncultured spirochete bdmA 4]
MRVSLVQLNSTIGDFKGNADRMLVIVNLILATFSASEKPDLIVFPDQSLCGYPPMDLLDQ
ESFLFGSVAALRRLHKLLPENIAVGVGYVDKNRERAGKSLVNTYSVLCNGIVLFTQAKTL
LPTYDVFDEARYFEPARTRQVFAQDCGRVGFAICEDFWWEAPDASQKYTIDPVKELMDAG
ADVLIVPSASPFIAGKLTTRLNLAEKAVRAGSIPVLYCNAVGANDSLVFDGRSFAMAEDT
SIRGVCGWGEEVLTYDTKTGVASRLVLDSPSQQLEAAEEDINPHINRYDEIRRAIIVGIQ
DYLRKSGFSRVCLGLSGGIDSALVAVLAAQAIGSRNVTCISMPSRFSSTGSLDDAVELCR
RNKLRLERIPIEVPFTAYLNALAVPFAGRPYDITEENLQARIRGTLLMAWSNKFNALLLT
TGNKSEVATGYCTLYGDMNGSLAPIADLYKTEVYGLAAYFN*
>SPBDM4_v1_40465|ID:27157770|lgt| Prolipoprotein diacylglyceryl transferase [Uncultured spirochete bdmA 4]
MVAMLDFPKWLSPEIIPGLPFRWYGLMYLLAFAVTWLLFRKESKRLGAPWSEDEAVNFFI
WSIVGLLLGGRLAGTLIYEPTNYYWIKPWFIFWPFDESGAFVGFQGMSYHGGFAGLIIAT
LIWCRVHKWNWFDWADLIAVSAPLGYTLGRLGNFINGELWGKVSTAPWAMIFPNAERFSA
KEPWVQEVARKVGIRLVSMNDMVNLPRHPSQLYEAVLEGIVLWLILWFVVRKHRTYRGKS
VGIYALGYGVARFIVEYFREPDSGLGYILPLGDPNAPTARFVSVFNFSMGQILSFLMIVA
AIIFMVYAHKHFKINPGDPVFVDASKTGPGTDAGANTGAQGTSRHTSKFDALEARKLRKK
IK*
>SPBDM4_v1_40466|ID:27157771| protein of unknown function [Uncultured spirochete bdmA 4]
VLALLAFVHPSNASVPVDLARTGGSILILILLDVLILGLLALDALRYHPPREGKPALGQE
AGSEKPQVSVHAHDDPEHEVS*
>SPBDM4_v1_40467|ID:27157772| exported protein of unknown function [Uncultured spirochete bdmA 4]
MRRSPFLLTAGILAFFRVFVLGIAAVSLGMGDYGGVSSALLRIFQLYNLLFVYIVILQYF
KPGAREFL*
>SPBDM4_v1_40468|ID:27157773| Carboxyl-terminal protease [Uncultured spirochete bdmA 4]
MKNIDTPVVRKRLISVLSFVVILALTFVTVSVPELYAQQTKTPDAKQYSQLLQNIFQFIL
QNYVDEPDPAKLYQGAIKGMMDSLNDPYSTFLDEDMMSDLMSDTTGTYGGVGLYISKQAT
APTEDTPRYIEVVSPIEDTPAWKEGIRSGDLITKIDGEDTAPLSADKASAKIRGEAGTTV
TLTFKRGSYEFEVTFTRSKIEIPAVKNAIITTAQGDVGYIRIIEWNPNTEPQMKIVLQDM
KKKGIDKFVLDVRSNPGGLLSSVVDVSDLFLSSGVIVSTKGRTISENYEYKAKPDLAIPQ
NNRMIVLINQGSASASEIFAGAMKDTKRALLLGEKTYGKGSVQQIFPLDKAGFKLTMAHY
YTPSDVNIDKIGIEPDISVAEPELSDKEFAEVQRLYDAGDIARYLQSVPQPTPEQRKEFA
KELAKKYAVPENILEKLVRDETERSQPARVYDLEYDVQLQKALDLIATPDFENKLKNTKT
MAQLHDADK*

>SPBDM4_v1_40469|ID:27157774| protein of unknown function [Uncultured spirochete bdmA 4]
MRQIVLPGNQSGKETCVLDAKTSHYLVSVRRMHRDDSFEAMDETGTRFTCTLLSDEPRGA
KVALVQASSPESAAHD*
>SPBDM4_v1_40470|ID:27157775| Ribosomal RNA small subunit methyltransferase E (fragment) [Uncultured spirochete bdmA 4]
LNAMGFEREHAVRILFHESEQQASLPLHTLLSAVQNRIVIGIGPEGGFSEEDCAAFAQMG
FAIHHAKGPVLRVETAATYAISAVRAITLEKNIWKV*
>SPBDM4_v1_40471|ID:27157776| protein of unknown function [Uncultured spirochete bdmA 4]
MESLNIPNAILDNVRIDFPSRFVLYALPYLWDDERNHQICFINTSQWQSAKNRDEYGSMI
ASTDIVLSRDRNLAERAHAHGKKALRSWSVPFIHQILLDALYESMEIENIDAHPMEKDLE
VYRPQKVLTLLLSAIEKRNGSIFLLGGSPITLIKAEKNIRATYPGITIVGSMHGMYRPQE
EIALIQAIQKGNPTLILAGDPIPAGERWIPRHMSTTRSGIFFYYGPILRWFAGR*
>SPBDM4_v1_40472|ID:27157777| protein of unknown function [Uncultured spirochete bdmA 4]
LAVALSKRFEVSIAGDLLELAAIMKEHKPAILLIGFASHDRAFNAYHLECVHRSILSAKI
GNQERSPPLSVFIILESEMHALMPGNSRLKALQSLPFCEVHVIYLPLPIERISSIIFSAV
EENLDSSCAERAEIQSFTCLFADECSPIIGKSKKFRRMISQIRSYADEQRPVLIIGETGT
GKELAAHSLHSWSKRKLNSFVALNCAAIPETLFESEMFGTERGAYTDACSRCGALEQADN
GTLFLDEIGSLSAASQPSLLRVLETGEYRRLGSTHSHSAQFRLVSASCLNPIDLAAENRF
RNDLLFRIADFVIEIPPLRERKEDIPLLARHFCTQFSKDIYDIEDGALDKLAVHDWPGNI
RELQSVIARACARKTAGEIGADDIEFLTGISRLAPHKS*
>SPBDM4_v1_40473|ID:27157778| Short-chain dehydrogenase/reductase SDR [Uncultured spirochete bdmA 4]
MSTANPEGLPVDDSTFHGRFALVVGGSGGIGRAISIELSRRGARLLVHGRHASERTQGMK
LGFHETFDAEFSSPGSFIHALDARLDKIGAEPDIVICAFGPFQEKPLAQCTTEEWEWLTL
ANLALPGALASRFLPGMIARKYGRFLFLGGTRTDGIRAYRKTAAYASAKTGLGVLAKSIA
AEGASHNVAAVLVCPGPTETEYLDNETKIRHASLIPTGSLAQPECVARTSLNLIDADPCI
ASGAIVSLDGGFAP*
>SPBDM4_v1_40474|ID:27157779| protein of unknown function [Uncultured spirochete bdmA 4]
MPSRLLSRIQKIPQPGSDAIISSQTSGDFPDCAVARFVGAVTDTNLEAVAQSFDAILSES
IHYLVVDFSTIEDIGPAGIGLMLVLRQRLRDRHGDIVLCAMRPRMERMQRILGLEGYFTT
AADSQSAIQGLKNVFDGTYPLSVRCPACDTLIDIEHPGRGRCQTCEAVITAFPDGAITLG
*
>SPBDM4_v1_40475|ID:27157780| conserved protein of unknown function [Uncultured spirochete bdmA 4]
MSTLPKIEQYLIDLGISYQEISNNSWLIEDENKGYPKMLVSLSDPLVIISADVMPVPKKN
AEQLFRMLLELNATDLLHGAYAISGNDIIVIDTLQQATLDKEEFLASIESISFALLEHYK
KLASYIQKAEE*
>SPBDM4_v1_40476|ID:27157781| conserved protein of unknown function [Uncultured spirochete bdmA 4]
MGMFERLKTVISSNINSLISKAEDPEKMLNQMIIDMNEQLIESKKSVAMAIADEKKLERD
MIENRAKADEWEKKAMLAVRAGRDDLAKEALLRKQEFEGYATQLAQQWEAQKQSVEKLKD
ALRQLQSKIEEANRKKNILIARAKRAEAQQRINQTMSSLSGNKSAFETFERMERKVDEIE
ASAEATKELDEASSGASLEKQFTQLESSPQAAETMLEELKKKMLTEDAGRAGS*
>SPBDM4_v1_40477|ID:27157782|murA| UDP-N-acetylglucosamine 1-carboxyvinyltransferase [Uncultured spirochete bdmA 4]
MDKYIIEGGYPIKGTLMLSGNKNAALPCIAASVLAEEPVTLRNIPAIEDVLVMFDVLRNL
GGTFVQLDAHTWRIDSRSISNYEVPEELARKVRASILFAGPLTARFGKAILPPPGGDTIG
RRRIDTHILALQELGARITLENMLTFSAAKLVGASVFLDEASVTGTENAIMAAVLAEGTT
VLMNAACEPHVQDLCRMLIQMGAEIEGAGSNRLTIHGKKSLGGTDFSISADYMELGSFVG
LAAVTGGDLRIEGVCADDIPPLKVGFSKLGVTWELKGTVLRPNTMGELRVVPDIGGQIPK
IDDAPWPGFPADLTSIMTVVATQAEGVALIHEKMFESRMFFVDKLISMGARIVLCDPHRA
IVYGPSKLVGNVLVSPDVRAGMAMVIAALCAEGTSVIHNVYQIERGYDNLVGRLSAVGAH
ISYC*
>SPBDM4_v1_40478|ID:27157783|pyrC| fragment of dihydroorotase (part 1) [Uncultured spirochete bdmA 4]
MFQIQDRFTIRMPDDFHAHLRQGGQLSAYVRGHAGQFGRVLLMPNTLPPVSDLARLEA*
>SPBDM4_v1_40479|ID:27157784| protein of unknown function [Uncultured spirochete bdmA 4]
MRGAEVEALARAGEVAGKYYPAGSTTNAAEGPRSFDEVEEALNAMEARKLVL*

>SPBDM4_v1_40480|ID:27157785|pyrC| fragment of dihydroorotase (part 2) [Uncultured spirochete bdmA 4]
VLDRERAFLPQVEHLLVRHPRLRVVLEHLSDAQSVGFVKTGPENLAATITAQHLLFTIDD
MIGESMHPHLYCKPVLKFDRDRDALRAAVLSGSKKFFFGSDSAPHPREKKECGAAASGVY
STPSALPALVGLFDSLGSLDSLIPFMTERGAAFYGLPLNSRTITLERSPWEVPKLLGGAV
PMCAGQTLEWRVARN*
>SPBDM4_v1_40481|ID:27157786| Xaa-Pro aminopeptidase [Uncultured spirochete bdmA 4]
MNTTKYFRRRAGLAAELGRHGLAAVMISDFEHGRDSSLRYLCGQPSDAMLVVDAKGVSVL
IAWDINMANRMGDADHILSYTDFGRQASDALRAALNLLAVPQGAKIAVPSSMPYPDYIEL
VQAFEAFDLVCERSGIDMFIREMRARKDADETGFYRRIASYTDQLMDMVEKEIAAGMLST
ELDVALFLEREARRMGAEGMGFETLAAGPGRSFGIHAFPAFTSGPFANAGLSILDFGARF
EGYTSDVTMSFVSDVLSQEQKTMIDLVEEAHRIAVEACGPGVPLLKVATLVDDLFKKAGW
FMPHSLGHGIGLDAHEAPMLSMRASPEALLQPGHIVTIEPGLYHPEFGGVRLENDVLITE
TGAEVLTHSRIVRL*
>SPBDM4_v1_40482|ID:27157787|argD| Acetylornithine aminotransferase [Uncultured spirochete bdmA 4]
MKERFMNTYARTGLVLDHGAGARLFAADGTEYIDFTAGIGVNSLGHGHPKLVAAIAAQAA
KLMHVSNYFMTPPSMELADRLTEATGFDTVFFCNSGAEANEGMFKLARKYGSLKNPDKNV
IVTLKQSFHGRTITTVTATGQDKFHRFFGPFTPGFAYVEPEDIGALDALLGPNVCAFVFE
PIQGEGGVRLISKGYLQAAEKMCRERDILFCADEVQTGIGRTGALLACERLGLRPDVTAV
AKGLAGGVPVGAILARGAAADVFQPGDHGTTFGGGPLAASAALVVLSELDSPGFLDEVDK
KGRHLMSLVEGLRHPLVKDVRGMGLMVGIGVVVDPHEVVDAARAHNLLVLTAGDDTVRLL
PPLVITMEDIDAGAAALKLALDDVEKAHG*
>SPBDM4_v1_40483|ID:27157788|argB| Acetylglutamate kinase [Uncultured spirochete bdmA 4]
MDDQFNADVLIEALPYLRRFSGKIIVIKYGGAAMINEGVRQTVIQDLILMQQVGMKPVLV
HGGGPEIDRMLKKFNKETVSVEGLRYTDDETMEIVQMVLAGKVNKDLVELFSRTGGKAVG
ICGSDGGLFMAKRLRKNGQDLGLVGDIQAVNTEMVRVLLDNGIIPVVASIALRSDEECGF
YNVNADTAAGALAIALKAEKLILLTDVPGVMEDKTDLGTLIREMDVQMAKTLMKKGVIKA
GMIPKVEGCLQAIAQGVSAAHIVDGRSPHSLLVELFTDTGMGTMIR*
>SPBDM4_v1_40484|ID:27157789|argJ| Arginine biosynthesis bifunctional protein ArgJ [Includes: Glutamate N-acetyltransferase ; Amino-acid acetyltransferase] [Uncultured spirochete bdmA 4]
MKQTRGGITAAKGFLASGIHVGIRKNREKKDLALIWSEVPCAAAAVYTTNRVKGQPLIVT
EEHLADGRAQAIIVNSGNANTCTGEEGMKAARRMTELVAQNMPVKAEDVIVASTGVIGQQ
LDVSVIEAGMPELVQALSKGGSIDAREAIMTTDTMKKEIAVVEEIGGVLVTIGAIAKGSG
MIHPNMATMLGFITTDCAITSPMLDKALKDVVRKSFNRVSVDGDTSTNDMVAVLANGMAG
NPPIEKEDVNYGEFRDALEFVCVELAKMIARDGEGATRLVECTVSGAVSEDQAEALAKGI
ITSSLVKAAMFGADANWGRILCAMGYSRDDFDPDKVDVSFESPRGYIEVCRAGAPLPFNE
ERAKKILNNQEVEIIVDLHAGDAEVTAWGCDLSYEYVRINGDYRT*
>SPBDM4_v1_40485|ID:27157790|argC| N-acetyl-gamma-glutamyl-phosphate reductase [Uncultured spirochete bdmA 4]
MIHVGILGATGYAGLELTRILLQHPRVDRLSASSVSFEGKSLEDVYPSMVNRQDSVALPA
LEKAEKVIAKAEVVFSCLPHGHAEEAGYQCVTASKPFIDISADFRFGPHEDTFKKWYGAG
YHYPDLHAQAVYGLPELHREAIESARLIANPGCYPTSAELGLMPVIQEGIVDLSSIVITS
MSGVTGAGREPSQTTHYPEAADAVSPYKVGAHRHQPEIDLLLSEMAGTSVQSVFTPILAP
MNRGIVSTICFRLARHLSVADLYAIYAKRYAGEPFVRVLPLGSTASNRFVKFSNYCDISV
HLSADGERAVIISAIDNMVKGAAGQAVQNMNILLGFEETEGLRFIPPAF*
>SPBDM4_v1_40486|ID:27157791|hppA| putative K(+)-stimulated pyrophosphate-energized sodium pump [Uncultured spirochete bdmA 4]
MLSSGVIVAVSTALVALVYSLITSSSIKKIPLYNSTVARISGYIEEGAHAFLTREYRTVS
LILPVIALLLFALNGGILKLQSISFLVGAALSASAGWFGMSIAVKANARTAEAAQKGVLP
ALKIAFSSGSVMGMTVVGLVLLGIGGILGLLLLIPNAETMLAETIFPIVSGFSLGASMMA
LFARVGGGIYTKAADVGADLVGKVEVGIPEDDHRNPAVIADNVGDNVGDVAGMGADLFES
YAGSIIGCMVLGIAVSASPQMQLRLAILPLIAAGTGVLASLIGTLFVHLSRAKRPQTALN
AGSLGAALIAIVLLLPVIRFTVGTGHFLSGTREAGWLGIYFACITGLVAGTAIGLITEYY
TGSDTPPVRKIAKSCTTGAATTIISGLGVGMSSTVFPILVIGLAIGLSNQLAGLYGIGIA
GLGMLLTLGIQLSVDAYGPVADNAGGLAEMSEMEPEVRKVTDSLDAVGNTTAAIGKGFAI

GSALLTSLTLIVAFLSAAGIPIETMNLANPSVLIGLFIGGMIPFFFSSLSMDAIYKAAFA
MIEEVRRQFRSKPGILEGTEEPDYRSCVDISTQTALKSMILPGVSAIAAPVLAGLIGGPA
VLTGLLVGATVSGGLLAIFMSNSGGAWDNAKKLIESRSEEGKGSAAHRAAVVGDTVGDPF
KDTAGPSLNILIKLMAIISLVLAPLFRTVWH*
>SPBDM4_v1_40487|ID:27157792| conserved protein of unknown function [Uncultured spirochete bdmA 4]
MANEKHLYMIVHPNHSLIASQLEPEHFIKHYVQGSTRYFEGRLIFTEIDPEFRNPYFDID
TAYAELKPHEDGRPKATKFIKSYRVMEHMDYGALGKLYISNSLGDYVELESKPYEPSIDT
DEFRIMLEVSPLRFIVLTKLNCHDFGQFITDPRNSKGAPKMAFTLLEFDAQEFLKEYEEN
PLIRCYVPGIHPARLVSAIEEVRTTPGKVVKGISLDCPIDRISYKYLKEGFMFAENGKPC
KFYPYLDLDTVERKFYKFWKSM*
>SPBDM4_v1_40488|ID:27157793| conserved protein of unknown function [Uncultured spirochete bdmA 4]
MELNVGLVAEKEIVVEEKHVASHLGSGGVPVYATPSMVLHMEETSRQAVDHLLGPDGATV
GAYIAVKHLAPTPKGMKVRIRSELVKVEGRLLTFKVEAWDEVEKVGEAEHVRAIISMAKF
SERLEKKKKAAAQK*
>SPBDM4_v1_40489|ID:27157794| Alpha/beta hydrolase fold protein [Uncultured spirochete bdmA 4]
MWLIIVIYAGLIILLVGMTYKFMFRLKPRSLEFTKQYSLAHGEIDEAFLDLDWKEWEFDS
PHGYVLKGQYLRGKEDAPAALFVHGIIWTRYGMAKYMRPFIEWGWNVAAFDLAGHGESAA
PRRFYPSYGFYEKFDVQAGVKELHSLFPEAPLYGLFGESLGAASALEYAPLAGSAASRGV
DFVVADCPFSSAWDELMEHYRMAHVPNFIAWPAAHVSRWLVRFLHGFDIEEASPKKAVMQ
TDMPILFTHGMEDRDVPTIMSVRMASARMANNVGMTELVLIPGATHAKGILTDKARWLAG
VTAFIDRVCSEKNSAHMSGQRGV*
>SPBDM4_v1_40490|ID:27157795|msrB| methionine sulfoxide reductase B [Uncultured spirochete bdmA 4]
MVYSEKDANTICYIQPTEELKAKLSPEEYAVLVEAATEPPFENTFWNNHRDGIYIDKIDG
TPLFASTTKFDSGTGWPSFWKPLDENALILVDDYSLGMHRIEVRAKKSGGHLGHVFDDGP
NPTGLRYCINSASLRFVPKEDLELEGYGALLSLFR*
>SPBDM4_v1_40491|ID:27157796|trpS| Tryptophan--tRNA ligase [Uncultured spirochete bdmA 4]
MEKKRILTGDRPTGKLHLGHYVGSLANRVRLQDDYECFFIIADLHTLTTKPEKENIEQLS
ENIHSAVLDYLSVGIDPKKSVIYLQSAVPEVTELSLFFENLITVPRLSRVPSLKEMAQNA
HIEEMPFGLLGYPVLQAADILLPRAHLVPVGKDNEAHVEVTREIARRFNFLYGETFPVPE
VMISEYGSLVGTDGNAKMSKSLGNAIFLSDDAKAVQKRVMSMYTDPNRVRADIPGTVEGN
PVFIYHDAFNPDKAEVEDLKARYRAGTVGDVEVKEKLASAINAFLDPIRERRAYYESQKG
LVEEIIYEGTMRMREEARITLSMAKKAMGLSSVWNRISRKAEDRRKRAAE*
>SPBDM4_v1_40492|ID:27157797| putative Diguanylate cyclase [Uncultured spirochete bdmA 4]
MKIDISTINALFTDSSLSGFFAETDLFYIDFQSGTARSCYTLRSLGYEDNDLLNITSPFS
VQEADKERADRLIHRLIANEADSVIDAFRVASKDGSSYHWLRFSTRVVSRDAEGRPEAII
GHVLDVDDLITSQEEIRDRLVEIDAMRELITAINKSLDFEETFLRIIEQLHRIIPFDRAS
AQSLENDRLTVVAGFGYREGELEGLTFPAHGIENPSVRALQEHKIVICNNVPEEFPGFIR
PNNDFITLSWLGIPLIYEKKVIGLLALDNQALNAYNDQHVRIVSSLADYIAIALEHARKH
QLVTYQAMTDRLSGLANRYGLETQGMEIFQKSIEQDQSIGVLMIDIDHFKQVNDTYGHAY
GDIVLRSITQAIREQIRSRDYAVRYGGEEFVVLLPGLSAREALIVAERLRIRISQTVIEK
ERRLPTVSIGIYAAVPGPLDILHEFIRRADLALYTAKEAGRNRSRVWSTSPEFYVNE*
>SPBDM4_v1_40493|ID:27157798| putative Methyltransferase type 12 [Uncultured spirochete bdmA 4]
MESKEGPKLKLDTTLVSRLSFWYDISVSLGLAIKKYKNVKIIGENVRALQRGLTGDRTRI
ASGYMDEQSLLQAYLLYYWAVSLFEVTMVLEELDARARLPELHSVLDIGSGPGPASFAAN
VFGARRAFLVDKSQKALATASEIAAEARREHSDFEILTRYANLEEFRVPAGETFDLIVAS
QSLNEMWHDVPDWLERRRDFVLRLLPSLRAGGILVIIEPSAHYTSIPLLALRDRVLESIG
RQEPHEIAAFTGGAQIACAGPCPHSGSCPMRTLKGRPCFSEWQWEAPPLVNELANFAGLD
RTSLKAAWVAFKKTPPETGRAKAEGARNMPDTPAKSGSLRGRIVSEPMRNKAGRIRYIVC
TDCGLLTTISAPQGDPEARSLGFFQLARGDLLEADGLEARGDAHLGIVRGTRIHITMKAP
RI*
>SPBDM4_v1_40494|ID:27157799|hndD| NADP-reducing hydrogenase subunit HndC [Uncultured spirochete bdmA 4]
MVNVKVNGIPVQVAEGSTMLEAAKKAHVKIPTLCYNPDLSPWAACGICVVKVEGSNKMLR
SCCTPVSEGMSIITNDADLVQTRRTVIELILSTHPDDCLFCPRNQSCELQTLAQEFGIRE

QPYKKMVRDIPTDTSTNSLILNPSKCIRCGRCVEVCQEMQGVWAIEFLGRGESIRIAPAA
DVKLGDSPCIKCGQCSAHCPVGAIYENDQTSLVWDALMKEGPDAKICAVQIAPAVRVALA
ESFGLPPGTDLTGKIYTALRRLGFDAVFDTNFSADLTIMEEGTELVHRLTEALKGGMQQA
TADHKLPLITSCCPAWVDYMEKYYSDMIPNFSTAKSPQQMMGVMIKTYWAAKAKVDPAKI
YSVSIMPCTAKKFENGRDESMYSSGYKDVDVTLTTRELARMIKQAGIDFLNLPDSQPDSP
LGPYSGAGVIFGATGGVMEAALRTAYFLVTKEELKDVNFTAVRGLSGIKEATVHINGIEL
RVAAAHQMGNIATVLEEVRKARAEGRETPWHFIEVMACRGGCIGGGGQPYGATDEVRKLR
MRSIYDHDEKSEYRCSHDNPYIKQIYAEFLEKPGSHRSHELLHTHYVEKPLYLK*
>SPBDM4_v1_40495|ID:27157800|hndC| NADP-reducing hydrogenase subunit HndC [Uncultured spirochete bdmA 4]
MAYKNFILVCGGTACESSHSEEIYRNLIKEAEALGVSRDVQVVKTGCFGFCEKGPIVKVL
PSESFYVEVKPEDAHEIIAEQVIKGREVTRLLYRKDKKSDTARLEDIEFYQKQFRIVLRN
CGVINPESIDEYIARTGYRALEKALFEMTPEEIIAEVKASGLRGRGGAGFPTGMKWEAAR
KSPGDIKYIVCNADEGDPGAYMDRSTIEGDPHSILEAMVIAGRAVGANFGYIYIRAEYPL
AIERLKIAIQQAKDYGLLGKDILGSGFDFDIEIRLGAGAFVCGEETALLQSIEGKRGMPR
PKPPFPAIKGLWGKPTVINNVETLANVPVVLTRGANWFSSIGTEKSKGTKVFALTGKINN
SGLIEVPMGTTLREIIFDIGGGIRNGKKFKGVQTGGPSGGIIVEKDLDSPISYESLTALG
SMMGSGGMIVMDEDDCVVDISKFYMAFCVDESCGKCAPCRIGTKQMHGLLDKISKGQGED
SDLAKLEQIGKAMTKASLCMLGGSAANPTLSTLRHFRDEYLEHIHDKKCRAGKCKDLVIY
EIDPAKCIGCGLCARRCPVPCITGEKKKPHVIDGSKCIKCGECYAACKFGAVIKR*
>SPBDM4_v1_40496|ID:27157801| NAD(P)-dependent iron-only hydrogenase iron-sulfur protein [Uncultured spirochete bdmA 4]
MAKMTLDHLRALRAQKQGELEKRNVEGKDIQVIVGMGTCGIAAGAKQTFDAIVAALEKHN
MTGKVVVRQTGCMGLCYVEPTVEVVMPDMPPVIYGKMTSDVAEDLVVKHLVEHSLLDSHI
FDRPAIDIVKH*
>SPBDM4_v1_40497|ID:27157802| Histidine kinase [Uncultured spirochete bdmA 4]
MHCTVGDFVLDVVQNSVEAGSNLIEVEIDDAHDGVLALIADDGCGMSPEERARALDPLRN
SGAKHPGRKVGLGLPFIQQAVEQADGSFSLKSEKGKGTEVRFFFPASNIDSPPAGDLVSL
FVATLSMAGACEMKLRRTRARDGLSYEVKKSELVEAVGDLASASSLSLVRQFLASQEEG*
>SPBDM4_v1_40498|ID:27157803|hndA| NADP-reducing hydrogenase subunit HndA [Uncultured spirochete bdmA 4]
MVAGDIEFSNELQSYIDEWSKKEGGLVMMLHRIQNEFGYVPRAAAEKLSLISGIPLAKIY
GVITFYHFFKTTKPGKHRIAVCLGTACYLKGGQDLLEETCSILSIEPDEMTDDGLFSVDE
VRCLGCCGLAPVLMIDNEVYGKLTKEQLPGIIAKYRSL*
>SPBDM4_v1_40499|ID:27157804| conserved protein of unknown function [Uncultured spirochete bdmA 4]
MANSEKNKVVSELNNRAVSLIDAGRYQEAIDLLTKALSLDPLRTGILFNRAEAHRLSGNV
EAARRDLLSDLHIVPDSPEVLHGLGLLAYEQDDFSLATDFYKKALDKDPAFTPAWNDLGV
VEFRAEHYDSARTFFEKATTLDAGFSDAWFNLADTYDELGMADKRAYAREQLRKARLRSG
EKPDSEEDE*
>SPBDM4_v1_40500|ID:27157805| putative Serine/threonine protein kinase [Uncultured spirochete bdmA 4]
VKAGDTIGSYVLERRLGRGVTASTWLARRAQPDSGSGAESPLVLKIFDIAETDSWKPLDQ
FKREADILRTLSHPRIPKYIESFDATLEGHFLFVLVMEYIQGETVEAYIASGKKFTEEHI
EQILAELADILAYIGSLRPPIVHRDINPRNLIAQPDGHVALVDFSGVQDAIRTALYPGAT
LVGTAGYIPVEQVSGRATHRSDLYGAATTTVFMLTGRNPAELPMRNLKIDLSGLLYMSPR
LSYVLDNWLDPDPDRRFLSAAKSASILRGESAIPSAASGASRSETSSRQSERAGQTPRSI
FTDILHSFEHQSEPREYPEELPSDSTLEIQALDPGLYIKMPRRRRGASSGFSVFPVVWIG
FIFFWTTMTIRMHAPIFFSIPFLIAGVAMVKSFLNPSITDTELFITPDGLLKKNTSFISN
SAQQFRISDVGKARVRRSSMQENHRYLKELAIEAGTGTLTFGLGLSDRELYFLEKRVNEE
LARLKQLLPPGTAS*
>SPBDM4_v1_40501|ID:27157806| putative enzyme [Uncultured spirochete bdmA 4]
MNMSANLNPRPVSVSFVEMDEIVQPSDLNINGSIFGGYLMSLMDKAGGISAFKHCGVNVV
TVSVDQLVFKNPAPAGTVLSVKASVNRAFHTSMEVGILVTGLKPGESKETKLCSAYITYV
AIGADGRPIPVPPSLPETPEQIRRFREAEVRRSARLALAERLAAQQ*
>SPBDM4_v1_40502|ID:27157807| Transcriptional regulator [Uncultured spirochete bdmA 4]
MENIEEHAFFRYLTYSDEDERWEMVCTDAGYTEVPPYTIYPPNKEDHPRMFQRVAVGRTL

NEYQIIYVTKGEGIFETSGRRYEVKPGSIITVFPGVRHFYKPVYEVGWMEYWVGFKGGHF
ELLRERGFLNPQQPFVEIGLQNDILDLYNEIIEEVRDQKPLYQIVASAKILSLISEINAC
ARRKAQTSHAAQIVESAKFIMAEKIYGDIDISSIASQLGISVSRFNDIFKTYTSMTPYQY
YIHIKIHAAKSLLEQGDLSVKEVAYRLGFEDQYHFSRLFKQKTGIAPSQWRVFMYE*
>SPBDM4_v1_40503|ID:27157808| conserved protein of unknown function [Uncultured spirochete bdmA 4]
MIGNSTVNPAVGLFGIGLDTYWAQFPGLEERLKGYQAETRATLEQAGARVIDAGLVDTVE
KARAAGALFREKRVSVVFLHITTYALSQTVLPVAREAGVPVVVVNVQPSKKIDYEWFNSL
GDRALMTGEWLANCQSCCVPEIANVFNRNGIPYHMITGFRGEAFVERELHGWVQAARVAE
ALRRTNIGILGHYYEGMLDVYTDVTRMAGVFGSQFDFIEMDYLAQLHKKVDPTQIEAKIA
QFRREFEVVPECEESELVRAARTSCALDALVNEKELGALAYYYEGTAGNVHQDIVTSVIA
GNTLHTAHHVPVAGECEVKNELAMKILDLLGAGGSFSEFYLIDYEDDVVLWGHDGPAHSA
IAEGKVKLVPLPLYHGKPGAGLSIQMSVRHGPVTLLSVVEGAGGKVFLLTAEGESVPGPT
LQIGNTNSRYRFPLSAREFSEAWSKEGPAHHCAIGVGHVASTLEKTAALFNIECHRIC*
>SPBDM4_v1_40504|ID:27157809|rbsA| fused D-ribose transporter subunits of ABC superfamily: ATP-binding components [Uncultured spirochete bdmA 4]
MADYILELRHISKFFPGVQALKDVHFQLKPAEIHAIMGENGAGKSTLIKIITGVYQPDEG
ELLFNGQPIHFKSPLDAQRRGIAAVYQHVACFPDLSVLENIFLGHESFDSVTHKIHWREL
RKQAESLLQSLDADFSPSATMGSLSIAQQQIVEIAKALSVDAKILIMDEPTAPLSRRECE
DLYRITENLRDKGVSIIFISHRIEDMYRLAERVTVLRDGRYIATWDVPGLEQARLVQGMV
GREITQYFPSRHATIGEEIFRVEGLSRIGFFKDISFSVRRGEIVALTGLVGAGRTEICES
IYGISPRNGGRFFLEGKEIAIHTPAEALAHGIGYLPEDRMKQGLVLDWDLIKNITLASLK
NFSRRGFMQPSREKDIALRLTDNLKVKTSHIYAKASTLSGGNQQKLIVAKLLTSKLKLII
LDEPTKGVDVGAKTEIYRIMNQLAEEGYGIIMISSEMPEVMGMSDRIVVIREGRKSTEFE
TPQATQEAILEASMTAHGRELHAIAG*
>SPBDM4_v1_40505|ID:27157810| Ribose transport system permease protein RbsC [Uncultured spirochete bdmA 4]
MQLQVKMNADGSSGGGGNGRPQSSEHQSSSLFSSLVKRLTRNSEAFRELGLLIFIVLLAA
IFQIRNPKFLSVANIKDLLANTAILSILAVGMMMVIITRGIDLSIGSTMALSGMVTSLTV
SANPDISPFLSLAQGMAIGLAAGLVIGVLVAYFKILPIIATLGLMNILRGLTYLISKGKW
VSAYQMSAGFKNLSTGTTFGINNLILFVIGIYILYTYFINQTRTGRYIYAVGSSPETAEL
IGIKRKRIILLVYALMGLLAGLAGVLWVSKFASAQGDTAVGYEMNVIAATVLGGVSVAGG
RGKVSGILLGSILFGILANALPLINISPFWQQFIQGLVILAAIISNVLLQRRNDRLTLKK
RVI*
>SPBDM4_v1_40506|ID:27157811|rbsC| ABC transporter periplasmic subunit [Uncultured spirochete bdmA 4]
MEKTSIDINKPRGLHQYQWEIFLIVLFLAVNVVNSFLSPYYLTLDTFVSTPMNFLDKAFL
VLPMTMIIILGNIDVSVGSIVALSSVLMAVSYNAGLPMPLAMVLALLVSTTCGFINGFLQ
IKFKELSATIITLSTMTVFRGIAYVILEDRSAGKFPSWFSFLAWGYIGKIPFILIVFALT
AIVFAVLLHRTRFGRMVYAVGSNRTACEYSGIRTDRIILGVSMLAGLMAGFTALFLTSRM
GSTRPNVALNYELDAIAMTVLGGISTNGGKGRITGPLISIFLIGFLNYGLGLRNVSAQVL
LIILGFLLILSVLVQNFTDRPASISPKKLSAPTHRSDAGDGSDIHENSAQGG*
>SPBDM4_v1_40507|ID:27157812| Sugar ABC transporter substrate-binding protein [Uncultured spirochete bdmA 4]
MKRTITVVALVLLALTLVIPSSVFAAEKAKFAIVFKNTGNPYGEKQLEGFKVGIEEQGFE
AILRSPDLPTAEAQIQIIEQLIAQKVASICIVGNDYDALTPVLKKASAQGIKVFSLDSSV
NPQARLTHVNQADSEKIGQTLIKAAYDMSGGKGEIAILSATSQASNQNIWIDFMKKELAK
PEYKDLKLVKIAYGDDLRDKSVSETEGLLRSYPNLKVIIAPTTVGIAAAGKVLTDKGLKG
KVFLTGLGLPSEMAEYINNGVCPYMFLWNPIDVGYLGSYVGTALVSGKITGKVGDKFSAG
RLGNYTITKAPDGGTEVLLGPPFKFDKSNIEDWKKVY*
>SPBDM4_v1_40508|ID:27157813|rhaM| L-rhamnose mutarotase [Uncultured spirochete bdmA 4]
MKRNVFAMQLKPGNEAEYKRRHDEIWPELKAELRKAGVSDYSIYLDPQTLRLFAIQSLAD
DETNDLLPQNPIVRKWWDSMKNLMETNPDNSPKVWALNEMFHMD*
>SPBDM4_v1_40509|ID:27157814| putative bifunctional rhamnulose-1-phosphate aldolase/alcohol dehydrogenase [Uncultured spirochete bdmA 4]
MNLDPIVQLSRNFGSNPDYVLAGGGNTSQKEGDFIAIKASGVQLGTIDAAGFVRLSLSKL
TALFNIELPSNSDEREKIVLEKMLAARLPGQTMRPSVETLLHALFPFRFVVHLHPALING
MLCSREALSAAQALFGSEAAVLPYITPGYTLADAVRRIFETRTLSGQSPYKILFLQNHGV

FVAADTAEEIKALYDHIFATLKAKVNALPAEDKVAESVSAEEKVLRSKVHTAVEACSTKF
NVRFFFTLNSVVGHFLASPQAFAPLTAPFTPDHIVYAGAWPLFVTQHQAQTSEHLEVQIN
AHHARHGELPKVLAVQGVGIFGLGKDIAAAERACLLFTDAAKIAWYAQSFGGVHSMESPD
IEFIRNWEVEKYRSSIASKNNIAASKQ*
>SPBDM4_v1_40510|ID:27157815|rhaA| L-rhamnose isomerase [Uncultured spirochete bdmA 4]
MSRTEQEYKAAREIYAELGIDTDQVLKTLSSIPISIHCWQGDDVIGFDGATSLSGGILAT
GNYPGRARNAEELRADAAFAFTQIPGKKRFNLHAMYAETGGEKVERSSLEPRHFNNWVAW
AKEQKIGLDFNPTFMSHPLAASGWTLAHPDNNIRSFWIEHGKASRRIAQAIADTLKDFVV
NDLWIPDGSKDMPADRLGPRLRLKESLDAIYAEKMPNDRVLDCVESKLFGIGSESYVAGS
HEFYLGYAMQKAIGICYDMGHFHPTENIADKISATLLYVPYVVIHASRGIHWDSDHIIIW
NDALTDVCREIVRMHVWDRVYLALDYFDSSVNRISAWIIGARSTQRALLYAFLEPVEAMR
SEEERGDFGARLGFIEESRSLPFAAVWRYYCENQDVPYGPAWIEDVKDYEKKVLSRRE*
>SPBDM4_v1_40511|ID:27157816| putative Phospholipid/glycerol acyltransferase [Uncultured spirochete bdmA 4]
MSDKENISLKKNRPDVKPGPYDVRASSSSTLSPAEDYDPEHEMNANRYAEIALQSKVPLF
RFLHVAMKEDSLFLLPRILIRTIKTMRRIPSTFFIKDGAELEHYVFRTIASWSKAVIKIT
KMKLDVRGTEHLKPNQTYLFVSNHRSPADIPVLFATIPQHAAFVANAIFQKIPAISYWMR
ASGSVFIDQGNPKSELRAFKAMTRRLKGGRSLILFPEGHMHQGKGLDTFNRGGIFSAVLT
GTPIVPVCLYGTDKVIRPGSFHINPYKRVIVDIGTPLKTIDLGRAEKKDIDAIVQDIIAE
KRSFYEKKYPAYKRAET*
>SPBDM4_v1_40512|ID:27157817| conserved protein of unknown function [Uncultured spirochete bdmA 4]
MAILYRGLPYPVIEWTEGDKCCSARWYSENVALPPKRVQVVDDRLPADIAYRLACEGTAL
LWRGDFQNARHLLKAITRRADRKSAKPGTTMSETFHLYRQAQSRKARVLGMLLIPLSADY
SIPLRRAPDVRQACLEAYGPKGEESVVSLRGLQGVIGAHEWHKKGVKIPTLGGLIHPWYG
VYAPIRSEYIDLVACVPLPTKVLAFDIGTGTGVLAAVLIRHGVARVVATDISLRAIACAR
ENLTRLGLRSQVDVVYANLFPEGRAPLIVCNPPWIPARPSSPIEYAIFDPGSSMLRGFLA
GAADHLEPGGEAWLILSDLAEHLGLRSREELDSWIEAAGLMVIDRLNVRPRHPKVKDISD
PLHAARAKEVTSLWRLVARSEK*
>SPBDM4_v1_40513|ID:27157818| MATE efflux family protein [Uncultured spirochete bdmA 4]
MQRYLLTDGRLFPSFMKFALPVLGSLFLQMIYGATDLVIVGRYADSTSVSAVATGSQVML
TITSIITGFTMGETILIGQLMGARRERECAGAVGSGIVFFVILALVLTAIMVPFAGNFAA
LMHAPPEAFAQTTSYIAICSMGFVFITGYNVVSAIMRGLGNSALPLLFVAIAATANIFGD
LLLVAVFHMAATGVAISTVMSQGVSLLLAIVILKHRGLPFKFYRSDIRFDARSTFTMLRL
GFPIALQDMMVGISFLIILMILNSMGLVASAAVGAVDKILGFGLLVPSAFSSAIATFVAQ
NMGAGKELRARHAMYYGMSGSLVFSLFFFFASQFAGETLVGFISRDALVIAAGAAYLKAY
SWDCILVCFVFSMTGFFTGCGKTSFVMVQSLIGTFLVRIPFSAFMSRRPGVTMFQIGLAA
PTASIVQILLCAVYLLSGKWRISSTERGSYGHSL*
>SPBDM4_v1_40514|ID:27157819| conserved protein of unknown function [Uncultured spirochete bdmA 4]
MRHEKIKIGEISKLFQIPQSRLRYWEDENLIHCERDATNNYRRYTLTNYFEVGDIVFFRD
LGVSINELKNLSSMELEDLSRLLEENEVKLDGRIAALEETKQKIRKKRHMIAEIGRLRAN
SFTLCSPDFEKVIADPLFENSRMMPPLQDVSSCAILLTQDRKEPRYGIIVSEVQPGNTVV
WKRAIGAQRYAECLLEISFDHPDNNNLATILHALAALGYRTGSLVGRYLASAFRTTWYEY
YQSWIEVE*
>SPBDM4_v1_40515|ID:27157820| exported protein of unknown function [Uncultured spirochete bdmA 4]
MNVMRREKFGLVSPLNHWNRKAYISFCLAFSAMSILLVFYACSTSSAVLPPPHPSGQTST
FNRADFEHALAGRNLDVLASYSSKDSGSLGANLNSADREKAARRYEALRQELLLIPASQA
ALAPDLPSLAALLAKPEARHFTSFERAKLDSALARYAAVESPASKYRTGIVDPRISRITK
TQLSNAARDPGHNLKAIVNALLSGESDPFLRIKLIHDWIADTVTYDVSMLSKNSVTGQQD
LASVLASRRAVCSGYARLFEKMAELAGFEVKTVNGYTKGLGGDFAFDVRNSHAWNMVRIG
DLWYFIDTTFDAGAFDSDGGSAQYHKRYSTDYLFPSPFQLRFTHFPDNALYQLSVTPTDK
EDFKSRVLVSPEFFKNGLTVQRMPGATASLSSFAYLQKTSGLFSLDIEATEKMLVDASLF
DAKGKELERYTFASHISPTSWRLIFAPPSTGRFKARIFAGPENPGNADSSPSILAGVLQF
ELEMSSAGSKASASARSANPPFPKIYGRYHEPAGELLESPLDGSLAKGSTTHFFYHSKSK
YVSIIYDNTFVPLKRGADGSFSIDFKVPATSAIKLGVSEDNVRYSIVLAWDVR*
>SPBDM4_v1_40516|ID:27157821| conserved membrane protein of unknown function [Uncultured spirochete bdmA

4]
MNGIIDSVWQWIDDLPSAIGAALILIVGWGVAKILKIIVPKLLAWLKFDVLAGKVGVNEF
LRKGHVQYAPSKLVGVFLYWIVMLFALAEAASALDQRAAESIRSWLLGAIPTILASGITA
VIGILIVNFFSNFVLTIMNNAGVGSGTLVQRLIRVVGYIIVVVMVVDQLGLGQSIISILL
LLLIGSVALGVAIAIGLGCKDMARQYVENIIAAIQERERASHGTDLEG*
>SPBDM4_v1_40517|ID:27157822| putative opine dehydrogenase [Uncultured spirochete bdmA 4]
VKKTDWGGIVEHLDRVASKGKKMVWCVAGAGNGGIAMAGHLGILGFPVQMYNRTDEHLNA
IRWYKGIDIEGAVTGFGSVRLASSHIEDAIKGADVIMIVTPSTAHYGFATLMAPFLKDGQ
IVVLNPGRTGGALEFRAALDRENCEARVVIVEAQTFIYASRMITRHRAHIFRVKNGVPVS
ALPSFLTPAVLKVLDVAFPQFIAGSNVLATSLENIGAVFHPALTLLNVAWIESTGGDFEY
YLQGISPSVAKVLQAIDDERLALARALGIRTVSAREWLYLTYDSVGADLCSAIRATTSYV
GIKAPRTIDHRYVWEDVPMSLVPMASIGAMYKVETPAINLTIDLANLMNGQDYRANGRTV
RTLGIEGLSVEELHRLVTEG*
>SPBDM4_v1_40518|ID:27157823| Cobalamin B12-binding domain protein [Uncultured spirochete bdmA 4]
MYKVSQNDVVGLVKPAIDAHTLGILSFAQILRDCGIRVEIADEAVGKEIDTLAERGSIDT
LTQWIRAKNISVLGFSYRLDPERGVEVFVRLVGLLQRARLLARDGGPLRALWFAGLPPAC
DRVVQRAPFVDAVFRGDESYQEILGSLGLPAHLVPEQASAELAYDKARLELGAEIVKNRV
YLDVAPVDRSGSPGFGTRGERVADRVRHGAVHSLPPLMRAHVGPYAEDRKQAVALFFDWT
SRLAKGGLLDVLSIGSSQLTQSNFGEDWSGLPNGGGVPINSAEEYRRVWQAARPMLVRTY
AGTKRVPHMAKMHEEALDICWHALSLWWFSKLDGRGENTVMQNLEEHFETLRYIASTGKP
MEPNVPHHFAFRGSDDVGYIVSGYVAAKAAKAQGIRTLVLQTMLNTPKYTWGIQDLAKAR
ALRMLVRELEGPDFAVILQPRGGLDYFSPDMEKARAQLAAVTMLMDDIEPDNEASPQIIH
VVSYSEGVRLADPEVVEESVRITRYALSEYRRLKKKGDMPEYGKLPIVETRTQYLYEEAR
RMIDFVEATIPNTYSPRGLYAMMKGGVFPLPWLSACKEEFEAAVNQPVQFMHGGVRTVDC
EGKPLSVSKRLFGIARNLERMGFAPSDFQHMGSLRHARKGQKEAHSEKD*
>SPBDM4_v1_40519|ID:27157824| putative D-alanine--D-alanine ligase [Uncultured spirochete bdmA 4]
MILQIIMKRNSDPIIAAENAVGYARMAGIYNGAGFEAYFSYANEIDDIVSIISENRPDLV
LCGMDHLPDKIGDVSISHNVHKWFEEHHVNYVGSDPETIERALSKAALKRRWEENGIRTP
AFVFVDGASQDYGSALERLVSLDAFPYIMKPENLGNSKGIDEDNIAWNADKLEPTLKRMR
SRYGGAILVEHYLGAYPDVREITCAMIQGVEAMQCMPAQISFLQPKRFHLITTNDKDGHR
TVMSPLESKMAASFVPFARAAFESAGMRDYARGDFFFADGKLWAIEINGQPMIPDRWFEG
AVQFAGLSEAQYLVGIVAAGYRRLRSKNRIAGSLPEGAQALLKGTVLGGF*
>SPBDM4_v1_40520|ID:27157825|pcm| Protein-L-isoaspartate O-methyltransferase 2 [Uncultured spirochete bdmA 4]
MITCNERKDERDFMVRSQIAARGIRNKRVLEAMRKVPRHLFAPLPYRAEAYDDYPLPIGS
GQTISQPYIVAFMAEALLLKGNERVLEIGTGSGYQTAVLSMLAKEIYSVERIAILLERAR
AVLESLEIDNVQVWLGDGFFGWAEKAPFDAIILSAAPFDIPKTILHQLVDGGRIIAPIGP
LGMQTLVRMTRRNAHFDTEALLDVAFVPMRPGVSEI*
>SPBDM4_v1_40521|ID:27157826| conserved protein of unknown function [Uncultured spirochete bdmA 4]
MHAARNLRLCTKDCICLFVCPTKATDTENGQVDFSKCLDGCRLCVDACPSHAIYLVNANY
AEPAPKARRVSEKAFALLDRKAGEEAMVKQMLVKAAVPGKDAVNTANASAALVLKALEKS
LRIVAEDAAREAAYMLPQSSSTQMLIETLLQDRPKCGEGQFPEQELRELKGLLSRT*
>SPBDM4_v1_40522|ID:27157827| Rubredoxin (fragment) [Uncultured spirochete bdmA 4]
MEHAYGPVFSNLAKACEKQFRTREAELLWKLSGYFDGAVPSVGEVLSSGRADRAGPSLDA
RKPASFQGLAAALAADETEHFDSIKTEATAAKDRGALRMTVWGSKVNAIQQSVVERYAKQ
GDALLQDKSVFVCEACGFIFIGQEAPEICPVCKAPRSRFSKIS*
>SPBDM4_v1_40523|ID:27157828|gltB| Ferredoxin-dependent glutamate synthase 1 [Uncultured spirochete bdmA 4]
MQKHKNELYNYPKRHLQTGSLYKAEDEHDACGIGIVAELKKKANHRVIIDALSVLRNLEH
RGAVGSDRKTGDGAGILCQIPDRFFQKQMDLGRFFANIKTAHDLPYGLGMLFLPAGHSDF
LKAQALISTVSAHRGFRILAWRDVPVFPQVLGERANASLPRICQAVFAKANIAEDQGDDV
QDADGHLERELFLLRKYLEKEAKTAGFSLDQFYISSLSSRTIVYKGMFVASQFASFYPDL
EDPLFESQFAIVHQRYSTNTFPSWPLAQPFRMIAHNGEINTLRKNINSMKARQATMKSTL
LGSDFFNDVPILEEDGSDSAIFDNIFELLVHAGRSPEHAFMMMVQEPFSERLSISKDKRA
FYDFHAAMLETWDGPAAMGFSDGHRVGAALDRNGLRPFRYSLSKTGYFLGASEAGVLNLD
ESEVEERGILRPGQMFLLDLDSGRFIKDTEIKTCISRQKPYRRWLEANHIGLRGLFADQE

LHTHGERPVSTEEMTRRASYFLYGQETIRILKPMMLQKQEATSAMATKKPPAILSRTSVP
LSAYFRQRFAQVTNPAIDSYRETVVMSLENYVGTQKNLLEESPEHCRQLKLQRPVFSNSD
IETLRSAKVPDFQVATVSMLFQIHGEHIDLEQTLDDLCAEAAQKIDGGANLIILSDRGVD
ADHASIPALLAVSALHTHLVDVRKRHLAGLIVETGEARDIHETAALLAYGASGVNPWMAF
ELIPSLAAQWNEGRGAEILADNYIEALNKGILKVMSKLGISTVSSYRGSRLFEAVGLSEE
LVARYFRGTESRFGGIDMEDVAQDILCRHRIAFAREYKDNGAYPTTPLSQKEPAALPWPA
ALAVKLTQAVRSGEPEAWRAYADDMDSSLRPPFALRDLFGFRQATPIPLEKVQPAEEIIQ
RFSVAAMSLGALSPEAHEALAAGANAIGSWSNSGEGGEDSERCSYKELGYSSENASKQIA
SGRFGVTARYLATGLELQIKIAQGAKPGEGGQLPGAKVNDYIARMRHSKPGVTLISPPPH
HDIYSIEDLSQLVHDLRCVNPDARIAVKLAAEAGIGTVAAGVAKAGADCIIVSSGDGGTG
AAPLSSLDYAGSYWEEALPEIRQVLAMNGLDINTVIQVDGRLRTARDVVIATILGAREFA
FGTLSLLAMGCIACGQCNLGKCPAGIATQEPEFRAKFKGRPEHLAALFRYLADDVRVILA
SLGAKSLDEIAGKYELLDFGARAATPKEKKLSLDRIVEALEIARRYPLLDTSASDEAGAP
AIPLPPAGLRNFRPGAQLPFSISEAESQLLQKCEPLFSWTVAPSRLDLALSICNSDRSVG
ASLSGQIIRAGLDLEPDTLCVNFTGAAGQSFGAFLVKGIFFRLYGEANDYLGKGLSGGRI
VVQPRPESRCEPELNVIAGNVCLYGATAGEVYLNGTVGERFCVRNSGALAVVEGTGNHAC
EYMTEGTVAVLGSTGINFGAGMTGGIAYVLDEDQLFDTRCNASDIDVTGVTRPEDIERLR
RIVERHYAFTASPRAKHILDEWDAYLPLFLKVTPLEH*
>SPBDM4_v1_40524|ID:27157829| putative amidase [Uncultured spirochete bdmA 4]
METGELDEFTVEALQKAMEAGELSATDIVEYYLQQIEAVDRGGPELRSVLYVNPDARVIA
KGLDEERHAGAVRGPLHGIPLLLKANINTADTMPTPAGSLALHGFIAPEDAPLVARLRSA
GAVILGKTNLSEWANFRSTHSSSGWSSEGGQTRNPYVLDRNPSGSSSGSAVAVAANLCAA
AVGTETDGSIISPSAISGIAGIKPTRGIVDAEGIIPISHTQDTAGPMARTLRDAVLLLDA
MLGQWYSEEFAQVQWIDRDRPLEGMRLGFAEKLSNFHPQVDQAMKTSVSVLTDLGAEIMP
IDIETNEEVRNAEYQVMLYEFKNGLERYLAKYVADRGRGDSEESNGRKKRGQWPLTLQAI
IEFNLAHADKVLPYFGQEILFEAAAKGPLEEPEYLKALAACQTFEREKGISAYAAKYGLD
AIIAASNAPAWKTDYLLGDHYVGGNTSLAAIAGCPHVTVPAGFAGELPLGLSVFGVPFSE
KTLIRVGLAFEHATCARRAPKFVPSFW*
>SPBDM4_v1_40525|ID:27157830| protein of unknown function [Uncultured spirochete bdmA 4]
MFVISMLLFLPLDLFGQVILVNPGIADRERLVYIERSGGTSQEIVQESRKIMRDGRAYFE
VTGTSDHFQSRLRIDTSITHKPLY*
>SPBDM4_v1_40526|ID:27157831| Transcriptional regulator, RpiR family [Uncultured spirochete bdmA 4]
MPLVSTKIKETLPHMNPNFQKIGSFILDHDNKVAFTSIYTLSDALGTSTATLVRFAKSLG
YSGYQSFKKELQEEIQHRLQPYEKVSLSKLGSLPEEKQLQRLIQNECNNLRITLNNLKLT
DMETMIHAVKNARRIFIAGFGISRHFAHILQTTFLASQEKDVFVITGSVSDYSPLLKSFG
HEDIMFLMTFPPYSAEVRHVADVTKGRGGYLCLFTDSASCPVYSHADVTVKCSTNSLLMS
NSFVGLVSTIQVFIHMLLLSGDHGGDNIRNGLEMQKLGYSIISNREETQ*
>SPBDM4_v1_40527|ID:27157832|iadA| Isoaspartyl dipeptidase [Uncultured spirochete bdmA 4]
MLVIKNAEIFAPAPLGKMDILIGGEKILAMEPYIDPAFLPGDIDELDASGMVIVPGFIDG
HQHFTGGGGEGGFQTRVPELTVSMNFRNGVTTAVGLLGTDSLTRSVENLYAKTQAFNAEG
MTAFMLTGAYWYPSPTITGSVARDMVFLQPVIGVKLALADSRGPHIDAKDLAALASDVQV
AALVANKPGIITVHTGIRTQGLDLIFEIVEKFEIRPSIFVPTHINRKSGKLSDQALALAE
RGCVVDATCHQELPEPDSPRLTAADFACIARDNGLFDHVSFSTDAGGSMPIWNQDHSRTV
GMGVGTPSSLMFELNLLVNKKHIPLEEALQPLTTTPARIYGLETRKGSLRVSMDADLVVI
DPASFSIRDVVARGAIAIKNKEAVKKGYFEA*
>SPBDM4_v1_40528|ID:27157833|yliA| putative peptide transport fused subunits of ABC superfamily: ATP-binding components [Uncultured spirochete bdmA 4]
MTATNLRMRDDELLAIKDLSLSFPGYYGYTQVLHNISFSVKKGETLAIVGESGSGKTVTM
RRVMRLLGNVRTDSGQILLRKRNNQIIDITNISHKDATAIRGTDLSMIFQEPMTSLNPVF
TIGNQMLEAILVHQRIPKAQAMQKVMELLELVRFPDKSRRVNDYPFQLSGGMRQRVMIAM
ALASNPQILIADEPTTALDVTIQAQILALIKSLQNKLHISVVFITHDMGVVAEIADRVVV
MYKGELVEENDVNTIFSTPSHPYTRALLKAVPKLGSMRGRSFPATLPVLEMSKELSRKAD
DAPPEEKTIDTADYTKQPLLQARNLTTHFVAEKTFFGRTTHVVRAVQNVNFDIYEGETLG
IVGESGCGKSTTGYAILKLVPAEGKVLYKGQDILALSESQLKHYRRDIQFVFQDPYASLN

PRIRIGAAIEEPLIIHNIGTAEERKKRVEYLLDCVGIPRAHKDRYPHEFSGGQRQRIAIA
RALATQPKIIIADEAVSSLDVSIQATVLNLMLELQKEFKLSYLFISHNMAVIERVSNRVM
VMYLGQVVESGSRMQVFENPSHSYTKKLLSAIPVPDPSHRSNFGMLSGEIPSTIHRVSDA
IPKIEYVEIEPGHFIASA*
>SPBDM4_v1_40529|ID:27157834|yliB| putative peptide transporter subunit: periplasmic-binding component of ABC superfamily [Uncultured spirochete bdmA 4]
MKAKHLVLAALMVLTIAAVPVFAAGKDIIYASDSTTKTLDPHNTSDTYSGAIERAICQGL
LGFDKNMNIIPLLAESYTYNDSATEFTFKLRKGITFQDGAPFNAKAVKVNIDRLMTGKYV
RSSLMDPVKELKIIDDYTVKFLLKQPFGAFLNALAHPGALMLSPAALEKYGDDIGSHPVG
TGPFMFQEWVSGSYVLIKKNPNYWRGTVKVDSIKFVPIPENGSRVAMLRAGQAQYIYPMP
AELLKLVENDPNIDVIKQPSIIERYLIFNTKSKVLSDERVRQAINYALDKKAIINIAWGG
AATEADSVFPAALPFFKKEGPWPYDLAKAKELMKAAGYPNGFKVVFLTPNASARLRATQM
VQQQLQAIGITGEIQSMDVASFYDMLSKNKPDTVGDQAFIAFGGWSSSTGDADWATRPLL
STEAFPPNMSNYGFFEDKTVDNLIKAGLTSADPKVRGTAYAQMQDYIWSKAPWGYLFVDT
LIAAKAKNIQGIYPMADGAFTVEEAEIVQ*
>SPBDM4_v1_40530|ID:27157835|yliC| putative peptide transporter permease subunit: membrane component of ABC superfamily [Uncultured spirochete bdmA 4]
MLKYFIRRCLSILPVLLVVSILVFLFVHLLPGDPARVVAGPDADEATINRIRTELGLDKS
LPEQYLTYMGRLFRGDLGKSLSSKLPVAQEIALRFPPTLYLALTSLAWSIIIGILFGAYA
AMHRGKWQDYTAMIVAVSGISMPQFWLGLLLMQLFAVHLGWLPVTGFTGKFKELILPSLT
LGASVAAIIARFTLSSFLDVFQEEYINTARAKGLPERVVMWKHAFRNALIPVVTMVGLQF
GFLLGGSVVVETVFAWPGLGRYMIESVSVRDYPVLQALLLLYSFQFVIINLLVDMLYAVI
NPEIRYK*
>SPBDM4_v1_40531|ID:27157836|yliD| putative peptide transporter permease subunit: membrane component of ABC superfamily [Uncultured spirochete bdmA 4]
MAEKTITPSMIFWQKFKKQKSAVVAGIFILLLVFVAIFAPFIAPYDPYAIDYGNAMMPPS
PQHWAGTDIYGRDILTRIIYGTRISLSVGISSVTIGAIVGVTLGLISGFFGGFLDEIIMR
SADVLFAFPGILLAIAVVAILGPGLVNVVAAVAIFSIPTFARIVRSNTLSIKESLYVRAA
RSMGASNNRIMFVHIMPGTLSSSIVYFTMRIGTSILTASSLSFLGLGAQPPSPEWGAMLA
ESRDYIGVADHLTIFPGIAIFLTVLAFNIFGDGLRAAFDPKLQ*
>SPBDM4_v1_40532|ID:27157837| exported protein of unknown function [Uncultured spirochete bdmA 4]
MKRILLYSAILMLIALVPAIAQNEAELKTAYENAVKLAAPAPQDYTLNWQAAQAARKYGD
YLVAHEVPGWKDTARAAAKEGIKYGEIAFKLNPTGIEGWYYYGLCVGTYSDCVSVVKALT
EGLKGKTQMGFENAYKFDKTYDNGGPILSLGRFWQVLPGIAGQDRKKAEQLFDEYINLFG
SSPDVNSDVWYFRGQLYKDTNRLAQARADLEKAASMGNNNAQKLLAEFK*
>SPBDM4_v1_40533|ID:27157838|deoC| Deoxyribose-phosphate aldolase [Uncultured spirochete bdmA 4]
MITIEELARSIEYTLLKPTVTKEMIELLCEEAKQYQFASVAVNPFWVSLCSKKLEGSEVA
VCTGIGFPLGANTTEIKVVEAKRAVMQGAKEVDVVINIGKAKEGDWSVVREDIAAVVAAV
KPAAVVKIILEACYLTDDEKIMASKAALESGADYVKTSTGFGSGGATIDDIRLMKSVVGN
RMKIKAAGGIRRLDDAMAFLEAGADRIGTSNGVSIVNEFKRRFK*
>SPBDM4_v1_40534|ID:27157839| Aryl-alcohol dehydrogenase (NADP(+)) [Uncultured spirochete bdmA 4]
MKYRYFGKTGLRISEISFGTQTFGWTTDKADAFRLLDYYVAEGGNYLDTADSYNDGASEE
ILGQWLKGRKRTDDVLIGTKVFFNEHAGPGSNEIGHSKKHIVHSLEKSLARLGIEAVDLY
QLHCYDRGTDIETVMQTMEDLIRAGKILHYGISNFIPSIIMKMVMLGHLRQCHRPVSLQL
EYSLLVRSPEWELLPLCEEEHIGTLAWSPLAGGWLTGKYQRNAPPPENSRVGRNDREYDQ
PGKRDGARTWAILDELRKIAERRNVPVSQIALNWLRRKSSISSILIGARNLEQLEANMSC
MRWDLSDEETRLLDQASDVNAPYPYSFISSYSRESYTREA*
>SPBDM4_v1_40535|ID:27157840|eda| putative KHG/KDPG aldolase [Includes: 4-hydroxy-2-oxoglutarate aldolase ; 2-dehydro-3-deoxy-phosphogluconate aldolase] [Uncultured spirochete bdmA 4]
MNSIFDEIKKYKIVPVIKLEKPSDAFRLGRALLEGGLPVAEVTFRTAAARDAIKLLRDQY
PAMMVGAGTVTTIEQVDLARDAGAMFIVTPGFNPRIVDYCLANNIIIIPGVNSPSQIEQG
LERGLSLLKFFPAEASGGVKMLKALHGPYSEVSFVPTGGIDASNLLQYIQLSNVAAIGGS
WMVKEDLISSGQFDVISRLCGEALTIVRQSHT*
>SPBDM4_v1_40536|ID:27157841| conserved protein of unknown function [Uncultured spirochete bdmA 4]

VLKMKIIDGENISLEPLSEYSETRLRIQWLGQAGFLIDSKLGRIIIDPYLSDSLAVKYSG
KRFEHVRMMPIPVNPAEIGSVRICLSTHHHTDHMDESTIRAIASRNPDCLFVVPAASRDN
SKLSGIPKENIVAADAFSPMDIGDIRIYPIPSAHEELAIDGAGHHVFLGYLVSIDGLTIY
HSGDCAPYAGLLANLAGFRVNIALLPVNGRDEIRKAAGILGNFTLNEAVQLAEAAGFDFS
IGHHFGMFDFNTIDIGQAEQMIQKKDAAHFELAKMGRMYEFR*
>SPBDM4_v1_40537|ID:27157842| D-isomer specific 2-hydroxyacid dehydrogenase, NAD-binding protein [Uncultured spirochete bdmA 4]
MENIIVTPRSLSKGNNPLLARIKDAGYNLVFPNPGKQPTEDELVEVIGDAVGYIAGVEPI
TAAVLEKAEKLKVISRNGTGIDNIDLETARKKNIKIRKADGANARGVAELTIGLILAAVR
DIVLSDRSLKSGIWNREKGFELEDKTLGIIGCGKIGQLVSRFALEFGMNVLAYDAYPNPD
FHPSGNFRFDALRAVLKNSDIVSLHCPPLPDKRPLIEAAEIATMKHGAIIINTARQSLVD
EKALLEALDSGILHCYAIDAFDKEPPDDLSLAKNERVIVTPHIGGFTEESIQRATEAAID
NLLESLQEA*
>SPBDM4_v1_40538|ID:27157843|pgiA| Glucose-6-phosphate isomerase [Uncultured spirochete bdmA 4]
MDFDAIGKYMVPFDVDLDLNEGILKGYTNHTIRRASSMWHYYQDESALETSINNGDPIHY
EVFEKTVPEEYGHLMMGVSKLYAGTVGQEFFMTKGHYHAVAQTGEVYLCLKGSGYLVMKT
KDGDFFALPMERGKVLYVPPYWAHRSVNTGTEPLISFFVYNAEAGHNYGDIETEGFSKRV
FNQNGKTIFV*
>SPBDM4_v1_40539|ID:27157844| conserved protein of unknown function [Uncultured spirochete bdmA 4]
MTVNRLERASRPTFYFIGVTTLKSSIMRVFPQWMKYLNFGDVEIRGIDCLIHDNPQTYRD
IVAFIKQDPLSLGGLITTHKMDLLAASKDYFEYLDPYAELLGEISSISKRFSQLRGHAKD
PITSGLAMESFIPQSYWQSTNAELCILGAGGASLALTCYIMKQKKRYEWPSRIIVTNRSQ
PRLDHMKEIHNIINPGIPVEYIHAREAVINDSIVNDLKPGSVVVNATGLGKDQEGSPLTN
DVIFPEHGIVWEFNYRGNLVFLDQANKQKKDRYLTIEDGWIYFIYGWTQVISEVFNSEIP
TSGPVFDDLCRIAAEYR*
>SPBDM4_v1_40540|ID:27157845|rbsD| D-ribose pyranase 1 [Uncultured spirochete bdmA 4]
MKKGRILNQKLSEAIASMGHGDWILVTDAGFPIPNDSRRIDLALEANIPTVQQVLSAILS
DFVYERCIITLEQKTNYPKLYSQIEAMIDRCPIETLPYSQFMSDFANKPKFFVRTGAFEP
WGNIALCSGVDAPRWFLKDKGIIVPDYYEERVNYKEKG*
>SPBDM4_v1_40541|ID:27157846|rbsK| ribokinase [Uncultured spirochete bdmA 4]
MQIENNSEISGMAPKIIVMGSFVADLMGRAAHIPVSGETVKGNFFKVGPGGKGANQAVAA
ARAGGNVAMITKLGMDSFGDMAFNNFVNEGINTDYVFRSREFSTGAALIMVSEETGQNSI
IVIPGACGQIKEEEVISAIDGLSPADILVLQLETNFEAIAESLKVGMAKGLKVLMNPAPA
QEIPEEYYSMIDYLTPNETEASTLSGIQVNTIDDAKRAARILRSKGAKNIIITLGEKGVL
ILDNEDHFLHIPSFKVNAIDTTGAGDAFNGAFALALAEGKDIVPATIFASAAAAISVTRI
GTAPSMAYRKEIDDFLSNNQPNR*
>SPBDM4_v1_40542|ID:27157847|xylB| Xylulokinase [Uncultured spirochete bdmA 4]
MQYIIAHDLGTSGNKATLYDEAGKLVAASTIEYPTTIGPAGYVEQDANEWWNAVVLSTKK
LLQISEIKNADIACISFSGQMMGCLPVDAEGNPLRPAIIWADMRSEKQAAQLRLAFDEKE
FYKIVWHRPISSYTLTKLMWIRDNEPEVYNKTEKTLQAKDFIVYKLTGAFATDYSDASGT
NAFDLIHKKWSDTILDSVRIRKDIFPEPYSSTTIIGHVTKEAAGLTGLAEGTPVVLGGGD
GSCAIVGAGIFGGQKAYSILGSSSWVAFESDTPLYDEEMRYITWAHLDERKYQPCGTMQA
AGLSISWLREVLAGYETYAEKHLGENALDLIESSAHVSPPGSHNLIYLPYLLGERSPRWD
PYAKGAFIGLTSKHTKEDIFRSVYEGIAYNLKTIINAFEENIYLKEITLIGGGAKSSLWR
QILADVWEKPVLVPRYLEEATSMGAAIAGGIGIGIFKDFSVAEKLNPTVNKIMPDKSNIP
TYKKLYPLFEQSYASLKDIFRKLED*
>SPBDM4_v1_40543|ID:27157848|rbsC| Ribose transport system permease protein RbsC [Uncultured spirochete bdmA 4]
MKAHQPVEQDSKEKKRFKLKTEGTLVLLLLVLAVFLTIFTRGFLTGNNLSNLVRQTAING
IVALGMTFVVISGGIDLSVGSVVGFAGILVALLMKSGVPIGLAVLASISISFLLGVANGF
MVFDGRIPPFIATLGSMSIIRGAIMLISRARMIAGLPKPFLDFAQTTYLGLPALFVVWLV
LILFTIFLTKKTPFGRNVYTIGSNLEVARLSGINIRFTTYMIYGLSGLMAGIAGILMTSR
LANGIPTGGQGYEMDAIASCVIGGASLSGAEGTILGTVLGAVIIATLRNGGNLLGIDPFI
LQIVNGVIIVAAVYIDQYRKAHQK*

>SPBDM4_v1_40544|ID:27157849|mglA| fused methyl-galactoside transporter subunits of ABC superfamily: ATP-binding components [Uncultured spirochete bdmA 4]
MYQDSMYQVGKMSDYIVEMKNISKSFPGVRALSNVNLSVKKGEVHALVGENGAGKSTLIK
ILTGAYQKDEGTIVFDGKEVDVKDPHFSKNIGIHAVYQDITLAMHLSVGENFFLGELPMS
SPFTVDWKKVYSTANDVLASLDLKIDPHMLVRNLPVAKQEMVAIAKAIYEKSKLMIFDEP
TALLTNEESEELFKIIARLKSEGIGIIYISHRLEEVFRFCDAVTILKDGRYVATMPTKDT
NEDELISLMVGRKIEEMYYFEHHNEPEVVLSVRNLTKKKVFQGIHFDLHKGEILGMFGLV
GSGRTDIVLSIFGATKYDEGSISFFGKPANVRKPREAIELGFGLVPENRRTQGLALPLSV
KININLLRYKKISHFGIVIDRNRELSNAKSFIQELSIKTPSEDAIVRNLSGGNQQKVVIG
KCLANQPKIMIFDEPTLGIDVGSKEEIYTLLEKLTKTGTSVIFISSYLPEVIGVSDRIVV
ISEGRQMGIIDRNEATEERIMKLASGIEENSAKIESEAV*
>SPBDM4_v1_40545|ID:27157850| Periplasmic binding protein/LacI transcriptional regulator [Uncultured spirochete bdmA 4]
MRKRVAIIVLLLFVIALIPTFAQTPKRVAVIIKATDSDFWQYVLVGATNYAKENPGKVVV
TTYGPPSEADIDKQVAILEDVVNSKPDAIVIASTSSDATVPAIERAVKLGIPVITIDNKV
NTNAVSSHLATNNIKGGALAADMLVERLKAMGKPLSGKVALISAMAGVQVLTDRDTGFLN
RLKEVAPNLKVLPTRYIDNDIMKALAAAEDLFTANPDLVGFFADNNHSGDGVARAIKERN
LGGKIAAAAFDSDPEEVQALSDGILYALILQDPYGMGYKGVDSALQALAGKTLPAYVDTG
ATAVTKANMNEPKIKGLLDPMTLKK*
>SPBDM4_v1_40546|ID:27157851| Transcriptional regulator [Uncultured spirochete bdmA 4]
MLIPADIDYFKNRPLDKSIPIPLYYQLIEVLQNYINEHDNSFPIPTENDLCKTYEISRPT
VRQAINELVAEGLIVRQKGRGSFINKKKIPQDFLLNIMSFNDEMQSKGLKPETKVLTFCS
RNPTPEVMKNLRIKSSDSTYFLSRLRSINGEPIVLVNTFLPSHLLKGILQKDMEKESLYH
IIEYDFGYKILRTLRTLEIRKAGKYEASSLNIKEGDPIHFIQTVAYIENDVPIEFSTAYY
SGERNKFTVDVRIK*
>SPBDM4_v1_40547|ID:27157852| D-tagatose 3-epimerase [Uncultured spirochete bdmA 4]
MRKIGIYYAFWTREWDADFFPFIEKIKNLGFDQLEINGGTFAVMSPPARKRLGDEAKRCN
ITISYGIGLTADHDVSSLDEEVRRNGIQFMRSMIEAVGKSGGGMIGGTVHSAWPSTLPKD
ATDKRPYLDQSKKSMREMVRVAEDNNVILNVEVINRFEQYLLNTCDEALSYVKDINSPSC
RILLDTFHMNIEEDSIGGAIRKAGRYLSALHLGETNRKPPGLGRMPWEEIRSALDAIGFD
GPLVMEPFIAKGGQVGRDVAVWRDLIPNPDYDKLAHDAASFVRKVLC*
>SPBDM4_v1_40548|ID:27157853| Sodium/hydrogen exchanger [Uncultured spirochete bdmA 4]
LIKEIVGMLDFTTLSPANFILVLAMILFLGTLGGFIFKLLRLPQVVGYIAIGILIGQTGF
HLLNSKIITTLSPISNIALSLIGFLVGAELKISVLKKYGKQFTAILLFESITPFITVAFL
VGLVSYLVTHQLKSSIALGLLLGAMSSATAPAATTDVLAENRTKGPLTTTLLGIVAMDDA
VALILFAIVSTIAGGLLGERTGSLLSQLLAIIYNITVSSALGALAGLLLNLLAKWIKNDN
GRILAFALGIILLLTGVGLYLQLDTILSAMAMGFFIVNFAKFKTDNLFNVVDRFTPPIYV
LFFVLVGAKINIWSISPFFAILAVVYVLARTFGKSIGSSFGAKITNAPDTVRKYMKWCLL
SQAGVAIGLSMSAGQMFPHTVGPTIIMIITATTFIVQLIGPISVKHGVTKAGEVGLDVTE
EDLIRESKVSDILAYEKKAPPLNDNATFEKVLETFSRQESLSSPVCSSDNKLVGIITIDN
LKEALLLGDVAEGLLAYDLMNPSEVTCKPDCSLLDVKEMFRTNNVDTIPIVGDDGTFVGI
IEQRSIEKYFHRKVLELHEKLSSLEKTTLQPAQG*
>SPBDM4_v1_40549|ID:27157854| protein of unknown function [Uncultured spirochete bdmA 4]
MRKIGIYYAFWTQEWDVDFSPFIEKVKRLRFDLLEINGGTFAIMAPPARD*
>SPBDM4_v1_40550|ID:27157855| Sugar phosphate isomerase/epimerase (fragment) [Uncultured spirochete bdmA 4]
VNEAEQCGITLSYGIGLTADHDVSSLNEETRRQGIDFMRTMIESVGHAGGGMISGTIHSA
WPRMLPKGATDKRPFLEQSKKSMREMAKIAKNNNVMLNVEVVNRF*
>SPBDM4_v1_40551|ID:27157856| D-tagatose 3-epimerase (fragment) [Uncultured spirochete bdmA 4]
MNIEEDSIGDAIRKAEGLLSVLHIGETNRKPPRLGRMPWQEIREALDAIHFDGLIVMEPF
ITNGGQVGRDIAIWRNLIPNADRDQLVRDAARFVRTTLCD*
>SPBDM4_v1_40552|ID:27157857| putative Sorbitol operon regulator [Uncultured spirochete bdmA 4]
LKTNKTKEVSEKAKIQLIVQICKLYYESNATQQQIADLYKLPRTSIVRIIEAGRQLGIVQ
TRIVDPYSSREDLEIKIQQHFNLKDVIVVSNFNPGNPVLPTLLGQAAVEYIMGILVPNQT
IGLGWGKTISSFVSLLGQQDILKYREFWVPLIGNLGETEIEFNMNQQVKSISEKTGGYFT

SIHAPALVDSKQMKNLLTNSSSIKNTIDFWSHLDIAIVGIGGLFTGRLSHMPLLHLKYYP
QEDRNEVFYSSAVGDILSRYYDIEGNICDISINSRIIGIDIEALRKVPLTIGIAGGAYKT
KAIIGALRGQYINTLITDETAAIGICDYVEKNP*
>SPBDM4_v1_40553|ID:27157858| Periplasmic binding protein/LacI transcriptional regulator [Uncultured spirochete bdmA 4]
MKKALLVMLLATTLVVIPAFAQKAPVLQSVAFTCQDLQNPFFNVMANAVKKAAQDIGGSK
VKVMAQSCDNDLNKQSQQIDDFIAAGAQIIVVNATDSNGIGPAVARAKAVGIVVIASDVT
AAGGVDATMTSDNRQAGVITAEYIAKRLNYKGNVVAIIGDPNSSIDRFYGFQDVMKKYPN
IKILSDTQNGHGRREASMTIMTDLLTAFPKIDAVWCINDPSALGAMLAIKQANRDKEMFV
VGVDGSPDAADAMKQPGSIFAASAAQNPYYMAYHAVEVGWQVMNGKIPANKTELIPVKLI
TQDDVKAGYSGWPSK*
>SPBDM4_v1_40554|ID:27157859|rbsA| Ribose import ATP-binding protein RbsA [Uncultured spirochete bdmA 4]
MDDLILEMNHISKTFPGVNALKDVTLKIHRGEIHSIVGENGAGKSTLMKILAGVIPPTKG
ELILEGKPVKINNPRHAISLGIILINQELSILPDLTVTENIFLGKDKNNYGILKRHEMRE
EARRLLDSIAAPFDTDILAGKLSIAQKQQVEIAKALSNKTKILIMDEPTASLSEKETENL
FNAIIKLKNKGLTVIFISHRLVEILKISDAVSVLRDGNHIGDLNKEEISEETIVSWMVGR
KLTDYYEHDSNTMVVPNAFVVDHYGDDAKTKDISFSVGRGEILGIAGLVGAGRTELCRLI
FGIDKKKHGNLYLNGKTIKIKAPKDAIQEGVAYIPEDRKLLGLFLYMPVADNICMNILDD
ISTLGVISNKKINTIASAAVKNFNIKTPSVLRNAVFLSGGNQQKVLISRWLQKTPKVLIL
DEPTRGIDVQAKSEIFKLIGKLSLQGVAIIFISSDLTEIIPLSQRILVMRSGKIVAELKD
RNEFKQETIIEYATGLRPDSYHYEYKG*
>SPBDM4_v1_40555|ID:27157860|rbsC| Ribose transport system permease protein RbsC [Uncultured spirochete bdmA 4]
MQTKEATNNNTVFKIILSKFGIFPILLLFVIGFSIISPNFRTFNNMMNIFRSVSINLVIA
TGMTLVILTGGIDLSVGSIVALSGVVALRFSVTNPGFAIPAALLVGVFAGLINGVLIAFV
GLPPFIATLGSMTYLRGIAFLLCNGNSIINNKLPFAWIGNSYLGPFPWLAILALLIFLLG
WFLLRGTTYGRRIYAVGGNVEASKNTGIRVWSILLSVYALSGLCAALSGVMISSRLYSAN
GLLGQGYEMDAIAATILGGTSFSGGKGNLTGTIIGALIIGVLNTGLTISGVSYYWQQVVK
GIVIILAVVIDIVRNKISE*
>SPBDM4_v1_40556|ID:27157861| putative PfkB domain protein [Uncultured spirochete bdmA 4]
MKISAAGSWIMDIIYNIDPNKLDIFKSYLINKDNPNGIKRGGACLQHAVESHYQKNIMDI
ISEFSDVSTENKKLGGVAIAAIINTAQVLFGENIDVSFYANLPNDENGEFLYDIIKKTPL
RINRIRRKTEKCPKTFIINENVDCLIQGERSFITVENAEALSLSLDELDNDFYKSDIVLI
SCPHWEREINNNLTSVLKKCKDEGAFTVISTAFNPIYANYSGKWTLGDSDEVFQYIDLLI
TDKEEAYVYSGANDLQNAKRFYHHKGVKIFVVTDGVNPVFYYSDGSIIQKQEGFISISEE
IIKEKESGLLSCGDTVGCGDNFAGGVVASIALQMKRQESLDILKAIRLGNVCGAFATTYY
GGVYPEKYPGEKKEKIKHYYNEL*
>SPBDM4_v1_40557|ID:27157862|fbaA| Fructose-bisphosphate aldolase [Uncultured spirochete bdmA 4]
MPIVSMRELIKDAQNRCYAIGNHNSIDYEAIKGIILAAEEMKAPVIIGIAQSQLDNLPVD
TLSSILLHEGKKAKTSIAIHLDHSTSFDVIKKVIDLGFNSVMYDGSQLPFEENITNTIEV
VKYARKRNVAVEAEIGKMINTENAKYNELTSSDNDKNDYTDPSIAQEFAERTGIDMLAIS
YGSIHGLHAKKPTLNFALLEKIHAAVSIPLVLHGGSGLDTSDYTRSIKHGICKINYYSFG
YNAVALKIKDAIMKQKDPIFYHDISAWAIEAFRDVFKETMVQFQSAGKA*
>SPBDM4_v1_40558|ID:27157863| putative Xylulokinase [Uncultured spirochete bdmA 4]
MPEDRELIRLGIDLGTGSLKMKAYCGEHSFSSSQKYDILSSRAGIAETAPDAWINALKLA
WADIRQQLSSAGRPSTITSSIGLSGQMHGFVPISKSGKALHNAILWADVRGAEYAEMYSR
LLNGAFDRLLNAPAAGLTALILLWLKHSKPDLYKRTAFILFPKDYLRYRLTGHIGTDAGD
ASATLLWDFKAHSWSQEAFNVLGLESSKLPPVLDSFSVGGFVTGEASRETGLPEGVPVAV
GSADKSCEIYGSGFFNDYFEHHTPRLAASGPGSPVVGVSVGEPATDADWQQPHIAQVSIG
TGIQVVIPVRGIPPYSPALNFFETCVAEVGYRMAAMLNGGLALEWVLSVFNGNWETIYRD
LDEGKTQLPQDLIFLPYLTGERSPYQNSKARGAWIGLGLHHSRTDMLAAALLGVACTIRL
GIETLGIAPEAKMYCIGGSTRYPVWMSIVSAMVGRRLLVSPEPDASVRGAAAIGFEAAAG
AGAAHPGDYTHQNMPEPLETSPVESEQPGWMNEYYKRFKMYYERLFGEEKTH*
>SPBDM4_v1_40559|ID:27157864| putative integron gene cassette protein [Uncultured spirochete bdmA 4]

MIFNIRAIRLEDAAVYHSLVDEIKDEGQYLFSTLRFSLEDTKKYIERHEEMSSPIWGAFD
EQGTLLGWIDFNRGGFSEIAHTAMLGMGVKKEFRGQGIGSALMDACIENAESLGIEKLEL
EVFASNTAARALYIKKGFFEEGIRLKKRKFEDRYDDLVCMGLFLG*
>SPBDM4_v1_40560|ID:27157865| NAD-dependent epimerase/dehydratase [Uncultured spirochete bdmA 4]
MRVLFVGGTGNISSACTDEALRRGYEVFHLNRGTHTEKEKPGVGLLRADIRDAAAAKSAV
RGLHFDSVVQFLAFRPEHVEADIKIFDGITDQYVLISTCSAYRKPSLTPVITEETPLENP
YWEYSRLKIACERVLTGRAGSATCEAGPADDDLCDRRPTDRSQEPSFPYTIVRPSHTYDD
GWIPGCFGSAGYGLAWRMLQGLEVVVPGDGQSLWTLTHASDFAVGLVGLLGNPAALGEAF
HITSDEHLTWDAVHMIIAEALGVRPKIVHIPSDFIAKVRPERGAGLLGDKAVSVLFDNSK
IRRFVPGFAPRVSFAEGIRRSLAWFDAHPERKTPDPAMNADMDAILERWRGLVGRIS*
>SPBDM4_v1_40561|ID:27157866| protein of unknown function [Uncultured spirochete bdmA 4]
VFANIITSKKTKQKNWRDALT*
>SPBDM4_v1_40562|ID:27157867| exported protein of unknown function [Uncultured spirochete bdmA 4]
MKALGRSAGVARKFSFLVVFAVLCGAMLSAQPYVDAYSSATEQAMKSMLTGDAFKAAAKA
IEAGSADLANLATSKAPGYTAPKSYLANVMSVNPDGSVGISTISQWQYIASDFGADHAIL
QLTWGQNAVNLAEVGHRGSLFIPGVSVNGKSYRFLIHLKVVNVEEMKYTDEAFNAGLFNA
YYSGAAGKKTQYTITCEVLAIEDVGNSVKLGMQMP*
>SPBDM4_v1_40563|ID:27157868| PAS fold domain protein [Uncultured spirochete bdmA 4]
MSDTQPENTVTAWQDEFSASITVIDRDFTILFMNEKSATGFAKWGGMTLLGKDVRHCHQE
RSVRIIEHILETGEPNIYTIEKNGVKKLIYQAPWRENGQIAGVVELSMEIPFEMPHHVRG
*
>SPBDM4_v1_40564|ID:27157869| TRAP transporter, 4TM/12TM fusion protein [Uncultured spirochete bdmA 4]
MRKFSGRLKTVIYIYVIAVGFFHLYTSVFGNFEAYLQRALHLSMILPLVFIWWPVSKKAP
KDKVPFYDWILAALSVLPGTYIAANYWAISTRIVQVDPVTTAQLVLGILLVVLLLEATRR
IVGLPLMIIASVFLIYMAFASKIPGLFQGISFSVPEIVEEVFLTDEGIFSSPLGVSATYV
MIFLIFGGFLEKSGVGDFFMKFAQAFTGTQPGGPALIAVTSSCMFGSISGSAVANVYGTG
TFTIPLMKRIGYPDYFAGAVEAVASTGGQIMPPVMGAGAFLMASFLGLPYRTVMIAAIVP
ALMYYGAVFLMVRLSAHKYGLKGLSQEELPSKREVLKQWYMVIPLAGLVYFLLAGFTPMR
AAVFGIILAWLVSFFRPKSEDSERRKRSVITGTVISLLIAVAFVFPDIMEPISRSKIGLW
GLVSAFVGVSFLNDGMRPKDVLDAIYNGVSGIPLVAIACAAAGIVLGSVALTGIGGKLVG
FILGFAKDFRFLGLLLVMIMSIFLGMGLPTTGAYILASALGAPILVRMGIAPLAAHMFVF
YFAVISNITPPVALACFAASSISGANPNKIGFQAVRLGFLAFVVPFAFCYDPGLLLQSTT
MANILAIVTAVVSVLALGYFWVGYIKHPIPIWMRAILLAAGVLALAPQLLYVGIAAAAVA
VCYILSATGKVKAPEKHLAL*
>SPBDM4_v1_40565|ID:27157870| TRAP transporter solute receptor, TAXI family [Uncultured spirochete bdmA 4]
MRKAVVCLLALVLVTGVYAQNKAGWPDQLKFMSGPPGGNWFALGTALSEMWSKSVLQTTS
SSGGGVSNILNADVKKGDFGFTVASLLGAAIAGEEDFEGKAVKNSVIMANLYTQYTYFIM
RKDFAEKNDIKSVDDMIEKNIPVRFATLKPGTASEFVIKALFKKGYKTDYDQLKKKKWTF
EFTSYDGGADLLADNHLDCFAFSVGKIASVVMNIESNTQFVILPVGQKALDALAAAYGTT
TFTINPGTYKSVTVPIKTIGDYTCIVIRKDLPDSLVYELNKALWANRDSLAMAVGDIAEL
NPKEALPAGLPSHAGSVQFWKSVK*
>SPBDM4_v1_40566|ID:27157871| Transcriptional regulator, IclR family [Uncultured spirochete bdmA 4]
MAEVNSVRKALAILDLLSDGKERTLSEIARQLSLAKSTAHGLLETLAAERLLEKDSGSNT
FSLGIRLIELGYCAQTGLDLVRIAAPFLKGLNIRFDETVHLVVLDNDEVLYIDCIESQRR
LRTYSVIGVRAPLYCTSVGKAILAYLPDTEVRRIVAERGLAPFTDNTITSEERLWQEIAR
IRQCGFAIDDREHEDHLRCIGAPVRNAHGKVFASVSLSGPAERNTMERIESMAPALLEAT
GEISHRLGFRNRELGRSRG*
>SPBDM4_v1_40567|ID:27157872| DctP family TRAP transporter solute receptor [Uncultured spirochete bdmA 4]
MKKAIIAVLVLLMLATPALFAQQKPVKLVFTSVSVPGDAHTQAMYVFKDEVEKLSGGQIQ
VDVYDSGKLFTQQGEQDAIRKGTVDMVYTSAQWLAEFIPYLSMFGAAYTFQSYDQMTKTF
NGPIGKKIFEEVAQKTGIRPLVAYYLGTRQLNLTSKAGAVTKPEQMKGVKLRVPNSPTWI
AMGKALGANPTPMAFNEVYMGLKTGSVDGQDNPLPTDKNAKFYEVTKYIVLTNHVVDSTW
PSINEKKWKSLTSEQQGWIMQAAEKARQFCDKTNLDNEKSILDFFRQQGITVIENPDRAA
FAAYAKNSYLTESKDISKDWDLALYDEIQKIK*

>SPBDM4_v1_40568|ID:27157873| conserved membrane protein of unknown function [Uncultured spirochete bdmA 4]
MPNWLRKTGKFLLDCIELYLPMLTFTVLFVSFMSQIVARYFFKPMVWPEELSLICFIWTA
LLGGLYAKRTGSHVAFTMLYDAAKPGVQRAMRIAGNLLLLVAFVIIFVPSWNYIQFMAYK
KSDALRIPMNWAFFPFMIFLADMIVRLVVDIVRDIVKAGGTK*
>SPBDM4_v1_40569|ID:27157874| TRAP transporter, DctM subunit [Uncultured spirochete bdmA 4]
MNLPLIVFFVSFVLMFLLRIPIAPGMMMSSAFYFFLSKNPASSLDMVAMQFLTNMNASFI
LIAVPLFVFMAEIMNSGKVTSMIFSFANALVGKRKGALGHVNVIASIIFSGMTGSALADA
SGLGSMEIKAMREHGYDDGYTCALTAASATIGPIIPPSIPMVFYAMLSSASIGSLFLGGV
LPGLLVGVALMIYNAVISQKRNYPEGDKLSLREFWKLTLRSIPALFSVVVLLGGIYTGVV
TPTEAGALAAFYALIISVFFYRAFGWKDFKQVLINTVRTTGTLSLLVGSAYAFSYIVTIE
KIPNFAAELMLGITQNKYIMLLLINVVFLVLGMFIDTTAITLVFIPIVLPIINQLGIDLV
HFGVMIVLNMMIGLSTPPYGMLLYVVSGISGTPLKKIIKEIIPMLFVIVAVLLLITYVPD
IVLFIPRLAGYK*
>SPBDM4_v1_40570|ID:27157875| Dihydrodipicolinate synthase [Uncultured spirochete bdmA 4]
VRYTMKNTIPDGVYPTMITPYQEDGRIDWAALEALVEWHIERGVAGYFAVCQSSEMFHLS
LRERLELARMSIKFARGRVPVLVSGHVADTLDDQIEEAKMMADTGADAFILVTNRLARRH
ESDEVFKRNLELFLSRFDRDILLGFYECPAPYKRLLNADLTRFVVGTGRFGFLKDTSCRM
GDIRAKIEAARGSNFKLFNANAATLLGSLQAGAAGYSGIMTHFHSDLYVWICRHWKDEPE
LARELQEFLGLASVIEYQLYPVNAMYALQLEGLPIKLYSRRADARQFTESMRLEVEQMMR
LSHRFSERYKE*
>SPBDM4_v1_40571|ID:27157876| protein of unknown function [Uncultured spirochete bdmA 4]
MQLPPKNRKASIFSVESQKGNVPNMNKQKISIHEVARLAGVSKSTVSRVISDKGGSVSHE
TLEKAQMARFDGVILNSVTDNIDLIKNLGVPAVLIGERSAAQNIDTVGTDTLQATRIGME
YLYETGHRKIALATSDHGSERYLSLHARAYRDFLQEKGLPFNPELLFSVNLDAEGGRELA
KQLLGLQNWAERMDSLFCGNDIVAITAMNELRKYGIIAGIDLSIIGMDDIPASSQTYPPL
TTVRKPRERIAVNPKREGLLWKAKKADLPCMEIAQLMREVRAITGVPAAPRFTDRPVALI
EWRDGSIIDTVRQLEAE*
>SPBDM4_v1_40572|ID:27157877| conserved protein of unknown function [Uncultured spirochete bdmA 4]
MRTNIVLDDSLVDEAFKYANNIHTKRELIEIALKEFVAVRKVKNLRDLKGKISFAEGYDY
KKMRVGE*
>SPBDM4_v1_40573|ID:27157878| conserved protein of unknown function [Uncultured spirochete bdmA 4]
MILVDTSVLIDFLKGNTNHVVQLFDDVLDRQIPFGINDYIYQEILQGARDRKEFNKLKEY
FETIPFYTLKKGRNSFEKAAIINFSCRKNGITIRSTIDVLIAETAIENDLYLLHNDLDFT
YMAKVIPELKIYTNKNDFL*
>SPBDM4_v1_40574|ID:27157879| putative PilT protein domain protein [Uncultured spirochete bdmA 4]
MTAVLDVSAGIELLLQKDKKDLFTATYEKASWIIAPDLYIAELSNVLMYYAVLARRNDAL
LMTNDEQLSILCKKLNIEVVI*
>SPBDM4_v1_40575|ID:27157880| conserved protein of unknown function [Uncultured spirochete bdmA 4]
MPLLQVRDCPEDIYKKIVLAAKRKNRTIAQQTVVLLEKSLGQEQPNIERRRRLLEKIKTR
QIPEKVKKIDAVALLREDRDR*
>SPBDM4_v1_40576|ID:27157881| protein of unknown function [Uncultured spirochete bdmA 4]
LYAAIKWQAYIQVRITCITLREAIRQTVGWYRSNFSV*
>SPBDM4_v1_40577|ID:27157882| conserved protein of unknown function [Uncultured spirochete bdmA 4]
MQWIKRFYESDPSALFKKGKVVILYGARRVGKTMLMEKLLAGTEGKIFKGSGDDFELVAI
LASRKTETYRLFFSPYDVIFIDEAQYVPDIGACAKLLIDIFPEKSVILTGSSAFNLSQTA
SEPLTGRSIQRFLFPVSLMELKLERTDFQIFQDIESYLIFGMYPEVLSLDNAPENTEYLV
NLRNSYLFKDILALEHIRNSQKLQDIVRLLAFQIGNEVSLNEIAVKTGLSKNTVERYLDL
LEKAFVIKKIGAFSRNLRNEISKSAKYYFWDTGIRNAVINDFRPIELRNDLGALWENFVV
MELMKKYEYQRRYASFYFWRTYDQKELDLVVDENGRLSGYEIKWRDDKAKIPHLWIETYK
GHTEVITKENLLKIMSEEAKAE*
>SPBDM4_v1_40578|ID:27157883| putative transcriptional regulator [Uncultured spirochete bdmA 4]
MELAKLTSKGQITIPVEIRRKLRLKEGDKVFFIEENGKIIFQNAAQTALSTFQNEMVGEA
TKAGFSSEDDVVQYIKNLRAQNGDKK*

>SPBDM4_v1_40579|ID:27157884| conserved protein of unknown function [Uncultured spirochete bdmA 4]
VRILVDTNIILSAALFPEGKVSRVFSYLLEAHTVVIASYSIQECKTVFMKKFPDKIDCLK
AFLLKIDYEPFKTPKKLDAHDFPPMRDASDLPVLASAILSDVDILLTGDKDFEGIGIKKP
LIFTPNQYFDLIQK*
>SPBDM4_v1_40580|ID:27157885| exported protein of unknown function [Uncultured spirochete bdmA 4]
MKKVFVILTLFALAGSVVFAQLPAQSSAMAAVPSSTTGEEFADVEGMSLSADEMVQVKEG
DLILSINRNKQTMKVISGSPLAIVNDKAEVYTVPVTTRVVTNAQARAGQDRDAMEGSSFV
GSGKDAYLPIQLPAGIYSLDYVSKNVGGYGPGIHIDASVLTTMLVGSPRNMNDFFVHWTD
KDNT*
>SPBDM4_v1_40581|ID:27157886|vnfA| Nitrogen fixation protein VnfA [Uncultured spirochete bdmA 4]
MNSAMIPDEKETLRFLLDIARTLDKHVELRSSLGPLLSLLEVRAGLVRGMVTLLERTTGV
LKIEEANGLSVEEKERGVYRLGEGLIGRVFETGLPIVVPDLSKEPRFLNRIGGRSKEDLT
GLAYYCFPIKAGGSVIGTLGAERRLSGELRDAWMEWDQRLLEQVASIIADSAKLRERILE
EQFRLRQELLSNESYQMVSPSTRFVHPGSSIIGTSTAMQGVYELIAQVATSNATVLITGE
SGTGKELVAQEIHRLSGRADQPYIKVNCAALPESVIESELFGHEKGAFTDAVAQRKGRFE
LAHRGTLFLDEIGELSPQVQVKLLRVLQEREIERVGGDRPIKVDVRLIAATNRKLEQEVQ
SGSFREDLYYRLNVFPIHIPPLRERKSDIVLLADYFVEKYAEKNGKLIKRISRPALDLLT
SYHWPGNVRELENCIERAVILSTDMVIHSYHLPPSLQSALSTNTEPTTTLDAALSRLEKE
LIVEVLKITSGNMAAAARRLGITERQMGLRIHHYGIDWRQYRTTKM*
>SPBDM4_v1_40582|ID:27157887| conserved membrane protein of unknown function [Uncultured spirochete bdmA
4]
VKRRMILLLLMGFGASLATAFGEETIESLTAATDMIWLVLTGSLVFFMQAGFAMVETGLT
RAKNTTNILMKNLMDFCFGSIAFWMIGWGLMYGSDKLGLIGFSDFFKGPMGDPKFYRDWF
FQVVFAATSATIVSGAMAERTQFKSYLIYTVFISAIIYPVSGHWTWGGGWLSQLGFHDFA
GSTVVHSVGGWAAFMGAIALGPRTGKYVKVNGKVTVKAFPGHNIPLAMLGVFILWFGWYG
FNPGSTLSGVSTDIARVAVTTTLAASAGAVGALIFSWLWFKKPDASMTMNGALAGLVAIT
APCAVVSPGASIVIGIIAGVLVVLSVEFIDKVLHIDDPVGAVSVHMVNGVFGTLAVGIWG
NMDGVAVGLLHGGGFHQLGVQALGVVTVGGWAAIMSWILFKLIKVTVGLRVSIKEEMMGL
DLAEHKSEAYSGFQIFSNM*
>SPBDM4_v1_40583|ID:27157888| Nitrogen regulatory protein P-II 2 [Uncultured spirochete bdmA 4]
MKLIIAYIQPHMLNDVKQELYKAEVYKISVTNAMGCGQQMGYHESYRGVDIEVNLLKKVR
IEIGVNDNFVKPTVDAIIKGARTGEIGDGKIFIIPLEECIRIRTGETGSVAIG*
>SPBDM4_v1_40584|ID:27157889| protein of unknown function [Uncultured spirochete bdmA 4]
VPKVAISSKKNVPGYFTGPIEGTELLIISKMALNGLLVRNRRLATIVSGACSVWGSVLDA
TFMWEE*
>SPBDM4_v1_40585|ID:27157890|glnA| Glutamine synthetase [Uncultured spirochete bdmA 4]
MHETFDLTENPVSSLYGSNCFSNAVMRERLPKNVYKEMLEVQAGEKELTLEVAEVVAASM
RAWALEKGATHYTHWFHPLTGLTAEKHDSFIAPTPDGKVLMEFSGKELIKGEPDASSFPS
GGLRATFEARGYTAWDVTSPAFLKQDPTGTTLCIPTAFISYYGHALDKKVPLLRSMDALN
VQAIRVLRTLGNTTSRRVTTTVGPEQEYFLVDKKYFDERPDLLLTGRTVFGSMPAKGQEM
EDHYFGAIKERVAAFMRELNTELWKMGVSAKTQHNEVAPNQFELAPIFTTTNVAVDANQL
VMETLNKVARRHGMEALLHEKPFAGVNGSGKHNNWSMATDDGINLLDPGENPESNARFLL
FTAAIIEAVDRYALLLRTSTATAGNDHRLGANEAPPAIVSVFLGGQLMEIFDHIAEGKAS
NGVAGGEKIKLGVTSLPSLPKDVSDRNRTSPFAFTGNKFEFRMVGSSQSIATPNTYLNTA
VAQVLSEFADKLEKATDKNAAIQAIVKESYGKHRRVVFNGNNYSDEWVREAERRGLPNVR
ATVDALPVLIQKDTISLFETHKVLSREEQESRYHIYLERYAKQINIEAGVMIDMARRSIF
PAVSSYASSLARDASALAALGAVSVPQEKRAKKIAELASELYDETDKLEKVLGDAQVIED
AFTQAKSYREKVVPQMANVRNRGDALEKLVAKDVWPFPSYEELLFKL*
>SPBDM4_v1_40586|ID:27157891| protein of unknown function [Uncultured spirochete bdmA 4]
MTHTWKFSWGENPIDNDVTIIDLGVQQPYMVVYSQKFIVTSYTQEGYQFIFEGQWETRDE
TSIPGSLVVVYSEDGNSLYFESENPEGFTLLGESYRLYRVPVDAPVVPIGPEGP*
>SPBDM4_v1_40587|ID:27157892| membrane protein of unknown function [Uncultured spirochete bdmA 4]
MDSRYAELNTVQKAILMPNLLWTYTIVVMWCMFIQYYSLTIAFISTVAVSVAVIAWLLAN
GMIRKVSRLLVWGIAVSVADILSTLWALLDPVVSANAVETNPLFSASAARHPVGALSVFL

LFKITLTLILFSSYYPLGTPNTMEQRLKEAMRAVDRRIEERMGGLELIIPRCCFALVFRK
ANVLTVEDMEELDSSIVLLMARRKIEYIAICALVLLASLSNIALILFYHLGYFRCAYWTW
PVAMGCIGLILLIVNVFDERIIRINTSIFNRRSVLCVWGGGAGNEASKFSLFYFFYDSLC
NCMCRSTSDQSFRGMVYSGR*
>SPBDM4_v1_40588|ID:27157893| protein of unknown function [Uncultured spirochete bdmA 4]
MCSSTISIEGILGEGDLTAAVLQKKILALHEENTRAILSIDSEGGSIDDVVDIVAFCRYF
GIPFDTCAKGKVYSAAFLLFCYGERRLATKNAEFWYHRPSHYVHGQEDDWAGIGCDLEFR
ESAVTHEVVRRALARQEESGTENSMLSIPFLMKAGVVTEVV*
>SPBDM4_v1_40589|ID:27157894| protein of unknown function [Uncultured spirochete bdmA 4]
MNTTVDWKRAIFMYGRVGVDEDDSTTPHVSAKEVTEQLAGFASGSDDVTLMLHSLGGTYD
DVMEILGFEHLVPFKTNVFSFGCCLGEAALLLFASTGNRIVSSGTRITTTDVFEDVGFVN
ASKIQGYARMFEQRRESLLHFLEQRSGTPREVLREKILNGNNRPILPSEALQLHLVDEVF
DVLQHN*
>SPBDM4_v1_40590|ID:27157895| protein of unknown function [Uncultured spirochete bdmA 4]
MKKEVFSAYGKEARMKFLITNKSLPDNPRLQDFDELVPLRHFWRSVTITLFVVVLIVILL
KLIVHYTLQVDPEYVLPYSKGSLNIRHAGRNHEVSDNTR*
>SPBDM4_v1_40591|ID:27157896| membrane protein of unknown function [Uncultured spirochete bdmA 4]
VHFVGKLKASAREFWREKILLFIALFIVVATIAFFAFSNTFESRNDFYQAHDLHPVQKNQ
SMFPLDVFKLSVPVLFILDTLIIWGWNSFVVLLNRYFAIMLVSPLFFFYQFIVKVPAFPN
LGMELSLFFRTSATALPAHDTLRLVVLVGYSVVEATLDLFWKLVCTKEIIVIIRKYRKYN
SKVWVEYLDGSHVKGMYLLYMLLLLPCAISESLCVHVLQ*
>SPBDM4_v1_40592|ID:27157897| exported protein of unknown function [Uncultured spirochete bdmA 4]
MKLQGILSFLFIFSYAAMAGSQPQINPSGVWSMWDWNMNYTKAFSWGENPVNNEVVVIDL
GAEEPYIDVAVGEFKVSSYRQEGYRFIFEGGWGTMSEELIPGNLVAVYSEDGNSLYFESI
KPEKFGAVMELGETNRYFRVPVDAPVVPIGPEGP*
>SPBDM4_v1_40593|ID:27157898| membrane protein of unknown function [Uncultured spirochete bdmA 4]
MKKEIVSAYGKALLLFLLIPWLINFFVPLSIFLPLSSTIERTRPSTTQMNIMGAVLSLIV
VLCYLLLGKYIQEGMRDADESPVERNDSKSVKEIYSSKKGDLLLYSLASLALPVLVYCLQ
NIFGVTEGPPSINLYQLPVVLAIGPVIEEYLFRKVLYLYCTKYGIRRFVFLSALFFTLIH
GYSVSMAAVVIFLGSFILAYFFLATLYSMYRIIYIPIQMHICWNFSIFMIGMFHVDTLAP
VAFFTISLFVLLYAIYRIMKTKRLMITEGPI*
>SPBDM4_v1_40594|ID:27157899| exported protein of unknown function [Uncultured spirochete bdmA 4]
MRSIYRETKRHAMSGRLRLCVACLLISVCGAKLSADGVDFSALESIVENGTKTYYGGIGA
CLFSDQGTLWRYRSGGGDNRIAFDENTEIHIGELSGQFTVLAVRALCAQGKIDIDSPVSR
YLPELFSGANANVERIGGLKIRSLLAEASGYADRPIPNMDGILDADSLAKRLSGAKNRFP
EGVRRSESALMMDVLGLLVEKASGRSFGDYVRDEVYGPLGMVASSFEKDSTPPEKSRFYH
AGGEPFLDSSSDMYNYLDPFFSMRSTLSDLAQCYSYLLRTEDEDQTKEKRAIDSEAPFSP
AIAGQLNRQGYESGEGWILTEPSLDYLGSVAWAEGSYLSHRVVVILARNQNVGIVLAGNI
YDQWGINALRDIGIKILKRYIEEQFKIPPPSFSRPEVREIPADVSPQPGLYASSQGVAIV
GVGNNLVTLECNRAYSEFAFSTGESFLPTTENEFRELKIIDSASFLIRWNTGTEALMRRP
SFTHYSFIVPPREGTYRPDAEASGGWSDFWVIASHGQYFTISGGDMKEYLLLDSSPTTAG
ILCDDGSVFFGKQVLIDPSGTVSIGFAKPDP*
>SPBDM4_v1_40595|ID:27157900| membrane protein of unknown function [Uncultured spirochete bdmA 4]
VHFIGKVKASAREVWREKTLLFIALFIVVATIVFFSFSNTFESQNDFYQAHNLHPPARTE
SLFLDIKLPIWALVIIDLIIIWVWNTFVFMMNRFFAYILIMPLIFFYQYIVKSPAIPGLG
MELSLFFKASATVFSAHATLRMVVWVVYAVVELTVNLFWILVCTKEVFATVRGYRMLKRE
RAPKETISEYLREDRLKGMHVCCILLLFFCAVLESLCGHVL*
>SPBDM4_v1_40596|ID:27157901| exported protein of unknown function [Uncultured spirochete bdmA 4]
MKLQGILSFLFIFSCAAMAGSQPRINPSGVWSIQGVDMTKTKMFSWGENPIDNDVVIIDL
GAKQPYIIVSSVKFSVTSYAQEGYQFIFKGQWETRDETSIPGSLVVVVYSEDGNSLYFESV
KPEDFVDIVDLGESYRLYRVPVDAPVMPIGPEGP*
>SPBDM4_v1_40597|ID:27157902| membrane protein of unknown function [Uncultured spirochete bdmA 4]
MKKEIVSAYGKALLLFLLIPWLINFFVPVVFTFPLSLGFGGAQPSRLFLNIYSTAVALIG
EFCYLLLGKAIEQNMVCKEENPAEEDQPKSLKKVYASGPKDIVLYSLASLTLPLLLYSLD

ELLGIAGELPKINPIFSLAALVVAPVIEEYLFRKVLYMYCKEHGIQRFVLVSALIFSLIH
GYSISVAGAIMFVVSYIHTYFFLATLYAMYRSIYIPIQMHICWNLTTIVVIGIFHGEKLA
PTAFFIISLLILLYAIYRIIKTKQLMIAEGPI*
>SPBDM4_v1_40598|ID:27157903| membrane protein of unknown function [Uncultured spirochete bdmA 4]
VHFVGKLKASAREFWREKILLFIALFIVVATIAFFAFSNTFESRNDFYQAHDLHPVQKNQ
SMFPLDVFKLSVPVLFILDTLIIWGWNSIVVLLNRYFAIMLVSPLFFFYQFIVKVPAFPN
LGMELSLFFRTSATALPAHDTLRLVVLVGYSVVEATLDLFWKLVCTKEIIVIIRKYRKYN
SKVWVEYLDGSHVKGMYLLYMLLLLPCAISESLCVHVLQ*
>SPBDM4_v1_40599|ID:27157904| exported protein of unknown function [Uncultured spirochete bdmA 4]
MKRSSFLFFIFFMILCATAFADQPQINPSGVWSIQGVNMTHTWKFSWGENPINNDVTIID
LGVQQPYMVVYSQKFIVTSYTQKGYRFIFEGQWETGDETSIPGSLVIVYSEDGNSLYFES
ENPQGFTLLGESYRLYRVPVDAPVVPIGPEGP*
>SPBDM4_v1_40600|ID:27157905| exported protein of unknown function [Uncultured spirochete bdmA 4]
MKRFVFVLIGIMMTGGMAAAEATGNESATQATVPVGSTIVMPVSEVSKEVVFDVEGVEII
AQAELDAVEGARQIRNDPNEGPYTEKEMQKERDLDREWKRERAEGLVTQADLGERAVANW
NQACKFLTLVSGPQLDSGRIWSDTKTIEVINYMMGKGWIREDFYVNNPNAIVNYAAKECG
FSTGDVTFKNNGKITPRYLEMVGITALKGDHTNLCNLEGKFIWDPYYGGEDRIIRRTNQR
FIRFE*
>SPBDM4_v1_40601|ID:27157906| protein of unknown function [Uncultured spirochete bdmA 4]
MRLFLLLLLFSETVDRESLRFAYSTEQGFDTSCGLTALSCLMDRYWGVPSDEVSLARDFL
AEKIVGGDLTVSFADMAAILKAKGFSCKAYQMNFGQLEKAVARYAPVIVHYDRPEGHFAL
VLAIRDGSVLTADPAEGTISREQAAFESLWSGNVMLAALPGGAVDLELIEEAKRSVWGRG
ELLERAALAGAGVTGW*
>SPBDM4_v1_40602|ID:27157907| exported protein of unknown function [Uncultured spirochete bdmA 4]
MVKAMKNIVRRVLSAMAAVALLSAIGAPAFAQTTAHQRVGVEAKDSLEATVGTTLEVFRS
DTAKTNGVEIDPTISLDLFFRRRFGLSFEVPALVWIATGHEAVPRAVGAVGDPEVTASYT
FRLSDWRLSAALSYTHPLGIWNYYEARERQIASGSGYPKLGALFSAVRYLDPIVAGTSLR
GETCFARPERSGTSTRPLILTANLFATEALNDVVALSASLSPKLSWPRYLNGVPDESGII
YSLTGSASLVFSEKDHTLRVRVSKLLSDYSSPVAFDLGFSHTFRKKE*
>SPBDM4_v1_40603|ID:27157908| exported protein of unknown function [Uncultured spirochete bdmA 4]
MRATACKTAFCVAAVVALFSIASCSNDPSSWYPSGKATIVSWYETSVGGSQVCMITLKIG
NTGQSTISVYTVSLSAKTDVRTYYKTIAESFVILPGKSVYVNAEIEYDSNTEALAADGLS
IVDEYYQ*
>SPBDM4_v1_40604|ID:27157909| exported protein of unknown function [Uncultured spirochete bdmA 4]
MKRFVFVLIGIMMTGGMAAGEGSASVSVGNIGIGSSNEPFPDIEGRPLTDKEQQQTDGGF
FKGLVGFFVSGGLEIADQLIEKGTVSNWGKVGIEAVIGAISAEIGGTTYKMSEVGVGLYR
RTEELIPIFRSAAASAFTWGAGRVRRIMWGND*
>SPBDM4_v1_40605|ID:27157910| protein of unknown function [Uncultured spirochete bdmA 4]
MAAAGKQKDRNGENNAKDSGDTIDDMWPDMSLFSFSPRRLLYEKGQQHTNKETYNYRLHL
*
>SPBDM4_v1_40606|ID:27157911| membrane protein of unknown function [Uncultured spirochete bdmA 4]
MKSIIIGFLVGVLLTFLVEKTSRRKTEEGHVRPHVIYCITGVLGIIFAITIFLLPSSGHN
TPFYILLIFSALAGALCIYAYFACSIDVSPDSISITSLFRKRAEVTIEDILRLKEVGFFR
LLIIETRNTGNYYFPIDAYNMREFIAMMQKDHPENHVGF*
>SPBDM4_v1_40607|ID:27157912| membrane protein of unknown function [Uncultured spirochete bdmA 4]
MKFLITNKSLPDNSRLQDFDELVPLRHFWRSVTITLFVVMLVVILLKLIMHYTLQVDPEY
VLPLLQRLVFICVTVILYEVCHMVFLLKYRKELYLVVQPNALLIGVGVEDFIGRQEMLMA
LAAPLIILSFVPYAAMMMLGHFVMNIFIVVCTNLYISTGDIVAFRFIAKNCERHDSVHYS
KGFLHIRHIDYNREVSNNS*
>SPBDM4_v1_40608|ID:27157913| protein of unknown function [Uncultured spirochete bdmA 4]
MIGWPLANKTALRYYESIETYENSKAALHEIYTKLYPDIY*
>SPBDM4_v1_40609|ID:27157914| membrane protein of unknown function [Uncultured spirochete bdmA 4]
MNGLWSYSFVVFQITSVIIFFILFNNTSTSVSKLVKNIVLLTSVVFFSLFLVSLYIPNQQR
DNLKAFLPFIIGAIASIAVLIIEYTKYYRGKNLFAFAKPSNVSITLYLLTLAFYIGMRLI

PAQVSGERIVIKATLLPMLLISSVNEEMYFRYYLDDILASIKATSNKTFRAILISLLFSL
VHMVSTFSIPYLAGRFAISFILLLVKRSMKNPIYPVFLHFVINMLVIS*
>SPBDM4_v1_40610|ID:27157915| exported protein of unknown function [Uncultured spirochete bdmA 4]
MRKFLLCLSIFISMVQVPSYTQSTDTVDAFTTEEFVLNDRHFSVNVDHFNTCLLVMLSYY
YPEQFSLTDTEVKSLIDGYVRGKDVGHRFVFNTNQKAALGAMLDPYTLESTTVNRVLKFS
FSKASNMVGLTIDLRQEGFEIPPDAYASISLFYDENSDAVHRFERDVISIVKRRISPLVE
FLDKEKQYNFLPHKPLSLYVTFANNIQSLHGMANDYAQNAVMAYSLASEHDDAVLFFHEL
AHLWASVNEVKANIISEVEKSDKKEKYEEILKDCAGKIVQDDELLSKGSIQSESIVDIVN
SIYDASLLGFNEFWAITASKYLFPLYSPAFSESACRFPAGLSGDKITWEHARDLFDWISA
TNYLDVVRLTADPQKVIYREFVLFVLCIGHGLDYALEKSGEFVQ*
>SPBDM4_v1_40611|ID:27157916| exported protein of unknown function [Uncultured spirochete bdmA 4]
MRSIYHETKRYAMSSRLPVLVACLLISVCGAKLSADGVDFTALESIVENGTKTYYGGIGA
CLFSDQGTLWRYRSGGGDNRIAFDENTEIHIGELSGQFTVLAVRALCAQGKIDIDSPVSR
YLPELFSGANANVERIGGLKIRSLLAEASGYADRPIPNMDGILDADSLAKRLSGAKNRFP
EGVRRSESTLMMDVLGLLVEKASGRSFGDYVRDEVYGPLGMVASSFEKDSTPPEKSRFYH
AGGEPFLDSSSDMYNYLDPFFSMRSTLSDLAQ*
>SPBDM4_v1_40612|ID:27157917| protein of unknown function [Uncultured spirochete bdmA 4]
LRTEDEDQTKEKRAIDSEAPFSPAIAGQLNRQGYESGEGWILTEPSLDYLGSVAWAEGSY
LSHRVVVILARNQNVGIVLAGNIYDQWGINALRDIGIKILKRYIEEQFKIPPPSFSRPEV
REIPADVSPQPGLYASSQGVAIVGVGNNLVTLKCNRAYSEFAFSTGESFLPTTENEFREL
KIIDSASFLIRWNTGTEALMRRPSFTHYSFIVPPREGTYRPDAEASGGWSDFWVIASHSQ
YFTISGGDMKEYLLLDSSPTTAGILCDDGSVFFGKQVLIDPSGTVSIRSVSSDP*
>SPBDM4_v1_40613|ID:27157918| membrane protein of unknown function [Uncultured spirochete bdmA 4]
MVCMILAYLLDLILLYAMVNVVPLVLAFIKKKDLATFYHDNINLLLLPGLAFVVCLLGHS
LFTKEAGLSLHVYIVWIPWGICLIDVVNAIRYLFRKGEHVPVENAIADRLEKWWRRHSGK
SGENK*
>SPBDM4_v1_40614|ID:27157919| membrane protein of unknown function [Uncultured spirochete bdmA 4]
LSIIFALILVLLFILLHVHSLWAKPFVRCSWGIVICLLLTLILNHVRDLTGQREFFLSAL
FLLACLEITIFYYLRIRETSIDEKRRGVGNLVLAAIIIFLIMLPLRKSSLPFVNNRPIDI
VFYSITFFLFAFIEELTFRYLPMNQFITLARFDGIIRTRPNIRDIATILLSSLLFSFVHF
DITPSVLVGHLFFGILMCLLYLTTKRLWLLVLVHGIYKIFAM*
>SPBDM4_v1_40615|ID:27157920| ABC transporter, ATP-binding protein [Uncultured spirochete bdmA 4]
MITVENVYKAYSNVQAVAGLSFEARQGEIFGLIGPNGAGKSTMIRMIMNILAPDSGTIRF
DGEPLSETDKDRIGYLPEERGLYRKAKLGEVLEYLAAIKGAKPADYRPSIDAWLERFGLS
EWKLRKVQELSKGMAQKAQFIGAIAHNPSVVLFDEPFSGLDPLAQDELLAVMVELKQKGV
TVLFSTHIMEHAEKICERILLINKGKEVVYGTMADIKAQYGRNAVQLEFDGDASFVAGLD
FVESVVSYPRWIEVELKDGADADLLFRAIAGRLKVRRFETVAPSLHKIFIRIVGGEEAQN
VQA*
>SPBDM4_v1_40616|ID:27157921| conserved membrane protein of unknown function [Uncultured spirochete bdmA 4]
MSRLKHVIRHEFRLTAANKTFVILTILGPFLIFAITVLPALLAQSPGSVASGKPLALYGA
PPAVEQALAAAFSGMDIEVERVDDVAAAKASVAEDARIAFLSIEPGWPDGGKAVWYSKSG
SEVALYSAASAVLEAAARETRIVESGIDPALVAKVLSPPSFEVIKLDKGGAEEARGSGSF
VEALMTVLVFIMLIYMTVLLYGQMIGRSVVTEKTSKTVEIMLSSVKSRDLLFGKILGLGL
AGLLQYAVWVSMAFLLKQFVGPLLRLSVPAGISAGNLAWLVVFFILGFFLYSSAYAALGA
ASEDEQQLGQMAWPLIMFLIVPLVMISTLVSNPDSTLVVVLSLFPMTSPIVMLARVLVSS
PPVWQLALCLVLLVAAVAGMAILAAKIFRVGILMTGKRPKLKEVLRWVTVK*
>SPBDM4_v1_40617|ID:27157922| ABC transporter related protein [Uncultured spirochete bdmA 4]
MIHIESMTFGYGKRPLFEGLDLNLEPGTAYGLLGLNGAGKTSLLKLLAGALCPQSGTIRV
FGEEPWRKKASFLADTIFVPEDPWVPAVKPSAWLDRYAVFRPAFDRERFAALSREFALEE
DKLLTRYSYGQRKKFTLAAALASGARLILLDEPTNGLDIPSKSQFRRALAQSVDPNRIVI
VSTHQVRDLERVIDPALIVHGGKILFRLGSDLMAERLGAAHVSELSPDVVYAEKDRVGYS
ALLELGHGTGADDVQAADLELVFNAAIASPDRLKTALSGAQLPPLDPADAAVREYGEKEE
AQ*

>SPBDM4_v1_40618|ID:27157923| conserved membrane protein of unknown function [Uncultured spirochete bdmA 4]
MKMTKETLRKPFSAARVVLLAKNRALEDLPAFGIGAGILAGLNLLTIIATRRAMMNDSNG
QSWAVIISIAGFFLAAAAFKGMHDGRAGTEWILLPATSLEKYTAALVFYAIIYPLVAAAT
ATALSALLSLVELIAGGPGGRIWNPLSAGFDGWVNYATGALIFAAGSAAFRKRAFIKTVG
VSVAYALACMGLLLAGMLLIRRVQGLPLSFTGSFQNEMSFSGNITPGAQRAMDVIEKIVR
YALIPLFAIFYGYFRVAEKEARDEVQ*
>SPBDM4_v1_40619|ID:27157924| Transcriptional regulator, GntR family [Uncultured spirochete bdmA 4]
MRFSDQKPIFSQIAELLENDIIAERIPAGARLPSARDLASSLEVNPNTAARALQSLAELG
VAQVERGTGYYVSEAGAAKVREFRKRSFLDEELPRLFRSMADLGVTLEDIEARWRGFIAS
ATEKKDGIS*
>SPBDM4_v1_40620|ID:27157925| SpoVT/AbrB domain-containing protein [Uncultured spirochete bdmA 4]
MLISVIPIGNSRGIRLPKAILDQLHVSDTLELEVEDQQIILKPVTKKPRHNWNEAFIKMH
QEQQDTMLLPETKVPEAFEWEW*
>SPBDM4_v1_40621|ID:27157926| mRNA interferase [Uncultured spirochete bdmA 4]
MGMVINQYEVYLVDLDPTQGHEIQKTRPCVVLSPDEMNHNISTIIVAPMTTKSHPYPTRI
EVNFNNKDGWIVLDQIRTIDKTRLVKKMGKIDRKIIQNVKKVIKEMLVD*
>SPBDM4_v1_40622|ID:27157927| Prevent-host-death family protein [Uncultured spirochete bdmA 4]
MAGHITANDLKTKGIGAIEQMTNDEREVIITVRGKQKYVILPIEEYNQLREFELDAAIQE
AKRDIAEGRYHTGSIEEHMNRVHHV*
>SPBDM4_v1_40623|ID:27157928| conserved protein of unknown function [Uncultured spirochete bdmA 4]
MYKLLFTDSYEERAREFLKKHPEIEKQYIKTLQLLELNPFHPSLRLHHFKTSNFEGYSVS
INLSYPISLEFLISEQEILLVNIGDHQDIYNKK*
>SPBDM4_v1_40624|ID:27157929| Alpha-galacturonidase [Uncultured spirochete bdmA 4]
MKTDLVIAYIGGGSRGWAWTLMSDLAMEGALRGVVRLYDIDRQAAETNARIGNALLSRPD
VVGKWHYEVADDLKSALRGADFVIASILPGTFAEMASDVHAPEQYGIYQSVGDTTGPGGV
LRALRTIPMYEVIGKAIRDYCPEAWVINYTNPMALCVRTLYEVFPGVKAIGCCHEVFGTR
KLLAAMVRERLGAAGAPGARGGSEVPGASGATMKDIEVNVLGVNHFTWIDQASYGTFDLF
PHYKAFVEEHFESGYEGTEGENWLNSYFASGERVKFDLFRRYGLIAAAGDRHLAEFMPGR
MYLRDPETVRSWKFTLTPVAWRIENAEKLKRKAARFAAGKENFELKHSGEEGVKLIKALC
GLGSFVTNVNFPNRGQIDSLPEGCVVETNALFSRDSVRPVEAGKLPPSVGELVAPHAIAQ
ETILRAAMARDAWAAFPAFMHDPLMTAPPGEAEALYTRMLRATEKYLTDYTL*
>SPBDM4_v1_40625|ID:27157930| Extracellular solute-binding protein family 1 [Uncultured spirochete bdmA 4]
MKRIGLVLFTLLMSATIVFAQSGSVTLRWAYWGSESRIKRSQQAIDLFMAANPGIAVNPE
ISGGTGDHFNKVDTQIAGGSGPDIVQMGGNISDYVKRGVLLPLDKYVGSVLNTSVIDPSA
IESGTIDGHLYGVSTGVTMPALVYNKSLLQRVGAPLPKVSMTYPEFRAYLVSIKSRLPAG
VYPMQDIGALSSNSTPFGYWTRYNGTPLYNAETNMTAVTSAAAQKYLELFKDYRDNGLVP
PADIASGFAETNADTSMIIAGKAAIGFIWTNQLNGYQAATTDELGLIEFPGAAATKALWQ
APSQFYTVNKYSKNPELAVKFINFLVNSPEAAKVLGNDRGASASSTARAAGSTNVNDQKV
LEYLNSAGPHSSKETTHLPNDTEFNSTLFLIYQRVAFGQISPATGGQQIYDLITRLIKK*
>SPBDM4_v1_40626|ID:27157931| protein of unknown function [Uncultured spirochete bdmA 4]
VTLPLCAKTMVALIKRVNNTNPILFIQNLLLK*
>SPBDM4_v1_40627|ID:27157932|yesQ| putative ABC transporter permease protein YesQ [Uncultured spirochete bdmA 4]
MKNRTARRISAFLSNFIIIVLGVGMLYPVVWLIAASFKESNTIFSDPGLIPKSFTLQNYI
QGWKGIGIIGFGTFFKNSFIICTLCVIANVIFCSLTAYAFARLRFAGKNFWFAVMMVTLM
LPSHVTTIPRYIIFRSFGWIDTFLPLVVPKFFATDAFFIFLLVQFMRSLPKELDESAMID
GCGKFGIYTRIVMPLTTPALITTVLFTFLWTWDDFFNQLLYLNSPSKYTVPMGLRLFVDS
SGMSSWGPMFAMSVLSLVPCFILFFSLQKYFVQGIITTGIKG*
>SPBDM4_v1_40628|ID:27157933| protein of unknown function [Uncultured spirochete bdmA 4]
MGRQHQGHHHHGEPEVLPRETETGKGIGSKGTKDDIGYDTKGANDKGVFKKSTEADNTDP
FPALDIIL*
>SPBDM4_v1_40629|ID:27157934|yesP| putative ABC transporter permease protein YesP [Uncultured spirochete bdmA 4]

MNRKARKKAIRENLSSYAFLVPWLLGFFIFTLYPMIYSLYLSFTDFNMLQPPKWIGLRNY
FVMFVGNDQFPRDERFLNSLFVTFRFVFISVPLKLVFALAVAMLMNQRLRLIPFYRTLYY
IPTLLGGSVAIAVLWRRLFAGDGVLNAILSGVFNVNNPPAWISNPKYALYTLILLSVWQF
GSPMIIFLAGLKQIPQEYYEAASVDGAGKTRQFFAITLPSLSPIIFFNLIMQMISAFQSF
TQAYIVSGGQGGPLDSTMFYSLYLYLKGFAFSEMGYASAMAWVLLVIIAALTALAFKGSG
SLVSYGSGE*
>SPBDM4_v1_40630|ID:27157935| protein of unknown function [Uncultured spirochete bdmA 4]
MEGQGALLINRAKYIIDGNRIYFVQPREFHSILPEAVEKPISYYAVLSGPEYRAA*
>SPBDM4_v1_40631|ID:27157936| conserved protein of unknown function [Uncultured spirochete bdmA 4]
MRYTDTWTAHPRIEQTRVSTVEILRCPGVMMQADVSFMEKVMKRDTIAIRDPFVLAVPEE
ETYYLYGTTDPDPWKQSGIGFDAYMSADLEDWRGPYPVFRPMTGFWGTHNFWAPEVYRYR
NAFYMFASFKAPRHERATQILRADTPLGPFEVHSAGPVTPSGWECLDGTFFVEDGQPWLI
FSHEWVQVNDGEMWAVPLSPDLRRTEGAPSLLFRGSDGPWTKGLVRRDGSGLEDARVTDG
PFVYQAGQGNLFLLWSSISPSGYALGVARSVSGRIRGPWKQEPEPLLEAGRGHGMIFRGF
DGRAYITYHYPNDTPNERFQFREIRLEGARLALKGEER*
>SPBDM4_v1_40632|ID:27157937| conserved protein of unknown function [Uncultured spirochete bdmA 4]
MSIDRHELVRRHNPVHHAFDPFSPLSVGNGEFAFTADITGLQSFFPTMEGATPLCAMAQW
GFHSYPETEGVARNPDLLRRSYFNAENGTGGYMTDPAGQEELFRDLRINPHRLNLGRIAF
LFGGKELSAQCVAGIEQVLDLWSGELRSTFLYKEQQVEVLTLCHPNEDILGIHIRSPLLA
HDSLSVQLSFPYGSHEMDASDWQAEDRHATRILGQAQRTCVSLLRVLDDDRYYVDAATHG
GERSRIAQTGRHAFEFATSGLSLEIVIRFSPYEIKEDLPSFYEIQNAAHQFWEAFWSSGG
AVELAESRDKRAIELERRIVLSRYLTAIQCAGSYPPAETGLTCNSWYGKFHLEMHYWHAV
HFIVWGKPEFFERSFGWYRRILESARQRAAEQGYEGARWPKMTDPSGRDSPSPIGPLLCW
QQPHPIMYAELLRRAHPERPILSEYADIVTATADFMASFAQWNETKKRYELGPPLIPVQE
NHRPEETRNPPYELEYWRWGLSTAIRWLGMLDRPVPQEWIQVAANLAPLPLDSRRNIYLA
HEACPATYEEYATDHPAMLFPLGVLPGASVNREYMSQTFDAVLRAWDLQSLWGWDFPAMA
MTAARLGRTRDAVDLLLMETPKNTYRPNGHNPQLPRTDLPVYLPGNGSLLLAIALMTGGW
GERTEAAANQAGPPERPAPTASRASGGAPVRACAYAPGFPDDGSWTVQVEDICPIP*
>SPBDM4_v1_40633|ID:27157938| exported protein of unknown function [Uncultured spirochete bdmA 4]
MKRKSILVAVGLVLGALCLASAASTADITGIWLGEGGAQLSVRAHGSSLHLELNADASRI
RVEGMEQGSDGAWNLRLAAGPAAAEWSGTLKLVFPKDSMLPGPMNLGISAQLKDNSILEL
SLPDAPFSTRRLHLRRSSGGKGLQGLWVMDEAPLFLRVSDEGRTMLFTFTGGNPVSLLME
DAKIVTELRQSGDGTWAGSFTRPSGRTFALVLTLENEKLIFGVSSFPFSRKTSFSRASGP
GQ*
>SPBDM4_v1_40634|ID:27157939| exported protein of unknown function [Uncultured spirochete bdmA 4]
MIRCSTVSLMVLVLAAAAGPLWSQASQTQGQDDEGLFDQGTLVTTQPQPTSPAEAAGGSA
PTAPPSDILLTSEVVTIGGSFDGKYTSTFSWNKFPDSFENLGSPDSQVFSDELGAQLYFD
ARPDANFRVYGKIELSYPFSFVSVEDASTYEKNPSIQVEELFADFNWDRKLFFRAGKQSI
KTGVGYFFSPADVLSIQPIDPTDPTKEREGPVALQVNYPFGLDNLYLYVLDTGVTDPAAL
AISPQAQFIVGTTELTVGGLYQKDHVPRFLLTASYTASDFKFFHESVLTVGSDRTFVRTS
AIPGSLETYIDDNTLYYTGTIGFIYSTSSPDFTLAVQYLYNGDGYEDPSVIRDNAPAVFI
LKESGDLSADDLYMRGLHYLAATASLKKIGGDNSPWDTSLFWIGDISDGSGKILPKLTWN
ALDFPSYTVSLSLGLPIYYGGTNSEYAPKGDTYAISLEVHLGKGDF*
>SPBDM4_v1_40635|ID:27157940| conserved exported protein of unknown function [Uncultured spirochete bdmA 4]
MKSRLIFSTLAFCAIAFATVSEPRAEPLEHFNSILAEIDKQSDFGTKDFAATNSVVVYRP
DKAPETIKAQVFRRDYKSQFVIVVLDPSYKRGQGYLQADENVWFYDPESGQFSHTNLKDN
FQDSDTKNGDFNKSTYAVDYDVTAETEERLGKYDCWVLELAAKNDAVAYPFMKIWVTKDS
HLMLKAEDYSLTKRLMRTVLYPSYTKVAEHLLPTRVIFIDNLTQGNRTEVTRSDLSLSSL
PDSLFTKSYLQRVNR*
>SPBDM4_v1_40636|ID:27157941| putative ABC transporter, permease protein [Uncultured spirochete bdmA 4]
MAASMKIALRNLSRQKGRTSMLMVAIGFGVMVVTLLNSFTAGIVDNVQGTVSRMLGGHIY
ITGQKIAEDGRIISGIPEDNELTKIIAETGIRAEDITRRSLANATIIFGSNSVTQRIEGV
NWDEEKTLRSSLAVMKGDLASIKNPSSLIVTEQVADQLNATVGDQVIVKLQTVSGQQNVG
DFIIGAVISDPGVEGMLSAYADLSYLNGLLNLDPQAYQQLSIFLRNPGTMEEDASRIYSS

LAALGPVYPRDSAASGGNNFLGAGGPGVLTFLRGFSVSTLDSDESPWQGVRYRVTTLNDL
TTQVQDIVSVLNSVSFVVLIVLMIIVMVAITNTFRMIVNERTAEIGTMRALGMQRTSVRN
SFLTEASLLAIGSVVAGILAASLVSNIAGLFTFSTDSALYIMTNQGRLTFTFKQDSIILD
ALLVMLMAIGAASSPARRAALLTPAEALRETR*
>SPBDM4_v1_40637|ID:27157942| putative ABC-type transport system, involved in lipoprotein release, permease component [Uncultured spirochete bdmA 4]
MLVILNMALRNLREHKTKSLIIGSILGLGAAVMVIGSSMIDTATAGMRESFVENYTGDLM
ISAKKNMSIFGTLGFDTGDSLPQISGYQKLETSVKAIPGVRSVTPLLTGRAGATFLEGKN
VPVQLFGVDPESYAAMFPGNIEMLSGRMLQPGEEGIVLSESVAKGIKSADGTPVQAGDSI
LLSSSNSATGVKVRKMTVRGIFHFIRSNQYLDTANLIDADDLRIMLGMTAAEESGANNMG
NTDGANGTEGAASTISAAGTENGDFEQDLFSGGLVQTGTLAVSPDIYNILGSGPREESKP
DPDGWHMLLVRIDDTADSGNVEQAIRSTLPDLGDKLYIDDWMMAAGPVGRMAEAIHIVFM
IILIVVAVVALVVIMNTFVLSIIERIPEFGTMRAIGAQKSLIFRMIFIESTTNCLLFGLI
GIIVGSLVILVLGAFGIQAPNEYFQILFGGPSLHPALSVASVGESLAAILIVGFIASIYP
TSIALKVRPAVAMGGK*
>SPBDM4_v1_40638|ID:27157943| conserved protein of unknown function [Uncultured spirochete bdmA 4]
MALVELQNVVKHYPLGKTEVEALKGISFSLKTGEFLSIAGPSGSGKSTLLNIIGCIDVPT
SGAVMLNGKVTSDLGDREITRLRHEAIGFIFQSFNLIPVLSVEENVEFPFLLGPTRGEYE
SKEKRSEWVNYLIEEVGLTAWKKHKPNELSGGQRQRVAIARALVTRPTIVLADEPTANLD
STTGSNIIELMKRINRELSTTFIFSTHDRKIVNAADRVITLKDGLIESDEGRKAPPEASP
CW*
>SPBDM4_v1_40639|ID:27157944|regX| Sensory transduction protein regX3 [Uncultured spirochete bdmA 4]
MDASVLIIEDDQDIADLIAMYLKDEGIRSDIALTAEAGLELSAASAYDLVVLDLNLPRMD
GYEFLQTFRRMTDTPVVIVSSRDADEEMILGLGLGADDFVNKPFSPRVLTARIRAHLRRS
HTPAMDQNREVIQFGDISMDLGALIVERRGEPLAMPTKELELLAYFVRNRGVALKTEQIY
KDVWGNMYGDYTTVAVHVQRLRKRIEPDPAQPCFIKTIYGIGYRFEIPTEASACPERGRA
S*
>SPBDM4_v1_40640|ID:27157945| putative Histidine kinase [Uncultured spirochete bdmA 4]
MKLRSQFILLDIFTGFAAIAATAALVIFASHINLLTGPGREMISVRERLEAAVDHGAERA
ELESILEESKSLKLLLEDSDFRLLASNFPEAARLASGEPAEIARNFGEFFMNRIGGGTEE
YYLVAIQLTPGGFYSSFITSTAPFVVLFLAILLLLPFLASFRLNRTLKAIKSLQLATERV
AAGDLEYSPIMEGPEEIVSLAHSFDAMRLALRDESDRRARFLMSISHDLKTPLASVRGYV
EALRDGHAEDSGRQEHYLSIIDQKSMLLESRIRELVNFASLSTSDWQLTLRPVRLREFFS
GIAEAFREEASVFGRIFAATINIPDDVTAMMDEQLFTRTLENLFSNSLKFTHDGGRIELD
CYTLEGFTDIAFSDDGPGIADADKPYIFEAFYRGDKTRTTQGMGLGLAIAKSITEAHGFS
ISVRDTQGPGTVIVIRIPHCQG*
>SPBDM4_v1_40641|ID:27157946| exported protein of unknown function [Uncultured spirochete bdmA 4]
MHDLMAKFFRSPLAIGIVCGNALLAVGAALAGIAPVLVVVPLFVLVTGGELALALLSKPG
AKAILGEQERERKEHDVDRLEETARLRKRLAMLRVENPEVKAAVERLVLAAGLYLESCAK
GNERDPLVEEAVQNAVATVDGYLHLSDAGRIRARIDSEHGNTRQDLEQKTILSLDTSTRL
IGEKLALPFGGIEGNHTVLDAMDGRQELEE*
>SPBDM4_v1_40642|ID:27157947| conserved exported protein of unknown function [Uncultured spirochete bdmA 4]
MKRNGFLLVMLVSIGLWGQPLAALEAQAESVEIFLTLHSDGKAVVRHSIVWDVSSGTMGG
FYFQGEQAPMVWDAERCWADLPNGTRTPLDIKNAGDKWDILLSQGKRTSGISTWVLTYGA
DLAAANMVGLTDKSDGEKLFYFHWAPPQWEQPLDHRSVTVVFPLEAKAGESGDSRSERLA
QLGFATEKHVNDENSIDWYAAEGSDGKSYLALRFHQRNPAAHATQDIQFYMSAENVGLAF
SPLFDALAGQAGPANPSEALQSGEQPGDVGAEPSDAARSGSALGWQRTPLLALFLCGGIA
ALGVFLYRKKIAGYPKAVAKAEGIAWAGDSWTPPRLSEGTYAIPGKVAEDLHPVEVALLF
ELPLPRVVAVMIKILEDEGVVRLAQEAPLRLEVLKAESADEYGYTFLGAFDTEGWVLSGR
MADFFETALKKLQEKTWDCDIEATKAYYRKKMEEAEAEEAKGGTTPTEATPRYACWPSFI
YVYSHPYYYGNLGLPKEFSGSYASFMASSACFSGCFSPKNLAGTAGACYSACHNACHSAC
HQACHSACHSACHSACHSACVSGGPH*
>SPBDM4_v1_40643|ID:27157948| Radical SAM additional 4Fe4S-binding domain protein [Uncultured spirochete bdmA 4]

LSPRTKRAANAEAADATAVARRPPGAAGVPASPSVAAPSNLSWIDDFWAQAGEHIFSRED
DGVLILPPNRVYKVNSTAASLIAHLKRGGSMRALQFPSEDAAHDTALFFGDVAALYRGDM
PVNGTVSSEPFSFPFTRLPVLGEIALTYRCNNACRFCYAGCGGQSGRPASAGSVSNHPER
STADDRRIIDIFADEAKIPFFSFTGGEPTLREDLEALVEYAYRRRLITNLVTNGTLIDET
RARELKKAGLGSAQVSIEGPNAEIHEFLAGRKGAFSETIAGIAALQKAGIPTQTNTTITR
GNLHVIQRMPAFLASLGVSRFAMNLFIPTIPGEETDRLFVAYEEIGPYVDAVAKEAKKLG
MTFYWYSPTPFCNYNPIARGFGNKSCAAADGLLSVAPDASVLPCSSWDEPIGNLLRQNFR
ELWFSETARFYKEKHFAPAPCRACDSFTACQGACPLYWRYTHAEPSGARKSKEPL*
>SPBDM4_v1_40644|ID:27157949| membrane protein of unknown function [Uncultured spirochete bdmA 4]
MNTAVIRYRIPGLGTTSRLLIFGAFGLGGILPQIFWRGPGTVIGTLLLLLPLFLLSAKPW
TNKPKDIGEEDWQPVTDAELDRIADAFKSTRHIHLPLWYRPGFGVPFTILLAILAVAFGS
ALTLPGLLATDALILLWPALNFLKIRLWVPREFEMVMRALQAARSVSLPPDIVCTPYLRL
DRDEQGLRIPENARLMLEPRQKPEDLVGVQMQVAINKGPNGTVPYLYAVALTHGQGATWK
KACGFRAPGYVIEPGGDASYGTVVIRQHTDNGGYQTSPQDCSRLVETAIALLKKSSE*
>SPBDM4_v1_40645|ID:27157950| 2-hydroxyglutaryl-CoA dehydratase D-component [Uncultured spirochete bdmA 4]
MTGYTCKYTPVELLYALGGKAELLNDEAEDFEYAEGLTHANLCCHAKALLEHSTAVDELI
LVNCCDSVRRVYDVLKSQGRHKFLFLFDLPHDDEACARQQVKQELLRLSELYCGQHNSSF
DRQLFFEACGASADPVPDKPFLAVMGARVSRALLLSMQEKVQYPVIDLTCGGNRRLEPLP
SHAETLDFVALMDWYAAALLQLVPCMRMTNVGGRRSLWENENLRGIIYNTVKFCDYYGFD
YATLKKETALPILKIESDFTPQSAGQLSTRLEAFAESLKARYDRLPRIKNRQSRLFAGID
SGSTTTKMVVLDQTAQVVASSIIRTGAKAQKSAQAALEEVCRQLGTDQDSFAAIVATGYG
RNNIVFASDTKTEITCHAKGAYFLNSDIRTIVDIGGQDSKVICLDKNGAVVNFIMNDKCA
AGTGRFLEVMALTLDLELVDMSIAGLIWNHDLTISSMCTVFAESEVISLIADNHSSADII
HGLNKSVATKTASMVSRVGGQAPYMMTGGVARNMGVVRELEKKLGASLFITEAPDLCGAL
GAALFALE*
>SPBDM4_v1_40646|ID:27157951| conserved protein of unknown function [Uncultured spirochete bdmA 4]
MNRIIKKFGDVVSKEVYANPGRARRLLTTAYSLNGWKDARFGGSGLAGAYAQMNAVVSRY
ICHAFQHPHNAAMVNVFFPCEILHAMDFTPMFPEALSVYIANTACEQVFADAAENADVPE
SFCSYHKIMIGLAETGVLPKPLLIANTTLACDANQLSFRRLAQDLQIPHVVVDVPNNIGD
DAVNYVAEQLRSVTAALEDVCHRKMDEDRLRAAIRISDRSIKTYREYLELRANCSMPVTM
SGELFSLVAVHVFLGTPQGEQFINAIREAAEEAPKNRSKKRILWLHTLPNWQDSMKAIFD
KSERCEIVGCDMTYDVLCDMNPDKPYESMAQRVVYNINNGGAQRRIEAAIEYAKKLHADG
VLVFCHWGCKQTQGMSQMAKKALEAEGFPVLVLDGDGCDSRNVSDGQMVTRVNAFLEQLE
GMR*
>SPBDM4_v1_40647|ID:27157952|bar| Phosphinothricin N-acetyltransferase [Uncultured spirochete bdmA 4]
MTSGSIRDVRVEDSARICEIYNYYVESTSISFEEEPVPQQEMARRIEAVSRNYPWLVYES
DGEVLGYAYLSRWKERSAYRYTAETSVYVAKGHTGEGIGTALMQALLERVKKLEVHALIA
VIALPNEKSVAIHEKFGFKKAAHFAEVGFKHNAWINVGYWELVL*
>SPBDM4_v1_40648|ID:27157953| putative Flavodoxin [Uncultured spirochete bdmA 4]
MKILVAYISKTGTTEEIAKRIGEILSAKGAETNIDVEVAPIDAVGSVQGYERLILGSPIN
GMKVLPQFKEFLSTKVAGSNIPTDIFIISYLFERGRNFWKNAIRKNVESIRTLAGASSVE
IFAGRLSEPLPSFARFIFGTPKDLPLDMRDWGKIEAWAEGIAGGM*
>SPBDM4_v1_40649|ID:27157954| putative Transcriptional regulator, LacI family [Uncultured spirochete bdmA 4]
LSVLTEKMMEYRTVTKLEDIAKETGFSIKTVSRAIHNHPDIKRETREIIMEVVRKRGFTP
NWAAQSLRNQKTNTVGYIIPNITNGFFGEIGIAVDAFFRAKGYSTLICFTSDSRENEIES
VRSLLAKNIDGIILAPIGNQGEYIDQIPNLDDIPLVLIDNRLSGPARNYVLQDNEGNSRR
LTAHILGHGHARIGFITGPLEESSSRERLSGFRNALKDTGIPFDDSLVRSVDWEIHSGYE
ATKDLLDQKGSSRPTAVIYGNSQLLLGGYKALREKGLSIPRDIAVASFEHPDIIDALSPC
PTTLEKVETRIGQAAAEMLLSIIESKEKTPPREVYIPSQLIIGESCGCPGRS*
>SPBDM4_v1_40650|ID:27157955| exported protein of unknown function [Uncultured spirochete bdmA 4]
MKKIGALMLVLFLIASAAGWSQTTTTPAKGGKTLIGVSVMELTAYTWFLGVIDGCKQWAA
EHPEANFTFQFEDSHSDVQTMLNNIDNLLAAGAKGIILFPADASSAIPTIKQNVAKGIPF
VIGDYKQQPAKPSDAVWATFVGHDMRALGVAAGKIAVDYLKTLNKSNPVCLFVSRPTSGQ

VSKDRFEGFRDTVLAAFPKAKVIEEGDVGAGSRASSQDLVENVLKREPVIDVVCGHNDAE
VVGAYNAAVSQKRTNIKFIGIAGDKDVLSFITRGNPAWIGEVLQNPVVLGYQATDAMNRA
LKGEKLPETYNLPQPEGITPANIKNYDWQTWSWL*
>SPBDM4_v1_40651|ID:27157956| putative ribose/galactose/methyl galactoside import ATP-binding protein [Uncultured spirochete bdmA 4]
MTSRFSADTSVQAAALPLVELKNCTMEFPGVRALDNVNFTLMPGECHGLVGENGAGKSTL
STCITGENRMTRGELFIEGKKVRLSGYSIKESQRLGLQVVHQEFQLMSDMTGLENIFIGH
YARKAGLIDWKTLNRKARELMDFLQCDVNLNVPVHYLRTAEKQIIQFAKAVQSESKVLIL
DELTAVLQEKDILNIFRIIGILKAKGMGIIYISHRLNEIFECCDSYTVLCDGRHISSGNV
RDIDQKELIKMIIGRELTQAYPTMNTEFGDVMLEVKGLTAPKAFRDIDFQVRSGEVIGLA
GLVGAGKTELVQAIFGTHPVTRGKIFIKGKEVKIKNPEHAIQLGLGYIPDERRTLGLNMK
FDIKENTVLPSMNRFRKFKIFQDHVAEARSAYDINEKLELNYYSLWQSVEKLSGGNQQKI
VIAKWMLRNSEIFLLDEPTRGIDVGAKFEIYKLINELTRQGKSVVMVSPELEELIGLCNR
IYILYEGSIMDIVEGERKTQEVIISSLLGVNGK*
>SPBDM4_v1_40652|ID:27157957|rbsC| Ribose transport system permease protein RbsC [Uncultured spirochete bdmA 4]
MNTFEEKAKRYGGGWRSFIRDWMILFLFVALVVIFSLTLSTFRTTDNIMNILRQASFTAI
IAMGEFFVILIGQMDMSISSIIGMVSIFFAGFVVKMGIPIWIAFLLVMAMAAVIGLFNGV
LVVYGKMPSFIATLVTMNIIKGLNYLYSRGLPISGLPDGFNFLGSGYVGIVPFPVILMFL
VAIILFVITGHTSVGRSFYAVGGNLEASKLSGINVVFTSILAFVFSGVLTAVGALGLTAK
TLAGNVSLGENLLFDVMTIVVLGGTSLTGGRGRIFGVVIGALFLQVISNAMVLMGINTYW
QWVVKGIILIIVVLIDSNTKRDVVTTSRKAKQASAEEK*
>SPBDM4_v1_40653|ID:27157958| Creatinase [Uncultured spirochete bdmA 4]
MHGDICIPNHEYKERVGKAAALMRREGLDALIVNGNEADYANTRYFSGFWPLFERCGVAI
SAAGDAALMVGPESRAFGGDRNKLDKLYVLKDYREGADPAYPELKADTFNDVFKGLGITG
KKLRIGVASYLDTSVIIMEGIKKAFPEAEIVRADHLMVELRSIKSRNEIACLKEGYRLAE
LASQQVIREIRPGMTELQMVGVAQRVIYENGAEYEGLPMYYVFSEASTRHAISRSSYRRFE
KGDIVQLNLSAKIDGYSASIGYPVILGKLTGKRRDIVLFGLEAHNWTQGQLRAGVIAADV
AKDFYKFFVENGYKDNFVYGPMHGTGLIEVEAPWVETVSNYAFKPNMTFQIDTFISTDTF
GVRWEKGVAIEENGVDVMSPQIGMLYELDF*
>SPBDM4_v1_40654|ID:27157959| protein of unknown function [Uncultured spirochete bdmA 4]
MKPIYVLFGVDMETDVGSFTPFYEGAKHGTPLLLDMLADKGVRGTFFFTGDCARENPEVA
KLVAKSGNEVGCHSLYHETVGDELFPIPGVKPLLPHEVKPRLKLCKEWVEKAAGVKTTSF
RCPRLWGSTAVVNALEELGFTCDVSYPMYFYREQFAPYHPDRKDWTKKGDSSVLEVPNFA
DMTMKSDDPGLERDRDQWPLFRTVGAEYIFGKSVSFLEFCGKKEIPPVLAFYIHPWEFWP
MEASYHFGEATVIPDEFITKNCGPVALRELGKLVDDLAGIGARFVTARELTEIFA*
>SPBDM4_v1_40655|ID:27157960| protein of unknown function [Uncultured spirochete bdmA 4]
MIHIFPEPKIVRELEGRTKPFDELAIDFGGLPEAEKADLLDIARMRFRNRKEVRITSSVT
EGDCIRVTPVRDLSGIEAENRDLFLKQGYCLKIEKDQILLKYQEKSGFIYAITTLKQLLI
RENGQSVLPCCEITDWPSVEHRAVAPTFSWYAGYGRLGFDMQLWGHDEWLEYLNVCIDNK
INQMNMVMYGYWPFELEEYPETVFRDVPVKIWNKENRRWLTVRYSHPNIEENFLADFIAM
AHRFDFKIFAYVGLNSYNGAYSIKHPEKRMVKPVGSQFMNDFDSLCLSDEGNIGYILASM
RKIARLGFDGFTLEESEEGFWFCTCEKCQKRWGATSASPVEAKHKANMWLLNLIYEEIRK
VNPRAVIGIRAFRQPPLEKDPAFLEACVRSMPADIKLFWAPALYVPPTEFKKWIAAFGRD
RIWGRDTEANAITSTMGRLFRVFESNMIRYADEPNAQVIERDIEQHMTSAAEGVSGINGF
MFEWYGLFMFQWAHGNYGWGSRMDKKEFFRLSCEAAFGEELGDRVLFALRSILTIHESQM
PFYTTPFPFQKNRITKADIPEIMRAKLRHPGLLEEIHAIQREIEKDPRLAEYIPHFARIE
NAERRNAVIYDMALASLKYEDAASPEEKERLLDEILRYNEQDFDLVKDMFFDINPVSESG
VKSCMYPYHEIKRLIHNIRHPEAPDDAIVCSGIEALGWLWL*
>SPBDM4_v1_40656|ID:27157961| conserved protein of unknown function [Uncultured spirochete bdmA 4]
MFSEQEQLILESPSFSASAFRYRSGVEALRIRAGRGEFIWLPYLGQQVWDWSVDGKSLKF
EGFVEEPSYGKNFLQNYGGFLIHCGMTAMGNPGPRDTHLHHGELPVARFDEAWIETGTED
GREEGLSLCGHIHWKVPFVAEYHCTPSLRISADGLRLRAEVCLENPTSRDLEYMYLAHIN
FAFVGAEKILSTVPFDAAHVTVREEILPGLALPGQAANPALIQRIDGDAAYEPELVAILN

HGDAAMPAGSVLVNKDGSVRWVRQDSPELDHHVIWITHNRDRGACGFHLPATAGPTGFAS
EKALGNLKRLPAGGSVTLRYECGFGDRLGDAPIAGAH*
>SPBDM4_v1_40657|ID:27157962| conserved protein of unknown function [Uncultured spirochete bdmA 4]
MTEQQRDMGRNQWVYPDLELPPPGDFPLKGHESLIILNMNAADASVALTLYFTDREPVVL
APLKIPARRVRCLRMDKVDDIGIQIPRETQYALRLNSDLPVVAQYGRLDTRQQNMAFYTV
MGYSG*
>SPBDM4_v1_40658|ID:27157963| conserved protein of unknown function [Uncultured spirochete bdmA 4]
MIGALRLGIVTIGQSPRSDVLPDFIDALGFLPAFEQLGLLDGLSCEGIASLAPRGPDDQT
LVTRLRDGTEVKLSEKSVVERLPLAVEEMERRSVDFIVLFCTGEFPPVESRVPVLYPSAI
VSAVVSAVFSSKKNGGARLCVIAPASEQIPMLARKWGTAGVTLLFESLSPYTSGDSELLD
CAGRVARLQCDMVVLDCIGYTEKVRAAFAQATGRPVILPRSLLARVAAELAAG*
>SPBDM4_v1_40659|ID:27157964| conserved protein of unknown function [Uncultured spirochete bdmA 4]
MLKQVIEAYELLDDARVNGSRVADFLREKGLENVVVHEIKGEKGKTDFIKAVIPGVSGKC
SGGGSATLGIIGRLGGIGARPERIGLVSDADGAIAALACASKLAEMMQKGDRLAGDVIVA
THVCPDAPTQPHAPVPFMGSPVDMKAMNAYEVDPDMDAILSVDTTKGNRIVNHRGFAVSP
TVKEGWILRVSEDVLDIVEWSTGDSPVVLPITMQDITPYGNSLFHINSILQPCTATKAPV
IGVAITAVSPVPGCGTGASREMDIEATARFCLEVAKSFTAGTCKFYSEQEFGRLKQLYGL
MTHLQTEGMTL*
>SPBDM4_v1_40660|ID:27157965|arlR| Response regulator ArlR [Uncultured spirochete bdmA 4]
MNILVVEDEHSLARMIEVELLLQNMNVEVCYDGKSALASAIEKKFDLVILDWILPDIEGI
EVCRILRSKGLQTPIIMITARQEVSFEVRGLTEGADDFIVKPFDIEQLVARIHAVMRRAS
AQKGALPKTVINSLVIDPATKAVYDNGQRVHITKKEFEILMLLLANRGKVVTKEEIYLSV
WGKDVHIEEGAIAVHIKAIRDKLKGLKIENVRGFGYMIENSEQEIPQENS*
>SPBDM4_v1_40661|ID:27157966| exported protein of unknown function [Uncultured spirochete bdmA 4]
MKKRFLAILAVVICFMGIVTFILSYFTAKGSIEQLVLERANTRLSIMVQGFSNIANDIDI
YSYINFPHESISIRKGSLSVFQYGTSRLDENSPRVKKIETQKGDYDFILYVDLDAEVSGY
IKPFRQIIAITSILYAVIFAVAGIIFINTVVGPISSLAASMAKITSQNLRERIPVPRKKD
EISQLVETFNSMLDEIEGAYNRLKTFVDDMTHDIVTPVQILEGYRQLIERHGKNEKIIDE
YLDVSKIQLFRLKEMTTSLKAAFLVEKRRRVEFADASEITSRNVAYYRKLYTSIHFDAFI
EKEVMFPIESMDLERIENILIDNAVKYGDEGGRIEIRLKKNELVVRDFGRGITDPTFAFE
KNRRDENEDEKKHGEGAGIGLSIIKRFSEEYGFTLDLESHAGDGCQFIIKFPERKQ*
>SPBDM4_v1_40662|ID:27157967| exported protein of unknown function [Uncultured spirochete bdmA 4]
MFLKKFGIAAVILFVSIASLMAETTLVVGQATDPIILDPPMYSDTPTHNVNLLIYDRLYD
LSSDGKVIPGLAVDLPKIEENGTKYTIKIKSGVKFHNGQPLTIDDVIYSFQRGAFNDKSQ
MKSIFAMMYDLKKIDNYTFQFRTGFFDKSKAPANVQDSDLKSFEDRAKYYKPAAFGSQLN
QLTWLGASIVSKATLEKAQQDGTLKDYGVTWAVGSGPFKFDSWKQGDSITLVRNKDYHDS
AIQTNVEKIIFKTIRDPSALKTAFLNKEVDLIMNVAPLDAREIEMAGGKILLTNGYFGYH
YLGFNMKSPRVGQVNADGSPDMDGEYDLSAPSAKLRLAILYSINPSDIVNSPDIMDKRGK
VTLQYVESLPFGHIDDPKGTRIEEGNLKTGYYNPAKAKEIFASLPDSLKKPGSLKCTALS
GSVYYVKEALVIKDQVKRVLGVDLINIEQVALSELATRRSKSDPDVWDLIINWTQTDDSYY
IFVAFDGFNTSLLHDTKFYTKNAQEWIEKGNSLPNGPERNIAYQNAQKEILTALPRVPLV
AIMGISASQQNISGVFLSPSGSFMLENVVKK*
>SPBDM4_v1_40663|ID:27157968|dppB| Dipeptide transport system permease protein DppB [Uncultured spirochete bdmA 4]
MTRYIFRRLLMIIPVLIGVTFIVFSLTYISPGDPARMILPQDATDADIVHMRHLMGLDEP
FFMQYIHFLFGFKSPSEDSSFSYKGLLALDLGRSYVSNRPVFKTILERFPNTALLTFLAL
ILSVILSIPAGIISATHQYSLSDMIVSILSLLGLSMPAFWLGMILIWIFSVQLKILPSLA
NPNNLKSLVLPVITLATMNTAVQARMTRSSMLEVIRQDYIRTARAKGLEEKKVIRKHALS
NALIPIVTVIGLQVGQLLVGAVLTETVFSYPGIGTIMVDAIQRKDTPQILASVVFVAIVF
ALVNLIVDIVYAFIDPRIKAQYEGMSKARSNRASLKGK*
>SPBDM4_v1_40664|ID:27157969|dppC| dipeptide transporter ; membrane component of ABC superfamily [Uncultured spirochete bdmA 4]
MDQQMRIANVPTSKIKENQKRKSAFQEFWRRFKKNKLAVAGLIVVLLYILMAIFADVVFS
YNFITQNQISNAFAKPLTKAEIDYQDKTTEHLYILGADNFGRDILGRIIHGARVSMAIGF

TTIAFACLIGSALGAIAGYYGGKIESLIMRGTDILLAIPNILFALAIVAALGPSFQNLLL
AIGITSIPAFVRLMRASVLTIKNQEFIEAARALGAGDFRIIFKHIMPNCMAPIIVQATLG
IGQSILSATSLSFLGLGIQPPSPEWGNMLSDARSYISYAPHTLLFPGLAIMTVILAYNLI
GDGLRDALDPKLKR*
>SPBDM4_v1_40665|ID:27157970|oppD| oligopeptide transporter subunit ; ATP-binding component of ABC superfamily [Uncultured spirochete bdmA 4]
MDELLLSIRDLTTQYITDENIIHAVNGINLELKTGGSVGLVGESGAGKTTTAYSIMGLLP
KLTGKIVEGNIFFQGKDLTTIPEDEMRKIRGNQISMIFQDPMTSLNPILTAGDQIAEVLE
IHGTADKEEALAKAGDMLEMVGIPRERMHEYPHQFSGGMKQRVIIAIALACDPKLLIADE
PTTALDVTIQAQVLRMIKELKTKLNTGLILISHNLGVVAQICENVAIMYAGKIIEYGPLR
QVFENPLHPYTEGLLNSIPRIELDKTRLSPIRGTMPDPSIVPQGCIFRPRCPYAEDICAT
KAPHITEKSNRKVMCLMHEGIIANKMRTEHA*
>SPBDM4_v1_40666|ID:27157971|oppF| oligopeptide transporter subunit ; ATP-binding component of ABC superfamily [Uncultured spirochete bdmA 4]
MHERSGAEPILQVRNLKKYFYTPRGNLHAVDDVSFEIQEGGTLGVVGESGCGKTTTGRAI
LRLIEPTSGSILFNGTDIVSLDKHRLRMMREKMQIIFQDPYSSLDPRMTVSGIIAEPLII
HRRVSGKKELVKHVQKLMEMVELEDRLYSAYPHELDGGRRQRIGIARALALEPKFIVCDE
PVSSLDVSVQAQILNLMQDIQDQLGLTYMFVTHDMAVVKHISDEIAVMYLGQIIEKSPAK
ELFKHQYHPYSRALISAIPKIDSKSSDTSTILRGEIGSPIEPKPGCRFAPRCEYCIDKCL
SENPLFKAVSPGRYAACWRAEEI*
>SPBDM4_v1_40667|ID:27157972| conserved protein of unknown function [Uncultured spirochete bdmA 4]
MDLSAAKSSLWDIIDKRKNEIVGLCSDLIRIPSENPPGNVDEIVSFISQFLAKNGIESRI
TGPAPEYPNIIAGIGKGPVLLFNGHCDVVPARDRARWNFDPFGGEITSTTIRGRGTSDMK
CGLGAALFAMSLFAQGKIPLGGRLEIHIVPDEETGGANGTKWLVENGYADNAFACIVAEP
TSSDTCEVGQKGQIRIHVTAQGKAAHGSIGNYVGENAIMKVATLLSRIEELRSMKGVYSE
SQTQVLFDSKKIAQEKLERPHVEDVIDHVTVNPGIIKGGIKINMVPDLCEADIDVRIPIG
LPAKKVIAQFEEIIQQLNLNNIQYRVEYSEANFTEVSEPLVHTIVNNAEAIWHHKVIPAY
QWASSDARYYRNKGIPTLQYGPANTEGIHSYNEDVDIEDVINATKIYSATICDLLVRR*
>SPBDM4_v1_40668|ID:27157973| conserved protein of unknown function [Uncultured spirochete bdmA 4]
MHRVLVGTSGWNYEDWKGSFYPEDLPKTKWLEYYARNFQTIEVNATFYHEMKESTYKKWS
EATPDGFCWAVKAHRFITHIKRLRDVEEPLKRFFGSVSALGDKLGPVLFQLPPSLAFDGA
VLEEFSATLHNAARSSAGLPKIMRFAIEPRHASWMRDDALACLRKLGLGFCISDTGGHYP
CRKAVTSDFAYIRLHGPTTLYASSYSDAQLEDWAARVATLDRDVYIYFDNSCMAYAPNNA
LDLKKLMDNFVPGSGS*
>SPBDM4_v1_40669|ID:27157974|chpA| toxin of the ChpA-ChpR toxin-antitoxin system, endoribonuclease [Uncultured spirochete bdmA 4]
MVGKKGYVPERGDIVWLDFNPQTGHKQRGRRPALTLSFRAYNEKIGLGLFCPIASKVKGY
PFEVEIVSGVIQGAVLSDQIKSLDWVERNIEFIEKIDSAKLQEVIEKIEVIIK*
>SPBDM4_v1_40670|ID:27157975| Transcriptional regulator/antitoxin, MazE [Uncultured spirochete bdmA 4]
MKAIVQKWGNSLGIRIPAVYAKELDLKNGVFVDIVKDGGKIVILPPKPTLEGLLSEVTKD
NMQDYIDSGSSVGKEAW*
>SPBDM4_v1_40671|ID:27157976| Type III restriction protein res subunit [Uncultured spirochete bdmA 4]
MPLNPTFPTSPYVILDPECRWAPSGKDLSESGYEKLLPPLVYKIRREVKAWRDTDYEGAS
PTSKALLNWWFDAGHSLEKSDGELFGFRYYFAQREAVETVIYLYEVAQVKDKYDLIRYDS
SGAVSSGMFEEDWLRFVIKMATGSGKTKVISLILAWCYFHKLYEPDSGLATNFLIIAPNI
IVLDRLRADFEGLRIFWRDPVLPDNGYEGQNWKDDFQLTLHVQDQLSGIRKTGNIFLTNI
QRVYTGNDTEPCFDDDNLEDYFLGKRPVGKTTDSKIDLGVIVRELDELVVFNDEAHHIHN
ADLAWFKSIKDIHNRLKQKDHFLSLQVDLTATPKHDNGSIFVQTVCDYPLVEAITQNVVK
HPVLPDAESRKKLQERQSSRYTEKYADYIALGVEEWRKVYAEHEKLGKKAILFVMTDDTR
NCDDVGQYLEATYPEFKDAVLVIHTKNNGDISESDTGKSKAELEQLRKDANAIDSWESPY
KAIVSVLVLKEGWDVRNVTTIVGLRAYVAKSNILPEQTLGRGLRRMYLGEDTTEFVSVVG
TPAFMDFVESIQSEGVQLDYRPMGTGTDPKAPIIIEVDQKNRKKDLDKLDIEIPVLGPRI
YREYKNLDALDPSTFGNKKLAYRHFTDEQNRKIVFRDITNGAIDHTTMLDSATVSDYRSV
VGYFAQVIMKELRLVSGYDVLYGKIKEFIASSLYTTPVRLDDLNTLRNLAELESTKTIIE

TFVRGINHLTVQDRGGVEIRDTIRLKDTRPFVVKEQSFYLPQKSIFNKIIGDSRLELSFA
SFLDNCPDVVAYAKNYFAVHFNLDYVAANGGISNYYPDFLVKLDDNRVVIVETKGLEDVD
VPHKMARLAQWCNDLNTAQKKTHFDYVFVDEEDFKTYTPDSFERLLRNFTKYK*
>SPBDM4_v1_40672|ID:27157977| conserved protein of unknown function [Uncultured spirochete bdmA 4]
MIERKKICITVKTYPTLSAKYGELVCTAGLLEDGSWMRIYPLPFRMLEYDKRYKKYQWIE
ADVERNLADARVESHKIFNIDTLKTLDTIGTEHEWSRRNALVFKSQKVFTNLAELIAVSR
AIDISLAIFKPSRVLDFIWEEVEREWPKEKLEALKAKASQLSLFQSPEEIAHELQIVRKL
PYKFSYVFEDDSGKRSTLMTEDWEVGMLYWKCLKAAHGDETQALEKVRQKCFARFVRDRD
LYFFMGTTKEYHGWALNPFIIVGLYYPPRKTQPEFVFDRGTDYAP*
>SPBDM4_v1_40673|ID:27157978| conserved protein of unknown function [Uncultured spirochete bdmA 4]
MISNSSQPPTFKRQKFLLAFIKSAGGTLSYMDFQKLLFLYLQRTNDSCYEFVPYLYGCYS
FQAARDIDTLGAMGWLDKKDESIRLLDSRVSSGSDDFFLESFHDEYRNIRGNALVRQVYR
EFPYFAINSKIAGELMDGDELAIIKQKKTDLKKNEAVIFTIGYEGLSLEKYLNILIQNDV
RVLCDVRNNPISRKFGFSKDMLSHAVTKIGIDYVHYPELGIASGKRKHLSAEADYASLFE
EYARQLPAKKSFVDVLFSIYMDKKRIALTCFEHDPAHCHRHVLSDYLATKYHVKVAHL*
>SPBDM4_v1_40674|ID:27157979| Nucleotidyltransferase [Uncultured spirochete bdmA 4]
MSERQVGRVVSVDNFRVFIRLDDDLTSSYKSGFKGLYEVARINSYLIIPVGADRIVALIT
RVKMQEEAEFDTDKHSITLPSSARYVTATMLGTIEHGDKSDRFIQGVYNFPVLGNPVWYV
TEEDLNRIFDYKPDDTDINYQEDFYLPIGKSPAFPDYDVKVCPDQLFVKHAAILGNTGSG
KSCTLASILHSLFDYQYPPDKGKKEKRYLRNAHFIIFDTNGEYKSAFIDKEERSGKDELF
HRINALHIDEKGLKVPYWFMNWDDFDYLFDPGPGTQAPILKRAIGLGKNNIQSSQAGIID
QVQESTLNEIINVTCGDEGWKFEAKRNKNKSYDANNEIIQLSTLLANYDNQILKDIAKYL
SILGKEKLSLEEKSSSIRMIKSLYSGYRETIASQNIVNDKNIDLPTWFEYQKLITQFIDA
AIDEHEGSNSKLSEYVSTLRLRLEGFLSDSRIAEPLLLNSKEEFDNSLSSFLSFIIGDIY
RYFVGKDQETDAFDPFMSYYSSSKCKKETDITKVSQLTILDLSLISYDVLENIVGLLGRL
IIEFVSHFKPDDRGKYPIVLILEEAQNYIPEKNRKEKESIAKRVFERIAREGRKYGISLI
VSSQRPSELSKTVLSQCNSFIVHRLQNPDDQKYVRGLVSSANSDILDQLPLLPQQHAIIM
GDCVRTAVQVKINTVSPKPNSNNPHFVEKWLNENNDFPDYKKVCDDWQKGIKEGDNKDDK
*
>SPBDM4_v1_40675|ID:27157980| conserved protein of unknown function [Uncultured spirochete bdmA 4]
MREIKCFIGGKEKNDNGNSDGMGEKTERVKLLIKKYLELNNVSVLTGAGTSFHLGAPVIQ
TIPQKIHEALMATEKVKDEYKKQLDELGGEFTTTPLETFINYLQASRFIFGIRGEDTSNI
DGLISAAQEKLFKLCNTEQIPLNQEYESDDHLKENKYFYHEKFVKKILQRPVNLRRINLF
TTNYDLAFEYAFDNTGVQYINGFSGFSRRSFHPETYDYDIYYPGQTTSGTVHRAERVIRY
YKLHGSLSWIAKEPGPGNYYGIEEIPVGREKPPHELIIYPCVTKKTFTLDLPYSELFRLF
SSAIKQDQSVLFCLGYSFNDEHINDIIYQSLSIPSFTLFIIDYRGTENPEIRRLRDLDDP
RIIIIEGEGAKFTNFVSDILPDLCEEDDDIKIAETMNMLSQKGECSEKEDIDV*
>SPBDM4_v1_40676|ID:27157981| Adenine specific DNA methylase Mod [Uncultured spirochete bdmA 4]
MKLTDAEQREVVKLVEAGKPLPDKYRFLLFDDKREVELVWNGKTDEVCNIVLPFQVIEQI
DEPRKEKPEDSSLQGELFDFRGRQKSGWTNKLIWGDNKLILSSLKNGPMREEIEKNGGIK
LIYIDPPFDVGADFSMNIEIGDETFTKEPNVLEELAYRDTWGKGADSFIAMIYERLILMR
DLLADDGSIYVHCDYRVNSYIRLCLDEIFGSDNLLNQISWKRTPFAGSSKARSQKYPINH
DTIFLYTKSSKYLFEQQYIPFSEEYISRFKNPDNDSHGPWQSVSLKTYSQETFNKLKEEN
KLIPPQREGAGWRYKWYLNETKGKQVEDIWTDINLTNPMAKERVDYPTQKPEALLERIIA
ASSNEGDIVADFFVGSGTTLAVAEKLGRKWIGSDLGKFAIHTTRKRLIGVQREMKASGKD
FRAFEVLNLGKYERAHYIGVNMNLREEERQKQLEQKEKDFIALILKAYKAEPVENFKCFQ
GKKAGRLVAIGPINLPVTRLFVEEIILECREKRVSRVDILAFEFEMGLFPNIQEEAKNKG
IDLALKYIPREVFDKRAVERDQVVFNDVNYIEVTPHVKSADRRKKDSRGAISIALTDFSA
FYSQDSVLNASASLGNGKNKIVVENGKIIKVSKDKDGVVSKEPLTKKWTDWIDYWSVDFN
FENKREIVRVKNQATGVEEEIWTGDFIFENEWQSFRTKKDRGLELETPYHECPAGRYKVA
VKVVDIFGNDTMRIVEVGV*
>SPBDM4_v1_40677|ID:27157982| putative transcriptional regulator [Uncultured spirochete bdmA 4]
MFDTKEELIEKIGLGEDSTIEFKSVRFRGNRVALIRDDIADEIAAFANSFDGVIVMGVDD
ETRTIEGIPLEKLDSVVDFITETSNDSIKPAVPIRVFRMMLPNAQGIDVPVIKLDIPKSI

FVHESPGGYFYRQGDKKRKLSPEALARLFQQRSQVRLIRFDEQAVPQTDISDLDEGLWRR
FAGDTGDAPKVVLDKLKLITKDDTGTLRLTVAGALMATPHPEIFIPNAIIEAVKYRGTGR
DANYQEDTRIVTGPLDAQVREALSFVKNNMKIMAVKNPGREETPQYSIRAVFEALVNAVA
HRDYSIAGSKIRLFMFDDRLELYSPGTVPNSMTIESMPLRQSTRNETISSLLNRCPVDAA
AQPEVKRQFMMDKRGEGVPIILQESTTLSGKKPVYKLIDDTELLLTIYAADLNEGSE*
>SPBDM4_v1_40678|ID:27157983| PHP family phosphoesterase fused to chromosome segregation ATPase [Uncultured spirochete bdmA 4]
MSGQDWTFKGAHWWEFDFHCHSPASLYPDYGRGDASLKSLPPREWLLACMKKQIDCIAVT
DHNSGEWIDMLKAEYQSMKTESLPEFRELYLFPGVEISVQGGIHLLALFDPAKTRDDIFA
LLSKSELGFTKSQLGSPDFVTKGSFLDIVACIENEGGVAIPAHVDRPSGLCNELTDGPTL
RTILGNTHIYAAEVVDSSYSFPGLCRDCKPSWTRVVGSDAHTPDEIGRRFTWIKMGVPSI
EGLKLALLDNDLSVKRCDSMPEGSDPNAHADFVIRSIEISKSRYCGNGQPEILQFNPWLN
CLIGGRGSGKSTIVEFLRLAFRRDKEIKELFSKSDFGENEQSELVKNLDDFYREPKGRMD
KGVLEPATKITVDCRLHGEEFRLIWQQMPDGSISPIQKWDGAHYAKSSGEDIAGRFPVRI
YSQKQMYEMAQNPKALLRIIDEDPQVDYREWKNRHDQIAAQFRSLRAKERELMVQIAEEE
NIKGNLEDIRSKLALFERGENAALLKEYQIRRSQIKEVEAYQASLQENYQALANIELGTI
TPDFSLFGDSAEDEEIKKLIVRYTETLTSLNTRLSAIRDECRKAASQFSKDIETSLWKKK
TQKILQEYQALIEELRKNGVENPDAYGLYVQKKQNLEARLLAVKGLKQQIEELEQQAQEQ
LTRLDAYRKELSQSRRSFLVKVLEGNKVVRMCLRERGDADSLEEDFRRIIGKEDETFKSD
ILVTEEGNESGILSALYKNYSDENLLKVKKTIRDLRKDGTHTSFGGKFINYVKDKVTPEM
MDELDLWIPEDSVDVEYCRDQEKKEWTPISQGSAGQKTAAVLAFVLSHGSSPIILDQPED
DLDNHLINDLVVEQIREKKPQRQIIIVTHNPNIVVNGDAELVHVMNFAGGQIRVKISGSL
QEKEIRREICQVMEGGAKAFEQRYRRIYIEGEHV*
>SPBDM4_v1_40679|ID:27157984| conserved protein of unknown function [Uncultured spirochete bdmA 4]
MAQILNFPGDRPGMNNEEKAQDMVYDALESDSPVQRKRLARKAIELDPFCADAYCVLAEE
YESIEKRRECFTKGIAAFKEKYGKKYFKENTGYFWGLIETRPYMRLCAGYGELLWETGEK
EAAIAQYEELLRLNPNDNQGLRYNLLNWLLAMGNLEKAGELLKKHDEGSAFMLFSKLLLI
IKTEPSNTKKIADAYRKANRQNPFVVEYLLDKKGLPDYEPEYYGFGDENEALTYCFDAKK
IWEEDDTAMAILGELV*
>SPBDM4_v1_40680|ID:27157985| conserved protein of unknown function [Uncultured spirochete bdmA 4]
MSDDINERFARFLRAVQLQDIRPMSLQSEVKGSMPPQGSELQLEWRQSFANGDPIVSAPD
LRIFRPKYEFTVKFQDAVIFVQISIFVISFKIIDPAAYGELWADEELRKIFMEKQIQKTI
WPLFRQHIHDGMSRLGMSPVTLPWIL*
>SPBDM4_v1_40681|ID:27157986| putative zinc finger/helix-turn-helix protein, YgiT family [Uncultured spirochete bdmA 4]
MSMQLSCPRCGKTRDVETVEREEKVTIRGREVPFTARFSRCMTCGEEFEAPGQLDANLDA
AREAYTRLYEAPKPEELVALRTRYGASQKAFSIILGFGELTMNSYENGATPDSTNRLLLK
LAAKPYIFKEMYTINKDRIGAIQRQRIEASKGFQSAMRWDGLEALSASLTALQCEKIEVC
AEKSGLSVPEQIARYVGCASFQDYTRLYAEARWTSGTTRQISATSMLANSVSGAA*
>SPBDM4_v1_40682|ID:27157987| conserved protein of unknown function [Uncultured spirochete bdmA 4]
MDRLLATPLFLRRLKPLLVAEKLEFKPRNWRKTMEFMLEEGLSEEDVYDIVAKLKPEHYH
QGPEPDDNGSAGEVMVFFYPYRRQAPPKDTILLYIKLKIWTDIGGDAGVVMSFHDEGNI*
>SPBDM4_v1_40683|ID:27157988| putative Cobalt transport protein [Uncultured spirochete bdmA 4]
MRVNTMKNAKRLRDFSPLGKFILMLAVIACTYLTDRLILIAGLIVLQGIVAWTSGAGRVY
RTTLLALAVGAITLTLFQIFSISEGATVFTLIPPLGFGRVTDKGLQMSLLMSLRMVSSVG
VISLVVALTPSTQIVSLVSGTFRLSPAYTCVLITALRFIPTFGERMGKVLEAEACRGYRA
DTANPFRKIGMILRLSLPLLVTCVRDVDSLALSLEARGFSPDSRTRPVTIKPDAFELAVL
GCALVAMGVVVMLGRI*
>SPBDM4_v1_40684|ID:27157989| Cobalt abc transporter, ATP-binding component [Uncultured spirochete bdmA 4]
MIHIEGLIARAGAFEILKGIDLDIGAGEFVALLGPNGAGKTTLIKHFNGLRVPSAGSVMV
DGMDTRRVKTSRLARSVGFLFQNPDHQIISNRIDDEIAFGLKQTGIPEPERGERVREAAA
ILGLEGLLNADPFTLPRALRQRVALASVLALRPKVLVLDEPTSSQDERGATMVMEIAREL
NAGGTTVIFVSHDMELVARYARRALVLVDGRILADRSVHALFRDTQPLNAAGLSMPGAYR
LAAALGIEPEEGRQFVPTSVVEAVERRLSEARPCA*

>SPBDM4_v1_40685|ID:27157990| putative Phosphonate-transporting ATPase [Uncultured spirochete bdmA 4]
VNALFSEKQGRVADENAAPSASSFSPRAAPAAPIVRLEHFSFTYEGAARPALADLDLEVR
PGEFVGVVGAEGSGRTTLFRVLNGSAPRHFRGTWSGIAEVGGLDACEVGHAELGVFVASI
FDDPDAQIVSLTVEEEVCFALVQRGLPSDIVAARMKEALAAVGLSGLERRATSSLSGGQK
QRLVTASALALEPKLVLLDESTASLDPRGARGLYSLLRAKCQKKGLAVVAVERDLELLME
YADRVVAMESGRVVMDAGRRDIAPNSRLLKRIGVRLPAWIETVDALGASGLLSAPLPATE
AEAAARIVELLVESKARTTRPHAIPPTISPAPTGGSLTGNMEENLAESLAERELPA*
>SPBDM4_v1_40686|ID:27157991| conserved membrane protein of unknown function [Uncultured spirochete bdmA 4]
MATTKKGMTTRDLVLIGILLAAGTVIKAVFPKLPVTPNFIISMYCLAVLLVRPNLAETIG
IGVVSAILAQLTTGAAVPFINFASEPIAIVVCYFLCKISFPKGTASNIVRPAVVSFITTF
VSGTIFVLILSFVLLARGSSAKYVMIYSVVLPTAVANMVITTLCYAPLRKVLNIKDEEPA
KVVA*
>SPBDM4_v1_40687|ID:27157992| membrane protein of unknown function [Uncultured spirochete bdmA 4]
MFMEAKKKSHPKASVVVAKKTVRHAPASRPPLKSARGIVALLFTAAGAFALLSMLLWTHF
PSIWKPLVVPGQVLLSVFSWAAYYLPLWLVWAGVLAFVPKFCPRSTYLLGISALPFVVVT
GFARLSSNPGAFFESHPSMAKVGLSSLYAALGFLLAASFVVVIAGYIKLSEWLKANGYIK
TRAEKKKNGTAQSFGQKLTTFLRNVSMKREERRKRKEEARLARAEALAEKNISLNIPDVP
PPGPLKSEEKTAAVAVADAQAMPEPALGTAGISFTSVPPARPALPGEEPKRAIVERILER
KAAKTYHVPIDGLLNTYPDGQYWIIDDKTRASAGVLKETLAEFGIEAEVTGIRKGPVITM
FEILPAHGVKISKITNLGDNIALRLAASSVRIVAPIPGKHAVGIEVPNERRSIVSLRELI
ESELFRQTKMEIPVALGKDITGEVQLVDLTQMPHLLIAGATGSGKSVCVNSIILSILYKR
TPEEVKLMLIDPKIVELKLYNDIPHLMTPVVTEAKKAFQALQYCICEMERRYSMLDSLGV
RDIRSYNRRVRERNLAQEKLPYIVVIIDEFADLMQITGKELEATLARLAAMSRAVGIHLV
LATQRPSIDVITGLIKANIPSRIAFMVASKFDSRTIIDMVGAEKLLGRGDMLFSGAQDPF
PVRMQGAFVSEEEVERVVAHVKTLGEPEYIDEEIFIDEEDSDEPTLFDEDGSDPLFQKAL
EIVLQQGKASVSYIQRRLKIGFNRAARLVEMMEEKGIVGPAQGSKPRDVLRNPDVFDGGE
ATRMREDKE*
>SPBDM4_v1_40688|ID:27157993|slyD| FKBP-type peptidyl-prolyl cis-trans isomerase SlyD [Uncultured spirochete bdmA 4]
MEIKKDRVVTIDYSLRDDTGRLIDSSAEAEPLVYLHGNENIIPGLEKELEGKNPGESIAC
SIAPEDAYGDRDEALVFKVQKKDFGENVEVAPGMQFEAHGENGVQIVTVVKIEGEDVTLD
ANHPLAGETLHFDVKVVDVREATSEELEHGHVHGDHGDGECDDDCDCGCEDDCDCDEGCC
N*
>SPBDM4_v1_40689|ID:27157994| conserved exported protein of unknown function [Uncultured spirochete bdmA 4]
MKFSSKVHVRFLDIVIFGLAAALVVGTSLLVLNRQSGTRYVQMTGETGEWIAPLDKDAEY
EIPGPLGITVVHIHDGKAAIEESPCKNKLCILAGAISEPNQWVACLPNKVFVRITSSSSK
DDGGVDAGAY*
>SPBDM4_v1_40690|ID:27157995| putative heptaprenyl diphosphate synthase component I [Uncultured spirochete bdmA 4]
MQVPTSPSCGNVRSRFANRTDVTALLAAFCFFLSAIEYMLPKPLPFMRLGIANLPILLAI
DLLPFKWFLVLAAAKVVGMSIVSGTLFSYVALFSLAGTMIAALVMRGVRRAGKGAISLIG
VSIAGAVTSNATQMLIARFVIFGEVAWLIAPLFLLTGLITGTLMGFFAEYFVAHSNWYSC
AMGESDVLIAEGGTGADTADTGPSATMATNPAGRGSGVEPQDAKKVHRSRTEKNAGRFAR
RQRYESLMEPWVAAILGVAIAVVFLLQQELLVKTLYLLLFILWTWLAGKRFSLVSTLVVM
AGIVAANLLIPSGRVLFKLGPLVVTEFALTEGLAKALTFEGLMYLSKAAIMQGLHFPGRF
GTIIAQAFQYYDRIIEYRGKVHAKTAVRDIDVLLQEVWLYAWLAPGVDKADAGGAPASAT
APRATTAESRSRILRSLLFAAVIAAVAALLPFALALIAK*
>SPBDM4_v1_40691|ID:27157996| putative Diguanylate cyclase (GGDEF) domain protein [Uncultured spirochete bdmA 4]
MKLNFNAISVIWMVSAVVVLFNAWISWSRSGTRGSKTFALFNIAIAWYSFFAAFELMSTG
IDQVLFFTHLEYIGLAAMPALMLSFAFEFLDVEFPHRRPLVVTVGIIGFIVMLFQWTTLR
HGLVYISPHVEAYGDLQVIHFGKGPVYILWTVILGMSVASAATLFIRAVGKADGLLRKQL
LLASVGFCFPLVVFVLYVTSVFSVPYDVNPFSFVVVVSVMTTVALFGTGMFNFAPVARERV

FMSMGESCIIADENGCIVDMNPRAKEILPILNAAVIGKSASDVLSMFPSLAWAIVRQEEK
VVEFDASSLGGPERVMAKISLIKDGSKQNSGYLIVFEDTSERHQMIAELERYATMDDLTG
LNNRRHFFKAIRPEVEKSIRNGMAVGLVMIDVDDFKNVNDVHGHALGDEVLRSCARTMAA
TIETQDIICRFGGDEFLVFMPDADIEKAQKAGERLRLAIASIQPPRCLEKQIHISLGISA
APIASFDEIEGLISKADSALYRAKRNGGNQISL*
>SPBDM4_v1_40692|ID:27157997|leuS| Leucine--tRNA ligase [Uncultured spirochete bdmA 4]
MAKYPFRDIEKKWQQRWEDEQTFRVVEDERFPKEKRRYVLDMFPYPSGAGLHVGHPEGYT
ATDIYCRYLRMNGYNVLHPMGFDAFGLPAENYAIQTGTHPAVTTYANINRFRTQIKSLGF
SYDWSREVTTCDPEYYKWTQWIFLQLFKKGLAYEAEMPINWCPSCKTGLANEEVRDGHCD
RCGTKVVRKRIRQWVLKITAYAERLLADLDTLDWPEPIKLMQRNWIGKSEGATVRFTIAQ
SVASASADCAKSSAEGDQLEIYTTRPDTLFGATYMVLAPEHPFVPQITTAGQRAAVEAYI
EEAAAKSDLERTDLAKDKTGVFTGAYAINPVNGARIPIWISDYVLISYGTGAIMAVPAHD
SRDWDFAKKFNLPIIQVVAGPDETGPFDKEPEACTDEEGVAVNSGEFDGLPTAEFKARIS
DWLEERGLGKKAVNYKLRDWLFSRQRYWGEPIPVVHCEKCGIVPLPEESLPLTLPEVKSY
APTGTGESPLAAIPEWVNTTCPKCGGPAKRETNTMPQWAGSCWYYLRYLDPKNPKAFADR
SKIDYWMPVDLYVGGAEHAVLHLLYSRFWHKVLYDLGLVNTDEPFVRLVNQGMILGEDNQ
KMSKSRGNVINPDDIVESYGADAMRVYEMFMGPLEVSKPWSTAGLVGVSRFLERIWALSE
RPIVEEPPSPELEKLLHKTVKKVTNDTATLNFNTAISQMMICSNELAKLESLPRAAWEPF
VLLVAPYAPHLGEELWVKLGHEGSVSKAPWPAYREELTHDEEKEIVVQVNGKVRSKFIAP
AGTSKEDLIEMAWRAEKIAEWLAGKEIVKVIAVQDKLVSFVLK*
>SPBDM4_v1_40693|ID:27157998| conserved protein of unknown function [Uncultured spirochete bdmA 4]
MQNPADFKLEAANGGAPGHDLRDPGTHGLSGTGRTAPQRKNRYTFIRVLIALAMLALGLG
ATELLARDEEHPAAGVMQKAAQRMARAEHAVAEKRRSLGITIDPALDPYRTGLIGVETNM
LTTTFGDIVAKRTTTNTAFAALVVRYFYELGLKPGDRIAVGSSGSFPGILLAVLSACAET
GVEPVVITSIGASEYGANIEGLSNAEMMAACREAGVLPYMPAAISPGADGDQGISSLYRL
EPSDDIARYARSAASALKVPFVGGDGFDASFSSHLDVYEKAGPIKAFVNIGGADVNFGSD
PASLRLKPGLILPSARKSGVEPDGSGGARGSANSSEIQPPGGLLGYYLARGTPVLHFLNI
KGLALQGGIPVDGDPEAPIPSEILHRRKKPAWTLVLGLLLAGAALAIRKPLG*
>SPBDM4_v1_40694|ID:27157999| CapC protein [Uncultured spirochete bdmA 4]
MAGTSIVVSIVLGFLFGELTGWLTGGLVAPGYLALYVDQPLRIVMTYAAAALAFFAVNLL
SRVTILFGRWRFMAFILAGICMGALLDAVVGWLPPTGSDLRAIGYVVPGLIANDTWKQGP
WKTLLASLVITLATRAVLLLFMH*
>SPBDM4_v1_40695|ID:27158000| putative Capsule biosynthesis protein CapB [Uncultured spirochete bdmA 4]
VIFFVVAISAALAAAAIVEYRIHITRIRAIPVRILVNGTRGKSTVSRLVAAGLTAGGLRT
YAKTTGSAARLILPDLREVSIRRRGSAHRRASIMEHRWFVREAHKNGAQALVAECMAIQP
ETIRTLETRLAHSTIGVITNVRLDHMDTMGTELTSVAEALAESVPERGRLFVGLEGMDEA
VKEVFARKAARRGAELRFVNCEEETQSLCGQFSYPMFAQNLALALAVCETCGVSREVALR
GMLGAAPDPGVRSSLELVWRGIAVRVVNAFAANDAQSTLAMWRAACGDSSQPDSRADGGA
GGGQSFRALVFNHREDRAWRALQLGEIAAGMSADAILVYGMGQRLARRTVRGYYRRNGLQ
NGSQAGAVPFPVVRSMRDADVGAVLETIRSIEGFSTAGESARIEILCAGNIKGQGLALTD
SFAATLPQETEKAFQGRA*
>SPBDM4_v1_40696|ID:27158001|gpmA| phosphoglyceromutase 1 [Uncultured spirochete bdmA 4]
MYTLVLVRHGESEWNKENRFTGWTDVDLTAKGVEEAISSGVALKKGGFAFDVAYTSVLKR
AIRTLNFVLDELDMQWIPVHKDWRLNERHYGALQGLNKAETARKFGEEQVRIWRRSFDIR
PPELTLDDPRFPGKDRRYAGLASSELPLAESLKDTIARFMPLWHNVIAPDVQDGKRVIIA
AHGNSLRALVKYLDSMDDEAIVDLNIPTGIPLVYELDEELQPVRHYYLADENAVKAAAEA
VANQGKAK*
>SPBDM4_v1_40697|ID:27158002| protein of unknown function [Uncultured spirochete bdmA 4]
MQEGDVKSEAQIGPRRAGLCIHNQLPDVLSGILAEPTLDLKKSPLFHIFKKVN*
>SPBDM4_v1_40698|ID:27158003| CoA-disulfide reductase [Uncultured spirochete bdmA 4]
MARYIIVGGVAGGASTAARLRRLDEHAEIILFERGPHISYANCGLPYYAGEKIKERERLF
VMTPEKFKAWLNVDVRVRTEVIAIDREAKKIQARELDSGREYSLTYDALVLSPGAEPIKP
PIPGIEDPRIFTLRSVSDIEHIKEYLETKRPERTIVVGGGFIGLEMAENLHARGSFVTIV
EALDQVMNPIDFEMAALVHQHLKQKNVELYLSSAVSKFEQAGSRVVAILADGTRLDADMI

MLSIGVRPETGIAKAAGLDTSPNGAILVNENLQTSDPSIYALGDAIAFPHPILGMAMPVP
LAGPANKQARIVADNIVKEPGTRKWHGAIGTSIAKVFDITAAAAGVAEKLLKKNNIPCIS
VITHGSSHAGYYPGAQPLSIKTIFTLDGTLLGAQVVGYDGVDKRIDLIADYIRRKAKVTE
LGEIEHAYAPPFSSAKDPVNIAGMVAENVLAGLSHQLQWHEVKAFQERGGFVLDVRTPEE
FSLGVIPGAVDIPLDVLRQRLGEIPRDREVLVYCGVGLRGYLAERILRQNGWTAIYNLSG
GYKTWEIATEPQSHKGIYKQGLAGLQSSQAKGAQELLHTTFAEGSGMPESLAAKRQTIVQ
VDACGLQCPGPIMRLKTEIDKLPEGGRVVISATDPGFVRDAGAWCKVTGNLLISMEESNG
KYTAMIEKTVKQSALAAAQAAAAAGGAPFIQMTPKGATIIVFSNDFDRALASFVLANGAA
SAGKDVTMFFTFWGLSVIRKPDAPRVAKDFMGKMFGMMLPKHAGGLSLSHMNFGGIGPKM
MKSRMKAKRVDALETMMAQARQAGVHLIACQMSMDIMGVKREELLDGVEIGGVATYMEAA
SEAGVNLFI*
>SPBDM4_v1_40699|ID:27158004| conserved protein of unknown function [Uncultured spirochete bdmA 4]
MNGTANAKGAPNTKTRRLLIRGALLVVYVLIMVLMIYSGRRHTILIDNKDAADGSYSAIN
GMEVSIDKQESSEYYPGDRDKAMVQGQKHTIRVNIFDDNKTIEKSFTVPLWSDVMIILVP
KMVAGIEPWIAPFTMAEQIQEAQESAPPAGETTFQSLGSMIPEGTEEAQQSP*
>SPBDM4_v1_40700|ID:27158005| ABC-type transporter, integral membrane subunit [Uncultured spirochete bdmA 4]
MSAQKKLSTFFSRYAVVIIFVIITLIAIPPSGLSIKYILQEIITRLGRNSFLVLALLLPI
YAGMGLNFGMTLGAMAGQVGLIFAVNYGISNWQGLVFALLVGLPISVLLGYVCGLVMNRA
KGREMVTGYILAFFINGIYQFFVMYMLGSVIPMGNKSIVLSRGYGIRNTLNLESVRQSLD
NIVMLRVGGFSIPLVSFIVIALLCVFVVWFKKTKLGQDMRAVGQDMAVAEAAGIPVEKTR
IVAIIISTVLACAGQIIFLQNMGNLATYNAHDQTGFFAVAAILVGGASVTHASIANVFLG
VTLLHAMFVVSPLAGQKLFGSAMIGEYFRQFIGYGVIAISLVLYAWRTRKAAAEARLSLR
GAQNGPGKGNGGTAVGNGGQA*
>SPBDM4_v1_40701|ID:27158006| ABC-type transporter, integral membrane subunit [Uncultured spirochete bdmA 4]
MRNFKTFIQDFGWPRIIIFFFLVALFIMAPFVGVRLDASFSDVLNRFGQNAIMVLAMVPM
IQSGCGLNFGLALGLVAGLLGATLSLQLELHGMLGFLGAIVLATPFSIIFGWFYAKLLNK
VKGEEMTIAMYVGFSFIMFMCIMWLILPYSNPTMVWGYAGKGLRTTISVENYWLKILNNF
LAIKIGQFFVFPTGLLLFVALFAFLMWLFLRTKTGTAMTAVGSNPDFARASGVDVDKMRT
ISVIISTFLGAVGIIVYEQSFGFIQLYTAPSPMVFPAVAAILIGGASINKASMVNVLVGT
ILFQGLLTMTPSVINSLLQTDMSEVIRIIVSNGMILYALTRKIKVSK*
>SPBDM4_v1_40702|ID:27158007| Monosaccharide-transporting ATPase (modular protein) [Uncultured spirochete bdmA 4]
MKKLVAVVLVLAFLVVGADVFAQSKAKFHIGVVTGTVSQSDDDLRGAEQLIKEYGIVKDG
GMIQHITYPDDFMSQQETFISNVVALADDKDMKAIVINQSIPGTAEAFKRVRAKRPDILL
LAGEPHEDPLVIQGAADLALSADFVSRGYTIIWAAKQLGAKNFVHISFPRHMSYESLGRR
RAIMEQACNDLGLNFYFDTAPDPTSDVGVAGAQQFILEKVPQWVQKYGNDTAFFCTNDAH
TEPLLNHLLAYGGIFVEADLPSPLMGYPGALGLDLSNEAGDFPAILKKVEAAVVAKGGAG
RFGTWAYSYGYTLTAGLGEFAKRVVEGNAKKDSMKDVFDALGKYTPGAKWNGAYYVDMGT
GVRAKNQILIYMDTYVFGNGFLPTTQQNVPENYYTIKMHNYFFFSFFFFFVFFFFFFFL
LCCFFLSFFLFFLVFGFFFRKLIYNGIIMANGSSLLSIEHVSNDFYGNVVLHDVSFDLLE
GEILGLVGENGAGKTTLMHILFGMPVIMETGGFQGSIKIGGTEVHFSSPFDALDAGFGMV
HQEFSLIPGFTTTENIVLNRESMRASILNDVFGDRMSVLQRDRMRERAHKAIGKLGVDLN
PDMLVSEMPVGHKQFTEIAREIDREQTRILILDEPTAVLTESEAEVLLKTLKRLASEGIA
IIFISHRLQEVLAIANRIVVLRDGHAVKDTPNENISMRDIASWMVGRSLGESGQRSEARG
FAESLFKVRHLWVDMPGETVRDVSFDVHRGEIFGIGGLAGQGKLGIPNGIMGLFPAGGEV
VLDGKTVPLNDPAKALAMGMAFVSEDRRGVGLLLDESLEWNIAFTAIQVHGQYLKKIFGL
FSQRDEKAMEDLCAEYVKLLDIKCTSTKQKAKELSGGNQQKVCLAKAFAIKPQLMFVSEP
TRGIDVGAKKLVLDTLHHYNRDFGTTIVMVSSELEELRSICDRIAIVDEGRIAGILPPTA
DISEFGLLMSGAAMEEAVNA*
>SPBDM4_v1_40703|ID:27158008| membrane protein of unknown function [Uncultured spirochete bdmA 4]
MQFSRKRFANELYYYSNQMVLFLVMIISLTSGSWKKALVTSLIVLTFMLIQTGLLSLQGH
IPILRLLYSLITPAAYSILRATLSDIAFTETVNILLWGSSIYIAVFQTLTLVFRKPFWKR
FSEIALSLGSALIFILFYAYLDLRISVTASFAKGEIDAETMAQALRIQSFLAVFEKFIRA
PQHLFALFGVISFDFLLLVARMRAITLEHRLTRIIEMPQEAKRIIDASRQESENKDSEES

PGERQPEPADTEPPESLPVRSPTAFFVTVVSSDIIGFTDLSEQLGRTEATALLNRYYGIW
TRCAGSFGGRIASLSADTVVTIFGLVDADRSADRALNAAYAFLEEMEGLKEDMVVKSLPN
DIKVSLGIHSGFVTAALLGPPGQQKMGFYGDTIAIAARLDSLCREFHQSLLVSHATYRRL
ALESQVTLERFSEALLRKSTRPMPLYAKKP*
>SPBDM4_v1_40704|ID:27158009| protein of unknown function [Uncultured spirochete bdmA 4]
MQSQFQKQQTFEIGSTGVKLPMKSSTNQKTAAFVKSILWVAISATAFIVSTSCSGNLDAT
LKTDGSVRAAVRIDIPEALSGRVRQFVGIGSREPLFSTDAVKNQFLGRTSINLVDVSSPT
PDILTSVVWVQNLDTLIADTSLVPPGMILYQKIPAQGSAPAMRELSVNLSRENAPYMIKL
FPGVDKRIIESLSPPALEPDPVTADEYRLNLEQVIIGKKNMPAFDACSVDIAITVPKAIS
SASGGNFSGPVFRAKLPLFNLLTLEKPIAFSMRWPE*
>SPBDM4_v1_40705|ID:27158010| protein of unknown function [Uncultured spirochete bdmA 4]
MAMIFTFDFHNHSCLSPCASLESSPSVMAARAAQKDIDFFALTDHNSALNSLAFALACGR
AHRRACARARLIPLFGLEINPFEEAHLLAIFPDPLSALAFSDRVFRYLPRLEVDPGQFGD
QVVVDPEERILAMPSAWYGNSLQESFTFFAKEAADAGALVIPAHVDRPQFSVYSQLGFLP
DGAYDAVEAMGADPDERLCGQHCVISGSDAHVPEHIGRRPSTLEIENERLVDDVSSGLAA
MLQAWEGARPGIEECVAPQTAAETGEKVCGAGLSSEIPGLAPLLAFLEEWYPCRESQALF
EEIRRSLRVGNAWSVYRRPRQPGAVLH*
>SPBDM4_v1_40706|ID:27158011| conserved protein of unknown function [Uncultured spirochete bdmA 4]
MTTRNIPELIHAEILQGDFEDVELTGVYTSDLLSDVLANGRDASVLITIQAHKNTVAVAT
VVDISLIILCNNRPVIDDMIDAAAQARIGVIRTSLSQFEVSGRLWQAMQTLPAERL*
>SPBDM4_v1_40707|ID:27158012| ABC transporter, ATP-binding protein [Uncultured spirochete bdmA 4]
MIELHNISKTYAKSGTKAVDTLTLQIPDGIIYGFLGPNGAGKTTTIRILTGAIAADTGSV
LIDGIDLDKNPIEAKQRFGLVPDTPDLFSRLRAYEYLNFVADVYRVPAAARTSVIEHLAA
RFELSDVLKSPISSMSRGMRMKLNLIASLVHAPHNWILDEPIVGLDPHSAFALKELMREH
AAAGGTVFFSTHVMEVAERICDELAIINRGKLIFTGNLEALRELRESRGAKSGTGEAGTP
VAAEASAAADDESLEALFLALVEDSEGPKL*
>SPBDM4_v1_40708|ID:27158013| putative Membrane protein [Uncultured spirochete bdmA 4]
MSRRPASPFMALLTLNLKSLFAIHLPEKKALASPKVLLKTFGWIVLAVFLVADFGFMFAM
MDIGMYNALAPLGMQSFMLLYDTVMSSVIVFLFAFITSLSFFSSAQYEALFLTMPLKPSS
LLTARMATVYAIEAPIAFLVMGIAAGVYGIKSHPGFDFYLYMLFNALAMPLVPLAVSYAV
LVPVMSVSRWLRRKNTILYVGGFIGLALALGFNFYLQRMMARIENPVLLQNMLTQNQLNF
ADIADWWPPAWLTMQAIAQGRTALGSIAATLANLALGVALTAAVAFLFGKSYVKILVNFG
ETSAVKGTLNQTQAGSIFRARPVFLSLVQRELRLMNREPMYLLNGPFVILLLPVIFAITF
IAQGDEMRGAVDQIRPYLTGQAEYLIPAGLGIFLATATSISCTAFSRDAKSLHFLKSLPL
RPRDIIAAKLVHALIFAALGIVLGTVGGAIMLGVSTLDAIVALLLAIFGSVALNMGGLAI
DTFWPRLSWENPMSALKRNPNAVIMILGSMGLIAGIGVLAATLPLAKYTFALLYGTIFVA
ACFALGFVLFRAGEKRVRQMEP*
>SPBDM4_v1_40709|ID:27158014| Holliday junction resolvase-like protein (modular protein) [Uncultured spirochete bdmA 4]
MKLMHDDMARWLFLKESSVYAECMNGFGVQSQSWYLSSWFIAVMVVFFAAICVAGVLAYR
AGKRRGLLEAQLAERDRMDLARKDAVERSRSALSGQVSEQIAPWLPDFPTSPADARFIGK
PVDFVSFCGADEGMVREIVFIEVKTGRSSLSTVERSVRDAIVQGHVRWVEYRIE*
>SPBDM4_v1_40710|ID:27158015|pckG| Phosphoenolpyruvate carboxykinase [GTP] [Uncultured spirochete bdmA 4]
MSTIMQQPSYVRNERLLEWVSGMKALCKPDFVYWCDGSDEEYETVCRSLVASGTFLPLNS
DKRPNSYLARSDPSDVARVEDRTFICSQRKVEAGPTNNWMDPKQMKGILNTLFEGCMRGR
TMYVIPFSMGPIGSPMSYIGVELTDSAYVVANMRIMTRIGQKVIEALGESGEFVPCMHSV
GMPLEHGQKDVPWPCNPETKYVVHFPETREIWSYGSGYGGNALLGKKCFALRIASVIARD
QGWLAEHMLILGVESPEHEKTYVAAAFPSACGKTNFAMLIPPKAFEGWKVTTVGDDIAWI
RAGKDGQMRAINPEAGLFGVAPGTSEKSNPNAMATIRTNTIFTNCALTPDGDIWWEGMSK
NPPEGLIDWQGKPWDPTSGKPAAHPNARFTAPIQQCPSVDPDWKNPEGVQIKAFIFGGRR
STVYPLVYQAFNWIYGVYLAATLGSETTAASTSAIGHVRRDPFAMLPFCGYNIASYFNYW
LRFGRSNGNPPRIFGVNWFRKDKDGNFLWPGFGENIRVLKWIVERANNHGKAIESPIGWM
PRYEDISWQGLDFSKERFNELMSVDRDLWANEILSHEELFMKLYDRLPKELIFVRELMLS
SLWRAPEHWEFATVSPHMYNE*

>SPBDM4_v1_40711|ID:27158016| exported protein of unknown function [Uncultured spirochete bdmA 4]
MRIFYIVLLIVVGIVAVVFAGQNSTPITVAFFGWSTNASMSLVLVITLAAGILLGVLLLL
PSVWKRTHALSAQKKKTREAENQLKNTGSIPEPKPSSPTESVTKENVSQDKPSVGGEAKS
*
>SPBDM4_v1_40712|ID:27158017| conserved exported protein of unknown function [Uncultured spirochete bdmA 4]
MTKNMGKTDKTIRLIVAIVIAIFILTGVLTGAWAWILGILAVFFAVTSAISFCPLYTVLG
ISTASKAEKEKPKEEEKK*
>SPBDM4_v1_40713|ID:27158018| putative Transcriptional regulator Crp/Fnr family [Uncultured spirochete bdmA 4]
VKCINCINTFCASLPSEISEEMCRVAVHSKHNAKEFFVKSVGPNGYLYILSLGTVIPYRL
ASNGNEIGVDCLHAGSILGLCDFLNGNADQITVYAKTNIELCAISVIKFDRLRSRCPAIN
DSIIKMLGVRLKHSYIKLGYTLGDSIEKMLYQLDELFEQQDVSINFTHDELALLTGMNRV
TVSRVIESLKQRNIIKPLGRGRFVYINSLDQEFGGPSFKKINRKESLTLVLK*
>SPBDM4_v1_40714|ID:27158019| putative Divalent anion:Na+ symporter (DASS) family transporter [Uncultured spirochete bdmA 4]
MKNVKRVFSVIAVLCIFGFKYIPAPEGLTPLSMEISGVFLGTIILWLTVSITWPSLLCIF
ALALSPLYTVNQALSASLGNWVPSFVLFSSALCYMLSKTGFLKRCAIWFITRPISKKNPW
MFLGLLFLAPLAIGSIMSPITTFIIFIPIIEQIFIELGYQKGDRVPKIITLCILCFSSVS
TMTTPIAHTFPILGMSLYSQFSGGAHIDFVSFTIVGVISSLILFVLIMLMIKYCFKPDLS
RIQNVDTEFLLKDMKPMGKEEKIVLTVFLSIVVIWMAPGLITGIMPQTAKFLDSLGPAIP
PLIGLIVLSIIKIEGKPIMNFAEVMSKGVPWSSWLLVATTIMLGSAITNKDVGLTNWLGT
FIAPAVSRLPPMICVLAIVSITVLVTNFATNAVTVTLVSSIVVPLVISGAIPNLNAPAMA
TVIAMGSCVAMATPPSTAPAAMAIGTDWLDTGTMFAWGMFMCAIASVVLTFIAYPLASLL
M*
>SPBDM4_v1_40715|ID:27158020| putative Peptidase, M24 family [Uncultured spirochete bdmA 4]
MSEKDRREKLFFPIPQAELERRHKAARAAMKTKGIDCLLMQNSTKYLGGYVRWFTDVVAE
NNFPVTVIFPMDDEMTMISLGGAPRPAFPPLWATYGVKEVLARPYYLSVNYTNTYDAKAA
VETIKKLNAKRVGYVGPAFIPMTFDKYLTANLPGVEFVDMTEEIDRLKALKSDVEVEYIK
KTAAVQDAVMLALPALIHPRMKVIEVHNIIQHILSDMGAEEFLLMLGTAPFGELAKHSSP
YLQNKVIGEDDYMNIMIETNGPGGYYTEVARVFCMKEPPRDLLDLWDLNVKAQDRTVAKL
IPGAELKPIYEQNNEFLVSCGDQPDTCVFAHGQGYDVMERPLIRPEEPIVIQPNMNIAVH
PRLSGPTSFTFCCDNYLTTQGVPIRLHKTPREVFMIPH*
>SPBDM4_v1_40716|ID:27158021| protein of unknown function [Uncultured spirochete bdmA 4]
LRHLRTVSTKPEQMQSQFQKQQTFEIGSIYLDMVTIFDYCSEGYGKILRFVLST*
>SPBDM4_v1_40717|ID:27158022| conserved protein of unknown function [Uncultured spirochete bdmA 4]
MDFLVASLQDMRRKNWAPGDLDAIIVTGDAYVDHPSFGAALIGRLLEAHGYRVGILSRPD
PDDPEAFRMFGRPKLAFLVTAGALDSMVSSYTANKKPRSKDEFAPGGDRSLCMRADGTLG
LGVRGRANARPDRATIVYANLCRQAYKGVPVILGGIEASLRRLAHYDYWSDTVRRSILLD
AKADLLVYGMGERQILETMRRLRAAGEKGTEVDLRGIRGTVWTVHASKLTEALGDRLPEE
RGGHIPGGAAPDAATLPAYDTIQPDTEEGRRAFAASFKIQYRNTDPWSAKLLVEPCGDRF
VIQEPPEFPLPRGELDEVYGLPFLHEWHPMYRGFGGVPALAEVKFSLVSSRGCFGGCSFC
ALSFHEGRVVTSRSIDSIVREAQSFAKMPDFKGYIHDVGGPTANFRGPACAKMAKHGACI
DRRCLAPQPCPNLEVDHREYRELLQRLRGLPGIKRVFIRSGIRYDYLMLDKDQSFFSDLV
RHHISGQLKVAPEHVSNHVLELMGKPPLESFDRFAEEYARLNRKFGKKQYLIPYFISGHP
GATLQDALELALYLKRFGFVPDQVQDFYPTPGTLSTAMWRARMNPLDGTPVYIPRGAKER
AFQRALLQFSKPENRALVREALRILGRTDLIGKGKECLVGAETNS*
>SPBDM4_v1_40718|ID:27158023| putative Transcriptional regulator TetR family [Uncultured spirochete bdmA 4]
MAMPKNSAKRNEILRCAYRLFTESGYTKVFLRDIASEAGISKSLLQHYFPKKNDVIQSIL
EETLSISFEYVENLIPAEGELFLSLSVYTRLFWESVSKDKKWDRFNINVISHRELLESWI
DIIYRWLRSMKDSHLEQIKERDLKIALTFSITGGMELYLYRDELQVEVISICEQITTSFM
EILNCQNDEIQATLKKTHEMLTSKNIDAFTVFCEDKIQWLNSP*
>SPBDM4_v1_40719|ID:27158024| 4Fe-4S ferredoxin iron-sulfur binding domain protein [Uncultured spirochete bdmA 4]
MDSVKVLPGKGKEITVERTAVAHINPKNCVNCGTCRELCPMEAISESQRVICRICPECTE
QDAMTFDEMYEFTTKAACTTGCPLGISPQGYINLTRTGKLEEAYKLVWDKNPLPSVCARI

CHHPCEQVCKRGVLVDEPIAIRSIKRYLGEKTTFRAEKYPRLYEERIAVIGAGPAGLTAG
QYLSRAGYEVTVFESATEAGGMLKRGIPEFRLDRSVVDEEVTNLENAGLAIRLNERINKH
SLEKIQKEYDAVIVAAGAPNSKELKIEGWRLSGVMTAMDFMEQVNHRQEIRRHPGQIFNL
GGEVVVIGGGSVAIDTARAALRMGATKVTAVCLECDEEVPCHPWELKEAEDEGITLIQSH
SPTRFVGTYPKLEGVEVCKVTSFSKDGKGKISCELDENDTMTIKADWAIVAIGQAPDTMW
SSLKGDRVYFAGDISKSACSVIDAMASGRKVALNVDTDLRGRTLKDPMDTHELHSAPLSE
KIYPYNRRKSIRPEIPMLAAESRIHSFDEVEGGFDDEAAQMEVLRCLRCGYQVVDEERCI
GCGICQRECPEGDVITMEPVAKGGEK*
>SPBDM4_v1_40720|ID:27158025| putative geranylgeranyl reductase [Uncultured spirochete bdmA 4]
MKRQVVVVGAGPGGCTAAFYLAKAGIDVLLIDKETWPRDKICGDSYLPALYPFFEDMGIM
NEMKAAAACNPKALRIIDPDEEYADFDVEPWLIAPRRIGDDIIRRGALTSGADFMENFQA
EELIIRRGVVRGIKGIYNNREIEVEADAVVIADGSHSALARQLGIYKEDPEYILYAGRAY
YDGVEGMEEGKFEEFYTPSTLPKPTYSPICLTWVAPLYQGKYASVGITIAEKYLRETGMT
LEGLFQWWCENTKFGRERMHNARPVEKLKGWRLPGSRKIEKNYAPGAVVIGDAVSSAEAA
FEYGIPSSMIGGRVAASFLPEVLSSGDFSEKKFSEFQTLVGKELNPGLEYNANFRDNILA
NKKTMKQFQLWAKTQPGYPHLIYDQSVVKFITEVLHKEIVLSDKKITQ*
>SPBDM4_v1_40721|ID:27158026| GntR domain protein [Uncultured spirochete bdmA 4]
LTRNIIRIDRVSISDQVAKQLQSLITSGEFKVGDRLPTENELCAQFGVGRSTVREALRVL
VAMGMIRIQPGKGAFVAKDEDNSFDAIKYWFTEKHAELSELIEVRMAIEPLAVRLAIQRA
SPESIRQIQEIHNAFKAAAQQKDHIELAILDESFHNAIIYASGNSLLIKIGKLIADAMKE
FRARSFAVSENISHATIPHEKIIEAILKKDENAGVAAMMSHLEISKEDMEKVVNI*
>SPBDM4_v1_40722|ID:27158027|dapA| 4-hydroxy-tetrahydrodipicolinate synthase [Uncultured spirochete bdmA 4]
MLQKEKYGKRLVPIVTPFKEDMSVDYDALLQIGEILIERDYADSFILTGTTGEFFTMNYD
ERVKIFEMMKKHFGSRIPLIPHTGAASTMEALALTRKAKELGYDLVMIVAPYYTKPSPKE
LVNHFSIIADSVDVDIMLYNIPIFTGVNIEPTNLKELIKKKNIVAIKEEAELRPKQMSEY
LLATPDNFIVYCGDDTMVLEALAQGGNRIGGFVSGGCHLFGDRMKNMINLFLSGDVIEAG
KIQLSLLPLLRSLTQNGRNNPVCLLKGAMNLIGYPKCLPRLPLMPATPVEMELVKDKMKQ
LGMLP*
>SPBDM4_v1_40723|ID:27158028| TRAP-type transport system, periplasmic component, predicted N-acetylneuraminate-binding protein [Uncultured spirochete bdmA 4]
MKNRVFSFLVMLMFIASAGAAFAADTGAQYTIKFSWADPADPLKQSTSAYALVFQQEAER
LSGGRIKVELYPAGQLGDQRSSTEQVARGTIQMTNISSGVLASLYFPKLEILDMPFLFSS
REHASRVLDVQTNPFMIKLDNDLIKATGIRLFNVVPFGPRHLTNNKRRIRTPADVKGLKI
RTMEIVPHQKLIESMGGTPVPIPFLELYTALQTNVVDGEENTIQNILAQNFFEVQKYLTI
SNHVMGVGATLINEKFYQSLPNDLKAALVEADHVAQMVYNGLAQVLDATGIEVLKEKGMD
IYYPTPAEYKAFQDVAIPYVRKYMEQKLGASFVKDFLESVDETGKEMKAEAVK*
>SPBDM4_v1_40724|ID:27158029| putative 2,3-diketo-L-gulonate TRAP transporter small permease protein YiaM [Uncultured spirochete bdmA 4]
MNEKKKSVFIYIIEIISNCSLAIMFLSVMAGVISRYILKSPLFWTDELSRYLMIYMVFFG
SALSFRADKHPSLTFIIDKLPKKWRLIWDLVIDLLLMAILVLLIWGALDMITSGPVGFTP
SLRIKFTWVYWAIPLGSISMLIEIIFRLYRRIKNLSHSYLSPSQEQKTGEY*
>SPBDM4_v1_40725|ID:27158030| conserved membrane protein of unknown function [Uncultured spirochete bdmA 4]
MSLALTLMLILFAFFMLAGIPLSFSMLLSSAVYLIMTGKPLYLLAHRMFTGIDTTTLMAI
PFFIMAAEFMVLSGTAERLLKFANVLVGRFRGGLAYVNVLASMLFGGCSGSAIADVAGLG
ILEIDMMEKGGYDRGFSTAVTITSSIQGPLIPPSIMMVLIGATTGVSIGALLIGSAIPGV
LVGLSQMLVIFLIGRKKNFPKSNIILSPKQKLLALLDAFPFLLMPIIIIGGITGGFVTPT
EASAVAVAYGLLLVFIYGRNKVKIKDFWKILYNTVIVAASILLLSGSSNIFGWILSTEKV
PNLISNAVMGLSHNKYVFLILMNIFLLLWGMIMDSLPAILILSPVLFPIAMRFGIDPLHF
GVIFGFNLIIGLITPPYGAALFTGTIISGLSMEKLTRNMLPFILSSILILILTTFIPEIS
MFLPRLFGLR*
>SPBDM4_v1_40726|ID:27158031| conserved protein of unknown function [Uncultured spirochete bdmA 4]
MQIRLSSGMMHETIEVSEKHIAGILCPNTSSAFAPLDDNMIRNAINHPVNSLNIENRTTS
SSKVAIVVDDATRPTPTSRILSIILKKLSGIGIPDQNISITIATGLHRNTTAHERTLILG

DDVISRFDIADNDARNPIAFAHAGTITGGKEIYLNKRILEADIVITIGVVKSHAFAGFTG
GAKSILPGVASQNTIHGNHCFYNIEYPRGVLGSCEMSATRKEMEATARLVNPFIVNVVLG
DSNQIIYAVSGDVVDAHRSAVDFYKKIALRTFPEKVDIAVVHGGLAGSINFYQALFGCNV
VKTTERPILKKNGIVILFAECKEGSGSKLLEQIMPTFSEPDEILKYLASNKVFDDQWAVQ
FLATFLRDIHIFLVSEGVSSEIAKNLKVKLFPNAKEALDAAIQTSDLDYRMAFIENPDVL
IVNLA*
>SPBDM4_v1_40727|ID:27158032| Electron transfer flavoprotein subunit alpha (modular protein) [Uncultured spirochete bdmA 4]
MHILVLIKQVPETDKVKMDPETGTMVRSGLESIINPLDLYAIEEALVLKERLGAQVTVIS
MGPPDAERALRESLAMGCDNAILLTDRAFAGSDTWATSRVLAEAIRRAGDMDLILAGERA
TDGDTGQVGPAVAAWLDLPILTYASALEIEPASAAVESPILSKTTPLAIRVHRLTEEGYQ
VLQATPPCLVTVVKEVASPRLPTLRGKKFARSAEIPHWGARDIGLDPVSVGLAGSPTRVV
KIFYPKVARNGLRIMATDEAGAEKAISHIMALFENRGLLGKATVEDGASAPSAPLAASSA
LRSALERSPVAGGAPGPDTAEASAKPEFWILAERRTDGLDVVSFELLARARALADNRGAR
LAAVVLTSSLSEEDAEALIAHGADRVIAVEHPLLRDFLCEIWAEALLRLVRGRKPEVFLA
AATTTGRTLMPYLAAKLGTGLTADCTELAIEEETGLLLQTRPAIGGNIMATIKTAHHKPQ
MATVRPHSMQPLPADSGRQGTIVRVAGAPLAGKDSPEPPVRILALERNAKDFENLEGARI
VVSGGRGLKKADNFRLIRELAHALGAVVGASREAVDRGWVSYPHQVGLSGKTISPEIYLG
AGISGAIQHLAGIRTAKTIISINSDPDAPIHAVADLAIVGDLFEILPRLTARLRQRSTGA
APHEPSILSPDTPAAAPPAENKASASSREARS*
>SPBDM4_v1_40728|ID:27158033| FAD linked oxidase domain protein [Uncultured spirochete bdmA 4]
MIYNHINKEIISELIQIAGERNVLVDPARIEAFSHDETSKEEYGHMPDVVVTPQSTTAVA
EIVRLANRTRIPVTPRGAGSGLSGGAIPVYGGIVLSLEKMNRVREIDYDNLTMTVETGIV
TNEINNLVKDKGLFYAGYPMSLETCTLGGNIAENAGGGKAIKYGVTSRYILGLEFVTPAG
EVVWLGGKLAKDVTGYDLVHLIVGSEGTLGIATAAIIKLIGLPAAKSDLLVLFHSPQEAI
SCVPIILSKGLIPTAIEFMDRRSVETSCAYLNENLPYAECGAMLLIEMDGRNPALVESET
EAVGDLCMEQGAIEVYVADNRTTQERLWAIRRNIAEAFKVYCPVQSLEDIVVPPAAIPAV
IPELDRISEQFGITIPCYGHAGDGNLHATLVKAPGMPLEDWQRIEPQALAALYEAITKLG
GKISGEHGIGLKRKEFMKKFMIPTELELLKTIKRAWDPNLIMNPGKIFDMES*
>SPBDM4_v1_40729|ID:27158034| dTDP-4-dehydrorhamnose reductase [Uncultured spirochete bdmA 4]
MKILVTGASGLLGRSLMKFLAERSDLEVKGVAFSRAVPPLEKLDLTDESAVALFFARHSP
DVVMHLAAERRPDVVDTALPKAQAINVDATQIIARSCAGRGAFLLLISTDYVFDGTSPPY
YPDSPVHPLNAYGRMKLEAEYAAEVALSGAVRSSEGRSVGAVLRIPILYGPVESLEESSV
TEVAFALKSSSPRKMEHWARRYPAHVDDVSSAILAVVDACRNGRCSYGPFPRFLLSGDHA
YTKYEMAQIMAKALGLDARHIQPDPSPPSGAPRPRDCRMDTSLLESIGWCQKKEFESEIG
GILRPFFF*
>SPBDM4_v1_40730|ID:27158035|pdxS| Pyridoxal biosynthesis lyase PdxS [Uncultured spirochete bdmA 4]
MEQKLWSSRQSEQWRIKVGLAEMLKGGVIMDVTNAAQAKIAEKAGACAVMALERVPADIR
AQGGVARMSDPKIIKEIQNAVSIPVMAKCRIGHFAEAQILEALGVDFIDESEVLTPADDH
YHVFKHDFKVPFVCGCRDLGEALRRIGEGAAMIRTKGEAGTGDIIEAVRHMRAVRDGIAR
LASLRDEELMTAAKELGAPFELVWEVAKTGKLPVPNFSAGGVATPADAALMMQLGAESVF
VGSGIFKSGNPELRATAIVSAVVNYRDPKILAEISEDLGAPMVGIGLDELAENQKIAGRG
W*
>SPBDM4_v1_40731|ID:27158036|pdxT| Glutamine amidotransferase subunit PdxT [Uncultured spirochete bdmA 4]
VSGPRIGLLAFQGDYEAHGKALARTDIASITIVEVRCLSDLEGVDALIIPGGESTVMGML
LERFGMFKALKTRIEDGLPVLATCAGLILLAKNIEGSNQARLGVLDVTVLRNAYGRQIDS
FHAPVQTSIPQTGTIDGVFIRAPKITALGEGVKVIATYRNEPVMVRQGSIVAATFHPELL
PDASLHRWFIQSFLLKTPQE*
>SPBDM4_v1_40732|ID:27158037| conserved protein of unknown function [Uncultured spirochete bdmA 4]
MYHNTEDYCLVLGGGGSKGVYHIGVWQALQQLGIPVGAFLGTSIGAVIAAFLAQGSGKFL
ERLGQEITVDNVINLPDGFARDGEIDIGLHTISSIPHFFASILNNKGLDTAPFRRLIGDN
LDEEALRKSGKDFGVVTLDVTNMKPREVFLEDMENGKVVDYIMASSAFPGFKNPQIDGKS
FMDGGMYDNIPYAMARKRGYRKIIVSDLSGIGRTRRPQIDGCITVYIKNSIDMGWTFNMK
RDFLNAFMLLGYLDTMRTFGRFTGYSYFLEPDNELEQVYAPDPSIPFDKKPQYMCYDRRE

LLVALECAASILNVEKIHAYSYGELEKAILKSARETEDKIVRVVQTSKERIISLMKELKE
AVLNRNFTECPYYYYRLVTALFQPSSSEILKKALERLNPELPIGVAWIACHVSRSPVAIP
AEFLAKRTE*
>SPBDM4_v1_40733|ID:27158038| conserved membrane protein of unknown function [Uncultured spirochete bdmA 4]
MYRKKLLYVAIYAIGMAYLEAAIVVYLRRLYGITDLMTSAPPFDPQISLIEVGRELATLI
MLLAVGLLSGKRLQSKIGFAIYAFGLWDIFYYVWLAVFIGWPKSLFDWDLLFLIPLPWWG
PVIAPVMIAALMVLCGSGLAALEDKERILRPTKAEWLLLVLGICLMLYVFMADAIAQMPA
SIETLARLRPSSFLWGFFVVGYLISAYSAWRILSAKSVGKPSVSNSGRENNVS*
>SPBDM4_v1_40734|ID:27158039| CDP-alcohol phosphatidyltransferase [Uncultured spirochete bdmA 4]
MKQKHNSQFDHVLFLISQGRNRTNLLKKQEQKAIAFLVQYIPSWVSPNMLTGIGFFGNVI
VSLSFILGARIDPRFLLLGVVGFAISWFGDSLDGRIAYYRNTPRKWYGFSLDLVVDWIGI
VLVGLGFIIYAEGFSKILGYGFIVLYGLEIILALLRYKISGEYSIDAGKLSPTEARIFIS
LFLVLEVIFHGTMTYMAGLANVVLAFSNFTEFKKLARIADQRDEEEKKKKNVSKISDIQV
N*
>SPBDM4_v1_40735|ID:27158040| GtrA family protein [Uncultured spirochete bdmA 4]
MHHSILTFSKAQISSFIGGVVDYGIMVLFTELLHVHYTISIAFGGIIGAVINFSVNRMWS
FYSKETPYKYTVIQQLTMFCPMVLGSIALKSGGTFALTSLTKIDYKFTRLAVDAIVSVFF
NYMLQRHWIFKKKH*
>SPBDM4_v1_40736|ID:27158041| conserved membrane protein of unknown function [Uncultured spirochete bdmA 4]
MVTEILVLFLLILLNGFFSLSETALVSSHKVRLQAEADKGKKAYKLALKARETPSRYLST
IQIAITLIGILMGALSGTTFSQWLAQWLAKWSFLEKYAASVSVALVVLAITVLSVVIGEL
VPKSIALHNPEHIVGLTIQPMHVLSVIFFPVVRLLSSATDGLIKLFGFSHGRESAITPEE
VKILVEQGEESGVFEAAEREMVEDVLSLDDRRVTLFMTPRTDVVVLDVENSCEAHIQAII
ENAEFGYLPVVDGDLDHTKGMLAVKRALKHLAEGRFVSVETCMEPPVMVPETFSGLQALG
ILRDAKKSAGLIIDEFGGVSGLVTIGDLLEAMANISEIEQEETPQIFRRNDGSYLVDGSM
PIDEFAENLGLNIESVSSEDYDTVAGFVLHCSGSIPKAGDVIEWPPLHIEVVDMDGKRID
KVLVKKAE*
>SPBDM4_v1_40737|ID:27158042| exported protein of unknown function [Uncultured spirochete bdmA 4]
MCVARIVILFAFASLMSVPLIAQQNASPSQAFPGTFDLEFPEGDTLRNITEPVFLGKATF
LQRLEERNGVQAPATATPSALRAPLGLVLCGGSARAYAHIGVLKALEKAGIYPDFIVASS
MGAIVGMLYAAGYSPDDIQMLIHAIPLESYFDIVIPTNGGLINTEIFRATFRKLVGKLDL
SETAIPIIVTAEDLKSRQQIWIAEGPFDRVMTSAFAMPAIFEPQPFGEFGLIDAGATIIA
PVEPALQISDHLIISTAFYDRTMNFSSPITVLNRAVDIGKTRAGMEEIGKSHAFVIRNDV
ENLSYMQFSDPDIIISKGEQSAQAALEKLNPNLRSQFENSPLPGFFEHRSLIHNSLVRTI
QQLQSGEMPSASFMIRGLPILKLFEPFSRAPGETGTEPRIGASLVFSISKFRAVVSYFAA
LNPEPGKEWAVETGIRANPLGSLNVWLTTRLWGEYGSTYILDHKPEYWEFAGLVKNTAVL
KNKKIGFQLGGDMLLKTAGTIATWQTLGLVEASSTKQFSKAGPFLQPWYSVEAGGFVENT
SGAQIASGIQSTLMGGINSTWVSPKARAFAKVSLNGQEFAESKFDGFRSAAPRGSALSDL
ITNAEIAVAPQDMYLDMAEALLFRNFELAPFFDTRWSNASGEYFHMSDWAAGLSFSFEAR
AFGLAPATISLYASYAGSKVFTLQIRAGVLLSEQ*
>SPBDM4_v1_40738|ID:27158043| membrane protein of unknown function [Uncultured spirochete bdmA 4]
MKKLKIAAVVFFMIMIAASPVLAQNVTNDVHAEKGDLVGFGGVGYGWRGFGISGGVESII
QKFTIPGFPLTMGVMGIAGLDFGTTLNFSAAGMATLHWGMKAYKDFPEFLRNFDWYIGLG
MGVGTVPGGFGVSSGGGLSYYVNDKLAIDLHSFYINHFVGSGSGFSETIGIRYALK*
>SPBDM4_v1_40739|ID:27158044| DNA-3-methyladenine glycosylase I [Uncultured spirochete bdmA 4]
MIRCPWCGNDPLYVRYHDEEWGTPVHEERTHFEFLLLETQQAGLSWRTVLGKREAYRDAF
VDFNPERVARYSEKDIERLLLDPGLIRNRRKLEAAVTNARIFLAIQEHHGSFDDWIWHFT
EGRPITNTWHTLSEIPVRTELSDSISSEMRKAGFSFIGSVTIYAHLQAIGIVNDHLVSCF
RHAEIAGL*
>SPBDM4_v1_40740|ID:27158045| Ferroxidase [Uncultured spirochete bdmA 4]
MVSQKMVDRINLQINREMYSAYLYLAMAAKMNELGYTGIGKWLTVQYHEEMFHAMKFAEY
LHDQNAVVAFAKIDTPEFKEKDVKPLFEHVLAHEQGVSASIREIMDLAIAEKDYATQTFV

QWYINEQVEEEKNATEILQNIDLVGNSAQGLFMLNTELGKRDPSVPLDFNKI*
>SPBDM4_v1_40741|ID:27158046| Threonine synthase [Uncultured spirochete bdmA 4]
MLMNMAVRYTSTRNAHVNVSIAEAIVQGMPQDGGLFVPVKMPKLASDALFEPNLSYAELA
WMVLSPWFDWPEQELRPLIEGTYSSTGETPMFDVQEIVPLAAAGSLDCDVRLPSASGAPS
LFLLELFHGKTCAFKDLALSLLGRLLRTSLEKRGIDEPMLILTATSGDTGSAALSGLGGQ
PGIRIAVIYPASGTSEIQRLQMTSVPTDGRFVIGLRGNFDDAQRAVKAIFQTASNPGSPF
YGLRLSSANSINIGRLLPQIVYYAAAWRDLFFSGVLEKGQAFDVVVPSGNFGDILAARYA
KAMGIPIRKLVCASNSNRILHDFFSTGLYDRRRKFSKTLSPSMDILVSSNLERLLYLALD
DNPELVAAMMSEFEHEGFFQLPEHSRTKIADFSAGWANDIQTLASIQHMWEAHGVLVDPH
TAVACSVAHESIKIARTSLRRRAGKELEASPPVVIAATASPFKFPAACLKALQGPLLSLR
ENTNDLDLAFELSRVTGIPVPAPIAALTTAKIQHHTIAEESDLAALLADFAHQPSKTAQG
KQLDSTRPL*
>SPBDM4_v1_40742|ID:27158047| putative Homoserine kinase [Uncultured spirochete bdmA 4]
MIEIEVPATSANLGPGFDCLGIALSLSNIVRIKRAARTYLTGCPSQWAGEDNLLLRSFRH
ACSILQTSVPEIAVEFVSNIPPARGLGSSASLAVAGAAAALLFHTGAEAGTEVDYSAFLH
RPENLDFLLRAAADIEGHPDNAAPAMYGGFTAAALAGRKIIVSHSSAPEHWHFEACIPDF
NLETSVARKALPDVYPRADVVHSLSHAVLTALAIQKADLTMLGRVCEDRIHEPYRRPLIP
DFEIVETACRAQNAAAVWISGSGPTILAVFDTSAPEKINKSLASDIAERALHSWHIIPLQ
ADNLGIRAHQVRQEEPSV*
>SPBDM4_v1_40743|ID:27158048| putative Methylated-DNA--[protein]-cysteine S-methyltransferase [Uncultured spirochete bdmA 4]
MNYSSISEDSSATLPCAPIFCAPLPSSFGDILILWEGQDASSLCRIYLPKDVVAQFDRAR
VDFPHLTLWEKGAALPSAIESIAELLHAILAGRPVQLPFAILEKSLRPLGVFQQKVLMLE
ATIPFGHISTYRELARASGNSLAFRAVARALSHNPLPLLIPCHRVIASNGSLAGYQGGTA
MKQHLLELEGIPFSSEAVDLKRAKLWNFSA*
>SPBDM4_v1_40744|ID:27158049| putative enzyme [Uncultured spirochete bdmA 4]
MSDETNIKAATVESSVIMPAIFAGHGLAINAALNNDFAKSLSDITARIPSPSAIAVISAH
WSTPEVHVTSAPRPRQMFDSIEFQQELHEISYEPQGDPSLAETICNLLLSSGINAKNDPK
RGLDTSVWGILVHMFPDAKIPVVEISLSYHVDTSKIIEVGKALAPLRSRGVLLMGSGGLV
HNLYEMSKNIDTKPPVWAIETDKKIAALVQSGDAAALSEFTLQNLGHSPAIPTPEHILPA
IAVLALKEPEDRVSFFYEAFQNSTVSMRSFVIEREQG*
>SPBDM4_v1_40745|ID:27158050| putative Ribosomal RNA small subunit methyltransferase G [Uncultured spirochete bdmA 4]
MNHEQLLARGLAALDFDVSSGISETLSRYLDELERWNPLYGLVNAEGEDLVIKHVLDSLA
PWRVLEELFADIESPVAQAGEVRVADIGTGAGFPGIPLSIAFPEHPFILIERLEKRARFL
ENIVVLLKLDNVEIRQSTVENEHEPLQCVVFRALKPFGDRRVFRSIWKKIQPGGALCAYK
GRIMHAKLELAELSDDPVLGGLVANARIVPVWVPFLEEERCVVIAKKVS*
>SPBDM4_v1_40746|ID:27158051|pyrG| CTP synthetase [Uncultured spirochete bdmA 4]
MRKLLFVTGGVCSSLGKGIAASSIGALMEARGFTVQMVKIDPYLNVDAGTMSPYQHGEVY
VTDDGAETDLDLGNYGRFTTAKLSRANSITTGQIYKSVIEKERAGEYLGRTVQVVPHITN
EIKQRILAVASNPEVDMTIVEIGGTVGDIESVPFLEACRQLIHEFGKQNAISVHVTLVPA
VSGGEIKTKPTQHAVKELQELGIQPDALILRSTKPLNEDLRRKISLFTNVEEEGVISGYD
APTIYEVPLVFYNQKLDLFLLRKMNVESRHAELTKWQTVVQAITKPNRKVKIAVVGKYMD
LHDSYKSIWEALCHGGIAHHAGLEIEKVDSSRLEDSNADLEAIFKGIHGILVPGGFGERG
IEGMIRAARYAREHNIPYLGICLGMQIMVIEYARSVLGWQDAHSTEFNKSTPHPVVCLLE
EQTRIKNYGGTMRLGAYDAITKAGSSIRAAYGTEQISERHRHRYEVNNDIRAELEEHHLT
VSATTPNGELVEACEWPNHTWSVGVQFHPEFKSRPTSPHPLFREFIKAALNHSGS*
>SPBDM4_v1_40747|ID:27158052| exported protein of unknown function [Uncultured spirochete bdmA 4]
MDGYKKALKGMFRIASVLCLVCVLSLLFPSCGQQVEENTSDAQAEVPDARFVDLTRKEYR
GGKLSMQISARLATWYEADQRLEIEGLAFTTYNTDDGSVSASGEADKAVFYEASGDAEFS
GHVQVRSAEGDVSFETKKITYKRALDIFETPNDAEVVIKAKEQLLMAGKGLLFDVKQKYY
EIRENVSGSVNQ*
>SPBDM4_v1_40748|ID:27158053| exported protein of unknown function [Uncultured spirochete bdmA 4]
MKTRAPLWLLVLAMIVGALQTGLAQATPPAAPVPSSPPAGPQTASGKKPISFSARSVQGV

LAKDKQDTILTGSVKIITGSLFITADRVELSGEDYVNVACSGNVTVNDTEKGFSLVAEKL
NYQRDTEIGLAQGKVAVNDTKNNTIIDAEWIRFDQQQSIFEAAVSVLVLKEDMSIRAEYA
HYNRDTEEIKLHGGAIAVTEEGTLKGDVISATSDWSNLQISGEVSGNITSKENSASQ*
>SPBDM4_v1_40749|ID:27158054|lptB| putative lipopolysaccharide transport protein B: ATP-binding component of ABC superfamily [Uncultured spirochete bdmA 4]
MSMATLEVSRLRKSFGRTLAVADVSFSMETGEVVGLLGPNGAGKTTIFYMIAGFLKPSAG
SVRFNGTQIDRLPMFKRAMLGISYLPQEPSIFRRMTVEENILAVLEARSELPLSGRKEKA
RTLMEEFGIAHAAKQPAYTLSGGERRRTEIARALAIEPKFLLLDEPFTGIDPIAIREIKL
LIRSLSAKGIGVLLTDHNVRDTLAITTRAYIISKGIIVAHGEPQSIIDNPLARETYLGQD
FEM*
>SPBDM4_v1_40750|ID:27158055| Hydrolase, TatD family [Uncultured spirochete bdmA 4]
MRYFDTHAHIGLIYDDPIEQLLVCQQAKLVGVSRIVSICNSLVDFKQVYENLKPAEHVYH
AVGVSPSEVSNPGRDWQRFIEESIKLPRVIAIGEIGLDYYHKFGDKRSQIELFIDQLEIA
ARLNLPVVIHNREAGRDVLDILKDRMPPAGAVLHCYSENAAYAADILDLGLDIYFSFAGN
LTYRNARNLHETVAFLPLDRVLLESEAPFMVPAEYRNKRNMPEYLPSTAQFLADFLNIDV
EEVAETTYQNACRFFRLNP*
>SPBDM4_v1_40751|ID:27158056|dus| putative tRNA-dihydrouridine synthase [Uncultured spirochete bdmA 4]
MKHSKPWPFPLGEEKWNAPYFLAPVAGYSDAAFRAVCTEFGAALCYTEMVSAEALIRGHS
KTRELLVRDPVEKQYAVQLFGSNPETLAKATEIVSAYNPIVIDLNCGCPVPKIIKAGAGS
ALLKTPERIGEIIRAMRNATSVPISVKLRTGWDQNSINYPETSSAAIEAGACAITLHGRT
RAQGYSGKSDWNAIRSLAEQTTVPVFGSGDVFSAQDAIDMRRTTGCDGVMIARGALGNPF
IFTELRRHSMQAAMEPSDAPPSPRTIAQTAAHHLELAIKYLGEKTACIEFRKHFCAYTKG
FAGGAGLRAQAVHCATFKEYEALFKEFGTIAGSPTLSGSSF*
>SPBDM4_v1_40752|ID:27158057| exported protein of unknown function [Uncultured spirochete bdmA 4]
MKKKMYKYISGLAGGLAIFLLISFLSGCDQSPQSALVWTDVPELVIAAQIFNKENDRFAV
DVEYKANVASEMKKTNKPPSLVVGKYLLSKSLTKKFVSLDVLFSKYYLDPNDMYPALLRA
GKQGAAHILMPLSFDCMVLVERKSENESAGVAVLNPDVLQSSSEAFTRIGNGEISAMGFS
PRWNLEFAEDWLLAGGTGFSLNQNWKSTSAPKPNEANSWPILWDKGNLEESVGALLSFNS
NVTTEQEDAFAFTYFNKPGYQLVLDNRVLYWPMKASEFFRLPYSAKTQLRYRFPAVNQKL
LLTADTRYMGIPKGAKNKKAALAFARWLFVAGNQEKVWKEMESQQLLPDYVGPFGGFSSI
MQMNETVFSKYFPEYAQNPLVPSALPSVTALPDYWGSFSRDFLRHWLESVLSDSASSGSI
DERFCASLEQYLGTMPDWRIEDR*
>SPBDM4_v1_40753|ID:27158058| putative Radical SAM protein [Uncultured spirochete bdmA 4]
VQELRDPKSMPRINWHSQCLKARYDEHVWRLGIDAGFTCPHRSPDRLQGGCRFCAPDGNI
SAYQKTQAPVPSMEKQIELALIFTRRRYHANAFFLYFQAYTGTNAATKTLTQVYENAIDA
FWRVYEKVGEAPRAAPETHAPKMGPLKGIIVSTRPDCFDQEKADLLAAYMEQGMEVWVEL
GLQSAHEETLSFIRRNHGVKAFLDAMEIAKRTHLKRTVHLMLGLPGESRTMMIETAQLAA
STETEGVKFHDFRIVKGSAFARNFLAGEITAMHPSRLPGLLADCLEVLPPSTEIMRISAD
FRPEESVNIHPPTDKHTLARLVEDELVRRNSCQGQRFAG*
>SPBDM4_v1_40754|ID:27158059| DNA polymerase III, subunit gamma and tau (modular protein) [Uncultured spirochete bdmA 4]
VPQSAWPYALCLLYPSMPPCYFSYMPFEVTATRKRPQTFEQLAGQNFVAATLISSLEQGR
IAHAYLFSGPRGCGKTSTARILAKALNCEHGPTPHPCGSCPSCLAISRGSSLDVIEIDGA
SNTSVDNIRQIKDEVLFAPNSGRYKIYIIDEVHMLSMSAFNALLKTIEEPPPYIVFIFAT
TEPHKVPATIKSRCQQFNFRLVPVEVILQLLRQAATETGIEAEEEALLWIAREAAGSVRD
AYTLFDQIASFSGTMISAQNIRDTLGLVGIDHLNDLFRAIVASDTKEAFSVLDEILGRGV
SPEQFLTDAVDYCRSVLLILYGVQKEGLLSAPRTVFEPAVLASLSKTQIEYAIALLLDTY
RHLKETIEPRYELELAVAKLCHIKSYISPPELFNALHSVQQAIKAKEIPPRGQREKDASL
PFQGRPGNSADAQSAPLLRDLHSRFAANQQSANPPTEPDEATEVHAPASGAISSSELKKK
IIAKLRGQNLFLASALEKSAEWTKKHNGFIIPVMNHVEMDIISRFSSLIAETGAEILGTP
CAIEPHFAKDQENHTPPSRQSESRTASPLQRETPQQAPHPPAASKSHEAAVQPEPDIEYN
SPTSFENSFPETELDTTAEFENTTRIQDEQALPETDARTIELIRKMFKGKVIATKLPSEA
PSPEPLAPPSFPPEDASESREFSEGSEELIDTEEDFD*
>SPBDM4_v1_40755|ID:27158060| Nucleoid-associated protein Spica_0202 [Uncultured spirochete bdmA 4]

MDVSNIFDMLKNPQALKAQAEEMQRKMNAIRATGEAGGGMVKITLSGNMELLECYISPEL
SEFKDIPLLQDLIRAAHHNAAEKIKEAMQNEFAGSMGGSGMPLGNYPGNFPGTEV*
>SPBDM4_v1_40756|ID:27158061|recR| Recombination protein RecR [Uncultured spirochete bdmA 4]
MKAIEDLVSLLTRLPGLGRKSALRIVYSLLKSDISFSETLAERIRTLRTTIHFCSECGSY
SESELCQVCADASRDRSIVCIVEQPQDVITIEASKEYRGLYHVLGGLLSPLEGIGPDQLN
IGKLIQRIHRSFATSVPIQEVVIATNPTIEGDTTALYLKKLLEKEPVRVTRLATGIPVGG
DLEYADRLTLARSFRGRSPL*
>SPBDM4_v1_40757|ID:27158062| conserved protein of unknown function [Uncultured spirochete bdmA 4]
MRATEIARIINAEIVAGEEIAYNLDIETACGSDLMSDVIAFVKERVALLTGLTNPQVLRT
ADLMDIRLIIFVRGKKPSAEMIQMAREQGIVLMKTHNTMFTACGELYQAGIKGDSSCGDH
*
>SPBDM4_v1_40758|ID:27158063| Anti-sigma regulator [Uncultured spirochete bdmA 4]
VVIIEYIIPRLDFSAAGKASTDMKHRLTQLGLPPALIKRVAIAMYEAEMNIAIHGDGGRA
EIEISQEGITTRFIDQGPGIADIELAMQEGYSTASEEIRDMGFGAGMGLPNMKKNADEFI
IQSEPNKGTTVQMRFRLPAGEP*
>SPBDM4_v1_40759|ID:27158064|ffh| Ffh [Uncultured spirochete bdmA 4]
VSFEPANQSPNIFQTFHSVLLDADLCVGCTTCIKFCPTKAIRVRNGKAKIFEDRCIDCGE
CIRRCPKGAKKAVSDPLFMMDAYDIKVALPAPSLYAQFGTKYSQSDIFNAIHSAGFDEVF
DVAWGALVVTTMTRSILAQEALRPRISSACPVIVRLIQQRFPSLIPNLMPILPPSEIAAR
EARRRLGSLSQKVGIFFLSPCTAKVTSVRTPLGYEQSAIDAVFSFGDIYASLKRALDPTP
SSAPHIDAISSGNASTHHLLPIMNNLEPGMGWARSDGELDALHIENAVSVDGISNVIELF
EAIENGNIDSIQYIEALACPGGCVGGPMAVENPHIARSTMRQRYQHPFSAYPGSKTAQDH
HRTAKERSKSLSPESSTADKEYLAFKWTRPLPPNPVLVLDTDLSKALQMAEKIEQIRNRL
PGIDCGACGAPDCDAFAEDIVRGLSSIEDCRLLASPPPARNEEENPK*
>SPBDM4_v1_40760|ID:27158065| conserved protein of unknown function [Uncultured spirochete bdmA 4]
LNKKDFPHIHFYDQDFVDVYDRTWAWIADCWTNGGSQTKISKTKFFYYPEQKRLDFLEQV
FSSFFLVYSNRIYPASNGLDALYALQEPDGAIHTAYDIETGEPILPKDNPHGVTLPLLAW
AEFNLYHKTANKKRVKEVMPMLSKHYKWLEANFKMPNGLFATPLSSTGMENTPRTDVRYF
IDFNAAMAMNALYLSALGDILNDKDASFQYKRDYFSIKTRINSKMWNAEDGFYYDLGEHE
NQIPTKTLGTYWLLLAEIPNEERAEKLVAKLKEPQCFGTENPFPSLSADHPAFDKKGQGF
RGSVFPWLTFMVIKGMEKYYFYELARDSAIRHLYYILDSMHSNEHEGNRHNNHPTVWSAY
QPMNEGKAVWDGHPEWPLQNYFPSNGLCTVTLVIENVIGLYISLPRKTVDWIIPNLEVMG
IENLSLKRNMITILSGKSGRGWEIHMESEKLYYFTINVLGKKKKTLPIPSGKCSMLIDKI
*
>SPBDM4_v1_40761|ID:27158066| conserved protein of unknown function [Uncultured spirochete bdmA 4]
MTGRVWNPGANLPFDPIAAAKAKELGLRVIFASGSNLENFDKILSEKSFIGTIID*
>SPBDM4_v1_40762|ID:27158067| conserved protein of unknown function [Uncultured spirochete bdmA 4]
MTIVISLGGSIVAPQEGPSGLFLLQFRNVLLSWLEAENRKAIIVVGGGSAARQWQKAYKE
FIEQAAERETSNDGNGEVEPAKALYLDESLDRIGIAATRLNAQLVREVFSDYCPDSVVTD
PSADISMRGEILVAAGWKPGFSTDYDAVLLAERFHANQILNLSNISQIYSADPKKDPGAK
PLAHITFDALL*
>SPBDM4_v1_40763|ID:27158068| putative Peptidase M20 [Uncultured spirochete bdmA 4]
MGKSDDPIDTQSLAIRLVKSASITNTSGEVSFPDILIPILREISFFNSHPEHIQEFYIEG
DEWKRRIVIALAPGEGTSCVVLTGHYDVVDTSNYGALEPFAFDPETLTKKMLQSLSSKEK
LSPPEILLKKDLESSFFVPGRGMLDMKSGLAAGIAALADFLGDDERSGNIVYMAVPDEEG
SSVGMKAAKKILSAFASEHGLRLKAIINLDAAVDQGDGAEAKAVFLGSVSKLLPFVLFVG
KPAHAGAPFDGFNPVMAASVFAREAECNSECLNIRTVFPGEEPPPPTILYYREMRSRYDV
TMPADVFCALNVLTFEKNPAKVFENVTLLVQKALDSSISLLYERMSAFSRKQNEHVVMQK
LSSEIIDLPELINRAEYVAPGILDRLRRVAEKSFPEDKVLQTAKIVHELLTYAQIEGPCA
IVGLAPPYYAKAELDSDRDQLFLAYVKDEINQFNRDHEESVKIRPYFPGISDMSFLAPSV
DAQSIEYIKQRSAVDQPDLDEYLKDSLDVPVINIGPWGREYHQCGERVNRHYAFNLLPEL
VFKICQRLLKENL*
>SPBDM4_v1_40764|ID:27158069| AMP-forming long-chain acyl-CoA synthetase [Uncultured spirochete bdmA 4]
MMKRTVLRMLKEAAAQYSNIPYALKKADSGYVGTTFLEVREQARKFAAWLLVNNFKKGDK

ATIIAEGSPEWLVSELGVLCAGMISVPLSIKLLADEIPFRIEHSESKIICTTHNQLEKIA
NALVIAHTPDMRIVYFDDDVDWGRSVLSKSKIPEDCIISLKEALSAGASLLAQNDEYGQK
LDLIEEETEENDVVTISYTSGTTGNPKGIMLSHLNYWSNCHDAIDLFDNPMYFRTLLILP
VDHSFAHTVGLYTAMICGISLYFVDSRGGGIATLRNIPANLKESDPIFLFTVPSLSGNFM
KKIIAAIEEKGGIIESIFKKGIAAGILWNGDGYHKPPFKDRFNAFFPYFIAKLLVFNKIK
KSLFGNSIRFCVGGGAFLDIKQQQFFAALGVPLYQGYGLTEAAPIISSNTPRVHKFGTSG
IMAPSVQCKIMDENGAEVSQGTMGHITVTGENVMLGYYKNSEATAEVLTDGRLWTGDLGY
MDEEGFLSVIGRAKALLIREDGEKYSPEEIEEAITMSTDVFDQLMVWCDHKKYTMALVTL
DETKVKNLITRKSIHSAEALLAVLIEEFYRFKNDPKAKKVQSAWVPSVFQILEEPFSEQN
GTINSTMKLVRHKVAEVYKEYIEYSYVSEGSSTINKKNLALVAKKFSLK*
>SPBDM4_v1_40765|ID:27158070| transposase (fragment) [Uncultured spirochete bdmA 4]
MNQDGSQEKIIRIDEVQVKRELNDIVRSTVEEPVNKLLDAEADELCGVGRYEQNPGRIDT
RAGSCQRNLDTQNIKMYLLASFRM*
>SPBDM4_v1_40766|ID:27158071| Carbohydrate kinase [Uncultured spirochete bdmA 4]
MKDESQFSDLYACIDLGTQSVRVGIIDEHGTLVACAEHEYPISYPNDGWAEQKPEDWYKN
FLLAWESALKDISPPRRKCIKALCACATSSTVFPVDAEGRPLSNALVWMDSRAVKEAEDI
NATGNPTLSHCGGEVSVEWLIPKVLWFKHNKKEIYDTAYRFVEQLDWFNWMLSGEWCSSE
CNATCKANYSHDDGGWNPHYFGNIGLDDYKEKIITQVTPVGGLIGELRADFCETYGLNKS
VSLYQGGIDAYIAVLGLGVVHEGILGAILGTSFVELCMTSNPLKIEGIWGPYKDAIIEGY
YTLEGGQISAGSITKWFREIFGNGKSIDFKSISSEAKNSGVGAHGLVALDFFQGNRTPYK
NPKSRGVIWGLSLSHSRGDIYRALIESVAFGTRNIISNFNLFGYDISSIVACGGVTKDEL
WMSVIADACDMQISLVNSSSYAGLMGCAMISAVSSGLFRDYVSAANSMIHEVAVVEPNHK
NKELYESAYDQYIALCGIMNSMTSKK*
>SPBDM4_v1_40767|ID:27158072| Short-chain dehydrogenase/reductase SDR [Uncultured spirochete bdmA 4]
MRERFDGKVGIITGSARGMGKEAAKMLASEGCAVVITDIDKTELEKTLLEFTNLGYKVKS
FQCDVSKVEQIEKLIAFTYEQYKHIDILINNAGILVSATIEETTNEIIDRTLDINIKGVL
YTIRAITPIMKAQKYGRIINVASITGKNGDNTTTFAYGASKGAVISLTRSVARQLGPYGI
TCNAIAPHAVMTTLMSYWSEKKKASMAKNIPVRRLGNEKDMAALMCYLASDEASFINGET
VNINGGYYMD*
>SPBDM4_v1_40768|ID:27158073|deoC| Deoxyribose-phosphate aldolase [Uncultured spirochete bdmA 4]
MDQNQIIQKIVEEVLSRMNSSTANSAPGGGASPAASAGGVAKTATPEDILLPSDLPKYID
HTLLKPEASEEQIDALCDEAAKYHFYSVCVNSSWVEHCVRRLGGTGVKTCAVVGFPLGAM
DRRAKSFEARTAVSNGADEIDMVMNVGAMKANDIKTVREDMLAVRRACRQGIILKVIIEA
CLLTDEEKVLACQIAKDIGADFVKTSTGFGKGGATVADVALMRRTVGPKMGVKAAGGIKS
FEDAIQMLKAGATRLGTSSGIALVSGQKASSNY*
>SPBDM4_v1_40769|ID:27158074|ywlF| putative sugar phosphate isomerase YwlF [Uncultured spirochete bdmA 4]
VSNESIEGPVDLVHSVAIGSDHGAVAQKEMLAKYLELLGYRVVNVGCFGTDKVDYPDIAE
AVCRKVVSGDCERGIVLDGAGIGSCMAANKIKGIRAAMCYNLRTVVNSREHNNANVLTLG
GPLHTPDELAEMVRVWLETRFAGGRHIVRINKIMALERGGSSN*
>SPBDM4_v1_40770|ID:27158075| conserved protein of unknown function [Uncultured spirochete bdmA 4]
MIVGRVVANVVSTEKHPQYVGYKLLMVQPLDAAGQPKGKPLLALDGVQAGIGDTVLVVDE
GGSARSVMGDEKALTIRTAICGIIDRIDIEVNT*
>SPBDM4_v1_40771|ID:27158076| Ethanolamine utilization protein EutN/carboxysome structural protein CcmL [Uncultured spirochete bdmA 4]
MKLGRVVGRVVSTKKMPCFDGLKLLLVQPLDHLKRNAGPVIVAFDTVKSGEGDLVFFESG
KEAAQANPNGWFNPGDAAIIGIVDAVNTEEIR*
>SPBDM4_v1_40772|ID:27158077|eutE| putative aldehyde dehydrogenase, ethanolamine utilization protein [Uncultured spirochete bdmA 4]
MNTNMANLTDDIRVVVEEVLKSPDIKALLSGNTPADDAVPEQSGIFPDVDSAVAAATIAQ
RELIALPLDTRKAIIEAIRRVVLWNNRDLSSQAVAETGMGNLADKQIKNALAANKTPGVE
DIEPRVCTDEHGLTLTELAPWGVIGAVTPVTNPIATITCNAIGMIAAGNSVVFNVHPNAK
KISCRLVSMFNDAIHMEGGPRNLLCAIGEPTIESANILMKHKGIALLVVTGGPGVVKAAM
GSGKKAICAGPGNPPCVVDSTADIVKAGRDIVLGASFDNNVVCICEKEVLAVASIADRLK
GEMRKNGAFELTGAQIDAVTKLVIAEPGGADHEGAPNKAYVGKSPIEIARAAGIAVPPET

RLLLMEVGREHPLVWTEQLMPVLPLVRMNDVDEAIDFAVVVERGCRHSAMIHSHDIEKLS
RMAKVMNCSLFIKNGPCYAGLGQGGAGYTSFTIASPTGEGITRPRSFTRERRCTLVDSFR
II*
>SPBDM4_v1_40773|ID:27158078| Ethanolamine utilization protein EutN/carboxysome structural protein Ccml [Uncultured spirochete bdmA 4]
MNLARVRGTVVATKRSDAVASPRFLLVEECNERGEGRGEYLVALDMVGAERGHLVLLTQG
SSCRWTYATDDKPIDTLVVAIVDQMDEGGVDVWKQE*
>SPBDM4_v1_40774|ID:27158079|ccmL| Carbon dioxide concentrating mechanism protein CcmL [Uncultured spirochete bdmA 4]
MVLARVRGTVVSTQKPQNMEGLRLLLLEKIDPVSLQGKGDFVVGIDSVGANAGEIVFFVT
GSSARMTETTKGKPSDATIIAIVDTIEKDGTFTYRKAVEQE*
>SPBDM4_v1_40775|ID:27158080|cchA| putative carboxysome-like ethanolaminosome structural protein, ethanolamine utilization protein [Uncultured spirochete bdmA 4]
MADTNDALGMIETRGFAAMVEAADAMVKAAKVDLIGYEKIGGGYVTAIVRGDVAACRAAV
DAGQRSAEKVGEVVSTHVIPRPHEAVDAALPLGRKPANPSNKKLASSAK*
>SPBDM4_v1_40776|ID:27158081| conserved protein of unknown function [Uncultured spirochete bdmA 4]
MAVDLRTYVFLDSLQLQNASFIATISKGYYPIGMQACCIIEIAPGIEINRLTDIALKATN
VTPGLQIVERAYGLLEVHSDSQGDARMAGEAVLRELGMTEENRIKPRVMTSQLIKNISDH
HAQLINKVRHGNMVLRGDTLYVLEVEPAGYAYYAANEAEKTSDINIIEVVGFGAFGRVYI
AGDEAEVIEARKVVEARLDGLGGRVLAANAARI*
>SPBDM4_v1_40777|ID:27158082|cchA| putative carboxysome-like ethanolaminosome structural protein, ethanolamine utilization protein [Uncultured spirochete bdmA 4]
MAEKTNDALGMIETRGFAAMVEAADAMVKAAKVDLIGYEKIGGGYVTAIVRGDVAACRAA
VDAGVKSAEKVGEVVSTHVIPRPHASVDEALPLGRNGVEA*
>SPBDM4_v1_40778|ID:27158083| putative Transcriptional regulator LsrR [Uncultured spirochete bdmA 4]
MAERGSRKIISRTDDSEFDLITRIAWMYYKEKLTQAEIAEKIFLSRQKVQRYLEKARDLE
VIQFNLKHPRVNLLGIEDELRKKFGLKDAVVVPSPHTNAEGLRKSFAMAGAHYLERRLAG
AGNCTLGLGWGNTTAYLADYFEPQNVEEKVNVVSLIGNLMLNVSMNPFLMGQKIAEKLDA
PFYNIWAPAIAQTKERADIFKSEPWIHDVLDIASKADINLISIGEVSRSASLFQMGYLSG
ADLKRLTDKDAVGDILSRFFDSEGTIIEDEIHDRVIGIPLDALCDERKLCIGIAGGPSKT
QAIAAAIHQKYINVIITDENTATELLRR*
>SPBDM4_v1_40779|ID:27158084| transposase [Uncultured spirochete bdmA 4]
VKGYKRITNKDREEIAVYYGRGETQEATAEKLGVSQSAISRELKRGTDRGSYNPFLAQRE
SQHQAICRRPMLKINAQTWKIIEHHLALHWSSYQIAAFLRQNVNDGTEVIVSEKTIYHYL
HFPMKGELQKLALQELRQKGKKRKKKGEETRGKLANIKFIDERQEEVNTRIVPGHWEGDL
IIGKDHKSALSVIVERQTRYGLIDRLENYTAPEVRKSLEKRLKTLKPELVKSIPYDQGKE
IAEHEMLARKIRMRVYFCHPHSPMVKSLDFWN*
>SPBDM4_v1_40780|ID:27158085| protein of unknown function [Uncultured spirochete bdmA 4]
MTTLLYFAGHTIWYINTDNIVALMDKFTHPLFYFMQQAARNYTWRD*
>SPBDM4_v1_40781|ID:27158086| putative D-ribose ABC transporter permease protein [Uncultured spirochete bdmA 4]
MKKRLLTAFFLLIVVVLASAQSKPLIGFSHVSMNNPYYLAMEKAAKETAAARGADIIVLN
AEEDISKQISDIESLLVKGVKGLIVNSTTEYGTMPVIKKAKAMGIPVVAIDRLLYGDYLA
YVGIDQWKAGELQGEYITKKLLPKGGNIVMIIGDPGDSASIGRGNGMLSILEKPENKGKY
NILGTYKASYNQMLGMQKMEEAIASFGNKIDLVYCANDSMALGAEAALANAKMDKVMICG
IDGQKEAYAEILKGGPYKSTVINNSWEITQKAVNILMDYITKKIEPEDKQVITGTILVTA
DNVKTYYNKDSVF*
>SPBDM4_v1_40782|ID:27158087|rbsC| Ribose transport system permease protein RbsC [Uncultured spirochete bdmA 4]
MKPKMSNFISDYGLFFILVVLVIMSALLSPLFFTYANLANFFTQVAYNGLLAIGMTFVIL
TGGIDLSVGSIVGFSSILYATLMHGSFFTFMPNQIFMYNGSPVLTPILPFPMDIIFVLAI
GALIGFLNGAISYKFRINSFIVTLCMMIFIRGLAVCYTNGQPLFGVPDAVSYLAYGVPFK
IPMPTIIWLLTGAIATILLRYTRFGRGIYAVGGDEEVARLSGIQPPKYRIFPYVVSGFLA
ALVGIIMSGRMGCGDPKIADGWQTDAIAAVVIGGASMSGGKGSIGGTILGVLILGLITNV

MNLLEIDAYPQQMAKGIIIIAALALQGTLSARKGK*
>SPBDM4_v1_40783|ID:27158088|mglA| fused methyl-galactoside transporter subunits of ABC superfamily: ATP-binding components [Uncultured spirochete bdmA 4]
MSNSSVLLEVKGVTKVYPGVRALDNVDFQVRRGEVHCLAGENGAGKSTLIEIIGGSYPKD
SGSIQIEGKEVVFENPRHAQKCGIVVLHQELPILKDLSVAENIFLGRQPRNNFGFVDYRE
MNRQAKRWLDMIQANIDPRALLGRLPVSKQQLVSIAKAISLHAQVIIFDEPSAVLTTAEL
ERLFQIISSFKSEGRGIVYISHRLEEIFEIGDRVTVLRNGKLIGSEPIAQLSRESLVKML
VGHDVSEEYIGSRQVSHNSEPSLVVCKLSRQGILTDINLRIARGEIFGIYGLVGSGRTEL
ARAIIGADPIDSGDMFLDGKRIKNHSPQEAIQNGFCLIPEDRKNQGVLLEKSIADNIALP
ALPRLKSSFLVDAKKVSNYASEYMDKLKVVAPNARQYVKFLSGGNQQKVVLARWIGMKLD
VFIFDEPTRGIDVGAKEEIRNLIRSIAEEDKIVIFISSEISEILSIADRIGVMHEGRLVT
VVDRKNATREKLISYSMGAKEP*
>SPBDM4_v1_40784|ID:27158089|rbsC| Ribose transport system permease protein RbsC [Uncultured spirochete bdmA 4]
MKDYMKRLNQQGGLSNIFRYGVLVILVIVASILAPGFLSMQNVLNLLRNFSFLGFISIGM
TFVIISGGLDLSVASTFALSGVLLGLFQHWGIYLGHVDALSLLAPTPVLFIIILAIGAFI
GFLNGFIITRLKVADFAATLGTMIFVRGLAFAVSGGRTIFNLDPIALYMGRGMIGPIPFV
AILFILAIIICAIVLRKTVFGKRLYACGENAVAARLSGINPDHYKTIAYMISGFFAALAG
ICMAGRLNVGEPRVAEGWELDAIAAVVIGGTSFAGGNGGVGKTLLGVVIIAMIRNILSLI
GILPDPQEMIMGLVLLAAVVFGSLGTEKGVLKRA*
>SPBDM4_v1_40785|ID:27158090| transposase [Uncultured spirochete bdmA 4]
MAQVKQFDKRSGITYVYESKAYWDKEKHQSRCTRTLIGRFAPDTGEVIATDGRGRKRKAA
EPDIKPTSGPISVTRTRRLFYGATSLLDQIGETVGITADLKQCFPDNYKNILSIAYFLIL
EEHSSLMRFSKWAALHRHPYGKDIPSQRSSELFQSITEEQKMKFFRLQGSRRAKDEFWAY
DSTSISSYSEHLKQVKWGKNKEYEPLAQINLVLLFGETSNLPFYYRKLAGNIPDVKTVNE
LVRELNILGYRNIKLVMDRGFYSAENINALYKTHYKFIQACGTSLSYARNCIKKEAEEMR
RLANYNEKYCLYMHSETISWDYTQERPYKGDTITGDRRMYLHLYYNPEKAVEDEMNLNRG
MLELQRELLTGQRNPDHEKDYAKYFEVTQTPVRGVRVKPIQEAMESAKERYGYFVLISNE
IKDPVMALELYRNRDVAEKAFGNIKERLNGRRTLVASDASLEGKLFVEFVALIYLAYIKK
KMQEKGLYATYTLQGLLDELDVIECFTEPGKAPIVGEILKKQKQIYIDMGVSVPLNPASL
C*
>SPBDM4_v1_40786|ID:27158091| ABC transporter transmembrane region (fragment) [Uncultured spirochete bdmA 4]
MPLMQFCLYGSMLLLSWFGAKEILASNNNPVTGLSTGELASLITYAMQILMSLMMLSMVF
VMIIISHASAERIVEILNEESDLKNRENPVYAVEDGSITFENVTFSYTQKADKPVLDGIT
LSIESGETVGILGGTDSSKSSLVQLIPRLYDAVSGKVSVGGVDVRDYDIESLRNQVAMVL
QKNVLFSGTIKENLRWGNENATDEEMIHACELAQADGFIREFPNQYDTYIEQGGTAVSGG
QKQRLCIARALLKKPKILILDDSTSAVDTRTDTLIRQAFQKDIPNTTKIIIAQRVASVQD
ADKIVVLDDGKIDAVGTHAELLQTCTIYREVYESQNKGGVLHE*
>SPBDM4_v1_40787|ID:27158092| putative ABC transporter permease/ATP-binding protein [Uncultured spirochete bdmA 4]
MSKILPKGDPKLFGKKGGRPPMQRFKPGTIKRLFSYMVEYKIQLIFVVVCILISAATGAA
SSLFLRALIDNYIVPLLGQAHPVFTELFKAILLMGMIYVVGVLATLFYNRTMVVIGQGTL
KKIRDRMFTHMQSLPIRYFDTHTHGDVMSRYTNDTDTLRQAIAQSLPQMFSSLVSVVAAF
FAMLYLSVGLTALVVLFAFVLLKVVRFLVGRSGAFFVKQQRALGDVNGYIEETINGQKVV
KVFCHEGKAEEEFDKKNDELCYCATKANTYGNVTMPIVGNMGYMLYVFLAIIGGAAGITG
VPNLSLTGVHALTIGTIVSFLTLSRSFINPIGQISMQFNMVVMALAGASRIFELMDEKSE
QDSGYVTLVNAKMENGEIRECSERTETWAWKHPHGDGTLTYTPLRGDIRFFDVDFAYEGN
KTVLHNITLYAEPGQKVAFVGATGAGKTTITNLINRFYDIADGEIRYDGININKIKKADL
RRSLGMVLQDVNLFTGTVMDNIRYGKLDATDEECIEAAKLANADGFIRMLPQGYNTVLKG
DGSGS*
>SPBDM4_v1_40788|ID:27158093| protein of unknown function [Uncultured spirochete bdmA 4]
LISIARAAVADPPVMIMDEATSSIDTRTEAIVQKGMDALMKGRTVFVIAHRLSTVQNADD
IMVLEDGRIIERGSHAQLISQMGKYYQLYTGAFELE*

>SPBDM4_v1_40789|ID:27158094| putative Xaa-Pro dipeptidase [Uncultured spirochete bdmA 4]
MLLNRPRLTSLMKKAGIPALISAKPENAIYLTSYHPMGSRTIRDRLTYVLYFADEHKAPL
ALCPSIDIRQMRELSWFPGDRIMPFVEFKTGKDQGLVTDKFGFLAEQIRNAEYDTDEIGI
ETSFLPETILNGFREALPNAKFVNSDPIIKRARAAKTPEEIERLRKACKATQEGCKKLIE
HASRLSYEDDAAAAARAESMMRGAETIGFCAVGSSLRSANVHNNPRHEPITEGSVFRFDY
GAIYEGYWGDLARTFVVGKPNREQQKYHDAVRAAQEAGLRAVKAGVTADYVYREALRAGQ
AYDPELRREHVGHGLGLEVHEEPLLRLGNEQVIEAGMVMCVEVGKYISDLGGFQIEDTVL
VTESGIEIMDSLPKDISIAIQ*
>SPBDM4_v1_40790|ID:27158095| conserved membrane protein of unknown function [Uncultured spirochete bdmA 4]
MAHKMSDIMDDVKTSGKRKLSPAMSKIAFALGICYAAFNLTHLLIHSFGPWVHLPVNLCF
ASILTLTCYNSLFEKRRKLAVAVDILLILMAISSTLYYAIEYQEMIFRQGSSSTIPDMVF
ATCIIIVSFEITRRASGVALSIVASVFLLYALFGKYIPGYLGHSGFSYERVMAYLYSDVG
IFGSALSAASSYVFLFILFGSFLEKLGGGQVFIDLATGVAGHKRGGPAKVAVMASALFGT
ISGSSVANVAATGTFTIPMMKKLGYKPHFAGAVEAASSTGGQIMPPVMGSTAFILAEIVG
VTYSTLALKAVVPAVLYFLAVFIMVDLEAVRTKLVGVPKDQLPNAGKIMREKWAFFLPIV
VIFFTLLVLKTSTSRAAVLGIAATFIGPIFFSKGTKLSAKKVCDSVHGGSMGVLNIIAAC
TSAGVIIGVLAMTGLGLKIGTLLVKLSQGNTFFMLIAAMVLTIILGMGLPTAAAYIIAAS
VVGPALTQAGIPMLTAHLFILYFAVTAMVTPPVALASYTAAGIAGCSASKVGWSGLKLSI
AGFIVPFMFVYGPGMLLEGSIFNILYSILCSLIGIALLAVASEGYAYFIGNLNVPQRFII
SAASICIMLPGFMTDAVGIAAAVVAFLWKYTANRMHNKNVQNPI*
>SPBDM4_v1_40791|ID:27158096| putative 31 kDa immunogenic protein [Uncultured spirochete bdmA 4]
MKAIKVTAALMLFGLVAVSTLAAAPSWNFSIGTSTLGGSFYIMGTPFAKVITEKVPNVIA
TVQATNGPSANIQLMERGEMKLAYVSSAAAYEGWNGLSWAKGVKYQRMRTMFTTYSSYFE
MVTLSKLPIKTIRDLAGKRVHMSMPNGTPDIAMRYCLELLNIVPKDKLYMQTEQALDQLK
DGKLDVVIFAMGLPTSMILDLQATHDIRMVDISPEDLNTVASQYPYFAKAVIPAGTYENM
PKDINTFAFWNYSVCDKDLPDDLVYQMTKAVFENKKDFEAASSYGKELFAKNIVNAVVPL
HPGAVKYYKEIGIDIPQNLLPPTD*
>SPBDM4_v1_40792|ID:27158097| conserved protein of unknown function [Uncultured spirochete bdmA 4]
VAKIVAEYIDRLINIEMRQTGGIPRGVTHRLYEAARKFHKEPLTYLAASKLIEAVGPKDH
VLIATGAGVAPWLPKGETDGPLGAAALARAIDLGLGGKPVLVGEGRCLPPIAATVEAASL
LVADDDLFAQRDHVAQVLEYPLGEKDGSLFAEELLEKYKPKAIITVEKHGPSASGKYHSI
MGVGRSPDNVANVKFLVEAAMEKGILTIGIGDGGNEIGFGNIHEDVRKIQKYGSKCQCPC
GAGIATVTKADVLVAAAISNWGAYGIAAMLAFLMKNPRLLHDKDTEYRMLEASIRAGAMD
GLYTNLSMYVDGTSCETQLSMITMLHEIVANGLSEQPRHW*
>SPBDM4_v1_40793|ID:27158098| putative Endoribonuclease L-PSP [Uncultured spirochete bdmA 4]
MEYKRRLKSAIMKKQEIVCDGIAKSANPLSQGLKFGNLVFLSGQLGRNPDTGKLENGVYN
QTVRVLSNLKSLLETEGLSMRDILKTTIFMADISKVAEMNKAYGEFFAAPFPTRSCIEVS
ALGGGAEVEIEVIAGK*
>SPBDM4_v1_40794|ID:27158099| YheO domain protein [Uncultured spirochete bdmA 4]
MGGYMSNDSNAIILETLVTIARGIANTFGPSSEVVVHDLSKPKTSVIAVFNNTVTGRKVG
EGIRDLILTVLRSPDFKDDMLVNYQSVTPKGKNIKCTTIVIRNNEKDVVGALCINTDLSA
FVATKKFFDDFTKTHNLTPPEDKVVNVKNADVLDILDHLIQSTIKETGKLVEQMSKEDKA
QVIKYLDEKGTFLVRGSVNWVAKKLGMSRNTVYSYLEQHRLSKNIK*
>SPBDM4_v1_40795|ID:27158100| putative Transcriptional regulator, DeoR family [Uncultured spirochete bdmA 4]
MAINQKEIDLAYRCADLYHNHDASEFEISQKLDISRPKVSRLLALARDLGIITISIKPPE
LFNQIELERSLTAQYHLQNVLIGIPKDNTDASIQSAIASRFLEAFQNLLKPDIRIGIGVG
STIYATSRMLRLNGPVPPRISLFPLMGLAGRTDPAYQVNNIIDLFAEPLGANRNYLMAPA
IFENTVQKDVFYKSQQVFEIVSMWEKLDVAVFGIGGPIERSAILFSSFPEKYLVQFVQSH
AVGDILAHFFDLEGKLVCPEAEAVLLSISFSALKAIPQRISLAGGLWKLNSIKTALRSNL
ITILVTDLLTAQNLVE*
>SPBDM4_v1_40796|ID:27158101| putative Alcohol dehydrogenase GroES domain protein [Uncultured spirochete bdmA 4]
MKAVMYEAPRVVSFRDVPEPSFDSNTILINISYSFICATDIKTYKQGHPHIIPPTILGHE

FTGVIERVGENVEGYAVGDCVTASPYINCGTCDSCIRGRPEVCSSRKFPSNGAFAERIAV
SSDYAKLGLIKVPKVLLKQAALSEPLACVLNSASSFNPKPAENILVVGAGFMGVLNALTM
KTIFGAHAFITDTNHDRLRLPKKLGIEVIEDIGEMHKKFDAIILAVPIPELIMQYESHIV
PFGHLVLFGGYAKETKAVFDPNLVHYMGITIVGTSGCSAVNFQTATKIIMNGTLKMDEFT
NEMYKFSSSDFQTAFQDAMEGRVLKAGFIL*
>SPBDM4_v1_40797|ID:27158102|lsrF| Uncharacterized aldolase LsrF [Uncultured spirochete bdmA 4]
MSFGKEVRMSRIFRGKNNMTVMLALDHGMALGPMRGIERPRELLNSVSGSVDSIMLNKGI
LVQCASPSSGAGIVLRISGAATIDGPDLTAERLTTTIEEALRLGVDAVATSIFVGTPNEH
ASIEACALLSDECDRFGMPLLAVTALGKDREKAFKSRYLALAVRVAAELGADIIKTYYCE
DGFDKVVEAAVGIPVVIAGGPLMDSTSQVLEVAEKAIKIGARGVDMGRNVWQSEKPIAML
KSLRDIIHEGVTAQEALLRYWN*
>SPBDM4_v1_40798|ID:27158103| putative ribose/galactose/methyl galactoside import ATP-binding protein [Uncultured spirochete bdmA 4]
MQEEIVRIESVSKDFPGVAALCNINLSLKTGEIHGVVGENGAGKSTLINILAGVYPPTSG
SLFINGEKVNSFHPSFAQSLGIAVVYQETVLVPDFTAEENIWLGRESSKYAVLDKNEQKK
RTKLLCKEYGINIPLSLPVSSFRVAEQKLVEILRALSMKSKVFIFDEPTEALSQQDVEKL
FKILSTLKSLGFAILYVSHHLEEVFRICDRITILRNGEEVGCYLPNELSTQSLIQLITNR
DMKDQYPVPPKDVKMNPLLQVENLSNPVLGLLNISFHINTGEVVAFFGMVGSHRSELMKC
LFGAARTKSGNIIFNGQKIRFHHPQDAIKVGIFLCPEDRKTEGLIADMSVLDNCSLPFLS
HFSRIGIVRKALQKDKVKKLILDLSIKTPSVNQKATFLSGGNQQKIVLGKWLIGNKGKLF
IFDEPTKGIDVGTKMDIYKTMQNLAREGAGVIFVSSDIRELLGVSDRLYVMRKGGIAGEY
ERKDFDQQTILLKALTEEKTVGVSS*
>SPBDM4_v1_40799|ID:27158104|rbsC| Ribose transport system permease protein RbsC [Uncultured spirochete bdmA 4]
MKQEIKNITQKYGTILTLIILIIVFSIATSTFLEVRNLLNILAHIAMLMIIASGLTVCMV
PGDFDMSIGSIASLAGILFTSLVLKKVPMFPSIVMVIIMGAVFGLLAGFLVTKVRVSAFI
ATLALGQIATGITFMYTRGQAVFGDFNSSFLFLGQGRVFKVIPTQVIIMLIITCALSFFM
EKTKAGKYMYAIGGSIRASYLSGIKVNLYRIIGLMISGSLAAFTGCILASRLGSGQPTAG
DSYLMDSIAAAYIGMTTIKVSRPNIPGTLIGVLFIGIIDNGLTLIGVSYFFQYIAKGAII
ILAVALSSRGSTVVEK*
>SPBDM4_v1_40800|ID:27158105| putative ABC-type sugar transport system periplasmic component-like protein [Uncultured spirochete bdmA 4]
MKRFFVVFLALAMVTFTTGAQQLTIGFVSPDQGASTQARVANKLEQVAKREGWKISISSA
TGSWEKMANIVEDYVSKKVSVIVIAMGQVNSLTPALQAAVKANIPVIAIDSEYTDLLTAD
ILTNNWEMGAKISTYMVDRLNHKGNIIVFKFDQFYGTRYRGEILDTILSEEPDIKVLDTH
FVPPTGFVEDAQSAMQAYLVKYGSKIDAVWCAYDDLAYGVSLAVKEKGFGPDDIFVVGID
GTDRNLRLIKSGTSPVVGTIIQPYDDAVEKAADLIKKLIIQKKSAKEVVGSVKVIYMSTP
LITSANVDDYLK*
>SPBDM4_v1_40801|ID:27158106| Pentulose/hexulose kinase [Uncultured spirochete bdmA 4]
MILGVDIGTASSKAVLIDNRLNIVSEERIQHSIEYVKPGFVEQDPEIWWADVCQLTKSLS
KKEYLSKVKAVGISSMGPTVLPVSSSGEALRHSILYGIDTRAFKEIAELNDSLGSDVLLK
TYSRFSSQSILPKLLWIKNNQSDIFKNTHSILAANGYIVFKLTGKSSLDYFTASSGGIID
FLTNSLYRQSFLASRIDENIIPKLYWPGEIVGNITPEASKMTGLPIGIPVIAGTTDAAAE
AVISGCVEAGDSAISLGSTYIYVTCRNNANIIKNVVVSNFLSPHSYILGGATGSGGILVE
WFAKNFLNSDPSRLSFSLPEKDFKKTSLIALPYLNGARTPVNDQNARGLILGLGTETKLA
DIYMALIESLALEIDMIVSEISQRNKANGKLRITGGGSTNEILLQAIAEVLKKDLEILPA
NMSSAAGAAVLAGIATKEMSMEEATKLVPIEKRVKYTGSYDEYFKKKKEIFGKAYSANKE
LFIDLGQMVKE*
>SPBDM4_v1_40802|ID:27158107|buk| putative butyrate kinase [Uncultured spirochete bdmA 4]
MKKILAINPGGTSTKIAIFDGDTLLYQKQIQHKMEDLIQYKHVIEQFEYRYTLVLSSLHE
SLTDMSSLNAVVARGGMLYSIESGTYSVNDIMINDLKAAKRGEHASNLGAMLAYKIANDL
GIPSFIVDPVSVDEFEDVARVSGLKELPRVSLSHALNSKAIARKAADAKGKSYELSNIIV
AHLGTGISVSAHKNGRMIDVNNAQEEGPFSSDRCGTVPSYRLVKMCFSGLYTENQLLKML
EGSGGLNSLVGMKDIREIELQANAGNADADLALRALTYQIAKEIGAIATVLNGKVDCIAL

TGGMVYSDRIVNDITRRVEFISNVLLFPGEQEMTSLASGALRVLNGEEAAKNYRGAAQC*
>SPBDM4_v1_40803|ID:27158108| protein of unknown function [Uncultured spirochete bdmA 4]
LPFSMETHYCIKNKFNIKWKI*
>SPBDM4_v1_40804|ID:27158109|ptb| Phosphate butyryltransferase [Uncultured spirochete bdmA 4]
MLKNFNDVIEKLRNIGHITIALAAAEDSESIEAIQKVASLADVKAILVGNSDKIIALCGP
KSLPENFKIINEPSSPAAALRAASLVRTGEADILMKGLVNTSDFLRAVLNPEQGLRTGHL
ISHLAAFEVPGESKLMFHSDGGMVVSPGLEEKKAIISNALDSLHCLGIQKPNIAILAANE
RVDAKIPATTDAREIVDAWKRGEFPDCVIEGPIAMDVAASEEAAKHKGISSSIVGNVDLF
IFPSIESGNIAGKILVHYAKAKMAGIILGAAKPIVLASRSDNSLTKVDSIALACLASRKS
*
>SPBDM4_v1_40805|ID:27158110| Mpv17/PMP22 [Uncultured spirochete bdmA 4]
MKKGDLVWGIALVAFISLFIFPSTASVLFKVTDKHPYIMGFSKFSVFATMGELLTYRMAK
GSWKIFTGFLFKALVWGIIGMLTVLMFNLYSSGVKTVAAAGLLPVGKGILKSFLLAFWTS
AIMNLTFSPVFMAVHRIVDVIIVKKMNKVAIHLSEIIQEINWEYFASFILGKTIPFFWIP
AHTVAFLLPSDYRVLFAALLSIILGIILSWGERKA*
>SPBDM4_v1_40806|ID:27158111| transposase [Uncultured spirochete bdmA 4]
MNVRRCTLKNAKGGTFTYLQLVHNYRVKETGKTKTNVLMKLGREDQIEPSYVQSIIKALS
AVIGESPSEAVTGFEFHASRELGGTWLLDALWNRLGIGKAIKALLKERQFDIPVERLLFA
MVAGRILAPGSKLSLEHWVSRKAYINDLPEVDVHNLYRAMDLLIASNEELQKQVFTEVAK
NASLDLDLIFLDTTNTYFETDEDTSDSGLLKRGHSKDGHPELPLVSIAFAVTKSGIPIRC
WVFPGNTSDQTIVEQVKHDLGQWNLGHLVMVRDAGFNSADNRRILLRECGDYIIGEKLRV
GTDGAAVEALHRKGRFKILENGLAIKDVILDAGTATERRFIIVKNPEAETRDRVIREQIV
EATKRKLEELSQYTGKAHTKAACALRSHSAYGRYIKQDEKGVLRIDTAKIASESLLDGKF
LVSTSSMKMDAADVVAGYKQLWAIERVFRDMKNILDIRPIYHHLDDRIRSHILICWLAMV
LVRHAENIADKSWYQIETALADITAGLIESENMSLWYCSDISDEAVDIFKRLNIALPKKV
LSTVEREAAAV*
>SPBDM4_v1_40807|ID:27158112| transposase [Uncultured spirochete bdmA 4]
MKATTAKQPGYQTVHIVRGVHYIYECVSTYDKEKKQSFNKQVCIGKKGPDGSFIPNAYYR
ELHQLATTGEKITVSSKVIGATETLTQVANTEGLERCLTLSLGKQLGSQALALAEYILVR
GSALSHFPEWAAHQKLPQGAEVLSSQDISRFLGTISDDMMEKFFTRWASNFSGHDTICLD
LTSIGSYSECNELVKYGYNRDREQLEQVNILGLFSASKMLPVAVRMLPGNIADVSTLVNE
LTHFSYLGLSNPVLLMDKGFDSEENLTRLLDRRLKFIMMAHCNRSWLKELEEQHRDSMRV
PSKLFHYQDDRYYAVTELLSLGSEKNRRCYAHIYYCSRLAEKRLDRFNERLHDYYDRLVA
GGGLETIPVDYQKYFSIKETPKRRRSVILDEAAAVATEKSFNVMFVILSNTEKDAHTALR
LYRERDAVEKFFDDMKNSMDMDRLRVHTSPRAKARLFLQYLSTILLYLCRNKLGFYESSN
TSVRGILEDLSGICEVTHSNHYGSIITESTSAQRATLAQLGIDTSSWLQK*
>SPBDM4_v1_40808|ID:27158113| transposase (fragment) [Uncultured spirochete bdmA 4]
LDKLPTALQEKAKGMLHDQYLAPTREEALKAFALFVESFGAKYPKAVECLVKDKDDLFAF
YDFPAMHRIHLRTTNPIESTFATVRVRHRKTKGNGTRKATLAMVYKLCREAEQRWRKLDG
SKLIPLVEAGIKFVNGEWVDEAVA*
>SPBDM4_v1_40809|ID:27158114| protein of unknown function [Uncultured spirochete bdmA 4]
VYCNARLEDERSCLLVVMGADSFGNKELLAVSDGYRERTQSWKEILLDL*
>SPBDM4_v1_40810|ID:27158115| protein of unknown function [Uncultured spirochete bdmA 4]
VSKTIIETRIEATPETMGALEIIVREGARKMLQAALEAEIEEHLSRFKNLVDEEGKRLVV
RNGVMPERTVLTGAGPIPITRPRVDDRALDARGEQRFTSRIHPPFMRRAPSIDTLVPVLY
LKGISDRRLSDGAGGDPWPPGERTFGEHRRAPEGDMDRGIRGVIETGSLGETLCLRLGRW
RVLQRPSRG*
>SPBDM4_v1_40811|ID:27158116| transposase (fragment) [Uncultured spirochete bdmA 4]
MSKRYDKEFKAKVAVEAVKGEKTIQEIAMRYQVYPNEI*
>SPBDM4_v1_40812|ID:27158117| protein of unknown function [Uncultured spirochete bdmA 4]
MSLNLFKIKQKLIAEGQRFAVATIAAESSSLINGAFPVPQIGSKIILTADGQVFGTTGND
TLDEEVMRKLRECLHNREDNSISITQSLKIFIERVEPAPALVIIGASEIGLAIAKLAANL
DFSIIIVDERADLANKQRFPMADSIVCESDLLKALSLISFDESCYVVIVFQANDDRAVRY
CLNKPWAYCGMLGSKNRIGAVWEKLRRDGAPEARLKAVHAPIGFDIGAQSPEEIAIATLA

EVLAVKNKKS*
>SPBDM4_v1_40813|ID:27158118| Ribose-phosphate pyrophosphokinase [Uncultured spirochete bdmA 4]
MSYSDPTHLGIIACPGAEVFANQVIRKLAGIYKHRFARKAQAIAGRYHISPELVIRKINF
ANDCNTGMLFLAGDTDHYRPPQFKINVRHTFFANGEIKTEILESIRGRNIYIFQDIENHQ
PVAFNDGTVHKVLSVNDHIFNLFVAIDAAMQAGAAEINLVLPIYPYSRQHKKKGREGLTA
ARIGKMLESLGVNRIITLDIHSKEIENAFDRLRMENLHASFQIIDKLMSIVDLAKEDMVV
LAPDTGAIDRSKFYANALQKPLALLYKERDYSKVSRNAAETNISEMRLLGNVTNKIVFMA
DDMIGTGGTLIQAMKYLKELGATKVICAVSLPLFSGNAIEDFEAAYQEGLFYRIIGTNAI
YHNELLKKEWYIQSDVTGLFAQVISLLHHNRSVSSLLDNRNVVARLIKRKLDITTNPKPQ
ANAPQYPQGEGSPSNDDDIVPYGAADKQ*
>SPBDM4_v1_40814|ID:27158119| conserved protein of unknown function [Uncultured spirochete bdmA 4]
MSIATFGEIMLRLKSPGQERLFQSPLLEATFGGSEANVAVALSRLGIPARFISILPENQI
GKECMRELRSHGVDVSGIKLKKGRIGIYFFENGANQRPSNVIYDRENSALSAISSEDINW
AAAFEGLKWFHVSGITPALSKNCADETMRAVRTAKEKGLTVSFDLNYRAKLWNYGVEARI
IMSEIAQYADILIGNEEDYQKSLGIEGPSAMGNGKIDIDAYRTMCERALRRYPKAKMAAV
TLRESFSADSNDWSGMLVTRDQRFVSRKYHIANIVDRVGAGDSFSAGLIYGLFTLKDPGA
ALEYAVALSCLKHSISGDFALIERGEVEKLLGGDSSGRVQR*
>SPBDM4_v1_40815|ID:27158120| Putative KHG/KDPG aldolase [Includes: 4-hydroxy-2-oxoglutarate aldolase ; 2-dehydro-3-deoxy-phosphogluconate aldolase] (modular protein) [Uncultured spirochete bdmA 4]
MKDLKELFYEAGLVPVIKIESVKMADGLANALCAGGLSVAEITFRTKAAPQVIEKIASFR
KDITVGAGTVTTKEEVNAALSAGAQFIVSPGFNPSICEYCLERGVPVFPGVNNPSLIEQA
MSMGLTTLKFFPAEVSGGVKALNALKSVYQQVSFIPTGGIQEDNIQEYLTLKNVLACGGS
WIVSTELIEAGKFDEIERLIKSARRTMMGFLPIARGDEGSRASSGSSGEVKRDRGFIEVR
TPSLKRTLAMLSPLGASVIPGSEIMKGQSLIGASIDIPGIETHIRLIE*
>SPBDM4_v1_40816|ID:27158121| conserved protein of unknown function [Uncultured spirochete bdmA 4]
MIVSMLSELSRYRGLSRNLDVAFDWLNEGAWRQLSAGKYPIQDDDVFALVQEYVTKEHSA
CRFESHRKYIDIQMLISGEEIIEALSLDSLKVVEPYKSDIEFFALPEDKKAHALSMNPGQ
IAIFFPEDAHRPCMKVGDSAESVKKIVVKVAV*
>SPBDM4_v1_40817|ID:27158122|kduD| 2-deoxy-D-gluconate 3-dehydrogenase [Uncultured spirochete bdmA 4]
MIFDKFKLNGKVAIVTGSSAGLGQAMCIALAEAGADIAGISHARSNETTREEIEKRGRRF
LSITADLAQMESIQPIVSKAVEMFGHIDILVNNAGIIRREDSINFTEKDWDDVMNVNAKT
LFFLSQAVARRFIAQGTGGKIINIASLLSYQGGIRVPSYTASKSAVMGLTKLMANEWAKF
NINVNAIAPGYMVTSNTAPLRADPVRSEELLARIPAGRWGMPEDLQGAAVFLASDASSYV
NGFTIAVDGGWLAR*
>SPBDM4_v1_40818|ID:27158123|kduI| 5-keto 4-deoxyuronate isomerase [Uncultured spirochete bdmA 4]
MEAREYVNSDYAKMMGTQELREAFLIEKLFVPGESIMVYSHVDRMIVGGIVPTKEPLALP
VSKELGTEYFLERREMGIINIGGAGTIEVDGTRYDIGPRDGFYISMGSKEVKFSSKDPGE
PAKFYFNSAPAHRACPTRLVTFEQAKHVAMGSDTECNKRVINQYIHPAVLETCQLVMGMT
TFSEGSVWNTMPAHTHERRMEIYLYFDMPQDRIVFHFMGKPQETRHIVVRNEQAVISPSW
SIHAGVGTGKYTFIWGMAGENQTFGDMDNVAMADLL*
>SPBDM4_v1_40819|ID:27158124| Transcriptional regulator [Uncultured spirochete bdmA 4]
MADIRVQSLDRTFDILEILAREPSGLTLAEISAKADLPRSTAFRLLAVLLQRDYARKASE
TNRYRLGPGFIELSGNYLNSLELKTESAPFMRELASTLGTIVFLARRQGNMMVYIDKQDQ
FTSLRKYAIIGEQRPLYCTALGKALILDMGDEEIRTLLSNTAFQKFGPHAHADIDSLIED
IHHCKARGWTHDNEEAEPGVNCVAAPIRDYRSQIISSISTSWFLESRPELEPEKAAIHVT
KAARAISAAMGFSGKE*
>SPBDM4_v1_40820|ID:27158125| Nitrogen-fixing NifU domain-containing protein [Uncultured spirochete bdmA 4]
MLFEEVKSAIENVRPSLMADGGDIELVSVSDDGVVKVRLTGACGSCPYSIMTLKQGIEAY
LKKAVPQVKEVQQD*
>SPBDM4_v1_40821|ID:27158126| Protein Soj homolog [Uncultured spirochete bdmA 4]
MARIIVFVNQKGGVGKTTSAINIGTSLALLHKKTLLIDFDPQGNLTSGIGGKLTHSNIYH
VISGLTEIKNIIQPLAIPRLFLAPSSVDLSGATIELVDRPDRNDYLRRAIAPIVDDYDFV
LIDCPPSLGVLTLNGLAAATEVVIPLQCEYFALEGLSLIIQTIGLVQKSINPNLKIGGIL
LTMFDARTRLSQEVVRQVTEYFKDKVFKTIIPRNVRLSEAPSHGLPAIMYDPTCIGAKSY

EAVTKEIMNRG*
>SPBDM4_v1_40822|ID:27158127| ParB-like partition protein [Uncultured spirochete bdmA 4]
VAKFGLGKGLGALIPEHQQFFEQSSQEIPESIVRLVSIETLIPNPDQPRKTFSQESLDEL
AESIRRHGLLQPLLVHEQEAGKYTIIAGERRYRAAQIAGLEQLPVIVRAQNDSDRHLELS
LVENIQREDLDPIEEAQAYVKLMEISGATQERVAEMVGKNRVTVANTIRLLRLPEDIQAA
IKQGTISSGHARALLSIEDQEERKKLFDRIREDNLSVRQTEQETQKLQSELQARKAKSRK
PHLAAHEGVEASLDPLLKELKEKLIERLGTKVEIQGSLDGGTIKILYYSQEDLQRMYDVL
KLE*
>SPBDM4_v1_40823|ID:27158128| exported protein of unknown function [Uncultured spirochete bdmA 4]
MKFWVQRLKKFLLPAFIATALIGTATFPLFAQQASGSPASIPISKISSQLPAAFQAALPQ
GVSFQPGSPILRTAEHIGGGLMLAPSLDGASTVVDHINSAGTASNLIIEALVFLPAPTAA
NPYSAGTQLDSLGLLFNQFSSLQGIQYWSASRKIMRTFYTDVHRLDNPKDRNKIEDPTSA
AELHTLLSRPAYIYQKDQTFDGTVTEVRCFMSQTSFLMTNTNATPLRLIGIPVLPVDGLR
TGFLVAPSPEGVLLYFVTSMKSPSIGRDRVFESASNKALALLHWFVEAASARQIIVPTNL
PWNFDDLPPEARLARP*
>SPBDM4_v1_40824|ID:27158129|eno| enolase [Uncultured spirochete bdmA 4]
MSIIEYVEAREILDSRGNPTIEVDVVLEDGTLGRAAVPSGASTGEYEAVELRDGDKKRYQ
GKGVLTAIENVNTTIAGEICGLDSLEQVDVDRTLIELDGTENKGKLGANAILGVSMATAR
AAAEYLGLPLYKYLGGVHTTLLPVPMSNIINGGKHADNKIDFQEFMIMPLGAESIREAVR
MNAEVFHTLKGILKAEGHNTAVGDEGGFAPNIGNEDALDYIVRAIEKAGYIPGEQIAIAL
DPAASELFEEGGGKGYRFWKSNPDKTFSADEMIELYTTWVNKYPIVSIEDGLDQNDWDGY
VKFTKALGKKIQIVGDDFFVTNTKRLAKGIEMGACNSILIKLNQIGTVTETIETINMAKR
AGYTAVVSHRSGETEDTFIADLVVGLETGQIKTGSMSRTDRIAKYNQLMRIEDEMEGIAE
YWGRKAFYNVHG*
>SPBDM4_v1_40825|ID:27158130| Membrane protein [Uncultured spirochete bdmA 4]
MLTRRLDEAKEAWKRRDPEAARRAHLPPAGIQAEEKHTTKGKYLKSIIYGGLDGTITTFA
AAAGVAGAALSPGVVLIVGLANLLADGLSMSIGDYLSSKSEAEYEAAEREREAWEVENYP
EGEKVELEEIYTARGMSKEDAHAAVEIIAKDKKAWVDTMMVEELGIMQSDESPTANAIAT
FISFGLFGFLPISAYVLALFIPSLDAIRFPLACVLTGVTLFSLGALKTIITGKKWLKSGL
EMLLVGGIAASAAYLVGMLLGGLA*
>SPBDM4_v1_40826|ID:27158131| DJ-1 family protein [Uncultured spirochete bdmA 4]
MKTACLLLAEGFEEVEALTPADYLRRADINVTIAGIAGRKVKGAHGIVVETDAGPEALER
DYDAIVLPGGMPGARNLAESSTVRNLISRHYAQGKLIAAICAAPAVVLHGACNLLQGKRF
TGYPGTEDSVQGAHFVPERVVVDGNIITSRGPGTAGEFAVAIIVALENSQKADEVAQHAL
LK*
>SPBDM4_v1_40827|ID:27158132|yjgF| Enamine/imine deaminase [Uncultured spirochete bdmA 4]
MNKQCIGAPNAPAAIGPYSHANRAGNFVFCSGQLGIDPKTGGLAEGIEAQDAQALHNLAS
VLEAGGCSPADVVKTTVFLKDIRDFSAVNAIYGDFFRKDFPARSAFQVAALPKDALVEIE
AIAYKA*
>SPBDM4_v1_40828|ID:27158133|patB| Cystathionine beta-lyase PatB [Uncultured spirochete bdmA 4]
VPYNFDSLIDRTKTHSIKWDTGLLPEDCRDAIPLWVADMDFASPPEVVEAIQDRAAHPIY
GYTNCSEEYYRSFIEWMKRRDSWDIKKEWIVFSPGVVPALNLAVLAYTQPGDKIIVQPPV
YYPFESAVRNNGRQLVENPLVIQDDRYTMAFDDLEKKIDSHTKLFILCSPHNPVGRVWTT
DELSRLVDICAGRGIVIVSDEIHSDIILGTEVHHCTATVSDKAAAITITLTAPNKTFNLA
GLQTANIVIPNARLRNAFVQQVTNIGLGLSNIFGMVAQGAAYDKGEPWLEELLIYLRENY
DTVRHFLAQRVPVIKVFPLEGTYLPWLDCRGLGLSDAQLHALFLKDAKLWLDDGTMFGTG
GSGFMRLNIACPRSILVKALEQLEKAVYTIS*
>SPBDM4_v1_40829|ID:27158134|mntP| putative manganese efflux pump MntP [Uncultured spirochete bdmA 4]
MLTYILVGFALAADAFAVSASAAACTDALPLLIGLRAAFMFGLFQFFMPIVGWLLGSAFS
KFIQGFDHWIAFALLAVVGGKMLYEAIRARKKADCPDPDESPKVNGIMKWDTLLVLAFAT
SIDALAVGLSYSILGESIFIPSLIIGVTTFGVCLLGIEFGKRLKNVLKEWTEIAGGSILI
LIGLKILIEHLARGQ*
>SPBDM4_v1_40830|ID:27158135| Flavin reductase domain protein FMN-binding protein [Uncultured spirochete bdmA 4]

MATSSIPEKYKEIPAGKAYSLQNPGGLILLCTRGSTPKSSALRYDFAPLAWCTPYEYDPV
SKLLLVCDTSHKTFRDIQENGQFVVALPSFEMRALIERAGAVSGFDVDKFELLEVPYFTA
QSIDIRIPEGVAAWMECSLERIIVEGTSGIVLGAVHKAFGVPESWKLRLHYLDDTTWYTP
GKRL*
>SPBDM4_v1_40831|ID:27158136| exported protein of unknown function [Uncultured spirochete bdmA 4]
MSKRTLFALSLLLFYGSMYVSASPGISFSLNSIKTNSDIIAGLPLPAGADLALEIPLGSM
TSLTLRGAAGYESRMILRDTTTSVPIAEPATIDGTNRFYWTKALAEIGLRQYLIRDDDAA
AWLFGLVRGRYESNATSFPTTLFTDAQNIESVSGTAGIAFDSSRWREINRKTGTYAELSV
EYSPQFASFTSTPTNYGRANLTAEAFIPLSAECNTLRNPSLIAPYLVLYGAGDYAAGSAI
PQEVLTSFGGLVGTEGIGDMVRGAQSWGYESPAKAYASAELRVAGPSLFKNLVVYPVGYI
FADAAGYAGLYGSPNGSSLADNSGVFASTGAGVSLSVLNFLWLGAYAGWRWPIHDPLAPI
YYSSPQGFFWNFTFVAHY*
>SPBDM4_v1_40832|ID:27158137| conserved exported protein of unknown function [Uncultured spirochete bdmA 4]
MKKFLIAALVLVMAGAVWAQTPGGISSAMSTRMEADFTKIVTDMGWGTVDTVHPVLAERM
WDYYFFYPVLNKDKQVTGYVWWAKNVRIASHYDDILAIINPDGTLQNLWVGANSRHADFY
TDFAKKSQGAAIQKFIGMDSKRDWNEATDAVSGSTFSAYKYFGELKAVLACFKIYVIDAG
KLIK*
>SPBDM4_v1_40833|ID:27158138| conserved protein of unknown function [Uncultured spirochete bdmA 4]
MQILDNVNNTLKDDLAATISKGDKLSIAAACFSIYAYEALKKQLEGIDELRFLFTSPTFL
REKAPKEKREFYIPRLNRERSLYGTEFEVKLRNELTQKAIARECAEWIRKKAHFRSNVTG
GQMSGFLSVVKPSETIAYSPINSFTTSDLGCERGNTIMNLVNRIDAPLAGQYVKKFEQIW
NDKSLLQDVTDQVVDGITAAYNENSPEFVYFVAIYNIFNEFLEDISEDLVPNEATGFKQT
AIWNKLYDFQKDAALAVINKLEKFDGCILADSVGLGKTFTALAVIKYYELRNRNVLVLTP
KKLSENWNTFRQNYLNNPIARDRLRYDVLYHTDL*
>SPBDM4_v1_40834|ID:27158139| conserved protein of unknown function [Uncultured spirochete bdmA 4]
MTGHIDLAKLNWSNYDLLVIDESHNFRNGGDYSGRGDDRRENRYLRLMNKVIREGIKTKV
LMLSATPVNNNFSDLRHQLELAYEGKPALINDKLGTSKPIDLIFRDAQTAFTRWSKLDPR
ERTTENLLHALDFDFFTVLDSVTIARSRRHIEKYYDIAKIGKFPERMKPLALRPKLTDLP
SAIDYDEIYEELMLLNLAVYYVPTDFLLDSKRAKYIDPSANIDRAGREIGIRRLMCINLLK
RLESSVYSFRITLARVRALIDGAIADIDNYADNYAHGTSKIIRAQELSEDEFDGDDANTD
FFVDGGKKLDIDLADMDYVSWRQKLAEDSETLALLMSMVEDITPGHDTKLQALLDLIAEK
IQNPLNAGNKKVLLFSAFADTVQYLYDHVAPHIKQRFGLDSAMITGTIDGRTTVKLPRAD
MNTILTLFSPISKDKSLLMPNNPAEIDILIATDCISEGQNLQDCDFCVNYDIHWNPVRII
QRFGRIDRIGSHNERIQLVNFWPNIDLDKYLELKGRVETRMKASVMAATGEDNPIDPEEK
GDLEYRKAQLERLQNEVVDIEDMQSGVSILDLGLNDFRLDLLDYIKDHDELDKVPFGLHA
IEKGNEDCPGGTIFILKNRNAGIKPRNRNQLHPFYMVYVRDDSTIHVDHLHPKKLLDAFR
QLCKGKAEPVLELCGRFNAETKDGRDMRHYSDLLHVAVKSIVDLNEGDSIDGFLSGRQIS
FATGKIDGLDDFELVCFLTVK*
>SPBDM4_v1_40835|ID:27158140| conserved protein of unknown function [Uncultured spirochete bdmA 4]
MFGLPKSTEINKPLPKKVVFDTFNLKPADRRLFDEQINRMTIVAEISPQTVSVAASGDVS
AVFVILVTLKVPECDKKNIALLSKLIDQRMIFALRYEGDVRLAVYRAGRVLLSQSKPLEE
WKLSLSGLDLGAVWEKIIAEIGGVDLAGGKDLDETIAASERRDKLTGQIAALEQKAMNEQ
QPRRKWEDAEEIKRLKAELEELNNG*
>SPBDM4_v1_40836|ID:27158141| protein of unknown function [Uncultured spirochete bdmA 4]
MDKPEKLAMQTPNLADGNFAALSRLFPNAVTETIDENGAVVRAIDAEVLAQEINTRVVAG
REER*
>SPBDM4_v1_40837|ID:27158142| DNA methylase N-4 [Uncultured spirochete bdmA 4]
VDFDTTENLYIEGDNLDVLKCLRETYLNKVKMIYIDPPYNTGNDFVYEDDFAGDADEFLS
RDGQFDEQGNRLVKNLDSNGRFHTDWLNMMYPRLRVARDLLTEDGVIFISIDDNEVENLK
KVCNEVFGEGNFVAQISVLSNPKGRSQDKYLATCHEFALIYSKSVLRKGALSILKDEDDI
KSNYPLTDSDGSYRLLELRNTHREFGKHNRPNLYYPFFVDANGGISLLPTAEHIIPVYPN
WNDGFEGCWTWGTNTALDNMHLLVARKVNGEWKIYRKAYALQDGEIVKKQLKTIWSEKIY
YTEKGQKTFSELFDSREKIFQSPKSVDVIKQMAEMVMKKNDIIMDFFSGSATTAHAVMQL
NAEDGGKRKFILVQLPELTDEKSEAYKAGYKNICEIGKERIRRAGKKIKDEAGLQAQDLD

IGFRVLKLDSSNMKDVYYTPEQYEKLNFDLEGFKDNIKPDRTDEDLLFQVMLELDIPLSA
QITQDVGTSNGSRTFSVNDNDLIACFGGVDTALLTEIAKKHPYYAVFRDSSFISDSALVN
FEQVFNTYSPNTTRRVL*
>SPBDM4_v1_40838|ID:27158143| conserved protein of unknown function [Uncultured spirochete bdmA 4]
VKFQKPPYTITEKAADYLAKIVETATRLEFGTEFKRDIRLHRQNRVRTIHSSLAIEGNSL
SLDEVTAVIAGKVVAGRQEEVKEVKNAYEAYDKIMTFDPYSIGDFLKAHELMTSGLVEES
GKFRSGDVGVFDGDKVVHIGARPQFVPQLMDELFGWAKTSELHPVLKSAILHYEIETIHP
FADGNGRMGRLWQTLLLAKWKSIFAWIPMESVLYQNRPQYYQAIEDARKANDSGVFIEFT
LFALLDSIVLQEKHQVKHEDKHQVERKVERHVEQLPPIALSVLKALENAALSRREVFAAI
GMYGDSRAFKRNIAPLLAGGYIEMTVPDKPNSRLQKYRLTDKGIAALGQAVTALSENDNH
RFANRGGKK*
>SPBDM4_v1_40839|ID:27158144| conserved protein of unknown function [Uncultured spirochete bdmA 4]
VKFQFKIQPFQTEAAESVVRVFAGQPNHGERKYRRDVGRREAQAVFAEQDSEFETAYRNA
DVELTAQQLLKNIRTVQTANEIKYSTELVPGLGAVSLDVEMETGTGKTYVYIKTMFELYT
AYGWSKFIVVVPSIAIREGVMKSFEITQEHFMELYGLKARFFVYNSSNLHQLDEFSQNSG
INVMIINTQAFNTTMNEDKNIEGRSGNEAARIIYSRRDEFGSRRPIDVIKANRPIIILDE
PQKMGGAATQNALKKNFNPLFSLNYSATHKTSHNLVYVLDALDAFNKKLVKKIEVKGFEL
KNLGGTNGYMYLSSIELSHNKPPRARLEFEVKHQNGIRPETHLLDVGDNLYTASNGLEEY
RNLSVAEIDPVRAMATLSNGAVLAAGEACGNINEDDIRRIQIRETILSHFEKEEKLFEQG
IKCLSLFFIDEVAKYRLYDDDGEEVLGQYGRIFEEEYSDALNERMTLFPSAYQEYLSGIK
TSETHRGYFSIDRNGHAVNSATARGSEFSDDISAYDLILRNKERLLSFDEPTRFIFSHSA
LREGWDNPNVFQICTLKHSDNATAKRQEVGRGLRLCVNSSGERQDVDVCGETFVHDVNML
TVVASESYAGFVAGLQSELKADLYDRPTKASVDYFNGRTVY*
>SPBDM4_v1_40840|ID:27158145| conserved protein of unknown function [Uncultured spirochete bdmA 4]
MSPQIALYSESIHKLVREIFDPGVLKDITVNGHDTKIRGNPLNANWSDFKELWERINKRY
AYTVEFDSAELVAKSIAAINAGLNVAQLVYTTTQGEQQGVEFSIERTETKKLDRAQGGFA
SYDLIGKIVEGTTLTRRSVAAILMGIDRKKLQLFRGNPEEFISRAADIINKQKASVVVEH
IMYAPSAEEPYSQDIFTMSRASDEYARAFKARHAIQDYVFTDGTAADSVERKFAWDLDAA
SEVIVYAKLPKGPRGLYIPTPVGKYSPDWAISFKKGTVKHIFFIAETKGTMDSLELRPIE
RAKISCAKKLFNEISTSGVKYHDVDSYQTLLQVMETL*
>SPBDM4_v1_40841|ID:27158146| transposase [Uncultured spirochete bdmA 4]
MKSISDILGGMQILLEVSFDIQAGFEEYLTKDQRTFLAILRVIEEHLRVPYEAESRYGRP
AYSLPPFIRALWAKSYFRLLTMDDLRKRLLSDPNLRMICGFTKVPSLATFSRRMSLLSES
SLMEKSFETMVSEYYAGSIVGDVARDSTAIAAREKPCNKKSDVALPKPKKYRRGRPRKGE
EHPRKAPPVIAQHTTMSLEEALHTLDTRCSWGCKKNSQGNVSYWKGYKVHLDVTDAGIPI
SVVVTGARVHDSQVAIPLERMTEARVTHLYSLMDAAYDSNTIRSFIQERGRVPLIDHNKR
KVDTRPPFDPASQRRYAIRTTVERTNSHLKDWFLSSPYFVKGIKKVTFQVMCGVLCLTAI
KILQFFIAPSLAKSA*
>SPBDM4_v1_40842|ID:27158147| protein of unknown function [Uncultured spirochete bdmA 4]
MNIMGLNYVLSATPLFMPLSLDESNLFYIRKPVKEMKKRKRTRNNIIIGGSFKSHEMSEF
*
>SPBDM4_v1_40843|ID:27158148| putative type I restriction enzyme HindVIIP M protein [Uncultured spirochete bdmA 4]
MAEKNTASIGFEQQIWAAADILRGNMDAAEYKHVVLGLIFLKYISDKFEERYRELEADND
DVEDKDAYAEKNIFFVPPSARWSVISEAAHKEEIGTIIDDAMRAIEKENKRLKDILPKNY
ARDELDKRRLGNVVDLFTNIQMIEHGTDKDILGRTYEYCLAKFAEQEGKRAGEFYTPSCV
VRTLVEVLQPFKGRVYDPCCGSGGMFVQSTDFVKNHSGNIGNLSVYGQDANPTTRKMALM
NLAIRGIEADLGGYNADTFHNDLHPTLKADFILANPPFNLSDWNDGSLNDDPRWKYGLPP
SGNANFAWLQHMIYHLAPNGKIGMVLANGSLSSQSGGEGDIRRKIVEDDLVEGIIAMPTQ
LFYTTQIPVSLWFISRNKKQKGKTLFIDARKMGTMVNRRLREMTAEDIAKIAATFEAFDE
GTLEDVKGYCAAVTTEDIAKQEYILTPGRYVGIEEQENDGEPFEEKMARLTGELSEMFKR
SHEQEDEIRKRLGAIGYDI*
>SPBDM4_v1_40844|ID:27158149| Restriction modification system DNA specificity domain protein (fragment) [Uncultured spirochete bdmA 4]

MAQTIFKSWFVDFEPWGGEIPSDWQVVEFSSFLAPRIEKSSDPTIPLFSVTDTGIYPRGE
KFNKNLSKADTKNKIVRETDLIFGMSREILNWGVMRSPIGGVSSAYNVFSVDSGINSKYL
ESFIKAHHSYFKDLIRPATREGQGVDKGALMLKSIYLPQNDALADYYAIEDALTAQIREK
EVESARLASLRDTLLPRLISGELSVADLGDAK*
>SPBDM4_v1_40845|ID:27158150| Integrase family protein [Uncultured spirochete bdmA 4]
MKDRLITEIQASMAPMLTQAQLEKLRRVLTHSLHNVEVTERKASDTQETAENGGLLEVFI
AAKRIEGCSEKSLKYYDATVRAMLKAVKKPVREIATDDLRQYLANYQKERGSSKVTIDNM
RRIFSSFFGWLEDEDYILKSPVRRIHKVKTEKTIKETFSDEGLELLRDACEEIRDLAMID
LLASTGMRVGELVQLNREDINFHERECVVFGKGGSERMVYFDARTKIHLLNYLDSRSDDN
PALFVSLTLSNERLLIGGIETRLREIGKRADMQKVHPHKFRRTLATRAIDKGMPIEQVQR
LLGHVKIDTTMHYAMVNQANVKNSHRKFIG*
>SPBDM4_v1_40846|ID:27158151| Restriction modification system DNA specificity domain protein [Uncultured spirochete bdmA 4]
MHQGWMPTRIGDICKTNQQTYSVSEKWPFVNYLDTGNITENRVSEIQHLVTGQDTLPSRA
RRKVAVDDILYSTVRPNQRHYGIVKEVLPNMLVSTGFAVITADKAVADSDFLYYYLTQND
IVDSLHAIGEQSVSAYPSIKPSDIESLEMLLPPLDEQIEIGRTLRALDDKIANNKAINHH
LEQMAQTIFKSWFVDFEPWGGVMPDDWREVPLGGVVEMSTKALNPQSCPDTVLEHYSIPA
FDETRLPVFEAAAEIKSNKYIVDKDCFLISKLNPTTKRIWRPCCISKHAVCSTEFIVYRA
KNPAHKDFYYSVIDSPAFTDFLLSHITGSTGSRQRAIPRETLSFSVIVPPDDIIEDFCDK
VAAIYAHFEQNHLESRRLRQIRDSLLPRLMSGELSVADLGDAK*
>SPBDM4_v1_40847|ID:27158152| putative FRG domain protein [Uncultured spirochete bdmA 4]
MKEERISSVNAFVDRITKLRSEIPAGNAEQWFFRGQKCSLWGVQPNIFRSNGLSQEHAII
ECAQRQNPLEFRDCVSNFEILTKLQHYGLGTRLLDVTLNPLVALFFATEQSVSYTKNANG
QYSYQEHDGKVLYRFVNGCALRDLQIRVALEIPFVEFGKGLSLEAFCLQLRDTKAISNGE
YESLIADNYEKAINVIQTNSFIVATNSNARLIQQRGAFLLSTAVNVKSNTDVKTSALSKA
RLDLDKEFEGYFIIPAKDKEEIRTELDFFNVNEATLFPELEHQMRYIQTQATVTVGTIEE
YSQYVRKTARLSTASKNLNKSEVETIVKALLSELDKKISDDVVGAIIEQQELDWQNKDSV
ISRIRRTINKILSNSFSAVEAKNKASAIVDNLLEVQ*
>SPBDM4_v1_40848|ID:27158153| Type I site-specific deoxyribonuclease, HsdR family [Uncultured spirochete bdmA 4]
MSYTEANYENAVIEVFRNTLGYRYVYAPDLARDYSDPLYMDEILPALRRLNPKLPEAALA
EAVYKLRNFEGGTVLQKNVQFMSYLQNGVSTNYYDKGEQQAALVYLVDYENVERNTFTVA
NQWTITENSEKRPDVIVFLNGLPVVVFELKSPSREETDASEAFLQLRNYMHEIPSLFIYN
AFLVMSDLAISKAGTITAGEDRFMEWKTKDGSYANTQYAQFDTFIEGMFDKTRLLDIIKN
FICFSGDTKILGAYHQYFAVRKAVASTANAIVTDGKGGVFWHTQGSGKSLSMVFYAHLLQ
ETLNSPTIVVLTDRNDLDDQLFGQFAKCAAFLRQTPQQAESRVHLKELLAGRQANGIIFT
TMQKFEESFEALSDRRNIIVMADEAHRGQYGLEEKVDAKTGRVILGTARIIRDSLPNATY
IGFTGTPISSKDRSTIEVFGNYIDIYDMTQAVEDGATRPVYYESRVIHLKLDEDILQLID
AEYDIMAQNAEPYAIEKSKKELGQMESILGAEQTIIALCEDILKHYEENRQYELTGKAMI
VAYSRPIAMSIYRKMLQMRPEWTEKLGVVMTSGNNDPEDWREIIGNKRHKEKMAKKFKDN
DDPLKIAIVVDMWLTGFDVPSLATMYVYKPMTGHTLMQAIARVNRVYKDKEGGLVVDYVG
IASALKQAMNDYTNRDKNSYGDTDIAKTALPKFIEKLEVCRSLMHGFDYSAFLTTESDLV
RAKTITGGVNFLSSIEKPKHGVVMDGEDWRYPIVAADPSPGYAESPSKKDLFIKESMLLR
QALSLCRSLLTAEQRFEAAYFEAVRTLLTRITGEGKPLSLKEINARVNELLKASIQSEGV
INLFSDVDTGFSLFDPKFLDEIAKMKERNIAVEILKKLLAEQVSLYRRTNLVKSEKFSEM
LSRAMKAYLNGMLSNEEVIAELMKMAKDMANAQAEGDALGLSDEELAFYDALTRPEAVKD
FYQNEELVAMTHELTDMLRKNRTIDWQKKESARAGMRRMVKKLLKKFKYPPEGMEDAIAT
VIGQCEMWTDN*
>SPBDM4_v1_40849|ID:27158154| protein of unknown function [Uncultured spirochete bdmA 4]
VGRELQGPVYLRTRIILPRPKVRRKECFVTTKPDADNLVKGIMDVLTQIRAWRDDAQIAK
LEIEKVTRPRTCHVARRSRSLYRSSLPSICVRGSDTDRHNTDTMRNTRKHIRPCMLCTHK
NTFKLENQVFSMNP*
>SPBDM4_v1_40850|ID:27158155| protein of unknown function [Uncultured spirochete bdmA 4]
MNESWMLIDTIRVLGTPKAQPRVKAFVRGGHASVYTPSTANG*

>SPBDM4_v1_40851|ID:27158156| protein of unknown function [Uncultured spirochete bdmA 4]
MKTPDIQEKRQTGSVSSPPQRLPLGSLPNARRSLTRLMISFYKGEMEASKFKTILYGFNV
LLGFLKEERLDQIEKRIKALEDAAKINTKGEVK*
>SPBDM4_v1_40852|ID:27158157| protein of unknown function [Uncultured spirochete bdmA 4]
MLNTSDDFEASVDTSGKLERERLDRLSNIKIVSGSELMQTKFEAPRYFWEGILPDAGLCV
MAASKASGKTMFLLQAADAISKGRDFLGIPTRLTKTLFVELELTKRKMQERLFKMGIVLD
DNLGFAFNGKQGDVWLQTIQDAVVEYGYKLVVVDVLQRLWPEKADTNSYQDTYALLGPLR
AIANELQCLIVLVTHCRKADVVDYIDGVIGSVGIVANADVILTLKRERGASEAALFIDGN
DIEHKELALNFNTDPLGFSLSNASPAEIGLTSERRDVLGAIRSLRGSARPAQIAELLGKD
EKAVSKLCIKLVTDGLLKNTKYGTFALFDPEKVGEVGEPGEVDRNASPTSTTSPGIFGGD
EAKLCVS*
>SPBDM4_v1_40853|ID:27158158| protein of unknown function [Uncultured spirochete bdmA 4]
MNIIPQLPDWMTEKLGKIAATTDIWALEQESITILASEIRTFPELAEIFPKTFRTKVQHG
KPRELEPLERASIIIAAYDWQAVLDLWKGSTIEEKVAAKFGALAFALRQGGEIC*
>SPBDM4_v1_40854|ID:27158159| protein of unknown function [Uncultured spirochete bdmA 4]
MAAANNTRAAELLEVAKPYLLKLFENAPAYGTEGIACVFHDGEITRIDVSASVQRKVAPH
AAREGGMK*
>SPBDM4_v1_40855|ID:27158160| DNA binding domain protein, excisionase family (fragment) [Uncultured spirochete bdmA 4]
MTEFLSVEEVAALLKTTRGYVYKLVCERRLPCYKPNGGRLLFDPVEIEEFIRSGRRSTHR
ELSDRATAILNSRRK*
>SPBDM4_v1_40856|ID:27158161| protein of unknown function [Uncultured spirochete bdmA 4]
MSTEFEREIGSSMMPNENPGDLVENEKWIEACKKRLTILRPETEEAKHEFLEEAARTLKI
GQEELITKAKKQFRDNYIEDDEKAIRREETKIAAYGKPQEYLKKAEEAAEALLAENTSSV
KPIDNDFPAILSILLITKQTRTSQAKRAEKALSKISEYDIMRTIRMMFENKDSIEELYKA
WGKPWGDINALRSEARRRGIREELLEVHAIQSGEADRLQEVGKKVVEGGRKGARVASYGS
EKSRRKDQVLKKYGEIMGQPHKMPESEVKKQIAKEFRISLYTVRRYLQNR*
>SPBDM4_v1_40857|ID:27158162| putative Site-specific recombinase, phage integrase family [Uncultured spirochete bdmA 4]
MSVRLRQKKGHLYLDTYERGKRSWEALHLTVPKDAAGRREVMALAEEYRRKREMQLFAQR
HALTDPVKNKMKLIDFAEEKAKGYDKAAHLPKSIKYLKPYASETLLADVDERFVDGYRAY
LLEQKTLGATSARHYLDAFRALMAQAVRERLIEVNPAKNVRTIKIKEAKRPFLTVDEIQA
LFNTPITGETLAEDCKRAFLLSCFTGLRLGDLKTLTWGDIQREPEPAISKIMNKTGEIIT
IPLVPSAWRILDDKQLHRRDELVFPALAKSTSEYKPLDRWRKTAGIDKKFGWHAARHSFA
MMSLDVSGDIYAVSRLLGHSDIKITTAYLHFMDARKRQILDALPDIDTQEKGEALKFKAA
E*
>SPBDM4_v1_40858|ID:27158163| putative Phosphoesterase PA-phosphatase related protein [Uncultured spirochete bdmA 4]
MALIESMHAAELHYIQQLQSFLGPGLRIPMQIVSLFASEAFIIAIVPAIYWCVHRKKGAE
LGLLILGSTFINLWIKQLLAWPRPYEIIPSLGLAKESTYGMPSGHSQMSVVFCAYAVQFL
PKKWRIPVLTGVPLLIGFSRVYLGVHFPSDVIGGYIIGAVFFMLFKLFGSDIKKALQHSE
WRLRVILAAVLSFIMNSLLPEDTMISGAFLGAAAGFALASRNIPIDQQDTVRQKIFRYLA
GLASTGIVYFALKFASNPLNALSNNSQAQLIHFVRYAIVGGWVSYGAPFIFLKLGLATRE
A*
>SPBDM4_v1_40859|ID:27158164|apbE| Thiamine biosynthesis lipoprotein ApbE [Uncultured spirochete bdmA 4]
MRFSKAFLLRSVAAAVLLAAGTVAAPFLYAEELSKTDFVLGTVCTIRLIDGGTNATLGEV
FSRLRNIENHMSVNKDDSEISRVNAMAGKEPVQVSEDTFYVVSKALEYARLTNGAFDPSV
GPLVKLWNIGNGGEKVPPEKEILAAKALVDWRQVVLDADTRKVFLKKPGMRLDLGAIAKG
YAADEVEKILADHKVKAAIVDLGGNIFVFGGKKDKSPWRVGIQNPESARGEYLGIVTGGQ
MTVVTSGVYERYFFENGKRYHHILSTQTGFPVDNGLVSVSIVSKSSIDADALSTSLFILG
IEKGMEFLRQFPDTYAVFIDKDNKVYLSPGAGKVFTLQDKDYQLAEK*
>SPBDM4_v1_40860|ID:27158165| Beta-lactamase domain protein [Uncultured spirochete bdmA 4]
MASTVWRLKVGPIGVNCYIVASEGAVLVVDPGAEPEKIHSFLQTQALAPTMIALTHGHLD
HTAAIPGLFEMLGTELPIAIHPADAHYLGEAGETTNRELFDAIGAPSYFHAFWKPLPKAA

VFLSEGMMVPGTSLRVIHTPGHSQGSICLYEAVLNAGSYGADEEAREDAAEKSLGWSCLI
SGDTLFRDGVGRTDAPDSDPVALERSLKKLAHLSFATLVFPGHGPRTTIGRELPAALRD*
>SPBDM4_v1_40861|ID:27158166| protein of unknown function [Uncultured spirochete bdmA 4]
VPDDTAASKFNEKNLEAFLEGLLEELKHPQDAKLLEDVRRAFRKKVPFHLRSYASALMIL
RAAGIYRPKSPKKVLQEPAPRSRNAMEQTNVEPKQELQNKAMVSLFVSMGKRHHLKPLEL
KKKIAERTGLPQDTLGRVHLLENYSFIEVPVQEAQRIISAMVGTEINGRALEIKPAKKRS
ESFPEGL*
>SPBDM4_v1_40862|ID:27158167| 3-dehydroquinate dehydratase [Uncultured spirochete bdmA 4]
MEKNDRPKICICLTGETIAEDLRILERYRSMVDYAELRADCLDPNERFYIRSFPEKAGLP
CILTIRRLQDGGKFEDGEGVRLVLFAKALSFPKAEASANYAFVDLECDFKAPVVEEACHT
FGTRIVRSLIYRDSLPENLDTAYDQLTLEEDEIPKLTVNPQNSADFIRFLEWSLQRPKRD
QILIARGPYGIVSRILAWRLGSMWTYASPLQYGMEMAAPGHLDPQDLVNIYNFDLVDPDT
VIYTLIGQHSIAQSLSPYLHNTAFRKMGKNALLVPTPVDSFSDGLKILEMLGGKGAAITV
PFKEDVLPFLAIHSTDVKRIGACNTLVWRDSAWAGYNTDADGFERSLLEFLGKQDISGCR
ATLIGAGGAAKSIALALFRKGAHALVLNRTYSAGRDLARKYDFSYTHLDDRAIDQISEYS
NLIVQATSLGMKGESDPINFYEFKGSEAVFDLIYHPSKTVLLKRAEAAGCRTINGYKMLC
YQAAGQYKLWMNEPPPDIYAQLEQPESS*
>SPBDM4_v1_40863|ID:27158168|era| GTPase Era [Uncultured spirochete bdmA 4]
MPKSAFVAIIGRPSAGKSTLLNALCGAKVSIVSSVPQTTRNAIRGIVTRPQGQLIFVDTP
GYHISEKKLNLKLRELVLQSLSEVDLILYVIDATRDPGKEEEAIIELLSKEARRIVIAIN
KIDHPEARPLSVEEFVIPRLPDAKRISISASQGTNLSLLLDALFSLAPEGPFWYPEEIYT
DQEPVFRIGEIIREKAISHTRDELPHAIAVEYRDSSKRADGVLFARFDILVERDSQKAIV
IGRQGSVIKRIREEAEADLKKLFDYPIKLQLQVAVDPDWRKSDSVLSRMIF*
>SPBDM4_v1_40864|ID:27158169|pth| Peptidyl-tRNA hydrolase [Uncultured spirochete bdmA 4]
MIRLCAFLGNFGKEYHNHRHNVAWLFLESTRIGAELWWSSKFKGKIAQADLGPETAYFLK
PHTFMNLSGESVIEAARFYRCKPEEILVVHDELEMQFGAFGYKFSGGLGGHNGLRSLERH
LGTRDFWRLRFGIGRPDHDDIAGYVLSPFTGEQLDFLTTNVFPEAEKTFYALFSEGIEGF
EVRYKKVLSISP*
>SPBDM4_v1_40865|ID:27158170| PHP domain protein [Uncultured spirochete bdmA 4]
MPLWKGNVPWCILKQMISNMHLHSRFSDGMSWPEDISLEAARLGLDLVALTDHDTMAGVS
RFARACESRGLTAVPACEIDVYESQIEYKSELLAYFPNSDAGDCAPQTKGLLRESLQKRR
ARLEFLLDSAQELYPEKELSFQDLFKDKTGLDFNDGLANEISWSKVDLFLYLKARRCILP
EMNYKAFKKRFFAGGLLKKYKLDKPDIATVVQAVHADNGFVVIPHIGHKWDDDIALLYRE
SEKLRILLGWFRRAGIDGVELYWYNSDFKSAKINGLVREIAKPQGFFFTYGSDCHGAGSG
KHTIMKFKGHFDGFPSRKSQP*
>SPBDM4_v1_40866|ID:27158171| protein of unknown function [Uncultured spirochete bdmA 4]
MFQLTDASLERIIFAMEDQSNELLIDLETGDFIEKSEYQEENPDTASERYVEPPSWDSRR
GFAILEAFAATVASPPELKLALNAALRRGKGVFKAFRQALSNDEILSRRFQDFKLRAMRP
IVEEWIESLQEEQRLARLSEEPEDIGDIVASEIEFKVLPIKDIPFRLENFLTDYAQETFT
SLPAAIRSWSLSVLLEKAEASKDSIFISFATVDGTNPLMFGIFTLQTSKALPLCVVEGVF
GSKESAMLSIDWPLVDTISAYASKIGTALMVLEGPLFPSPISEEASAHGFVQSGSVFYKT
L*
>SPBDM4_v1_40867|ID:27158172| Malonyl CoA-acyl carrier protein transacylase [Uncultured spirochete bdmA 4]
MKTCFLFPGQGAQYPGMAKDFYETSKAVRDLFQSASDAISMDLKKLLFESDEETLRQTKN
TQIAMVTAEVAAALSAREHGIKPSGAAGFSVGEWPALAEAGVISLYDMFRLVAERGRLMD
EAGNHAGGSTMTAVLYLSPEKIEQVIQEAGLKHCWIANYNSPVQCVISGTEADIAIAEEK
LRQAGARRVIRLKVSGAFHSPLMQEAYEAFRELVEATAFSDPKISLYSNVTGKKMLTAAE
ARKYASLQIISPVRWIDEELNITADGFDQCLETGPGTVLTGLWKASGSPVSCVAVGTLDA
LVSFSL*
>SPBDM4_v1_40868|ID:27158173|fabG| 3-oxoacyl-[acyl-carrier-protein] reductase [Uncultured spirochete bdmA 4]
MRGLLSDQVCIVTGASRGIGLAIANRFREEGALVYGLSRTKPHADISWISCDITDEASID
AAINSIVQKESRIDVVVNNAGITRDGLIMRMKTEDWDAVLTANLRSAFLLSRTVSRIMLK
QRSGCILNMSSVVGLHGNGGQANYAASKAGLIGLTKSLAKELSSRNIRVNAIAPGYIETS
MTSVLPETAKESLKAAIPLQRPGTPEDVAEAALFLCSDRASYITGVVLNVDGGMGM*

>SPBDM4_v1_40869|ID:27158174|fabF| 3-oxoacyl-[acyl-carrier-protein] synthase II [Uncultured spirochete bdmA 4]
MSKRRVVVTGFGVVCPLGNDVPTFWSNIKANKSGIGPITIIDSSDLAVKIAGEVKGFNPA
LRIDPKDSKKMDRFAQFAVYAALEALESARLQKASLDPVRTGVCLGTGQGGSSTTEEAGV
RLAERGPGRVPPMTVAKGLANFAAAQIALSLDIHGPNMTVVTACAAATDAMGQAMHLIRD
GHADIMFSGGSEASIVRMCMASFINIQALSSRNDAPEKASRPFDKDRDGFVMSEGAGILV
LEEYEHAKARNAKIYAEIAGFGATCDAHHLTAPDPEGLWVSKAIGLALADAGMKPEDIDY
VSAHGTATPLNDPIETKAIKHAFGQHAYKLKISSLKSMIGHCIGAAGAIETIAAILGMNE
GYIHPTINLDNPDPECDLDYVPNKGIHMPVRAFVKESMGFGGQNAVLVITRP*
>SPBDM4_v1_40870|ID:27158175|glgX| Glycogen operon protein GlgX homolog [Uncultured spirochete bdmA 4]
MNSHWRTLPKELHLLPGNPLLPGAVAHPQGVYFSVFSRHATGMILCLYENPHDDTPTVSL
HLDPVGNRTGDMWHCYVPKLRAGTLYLWRAEGPFAPQEGHRFNPNKVLIDPYAKALTDGT
LDLCSALAYDDSAPEKDLSFSDEPNDRCMPKCVVVEDDFDWEGDTPLNYPLKDCIIYETH
VRGLTRSSSAKAKHRGTYRGVAEMIPYFKDLGITSLEFLPVQEFDSSERFRRNPITGEKL
SNYWGYAPIAFFAPKTSYAFPEGADHDSFDPSFPVKEFKYMVRELHKAGIEVILDVVFNH
TAEGNELGPTLSFRGLDNSIYYMLADDKRFYRNYSGCGNTLNCNHPVMRTFIRECLRYWV
VTMHVDGFRFDLGSILGRDSQGNLLPNPPVIESIAEDPILRNTKIIAEAWDAGGAYQVGS
FPGGRWAEWNDRYRDEVRRFWRGDDGFAPPLATRITGSSDLYLQNGKRPFHSINFITSHD
GFTLNDLVSYHQKHNEANGEQNHDGLNENYASNYGIEGPSDVPFIEEIRNRQVKNFFATL
LLSIGTPMILGGDEFRRTQNGNNNAYCHDSELTWYDWSLLTQHRDIYRCVKTLIAFRKNH
SAFRREDFFRGLSDERPQSEHGIAHADGIAGTNPPMGPDASTARLAEETPPDILWYDEQG
NPPHWEKLGKVLIARINEHTVAQNLRHSYILAFNASPEAIQIHLPHDHQPDTWGRIADTS
LPSPHDVEEGDYLVLSDQQDYVMAPRSFVLLISSIEATEENPAASG*
>SPBDM4_v1_40871|ID:27158176| putative SEC-C motif domain protein [Uncultured spirochete bdmA 4]
VKTISGADKASFFLRKGLYELAHHRPARALVFLRKSVELIPPSCESELSKALYWLSIALI
RLDQRDLAVKSLASAQKLRRQSYARKLYVRNVNAYGMIKRPTNELDDLYAFVSIQLSSYL
LKRSNHRFGSEAEHSAVLKLILETWKRLNESEEFQNLECGEKLHLFKKLKIDFPSFSAYT
VGLRYAERQMLRANIDNVQTCSCGSGLPHMQCCGRIRGLSEL*
>SPBDM4_v1_40872|ID:27158177| RNA polymerase sigma factor [Uncultured spirochete bdmA 4]
MTEKKRVRETQFSADFDDENVLSMYLREINRIPLLSREEENDYATRAAAGDEQAKAILAK
SNLRFVVNVAKKYQNQGLPLADLIAEGNLGLLNAIDHYDVTKGYHFISYAVWWIRQAILK
SICEKSRMIRLPLNRANELVQIEKARALFEGELSEDAEIREVAEFLEMEPEHVKDIISIS
RDLISLDAPVYDEKNSTTVGELIENEHHESPEQTAIQSSLRDDINKVLGTLTVKEREIVE
YRFGLNGKRPMSLKEIGDRMHLTKERIRQIEKAALKKVSVPETMEILQAYVA*
>SPBDM4_v1_40873|ID:27158178| putative Short chain isoprenyl diphosphate synthase [Uncultured spirochete bdmA 4]
MNSSTYASFPVFAKLTKKAISERIAAIFQENRAMFGNLPEMGDRLAADMEDFACSGKMLR
GALAFVGSRLFRGQEESANAQTLSLAAGLELLQAGLLVHDDIMDHDEKRRGKPTFHFRIK
NWLVNDHFQIDEKLALDMAEAQGICVGDLFFFIAWQEITKLTPQVSSLVSREHAIVTLAQ
MRDIAMGYDEQFPDIDDVIDMYHHKTARYTVALPLLAGASMCQRTDAEVLRLLETFGEAL
GIVFQLQDDRLGLFGTEQSIGKPVGSDIKEGKKTPYVLAMLDRINDEERTRFYSMYKSPD
IDSGDIAWLRGLMITNGVDAYIQEMIEGHLSRARVALEELSKLLTERAESIRLLESFIDY
SGQRKS*
>SPBDM4_v1_40874|ID:27158179|gyrA| DNA gyrase subunit A [Uncultured spirochete bdmA 4]
MMAESSNIIPVPIEEEVKTSYLNYAMSVIVSRALPDVRDGLKPVHRRLLYAMEELGLHNN
APTKKSARITGDAMGKYHPHGDLSLYDALVRMAQDFSLRYPLVQGQGNFGSIDGDPAAAS
RYTEAKLSKLGEEMLADLDKETVDFVPNYDESLKEPSVLPSAIPNLLINGSSGIAVGMAT
NMAPHNLREVGQAIEAYIDNHEITIDGLMNYIKGPDFPTGGILYGMQGIRDAYETGRGRL
TVRGRFVIETMKSGREQIVFTEIPYALNKTTLVTRIAELVREKQIDGISDLRDESDREGI
RIVLELRKGAITKIVLNQLFMHTPLQSTFGVINLALVDGAPKCLNLKELIVYFVQHRFEV
ITRRSQFELKKAEERAHILRGLVIALRNIDEVVAVIKSSKNIDTAKARLRERFDLSEAQA
QAIVDMRLGRLTSLETEKILAELKEIETRIEYLKSLLADPGAIYQVIKQETHDLAEKYGD
ERRTEIVPNQVEQINLEDLIKPEEMVILISNKGFIKRISVNQYRSQGRGGKGSNSASLLD
DDFIQQLFIANTHDYLLFISSWGKAYWLKALEIPEASRQARGSHVRSLLAISEDEEITAV
VDFSDFSEHEFILMGTLNGVVKKVATKEFANAKTRGIIGISLDEGDRLVSAILTGGSDEV

VLISRNGLALRMEESQVRQMGRTARGVRGISLQEGDELAAMLRVDSQESMLIITQKGSGK
RVRYELFTPHARGTHGQIIYDPDEASGEVIKAVSVREEDEVVAITSTGKTIKLSVSSVRE
MGKAAHGVRIVNIDPPDSVIGMDKIIQQQE*
>SPBDM4_v1_40875|ID:27158180|gyrB| DNA gyrase subunit B [Uncultured spirochete bdmA 4]
MESTTSNYSASTIQVLKGLEAVRKRPGMYIGSTSIEGLHHLVYEVVDNSIDEALAGFCDN
IVVALERQDIVRVEDDGRGIPVDIHPTEGISALELVMTRLHAGGKFDKKNYKVSGGLHGV
GVSVVNALSSWLEVYVYTGGKEYYQKYLRGVPEKPVVMIGETDKRGTLVRFQADPEIFEE
TVYSFDVLSNRLRELAFLNKGVKISLIDERLSQLKRIEFRFEGGLREFVEYLNKNKSIIH
KDVIYFSGVRDEVEIEVGIQYNDGYNETMFSYVNGINTREGGTHLIGFRNALTKVINDFF
KKSKHNKKIEENLSGDDVREGLTAILSVKVMEPQFEGQTKGKLGNSEVKGIVEGFLSDQL
DLYFQKNPDVINIILEKCTTAARARIAARQARELTRRKSLLDASSLPGKLADCQEKDPTK
CELFIVEGDSAGGSAKMGRNRVNQAILPLWGKMLNVEKARLDKVIGNDKLQPIIASLGTG
IGEDFNVSKLRYHKVVIMADADVDGSHIRTLLLTFFYRYMTELIEKGHVYLAMPPLYRVV
YDKEAHYAYSDEEKDQLLSETNKDSAKISVQRYKGLGEMNPEQLWETTMDPERRLIKRIT
MEDAILAEEMFVTLMGEQVEPRRLFIEENALAVSNLDV*
>SPBDM4_v1_40876|ID:27158181|dnaA| Chromosomal replication initiator protein DnaA [Uncultured spirochete bdmA 4]
MDDDGNEIIWQKALEIAKSQVPESEFIMWFRLAYLGFENGTIRLRANNMFLRDQFVRKYS
KFMKEIISSLVGMPTELSVEAQPAPIEQHFVQQKTASQANKPSPKSEKAELFQPDAASLN
SRSKAEEDFAIETAYASPSARMHEPRSSNLQPRYTFESFVVGENSNFAFNAAAAVAEIPG
TSYNPLLIYGGVGLGKTHLMHAIGNRILERHPELHIICVTAEDFTNEFIKTIHDRTTNDF
KNKYRNIDVLLIDDIHFFQSKHGVQEELFHTFNALYDSNKQLVFTCDRPASELKDFSERL
KSRFMMGLKTDLTTPGFETRAAIIRKKLDLYKQSFSEDVINLIAKSIETNVRDLDSCIHQ
IIAYADLIKVEPTYEIAQNVIKQLTNAFKPPSIDVSSIIKTVADYYKLSISDLKGKKRSK
NIALARQVAMFIIRETTDYSTTEIGTEFNGRDHTTVMHSCQKIEDLIKFDSLFASAVNRI
TQECKENSVS*
>SPBDM4_v1_40877|ID:27158182|dnaN| DNA polymerase III subunit beta [Uncultured spirochete bdmA 4]
MKFICDRDTFAKEIAIAQDIIASKNAFSIMSNVYLEVMDSKLFIRATDAKVGFETEIPIS
DAEPGSLTVFCDKLLAITNSIPEGDMRFEQHESSIEIRPLSKKVRFQLKTIAGDKFPEIP
RIGESHYFALQAHDLRKMISQTIFSVSNDETRFFMNGVFMEKLPDGELAMIATDGRRLAF
ISTQEADIPDFQSAIIPPKVLSVILKRASDEGQILMAVNEKNVFFRFGNYQLTSYLIEGK
FPNYQKVIPKDQHFKFIVARDELQAALRRVSLFVEKSNKVIFNVSSKGLVLESEETELGA
AREELSCEYEGDDVVILLSLRYLEDPLKAMDSDNLAIEFSDPSRAITLRPEPDSSYFHII
MPMQQP*
>SPBDM4_v1_40878|ID:27158183|recF| DNA replication and repair protein RecF [Uncultured spirochete bdmA 4]
MAFEQIRIARFRNIEETELKLGAGNIFLLGENGQGKTNFLEALYCLSYGNSFRTHQDREL
PMHGNSEFLLRAAWSELNSPAQEQISLRYSIEGRKEVRLNEKPLTDRKELVAHNPAVVFC
HEDLAFAAGVPEERRFFFDQCAGMLWLEYIDLLRFYRRVLKHRNMALKTDTFSILDTLDE
QLAHYGQEIMAYRKKLSQLFAQRFPESYERVSRLGTEVAIHYVSSWPEEASVSEIIEVLA
ERRSKDIEAATSLSGPHRDRWIFIREGRNFSTFASTGQLRLASLILRIVQVHLYAELSGS
EMHRFPVLLLDDVLLELDVAKRRRFFSLLPEQKSGAQAVFTFLPEEPWHEYADDTTIVYR
VSDGRFSREEGL*
>SPBDM4_v1_40879|ID:27158184| protein of unknown function [Uncultured spirochete bdmA 4]
MDDFRAKKASEILSRFFDDTTLQAATQFEMFRSTWKQIVGQRLADHSKPKSILHRTLLIS
ADHAGWIQLLQLDQERILARIAKHYPELEITSLAFTVEEQGDVLPAEALPSAEPATKASA
AADNGPEATSKEAQSGPEMKTPLPEPLEEIFLRMQRARVSTRRTKGN*
>SPBDM4_v1_40880|ID:27158185| protein of unknown function [Uncultured spirochete bdmA 4]
LTKDTIAVYTPPPDCAIFRLQSKKFGGPMQPFVTRRMDHETHLSAEPS*
>SPBDM4_v1_40881|ID:27158186| protein of unknown function [Uncultured spirochete bdmA 4]
MNDIFEQQTNSKEDRRRTILAVVLSTLIVSVGFMVQNALFPPSEQQTTQAPAQTSSQGTE
LPAQNNQTLPGAVAIVPAPVPVQAMGTAAGSTIPVPAAEQEYIVETDVVRATFTNAGGEL
VSLTLKKHKDMSDSRGGVDMVVPGSQGSEGLSLSFGSTPSPMRELMNTQYLGENKNTIAF
SRMFEAPLAGSDQKVPFVLKKIFSFNDGEYMFGLAVSIEQPDGKPIVLGSNGIAYRIDLG
PQIGPRFDQLPKNADFRKYIAEVDGKKKTESPKANTATVIAPSASWLSLSGKYFAFIAVP

KAPLSGYEIETNQDPLIKQTNTLGLLRSSFAGASTTDTYYFYFGPKTSGELGKYE*
>SPBDM4_v1_40882|ID:27158187| membrane protein of unknown function [Uncultured spirochete bdmA 4]
MEGSGIFGWLENFLKLLLNLFYSIIPNYGVAIILVTIVIKALFFPLTKKSSFATARMAEI
QPKMQELQTKYKNNPQKLNQEMAELYKRENYNPMSGCLPMLIQFPLFIAMYNLFNNHFDL
RGAMFIHGWITDLSLPESIINFGNFRMPIVGWNDLRALPIIYVLSQFLYGKFTQTPQSAQ
MDSQQANQMKLMTYGMPLMFFFILYDVPSGLLIYWITNNILTIAQQVVINDLMKKHKIAR
AEVAVAGGPAGSAAAGAPSKLAGGKSSAVDTKAKPTGRSAPKEGFSEKVTKWLEEKAGEA
EKAGKSGSAGKSGSAGKRGGSGSSKASRSRKNSPKQ*
>SPBDM4_v1_40883|ID:27158188| Single-stranded nucleic acid binding R3H domain-containing protein [Uncultured spirochete bdmA 4]
MIYEFEGKTEREAIELAAQELGLDTSSFDVEIIENQGGGLFKKGKVRIRVHTKDSPIPIA
REETETKGEASEQRQVEPIPMDDFERKIVEWTKEVITRMGYTAELSVAFREPNKLGLRID
TESASILIGKKGRNIDALQLLANVYAGTLGHNDIKVVLDSENYRLRREEALVRIAYETAE
EVRRTGRSILLEPMNPFERRIIHTTLNDIIDIETKSEGEGLMKQVRVMMKGRK*
>SPBDM4_v1_40884|ID:27158189|rnfC| Electron transport complex protein RnfC [Uncultured spirochete bdmA 4]
MSIRTFRGGVHPPERKDRSQGTPIERVTSVPQVVIPVNQHFGAPIQPLVKVGEYVKAGQK
VADAEGRMTVPVHASVAGVVKKIEPRMQSNNTEGLCIVIEPKDPPEGESAQDYMLPLDPF
ACSKEEALARIRAAGIVGMGGAGFPAHVKLAPTKPISYVIANAAECEPYLTIDERALLEI
PEKVVDGVVIVMKTVNAPEGVIAIEENKKHAIPYVQKAIDENPQVKAGMKIRIQELRTKY
PQGGEKMLITAITGRQVPSGGLPMDVGCVVSNVGTLCAIAEAFREGKPLIERGFTVTGGA
CKTPKNLVAPVGTLVSDLAKAGIVEIDDQSLGKVIFGGPMMGVALPSYEIPVQKNTSGVL
LMSEQEAVQYEEDTCIRCGRCIRACPTSIFPALLAIAIESGNYAEAEKIGLMDCIECGTC
SFVCPAHRHLVQRFRVGKQVLRALKQKEAQNVR*
>SPBDM4_v1_40885|ID:27158190| Electron transport complex, RnfABCDGE type, D subunit [Uncultured spirochete bdmA 4]
MSDRKMRLSSSPHAFSPVDTPRIMLSVVIALMPATAYGVYLYGLPALGVVVTSIAAAVLS
EFLFRKIIKASTTVGDFSAVVRGLLLALILPPSTPLWMVALGAIFAIVVAKEFFGGLGAN
PFNPALIGRAFLLMSFPAAITSWTMPKGLSLAADAVSAATPLNLLKQGNALTDVAKYFGA
NDTGAFYRQLFMGFRSGSIGESSILLVLLGGLFLLAIGVIQWVVPLSVLASTFVFSWILG
MDPVLGLLTGGIVFGAFFMATDYATRPMTPYGQAIFGIGIGLITVLIRKYGGYPEGVTYA
ILIMNTLTPFLNKLRMKKYGFVPPPKPARPSKEATK*
>SPBDM4_v1_40886|ID:27158191| Electron transport complex, rnfabcdGe type, g subunit [Uncultured spirochete bdmA 4]
MKKDMTKLGVTLMLFASLACASLAVVYAFTKESIDKQSEIQLQASLKELFPQATAFEPIQ
DLVSPDPNIKFEAAYEVKSDMAPLGLAMKATGPSYGGAATLLVGIDLHRSIAGVRVMALN
DTPGLGMNASSPTYYVDKTKKVTFLGQFNEKFLTDPFEVKKDVVAITAATISSRSLTKII
KLAGDAAIAYMDQKAISAPAENGASADAGTDANAQPQAGGK*
>SPBDM4_v1_40887|ID:27158192| Electron transport complex subunit E [Uncultured spirochete bdmA 4]
MKKLYSIFKQGIIFDNPLLMLMIGLCSAIAVTTNVSNGIGMGLAMTFVLLFSEIIISLFR
KLIPSMIRIPIFIIVIAAFTTMVDLVMKAYFPDLSKSMGVFIPLIVVNCIIMGRVEAFAS
KQNLASVIADALGMGLGYTWVLIAIAAVRELLGNGTLAGIQIMPASYQPILFFILPPGGF
FVFSLFISLNIWFKKRMEASARNRELVAKKEAA*
>SPBDM4_v1_40888|ID:27158193| putative inner membrane subunit of an electron transport system (fragment) [Uncultured spirochete bdmA 4]
MMELGKIFLLALLTNNIVIMRFLALCSYIGMTSDVGQSIGMGFAVTFVTVLATAATWPIY
HYILVPLNLTFLQILIFILVIASLVQLVEFYLKKNVPGLYSAMGIYLPLITTN*
>SPBDM4_v1_40889|ID:27158194| Electron transport complex, RnfABCDGE type, B subunit [Uncultured spirochete bdmA 4]
MIETILLTIGFSAVLAFFIGIALAFFSQKFKVERDPKIDEVRGALPGANCGACGFPGCDG
YSEAVATGRAPTTKCSVGGSATAETLAKIMGIEGGAAEDRVAVLLCQGTVNKTVSKGQYS
GIPTCRAAKLSTGSVKTCAWSCQGFGDCTKVCKFDALAMEEDGLPHVNYDNCTGCGLCVE
ECPQKILTLVPRTRVGSIVLCSNRSTVKASVIKTCKVGCIKCELCVKSCPEGAITMVNGI
PVTDYAKCTSCGICVQKCPTKCYKMLETDVFGTKKEVPVVEEAQA*
>SPBDM4_v1_40890|ID:27158195| protein of unknown function [Uncultured spirochete bdmA 4]

MFVQMLSDNVDMGACHVRVGGWPGGHRRLTSVCFFLLFLALAFQPAFGVVSAPPLLGLAS
SSARQSVLNAAESYLGVPYRFGGSTKSGIDCSGLVYVSYLQATGIKIPRTVDTLARWSLV
IPVGELEPGDLVFFDLQAKGGASSAQAPTSVSQAASFLSKADHVGIYIGDGFFIHAASSG
PHTGVIQSSFNDSEWKQRLLFAGRALPTSTLSGFAFDWGASVSFAQLETLEGGPLAFVRG
LSGWGEVSFPLSKNFSAGLRAGADWDRYLGVVRVPVELDIGQISGFSVFAGPALTLGSPD
MDGRLYSPGDFFIATGGLRWSPLFFSSGAQRFGTYFELRYDYYVPAEGQTEAPQTDLRSC
LNFSIGIRLRTVKY*
>SPBDM4_v1_40891|ID:27158196|metK| methionine adenosyltransferase 1 [Uncultured spirochete bdmA 4]
MTQPLLRPFTSESVSEGHPDKVCDQIADAVLDACLAKDKHSHVACECFATTGMVLVGGEI
TTRTYVDIQEVARSVVREIGYDNPDYGLDWKSMAVLNTIHNQSPDIAQGVEGRGLKEYEG
RQGAGDQGMMFGFACNETPELMPTPIIFAHKLLQKASALRKAKEISWMRPDGKSQVTVEY
DGYKPVRISAVVLAQQHDEKNGSGHYLTYEEIRETLIEKVIKPVLGPSGLLDDNTKYFIN
PTGRFVIGGPAGDTGLTGRKIIVDTYGGMGRHGGGAFSGKDPSKVDRSAAYMARHIAKNV
VAAGLADRCELELAYAIGVPEPISVLIDTFGTEKVEETAIEQAILKVFDLTPQGIVEALQ
LKNPIYRATAAYGHFGRDNFSWEKTDKVEALRKAIR*
>SPBDM4_v1_40892|ID:27158197| conserved protein of unknown function [Uncultured spirochete bdmA 4]
MMIDTIRGAIASINGQDAVKISDWRLVERKLQLQERYYIAAKAEQARVVDECSYSLTVYV
DTHEDNKQYRGEATITIQPTFSLEDCTAKIRQAVFAASKSHNPWFGLPTPSALKVTLPVS
GFEALSPSDQVEVVRKAFYSAAVPFAGSARINALELFIIKEQKRLINSCGVDYSATIWRG
YSEFVVEADSPNGPVELFDDIEFSEPDTARLADTISARLAQVRDRAHAAPMPTLQGLSVI
LRGKEAEEVFGWFFHNSRTEMIYSKASAFGLGKNVQKASDSEEVAEPLDIWAEPVISGLP
SSAAFDIDGFPLERTTVIEKGVLNTLIGSVRHADWLGVPRKGAFPLFSVSSGSMSLEAMH
AAPYLEPVMFSDFQLDPVTGDFGAEVRLAYYFDGEKVIPVSGGSISGSLGALRASMRRSA
ETAVATLSRCPVAIMLQGAIITGMN*
>SPBDM4_v1_40893|ID:27158198| TldD/PmbA family protein [Uncultured spirochete bdmA 4]
MQKRSHSPFLLAAKPLLAKIVGHLRPHFDYISVLATDDRGLSYFALPGETRTAEPMWVQR
GFVFRAQRDGKVAEYACAVLPGDSAAKAAADIRARLEALLTDPTLITFPQIPDEPATSEY
LGEIEEDPFAANPEAILIRLAALREKLTDGKVVVMAQSRYECVDVSRLFLSPNRSLMQSF
LWSQAYLFGVARRGQMSKMSYRPASGRKGLEILRTLETSVPELARELSDLLDAVPMEPGE
YEVILDPDMAGTLAHEAFGHGVETDMFFKKRARAVEYLGKRVGSDLVTMYDGAAGVDQTG
SYLFDDEGVFSSKTCVIEKGIFKSGISDTLSALALKIPRTGNGRREAYSHKAYARMTNTY
FAPGASSLGDMIASVKHGWKLSQLNSGMEDPRNWGIQLICMVGREIKDGKFTGRVASPIV
CSGYVPDVLSAVSMVSSDFELGGSGACGKGHKEYAKVSSGGPYVKTRMRLG*
>SPBDM4_v1_40894|ID:27158199| Type II pantothenate kinase (Pantothenic acid kinase)(PanK-II) [Uncultured spirochete bdmA 4]
MTIGIDIGSTTTKAIVVDHEDIIIKAKTRAEDPLTAATGIIGKITQENNLHINDIKKIIV
TGVGSSGIKNNIFDIETERIDEISAIGRGGVFLAGIDPIMIVNIGTGTVIVEYDHGESRH
FGGSGVGGGTIIGLSKALLNITGFSEIMDLASKGDASRVDLQIQDIVSETISFLGKDATA
ANFGKMEEGASREDIASALINMVYQVIGMLAVFAGRAEKMDKVILTGNGSNNMLGRKVLE
AITAMYGIEFIYTQDAEYATAIGAALSNYPHHVTYKVS*
>SPBDM4_v1_40895|ID:27158200| PilT protein domain protein [Uncultured spirochete bdmA 4]
MFYLDTNTCIYFLNGTCASIKKKIMETPPSEIAIPSVVKAELLFGAYKSKRKKENIEKIE
QFLEPFEIVPFDEVMTYVYADIRYNMEMKGTLIGPNDLFIAAIVKFHEGILITNNEKEFK
RLEGLKVENWIE*
>SPBDM4_v1_40896|ID:27158201| conserved protein of unknown function [Uncultured spirochete bdmA 4]
MPQISLYIDDKTLKKVQNAAKQHHTSISKWVAEQLKMKVDPSYPAHFEELFGSIKDITFK
EPEEIHLSADLPRESL*
>SPBDM4_v1_40897|ID:27158202|lpd| Dihydrolipoyl dehydrogenase [Uncultured spirochete bdmA 4]
MYDFNLLVIGAGPGGYEAAIRASQLGLKVAIVDKQWLGGVCLNVGCVPSKALLKNAEVAY
TLRERGKEFGFSFDNLNLDYAAAVRRSRQVSDRLTRGVGFLMKKNNIAVHMGAARLLDKN
TVEITDKTGQVQRVSAENILIATGSQPTMPPAFPVDGEKVLTYREAILQEKLPTSVVIIG
AGAIGLEFATIWSAYGTEVTLVEMLPRIAPLEDEEVSAELTNAFNKRGIKTLTGTRVLAI
RTTEAGVKVSVAGPNGEQTLEAEQALVAIGFKPNTANLGLEAIGVALTEKHFIQVDDRMA
TNIPGIWAIGDVTGKLLLAHVASAQGILCAEAMAGKATKPIDYSMVPRATYSQPQVASFG

LTEAQVREKGLAYKVGKFPFMANGKALGLSDSGGFAKLLTESQTGEILGAHLIGPEVSEL
LGELTLAWQNELTVTEIGRNIHAHPTLSEVVMEAAENAEGRAINI*
>SPBDM4_v1_40898|ID:27158203|acoC| Dihydrolipoyllysine-residue acetyltransferase component of acetoin cleaving system [Uncultured spirochete bdmA 4]
LATEILLPQWAMGLMEGTVAHWLKAVGDYVETGEPLAEVEEAKVTGEVSAPAAGYLLKIL
VQEGETVPVRSPICLIGTKEELQAQAQTPQETSQTTSPPTAASAAPSPALTKTPEAQPGG
SKQVTPIARKLAAEYKLDLSTIQGSGPGGRITEADVRQAYAMHGASKETEIPLAGMRGAV
AQRMAESLHNSAQLTITMQADVTELLRQREALKGSEKSNTAESGGGAVNVTLTDVIIKAT
TLTLKKHPRLNGWVEKEKIRLPSEIHIGLAVALDEGLIVPVIRNADRKTLREIAVESKQL
AQKARNKALTPDETFGSTFTITNLGTYGVGFFTPIINPPEIAILGVGAVNDCPVSQNDVL
AWRKMLPLSLTIDHRAIDGAPAAAFLRDLKDCLEHLDLQTL*
>SPBDM4_v1_40899|ID:27158204|acoB| Acetoin:2,6-dichlorophenolindophenol oxidoreductase subunit beta [Uncultured spirochete bdmA 4]
MHEITYLQSIDEAQREEMKRDSRVFVMGEGVAHGVMGTSVGFLKEFGKERVRDTPISESG
FTGVGIGAALVGMRPIVDFTIASFVYVAMDQLISMAAKCTYLYGGQAKVPIVFRACMYYG
VSNAAQHSDRPYPIFMGVPGFKIIAPSTPYDMKGLLKTAIRDDDPVISFEDINLWGIKGT
VPDEDYTIPFGQADVKRKGTDVTVVAIFSGVPMALAAADELAQEGVSVEVVDPRTLVPLD
KAAILESVKKTGRLVVVDPAHKTCSVASEISSIVAEEGFWSLKAPIQRVTTDDIQVPFST
ALEPQIYPSKEKIIAAVHKTLQ*
>SPBDM4_v1_40900|ID:27158205|acoA| Acetoin:2,6-dichlorophenolindophenol oxidoreductase subunit alpha [Uncultured spirochete bdmA 4]
MNLTNEQLIEMQRRMLRSRRFEEKVRDLVAKGELPGAAHLSIGEEGEIVGACMALRPDDY
EMGTHRSHGHPIGKGASLKPLMAELLGRETGVNHGKSGSMHLADFSVGSLGETSIVGSGL
PAAAGAALGAKMQGTGRVCLCFFGDGASNQGTFHEALNLAAIWKLPVIYLCENNGYAMTT
AASYSVSVKNIAERAKGYDIPGVVVDGQDPIAVYEVVSQAAERARAGLGPTLIEAKTYRY
REHAEGAMFEALAKSLGYRSDEEYERWLKRDPIVLFRAKLIESGVLTEAQADQLDQEVQE
EVLAAEAFAKASPYPEAHEAYEGLYTNPISA*
>SPBDM4_v1_40901|ID:27158206| NADH oxidase [Uncultured spirochete bdmA 4]
MDFSMSMSPIKIGTMEVKNRFVVPAMGTNFANPDSSVSQQLIDYHVTRAKGGYGLIIVEV
TAIDPLGKAIPFEPGIWDDSFIPGWKKLADEVHKYGCKIAVQLHHAGRQTTSGTLGGNQP
VAPSPIPCPVDKEMPRELTTEEVYELIEKFGNGARRARDAGIDAVEVHGAHGYLVAQFMS
AYSNKRMDEFGGNLSARMKFPVEIVKNIRRKTGNGYPIIFRMSGDERVPGGRTIDESRAV
ARIMEEAGVNAVHVSTGVYGSIAWIIPPAAVPTGFNLYAAEEIKKSVSVPVIGVGRINDP
LLAEDALRTGKADMIALGRASLADPEFPNKVASGELESISPCIGCIQGCIGYIFDPNHGK
TSCLVNPFVGKEGEMKIEKTNKPKKVMVVGGGPGGMEAAWIAAKRGHRVTLYEKEKILGG
QFRIGAISPTKQEVLKSLRYYLYEGEKYGVEFKTGVEVSPDLVKAEKPDVVILATGGVPL
VPHIKGVDNHGIVRAIDVLEGKVTVGEKVLIVGGGMVGVETADFLGEHGHKVTVMDMLPG
IGMDEEAAVKVFLFERLKNYGVASITNATVKEFLDGGVVYEKDGKEEKIQGFDTIMMALG
VKSYNPLEKEIKGKVPEVYVIGDAVKPRKALEAIAEAASTAVKL*
>SPBDM4_v1_40902|ID:27158207| conserved protein of unknown function [Uncultured spirochete bdmA 4]
MEELFRTLGMNDNERDVYLSVIKAGKISANQVAKETGINRTTVYSIAKKLEKMGVLAFDL
GQKVGYLVAIPPENLVTIFEKEEKKLAEKKQVAIELAKELSSLTHDKRYSVPRIRFVEED
DLEEYLYAVYPRWEESSSNSDNVWWGFEDDSFTTCYEKWIKWGWHRPNEGLRVELFFNEA
QIEKQLTKEYSGRKMKTLPNHISFDSTLWIVGEYLIMVQTRSRPHYLVEIHDPVLARNQR
ELFKGLWALAPELSCT*
>SPBDM4_v1_40903|ID:27158208| conserved protein of unknown function [Uncultured spirochete bdmA 4]
MPEFASAFSGQKSDRKLTHQELVRAIRYMIAAEYEAIQLYTQLAESIDNKLASEVLEDIA
NEERVHAGEFLRLLRELAPDEAKFYAEGAGEVEEEIEKFGKSS*
>SPBDM4_v1_40904|ID:27158209|cobB| NAD-dependent protein deacylase [Uncultured spirochete bdmA 4]
MRHIVFLSGAGISAESGISTFRDAGGLWDTYNVMDVCSAEAFRRNRQFVLEFYNKRRREL
ADKTPNAAHRMVADIQREFPDRVLNITQNIDDLFEKAGCEGTIHLHGELTKARCEVCGNI
WDIGYADLVGTELCPRCGASRVRHHVVMFNEQAPEYMRLYESLDELHANRGMLVVIGTSG
LVLPVEEFARYASYSVLNNLEPQAAIDDRVFSVRLYEKAGTAAPKIAELARRFLDSDQRG
PRDGRAETAS*

>SPBDM4_v1_40905|ID:27158210|gltL| glutamate and aspartate transporter subunit ; ATP-binding component of ABC superfamily [Uncultured spirochete bdmA 4]
VEKYIEIIDAVKNFDSVKALQGVSITVKKSEVVLIIGPSGSGKSTLLRSVNRLERLDSGR
IFIDGESVTDADADIRHIREEVGMVFQSFNLFPHITVLDNITLAPICVLKKNKDEAIDSA
RKLLAKVGLSDKEKAWPEQLSGGQQQRVAIARALAMNPKVMLFDEPTSALDPEMIKEVLD
VMTELAREGMTMLVVSHEMGFARAAAHRIIFMDAGQIVEEAPPDTFFTSPREERTRKFLE
HIL*
>SPBDM4_v1_40906|ID:27158211| Polar amino acid ABC transporter, inner membrane subunit [Uncultured spirochete bdmA 4]
MMKGSPFSDRNSKNSDKTTINVTDGTLIPLKDEGSLLSAWNISFFGAIALLILLPVLSPD
PYRRILSFVPDGLIATFSVTLISIFFALLIGLFAGLGRISQKRFVNRIATVYVEIVRGIP
LLVQLFYIYYALGPLLKLNGPAAAVVAMSICYGAYMAEIFRAGLQAVPRGQMEAALALGM
SRSMAMRKIIIPQTIRIILPPIGNEFIALLKDSSLVSILAVSDLLRRGREYASTSFRYFE
SYTVIALIYLVMTLFFSRLVAMMEEKLRHRGKVH*
>SPBDM4_v1_40907|ID:27158212| ABC-type transporter, periplasmic subunit family 3 [Uncultured spirochete bdmA 4]
MKKFWSVLTSVTLVAIVALGAVSLFSCAKKPAQITVATDATWPPMEYVDENKNIVGFDID
LVNEIAKAENFKVEIKNTAWDGIFAGLAAGNYQVIASSVTITDERKNTMDFSDPYVNAGQ
VLVVRKDTTGVTTLADLVGRKAGAQIGTTGAIEIGKVQGVELKTYDEVGLAFEDLVNGNI
DGVVADSPIAANFALQNATYKEKLMIVGQPFTDEWLGFAFKKGDTKTQKLFNDGLAKVKS
SGKLDELKKKWLQ*
>SPBDM4_v1_40908|ID:27158213| conserved protein of unknown function [Uncultured spirochete bdmA 4]
MNSKHAWQIDRELRLSAVPLDRRTHQSNWYTSETTFPVGDDLIALFWFSSVPGSGAPEIP
YLEMVQAMGNKGYDVSAAERLLYEGMEEARKCGILDRARFGSRESDAAAQANFEQPAYPG
SRENATARAPNPAVSPELRALTAELLHEIHCAPRDPSHPYWNYVHPAEWEAVRAEMPAAE
TSVGRRGAVPESSAIESPAAQRPPLPDNPEARIHAGWLGQLAGGAFGTAIEGYHSAQLQK
VYGRIDAYITDPETVNDDVVYELAFLDAFERCGRDITSRDIGREWVKQIPFGWSAEWVAL
HNMAEGILPPESGSWHNPYSDWIGAQMRGMVCGLVAPARPMEAARLAHIDGVVSHAENGV
YGEMYAAVLTALAFTEGEPRALLREAARFIPKRSEYAAILEKCIDTAARARSAEAAWDIL
DKYFERYNWIHAYPNIAAGITALWFGNGDMTESFRILAHAGLDVDCNAGLVGTVLGIMGG
VPEKWAQPLHDTLETYLAGKERLGIRELAARTARLAAL*
>SPBDM4_v1_40909|ID:27158214| putative ABC transporter permease protein [Uncultured spirochete bdmA 4]
MNIIVGLITGMISGATPILLAALGGAFTFYAGIFNIAMEGMMLMAAFFAVLGSFLFHSWL
VGLLFAIAGAMILALIFIFFSVTLNTDEFVTGIALNLFALGSTTYLLRQIFKVKGVFTSK
LIDSIPNIHFPLIEKIPLLGPVVSGRNLIVYLAVIATIFCYYLVFKTRFGLRLRAAGYNP
NSLDSSGVRTSRMRIQSLLLCAVFCGLAGAFLSLGYVTLFAENMTAGRGWISLAAIILVD
GHPYGIALISLLFGFFDGLGLFLQGYGVSSQFTSMVPYIATLGALYVYARRNRERERNAL
*
>SPBDM4_v1_40910|ID:27158215| putative ABC transporter permease protein [Uncultured spirochete bdmA 4]
MTRRILSGVLGVVIALVVGAIIMLIEGYEPLATYGALAQFSLGGIGPLTTTLKNSVPLVL
TGLSAAIAFASGPVNLGQPGQLVFGALAATLGGLYISLPPVLEIPLLLLLAIAGGALWSG
LAGLMKQAFGMSEFIVTLMLNMIADFFTAWVIAYPLMDRKAFSPMTPPIARSGWMPDIGP
LNSSVLLMLAAVVVMWFVFQRWRAGYEWRITGQNSLFARLGGCDVRGNFMAVMLVTGALA
GLAGGLVVMGGPHRFLKGLGANYAWDGIMIAIMANNGLVETLIYGVFVAAIQTGALGMEL
ITNVPSEIAQVLQAVLVLVIVATREYAAVVFDRLAARRQARERAA*
>SPBDM4_v1_40911|ID:27158216|tsgD| Glucose import ATP-binding protein TsgD13 [Uncultured spirochete bdmA 4]
MDDFISLEHMVKVYPPRVLAVDNVSVGFQKGEIHSIVGENGAGKSTLMKLLYGLVPFDSG
VVKINGAAVHFRKPGDAIAMGIGMVHQEIELISQYTVWENVVLGAEPVRGGIGGTLDSAR
AIELVRRKIDEFQFNLDPSAQVERISVAALQKVEILKLLYRNVSVLILDEPTSVLTPQEI
PQLFAELKRLRDKDTTILFISHRLDEVLELSDRITVMRKGKKIATVAASETNKEALAQMM
VGRQVIFTSKRTPHPRGDRVFEAKGLSVTRHDGLKLLDNVSFSVHAGEIVGIAGVEGNGQ
FELVNSVMGLAAPTSGSITVNGGEITKLPILERRKLISFVPQDRARMGASLAASILDNAI
MTHHRLEPSFSAWRGLVLNYRNARAFTDTLAKRFSVQMASRQSPFRSLSGGNQQKLILGR

EFLLSTPFFLMDQPTRGLDVGSIEYVHGEILSMREANRAVLLISADLEELFLIADRILVM
HRGRIVADLVAEQTNIEEVGRYMLEGKITA*
>SPBDM4_v1_40912|ID:27158217| putative basic membrane lipoprotein [Uncultured spirochete bdmA 4]
MKGRKYFAVIAAVMFLAAAMSAFGAGPSVKTDRVVYLINGALGDNAFYDSGEAGVKNIEK
NYKLQTRTIECNFDAAKFQPALDAAVQYADVIFVISYGFEDQLKAIADKYPNKIFVNIDT
IVENPKHTITSVFFIAEQSSYLAGMTAALATTTTSLKGINKDKVIGVVGGDTDPIVSAFV
FGFQNGAKAVDPGIKVLAKSLGGAWDDSAKGKQAALQLYDQKADVVYQVASAAGIGVLQA
AAERGLYAIGVDTNQNDLEPGHVIASAVKNVGATIEEVFKTIKDGTYKAGEVINADLASG
GIDLVFDAKQQVLPKSLVDKVNAARQQIIDGKLKVQLYNGENVWQQ*
>SPBDM4_v1_40913|ID:27158218| putative AraC family transcriptional regulator [Uncultured spirochete bdmA 4]
MNPVREIVYATPGYSFCIQYFDDRRLHFNWHYHEDIELVLIKKGEGQIHIGDLVKYYKAP
AGFLIGPSLPHGLLSIGSLQGWIIQFQEKQIKYPNAPSEFTNITNAISESKKGLSFSPPV
ITGCLPLMDDLNGSNGLEKWLLLLKVLNTLSQDKSRELCSLLPHNQDVQTDRFEQAITQI
FNEIDKTYNLEEVSRKVGMKVSSFCKTFKKRYGLSFIEYIHSIRINNAKKLLIQTKFYID
DICYESGFNNVSFFNRKFKEATGLTPSGYRKRYKEA*
>SPBDM4_v1_40914|ID:27158219| conserved protein of unknown function [Uncultured spirochete bdmA 4]
MAASTEPIQSVLVGAGNRGAEVYGQFALTHPWLFRFTAIADPNPARRTKFSRQHGISETR
SADSWERLLDIARPSDLVFICSPDRFHYEQVLAFMEKGCPIVLEKPVVVNPRQCATVSKQ
ASEKSARIIVCHVLRYTPFFSTLKKVLDEGRIGRAVSFNLQENIAWYHFAHSYVRGNWRN
TALSSPSILAKSVHDLDILYWLAGAPAESLVSMGGLRWFREENAPAGAPERCLQGCPHAS
ACPWYAPDLYLTENTDWPTSVISDDKSLAARRRALEEGPYGRCVYRCDNDVADHQDVLIR
FRNGVSASFSMNALTYDKTRVIQVTGSEGEIIGDLDGGWLEVRGFLRGERERIQIGAAVG
GHNGGDVGLMHDVARIFHNAVDAAGAFKSKSSIEASLEGHWMAFAAEESREKGILVDLDA
YRNNVVSTRETVNI*
>SPBDM4_v1_40915|ID:27158220| putative Extracellular solute-binding protein family 1 [Uncultured spirochete bdmA 4]
MTKSRKTIIAAFLLLIGLVSPFFVMAQGSQKGEKIKIVSQMFYDPAQQQYIKEQILPKFT
AETGIEVELQVVANASELYKVLQAQQQTGKWSTDILIAHDSTAVPVVQEYHAVQAYTKIP
AGTYITQFDDNFVQGGKRYFVPLQADVYLTIANKKALPYLQKLGYDINNLTWEQLAEWVR
LIKKETGLPKYVFPALAGKFATYEFNSVQLSYGDKYVPAFNTPASQAAFKVIASMKDGIL
PSSPTIDFPTASLATEEAWITVFHQAYANASFSQAPDKFVVAPVPIGASGKRGTIIGGHG
IGIVTGSTHKAAAEKFVEFFLRDDILYAVMKNTGPWIPSKAEITSKLKDDPSDQIMKMGL
ATLTGPTLIDRVRVAEYQDWGQVKRLYEQVIGDILAGKDINKQYLDQKQKELESLKVE*
>SPBDM4_v1_40916|ID:27158221| conserved membrane protein of unknown function [Uncultured spirochete bdmA 4]
MHVSRRTGFFKSPTWFLVPVVIYYAAFWIRPTISVIIESFTDKAGAFSFSNYVRLFSQEA
IRTAFLNTVLFTVGSAIIQFVLAFALALWLNKKFRFSNLVLFITLIPMAFPPAAVGILWK
TGLYRFGWINSFLCAIGLMNPANPVDWMSFRNLNAVMLLIVVDTWTVLPSVMIILLAGLQ
NFNKEFEEAGWVFGANKFQTLKDIVFPIMKPTIITAMILRMIAAVQVWLIAVMIFGYNVV
PFLVERIAYNVDVITLAKYARKDAYTISVIVAAIVLISVSMYLRVSGDKQKGGETT*
>SPBDM4_v1_40917|ID:27158222| ABC transporter permease [Uncultured spirochete bdmA 4]
MSVRRGKLWSRTVFLLILATVAGVVLLPVFYFLTISFASNYEAYQFPAKIFPTFSYPAQL
RYNADKENYTLLIKKGADFEAVKTTSDTSDFSLYCKSQLNVLIASSEVQALFDKAKGADH
AIPIKLKKAFFRNYVVFFILAEGTMRALFNSLKAAGWTILISLVLGGVTGYILARYKFRF
SNTFSTGLLVVRMFPAVALSLPLVVYIVRMNLYDTPFALAIVYSVPNIALTAWITSSIFK
GISVELEEAAMVFGATRLKTLLTITFPLAFPAIVASSLYAFLAAWNDSITALIMTNDNPT
LALLVYRTVGSSTIPNLPAAGAVVLLVPSLVFTFIIKNYINQLWGNVAL*
>SPBDM4_v1_40918|ID:27158223|ugpC| sn-glycerol-3-phosphate import ATP-binding protein UgpC [Uncultured spirochete bdmA 4]
VSYLELNSLKKTYPNGVTAVKDIDLEIEKGEFVVLLGPSGCGKTTTLRMLAGLEEVTEGS
IILDGKDITHLPPRQRDISMIFQSYAVWPHMTVAENIAYPLKLRKMDKQAIAAKVQEVAK
ICKIVDYLNRYPAQLSGGQRQRVAVARALAVNSKLSLMDEPLSNLDAKLRVSVRTFLKEI
HRNTGATTIFVTHDQAEAMALADRIVVMNEGRIEQVGSTREIYNECESLFTVQFMGTPPA
NIQKVALASSQGRLVAAAGGGEGAMSLDLGDQSAFPGIERLMGEEVFLAVRPENIGIGEP

NGINETSIEIVEPQGAYTILVTNVLGGEWKIMLEGDVDIRIGQKVSLEIDPGKVMLYDTQ
NKKRISLT*
>SPBDM4_v1_40919|ID:27158224| conserved protein of unknown function [Uncultured spirochete bdmA 4]
MRTQQAWIERCSEVLRGNLPDQKVLEKLLYRYTCPAREKGTYLPQWLWDSCFHAMAYRWF
DPDMAWEELQSLFVHQFGEGPDAGMVPHMSHLEESGALAAQRLFRHKDCSTITQPPLISV
AARAVHRKAPDKSILAKLYPKLRAYHDWFDRRRDDDHDNLVAIVHPWESGWDASQRWDGL
MGLDALGAGALRALEQKRNDLVSVCAVHGYDAEVLAQVPDAFYAEPADFNAIRAADLLAL
AEIADELKKPSESRELEARARAVQRAVHDKMVSFGDGELRAHDLLGPAEEKSPTDHAGKF
VLMFGHCLLQAEADALSSELFDPAGAYATPYKVASTSRFDPLFDPKEYWRGNVWLSLNWL
VWKGLLAYGQEGAAAAMVRDSLALVEKSGFCEFFNPLTGEAGEKYGRICPRNQSWSTIIL
DMLQGTTDE*
>SPBDM4_v1_40920|ID:27158225| putative sucrose phosphorylase (Sucroseglucosyltransferase) [Uncultured spirochete bdmA 4]
MNESHLKKIADLLVFLYGREQGARTFKSLQRLIPRGLARGGPADHPEPLSEKDACLIAYG
DILSLPFDESSSNGNEAPRSSAAASMPTALARLREFLEHRDHGGFSYLHILPFHPYSSDD
GFSIIDYREIDPRLGNWGDIASLAQEYKLVFDLVLNHGSVQSPWFQSFLKNEQSHRDWYI
TKPADFDVSSVFRPRTQPLLTPFLREDGTTVYYVWTTFSADQADYNFANPGVLLEFIKIFF
EYVRHGARMVRLDAVAYIWKEDGTSCIHHPKTHAIVKLLRALIDYLELDVLILTETNVPH
EENLSYYGKGDEAHLVYNFALPPLVLHAAVSEDAGPLRDWARTLPAPGEGPVFFNFLASH
DGVGVLPAKGLIDDRSFQKTLDTVLERGSLLSYKDTKEGPVPYEINCSYLSAVAPPSLGT
VQERARAFLCCHAVLFALAGLPAVYFHSWIGSEQWTEGPRLYGYRRAINREKPYIDQVEK
ALSDEHSLRADVYAGFEKMLAFRRNEGPFAPEAPQKILPAGGPVFALVRGPDRGGRYVLC
LQNFGSRPETCRLDEAPAFEMLVRGGMGREILLSPRETRWIGCGGSRNISVLRI*
>SPBDM4_v1_40921|ID:27158226| Extracellular solute-binding protein family 5 [Uncultured spirochete bdmA 4]
MKRKGMGLVGLFLFMAIPLVMAGNIPKDTLVMAMNTEIFKTFDPGVCFEVEPSAFVKNIY
ANLVTLDIKDGAFTPVGDLAEKWEQSADGKLWTFYLKKGVKFANGDPVKASDVEYSYKRA
VKLNKSPAWLFTDTLGLTEDSIKAVNDTTVTITTNGAPANVVLSVLAANIGGVLNEKEVK
AHETNGDMGLAWLTDHSAGAGPYVLKEWKRKTQIALTVNKNYYGDTPAIKTILLRDMPEA
SDRYLSLKKGDVDVAYQITVEEAAGLRDNKDESVQLFTTPGQTLEYVAMNASWGPFKDAR
VRQAVKYAIDYDAIINKVREGFAIKNQQFLAIGYFGYKESNPFTRDVAKAKKLLADAGYP
NGFEVEIVTNESEIRRAEAVVVQSNLADIGIKSNIIIMQAAQMYDKYRNQGLQMIVAGWG
IDYPDADALAKPFADYTVKQLAWRNAWLDDKAASMASAAGKEINEKKRAQIYYDLVKYWQ
DNGPFAMMYQPIEFWGIGNDIKGFDKAFDGYNVHCDFTLISK*
>SPBDM4_v1_40922|ID:27158227|ddpB| D-ala-D-ala transporter subunit ; membrane component of ABC superfamily [Uncultured spirochete bdmA 4]
MELGSFIIKRLALMVLVIFGVLIIVFIVTRIIPADPVGAILGGNAPPATVDAMRHQLGLD
KPIFAQLLDYLGGLLRGDLGTSLVSSRPVLTDILEFLPATIELAVSSIIFAVVLGIILGL
LSAVFRNHFIDHFSRVFSILGISLPGFWLGLILILIFYYKLGWLPSGGQYDMFLFPTRTT
GLILLDAMIHGEWNVFWNGIKHLILPTFILGYSSTASIARIMRASMLDVLHQNYIRTARA
KGLPRKMVILRHALKNALIPVITIIGLEFGSLLSGAVLTETIFAWPGLGRYIVNSLLTLD
YPAVSGGTIFIALIYSVVNLAVDILYAAVDPRMRS*
>SPBDM4_v1_40923|ID:27158228|ddpC| D-ala-D-ala transporter subunit ; membrane component of ABC superfamily [Uncultured spirochete bdmA 4]
MKRTSSAAGYAKGSSWKKFLYERKPMMDDMKHSVYLWKKTPLALIGTVIIVLFLLVALFA
PLLAPYDPIATDLSRKLEAPSAAHPFGLDQFGRDVLSRVIMGTRIEVQIIFIISIISITI
GLIVGIVAGYFGGVIDEILMRITDIFLAFPRLVLAMAFAAALRPTLTNAIVAISLVEWTV
YARLARAEAIKVRSQPYIEAIRALGASDFKIMFFHVLPMSISPVIVQLTMRMGTIILTAA
SLGFLGLGAQPPLPEWGAIISDGRSYLMNNWWITAFPGMAIAITVLGFNLLGDGIRDILD
PRIRR*
>SPBDM4_v1_40924|ID:27158229|oppD| oligopeptide transporter subunit ; ATP-binding component of ABC superfamily [Uncultured spirochete bdmA 4]
MQYRRGDLLDVSNLRVVFDTYAGQVKALNGVELWMNHGERLGVVGETGCGKSVTALSVMR
LIEEPGRITEGSILFEDKDLATLSENILNTVRGKDIAMIFQEPVAALNPVMKVGAQIVEN
IECQRKSAVLAQGARKAGPVTDSGLSQKHRTKETGSVEIMRDMLGRVGLDWQRTENLYPH

ELSGGMAQRVMIAMALSSKPKLLIADEPTSALDVTIQAQILNLLNNLVRETQTAVLLITH
AMGVAAQFCDFIAVMYAGNVVEYGSVQSIFKNPLHPYTKGLLRAVPKIGRTDELQSIPGI
VPDLISPPSGCRFHPRCPHRRPECDKNQPRFEPVREELEADRHHVACFLYQKDL*
>SPBDM4_v1_40925|ID:27158230|dppF| dipeptide transporter ; ATP-binding component of ABC superfamily [Uncultured spirochete bdmA 4]
MQASADREDSDILIQAEHLKKLFAMKKNLYSRPLYVKAVDDCSFSIRKNTVFGIVGESGS
GKTTLGRVILRLIEATDGSVVFDDTDLMSLSNKELRLFRRRMQIISQDPYNSLHPRKVVK
KLIGEGLQIHFQLSSQEVDERVKNILTLVGLREEHMYRYPHEFSGGQRQRIAIARALVLK
PEFIVLDEPTSALDVSVQAIILKMLKELKRDFSLTYMLITHDLAVIDYMADDVAVMYLGQ
IVELGSKDDIFLRTAHPYASLLIDSVPPPTPSEHWSGKILNGEIPSPIHVPSGCRFHPRC
PQVQEICRTKEPVLSELQPGHFAKCHFPLNS*
>SPBDM4_v1_40926|ID:27158231| putative Beta-lactamase [Uncultured spirochete bdmA 4]
MRPTEIANAIHFIENYVPDAVHETKTPGCSLAVICGENVVYESGFGWRDIGRSLPATPDT
LYGIGSCTKAFVATAILLLSEQGKLSLDDPVDRYIPFKLRSEKGPITIHHFLTHSSGLPA
LSTSEILIQKGLGVDLGFPLASGGDFYRWVNGAQKEMTPGLGEKFFYSNESYRMLGHIVQ
TLTDMPFHEFVTKNILGPLGMTRSTFVRSRYEDDADRMQPYLTKPDGTVVPASFPYPDVT
SNPEFSFMLAAGGLISSVREMAKFIASNLPTSKARLLKPESLELMQEPYIARPSSHYGKN
GYGYGWTITENFLGIPMVAHGGSIDVSTAYTAFLPQKGVGAVMLANTAGLPHAAIVEGIF
AALLGLDPFKVIPPLGIKKRMNVLAGDYEAYRGATRARVFSKAGLLYLEQKDDFQDLVVP
LIPEDDNMARYRFYILTEGARQPVEFRSTPDGLDLYVERNRYHKVR*
>SPBDM4_v1_40927|ID:27158233| protein of unknown function [Uncultured spirochete bdmA 4]
MMTNERAKKKYEAVQTFLRSKIESGVIVPGEKLPSESELMAQFGISRNAVRQAINELIKE
RLVESRQGIGTFCVKRTQGTSMLIAMVAFRISSYIFPRLVSACNQSIQRDGYTLLLNESL
YNVAKERSVLLDLRKRNVDGIVITPVLEGSKQYNADLLNEFEAEGIPVVLLDGEYPDYAF
TSIVIDEVAAGRKAAEYLWERGHRDIGMIYSSNYRPKVLRRDGVLGFLSSVGACIRDEWL
VGIHGQISSLETYGQIRALLAQKKQLPSAVICSSDDEALMFMRQAQRHKIRIPEDVSLIA
FDNSDMARYSSPRLTTLDHPSEYMGELAGKLILDRIYHPDVKSRTRTVIDTNVISRDSVC
SLNFSVGAHNS*
>SPBDM4_v1_40928|ID:27158232| protein of unknown function [Uncultured spirochete bdmA 4]
LNALITGGDQTGENIRRDPERYHGDKHGRTLRPLDTKGSNALPALNKPLFDQFIDCLANS
IAGYTELGHQLRF*
>SPBDM4_v1_40929|ID:27158234| Sugar phosphate isomerase/epimerase [Uncultured spirochete bdmA 4]
MRVGFHTDAFNSAAFSFEKCLQWARDNDVHCIECGAIEGVSWIHGLGYFPHISLVEDPVL
LREKMDRYEVSFSQIDAAYPLSGKDGPIFGVPYVLKTIPWAKLAGCPNIATTDGLYKPEG
LTDDEAMEAMKRSYGMIVDTAEAYGININIELHGYFTTNPDMIEKMLDFTKSERLGINFD
TGNSFIAGNDPVAFCRRVLPRIRHVHIKDVSKSLADAARGKATGIGISHSAIGDGVNAEN
IRQILTLLRDYGYSGTLSLECEGQGGPLIERSLGWLRGTLKALNIVEER*
>SPBDM4_v1_40930|ID:27158235| ABC-type sugar transport system, periplasmic component [Uncultured spirochete bdmA 4]
MSRKLLIVALAALMVLGGIVPATAQSSGKQYVVGYVTPGPDTWYKKDVDGFVYAAGLAGA
KVIVLNSNYNPETEIANIDSLINQGVDGMCVFTFNQNGANIAAQKCAAAKIPLVVTDNVG
LVLKSGYDVVAAIDFDWVGMGKDVAEYAAKQFPGQDIAVITGLSEHVPVQMFMSSFKPTV
EKLGKNKIVAIRDGKYDPTVAANAAQDLVQSGTKFSLLFVFNDEMGAAVVRVLKAAGVLN
NPIKVLTTNGAPYGIDSIKQGGISYSISTSPGWEGMVSFLALHANKTGKIKTLNQQLLLP
NTAITPSNIDDKMQVVPWDIDPVWKNLTAKYFPQFNGMY*
>SPBDM4_v1_40931|ID:27158236| putative ribose/galactose/methyl galactoside import ATP-binding protein 2 [Uncultured spirochete bdmA 4]
MQRNFVTNNEPILSAKQVSKTYGGHCVLDKVSFELHSGEVLCVVGENGAGKSTLIKILSG
AIRPDSGIIGVHGGTYSALNPRESMQLGIATIYQEVELIDSLSVSDNIFLGNETLGRFPL
VIDADSQNKKARQILDMLHIDIDEKMLVEDLSAAQKQNLQIVKAFHQDARVLIMDEPTSS
LGVEETRSLMQLVHDLRGRGLGIIYISHYLEEIFEIGDTILILKDGEQVGLYPRADIDVN
FVIHNMVGRDASMFFVRERVPIGADQIQVENISWKDAVKDVSFNVRAGEVFGIGGLVGSG
RSELAASIFGMVRKDAGTIRISGQELSINNPKDAIKNKICFITEDRKKYALFDSMDVQEN
IVIIHTEFGKSHLLKQKKENSLAKSMIDKLHIAVDNTEESVISLSGGNQQKAVLGRWLLN

DATLFIFDEPTKGVDIGAKEEIYKLIIELARKGKSIIMISSDMPELLSMSDRIGIMREGR
LIKILDNQRITEEDLIHDFIGI*
>SPBDM4_v1_40932|ID:27158237| Permease component of ribose/xylose/arabinose/galactoside ABC-type transporters [Uncultured spirochete bdmA 4]
MERLMKNGKLHKILHTQWFTLLVVIIVISVVTGMVNPRFFRLANIINIFEQISTLGVVAS
GAAIMIISGNFDISVGSIIGLSASVMAMMIKAGVPIWVVVPCGILIAIVCDAFVGFTSIV
FHAPSFITSLASSSIFKGIALALTSATLQTIFGKFSFLGSARVFGVVPLMFILGIIVYLV
VYFILKLTRFGRRVYAIGSNPQAAYLSGINVKGSKMLFFIINGTLVGIAATMLLSRIGAA
GASTGAGIEMQAIGAVVIGGTPISGGKGGILGTFLGVLLMGLISNSLNMLQASPYLQELA
TGALIILAVAVSALSNRNGKASM*
>SPBDM4_v1_40933|ID:27158238| Sugar phosphate isomerase/epimerase [Uncultured spirochete bdmA 4]
MTVSNKSASATSKDQNFEKKNAEIRIRYLHAKRTNPAFFSRRIDLSWSIWMFGLEPIGAS
LDRLTRNDIRYVELKGDHWTSDSGIRSHELSAALAAAGVRVSGACGMFSQDNDLSSPSAY
ARQNAIDYIRRELDFLAEVDARYLIVVPTAVGRPVAKDTSEVQRSAEALRRCGDDFARTK
IRAAIEPIRASEVSLVHTVSDAIRYLEVVGHPAIAHINGDVYHMSLEERNVGEAILLAGD
KLANLHLADTNRDALGTANLDLDTVIMAAYLAGMNQEGRFLTPEPLGPFPDPYVLSNSPC
NVSIMDELVRKTVSYFREREEIVRSLE*
>SPBDM4_v1_40934|ID:27158239| conserved protein of unknown function [Uncultured spirochete bdmA 4]
MIEKVHQFSRIDSGVMEKIIDEDHVNINHIIVAPGAAVPVHVANSHVHQIVIRGTLSLSL
EDGPEHNYPAGNIVAVPFNTKMAIRNGGIEPLEFFVVKSPNPRSMPPTKKI*
>SPBDM4_v1_40935|ID:27158240| conserved protein of unknown function [Uncultured spirochete bdmA 4]
VPPLPFPRIDLTAICVIIEAMNQHEIISPIELLDDRGHIVEEGWARRQLWRYDRSRIRAS
WYRIKEWDYYCVLSQKREYGIAFTVSDLGYAGLASIVWLDFAKRTFVPFDSLKMFTRGKL
GFAPTSSRGDLRYKDNKLEISYILRKGERRIVARCPSLGDGRELQCDLVLAQDPEADSMN
IATSWKENRRAFYYNQKVNCMPARGEVTIGGEHYLFEPDDSFGVLDWGRGYWLYRNRWYW
GSASGLYEGIPLGWNIGYGFSDRTPASENMIFYGGRAHKLEEIAFHINPNDYMAPWKITS
SDGRFEMDFEPIVNRHSDMRLVVISSLQNQLFGYYSGQLILDDGRTLKIDKMLGLAEDVQ
NKW*
>SPBDM4_v1_40936|ID:27158241| putative ABC-3 [Uncultured spirochete bdmA 4]
MRELLQIPAFSRAIVALFACGTAFPLLGIYILSLDLIPVRFGVMHLSLLGATVGLIFGVD
PVFFAMLFSVAAGFAIAGLSSRKSGGTSGGAMALLMTASLGVVFILFYKTNIHAIEAFNL
FWGNVLALDRMEVLLVVLVSALILAGTIRFLRPISAVLFDRETAYAFGFPSTVVYTGILV
MVCAGIGLGIRITGALMVDAVTLLPALAAQSLKKNFKPTMLLASVFGVCINLGGFALAVV
FDLPTSPAIIVVGTVVVLVCKLAARLQTKKKEKGVSWRASSSISAH*
>SPBDM4_v1_40937|ID:27158242| putative Zinc import ATP-binding protein ZnuC [Uncultured spirochete bdmA 4]
MSAGAADKAPCDGQSILSAKMLSIGYRRAQPVVRDICVDFPAALRKAWTGEKGQRDNVFG
FVGPNGAGKTTLLKTCLGLLSPLGGELRLLGVDTRKACFAETRKKLAYIPQNRPEGSSGQ
LRISVREAVSFGRLGKCGLTGRFNRADREAVEAAILYCGLETLADKAVQDLSGGQFQRVA
IARAMAAEPALYLFDEPGSYLDEEGQAAMQNLIRSLAESGIPLIIVSHDRALIALCDTVL
LFEKGTARLLDAGAFLSKMAERDI*
>SPBDM4_v1_40938|ID:27158243| putative cation ABC transporter, periplasmic binding protein [Uncultured spirochete bdmA 4]
MAIRHSHHGAQVLLALLCLLVLPLAAQEQPVIASTSWVQAYVIAAGAKNVVTIAPFELQH
PPEYEIKPSDLLAVQHASLVVYSGYEKFAKKLAETAQNKNLRILAVYTDNVPSTIIAESK
KIAEALGTTEAQAQWAQSFTAFTKNLRSEVASSLPDKRVVVQAYMKTFVDWLGLDVVGTF
GPGEPSPAVVLDLIKKKPAMVLDNYHNPGGKALAESLGVPYVLLINFPGKDGTRTIEDVF
LYNEKAILGRGKE*
>SPBDM4_v1_40939|ID:27158244| conserved protein of unknown function [Uncultured spirochete bdmA 4]
MQKKPIIIVVDGLGGGIGAQLCTKVRHIFAQRVEILALGTNSTATEQMLRAGADRGASGE
NAIQVSIDLGQVILGPIGIVLPDSMMGEITPAIAQTIMRANGKKILLPVNQPHFTLVGVP
PSPLSRNIDEAVGLLASELADMVEREQE*
>SPBDM4_v1_40940|ID:27158245| Ferrous iron transport protein A [Uncultured spirochete bdmA 4]
MNDRLRDYNVGDSVVILGYAGGDHGYRNKLLAMGLTKGSVIKLVKIAPMGDPVGIEVRGY
ELSLRKAEASVLVVGRKE*

>SPBDM4_v1_40941|ID:27158246|feoB| fragment of fused ferrous iron transporter, protein B: GTP-binding protein ; membrane protein (part 1) [Uncultured spirochete bdmA 4]
MNGKGTTIAVVGNPNCGKTTLFNALTGSDQRVGNWPGVTVEKKEGLLKFGKEKVTLVDLP
GIYALFASSEDERVARDYILSGEPGLVVDIVDATNLERNLFLTTQLLDLQLPLIVVVNMV
DLAKARGVSIDYEALSDRLGCPVVGTVASDKHEAAKAVGAIEKAWKAKRKPTTLVEAPYQ
AERFAKDWRERLSAVAQKLGTNGRWLALKLLERDAWAIEQVVSAGVATEAEIEAAIAEVE
KLLKDSADVALAEARYAFIQMVASKSLKHAGHKRTATEVIDKVVLNRWLGIPVFLLVMYL
LFWITINVGGAFIDFFDGVGATIFVDGFGSLLERLSAPAWLRVILANGIGGGLQTVITFI
PQIFVMFFLLSLLEDSGYMARAAFVMDRFMRWVGLPGKSFVPMLVGFG*
>SPBDM4_v1_40942|ID:27158247|feoB| fragment of fused ferrous iron transporter, protein B: GTP-binding protein ; membrane protein (part 2) [Uncultured spirochete bdmA 4]
MATRTLENKRDRYLTIFMNPFMSCGARLPVYALFGAAFFGAAAGTMVFSLYLVGILVAIA
TGLILKRTLFKGEASFFVMELPPYHKPMMGNVLRYSGRRLMAFMTRAKYIVPIVTVLAVL
NSLGIDGSFGNEDSPKSVLSQAGRAITPVFEPMGVEKENWPASVGILTGVLAKEAVIGTL
SSLYSQNAAADKEASAAIEETPVADNKGFHFWDGIVAALATIPENLVGIGGSFDDPLGAG
VVSGDQEAVAEEVGADTSVYAGLRANFTGGPFQAYAYLLFVLLYLPCVAAFGAMTKEMGL
KYTLIATAYIAVTSWSAATLFYQITLGHNIFWIIFAVVLVAAMIFVLRALGKQVEEESSG
LSVKEAAA*
>SPBDM4_v1_40943|ID:27158248|aldB| aldehyde dehydrogenase B [Uncultured spirochete bdmA 4]
MRKFIDENYKLFIDGKWVDGGGKKFETHCPANGELLATCAEADKGDVDRAVSAAWRAFET
WKDVSPQERQRILLKIADLMEANAEKLAMVETFDNGKPIRETRAIDVPLSIDHFRYFAGV
VRAEEGQATMIDKDTMSLILREPIGVVGQIIPWNFPLTMAAWKLAPALAAGDCVVIKPSS
ATPLSILELAKLLAEVLPPGVVNVVTGRGSTTGNYILEHPDFRKLAFTGSTEVGYSIAEA
AAKKIIPSTLELGGKSANIFFPDCPWEKAIEGLQIGILFNQGQVCCAGSRVFVHEAIYDK
FLKEAIAAFERVKVGMPWEESTQMGSQINEAQVEKILSYVEVGKKEGAKVATGGYRLTEH
GLDKGCFIAPTIFTDVDNKMRIAQEEIFGPVVCFIKFKDEAEVIKMANDSEYGLGGAVWT
KDINRAIRVARAVETGRMWVNTYNSLPAHAPFGGYKKSGIGRETHKMMLEHYTQKKNIFI
SLSEGKVGLY*
>SPBDM4_v1_40944|ID:27158249| conserved protein of unknown function [Uncultured spirochete bdmA 4]
MVVTKDTQVEEVVKIKGVISYFIQRGVSPISCSGAFPQSLGNLLSIKKVADPDAFIEGLN
EYIASQSQELKDKTDD*
>SPBDM4_v1_40945|ID:27158250| conserved protein of unknown function [Uncultured spirochete bdmA 4]
MADDVSLKDELIQKAIEVFSRKGYEATNLTDITDALGISRGPVYYHFKDKYGLYVAAFNL
WASKFQHDHDLILAQDKHIIKILEDSVYCCIDMYKHFRPNFFVGIDTVEELSELKDRYYL
LVQHIYNKKVETVKRAIARGEIRRSISPELVVDMIYVLYDGIKDGLERPLVKIKESELKT
IVTVQLLGIERFCCD*
>SPBDM4_v1_40946|ID:27158251|folD| Bifunctional protein FolD [Includes: Methylenetetrahydrofolate dehydrogenase ; Methenyltetrahydrofolate cyclohydrolase] [Uncultured spirochete bdmA 4]
MSIIMKGSEVAAIIQQELLKQIERLNQYGITPTLAIVRVGARPDDLVYELGVKKKMRTLG
IKCPVFAFPETIDQADFEKNFSSLNLDSEIHGILLFRPLPKHLNEKTIIDTINPLKDIDC
MSPVNIAKVFAGEETGFAPCTAEAVMEVLNHFGIGLEGKRVTVVGRSMVVGRPLSMLLLK
KNATVTICHTKTKDIENTCRNAEILIAAVGKANMITDHFVAPDAVVIDVGINVDSNNKLC
GDVDYDDVAPKAGFITPVPGGVGTVTSSVLAVHVVRAAKYLNKIEER*
>SPBDM4_v1_40947|ID:27158252|fchA| Methenyltetrahydrofolate cyclohydrolase [Uncultured spirochete bdmA 4]
MFMEKSGNEFLNLLSSAAPVPGGGGASAFVGAMGVALASMVANLTSGKKKYADVQADIDR
IIKKADGLRNELTSLVEKDAEVFEPLSRAYSLPKENEAERKIKDETMESALKSACEVPLE
IMLKVVEAMDLHEELAKKGTRIAVSDVGVGAAFCKSALMGASLNVFINTKLMKNRQYAEE
VNQKTEAILSEGIAKADNIYRQVEAAVR*
>SPBDM4_v1_40948|ID:27158253|fhs| Formate--tetrahydrofolate ligase 2 [Uncultured spirochete bdmA 4]
MEIKSDIEIAQASDMQPIENIAQTLGIEKKDIELYGAYKAKVNYNIFKELNKKKDGKLIL
VTAITPTPAGEGKTTTTIGLGDSLAKLGNKTMIALREPSLGPVFGVKGGAAGGGYAQVVP
MEDINLHFTGDFHAIGAANNLLAAMIDNHIYQGNSLEIDPRNITWKRCMDMNDRQLRSIV
DGLDGKANGIPREDGFDITAASEIMAVLCLSRNIEELKENLGKIIVGYTRDNKPVTAMQL
NAQGAMAALLKEALKPNLVQTLEHTPTFVHGGPFANIAHGCNSIMATTMALKLADYVVTE

AGFGADLGAEKFIDIKCRLAGITPSAVVIVASARALKMHGGASKAELGKENLVALEAGLP
NLLKHLENITQKFGLPAVVAINRFPTDTEAELRLIEMKCKEHGANVALSEVWEKGGAGGI
ALAEEVVRLTQVGNGKFRYLYENELSINEKIEKIVREVYGGDGVDYSSAAQKEIHRLKEL
GFGNLPVCMAKTQYSFSDDATKLGRPKNFRVTVKNVKVSAGAGFLVVLTGDIMTMPGLPK
VPSAEKIDIDSNGKISGLF*
>SPBDM4_v1_40949|ID:27158254|focA| putative formate transporter 1 [Uncultured spirochete bdmA 4]
VEKAFLSPAETAMSFIETGLKKTTLPVFKQFLSAILGGAFIAFAAEGSNVAVHTIASVGL
SKALAGAIFSTGLMMVVIGGAELFTGNTLIVIACGERKISLGVMLKSWLCVYLGNFVGAM
IIVFLILYSGQLDFSNGLLGGFTIKIAAYKTHLAWGHAFFLGILCNWLVCMAIWMASGAK
DTAGKILAIFFPIWLFVTSGFEHSIANMYYIPAGILAKANHTWATAALALGATQEQIEGL
TWGSFIVKNLVPVTLGNIVGGGLFVGIIYWFIYLHKAKEN*
>SPBDM4_v1_40950|ID:27158255| conserved protein of unknown function [Uncultured spirochete bdmA 4]
LKLNLPIIAFKLADRKPNLHLCTNHGNNLLSARLLSPGIVHFDPYAVYIGTAEEIRTIDQ
ASLPSDVIVVGEEAAQALSETECNWLSFEEGDVPEIFNAVQQVFDFYNQIDSELYDAVMQ
EQDIQSLLDICSRFFDNPVHLVDSSLRLIAHSSELDDDIHDESWMEMLSTGYASVTVIDA
LKKANLLDELNRGREAMLVNLGGFILPYMSANIFAHDKRIVSICVREHSHHLEEAQLGLL
DHVATVLTNVIAKRQNLHYLHLGYVERIMLDLIKGNSVDTGILRFGLSQIGWNVEDSYYL
LKIIPDFKDVSGGTIEYTGAMIKNLFSGSILLSLSDVLVLIVNLKYCTKALPNIFGTLET
FLCKRKFKCGVSLKSHDLSALHGQYMLAAAAIKNGTLIEKDKYLFFYENYVASHILELCA
KMIDVRILCHPEALKLYEHDQKNEGTLLYTLYVYLMKEKSLVMASQELNIHRNTLVYRLS
KISEIIDADLNDGNTRLHLIFSYEILRYLDYFK*
>SPBDM4_v1_40951|ID:27158256| conserved protein of unknown function [Uncultured spirochete bdmA 4]
MNENYESLHKIDMNKVDIKHDPQLRFTPFVPYLPAAQPYVGGRLLMNSWGIATPVWYSGW
RDETLSWHQTCSLSAVLNPTSATIIKGPDAKKFLKENFVNNVDNFPIGIIKHGLLCLDNG
LLATHGVLMHTAEDTYEAHWHAPYINYLFSKKNYNAELIDISLKQYIFQLQGPRCLEILE
KVTGDDLHDIAYRHFRMSQIDGHAVRILRFGMAGTLGYEVHGEAEYAREHYKKILETGQS
YGIRPIAWFSYNMNHIEGGFPQLYEHFVSAMSLNEDFVEYLKKTPYTGGNTYNTSTEGIE
FLGSVGPDPHNYMYNPFELGLGRCINWNHEFPGKAALQKIKDTQDIDTVMLKWNVEDLCK
IYASQFVPGEDYYKQMDLPADSASVALFGHFLFSTDQVFDENGNKLGRSYGRIYSHYYRA
MLSFGALSAKGRELGKEVYILWGEPGTKQMKVRATVERFPYNDHLVNDGFDVETIPHPVF
DR*
>SPBDM4_v1_40952|ID:27158257| Aminomethyltransferase [Uncultured spirochete bdmA 4]
MKEIPITLNKVSELKYVPQYHDVKTYANIFGGIFQWEADDWESTSLSWKKSCYIHAGISG
VEVTLKGPDAQKLVSMSAINNCYNWKVGFAKHLVMCDDNGLISSHALVNRTEADAFVMSA
ANPWPWIKLLLNTGKYNAQPFFRDIFVFQCAGPKSLTCIEDLTQTDLHDMKCLEFRPVKIP
GLDAKVEVERIGMSGTLSYELRGAGEDGPAVYDAFYKLGKEKYDMKRLGWRTYFVNHTEG
GYPQTSCSFSQACDYDPEFVSSGMNLLPPVYTGSYDPANYRARLRTPGEVGWMWMAKFDH
EFVGRAAVEAEAKAPKRTIVTLEWNTDDVVNIYDSQFRDGEPYKYMEMPIAPQQPAGGHA
DRVLTVDGKEIGVSSSVVYSKYFHKTISQCTIDVDQADIGNEVIVEWGDFGGKIKKVRAT
VARFPYLDLVRNEKYDMNTVPSGVIER*
>SPBDM4_v1_40953|ID:27158258| protein of unknown function [Uncultured spirochete bdmA 4]
MGIDIKSGIKPPIAMPVINNPIHSSAPCGESHANATPNAEIKPNTNIKGFIFPSFVAIRG
ANRHPTKNPSVMAVEKAPAATELMPRPPRAVGRNELIVILLARMHIRQSPTIQTGELFAT
TFNDSEIGREGGTLSAPVLTGFFIKAIQRTAAKTLTTAAVAYALVIEGNRAVVPVPTVMA
MVEAAKNIPIMEEIFSGGNQPPKILAAFAFRNAAPKPVMII*
>SPBDM4_v1_40954|ID:27158259|folE| GTP cyclohydrolase FolE2 [Uncultured spirochete bdmA 4]
MPDVQNMMKDERLMAIQKVGVKGLHYPIVLLDKVNSVQHTTALVNLYADLPYEFKGTHMS
RFIEVFDNYRDDLSMPQFLRMLDGIRTALAAETSYADIHFPYFLEKKAPVSGRSAMMSYE
CAYRGKVGSGGKEFDVVVSVPVQTVCPCSKAISACGAHNQRGIVTLTVALGPFFWIEDLV
SLVEDSASCGVYTLLKREDEKFVTERAYNNPRFVEDLVREVYLRVSALGQFPKFSVEAEN
FESIHNHSAYAYVEYPEG*
>SPBDM4_v1_40955|ID:27158260| conserved protein of unknown function [Uncultured spirochete bdmA 4]
MISNRFIITVIGSDRVGIVARTTTVMANFNVNIVDISQTIMQGIFTMIMLAEAPQEGFDL
AAFQEGMAAEGKSLGVEVKVQHEDAFRYMHRI*

>SPBDM4_v1_40956|ID:27158261| conserved protein of unknown function [Uncultured spirochete bdmA 4]
MLMLINPNEILETIRMVRMEHLDIRTVTMGISLRDCAHPDIRIFNENIYKKITSRAKDLV
RTTNEIQNLYGIPIINKRISVTPIAIVAESCGAPDFVSVAKTLDEAAKKVGVDFIGGFSA
LVHKGTTPGDLKLINSIPEALAETEKVCASVNVATTKAGINMDAVGLMGEVIRKTAELTA
ERDGIGCAKLVVFANAPEDNPFMAGAFHGIGEPECAINVGVSGPGAVNAALRELERPNLT
EISEVIKKTAFKITRMGEMIGRVVSERLGVEFGILDLSLAPTPEIGDSVAAILEAMGLER
CGAHGTTAALALLNDAVKKGGAMASSRVGGLSGAFIPVSEDAGMIDSVEVGALTLEKLEA
MTSVCSVGLDMVVVPGDTPASTLSAIIADEMAIGVINKKTTAVRIIPAPGKRVGELVEFG
GLLGRAPVMPVSGFSSEIFVKRGGRFPAPIQSLTN*
>SPBDM4_v1_40957|ID:27158262|trpB| Tryptophan synthase beta chain 2 [Uncultured spirochete bdmA 4]
MMTKAILTEDEMPRQWYNLAADLPGPLQPPRGPDGKPVTPEQLAAVFPMNLIEQEMSSER
WIDIPEEVLAILFKWRPSPLQRAYGLEKALKTPARIYFKNESLSPAGSHKPNTAVAQAYY
NKAAGIKRLTTETGAGQWGSALAFACAQFGLECKVYMVKSSYEQKPYRKYMMQTWGGTCV
PSPSDTTEAGRAVLRDDPKSPGSLGIAISEAVEEAVSDSTGQTRYALGSVLNHVMLHQTI
IGLEAQKQLAKFGEKKVDTIVACVGGGSNFAGLAFPFIKEKIEGADIEIIPVEPASCPSL
TKGKFIYDLGDVAGMTPYLAMYTLGHTFMPHPMHAGGLRYHGMSPLVSQAAAEGLLSPSA
VPQLECFEAAVTFARSEGIIVAPETSHALAQVIREARKAAEEGKEKVILFGLSGHGLLDM
QGYADYFAGKLSNLELSEAEIEKALARLPKLETVG*
>SPBDM4_v1_40958|ID:27158263| ABC-type transporter, integral membrane subunit [Uncultured spirochete bdmA 4]
MNQQQGVQMRGKRGKKPIRPGTILGKAVAYLVMGIFAFMTIYPLVWLFFSSFKTTTEFRT
NMMGLPIDWTLENYRSAWKIGEFDKLIGNSVLYTLGSTVGITFLSLLTGFAFAKIKSKAT
GPIYGSFIIGILLTIQSLMVPLFLEVTSIDRGIGDFLRSLGLVGSGEFRLFHNTRFGVLI
IYIGSALPVGIYMCTEYIRGIPSAIIEAAQIDGAGYFRIFRSIIVGMSKPIAMTLSILNI
PSLWNEFALINILVSETNLKSLPLGIYKFSGGLSSDYGKQFAALVIGMLPMLLFYIAFRK
QITAGVSMGALKE*
>SPBDM4_v1_40959|ID:27158264| ABC-type transporter, integral membrane subunit [Uncultured spirochete bdmA 4]
VNGTATKAAKDRRAYWAMVLPSFLIYLFVFGFPIVLAIVLSLSNYGGGKMFGGTSWGITG
FRQYQRLFADPYFWNALKNNIYIVLVSVLGQLPLGFIFAYLIYRKTVKFEEFWQGVLYVP
AMISTIVVGILWSIIFSPYGPIADTANHYYASAYSAKISGIFQTTGGLSDPDELTDQIIK
ASPSTINTVFSDPSVDLKDFLLSYEPGDTATLQKDLVNLLAPRWNADFLSKNNVAMIPIC
FVTLWMWVGTYLLIFLANMQKIDRGIIEAAQIDGASEMQIMGRVVLPSLSKVIANSAILC
ISGSLSGFALILAMTGGGPARVTQVLSIYMYDKAFMGAPDYPLANAIAIMIVLFSLALVG
IMFALERRFGGKE*
>SPBDM4_v1_40960|ID:27158265| Extracellular solute-binding protein family 1 [Uncultured spirochete bdmA 4]
VKKKICSALMFLVILSMGAYAQKTTLTVFDYIDATAPGYAENEAIWQRFIDENPDITIVK
EELSNEPFHQKLAAYVAAGTLPDVMYMYPSGRSTMLHEQKLVKDLAPLLGKDYLANFIPS
VTDPSGQKGKYLAELPQSISYTTVMFTNTKLLSDLGLRVPSTYAELKAMLPKLKAKGIRP
VLMANKDDWVMQSCLFSTIVGRMLGDEWIDNALAGKVKFTDKEFIKALDFVETMYKDGVI
GRDTIQISYGEAPALFAEGKAAFFIDGDWRQNAFLTDPSSGKALIDPKRQQTDFEYFNFP
AIPGEKNPGVASAVLGCGYGISASIPAGSDKEKAAVRLLKYLYSADVQRQYLELGRYITS
RKDVKSDKLEPFTVKMAAYHDAVKKTCYVLDGALDTSIYTPLNLGLQQIGLGTKTPTQVA
QSVQAAVDAWRASH*
>SPBDM4_v1_40961|ID:27158266| ROK family protein [Uncultured spirochete bdmA 4]
MPKTKNSSRILRTIWQYPESSRAEVASHLGLDKSTVTLEVANLIEQGAITELPQGPSGKN
GGRKPIPLIINKDYGYIIGIAIQSGHYSAVAVNLAGEILEVKEEDESITKENLAPSIQAI
YWELKSRLGKYPGAILGVGVGVGGLINPNDGTVEFSVPLKISEPVNVVETLSKKLNVPFF
IENNANCCAWSELAFHKNTELKNILFALIEFRQALVPHAEYGGVGVGFGIVLNGKVHYGS
NYFAGEFRSVLCTENNGIQVSCSKEELSQILNDFSVLEKFVSELARNIAMLVNTLDCDSV
FIGGDIEDSDLDFCRILEKAIHDNWMYPVKRHVRIQYSSMGAEAVAFGAAGMLLNRLFAT
NKFPVGPREQTELSQ*
>SPBDM4_v1_40962|ID:27158267| conserved protein of unknown function [Uncultured spirochete bdmA 4]
MTYYRKGSDMAQCMIIPTYWSTPDIPSWKIFDHPIPIGETGTLGRTLDNIESTGYPDPVI
IFPAPTDSHIEAKVKEISRNRALDIHVFSDSDLISIRQRLRAVGFPAPLLGDISMDSYGG
VRNMGLLYAALHGFDTVIMIDDDECIDPHYRDSALKFIGDSWEGKTVLGKTGCVVDSKGN

KFYDGQKSDALRGWPKDELFNKEVVEALDKKDQLSESKIAFGGNMVLNSGLFLRVPFDPY
GTRGEDDDYILNARYCGLPVFFDQELLLLHLPPERICNYWTRQRQDILHFRYMREKVRIF
GFEPESLGGFLGYFTQDDLEYKAISSSIQAALHFIDTDRNEFTEFLNNAIAAETPPVVQL
RSRVNAFLRFMDAWQSVLPKIAD*
>SPBDM4_v1_40963|ID:27158268| DNA and RNA helicase [Uncultured spirochete bdmA 4]
MRIIADLHIHSRYSRATSPKLTLSHLDRWARIKGIDLVGTGDCTHPTWLAELMEQLEPAE
GGFFQLKSTLRREFDSGEALAEGLPAPSGENPVRFVLTGEISTIYSRDGRTRKVHHVVIL
PDFAAAAAFQARLERMGNISSDGRPILGVDSRDLFAALLDADERSILVPAHIWTPWFSAL
GARSGFDSIEECYGDLAPRIGAIETGLSSNPPMNWAVSALDRFAIISNSDAHSPEKLGRE
ATVIDMENSFVGLAGALTGGGPAGRIAGTVEFFPQEGKYHYDGHRACGVVLSPAESAAYG
GLCPVCGKPLTPGVMRRVAELADRPVDETASCPPDYAGTNRRPYKSLIPLPELLGELLGV
GSGSKKVAAAYGALVERAGSEFALLLDQSEAEIETLGTAGVPGELLAMAVGRMRSGQVSI
KPGYDGEYGVIHAFAPGERIGVKAGAGLFDDEVGTGEEETPPPDKAKAGTPAASEPAGGK
KRATPPAASDKAPKAVPESGTAAFGLDPAQEAAVNHPGGPALIVAGPGTGKTTVLALRIA
RLVEGGLDPSSILAITFTNKAAAELRGRIEGTIGGERAARLTTATFHSFCLSVLREHTAD
ASLLSSFGVLDEEEKTAFLKLAVGPSEGRAGAGDAQMDSTHAVPMQARPQKIRRLASYIE
ERKRFLLLPGDRVPRLGPGAPGGLAELAAELGVPPLDVDLDVAYARYRDELKRAHALDFD
DLVAGAARLLAARPEILSAYRARFRAIFVDEYQDVNFAQYALLRLLAPGTPAAPGTLGPR
GSISTELCVIGDPNQAIYGFRGSDRRFIERFLADYPGTAIYRLVRSFRCAPGIIGAAGRL
VGAELGGSGTVVALSRCEFPTDASEAEGIAREIDRLIGGTRFFAIDSGVVGSVGAGSGAG
LAEAHTASREGPVLSSLGECAILVRAAALAPPIEKALRDHGIPYRFIGEKPWWEEEPARS
ILSLVRAAARTDVRVDVSSAGNLKEAAGATVDIVAGPHCGSPNTPQGQAPVGEALRALLL
GLSPAEAVSAAIELLDVEADAGASKAVAAGVPREAFGTRPGKSLERLVSQASLYPDLASF
LDDLALGSPQDGYEAHRENVSLMTMHAAKGLEFDYVFVAGLEEGILPFTLFEGRGAGAGD
RKGETSEEGGDRSGRATGQDNALLRERIEEERRLLYVAMTRARVGLYLSWARSRHFFGRK
LTLAPSRFLAQIEDLVPMVERAAPKKPKDSQLTLF*
>SPBDM4_v1_40964|ID:27158269| protein of unknown function [Uncultured spirochete bdmA 4]
VKKNKESVDPSEACADATELAGKTSTQLAPSVSEVADILVRRYRPWPDESAGPVIDQLVW
FLLSTRTTVENCNAAYAALRGCFSGWDEVAEAGEEALDAHIWRVTRRLGWHNFPGETPSR
RGADHLNALMPAGLDVLSLHVNLIRLGREFCPAGKPRCGECPLGAICATAATKRPGTSAR
HALDQVHGFKKSRPEL*
>SPBDM4_v1_40965|ID:27158270| Toxin HipA [Uncultured spirochete bdmA 4]
MSSLAEVRLWDTRIGAVSLRDGEEVAEFQYSDEFLKSGIQLSPLVMPLSSRVYRFPTLPR
GTFHGLPGLLSDSLPDRFGNALIDVWLARQGRRPESFNAVERLCYTGKRGMGALEFVPST
GPEAKQAQRIDVENLVRLASQVLQEKEGLRVALSGDKQGGVGRTEGARTFSAAGAGNDDK
ALLQILRVGTSAGGARAKAVIAWNPRTNEVRAGQLDAPPGFEHWLIKFDGVKSNRDKELD
DPKGFCTVEYAYHLMARAAGIVMTECRLLPEHGRRHFMTRRFDRTVDGGQKIHMQSLGALA
HFDYNRAGAYGYEQAFATIRALGTAMDDVEQQYRRMVFNIMARNQDDHVKNISFLMDRGG
RWTLAPAYDLTFSYHPSGEWTGTHQMTVNGRRDGFIWGDLKAVAGVAGLKRGRPEDIARE
VHDTVARWPEFADMVGVAPAQRDAIFANLRLSGELLP*
>SPBDM4_v1_40966|ID:27158271| Helix-turn-helix domain protein [Uncultured spirochete bdmA 4]
MKLNNALTDVTVLEELGRRLARARIAASLTQADLADRASVGKRTVERIEAGGSVQLVSLI
RVLRALDLLETLDVMTPDEGPGPMEMLERQGKVRRRVSSSRKAGVPGPTATPWVWGDER*
>SPBDM4_v1_40967|ID:27158272| putative ABC-type transport system, periplasmic component/surface lipoprotein [Uncultured spirochete bdmA 4]
MKRLVLMMLALCVAFGSVYAVPKNKIKIAMIVESTVDDKGWCQSMHDAIMAVQKKYGSSL
VEYSYSEKMKPVDAGSAARQYVAKGYDIIIGHGAQYKNLMLEMAEEFPKTTFAFGTSGEI
GPKNVFTYMPESEETGYINGVIAGLVTKTNIIGLVGPVDGGDAARYNRGFVLGVRSVNPK
AQVQVAYTGSFGDYVKAAELAHTQIKAGADVLTGSAQQALGALRAVAEYKDKAIWWVGQD
TAQLGIPEGYKVIAAASYAYEAVVENLIDKRQAGVLGGECIPLNFANGGFVYQFNDKVGP
VLTADIRKSAEKAKADISSGKLSIDWQSVK*
>SPBDM4_v1_40968|ID:27158273|tsgD| Glucose import ATP-binding protein TsgD13 [Uncultured spirochete bdmA 4]
MGAMNEKIRELRVEGITKRFPGIVACDSVDLAVGENEILALVGENGAGKTTLMNILMGLY

QPDEGRIFINGRPVQFKAPADAFAAGIGMVHQHFMLVPNMTVAENVALGLKESHKYLRLD
LSGVKKRIAEVSERYNLPVQPDAYVWQLSVGEQQRVELIKTLCFGARFLILDEPTSALTP
QETDELIVLLRRMASDMSIIFISHKLAEVTALSDRVAILRRGRVVFKGTTAGQTAAGLAA
LMTGHEVVLPRNKCIDGCGEERLVVRDLKVKSDRGFLALDGLGFSIRSGEILGLAGVSGN
GQKELADALAGLRKVEAGGISLMGMDLVNRSPREIIDAGLGYIPEDRNNEGIVSSFSVKE
NLILKDFASSGFSRGVFIKSREEEQNAVLLQERFDIRCSSLNNATGSLSGGNIQKVILAR
ELAREPKALIAVYPTRGLDMGAEEFIHTRLLELRARGAAIFLISEELDELMNLCDRIAVI
FKGKILDTLSSRDATRPKLGLLMAGIKPEAEEATAEAGTEAVTKQGFGARA*
>SPBDM4_v1_40969|ID:27158274| ABC-type uncharacterized transport system, permease component [Uncultured spirochete bdmA 4]
MKTPLRVAYVVLVSLVAALAAVGIGAIVLTILGADVFQTYKVILTEPLKDLFGITEVLVR
AVPLILVALGIAISFRAGILNIGAEGQIQMGVISATAVALVWPDLPKGILVPLVLLGGAA
GGALWASIAGYLRAKLGVNEILSTVMLNYIAAQLYGFLLRGPMIDPAELQTGSGTPQSMR
LPKSAWIDRIIPGTRLHWGIVLAVVLAILVYILMWHTTWGFKMRAAGASPKATRYGGINV
ESCLFSAMVLAGAFAGLAGAVEVTAVHRRAIEGISSGYGFSGIVVALFGGLHPGGIIPAA
LFFGVLLVGTDMTQRIMSIPANMVSVLQGAVILSIISAKMVINDPYAREKFARRFLHSKD
KERGPGGEGIPEEHGAQNEETVNIEAKQ*
>SPBDM4_v1_40970|ID:27158275| ABC-type transporter, integral membrane subunit [Uncultured spirochete bdmA 4]
MTILNIVLNILSIGIPFSVSLLLASLGEMFNQRAGIFNLGCEGIMSMGAFLGFMSAYYLQ
NTAMVPYANLIGIAAAGIAGALLGLLFALVTVTFHAPQGIAGIGLQMLGTGTAGTLFRHF
VGGVTGVPGLPSYPIPLLSKIPFIGPVFFNMNILAYAAFLFVPVSHYILFKTPWGLKVRA
VGTSPRSADTMAINVNRTRYQALAAGGLLAGIAGAYLSLGQAKMFADDIVAGRGFIAVAL
VYFGRWDPYWILGGSLLFSIAQSLQLSIQVLGIKFPYEFAVMFPYVLVIVVLAFTRNSAI
KGPSELGVPYDREKRI*
>SPBDM4_v1_40971|ID:27158276| protein of unknown function [Uncultured spirochete bdmA 4]
LDRETGKALVFANRPGDFEKMMRIVGKHFEPEERVPLNASMMVLTIAYDILGAADEIDVW
EKPFGHSDEASRSG*
>SPBDM4_v1_40972|ID:27158277| protein of unknown function [Uncultured spirochete bdmA 4]
MPPKNDIESGKLKIGDNWNAISIIALSQNNPLKAIAEFVENSIDAGAKNVTIVRGKQKND
HYIKVIDDGCGIKDFRYVATHIGDSIKRELKRKGGTDIQGEFGIGLLSFWTVGETCTITS
MGEDGVVRSMRLVKGNPSYAIRETGTLFGRQGTELIISPVLAGVRSLSGDKIQNFLASEL
RDRITKSGVRILILDHASRRELVVEPRKFKGRLIHQLPEIRCPFGELYLELYFAESPQDA
VVGLQKRGTRVLSDISRLDYFARPPWDSRQIEGLIDAPFFQLTPGTRDGVVLDEAYDSFC
VAIAPVEAALNGLLDEQRRADEEEASRQVLNRVTRAFREAFLHLPTEDYSWLAARTSDAR
RGARAGAGGEGGAGADATGNGEGQTTPAISGGPAENSGTEASFGEYIPNPLEQKEVQRDF
FEYAGPLYKLMIFPASSVIGVGERKKLKAVARDRAGRMVEGDIAFEWHIADGGGALEGTD
SVYAEYVAPEEPCVAAIELKAVQGETVLTASALVTVTADLVKRSAGAGAGIGGAIRRGLP
GYTYQKAPGQLWRSRYDMEQSVIVINNAHADFIYASRQPMTKLRYITRLFAKELVLANFP
EATKEEILERMVELTLYTEENLK*
>SPBDM4_v1_40973|ID:27158278| protein of unknown function [Uncultured spirochete bdmA 4]
MRKRYDKEFKAKVAVEAVRREKTAQELATICRVHPNMIAFGPVTSNIFQ*
>SPBDM4_v1_40974|ID:27158279| conserved exported protein of unknown function [Uncultured spirochete bdmA 4]
MKKIVSLLLALTLTAGSVFAQASGAMITFGLISDTHVCDKPDQSTTIALNASPRYFTGGL
AKIEAFATAMNAADAEFVAELGDFTDNPATPGLTPARRREVAMGFMRAAEAKFALFDGPR
YHVFGNHDTDQVSKADFMSVVRNTGISPATTYYSFDKSGVHFIVLDASFRGNGASYSGIP
GEPGAGYTWDDANIPAEELKWLAADIATAKSPVIVLTHQPLNNQELIDPAYDPSHTVRNA
SVVRGILEASGKVLAVFSGHYHDGGYQEINGIKYIVVQANAAYGNDVSYHNQYSTVKIFA
EGNKYTLALSGNGLQKSYAFSKVLY*
>SPBDM4_v1_40975|ID:27158280| protein of unknown function [Uncultured spirochete bdmA 4]
VEFEENYMGTTRYSEAQKEKILKTASKLFIDKGYKGTTTREIADVARINKGLLHYYYNRK
EDIVSDIYKAFVEGLIGFVENRYGHELKGLDFFALLNMLFFKVATSERQYIEILLDVLSN
TKLTKYKIDRSMECCLDVLLENGEQEKRSQLLIAITISVGAESQLMLAIEDGSVDMTYRE
LAITVLHVCLVMMEIDEAETEAIIGRALQKADTIDARAILDYIGQRCAWAGLPQAM*
>SPBDM4_v1_40976|ID:27158281|fldZ| 2-enoate reductase FldZ [Uncultured spirochete bdmA 4]

MKYQILFTPFKVGQVTVKNRFCMAPMGNSIGFGAKGEFSQNGVDYYVERAKGGVGLIFTG
ALITDMQVDPFSPVEGLSPLYSPMNFRRTAIALNERVHSYGTKIFPQITMGLGRNYPGLY
APSELPVFFDPNQKSPALTKDEIKKKIDAMVQAAALMKASDFDGVEVHAMHWGYLLDQFA
LAITNHRTDEYGGSLENRLRAAREIVEGIKQVCGSSFPVTMRLGLKSYIKGLFHASLTGE
DEAGRTLEEGVEICKLLESYGYDALNVDAGMYDSFYYAAPPMYQPKGFTLELAKAAKKAV
HIPVLAGGSRIDDPALCAKAIEEGEADAIVIGRALLADSQLPKKVESGQIEDIRPCIGCN
ACMNRAFTTGDALCAVNPTVLREGVYGLNKTVESKKIVVVGGGLAGMEAARVAKMRGHDV
SLYEKSDKLGGNLVPGAKHSFKADMQRLIEWYQHELKKLQVPVHLNTGLDADAIKKMNPD
VVFLAVGSTPVMLGFKGNDDPRVISCIDALLGKKKVGQRVVIVGGGQVGCEMAYEYAKEG
KTVTLVEALDAVLSTGPAVPIMNKMMLNDLLDFHKVKVYTGYTLSSVDKNGVVIKSTKDT
PESIEIAADSVIISVGFKPVPTMAKDLYGSGFEVYEMGDGSKVGNVMSAVGDAYEIARSV
*
>SPBDM4_v1_40977|ID:27158282| conserved membrane protein of unknown function [Uncultured spirochete bdmA 4]
MKKLFAVIALLCMATVFATAEAPVGNTSRPHADVGDLMVNVGANMGWGSFGLGGGVEYIF
AKWDIPNFAPLTFGTAAKAGLFFGNSLHTDIAALGTMHFGLKTFSSLPAFLRNFDWYYGL
GVGFGIGDTGGFGLSTGTGISYYLNPKLALNADYFYTNYFGAGAGSSGTLGIKMEL*
>SPBDM4_v1_40978|ID:27158283| conserved protein of unknown function [Uncultured spirochete bdmA 4]
MELKEAFKTAIKGEIEGRELYRTAAEKTTDKKAKKNFALLADEEQKHLDALLKLAQEYNE
GKELTIPSLPAPTSFQDAESPIFTKEFKETIANKDFEMSTLSIGIKLEIESEKFYREMAK
MATGPDLKKFFEYLADWEKGHYEYLSKQISFFENYYRTKYSMARF*
>SPBDM4_v1_40979|ID:27158284| Alkaline phosphatase [Uncultured spirochete bdmA 4]
MRKDSKKGIILAVLILLAGMQDFAQSVIMLIPDGMSVAGTTLARFYKGAPLALDPIACGL
VSTWSSDGTIADSAPAGSSYATGWASQTGNIATIGKSYVLPGVEIPDYEAQRPVATILEA
ARLSGRSTGIISTSEFMHATPADFSAHDPSRSNYDNLAEQIAYNGIDVVLGGGTPYLNAD
KRKDKEDLKAIIDANGYTFVDNTAALKSAPDGKLFGVFGKDKSATAMSYDIDRDPAVEPS
LAEMTAKAIEILSANKKGFFLMVEGSKVDWAAHANDPVAFVTDILAFDDAVKVALDFAKQ
DGNTVVIAVTDHGNSGISIGDRSISSGYDKTPWTTFIDPLKKAKVTGEGLEAKLASLIKV
DGTYEPSEAPVIRQTVARYLGITDLTDAEVEAIGKTKAGSMNYTVGPMVAGRAKIGYTTN
GHTGEDVVLYCYDPFDKRITGLVRNTDVALYMASTLNVDLGQATSRLFQDAASMFAKKGA
TVSMDTTDPENPVLVVTKADQTLRVPRNKSYAYLNGKAIASDGVAVYNGTKWYVAKNLID
LID*
>SPBDM4_v1_40980|ID:27158285| Anti-sigma factor antagonist [Uncultured spirochete bdmA 4]
MDFVIEEKDTTATYSVVVVSGRLNGASAPEFKHRIKQIVEAGKSRLILEMGGIVFIDSSG
LSALISGLKCAREAGGFFRLADLRDQPASVIKLMMLDRVFDIYADVNAAKP*
>SPBDM4_v1_40981|ID:27158286| putative glycosyltransferase [Uncultured spirochete bdmA 4]
MKSAHHHDTRLLLSRAIGLAACALGLNYIIWRYTSSLNMQALWFAIPMVIAETYGIVDML
LFVFMSWRKPKRDTLPSPEKADVDIFITTYNEPEELVATTAKAALRIDWPDKKVYILDDG
ARESMKKTAEEIGCGYISRGEEWTEKPRHAKAGNVNNALLETSGEFILILDADQIPAPAI
LRKTLGFFADPALAFVQTPQYFYNLPPGDPFGNEASLFYGPIQQGKDGWNAAFFCGSNAV
LRREALMQLGIREYVETVEKREREMVDKLTKRVKKTSCDDEETCAAVKRLTEGLKAAREA
LDDQAPLEHVSDLVIEAVRDAEFLVSKKEVGEIAGAMNELAESGDAEAAAVYRHIIGNLD
SIARKTPPSFEALRDSEEYVEALNLARSDEAVPVQALATISITEDMATAMRLHSLGWKSV
FYPEVLAYGLAPEDLGTSLKQRLRWAQGTVQVFVRENPLFKKGLSLPQKMMYFATMYSYF
SGFFNLVLLLAPIIYFFTGIAPVNSWSTDFFVRFIPFFVMNKIMFRFVASGIPVWRGEQY
NLAMFPLNIRAVLSVLSGKKLGFVVTPKQKEGGHDFRLIWPQTTVIVLTIAAATYGIIEF
FLGRGIPPVGLAVNLTWSIYNIISLAVVVKALYYQPPDGWSAQLPEFARGDARSGHAPSS
DMPALDDAGKPDSAGDPGAEKKS*
>SPBDM4_v1_40982|ID:27158287| putative Phosphoserine phosphatase [Uncultured spirochete bdmA 4]
VPAKNANGTESLIDRWNEENYRLLAENSTDLIARISIEGLCLYVSPACTELLGYTPEELR
GRRVFDFIHPLDRRTVADSLESEVLFRFHKKIVCRLRRKNGYYGWFESKFQFFSDVGTKD
LQVVAIARDIGDRIRAERFSTVRHTIAALKPSEADLEKDFASLLGTICTTLTWDLGEIWL
VEDATSRLKLCSHWCGPSPRLKRFSETSSRMAFAPGVGLPGSLWSRGGVTLIDNLPAMYS
AVRRKEYEEAGLKAAIGTVLADESRTYGVIVFISRRSIQRNQELLDMIEETGLELGTFIA

KFRARESLRAESEKLGTLVEESTARIRELQSEINRQQRIEQDILMAAEVQRNLLPVGTPV
LPGFDFSFAAIPARYISGDFYDFSKPSPSLCDIIIADVSGKGISAAMMTMAARAFFRHDD
TVGISPAALLGAMNGALFTDLDHTEMFLTAQLIRIDSGRGIITYASAGHTEAVRFNPSSG
ECVCFPSTAPPIGILRDIEIGEMEIRTRPGDFFVIYSDGVTEASDESGGLFGMERFVRIL
RSREFVSSADIVQTVLAEVRSFSGDQPLADDLTLIAIGASQRESRLQIAATMENLDPAVA
FVRDAALPYGSKIADDMELVASELVTNVIKHANPESAVAGNHANAAARDTMSMDISLRLE
SGRIIFDLVYPGPPFSPDTRARTLPDPFQEGGRGIHIVRALVDELEYSSTPMNHWHVVKR
VSAEGSI*
>SPBDM4_v1_40983|ID:27158288| Peptidase S9 prolyl oligopeptidase active site domain protein [Uncultured spirochete bdmA 4]
MKPIAIDHFCNYLFPSSLKSNENGRIAFIGKRASLEDNKYLSDLYLLENGKPVKLTASGG
IQAFYWLGDSLVFPDIRKEKDKAYTEKKLPLSVLQRLSPGPGEAQEFVRFAMTLTDMAFV
SENEFFFTAVYDPRIERLPADDEEKATKTLKEENEYIVFDEIPFWRNGEGVTNKKRNRLY
HYSGGTTEAVTDPFTDVLKFSLIPDSGRVIFISTRFTDKMDTSNELFLHEKDGSLKNISF
EPSGFDHQNFISAGADTIVLQGTDRKEYGLNQNGDFFLYNYVTGEKTVIDDSHNHNGYNS
VGSDVKMGGGQGPEWQIDKTGIVYFTETIDDSSHIMSIDPGTRKLSRITAENGLVQEFIL
SGGPDTNEGSGDSFIAIAMRGNDGPELYRIEKNGRETRLTTMNAGAIEGYAVSTPESFTF
RNEAGDEIRGWIIKPSGFDPVAQYPVILDIHGGPKTVYGSVFFHEMQYWASCGYAVIFCN
PTGSDGKGAKFADIRGKYGTVDYNDIMTFVDKCLERNPWMDADRLGVTGGSYGGFMTNWV
IGHTQRFKAAAAQRSISNWLSLSNTTDIGYYFSQDQIGEDLWSGTDKLWWHSPLKYADRV
RTPTLFIHSDEDYRCWMAEGLQMYSALKYHGVDARMCLFRGENHELSRSGLPKHRIRRLD
EIMKWFDKYLKCT*
>SPBDM4_v1_40984|ID:27158289| Formate C-acetyltransferase [Uncultured spirochete bdmA 4]
MNERIKRLREESFNAKPSFSAERALLTTVFYKEWLGRVSTPVLRAMNFYSLCEKKTIYIG
HDELIVGERGPKPKAVSSFPELTCHSEEDLRILNSRPMTRYSVSAEDIEMYQKTVIPYWR
GRSMRDRAFAEMPQAWKDLYEAGLFTEFMEQRAPGHTALDGSIYRKGLNARRAEIAAARA
ALDWGKDPEVLAKDEELKAMDIACKGAILFAERHAELAATMAASEPDPKRAAELRQIADV
CRRVPAEAPRNFWEALQMYWFVHLGTITELNGWDAMSPGHLDQHLGPFYERDIAEGRLDR
DKAKELLSCFWIKVNNTPAPPKVGVTAAESGTYNDFTNINLGGIKPDGSDGSNEVSYLAL
EVLDELQLLQPQANLQVSAKTPERLVKAACRVARRGSGYPSFFNADEVVMAQVGMGKRIE
DAREGGTSGCIETGCFGKEAYLLHGYLNSPKILELVLNDGVDPLTGKVIGLRTGDPATFQ
KFEELYAAFERQLAYVVDTKMRASNYLDRMFAEYAPAPFLSAVVADCIASGKDYYNGGAR
YNTDYIQCCGLGTTTDSLSAIKTHVYEKRDVAWDELLRALSSDWEGREALRLLLSNKTPR
FGNDDAAADSIAQRIFGSWYQAIDGRPSPRGGTYHIDLLSTTCHVYFGLKTGATPDGRHA
LAPESDGASPAQGADRKGPTAVATSLAKIDVAKTGGSLLNQRFLPQTLEGEKGIDSLANL
IKTYFRMGGHHIQFNVVDTATLREAQKKPEDYRSLLVRVAGYSDYFVDLDRNHQEEIISR
TAQEAF*
>SPBDM4_v1_40985|ID:27158290| Glycyl-radical enzyme activating protein family [Uncultured spirochete bdmA 4]
MVSGIVFDIRKYSVHDGPGIRTAVFLKGCPLHCLWCHNPEGLSFSPEILRRPERCLACGA
CALACPLKLEPRAEAGGQKCAACPAFGACVGACPAEALQLVGKRMSVGEVLDAIREDLPF
YEESGGGVTFTGGEPLAQPEFILELLQACRAEGIHGALDTSGFAGRDLVVKAGTLADIVL
FDLKLLEDGRHKTVTGVSNAPILANLAALYEAGANVILRLPLIPGINDASVDLENMAHYI
ASLAAEAGPQKAHILPYHSAAKGKYSLRGENYAMGDTPVPSAEAVNKAAAIFEAAGISVT
IGG*
>SPBDM4_v1_40986|ID:27158291| Transcriptional regulator [Uncultured spirochete bdmA 4]
MNRERDATSEVASLSSAVYEELKRRLNAGTLRPGQFIDLTALGRELGMSRTPLRDALIRL
EIEGFIVVYPRRGVMVRPLQPSDIRDMYQIIGALEASVIEEIPRRFRSSDADRMDSFRTD
MTNALAADDFDDYYSANLAFHDVYLELSDNRKLIDTVHTLKERLYDFPRRSTYVKEWERD
SMAEHTEICSRLREGDFKGAAAYIRDVHWSFEVQERYIRAYYA*
>SPBDM4_v1_40987|ID:27158292|appF| Oligopeptide transport ATP-binding protein AppF [Uncultured spirochete bdmA 4]
VNELNQQTTVDSKILSVKNLKVYFPVEGSKKSAHKYVKAVDGVDFFINEGEILGLVGESG
SGKSTIAYTIMGMNKPKEGSILFKGEDILKNNNKRSISFKKDVQIVFQDPGSSLNPFQDI
RQILKLPLKVHKIVPKDQLDEAVAKALDRVELPRNFSYKTSTSIGGGEKQLVSIARALCS

EPKFIILDEPTSSLDVSIQAKIINMLLKLHDEQKLTYLFITHDLSLMRNISTRVAIMYLG
KICEVAPTKIFYTSPMHPYTQMLLSSIPVISKEEEELKPKKVVSVGEIPSPVDIPTGCSF
HTRCNRKIDICSQKDPFMREIEPGHFVRCHLA*
>SPBDM4_v1_40988|ID:27158293|oppD| oligopeptide transporter subunit ; ATP-binding component of ABC superfamily [Uncultured spirochete bdmA 4]
MNEHLLDIRNLKTWYRTYQGYAQVVDGVNLTIDAGKKIGIVGESGCGKTTTMKSVLRVLD
ERKAYLPEGEILFKNQNILKMRETELQQIRRKNISMISQEPSAALNPVFTVGQQIFDVIS
YSKQDDTAKNKKELTDIAIKAIRDVFIPDPERILECYPHQLSGGMKQRICIAMALVTPRE
LLIADEPGTALDVTIQDQIHRLLRALVDEKQTALIMITHSLGVVRELVDQIYVMYAGNIV
EVATTNELFKNPLHPYTRGLMDCVPRLSGGGISAGIYGYVPDYVHPTPGCRFSPRCPYVK
EICRVQKPLMLEVSEGHTVACFRYTDSFTEQHRQEESIGERA*
>SPBDM4_v1_40989|ID:27158294| Binding-protein-dependent transport systems inner membrane component [Uncultured spirochete bdmA 4]
MTKAQLKEDLRLYWYKFSRNKLSVVGLCIVLLSILCAIFAEIITPYPQHAKEFVDFNQAS
LAPCRAFICGTDIFGRDIFTRLIFSCRGALSMTVVVLSISVPIGVFLGLIAGYYHGTWAD
TIIMRTADVFLSLPSLMLAMAIAAVLKPGMRSSMIAVTAMWWPWYTRQVYGMASSLRNEY
FVKNAELIGASRLHILFREILPNCLSSVFTKMALDVGWVILIGASLSFVGLGEQPPTPAF
GQMISEGARYMPDLWWMTVFPALAIAFIILGFNFFGDGIRDMLERGR*
>SPBDM4_v1_40990|ID:27158295| Binding-protein-dependent transport systems inner membrane component [Uncultured spirochete bdmA 4]
MLRFVGKRLFEGIFVLIGISIMLFIIARIIPGDPARMSLGPRAPQFAVDELRKEMHLDKS
LPVQYLYWIKGVLQGDFGKSINTKRPVSTDIKEFLPATLELVLFSGIFLIILSILLGLLA
AKYRDTLVDNMIRGLSYIGIAIPAFALAILLLLLFGYVWQVIPVLGRLSSGVAAPIHITG
LFAFDSLITGNFTTFFDTLGHLILPALALATGPLFQEARLLRASLVDNMGKEYISVATGY
GLPSRVIMTRYLLRPSFIPVVPVMGMDLASLMGNAFLVEKIFNWPGISRYGMNALLNKDL
NAISAVFLVFGAIFLLVNIIVDIIVASLDPRIRLGAKS*
>SPBDM4_v1_40991|ID:27158296| putative hydantoin racemase [Uncultured spirochete bdmA 4]
MKMEILVPVATNFRNAEILYEAKKTQSSNVDIGINNIKQGPTSIECDYDSAFAENYVVEM
AEEMEAGGANGIVLYCFAEPALSACKEKLNIPVVGLREASIAIASLLGDNIGVIAPMENT
KKSFSRALGKKVRQIISLDLPVLEYADQEKVRTAIESKIQQLVQNDCDVIVFGCGSILGL
DIEHLQDKYHLPIIVPIHAAVAVCKYLMQHKLMQSKIAYPSPALGKVIH*
>SPBDM4_v1_40992|ID:27158297| Extracellular solute-binding protein family 5 [Uncultured spirochete bdmA 4]
MYVEGRTNENEWRKIGSCMHQEKQHSIEYSKHIKSKTFSKPHYIKKLLLLLFVFWGIVLT
NSSAAPQDAKNEKIIRIAVSGTPVIDPAVGLYVSSSIALVNIYDSLVFPSASTAEMEPAI
AQKWDISKDGKEYTFYLRKGVKFHNGDELKASDVVFSTKRLLRIGEGYAYIFTDIIKDVV
ALNDYTVKFFLKKPFGPFLNTLNRLYILNERQVKEHIKPGNYQEWGDYGRDWLITHDAGS
GPFMVKELVQQNYLYAVRFEEWYGGKWDPNAPTAFKEIDTTEASTIRTMMRNKELEITDM
WQSSENLNAMSKLPGVEIVMYSALANQNMYFNTKKAPTDDVNFRKALACLFDYDMIIKNI
FPGSSRSFGPVPIYVAGHVNTNQYNFDLDKAKEYLSKSKYANQLDQYPVEILCNSDVADH
EKVALAFQAAARKIGIKVEITKAPWISIVDRVAQISTTPNLVSININAQYNEAGSMLETR
YSSKSSGTFENGEWLLDKNLDSMIEDSLSTSNKEERYKKYANIQNKIVDEICPTAWLGDT
LDRVACQRDYVYWPVAEASRKGKYVSNIYGYHYFFFDMRVYPDKK*
>SPBDM4_v1_40993|ID:27158298| Asp/Glu/hydantoin racemase [Uncultured spirochete bdmA 4]
MRRILWLNPIAHSDHDPIMKKMFQREALPDTEVMVKSLTNGPWHLGYHYYGTLVLPESIA
IFLNAEREGYDAAVMGCFYDPGLREIREVLTTMPIVFPEETCTHLAATMGEKFSILVAEK
KCIPAMSENLDRYGLSKKLASFVSLDMNVLEFQKDPAVTQSRMMAAGKKALDDGAEVLIF
GCTMEYGFAPKMQDTLGVPVIDAAITSFKYAEFKADLRQRYGWSHSKRCAYESPKNEEIT
AWGLDKNAFDTQII*
>SPBDM4_v1_40994|ID:27158299| conserved protein of unknown function [Uncultured spirochete bdmA 4]
MYDLELVGASIKLLRDMFGLQKGESVVITCDTESNYDVVDATAQAAYALEGKPIIVKTPA
ARGVGKAADPDLPVKELIGLLSAADIWIEYNNEWIFYSTVFDEAFRKNKNLRYTNLVGAN
PDLIMRNIGRVDIPLLREFILKVEDISKKAKHIRITTPAGTDVEFDNDPNRAFYTADGYI
RKGEIKMMPGQISWTPLFKTINGTIVIDGTLTPPLGKVNDPIRLTIKNDKVVDVTGGQDA
KVFKTWLESFKDDRMFYLAHLSYGFGPGAKLTGDIVEDERVWGSTEWGFGNIGPMLVPDI

PGGIPASSHSDGICLNSTVYLDGELLLKEGQIVGPTEEIVALARKLGK*
>SPBDM4_v1_40995|ID:27158300| conserved protein of unknown function [Uncultured spirochete bdmA 4]
MRDRLIKNLCELVKIDSESGEEQEFLHFLGEKLASELHATIQFDAYGNLIAKIPAKHCSI
HEPLMFAMHGDTVKPGKGIKPVIKGNIIYSSGDTILGADCKAGIAEFIEATLEAPHHPPL
EVVVTRQEETGMFGVKNLDFSLISARKGFLIDMDTIDAVVIGTPSRMIINVDITGKPAHS
GMEPEKGISAIKAAANAITLLKDGRIDFETTGNVGIINGGLIVNSVPEKASVKAEVRSLN
HDKCVHISETWKEIFEVSAKALGAKAEVSFTLDCKAARMSAESPMVQVTCDALRSIGMEP
KLMVITGGLESSIYNEKGIETVPIGNGVKAEHTVSENVSVEDMEKVIGVISFLFSHFC*
>SPBDM4_v1_40996|ID:27158301| putative Transcriptional regulator, PucR family [Uncultured spirochete bdmA 4]
MAVTVRQIMKSEIMGKARIVAGESGLDRVIRRVSFIDSPFTAEVLNEPGILMPGDFFISS
FFVVKDNPDQMMEMLKLLLASESSGLCVISKLFSELPQVLIRFADEHNYPIVFINQDYPY
GDMIRDILMLIVQDKEGMLIEMKLNDIVGLGKDEDKKVEEKMLKINNHFAKYVVAVYCRS
FDLEDITTLVFLKNRINVIKEWLCVKFRDNILIILTFGRSDQDNINTKLKYVFNQLKQVS
KRYMAGISTVGDLGKANRVIMEAMIASELKPRHDQDTVCYRDIGSYKFLLPLKDQPELLE
FHDEIILPILEYDEKYKQNLMKTAVCFVDNDGDFSKTAEKMYLHENSVRYRLNKIMEILN
FQDKNMAFYEQLFIAVKLHRILKGFEGDQTV*
>SPBDM4_v1_40997|ID:27158302|livF| leucine/isoleucine/valine transporter subunit ; ATP-binding component of ABC superfamily [Uncultured spirochete bdmA 4]
MDTLLEIQNIVLDYGAVRALDGVSVTVGENEIVAVLGANGAGKTSLLKAVSGLRKPTSGQ
IIFRGKDMANTPAFELAGQGIAHVPEGRRVFSTLSVRENLMLGMYGVRRARDKKQLSTKE
ERIYSLFPILKERRNQLAGTLSGGEQQMLAIGRALVSAPALLLLDEPSLGLAPIIVKEIF
EMIQKIHEEEKVAILIVEQNARKALKVASRGYILELGKVVLEGNAPDLTSNPKVRAAYLG
GGMEGCD*
>SPBDM4_v1_40998|ID:27158303| ABC transporter, ATP-binding protein [Uncultured spirochete bdmA 4]
MNITKTVLDYVTPAGQNKGRGPLPDRKRPGLLTAAIVSIAIAPFLPFIDNYWIDIGFYFG
IYTLLGLSLNIVLGEVGLFDLGHTGFYAIGAYVTAILNTTFHIPILVLLPVSALVAGLFA
WMVTAPVIHLKGDYLCIVTIGIGEIVRLTMINNPFGLTGGPNGINGIDTPVFIVPIVSSR
QFYYLIWALIAIVIFGLVRLQRSRVGRAWNYIREDEVAAEALGINVRHYKLAAFVLGAGI
AGMAGNIYASKQMSVSPESFTFMESSLLFCIVLLGGLGSIPGTVLGALAITIFPEIFRPL
AQYRLMFFGLALLVMMIFKPAGLMPRQRESGEKLRSALSKKRREAKGRLTGNDGCRKDMP
PPGRAENLLEVKDVHLSFGGVTAVAGIDAEIEAGKITALIGPNGAGKTTLFNLITGIYKP
QAGSIRFNGEDISGQSPHAIAGKGIARTFQNIRLFPSLTCLENVLSGQHCRGKAEFTASL
LHLPSQQDEETQMLDFSLQCLDRVGLSDSTDKLAASLPYGKRRYLEIARALALRPELIVL
DEPSSGLNDAETEELAQLLLSLLHDGLSVLLIEHDMHLVDRVSDHVIVMQSGKKIAEGKM
CDMRKNPLVIEAYLGTEED*
>SPBDM4_v1_40999|ID:27158304|livH| leucine/isoleucine/valine transporter subunit ; membrane component of ABC superfamily [Uncultured spirochete bdmA 4]
VTILLEQIVNGLTIGSFYALVALGYSMVYGVMKLINFAHGDLFTLGAFLGYTILASTSGV
VTQNFGIWGGIAFTAAIVGMSVALAGILVERVAYRPVYPSGRLSLVVSALGMSIFIQNAI
MAIWGARPRAFPASVVPSARLVVFGVPVTVLQIIILAVSFILMAAIWFIVEKTAFGASVR
ASALDRETATLIGIDVRKVIFFVFALGPGLGGVAGLMNGLYYRSISFNMGWNYGLKAFTA
TILGGIGNIPGAMLGGLLLGILESLLAGYVSGAWKDVFVFVILIVTLIFRPTGILGEKTA
EKV*
>SPBDM4_v1_41000|ID:27158305| Amino acid/amide ABC transporter substrate-binding protein, HAAT family [Uncultured spirochete bdmA 4]
MAKKTNTGRRLVLEVCCVVLVSTVLILAGCGTSKSEKTIKIALQAPITGDYAYEGQMAKQ
SVDVAAELINKAGGVLGKQVEIVVVDDGSNPKDSALAAQKAVSQKVVAVIGSYGSSVTEP
AADIYEKNKLVSVGYGCTAVRLTMDKERKYFFRTCGRDDAQGLFFGKYAVETMGAKRIAI
MHDNSTFAKGVADEAKKALDPYIAEGKTEIVYFDAITPKEKDFSAVVTKLRETNPDVWYF
TGYYPEAGLLIRQARDAGLTCPFIGGNAAINDDFVKIAGLDVAKGAMMTQEPLVTDVTTD
IAEQFKSLYMEKYNELPSSPWPVYAADALYAITGAITKSGKTDSNSIAETMRTKMDGVEG
VTGPVLFTDRGDRKDVPYKMYIVDPEGKLKVYNQ*
>SPBDM4_v1_41001|ID:27158306| protein of unknown function [Uncultured spirochete bdmA 4]
VRPASTWWAVTVSRPTDGIIDTFLNEFSLIYVFIVPMKKARQALTLSICLDP*

>SPBDM4_v1_41002|ID:27158307| conserved protein of unknown function [Uncultured spirochete bdmA 4]
MIKKVFLDSDIILDVATGRMPFVEHSTSVLASIENGKVLGYISSNSVKDYKK*
>SPBDM4_v1_41003|ID:27158308|bfmBC| Dihydrolipoyl dehydrogenase [Uncultured spirochete bdmA 4]
LKRRCSLGQKTKNKEIGNHMGFDSDVLILGSGPAGYVAGIRAAQLGMKALVVDKVKSGGV
CLNRGCIPTKALINQAEDFLLLAEVEKTGVKVDRSGFQYKSVFETSRKASESLSKGVQYL
LKKNGVGFIQGTARISGPHQVTLDDGRVLTGKNLLLATGSRPRQIPGFEFDGTDILSSDH
ALFLEELPARMLIIGGGAIGCEFAHVMNAFGVEVTVAEMMDHLLPQEDPDTAAIVERAFR
KRKIDLRTGARVAGYQRREGELVVPVTLKDGTAEELVTDKILVVTGRAQNTDDLGLERVG
IVTERGAVPVNAHYQTVVPSIYAVGDIISSPQLAHLASAEGEAAVEHMAGKGPEHAVDPL
LVPRGVYTDPQVAGFGLTETNAKAQGKRYAISLFSYRAAGKAVATGKTDGQVKLVYDPDT
KEILGAGIVGADATEIIHEVLLAKKSGLKTGDIAGMIHAHPTLSEAIMEAARGAEGWMIH
A*
>SPBDM4_v1_41004|ID:27158309|gcvH| putative glycine cleavage system H protein [Uncultured spirochete bdmA 4]
MAEEYEVRAIPSYTKEDTWVRIMPDGMVKIGITDFAQKMLNEIVAVSLPDVGSEVRQMQI
FGSVESVKSVSDVYSPISGKVKEVNEKVLEEPAILNQDPYDAGWLLVVEPAKLDEELKNL
LNADAYKALIQEKGH*
>SPBDM4_v1_41005|ID:27158310| putative CoB--CoM heterodisulfide reductase 2 iron-sulfur subunit D [Uncultured spirochete bdmA 4]
MLEKYKQAVTVCSRCGMCIVGENGYICPVQQHSGGFDPSVARGRNQIARAILEGRLKYSD
ELMESTFTCLGCDNCHEQCGRVDPRTGEKLIDEGKITRALRVDLVKAGYGPPEPLKAVDA
NMEKVHNPFGEPEGRRSAWAAGLNLPVLGDTVYFAGCYAAYRNPKIARATVAVLQKGGVN
VAYLGENEWCCGVPELWDGNAPLSEEVIQHNVEALKAAGAKRVVTSCAGCFHALKSDYPE
IIGKLPFEVVHSSEVIADLIASGKITLDTEVATTFTYHDPCHLGRYEKVYEPPRAILKSI
KGAKFVEMPRNKNNAWCCGGGSVVAVAFPDLARDIADDRVQEAKGTEAEAIVSACPLCEN
GLTQSARKAKMEVYDLSVVVANAMGMHV*
>SPBDM4_v1_41006|ID:27158311| putative D-lactate dehydrogenase (cytochrome) [Uncultured spirochete bdmA 4]
MVTMAIRHHHLHDPLAAIVGSKYVADEDFVLETYSRDISAFPGSMPGIIVRPGTTEEVSE
IVKLANRTGYPIVLKGGGQGGSGVTKGEPTRNIVIDTGRLDKVEVDIANLKATCGAGARN
SRIDDALRPYGYYANTVIGPYFTATIGGVTSGIAGAGFGKNVSSVGCNWGHILGLKVVLP
TGDIITTGAGPDSNIYRKGIEFREVTGPDLTSLFIGSGGALGIITEVAIKIYPTPKYSKA
ASYVFDTMENIWAAQLKMAESPTALYTNLIMFEMANHMVRAMAGDMHGYGALFMAVEGDS
EADVDLRLKEIEKICTGFGATKGTPALDHYAATGATGTGAFVHDVCAMSCPFMTWESMST
RSQSLEYAKGMLDILNNQFKEENAKYHTSGGLYTVPIANTMLIGITIHWDDTVPGVEEHN
RKLWKAGADFMLKNGTFSAYAQGNHSSSISSAWSPTYYKVMSGIKKTLDPNNILNPGLWN
L*
>SPBDM4_v1_41007|ID:27158312| protein of unknown function [Uncultured spirochete bdmA 4]
MKLSMWILADWLGEFAPSLHIIEGKAGLTGTRLYSAGSPEQDPSAAIIGDAAQMLPRGSR
ARTDAVLCLNGEDWLLFDHGDVGVILNACLEAFEFYNAWESAIKDVAYASPDAPFEKLLD
LSSPVLKNPILVADWKGEVLAISRNHKPIADRETWGHWVDQGYFPSYTYDRLKNYPQFLE
DMNRSEEIRIFDFPQFEYRCIHFSVRYNNETSLYVHIIEDDTKLTEGLIQVASVLKQTIA
IVLDRSNVMAKTNQIVSLFSDIIAGKYPERDALVWVRSQLGWDKSKTWYLVIFRNFLPES
FSETALLEVLKKQVRKGCSFIWENHPIMIVDCEEWSVAFPKIKKLLGGSGFCCGVSMPFD
KQKDLPFALNQAKLSMELSDGKNTIAMCEDCAWSYIVKEIKSQITDMQMLHPAVKVLQEH
DRQTGGQLTRTLYEYLRHERSADATSKALFIHRNSLRYRLDRIEELIDADLNDPDTRMYI
ILSYEIKEMTSS*
>SPBDM4_v1_41008|ID:27158313| protein of unknown function [Uncultured spirochete bdmA 4]
MERTATGNLFFERPILNSPYEYPKRHWELDKAKIVITNYHAFIPRERMELSKGGRSLLQG
TGAPLQTLETEGQVLQRVMPELMGLRNVIAINDEAHHCYREKPDGEAKLSANEKKEAEEH
NKAARVWISGLERVNRKVGLARVFDLFGNADQLDPLSIPEELQTALEALYGHYRKTFDLW
EAAGIGGTSMSGGEGTLVGALKNSSFPLFHTTE*
>SPBDM4_v1_41009|ID:27158314| conserved protein of unknown function [Uncultured spirochete bdmA 4]
MNPKVDEFIRDAKKWREEYEALRTIVLDCGLTENLKWGVPCYTFNEKNVIIIHGFKEYCA
LLFFKGALLADTDRLLIIQTENTQASRQIRFTDVREIVEKEPILKAYIHEAIEAEKSGAK
VNFKKTAEFAIPEEFQRKLDSTPGLKTAFDALTPGRQRAYILYFSSPKQSKTREARVEKS

TQRILAGKGLNDQ*
>SPBDM4_v1_41010|ID:27158315| protein of unknown function [Uncultured spirochete bdmA 4]
MNQTLIHNLSIIVEDACKSDANVFGYGIWSNHIRPMIPVARRLADEYQADAMVHIQEIGI
K*
>SPBDM4_v1_41011|ID:27158316| exported protein of unknown function [Uncultured spirochete bdmA 4]
MLMNRTRNAKLTFGIAIFAALALFLSVLPMGATESSSYQAGFAVGSSQLAQIEENIAEMK
LAAQKRGVDYTTVLAAAREREALALQILPEKVEWMHGVADGSGISYEDVLVYNTSDRLMT
GFVGECTTFIANGKALAAGAGSIISKNRDLGPNTISEVAKEEGRDYPSTELYKAAYIDIP
QASRTYSFVGSRSAGRWGYGMGINEFQVIVSDNDAPTRDELAFTDGLHDNDYVRLVLERA
KTAREGVQILTKLTEKYGQAWNAIMFEIGDPQELWVVEITGRRWVARRYQDTYTARSNQF
QITDDYEICSHDLLTYAQSQGWIGADVTKINFRAIYGTTELYPSDNNDIASRPAVETLYN
TEKRYERAMELLQGIAGNISAQTLMPCARDHYDTYTLPSGEVLKLNQVPYYSTDYVYDRQ
EWMTEPPTKDTVEVSIFPRAICHHAFEGTTAATGILIARPDVPNELGLMIHAYDQPCNSL
FIPFYVGASSVDTRYSTPEAGSRFLTISKLAFGCYTTYHEGIRSVFDPYEKALFTEMPAM
EKNYVDLKAAGKTMAAQAALDAFCAQHWTKGFELADTALKTMVEETAASSAWGR*
>SPBDM4_v1_41012|ID:27158317| Peptidoglycan-binding lysin domain protein [Uncultured spirochete bdmA 4]
MRRRVYAVVVILFALTTSFAFSQDLKSTEFVVYIPNEGRMVPATVCLPIGAPGETFPAVV
MLHGTGSSRDEAGNGYKMLAPYMAQNGIASIRFDFAGQGDSKADYVEYCYTSGVSDAVAC
TEFLKNLKVVDPSRIGIMGWSQGGSVAILAAARNPQYKALLTWAGALDMFGFFGDSLYQK
AKVDGFVKIDLGFRPPLNLSLRWFEEAKDISLRDELKKFKGPVLAIAGSADTTVPLSNLD
DIVANAGGTDKAKYLIDGADHTFNVFTGDLSKFNQLKEVTTDWFKSKL*
>SPBDM4_v1_41013|ID:27158318| AAA family ATPase [Uncultured spirochete bdmA 4]
MIARQAADTLKRLEKGFPVLCVTGPRQSGKTTLARATFPDKPYLSLEDPDIARLAREDPR
GLLESYRDGLILDEAQAVPEIFAYLKTAVDTDPRPGRYIITGSQQFGLLAGAPESLAGRA
AFVSLLPFSGEELKNEGKLPSDPFEAMVRGFYPPLYDREVSPYDWYTNYLASYVERDVRS
ILNVKDLGQFQTFVKMCATRIGQLLNLNSMAVDCGLSHNTARAWLSVMETSGIVYLLRPY
HENYGKRLVKSPKLYFVDTGLAARLLGIKTAEELFVHPNRGNLFESFIVSELLKARYNRG
RDPDIYFWRDSAGTEIDVVYDGGPKVKAIEIKSGKTFTPEFTKGLEAWMRYSQAKPSDCA
LAYAGERRMKWKDITLVPWASASEVVKE*
>SPBDM4_v1_41014|ID:27158319| conserved protein of unknown function [Uncultured spirochete bdmA 4]
MTGREASIRARLKVVAAQKGITFQYATMLYMHEGFLRRLSASSYRDRFILKGGLLLQCLS
ESSPRTTKDIDLLGRGIPNDAAQMKVTVSEICQTPEDDALCFDHESLKFEDITEDNEYHG
IRISIPCYLGNIHNTIQVDIGFGDAVEASFQKIDYPILVDGRSFSMLSYPLASVVAEKFE
AMVALADINSRMKDFWDVAYLLEHQSISDEELLKALQATFQQRGTPIPAEPTVFSSAFAS
SDMALVRWRAFMRRSHLPFREWADVLSIIRARLEHLYRDIQK*
>SPBDM4_v1_41015|ID:27158320| conserved protein of unknown function [Uncultured spirochete bdmA 4]
MTRETEQKVLSIIAAKGGYARAGALRAEGIHSSQLSALVESGALVRLKRGLYALANGIER
SELVDIQKAIPGGIFCLGTALSIHGIGTWEPPEIQLAIRRDYRIAIPDFPPVRLFSFSGT
RFELGIIEKSVGTGTIRVYDREKTICDIIRFRNTLGVDIAMEALREYLKEHTRNIPRLLE
YSKLLRMEGPMRGYLEALV*
>SPBDM4_v1_41016|ID:27158321| Helix-turn-helix domain protein [Uncultured spirochete bdmA 4]
MSISFGQRLKAARVMAGLSMDGLVEKINNKVSKQAISKYENGLMMPDSSVLIAFADALGV
GVDYFFTESKVEIADLNFRKKASLGKKATDSLDERVRDALERYIELESFYQSAPTFENPF
AGLVVSDRDDVERASATLREAWGIGPGAAVSHVVDLLEEHLIKVIELEDDDAFDGLSGRA
NGNPFIVLNARMPTDRKRLTALHELAHLCLDFDPGLRKAEKEKLCHSFGSSFLLPRAVLE
RELGTKRSEISLFELAALKRHYGLSMQAIMYRAVSCGIISDYSYEQFSKMISAKGWRKTE
PVIYPVPEQPRRFKQLLYRALSEDLISISKAAYLGRMSIDELRREQALEDVFSHS*
>SPBDM4_v1_41017|ID:27158322| conserved protein of unknown function [Uncultured spirochete bdmA 4]
MPEESSGKLRHLIVEGFSETENYKRPRGGSSVKVPEQDRALHSDHLLGQIKTISNDMLSA
IQIQKKTGLEDDLGITIEFESFPEIKDAFDDLSFGRTGIELLNLRQIGLSTFATVFIPEG
ELQYFIKKIDAYRLYGKDKNGKPRDNQKLVDTIKEIRTASLRALWTDDENCFPAESEPAL
WWEAWLPLRGDREKTVRLFRTMAAKLGLPTSKSDMSFPERTVVLVFADREQMESAITILN
TVAELRRAKETAEFFVELQPHEEKTWVDDLLKRTDYAEATDDVPRICILDTGVNRGHPLL
GKALTSDDLHSVDPEWEFADQDGHGTAMAGLALYGDLSEPLSSREHVSLAHRLESSKLLP

KDGANTGDEHLHANLTLQAVSYPEIAFPHRKRVYELAVSTDDYRDKGRPSSWSAAVDQLA
ADRDNDEKTPRLFVIAAGNINDIHKWERYPTSKDEEGIHDPGQSWNALTVGAYTNKVEIR
GERAQGYTAIAPRGDISPFSTTSAKWDQQWPLKPDIVMEGGNAGRDLFGAACLDSLSLLT
TNHIPSRRLLTTVNATSAASVLVARMAAQIMAHYPELRPETVRGLIVHSAEWTKAMKLHY
GIDENSSKDAYANLVRHCGFGVPSLERALWSMSNSVTLVVEDGFSPFREGETSICMNEMQ
YHTLPWPAEALSSMGETPVEMRVTLSYYIEPNPSSRGRSKYCYESHGLRFYVKQPSEKMN
DFMRRKNGEFREEGTGYTSANDSTFSWLLGEKARHHGSLHSDIWRGTAVGLADCNAIAVY
PTGGWWQKRPALGKYSSAARYSLIVSIKSPMADVDLYTPIETAIKTRIASKVEIG*
>SPBDM4_v1_41018|ID:27158323| ATPase central domain-containing protein [Uncultured spirochete bdmA 4]
MANAAQMVALLKSHLDGDDEHFYAVALQVAANEAKQGHDKLAIEIRQLIDEAKRSKAKRN
IIPLNHPRGELTNLLLVSYPEYRLSDMVLGEQLALQLKRIIKEHRNAVKILEHGLSPRRK
LLLIGPPGTGKTLSASVLAGELGLPLFQIRLDGLITKYMGETAAKLRQIFDVTTETRGVY
FFDEFDAIGSERGLANDVGEIRRILNSFLQMIEQDSSNSLIVAATNHPEILDRALFRRFD
DVLKYELPDERQAARLLQERLANYSQNYIDWGPIGAIAVGLSYGEITRACQDVIKDALID
GKTLIAGPQLVAAIKERHELVEKLKTIV*
>SPBDM4_v1_41019|ID:27158324| conserved protein of unknown function [Uncultured spirochete bdmA 4]
MARKIAAPKTTGGGGFVFEDDVCAWLMACILAGKSVFNPEYGPPVRLDFQTKPAGWFLDD
VLVTTKGDTTYHIEFSLKSNPQFTASKAPRDFVKTIWEQWLHIGSEVFDRSLDLMGIITA
QLSTAAYTSVRSLIAKIRVADPDSFPRYIGNPNWANKNERNLFSSFACPTQLTADRYITA
SDTVRLLQRIIFYQHDFGTTPSQSLNDSLGICRDTLRNKSAIEAEKLWNELRSIAAEYRP
HAGSIGRNELVEKLRRHFVFVDYPDHAADWVTLEGYSRRSMQLICDSIAGRIHLPQESTL
AAISEVLSIERLIALTGDSGVGKSAVARSLFESRVSVGGRTLWFDAHSFECPDMHTIEAN
LHLRYSFENLFTNTSTSDSVLILDGLDSLYPDTAFRNVAMLLHIAYQEAPTAQWRIVLPC
QSNEWPRVLECIRRAGFIDGQWKQIELKRISNKELAKVRDAIPSFTKVLLQPRMADLLGN
LKILDLVARHVIEGSVIDASSWVGESSVADWFWRAIVDVGQNATIKVRFVRCLAQIQGDN
LSRSVSIDDFPSEIDISPLDLLQKDQICIRTEDDLISFSHDLYGDWIRLHILLGHHTDLA
SFLRSRRESPIWQRAIRLLGVHLLEHSGGIEEWRTILESLDTKEDSPIYDLLLDAPIFTT
NPLPLLEAIEPDLLANHGQNLRRFLSRFLVFATVADPMVLAFAKVEGMDENVARATHRIP
YWPYWLDVIRLLHTHKAEVVAVAPYEIACVVELWLTFKQKGRILRSEVAELGVLLGRYAL
ESAEVYGGPDLHVERQKFFECALLSACEWPDEVAEIALRAANRKQRLIGSSVASEESRII
SEDQRIERFVHSASNIKVWPDGPQTRIDDAFQDAVLKSQVILALFDVRPDIAREIVLATL
IEPPREVYWYDDMETLEELAIVEPHGWHPAFYTDGPFLSFLQMNFKEGLELIARLVDFAT
SRWNEYEEKNNRDEQAYAQTQGEFEDEQDQQFSPSIYKGLMVGIEEEAREFYGDMSVYGW
SAGLGNPPAAVEASLMALEQYFYIQLDKGKDVTSEIEAVLARVKSVAFLGVLCDVGKRQH
ALFEGPLRPLLSKPEFYEWDINKMVLGREHLMIDAFLKGNWFMEIAPKFHQLEHRKEDLR
NIATFLMLQCPAMREYFNRVRIYLEASRAPDSSGPISPMVDQLITLLNPSNYQLYRNQDN
SVELVNVEELRLQQKHAPELRLINNNMLILTFPMQCQKVLEEEQKLANEELDVMYKKWKR
ICGLTKRDGEFSGDKELSENRYVSAMLAGIAVFLHYPDWCAQESSRNKEFITILRAVLEN
PPDIDKYDSPYSVSKETWDCFAADAVVTLWAREPANVEWRQAVAEMVFAQHYAAVALLFA
RCASMRTLFRTDFSQLRRLAIEWAYIRKFMEAVPNLSRDIQDTNGAALLERMPDAIADWA
QERVAAFVSGSIMPMLLDWDECDNRRKFSEIASTYLQGRPLPAIDLHLVRCVYDWLPLPE
KALDENERHDWIQFWHSALKEVIKPRIATREPAHRRYPNEDERWFLDSIAAVVLQLRDDE
HADFFWQAIFDLYGEGHNWSELFIQALHRHALTQDQPPQSYVSLLHRMVSYALTDIKGKT
RWPWYERVWDALIGVDMFSIRLWEPRHTVVIKNLSDTLDLWMAQVSSDENRLVNFASWLA
RPAAEPVRLHCLGWFLNHLPVRRQSRSSDFNSAADAIASLLNVTWTKNEIQLRNDEYAFR
SFRKLLTWSGDQQNELGLDLLSRIGSL*
>SPBDM4_v1_41020|ID:27158325| DNA methylase N-4/N-6 domain-containing protein [Uncultured spirochete bdmA 4]
MANHTSKKSVETFRHGEATRTNIPTAEYQSIMNAKEETPLRVAYERRNRDLDPQLVWRGK
DSQDWSDLVVSAPPLYIQEKIHPKVLIDDLMRRTEEAKKSEDSQPELFSDFNGLLDENAR
TEFYQHEGHWTNRMILGDSLQVMASLAEREGLRGKVQCIYMDPPYGIKFNSNFQWSTTSR
DVKDGNPDHITREPEQVKAFRDTWRDGIHSYLTYLRNRLTVARDLLSDSGSIFVQIGDEN
VHRVRCLMDEVFGEENFISQIGFLTTTGKASAALDSTCDYLLWFGKSVEHLKFRQPLFLR
DVKQDSNFRYDFTIEGNFISQSEKPYRIARMNPLTSQSGSTTTLFEYQFQNQLFKPGKGG

WKTNLEGMERLQKSNRLTSSGKTLGFVRPFLDFGAQSYANIWDDSRQSGFGDEKKYVVQT
ATIVIQRCLLMTTDPGDLVLDPTCGSGTTAYVAEQWGRRWITIDTSRVALALARARIMGA
RYPYYLLADSREGQLKEAELKGEAPSSQPVFGNLRRGFIYERVPHITLKSIANNTEIDVI
WEKYQTTLEPLRTALNEALGKSWEEWEIPREAGTSWSTAAKEAHRKWWESRIARQKEIDA
SIAAKAEFEYLYDKPYDDKKKVRVAGPFTVESLSPHRVLDVDEDGEFIDTTSEAEPGYGG
ARNFTQMILENLKVAGVQQAHVDDKIVFTSLTPWPGDLICAEGRYVEGGSQASKNESVRP
EKRAAIFVGPEFGTVSRPDLVAAAREAGDAGFDILITCAFNYDAHSTEFSKLGRIQVLKA
RMNADLHMADDLRNNGKGNLFVIFGEPDVEILPAPDGQIRVRVKGVDVFHPNTGEVRSDG
PEGIACWFIDTDYNEESFFVRQAYFLGANDPYTALKTSLRAEVNEDAWSTLRSDTSRPFA
KPASGRIAVKVINHLGDEVMKVFRV*
>SPBDM4_v1_41021|ID:27158326| Type III restriction enzyme, res subunit [Uncultured spirochete bdmA 4]
MEQTATGNLFFERPILNSPYEYPKRHWELNQDGQPTQKIIESRRRAEFITPIPKPRVHRG
AAGQAQLIFDEGKGLSTEKQQYDPTAVINQVRSHVDEWRQLPDKNDWHVTPETARLLEHW
RHHQFSGLRPFFCQIEAVETAIWLTEVAPTQGKTGDTFLEYLAVASEDANPGLPRLALKL
ATGAGKTTVMAMIIAWQTINAVRHPQSRRFTRGFLVVTPGITIRDRLRVLKPNDPDSYYA
SRELVPSDMLPDLDKAKIIITNYHAFIPRERMEVSKGGRSLLQGTGAPLQTLETEGQVLQ
RVMPELMGLRNIIAINDEAHHCYREKPDGEAKLSADEKKEAEEHNKAARVWISGLESVNR
KIGLSRVFDLSATPFFLSGSGYAEGTLFPWTMCDFSLMDAIECGIVKLPRVPVADNIPGG
DMPKFRNLWEHIRTRMPRKGRGNAGELDPLSIPVELQTALEALYGHYRKTFDLWEAAGIG
VPPCFIIVCNNTSTSKLVYDYVSGFWRETGDGRKLFENGRLPLFRNYGEDGKSIGRPHTL
LIDSEQLESGDALEKNFRDAASDEIDRFRREIVERTGDIRQAENITDQDILREVMNTAGK
PGRLGDSIRCVVSVAMLTEGWDANTVTHVLGVRAFGTQLLCEQVIGRALRRQSYEMDEDG
HFCVEYADVLGIPFDFTAKPVVAPPQPPRPVIHVRAVSPERDALEIIFPRVQGYRVELPE
ERLTAKFNEDSVLELTPDLVGPSVTRNSGIIGESVDLRLDHLETVREPTILFKLTQRLIE
TKWRDPGEEPKLYLFGQLKRIAKQWLDTCLVCSGGTYPAQLLYQELADMACERITAGVSR
AFADERPVKVVLDPYNPTGSTIHVNFTTTKRLRWETDARRCHINWVILDSDWEAEFCRVA
ESHPKVLAYVKNQNLGFEVPYRYGSETRTYLPDFIVLLDDGHGADEPLHLVVEIKGYRRE
DAKEKKSTMENYWIPGVNNLKSYGRWAFAEFTDAYRMEADFETRTGEAFSAMIDGAVSQG
GKK*
>SPBDM4_v1_41022|ID:27158327| conserved protein of unknown function [Uncultured spirochete bdmA 4]
MNPKVDEFIRDAKKWREEYEKLREIVLGLGLAEDLKWGFPCYTLQNKNVVLIHGFKEYCA
LLFFKGALLEDPEGILIIQSENVQASRQIRFTDVREIVEKEPILKAYIHEAIKAEKSGAK
VNFKKTAEFAIPEEFQRKLDSTPGLKTAFDALTPGRRRAYVLYFSAPKQSKTRESRIEKC
VPRMIEGKGLNEWTSMKEARHHDTKSDGQ*
>SPBDM4_v1_41023|ID:27158328| conserved protein of unknown function [Uncultured spirochete bdmA 4]
MIRKATVNDASRIAEIQIFGWRNAYRGIIDDKILFAKLNIEKKSQSLRKVLEEGTDEWYV
FEEKEIVKGAMVAGKCRDADKPEAFELWCLYVDPFMMRKGVGSQMLEYCEKMARDRGYKE
NILWCLEKNGIGTGFYEKRGYRRDGKAQEIEHLKATEIRYRKEL*
>SPBDM4_v1_41024|ID:27158329| conserved protein of unknown function [Uncultured spirochete bdmA 4]
MDELLVKLLDGEGKVKRWPKKNAEKEAVLHYIQGKIGKNKIYSEKEINEIILSWHTFNDY
ALIRRMLYDNFLLERTPDGRQYWVEDKREDAKG*
>SPBDM4_v1_41025|ID:27158330| Putative transcriptional regulator (modular protein) [Uncultured spirochete bdmA 4]
MRRGRRESNPLHELGKLSKARIIQNQRDNKSDALNPIRYIVDINPIGYIIDIYLMGDIMQ
TTIFTSMQLATMLKARRKTLRLTQKDVAVLVGLLPKTVSALETDPDRCSVESLRMLLSAL
KLELTLSPKDESSRIVREAEW*
>SPBDM4_v1_41026|ID:27158331|hipA| regulator with hipB [Uncultured spirochete bdmA 4]
MGRKSKTKGLVVWMNGERVGVWSILPSNIHEFRYDGSWLEFPDVRPISLSMPIVTKDYIY
KGQVVEAFFENLLPDSVDIRRRLQRRFSVETDSAFDLLAQIGRDCVGAIQLLPEGESPQD
VRTISAEPLDEAGVARILRQVVASSGFGQAGDDDFRISIAGVQEKTALLWNEGQWCKPMR
STPSSHIFKLPLGFVGVMQADMSDSVENEWLCAKIVEAFSLPVAHCDIADFEDQHVLIVE
RFDRRLSADRRWWIRLPQEDMCQATGTPPGAKYENEGGPGMVRIMDLLIGSRNALVDRRN
FLKAQVLYWLLAAPDGHAKNFSIFLEPQGRFSLTPLYDVLSSYPILGHGNNKLAPQKLTM
AMSVFGKDTHKKWAHITASHWRTTASKCRAQGEIEGILHEVIEETPKAIEAVSSALPDGF

PSTVSEPILEGIQVAVERLRKGIVPEV*
>SPBDM4_v1_41027|ID:27158332| GntR domain protein [Uncultured spirochete bdmA 4]
MSQSKKALSPVQKTTVVNQVMDQIRDLIASGVYKPGDKLPTEKELAEQLGVGRSSIRETM
KVFNYLGVLESKSAKGTFVCSRSSISREVLTWALLLGEDDIKMVIDLRAAIELWSYLQLT
ERFRTAPEASTTIVKDLNEILDTMSKAIADQNAPAVIQADYDFHRRIIAGVSNSLFVEFY
DILRSFLLKEIDASQSRYMDRSKILEEHRALLDALESGDLQKAASTYQDHINNIKNLLGV
KDTF*
>SPBDM4_v1_41028|ID:27158333|gap| Glyceraldehyde-3-phosphate dehydrogenase [Uncultured spirochete bdmA 4]
MAKVKVGVAGYGVIGQRLADGVALQGDMELVGVADVAPTLSVRALREKGMPYKFFLAAPD
KREELEKAGIPISGSLEDLVQRVDVMLDATSAGVGAKNRALYEKYGKKGIFQGGEKNSVA
DVFFHGYANYEKGLGAQFLKLTSCNTTGLIRAVDCLDRKVGVEKVAITIIRRVADPGDYH
RGMTNALQIDKAPSHQALDMMTIMPQVEATGILVHTPVTHGHIITVVATPKKSVSPENVI
EFFREHPRIRVVSLAEGFLGNASLFRYARDLGNPRGDMYEIAAWEDSVVMSGKDVMFAIN
IPQEAVVIPENMDGVRACMRMEEDRIEATDTTNRYLGIGKWRNLG*
>SPBDM4_v1_41029|ID:27158334| Phosphoglycerate kinase [Uncultured spirochete bdmA 4]
MEGPRLKIRPFTELALAGKTVIFRPDINSPVDPATKRIVNTNRIEKTVPTLNLLLERGAK
VALIAHQGDTLDYQNLIPLAEHAEILSRLTGKRISYIDDVCGPAAQAAVKALSPGEAVIL
GNLRYLAEEISTFETVVKLSAQEMTKTWLVRSLAPLADYYVNDAFAAAHRNAPSMVAFQE
LLPSAGGIQLMEEYTALKSVMDNPKRPCVYVLGGAKISDAFDMMRKVLTEGSADHILTAG
VTGIVMHLARGEDFGPVVTKFLADRSLDVFIPEAKALLEEFGDRYVLPVDFAYDANATQS
TAGASPQRRAEAPIASLPKDRMFPDVGHRTIELFKEYIAAAGSIFVNGPAGMYEHEPWSD
GTRELWRAIAAAPGYTVIGGGDTISAATKFTDLSKYGYVCTGGGAMVRFLAGKRLPLIEA
MEKAFERDLG*
>SPBDM4_v1_41030|ID:27158335|ytsJ| putative NAD-dependent malic enzyme 4 [Uncultured spirochete bdmA 4]
MDIDLDLANLGEAFPADFSPEQKAQAQTLFLKKLALSAHEFYGGKMQTIPKCGFYGFSWF
NTWYTPGVSAVSTAIRDDNDLSFSLSNRGNLVAVVSDSTRVLGDGDCTPPGGLGVMEGKA
MLMKYLGGIDAVALCVDSRNKEGVHDPDKIIEFVKMLQPSVGAVNLEDISQPNCFKVLDD
LRESCEIPVWHDDAQGTACITLAGLLNALKLAGKKLSEAKIVLLGAGASNTTIARLILAD
GGDPAKLVLFDSKGSLHAGRKDIEEDRRYYRKWELCVATNPRRYASEVEALKDADVLIAL
SKPGPNTVKREWIRAMAPKSIVFACANPVPEIWPYAAKEEGAYIVATGRGDFPNQVNNSV
CFPGILKGALLVRARKITDGMAITCAHSIADFAESRGISTDNIIATMAETDVYAREAADV
AVQAVKEGVARRNITWQEAYDSAKADIAASRAMFDTMQAQGHIGVPPASMLNEAFAWAVQ
QVRG*
>SPBDM4_v1_41031|ID:27158336| conserved protein of unknown function [Uncultured spirochete bdmA 4]
MRIDLPYRNGKIGLDLPDSTEVIEPNNKETDDSPRDLVRQALEKPLDSPDLDRFFVGAVH
PLVIVNDGTRPTPTSVVLDVIGDGLEACGARFIVATGAHRAPTEDEYRFIFGANYGRFRE
KTEAHDARRRESLVFCGTTRAGTPLWLNRRVLEADRILVIGSVEPHYFAGYTGGRKAFLP
GVAGYETIQANHKLALQPQAHSLALDENPVHRDMEDALSSIKAPVFSVMTVLDKDQRIVY
CASGDLTASFMAAVRSANEVFAVPLRRRFDVVISVARSPMDINLYQAQKAIDNGALAVAD
GGVLILVASCWDGIGDSAYLDLLGSASTPQEALDKIAAGYKLGYHKAAKIAQAAERIRLV
AYSELDRATLKRAFITKADSLQESVDTALAGRGPGAKVAILIDGTLTVPIIE*
>SPBDM4_v1_41032|ID:27158337|tkt| Transketolase [Uncultured spirochete bdmA 4]
MNKEALKKIALSVRSLSMDAVQEANSGHPGLPLGAAELGAFLYGEAMKYDPSDPIWIDRD
RFVLSAGHGSMFLYSMLHMAGFGISLDDIKCFRKVGSPCAGHPEYDADLGIEMTTGPLGQ
GVSTAVGMAMAETMLAAKFNTEKRKIFDHYTWVLVGDGCLQEGIASEASSLAGHFKLGKL
IVFYDSNDVTIDGPTGISFTEDVAKRYEAYGWKVLKGDMYDFAGIEKLVAEAKADIDRPK
LIILKSMIGKGAPTKQGTNKVHGSPLGEEEIARAKETLGIPKEQKFWVAPEAYEYFGAYK
QGLESRHARWTEEFEAWKREEPALARELDVWYSGDPLSEVKMPTFAPDEPLATRAASGKV
LQAIAAAWPNFVGGSADLTSPNVSALNEVADYTPENRSGRYIRFGIREHAMAAIGNGLML
HGGLRPFVATFLSFVDYLRPALRLSALMKLPLVYVLTHDSIYVGEDGPTHEPIEQLASIR
AIPNVLVLRPADGEETAEAWSIAMKRKDGPTVLVLGRNNVPFLKKDDPDWRADMAKGAYI
VRDTKKTPDVVIVATGSEVSLALEAAKLVPAKSVRVVSMPCREAFLRQPERYQAELLPKT
AKIVVAEAGVAQGWGEIAQRANIFSIDRFGASGPAGDVAKHLGFTVERLSEIIAR*
>SPBDM4_v1_41033|ID:27158338| conserved membrane protein of unknown function [Uncultured spirochete bdmA

4]
VAFTPDICLLAIGIEFFCFFIKGLAAFGDPLISTPLLSMIMDNRVISPMNLLLGTPINAH
MAWKNRKAFTAKTVVPILIAILCGIIPGVVLLKYATSWTLKACLGALVIGIGVEMLVRDR
TKPAKNSKVMMIVASFFSGVTAGLYGINLFFVAYVERTTKTREAFRGNVCFIFLVENIFR
LIVYIISGVLTREVLTLALATMPGAFLGFFLGSRVDKRLSERAIRYIIIAMFMAGGVSIL
VKALVLKS*
>SPBDM4_v1_41034|ID:27158339| Transcriptional regulator, XRE family [Uncultured spirochete bdmA 4]
MPSNPTDFEADNVKVTLGARIRAQRTKHNMKISDLAELTGLTSSTISQVERALISPSIAT
LKKICDAMDITIGSLFEDKDSFNVPAEPEEQKAPSENLAETELASHLKLYTSVHLMGLSP
VVHKENRKFLSPSPGIRFYLLNPNLSGPVELIYNEYDPGTSTGPALYTHPGSECGLILSG
ELVVQIKDATYVLKEGDSITFNSSEPHSKRNDSDTMCTCIWANNPPWF*
>SPBDM4_v1_41035|ID:27158340| Xylose isomerase domain protein TIM barrel [Uncultured spirochete bdmA 4]
MNIWNYALSSADRAPDTAPILLRGDICHNLKTAAVLGYQGLEVHTREDVSLDYDEIGRTA
AECGVCVSAVVTGRLNTQGGVNLIDDRPYISYAAIEGLRKYVQMAARLKTNLIIGWIKGQ
IPEGTDPYPYLERLAGNLTVVCAEARDQGVKIFIEVINRYEANIFTTAQETIDFLEKWKI
PNCYLHLDTFHMNIGESKPVEAIRACGGRLGYFHVADNTRLYPGSGTLDFKSYFSALKEI
GYKGFVSVECLPCPDGETAARAAIEYLRECESDIKTEK*
>SPBDM4_v1_41036|ID:27158341| Creatininase [Uncultured spirochete bdmA 4]
MSKWQIPARGGYMESNRGVYYQNMTNKDVAERLEKNDVILIPVGSTENHGASAPYGEDTY
LDTRLCEQVAVATGCTVAQPIWYGSHPYHHLGMPGTIMIPEETLADYLCYVFAGFWNTGF
RKMIVVNGHGQDYVIPLAIHKFGKKFQVPGIVLYVHFWNAAKNPLTGLTDLATKDQGGVY
DAPFIHADEVEQSYALALFPELCRQENAVATKPSPMLPPGHINNSAENGVGPIKWYNAYG
SVGMECICSPEGVIGDPTKANADKAKAGIEHILDYMERLVKDILTDYPAGKLPPIEKVTQ
RRREDIEAVIKGPSNGGRHIYTLTY*
>SPBDM4_v1_41037|ID:27158342| Alcohol dehydrogenase [Uncultured spirochete bdmA 4]
MDKTMRALVADGPGSFSIQQIPVPIPERDEVLCRIKSVAICGTDPELFAGKFVDIGWPPK
YPFVFGHEWAGEVVAVGGDVIDFAVGDRVAGEAHCGCGHCENCRKGDYTVCLNYGKMAQG
HRHYGFTVQGAYAEYGAYKTKALEKMPDSVSFDEATMCDTAGVALHGVELGNVGSCQTVA
IIGPGPIGNLAMQICKAKGARTVMIGRRLRLECAKECGADHIINYESSNPVEEVKKLTNG
KGADVVIECAGTPAAISESLFCTRKNGNDVLISLPKVRDIPMSILTIVQNQIHVIGSKAN
PNCSRQVLQMIGSNMINAKRLITHQFDLSEMEKALDIFVNKKDGVMKVIIHP*
>SPBDM4_v1_41038|ID:27158343| exported protein of unknown function [Uncultured spirochete bdmA 4]
MRKKILGVVLVLLMACSFVNAQGTAGKKNIRVDFLVKKLSSAFWTDMVNAAQAQVDKYGW
KMKVLCPITADSNEEQIQLLEQALLDPPDVFLIAAADSKGIAPAIEEINKAGIPIIAVSA
KIIGEGLRYETFVGVQFEDLAVSAAQALCQKLNGKGNILYLQGVTGASSAQDVDKGAYAT
FKKYPGITVLASEPADYQRQKGMTVTQNLLQKYDKVDGIFAANGESALGSIEAIRQLGRK
GIIVTTINSSEELVRAIVDGKLYALADDVSWKVGQQAVVAAKKYLDGETLSKDNFQQPVM
VTRDSANLAEYKTKYGIK*
>SPBDM4_v1_41039|ID:27158344| putative ribose/galactose/methyl galactoside import ATP-binding protein [Uncultured spirochete bdmA 4]
MNGDQQATLLSIRNVTKVYPGVKAVNNLSLEIRKGEIHIIVGENGAGKSTLVKIIAGLIK
QSTGDLIFEGEKYSPNSVLDAQKAGINMIHQELNLMQDRTVAQNVFVSREPVKTRLRVVD
TKKMNSSCNKILADLGLCFDASMLIKDLSIAQQQMVEVAKAVSRENKLLIMDEPTSSLTQ
KEIDHLFAITRLLKSQGTSIIYISHRMQELMEIGDRITVMRDGALIATKNISEISMPEII
KMMVGRKIENVFSRTETTVGEKVLSVNNLTGLRFRNVNICIREGEIVGLAGLIGAGRTEL
AKAIYGKDPIESGEVLFYGKKINRRNYTTRNAVDLGISFIPEDRKAEGFFSGMSIKTNMM
EASWRFLFRRGIVKNSVLDRTAEEGVKRLNVATPSVEKNVDELSGGNQQKVIISKWLVHD
SKLFIFDEPTRGIDVGAKAEIYSAIDELSKKGGAVLMISSDLPELLGMADRIYVMKDGEI
CGEVRRDAPDFTQEKVLSIAIGESQIA*
>SPBDM4_v1_41040|ID:27158345| putative Ribose transport system permease protein RbsC [Uncultured spirochete bdmA 4]
MNDILKKVPGLMWGSLLMVMFFVIFAPNYTNQRNIINILQNSAIIIIVSMGMGLTILSGQ
IDMSIGGVMTASAVISAMYFAHIQSPTALDILVTFLIGIGVGSVFGFLNGILIARYKFNF
WLVTFAMMSISYGIAQVVTGGKVISGFSKSFRNVTRFSLEGIPMVVYIAVIVSLLMFFLT

YKTKFGMHMYAVGASEKCAAQSGIRVERIRFLIYLLSGLLSGFGGVLLVSKTNFASANVA
SGQEFTAMANVIIGGISFDGGKGGLLGAFAGSIILTAILNGLQLMGLSNYWQQVFTGIFI
LLIIIIDVISQRMKKTKNTRRVYKHA*
>SPBDM4_v1_41041|ID:27158346| Branched-chain amino acid ABC transporter, permease protein [Uncultured spirochete bdmA 4]
MLNKTIHRHLNKYSRLLLLIALFIFFSIFTDSFWSPTNWSNFANIMFQQAPFTVLLSLSM
TISIILKGFDLSMGADIALVSCIVGFVLRASHSPLLAILAALGMGALIGMGNGLLITKVG
IQPFIATYSMNWIIRGIALVLLGGSQIYDFGPTFRPLFLNSPYTFLFIAAVICAIFSLIF
SKTIFGKYIYAVGTNMGAAELSGINPNFVQFVAWTLCGVILSVDAVMYTANLGVAEPVIG
DSFVMTAIAASLIGGTSVSGGVGGVYNAVVGALILVVLTNGMIHLGVPSAWQQVVVGFVI
VLSVFFEHLMNRKED*
>SPBDM4_v1_41042|ID:27158347| putative 2-(hydroxymethyl)glutarate dehydrogenase [Uncultured spirochete bdmA 4]
MESVGFIGLGKMGSRMAKRILDKGYALYINDVIRDNAEALVKQGAIYVSNAEELVRKTNV
IFTSVPSGSNLKEIIVGKNGLLNSASQDTVLIDMSTIAPKDSAEIAAILDTKKIGYLRAC
VTGSTAFAEQGTLGIMVSGSKEIFEKELHLLKIIGNRQRYLGKEEQSRYMKISINMMIGT
AMQMLAESLVLGEKAGIDWEMMVECIADSAAATAMIKAKEVPLKKRDWTAMSTVEMMEKD
MSIALEIAAEEGLALPVTAITRQFYSAMSSSGYGKVDYSGILLVNEKLNNIGKAEK*
>SPBDM4_v1_41043|ID:27158348| Alcohol dehydrogenase [Uncultured spirochete bdmA 4]
MVSMMEALVLTAPKEFEIKQIPVPGIGPNEVLCRVKSVAICGSDPGFISGKTAGVWPPHY
PFTIGHEWSGVVETVGSNVTMWGKGDKVVGEAHNGCGICRNCKEGRYNLCLNYDNPKTEH
RHYGHKDQGAFAEYGIFNQKCLTALPENVSFDIGAIVDAAGTALHVETLTDITPGGTVAI
IGPGPIGLIAGKVARALGASRIIVIGRGPRLAKAKELGIATETVDFESQDAVKAIEDLTG
GLGCDEVFECSGATGTLDQAIHIVRRGGKIGLVGIPAPGNMESIPNRMVVIKEITIQGSR
ANPNVTDKLLSMLAAGTVNFDDIVTHSFRLQDFRRALDTFLDRSTGALKVIVHP*
>SPBDM4_v1_41044|ID:27158349| Myo-inositol catabolism protein IolB [Uncultured spirochete bdmA 4]
MLDNPAFENGQKTLCQMNGKNAEMLMNIYVKRCRKGEKIEFLESRNETAILLLRGEVRFS
WGDAAATGKRESPFEWKPYCLHFSKNTKASVNALADSEIIVQQTDNAKEFPAVFYTPETC
LYQEFGKGQWNGAGHRIVSTMFDLDNAPYSNMVMGEVFNQPGKWSSYPPHHHPQPEVYYY
QFDHPEGFGACFIGDNVFKSTDGSFAVITPGNAHQQVVAPGFTMYYVWMIRHLDGDPWDK
TRIYVPEYEWLAKAQ*
>SPBDM4_v1_41045|ID:27158350| putative Histidine kinase [Uncultured spirochete bdmA 4]
LNRRIILQLEDIHARYTNIDALNGIDFNLFEGEVHGLYGEHGAGKSTLAKVISGAISRVS
GNYYLEGKKNEKMTIRNAIKNGIIMVYQDTLNITPHMTVRDNIFMRNFYQVNYFGKKYTE
FDKKLSSFFQKYSIAFDTSKYASTLSIQEQYFIDVISVLVNTPKIVIFDEISNRLQHDEF
QIISRIMLDLKKSGTSFIFITHNMDEILAFSDRITILRNGLRIATEKTDDLDKSKLFQLS
FSAVYKQSTLTKSNATESFIFETLQNVTNHLSEGLIIFDENFSLKLINFSAIEIFGINDT
FGYNNILSIIKEKLPEYSDSIIEKILNRNPFEVHEVFSNAKRLSIRCIPIDLFTQNSTYF
CLIVEDLTMKFQIQGYLLDAEKMTSVAEIAAGIAHEINNPLFIIKNYIELIAINLHDREP
LSYIEKINNEITRIMRICSGVLSFSKIKKTEKISANVCDLVDEALLLLDYQIKSKDIKVI
KTYFDSDLNLLCDETALTLCFVNIIKNSVEAVLDKGQIEISVALVGGNVAIEITDNGYGI
SPENLEKIFSPFFSTKVGKKNAGIGLSISKNIIESLNGSIDAFMTSDSKTCFRILLPAER
CLG*
>SPBDM4_v1_41046|ID:27158351| Transcriptional regulatory protein [Uncultured spirochete bdmA 4]
MTENIRILIVDDEEEVCISLSKLLETKGFSVVYSFDTVHLASIIERNQIEVVLLDVNMPE
KDGINVLQEIHLHFPLMPVIMISGFASVNNVLKTMRLGAINFYEKPIDIPSLVNEINTLI
AAKRKKHNFVAEKNVITRNKEVIHLLDLVNKYAVTDAPVVIMGETGTGKEVFADLFHRLS
RRSAKPFIKINCAAIPDTLLESELFGYEKGAFTGAMDQKKGKLELANQGTVFLDEIGDMS
LNTQAKILRVLEEKQFMRVGGLKNIQIDCRIIAATNKNLKNLIERKDFREDLFYRLSVIT
LELPPLRERREDIIPLAQYFTKVFSDMYGKNIENLSDAVVAGMLRHDWPGNIRELRNFVE
RAVIFTDSDTLEIDNCATLYSKRVDFENKQVNPRRLSREVIREALLQSDWNKIKAAQLLG
ISRTTLYNKIEEYSIEP*
>SPBDM4_v1_41047|ID:27158352| putative N-acyl homoserine lactonase AttM [Uncultured spirochete bdmA 4]
MKKAKVHILYYGSHMSPLSDLISGAGKNGEKILTPSLGIFIDHPEAKIVVDTGWNDDLPQ

IGKDMGHIYSEEHTPIGQLRKLGVNPAAIDLVVLTHMHFDHVGGIRHFPGKTVLVRRAEL
EKAFVPPRNSNFLYLRSDFDVPDVRYETIDGDMDYQLCPGVLLLATPGHTLGSQSVLIDT
EEGSVLYAGDAIYLFDNWEKHLMPGICYSQPLQMETMSKLRGLGRFHLIPGHDPKVDVNK
IYG*
>SPBDM4_v1_41048|ID:27158353|rbsC| Ribose transport system permease protein RbsC [Uncultured spirochete bdmA 4]
MTHDSTMKRLSGKLGFLKGKGVLFAVIVLCVFLSICSPNFLTVGNLMIVLRQSSYIMIIA
FGMTMVIGMGDIDLSVSAIAALSGIIAAKMLVAGSSTFLSCVTALAFGFTLGFFNGFLIS
VLGMTAFIATLGTMSICRGVAMLITRGIPIFGLQFPSFQFLSQGFISIVPFPVVLSIVML
ACAAFLIKKTALGRYILSIGSNVEAARLVGINIKYVRVLVFSICGLLSALAGLLLTSRLE
AASPVAGDGYEMDVIAATVIGGTSLVGGKAKMSGTFIGALVMGIVRNGLTLLSINVFWHR
VVLGAIILISVEIDILSQRKKD*
>SPBDM4_v1_41049|ID:27158354| putative ribose/galactose/methyl galactoside import ATP-binding protein [Uncultured spirochete bdmA 4]
MERQYGEVPFAMCLSARERVVMHTNRKCSNAPIVSVKNLCKTFPGVAALKNVSFDVYPGE
ILILLGENGAGKSTLMKILAGAYRKTSGSICVGEKEVEIQSIKHAKKLGISIVYQEQALI
PDLNAVSNIFLGKEDRKILKKNYFGLVDGKKMKEKALALLSEFSVPIDLNTDVRDLPLGQ
KQIIEICRCLADNATVLILDEPTAALEEREREYLFKFIDRLVEKGVAIIYCSHILEECLK
IGDRVIVLRDGEKVNDSDIGQVTLGGLIDKMVGQELKEQYPKEHIALSDREVLSVKNLSN
KHNFKDVSFSLYESEILGIGGLDGSGKYELIRSLFGVNRYLDGEVSVHGDTLRANNIINS
MKEGFAFLPADRKSEGLFLDQPIGFNLSVANLKKVSNGWINSKKEKDFIRKFVDVLKIKC
SSEKQLALDLSGGNQQKVMIARWLFREPTILIFEEPTRGIDVKAKTEVYHLMGDFVRKKG
SIILVSSDLPELSEICDRVLIMFEGKITKELAGKDLTQEKILEFSISGRKNS*
>SPBDM4_v1_41050|ID:27158355| putative ribose ABC transporter, ribose-binding protein [Uncultured spirochete bdmA 4]
MSKFRVGILCLLVLSMVSLGWAQEQRAADQQLISVKEDGTFDKTGLEVPPMKKAHLKIGF
SPNAMNTQYDMVISGCKEAISKLPDPSSVDFVIQAPTNHSDTAEQMDIIESWIQQKYDVI
AVCSVNDMAMYPVYRMAAAAGIPIFHFNTPSSSLVDPFFVSTVTCNQVMAGQAIGEAFVK
RYGKKDTNIVVIEGLVGTPHNTERINGFKKAIAGVNNFHIIDMQAADWVRDKAQTVMEDL
LTKYGKKINVVWGMYDEMALGAVSAIKSRGLTKQIEVWGYDNTEDAYNSILRGEMFGTVD
TASKQSGRDLINSIVKYCMKGEMIPKQVYVPPVVYTRDNIKTFNTSEYK*
>SPBDM4_v1_41051|ID:27158356|adh| Alcohol dehydrogenase GroES-like protein [Uncultured spirochete bdmA 4]
MKAAVFTQFRTPLSISEVPDPEVPDDGVVLSVDATGICRSDWHGWQGHDPDIRLPHVPGH
ELAGTIVEVGKNIRNWKRDDRVTMPFVAGCGHCFPCLTGNQQVCDHQFQPGFTHWGSFAE
FVAIHYADMNLVRLPDTIDSLTAASLGCRFATAFRALEAQAKVRAGEWVAVHGCGGVGLS
AIMIAASMGARIIAVDIQDDKLAMARQFGADIVINAREVPDVISAIRDATDGGAQVSMDA
LGSRQTCFNSIACLAKRGRHVQVGLMLADQRHPEVPMDLVVARELEIYGSHGIQAHRYGV
LLGMVASGRLHPERLVTGRLSLAQGIDFLQKMGQFPGMGINVITTF*
>SPBDM4_v1_41052|ID:27158357|ymdB| conserved hypothetical protein [Uncultured spirochete bdmA 4]
MKELYRSPDGCCIVSVGDITTLACDAIVNAANSSLLGGGGVDGAIHRAGGPEILAECRAL
RRGPLPDGLPAGQAVATTAGRLPAKRIIHTVGPIWRGGTHHEDETLVSCYRESLALAARE
GLESIAFPAISTGVYGFPRERAAKIAWRTVRDFLESALKPPCGPSPSTPGTSCPRTVWFV
FFSDEDARLFLKTNQLS*
>SPBDM4_v1_41053|ID:27158358| Response regulator receiver protein [Uncultured spirochete bdmA 4]
MAKFSNKVRTFSALEVARLCGVVNQTAINWIRGGHLKAFSTPGGQYRVYSEDLVSFLKER
NMRVPDELRDDLTMPVDPGLALIVDDDKDLNTILKRLLERKIEELRVAQAFDGFEAGRII
AERHPAIVLLDLNLPGVDGVSLCKKIRSDESFGQPAVISMTGIAEEEAQPQMMEAGADAF
FAKPLNFDALIAKIRELLEKHKNPRAEDEG*
>SPBDM4_v1_41054|ID:27158359| conserved protein of unknown function [Uncultured spirochete bdmA 4]
MRPPAPKFLQFSATSRNVTLIMDSTTTFDAGTIEALSVDELAIAQKIAGELGVRLSQVQA
VLSLSAEGCTVPFISRYRKERTGNLDEVQVRDCIQKFESNRNLEERRLEVLKGVSAQGKL
DAFLYGNIRNAATLTELEDLWAPFKKKKKTRGMLAQEKGLGPLADLMAKAAIEEVVAAAP
SFIHSDAEHPELSVADADESLAGARDVLAERLSQDTEIRAYVKQVVLQNGSLSVKGIGDD
EKREQSTYQMYWDYREPLSTLKHHRVLAVNRGEREGELDVSIEIDDLLVEDRVLERVRPA

NAQHKEAVADGLARLLLPAVRREIRSGLSENAESHAIEVFSTNLRNLLMQPPLRGTRVLG
IDPGIRTGTKCAALDETGKFLDYFVINQETKPEQGKKNIAAAVAKHRLSVVAVGNGTGSH
EVQKLVAETVTENRLECRFAVVDEDGASVYSASDLAREEFPELDLTIRGAISIGRRLQDP
LAELVKIDPKSIGVGLYQHDVNQKQLAGQLDEVVGSVVNQVGVNLNTASYSLLRYVSGIN
ASLAKKIVKFRDASGVIRNRSLLKEIPGLGEKTFEQCAGFLKIPESGNSLDNTWVHPENY
ALAAEIQPIVKSGRDPSREERVLLKEKYGVGDITLDDIIVELKKPNRDPREDLPPPLLQQ
GVLSFEDLQPGMKVKGKVKNVVDFGAFVDIGIKESALIHVSEMGDRFVKNPMEVLRVGDL
KEFTIISIDPLRRRIGLSLRAQAKAQGAQAAEGSGTRQSAPDAAPSGRAGNAPRDGNPRQ
DRPLAGRPAPRGHPQHGGEDDGMTYNPFADLLKKRK*
>SPBDM4_v1_41055|ID:27158360| Acetyltransferase family protein (fragment) [Uncultured spirochete bdmA 4]
LRLKTNIVRVEAHRFYENMGFERTKTQYTYVKKLAPDSKAR*
>SPBDM4_v1_41056|ID:27158361| Metal-dependent hydrolase, beta-lactamase superfamily (fragment) [Uncultured spirochete bdmA 4]
MAIIFEPLAGNTFVALGPTNLGLYVFRQGDAARAVAIDSGGDEDAGRRIVRECERLGVNL
SVLLNTHSNADHCGGNAAVYKRTGCAIASTEIEAAFLQ*
>SPBDM4_v1_41057|ID:27158362| Beta-lactamase (fragment) [Uncultured spirochete bdmA 4]
MAPASRATHLLEPPCELALDEKGSIDIRSVPVMVPAGLRDAERARSDLVPVAGTEGLHII
SLPGHYFGMVGVMTPDGVFFAADALAGATILEKYNIFFFYDVAAELETLSMLERIEAEWF
VPSHAEPVRDIKPLVALNRAKIFEIAEVIVGLVRGDSSLIPEKRARDGASLEDVVAGVCN
RYDIILNADQHVLVGSTIRSYLSWLCDQNQLEYGFEQNRMTFRVKQ*
>SPBDM4_v1_41058|ID:27158363| exported protein of unknown function [Uncultured spirochete bdmA 4]
MSRKKPAIQARRAQKNGALSKSLRGVVFLVVLFYMLLGGAGIGTAFAQGHLAVELGHPVY
AVIETAELRGVVTRLSSVKPYTSQQVAELLATMMGHMEAFSPAERALIRQYAQEFKAGTG
VEQSLWEDSSGKARAGIRVEATTKLDAGGLYDMIDGSSSTALKDMWHLNSRWVPYLEGDP
IPWLSLKGEAGFTFDKIENDLYLPYDYTKEWDATHIWFGTPRNSDGTLDYPTASYDIRED
IAAETDNGSLMVRLSRFRRDWGIGSGSFSLSGTARPFVGFETTFRPSKFFAISGLVGSLT
NWEEGSDEKSTAVTYVDTNGDGVADNKDTYFYYSALSYQKMLGLQRVELFPFDWLTVSAT
STLVGAKRFELGYFSPLLFSVMYQNQLADVDNLGVQVDGQILVPRVGKFYASFYADEMDM
TNLSELFTKARNMFALQGGX
>SPBDM4_v1_50001|ID:27158364| protein of unknown function [Uncultured spirochete bdmA 4]
MIDGSSSTALKDMWHLNSRWVPYLEGDPIPWLSLKGEAGFTFDKIENDLYLPYDYTKEWD
ATHIWFGTPRNSDGTLDYPTASYDIREDIAAETDNGSLMVRLSRFRRDWGIGSGSFSLSG
TARPFVGFETTFRPSKFFAISGLVGSLTNWEEGSDEKSTAVTYVDTNGDGVADNKDTYFY
YSALSYQKMLGLQRVELFPFDWLTVSATSTLVGAKRFELGYFSPLLFSVMYQNQLADVDN
LGVQVDGQILVPRVGKFYASFYADEMDMTNLSELFTKARNMFALQGGAKVPVPWLAFTTL
TAQYTKIEPFVYSHYPTWYPDYRLRVDTSYTQDGENLGYYLPPNSDEFLVKFEVTPAPDW
RVAMKYRFVRHGDNPGNVGDSVIFGDVNKWLEYTPALLYMDKDFLHDGIYDYNHIGNIDV
TWRPADPPKLLGATIPFELGLGYGLSYTWYEDGTGADRTVSSPEWNNVLSLSMKLFL*
>SPBDM4_v1_50002|ID:27158365| putative Acyltransferase [Uncultured spirochete bdmA 4]
MRYRKGGPLTSDTPFSRLIWDILIVLIRLLGWPLSRAMYNIKIEKVDAMAGHRLPSRAIL
ISNHCMPLDPLSHGLAIFPRRTYFTLLEETCEAPVLGSFVRLLGGIPLPRSRNRLQDIEE
AVSHGLSTRGLVHFYPEGECFVGNQNIFPFKAGACYFAIKFGVPVVPIVTILNRRNGRKS
NRTQVTVHMLAPIMPPSPGSPPHATLVRSIQFSNQVHDLMQIEIERTGGDKSLYRGPMPR
IKGVND*
>SPBDM4_v1_50003|ID:27158366| putative Glycosyltransferase, group 2 family protein [Uncultured spirochete bdmA 4]
MSTTILTVYGVITFAACVFFFFITVSNILWLVHTLGTEPKQAGPPVAVLIPARNEALRIR
PCLDSLLQQDYNSYQIYVIDDNSTDETWEILRDYMLRFPRKIKAFKAEPLPEGWYGKPHA
LQELSSHVQEDYILCTDADTTHTPDSIGKAMAVAEKYNADLVTGYVHHLMPTFAEASVVP
SMYILTMFGMPLYLIPFIKSPLISHAIGQFMLFRRSFFERIGGYEKVKHQATEDVKLARV
VKQHGGRIAFVDLRDTVECRMYSTYHKAVQGIAKNAFDYLGKNFFFLFLGTVAVPLVFFV
PVIVLFVNIPWLGPALPFLKASAIITFYTWALETIDRRLPWYVPFIYPLIFVNTLSALWR
GFRLVNKEGGVEWKGRKVK*
>SPBDM4_v1_50004|ID:27158367| exported protein of unknown function [Uncultured spirochete bdmA 4]

MMVTERHFTALRSIALASLAILIAANSFVSAQTQPSPPKNPSSVGDFQLNNAGALAKNQD
ILPGVPFPPAMSQSAVQAFFELRDSVYSGVAPESVDAQATSLLKSIGGQQLSENDRALLE
ARIAYLAGRSWNDHKNKKKAAPWFEQAVDAAHTMIALDGETPTALVALAEPLGELSLLND
LGFLVSNGPKVGQYANKALESEPLNIKAWLLKASALAYPPPIWGGNYKKALETYAAILPM
AETGLPKDVLFDIRVGIATAYANLKLSEHAAWWFKAALELYPQNPYAKSELVKLAP*
>SPBDM4_v1_50005|ID:27158368| putative Peptidase U34 [Uncultured spirochete bdmA 4]
MCDTLVLHDSAGKPLIFGKNSDRDPEEPQALVHLPPREPGTTFIREGIGFSDEGFAMLLS
KPSWMSGGEMGINSKGLAIGNEAVFSRFKPDENGVLGMDILRAALSACATAKEAVDFITS
FVEKYPQGGNGAYKGKLVYNNSFLAADPEEAYIIETAGKRWAWRKAMVADAISNAYCIED
DYKRLDTQTRKEIAPVNERAACSDEADPGRKGQRNSWKKHVEDAKYLFFTKGEQRRSNSL
GGLMRLAASGAGTGDASSVSGARAGMEALFSLLRSHEGAPKPGPFLNRMKNLCVHPGLFP
KSATTASMAVEYLPGGVIVWYTNSSYPCVSLYKPAILKDGRFHSLWKPFAGETKADEWYA
YWRARKTWVERSHHLGLSNQEAFVQSRNEAQKSIIKVARQAFGSILKEKSSPERVLSVYA
NEVAAIVGEWEDRWGD*
>SPBDM4_v1_50006|ID:27158369| exported protein of unknown function [Uncultured spirochete bdmA 4]
MDRVRLSKPVVLLFIATLVLAIVTVVVVQVLAHSQPDVQSQFTLYMEKLEPQSMVVAATT
QERYEASKEFTAKLLAIFNIKAKIRLSAMADITYVIPASDPSAWSIQWDARARKLVISTP
APDCLLPAVHTDTIEIVTENSNLLTNTMFRLKEEAARMQDELSNDLLVRAKATLEEPAVR
AVIEDGVRRFAGTFCESAHLGKPALIEVQLGRLQKTGQNQ*
>SPBDM4_v1_50007|ID:27158370|ggt| Gamma-glutamyltranspeptidase [Uncultured spirochete bdmA 4]
MKRLTLALVLFVAALGAVSAQLPVNLYGRAAMGANGVVAAAKPEASQVGIDILKQGGNAV
DAAVATGFALGVLEPNASGVGGGGFMIIKMKDMVEPVVIDFREMAPGKATPTMYLGSDGK
VIPKSTYEGGLSVGVPGEVKGLLYALEHYGSGKLTRADIIQPAIQWALQGIPVTVNLESI
TKDNFGKLVQYENGAQIYLKDGLPYEVGDVIYNPDLAKTLAKIVKEGEDAVYKGEIAQAI
VDEVQKRGGIITLDDLANYQVKIRKAVEGDYRGYKVYSVPPASSGGTHLVEILNILENFD
VKKIGFETPQAAHLWSEILKLTFADRSKYMGDTDFVKVPLAGLTSKDYAKEQAARIDLNA
SMNPPQAGDPYKYESGSTTHFSVMDKEGNMVAVTKTINYFFGSGVVIPGWGFIMNDEMDD
FVATPGSVNSVEPGKRPLSSMSPTLVLDPQGRSFMTIGSPGATRIFPTVAQVISNVIDFG
FPIQEAILAPRVYQAASGDLQLEGRYPVSALEGVKKLGHNVNIRGDWDAYFGGVHAIVYD
YDEGLLYGGADPRRDGQAAAF*
>SPBDM4_v1_50008|ID:27158371| conserved exported protein of unknown function [Uncultured spirochete bdmA 4]
MRRSGRIVIIVAAVMAACLPLSLAAYNLKAPIAAQPALLTSVGQSADVEMAKAIMGRAKL
SFTMDSLVKAQGLASANAKTLIIVVGGSSKGLGAAGISADAELERTKALLTEAKKRGMKI
IGLHIGGEARRGELSDRFINAAVPYCDYFIVVEEGNNDGLFTKLCGTKIPLDTVQKISQV
GEPLAAAFTK*
>SPBDM4_v1_50009|ID:27158372| conserved membrane protein of unknown function [Uncultured spirochete bdmA 4]
VSQEFWLFILMVGVFAAGCFALKWPVSVSMLLAAVAGALAGGVQNPIRHLVEGTFGYVDT
ILTIATAMMFMTGFRDSGALEALTAAVIKKFNKLPGILMILLTFVIMVPGMITGSSTASV
LTAGAVVAPIFASMGMTAAESGALISIAAILGMIAPPVNIPAMIIGGGIDMPYVGFEVPL
LLLTFPLAIVFALWFGWRKVRKVDRSKVLASLDFSTLDQYGGLRLFSPLILIFVLMILDK
AVPRVFGLGMPLIFILGTMLTWVTGKRFNILQSAKKSIEDVLPVLGILVGVGMFIQIMTA
TGVRGFIVVNALSVPPALLYVMIAISIPLFGAISAFGSSSVLGVPFMLALLSQDQIIAAA
SLSLIAAIGDFMPPTALAAIFAAKVVGLEKYGAVLKKLVVPALIIIVYAILFIVFSKQIR
AVL*
>SPBDM4_v1_50010|ID:27158373| conserved protein of unknown function [Uncultured spirochete bdmA 4]
MFYIYLAILAVVLALTVWVLFDTKKFSLQVTAAMVIVPLLLRLLMIK*
>SPBDM4_v1_50011|ID:27158374| conserved exported protein of unknown function [Uncultured spirochete bdmA 4]
MRKHTITAIICLAGALVASAVAGASFLAMRKPDVIVNGPGVSEKKMLSDWFPGLKGTPGD
TEVYILKGADDGASMLILGGTHPNEPAGHLAAIVYIENVVPKTGTLYVIPRANASGFTAN
DPQEGAPQRFTIQTPNGPRVFSYGSRATNPVHQWPDPNVYIHASSGQTLSGSETRNLNRG
YPGRPDGTLTEKVCYGIVQLIKQQNVTITVDLHEASPEYPVINAIVAHPKAMTLASDTVL
GLEMQDIKISLEPSPVNLRGLTHRELGDFTDTLPLLMETANASQGRLRGRTDEALVLTGK
DKWYVRAQKLGRLYVPYDEKGHPIEERVGRHVTAIGEILTSWNLEHPEEPLEFENIPGYE

DILAKGVGAYLLPAPAK*
>SPBDM4_v1_50012|ID:27158375| NADP-dependent oxidoreductase domain protein [Uncultured spirochete bdmA 4]
MLYRRLGSAGIKVSVLSLGSWVTYGSQVDVDAAMEMLKYAYDHGINFFDNAEAYAGGKSE
TIMGQAFRKLGFRRGSYLVSTKLFWGINEGPNEKNTLNRKYLLEGIEGSLERLGMKYVDL
LFCHRPDPDTPIEETVWAMHDIVSSGKALYWGTSEWSAEQIREAWEIADKRNLRKPQMEQ
PQYSMLVRTKVEKEFARLYQGTGLGLTTFSPLASGLLSGKYKDGIPKDSRFALPGLDWLK
DRWYHDEVIATIEKLRPIAESLGCTMSQLALAWVAHNPNVSTVITGASRLSQLEENLGAL
EVLPKLTPAVLKKIDDALEPLLTMSN*
>SPBDM4_v1_50013|ID:27158376| putative Nuclease SbcCD, D subunit [Uncultured spirochete bdmA 4]
MPTTSKIFKLVHISDLHLGKSLRSQDLAEDQAYVLGQIVRQAEKLAPDLVIISGDVFDRS
IPSESAQTMFGRFMADLRRALPGQARSALRGQARILVIPGNHDSPRRIAFAAELFEAVGI
YLVSEVRLAPALVLEKGGKRAAVWALPFVTHGAYHEFRRNLPEGTPVDALGDGSMAERMA
SIIANIRPHFGEYDLNILEAHCYVRGAALTESDTAFIGGTEAVPASLFDAFDYVALGHLH
RMQALSPKMWYSGAPMAMSFGDGGSEKGFLYTELGAKGPAKIVPVILEPLRAFRRVRGTF
EQLTAESSTLDSDYVEVILTDADPVYNAFNELAARFPNLTSVRQEAFEKMAAGTGFDGLP
VDEARLDTENRQALGLSHEDTRHAAALRDFASFARCILEEPPPEEMMQAFESLLAEAEQE
LA*
>SPBDM4_v1_50014|ID:27158377| putative Nuclease SbcCD, C subunit [Uncultured spirochete bdmA 4]
MRPLTLAFEHFGPYRQRQDIDFSTLDEFFLIYGKTGSGKTTIFDAIAYALYGEAVGGRSN
LERELASRFSPAGSKPWVEFEFFASSARWKVYRSLPYRRQGRKGKESEATSEVALYRMNE
AGAYELLADRPTTANQTLLGLLRLSADEFSKIVLLPQGEFQQFLEMKTTDRVQILEKLFD
VRIYDEVTEAARRKVMQLDSSLKSKHEELERISNELGTEPDARMSASGKLILEFDANIET
AARDASRLETEIAQKNERLAFWQSLLAAWKAFSEIEQSKPAFDEREKKLEAVKTLAALGS
LARQAGSQREEVATALEKALRTGTELARLESDRGEIEATAKTLPHLREEHDELQRRAALY
QKALEAWQQKAELESQRLEALEKLKTSTEQCARQEQGILSLKKGIAELDAVVTGEPETQE
KLQKNLMTGEALKRIRALAEQKEKTEAQRSEILRKIQTTEDLLLSSDRDLADAEKNRARM
DEEIRHAQAGLLAASLISGEPCPVCGSREHPKPTSLPEHAPDEEAVRKAREAVETIKSSR
AALLQTKEDQEGRLQEFVQELEIARQKISDEWRAHAGAFPDGEAASPLDEVSRGAIESAV
TDLQSQRQNLEEKLKNYRDARESLRQMRRTLEKAERDYAQIQKEKAALEITRTELDAHLS
SLIEQVGNEDPQPRLQEVRERLAALQREIFRMETRARDWLEAWSTANAETMTHLETIASK
GKALATGFEQFFTEAEKADVRGLFQTLQSPAGTAQASQPQRRPQSQHQNASNGSIHTALL
DLARNLPHTEKFGPSSNRLTGLAKSLETIFSARATSEAETLDLIEDILSSVWPQDRIREE
EKALATFRESYAKARTSYEVLASRAQPLSPLELEQEERALIAVVTALAERKKALEASISE
LRSERARLSAELEQLRHLLSRAESLRSEYSAAASEFGKQEKLARLLSGSLNPQRKMPFKN
YVLGLQFREIAARASERLYRMSSGRYIVEADILSGSGNQKIGLELFVIDAWNGARRPVGT
LSGGEKFMLSISLALGLADSIQERAGAQRIESLFIDEGFGSLDEESLSLAISVLDELRGD
KTIAIISHVDELYSRIPSRIVVKKGVSGSHLEFERD*
>SPBDM4_v1_50015|ID:27158378| Alcohol dehydrogenase [Uncultured spirochete bdmA 4]
MYEFYAPTKIIFGEGCLSETIPQIKKMGATCALLVTGRSSTASSAGFKSLCKELDAGGLR
WAHFAEVSADPETTIVDRGAEIFRAKGCDAIIGFGGGSPIDCAKGIAASVAEGRSIRDFV
GTGLAFSAPVSPLVAIPTTAGTGTEVTNAAVFTVVDENGHRSKKGTSSQFYFPRLAVVDP
LLHASMPPSLTAATGMDALTHAVEAYVSRFHTPLSDMYCLEAIRRIGRSLRTACRGSGID
AVAGARSDMAMAATLAGVALSQAGLGMVHGFAHPIGAMTGLAHGLANAILLPFVMNALIP
DAGERLSSIGQALTGKSGISAKESVRAIARLGRDIGIPENLEAAGVPETYFENILTDALS
YRRRKASPHAFTDAELRALLERMYGGNVEA*
>SPBDM4_v1_50016|ID:27158379| ABC-type transporter, periplasmic subunit [Uncultured spirochete bdmA 4]
MKRSLFVLLALLAFAGSAFAQDFIIANGAEPSTLDPALMQDTTSNQFYLAMFEGLVQYDP
RTSKGIPAMAESWTTSPDGMTVTFKLRDAKWSDGTPVTAQDFVYGWLRTLAPETASNYAY
MLGMVVKGADDYNAGKGKAEDVGVKAIDSKTLQVQLVGPAPYFVDMTAHSAFAPAPKWTI
EKYGDQWTKPGNIVVNGPYILKEWKPQDYVLVEKNNKYWDAKNVKLRTIKYLASDSMSTN
YKMFKAGAVDWMNGIDVSMIDEIKLRKDYQASAQFATYYINLNNQREPFNDVRVRRAFAA
AIDKKTLVDKVLKGGQIPTDEFSPPMAGYTPQPGQGYDPAVAKKLLADAGYPDGKGFPSL
TYIYNTNAGHKLIAEYVVQQQLKTNLNVDIVLQNMEFKTLIEMRNKHDFTIARDGWVGDYL
DPNTMLELFISDSGNNSGDHRNPAFDKLMEDARMAKGDARMKMLEQAEKILLQNDQAIIP

LYHYANQDMIDTSRWGGWFANPIGYHPPKFIYKK*
>SPBDM4_v1_50017|ID:27158380|oppB| oligopeptide transporter subunit ; membrane component of ABC superfamily [Uncultured spirochete bdmA 4]
MSRYIIRRFLSLIPTIFVIITLSFFLIRFAPGGPFSSEKNVPEQVIQNLMKKYHMDEPLF
KQYLSYMGDILHGDLGPSFRYRDSTVNELIGQSLPNSMLLGTIALVLATVAGIAVGIISA
LKQNHWQDYTFMSIAVVGISVPLFVVGPILQLVFAMRLHVLPLSGWITGREGIKALIMPA
ITLSFPYFAYIARLSRASLLEILRSDYVRTARAKGLAERTVITRHVLKGGLLPVVSYLGP
AFSGIITGSIVIEQVFAIPGVGRIFVQSALNRDYTLIMGEVIVYSLILVLMNFVVDIVYG
FLDPRIAYN*
>SPBDM4_v1_50018|ID:27158381|oppC| oligopeptide transporter subunit ; membrane component of ABC superfamily [Uncultured spirochete bdmA 4]
MSRKIGQAVNEFNQQMKPVSLWADAWKRLRRNKMALISLWVVGFYALVALTAPLFPFLDY
RTQVIEHANLRPSLQPAGKLVIQKLEAHRAVLQERINEGMDNLKPDLERTEADIQYAKDH
LNDPVNKKVYIFGTDNLGRDMLAKTIYGGRISMMVGLIGTLTAVFVGIIVGAVSGYVGGW
LDNLLMRFVDIMYSLPYMLIVIILMAMFRKEGQSSIFILFIGIALVSWLTIARVVRGQII
SLKNSEFVEAARSMGASTGRVIFRHLLPNTLGVIIVFTSLSLPSFIMNESFLSFLGMGVS
APLSSWGSLVSEGANTMELYPWQLLAPAIEMTVFLFFMNFLGDGLRDALDPQSKNRT*
>SPBDM4_v1_50019|ID:27158382|oppD| oligopeptide transporter subunit ; ATP-binding component of ABC superfamily [Uncultured spirochete bdmA 4]
MSDEVILQVKDLKTYFTVDEGVVKAVDGVSFELHRGETLGIVGESGSGKSVTNLSIINLI
PMPPGKIAGGQVLFHGKEMLTMSPNEIREIRGNKISMIFQDPMTSLNPFLRISTQMVETI
VLHQGLSKEESKTKAIEMLKLAGIPAPEKRIDQYPHQFSGGMRQRVMIAMGLSCNPEILI
ADEPTSALDVTIQAQILELMQELTRRLGTAVILITHSLGVVAGMCNTICVMYAGRIVERG
RTEEIFESPRHPYTMGLIRSVPRLDQETRGRLYSIPGQPPNVIDLPDCCPFYPRCDKVMD
ICKKKYPPTVPFEKEQSAACWLYAKEAQNA*
>SPBDM4_v1_50020|ID:27158383|oppF| oligopeptide transporter subunit ; ATP-binding component of ABC superfamily [Uncultured spirochete bdmA 4]
MPDHEILLDVRGLKMHFPIRTGFFSKAKNFIYAVDGVDFQVRKGETLGLVGESGCGKSTT
ARAIAQLYKPTAGEVYLNNLDLTKANGQELLAARKNMQMVFQDPYASLNPRMTAGDIIAE
PIRIFKKNGLMEASKDEIDDRVEQLMEKVGLSRFFKNRYPHEFSGGQRQRIGIARALALN
PELILCDEPVSALDVSIQSQILNLFRDLQDEFGLTYLFIAHDLSVIKYISTRVAVMYLGL
IVEIADAGDLYKNPLHPYTEALLSAAPIPDPKIEAKRHRIILTGDVPSPDKQRPGCNFYD
RCPKHMDICKKVRPALKETEPGHQVSCFLYHKPE*
>SPBDM4_v1_50021|ID:27158384| protein of unknown function [Uncultured spirochete bdmA 4]
MSAEAATPHSRLCFVRSGSTEPLRLYSKKYKRVSDFPVGSTTAIPNDPTNERRALILLQA
KGLITLKPDAGFEASTPCRIRSSPKAESRPTRK*
>SPBDM4_v1_50022|ID:27158385| protein of unknown function [Uncultured spirochete bdmA 4]
LQRPRRCKGKSQRKLVELLGLSLKAVESYEQGWRSVSSNVERMLYFLLFKLNQKAFEAEF
PCWEATSCPEERRGHCVAYVAQEGYFCWFFTGGLCASAKALGEGERHCYGCTVFIRRRSL
AEASAAGGDASGERRDDASR*
>SPBDM4_v1_50023|ID:27158386| protein of unknown function [Uncultured spirochete bdmA 4]
VANDYNKIAFGHYLDDAVETLLMNMLYKSEISGMLSVLRYKKYPLSIIRPLTLCEERQTI
AYAEEKGFRSGRSAPVLLGAIPSGARSEQRLRPSPGA*
>SPBDM4_v1_50024|ID:27158387| NAD/NADP octopine/nopaline dehydrogenase [Uncultured spirochete bdmA 4]
MHRTWAIIGGGNGGQAMAGHLALLGERVRLFDVFSDTVNAISRQGGIKVTGAVNGFGPLE
FATTDMGQAMEGADVVMIVLPSIYHEEMGRNCIEHLRDGQIVFLHPEASCGALAFKKLLV
EAGNTADVTIGAANTLLYSTRIITPGTVDIFGVKHSLYMAALPASRGTRLEEAIGSVFPN
FTLCPNVLYTSISNNNAMMHPAPTLLNVARIEAGQDYFYYTEGITPSIGAFVEQMDDERI
ALGEAYGFIMKTLRKSYFDMYNSGDMSESDSLSALCKKVVAYREIKAQKTLRTRYVLEDV
PYSLVALQSLAKIAGVSTPCIDAVVQLAHGMLHGELDEGRTCEALGIAGMTKADLLRYVS
*
>SPBDM4_v1_50025|ID:27158388| conserved protein of unknown function [Uncultured spirochete bdmA 4]
MTNGKPLLSMKGIQKDFLENRVLEDINLDLAQGEILGLVGENGAGKTTLMNILFGMPVIQ
QTGGYKGTITLDGEEVHFSNPNDALMAGIGMVHQEFFLIPDFSATENIFLNREVTRYSFL

VEIFGERLRTLDRAAMNERVVQLIKELGVDINPDTLVSEMPVSYKQFTEIAREIDRAKIK
ILVVDEPTAVLTETEAEALLTVLRRLAAKGVGIIFISHRLHEILDICDRIVVLRDGKLIR
DVPSRQSDVKSIASWMVGHDVGDFVFQPKQGCGEKESFLSIEHLKVLMPGEAVKDVCLQV
RKGEILGIGGLAGQGKLGIANGVMGLFHAEGSVTLGGKSLKLNSPLDAFRAGMAFISEDR
RGIGLLLTQSIGLNIAFPLMQVKGGLLKKIAGLFTWEDEKKIREITTKYIERLDIKCTGP
DQVVMELSGGNQQKVCVAKVLALSPQILFASEPTRGIDIGAKHLVLSVLKEINEQEGTTI
IMTSSELGELRSICDRIAIVSEGKVAGVLPAEAPVEDFGLLMLGE*
>SPBDM4_v1_50026|ID:27158389| Monosaccharide ABC transporter membrane protein, CUT2 family [Uncultured spirochete bdmA 4]
VKNEDRKPKGRVGEFIANFGWPRIIIAIFLLLLFVIAPFIGIDIGTSLSDMLVRVGMNGI
LVLAMVPMIQSGCGLNFGLPLGIIAGLVGAVTSMEQNWQGLFGFGMALLLAIPLAVVLGI
IYGYFINMAKGDEMMIGTYVGFSIVAFMSMMWLRLPYKNPLLVWGFSGTGLRTTISLNGF
WMKVLDNFLAIRIGAHFFFPTGSFIFFALCCVLVWAFFKTKTGIAMSAVGSNPQFARAHG
VSVDRMRIISVVISTVLGAVGMVVYEQSYGFIQLYMGPLYMAFPAVAAILLGGATINSAT
ISNVVIGTFLFQGILTMTPSVINGMLKTDMAEVIRILVSNGMILYALTRKGKGMPK*
>SPBDM4_v1_50027|ID:27158390| Permease component of ribose/xylose/arabinose/galactoside ABC-type transporter [Uncultured spirochete bdmA 4]
MKQKYSFKGALISNLVPIVFLAFAAFAVPMSGLSIQYLLEEMITRLARDSLLVLSLIIPV
LAGMGLNFGMVLGAMAGQIGLVLVVDWGIGGMPALLVAALVGTPISIGLGYFCGYILNLA
KGREMVTSYIIGFFMNGAYQFLLLYCMGSIIPISNPELLLSRGYGIRNSMDLPNIRQVLD
RLVSVNIFGMNIPIVTFAVIAAFCVFIVWFKGTKIGQDMRAVGRNRAVANGAGIPVEKTR
IMAIIISTLIACYGQIVSLQNLGTINTYNSHEQVGIFSIAALLVGGATAENASIVNVFLG
VFLFHLMFVVSPMAGKNLLGDAQLGEYFRVFVSYGIIAVSLLLHAWKKRHEMLERRLLEN
SVKE*
>SPBDM4_v1_50028|ID:27158391| exported protein of unknown function [Uncultured spirochete bdmA 4]
VNVKLRRNLLRVALCLIAVAGGFLLSLVGMYHSIILDNSEFSSAGAVLPPLEGLRVLSEA
EPVEMSPDDRILVSTKGPSTTLVFEITSPETQQSREISYTLHMGFKKQFFLSVPAIVGGE
KNPFLVE*
>SPBDM4_v1_50029|ID:27158392| Amidohydrolase [Uncultured spirochete bdmA 4]
MMELYGKLGGKTDAWIEGHLAEYGRLSDQIATHPELSNQEYETSAALCRALEAIGFSVEH
PYAGYATGFNARYEHIVGSSRPVVVVGLLAQYDALPEVGHGCGHNMCGPMSNLAAGALKDV
LEAGNISGVVRVIGTPAEETTGEKVPMAAAGLFDDCSFVMMAHPTTASSFVWFNSLAADP
YEFTFMGKAAHAAAAPWDGVNALNGLQLFFHAVDMLRQHVRPEVRLHGIVVEGGSAPNIV
PSRASAHFLLRAPWRNYLNVVKEQVFNCARGAAIATGTTVEWKMFGNAMDNMLRNTIAEN
TMTHILQDELAEPINSNPTLTGSTDMGAISWRVPTIELQIKVSDKEIPPHTVEFAQAARG
PGALSPMAKAAKGLARMGLRVLLDEPFRRTVHEELDQRKKDLL*
>SPBDM4_v1_50030|ID:27158393| conserved exported protein of unknown function [Uncultured spirochete bdmA 4]
MKKGLVLALVVGLWLGFSAGAAFADAEFHIGMVTNTVSQSEDGFRAGQAFLKKYGDVANG
GMVKHITMPDNFTTEMETTISQIVSLSDDPKMKVIVVDEAVIGVTEAFRRVRERRPDIVL
LAGEPQEDPNMITAVADMCVMGDHISRGYTIIYGAKMIGADTFVHISFPRHLGIELLSRR
MNIMKEACKDLGLKFVQETAPDPTSDVGVAGAQQYVLEKTPAWIEKYGKNACFFSTNQVH
LEPLFIQIAKYGGYFLEGDSPSPLKGYPGAFHLDLSNEKGDWPAIMAKIEKAVIAAGAGG
RLGTWAYSATYSMTLGLGEFGKAIAEGKMKASDTKALVKAMETATPGVTWKSTYYRDKNT
GVQKKNYVMLMEDTYVFGKGYLHLTDLEVPEKYYKIK*
>SPBDM4_v1_50031|ID:27158394| Peptidase M24 [Uncultured spirochete bdmA 4]
MFHSVRLTKLIDVLRERGLDAIFLAPSSDLEYLTGMRLHPDSRFKGVMVSKEGKAFSLCP
SLYKAEMSKSLPEAQLCEWKDGEGFRDAFLKGCTALGLMRGKVAFNGGVRAADMLDATAD
TSIRCVNGATLLSSLRRCKDAEERELLRRASKKNDSMMEKLSHFIRPGKTEREIAKFVMG
VHEEQGGLPRYPIVGSGPNGAKPHYSGEENRTIQEHDVVVVDSGAWYESYNCDMTRTFIM
GSPSSEVRRVYDIVFRAQNAGQAAAGQGAIPEDIDRAARSVIEQEGYGEAFFHRLGHGTG
RDPHEEPFIVQGNRVPLQEGNCFSIEPGIYLAGRFGVRIENLVLISEKGTEILNSFTKSM
IIIE*
>SPBDM4_v1_50032|ID:27158395| conserved protein of unknown function [Uncultured spirochete bdmA 4]
MLKRANRSTLHGDIMQQMIKAIQEKQWAPGSKLPSELELAETFGVSRTSIREVLKALAHS

GVLEAHPGKGTFLSPSAEEILSGTKLTAAMFSEYSYTELIEVRRLLEGQAVYWAAERASP
EDIARLEEILKGNEGETSFDIHDKFHTAIVEMSGNRLLIKLMASLRDEIVAQREFHNVVL
PDTDRQQQWQIFEAIKSRSPKKAFDAMMKHINVFWEKLA*
>SPBDM4_v1_50033|ID:27158396| putative ornithine cyclodeaminase, mu-crystallin [Uncultured spirochete bdmA 4]
MIEDTMLLNQKDVQSLITMEDVIDCVDRTFQGMGNGTVVNPTKVNLDLGETAAYPSYSGF
MNAMPAYIGWSDSAGIKWAGGFLGERKKLGLPYITSLILLIDPRVGNFRAVMDGAFITNY
RTGAQTAVALKYLYGKGKKIRLGLYGAGVQGHTQTMAISALFEITEVKVFDMNREAAEKY
ARDMESCVKGPIQIVPDARSAAVDVDVAVCVTQAKSEFFRNEWFKPGMLLFPMGSYQECD
DACIETADRIIVDHIGQALHRGALAKLGTSGRISEKNIYATIGEVAAGKKAGHFSGGERV
ICVPIGTGAMDISVATLAWQRAKEKGLGGRFKFV*
>SPBDM4_v1_50034|ID:27158397| putative ornithine cyclodeaminase, mu-crystallin [Uncultured spirochete bdmA 4]
MVADTMLLCQDDIRALITMKDVIECVDRTFQGMGDGTVINPTKVNLDLGARAPYPPYEGS
LNAMPAYLGWCDSAGMKWAGGFLGERKRQGLPYLTALILLIDPRVGNFRAVMEGAFITNY
RTGAQTAVALKYLYGRGKKIRLGLYGAGVQGYTQTMAIAELFEITKVQVFDIRRSAAEQY
AEDMKNIVRGPIVVTSRPEEAAVDVDVVVCVTHADDGFFRNAWFKPGTFLFPMGSYQECD
DQCILTADKIVVDHVGQTIHRGALAKLGASGRITEKDIYATVGEIAAGKKPGLFHGDERI
VCISIGTGAMDVGVATLVWQRAKEQKLGSMYRFV*
>SPBDM4_v1_50035|ID:27158398| L-serine dehydratase, iron-sulfur-dependent, alpha subunit [Uncultured spirochete bdmA 4]
MDDTNRLVSLFDTLGPIMTGPSSSHTAGVLRIGRMGRMLFGGTPEQIDLYFYGHALAQTY
KGHLGDSAIVAGLLGHSEDYPALKYSLQEAEHQGISVACHTELASKRDPNTVDMRLRRGD
KEVRVVGITLGGGEIEITELEQFPIHLKGDEVGVLILVNSYFDDAQLHKLYEGEHRRISV
SKKGDETLYTCLLDTPLLPEEIEHLRAWNGVVAVYALEPILDYRLRDAVPLFSTVEGMLA
MGEKEGASIPQLAIMYERRRSGLSESEIRARIGKIWQKMKESLAAGAGGNNTMLAGFIPG
NNGWKMLRAVDEGITLSGPTLGKAIGRAIAVMETNACAKCVVAAPTAGSCGVLPGVLTTA
AENLKADETTVHDALLSAAMMGTLVSMRASLSGTKGGCQAEIGVASAMAASALVQLAGGS
ARQVTHAMALAIKNILGLICDPVAGPVEIPCIKRNSIGVANAFATADMSLAGIESAIPPD
EVIDALVNTQKLLPSALKGTMIGGLGSTPTAQRLKKEWDARVRVARE*
>SPBDM4_v1_50036|ID:27158399| protein of unknown function [Uncultured spirochete bdmA 4]
MEAPSDGAKGNHYVVCKKITDLIGNTPLVQVLAPEAAQGDNRGGNLARYRWESGHRRGRR
GYRRHDPRHRRGIRARRVASRPSSAEKTIVAILPDTGERYLSTWPFEEGGEANL*
>SPBDM4_v1_50037|ID:27158400| protein of unknown function [Uncultured spirochete bdmA 4]
VDIVVAGVGTGDTIPGIGAGFVPAG*
>SPBDM4_v1_50038|ID:27158401| putative Methionine synthase [Uncultured spirochete bdmA 4]
VSPRELSGIAISGATDSLAAGQVSNPAVQGARPDEAKLRSLIGGRLILADGSTGTALEAL
ASDAASGGRIPLLPLERPDVVETLHEAYFRAGCDLVETATFSASARDLARFASKDEGGAE
GLSEAVNKAAAAAARRAADKASAIDKRIRLVAGSIGPGDAPPSLGTSTYGELFLSYLPQA
KGLTEGGADLAIVETCQDPLQIKAVIAALRSQQGGRGLPFIVSATVDARGRMLAGTDIAA
FVAIVSPFRPLALGLNCSGGPDELEEPLKELASLSPFPVCFMPNAGLPCTEDGCVSYPFG
PELFAEKVESLARRFGVAIVGGCCGSGPGHMAALSARLSDRPNVPKRPPLRPALASLSEA
KFIGPGLFRIGERANSAGSAAFAALLDAEDFEGMAEKALKQESSGAEALDLHVSRPGRDE
AKDLERLVALLAPRARAALCLDSGDPEVLARVLPSVGGRPIINSASLEDPAKARRVFGLA
AEFGAAVICLAMDGAGPARILEDKIRVCRRLYEMATGEFGLPPQSLLFDPLTFTVAAGGD
ASTTINAISAIKKACPGSLTVLGIGNVSYGLPKSARPAVTALFLDAAVRAGLDAAIMDTA
GTPAPESIAPELREAAEQALGIAGRDWSIGCRRGGSHGGSRPAGDDSSRRDAVDPLGPLL
AWAEARSGKNTEAGAKSPGAALLAGTKTPDSEAADPTAALSAAVLRGDTAGAEKAGQKLG
ETGGPEKLAAAVTAAMAESGRLWNEGTISLPLVLRSAEAARRALAPLASDAAKTAKGSIV
MATVKGDLHDIGKNIVSAILACSGWRVVDLGTDVSASAIVEASHSQGALAVGLSGLLTRS
LVEMKTVCEALETAGSECLVLCGGAAVDPAFVTREIEPKHPGLVRACADAFGAAAALNDF
LENGARLRTPADRTSADRAHSFGPVGGGRKSTGTPILPKASPAFEPPFLGSSPPLAIPFS
ELLPLVDQRALFASRWNYRRGDWEEAQRHLGILLPEAERLSTPAGVYGYFRCRRAGERTV
SIEVPGGRSFDLPFPVEAGGHHRTLAAHFSSAGDAIAFFAVTTGPEIARAALALQARGKL
ESYWRLHGLGSSLAEAAAQWMHERIARELSACGAGGRGRRYSFGFPACPGTKYQNTLLEL
LGAHRIGIRATTGHQLVPEHSVTAFIIARPDTVYFEA*

>SPBDM4_v1_50039|ID:27158402| putative Methylenetetrahydrofolate reductase [Uncultured spirochete bdmA 4]
MPATSYCGVTMHNDKPSVNTSRARVLDTLASGLASRPVSGRAPRRCISIEMVPPPRGGDL
DSIMAAMETIAPHRPSFVSVTDHPGGKAWVDSGEGPKRVALRTKPGTLGTAVALRDAFDI
TAVPHIVGGMADRLDVEDLLIDLHYANFRDIFVVMGDDRYAPAALPPGGRAGRMGNGPDG
YNHARDIVEHVVRLNRGEYTPPSEGKPTDFVVGVAAYPQKHFAAANPETDFEHLVEKIRA
GASFVITQMVFEAGPYADLVRRLRDAGVGAPVLPGIKPILKASSLDMIPRTFFVDIPPLL
VAALREARSPAEERAAGIAWTVKLCQDLLDAGAPGLHFFTMGRGVGTRWILDALFGTPGG
KV*
>SPBDM4_v1_50040|ID:27158403|metA| homoserine transsuccinylase [Uncultured spirochete bdmA 4]
MPVRIPDDLPAAKQLEEENIFVMTKERAFHQDIRELHIAVLNLMPTKMVTETQLLRLLGN
TPLQTDIRFIRMTSHDSVNTPPEHLDAFYEPLDEILDEHFDGLVITGAPVETLPFEEVDY
WSELVRLMDWSTTHVWSTLHICWGAQAGLYHHYGVGKRPLPRKMFGLFRHKALDLKEPLL
RGFDEEFLAPHSRHTEVCADAIAAVPHLKLLAVSEEAGVYLVASQDGRRVFVTGHPEYDR
LTLKAEYDRDLAKGLPIAPPVNYFPGDDPAREPLMTWRAHAHLLYANWLNYCVYQETPYD
LDSIGGPNA*
>SPBDM4_v1_50041|ID:27158404| Aspartate/tyrosine/aromatic aminotransferase [Uncultured spirochete bdmA 4]
MPDISAKSRSFGESVIRRMTRVALRHGAINLSQGFPDFDPPRALLDAAARTAVGGPHQYA
VTWGAQNFREALAAKQRRFMGLPIDPDREITVTCGATEAMMAAVMTAVDPGDKVIVFSPF
YENYMADAVLSGAQPLCVPLRRDSSSADDFTFDPDELEAAFEAGAKALILCKPANPTGKV
FSRTELETIAALAERYDAFVITDEVYEHIVYEPYKHTYFASLPGMFERTISCSSLSKTYS
ITGWRLGYVIAPATVTEGVRKVHDFLTVGAAAPLQEATVTALGFDDEYYKELGTLYRAKR
ELFLSYLDRAGLSYVRPQGAYYVLVDIREFGAKDDTAFCEWMAEKAGLAAVPGSSFFKDF
EGRYIRFHFAKRGETLVAAGERLLALKSRWEESGQRVARRG*
>SPBDM4_v1_50042|ID:27158405| O-acetylhomoserine/O-acetylserine sulfhydrylase [Uncultured spirochete bdmA 4]
MAEKRTPSIETIAVQAGHSPDPATLSRGVPVYRTSSFTFKSAEHAANLFALKESGNIYSR
IGNPTNEILEARVSLLEGGAASVAVASGTAAVFNTVITIAKAGDEIVSASNLYGGTYTMF
DAILPDFGITTKFVGDLDPASFDAAVTDRTRLIYIETIGNPVLDLADIDAISAVARKHHL
PLVVDGTFTTPYLLRTIEHGADIVINSLTKWLGGHGTAIGGIATDAGTFDWSDPKFVLFN
RPDPTYHGLRWAADLPPELRKIAFAMRTRTVPLRNLGACLSPDNAWIFLQGIESLHVRMP
RHVENALAVAKYLKRHPKVAWVRYPGLEGDPSYELSKRLLPKGAGGMVVFGVVGGKPAGE
KFIESLRLFSHLANVGDAKSLALHPASTSHSQLTEEQQKASGLTPDLVRLSIGIENIDDI
IADLELGFAAV*
>SPBDM4_v1_50043|ID:27158406| protein of unknown function [Uncultured spirochete bdmA 4]
MEIPLRLVTRKDGSLFYGIPISFRIMMGGMLALIGGAIALEEFHTGPLGWIAVILLVLGF
VYKEDWLFDASGKTISGLVGFYPVLKKTNLAFEEVAYIQLAAFSKGTVPGSPEEKSADRE
AFNEMRGDVSRSQMKGGFDIFQRKKLYIAMLIITKNEEHYLLDMVPARRASRLSIAGRAL
AALIGCSFLENLPE*
>SPBDM4_v1_50044|ID:27158407| protein of unknown function [Uncultured spirochete bdmA 4]
MRACISEMSQALEGADAALIEQFFYQLFTAAEADEMAKRWALVKELAKGTPQRKIAAQLG
LSLCKITRGSRELKKPGSSFRRLLDKLPPTSEHESSGSSPKPGIAKA*
>SPBDM4_v1_50045|ID:27158408|gap| Glyceraldehyde-3-phosphate dehydrogenase [Uncultured spirochete bdmA 4]
MSKVKVGVAGYGVIGQRLADGVALQGDMELVGVADVAPTLSVRALREKGMPYKFFLAAPD
KREELEKAGIPISGSLEDLVQRVDVMLDATSAGVGAKNRALYEKYGKKGIFQGGEKNSVA
DVFFHGYANYEKGLGAQFLKLTSCNTTGLIRAVDCLDRKVGVEKVAITIIRRVADPGDYH
RGLTNALQIDKAPSHQALDLMTIMPQVEATGILVHTPVTHGHIITVVATPKKSVSPENVI
EFFREHPRIRVVSLAEGFLGNASLFRYARDLGNPRGDMYEIAAWEDSVVMSGKDIMFAIN
VPQEAVVIPENMDAVRACMRMEEDRIEATDTTNRYLGIGKWRNLG*
>SPBDM4_v1_50046|ID:27158409|fsaB| fructose-6-phosphate aldolase 2 [Uncultured spirochete bdmA 4]
MKFFLDTANLKEIREIAEIGLIEGVTTNPTILSKEGSGWEKGIKEILTIVKGPVFAEVFS
TQAPDMIREAETISSWNDRLVIKLPMIPEGVKAINALSKKGITTCATLIYSPGQAVVAAL
AGASYVAPFVARSFEIAMDGIQLIEDISNIFSMQEVETEILAASIRTPHDAVRALLAGAD
ILTLPYGVLKQLMGHPLTDVTLKKFMDDWSAVNR*
>SPBDM4_v1_50047|ID:27158410| Glycyl-radical enzyme activating protein family [Uncultured spirochete bdmA 4]
MSSGIVFDIQRFSIHDGPGIRTIVFLKGCPLRCLWCANPESQRLEPELFYIPGRCIRCDA

CFEACPAQAVRRDDYTEGRIDRGACILCGACVEVCPTGALSMKGKRMGVDEVYGEVLKDR
QFYASSGGGVTFSGGEPFLQVDFLQALLERMKAGGIDTVVETAGDVPWCAIERCLPFLDH
ILFDLKAFDPERHRRIVGTGNKRILENLEKLSSADVDMAVRIPVVPGVNDDEEDMKGLAR
LIASLSIRSVEFLAYHELGVTKYGYLDRVYPLEEKADGDPEKSIRRLAKLFGDYGVKASG
GSGG*
>SPBDM4_v1_50048|ID:27158411| protein of unknown function [Uncultured spirochete bdmA 4]
VICEEFHAPNRKRRDQPCEPFHVFLIVIHSGDDGNPYGHIHVGARELLEVFEYPLVSGPN
DSSMPFGIEGLEVEKDMIEKRQAAFDRAPRDVSGSLDYGIDASRLHPLKQRLKEIDLEKW
FPARKSYSPPGGGVKLAILQHFAVHLIDTHPFPFHRERTSGADLDAGTTQDTGAAIDPSL
SIIVAADGLRGAGFETGVASDTTPRDIEQFGFEPLRFGVCTPQTAERTTFEKDDGPDSRT
VMD*
>SPBDM4_v1_50049|ID:27158412| Pyruvate formate-lyase [Uncultured spirochete bdmA 4]
MNPTIGEYPNPSSRIVALKQKFLDYESFMCCQRARIYTEVYRETEHQPMIIRRALALCET
LRQIDLFIEDGELIVGHPASRPRSAEVFPEVSFHWMKEIDEFETREYNRLKVTSEVKAML
AEIHPYWEGKNPTDKFRARRPEETKRAFAAGLLSNPHEWSTFAHVGLDYRKILTKGINGL
LEDIKKQKASMRITDPDYHGRSVFYDACEIICEGVVDYAHRYGELVDSFIARTRDPVRAS
ELQGMREVLSRVPLYPARTFHEALQSFWFVQLITQIEGNGFSISPGRFDHYMNPYLEKDL
EEGRLSVERAIELLDCTWLKFAEIMRVDDKKAAEVNAGYASGQNLVIGGIDSKGKDSTTL
LSYLCLIANYHVRLHQPNFTVRLHRGTPEEFLEQVVLSISSGNGMPQVLNDEIIVPSLTG
RGLPLAEAREYIPVGCDEITVPRMWGRCNGGYLNFAKVLEMTLNEGVDMKYGIAFGEPPS
LRMIGSWDEFHQAVLEKIDYAVERQVSEANLTDRIHEEILPLPFLSMFLDDCIETGKDVT
AGGGHYNTTGLVGVGSANIGDSLFAIKRLVFDEGRLTLAELRSVLLADFEGNEPLRQYIL
NKIPKFGNDEDEVDQYVVGVTNRFFDAVDRYRNNRGGPFYPALYSVTAQVGLGGATAASA
DGRKSGLPLADGLTPMYGQDVHGPTAALKSVVKVDLVRSPNGVIVNQRLTSGILGTPKGR
EKMELLLRSFVDIGGFHWQFNIVDNKELLEAQVHPEQYKGLVVRVAGYSAIFIELSKKAQ
DSVIARNAASL*
>SPBDM4_v1_50050|ID:27158413| TRAP dicarboxylate transporter, DctM subunit [Uncultured spirochete bdmA 4]
MAVALLGILLLLILMSVPIAFSIGISSLFVILMEGNLTPWIMIQRTFGGLDSFVILAIPL
FLLTGEVMNRAGITDELIDFSSTVVGHIRGGLAHINVFVSMIFAGISGSSTADTAGIGKL
LIPAMIKRGYSKEFTVAVTAASSTLGQIIPPSIIAVIYAATVGISVGALFLGGAVPGILI
GLAQMVVSYIFARKYNYPAEERSSFRKILHAFKKAILPMLSPVLLIGGIIGGIFTATEAA
VVSAVYSILIAIFVYRSIKLKELWDIFVTTALNASVTIFCIAIAAVFGYLLAYFHVPEML
GSVITQIASGPVSFLLIVILLFLIVGTFMDATPAIIILAPMLAPIACTFGVNPIHLGVII
VLTLALGLITPPYGLCLLVAAQIANIPVNFKLMRTMSIFILVSLLIIILIALFPDIALFI
PKIFAPKMMGVTL*
>SPBDM4_v1_50051|ID:27158414| Tripartite ATP-independent periplasmic transporter DctQ component [Uncultured spirochete bdmA 4]
MNKISTVLEKIIRFAVGVLLIVLSANVLLQILSREILHIQTVWTDEVARFSFIWMTMLGA
SLQVKKKSHFAVSLFSLKGTVRLLHSLLVYALMAVLMVVFIWFGYKYTIQGWSRYSTSSG
IRMAWIFAAIPVGGLLMLFFIVELAIADIREARKSSISAKGGD*
>SPBDM4_v1_50052|ID:27158415| putative Tripartite ATP-independent periplasmic transporter solute receptor, DctP family [Uncultured spirochete bdmA 4]
MKRIALVLVCVLLAGGVVFADSDTTKAGVIKLKCGIMVSKDYSQAKAAKYFAEEIAKETD
GRVVIDVFTDGVLGSEPEMWEAINAGALDMAVVSPAYIGNFVPEYQLYDLPFLFKSRAHR
DAVVDGPIGAQLDKLLEKKGNMVVLGQFGGTGRIMISRNKKIETIDDLKGLKMRVYASPV
VVSTWKALGTLPVTVAYAEAYTALQTGVIDAAENETSTLITQRWYEPCKYISMTEHNINT
RPFLIGAKQFAKLPPDIQAIFRKVGKDAAQYAVKVEREDDARYIEQLKSFGLQILELKDK
DKWIAATRDLRMNFVKKYNLEQTLKDIETTAQ*
>SPBDM4_v1_50053|ID:27158416| protein of unknown function [Uncultured spirochete bdmA 4]
MKSVKIGTLYLDPVRNPAILDTATWDIPAAIRLFKQMDATEWASLRASLADGRPGGIVQK
RGAAFYLSSTMSFEGGICLLATLPDSQRAILHIYAPGAGEALDGSHSVFRDDSMSVSLYA
AEYETLHTLIRRLNPLKGPRALGSVPRIGIGSRNSTLAWPAVYRSMKSGGFAANSIQNSM
REIGTLQEVSTNEAVDRSYLYGFGEVPPAYTGATFEGVWTYGVLEALSSDFLPIFGADAD
HLQLKSDVRSVERMKKILNAARHYSFFTMDISQIADYQAMFRTSLSGAAELLDRHIPQDA

LRKDVLMWHRQRYWFNGREYRFDEALLGRLVGKYWEALSLLEKYFHLIRDMKQGEPFDFE
LTIDEVPPGIHTHEIITGEEELLFLLNEAERRGLSLTHVAPNFGIEKTTDYCGPDGLEGL
FRRVSNLHAIAEQYGLMLDCHSGDDLSDETRHVFGRATGGRIHYKISPSIGDVFAETLFD
YDRERFMVWWNDTMEFARKCAAEGSGLATALLASRDRRGNGTPNPKDFLFKEFKFTMPGR
KDSTGQMDGREFFYDLPEDFKNEYSARVEKWILQVSRSLFDYQ*
>SPBDM4_v1_50054|ID:27158417| Transcriptional regulator, DeoR family [Uncultured spirochete bdmA 4]
MIISTIMGRVTDIKNYLRQQEIVRMLQTEQIIRVSELINRFSVTPATIRRDLSVLERAGK
IQRTRGEARIVQSQSIVLPFFQRSELQTSEKIAIAKTARDLIADNQAIILDSGTTTLAIA
QQLFNKKQLTVITNSIPISYVFANQDIEVRLSGGIVLPAHMALIGPDAEAYFRGIQVDIC
FIGASGVRSGSGFTTSSPFECSIKQQMMRAARQVVAVVDSSKFSISRIIEFARFEEINKL
ITTYPINDKESLSRLEKLGVEIIYAQV*
>SPBDM4_v1_50055|ID:27158418| protein of unknown function [Uncultured spirochete bdmA 4]
MAKAKVGVAGYGVIGQRLGNRLWRLMPAADTP*
>SPBDM4_v1_50056|ID:27158419|iolE| Inosose dehydratase [Uncultured spirochete bdmA 4]
MEKTIRLAIAPIGWTNDDMPELGGEIPFEQCVSEMALAGFEGSEVGNKYPHDPEKLNKAL
KLRGLTICNAWFSSFLTTKPLKEVEHEFVQQCDFLYAVGARVIGAAEQGNSIQGKPLPVF
DAKPRFTDDEWKRLTDGLNHLGAIAKQKGMQLTYHHHMGTGVQTTEEIDRFMEMTDPELV
GLLYDTGHLVFSGEDHLAILKKYVKRIKHVHLKNIRFDMLEKAKREKWSFLQAVRNGAFT
VPGDGGLDFTPVFETLKSAGYAGWWVVEAEQDPAKSNPLEYALIARRYIREKAGI*
>SPBDM4_v1_50057|ID:27158420|iolD| 3D-(3,5/4)-trihydroxycyclohexane-1,2-dione hydrolase [Uncultured spirochete bdmA 4]
METVRLTVGQAIVKFLDNQFVEFDGKVEKFVGGVIGIFGHGVVVGLGEALAAPGHGLTFI
QAKNEQGAGHVAIGYAKQNNRRKIMAVTSSIGPGALNMVTAAGTATVNHIPVLFLPGDVF
ADRQPDPVLQQLEVPHDYTISANDAFRPVSRYFDRITRPEQVMTAFLHAMSVLTDPAQGG
AVTIALPQDVQGEAYDYPVEFFDQRIHHIERRPPTSAQTSRVADLIKASKKPYIICGGGV
RYSEAGRALADFCKKFGVPFGETQAGKGSVPWDHPCNLSGIGVTGSLAANRLAKEADLVI
GVGTRFGDFTTCSKWLFQNPRVKFIAINIATFDAYKMNAEPIIADAKLTLAAVGRALGAA
GYHANWGEAIARAREEWSREVDSLYTQEDPAGLSQIRVIGELNDVLLPKNAVVVSGSGSI
PSDMQRTWRTRVAGTYHMEYGFSCMGYEIAATLGCKLAMPERECVAIVGDGAYTMLHTEL
LTAVQEGKKIIVVVCDNSGFQCIDNLQHSQGIAHFANEWRARDPETGLLAGDQVPVDFAK
NAESWGALGLRARTVEEFRKAVRRALRAKRPVVIHARVSPKSMTQGYESWWRVGTAEVSA
NPQVVRAAREMKDELKKARKF*
>SPBDM4_v1_50058|ID:27158421| Myo-inositol catabolism IolB domain protein [Uncultured spirochete bdmA 4]
MVLHHSGDYADGYELLVLRDSEKANMMMDFASLSLKEGAIWRSNSKDERALLLAEGSVEI
MVGEKVEWIGAGEGRTPDRSPVSVESLAEKGTRIRASRVSLLEENPAVLHLPAGTDVAVK
ALSERALLYITATVNGTRFAPRLYLPYECQVEYRNEGTMQGTGTRIVRTVFNKNNAPWSN
LVLGEDINLPGRWSSYPSHHHPQPEIYHYRFFPSQGFGMTALGTEAYQLHDRDTLLITDN
KDHPQVAAPGYTMWYLWVIRHIEGNPYISPEFNPDHVWVDQPGAKIWDMKRTGR*
>SPBDM4_v1_50059|ID:27158422|yrbE| Uncharacterized oxidoreductase YrbE [Uncultured spirochete bdmA 4]
MANKITVGVIGAGRIGKLHISNIVYYMPEARVKTVADANLTPEIESWVKSMGIPNVSKNA
EDILSDPEISVVMICSSTDTHADYIIKAAQAGKNIFCEKPVDLTIEKVKAALAAVKKAGV
RLQIGFNRRFDHNFAHVRELAKSGSVGQVQLVKITSRDPAPPPPAYVKVSGGIFLDMTIH
DFDMARFQAGSEVEEVYAAGAVLVDPEIGAAGDVDTAIITLKFANGALGVIDNSRKAVYG
YDQRVEVFGSAGAAAAENDTPSQVRLSDANGVHGDKPLYFFLERYREAFVAEMKAFFEAI
RTGTETPVTGNDGLMDLAIGLAAKKSLAEHRPVKVSEVM*
>SPBDM4_v1_50060|ID:27158423| Xylose isomerase domain protein TIM barrel [Uncultured spirochete bdmA 4]
MIESKRFALNRIAAPALGLAEFFEAATGLGMSSVELRNDIRDGSVTDGLKGTEVLRLAKA
AGARIITINALQQFNLPGARAKALSELEKLLALCVEIECPALVLCPNNRPDDVRTPEQKY
RDTVEALNTYAPMFSTHGILGYVEPLGFGISSLSSAETALKAIGESGASCYRVLIDTFHS
YLGPDKPEFFSDAAVIGKIGLVHISGVEAGIAKSEFADAHRVLPGPADLMQSAALMGRLE
KEGYRGLYSFEPFSSEVQSLGKKELVEALRASMNFLAKA*
>SPBDM4_v1_50061|ID:27158424| exported protein of unknown function [Uncultured spirochete bdmA 4]
MRNHILNGAFSRIGTMRRMAIATTMLTLLLCGQPLTAQNQIPSPGDPNPGGDRLKSALMN
ATMRAIGFEIQMYEIRLNAAKNGPGDPANIPVFEAEIATLKDELAHTSQMAPSDFALPPK

EIVHITVTQPYTYGSMLEVDNMTRSGPFYHVTGITNDDFSLLEPGKKYALAIYVVRPRDY
VLPFAENAYVYVQIGDQRTGPSEATADQQLIPESPTPSPPPAIPPTPSFGILSLSPRQTG
QEILLAINLGANSFTIRTASDGCTFKSSFRVDIHKAEGISPKLPHYDLTIYRIQPDECKM
ILYDGVEITFDLAEDLSIAGPCTYSVTNWVYRSAERPQ*
>SPBDM4_v1_50062|ID:27158425| conserved exported protein of unknown function [Uncultured spirochete bdmA 4]
MKYTKYGAFLLVLCIGAVPDLFADKGQMWPVPVNLSEPSQNAIIFHNRKEEILVLGTELQ
ADHQVDILEFIPFPSEPKVSIAQGNPFEAAEMLIKNKGLEFVEYSKGLAGPSPVSIVFEG
RLGPHDVTIVQIRDISGFADWTHAFFMNKGIVVAASLDAATANAADYMRRGFTYFVFDYV
HLSANSGAIAPLVYQFKSKQLYYPFKTSNITGNEGTANLILILPGSLGLLQDHSDIKALQ
RVSSSLFNWDLSSSAKLYPREIADIVPGAHDFFGDQKIYLQVLRYSGPYYFEDDLLFDMT
DIAPFAYKHVLYTPDYGNPLGGNYSLVGNLSKDELADIAEAYKLGANTWFFDPDILSLIQ
*
>SPBDM4_v1_50063|ID:27158426| conserved membrane protein of unknown function [Uncultured spirochete bdmA 4]
LGQSAPLIDADKVHVAQYAIMLVEKRFLPLYSGLFGIFALFGISMTVIGATLPKIFADFG
WSYTTAGAVIAAGSIGYFLSSYMAGIFLSTVGLRLTISVALLLIFVGLEFFAATSIPWLN
MLLYFIVGIGQGGIELSVDWATLRMEGPGGGRAMSLMHGAFSIGAFVGPLIIAILISAHL
QWTLIYRGIGVLFLAIAIFMQFMPLAALGKDRGHIAGGSRRDLFRHPAYWLGFFALFVYV
GVEMGISNWVAEYFVSVFKAPIPTGSFMVSLFWAGLLVGRFGAPFFQAVIKREKILVALS
ALMAFSVIALAVIGFVGTGRIASAIAAMLVIFAGLGCSIIYPNVMSIIGDFFPHTQSEAV
GFAAMGGGMGGFVFPYLMSAISAAWGIRMGFMTYALFSLVIVVLNVSLVRAENRGGLAKD
A*
>SPBDM4_v1_50064|ID:27158427| Transcriptional regulator [Uncultured spirochete bdmA 4]
MTSIKDIARLAGVSPSTVSRVLNEREYVRAEVRQRVLDIVKEKGYVQNHVARSMVLRRSF
TIGVVIPYLFNMFQRQLFASMEFFLEQHGYKTQFFFLRWSDSSEQAFLRRLKGETLDGII
FIHELDNPEIYQYIQERSLPVALCTFEKREFGFPAVHIDEEASAELAVDYLIQRGHHSIG
CITGEHFSFGRQREDGYKRALAAHGISYSPELTVRVPSYNPEAGHTGMLSLIRQKQPLSA
VFAATDELAIGAIKALCESGLKVPEDVSVIGLDDIDISAFTTPGLTTVRQPIFEMGKKSA
DLVCEWIARDHIQEKVESFTTTIVERESVRTLH*
>SPBDM4_v1_50065|ID:27158428| protein of unknown function [Uncultured spirochete bdmA 4]
MDELMQALKNAMKSEIDSVTIYSEAALRAESDVAQFFASRAEEEKRHYNWLLSYYKELLG
GQMPTINLAAEIQASQHKSPIFSQDFLTRVASDRHVSAAVSSAVLLELNATQHYQKMADL
AEEPHLKAFFRSLSEWEMRHYEELLKIQEDSRHYWFEEQNFEPF*
>SPBDM4_v1_50066|ID:27158429| Adhesion protein [Uncultured spirochete bdmA 4]
MKYIHRSADVRRFSKVFFVLFFVLAAAGTSFAQPKKPALTVAVSIPPMREWVNRIAGDRV
AVTLIMPPGSSPHAFEPSPRQLVELGKAQVWFSIGVDFEYSLRPKVSAMYPRLAIVDVTK
NVKFRTLRPGEQETEHESTDGHIDPALTNRDQHTWLGYEQARTEIAFILDTLIARDPAGA
EVYKANYENYMKEIDAVYVSLKAKLAPLSGSQVFVYHPAFGYFLDMFNITQVAVELGGKE
PTQKELMALVDLAKKEKARAIFTQAQFSESAAKAVAGAIGAAVVPIDPLAADWLENLSRM
GQALLQAVPGGK*
>SPBDM4_v1_50067|ID:27158430| ATPase component of Mn/Zn ABC-type transporter [Uncultured spirochete bdmA 4]
VSDIIQNAQQAQGVNEVHMPHPPENVAIWCQNLAFHYREAEVFNNVSFHVHRGEFVALTG
KNGAGKSTLLKLIVGLLEPTRGQVLVFGAPPRGRIESIGYVPQHTGFDAAFPIRVEEVVG
MGRLSGLSPVAKHQKQQEIEWALRQAEVCDLRERPFSALSGGQRRRVLVARALVGHPKLL
VLDEPTTNMDAESEKKFYDVLARVKGNTTVLIATHDSDFVSALAETVLCVGQDIDHPHSV
HRHAIEQVVNVPEDIYGGKVVRVRHDIELPDDACCEGKE*
>SPBDM4_v1_50068|ID:27158431| ABC-type transporter, integral membrane subunit [Uncultured spirochete bdmA 4]
MNGFLQALTNPSVPFVRNALIGSMLAAFLFGVLGSLVTVRRIAGLAGAISHTVLGGIGLA
LFLASRGAPKWVSPMLGALVFAVLAALLISLVSLKAKQREDTVINALWAIGMSLGVVFLA
KTPGYADPMSYLFGNILLVTEQDLYLLAALDVIVLLLVVRYYRHIEAASFDEEFAATKGV
PVTKVYLVLLIVIAVAIVLLQTFVGIVMVIAMLTLPAGTAGYFSRNLAGMMVNATILSLI
FSVGGLVVSWHFDLPAGAVIVLLSGAVFIAFSAGKMLAGRRLAKS*
>SPBDM4_v1_50069|ID:27158432| Fe2+/Zn2+ uptake regulation protein [Uncultured spirochete bdmA 4]

MTKARTHVLEAITQAKEPVSARQLCEKFQSDYDPATIYRALHYLEGKDRLDSFILHCSEH
GTERYYVLHSNEHRHWFHCERCHRFTDLGLCRFDDLVAQMSAEKGLAITSHTFYATGICP
ECRQKI*
>SPBDM4_v1_50070|ID:27158433| NQR2 and RnfD family protein [Uncultured spirochete bdmA 4]
VIKFLKQPMMRKVLIALAPIYLFSLWMYGLRVLAAALVVFVCGIGTEWLFERKRQGKVSE
AVLVTCALYTLAWPPATPLWILAVGIVFAVAMAKGVYGGFGRNIFNPAIAGRAFVYISFA
IVLSRAYTSFGNFGIGAVDVLSSATPLAQMRAHTPVPISNLLFGLRPGAMGESMTLLIVL
AAVYLVLTKTASWRIILATLAGGAVTNLIILATGAQKALPMESLLAGSFLYMSVFMATDP
VSAPKRQPSHYIYGALIGATAVIIRTFSAFPEGTSFAILFGNTFANLIDIAVDSFKKKAP
APDQKPAGSDASNPPAQPAAPRTKGGA*
>SPBDM4_v1_50071|ID:27158434| FMN-binding domain protein [Uncultured spirochete bdmA 4]
MKLNRNSIIYAAVFTFVVCIVFVVILAIANQVTISRVNANKKIESQTAILKAFGLADGST
PASEIDSKYTQFITEKTVDKATAYIATIDGQPYIGIKLTMPGLWGPITAVLVTDTKGTVI
RGFEVVDQQETPGLGGRISEPWFSAQFKGKQTLPDGTIAFVQGSGKGNFDPANGTVDAIT
GASRTSDFVKALVNRELALARQIGGTL*
>SPBDM4_v1_50072|ID:27158435|nqrD| Na(+)-translocating NADH-quinone reductase subunit D [Uncultured spirochete bdmA 4]
VSAPSKVFKDGLWYNNPIFVQVLGICSTLAVTNNLRNTLIMVLGVTFATSMGSLTLSALR
ELIPRKVRMIVQVLVLSFYVIIIDIVLRAYQPAISKQLGPYVGLIITNCILMGRAEAFAA
SNPPGLAFLDGLANGLGYGWVLLVIAAIREFLGFGSLLGFKIVGDGFTPWTIMVMAPSAF
FMLAIALWIANNHKAKAEAAKKTAAPGAPAGASGAPAPQKS*
>SPBDM4_v1_50073|ID:27158436|nqrE| Na(+)-translocating NADH-quinone reductase subunit E [Uncultured spirochete bdmA 4]
MIDAPNVGLLSLFLASILTSNILLANFLGTCSFISISKDFKSSMGLGIAVTLVIGITSAV
CWVVLRYIIEPLGIEYLSFIIFIIVIAGVVQMLEMIIERFSPALYMALGIFLPLITVNCA
VLGVILFMQIRKYNFPQAVIFGFGSGAGWWLAIMLLASIRRKLDSNNAIPAGLKGTGITL
ITIGFMAMAFVGFSGMIAVQ*
>SPBDM4_v1_50074|ID:27158437| Ferredoxin--NAD(+) reductase [Uncultured spirochete bdmA 4]
MNPILVGPLTVAGISTVLALIIAVLDKFLNNYGEVEISINGGEKKLQVKGGSPLLGTLAA
ENIFVPSACGGRGTCGACKCKIVSDVGPHLPTETPFMSQKEIESNIHLACQVKVRKNLEI
ELPRELFSIKKYKSHVEKIRDVTHDIKEVLFSLDDGEIEFVSGQYVQLVVPPYGEIKESV
QRAYSMSSKPSERKHVELLIRLVPGGIATTYVHKFLKEGQAIELVGPFGEFRVHDTPAAM
ICVAGGSGMAPFKSIFHSLHETGAFPEKDIWYFFGARTSKDMFYLDELRELQAKWPRFHL
VAALSEPSPEENWQGDTGLITDVLDRYLQTTVPKNDKGWEGYLCGSPGMINACINVMKKN
GITEDKIYYDKFA*
>SPBDM4_v1_50075|ID:27158438| conserved protein of unknown function [Uncultured spirochete bdmA 4]
MKQSPADKQAFQKMIPGVITAAGFLGDDDRSPERIIEEDGIFFDELRLDFDVVSDKLEEL
QHRGEAGLGEPITVGKLLVQSGDARGMLPCPWSDGFFHKNAVSVRPADVPIESCVEGEDM
LIYSELSVHMLRVHHFCQGRGSPFRLEPALIKRLLF*
>SPBDM4_v1_50076|ID:27158439|pyk| Pyruvate kinase [Uncultured spirochete bdmA 4]
MEKRTKIVATTGPACSNLATIKELIREGVDVFRLNFSHKTYDEANQDVTMIREARKALDR
PAAIMADIKGPAVRLYGYTEQIALKPGAELTIESADSAIIEKLSSKKPMQVYTNLPDIDR
ICAIGQKAILMDGYFTGKVIRKVSKGIVVVVDNEGNLRPKAHLTLPGVDYPIPFLSDKDI
ADIGWAVEQDIDYIALSFVRCATDVEEVRRLVQRTALSQGKNTFIKLIAKIESARGLENI
DEIIQTADGIMVARGDMGVEMPIENVPIAQKQIIRKCYLAAKPVITATQMMESMMENPMP
TRAEVSDVANACFDSTSAAMLSGETAIGKYPVQVVRMMRTVIEAVEKEFDYLDFHHDVPP
EVQNGDIPAIMSYNAVSVAYRCDAKALIVLTETGHAARLLSRLRPRMPIYAFLTNERLYH
QMAINWGVVPLIHSGKGTRLDAVVNEAISICKHKALLKKGDRVVIVAGLPLAHQGTTNMI
RVETIE*
>SPBDM4_v1_50077|ID:27158440| putative enzyme [Uncultured spirochete bdmA 4]
MNAPGQPGEAIHGAAPACQAVPACQTTHSEPNLDLLAKLKEKNLAAQGLMVLEGRIAIER
ALDAGIAPRFLVCTEAEEEYWQARARKAAVTPDSAADPDGAAAPREDAAPQVIAPQAAVP
SAFPPAFPVRVMSHEALCDLVDFKFHRGAIAVADMPRIVPFEAEAAYAAPREVFLCLWDV
TDPSNLGALVRTAAGLGLTGVLLGPGCASPYYRKTIRASMGNVFSIPIRSASLGTLEFLN

RNGARILAAALTAKAVPPEELGLHSLGLAPDSFGRPENRRSPIILILGNEGYGLPDEVLD
LCTDEVRIPMSRGVDSLNVAVAGGILMYELLSLAYLPSRE*
>SPBDM4_v1_50078|ID:27158441| putative xylanase/chitin deacetylase [Uncultured spirochete bdmA 4]
MPIKKVNRFFSSEFTATNRALAAAAFSVLALLLAGCQTPPVQSNQSNIVQQAIPPSPTDQ
ENDSSMVEEPSSLDPVRWWDQGKQSPIRELVIETPEQLAARVPKRADPKIVILMYHNIVF
GRTGGEYNRDLYNFDHDLVFLRNRSQIIGIDDLPYIKSGEKKLDTDASILTFDDGDLSIY
AIVFPLLKKYDIKATFFIITDFVGTVGYVNWEQLKEISDYRNARGEKLFSIGSHSLDHKR
FDGIPAEQIPHELTESKFIIEKKIGSPVWYFALPFGAGAGRKEIIDTARNAGYRGIRSSR
KGVVHPGSIDLYNLPAFYMSNERADILAQQIYSLLGR*
>SPBDM4_v1_50079|ID:27158442| putative Major facilitator superfamily MFS_1 [Uncultured spirochete bdmA 4]
MEQSYRVFAYRWAVLAATMLVNLTIQVLWISYSPITSAAASYYGVSEMAVGFFAMSFMIA
FIPLSIPASWLIDRFGFVRTVGAASIAVGILGVARGAAGQHYAPAFAVTCGIAAAQPFLL
NSWTKIPAAWFPARERATAVGLITIASILGTAVGLVLTPILAVSMSIARVQMVYGAAASV
AAVVFVFVAREKPKLPPDESAFRERAFMIEGLKHALKTPSFLRFLVIAFIGMGIFNGVTT
WIEGIVRPRGIGSDQAGILGAIMLIGGVVGAIVIPLFSDRAAKRTPFIILGLSGAIPGLV
GLALAPGFGLIAASSFVLGFFLIAVNPVGMQYAAEAARPTPEGTSNGLVTLAGQISVLLV
YAMEAINNITGTFTASLIAAAVLLGFSVLLATKLTEKGEPDQQRLAKA*
>SPBDM4_v1_50080|ID:27158443| exported protein of unknown function [Uncultured spirochete bdmA 4]
MKSKSRKLFIVLAMGALVLSACQLSTGTVKSGSASDKGTVTISIPRVNPYIVASQGSVSK
GASSKAFAYVDAIEVDLYQDGKFVQAVSLGDAYYDADANTITGNISVLAGTYSRLIVSVF
NNAVSSTEVVVAGQTDGSFVVPSGGSVDLSITLYPSNPVSLVKDTYSNQTKLSQYGEKWY
SFEAPGTIATVTLKTDSGDLDAYIFGPDGKPLSGISGSGAEESDQFETTAYDIYYVCLIS
PKETSTGEVKYENSSGMVNIAID*
>SPBDM4_v1_50081|ID:27158444|glpK| glycerol kinase [Uncultured spirochete bdmA 4]
MDRNASYILALDQGTTTSRAILFNHDGIIIGVKQIPFRQIFPKPGWVEHDPEEIWQTQLD
AAHGAIEAAQIEATQIAAIGITNQRETTVVWERATGKPVYNAIVWQCRRSSGICDELRAL
GAEASIREKTGLVLDAYFSGTKLTWLFREHPELKVRAERGELMFGTIDSWLIYKLTGAHA
TDPSNASRTMFFNIHERRWDDELLRLLDVPARILPEVKPSSGFFGMTKRELFGTEIPVAG
VAGDQQAALFGHACFEPGDVKNTYGTGCFTLMNTGPFPVESKHNLLATVAWDLGRGYTYA
LEGSVFIAGAVIQWMRDQMGLLADSAASERLARSVEDSQGVYIVPAFVGLGAPYWDSEAR
GTVVGLTRGTSRAHFVRAALEAIAYQSMDLFEAMEKDSGRELSCIKADGGASQNSFLMQF
QADITGTRVILPEVSETTALGAAYLAGLAVGYWESLDDVRKNWRMRREFVPRMADDVRAG
LVAGWRKAIATARTFR*
>SPBDM4_v1_50082|ID:27158445| C_GCAxxG_C_C family protein [Uncultured spirochete bdmA 4]
MTRKERAVTMHTAGSACSQAVFTVLAEDLGMSAPQAHRLAGGFGGGIGRMGLSCGAVTGG
IMALSLALGASESADQETKLALYGVVADYIERLKAYKGTIECRALLDGADLWTQEGRDMV
STRHLADSVCNPMITRAVEEAERILREKGKLGV*
>SPBDM4_v1_50083|ID:27158446| SagB-type dehydrogenase domain protein [Uncultured spirochete bdmA 4]
MQYERFEGRWFLKSNWHLVKSGQSDQSKGVPVPLQEEPPAPEDCIIDLPSPDSLFKGEVA
DAVSRASENEEGPPSSEGPGVPSPAETGTKSSGLARAAVLYGLLHSRKSRRKYSADPLAL
GELSYLLWTIEGVKENRGKFSFRTTPSGGARHPLDIYIFAHKIRGLNVGLYRYLPVEHQL
VLERQGDDSEALNEALNGQFWNAACVVMWAAVPYRSEWRYGKAADKLVALDAGHSCQNLY
LACESLGLGTCAIGAYDQEKLDAYLGLDGGDMFALYVAPVGRLTPS*
>SPBDM4_v1_50084|ID:27158447| conserved protein of unknown function [Uncultured spirochete bdmA 4]
VGQSHFIKTVEDIAEIMMCSVPGVKFGLAFNEASGPCLVRTEGTDPTLIEVAQHMAVAVG
AGHTFYLVIGPGAYPINVLDRIKAGPETCRIFCATANPVEVVRAVTGQGAGILGVVDGFS
PKGVEGEDDKAARKAMLRKFGYKF*
>SPBDM4_v1_50085|ID:27158448| ABC-type cobalamin/Fe3+-siderophore transport system, ATPase component (fragment) [Uncultured spirochete bdmA 4]
MSPKHEPRHTEQRQSALPPIGPSANLPVVEASSLAIGWKQGRNLQILTRNINFQVAAGHL
IALVGPNGAGKSTLLRTIAGLQAPCGGSLSLLERDITLISAEERASLLACVFNERIEAGY
LTVWEFVAFGRYPYTNAHNRLTDEDRRHIATALALVGMERFAARTFSSLSDGEKQKVQIA
RAVAQNTPLLMFDEPTVFLDAPSRIEIFKLAERLVQEEGKTVLLCTHEVDLALKTADELW
VIDREHRFSAGAPFVVARSGAIGRAFDLPTVAFDVISGTFRAREPQR*

>SPBDM4_v1_50086|ID:27158449| Transport system permease protein [Uncultured spirochete bdmA 4]
MNKNTEPESRQIGDRAPHKSRARLIAGLGACTLLLALLDISVGSVRIPFAEVWKALIGQA
SRQEWATIIHVFRLPKMLTAMLAGASLSVAGLVLQSIFRNPLAAPDSLGIGAGASIGVAV
LMFAGGALGALGAGAQGIGTQGSAILGNLPPLGYTLLVIAASLGAGVVVSIILLISRKFE
QVVTVLIMGLLVGYLTSSLVSLLVYFGSPQKVQLYLSWTYGSFGGVRTNELPYLAGATLI
GFALVGSDIKALNAFLISERFASTIGIRVRASRTRLLIAASILAGSVTAFCGPIAFLGIA
APHAARLLSRSAEHRILLPITALSGMCFALLADMLSSLPGHGAVLPINPLLALIGSPIII
SMFFHRDRGSLVEEME*
>SPBDM4_v1_50087|ID:27158450| Periplasmic binding protein (fragment) [Uncultured spirochete bdmA 4]
MRRIVGIFLFLFLISISVAPAHQANFQIEQKNGYKILTVSKLWPGANSTRTYVLYPRGSQ
APGGVKADLFIQVPVQRVVLYSTTYIPAIEAIGELDTIVGVDNANYVYSPALRARIDAGK
VVETTKNWMPDIERLIALKPDVVFNFGVGNEWDTFPKMQEAGLPVVLLGDWNEQDPIVRA
QWAIFIAAFFNKEARAQERVNAIAKAYDTLKTLAAGAPSRPRVLVNGPFQGIWTVSGGQS
YMARLIADAGGDYLWADNKESGGLNLSIEAVFERALRADVWLNPVYGANRLSDVRGLDPR
FSALPVLKKGQVWTNELRMSPGGGSDYFESAVMNPNLVLEDMIAIFHPELLPDHKFNYYK
KLNE*
>SPBDM4_v1_50088|ID:27158451|proB| gamma-glutamate kinase [Uncultured spirochete bdmA 4]
MDLSETADAASARAALANANRIVVKIGTATLTRTFGADGKSFLSEKGAPGTSPPRTPPSQ
SAKGTIDTAYIYHVAEQFANLVQDGKQIILVTSGAIGMGARELGLTKRVTEVRMRQACAA
IGQPILMEEYRRAFGVFGLSAAQLLVTRDEWDDRASYLNLRKTVETLLESRVIPVFNEND
SVSTAEIGNAFGDNDRLSAYVASKIDAELLIILSDVDSLYDADPRENPDARPIPYVKELS
DEHLAAAGGRGSEFSTGGMKTKLAAVAIARDAGCRVVIAHGREPDIIQRVVSGEALGTLF
DAAHALKNRIRWLKNSQPRGRLTIDEGALAAIRGRNSLLPRGVVAVEGDFGRGAVVLVND
IVKLISNFSSAELRAVLRKRSEEIDAVLGENAPHLVARPEEMAFLDE*
>SPBDM4_v1_50089|ID:27158452|proA| gamma-glutamylphosphate reductase [Uncultured spirochete bdmA 4]
MNMQERLERAALAASLLLSAGTEVRNAALAHIARQLEAHRDELFHANQKDMDEARAGGLA
APLLKRLLFDEKKLSEVLSGIRGLRAMPDSVGKVLEARLLDEGLVLQRISCPIGLIAMIF
ESRPDALVQMASLAVKSGNAIVLKGGSEARESNQALASVIARAGEEAGLPTHWLTLLETR
EEISELLTFDQYVDLVIPRGSREFVAKIKATSRIPVLGHSDGVCHVYVHEDADPAMAAKI
AVDSKTQYPATCNAAEVLLVHERYAEKGLVPLLRALSRVGVVLDVCERTAAMLEKARAEG
EIVPHSLKSDADWSVEYLDLRMAVKIVDSLDEAIVHINRYGSGHTDTIVCTAFEAARRFL
TEVDSASVYHNASTRFADGYRYGLGAEVGIATGKLHARGPMGLQGLLTYKWLLEGQGHIV
EDYASGRRHFLHRDLPLD*
>SPBDM4_v1_50090|ID:27158453|ykwC| Uncharacterized oxidoreductase YkwC [Uncultured spirochete bdmA 4]
MPQQTIGFIGLGVMGGAMAGHLRASGERLLVYTRTKSKAQLLFDAGAEWRDTPKKVALEC
DVIFTMVGYPSDVEEIYFGPEGLIENARPGTILVDTTTSRPDLAVRIYEAAKAKGLGALD
APVSGGDVGAKNASLTIMVGGDEETFQRAKPILEEMGKTVIRQGGPGAGQHTKMANQIAV
AGNLLGAVEAVSYAKKAGLDPRHMLLSIANGAAQSWQLSNNVPRMLEGDFGPGFYVKHFL
KDLRIALDSAHSMRVELPMLALAEQLFAKMVAEGMGDLSTHAIYLLYERGLA*
>SPBDM4_v1_50091|ID:27158454| protein of unknown function [Uncultured spirochete bdmA 4]
MNQDKPENSKYISQTGNLWQHIFNTQQAGIVVSINGIIQQMNSTAVKMLCGQSSSDFIGK
RFHETLISSEYIECTLERERQLRGGQPTVPPIEIRVRHLNGGESDALISATSWFKENDII
VESMLIDITPLKQRERLLEAILGLMAIAGQEELSTHATPVLNHALNAMQKLYAEHHNCGY
IGFSLHGNGFWEANSRMSSPGAPFLISYEPLSDMTRQWILDRDKMIAQCEPRKDTLYTIP
IAPGQWGSILLAPLVLDNEPKGYFFWIFAAAFLPEEIEADAERRNTFVLAALTTVKTFLT
GRDNARKSTDLAILHRATLEIGTMNSLQQIADVALEILEKEKGWHPSVIRFRSRAGEMLE
TAAYRGSPTMSPEENRTRMDFLNKAIDRPGKGMIGHVIEQGKPIRSLDLPSDTHYIETAP
GIKYGIYAPIVIEGNTEGAIGVESKDYSFTESDLRLLSSIGEIVGMTVRSLRLIEMLRER
VKWLEILHQINQQIGIEAKSRELYQILVDKAIEATGAEAAALLIYNPDKNILQKEVACGW
LGTVFDRPLTPDEGISGKIFSMGQPHLSPLVSEDPLLIPRNRKLVPSDRANIGVPVVAEG
IVLGVFHIAMKAPAHFSREFVDIVEMFGSYAGIVIGRMQLIEALRNADRQMQTAYDETLE
GWARAIGLRDDDTLRHTMRVVKIAVAIGKHLNLDSKTIENLRRGALLHDVGKIGIPDTIL
RKPGPLNDEETAIMQTHTSFGYELLRPIQYLEGALVVPDCHHERWDGAGYPRHLQGEKIP
LLARIFAVADVYDAMTSDRPYRPAHRSQEAIDYVRSQSGRHFDPHIVETFLAIIDEIEK*

>SPBDM4_v1_50092|ID:27158455|thyX| Thymidylate synthase ThyX [Uncultured spirochete bdmA 4]
MAHCSIPEAEAILDKEFPVLDKGFVRLVDYLGGDRRIIQAARVSYGEGTKSYREDAALID
YLWRHEHTSPFEQVILTFHVKLPIFVARQWIRHRTARVNEISGRYSILRDEFFLPEPEAL
APQSDDNKQGRSVTPFAAQTASQVQEILKEGSQISYARYKQLIDLGLAREISRIALPLSL
YTEWYWQIDLHNLFRFLMLRLDSHAQREIREYAKVILEITRAVAPAATASFERHTLRGVR
FSGPEMAELRRRLGIEVSDAHDQPDESAGMPPGLSKKELERFEEKLRSGRQL*
>SPBDM4_v1_50093|ID:27158456| protein of unknown function [Uncultured spirochete bdmA 4]
MSITYFNSKPSAKLGHLGPGETHPSQRMPLEARRRRGRHRPRYFKYHLCILADFALRVRI
EPQHEEAEQVVQIDLPIPFGIERERQGDARDLPGKAEIDQLFVARVGNLRTFFQNLLNLR
RGLRGEGSDGTTLFVVIALRSERFGLGQEKFVPQYRVTAGDFVYASGAVTNPLPRHEDRQ
FDVEGEYDLLEGARMFVPPEIIDERGIFAVALRAFAVRDPRRLDDAPISAKIVDKAHEAF
VEHRELFIEDRFCFGNTAMGHERIVAEIQKVESMRLSHNFSRHDTMGLMNSKIEVTFQRL
PGDVLKIFFRACEEIHCDEPLLELGEFEVSALCHALGNECRDAEATSGVQAAKLAEPRSR
FVLVNGWQSWSFAGELAGGERPRRACYKRALNLFVDHPAEVALRQRARRFGHRHDHISHF
MLGLRAADLRLMLVSDNAARYQGALQAGGISGAAAADRVERGTQQARDASTGASAGHMEG
RPAANTTLDAYLPPLSFLLRDKTIRVFAYAEGASFARGEIIARIAVLIAPDYFALKDRLG
KLWGAPQRFDDLRWLSSGAAYTPAGVPAPAAPEQAVKPEMPAPAAPFKGVIGGYVSWYNH
YTAIDERVIGEDLESIKANDNIVNTYFIERGRPTVFQIDDGWELAIGDWQAHPEKFPDGM
ARMASRIRGKALIPGIWLAPFLLMPGSDTAKAHPDWVLRDTSGAPVKAGWNPNWGGDVWT
LDLSLPEVEEHLVGLFDTVVNGWGYRYLKLDFLYAGLMRGAFAGRKGGAWQHYARVMARI
LEFSYAADGSPVAFLSCGAPIESTAPFMPLMRSGADTREHWEWPQAKLIGHQGRPSAKIN
MEDSIGRAILDKTLLLCDPDVIFCRTGRTSLRDTEKFLVGMLAAMFGSQIMSSDDPAAFG
KNPPSSHQLAEETFTQQLMDWYGRMGGKEFGVERSHLRARDVYHFFTRDRSIYGGINLSD
REGMFMLSGASGDTGGSEPDAAAGESARPSTAAPLPRHSLLIFGA*
>SPBDM4_v1_50094|ID:27158457| Inorganic diphosphatase [Uncultured spirochete bdmA 4]
MSKTVYVIGHKNPDTDSVVAATAYAALKCAQGQAHVKAARAGAVNPQTDYIFQRFGVALP
EFIPDLIPKTEYYIDEAPPSIAENTPLWEALALLEKTPRQVMPIVDGEGRYRGLFYYNAF
AKNILAKINPHRKAVIPTSIRHLVDTIKAQPLVAGDLDSKFNGRIVVASLSDKRFKDYIF
AEPAHNKVVLVGDREEIMRIAIDAGVRALIITNGFIPSKEIVRLAESKGVAMLISSYDTS
STSLLALYSTPVMSVADTSVKPLGARDLMKTAKHAISESAARAVPIVDENDRVLGLLTEG
DLIREPNIELIMVDHNEFTQAVDGIQNYRILEVIDHHRIGTFSTTYPIMFINRVVGSTST
IVTSMYHETKTPLDRNIASVLLCGILSDTLVLKSATTTDTDREMADYLASITDLSIEDLG
RDIMASASLAARLPIDQLLRMDRKEYEVQGKKLTVSQIELTNSQELLARQEEVLQGLARI
RAETGTYVAALMATDITKLESLLYINAEREFYPYLSYPVQQPGIYILKDVLSRKKQLMPA
LTEMVQAALS*
>SPBDM4_v1_50095|ID:27158458| TraB family protein [Uncultured spirochete bdmA 4]
MPQTIRTIQLGERQIILVGTAHISKESIEEAKETIRQEQPQRVCVEIDMGRYQSIQQDSR
WQDLDIIKVLKEKKGFLLLANLALAGFQKRLGADLGTKPGEEMMAAIQTAEEMGIPWSAI
DREIQVTLRRAWAKSNLWNKAKLLASLIESAFSREKLSEDDLEKLKEGTELEQMMNELAD
FMPSVKEVLIDERDRYLATQIFLSTERKVVAVVGAGHMNGIETWLEKLARGEVSPDVSDI
ESLPKPGWFAKSAGWLIPALIVALIVIGFFRSGTQASLTMILRWVLLNGSLSALGSLLCL
AHPLTILGSFVLAPVATLNPVLAIGLFAAVIEAHFRKPTVRDAETLADDVTSIKGFYKNR
ITHILLVFFCSSIGGMIGNFIALPVLASKAIG*
>SPBDM4_v1_50096|ID:27158459| putative DNA polymerase III, delta subunit [Uncultured spirochete bdmA 4]
MAEALIPPVWILAGPEIGLKEAFVADIVKKVRDLGGDEPEIHRLYAGDASPSDAISLLRN
SSLFASWVIVEFRNVDQISQKADIDALARYCGAPAEQTVLLLETEGYSAPKAIEKTVPSA
CRKTFFELFENELAGWVRRELGQSELSLDGEALESLLELVPHDTASLKAACLILNSSFPA
GSALSAENVEAAILRSKPEDAFTLFEKIGQGELSLSLEVLDTVLDSRRGDANQIIAALVW
SFRRLQRIGDAVAHGERFEDVCPREGARSKTVQRQLRAALQHYKPEDCRRIILALSETEA
ATRGPLGSAFDRQLLHLLITSIITEKGQGLAAAGWSEQGIYPHLVL*
>SPBDM4_v1_50097|ID:27158460|lexA| LexA repressor [Uncultured spirochete bdmA 4]
LKDLTARQKEILGFIHEYIQTNNYPPTVRETARAFSISVKGAYDHLKALEKKGCIKTSEK
RSRSIELVAPPEDDTPVVQIPLLGEIAAGKPIFADENFERTILVPADIVANRVPHFALHV
KGDSMIDAGILSGDIAIIERSETAKNGDIVVALLEENVTLKRFYIENNRFRLQAENPRYA

PIYTQDLRILGRLRGIYRSY*
>SPBDM4_v1_50098|ID:27158461|ptsH| Phosphocarrier protein HPr [Uncultured spirochete bdmA 4]
MVELETTIKNRAGIHARPSALIAQTAIKFASRVYLEKAGNRINANSIMGIITLAASFGTK
IKIIADGPDEQRAADTIKALFESGFNEELG*
>SPBDM4_v1_50099|ID:27158462|hprK| HPr kinase/phosphorylase [Uncultured spirochete bdmA 4]
VKEQEPVNFTVLDLLSLELKEQNDLKLRCVAGRAGLNREITVPDLNRPGLALAGFYDSFA
WERLQIFGRGENAYINKIEADGVAEQIYEKLFSYQIPCCIFTNNIEPTTNFSAAADAAGC
PLLITELSSVEFTIRVIRILHDIFAPKIIMHGVLVEVFGIGILLVGDSGVGKSETALALI
ERGHRLVADDVVEIRCLNGTLLMGQGTNKVIGHHMEIRGLGIINVTHLFGVGAIRDKKQI
QLVIQLEDWDPNKVYDRIGIDELTMEILEVKVPKLEIPVKPGRNIPIIIETAAMNERLKK
MGYHSAREFNQNILRWLESESARTMYFSRDDY*
>SPBDM4_v1_50100|ID:27158463| Ribosomal subunit interface protein [Uncultured spirochete bdmA 4]
MNIDVRSVHFDLSDQSRQYLDTKISRIDYAKDMIVDLLFVFTKDAGRFRLEVTANFRWGK
QAHIVETSQELNPGIDMLIDKLEQKIVKEKEKIQEKK*
>SPBDM4_v1_50101|ID:27158464| RNA polymerase sigma-54 factor [Uncultured spirochete bdmA 4]
LQIQQRPVLAQQQQLKLSPRMLQAIRLLALPLQELTEQIHQELEENPALELLSDSGEVSL
DVMEASIDTIKDSDDNLREAEYSDDAFGFNGEENDRSAIIEQTIATEESLQDHLLGQLGL
LSLREEERRIAERIIQNTDENGFHLSSPSEVCANCDEETLARILSLLQRLDPPGTCTSDY
RESLIVQAEIASNAPPKTIEVLSRHFENLQSRNHGTIKKALGLDDRQLDRIIEFIKTLNP
YPGRIFSKARPRYIVPDLAMWLEDGEIKVRLNDETVPKLGINRLYTELAARSTRGEAEKF
ARERVENAHFFINSIKKRNETLLKTAFAIAEAQKDFFTAGPKSLKPLALKEIAEKIGVHE
ATVSRVVNGKYVQTDWGTFELRRFFTNAVNPENGENISKEGAKAEVLQIIGELESKGEKI
SDRIIAETLARRGINVARRTVAKYRSELGK*
>SPBDM4_v1_50102|ID:27158465| ABC transporter substrate binding protein [Uncultured spirochete bdmA 4]
MKKLGIVLIIALLSVSMAFAQGTSTKDLSELITAAKKEGQLTVIALPRDWVNYGELIDTF
SKKYGIKVNELNPEGSSGEELEAIRANKNNKGPQAPDVIDVGLSFGPLAKQEGLITPYKV
QTWNTIPNEVKDSDGYWYGDYYGVLAFEVNADIIKNLPKDWPDLLKPEYKGKFALSGDPR
TSNQAIMTIIAASLSQGGSLANMEPGLKFFDKMNKAGNFVPLIAVPGTVASGETPITVRW
DYNALSNRDKSNGNPNVAVIIPETGVIAGVYVQAISAYAPHPNAARLWMEFLYSDEGQIL
WLKGYGHPVRFNDLAKRNKIPADLVAKLPPSSAYEKAVFPTIEQQDTAKKYIAEQWDQIV
RVEVMKK*
>SPBDM4_v1_50103|ID:27158466| ABC transporter permease protein [Uncultured spirochete bdmA 4]
MRDSLQKERFGRKGAASPEKAGGALLVHAAPRSKSKAWLGLIPFFVFIFAFMIIPAGSLL
IGAFQRNDGHFSLVNFSKLFRPNIISSYVVTIRLSFTTALGGGLLGFFIAYGICLGGLPK
GTRSFMSTFSGVASNFAGVPLAFAFISTLGRMGLVTVFLRTVLGIDLYASGFNLYSFWGL
ALTYIYFQIPLMVLIIQPALDNMKKEWREASENLGASPLQYWLHIGIPVLLPSLLASFIL
LFGNAFGAYATAFSLTGGLINIVPILIGAQIRGDVLHDPNLGYALALGMVFIMTLAILAY
SWLQKIASRWMK*
>SPBDM4_v1_50104|ID:27158467| ABC transporter permease protein [Uncultured spirochete bdmA 4]
MKNGTATRKAMSWFWLILGLLYFFIPLISTFLFSLRGKKGVLSFVAYQHVFADPKFLQSF
LFSLQMSVYTIIVGLVLIVPTAILINLKIPKAKPIIELFSLMPFVIPPVVLAFGLIKFYA
NPPLTLVSSPALLVAGYVVISFPYIYRSIDTGLTSMDLKVLTEAAQSLGAGWLSILFKII
LPNLRTALLSATFLTFATVIGELTLAVLLAWPAFGPYMAHVGRDLAYEPAALAILSFMMT
WGSISLIQFLSHGIPGAKSEIGGLH*
>SPBDM4_v1_50105|ID:27158468|potA| Spermidine/putrescine import ATP-binding protein PotA [Uncultured spirochete bdmA 4]
MGFIELHEVTKSFGNNTVIKNLSLSVEEGSFLSFLGGSGCGKTTTLRMIAGFEIPTAGQI
LIEGKDVSRVPPAKRNLGMVFQNYALFPNMTVRKNIAFGLKIAKITKQEIHKRVDEMLAL
IHMESYGDRYPHQLSGGQQQRVALARAIAVRPKALLLDEPLSALDAKIRVQLRDDIRNIQ
QELGITTIYVTHDQEEAMSISERIAVMRNGRIEQTGTPFEIYNNPATSYVASFIGTLNLL
EGPVVDAARGTVELNGTMVRTVSDLRGLEGKKVKLTVRPEALSVARDADAGSYPDVNTIA
GTVEMVKFLGSTVRFVMKFGETFMFMDRFNNSQLVLPKKGDPIRVSFAIDACRLLPEGDA
IAMPGSGDVEEMVH*
>SPBDM4_v1_50106|ID:27158469| putative Protein GrpE [Uncultured spirochete bdmA 4]

MSKHKHEQGQSSNEMKPENEQTQNQESQPLVPEVEVQPGAEEQQGNHPGAVNTPEAQLAA
AKTEIELLKTQFAELNDKYLRTLAEQVNFRKRVVKEKEEFQQYAVSTLLNDLIPVLDDFD
RSLEAVAQHQNDASKVVDGIRLIQKRLYDTLSNKYGLSRYESNGGVFDPRLHEAMFSDQG
DVPEPTVTQEFMPGYKLHDRVVRTAKVKVTMPANQNGAASQSTETPADEQNASSDESASE
DNAS*
>SPBDM4_v1_50107|ID:27158470|dnaK| chaperone Hsp70, co-chaperone with DnaJ [Uncultured spirochete bdmA 4]
MGRIIGIDLGTTNSCVAVMEGGEPIVIANSEGQRTTPSIVGFTAKGERVVGQPAKNQMIT
NAENTVFSIKRFMGRRFGEVLNETKLVPYKVVDNGNDVRVEAGGKLYSPQEISAFILQKM
KKTAEDYLGEAVTEAVITVPAYFNDSQRQATKDAGRISGLEVKRIINEPTAASLAYGFNK
DQKKEKTIAVYDLGGGTFDISILELGEGVFEVKSTNGDTHLGGDDFDRRVMNWLVEEFKN
DTGIDLSQDRMALQRLREASEKAKIELSNMQQTEINLPFITADASGPKHLQKTLTRARFE
QMCMNLIDRTREPCMKALADAGLTAAQIDEVILVGGMTRTPRVQQMVREIFGKEPSKGVN
PDEAVAIGAAIQGGILGGEVKDVLLLDVTPLSLGIETLGGVFTRLIPRNTTIPCRKSQIF
STAADGQTAVSIHVLQGEREMATQNRTLGRFDLVGIPPSPRGIPQIEVTFDIDANGIVHV
SAKDLGTGKEQKIRIEASSGLNDTEIDRMVKEAEAHAEEDKRERERIDARNEADSLIYTT
EKSLKEVGDKIGAKEKTAVESAIKDLKSAMEGKDTEIIKQKTEVLKQASYKLSEELYRHA
SAQGGEAAGGTAGGASGATGGAEGEASSSGESNDQGQNADDVNYKVVDDDK*
>SPBDM4_v1_50108|ID:27158471|dnaJ| chaperone Hsp40, co-chaperone with DnaK [Uncultured spirochete bdmA 4]
VAKRDYYEVLGVPKTATKDEIKKAYRRLAIQYHPDKNPGDKTAEERFKEATEAYEVLGDD
QKRQAYDQFGFAGVEGMGGASQQDYSHVFHDFEDIFSGFGDFSSIFGSFFGEGAQQRGGT
HVNHGANLRYDIELPFEKAIFGTTIEISYNKDDVCKTCGGTGSRDSQGRKVCSMCKGTGQ
VRRSSGFFAISQPCPVCHGEGTVIENPCPDCGGAGVVKKKQKIRVTIPAGVEDGKRVIVP
GQGNAGANGGPAGDLHVFIHVRPHDVFERQDDDLYCAVYVDFVTAVLGGEIVIGTLEGRK
LSVQIPAGTQNGKLLRIRDEGVPVSGNRRGDLYIKVFVKVPARLSRRGREILEELKATEG
ETSQPELIRLKDVPH*
>SPBDM4_v1_50109|ID:27158472|copA| fragment of copper transporter (part 2) [Uncultured spirochete bdmA 4]
MKKLLKIEGMSCGHCVMHVQSALEDIPGVKSAKVDLLERQALVEGDALDDQALRAAVADA
GYRVLQIIP*
>SPBDM4_v1_50110|ID:27158473|copA| fragment of copper transporter (part 1) [Uncultured spirochete bdmA 4]
MSVHKTFAIKGMTCASCVAANERAVSKIKGIARVSVNLATERMDVDLDPAEVDVSQIEAA
VKKAGYEAELLEKPANAPLPTYESYGWKRFTISAIFAAIILYIAMGSMLGLPLPGFIDHR
LHPLAFALAQLILLIPVLIAGKSFYIHGFKSLFNLSPNMDSLIAIGTSSAILYSLYSISR
ILNGDHTAATQLYFETAAIIITLIMLGKNLESQSKRRTSEAVRKLMKLRPTTATILDGEV
ERKIPIDAVKPNDVLLVRPGERLPVDGRIQSGISNVDESMLTGESMPVEKTDNAKVYSGT
MNLNGSLVMVAEKVGEETALAKIIHLVEEAQSSKAPIARLADQVSAVFVPTVMAVALIAA
FGWIIAGHSITFALTVFVSVLTIACPCALGLATPTAIMVGTGKAAELGVLIKSGEALETV
HKVNTVLLDKTGTITKGKPELTDVIAAPGFSSDTVLGLAASAEIASGHPLADAIVASARE
KGLVLSRPERVETVAGKGIIADSGSIAVGNAALAESLDISLSNQAPYLFNEAARLAGEGK
TPMFVLQNAAPIGLIAVADQPRAESRAVVAALKKLGIDVAMVTGDSRTTADAIAARVGIT
EVYAEVLPSEKASIVKRIQASGRKIAMVGDGINDAPALAQADVGIAVATGTDIAMESADI
VLMHNNLDDVATAIHLSRQVIKNIRQNLFWAFGYNVLGIPIAAGILYIFGGPLLSPMFAA
AAMSLSSVSVVTNALRLKGYRKPQGFGEQTAL*
>SPBDM4_v1_50111|ID:27158474|acyP| Acylphosphatase [Uncultured spirochete bdmA 4]
MKAFHAVIKGEVQGVGFRMSAVIRAERFGLTGWVRNTPEGGVEVWAEGEAQSLEQFYEWL
QIGPSSARVDEVLKTDEQPRGFYKRFSVAF*
>SPBDM4_v1_50112|ID:27158475| Bifunctional protein: zinc-containing alcohol dehydrogenase quinone oxidoreductase (NADPH:quinone reductase) Similar to arginate lyase [Uncultured spirochete bdmA 4]
MVSVMKAMVIRRTGGPEVFEQAELPIPQPGANQVLVKVKATSVNPIDCKVRSGAVAILPP
FPAVLHGDISGTVVGVGQDVYHFHEGDEVFGFVGWTGGEGGALAEYAVCDAQLLSHAPRS
LPLEEAAALPVIALTAYQGLRRKIRLGKDTLVLVQGGAGGVGHVVVQMAKAFGAEVAATA
SSDEKIAVVVSSGADWTIRYDQEDVKHYVGRITDGKGFDVVFDTAGGANLPKSFEAARMG
GDVVTIAARATVDLSLMHSKGLNLYVVFSLLPILTREGRDVIGQDLAAIARLVDDGHLRP
IIYPKAFTLDTIAEAHRFLETQSHYGKILVRVG*
>SPBDM4_v1_50113|ID:27158476| Polyprenyl synthetase [Uncultured spirochete bdmA 4]

MTDYWKNFPHIADFLRDVEALILSILEDNAFPLGKDIEKLVESGGKMLRPALVFVGSEFG
LDRNPKKWTKKQKDKLVHVAAAIELLHTATLIHDDIIDRASHRRGIPTLHQTLGTTNAIL
AGDWLLSRTYRIIAQYVHPNRAVLLTDSVSAICKAEINQDLVKYSFVSSRRRYLQTIAGK
TAALFALALYIGASETGCSVFNSQALRRTGYNLGMAFQIIDDILDYESSLEELKKPVGND
IREGLCTLPLIYALQENGKEIRPLLSDIRTQPNLVESVLGLVLQTKAIERARSDAAHYTR
LAHAEIERLPAGKPRHDLELIVNSLLVRTY*
>SPBDM4_v1_50114|ID:27158477| NADH dehydrogenase [Uncultured spirochete bdmA 4]
MATKKILILGGGYGGVWAGKILERHFRKREGVEITLVDKRPFHTLMTELHEVAGWRTEPE
SVQVLFKKIFGAKRINVVLDTIEKVDFEAKKATGKVQTYEFDYIILGVGAEPEFFGIPGV
AENSFTLWSFEDAIKIRNHIEEKFYEASLETDPDKRKRLLSFVVAGAGFTGIEMIGELLD
YRDVMCRKYFLDPKEVRVLNIEALPSILPILEEPLRAKAEKYLQKRRCDVMLNSAIIGAE
PGKVLLKSGEIVETETFIWTCGVKGSSFAGQLGLPTGKRNRIDCDMELKSTKYPFVYVVG
DISGLMLEGKPLAQIVETAHFTAEAVAKNIIADIDGGERHQFKPNYHGFMISIGSHYGVS
NAGGMKTSGFLALFIKHFINVWYLLNIAGIDQVWEYLRHEFLDMKNRRSFIGDFVSYKVR
PYWPLLLRMWLGLMWIFEGINKIGEGWLTFSAGSKSGWMFSPGVIQAGVKTAEATSGASA
WESAGEAAATAGQAAVAATSAASAAVSAAAAAPAATSAASAATSGVTEATTSTFKAVWDL
TKPIFDPNSGIVTWFRHTFLDGVFAHLPFTFFQVMIVVMEIGIGLALFGGCFTWLAAFGS
LGMCLIFTMSGMFAWNQVWFVFAAILMLGGLGRSFGFDYWLIPAIKRWWNGTRFARKWHF
YAGDPTK*
>SPBDM4_v1_50115|ID:27158478| protein of unknown function [Uncultured spirochete bdmA 4]
MERFLQRTGYAEDQILVSFARLIGDQFFGENRDILVNDESAEYRAPLVAAMIGSFLEGSE
ATDQNERGRLRALILAEHEKTLSALKQLFTEAHIGSAIVGSEEAVKEAGERLCSEPVVAM
PASLFLAAVENGCFKPRDFGFLIIEGADIFSEQPSEVQRKIWGHLLPPWERRTALFAEHI
GIRAKNTAIDFARNPKTIVLKKAQASLSAISTSMYRVSSEKKFRVLLSLIQEERQRGRKA
VVVFCNLRQTSREVEARLRLNHIHAEHVSAAAPKVNNEALLRRFSELAGSEKERGGYEER
EHLVFVVSNDNLEVLPSEFAPVGIHYDIPLDADVYLERVRTMHLRNATMAGLVCERYEVG
LSAITSRFGIQFELKEPSPEMQECRDASEGVSLDLERDHPADRQTPPRRNEWRASEAPPG
GSDRRNRHNQESRRPRSKHTKRSHRAPALHNSDETGQDQSLYSMSTEERLAYFRNKYRNI
LKTPIQPPPQQNSTPKTNETAQDNREPDNNSIVKRFIGKFMSGGKGSGQDREEH*
>SPBDM4_v1_50116|ID:27158479|glgP| Glycogen phosphorylase [Uncultured spirochete bdmA 4]
MKIVSHTIKPALKGRLARLDTLARNLWLSWNFDAVSLFIRIDSGLWADSGQNPVKMLGTL
SQEKIDELASDQSFLSDLDDVYARFQRYLKNRPWYRGPVDKTIAYFSMEYGLDVSLPTYS
GGLGVLSGDHMKTVSDLGLPLVGVGLLYRQGYFKQYLNADGYQQETYPENDWYNMPVERC
VDQDGTPILIHVDMAGRTISAGIWRVDIGRAQLYLLDTNIHENQPEDRTITATLYGGDKE
MRIKQEILLGIGGIRALKALGIEVAATHMNEGHSAFLALERIRALMEEYHLDVHAAIQAF
TPTNIFTTHTPVPAGNERFGIDLMDKYFHSFVSGLGLDWNEFLGLGRENPFDSQESFCMT
VFALHLSAKSNGVSRLHGEVSRKMWKGLWPDLELKEIPISHVTNGVYPRTWISHDMLDLL
DRFLGPKFYFEPDNAEVWARLESISDEELWRTHERRRERLVATCRQRLAASYVRLGMPDG
EVVRSSDVLSPYVLTLSFARRFATYKRGTLLLKDPDRLIKLLSNKEMPLQLVIAGKAHPH
DAAGKDLIRELVHFSRREDVFGRIVFIEDYDMAMGRYMTSGSDVWLNTPRRPLEASGTSG
MKAGMNGVLNCSVLDGWWAEAYTSDIGWAIGSGEEYHDEELQDKIEAEDLYDLLEQEILP
LFYSQGRDNIPHGWLKKMRASMKAIGSQFATHRMLKEYYTEYYETALAESRALEADSYKA
SVSLARYIEKVRRAWQGVKIVEFVDDGAPVVSRGTAITVRALVDLAGLSPDDVAVECYSG
RLSSKGEILDGVRVPMSLVGQEGQHYRYQYVMHGEMTGQIGHSVRVLPAHPALDGRFIPG
LVRWAQ*
>SPBDM4_v1_50117|ID:27158480| FAD dependent oxidoreductase [Uncultured spirochete bdmA 4]
MEQFDVAIIGAGVCGANIARKLSQYELGVVLLEKEIDVSLGTSKANSGIVHGGFHDHIST
LKARLELRGALMFDRLHEELDFPFERCGILVAALHEDEMRAIEQLYLQGVENGVIGIEMC
SRERMLELEPKLNPDVVGGLYAPSGGILEPYRFVFSLVESARQNGVHVKTEFEVASAERQ
GDCWQIRAKDGQEIRARYVVNSAGLHADTISAAFGAERFTIMPRKGEYYLLDRTTRAKPS
RVIFPVPTEVSKGILVIPTVEGTVLLGPTASSALGKEDFATTRDQLEHILHSARMMVPAI
SENDVITSFAGLRAAYGSDFYIAISEKVPAFVQVAGIQSPGLTASPAIGEYVKDLLKKAG
LRLVEKPEWNPSVHKVPRARDADPFALDTLIAQDPAYGDIVCRCERVSEAEIVAAIRAGH
TTLDGIKYYTRAQMGRCQGGFCTYKIIRILMRETGMSFDQITKRGGDSVILKGSL*

>SPBDM4_v1_50118|ID:27158481| FAD-dependent pyridine nucleotide-disulfide oxidoreductase [Uncultured spirochete bdmA 4]
MKQLTFDAVVIGGGAAGMAAALELDARGHSVLILEREDTLGGILMQCIHNGFGLIEFNEE
LTGPEFAQRFEEEVGSRHIQASLRTTVLDIREEGGLKAVYCVSPAQGMMRVESRAIILAM
GCRERNRGNIRIPGTRPAGIYTAGLAQRLVNIEGYIPGRDVVIIGSGDIGLIMARRMSWS
GCKVHAVIEILPYPSGLTRNIVQCLNDFGIPLHLSHLVTDIYGEDRVEGIEVTPIESGAL
MHEKAFKIACDTVLLSVGLVPENELSRNAGIEIQPTTNGPWVDSALMTTMPGVFACGNVL
HVHDLVDYVVEEARRAGANAAAWLSGYRPAREIRAKTGSNIRYVMPMRINPERENRLYMR
SLVVKNDARLEVRLDGNVVKSRKLAHVQPSEMLSLTLIPEDLAAAGRDSTVEIALV*
>SPBDM4_v1_50119|ID:27158482| conserved protein of unknown function [Uncultured spirochete bdmA 4]
MRELICITCPMGCHLAVDTDERGEMKVTGNRCVRGEQYAREEIFNPKRVVTFTCAAVLPD
GSVPGPESNLPRRVPVRTTIAFPKERIPELLGALRGMTIRLPAVRGSVVLKKALDTEVDV
IVARSIASE*
>SPBDM4_v1_50120|ID:27158483| Heat shock protein Hsp20 [Uncultured spirochete bdmA 4]
MNKRPYMDIGSIFDDIFEAAKDFGERMKNFAPGFEGPGDPDEHDEGSGKRPGCHGMGGGA
WFEAHGDDNVDYYPNYSYPPLNVYMLPDKSLVFEFAVAGFDEKNMSLSFQGDYMVFSAKI
DVTEQPEEGVRYFKRRLKLKDIDKQKYYVPADKFDQEKVKAAFRNGILKVTVPPKDVVDT
PEGIKIEIVKEGE*
>SPBDM4_v1_50121|ID:27158484| 2',3'-cyclic-nucleotide 2'-phosphodiesterase [Uncultured spirochete bdmA 4]
MKRFLVILCALLAMTMSLAAQSKSVDLTFIETSDIHGAIYPYNFITAKPAATSLAQVASL
IKEERSTSDSEVVLLENGDSLQGQPTVYYYNFEKTSGPHIWSQAVNSLGYDAIGVGNHDI
EAGHAVYDKLYEEMQAPVLCANAVNPDGTPYFQPYAVINKGGVKIAILGMITPKIPDWLP
PQFWTGMQFEDMVQTAKKWVPIIQEKEHPDLLVGLFHAGVDYTYGNVTRDTPNNENASQV
VAEMVPGFDLVFVGHDHQGWSGQGWDPVNKKKIDVKDPNGKVVPIYGPLNAARNVAVVKM
NLTWNDQTQSWDKMVRGGLVDMTKYQPDPDFMAKFQPMFDEIKTWVDRPIGKMDGVISTR
DSMFGDSAFVDLIQRIQLDLSRDPSLGLSPADVSFAAPLSADAKIPSSPDGTLYVRDMFN
LYVYENFLYTMTLTGQQIKDFLEYSYQYWFDTMPNDGDHLIAFQKDKEGKLVFDNRTNMP
MTATRYYNYDSAAGINYVVDVTQPPGNRISISCMSDGRAFNPDETYTVAINSYRGSGGGG
HLEKGAGLDKDTILSMKLVNGATTKDLRYFLLKWFEKQEGPITVMPIGNWEVIPSDLAAL
GEKTDFPLLYPSK*
>SPBDM4_v1_50122|ID:27158485| NADPH-dependent fmn reductase [Uncultured spirochete bdmA 4]
MLKIAILTGSTRPGRNNEAVAQWVLDQAKSRSDAEFEIVDIKDYNLPLLDEPMPPSLHQY
TKDHTQKWAQKISQFDGFIMVTPEYNHGTSAALKNALDFLYAEWNNKAVGFVSYGAMGGT
RAVESLRLVAGELQMADVRTAVHLSLFTDFENFSVFKPAPIQTDTLKTMLDQLVLWAGAL
KSIRA*
>SPBDM4_v1_50123|ID:27158486| conserved exported protein of unknown function [Uncultured spirochete bdmA 4]
MRPRRLAMLMILVLVLASLFGAGAQPVVSTKQDIAVFALGYYGWFIPSQALGTIDLKIQK
VFSDIGRFTVFGTTQRLSSDGLQQFIDALRQTKQADFVLPEQYQFGEAFLTKAEFERLTG
AFIVVVPVVTNFAVYWNSKAGTWNCNITTGITFIDVAHDATVLSIESIDTSGSDKTNHNN
AIMSAINAIPSELEYRIRSIPEFQINTRILEAKGSTVKIQLGANMGLKKGDEYAIIEEQT
VGGIEDTREAGLIMITDVSQQLSTGRVLYRSGALDANTQLKEIARRGVDLDLYFHTSGTE
ILPGFRATASRGYFPLRPYVGVQMPVSLAFNVLTVTVIPVNAIFGAEFMMPMGRLHVAPY
AGVGFTYAHLTEALSGYETDYLSHIGFQAGGRVGYLINRNMRLFADAGIDYWLSLGWPFS
DYGGLTLGAGVSFKL*
>SPBDM4_v1_50124|ID:27158487|feoB| Ferrous iron transport protein B [Uncultured spirochete bdmA 4]
MQDTRALTIALAGNPNAGKTSIFNALTGAHHKVGNYPGVTVEKREGSLEFQGWNIHIVDL
PGTYSLTAYSLDEVVARDFVLNEKPDIIVDVLDSTNLERNLYLLLQLIELGLPVVAALNM
SDEAAEKGIVIDHTILSKTLGVPMVPTVGSRGEGARELLETVISTFEAHTLPRMPSYGAD
IEAHIAALVELLKEDKDFAAAYSPRWMAIKLIEKDPDARKRIEHHARAAKIHAALEAAWH
WITLHYGNDAEIILSEQRYGYIHGAVSEAIHRKRLRGISLTERIDRFVMHPIVGLPLFFF
IIWAIFKLTFVVGEYPVAWLEQLFGYLEAVVQHSTLPAMIQDLLINGIIHGVGGVLSFVP
LIVILFFCISVLEDTGYIARAAFLTDHFLHTIGLHGQSFMPLMLGFGCSVPAIMATRTLK
SPKERIATILAIPFISCGGKLPIYVLLAGTFFPKNASTVVMLMYAAGLVLSILSTFFLRR
TVLRGESMPFVMELPPYRMPTLKGVLWHVWEKTWSYVRKAGTIILAVSVLMWIITSYPKA

KGIEEFASGAQASVAEALPELSDAAITARVEVLVRQYQLEHSIAGQIGKIIEPVIAPIGF
NWKIGIALIPGFTAKELVVSTLGVLYGAPVDESGESPSLRQSLREAPDWSPRIALALMAF
ILVMPPCFASLATIRAEAGNKWLAFQVAYSLVLAWLISFLVSMLGRAAGM*
>SPBDM4_v1_50125|ID:27158488| protein of unknown function [Uncultured spirochete bdmA 4]
MLLAELGPGEKFKVMRVVFGGEIGKRLADLGFISGTEGEVVRAALFRGPMQIRLGDYDLI
MRRHEARLVEVELLPQTAAKKPTPEARTHIDLRPHFRHGLSHMAHGIRSRRPVSGKKPHS
CH*
>SPBDM4_v1_50126|ID:27158489| Ferric uptake regulator, Fur family [Uncultured spirochete bdmA 4]
MNELVKFENYLRAHGQKMTRPREVILEAFLKTEGHLSIDDILREAKKIDAGIGQATVFRT
IRLIADAGLAREALQEDGARTFEHLADHPHHDHLLCVGCGKVIEFLSPVIEREQQKIFVQ
YGFAPRGHMMELLGLCPECQAKERGEH*
>SPBDM4_v1_50127|ID:27158490| conserved protein of unknown function [Uncultured spirochete bdmA 4]
MEKLYYTYQMIHKLAQSIAAQIEESGFNPDVIVAIGSGGFIPARILRTYLGKPILAVGIA
YYDLNDKPMDYPRKVQWIEEAEKKLAGKRILLVDEVDDSRVTLEYCTKELLAYHPAALAV
AVLHDKHKEKRGAFPPGVSKVFVGEALGDVWIVYPWDAHDIDEQDRNAALERGAET*
>SPBDM4_v1_50128|ID:27158491| Peptidase M16 domain protein [Uncultured spirochete bdmA 4]
MRRWNVAPKHDTIRKDAGWFDVSAGRRNKHYMKQKKILWDFILVLMMALFSPNLAVAQAS
TAQETGLASNVVRNATLDNGLEVFVVENHAVPLVTVCVAFRGGALAQTPETAGFFHLYEH
MMFNGNDKYPTKDAFTAALNRMGTTSWNGATGKEYINYYITVPSEKLADAVDFWAHAVMN
PTLNYAVLENEKLVVLNEIRGYHSDPAQIASNALESRMFNTFPWRKNIDGPESNVANATV
AELRDMQKTYYVPTNTALLVGGDTTLEQVQALAEVSFGKWARAPQPVLAEPAQGPIPDGI
RLVTAEDQFYRGIAQVQFRWRGPDVTRQTFDTYVSDVLLFLLSSPSGPFKSEIMRKVFGL
YDAEYIDFIYPTARDGGNYIFSTFMLVQKPATEEPVLQRVENLRKTVLDEFAQIARDPRA
YFGDEALEEAKTKLVDQNIYALESAGSFVTDTLTFWWSTAGTDYFFNYEANCKKVTWDDI
SALIRKYLTGEAGQGSAVVAGSAPGSSVSTSGAPSASSVPPAATLVRLRTSTFGADPRME
AKIGELGYTRATAQNAFWWQQR*
>SPBDM4_v1_50129|ID:27158492| Peptidase M16 domain protein [Uncultured spirochete bdmA 4]
MNNFMKLHRSGAKRPSVCVHLARFALAATFVLVVVAGAGAQTAFVRENAPTFMKTVLSNG
IPVYMKVQNANRVFHVSLVLNGGSLVTPPGHAGWEKIALNTMARGSQNYPYEKAASLLDR
TSSNIAAAVQFEYSTFGLTTLDKYRDELLDLWGDMLTAPSFSQADFDRTKEDASLALQAM
DQDPWSMTQKVMNQAYFKDHPYGVNPEGTEQSIGSMMAGDARQWYKDHLSADRIFVVAVG
DFNPAELARELEALLGSIPNLRLGPVSRPADFPTGPSGSLITEAHDQSQGTIYLRGDFAA
PAPGKDDYFATALAARIFSDLLFSVVRDKYGAVYTPSAMIRVFSANYGSIMMYKSNAPDK
IKEYIDEAAGLMARGRVVSVDPTKTEGDGYMSITDAIDTYKQLYMNDYFEAVRNNAGIAS
LMIRSVRLTGDPADWLKDQGRIETLDSLAVRRAFDTYFMKGAFLWVAVGDQALLDKLNPR
AFDSMH*
>SPBDM4_v1_50130|ID:27158493| putative M16C family peptidase [Uncultured spirochete bdmA 4]
MPFVLQKETSVPELDSILRLYRHEPTGARLLSVINKDENKVFGVTFRTPPQRSDGVAHII
EHSVLCGSRKYPVKEPFVELMKGSLNTFLNAMTYPDKTCYPVASTNLKDFYNLIDVYLDA
VFYPNLTKHTFMQEGWHYEVDPATKKLSYKGVVFNEMKGAYSDPDDQHDDLCRRSLYPDT
PYGLDSGGDPLEIPSLTYEEFVGFHKKYYHPANSFIFFYGDDDPEKRLAMMDDWLTSFGQ
ANIESLPSAQPEFSEPRHLEYRYQASEQDEPKAYTAINWALYEHGDPVLSMKASILFHIL
IGTPASPLRKALIESGLGEDLAGFGLSEELRKTAFSIGLKGVAPDKVGDVERLILSTLEE
LSRDGIDSDTIAASLNTIEFALREKNTGHFPRGLAIMLDGLNDWLYDKDPIESLSFAGPL
EKVKKTAAENPRLFSELIDSLLLRNTHRTTVALLPSPDANREREEAEKTMLARAQAALDA
QKLQEIERTAEMLKVLQDTPDSPEALATIPVLSLDDIPKEAPVLPSDPLDLGNGGAHGAP
DEARAYYHELATSGILYLDLGFDFGALESKLLPYVSLLGRFLLEMGTEAENFVQLIQRVG
MHTGGIRSTSVSATRWDDRKPAPWFFLRAKALPEKAAILAGILVDVLARARFDDRERVRQ
IVLEEKAQAEAMLVPASARIVGLRLRSRFNVADWASERLYGIEHLFFLRDLARRIEEDWP
SVLDDMERVRGALISRDNLVVNVTSDRATLAASADAIARIAGALPARRALAAGGAVDSDM
PWIDEALAACGVSDGSAPASPSGGLRPSLETLELPTQVNSVGMILPLRHLKKVAGGAYVA
SKYLDAVYLWGQVRVMGGAYGGYSSLDPASGLLMLLSYRDPNLERTIDVYRKVAAYLRSC
EVPREEIQKSIIGTIGDVDAYQLPDAKGFNALMNELTGYTQETRQSIRDQILAADIGDFH
RLGELIDEAARRSMIVALAAPERIGKALPALPSPIERLSVH*

>SPBDM4_v1_50131|ID:27158494| conserved exported protein of unknown function [Uncultured spirochete bdmA 4]
MISRYRLVTFVLLLMLSLGGVSGVIAQTTDTSTETDELQNDITTVNAAAELDQTALLIRL
SAQFQVELAVLENLSAQGYTAGQIWLALEISQQTGAGLAVSIAQAASIGGDGHGWGVLAQ
ALGISPGSPEFLALKAQMRNRTRTMASDIAKEHVKDPDNSDRVSAEKDNSKGNGGQNGKH
*
>SPBDM4_v1_50132|ID:27158495| putative RNA polymerase sigma-H factor [Uncultured spirochete bdmA 4]
MLLGMQGHADTLGHAEPDSMKEAAERNREDSIIARVLDGDIDSYRFLVNKYSARVLAFCR
MRMRSEEDAQDASQEVFVRAYRSLASFKRGESFSAWLFAIAANNVRTHIRLFASRKQREV
AFVRQQVTNPPDDPAGEAERACEFQALREAVSSLPVDLRKPVELYYFAQLSVEETAKVLR
LGQEAVKSRLFRARKQLREALAENVQPKRPPGGISL*
>SPBDM4_v1_50133|ID:27158496| protein of unknown function [Uncultured spirochete bdmA 4]
MMNCTEARAFVDAWERGERGMMTSSLDEFRMHLADCETCAHEFRALLPFIERDANTEANI
AADSGVGSARRSGTLGSVPSEFADGVMKAIGGEKRPSHIIRSRPALVAAAAAIFILGLSL
GLYFGTRTKDTVSVKFVLYAPEASTVELAGDFTSWNPGTYVLKKASVDGMWEVLVPLKKG
RVYVYNFVIDGDRWISDPKAAVQVDDGFGGSSSLLRL*
>SPBDM4_v1_50134|ID:27158497| exported protein of unknown function [Uncultured spirochete bdmA 4]
MEKTRPKVFTPKFMRLFSLLLFLSAPSVLFAQTVAAWLAKPSNVAVYGTIAPEVNTMVQQ
LEKLGLSDSILVVRLEEGAQKQVNPEILVASLRVDIQRVVRLAAILRANGIFPSNRNTAS
SAIEQMLIFIRAGLTEAEVQKALEEGVAKSGAKQKATARALAALSVVTSAKAQFGLGEED
RLRLASVLIASDLDEDGFGSILVSVEEFTSAGHSAAEAVSKALEKVSLSGKKNQGDISQN
AEGQSDSGKSQSGKSESGGQGNQGGHGKK*
>SPBDM4_v1_50135|ID:27158498| exported protein of unknown function [Uncultured spirochete bdmA 4]
MRTSRKAITLLMVGAMFVAVTAGAFAQGFGQTGGPGATTPGYGYNYGYYWFGMQNEAPGF
GPYGHGQQRGQGSMNAQPGFMGPQGAQRPQMPQGSMMGPTGMRGQSGFGAFSYYQYQKLS
AEDKAKVEKIVQDAASQILPLQNEVRSERLLLNNLLWSASPDKAKVDESISKISDLQKQI
QEIRADSIIEVNKIFQANQ*
>SPBDM4_v1_50136|ID:27158499| exported protein of unknown function [Uncultured spirochete bdmA 4]
MKSNRAKTLALLFLIAGVAFVSAQGGPGMGMGNQGVMGPGAVREWFRSLNLTDEDLAKLE
KILDAREIELAKAQNEIRIYQTQVANMLLDPNPDMNSIEDAISKSLGYEKTVRIIQIERQ
VAIRKIFGEERWQSILFLVREARMSEKMGQFSNSFSAKGFSSQEADRYGRLLIILRRIM*
>SPBDM4_v1_50137|ID:27158500| putative RNA polymerase sigma-H factor [Uncultured spirochete bdmA 4]
MISDEILVRTMSAEKKESGPLTPATPKDASKSAATGDDPLDLELAANASRGDKGAFERLM
WRWWDRIRGYCAAFVAFDPELAEEAAQESLIRIYKALPRWRKESPLGGYLYGICRTTSAD
VMRTHARHHARNVQVEEFESLSLESPHASGEANVLRQEANQLLAIAMQKLEPVDRSMLYL
HEVEGKGLSELGAMYDLPLGTVKSRLFRARDKLGMLLREMGYELS*
>SPBDM4_v1_50138|ID:27158501| protein of unknown function [Uncultured spirochete bdmA 4]
MSSAEKKTIDLKAALKDVAMTSAPSFPDFARVYQEAEQRPRKVRRFALSAALMALAAAAS
FWVGIYWHSQSSRDVALIESWSSSSSSAPSAVSIGTSNGEQSQVVLASSQNTAVNLFVQD
LWESSSSGGL*
>SPBDM4_v1_50139|ID:27158502|proS| Proline--tRNA ligase [Uncultured spirochete bdmA 4]
MAEKITPREVDYSQWYIDIILNAKLADYSPVKGTMVIRPRGYAIWEKIRDEFDRRFKETG
HENAYFPLLIPMSFLEKEAEHVEGFAPELAVVTYGGGEKLEEPYCIRPTSETIIWAMYRN
WIQSWRDLPLLINQWANVVRWEKRTRLFLRTSEFLWQEGHTAHETEKEAREETLRMLEVY
RDVTERYLAVPVVAGMKSESEKFAGAVDTYTIEAMMQDKKALQAGTSHFLGQNFAHAFDV
QFQNRQGELDYVWATSWGVSTRLVGAVIMTHSDDKGLVLPPTLSPEDAVIIPIYRNDTKA
AVSAYAEKLQAGLKAAGFKTVLDADDSSSPGWKFAEWELRGTPVRIEIGPRDMQSNQVVM
VRRDTGEKLSVPVGEVPARLKELQTLIQHSLFERAKAFRDENTVPVSSLDELISFFGVET
AGTTGAKGGFAEALWCGSAECEASLKEKTKATLRCLPLGRQEHVEGKCAICGAPARYRAI
FARNY*
>SPBDM4_v1_50140|ID:27158503| membrane protein of unknown function [Uncultured spirochete bdmA 4]
VTTRNFRLTYWILSSLSLVMLLGTIVLATRIGTDKRLPFFLADTRLYTEYFFFGKPVGIQ
LAAALEMCVLALGVSAVLMVVLFTFQKTVSAEVYLIALWVFLVDVETLRLLMLVIAQRST
SIGSLVSLTRVVYGGRFTGLILLFISGLYAVGIGHERPQTGALLALLFGTMLAMLMPISI
DRFQSSFMLQAGYRNITMIVTTSLIVINCVDYFVAARLKEDMSYAYAGIGLTAAASAWVW

LWDARSLVGGMGVALFFVLCVLVATERLHKIYIWK*
>SPBDM4_v1_50141|ID:27158504| Phosphatidate cytidylyltransferase [Uncultured spirochete bdmA 4]
VRIDHVVPRAEPLEHASHNRRLHFRSPAEDLRIEVTRKSLHLLILFCIPLLDISPTLPIV
CLLAGLLVYSVSELLRHHGITVPVIAPLTERAARRRDGNRFVLGPVTLALGAMLALLIFD
PLQAKIAILALGIGDSFSSLIGKSFGRIPMPLSDGKSVEGSLFCFATVLLAVFLLTGRFI
PACIIALTTALVEFWPTKDWDNVLIPLAAGVVASLVI*
>SPBDM4_v1_50142|ID:27158505| protein of unknown function [Uncultured spirochete bdmA 4]
MQGFSRHLDAQVLSGRTKVKAAVVRCVFEGLCARYYVIDSHIQPLSVLSLTCGGLLC*
>SPBDM4_v1_50143|ID:27158506| Major facilitator superfamily MFS_1 [Uncultured spirochete bdmA 4]
MRPIQRYFNAYRGLPSSLYVLFGATVINSVGMFVFPFLTLYLTGRLGMSQKEAGLFIMIV
NFAYIPANFIGGKIADTFGRKKLMVTAQVLSGLCYIPCGFPGIGGNVRWFLLASVFFDGL
TDPARTAMMTDLTTPETRRPAFSLTYLGHNLGFAVGMMIAGFLFEKATSWLFWGNAIAIL
AAISLVALKVPETKPTNEQVEASFGSGREDEGHRGNIVQALLSRPYLIIFTVLTGLYGFV
YAQHRFILPLQTKEFFGMRGSVIYGTLMTLNATMVIVLSTPIMTLTQHWKPINAVALAGL
LFGLGFGLIGLAPSVPLMYITTAIWTLGEIVNATNEGTYVANHTPISHRARFQAMLPLLG
GFGWSISPPLVGAFTDRFGLRTAWPFLGGIAVIASIAIWCLGIIERRPTRKENAATD*
>SPBDM4_v1_50144|ID:27158507|gtaB| UTP--glucose-1-phosphate uridylyltransferase [Uncultured spirochete bdmA 4]
MKGIIVAAGYGTRFLPVTKTVPKELLPIGVTPSIAYIAQEFVDSGIADIIIITSRRKKAL
EDYFDREIELEELFKREGRGDRLEKIRPFPARVSFVRQAEMKGTGHALMQVSHLLGGEPC
VVAYPDDIVLGPKPLARQLIDVYERTAKCVLATIFEPGDVSRYGVVDPAADGMGVRGFVE
KPAVGAEPSHEVSIGRYLYTPEFFAWLEEGWTHHGSGEYYHTYALDRMIEAGKVAFARVE
GRRLDTGEPAGYFEAQLLNALGDPALKKVLAQFVAEHGPELE*
>SPBDM4_v1_50145|ID:27158508| Betaine-aldehyde dehydrogenase [Uncultured spirochete bdmA 4]
MAKMIIGGKRVDSSDKAVIEVTNPATGEVIDTVPAATKTDVDRAVHEAIVGQKAWARVPL
HKRGDVLNTFVDLALAHKEELARILSMETGKPIIEARIEIDNIPTLFRGFVEKAKHLYGS
VIPNGTEPGQEHTIQFTVREPVGVVACIIPFNFPCDLFDQKVAPSLIMGNAAIVKPSTDN
PLTLIRLCELLMEAGVPDGAIEIVTGRGSTVGSWLCSHPDLGLISLTGSTSVGIETAGVA
AKHLTPCTLELGGNDAFIILEDADVDLAVEEVMVGRMYNTGQVCCASKRFIVHNSRKEEF
TKKLVDRLGTLVQGDPRDERTTIGCLISEKAAIGVEEQVNKTVAQGARILLGGKRRGAFY
DPTVLADVRPEMDVAKDMEIFGPVVPIIGFETTEEAVKIANASVFGLSGCVFSRNINEAM
KVASYLECGGVVINGSSFFRSCEMPFGGYKMSGIGNEGIMCSLDEVTETKTIVLKNVLT*
>SPBDM4_v1_50146|ID:27158509| conserved protein of unknown function [Uncultured spirochete bdmA 4]
MLQANVGSSIAADAREAGKEAATAATVKGAPLAFVYSSCDYDQSKLLEGAAEILPDAALV
GCTSYTGVLTPSGFMTGPKGYVAVMAFRDDDVTLGIAGEAKKGDARETGRRLARAAMTAA
KKDVPPAYFYMVAAPGEEESYLKGVEDVIGRVPFFGGSAADNSVEGKWKLLFGKDAECKA
FSEGLILVFFYTKKAVGTCYTGEYAETRTAGIITKVEGKRRLVEIDGEPALKKYASWRGL
DPEKLKGLNLLGETICHPLGVKDRLGDLVAIRHPMVGNEDYSMNIGSDIAQGTAVVLMEG
NAESLIASTGAALRKVDERLGSKPGAYLLVHCGGRRGGIGGRMDEAYREIKRAAGDVPFI
GVFTFGEYGYEDWGANTCGGLMLSFAGFEK*
>SPBDM4_v1_50147|ID:27158510| putative HTH-type transcriptional activator HxlR [Uncultured spirochete bdmA 4]
MPINLRDDLNDTIKAGGLHCEKELTMSIISGKYKVVILWHLGHDGALRYGELRKLFKDIS
NRILTKQLREMEQDYVVSRTIIAESRLKVEYSLTDIGRSLLPIVDSIYEWGREHLAFYIE
KEKIETERGREEGPRSLRIASAAKGTKGDVL*
>SPBDM4_v1_50148|ID:27158511| protein of unknown function [Uncultured spirochete bdmA 4]
MHAPHDKREALSKVGRVLKGPPFIAAFVYGFNETGANAKPISEVTDF*
>SPBDM4_v1_50149|ID:27158512| conserved membrane protein of unknown function [Uncultured spirochete bdmA 4]
MTATPTSVTVGTTLGALAALLFLIADLYVILHMVHTIFAPKSKWPWLENMGKKWHPIHYF
GNIALVIVMIVHAIIMAPYTGFWNWLLFALIVWMGFAGIMIRFSHISPKAKASLSRFHAR
WYMILIVLVLLVVAQLVSLQTFPYVLG*
>SPBDM4_v1_50150|ID:27158513| conserved exported protein of unknown function [Uncultured spirochete bdmA 4]
MNYSIARISSGRVRSFLSSLISFVLLLSLGACSTAGPNSFIISSTAADLPPPRYPDTRFM
VLSDTHLYDTSLGTIGEAWDAYMAEDRKLLPDSAETLSVVMDRIKSARPDFVLITGDLTK

DGEKQCHEQFAAALGELNKEGIKTYVLPGNHDINNPAAARFLPSGGTESIPTVNPEEFAR
IYSASGYGEALYRDPSSLSYVAEPTPGLWILALDSAKYENNAGKKWSETSGAIRESTYQW
IEARLAEASQKGIAVIAAEHHPIMEHIDGMKDKYPDYILDDNWRLASLLASYNVRLLFSG
HFHASSIVMHRWKEDAPAQLRGKYIVDIETGSLVTWPCSYRSVSLSSQDDTVTISTARVD
QLSSYAAEGRSFDVEGKKVIERGIGNIASATMKRYHVAQRDIDVVTPEIVAAMMAHYAGN
AHFEGEDMLSTKGLGLMGRAVVASYNKFVHGLWKVKAPPNVELMEDNNLTIRADGSWMTI
PSVEGF*
>SPBDM4_v1_50151|ID:27158514| protein of unknown function [Uncultured spirochete bdmA 4]
MLRITFINVGYGDSILVEELKSGRRIFSMLVDGGNPYDQRYKHAYDSHAGQTPAWRYLQG
RGIERLDLVVLTHFHLDHIGGIPDVVKTFPCGDLWSNYVFSGRPPAIDVGKKRRYRPESR
MMVHSLRLLAELSDFAGHQNKEIHEMRRNEFNLPLTSELSVDRFGVDEAIYARMDGLVRD
ICAKGGARGATVGSGDCAAEDVEAQLFGLDKILNASCAALRLSYRGRSVLLAADLPATYW
YPILDAGHTIGADILKFSHHGQKDGVSKRFASAVNPSNVVFCVSEDNPFHCPNPSVFSFF
DATTRFDATGIVPLPPAWKPHPAHSAVVFEIGDDSSIRLMLQ*
>SPBDM4_v1_50152|ID:27158515| putative Catabolite control protein A [Uncultured spirochete bdmA 4]
MTIDHIAARSGFSKATVSRVINHEGSVKPSTAAKIEQVIQELGYTPNTIASALSGGKSRT
IGVLLPDIITEYYSALLMGIDTVAEEKNYNILLKTRNSRKVLMDLAQSNRVDAFIIRNNG
LQPIDHDFLVTLRRKGIPFLFIGKPMEEHCPAILIDNVGGARQMAHHYAEHGFRRILFIA
GPEENLDSNDRIYGFRLGLSEKGIEPEQFDVVHGDFAKNSGHEIAREMLEDKKYDAIFAA
NDHMALGAIVYCRSKGIRVPEDLAVTGFDDTFFAQFLLPSLTTVQQPMYEIGTQAMESII
QLIERPVSHEHRIILPTRLQVRQSCGCCQENVDI*
>SPBDM4_v1_50153|ID:27158516| conserved exported protein of unknown function [Uncultured spirochete bdmA 4]
MKKFLALLCALLATVSVFAKGAQDDTVVVYAALDEKTANELAAAFKEATGLNAEIALQIE
EAGTIAARIKAESTHPRADVFIGGNSNFHTDLASGGYLEKYTSPVVKEAKIDSSFMDPDG
YWTGWYLGALCLIYNNRLYEQKVKPLGLNPPSTWDDLLNPVYKNQVIASNPATTGGAYLM
LCAQIFRLGSESAGFDYIRKLNPNVAQYTKGANGSIPLVAQGEAMVGFAWGHDALKQKMQ
GNLPITVVFPKDTGFEIGCGSILKGAPHADAAKKFIDFLLSPQAGKINAKNGFRYPVRAD
VEMPAGVPPFDQLKLAPWDLKAAAVNVDRWKKQWSEITGK*
>SPBDM4_v1_50154|ID:27158517| Binding-protein-dependent transport systems inner membrane component [Uncultured spirochete bdmA 4]
MKHREREWPLSLLIFFIVFCLLAFVVYPIVEVFLYPKANDFVSIFHNARYMRTALNSLMM
LVLSTLSATFIGFIFAYTVTRTDVPGRKAFKFITLLPLFSPPFMVSFSYIMMFGRNGLIT
KGILGLDVNIFGWHGLWLAQTIAFFPVAFLVMEGVLQSISPMLEYAGRNLGANGFTLFRT
VTFPLARPGVAGAALLVAIQVLADFGNPIMISGGFSVLATEAWMRVEGWADIAGAAVLSI
MLLVPSLVLFFVQRYWVGRKSYVTVTGKMAQVNIQKTGKVMKWVLFTVCAAVSLLVLLVY
VGLIVGAFVNGWGYDWTFTTRYITEIATRSKELVHSLSYSAIAGFSSALFSMTAAFLVSR
KKFPGRRFVDFTCMLPAALPGIFLGIGYSIGFTRPPVDLYGTATIIILSMIFWNISTGYQ
TGIGAFQQISPSFSEAASNLGAGSLRIFREIEIPLIKGPFFSSFIMSFIRAITTLSVIVF
LSTSKNAVATFSIMNLVSDGFYGKAAALTCVLLVISFGTIGLAKLIMGKQVQFFKV*
>SPBDM4_v1_50155|ID:27158518|fbpC| ferric transporter subunit ; ATP-binding component of ABC transporter; CP4-6 prophage [Uncultured spirochete bdmA 4]
MAQAHVIIREVSKCFGKTKVLDTISLEIEKGSFTTLLGPSGCGKTTLLRTIAGFYDVEQG
EIFIGEKKVNDVPSHLRNAVMVFQDYALFPHMNIRENITYGLRIRKMPQDEIEKKLETTA
GYLDIKNLLDRTPGEISGGQQQRVALARALVMEPEVLLLDEPLSNLDAKLRVSIRAELRQ
LQQRLKITTIYVTHDQAEALAMSDTIAIMKGGKIMQVGSPSDVYYRPDNAFVASFVGTVN
FIDGTVRSVSDEGVEVETGGTTITVRERQPAGKPAAHPPLPGEKITLSIRPESIRIVEHP
APAAAFRDENVFRGTIKDYIFEGSSIRYWGEALGKELLIDVFNPVENALHTGDVWFRIDP
ERIHVIPQSIT*
>SPBDM4_v1_50156|ID:27158519| putative glutamine-fructose-6-phosphate transaminase [isomerizing] (Glucosamine--fructose-6-phosphate aminotransferase) [Uncultured spirochete bdmA 4]
MVTMCGIVSLAYKDENPSMGKEAEALLKRLEYRGYDSTGASFIGDDKRISLRKQVGAPSK
VCPLLHIDTFSGQRFIGQVRWATYGSVTDINSQPHHVSCKIELVGAHNGNISNTDTLKTW
LSARGHAVVSDNDGEIIVHLIEEHYAAAQSLASSELAYLRKAYANAGLAEGVPDGVLRMI
EAIRKAESLTEGSYAAAIADPELPGVFAIKSGSSLYAGVGSDSHGDFIVLSSDLTSVLSK

TRALIPLSEGEGIWFTENQYLVFSLQGQPFFSHPRLKRSKLSIKDTQLSPEYQHYMEQEI
SSCPNNITQVLRYYFRDPSLEPLSELFEERKDDCKETSDIIASLSERFGNDALLAGMHDV
FNSSAWNEIEARVQNAGAAHFVSRNFISDETELLKELAALDPQARDKLLLMDKVMVWRKR
RAVLRYTGELKNAIHEAARSGGRIYLVASGTSYHAALTAAYFFNEMAHIPVYPCNPGTFR
SMYFNCLGANDLIIGISQSGETKDLVDIFQDVKKANASVRLVSIVNNENSRIPQELSDFY
LPLLCGPEIAVAATKSFTSQVAILYLIAASFSMSEQKIRENLLKAKDLMTETIETRMGDI
ETVAAHLFAASSIHILGTGLIGLAREGALKIREVVLNHTEGYDAAEFKHGPNTILGRNTI
LSLADLAKLAGKEPVSGITNELFEKLTNNYPLVFICPPEERDRRITISQIHTHKIRGADI
VLIAEPQQELALAVEGKPMGMEKYWSKYIPLPQSGEPCLFVFSATIVLQYLAYRMSVRKM
EWLDSLGIQNHGVHPDAPKNVSKSITVD*
>SPBDM4_v1_50157|ID:27158520| putative Acyl-ACP thioesterase [Uncultured spirochete bdmA 4]
MIKPLEIQYVIRGYDCGYGGPLKPFALANFFQEAAGAHASQLGIGMEDMWASGLTWMLSR
IDIRIDSMPQAGQTVVARTWPVGTRKLFALRCLELVDGHGTKYAGAMYEYIVVDMKTRRV
MRPERTLPLDLSTDYPWPFDDLAPGIDDPSFKALSQHIASEGGATEGIPADEKTDNVVAG
GFRQSFSIESRTRHIDQNGHVNNAHFINWLCDAAPLREGQRFSRIKVDFVHEIMKGENVR
AWAGELASSPEDSEKLTDSVTEYPRETAWLTALTVERSLAARGSLKIAPAED*
>SPBDM4_v1_50158|ID:27158521|tpx| lipid hydroperoxide peroxidase [Uncultured spirochete bdmA 4]
MATITFKGNPVHTIGTLPQKGTKTPDFKLTGADLGDVGLATYAGKVKILNIVPSLDTGVC
AASARAFNKAAASLGDVVILTISRDLPFAQKRFCEAEGIDKVVTLSELRDREFGKAYGVE
MISGPLAGLLSRAVVVLDRGNTVTYTQQVPEISQEPDYESALAAAKKAL*
>SPBDM4_v1_50159|ID:27158522| putative UspA domain-containing protein [Uncultured spirochete bdmA 4]
VYKKIMVPLDGSSAAETALPWTIYLANAFQAGIVLFHAIEKNPPEQIHGDAHLANMEEAS
AYLESIKNSLHNVFHFKGDISTHVHLEETTDVAASIAMHMKELRPDLVVMCSHGRSNFTQ
AFIGSLATKVIGLGEVPVFLVKSHESEKSTGTDAVGPSISNVVVALDNASIHDKAIRYAE
EFVQETGARLVLFSAVPRFGSLKGKDGGWGLMAPATSSALLDIEEEKMRQHLDEHQKSLA
ARGVSSSNAVVRGDPARKIAQETKKLEPCLIVLGTHGRSGMGAFWKGSVAAKVVSLTDSP
ILLVPLHE*
>SPBDM4_v1_50160|ID:27158523|mntH| manganese/divalent cation transporter [Uncultured spirochete bdmA 4]
MSQKTNQKSTGVPRAPHEPSRSDSKTIESATEVLEGTSKKGFVARILPFMGPAFIASVAY
VDPGNFATNISGGAQFGYLLLWVIVASNLMAMLIQALSAKLGIATGKNLAEHCRLRYRRG
VVIGLWILMELVAMATDLAEFLGAAVGFNLLFGIPLWAAAILTGVTTFIILGLERYGFRS
LEAVITGFVSVIAISYIIEIFLGKPDWGQVAHHAVIPEFKGQSSIILAAGILGATVMPHA
IYLHSSLMQNRVVVHEPALLKRLFRYEIIDVVVAMGVASLVNGAMLIMSAATFHAQGMTQ
VGTLEEAYRTLEPLLGKAASWVFAISLLFSGLSSASVGTMAGQIIMQGFMNFEIPVWLRR
IITMVPSIVIIALGVDPTKALVISQVLLSFGLPFAIIPLISFTSDKKLMGVLVNRKSTKI
LAICVAGIILALNVYLLYVTFTGG*
>SPBDM4_v1_50161|ID:27158524| conserved exported protein of unknown function [Uncultured spirochete bdmA 4]
MAKAVFSVALAFALSAVPGFSQSWPEGKTSDRFDTGNGELEIVFLGHGSLILVYKGSYIY
IDPVSQYGDFSKYPKADLILVTHEHGDHLDPKAIAALSKNGTRILLPEASRKKLGAGEAL
QHGRPLDVAGVTLLAVAAYNVTSGRMIYHPRDRLDNGYVITADSLRVYVAGDTEPIPEMA
NLGHVDIAFLPMNQPYTMTPEQAAAAARVIKPKILYPYHFGSTDTGALTKLLANDKKIEV
RIRNLQ*
>SPBDM4_v1_50162|ID:27158525| conserved protein of unknown function [Uncultured spirochete bdmA 4]
MDKITTGLFNDGYMPIIDGVTLTVRNYAYWLRKKLGPTYVITPFVPNYKDTDPFPVIRFL
SLPTVVRPPYRIGFPDFDIRLRSVLKNRDFDIVHAHSPFAAGKLALRVARGKHVPIIATF
HSKYRDDLTRAIAIKAIVHDQIKRIVEFYYNVDHVWVPQESVAQTLREYGYKGPYDVVEN
GIDFEPLDDIAPYRTSGAEHLGLPEEYLVGLFVGQHILEKNLELLVRSLPSIMDALPNFR
MVFVGKGYAKESLIDLARQLGISNKVIFHEVVYDRELLKAIYARADVFLFPSLYDTSGLV
VREAAAMKTPSILIQGSDAAVFVHDGENGFLSAEDTVSYAQTVVSALSDKEALAEIADHA
QKTLCRTWEDVATEVAERYRSILNRWA*
>SPBDM4_v1_50163|ID:27158526| Deoxynucleoside kinase [Uncultured spirochete bdmA 4]
MSRKYVVVAGNIGAGKSTLVSLLAEHLGFIAYFEPVLENPFLKDFYADMRRWALQSQLFF
LSHRATMHQKLSADTHSVVQDRSIYEDAEVFAKNLYVQGNMEKREWLVYQDLYRTLTEIL
PHPSLVIYIRASVPTLQTRIAQRGRDFESAIPDAYLEGLNVLYEEWISSFTMAPVLTIPG

DSLDFVAESRDLQVILRTVTKRLEDNQPYLFPYEM*
>SPBDM4_v1_50164|ID:27158527| conserved protein of unknown function [Uncultured spirochete bdmA 4]
MWQLHNRRAFWDTSAGQQILTLDLIFRKISDAQRIFVGRAAQRGVELSCPPDCGACCHGF
MPDVLPVEADYIAFFLLINQNNPGDGPEGHFSLLSPSTPASPSPCPFYAADRPGANCRIY
EARPLICRLFGFSGTKTKNGGTAYRLCRHMPTPRGFPGRSPTQEALQRAIGTLPPLMSDF
SIGILAIDPSRASERKPLVEALPPALARISATLHYCPEEPNAA*
>SPBDM4_v1_50165|ID:27158528| protein of unknown function [Uncultured spirochete bdmA 4]
LILNKYGKGWYHAVRQILPFFQKNSRASAGVLHVAIAQQAGVLGYIGRSTNSYVRFNLS*
>SPBDM4_v1_50166|ID:27158529| exported protein of unknown function [Uncultured spirochete bdmA 4]
MKRFAVFAFVFIAAIALLGAQESQTPATFYADAAQQTKEALDASRLLQSKGQWSKAWNLL
AALDPENKDGYVLAEKIRLALEGNVANGMLVSFGFVDLAEGQNLDEMRTNPPEKQTIADF
NPVDLADALEKSGEAVPPVLSYELGNYLYTVNHEYGDNWILESQAVQQKAVENYDRALAY
DTYTEKSLSNQAELLLNLQQYDAVETVLRKAIAAYPENPNFSVSLASALNDQGKFDQVYP
IVDEVLANPDASDATYNAFVEGIKAGLNAGDVQKTDAYVEAMIARFPDEYVPMLIQHLIE
VRMGTPEKANAVADDVTAKFNVDPNVVRSLISTWLNVQDPQSGFDYLNRSLEKYTNDDMA
VGTLAFYRALMYAQTAQSVDDLKLAMADIEEAEKRFGTVYDKDNQVFLVISQLKDEWNQF
LQQQSAPDASAVSGDQSQQPAETPEAGSSQTDDQTQQPAETPEASPEASSDATSGATE*
>SPBDM4_v1_50167|ID:27158530| N-acetylglucosamine-6-phosphate deacetylase [Uncultured spirochete bdmA 4]
MGSAFCIHNATVIAGYARMDRSAVLVEGNLIADIFSERRFAQKTFAPDVELIDAEGAYLT
PGLIDTHIHGIGGYGTEDMSADSILGMSKMLPAWGVTSFVPTIYPMPEEDMLRAVRAIVS
AMGREEGAEIIGVHMEGPFISPAQLGVQRVEFVRPVDLGLMQRLWDASEGHIVSMTVAPE
LKGMRELALFCNRLGIVLQAGHTDASYENMIEGMQAGIRHSTHMFNAMSRLHHRNPNAVG
AVLIQPDLSCEIIADGLHVHPDLIRLLFRDKPEGNIVLVTDSLKPTKQATDAPMFANEEE
VYLTNNLFFRASDGVIAGSSLTMIEGVRNLISFGVPLESAVKMASANPARIFGLRRRGMI
SPGFQADLLLANNKFEAQMTIIDGIIKYRRSAR*
>SPBDM4_v1_50168|ID:27158531|nagB| glucosamine-6-phosphate deaminase [Uncultured spirochete bdmA 4]
MRVIVQNNYDFVSRWAAHHVVQRINEHQGNTPFVLGLPTGSTPLGMYNELIRLNKAGVVS
FKNAVTFNMDEYVGLPSKDERSYHYFMWDNLFSHIDIKKENVHIPDGMAPDLDKECRRYE
EAIKQAGGIDLFVGGVGADGHIAFNEPGSSLSSRTRIKTLTRDTKLANARFFDGDPEKVP
SHALTVGMGTIMDAREVLIMVSGIEKARALRHAVEMGVNHMWTISCLQLHPKAILVCDDD
ATSELRVGTVRYFKEIEAPNLTNWD*
>SPBDM4_v1_50169|ID:27158532| Methylated-DNA-(Protein)-cysteine S-methyltransferase DNA binding (modular protein) [Uncultured spirochete bdmA 4]
MGTLYRKKVFRVRGPRGRRKPSGVSVRPPAACYTTVMPSEQTLRIIRAIRAVPPGKVASY
GQIAVIAGHLRGAGGARDVVRILVSMSEKENLPWWRIVRKDGTIALSPGDGAELQHALLV
REKVRFDASGKVVAACFWGGRSET*
>SPBDM4_v1_50170|ID:27158533|yjjK| fused putative transporter subunits of ABC superfamily: ATP-binding components [Uncultured spirochete bdmA 4]
MPTTDDKKIIYSMYRLSKRYGTKQVLKDISLSYFYGAKIGVIGLNGSGKSSLLKIMAGLD
TEFSGEVACAPGYTIGFLPQEPHLEPGKTVREVVAEGVQEVMDLHAEFERVNEAFGDPDA
DFDALAVKQAEIQEKMDALEAWDIDARLDFSMDVLRCPPADQVVDTLSGGERRRVALCRL
LLQKPDILLLDEPTNHLDAETIAWLEQYLRLYQGTVIAVTHDRYFLENVAGWILELDRGE
GIPWKGNYSSWLEQKRQRLEQEEKGESMRARTLGRELEWIAMSPKGRHAKSKARIDRYER
LLAEDTKEKIRDVRLVIPPGPRLGDIVIEAVGLFKAFGDTVLFEDIDFSVPPGAIVGIIG
PNGAGKTTLLNMIAGVEKPDSGSLRLGDTVKLAYADQMRARLAPDKTVWETLSDGHDMIS
LGGRTEVNSRAYCAWFNFTGADQQKKVGILSGGERNRLNLALMIKEGANVLLLDEPTNDL
DVNTLRALEEALDDFAGAAMVVSHDRWFLDRVCTHILAFEGDSEVIWYDGNWSEFAEWRR
QRLGIDADRPHRIVYRKLER*
>SPBDM4_v1_50171|ID:27158534| conserved membrane protein of unknown function [Uncultured spirochete bdmA 4]
MKRDIKGIMAMVATALCWSLGGLFIKLVDWHPVTIAGARSLVAALFILAVVRRPKLNFSK
NQIIAAVAYSFTMLLFVYANKHTTSANAILLQYGAPVYVMLLSGIILGERPLPEQIGALV
AIVVGMGLFFADSLSLGHLVGDIAAVLSGMTFAIHILFMRRQKAGSPIESLLLGHGMTAV
VALFISLFLPAPVMNAKSIAAIFGLGIVQIGFAAVFFSFAIVRISAIQSSLIAIIEPALN

PVWVFLAIGEKPTGRAVLGGVIIVAAVAVSSVFSVSREARVSGKEAAA*
>SPBDM4_v1_50172|ID:27158535| conserved membrane protein of unknown function [Uncultured spirochete bdmA 4]
MKQSFLSVTILLLILITDPLGKIPLFIASLKNVPRSRRPLVILRECLIAFLVLVGFLFFGE
VFLSALGLSDDILRISGGVILFLIALNMIFPGTGGKLVEDEIEGEPFIVPVAIPLIAGPS
AITYVMLLMKSDPSRSLEWVGAIFIAILVTMASFFMSDKLKEWLGPRALSAIERLMGLVL
TAIAIEMLLDGLSQYLGRQ*
>SPBDM4_v1_50173|ID:27158536| putative Histidine kinase [Uncultured spirochete bdmA 4]
MHKRNAIQRNFLDHVRQLRTTEQIGFFIVFLLLVGFVTVGAHFQWQSILRDKKSYIELIG
QSKIGQLESWYDSQLSEAKELPASPVFIDIVAQAIEKPSSQNLARIDDYLRPILRSYNYA
DSAVLTPDFHVLVSLTQLAESNCPEVCEEISRKDSLAPFFTTLHLTSPYGKPGFHLVIPL
VKDRQKAPFAYVVQTFFAGDYLYPMLARWPGNEKTGETLLLERTDDMVQVLNPLKLVNIS
AFSLQVSVSDKNAVEARAGLGQTGVMVGKDYRGKRVLAVVNRVPELGWTILSKMDYAETM
SSWIPTLVIIIVFFFVAFAAILAGSYVLFSTRALSASRSRLELLQRTERSEALLSAILEH
VDAPVVIIDKSSAIQFSNRAFKEHFNDILPNDLKALQPSLDKDAGVLSHKLELSDVRGKA
LQLYVMPIHILLEKQESLFGYVMRDVTELESALEQVQRLNRDLAQKVEAQTKRILEANEE
LRTIASAISHNLATPLRAVESFSELLETKAYDQLDAETSGYVLRIRKASANMATLTDDLM
TFLSLDSIVLAHDEFNFSLAAQDITSDIIRRSPKRRYQITIMPSLKVHGDGALLKTALRN
VIENAFQHCSDNTVASIEIGKYGESGIFVKDNGIGMTSDEINSILKPLSKTETENYVPGL
SVGLTITKKIVELHGGSLEIESELGKGTTIRFKF*
>SPBDM4_v1_50174|ID:27158537| protein of unknown function [Uncultured spirochete bdmA 4]
MCADSDEANEKEKDNKESYLLSSPQLPDMVEEIALYCIPFVHFGILPVLDPRADP*
>SPBDM4_v1_50175|ID:27158538|tkt| Transketolase [Uncultured spirochete bdmA 4]
MNIPALQSIALSVRSLTIDAVQKANSGHPGMPMGAAELAAFLYGVELKYNPKDLKWLNRD
RFVLSAGHGSMFLYSILHMAGFDVSLDDIKAFRQVGSKCPGHPEYGVTPGVETTTGPLGQ
GLANAVGMAIAERKVAAHFNRPGQEIIDHSIFVVAGDGCFMEGVASEACSLAGYLGLGNL
IVYYDSNHISIDGSTNIAFTEDVGKRFEAYGWQVLRGSMYNFEELAALTARAKADRTRPS
LIILESIIGKGAPSKQGTSGVHGSPLGEEEARKAKEGLGIPLEAPFWVAPEAYAYFEAHR
KTLDQEYQAWQARFEAWKKANPELARELSTWLSGKATRELDLPAFKVGEKVATRNASGKC
LGAVAGAWPNLIGGSADLTSPNVTQLPPNADGTPLVLTKENPAGRYIHFGVREHGMAAIA
NGIALYGGLRPFVATFLVFADYLRPALRLSALMKVPVIYVLTHDSIYIGEDGPTHQPVET
LASLRAIPGLKVLRPADAEETAVAWRIAMERLDGPTVLVFTRQNLPVLEKADPDWPYTMA
LGAYVVQNPARPPEVTLVASGSEVSLAIAAADIVRAQRPKTAIRVVSVADRQAFYAAPAP
VRDAILSPGSLVFVAEAGIAQGWERIAPSENIFSIERFGESGPGDKVAEHLGFTAQAFAQ
MVLAKFA*
>SPBDM4_v1_50176|ID:27158539|uvrC| UvrABC system protein C [Uncultured spirochete bdmA 4]
MDIMKRESTEAEAERRSRLKTLIKEAPQSPGVYLMRDETGEIIYVGKAKSLKNRLSSYFS
GKKDIKTRHLVSRIHNIEWVLAAHEYEALLLENNFIKEHTPRYNINLKDGKTYPSIRITS
EEFPRVFRTRRIINDGSEYFGPFPSAEIIDTYLDLIKRMFPLRRCVKMKKRASPCMYYHI
GRCPGACAGKISHEDYMKRVGEVRKLLSGDTESLVVDLGREMKRAAAELRFEEAAHLREA
IKAVQSFMGHSNVVDFNSLDARDYIAWEADGDQTSYVVFQMRDGRLKNRDSFMENLVDEL
EALDSFLARYYDGGRVPPPSIFVRKAENTASIAHYLSDRAGVPVEVKVPDAERHWAVMNL
AVQNAREELAKRRRMHGDIAALVELKEKLGLGAVPTRIEGFDIAQLGGKHTVASLVSFKN
GVADKKNYRYFKIRSVEGQIDDFAAMREAVARRYTRLINEEAELPDLVLVDGGAGQVSAA
KEILDDLGLDVDLAGLAKRNEEVWLPGESSPVVLPKDSPALRVLVAVRDETHRFATGLSR
KLRMTDATFRVLQEVPGIGDVRAKSILREMGSLDAVAQAEPGYISSIAHVNEETARKVIS
VARRTVDIDTVDIDEGLEDAD*
>SPBDM4_v1_50177|ID:27158540| GTP cyclohydrolase II (modular protein) [Uncultured spirochete bdmA 4]
VDIPSIEQIIKEDIEFLRHCPNKDHCAACEESVCVMVASVADFPTAYGHFTIAGFVNNKD
NKDHIMIIKGDIGDGENVLLRVHSACLTGDALGSLRCDCGPQLHHALERIEKEGRGIVLY
HQAEGRGIGLVNKLRAYALQDAGYDTYDANVALGFPPDARDYEIPAEMLKKIGVKSVRLL
TNNPGKVAELEQYGIQVTERVPHELPPHMHNREYLRTKKERFGHLLELDHGRE*
>SPBDM4_v1_50178|ID:27158541| putative enzyme [Uncultured spirochete bdmA 4]
MDSVRWGVLSVSNHYRLRVHHQLIGSGVTKVLGIASRDAGRAKQAARELGLARSFGSYEA

MLADPEIDAVYIPLPNHLHAQWINKAADAGKHVLCEKPFAMDAAQAAEAVRYAESKGVRV
MEAFMYRFHPQWAHTKQVVQSGEIGKVQFVQIQFTYNNKDPRNIRNILDTGGGAMYDIGC
YACSSSRFVMGKEPERAISIVRRDAEFGTDTLSSAMLDFGDARALFTVSTQSFPVQQVDI
FGTSGTITIFIPYNMYDDVPAEVRITTGIGPRMVRLGPAGQYRLMFDAFSKSIIEGKPVP
TPPEDAIANMRAIDALFRSEKSGLWEKV*
>SPBDM4_v1_50179|ID:27158542| Major facilitator superfamily MFS_1 [Uncultured spirochete bdmA 4]
MDEVLNNQKISLRFGLNYFMLFAIYGISSPYLQLMVQRLGYSPAAVGLFLGFFELIGITG
PIYLAQIADRSGKLKPFLFASTVMILAGLGLLVSFHIPLVTLLSLALLSLGIKTPVPVLD
TSLLRTIEFASTKGTKTPNYGLLRAIGSVGFVVVALIAQSIPGFDSSPTGTMAFALGILA
CLFLVSLFALPEIGTTQARKEKIAFSFKWLDSTYLLGLGVIALGRLAMAPIGSFFSMYLT
EELHWHAVGAMWALSASCEIPFIILSWKFIQKKNPMLAIAISSGAIVVRLFIYALFPTPA
GAITGQILHSLCYGLFQPAALAFVNLKTPPAARTTGMALYMGVGIGLPTFLGSALGGVVV
ETFGYRWLFASYTLFALASLALFWKFRARLTAVR*
>SPBDM4_v1_50180|ID:27158543| putative gamma-glutamyltransferase [Uncultured spirochete bdmA 4]
MNFDPGYLPYPSTRYPVYARHGMVAASSPQAAAAGLVALRKGGNAVDAAVAAATALTVVE
PTANGIGSDAFALVWIEREGKLYGLNASGWAPADISIEKVLARQGSATGGRVEAISGALP
AAARMPVYGWAPTMVPGAPKGWAELVRRFGALSLPAVMEPAIDYARNGYPVSANLARMWG
RALEKYRKTCSGPAFEEWYRTFAPDGRAPSAGEIVRLPNHARTLELIAESRAEAFYRGEL
ADRIVADSEEFGGYFSRADFAEYEAAWVEPISVNYRGYDVCEIPPNGQGIVALMALNILK
EFSFAERESADTFHLQWEAMKIAFADGMGHVTDPKSMRVRPSDLIRPEYGAARAREIAPD
GPAGLYGAQTPPKSGTVYLCAADGEGNMVSYIQSNYMGFGSGVVVRGTGIGLQNRGADFS
LDPTHPNCLAPRKKTYHTIIPGFLLKDGRALGPFGVMGGYMQPQGHVQTVTNLVDFHLNP
QQALDAPRWQWLHGKKFAVERAFRPDIVGALAARGHDIEVSEDSTPFGRGQIILRLPGGA
LIGGTEGRTDSNIACW*
>SPBDM4_v1_50181|ID:27158544| Oligopeptide/dipeptide ABC transporter ATP-binding protein C-terminal domain [Uncultured spirochete bdmA 4]
MTDRPVMLELKKLKKYFPVKRGLAIHALEDISFKIYEGEKFGVVGESGCGKSTLGRTILQ
LYPPTSGACIYHGKTLEEVSPKYVVKREIAELLNYQKRACVFYQKSLEVEAHIAKLDKEKD
ASTIAALQSRAKELRKDASRQLRDGSRTVGSLILSEKLPEIAELFSLSEQESERAHAGIL
ELAKLEAEELEERASHVASDEIARKIGECRAFIGEHIKKSQGYREKAFEYRGKHVLPITE
RCLDPAYQAKLDHNYERGINLGKLTGEEMRALRRDMQIVFQDPAASLDPRQTVGKAIEEV
FIINTDYSPTVRWEKTVALFEKVGLKREYCDYYPHQLSGGQKQRVGIARAIALDPKFVVL
DESVSALDVSIQAQILLLLDALSKEKNLTYFFITHDLGVVKHFCDRILVMYLGNVCELAP
SKTLFRAPLHPYTTSLLEAVPRPVVREQSKKERILEGEVPSALAPPPGCPFHTRCPKRMV
ICTHDKPELREIEPDHFVACHLYDRSDA*
>SPBDM4_v1_50182|ID:27158545|oppD| oligopeptide transporter subunit ; ATP-binding component of ABC superfamily [Uncultured spirochete bdmA 4]
VPADAEADALLLEQETDSPMEKILEVKDLVTSFRIAKKDYQVLRGISFDIHKNETLCMVG
ESGCGKSVTALTIMDLLPLNARVESGNIKLDGQELTTLTHRERNALRGEKMGMIFQEPMT
ALNPLLTIGYQLTEGLRYHKRIDSKTARETAISQLKKVGVANPAERLRQFPFQLSGGLRQ
RIMIALVMSLQPSLIIADEPTTALDVTIQKQVLALLNALKKDMNAGILFITHDLGVVAEI
ADRVIVLYSGTKVEEGPVATIINSPKHPYTLGLLNAVPNVDKEDFTIQAIPGTLPHVTEE
IPGCRFHPRCPYATERCRLEIPREFAYAAGHFVACHKVEEEARK*
>SPBDM4_v1_50183|ID:27158546| putative ABC transporter substrate-binding protein [Uncultured spirochete bdmA 4]
MKRTRSIFIAFVLIALMLAATGSAFAANKKVTGDKMFNVALTAAFTGFDPLRTNDQASTY
VNAQIYETLYRIGPDGDYIPLLAESLPDFSADGKSATVHLRKGVKFHDGTPFNAQAVKYT
FMLIKDPKFGSARASLAASIASMDVLDDYTIRFNLSYPDGVLLAKLAHTNSAIVSPTAQA
KQDLMIKPVGTGPYKFVSSISGSNVVLTRNDQYWDKPPVIKNVTMTIIQDESTAVARMET
GEADFMPELSVPQIDRVKSIPGVTVGTSESARITYVMLRPTSYVNPLMGKKEFRVAIAKA
LDTRGYTETILEKYASPTGSMIGPTVFGYTPQAENYGYKYDPEAAKAAVKANHWENEKIK
LFVPSTPVYTKLGEYFQANLKDAGFNNVQIEMIDWSAWLTESKAPNRFDISLAGWANVTR
DGTELLEPNWESKVSARTKINNPEFDKLVYEGKTTTNRAVRIKKLDAANKLLLEEAYAVP
VYNAVNIFAYNSAYTDVTNDVSGTFYLCDFTVKQ*

>SPBDM4_v1_50184|ID:27158547|yliD| putative peptide transporter permease subunit: membrane component of ABC superfamily [Uncultured spirochete bdmA 4]
MSAHHKNWMGSALRKLLKNKLATACAVFLVLEIAILALAPWIAPFSPDDTNISIRFAPGF
WARFSHDPDVAAKYVPGHIFGTDHLGRDVFSRLIIGGRVSLTVGFVSTALGLVVGTVLGL
LAGFYKRLDNPIMRVMDLLFTFPGILLAMLIVAMMGVSTVNAMLAISIWSIPNFARMVRG
KVLQVKEEDYIMAIRATGAKNARIIFVHILKNCLPLIIVIATMRMASSIISISTLSYLGM
GVPPPAPEWGSMIAQAKEYMWKMPSLIVVPGVAVMLTVISFNILGDKLRDILDPSLKD*
>SPBDM4_v1_50185|ID:27158548|yliC| putative peptide transporter permease subunit: membrane component of ABC superfamily [Uncultured spirochete bdmA 4]
VANYIGKRIALMFLLLLALTFIVFASLYIAPGDPAQMVAGLNATEDDIARVRTALGLDQP
FIVQYWIYLRNLLTLNLGTSYSTHQPIAQEIIIRLPNTLNLALASIILSILVGIPSGIIA
ALRKDRLADNILTTTSLAGISIPNFWLGTMLIYIFSVKLRWLPSGGMSAPFWTPQGFRQI
ILPASALSTATIASFTRIGRSSMLEVLQSDYIRTAKSKGLSGWLVVMVHALRNALIPLVT
QFGTSFGGLLGGAIITEQVFVVNGIGTYLINAINTRNYPVVQSTVLIIAAMFILINLGVD
ILYSFIDPRIRYD*
>SPBDM4_v1_50186|ID:27158549| conserved protein of unknown function [Uncultured spirochete bdmA 4]
MNINDDKELLQSIEEGEWKTVSNFEEIKKNLMAAAAETAIKDYRINVRISKRDVEALKTK
ALEDGIPYQTLVTSILHKYVTGRLQEMHPKETKETSFA*
>SPBDM4_v1_50187|ID:27158550| conserved protein of unknown function [Uncultured spirochete bdmA 4]
MSFYWDEDKNTLLKQERGISFERIVVAIEERHLLDVLEHPNKEKYGNQIILVVEIEGYAF
CVPCVPEENGNYFLKTAYPSRKYTKQYRGGNNEHK*
>SPBDM4_v1_50188|ID:27158551| membrane protein of unknown function [Uncultured spirochete bdmA 4]
MSASLWFLAVSDALKLQALVSFVLLAVDSVFPLAGALAIYLSAFALSLLLRQRRLAVYVH
ALAYVLGWGFAGALLEAWVSGQALSPAALVQTARANPVVFGAMLAAVTVFWSRGAWLAAK
AKTHAFAVLRFDEGLAAFLCIFLIAAFLRVRALTAEKLVIPYLAFSIIALGLSKSEHARR
GGLSPASTRRALVPVVAVVALVAVGVFFLMPALTEPARSAAALLKTGSLTLLSYIAAFLK
WLFGTRRATFPGEEGASHLSQNQQFEEKSNSLFGTIMMYILGILVALAILALLAFLVVSI
WRFLKRRVGKEGSGVRNLPSLGERLARLLAGLRRFLAALRAWKSRFFEKRSEAVRGFMRL
RTSGRAAGLVQKRTETPREYASRLAARFPAAAASIELIISSLEKEIYGGVSLTMETRRAL
SAAARHFHRYTFIAERLRHHQ*
>SPBDM4_v1_50189|ID:27158552| protein of unknown function [Uncultured spirochete bdmA 4]
MNENESSYFIYPISLVGLGAIAFFAAANRALPLASFALISLALMLAIRLWGKLSLRRLDI
SLACDAQKLFAGETLSVRAQIANRKALPLWLRLEIAAPVGGAGIESLAGADSELSGETGL
LPFGEISGAWQFRALRRGVYTLGPAFAQGGDLLGIYRCEKKLPDTWDIVIYPRRVALRQF
DLPFRDYFGIHPSKGIIEDPAWYEGTREYTGNRPARTIHWKASARLGKLQEKIFEPTSHQ
KIFFLFEGLGFRRAEDRMGFERALEVLGALANRFAESGASFGIATDCAVKGFPAILPLGR
GPEHLGMLLELLARCEFERGQSIAELIGSVGAAGAGFLIVARAPDESTDRFFALPSARRN
RVYFVFARANDGTEVEGELEARTEGGAVPSMGAAPDDTYTDEATEKGLRYPALYFDDLVR
EEAQRPLSEAGRLDLSGYSGEAGHTGEAAP*
>SPBDM4_v1_50190|ID:27158553| conserved protein of unknown function [Uncultured spirochete bdmA 4]
MANDLLQNKDFLQACKKATLSAFKNKIDDAKLNSLLEHMPSPAAGPAKNVLRVSGGSGAI
QAEASIVSAVIYAKVKCYPDNKSWKFEESAWGPGIGGGECIGFMYTAYTGDNAWDTFFND
ATAYHAQGIAEAGGIFQINWFRSDGTPIGQFNGALGGAGVFEIGGSGKWKKK*
>SPBDM4_v1_50191|ID:27158554| protein of unknown function [Uncultured spirochete bdmA 4]
VLAVLEGRQEEAGGLDKSAIYRRLLRAGPNSLAI*
>SPBDM4_v1_50192|ID:27158555| conserved protein of unknown function [Uncultured spirochete bdmA 4]
MNKKTVYSDAPDDLGEVLLKGKKVDDFLPPPDQLVRRVPKVKVTITLNQQSVEFFKKSAK
KNKVKYQTMINEVLDKYVEKYSDTL*
>SPBDM4_v1_50193|ID:27158556| ATPase [Uncultured spirochete bdmA 4]
MERLIENYRRKIAAIQVKFIRSIAGTVAWDARLVGIRGARGVGKTTLLLQHIKQAFGDNL
NAALYVSLDNLWFAEHSLAELADTFVKRGGTHLFLDEVHNYPQWSQAIKNLYDDYPDLHI
VFTGSSLLEILNARADLSRRAVVYTMQGLSFREFLNLQTGKDFPALSLQEILQDHTTLSA
DIVGEVHPFQYFPAYLQTGYYPYFLEGLDVYALRLEETVHMILEMELPQLRHVDTAYVPK
LMHLLSIISESAPFIPNISKLSDRIGLNRHTLLAYLQYLSDAKLTYSLYRDAHGISALQK

PDKLYLENTNLMYLFRGKETDTGNIRETFLANQLSYAHRLEFSVSGDFLVDGQYTIEVGG
RSKTRRQIQGVPDAYIAADNLEYGNDRKIPLWLFGFLY*
>SPBDM4_v1_50194|ID:27158557| putative ATPase (AAA+ superfamily) [Uncultured spirochete bdmA 4]
MVRRELYLDKIRPFIGADVVKVLTGIRRCGKTVMLGLLREELLRRGIPSSRMFSINFESG
ADPEARSQESVYARIKTMAANAKGKLYLFLDEVQEMEGWERLVNACMIDFDIDIYITGSN
AKMLSGELATYLGGRYVEITLYPFSFKEVMESYAQAGNPPDEAEAFARYLVLGGMPFLYN
HVLDETSAKQYLSDIFNSIILKDIAQRNKVRDIERLERLILFFIANVGNTFSAASIVRYL
KNEKRSLSTETIYNYISYCKEASLLHLVPREDLIGKKALSFQEKVYLSDHGLREAVYGNN
QRDISQILENIVYMELLRHGYDVHVGKVGSAEVDFAAEKSGEKLYIQVAYLLADERIVER
EFSPLEAIPDNYPKYVVSMDELDRGRNGIRHMNIRRFLLEQSWV*
>SPBDM4_v1_50195|ID:27158558| protein of unknown function [Uncultured spirochete bdmA 4]
MNLGSLFIPSQSCYPVGKHGMSVKSVTMGTVDKKDESRGTFRSGAKFLIRLNEPEAAQGV
AIVGHRLEPFRNYETPTEKIVFEDAAGKVLRQRLVLMEAEEALQFFTLYGELRMLPRIMM
EDASNIEAFQQPGFTPEDKRVRVFALDLKAIGNGVSLAAGDYLAVTMLDAGGTRFKIEPL
PIARFDTTLTEPWRNALAEAFREVFATEEPSLNPIELMARVYRAAVPLVMERPGGAYSEF
FNDSGILGTFWWQGVPVLWDKDAAPKAVAKKMDTAYSSIIDDLSKAELSVLRELAVDLRE
SEEDYGDDFYSLAKEYDLSVDSSDIELFLTVELSRYPGTTKLTRKALASNAALRERVTER
AVDRVLVGLRLTGIPESGAKALRASALDAASDILDDFDLSIIKNPAVNNLREALMDIYER
FLLWMRIGREDLAFAPEYDQERFEKKLMKMNELLNVSYMLNELELPDDKLVKDLLKPGST
GLAEYWETLKDLESLMPSRRAKKIPERGRISSRAVLTKQGYEASRYRYELEVVLKGSNPR
IYRTLLIPGNRTLADLHRCLQDAFGWKNYHLHEFSFDHMVFGEPSNEEDRVAIAEDIVSL
DDLALKVGHKIEYVYDFGDTWVHSIKVKSREKLEGEGVWTSLCACADGARAAPPEDCGGI
EGYKEMLAELEPSLFASGKKRTRWDPESFDKEAVDKKLARR*
>SPBDM4_v1_50196|ID:27158559| conserved protein of unknown function [Uncultured spirochete bdmA 4]
MGYTLAMIRRQYWEERIHSLWKSRSIVWLSGVRRVGKTVLCKSIEGIRYFDCELPSTRRE
MEDPEGFLKSIGSGMIALDEIHRLGDPAPLLKIAADHFPDIRIVATGSSTLGASAKFRDT
LAGRKLELRLTPLCEKDNSFPGYGDRNRRFLNGGLPGFFLADTRDDREYSEWMEAFWARD
ILELFRLERRASFLKFAELLLAQSGGQFNAASFAASCGVSRPTIMNYLGILEATHLVRVV
RPYHGGGSREITATPRVYGFDTGFIAWSQGLHEIPAKEKGFFWEHLVLNEMDAGLRDDTP
LTWRDKEGHEVDFIWAPRGAEPSAIEVKWSTGAFEPAGIRAFRALHPKGRNFVVAADLEH
PYERDVGGLRVIFCPLGELIPQLLTKSAE*
>SPBDM4_v1_50197|ID:27158560| ATPase [Uncultured spirochete bdmA 4]
LLHAVWPEKTYASLENPDTMDFVQADPRGFLLQGEESGMVIDEAQRYPPLFNYLQGYADR
SSPGRYIISGSSNFLLMEKIGQSLAGRTAVLSLLPFSAAELGREALEANWENAAWRGFYP
RVRCTSLPPDLFARDYIATYVERDVRLVRNIGDLESFRRFVRLCAGRAGQILSIASLAGD
AGISVNTAKSWLGLLQAAWLVFLLPAWHRNLNKRIVKSPKLYWHDTSLLCFLLDVRKPEN
LAFHPLRGAVFENLIIAERFKAATHRGRTPSLHFWRDSTGREVDLVENAGDSGSTGKTGG
ATSEGRAWECKSGMTVPGDFFRHLALFGEEAGVPPERRILAYGGMESQTRSDARVLGWQD
ALLEP*
>SPBDM4_v1_50198|ID:27158561| conserved protein of unknown function [Uncultured spirochete bdmA 4]
MERKKLADLEKWRQKPDRKPLIVHGTRQVGKTWLLKEFGKRAYKDVAYINFEANERMAAL
FSSNLDTQRLLTGLRIESGCSIEPGTTLIIFDEVQENPQAQTSLKYFAEEAPTYHIAAAG
SLPGIAMHRGTSFPVGKVEFLDLYPLSFEEFLWALGEAELAGLLSGHDWQMLELFRDKLI
DYLKYYFCVGGMPECVGSFAAGRDLDKVRETQGRLLSAYEQDFSKYAEPAMVPRIRAVWN
SIPSQLAREQRKFTYGLVREGARAREYEIAIQWLCEAGLLHKVYRVSKPGLPLRAYQENS
AFKLFMVDTGLLAAHAHLPTRAVLNGSGIFEEAKGALTEQYVAQELRLRDDFDVFYWSSG
TSQAEVDFLVQIGESVYPLEVKAAENLQAKSLRVYRDKFSPPRSYRTSLSPYRAESCLTN
IPLYALAAIPTG*
>SPBDM4_v1_50199|ID:27158562| protein of unknown function [Uncultured spirochete bdmA 4]
MNRLTKNIIAHIPNPLFTAKELAVLEPSSDNVRYALVKRAIADGDILIEEDQLSEMTHAD
FDELEGNYSSRRVLRFLAGLKQEIVP*
>SPBDM4_v1_50200|ID:27158563| conserved protein of unknown function [Uncultured spirochete bdmA 4]
MQRTLMQKLVEWKDRTSRMPLLLYGARQVGKTYLLQEFGQQYFRDTIYINFETDAALASE
FSRDIDPVRLLNTLEIFFNKKIDPDSTVIILDEIQACERALTSLKYFSERTPQYHIVAAG

SLLGVTVNRNQHSFPVGKVEPLTLYPLDFEEFLLALGQEALISAIREAYAADSGLPEALH
AKALELYKTYLVVGGMPGAVWAYLKERRMLDAAPVQNLILSSYVADMSKYASPAETTRIM
ACFDSIPTQLAKENRKFQYKVVRKGGSATLFGPSIDWLTAAGIVLKCARIEHPFMPLAAH
LDLGAFKLYMADTGLLVLKSGIPAQLLLSGWENNTFSGAIAENYAATNLKAKGYQLYYWE
SGSTAEIDFVLQQNADIIPLELKAGIHTKSRSLAVYRERYKPAHAIRISQKNFGLENGIK
SVPLYAMFCL*
>SPBDM4_v1_50201|ID:27158564| conserved protein of unknown function [Uncultured spirochete bdmA 4]
MHGYIERSLTPAILNDIKLFPAVAILGPRQAGKSRLAAHIAERSPASLRLDLENPRDRAK
LTDPMLYFENKAGLMICIDDIQLMPELFPVLRSIIDDDRRPGRFLILGSASRELVNRSAE
SLAGRIAYRYLTPFLPTEYLSGTPPSILDTLSRGGFPESALAENDAASYRWREAFLKSYI
ERDLSMLGLGASSIATERMLTMCAHLQGQILNMASLGSSLGISGPTVRARLEFLQEALLL
RLLPPWSGNLKKRLIKSPKLYIRDTGLCHTLLGIHSVDELMGHPVFGPSWEALCVETLCA
SFPGAMASYYRTSNGAELDLILEKGARRFVFECKASSAPQLTRGFHTALRDLQPEQAFVL
CPIEDSYPLGPGITACGPVQCVELLRDLGEDF*
>SPBDM4_v1_50202|ID:27158565| conserved protein of unknown function [Uncultured spirochete bdmA 4]
MIYRDIIDRIRPWLSQDTILMLKGARQVGKTTILHYLQKEIEGQGGMARYIAADLDFADP
SFGDPRLFILRLADLFQGKTGTVLIDEFQSIPNAGLFLKTVVYDQTKDRYHFIVSGSSSLE
LTKNAESLAGRKKEFIIRPFSFREFIRARFPDIPLKCFGLDELRAIEDFAALYGTLLKEA
YAEYLTIGGYPGPVLSPPELRVDILKELLSTYLHKDVAGYQKIENVTGFNNMVRLLSAQT
GSQVNKSELGATLRLNAETVNRYLDILEGTFVFSLVPPWFSNPRKEVSKMPKVYVNDPGI
LLAAGTRPAVRPAYELLDGHLVENAVYLSFVARAEKIRYWRTIGGAEVDFIVDTETGALP
VEVKFTAAKPTEPVAMRNFRAAYTEARKGIIVSRDTISNDGTVVIPAYLLDFLTGLTE*
>SPBDM4_v1_50203|ID:27158566| protein of unknown function [Uncultured spirochete bdmA 4]
LRNNASDHSARLFPFFTHELAGNPLFVHASTDECLQRGWYPPLFDRPFIPSDWYELYVAL
YLEKDLSRLINIKDLSQFHKFISLCAGERFSFWHAPSAGEVDLVVEQGNDIRAIEIKSSA
TFRPELSTGLERWGKLASLPPERLSIVYDGVEQFTFKGMRVVPWRTRL*
>SPBDM4_v1_50204|ID:27158567| conserved protein of unknown function [Uncultured spirochete bdmA 4]
MIPRKDLDKLVRQADNHSASIIIGARQVGKSTLLGMIRDSLARPSEMFNLENPLHLALFN
EGYTSFIRTVRTNTIFIDEFQYCKDISSVFKAVYDLNPQVKIYATGSSSMEIQAHLKESL
AGRKLETILYPLSYGEWLSKESPRERPLLSSDITEVTGPDDLESHRKGLGEFLRYGALPG
LLALSDDLEKREYLFGIYQTYIAKDIKAFLKEESVLSFNKAITWLALHNGAQLNKSTLSA
VAGISSRQIDRYLDVLVGTFVLALLPPLTNNRGKELTKTPKFYLYDQGIVNSIIQDFRSI
DLRPDAGMLREQFVFWELKKSIDIRFSLHYWRTTDGKEVDFVLRKDQELFPVEVKSSWNP
PALPTGLRHFLTFYPETRKAVVLYDGPERTSRHGPCVVHFAPLHKAFALPGFLAG*
>SPBDM4_v1_50205|ID:27158568| protein of unknown function [Uncultured spirochete bdmA 4]
MSKYVIIRSMKNRLRTLDGLEVDLLVELAQGYLAFEIKSAERVSPVDARHLRGLGEFLDK
PLLHSFVLSDDPETKSLAPDITAIHAAYFLG*
>SPBDM4_v1_50206|ID:27158569| conserved protein of unknown function [Uncultured spirochete bdmA 4]
MSVAIIRDRLATYDCKSEIEEEQAVREITQELILAALGRTEFFKEAAFQGGTCLRVLYGL
DRFSEDLDFALKERNPAFEWEKYLGAVTEELAAYGYRIEIQDRSVRDAMVKKAFIKDDAI
GKVLNFQYAGKTGVLGKIRIRLEVDSNPPAGSRYEIRYLDFPFISSVVAQTLPSLFAGKI
HALLCRDYLKGRDWYDFLWYTARKTPINYTLLSSALAQVGPWKGVDLSVDYSWCFEALRN
KIETTDWHSAAEDVRRFVRVRDQKSLDLWSRELFLRQLEKIPRE*
>SPBDM4_v1_50207|ID:27158570| conserved protein of unknown function [Uncultured spirochete bdmA 4]
MNRLTKNIIAHIPNPLFTAKELAVLEPSSDNVRYALVKRAIAGGDMLKIRRGLYALSPLY
RKTGINPLSAAQLVYGPSYISLETALSVHGWIPEGVRDITSVALRPPKDFDTPIGHFSYV
RVPQKTLYAGVNRGTGDEGQSWLIASPLKALADYVYCHKLDWTSKNPLIDSLRIEEDQLS
EMTQADFDELEGNYSSRRVLRFLAGLKKEIVP*
>SPBDM4_v1_50208|ID:27158571| conserved protein of unknown function [Uncultured spirochete bdmA 4]
MQVQMPDLVDMLWFARKEAGLRYPKAAASRLAQKGELIRLKQGLYLRSGLEHNTYALGSA
ANRLYGPSYISFVWALRFYGLIPEDVPHVTSATFAKGRSKRFDTAAGGFFYHDVPSAAYP
LSLTFLGDGEQQFLVASPEKALCDELYLKPQVRALKEIPALLFEDMRIDEERFFALDRAL
LCSLAPVYRNTTLHTLIRFLEKEKT*
>SPBDM4_v1_50209|ID:27158572| conserved protein of unknown function [Uncultured spirochete bdmA 4]

MIEAAVQLFEDRAEKNGGLMERALAETIQYIALLGLSRRDFFYKSALYGGTALRMLYNLD
RFSEDLDFSLLDSDRNFSLDPYVSFVQNELDSYGFHTEVFVKEKNVQTPIESAFIKAGTK
LHIVEARVPPSLTRRIAGNDVCKVKFEVDTDPPAGIESDISYIDSPISFPVRVCDGPTLF
AGKLSALLTRGWHQRVKGRDWFDMVFLINRNIPVSLSHLESRLRQAGFYTKVSHLSETEL
RELLEKRIQDVDIELAKKDVIRFIRNPKDVDVWSKDYFRYVAGKLRVQ*
>SPBDM4_v1_50210|ID:27158573| PilT protein domain protein [Uncultured spirochete bdmA 4]
MNYLLDTHTFLWSLGQSSSLPERVLKELRNPKNEVYVSAVTFWEISIKTRINKLDLEGIP
FHDLILLAKKIDFQCIDLTSEEANSYVDLKEDTRNDPFDRILIWQCICRNLTMISKDSAF
ERFKDYGLKLLWE*
>SPBDM4_v1_50211|ID:27158574| protein of unknown function [Uncultured spirochete bdmA 4]
VTREMYGYLVNNSLKAYEFFFGHFEIIGEVYGAFPIKDA*
>SPBDM4_v1_50212|ID:27158575| Prevent-host-death family protein [Uncultured spirochete bdmA 4]
MKTLPVGVLKAQFSEVLEKVKQGEKFGIVYGRKKKPIAMLVPFHDTETKKERKLGILDGK
CTIHFADDFKMTEEELIGLQ*
>SPBDM4_v1_50213|ID:27158576| conserved protein of unknown function [Uncultured spirochete bdmA 4]
MQGSVDTKVSECFIFRNFPRRAIIPRMSEKNETSSIIDNASKVVVGKRRALELMTVALLA
GGHVLLEDVPGLGKTLMAKALARSIGGEFRRIQFTPDLLPSDVTGFSVYNQKSGEFVFHP
GPVMCNVLLADEINRTIPRTQASLLESMGEFQVTVDANTIDLPRPFFVVATQNPIEMDGT
FPLPEAQLDRFLMKIDLGYPDRDEELLILDRFLMDDPLVELAPVTSPERIVELQKKRGGI
TVAAPVREYIAALVGATRTNPKVRYGASPRGSLGLIKCAQALAFARGREFVIPDDAKELA
GPVLTHRLILKHEERAKGATAQAVVEEILGKVPVPRI*
>SPBDM4_v1_50214|ID:27158577| Prevent-host-death family protein [Uncultured spirochete bdmA 4]
MKTLPVAEIRTKLSSLLKEVESGKEIGITFGRKKETIAVIVPINEYKKIKARKLGTLEGK
AKVTFSEDWEITDEEFINS*
>SPBDM4_v1_50215|ID:27158578| transposase (fragment) [Uncultured spirochete bdmA 4]
MHRDLKAEATRPPGKNMLAQQRKFNHFREEYNTIRPHEALGMKTPAEVHTWSNREFPRRI
RDWDYEKDIMPKMVTVNGAIRWKNKGFAMISTALGGKYVGLSAVDDGLWLVYYRHVALGY
FCERTMKVYELNDFDF*
>SPBDM4_v1_50216|ID:27158579| conserved protein of unknown function [Uncultured spirochete bdmA 4]
MHAGTSFPVGKVEFLDLYPLSFEEFLWALGESSLAGLSRGHDWQMLESFRDKLIDYLKYY
YCVGGMPECVNNFIATRDLNRVRDVQGRLLDAYEQDFSKYAEPAMVPRVRAVWNSIPSQL
AREQKKFSYDLIREGARAREYEVAIQWLCDAGLIHKVYRASKPGLPLRVYQENNAFKLFM
VDTGLLAAHAHIPMKTMLQGSDLFREAKGALTEQYVAQELRLRGDFDVFYWSSDRSQAEI
DFLIQAGESVYPLEVKATENLQAKSLRVYRDKFSPPRSYRTSLSPYREESWLTNIPLYAL
SALPV*
>SPBDM4_v1_50217|ID:27158580| protein of unknown function [Uncultured spirochete bdmA 4]
MSEKNEASSIIDNASKVVVGKRRALELMTVALLAGGHVLLEDVPGLGKTLMAKALARSIG
EFQVTVGANTIDLPRPFFVVATQNPKVRYGASPRGSLGLIKCAQDYPQARGTGEGRHRSD
RQRRQRIEWNIGQPALFPVWRK*
>SPBDM4_v1_50218|ID:27158581| putative Polysaccharide biosynthesis protein [Uncultured spirochete bdmA 4]
VHAIDAIKQKLKGLSGDNKSVLTLVGGTVVAQGLSFLFSPITTRLFSPEVFGDLSVFTSI
TGIVGVVVCLRYELAIVLPQDDDEGFSLLKLCFIFTSFVSVVTGLVLLFGGEAIYTKFGA
KNLAGYWYYVPISLFLTGIIQASNYWLTRTRQFTVLSWNKVVPVIAVNLVSIGLGFAGNR
DIGARLFAILVSNIANIAVIARVVAPELKPKKHARKYSYRELIGRYKNFLVYDIWGALLN
NLSWMLVPILMNAYYGSNAAGQYSIGMRVIQMPASLIGASISQVFLKNASEKRYSKTLYP
YCIETTKKLFKYTAPIVIVLLLLGKPVFHFVFGDKWDLAGVYTQILAPWALLWFCASPMH
SVFTITQKQNVYLVFSIVNLATRFLSLYLGKMMNSDIWGIAFFSISGFIVYGISLILALR
EAKKSDLSPANS*
>SPBDM4_v1_50219|ID:27158582| conserved protein of unknown function [Uncultured spirochete bdmA 4]
MDKTQFTYALIGCGRISYKHIEAALKNRDRIRLVAVCDSIFERAEERVRDYQKGIPDAIV
AIYGDYREMLEKEHPDICTIATESGKHPQIAIDCLQSGAHVICEKPMALSIADADAMIAA
ADNAQRKLAVCFQNRFNAPVQKARKAYEAGRFGRMLHGMIQVRWNRDKAYYAQAPWRGTW
ALDGGTLMNQCTHGIDLLQWMMGGDAVRVQAATRKFLRPIEAEDFGSAIVEFKSGAVGII
EGSADIYPANLNETLSLFGEKGSVVISGLAVNKIETWRFSDAETVGDTEDIVLNPNEKDP

PTVYGFGHSALYADFLDALENDREPLVSGARGKKALEIILAVYKSQKTGRAVELPLVDFS
TLEML*
>SPBDM4_v1_50220|ID:27158583| Acetyltransferase [Uncultured spirochete bdmA 4]
MASPIERHIAICDVQFGKNVVVIEPANLYGCSIDDDCAIGPFVEIQKDVHIGARTKVQSH
TFICELVSIGTDCFIGHGVMFINDTFQDGHPAHRDKRKWKSTYIGNNVSIGSNATILPVK
ICDSVVIGAGAVVTKDIIEPGIYVGNPAKKFK*
>SPBDM4_v1_50221|ID:27158584|rkpK| UDP-glucose 6-dehydrogenase [Uncultured spirochete bdmA 4]
MAKNICVIGTGYVGLLTAVGLADFGNQIIGVDVDGNKINSLNEGKSVIYEPGIEEYLTKN
ISAGRLRFTIDLQSALEKSEVVFIAVGTPSLETGEADLHYLYEVIDSIADNIKEYKLIVI
KSTVLVGTNRNIYNRIKAKTSTPFDIVSNPEFLREGKAVYDFFHPERVVIGYNSNNAKEI
IEDIYKAFNRASVPFVWCNWETAELIKYASNAFLAIKIGFTNQIASLCEEVGADVRIVSK
AMGMDGRIGSKFLHSGPGYGGSCFPKDTRALASVGKKKGVPITIVEAVIEANENQKKRVV
KRLEKRLGDLKDKEIGILGLAFKAETDDIRESPAIDIVSSLFSLGARIQAHDPQAMSNFS
VLFPAIKYMNSAYDVAKGCDALLILTEWNEYRSLDMVKIKEYMANPYIFDTRNIIAEDEL
KKYGFSYDFIGHLGSS*
>SPBDM4_v1_50222|ID:27158585|degT| Pleiotropic regulatory protein [Uncultured spirochete bdmA 4]
MDNIPFNNLYEGINPIYSEIMDKIGELIKTAEFIGGKEVELFEKEYAEWTHTKYVVGASN
GTDAIIIALKALGVGPGDKVLVPDNTFIATAEAVTAVGAEVDFIDIESDYYAMDPIQVEE
YLKSTKGRNVKAIIPVHLYGQMANMPVLREIADKYGIKVIEDSAQAHGSKLLGNQPGYWG
DIATYSFYPGKNLGAFGDAGAIATNNPDLYKKCRMLVNHGRWHEKYTHEIEGYNMRLDTI
QAAILRIKLQYIDEWTQNRKEKVSKYLHLLAGIDSVISPRVRREAEPVWHIFPVLCKDRD
STIERFKKAKISYGIHYPVPLHLQPAYNYKSYRANDYPVAEMISSRELSLPLWPEINEDI
LKYITECL*
>SPBDM4_v1_50223|ID:27158586|degT| Pleiotropic regulatory protein [Uncultured spirochete bdmA 4]
LNVPFYTSTREYHDHKDEFDAAVQGVMERGDFILGNEVAEFEKEAAAWLGVKYAVGVASG
SDALVLGSDILGFKDGAEVLTPTFTFFASTSCVARLGGKPVLVDMDEETLEMDVAQAAGK
VSDKTVGIIPVHLFLQPTPMQEVMELARTYNLKVLEDAAEAWGMESRVDGVWRKAGTIGD
IGGFSFFPTKTLGSYGDAGLMVTNDEELYSKIKSYRVHGSSVKYHHDYIGYNSRLDSLQA
AVLRVKLKITNEAIAARARHAKHYTERLSGISGLRIPLIGQGSRGVYYVYNILVPRRDEL
AATLKGKGIGTSIYYPIPLHLQKCFAYLGYKEGDFPVAERVCRQILALPIFPELRDDEVD
YVCDNVEDFYITKKN*
>SPBDM4_v1_50224|ID:27158587| UDP-3-O-(R-3-hydroxymyristoyl)-glucosamine N-acyltransferase [Uncultured spirochete bdmA 4]
MKLSAIKNIISDIVIVRDAEFYELGLTATQCSVSMLSFLENTKYIDELLSNATISCAIVN
PETAEMIKDRTTIGIAVSESPKHSFFFLHNYLIENTDFYGQSDGSIIDPSADINSTAWIS
KTGVSIGAGTIIGPHVSIYPNVRIGNNVTIGANSIIGGEGFECFRFQDKAFMVKHAGNVL
IGDNVDIQASCCIDKGLFKNSTIIGEYTKLDNLVHIAHNVILGRRVFIAACAMLAGRVTV
GDDAWIGPSAALRNGITVGNNGYVSIGAVVTRDVLEGQMVSGNFAIGHDKFLQFIKSIR*
>SPBDM4_v1_50225|ID:27158588| protein of unknown function [Uncultured spirochete bdmA 4]
LQKLVMPNCKISRDHLPFKDITSNDSSNRYIAIITDSNTITKRSTWPNPSIIPYSYTPRK
HSTCSNKDTSTKNYIMSYMYQIIELSIFTNYS*
>SPBDM4_v1_50226|ID:27158589| conserved protein of unknown function [Uncultured spirochete bdmA 4]
MVFTYDTYKYLMSYLLKNDYEPILIGKEKGSVSKKQLFLRHDVDTDYLGVLPLANVERGL
GIFSTWYFLPDSSIYNLLCHELGQIIIELDSMGHQVGLHIDASQYESVEQLEADIERIYA
FLNVRLPLSKTVSFHKPASWLLNDIVIDGWTNAYEKTYYSNVVYVSDSNRRNFMDEDRLS
NAVQQGKSLALLTHPLWWHERPEDFDETFSEACRCLGYDRIHNYLANTARSYAEKGIRGI
VR*
>SPBDM4_v1_50227|ID:27158590| protein of unknown function [Uncultured spirochete bdmA 4]
LLFSIIILKKLHEGLSWVLLWIQNMPSFHLTMQFLANKHNSIIPRSIDPNFP*
>SPBDM4_v1_50228|ID:27158591| putative UDP-3-O-[3-hydroxymyristoyl] glucosamine N-acyltransferase [Uncultured spirochete bdmA 4]
MISCELSKEVLQQVTGLLIPREGDFKYHHICSFDECYDNSIYFLGKKINGINLLCKNAED
SIYFVEKTVSFNNASSDSIIIYVENVRNIMMKILLYLEEGTLDDFIDEDYYITDKKSKIH
KTAVVDNNTAIGSEVIIGPHVYIGRNVAIGDNTRIFSGVKIVRNVSIGKNCLIRENSVIG

GGGFGIEKDESGNNIHIPQLGGVVIGDYVEIGALNTVCSGTIKPTYVGNYVKTDDHIHIA
HNDLIEDNTSITAGVIISGSVIIKQGSWIGINSSIKQGVKIGENSLIGMAACVNKDVQEG
TTVIGNPAKEISEFIKVQNKINSIL*
>SPBDM4_v1_50229|ID:27158592|wbpA| UDP-N-acetyl-D-glucosamine 6-dehydrogenase [Uncultured spirochete bdmA 4]
MTEQVDSLLERIEENSIIIGIIGLGYVGLPLAVSFAKKGIQVLGFEKSVAKTDRVNAAKN
YIADIKDEDFTKVVNEKKLSATGDLGRIKECDAIIICVPTPLDKFKKPDMSFIESVCVEI
GQNMKAGTFISLESTTYPTTTEEFVKPIIERESGLKEGVDFWLCFSPERVDPGNKTYKTE
NTPKVVGGLGKEAQTIAMALYGKAIQNLYPVSSPRVAEMVKILENTYRLVNISLINELAL
LSGKMGINIWEVIEAAKSKPYGFQAFYPGPGIGGHCIPLDPFYLEYIAKGYNFDLTMIDA
AGRINNLMPYRMMNKISYALNRQGKAMNGSKVLFLGVAYKPDIDDARESPALLVMHEVAR
KGAEVLYYDPYIPEVIDEYGKKWTSTPLADDLIEKVDCVIFTTNHSAFDVEHIVGKAKLV
VDTRNAVKSVEIEDGKVFKL*
>SPBDM4_v1_50230|ID:27158593| conserved protein of unknown function [Uncultured spirochete bdmA 4]
LRKIFFISASIIEYDGRLRELIEVSKSIGETYYLTRSNSVTPIDEHHIIFTSRGPSTYIN
FLLFCFKTARKIGKFDVIWADNRKALIPSFLIARLRKHPILIQDARELYMFGDVRHFIGK
IGCLVEIMLNRKFDIIICANKERAEIMKRYYHLRMMPLVFENVRKLEFDGTVDDAAFEER
YQMSKDKARWKIISTSGCDLDRGIEQLVEAVGRLGEEYSLYIFGNSSMKQEDFIERKIME
IEYKNIHIFGPLKQDELKYAIGMCDIGVVNYHAKDMNNKYCASGKLYEFIFEGVPVITTE
NPSLKALCERFEVGISCNDYQSAIRKLTEEYEYYQGKAREMSKKISVKSNRAGLKHHVVK
ALETIERDRSGKYPEVV*
>SPBDM4_v1_50231|ID:27158594|rffE| UDP-N-acetyl glucosamine-2-epimerase [Uncultured spirochete bdmA 4]
MLIFGTRPEAIKMCPLVNELRTRIGIRVLVCVTGQHRQMLDQILKAFGIRPDYDLSIMQD
KQTLFGITSNILNKIKDVLEKEKPDVVLVQGDTSTTFVSALACFYLQIPVGHVEAGLRTY
NIYSPYPEEFNRQAISIIAHYNFAPTELSKQNLLKEGKKPKTIFVTGNTGIDALKTTVRE
DYWHPELEWAMGSRLIIITAHRRENLGMPMHNMFKAIRRIMDEYSDVKAIYPIHINPVIR
EIAEEELGGCSHIHIIEPLDVLDFHNFLARSYLILTDSGGIQEEAPSLGKPVLIMRDTTE
RPEGIAAGTLKLVGTDEKMIYESFKRLLDDRAEYERMSKASNPYGDGFASKRIADILCAN
*
>SPBDM4_v1_50232|ID:27158595| putative Glycosyl transferase protein [Uncultured spirochete bdmA 4]
VKLLLMIPYCSQKTYSAVFINASIQTNQQFHKYFRLFTAGLIANGLDVVVGCQFDVNTTS
TKQRVISMPDESINGVRFHYFKTYNISIIGRICKFFQSLVYTFSFCMQNRGTIVICDPNK
VASAFGVMMGTKLARGNLVYILSDLPSINTGSLEEKQSLRQKLADLIISKADGYYFFTKF
MNDLLNKQNKPYVIIECLVDEKLENIRCNLADKYPTRVMMYTGGIVAHYGLEQLLEAFEL
AGVPNTELWIYGGGPYAKSLTELCAGKDTVKYLGILSNEEIVKEQMKATILVNPRLTNYE
YTKYSFPSKNMEYMVSGTPTLTTRLEGIPEEYNAHVFFFDSESVEGMAKTIRDIYSHSDA
ELYLFGQETKEWILRNKGYTAQLRKFYERVLTTL*
>SPBDM4_v1_50233|ID:27158596| membrane protein of unknown function [Uncultured spirochete bdmA 4]
MEAGSNLKPISRLPVVDWIFIIFWCQEILIDYLLQIYRRIPIIGDITYQAVIPLIFFLLF
IITLYARLKVKLIDIVFVIMAMLPCIFALVQYQEAAANELPTFFLKVFPMFFVGLFLNED
KKVWKSYSLFQILYILAITNLIANFLYTSYYLSSRVLEGYSMTWAYSILPSVLMIIFWGV
KGKRVIADILSVFGFLLILSYGSRGPIVCVLVFTVLLIAINMVNRKKYSIIVLFCILIVI
IMNTNYVSLIVSYLQGIISRTGFSTRVFDMMLRNDILSDTGRSPIRAALLSAISERPLLG
YGLYADRYLSSSGIYISGMYAHNFVLELWTQFGVVIGSLIIFAIGALVINAVVKAKENHS
RLCMLLIFICVGVVKLFMSGSYLQEPYFFLLIGYCTKIIREKRSTGRVIKYANSLAM*
>SPBDM4_v1_50234|ID:27158597| conserved protein of unknown function [Uncultured spirochete bdmA 4]
MRILWLCNVALPFISIDAKLPTVSVGGWLTGAYEGIKNCSDIVLGICFPVDISEPVKKGT
VENITYYCFRRGNQRLTKYCSVYESIFRGIIADFQPDLVHIFGTEYSHSLAMTLAFSNPK
RTIINIQGLVSITAQHFRADVPSRYFYGGSLRNVIKCDSLFEQRKQLEHRGQFEIAAIKN
CGHIIGRTDWDRACTEQINPTATYHFCNETLRPVFYKNRWQYEKCEPFSIFVSQSATPIK
GIHYLFRAMPEILRQYPKTKIYTTGRSPFKTPWYKRSNYEKYLMRLMEEGRFKDNVFYLG
SLNEEQMCNQYLKSNVFVSSSTIENESNSLSEAKILGMPAIASFVGGVTNRLEHNIDGFL
YQYNAHYMLAYYVCRVFSDRKYAENLGDNARIHAQKTHAIKENAEVLVKIYREVLA*
>SPBDM4_v1_50235|ID:27158598| putative Glycosyl transferase group 1 [Uncultured spirochete bdmA 4]

MKILHLCLNGPYTDNWGYQENILPKYHRLAGNNVTVIVSNMKHLPSGEIVEVDGGISYTE
DGVKVIRVAPKILISRKCTELLKPYPIYKILYREEPDFIMVHGLMGSISELSVKRYVRNC
NPKCIVCADLHEYYLGNRGGNISIKLALIKSYYRSVNKLMYPLYKKVFCITPNCMDYAKD
YYHVPKAKLELLPLGYDPQKIASTNRNSIRHVIREKYHIGDGNILIVHGGKIIPVRKTME
LIDAAAMLDDPRIRLIIFGGADSECMLLLQERMQRYAKWIRYTGHLEQKDYYDLYLASDF
AAFPGRQSTIWQEAIGCGLPLLIYRWDGVEYLDRGGNIAFIESQESAAIKRGLEGMLEDN
KYIEMAEVAKQKAAPFFSYERIAQQVLDSSMK*
>SPBDM4_v1_50236|ID:27158599|capD| UDP-glucose 4-epimerase [Uncultured spirochete bdmA 4]
MMNKYILMITGGTGSFGNAVLRKYMDSDAISEIRIFSRDEKKQDDMRHLYQSEKIKYYIG
DVRDMRSVMSAMKGVDYVFHAAALKQVPSCEFYPVEAVRTNILGADNVLTAAIECGVKRV
VVLSTDKAVYPINAMGMSKALMEKVMIAKSRDLDEKKQIVCGTRYGNVMASRGSVIPLFI
EQIITGKPITITDPEMTRFMLHLDEAVELVEYAFKNAHNGDIFVKKAPACTLADLAEALK
ELLEADTEVRVIGTRHGEKVYETLVNREEMAKATDLGGYYRVPADTRDLNYDKYFSAGNE
KVKQAAEYTSHNTRRLDVEETKKLLMTLDCVHEARRQRGLE*
>SPBDM4_v1_50237|ID:27158600| NAD-dependent epimerase/dehydratase [Uncultured spirochete bdmA 4]
MKVLVTGAAGFVGQNLSIRLSRENDITLLPFDIDNTEAELIDALSVADAVVHLAGINRPL
DPSEFVTGNVGLTSTIADALEKFQRPLPIIFSSSIQAEQDNEYGKSKHAAEERLKSYAEK
TDAGVQIFRFANIFGKWCRPNYNSAVATFCYNIAHDAPINIRDPDAPLRLVYIDDVVEAI
TAALRTEKKGFAFCSAEPVYETTVGAIAATIRDIHDTRADARVGDYSDPFYKKLATTYLS
YIDIPALATAPELKSDERGWLFELIKSDSAGQIFVSTTKPGKKRGNHYHDTKVEKFCLVK
GQARISMRKVNGTERHDFDLDERDIRILDIPPGYTHSIENTGSEDCIILFWANEIPNPVH
PDTFRLEV*
>SPBDM4_v1_50238|ID:27158601| UDP-N-acetylglucosamine 2-epimerase [Uncultured spirochete bdmA 4]
MKDKLKVMTVIGTRPEIIRLSEVIRACDKCFDHVLVHTGQNWDYTLNEIFFKDLGLRAPD
RYLNVVGDDLGETMGNIISASYKLFLEEKPDALLVLGDTNSALCAIPAKRLKIPIFHMEA
GNRCFDQNVPEEINRKIVDHIADINLPYTEHSRRYLLSEGFRKEHIFVTGSPMVEVILKN
RAKIESSKALEEYGVQKGDYFVLSMHREENIDIEHKFNELVTALNSLAERYNFPIVFSTH
PRTWKRLEANHISLNPLIHSVKPLGFTDYCRLQCDAKCVISDSGTLSEESSILRFPAVLL
RNSTERPEVLDKGTIVIGGYTAETLLQSVTMAMDLFDGEDRRPADYRDSNVADKVVRIIQ
SYTPIINKIVWNK*
>SPBDM4_v1_50239|ID:27158602| protein of unknown function [Uncultured spirochete bdmA 4]
MKIEATKQQLIEFLESHVLTPVEHHIGADETIKRKVRATRMHLNNLRSAEEVEDFFWNTM
ASDHGIDSYIRIRAIGGITFEDVRQEFKSPYGRTKANYFNK*
>SPBDM4_v1_50240|ID:27158603| Glycosyl transferase [Uncultured spirochete bdmA 4]
MISVIIPCYNEEKYIGPFLESLLHQTIGIDNLEVIIVDGMSNDGTRKILKNYLNNQNILL
IDNTERTVPFALNKGIQYSHGDYIGLMGVHCKYPEDYFEKLYSAIRQYNADCVGGASITR
PARNTSLCSAIAIAGSNIFGVGNSRFRIGVTDIQEVDTVAFGLYRREVFDKYGYFDTALT
RDQDDEYNARLKKNNRKILLVPNVRIEYYARDSKKKLFQMNYQYGLFKPLVAKKIGKPIS
IRQIVPFLFVCWLLIGSIISIFSKAMMIINVGIALVYIGISLAISLKEALHVHDMKLIFI
LPQIFFIQHFAYGCGYLSGIVYFLLLGREKVDLKVSR*
>SPBDM4_v1_50241|ID:27158604| conserved protein of unknown function [Uncultured spirochete bdmA 4]
MSVTDKTRKLIWVKTGNRCAICRQKLTIDETSKDSDSVVGDECHIVSESENGPRFDKSYP
EERIDSEENLIVLCKTHHKMIDDQVDTYTTEVLRAIKKNHEKWVETKLSQNGEIKPVRIV
RHKNKIPKKLSRVTDGQELFNIASSYLSYYHDFPDNLSTEEAELIGSFLQNVTDWADLSD
AIEPIDKVRAVKSISDNLDRLQDYGFLVFVAVEEQEIEGGIGPSSIWNALHLTIVQDDDP
RIQK*
>SPBDM4_v1_50242|ID:27158605| DegT/DnrJ/EryC1/StrS aminotransferase (fragment) [Uncultured spirochete bdmA 4]
MPKDYLLGMSDVQKRAAVKALTKLPRMNALRKANGALYTSHLAQAGLNHVDPSLHENHLF
LNYPLLVHNRAAFIRKAREANIPLGDWFCSPLHPVEGNLSQWKFDPDRYPNAVFAASHVV
NLPTDTRKPEKVLDFIDKNSSLIMDMTLGGRITNP*
>SPBDM4_v1_50243|ID:27158606| UDP-4-amino-4-deoxy-L-arabinose--oxoglutarate aminotransferase [Uncultured spirochete bdmA 4]
MITQRTIAFSPPDISDEEIQEVVEALRSGWITTGPKTKLLEQGLASFCHVPKAVCLNSAT

AAMEMTLRLLGVGPGDEVITSAYTYSASASVIDHVGARIVLVDSGMDSFHIDYEAIADAI
TERTKAIIPVDIGGVMADYDRLFDIVESRQQLFRPSNELQEVFGRVIVLADAAHSVGAVY
KGKMSGEVADFTSFSFHAVKNLTTAEGGAIAWRERDGLDSEALYRRLMLLSLHGQSKDAL
AKTKLGAWEYDIVGPWFKCNMTDILAALGLAQLRRYQGILQRRREIIERYDALFEDAPVA
SLRHYTGESSSSGHLYLVRLLGKDEAFRNRVITRMAEKGIATNVHYKPLPMHTAYKNLGF
DIRDFPHAFEMYRNEITLPLHTLLSDEDVEYVAVSLKETLRELAH*
>SPBDM4_v1_50244|ID:27158607| Sugar transferases involved in lipopolysaccharide synthesis [Uncultured spirochete bdmA 4]
MLLKAWDAMPARMRNAPVRGYYELLRQKRAALVLKRLFDVLASIILLAILLPLLLALIVL
IKLDSEGPVMFRQTRVTQYGRQFRIYKFRTMVHHAESLGTQVTTRDDARVTRAGRMLRKL
RLDELPQLFNILVGDMTFVGTRPEVPKYVARYSDEMLATLLLPAGVTSEASIYYKDEEQL
LADARDADATYLGEVLPAKMRYNLEGIRKFCGSYELRVMSKTLVAVTKK*
>SPBDM4_v1_50245|ID:27158608| protein of unknown function [Uncultured spirochete bdmA 4]
VPKYDLGESISRLERGVVKYEVLKAICDTYFVFKRENATSHRFYEDPYAGGIVNIQSGPS
GDAKRYQQRQVAALLKRRNEEG*
>SPBDM4_v1_50246|ID:27158609| conserved protein of unknown function [Uncultured spirochete bdmA 4]
MDIIDKYEFHLHYSEEDAAFISSVAEFPYLSADGTTPEQAYAAIRSVVEEAVGILAEEGR
EAPIPLSEKEFKGNISLRLSPETHRMATMRSRQEGCSLNQFLTSLIERNLYADSIDKAVK
QLSLATLTLSSQFEGGPWQQPTQVASIWAKNSQPSYSDAFAANSASYAIVQMNVSTVAPS
LGAELCK*
>SPBDM4_v1_50247|ID:27158610| protein of unknown function [Uncultured spirochete bdmA 4]
MQIIDLRSTLIDLFPVDSLDFMALMLDSFSTAIINKYGCAKLNVPPPSGSVGLVFTLGKV
EIHGKVKIIDRIVVEERRIIVTIGGSSDEARVLMKDLILLIASCETRHEIRPISPVLEID
ESQSTVQLSIDFSRLVSGSIAESIPESLSSRTVIAPTSMRAAITPAGLRFKIRYLGENED
LKNHSITMADKYLSIEAREGIGIEAKIFFASAPVKSDTLLELLRSFE*
>SPBDM4_v1_50248|ID:27158611| ATPase (fragment) [Uncultured spirochete bdmA 4]
MRSLSRNISTLASNETIRADIAMSDEDENISDKTILQYLRALNRIFVTDNLPAWNPALRS
KTAIRTSPKRQFADPSIAVATMRLSPGRILDDFEYFGFLFESLCDRDLRVYAEANDGQLF
HYRDSSDLEADAVICLHDGRWAPIEIKLGKKEIEEGAKHLIELKSKINTTKMKEPSFLMI
VTGTEVAYRRDDGVFVVPIGCLKN*
>SPBDM4_v1_50249|ID:27158612| putative transcriptional regulator [Uncultured spirochete bdmA 4]
MSKSYDSIVRGLEEIKAHTEGEIQLKTTRVEIHLPPHCDASTVKELRKDLRLSQHAFAKV
MGVSPKTVEAWESARNVPSGSSCRLIEMIRNDKTILKRQKIVVVS*
>SPBDM4_v1_50250|ID:27158613| protein of unknown function [Uncultured spirochete bdmA 4]
LCHKEEQLRADARDASKDLRSLVVGKKPLDLLKKSNPEAFYYWYRIEKLREEREPIHTLD
E*
>SPBDM4_v1_50251|ID:27158614| conserved protein of unknown function [Uncultured spirochete bdmA 4]
MKATVSERGQITLPKAIRTKLGIRPGTIIEFVLENDKIIGTKKEPRDALRTWRGKGRLPD
GIPDVDAYLDEVRG*
>SPBDM4_v1_50252|ID:27158615|vapC| Ribonuclease VapC [Uncultured spirochete bdmA 4]
MTTAIDTSVLLDILTDDPRHASSSEAALAEARASGRLIVCESVIAELRPALDSDQQIEAF
LQDMGIEFLPASLESALLAGSIYASYLKNRGPSRRVLPDFLIAAHAQLHADCLLARDRGY
YREYFRELNVMDHVNK*
>SPBDM4_v1_50253|ID:27158616| protein of unknown function [Uncultured spirochete bdmA 4]
MTRGELWRADFGFPFGRGPGFRRPILIVQDDAFNESKINTIDFEEACAREQGEDSTRVAV
IVPSLTFIADISAWFS*
>SPBDM4_v1_50254|ID:27158617| conserved protein of unknown function [Uncultured spirochete bdmA 4]
MSENIRFKYVFPDDYNPVYVNGAYGGVGPRGEIIMNFYLERSPVPREEFRTVNEDGTISE
QIKIEPEDLSRTIVRYVSSGVIMSPETARLIHTWLGEHLKRIGEGGKA*
>SPBDM4_v1_50255|ID:27158618| protein of unknown function [Uncultured spirochete bdmA 4]
MSDACIMGSKIIRYQQFFIGTGYANGTILPAGFCDAASADTGNVDSAEKQGKQMLNRRID
SIAGLKDDWNGYGAVSFSSAVVGNARALVSLLPCKAKVFPTAQSSIQFEFDTVPGKYLEV
EVFSDHYAMFFDDSTVQEELDSATQEYILQRIAEYNAK*
>SPBDM4_v1_50256|ID:27158619|pglF| UDP-N-acetyl-alpha-D-glucosamine C6 dehydratase [Uncultured spirochete

bdmA 4]
MIKESERKWNSMLKIFDKIDRRFLNFCVIAVVSLVTFFWTFFSFHKPFIWVLVLVVIALR
MLASFFIFRDYASSWRRSTQVTYLLKSFVYTVAFVVYLPIFYGKVEVALLVSELLFFIFC
INFLVAVYSYIMNKTRRPKTKSVVIYGAGRAGTKLREELVNSEYKVEYFIDDASSLHDRN
INTVRILSREEFKKRARGEPKFSMLLIAIPSASRAQLNSIYEEVGRFFNEIKILPLMGHI
VETSGFSGQLRDIQIEDLLARDPKDLDKHVIERFVKGKRVMVTGGGGSIGSELVRQCVKN
GAKSVVVVDHSEYNLYRISEEFPDDSVRKVLASVTNDRDLEDLFRETMPEIVFHAAAYKH
VRFCELNPHAAVYNNVVGTKNCIDLAIKYGVRKFVLISTDKAVRPTSVMGSTKRVCELYA
QNVDPGESEIVAVRFGNVLGSSGSVVPKFRKLIKENKPLPVTHPEVKRYFMLISEACQLV
LQAASLGKGGEIFILDMGEPVKIVDLARKMLRLAGKSEDQIVFTGLDEGEKLYEELLLDD
KAKDTKYPSIFVAKKTPRDLGKLKKEIDELVTLKEKSQIVEKLGMLIEHL*
>SPBDM4_v1_50257|ID:27158620| protein of unknown function [Uncultured spirochete bdmA 4]
MNLDETELKLLEKLYLSQEDDSPLTQRELAGDTGLSLGMTNILLRRFAERGWITLTHLSG
KKLRYALTARGMEEIARRSVSYFRRTIRGALLYRERIESYVRHLFRKGYSALVFEGPAEI
DFLFKSACEEVGIAFYANLSEADRKKLISLERTMVVVEGKQSEDDNGMPRTDDFTESDVD
MVRLSDILFNDYA*
>SPBDM4_v1_50258|ID:27158621| exported protein of unknown function [Uncultured spirochete bdmA 4]
MKMTKKIIAWLSAMVFSFVLLAGAGAQTTDQAAIAQALSAMQGGTGGSLSGSLTGTAPTA
ASPQPTVNQLTAAPSAQVSEAQTPNGQAVGTGQAAGQTAQLSSIEQMFATMASSLGTPAQ
NLTQFGYSLFGNPTAPSLAAIGDDYTLGPGDGLVLYLWGDPVDIKELSSSYVLTVDRNGS
IFLPPVGQVSVWGQDLGTVKDTLKGMLDRRYKRLEMNLTLSSLRQFPVFVSGYAGNPGTV
LANGADTVFTVLSRAGGILKTGSLRSVTLTRQGKGGAAERLDIDFYASLLEGKPIDLRVR
EGDSIFIPGIGSVVALAGELKRPGIYEIKSEASIGQALALAGGALPSVRSGSVSLVRFDA
AGKVLRTGDIADKTFAELPAGDGDFVYFGRVSDLLLGQAQVSGAVKYPGRYDVSSFKTLR
QLLLKAQPLPETNLFYGRVYRIDPSGRDKSFAFSPKDVLASVGGASDAPLTEFDKVVLYR
YDDTAIDPDFDRFADTVVISGPVKYPGFYLYKEGMRLSELLSNNTLLLDTNHSYAELTRR
TPEGKQEYYTFSPDSVLAGKTDLALARYDSIRFVRRGEQAAGHDFDKFPDAVTLTGNIAR
PEVYALMGGMKLSQVVVKDQVLLDTNLNYGEITRLRSDGKNEYVTFRPAEVLSGAWDFEL
ASRDRVNFVKVGYAPERPDFDRFADAVEVRGPVQFGGMYAWKEGMGLRELLALAKPLLEA
NQYYAEIVRGGENGRNEYLTFAPREVASGAFDLALRAKDIVRLYTNMPVGGVAGKAEGSG
TVQGVAPTGDSAGANVPTSTVTSPSAVVPNAAPSSSPLAPGSSVQSGPTTGASVLPLVAQ
QAPQTVSQTAASQQATVTYGPSDAEKALFNEVVTVSGAVRYTGPYARTPSLKLSSVVTAE
QILEETNLEYAELTRLKADGTPEYHTFAPKEVLEGKYDLPLQAKDVIRFVKKTSFGGTGE
KPNLEKFSDVVQLTGQVARPEVYALRPEMKLSQVVVKDQVLLDTNLNYGEITRLRSDGKN
EYVTFRPAEVLAGSFDLDLGPRDVIRLVKVGYMAPAADAERFGQAVTVEGPVEFAGSYAW
REGMGLRELLVLAKPLLETNQVYAEIVRPLGGGKNEYVTFAPREVASRVYDTTLKARDRV
RLYTTIAPKIAFTAQGAAETAAPLAGPEAGQSPAGVLPGVSGAAAQGSVPGAAAASTPAT
ATAAPLPAPPSAEATNAISPDLGLFLEVVTVSGAVRYTGPYARTPSLKLSSVVTPDQILE
ETNLEYAELTRLKADGTPEYHTFAPKEVLEGKYDLALKAKDAIRFVKKTSFGGTGEKPNL
EKFSDVVQLTGQVARPEVYALRPEMKLSQVVVKDQVLLDTNLNYGEITRLRSDGKNEYVT
FRPAEVLAGSFDLDLGPRDVIRLVKVGYMAPAADAERFGQAVTVEGPVEFAGSYAWREGM
GLRELLVLAKPLLETNQVYAEIVRPLGGGKNEYVTFAPREVASRVYDTTLKARDRVRLYT
TIAPKIAFTAQGAAETAAPLAGPEAGQSPAGVLPGVSGAAAQGSVPGAAAASTPATATAA
PLPAPPSAEATNAISPDLGLFLEVVTVSGAVRYTGPYARTPSLKLSSVVTPDQILEETNL
EYAELTRLKADGTPEYHTFAPKEVLEGKYDLALKAKDAIRFVKKTSFGGTGEKPNLEKFS
DVVQLTGQVARPEVYALRPEMKLSQVVVKDQVLLDTNLNYGEITRLRSDGKNEYVTFRPA
EVLAGSFDLDLGPRDVIRLVKVGYMAPAADAERFGQAVTVEGPVEFAGSYAWREGMGLRE
LLVLAKPLLETNQVYAEIVRPLGGGKNEYVTFAPREVASRVYDTTLKARDRVRLYTTIAP
KIAFTAQGAAETAAPLAGPEAGQSPAGVLPGVSGAAAQGSVPGAAAASTPATATAAPLPA
PPSAEATNAISPDLGLFLEVVTVSGAVRYTGPYARTPSLKLSSVVTPDQILEETNLEYAE
LTRLKADGTPEYHTFAPKEVLEGKYDLALKAKDAIRFVKKTSFGGTGEKPNLEKFSDVVQ
LTGQVARPEVYALRPEMKLSQVVVKDQVLLDTNLNYGEITRLRSDGKNEYVTFRPAEVLA
GSFDLDLGPRDVIRLVKVGYMAPAADAERFGQAVTVEGPVEFAGSYAWREGMGLRELLVL
AKPLLETNQVYAEIVRPLGGGKNEYVTFAPREVASRVYDTTLKARDRVRLYTTIAPKIAF

TAQGAAETAAPLAGPEAGQSPAGVLPGVSGAAAQGSVPGAAAASTPATATAAPLPAPPSA
EATNAISPDLGLFLEVVTVSGAVRYTGPYARTPSLKLSSVVTPDQILEETNLEYAELTRL
KADGTPEYHTFAPKEVLEGKYDLALKAKDAIRFVKKTSFGGTGEKPNLEKFSDVVQLTGQ
VARPEVYALRPEMKLSQVVVKDQVLLDTNLNYGEITRLRSDGKNEYVTFRPAEVLAGSFD
LDLGPRDVIRLVKVGYMAPAADAERFGQAVTVEGPVEFAGSYAWREGMGLRELLVLAKPL
LETNQVYAEIVRPLGGGKNEYVTFAPREVASRVYDTTLKARDRVRLYTTIAPKIAFTAQG
AAETAAPLAGPEAGQSPAGVLPGVSGAAAQGSVPGAAAASTPATATAAPLPAPPSAEATN
AISPDLGLFLEVVTVSGAVRYTGPYARTPSLKLSSVVTPDQILEETNLEYAELTRLKADG
TPEYHTFAPKEVLEGKYDLALKAKDAIRFVKKTSFGGTGEKPNLEKFSDVVQLTGQVARP
EVYALRPEMKLSQVVVKDQVLLDTNLNYGEITRLRSDGKNEYVTFRPAEVLAGSFDLDLG
PRDVIRLVKVGYMAPAADAERFGQAVTVEGPVEFAGSYAWREGMGLRELLVLAKPLLETN
QVYAEIVRPLGGGKNEYVTFAPREVASGAFDLALRAKDIVRLYTNMPVGGVAGKAEGSGT
VQGVAPTGDSAGANVPTSTVTSPSAVVPNAAPSSSPLAPGSSVQSGPTTGASVLPLVAQQ
APQTVSQTAASQQATVTYGPSDAEKALFNEVVTVSGAVRYTGPYARTPSLKLSSVVTAEQ
ILEETNLEYAELTRLKADGTPEYHTFAPKEVLEGK*
>SPBDM4_v1_50259|ID:27158622| protein of unknown function [Uncultured spirochete bdmA 4]
VKKTSFGGTGEKPNLEKFSDVVQLTGQVARPEVYALRPEMKLSQVVVKDQVLLDTNLNYG
EITRLRSDGKNEYVTFRPAEVLAGSFDLDLGPRDVIRLVKVGYMAPAADAERFGQAVTVE
GPVEFAGSYAWREGMGLRELLVLAKPLLETNQVYAEIVRPLGGGKNEYVTFAPREVASRV
YDTTLKARDRVRLYTTIAPKIAFTAQGAAETAAPLAGPEAGQSPAGVLPGVSGAAAQGSV
PGAAAASTPATATAAPLPAPPSAEATNAISPDLGLFLEVVTVSGAVRYTGPYARTPSLKL
SSVVTPDQILEETNLEYAELTRLKADGTPEYHTFAPKEVLEGKYDLALKAKDAIRFVKKT
SFGGTGEKPNLEKFSDVVQLTGQVARPEVYALRPEMKLSQVVVKDQVLLDTNLNYGEITR
LRSDGKNEYVTFRPAEVLAGSFDLDLGPRDVIRLVKVGYMAPAADAERFGQAVTVEGPVE
FAGSYAWREGMGLRELLVLAKPLLETNQVYAEIVRPLGGGKNEYVTFAPREVASRVYDTT
LKARDRVRLYTTIAPKIAFTAQGAAETAAPLAGPEAGQSPAGVLPGVSGAAAQGSVPGAA
AASTPATATAAPLPAPPSAEATNAISPDLGLFLEVVTVSGAVRYTGPYARTPSLKLSSVV
TPDQILEETNLEYAELTRLKADGTPEYHTFAPKEVLEGKYDLALKAKDAIRFVKKTSFGG
TGEKPNLEKFSDVVQLTGQVARPEVYALRPEMKLSQVVVKDQVLLDTNLNYGEITRLRSD
GKNEYVTFRPAEVLAGSFDLDLGPRDVIRLVKVGYMAPAADAERFGQAVTVEGPVEFAGS
YAWREGMGLRELLVLAKPLLETNQVYAEIVRPLGGGKNEYVTFAPREVASRVYDTTLKAR
DRVRLYTTIAPKIAFTAQGAAETAAPLAGPEAGQSPAGVLPGVSGAAAQGSVPGAAAAST
PATATAAPLPAPPSAEATNAISPDLGLFLEVVTVSGAVRYTGPYARTPSLKLSSVVTPDQ
ILEETNLEYAELTRLKADGTPEYHTFAPKEVLEGKYDLALKAKDAIRFVKKTSFGGTGEK
PNLEKFSDVVQLTGQVARPEVYALRPEMKLSQVVVKDQVLLDTNLNYGEITRLRSDGKNE
YVTFRPAEVLAGSFDLDLGPRDVIRLVKVGYMAPAADAERFGQAVTVEGPVEFAGSYAWR
EGMGLRELLVLAKPLLETNQVYAEIVRPLGGGKNEYVTFAPREVASRVYDTTLKARDRVR
LYTTIAPKIAFTAQGAAETAAPLAGPEAGQSPAGVLPGVSGAAAQGSVPGAAAASTPATA
TAAPLPAPPSAEATNAISPDLGLFLEVVTVSGAVRYTGPYARTPSLKLSSVVTPDQILEE
TNLEYAELTRLKADGTPEYHTFAPKEVLEGKYDLALKAKDAIRFVKKTSFGGTGEKPNLE
KFSDVVQLTGQVARPEVYALRPEMKLSQVVVKDQVLLDTNLNYGEITRLRSDGKNEYVTF
RPAEVLAGSFDLDLGPRDVIRLVKVGYMAPAADAERFGQAVTVEGPVEFAGSYAWREGMG
LRELLVLAKPLLETNQVYAEIVRPLGGGKNEYVTFAPREVASRVYDTTLKARDRVRLYTT
IAPKIAFTAQGAAETAAPLAGPEAGQSPAGVLPGVSGAAAQGSVPGAAAASTPATATAAP
LPAPPSAEATNAISPDLGLFLEVVTVSGAVRYTGPYARTPSLKLSSVVTPDQILEETNLE
YAELTRLKADGTPEYHTFAPKEVLEGKYDLALKAKDAIRFVKKTSFGGTGEKPNLEKFSD
VVQLTGQVARPEVYALRPEMKLSQVVVKDQVLLDTNLNYGEITRLRSDGKNEYVTFRPAE
VLAGSFDLDLGPRDVIRLVKVGYMAPAADAERFGQAVTVEGPVEFAGSYAWREGMGLREL
LVLAKPLLETNQVYAEIVRPLGGGKNEYVTFAPREVASRVYDTTLKARDRVRLYTTIAPK
IAFTAQGAAETAAPLAGPEAGQSPAGVLPGVSGAAAQGSVPGAAAASTPATATAAPLPAP
PSAEATNAISPDLGLFLEVVTVSGAVRYTGPYARTPSLKLSSVVTPDQILEETNLEYAEL
TRLKADGTPEYHTFAPKEVLEGKYDLALKAKDAIRFVKKTSFGGTGEKPNLEKFSDVVQL
TGQVARPEVYALRPEMKLSQVVVKDQVLLDTNLNYGEITRLRSDGKNEYVTFRPAEVLSG
AWDFELASRDRVNFVKVGYAPERPDFDRFADAVEVRGPVQFGGMYAWKEGMGLRELLALA

KPLLEANQYYAEIVRGGENGRNEYLTFAPREVASGVYDTTLKARDRVRLYTTIAPKIAFT
AQGAAEGRMAPGGLEAGQTMMTPGAAPGAATGQAGATAAAPVAAPTIAPSAPNGAAGPGA
TAASAGTAPAAGATSIETDTGLYLEVVYTSGSIRYEGPYARTPSLMLSSVVTSDQILQDT
NLDYAELTRRKADGSWEYLTFSPREVLGKTYDIPLRAMDSIRFVPVKYLPEKIDFDKFGN
AIAIEGATNFPGLYSFSTPQMLSEILTSDQLLSTTDIFYGEIERWQASGRIGYLTFSPLA
VLQGYQDIQIYPRDIIRFVTAGDTGENHDFSKYPDTVLLKGSIRYPGRYAWYKGMKLSDI
LQESDLLIDTETSYAEVRRYSATSDSIINFAPGQVIARKTEVELQPRDTVFFYPRYYQKP
ISIAGEVADPKVIPYYDQIELSSVLRSVTLLQSLMDLKAEIKKTTGETTVVYLEDYLKRQ
PNSKVLLSPGDAITIRTLLPDEHLPVIVVRGQVRQPQTIAFSEGMRLSDALVAAGGYDST
AYPKGLVLIRKSVATAQQTQVDRLIAQLEAATLAGTSLPTSTDTTLNSASAVIANMQIEL
AIQKAKLGNLKQLYKEGFGRISLEIPDSLEALAGSLSDIRLERDDLIYVPKVPTYVLVSG
EVSSQNVVLYREGMTVRQAIAESGWMSGEADLSHAYIIRASGKLDSTEGKGFLFFRPNIL
NYTLQPGDTVFVPTKSAKVSVAWAYMRDTFVSIGSILTSALTAKTLLGL*
>SPBDM4_v1_50260|ID:27158623| protein of unknown function [Uncultured spirochete bdmA 4]
VAEKNENSDEISFIDLVATIWRQKWLVIVVTVLAAVLSVAYALMQPNMYTATSTVLPISG
SSSLLSQYAGLAAMAGVSLPGSDSSNPSVKIQAILNSRELAVKVINELDLVPKLIKEPEK
LKDVSPLATAVGIFQKSVFSVSVDSKTSLMKVSAKTKSAALSAQIANTAIDLLQQDLSSR
VLTASGKNIVVLEQQVADQEKKVRELQSKMEDYQRKNKLVAPATQSQQGLDLYRSLIQQK
ITLEIQISSLQNALSADNPKVTAAQAQLAAIQKQIDNFERTGGGVGPSMNETPKALMEYA
NLQAELELATKIYGGLLTSLENLRLQDATDKVFVEVIDTAVPPEQKSEPSRSMICVVGTL
AGGLVGLLLAFLREALQKVIADPEVRAKFAKKKEM*
>SPBDM4_v1_50261|ID:27158624|ytfG| NAD(P)H:quinone oxidoreductase [Uncultured spirochete bdmA 4]
MIYAVTGSTGAFGSLVIQRLLDFKVPASSIVALVHNQSKTAHLVNLGLETRLADYDQPST
LDRALQGVDRLLLVSGSEVGKRLRQHTNVISAAKKAGVKLLVYTSLSHADTSVSPLAPEH
KGTEEFLKTSGVPFVILRNNWYTENYSTDLKQARTTGIIEAAVGTGKVASASRSDYAEAA
ARVLIGEGHAGRTYELAGETWDYNALAKAASEVLGRPVVYKTVNAAQRTQSLMAAGLSQD
AAGFVVALEQSIEAGSLAQAGEDLEKLLGRKPKSLTDGLKASLELT*
>SPBDM4_v1_50262|ID:27158625| protein of unknown function [Uncultured spirochete bdmA 4]
MLSQTLKVLEKEGLVHRQEWDGKPPRVEYSLTESGQRISKLAWALSGFLRVASPDFGEDK
SLARIKRS*
>SPBDM4_v1_50263|ID:27158626| exported protein of unknown function [Uncultured spirochete bdmA 4]
MMVFSLSIRRFKLCTLAALMALLAAPLAMFAQERAPASDGLSRVALVIGNGNYEGTTKLN
NPGNDATDIGDMLSRLDFDVDVLTDADLYTMEDAVLRFRDKLSDSPDSVGFFYYAGHGVQ
SNGENYLIPVDARLNSESLLRTRAIPLQFVLDSLGEARNRLNIIVLDACRDNPFSWARSG
ARGLAVVGQLPPASIVVYSTSAGKVAQDGTGRNGAFTEELLKHLPTPGLDITEVLRRTGE
GLQKKTDGIQIPAIYSQFFGFLQLAPGEGAAGTGAGSGPENGSSLDYLPAFFDEAPMDAK
LVIAHAEELTWDGLWLSAWNVLAKVDPNDRDPYILAEKIRTVLDGNTYTDLYQGFWLADL
PPDGDADAAREAGTLDEEYFAFDVHMAVQRMLDRGVEIPPVLASALGDFYYGAYDYCGDG
YVLSAEDTKAEALRWYDMANENYILVDLVSILHYAELLIDAGRADEAVAVLKDETEWEAD
DAGLRLRLVDAYVVTGETDSAFAELDGLIQRAASKDEARDYYAKAIDIAFDQNNRQALDR
YLAAMEANYPDDWLAGLTRHKVAVRAGDNASAQNIADDLLTKFPLHTNVLEGVLTDWLEN
PAGADGGLAFLNRWIGKSKNDALALGVFYLYRSLYRYYSIESSPSSSLKKSSIQNALADL
DSAEKLLTQSPDADKTTVEAIQYIRKELQDALEQ*
>SPBDM4_v1_50264|ID:27158627| ADP-ribosylation/Crystallin J1 [Uncultured spirochete bdmA 4]
MREQKSAGGRPMQDLSGPPPREFSPELLRGILTAYSVGDAMGMPTEFMTRPEIRAKFGLV
DRLLEPSESQNHPNIPRASVTDDTEQVCALLEEYAERDRVDARDTALRLLRWMRESGAIE
KKYIGPSSKTALEAIEGGAAPEDAGLGGTTCGGVMRSPAAVLFAAIRQKPLAECVRACLL
PTHNTHLALEAALGYAYALRAAMRGESVGQILDEMETGAAEGARLAPYPMCGPSLAARVR
HFMARQFQAQSASFSGADEVLDFIYNVYGTTLASVDVASAALCIFLAARDDVWLAIRMGA
SIGGDTDTIAALEGALSAAYALSHDRAINIPRAIVREVTEKNKLDFDSLIERLLA*
>SPBDM4_v1_50265|ID:27158628| Xylose isomerase domain protein TIM barrel [Uncultured spirochete bdmA 4]
MVDTVNRYSFSTAALYPRSASESLRLIAGAGFPHAELMPQCFADASESFAREAERIDIHV
ASVHYPLAMFSMLYNASPGMVAEARAFGVGLVRLCKLLGAEVLVIHPHEVQQDPRLKELI
EHPIRGNILHLAEQCDKAGVLLVMENSPKGVGRTPEGLLSYIESLGAPLVRPAVDTTESC

ESDIDPAEFIKKAHPAHLHLSDHSGDKKHIPAGEGDTNWRAVAAALDACGYGGYYTLEPL
YRYYLDNPEEKLARAHAFISGLIGD*
>SPBDM4_v1_50266|ID:27158629| Extracellular solute-binding protein family 1 [Uncultured spirochete bdmA 4]
MKKSVMICVAMLALVAVAIPVGAQTKLKVWFAVSGDSGEAFKALLEKYKVANPDVALDYS
YSGNYGDTATKVSAALISNTAPDVALMAAGPLYTGARNDFFIETKLNDADLNKDDIYPGV
WDYAKYNGRVCAVPYGISTPVLYYNKAILAKAGIDIKNPPKTWAELLQLAKTAQAKGNVS
GSPDFWGFDATDVAWLFKTMLYQNENTVISVKGTKISPAFNDEKGVEVADFWKKLTDEKV
MPVGQHSNAEKKFLAGNLAFLVATSARIARWSSDPSLQIGAIPMPYFKRPSVALGGNVLV
ILSKDQKTKEAAWKLVKFLASKENQTTFALKTGYLPIRKSGLSLPEAKEAVTSNPMYAIA
FDQLDTSWSYWHFEQMGTMDQLLAQAIDALERNVLTPKATLDKAANELQAEIDG*
>SPBDM4_v1_50267|ID:27158630| Binding-protein-dependent transport systems inner membrane component [Uncultured spirochete bdmA 4]
MSKHLRSRYPFTTLFLFISAALMIFPFAWMLLSAFKTPADVYNYPPKWIPSSFKFTNFAE
VFKKIPFIRFYANSIFVSVVQTVLQIWISAMGAFAFAKLDFPLKKKLFAFMQSSLFVPEI
VTIIPLFLLAAIAGLVDTYTGLIVAELNCAFTTLLLLSFFQTIPNELIDAAKVDGCGYFR
VFLKVILPNSKTAIGTASLFAFLTHWRSYMWPLIITNNVKMRTLPIGLKYLVTETGTEYQ
VLMAASLMAIVPVLVVYMFMEKEFVQSITLTGLKT*
>SPBDM4_v1_50268|ID:27158631| Binding-protein-dependent transport systems inner membrane component [Uncultured spirochete bdmA 4]
MVMKKKEATKVYSLKKEQQRAAAVMMTPLLITLILLFLLPVLLVLIMSFTNWSMTTTTRS
YVGFRNFLFVLNDCRFWNAFGNTLFYTGIKLVADTGLALFIAVLLDKKIHFRRFFRIAHF
APVVVPITASSLIWLWFYDPGIGPLNQILSWLGLPSMQWLYSEKTALLSVILFSVWKGLG
YNVILFLAGLQSIPESYLEAARIDGASERQVFRKVKLPLLAPITSFIVMMGIINCFKVFT
EVDVMTPNHGPLESTLLMVSYIYDQSFTRGKMGRGAAAAIILFLIIFVFTMLQRRLGRKE
VSFD*
>SPBDM4_v1_50269|ID:27158632| protein of unknown function [Uncultured spirochete bdmA 4]
MKVGVIIGSASGKGSAREVITSLAARLQSCEVAVCGGAFGCDGAFGCDELPPSWSRLSPG
LAGALPQAEGYVENLRKTVASLLGWGSDALICVGGDGLASYVLDAMAGLGKKVPLLGIAS
GTINVGPVVAFKTEDIPTLDISRLPLRSIDAVEVLVDGRHLAYALNDVVIGNSFLGTAGG
EVVNLSTRVLLRSGEKLKIDPSPHIAGAAFRLSKNGAPVATSMAQPAQIIVSPLGKREFF
GRAIAGVLCNAAFMDGAAALGLFDTVLVKASRPSRGFGDTARSEHLLFEKGDRLEIVGLA
DDADIIVDGNPYAREG*
>SPBDM4_v1_50270|ID:27158633| protein of unknown function [Uncultured spirochete bdmA 4]
VKKGFYDIVIIAVNKIELVSEYITLTSDAGTVLMFSGLSKDEFVSVEAYSIHYRQVSLKG
SFGYAMHHFKEALSLIDAHKEMFSRIITHRYPLERGKEAFDLLASGDALKIVLKP*
>SPBDM4_v1_50271|ID:27158634| Alcohol dehydrogenase GroES domain protein (fragment) [Uncultured spirochete bdmA 4]
MKALMYNGIGQLALQDVPDPESDFVVKVLGCGICGTDLKTYHKGHHLFPPPAILGHEFYG
LVDKAPRVCGYSVGDMVVVAPYAECGVCDVCQRDAGSLCSHNHYVEDGAFCERVGISLDY
IGRGVFPLKEYYDVYTLVDPLACVLNGVEQLNLKPTSRALVVGAGPMGTLFALLFSARGV
PVTFFDPAVQRCYSIASWGIETCEPC*
>SPBDM4_v1_50272|ID:27158635| Transcriptional regulator, DeoR family [Uncultured spirochete bdmA 4]
MKTGPDISTIYRIARYYYADGLSQEEIAAKEGFSRSQISRLIDCAIVLGLIKITIVPPAS
QHTEELSEMLVEKLGLRTAIVVPVRKSSPQEEITKAIATRAADFLSEELPSFKVVGIGWG
KTVYETAMLLPRRAEQAAKPFFVPLIGLSGDTNPNLQINTIIDRFSTSFQSRGLFVNLSS
VRDKGSALSGIEEQRIHALRDYWSQVEVAVVGLGTTPSSSVSLIDELPERYKDELKKSAS
CGDILAQFFDEKGTIFHSEHEYDLLAFDIYGLPCLRRSICLAGGELKARGIIAAAKARFI
SDLITDEQTAHTILKFLREHSSAARYGAAAKGERA*
>SPBDM4_v1_50273|ID:27158636| putative Fructose-bisphosphate aldolase [Uncultured spirochete bdmA 4]
MTDGLIRNTEKLFDEHGHTFLLALDHAQSGVMPGLEHTPALLSALADAPLDGFILNIGLA
AHLARAPFLRKKLLLRSSLGGSNLADAYAPAHLNHVSPETSLAAGADAVLMMAVIGGEDY
QSLQTLAADIDAFHQYSIPVVVEILASDFSKTATFDVQYHGARIAAELGADAVKAFYVDN
FEKVVDCCPAPLILAGGPKDRDINAVARHAVECGARGFAFGRNIFQASDPLAIIQSLREI
LG*

>SPBDM4_v1_50274|ID:27158637| putative MlrC domain protein [Uncultured spirochete bdmA 4]
MRIVVGSFQQESNTLVSRKSGREDFDIFRGEEMLSHISVTDYFASMEAEIVPTLYAHAVP
GGCLRETDFLAIARDLISCIPSQGIDGIWLYLHGALEVENLGSGELALMRMIRRKVGDDV
PIALALDFHANNTAGLMKLVNVIAGYRTVPHRDMEETELRAAKLLVRCIEGHFLPEPQMA
RANVVAPGDCVLTDEPPLKEIIAEAAALEKTPGMLVCNVFNGQPWVDSPNMGPSMVCIHE
RDKAAAKAAAERLAKMFFEARHEFKFSVDACEPGEALNKAWAENRRPIFVTDSGDNTTAG
SSGDNAFLLKLAQEKGMRGILIGGITDSPAVVQCSNIRQGQSVEFDVGGSIEPSSTKTHI
RGELLYKGDVEGWYGENAGPCVVVRSGGIDIVITARSCAFTKPRIFEGLGLDIMTYRFVV
IKLGYLYPDLAAVAARAILAFTTGSSTERLQDMGMKRIRRPMFPLDDNFTPVFD*
>SPBDM4_v1_50275|ID:27158638| putative Ribose transport system permease protein RbsC [Uncultured spirochete bdmA 4]
MNLQKANRTSPLQILFDQTVLFILFGFTIFMAFVKPSFLSWWNIQNVLIDVSIYGVVACG
MTILIISGEFDLSASSQYMWAQILFVSLLNMTKNPFVSIIITLLSGFILGTINGLIVTKL
HINSFITTLGTMTMIRGLCLVFTSGKMVSTDNSFMKAAGSFEFLGLSSFFYIYIGVLLIL
SIILAYTNFGRRFYATGGNLDVARLAGINTSFQKTAAFSIMGILCSIGGMMLVVQLRAGS
TQYGNDIALSCVAATVIGGTRLSGGAGNALRTALGMLVIVMLYKALVYLGFQAYYQNLVK
GLVLILVVMFDLFMSRRGATAR*
>SPBDM4_v1_50276|ID:27158639|rbsC| Ribose ABC transporter permease protein [Uncultured spirochete bdmA 4]
MSGKPEIENLKARIAKLLRSQGVLIGLVLIIIVASILEHSFLTASNLLNILRQSSALGIV
CLGVTFVMMTGGMDLSVGSIVSLCAVVAVSIMNRYGQGSNSNGTALLAILATIVIGFLVG
TINGSLIVMFNGRLGETFIITYGMEIVVAAIALLYSGGQFMAGTFAPGLYEWLSSGTVPI
IIFLALAAIMHVLLAHTSFGKQVSFIGANMDCARMSGIKVSGIRVLVYGMCGVCAGLASV
IVTSRVYSASPLQGNGYELDAIAAVMVGGTSQTGGTGGIINTILGVLVLSVLGNALNVIG
VDANAQLLFRGAIILAAVGLDVWNKNISMKEAAK*
>SPBDM4_v1_50277|ID:27158640|mglA| fused methyl-galactoside transporter subunits of ABC superfamily: ATP-binding components [Uncultured spirochete bdmA 4]
MGQYILEMQNITKIFPGVKALDNVSFAVRRGEVHGLVGENGAGKSTLMKILNGVLAPDSG
NILVDGRPITFKNTKQAQACGISLIYQEFNLVNTLSVAENIYLGFLQTDRAKFVDWKKVR
TEASELIKRLGFDFNVREKVENLSVAEKQLVEIAKALSLNAQLIAMDEPTSSLTANEIDK
LFYIIQGLKERGITIIYISHKLDEVMAICNNVTVLRDGRIIDTKMVGETNNPEIISKMVG
RPVEMAYPRRPLATNSEVVLEVRNLSCAGFVKDISFTLRKGEVLGIAGLVGSGRTELVEA
IFGARHVQNGEVFVHGHRTKIRSTADGKRCSIGLVTEDRKETGLALGYNVTDNIIITNLQ
KVRAGLLVSKNLIQSSVGRYVDDLGIKTPSIRQTVVNLSGGNQQKVVLAKWLFSDVEILI
LDEPTRGIDVGAKYEIYLLMNALVEKGKSIIMISSELPEVLGMSDRVIVINEGRKKGELQ
GQAMTPEAVMSIAIQ*
>SPBDM4_v1_50278|ID:27158641| exported protein of unknown function [Uncultured spirochete bdmA 4]
VKKVVVLVMALTIAVGAGFAQGTKPLKKIILSNAYYTAPYCAAFNPAAIAKAKELGYDLQ
ILDGEGNQQKQMEQARLAISEAAGFIYFPADVAGSVPIVEALNKAKLPYVIVNNYTLDAL
KANNVPCYVGTNVKQHGHNMATLVKQLLPSGGNVVAIEGTAGHAQTIAFNEAFDEDFKGT
NIKFLDKQPADFDADKALNKMNDLLTKYGNKIDVIISHDGGMLAGIISALRAAGKLGKIP
IVCAGSNKIIYDAIKSGYVYGTSTQDPGVEGALAVSTLIDIINGKKVPQWVELPIKPATK
ATIDNYNWF*
>SPBDM4_v1_50279|ID:27158642| conserved protein of unknown function [Uncultured spirochete bdmA 4]
MSIGKKRWVFADGDLPPMPEGLDGPKAHEALMVVNNGDEEARLSVTLLFEDSEPKKGFVL
IVPPRRVKCFRMDQPIWGTDYVIPFGQYAFIVESDVPVVAVFGRLDRRPGMAYYPVAPFA
E*
>SPBDM4_v1_50280|ID:27158643| conserved protein of unknown function [Uncultured spirochete bdmA 4]
MAYFDLNETKARLEKVRAILRNKQLDAALIYYDELNVANGWYLTGWCPQFEKGCILVPAN
GEPLLLGGPESEPFAKMSSAIPDTRNFPPFMVPDEEYPNAKISTFAMLSEELEKKKLPMN
TIGIVGTASLPHKVYEQFVAGFAGIHLVDITDEYERLRSVKSPWERTNIRKSFGLTYKAY
EVMKQKIAPGVIEYEVAAEGEYVCRKAGANNFAFSCIVGSGERSNAVVPTAWNREMKAGE
LVMIGIAPRFSGYAGVMGDTLPVSRTYTERQKECINHLREAFRLTRAMLKPGMKGREIDV
PARNYFVKHNLIQYLVCPFAHTIGLMEAEAPFFGPNSDDVLVPGMTVCVDVSFFGHPEFH
GARIETGFEITENGCTPLCPEMNDLFSQNLK*

>SPBDM4_v1_50281|ID:27158644| putative Ribose operon repressor [Uncultured spirochete bdmA 4]
MKPKRITIKYIAGQANVSFSTVAKALHNDPVINEKTREKVQKIAKELNYRPNVLAKGLRN
SKTKTIGIILNDLQSPFYSEIYKAIGNVINKRGYTMLLSDSSYDEELEMKNVSTMISQGA
EGIIISPVTRDSASIRALIDDPIKAVFIDNRPSNPNINCVYVDHEEAAKIATEYLILAGH
RNILLLNGPEHLPSSQDFRKGYVKTLIFHGIEVDERLIKNNPISIEKTCIQMRAIFSGED
LIKRNEFTAIVCLSDVIAIGVYESALQLGFGIPDRYSLIGYDNILATKYLNPPLTTVHQP
KEQTGLYAINLLLDEIENSHNEHRQIILNPVLINRASVRDIRQSL*
>SPBDM4_v1_50282|ID:27158645| putative Transcriptional regulator, LacI family [Uncultured spirochete bdmA 4]
MTVSRVVHGGTSVKEETRKRIVEAMIRLGYVPSAAAQALRSNKDLTISPSSLFALIFGSG
TEYSVSFFHDITRGVEKAALELKLCPVYISIYKDMEELWMRLQGLFSAYSLSGALLVGQF
TPEIIQFIRENVKNIVMVDGPAQSGEGIGVVESGNMEGSLLALDHLISLGCRKIKIITVE
QNHYFARAMTMAAKIRRSKDVDIELWYNCDSCEKAEELVMTNWNNGNHFDGLYSNDDFAI
GAMKALQTLNIKIPKDVKIVGFDDILYASFTVPPLTSVRIDKYLLGSEAVHTLVAISKAS
NKAENITKVIRPSLIIRGSA*
>SPBDM4_v1_50283|ID:27158646| conserved exported protein of unknown function [Uncultured spirochete bdmA 4]
MRRKGLFLLIVLMIMLIGVTNVFGQATKPQDTQTVGPQGEKPTWYSDLKLTDAEKETIRK
GHYKAAYLLHTTSDFTNALLAGAKAAFKDLGIDLVVTTDAAMDSTKQKTDVETALALKPD
IILTLVIDPVSGAEAFRPAVQRGVKLVFMSNLPVGYVQDRDYVSIVTDDLYGMGKAAAEM
LADSMNKKGNVAWLYHDADYYVTNQRDKAFKSVIQSSYPNIKIIAEKGIANPADAESIAS
ALIIQHPEINAFYVPWDTPAEGVVAACRAAKRPDIKVVTLDLGANNGLDMVKSGNVIGIA
ADLAYQLGYTKAICGAYGLLGKKAPAFVIVPAIKVTKKNIVEGWKQSLNVEPPKEIMDFL
SKK*
>SPBDM4_v1_50284|ID:27158647| conserved protein of unknown function [Uncultured spirochete bdmA 4]
MVLDSPVLEAVGICKAFNKVSVLSNVDLRLYPGQVHAIVGQNGAGKSTLMKILNGVYRKD
SGEIKINGQSCSYNSHAEAARQGISMVFQDFSLVPSMKVYQNIFLSQVMSRKGGGFFDER
TMKEKARELLKEVGVDINIDVEDLVENVSVGSRQLVEIAKALCLESKILIFDEPTASLST
AEITRLFFTVSRLKSRNVAIVYITHYLQDVFKICDSITVLRDGKVSLDDAIANVDMGKVI
HAMTGKVISVEKSARAAKDSSQPEVLRLESVSTKKIQNMSFTLRKGEILGLAGLLGSGRT
ELLRALYGLDKIISGEIFVDGKRVNIHATRDAKKSRIALVPEDRRTQGLIMDFSLYDNIV
VGVLPMLTRHGFVQKSKGESVARSYISDFKIKANGISDGVRFLSGGNQQKVVIAKNIADQ
PEILLLDDPTFGIDIQSKTEIMEIIRKYVEQGNAAILVSSEFNELIELCDRILIIKKGTI
SQELEVSAQSAEEALLQLAQ*
>SPBDM4_v1_50285|ID:27158648| conserved membrane protein of unknown function [Uncultured spirochete bdmA 4]
MKRFFHGISVGKIKNYIVYISFACIFAFFAIMLANRGFLTGANLMNIARQTTMISVMAVG
MTFVLSAGEIDLSIGSIVALASLTTALGLRNWGLIPGIVIGLLTGFVFGTINGLFVAKIG
VPSFLVTLGMTGIITGLARWITNLQSVPIVNDTYNFIFGSGDIGPVSILFIWTVVLLVIG
QIGLKKTKFGWSTLAVGGNKVSANYSGISVNGIKMKVMVLNGVLAALAGMLYAGRLHGAR
YTLGETDLMTVIAAVIIGGTSMAGGKGSVIGSVVGSIIMGMINNGLILMGLSVDQQMIFR
GIIIIVAVSLTMGEKQS*
>SPBDM4_v1_50286|ID:27158649| conserved protein of unknown function [Uncultured spirochete bdmA 4]
MGDQNRISQKAHARYREKAFSKKTRIAVAIAPLIGQYPLEIIRTAIPGCLLLPLPFENFE
ASMKDGNIEADLAVCIEDKDGLVPEGAFSVKTGLDWGIPLVVVSGAGVAGAIYGVRELFE
RQIRKNADGGFILPSLEIEEAPGLPYRIFWTWDHSTNWYLNQTGLQDIGAMNYYLKPKSG
FLEDYKRLVDFMSKNRISAVTIYGFLRDNHGGVEAAKELCMYASQRGVRILPGVGINAYG
GIYWEGESPYNLSNWLRKNPRLRAKLGTRAAFHIADIPELWFPENQYIDAACPSKPENAA
FHEDAIAWLAETFNIGGINFETGDYGTCHCDECTARRKENTTWSFGDMALLYPRLFSAAK
RSGRDLWLVVEAYWDTILDARSLAPLSSLPDEAIYQYCVNRSYWPRLRNELTKEYVKTLP
RSKNIFRTHMGSQWNGERYSLVADRFANMASLAYQTGMKGMTIFGEVSSFNPANEINYLA
FSRFSWNPLLGWDDFLRNDLAPILGGVSEAFKFLELLEVKEDRASLLRALNEAREIARLT
NGDVYRRWVQLQDRLYMKSEMLGTA*
>SPBDM4_v1_50287|ID:27158650| conserved protein of unknown function [Uncultured spirochete bdmA 4]
MKTLQDCLREEKIEVDEAIIATIASMDIEHKAGQLFMLAFGGDDPKRACRMIENHYLGGC
YISDANASTPEKAALLSSALQRCAISSSGIPLLLGVDQEGAWGVMMSASTTGPGNLGLGA

THDPGLTWAMYGVIGEELEAVGYNTLLAPCADVNSNPANPIIGMRSFGGDAAEVARHVKA
AVHGASETGVITTVKHFPGHGNTSEDSHRGIPRVDRDMKTLFEVDLAPFAAGIQAGVDIV
MTSHILYSAIDPAMPATLSRPVITGLLRGQMGFKGVVLSDSMNMRAMRRSYEPGRAATLA
LAAGVDMIMLAEEHYDHDAEKYEIVQRATIQGVIDAVKEGYLEESAVDMAVYRILKLKKR
HGLLGRQNFILKPETVGSQAHRAVEEAVCSRTICVMRNSNECWPILPDSSVALVRVVPAS
AYAILTSTRGIGPNQEVSAFDTFRESFLRIHATTRVIDFYGERNSMPVDLSILEKFDVIL
AVTEDYPLPGVDFDTASQKRAVLRLSGKFGHKLAVVGLRTPYELAEYPKVKAYVCSCSSR
PCAAKAAAYALLGIVEAVGISPV*
>SPBDM4_v1_50288|ID:27158651| transposase (fragment) [Uncultured spirochete bdmA 4]
MLPMVLGYVEGVTHDYIRHGTITLFTALNVLDREVLAACKPRHRHQEFLSFLRNVENSVP
PDLDIHMVVDNYVTHRHPKVKAWIAARPRWHVHFIPTYSSC*
>SPBDM4_v1_50289|ID:27158652| transposase (fragment) [Uncultured spirochete bdmA 4]
MVLDGASSHRSKDLVIPENVALMQLPPYSPELNPTEQIWNILRRSYFANKVFDSLDAATT
QAESEGIYLSDQILHRFE*
>SPBDM4_v1_50290|ID:27158653| conserved protein of unknown function [Uncultured spirochete bdmA 4]
MTLHQKIVKPKVWFLELGKQLRNVAEECRIMGYSRDAAIEETLIEMTFEYPAYGQVRVSN
ELRKEGIFISSGRVRCVWRRHDLEVFQKRLTLLEAKAARE*
>SPBDM4_v1_50291|ID:27158654| protein of unknown function [Uncultured spirochete bdmA 4]
VYQQTYLDTYCNYAIAKLYTNKTPLMAADLLNDRVLPFYNANMRYHSEFYQVAFRKKVYA
SLESL*
>SPBDM4_v1_50292|ID:27158655| Threonine synthase [Uncultured spirochete bdmA 4]
MDFICSRCGKKAPVTTREALCECGGLWKLDFQPPLFDPDLIDRREWSLFRYRAFLPLEEL
EGAKEENAADTRGPARAARAQHDGWRAVSLGEGMTPIVRLDEDALLKMDYFMPTLSFKDR
GAAVLIAHCRAIGVDSVIQDSSGNAGNSIAAYSARAGIQCEIFVPEGTSPAKIAMIRSHG
ARVTIVPGSRDQCAEACRAKVAQEGIYYASHVYNPFFYEGTKTYIYEVWEQLGRIPAHLF
VPLGNGTLFLGMVKALEELLAADIIAKMPQIIAVQSEHCDPIARAYEDGATSPAHITPLP
TLAEGIAIGGPMRGEEILDYIYKYKMKVITSPESRILEARAALAGRGIYCEHTSAATYAA
YLAWCEKHGRTYDSLIPMCGAGLKSDQ*
>SPBDM4_v1_50293|ID:27158656| exported protein of unknown function [Uncultured spirochete bdmA 4]
MRKSYVRVMERALLLAVALLVSACSASIQNMSLEQPHNSDAESKAVELTPSDPIEYDTSM
PSLPVLQSVGSGTAAPVLILLYHNITTLTDPGTYDRRLQDFDNDLKYLQDNNITIIRLPD
ILKIQSGEIVPQEGQRFAVITFDDGYVSAYARAFPILKKYNMPATFFIITSFVGTSGYMT
WEEIEEMASYSPDNGRQPLYEFGSHTVDHRSLAYDASAFPDRNDYIMFLNMEMNQSKNAI
LQHVNQTDIFLALPYGDGAYEQEVTYAAIRTGYTGLRTSIYGAFDAYNTNWNYHLPSLAI
LGSTDIGVLPDYYEQLLPETGMLMLSVR*
>SPBDM4_v1_50294|ID:27158657|bfr| Bacterioferritin [Uncultured spirochete bdmA 4]
MKSDKRIIDALNGLLADELTAINQYMVHSEMCDDWGYEKLHKNFEKRAIDEMKHAEKLIG
RILFLEGTPIVSELKKINIGSSVDKQLANDHAAEEIAIKGYNEAIALVAGLKDSATRDFL
QDILNDEDAHMDNIEALQSEIEQMTLPTFLSTQVEK*
>SPBDM4_v1_50295|ID:27158658|uxuB| Uncharacterized oxidoreductase UxuB [Uncultured spirochete bdmA 4]
MTVYLHGNTAVVTGGSGVLGSAMCVGLAKAGAQVAVLARGLEKAQHLVDTIEAEGGTAMA
VSCDVLDAEAVAHSAELVTKTFGRCDILLNAAGGNNPKATTIKERLDAEDIERIMSPATG
NREQSANASTFFDLDPDGVRSVFDLNFMGTFIPTQIFASHMMGMSVATIINMASMGSYAP
MTKVPAYCAANAAIVNLTEWLAVHLAPAGIRVNAIAPGFFLGEQNRRLLQNEDGNLTLRG
QKIIARTPAGRFGVPDDLVGTLLWLCDATASGFITGTVIPVDGGFNAFTGI*
>SPBDM4_v1_50296|ID:27158659| Mannonate dehydratase (fragment) [Uncultured spirochete bdmA 4]
MGRIPFAHVRNVMHTSGQGSHTTFYDAQHLSSMGSLDMYEIMRAYSDIDFEGYIRPDHGR
MIWGESGSPGYGFYDRALGVSYLLGLWEALRKGKGMRA*
>SPBDM4_v1_50297|ID:27158660| Mannonate dehydratase (fragment) [Uncultured spirochete bdmA 4]
MNMVFRWFGEGFDPVPLEYIRQIPGVSGVVPMLSDIPAGEVWPLEKILAMKHRIEAYGLA
MEVIESVNVHEDIKLGLPDRDQYISNYFATLENLGKAGVKVVCYNFMPVFDWVRTDLSHT
LDDGSKVLYYDSAFVDGVDPEAFAKRFNAGSNGFSLPGWEPERLETLRRLFEQYKGMPEE
ELWRNLEYFLKAVIPAAERAGIKMAIHPDDPPWSVFGLLRIIKNE*
>SPBDM4_v1_50298|ID:27158661| Oxidoreductase domain protein [Uncultured spirochete bdmA 4]

MTKIGIIGAGAIASVHIDSYNRLSEHAKVTAVCDTFRQKADGLIAEKALDATVYDDYEKL
LADPVIDAVSICLPPNMHCSVAVIALKMGKHVLVEKPMASSLEECDRMIEASRVSGKILS
VVSQNRFNTPFARVKNMLDIGVLGSVVYANFESLWWRGQLYYDLWWSGTWEQESGGCFMS
HAVHYLDLMHMLFGMPKRVQSVMSNVVHDNSECEDLGFAVFDYGDKLVQFTCSLVSHGER
HGISIEGEKGSISIPWAVSASRALPNGFPEEDTAQREKLEQTYHSIPELPLEGHDAQISN
FLKAIRNEEPVVVNGSEGRKTLELVMGIYNSSVSGTSFAFPVATDDAFYRKETMVAAMPK
FHEKKKSVDNFSTSKITLGSNMGR*
>SPBDM4_v1_50299|ID:27158662| TRAP dicarboxylate transporter, DctM subunit [Uncultured spirochete bdmA 4]
MWQIFFVFAILLAFIFAGFPIYMAIILTTISFMVGMHLPLNLVVIKMFGSVNSFSLMAIP
FFIIAGNIMTKSDITDKIVDLSDSLVGQLKGGLGHVNILASMFFGGIQGSGIADASAIGG
MLIPAMEKQGYDKDYAVAVTAGSSMLSPIIPPSIAMILYSYYTEIPVSKLFLGGIVPGFG
IALLQMIWNRIEYSRRGYNIPTHTFSFRRLGHTLAHSIGALLMPFIIIGGIISGIVTATE
SGVLAIVYGLVYGFFISKKLKLRDLVGILKDSAVTTAVVMITIAATGALSNVLVRMRFQS
NVLIYVVNTIGNPVLGTLFLMFVIFFLGMFLDPTVLIAMLAPTILCIGNAFGFNPIHYGV
VMVIMMHVGAITPPVGSFLFVSCAIGNLPIEKATVPLIPFIVTVLLTILISFFVPQIVMF
LPNLL*
>SPBDM4_v1_50300|ID:27158663| Tripartite ATP-independent periplasmic transporter DctQ component [Uncultured spirochete bdmA 4]
MAKTFYVWFDRIRTVMLISISSFTVILCFIQIVLRYFTFLSLRPFAWGDEVLRLCAIWVI
FLGISLGVRKNAHFRVDLFLNKIKSPKARQVVDTIIDALVMMVLILLTYYGGIYAMSNIT
SSLQNIDMSMAWFYAAIPVGCIFEIIEYIYKLAYGKDYPEKMFPQKQRVTLANSSTK*
>SPBDM4_v1_50301|ID:27158664| Extracellular solute-binding protein, family 7 [Uncultured spirochete bdmA 4]
MKKKTLVIFLIIVLGVASVYSQGKVYQLRTSTNLAGTGTIGRGLTYFVDLVNKKSNGRIK
ATANYGAELGNQSEQVEMCRTGSLEMVVAAPGTGPGTWVPQLMMFEFPYLFKDNDHYRRV
LKGMEGEVSRLVQPYNFVAVAGQSQGSRDMLTKKPVEKLEDLANMKMRGPNAVYISMFKA
LGAAGTTMDWNEIYTALQTGVIDGMEASPSMINSMKFQDVAKNLTITDHIIACVYYFFND
KWLSSLPSDLKQLVLDCAQEAAQYQAKIDDEDQIASLNKMQAGGVTIHQLKDKDKWVAAC
KPMLNEYRAKGKEWNDFIDKLLAIQ*
>SPBDM4_v1_50302|ID:27158665| Transcriptional regulator, GntR family [Uncultured spirochete bdmA 4]
VDIPSRLPKENAREYAIRILKSNIISLELAPGSVISENEIASQLGLSRTPVREALIELGK
ANIVEIMPQKGSRVLLIDYSLVEESRFLRLILESAIIKEVCELKEPQDYSILQENLTLQE
FYLKNPSPSKLLELDNQFHRELFRLANKMRCYQWMIDGMEIHFDRVRSMSLSTIKDIKIV
EDHKAILQAIQKKNASQAVAAVTKHFSRYKIDEQAIRTKYPDYFLS*
>SPBDM4_v1_50303|ID:27158666| NUDIX hydrolase [Uncultured spirochete bdmA 4]
MKNPEPWIETSSREIADCKIFRVRESMRRTRDGTVDGAFYLVDAPDWAGIIPVVDAPEGR
KFVMIRQFRHGTGKISVEFPGGTIDPAEDPCKAIARELLEETGYRAEMILPLGDLSPNPA
FMTNTFHAFIAEGCYLDSMQHLDAHEEIEVVLVPERDAVDMIGGEEYGHALMTATLFFYM
RYRGYSD*
>SPBDM4_v1_50304|ID:27158667| Glycosyltransferase, MGT family [Uncultured spirochete bdmA 4]
MGTGVFFNLPAYGHVNPTLPVVRNLVARGERILYFDTLEFKEKIEATGAEFRDYGFGMPA
VAENRRKGGNFVQLAGVLLGAAEKGLPHYIEEVERLAPDYIIHDSMCPWGKYIAGYLGIR
AINTTSTFVFTKGTLGKAGGFGGKVVKMARESGAAGVSAVLRSKKKLERQYGLKIGIMDI
FRNTENLNLVFTSRYFQPGGDDFGAEYEFIGPSIAERNDAPGFAFDFAAADPLVYISLGT
LENDRRDFYVRCIKAFAGMGVHVLISIGRNVKREELGSIPPNISVVEYAPQLEVLKHAQL
FLSHAGMNSVNESLYFGVPLLLFPQQQEQVMVARRVEELGAGICMPADGADSGDIRAASR
TVLESRNFYENARKVSDSFKASGGYERGAELILRYVSTG*
>SPBDM4_v1_50305|ID:27158668| putative PEGA domain protein [Uncultured spirochete bdmA 4]
MRKKILLGVLVFLVLGGLTFAQAKVSLTIQCNQSGAQIYLNDNLVGYTAPNFSLSIFPGT
YTIRVVKDGFPEFRTKVVAAQSPITIVANLGGATPQTPQAPAPGPIQLMPPTPSSPPPKP
PTPPSPVWQLSIDANINGARVYINGSYAGTTPFAISLRPGTYSIGVSLGGYEDYTTTLRL
NGPYHIYARLSPLQLPVYIDAVNTPGANIYRDSAYIGTTPYRGTWPRGTYVVRITAPGYA
DYVERVFVDGSTNLQVSLSPLPVEYEIKLPDNFSNSWEKPGKFNEMELFIDGVRLNRLRG
TIMPGAHTLTMTYRDLRFENEFTVGPGKPATIELFLGVRVY*
>SPBDM4_v1_50306|ID:27158669| exported protein of unknown function [Uncultured spirochete bdmA 4]

MKRRLKTVLIIGVALALLLSACTIVIGYSRVLYDDNGSNSGSVPIDSSRYRYGDTVLVLG
NTGNLTKNGSLFVGWNTEPDGSGRAFSPGDAFSIGSSSVVLYARWMPWTSIPGEWTLSYK
WDTDPQWRSAIWTIYSDKTFTDNYGGAGVWDISGNMIQLLYNNGTYFYSYSGTLYTSSNN
TWNSMQGTMSGPDIYGATHNGTWSAERGVKASRAPELPSGGLTPSGEPVK*
>SPBDM4_v1_50307|ID:27158670| conserved protein of unknown function [Uncultured spirochete bdmA 4]
MKKIKEAEGRGRPRDVTRDTVILEVTIQGLVELGYERLSINEIASLAKASKSTIYRRWSS
KATLVADAVAYWMEKSVPLETPNTKSLTDDITLIVSVVPDGDAFRNAVTVFLDLASVGIR
HEELRSAISHEILEGRRQQLREVINNAIARGEIAASAKTAQIPNMVIGLILVHMLTDRLC
GRDDIHRLVESVIEPLRKKS*
>SPBDM4_v1_50308|ID:27158671| NAD-dependent aldehyde dehydrogenase [Uncultured spirochete bdmA 4]
MTNSDPKKMQHEERLLINGRLTHAKAGGTFNNINPATEEVMGVVADASAEDIDQAIAAAR
KAFDETDWSTNHAFRKHCLEQLHAAIMEEKELFRAELIAEVGCPVTLTFGAQFDWPISDG
LLWPAHMIDTYPWYRKVSDQHILGGRPTYRWVWKEPVGVVAAIIPWNYPFEILINKLGPA
LATGNTMVVKAASMTPWNATRVGRLIAEKTDIPAGVVNVVASQNHQVAQMLFSDPRIDMI
SFTGSTTTGRMILEQSAPTFKRTLMELGGKSATIVLDDCNLSAVLPQAVMTAFMHAGQGC
ALPTRLLIPESRYDECISTIAAVVRMVGCGDPNDVGTTVGPLVSEKQRDKVLGYIEKGKA
EGARLLAGGGRPKHLKRGWFVEPTVFVDVDNMSTIAQEEIFGPVLSVITFKDDEDAIRIA
NQSKYGLSGFFWSASRERALKVARRVRTGSMSINGGQFYGAESPYGGYKSSGIGRQGGIE
GMEQ*
>SPBDM4_v1_50309|ID:27158672| protein of unknown function [Uncultured spirochete bdmA 4]
MKDTTDMWGYRGKTVVIDGAASGMGMAATKILIDLGAEVYALDIKE*
>SPBDM4_v1_50310|ID:27158673| protein of unknown function [Uncultured spirochete bdmA 4]
MKRGEGAIAIISSMAGMQWRFHGLYYQGLIDTKTFEEGVAWIAENGVKIKTEKGGSYSFS
KECISAYGKAKAAELAPRGIRFNVLNPTTTVTPMMKDFQKNVGEMLNNYKVSRFATAEEM
GYPLVFLNSSMASFISGQELTVDEGLSAIMELAPVTQS*
>SPBDM4_v1_50311|ID:27158674| conserved protein of unknown function [Uncultured spirochete bdmA 4]
MYRKTEFLAALRKRGLDESAIQTHLQAIAGLDSSLAARGLSLERPKLAAVEAWLSERLRE
GESIYDFLIPAARYFTAARQETVAIRLMAYLLPIGVLPAMADRLTTLEGEAVRSHVMSGV
TIPAEGSPPEAYPDATARFVEALEAEIGPEKARRVLAWNVHEIPTAAFALDRERFLDLGS
IDAWLLDHHRRQVEILRRHAKDGTLWFEQRITQAVVDFVEARPEILGGVREGDIIYTTKI
PYDPDGYLQATDGLERRRLACHCPLALASITERDAGVPPLWCACSAGYTKFMFDVVFGEE
TETVVLESVLSGSERCRFAIKIPPSVSLGA*
>SPBDM4_v1_50312|ID:27158675| Transcriptional regulator, GntR family with LacI sensor [Uncultured spirochete bdmA 4]
MSYKKYEMVTRGILGKIESGEWSPGTQILAERKLAELFSVSRITVQKALDDLVERNILER
PAGRNGTFVRSDPMVSQRTTARKESRLVGVAIDDISDSFGAHILRGIEDCLWNRRYHTII
CNVDRNFRKVEDYFFSLSEQNIDGVIFSPVIESEAYEEKNRSIIERIERQETPLVLLDRT
IADTRKNFVSTNHREASRRLTADFIARGNHRRLILVTGVSCSSIAEREEGFLDAVRAAGL
DPGEQHIVRLNDNLLFPELQPNSPYIEEIRRAIGDVDGATGFVALNGRLLRGASLALCGM
GFPLGRFTRSDLHGQFAVPLDGTGDTLVSVQPAYQLGYEAARLLLHSIETPGSATMQIRL
DADITLFGE*
>SPBDM4_v1_50313|ID:27158676| LysR substrate-binding protein [Uncultured spirochete bdmA 4]
MKRTFILVLLVALAGLMPLAAASNTVTIYTMSGPEITTPILDALRAKYPSINVNLVKAGT
GEIVARLQAEKNNPAADILWGGDNLAVFDGNSDLFAAYDSPEDKYMVSRDPNHKWHAFTI
FCQAILVNTNLVKPADYPKNAKDLLNPAWKKAGGVALADPNKSGTGYTIVSGLSSAFGWD
FIEKLVQNSVVLPGSDPMFKAVKDGELPVGFINEDLGATWKAQGLPIEVIYAKDAVTVQM
DACGLVKNGPNPELAKKVLDFLCSKEAHTIAVKVINRRSARTDVAPPAGLPDLGNLNLFT
ATEPREVVNAKFTKIYGK*
>SPBDM4_v1_50314|ID:27158677| Binding-protein-dependent transport systems inner membrane component [Uncultured spirochete bdmA 4]
LKFSRIHAFKDPWKFFTLIIFVVLTVLTVVSQLWILKTSVQEKGGAFTLRTGQGDRPAEI
EGETLSGFTLRQETPSVITLARSGRDFFSIDSSVSGQIVLSSQSLTFGEPALDEDGKLKI
PYGTSTGGDPARRSGTGSLQIEQKVFGTLFQSSAGKRLEVECLSPGSLRIYHAPKAYLNF
SDSSVRFGLANYITFLTTGKYLDAIVNSLLIALTATVVAGVLGTTLAYLYARYKMPMTSA

VISLVTMASVSPPFLGAYAWRMLLGSSGIITRFLGINWTIMGMHGVIWVIIWLIFPVVFL
LSYDSFVSIDNSMRECSYSLGADRRRTLFRIELPLAIPGIVTGLYMAMMTAFTDFGTPYI
ISLDLNTLPVLIYKEFMSEVGGNYSIASTGSVVMVLLSSMILMAQRIYLAKRSYASIKTQ
QPSARMPSAGKKVAIEILTFVLIAMAFVPHVTVLVTSFFKWEAGILSSRLTWENFSRLFN
LNLNSIWVTLSTGTAATFLTFSLGIGIAYVIVKKRYPFFGQALNLLVMIPYIIPGTVLAI
GFILLFNKPPILLTGTWLILVLAYFVRKLPYAVKSAESALYRIHPALEEAARSLGATPTK
SFWDVTFPLMIGGVISGATLSFLQIMTEISSTIMLYRPPWKPMTAVIFENTIDAGADFGV
ASAMTVVLMVMLYVPLYFITTKTRKPKETRIEEL*
>SPBDM4_v1_50315|ID:27158678|fbpC| Fe(3+) ions import ATP-binding protein FbpC [Uncultured spirochete bdmA 4]
LKNYERSDDAQAVSVTIENVVKKFGSFQALGGVDLSIRSGEFFTLLGPSGCGKTTLLRCT
AGFERPTSGRILFNDADMTHIPPWEKNIGFVFQNYALWPNKSVYANIAYGLEIRKKNTSF
IREKVEWALDLLGLPGIGEKMPEQLSGGQQQRVAIARALVIDPAILLLDEPLSNLDAKLR
ISLRFQIREIQKKLKITAIYVTHDQEEALEISDRIAVMNAGSVLQVGTPEDIYERPSQRF
VADFVGKANLVPGSREGDVFVCSDRFKLRLSPEDHERMAESQDAEIMFRPEALREIDGRG
HADIEGRIIDFLYHGNLRRYMVDAGCDAPVVYETNIPHTVGDPLRLAITQYRLYA*
>SPBDM4_v1_50316|ID:27158679| Glycogen debranching protein [Uncultured spirochete bdmA 4]
MKKSEFFSRLPAMDAQFGNLILIRDKADRKDVPGAQECFLSSVVQFQPLARLGICGPAGA
IPLAPVSSSSDPIRARKVFEGGGLRLFQELVVSGDEASLALKIEKMGEGGGGPSGDTGRS
VFTLTVDGACLFSPQGVPYHWRDESLLSVRRAGGEENGLDGEWSYGRLPFSLRSSAHFDN
FKIRRVADDEIIRTHYFTSFNLKKRYTYWVEAEGGTSAGAAPSADSTVGNGASPVTARNV
GYEASIDLGDLREAREIVLAFGIGGAAEKTEASAGRHAELIRGVEADWDGFLEGVPEFRA
GDKALERMYCTSWFILKANRVHFDDPRFPYPFTSVNKHHYYNQFFWDSAYQAIAWLWYNR
AEPAQDELRNFVTNQWRNGMIPYELFMYPVNGREWMDGDGTSTGMTQPPVIGIALSEIYK
KYGSSEYLRFFYEPLLRYEDWLTLYRDLGKRGLSCYLNIWETGWDNSPRLDASARNRVLD
PAIESADFNAYIYYMRRTILEMAAALGEKVPPRVSGRMEATKRAMNTLMYDVADGFYHDV
VAGTDERIPVKTAAGLIPLVTDIPDRAARGRIVAEYVESEKEFMTGCPVPSVSRSEPTYN
PVDFWRGANWPQITWTVIFSLADSHPEAAGEILRRFLASTKGNSTCYEYYDAETGKGAGM
PFQGWGALYTDMIMRFVAGIIPENAGMRFHPLSAEIGDFSVDNVRLKDLALDIGRRGDTW
TFAFHGFGGFAMEGVHEFRALPSADGARLKIKFGPGCDRNLVMPLPGENRLEFRFV*
>SPBDM4_v1_50317|ID:27158680| putative Glycerophosphoryl diester phosphodiesterase [Uncultured spirochete bdmA 4]
MELFNIYDEHRATPLITAHAGCQGTEPNSRESIVAAFNSPADIVEVDIRASADGVIFLMH
DNVLALDDGENVPLGNLSWNEIVHTLDSRQQRVILRFEAFCDFVDELEQGFPRTTAPKRP
FFNLDIKDPAALPATAAIARSREIMPRVIFSGLDEEGIALASRRIPDLSYLFNADGMLPG
REKAGTEEVGMKEACSLALRCGCKGINLEWTLASAALVHHIHERGLFVMLWTVDDMPAMR
CVLEYGPDSITTHYPDRLALLLEDRRKSRPGTR*
>SPBDM4_v1_50318|ID:27158681| Binding-protein-dependent transport systems inner membrane component [Uncultured spirochete bdmA 4]
MASYVTTKHSPLFPILMIVILLITLFPIYWIVVTSLKTPVENILPVPTLYPHKFTLDNYQ
KVLLGGSLRNLFNSIFVTAVSTLLSLAIGFLAAYALVRYKFPLKFNVVFLVWIIIVKMLP
PVVLAVPLYEMFSKMHLINNLWGLVVVFQVYTLPYCIWMLFGFLKTLPLEYEEAADIDGA
SRLQIVRLVVFPLVQTGVVATAIFSIIVAWDEFLFSMLFIRSPELQTLPLAIVGYIGEFE
TLWGQLMAISVLAIIPIIIFVRFVYREMASAYSLGLK*
>SPBDM4_v1_50319|ID:27158682| Binding-protein-dependent transport systems inner membrane component [Uncultured spirochete bdmA 4]
MKSIGKTRGKTLFFWSILLPALIVMLWLTVYPMITVFLTSFQNYNYISGRRRWIGLGNYR
NILTDQVFLTSFKNTIVYCFSTTFLEVFLGTALALVFYGNFRGKSIFTILLILPMMLSTM
VVSSVWKTLFHYDIGLLNTILSQIGLSRVGWLIDPKMALWSLVLVDVWQWTPFAFIVMQA
GLSSIPQEVFEASVVDGATYRQRLFRITLPILRDQILLLCLLRTIDTFKIFSKVFALTGG
GPGNATETTSFFVYREAFQYFNLGRSSAASMITLVAVVLMSIFYLRKMLNPREA*
>SPBDM4_v1_50320|ID:27158683| Extracellular solute-binding protein family 1 [Uncultured spirochete bdmA 4]
MTKKTALLLLSLCLVLSGVSAQKQVKLVIAGRDGDYGNAMQVAVDAYMAKNPNVKFDVLK
LSGDDVYQKTVIDLKSGTGTYDLILIDDPKVLQYQRAGWLTDLDAMFKKSGKTVDSDFIS

TVMDLCRYPNNAKGKLYSLPFVGNVSLFAYRADLFAKYGLSAPKTWDDVLVAAKTISEKE
SGVDGISFRGVKGNPILTAFLPIFWSFGADFTDASGNPTVNSPEAVKALKFFLELAKYGP
KSTPMNNTAQVRDGLLAGTVGIAPEVWPAWLGKIDDPNESKVAGKVKITKHPEEVKKSSP
MIGIWHIAIPAASRNKDAAFDFLTFLTSRETQKLMVLKAGLPPSRVSVFEDKEILAKYPW
YPAISDALENGVARPRTLYWAEIENTMGGLIQEALIGQKTAEQALNEANAKIAEILKQ*
>SPBDM4_v1_50321|ID:27158684| conserved protein of unknown function [Uncultured spirochete bdmA 4]
MDTEKRASKHRLKQKDLVALLLALFSEEYDKAQVDSITDILLPMSAIKTYLQSKVGISYT
SDAWIITQVRKYEEEIDTVLFRKTASQEGAALGLCQNISTYEQKRHLYVTQKIRTANGVF
DLMRNSVEKRPSGNTISILLEAGSTVTRVAEIIAQNLNSLPLKWDICTHNLGVIECFGKT
SPAYKNVSISVPQGKFDPVTNLILSDKLELYMDREFDWIIQGTSFLADGNLYVEKPDESR
LKAKILHDCRGTKVLVLTGHEATLHLSCPTEPFGVISDYDYIVYPALAGDSAAAKKMNKE
MAQFAETLPVIIRNWSYVIMAHI*
>SPBDM4_v1_50322|ID:27158685| conserved membrane protein of unknown function [Uncultured spirochete bdmA 4]
MNTAADAKLMGDVLRFQREEITGSLLYGKLAKKCRDEHNAAVLSDMAAAERRHYEFWKSI
SGQDVAPRRGLIILYTALGRLFGLTFALKLLEKGEEVGAADYARVAPFHPGAEKMGEEEE
NHEDALLGMIDEERLSYVGSIVLGLNDALVELTGTLAGLTFAFQKGKLVAVSGIITGIAA
ALSMAASNYLATKANDDPKAKKAALYTGGAYLITVMLLVLPYLIVPQTGFWIYVSLGITL
LIAVGIIAGFNFYVSVAKGQEFKPRFLEMAGISLGVSFLSFLVGLLVRSVFGAE*
>SPBDM4_v1_50323|ID:27158686|garR| tartronate semialdehyde reductase [Uncultured spirochete bdmA 4]
MKVGFIGLGIMGKPMSKNLIKAGYSLVVNDVNGATVAELAALGAEAAGNARELTEKVDVV
ITMLPNSPHVKQVVLGPNGVIEAARPGLALIDMSSIAPLVSRELSLRLAEKGMDMLDAPV
SGGEPKAIDGTLSVMVGGKKEVFERYYDLMKAMAGSVVLTGEIGAGNVTKLANQIIVALN
IAAMSEALVLAAKAGVEPELVYNAIRGGLAGSTVLDAKAPLVMDRKFNPGFRINLHIKDL
NNAIETAHEVGAPLPLTASVMEILQSLKVDGMGDLDHGAIACYYEQLAKTEVRRRN*
>SPBDM4_v1_50324|ID:27158687| PfkB domain protein [Uncultured spirochete bdmA 4]
VGALCLICGNITIDDLVFHDGRVRMGVLGGDGVYSALGASLWQKDVKISAVAGPDYPVQS
LAEMYGLDVSYVTYKDKPSLRNWGLYEKDGTRQFVFRNEKNQWSEYSPAPSDLPPEILEG
AFVHCAPMPWDKQIAIVRHARQCGATFISLDPPFQFMEGMDKEKLGEGLGFVDAFLPSCQ
EVRALFPDLSPNDALERLIREFPHLKAIVIKLGADGAIGYDRITGEKFSVPAYKAHLVDP
TGAGDSFCGGFLAGYLKTANARMSALRGVVSASFIIETEGTFGMNFDTQTAEKRLQEVTT
EY*
>SPBDM4_v1_50325|ID:27158688| Glucosamine 6-phosphate synthetase, contains amidotransferase and phosphosugar isomerase domain [Uncultured spirochete bdmA 4]
MHYEKKVMLEEILLQPSFLKENGKAILENLNSSLKKHDISAITHLYILGCGDSYYSALAN
RMSLMEMTGLYVESIEALEFSRYVVVNYMPTGSAVIGVSNSGTVSRTIEGVKRAREKGALT
FAITTSMTSPLAQAVDIPLIVNSPPNIKERPDGPPVVTPGSVSYIASLLGVYCVGIAFGK
KLGRLTDENVQSRLAELDGLADNILATIKLLDPIAREYAEKLPVERRLMILGGGPNYATA
FFSVAKLFEGLRHPASAVEMEEWAHEEYFISDKDASVFVIVPPGESRSRALEQMRAAHDM
GAEVIAICSAADVEIEKYADVLFPISGAIREELTPFIYCVPFELFSCYLSSVRNKFFLGF
DDPKRREVNFRQIFNSQIKDLQGKK*
>SPBDM4_v1_50326|ID:27158689| conserved protein of unknown function [Uncultured spirochete bdmA 4]
MSKILDRMHAGEKIAIGMVQLTSLPGSSTFRGGSLKTMIADALGEAMALEKAGFDSVMIQ
NLGDLPVDHKVSAPQLAWVSRIVFEVANKVSIPIGLNLMENDAEAMISISSAAGLDFVRL
KVFIGVMVTPFGLVEGCAHIANKTRNLVDASDVAIFADVHDRTGINLGGREIDPDIREAI
DLGHADGLVLTGMDYGESMEFIAKAKRKFLRTPVLLGGGCNEDNLANTLKLADGVIVSSA
LKDTNSAFGKINSEKAKRFMDLFKKMG*
>SPBDM4_v1_50327|ID:27158690| ABC-type transport system substrate-binding protein [Uncultured spirochete bdmA 4]
MKFRKMMQIALVSVAMLAAAGSVWAAPALKVAMITSESGLGDRSFNDMMYQGMLRAEKEL
GVKVNVIQPKSISEFKSTLARAAELGFDIIIGSSFDMIQPMQEVAAQYPKQKFGLVDVGA
DSIAPNVVSSVTKDWEGSFLVGVIAAKTTKTGIIGFVGGKDIPIIHRFYLGYYYGAKTVN
PNIQVLESYAGTFTDPAAGKEYTLALINKKSDINFAVAGATSAGVIDAAKQTKTFAIGVD
SNQNYLAPGSVLTSMVKRVDNQAYDMIKLVVDGKFKGGVVLDYGLKEAGVDYAMDEYNKG

LISDTTLKLAEEYRQKVMSGAIVVPNYMELKEGQTMVGNPPMKNPYVK*
>SPBDM4_v1_50328|ID:27158691|yufO| Uncharacterized ABC transporter ATP-binding protein YufO [Uncultured spirochete bdmA 4]
MTEALAMKGIVKCFGTLIANDHVDFSAYKGEVHALVGENGAGKTTLMNILYGVLTPNAGT
IEIDGREVEIPSPSVAIKLGVGMVHQHFKLVPSLSVAENVVLGQEPGRRFLLDSVLAKKQ
VKEIATEKLGIPIDPDAIVADLPVGLQQQVEIIKMLYREAQLLILDEPTAVLTPQETEEL
FKTLKKLADSGRTIIFITHKLNEVMSVSDRVTVMRAGKLVTTIDTLQTSAEQLAEKMVGR
SVLFRVEKKPSVPGQVLMEVRNLRVNNSRGLEAVCGLSLRIHENEIVGLAGVQGNGQDEL
VEAISGLRQISGGNIFLEGKNISSASPRQIRDMGAAYIPSDRTTIGLSVNDTIWKNMILG
HHSSKEFGRGLSLDKRKANAFTEEMIEKFDIRGASPQAAAKSLSGGNQQKIVLARELSRG
GKIVIADQPSRGVDIGAIEFIHRQLVAMRDLGCGVFLISADLDEIFSLSDRILVIFKGRI
MGELPSSEASVEEVGRMMAGIAIGGDTQIG*
>SPBDM4_v1_50329|ID:27158692| Sugar ABC transporter permease [Uncultured spirochete bdmA 4]
VEILKLANSTNSVAVETRRASRSATAKKITVEIGRPIITIFIALLIGGVIILLMGEDPIF
AYGMLFRGAFKGMSNIIATLQNTTPLLYTGLAVSLAFRSNVVNIGVEGQMLFGALAAGIV
GYYVHLPWFLHLPLAIAAAGLAGALWAYIPAILKAKLNVNEMVVCLMLNPIAQLITSYIS
SYPLKAPGPTNKTYDILQTAKLPQFSKSSQFNAGFFIALVLLLIFWYVNNHTKKGYEWEL
IGQNPSFARYGGINVEKNIVQVFLVSGMIGGLAGAEQALGVYGAFYNGFSPGYGFDGIAV
AMLAKFNPLVISLSALLLGALNSGSIVMQMMTSVTRDLVKVLQAIIVLLLAANFTLEMFR
KKRKDDETLKKRPKTERKNHE*
>SPBDM4_v1_50330|ID:27158693| Sugar ABC transporter, permease protein [Uncultured spirochete bdmA 4]
MNDILTLDLITTIIRISTPLAFAALGGLLTFKAGILNIAMDGFMLMAAFVGVGTAYFTGN
LYISVVAAVLSSMILSALFGYFNLKFKANIFIAGTAIGILASGLTAVLLKGVFGQMGVFT
AGKVLRFPQYNIPIIKDIPVLRELFSGYTILVYMAYILTPVVSFVLYRTKWGLRVRAVGE
SPDAAASLGINVFSIKMQTVILSGFFCGLAGAYLSLDYVSLFSRDMTGDRGLIALAAIFF
AKGDPVRTVAVTLLFGTAQGLSVRMQLTAGIAPQIMQIIPYFVTMLALIIVGVQAKRKHF
NIEATTAR*
>SPBDM4_v1_50331|ID:27158694|udp| Uridine phosphorylase [Uncultured spirochete bdmA 4]
MLDASKPTSEAGKQYHIDCSKGDLSRYVLLPGDPFRVPIIGSYWDSCQEISHHREYRSAT
GEYRGVAISACSTGIGGPSTEIAVNELAVIRCDTLIRVGTAGGLRPDIACGDMIISTGSV
RFDGSSGHYVWPEYPAIANYEVVMALIEACEVLGFTYHLGVTASVSSFYAGQARPSFHDY
FPSSKRHLIEDLIAAGVVNFEMESGTLFTLAGLFGLRAGMVCAANANRITNEWKVGDNEE
NVARVGTEAVRILAGWDQKKTESNRKYLSPSLFRD*
>SPBDM4_v1_50332|ID:27158695| putative Asp/Glu racemase [Uncultured spirochete bdmA 4]
MYTKTKKIAILPPIPVLEFERARRQNQYEKFAAPGTSVDVRILKGGPELTDREYELFWST
GFMILEAEQAASEGIDGILIDCTADPGIEQIAEAVDLPVIGALSAGVHLALQVGRKFSVL
ALDAQWERMVRSRLRDYGLTGHLASIETVGKHVYQPARGRNMVEGEAAAFINLLRAAGEK
ALTAGADSIILGSTTIIDGIAELENMLRIPVIAPGIAALKTLEMMIELGIRQSRRAFPKP
AIAYGTKMAGLLHIQ*
>SPBDM4_v1_50333|ID:27158696| 5'-nucleotidase [Uncultured spirochete bdmA 4]
MKVYETILFDLDGTLTDPKQGMVKSFQYALSFFKIEDPDLSMLEKCIGPPLQDSFSEYFG
FSRKDTEMAIKKYREYFSERGLFENEVYAGIPELLEALREDGRMLILATSKPALYSRKIL
EHFYLRKYFAFISGSTMSGSRLRKADIIRYGLKKRPPATSAAVVMVGDRKHDVIGAKEAG
VDSIGVLYGYGSRAEMEACAPDAIIPSVDALKKFFLEDGRP*
>SPBDM4_v1_50334|ID:27158697| putative D-hydantoinase [Uncultured spirochete bdmA 4]
MKKLVRGGTVVAASGIFMADVLIEGDKIAGIGHFDDLGDSASEKELEIIDAQDCIIVPGG
IDAHTHIDFPLKGFRSNDDYWSGTCSAVAGGTTTIAVFLPAPRTDESLAENIRGARIAGD
ENVVSDYCFQQTLSSLSPSMLGEIPRLVAAGYPGFKVFLGSDIAMNDRDILRVMEILRKC
GGQLLVNGGNLGMEQFATGALTSAERADLSNYGRNRPEVIEAEGLSRAAYLARLTGMPIY
AVHISSAQALGEIKKARAAGCSVVAETCPQYLLLSDGKYKLNYQEALKYTLNPPLRDRSN
FEPLWSGLAEGSLDIVSSDHCPFNLHGQKDQSPSDFLKVPNGLPGIEPRLLLLYSEGVCA
HRLSLRRFVEVVSTNPAKYLGLYPRKGTIVAGSDADIAIIDTRPDVIFSKRSMFERNDYT
PYESMPRHGTIRATLVRGEVVFDAERGIIAQKGFGRYLPRFFGKST*
>SPBDM4_v1_50335|ID:27158698| putative L-hydantoinase [Uncultured spirochete bdmA 4]

MINLFVKNAKVVDGRGISEENICIDKGKIVGKVSRSLQIEARTTIDAEGRFVVPGAVDSH
CHIGQIPGTGHYRPQASKEENFRTETASSLYGGTTTALNYMFSESSYETTIADYQKWVKE
DSVIDIKLHGGLLSQLHIDNLPLYVKKLGLTSFKIYLPYKGEEAHKLGGLTSLSDGQVLE
AFAALKRFRGLPIVHCENPDLIEYFMAKNQRNSEQSLAAWESTRPSIVESESANKILYFS
TKIGNRVAIAHVSASDTVDLIEKYGAISPVLETCPHYLALSNDMDMGSLGKVSPPIRSKK
DQERLWEAIARYPLVMIGSDHNAWLRRHKSELWNGFAGLPDNAFILPILFSEGFHKRRIP
LTRIVQVTSEIAARQVGLYPRKGSLSVGSDADIVIMDTGIKKFVDPAELGSISDYSPFSG
YEFSAWPHTVIVRGEIAVDSGRTLGLRRKANLLNAIRK*
>SPBDM4_v1_50336|ID:27158699| Deoxyribose operon repressor [Uncultured spirochete bdmA 4]
MRKIDPRMERIRKITEILKANKASSIHDLATSLSVSEMTIRRDLVVLEEKNIIKLIHGGA
VFNKPSGSIRGLNASRYYVDRDDDLFLDQKIRIAKKAVSLIQPKDFVFLDAGSTTERMCE
YLPNDITVICHAINILESIYSQVECNIVFGGGYFHESTLIFESPQTVQLIRENRTNKAFI
SARGADLKLGVTTSNRYETEIKRAAMESSQERILLIDSSKLGKVRPVFFSDLRDFHAIIT
DDGIPQEYVDAIRESNIELYIA*
>SPBDM4_v1_50337|ID:27158700| B3/4 domain protein (modular protein) [Uncultured spirochete bdmA 4]
MRRRLVPHRCQIFPRWFFAETPTQTCAAPASKNYFLLQLTIMKTELQFADDERLRKVTLA
LVELEGIDWGRAAEVAPEALFEKILGEARASGTEVFGPRIKAARDMLRNGRYKPAGRAKP
SSEYLLAAALEGSFPRVNPFVDAVNLASLRYMYPMSIFDADKAGRALLCRLGAPGERYVF
NSSGQEIDVEDLVCLCAAGPAFALAADLPPQPARGTPIVNPVRDSMATKLFEGAHNAVVV
VYAPGGPYGAEGTAIASAARDLAEWCGRPSESVAVRFASLS*
>SPBDM4_v1_50338|ID:27158701|sua| Threonylcarbamoyl-AMP synthase [Uncultured spirochete bdmA 4]
MEILSTNDADLARAAQALREGKLVAIPTETVYGLGANAFDEVAVARVFEAKARPTFDPLI
VHIARIEDIGLVAREVPESARSLARALWPGPLTMILPKKSEVPDIVTAGLPTVAVRFPKH
PIAQRIIELAGVPVAAPSANPFGYISPTTAAHVISMLNGKVDFIVDGGPCDVGVESTVID
MTGARPVLLRPGGMPLEAIEAVVGPAVIPPRILHPAGEALSSPGQTLSHYAPSTPLYLFE
ANSLPAAALATGIVHPSVALVYTPARAREIEALHVFDEIEVLAPCGDMREAAARLFSLLH
EFDARQFRAIYAERVPEVGLGRAINDRLYRASKK*
>SPBDM4_v1_50339|ID:27158702| exported protein of unknown function [Uncultured spirochete bdmA 4]
MKCIYGLSLALLLCGAPLSLAAQDSEPVPSPTSPQTGDITQTLGRQTPPPLPLMLPVLGD
PADPSGFFFAAEQMCTQAQNTGLTPDQQQLAEFMYTLAFRFAKNPAAQEAGKSLFNLLSN
QGRLAEAAIIAREWIQVFGPDWGMYRNLYDSLMAQGSFSEALSLVPELSKALPSAAKSRS
TELSWMEYNARSAMQDFSWAENGLPFINAKTPDAYIAKVYRLYAAAPNVPQNIAALALFR
AAATEKKYAEAVARATPILPTFAMSDTPRAWISELGRSFYGAGTWRQGLDFFSIALGLEI
RSPEVPHTMMPDSMEAASTDATTEDATAAQQPTTPESALPALDRARIPSELSWVMAFYLA
RMYQGMGESQTAASIFIELVPLAFSGEDSDSALWYWLDITMDSIATTDSLLGDLGQDEAN
AMHAKRALEISALSQAAALWKSPSYFEDIVADYMRRLLREGAWDDVVRLCALMSQKLTVA
MRGPLLYLSGRLIETGRANVDLSSESDFWKWLAPGPQTSAPSAFADAQNGNQGHAASPQD
APQNAAPLFYQALLREKGIEEHYRTLAAWRLGIDPPVLANAPILDKNFDIITAISSLYPD
SEDPPVSDDKLKDVLDFIGQSLDYGLETLAADQVAALSPFSTDALLWLAMKFVQHEQYYP
ALRIGREALNRKSAAKPELGYALLYPWAWPEHFGELTAPRNIAEPLAYAIVRSESLFNQS
AVSRSGAMGLSQLLPGTAAETARGFKMSQYALFNPKDNLTIGLTYYGYMLQRFDSSPVRA
IAAYNAGPSRMAQWARDWGNLEDDILIELYPLAEPRQYTKNITAAALFYGKLYYGISAKD
MLDFIYGIKPLPQQALPAEATNQTATATSDAAGTAPQQIPAQNVNGSPPNLSP*
>SPBDM4_v1_50340|ID:27158703| Methyltransferase type 11 [Uncultured spirochete bdmA 4]
VNKFIRNKKLKGNFSCGTQEWFQDESFWDEFAPFMFDETRWSMADAEVERIVALSGIAPP
AKVLDLCCGVGRHSLAFARRGFQVTGVDITNSYLEAARESAAAEGLAIECIREDARAFQR
PGAFALCVNLGASFGYFSDARDDWLILRRCRENLASGGVFVLETLGKETAARAFVETETI
ECDGWKVGARYSIVGDWEKLGNVWRAQKENTVYERSFAIRLYAGTELKRLLLRAGFDEVA
LYGDLDGRAYDQRAEMLVAVARKADIGRRALHTIGKNRRAHSESRIHRE*
>SPBDM4_v1_50341|ID:27158704| protein of unknown function [Uncultured spirochete bdmA 4]
MKPEIKAMNDLLDALSGLAARDWTAQEISKALGRALPAIPRPAIEILIGLTEEKFRSNPS
RGPLVIVTFAGVLLRQYDGTPLTLSEWRELRDLVSDCADELDMDTVTYAMSLIMDYGAL*
>SPBDM4_v1_50342|ID:27158705| membrane protein of unknown function [Uncultured spirochete bdmA 4]
MADPSAKRFSTYIALLLACIAVWADIIFSIPQPTAFSIAWFIVSALKNIVRIVFILYIAS

QLSFFSMRQWKHALGHFPPRPEMKKALFVMLLSMGCAGIGMVVSWLSGLSNPLLIAHRHV
SVLVLLPFIVFSSISVGYAEELFFRFFLIDGLAEAGTSINTAAFVSTLIFALSHYAQGIF
GIAFAGALAAVYTSLRFRGYGLHSLALGHALYDAIVLLIALA*
>SPBDM4_v1_50343|ID:27158706| membrane protein of unknown function [Uncultured spirochete bdmA 4]
VRLFYEGIVANLKWGVRILWRNIERRGPPVCLAALGLWAGLGLGGGGTLQLGFAVVFGAF
AVSWVELCAAAAKRGRILLGALCAGCALSAGVLRFQIQQEAVPGAVASADVRGLHIDAFE
GRLDIDSQTTARKNRKLELKVRALEFVGLNFRVRCEWKRPAFSQFLLSGAGPRLSAGTMI
RAESVRGGDFFWVDARDIKRLEDARFFARLRAVLASHFAQGISTAAGKAGPLAQALLLGV
KDELDTEFKGLFQEAGCAHLLALSGQHLSIICALVTLVGRRIVRKEKRVRRVSLGFAWFF
VWLAGPGPSLLRAIFMLRIAEIGKALDRPQSSFALLSCAAVLLALLAPSSINSLSSIYSF
SAMAGLMLFAHRFSGFLKPYLPNSIAQALSASLAAICGTAMVSVLTFGTLIPASVLSATA
AAPVMLAFMWIALSGGLLAGFFPVIGHITAPALELLQTVLTSILAFGASLPAIHLESDMA
GRIAACVLIALIIALIYAVPWLQWRNSRRTMEMLDAKNPSLRARNRFPLFEPRKE*
>SPBDM4_v1_50344|ID:27158707|rsmA| Ribosomal RNA small subunit methyltransferase A [Uncultured spirochete bdmA 4]
MNFRKRLVIYMGYNELNYDSPASIHAFLENNNLAMSRRFGQNFLVDRQARERIIRALEVE
AGMRVWEIGPGLGAMTELILEKGALLTAFEIDYGFVRLLEGLFSSCDNFSIVKGDMLKTW
RIQADRPDRIFGNLPYNVAFDIIMDLLKHGCVPPRMVFTLQREAARRMTARPGSKDYAAF
SVLCASVCDVRILFDIGASAFWPQPRVTSSVIQLVPKREPVPFEQRKDFFDFVREAFSSR
RKTMRNALYLWARATFPSLDEGEFEGRLQESLKSIGFGADIRAEVLNPEELFVLYRALML
PGRPDRH*
>SPBDM4_v1_50345|ID:27158708| conserved protein of unknown function [Uncultured spirochete bdmA 4]
MSEEEVKLSVVDEEEVDTVLGSEIEFEGDIESSKSLMIKGKVSGTIRCGAELYITEQADV
HSNIHAATVVIRGKVYGDIAADSLIAVLDWGHVEGSLAAPDIYLSPNCFFKGTTVITS*
>SPBDM4_v1_50346|ID:27158709| putative Tetratricopeptide repeat protein [Uncultured spirochete bdmA 4]
MRHTSKSIARIAILGVFICIVCASEASAQTLIAAINVPSGMTGSDILRMSQEGKHNEVAL
IAPEILKKNPQDIEAYIGYAWSLNALKEYQKARDIAQAGYAKFKDPRLAQALGESLYYLG
ENETALANLQEYLAKFPEGNKAAVTFYLCGELFVRMGKFNHADIAFSTALQYNPNNARWW
TRLGWVREKLSRYLPALKAYEAAVNLNPNLQDALSGKTRMLAKIQD*
>SPBDM4_v1_50347|ID:27158710|mtaB| Threonylcarbamoyladenosine tRNA methylthiotransferase MtaB [Uncultured spirochete bdmA 4]
MTLLRVAFQTLGCKLNQLETESVADKFLDAGAEIVPFHLSADLYVVNTCTVTSKAEQKAR
HDIRAALAKVPHAVVVATGCYAQMEPDALAALGERVVVISGDLKGSLLLLARWLSDNWQG
HGELLHAVLEWQEEIKGCAKAAADTAFASFDRFVFHPERFAMHSRPALKIQDGCDNRCSY
CRVCLARGPSVSLPAREALARVRKLEESGKAEVVLTGVNLAQYRGDGLDFAGLLAFLIKE
TNSIKFRISSYEPERIDEAFLATFANSRVRPHMHLSVQSGCTDTLHRMARPYGAGQVRHA
VRALRTARDDPFLAADIIAGFPGETDAEFETTFGLLSEFDFAWIHAFPFSPRPGTRAATM
RPHVPERVMGERVERLLALARRGKHAYIGRWLGREVEMVVEHETADAPRIIGTTENYLKA
EVISNSIGDIHPGEAVKIVIRDESKSGHAEIRAYLAGI*
>SPBDM4_v1_50348|ID:27158711| exported protein of unknown function [Uncultured spirochete bdmA 4]
MRRGYVAYAALLAMVILLFTGCDALIGNAFKAANLGQPSVEKIKELDTAALVAQAGITSG
SVSDTFIQTIISDDATKAAVLATLQTTVDTGSASDAQAAQALILDIKLADIGADEVISNV
NGAIGELASLSDSEDVKPADIINTLLPADLTGEALADFIDEIGALGTDVDTLAQKITDNG
GTVTEGLDVATLAQTAALVKFVNIVDPADGYSTGEAVAQAVADLKADPDADVTKYFDKEP
DISSLADDATLKTLFDAAGLGDLLDQLSGSGS*
>SPBDM4_v1_50349|ID:27158712| conserved exported protein of unknown function [Uncultured spirochete bdmA 4]
MKRKALIVILLITAVLAASAQIQFSEIVPKDARSMGMGGTFRVFSQGYSSFFGNPAGFAG
ATQSFTLADVSAWAYLAPTSENLGRVQGIINDTASDTDILGYAGDWLASNNGLGAGASVG
GGWVGKKGLAIGVTLVTDELAYGDSLLGAKLVSATQANGIIGFAYPINLGPVWLKVGADG
RVFYRLQSDPTTGWPFNTILTDVLNDSFSPSSLDLFGGYGFAADAGLVFGVGPVMLGFTA
RDLGLEFKVGSLTAQNLIDSNVSALPTSGTTAAALTPTYTAGLGLRLFEDSKFEPSVYAE
IDDPISLFSSTDIVTDVFTSLHAGAQLRLFRFISVRGGLNKGWYSVGAGLDLSFVEIDAA
IFTEEVGLYPGDRGRSGLSVQAAIRFGR*
>SPBDM4_v1_50350|ID:27158713| conserved exported protein of unknown function [Uncultured spirochete bdmA 4]

LIVHCRRLAAIILFAAICSISGFAQSSLSNLLPKDARSMGLGGSSLVFAEGYGALWGNPA
GLANKKSLTLIDSSTWAYIKPTPLNIKNILAILNQQMSHDETASTLDGFIAENGFGGGEY
LGFGWIEDGMGLGITSITDAVVSGSALSDSAVDIRSQTNAVLGMAWPIHFGPVEFDIGAN
VRGYYRMETASGGWAFDPLAEALMTNSDIYSLMYPNKVKGGFGFSIDAGATVSYGPFALG
FMVRDLADKFAMKDSSIEEIANSYMVPSGGLDYYAVSPVYTAGLSLKLNQGSSFSTTFFV
EADDPFSLVSLLANDIEAIPSKLHIGAEIGLLKFLALRVGYNQGYLSFGTGLDVLFLEVN
AAVFTEPVSVAGETVGRTGIMVQAAVRF*
>SPBDM4_v1_50351|ID:27158714| protein of unknown function [Uncultured spirochete bdmA 4]
MGQPLPGGILVCRPGPDEFFADEVLSKVGRVLKERPSIAAFAYGFNETGANAKSISEATD
F*
>SPBDM4_v1_50352|ID:27158715| Calcium-transporting ATPase 1 [Uncultured spirochete bdmA 4]
MKSWHTRTIEATLEEFRTNAELGLTSEEAERRLEEYGPNTLKGKKPKSLFRRFFEQFNDF
LIYILIAAAVVSVLLGEISDAIIILVVVLINAIVGVIQESKAEKAIEALKKLSSPRALVV
RDGRQTEVKAEEVVPGDLVVVDAGRVLPCDLRWTETVNLKVEEASLTGESVPAEKDASFL
GEEEAPLGDRLNMGYLSTTATYGRGRGIAVATGMSTELGNIAEMLEAEPEEATPLQKKLD
DFGKKLGTGILILCGAMFLLGLGEELIRYGAIAKGSVFELFLTSVSLAVAAIPEGLPAIV
TIVLAIGVQLMSKEKAIIRRLPAVETLGSVTMICSDKTGTLTRNKMSVTAFSSDGQAGTA
AELDPAREVHKLLLDAMTLCNDATIGAGLAGSQAGGSASHVTQATGDPTEIALLELAKEH
GIEQTDLLLAAPRVGEVPFDSVRKMMSTVHEKDGARFIMTKGAVDRLLGKCTRYYRDGRE
LPLDDAARSAILASSDTMAAQALRVLGAAWRPLADGEEVSGESVESDLIYLGAVGMMDPP
RLEVRDSVAACRKSGIGVAMITGDHKATALAIARELDIASDENDALSGTEIDRLDDAQLA
EKVRHVRVFARVSPEHKVRIVKAFRSIGHIVSMTGDGVNDAPSLKAADIGVAMGITGTDV
AKGAAEMILADDNFATIVRAIETGRKIYNNIRKAVLYLISCNAGEIIVVFTAVLFGFPIP
LLPIHILWVKLITETFPALALGMDPGDPAVINEKPRDPKESLFARGGTSSLIMNGIVIGA
LTLSAFFIGMKRATVEGLTGDAALKLARTMAFATLSLSQLFHAFNLRHPTRSLFSIGIFG
NRYLVGAFDAGCLLQVLVIMIPPFAGFFKVTGLGAMEWLIVLLLAVFTITLNEVVKVFKR
RKL*
>SPBDM4_v1_50353|ID:27158716|malQ| 4-alpha-glucanotransferase [Uncultured spirochete bdmA 4]
MNSSSVSSECIDYAGLFAINSYLMDTAWCLFKTNVFQQFQSDFGLFCAINAVWLDDYALF
AAIKETEHYHAWNEWPQELRFRKRSALSRFSLDHVDHIGRVKFSQFLFFSQWQALKDYAH
AHGVNIIGDIPIFAAYDSADVWAHPELFMLDYEGLPTHFAGVPPDYFTATGQLWGNPLYN
WPVHAKQNYAWWIERVRHSFRLVDALRIDHFRGFEAYWAVPAGEPTAEHGRWEKGPGYTL
FNALKGALGELPIIAEDLGFITPEVIDLRDGLGFPGMRILQFAFEPEEDNPDYPHNYPQH
CIAYTGTHDNDTSTGWLESASPAARARALAYTNGSPRSFCWDMIRTVWASPACIAVAPAQ
DLLALGSEARMNFPGRQNGYWTWRMRENALSADIARRLRSLSETYFRVSGV*
>SPBDM4_v1_50354|ID:27158717| exported protein of unknown function [Uncultured spirochete bdmA 4]
MRNKILFFALMWAALAMSVSGQTVSLVDSSVTPSTVTYDSYSGSDTTGPQFTFTVKLSSQ
PSKTVYFSAVINGSSSPSSRALTSSSNTLTVGFYQDSSYNTEIKSTSDYPPYYYISGSFA
RKTNILTQTFTVYPLLAKGQSVPWGTYTGTFSIRLYQSSTPTGTLRDSETIYYTANVDQT
IDVRVGSSGGSYDTGESSYNIALGDLSQGPTGSFGIFVRGNTAYTLYMNVTSGGYLTSTT
TSDKVRYSLTINGISYTLGPSVIIDRQTSKAMYSKVLLGEISVPSGQDVEAGQYSDIVSF
SVVAN*
>SPBDM4_v1_50355|ID:27158718| exported protein of unknown function [Uncultured spirochete bdmA 4]
MVFRKTSLFLVLSFVWGITMLFGQVPGVRKTNGQPAFPNVPIAHDTIMPELRISFALTIN
GKPMGEIDAGVWHNEAILEKEPAARLLIDYLRPDIYSTIFDTIFKGLTWLSAEDFAMAGM
TFVFDSSALTISITIPPEYAPVVDIDFVPEQVPNYKPILRPAPFSGFIQNESNLQVSSSS
DSTLFFSTKLYSMIDIMGTHLFGSGYVSLSQTSSSYYFDSIYALWNDNARKLQISLGMIS
APGTGFQSQPSLYAISFASVENQRYLVRQGFIDDKTEFTIHKLAKVTVEVNGRPIRQVLL
APGNYRILDLPFTTGLNEFILRIEETDGNVQVLRRIVPRDGNILQVGTSEFAFSAGTSTT
DWSEFFASGYYLYGFSPTFSGGINLQSDKRSAMGGLTWVSALPIGTLNGSASVVGRWDGW
GEMFAPAASVSYLFSMPEKDYIPSLRISANYRGLGFESPGTSAPTGSAPDAFFSLSANLY
SKIFARTGTSIGYSFTLTESAPPTMTHGLYASISQSFIRGGNVNLSGQLSFPASGTPTFS
AILAFSIMPKDISQRSFNYLQTSEGSTSLGILDKTSAFGQVFDVNLNASNLLPGSKNDSS
VGLGIRNNSDYIDVSASGTLSYANSTNTYSGSGYAQLRTTMAFVGNHVAFTRQIPDSFLL

ITSSPAMKNGAVSYKLSSGSQYLAKHGQNVVVPLTSYTTAVLSTDLVEASLNLNPRHPFV
VVSPAYRSGILFESDVVKRYIITGRLIDQNGKPIAYLPGDVYDIGGSLETSTFTDELGKF
DIYDILPGMYRVEWPEGYGSTQFELPETESDSIDLGDVMIHIETK*
>SPBDM4_v1_50356|ID:27158719| conserved exported protein of unknown function [Uncultured spirochete bdmA 4]
MQNILRHYKSLAFASLFMILGTAVFAFTFMPMSISISPSGAQSIASFRLTNDAAQQIAIS
VKAMTRQIDENGNETNEPADNEFTIFPTRVVVQPNSFQNIKVQYKGSPKLAKEVAYRIIA
EQVPIDFSQPQSSGVKVLFRYIAALYVAPSNVKDTLSVSKVEYAEQDGKKGFLVTITNSG
TRHALINDPVLKISGGPSVSVTLSDDLLKAIQGQNLLAGNVRKFFIPYDNVSPNANYSGA
LSATIE*
>SPBDM4_v1_50357|ID:27158720|nadE| NH(3)-dependent NAD(+) synthetase [Uncultured spirochete bdmA 4]
MSEEKERIIEFIKTQIAAYGFGGAVVGISGGIDSAVVGKLLTEALGKHNVFGLLLPERDS
SKTTIADSVMVCEFLGIHYSVRNITPLVRKLGVYRTKPPAFLFPRKVQEEYARAQWLKTA
NPYINDLKNSGGETNRRNLAYYRTKHRFRMIELYYEAEQRGYAVVGTTNRTEYLTGFYIK
WGDDSSDIEPIMHLYKTQVFELAKELGIPQRIIDKKPSPDIAPGITDEFAMGITYGDLDR
ILQKIELDQPLSDEKQDVVKKVREILASAKYRNIRNINLLQGET*
>SPBDM4_v1_50358|ID:27158721|galE| UDP-glucose 4-epimerase [Uncultured spirochete bdmA 4]
MKVLVIGGAGYIGSHVARLLADKGHDISIIDNLSSGTEENLFPKYAFSKGDILDYPWLVS
VMKQGYDAVVHLAAAKAAGESMIKPEKYATQNLTGTINILNAACEAGISRLVFSSSAAIY
GEPKYLPIDEAHPKDPANFYGFTKLEIERCLAWYDRLKGLRFAALRYFNAAGYDPEGRVR
GLEKNPANLVPVIMEVAVGLRPHLEVFGTDYPTPDGSGVRDYIHVTDLAEAHCAALMRLV
KEPQSFAVNLGSEHGLSVLEILETARKITCQPIPAVITGRRPGDPAKLVASSAAAKKLLG
WEAKHSDAETIIATSWRAYAQHFAHERERP*
>SPBDM4_v1_50359|ID:27158722| Diaminopimelate aminotransferase [Uncultured spirochete bdmA 4]
MTTLAQIQQFLDSQEKGIIELETLLTSHPALAPESDGTGEIEKAAALETWLKEQGIVNIQ
HFDAPDSRVPSKIRPNMVATIPGKRQDSPVWIMSHLDVVPEGERGLWESDPWKVIAKNGK
LYGRGVEDNQQGLVSSVFAALAFFKLGVVPERTIKLLFIADEEVGSKFGIQYLLEHHHLF
GKDDIIVIPDGGSSDATEIEVAEKNICWLKVTTKGKQTHAAMPDKGANAFLAACDLALRI
HRLEKSIFTARDSLFSPDRSTINPTKKEANVPNVNTIPGEDVFYVDMRILPVYPVAAALE
EVSRLAHAIEAEYNVKIQLDVVQSTESRATPAGSPIVGLLAEAVRQVYGVEAKPIGIGGG
TVGAYLRKAGFDCVVWSRMNETAHQPNENADIANIIGDAKVFATLAMMP*
>SPBDM4_v1_50360|ID:27158723| Lytic transglycosylase catalytic (modular protein) [Uncultured spirochete bdmA 4]
MTKIRYIGILVSLCIAVSFASAKGVEGATSLPAVSSDVLGSSASSEMSAGGSPAAIPAPL
PQSVPETQPTTVSPDAAAEIASLESPSSESPEEPQAAPLEENGEEPDLSQLQPEELTIEE
QAKNLQYDIPMPWSHELFEKYRQYYLSDKGRKILLSTMEKAAPFMDYIQSKVTEYGVPQE
LAYLPVIESAFSPFAVSRSGATGIWQFMRNSIGGYGMTITEWVDDRRDFMKSTDAALNKL
IDNYNDLKDWPLAIAAYNAGLGALRRAVDAAGGDTDFWNIYDKGLVSRQALDYVPKFLAV
ASILRYPELYGLELPSSHIRWEAIPLDRQVDIKLLAAKAEIPLDTLKLGNAELHYTITPP
ENAHLLKIPADKSEEVKAILSDPNAPLVRYEIYKVKEGDTLTAISKRYGISISMIAKVNA
GLNPDRLKIGQKLMIPLSLQPLQPSSGSAGGAGGARTAIHTIQKGDTLYGLARRYGTTAQ
KLANLNGIYLDSVLRIGQKLKVPAAP*
>SPBDM4_v1_50361|ID:27158724| conserved exported protein of unknown function [Uncultured spirochete bdmA 4]
MRKAQKGRATILCMLSILIVLSLSACNSDLSVPFFSRAHEFSVSNKAQEKYLRDALEIFH
SPELKKSGYYPVQRMLAARRISTILIEDGQEARAARFLSSLSDEHDAYEAWYLFAAGAVY
ESMNSGPVARLFYERILNTLPDMTIEGQSIHRTCLAKLLESDSPPAKKIGWYRDFIQRFP
DADEVGPFHFLLAKEYEKLGLWSQAIEEYRKFLPYFGVSVPGYSDAHDYARKMVEFNDSP
KDWTYANLDDLVKGVRKAIAARSPQTLRKYMSKAGFFAMSWYKEGGEDSNSNVLFNFSNF
MTRGSIYVAPSLDPSLNDSEAFLRTSGWTGYIPVWYLCFRKVNFPADPKVHGNWEWAGIY
FGEKMQ*
>SPBDM4_v1_50362|ID:27158725| exported protein of unknown function [Uncultured spirochete bdmA 4]
MKIRFRFSAAIRNIFLVSIALAGMLALVVPGSGYAQQAMPKIDTEQLKKITSESSDLEAL
IVLATPQSIAQAQARIEASQYLSAPDKVALDAFARNVGLIVYGAKDTRLEIPAAAQGASQ
KYITCLVGLADASSGEIPRIFQNAACSTMSEFISSLTFFRSSRNETRDQALAALGRFEAL
GSVSVVPMLIKGQIALESKQYGDSAKNFQQALALDQQSQKAACGLARASLALGNPEGAKK
ALDPIVTRAGAGTADIAGQAGAATRPGLSSDYKSLYGMTLYSFNDILDAEPWLAAALKDD

PTRTDVLVPLAKAAMQRRDYPAAWRYLESAAKTASQDRTWLVLKSQYALENSRQYDAERF
ARSAVRYFPKDPVAIAQLILALQISPDQARHLEAAELAKVVLELTNVQNANLTPFEQARR
AQARDEALQFLVSESYNQQDWAAAANYIHEAGSAPLDKEMVATILRKSGNVQAAVQFASS
WYARDPNSEKAVEAYLRSLAMATGGGLASAAPASDVHTGLGIAFSALGVGQGGSANSAIL
DMVVKFIAAPFSKELKSFLYYMSASLQDDENKAIDQLKDALAERADNVEALVSLASIYLD
RYNRQSDKSDTTNRDKALRYLTQAKALNPTDNDLRARISQLESQIK*
>SPBDM4_v1_50363|ID:27158726| conserved protein of unknown function [Uncultured spirochete bdmA 4]
MMKSSRNNFSIEEWGENREKVNSAVRGGPLKIESYAANDRPPAGYDPDIACFLEEIYAVC
NRFERLELDPLAVVRRYSAPGDIEVAGLVCSTLAFGSVELIMRACETALEPLGAQPAEAL
CRMNQGDIERVWGSFQYRFCFPKDMIALMWAIRKALREYGSLETLFMKGDGGLMLGAASA
FVRNLRRFAREALPSGLRKNLLPDPADGSASKRLFLFLRWMVRRDEIDPGCWNQVNPARL
IVPMDTHMIRTCTERLRFLPGRTAAAGGGAGGRGALNRAPAPSVNLRAALRVTEAFRLYA
PDDPVKYDFALTRPGIDPRPGDERFGCR*
>SPBDM4_v1_50364|ID:27158727| protein of unknown function [Uncultured spirochete bdmA 4]
LSDISFALTTALSPCYFIQAFKRHIRAIAQVVSVQVWGTWGRQFESGLPDYYFVF*
>SPBDM4_v1_50365|ID:27158728|alkH| Aldehyde dehydrogenase [Uncultured spirochete bdmA 4]
MVIPDKRHLVELFESQAERRWQLAATGPAERIARLKKLRAAILARSDELYAAVKADYGKP
AFEAWLTEIFPSIEEIDCVTKNLASWMKGKRSKGTLILPLSSTRVLYEPKGRVLIMSPWN
YPFQLLIAPLVDAIAAGNCVIAKPSNKTPHTAAFLADLLENVFPPEEVAVVEGPGSTIGN
LLLELPFDHIFFTGSPSVGAKVGEAAARVHAGLTLELGGKSPTIILAGARMEETARRIAW
GKFLNAGQTCIAPDYLFCPKDSVRDFAAALQKTVESFYGKTEEDREASPDLPRIVDRAAC
SRLQNLVSDAVSKGARLEFGGRFDIERRYAAPTLLTGVRPDMDIMAEEIFGPILPVLPYD
SLDEAIAFIRARPKPLALYVLGRDKKSAERIIGSTTSGSAAVNDLLVQIEKLDAPFGGVG
MSGTGNYHGFYGFRTFSHERNVLYQGPFSAVEKFYPPYDSPAQKRLWGALKAVKRLNR*
>SPBDM4_v1_50366|ID:27158729| HD domain protein [Uncultured spirochete bdmA 4]
MEGRDLFVSVLEQMPDGLIVLDNNDKVVFVNKAAEKISDIKKESILGVDVYQCHPEHEKE
KLGKALDYLKNKKHEVRRIFHNETNNTTQENLYRAIFDEEGEFLGSLVMTRDISEKYDLD
EKKLLLIDQMDDKIKDLRARFQNLFLSSLETLVNTLEAKDYYTKGHSMRVASLCERFLER
ESLMVPLKQNIIVAAKLHDIGKIGIKESILMKNDKLTAEENEQMQQHPIITAKILEPMDE
LKDVVEIVRHHHERYDGTGYPDGLKGNAIPFGARLLALVDAFDAMTSVRPYRDSLSFEEA
VSQIRNNLGTQFDPQLGEKFLLHLELGDLDLPDFF*
>SPBDM4_v1_50367|ID:27158730| putative ABC transporter substrate-binding protein [Uncultured spirochete bdmA 4]
MKKSRIFALCFLIVCLFSTAVLSADEVKLTIWGRDIPDDSADHAYVHALINGFQAKYPDV
KIDYIALGDPGLADKTKITMATGSGLPDIFQTWGGSVMGGYADAGMLFDLTNELKGIPCS
DAARQAMTWKGKIYGVAPFFAVAGIFVNEGIFKANNLKVPATIEEFDKVCDALKAKGIQP
FACGEKDKWPALALYMYLTNRYGGDASAKAAARQIPYDSDAFVKAAQLYQSWIKKGYFGA
TPLGEGYGDAQQLMATGKAAMHITGSWMCAQYANSEFTDQTIGFYAFPAFKSGGVGTVND
IMGQTDIGFAATKVAASKKDAVVKFLKYAMSTEACEAEPGRICSVPGVKAKSSLTAQAAA
VFATAKTVTFWWDQDLPPSVTSPLNDMLQTFFLPGADVKKSLSSFEALCVENIGPVKK*
>SPBDM4_v1_50368|ID:27158731| conserved membrane protein of unknown function [Uncultured spirochete bdmA 4]
MEIVEGKTSFAGKKKHKKKDRNRSVFIFFCILPAILFLVIFLYYPIEETFRLSLTKTTGL
SKEVFVGTDNYQRLFHDEEFFAGLKHVFSWAFWSIVIQLPLSFFIAFSLTYYSNRFTRPL
QAVFFLPNVLPSAITAMLGKFVFAPNYGLIVSLGKVLGWKWLASIDFLGNPNLAFWAIFA
IATWAYSGFYVIYLMSRIEQIPRELREAAELDGASMWSYARRIVLPELNYPLRILAVLCT
VGSLKLFDLPNMMTTGGPGNATVTLGITLYRHGFINWNYGKAAAIGVVIFVLSLVFTILQ
FSLGRKKDEAI*
>SPBDM4_v1_50369|ID:27158732| putative ABC transporter permease protein AmyC [Uncultured spirochete bdmA 4]
MRMRREQSAGRGRSSSRVHYGMSEIIITIVLVVLAVITIFPLVVPLLFAFKTQLEFAYHP
WAIPEALRWNNFAEAWKAVRIDQGLLNTLIVCFGSILCTIPSAVLAGYVFARYRSRVTEI
LFYAILIGYFVPVQMVLVPLYRMEVSLKMNDTLYGLFLPMAAFGIPFWTLIYRSFFKSLP
RELAEAASIDGAGHTKTFFRVMLPLAQPATVLAVLQVFMGAWNDYLLSLILLSDQNKFTM

QLRVASFLNSYGTDRMPRYAAAVIISALPTLILYLVGHRWIIKGMLSGAIKE*
>SPBDM4_v1_50370|ID:27158733| Non-reducing end alpha-L-arabinofuranosidase BoGH43B [Uncultured spirochete bdmA 4]
MKYRNPVLAGFHPDPSICRVGNVFYLATSSFHYFPGIPLWRSDNLVDWELAGYGISRPEQ
MDLAGTRASRGLYAPTIREKDGRFYIVCTEVDRLGNFAITAPAIGGPWSDPVKIAVDGID
PSLFFDDDGVIYVCASCDVGGRRGITLSVIAPGSGTVRGGPRLITRGSGGRWPEGPHIYK
RNGLYYLLLSEGGTEYGHMLAVFRAISPWGPYESCPRNPVLTHRNLARSPIQCVGHGDLV
DDAAGNWWLVCLGVRPIGSLLLHNLGRETFLAPVSWDPDGWPIIGDNGLISLEMDGPLPG
MPQNDGPAGVIQPDRRGGDGRAWNWRDEFDGARLSPEWNFIRARQPSVATIEKGGGLVLS
GGEAGLSEPLAAPAFVGRPQQSFDCLFEAAVDFDPASEKSEAGITAYYDDSYHYEAFITR
HAGSRVIVLRKHVHDIEVAVATVAIPDTGCVRLSVDANRERYRFSYSCNDVGGELGLGMT
AGLCSEGTWRMSFTGVFFGLFCVQGKAFFHDSSCVDLEGTSCYPRQ*
>SPBDM4_v1_50371|ID:27158734| Histidine kinase [Uncultured spirochete bdmA 4]
MLSSTMMQDKRKKSSQHVPTVGFLAARFDEAYQYAVWSGAADEAKSLGISLVFFGGQRIS
SPIGYEALDNIAFDLAKRCDIEGLIVMSNVIGTYISNKEQADFLHHFHDKELVTIGVEFK
GIEGVCVDPSGGMSSIVEHLVHVHGRKRFLFLAGPKGHIESDERKAEFLQRLSCLLPEAK
PSIVHGDFTDDDACARVNEFLDAGGTADAIVAANDQMAFGALASLSAHGLSVPSDVSLTG
FDDTEDSRFSDPSLTTVRQPVAILGHMAMRHMAAKLGLVPKEPSVRLPVSCIIRESCGCR
YSPDQEETRPGGSALPLESGEEKLPPNTEQECRHFVSQRLIVEKRAAILSEIEASLISSF
SMEDILREIARSTRELGISSCWLSLFDSSDFPPVWSRLFLAADKKTVRILVPQGMCFRTA
ELVPGGLPEKWDSYVCEPLCFGNDRLGYLICTADSTDRRMYAALRDQVSSAIKGAMLMTA
ERNKEKDLERSVHLRTLELSTTNSRLVEEMERRKSLERELLDISNTIMGDIGRDIHDNLC
QDIAVLGIMAAVLEGKLARLGLPAEAGEASAIARRSGEIAAKAKDMARGLYPAEVEAKGI
ISAVESLVVMARERNECRIRFEVTKGFFVRDSQKALQLYRIVQEALSNAVKYAKASEIKV
GLYADRENITVEVADNGIGLPKNAGEEGGLGFHILKYRASVIGGELRIRSSGGEGTTVTC
RVAR*
>SPBDM4_v1_50372|ID:27158735| Two component transcriptional regulator, LuxR family (fragment) [Uncultured spirochete bdmA 4]
MSLHPTFIVIDDHPLYRQGVMTLIRQELHLECVGEAGTISDALELLGRTKPRLALVDISM
QNQSGLELIHLIKREYPETLMLVVSMHEENLYGERALKAGARGFVMKHENPSVLLEAVQK
VLAGHIAVSEDLRQRMLERIVVGKQDVDIVSRLSDRELEVFVLIGKG*
>SPBDM4_v1_50373|ID:27158736| Xylan 1,4-beta-xylosidase [Uncultured spirochete bdmA 4]
MAMAVRVPEIDYYSLHGIWGAYASLVLGRIGKGAGVVVGDVRPPDRGLFVGYRIGNDEPR
LLPFASGKDIAAGASAYHADEDMPAREESGLRYLSYFKPEEMERRLSFSGEEWRAGALSF
RVISFFGEIPDPQSMTPASARSALRPAIFLQLTFDNTANENQMIGLFGMQGIRRPLSDIT
NGGLLGMACNNCLGFAIRPDIGIDEVMDWSVIDAAFNGNRPLRRLAQEGGLRFRIAPGQK
AQYVIALGVFREGIVTTGRPAHAYYTCLFKDIEDVLENALDEQVESLFRAERLDSFLDNS
GLSDDRRFIIAQAAHSYVANTELLLSQSGEPVFLVNEGEYQMMNTLDLTIDQAFWEARFS
PWTLRSELESFGEYSSYRDSLGIAFAHDQGVDDCFTPSGCSVYEMPGLTDCFSYMSYEET
LNWLLTACLYVNLTGDWNWAKERRKLLSACLQSVRVRDGNNDGVMDRDSDRCEGGAEITT
YDSLDPSLGQARNNLYLAVKSWSAFVCLGALFRRIDRGESPDAQETDRAAALVAATLTQK
MQGRKGYIPAIFESDNQSSIIPAIEGLVYPVFCGASEAIAPDGPYGKLVQVLREHLDTVL
VPGRCVDAESGAWKISSTSRNTWLSKIFLNQYVAEHLLGFKDERVTDDHIYAYWLRTGSA
DFAATDQVDSSSGKALGSRFYPRLVTSILWLGTPLSSGSSRESEAAMR*
>SPBDM4_v1_50374|ID:27158737| protein of unknown function [Uncultured spirochete bdmA 4]
MPYQFLLSGIGNTTEEGVVVAWFKREGSEVQEGEPLLEVQVAKTSYEIPAPVSGRLHRIV
APRDTIVKEGQLLALLLLPGEEPQKE*
>SPBDM4_v1_50375|ID:27158738|acoB| Acetoin:2,6-dichlorophenolindophenol oxidoreductase subunit beta [Uncultured spirochete bdmA 4]
MSNERELTMAKAIAEAISEEMERDEHVFVMGEDVGVYGGIFGATQGLLSKFGPKRIMDTP
ISETAFIGAATGAAAEGMRPIVELMFVDFFGVCMDQIYNHLAKNTYMSGGNLHLPVVLTT
AIGGGYGDAAQHSQCLYSVFAHIPGLKIVVPSTAYDAKGLMIEAIRDDNPVMYFFHKGLM
GLGWMRYPERSVNVVPERSYTIPFGKADVKRPGTDITIVCLAMMVHCALDAAEKLAKEGI
EAEVIDLRTLVPLDREAVIASLKKTHKLLVVDEDYLGFGLSGEIAAIAADEAFAYLDAPV

KRLAVPNVPIPYSRPLEQFVIPNAEAIAEAARSLAR*
>SPBDM4_v1_50376|ID:27158739|acoA| Acetoin:2,6-dichlorophenolindophenol oxidoreductase subunit alpha [Uncultured spirochete bdmA 4]
MTPSTEQLLEMDRQAWTIRLFEEALARVYMEGKSPAFDIGSGTIPGEMHLSAGQEPVAVG
TCAHLGQEDTVVGTHRAHHFAIAKGVDLKKMAAEIFGKETGLGKGKGGHMHLFDPEHKFS
CSGIVGASFPVACGAALAAKKKEKDWVAVAFAGEGAANQGSFHESLNLAALWKLPVIFII
EDNAWAISVAKSKSTAIPYNSQRAAAYGIPGVRVEKNDFLSVYDAAGEAIARARRGEGPT
LIEVKTDRYFGHFQGDPELYRPKDEVKGLKAADPIGRFEQTIIAKGLVSRAQLDKERETA
VKAVDEAIEFARESPYPESEEALEQIFA*
>SPBDM4_v1_50377|ID:27158740| putative Transcriptional regulator, IclR family [Uncultured spirochete bdmA 4]
MAMAFQQIPSLSAQTQKMKNDIIGSVRRALGIMELLATCPQGLNAKQISYRLRLNVGTCY
HILNTLIVSGYVVKDPDTLLFRPSGKIAYTLCKAVSPAQIVSYLMPHVQSLQEKTHETTY
LSVWNGKDIFLAAIDESPLSVRVKSLTLGYDEANHAIALGKAILAHLDESQLDNYLIDHG
LERYTSNTITSDDLLKEHLMDVRRHGYSLDSEEFLTDVCCIGAPIFDAQGQVIASIAISL
PSTRYHNLGDTLIPYVRQSAKAATRALSILGYIRPTVASEGENRN*
>SPBDM4_v1_50378|ID:27158741|ppiB| peptidyl-prolyl cis-trans isomerase B (rotamase B) [Uncultured spirochete bdmA 4]
MSNAFVKFETSKGPIVIEVFEDETPITAANFIEYVKSGFYDGTIFHRVIPGFVVQGGGMV
SGMEQKTTRPPIANEASKAPKNSRGTLSMARTMHPDSATSQFFINLVDNRSLDYRGPGPG
AGYCVFGKVIEGMEAVDAMAKVKTGQRPPHADVPLEDIVLVSARVVEPVRN*
>SPBDM4_v1_50379|ID:27158742| Glyoxalase/bleomycin resistance protein/dioxygenase [Uncultured spirochete bdmA 4]
MQFCWVTINVHDMEKSLAFYRDIVGLPLNRQFSPMPGDHIAFLGSGDTEVELIHNEKNAP
APFSQNISLGFKVESLEKTMEMLKAHGIPVLAGPFQPNPSIRFFYVHDPNGLRVQFVQDV
K*
>SPBDM4_v1_50380|ID:27158743| MscS Mechanosensitive ion channel [Uncultured spirochete bdmA 4]
MNSTILPDFINNLFKDAFTPEFWRKAFSAIIAAVLILGAFRILQAIATRTMKKNMPEQKA
QLVKKAIHYTGWAIAIISVLESMGVNLSALLGAAGIAGIAIGFAAQTSVSNLISGIFLIS
EKPFQIGDVIQVGDITGIVMSIDLLSVKLQTFDNRFVRIPNESIIKLNVTNNTRFPIRRL
DVWVGVSYNSNLNKVTEVLKDIAANNLYALDNPAPLIYIDKFDNSSINILFGVWFEQSKL
VDLRNSIIIDIHTRFAEEGIEIPYPKRDVYIKGIVDTQIPDVRKDMREQNVGTNT*
>SPBDM4_v1_50381|ID:27158744| conserved protein of unknown function [Uncultured spirochete bdmA 4]
MMYRMWSFFEPKDSQWYRWKLDGASLWMHKNGEEWRFALNSLPFKHLQSDASGPEPAEPP
ADISVSLAVAAGRKAALHPHPSPMPYLVSARNDVTIHPGTEALFTIALPPVLRVELESGY
ALYEGSPFTTTYTWFGDKTSGNLCLSLPIELDPECKNEQRAFDSASGAPSATNPVYPSIE
EPLRRAAQYLACQSLIHCKIVVKNKSKEPLEVKRLAIFTDLMNFYEKAGTLVSDMVFITG
TSDGSLQTNIDDTGFKNLHKIHTASKTGLNEVLIMRGVSFLRNIAGL*
>SPBDM4_v1_50382|ID:27158745| Class V aminotransferase [Uncultured spirochete bdmA 4]
MKGKRLLMIPGPIEFTDEVLDEMSKPTLSHTDPQFIEEFGQALEKMRAVWLAPHGQPFAI
AGSGTLAMELAAANITGPGDNVLIVHTGYFSDRMADIFRLHGANVDIVPGAVGDLPKVEA
VRVQLKAKKYKTLSITHVDTSTGVRADVRNLAAAAHETDTLVVVDGVCAVAGEELRMEEW
GVDIAFTASQKAVGVPPGLALFVASTKAIAAFKARKTPVRAYYCDWQYWLPVMQAYEARK
SAYFGTPAVNLVRALNVSLGQILAEGMDARFKRHSRNARAFRAGLSAIGLKYVPVREDIY
ANTLTAAYYPEGIDASILPRIAEEGVMLAGGLYPSIKTKYFRIGHMGMSDASEILATLGA
IERGLAKAGYAFGVGAAVAAAQAEYKKS*
>SPBDM4_v1_50383|ID:27158746| Dinitrogenase iron-molybdenum cofactor biosynthesis protein (modular protein) [Uncultured spirochete bdmA 4]
LRSPLCLLSAAQRLRHKSMPRPCCVRRIASEIRACSIVPFMEDAELAQKLFGGAELIVLE
PDEVEALRLADLESLYQEQAARMMGISRQTFGRILERVHRKVADALLNGKALKLESRTQN
SIQSLKEKIMKVAFVSDDGKTISRHFGRAAYYVVLTVDDGKVVQKEQRPKLGHGNFAGQQ
EAHHEHGATGGHGFEPGAQERHAGMVQAILDCKTLIAGGMGAGAYASIQGAGIEPIVTDM
QSIDDAAAAYLAGTLANHMEYLH*
>SPBDM4_v1_50384|ID:27158747| ATPase-like, ParA/MinD [Uncultured spirochete bdmA 4]
MSLIPEKKQNPSVKRVVGVVSGKGGVGKSMVASLLAESFASRGLKVGLLDADITGPSIPR

MLGVDSFRGESDGEHLYPVENEEGIKVLSINLFNEKEDEPVIWRGPLLAKAIDQFWSDTI
WGDLDYLIIDFPPGTSDVVLTAFQTITFSGIVIVATPQDYVSMIVRKSINMASMLKTPVL
GVVENMRTMVCPHCGSEVALFDDGTQNGAQRMGLPLLASLPWRKDLAQSRALRWSALGDA
IRKDADSLANEVELALASVSAVGSAAPQA*
>SPBDM4_v1_50385|ID:27158748| conserved protein of unknown function [Uncultured spirochete bdmA 4]
MARYRNLVFKGGGVRGIAYLGALKYLYEQGFMRTIERVAGTSAGAITALGVALFPSDFAQ
FKHMADSLDYRKVPSEEDEHTNKLANKRLLSLTEHYRELALFKNLQCSIRLLQEKGWYSS
DYFYQWLRSIIAERFTVAKEAYTFADFSNPEIHKDGQVFPQLYITGTDVTNRTTRLFSSE
TTPDMEVALAVRISMSIPLFFEAVQYQFPGTDEPQFYVDGGLMWNYPVNMFDDRKYARKL
ADGANEETLGLFLYSSSEKTRYKPVKSMIDYMEALVESASLVQEHLVVRTEKNYSRTIFI
DDCGIESTDFDIELDDPRYKSLFDSGYSATSEFFESRSEWSKFFSSLKERLGWKE*
>SPBDM4_v1_50386|ID:27158749| Regulatory protein TetR [Uncultured spirochete bdmA 4]
MEENHPLTKRKLQARETRKRILESALSLFREKGFDQVSIDEITSTAGVSKGSFYTYFQTK
SDIIIEEFRLIDYFYEKKEAVILRNPDAASRLIAFTRHQLDYIHKNMGFRTLSILYINQM
STFYDQKILANRERALVRIVSKIIAEGQAAGQIRQGDPVELAEWMNRCMRGFFLDWAISK
GAIDIRKDGMRFFSEFVLPALCTKQMHA*
>SPBDM4_v1_50387|ID:27158750| conserved protein of unknown function [Uncultured spirochete bdmA 4]
MKLKEVVAVVTGGASGIGETVAKYFASNGAKVVIGDVVQESIDRVVAEIKAAGGEVVGLK
TDVTKDADVAALMDAAVSSFGAINVVVPCAGIIRDGVVINTDRETGKVKRVMETDQFRTV
IEVNLIGSFITLREAARRMVDNKWQGVLFTISSVNKVGQVGQINYSSTKAAVAIWPHILA
GEFQMKGIKGIRVMSIAPGYVGTPMVKGMNQDALNEILKDVHIARLIEPEEIARAIAMVV
ENEAFDGTCLEITGGVTYGARSIAK*
>SPBDM4_v1_50388|ID:27158751| Zinc/iron permease [Uncultured spirochete bdmA 4]
MKVENPILQALLATLFTWGVTALGAAMVFFFKEINKKVLDAMLGFAAGVMIAASFWSLLA
PAIELSEAMGLIPWLPAVIGFLLGGAFLFGVDHLLPHLHIGANMNEAEGIHTTWKRSVLL
VLAITLHNIPEGLAVGVGFGAVAAGIPSANLSGAIALAIGIGLQNFPEGAAVSIPLRREG
LTRWKSFVYGQASGLVEPIAGVLGAALVLAMRPILPYALAFAAGAMIYVVVEEVLPEASR
ESNGHLATIGTMIGFALMMLLDVALG*
>SPBDM4_v1_50389|ID:27158752| NAD-dependent aldehyde dehydrogenase [Uncultured spirochete bdmA 4]
MNNGIFHVPTLGNEPVFGYAPASPERAALKKEIERLRATHFDIPLIIGGKEVRTGKTAPI
RRPDAHKEVLGVYHCAGEAEARMAVEAALAAKAEWAAFPWEERAAVFLRAAELISVKHRA
LMDASTMLCQSKTAHQAEIDAVCEVIDFLRFNPLYMEQIYAQQPSNAKGEWNRSAYRPLE
GFVYAVTPFNFTSIAANLPTAPAMMGNTVVWKPASNAVYSNYMLMKLYMEAGLPPGVINF
LPGSGGVISSVALSSPDFAGFHFTGSTEVFRSLWKQISSNLERYKVYPRIVGETGGKDFI
LIDPSADLQSTVAAVVRGAFEYQGQKCSAASRLYIPASRSREFTDGLAAEITSIPMGEIT
DFRNFMGAVIDEAAFNTICDYIGRAKASGNAHIVAGGRCDKSKGWFIEPTLILTEDPHSE
SMVNEIFGPVLTAYVYDDRDMEAAYKLVDATSPYGLTGAIMATDRQRINRALLALQNTAG
NLYINDKPTGAVVGRQPFGGARASGTNDKAGSYLNLLRWVSALAIKESFVPPSSWRYPFM
MGND*
>SPBDM4_v1_50390|ID:27158753| conserved membrane protein of unknown function [Uncultured spirochete bdmA 4]
MVQKTKTLNAFVVVVMLGLFLAGMIEGNIIQTFQGFFRLQVTPARLVSDFIAVEGVGPTL
LNVAAVGLLGYLFVILNGLQITGAEVAALFTMMGFAFFGKTLFNCIPIMIGVSLSALIVK
KKPRDYALIAMFGTAMGPLITFIAFELGMRRIFALPGSFVIGIGIGIVLPPIAIAMLRLH
QGYNLYNVGFSAGFLGLFTASLSHAAGADILPLSMWSTAREPLLVAILPAIVLIALLCIV
AENPRHVGTGFGEAFRDFRKILTMSGRLPSDFADYVSSKGSLLNAAIMGILFWLGVVLLD
APLNGPVLGGLFTIVGFSFFGKHPKNVFSVVLGIVVAIIIFGKDIYTPGPLLAILFGTAL
APLAGEFGPIVGFIAGFLHLVIVDRTGAWHGGMDLYNNGFAAGLTATLIVSIIEWYRSAQ
DE*
>SPBDM4_v1_50391|ID:27158754| conserved protein of unknown function [Uncultured spirochete bdmA 4]
MKRQELYVQLISELANYLLHGNPKKMVISMHLEPDGFHIAGIDDRPRTDKEIADIEHSLM
MHQRPELASYYGMMVGHDLLGSSRLELIGFQIKGVKVERIPEGGVMINLWIGSEGFDPAM
FTIDQEPQGS*
>SPBDM4_v1_50392|ID:27158755|sbcB| Exodeoxyribonuclease I [Uncultured spirochete bdmA 4]

MPATILWYDLETFGRDPQHDRIAQCAFIRTDEALEEVGPPLVLYCRLPPDYLPDPEACYI
HGITPQEVEQKGLSEYEFALRVMHEMSVPNTTVAGYNSIQFDDEFIRRLFYRNLLDPYTR
EYRNDNSRWDVINLLRAVHDLKHEGFHWPANADGNPSFRLEELAKANGIAHENAHDALFD
VRATIGLARKVRTAYPRLYQWYFQHRRRENLTPLVNLSLRENMLVHTSQLYTRANGCSTV
ITPLGLIEADRHALVAYDLRFSPGPFSPMSEDELRDLLFTERLENGPARPPITMIRLNSC
PYLAPVNVLNKQSAKRLKIDIETCRQNLLELEAVPGMKERLIAAYRYEKKPLDELDPEYA
LYGGGFYPDEDRRELDRFHEVLQSRGPHAAKNEFLRVQFRDSRILTLMGRLLARNFPETL
TAEQHLSWRKHAYGWVQLPLEKGATALADYARLEEQLREMSAEDPHRRIASALLEWKEKI
EKFIREQ*
>SPBDM4_v1_50393|ID:27158756| conserved exported protein of unknown function [Uncultured spirochete bdmA 4]
VKYRYGLVIVLVLVVAGIAAAQTVPSDKTMLIKLERTAVRTAPSFASPVLVFAAYRTPFA
VIRTENDWVFGYVQGSSVPGYVHVSALSPVKLSLSADAVSEPPALQESEIVLAGKGFSSS
LETALKEGNAFNFDAVDDMVKLTYSYAECLAFIQGTDISPGEL*
>SPBDM4_v1_50394|ID:27158757| putative Peptidase, M48 family [Uncultured spirochete bdmA 4]
VYKGAARSRMLAVSLIILLTASFLTSCTVSPEALQLFLNQALLQGMVTPEQARLIREAAR
SFQTESRPFTYEEEYAIGRVVSAAVLSQYPVYNQPELTAYVNKIGQGLAFFSARPLLPQG
YHILILDSEEAHAFAAPGGFVLITRGLLRLATSEDMLAALLAHEISHTALGHGLVSLASY
RMGDLAMYYAELSTQRANSDNKEAAALFSAAVHDFVSLISVQGYSQTMEFEADAEAMRML
YEAGYRNSALKDFIHVLANSQDERIQQYSMQHPLPAERLTRIGVIERQYAQAAAHAARQE
RSRRAASLLRAESESMDIGYPAAARSVFELGAEMADGEATRVAPLELIRQRRFEAMQQLF
*
>SPBDM4_v1_50395|ID:27158758| putative Adenylate/guanylate cyclase with Chase sensor [Uncultured spirochete bdmA 4]
MARRARSLALKSRSTMNRRARFLCALAVGIAATLVFLWIGASEKLGMAVYDSQMRALVRA
KGQSVAAPSQTLRKIQSTSNSQNSIAPLTNTTSIVLVMIDQSSLDWVQKELGLGWPWPRE
LYGVMASYLRDARVQAYDILFTEPSTFGPEDDARCSKAMTEAGNVVLATLVNKKAVLDVP
NAAFGHVAALVDADGICRKYQVWFDRDGERIPSLGLAAMEAMGAGDAIPAHGEALLKFEN
PSRFERYSAAQIIAASFHGSDKSPSIDVHDKIVVIGLTAPGLMDRQATPIDSALPGMEVH
ATFISDALSSSFMERLPLWVEILTAVVAAALVGYFPLLRRKSLAALGIAAAVCIPIALSL
LLFRYQIFYNPVLALASELFAFIAAVALGYGAEGRQRAYLRRAFAQYLSPEVISALVERP
ETLRLGGESKIITTLFSDMAGFTSISEKLDPERLAQFMNEYLGIISEEVLAQGGTLDKYV
GDAVVAFWNAPLDTADHAYRALVAAVHIQARMRMMSHEFEQRFGISPRTRIGVATGQAVV
GNLGSSRRFAYTAVGDSVNVASRLEAANKAIGTSILTMQDTVVSAMGAESTGQLAPEVKS
KLSEGGDLILRRLGPALVEGKMQPIELWSVEMIDPDGERPLVSIEPWVEIRHFSK*
>SPBDM4_v1_50396|ID:27158759| putative Energy-coupling factor transporter transmembrane protein BioN [Uncultured spirochete bdmA 4]
MANRLANPFAFEPGKSALAALHPLAKLGFLLAATSVAMQSHVGVLLGLFTVGFLFLIRVP
RVAGGALFSILVLVAFAALVRGALPGDGRLFDAATLPDSGVYALRLFTVYVYSRLFYATT
RVSEIGDWMTAGIRAIRRPFRGRTKAKKSEPGILGDPGMLFSLVLLFLPRIFDTYQRIRE
AGEVRGIMLSGRNLRRSLVMLEQLIVGSMVQAWRTAVAMEVRGYSPRRSIWLSAFAGRDW
GLLGLATALLFLKMI*
>SPBDM4_v1_50397|ID:27158760| Polyamine-transporting ATPase [Uncultured spirochete bdmA 4]
MPELFRAEALQHLFPDGKAGIENVSLSIDEGESVLVAGKNGAGKSLLMRHFIGLSKPTSG
AIYYRGEPIASQIPLIRREVGYVFQDTDAQIFGQTVEEDLAFGPANLGIHGKELEEAVRS
ALHDAHLEGMSNRRPGTLSGGEKRRLAIAGVLAMRPRCVILDEPFVNLDFPSVQEIIEVL
KTLRAEGKTLIVLTHEIEKVFALASRLVILDRGNIVFDALPSRAKKEDFMQSGLLCPYRE
CYPWQID*
>SPBDM4_v1_50398|ID:27158761|bioY| Biotin transporter BioY [Uncultured spirochete bdmA 4]
MESINERLTKAIMACLFAALISIGAYIAIPIPGTPVPIVLQNMFIILAGFILGPWWGLAS
VIFYLMLGAAGLPVFAGGTGGLVKFAGPTGGYLLGYIPAIIVFGLVSKLGKKTWYFNILA
GIVGMAIVYAFGVARLKAVLNVDWTKAFATGLLPFLPGDIAKIVIAAVLAPPILKAIPYI
EQQPNDA*
>SPBDM4_v1_50399|ID:27158762| protein of unknown function [Uncultured spirochete bdmA 4]
MKSFAAVDVTGTTIGASLERFQKKLAERGTSFSEFLLDAYIRKWTGEELAHALKIRTALV

LGLQEFLVSRGILNIDRVSLSPVTDPLCHSVEHVPVILFQDTPYRTTHSMIYSKMLACMS
PHVPGVYIDSPNIRLELPSDTSRHKYLIDFSQMDIELKRTQMLSEEEYFDRPQETAMTLT
QEWHLALDFFEDLIIYSVKKILALASDDLTALGVTLAVPQKPFPRFYKDEEEDHETSSLE
RRLGEKAGVQFFWVLGLLRENYDLVYPYLSKDGKRSARASISSRQIFNYDLCAAPLYTDG
TLGSAYEVLSGGLREWVYDAIVARLLDNDILREAPRFDEKGNLLNMAALEGYGPFLTVAH
MRDAKGRGLFPQVFGGGLGIERTLFALLHGPHVKDIDEITYFGKNPDKSLPFLF*
>SPBDM4_v1_50400|ID:27158763| Cation efflux family protein [Uncultured spirochete bdmA 4]
MENRTAIIQKASWIALVGNFILAFSKIFIGILSGSLAVLSDGIDSATDVLIAAMTLIASK
ITAKPGDREHPYGHGRIETVATAIISFILFFAGSQILFNAIGGIFSGVPEKLPSTMAIWV
TIVSIAGKVGLAWSQFHFGKKSGSPMLIANAKNMRGDVVTSAAVLIGLGLSYLTGLAVMD
KILGALVALWILKNAIEIFMEANTELMDGTKSREPYKGIFAAIAKIPSVDNPHRVRLRRV
GSMLIIDLDIEVDPAMTVYESHEIALKVEKSIKESLPDVYDVIIHIEPRGNVEDEKFGLT
EGDVKR*
>SPBDM4_v1_50401|ID:27158764| conserved protein of unknown function [Uncultured spirochete bdmA 4]
MDRFDVIIVGTGPAGLGAAFELLKKKPSLRILLLDKLQVSSGGLRNDCKMNFTWPIGFPF
ECWDEALGNFYLKRVEAFLQPHIMEKRNIDIYAKRAEKIGVKLLDVRQSHLGTDGGLELI
KALTARLFDLGAEISLGEEMLSVEQSSKTVTTDKRTLQYGSLIVAPGRGGFAFLQRLMEK
LGIDYHDNVVDIGIRIETREEHYPIVRDYYDPKFLFPKKTRTFCTNSRSAHVVQERYGDQ
KTGHWYSVNGHAWSEQRAANGLVNFAILKTVTLTQPLASGQEYAQMLGRLAVLLGGGRPI
MQRIGDFRLGKRSFADSFTGDLYDFHPTLQTCTPGDISLCIPAKTMRAIWNALKLLDTVV
PGVMHPSTIMYYPEIKLYANRPAFIDKYFQVMPGIYLVGDGAGTSRGITAAWASGLRAAD
GILKSL*
>SPBDM4_v1_50402|ID:27158765| Extracellular ligand-binding receptor (fragment) [Uncultured spirochete bdmA 4]
MKKAVGIVLLTVGIAALIVACGPAAPKTVKFGVFEPLTGANAGGGALEVEGVKLANELYP
TVKVGDKEYKIELVIADNKSDKVEAANAAQRLVDKDKVNVVLGSWGSSLSMAAGPIVKNA
KVSAIGLSCTNPLVTKDNEYYFRVCFIDPFQGTVMANYAFKNLSAKKAVIIREISNDYSV
GLAKLFADAFKQLTGDENAILAELNYNTGDTDFSAQLTEVKKFKPDVIFAPGNYTESALI
IKQAKELGIAVPFLGGDTWETPEFIDVGKKAVEGVVFSTFFATETPITDTSKVFLDA*
>SPBDM4_v1_50403|ID:27158766| protein of unknown function [Uncultured spirochete bdmA 4]
VARDAIQRAGSLDKDKVREAIAVTKDYPGATGMITLNEDGDAVKSAVIKTVKDGKFVYMA
TVQPY*
>SPBDM4_v1_50404|ID:27158767|livH| leucine/isoleucine/valine transporter subunit ; membrane component of ABC superfamily [Uncultured spirochete bdmA 4]
MTLSLLFQHIVNALSLGSLYALIAIGYTMVYGILRLINFAHGDIFMLGAYVAFYGITLVS
LPWWAALLVSIVFTAFFGVGLERVAYKPLRDSPRISIMISAIGASFLLESLGVLIFGGRP
KGVPVPDVLSRVIHFGSVSMVTISLIIPLFTFALLGDLLWIVNKTKTGMAMRAVSTDIEA
ARLMAVDVNKIVSFTFGIGSLLAAFGGIMWSFKYPQLNPLMGVMPGLKCFIAAVIGGIGS
IGGAVLGGFLLGILEIMTVAFLPTLSGYRDAFAFILLIVVLLVKPTGLLGKRQAEKV*
>SPBDM4_v1_50405|ID:27158768| conserved membrane protein of unknown function [Uncultured spirochete bdmA 4]
MNEATRKKTIALPRNRLYSVIAIAAFVLFAFIADRFFDAFTLRVFNLCAIYIVLALSLNL
LNGFTGLFSLGHAGFMAVGAYVSALLTMSPQLKDMNYFLVPIVPWLHNVTIPFLPALIIA
GLVAALVGFLIGAPVLRLRDDYLAIATLGFGEIIRVLITNAQPITNGAQGLKGLPRFSTT
FAVWAVAGLMILFMVLLMRSSYGRAMKAVRDDELAAEAMGINVFRVKVTSFTVSSFWAGV
GGALLGHMITTIDPKMFTFMLTFNILLIVVLGGIGSISGSVISAVVVTILMEALRFLDEP
MNLVIFKTEGMPGLRMVVFSILLMVMIIYRQRGLMGNEEFSWDMLRGLFVKKGRKGGDHV
*
>SPBDM4_v1_50406|ID:27158769|livG| leucine/isoleucine/valine transporter subunit ; ATP-binding component of ABC superfamily [Uncultured spirochete bdmA 4]
MSEQTSDLMLSTSQLTMKFGGLTAVSDFNLEIRKNEIVGLIGPNGAGKTTAFNVITGMYR
PTEGRVLFKDRDITGLKPHSITSLGVARTFQNIRLFKEMSVLENVLVACHMSINTGIFGA
TLHLPGYRARESEARRFSMELLGQVGLGHLAYERGTSLPYGQQRRLEIVRALATKPKILL
LDEPAAGMNPQESFGLMDFIRKIRDEFDLTIFLIEHHMQVVMGVCERMYVLDYGVTIAHG
DPASIQKDPKVIEAYLGVD*

>SPBDM4_v1_50407|ID:27158770|livF| leucine/isoleucine/valine transporter subunit ; ATP-binding component of ABC superfamily [Uncultured spirochete bdmA 4]
MLKIKSLSVHYGGIHALQGIDLSVPEGKIVTLIGANGAGKSTTLRSIVGLVKPTGGKVLL
RDAVISGKATQEIVQMGVVLVPEGRRIFPNLTVEENLLLGAYARNDKEGVLKDINHVYAL
FPRMKERRTQKGGTLSGGEQQMLAVGRGLMSRPKLLMMDEPSLGLAPLIVKMIFEIIKQI
NAEGTTVLLVEQNAKAALEVADYGYVLETGRITLQGKGQDLLQDDRVRKAYLGEVQ*
>SPBDM4_v1_50408|ID:27158771| membrane protein of unknown function [Uncultured spirochete bdmA 4]
MKFLRLIVPWLLVIVIILAALYLPDLGPENYKDFDSLFANERVRQGIAGYEIAIIKDGEI
AFNEAYGFDGQRKPLEKSTPLYLGPASEILTGTLLCQFVNEKKIALDVPIAQFIPDLASL
KAKRSGAASLDMPLTLRQLAAHRVAFPEKDLPDYDPEATGLEAGLPDPELFLRSHFPTEN
YTRSRLSYRIVGAILEKVSNRKYSDLLESMITIPLGMHLTTARPSSIENIAVGAGSFFGL
TFPYLEKVPYDAAASDGIVTTSEDMAKFLKFVVSPERGQNIPGLASSQTPLLYQALYKDG
DTGFGWRIVDSKKGRYIFQGGSIRGYSSRIVIYPERNAAIVILTSQDGVIISNFLLPTLV
SCAEQILFNGESRRPFPTQRAELVAGFVFLIYLLSKILQIQSSYSWARDLLKYRETGISQ
LYARLVLVRTIIGLLLRILIVVLAPVGALALVGRPVSYQELIAFEPGVSSMLMTALLFGA
FRNISRLVMIVRLRRS*
>SPBDM4_v1_50409|ID:27158772| protein of unknown function [Uncultured spirochete bdmA 4]
MPSFTPSMSEIEIAVPNLLLGLSRALLRAEEPGDAIGDFLSELSDILHGQAYAACGRTSH
DDILRYVAIGGKEITLFASVAELGQEVRLGSLPCGLALEADEFIQKKLNGSLLLEAPAKQ
QGIIVFVLSFLLPQNAEPDVAWVASSALKSATQIIEEFFALRVRLAEQMENDSGRPISAI
PPDMDESPVSWRGIVGNSDVIREVFTMVKQVAGTDATVLLLGESGTGKELIARAIHQESS
RAAKPFVAVNCAALPESVIESELFGHEKGAYTGAYEQRKGRFEQAHGGTLFLDEIGELSP
GLQIKLLRFLQDHRFERVGGNTSIEANVRVIAATNRNLQEAVDQGSFRADLYYRLNVFPI
NVPPLRERGADILLLADHFVVKFNKENTKKIQRISTPALDLLMIYHWPGNVRELENCILR
AAILSVDGVIHAYHLPPSLQSAVSTGTEPATGLDAAIARLEKELIIEALKLENGNVAATA
RRLVATERRRIRYAVQRYKIDTKRFKIKL*
>SPBDM4_v1_50410|ID:27158773|asd| Aspartate-semialdehyde dehydrogenase [Uncultured spirochete bdmA 4]
MINNQSRTVFSPKKKIPVTILGATGVVGQRFLRRIADHPWFYPAFLAASDRSAGKKYAEA
CQWHLPGLPYAGCAGTVVVPCSPGAAFSPIVFSALDAGPAREIEPLFAAAGAYVFSNASA
FRMDEDVPLLIPELNPEHFGLLSVQQAKRGWPGAIVTNPNCTTVMLASPLAALQSRFGLD
AVLVTSMQAISGAGYPGVAALDIVGNVVPFIRKEEPKVESESNKILGELVQSGSAASVVP
APFVVSATCTRVPVIDGHTLSISVRLKTKASLGEVAEAFRAFEAKTARYGLPSAPSQFLT
LLDAEDRPQARKDVEEDGGMRISVGRLRACPILDFKFVSLGHNTERGAAGASVLNAEMAL
AMGMLYGIV*
>SPBDM4_v1_50411|ID:27158774| Aspartokinase [Uncultured spirochete bdmA 4]
MTTLVMKFGGTSVGSAARIREVARIVQSRQGSKRVVVVSAMAGVTNSLFEMAEAARNHDL
DRAVQLLSKIEQVHLEACRSLEIATPEVVGKIKEIVENLQRRVHGIDLLGELSPRTMDEI
ASAGERLSSILVAGFMDCPLLDARKVIRTDSHFGGAHPRLASIKKFAALNVVPLLESYDI
IVTQGYIGSDDADDTTTLGRGGSDYSAGLLGAAIQADEIEIWTDVEGILTADPRIVPGAR
TVEILGYDEAAELASYGAKVLHPATVRPALDAGVPVSIRSTFRPNGMFSTISPGESSGRP
CVAIAMRRNVVIISVTQESMTDQSGFLAKLFDVFGRRGISVDLVSTSEICVSVSLDKNAP
LDALTQDLGKLGQVSIAQDRAVIAVVGDLLRKTPEVLRTTFVAIDGIPVDFISMGANAIN
LSLIVQEQDAERAMRNLHTAFFEGGSR*
>SPBDM4_v1_50412|ID:27158775| putative 4-hydroxy-tetrahydrodipicolinate reductase [Uncultured spirochete bdmA 4]
VNIRIGIFGAGKLANAILEEAQAVNAKTASASRSPQFLIEWVVDRGDPIPAHAIDVAIDA
SMPDAVESHLVWAIETSTAFVIATTGWDIPDMETRIGNKTAVLVSPNFSFTVTFMRRIAA
QLAQFADWYGEGDLAVFEHHHSAKKDAPSGTAKALAQAIVSGSKRYTAWNASGWDAWDPG
KVAIASLRAGSEAGVHEVVFDAPHEQLSIIHRARDRRVFASGALKAAQWILGRKGLFGMD
DVLESMLGSQDAISQEV*
>SPBDM4_v1_50413|ID:27158776|dapA| 4-hydroxy-tetrahydrodipicolinate synthase [Uncultured spirochete bdmA 4]
MMDNIQGLGVAIATPFDDTFNVDYTGFERLLDFLCGRDFEASAGEYARQAFSDEARRGRF
RAFWEKESGGTDFVVVLGSTGEGATVESEERRELIQRAVERKLGVPIVVGTGSNSTKAAV
RMTEEAVDLGADAVLVVVPYYNKPTPDGLYAHFETVAHAAHGKPVIVYNVPGRTGLNLAP

AVLSGLWNIGNVTAVKESSGNLGQIGEICRTLPEGKTLLSGDDGLALPAIAVGAEGLVSV
AANVLPRRFKALVDAARAGHRQEAKALHAQLLPFMGALFLESNPIPLKATLKMTGLCGEA
LRLPLAPAVRGTRETLEGVLAGFGARVVA*
>SPBDM4_v1_50414|ID:27158777|dapD| 2,3,4,5-tetrahydropyridine-2,6-dicarboxylate N-succinyltransferase [Uncultured spirochete bdmA 4]
MKIAHTLEQAFAEQGGFDEAQWSAFLEALERGDIRVVEQQTDGEWQVNAWVKQAILSGFK
KGGLSEGHWPAEGFFDRPAFPPRRFSSADGIRMVPGGRSVRRGAYVAPGVVIMPPSYINV
GAFVDEGTMVDSHALVGSCARIGKNVHLSAGVQIGGVLEPPQASPVIVEDEAFIGGMCGI
FEGIVIRKRAVLAPGVIITKGTKIFDLVNGRAFSGEVPENAVVVSGTRPAHGDYAVRLGI
SLSVPCIVKYRDEKTGTAVVLELALRG*
>SPBDM4_v1_50415|ID:27158778|proC| Pyrroline-5-carboxylate reductase [Uncultured spirochete bdmA 4]
MQYKLGIIGAGKIGEAILAGSLAKGLLLPSEVILSVRTEQHRQNLEDTYHVTAVLHNRDV
AKQSEIILISVKPRTIFDVLQEIADVISDESVVVSTAAGVTLESMQTKLTRKIALIRAMP
NLGVSVGEGMTALAPARFTEEDKTQKVKRLFEAVGRALVLDEQYMDAVTGLSGSGPAYVY
LIIEALADGGVKMGLPREVSIELAAQTVLGAAKTVLVTGEHPAKLKDQVTTPAGSTIDGL
MELEDGGLRVTLIKAVVKASERAHQLLHK*
>SPBDM4_v1_50416|ID:27158779| protein of unknown function [Uncultured spirochete bdmA 4]
MLIEHLAQMYVHWGNPGQTGEQGRRKDQSVLALHQFLEVIEETSEDRKALFTQSQQSRRK
AMKLSYLLLASETEPEAIFDAFLRSGLLSSGQRSNKPEVLLWLAGTEAIARGIHPYIVFL
IMSAYFGLAIAESEFRWLRERSKSSNIKLEEFIVPGDLTDTIGEALKEPRRLQRTIRVAG
MPLSASAFAGCSLYYIEKILALVGPIGALILTEMIISARKRLVSDEISTAQQAFLDLFTQ
EETEPKMPEEERKTGLEAFEEQGLPDPDLIRTTTNIVMYAEAKILKSTLSSMSDSEIASL
LRCLEAIAHERLLNLISPGRQKRVLMVIQKTEDVVHGQMLRDAQLFTQKLLAAYAPKKLK
PGVTLSIPEEVRKLISSLLSRE*
>SPBDM4_v1_50417|ID:27158780| protein of unknown function [Uncultured spirochete bdmA 4]
MIYWNADLAAKISCSRQEMETLPPYIDHLIEVSKAVGQSGIQPTADQFSSEKDAFFRYGL
LLVSEGLAGEILEEILTVLLYVSDVKGIEFLRQCVAAEAILSIANGEDQDMMLRRLLPYC
GIDKALTAMTKRKSDHAD*
>SPBDM4_v1_50418|ID:27158781| putative GTP cyclohydrolase 1 type 2 [Uncultured spirochete bdmA 4]
MTITDFDKWARSFLEIDQLRQIDDSLNGVQVACSASKQIKKLAVAVDACAESIRRAHDAG
ADILFVHHGMFWGKPEPVSGSLRGRLKMLLDFDMALYACHLPLDRHPEVGNNARIADVLG
VTERKPFGVYHGISIGWSGNLPKPSTLDEITAKILPDRSAPKSIIAAGPREVKTVAIVSG
GAPFESLEALAAGIDLYITGEPSHSIYHYMLEGGMNFIAAGHYATETWGVKAVAEKAQAE
LGLETLFVEVPTGL*
>SPBDM4_v1_50419|ID:27158782| Lipoprotein signal peptidase (fragment) [Uncultured spirochete bdmA 4]
MKGMKVWFPLVAAAAIIIADQVVKIFIVTHVPEGRIFSRMFGDFIWIVHARNTGAAFSLG
ASSAPLARFLFFIAFPVEVLGGLLVYYFRAQNLSTPLRWSIGLIMGGGTGNLIDRIFRPE
GVVDFISVNM*
>SPBDM4_v1_50420|ID:27158783| protein of unknown function [Uncultured spirochete bdmA 4]
MNKTPANKGKVELFVIGATVANILLMEICFVLLMLLYSAMLSKILPAEALIWAIAVAFIL
ALLMSTLIYRRLLKLLRERYHLDEYLGLNKK*
>SPBDM4_v1_50421|ID:27158784| conserved protein of unknown function [Uncultured spirochete bdmA 4]
MVAGRGRLERIGVTVPEIYLPGAGTDYRKWAVVACDQYSSEREYWEEVDREVNGNPSTLR
LIFPECYLGDSDKDTRIATIQKTMQDYLDEGILSPRGHGFMLVERTTPFEEKPRLGLVVA
VDLERYQYGKDSKSLIRPTEGTIVERLPPRMQIRRGAPLELPHIMILIDDPRKTVIEPLY
QKRLYFERAYDFDLMKNSGHVRGWFIKEEAEFARISEAFENLADPEAFRAKYGVDEVLLF
AVGDGNHSLATAKAIWEETKAAFAGHPDAETILASHPARYALVELVNLYDEGLPFHPIHR
VLFNIEAQDFLGMLAGAGAEIQYTSDAAQAFAIVDASATGAQTAGCTHRFAFVTENRAGT
ITFEAPRAGLAVATIQEYIDIYRKDHPEAGIDYIHGTASTESLGRKKGNLGLYLPPVDKA
SFFSVVIHDGAMPRKTFSMGEAPEKRFYVEARKIVRGQD*
>SPBDM4_v1_50422|ID:27158785| D-isomer specific 2-hydroxyacid dehydrogenase NAD-binding protein [Uncultured spirochete bdmA 4]
MVILLADAFAPDLPGRLAEFGEATSDMSRLGDAEVLIVRSKTKVDKAMIDAAPKCRYIIR
GGVGVDTIDVEYAKSKGISVDNTPEASSLAVAELAFALMIAMPNHLVRADVSMKEGKWLK

KELERTELHGKTLGLVGIGRIGRELALRAKAFGMSVLAYDKYVTSSDVAKLVSLDEVFAK
ADYISLHTPLTDETRGMINASSISKMKQGVFLINTCRGQVVVEQDLADALKSGKMSGYAT
DVYNKEPPEGSPLLGAPNVLMTPHLGASTEENLLRLGDSIVARLRKYTGR*
>SPBDM4_v1_50423|ID:27158786|serC| Phosphoserine aminotransferase [Uncultured spirochete bdmA 4]
MKRVINFNAGPAAIPMEVLQKAQEEMLDWNGTGMSVMEVSHRSKEYEAMHNEAQDLFRKL
AGMGPEWKILFLTGGASSQFFMIPMNYLFDGRKAAYIVTGHWGKAAIKEAKHFGAFDVIT
TENPDGTFTRIPKQEELRIDPAAAYVHMTSNNTIFGSQWHYWPEVGKVPLVCDMSSDIFS
RPFPADKFSLIYAGAQKNLGPSGVTLLAIREDFYSLAQEQAKLPTMLSYRTHGENNSLYN
TPPCFSIYILNLTLKWLLNTVGGLDKMAKINEEKARILYEAIDKSEGFYRGPVEKDSRSQ
MNVVYRLKTPEMEETFVKEAKAAGIVGVKGHRSTGGIRFSIYNANLVENVKKAADFMDEF
RRENR*
>SPBDM4_v1_50424|ID:27158787| conserved membrane protein of unknown function [Uncultured spirochete bdmA 4]
MRRELNAKIDQPESIEPPPPFHPQAASRWFKRRRITIAESYLMVVRDLDSRHSSARLDAL
RKLAEVAFQSTNIDYPLNTARVQSALVKEVVKHRSNKRRQLELLYDFSMSTRGQHQVIRK
LCDELNIVELPEKGVQIGGLSYGWDGHVHDTATSGRKNPTQLIIDAFIKGISSLTVAYGS
VSDLEMMEEALEAGNILGLKVNIGLEFSVMVEGSRYHFMAELPHFSTREELRAFFGKYAI
SLDSFFQGLDTNRENRLDAVRRLLDVFNQSTLLKINEGFEGKPEYCLAPLSLDDLLATIP
NMNITPLHLAEFMYLQYRPVLQKRVWYFKVLREKTRRESQSDTDEKAEKIAIESKYSELR
KELRELSTDMLLSEYFEDPHAISYQTVFEDLQSLSHLLHGAGCTIKFIHPLEYGLEKATS
VFGQFEECLDSIEIYNTQDCINREPEEIDTFARVVNEHNRRAALENKKFLQPVCGSDATG
RNPKIPGMGFIFEDRITGRLRQRYIRRHVALTPLVSAMIRARDVVVDEASFQKTSVPRII
SMGKISSGEGYTSTSNDEKISPVRAWRYFNPAFKNTIRALIGFFVATAFVGPGYALLWIG
ITGFRNSIADLISYRGPRLNQWRLKSINFDNVAQSLFWTGFSVPILGFVKANFDVVWPLA
HGTFLFNFAKFFFISFANGFYLAAHNTLRGFDKNVVRANIFRSILAWPLATVFAPLGDTL
SIPSIVQTKIWSDVIAGFIEGGNKYRKVLRQRQKILEEIIPTIIHTKGNTQYIAMLDVLY
LFSQEPRTKSIIKAVLSPYRLFTWRLRKNSSLRLNLLVDLHTTMCDERLWTDLVDYVVAT
YDEEMADDLVDLVVDELPDLQDWLGRLIEKYSKKSGLMSKLASRKKQEQPPTHQ*
>SPBDM4_v1_50425|ID:27158788|mdh| Malate dehydrogenase [Uncultured spirochete bdmA 4]
MDSEVTWWKFDEVEAFMKAGFEAVGVAPEVAAVCADVLISADKRGVDSHGVGRYKPIYLD
RIWAGILNPKTIFDVVRETPTTAVIDGHNGMGHYIAKRAMEMAIEKAEKYGLGMTVCRNS
THYGAAFYYARMAVEHGMIGLTTTNARPAIAPTWGVEPMIGTNPLTWGMPSDEDFPFMLD
CATSTTQRGKIELYDRLGKELPDGWVIGQDGKYRHDTHQVLIDLTQDKAALTPVGGLGED
LAGYKGYGYSMVVELLSSALSQANFMKALADIGPDGKKKSIELGHSFLAINISAFCDLDD
FKHHVGEVSRQIRASKKAPGATRIWTPGEKEHDTWLYRKDKGVPFNPPLKKAFQEVKDRC
KLDIELPF*
>SPBDM4_v1_50426|ID:27158789|trxA| Thioredoxin [Uncultured spirochete bdmA 4]
VTENLTKKTFLEKVFNYEKEKDWKYNGELPAIIDFWAPWCGPCRMVGPVLEKIAEEYSGK
LVVYKINTDEEQELAGAFGIQSIPSILFVPVSGQPQMAVGALPKSTIVDAIKDVLGVEAA
*
>SPBDM4_v1_50427|ID:27158790|hutU| Urocanate hydratase [Uncultured spirochete bdmA 4]
MKTQPYTVSLGLDELPPDPVFEPGIRRAPRREAALSPAQERLALKNALRYIPSQWHERLA
PEFLQELRERGRIYGYRFRPQGRIRGLPIDRYSGRCIESRAFRVMIDNNLDFDVALYPYE
LVTYGETGQVCQNWMQYRLICKYLEILDEDGTLVVESGHPLGIFASHPYAPRAIVTNGLM
VGMFDNLKDFNIAAAMGVANYGQMTAGGWMYIGPQGIVHGTYNTILNAGRLRLGVPADGN
LAGKLFVSSGLGGMSGAQGKAAIIAGSASIVAEVDHSRIETRLSQGWVSCEANSLEEAVE
LAVSARDKGEPIAVAYHGNIVDLLEYLDAKNVRVDLLSDQTSCHVPYDGGYCPAGITFDE
RTRLLAEDRTYFRALVDASLRRHFEVIMRLVRKGTYFFDYGNSFLKAVFDAGVKEISKNG
VDEKDGFILPSYVEDILGPELFDYGYGPFRWVCLSGEKEDLRKTDQAAMDCIDPNRRGQD
RDNYIWIRDAEKNKLVVGTQARILYQDAFGRMKIALRFNDMVRRGEIGPIMLGRDHQDTG
GTDSPFRETSNIKDGSNVMADMATQCFAGNAARGMSLVALHNGGGVGIGKAINGGFGLVL
DGSEKVDNVIRMALSWDVMGGVARRSWALNPHAMEVSEEFNREHAEYGTVTLPNVANEEL
LDRVLGAR*
>SPBDM4_v1_50428|ID:27158791| conserved protein of unknown function [Uncultured spirochete bdmA 4]

MTNDIRILQETRVGKGFIPLQYLGPGENEYRIRDEQLRSLGKEPGAFRPLSTHELEVLVR
NNNTCSDWSLFLVKDPFCPELLRNNLFFGLIRISRLEHVTLEHHDISMETGIFNSQIIAC
DIGDNCVISDCAYLSHYIIDDHCILINNAEIQVSNHSKFGNGIVMESEDESVRITLDVMN
ENGGRWIYPFDGMLSGDAWLWAKFRDRTVLMAQFARLTDTKFDRRLGRYGVIGRNSVLKH
NRIIKDSYIGESAYIKGSNKLKNLTINSSADAPTQIGEGVELVNGILGYGCRVFYGCKAV
RFVMCDNSALKYGARLIHSVLGENSTVSCCEILNNLIFPAHEQHHNTSFLIASLIKGQSN
LAAGATIGSNHNSRAPDGEIEAGRGFWPGLCTSVKHSSRFASFCLLSKSDYKHELDIKFP
FCLVDSDETQGKLILVPGWWWHSNTYALMRNEKKFEERDRRIDRSIQFIYSPFAPDTMSE
IFEGLAILERATGDAAMEALVDDETNRSLLGDRVFELMDEIRASAEKSPERRSLSKRSPE
VTRKLGVHILANAKDALPFEVRVSGFENSARPEVVIKPCKSWNSYREILLWFAVQTLLPV
FENLKRNAEEPVDLFEKAQALYQSENGAALELQWENLGGFLVRHSKLENLLAHIETGDIQ
SWDDLHDEYRKLSSEYEQDVRAFAWGILGYLSLDPESASAINRGISHSDEAESARSWPDL
TKDALVAQLADARRITSEITKRVYATRAKDWSNPFRKTMFRNEAEQIAVYGRIEDNDAIQ
VVRREMESTQQAIDRVLAWLSVE*
>SPBDM4_v1_50429|ID:27158792| protein of unknown function [Uncultured spirochete bdmA 4]
MAMDFVDSARVDTLVTESEKKVFELFEMMVKSTGQERSRSAVALANLLGNPGEFSFYIDC
TEDQRLIRVFHLLRVFRENMTLLINKTWADGMENVTQEQLLGDLARFIEEYRDGRIVSAF
RTFVGISRQIPSLLFGSLGKANDFLEYAFRIDPKFGLFFWYIGEIDLQLRNIDSIPEHRE
LFELEVLIGAFVLSCF*
>SPBDM4_v1_50430|ID:27158793|fusA| Elongation factor G 1 [Uncultured spirochete bdmA 4]
MSLETIRNIGIMAHIDAGKTTTTERILFYSGKTYKIGEVDDGEATMDWMDQEQERGITIQ
SAATTTYWRDHQINIIDTPGHVDFTAEVERSLRVLDGAIAVFCAVGGVEPQSETVWHQAE
RYHVPRIAYINKMDRLGADFEAVLQDMRSKLGAHPVPLNIPIGAENSFEGVIDLVEMEEI
RWHEDGSEMTRSPIAQERAEMAHRWHENLLDAVSAQSDIITNLYLEGAPIPKETLRAEIR
KACIARHFVPVFAGASRRNMGVQPLLDAIVDYLPAPDEVGAIKAFHTKKEEDVEIPCQIN
GYPLGLVFKVQYDREAGSLCYVRMYSGSLHTASTVYNTSKKKRERITRLLRMHANKSEPI
DSVSAGDIAVFVGLKLAQTGDTLGTEGYPVLLEKMHFPEPVISVAIEPKTLSDRDKLKEA
LEILAREDPTFAVNEDQETGQIIIRGMGELHLDVLVTRVLREFKVQAKIGNPQVTYRESI
STKFRHTEKFHKVIAGKENTAGLTIEVEPLPRGTGNQYRKALRAHDVPEEIFDAIERGIT
SAFSSGIQYGYPAVDIGVSLVDIDYSELTSTAFAFEACANMAYDAACRAANPVLLEPIMK
VDILAPKEFVGEVMSLVSQRGGLIHGSETKNVSEVIHAQAPLATMFGFTTSLRSVSQGRA
SFSMEFSHFEPKRG*
>SPBDM4_v1_50431|ID:27158794| protein of unknown function [Uncultured spirochete bdmA 4]
VGWPVFPYGMKGHFSCVVFYALFLYIFIAWFQGTPSFKGFQSLHLPRSNP*
>SPBDM4_v1_50432|ID:27158795| Peptidase U32 [Uncultured spirochete bdmA 4]
VKRIELLAPAGSSEALDAAVGEGADAVYLGLRSFNARMRTTNLAFNQFEAAVYACHERAR
KVYVTVNTVFEEREADRLYQLLEYLVSVGPDGVIVQDAGVAKMVRDHFPSLSLHGSTQMN
VASSAGCNQLSRSGFKRAVLARELSLEEIKQIRQYTSLELEVFVHGALCVSASGLCLFSS
YLGGKSANRGMCTQACRRLYESETGTGYYFSPDDLQLIEYLPALIAAGVNAFKIEGRMKS
AEYVGTVVSAYRYLIDNYETDQDRAILKSSAMLQADFARRKTSFFITGTPDDYIHPDQAG
GTGIHLGRIRDARTIDDKRWALMNTYEGLAERDSVRIHRKDDSGRITAKIQAVKYGVDGM
LLQIDGEWRQNDDVYLIQTASMTRRYRPILPKNLDRFHKFPSSHTAPKPTLPRLDGRKLD
TIFEPGTYVLAGKVADLHAALTFRPKKAMILFDKLNAETMRRDEGNLPFKRDRLILWLDP
YFAESDAAWLQTELEYWIGKGVSLVVVNNQAHFTLLRGKNVTMVVGPWLYTFNPWALSYY
LEQGVQAVIPPYEISRQDLYRLTGYVPAQFFAPIIFAYPDLFRIRADLSRVYGQSHFSDR
EGNLFTLVGRHDYSVVIPDKPFSLIDLVPNLKKQGFSRFILDLSNAEPARGLYRDIARAA
DQCKPLSNTSRFNWKDGFWSEETVRREENMRNRDDNRREEAVRFGGGTRSMKNPRQPVAK
SQVRGTKSPVRATKNAQGAKRSRKQ*
>SPBDM4_v1_50433|ID:27158796| putative Acetate operon repressor [Uncultured spirochete bdmA 4]
MSESAGKALDILFYLANVGEGVSLARLSKETGMNKATALRYLNVLESKGVVERCLSGWTL
GLSLFELGSKVPVRQLVAEKVRPIIERLARESGESVNLAYLAGETAIYLDRAEANRSLRM
RSMPGDRLPLYCTGVGKAILSQLSEERIRAILGPGPLPKITESTYTDPDEIICEAGRARE
SGYGIDREEFEIGLTCYAMPLRLPGSDFVGAISISGPTARMKNPEIRDRFLALLAQAVQD
ATDVLSPSHHFEDPPKKPEGCA*

>SPBDM4_v1_50434|ID:27158797| Single-stranded DNA-binding protein (fragment) [Uncultured spirochete bdmA 4]
MNHLNSILVEGNLVRDPNFRSTPSGNQVCDFTLATNRSYKVADQKYENEVSYFDIEAWAR
LGAACAQNLKKGRGVRVVGRLKQDRWTDPEGKAHARVKIVAEHIEFKPMNKANREDGSKS
DDAFVEASKPSEASAAVF*
>SPBDM4_v1_50435|ID:27158798|purB| Adenylosuccinate lyase [Uncultured spirochete bdmA 4]
MNSFDQFQSPFSWRYGSEEMRKIWSERNKRRLWRRIWISLAKAESLYGLVNSGQIAELEA
HAEDIDFEKAQEFEKSIRHDLMAELRTYASQCPSAGGILHLGATSMDIEDNADALRIRDS
LRLLKKRLAGLLDVLAEKIERFAATPAMAFTHLQPAEPTTLGYRFAFYAQDLADTYERLS
ATIDAVKGKGFKGAVGTSASYAELLGIAQLEDFERVLSEELGLPFFEVATQTYPRVQDYR
ILVLLAELAAILHKMAFDFRILQMPLLGEISEPFGAHQVGSSAMPFKRNPIECEKIDSLA
RLVASYPGVAWDDAALSGLERTLDDSANRRVILPEAFLAADELLATMRRIVEGQVIDEEA
VQCTVESYAPFAATERVLMALGKLGTDRQEAHERLRDHAMLAWSAVRGGKPNPLKTLLQS
DEFFASRLSSDAIEMLFDVDTYIGAAEQRARATAARILRLALDEP*
>SPBDM4_v1_50436|ID:27158799| putative tolQ-type transport protein [Uncultured spirochete bdmA 4]
MLDFISKGGPILWVIMALSVVALAIIIERLLYLRRISIDEEKLFLRIKTSLMEGHYSEAL
AICDQNVSPFSTLLKVGIENRNQPEYLQRELLKDAAAMESVNLERGMTTLGTISNISTLL
GLLGTVTGTMKAFGVLGKFGAVSDPAALASGVAEALITTVGGLVVAIPVIMFYNYFASRI
NLILTRLETQVNGLVNLVSSYSDKENGKNEEKPSKEI*
>SPBDM4_v1_50437|ID:27158800| conserved protein of unknown function [Uncultured spirochete bdmA 4]
MIRDRKLSPKINFDLTPLIDIVLQLVIFFMITTTFKTSPGIILQLPASQTAQSVATPELR
VVVVSSNEIYVDKTKTNLAGLEPILRKRVSGSDAKQIRAVLEGRSSAEYQLIISVLEALR
LNGIENVGLITRKDKVVQ*
>SPBDM4_v1_50438|ID:27158801| conserved protein of unknown function [Uncultured spirochete bdmA 4]
MTEAHTYAEREQRKRTIISYAIALGLYALLFGAGFLFDLFHPQNLDFSNKTLIVNIQGPV
LNDVGKGSPVEKKEEAVVAEKPAPPPPAPKQQTPVAAQPAPVTPAPAPEAEPTPAPVVP
AVQEPVTPWTPGERGPGSRISSSESSVLSPGEGQVPWGTGEAVRITKAEKGNTVETTLGG
SSETVGQSLYVPIYLNMPLPSVVPAGLFNAIPNEIIPPSTLIASAESRKRAFLNYYTKSG
NEYRLTNAVPLEVREKLWGILEDAGYNAEQADYKTGRTLNPVVIGFSVTKDRQLRGVELL
QSSGDSDIDASVLYGFKRASFWNKSGETIQGRFVYRF*
>SPBDM4_v1_50439|ID:27158802| Response regulator containing a CheY-like receiver domain and an HD-GYP domain [Uncultured spirochete bdmA 4]
MNTDQKRMVLVVEDNAPMQKVLSLSIQKMDYDYAIAKNGREALDLIESGDVSPDAILLDI
RMPVMSGLVVLPKIRTIIPSVPVIMLTAFGDLETGLAAMKAGAFDYLVKPSSLEKIRETL
EKAFSYREIMEMKAEEERKREQQRLELEKRVESSYQELGDVYGKLKKMNLQTAYALAETI
EAKDRYTQGHCERVRWLSSRIGKAMSLADEEIEQLEYAALLHDIGKIGIPDSILNKEGPL
NERERQIIRMHPIIGAQILSTVEFFEKAASAVRHHHERWDGKGYPDGVEGERIDHLARII
SLADTLDAMATSRPYRRALSFDEVLQEIHLMRGIQFAPDVVDVFFAADLDKAYLEKFREE
VRKKKDKP*
>SPBDM4_v1_50440|ID:27158803| membrane protein of unknown function [Uncultured spirochete bdmA 4]
MHDIWKIREYYSIVVCRMVSIISIFFGFVLLMLAEWRYVRFSQKNQGKNKPEHHVPPLPG
HTALQVPGYVLIVLGMCSALLHSEQNMKLPEYMRTVCLVLALTGGGFLVWTVFLEIPLGA
KKSKVPRGHAYARGSYGKCRHPGFWWFLIFTLGIALWKSDFYAFLLFFFENALNLLLILL
QDQYTFPIQFVDYREYAEKVPFLVPHFRKQHR*
>SPBDM4_v1_50441|ID:27158804| Multi-sensor signal transduction histidine kinase [Uncultured spirochete bdmA 4]
MILDVSSLIPALSFCIYIPFIIFGLANKKERVNFSFLQYMGLMALWSFGSFMMHANTGLF
TPLVWNRVMLVGMLGGPITIFATLIYFSKTEKHRYRIFLYFGYIIYIFSLYLNFSGLIVT
DAGFVDNAFYYTLGSGAIVAYSLSYFYLILAILLVLWELRTSKDKFLKRSLRLVVAGVVI
MLLGIASNLYTPLGRYPIDLLAATINAVIIFFSVYKYRIVHYSAVVLNIFLTILVVIFSS
LIYMFFFVLVFKLERLIPFSELTLMSVCLGIVSSLFLAPLRTATQALLERLYGGKSFLYY
QELRKFSADLTSIVSLEDLAKLTVDNVMSTFKLDWAFVLINDYNARHFRIIAAKGLDFAG
EGEQGPKEHVIVPRTDQFIQSYQMRTIQQSQTSAQRSISIRLTKGEEHEMVAASLVLPLK
FKERLNGFIVLGPRIEKDYYNQYDLEILQFLTDQASVAVENAISFERLRQQQKRLQETNE
ELALSRNKLEAFFDGIATPISIQDINYNIVTANYAAQRYFEKPQEELVGSKCYKAYFNRD
RPCVECLAQDCLHTKLPFSSEKQDDRTQLTFSLNFYSIPTPQGSIGSFIEFFQDITKQKT

LQEELIQSEKLAGIGTLVSGIAHEINNPLGGILGTADLMLPETPEGSPLREYTSDIIRYA
QSAADVIKDLMIYSRKTRTSAEMLSIVNILENSLKMAMRGIDFGTIVVRKSYDPVHEILA
NPTELQQVFLNLIVNAVQAMNSDGILTLSCRQEDDDVAVTVQDTGMGIEKENLDKIFNPF
FTTKETGAGTGLGLSIAHHIVTKYGGRILLDSQEGKGTTFTVILPAADQDKDRVHLVHAR
ELRQFEDSFYLQRKVLVGEKGYKEETIRRKSDEVAYHILAYKGLQPIGTVTLHLSEEEGH
VPIEENFSIARYLDGSLYAEIDRLAVSKEERGSLIPFSIMALAYLYARGRGAKRIFLDVF
ADEMKLIKMYEKLGFEIIGSYSKPLPCTVMMMDHVSKYEQEVSRMEHFVKPFFSRLVPKI
DFSGEDMHYVMKAIDEISAKFSKQSTEQGPLDEAI*
>SPBDM4_v1_50442|ID:27158805| Diacylglycerol kinase [Uncultured spirochete bdmA 4]
MKPDDLAEGLNTIFAHSPVFQDKSLIVDVIANPKAGGFSRIHHSKKRFNELKEIVKRSLS
LPERTSPFSLKMHLTERCGHAAAIVQRILDRSPSNGKDSYHLIVTAGGDGTSLETAERLT
RLPESEKNRFGIVRLPFGTGNDGSEGRNLLVALERFLGPAMFERRPALRVTPSEEGGSLP
RYSFNIASIGLDAYVADMTNRLKHNFPGDSYKFWVDVGTLFYDRVYNVVDMGLKAWNEEK
LMVDSNEPRLLIAMGASGNRQYGSNKNILPNEYNCVAVAQTSLFRKLLIKGPIEHGRHEK
IPELMHFTADKLLIEYSERIPLQCDGETVILAKCDFPLIIERLHDVYNVLVPA*
>SPBDM4_v1_50443|ID:27158806| putative 2'-5' RNA ligase [Uncultured spirochete bdmA 4]
MRLFASLPFPAHVQRQIADYSHSLVPAFGRAHPSWVPQENLHLTLHFFGELEAGTTAKLQ
TLLAESAPLCPPLQLATGKLSVLPSLRAPRVLYISIEIQPVEPLAELIGRMRDIAAQIGA
ETDTRPWKAHLTLARLKIFWIPEFSSLAAPPKLTFVVDSFELMQSKLDRSGAVYSCFRRY
PLAGK*
>SPBDM4_v1_50444|ID:27158807| PP-loop domain protein [Uncultured spirochete bdmA 4]
MKDARTTLSKLTEIALRHYEMIQPGDNILVAVSGGKDSSCLAWDLALKRQWWDVPFEITA
CHVATDLADKGYAKPIDEAWLASRMTEWGIPYVRLDVPVAGRLKPGETMNCYWCATQRRT
ELMKYAQAHGFNKLALGHHMDDILETLFMNMMKKGEFATMPPVMPYKKYPLTVIRPLSLC
EERQIIGCADELNLTSFTCSCSFNLEGERKKTRRLIDQITGDSSHIKRNIFASMSRVRPD
YLA*
>SPBDM4_v1_50445|ID:27158808|msrA| Peptide methionine sulfoxide reductase MsrA [Uncultured spirochete bdmA 4]
MNGHEINTEKALLAGGCFWCMEALFKRVPGVVQVKPGYCGGSVSNPTYEQVCQNTTGHAE
TVEVTFDPSRISYQGILEYFWKFHDPTTLNRQGADVGEQYRSVIFYMNESQREIAQQSKS
SAQKAFDTPVVTSIEPVGRFWPAESWHQDYYAHNPDAPYCRFVITPKVAKFEA*
>SPBDM4_v1_50446|ID:27158809|udk| Uridine kinase [Uncultured spirochete bdmA 4]
MPPVKIIGISGGSGSGKTTIVRKISEMVSDFVFLPQDNYYKSAEFINNRNITAFNFDHPD
AFDNELLIEHLGALKNGDSIDMPVYDFVHHRRTERTIHIQPSKLVIFEGIMIFTNKQVRE
LIDLKIFVDTPDDIRFIRRLTRDIKERGRTVDSVVEQYLTVVRPGHYEFIEPTKAYADII
IPEGGFNERALDVLVSFINSVIYPLEGREKLSTGFDNMPGVDGPAGTTI*
>SPBDM4_v1_50447|ID:27158810|ispH| 4-hydroxy-3-methylbut-2-enyl diphosphate reductase [Uncultured spirochete bdmA 4]
MQIIRARVLGMCMGVRRAEELAREAATKAFQEGSRVYTYGPLIHNPQAVADLESLGVHVL
DAKAIETGTGELPDLKNAAVVIRAHGASLAALSRLHELGASIIDATCPRVIKSQRLARHY
EKLGWQVVLVGDPHHGEIAGILGHTSNALVVDSPKMALQMAEKLKDHPCALIAQTTIRQE
DYDAVIEVFQAHVKNMAAEKTICPATRERQRALIDLCGQVDAMLIVGGKNSENTKRLYAS
AIECGVPAWHIETASELKTEMVKFARIGITAGASTPDFIVDEIQETLSNMV*
>SPBDM4_v1_50448|ID:27158811|cpkA| Carbamate kinase [Uncultured spirochete bdmA 4]
MAARKTIVVALGGNAIIEEGTEGTIEQQFENTRKSMKAIVGMIAEGHKVVLTHGNGPQAG
VHLIRNEAASNQVPPSPLNVIVADTQGSMGYMIAQSLSNALHAEGIEKDIVTVITQVEVD
PDDPSMQNPSKYVGPFYKAEQVEKLRDRGWVIKEDPMRGYRRVVPSPTPLDVVEKDIIKD
LIDDGKIVIAVGGGGIPVKREAGGTLSGVDAVIDKDRASALLANLIDADELIILTGVEKV
AINFRRPDQEVFDHLTVEECDKYMAEGQFPKGSMGPKIEAACDFIRRGGSKVIITSMENA
TAAVDGKAGTVITA*
>SPBDM4_v1_50449|ID:27158812| conserved protein of unknown function [Uncultured spirochete bdmA 4]
MNGIQNGLTLLQQTTATNSTGSLVSTIVTFGLVFVIFYFLIIRPQNKKQKEMQQMIAGVK
KGDRIVTIGGIHGVVASVKESTVVVKVDDGTRIEFSKSAISSVSPVKAEEKTEEKAKPAD
EPEEKTEDQ*

>SPBDM4_v1_50450|ID:27158813|secD| Protein translocase subunit SecD [Uncultured spirochete bdmA 4]
MSKFRRLLIVIAVLAVAFMFLFPSIQWYFLTPKEDQAIAVGSREQIREYSRRMTYLIITD
LKQKAVAKDATDLSGQKAYKVVVESAKKAYSLAKKPAPKSWDAIAILSGFPGERSMFDSI
ESRYREHVLSLKNVHSNAVQLGLDLAGGMSAVIQADLKGLSEKLGHDLSQAEKEDAMKRA
VEILNSRVDKFGLTEPVIRRQGEDQIYIEIPGTPDPERINSIIMGKGNLAFYIVDSEAST
VVNSYLSTNPVGIDEQTMIVDKPGLVPEGEIVRKVYKKDSYGLDEFTGEYMVLQGKPGLD
GSHIQSATVSTDSITGKPETNFVLDKEGGDTFYALTSDNVGKTMAVVLDDRIKSYARIQE
PIRESVRITGFDADEAESLALLMRTAALPISLSVVSQQAIGASLGEDSIGQGRTAILVGL
LSIFIFMFAYYKWAGLNATIAQGLNLYLMLSVLTAFKLTLTLPSIAGFVLTTGMAVDANV
IIFERIKEELRAGKTRAAAIEAGFHKAFWAVMDSNITTIIAALFMAQLGTGPIQGFAVSL
AIGNMTSLFTSLFVSRLLFDFETDVLKATHMSISWRVR*
>SPBDM4_v1_50451|ID:27158814|secF| Protein-export membrane protein SecF [Uncultured spirochete bdmA 4]
MKRLIRFSKFFLPAMIISSTIIVLGLIGYFTKGFNLGVDFQAGINETIQLAFPAGQVTFA
GRGNAELTVSENQLTLVFSGAEAQKRTVTLDYKTYPTIADIKAALEKEQGIAVTLADGSD
TLPSTSLVPTYQGNSLLSTNPVKLYRAPVNDSERYASIDVVREALKSLGQVSVQTINPAS
LQRYLVRVRDDGTDPQFTTTVTQTIRQLIEDKVGKDRVIVVKTDYVGPRYSQTLGRQSVW
LVLVTLLVILLYSTIRFKIEYAIGAVLAIMHDALIMVAFIVWTRMEFNTTTIAAILTILG
YSINDTIVQFDRVREERKMHPSDAFVDVLNRGLTLTLGRSIITTLTTMLAVLALFLFTTG
SIKDFALALLVGMTSGVYSTIFIASAFVLLWENRKSKQKPKAVKETKGKVVAPAKS*
>SPBDM4_v1_50452|ID:27158815| conserved exported protein of unknown function [Uncultured spirochete bdmA 4]
MKKFFLVVLAVLFVAVPAFAATSISTTLQATVGASLSITTTIPGTKALDPTLTSATLGNV
TITSNVTNWTITIHSANGGKMIRSGSADIYPYLLNFGSETGINLTSDHVITKTAPQTAQT
TSVSVTYDTAATLGISAGTYEDTLTISLVAL*
>SPBDM4_v1_50453|ID:27158816| protein of unknown function [Uncultured spirochete bdmA 4]
MTKEKIAKAQSASEKTIGGTLPLLLSEPRIMFIIQPNHHITNKNPIAMGTKVNIELFS*
>SPBDM4_v1_50454|ID:27158817| exported protein of unknown function [Uncultured spirochete bdmA 4]
MNIIRGSESKRGRVPPIVFSLALCALAIFSFVMLRDSAYRSESALLKAASEKIALRVSAK
LQAMLDTGEQFRQLLRTEGGRTYAALRPLAEDSLARSSYIDSITIAPGAIVRYSFPEDRA
SASIGHDLLNNPERMQALVEAVREKKAVLQGPSVSAEGEDLAFLRIPVMDKADLWGFVSI
GFDIDRMLADLDISSEFPGLFVAIASSGTDGRGRQVFWGDQRALQGYEVAVTAGSEDFPW
TVYTASSYPVVRVVWWGAGLLALSIVSLVLFMQGMVAGRQRQKRTLSAPSLKEASFVVTP
FVQKAEENEGPLPEVAAQDQAAPASTALASSEEGARAAVRTPVSVLVVDDSEVNRELLFR
MLTLKGYEAQSVSSSEMALDAIKERSFDVLLIDCIMPKTDGYALASAIRNIIGEAPSLMI
AMSPRHDPQETEKCRAAGFDSVLIKPFTMTSLDQKIQETLYHKGDKVPGG*
>SPBDM4_v1_50455|ID:27158818| conserved exported protein of unknown function [Uncultured spirochete bdmA 4]
MKKFALVALFAVAAFGMAFAANPSGTVQFTGAVAESFSLTVPQAFTNGTISNDSETTWSL
GDVKVISNVKNWTIALSSAHSGNLVLSSDNNETIAYTVKLEGLLSDVSLSGAQTSQAQAR
TAKAGNAYALSVKIPASSNFYQAGTYSDTITVTIIHP*
>SPBDM4_v1_50456|ID:27158819| Rhomboid family protein [Uncultured spirochete bdmA 4]
MYSNSVLRRPFRYRFFNATLYLIAANVIIFALGYVWPMLTYILALNPRAVMAGWVWQVFT
YMFAHANLTHLLVNMLGLFFFGRPVERTLGSYEFLLYYLLSGLFAGIASFAIYAFSGAWN
TMLLGASGAIFALLLAFAVLYPKAMVYLYGIIPIRAPVMVIGYTAIEIVSSFLGAASSVA
HLTHLAGFEAGEAYFPVRLGVNPFKRLREK*
>SPBDM4_v1_50457|ID:27158820| Phospholipid/glycerol acyltransferase [Uncultured spirochete bdmA 4]
METIKSRYAPFITELIAHVPREAQVSELNVYQEGIASILPYIDTIIHDYLDDCSTIEGLE
NLGALHDDAQKGESALILMEHYSNFDLPVFHYLLRKLGAAGDAAGESIASNIVAIAGIKL
NEENPAVYAFARAYTRIVIYPSRSMQIIREKFNDPKELYQEIKRSISINHAAMKALSAAK
TSGKMILVFPAGTRYRPWDPSSKRGVREIASYIKSFSKFCLVSVNGNILRLNPSGGMEED
ILQKDRVIYKVSPVHDSKEFLASVKYDLHFGDDKKQAIVDTVMDLLDAMHKETENRCRD*
>SPBDM4_v1_50458|ID:27158821| conserved protein of unknown function [Uncultured spirochete bdmA 4]
MATVVSASRRSDIPSFHAEWFYGRLRAGCVDVANPYNNAQVRRVSLRPEDVACFVFWTRD
ASPMLKGIDALEGYEFYFHVTITGYGPPLEPSGLPAKEAIEKLQALAAKVGASRLVWRYD
PVLLAGRYDAAWHAENFSRLADGIGGSVRHCVISLYDAYRHSDSRLRKAGILADSASGRM
VNWADSQGSVVDMMQTLAEIARARGLPVSFCAEPELEHRIASENLACIDARIIRELTNRP

FSVRKDKNQRSGCNCIESVDIGSYGICPRGCLYCYANRRQNVLI*
>SPBDM4_v1_50459|ID:27158822| HAD-superfamily hydrolase, subfamily IIB [Uncultured spirochete bdmA 4]
MNVPGISPISALSREEAHTIRYVLMDIDDTLTTDGKLRSESYAALWKLHESGLVVVPITG
RPAGWCDLIAREWPVEGVVGENGAFVFWEGDGHRLATLTHPNAIPNSDPRLVALRDRALA
KVPAARLSRDQFSRLYDVAIDFAEEEPVLPLSIAQQIKAIFEGGGAHAKISSIHVNAWMG
EYDKVSMAIHYLTKRFGYDDLHDADSVMFVGDSPNDEPMFAHFPMACGVANLLEYRELFA
HLPHFVTSRESGAGFSEFAQVLLERRSS*
>SPBDM4_v1_50460|ID:27158823| exported protein of unknown function [Uncultured spirochete bdmA 4]
MRIKKNGIIHAALLLILLGGISGRVQAQLLGSLEPVLHFQTESFDIYAPPNMEPEAERLA
GFADKTYAMLCEFFGVKASARRIPVLLSDIEYSLNGYTTLYPSNRIVLLLASADPRSQLA
TMQDELYSVFLHELVHYVTLNERAGGWRALAWLSGDWIAPEVWMMPQALVEGTAVWVESR
LGEEGIGSPGRLNDPAALETVRLERARGQSRSLWDVSGLVDFYGAGNLPYLYGGLFADFL
SERYGPDMLGRLWRASSDGNIFRGFDGTLTSRGILERETRVPPRQLWQDFLAWVDDGSSS
VEGGGGAIWTGGAQELFSGYVGVAGAGEGVLYFVDLERRGLYALPVKAMAEGESAQENGI
EKSEGEGEKKNVRPERLFAVDGMLRNIFFNVKSEALELDWIRIDAQNQEIPARYRYDLKN
RTLTYEYDLPVAAPGSALQALHDESEQNIFLYDSWQDSETSIRYGLARIGTAVLPARQLP
EGRIEVANIPNNAMRWLSPGSRDQSESPDSVRFALSTIPDKGLSRLAVLEEKKGSWQLSI
ARDAPQGGVHQPIFIDASHIVYRESKENGQAALCLLDISEKRQGAAVSDRFSAPIPIDWI
LPSEWLALYAPEIHTALPGPSDQGHPQKSRSTLFPALFSTSRIPYANGSLIGLDIIASDL
TERLAWVVFAGWDFSTARPAASTELQLGAGAWQFNVSASDQSVLTTPVARKSTLGTTVTW
HRTLLPSFRSISANSHAAFAGVQNNYSASGILNVVPDYYAWATGIGLDFSSAYASRKSPY
GTLGFSLSGNVEYESASTAGFGGMSLSGSASLKGRFDASLYGAVAPAGGVAFAPAARYLE
SGGSYVLSAADIPYPVYQEYRSFLNPSPWYVFGEAQYRLFSLETGWHLRLPFMPAFALRR
IVGNIGLRGAGLDVSGAPVILSSAFAQADFDFALLAGLAAETHTHFTVEAAWAFQPGKAG
GRPTHISVGLQTSLE*
>SPBDM4_v1_50461|ID:27158824| NADH-dependent butanol dehydrogenase a [Uncultured spirochete bdmA 4]
MQNFEWVTPTKVIFGSKILEKLGKETAGIGKRALFLYGKESIKKSGLYVMVVAQLKGEGV
FFVEHGGVQPNPRISHAEEGARKIKENHLDVIVAVGGGSVIDEAKAIAVAGCHEEPLWDF
YMRKISFEKALPIVAVQTLPATSSELNGASVMTNETTHEKFSIRSETIYPKVSFLDPSLT
LDIPVQYTAYACTDILSHLMEGYFTASSDFPLQDGMVEGVCRAVMGALEIVLTDPRNLEA
RSTIMWAAALAWNGILKSGVEGASIPNHVLEHPLSGFYDVPHGAGLSIVIPAWLKYMKPS
IAHRIVLYGERVMGLGKKLEGKTPPEGADIVIKELENWYRHIHTPVRLKEAGIISLDIDA
CTKQAMALSVLWGVPGYDAEDIRAIYALMN*
>SPBDM4_v1_50462|ID:27158825| Iron-containing alcohol dehydrogenase family protein [Uncultured spirochete bdmA 4]
MDSFSYYMPARLIFGPGKLAELATTSYFPWGKALLVIGSGGAMRRSGTLARVQSLLASRG
VAVLVYDKIKPNPEMEQVEEAARIARKADCDVVVGLGGGSTIDSAKSIAMLAKNPGRYWD
YIMSGTGGRKIPVNGALPIVAITTTAGTGTESDPWTVQTNPQTREKIGWGTDCTYPTLSI
VDPELMISLPPKQTAYTGMDAFFHSAEAYLATCHQPASDLLALDAVSRLTKWLPVAVADG
SNIEARTSVAWANTEAGICESLSSCISHHSLEHALSAFYPAVAHGCGLTMLSVAYFGDVA
ERNPERFADLARAMGEDVDALADAERPFAFIKALEKLIAAAGLAGEKFSDYGVKKSDIPA
MAQNALTAMGGLFACTPVKQEQADVERIFERAYR*
>SPBDM4_v1_50463|ID:27158826| putative DinB family protein [Uncultured spirochete bdmA 4]
MVEVFQYYSKCNKVINAEMMGVIGGAKEDVFDAPVDGYFKSIKELLDHIYGADVRALLSF
RTVREYAIFKDPFFKTPPDPKAMVSADFASFRENREHLDALLVQLTDEIAEADLSKYVTR
TTRQGEKQQKLFWKTLMHTFNHQTHHRGAVSQILDQFKVENDYSGMIRID*
>SPBDM4_v1_50464|ID:27158827| exported protein of unknown function [Uncultured spirochete bdmA 4]
MRELKKARRAIVGIVLAALFIVAPCGLAFADEIKAAEYALSCLNISPGANASELNFAWLT
PKLGKTIVQMAKATDKTSAEFPADKAVSFSGSQAIVTATTFNTDDVTAPTGLFSNKVTVN
GLANSTEYVVYRVGDGSNWSDTYAVSTRDPKAFGFLVVGDPQLGSKATGPKTLEADTAGWT
SLLNKATALYPDSSFMLSLGDQVNDYSDRAKQDAEYLTYFSPKQLRSLPVATVDGNHDFA
MGEYYGYHYNLPNLSSQYGVSYGNDGDYWFRYGNALFLMLNSNTESVYTHDLFIRDAVAK
NPGTTWRIACFHHAIYSEADHVNDPDIIDRRASYVPVLERYKIDIVLMGHDHAYTRTFQM
LGGKPQKTQVQDTSGTVINPTGILYLTFNSGSGSKFYDWKNSSAPEAYSAARWQGKVPSF

GHISISGGVFTFTEYRADDMSVIDSYSMIKTE*
>SPBDM4_v1_50465|ID:27158828| protein of unknown function [Uncultured spirochete bdmA 4]
MINIDTAEGFIRKNGTEIERARLSSILGSMFDTQKALSLIQSLQNKDGGFSLVKDRESNI
SDTGFILTWLSDLKALHSNIAEKAIVYLESLQNKNGSWNETLPTDDTETPEWQKPENHRA
MLFHTANTLFWLNKYSRNSICIEKGKRFLNENYKSEQEYIHTKWLFASIMSEKHSWRSSI
IREIVKEIYEEITPEMPSSILTWMLCAFSVYEIPRDMEYIPAMMRQIHQEGDGSIESEDD
DSYKVNATLEAVKVYKYYIE*
>SPBDM4_v1_50466|ID:27158829| conserved protein of unknown function [Uncultured spirochete bdmA 4]
MWIVDATEEYLEWFREQEPDVQSAVLAKVLLLETFGPQLGRPHADTLKGSTIKNLKELRA
RTSVHVLRVLYYFDEHRQALLLIGGDKKGKNEKEFYKSLIQTAETLIERYRQ*
>SPBDM4_v1_50467|ID:27158830| conserved protein of unknown function [Uncultured spirochete bdmA 4]
MATVKNAVKAMEGMMTEEQIRRARSTAECEILAIRLAELRERRGVTQAEVTAFSQTAVSK
LEKRKDMKVSTLVEYLEGIGMGLELRVYPKGSKGKTQGEILLKV*
>SPBDM4_v1_50468|ID:27158831| Transcriptional regulator, XRE family [Uncultured spirochete bdmA 4]
MSNLSKYIEARKKVDPAFAVGYDAGYEEFKIGVLIKELRLKEGMTQEELAKRLHTKKSVI
SRMENHSEDIRLSTLGKVAEVFGKRIQIAIEDLAH*
>SPBDM4_v1_50469|ID:27158832| protein of unknown function [Uncultured spirochete bdmA 4]
LPFHTEKLTYLLTCVKLLAFKESMAKYIHFYNSERFHQSLGYETPAARVRSPLGIQECNM
EEVIRFKTLDKWAHLRPF*
>SPBDM4_v1_50470|ID:27158833| exported protein of unknown function [Uncultured spirochete bdmA 4]
MKKWSMALVLLFCGMCLFAQSLFDVVTTGTPEQVQAAIKAGARIDDRDNWFSDTPLMWAA
FLNENPDVIMALLNAGADGTIKSSEGKTAFDYAQQNQKLEGTKAYWDLNNARF*
>SPBDM4_v1_50471|ID:27158834| putative Cro/CI family transcriptional regulator [Uncultured spirochete bdmA 4]
MQIVEKTRHIRVNLTGEGAEKVAALVKERFPEAMLIDDDDEVVEWESSSLVREIKAKKTP
GKLVRAYRERAGMTLVELAKAVGTKYPNLSAIEHDRRAVGLGMARKLGKVLGVDYRKFIE
*
>SPBDM4_v1_50472|ID:27158835| protein of unknown function [Uncultured spirochete bdmA 4]
MMVPDSNPSPDDLIQKLEEAIHEPVISFSIFSDTKMPSNWHIYNAEPLETYMADPQRYTI
VLYIPKKYEGYLCSTSIAVFDRTKDKYIAFACCYDEG*
>SPBDM4_v1_50473|ID:27158836| protein of unknown function [Uncultured spirochete bdmA 4]
MTKSERRIKRLEQQSAQDDDAPAPPEAIRLIEAMMGEKMSEEAKRRPQKRGGPLDPELKA
MIDKIIA*
>SPBDM4_v1_50474|ID:27158837| protein of unknown function [Uncultured spirochete bdmA 4]
MQGDSSIVAEKKLPRIRRYATLGAIRREVLRCYEELLAKPNADPVEIQRSRALGYLLSTA
GELLKSEKLDEIEKRLDALEAKRDDKETRQ*
>SPBDM4_v1_50475|ID:27158838| conserved protein of unknown function [Uncultured spirochete bdmA 4]
MTDASLVDDIIMHFQAGTTETAAGLLRQLSDIDYEQHRTPLAEAAGVRPAALDKLRRQAE
KKESGEKMQGRALTLPDITPADEPQDGATLLDDLARHVRRFVVIDETGIAAITLWTVFSH
LAHDAPCCPNLVFSSAVKACGKSTALDVVSRLVPKPLATANVSPAALFRTVEMLSPTLLV
DEADSMFRNNDDLRTLVNAGFTRTAATVVRIVGDDLEPRLFSVFCPKAIALIGTLPDTIE
SRSIVIRMRRRLPDEPIERLRSDKDQGFAPLARRIARWVSDNKHLILAEEPELPESLSDR
QADAWRELLRIADIAGGEWPTRARNAALELCSKLDEDADISTRLLSDIEKIFNDDRERTK
WPSQTLVDALNAMEESPWAEFARGKGLTTNRLAKMLARFDIRTRTLRAGTGTPKGYTVES
FAEVFSRYLPQNRNTATFDDNTLQNNDLKCCGNVSVADAKRNISDETGQQEGAQDEFPLF
*
>SPBDM4_v1_50476|ID:27158839| protein of unknown function [Uncultured spirochete bdmA 4]
MSALTANADALMAVLKPELERLCRNAPPFGEITLKASIHDGDVGRVSLGIEEARKIAPRS
GREGGRL*
>SPBDM4_v1_50477|ID:27158840| protein of unknown function [Uncultured spirochete bdmA 4]
MENGEKRFLSRQEAADVLGISLVSLARRLRDRSIPYVKLGNRTLIPSAYLDELVEKALSG
QGAK*
>SPBDM4_v1_50478|ID:27158841| protein of unknown function [Uncultured spirochete bdmA 4]
LEEDSQGWETNVYTWQRGDTGRKKSSSKSLAGTGFAYTGRGRLGTDLVN*
>SPBDM4_v1_50479|ID:27158842| Toxin of toxin-antitoxin (TA) system [Uncultured spirochete bdmA 4]

MWEVVYTKQADKDSKKIAACGLKERVQNLIEILKEDPFKNPPSYKKLLGDFNGAYSRRIN
IQHRLVYQVFEKEHIVRVLRMWTHYE*
>SPBDM4_v1_50480|ID:27158843| Antitoxin YefM [Uncultured spirochete bdmA 4]
MAVLTASNARANLYRLIDQTNESHEPVVISGKRNNAVLIAEEDWDSIQETLYLTSVPGMR
ESIINGMKEPLAESIKELSW*
>SPBDM4_v1_50481|ID:27158844| Site-specific recombinase, phage integrase family [Uncultured spirochete bdmA 4]
MARNPVSISKRPTLKKGELRYYVRFWNENTGKYSNARSVLSIVQELGLNEKEYPPTSRTG
ALLIGQELLKRGGALSKKNDPLFADYCASMWDWDTSPYIQGRIARGLRIGRGHALHSKRF
IENYVHSAFPALKLCALRPYMLETFILGLKKNSRLRNRSINSIIDAIKTPLKEAVRLGII
AVDPSASLQKLGDDRKIKGIPTEKELSGLLSLNLDPRIRCAILLGAACGLRLGEVQALKL
ENIEGNTLRVAHSWSRFEGCKSTKTGKERVVPLPEIIRTELLKLADMNPHGRDGFLMYGL
LPDVPFDCRAIARGFMNALAHLTLGEKYSTATRAEKTAALQPWKERNITFHSLRHFANAE
LRGAVPDETLRKLTGHSTEAMTDHYDHTTEADLEALAKAQEARIVPFIRTA*
>SPBDM4_v1_50482|ID:27158845| putative RNA methyltransferase YpsC [Uncultured spirochete bdmA 4]
MHTSRVANVMRAIAVCAIGLEKVLARDLAHLGFRDVGRSAGRVFFNVGADSLAEDLTKAN
IGLRTADRVLLVVGEFKAFDFDGFYQGVASVPWERYCSKETKVLIERARANRCKLHAQAT
LQSMTQKAIYTELMQQFRVRKMPETGNTLEVRIYGDNDWWQIVIDTSGEALSRRGYRRFT
HAAPLKETIAASMLFLSGWSRARPLVDPFCGSGTIPVEAALYACNMAPGLKRHFAFELFP
EMDGNKIEDTRVFFRQQMRKDIRTDIQASDIDGQALELARNNAKLAGISEFVRFSSVDAR
ELVPSGNRGVLLGNPPYGQRLASQEEAHALYRELSPMLERFLNADWECGFLSADKEFAST
VGFEPASVREIVSGQETIYFNWFSGKGGRKNETDSGSGDA*
>SPBDM4_v1_50483|ID:27158846|zwf| Glucose-6-phosphate 1-dehydrogenase [Uncultured spirochete bdmA 4]
MNIADDSPYCRLDTTRFSPEPALFFVFGASGDLASRKIFPALYDLYLEKQLPVGLLMIGA
ARREYSTEQFREIMRTSCMAHSRHKAEALHDEAWHDFSQRIFYLRNDVEDTGSYATIRKL
VVERDRSVLAGLGPGAELPENTLYYLAVTPEFFPVIAENLGRSGCGSDASSQGWRRLVVE
KPYGKDQQSAARLTESLHRWFEERDIYRIDHYLGKEAVQNLLHFRFANTIFEPVWNRNYI
DRIEITVVEQEGIGTRGGYYDGFGAARDMLQNHLTQLLCLTVMEPPASLSPEHIRDEKVK
VLRAIPEYSKEQILARARRGQYMAGTDAQGRAVPDYRAEHKVRPDSATETYASLTLSVEN
WRFSDVPITLRTGKALAEKYSEIVLYFKRPPSALFAALCGDRLATNSLTVRIQPDEGIWL
SFNAKVPGEPAIRSNSLRFSYREVTDYFPEAYERLILDALSGDSTLFIRADESELAWKVI
DRLEAAWASVESGSNPEEGGLLRYSAGTSLKALRHQIEESPRLEGIS*
>SPBDM4_v1_50484|ID:27158847| putative 6-phosphogluconolactonase [Uncultured spirochete bdmA 4]
MILQIYTSNDEWLRAALDLILSTQDGGERRRSLHLCLSGGTTPKPVYESLARSETFKAIA
ASKAVHLWVGDEREAPVHSGLRNSEMIAGIFVAAGFKNFAFREPKNSALKIQIPAPPIIK
NLDSAAPGLILHAWPTGPRENAAAAYDRELAVFAGETIRADRPFFDLTILGMGEDGHTAG
LFSMEDVRRGAGRLVILTEAPQEPKKRMTMAPHVLLSSGKILVLLRGLPKARLLMANLFG
ERADPIKYFLLDRCEVVAQI*
>SPBDM4_v1_50485|ID:27158848| protein of unknown function [Uncultured spirochete bdmA 4]
MRYRNILLYMELFLALHGTISAQIRERIKFSGFDWYVRKTTEPEGPMNNLFGGLGTSVDT
LPDGALRLSVVYRNGTWYSAEVWTTRSLG*
>SPBDM4_v1_50486|ID:27158849| protein of unknown function [Uncultured spirochete bdmA 4]
MILGLFTYRRTLWYHNREIDIEFSAWGTDTERLNGQYVVQPHDKAGHLYAFPAAAFAGPS
TQQFTWLSDRIEFSSWSGYGEKPPPGDPRLINSWVFSDAKSIPRPSAPIHMNLYLFESPP
SDKKEGSLVVILDGFEFAPAKK*
>SPBDM4_v1_50487|ID:27158850| conserved protein of unknown function [Uncultured spirochete bdmA 4]
MLKEEFIQKSPVRIFEKSIEGGLHAGNIGVLASKKGIGKTSVLVQIALDKLMQGEKVIHV
SFNAHTSYVISWYENIFNEIARRKNLENIDDVKEQLVRNRMIMNFTQEGVTVDQITRTIN
ALIVDGGFNAKTIIVDGFDFTRAPAERFEKVKTFLAEMQLEAWYTCTLAETEPVLDTQGI
PIVLRSIEPLISVIVVLEPKKDFIHLRVVKDHERRNPSDMNLMLDPRTLLIAET*
>SPBDM4_v1_50488|ID:27158851|fusA| Elongation factor G 2 [Uncultured spirochete bdmA 4]
MTFDIHKMRNIGISAHIDSGKTTLSERILFYCRKIHAIHEVRGKDGAGATMDSMDLERER
GITIQSAATNVQWKDYFVNLIDTPGHVDFTIEVERSLRVLDGAILVLCSVAGVQSQSITV
DRQLKRYHVPRLAFINKCDRTGANPFKVKTQLAEKLGLNPAFVTIPIGLEDKFEGVIDLI
DMRAVYFDGPNGEELRLAEIPAHLQKDADRYREELLEAASMFSDELAEAYLEGEVTKDLV

LEAIRKGTIEEKFVPIFVGSAYKNKGVQLLLDGVVNFLPDPSERRNIALDLDNNETETEL
VADDQAKTVALAFKLEDGQYGQLTYVRIYQGMIRKGDELYNTRARKRFKVGRLVRMHASS
MEDIQEGNCGDIVALFGIECASGDTFCNPDLNYAMTSMFVPEPVISLAINPKDTKSSDQM
SKALNRFTKEDPTFRTYVDPESNQTIIQGMGELHLEVYIERMRREYKCEVTTGMPQVAYR
ETITQRADFNYTHKKQTGGSGQYGRIAGYIEPLKDETYEFVDMIKGGAIPNEFIPSCDKG
FRAAMEKGTLIGFPVTNVRCLINDGQSHPVDSSDIAFQFAAIGAFREAYEKAKPTILEPI
MKVVVETPNEFQGNVFASLNQRRGVIMANTEDGTFSRVEAEVPLSEMFGYSTVLRSLTQG
KAEFSMEFEKYAKVPTSISEQLRKDYLEKRKKDQK*
>SPBDM4_v1_50489|ID:27158852| OsmC family protein [Uncultured spirochete bdmA 4]
MTKEIQMNWLEDMAFQTDLDGHRLVVDADDSVGGKNRGPRPKGLLLVSLAGCTAMDVISI
LKKMREPVGWFNLKVSGNLTEEHPKKFTDFKVIYQFKKSDGLNPQNVQKAVELSQNKYCG
VSATLRDAHDVAWEIEYI*
>SPBDM4_v1_50490|ID:27158853| Major facilitator superfamily MFS_1 [Uncultured spirochete bdmA 4]
MEQWRKSFSTLMIAEFLAIAGFSTSNPIIPLYLRDLGVTDTAALNAWTGAINGLAAFVMA
LAAPIWGALADNYGRKLMLLRAMGGGAILMGLMAITTSPWQVLVLKGIQGAITGTVAAAT
VLTASLVPTNQIGYYMGLLQMAVFAGNSAGPLIGGVVTDIAGARVNFLVTSALLAIAAIM
VHRRVVEIDIPKPKQRSVIRNAIPDFSVLKGPSVLKQIFIVIFFVQMGNAITGPIIPLVV
LALQKRDVFAGSISGILIGVTSVAAALGSVVTGKVSQRFGYGRALLVCVTGAFLFYIPQG
IVMNVWQLLVLRFISGFFIGGTMPTANALIALHVTKEKQGSVYGLSSAVSNMGGSFGPVL
GAAAATLAGYQSVFFLSALMFFGVSIGVWRLVRKPKELVGHNVE*
>SPBDM4_v1_50491|ID:27158854| membrane protein of unknown function [Uncultured spirochete bdmA 4]
MRGILRSIAKTLLVLVLVTGALSSVFAQTSNKTSATIQLAVYVPPILTLSVNFASGGVTQ
ITGHIGNTPGTYNNGFELRPNSIFTLGAARIVSNLTSSYSIVIQSMNSGTLKNQDSGSEI
AYDLLIGGIPAARYGDAFRVVSSIKTPRDGIEFPVSIALGNIPPNASSGLYADNLLFNIM
AN*
>SPBDM4_v1_50492|ID:27158855| Peptidyl-prolyl cis-trans isomerase [Uncultured spirochete bdmA 4]
MNHRIILFALLLPLFSIHFISCNAQVSRPDGMYATIKTNKGDIVISLEFEKAPMTVGNFV
GLAEGTLDVTKGKPFYNGLTFHRVVADFVIQGGDPAGDGTGGPGYRFPDEIVPDLKHDGA
GIVSMANAGPNTNGSQFFITLGAAPWLNGKHSVFGKVIEGMDAVKQIQQGDRIESITIQR
YGAKAKAFDSSQKAWDQRLSAAYATLNALSRQKRDADMALIAQKWPDIKPDDKGIFQKVI
RKGFGAVPNPGSKVSVIYKGMLLDGTVFDQSALSGGPFSFTIGKGEVIDGWDIVVSTMQK
GEKRLVILPPELAYGSQSIGDVIPPNSFLVFEIELVSIEQ*
>SPBDM4_v1_50493|ID:27158856| CMP/dCMP deaminase, zinc-binding protein [Uncultured spirochete bdmA 4]
MPEYRRPTWDEYFMEVANAIAKRATCDRGRSGCVIAKDNQILATGYVGAPAGLPHCDDVG
HQMRKMLHEDGTITEHCVRTVHAEQNAICQAAKRGVAINGATLYCRMTPCRTCTMLIINC
GIVRVVSEYRYHDADESESMFKMAGIQLEYVHNEVLKYNRQ*
>SPBDM4_v1_50494|ID:27158857| conserved protein of unknown function [Uncultured spirochete bdmA 4]
VAITIVFLGEIVGKTGVFTIKSTMPEIRRRFNPDFVCANADSATGGAGLGVQHAVYLRKL
GIDCITMGEASYYKPDMTEFYPKAGWVLRPANLPEGDPGRGWKVFQKDDKKIAVVALLGQ
SGFARVHADNPFLAVDQLSHFLHRDTSCIIVDFHAATTAEKLSLARHADGKVSAILGTGG
KVLTADARILQKKTAAITDLGRTGSILSVGGLDPEVKVREFLTGVPAWSRDARAQPEAQG
VCVRFDEDGLALSIESFHIGGKEIPDEGSSDSKRD*
>SPBDM4_v1_50495|ID:27158858| putative Positive regulator of sigma E, RseC/MucC [Uncultured spirochete bdmA 4]
MKEVATVKEIKGDMVTVAIQMQEGCGVCGNNGACKIRRSNLLVYNKSRIDVKEGEEVVIE
VPGIEQAKSAFWVLGLPLIMLFVGYGFGALVFHAATEGPSVASAGIGFVVALLVGMLVQR
RNRLESFPYILAKEGEGVY*
>SPBDM4_v1_50496|ID:27158859| DL-methionine transporter subunit ; ATP-binding component of ABC superfamily (fragment) [Uncultured spirochete bdmA 4]
MFHAERPEEAADQALIAQPSYNAPSRGDLFAPSRAAFQAISVPTQAPPPNVMRVDGISKA
FGSHVVLSNVSLGVPKGSIHGIIGKSGAGKTTLIRIAGLLEKPDSGSVRYEGIETPVHLL
RGRALLEARRKTGFVFQSFNLFASRTAGENIAFPLEAAGWPHAKAHARVAELLALVGLSD
KADSPSMHLSGGEKQRIAIARALANHPSILFSDEATSALDPETTRSILELIRSLREMLGL
TVIMVTHQMEVVRRICDTVSVIEGGKIVEQGLVADVFTNPRSDAAKAFLKEDRND*

>SPBDM4_v1_50497|ID:27158860|metI| DL-methionine transporter subunit ; membrane component protein of ABC superfamily [Uncultured spirochete bdmA 4]
MTDALNLLIQPLGQTLLMILVSTLIATIFGGALGILLFALSNDRFAGKHKGARFAKHVAD
RAINVLRSFPYIILMVLVLPLSRLIVGTSLGTRAAIVPLSIAAIPFLARIVESSLSEVDA
GVIDAAISCAASKRRIVFAILIPEALPSLVSGLTLTMISLLGYSAMAGVIGGGGLGDLAI
RYGYQRFRGDVMAVAVITIIILVELIQFAGTALANRIRATR*
>SPBDM4_v1_50498|ID:27158861|plpC| Outer membrane lipoprotein 3 [Uncultured spirochete bdmA 4]
MKNSMIHGASKTTSPKRAPRAAFREGLVLFFLVAMVAGGVFAQTGGKLVTLKVGATPIPH
GDLLQLIKPDLEAQGIKLEIVELTDYVTPNILLADKQLDANFFQHLPYLNDFCADRKLQL
ESAGQVFVAPLGLYSRKYKKLEDIPAGSVITLPNDPTNEARALILLENKGLIKLNPKAGL
KATIRDIVENPKRITFREIEAPQLPRTLDDAAAAIINGTWAMQTGFVPSRDSLVLEGAES
PYANIVAVRKGDANDPRVVALVKALQTQKVKDYLLNKYNGSFVPAF*
>SPBDM4_v1_50499|ID:27158862| conserved protein of unknown function [Uncultured spirochete bdmA 4]
MEKPGDFKESSRAEDADSAPTVAAEEGPESPPAEGASNHFDEAFIGAARDYRMLLDKEYP
VDASLKLVGDRYRLSHNGRMMLYRGILSRSASQIDLAKLVRNPWEGATLGIDGYNILFTL
TNYMHGHPMFIATDGFLRDVGGAHGRIADHRGFERMAGLTVDILATLNLDQITFFLDAPV
SSSGRQAGFIREAFARRGINAEVRLENGVDHFLAYWEGELIATADSAIIARTKAHVFDLA
RYILERQFSARFPQLAFFLE*
>SPBDM4_v1_50500|ID:27158863| Alpha amylase catalytic region (fragment) [Uncultured spirochete bdmA 4]
MIPETDAIYQIYPRNFTKEGTLRAAIPQLGRIEAMGFDWVYLTPIHPIGKAARKGSLGSP
YAIYDYRAINHELGSEADFAAFIDAAHAHRLKVMIDVVYNHTSPDSVLAREHPDWFLQGP
DGRPGRKCGDWSDVVDFDYQASPHLWVELIDTLSMWRDRGVDGFRCDVASLVPADFWKQA
RVRVNQYDPGARKERAPLVWLAESVHPAFLRRMRQDGHGAWSEPELHAAAFDLTYDYDGW
ERLEVKIGV*
>SPBDM4_v1_50501|ID:27158864| protein of unknown function [Uncultured spirochete bdmA 4]
VQETLYPKDAKKLRYLENHDQERAAHRLAADGPLRAWTALYQFLPGVSMTYMGQERALDH
RPDLFERDPIEPERGDMEFERYFRESLRAAKEAKQRAPFFSWSVAEDGTVYCLRSTEAAL
NPHPVDQTLLKKGNYLLVAQPEGEATRGNATASAAPFSFGGTDLLSGEKVRVSGGATPAY
APAMLIRLGD*
>SPBDM4_v1_50502|ID:27158865|nagD| Ribonucleotide monophosphatase NagD [Uncultured spirochete bdmA 4]
MKATEKTLKHIRKKQAFIIDMDGVIYHGNVLLPGAAQFVDWLKREGKRFLFLTNSSERSP
EELSQKMARLGIEVAPEHFYSSALATAAFLASQKPNGSSYVIGEPGLIHALYRVGYTMNN
VNPDYVVVGEASSYNLDTLIKAVRLVMGGAKLIGTNPDLTGPGEGGLVPACGALVAPIEL
ATGRKAYFVGKPNPLMMRHALRTLGATREETVIIGDRMDTDTIAGIESQIETVLVLTGVT
AESDLPRFAYAPSHVLEGVFEIPDKEPKKIA*
>SPBDM4_v1_50503|ID:27158866|ppk| Polyphosphate kinase [Uncultured spirochete bdmA 4]
MNKLPTMNRELSWLDYNARVLAEGLKPSTPLLENLGFMRIFASNLDEFFMVRVAAVKAAA
QIGQTNEEWAGMDPQTLLKAINQKVQAHYDMLYKHLHATVLPGLAGHGIQLISEKAWTSR
EWRYLERFFMENVYPLLTPLRLEPRMELRREQDYFPSTGSLQVHVAFRLEEDGSIAVVQV
PKNLDRFIVLPQRLSIEPEPATSQPQENVLRLALLEDVILAFGSKLFAGLKVLHGIIFKV
NRDADFSVDEDRDDDFLTAMEEVLAGRQNSTPVRMVYSGEDQILLGALMQSLSLEPLDVY
HVEWLIDLGRAMELCDPEFYRVHGITPDPSLFFPLWKPARTFDEKGSIFDWIDEHDRFVN
LPYESFDVVQRFVEESASDPSVLGIKITLYRTSGMNSPIVHALAKAARSGKQVVVVLELK
ARFDEEKNISWAAELEQAGAIVTFGVARLKVHAKAALVIRRRQDGSIARYLHLSTGNYNE
KTARSYVDFSLFTANPSLCTDIALFFNILTGYSSIQSLSLIAVSPFDLKERIIALIDREA
AQSSLESPGCIIAKLNSLSDPDIIGALYRASAKGVQIKLNVRGVCTLVAGLPGVSETIEV
RSVLGRNLEHGRMLYFRNGGSEELYLSSADWLNRNMKKRIELLFPVLDSEIKARCKDILS
AYFSDNTHSYKLEQNGDWTNLSLTRRPEEPAICVQEKLFKMARKAVRNEEDRQEILQVRR
SKGKFFEPGTIS*
>SPBDM4_v1_50504|ID:27158867| Ppx/GppA phosphatase [Uncultured spirochete bdmA 4]
MSDAQPQKSPQSKESIPSSSSSIQGLHAAIEIGSTGIRLIVAEIDSKGGLKIIDRASKQS
RLGRDVFTMGSISRDGLRETIAILSSFSELLQGYGLQPSDIQVIGTSALREASNRDTFID
RINLQTGFHVRIVEDIEENHLMYLAVQKTLEDERSFLTRSNAMILEVGGGTTEVMLLKKG
QMVSSHSLSIGTLRMDEQIRESARQPRQFIEMYLESNIKTACDVLAEDLPLDSVRTFVLI

GSDARFAAYCLSGKNFSHYAVLDRAQFIEFADSVAGLSPEECMAQYHLSWNETESYAAGL
TIERMFLEKTGADIIIVPNVSIREGILLAQVQGMDQKIEQELRRQVVASARSLAKRFRYD
EAHAAHVKNLSLEIFDALKKEHGLGKHERMLLEVAAILHDIGTYLRTSGHHRHSEYIIAN
SEIFGLNREDINIVSNVARYHRKTPPLSTHVNYIALPREARIIVLKLAAILRVADALDRS
HSGRVRDLSFERTEDRFIIRPSQSLDFSLEKLSLAEKGDMFEDVFGLLPVLM*
>SPBDM4_v1_50505|ID:27158868| putative lipoprotein [Uncultured spirochete bdmA 4]
MDSTTGRKKGAAAKLFLVAGLIVILFSVLLSGCARREGWGMVLWSVKGTAVKGGAVVPIY
LKSNISKSYVIGLPDDNKTKIEVPFPSLEFFSSKSAAETRAREFAPYAALYLSAGRDGLP
IREKPSVNARRVYRLRLGESVKVLSVADGEAVFTGGKALPGEWYFVQAIDGTRGYVFSNT
MVLYEEKEGTAAPVIASSPAPSASLLDIIYAKPWRPAYYQIMVDDSTIDPDLFILRYGLF
ADAKNSQVRIELPGYSGAFSFTSVSQSGDWLVFEKSKLRIRFENESTIVADWSGTDQVLA
DEGWSQGGQAARFVQLDASIPVLLAGENSRRESELKAFFQRSSQLQSNGESTISFLSDTA
GILSVDLKGTFEWSHTDQLPAGFAPEIPAEARDNVIKGEIRFGLHLGTAISSAWMGGFSL
LVGDNPDRQDFVYRFENGQLVIAKADSVTLRGTTESLDTRFSSVNYYLVVK*
>SPBDM4_v1_50506|ID:27158869| ABC transporter related protein [Uncultured spirochete bdmA 4]
MSFIQLGGISLSFGARDLIRNATLNLQDGSRAALAGPNGAGKSTLMKIAAGIVKQDSGEV
ITTKGARVVYLPQTGIRFEEGRVHDIAEQAFSYYLGLLDEQEKIGRALESEHLSEKETAR
LLEQHHEIGETLEDANYWRREERIAEVLRGLDFKTGDFERPASELSGGWQMRLALAKVLL
EDPDIMLLDEPTNYLDLEARTWLENFLRDYRGAVLVVSHDRYFLDVTVREVYELFNGTLT
RYAGTYSQYEVRRSQELAALFEAWERQQEEIQRIEEFIRRFRYKESKAPQVQSRIKMLEK
ITPIEIPEGMKRIHFAFPPAPRSGQIAVRIRDLTKAYGSMQVIGRFSLEVERGQKLAFVG
PNGAGKSTLMRIIAGAESSFEGEMTLGANMLIGYFSQESAELMESGSTVEEEAESVCPFE
MLPKLRNLLGAFLFRDDDIEKPISVLSGGERSRLALLKLLLKPSNLLVLDEPTNHLDLTS
KDILLEALRKYEGTVIFVSHDRQFLDELADRVLELSCGLPPRLYHGNYAYYLEKKVQESS
AETQPEHPDPNKNRESGAPATAAVQNSDQPSVTRDWGQEKARKAQVRRLKRREEEISARI
EAIAVGKARLQEELAHPNTYINGEKTRRILADLETLDQEAECLNQEWLEIAESLAGEVT*
>SPBDM4_v1_50507|ID:27158870| conserved exported protein of unknown function [Uncultured spirochete bdmA 4]
MRRSLIRVVALLMFIFVSVLFFSCASSPAPVPQDASAQKIIQLAQERYDAYDLNGASYYY
QVLLDRYGSDPNYMLNAKYEMAFIEYKKGHKDQAVAAFKEIVARYDAPDASSLSPTWKVL
SEKMIAELTGTK*
>SPBDM4_v1_50508|ID:27158871| conserved membrane protein of unknown function [Uncultured spirochete bdmA 4]
MIATVINAAAIILGSLIGLLIRKGIKDEYRKVVFTAAGMTSLTIGIQMALKTTHILAFAL
ALMIGGLVGTMLDIEGGIERLGERLKARFASKSEGAFAAGFLNGSILFCTGAMAILGSFK
AGTEGDYSLIFTKTVLDGFVSIIFASAMGIGVAFSALSVFVYQGALTLLSVYIKPYVSDL
MLAELTAIGGALIIMIALGLLEIKNFKTGNFLPSILVIVVLVLVMPFLSFL*
>SPBDM4_v1_50509|ID:27158872| Elongation domain-containing protein [Uncultured spirochete bdmA 4]
MRQDHREAHEQRFVHTRREDEAGEWYALDNAASIMPAVANGIMTSLFRFEAELDGPIDLE
AIKGALDLTVQRMPYFNVTLRRGLFWYYFEPCGSLPPVYPDSPSPCQQWDIHRHGTRMFR
VRCEQNRIAAEFSHAMTDGTGGLSFFRTLLANYFLLKGVDPEARLKEGEWSGILASESTV
QEEFEDAYQRYFPEGLPFPERGLKAFHLHSKALPRGQYRVICGTLSTAEVLAEAKRRNVS
LTELMVAVYLDALQQIWHETKPHPRAPLVAVEVPINLRVLFPSRTFRNFSLFILISEDMR
LGMRGFEELVRRTHHQMKLEYDRMSIEKHLSRNAGSARRLVVRIVPLWIKDVFARIFFAR
FGESMLSGFISNLGPVKMPPGFVPHIHTFGFIPAPSLTTMTNASMVSWGDKLVIDFGSLA
QSRDLERLFFRRLRNLGLHVSVSCRLDHAKEGTHVILP*
>SPBDM4_v1_50510|ID:27158873| conserved membrane protein of unknown function [Uncultured spirochete bdmA 4]
MSYCPDCGAEIGDAPQCPLCGARNPRAGTGPDSEKSCAEPTSEKFSDLRFMLKDAEEEFS
GAEKRTIEWEVLSVAFCIAILVLGAVNLFESRQFSWSLYPIVSILLVWVEATSFLVLRKM
PVFRVLLSVLAPPVFLMVLGFISKSPRWALGLAVPIAVLVESLTGAVSLVIGKSRQKGLN
LIACVLVAIVALCIGLEVFIDLFSRGAVVLEWAPVCAIALLPIAAFLLYLHYRVIKITNL
RRLFHL*
>SPBDM4_v1_50511|ID:27158874| conserved protein of unknown function [Uncultured spirochete bdmA 4]
MNAIERAGYTEPTPIQAQAIGPILEGKDLLGLAQTGTGKTAAFALPILQHLLSGERCTAR

ALILSPTRELAEQTRKAFETLGKETGLFALSIYGGVSVSAQLRSLKRDMPEIIVACPGRL
LDLMGQHAISLKDIEILVLDEADQMFDMGFLPSIRKIIGSLPEKHQTLMFSATLPTEIRG
LAREFQKEPVRIEISNSQPVETVNHIVYPVMQADKYAVLSVILKDSATGQALIFARTKHR
AQKLATRLSEEGHLAASLHGNLSQAQRDKAMQKFRSGKVRIMVATDIAARGLDIRGISHV
INFDMPDTPEAYTHRIGRTGRMMHSGIALTLTTPEDRSMLKIIERLVGKPIERKIVSLGD
KEILNSMDDRADTMQEPAAASNGMRDQRSTMHNRTHEKTSHRPPATHATNSFAHRAKPDS
TRRSVHGSPQKSVRHTSGRHPKEYASIP*
>SPBDM4_v1_50512|ID:27158875| protein of unknown function [Uncultured spirochete bdmA 4]
VVCERLTPEEWNDSIKNPDKWIQGMPDEEPEGYILRLKNVRLP*
>SPBDM4_v1_50513|ID:27158876| putative heat shock protein [Uncultured spirochete bdmA 4]
METSITRSGNKMIVPPAQIREDESEVIAQLEMPGVTKEGLDIKIDGHTLTIDGKRSDDIP
SGKYLIRERRHYDYHKAFTIDESIDREGVSADLTDGILTLRLKVVKETAKPRKIPVK*
>SPBDM4_v1_50514|ID:27158877| putative heat shock protein [Uncultured spirochete bdmA 4]
MDHANLVTRKRQAPTTLDLWDAFDDLRREFERTFGDLEFPSVSGLLDWPAGPDIDLVEGD
DEILVLADLPGVRKGDLELSIQGNLLTIKGEKKREEPTQSHKVVRTETWVGSFSRTINLP
DSVNPEKVEAELRDGVLRIRIAKREEAKRRAVQISVK*
>SPBDM4_v1_50515|ID:27158878| Cpn60 chaperonin GroEL, large subunit of GroESL (fragment) [Uncultured spirochete bdmA 4]
VVEYQIEYVEGMQFDRGYTSPYFVTNRDSMTTVFENPLILIHDKKISNMKDLLPLLEKIA
QTGKPLMIIAEDIEGEALATLIVNHLRGTLNVCAVKAPGFDPAKVTRSALQNAASVAALL
LTTECAITDLPEKEKPAAPNPGMNY*
>SPBDM4_v1_50516|ID:27158880| Prevent host death protein (modular protein) [Uncultured spirochete bdmA 4]
MTVITKHYRVVRRIEIRLTTSLSLSLTSGSVLTRKLLTRCGLSPRSRSMGFTALCDSLTR
LVVHALVRWRSSALCLTFQSICGIIYALEDKMAINLKEDIKPISYIKTNAADMMKYVTEV
KNPIVITQNGEAKAVLLDIESYQNIQNAFSLLKILQLSEKQIEKGHYKSSEQVFDEIETR
LSLKQ*
>SPBDM4_v1_50517|ID:27158879| protein of unknown function [Uncultured spirochete bdmA 4]
VRLSHNAVNPILRERGLKPHLVKSFLVSTDPDVRDKLKDVVSRISILRTTR*
>SPBDM4_v1_50518|ID:27158881| Plasmid stabilization system protein [Uncultured spirochete bdmA 4]
MKQYEVIVSHLAESDINEIINYFIPINKEYVIKLYQTFKDRITELHTLPNKGRIVPELEK
KGIHDFRELIEGNYRIVYSIHATSVHILATVDSRRNLDEILVNKIIDFFNEE*
>SPBDM4_v1_50519|ID:27158882| exported protein of unknown function [Uncultured spirochete bdmA 4]
MRRAIVLLVFLSLIAPAFAQSGSAVVGSGSVVVSGARPGDPQESIYVRKVGHGPRTIVLV
PGNNTSGAIFEGILQYFRSVEDLNNAYTVYAFDYRGSGFSSYNKEITGLKDFALDFEKVM
DKLEGFPKTGVALVGYSMGFGVALEMYIANPGRYSSLISLAGIGTRGVRISIANASQAGS
DALGQQWAVGDWITVTNDTKGIAGTAFQQSSWQGEARNLAGIQFVWDLLVFEDALKYDIS
SYKVGDVGPKAQPGYMGALLDGLSVQYMPESLYYCHKFNVSASDLTHKNGDGTVVTIPGD
NRLGRLLAGKKVLLVKASTDFQNWRGDQVIYDNYTATTKYDLKKAGADVTAILIDPNQGF
DHGFPVLHPLETVHLIDSFLKGNLKASTVSAAIGGTAKFYDSAETTWETNNFTGF*
>SPBDM4_v1_50520|ID:27158883| protein of unknown function [Uncultured spirochete bdmA 4]
VTPIAIVTAILEAMTSVCSVGLDMIAVPGDTPASTLSAIIADEMAIGVVNKKTTASESAR
FPERPSEIRWSSAAF*
>SPBDM4_v1_50521|ID:27158884| Oligopeptide/dipeptide ABC transporter, ATP-binding protein [Uncultured spirochete bdmA 4]
VTNKPILDIHNLNVVYETPSGPIVAIRDLALELNANEALGIIGESGCGKSTLSYAILDYL
PSNARRSGSIMFNGEDLLLKTEKEMQNYRGDRIAMVYQNPYSALNPSLTIGEQLDEVTRI
HRGFSRTQARKESIQSLQDLSLGEAEGIVRRYPHQVSGGIQQRICIAMALLCQPDIMILD
EPTTALDVTTEASILDIIGELKERHRMSLIYISHDIGIVNKISDRIAVMYRGEIVELGPQ
EELFNHPKHPYTRALINCMPRAGVKKETTRLNTIPGYVTRRSADERGCPFASRCEKKNAV
CEEKYGLREIESGHLAACDRAYATDVPDKSTFAPLIPKANARPGADQTDNASSGIEPGIG
PAPLLELHEVYKYYKNRKRTVRALDGINAKIEKHDVLGLVGESGCGKSTMGHLVAGLLEP
TKGNIHFDGKDISLTWKHRSRDTIRDIQLVFQNPGRSLNPSFSVEQILDRPIKKMLKIRS
RRERRAVMINLLKKVDLGEEYLSRPSTRLSGGEKQRIAVARAFVTSPRLIVCDEPTSALD
VSVQASVLNLLGELQEQSHTSYLFISHDLNVVNYISDHILVMYLGRVCEYGLRDEVVNPP

YHPYTEALLSAAPDVNPSHKQKPIRLEGAPPDPSAQIVGCPFAGRCHKKIDGLCDTTPPP
LKRLSETHYIFCHLSEDRLRGVAHL*
>SPBDM4_v1_50522|ID:27158885| ABC-type dipeptide/oligopeptide/nickel transport system, permease component [Uncultured spirochete bdmA 4]
MQRIWYMAKRYPLIFPALIIILFWFFIATFGPSLMPFSYTAMDMDHQMTAPGVGEHLMGT
DSLGRDIWSRIVYGSRSILTIALLTSLFSSLAGVILGFTAGYFGGRIDALFLRVMDIMMA
IPPLVLSMVVLGVLGNSNILTLTLIVSIAYTPATARVARGTLLSCKNIEYVDAARLRGES
HLYIMFREILPNTMGPIIVEITARFAYSIMMVASLGFLGVGLQPPTPDWGMMVIENKTII
KAAPWAVLYPSLAIASLVIAVSVFSDFLSKVVIRDQ*
>SPBDM4_v1_50523|ID:27158886| ABC-type dipeptide/oligopeptide/nickel transport system, permease component [Uncultured spirochete bdmA 4]
MTFFRPFFRRLGGMAITLIAMSILIFLLVELMPGDAAQRVLGQSATPQAVAALRESMGLN
DPVWVRYGRWATGMLRGDLGTSLYMRGVPIASIFWRKVGNSLVLALLALLFYVPLSIVFG
ILAGVKAGKIIDSIISFFGLATMALPEFVSGIILMTIFAVKLAWFPITSVIPIGESIWSN
LNILVLPALSITLVMFGYVSRMQRASMISVMNSDYIRSAILKGLPMRKVIFKHALKNALL
PTITIIGMNMGWLFGGLIVVETLFGFPGLGSLTMSAVKTQDTPLIEVCVLFITAVFVVST
AITDLLYGFLNPRIRYPGGS*
>SPBDM4_v1_50524|ID:27158887| ABC-type dipeptide transport system, periplasmic component [Uncultured spirochete bdmA 4]
MKKKLYFFLAVGIVFLLAFSGCSGKDKQASQNKAIRIAEQVPNLITPGVWDGQAFSLDSS
IYEYLVDIDTEGNLVPALATSWETPDGKVWTIKLREGVKFHDGSDFDSQDVKFTIMRTQD
PTVGHLKAQDFSVVDSVETPDKHTVVINLKEVRPTFIYQMTDYNMAILSSSYDYAKSGET
KPMGTGPFKLEKLIQKESAKLVRNKNYWDKDYPLADELLIYFVPDIDSSVEMLEAGKVDI
VPQVTPLIKQRLEKADGFKVVSPYQEQRFLSMASDRTPFNDNRVRLALKYAMDPEILAKA
CQGTLNTDVFYNETPIMNSLAQYHMLPSRGRDIEKAKSLLAQAGYPNGLSVELYYASDHP
YSPALAQTVKELAEPAGFNIQLKGYPRDVYLSQYWMNAPLSITGWGGRIDPSVLLNLAFT
SQGPWNESHINDQEVDQIITKILKEVDDSTRQTLYDRLQEVFYERGTLINVQVPYLVAMK
DTVEDYRQPITMLPQLKYAHLKDVK*
>SPBDM4_v1_50525|ID:27158888| Transcriptional regulator, MarR family [Uncultured spirochete bdmA 4]
MDVNVSEEVLSTLRKISHAVELYSRILFREYGLTSPQLTILTAIFRSGPLAVTDIARRVS
LSQATVTNILGRLEQQGFILRARSTQDRRMVYIELTDKTRKILEKNPSPLHTDFLSRFDR
LQDWEKTLLLSSLQRIAKLMDVEQLEEPDIEHMQGKALKE*
>SPBDM4_v1_50526|ID:27158889| exported protein of unknown function [Uncultured spirochete bdmA 4]
MKLVKVIGGVLVALLLFSCGAPKLAQTDLDAANAAFADAQNAKADAYAPDAFKAAQDAKA
ALDAELAAQDAKTSGKSYKQANDLIKAFADASNKAKEAAATNMDQVKGEVAQLITDIDTQ
YANVQALAQAASKDAKKAAKAKLNVKDIEAKVAAAGQAITDAKAANDSQDYAGARDQLNA
VKSTLDELKSTLEAAGFTAQ*
>SPBDM4_v1_50527|ID:27158890| putative Lipoate--protein ligase [Uncultured spirochete bdmA 4]
MQIVAQSYKVVADSPLDIISFEECLVSSWTGNKIILLFYVNPPSVIIGRNQNYWREVSPS
CKVPVYRRSSGGGAVYHDTGNLNWALIVPRAIHSQNDELTFIAKAISELGVYARPGDRGG
IFVSMGDGETKGKISGTARRFGTRNVLHHGTLLVSADIQALKASLGGIQMFEDVSIASVP
ARPVNLTRFVPSLSVDEVMSQISGVLSDVELKTIDLEGSWDEHSSRDDSLFKNPEDLGEG
PDFCIEREDFDAFKCQFGSREWIIDRSPPFSVLVSSDMSRAVVRIEQGKIAEIQPLEAED
ERSAVYAKHLHTRFSGTQFDFKVPEILEKEHVWTNMRY*
>SPBDM4_v1_50528|ID:27158891| conserved protein of unknown function [Uncultured spirochete bdmA 4]
MDKYAVLNEVRSLIDEVKFGVLATVDADGYPHQRWMSPVLLPRFPGSLFCVTSEKFGKIA
QLEKNPQVSWIFQSMGLDRVATVQGRVEIVRDPMLAAEVQEAIGPRLRVFWKYAEDPSKL
VVLETTIEEYQFFAPLKSINEYVKAKAVSYES*
>SPBDM4_v1_50529|ID:27158892|pdhA| Pyruvate dehydrogenase E1 component subunit alpha [Uncultured spirochete bdmA 4]
MKAEFAHYKKEELMRMLTIMQTIRTFEEKAGMMYGLRKIGGFCHLYTGQEAVAAGAISSL
DLSKDYVVTAYRDHGHAIACGMDPKKAMAELFGKVTGCSHGKGGSMHLFDVEHHFFGGNG
IVGAQIPVAAGVAFAQQYQGSDGVTLCFFGDGAFHQGAVHETFNLGKIWDLPIVFIIENN
QWGMGTNWKKVSAVDDFSVTAGAYGMKGSSCNGLDVLDVRATIEKAVAEARLGRPSLVEA

RTYRYKGHSMSDPQKYRTREEIDEYRKRDAIQSFRSRLSEEKILSEEEWSALTQRINTQI
EEAVAFAEESPEPSLSELYTDIYA*
>SPBDM4_v1_50530|ID:27158893|pdhB| Pyruvate dehydrogenase E1 component subunit beta [Uncultured spirochete bdmA 4]
MAEMTYREALNRALDEEMARDDRVFLIGEEVGEYDGAYKVSRGLLSKYGPKRVRDTPISE
LGFTGLGVGAAIAGLRPVVEWMTHNFALLALDQVVNNAAKLRQMSGGQLSVPIVFRGPNG
PAEFLAAQHSQALGSYWMHVPGLKVVAPATPADAYGLLKSAIRNDNPVVMLEAELMYAWK
GEVSEQEFLVPIGKARIAREGVDVTLITYSKPIRVVEEAADRLAEKGVQAEVIDLRSLRP
LDEETIVNSVRKTGRCVVVDEAWPLASPGATIAHLVDSRCFDVLDAPVELVSGEDVPMPY
NHTLELAAQPSVEKVIAAVAKVLYREDV*
>SPBDM4_v1_50531|ID:27158894| Pyruvate/2-oxoglutarate dehydrogenase complex, dihydrolipoamide acyltransferase component [Uncultured spirochete bdmA 4]
MAEVLRMIALSPTMSEGRIAKWKMNEGTEFSSGDVLCEVETDKASMDYEAPTSASLLKII
LQAGGTAKVGDPIAVIGQKGEDYASLLKERASEAGVAEKKAPGQQPSKEVSQKEKAVPGK
TEQEPGSTPESAPQSDTENAPPRAPKASPASVGAAPRQTPQAPASVPPSSPLARKLALEK
GIDIRLVPGSGPNGRVVERDILEFAERPKNTVVPEVAPQELRQGPTVIQPNMKRLVIARR
LSESFFTAPHYFLKKKINAEALLELRATANAGRPAKLSFNAFLMKIVAEALVRHPEINVY
WRVPAAASAADAAASPEPPTAMRPILEQRHSIDVGLAVALPDGLITPVVRDCNLKSASDI
DAELAQLIEKARGGRLAPEEYEGAGFTVTNLGSFGIDEFTAIINPPGSAILAVGAIVKEP
IVGDNDVIRVAQTLTLTLGCDHRSIDGAVGAVFLADLARMLENPGMALV*
>SPBDM4_v1_50532|ID:27158895|lpd| Dihydrolipoyl dehydrogenase [Uncultured spirochete bdmA 4]
MESKQYDFVVIGAGPGGYVAAIRAAQLGLTTAVVEKNLVGGVCLNIGCIPSKSLIHNARL
FAEGKKLLETCGAKVDLSGFDYAQVWKASRLAADRLSKGVSYLLKKNKVELVQGKATLSG
AGTVDVETEGGARQLKAKAILLATGSRPRPIPGFEFDEKKILSSTGMLMSEKLPGRLFIL
GAGAIGMEFAYVMNAFGVEVTVAELLPRVLPLEDEEASKIVEKEFRARGIAIHTGAKASG
AEVKGDHVVVHLTDAQGSPLEIETDAALVSVGRTTNIDGLGIEKLGMRVERGYIITGEYH
ETSCAGIYAVGDITTYPQLAHAASKAGEIVAERVAHLLKGAPNPREHVLDRLHVPSAVYC
EPEVASFGLSEASAKEKAIQYEVARFPYRGNGRAVAVEAPEGQVKIVFDPDTKAILGASI
VGESAADTVHELLLASKAELTAEDVAELVHAHPTISETIMEASKAVLGHAIHI*
>SPBDM4_v1_50533|ID:27158896|arsC| Protein ArsC [Uncultured spirochete bdmA 4]
MVRALFLCVHNSARSQMAEAFCKKYGGENFIAESAGLEPGTLNPYAVRAMAEVGIDISKN
QTKSVFDFHKEGRFYHVVVTVCSKEAAERCPVFPGAPITLHWPFDDPSSFHGSDDEIMTK
VRFIRDAIEKKVQEFVEAWPRVIE*
>SPBDM4_v1_50534|ID:27158897| putative NADH dehydrogenase/NAD(P)H nitroreductase [Uncultured spirochete bdmA 4]
VDLNETLRTIFSRKSVRAYAEGTIPKEKLEVLARAGMAAPSAVDQRPWEFIVITENAMLG
DLAARLPYAKMAAHASAAIIVCGDMRRQWGGSESVMWVIDCAAAAENILLAAESMGLGAV
WTAVYPHPDRIHPVRELLGLPDYIQPLCLIPVGIPRGGEKAKDKWNTERLHWERW*
>SPBDM4_v1_50535|ID:27158898| Regulatory protein ArsR (modular protein) [Uncultured spirochete bdmA 4]
MVNILTKVFQHCNIAIMKSIERKYERRAEIFKALSNPLRLIMLEKLEEKPWCVCALAAEL
GIDKSIVSKYLSQLKSVGLIEDSHKGTLVEYRLVAPCVLRLASCAEETIQKQRRKLLSED
*
>SPBDM4_v1_50536|ID:27158899| Redox-active disulfide protein 2 [Uncultured spirochete bdmA 4]
MKIQILGTGCSKCKLLEQHAREAATELGIQAEFEKVQEVEDIMAFGVLMTPALAIDNEVK
AVGRVLTKDQIKALLS*
>SPBDM4_v1_50537|ID:27158900| conserved membrane protein of unknown function [Uncultured spirochete bdmA 4]
MSTKGWTSNKKLLVLFGVFLAAYFIPFHSPRVSGAVNEAFLMLSEYARQHVVLCLVPAMF
IAGAITVFLNQQSVMHYLGPDAKRLTAYGVASVSGAILAVCSCTVLPIFKGIYKKGAGLG
PAIAFLYSGPAINILAIVLSAKVFGLKLGLARAVTAIVFSIVIGWLMALIYRKDDEKRAA
DVRLFSAPSDGEPRTLGQTAIYMASMIGILVFANWANSRGGSPVWDAIYSAKWWITGGFG
LILAYAVIRWFNADERIAWVVATRDFALQILPLLFGGVLVAGFLLGRPGHEALIPNAWIA
GLVGGNSLFANAFAAIAGALMYFATLTEIPIVQGLLGAGMGQGPALALLLAGPSLSLPSM
LVIAGEIGWKKTLTYVGLVVVMSTLAGMLFGTLA*

>SPBDM4_v1_50538|ID:27158901| Cupin 2, conserved barrel domain protein [Uncultured spirochete bdmA 4]
VFGKHSNTGFHETLPGNHIKTLCYGAHTLLVEVRLEKKAQLPEHKHPYEQTGYLVSGRIR
MNIAGNVRELESGDSWCIPMDALHKVDVLEDAVVVEAFSPTREEYIKYKNDNDIVS*
>SPBDM4_v1_50539|ID:27158902| HAD-superfamily hydrolase, subfamily IA, variant 3 [Uncultured spirochete bdmA 4]
MKSNSSSHAGRSAFRAVLFDMDGVLVDSERLIAEAARRMFAERYGLEVQYEDFTPFVGAG
ENRYIGGVAERYGLSIDIVSAKAWTYEIYGQLARQHGAGMRAMPGAVDYVRACRAYGLKT
ALASAADRVKVLINLDYLGLTPDEFDTMLTGGDVTHKKPHPEIYLSAAERLGAAPSACIV
VEDAVNGTIAGVRAGARVLGITSSFPEGALRKAGASWTAPDLARAPLPWKLTAVPVVGEP
SGGGL*
>SPBDM4_v1_50540|ID:27158903| Fe-S oxidoreductase [Uncultured spirochete bdmA 4]
MNTISYAVGSTALNVTLRYLNRNPEKNIPKIADVLMPFTYREDMKRQIATAKKVMQDPAN
PYRGLIMRAFSELAPNVRKSFLSNFVLNSAMVGADRTSMLKVKYKCNIPWAILMDPTSAC
NLHCKGCWAAEYDKTDSMDYALLDRIIREGKELGVYFYIYSGGEPTVRKDDLLNLAKVHI
DCIFLAFTNGTLVDDAFAKALAEVGNFGLAFSIEGFEDATDFRRGNGTYQKVIRGMQNMK
KYGAPFGFSACWHSKNTEAVGDEKFLDFLIEQGCAFGWYFTYMPLGKDADTSLLAKPEQR
EYMFHWVRRMRDTKPIFLIDFWNDGQYVNGCIAGGRSYLHINAAGDVEPCAFIHYSNVNI
HDVSLLDALRSPIFKQYAAHQPFNENHLRPCPLLDNPGMLAEMVKASGAHSTQPIDKEDV
DDLTAKCAPAAAKWTPVSEKLWAGELPHYLEAQKRKDEEQERNRAILEKKLKKTKDDASL
EAAS*
>SPBDM4_v1_50541|ID:27158904| conserved protein of unknown function [Uncultured spirochete bdmA 4]
MPKMVDHEERKRKIADLALELFAQRGYYAASFGEIADACGLSRTNVYNYFKNKDEIFHYA
VSELLDTIAKKIELIIRQQELSLAQKLQKIYQVFTNDIGEGKYTSIILDLALRLKRENTH
LAEALDEATKALRNKIEMLFAGETSLLPSSQTAFATTLFFSLIESSIMHSLFTDGAFIQN
NIGSILHMLGT*
>SPBDM4_v1_50542|ID:27158905| conserved membrane protein of unknown function [Uncultured spirochete bdmA 4]
MFSIPALIKILVIFSLVVIATAKHVHLGLAAAIGGIVIALWQGLTPAAVAVAAVRELLNP
DLILLVVLLAAIMAFSAAMKKSGAMDTFANAIAEAAPSRRVALAITPLLIGTLPMPGGAI
ISAPLVGAMNDDDSRSPETLSAINYWFRHILELIWPLYPAFILTAGLTKLPITTLMVLNL
YAPITLFTLGLIFILPRGRESTAHPRLEQDSAPRKLEKTKLHRLVRGIAPLGIVLGSYIV
LDVIWELAAPHLGLDATTKSLVGRYAPVLCGIVIGSLYLVKIAGGPQIFKKSITKSTLGL
IAVVIGIRIFSALMNAADLAHAASIELASAGIPSIIVIALLPFISGIVTGVGLGYVGLSM
PIVLGLISSSGIPFKAGVVIAGAFGYAGMMFSPLHVCMVVTVQHFKATLPSTIRKFALPL
GIFVAIASVYGAILMTVMK*
>SPBDM4_v1_50543|ID:27158906| protein of unknown function [Uncultured spirochete bdmA 4]
VFSSLRIEGGTTPDKDKIMNIFAPLIGKAYSREELENALHAAYAAGDFSLVKFDIEQLQG
AGAPGALRGVVSIVPQKPVENEFFASLDFQGAVSNNISGNLVASLAFLAKELTGPGSALF
SSVSFVNKTSASMEYFQPFGPFFILPWTRFRFEYDMSASESVPVAIASKFRTYGAGVWGG
LVLGNIADVMAGYSFENVLTGDDWTSLVTQNAGALRVALRLDTRDRTAFPRKGIAFTAYG
RWFSPNFGGQFSFAQMEVNASAAIPVRRTDALHLSLFGGTDFVGLVTGAQPAKISYYSTL
SQPVMFYGLGYIPTSCAGNSVLAGSLEYRHRVKVINELTGGDIYIFANGSAGAVVQYDDP
STYAMWPLKWSATLGASARLTRHYGILVGASVLGNIDDTDAIMPAFVIQVGSFNHSSVID
RR*
>SPBDM4_v1_50544|ID:27158907| putative Uncharacterized NTE family protein YlbK [Uncultured spirochete bdmA 4]
MGCAMNTYRMHSPFRQHRARVLFVCLLLNLAAAAPVAADDSTAQISDQISEPARPRIAVV
LSGGSAFGIAHAGVLKKIEEAGIPVDMILGTSMGSIVGGLYAAGYSPQAMQDLISTIDWN
SLFMDPKELPQNMFERAVESMYALRIGFDSKGPNIGTGLLEGQNILSFFTARTIHMAAKP
DFDAFPVPYRAIAANVLNGDKVVFDHGSLAEAMRSSMSIPVVFMPYEYNDQLLVDGGVVD
NLPVDIAKQMGADIVIAVVSRGKAPDSIDELNTSVEIGSQTGNLFIMQNMQPNIEAADLV
ITPNLQEFTTASYARAKEIIARGEEAGDAAMPQLRELAAKIA*
>SPBDM4_v1_50545|ID:27158908| putative oxidoreductase [Uncultured spirochete bdmA 4]
MIPQAPFGRTGHNSSRLLFGAAALARVTQAEADTTLSLVLDSGINHLDTAASYGQAEERM

GPWLKVHRNKVFLATKTEKRTKKEALEELERSLRLLRTDHVDLWQMHVLVKDDEWDVAMN
EGGALEAFVEARQKGMARFLGVTGHGKAAPAMHLRSLARFDFDSVLFPWNWPLSNDPAYA
AAVRTLLYICKTRNVAVQLIKAFLRRPWGERPHARATWYEPLDTPQDIGMALGWAWSIEG
AFVNSAGDIHLLPTIIKEAAKAPPSPSGEAMAEMAQRLDMTDLFA*
>SPBDM4_v1_50546|ID:27158909| Ribokinase [Uncultured spirochete bdmA 4]
MRILNFGSLNIDYVYRVDEFVKPGETKAAQTLSVFAGGKGLNQSLAMARAGLNVVHAGKI
GAEGEFLVKTLNDGGVDTSRIEIAKVATGHAIIQVDDKGRNCILLFGGANQDINEAYIDR
ALAGFGPDEMLVLQNEISSMPSIIAKAKTKGMTIVLNPSPYSNLVPSYPLELVDVFIMNE
IEAAYLSGESAPERAANLLMRSFPQARIVITLGEAGSMCAHKGEIVRQKAYRVRAVDTTA
AGDTFSGYFLAGLVEGMDIAAALDLAAHAASICVTRKGAADSVPWRKELETREQV*
>SPBDM4_v1_50547|ID:27158910| conserved exported protein of unknown function [Uncultured spirochete bdmA 4]
MRETNNVMRGISKNRRPMGRRAFFSVFLLGIFGVCGFAPVGAQQNGPSQESPMTLEGDWS
GLLRVGATSLRIVFHVTADGTQYSATMDSPDQGARGIPVSAVRIDGRNVAFEVAAIGGKY
SGTLNETGALEGTWKQSGQSLPLNLERQSAQTPQKPAAASGEPSPPFPYIVKNVEFSDEK
AGIQLAGTLTYPKGKGPFPGVVLVSGSGLQNRDEEILGHKPFLVLADYLTRRGIAVLRYD
DRGAGGSKGSFQSATTFDFADDAQAAVDFLARQTGIDAHHIGVIGHSEGAIVASILAARG
AEDTQDAKVAFIVLLGGPGVRGDELLLMQNAALGRASGLSESQIAQANKINRELYSIAMS
EGDVSTLRKKIVDTMNGVIESTPSLTAQQKEAQKAQVEAQADQLLSPWIRTFLSLDPSEY
LRRVQVPVLALDGSKDLQVPAKENLAAISAALNAAGNHSATMTELEGLNHLFQHANSGLP
SEYGEIEETFAPEALKQIGDWILSVGAR*
>SPBDM4_v1_50548|ID:27158911| conserved protein of unknown function [Uncultured spirochete bdmA 4]
MMIAGTACGHFTDESGRWLMLRGVNLGGSSKVPTVPDGRSHAREGFYDGAHVSFVGRPFP
PEEADEHFSRLARWGQNFVRLVVPWEALEHAGPGLYDSVYLEYLEGIVEAAARHGISLYI
DPHQDVWSRWTGGDGAPLWTLEAVGFEPRNLHASGAAMLHQEMGERYPQMQWFSNHLRLA
CATMFTLFFAGNDFTPGIEVAGEPIQDFLQGRYIEAFGMLAERLAAYPNVAGFGSMNEPG
EGFIGIGDIRAPRNDLLLPGLAPTPWEAMCAGEGVATTAEHIEVKGGRMRSVGRVSLGTP
GTRAWKDGEACIWRRVGIWDIEHGEAVLKKPQWFARAGIGSNEASSSQNPRFHRIFNQRY
LKPFTAKFAARIGSVAGNMNRFMIFVESSPLGEMPAFTDASLETPPQETGNAPEVSTLVN
ETHWYDSLTLTLKRWTGFLAYDSTLQKVIIGPRAVRRNFREALARIVRYSRDFMGNAPTL
LGEFGLPFDLNGRKAFRSGDFGVHQRALSAYYEALDANLLSATLWNYTADNTHAYGDGWN
GEDLSVFCVEDGGGRALAGFVRPYAMAVAGNILRMQFDQGHGRFILEYAPDTSISAPSEV
FVPMLQFPRGASISVSGATVAEPSLKIEGKTHCALAPTEPERYFMLRLDAKPDAQRCRLT
ILRL*
>SPBDM4_v1_50549|ID:27158912| ABC-type transporter, periplasmic subunit [Uncultured spirochete bdmA 4]
MKKILFVSVLAVALLFSATAQAKYGPYADKVIYDVRMDQTIAIKDTAEGKTDVFFTGIDG
KTYKGIQSADLDKLSTYAVPSGSWSLLLNPIPNEAPYTFQAKDGRTIFNPLAIRDVRYAI
NWLIDRKKIVDEILLGAGEPAITAQTPGQPGTYRFNLVPAKLGMTVRGNEKKALEDIDTA
MKEAANLPANKGKLVKSGQFWTYNGEPVTIRFMIRVDDPTGRLLEGRYIADQLEKAGLKV
ERLEYDRSKAGNLAYGSDPAAWEWSMYTEGWGAGATRAWWDVTISQMYSPYYGYMPGGQT
EGFWNYENKEIDRIAQKNINGWFLTSDEYWNDNMKVQEMALKEAVRIYVCSQVQYYVANK
ARFDARMLYGLGDGLNDWSVRSADIKPDSKGEKVLHITQYSARGGLFMSSWDPVGVDGFS
DVYSAAIFGACTDPSTFESPNAAKDTPLRVKYDLKNVETKVAAGKEGEPPTGLIDVDKAA
LIFNSKTKKWETGVEYKDVGDGTYDYVKNSAMKAYSKLSGVQYIYGKWHDGQPVTIADIM
YAIAFTYEWANKDGDGDKYYDEAYASQYQSSLPVSKGVVLNKDGSFTSYFDFNWPMDKDR
VAATGVVSPKAGNPGRSTLVSWEIYEALAKLVAEGSKSGTQYTFAYDPSVTEVDVINPKC
VADIKAKLQDFAAAKYVPDSIKQWVTPAQAVARYNAAIKFIDTYGNAYISNGPFFISKVD
YNANYIELSAFRDYPYRADYFPKLFRTTLTRIDDVKVPPTAQRNADAKIDVQISAVTYPD
DTAKAADNKAKVTVTLILADNTEKVYTAKYVSAGSFQAVIPAKDLGALKSGAYTLVVQSV
FNTESPAVQASSLVVF*
>SPBDM4_v1_50550|ID:27158913| ABC-type transporter, integral membrane subunit [Uncultured spirochete bdmA 4]
MYKWFALKRVLKGIFTYVIIIFIMSVLFNTVNEQTMRSNIEEQVRAETMRLKNMQPAAIQ
RFQQQRRDDLIHLYRLDRPFMERILYRTWNTLTFNFGKSTIIKSSRGEREVLTIVGEALP
RSLILFTLAAVIEIIVGIILGLKKAQKPGGSLDRTTSLLTMIVYGMPTFWVAMILIMFFV
YQLKIFPSAGMHSVPPPQGIMYFIDLLWHMTLPLLTLVVIGFWGISYVVRNIVLNILQED

YIMAARARGIPENKVLFGHTLRTAAPPLITMSVLSLLSSISGAIIFEGIFSWPGLGNLYW
IAVQQKDIPVLMGDLAITVGIYQIGLILLDLTYGFMDPRIKVGGKA*
>SPBDM4_v1_50551|ID:27158914| ABC-type transporter, integral membrane subunit [Uncultured spirochete bdmA 4]
MNLGDTKYRISEFWSEFRKERSGLVGLAILILSLLVVIFEPAILPWKEANSKWRSIDYWQ
DNSASAPPAWTNAFTKLKAPVTVRLDDGEKEEAYLDGGVKLVTYTFDYHYTADKAPLDVI
FHITGHGDIPFQVSVERPDGTTLDLGQRFEQGLAGQDIRISVDNDGREAAFTFIKNYESE
GALEQYSGKSLRTNDILFNTAREGMAREFRPLKGNYKLMVKAMLLDPENSDVQKPYVVVT
GSVSGLLGTDDSKRDLFSGLVAGLKWALLIGLLTSAISVLIGVMYGIISAYFGGTVDSVM
QFIYQIVNSMPVLPVLIVISAIFKPNIWMLIAVMVLFFWTGSVMTVRSMALQIKEETYIE
AAKALGAKKSRIIFKHMVPILIPYSFASMALSVPSAIVYESSVSLLGLGDATIVTWGQIL
HDAMQGAAILKGIWWWVLPPGLLIAIMGMTFAFLGFSMDKILHPKLKTR*
>SPBDM4_v1_50552|ID:27158915|oppD| oligopeptide transporter subunit ; ATP-binding component of ABC superfamily [Uncultured spirochete bdmA 4]
MNSLLKVENLQMHYFTSKGAVRAIDDISFELTPGETIGLVGESGCGKTSLGTSILRMPTP
PGRYISGKILVDGVDVIPLPEREVRKDIRWQKISMVFQGAMNCLTPVYTIGRQMMETLNE
HSDMSRSDAESLITEYLGYVGLSPEIVQRYPHELSGGMKQRAVIATALFLKPEIVILDEP
TTALDVIVQAQIINLLKKLKKQFSLSFIFITHDLALEAEISDRLCVMYAGKIVEMGSNAQ
IYGAQGPGHPYTERLLGATPRLFKKVEQLQFIPGTPPDLIAPPKGCRFNPRCDKAFDRCF
QEEPPLIEIEPGHMSACWLHQRG*
>SPBDM4_v1_50553|ID:27158916|dppF| dipeptide transporter ; ATP-binding component of ABC superfamily [Uncultured spirochete bdmA 4]
MTDTILSIKNLKKYFEPHQSFTQSIGGGGQKKPIKAVDDISFDIRRGEIFGLIGESGSGK
TTTGKLVMKLIEPTSGKIFLNGEDVTHLDKEKLIAYRRKVQMIFQDPYASMNPRFKIKDV
LEEPLIIHKVKGTIEERTKMIVKALEEVKLTPPDEFMGRWPHMLSGGQRQRVATARTLIL
NPMMLVADEPVSMIDLSTRAEILHMMKDVQKELGLSYLYITHDLSTARYFTDRIAVMYLG
RIVEIGRADDIIDNPLHPYTQALIEAVPEPVSGKLDIIKELPIFGEIPSPANIPQGCRFH
TRCPYATDACKSEPEPELTLAGEEHYHACSRAYEIPAERKHKQAG*
>SPBDM4_v1_50554|ID:27158917| conserved protein of unknown function [Uncultured spirochete bdmA 4]
MEIIDRRIFPIMRALSDEKAILVLHGARQTGKSTALHWWLQEERNRGRQTLYFDLEDPAM
LSLCESGVEAFIRYIEARGYPRQNLSVALDEIQYLSDPSRFLKLLFDHHSDDLKIAVSGS
SSFAIKSKFRESLVGRTIPLELFNLDFGEYCRFVGRQYDFSAEWPEQLDLEVRPVFQHFA
ETGAYPGLARTKEIATKSRRIRQIIQTYIQTDVRELGKIRYPDRFESLLRFLADQAASLA
QVSELASSLRMARETVEEYIFLLEQTYIIRRLRPFSGNARSELTKTPKIYFEDNGILAMC
RAYEFLPLDGALFENAVFTELRKSFGIERLHFWRTTEGQEIDFIIDEGRVAVEAKLRPRP
ADCKNLFKFQKRYSAKRLVICGMEKPVSLPEGVEFRYPWHLADSVGQQ*
>SPBDM4_v1_50555|ID:27158918| SufBD protein [Uncultured spirochete bdmA 4]
MAEQAIVDKEALLEELLASINQHKFTPDVAHVEIHGNQVLNKNLVDGLMVEAESIEDGVK
VHIRVKSGVTLKNPVNFCFGLIPENGVQRIIIDTVIGEGAHAKFIANCTFPNAVNIQHLM
NATITVEKGASLSYFERHVHGPNGGVTIVPVTKVLVGEDARFSTEFELIKGAAGVVELEY
AAEVTKNATVDMMTRIFGREGDRIHIKEAAHLSGEGSTGVLTSHIALKNRASATIENELI
ADAPYARGHVDCKEIVQDEATARAIPIVQVNNHLAHVTHEAAIGSVDSKQLETLLSRGLT
EDEATDLIIQGLLS*
>SPBDM4_v1_50556|ID:27158919| conserved protein of unknown function [Uncultured spirochete bdmA 4]
MDLLEIKNLSLFKEGRPILNNLSMDIWEGHVHAIIGPNGAGKSTLANTIMGLSGYRDFEG
DVLFEGKSIKALPVDERARKGITLLFQEPARFEGLGVSQFILAGAKEKSRAVVEDALQHA
GLSPEKYMRRAVDKTLSGGERKRIELASIYAMKPRLVLMDEPDSGVDIDSVKYIFAVIKE
LRHRGSTFILITHSPEVLKHADHAFLICAGTLVDKGEMSRMFDYFNGKCVPCVHVGQPTV
DAAVQPLAAN*
>SPBDM4_v1_50557|ID:27158920|ltaA| L-allo-threonine aldolase [Uncultured spirochete bdmA 4]
MNYIDLRSDTVTWPTSAMRKAMAEAAVGDDVYGDDPTVNELERLAAEMTGKEAALFVPSG
TFGNQLSLFTWCPRGSEVILGEQCHIIQHEAGAASVIAGVQTRPVFAPDGILPIEAIEER
IRGNDIHFPPTSLVCLENAHSSGRVIPLSYMKNVSELAHRYGLPVHLDGARLFNAAAALG
AGAAEIAGTVDSVMFCLSKGLCAPVGSMLAGPRDFVERARRKRKVMGGGMRQAGVLAAAG
LIALKDMTRRLAEDHKNARYLAGQLGTVPGLEVDAEALDINMVFFRLPAGSDGDALVSHL

KAHEVLINPPELGLCRFVTHYWIDREAIDKVAALMADFFKRG*
>SPBDM4_v1_50558|ID:27158921| conserved protein of unknown function [Uncultured spirochete bdmA 4]
MNRKDIAIEDFRYDPAQLRDHWLSLFAGDFNSGAYNAMTISWGSYGQIWNKMFFQVFVRP
TRHTYEFMERYDTFTLNAFEPKYKQALSILGSKSGRDGDKIALAGLTPMASREVAAPCFR
EASLVIECRKMYWQDIDNTHFLDSGIEGNYAKKDYHRVYFGEIAHVSRA*
>SPBDM4_v1_50559|ID:27158922| Uncharacterized ABC transporter ATP-binding protein TM_0288 [Uncultured spirochete bdmA 4]
MAYETTSSIQDVRTSFQRRPGRPTRFEKASDPRKAMIRLFGYLKPFKKPLASVVCFVLLQ
SLLGLAGPYLLGRAIDTSIGGKDVRSLLNTAALMLIAYVLSNGFNILANWRMARISQNAL
KNLRGDLFAHIQSLPMSFFDTNPAGGLMSRLTNDIDAINQTVSQNIIALLASIITMLGIL
VSMFVLNHWLALASLVVIPIMYWFSNFVAKYTRKGFRELQKDLGNLNAVAEETISGFKEI
KAFRRNDSAIEAFRQKNQAVFKSAVYANSYAMLLMPLTGVLGSFFVIVLASLGGWLALKG
LVSIGMIATFINYAQNFTSPLRQLSNLYNSIQAALAGAERVFEIIDMESETAGRDETNSR
HKTRADGQAAEAGAPRPRVPFHLDGFQGNVTLQNVVFGYGPDNIVIRDFSVEIVAGQTVA
LVGPTGAGKTTITNLLTRFYEIQSGRILFDGVDIREIPKKDIRRAIGLVLQDTFLFADTV
LENIRFGRLDATDEECIEAAKMAEADHFIRQLPDGYHTNLSERAGNLSQGQRQLLSIARA
ILADPRILILDEATSSVDTRTELRIQKALLRLMEGRTSIVIAHRLSTIRDADSIVVINDG
EIVEQGNHEALLGQRGFYYHLYMSQFKGNEI*
>SPBDM4_v1_50560|ID:27158923| ABC transporter related protein [Uncultured spirochete bdmA 4]
MNEFKKLLHFARPYWKLAVFSLVMLVAMVGFDLAVPRLVGRIIDKGIRQKDLTVVMTTSA
IMLCISMLSALVAVLNSISSIRVGENIARDLREAIFVKIQNFSYGNIDRFSTAKLMVRLM
SDTAAVQRLFQMSLRIGTRAPLSMIGSIVLMFVTSKTLALAMMPILVVSGIVIVFFSTRL
EPFFRTVQQRLDRLNTVLQENIAGARLVKAFVRGKYEADRFDTANASLAQENIHVMQLMA
SMSPILTLLINIGIVLVVVWLGGLQTIHGNLSLGQIIAFTNYLMATLQPLTMMTQLSNNWA
NGLASAKRINEVLDAEPEVAELPDATSLPSPIQGEIEFDHAGFHYNGDMDIAVLENICTR
AEPGKTTAVLGATGSGKSSLVNLIPRFYDASAGSVYIDSQNVRTLKGDSILEHVSIVPQD
TVLFSGTVRDNIRYGRPEATEEDVIAAATAAQAHGFIMNMPAQYDTHIEERGVNLSGGQK
QRIAIARALVCKPSILILDDATSAVDVETETKIHTAIAKSAQGTTVVMVAQRISTVLNAD
KIIVLDKGRIAAEGTHRELLRTSPIYREIYDSQLGAGVRHGL*
>SPBDM4_v1_50561|ID:27158924| protein of unknown function [Uncultured spirochete bdmA 4]
MDESETKLDAVVENLLEVLPSVWDRIRSNFRSAGTSKFGISMEQFHTLRHIYKGYCHGGE
IAEKRQVSCSAVSQAIDALVAKGLVTRIQDSADRRQVRLELTVHARHVLDENAEENRSYI
RQKMSDVEPEEREAVMRAMGILKNTFLST*
>SPBDM4_v1_50562|ID:27158925| Transcriptional regulator, AraC family [Uncultured spirochete bdmA 4]
MKINDAVYVYRLISGERLAWHGRYHAHGPGEYEFHYFMEGQGALLINRAKYIIDGNRIYF
VQPREFHSILPEAVEKPISYYAVLFRPEPGNPVDEEIVGLMGHLWHARNRALPADAKDRF
FLDDLYRLSKSSSERSQKSAEHALISILYRWFDHFDSTSEDVHSGGTKNEHVERALALMM
KHVREKLSSDDLAAMLGLSEEYFIRLFRHHLGMPPFQYFTRLKIEAASAVLVDNQLTISD
IAYRFGFENPFHFTKVFKKCTGLSPREYRKIFFEAGKPPQTAAPLT*
>SPBDM4_v1_50563|ID:27158926|xylB| Xylulose kinase [Uncultured spirochete bdmA 4]
MKSVCGIDLGTQSCKIILYDPGAKQIIAKSQSSLDIIARNDGTREQRAEWYEEALLACFQ
AISPELRATITAIGVSGQQHGFVPLDGQGRAIRPIKLWNDTSTAAECAELTRRAGGEASL
LSETGILMLPGYTAPKILWLKKHEPENYARVRHVLLPHDYVNFLLTGNDVTEYGDASGTA
LFDVRVRRWSRRICDLIDPSLIDYLPSLVEPDKPAGRVNAAAAAHFGIPEGVLVAAGGGD
NMMSAIGTGTVRDGFLTMSLGTSGTLYGYSSRPVVDPEGNLAAFCSSTGGWLPLLCTMNC
TVASEQIRALFGMSIDEMNVRAASAPVGAEGIVVLPFFNGERIPNLPQGRAGILGATAAN
FTKENIARAAMEAAIFGMRIGLESFRKLGFTAREIRLVGGGAKSGLWREIAANVTGLPVR
VPKEEEAAALGAAIQALWCFECNAEAAVPIDIAALTDAHVSLEGGTSIEPEMTLVSRNEA
AYDEYAKYLAALSPLYR*
>SPBDM4_v1_50564|ID:27158927|xylA| fragment of D-xylose isomerase (part 1) [Uncultured spirochete bdmA 4]
MADYFVGHREYFPGIGKVRYEGPKSDNPLAFKYYDPDRKVGDKTMREHLRFAVAYWHSFG
ADGTDPFGDATHIHPWTTDATTAIEAHEHKLDAAFEFFTKLGADFYCFHDRDMAPEGDSP
GQSEKNLAHMVALAKERQKATGVRLLWATANLFSHPRYMNGAATNPEFGVVAQAASQVKA
AIDAAIELGGQG*

>SPBDM4_v1_50565|ID:27158928|xylA| fragment of D-xylose isomerase (part 2) [Uncultured spirochete bdmA 4]
VFWGGREGYMTLLNTDLKREKDHLARFLTMARDYGRTHGFKGVFYIEPKPMEPTKHQYDF
DVETVAGFLRAYGLEKDFRVNIEANHAELAGHDFQHELETAASLGIFGSVDANRGDPRNG
WDTDQFPINAYDTTLAMYAILRAGGFTRGGLNFDAKIRRNSVDPSDLFEAHIGGMDAFAA
GLLAAQHIIEDGKLEAFVRQRYASFDGGDGRRFEEGRMNFEELATLASSPGAANADKVGR
TSGRQEHLENLIMQYVLGVK*
>SPBDM4_v1_50566|ID:27158929|chvE| Multiple sugar-binding periplasmic receptor ChvE [Uncultured spirochete bdmA 4]
MKRFVLVVVALMLVLSYSFAQSKNVLIGVAMPTQSLQRWNQDGANLKAKLEAKGYKVVLQ
YANNDVNAQLQQLENMITQGAKMLVIASIDGSVLSDVLKEAKSMGVRVIAYDRLIMNTPN
VDYYATFDNFKVGVIQGQYIESRLGLKEGKGPYNIELFAGSPDDNNALFFFNGAMSILQP
YIDSGKLVISSGQKNFTTIAIQGWDSGRAQSRMDNLITANYASGKKLDVILSPNDSLAIG
IVASLKNNGYGTASKPYPVLTGQDCDKANVKAIINGQQSMSVFKDTRDLAAQVVEMVDAI
MSGRTVPVNDTKTYNNGVKVVPSYLCTPVFADKNNYKQLLIDSGYYKESDIQ*
>SPBDM4_v1_50567|ID:27158930|xylG| fused D-xylose transporter subunits of ABC superfamily: ATP-binding components [Uncultured spirochete bdmA 4]
VENDLILEMKDITKQFPGVTALKNVTLAIKQGEIHALVGENGAGKSTLMNILGGVYPHGT
YSGHIIFEKVERTFREVRDSEKIGIGIIHQELALSPYLSVGENIFLGNERARHKIINWDT
TYSDAKGLLDLVGLKENPHIAVKDISVGKQQLVEIAKALSKKVKLLILDEPTASLNEDDS
EHLLNLLLDLKKQGLTSILISHKLNEIAQVADSITILRDGEVIETLEKGKDPIDEDRIIK
GMVGRELVDRFPKRIPKIGDISFEVKDWNVFSPLNPDRQVIKNASIKVHKGEIVGIAGLM
GAGRTELAMSIFGRSYGLHIRGTLIKDGVELQLKNVRQAIDHKIAYTTEDRKTAGLNLIG
DIRENITIAALGSISSNYVINNEKEIKVSEMYRKKLNIRSLNIFQKAGDLSGGNQQKVVL
SKWIFAEPDVLILDEPTRGIDVGAKFEIYTIINQLAEEGKSVIFISSELPEILGISDRIY
VMSNGKIVGEFMKAEATQEGIMKCIMQSIGGNSNGEY*
>SPBDM4_v1_50568|ID:27158931| Inner-membrane translocator [Uncultured spirochete bdmA 4]
MENIKYFLKNNIRQYGMLIALIFIIIFFQIMTNGILLVPMNVTNLILQNAYVFILAIGMT
ITILTGGNIDLSVGSVVAFIGAICGTFIITMKMDVGIALILALLIGVLIGVWQGFWIAYV
RIPAFIVTLAGMLIFRGLTVFMLKGLTINPFPESFQFISSGFLPDIAKNAFGKYHATAII
AGIAIVVFYILSQIKRRAAQRRNGFDVMSGPLFLLQLIIVSAFIMLIAIWLAAYKGLPVI
FIIIGILILVYSFFTTRTVPGRYIYAMGGNEKAARLSGINTNKVLFFAYVNMGALAAVAG
IAYTARLNAASPQSGLNFELDAIAACFIGGASAYGGVGTIVGSIIGALVMGVLNNGMSIL
GIGSDVQQIVKGLVLLLAVAFDVLSKKKAH*
>SPBDM4_v1_50569|ID:27158932| Oxidoreductase domain protein [Uncultured spirochete bdmA 4]
MIEGSAGAKTSRKLRYGMVGGGKGAFIGDVHRRAIALDGLAELVAGSFSQSFENTLETGA
SLGLSRDRLYATYSEMAEKEAARPDGIDFVSIVTPNALHYPVAKAFLNAGINVICDKPLT
FEESEAKELDELAKKKGLLFGVTYTYTGYPAVKHAREMVRRGDIGEIRFVNAEYPQEWLA
TPSEREGSKQAFWRTNPALAGKSNCVGDIGSHAENMVSYVTGLKIKSLCARLDTFVEGRL
LDDNATIMVEYEGGAKGLYWASQIAIGYDNGLRIRIFGTRGTIQWSQENPNYLVVSKLGA
PTEVLSRGRDGFYPHAQSYSRIPSGHPEGYFEAFANLYKTYISALAKQKSGEKLTSDDLD
FPSADDGVLGVRFIGKCVESSKKGAVWVKF*
>SPBDM4_v1_50570|ID:27158933| Xylose isomerase domain protein TIM barrel [Uncultured spirochete bdmA 4]
MARPVTIFSGQWADLPFEQFCEKVKSFGYDGVEIACWGDHMDVKRAATDPKYVAERKAIL
EKYGLKCWALGAHLAGQCVGDLWDPRLDGFAPAECKGNPAKIREWAIQEMKYAAQAARNM
GCYVVTGFMGRPIWKYWYSFPQTTDQMVDDGFAEIVRLWTPILDEFDRCGVKFALEVHPT
EIAFDYYTAGRLLKEFEDRPTLGFNFDPSHLIWQGVTPHIFLRDYAKKIYHVHMKDAAVT
LDGRAGILGSHIAFGDTRRGWNFRSLGHGDVDFENIIRELNAAGYQGPLSVEWEDSGMDR
EYGAKEALEFVRDVDFAPSTIAFDEAMKK*
>SPBDM4_v1_50571|ID:27158934|rbsA| fused D-ribose transporter subunits of ABC superfamily: ATP-binding components [Uncultured spirochete bdmA 4]
MEVIVKTEGIAKEFSSVRVLNDISVEIRAGEVFGIIGENGAGKSTFIKILAGLYQPTAGK
LWFDGKAVEIRNPADARGIGISLIPQEFNLVSDLCVYENIFLGEELVQKNGFLDIKAMVS
RTRQLLSELDVSSIDPYARIQDLSVAQKQMVEIAKAIAFDSRFLIMDEPTTMLTSKEVEI
LFRLIERLKAHGVTIVYISHKLKEVKRICDRVMVLRDGEFISLEEIANIGIDEMARRMVG

RELKEIFPEKKPAAGEVVLSVHNLSVEGLLHDISFDLHVGEVLGFAGLIGAGRTELAETI
MGLRRAESGEISLRGKPLQLRKPWDAVHAGLSYLSEDRQGSGIITSFDVVKNITLTSLRE
YAKPFISQKAEIEKSKFYVDRFSIKIPSLFAKLEQLSGGNQQKVSLAKSIDTQPKIIIID
EPTRGIDVKAKRDIYVFVRGLANAGISVIFISSELEEIIGMCDRVLIMRSGRIVGELPSE
KLSEEEIMYFATGIKGGY*
>SPBDM4_v1_50572|ID:27158935|rbsC| D-ribose transporter subunit ; membrane component of ABC superfamily [Uncultured spirochete bdmA 4]
MAVSIPKKLSLDKYGPLIALVALVIVSTILYPRFIEIRNLLNILRQVSYTGIIALGMTFV
IISGGIDLSVGSMTALAGAFAILTLNAVTQGLMMFSPGWLGTLLSILAAAAVSIVIGLAA
GFVNGFLVTKGRIAPFIVTLGTMAIFRSLTLYIGKAGEFRSVSSSYGDMGMSVILGIPLP
VWIFFILAALLSFVLNRTKFGRYVCAVGSNEKVARYSAINVDKVRLISYVIVGLMVGITA
VLISARLNSLSSTNAGKDYEMDAIAAVVIGGTAMTGGKGSVWGSVIGAMILGIINNMLNM
LGVSPYLQGLVKGIVIIGAVSIQRGWFNNGRTEK*
>SPBDM4_v1_50573|ID:27158936| ABC-type sugar transport system, periplasmic component [Uncultured spirochete bdmA 4]
MKKALIAMMVLLAIAVMPSFAQKVKIGVSIPTADHGWTGGINYYAQKAIKDWQAKDKNIE
FYLVTADSPAKQVNDVEDLMVKGIDALVILAHDSAPLTPVVKKAYEKGIYVVSVDRGLTE
EVQDVYIAGDNPGMGRVSAEWLGKALGGKGDIVVLEGIPCVINSERVDSFKDVMAKKYPG
IHILDSQPAYWDTQKGLEIMTNYLQKYKKIDAVWAQDDDVLVGVLQAYKESGRKDVKIFL
GGAGSKIMIKKVMDGDPLVQADVTYPPSMIGTGISLAVYGSRNQPLPGFYQQKIPSKIIL
AAELITKQNAKDYYQPDSIF*
>SPBDM4_v1_50574|ID:27158937| exported protein of unknown function [Uncultured spirochete bdmA 4]
VKNMKLLRRNGVIAFMLVFAVSAGAQQQAPDFADLPAVLDHMLDNHYMPSVQVGMGSFTY
GDTQLPTPFARWFEDELRLGLAQTTRMKLFDRQVAAAMDPAIRAQYSEFFGQDRADSILY
GKYAEDAGGVAVTLSLTDLATGTLIAEKRYEVPRSAIPSDVTVQPSLKTVQTAMALSQLV
PGSGGQISGVPDLMLTLSTDRGVGAVYRDGEKLTLYITSSKDAYLKIYHVDVNGIAQLIW
PNRFGGSGKVKAGEALKFPGPSDKFQYVLGRPYGTEYIKAVASTRPFATMEADFSDLQGA
AAEAITRGLSVASSDTTRAEALAVYEILP*
>SPBDM4_v1_50575|ID:27158938| protein of unknown function [Uncultured spirochete bdmA 4]
MKTNFAHLTLEEKSIVARLKASLALRLAEESYASDTELQKICPSRGISMKLSIRGAGPSL
HLRVPQSSSRRPSLLLFFPNIESAVRVLSGAKGTAVPIPLDAGALKTLAFFRRASNRATE
LLRAADTPAAIRARLLLAATLYGLEAIAGESYLERRMQIIPDGVVGVRAGEIAYRVAKEG
RTIHVDRAVPAEADGVVSAVNSPRPDAVLSFAHYQSAIDVLSGKRQAVVALGSGEVRIGG
LLPLVQGLFAVLDRLSWYMGVEV*
>SPBDM4_v1_50576|ID:27158939|fumI| Aminopentol aminotransferase [Uncultured spirochete bdmA 4]
MSDISGEESGAAGGISGRGADTQDSRFAASMAYFERAAKVIPGGIYGSKSPGFLVPGHFP
YYLSRARGSRIYDADGNEFIDYLCGYGSQIVGYGNPAVDGPALEQARKGDLLNQPHPVMV
ELAERLVGLVNDMDWAVFVKNGTDATTLATSIARADTRKQVIISAEGAYHGAANWCSTNV
FPVFMQAEQRDVRFFPYNDIQALEALFAANRGNIACIILTPYHHPTYKPQQLPTPEFLTA
VERLCRAESAYFIMDDIRANFRLDVHGSHTFFGAHPDLITMGKALANGYPISVLLGTDGL
RKTASSFFITGTYWMSAVPMIAAMATLDEMERMGGTAQLARHGRMLKDGLESLGREAGFR
ARVSGPLAIPYMTFDEDPDLYLNQRFCAAMADRGVFMHPHHNWFISLAHTEEDIARTLEA
ARSAFEELRLFSSSERF*
>SPBDM4_v1_50577|ID:27158940| putative integral membrane protein [Uncultured spirochete bdmA 4]
MTDVLLILFVVLFPVALLYLKEHVALMAKWSTLIVCYIAGLVLGNIGILPLSATGLLDTI
SSVAVAISIPLLLFSVDFKKWRELSGGAILAFLLAAISVSLVAGIAYAFFRAKNAESYKV
AGLLVGLYTGGTPNLAAIKTALNVDKNVYLAVHTSDIVLSAVYLLAVMSVAKPILKHFLP
LKNWDTEVASPTGLNFTTQFSDYFKKGLWKKILMGFGLAAAIVGVSLGLSTLVPADSQTM
VTILLVTSLALAASFVPSIRALPMTFATGEYFLYVFAVAVGAMGNIMQIFNSAGTYFIYV
AIVLFGSFILHVLLCSLFKIDVDTMLIVSVSAICSPPFVGVVAVSLKARKLILPGITTGI
IGYAAGNYLGIALAEALKALGG*
>SPBDM4_v1_50578|ID:27158941| Oxidoreductase, short chain dehydrogenase [Uncultured spirochete bdmA 4]
MHQTLAGKVVWITGASSGIGRALAAEAARRGARLILSGRNEGALRETAVLCGGYRTAKAL
ADGAPRLPADALLPFDLADPGARRKAAQEALVLFDRIDTLVLNAGVSQRARFAETSPEVF

DLIMQTNFGAAVDLTRAVLPQMRDRDSGMIICISSIAGLMGAPWRTAYSASKHAQAGFFS
SLRAELYGSGIHIAMVYPGFVRTSISENALSGDGARHGELDPLQKSGQEPSETARIVWDK
LETGALDIKVAFDFKAHLGVFLARYFPKLFVRSISKHGGL*
>SPBDM4_v1_50579|ID:27158942| putative signal transduction protein with CBS domains [Uncultured spirochete bdmA 4]
MKVGQRMTRNPITITPDMTAPEAQAIMRREKIKRLPVVDYNGRLIGIVTSLDLIHASPSP
ASSLDIYEMHYLLSKLKVEKVMTKNVITVTEDLPIEEAARIMADNSISGLPVMRGDILVG
IITETDLFKLFIELFGARHKGIRLTLLLPEKKGELAKLSNAITKSGGNIVSFAIFEGEDP
TNGYCTVKVTGVEKDVLLEAIRPLVEKIVDVRET*
>SPBDM4_v1_50580|ID:27158943|livF| leucine/isoleucine/valine transporter subunit ; ATP-binding component of ABC superfamily [Uncultured spirochete bdmA 4]
MALLELKDVRTFYGNIQALKGISISVDEGEIVTLIGANGAGKTTTLMSICAITPLRSGQI
LLNGKEISRVSPNKIVKMGVSQVPEGRRIFPQLSVSENLDMGAFLRTDKEGIKRDMEEVF
AIFPRLAERRNQLGGTLSGGEQQMLAISRALMANPHLLLLDEPSLGLAPLVVQNIFEVIQ
KINKERKTTILLVEQNANMALKIANKGYVMQNGVIKIADTAAHLLENEEVRKAYLGL*
>SPBDM4_v1_50581|ID:27158944|livG| leucine/isoleucine/valine transporter subunit ; ATP-binding component of ABC superfamily [Uncultured spirochete bdmA 4]
MSTQQLQSKDAILEIDNLVMTFGGLRAIDGVSMHIDEGEIAALIGPNGAGKTTIFNCITG
EYRPNEGTVKIRNREGHVEEIHGLKPNVINQKGLARTFQNIRLFSNMSVLENVMIGRHNS
LKAGIFKAILRDASTREEEQRVIEESYLILKKLKLDMYVNETAKNLPYGEQRRLEIARAL
ATDPFLLLLDEPVAGMNAQETKELEETINIIRDQEHITILLIEHDMSLVMDVSERIYVLD
YGKLIAEGPPHEIKRNPDVIKAYLGE*
>SPBDM4_v1_50582|ID:27158945|livM| leucine/isoleucine/valine transporter subunit ; membrane component of ABC superfamily [Uncultured spirochete bdmA 4]
MKVRSILADVRKSLGIAVWAMVLLFPLMVMKVSVTRGVAQVQFRWNLLPFVGIAGFVLSF
VWRIALEWKDRRGNNKSEGAFSRVKAWGGQYFARPAVRKSSLVALLCFVIAYPFLFGMYH
TNVMITAFIYVILALGLNIVVGLGGLLNLGYAAFFGVGAYTYGLMWKYLGHSFIAAGIDP
GWLFWISLPLAGIMASLFGILLSLPVLRLRGDYLAIITLAFGEIFHMVMQNSSDITGGAT
GISLIPRPWFFGHKLPPPQAATYIYFIAIVLVIITIFIVRRIEDSRVGRALEAMREDEVA
CEAMGINLVKNKLITFALGSFWAGIAGVIMAAQTTYINPDSFTLWESIIILMAVVIGGTG
SIPGVIGGAILLKLLPEYFRALAQYRMLIYGIAMVLVIIFKPDGLIPRKRKQYTFEEKEL
SK*
>SPBDM4_v1_50583|ID:27158946|livH| leucine/isoleucine/valine transporter subunit ; membrane component of ABC superfamily [Uncultured spirochete bdmA 4]
MEYFLKLFLSGTAKGSIYALIALGYTMVYGIIQLINFAHGEIYMIGGFTALIFGGFFFSK
GMPVGFVLLFSVLLSIIYAAAFGYTIEKIAYRPLRGKPRLSALISAIGVSMVLQNFVLLA
QTEKFLSFPSYLPEFKWLQPYKEYINSTQLIILGVTAVIMVLLTFLIKFTRMGKAMRATA
QDMHMAQLVGVDINQVISVTFILGSALAAIGGVLICSYMGQINYYIGFIAGIKAFVAAVL
GGIGSIPGAVLGSFVLGWTESLGTGYISSDYEDAFAFIILIVILLIKPEGILGRTQRQKV
*
>SPBDM4_v1_50584|ID:27158947|braC| Leucine-, isoleucine-, valine-, threonine-, and alanine-binding protein [Uncultured spirochete bdmA 4]
MKRRNMVLAVFAMVSLLALFGTQSVFAAPIKIGVAGSHTGDLASYGLPTVNAAKLVVKDI
NDKGGINGQKIELVIEDDQCKPELATNAAAKLVSAKVVAVIGHICSGATKAALGIYKDSK
IVTISPSATNPPLTQSGEYPNFFRTIAPDDAQAKLAATFLAKTLKLKKIAVLHDKGDYGK
GFAELVKQYAEENGIQVALFEGINPGAPDYSAVINKISNAKVDGVVWGGYHPEASKLVQQ
MKDKGMNIPFISDDGVKDNTFIEVAGKYSEGVYATGPTDTSTNPLAIKAIADHKKEFGAE
PGAFFLNAYAATQALLNAIAKAGSTDYNKIVAALQSEYVDTPLGHISFDKRGDIIGFGFS
VFQVQNGKYVELK*
>SPBDM4_v1_50585|ID:27158948| ABC transporter substrate binding protein [Uncultured spirochete bdmA 4]
MRPTTKLFAAIGFAGMLLFSCASKNAKIVGVAKFVSHPALDAIEKGIVDELSSSKPDYKI
DLQNANADMNTAAQIAQRFKQQKVALAVGIATPTAQALANQIKDRPVIYSAVTDPVSAGL
VASMDKGGANVTGTSDMTPVREQLDLLRSLKPDIKRVGNIYSSGEANSVALAAIVRAYCE
ENGLEYVESTITNSSEAKQALLSIAGRIDGLYLGNDNTVFSALSGIAEVALEKKIPVVTA

DPSSAETIPVLAALGYDYYRMGVATGKIVVRVLNGEKTADIPAFLPKDSEDMTFVLNLDT
AKNIGVTVDQSIIDRAKVIISDGQLTRK*
>SPBDM4_v1_50586|ID:27158949| ABC-type transporter, integral membrane subunit [Uncultured spirochete bdmA 4]
MIEGILVEGLIYSILALGVFVSFRVLDFPDLTVEGSFPAGAAAGAIVASSLSLSPNAFAK
ALAVPAGMLAGFVAGGVAGFVTAAVYHRLKVPPLLAGIVTMTGFYSINLRILGGKPNLPL
ITNNPMLKLARSFMSGILSPEWSLLASCILICLVLFGLLDLFFHTEIGIAMGALGDNENA
VIQAGIQPNRLRAWGIMLANALPGLSGAMAAAYQGFADVNLGQGVVAAGLATVMLGELVV
HSQFIGVQLARVFIGSIIFRALMYAARSWGYVAGITPNDLRLLTALLIIVSVAISRYGRK
HR*
>SPBDM4_v1_50587|ID:27158950| Phosphonate-transporting ATPase [Uncultured spirochete bdmA 4]
MIRLHNIEVSFDLGGGQEHTVLKNLSLEVERGQTVSVIGSNGAGKSTLLNAIAGTVPVRA
GKIFLDDSDVTPLPEWARAMYVGRVRQDPLAGTAGDMTLLDNLALAYRKGPRRFRIATPP
KFAREMADKVAELGMGLEDRLRENVSRLSGGQRQALTLLMAILSRPSVLLLDEHTAALDP
ANAEIVGELTRRFIAEFNLTALMVTHDMKRALEQAGRVVMMHEGEIIADLSGAEKEKMDV
AGLVRLFKKVRGAEYAEDRDLLI*
>SPBDM4_v1_50588|ID:27158951| Sugar ABC transporter [Uncultured spirochete bdmA 4]
MKKRYSAVFAILLILLTVVPAFGQTKKQLTFLFMPGVQDPFYTTMEKGVRAKAKELGVNI
IVAEYPKAWGPEYQVPILQAYAQRGGFDGLLIAPTSVDALKAPLKAIYDKGIEIITVDTF
LGDGDYSKPNTDYNFPLSYIGSDNELGGKMIAEHLAKLVGEKGKVFCEATNPDVSSVAGR
VKGFTEGVAEFPNMKLVGVEWCLDVQQKAQEQTLAALQRDKDIVGIFGTNVFSAQGANQA
VVNAGLVGAIKIASWDATITNIDNLKKGVVDLVLAQKPAEMGSLGVEWLYKYLTQKVQVP
KKVIPGFEFFTKDNVNDPKMQQYIYQ*
>SPBDM4_v1_50589|ID:27158952| putative Ribose transport system permease protein RbsC [Uncultured spirochete bdmA 4]
LADKGRPLAGIPGGGSPSSKGPSSISDQTRGGFFQTTFGRQTAFGQFTARNWALLFLILM
IVIFSFAGPGFFDIVNFQNIIHLSTGTFLLAAAETLVVITGGIDLSIGYVYGLASVLGAK
VMQILGAGGMPVPLVIVLGSLAALLVSILPGLGNGVLITRFKVPPFIATMGMWGICNGVT
LFLSDGFMPVMGAPSQINDIGNSYFLYIDPSKTVSFFSKPPYITLTSIRNIRRLIPNSLF
LTLLVLFILGFVMSRTRFGRHTYAIGGSMDAAVRAGINVDRHLVMIYVISSFVSGIAGLF
NLFQTGIGNYTPSGANYELMAVAAVVIGGASLTGGKGRIMGTAVGVLVLAVLENGLQIAG
ISAFYRYIGVGVLLTIAVIIDRAFPDMF*
>SPBDM4_v1_50590|ID:27158953| putative Ribose transport system permease protein RbsC [Uncultured spirochete bdmA 4]
MHADSVNKTSSLQERLGRNWIAIFIVLLVVLFSVSARAFFSVDTAQLIFFNGTEVFLLAI
AELFVIITGGIDLSVGFVMGFSTVVSSKLIVMFAGMGLSPLASILAGSIVMMIIGLLPGL
VNGWLVAYLRVPAFIATFSMLGVTHGISELMTQGIPTKNLPSLAGIIGNGSFLYIAPNGA
LSFFARPQVARGQTVIAILPNMVVFAFDFILLFAFILGKMKFGRHLYAIGGNIDAAIRSG
INVKRDLIKAYVISSFFATLAGLSYVMKYITGKPDAGANMLLEAIAAVVIGGASMAGGSG
TVGRTILGALVIAILETGLRIIGMQTFMTYILVGAILILAVIIDQVFPNRNR*
>SPBDM4_v1_50591|ID:27158954| ABC transporter [Uncultured spirochete bdmA 4]
MAHLLEAKNITKRFGGLTAVDDVSFYVDEGEVVAFVGDNGAGKSTLIKIISGVHKPDIGE
IFLDGEKIHIGSPIDAINYGIETIYQDLALAENMDVPSNIFLGRESTKKIFGVVPVVNHE
HMMRESHKVLDRLDIDIPSLKSNIRNLSGGQRQAVAISRSIYWKARVLIMDEPTAALGIA
EQKKVLELVSSLRKQGIAIIIISHQMYDVFSVADRIIVMRRGIKAGERLVKNTTSEEIVS
LIVGAESVEKRSQ*
>SPBDM4_v1_50592|ID:27158955| putative Histidine kinase [Uncultured spirochete bdmA 4]
MRFFARTFNSFISVIVLQAILVILLVSGSVARSQQEDSKEELKVEALNIYDNFNSWKRVL
WGSIVDLGKSGQLKSMIASQRHITLDDALESAVKKAATQAGCEFLIMKNSWSAFTVVRPL
TEKPVPAPKPQDFSIDRPHPYVEMLLSDGILYFTGAVRVTADNGRYLDIFIAKRVDEVLM
RQLSFNQRIKALVSLNSHFAVGSIAGTAFSEWMRGKTFNTSYTVVDELNEDNVPYSVVIQ
QSGNARLAEGGQNTEATLYVCTFLSLTDYRARVNFINRSILLVSLFVVLFTILLSAGMSK
AVTDPVRQLRNAMIRLKSGDKPVKVMGPKTGEIADLFHGFNDMSARIEEDKRALSSHIQE
ITRIKEYNDKIFNSIQEKILVINAVFAVEQANRAFLEYCGQTEKMVLGRNIDELSPALFD
EPVHASIRAIISGGKAFDTQIRRASSGLSFEIKFYPLLEHDTASTSIHCIMVIEDVTARV

AYEEKIRQAEKLASISMLSAGVAHEINNPLSSILSNVQNLIKAEKEPERLEDLRLVEQET
KRIARIVRNLLEFSSSSPVEKPRNDINACIQKTLQLVDYSLKKESGVSIVVRLSPDLPSA
AIGEDECKQIILNLIKNSLEALGSSGTIRIETAFLSSESMVQCRVSDTGRGIPKALLPRI
FDPFFSTKNEEGNSGLGLSVVYGLVSKFKGRIEVESEEGRGTMVRISLPVARPGT*
>SPBDM4_v1_50593|ID:27158956| Two component, sigma54 specific, transcriptional regulator, Fis family [Uncultured spirochete bdmA 4]
MKTATILIVDDEEGIRHGLSRFFEREGYAVESAEDAVHAETIFESRKIDIAILDLRLKGP
VSGLDLLVRLKREDSDLPVIIITGYGSIESAIGAMKHGALDYILKPVDNEALLVLVRRNL
EVAQLRRDNRYLKKELYQKEYQRQIITKTPAILDIIARLDSVKDSPAPVLVMGESGAGKE
VFARYIHFTGNRSAGPFVSINCAALSEDLLLSELFGHEKGAFTGAIERKTGKFELAHGGT
LFLDEIGDMSPAIQAKLLRVLEENSFERVGGTKRISVDIRIVAATNHDLHELLRTEKFRS
DLYYRIATVEINLPPLRQRAQDIPYLAEFFINLYAERYHKRIDGISPAVMQRWLSYEWPG
NIRELQNVIHQAVLLCTGNRIEIDTVLAGCGSGEGQRAPENSFREARTATESSVRATQSE
VAPDQGVPHPAGPDPVRSASFQPGRYTSLKEVAEAAAAFYERQRIESEFVLAKGNKSQTA
RALGITRKTLMEKMRKYSLMQSGPKEGTAPPSPAYEPKVIARPRKGAEPRGGSTC*
>SPBDM4_v1_50594|ID:27158957| PfkB domain protein [Uncultured spirochete bdmA 4]
MLTISGTGCALMDYLHADIRLDSEAFLRYRARRGGDGGLEPGKLVFVEDLERFAGEPFSR
ILEALRGGQEPDRANLGGPSIVSLIHAAQMLEGHQAQVQFFGARGVDASSDAIMHIVSRT
PLEVSGYLAFAGHTPFTQVFSDPTYDHGHGERTFVNERGVADLFAPGHLPERFFDAEIVA
FGGTALVPQIHDNLDLLCRRAHQGGSIVVVNTAYDFQSEVRRTAADRALAGSYEENGARW
PLGARDGAYRHIDVLVADREEALKLSGADNVGAALAFFRSRGTSAAIVTDGARDIHFYSD
GRLFAPQEEKILPVSAEVRRALGSPSRPKGDTTGCGDNFVGGLLASLALQLEAGQKRGAF
DIIDACSMAIVSGGFSCFYLGGTYLEEMSGEKRRKIETLYGLYLQQLSAGS*
>SPBDM4_v1_50595|ID:27158958| Mannitol-1-phosphate 5-dehydrogenase [Uncultured spirochete bdmA 4]
MQTLVQFGAGNIGRSFIGQLFARNGYEVVFVDVSAPLVEALNERREYRIVIKRNGVSDEE
LVVQNVRAIDGREHASVADAVAGADYVATSVGLGALPDIIPVLVRGIELRALRSPNRLLD
IIIAENIHDGAQYFKESLGRHLPQGLPLADHVGLVETSIGKMVPLMRKEDLAADPLLLFA
EEYNELIVDKHGFRGPLPQIPTLNPVENIRAYVDRKLFIHNLGHAATAYFGFSQSPDSRF
IWQALELPGVAVRVRSAMGESAAALALEYPHDLKPGALTAHIDDLLVRFANRALGDTVYR
VGRDLYRKLAHDDRLIGAALLAARHGLPCDTIADAIRAAVGFRAADEEGKLYPRDADFIA
REVPKGLEGILRDVSGLAMEDPVERGVAVRILSSRQ*
>SPBDM4_v1_50596|ID:27158959| MATE efflux family protein [Uncultured spirochete bdmA 4]
MNLMFLHQSTEQRRDFILNGPTSRTLMFLAAPTLMLGIVQALMPLMDGLFINNIAGTLVA
SSVTFSEPVINMAMALAQGLSVAAMAIIGQMNGRGNFEESRRTSTQIVVMGSILGCFSAP
LLILAAAIISSRVNPQISHNVFLYLSLYALVLPFSFLESIYNGIKNANGVPEAPFIRMIL
MFVLKVIFNFVFIYVLRMGIVGCVLASLAANILITVWMFWELFIQKGSDRLELRGFKFDP
AAARQLFHIGAPAMANTFILNLGFFLINTEVEKYGPVVLNGQGIANNITQVAFILPGAFS
SAVTTMVSMNIGAENPEKAKSSLVQGTILSAITAAIIIAVIVPLSPHLTILFTRRADVLE
IANRALHIYTFSVVGFGITITIQGAFIGLGKTKVPLVLGILRIWFLRYLFILATEKYLQY
YAVFWGNLFSNYAAALIAIFLISRTKWESVIPGSEKNKVVIV*
>SPBDM4_v1_50597|ID:27158960| conserved protein of unknown function [Uncultured spirochete bdmA 4]
MEESDNIIIEKRSLAEQVVYMYLCNSISNGKLKYGDTINTKRLAEELHISMMPVREALKRL
EMDGIVEIKPRSVCLLRTPTKEAIMDAIAAREMLEIYSVRSMYASIDVARLDVLREIIDK
MKAAIAEDPINLRKYIKYDWQFHKELCNLSGNQFISKFYKELNIRLNMDYMYDIGIKPNV
SQTFKDHIDLVEALAAHSPTAVEIIEKHLRISRQNILNGRFFSVQDAAG*
>SPBDM4_v1_50598|ID:27158961|fabG| 3-oxoacyl-[acyl-carrier-protein] reductase FabG [Uncultured spirochete bdmA 4]
MELQEKTAIITGSAKGIGFEIARTFARQGACVVLVDRSGAEKAAESLKNQHYDAIPVSLD
ITDMKAVNNAVEAVANAKGHIDILVNNAGIIARGDILDLSREQWLQVMDVNVNGNFYFCK
AVVPYMIRQHSGSIINITSIAGKMGDITAAPVYGTSKGAINTLTKSLARQLAEYGIRVNA
VAPHAIETDMSADWTNDKRQEVIEAIPLKRMGKPEEVAEAALFLASERSSFITGEILNVN
GGQLMD*
>SPBDM4_v1_50599|ID:27158962| Transporter [Uncultured spirochete bdmA 4]
MLLVGILFLFFLAIGMPIAYAIGISGVAFFLQHPELPLTMIAQLPISQTQNFTLLAVPLF

ILAGNLMNASGITNRLVKLASVLTGHMRGGLAQVNVVLSTLMGGVSGSAIADASMEARIL
APSMIKQGYSRGFSANCTAWTALITATIPPGVGIILYGTTGEVSIGRLFAAGIFTGIFLM
AVYMFTVWIIATRRGYKPERTKASFRERMAAIKENVWALLFPVLLLVGLRSGIFTPSEVG
SFACVYAVLVGVFAYKELTFKNLVQTLASSVLDVGAIMFIIALSGIFGYGIPFERIPDLL
SGVILGLSTNKIIVMFIIIFILVILGMFIDGSVVILLFTPIFLPLAVKVGWDPVHFGILF
CTVITMGNMTPPVGLAMNAVCTVLDVPISEYAKEMWPWLLATLLAVGVLVFFPGLVLALP
RLLF*
>SPBDM4_v1_50600|ID:27158963| Tripartite ATP-independent periplasmic transporter DctQ component [Uncultured spirochete bdmA 4]
MKKLYHFVCKAEVFLAATCFVVSCALIFVAAIARTVRHPINWSQDMSLFLFAWSVFLSAD
AALRADKLVNIDLLVTHLPLKARTVLNTIIYIIIFVFLVLLFYNGIKLSLFSRRRVFQGI
PGFSYSWVMLSIPVGSLLQMITVILKIKNLYVRNEN*
>SPBDM4_v1_50601|ID:27158964| TRAP dicarboxylate transporter-DctP subunit [Uncultured spirochete bdmA 4]
VKRKFMFFVVFFLIAGILLVSAQTEKKYVLKFNHVLTTQDPYHEAFLKWAEAVSARTNGN
LKIEVFPSAQLGVEEDILEQIRQGANVGQNTDAARLGQYVPAIAVMNGPYFVDSLEEVEK
LNTLPSVAAWKAELERTQGFKVLSFMWVQGFRQMITNKPVRKPSDLAGLRIRTPPAPIWQ
ESIRAIGATPVAMAYGDMYSGLQTKAIDGVELSFTATASGKFQEVTKYACETKHILLINF
EVVSSKWFNSLPVEYQKILEEECNKAGLEVSRRYLNEIDPKNLNVLKAAGMTIIPESEID
MAAFKAAGEKAYQKLNLLAVRDQVYKELGKTK*
>SPBDM4_v1_50602|ID:27158965| putative glucose-6-phosphate 1-epimerase [Uncultured spirochete bdmA 4]
MHGIHEYLHSKLPECVNVEEGAGGLPLLHVRNRYAECSIYAYGAHVVSFKPHGERDLLWL
SPYSRFSEGAPIRGGIPLCFPWFGKHRTRDDLPLHGFVRTRFWNLESAEVLVDGRTKIVF
FVENRGPTSEARPYRFRLELSTIVGETLEMALTVQNLEHLPIICEEGFHTYFKVEDPRRC
EVIGLDGIEYIDRVQGDSRATQRGAGYFEGETVRAFMHVPPVLELLDLGHRRIHIEQVHM
NSAVLWNPGEKAAAENPEIRETWKQFVCVESANCLDCQLEIPALGYHRSVLRLSAVNFDE
QKQGQECSKGALG*
>SPBDM4_v1_50603|ID:27158966|mdh| Malate dehydrogenase [Uncultured spirochete bdmA 4]
MSHKIRIERMKLEQFCAAVFMKLGLSPDEAQDSAEILVAADARGIESHGVGRLWRYKNGL
QRGIMAGSACATELRKTPLSLVLDANGAMGLSLSKRTMMRIIEKAKIGGAAFSSVRNSNH
FGIAGYYTEMAARNDMIGICMTNTAALGVPTFGRKAMFGTNPIAFSIPAHDGRMFTLDMA
TTVVTRGKIEVYDREQKPLPGGWAVNTKGVGTSDAHSLLEDMLYQRGGGILPLGGEGELF
SGYKGYGLAEVVDIMTAILSGGVFGQAVMDSKATSARVCHFFGAIRLDLFRNPEEIKSDM
DRLLSELENAEPAEGCERVYYAGLKEHEAEAESARIGVPLSEKVAAQLRQIGEELGVAVP
FTF*
>SPBDM4_v1_50604|ID:27158967| Pyridine nucleotide-disulfide oxidoreductase [Uncultured spirochete bdmA 4]
MRIVIIGNGIAATSAAVSIREYDMENGITMLSDEHAPFYSRPRLTEYLAGKVAFEKIVIR
DETWYAKNNIELMQGVRVESVDPGHRAVTGSFGLLNYDRLLIASGASAALPSFYNSHLEH
VFTLRTKEDADRIAAVAARSRTAVIIGGGLLGIETAYALAERGLEPTVIEVFDRLLPRQL
DAESANMLQDMLAQKGLHFLLAQQTASLSNEYGMVKISFKDDSALSADMAVISAGIRPNI
SFLKNSQVEVGHGIIVDTRLRTNIPDIYAAGDCAEFKGKIYGIWPAAKEEGEIAGKVIAG
QEASYHGSLMSAKLKVASIDMASVGDITIGSQTRAESRRDGSSFRKLFYENEKLKGAILI
GDTTDYFKLQREIAQSAG*
>SPBDM4_v1_50605|ID:27158968| putative peroxiredoxin [Uncultured spirochete bdmA 4]
MDEKRFSMPLLGDDFPEMELVTTQGVLKLPQDLKGSWFLLFSHPADFTPVCTSEFVSFEQ
HAKEFEDLGVKLIGMSVDQVFSHIKWVEWIKDKLGVNISFPVAAANDSIAEKLGLLHPGK
GTNTVRAVFIVDPKGKVRLIVYYPQEIGRNISEFVRAAKALQISDTGVAVPADWPNNSLI
KDRVIIPPPKTVKEAEERLKKYEGFDWWFCHRPLENN*
>SPBDM4_v1_50606|ID:27158969| Transcriptional regulator, NifA subfamily, Fis Family [Uncultured spirochete bdmA 4]
VSTNISVMDSDAMLELILQSIQKLIDYELAVILRYDGQQTLSVTKAAGPLADATLTGHTI
SLKNRKDLAAILEGRKPYLFSESEDHIDTYADIINLPLHHSCLVSPLILGDEIVGLLTFD
HRKCNRFNPQIIGFIEMISTLVAALLVQFDVSNELIERTAQLLNERNNLLASESDAFRNL
IGSSPQWIRVLDSIRLVAAAEAPVLIMGETGTGKEEVARAIHRLSSRAERPFVPVNCSAL
STSLAESELFGHEKGSFTGALALRKGRFELADKGTLFLDEVGDLPMELQPKLLRVLSDGI

FERVGGEKPVKVDVRIIAATNVDLAKAMQDGHFREDLYYRLSVFPIALPSLRERTSDIEL
LALHFLHSIKERTKNSGLHFTEQAFHAILVYSWPGNVRELKNVLERAAILAGSGEIREEH
LGLRLHSLTRHDAAGFSAKSAAVTEKQAVYAGSADVDDGALPLQSIIKKRIEEALEASGG
RIYGKDGAAAALNLKPSTLQSKLKKLGIRREWFLSPRGEH*
>SPBDM4_v1_50607|ID:27158970| putative Membrane-bound serine protease (ClpP class) [Uncultured spirochete bdmA 4]
MARGNKFHGKENEPEGLMCTVYYAFLFCMLFASLGASGAQPRDIEFGGIPWGLRQTAGPV
DPGPNTFSNAPDQVWVDAQGRAHLTLQKRGDVWVASEMMAKRDAGYGTYRFTVSGALNEL
DPNIVFGFFTWDKSPEAFNREIDIEISRWGDAGGPNGWFTVQPYDVPGNQHSFFLPGADA
YTFEMRWEKDAVEYALFCGGNVCERWRYSGTVPEPGRARLRINLWLFHGRVPLGPGPYEV
IVSDFSYSK*
>SPBDM4_v1_50608|ID:27158971| Dihydrodipicolinate synthetase [Uncultured spirochete bdmA 4]
MTDRSSLIATFFPGGIPTLWCPPLTHFKPDGTIDKARIHAHLRFMRPYVPALLVPGSTSE
GWELSQQEEAHLVDIALDCAREMDFTILVGILRPAVGVAKADIEVTVARLFGDAHPDPAG
FHERQLCGFAVTAPKGKELSQRAIYQELDAVLELGYPTAFYQLPQITENEIAPETVADLA
SRYENLYLLKDTSGGDKVVTSGLDFGGVFFVRGAEGNYAEWYKKAGGLYDGFLLSSANCF
PRELREVLDLLEQRRTAEADALSARVSNVVLRTLEAAGALPFGNAFTNAAKAIDHFIAWG
PDADISAKIYVKSGATLPQSLLETAREALDREGLMPHEGYL*
>SPBDM4_v1_50609|ID:27158972|aroQ| 3-dehydroquinate dehydratase [Uncultured spirochete bdmA 4]
MTRLRIAVIHGPNLNMLGTREKDIYGTKRLQDIDEEILREAEKQGFEVTFFQSNSEGGLI
DYIHGCMGKIDGIVLNAGAYTHYSIAIRDAIAAVNIPTVEVHLSNIYKREEFRHSSVIAP
VCVGQISGFGAYSYILGLHAILNVYATS*
>SPBDM4_v1_50610|ID:27158973| exported protein of unknown function [Uncultured spirochete bdmA 4]
MKTKKLIIAAIISLFMLGSAFADGADPKTVTPAWVTEKQLTQQAQAQEQAEAEAPKPKNF
NVFFIGYDYPTLSGPLASHLTGWNSPVNFSLGIESSNTSGSSFLSGLELEFFITTNDQGM
RLQMNDMVMAGYSFNIKPVRFNVGARLGLSLLDVTDDDPANPTYMALGGIIGPEASLYAE
LAPDFWLWARGRYSMAYYFTIDSSGGANPIDSGDNTLNCVSLEAGLAFRM*
>SPBDM4_v1_50611|ID:27158974| exported protein of unknown function [Uncultured spirochete bdmA 4]
VKYAKLALALFVAFFVAGCASAPPPTTSTTSPPGAAPQASAPPQGSAPPSIAPPKNDTWP
KIYTDETYLTSFDSSWSEAEAVGAIKNLQNSFVEFNAGIPLSELDVDSYGLRAKWQWVEN
NSFIKSASFIIPFDQVTSILLEYYPALNKDYKWGLIVNLSGSSAVSVRTPSRDTAERLGK
AILVLAKARSAKLSIPNLRFGAALSALSDAQAQAAGILKTSGVIVLWVFRESPAEKAGFS
PQDIITGVGGKPVHSTEELFAAIDAAADAGAKELKIDGIRRSYRIEDKKYVEIFVPLTYT
LAIDQAGGTK*
>SPBDM4_v1_50612|ID:27158975| conserved protein of unknown function [Uncultured spirochete bdmA 4]
MNEESTEQKALRGASPGHASPRRRPAWYSGLKRRARKLKRQIWALSLALKDPRTPWTARI
IIGFTVVYAVSPIDLIPDFIPILGQLDDLVVLPGLIALALRLIPRDVMARCRREAWRHLE
AGDRVKTPAATAASIIFVLIWVLLILWIVLRIV*
>SPBDM4_v1_50613|ID:27158976| Major facilitator superfamily MFS_1 [Uncultured spirochete bdmA 4]
MEDRKKAAYKFLVLMGIVSLFSDLTYEGARSILGPYLLLLGASASTVGFVSGLGEFIGYA
LRLVTGYISDKTKRYWPITIIGYAINLIAIPALALAPGLGWVYACGLIVLERFGKAIRSP
AKTTLASFAASEVGAGKGFALQEVLDQIGAFIGPLMLFIVLTVKGNDNKLSAYALGFAIL
AIPAAVTLILLLVARNKYPRPHEFETTPAPVTGDGLRSAYWYYLVGIGFLALGFADFPLM
AFHMTKTKLFPDNVVPVLYSVAMGVDALSALFFGRMYDRKGMTAIIISSAISMLFAPLVF
LFRAPALAVAGVVFWGIGMGAQESILKSAVTTIVSKERRGTAFGIFNAGFGAFWFVGSWI
MGLLYDRSLPLLVIFSVVSQVASLPFFFKTRSLMRHAFEDVKVSG*
>SPBDM4_v1_50614|ID:27158977| ChrB domain-containing protein [Uncultured spirochete bdmA 4]
MQLVLHSSYEYTYSMEDHSTWLLFAYQVPASPSTHRSYVWRKLKALGALYLQNSICILPS
IEPVEETLRQLHAEIINRNGAAIILHVNLLNEEERTEIIARFRRQMEDEYGEFIEECGEF
HAELTKERARHQLTYGELEENDVDLGKLRLWLPKLRVRDFFQVDTATKAAEALESCEADF
SQFESEVEEASRG*
>SPBDM4_v1_50615|ID:27158978| Alkylhydroperoxidase AhpD faimily protein (fragment) [Uncultured spirochete bdmA 4]
MLAVTEVNGCEICSYVHTRIALEKGLSDEEIQMILGGNSEKIPEQEVVAILFAQHYADTR

GKPTQKTWNTLVATYGEQKSYHILGIIRMMMVGNIFGIPLSALKNRIKGKPNKKSNIGYE
LIMMVLPIPFIPITLLHALVSELLRIPSITFSE*
>SPBDM4_v1_50616|ID:27158979| putative transcriptional regulator [Uncultured spirochete bdmA 4]
MSQTERIFYIDRTIRDHGGITALQICRKFEVCERQAKRDIEYMRNRLDAPIIWTPGRMRY
EYSEPWDGLRFADETSFLAFAFLRAILGRYSYVPVVSEEITALLREKIAGRYAAISDKVR
YELPDMETIDGETAYTMCQALLEAGTLDISYTDSKGEQSERTIAPLRLINYAGKWYCVAF
DSKSMELRTFAVSRMQNLRPADTPHFPIPADDEVERYISSSYGIFKGEVLGTATLRFYGG
AARAVRTQQWHRDQKISAVPRAADSDAIDLSLPVHDWTELLGRALRCGANCEVVGPPEFR
ARWREEIERMKTLTDKSTL*
>SPBDM4_v1_50617|ID:27158980| protein of unknown function [Uncultured spirochete bdmA 4]
MSAKAIYLIQFEKDYVELFSESQSGAFDFAGVVSGDGAAAKARARAKDFIEEAPETELYF
QNHFGKPPVYEASASHPTVRRMGLPGAPEESVLAFLYRYPAFRQAELRGDFIGAGTAVGR
SGKLKSHRPLLRYLAERDLFLIFALYWLWAFDADKTARLVELFDGKITDGAGDIWAELYD
FAVPGIDARDLPAFRAEVKAKLSEYLFDRGGRLVLPLVGDNFGGAVVEMRRRSVTALAEG
RRRALVEGLSGTEDRVRAWLESIRFTLQPEPWNKADRDAMSVLATFPEDGRTDLAGYLRR
DIAAYLTPLAREGRNFEARLFCLDEDSLELSIRAARPPD*
>SPBDM4_v1_50618|ID:27158981| putative prophage exported protein [Uncultured spirochete bdmA 4]
MKKIKEFPGKSLFTVLMFVFAILSVYPQTPNQYHDLVAALQRAAAEKDDTQRLALYDALV
RDFGIAEAPAATALTTPQEPSKWVFDQRADPLTDKQRYFFILTADSGANEYGDKPTLIVR
SDGEDLELYINWKTYLGNDTDDYKYEGKYITTRVDSDQPTTSLWDNSTDSKASFCPWNDV
PDLVRRMGEGTKFVARCTPYDANPITAVFDIRGLKAVSMPYNEALGWWK*
>SPBDM4_v1_50619|ID:27158982| CRISPR-associated protein Cas5 [Uncultured spirochete bdmA 4]
MSTYNKSYKVNFEIAGPAAMFTRPDTGAAPVSYPVPTYSAAKGMFESIARLNSAYIRPTE
VEVCSPIKFQRYTTNYGGPLRKDNQMKGDTSYQLIATILIDVCFRIYGEVEELYKPLDVK
TNHLHHLQEMFQWRLERGKCYYMPCLGWKEFTPSYFGPFREETKVDESIQITIPSMLFSV
FNNEGKYNPVFKQNVEIIAGRLKYAE*
>SPBDM4_v1_50620|ID:27158983| protein of unknown function [Uncultured spirochete bdmA 4]
MLNEGYTLYKSLERCGISLMPHHPDLKKPGRKEGLIVGLDTRGQVANIEYRGSDKMAELW
TTSEGNHNSFPVLKLQHPLWRVDPNDAVRERIKELKKDEIKKRQFLMQQYYDINITSTDI
NWWARVHGRVDEMSHFFHTTEREYVALYNLMGRFLEANDLKRFLQELLNQLKKHDEIPYS
FIENILIGNKWDKKKHEFRSEVPLVMDLCDWGNYPTRIASQKLESFVSECLFKMQKPSQT
NGISALSGRIIEIEDKKFPNPTLPIIGQTYLFSVNDQTPCQTRYKKTSTSIFPAGRNEAN
VIQESLRWITSEERRGKTWYPVPGIKDSETDLLIVYLIDKPNTNLNKARLLGGVSSTDLN
ESTFEEIAAPIIDAYRGQHILKASDKIRFFVLRQADPGRKQVVVNGDFTASDVVKAAEVW
QQAGGNVPPFALLFPGAKDENAKILTPRCPFPADLVRLTQKQWVRLGEDYSNKVPGIALG
KVYDIFFDQGGKNKAAVYALLRIVLRRTEPLLIGLVGAMHKNTLEKFTPESRFTLLLAIS
VISICLYKLGISKEKYMSDTFFFVGRFLSLTDTLHMEYCKNIRNGNIPPQLLGNAHLNLA
LKNPAAAFALLSQRIGVYQAWTRKEQGEKVKLARWAVGEMGEIALSLANEGLPTTTDDAA
KAQILLGYLAKSGKKESIGTDTDSVK*
>SPBDM4_v1_50621|ID:27158984| conserved protein of unknown function [Uncultured spirochete bdmA 4]
MTATANDMIKRATGLLVIEVINSNPNGDPDRESDPRIRDHDQRGEISPVSFKRKCRDLVE
RKDGPVWQTISNELDLSAEKYMISESRETKLKDIRDLDENKFTERFWDGRVFGNTELEKG
RDIPVKTGVVQFGLGVSISPILVERLTTTNPSVQEDKSRGMAPLAYRIVQHGVYCMPFFV
NPSAVRKTGCTKLDIDLLLRIIPYTYTHTASYIRPQVFIRHAWYIEHKSPLGSCSDFDLI
TALTPIRKSELEQPSASWADYEVPVKLPDDLQIKVEPLIDLMNSI*
>SPBDM4_v1_50622|ID:27158985| Helicase-like protein [Uncultured spirochete bdmA 4]
MNTKWLAHSVGKKPSVPAQSYSEHIADVVGRVDHNLDHLLKYYNGDVNFFRTTVRTAALY
HDLGKLDDENQKVLSQGGSRHLPTNHVDSGTAYLLDKDFVESSLLVYSHHIGLPSIPAEQ
AKEGLFLRDPELKNITEENLSKYLLRHIESGFDDLVEIQDKQSDWNGLSRRIALSCLVDG
DYGDTAKNYGKEFPVSCPKPRWTERLKALDRYVEGLAKDLNVAPERILLRQKLYQASREA
DTTPSFFACDSPVGTGKTMAVMVHLLRTAIDKNLRHIIVVLPYTNIIKQSVEDYRQALVL
PDENPDEVIAEHHHQVDFDEVEVRQLSTLWQAPIIVTTAVQFFETIANNHPSRLRKLHEL
PGSAVFIDESHAAIPNYLWPQTWTWLNDLGEKWHCYFVLASGSLPRFWELKDLITVPERL
PQLICDDLRKEAVRQERERVVPTRHSDILNVDGLIKLVLSQAGPRLVIMNTVQSAAVIAH

RLASQIYGENYSIDLETSQVLHLSTALAPIHRDKIVDIVKQRLSLARENYDTNFTLIATS
CVEAGVNLSFRSAFRERAGAANLLQVGGRVRRHGENFKPILIDFRIEDDLISHHPTFDIS
SQVLERLFDEGRVANDSPSDLVTEALRRELMSDTNVQYKRLQKYEEAGDYPAVTDLYRVI
SGDTKLILVDQEIINKLHEGEKIDPKELVRRSVQMWTSKIGKTCAYQIDGFPDLFGWPED
GYDEFFLGYMKGVLPILQLEEKGFGII*
>SPBDM4_v1_50623|ID:27158986| protein of unknown function [Uncultured spirochete bdmA 4]
MRSDYRPRALDLGPTLEQMSVFLFGPRQTGKSSFVREKLKPEPTLSYNLLDGGLRLRLLA
DLTLMRQEMETCDIPAFARFLQTIL*
>SPBDM4_v1_50624|ID:27158987| CRISPR-associated protein Cas4 [Uncultured spirochete bdmA 4]
LYSEDDLVPISALQHILFCERQYALIHIEQVWEENRFTAEGKVLHERVDVEHHESRRLFR
QEYGMAVRSLERGLIGKCDLVELYLAPNGGVADAVPVEFKRGRNKEEDVDRVQLCAQAFC
LEEMFKVPVAVGQLYYLQEHRRTTVDIDGPLRAKTLQLVERIQQLQSAGTTPPAVYEKRK
CDRCSLLELCMPESAGSGGKRVDRFVQSQIRATREECGQ*
>SPBDM4_v1_50625|ID:27158988|cas1| CRISPR-associated endonuclease Cas1 1 [Uncultured spirochete bdmA 4]
VRKLLNTLYVSSQGSYIHKEGETIVVEREQQKLLQLPVHTIGGIVCFGNVLCSPFLLGFC
AEHDIAISFLTEYGNFLASVRGPVSGNVLLRRGQYRMADDAEITRKIAANIVAGKLANSR
LVMSRAVRDHGEKIDAASIKSASASIDRLMNELPHAVDSDGVRGIEGMAAAEYFSVFNHF
IVDQKEDFVFNDRNRRPPLDPVNALLSFVYTLLSHDLRSAIETVGLDPAVGFLHRDRPGR
PGLALDLMEEFRPVIADRLVLSLINRRQVSPQGFKQAENGAVMMDDATRKTVLVEYQNRK
QDEIYHPYIEEKIPVGLLYFVQANLMARFIRGDIDGYPPFFWR*
>SPBDM4_v1_50626|ID:27158989|cas| CRISPR-associated endoribonuclease Cas2 [Uncultured spirochete bdmA 4]
MFVLVSYDVAITSPGGKRRLRRVAKTCLNFGQRVQFSVFECVVEPAQWVTLKSQLETIID
MEHDSLRYYYLGSNWKRKVEHVGAKPTLDVDDPLIV*
>SPBDM4_v1_50627|ID:27158990| protein of unknown function [Uncultured spirochete bdmA 4]
MYISNYYHKNKYQGNSAILSGPDSKIYGFPFPMFSKIDLIKNYVLRDSTGVFCVIYVRTS
IWPGPQFPATGTSKRRGLIFR*
>SPBDM4_v1_50628|ID:27158991| conserved protein of unknown function [Uncultured spirochete bdmA 4]
MNVAPPAGAWIETTEKWTVVAVADVAPPAGAWIETRYCRNIQNIHHVAPPAGAWIET*
>SPBDM4_v1_50629|ID:27158992| conserved protein of unknown function [Uncultured spirochete bdmA 4]
MNGMWVRSWDASVAPPAGAWIETMSVILIRKHGLVAPPAGAWIETTDATEWEIADIKSRP
PRARGLKPVMVDGTKYDSSRAPRGRVD*
>SPBDM4_v1_50630|ID:27158993| protein of unknown function [Uncultured spirochete bdmA 4]
LKRLDKLKADIQSVSRPPRARGLKQSVHTAYDQMGAVAPPAGAWIETEAERRTAGVAQVA
PPAGAWIETMKPSYSSWCF*
>SPBDM4_v1_50631|ID:27158994| protein of unknown function [Uncultured spirochete bdmA 4]
MLVAPPAGAWIETDTDRQEKVRILTSRPPRARGLKLVNSADTWQLERSRPPRARGLKQSG
TLTEERGIKVAPPAGAWIETSILPEGFRRCPSRAPRGRVD*
>SPBDM4_v1_50632|ID:27158995| protein of unknown function [Uncultured spirochete bdmA 4]
LKRRTRRSGRSRISVAPPAGAWIETTDATEWEIADIVAPPAGAWIETAMESLYSAQAAGR
APRGRVD*
>SPBDM4_v1_50633|ID:27158996| conserved protein of unknown function [Uncultured spirochete bdmA 4]
MVSIHAPAWGATSLYRDFCDFIKGFNPRARVGRDSIKRSCNKQFFRFNPRARVGRFRLLS
ILDSFNPRARVGRDVISARAMPA*
>SPBDM4_v1_50634|ID:27158997| protein of unknown function [Uncultured spirochete bdmA 4]
VIVPKHQREFKSFDDNILSMYARGMTTREIAGHLKEIYGTDVSPELISRATDSVKGLLED
WRNRTFYPILFLDALVINVRENGKVVKKSTYMALAINQEGRKEPLGLWIDQAEGAKFWLK
ILNELHNQGLQECWLPPSTASQDFQTPLPRCSRRLKCNCVLSTWSTIP*
>SPBDM4_v1_50635|ID:27158998|blc| outer membrane lipoprotein (lipocalin) [Uncultured spirochete bdmA 4]
MKRTLGFVMTLIIGMAFFSSCATIPNGANVVDHFEKEKYLGKWYEIARFDFSFEKNLNNT
TAEYSIRKDGKIDVKNRGYNYVTKKWQEANGKARFRGDDTLAALEVSFFGPFYAAYNVIA
LDKDYQYALIAGSNLNYLWILSRTKTIPDEVRNEYLMIAKSAGYDVSKLIWVEHDK*
>SPBDM4_v1_50636|ID:27158999| protein of unknown function [Uncultured spirochete bdmA 4]
LKEQPFIAAFVYGFNETGTNAKPISKVTDF*
>SPBDM4_v1_50637|ID:27159000| Radical SAM domain protein [Uncultured spirochete bdmA 4]

MSLKARIAKSIVMSKIKYIREDPDTRLPEILDSARKIDRTGRFDSAFDTLEPIAKDRNNN
WNRMVRSLLAETDPVCLEKLIGNLAINTELSSLHIRERAMKKYGCNIPWAILMDPTAACN
LHCIGCWASEYQKTANMDFDLLDRIITEGKKLGIYMYIYSGGEPLVRKADLFRLAQKHSD
CYFLAFSNATMVDEAFAEESARVGNFALAISIEGFEEETDMRRGKGTYAKVIAAMDILKA
RGIPFGFSACYHRYNTDVVGSESFVDFMVEKGARFGWYFTYMPLGRDAKTDLLATPDQRE
YMYRKVREYRETKPIFVLDFWNDGEYVKGCIAGGKSYLHINAAGDVEPCAFIHYSNANIH
NMSLIDTLRQPLFREYAAGQPFNENHVRPCPLLDNPEKLRGMVERSGAHSTQLLDEEDVV
SLTAKCEDAANNWSARADTLWKERNEEKARQEAERAARNAARQGAKNKKTEEEIYQP*
>SPBDM4_v1_50638|ID:27159001| conserved protein of unknown function [Uncultured spirochete bdmA 4]
MKVNDAVGKWLGWIGIVLGVIGFFWASIWMGIGAIVLGLVGIFSSQKVLNGVAMGAGVIA
LIIGLV*
>SPBDM4_v1_50639|ID:27159002| protein of unknown function [Uncultured spirochete bdmA 4]
LYGFEGCTLAGFSLIDIHPELLNQYYKLGMIDYYLVLEVTKDASADEIKSHYRRLAMQWH
PDRNKGSKQAEEHFKAISEAYSVLSDPASKAAYDESLRMGTDYRAGQQQGRPEESGAYGP
FGFGFGFADGFGSSSGQERAGAFEWASGLSKERASNMFMDEMYNLAIELTMQNIPWRDIA
QELIKRGCPESTAREIAKKIETQRKAMVRNRAKPYFIRSALSGALGLGMFATFGGFGILG
FIGLIMSLNGGYNLIRAIYFLTTGVAPKGGVM*
>SPBDM4_v1_50640|ID:27159003| putative 5'(3')-deoxyribonucleotidase [Uncultured spirochete bdmA 4]
MDESSFIFGVDLDGVVGDFYGAMRTIAAEWLDRPIESLTPEVSFGLSEWGLDEYGGYEKL
HRFAVTQRGLFRNMEPVRDAPATLRKLSVRGVRIRIITHRLFLKYFHREAIAQTIEWLDA
HDIPYWDLCFMKDKGAVGAHVYIDDSPENVESLRAQGCHTIVFSNSTNRAIPKPRAETWK
DVERLVLEAYEEWRTGTLGLFGASGYDPGSVRG*
>SPBDM4_v1_50641|ID:27159004| protein of unknown function [Uncultured spirochete bdmA 4]
LTESGNGFIVNSSKRFDDLMEAKHEEEKNCIDDGAPSFGGSPGIAVGAAQDDAYRAVVQR
RE*
>SPBDM4_v1_50642|ID:27159005| Extracellular solute-binding protein family 1 [Uncultured spirochete bdmA 4]
MRKKKIASMMVLLLLAVALGSLWAQPKTTLTVLWFNDANESDVFMATMADYQQDHPNVTI
DMQVIPFKDYENKLRLMIAGGNPPDVARLASAHVPLFANSLEPLSGKPVFDELAKGYFAS
SLALGQDDNGRVMVMPTEATANGMIVNKTYFKNAGIDVDKLSQTWTWDQWVDAMKKVLKA
NPKAKYGITVDFSTHRFSTFLYEAGGRFLSSEGKSMNFNTPETLDALKFFKMLHDEALAP
KSVWMGSENPQELFQSGLVACHVGGSWLINAYSRNIKDFEWGVVRMPSRKINSSVPGGKF
VGTFKNSPNKKEALDLIAVFSDKAHNEQYCRDTFNLSARKDANITYASRSKDFAVFTEDL
NLTPAYTADDWKSDVVAKLNSYAREQIVEGLLGKQTMEQTAANIQAKGNSYFK*
>SPBDM4_v1_50643|ID:27159006| ABC-type transporter, integral membrane subunit [Uncultured spirochete bdmA 4]
MKSRQRSHGTFEQTRAPLLFLAPNLIIFSLFIILPAIMGLRMSFFDWSILDGSKYIGFKN
FRDILADSMFWLTLKNTLIYVVAVVPLLSISALALALPLAGEGKGMGVFRAIYYVPTMLS
MIIVGIAWRWLLGYDLGIINYAIRLAGGKAVSWLTDGTMANISLIFVTVWTRVGYFMMMF
IGGLQAIPKTYYEAAEMDGAGRFTRLRRITIPLLKPIILVVVVLSTIEAFKAYELIFVMT
QGGPGTSTKFLVQYIYQAAFEEDRMGYGAAMSVILLVIIGIFTAIQFGARREEYTNE*
>SPBDM4_v1_50644|ID:27159007| ABC-type transporter, integral membrane subunit [Uncultured spirochete bdmA 4]
MNKFLKYTLLLIVSFVFLFPILWVLLSSFKPQSELFTYPLTILPHRPTLENYVNAFASGD
FITYYKNSLFVSVVATLITITINVMAGYALSKFWFRGRDLIFYVMVGTLMIPLQAIMIPI
FILLKNLGLLNSLWGIIIPPAATPTGVFLARQYLQTIPDSFIEAARIDGAGEFKIFTMII
VPLAKPIIATIAIFSFMWRWNDFLWPFIVVSANKKMTLQLALANFVGQFQINWGNLLAMT
VVTMIPMIIVFLALQKYFVKGLTAGGVKA*
>SPBDM4_v1_50645|ID:27159008| Glycoside hydrolase family 31 [Uncultured spirochete bdmA 4]
MLHALKNLDGLRVEGGKAYLSGENGRLEVTIHAPHVVSVFYVFDESIVSPASNIPHAGLF
ADYAPGGLPDAGGIHGGPAISWDSASESGPGYELRHGKTEVLIDKATARVSVYENGVLVH
GGEIGHKDLVVPQYPLRVQQGAAQGSVRGKFNFRLEEGDAFFGLGEKTGRLNKRNRSFKL
FNRDALGYDAESSDPLYISVPFFMKVNRGRGAFCGLFFPNLQVEEVDFGVESMFYYDVVL
DGGPFGYYVMTGGRYRDILSSYCKLSGMPALPPLFSFGYLTSSMGYTDPDNAQERILEFF
DRVEKADIPCEGMYFSSGYAKADNGERYTFVWNREKFPDPGRFIRSLRERGYRICCNVKP
GILSGHPWYKRLAAAGVFIPGADGAPLKSYYWANSASFVDFSRKEGFDWWGKALTEHIID
KGVAGVWNDNNEFEIEDDSLPAQTYKKFLPVKMAQASFEALRKANPGKRPWLISRSGYAG

LQRYARTWTGDNVSSYETFRFNIVMGMNLGLSGLPMYGHDLGGFVGPSPDEELLLRWCQS
AIFQPRFVMHSWKPDGSITEPWSFPHRLDAVRHFIRERYRFLPYIYDLAIRASETGIPLE
SPVALDFPDDPSLSYESLDRMAGDAVLVPGPPGRGETAVRLRFPAGADWFDPAKEVLHRG
GTLLEFNYPLDAVRYFFRCGTVIPTSLKTDPVGKTAPDEYEFLVVPPADAGDISPLHAAC
VHSEDDGESDFVAGSYWRWRMDFEMPREGDYVLNITRTASACATDYRKYWRFSVPKGFVI
LDGNRYADGRSVEFRFQSAPEHIRLVLRGAYRTNGT*
>SPBDM4_v1_50646|ID:27159009| Transcriptional regulator, LacI family [Uncultured spirochete bdmA 4]
MVTIRDVAIAAGVSVSTVSHALSGKRPISQATKDKIHDAIERLGYEPNPAARALRTTTSG
VIGFFAFDITEVFAARIIHGAEKIAREKSSYLLFTSGVEFNNDITSAIDFLRKRRVDGII
AAYGVRQAIRPEMRVAFDLPAVTVNTFIYDSIPSVQPDDFAGGREAALHLVARGARRLAV
IAGPEMRLASMERLAGFTKALEECGIGFDPSRRVVHGDFTAESGAHCMDVLLEVCPDMDA
VFCANDYMAAGAINRALARGRTIPGDLRVVGYDDREFSSFWPIPITTFALPLERMGEVSA
AMLFERIEGREPDPMRVLLPSRLIIRQSS*
>SPBDM4_v1_50647|ID:27159010| Ser/Thr protein phosphatase family protein [Uncultured spirochete bdmA 4]
MKRYTIFLVICLVFAQAVFGGGTGDSPASQNPGGQQCMAEGIGFTLSAYNPRGSFEKLYA
VREESGSHSAPEELTRTDALETDMNLTVFHFNDLHGHITDQDSKRGDTHRVAQMVKIVDA
ARKNADERNIVLFFSAGDEHTGTPFDELLGWNAQEFIVDPAYRILSAAGVDATVVGNHEV
DRGYELLAKGARQDAAFPVLSANIVGSRWATPDVIKPAAIGIAKGLRIGLIGLTTPEETR
EATVEDPNVRIAAPLESIEYYLPRMEPFVDVFVVLSHLGFEGEERHRVSVGDRAIAEAAS
RCTGKPVLVIGGHTHSVLNKDGLEAANIVEGIPLLQAGSYGAWLGRMDVSLARKEGEYAA
TDMKAALLPIKSRDDRVASDQKGWANLEHEGDYDGRFEKENVEPILTRLAGKMKERLGRT
AATQGFSTERILAERYVGECGMANYMNDMIVARSANFPGGPVDLAVFNASGIASGVDGNT
DVTFGDWFKVMPYADNIVIISLSGAQLLDVLRSNAQRLVRPEELAGTKPIALDGFVSRGF
LHFSSDLRYEIRLGANAEAATVENASFRGVDLRKTPDKIFRIAFSTYIANGFEGWKTGPV
GAGLPQGVLAWDLAALDKLDTGLVYRNEIIAGIKEWGRIAPFGGAAGPLDGRVTIRQ*
>SPBDM4_v1_50648|ID:27159011| Band 7 protein [Uncultured spirochete bdmA 4]
MTTFIIVIAVVVVLFFSGIRIVRPTHRGLVEVFGKYRSFAEPGFHWIFPVIMRMFQVNTT
EQMVNAEPQEIITNDNLNASVDAQVYFRVLPDERSVKASQYNVNNYQLQIVSLARTTLRN
IIGTMTLKSANSERSRINTELLQTLLKETEDWGISIVRTELKEIDPPKDVQETMNKVVKA
ENEKIAAIDFATATETMADGQKRAEIKKAEGIRQAEILKAEGEAQAIQLVNEAADRYFVG
NAQLLKRLQTVEESLKNNSKIVLPTNGELVNVIGDLAGVLPLKDKKPQ*
>SPBDM4_v1_50649|ID:27159012|mtnP| S-methyl-5'-thioadenosine phosphorylase [Uncultured spirochete bdmA 4]
MDKATVGIIGGSGLYEIEDVEVLEEIRMDTAWGAPSDAITIARIGGIRAAFLPRHGRGHF
ILPTEVNSRANIAALKMLGVREIVAFSAVGSLKEEIRPRDFIIPSQIIDRTKDRPGSSFF
GEGIVAHMSFADPFCGRLCKIIEPFVVARGLRLHTEETLVCMEGPAFSTRAESRLYRSWG
AGVINMSALPEAKLAREAEICYAMVCMATDYDCWKTDHADVTVDMVVENLNANSAHAKAL
IKEVVPALGGGEDCSCRSSMRYALMTAPAKRNPETLAKLKTILPELR*
>SPBDM4_v1_50650|ID:27159013| MATE efflux family protein [Uncultured spirochete bdmA 4]
MEQKDPRYTKMVQARIGTLILSLSVPTIISMLITSIYNMADTYFVSKLGTSASGAVGVVF
SLMAIIQAVGFTVGMGSGSLISRLLGAHRTDEASKVAASGFYMSIFFGGLVAAAGLLFLD
PLMQSLGATDTILPYARDYARYILFAAPVMAASFVLNNILRAEGQARLAMVGIATGGILN
CGLDPLFIFVFKMGISGAAIATALSQLISMLILLSCFVRGKTVCKLGLSKISRTPDTYLQ
ILKNGLPSFCRQGLASISTVALNVNAAVYGDAAVAAMSIVGKIFMFIFSFTLGIGQGYQP
VLGYNYGAKKFGRVREAFFFTLKTCMAIMTVCSIGGFAAAPFLMKLFIATDPAVVAIGAK
ALRAQCLAMPLIPLGVMCNMTFQSIGKSWTATFLAAARQGIFFLPLILVLPRFLGLTGVQ
ITQPLADLCTFLCCLPFTFHFFRELKAEKTVAT*
>SPBDM4_v1_50651|ID:27159014| exported protein of unknown function [Uncultured spirochete bdmA 4]
MRQHAFMLALLFAASGVFAEGWDEIYTARLNNSATYLESKLTLKSAEADYAGYTKPYIPT
VSISTGTSGSDDTYESGLVVGSDGATSGTLIPSITFEKLLGGADLSFKAPVNIASTGEVS
MGNPALSVSRTLFPETAANQLDAEAALISAQASLQAIKNDIRIELATDVLDTIYYRRLLE
SSKENLEILEKVRKATVDTTTQRELDKSILEAQKSILTATSALADIKDDVKNNADALYED
ITRLQSGWLASINGEEPKSSMTIRSLELSLAAAEKRKSFSILPYLPNPTIAASVYYNVDT
SKVDWALSFKLSYDLVNKNENALSALKREEYPKIYSIKLEEAQKGLKDGLRQINDTLESL
DLDKKIKDIDLADSDDDMARQEKLYNGGFISEEDYVMAQIDLAILKIEAQKIDFDILIQK

LKLAQYYEESQ*
>SPBDM4_v1_50652|ID:27159015| exported protein of unknown function [Uncultured spirochete bdmA 4]
MRSRGSFFLALCALSLVCSTVPVAAGTLEELFTAATKSNQDYAVYKLDLEIANLKKTKGE
IEAKVELDRVNAQYTYVTSLADYQSLVLSFYDKVIDAVFDAATADLDDQSAALSLENAKE
DKKYADVRYQNGLISEDVFKEIGIAYDTAVSDKQLSEYTLQDAKDHVRLATGLEWSWDLL
PEIPSFEPSATSEEWVARDMTLEQANLSKKIADLTTAALATNTSVYDRRIQETENVKAEV
AVTNAENNAKRSYESTLSSIKNNAALLQIRKDEYALKETLYKEAQRQFENGTISLGSKNE
KAIDVLSARQNLLTAQKNYIEAVASYLSAMGETPLGL*
>SPBDM4_v1_50653|ID:27159016| Acriflavin resistance protein [Uncultured spirochete bdmA 4]
MSVVRKSIDHPVTIVMVFILICGVAAIFVSQIPVSLNPETDMPMLSVSTTYSGAGPEDVE
QNVTKVLENALSSVEGLEKMSSTSAQGSSTINLEFGYDVDLDKAQSEIESDINGVLDSLP
DDAETPNVRRFNMSSMPIISLAIKGDRPLKELQQIGEDTIQPQLERIKGVASASVVGGSD
EIISVEVSQNRLAAYGLTVTDVASALASQNVLLSGGNIVRGTMEYQIRTHENLSSIDEVK
NLAIKTVASDDSGNNRVIRLQDLADIKEGEDDPDRLVYIDGQRGINVQIVKESGSNAVQI
SRQVHSVLKSINKSLPAGVTVEVLSDDTTLVDSTLKEVYNSAWQGILLAVLILFLYLRNL
KATFIIAISIPISILITLLCMYFSGLTLNTISLTGLIMGLGMVVDASIVILDNIYRYRER
GTKSRIAALLGSQEMITAITASTLTTLCVFIPIILFRNKLGMMGQLFNDLVFTVCFSLAA
SLIVAVTIVPVLSGPVMHLETRTQKPVKNPFLKKIDDKLEQFFSAQEQVYKKALEFCLRN
RLLIVALVAVILVVSLLYLSSFGLNLFPRSNTDDSLTVNLTFPLGTTTAHTQEVLEDLQA
NVQQDVKGFKSLILTVGGSGGPFSGNYTNEGSLQIMLPEPKDQTDTPTSIKTKLTPYLES
IPGASFSYTAGRQFSSSSAVDVEIRSKDQDASLAAAQEIKRILATELPEVENFSISLEEG
SPELLIKIDREKAAMYGFSVSQVAQEVRNAVYGVTAATYNSAGDMIDVVVRLREEDRRTL
SDIKSLFLITSAGDKIPISSFVTISNSTAPQQIKRENKERIIHVEGDLPASSKVTSTEMA
QQVQKVLDEKYVPRDGVTVSLGGESMDVGTYLPVFILIIAVAIFLVYGVMASQFESFVDP
FIIFLSIPLMLIGVVWIYKLTNDSFSLFSMIGIVALAGVVVNNGIVLVDYTNTLRARGYG
LFEACSEAGRHRLRPILMSTQTTLLGIMPLALFPGEGTETIQPIAKTMFGGLLVSSIMTL
FLTPVLYNLFNSRRERKNVKKERETRNLLKEAAAMKAAEDKETNDGAN*
>SPBDM4_v1_50654|ID:27159017| putative Efflux transporter, RND family, MFP subunit [Uncultured spirochete bdmA 4]
MKKAVSQHVPQIVITIVIVVIAAVIILKALGISFTKSSSPSAKELPSSPSVLSGQLLQTG
GEETRTVAVRASTIALGSISNYTKLQGDVVSNNEIKIYPNIGGKLLTREVSVGDRVSIGT
TIAFVDPSKVGEKYMPNPVESTVSGTVLSLPVHEGDTITTSTVIATVGDISRLKVSTAVP
ERFLANLKIGSSAEVSFDAIPGIVYTARISEMNPVLDTTSRTLKINLTLDRPDSKVLVGM
SATVTLVTEHRGKVVVVPRSSITTDTNEDYIFVVKSDGSVEKRSVALGLEGEEFFEVKKG
LSVGEQVVTEGKNSVTNGSKIKIIDGNSETEAGVSQ*
>SPBDM4_v1_50655|ID:27159018| membrane protein of unknown function [Uncultured spirochete bdmA 4]
MASLPSKPHRERDVSRIILMTLFFLFHFMLITQPISFRRGPEGESPADFSLMSALNIAII
GFWFILEVFEFLAWNYKWKNLKYGVTITAVRCIPVLALVAFFPRLIPGMMGFMAPLLTFY
LSLVFSQVWSYVVVAIFWLIQLLLFFTAGLSATPPKSDRSYGVVILLYQLMSMMLMFLFA
QFWKEDKKNRERQAALTAEIQASQEELKRYASKVSHVVALEERTRIARDIHDNLGHTLTA
ISIQLNKAEAFFRKDPEVSIQAIVDARSSMHEAMLDIRSTLDTLNAQVEGFDLLSQVQKP
LESLRQTGISVLSDIKGSTEGYNISVLLALYRFVQEGVTNILKHAEAHEVNLEIRLDGDQ
AFAELRDDGRGFDPRAMKNRKNDASGYGLTGLTDRITLVRGTFSVESAPGQGTILKACMP
KDPVALIGKENTDGSDR*
>SPBDM4_v1_50656|ID:27159019|yfiK| Uncharacterized transcriptional regulatory protein YfiK [Uncultured spirochete bdmA 4]
MAPIGKKIKVLVADDQRLVRDGIASLLGLDEEILVVGTAENGKEAVALARQCRPDVCLLD
IRMPIMDGISALEHIRAEGTSQYIVMLTTFDDREYIVRSIRSGAVGYLLKELPREDLVRA
IKQVVKGVFLANGEVMNKLLGSLQSLQDEEKELSSDTMALEQCSYLTERERHVLALVGEG
LTNSEIAETLSLSEGTVKNYISNILTTLGFRDRIQAALFAARNGLVRK*
>SPBDM4_v1_50657|ID:27159020| GCN5-related N-acetyltransferase [Uncultured spirochete bdmA 4]
MKVEISDINFTSFADLFSPCNRCIYWEAPEQFGGNEHARRSLSGKEALGIKKAWFKKTQE
IFGRCGLILYAEGETVGYAQYAPPALLPNVGGYAQSIAPPSQDAALISCLYIRAEYQRQG
LGKMLLQRVTGDIQEKGYRAAETYARDDSPDNCSGPTAFYLSNGFTLLKTKKWEQATFSL

LRLDLTE*
>SPBDM4_v1_50658|ID:27159021| conserved protein of unknown function [Uncultured spirochete bdmA 4]
MKLDWKKGEKIYYLPKEQPELIDIPEFKYFTVRGRGNPNDPFFAEYISVLYSLSYAIKMS
PKQNCAPPDYVEYTVYPLEGVWDLTEEGRNDYRGTLDKNKLLFTLMIRQPGFVTEEYAAE
TIEKTKRKKPDKLLEEVHFERMLEGKCIQMLHIGGYDAEPASFRMMEEYAKSLHLERINR
QHKEIYLSDPGRVEKNELKTVLRFRVQ*
>SPBDM4_v1_50659|ID:27159022| exported protein of unknown function [Uncultured spirochete bdmA 4]
MKKSVIVLLIVSLSAIGAFAEDTTASRKVVLETNLAYLGLSAFSTNGSNMFLVIPLEAQV
MISDRVSVNPSLTFLYFGNSGGISDGSMLLGECGIGYHSGREALCGWSAGISPGLAYAFD
SKLFAFVLSGELGYQWILGKGLFLGLSGGGKYIWMNGNLVIPDLKLRIGYAF*
>SPBDM4_v1_50660|ID:27159023| Peptidase S51 dipeptidase E [Uncultured spirochete bdmA 4]
MLVLIGGGEIANGETLAIDRYIVESCGKKRPNHLFIPTASGEPEGYIATVQKVYESLGCR
CDSLCILNTGVACDEIKQKIDDADIIYVGGGNTKFMISEWKKVGIDKLLKNSWQTDKVIS
GLSAGSICWFEYGISDSESFGNNADWDYSYVRGLGALRGYHCPHFDDREKESRFNRFVEL
NKKSFLAIENKCAVSIQKDEYTIVRSDKDKNAYVVAFHARGMIKTKVEDSGDIKSILSRC
N*
>SPBDM4_v1_50661|ID:27159024| protein of unknown function [Uncultured spirochete bdmA 4]
LPEAEERISYNMPSFYQNGTVRKIVEFRLRENLAKTKGP*
>SPBDM4_v1_50662|ID:27159025| conserved protein of unknown function [Uncultured spirochete bdmA 4]
MIRKSRPEDASRMADIQVFGWRYAYRTIVDDRILFSKLNIEKKAQSIRKTIENDTEEWLV
FEEDEIIKGIMVIGRSRDEDKKDSFELWCIYVDPLMMRQGVGREMLEYCENEARRRGYRE
NSLWVLERNTIGKNFYEKNGYVADGKRQELEKLHAYEIRYCKQL*
>SPBDM4_v1_50663|ID:27159026| protein of unknown function [Uncultured spirochete bdmA 4]
LAIILFTLIILDYLERYIFPPERVKVIHLYFHGAGIDTKKIKKALSAYKIGIQSIDVVQS
IQKEKVQVNVLARTPVSLNINEFYKELQNLPDIYKIKMEESQ*
>SPBDM4_v1_50664|ID:27159027| putative GCN5-related N-acetyltransferase [Uncultured spirochete bdmA 4]
MALFRKADLEDFDAILPLKNEVHQKHVQAEPDFYRNLQNAITKEEYAQELQMHEVYVLED
NNKIIGYSFCYVMEIKDHPLILNQKIFFIDDYCVARREKRKGYGRAMFEELEKLAKAQGC
TSIELNVWDFNEEAKTFYKSMQMRRTRIRMQKALG*
>SPBDM4_v1_50665|ID:27159028| conserved protein of unknown function [Uncultured spirochete bdmA 4]
MEFIIRKKQNSDNAWITELLRRDWGGDFITTRGVKYSPRDLRGFIAENKQKVVGICLYNI
KNEECEIVLLEAFVQYQGIGTGLLEKLRDQNQEKSS*
>SPBDM4_v1_50666|ID:27159029| conserved protein of unknown function [Uncultured spirochete bdmA 4]
MVRKTFYGDVYSTINEQFAHIINVQNYYLAQIGTGIETGNDLLSSRRGFIEQLRSKYLAE
GNRLYKNEEESWTIKKIARRIIWHDRIHAKAIGRMEKRKSADAGTASH*
>SPBDM4_v1_50667|ID:27159030| protein of unknown function [Uncultured spirochete bdmA 4]
MRVFSFEKLASPDTKEKYGFVRELLNTGKERPRDLYPYLPQIVTLLDSENNIIQWAGIDL
LGLLITVDRDQGIPPLLPRLYRCLNTGKMITANHAVWALFEIAKAESDSQNEILEQILRT
QEYQYDTQECKNIVYGNIIKGARALYPGIRDESTKKKIYDFIEAQTSNSRNSTKKKAEEF
IKKYAHFEYNWE*
>SPBDM4_v1_50668|ID:27159031| protein of unknown function [Uncultured spirochete bdmA 4]
LRLTALSRGTRDEIGTVFDRLEPVLQNYENEGFTWKNTGSIENACIQF*
>SPBDM4_v1_50669|ID:27159032| conserved protein of unknown function [Uncultured spirochete bdmA 4]
MSLERAREHLQKWDKGEEIKELNVSSATVGLAAQALGIEEARIAKTLAFMIDARPILIVT
AGDTKIDNKKYRTLFGTKARMIDKDLVEKYVGHAIGGVCPFGINEDVSVYLDVSLKRFAT
VYPACGSANSAIEMTCEELEEIGRAEKWIDVCKTEL*
>SPBDM4_v1_50670|ID:27159033| Ribosomal-protein-S5-alanine N-acetyltransferase (fragment) [Uncultured spirochete bdmA 4]
MRYETQRLVMRTIEPDEAHLYQRYLLDNKVFLSEWEPERENSYYDEENIKRMIHSGTLSP
*
>SPBDM4_v1_50671|ID:27159034| conserved protein of unknown function [Uncultured spirochete bdmA 4]
MDQFSVIEKYYNDNVEAEWNRFERHPIEFEITKRILSGNLPQPPARIIDIGGGPGRYSIW
LAGQGYDVSLFDLAQANIDYAQKMANRANVRLREHITGNALHLSQYVENDTYDCALLMGP
LYHLIEEEDRASVIRQTLACLKQGGSLISSFISGYAPIIDILKNYPDKMNGNIQEYLAYL

EDGRNIVSDDNPGFTTAYFMDPKKIDAFMNNFNITKKGLFTVESILGPYENLLKSADTHT
FDSCVELAMHFVEDPSCRACGEHLLYIGIKK*
>SPBDM4_v1_50672|ID:27159035| protein of unknown function [Uncultured spirochete bdmA 4]
MLFSSVDIQKQKARADGPIVFPFNTAVDGTIITGSPVFRSNVLDLFRRANNHIQVEDAIL
PDGTLDATMPLSEDTIGDSLSVCCKNALPHCNKKQWFMENRDNVLGFIGYRNSVPVAILE
IMRSEACPFTGITQNQHTAAILCFYSNPDDYDYKYDLLRKSVDTIKDMGIRELEVIVGER
GYYPNGSLRQFEDYGFVRQKALGRTYLLNRGYDALWLIRKTL*
>SPBDM4_v1_50673|ID:27159036| protein of unknown function [Uncultured spirochete bdmA 4]
MQDHILGAGFVGVNNTETFDYIPAFIILGSSHIALAFCQSFFVGFISNVSLFM*
>SPBDM4_v1_50674|ID:27159037| putative Uncharacterized methyltransferase Clos_1076 [Uncultured spirochete bdmA 4]
MRGELPATFASRLICTLGWCRGQDKGKMIKSACHDNQAEHYDLQVKNSQNDTSDYIRENY
ATIHQTALHMLELNPRDRLLDIGIGTALLEEKITTKLSFYGIDISERMLAKAQEKGLSIE
LKKGSFLSIPYPEKYFTKIVTCFAFHHLDDTEKSVAIDEMFRVLTSNGILVIADFMYKNK
TEKANLTKRFTTEGRKDMLEEMEEENFTDIEWLTGIIKRYGRKIYTLQGSTISWIVKAA*
>SPBDM4_v1_50675|ID:27159038| protein of unknown function [Uncultured spirochete bdmA 4]
LYTSASVLFKLLLKLGKTDEACVQVEFLFIYSTIIYLQPRPGIEPGTCALRVRRSAR*
>SPBDM4_v1_50676|ID:27159039| Fructokinase [Uncultured spirochete bdmA 4]
MLIGAIEGGGTKFICAIALFDPDDSAEKKPEIIEQIRIPTEGPEVTLAAASDFFRAAQDG
RRESRQGLKVRGGLNIEALGLGMFGPVECDTRSPRWGYLLNTPKPRWSDIDVAYRLQREL
GVPVRLETDVAAAALGEWKWGAGRGAKVCVYVTVGTGIGAGILVDGAPLRGHFHPEVGHM
RVPKIVKDLGAKASGGHSSLRGRGESAGSGQFADTGFEDEKFKGVCIHHGDCLEGLASGP
AIKARWGIAPEDLPADHPAWRLEAEYLALAFSNIALTVAPDRIIVGGGIGLHDGLLDMVA
ERMNGLLGGYIQALDSADAIRRYIARAELGADAGILGAAQLGWEAARP*
>SPBDM4_v1_50677|ID:27159040| putative GCN5-related N-acetyltransferase [Uncultured spirochete bdmA 4]
MFSSFNFKEYESVMSNNGAFKGVEVVLMIEILKDWQQAPGQPYTLLELRDGKTLVAFAII
HQVSGRNFTFDIRFIVLDRDYRSSTAIQHLFEMIDEELLKKIPFAVIRIEISSVKKESLG
ENALENASYCQIGHIAAYYGENDDFFFYIKAIFRNPPDFIKISKPFEDALSSAAPLEEIH
TQEENG*
>SPBDM4_v1_50678|ID:27159041|kdd| L-erythro-3,5-diaminohexanoate dehydrogenase [Uncultured spirochete bdmA 4]
MPMGNKYGTHRVLEPAGAMPQTAQKLDNDMSKLYDNEILVDVLALNIDSASFTQIEGEAD
GDEEKIKAIIMNIVATRGKQQNAVTGSGGMFIGRVAKIGPALAQRDLRPGDKIASLVSLS
LTPLKIRRILAVHRDIDRVEIEGQAILFESGIYAKLPGDMDERLALAALDVAGAPAQTAR
LVKPGDRVLVLGAGGKSGLLCCYEAMKRGGPTGHVVANIHSERSRTYIQEMGLAHEIVVA
DATKPVEFLEKVLAANKGQEFDIAISCVNIQGTEMAAILPVRDGGTVYFFSMATSFTRAA
LGAEGIGKDVLMIIGNGYTRDHAEITLWELRENQKLRKFFEQQYVK*
>SPBDM4_v1_50679|ID:27159042|kat| 3-aminobutyryl-CoA aminotransferase [Uncultured spirochete bdmA 4]
MMPKEFPRLKLDASMRMYAEAREVCPGGIMGIRRPYNFVEGEYPIFLERGYGGHIVDVDG
NDYIDMLCAYGPIILGYNEPEITEAVIAQLEKGFCFSLVQPIQNELERRLVSLIPCAEKA
ILVKTGSDATSLAVRIARGYTDRNYILRCGYHGWHDWCVEIHGGIPEEISNLTVEFEYGR
LDDLEAKLAAHRGRVAGVIITPVGHPNAKPVMAPPPGYLEAVKELAHAHGAALIFDEVRT
GFRVSMGGAQSRYGVTPDLATFGKALANGYAISAVVGAREIMQVAEKKVFVSSTFFPNSL
EMVAAMKCLDILERERVPDSLWERGTKFLGRLRDIAARSGMPVTVSGIPPMPYLTFNPHP
ADKEKTYRARREYFYTQTIRRGLFVQPYHHWYIAHRHTDADLEKALEAIDEALELTARRF
PVGS*
>SPBDM4_v1_50680|ID:27159043|kce| 3-keto-5-aminohexanoate cleavage enzyme [Uncultured spirochete bdmA 4]
MERKIVITCACTGAETVKAQNPALPVTPEEIADAAFDAWRAGAAILHLHVRDEEARPTMS
PAVFKKTMELVRRKCGIVIEITTGGAVGDSPEARLAPLVELEPEMASLDCGTVNFGDDYI
VNSLPIMRHFAEAFRAHRVRPTLECFDLGHVYASRMLIDEGLVEAPFHYGVVLNVPGSVS
YSIENLLAFQRAMPKGAYFTVMGIGRSSLLAQYGAIAAGGWIRVGFEDNVYYAKGQLARS
NAELVERAARIAKEAQLSVASSDEVRAMLNLRRG*
>SPBDM4_v1_50681|ID:27159044|kamA| L-lysine 2,3-aminomutase [Uncultured spirochete bdmA 4]
MQEFPSFDWKRIPLFKEVTPDEWYDWRWQMRHNIRDIDALAQVVPLSGQERHDIEEVLRI

FRMAITPYYASLMDPANPACPVRLQAIPRIQETHFLASDQADPLHEDVDSPVLGLTHRYP
DRVLLLATNVCSMNCRHCTRRRFVKQEDAHQSEELIDAAVEYIRKTPIIRDVLISGGDPF
VMSTERLERIIAKVRAISTVEIIRIGTRTPVVMPMRVTDELVNMLKKYQPIYVNTHFNHP
KEITAEAKEACERLADAGIPLGNQSVLLRDINDCPVLMKKLVQELLMIRVKPYYIYQCDM
SLGISHFRTTVSKGIEIIENLRGHTSGMAVPTFVIDAPGGGGKIPVMPDYLITSNEKRVI
LRNYEGVITSYEEPESYKEDCGQCRICRDEPWLNPTCGVARLLSGEEKALEPKNLARLNR
HKAETGG*
>SPBDM4_v1_50682|ID:27159045| exported protein of unknown function [Uncultured spirochete bdmA 4]
MPLDASSFIGNLSFFAGFSAASGKTMAMKAALACIRGVGARAAILCAGHEGERPGSGAGG
TSVEVWEGEIFSTAASFAGRLDCAPEIVAGELGTSALGPIVLVRAGRRGSGILVGPSESA
ALAAVADEAMRAQYPHHTVLVDGALDRLSPISALPNAQLFCTVRIDRANLMHMSQRMADL
HRRSHLPLWTDEQLRRGAHIILDGPLSRMVLEKYAVQEHAAFVVKDFAHIFLRPEELALL
AAQNKLFVQKEIDFHGFIVALCDVGQEECGARLPPEVFASILSWNPYEDEKHEHGLP*
>SPBDM4_v1_50683|ID:27159047| protein of unknown function [Uncultured spirochete bdmA 4]
MHDIVPIPPHVASFCRLNSMLSHIVPLSPYGRDRVAEIGLVTDAGILESLYDDIEAAGAF
LAKLDEVRRDRLFWHFGKLPRIPQLNVAAPCGLMELFLFKKFLLHYRNICDLVSEEFRDH
FGIVFESSDLLEALSAGSRDAESFAISEGHEPGLGPIRARIAEADQTVAQRRQTLEAACQ
EAYGFDFAGREFIVISVDRALAAIEKGRRGEAPSLAAEPYDAHSMIVRIIPDAVLLGAEE
DRRRAREEERLLEQTAIARLSGLVAASGGALARYCSAVKRFDYALAQWKFVRQYRLVRPA
LLKNAAGDVHATGADRSDRAPAGNAASLSCRGARFIPLEEECVSRGVPYEPLDFTLDRPV
GILSGSNMGGKTCVLQTLVFLQVLAQCGMFVPASHFETPVFQWLDVVGEAGETAGRGLSA
YGFEIRRLIDVLKAARDAPGLAVFDEFARTTSAGEAEALMQAIVEHLASFSGTLCIFATH
IQCSPAQGKGRAFRMAGFDAGQARATLNSGKAKGEETLEALLARINSLMRYRVVPRDESG
EVSDSDALAVARLLGLDATLVDRAEELLNLGARRDAHDKRRMARSNEEH*
>SPBDM4_v1_50684|ID:27159046| protein of unknown function [Uncultured spirochete bdmA 4]
LNVRRKNAERARERSQMLDDGLHQSLCLARARRARKFIEDRKARGVPRCLQHIDEPADLE
AIGRKPPARCLARFTDDIEPLKHRCFKVRRGHKHAALCQHLKEHKSLQHAGFAAHIRTAE
YSDGSIQGKIERLVGYSARDAFFFERYKARASAGEGSRVACRRSVAAVGPRSMDIPGGVF
EKCRPHQAILPDKLPLGERIVKALYGAAIPGKRAARSGDEAGEPCNCRLLKETFFFAGPA
AVFLRPEEDGIGDNAHDHRMGIIWLGRKRGGLAPAPFFYCGKRSVHRYDNEFPAGKIKAV
RFLACRFESLPALGHSLVRLGYPGPDWTKTRLMTLGNRKTFRVAAARGECLEQVGGLKDN
AKVITEFFTDKIAYVPVMQEKFLEEKELHEAAWCRDVQLGNTGKFPEMPEKPVSSDLVEL
RKKGSRCLDVVIERFENPSISDKADFRDAVSSIGTERHYVGQH*
>SPBDM4_v1_50685|ID:27159048|kamD| D-lysine 5,6-aminomutase alpha subunit [Uncultured spirochete bdmA 4]
MTHNKLNLPGEKIDRARELAKNICAPVLDFIDVHTTVTVERATLRLLGVDGVSSVGVPVP
NLIVDALSDKLGEGAAIWYSNALLQTGLDQSELDKRIARGFNIASLPMGDRARAQEYATR
LAQRQFARIDANRAYREARLEEYRGNSHSPLLYLIVATGNIYEDVKQAQAAALAGADVIA
VIRTTAQSLLDYVPFGSTTEGFGGTYATQENFRIMRRALDEVMEREKRYIYQTNYASGLC
MPEIAAMGALERLDMMLNDSMYGILFRDINMYRTFVDQRFSRMINAYSGIIINTGEDNYL
TTSDGFEKAYTVLASQFLNERFAYAAGLRPWQMGLGHAFEMDPSIENGVLYELAQAQMAR
EIFPEHPLKYMPPTKHMSGDIFRGLVMDSMFNFVSKATGQDIHLLGMLTEAIHTPFMMDR
HIAVESALYVMNGVASIADEIEFKSDGLIVRRAHDVLDRTVNFMEEIGRIGLMESIERAM
FADISRSRTGGKGFEGLVEKGENYFNPVESTLTRSLGLA*
>SPBDM4_v1_50686|ID:27159049|kamE| D-lysine 5,6-aminomutase beta subunit [Uncultured spirochete bdmA 4]
MAKIIKPYGDAMDDGKVQLSFTLPVPAGSRAMEAARRIALAWGFHEAEVVHMAPMSDEFT
FFVVYAATSRGIDWDTVEGDRDDGEKPMSMDEINAFIRARIGRKIVVIGACTGFDAHTVG
IDAIMNMKGYNHHYGLERYEMIDAHNLGAQIPNEKLIEYAETHRADAILVSQVVTQKDSH
IHNLTEFMELLEAKGERGRFICIAGGPRISNKLAAKIGFDAGFGHGTYAEDVATFIVKKM
AQERRT*
>SPBDM4_v1_50687|ID:27159050|atoD| acetyl-CoA:acetoacetyl-CoA transferase, alpha subunit [Uncultured spirochete bdmA 4]
MIEKTVIRPAEAARLINSGSVVHIGGFLGCGAPDGIVAAICEAGIGDLTIVCNDTAIYDP
KTGRTTGLAPLILKGLVRKVITSHIGTNAETQRLLCEGKIEVELVPQGTLAERVRAAGYG
LGGILTPTGVGTEVEEGKRKFRLHGKTYLLELPLPGDVAIIKAKKGDKAGNLIYAKTARN

FNPLMAGACSLVIAEVEELVDIGGLDPDQIHTPSIFVDYLVVV*
>SPBDM4_v1_50688|ID:27159051|atoA| acetyl-CoA:acetoacetyl-CoA transferase, beta subunit [Uncultured spirochete bdmA 4]
MPDYTENKEIIARRIACFFTTGDVVNLGIGLPTLVGNYIPENVTIILQSENGLIGLGPAP
AAGHEDKDLTNAGGKPVTILPGGCFFDSATSFGIIRGGHVDYTVLGVLEVDQEGNLANYK
IPGKLIPGMGGAMDLVAGAKHVIAATTHFEKSGASKLRRRCTLPLTASHEVDYVVTDIGF
FAVSGHSFALKEAFVPYTPEWIIKHTDADIELALDFRIWNSPEACR*
>SPBDM4_v1_50689|ID:27159052| SUF system FeS assembly protein, NifU family [Uncultured spirochete bdmA 4]
MSDPFAAVYEEIIHEHYKKPKYRRILEGLPFAENPSCGDRVRVSITLGADGRIAGAAFDG
SGCSISMSSADILAEDIIGKTPEEARRLIETFLAVLRGELDVDELDAFGDAVAFKGVARL
PVRVKCAALAWRAALSQLDSIDRPQENSKS*
>SPBDM4_v1_50690|ID:27159053|sufS| selenocysteine lyase, PLP-dependent [Uncultured spirochete bdmA 4]
MRAQFVAHAPVEENSSIAGALKREFPIFGERPELIYLDNAATAQKPLSVLDAERDFYLRS
CANVHRAIHAIGEEATARYEEARRRMADFIGARPREIVFTRGTTESINLVARTFGETLQP
DDEIILSVMEHHANMVPWQQLTERRGVSLKFIPVTEEGELDLAEYEKLFSPKTRLVAVTM
VSNVLGTINPVDKIFAAAHGAKVPVLLDVAQAAPSMPLDARALGADFAAFSGHKMYAPFG
IGVLYGTERMLDAMPPFMGGGDMISEVRLEGFSVNELPYKFEAGTPPVAQAVGLEAAADW
LSSVGIAALGEYESALARRFMDGIEGIPGIRILGSAARRAGIVAFTLGGAHAHDVAAYLD
RFNIAVRAGHHCAHPLARRFGIVSSARASFGAYNTREDVDRAVAVLAGAGEVL*
>SPBDM4_v1_50691|ID:27159054| SufBD protein (modular protein) [Uncultured spirochete bdmA 4]
MSDTHTLERPKGLLTGSASGGQPAGSPDIEAARPGAPEVFGGGNATEAARLVFPAGDKSD
EVRLVQIEVSPPARESLHYRATIELGEGAEAKVLVRFAGSSAGIAVPPRPILLTTALEIR
LAAHSRLKLYVVSELSVPYFREAFDRAVLQEGAKLEWTMIGFDENNGIYSANIDLSGAES
ELDLAGAYGAHRNTEQEHIISVHHSAPRTRSRTNLKSALKDSAHLIFRGLIHVEATARGT
DAYLSDRNLILEDGARAESLPQLKIETDDVACSHGATTGGPREEALFYLMSRGLDRDAAK
NMLVLGHLGSVFSRLPADLAEEMEGAAASSLGIGDERSCP*
>SPBDM4_v1_50692|ID:27159055|sufB| component of SufBCD complex [Uncultured spirochete bdmA 4]
MRTDAAVQREEIRNIGEGYAERFGFTMPEHSIFNTGVGLTEETVRAISAAKDEPEWMLKF
RLRAFHIFQNMPMPTWGADLSKLDFDRMTYYSRPTETAQDSWDALPDDIKKTYERLGVPE
SERKFLAGVGAQYDSEMVYHNIREDLKKKGILFTDIETAVREHSDIVKRFFGTVVPPDDN
KFAALNSAVWSGGSFVYVPPNVHVEIPLQAYFRINSQAFGQFERTLIIADEGSFVHYIEG
CTAPVFSKDSLHTGVIEIIALPHSRIRYSTVQNWPNNMYNLVTQRAFAYDHAVMEWVDGN
IGSKVTMKYPAIFLMGPGARGEVLSIAFAGKGQEQDAGAKIFHFAPNTSSVITSKSISKD
GGIASYRGFVKVEAGLSDIKSKVECDALILDPHSRSNTYPVMDIRSEHVAMEHEAKVSKI
GEEQLFYLMSRGLTEEEAAIMIVNGFIDPLVKELPMEYAVELNRLIELEMEGSVG*
>SPBDM4_v1_50693|ID:27159056|sufC| component of SufBCD complex, ATP-binding component of ABC superfamily [Uncultured spirochete bdmA 4]
MQNGLVIRGLSAGLDTKRIIDGLNLEVPDGEIHALMGPNGHGKSTLANILMGNPGYTVES
GEACLDGDNLFELEVWERARKGLFLAFQYPAEIPGVTVSKFLKRVADLRRDPPKNTSAFL
AELKANFAMLDIEPSFANRYLNDGFSGGEKKRMEILQLLTVRPRFAIFDETDSGLDVDAL
KVVAGGINQMRGPGFSALVITHYRRLLDLVKPDAVHILEKGRIVASGGFELAELLEREGY
DGIRRVVVDQKTREAAYEN*
>SPBDM4_v1_50694|ID:27159057| conserved protein of unknown function [Uncultured spirochete bdmA 4]
MAKKFSQNELLEILQGVEDPELGYSVVDLGLIYRAEQTEDGIEVDFTLTYIGCPLEDQLK
RDIVRTLRDATGIHNIRTKLVWDPPWTVDRASAEVRLDMGYPIW*
>SPBDM4_v1_50695|ID:27159058| NAD(P)H dehydrogenase (Quinone) [Uncultured spirochete bdmA 4]
MNALVVLAQPKKPSFNSAIAEKVTESLESQGCSPVELIDLYNDNFDPVMPQEELPRKFSF
DETTLRYQERIQGADRVVFVYPDWWGGPPAIMKGFLDRVFRPGIAYGFRETDFRNDDAPG
LFTGKRFDVFVTTDAAAPPDGAIDTWPPALVWKRNVLAFCGVSDAVVHVFWNLRHSSYAQ
RKAWLDGIPARLSIRPKSAQD*
>SPBDM4_v1_50696|ID:27159059|pepT| Peptidase T [Uncultured spirochete bdmA 4]
MNRFISYVKVDTQSNRHGEETPTTKSQWDLARKLERELRELGVPSVELNDHCYIIAHLPA
SPGAEKAPCIGLMAHMDTASDVSGSGVHPRVIKNYDGKAVQLSDQYVLDPAEFPDLVDHV
DDTIIVTDGSTLLGADDKAGLAETMTAIAWLIGHPELKHGPIDIYFTPDEETGKGMDKFP

LQKAKAVACYTLDGGKAPEVEAECFTAYAVKAEFFGKVIHIGAARGKLANAVAMAASFIG
MLPRSESPEATDAWYGYYCPIEVSGTLDHAWTEVYLRDFSSERMQERIAALKAFAGAVES
QFPAGHVKLEITRQYLNMKQKLDARPEVLEKLNRAITTAGYEPVMKPIRGGTDGSRLTEM
GIPTPNLFTGGYNYHSRTEWASLSEMALAVETVVNLALIWAE*
>SPBDM4_v1_50697|ID:27159060| putative ABC transporter, permease protein [Uncultured spirochete bdmA 4]
MASRGLPRKWTHRILVAKARPEYLNAMIIPTYPEFAPLELDARDSLYPGLNLLSDGISEF
TFAGLYLFRHAYGYQISQLPGGSLVISGRQKEKTFFYLPCCFPSLEQFDELMQRFDYMRH
FSESQARQYRIELEARGYLVYEDRDSFDYLYNREDLATLNGRAYHKKRNLVNGFVSTYAC
EQRPLNNSRVRDAIAVLDEWRSTKGVEGDYAAALDALEHFDVLGLRGAVYYIEQQPVGWC
LGEPLAKGKAFAVHFEKACDRFKGIYQFVNQAFAQSLPKHYQFINREQDLGDLGLRQAKM
TYRPVGFVKKYRVVHPDSVDFTPAPPEPPAECGPGTIHECSDD*
>SPBDM4_v1_50698|ID:27159061| conserved protein of unknown function [Uncultured spirochete bdmA 4]
MTSAIPQPRFEILPLPEVPADEPLRRSLLAALPPLSGRDEAGAISITFLAGAGTRWKTSL
LEAKRAPGAWPAGDVAEAFPLEAPRGLFPVPDFIHGEQGEGRIAMAAYAFDAVRGIGRHI
VVIRGWEDEIDAQGLKPAGILPERRIFFTQETGPDGQVSGHGDATLQCMGLWQNAHYVVT
NFAGDANSWLTVELAMRAFAQFDARGMEIGVLIPVAHTEKPSYPVYLDETGLPAGFWHEK
LAGSRPKAAPTSLTNVGIRVYRADWLKRALLELRAKYYVSGPDREAGWHIPGNDPAKHEC
ALDNVDNLLAGKGLARVMPTSLPRELTPLKALGEYPAFVRAVKEVQQEINAARRGAC*
>SPBDM4_v1_50699|ID:27159062| Chromate transporter [Uncultured spirochete bdmA 4]
MEILNVSLTFLKVGVVAFGGGWSVVGLIKQEIVPRWMSPDAFNSLIAIAQSTPGPIALNA
ATLVGWQRAGFWGALAATLSVIAFPLLAMSLALAFSKYIPLKKDLADESLRSGSMAMILM
TLWTLLPHTSLPMPWIFAFAAFGITAFTKINAVWVILGAGALNMLLGVLH*
>SPBDM4_v1_50700|ID:27159063| Chromate transporter [Uncultured spirochete bdmA 4]
MKTPAKAEGIAKTVSPTPWRIFLTLARIVSLTLGGGYAIVPAIGNAFEKQGWIKEEEFYR
VFSRAQVFPGPIALTTSFLCSYRIAGARGAVAAVFGVVLPPFFALILVGGFISLYGDTEL
FKRFLHGAGAVVPGLVGSILWRTARNRKWSVRIVIEVVTLAVLLAVFPSSAFFILIGGII
VLYVGRLVWKS*
>SPBDM4_v1_50701|ID:27159064| protein of unknown function [Uncultured spirochete bdmA 4]
MPPYNLIEALKLDEFQRDAARTEKTAVVTAGAGAGKTRALVGRFLYLVLGKGVDPENILA
LTFTRKAAAEMSQRVHEALGAPESPRLDLADHLASVHIQTLDSFCREIVAASAAQYGYTP
DFQIDEVACAQTAAKAAYRYVLDNRESGGLRNLLGAFGFDVVVRELFASFGSANVGPHWK
GRHICRASARLGEEEFKKTAWQLAEETREIAARILERENDVPPSGFREGTAQAIRAAKAF
PSSEASASSDLEGFFAYMQRPENAINLRSVGKNETESAIKDLAKALRDESRLKKISQIVD
FEKFLPDYYDIMDRLDEYADLLCQEKRRANIMNYNDLGALAVDILSGQEDVRRYWASRFQ
YILIDEFQDNNDLQKRLLLLLSKEEEKVRRGKLFFVGDEKQSIYLFRGADVSVFKSLAGE
LEASRFSLVRNYRSARRLIAFFNAAFNVIMQPADSQNPQRFEAAYQDMESTPVPEPADFM
SRIEYRCVTNEVAPGDFGGALLSAKETMAYRLALWIRSAVESPSPLFVRSEAQSAASGEG
KQELALRKAEYDDIAILMRTTSRQYELEKYLRMLAIPFVTDTTASLFSEEPVNDLYYLLR
LLLDAHDLFATAAVLRSPLCRISNDGYVAILAEGKSLAELRTRIPEALGAADHESVQNMI
LFYNELRAISDHMPLMELVAHVWEHAHLELSILSDPQRLPYLDHWNAIRAIAADIEARGG
HLQAFLAALRGYIHEEHSFDANAVPQEDTRGVHLLTIHKSKGLEFPIVVIPWMETAPNKN
SGSELWGVLESAQTQGKTRFYTVDIGFHDRAEGGSNILQTQAQDLRRAKERAETRRLLYV
ACTRAIDHLIMFDAVPEKRSYDQDSFHALLFQSTERAHPMQGETEAQIPPHLSHLRFFFE
PLASEQEVQMARKGKTARAQQSYAEDIETLKPDERNFMRPPRRLTASTVNAMATKDSLAA
TTAGGLSAHIVRERLFEPARPDESSDGPDAASTQGADHSGFEMRSDPTAYGSLVHEMFAH
LVAGKSVGNFAASPSVAREIRSGLQLSDGGSGAPGEPATYSVIELASRQLMPLLQSRAFS
DILHNKTLKFEFPFLLALPPWSIEGRMDMLAESHDEVIVLDLKTDRQFSPHEYALQLGIY
RIAARALFPAKQVHTGLLYLHFGEIAWLKSELHEGTLLQICDTISYNHTKQ*
>SPBDM4_v1_50702|ID:27159065| protein of unknown function [Uncultured spirochete bdmA 4]
MERYAALIGTPLGADIRQHLGEPDTIFVFPSQDSADSWAQALVKSEACKAVALDRFFGFE
NFLRHCATLREHEDFRTVQQIDRWAWALEVLDNRAGTSDPERGLPLKRLLPPEETSTMHL
SRLVGLIPGLYEVEALKRAEGAIRTVSYDFLREEFEEIESLARNYRNYLDRNHLFDGHLA
PHVLPAEVKVRAYGLKEDFTRLGLIADTQNSQLAFPDIVPHGDVRGKAFNSYEYYEFDSF
LAEIASVFTEISKEIEQGLDPEDIAISVCRLNAQKAAWIRQAAEDVGIPVSIRWGEPLSL

TAFGRLLHAIQNAAREGLTLDSLDAFSAIESIKSRNPEGWNALRDTALRAHIPSPSPNAS
YVHRLWKESGQIGLCTPSCAQMYQRLWQDITGIARAESFPQLYAQVLGFLEHWVDTSRFS
ANVHTDRSMRMALDELQSWMEREDAMQRGPFLPFELYMTALGTKSYIPLLEGNAVHVYDF
KTASGLAAISQYVVGASRAGLASSLEHQSALPAELSALTGSKMAADVEEILAMHGIARTR
YSFAREGFEGYEVAHPLFAPPSERPARAKRRRRISAGTASDSAPRAHCYSNPSPIDPAVS
ARFLRSTQSFDFEKNLAVFSPYSLRDRARCAFRWFAQRINIEDVYSNDDTARIIGDFLHA
TYQRTIKNLPQRLSESEAPARFREAFEIALRATLEKIFAEHGPGLRPTLVTLIDRARHRL
GELWKFEREAFSAYEREGFEIPVSYAFEEEGAILNGRIDCVFRRTDESLGNVKCYIIVDY
KKNRIPSLSEMKLRAQNAVRDDLESTGETESETEEGALTVREIQIPSYALMLELSGGIVE
GALYWSIEKSEAVAYIKPPAASSLHQIRPAYTRREDTAPVRAAVRDMLARAATAVQGGEL
LDPAPDRGACSDCAFKPLCRYWYFLEL*
>SPBDM4_v1_50703|ID:27159066| Alpha amylase catalytic region [Uncultured spirochete bdmA 4]
MALQINRTLRSQNTPPKHLWMPSGAVAIPDAVSARLVARKFQLPAGELRALSLIDDASRI
IIDVYRKQLSKTLDQIASPALTASGDRAALLQVLQELTLEFPPAPMYDGLAEPGDWLESS
TADKGGSGRPHRELAIEQFVLIRLFNENPACGPYRILFDDGVSPAGATSPGTISAKTPYL
KVFARLEEALKTLPGLSHEKGKTLDLISFLREPAKRAPHSLKAQLEWIIQNWGELLGDFR
LALLAGIDMINEETAPRFPPGPGPVRAYQYRSSLHEYEKFSTDRNWMPSVVLLAKNALVW
LHQLSKTYGKEIARLDQIPEEELITMAERGINGLWLIGIWQRSPASEKIKKLCGNPEATA
SAYSLFDYEISPELGGWEALDAFRESCGRFGIRLAADMVPNHTGIDSFWVRTRPELFMSL
PYCPYPGYTFNGPDLSSDPSVGIWLEDHYFNRTDAAVVFKRLDRHTGEIRYIYHGNDGTG
MPWNDTAQIDFLNPAAREAVKERILHVASHFSIIRFDAAMVLAKQHIRRLWYPAPGEGGA
IPSRSEHAISDEAFDKAIPNEFWREVVDLCAEKAPDTLLLAEAFWLMEGYFVRTLGMHRV
YNSAFMNMLKDEKNSLYRLTIKNTQEFDRDILKRFVNFMSNPDEETAVAQFGKGDKYFGV
ATMLATTPGLPMIAHGQIEGFTEKYGMEFKRSYWDETPDRDFIARHEREIFPLLRLRPLF
SEIEHFYLFDYMRGDTTIDENVFAYTNGQGGQRTLVFYNNHWERTFGRIHTSCAFAQKTP
QGGKHLETTSLANALGVEASPDNYVIMHEIRSRLWYIYRSEDIAADGLSVPLEGYQSKVF
LEIANVLDTEGRYARLYEIVDSKGIADLDDALLEADQPDLFRALHNAISSLSSVEAVQDL
SKAEAIQRAAIFSEMFFSRLCAIAGSDDTGAAYRSEAVRNAIAWLRTVLAALYRTGVSDV
SAEGLALQFENPRHRLILLVYSFVAALAKSFAADGLKEEIGRMIDEYRIAKKLKEFAVEL
SRTMPEDEKDYDASMSAEVAIAWALRKEKQLTELEMPPASATSLKLHAHEILAWAFSDSL
MRMALGINHYRGIEYFHKERFETFAWLLPACAWIDSIGPSEPRMDLKALASWKALAQLLV
EQAADAGYMTNVMLEDTAAAARE*
>SPBDM4_v1_50704|ID:27159067| Transcriptional regulator, TetR family [Uncultured spirochete bdmA 4]
LSGQIAHEKRKQEILEKALDVFIEEGYEDTTFQKIAERCGITRTILYLYFDNKKQIFNES
LKRFLGSLESEIAIVAHDEHLNSEEKILKIGEIVVEACEKESRLLSIVMDYLLRLKASGG
NPDEKVRRRTVRMRHILADIVIEGKRRGEFNEASSVKGTVEMFFALVEAGVFRLVVLGRK
EATGLREALSPFVQSMRGPAGTQKM*
>SPBDM4_v1_50705|ID:27159068| exported protein of unknown function [Uncultured spirochete bdmA 4]
VQSEKKISSITRNKAARLVIAASFCIASFFLISSSATLFETRAFWNSKKNVSSILFSINI
GDTDIYDDPILKLYTNAKTKNQVLKFFSAITNSERVAKAILDNSVEHNVRPSLAFAVAYV
ESRYNPKARSKNSSGSLNSGLFQLNSSVFGMTSETLMYDPYHNAKLGLSHLEEYLNLSDD
EFSALAAYNAGITRISEQGIPGMTFKYISDVSIMERKIMNLFVASMALNNNAL*
>SPBDM4_v1_50706|ID:27159069| Asparaginase/glutaminase [Uncultured spirochete bdmA 4]
MNPESIRVIVTGGTFDKHYDEIKGELTFIDTHLPEILKLARVSLPVQVELNQLIDSLRMQ
DENRRSVFESCARASEKHIIITHGTDTMAETARLIGQAGLDKTIVLTGAMIPYKILDSDA
LFNFGTAFSAVQLLPRGVYISMNGRIFNWDKVRKNKAQGIFEEI*
>SPBDM4_v1_50707|ID:27159070| putative Transcriptional regulator, TetR family [Uncultured spirochete bdmA 4]
MARVKTMEKRALILETAKRLFATRNFDVSMSAIAEEIGIPVGSIYTYFDSKQALVETIIE
EGWSEFRTWTEDGLKVSKTAERSDDAHAQSLKALSFLVNGALPRLFSDVELIMLLLAEAG
STAKLEEKLQYLAALIAELVSACMLPGVSHVDPEQYDTGITVLLLGALETLRIASKTELK
ISAADIQAFFKQTVENALGQPLPDMD*
>SPBDM4_v1_50708|ID:27159071| putative Nucleoside-diphosphate-sugar epimerase [Uncultured spirochete bdmA 4]
MRVFVTGGFGNVGMSTVRALLSFGHDVCIFEHPSARSRRQKRLRELLRDGRDKLRIFYGD
ITDAGALSEAMACAGGPDAVIHLAGIIPPLADREPKLASHVNVGGTRALLEVCKLLDRPP

RVIFASSVALYGDRLANPWISIQDPVAPNDTYSVTKNECEALLRASGLEWVILRLSYVVS
SDWLPFSPQLYDVPPTTRLEVVHTEDAGRAFAKASDSTQSAGRIFNIGGGPGCRTTYRAY
LDRLMRYFGLGSSSFLPDGLFAKGNFHCGWFTDSDAAESLLHFRSKTIEDYYSEVSWRMR
AVRPLGSIAVWAVKPWIRSLSPYQAKAGRVRSPQFV*
>SPBDM4_v1_50709|ID:27159072|alaS| Alanine--tRNA ligase [Uncultured spirochete bdmA 4]
MITIHELRRKYIEFFRARGHAEISGKSLIPENDPTVLFTTAGMHPLVPYLLGEPHPAGRR
LTDYQKCIRTGDIDAVGDPSHLTLFEMLGNWSLGDYFKKEAIRWSYEFLTSPDYLGIDPE
KLSVTVFAGEASIPRDSESAEIWRSVGIPDERIYFLPREDNWWGPAGETGPCGPDTEMFV
DTGLPACGPDCKPGCHCGKYFEIWNDVFMQYNKTSAGAYEPLAHPCVDTGMGIERTVAML
QGKKSVYETEAFTPILAVLESITGKKYAAPDNDPEVDRSFRIVADHIRAATFILGDPKAV
LPSNIGAGYVLRRLIRRAVRHGRKLGIDGIFLAKPARAVIDIYRSPYPELVENSARILDE
LEREEQKFLETLQKGEHEFEKMLPNLLRNPERIMSGRLAFRLYDTYGFPIELTEELAAEQ
GLVVDRAGFDEAFKKHQELSRAGSEQVFKGGLADHSEIATRYHSATHLLHKALRIVLGDH
VAQKGSNITAERMRFDFSHPAPMTREQIAEVERIVNEQIARDLPVTMEMMPLEDAKASGA
IALFGEKYEPIVKVYTMGDFSKEVCGGPHVARTGELGKFKIIKEQSSSAGVRRIYAVLE*
>SPBDM4_v1_50710|ID:27159073| protein of unknown function [Uncultured spirochete bdmA 4]
MNIALRILHLHRALHYVPIEKPAHFDETALAKLASQFGECLLVFQKSAFFTMTGDGPRVS
GNPEPDAAYRIAAQETPSPAGMGGQNSAGGAPFGFLLAAGDYVFFQWHAWHETEKSARPS
YAAMLEEVVREIWWQRLECEGPWFLRLVFEDDKVAVQALRALKKAP*
>SPBDM4_v1_50711|ID:27159074| conserved protein of unknown function [Uncultured spirochete bdmA 4]
MDYILDEVERFYKIIKLKPFRRTPGVAFDILPMQFLPHIEGIDRVIHQRRALSPGSVGNV
ERPWYMHPHQEDNLIVLHGVRTVDIYTLEHRRVETFVVEPERIVHNGKVIFEGPAMVVWP
THVFHRIVSGDEGSASLNFAVRLPGFDPETNFSIYDLNTKTGEYREIRRGSLDQSGDV*
>SPBDM4_v1_50712|ID:27159075|dxs| 1-deoxy-D-xylulose-5-phosphate synthase [Uncultured spirochete bdmA 4]
MSLLDRISGPADLQGLSISELHRLAEEIRLRIIETVQRNGGHLASNLGVVELTIALHRVF
DSPVDAIVWDVGHQCYPHKILTGRAVRFGTLRRKGGISGFPKRQESEQDIFDTGHSSTAI
SSALGLLQARMCTGQKGRVIAVVGDGALTSGLAMEGLSNAGQLSLPLILILNDNRMSISR
SVGSISRYLSKLSASVRYQSFRTRVDAAVLRIPRIGNLLFSLINRGKRAIKAIFFKENLF
VDYGFEYVGPIEGHNITALIDAFLHVRELNRPVVVHVVTKKGKGLGKAEEDPESFHGLGP
SCPDIPGPGTTSHGESFTTSFADALVRIAQQDHRVVAVTAAMGSGTGVAKLAERWPRRVF
DVGIAEQHAVAFAAGLAQGGLRPVVAIYSTFMQRAVDQVFQEVALAKLPVLFALDRAGAV
GEDGETHQGIYDIAIFKSLPNLVLFAPADSEEVSAFMDFALGLDVPCMMRYPKAYSPCPS
PQPMLPLELGKGIFMRKCAGAKVLVLAVGPLAYTAAEASDRLEKAGLSVDVYNVRFLSSI
DENYLATLCAQYEGLVIMEDGVRTGGFGESVAAILAERGVNVKITACGFERNPLDQASRD
ELLARAGLDAKGIQKVLSEMLQSLEPSPQQVASTPGVARIGPRAVSGEVQHVR*
>SPBDM4_v1_50713|ID:27159076|folP| Dihydropteroate synthase [Uncultured spirochete bdmA 4]
MSGSAGPAATEKPQRLLKLPRGRTLDLGGLPDDLPCIMGIVNVNQDSFFEGSREPDTGSA
VKAALDMVGQGAHIIDFGAESTRPGSTEIEVKDELARILPVIESFRAVSDAVISVDTRHP
EVAAAVLGAGADIINDIEALARTGMAEIAARSQAAVVLMHMQGTPQTMQCVPSYANCLGE
VFSFLEGASNRALEAGIDREAIILDPGIGFGKLKEHNLALLKGIDRLKTLGYPVLVGVSR
KRLIGELTGKDVQERLAGSLGAALAAWLNGADILRVHDVSQTVDALRVFREVRQ*
>SPBDM4_v1_50714|ID:27159077| conserved membrane protein of unknown function [Uncultured spirochete bdmA 4]
MNASSLLNFLTTYVRPVLDVGILAFLIYQAYQLLVMTEAVYLIRGILILVVVYGAAFFLN
LSTVTWIMNILAPGLVVSLAIILQPELRRIFIQLGRQSIIRKKNAARTSWIDAAVSATVY
LSGKRRGALIVIARKVGLSSYIEKGIPLDAIISTSLLISVFEHDNPLHDGAAIISQGRLA
AAGCFLPLSDQQDIRKTFGSRHRAALGIAEESDAVVLIASEETGALSLSYDSKLYYDLSP
DEVLERLRTLFSENGVSSQTGEVDNEP*
>SPBDM4_v1_50715|ID:27159078| putative YbbR family protein [Uncultured spirochete bdmA 4]
MNLDSRISKLLENWPAKVISLVVAIFIMFLYNLTRLDQRLITVPLNISEGRSYVPATEYP
KTVRVTIRGDRDQIYRIREADIVASLDLTRYESEGVFRVPVKLERRGDARDIDPLELRAD
PAEIPISFERLAAKRVAISPTFKGFLEQGYELNSYEIVPPEIAIEGPTSLINGTKDISTD
VIELSGKTGDFSLTVPLVKPSDLINLIDVNTVRFTARIQKQERRITLNNVPIKAVNLDPA
LGFAETMPTGKMTLLPRQGQDMATATNALLEADFKDITKPGQYSIPLTPIPPEGFDVEAY

DPLVITVRLRIAPAPASNSSGGAGLQGQNQAQGSTSQPVVP*
>SPBDM4_v1_50716|ID:27159079|acpS| Holo-[acyl-carrier-protein] synthase [Uncultured spirochete bdmA 4]
MISGIGIDIVHVDRIRRWVENPSILERFFHPAEIETARSRNKGMALSLAARFAAKEAFGK
ALGTGLAHFALREVAVVNDSLGRPHLQLEGSARREFERHGGGTIHVSMTHEGDNAVAVVI
LEKGN*
>SPBDM4_v1_50717|ID:27159080| tRNA pseudouridine synthase A (modular protein) [Uncultured spirochete bdmA 4]
MQRESSGRTQPPSQRETSGQERVYSEDPSTSRARADFSAKSSVRAVKLVLAYDGSDFSGW
QRQKNGRSVQEELEKALAKMHGHEIRVTGAGRTDAGVHAMGQVAGFYTDIKSIAADRFVF
ALNKLMPRDVRVLSSEEAPPDFHARFDASLRRYRYFLLCGGTPDPFALRYAHSLSYYPRV
AVLNAMAAVVLGEHDFSTFASAQDASESRSRHISESIFFFERERLVYQVAGNAFLWRQVR
SLVGTFLELERIAESSAHGEALMREMLESRDRGRAGTTAPACGLFLWNVEYGERIHGHTR
RRGGAEGSGGRRSESSESKDTRARTTEAEPNPASGGSRLVPGLGWIDEGE*
>SPBDM4_v1_50718|ID:27159081| Pseudouridine synthase [Uncultured spirochete bdmA 4]
MKANKKRNASPESPAGISATSSHSFWEGVASPPEPMRADRYLSEILKLMTRSQLKARNAR
LFCNGKEVKLSHKLKHDDSLSLEWTEEPPEEIVPEPVPLKILYQDDGVFVIDKPQGMVTH
PAAGNWHGTLANGVLWLSGGSRPVSESQELLVPPHPPRAGIVHRLDKDTSGVIIVARTAS
AHEFLSRQFRDRQVRKEYFAIVRGRPPASEGRIDTWLARSPRDRKKFAVSQPGRGKHALT
LYKIRAVWNLSAANANGSPSAAASGPGARHRASGSKASAKSATYSLLALYPKTGRTHQLR
VHCAHIGCPILGDPIYSKKDTLFPDATLMLHARRLKIRLPSRSEPSVFKAPVPKRFRDMI
TLLNTGI*
>SPBDM4_v1_50719|ID:27159082|rbpE| putative RNA-binding protein RbpE [Uncultured spirochete bdmA 4]
MSKKIYVGNMNYNTSERQLQDLFAQYGDVTTVNIIVDRFTGKAKGFGFVEMENPEAADAA
IAALNGQEFMGRQLRVNEAQEKPRYEREESGGGYRGRRY*
>SPBDM4_v1_50720|ID:27159083| protein of unknown function [Uncultured spirochete bdmA 4]
MHCFSFRSTGQLKAADAHAGFSPLRFSFGKTNNNQNSTLISPLKTVGVVLCFLVIASDSP
*
>SPBDM4_v1_50721|ID:27159084|rny| Ribonuclease Y [Uncultured spirochete bdmA 4]
MTPFVWYIVLPLAGLCLGWMIRWLYARFQLSSAERESERVLQDAVKEAEAKKKEILVEAK
DQIIRERNQQEKEIRDRRIELQKYERRVLLKEENLDAKLASIERIEANLKNRENSIAEKE
AYLAGQEERYTAELERVSGFTADEAKKLIIQNMEQEARRDAQVLINKIDQEAQLTAEKKA
KDVLITTIQRIATESTAESTVSSVSLPSDEMKGRIIGREGRNIRTLETLTGVDIIIDDTP
EAVVISCFDPIQREIAKISLERLIADGRIHPARIEEMVQKVTREITQKLYDEGEKVVFDL
GLHNMAPELIRAVGRLYYRTSYGQNVLMHSKEVAVIAGLLAAELGLNREIAKRGALLHDI
GKGIVTDSDKNHAEIGTEIAKKFGEDARIINAIASHHNDVEPNCPESVIVQIADAISAAR
PGARRETLENYIKRLEDLEAVAESFSGVEKAFAIQAGRELRIIVNNEQISDDQAKELCKN
IAKKIESDLRYPGRIKVTIIRETRIVEYAR*
>SPBDM4_v1_50722|ID:27159085| conserved protein of unknown function [Uncultured spirochete bdmA 4]
MSAGTANVLMIGDVVGAPGLRALFTFLPSLIKRTEADLVVANGENALKGFGIGADEIAAM
RSYGVDVITSGNQVWEHKEAPELLEAEQSLLRPANYPKGLPGRGTAYIEGGGAPGQPAPT
RACGASGRFEWLVVNLQGRRELYDIDCPFVKADQILATAAREHPNALIIVDFHAESNEEK
EALAWHVDGRAAVLAGTHTHVPTADERILPKGTGYLTDLGMTGPVDSVIGMNGDICIRRF
LTQIPYKMETAEGSAAIMGALFRLDPESRRCVSIEHIFESL*
>SPBDM4_v1_50723|ID:27159086| protein of unknown function [Uncultured spirochete bdmA 4]
VARAPFVFFRRGHKVYVQFWNDEKAGYGTARSTGMVTENEALKVVMEWMKAGDPPLARRS
IKRKSGFQMTACGYLSDFWKAGSPYVLGKQARGATLLACLCGFRLGEVRGLQWEDVDFAN
STIRLCHNLPNSERAAEGLKSPKWGSSREVPAPD*
>SPBDM4_v1_50724|ID:27159087| protein of unknown function [Uncultured spirochete bdmA 4]
MFPKAAQKSFESMLRSIGITEEMQKARRIVFHSMRHTFISLSRGAGVPDFVVQRIAGHKT
MNMTNRYSHASEEDIKNAKLLIEKMFHEARGQCSR*
>SPBDM4_v1_50725|ID:27159088| conserved protein of unknown function [Uncultured spirochete bdmA 4]
MAGELAMGEKELIRAKVMEQVVQQQLTLKEAEKLRECHGIEIDHETLRRWLRAAGLWERE
RRGSPCCLMNMVDDATGITFSFFCEEETTSDAMRLLWRWIEQYGIPQALYCDKKNAFVLT
REPTIAEQLERNHGVYQDRLIKELRLAGISTIEEATRFLREVYLPKINTKFAKTPAQPED

AHVPLIHPTSLEDILCYETPRVVSNDYVVSYKCRPLQMQRHNRLLPTPKAQVTVRELLDG
RIKLLYHERELEYVELEKPFRKEELASRSA*
>SPBDM4_v1_50726|ID:27159089| protein of unknown function [Uncultured spirochete bdmA 4]
MDRYSIKEIAEILKIHPKAAKSRLHRAGILPVSYVGQAALYSPDAIEAIRVVRGRGRPKQ
NKKQSQGC*
>SPBDM4_v1_50727|ID:27159090| protein of unknown function [Uncultured spirochete bdmA 4]
MTDSQVLMASRTVDPRRLEFDEEDFIAFAAPFLRHGWRLAAGHNSYNHYLRVGEKPYYSH
AVLLFRMNYGVVQYRILDDDQNGANSIPKKITIQTKDERDKYYNGYSCGYLFAHFATVFP
GVKPVKGEQQLYGIEPDGKGEKWPEDDVSEETITKVIAGGRKYAESNFEA*
>SPBDM4_v1_50728|ID:27159091| protein of unknown function [Uncultured spirochete bdmA 4]
MPKVTSKPEKGLDVNFFAAFDELSKMYGDDVAFKPLQDAVAIIRTRREKGEEPTEEERER
VRYLIGIVFRNSLPLRAGT*
>SPBDM4_v1_50729|ID:27159092| putative cytosolic protein [Uncultured spirochete bdmA 4]
VNYVVRISKQADKTIAKAPRAIQQKFALLIDDLEKYGPIRKDWPNFSPLGNDLYHCHVSY
SWVAVWRNEKGTVLVEVEYAGSREKAPY*
>SPBDM4_v1_50730|ID:27159093| protein of unknown function [Uncultured spirochete bdmA 4]
MPVAEKKRHIKSQRKLISVSAKEASTILAYAKKKDPQAHIVVSERNATDAVLPADSKWYQ
EVKASWHPGVTLRIRRENAGLTQAQLADMTGIAIANISAYENGRRTMGLSVAKRLAEALK
RPVSEFVEPESTKA*
>SPBDM4_v1_50731|ID:27159094| protein of unknown function [Uncultured spirochete bdmA 4]
MAFKVMIEDNNHLGFNDDENHPYSAGVFTAEAEAITICEAIVNDFLTKSLYPGERAKELV
KQYYTYGEDPYIDGTEGDKVKQGFSGWGFAEERSVILCTKDNPNNLRRVEVTKTP*
>SPBDM4_v1_50732|ID:27159095| conserved exported protein of unknown function [Uncultured spirochete bdmA 4]
MRISERLLRKNLWQEAIFLSLFFLSILATAAPQDLSMHLAGNYSTETPNPLFADGSLGIS
YHNGPFGLTWDITADNRGTYVPIASSVSNPFSLDIRNAGFTYDAGSLSLFLGKLPVKDEI
ESPYSLFLSGASPSLMTGGFRYHSGVFSFSDRWIGLNRNIRSGLYQTSIQNIYRDRGAVL
KSYTFDAGPVRIGYQDATIFPGSYFDVDIFANPVQSTLVQAVLTAAGQPSSRSGNYNSLM
GFFGEYRGEGWNMFGQLMIDDFNLNRFLNPTSYQNPDKLAWSLGCNVALDRGKLGIYTAG
ATRYTFESTSWEFYSYTLYPGSAIISGGEPIAIPLQDQMLGYINGENNLAFMATWSMPIK
QMNLETGLECVLSGSKSPTNPWHNGEAWASLGTQLLNDPVLNIKLLFNARISRTWGDMTL
LVRTTGGYVFNRLNPVYPGDPGSPFAVITDDGKEMHEPVFVPAAGDSGFVVEVSVGGIWN
IGD*
>SPBDM4_v1_50733|ID:27159096|araD| L-ribulose-5-phosphate 4-epimerase [Uncultured spirochete bdmA 4]
MSETARELENLRRDVLAANLELFRLGLALFTWGNASAIDRERGLVVIKPSGVPYDTMRPE
DMVVVDLEGRHVDGALKPSSDLPTHLVLYKVFPLAAGIVHTHSTHATAWAQAGLDLSAEG
TTHADHFYGPVPCTRCLSDAEIAGDYERETGNVIAETFRNRNIDPASVPAVLVHSHGPFI
WGSSALDAVHNAAVLEEVAKIAILSRMACMASKDAPPPMQQSLLVRHFERKHGKDAYYGQ
K*
>SPBDM4_v1_50734|ID:27159097|araA| L-arabinose isomerase [Uncultured spirochete bdmA 4]
MKTYTSHKFWFIVGSQLMYGPEVLEQVRAHAKIMTDFFNQSDAVPAPVIQPGIATSPENI
VALFRKANADPECAGIITWMHTFSPSKMWFQGLVENTRPLLHLHTQFNRDIPWDSIDMDF
MNLNQSAHGDREHGFIHARLGTARKVVVGYWQDPEVLARIGAWMRAAVAFADGKAMRVAR
LGDNMREVAVTDGNKVSAQIQFGWQVNGYGVYEVAKRLSSVTQKEVDSVIREYEARYDIA
EEVRENEGAMRKVREQAAIELALRRFLADTGAGAFTTTFEDLEGLPQLPGLAVQRLMEDG
FGFGAEGDWKTAALVRAMKVMGRGLPGGTSFMEDYTYHLEPENEMVLGAHMLEVCPSIAA
ARPRIEVHPLSIGGKDDPARLVFDAQPGMAVAATVLDLGDSFRLVANEVEAVPLPHSMPK
LPVARALWKPQPDFKGALELWLIAGGAHHTSFSYAVTTEMLEDWARMAGIECLVIGRQTS
VPEMRRQLERARLQFQR*
>SPBDM4_v1_50735|ID:27159098|araB| L-ribulokinase [Uncultured spirochete bdmA 4]
MTDTTKQYVIGIDYGTDSCRAVVTDAQDGREIASAVAQYPRWGEGRYCNASNNQFRQHPQ
DYIDSLVAVMDEIAGKTGTEVLPFIKGIAIDTTGSTPCLVTARVEPLAMLPEFADNPNAM
FMLWKDHTATDEAFEINALAHKWKIDFTMYEGGVYSSEWFWAKLLHIARTAPEVMRAGVS
AIEHCDWMPALLTGIRLLGGIKRSRCAMGHKAMWHESFGGYPSDEFLAQLHPELARIKKT
LGTETWTSDKAFGNLTTEWAERLHLAPGLPVAVGAYDAHMGAVGGGVEPGVFIKVMGTST

CDMIVGPGSAGTEHLVKGICGQVDGSIIPGMLGYEAGQSAFGDVYAWFKDLLMWPFEHVL
PKDSLPESYREETASALMPALEKAASSIEPGASGPLALDWLNGRRTPDANQKLKGAIIGL
TLGTTAPMIYRALIEATAFGARAIVERFVQENIDIKKIRAIGGVARKSPLVMQIVADVLG
KPIEIVASDQSVALGAAMFAAVVADIYASVPEAQAAMRPAIEAVIPPEPGHVEVYNRLYQ
DYQKLGAFIERELT*
>SPBDM4_v1_50736|ID:27159099|rbsA| fused D-ribose transporter subunits of ABC superfamily: ATP-binding components [Uncultured spirochete bdmA 4]
MAEPLLQLNRICKSFPGVRALDDVDFSIQEGEVVGLLGENGAGKSTLVKIISGVYMPDSG
DMLWRGNKLNIKSIMDAQERGITIIFQELNNCPNLSALENLFLGREIHSKAGMVDFRTMK
KKAEEIFSYLGVQIPLNIPVGKLSIALQQMVEIGKALLTKAKLIIMDEPTSSLTERETQK
LFQIIKELKAQNITVIFISHKLEEVFEVTDRLVVLRDGRRVGELETKSATRDALVSLMVG
REIHSFFSSRTKAAETDVLLKIEDFSGPPNVSGVSFDLHRGEILGLAGLIGAGRTELALL
MFGAEQKTSGRIFLQRKEIQVRSPWQAIENGIAYLSEDRKTKSLILPMMVRENITMSIHS
TLSGFLGTLNRKKEREIADSFVRLLEIKTSGLEQVVNNLSGGNQQKVVIAKWLATKPSIL
ILDEPTRGIDVHAKAEIHKLITDLADKGTSIILISSELPEVVALSDRVLVMHEGKIRKVL
DKPEISQENIMGAVFAS*
>SPBDM4_v1_50737|ID:27159100|rbsC| D-ribose transporter subunit ; membrane component of ABC superfamily [Uncultured spirochete bdmA 4]
MAREQKSFVAKLFSYRESAIFLALILLMAAVTIFAPNFMSGSNLYLVSRQISFVAIVAYG
ELFVILTGGIDLSVGSIMGLSGVISALAMASQLPIWLSVLLGLLTGIACGLLNGLLISYV
RIAPFIVTLGMLSFARGIILIITKGWPVTNIPKPFLAVGQGDLLALPIPVWVMIVLGVAA
HFVLSKTTFGRRTYAIGGNEQATFLSGINVKKIKVFLYMVSGAMAAVVGIILVARFNSAQ
ADTGSGWELDAIAAAVIGGTSLSGGSGSILGVIIGAAIMGVIRNGLVLMRVSAYWQTAVI
GVIIVLAAVLDRLKNK*
>SPBDM4_v1_50738|ID:27159101| Periplasmic binding protein/LacI transcriptional regulator [Uncultured spirochete bdmA 4]
MKKLLVFVLALTVLLPFGAFAQKKPVFVMVPKGVHPYYEPCYEGFKAAAAKYGVIVEKVD
PQKFELPLQVKVIEDLIARRVDGIAISALDDAGLVPVIADAMKAGIKVITFDAPAPSSAA
LTYIGTDNETAGYEAGKKMADLMNKEGEIIILQGGLGAANLNLRTKGFKRAISEAAPKIK
VLDVVDVQGDFAVATNKTEAILQTYPNLKAIFAVSAEGAPAAANVLKQQKKAGKIILAGF
DDLKDTLEGIKDGSVSFCLVQKTYKMGWLSVEKLLDAMNGKPIPKVIDTGVLFVTKGNVN
SYMDEMKKEFAQ*
>SPBDM4_v1_50739|ID:27159102| Transcriptional regulator, LacI family [Uncultured spirochete bdmA 4]
MTVREIAEKAGVSIGTVDRVLHNRGRVSPKTKASILAIIEQSGYTPNPIARHLKLNKKYP
FAVIMPVSDEDSGYWRIACAGIQKAQKELAAFGVSVKRFEFNRYVVRASFAKAMASFDPMQ
FAGLLIAPVIPDETTALLSRLPANYPVVFFDAQIPTFKPLTRIGQNAYQSGLLAGRLLEA
FSSRSGPYVIVGTHSEDYHIRRRIEGFNAYFEHLAYEVHLRECFDIEHREKREHFTRLLL
QEFSSIDGVFVTNASVHGIAADLNRRPGQKHITVVGYDLVPENIRQIEARTIDCIISQRQ
EQQGYQGIYQLYRSVVLGQSVEPEIEMPIDIHLRENLPTESHGETQKAEVHVSKHSESSL
CEEA*
>SPBDM4_v1_50740|ID:27159103| putative Sugar transferase [Uncultured spirochete bdmA 4]
MPQNLKRRQFFMVFVDVVLMYLALVLALVVRKGTLLSSDYLWEHVIHFTPVFAIWVVIFY
ILGFYRLDRTFNTYNYLKLFISGLLVAGLASVISFYINPNAYIAPKLLLVLLLIIYGLLL
WTWRYLYEKLWSQNHKYRVGVGFIGVTQEVGQVIRELNRHTLVGHDARFIYSEDPHVQTL
DDNLPLIRDSGMIRTTVEKTNADLIVIAAGQKELSSELIRELYGLLDMKVRFMRLPDFYE
MIFRRVPINAINESWFLENIDLRAAVPYETIKGIFDRILALILLLICLPFIPFIALGIKL
ESKGPVFFKQERMGRNGKPFMMIKYRTMRVEGNDFSPTEKDDGRITRLGSFMRATRIDET
PQVLNILKGDMSFIGPRPERPEIARKLAEAIPYYQQRHLLKPGVTGWDQVCGEYHSPSIA
DTKKKLQYDLYYLKNISFSLDVSIFSKTIMTMLRRKGR*
>SPBDM4_v1_50741|ID:27159104| conserved protein of unknown function [Uncultured spirochete bdmA 4]
VRILHVVTNADLGGAPRVVTELANRAVRDGHICAAASVPAGPFWAHLDPRVERMPLRHLR
RELHPVQDPSAVLELVRLFRRWKPDIVHLHSSKASVLGRFAALLANFPAIRRIPTVYTIH
GFDTILKTHRKFLPLERIMARITSAIVPVSAYDRRNLEEAGIHGNVRLIHNGASDRFGMT
PDPAVTERIGVARTEGATIVLSIARLEAPKRFDLFLEVARAFAGTGAGQPADSAAKVAFF

WIGNVQSIDQTSLPPNVEMLGEVPEAGNCINLCDVFLLLSDYEGLPMSVLEALSCGKPIV
ASDVGGIGEAVGVAGGTEAGILVPNERSAASMATARLVGDPDLRARLGASARRRYEQGFS
ADSMWQAYLRLYMGLR*
>SPBDM4_v1_50742|ID:27159105| Glycosyltransferase, family 11 [Uncultured spirochete bdmA 4]
MKIVKVAGGLGNQMFQYAFARALEERTGDEVYLDLSTYEYMPAHNGFELDRLFAIQYRAA
RKEDVERLATHPRNSLSQIRRKYFTKRTHHIDRVFRYDPSVFELKGDLYFEGWWQSEKYF
APFAESLREEFRLVGNPGERNRRLVETMADIGAGSGKEGEVACTDKSHFVSVHVRRGDSL
RLPETQVCTETYYRNAFSLVRERIERPHFLVFSDDLPWCRAHFGLDAAEVSFVDWNRGSD
SWRDMWLMSRCRSHIIANSSFSWWGAWLDPCLGKLVVAPERWSLASPGRFAYYRYDFGDI
VPETWVKVPLH*
>SPBDM4_v1_50743|ID:27159106| conserved protein of unknown function [Uncultured spirochete bdmA 4]
VVAAEYDLVVCAIERDLPVLEIAIPYMWRFLDPRHLVLIASSPCLDRFSTKYHDAEHKND
LCLVDEDKVAPGLTLNYVRSLLAARGANPARGGWYFQQMLKLCYSLNSMADDYYLVWDAD
TIPLRPMDFFDETGRVFLEKSSEFNPSYFTTMKRLIGLDKSKPYSFISEHMMFEKTVVTQ
LLALVCGQDGPSGKVVAERIISAIDVGPLNASGFSEFETYGTYVMSHFSEHYLVRALPSQ
RQGVLIYGRQPKPQDLFALSRRYYWASFEAWPVTSVKTRVKRFAWSALGSVEAWAARRLD
SHMYKEFLDITKKDEA*
>SPBDM4_v1_50744|ID:27159107| conserved protein of unknown function [Uncultured spirochete bdmA 4]
MKTFVISLSGDDVRRSFMRRQLDALGIDYEFFDAIRGSERIGDLRWYDDVSVHHLEGRSL
RPGEVGCALSHAAVYAEIVKRGLNWALILEDDGLLHQDLPQVLSTIEQGAGAQGQLISLS
RCDAYLPWASKNLAGEFRLVTPILIKEGSIAQTVGYVISLKAAKAIAKINIPVKFPADSW
GYYRGIVEFKGVIPTLTLVTQNVDLGSTTLESGKRQVFTPYSIKDLLLHGFTTYNSFGRF
LKRSAKHLFGS*
>SPBDM4_v1_50745|ID:27159108| putative Glycosyl transferase group 1 [Uncultured spirochete bdmA 4]
MQRKHIVRVFIPGIFAYIGGAEKYTATIVQYLVNAYRNLDIGIVSYRHNGAGKANDIAIL
NENYDLALPPAIKTILLDDEGKGIWGRFIAHQRLRNTSHNVDLYMNCFHNVQFFKAAKNV
HIVHFPAQRRVLGSPTFGGKPLLKPLADALDGKYKDCYDLFICNSRFSESWLQEYWGIEP
ERRAVLYPPVAHDATWSPDLYDQKKNIILLVSRFDPRKNMLEAVEYFVTNENRFSAWRLV
VAGSCSDNDKEYYNRVALASRGHRVDIRPNLPKPELDELYQGASIFWHAMGLATDENSCP
IEVEHFGITTVEAMSAGAVPVVIDKGGQREVVDDGINGFRWRTLDELGNVTQKLIADPAL
RRALAVSAVEKSNTYSLEAFYHAIDAIFMEHRLIPEEYRK*
>SPBDM4_v1_50746|ID:27159109| putative Glycosyltransferase group 1 family protein [Uncultured spirochete bdmA 4]
VMEKLLFVGLPEHKRTASFEFFTQLLENTYQIEYTKITIDKNWTNTTYAYNRIIFWQRLP
PPEHLRAVSPTSVVYVPMYDEARNYNRQVIRTWAPFKIISFCRSIHEDLTAWGFNSKYVQ
YFPKPMVEAPLPLAVHKAFFWKREGSYINEAIPDAMIPLLLHDSGMKLHYHSETKPVCEK
EFEDMVASRSSWLRSKEEMISLLRECSLYIAPRESEGIGMSFLDAMANGLAVAGADNPTM
NEYISDGQNGYLFDLMKPLPINLENLQQVREKSLESCQAGYEKWEKEKWSLIDFIEEPHP
SSLLVQVPRGFEYRSLKQKSMSFRAKRLVKRLAGK*
>SPBDM4_v1_50747|ID:27159110| protein of unknown function [Uncultured spirochete bdmA 4]
MRLKRIFAVFVKTPFKFIPLAAVALNAGENKLFFAPFRWKILQLLEREGKVGRKHGCRPS
VYRYWQKRYQIENQHAVAESLEYLNKTTASPSFAYIVEPEANFFATCSTINSLRHQSYKN
IEIIIRNNATRPLRSRLLQNVAGNMAHIVGKNENIKAYLDKASFVLDISPGDKLSQQALL
EIGLYMERSDRSWDIAYFDEDVQSSFGRCRFPFFKPDYSPLYNSKVNYISGACVYKAGLF
PPWKDKCLKDDICLQIPESLRREKILHIPEVLLHKREPRAISSMGKKDYQYLIINQADSA
KDNALVSIIIPIKDNVSYLNQCISSILDKSQSDQVQIIVVNNKSEKQETLDYLKKLSEND
RFRILEYPYDFNYSAINNFAAREAEGQYLVFLNNDTTIISAEWLKHMKYWLSKPLIGCVG
VKLLYSDQTIQHVGIIVGASMGACHYNVGDLQEDYGYFGINTFPREVSAVTAACMGVRKD
NFVNVGGFDEKIPVAFNDIELCLRMMNAGYVNILDPRIQLFHYESRTRGRDDDIFKLTRD
RKERNCLVKNSMTDIRFDAYYNPNLSLNNTYCLSFPPRTVSMIKSYEMKLKRLVVLVGSI
QRYGSALEAIMKEHERLLKGQGYSIIWGVDIKCNVTTKGDTPFHLVKNEWDAMDMIRKMS
TAYVIVYSEPYQKIAWMMPREIKVFYFHGKTGWTEQKGTTMELSVLDREAAETYAYPIVS
IEEFFAHTRSC*
>SPBDM4_v1_50748|ID:27159111|rmlB| dTDP-glucose 4,6 dehydratase, NAD(P)-binding [Uncultured spirochete

bdmA 4]
MRLLSSILVTGGYGFIGSNFIRYFFDQPDLSSKIVNLDALTYAGNPENLKDIASQYPDRY
FFEKGDIRDRKLVESTFRKYSIDTVVHFAAESHVDRSISGPEAFISTNVLGTFTLLDVAR
TFWKEKFGDAWESKVLFHHVSTDEVYGSLGDTGYFREDTAYDPHSPYSASKAASDHLAMA
YHYTYGMPITLSNCSNNYGPYQFPEKLIPLMILNMLEGRPLPVYGDGKNIRDWLYVEDHN
AALWLILQKGKSGEKYNIGGENEWENIHLVQRLIDLVTEESGIRQRDIDNLIVYVQDRPG
HDRRYAIDCSKIKTELGWKQQSTFDEGLRKTVRWYLTNKEWCDRVRSGEYQRWIEENYRK
RV*
>SPBDM4_v1_50749|ID:27159112|rmlD| dTDP-4-dehydrorhamnose reductase [Uncultured spirochete bdmA 4]
VIWIVGNKGMLGSELSQILENRGIECVGTDKDVDILDPMALVAFAANKDISLIVNCAAYT
AVDKAENEESLALRLNAEGPANLAALAVKRHAGFIHISTDYVFDGEGSVPYKENDPVAPL
GAYGRTKAEGEKRIQAIDPDAIIIRTAWLYGQYGKNFVSTMLGLMNGKSELSVVADQYGS
PTWAHDLAEAICAIADRTAPTAGIYHFTGEGTTTWYEFACEIQRLGLEFGVLTTPCKVKA
ITTDLYPTKAKRPAYSVLAKDKIKEAFGIYPPEWRDSLRKYFESIYQVKTGGEG*
>SPBDM4_v1_50750|ID:27159113|rmlC| dTDP-4-deoxyrhamnose-3,5-epimerase [Uncultured spirochete bdmA 4]
MAFKFNETPIRGLMVVEPRAFTDDRGFFMESYKASDFISAGITAQFVQDNHSRSCYGTLR
GLHFQKAPHAQGKLVRVTQGKAWDVAVDLRAESSTFGKWHAVELSAENKLLFWIPEGFAH
GFVALENGTELQYKCTSEYAPESDLGIIWNDPELAVEWPIKDVILSEKDMKLQSFAAYKR
TIQ*
>SPBDM4_v1_50751|ID:27159114|rmlA| dTDP-glucose pyrophosphorylase (glucose-1-phosphate thymidylyltransferase) [Uncultured spirochete bdmA 4]
MKGIILAGGAGTRLYPLTYAVSKQMLPVYDKPMIYYPLSALMLANVQEVLIISTPRDVPA
FRQLLGSGDRLGMKFQYAVQTYPRGLADAFIIGEDFIGEENIALVLGDNIFYGQGFGKSL
ERAGSIVEGAAIFGYYVKDPSSYGVVEVDKNGNAISIEEKPKEPKSHYAVPGIYFYDNEV
VNIARGVKPSSRGELEITSVNNEYLARGKLHVELLGRGMAWLDAGSYDGLLEASNFVEAI
QKRQALYVACLEEIAFRKGWISRERLLESASEIHTDYGLYLKYIAESD*
>SPBDM4_v1_50752|ID:27159115| conserved protein of unknown function [Uncultured spirochete bdmA 4]
MNFASIRQTISIFIKSPGKFRIVRKGISALVKEGIPGLKKGIKGKIDFYERLSSVHTDEA
KVQQFFYSQQNQFSKNSMGEMETHFKDSPLISIIMPTYNTSERCLRLAIESVIRQIYENW
ELCIVDNGSSKSEVYNVLESYSKKDPTRIKVKFLSINTGISTASNIALEMASGSYVALLD
HDDELTPDALFWVVKEINDFPESDFIYSDECKIDNTEERKLFHFFFKPEWSPELLLNGMY
TGHLTVYRTSVINEAGAFRSKYDFSQDYDLALRISEKAKTIRHIERILYLWRSVPGSAAQ
DGKPWARISNLDALKDTMKRRNIDSYILPYPYTNRVKIRRATNAKVSIIIPSDSLRNLYD
SINSLLAKTAYVNYEIVIVTNSVLIEKLKEQWRPFQHIIFVPYNKNFNFSDKCNEGAKHA
FGKILVFLNDDVFPRDESWIENLIEYLYLQDIGAVSPKLIYEDGIIQYAGMFVGAPGFVG
TAYSGYYENEVDEFNTLHRWVRDVSILSGACFAIKKSIFDELGGFDPDNTPNGHSDVDLS
FRVREKGLRIVYTPYVILIHKGNHSWHEADKCTRPDIYLMKRWADYLISDPYFTNSMKKF
IYRDFPYEFKFFGKRIKEKSKRNILLVSHEMSETGAPLVLLYLARIIRGNGDYPVFVSPI
DGVMRRNLYNEGFHVIINQNIYGNDLLFKKFAENFDLIIANTLLSLPVVRQLSGMRIVWW
LHEGEFALNNYVEINKNVISDMKNLGNNSNLQILCVSEYSKEIFTKRVGFNCDQIFAFGI
PDRRPDLRKKQHSDIRFIMAGSIEKRKGQDIFVTAIAQIPVVIRRNARFVIIGNSLDAQY
YQDLIVKTADIDEIVVENALPHLKLMKEFEDSDVIICPSRDEPFSITLLEGMMLSKACIC
SNKTGIANYIKSEDCGWVFDVNTPEELTEIMNNIIMGKENLEEKGKNGRKVYEENFTMEC
FKKRALKQLSAVEDHNNSVQPENKLSLDCLGE*
>SPBDM4_v1_50753|ID:27159116| O-antigen export system ATP-binding protein RfbB (fragment) [Uncultured spirochete bdmA 4]
MNTVVKVEHLSKEYRLGVIGNGSLWKDIQSWNAKLQGKPDPWTRINEESRIGKRDHFWAL
KDVSFEVQRGERLGIIGRNGAGKSTLLKILSQITAPTEGTVKMRGHVASLLEVGTGFHPE
FTGRENIYLNGAILGMSKTDIDRNFDEIVAFSEIEPEFIDTPVKRYSSGMYVRLAFAVAA
HLDSEILIADEVLAVGDVKFQEKCLGKMKDVSENEGRTILFVSHSMGAVKKLCQMGVLLE
HGEITAQGKIDDVASSYERLLNN*
>SPBDM4_v1_50754|ID:27159117| ABC-2 type transporter [Uncultured spirochete bdmA 4]
MTSTDNETWDVVITAKRKWLGLDIKELRQYKDLVNLMIRREFVSVYKQTIFGPLWFIFQP
LLSTFLYMFVFGNIAQMGTDSIPQPLFYFSGTMLWTFFATTLQKCSDTFVNNAGLFGKIY

FPRFTMPIAYVANAFFTLCIQFVVMAVFYIYYLLNGVQFTISWWILATPLYIVQLALLGT
GFGIMTSALTTKYHDLRNLVTFGLSVLMYATPVVYPVSSIPIKWKLLFQFNPVTPVIEMF
RYSFFGKGSCDLNMWAYSLMVSVIVFLIGLMTFNHNEQTFVDVI*
>SPBDM4_v1_50755|ID:27159118|fcl| bifunctional GDP-fucose synthetase: GDP-4-dehydro-6-deoxy-D-mannose epimerase and GDP-4-dehydro-6-L-deoxygalactose reductase [Uncultured spirochete bdmA 4]
MNTHDKIYVAGHRGLVGSAIVRRLKAGGYDNIIVLGHAELDLTRQAEVEAFFEREEPDYV
FLAAARVGGIGANSYYPAEFFYENMAIALNVINASWKNGVKKLLNLGSSCIYPKMAPQPL
KEEYLLTGPLEPTNEGYAVAKIASIKMCAYYNKEFGTNYISLMPTNLYGTGDNYDLFTSH
VLPAMIRKIHEAKEGRQPVVLWGDGTPLREFLHADDLADAAVMLMEQYNYKDIGELINIG
SGQELNIRQLAELIADVEGFRGEIQWDTSKPNGTPRKFLDSSRLFALGWKPKIGLREGIE
RAYREFLSFNTENRR*
>SPBDM4_v1_50756|ID:27159119| Mannose-1-phosphate guanylyltransferase (fragment) [Uncultured spirochete bdmA 4]
MMVNLILCGGSGTRLWPLSRKKTPKQFVRLFKDKSLFEEALSRNRPLCDKFVVATNIEHL
PLVERQMARMGIDKVEGLLEPLGRNTAPAIALILMGLPRDELVLVSPSDHHITKLDQYTE
AVRRAAALASQGFLVTFGIKPQYAETGYGYIEADGEKVISFREKPDRKTAEHYLASDRYF
WNSGMFVFSAGTFLDELSSYAPEVFTAAARTYERAEKGDLLRPRPEDMSAIPSISIDYAV
MEKSKRVKVVACDPGWSDLGSFDALYDEARGDADNAALADNAMFLNSRRCLVVGGEKPII
LEGVEDLIVVQTDETTLIMRRGQSQNVRDVLKAVADRRPDLL*
>SPBDM4_v1_50757|ID:27159120| conserved protein of unknown function [Uncultured spirochete bdmA 4]
MSSVSVLIPAYNAEGTLRKSVESVLGQKMDGSQQFEIIIVDDGSTDSTACVAESLADRGR
GLIRIIHQKNSGPAAARNAGIRAARGDYVAFLDADDQWLPGKLGTQLEILEADPNLDLVC
TAMNGKRFLLRPERFALNFMDLLPNNIVYTSSVVVRKSTLTAAGCFNEARMYSEDFELWL
RIAHRGKIMVLNKPLIRYMKGRGISAKFWPMEKGELETYAIMLREGWISARRQTTLRWWS
LFKYLIRRIR*
>SPBDM4_v1_50758|ID:27159121| putative UDP-glucose:polyglycerol phosphate glucosyltransferase [Uncultured spirochete bdmA 4]
MKVLFVINDAHGWAGTERVTNLIANGLSARLEVEVLSLRPRPQNACGYPYNPEVKLSYLP
LAGGIRTFLASNVRVFRFTEQASPDVIILSGVGEIKNFIKVASPRRRHRPRLFAWEHFNA
AYASRHLSRRIAARYCDVIVSLTKKDAEDWRQMLRPRAVVSIPNPVPEFPERAASLDTNR
ILALGRLEDQKRFDLLIDAFALFAQSHPGWYLRIRGSGSKEAELRDKICVLGLQDKMEIF
PPTHDVAEEYAGASMYVLSSGYEGFPMTLVEAMAAGVPCVSFDCPDGPSEIIENDHDGFF
VPLYDVVALAEKMGRLADDVTLRRQMGAAARQNIRRYEAGPILEQWSALLHGLGAGE*
>SPBDM4_v1_50759|ID:27159122|gmd| GDP-D-mannose dehydratase, NAD(P)-binding [Uncultured spirochete bdmA 4]
MEKTKTALITGVTGQDGSYLVQLLLEKGYTVHGIKRRSSSFNTQRIDHLYKDPHDPDNHF
RLHYGDLTDSTNLIRLVGEIEPDEIYNLAAQSHVKVSFDTPEYTANTDGLGTLRLLEAIR
LLGLEEKTRFYQASTSELFGMIRETPQRETTPFYPRSPYAVAKLYAYWITVNYREAYGMH
ASNGILFNHESPVRGETFVTRKITRAAARIKYGLEPKLYLGNLDAKRDWGHAKDYVEGMW
RILQQPEPDDYVLATGRTTTIRDFVTMAFRHAGFEMEWRGNGLEEKGIDSATGKILVEVD
PVYFRPTEVDLLLGDASKAREKLGWVPQHSLEGLVHEMMEHDLAEAEKERYLKDGGYRVF
EPRE*
>SPBDM4_v1_50760|ID:27159123| exported protein of unknown function [Uncultured spirochete bdmA 4]
VKSVRTAFPLAFLMTLVISVVSLTAADADSGYAGVFRNLSAAEASMLERGELVIRPLKAS
DSLSLAREDSGAKEIVKRLRDLHPNYTVEFMAIVPIKNAAKTESTLNRIAEALSDVQGYV
NIPYWSQRQKTSYALFDKMEVLDRKTAGTGNAESIEVRQHMEPFDEFRARYDYRLEAGAL
RFSAVNLDTIVYTYQHFRAVAPNNMAWELYIFLQEDRFYVYGIGAVKAFDLFGLFRDRLE
PSLMGRAESFFTYITQKISPLEGNE*
>SPBDM4_v1_50761|ID:27159124| conserved exported protein of unknown function [Uncultured spirochete bdmA 4]
MHKPGFFLVILLIICAPFAFPEGESPSEGESRVVVYTVRSVSFKITGSTMERVLRQKANID
LGTEFATLEEFESYLEQRRQVLLNERVLAEVKAEYTLEKGDDGRVFIDITFITKDSWNVV
ALPYFKYDSNDGLVLSARGRDYNFFGSMQVLILNIDYAIDPIGRHSYGGLASMNLPFQLA
GYDAGFSVYEDLAVHADGRPTSSISNLGFSVLLPTRSYPINLSANQGLQFNPDEVDNDSD
PYFLVSSLTASSSLPTGFPVGRYGSVSYVPALTASLNWRPGEMVRDDRKGFKLTFSHGLH

FSRIDWINNMRRGMAASLTNTDAYNLMTAVPQIDIDANFFYHATSKGLIGFEARFVGFHS
FTNTIRTDLGSYMRGIVDARLWGGSAAFLNLQMPLKLFDFPTHVIIGKNWFDFELQTTPF
VDLGYCRKNLGDTFLDSAWYTAGLEFSVYPLRMRTFIVRASAALDLDAVIRNKSLTAPSP
RDGGSPYEIFFGLGLFF*
>SPBDM4_v1_50762|ID:27159125| Peptide methionine sulfoxide reductase MsrA (modular protein) [Uncultured spirochete bdmA 4]
MKFRIYAVLSIVLSFFLFSCASKGSDSIESMNSALASAKNAKPPLLGPRTTPAQGKDGPR
IEEQFGMPVNIVRTDGLQYAILAGGCFWCLEGVYELVPGVEDVISGYEGGSAPSPTYEEV
STGTTGYAESVLILFDPAKVSYGELLDIFWHIHDPTTKDRQGYDVGPQYRSAVFYLNDTQ
KAEAQKSIRAQQEEWPVPIVTEVRPAGLFWPAEDYHQDFYSNNPDYGYCQVIITPKVEKV
FQAK*
>SPBDM4_v1_50763|ID:27159126| protein of unknown function [Uncultured spirochete bdmA 4]
MSRERFFADEHRIIDAAISLICENGYEKFSTRRLAARLGVSPMTLYNYFSNKEEIVQVTI
STAYEKAFEAIQDDLRGYFERESACPLMGFVEMGRKLFAFSKQYPQMYALVFVMNFSPYR
DQPSLVECYNYTFKKVFERLVDKNIEEELHRHIYLFQVLVSALVRNIYNKLGPTDEETFE
INLLLAYNRLLKPFERYFAVCQGIQ*
>SPBDM4_v1_50764|ID:27159127| Esterase/lipase [Uncultured spirochete bdmA 4]
MIFMNHEPQSVRSMLVEQVFRSAQVSHGQDSSPPRFIVRQYTKSLLAAEPPLVIRARLDV
KSFSIAESRVHVLIPRFEGCGRCILYLHGGSYVATFTRQHWNFLAKIAEKTKSVIIAPDY
PLAPEHHWSEAYRMLFALWERMGEFLPRKSIVLMGDSAGGGLALGFVQALRDAHLTMPAS
VIMLSPWLDVTLENPQIGELSPVDPFLNVEALRTAGKAWTGGSNPRRMEISPIYGHFENL
PPLSLFIGTKDVLLADCRKLWNICSVTEVPLDYYEYEHMLHVWMLLPIRESGHAVAQIAG
ILATQAQCADIDAVLEFSKEDEA*
>SPBDM4_v1_50765|ID:27159128| putative 26 kDa periplasmic immunogenic protein [Uncultured spirochete bdmA 4]
MRAGRTAKRTGLWGTGLAMLVLGLFVVTTGAAAQGMDAPGAGSQFQKRSIQVQGQATMSV
EPDVAYLRLGVETQGDTLAKAQGQLAERASKVLDALSQAGIDRTNMKTSSYRVSPVYSTR
QDKQNVIIGYRAETTINVEVADLSSVAGLVDAAMNAGANVVRSISYDRKDMQTFKVQLIQ
AACMDAKMKADAAAAAFGAQVGAPVSVDIQDSFSAREAGNVMMMKAAAPDFLSPGELDIG
VSVSVEFDLRVE*
>SPBDM4_v1_50766|ID:27159129| putative Glutamate racemase [Uncultured spirochete bdmA 4]
MRAPCDIVYVGDNANMPYGNRPFSEIVSLAGRMLDMLSEHEVEVVAIACNTISATVEHLR
PLSKVPLVDIISPAAEALARRGEPDVGLFATEFTVKSGLHAKRVGELNPVVKVHGVSSPR
LAALIDGAIGDEKAIRDEIASMLEKLASLHPVTTVLLGCTHYPIVMEIFKSLAPDIAFID
PADLQADEVMRLLRHDGWGSAVALNMAHAEVSGAARAAAPSLEIITSGIPDSYRRIMQRL
GIPEAQSIRRLEA*
>SPBDM4_v1_50767|ID:27159130| conserved protein of unknown function [Uncultured spirochete bdmA 4]
MKYVKIRDVTLRDGQQSQFATRMTNEQVKAVLDDYRQSHFYAMEVWGGAVPDSVMRYLGE
DPWDRLATIKKGVGDASKLTALSRGRNLFGYTPYPDSVIEGFCKNAIATGIDIMRIFDAL
NDIDNMKSSIRFVKEAGGLADCAVCYTVDPRFTFSDRVNALFSGKKIPPKLFTVDYYVKK
AKELASLGADMITIKDMAGLIDPSAAAELIKALKNELAVPVDLHTHCTPGFGVASLLAAM
VNGVDIVDTAVLSFSGGPAAPAYEIIRVFADRLGLSTDVDNAAVARIDRRLRQIRGELSA
YDQYKKLPPELDLSKDSLPPHLSKLFDDALEAAVGGKYQKSLVICQEIEKAFSYPDPDEI
VRHAQIPGGMYTNMMAQLKEAKLQQHLGEVLRLVPRVRLDSGLPPLVTPTSQIVGVQAVN
CMISLHQGKEMYENVSKNFADLILGSYGKTPWPVDPEFRNKICGTREEKPYDTSKYQAQE
NPPVAEAGGALLAQNEKEQLLLELFPSVATKFLRNRRMEEWKTAHPEEETPQPGAQAATA
PPSPYEAADYEPSDGEPPSFWEDAPGWAGWTDAIKPQGGVYSEPQESQVFA*
>SPBDM4_v1_50768|ID:27159131|korA| 2-oxoglutarate synthase subunit KorA [Uncultured spirochete bdmA 4]
MVEKVRLMQGNEACTMAAIAAGLEFYAGYPITPSTEIAEMCSEELPKVGGKFIQMEDEIG
SISACIGASLTGAKAMTATSGPGFSLMQEAIGYASLCEIPLVIIDVQRVGPSTGMPTSPA
QGDVMQARWGTHGDHPIIALAPGNVKECYELTFRAFELSEKYRVPVIVLTDEVIGHMREK
VVLPDPGSYKIPMRAMPQNPAGYKPYAADPNGGVPLMAPFGMGYRWHCTGLFHDENGAPS
SKPAVASVLMRRLNLKVENRADDLADYFAEGLEDCDLAVVSFGSSAMAALSAVRRARSEG
YKVGMFRPKTIWPFPTATVRRLLAPAKRILVPEMNLGQLALEIERAAGCERPVIRLGKVN

GELFHPDEVFAAIKEAL*
>SPBDM4_v1_50769|ID:27159132|korB| 2-oxoglutarate synthase subunit KorB [Uncultured spirochete bdmA 4]
MAEFLSYFRQDRLPHIWCPGCGNGTVTGALVRAIDKLGLDKNNIVVVSGIGCSSRAVGYL
DFDTLHTTHGRAIAFATGVKLARPRLKVIVITGDGDSAAIGGNHFIHAARRNIDITTIII
NNNIYGMTSGQASPMTPHGMLGTTAPYGNVEYNFDLCELAKAAGATYVARATTYHYVLLE
DLIAKAIMHKGFSVVEALSQCPTYFGRKNKIGGAVDTLNWIKDQTINVKAAAALPPEKRE
GKILIGELHSIEKAEFTQQYLEVIAKVQRANPSYARNKGVPGCELNSD*
>SPBDM4_v1_50770|ID:27159133|korC| 2-oxoglutarate synthase subunit KorC [Uncultured spirochete bdmA 4]
MRVEFRLSGSGGQGLLLAGIVLAEAAIRDGKNAVQTQSYGPEARGGSSKAEVVIADGDID
YPKATAPDFLLALTSDAYRSYGLSMKKGIIITDSTVKIDPPAGVPVVALPILETASKKLG
RAVVANIVALGVLGGISGIASPKALKEAVRSRVPKGTEDLNLAALEEGLALAAQAKAAH*
>SPBDM4_v1_50771|ID:27159134|yliK| fragment of methylmalonyl-CoA mutase (part 1) [Uncultured spirochete bdmA 4]
MVDFEKLEQDFNVWKARVEKANEKYPERKKQFVTGSNAPVDRVYTPLDQVDSDYSRQLGL
PGEFPYTRGVQPTMYRGRLWTMRQYAGFGTAEESNARYKYLLEQGQTGLSVAFDLPTQIG
YDSDHPLSEGEVGKVGVAIDSLADMEVLFGGIPLDKVSTSMTINAPAAVLLAMYIVVAEK
QGVGAEALNGTIQNDILKEYIARGTYIFPPAPSMRLITDIFEYCAKNVPNWNTISISGYH
IREAGATAVQEVAFTLADGIAYVEAAIRAGLDVDEFAPRLSFFFNAHNDLFEEVAKFRAA
RRVWAHIMRDRFGAKNPKSWMLRFHTQTGGSTLTAQQPDNNIIRVTLQALAAVLGGTQSL
HTNSRDEALALPTEEAVRIALRTQQIIAYESGAAETVDPLAGSYYVESLTDRIEKEALQY
IKRIDDLGGAVKAIEQGYIQQEIQDSAYAWQMDVEKNERIIVGLNKFQVKENPPKGLLRV
DPAVGEHQRQKLASLKANRNQNNVDSALASLRKAASGADNLMPPILEAVRAYATLGEICG
VLREEFGEYKPNVMF*
>SPBDM4_v1_50772|ID:27159135|yliK| fragment of methylmalonyl-CoA mutase (part 2) [Uncultured spirochete bdmA 4]
MERKIRVLVAKPGLDGHDRGAKVIARALRDAGMEVIYTGLRQTPEQIVSAAIQEDVDVVA
LSILSGAHNHLFPRVVELLRERGADDVLVFGGGVIPEEDIPFLKSKGIAEIFGPGTPTSK
TIEFIRANLKRPLN*
>SPBDM4_v1_50773|ID:27159136| putative enzyme [Uncultured spirochete bdmA 4]
MNELVRRCIEGDVRAAARLITLLESQDRDAYAALYRLKDHAGRAQVIGITGPPGAGKSTL
ADKLITQFRARGLRVGVLAVDPSSPFSGGAILGERLRMQSHATDTGVFIRSLATRGSLGG
ISKATHAAIRVLDAAGYDIVLVETVGVGQSEVDIVRVADTVVLVSVPGLGDDIQVIKAGI
MEIGDIFVVNKADRDGADRVVRELRAMLETQVQLRGGQAPAGGPDTLAARDPDFMHHAGL
ERAAPAAPTGPGVPAVPFPPVLKTIAETGVGVPELADAIARHFERLKNTGELERKRLESI
RYQLGQFVFDEITHLLSLSELSKVRDDLAQKVISRELDVYSAGVALFHSIPEQGAPREHP
QT*
>SPBDM4_v1_50774|ID:27159137| conserved protein of unknown function [Uncultured spirochete bdmA 4]
VNTLRLDHIGIAVEKIDASLPVWEGILSLPLHGIEEVADQKVKTAFMPIGESEIELLETT
DPEGPIGRFLAAKGQGVHHLAFRVANIDDALAELKAKGIRLIDETPRHGAGGARIAFIHP
KATGGVLVELCERA*
>SPBDM4_v1_50775|ID:27159138|pccB| Propionyl-CoA carboxylase beta chain [Uncultured spirochete bdmA 4]
MEEKLRELEERRAKVLAGGGPKRVAAQHEKGKMTARERIEMLLDPGTFMELDAFVEHRAT
QLGMDKIDAPGEGVVTGYGLVNGRNVCVFAQDFTVIGGSLGEMHAAKICKVMDLAVKIGC
PLVGINDSGGARIQEGVDALSGYGEIFYRNTLASGVIPQISVIMGPCAGGAVYSPALTDF
VFMTEGTSNMFITGPQVIKAVTGEEVSAEDLGGARVHSEVSGVSHFSFPDEQSTIEAIRK
LLSYLPQNNVEDPPVMDIGDDPNRTDDALADLMPDSPNKPYDVRDIITRIVDSADFFEVQ
PDYAKNIVTGFARLAGRSVGIIANQPKHLAGVLDINASDKATRFIRFCDSFNMPLLTLTD
TAGYLPGVGQEHGGVIRHGAKLLYAYSEASVPKLTVILRKAYGGAYIAMCSRHLGADQVF
AWPTAEIAVMGPEGAANIIFKKEIEESADPATMRKDKIEEYKRSFANPYMAAARGYVDDV
IEPSHTRMRLCNALNTLLGKRQSTPSRKHGNIPV*
>SPBDM4_v1_50776|ID:27159139| protein of unknown function [Uncultured spirochete bdmA 4]
MTNPWMIMLLGIITVFVVLFLIILFLLGFPGFFGALKRREHGKPAPLPQIIEVTKKTVLA
ADGSASQTAGEFSAQEEETLVAVLTAAVAAASSSEIGSFAITQVRQAQAESSGGFNTPVW
GRVERLARK*

>SPBDM4_v1_50777|ID:27159140|gcdC| Glutaconyl-CoA decarboxylase subunit gamma [Uncultured spirochete bdmA 4]
MKQYRITVNGKSYDVTVEELGGAKHVPAAAPTPVISAPAPNAAPAAPVQPAAPANGGSII
TVKAPMPGTVLSFKVIVGQQVKRGDVLLLLEAMKMENEIVAPQDGTVVALRVPASASVNT
GDPLVDLA*
>SPBDM4_v1_50778|ID:27159141|gcdB| Glutaconyl-CoA decarboxylase subunit beta [Uncultured spirochete bdmA 4]
MEAFFSHFISSTGFIAMTWGQLLMLVISCVLLYLGIAREYEPLLLVPIAFGMLLANIPMS
GITDGPKDGLVGGLIYYLYQGVKLGIYPPLIFLGIGAMTDFGPLIANPWSLLLGAAAQLG
IFIPFIAAYYMGFDLKSCGSIAIIGGADGPTAIYVTSRLKPELLGPVAIAAYSYMALVPL
IQPPIMKALTTKKERQVKMAQLREVSKAERIVFPILVTLLIGLLLPAAVPLVGSLMLGNL
FRESGVTDRLSDTAQHALINILTIFLGVSVGSTASAAKFLNPQTLMIIALGLFAFCCGTA
GGVLFGKIMYRVTGGKVNPLIGSAGVSAVPMAARVSQVVGAKEDPTNFLLMHAMGPNVAG
VIGSAVAAGVLLSVLG*
>SPBDM4_v1_50779|ID:27159142| Aspartate kinase [Uncultured spirochete bdmA 4]
MKILKFGGTSVGSPEAVRALVTIAKDSGERVFVVSAFSGITDSLLQAAQCASEGGEWRLL
FETISERHHSMIAALLAPPLEATATASTNALLGELSSLLLGISLVSDLSPRTLDLVASFG
ERLSAGILAQTLLQSGIEAEAVDARQFIVTDDKFGAAHWLEAETTRRVSTFFENFPHIAV
VTGFIGSTEDGLTTTLGRGGSDFTAGILGACLNAEEIQIWTDVDGIMTADPKAVPDAFVI
PEISYVEAMEMSHFGAKVLHPPTILPAMSKGIPVRILNTFNPQAPGTRIVREAVPSTWPV
RGIAAISSISLLLLQGPGLPGVTGIAGRMFAALAAARVNVILITQGSSELSICCAVLPQD
ADRGVRALREEFRYEIGTGTIQMPAVEKDLSVIAVVGEMMKHRVGISGRVFKALGRNGIN
VVAIAQGSSELNISIVVSAQERNKAMSAIHDAFFLAGVRTVNVFLVGTGLIGGTLLSQVA
AQHRKLFQDRSIRVRIAGIANSRNMLIDSKGIDIDHWQETLTAQGHKTDLGHFVQALLKL
NLPNACFCDCTASDIPVQFYESMLSASISVVTPNKRGNSGTLQQYRRLIDLAREMDVPYR
YETTVGAGLPVIGTIQDLVASGDEIFKIEAVLSGTISYIFNNLGPGLAFSELVKQAKQLG
YTEPDPRDDLSARDIARKAIILAREAGYAFEGSTVQIEPLISERISRAPSIDACMALLPE
MDGVLEGRRTAASQKGTVLRYVSVITQRGCRLSLCEYGPDSPFYSLTGTDNMVVITSKRY
SQNPLVIRGPGAGADVTAGGVFADILKTAESYL*
>SPBDM4_v1_50780|ID:27159143| TRAP transporter, 4TM/12TM fusion protein [Uncultured spirochete bdmA 4]
MSDSQKIQQENAASDAQEVLKKFDKEANYRTYAGFFAKVISALAIAFSVFQLYTATFGVL
DAMIQRSIHLSFGLTLIYLLYPTSKKWSHTKLHPVDAVLAVLGALAPMYIIVNYQKLVLR
AGTATPLDIVFGILGVLLVLEAARRVVGVPMVVIALVFIAYAFAGPYIPGKLAHRGANFE
TLIQHLYFTTEGVFGIPLGVSSTFIFLFILFGAFLERTGLGQLFIDLANAVAGWAAGGPA
KVAVLSSALMGTVSGSSVANVVGTGSFTIPMMKRLGYKPEFAGAVEAAASTGGQLMPPIM
GAAAFLMAEFTNIPYARIIGAAVVPAILYYFGVWAGVHFEAKKSGLKGLSRDELPKLKNI
VLERGHLIIPLVAIIYLLVTGYTPMKAALWAIILSIVSSWLKKSTRIPPIEIVRALEAGA
RSALGVLAATACAGIIIGVVTLTGLGLKLGSVLVDLAGGRLVPTLFFTMLTSLILGMGVP
TTANYVITSTIAAPAIIMLLSRQAGLDPYAVAPASIILPAHMFAFYFGIIADVTPPVALA
AFAGAGIAKANPMKTGINATKLAIAAFLVPYIFVMNPQMLLFNVNAISFIWMLITSVVGI
ISIAAAVNGWLMTTTLWYERIVGFAGGVLLIYPGLTTDLIGMGLVAIMVISQVTRSRKAR
RVAASQ*
>SPBDM4_v1_50781|ID:27159144| exported protein of unknown function [Uncultured spirochete bdmA 4]
MKNTRKRSGILILIAVIAIVAILVFASHIRLVPVLIIKSDRGATIARLPLYDGQFIHHFI
HSVHKTPVDEYFEVHGTTLELTKVKYDTYGVGMPSDGGEAFTIENGRFVVTMHRTFSHIP
IRVSIVPDHGVIVGGTLFPFTRWVPQESAITLSAGRMLIFHNGRTSPK*
>SPBDM4_v1_50782|ID:27159145|bcsP| 31 kDa immunogenic protein [Uncultured spirochete bdmA 4]
MKKIAAIVFCLLLATSAFAQQQFITIATGGTAGTYFPLGGALADIWNKNIPKMNATAQST
GASIANINLLKGGNVDVIFTQNDVANYSYNGTELLKDQAYKDLRGIVCLYNETIQLVALE
SSGIKTVADLKGKRVSVGAAGSGTEANARQILEAAGLTYNDIKVQYLSFGESANNMKDGN
IDAAFNTAGIPTAAIQDLSVSRKMVLVPIDGAVAAKLMAKYPFYALQVVPTETYKGQTKE
VTTVAVKSMLAVSSKLPADLVYQLLETLFNNTDRLIAAHAQGKNVKLETALEGMSIPLHP
GAEKFYKDKGVLK*
>SPBDM4_v1_50783|ID:27159146| protein of unknown function [Uncultured spirochete bdmA 4]
LISFENWKRGFLITVAGQRLLWHTTKRPAVFVHRTGEMKSAWRGAGTCMSAIVLDGGTSF

AFSSKLVMREEFRGLGTFAAIFSLYFSSPDPEIDSLRLRFHDVPRAQWNGLGPRASHVVH
GRIELPSQVQTAAFALDGRFLQCQSTGARSLYFGDGSFVLTLDLPAEVHVGYAHNPAEAC
SLWHGWDQRVQSQRGGTGPLTHGASQEAVRIMSKDAAGLLGSLSFSGAEPEAFQHLNLSI
AKSACGQEDWKIADLMLCQPDLTLPPIFRREIPQGLNMSGNRMRAQNRYARIRSGFAQ*
>SPBDM4_v1_50784|ID:27159147| membrane protein of unknown function [Uncultured spirochete bdmA 4]
MTIVAAYFTALFGNFLLSLGFSLQKKHISWLSAKRRGEKVRRGEIIGWTSGFILMNMQPV
FNYLALGKLAPNIVAAISGSNIIFTILLSYFMLGEQIPVGKIPWIVLMTASLAYAGFVGQ
ESSQHFKADAFWIAFLIPACFAMLMLLASRRMRPKQVGIFLGSAAGALGGFQLLALEALR
ITHGADFLSWILSPYLYVYIFCGIASFFIKQVAFGRGKMTAVAPSFYGLLVLYPSIATYF
VSEVPLHVPQLFAFVGISLSIVLISL*
>SPBDM4_v1_50785|ID:27159148|ispF| Bifunctional enzyme IspD/IspF [Uncultured spirochete bdmA 4]
MSPSAPRPVRAPTLPIGRRAFALVTAAGLSTRYGGTKKEFEILDDITVLERAIEPFIEFC
SGIVVTCPIGAKPEFEAFFTKSTLMPSLSRLNAGFAIVEGGAARQESVRLGLRALAALGC
APDIVLIHDGARPWISRELILKTLECAQARGASLPLIPLTETPKIVEGEYIHTHPARTHM
MTAQTPQAFSFPEILAAHELALQEKYSATDDAMIWDHYVGPVFWVEGERQNRKITFKEDI
MNGSELRTGIGYDIHPLVEGRPLLLAGVRIENRRGEAGYSDGDVLWHAIIDAALGAAALG
DIGTHFPPDEPAWENADSTELARRALKMVKKQGWVIVNLDCTVVCETPKLSPYREQICAS
IAEILGLPSGAVSFKAKTKEGFDATGRGEAIEAQAAVLLVRR*
>SPBDM4_v1_50786|ID:27159149| Transcriptional regulator, CarD family [Uncultured spirochete bdmA 4]
MPNDQTVTFLVDQRVVYPSQGVGRIMEIREKIFNGQKTLYYIIYLEFSDMTVMVPVDKVD
ALGIRPLVSREEAETALNYISQAASTIPSDWKLRYQMNLDLLKKGSIMDIAAVVRSLYQR
SKIKELPILERKLYESALNLLQDEIAFALGRPKEEIVAIIRENLDK*
>SPBDM4_v1_50787|ID:27159150| conserved membrane protein of unknown function [Uncultured spirochete bdmA 4]
MVMPSMEEAKKDEEREPLTLGSLAFPILLVVLVIAGFLLKDKLAPLLSKRETMRTWVLSF
GAFGWLVFVSLQILQVVIFVIPGEVMQISGGFIFGFWGGLGLTTLGITIGSALNFLLGRL
LGPRFLMTILKRGQYEKLRQSADDARTFAGIILLFVIPGIPKDVLCYFAGTGSRSFLPFL
IASMIARMPGIIGTTFAGSAVYREKFGLVITLAVFTVILMIVLIAFRSQIEAIIKKHMQE
EKP*
>SPBDM4_v1_50788|ID:27159152| protein of unknown function [Uncultured spirochete bdmA 4]
MKRRERALHRRYWIVGILCSLIFHGLVIFLPLRFSSGRDALPRSLNIGLIGQIAPESRLE
TQQVPRNLKKAETESPAQKGASQQKPNPESSVNGTTADRKVVPESVAEEGPKEAPQTPHV
ASVLPAGPASVADVHAAEVEPPAGAQASLPVSAPVTQEPVNMQSLYEESAVLRELGSIAE
VPLAGQDSADSSDRMPKDALSKEASNSASAPPPVAARAEEAKKEVSPHDTAVPAPIHLEE
TAKLVETNTPAASRAPAAALASASREYFAPSGDLFVQARNLSGSSPSKVSVADSTSSPRS
DSAEAQAALHETVPLQSEQMAELAAERIDAYHTIRYQERAVSGISQVQSPQQNAGPRDPA
RHELTSQDQSEREVERLAEISPAVPPEENSAPFEIAAPAAQTALRPDAEMMPPAVSPVVP
PSATALGGTPPGERPLSDAVSDQSTPAQPPARQDTASSVAQLSLADSNPPHFDTAETLSR
RIIAALSAQKEYPVAALKRKTEGTVRLSLDVAPNGSLVMAKIQTRSGSAILDEAALRLVR
GIFPLSVRLASAVSLVVPVEYRIPK*
>SPBDM4_v1_50789|ID:27159151| protein of unknown function [Uncultured spirochete bdmA 4]
VLSATLTFDGEEPDKFRACTNKSPDGAKYSLDALASAAAGARLAAGVFVSTNFAVSSRWM
GAGTAVS*
>SPBDM4_v1_50790|ID:27159153| exported protein of unknown function [Uncultured spirochete bdmA 4]
MKKFLSKEQRSIFAVGLMLVLVTLTGCNLLLPTQGSTADVFAVDSKNGSVYEIDTDNAQS
ASVALVSTQQNSTGEMIIRGTKAFIAVGSYSNTSPGLYWFDLSSSNPTTELIGDKLSAQY
ICIVSDTKGYVSSSDYMGTYSNTVYSFDPSNPSAALGGEVTGFDSGFHPQDIAYVSDGGD
GRVFVTDNGNGKVYRLNAAGTAVELTFTTSAGGTTGLLSGEYDWDNNGSEDAGIFVANTG
GYDASWNALPGSIDFIPLDASGSSDITSVQTDFSVGRLAAFDTAHLIGTNYGGTWIIDLT
KSAGDSGRLTEIKNSQGESFGSLDVNMYDGYSYVPDGTNTVYRISSSGDVTAISVGKSGE
MITNVAVRE*
>SPBDM4_v1_50791|ID:27159154| exported protein of unknown function [Uncultured spirochete bdmA 4]
MQPSRPTRALLALCLFNLCFPAAFSQTVELPSVIVEGVLPMSDLAPSAGTTSIDHAAIEA
SAAPDLTTLLSSISGVIISPTGADGAQSTISLRGSTSNQVLVLVDGVRVTDPATGQTDLS

RLDIPLDQIERIEIQRGALSAQYGADAVGGVIHIHTKKKTAAPSIDISIQNNAFLPSSVT
VGSGFAATTVPFVGSALVDGQSVDISANMKNLSAWAKAERATNGYPYYDTNGQRRQRTNA
SLLATAGGFQGSFPLSIGELSTTARVNYRSMGVAGTEDSPTPEARQEDWNANLSTHFSTD
ALFAGALALDVNPYAQVGGIQYRESDTSGTDTHHSYRAGAESSFSWLPPWQGELKGGASF
RYDRLESSVVKASDGGPPQRFSAGTFVEPHFEIGKWTAIPALRYDATSDFPSGVSASLGL
LYDLAKDTVVRSSISSAYRAPSFDDLYWPASGGTEGDPDLEPETAFSGDLSVAHRDEQKT
WSIGAFGRYVQDVILWQPDASGTWKPTNYGNALYPGIEAEASSNIGTWQISGNYTFLYSF
VLSGNLTLADDKRVPYVPVHSASLSAVRSSRNFKSAFMFTYKSLRYTTTGNLAYLPATLI
ANAKLQWALSPRTDFELSFQNFFDERYEIVQGYPMPGFSVSAKFTMHIQGQKAPVQ*
>SPBDM4_v1_50792|ID:27159155| conserved membrane protein of unknown function [Uncultured spirochete bdmA 4]
MADKMHVWRFRRIGGMDQVVLKTGEDIAALSELDPKLWAALAMPTSQHRCKEALSLLDED
HDGRVRVPEILHAVEKFRMAFTTLDILFDENSAISLDRLKDGHLREACVNAAHIAGAEVE
GVDAAVVEKAISLYNERTFNGDGVVVPESAKDPHTAGIIETLIASGYQAIDSSGKNGVDN
VALDAFAHDANIVLEWNAKGESIRRTLPDLPFDAILPAFRTISDVMDDYFRRCAVLDLAG
TPDALKNLETQMAAALSTPLGNNAEALQALPLALPRADGLLALDAAYNPLYETEVKSFLD
AVKTPYGLEKELDKTAWSRIAADCRMYAAWTDSKPPVGAESIDPSMLSDILNGGSLDAIR
TLITRDLAEAAHAEALHELHNILVLKKDLLKILRNFVTMDEFYGSREGIFQSGRLFIDGR
ELELCLDVRNSTAHSSMAGLAGMYLIYCDITRTTGEKGSVVAALTAGDADRIYVGRNGIY
FDNDGLDWNATITKVVIQPISIREAFLSPYKWLARTIEEFTAKRAGTAEAGRQSQLKEFA
QKAVEQPTKAPQAAEQIAPKKIDVGTVAAIGVALGSIGVMITSILSLFIGMGVWMPIGIL
VVFLLISGPSMILAAIKLRKRDLSPILNAEGWAINGRLKLNLIFGAALSHLGNLPPNSIR
MLNDPYAPKKKPWALYIVILIIVVAVVAWLSGWLDPVFAWLKG*
>SPBDM4_v1_50793|ID:27159156| protein of unknown function [Uncultured spirochete bdmA 4]
MEAKKADFIESAAGQTVEVIYKVEPKRDILRQTIQVSPEEK*
>SPBDM4_v1_50794|ID:27159157| protein of unknown function [Uncultured spirochete bdmA 4]
MTYHREEICINNAGLVLCNPFLPRLFSLLQLTGENGFKDRSSAERAVLLLQYMLFESTDT
NEHEPMLNKLLCGLEVVSRSGLL*
>SPBDM4_v1_50795|ID:27159158| Single-stranded-DNA-specific exonuclease RecJ [Uncultured spirochete bdmA 4]
MKWTKKEIDPALVRTIAHRYQVDALTASIFVRRNLIEPEQIQFYLEDDLQLLHNPFLFTS
MEDAIDRLIMAREEEEKVLVFGDSDTDGITSTVLMTDALKNFGLEVFQKVPEGEEPYGLS
KAAVDSAEENGISLIITVDCGISNHEEVVYAQQQGIDVIIADHHHLQAQTPPEAIAVLDP
KLPDCGYPFSDLSGCGVSLKLAHALAIARLGMYKEPLALLYAGKTAEAETNSPAENGASE
TKSASSLVLEAVKLDNLIETSRLRLLSDSEGSFPADTLEKLEKFLRGRVIISWNKKEIND
FFRNQFNGSADLDVMDLSQLASTFWPGMAKSSFAELAQASRLKKYARGVHTAADTLKNIF
NAYVLQALQTQGLLTGRMFQLAALGTIADLMPLKDENRLIVRRGMEGINTAPTDGIRELK
LSLNLARPLGATEIAWQITPTINAAGRLGTPSLALNLLQADTIEHAIEAASKLVQANNER
RRLGTEGWEIIRDRLNESLEKSGGKFAVAGSAEIKSGITGLLASRAANMMKVPVIVAVFK
ANGTCTGSIRGGAAFPLTRLLAYCADLFLDYGGHDSAAGFTLKADQWQVFLDRLYEFMYR
TEFSTEEPEISIDAELPHAYVTPDLLHLCHHFEPFGEENDPLVFCSKKVPMVDAQVVGKN
GKNHLKLTLNFGTYKWPAMLWDGAERLERDFSFRNNDKVDILYKVTTNYWNGEERPQLEL
YDIHRTE*
>SPBDM4_v1_50796|ID:27159159|pdp| Pyrimidine-nucleoside phosphorylase [Uncultured spirochete bdmA 4]
MRAVDVIMKKRSGDPLNDEEIRFIIEGYVKGEIPDYQISALLMAIFFRGMTPEETAKLTE
VMLDSGDRMDLSGISGPFVDKHSTGGVGDKISLPLAPMVAACGIKVPMMSGRALGHTGGT
LDKLESIPGYFTSLELAAFRKGLIEDGFAMTGQTARVVPADKKLYALRDVTGTVESIPLI
TASILSKKVAEGAEGLVFDVKCGSGAFMKTFDDAKALAKSLVATGTAMGKRVVAVLTDMS
QPLGYKVGNFLEIEETIDCLEGKGPEDVMELTYRLGAWMLMLGGAAKDIDEGRARCAEAI
DSGAALDLFYRNIVRQGGSIGEMLARRGAWRSEFSREIRAEGEGFIESIDALKIGLAGVY
LGVGRNRTEDSVSPTAGFIFEKKRGAHVKKGELIAVAYGKDVESLDPALLLASGAISISD
TAPGSSPLILEEITAQ*
>SPBDM4_v1_50797|ID:27159160| protein of unknown function [Uncultured spirochete bdmA 4]
MAERNERYRGMFLHAKDTARGDRIVTFLCNEGLLSLFVFGGPKSSLRSAAMPFVSAEIEA
YRDKRSEFIKLTGVNILETYEGIQKSFSHMQTASGAAEFVLHTSAFGGEYGQALTMMTAL

LRELSKIDERHAVLALHVFLWKALMPLGLAPDISTCERCGRSFTGKGALPGRLLMGDRSF
LCAGCAEERRQQGFPASAMISFDAETLGCLHAVLQLPYGEAAHLMGSTRPSSNVMAIIES
LAESAGEGPLQSLRLFH*
>SPBDM4_v1_50798|ID:27159161| Peptidase S54, rhomboid domain protein [Uncultured spirochete bdmA 4]
MIPIGDDNSGRRGKPVVTWILIGLNILVFVFLQKFALDDYATLALAAIPEEILSGHRLFT
LITSQFTHAGFMHIIGNMLFLGVFGDNVECRIGKLRYTLLYLISGTIGILLQIFFAAIAG
GAALQMPLVGASAAISGVLAAYLALFPGNKVVVLLFYFIPTRLSAWLVIGFWFFLQVLGG
LSGLTSLQDGGTAYFAHIGGFLSAYIWARMYKRKELEHIHKWRRRNPSGASDGFNWWIVD
DN*
>SPBDM4_v1_50799|ID:27159162| HAD-superfamily hydrolase, subfamily IA, variant 3 [Uncultured spirochete bdmA 4]
MICALLFDLDNTLYSEASGLETGVAQRMNQFIANMFDLPVPEAIKFRREHAKPYGTTLEW
LMRERGFHEPERYFQYIHPEGEEDCLEPDHVLRLMLNEIHLPKAVLTNAPLEHAQRVLKR
LDIADCFTGVYDIRFNNLVGKPNPDAYLRVLRATGFSLKETLFVDDLPKYVKGYIDLGGQ
AVLKDEMNRFADFPCRRIKTVYEIAAVLEQLGLPV*
>SPBDM4_v1_50800|ID:27159163| putative ABC-type transport system, permease component [Uncultured spirochete bdmA 4]
MSGTAAVLADILKYTIVAATPLLFASIGGLFSELAGMLNIALEGLMGIGAFFGMAAAGLT
GNVFIGMLAAIGSAVAGSLLMGTVTIKFKANLFITGLAINLLAGGITAVLSQWWYHTKAV
VAFTLPPPVPAFPAALSQIPFVGPVLFSHSVLTVSAWLWAIVAAFIIKNSRHGIRIQATG
MNAQAVSSLGYSPDRARMAALVWSGVGSGLGGFALGVSVSAFVPNIVSGRGWIALVAIYL
GRKKPLWIAASCLLFALSESLSNYIQGFSSIPPLLVLALPYAVTLVSLIASGNKR*
>SPBDM4_v1_50801|ID:27159164| putative Glucose ABC transporter permease protein TsgB13 [Uncultured spirochete bdmA 4]
MPAERELHSPARVMLAVGVSIALSAVVLALFSKDFGSALEQFFLEPFSNRYFLGNYLADA
VPLMIAGLGVLIAFRSKNFNLGGEGQLYASALAAGVLALLLPAGTAPFAVWFLAALTGVL
TGAAIGALSGLLKAVRHVSEMISSFLISAIVINISDFLITGPFQDPASNFQTTRQIAPQF
LLPRILAPSKLDISIGLALVLIVAFMIVDKRTKIGFELKICGENEEFARYCGIPVAAYQI
AAMAISGGMYGLAGALLVIGPQGRVMRGFSSGLGWSAISVALLAQGSGAGIVPAALFIAF
LGTGSDHVMIGSGVPSEIISILQAAAMFFITARSLPRIWKKRGQS*
>SPBDM4_v1_50802|ID:27159165| putative Uncharacterized ABC transporter ATP-binding protein YufO [Uncultured spirochete bdmA 4]
MQPMTAVRMREISKRYLPSGVLANDGAELEVEQGSIHAIVGENGAGKTTLMKVLAGLELP
DSGDIEINGEPTHISSPAHARALGIGMVHQHFLMFDELSAAENIFFGLEPVQKPRLLRLL
GIIDSSGLNEKAKSLANEYGFSIDPSAPVGALSISTRQQIEILRQLAREMRILILDEPTS
VLTEQETEALFEKLAEIRHMGRTILLVTHKIDEVMQIADKVTVMREGKTIGTYSVASTSA
NELASIIMGGVPSEEIQIHKEHAIKGASVMRIEGLCTHRHHRGGVGLSEIAFDVHAGEVL
GICALSSNGLDELEDVLAGFLVPSAGRCAFGGLHFDVRKVHEYRRFLKEGKISYLPSDRM
RRAAALSLPVRDNFIALSRRNYFSRGWIRSTLARRATQEAIHAFDISAQPEQMTAELSGG
NIQKLAIARLFSKEQPELFILCEPTWGLDIRSTEQVHSRIREVQNAGSAVLLLSSDVDEI
LTLSDRVMVLYRGKQVLTRENSGYLNRALIGEYLLGTRSA*
>SPBDM4_v1_50803|ID:27159166|tpn| Membrane lipoprotein TpN38(b) [Uncultured spirochete bdmA 4]
MRRYIIACIITIVAGTALWAQAPRIGVFVPGAREGSPIYDNLAKGAERLASEIPGASVRV
IEAGFNQAEWEEKLTSFVASGKFDIVITSNPSMPELVNNISKSFPKQKFICLDGYLAGNP
NLYTALYNQLEQGYVTGYLAGLVSISGMKGTNQDKKIGMIIGQNYPVMDKMIIPGFAQGL
KAVDSAFQLDVRVLGNWYDAAKTADLSRSLYAGGADIILPICGSASQGSVKVAQETGKYL
VFFDDDEFARAPENILGCAVLHQEELAYRSLKAALEGTLPFGKADVVGMKEGYIEFLNKN
PSYIRNVPESIRKKVDAVIQSIKSGQLTFQVPSL*
>SPBDM4_v1_50804|ID:27159167| putative Mercuric resistance operon regulatory protein [Uncultured spirochete bdmA 4]
LTDQLLKIGELASLFGVTARTIRYYEELGLIEASNRTEGLHRRYPSDVIIRLKRIGELKS
LGLTLGQIREFFILCAEDPSGERCRLLLLQIYEDQKEEEMAKIREAQQHIARIEKNIVAT
KEKKSFFSCPGEECETCDLDGFCLDSVYNHDKE*
>SPBDM4_v1_50805|ID:27159168| Theronine dehydrogenase-like Zn-dependent dehydrogenase [Uncultured

spirochete bdmA 4]
MKAAVLIAPGKFNIEERPIPEPKENEVLIKVEACGICTLEQRLFSGAMRLPLPLVPGHEA
SGRIEAIGSKVVEEMVPGQRVALDLVERCGECYYCRIGKSNLCLNRYKDAARMLGGFSEY
IVVRPNQIFSVSDSVSYEEASFAEPLACCIHSLKRLKVAMTEDLLIVGAGTMGLLHLLAG
RSMGLRITVSEPNSARREMAAALGADFVIDPKATDISGFGRDITDGIGFSSCVVTSPSPS
ALEPAILAMAKAARINIYTAYEDVMHIPLDLNTIHRNELLITGTEGRGQEDFFQAVRLLS
FGKIDVNPLISARTSFSGLEEGFRIALSGDAYRVLLMHSML*
>SPBDM4_v1_50806|ID:27159169| Pentulose/hexulose kinase [Uncultured spirochete bdmA 4]
LILAVDIGTTRVKASLFDPEGRCAGLAAVELTESTSNSYNEIDARLWLRALSDISSQLLS
ESVRRELNAIVVSGNGPTILPIDARGEPLANAITWLDRRAQEEAKEASVALSYPLDAAFN
LPKILWLRRHSPQVYASSRFFVSCPEFVIGHLTGEWTTCLPNEGYTRIIWDAVALEALSL
ETEKFPPFAGIGEIVGRVRASAADEFGLPQGLPVVMGGPDFIASLLGTATVRPGRTCDKG
GTSEGINLCSEKDTGDAGALLVMPHVVKPYFNISGVISTSGKALSWFKSRFSPAEDFDAT
YRRVAATKPGAGGLVFLPYLAGERSPHWDPDARGVFLGLGLHHDLGMMERAVLEGTAFAM
REVLDVMETAGARVEDMRATGMPSLSPVWNQIKADITGKRVMVSDFSEPELAGCLAIGRF
ALGEESSLAAAAERVFVPAHIFEPHKEMAPMYDDLFAIYLESYRRLAGLFPAVSKLQRQM
EVPVRHVEEVEP*
>SPBDM4_v1_50807|ID:27159170| DhnA-type fructose-1,6-bisphosphate aldolase-like enzyme [Uncultured spirochete bdmA 4]
MNEGATLRLRHIFSEGGRAVVVAADHGAIAGPLRGIESPLELVRLCAQGGADAILAHKGF
VRAGLEGWGRGVGLILRMSGGFTTLGGRFEEELLYDVDDALMWGADAAAITVKFGHAREG
EFIKAAARLVSGCERRGVPVMIEAMAFEKGRQSLRADALAVALRAAEEIGASFIKAPMPE
NIEDFARVARGSHVPILLLGGEQRDSLQALFENVARALELGAAGVAMGRNIWGQPDALGV
LEAVVGLVHRGWGVEKALARVRNIA*
>SPBDM4_v1_50808|ID:27159171|pduL| Phosphate propanoyltransferase [Uncultured spirochete bdmA 4]
MPETKVIVNLSNRHIHVSHDDLETLFGKGYQLTKTKDLIQPGQFACAETVTIKGPKGQLE
GVRILGPERKETQCEILASDVFKLGVPGCPVRESGQLEGSFPFEVIGPKGSIKKERGLII
AMRHVHFDPEAAVRFGVVDKQIVALKVGTERGAIFLNVVCRVNAIYALECHLDFDEGNAV
GISSGVMGEIVQV*
>SPBDM4_v1_50809|ID:27159172|upp| putative uracil phosphoribosyltransferase [Uncultured spirochete bdmA 4]
VSKVILKAEDLDGYLNAGDLEYLSRMETLYRHAMVSFNILATSTSESQKHREEQTLIDLY
NRMGSLMQEICAAEPRIQVYSFVSPQETHGEVSRLIAKLRDVDTGRQEFVVYYIQRAFELL
FTLAFGGSDKASKNYLIVRTPVTIPVQNFAVHKIPNVDEAVRDTVMCVMLRGALLPSMIL
SKEIQEYSSSGYLTPFALFKIKRDDTKSETTMEYVLDLDHSYFDLAQLDGKHLLFADPMN
ATGGSLVTVVKYLLDQGVRPASVKFFNVISALKGSLRVVRAIDNITIYTLWMDPSLNERA
YIMPGLGDAGDRINGTDEDVYPRDMLRLVADYGTNIAGLYRSQLRIIEETVLRHR*
>SPBDM4_v1_50810|ID:27159173| putative NAD(+) diphosphatase [Uncultured spirochete bdmA 4]
MTSLPRRFAVFQGQDCLMPRHIAGHLQSSADGSAVSSLLPETVSWPASEIPLELFSGMPE
GYSYSAFAFENIVYGAVLLKDDEAHFVESAGLKNGVRFPVRNLALNFSSRFGRFVLFAKA
HAQWASVSRFCGACGSPLVDANGNDEVREPLDDHAHGARFCPRCKRIFFPRMSPAVIVLV
RKDNAILLEHNVRFPGNRHSLIAGFVEIGETLEEAAVREIREEAGIEVKNLQYVRSQPWP
FPDSLMLGFIADWASGEARPDGVEIDHLDWYTANNLPEMPMRGSIARYIIDTFAAGGFSA
T*
>SPBDM4_v1_50811|ID:27159174| Na+/glutamate symporter-like protein [Uncultured spirochete bdmA 4]
MDFSWKIIIDAGIISAGLVLATFLRSKIKFLQKYLVPNALTAGFLLLPVYNYLLPPMGYA
TNRLGDLVYHLLNISFISMSLRSSPPKIKGSRSNGGVLGMSAVILFGYASQAILGLLLTL
LFLPKIHPAFGLHLPLGFALGPGQAYAIGKGWESMGFEGGSTVGLTFAAIGYLWACFGGM
LLIHKGIRKGWMKPEQISIMTDKALLTGLIPRDREKPSAGRLTTDSEAIDSFSFHAAFVI
FVYLLSYLFLKAVTFFLGFAGKTGMELATNLWGINFIFSAIIAIIVRKIVDALKLGYMID
DDSLTRISGISVDYMVAGALGAISLVFVGKYWLPILLMSTLCGLMVYYTIPWVSSRIFRD
FRFERMLMLYGVSTGTLSTGLALLRVMDQEFKTKVSSDYMLSAGLTFVLALPFILAINLP
AKAYTTGSMTPFWMFVLLAVGYLIFVSVIYWILARKRRFATPGENFYRPGK*
>SPBDM4_v1_50812|ID:27159175| protein of unknown function [Uncultured spirochete bdmA 4]
MILKRRYHPTNFKFFTWLLRHTYGIWLRWTYRITAINAELFKTLKPPFIVVGNHTTLLDP

FIANSFVPFPIHWVASDGNMRNPIMRFLLLKLVGSIPKSKAIPDIETVNWIVDIIRRKRS
VVGMYPEGQSSWTGTPFPAFFSSAKLLRLLRVPVVLAKTNGGYLTKPRWSHVRRPGKVEI
SFSVLFTPEQLHKMPLVEIDVALNNALSYDDTAWCRQKGIQYKSSRGAENLELALYICPS
CGGKATMHGRGNVFACDRCGFSVEYQADGSFALNPEEPQRALISPYDALPFLDSIAVWDN
SQSAFLSKELS*
>SPBDM4_v1_50813|ID:27159176| Smr protein/MutS2 [Uncultured spirochete bdmA 4]
MIAVDSSQNKKARAACQAIFSSSPVKQIGFCDMYSGNMDFGKIYEQWEKQQTSRKKSSER
APGDKSGAEAGAFRETLEQWLEAHEPIDKDASTERRTGNDKSAKISAYTRFKRLQPQDEL
DLHGMRETEARLAVQSFIARCAAKGLEKVSIIHGKGNHSREEQVLVRVVREELERNPHAG
KFGFADNQHGGRGATWVRVRQAISRDK*
>SPBDM4_v1_50814|ID:27159177|infA| translation initiation factor IF-1 [Uncultured spirochete bdmA 4]
VAKEEAIEVEGIVKEALPNTMFRVELANGHIILAHLSGKMRKHYIRIVPGDKVKVGLSPY
DLNRGRIIYREK*
>SPBDM4_v1_50815|ID:27159178| Transcriptional regulator, TraR/DksA family [Uncultured spirochete bdmA 4]
MENAFIERMKNLLLAQRQEILDTIAESNEQLRTVLSETDPKDSADIASDDVDRIMIEALS
SQDLKRYRAIESALLRINQGRYGLCAKCGKKIPQERLEAIPYAVLCVECQKSDERKNR*
>SPBDM4_v1_50816|ID:27159179| protein of unknown function [Uncultured spirochete bdmA 4]
MLVRPDVRWSMGVISNGYSYALVMHSLQDACRRIRFTVLAAGDPAGIYFGDQVFLRGEVY
CLKCRVDIGWILLVEPSPICIILFYQIKVTDYIEPATFNHFGHILPIPPPNSSVIAIRCP
MANFKGKLLLRLIDGEEGFPGSGNVMDRANAVIEMERFIRGVVRHRYPGSVVGLKAQEYL
HPSWRIFLGERAHRRNIGFCTLPRQIPPRFERNRCMGRKAQMRIMMFESGIRHCAGVCLT
IAERRVSVKTKGHGPSYHSARIDATGALFYYEAMDEPAVRTIALLISDREKSEKIARFLS
RRKAGVKIFDQERTAAPGSAECVIAEAALLDSGGARELLDASVALIVLGNMGKSQKHLQN
SPLLRIMPARSSSTSLYGAMLQLCAVRDLIFASRTVVNAVQNQLRTLLSSVPDIVYILDE
RGNFIYLNESAVLLGYEPSELIGKHFSIIIHPEDKPNISRDIVVEKIRQANKFPDIPPKL
FDERRSGQRMTRDLEVRLVHKDGHVVYALVNAYGERNFDMPLLSDVVGQNPQTIGVVHDV
SAMHFYQQSLEDSLAAKERLLREIHHRVKTNLQLVASLAHLKQTDGASSASAEVLRELEA
QIKSIALVHEALYQSEQVDRVSAREFFAQFCHAAEEALETVGSTVHLRLSADDCSLDPDR
LVPLSLALLELLGGAYQTAYEKRAEVTVLITFRCADGKRRVLEVQGEGIMRKADSPIMRA
LLKHANADFEVIEGKSPHECCRLTIERQY*
>SPBDM4_v1_50817|ID:27159180|trxB| Thioredoxin reductase [Uncultured spirochete bdmA 4]
MSQLYDFAIIGAGVAGLAAAQYGARANLNTLVIEEMAPGGQALLIDNLENYPGIAEPING
YDLSDRMRTQAERFGSSLMDASASNLRKENGRFIIETSNGPVESLSVLVATGAKHRRLEI
PGEQEFIGKGVSYCATCDGPFFKGKRMLVIGGGDTACDEATYLSKLSGQIVMIHRRDRFR
AQKALAQRVLTNPAIEVRFNTIAKEIRGDTKLRSTVLENVVTHEIAEEPFDAVFVFVGSI
PQTAFLVDTGVQIDETGYIITDCTMQSSIPGLFAAGDVRATPFRQLITAAADGAIAAHSA
ANYIDEQRGQAYR*
>SPBDM4_v1_50818|ID:27159181| protein of unknown function [Uncultured spirochete bdmA 4]
MWVNDRIASCQKAIRRFLRERECECVRASEELHRCLLSPGQQSVFSVYIHLPEECAPSFD
RVDGLAIQDPDGAVCVLLPRSSQKDLELAARLKLRGNVYKIAGIMQDVRRLVLAVDEGEW
NNQSFMLMRARGEDIPFGVAEEAGGPTRGSRVVIRNATIEDLDALLSLHMAYEREELSLE
STQAETEMRMTSLLRQQIVAIAFCGDEPIGKINTNARGYFYDQIGGFYVKPECRSMRVGG
SLFFYLLHKIAAERRDPVLFVRHENVPAISVYRRAGFQPAGEYGMSTMSSTRRLS*
>SPBDM4_v1_50819|ID:27159182|rnhB| Ribonuclease HII [Uncultured spirochete bdmA 4]
MKRSTNFCTLDSVLSIPVELVCGLDEAGRGPLAGPVTAAAVILPADFPMALLDDSKRMSA
RRREDAYEEIVERALDWAVGWATIEEIERYNILGASLRAMERAFAGLTQAPALVVVDGIF
APNLWMNGTCVEAATMAKADGLVPAVMAASILAKVARDHLMDCLDTLMPEYGFRSHKGYP
TKAHRKAIERCGPSRFHRQGFQLLPQPSSLLFDSFESED*
>SPBDM4_v1_50820|ID:27159183| Phosphoglucomutase/phosphomannomutase alpha/beta/alpha domain I [Uncultured spirochete bdmA 4]
MPLITHPLLGLAVGDPTKIPLARETLPSPQEVQLAAHSLILSASGWRTICANASATASYA
SWDLDHSPENSLSRDLSPADTAIVAGMALVFSAFMKKLCKGRRPVVLLGVDTRPTGPAIA
DVFARVLIGGGCEVRYLFIVPAPEIMAYSAQTTSLPSGHEFHSDGFAYISASHNPPGHNG
LKFGIGGGVLTGEQIAPLITALKAFIANSEAPELALSAIEKTDLRELAACYGNVAHWKRY

SQSAYMLFAHRVFTDESELDAQEKMLSEIAAACAERPLGVIAELNGSARTQSIDFDWLSA
LGIQVKVLNAEPGVFAHRIVPENESLQDCSESLNEAHAENAAFALGYVPDCDGDRGNLVY
YSNALQGSVPLEAQQVFALVCLSELAYLRWKGETRPIAVVVNDATSMRIEAIARAFGARV
FRAETGEANVVSCAERLRSEGWVVRILGEGSNGGNITYPSKVRDPLSTIGSMIRLLRLGQ
AKKGNTCFSLWLEAIGAPERYRPDYGLDDVIASLPPWATTSAFEPYAALKTAAKDKIALK
NAYQSLFLEAWPQMLPELTDRLGIASWRAFATIGPREHEIGADFGASENGGLRIVLYDEQ
GASCAFLWMRASGTEPVFRIGVDIKGGTGADEAWLRGWHTNLVTQADLLVAHSQNVVG*
>SPBDM4_v1_50821|ID:27159184| conserved protein of unknown function [Uncultured spirochete bdmA 4]
MIGVFEHALFLLRGVKTYALVGSSGTGKSFRAKLVAQKYGVDLIIDDGLLIRGDQILAGR
SAKKDPTYLGAVKTALFDDREHREQMSRALQHERFRKILVIGTSERMVQKICERLQLPHP
IKVIKIEEIATKAEIEKAVQSRKVEGKHVIPVPALEIKRNYPSIFYDSVRVFLKRSFGVG
ATSPKLYEKSVVRPEYGKRGRVAISEAALSQMVVHCVNEFDPNVRIMRLAIRNDTQGYRI
TVMLEVPFGTRLASKIYALQEYIVDSIENFTGILVAEVNIVIDRLAARPQKEQKWSRAID
AHRQSV*
>SPBDM4_v1_50822|ID:27159185| protein of unknown function [Uncultured spirochete bdmA 4]
VNQNLHENKRLSLATKGQPPFRTIGAARLFAGFMPVQSSFLFSLVRRYFLELSLSTTGHL
YSPWKRLLQNYC*
>SPBDM4_v1_50823|ID:27159186|buk| Butyrate kinase [Uncultured spirochete bdmA 4]
MPFIVLVINPGSTSTKFAVFSGDTCIFDQSLSHSVADLAGFPTVASQYEFRERAIRHALE
ERHFDLKTMNAVVGRGGLLHPIPGGVYRVTEAMKSDLLSAQYGEHASNLGGLIADAFARQ
LGVPAFIADPVVVDELEDVARVSGHRLFKRISIFHALNQKAVARRYAKEHKCRYEDLNLV
VAHMGGGVSIGLHRGGRVVDVNQALSGEGPFSPERSGTLPAGDLAKLCFSGRYTQQEVLK
MIAGKGGMVSFLGTNDMREVSRMREEGNREADLYYRAFVYQVGKSIGGAAAAAHGVVDAI
ILTGGIAFSKDLTDRITEMVSFIAPVTVYPGEGEIEALALAGRMALSGETEIKEYLP*
>SPBDM4_v1_50824|ID:27159187|ptb| Phosphate butyryltransferase [Uncultured spirochete bdmA 4]
MNHISEIIEKAKTLGRRKLAVASAQEASVLEAVADAYREGIAESILVGDTAAIAAAAREA
NNGKGVDISPFRIVEESNPNAAAAEAVALVRSGEADFLMKGIIDTSLLLRAALNKETGLN
AGRLASHVAVMEVPTYHKLFVITDAALNIAPDLPAFVDIINSAVMVANALEVKTPKVALL
AAVEKVNPDKMPCTVTASILTQMNRRGQIKNCIVDGPLALDNAISAESARIKKIQSEVAG
DADILVTPDIEAGNILYKCLLDLAGAKGAAVIMGASVPMVLTSRADTAETKLASIALASL
LGSKGLRA*
>SPBDM4_v1_50825|ID:27159188| conserved protein of unknown function [Uncultured spirochete bdmA 4]
MAIKGSVEIDRERCKGCQLCIRACPTKVLGADTEPNSWGYFPAKAIAADKCIACGNCFAV
CPDVAITVYKL*
>SPBDM4_v1_50826|ID:27159189|vorB| Ketoisovalerate oxidoreductase subunit VorB [Uncultured spirochete bdmA 4]
MAEEIRLMKGNEAIGEAAVRAGCRAYFGYPITPQNELTAYMAKHMLAKGRIFIQAESEVA
AINMVYGASSTGARAMTSSSSPGVSLKQEGISYLCGADLPAVIVNVARSGPGLGGISPSQ
GDYFQATRGGGHGDYYTIVLAPKGVQDAADLTYEAFSISEKWRVPVLVLADGLIGQMMEG
VVLPEPIDPATLPRWQWAVGHSVEMGRPYNHVSSINLVPDELEAANRKRFARYGQIKKEL
LRYEEIEVEDADVVFVAYGTSARVSHGALEMARAKGMKVGLFRPVTLWPFPYARLLELAE
KGKRFLCVEMSMGQMVEDVRLAVNGKAPVYFYGRCGGNIPSQEEVFAEVARLMGK*
>SPBDM4_v1_50827|ID:27159190| 2-oxoglutarate synthase [Uncultured spirochete bdmA 4]
MELVYGHPKSLKPIQTKYCPGCGHGVIHRLIAEVVDEMGLQTKAIITNPVGCSIWADLYF
DFDSVQPAHGRTPAAATGVKRMLPDHLVICYQGDGDLAAIGTAEIIHAANRGEKFTTIFV
NNAIYGMTGGQMAPTTLVGQHATTAPEGRDPIVAGMGYPIRVCELLSSLEGTRYLARGSV
NNMVNIRKTKNYIRKAFEAQMRGEGFTMVEILSQCPTNWQMNPVESVEWLEKNMITFYPL
GEIKNTLSTAPAPAQAH*
>SPBDM4_v1_50828|ID:27159191| Pyruvate/ketoisovalerate oxidoreductase, gamma subunit [Uncultured spirochete bdmA 4]
MTEKTFMAGFGGQGIISLGQLWVYSAMKEGLKVTFFPFYGAEKRGGIARASVIVSDSEIK
SPLVTSADSVVVMNQDSVEIAEKVCKEGGTIFVNSSLVKTDPARPDAKIVHVPCNDIALK
LGDVRIANMVMMGALGKVTGALKLDKLESILKGFFPARKHSLISLNIAAMEEGKKAVT*
>SPBDM4_v1_50829|ID:27159192| Glycosyl transferase family 51 (modular protein) [Uncultured spirochete bdmA 4]

MKIHSSAGGKNTERPKPKRPIVRVLRIVLLSILIEHGVYIGITSALIFVYKFANPPTTVL
MIYRSLVSHWKVQKPIPLTTAQIPTYVRRMLVSVEDGKFYEHHGVDMEAFKRARELNQKI
GQPMYGGSTITMQVARTLFLVPDKSYVRKYLEVIAALELELILSKNRILELYFGYAEWGE
GIFGIERASRVYYGKGIASLQVDQTARLIALLSSPIKYKPDSLYRSRILRQRYEFLVQRY
VAPIAETTLAPDNPPPGGLEPSADIETPEDVFSSEPAENGQEILPAPETSTQEVTPQETT
PQETTPQEEMPQDTVPPGVLQ*
>SPBDM4_v1_50830|ID:27159193| conserved protein of unknown function [Uncultured spirochete bdmA 4]
MARHKRRASIMSAGMDKSPENNPVDGQNAHGCHTLTIWADADSLPKDIRPFLIKRAWTRR
DYEDIRVLVQFVAARAPADIPREFLVLVEPGEGAADRYIEAHAAPYDIVVTRDIPFAERL
VGRSVHAMNDRGDTFTTENIAERRSLRDAMVSLRSAGLAPPSPKNSQRTQTETKHFADAL
ERLVVRAVRGKKRNDQD*
>SPBDM4_v1_50831|ID:27159194|manA| Mannose-6-phosphate isomerase [Uncultured spirochete bdmA 4]
MQKDHQRLPCLAHLENAVQQYAWGDTQGILPFIDAHAGPGYPIGEVWMGSHGRAPSKLRL
DAGSLPLDELIRQAPRHWLGDRVADQYHELPFLFKVLAAGSPLSLQVHPTLREAREGFAK
EERAGIPLSAPERTFKDSNHKPELAVALTPFQAMAGFRSPGEISALLGPDLCDAFNFKGQ
KPEELRSFVRQVFSIKGDAYQTYETGLRVRSEGLLHSNSGKEREAAAMVLDLQSLYPHDP
GQFGPLLFNILNLEQGEGLFVPAGVIHAYVKGSILEIMACSDNVIRAGLTIKYIDVDLLC
DILKPHAKPALIRPEYERFDWGSRAVYATPASEFRLECLEVQANSPSTGISYSPFGPEIL
LCTEGGFHLRAEEELELRARTSCFVAGSCAKITLEGQGKVWRATSGGQAS*
>SPBDM4_v1_50832|ID:27159195| Helicase c2 [Uncultured spirochete bdmA 4]
MGKKHGAVVDAGTRFSSETQDKLRDAILGMEGREVFAIGSLDASGMVQNLEIVARGTEGA
VAAPFRYRTHAQVLIHNHPSGTLFPSDADIAVAAEAGAEGIGSYIVDNEVSQVLVVAEPA
RFKTIRPLNAEEIAAVLDTDGKLSRVMPEFEARASQVDMAHDVTQVISDGGILVAEAGTG
VGKSFAYLIPALAWAIGNSERVVISTATINLQQQIFKKDFPIVSSLFKKPAKAAVVKGRG
NYLCRRRLYEAIEEDALFSDSSIKLRQILEWDNNGGSGDKADLSLPDDDPIWNRVCSEGD
YCLSSRCPHHDKCHIMRVRLEAASAQLIIANHHVLLADLEAKRTREGSLNTILPSYQALV
LDEAHALEGSATGLFSETFSKRSVQRLLARLFRRKKRAQAGILPSLYRLPDVPPAFIDTA
RSQAEKAGAAVDAFNAAAIAAFPEKESSLLVRSLNGLNRTAFLSALQNLERDVVMLVTRL
DEIFEAISPELEDEESVIDLRLTMRSLQEAAALIPRFKTPEAEPGTIFWLQIDHRNPRET
IVICTATPLEVAPLLAERLFSKIRSCICTSATLTINGSFRWWRSRAGLPGKHVEATYLSD
TMSEEPSLLQNTHILEKSYPSPFPYSRHAMLAIDTSAPLPDASQFQSYLNDAVTKLLHAS
RGRALVLFTSHKVLRDTYDAVAPLLEKEGILALRQGQKDRYSLLHTFIHDISSVLFATES
FWEGVDAPGETLSLVIITKLPFRVPTDPIQSSRAAAIDARGGNSFVEMSIPEAVILFKQG
FGRLIRHSNDRGVVAVLDVRIAKKTYGNLFVASLPKCRLETGPLDQICTSVAEFLDDER*
>SPBDM4_v1_50833|ID:27159196| protein of unknown function [Uncultured spirochete bdmA 4]
MARYVQPSWRSMPAEDSYRHSRNEEKDSYVRALAKLEQEDEEISLPSERGPIVFRDGVFQ
IDMSRVEEKGDIDVELKKLIDSIMGSKEGT*
>SPBDM4_v1_50834|ID:27159197| putative ROK family protein [Uncultured spirochete bdmA 4]
MTQEEQHPERAHLIRERNQRLVLRLIFRLGSLSQSKAVQCTGLKAPTVFRIFSSLEQRGL
IEAVPVPDAYASKHRRKGRQPVYYRIRANAAYVVGVDFWARSAAAIIQDFAGNLIAERTV
CFASPPSAEGALELIANLIRDMLAESAIESDKLVGIGVGAPGSVRATTGVVQYYARIPGM
TEFPLAQRLRKRFSVPVVVTNNASIVALAEHRYGQAYGVSSLFLFLVRAGVGGAFIQNGK
LVSDRSRTAFEVGHLSVDPAGPACSCGNRGCLELYLNEDTVCSALSHIGSCTGIEDAERI
LSENRRGADAALAPILEASTHAVRDIRRLLAPEAVVIVTRSKCLSERLAKDASVDFMRND
QRFGPPEAKIIASEYNPTLACKAACDLIYEEYFAHGYEFRNSGMTAE*
>SPBDM4_v1_50835|ID:27159198|exoA| Exodeoxyribonuclease [Uncultured spirochete bdmA 4]
MIKSYSWNVNGLRACAQKGLLEWVEASDADFICMQEIKLQEPQLTEDLRHPVARSGVRYH
SFWAFAERKGYSGTGFYALREPVSVSGLGIPEFDLEGRAVVADYGDFVLVSAYFPNSQEA
GARLDYKLRFCDAMLKFCDRQRVKGKHVVVAGDYNIAHTPIDLARPEQNEGNAGYLPEER
AWMDQFVSEGYIDTFRWFCKEPGHYSWWSYRAPKAREKNIGWRLDYHCVDPEFAPALAAA
DIHPEVTGSDHCPVSLFLDI*
>SPBDM4_v1_50836|ID:27159199| Peptidase S54, rhomboid domain protein [Uncultured spirochete bdmA 4]
MRIRYNAPVTLTFTLLAAIVLILSQTIAPNIILSFFSTPAPFRTNVFIDYIKLFTHVLGH
ENIQHFISNFTMILLLGPILESVYGSGFLLLSILITALATGLSNALLFPHVVLLGASGVV

FMMIMLSSITNFSKGEVPLTFILIMIVYLGGQVWDALTKQDNISQFSHIIGGLVGSALGF
YHKK*
>SPBDM4_v1_50837|ID:27159200|kbl| glycine C-acetyltransferase [Uncultured spirochete bdmA 4]
MYGRLKEELTQKLDTIRADGTYKDERVIVTAQGASIRVSDGKEVLNFCANNYLGLSDDSR
IRAAAVEAMQKYGYGLSSVRFICGTQAQHKELERSVADFLGTEDAILFSSCFDANGAVFE
PLLDEDSAIITDSLNHASIIDGVRLCKAKRWIYKHADMRDVEETDPETGKSLKGLERCLK
EARGSHVIMIATDGVFSMDGDIANLNAICDLAEHYDALVMVDDSHATGFMGAHGRGTWEH
CGVAGRVDIITTTFGKALGGASGGVIASRKEIVEYMRQKGRPYLFSNTLAPAIVGGTLRA
LRILTESTELRDRLETNTKLFRGLMTEAGFDIRPGEHPICPVMLYDEKLAHRMADDLLDE
GIYVIGFSFPVVPKGKARIRVQISAAHSETQIRAAVGAFKKVGKKLGVI*
>SPBDM4_v1_50838|ID:27159201| conserved protein of unknown function [Uncultured spirochete bdmA 4]
MLFRDRCDGTRVTGQHALNALMPYMMRGRNESAVYYEKEINIENALAYLKALKMTQEEKA
DQDRFTLFGLIITAALKTMALYPRLNRFIHRRALYQRKHMTFSFIVKQSFDPDAPEVNAK
IFFEPDDSFQTVSSKINAAILDAREHGEGDGERFANIFHKIPGGKAILMALYRFLEHFNI
APWSLIRMDPLYSSIYFANLGSIGLNAPYHHLYEWGNTSIFMVIGKMFSKELWHGGVRTR
QRYIDVRLTLDERIADGYIFAEAASAFYRMLSNPKLLELPLDMLRNALG*
>SPBDM4_v1_50839|ID:27159202| Ring-hydroxylating dioxygenase, large terminal subunit [Uncultured spirochete bdmA 4]
MIPNQWYVVASSNEVSIRPVGMTRFGEKLVLYRTSSRFLVCLSDRCCHRGAALSKGKVCN
GDQIQCPFHGLEYDPSGKCTVIPANGRSSPVPSNFKVRSWPVYEAHGFIWVWYGTDLPVP
EIPEFFDDIPDGARYATVADHWKAHYSRVIENQLDCVHLPFIHHNTIGKGNRTLVNGPAV
EWISDHKFFMYVYNEVDQGQKPKKPNEVSVPSPSGYKIEFLYPNLWENRIADKVRVLAAF
VPVDDENTLLYLRFYQAFARIPLIGNLVARAAMPFNVYVAHQDRRVVQTQEPKASALKIG
ENLFQGDFPILEYRRRRQELQDKGRGWGPLKHLGRPSRN*
>SPBDM4_v1_50840|ID:27159203| Cupin 2 conserved barrel domain protein [Uncultured spirochete bdmA 4]
MIIKASERVFETRHEMRGGKGEVSLAQLPGDALAPHLRLLSELTIPPGAGIGTHAHSGET
EYFYILEGIGTVNDDGAIAKAQPGDVVVTGGGAIHNIENHTNAPLRLIAVIVTEA*
>SPBDM4_v1_50841|ID:27159204|racX| Aspartate racemase [Uncultured spirochete bdmA 4]
MNPILPAGKGKNLDYYMRDLETICTEPVVVYNSAMGNGVIGVLGGMGPDATAAFFSMLVR
LDVAGSDQDHVHIIVESDPSIPDRTRCLLEGGPDPLPAMLASARRLVAAGADIVGIPCMT
AHAFLPRLRRNCTLPILSAFEEARKALDAFSPPIRSLGILGTMGTRRARLFETTMPDKTI
LWPSEEDHRSLVMEAIYGTHGIKAGNLGDEPQRLLVKAAHLLIDRGAQAIVAGCTEVPLV
LSQKDFDIPFIDPMHFLARALIAAARKGVPE*
>SPBDM4_v1_50842|ID:27159205| Sodium:dicarboxylate symporter [Uncultured spirochete bdmA 4]
MAEKKKRGAFDWYFKTNLLARILIGLVLGAIVGIILGFFPSSVKPFVDNSRFFGDLFIRL
LKMIVVPVVLFSLIAGAASIAPSRLGRVGIKILVYYLLTSAFAVIIGLVSANIFQPGAGF
NIIGDAAVQGKQAAPPTIAQILLNIVPTNPFESIMKGDVLPIIFFSVVFGIGLSYIKDSK
QQALAKSGAVLLDCVNAAAETMYKVVRGIMQYAPIGVFVLIAQVFAQQGPKAIGPLLMVT
LAVYIGLIVHLVVTYGGILSVYKLGFGKFLKGANEAMITAFVTRSSSGTLPVTMRCSEEN
LGVPRTIGSFTLPLGATINMDGTAIYLGVCAMFIGFATNQPLTLNQQLTVIITATLASIG
TAGVPGAGAIMLIMVLESIGLKITEGSSVASAYAMILGIDALLDMGRTSLNVTGDLVGSS
IVAKTEKELDMSKWK*
>SPBDM4_v1_50843|ID:27159206| conserved protein of unknown function [Uncultured spirochete bdmA 4]
MRLPSEGSILELVAVLPMNIRGQGIAWDRSDRGVIYGIIRATSQEKAAGGNNKVTVFRLE
DRASK*
>SPBDM4_v1_50844|ID:27159207| putative tagatose-6-phosphate kinase (Phosphotagatokinase) [Uncultured spirochete bdmA 4]
MALHHATFLCYILYMLQNKDIPRTEIFTGTSYGLSQIDKSPSFLCVCLNPVIQKTLVFKE
IAPGEVNRTANWRIDAAGKGIIPTRILTQLGEKALHLTQLGGPTREWFLSMCAEDNIAIE
WVESGAPIRFCTTLIEEAQGHATELVEESLPVAPCTSDALVDRFTSRVKAATAVLLSGTV
ATGIAPGTMARFARLASEAGARLYLDIKKQDLLECLKFHPVCVKPNLEELAQTLGIPYAE
VRDEVPARALVTETGKRFFGEYGTYLVVTRGARPTLYWNGQTLCEEPVQPVAVKNPIGSG
DAFGAGVARTLERGGSIDKAVREGTRLGGLNAAQLKPGTILS*
>SPBDM4_v1_50845|ID:27159208|argG| Argininosuccinate synthase [Uncultured spirochete bdmA 4]

MKKKIVLAYSGGLDTTVIVPWLKENYDCDVIAVCGDVGQEADFEAIGKRALASGASKFLR
LDQKEEFVKEYLWALVKAGTPYEKKYLLGTSAARPLLAKGLTEVALAEGADAIAHGCTGK
GNDQVRFELGVKAFAPEMEVIAPWRIWDIQSREEEIAYLEARGIPVPMKKSDSYSRDDNL
WHISHEGLDLEDPANEPHFEGMLKMCVTPEKAPDKPEYVTIGFENGVPVSVNGERLDPVS
IVRKLNKIGGANGIGIADLVENRVVGMKSRGVYETPGGTIIMEAHDRLEQLCLDKKALSF
KMTVAQRFAEILYEGEWFSPLSKALCAFVDSTQNVVAGQVTLKLYKGNLIFAGAASPYSL
YEASLASFTTGPLFSHKDSTGFINLFGLPTKVRARLGERLKKFGIAAGPDVVKPGSSGYT
APAGD*
>SPBDM4_v1_50846|ID:27159209|argH| Argininosuccinate lyase [Uncultured spirochete bdmA 4]
MSRLWQTGEGEGLDPAVEKFLSSIEVDSRLVFEDIECSQAHAIMLGEQGVIPTQDASTLV
DELAKIRKELEAGLLQVDPGAEDVHSFLEDELTRRLGDVGKSIHAGRSRNDQVAAAFKLH
VRGACRRTRGLVQKAIAACLDVAEENIETLMPGYTHLQRAQPVTLAHHLLAWCAGLERDA
GRLADAAARADESPLGAGALAGSGLSVDRERTAGLIGFARVSTNTMDAVADRDAAIEYAA
ASATLMVHLSRACEDIVLWASSEYGFAKLSPRASTGSSIMPQKRNPDPAELIRGKAGRIF
GNLQALLVMEKGTPYAYNRDLQEDKALFFEIEETVNGALGTFCVLVKSLEPDVARMRAAL
DEGYLEATDVAEFLVKRGVPFRTAYQASKLLVARCIEEKKSLRDITQADCTVHEAFGRAG
VGAAELVQYLEPQACVARRMQTGGPAPLRVREQIERLRTWLASAG*
>SPBDM4_v1_50847|ID:27159210|yugJ| putative NADH-dependent butanol dehydrogenase 1 [Uncultured spirochete bdmA 4]
MDDFVFQNPTMIYFGKSHETEVGRIASQYGRRVLLHFGGQSAERSGLLPLIRKELAGAGL
TYFELGGVQPNPRLSLVYRGIELCRENKIDLILAVGGGSAIDSAKAIAAGVPYSGDVWDF
YTGRAAPEKALPVGTVLTIPAAGSESSPGSVITKEEGLLKRAVNADCLFPRFSILNPERA
TTLPSAQVANGVTDIMCHLMERYFTNSRPVEFTDRLIEATLRTVIASAPRVLANPTSYDD
WAELMWAGTIAHNNLLNTGREGDWASHDIEHELSALYDIAHGAGLAIVTPAWMKYALKHD
VARFAQYAVRVWGAEDNYFDPERVAREGIARLEAFWRSVGQKVRLSEIGIDDSRFTEMAR
KCTDGDTHSVGHFVPLHSKDIEAIYRLAL*
>SPBDM4_v1_50848|ID:27159211|yggP| putative dehydrogenase [Uncultured spirochete bdmA 4]
MKTKAVRLYGVNDLRLEEFELPPLKDDEILARVVSDSICMSSHKLSMQGDAHKRVRAPLA
QTPVIIGHEFCGEIVEVGKKWAYKFSPGMPFAIQPALNYKGTLWAPGYSYQYIGGDATYI
IIPNEVMEMDCLLEYKADAFFMGSLSEPVSCIVGAYHAQYHTKPGSYVHEMGIRGGGSLA
LLASVGPMGLGAIDYAIHGGIKPARIVVTDIDQVRLDRAQKLISIESARAEGIELIYVNT
KDVADPVATLRDLNGGKLFDDVFVFAPVKPVVEMADKLLGHDGCLNFFAGPTDPSFSAMF
NFYNVHYESHHMVGTSGGNTDDIRESLALMAKGLLRPESMITHVGGLNAVADTTINLDKI
PGGKKLIYTHKKLDLVAIADFAERGKTDPLYVALARITAQHDGLWCKEAEDYLLENAPEI
*
>SPBDM4_v1_50849|ID:27159212| Carbohydrate kinase, FGGY [Uncultured spirochete bdmA 4]
MTDAIAVLDIGMTNKKVVLYSTELSMIAEKRRIFPPLVREGLEIHDLAAMEEWFLQVLRE
YSREFAIQALAVSTHGATFVCTDERGNPVAPCIYYTHEPSREFHDRFFALAGDRKELQAR
TGTPDLSALINSAKGLLFLRERYPEEFASARWILPYPQYWGMRLTGKPSAEGTYIGCHTF
LFDWQQESYSSVADALGIRDKLPLPIQQAWDVLGTIAPEVAARTGLSPGTPVTLGIHDSN
ASILPHLASSGGRDFVLNSTGTWCVLMHPVEKYGFEPDELGKVVFFNRSAWNTPIKTAIF
LGGKEYETWIQIIAVLQAKAPKNLLQPTEEDYERVLKERKYFILPEIVPGSGQFPGSAAR
AVEGSLQCTLSDVEQGKNIPSFLRDPKTAQAVLNISIVLQTEVALHRTGLKKSADILTEG
GFRNNTDYNRLLASAFPDNSVYLTDLQEATSFGAAMTALAALKSIDPMEFSRLIHIEKAL
VPPMLPSAALNAYRDAWRALI*
>SPBDM4_v1_50850|ID:27159213| Short-chain dehydrogenase/reductase SDR (fragment) [Uncultured spirochete bdmA 4]
VPLEPLDRRYRTAIQAPGDESLLASAARASVVRGKIAFVTGGAQGFGAEIARGLAQSGAV
VWIADINLPAARSFAQKLAAETDSPAVFAVDIDVSSETSVQRAFETVAQTTGGLDLVVSN
AGILKAGSILDQPVGDFRLVNEVNYTGFFNVAQHAARLLRLQWLGAPDWHTDIIQINSKS
GLEGSNKNGAYAGSKFGSIGLVQSFALELVEYRIKVNAVCPGNFFDGPLWSDSQRGLFVQ
YLNSGKVPGAKTVEDVRAYYEAKVPMRRGCYGPDVLKAIYYLVEQVYETGQALPVTGGQV
MLH*
>SPBDM4_v1_50851|ID:27159214| protein of unknown function [Uncultured spirochete bdmA 4]

MKTTVSPSSIAPILRGLILSEASFSCMVKVRAEDQPCPKESLRFVADGILPNAEIDDGEL
AAALKKALSPH*
>SPBDM4_v1_50852|ID:27159215| conserved protein of unknown function [Uncultured spirochete bdmA 4]
MNDLNDAKMIVADAGRPPERRLEALAEAARALGRPEKRSGESNNHIHTCYSFSPYTPSGA
ALAARVAGLDVAGSVDHDSYAAAGEMRAACALLGIGAVTGFELRVSLKAAARQFPEKTAA
LLTGRKLNNPDSTGIIYMTIQGVPASARMEVETFLAPVRLARFKRTERMVERANSLLSSF
GLQRIDFEKDVVAASKFAEGGTITERHLLAAVSRSILDSVGPGPALPTWLAGTLGLRLSN
AQRRMLSDPANPYLMYDLLGTLKAGFLDKIFIQPEEECVDVRDAISFALRVGAIPAYAYL
GDVAESPTGDKKAEKFEDEILDDLMPILRDIGFPAITYMPPRNTVAQLLRLQELCGRYGF
MEISGVDINQPRQSFNCPELLQPEFRHLDDATWAMVAHEALSAVDRRFGLFSNENPLSHL
SIRQRIGIFARAGREIDFFDPSSLEKRASTLFQEKNA*
>SPBDM4_v1_50853|ID:27159216| Pyruvate dehydrogenase (Acetyl-transferring) [Uncultured spirochete bdmA 4]
MPKSLPIEPNEVRKSGTLKIAEIPINQYRRNFEQEKERFGEVGLLAMLHDMIVVREFETM
LNTFKTMGAWEGIEYNHKGPAHLSIGQEAAVVGQAAALDPDDFIFGSHRSHGEILAKCLS
AARKLDDAKLVPVMEAWLGGETFGYAKRMPHSDTFSLARNFIVFGTLAEIFARKAGFNRG
MGGSMHAFFLPFGSMPNNAIVGGSADIALGSALFKRINKKPGIVIANIGDASMGCGPVWE
AMTMASMDQYRTLWPESAGGAPPILFNFFNNFYGMGGQTNGETMGYGVLARVGAGVNPDA
MHAERVDGLDPLAVADAVLRKKKILLEGKGPALLDTITYRISGHSPSDSSSYRTKEEIEE
WQNNDSIAEFSKYLMENGILGEAELGTMREGIHRTLFEAVKLATNEDACPRADGRFIESV
MYSNKKVLKLEDRASEMLQAGDDNPRVQSIARRARFALDADGKPLPKNKVYQLRDGIFEA
LLYHFKQDPTMAAWGEENRDWGGAFAVYRGLTEALPYPRLFNSSISEGAIVGSGVGYALS
GGRAVVELMYCDFLGRAGDEIFNQASKWQAMSAGLLKMPLVIRVSIGNKYGAQHSQDWSA
LVAHIPGLKVYFPATPYDAKGMMHLALSGTDPVVFLESQLLYDMGEQFEKDGVPQDYYEI
PEGVPAVRKAGKDITIATYGATLYRALKAAQILEEKYGVSAEVMDLRFIAPFDYEPLVES
VRKTGRLVLASDAVERGSFLHTIASNVQGLAFDWLDAPVAVVGSRNWITPAAEMESIYFP
QPEWIIDAIHERILPLRGYTPSTAQTSQEIARRYRAGV*
>SPBDM4_v1_50854|ID:27159217|lpdA| Dihydrolipoyl dehydrogenase [Uncultured spirochete bdmA 4]
MAHYDVVILGAGPGGYLAAERLAAEKLKVALIEKTDVGGTCLNIGCVPTKTFLNSAKLYA
HAREGTAFGVETGDVRVNWPRVVAWKDEVVTRLRRSLELTLKKGGVEIVRGQGKLTGAHS
VAVENGASEPVLLDADSIIVATGSSPIIPPIPGVKGNPAVLDSTGMLSLKDIPQRLCVIG
GGVIGVEFASLFSSLGSKVTVIEMMDEIVPVMDKQMAMMLRNALKQVAFMLGTKVTSVDG
RNVVYETNGGEKGSLEADAILMSVGRKANIEGWGAQETGLEIKNRAVATDAHQRTNLPGL
WAIGDVTGKSQLAHAAYRMAEVAVADILAARRGERSVQEFVPETVPWALFSLPEAAGVGY
TEHEAKAKGYDIDVIRTPYGVSGRFIAENGFSAPGSIKIILEKTSERILGVHMLGAYASE
HIWGAALALERKLPLSALQNMVFPHPTVSEVIREAAWSAR*
>SPBDM4_v1_50855|ID:27159218|pdhC| Pyruvate dehydrogenase E2 component [Uncultured spirochete bdmA 4]
MATKVIMPKLGNTVESSVILSWKVKPGDHIDKDAVLCEIETDKATMDVPAGVEGTVLALL
HKEGDDVPVLEAIAVIGNQGETWQEGPETAQNPAVGETQAIPAASEPRESAPTASPPPSN
AQKTTAAEVSATMSSSMDKHASPRARQAMSQLGVNIGDIARGSGPNGRILERDVVAASQQ
EAIPYASAPQSDKGPGQALVEPAQEFEEVPLIGIRKRIAARMLNSIQTTAQYTEHSSADA
ERLLSLRARLKAQKNPDVAGITIGDLVLAAVVKVLPEFPEFNAHLVDGTLKLYKPVHLGV
AVDTPRGLMVPVVRNAQNLSLTDISKEVKRLAQACQNGTIAPDSLSGSTFTVTNLGAYGI
DQFTPVINAPETAILGVCTVRPTLVQTDAGVETRMRIGLSLTSNHQVIDGAYAARFLKRF
GEVIADIDFLFMSF*
>SPBDM4_v1_50856|ID:27159219| Transcriptional regulator, DeoR family [Uncultured spirochete bdmA 4]
MLFDLNDREKAILELLADRSFISVSELANALDVSEVTIRGDLSTLEERGYLLRTRGGAEP
SMHRSILEHQKLHMEEKQRIAKKAAELVGDSDRIMIEAGTTTALISRHLVGKKDVQIVTN
SMLAFSYSRMNPLINLILTGGTFRRETESLVGPIAVQSLGSFNARLAFVGTDGFSVKRGM
TTQLTEGAEIVRAMSKRAEITWLIADSSKFDKVGFVSVLPLNAVHGIISDTGLSKEAIEA
LQAEGLEVLLA*
>SPBDM4_v1_50857|ID:27159220| protein of unknown function [Uncultured spirochete bdmA 4]
VHFAGTKVELHILKSLNTGKSLTEVSYDNDGFCHASRLAFMYTFTFDVFIFMALFNSVKS
ENANDYINF*
>SPBDM4_v1_50858|ID:27159221|rbsA| fused D-ribose transporter subunits of ABC superfamily: ATP-binding

components [Uncultured spirochete bdmA 4]
VAEPIIVVRNLSKAFPGVQALQNVQFDLCPGEVHTLVGENGAGKSTLMKILSGVYQRDSG
EILIDGKPVEIHNPRAAQKLGISIIHQELNLMNHLTAAQNIYIGREPRYAGGFLLDERTL
NAKAKELFDMLHMDFDPRTKVGELTIAKQQMVEIVKALSFNARVLIMDEPTSPLNEAEVE
ELFDIIRHLRSQGVGIIYISHRMDELFRISDRITVLRDGQYMGTSRVSETNLDQIISLMV
GRKIDETARRIPKGKPGEVVLDVRNLNRGRLVKNVGFTVRKGEILGFAGLMGAGRTEVAR
AVFGADPIDSGEVYIKGKKITIRHPRDAAKHGIGYLSEDRKRYGLAVGMPVLDNICMTDL
PAFMNAFGVLKPKKMQKTSAEQVKALSIKTPSLLQKVKFLSGGNQQKVVVAKWLVKNCDV
LFFDEPTRGIDVGAKQEIYQLLNELASLGKAVVMISSELPEILRMSDRVVVMCEGRITGE
LDGATATEEAVMKLATQRETMVKTA*
>SPBDM4_v1_50859|ID:27159222|rbsC| D-ribose transporter subunit ; membrane component of ABC superfamily [Uncultured spirochete bdmA 4]
MANKPDDAAAATSLSRKTPETRGISARQKLLAFSSLVLLIVVFSILSPNFFQPYNLIAIM
LATAVNGVLAIGVTFVIITGGIDLSVGTMMTFTAVMAAQTVAVWNLPVFVGVLMALVAGT
ICGLASGIMVAKLKIPPFIATLGMSMLTKGLSLIIARSKPIYFTNAPSFSKISMGTMFPF
IKDFQIPNAVIILFVLAILASFALNKTIFGRYTFAIGSNEEAARLSGINTDRWKIAVYAV
NGLICGIAGILIASRLNSAQPALGQGYELDAIAAAVIGGTSLSGGEGTILGTIIGAFIMS
TLTNGLRILSVPQEWQIVTTGIIIIFAVYMDILRKKR*
>SPBDM4_v1_50860|ID:27159223| Periplasmic binding protein/LacI transcriptional regulator [Uncultured spirochete bdmA 4]
MKKRTVVLALAFMLIAASSLAAQQYYIPLISKGFQHQFWQAVKQGAMQAAKDFNVNITFE
GPETEAMVDKQIDMLAAALAKKPNAIGFAALDSKAAIPYLQQAKTAGIPVIAFDSGVDSD
IPLTTCATDNVAAAAYAADKMAELIGGAGEVAVIVHDQTSRTGIDRRDGFVNRIKSKYPK
ITIVSLQYGGGDQLKSTDLAKAIIQSHPNLKGFFGANEGSAIGVLNGVKEMGKVGKIVVV
GYDSGAQQIAAVRSGEMAGAITQNPVGIGYKTVEYAIKALKGEKFPKFIDTGFYWYDKTN
IDDPKISAVLYQ*
>SPBDM4_v1_50861|ID:27159224| Fructokinase [Uncultured spirochete bdmA 4]
MIAACGESLIDMVPSEQGRDLFEACPGGCPYTSAIAAARLTVPTWFVGKTSKDFLGDKIV
SKLQDSGVDTSLLIRNDQAVTLAFVERDAAGNANYAFYSADAADRFLSPSDLPAKLPEQV
VFLLVGSISLVQEPSCSTILSLIEREHGRKLISFDPNVRPSLIQSKSEYRARFEWICQRS
AIVKASDSDLEWLYECPANEAANKVLGLGPELVALTMGEHGSLFLTRRYRIEFPAKQVKV
VDTIGAGDSFHAGLLAGLGWLKVRDRDSLASLSENNLRTVLRLATSAAALDCTKRGAGPP
TLHELAVFDPEAVRVAPGFIPTFNTKERIC*
>SPBDM4_v1_50862|ID:27159225|fucU| L-fucose mutarotase [Uncultured spirochete bdmA 4]
MLIGIKPCISAELLSVLYRMGHGDEIVLADAFFGGDALNSRVIRADGLRIPDLLDGILRL
INLDSYVDAPVIMMQPSKGDALDPEVERRYRQVIDRYWPDIPPIARMERFAFYERTKKAF
AVVMTGETVKYGNIILKKGVPPVMD*
>SPBDM4_v1_50863|ID:27159226| putative Auxin Efflux Carrier [Uncultured spirochete bdmA 4]
MEQAAGSLLAIAILMVAGYVLAKRGWLGERAVEGLKKLITNLTLPLLLFRTFLQLRPDGR
NVLLAAGIFVACAVMGLLGALLSRLAHTPKPETRLLFQGFEAGMLGYALYAGYHGSEHLP
SFAALDMGQVIYVFTVLMVQMSMGTDVHSGENGGKPKFPWRDIANSKVLWAIGGGILLSL
LAPGFSEVLAVRSNLTRAVFDMVGGLTTPLVCLVIGASLSAGIVVDKYLVRVVAARVVVA
VGIGTVFAYLIVPALGFSEWHMRAAMMLFILPPPFVIPVYHKGNAQFVGSVLTLSTLVSV
VLIAVLAIAGVA*
>SPBDM4_v1_50864|ID:27159227| Transcriptional regulator, LacI family [Uncultured spirochete bdmA 4]
MAHSTITIRNVAQRANVSTATVSRVLNNDPRVRDATKNVVLEAMESLGYKINFVARSLKT
SRTHTIGVLAPDLSGDFFMFIAESMNRELTLRGYSLVVSTSRDSEEEEAKRIRHLAERLV
DGIVLIPIGSSSAYLPQISALNIPIVFVDRALKGYETDQVLVDNETGAYEAVRALIREGH
RRIGFLGGKPEVTTAGERFAGYCRAMEEEGLEIEQQYVRFGHPTLPFGYRTMEEIMKDPH
SPDTWFIVNLFTHLGATNYLVSEGGERARQTTFAAFDEMPYSPLLRYCRYAVQQPIAEMG
KAAANLIIDRIEGKGPPEPQILRFKTRLIQHTPAR*
>SPBDM4_v1_50865|ID:27159228|fucI| fragment of L-fucose isomerase (part 1) [Uncultured spirochete bdmA 4]
MEQAKDFALQQARNRLKGIAPKIGIRPVIDGRRRGVRESLEDVTMGMAKAAAKLISGNLR
HPTGEEVECIIADSTIGGVAEAAACAEKFAREGVAVTLTVTPCWCYGSETMDTDPLTVKA

VWGFNGTDRPGAVYLAAVLAAHSQMGLPAFGIYGHDVQDFKDIDNIPADVAEKILRFARA
GIAAAWMRGKSYLSVGSVAMGIAGSIVNPDFFRE*
>SPBDM4_v1_50866|ID:27159229|fucI| fragment of L-fucose isomerase (part 2) [Uncultured spirochete bdmA 4]
MRNEYVDMSEITRRIEEKIYSEAEFHRAMEWVKTYCVEGPDNNPPAQQHSRARKDKVWEM
SAKMAIIVRDLMVGNPDLRRKGLIEESLGHNAILAGFQGQRQWTDHFPNGDFLETILNSS
FDWTGIRQPYLVATENDSLNGVSMLFGHLLTGSAQIFSDVRTYWSPEAVQRVTGWKPSGL
SENGFIHLINSGATCLDGTGCERLGGQPAMKPWWHVESGEAKAALKATQWRYASLGYFRG
GGYSSDFLTEGGMPVTMTRVNFVRGLGPMIQIAEGFTATLPREVHDTLDLRTDPTWPTTW
FVPRLTGSGPFKDVYSVMANWGANHGAISYGHIGADLVSLASILRIPVAMHNVSVEKIFR
PSYWNAFGMDPEGADYRACSALGPLYK*
>SPBDM4_v1_50867|ID:27159230| putative ABC transporter (Membrane (N-terminal),atp_bind (C-terminal))
[Uncultured spirochete bdmA 4]
MAKKSTQNDYLHTIRGYLSRYRKLALPGLGVMILITLGQLAGPYILKQIIDVAVPKEDTG
LLLKYAFAFLGIVAVTGALSYVGMILLARLGLSIVTLIKQDIFSHLLKLPMSFFDKHPIG
ELMSRTETDTERVRDMFSNLGANLIVNIFTMIGIFAVTFTLVPKLALIMVGVSAVLLTIL
IVFFSKIFPMYEKARSFYASITAKVTELIQGIEVLKAYGRTGWAEAELDAPAKQKVSLEV
RISLIEYSMMSALNSLIGPLFIVALLLLYAPKVLTGTMTLGTILLFVEYGSRLLRPIAEI
AESLRSLQQARTSLSRIRKIMATPEEPNRGSGKLPTLDHEIRFDHVWFAYEGEEWILRDL
SFVIPKDSFSAIVGASGSGKSTTISLICGFAHPQKGHVLIDGIPLDEIDIIAWRKHIGLV
LQESFLFPVSVLENTRLYHEEITESEVRAAISVVHADSAIDTLPEGLSTNLWERGGNLSS
GERQLVSFARALAANPELILLEEATSNVDMTTEKHIKESMDVLRKGRTMIVVAHRLSSVL
RADTIFFLSEGRLIAEGTHESLYENLPAYRLLVDQQFPEGKKGHA*
>SPBDM4_v1_50868|ID:27159231| putative ABC transporter, transmembrane region:ABC transporter related
[Uncultured spirochete bdmA 4]
MNSSRNKNEKKSSARRTGKGAPRRSASPRFSHIRWIREVWRERQGLIWLLLFLTLLSSAV
AVAFPLLTKQLFDLLEQAIDTKMSEDAAMAQVKRIALYFVAIGIAGFVAGTFPGIRGALN
VVFDYIVRKRYFGEVVQKDMRFFSKFRSGDVITRLTSDISDFPKLSWFLCSGIFRAVESA
SKVTFCIAAMLSLDWRLALASLASLPIMLFVFTRTQSAIYDRVNKNEQAISAINNQLEMS
FSGVRVIKAFASEGKYTKFFQDALARRFETEMGVAKLEAVMQLIYQYIDYVAQIALVFVG
GLMVTKHTISIGTFYAFYNYLNMLIYPMLDIPQLFVFGKRAFVNIDRLEEMRNYQPEMQE
GERGREGGATKSADQDSAERATREVPLRNGPHDGSAKETLKKAPETTKQLPPLRSLRFEK
ATVYYEHKHKPAIDDIRLEIFPGEKILVIGPVGSGKTTLVKTALGLLEPRTGRVTINEKS
VRALDPEERRGLIGYVPQDPLLFSGTIRDNILFGCREGQSLPESELARLIEVSRLSDEIA
AFPDGLETKLGQRGTSVSGGQKQRIAIARALAGYPALLLLDDMTASLDTNNEEALWRSLA
EMPEFARAAVIAVSHRLSSIQYVDRVLFLKNGRITGFGEHSELMANNPAYREFVAEHVQR
PGE*
>SPBDM4_v1_50869|ID:27159232| conserved protein of unknown function [Uncultured spirochete bdmA 4]
MQEKSSGQFYAPKGKAAPVCAQGECNIGVIGLDHGHIYGMCNGLTEAGAAIAMVYDRDAA
NVARFREVYPDATPASSIDEILRASDIKLVASAIIPAERGPLGMRALEAGKHFFSDKPPF
TTRTQVDLARRTVAETGKMYAVYYSERLHVEASVYAQRLIEDGAIGKIVHISGWGPHRIS
LDQRPEWFFDKANFGGILVDLGCHQIEQILFYAGARDASISASHTANYKFSRYRQFEDFG
DASLVMDNGATGYFSVNWLTPDGLGAWGDGRTFIQGTDGYIEMRKYIDVASDPEGDHVYL
VNHRGEYHFRAAGTCGFPFFGQLVRDCIDNTRTAQDQELTFRATELAIEAEEKAEKLRAK
I*
>SPBDM4_v1_50870|ID:27159233| conserved membrane protein of unknown function [Uncultured spirochete bdmA
4]
MKPNQSTLKVLSIASALAFLAVLVVNALANALPLNGVTTGQLSDEIPNLFVPAGLTFSIW
GVIYLLLAGYVLYAIREAWRKNGDGGGWTARDAVLFLLNAAANIGWIFAWQWRHVALSMA
LMLVILGTLIALELSIEQKMKKGGALSLAEPLSPGEPSAQPRPQRSSRARHLRRFFLSVP
IRVYLGWISVATIANATALLVKLGWNGWGLDPRAWTVIVIAAGLAVALGFSVGQRQIAAP
LVVVWAYAGIVIKRTQVDAAYSRAVWIAALVSAVIILLSFVVVRIVRPGTAGSGKAAAGQ
S*
>SPBDM4_v1_50871|ID:27159234| protein of unknown function [Uncultured spirochete bdmA 4]
MLDSRDTMKGMEEDKTMSKKSILVMVFLCSLLLGAGAQVIVFGMGDPDIDAGLNELNASA

KLNMPGFQAEVALAWGQPAASITVALSQGLSPGEVYLAAALAHFSGKPLNVVIELYKKDK
AKGWGALAKELGIKPGSKEFKLLKETLEGSRKKVKGKK*
>SPBDM4_v1_50872|ID:27159235| exported protein of unknown function [Uncultured spirochete bdmA 4]
MKKTKYPIILIAGLIVFLWSCQTSPSDEELSSSEEEFVAEQTAASISTDEGGALYTTKAL
SDPSYYENLEDEAEYDATTGTFSVTTSIDLTYKGSTSVNMSYKLYDTDGNVQSSFDSATT
YRVEATMQFSHDVNTNRYEAHTSKSTNVTVTGLNTDTVVVNGEWLAERTAQTSSSVPQPH
ETTLSHHLLLRNITYTKESETGQYLLSGGSASSQIEGSINNKSFTRSVQWTFGDSNTATM
TYQGETMTVNITTGVIVD*
>SPBDM4_v1_50873|ID:27159236|ompR| Transcriptional regulatory protein OmpR [Uncultured spirochete bdmA 4]
MEKVVIVDDDEKMSALLVRYLGEFGFEVVSMSRPSEALALLGQSRFDIAILDVMLPEMDG
FELLKKIREIDDLPIIMLTARGELHDRVLGLELGSDDYIQKPFEPRELVARIRAILRRRK
ANGDVVSDSSVIKVAGIVLNRATFSVTVDGVPIELTTSEFQLLDIFLSSPRIVFPREVLM
DKLRGSSIGAFDRSIDVLVSRLRTQLQDDSRNPRFIKTIHGIGYVFMAE*
>SPBDM4_v1_50874|ID:27159237| exported protein of unknown function [Uncultured spirochete bdmA 4]
MRWRKSLFTKTYVTIVILALISALAVGLFVFGQYRLRFKSNSVSALPQFYKRNSMDLGIS
PDPEKGRILAERLGVDIFYFGKSSH*
>SPBDM4_v1_50875|ID:27159238| putative Histidine kinase [Uncultured spirochete bdmA 4]
MENISKRGRDIQGYRDRKLYVGFSDERGYFVFYGNPLFNIERLPVVLGTFLLIGSLPLII
TFFVIRMLLLPLKRLEQGVSELNAGNLSFRVSPVRDDELGALAKAFNHMAEQIQQMISDK
EQLILDVSHELKTPITRIKLSLEFLENEALRKSLEEDVDEMDLKVQELLTSARLNTPYGS
PKREKTELAAFLLWIIGQYGKDPPGAVLLPGDPAVFVEVDQGLFQTACKNVLENAIRYSR
PNAPPVELWYEVGASFVEISFRDHGVGIASEHLDRVFEPFYRVDRSRDRRAGGYGLGFYL
VKKIVTAHDGTVLIESEVGKGTLVTIRIPRNNSSN*
>SPBDM4_v1_50876|ID:27159239|ygeW| conserved hypothetical protein [Uncultured spirochete bdmA 4]
MNEIRSLIEQLRVKRIDMAGKDFLLTWEKTQDEIEATFLVADILRAMRERDISPRVFDSG
IAVSNFRDNSTRTRFSFASACDLLGLYIQDLDEGKSQIAHGETVRETANMISFLTEAIGI
RDDMYLGEGDRYQREVGKALDDGVKAGVLPSRPAIVNLQNDIDHPTQSMADFLHLAHYFG
GLDKLRGKKIAMSWAYSPSYGKPLSVPQGVIGLASRFGMDIVLAHPQGYDLIPDVLEMTK
KQASASGGSFTYTDSMDEAFKDADIVYPKSWAPYDVMLRRVPLLHAGDKDGLKALEKEAL
ANNSKFVDWECTEEKMGLTKNGKALYMHCLPADITDVSCEAGEVAASVFDRYRDDTYREA
SYKPYIIAAMILLGKFANPAALLESLLDRGKQRIFGA*
>SPBDM4_v1_50877|ID:27159240| 8-oxoguanine deaminase [Uncultured spirochete bdmA 4]
VILLKDCFAVAVPAATRGTETGADGAAGATQTAKAVRSARRRFDLLRDVDILVDGNRIEK
IAPTIEAPEGATVIDASRHVVLPGLVNTHHHFYQTLTRNLPAVQDAKLFDWLVYLYEIWK
YLDPEAVYWSSTLAMAELAKTGCTLTTDHHYLYPTGFGGDIPSLQFRAAADIGLRFAPTR
GSMSRSKKDGGLPPDSTVQDEDSILAHSEETLRRFHDPAPDAMRKVALAPCSPFSVSERL
MKDSAALARRYGARIHTHLAETSDEDDYCVQVYGRRPLEVMRDCDFVGPDVWYAHGIFFN
DEELDFLAKTGTGVAHCPSSNMRLGSGICRVHEMLGRGVPVGLGVDGSASNDTSDMLGEA
RQALLLQRIKYGSAGLTAGEAMHIATAGGARILGFEEAGEIAEGRLADIALFDVMKLEYA
GALSDPLAALLFSGYNHGVDYLIVNGKQVVRHGLLVGADEETIRRNALSACERMYKKAGI
L*
>SPBDM4_v1_50878|ID:27159241| Pyridoxal-5'-phosphate-dependent protein beta subunit [Uncultured spirochete bdmA 4]
MIDLNIHDAQRKKNIERCKQKGILLPTFAQMRDPSRIPSSVKNELSNIGLWDVHPRNLFR
VTWHNEPKEFGGGYGSVNYIEIPRAITGTKARIVGLAGKWFPTGAHKVGAAYACLAPELV
TGRFDPTTKKAVWPSTGNYCRGGAYISRLLSCPSVAILPAEMSRERFEWLKTMAEEVIAT
PGCESNVKEIFDKCVELQKTRSDVVIFNQFDQLPNHLWHYAITGPAMEEVFRAVGGPNSH
VGGIVLSSGSAGTLGSGSYIKEKFPGAKLAVGEALQCPTILENGFGGHRIEGIGDKHIPW
IHNFRDTDAAVGVDDELPMRFIRLFNEPAGRKALIDAGADPAVVEKLEWLGISGVGNLIA
AIKFAKYYELGEDDIVFTMFTDSMAMYQSRLAELTAERGAYDQRQADRDLDRLAGLSVDH
VFEMTHVDKRRAHNLKYYTWIEQMGKDLSELRDQWDNYRTYWGGLHGQIGALDELIEDFN
AEVLR*
>SPBDM4_v1_50879|ID:27159242| PIN domain protein [Uncultured spirochete bdmA 4]
MNIVDSSCWLEFFAGSRVGDEVAPIIEDTENLLVPSITIYEVFKKLLLELDEDKALFAIA

HMKQGNVINLDTDVAIYAAKTGKENKLPMADSIIYAINQKYDAMLWTQDKHFKDLKSVHC
FEKA*
>SPBDM4_v1_50880|ID:27159243| Transcriptional regulator, AbrB family [Uncultured spirochete bdmA 4]
MKTVTVSSKYQFVIPKEIRNTIGLHVGAKLEVLTYGSRIELVPIHPMKTLKGAFQGINTK
IEREEDRV*
>SPBDM4_v1_50881|ID:27159244| putative threonine synthase [Uncultured spirochete bdmA 4]
METRWMYRCPTCGKTYPIEPGRYLCDECAKKQRPDEPLRGVLECAWEGEAPARGSVPLPV
EDRFFPPIPVGQTPLWAPERLRSELGMPNLWLKDDTCNPSGSYKDRASWLVAAFARKFGI
REIVLASTGNAASSMACVGAAADIKIRVYVPKSAPIAKRVQILQYGAELVEVDGTYDLAF
DQSLAYSRATGMLSRNTAYNPLTIEGKKTASFEIARDLATAKKAAGADVRAMSAAPAYLA
PDHVFVPTGDGVIIAGVIRGFEDLVKLGWIERMPTVWAAQAEGSCAIARALETGIFEPRP
SSTIADSISVDIPRNGYFALDRLRRHSGRAVVVSDGEILAAQRRLSRSSGLFAEPSSACA
FAGFLKVKEQLAPGTRVVVMLTGSGLKDIKSAAKAVGLTI*
>SPBDM4_v1_50882|ID:27159245|ygeY| putative peptidase [Uncultured spirochete bdmA 4]
MNSKKILEKAREYRDYSAQNLSKIIQVPAFSTTEKDRIVLLKQLCEEAGMEDLWIDGLGS
LLGRVGSGSRKIVFDAHIDTVGVGDESQWKTPPFSGLIQDGLVHGRGASDQLGGAASMIT
AARILKDLAYKGDFEVWFSFTTIEEDCDGMCWKYLIEEEKFVPDFAVSTEPTSCRLYRGH
RGRMEIQIDIKGVSCHGSAPERGDSAAYKAARAALAIEKLNERLHPDDDNFLGKGTITVS
QINVHGPSQCAVPDQAMLYCDRRLTWGETDELAIAQVKDALKEAGVNNFTVFMPEYRRSA
WTGTEYHQELYFPTWKIPADHVLVRSGVDAYSELFTKPPVVDKWTFSTNGVAICGRHKIP
VIGFGPGDEAQAHAPNEITRIDDLEIASAFYAALPYVLGAKAK*
>SPBDM4_v1_50883|ID:27159246|ssnA| putative chlorohydrolase/aminohydrolase [Uncultured spirochete bdmA 4]
MILFRNVKIMELEPPAVSEPTDVAVFEHEEGDRGGTVAAIGQKLQTQYSKAKVAGEGGYL
SAGLVCGHTHLYSALARGMLVDIAPSKDFAQMLNHLWWRLDRAIDPAILRASALAGCSDA
LKAGVTSLVDHHAGPECIDGSLSVIRDAYEAVGVRGILCYETTDRNGMDGARAGVAENVR
FAKEIDELRASGEKPLTEAAIGAHASFTVSDETLGALAEAVHTTGRGLHIHLAEDKFDAV
DARHRFGKDPVERIEAAGALNDHSIIGHGLWLSPSEVELMNARGAFLAHNARSNMNNAVG
YNNLLPTFANVVLGTDGMGADMLEEFKFAAFRHRESQGPWWPGDFLKVLYRGNALIGRYF
DRDYAPGYPGFGTVKVGAPADLVLWDYDPPTPLTGDNIAGHFAFGMSSRSVRTVMVAGAL
RILDRRPLYDDSGIQTEAREQASRLWKRMEER*
>SPBDM4_v1_50884|ID:27159247| conserved protein of unknown function [Uncultured spirochete bdmA 4]
MSKVMKTTSLSVLLERITGEYLAKGTIFEIPKATWLDVFEQESESTGLGIMSANVSIPLG
PAAGPHTQIAPNLVAAFLSGARVFELKTVQENDHLDIDKPCIDALDEGHNVEWSTELTLE
QARMEYLNAWIAINLLARMWSHKPSDFIFNMSVGYTLDGIKSEKMDAFIEGMRRPEAGDY
WENAIEELQEFIESPKFIETFGQAALERARSIAEHMPVRPVHSVTLSTMHGCPPDEIERI
GRYLIEEKGFDTYIKLNPTLIGYEAAREILDRLGWTDIILRRESFEHDLQFDMALGLIQK
LTEAAVSRGRRFGIKLSNTLANVNDGCCLPGGERYMSGRALFPITVHLAAKLARAIPDFP
ARFSYCGGVSAFNAADLIKAGLGPLTIATDILKPGGYQRMAHMVKDVLAALQYAPAKPDA
EALDALAESVFARPYYRKEWKEGTASIGRPLPLFDCFAAPCVEACPVKQKVSAYIAATGA
GNADKGLSIILSDNPLPTITGVLCDHVCQEHCSRVDYEGAVRIRDVKLAAVRTAGTGLAA
EVQAAWPRESRGRTAVVGAGPAGLACAWHLAQYGQKVVVFEKSPVPGGVPSNIIPSFRIA
REDIAADIARLEKSGVEFRFGAEAASPKRLKDEGFDNIVIAHGAHGARELELKGGGVSVV
HALEFLSVCMKVGPSHFEGSRHILVVGGGNTACDAVRMATRIPGVKSFRWSYRRTRREMP
ADIEELTNAVREATERNTGDPIGAPLKDTTKESPILGAGSLFDPAGPVLLELTLPETIEP
GKAILRRMKLGEKDASGRRSPLPTEDTLELSCDLIITAVGEKPDAALLEDFGVEVGKSGL
PVVDGETMESAVPGVYVCGDARRGPSSIIASEADGRTAAHSILRKQGIEPAEVDYRAPAP
SSAARASRGKIFPPLSPEDPEFAKRESERCLSCDTACLRCVEVCPNRANMVIETDRAFDQ
PEQILHVDRLCNECGNCGLFCPWEGEPYTGKPTLFDSQKDMVASNNAGFTFIGNRERPRL
LLRTEKSGEIVELPYFAWDGTISLPDHRQMVTLARTVWNEHRYLVEVHE*
>SPBDM4_v1_50885|ID:27159248| putative Allantoinase [Uncultured spirochete bdmA 4]
MIITNVAAALPGERDFRLVDIFIKEGKITAIKEAGTLKGAGGPNVDPARSADEEILDAKG
LLAFPGAIDPHVHFDEPGFTHREDFMHGSAEAARGGVTTVIDMPCTSIPPVTAPHALEEK
LSIVREKALVDFAFYGGINGHMKPEDIPQVVESLAPNVVGFKSYFISGMDTFPAVDEAQF
AAAAQACAKGGRPLLLHAEDPDVIAEATRERAFIRGTQKHQWIDYYASRPMDAEVAAVLK

ALKLAGQYAKNIHIVHVGTAKAAIAVSERGATCETCAHYLAFDETDFARLGAALKTAPPV
KAPEQKAMLWHLLADGQIGFVTSDHAGAPEYEKYTDDPLTAYGGIPGTGTLFPYLLSEGL
FAKRLPLERFLEATSGGAARRYGLWQAKGSLLPGKDADFVLVDPDHTSRVIPSNMMSKSA
ITPFAGMLLSGRIEGTFVRGSCVYASVRLAAALGKKSDTIVNEAGMILARPGFGKFLTWG
YR*
>SPBDM4_v1_50886|ID:27159249|xdhA [H| Xanthine dehydrogenase, molybdenum binding subunit [Uncultured spirochete bdmA 4]
MKTEDTRNMNKTRIEEASPVHAMRAAASVDPSVIVPESLPNVLGQTQYIDDLPRSSGCLQ
AVVRLSDSAHARILAIRTEEALALDPSVRVILAKDVPGTNQIGFNKPDEPLLPEEEWEYW
GQPIAIVLASTRSLARKAANLLRIESEPLPVITDPREAAARGDFIFPPRTIACGDTGEAF
SQCAYVVEGKVDSGGQEHVYLETQGAIAQVTDGRRMHVISGTQGPSGVQKAVAQVLGLPM
NLVEVEARRLGGAFGGKEDQAANWACMAAVASWVTGKPVELYLNRKDDMRATGKRHPYSS
DFRIGADKNGKLLAFEADYYQNSGSSCDLSPAILARTVLHATGAYNIPNVRVTGYMCRTN
LPSFTAFRGFGAPQAFFVIESAMDALAQKMGVDPVELKRRNLYREGDQTYYGMTLERVRA
SDAVERLLDKVDYPSLKARIAQFNATHRFEKKGVGLIPVSFGISFTKLQMNQAGALVHVY
TDGSVVVSTGAIEMGQQVARKISLVVARALGVPVAKVSIQRTTTLTVANTVPTAASTGSD
LNGMAAKIACQEIRGRLVKKAAELLNAAPGEIDIREGRVFLNGTQSELTWQALIEAVHAA
RQDLSAHGFYATPFLEYDMRTERGRPFAYHVYGAALVEATVDTLRATYSLDSVSIVHDIG
ESIDLSTDRGQVEGALAQGLGWALLEDLRFGADGRPLSDTLSTYKVPDVTFMPPSIDIEF
MPPIENLSTPYNSKAVGEPPLQYGIAGYFAVLNAVRAALDRPLSSYNIPLIPERVSDLLS
SKSEAEGTEL*
>SPBDM4_v1_50887|ID:27159250| xanthine dehydrogenase, Fe-S binding subunit (modular protein) [Uncultured spirochete bdmA 4]
MASLHVKINNIWQDLRSSEGMPALDVVRGELGLTSTKEGCREGDCGACAVLVGEQVGDMV
RYRAVPSCLLALGELRGKHLVTLEGLVEGAKDGLTPVMRAFLDENASQCGFCTPGFIIAL
TSWLAEPQVPDLAGALRAIDGNLCRCTGYGAIRRAAARLVERFKDLPLESGARLKTLVEA
QVLPPSVLEFMAESAGKPEADSVSRKISGNAPARGGNADNAVPVAVVGGGTDYYVRNPDP
EEGFSPFLTRMLPEFGQIRYVSDREDRWLEVGAAVTVRDFFSSHVVRHEVPGIERFETMF
ASTLIRNLATVGGNIVNASPVADITSMLLALGARLVIVPEADAGNKNTPLRLCPLEQFFL
GYKKLNLGPGEVLKAVWIPVLADPSSRKFSFEKASKRRNLDIAAVNMAISFRIDKGRFRD
VRISLGGVAPVPLLGIGAQEVLEGAPCDVSDKKSLAGLAKNAARRAESAISPISDVRGSS
DYRRRMVHQLMLAHFIRLFETSGVAEELFP*
>SPBDM4_v1_50888|ID:27159251|ade| Adenine deaminase [Uncultured spirochete bdmA 4]
MNREIIDAATGKTQCDVNYTDCTVVDVLSGRFLPNSTVSVKGGYVAGINDGLTANETVDL
EGMFLAPGLIDAHVHIESSLLTPAEYARVVVPRGTTTVVADPHEIANVMGYDGMRYMLNV
SQNMPLDVYFMVPSCVPATDFDTAGAALYASDMHQFLQEPRVLGLGEVMNYPEVLAQNPQ
LMDKIALFKSNGRPIDGHSPGLRGANLSAYVAAGIGSDHECTTPEEALEKVGKGMYIMLR
EGSTAKDLRVLLPALRRENAARFMLCSDDRHCNDLRDEGHMDFSLRLMLDAGIDPIDAIR
VASSNAARWFGIPGQGAVAPGYKADFIAFPSFEYFETKVVIKNGGIVARNGALVQDFATA
PTTIRDSVNIKWLTKEDFAIPDKGKAVRVIEVHRESLLTGHGIARIPAEDGLLVPDLEHD
ILKIFVIERHTGSGNIGKGFIRGLGLTRGAIGSTISHDSHNMIIAGVDDVSIFKAARHLN
KIRGGLVYAVGDKILLDLPLPVAGLMSDKSADFVIDRLSAFEKLFQEAGLMATSPLMTLS
FMALPVIPSLKITDRGLVDVDRFEKISLYVD*
>SPBDM4_v1_50889|ID:27159252|tsgC| putative glucose ABC transporter permease protein TsgC13 [Uncultured spirochete bdmA 4]
MDFLTSVLTITIRAGTSLTIATIGEILTERSGILNLGVEGIMLMGALSAFATVFYTHSIA
LGFLVAIGIGGLLALLHAFLTTTMRANQVVSGLSITLFGTGFSSFLGQRLGPASNNFRLV
GLRAERIAPLAPDWAEKIPILGAFLNQDIMTYAVYLLIPIAWFFLYKTRPGLWLRSVGED
PQTADAMGIDVIKTRYFYTIIGGMLIALGGAHLSLSYTPGWSENITGGRGWIVIALVIFS
MWNPARAVWGALLFGGINAVQFRLQASGTSIPASFLNMLPYAGTIIVLVVMTWWEALSKR
VGAPAALGTSYMREDK*
>SPBDM4_v1_50890|ID:27159253| Inner-membrane translocator [Uncultured spirochete bdmA 4]
MKLLFEKRKASSVPALILVPFVSFLVSLVLTALLLEIFGANPFKTFAAMAVGAFGSSHGF
AETLVKAIPLMMTGLGVAIAFRLRFWNIGAEGQLTMGGVAAAGVALFMERFFPGRSLLVG

ALLGGLLAGALWAGVPALLKTSLKVDETLVTLMMNYIAILYSEFLYYGPWRDPKGYGFPG
SRMFSADAWLPRIVGRAHIGIWIAVALAIILWIVLKRTRWGFELQVIGASQRAARYQGIA
VERNIVLAVMLSGALCGLAGAFEVTGISHRLQQGLSIGYGYTAIIVAWMSQLNPLAVPFV
AIALAALAVGGDQVQMVMGLPAAMGVVMQGLILFPMLAGSLFTEYRLSVVRPESKASCPD
PVEE*
>SPBDM4_v1_50891|ID:27159254|yufO| Uncharacterized ABC transporter ATP-binding protein YufO [Uncultured spirochete bdmA 4]
MNDTVVPKVEMRNISKSFPGVLANDCVDLKLHKGEVLALLGENGAGKSTLMNVLCGLYRP
DRGEIYIDGKYAEIHSPRDAQKYGIGMVHQNFKLVDSMNVLENIILGLRDEPFVPDLKRV
RSRLVDLASQYHLDVDPSASIWQLSVGEQQRVEILKLLYRNAEILILDEPTAVLTPQESH
ELNRVVHVMKQEGKSAIFITHKMEEVMEFSDHVMVLRKGKVVDETSTAETTPKELARMMV
GREVLFQIEKKPMAPGKVALELSHIEALGDRGLPALKDISFRLHAGEILGIAGVAGNGQR
ELAEVVTGLRRSSKGKLQIDGKDMTNKSPLEIIRRGVAHIPQDRVSVGAAGDLSVASNLA
MKQYRSRPLAAGIFLLQNKILDFARTLIEKFRISTPSPKTQVKFLSGGNIQKTILAREIG
AARTLMIAVYPSRGLDVGATETVRRQLVSQRDAGLGVLFFSEDLDELLQISDRVAVLFEG
KMMGVFDTNTVNIDHIGMLMAGMDREAGA*
>SPBDM4_v1_50892|ID:27159255| Purine-binding protein BAB2_0673 [Uncultured spirochete bdmA 4]
MRKIILGLVTMLMLVSLVLPATAQAKKPLKVAFVYIGPPGDLGWTYEHERGRLALQKYFG
DKIDTKYVENVPEGPDAERIIRQYAIQGFDVIFTTSFGYMDPTYAVAQEFPKIIFEHCSG
YKTAPNMATYFGRIEEARYLTGIIAGRTTKSNKIGYVAAFGIPEVVRGINGFTLGVRSVN
PNAKVQVVWTNTWYDPVKEREAAVALLDAGCDIIAQHQDTTEPQKAAQERGKLSVGYDSD
MGKFVGDTVLASAVWNWETYYIATIQNVLNGTWKTHEFWGGLKDNIAKLSELSPRVPASV
KKEVEDTQKKIMSGWSIFTGPIKDQSGKVVYKAGEVIPDDKQLSMDWFVEGVVGKVQ*
>SPBDM4_v1_50893|ID:27159256| protein of unknown function [Uncultured spirochete bdmA 4]
LRPPISFIAIIAVIPRETNSLRSLSAVPFSEKLPTWIQYSEAEEEALGGEGGFDGLGAVL
EGVVVVEPAGLVVVVFAGADEGEGTGTGVDGFSVVA*
>SPBDM4_v1_50894|ID:27159257|coaE| Dephospho-CoA kinase [Uncultured spirochete bdmA 4]
MQIIGLTGGYCAGKNEVARILEKYGWEVVDVDKLGHAALLQVSDQLIKIFGESIKKEDGT
IDRKRLGTIVFSDKNRLDALEATVHPAILSLLNEEIEKAKHNTVEKFCINAALLYRFPQT
HLCNAVIEVRAPLYQRILRAQRRDHLSINAALERIKSQKYLWDMRPKEGVPIYMLWNSGS
SAELKISTEALLNKIAASGGK*
>SPBDM4_v1_50895|ID:27159258|polA| DNA polymerase I [Uncultured spirochete bdmA 4]
MENKEPLYILDVYAFIYRSYFAFIRKPLRNSRGENVSAVFGFFRFLFSLVQQKNPTLFAA
ALDSVGPTFRHDQFVAYKATRQKTPEDLTAQIPKIESILQALGVPTLRCEGYEADDIIAT
LARQCAKEERPCFIVSGDKDLLQLVGNKTMALRPSENFSFRETDAAGVMGEWGVRPEQIG
DYLALTGDASDNIPGVKGIGDKTAQKLISQFGSLDAVYGNLQNIEPDSLRKKLEEGRESA
SISKKLVSLCYDVPNCPGNISSLRLSLNLAAAAPLFLNEDIKSLVPEALRKQKTFQTSAS
PVMDDQKSSTLEKPASDSASEPEIAQTEKKSAMYESILDGKKLDEIIDAAILKGVCAFDT
ETDSLDVHHAGIVGFSISFEPKSSWYVPLISPDAECIPKGQALSSLSRLLHAEHMLLVGH
NLKFDINVLAAAGMDVSVPCFDTMIAAWVLDAEAPSFSLSSVAARFLDLHGIEYEEVVPK
GQTFADVPLERATQYAAEDADFSFQLYLALSKEIESAGLHNVFFDIEMPLLPILAEMERS
GIRVDAGRLRSYGRELEGELASIEAKVYQLVGHDFNLASPKQLAEVLFSERKLPVQKRTK
TGFSTDTSVLEELASLDPVPQLILRHRLLTKLKNTYIEPLADLAEARGRVHTTFVQTGAA
TGRLSSRDPNLQNIPIREEEGRKIRAAFVAEPGHSLISADYSQIELVVMAHLSKDSNLMK
AFREGVDIHKRTASLIFGISEDQVDADRRRIAKTINFGVIYGMSAFRLARDLGISNTLAR
NFIDSYFSTYAGVAEFIKKTVGHAQESGISSTLFGRRRRILGIDSRNKTEQQAAQRVAVN
TPIQGTAADIVKIAMIKVHRSLREKLPEVKMLLQVHDELIFEAPQASVSKAMDLIKDEME
HAVRLDVPLRVSVESATSWGDMHL*
>SPBDM4_v1_50896|ID:27159259| putative Heat shock protein DnaJ domain protein [Uncultured spirochete bdmA 4]
MVDQIFDRLEMLVRAWMSSAFGEGLGHTNYQDARAFEDQDMADAWDELESYLDPSKTESE
RKAGSQRRASSHRHSYGTQNYDEDIERRAVEDAYRFLGLEPYTSFSRVKIRYKELLKKHH
PDRHTDSPENMQKATVVSAKINTAYQLIETWEESRMEHPNGK*
>SPBDM4_v1_50897|ID:27159260| putative Kinase, PfkB family [Uncultured spirochete bdmA 4]
MLQIDKRIKPYCVVVGGINIDIQAFCHSEYIERDSNPGYIKRSLGGVGRNIAENLVRLGL

QTEMITILGDSPGWKKLISHTEHAGIGLGHSPCLPTVPLPTYLCILERDGGLVGAVADMR
AIEQMQIDHLEKQKPLLDEAAAIIVDGNIPQTCIEWLAERYNANGDRASFNVNTAHKNRD
ATKPLLVADPVSSSKAERFKSCFGKFDIAKPNIAEAAVIAGVETKDSLQNIISAMRKASN
LPGELYVSMGEKGMEVIEHNSMTNISLPFPKLRPHSINHSGAGDAACAALVWISIYERII
ARELKRKPGNLCPQDKAKMALASALYAASSKYSVNPKLNDYRLCETAANCYPELSEIIDD
IRHGGEM*
>SPBDM4_v1_50898|ID:27159261|yeiN| conserved hypothetical protein [Uncultured spirochete bdmA 4]
VNHYLDISEEVADALRINMPIVALESTIITHGMPWPQNLETALEVENAVREEGSVPATIA
IIDGRLKVGLSHDELMRLADGSLHPVKASRRDIPILIARHGNGATTVSGTMLIAERAGIR
LFATGGIGGVHRGAEKTWDVSADLEELAHSSVAVVCAGAKAILDLPKTMEYLETKGVPVI
GFGTSELPAFYTRQSGIDLSQHADTPGEIAQLIYVKWALGLSGGVVIANPIPVQYAMNKS
IIDAAIGKAIEESEMKGLKGAELTPYLLARVAEVTGGDSLEANIALVQNNAHVAALIAKE
YERIGLEAEA*
>SPBDM4_v1_50899|ID:27159262| conserved protein of unknown function [Uncultured spirochete bdmA 4]
MRIIRCEYEGKLFYGRIDGDNVMLSYDVPKIRMSHGGKEVYWESEEKSVPFSKVRLEAPC
APKKAVCVGLNYRDHADEFGQTIPSAPVLFIKPSTSLLGPGKTIQYPSMSKRVDYEAELV
VIIGRLAKNVTAHDAFDYVLGYTCGNDVTARDLQPQNGQWTIAKSFDTFMPLGPWIETDI
DPSSLQIKALLNGEVRQSSSTTNLIFNVADLIAYISQIMTLEPGDVIMTGTPSGVGPMKK
GDRIVVEIEGIGHLENIIG*
>SPBDM4_v1_50900|ID:27159263| putative Malate permease [Uncultured spirochete bdmA 4]
MTLSRIVSIFLLLVVGYIARKRKVLDADSIRSISDFVLSIALPFTIISSFDRSIPLTAAK
DLLKVALYAIGIHLIAILVAKISFSHVADSKRKILTYITVFSNCGFMGFPVAESVFGRIG
LMYASVYVMIFNIFVWTYGVALLSGDGGGKKGLFRVKDIIFNPGNVAVILGCVIWLLPFS
LPETINYAILLIGNCTTPLSMIVVGATLAGISIKGLFSRTEVWIGSLMRLLIMPMIIFFI
MRIFKENDASARVANFLVAMPAAAQTVIFAERYDADVSLASGIVFISTVFSAITIPLAAK
IISA*
>SPBDM4_v1_50901|ID:27159264| protein of unknown function [Uncultured spirochete bdmA 4]
MRRAKIAITVCEPVLYTEFYPIFEEAPFLIIVDEYNRIQKYSAEITAKGITRGRTEWIIS
RGAKILVTGSIDNDSYQKLKQAGIPVKWETFGDVKSLVERARRFAGYLIEMLGKERVVRT
RFDRRLRPKNIAAPYVIPYMGDDPEFLKDLERKEKREGKKRLLRPKNDEEANEDDYDTEG
DSFSGEDDDD*
>SPBDM4_v1_50902|ID:27159265|parC| DNA topoisomerase 4 subunit A [Uncultured spirochete bdmA 4]
VAYIKKLLNDNFLYYASYVIMDRAIPELDDGLKPVQRRILHTLFEMDDGKFHKVANVVGH
CMKYHPHGDASIGSALVGLANKGLFIETQGNFGNPVTGDEASAPRYIECRCTTFAKEVFF
HPKITQYIDSYDGRNREPLVFPAKIPVVLMLGTEGIAVGMSTKILPHNSVEVIQAEVAFL
EGKSFALTPDFPTGGFIDASDYADGNGKVRVRALLDTSDPKKIVIKEVPYGSTTESLIGS
IEEGVRAGHLKIASISDFTTDKVEIELKLARGVYAKEVVDSLYAFTECERSISCNCLVIK
DDKPTISTVSDIIRYHAGKLLNLLRQELELERDELQAELFARTLERIFIEERIYKEIETK
RTSDAVIKAVLDGFRPFAKELSRKIGTEDVERLLKIPIRRISLYDIEKAKKDMERIQQQL
AQNKEHLNHLVQYAIEILKTYIKTIESASSRRTRIKSFKKIEVKTVARHDFALRYDSEGG
YLGTEVSGEKILEASKFDKILILRKNGIYTVIPLPDKFFAGQNALWITVAEKEALSSAII
TLVYSLANQKGVYIKRTTIDSWLTGKDYSLVPEKAEILGFSIDTAYEFQLEYKPKPRSKK
TKQIFHAKEYPVRKRQAQGIRLADREVIQLEVLHAQKANIINSPSAHGGGLLEAALKKHK
K*
>SPBDM4_v1_50903|ID:27159266|parE| DNA topoisomerase 4 subunit B [Uncultured spirochete bdmA 4]
VGTKSIYDESKIKTLSSLEHIRLRTGMYIGRLGDGSSPDDGIYVLLKEVIDNSVDEFIMG
AGNQIAITIDGKKVRVRDYGRGIPLGKVIECVSVINTGAKYDDEVFQFSVGLNGVGTKAV
NALSKDFRVASYREGKVFEAKFSRGILTDSREGNNHEKTGTLVEFIPDEEIFGEYSFIPE
FIEKRLWNYACLNPGLTLTLNGKDFHAEKGLHDLLSTELGGSALYDIGWYRGDRIEFAFT
HTKEYGEEHFSFVNGQYTSDGGSHLSAFREGFLKAINEFFQASYRGEDVREGLAACIAIR
IKNPVFESQTKNKLGNSDIRPWILQEVKKGVDEILRRNPQAAKTLQEKILTNEKLRTELN
IVRKEAKEAAKRIELKIPNLRDCKAHLGDGDLGDRSTIFITEGLSAAGSMVSARDVHTQA
IFSLRGKPENMFDRPRSAIYKNEELYNLMMALGIENNVENLRFARIVIATDADYDGFHIR
NLLITFFLTYFEELVVQSRVYLLETPLFRVRTKKETLYCYSVRERDEAIQRLGQGTEVTR

FKGLGEISPAEFGQFIGKDIRLVKVDIQTLSSVPELLCFYMGKNSPERRQFIMDNLVTEL
*
>SPBDM4_v1_50904|ID:27159267| Ribulose-5-phosphate 4-epimerase-like epimerase or aldolase [Uncultured spirochete bdmA 4]
MSYLIKDEFKRAVVDGGMELLQQGLTVGTWGNLSVKDPETGLVYIKPSGMPYTSITPQDI
VVMDERGAIVDGHRKPSIEFHLHLSIMRARSDVFAVIHTHPIYSSVFGVLREDIPAISED
FAQIVGDRASCCVYALPGTEELAKNVVVSLGEGKAVLIPNHGTVCVGHTLDEAMKVAAVV
EKTAHIYLLARSIGTPHVLPHEDIVVMQDFMRNSYGQGK*
>SPBDM4_v1_50905|ID:27159268| Putative signaling protein (fragment) [Uncultured spirochete bdmA 4]
MAKEWIHRLSLLDIFREYFGSGRDFNAAVLKILRIIRETLHADSICLYIQNADGSLTSKD
YDGFLSDLIRDATIPPGQKNVGLAAEERRIIAADDLATNPDYVPHFRDLIEREKFVTQYC
IPVILANEARAVLELFFRAPFKPDETWLAFGQAAAYQIGIALQMQNIIEELDKTYRDLQR
ANESIIEGLSSALEFRDEETEGHTLRVTALFMSFASRFIQDENELKKLRIGSLFHDIGKI
GISDAILNKPGPLTFEERIEMQKHPLISKAILSRIPSLHEYIDIPLYHHEKYDGTGYPFG
LKGEGIPLSARIFAITDVYEALTSDRPYRKAWSKEKALEYIRDNAGTHFDPELALCFVEM
NMD*
>SPBDM4_v1_50906|ID:27159269| transposase [Uncultured spirochete bdmA 4]
MKSISDILGGLQILLEVSFDIQAGFEEYLTKDQRTFLAILRVIEEHLRVPYEAESRYGRP
AYSINPFIRAFWAKSYFRLLTMDDLRKRLLSDPNLRMICGFTKVPSLATFSRRMSLLSES
SLMEKSFETMVSEYYAGSIVGDVARDSTAIAAREKPCNKKSDVALPKPKKYRRGRPRKGE
ERPRKAPPVVAQHTTMSLEEALHTLDTRCSWGCKKNSQGNVSYWKGYKVHLDVTDAGIPI
SVVVTGARVHDSQVAIPLERMTEARVTHLYSLMDAAYDSDTIRSFIQERGRVPLIDHNKR
KADTRPPFDPASQRRYAIRTTVERTNSHLKDWFLSSPYFVKGIKKVSFQIMCGVLCLTAL
KILQYFIAPAMR*
>SPBDM4_v1_50907|ID:27159270| protein of unknown function [Uncultured spirochete bdmA 4]
MTILPENTDLNVLIIEDNKDDTELLVYQLKKAFKSLHYTHIQNLKEYLEALRQVAESESC
TEPERHNVILSDWAVPGYDCFAALEALHKIKQIIPFIIVSGAIGEPTAVALIKAGAYDFV
LKEQSSRLSYIIPKALEWAEQRKTDETQRLQIELQNKALQASPIAIAILNGQGAFEWVNQ
AYEMLTGWAIQELKGRYLWGFCGEVSSKNCMQVFQELKNKDIHQWEGTGTRKDGSRYHEF
RQMVKLSDADNAVQQYLLIRQDITSLKQYRSRLEIDAELPLILANCQTETSIYEESASYI
KRAFGIQRAGFIRIDEGGEKILRWLGDDMEESLSREGAHGTQYPVLVDGKSRAICTVDWN
AIPIEGTNILISYRGSFKSHEMSEF*
>SPBDM4_v1_50908|ID:27159271| Response regulator receiver domain protein [Uncultured spirochete bdmA 4]
MELNAPHVTILIVEDNPDDAELAVIALRQKGIKTPYRIAKNADEALEIALGRRAAESESD
AVNSGNTAVTTPSLVLLDLKLPGRSGIEVLKELRAAPTTRYVPIVVLTTSVLEEDMIQSY
ENGANSFIRKPVDFSTFLREIDLVCHYWLETNLQPKKNSV*
>SPBDM4_v1_50909|ID:27159272| putative Histidine kinase [Uncultured spirochete bdmA 4]
LVTTLFQNVTILISIYVLYHFISRSFKGKPWAISIINGIFLGATAILAMLTPFKIEEGIF
YDARSIVIGIAGFFGGPLTGTIAAAFALAFRISLGGAGVIPGSLSIISAMILSTIAARLR
PRYQPFAEKHKFATPIGTWFLGFLIHVCVILSQFLLPDGKWHEVIPLMLFPYLVIFPVVF
SLICLLFLDNERLAQASKDLAESEARYRSLFQNQHTAMLIIDPPTGRILDANPAAEAFYG
WDRATLTTMRVQDINTLSEAEVKVDLDLARIKNKNVFYFKHRRANAEPVDVEVYTGPISI
HGKELLFSIVHDISQRVHAEQELKALTETLEQQVKKRTEELEAKTQRLTELNREHESFVY
SVSHDLRAPLRAIAGFSTILGDLLHENLPNAPHQQETFKNRIEEINHLLNRIQENANRMQ
KLITDMLMLSRAGTRTLNPRSIDLSLIAQEIINEETEDKKERHLECNVQKNLHAFADPDL
ARILLANLISNALKFTQKRDVARIEIGSAQREGKNVFYIRDNGAGFNVEAAGDRLFAPFQ
RFHEAVEFEGTGIGLSIIKRVTARHGGSVTVESVPGQGTTFYFDFGEAQ*
>SPBDM4_v1_50910|ID:27159273| Biotin/lipoate A/B protein ligase [Uncultured spirochete bdmA 4]
MAYEFRLLETGFHRAAFNMGLDEALLRSVAEGRSLPTLRFYGWAPPAVSIGYFQGLHEEV
DAAACKTAGIDVVRRITGGGAVFHHHEVTYSIVLPLSHPLARPNILDSYRLLLGGIIEGL
SILGIEAEFAPINDIIAGGKKISGNAQTRKLGCLLQHGTIILDVDVDRMFTVLKVPQEKT
RGKLIVDIKSRVTSIKHCLPQSESMSFETLYATTIQSLKKGFAKALNLALVSSVPTEAEL
QLAEQLAQEKFSSQTWITLR*
>SPBDM4_v1_50911|ID:27159274| conserved protein of unknown function [Uncultured spirochete bdmA 4]

MRLELVGKPEGCKLLRMTIDVEPPLVPSSRIRSLSIRGDFFAVPEEAFDELEHKLEGTTL
EELGKKFDGLVRQNGLECLGITGSGLDEIIQKELHNGI*
>SPBDM4_v1_50912|ID:27159275|lipA| Lipoyl synthase [Uncultured spirochete bdmA 4]
MQSAQFKPKPSWLKVQLPHGNEWRHVEEVLASHDLHTVCDEARCPNKGECWGAGTATFMI
MGDICTRGCRFCAVKTARRGGPLDPEEPRHLAEAVKSLGLRYAVITSVDRDDLPDRGAGH
FAACIHAIRELSPDTKIEALIPDYTGTELAQVLAARPDMLAHNIETVRRLQSIRDHRASF
NKSLQTLRAAHAAGIPTKSSLLLGLGETEDEVVEALEELQGAGVSSVVMGQYLQPTPKEI
PVVEYITPEQFADYARKAKSLGFASVISAPFARTSFHAREAWVASREEAGA*
>SPBDM4_v1_50913|ID:27159276|mtnA| Methylthioribose-1-phosphate isomerase [Uncultured spirochete bdmA 4]
MHEFRSVALDDTRDELVILDQTRLPNEIVYLRLSRLEDVWEAIRNLRVRGAPAIGIAAAY
GAYLAVRNGGAETLEEAVALFNQAKQHLETSRPTAVNLFWALNRMAQRLAVALHSEPRPA
SSSASVANLINALKEEADSIHDEDSRMCRAIGEHALSLLQKGWGLLTHCNAGALATSELG
TALAPIYLGNQRNYRFKVFADETRPLLQGARLTAWELSEADIDVTLICDNMAATVMKQGL
VRAVLVGCDRVAANGDAANKIGTLGVAILAKHYGIPFYVLGPTSTIDLECPNGASIAIEE
RAGDEITAKWYISRMAPQNIKTYNPAFDVTDASLITAIITEKGIARPPFEKSLKEFVQ*
>SPBDM4_v1_50914|ID:27159277| putative DNA modification/repair radical SAM protein [Uncultured spirochete bdmA 4]
MDRLDTLRILSRAADWDAAAPSARIEQGVPPHACREDTKDQPAAVMPCPNPQVREEAAYG
ICRLKRLGGGSVPILKVLLSNACSYNCAYCVNRRSSNVHRASFQPEELARTIADFDARGV
IQGAFISSGVLGTPDETMERLIWMARSLRERYGYRGYLHLKVIPGASPELVRLAMRYATR
VSVNIELPSEESLLRIAPDKNPDAILTPMMTISDRLCELAEIKCQRVSSQSPKYGRQREA
SEIAASGPFSVMSAGQTTQLIVGASPEPDSVILGLAQYLYKSFNVRRVYYSAFRPEGTDP
SLPHPATPPYMREFRLYQADMLFRWYGFEPQELFESCDSLDEDLDPKTSWALRNIAFFPV
ELMAADFTQLVRVPGIGPNSARKILELRRAGRLNSSSLRKLRIRLKYASWFITLRGKAPS
ENLQFSSEAPANCTLDHPEILHEFLREKKPAPPPINPELDFLS*
>SPBDM4_v1_50915|ID:27159278| conserved protein of unknown function [Uncultured spirochete bdmA 4]
MAEWHDEKYKEVFDGTVSGLHRRRAVDPNFSIEEAENQLAHLYILDGNDWLGRGALGDIV
SQATIAAYELFIHEWKVEGGGKNGG*
>SPBDM4_v1_50916|ID:27159279| conserved protein of unknown function [Uncultured spirochete bdmA 4]
MAVEIRKVDGSKDLRQFIDFPVRLYRDVTNYVPAPRLDEVRTLRKDKNPAFEFCEAEYWT
AWRDGRLVGRIAGIINHRFIEKWGNKWARFGWIDFIDDSEVSKILLETVEQWAASKGMSA
VHGPLGFTDLDREGMLVDGFGERATIATIYNYPYYPQHLEKLGYRKDVDWFQFQIFTPKE
IPEKVQHVTELLAKRSGVHLYEWTDKRVIVKKFGRELFRLIDETYSTLYGTTPLTERQVD
TYIKQYLGFVDPRFTKILVDDQERLVGFGISMPSLSEAFYRSHGRLFPLGWYRVLQALKH
PRVLDLYLVSVKPEYQARGVLAIVMNALQRSALEAGVEYAETNLELEDNVKVQSIWKDYP
KRQHKRNRVYLKEI*
>SPBDM4_v1_50917|ID:27159280| putative Peptidase M22 glycoprotease [Uncultured spirochete bdmA 4]
MIVLSIDTSCPTLKIGVCRERLLASSILEPPVTHAETIVPAMERVLQSAGIGKKEIELLA
VAGGPGSFTGLRIGIATAKGISLGLGVPMVLVPTLDVYGMVWRDLHGIVVPVLDARKHRI
YCALYHRGEKIGRWMDIELTDIIAKLQAEEEVHFVGPDADFAETTCLERPGWTIHSPEPE
KEIEALAMLGAGIYKEKGPAEDSAGPMYLREPEIGEPLAR*
>SPBDM4_v1_50918|ID:27159281| tRNA threonylcarbamoyladenosine biosynthesis protein TsaE (modular protein) [Uncultured spirochete bdmA 4]
MSAKHSIKTKVQHKGGSVLIFLSSPHVHRTLAVGRAIGHAAPDGAVISFRGDIGAGKTTL
AKGIAEGLGIAEPVISPTYTIISEYYGRLHLVHIDAYRLSGEDEFLQTGGEELLGAPGTL
SLIEWSERIADILPPESQRITITVEANGDRLMMIEGDWIEKIDWKRFAIRREVIQ*
>SPBDM4_v1_50919|ID:27159282| Alpha/beta fold family hydrolase [Uncultured spirochete bdmA 4]
MEKFSASDKKELAYLRFDPKGGPKAVVLIGHGMNDHKERFIPLAEALASIGVSCWIPDLR
GHGDTDPDVNKGYLADTDGFERVVQDLIEMGDYASKSLGELPLFYFGHSFGALLGLALIA
TNGKYLEGAVLSAPPERQSPVMDKLGGWIVNLGGALKGLHAPAHLPNQMTFGQYAKTVQN
ARTRFDWLTRDSAVVDAYIADPKCNFVCTYGFYRDLINGLRKVYGEGFLESIPPTLPLFL
FCGSADPVIGMKEGYEKLIQEFKLLGIIDFESKCYEGGRHESLNEINREEVLDNIVDWFS
RHIR*
>SPBDM4_v1_50920|ID:27159283| Poly(A) polymerase [Uncultured spirochete bdmA 4]

VRIRYYRNANGGLQAKARVFDQTEHVIDVAGVDREAITIVKRLASHGYSAYIVGGAVRDL
LLGNRPKDFDIVTDALPNRIRKIFSNSRIIGRRFKLVHVYANRKIYEVSTFRSLSTGTIG
NEYGTIDEDVQRRDFTVNALYYDPLASEVLDFLGGVDDLRSKRLKAIIPLDRIFREDPVR
MVRGVKYAAVNHFLVSISLRFAIHRDAALLTEVSPSRLAEEFYKIIASGKSAAVIQALDK
FGLLQYLLPEVYASIHGDKTFREAFFQNLSSLDLSTQAPEDTSLLNSASAAEIVQSDRRL
ISPFLGWFIRQRLRSFAKQENVSRDPPLFRQEAFSDIRAFLTPLNLPRVALEEAMEQVLQ
EDALLPAPLPPKRRKRGPRRKKPKLHAESAHLDVPDANLQ*
>SPBDM4_v1_50921|ID:27159284|gsiC| Glutathione transport system permease protein GsiC [Uncultured spirochete bdmA 4]
MNFIKYAAKRFLRLILTIFIISTIIFFVIRVIPGDPALVIGGVDASPSDIQAIRAKLGTD
RPLSVQYVEFLWSILRLDFGRSMISDEPVISLILQRFPLTFTLAVLGLLIGILIAIPLGV
LSAVKRWSGWDYLGLVFSQLGMAIPSFWLGILLLLLLAIKFPIFPLFGADSPAGLVLPAL
SLGVGRAAILLRHTRASMIEELSKEYVVAARAKGLPESVIRYKHALKNALLPIITIAGIE
FGYMLGGAIIIEQVFSLPGLGRLFLYAIYQRDFPLIQGGVVFIALIFSLVNFVADMLYSI
VNPKIRLE*
>SPBDM4_v1_50922|ID:27159285|dppD| dipeptide transporter ; ATP-binding component of ABC superfamily [Uncultured spirochete bdmA 4]
MNTTEPIITIENLRVEFTQERQKAFALRGIDIAFYKDEIHGLVGESGSGKTVTAMSIMGL
LPKPAATILSGSIRYEGKELLSLSEDALRTYRGSKIAMVFQEPAKYLNPAFTVGEQIREM
VMLHKGLNKHQAEMRARELLGLVGLGKDGRALGAYPHELSSGMKQRAMIAIAISCDPDFL
IADEPTTSLDVTLQLQILKLLKILKKMKRMGMLFISHDLSVIREIADRVSVIYAGKIVES
ASVARLFEHQMHPYTRLLLLSIPDARHRGKKLATIAGQVLDATNDPQGCVFAPRCPLAKE
LCYRESPKLIAHEQGHTSACHFAEEAWDL*
>SPBDM4_v1_50923|ID:27159286| Peptide ABC transporter ATP-binding protein (fragment) [Uncultured spirochete bdmA 4]
MGPLIQCRNIVKIHRGRGIDGAKRGFKAVDDVSFSIEKNGNFGLVGESGCGKTTLARAIL
YLDPPTSGKVLFDGVDLGSLRSRNLRAFRRRMQIVFQDPHSSLDPRMTIYDSLSEGLVNI
GMDRAARTKKINRLLDLVGISGAMGSRFPHEFSTGQRQRIVIARALTMDPEFLVLDEPVS
NLDVSIQAQVINLLLDIKEELSLTYLFISHDINLVAYMCDTVAVMYKGKIVEQEPTEELF
ANPKNEYTQTLLASIPGGAGRISGASTNIAKDGTMGNTASAVRELSKGHVVSNTTGRTGD
KPDLTGGTR*
>SPBDM4_v1_50924|ID:27159287| Dipeptide-binding ABC transporter, periplasmic substrate-binding component [Uncultured spirochete bdmA 4]
MRRVLKHCILPGLFACIFAGVLFAQGPGTLVFGLSGNPDTLDPQKTAGTLTFQVVKSFYD
TLVEPDQSGKIVPALAESWSVSPDGLTWTFRLRRDVVFHNGQQFTSKDVKATLDRIVDEK
TASPKRSEFSAIKEIRTPDAYTVVLSLSQPYAPLLASLASGWGAILPSGLIASGHNFGAE
PVGTGPFKFEKWIRDSRISMTRNDKYWIKGLPKLAKVEFQIVPERAVQVQGLGVGSIDAL
EFVDPDDLPILQTNPKVTIKKELTSLIMVMAMNCSREPLNDLRVRQAVNYAIDKQVVLDV
AYGGGKIGSTFLDTGNAYYTDFSNLYPYNPEKAKQLLKDAGVGNREFTITVPQNYPLHVK
AAELMQQMLAKVGMNVKLQLVDWSTWLSSMYSGGNYDFTVIGHTGKLDPDGTLAGYGAGR
YVKWYNATVDAKIKEATTISDFAARKKLYTDALEIMANEVPFVYIGTSYRYVGTRSNVFD
FRMTPNLDTFDFRWTEMR*
>SPBDM4_v1_50925|ID:27159288|gsiD| Glutathione transport system permease protein GsiD [Uncultured spirochete bdmA 4]
MVLRKTRRILFSLASLYLLVLVALAIAAPLVSPYSPTEQFLDRRFHPPGPTNFLGTDNFG
RDILTRIIYGSRSALVVGIVTVSVALLLGGAVGLIAGMGPPAIDSALMLLMDSVLSFPTI
LLAITIVSFMGYGLVQVMLALGIISSPIFARLIRAETLSIKTEGYIEASRALGTGPVKIV
FRHIIPNIMSKVIVQCSLTFAQAVVIESSLSFLGVGIQPPAASWGLMLKDARNYLVQAPW
MAIYPGLCLALTVLSFNIIGDSLSERFNPRLNDLV*
>SPBDM4_v1_50926|ID:27159289|fhs| Formate--tetrahydrofolate ligase [Uncultured spirochete bdmA 4]
MSVIRINPVPDDVDIAQSADIKSISDIASRYGLGEKYLEHYGVDKAKVRLEFLAERRGQT
KRAKYIDVTAITPTPLGEGKTTTTIGLTQGLGYIGKKAIATIRQPSMGPTFGIKGGAAGG
GYSQIVPMTDFNLHLTGDIHAVSAAHNLCAAALDARMYHESRWSDSYFEKLGLKKLNIDP
YSVLWRRVVDMNDRTLRSMIIGMGGQENGPLRETGFDISVASEVMAVLALATSLEDLRRR

LGKIVVALDKAGNAVTTEDIGVAGAMTILMKDALKPNALQTLEEQLAFVHAGPFANIAHG
NSSIAADMIATQIADYVVTESGFGADMGFEKFMDIKCRASGLMPDAAVLVVTVRALKMHG
GGPKVTPGKPLSSAYTQENLELLQKGLANMVAHIGIIKQYGIPAVVSINAFPTDTEAEHA
LIQEAALRAGAADAVISKGWALGGEGTAELAAAVDKAASLPHEPKFLYPLEMPLKEKIET
IAREVYGAGSVVYTPEADAAIEKYTALGYGSSPICMAKTQYSLSHDPLLKGVPKGFEFPV
REIRLSAGAGFVVPMTGEITTMPGLPSKPAYIGMDIDTATGRITGLS*
>SPBDM4_v1_50927|ID:27159290| protein of unknown function [Uncultured spirochete bdmA 4]
MLLTSLGLLFHYTHVDAEKLSLLSDEELRLLAEKMGIYVPEGLDRVFLIEEIIDAFEDDT
AEKSFSHDAPDHVEEKKLTGTGFIPGRLEEIAIPDHYNETYIVAIVRDPLWIFSFWDIAE
SVREKIIAEMDEPKLLLRVSESNEAESAFHYEISANFDDRKWYINVPYAKRSYRIDLCVA
KFQKIKVLARSNIVRMPSQYMDMSVKLPQMTKSLLILSGSEELHLIEPKEENSMRILSID
GE*
>SPBDM4_v1_50928|ID:27159291| conserved protein of unknown function [Uncultured spirochete bdmA 4]
MASPPLIALVLNAHLPFVRKPQFTQFYEERWLFEVISETYLPLLRMFRKLELENIPFKVS
MVFSPTLLAMLSDSLLCERYVTYLDNQIELAEKEKNRLAGNAVFEPLANMYYDMYRRNRD
EFENLHGRNITRAFDYFSKKGYIELMTTAATHAFLPIYADFPEVIDAQIETAIITHRAEF
GKNPAGFWMPQLGWYKGLEKHLRAYNIKYTIVTTKGALLGTPLPFYGSFSPVMTPSHVAA
FIRDAGATKAVWSETEGYPAHPVYRDFYRDIGYDLDTAYLSPYLTGIDRGYTGFKYWAVT
GKTDIKRPYEPAAASAQAVSHAHEYLSDRKTQARAASFWMKDRPPLIVCPYDAELFGHWW
FEGLQFLEAFFRASSRKDEDNPIRLVTLSEYLAEHPECPESEPEFSSWAEGGYAEAWLDG
RNDWVHRHTRKAAERMRELTIRFPNESGLRERILNQAAREVLLAMSSDWALLLRSGRSAD
FAGRQIRESIYNFNHIYEMLSAHTVETEWLTNLEKRHNIFPHMNYRVFSPKK*
>SPBDM4_v1_50929|ID:27159292|purA| Adenylosuccinate synthetase [Uncultured spirochete bdmA 4]
VNVVIIGAQWGDEGKGKIVDFLASDAQIVVRFSGGANAGHTIVLGGEKYALHLVPSGILY
PDKTVILGSGMVIDPEALFKELEDLEKKGVDWKGRVLISDRAHLVLPRYREMDREAERHR
SKPIGTTGRGIGVAYSLKASRDGIRLADIDDEERLVNITDEDRAWLSLWKDRLLPLKIDM
VAYLRQHRNTWRLFEGAQGALLDIDTGTYPFVSSGMSCAAGAAAQGGIGPRGIDRVLGVF
KAYSTRVGNGPFPTEFADDSAGSLSQYIRTVGNEYGTTTGRPRRCGYLDLVALRYACTVN
SIDRLVLTHIDVYDGLEEFEACIGYRIDGQMREDFPASVRNLERAEPVLRTFSGWKTALG
TCRTYEELPLRAREYIEFIEEFTETPISIVSVGSDRNHTIVRESPWIK*
>SPBDM4_v1_50930|ID:27159293|guaA| GMP synthase [glutamine-hydrolyzing] [Uncultured spirochete bdmA 4]
MDKIIILDFGSQYTQLIGRRIRELGVYSEIVPGERVADAALLQDCVGIVLSGSPYSAWRE
GSPAPDLNMLESGLPLLAICYGIQWLTIHAGGSVERCDTREYGSMPVHLLPAARGTEAWG
AFTDGLPERFVSWMSHGDSIVRPGNGWETIGVSEAGVPAILAHAAKPWFGLQFHPEVSHC
EHGMEILSRFVFNVCGARRGWSMEAYLEQEARHLRARVGTEPVLLLISGGVDSTVAGAFL
LKSVSPEQVYLLYIDTGLMRKAENEEVMRNLSRLGARHIEGVDAEAQFLGALSGVSEPEQ
KRHIIGDMFMKVQEEAVARLGIPDAFLAQGTLYTDLIESGKGVGNKAQVIKSHHNVRSPL
IEKKRAEGRVIEPLDRLYKDEVRALGRMLGLPEEIVGRHPFPGPGLGVRILGDITKEKCD
ILREADSIFIGELRDRGLYDRIWQAFCVLLPVRSVGVTGDERAYKYVVALRAIESEDGMT
AQPYAFDSKDLFEISAAITNRVPQIGRVVYDISSKPPATIEWE*
>SPBDM4_v1_50931|ID:27159294| putative L-lysine 2,3-aminomutase [Uncultured spirochete bdmA 4]
MQDFVAEGGLPYRATPFWLSLADEEPKACARNAHGVPVDPILAQTLPSPAEALISPLELR
DPLGEAGNTIFGRAVRQYRSRILVRATGQCFLFCRHCYRRALLPSELHFLDEAAIAALSE
YLAIHPEVREVLVSGGDPLTASDAQLAVLFEAIRSVPLPVLIRICTRAPIVLPARVTPEL
VDLLARARPLTMVLHINHPKELSAPFLDRAEALMKAGIPLHSQTVLLRGINDAPDILIEL
FSALSLRGIRPYYLFQGDLAAGTAHFRVPLSRGLAIYETLRKELSGLELPRYAVDAPGGG
GKIYLPEGIVERKAKSWVLRASDGSLHEYPEEE*
>SPBDM4_v1_50932|ID:27159295| conserved protein of unknown function [Uncultured spirochete bdmA 4]
VKSITIHGIDAALDKKITEKSKEYGLSQNRTVKAILQNSLLPDQKTSKREMFADLFGKWT
ENEKKAFDTRIEDLETTDVSDWTT*
>SPBDM4_v1_50933|ID:27159296| PilT protein domain protein [Uncultured spirochete bdmA 4]
MKKILLDTNAYSAFMAGDTCVFDSMIEAEVIYISTVMLGELFAGFYGGQKFNQNNDELRR
FLSKDSIHVIDVTIETAEIFGEIKSDLVKQGTMIPLNDIWIAAHTIETGSKLITYDEHFK
HVNGLRVWEELR*

>SPBDM4_v1_50934|ID:27159297| CcdB antidote CcdA (fragment) [Uncultured spirochete bdmA 4]
MLVFYNIDAPKKPTNVTINSDLLLKAKELKLNISSVLENALAEKVRQEKRKEWLKENADA
IASYNKVIEDNGVFSDKVRTF*
>SPBDM4_v1_50935|ID:27159298| CcdB-like toxin protein [Uncultured spirochete bdmA 4]
MAQYSVYINTNQSSKKLYPYVLDVQSPVLDALDTRLVIPLALKGDFGDKEIHHLTPSISV
KGAEYLVLTPQMAAIHRQYLGEFVRDCIEHRQDILASIDFLITGF*
>SPBDM4_v1_50936|ID:27159299|argR| Arginine repressor [Uncultured spirochete bdmA 4]
MKERTERLKAIKAIIKENRVDSQEKLLQLLEDHGYSVTQATLSRDLKYLKVGKLSDGRSG
YYYSIPSEEERRESERHYIQDFLRGYVSIDWNQSIAVVRTFSGHSDSVALAIDNMGFDEV
IGTISGRDNVVFIALKKGVNGDDFIHSLKEKIPDFEEE*
>SPBDM4_v1_50937|ID:27159300| conserved protein of unknown function [Uncultured spirochete bdmA 4]
MDENNERSQARTQTTERPPLPKDFRAIVMYGMSKEEALAIMKAVKAISPSMRDTAFAMTT
DTNIAWPLGQLIAELGEEHRMMRKWTDAQRTKPE*
>SPBDM4_v1_50938|ID:27159301|clpB| protein disaggregation chaperone [Uncultured spirochete bdmA 4]
MNYEQFTLKAQEALRDATSVAQKNDHSSIEPEHLLASLMEQEGGVVPSLLERLGIDRARL
EHGNAELMKKLPKAYGEAAQVYFSPAMQKIMAKAEAESKAMKDEFVASEHLFLALIAVES
RVAALMKSSGITREGALAVLKSIRGSQSASDENAEEKYQVLEKYTRDLTALAHEGKLDPV
IGRDEEIRRVMQVLSRRTKNNPVLIGEPGVGKTAIVEGLAQRIISGDVPDSLKNKKLLAL
DLGSLVAGRKFRGEFEERLKAVINEIGKSDGKIILFIDELHTLVGAGAAEGAMDASNLLK
PALARGELRAIGATTLDEYRKHIEKDAAFERRFQPVYCAPPSVEDTIAILRGLKERYEVH
HGVRIRDDALIAAAELSDRYITSRFLPDKAIDLVDEAASRLKMEIESQPTELDQIQRKLI
QLRMEANALKKENDPASVERRGKLEKEIADLSEKRDTMVARWQKEKQVIEDIRQLKQKIE
EYKVEIDQLVRDGQLAKASEIKYGRLVETQRKLEELSKKVQEEKDTPRLLREEVSDEDIA
RVVSLWTGIPVSKMLSSEMQKYVELEKQLAKRVIGQEGALRAVADAIRRNRAGLSDPRRP
LGSFLFLGPTGVGKTELAKALAEFLFNDERALTRIDMSEYMEKYSVSRLIGAPPGYVGYE
EGGQLTEAVRRRPYSVVLFDEVEKAHSEVFNVLLQMLDDGRLTDGQGRVVDFRNAIIIMT
SNIGSEFILTMKSMDEVRSRIDDLLKNYFKPEFLNRIDEIVVFDRLDESMIRKITDLRLG
ELAGRLAQRNIALTVDDAARDYIAKEGFDQQFGARPLKRAIQNLLENPIAKKLLAGQILE
GDAVRVGMSGDELAIGTERTA*
>SPBDM4_v1_50939|ID:27159302| putative Hydrolase, TatD family [Uncultured spirochete bdmA 4]
MSGVFTDSHAHLEMVAQRLGVFALDGVVAAYAAQPGELIFDIGVDPGDLGRRRALVEKSL
AGMAEEPGAGPARAIELPIRWAAGIWPGREALDNPKASLEALERDIGAEIDALGECGLDY
HHMDAGPEAQRELFGAQIELALSHRLPLIVHSREAFQDTFALVQSCARHIPTIIHCFGYG
PEEADSFLRAGCYLSFAGNITYKKSQPLRDALALVPEERLLLETDAPYMNPMPHRGKPSS
SLDIERTYQVVSTLRGLDIEALRISVSSNLRRILGMSP*
>SPBDM4_v1_50940|ID:27159303| Metallo-beta-lactamase domain protein [Uncultured spirochete bdmA 4]
MKIIMLVENVVYAGGLSAEHGLSLYIEHGGHRFLFDTGQGDRFLKNAQALGVDIEKVEAL
IVSHGHYDHAGGLKAFCRANTSAKIFIKEGFFEPKYNKDKKFIGIPYDKELFEDRLQRVS
HPKEIFPGVHLMTGIPLANVWDTHFDNLFVMRNGDLEPDRFADEQFLVIEEEDAISVISG
CSHRGITNIVRAAAEAFADRSARHSAGPLAQGDSPGQGPKFRLVLGGFHLNNADGELADR
VIHTLDEFHIERLGCCHCTGIENYARIKSVFGQRAFYSWTGTELAF*
>SPBDM4_v1_50941|ID:27159304| protein of unknown function [Uncultured spirochete bdmA 4]
MRLCNGLREYGAKMMRGGKEFRFGPTFTWYILSAMLLSVFLAGCDRAPIHATVDEAFALV
YPELSSKVVKEFPPIPINTREGAKNSDAYLPYPLQPGRIEALVNALDDKNRPKASERAGS
ASPIQIFVTSPAVAETFPPSRLDAHTVGINLLAPADSFFSVEWDSEWAYRELGLIAGYRL
ASLQKKEDAAASAAILFSRGTGRGQKELDAFTQSFEKGFRLAGSRASAPLPPAGALSVFD
VESMGLPGNHLEQVLSALRQAEDRKPRLLVLASGSRLALEKAIGMKDIDIMADVRSLGPD
LPVKKIFAAIGENTPALISAIRNLAKKIEEGDAALETVRVYPTIVLSKEAKHIRAIVDEE
TPKAAK*
>SPBDM4_v1_50942|ID:27159305|fba| Fructose-bisphosphate aldolase [Uncultured spirochete bdmA 4]
MTSYKELGLVNSLEIFRKAMKGGYAIPAYNFNNMEQLQAIIQACVETKSPVILQVSSGAR
KYANATLLRYMAKGAVEYSKELGYAIPIVLHLDHGDSFELCVDCIENGFSSVMIDGSHLP
YDENVALTKKVVEFAHAQKDYVVTVEGELGVLAGIEDAVSAEMSHYTRPEEVEDFVKKTGV
DSLAISIGTSHGRAKFKPEQCTRTADGVLLPPPLRFDILEEVQKRIPGFPIVLHGSSSVP

VEYVRTIEQYGGKLPDSVGIPEEQLRKAARSAVCKINIDSDGRLAMTAMVRKVLSEHPEE
FDPRKYLGPARDELKKLYMHKNIEVLGSANHA*
>SPBDM4_v1_50943|ID:27159306|infC| protein chain initiation factor IF-3 [Uncultured spirochete bdmA 4]
LAEKDLRINEQIRVREVRLIDEHGEQRGIVSTLEALRIAREAGLDLVEVAPQSVPPVCRL
LNYGKFKFEQEKKVKDAKKRSKVTELKEIRMQPKIAEHDLDFKANHVRSFLDEGNKVKVT
IRFRGRELAHTEIGEEILQRILAKLENSFVLERPPSMEGRFMSMVLQPKAGAAKKQPEGD
TATTAPGSEH*
>SPBDM4_v1_50944|ID:27159307|rpmI| 50S ribosomal subunit protein L35 [Uncultured spirochete bdmA 4]
MPKMKTKKAAAKRYTLTAGGKIKYKKMNLRHILTKKTTKRKRNLRKAGFLDAGPVYQIRK
KLLPYG*
>SPBDM4_v1_50945|ID:27159308|rplT| 50S ribosomal subunit protein L20 [Uncultured spirochete bdmA 4]
MPRAVDGTRRKDHRKKILKIAKGYWGRRHSNYKVAKDAVAKALSDAYKDRRDKKGVYRRL
WIARINAACRMRDLSYSRFIEGLAKAEVKIDRKMLADIAVRDPAAFDVIVEKAKAALQG*
>SPBDM4_v1_50946|ID:27159309| protein of unknown function [Uncultured spirochete bdmA 4]
MLNLEQVRALEARVEKAVVLITKLRQENADQERRLADAAKSEELLKSQNAELERQLAVQA
RAVTENSSRLEGLVARAKEAEEKAAQAELRAAEAEERAAAMERKAQAADDEIAHYRERAL
TAERRVAELESKAEELKSEQDRIEQGLIQALSKLDSFEDMVLEMSLGSDSESAELPAQEQ
ETASRAPEETRLGQQPPGRKAQDTSESDQGGGSVQEPDENSQPDLESQDAPFRGGENELD
IF*
>SPBDM4_v1_50947|ID:27159310| protein of unknown function [Uncultured spirochete bdmA 4]
MSSISSNQPMHIEILGQSFSIKTDDSPEYIQNLVDELKQRYARISSRMNVADPLRVAILA
GLFLLDDVKKMQNQTLPGRGGRTAANGSPAGHASNTSRTGNESGNPQPSNVNFDEEQILA
QLDKRLGELGL*
>SPBDM4_v1_50948|ID:27159311|rpoZ| DNA-directed RNA polymerase subunit omega [Uncultured spirochete bdmA 4]
MIMPLESLINFSGNAYELAAAVSMRAYQLAVLRTPEVEKNNGKVVSMATQEILDKTIGYK
LIAG*
>SPBDM4_v1_50949|ID:27159312|miaA| tRNA dimethylallyltransferase 1 [Uncultured spirochete bdmA 4]
LPVDAVILAGPTASGKTDLLDAVFGAGASTWVPRLRDAWQTDFFSAEIISADSMQAYRGM
DIGTAKPSKDLLSRLPHHLIDIKNPDEQYTAGEFVTRADALSSELSGRDVLPIISGGTGF
YLMNYICGLPASPPSSPEIRAQVAADLTRLGPAALRAELEEADPATASRIHEHDLYRLTR
AVEILRTSGEAPSRFAPSKMVRPGKRFLILGASRTREVLHARIHARVEAMMRAGLAHEVE
CLVSRGFGPEDAGMQAIGYREFFELAGSSSETIAEAIELHTHQYAKRQMTFFRALPGLQW
IDPDPEHLFHALTNFA*
>SPBDM4_v1_50950|ID:27159313| putative membrane protein [Uncultured spirochete bdmA 4]
MKTPLLMTILFSILPISELRGAIPYAVYNGFSIPLAAAISIAANVCVPLIAFLFLESFHK
LFYKIKPYKKFFDRFVENARKRVHGKVEKYGYWGLLIFVAVPLPVTGAWTGALGAWVLGL
SYKKAFFAIAGGVVVAGIIVSVLVALWGVGTQTIFFKTMH*
>SPBDM4_v1_50951|ID:27159314|pcrA| ATP-dependent DNA helicase PcrA [Uncultured spirochete bdmA 4]
MHIDFQAELDPEQYAAVTAIDGSVLIIAGAGSGKTRVITYRIAYLLAKAVPQEAILALTF
TNKAAKEMVERAHGLTGLALKNLMVSTFHSFGAWMLRKEAGVLGYRSNFSIYDEQDRIHA
IRESARELGMSNEQLDPARLSQLFSARRAGYGKQGELSERELELYDLYRKTIKVYNALDF
DDLIALPLELLREHEDVRERYRSRFHYIMIDEFQDTSLQQYEFIRYIESGNICVVGDDDQ
SIYSWRGADFRNIERFELDHPGLAEIKLERNYRSTGSILTAANTVIAHNRRRKKKQLWSP
QGVRGTPITLFEAEDDEDEAERIIARIRELRFRERADWDDFGILVRTNSLAEGIEDRLVE
HNVPYRVAGGPSFYQRREIKDIIAYLKAAVNPDDDVSVLRVLNVPRRGIGRATVEFLSNI
AKAHSASLNCAAETARQPGFVQTKDGHLQKALNEVFAFFDYLAQMRTALLEQKMSVSVAI
RKVVEDTGYWQYLLEEHKKNDKAAVWKYRNIELLSASAERWEKDPDTFEKGIFAYLARIS
LSMRDDANEEEKRLNLLTIHAAKGLEFDYVFIPGCENGILPHARSLEDGDGDIEEERRLF
YVAVTRAKKQLFMSRAMQRKKNGSFQETAPSPFLNELPQNLIVMAEAQVQPQSEDDLRKE
FFSRLKSRFGA*
>SPBDM4_v1_50952|ID:27159315| Methyltransferase [Uncultured spirochete bdmA 4]
MQGYVFHAADGFLNHLLEEVPTVTRIDDELVSSWNLYGPAPFDLRTRPVFWTRNTWLEPF
LLEFGSISEAARALKALQRNWSPAPLRFARRTVLIAEQLPYVSSKPHTFPFQAPEAPMGS

FTLLEEHLLLGSARCTSPFPNGAFDFEENKDDPPSRAYRKLWEAFVLAGQMPAPGERCID
AGASPGGWTWALASLGASVLSIDRAPLEPRIARMPNVKWQKHDAFTLRPQDIGPVDWLCS
DVICYPEALWQWISKWMDAGLAHNYICTIKMQGDTYDRATTLKFASVPGSRVVHLWHNKH
ELTWMKLAAWS*
>SPBDM4_v1_50953|ID:27159316| Metal dependent phosphohydrolase [Uncultured spirochete bdmA 4]
MSEISLLNALIHRFDVVVRDPVWGDVHMDASLHTLFQSKSFMVLDRIRQLGPVALVYPGA
THTRKAHSLGVYHIARRMALALVEKGEIDFVSKTGLRSFLVAALCHDIGHFPYAHSLKEL
PLARHEALAGEMIVSAPLREYVSDCGADPEQTAAIIDADRSAEESRETLLFRSFLSGVLD
PDKIDYLTRDAFYCGVPYGIQDADYIMRRLVVHDDKLAIDMKGEMSIEALIFAKYQMYRA
VYWHPVVRAATAMVRKAVYLGLASDVIQPASLYGLDDTSFGRLINHAKFAPFRLAREAEE
KHIYRLAAEHPFDEENPLHADLLDISKRIEAESTLAQAAEMAENDVVIDIPERIKLETDL
MVKADDYFEAFDQRSTLFQPETVEKVASALRTIRVYIRPQSNADFKKIASLVEALLE*
>SPBDM4_v1_50954|ID:27159317|mraZ| Protein MraZ [Uncultured spirochete bdmA 4]
MKLLTGEFKNTLDEKGRLSLPARLRTDLPLSLVLTQGVDTCLWLFAPEEWEELSQRILAS
TSLFQARSRLIQRRIIAPAQEVDVDKLGRISIPQSLRDWAGLSKECIILGISRYVEIWDS
QKYQAYVAENQSEFLSAAEELGGLTLS*
>SPBDM4_v1_50955|ID:27159318|rsmH| Ribosomal RNA small subunit methyltransferase H [Uncultured spirochete bdmA 4]
MPAVRMVKRPDSEQHKGGSTEFSLHVPVMLKEVCVLAASVYPLSLYIDCTLGAGGHAEAL
LSEHPQLCCIGIDADPDACARASARLSPFSNRLSVVNAYYDDALADLCNAESQKKMIGTG
VGGACGGSGEVRASFILFDLGLSKYQLSSSGRGFSFSADEPLDMRFSPDTPASAEDIVNQ
LSEMKLADLIYNYGEERYSRRIARAIVETRKKEHIRTASRLASIIAESVPPQYRYGHIHP
ATRTFQALRIAVNDELGRIERALADALNLLSPGGLCAVIAFHSLEDRIVKHFFAEKAKTG
QFSLEWKKPKEPSEEELRRNPPSRSAKFRAIRARGNEVKA*
>SPBDM4_v1_50956|ID:27159319| exported protein of unknown function [Uncultured spirochete bdmA 4]
MNARRFWTFAIITCLFFFGLVFQTSRYTALKSNVLSLERMEMDLLKTGKELDANIAKLAN
LERIEKAALQNGMQIAAPEQRIIVTDIQAGKGSAQEGYPNGKQ*
>SPBDM4_v1_50957|ID:27159320| UDP-N-acetylmuramoyl-tripeptide--D-alanyl-D-alanine ligase [Uncultured spirochete bdmA 4]
MESNRETPGQQILFRAGELAGLIGGTCRGDLDAPICSVVADSRAAAQNSMFVALRGERTD
GHRHIPEAVQNGARTILAEIAQKDAVLASIHDLDEARASCLIFVESPRDGLQQAAREYRK
RNTLLKIGVTGSSGKTTTKECIATVLSACYPAGTVAMSPGNLNSDIGLALALFDIRPGHT
IGIFEMGINRRGEMDELANMYEPDIAVVTNIGTAHIGIFGSREEIAKEKSKIFACFDGRQ
KALLWENDEYKTYLASKVRGSVEYFGLQSTEGLGQVENLGLKGWKVEWKNQRIDFALPGQ
HNLLNACAALSIANLLHLDPVCASRGLSSTRPLFGRSEIREGKITVLQDYYNANPDSMAA
AIEFFAEIPSRHRKILVLGSMLELGKESARAHEKVGLMAGAVGSNAIFLFGDDMIAAETA
LKSSAFKGTLFWTSDMDELSDAILNFVQEGDLVLVKGSRGMALERLTRAFEEEKLIAPSV
SKVGGPHAS*
>SPBDM4_v1_50958|ID:27159321|mraY| phospho-N-acetylmuramoyl-pentapeptide transferase [Uncultured spirochete bdmA 4]
MLREIFLPLIKYFTPFNIFRYLTFRSVYAAITALVICYLVGPWLIERLRVLKFGQSIRTD
GPETHLAKTGTPTMGGILIIFSVVVSVILWIDIKSEYCWIALLSLVGFGMIGAADDLLKI
RKHSSDGLSPIQKLILQFIVSGAVVYAIYWTTGSPATKLYVPFFKVHVIDLGVFWLPIAM
IYVTAWSNAVNITDGLDGLAVGLVIVAVLAFSVLTYVTGRADWSQYLGVPYIKQASELTV
FNFALLGACVGFLWFNAHPAEVFMGDAGSLALGGVLGVLSLMVKKEILLLVIGGVFVVEL
GSVMLQVLYFKLTKGKRLFRMAPLHHHFELKGLKETKVVVRLWILGGIFALIALSTLKIQ
*
>SPBDM4_v1_50959|ID:27159322| putative Lipid II flippase FtsW [Uncultured spirochete bdmA 4]
MKKERISVQHRLESHQTDWQMIGAILVVSLIGLCALWSGSIGFAIRNGGGPYSIIGKQVI
LYVISLACMGIMAVIPIKLLRAVLPIAVVVGIVGLALPVVTPLGVTINNARRWIRIGSFT
LQPSELWKPVMVLYLASFLEKRKERVAESGVATIPAILLVAASVVLIYLQQDFSTSILVL
AIALAMLFLAGTPIVFIGSLSLFSVLYGALMIFSSQYRVQRLVGFLMPDHHVHTVNYQMY
RALRAIRTGGLWGKGIGLGTLKISSIPEVQSDFIFAAFVEEVGFIGVVVVMALWAFILVR
AGKRLSRADYFSKLAGFGLIALLSLQILLNLSVVTGVMPTTGLALPFFSSGGSAALMSAI

SCGILINLSRGAELAINPGMAFAMKGVAHD*
>SPBDM4_v1_50960|ID:27159323| protein of unknown function [Uncultured spirochete bdmA 4]
MTDVKVQEHTASESRIQKKKGERSLLSVSDTYSVNILQATARRETSMPGTGTGTVAHQGY
ESKPKAENRKTRQAGKQPLTGRIAPATIALSASAEKLSEAVKPAIQPKKHTGIRILMMVL
VLCLLAAGTAITLPRVTKIETIRISGLETLSENAILASVGNIGNESLFSLKLNELERTIE
QNPRVAHARAYRVLPSSLAIDIQERSAVASIMINSEQGTKLVLVDGEGIAFASIDAEDSR
NPELPVVSGIQFEHFSPGQRLPDLLHPLFAELATIKRESPELLRAFSEIRIERISGNSIE
LLLYPVHMKTAVRLPLKFSADALRNALVVLDILRSRGLGDQPSEIDFHAGTVVYQAKEAV
SG*
>SPBDM4_v1_50961|ID:27159324|ftsA| Cell division protein FtsA [Uncultured spirochete bdmA 4]
MSGADIKAVSLALSGANIESFNTNGQIAVTGRGREITQEDIARVHEAARAVSISMDREIL
HVIPRSYTVDDQKGIRNPLHMTGVRLECGVHIITGSVSTVQNMFKCVTRAGYRVEQSYLG
ILGCARTVLTEDERDLGCLLIDIGGGTTDFMIFAESEPVFTSAVPAGGSQITGDISIMLS
IPVDAAEHLKKESGAAWTEAIDPDETVIVPGFGAREPVELERRKLVSIVQPRVEEIFEMV
NERLVKNGLKELIKAGVVLTGGSALLPHIDECARSVFGVPVRIGTPAAMSGLDAEYRSPA
FSAAVGVALLEAERTGATTDPGGRKKTRKGKQGSFSLIKWIKDRFF*
>SPBDM4_v1_50962|ID:27159325|ftsZ| Cell division protein FtsZ [Uncultured spirochete bdmA 4]
MAIELVDDNGIGSPTVIKVIGTGGGGSNAINRMIMAGLKNVQFIAVNTDLQDLGRSKAEI
RLGVGSKITKGLGAGGKPEIGEKAAVEDRDKIEQALRGADMVFVTAGLGGGTGTGSAPII
AQTARDLGALTVAVVTRPFIFEGKVINRIAEEGFSKLKQAADTVIVIPNQNLLKVVDKKT
PLKEAFRIADEVLRQGVQGISDLITLAGDINIDFADVRTVMEGQGDAIMGIGVGSGENRA
QDAAEKAVNKPLLDDARIEGARNILVNVTCGEDFSLTEYQDVIDLITSKAAEDAHIIAGV
VSDGSLKDEVRVTVIATGFGSAHQKFSDKAPSKNGVSMPLGSARSSEFLGPNEWPFREEK
PAQDRDRPADNRDRPAPKENFGKVSFDPEGDGDEFDIPTALRNKRLMPEDFGGRR*
>SPBDM4_v1_50963|ID:27159326|xerD| Tyrosine recombinase XerD [Uncultured spirochete bdmA 4]
VSILVRRFQAYLLSTRRRSVLTADVYIREIEMLENFLSRYGKTPLDATGEDLLTYLVNRS
QSGLQRTTMARIVASLHSFYRFCLSEKLRSDDPSVQIRTPQQDRNLPDVLDTESIERVLE
SIDITTPNGLRDRALFELIYSCGLRISEAAGLTFQQLYLEEKLLRVLGKRRKERLVPFGE
EAHSWMMRYLTEARPLLEKHGKSDFVFLNQEGRGISRKGIWKRFSQIRAKSGVKAKVHTF
RHSFATHLLAGGADLRTVQELLGHTDIATTQIYTHIDEASLGMYHKEYFPRK*
>SPBDM4_v1_50964|ID:27159327| protein of unknown function [Uncultured spirochete bdmA 4]
MKAACVVRRVNPCGGSSKMAHEKPHKVRSALIQAELDRISTALPPLDSFSLPFKLAMQRL
HFLSWQERILLLEQIQSRENFLSLGLAEIESFLYRSVHARLPDMARVWEQAERDAEFLRR
FGAHFICVTEKEYPPLLRETYSAPFGLFVRGDFRSLCWPPVTVVGTRMPTWTGVRVAVRL
AKEAADAGLSVVSGLARGVDAAAHRGALQSGVSPTIAVLPCGLDRIYPPSNAPLAAAMLD
AGGCLVTEYPPGVSLDRYRFPERNRILAGFSKLTLVVEAPEKSGALITAELALSEGRDVA
IAAACLGSSRNAGADALAREGACAVHNGDEVVALLKGAGAWNWVFETGSTARCSRRSFEW
Q*
>SPBDM4_v1_50965|ID:27159328|topA| DNA topoisomerase 1 [Uncultured spirochete bdmA 4]
MAVAKKKEKNTSGATLAPGVSKPKTLIIVESPAKARTIEKYLGSHFRVLASNGQVIDLPK
SRMAVDIEHDFEPEYITVRGKADKLKELTEEAKRSSAVVLAADPDREGEAIAYQIGKYLK
EKIKQTPISRVTFNEITKPAVQEAMSHPRDINMSLVEAQKARRIIDRLVGYTLSPLLWKK
VKSGLSAGRVQSVALKLICDRERDVESFIPEEYWTIEAHLHAHQHAVRAELALLGGEKPS
IKNEAHAKQIIASLSGKPALVSDIQFADRTVKPKPPFTTSKLQQTAANRLGFTSTKTMKV
AQQLYEGVDLGHQRLGLITYMRTDSTRISESALGETHDWLRQYFPAQTPDAPVRYSVSNA
AQDAHEAIRPTRVDITPDEAARYLKGDHLKLYTLIWERFVASQMKSAVIRTSTADIQIGD
GLFRTSASSFLEEGFYKIIRLGASKEERTSHQLPFEKGETLHVDKIDGVQHFTQGPSRYT
DASIVRALEELGIGRPSTYAPTIETLIERYYVQRDKRQLIPTALGKIINDILSQNFPEVI
NTSFTAKMESMLDKVEEQSVDWVSEIKGFYFPLKGKVDDVMHALEDMHGALDEKTDEK*
>SPBDM4_v1_50966|ID:27159329| protein of unknown function [Uncultured spirochete bdmA 4]
LSCSGFPECTFTKSVPLAKCPKCGGDIIPRVSTRGRRKKFYGCSNYPECDFMTLYKPTNA
TCPQCGWFLVEKYDKKTGVHKACINPDCNYLHSSDESKEDKGGE*
>SPBDM4_v1_50967|ID:27159330|xerC| Tyrosine recombinase XerC [Uncultured spirochete bdmA 4]
MDARFLDYLDYLGAVRGLSPKTVEVYKRDLAHYEVFLEGKDVDEADASDIRTFAGTLVME

KRAPASVNRTLSAVRGFYRYRLRFHADAKDPAREVENVPAGRPLPSFLFEEETKIFLDGI
DGEGFRDVRDRALLEVMYSTGCRVSELCGMQLARLNLSADSIRVKGKGSKERLVFLCDSA
VQAIKRYLPYRAALMRRLGIEEHNKIFINAKGHPLSTRGAEKIVEKRRLQAGIKKHLTPH
TFRHSFATHLVAAGADMRVVQEMLGHSSISTTQVYAHVDMERLRRVYEQAHPHGSKSK*
>SPBDM4_v1_50968|ID:27159331|hslV| peptidase component of the HslUV protease [Uncultured spirochete bdmA 4]
MEMHATTILAVRKDGHVAMAGDGQVTMNNTVLKSNARKIRTIYDGKVLCGFAGATADAFT
LFEHFESKVQEYGGDLTRASVELAKDWRTDRILRKLEAMLLVADQTKMFLLSGTGDVVEP
SDDAIAIGSGGTYAYAAAIAYLDASRRVEEAARAQGTEEPTKLPGYFSAREIAQKSLEIA
SSICIFTNDRIVVEEI*
>SPBDM4_v1_50969|ID:27159332|hslU| molecular chaperone and ATPase component of HslUV protease [Uncultured spirochete bdmA 4]
MMAEDDTLTPHKIVEELDRYIVGQNKAKKAVAIALRNRIRRQRLPAEIREEIAPKNILMI
GPTGVGKTEIARRLAKLCGAPFIKVEATKYTEVGYVGRDVESMVRDLMASSVAMVKEEME
SQVTEEAKRRAEDRLLDILLPGITSSDEPVGESQATVIVPRSGTETKEKFRGLLREGKLD
AREIEITVPAQGPQIELFAGSQFEEMNIALGGLQNFLGGKKKKKTVTVAEALPLLEAEER
ERLVDQEKIVQEARRRAQESGIIFIDEMDKIANRDGKGSSGVDVSREGVQRDMLPIVEGT
TVNTKWGPIDTMHVLFIGAGAFNVSKPQDLIPELQGRFPIRVELDALSGDDFVRILTEPK
NALVTQYIQLLGTENVQLEFSEEAIARIAEIAAAANRQSENIGARRLQTVMERLLEDVSF
DADKYADTKVTIDKAYVDEHFKDYVQQQDLSKYIL*
>SPBDM4_v1_50970|ID:27159333|whiG| RNA polymerase sigma factor WhiG [Uncultured spirochete bdmA 4]
MDYASLEENRLWELYHRTADIHIRDELVMRYAPLVKYVAGKVSVTLPSMVEYSDLVGYGN
FGLLDAIEKFDPKKHVKFKTYAVTRIRGAIYDHLRELDWVPRSIRRKTKQIERAIASLES
HLARPATDDEIAAEMGIDRRELLKIMAKVASSSILSLQEIWPMSSNSEGGTIGDTLESLH
TENPDAALEREEIRRVILEALQELPDKEKKVLVLYYYENLTLREIGKILEVTESRVSQLH
TKAILRLRAKLSNQRRGIV*
>SPBDM4_v1_50971|ID:27159334|atoC| fused response regulator of ato opeon, in two-component system with AtoS: response regulator ; sigma54 interaction protein [Uncultured spirochete bdmA 4]
MRATLLVVDDETNIREGLAQLFSMEGYEVRTAKDGIEAKTIVDRDDIDVVITDFKMGRMS
GLDLLQYIVSTHPGIPVIILTAHGSVDKAVEAMRQGAFDFIAKPPNTDHLINLVHRALET
RELYRTNIELKAEVESQRARSYIIGQAPSIKRIFDLIRKVAPTRASVLITGESGVGKELV
ADAIHNLSPRKDMPFIKVHCAALAESLLESELFGHEKGAFTGAAARKRGRFEMADGGTLF
LDEIGEINQNVQIKILRVLQDRNFERVGGEDTLEVDVRLIAATNRDLKKEITEGRFREDL
YYRLNVVNIHVPPLRERREDIPLLAMAFLKEFAEENGKQIEGFDPKARQALYSYDWPGNV
RELRNCVESAVVMASGKLVVLDDLPPGPRSSAETRVISIPAFSSMEDAERILIAETLALA
GGNKSKAAEILKIGRKTLYQKIEQYGIVTQDIDAATSSKKTEVPQDT*
>SPBDM4_v1_50972|ID:27159335| PAS/PAC sensor signal transduction histidine kinase [Uncultured spirochete bdmA 4]
MKAVYENALKRIDRIPPEGLKQIIKNLSAENSIYEAALHSTLDGIIVCDLEYKPLFINRS
AEKIFRLVSWTQEFALWEQLNDDNLANFFRKALVADETVLNREISLNRHGGTRVISISIS
ALVSEGKISGNLIHLEDITDKRRREAQLRRAESLAALTTLAAGVAHEIKNPLASIRIQLG
IIRRILEKNRTKKTESIFHNISLVEQEIDRLNSIVVDFLFAVRPMDITLILDRAEEVVQE
VVELMSHEAEDQHISIVTKIEPNLPDVMIDRKNLKQALLNIVKNAMAAMPDGGVLTISVF
VRNDELQIAVSDTGIGIPEELMAKIFEPYFTTKESGTGLGLTITFKIVKEHNGEITVESA
PSKGSTFTIHLPLPQPEQKSLPPWEDGVQIEGSHMED*
>SPBDM4_v1_50973|ID:27159336| protein of unknown function [Uncultured spirochete bdmA 4]
VFENLLFQDRAKEQLTSMIAGEKVPPALLFTGPEGSGKLTAALECARVLSCEKEGLWNCE
CGQCNRHRSLSHPDLLLFGPRNLPQEPSVTAEYFMHSPNLTSYYAFVRSIRKLLKHFDPV
LWAGEEARLSKAIASIESLEEQLQEIQTLVSRSKIQTLDTIVNRVVETASSLEAFFPDGI
PIFMVRNMAAWAVIAPVGKRKTVILQNADTANESTRNAMLKILEEPPETVRFILTSSRRA
AVIATILSRSRLISFEPRTSAQAQHIVSRLFRSQEKVENISEFFHKNSPFPPDKAEDEAS
LFVGALLDRAMGKDAAMTGDRASNLARRARDQKGSALEVLRAVAEQTGSFGSKNTKFSNS
FFVFMRAVAQQLASIAADPTSSVALIMYVDRLTSKLREMGVQYRSFNRSPELLLEAFVDM
FGEYHESSV*
>SPBDM4_v1_50974|ID:27159337| putative CvpA family protein [Uncultured spirochete bdmA 4]

MSTIDWVFSALVIILAARCFSRGFVHEVLTVASIAVGILAGLLLSNTVIVQILPKVGAST
LPYQVQYIIAFLLCFIVGFILMKIIERMIREGLEAISLDVFDRILGLVLGIAEGFIVVGL
FIVILQIQSLVDVKSILANSMYVKLLGPIIEPAIGSSLAPILKNENIPNIIQNFKGK*
>SPBDM4_v1_50975|ID:27159338|murG| UDP-N-acetylglucosamine--N-acetylmuramyl-(pentapeptide) pyrophosphoryl-undecaprenol N-acetylglucosamine transferase [Uncultured spirochete bdmA 4]
MSESKTTAEKNVLDNTSKDMSVARPCIAFTGGGTGGHIYPGLAVIEELRHLGFEGRIAWI
GSEKDLDRSIVAEQGVEYFAIPSGKFRRELSLRNLTDLWRIVAGYVKAKSILKNLRPSLL
FSKGGYVSVPPCRASTSLGIPVITHESDTSPGLATRLNSKRASLILTSWEATADYFPEPM
RAKIVRTGNPVRPALFLGDRTRGLDMLGFSAEKPVLFVLGGSQGAKEVNDLVLASLPFLC
TFVQIVHQTGKANFDEVVSKIPDDPVVRASYRPLSYIGPEIADIYAASSIIAGRAGAGTV
WEAASLGKPMILIPLSGPGTRGDQVENAALAARAGAAAVLEGAQLSPATFADAVKKYLHR
ETYDSAVRACYSIAGIPAQTVRALLGDRDLSSEKQMPSAEFIARLLLARFEQQAKEGT*
>SPBDM4_v1_50976|ID:27159339|pgi| Glucose-6-phosphate isomerase [Uncultured spirochete bdmA 4]
MVNFKGLDACDAFASLKKISKIRLHDVLTPARIENCWIPHAAELSYCYAFAPVDDLIVDR
LQALADEQQLIEKYRMLAEGALMNTGEGRMVLHHLARGRLGAPVIWNGQDMEIFYRDEKL
RLYKFAEKVRDGRVRGSAGRPFAQVVQIGIGGSDLGPRAASIALSRWAITRKRAKLTPWF
VSNVDPDDADWVLSHLDLPESLFVLVSKSGTTQETLANEMLVRERLKAAGLDPDRHMVAV
TSKTSPLAGNPHYLDSFFIDDFIGGRYSTSSAVGGVIISIAYGPETFEEFLRGAHAADAL
ALEPSLKRNPVLMDAMTGVYERNVLGWPATAVLPYAEPLSRFPAHLQQLDMESNGKSVSR
AGQPLSYSTGPVVFGEPGTNGQHSFYQLLHQGTDIVPLQFIGFLKNQLSSDMSSEGSSSR
QKLVSNLVAQMMAFALGKEDENPNKAFPGGRPSSLLVGDELGPEQLGALFSHFENKVMFQ
GFVWNINSFDQEGVQLGKVLAKKALGAISKGDSSGDVRLDALIAAVYENTRRRDRADR*
>SPBDM4_v1_50977|ID:27159340| protein of unknown function [Uncultured spirochete bdmA 4]
MEDTGTGMSAVKGPDLVAALARPASPAGAEDPIEVFARERFHVPHLMPLQSFVIANILEN
TGYPESAPDEGESGPGTNREMLPCDESHETYNQLVLFPTGFGKSICFQLPALLLEGLTLV
AYPLLALMNDQKRRLDEAHIPCALFRGGLGEAEWRAQEELISSGKATTVVVNPEILGAPR
LRRFLSRFTIAHFVIDEAHCISEWGQTFRPSYLTLGESAQALSPRILSAFTATAGPAIVE
SIEKTLFLGKGYRLVTAEADRPNILYGVVHTLSPTRTLRQLLLSCKKPAIIFERSRPGTK
IKAGYLKSIGFPESRFYHAGLSRLERKDIETWFQTSQDAVLVSTNAYGMGMDKKNIRTVI
HTSLPDSAEAYIQEAGRGGRDGKDSLAILIDDLASGSAALGRGSRQEGEQSVQSLRRASF
SPYPALGTCRREFLLHLLGEKETPVCGRCDNCLEKMQVSQNPATIGDGAGLSAEWRHLSG
AEGFLETLLLCRAHQRSWTKAECIRMLGPYGRGKAKFAGLLFGWTSEERKELVQALIELH
IIHIIDCGPWKNMISLSEKGKILSRYAQELLVRA*
>SPBDM4_v1_50978|ID:27159341|nth| Endonuclease III [Uncultured spirochete bdmA 4]
MKKEPSETPGAAQSTALLLEEVHRRLAPIWPDAHPLLHYHGCFELLIAVILSAQTTDEQV
NMVTEELFDRFPTAGKLAEADIAEVEQIIHPVGFFHVKARHIIDTAKVLEERFGGKIPPT
LEQMLELPGVGRKTANLVASACHEVPGIIVDTHVLRVLLRLGVCPKKDAGLAESIVRVNL
SPEKHTQFSYSVNRHGKFTCTARKPACTQEGMSCPLEDICPKIGAKG*
>SPBDM4_v1_50979|ID:27159342|argF| Ornithine carbamoyltransferase [Uncultured spirochete bdmA 4]
MNPSTLKGRSLLTWIDYTSEEVRYFLDLSKKVKEEAKKGLHKTRFRGKTIALLFEKRSTR
TRCAFETAFGEEGGHPVFLSNQDIQLGAKESIEDTARVLGRMFSAIEFRGYKQEYVEILA
KYSGIPVVYNGLTDSYHPTQALADVLTIEENFGNCRGKHLCFVGDGRNNVARSLMVISAKL
GMHFTIVSPKELWPDDELKNLCETFAKESGSLLSITDNVEEGVKGADAIYTDVWVSMGEE
ALKAERVKMLAPYQVNAGLMEKTGNPGCIFLHCLPAIKGEEVTFDVIEGPQSKVFDQAEN
RKHTIKAIMLATL*
>SPBDM4_v1_50980|ID:27159343| DHH superfamily protein, subfamily 1 [Uncultured spirochete bdmA 4]
MKPVPKVLLEFIDQHDCFYILGHREPDGDCIGSQLSLASMLQGMGKRVHVLSSGPFNRIE
ILPFESRFKSEVPAERDFERTAALVLDCSSMSRIGNIAERMPNIPAAFIDHHATGGASGP
YDWLDDSASAVSAMILVLMETMGHVPTKDEAELLFFGLSTDTGFFRHLDEHGSETFKIAA
RLVDVGVSPKRIFMAINGGRTLASRRMLGELLLRVQPYYEGRLLVSWVTIDDQQKYGMSS
RDSDLLYQLMMGIVDCEVCFVVKQETDDMCTVGLRSRDSVNVAKIAEQYGGGGHRLAAGL
SMNGKVEKVVHTLVEAFSEVFSDEETGK*
>SPBDM4_v1_50981|ID:27159344| protein of unknown function [Uncultured spirochete bdmA 4]
MDSKNYLDLVRQMYAHPKLYGFFNEDEISDALHHFRGRIEGILDRAEKESRTREAYLLSS

MRFVAKSVHRQNFSMNLCENAYVYSHFSEEVVLGPPADTFIDESPKEESIDAQGMIGLSP
QIFLRKLTPERKRLLYLVIKCAWDIDEELLKKCSFALGMPEQYLFSLIELAKRKTEGSRK
QIYELNAKLNTLWIRLRVLELRLETSLLKNEKECIACSLDRCRQRYIRLLEKRKHQKSLV
SNELISELLGVPKGSVDSGLFYLKKNVNSGQTLADYRKLG*
>SPBDM4_v1_50982|ID:27159345| Non-canonical purine NTP pyrophosphatase [Uncultured spirochete bdmA 4]
MKLLVATNNLHKLEELKPLFSEHELLRPIDLGIHDFNPEENGRTFFENAFIKAEALYHLA
GRPVIADDSGLCVEALHGRPGIESARYGSNNGRLLSAAEKNSLLLSELENMKNRHCVFVC
CLVLYYGPQRFLCVQETLEGEIGMAPRGDRGFGYDPIVYLPRKAKTVAELSQEEKNRISH
RGKAAQKMAAILGITPIEGTV*
>SPBDM4_v1_50983|ID:27159346|rpmE| 50S ribosomal subunit protein L31 [Uncultured spirochete bdmA 4]
MKKGIHPKYEHTTITCACGNVIETRSTVKNIQVEICSACHPFFTGKQKLVDTAGRIERFN
KKYGIKPAEEK*
>SPBDM4_v1_50984|ID:27159347|rho| Transcription termination factor Rho [Uncultured spirochete bdmA 4]
MAVYRRKKSISSQTEPFNNGNQDSTESKKEETNMNQSQVEEKSSEHNGSLFDGYSEGPVQ
DTETSIHETHVGTPAKSQSHKNRRPVAKIRLKKKATQPAEEENQPSQPAEEPEIPETQPP
NGFVVASLPADDRQHQKFAEASRLELENRAEQRNETRARLSINDLSKMGIVELRELGAKY
GINHDLLITLKKQELIFNTLKAHTEHGGIIYAYGSLEILPDGYGFLRSPQNSYLPGTDDI
YISPSQIRLFNLKTGDTVYGQIRSPKEGERFFAMLRIEQINFDEPSVAQNRVPFENLTPL
YPTQKLNLETTTEEISTRIINLFCPIGKGQRALVVSPPKTGKTILLQKIANAITANHPEV
YLIVLLIDERPEEVTDMERTVQAEVISSTFDEQATRHVQVAEMVLEKAKRLVEHKRDVVI
LLDSITRLARAYNQTVPTSGKILSGGVDSNALHKPKRFFGAARNIEQGGSLTIIATALID
TGSRMDEVIFEEFKGTGNMEINLDRRLSDRRTFPAFNIKKSGTRKEELLLTEEELQKIWV
LRKVINPMDDIEIIELLIDRMMKTKNNEAFLKSMNTPYQGME*
>SPBDM4_v1_50985|ID:27159348| protein of unknown function [Uncultured spirochete bdmA 4]
MRRLNFEPPEPYATYIGPAVILNGKLRSNLPLDLRCVLHGSLEGTFIRIDHNADIETGMC
TAQHAVIAGRFTGSMNVADTIVILPNANVLADIKAANIRIFEGAKFEGDIEITGISPTNY
VRD*
>SPBDM4_v1_50986|ID:27159349|hup| DNA-binding protein HU [Uncultured spirochete bdmA 4]
MAEKLTKAELIDALYESLSPSSRTTRKEIHELIDGLFSEIKSAILEGKIVELRGFGTFEV
KLRKGRSKARNPKTGEIVSVSDHGVATFRPGRELKKAAWEMDTTRIPKSFRKRD*
>SPBDM4_v1_50987|ID:27159350|rpsT| 30S ribosomal protein S20 [Uncultured spirochete bdmA 4]
LSARNLSAEKRQRQNEKRRLNNKSAKTAIRTAAKKVVVASEKKDTAVAKEALREMIKRID
SAARKGIVKKNTAARKKSRMQKLVNRLS*
>SPBDM4_v1_50988|ID:27159351| 1-acylglycerol-3-phosphate O-acyltransferase [Uncultured spirochete bdmA 4]
VFVNAAIVFLVLSMLFGLEFVLVVKYFFTPKRSRDHLCATYEQFGTKIIFKLFRVFRGFR
ITVLNPKKLNIPNHCLVISNHQSLLDILVLIQLLGIERKPRFVAKKELQYGVPLVSFTLR
KGGHCLIRRHGAPLETMRAVSKMAQVCMQERTCPVILPEGTRSRNGELGVFHSAGVRKIL
DVEPIPVAAIAIEGGWRVATVRDFLRRFGKEPYVAEIVKIYDTPRGKHEIEKVLADAHVQ
IAARIDALRRR*
>SPBDM4_v1_50989|ID:27159352| conserved protein of unknown function [Uncultured spirochete bdmA 4]
MPDDFHTQAEQDFYRAKTKTLASRIFRMLKPSLSELMPFDQAKQLIRPQSETYRGVTPVL
LEKIAGSEGRYRDFNRFFFPKKEHLKARWAGIDELQYKDVILPPVILYEMGGVYFVRDGN
HRVSVARAKGQEFIDAEVISLQSEIHLDPEMSIQDIKRAVIKYEKTRFYKETNYPNIVGT
DDLNFSEPGRYDTIKEHVLVHKYFLNQNMSEEIPFHQALFSWHENVYQPICHAIEAENLL
SLFSGRTVSDLYLFLVAHWDELKRKFGRFVEIEEAAESFKSQTKRDRIGFFDQVRKIFGC
NPQKK*
>SPBDM4_v1_50990|ID:27159353| Metallophosphoesterase [Uncultured spirochete bdmA 4]
MKKPLHILCVADEVDLLVYSAQICERFSDIDLILSAGDIPNEYLEYIVSMLNKPLISVAG
NHDKSDDPIGRGVLQYNNEQRGGLGRIRFSIKKENGIHVLGLPGSIRYNDGQNQYSDLWM
TFRIIRMLPRLLARRLLFGRSVDIILAHAPPRGIHDGGDPAHIGFSAYRWLIRLAKPYYF
IHGHVHLYDLQALREMNYFDTTVVNVYGHRVITLPKEDDNAR*
>SPBDM4_v1_50991|ID:27159354| putative DNA mismatch repair protein MutL [Uncultured spirochete bdmA 4]
MMGKIRILPPETSRRIAAGEVIDRPASALRELLDNAIDADAHDISVSITQGGIEEITVSD
DGYGMSREDLALSIKEHATSKIYAPDDILRARTLGFRGEALASIAAVARLEIASRPRGLD

EGWRLMSSPLQEPTVEPFPCKEGARITVRGLFESYPARKQFLKRPQSEAFLCRTTFVERA
LSHPDITFRWKSSNDLDVIMPSPQRERIVHCYPELSHIHMFDTSSKMDDMQVHLVYADIS
SYRRDRKFLQIFVNRRRVPEWGLINLMEYEFGKYLPGGAHAIAFLFLEIEPSLADFNIHP
AKKEVRLKNPAQVRTSVHALLSNELNARYKGATQALETNFPAFGEIAFTSEETAFSPKFD
ESQPFTVEEKPGKWVNGAAFGKSSQNANMHQSGPPLPADFWQKVKQGQLSYPRYIGKGPG
PFLLFELGDTLFILDQHAAHERILYDRIKSKKGESQSLLVPYVLEPHENEHYLKESEQNL
DYIGYRIKKEADTWIVEAVPAIAAAQALEALVEWLSRPSSDATPIDAIAANLSCKAAIKD
GDMLDQPSAERLIAEALALPEPRCPHGRPIFLAIPKEKLYAMFGRIIE*
>SPBDM4_v1_50992|ID:27159355| conserved exported protein of unknown function [Uncultured spirochete bdmA 4]
VKKVLLLLALLVVVLPVFAQDNRGTGFNFAAGLGTDLLPNPSDATKMESWSKLALQPEFS
MGKFGIGLDLMVRFKLATSGTAAFELYEPDWVPQNGQSIFDVYLPKILYIRYGYQWEDPF
YIKMGSISDFTLGNGLIVDNYSNMRFLPQRRIFGMQLGIDGSLFGFPYVGLEALTGNVTK
LDVIGGRFYVRPLAFFGKGLLGKLQIGAIAAYDRDPYLYVDDATYGTSYESAGSPPSEAT
YVVGADITLPVIQSNAFSLTTFVEGAREKNKSMGAITGIRGKVLRIVSYGAQFRYFQEGF
IPSYFDANYDLYRADRFVYTETTAAGTDFNPSWLASLGFDLFKSTLSFKATLDAPFGALP
SAPGTDNPAEYPHMLGTLGLARGLIPNVGIAARYEKFFLGKKSNNVFSDLADLTDASIGM
TVSYKAGSAVVAMDYAYSWNPTKDDFDVVSSLSVGFEL*
>SPBDM4_v1_50993|ID:27159356| Orotidine 5'-phosphate decarboxylase [Uncultured spirochete bdmA 4]
MEVHGRAGCAQLALDRNLRIIEATSPYAAAFKPNIAFYEALGEPGMATLRKTLSSIPDDI
PIIIDAKRGDISNTAEAYAQALFGELGADAVTLSPYMGLDTLGPFLKWEEKGVFVLCRTS
NPGAGFLQDIMVDGSFLYLALAKQCGALPKEVGLVVAGNDLEALRKVRQAAPSAWFLSPG
IGAQGGQADRAFALGARSDGKGLLVAVARAIADASEPSGAARELRDVMRKARDIHLTAEK
ASQGDMESEGLAVLKHDFVHALLSTGCFRLGEFMLKSGKKSPFYIDLRRLVADPDAMKIA
GRAYAFLASKCTYDSIAGIPAAGLPLATAASLEIGKPMIWPRMPAKEHGTGNRIEGSYVA
GECALLLDDLITTGASKLEAIEILRSEGLQVHDVVVLIERGNEGRLDMEGAGVRLHAFIH
VLELFDVLKQSGKIDEQQYQTLVEFTKNT*
>SPBDM4_v1_50994|ID:27159357| Transcriptional regulator, BadM/Rrf2 family [Uncultured spirochete bdmA 4]
MRMTTRGLYALKATLTLAELSTEQKRVSLQRIAQIEGLSPEFLQQIFYKLRKAGIVKAFR
GPGGGFSLNKKPEEISAYDILFAVGETLEIVPCAFERNEKKPCPKYECCDAGNFWMGMER
IVMEYAKSRHLSDIQQGMNR*
>SPBDM4_v1_50995|ID:27159358| protein of unknown function [Uncultured spirochete bdmA 4]
MQPYRIQLVDLTSGDTYLAAQRPKDRKDIPFDFCPESFAHIRENCIRSLDQTTQEIKIQT
AQSKTLYIIDETGPLGHERNKGHLEFLKVVLDSALADALVLTVRADLQGVLLSILKKHDI
PTADIHLLEIDKQFSMDAQKAASFFIETI*
>SPBDM4_v1_50996|ID:27159359|rex| Redox-sensing transcriptional repressor Rex [Uncultured spirochete bdmA 4]
MKLNYAPSIRRLPSYLHIIRAFQRTGEPYISGTWIANELNLEPIQVRKDLAITGIVGKPK
KGYPVEQLIAAIEHYLAWDVEQKAMIVGAGNLGSALIGYQEFRNHGLHIVAAFDNDLSKV
GHSIHQIQVYGMEELKEVIEKKNITLAILTVPSPYAQSSCDAIVTAGIRAIWNFTNVKLK
VPEDVLVQREDLSSGYAILSVMMRTRAKAMS*
>SPBDM4_v1_50997|ID:27159360| protein of unknown function [Uncultured spirochete bdmA 4]
MSSLLKLMRILEWADWEAPPAEMNCEMPFKTIISTIADLVPDIASAEEPLNSLAPQQALR
LLNKTIRFYVLIPSIVNVLLNYKICVEHGLPLHPTVYYELKEARKYRISHSLGEIQRANE
LYWKSIDVARECCRFAPHATQDLSLLLSEAPPSVSSFVYTAVQDPYTWLGSKPSLLSTLA
EKIKKSYQPCLLLAAAHGSIMPALVLSELLDIPLYFIRFSMFKRHDEEPIVSLSDHAWLF
DFRGKNVLLYDEDVAGGRTLELFLKRLSPLFGQVKTACSIRHAGSSLHPDFFGRMWWD*
>SPBDM4_v1_50998|ID:27159361| protein of unknown function [Uncultured spirochete bdmA 4]
MSQRHDIVHEAEISAFCDPEFVFLNINDPASFETAPGKAARHGLFRNAQV*
>SPBDM4_v1_50999|ID:27159362|tatA| Sec-independent protein translocase protein TatA [Uncultured spirochete bdmA 4]
MFGRIGPMELILILVIALIIFGPKKLPEIGKAIGDAFKAFKKTQEDVTKEADKIVSDASA
KSGEPKEMAQKDADADKGEAAKS*
>SPBDM4_v1_51000|ID:27159363|tatC| Sec-independent protein translocase protein TatC [Uncultured spirochete bdmA 4]
MTYIEHLTELRKRLLIVLVVFLGFSILCFGWSKQLATFLVSPVQPVEFVFLSPPDLFMTY

LSLSLYAGFVLSLPVILYEIGMFIWPGLEKKERKAIFLSLLLGALLFIAGVAFGFFIMLP
YMLKFFLGFGTEGIKPMISIRDYLNFVGQIVLSFGIAFELPVVTTALAGLGVINAAQLKS
ARKIAVLVIFIVAAILTPPDVVSQLLLALPLLVLFEVSIVLASFVEKKSKAKVQEAT*
>SPBDM4_v1_51001|ID:27159364| Glutamate formiminotransferase [Uncultured spirochete bdmA 4]
MDKPLVECVPNFSEGRDHAKIEAIANAIRSVPEVSLLDVDPGSDTNRTVYTFVGAPEAVK
QAALEAARMAHAIIDMRAHHGAHPRIGALDVCPFVPVSGISMEGCAQLAKDFAAQLAEEF
RVPVFLYEKSASRPERQSLADIRAGEYEGLADKLADPAWQPDYGPARFDARWGATVTGAR
EFLIAYNVNLNTKDKKLAHDIALSIREAGRSIKDAEGNTTRVPGRLRAVRAIGWYIETYQ
CAQVSINLLDFHETPLYAVFEAVKEEAEARGLYVTGSELIGLIPLEAIRACGQYYRRKMG
RSPALADRELVDLAARTLGLRSVQPFESDKKIIEWAIGRAGSLVSMDVCSFVDTVSSDSP
APGGGSVAALAGALGAALGAMDANLTVGKKGYEAAAESLSVLGIKAQGLKRALLDIVDED
TEVFNAVMEALRLPKCSDEEKIARNAAIEEAEKNAARVPLKTAELCLETMKLCADTAKSG
NANSVTDAATGCNMAYAGVLSAILNVRINVQTLIDEQFKTAMRHDCEVLEDQAAIVRQEM
DKTISDIFRD*
>SPBDM4_v1_51002|ID:27159365|potA| polyamine transporter subunit ; ATP-binding component of ABC superfamily [Uncultured spirochete bdmA 4]
MKGADVDILGVSKFFGSFQALNNINLSISKGEFFSLLGPSGCGKTTVLRLIGGFEDPDSG
SIAIEGKNVVGLPPDKRHCNTVFQSYALFPHLNVFENVAFPLRIRKVPQQLVREKVIHYL
ALVQLETHALKKPSQLSGGQRQRVAIARALINEPSILLLDEPLSALDAKLRQHMLMELDA
IHDKVGITFIYVTHDQQEALSVSDRIAVMNMGEVLQIGTPRQIYENPATEFVARFIGETN
VFNLKIISIDGTRIYGETEGLGPMSVEDETEANPGERVLATIRPEKIRISTDIPNTNGGH
INILHGYVAEPIYSGFQTKYVVQLDTGMMVTVYRQHANWSEGIPDIEWKDEVYLSFSAAD
MVIVEMHKQ*
>SPBDM4_v1_51003|ID:27159366| ABC-type transporter, integral membrane subunit [Uncultured spirochete bdmA 4]
MKKQVGLLYSLPQILWLLVFFAAPLCIIIVYSFLKKGLYGGVEPQFSLEAYTALANPNIL
LVTWRTLKISIIATALTLLIALPCGYSIARSKNQAARLFLVIVPFWTNFLVRIYAWIAIL
GNEGFLNDILRALHLTTESVQFLYNQTAVILVLVYMYLPYAILPLFSTIDKFDFTLLDAA
RDLGASRFQSYVRVLLPNIKSGLMTAGLFTFVPIFGAYAVPLLIGGKDSYMLGNIIADQL
TKTRNWPLASSISMTVTILTTGLVFLIAMRKPREQKQVFDADPAFLQAVPAGKR*
>SPBDM4_v1_51004|ID:27159367|potC| polyamine transporter subunit ; membrane component of ABC superfamily [Uncultured spirochete bdmA 4]
MLRKGGIAARKFMPAGSSLSNVIYWVVIGFLFLPLFVLVLYSFNSGRQTTWQGFSFVWYQ
KLVMASPELWRAFFNSITIAFGSALMSTAIGTLAAVGTTRYSFKLKNLVSTMSFVPMILP
EIVVGVSLLIFFAGVGLRLGLLTVWIAHTTFNLPFVYLLVSARLEESDPSIVEAARDLGA
NEMQILFRIILPMALPGILSAFLTAVTLSLEDFVITFFVTGPGGTTLPLYIYSMIRFGVS
PVINALSAVMVAGTVLLVYPMRNFLKVFAAR*
>SPBDM4_v1_51005|ID:27159368| Extracellular solute-binding protein family 1 [Uncultured spirochete bdmA 4]
MVKLRKVLLNLSAFALICTLLVLSGCGGGGQSKTLYIFNWTYYTPDSVIQKFEKEYDVKV
VYDTFASNEEMFAKLKAGGANYDIIFPSGDYVSIMIKEGMLEKIDKSKLKNFANIDPQVL
ELCDFDPGNQYSVPYYMGAAGVAVNKTKVQTYDRSWSIFARKDLGNHMIMLDDMREVMGD
ALKYLGYSVNTTDQKQIDEARDLINNVWKPNLLKFDAEAFAKSFASGEVWVAQGYAESIF
AEVDKANWNTVDFFIPKEGGPSYVDSMVILKGSKNKDLALKFIDFIHRPEIYAEFCDYFG
FPATANVPARALKKGESWYRPEDLLNCELKEDVGADLEKYNAAWQTIRVGK*
>SPBDM4_v1_51006|ID:27159369| Rhodanese-like protein [Uncultured spirochete bdmA 4]
MALNPIEELINNGAVVVDVRTEEEFGEEHYPNAICVPVNEIMQRADEIGQKDKPVVLYCA
SGARSAYAARILKSLGFAKVVNAGGLYDMPNY*
>SPBDM4_v1_51007|ID:27159370| CoA-disulfide reductase [Uncultured spirochete bdmA 4]
MKLVIIGGVAAGATAAARARRIDEKAKITILEKGPYVSFANCGLPYRISGDIQKRGRLIL
QTAEGFFARYRVDVMLNTEAIGIDRQNKQVRVKSKDGESVVPYDKLILAQGGTPIRPQID
GLDSPNVFNLWTIPDTDKIEAFIKEYDPKSAIVVGGGFIGLEAAEAFNNRGISTTIVELM
NQVMPPADPEFGAQIAEALAEHGVQAITGKSVVRIDWNARRATLSDDSTVSADMVLLAVG
VRPNLELAKQAGLATGSANGLVVDEYLRTNDPDIYAAGDMVEVVRVPDGTKVRIPLAGPA
NRQGRLAATNALGGSMKYAGAMGTSVVKVMDYTFSMTGLSEKAAKAANIDVRAVTIHKAH
HATYYPGSEDLSLKIIYQKSDGKVLGAQAFGKEGVEKRIDVLAVAVHAGLSLEDIAELDL

AYAPPYSSANDPMQMASFAALNDMHGFSKFVTAQEAMQPIRQGTATILDVRTYAEYLNGH
IKGSVHIPLDELRDRIEEVPSEQILIVSKAGFEGHLAYRQLIQNAKNDIRYISGGYTSLR
LLQEAQNIIEEGE*
>SPBDM4_v1_51008|ID:27159371| Aminotransferase, class I/II [Uncultured spirochete bdmA 4]
MLRYNNHMHDFFSKRSEAGEAAPNVLALIREQFSGEQRPLKDLSQSNPTKVGLSHAPELF
SLKNPENAVYTPEPKGLFHSRAALSEHLAREQRKISPENIFLCASTSEGYAWLFKLLCDA
GDIVLIPKPGYPLFEHLAALEHVRTMAYPLEYAHSSGWHIEVAEMESMLASEYGGRIKAL
IVINPNNPTGSYIRKGERSAILRLCERYGMALIADEVFFDFPLDDAAERESFIGEERVLT
FVLDGLSKRLGMPQMKLGWIAVSGPPQEALRAKKRLELIADTFLSAGTPIMNALPSLFAQ
ESAFLQELLTRIRENYLIYRTILEQQGSPHRVLACQGGWTALIESPAFFEEEKIAGLLLQ
QKGISAQPGFFFDMERGVHFAFSLIIPQEWATSWCREYKEFFDALQTQ*
>SPBDM4_v1_51009|ID:27159372|folD| bifunctional 5,10-methylene-tetrahydrofolate dehydrogenase and 5,10-methylene-tetrahydrofolate cyclohydrolase [Uncultured spirochete bdmA 4]
MSAILIDGKKIAEDIRSELAEKVASLCEKGVRPGLAVILVGDNPASVSYVTGKEKACAEI
GIQSFETRFPEDVAEKVLLEKIAECNRDSNVHGILVQLPLPRHIDERRVISAIDPAKDVD
GFTPVNLGRMLLEEPCYIPCTPRGIIELLKRSGVPTNGAHAVVVGRSNIVGKPLENLLIR
KSVNATVTICHTGTKNLAAHVKEADIVIACAGKPGLVSVDMIKPGACVIDVGVNRVPDST
KKKGYRLCGDVDFDDDSKVAGWITPVPGGVGPMTIAMLLSNTIDAASRLSDIQW*
>SPBDM4_v1_51010|ID:27159373|folE| GTP cyclohydrolase FolE2 [Uncultured spirochete bdmA 4]
MVDVQSFQDDRHIAIQKVGVKGLRYPITLLDRSEKYQHTTALVNLYADLPHEFKGTHMSR
FIEVFEEYRQDLSMPNVLRMLKKIRIELDAQTAYTDIHFPYFIKKAAPVSGQTAIMSYDC
FYEASSSEKGHRFIAGVTVPVQTVCPCSKAISDGGAHNQRGVVTLQVSLGPFFWFEDLIR
IVEESGSSELFTLLKREDEKFITEKAYGQPRFVEDVVREVYSKVDALHRFPWFSVEAENY
ESIHNHSAYAYVEKSERSKIEPVNGDYGKFDTLGPM*
>SPBDM4_v1_51011|ID:27159374| membrane protein of unknown function [Uncultured spirochete bdmA 4]
MESNYPVDPKNLSDKGTKGVMSTAAGIGMLGVNALLGVPVVGTVISAGLLALGAIGLFGK
TKTDKASGTVLAAAGIAGIATLFVPGVAHSLLSLGGIGLLGYGVFNLFGFLKGLHRKK*
>SPBDM4_v1_51012|ID:27159375|miaB| (Dimethylallyl)adenosine tRNA methylthiotransferase MiaB [Uncultured spirochete bdmA 4]
MKKYHIETYGCEMNKAESAAMEATLREHGWERSPEEDAQLVLLNTCTVRTTAENRAWNRI
HQLSARKKERGFVLAVVGCMAEQHKGAIQKKAPGVDYVLGTFQKQSFGLMLDIISKGQRI
EVLEETPTYVFDASHHEPGAFRSFVPIMHGCNNFCSYCIVPYVRGREVSRKPADILHEID
QLEDLGVREITLLGQNVNSYRWEADGQVLNFPGLLRMIAGHLDNRHAIARGAPADPRQFG
GRIGWIRFLTSHPKDLSSELVEAIVDNPIFCRQIHLPFQSGSNAVLAAMNRKYTREEYIC
LVEKLRKEMPELTLSTDILVGFPGETEEDLDETLDLMRRVRFSYAFMYHFNTREGTPAAR
MPNKTPDKVKKQRLARVIALQKEITASLMEERIGQTDEVLIEDISKRSSKEVLARTTRDE
MVVFAASQKRIGHFARVRLLSVAGNTFRGEEV*
>SPBDM4_v1_51013|ID:27159376| exported protein of unknown function [Uncultured spirochete bdmA 4]
VNSTRREKLKGPVRFSGIAFLVVSLLMGMPAPSAWGQTYKNLAQARQAATGAKSQDALKT
VLLSALPQLSVRDGIMLCEEYESKVGTAFRAELRSTVGGLYLLLGQTEDAAMWYTKAASL
DSKYVMEALRLSIAVGDQKSTLQLLKNEALSEESRAMLDVWLSLYDNDYASASAKAKDAL
ARVSDQQVRRELLFLQYIADFGQFGTSHSSLTKDFPSSIESDLVTGKVFPSSWFVLSLGL
SWLGTPALLFDFPKKNLPNEENKNNTVTQWLQVGYFSSKDNAERLSKTLTAKNFQTRIVE
LKNENGDLRWAVHVAAKEDWQKTQSTLKDQGYESYLIGP*
>SPBDM4_v1_51014|ID:27159377| exported protein of unknown function [Uncultured spirochete bdmA 4]
MLKRTYIAAFLVASAFAAWLVALQRQENLQAAVPIENRTVMLEVPSGKNEVPSQNTIQQR
AKITPLPGEIFLKTIDVNLDDDEDFEQVILSKKSTASTSLEIVVADFSQALGVYFRHFEG
PIAATKLDSIIIQPMDVTADGLSDLLVQGLDASNDQTLTIFRRLSDRGYVRVFSGAGSEV
TLQDPDNSGGASAASIVVQTPSDSPGMVVQTAYTWNNRLSSFEKKSETSVRQNNAFALGS
GGTDAQVFLSWLNRLWTRNTDASDPRSLFLDVKNNALIFGDQQIQQRWIIKSAERNENRL
YLTCSTSEASDLDRVVVIDAKAQDEIAVGIIDQQVSHFRRDEGWSGTYSGVAPVAALPQS
IMRSEPNFDIFFGRYLGNDDSVLVLGPSKSIIVIDKKYREGIAKFYDYHGYQVLDFLQIK
PNGLAGERLLFVVSAEQSGDGAIRRVRLEPAQVGPDGVRIDYRPPYEFTKTS*
>SPBDM4_v1_51015|ID:27159378|rocD| Ornithine aminotransferase [Uncultured spirochete bdmA 4]

MNSRDYIALDNRYGAHNYHPIPVVISKARGCKVWDPEGREYYDFLSAYSAVNQGHLHPHI
VEAARAQLERVTLTSRAFHNDKMGPFLQKLCEYTGYEKALPMNTGVEAVETALKAARRWG
VEKKGVENGKQSIICATGNFHGRTLTAVSMSNDPESFINYGPYVPGFIKVSFGDATAVRS
ALEKNTVAVLVEPIQGEAGVVVPPEGYLTELRKICSDAGILLIFDEIQTGFCRTGRRFAW
HYESARPDIMCLGKALGGGIMPISAIVADHEVMDVFTPGTHGSTFGGNPLAGAVGVAAIE
VLEQEHLEENAFRLGERFRQAIGTVESARIRLVRGKGLLNAVVFEEKFDAYPTCLALKEK
GILAKQTHGNIVRFAPPLVISAEELEDALERIMSVLGNV*
>SPBDM4_v1_51016|ID:27159379|ddl| D-alanine--D-alanine ligase [Uncultured spirochete bdmA 4]
MKTIAVLYGGKSGEHEVSLISASSIVAHLDKAKYSIVPIGITKKGEWYLQHLPEWAYTPP
EQGLMRSLPEVENAERVVAVPGDGLWLETENCLQNLNIDIVFPVLHGTFGEDGTVQGLLE
TASLPYVGADVLGSAVGMDKEISKRLWLSAGLPVVDYISVNKEDIAEENFQALVRKIETR
FGWPCFIKPACSGSSVGTSKVNHPDALKAAILSALRWSERALLEAFVEAREIECAVLGND
KPVAFPPGEVVPSHEFYDYEAKYKDPNGALLLIPAPLSEETKNRIMSLALSAYKYASIKG
MARVDFFVEKITGSVFLNEINTIPGFTAISMYPQMCTSGGLQYPDLLDKLIGLGLDQYAT
RRALEYDFSKV*
>SPBDM4_v1_51017|ID:27159380|murE| UDP-N-acetylmuramyl-tripeptide synthetase [Uncultured spirochete bdmA 4]
MSKRLSDIISGLDFLDIQGNIEQSVSGLSYDSRECKPGYLFFALPGIHTDGKQYIRRAVQ
HGAIGVVYEGSLDQGAGEPSPWTGAKTRSENIAFLRVADCRWAMSAISGKFYDDPSDSLC
VIGVTGTEGKSTTVSLIYQLLQFAGYRAGFFSTVTSDTGDGERPNSQHQTTPESTAVQEM
LAKMRDCGLSFAVVEASSHGLSPRTARLAHVHFDIGLVTNVTHEHLEFHGTWEQYRSDKA
NLFRRLGEAGPKRLACTNACIPLGIICADDPSAAYFAGESAAPCLTYSSKGNLADLCARD
IEGDPEGSNFTIEGPSGTVQAQGAAPHAEARGAEGNVSRHALPARLNLPGTFNVQNVLGA
ILAASAATGLHWSRLVPYLPKLRPVKGRMQRISEGQPFDVIIDYAHTPSSFKEILPPLRR
ERKGKILCVFGSGGERDRAKRPQQGRIAADNCDIVILADEDPRSEDPMALLEEIAAGCPE
LPREERLFLIPDRPAAIRKAFSLAKTGDLVLLLGKGHENSIIYADHSIPYDEETTARSIL
ADIGFGAEGKGK*
>SPBDM4_v1_51018|ID:27159381| 6-carboxy-5,6,7,8-tetrahydropterin synthase (fragment) [Uncultured spirochete bdmA 4]
VWVSGKELGEGGMLVDFSIVKAALKKLIDEALDHRDLNGLPEFEDDPSAERIAKFIYDRL
RGVLPEVPLSAVDVFETDTSMARYVPDSVERF*
>SPBDM4_v1_51019|ID:27159382| putative Radical SAM domain protein [Uncultured spirochete bdmA 4]
MMREGRIVTRAVAADMANPRFEDARCRILIVRLSPYRDIEVSYSHLVLFDESRRALPDAF
IDFAFLPPLQDRKTLTALSRPWFFGRASGKSPAEFDIILISCAFTLELINLPWLHSQSGI
SPSRGERLRAPTVPLLFLGGSSTVTAGSLLKMEKDEVLESLVDAFFFGEGEGRVQDIVQL
ASNGLQGGAPKSTILTEIASEVEGFWPCASQFTAMRALSRQRPLPLVSPLVLNGENAGHT
KLAITAGCSGHCAFCLEGWDRRPYREKSLQELSEAALALKRATGASDVELFSYNFNMHHE
ILDLIPTMGRYFFRVSLMSQRLDILAKNPELLSAEFAAGKRSFTLGIEGISERLRQYYYK
GISGAQIWSAVSSILKQKARELKLFFIISGFENDADLNELGAFCGEMARFRDEMHSTTRI
IVSAGYLVRLPFTPLQFAPLSHDRAVLERIVSRFEALCAENNLEFRLASSFEDYWVDQLL
SIAGPLAHDWLSACPEHGFVYDVHVPRAALKSLDECLIKTSDFSMLLEEKPGDYRPCFSF
VESESHWNLLRAHYERSVKYLHEQTKLNTLQRQKSDLEGEAPVRPPAQFSKEADARRALA
LIAAQEESKAHFPHIFVKVSEDDALAFSTEAYECAWLVRTLSALVPNAERALFNCRQVLP
HDQWTNVFPNATAPHFGLSGEKFFALYGPDVAILEKIVAHASKHSVSGSPALLESIEQAN
MPPITETCMLLCTISGMSGGRLASILRSWLDTQNLHFTLNSTENGHEFHISKSSSSKKSV
LFVQYHTYSSNIELRVRVGKRANMQTFADLLHKEFPNEEILFRIEGWA*
>SPBDM4_v1_51020|ID:27159383| putative DNA/RNA helicase, superfamily II [Uncultured spirochete bdmA 4]
MNDFLSLGLDERLGLALHDLNLGTPTEVQKQAIPILLQKKDLAMQSETGTGKTLAYLLPI
FQALLASKPRPSETHWPKALIVCPTQELAVQVARQTALLAQAASLNIKNLALLGGTHFSH
QKEALKAHPDIIAGTPGRLADLTNMRFIDLSRLEFFVLDEADRLFSKEYLEPVEFLLSKA
PQNCVHIMASATIPEKTLRKAEPWMPSPSILELGSEGILSDAIEHWVFYAEHRKKVDMLK
KIIGAARPKRCLIFASETYRVQRITERLASSGLKCTSIVSRMEKQSRYSAIEHFRQGSVP
FLVTTDLAARGLDIPDITHVISLDLPEESNAYVHRAGRTGRAGKRGISILIADRIELERA
SRTAVRFGFVFRTKRLEYGHVIEPTVEEFLEHIEKMDADGSNRAHS*

>SPBDM4_v1_51021|ID:27159384|rnz| Ribonuclease Z [Uncultured spirochete bdmA 4]
MNLEAFVLGSGGMMPLPYRQLTSMLVRREGELFLFDAGEGTQVSIRRLNLRWKKITAIFI
SHMHADHVTGLPGILMLSSQVDREEPLYIFGPPKLADYIDQNRRLLDMYINYDIIVQEIS
ERTVVWNGDGYSIRAFPLRHTKMCFGYSLEEAPRPGVFYPEKALSMGVPRGPLWSELQSG
KTVTLPGGREVHSSDVMGEPRSGRKFSFVTDSLYFPEISQEVSDSDLFICEGMFEQGLIE
SAIDKRHMTARQAAQIARDAGNVKRLGLIHYSPRYADRELKVLLDEAREVFPNTVLTKDR
MCFPVEFVD*
>SPBDM4_v1_51022|ID:27159385| membrane protein of unknown function [Uncultured spirochete bdmA 4]
MKRTLRIILFIGTLVLIAALVGFVALKIGEFRLSGEQSASAEYAILRNAVSPITSEQEFG
DQFVRDRLKSLYSASAHLLAVQVLDRNGLVLWKIPDDSPYFASPATSPGSVFHAPGMSTV
IYMTPLPDGMKLMALYSILSQKDIANILLVPIIILAAWIILLVILQIVFKGKPVEVAKTE
PVPAKTVEPPMAEEIETQPEEPSGHEEQQKTAGHIEGNAEENIEGNIGENVGEAAPEEAS
MEAAELDEEIEEILQEEELPPEEAPFAAVPAPENPELKPGAEAVIPSQPEMPPEKEVLPV
REEPPQRKIISEETSPAESEYDFEEAEFVAPPEKTEETPEPEAFPSSLSLDMLKRTLETE
LQRSQETSLLLIQCMFSGESDPSALALGVTIRDYFASESLVFELAKGCYAAVLPGVDAGS
GLKLAVDLDDVLTTTASLYKDLSEEPPFYFGISALYDRTISPERLYKEALAALQKAHESG
SRILAFKPASPTA*
>SPBDM4_v1_51023|ID:27159386| conserved protein of unknown function [Uncultured spirochete bdmA 4]
MSMDLKKRKRYLPAGWYPDNAAEIRSLVSDWTKLQNNLSAFAIVAPHAGWYFSGDLAAKA
VWSLRDCDTVAILGGHLRRGDPILFAEEENFDCTVRDAANDAQVLNALNSELKDAGIQEI
APDHDIDNSVEIMLPLAALRFPEARLVWLRVPPDYKARELGSALARAASTCRKKLAVIAS
TDLTHYGPNYGFMPHGVGESAVDWVRNENDRGFINAVLSMDTDKVLGHARDRFSACSSGA
VAAAISFSLNNGAKRGVLIGHKLSFDMHPDRSFVGYAAIAFVP*
>SPBDM4_v1_51024|ID:27159387|murD| UDP-N-acetylmuramoylalanine--D-glutamate ligase [Uncultured spirochete bdmA 4]
MSIIDAIDEFRGGRITVMGLGLHGGGIASARFFAKAGAEVTVTDLRGPEVLEPAIRQLEA
FHIHYVLGVHHEEDFAKADIVIKNPAVRRDSPFLRLAKRIETDISIFLRYSESPLIAVTG
SKGKSTVASAIWYVLSQYHEKALLGGNISVSPLDFLDETGPDVPVVLELSSWQLGDLHNM
GILKPKVAVLTTIFPDHLNYYDSMEAYVADKRVIYEGQDEHCYTICSADQDWGRSFARET
RAQVLWYSEKPLAELAEGDSQGGWLAKDDSAQALAGSSPGFFSGPESFSGFGKFSQHGPI
ELLLPHQVFVPGLHQKKNLLAAGVALRAFGVPSPDIALSLGNFPGVPHRLEFVAQVRGIR
WYNDSTATIPDAAIAAIESFSSPIVLIAGGSDKMSDFSAFIQKAKKMKAIILLAGSGTER
IIPLLNENHIAYEGPYHSMRDAVDAANKAAEEGDVVLLSPGCASFGLFLHEFERGDAFKN
EVARLAAPTID*
>SPBDM4_v1_51025|ID:27159388| Pyruvate/2-oxoglutarate dehydrogenase complex, dihydrolipoamide dehydrogenase component [Uncultured spirochete bdmA 4]
MSRIEQHPILSVPKDRPCITFHFDGEEIPGFEGEPVASALIAAGHPVFSHHHGDGAAQGL
FCANGQCSQCTMLIDGVPKKSCMTPVAQGMDVRTLRGLPKLPAQDEPLVQAERRSIRTDI
FIIGAGPAGLAAAAELGTMGYQVLVADDKPRAGGKLVLQTHKFFGSESDCYAGTRGIDIA
RLLQDEATRHPSVTVLTNAPVVGLYKDRKAGIFLNYESYLLVEFTALVVAAGAREKALQF
PGWDLPGVIGAGAFQTLVNRDLVKACRKILIIGSGNVGLIAAYHALQAGISVAGIFEAAG
RISGYQVHADKIRRMGVPIFLNTTILKAEGAEKVERAHIAAVDEKFQPVPGTTRVYEIDA
VLIAVGLSPCNEFLEQARRYGIMAVAAGDASEIAEASSAIYGGKTAAVNLARMLGKKIAI
DPEWEKTRQVLASRPGDSYERSPIIPRLEWRPVFFCSEEIPCNPCATVCPVNAIRLKPLR
GTIMDLPYFAGNRCTGCGVCVAICPGLAITLVRRIADDKAQIVLPWEFNVDFEIGAEMDL
VDQKGGFVERAAIISRRKVSSRNTWLLTFNVNVESASRIAGIRVQPGEVAASLRRGNDAR
VAESTDVLDTVLCRCERVTLGEIVEFIKKHNVRDTNQLKSLRVGMGACGGKTCSQLVAKA
FALAGIDPADVEPPTQRPLFMEVPMGSLVNEGLHKLGGKDAGARGAP*
>SPBDM4_v1_51026|ID:27159389| putative oxidoreductase [Uncultured spirochete bdmA 4]
MKHFDVVVVGAGSVGLPLSWQLAEKGLNVAVVDEEASWGRGQNRAAIGGVRATHSDAAKI
RICLESIEIFSTLQSQYGFDVEWRPGGYLYVAYDAETESQFRSLLEKQKVAGLNIDWVNP
DTIGRMAPGILMDDLRGGTFSPNDGYASPLMAGSAFHSLALEAGVSFFFNSRIERIRREG
NRVASISVGDEEFAAGLFVNAAGAKAADIAKVAGFDIPVFPDCHEAGVTEPVERFLEPMI
VDIRSDAESGNYYFYQASTGQIVFCITPRPQVWGRDKDSTSTFLPLAVRRMLELYPRLRN

LRVRRTWRGMYPMTPDGLPIIGYPDSAVNFLQMTGMCGQGFMMGPGLGRIIARTILAGGC
HEKPAGATEYGFVFEELSINRNFDHVELLK*
>SPBDM4_v1_51027|ID:27159390| Rrf2 family protein [Uncultured spirochete bdmA 4]
MFQVPTKTQYAIRALVHLVRTKSASVASIAEAEHIPFKYLEAIFGQLKSAGLLIADRGRS
GGYRLAKAPSLISMSEVVRATEGEIRPVECLDNATLCIVSEGCLPRRFWLGLKRTVDAYL
ESVTLAEIAEPGFSAAEELAREETVVARD*
>SPBDM4_v1_51028|ID:27159391|iscS| cysteine desulfurase (tRNA sulfurtransferase), PLP-dependent [Uncultured spirochete bdmA 4]
MNTRYVVYMDYNATTPLRPEVRDLMVKSLDIYGNASSMHELGRKARAEIEGARANVAALIG
TKDPSTVYFTSGGSESNNTVFNTMFLRGRNQTRHKIITTAIEHPCVLNAAAHLRDEGFPV
VFLPVDRDGRIDMDAYRAELGPDVLLVSVMAANNEIGTIEDVGEIARLAKAQGAFVHTDA
TQAVGKIPVSVEDWNVDYLTLSCHKIYGPKGIGALYVRRKTPIEPLIRGGHQENGVRAGT
YNNLGIIGFGEAARLAQEELSEYRQQVGSLRAMLRDLILERIPMVRINGPAAEVLPNTLN
VSFPGAEGESILLSLDLEGIEVSTGSACASGSLDPSHVLMAIGLGPELAHGSIRFSLGKY
TTEDDVRYVADRLPPIIARLRKMSTIPLEQLAQGIG*
>SPBDM4_v1_51029|ID:27159392| IscU protein [Uncultured spirochete bdmA 4]
MDAFQWLYSDIVKDHFTNPRNVYDTDRDKDFRPDAEGMVGNIKCGDQMMFMLKIENDLIT
DVRWKTYGCASAIASTSMLSETIKGMSIRDAYHIKPADIADRLGGLPENKIHCSVLGDKA
LRAAIDVYLERKGRAGEFSGEDAQIICKCLNISDKDIEEAVRQGARDWYTLQEATKIGTV
CGSCKTKAEELLHEFVHIYS*
>SPBDM4_v1_51030|ID:27159393| A/G-specific adenine glycosylase (fragment) [Uncultured spirochete bdmA 4]
MNLSISTAEDIAESRRFLLEKGRACYRELPWRGSVSPWAILVSEIMLQQTQVSRVSAIYP
RWIESFPVPSALARLPVSDVLAAWSGLGYNRRALSLHKTAKILAEEYEDKVPASEEALRN
LPGIGTYTARAVLAFAFNLPSVFLETNIRTVFIRHFCSAEEHVGDKGLERIGEALLDVEN
PRQWYTALMDYGAWLKVHESNFGKKASMYRPQSPFKGSERELRGAILKKLLQTSPQTFVD
LAYQVAADVERVSACAEKLEKEGFVHILRDDSFVSDSEGTLVGLADSV*
>SPBDM4_v1_51031|ID:27159394| TRAP dicarboxylate transporter, DctP subunit [Uncultured spirochete bdmA 4]
MRRSKEAVFALILLLIPSAVFGQITLKLAHLNPQQPYDVASAAMAAVFKSEVESKSNGQI
KVEIYPDGVLGKEAETLIQVRSGVVQSFISSSGGMAQFYPLIDVTNMPFAFSSYNVGYKV
YDGEFGKELAADIEEKTGFHVLGFGESGGFFAITNSKRPIKSPSDMKGIKLRTMALPLHQ
AIVKALGAYPSTVAWAEVYTSLQSGVVDGQMNPVSIIAMAKLQEVQKYITLTNHLYAPYV
WVVNPKFYNGLPDQLKGVVDDAARTAIIAGRGLSRIIDSTDKGLPALAEKMQVYVPTKAE
MKQFRDATVPAARDFMLAQYKEEGRVWVDKFFDAIDAAEKELGY*
>SPBDM4_v1_51032|ID:27159395| D-alanyl-D-alanine carboxypeptidase [Uncultured spirochete bdmA 4]
MKRNYGAARQSAFSHATRIFAGIFFIFIAVSGLSALSRNEFEQFGITIPAPPPLNARSAI
LIDAATGAVLYEKEADLALPPASLAKLVTLHVVMEEIKAGRLSPDELIEINAKDCSPYIP
YGSSLMYLQPGMKVSVRDLMLGAAVVSGNDAAYALARRFAGSNERFAEMMNSAVRKLGFD
RMTFVEPSGLSERNLVTARDFALFCKKYIELHPEALKELHSVKSIHFPRPEHATAQYHPD
GIIIQYNRNPLIFSYEGADGLKTGYIIEVGYNMAATAMRNGNRFIAVLLGGSSTSYANGT
TLRTKDTKALLDWAFANFSTARPGYIMPKPLRVWFGAPIHVQPEPYGEAAVTVPAALASR
VTARIDTPKSVKAPLHKDDKIGRIVYEIDNKEIASVDLVSPQEIARGGFFRTVLDSVLRF
FAYIFGKV*
>SPBDM4_v1_51033|ID:27159396|asnS| asparaginyl tRNA synthetase [Uncultured spirochete bdmA 4]
MTLLPTIKQLLAMEPEGQKIVVRGWVRTKRETKQSVFVEINDGSCFSNIQGVVDASLLAK
DTVKDVLPRISTGASVEIRGALISSPAKGQKAEVAVQDIELIGEAPADRYPLQKKAHSLE
FLRELAHLRVRTNTFGAVARVRNRMAFAIHQFFQERGFYYVHTPIITASDAEGAGAMFQV
TTLDLEALAKSGKPVDYEKDFFGKRAYLTVSGQLNVETYCQALSRVYTFGPTFRAENSNT
TRHLAEFWMIEPEMAFVELPEDIALATEFIQFLVSTALKDCAEDITFFDQRVQPGLRESL
EKVARTPFTHMTYTDAVRELEASHAEFEFKPYWGCDLQSEHEKFITEKIVGGPVVVTDYP
KEIKAFYMKQNGDGKTVAAMDVLVPRFGEIIGGSEREWRLDLLEKRIEELNLSKEAYGWY
LDLRRFGSTPHSGFGLGFERLLMFVTGMTNIRDVIPYPRAPRQADF*
>SPBDM4_v1_51034|ID:27159397| TM2 domain protein [Uncultured spirochete bdmA 4]
MVKSRESGPIILMAEQSVRYSLSTAYLLWFISGFGALGFHRFYLGKTGTGVLWLLTGGLG
GVGSIYDLVTMPGQVREANIGYAVREALGSGSDAGFVPRVRREDSPERIILRLAKGNNGM

VTTGEVAIEANISIDEAQRQLDSLAKKGIAQVRIRSSGVLVYFFPEFSKENTDFVD*
>SPBDM4_v1_51035|ID:27159398| putative Peptidase C14 caspase catalytic subunit p20 [Uncultured spirochete bdmA 4]
MRSEQKKEPRGENPGPGHRRMFLRAIALFVCAAIFALAFSGCEFFPTNASTLGGNKYAVV
VGINDYINGSDLNYCVADAESMKKMLEDAGWTVNPITAESGEAIKKNATKSAIETALKSV
PADTTTFLFYYSGHGSIDYSDDAYIVPSDYDGSVSSLISATEFSGWLDSVTATNKSVILD
SCYSGGFVDAGDSVDSITDVGVWYPDGIYAIMQTSTAADMFFRFGELLAQNAAASSTNPS
TAPLVISAAGWAEESAEPLPTDNPNVGHGFFTYYFLDAAAAGTNGHMKGDSDGDGVLSCI
EAYDYAKNALEQANYGYLPHITGGLRDFALIDNR*
>SPBDM4_v1_51036|ID:27159399| exported protein of unknown function [Uncultured spirochete bdmA 4]
MKNTKFVILMLFLGFLGAKKAAALPAFNFYAGFANAWTSTSTTDVGYHVGWDASADVNWN
IRVLLPHSASRIRSSTASILISPGFRGQFLDLAASNWLADGSRYRAWSAFGLGPEIGVGL
NISHFGISRGQTFLLSTALLGNVAHYTQTSLYNAYMSWLIKASWDLELKHGWAVSTSFPV
EFAYRADGRSIVSGLGIGVKYAF*
>SPBDM4_v1_51037|ID:27159400| exported protein of unknown function [Uncultured spirochete bdmA 4]
MRKIILILSIILASLFISGSIASCKPRIRLPEDNSLGGSNQWLVISSLYCQLKSEPSGAS
HDLGILRRGTILKIMESKFSTDEGDQGTLWFKVQNAGQSGWVSIREAQTYSSETQARNAA
LRME*
>SPBDM4_v1_51038|ID:27159401| protein of unknown function [Uncultured spirochete bdmA 4]
MDFFKTWILPPLIGAIIGYFTNWLAIKMLFRPYKTIRIAGIRLPFTPGILPREKDKLAGT
LGNTVAKELLTPQVITKRIQSPEIQKAAAGAVKTALEGFLAQDIGRLFSSRQGSSLHDDE
TLHESSDVSNYLSENELIDQSDASPLDESSLSSYVMESFRRLVSSVGVQASIKGIFAEFL
ARVGEMEVRDLVSKEQFVGTILRAVPSFEDKDEDFPGSMRPGSEPKPHHLLHALAELPPD
ATIRALSDALVPKAYNYFLPHIDSFLHDDEFRARLEVEAHAFVKKALNRLGTVQRLFVSI
AGYDTKIAQAMPGIIEDLVQTIERLLREPSTPEKISEALCATLIAQRSKLDAQTPDAPES
VRSEKSKEDKAILDVALLSLKDSSEELRQRAEKSYDRLSELQIKELVGISVSAEDISGFV
LSAFVRTTGAHGDSGRSSTTIGALFVQVLEESSKDKTMAEFLGVRDEEIEQISLTLADAL
LKLIETRIPALVDAIDIRSMVSEKIDSLNMREVERIVLQVVRKELAWITWIGGILGAMIG
IVQSIISIL*
>SPBDM4_v1_51039|ID:27159402| putative Folylpolyglutamate synthase [Uncultured spirochete bdmA 4]
MHSMSFLSDPHLDKSRCLAHALCVSVGHFTSIDEVYDFVLGYVNVEKGQATEFKLDRMRW
MASELGNPHLGRVTVHVAGSKGKGSVATLIAKTLQTTGARTGLYTSPHIISWKERITESG
QEIPYDIILKASEEVFALVQGKTAETFYGGELPTYFELTTLIAFCAFRLAGFEAQVIEVG
LGGRLDSTNIVSPDVCVITPIELEHTQFLGSTIPLIAAEKAGIIKKNIPVFTIQPKPDAL
GVISGRAKDMGSPCFVVGRDILFSSVNVDVTGTCCRLEATETSPPALRALMIENSLEVRT
PLVGSVYAGNMALAALALSQLPVSLNAGHIQKGFASASMPARFEIVSRSPFIVLDGAHTP
ESIRTILTTFLQLSPAPRMLLFACAYDKRHDEMAELLSPHFEEIIVTRPGTFKQSAPDLV
FASFQKRKPATLLIESTEDAVQKAICGANEKGAGLLVTGSFYLCAEFTKSKLCSEQCRSS
HPEFRRSK*
>SPBDM4_v1_51040|ID:27159403| protein of unknown function [Uncultured spirochete bdmA 4]
VRIVHAAEVGQLAKPMIHRRSRGAGFSQKVATPSVESTGPDRPLHLHDKQVIVIIESEGA
VFDINRYWHETAYLPSFIGCFGSNVDPSIVGEIWRTVALETSLRGEHPLVILLAALRVLN
VLFPSMQRAVIIKTLEKYSLSEKTFDDLFKLDEKHPVEMMIIDWFSMAEGLIQELGRVPH
FSTAEEFLRSAKYIAPRSEVLVYSYLAEMSAMRMWQNAGLGECFYRIAGKERGRPGEYLR
AALSAGFERSSIMAIGSSAEMFRASQLAGIRFLPIVPGQEEESWKILAEQWFPAYLRGEA
WKIDMQAEPFYEQMCSEFNVVRTAGNIAARLELPNVSNDRR*
>SPBDM4_v1_51041|ID:27159404| Replication-associated recombination protein A (modular protein) [Uncultured spirochete bdmA 4]
VAGQRNSMPSHPLFGPGEVGPSESAEPLASRMRPRTLDEFVGQEHIVGEGRLLRRAIQKD
QLSSVIFAGPPGTGKTTLARIIANTTKSHFITLNAVLSGVADLREAIEQAKTYRDMYSRK
TILFVDEVHRWNKSQQDALLPWIENGTAILIGATTENPFFEVNRALLSRSRVFVLKQLSR
DDLFKVARQALEDRERGYGQYIIQFEAGALEHIVDTADGDARSLLNALELAIETSTPKWP
PSEGSTLSISMSDAEESIQRRAVLYDKDGDYHFDTISAFIKSVRGSDPDAALYWLARMVY
AGEEPDFILRRLLILASEDIGLADPNAISVVNSCAQAFDRVGLPEGQFHLAQATLYCALA

PKSNTTLGYFDALKAVQADQAEVPDHLKDANRDKAAFGHGEGYQYPHAYRDHWVAQQYLP
GSLAGRLFYFPGSLGFEGTRRTDVLVRREAQLAVLPDDIEEEQEKNLVWSKEGEARRRWR
VRSESSATERLIAIRAAIFDELSPKPIDNMVVADPRSGFYAMEALRRTAEGKAFIVLNDE
AARLQLDNLSKTIQDVLHPEIVLYENLIETPQKTGILEAYLAEAKPNHMLLCEAARIPLE
VSASLLNAVSQIKENAPRIVAFDIDAARSSMLSDALSSISSLSEDESEFFGRFSEFEQAL
GHGPTYMKTTAASQPLRGWLGDLKAKLPQLNAHKIDLRASYPRSFDEKELEEWLDPSNPY
GAGFRARFSEHDHKLLQSLMQKYNKAPIWPIVITLFEFQITEPAIEKHISG*
>SPBDM4_v1_51042|ID:27159405| protein of unknown function [Uncultured spirochete bdmA 4]
MREYIARLAENSLPKAGKVKSGRTPHAVGLIVAATFILLAFSGCDNTFSVFQTISQEEKQ
IGADLFKNTTVRAMADDSSNYYALLGQVVWRPKSTTSEGSWDVFSVNGSTDYFAAGLAGD
GTNIYVAKADKNNVLDDIYTTSDSGSSWTAMGAVTDIGSDAFVDWLKCANNTLFVAVHNS
ASKYSLFYYDGSSFVSAGAAVSGIDAPLVDILYDGSSSYWLVSDSKVYKGTTLGAIAEDS
TASNPDYDRYLLGVASDGAGRVIVSRKDGYIYSYASSVWTPIQIKSSTKLGPVFFLTQPA
STPRILIGKGVSSYGYMEYNESTPTLKENGSSFISTSSSIYYSTMLSKQVQAFWQPVNEY
DATKKTLFILLASGGTDTYALYRNDYTISTGSWSGWTAE*
>SPBDM4_v1_51043|ID:27159406| membrane protein of unknown function [Uncultured spirochete bdmA 4]
MSKKLLAVIVLIASVNALTLGAQEKTSDPFAAARKKGDQSITILAGGFIPLFVLDNNGAL
ISPSNMYPGASFGVQYRYMLGKSIGIGGSIAGSFVTTVGGGTLFFAPIGVTAAWIGGGEP
MEYEISTELGMNILRLYGNGLIEPYAKLGAGFSRYVSSSWSIGAKVSWCFMPEFHLGTYS
SLNSYANFLEFSVGAVYHF*
>SPBDM4_v1_51044|ID:27159407| protein of unknown function [Uncultured spirochete bdmA 4]
MERFVLVARPHGNIRQQLWKLKREAFDWNRDDASMAMPEGFICGWFDASKFSTGLSKSAF
SEHVQRTLTRYADEICAVLPDTFRFSSAIEARDRIMLKLDDELDIPKLKVTLERFAQDAG
LEPCADALTPDNEAYRGIWLGSGTISSAPFPLSFKKYELVLYLAELPETPFSGFQFRTVA
RIHRKTKAHPRTERE*
>SPBDM4_v1_51045|ID:27159408| conserved protein of unknown function [Uncultured spirochete bdmA 4]
MKRKSEDILSFLIDTISRWPGVDCICVDRRSQQDELDPHYALVFDVYYRRSVPAMDKRQN
LFDNPGAFESAPGGMKDRFFLDEIPIRVEYKHLPGVQDLVEHPLRHIKLLKNAGTYPLYR
LLNFPLVFSRSDWIERMRAELNNFPQEAWRGLFDSFSAKMEHYLSDFGAAAVSENQFFRL
MSRAGFLRFAGASVFMFNHVFEPSHAEYESQLKTQPVLPANFASLWDSIAGERNDLSEFK
KFELARLLAREIFELQ*
>SPBDM4_v1_51046|ID:27159409| exported protein of unknown function [Uncultured spirochete bdmA 4]
MRHTLRRQPGAGSCKVFLFLLFVSVFLCFFAVSCKGTEVYSFGWFDERGSRLAFSSSVAG
SDVSSFSRQGEKRRYTLAKAVEVPEGYSVRLTFSALSGGTEFQVGYGDEKGKNTSVLCSI
PEPGQFAFVLPFSGPNALRWVEVKILKMPQQISSGQDGSVQSSQNGSDAQALNSHETLSP
PLSLQEIRFVSPMRGMRHENNTVELSPHFSFETKNGSGHYEIRGPFSDLPSGIMDSMQLE
VVLEGGSGIAALFWGDQKLTYRHGGEKSAFSLPCSLFADTPDRIALDVKDDIRVISFCVV
QSKATTELARIDPGLLVYHAPLDDKPYYLARWDLRPEVLLFLFKDYAIQDRYLKRLAFFV
EKIGFAGRLAQDSEIADLHGWNAHDYRPEDLARFFDTAAAQKFPLSAEEIELRELLIKEG
VILKNGTRIVPGKGAIVSITQESPAYLQYRFLAHELSHALFFTDGRYRDLVLSLYQKMTD
DEKWFLIRYFRWMRYDVDSSYLMANEMQAYLVQQPLKDLEKYFSDTLGDRLAKEHPELAD
AIHGYMQQHLSALVAIARQLDSYLQASYGFETGRLYRVR*
>SPBDM4_v1_51047|ID:27159410| protein of unknown function [Uncultured spirochete bdmA 4]
MGKNANKSPPGKNSDDVLEGLFISPLENSESFEKMEAEEAKILSPVKEPDLDESADASSA
SISESREQELSEVKSSTKPASLAQEQVILRFAGELKSIKQDLLSINQHFEAMKKPQPLQS
LQPGTSSSPGANAIEQTRGASETNQDTLQLLDDIRKLLLYLDRLLESLPEDKIEEFANSE
FFNLYRHVFEKLGIS*
>SPBDM4_v1_51048|ID:27159411| exported protein of unknown function [Uncultured spirochete bdmA 4]
MPKFLLMYSPQRMPQKSRQLLTSLSYGLFSFLIAYCCISAVAGKAGLLAYQDMASQQQQI
QKAIEYLQVQNQDKTHTIDDLKNNSLAAAERAATLGYIREGEMLIVLPEAWRNEDTRGDD
MRLPVVMRDSTGLPDALIRLMAAITGVFAFLAIQLFHFKPRAQSVQRMSFEKQTEAP*
>SPBDM4_v1_51049|ID:27159412| Penicillin-binding protein, 1A family [Uncultured spirochete bdmA 4]
MYVFIALILALAAVLGIGLGLALSGTANTIRTENFTEFDTALPTKIYDINGRLITEFFAE
EQRIPISIKELPDHLIEAFITREDQSFYSHHGFSLRSIMRAATGQILGRNLGGGSTITQQ

LAGTLYADRRIVSIQRKLKELWWALQLERRFTKEEILEMYLNRMIMGPAVYGVEAASRYF
FGHPAKDCTPEESAILAIQLSSPSRYNPFRNPMLARDRSKEILDQMVARHVITRADADES
FDAYWASFDYSRVAVSAFYNREDKAPWFSEYVRRQLEDLFYGSIDIYSDGLSVYTTLDLD
KQAAADLYMKRGIETANASFKSTMSQRLSVADTTYVPIVELLGLAFDLPMLYAPQSRSES
DSQNYFLNVVNPVLDAASLMLNIASLKPYVGAASAKIKTELEKTTVEGALVTIDNSTGYI
LALVGGSDYNQANQLIRATQAKLMPGSTFKPLYYSAAIDSKKITEGTYILDEPTTFYNED
GTPYSPQNFKGEWTGSVLAWQALAHSMNVPSVKVLQTVGFDAAISRAAALLDITDPIEIR
KTFPRYYPLALGVIGVTPLQMARAFSVFANGGKSITPIAIRYVEDRYGNVIAEPEKDAVQ
AVRQRSPQVISPQNAAIMIDMLKRVVSSGTLAGATQSGSIFKQTSPDGKSYVIPIVGKTG
TTQNWADAWTLGSSPYYTTAIWLGFDRPGNSLGVSQTGATVAAPVWANYMRDIHIGLPYK
NFSKPDSGLVSVSVCAKSGMLPTEYCSDGTVNLMYLDGTQPTQFCTLHGPSITPETLGPD
QSQSENPLGPSIPRPEESPIFQNR*
>SPBDM4_v1_51050|ID:27159413|gapA| glyceraldehyde-3-phosphate dehydrogenase A [Uncultured spirochete bdmA 4]
MKVAINGFGRIGRLVFQSIVDQNLLGKGKDKIDVVAVVDIVTDAAYFAYQLKYDSVQGKL
KAQVTSKKSDPAKSEDDVLVVNGHEIACIMAEKELKNLPWGKLGVDYVIESTGLFTDEKS
FGHLEAGAKKVIITAPAKSKAEDKKIPMLLIGVNNEKYNPAKDNVVSNASCTTNCLAPLV
YVLLKEGIGIETGLMTTIHSYTATQKVVDGVSKKDWRGGRAAAINIIPSTTGAAKAVGEV
LPEIKGKLTGMSFRVPTPTGSVVDLTFRSVKETSIQEIDALLKKASQTYLKGILGVTEEE
LVSTDFIHDNRSSIYDSLATLQNNLPGEKRFFKVVSWYDNEWGYSNRVVDLLRYMASKDN
F*
>SPBDM4_v1_51051|ID:27159414|pgk| phosphoglycerate kinase [Uncultured spirochete bdmA 4]
MIKTVRDVSLTGKRIIMRVDFNVPMKDGKVQDDTRIVAALPTIKYILDQKPRSLVLMSHL
GDPDKDAAKAKEKAEKEGKPFDLKAYLDGKHRMKPVAEYLSKLLGREVAFLPSCFGQKAA
IDALPYGYVAMLENTRFHKEETAKEPAVQEVLARELASYGEIYVDDAFGTAHRAHASTAT
IAKFMAVRVGGFLMEKEVANLEPMLHNPPKPMVAIIGGAKVSSKIAVLENLLKNTSALMI
GGGMAYTFLKAQGVPVGKSMVEDDFLDTARQLLDHAKAQGVEIVLPVDHIAADRFAPDAT
PVAVDSRAVPDGLMGLDVGPKTLRLYAKVISTAKSVLWNGPVGVFEFDAFAKGTEQVAKL
VADATGRGALTVVGGGDSVAAVNKFGLAAKMSHVSTGGGASLEYLEGKELPGIACLEQK*
>SPBDM4_v1_51052|ID:27159415|cysK| cysteine synthase A, O-acetylserine sulfhydrolase A subunit [Uncultured spirochete bdmA 4]
LRIYENITELIGNTPMIYLNKIGTSLPAKIAVKLEFQNPGHSVKDRPALAMLLEAERGGL
LRPGMAILEPTSGNTGISLAMIAAARGYKCVIVMPESMSLERRAILKAYGAELILTPKSQ
GMSGAIAEAKRLLSTKPDEYFMPMQFENPANPQAHRTSTAMEILEDTDYGLDIFIAGVGT
GGTLTGVGEVLKEKKPAVQIIAVEPAGSPVLSGGTAGPHGIMGIGAGFIPSILNTAIYDE
IIRVSDEEAYSMARRAAKEEGLLVGISSGAVIHAAIKLASLKENKGKLIVGIVASSGERY
LSTPLFSDLIAG*
>SPBDM4_v1_51053|ID:27159416|cysE| Serine acetyltransferase [Uncultured spirochete bdmA 4]
MKMKMKMKENNPKNRSTYNNERGSDIEAVLHRDPAARTKIEVILAYPGLHALWMHRIAYF
LWHHGSKLLARLISNAARRLTGIEIHPAAKIGQRLFIDHGMGVVIGETAEIGDDVTLYHG
VTLGGTSLERKKRHPTIGDRVTIGAGAKILGDISIGSDSRIGANAVVVKNVPPNSVVVGI
PGQIVHRHIPHTASDEPDLHHERIPDVLGQRVHELTARVAALEEVIANMKSGCEDKRQAG
DVYS*
>SPBDM4_v1_51054|ID:27159417|tpiA| Triosephosphate isomerase [Uncultured spirochete bdmA 4]
MKRYFIAGNWKMYKTIPEAVSLASELKDKLSDCSEKLMIAPPFTALQAVAEVIRGTNILL
GAQNMGPEESGAHTGEVSVLMLKDLGVRVVILGHSERRHTYGETDALINTKVKLALKHRL
EVILCVGETLREREQGVLETVIRRQLAEGLQGVEPSALTEVTVAYEPVWAIGTGKTATPE
DADAVHAFCRKVIATLYGQEAAKQILIQYGGSVKPENAAALMAKQNINGALVGGASLKAD
TFVPIAKFR*
>SPBDM4_v1_51055|ID:27159418| Preprotein translocase, SecG subunit [Uncultured spirochete bdmA 4]
MGFFGVLLLVVFIIVCLLLIFLVIIQDEDSDSIGGIFASGSQSAFGSRSSNVVVRITYVL
GTLFFVTAFALAVVNKSPTGNVQKAVEQNSAQTATDEWWNNGGEGNQPAQDQTAPQSQAP
QTEQPSAPATK*
>SPBDM4_v1_51056|ID:27159419| protein of unknown function [Uncultured spirochete bdmA 4]

MVRRSFAEMGHEAAIVSDSPYGLSAYDFLVFMTESKGMLGGLLTFVPQKLARQDGLIGKR
CLAMVRKSGLRAGYTLRKFMGALEHEGLIVVEGEIFSDANAAVQIARDAPLKRG*
>SPBDM4_v1_51057|ID:27159420| putative DnaJ-class molecular chaperone with C-terminal Zn finger domain [Uncultured spirochete bdmA 4]
MMKDYYEILGVHPDSSPQDIKSAFRKQAKRFHPDMHYSTENTEARESPATIRESAMRLVL
EAYKILSDAEKRRAYDRELRRREKENKGFDYREFLKQRSDDPESQAKLIVYDLLHDLDEE
ALAIYERSKAFPDFRLERWLDRGEAMDSEYCIAEEYEKRGKYIKAYQIYKKIIKMELEKP
WFRYYFDVVALKFRFLILQKLPGRIDEEDYLDRLDEAIKLEIAPRETAQYLRKKVEMLLH
RGDAEAAFEVLQQISQIYPKLAGFAALRTKVEHARDQSVAENRVS*
>SPBDM4_v1_51058|ID:27159421| putative PpiC-type peptidyl-prolyl cis-trans isomerase [Uncultured spirochete bdmA 4]
MKKMTTWLMVFTFAAMPLFAQTSIDKPAATFKLTRQEVISVRQLRADIDRLENATGAKLT
VDQRKDVLDARINSMLFLQFCEREKISVSDAQVNAAVAQLRSQLGSNATDADLEKSLRAS
GVFVDPAIYVRQRLLFEMYVRTQKQDEMKTVLKAPTADEILKAYDLAKVSLVRPDTIRIG
VIYADTKGKSDADIAKAKDLMNGISTALKANPSKFDEYVLRASESAGYKAIPSLYLEKTQ
QSKSIFGDAFFNAAFQAKTGEITPVIQTPTGFRIVRVNEFIAQKQLTLSDPVPGNQNLTV
QEFLAMQLASEKEQTFLNKTEADLIDSLRKEATIKVYAENLTW*
>SPBDM4_v1_51059|ID:27159422|nusB| N utilization substance protein B homolog [Uncultured spirochete bdmA 4]
VASRRKARILAVQALYAWDMSGQSLADLLSFEWIDEDKREHYETDILDFSKLMIAGTIEQ
IDVIDSMIKKHLEHWTFERLRKVDLAILRVGAYSIFYQKDIPAQISIDEAIEIAKEYGSE
DSYRFINGVLDGIRKDIVEGTHDTREK*
>SPBDM4_v1_51060|ID:27159423|map| Methionine aminopeptidase [Uncultured spirochete bdmA 4]
MIRVKNERQIEGIRASCQMLSDMYEELRPLVRPGITPEELDTFAYDFIIRNGGKPAFLGY
EGFPATLCVSVNEVVIHGIPDDRPLKEGDIVGIDSGIELNGYFSDAAMTLPVGTISDEAQ
RLLNVTRESLNLAIARVGPHARISDISRAVFSYATKNGLKVVRQYCGHGVGLEIHEDPQI
PNYVSVGPNPRLMPGMVLAIEPMINIGTSDVRVLDDDWTVVTMDGSLSAHFEHTVLVTDS
GCEVLTHW*
>SPBDM4_v1_51061|ID:27159424| Protease Do [Uncultured spirochete bdmA 4]
MSIAKKLHSRNFFIANLVILGIVIGFALAFMFRANPSPNSASLPVVKAETPSMIAGSDTE
AAISTAESVQNAFRNIAKTVLPAIVELDVVEGGQKAQQQQTPQFPFDFFFGPDNSTPQQ
FQPEEGLGSGVIVRRSGKTVYVLTNNHVAGNATKITVKLSDGREFDGKLVGFDERKDVAL
VKFETDDTDIAIAKLGDSSKLQVGDWAIALGSPFGYVSSVTTGIVSALGRSGGPDGNIND
FIQTDAAINKGNSGGALVNIRGEVVGLNTWIASPTGGSVGLGFAIPINNIKGTIDDFIMH
GAVKYGWLGVSLSNITQDKASAKELGLEGKKGALVGHVFLDGPASKAGILPGDFITAVDG
KAVQSTDDLVRTVGDIKAGTTARFDLIRRGISMTISAKVDLRNNTVAANNANIFPGVTVI
SLKSDSVDQTQLPAGVRGALVVDILAKSPAATMGLRTGDIITKINGKSVNDLKDFYEILN
KESGKKLVFTVNRDGQTAETLAYVGK*
>SPBDM4_v1_51062|ID:27159425| Phosphoribosyl transferase domain protein [Uncultured spirochete bdmA 4]
MHKEFVPYDMIRDNAIKLAYKIYKDGFIPDVIYVSLRGGAYMGNVISEYFKFVKGNGRPV
FYAAVVARSYSGFDQQEMIRIDGWTYNPDFLRHGDKVLLVDDVFDSGRTINYLAKVIMDR
GLPRSDVKIAVHDYKVREYMPQYLPIAPDYWCRKIEIKSREDEIWIHYMSHELEGLTEKE
IADHYLKDDPELAEAMRLVVQH*
>SPBDM4_v1_51063|ID:27159426|glgC| Glucose-1-phosphate adenylyltransferase [Uncultured spirochete bdmA 4]
MAEILSIVLGGGKGTRLFPLTKERSKPAVPFGGKYRIVDIPISNCINSGFKKIYILTQFN
SASLHLHISHSYNFDHFSKGFVEILAAEQTPIHSGWYEGTADAVRKNLIHFRPHKPTHYL
IVSGDQLYRMDLGEMFRKHLESGCRLTIAGTLVSRDAASSLGIIKIKADGSIDSFMEKPG
PTKDINDFSVPEGLKPGDADPQKPYLASMGIYIFDAPLIEDALQMDANDFGKEIIPSIVA
KEKVNTYVFGGYWEDIGTIRSFYEANIGLTDINPKFNFYDEHRPIYTHARNLPASKLNYC
TLNQTLASDGCIITNASITSSIVGIRTVIESGASLDGVVCMGSDYYETEAEKAENRQKHV
PNIGIGGGSIIRHAIIDKNARIGSNCRIGIDPLPRDDGEYSTHYIVDGIIVIPKNQVIPD
GTVI*
>SPBDM4_v1_51064|ID:27159427|yprA| Uncharacterized ATP-dependent helicase YprA [Uncultured spirochete bdmA 4]
MFTVYPMNSETANAICDELASWLAGQSEIAWSTKLPARAPDIVPLPEDLSPVLVQALNGR

GIKALYRHQKKSYELARARKDFVVVTPTASGKTLCYNLPVLQTLLEEPEGRALYLFPTKA
LSQDQQSTLNEVMLAGSIGLTINTYDGDTPADIRTKARTSGRIIISNPDMLHAGILPNHT
KWVKFFSNLKYIVIDELHAYRGVFGSHVASVLRRLLRIAKFYGSSPVFIFSSATIANPRE
LAEHYIERDVALVQENGAGSGEKIVFCVNPPLVDEVQGIRRSSSLEAESVMLWLLRKGVR
TILFSRSRLQVELLASYLNQKLENPYNRDFGLVVKPYRSGLLPSERREIEKGLRDGRIHG
VVSTNALELGIDIGGLDAAVITGYPGSIASFWQQAGRAGRTFGVSLAVYIASSSPLDQYF
AAHPEYFLSRSAEQAHIDAYNPYIFTDHLKCAVFELPFAEGEAFSPDPSNHKAAGLTVEA
LEYLEEAGIVRHTAQRYFWSAEGYPGEKISLRSATTENVIIVDVTQGRHDVIGEMDRPSA
KELLFEKAVYIHLGTQYQVKNLDLEKRLCLVERSDVDYWTDSIVKRDIEVLSEDSTAPHE
QFDLMLGDILVRSQVEKYKKLRFNTHENIGYGEIWLPPEEMQTRSLMVVLKAESQSGRLL
RQLAPERADGILYSVTNLIRQLAPARILCDLHDIGIASRVRDLFFKGPAMYFYDMYPGGT
GIAEALHENLKETMASAIERLSNCPCSAGCPSCIGVEIAGSENKALSLQLLRLLHGSPGF
*
>SPBDM4_v1_51065|ID:27159428| protein of unknown function [Uncultured spirochete bdmA 4]
VNLKDRLALIKSDREKNHQMPHGKRSMPEGWTEISPFVWMREEDRPFIEVPLFFSPHLMR
ATVPRGDQDNGEFQSVQVSPIPADRIAFFDLETTGLSGGSGTIAFLSTVAHFEGTDLVLR
QTFLEDYPGEHDFLVAVISQLANVDWIASYNGATFDVPLLQARCVLNRIAMPLVRHIDVL
HDCRRFWGRMVLSCSLTSMEALVLKKERESDIPGALVPRVWLDYVKADVPREDQQALISL
VWQHNVQDVASLAELFLVVESAYRRPSNAVVRYAIDPSGLARNLLKMGRSGEAKSILLMV
RDNAEMFELTSGARMRAMRHLASIAWKERNRTLYVETVLAMDDESLYGCVAKAKLCEHFF
KDEKSALAWAQKARDIAGKGSQADRRNTQGLDLESLDHRIARLERKIAKKNSLAEL*
>SPBDM4_v1_51066|ID:27159429|rlmN| putative dual-specificity RNA methyltransferase RlmN [Uncultured spirochete bdmA 4]
MNLSSPKSRNVVYPSGLSPDELAALLSDEPLYRVKQAFSWFSKGIASFGAMTNMPQPLRE
RLSAHFDGHMRSSGVENKLIDEDGSLKLQIRLRDGAAVECVLLEDIEGRKTACLSSQAGC
PMGCVFCKTGTLGFLRNLGPDEIVEQFHYLRDMRGFISNVVFMGMGEPLLNLDNVRKAIA
ILSNPHGIGMSMRKITISTSGIVPGILDIAKDGPIVRLAVSLTSANPELRTCLMPVNRTW
PLDKLKAALLEYQKITNDRITLEVVLIGNVNAREKDAQELADWIHPLKTQVNLIAWNPIP
GMPFETPSAKQVRQFSEILEQRGIVTVQRMSRGRGVMGACGQLGDTLKAKQ*
>SPBDM4_v1_51067|ID:27159430| Tetratricopeptide TPR_2 repeat-containing protein [Uncultured spirochete bdmA 4]
VSDIPSLPYNDDFFEVPEEIQSENTEIAETSKKGYQLLKENRFDEAIECFVQILEKDKEN
NYALVGMGDAARKRDHFKEAADYYRRCLVFHPGNSYALFGLADCYKALNQFQKAIEIWEQ
YLLHDNTNITVLTRVADAYRKVHDFRKSKSIYHRVLELNPDNHYALIGLGHLHYDFKEYK
EALTYWQRMVELEGDNVDIRVLTAIGNCYRKLKHFDKGIPYFEKALEKEPENFYALFGIA
DCYRGIGKQNMSLVYWNKILEKDPKNKVILTRAGDAYRNMGDFEKASEYYQNALNIEFDI
YAVLGLAVIARQQGKYDEATASLRSLLQNDPKNYRIYVELAQTYLMNNQRQQAIEVLTEF
QQMGIKNPVIQEALSKLAGKT*
>SPBDM4_v1_51068|ID:27159431| putative Methyltransferase [Uncultured spirochete bdmA 4]
MRITGGKLTGQIVRMPEGDLKIRPAMDRMRESVFAVLGDLSGYSFLDLFAGSGILGLEAA
SRGAGPVVCVERDRKKFPVLLQNATISQPPVFCRAMPAETFILRNKAAFDIVFLDPPFDY
RFKPQLLERLDQSASIHEKSIVLIHFPHEDHLPEIVGNLQGYDIRSFGRSIVRFYRKIQ*
>SPBDM4_v1_51069|ID:27159432|smpB| SsrA-binding protein [Uncultured spirochete bdmA 4]
MRSQEPVKIIAQNRRARFDYEIEESFECGIALQGTEVKSIKGGRVSFGDAFAEIKGQEVW
LSNFHIAEYAHASAFSHDPDRPKKLLLHRQEIKRIDRRVREKGYTLIPLSIYLKHGLVKL
ELGLCRGKKAYDKRADIKARDLDREIRREFRLKDW*
>SPBDM4_v1_51070|ID:27159433| putative PEGA domain protein [Uncultured spirochete bdmA 4]
MKETEKISEQDIAQAKVTLKPVFGIRPRVYVPILYLLGILIILFFILINPGLRNPGAYLV
FQGAPESAAVYVDGSYAGNTLDGMHAKPGTHKVEIRKRGFSSQTLNTDVPHRIFATLIFR
PTVKISYQLNPEPLEAIIIPSFKEFANWSLSGKPSAIYQLPMVLSEAANDAASASSADRS
SFARPLLAAGLSVAENSTSVRDVIFASAALAAPGGSPLGLVAIARNAAALLGTSKSYTAA
IMETMPEKAGDNIRKAFSSLKEEMSAETLQQASAAGVRAAGPHSFIMFGGGQTKNVSSTP
GGTKMLYLDRVPEFGLASTEVTQRQFARFLEEKPEWKPENRTALIEKGFADDAYLVDFDP
SKADNRPITGVSWYAANAYCAWLNARAPSGYEVALPSEPMWETAAASASRDVGALGAFNN

RASTGPLAVGSMGRDALGFSDLFGNVWEWTSDGFRPYAWIQDDSSAFGELTEAIDSKTVK
GGSWANSADQISIASRGPVPAAHASEFLGFRPALIKK*
>SPBDM4_v1_51071|ID:27159434| Signal peptidase I [Uncultured spirochete bdmA 4]
MKRADFFDRLVIFSADMLTRRKQKSLRAKMKQQKRHPVLDWAYAILWAACVVLVINQYIF
QNYRIPSGSMENTLLVGDMLFVDKLSYGPELLPGVLKTPGISKPHRGNIIIFENPSYLSR
GPVYTVFQQLLYMVTFTLVDIDREPTGEPRVHYLIKRAIGVEGDTLRVRDGEVFIKPRGS
PDFFDERTLMTALALPVKTQRLVASSEYPEIDKVGVASAYSELNLPFPASLGTPSVQSAN
KDAFQYDMSRVTTLRDANPSNARNAQLAQRYGNGWFIADSRIFPMGDNRDNSRDGRYFGP
VAEKKVLGHALFIYFPFSRIGGVR*
>SPBDM4_v1_51072|ID:27159435| protein of unknown function [Uncultured spirochete bdmA 4]
MFSRRPVRYAEKVERRKKRNKILSYIVIFIILCVFIRIFILQTWQVHDDLMRPALRKADI
VLVMPYILLGSKTPVGIAGSPQKNSIVLVSDGAEDIIPYRRKAEDAILRFITFQRFSPIS
KEFGKDFNVPSVMRVRGIVQKKDGLQSKTFYALATDVQHNADRTLSVREVSAARIKGRVV
FRIWPLARIGLIK*
>SPBDM4_v1_51073|ID:27159436| putative Oxygen-independent coproporphyrinogen-III oxidase-like protein YggW [Uncultured spirochete bdmA 4]
MSSEAPQVYLVLPETKPFPINSLYVHIPFCAGRCSYCDFFSVSARNCPQVFSLEYESSYV
DAVLEQTQLWSEKFDAGPFSTIYIGGGTPSVLNEGVLERLVKGLEPYAASGCEWTVEANP
ESVHKSLLDMLAKTKVTRISLGVQTLSHDEWPALQRVGSVEDSKRSIDLVREYPFELSVD
VLAGIPQPQGRTEAWQSILLGSLEYLAERVSHISLYDLTLEEGTPLQRQVASGALVSPNA
DAMAEARDAANALLASRGFQRYEVSNYARSGHECRHNLAYWNMRPYLGVGSGAVSTIQYC
DPEEEPMLGTMVRITGRKDSARYIAKPAEIPPEIEYIDRKTALFEFLMMGFRTARGVDTQ
RISSLFGVDVAQIIPSALARWKPGIVRGRHCIALHTRNFDILNRFLVECLEELDKGGLG*
>SPBDM4_v1_51074|ID:27159437| conserved protein of unknown function [Uncultured spirochete bdmA 4]
MTLFQYAVVASILHDEKQKRRLLGNFESALEKAGGELQSLGSESVHPRNDTLPLFIFVLT
GGTEADAMRLVVQEKMLQRNEPVVLLAHPSQNSLPASLEILAKLKQDGGKGTILQADAAG
VFDEEALGDIVAVSQSMKALRASRIGVVGEPSDWLIASHHDSKIVQDAWGPELKDIPFEE
LLEIIEGIRSKPEKDINLPGLRGKAEFFSEANEADMRKSMEILYALKTLVSRHGLSALTL
RCFDLVLRDHSTGCLALSALADEGVDAGCEGDIPSIIGLHWVRTLTGQTGWMANPSRITV
QKEDDRAEMLIAHCTAPRSVLSRYGLRSHFESGLGVAIAGIIPEGDVTLVRIGGKNLEKV
WFSPARAIASPLKEGLCRTQAVLTMPSDKAQELLTDPLGNHLVMIQGNWVRRISAYLRLW
KSA*
>SPBDM4_v1_51075|ID:27159438| Metallo-beta-lactamase domain protein [Uncultured spirochete bdmA 4]
MKIYQHYSIFGFSNSYIIGNEDLGKALVVDPAELTPTMIEKIEQHHFDLRGILITHNHTH
HINGLSTIMKIYSPTIYASNAKLLGFQCRKVKDGDIFREAGYEINAIAVPGHSQDSIVYL
LAGLFLFTGDVLHAGLIGKTSSAFNTNALAARIQQKLFSLPDNTVIFPGHGPPSTIVTEK
NANISYQEGFAEHIRSNYDFFV*
>SPBDM4_v1_51076|ID:27159439| protein of unknown function [Uncultured spirochete bdmA 4]
LKSQFSKFTVFRSHNKNLELYKLEPENVQSTNIDPLKLVWISSEFSKLQSMNEDDPNEQK
STSAPEKSHEIKLDRSRKQRWKEAPVNIECWIFAFLKEQPTKRYREKSRPMNEASSKTRL
RMMFWACNS*
>SPBDM4_v1_51077|ID:27159440| conserved protein of unknown function [Uncultured spirochete bdmA 4]
MCYACGESLSYGYKPGFNDVCDTCGKDLHVCLMCRFYAPGAHWDCAEDVEGPVVDKEKRN
HCEFFMLAEKYIDKGKSRGIISGSDAKRKLDALFGE*
>SPBDM4_v1_51078|ID:27159441| putative Glutamate racemase [Uncultured spirochete bdmA 4]
MSIEESNVRVCVFDSGIGGLPFLKAIQEEFPFLEINYVADDAGFPYGTKSPEAIHDILFE
RIRRIRARLDPDILVLSCMTAVQIGLKDLQAAHRSMHIIGAFPPIAAAARESQSRRIALF
TTARAAEDAFLDNMIARDAPDIEVIRIPAQDLVEYVEQQLPFATLASAQASVEPYIKYVR
DEGTDRIVLASSHFVLLEDAIYGLLSEQGISNVRCLDSRKTVTSELRRLVHTERRVQTAI
QNPGFFLTGDRKTAPSYITWAGRFNLSVPQLL*
>SPBDM4_v1_51079|ID:27159442|rsgA| putative ribosome biogenesis GTPase RsgA [Uncultured spirochete bdmA 4]
MSRSRGTTAMRGVVLSFSNQHAKVKCEDDQIRLCTIKGKRIRALDGWYNALAAGDHVSVQ
PLSSAEGVIESLLSRKNVFGRYNEKGSADQAFAANIDIVVCVTSTKTPPFRPRFVDRVSV
LAEQCGAPLLVVCNKVDLGLDGVVEKRLSVFESLGFRVLRSSVALDLGIDELREALVGKI

SVFVGQSGAGKSSLINRLIPGTTQRIGEVSEKYNRGRHTTTLATMLFSQSGDLSIIDTPG
LRRLAIRNINLNDLSAYFPEMVPFIGLCDFGASCTHTHESGCAVRQAVSGGRIYHDRYES
YLRIRAELEESKQWKKAETGKPQKAAKYRFEDEE*
>SPBDM4_v1_51080|ID:27159443|mutS| Endonuclease MutS2 [Uncultured spirochete bdmA 4]
MITHEHSLARLDFFRLRERLEGYCLSYEGQALMRSTLPCSKIESVKRLKQDLAALAEGLN
NKEIPSLPFPNIETLVPKAQKEGMLLEADELFAIGLWAKSYEELFVFLRSTLRASQARTS
GVHGQKEKGQSGAGNSSDKSDSETGHLFLNECADMEDDECWALPGIERIAFSAPSLLQIQ
EKIFAVVTMEGAIKDLPRTRSLRTAIQKAQTENHHLVNQYLSDPNLQDALQSNAATERDN
RTVIAVKANFKGRIKGIVHEFSSTGQTVFIEPLELVERNNRLLELETKLGEEIRAVLRET
ANALRPLLPGLVEGRRFLAAADIRLARAIQAKREDLAIAQVLESGVHLYQAKHPLLGKKA
VPIDVDIPEGTNVLIITGPNTGGKTVSLKTIGLLALLNQYGAGIPALSTSGFAVFDTVFA
DIGDEQSIDQSLSTFSGHMRVISDIVRAATGRSLVLLDELGAGTDPEEGCAIAMALLDYF
LERGCMTIVTTHHGVLKNYGYTKSGCLNASMEFNAHSLSPTYRIFMGVPGESRALEIAKQ
TGLSSQIVDTARGYLDDERSDYSALIRSLGEKQRELDLLERERRKQLQGALESRRKADLE
NLKIRQKELELRRQGVAELTRFLSESRKTFENLVREMRESGRKVEEVSAGRELLQEISDE
IQQHSDGLARFEEETDVLERSMRSERDGVQSVDANILKPGERVLFQNREAVFLRMLDDKK
VLVQIGALRIPVAVDALSIPFAKKRKDGETRMSYQVELSGATGGGATASAELDLRGMRLA
EALEALTRQIDAASLAGLGTFSVIHGTGEGVLGRGIHDWLKTQAAVADYYFARPEDGGFG
KTWIHLKN*
>SPBDM4_v1_51081|ID:27159444|yebC| conserved hypothetical protein [Uncultured spirochete bdmA 4]
MSGHSKWATIKHKKGALDAKRGQLFTKLIKEISIAARMAGGDIEGNPRLRTAVLKARAAN
MPKDNIDRAIKKGTGELEGAMYEELFYEAYTPGGGAMLIEVLTDNKNRAAAEIRNIVTRS
GANLGTTGSVAYLFKRKGVLTYDGEKYTEDQIMEAALDAGADDVINEEDSIVVYTDSASF
EDVLNAMNGKGFETLGAEVSMVPDTYISVDVETAQKIQKLIDKLEENEDVQNVYHNIEMP
EIEE*
>SPBDM4_v1_51082|ID:27159445|ruvC| Crossover junction endodeoxyribonuclease RuvC [Uncultured spirochete bdmA 4]
MSARRSIVAIGIDPGIAAVGYGLVGAAEGKLNLIAYGCIQTESPEPMGKRLAYIFHEIEK
LLRQYAPDVGGIEELYFFRNVSSAFPVAEARGVIRLAFEEAHVELMQHTPNAIKKSVTGS
ARADKNQVQEMVKMLLGMPQIPKPDHAADALAAAICTLHSIGPAGYTL*
>SPBDM4_v1_51083|ID:27159446|ruvA| Holliday junction ATP-dependent DNA helicase RuvA [Uncultured spirochete bdmA 4]
MFNRIKGIFSGHTEEALHISTGAIEWEIFVPVRDPSFFIRTGEETELFTWLHHYEDGMRL
YGFFTAAERQLFLDLQKVEGIGPRQAFKILQGIAPRTLATMLDNSDLASLQKIPGIGPKT
AQKMMLALKGKLIGLEGEAAIGVPVSQGRFRDVARALVEMGFDRKDVERVVAQLGADLQE
NPESEKELFRKALLELSTGAGA*
>SPBDM4_v1_51084|ID:27159447|ruvB| ATP-dependent DNA helicase, component of RuvABC resolvasome [Uncultured spirochete bdmA 4]
MNFDDMDESSPHDPMNPTYDPGEDEKEASLRPRYMSEFQGQERIKENLLIFIKAARERGE
ALDHLFLMGPPGLGKTTLAIVVASEMGADFKPTSAPAIEKPKDLAGILTNLRPGGVLFID
EIHRLKPAIEEMLYIAMEDFELDWIIGQGPAARTVRIPLQPFTLVGATTKAGMVSAPLSS
RFGISLRFDYYAPEELASVIHRSSHILDIKIEDDAVELLAGTSRGTPRIANRLLRRMRDF
AQVKGAGTITKDVVHDGLGRLEVDYLGLERLDREILKAIIMRFGGGPVGAETLAVSVSES
VESLEDFYEPYLIQIGMLARTPRGRVATPAAYRHLGMEQPNENGRLQF*
>SPBDM4_v1_51085|ID:27159448|queA| S-adenosylmethionine:tRNA ribosyltransferase-isomerase [Uncultured spirochete bdmA 4]
MKTDDFNFELPETLIAQYPPQRRGESRLMVLDRAEGKTVDSMVSDIASFIEPGTLMVFND
SKVRKARVYGICEQTGARVEFVFLTPVEQESLIKIEELILHKGDQENVLVAPAQRIPSVS
RLWRAVCSKAKRQKAGRSYAFPGDIRGILAEDIHDEKIIEFSAAIGDDYFDRYGHVPLPP
YIKREDTKADEQRYQTVYARRTGSAASPTAGLHFTPEILSSLRSKGIGIEYITLHVGLGT
FLPVRTENIEDHRMHEEWFEITEDVAKKINAAKSNGRKVLAVGTTSLRALESAWYGGAIH
SGSFSTRIFIYPGFEFHVADRLFTNFHTPKSTLLMLVSAFAGRETIMEVYRSAIEKRYRF
FSYGDAMLIL*
>SPBDM4_v1_51086|ID:27159449|tmk| Thymidylate kinase [Uncultured spirochete bdmA 4]

MSFYNTDEFIKRFIVIEGIDGAGTTTQLQLLTDNLGARGIPCVTTAEPTKNPIGLMIRAI
LSGEMQAAPTTVAHLFAADRNEHIYGKQGIIENIEAGSIVISDRYALSSLAYQGVTCGPE
LPWLLNSAFPAPGLTLLFEVDPEVSIRRIHSREKKEIYETLEFQKRAHEMYEKMADRLQT
RGWRIERIDAEKDTESVRRDIESHVFAFLGIQA*
>SPBDM4_v1_51087|ID:27159450| exported protein of unknown function [Uncultured spirochete bdmA 4]
MRTKKPGIIIALFSLVLAILATVTILDIVRREVQLATTHTLDSVQALVMKTTGLRLKYDY
LSIDALNRFSMHGIELLRENPAPPAMSESSSPESDPDHIRAPIQLQRLSLRVSFWALLTG
HNSEVLRDIVADDLRISLLMPEDESIINKILENFSGSGSASLPHFMLTVGPVNVRVQEQS
TASGIPIQGGGLKEGTLKIDSLQFSSLSGSPEIVAPSISAEINGLFGLPRDLSIKVGLQG
SGAPDFSTFDVTMSASARTPQWLLMPQRLEVKKEGGRIEVRREGALAVRGWYEGGAWGGE
GQMEGYRPGEDVVVEGSWGEVVRGVTVSGSVSAQGEGGLLGGYKVAVGVEAGEGXX
>SPBDM4_v1_60001|ID:27159451| Endonuclease MutS2 (fragment) [Uncultured spirochete bdmA 4]
MRVISDIVRAATGRSLVLLDELGAGTDPEEGCAIAMALLDFFLERKSLTIATTHHGILKN
YGYTKPGCLNASMEFDAQSLSPTYRMCVGIPGESRALEIARQTGLSDEIVATATSYLDDE
RTDYSALMRSLGEKQRELDRLEQEKRKQLKGAQESRRNADLQNLKVRQRELELRRQGVAE
LNRLLSESRRTFENLVREIRESGRKVEEASSAREFLNELSEEAKRENDELSRFEAETKTL
ARSLSDGNAPSRRIDPASASVGEKVLYAGREAIIVRKLDEKRVLIQAGSLRIPVAMEDLS
IPPLHKKPDSETKLVYQVELSSPGEGSSKPLPEIDVRGMRLAQALETLAHQIDAASLGGL
GVFSIIHGTGEGILGKGIHEWLKTQPSVADYYFARPEDGGFGKTWVHLKP*
>SPBDM4_v1_60002|ID:27159452|yebC| conserved hypothetical protein [Uncultured spirochete bdmA 4]
MGTSETIKDLQLTPIYAAWYRYAAFLSAKGVFMSGHSKWATIKHKKGALDAKRGQLFTKL
IKEISIAARMGGGDPEGNPRLRTAVLKARAANMPKDNIDRAIKKGTGELEGTIYEELFYE
AYAPGGGAMLIEVLTDNKNRAAAEIRNIVTRSGANLGTAGSVAYLFKRKGVLTYDGEKYT
EDQILEAALDAGADDVVNEDGSIVVFTDPASFEDVLNAMNAKGFETLGAEISMVPDTYIS
VDAETAAKIQRLIDKLEENEDVQNVYHNIEMPEIEE*
>SPBDM4_v1_60003|ID:27159453|ruvC| Crossover junction endodeoxyribonuclease RuvC [Uncultured spirochete bdmA 4]
MAARRAIVAIGIDPGIAAVGFGVVRTREGSLELIAHGCIQTESSVPMGKRLAHIFNEIKR
LLQQYAPDVGGIEELYFFRNVSSAFPVAEARGVIRLAFEEANIELMQHSPNAIKKSVTGS
ARADKLQVQEMVRILLGMKEIPKPDHAADALAAAICTLHSIGPAGYTL*
>SPBDM4_v1_60004|ID:27159454|ruvA| Holliday junction ATP-dependent DNA helicase RuvA [Uncultured spirochete bdmA 4]
MFNRIRGILNGHADDALHVATGAIEWEIFVPVRDPAFFSRVGEEIELYTWLHHYEDGMRL
YGFISAPERQLFLDLQKVEGIGPRQAFKILQGIAPRALATMLDTSDLVSLQKIPGIGPKT
AQKMMLALKGKLIALEGEVGVPAPAVQGRFRDVIRALIEMGFDRREVERVVAQLGADVQE
GPESEKELFRKALLELSTGAGA*
>SPBDM4_v1_60005|ID:27159455|ruvB| ATP-dependent DNA helicase, component of RuvABC resolvasome [Uncultured spirochete bdmA 4]
MNFDDMDESSPHDPMNPTYDPGEDEKEASLRPRYMSEFQGQERIKENLLIFIKAARERGE
ALDHLFLMGPPGLGKTTLALVVASEMGADFKPTSAPAIEKPKDLAGILTNLRPGGVLFID
EIHRLKPAIEEMLYIAMEDFELDWIIGQGPAARTVRIPLQPFTLVGATTKAGMVSAPLSS
RFGISLRFDYYAPEELASVIHRSSHILDIKIEDDAVELLAGTSRGTPRIANRLLRRMRDF
AQVKGAGTITKDVVHDGLGRLEVDYLGLERLDREILKAIIMRFGGGPVGAETLAVSVSES
VESLEDFYEPYLIQIGMLARTPRGRVATPAAYRHLGMEQPNENGRLQF*
>SPBDM4_v1_60006|ID:27159456|queA| S-adenosylmethionine:tRNA ribosyltransferase-isomerase [Uncultured spirochete bdmA 4]
MKTDDFNFELPETLIAQYPPQRRGESRLMVLDRAEGKTVDSMVSDIASFIEPGTLMVFND
SKVRKARVYGICEQTGARVEFVFLTPVEQESLIKIEELILHKGDQENVLVAPAQRIPSVS
RLWRAVCSKAKRQKAGRSYAFPGDIRGILAEDIHDEKIIEFSAAIGDDYFDRYGHVPLPP
YIKREDTKADEQRYQTVYARRTGSAASPTAGLHFTPEILSSLRSKGIGIEYITLHVGLGT
FLPVRTENIEDHRMHEEWFEITEDVAKKINAAKSNGRKVLAVGTTSLRALESAWYGGAIH
SGSFSTRIFIYPGFEFHVADRLFTNFHTPKSTLLMLVSAFAGRETIMEVYRSAIEKRYRF
FSYGDAMLIL*
>SPBDM4_v1_60007|ID:27159457|tmk| Thymidylate kinase [Uncultured spirochete bdmA 4]

MSFYNTDEFIKRFIVIEGIDGAGTTTQLQLLTDNLGARGIPCVTTAEPTKNPIGLMIRAI
LSGEMQAAPTTVAHLFAADRNEHIYGKQGIIENIEAGSIVISDRYALSSLAYQGVTCGPE
LPWLLNSAFPAPGLTLLFEVDPEVSIRRIHSREKKEIYETLEFQKRAHEMYEKMADRLQT
RGWRIERIDAEKDTESVRRDIESHVFAFLGIQA*
>SPBDM4_v1_60008|ID:27159458| exported protein of unknown function [Uncultured spirochete bdmA 4]
MRTKKPGIIIALFSLVLAILATVTILDIVRREVQLATTHTLDSVQALVMKTTGLRLKYDY
LSIDALNRFSMHGIELLRENPAPPAMSESSSPESDPDHIRAPIQLQRLSLRVSFWALLTG
HNSEVLRDIVADDLRISLLMPEDESIINKILENFSGSGSASLPHFMLTVGPVNVRVQEQS
TASGIPIQGGGLKEGTLKIDSLQFSSLSGSPEIVAPSISAEINGLFGLPRDLSIKVGLQG
SGAPDFSTFDVTMSASARTPQWLLMPQRLEVKKEGGRIEVRREGALAVRGWYEGGAWGVE
GQMEGYRPGEDVVVEGSWGEVVRGVTVSGSVSAQGEGGVLGGYKVAVGVEAGEGVEVSGY
KVGGMEVRVTGAGDGERYWGTVEGGQKGYRVRYEGVVGYRGMSVEGVYEVGGPGVAVKGE
VRGGEGEYRVGVGGGEVKGIELGVGQGRVKMSQEGYEVGYAGAVGGGGVSVEGSIGKDGG
YEGVVEARGIGAGSIGGLVGLKGVEGRVSGRVYGLVRGGKVSWALNGGEYEGKVEGVGVK
ARVEGVGDEVGYEVKGAEVSVGGVTVGLKGKGSYKGKVFEGEVSAGGLAYGVEVTVAGER
IGVKVGQTLVGGVTVAGGRVEGEVTLKDFGVGTGGGVVWLQGDVKGWYERGAWEGTVERL
GVRYEGEGEYPEVLVSGTVDAKGANLAIDSIKYAGKQLKGTINVQYSSLMNPLKNMQALY
SFTSIEDERAKIEGTVQALEGKITAAITGTSIPIEQFLSSSSNIAGDVQFQGTASISATG
GKLSWADISLAELKFNCQKAEIKGIPFSAAGTISLKDNALSIHSGAFSYQNYKIDNIEAM
YDLSKQKLEYALNAKIVIAEKLLSAFLKGTGDINGSIFDKEAFKNAQFSGEITEAKFEST
VLDPITYVFSIADNNLDIHFSQANGDNAHASIRNMTEFELTIANLFNVNGSARGTIQANN
IEADINLDSMDXX
>SPBDM4_v1_70001|ID:27159459| Endonuclease MutS2 (fragment) [Uncultured spirochete bdmA 4]
MRVISDIVRAATGRSLVLLDELGAGTDPEEGCAIAMALLDFFLERKSLTIATTHHGILKN
YGYTKPGCLNASMEFDAQSLSPTYRMCVGIPGESRALEIARQTGLSDEIVATATSYLDDE
RTDYSALMRSLGEKQRELDRLEQEKRKQLKGAQESRRNADLQNLKVRQRELELRRQGVAE
LNRLLSESRRTFENLVREIRESGRKVEEASSAREFLNELSEEAKRENDELSRFEAETKTL
ARSLSDGNAPSRRIDPASASVGEKVLYAGREAIIVRKLDEKRVLIQAGSLRIPVAMEDLS
IPPLHKKPDSETKLVYQVELSSPGEGSSKPLPEIDVRGMRLAQALETLAHQIDAASLGGL
GVFSIIHGTGEGILGKGIHEWLKTQPSVADYYFARPEDGGFGKTWVHLKP*
>SPBDM4_v1_70002|ID:27159460|yebC| conserved hypothetical protein [Uncultured spirochete bdmA 4]
MGTSETIKDLQLTPIYAAWYRYAAFLSAKGVFMSGHSKWATIKHKKGALDAKRGQLFTKL
IKEISIAARMGGGDPEGNPRLRTAVLKARAANMPKDNIDRAIKKGTGELEGTIYEELFYE
AYAPGGGAMLIEVLTDNKNRAAAEIRNIVTRSGANLGTAGSVAYLFKRKGVLTYDGEKYT
EDQILEAALDAGADDVVNEDGSIVVFTDPASFEDVLNAMNAKGFETLGAEISMVPDTYIS
VDAETAAKIQRLIDKLEENEDVQNVYHNIEMPEIEE*
>SPBDM4_v1_70003|ID:27159461|ruvC| Crossover junction endodeoxyribonuclease RuvC [Uncultured spirochete bdmA 4]
MAARRAIVAIGIDPGIAAVGFGVVRTREGSLELIAHGCIQTESSVPMGKRLAHIFNEIKR
LLQQYAPDVGGIEELYFFRNVSSAFPVAEARGVIRLAFEEANIELMQHSPNAIKKSVTGS
ARADKLQVQEMVRILLGMKEIPKPDHAADALAAAICTLHSIGPAGYTL*
>SPBDM4_v1_70004|ID:27159462|ruvA| Holliday junction ATP-dependent DNA helicase RuvA [Uncultured spirochete bdmA 4]
MFNRIRGILNGHADDALHVATGAIEWEIFVPVRDPAFFSRVGEEIELYTWLHHYEDGMRL
YGFISAPERQLFLDLQKVEGIGPRQAFKILQGIAPRALATMLDTSDLVSLQKIPGIGPKT
AQKMMLALKGKLIALEGEVGVPAPAVQGRFRDVIRALIEMGFDRREVERVVAQLGADVQE
GPESEKELFRKALLELSTGAGA*
>SPBDM4_v1_70005|ID:27159463|ruvB| ATP-dependent DNA helicase, component of RuvABC resolvasome [Uncultured spirochete bdmA 4]
MNFDDMDESSPHDPMNPTYDPGEDEKEASLRPRYMSEFQGQERIKENLLIFIKAARERGE
ALDHLFLMGPPGLGKTTLALVVASEMGADFKPTSAPAIEKPKDLAGILTNLRPGGVLFID
EIHRLKPAIEEMLYIAMEDFELDWIIGQGPAARTVRIPLQPFTLVGATTKAGMVSAPLSS
RFGISLRFDYYAPEELASVIHRSSHILDIKIEDDAVELLAGTSRGTPRIANRLLRRMRDF
AQVKGAGTITKDVVHDGLGRLEVDYLGLERLDREILKAIIMRFGGGPVGAETLAVSVSES

VESLEDFYEPYLIQIGMLARTPRGRVATPAAYRHLGMEQPNENGRLQF*
>SPBDM4_v1_70006|ID:27159464|queA| S-adenosylmethionine:tRNA ribosyltransferase-isomerase [Uncultured spirochete bdmA 4]
MKTDDFNFELPETLIAQYPPQRRGESRLMVLDRAEGKTVDSMVSDIASFIEPGTLMVFND
SKVRKARVYGICEQTGARVEFVFLTPVEQESLIKIEELILHKGDQENVLVAPAQRIPSVS
RLWRAVCSKAKRQKAGRSYAFPGDIRGILAEDIHDEKIIEFSAAIGDDYFDRYGHVPLPP
YIKREDTKADEQRYQTVYARRTGSAASPTAGLHFTPEILSSLRSKGIGIEYITLHVGLGT
FLPVRTENIEDHRMHEEWFEITEDVAKKINAAKSNGRKVLAVGTTSLRALESAWYGGAIH
SGSFSTRIFIYPGFEFHVADRLFTNFHTPKSTLLMLVSAFAGRETIMEVYRSAIEKRYRF
FSYGDAMLIL*
>SPBDM4_v1_70007|ID:27159465|tmk| Thymidylate kinase [Uncultured spirochete bdmA 4]
MSFYNTDEFIKRFIVIEGIDGAGTTTQLQLLTDNLGARGIPCVTTAEPTKNPIGLMIRAI
LSGEMQAAPTTVAHLFAADRNEHIYGKQGIIENIEAGSIVISDRYALSSLAYQGVTCGPE
LPWLLNSAFPAPGLTLLFEVDPEVSIRRIHSREKKEIYETLEFQKRAHEMYEKMADRLQT
RGWRIERIDAEKDTESVRRDIESHVFAFLGIQA*
>SPBDM4_v1_70008|ID:27159466| exported protein of unknown function [Uncultured spirochete bdmA 4]
MRTKKPGIIIALFSLVLAILATVTILDIVRREVQLATTHTLDSVQALVMKTTGLRLKYDY
LSIDALNRFSMHGIELLRENPAPPAMSESSSPESDPDHIRAPIQLQRLSLRVSFWALLTG
HNSEVLRDIVADDLRISLLMPEDESIINKILENFSGSGSASLPHFMLTVGPVNVRVQEQS
TASGIPIQGGGLKEGTLKIDSLQFSSLSGSPEIVAPSISAEINGLFGLPRDLSIKVGLQG
SGAPDFSTFDVTMSASARTPQWLLMPQRLEVKKEGGRIEVRREGALAVRGWYEGGAWGVE
GQMEGYRPGEDVVVEGSWGEVVRGVTVSGSVSAQGEGGVLGGYKVAVGVEAGEGVEVSGY
KVGGMEVRVTGAGDGERYWGTVEGGQKGYRVRYEGVVGYRGMSVEGVYEVGGPGVAVKGE
VRGGEGEYRVGVGGGEVKGIELGVGQGRVKMSQEGYEVGYAGAVGGGGVSVEGSIGKDGG
YEGVVEARGIGAGSIGGLVGLKGVEGRVSGRVYGLVRGGKVSWSVSGGEYEGKVEGVGVK
ARVEGVGDEVGYEVKGAEVSVGGVTVGLKGKGSYKGKVFEGEVSAGGLAYGVEVTVAGER
IGVKVGQTLVGGVTVAGGRVEGEVTLKDFGVGTGGGVVWLQGDVKGWYERGAWEGTVERL
GVRYEGEGEYPEVLVSGTVDAKGANLAIDSIKYAGKQLKGTINVQYSSLMNPLKNMQALY
SFTSIEDERAKIEGTVQALEGKITAAITGTSIPIEQFLSSSSNIAGDVQFQGTASISATG
GKLSWADISLAELKFNCQKAEIKGIPFSAAGTISLKDNALSIHSGAFSYQNYKIDNIEAM
YDLSKQKLEYALNAKIVIAEKLLSAFLKGTGDINGSIFDKEAFKNAQFSGEITEAKFEST
VLDPITYVFSIADNNLDIHFSQANGDNAHASIRNMTEFELTIANLFNVNGSARGTIQANN
IEADINLDSMDLSLLQIFLPQEDIKDISGNGSASMHLSGDISDPKIDGKILLNSVSFSSN
AYLLEKVGPFNAEITINEGIIELSPTIVSIGTGNISVAATASLARWAIGDIKAFVSTGET
ASVKFKGTIAGLTAKDISLKADIKANISQSKLEISGNILLDNGILEVNPTGFIASEDTGQ
AAQPLSLNLAVTLGKNVELYLPSQDIPLVRGMASPNSALNILYDEASGALSVNGSVQLRS
GYVFYLLRNFFIKQCSIDFSENQTKFNPLISTTAELREPSKDGIITITLSADRTPFENFN
PRLSSIPPKSETELLALLGGGLALSDLADNNPLTLREAVIAGSEFLTQNSLFRSFEQRVQ
KALGVDVLYIQSSFIQRWLLDITDQTKSGQTPLSEYLTGTELFAGKYITDLAFAHFSLRM
VQDPLEGTGPLQLDSELGLELQAPFGLLQWSMSLGKEGTPLNNQTLSLSWRINY*
>SPBDM4_v1_70009|ID:27159467| Outer membrane protein assembly complex, YaeT protein [Uncultured spirochete bdmA 4]
MKYRRSFFIAAIMLFIAFHGVLAQDTSQATSQTASDDWFWDKPIAGFQWEGLHYANRNDL
DSLLRAYIGTVFTDAVWTDIQAKLYALDWFETIEPVALPAGDSKEKIVIKFIVVEKPSID
SIRIVGNSALRTSELLDAASSKNDAIYNEDKAKADTLAMQKLYLQKGYPDATVELETTFS
PQNSSRIILTFKVSEGSAVVVRKILFSGNAVFSSQTLKGQVTLKEAGLFQKGAFEESKLA
ESRTAIEDYYASKGYVDAKVVDILKNYTKDEKGQKNYLDITFVVTEGKQWMFGGVSFEGN
TIFSTSRLASLISLKQDDPLNLKRLLADKQKVDDLYYESGYIFNSIAMTQNRDEAAGSIS
YKISIVERDRAHIGSITFKGNTKTKDYVIAREIPLEVGEVFSKSKVIDGLRNLYNLQYFS
SIEPEIHQGSVEDLMDLVINVEEMSTAQIQFGLTLTGVGQSSTFPVSGFVKWNDSNMGGT
GKDLQINMTLSPDEQSLVTSFGQNWLLNKRISQSLSFTIKHAITQTAQDSVAPIFSTEDI
PDPFIAEGSGTNEWNGLLSSVPDAYLMDYEDYTFSLGYSLGYVFKIPKGDIGVAGGVSSG
VGMDSYDATKYRPYEQEMRDYNGIWVLKDSIFGRVYLNHLDYWYNPGKGYFLSQRFTYTG
FLPVERQFYIRSDTRAELYATLFSIPFSSTWVFSPIIGIHSGVQALLAQPWTPLSVTKDW

VYLDGTFNVRGWNSLYGSDGTMTWENWLELRLPIVPQVLSLDGFIDAGAMKTEDGWLDMT
LNSPSASAGSLDWKNFAFSTGFGARFVISQFPFRFYLAKRFVFDGSAITWKTAAGSFDFV
LSITQPLY*
>SPBDM4_v1_70010|ID:27159468|mutS| DNA mismatch repair protein MutS [Uncultured spirochete bdmA 4]
VLQEAAFYNVPMPESSSMLDQYRRIKARHRDEILFFRLGDFYEMFNEDAQEASSILDLTL
TKRQGQPMCGIPYHAAKTYIARLLKAGKKVAICEQKPLAGKRGLMDREVIEVITPGTAVE
DDYLEQTANNYLADICLLNNYLCFSYLDVSTGEFKAHSIVYRNGESSEAIRTELYRLSPR
ELLVQQSILDQPRLFQVIRESGAMIEPQQDWTYDLVHSLEILKKKFGLASLKGFGFDDQD
PALAAAGHLLEYVQEMIRQDCPHIRALLRYEQQEYVLLDEATKRNLELVKNLNDSSRRDT
LIEVIDRTKTAGGTRLLRQWLFQPLRKREKIEARLDAVAFLYHDQIFLGDVRKILYGVLD
TERLISRLAIDKAHAKDLRALRDSLDAGLNLFRVVEAAQPPCALKPGLSAEHAEDCKHVI
DAIDEAILDDPSVLLTEGDLIKDGYNAELDDLRNMHKNTQAVLERYLEEEKQRSGIQNLR
IRYNRVIGYYLEVTKGNLANVPGHFIRRQSLVGGERYTTDRLAELESKINGAQERIVELE
KQLFLEVREGIKKYIPALLECSQSISQIDCLASLAQAATEHGYARPEICEDSEIHIINGR
HPVVEMCLPYGDFVPNSISFSRENQWFVLITGPNMAGKSTVLRQTALIVLLAHMGSFVPA
DSARIGLVDKIFCRVGAQDNLARGESTFLVEMHETAFILNTATEKSLVIMDEVGRGTGTL
DGVSIAWAVSEYLIHHIGCRTLFATHYHQLTTMAIPGVRNMKMAVIERDGNVSFPKRLEE
GASSGSYGIHVARLAGLPDEVIFRASALESHFSTLERGLVEGNFKEANVPAQTAFSIPKD
NQRNNALTPYEKNDLSPRHEAYSGELFSQEDLVIAELKNMDTDEITPLKALQILSELVGK
LKQ*
>SPBDM4_v1_70011|ID:27159469| conserved protein of unknown function [Uncultured spirochete bdmA 4]
MHTLIENLVSASLDAVFPQRCILCGSIVSRVPWSPAPLCVSCEHTLECIGGPRCRRCGRR
LISEQDYCVECRSEENALFRILPLFEFRGGAASLIHSYKIEERASLAYYFAYKITKLEAL
FDDTFGDFSIVPVPPRPEKIRTGKLDQVGVLAEVLAQFGFQYKRLLVRLPGGRQQKLLDR
AERLKNASASYALRSPSQVPNRIMLLDDVCTTGATLEACARLLAREGTEVAGAIVLAAD*
>SPBDM4_v1_70012|ID:27159470| putative Ribosome maturation factor RimP [Uncultured spirochete bdmA 4]
MSDKELEKEIDEVLKAGGLDLLEFSASRHRGSLHVKAVIYGKNGTGTDECTKAYRLILPR
VQMQLGVQNPYIEVYSPGIDRVIRTAREWQAFTDRFILFLTADRPDWCRGRIIHFENGIV
QIETDDTVSNIPVASILKAKLDSSHEGEKAHGI*
>SPBDM4_v1_70013|ID:27159471|nusA| Transcription termination/antitermination protein NusA [Uncultured spirochete bdmA 4]
MASEMAEAIRQLISEKGIPEDLIVKTLEDSILAAYKKKFGTSENAVVRFKENYEGIEIYA
KKTIVSDEDFEDEVLEIPLEEASKLAEEAEIGDILEIPIDPSEFDFQSVMLAKQTARQSL
RDISRDTLYAEFKSKVGEIVIGYFQRERNGTIFVDLGKVEGVFPHKYQSPRETYHVGDRI
KALIVEVEKTKTGFQVVLSRTHAEFVRKLLELEIPEIYDKTIEIFKIVREPGYRTKVAVY
TKRTDVDPVGACVGPKGMRSQLISQELEGEKIDFIRYDINPKEFIQNALAPAQIKEVYIL
DEARHMALAVVDEHELSIAIGRSGQNVKLANRLCDWNIDVMTEEQYLQDSRNIELKKAAD
SLFSTEEEQGEELLIKDLPDMKTDWITALEAADIRSIEQFLGLAESEISQRTSLSPDDVA
AIRSIIEENVEIVQEEVEEEEPESYVCPECGAPITPDMDHCPSCGVALSFEVDEGQDE*
>SPBDM4_v1_70014|ID:27159472| translation initiation factor IF-2 (fragment) [Uncultured spirochete bdmA 4]
MGGSQYSGGTGGGGRTGYPSGRPGQPGGSRPGGYGGSRPGPGGGYGARPAPASMPPAEGS
RSSSRRSPAKKKGSFGKREEELELEKQIQLKKKAEAKLASVPKSVDIMENISISELAKKM
NLKPADLISKLMSLGVMATINQRIDADTAAILAAEYGCEVKVVSLYDETVIEKAADKPED
MEHRPPIVTVMGHVDHGKTKLLDAIRKTDVVSQEFGGITQHIGAYMVETPHGKISFLDTP
GHAAFTKMRARGANITDIVILVVSAVEGVMPQTKEAIDHARAADVPIIVAVNKIDLPEAN
PDRVKTQLSELGLIPEEWGGTTQFVEVSALQKKGIPELFDAILLQAEVLDLKANFDRLAE
GKIIESRIDQGRGIVSTIMIQNGTLRIGDSFVAGIYPGKVRALFDDKGQRVEEATPSMPV
EVLGFEGMPEAGDPFEAVEDEKFARQISAKRQELKKYEEGRNVKKVTLDNLYETISQGEI
KELKVIIKGDVHGSVEALKGMLEKLSTKEVHLNAIRAAAGAITEDDVMMASASNAIIIGF
NVRPTPNAKQIAEREKVDVRKYNIIYRAQEEIKQAMEGLLAPELKEQEVGKAEVRSIFRV
PKVGIIAGCFVTEGVVKRSCQVRVIRDSIEIYQGNLSSLKRFKDDVKEVAAGYECGIGIE
NCNDLQEGDILEFYEMVEVARTLDSGNDNGSDTKASS*
>SPBDM4_v1_70015|ID:27159473|rbfA| Ribosome-binding factor A [Uncultured spirochete bdmA 4]
MDQIRRRRLEELIREEISRMLALGEIKDPRVNSFLSITRVEAAQDGSHARVWVSSLEDEE

ARLDEAIAGLSHAAGFIQALIAKRVRLRLTPVLTFVPDRGIKEAFEVTEKLKDLLK*
>SPBDM4_v1_70016|ID:27159474|truB| tRNA pseudouridine synthase B [Uncultured spirochete bdmA 4]
MQRGGFFLVGKPAGPTSNRVLQDIRRSFLNEPGFRGLKFGHAGTLDSFASGLLVVLVGRM
TRLTPWFMHQAKEYEAVFRFGEETDTLDPLGKVIAAARTPTLSELENVLPEFRGSIYQIP
PSYSAVHVDGRRSYKIAKSGEIPQLSPRPVSIDKLELRTFEGNEASFTIRCSSGTYVRSL
ARDIARACGSRAFVQALRRTRIGQFDVAAACSPLGCTVARLCEFTPDIARDIGLGTGILH
KEFFIQFQHGSLLPTEAVTLRHRGTSVVALFTEGGAFIGLVEKREEEWGARMVAAEELQQ
*
>SPBDM4_v1_70017|ID:27159475| putative riboflavin biosynthesis protein RibF [Uncultured spirochete bdmA 4]
MKVLTWQEFNRDPPQEGLAATVGVFDGLHLGHQALIATVKQEKPHLFPCVITFRENPKKF
LHPSSYRGSLLTIEQKLSAIQSEGIEYCVLIDFSDNFATLAGREFLAALYNANVRFVAVG
ENFQFGYKLDTDAAKLEALSGEIGMRSCSVKNVMYRGHPVSSSRVRHAVLENRLREAEEM
LGRPYQIVARRERRGEHDMRELFVPEDDLLVPPDGEYDVFVRDDETIEQTKVNVFHRHIA
LGLRMQSERIRLAFVDKATQKENLVWL*
>SPBDM4_v1_70018|ID:27159476|rpsO| 30S ribosomal subunit protein S15 [Uncultured spirochete bdmA 4]
MALSKEDKAQVVLEYGKDPKNTGAIETQIALISSRISYLTEHFKTHKKDTNSRRGLLKLV
GQRRKLLKYLQRTNLESYRAIMEKLQIRK*
>SPBDM4_v1_70019|ID:27159477|pnp| polynucleotide phosphorylase/polyadenylase [Uncultured spirochete bdmA 4]
MINKLECKIGNETLVLETGRMAKQANGAVFATYGGSAVIATACCSSTPTEGLDFVPLTVE
YSEKYYAAGKIPGGFIKRETRPKDREILVSRIIDRPMRPLFRKEFGREIQVVPTCISADQ
INPPDVIAANAASAAVHISDIPFDGPIGCVRVALVDGSYIVNPSYDQIERAKLEIVVAGT
AVGITMVEGGSHESTEEEMIQAIETAKEPISQICATIEELRRLAGKEKLALAPLLVELNN
KEEIRVYARELLSSALFTKIKQERAKAVAQAIAAVKEKFKDSLTDDIQSKLFSKLMDDLQ
YEILRSSILDKGLRVDGRGLEEIRPITCETGVLSRTHGSALFTRGETQVLAVTTLGTVFD
EQIFDDIEGDRRERFMLHYNFPPFSVGEVGRLGTGRREIGHGDLARRSIEPMLPPKEKFP
YTIRVVAEVLESNGSSSMATVCSSSMALMHAGVPVSRPVAGIAMGLITDETRYAVLSDIL
GDEDHLGDMDFKVAGTKDGITGFQMDIKISSVSTEILRKALDQAKRGRLHILSIMEKTIA
APQSEISPLAPQVLSARIDPEKIGLVIGPAGKNIKAISEKYGVQINIEEDGSLTVYGKDQ
KSAFDARDAILGMVEEPEVGKVYTGTVKRIMDFGAFIEILPGREGLCHISRLAKGRVEKV
TDVLKEGDTIQVKLMEIDHLGRLNLAAVDSLDENGEAPQRPPRSEGRPSDRARDTRPPRR
ESYRDR*
>SPBDM4_v1_70020|ID:27159478| protein of unknown function [Uncultured spirochete bdmA 4]
VFDKMKKAPVLRLFISGTIGSQILIETLDVFGIVSTAGIAPTADFSRRGIIENLTCPDTI
LFAEVLYLARDRTAAPRSVADAGELRHSHQVNRRRRG*
>SPBDM4_v1_70021|ID:27159479|dut| Deoxyuridine 5'-triphosphate nucleotidohydrolase [Uncultured spirochete bdmA 4]
MIKSSLRNKNLLTSAQSSTIQSEGHAMDVQISLSEDARIPQYATEYSAGADLFASLKEAV
VLQPLSRALIPTGVKIALPPGYEAQVRPRSGLALRHGVTCLNTPGTIDSDYRGEIRVLLI
NLGDKSFTIENGDRIAQLIIAPIMQAHFSISEKLDDTSRGAGGFGSTGR*
>SPBDM4_v1_70022|ID:27159480| putative Permease YjgP/YjgQ family protein [Uncultured spirochete bdmA 4]
MIPRAARAVLALQAVEMKGKPPFTLWRMMFQELMSSFLGAFFFFFVVFLINQVLLFAEDI
LSRGADFFSVVKLLFYSLPTILAITIPFSVLAAALMTSSRQNADNEFLASSTLGIRPLWL
YIPFLIAGLGIAIGSFYLNDWSIPRAAQRYKHVYAELIRKSAKIELTPYSIKKYGEKLLV
TGPSKAGRIQNILIIDQKTGYDSNAVTANNVGIEFSADSLAAILSMDNVTEEKRLQNGNE
GDFSITQAQSADIRIQIQEQMPNYSGAAPSEMSLAALAHQIKQKEGRLAIRQEENRSQQA
TALDSLRLSYGSLQVQREPADSVSLKSQSPSKSENVGSVLNALKSLRNQKIDDTSLQIYR
LEYQKKFVIPSACFFFALLAFPLGIGSKRAGRTAGFGIALLLSVIYWALLFAGQTFGYRQ
NLDPVLSMWMPNLIMFVATAVLWIFRKITKGHFL*
>SPBDM4_v1_70023|ID:27159481| putative Permease YjgP/YjgQ family protein [Uncultured spirochete bdmA 4]
MMSTEKEMHAFPHGQRNVQGAPVLLWKTLSKNILLWTAGGTLFLSLVISLVELFSLLWKF
LVRNASFGDILQWIGLGAPKHIAESLPVAFLFAIVFTLSNWHANNELEAVFSAGISLQRF
LLPLFALSIVFCFAEFYLTDAVSIPFLRIRNTLQSEILKESDSRYTVPGLIVDKGRQVYT
YRYFDEKNLRLYDVSIVERDPNGNLIRKVSAQNARWEDGTWYFVNATVYQKKGGSWEFSK
IAKFADPAFKEPPSGFSRPTMDVRLLNTRELQAQVSFLKASGLPSIDADVEKQRRLSFSL

TPLIVIGLAGVFAGRFKKSIFLLSMLFSLSSATLYYVAQMIASLAAKAGMVSPAFSIWSV
MVGFAIVSVASYLRAKT*
>SPBDM4_v1_70024|ID:27159482|tgt| Queuine tRNA-ribosyltransferase [Uncultured spirochete bdmA 4]
MSEPIFHEIHRDNSCRARTGILSLPHGDVQTPAFMPVGTNATVKAVEPKDIAEMGFSIIL
ANTYHLFLRPGPEIIAKAGGLHGFSGWKGNFLTDSGGFQVFSLSQFRKILVEGVHFKSHI
DGSSHVLTPEKVVDVQVAFNSDIQMALDICAPWGETEKKAYHAAMLTYDWAKRAKSQWLA
QRDHGYQGYLFGIVQGNFYRELRKTSAEQIGSLDFPGIAIGGLSIGEPKDAYIEYLEYTA
TLLPSEKPHYLMGIGTPDYIIEAVRNGIDIFDCVYPTRTARNGLLFTSKGQITIKKAMYR
EDYLPIDPECTCHVCRTYSRAYLHHLFRNNEILYSMLASQHNLHFMAQFVSDIRHAIEED
KFELFAGNFLHVYEKMSSQD*
>SPBDM4_v1_70025|ID:27159483| Transcription elongation factor [Uncultured spirochete bdmA 4]
MAESPLNEKLQLMLNEEKWTRTLMANYSVVSLKELDRFIDETTAQGITDDIIKTCTDHLS
QTKNSVIALYVAGILNIMRQPADESFLVQLMDLFADSKKNNIIEYICNRMLDFGENRIAL
MRLAECYTDENKIEQRYGVWERLVRIDTEEAELAQLIAEWKEKNGQFEDAIDFYKKALIR
YINKGLFANIKEIWLKLIELSPEDMDFFLHAQKKIAKQISPEKAAMLLTDLYAWYKKSEK
WETALNILKLIIEYDERNPALRKEFLDCYTAIYSGHSMFDECVRLSNLTQTYRSLHEAIA
DFEKRIAFDKGNYVFHRTWNIGRISNITNDEVVVDFAKSRNHHMSLKMAFDSLTTLSRDH
IWVLKATWPREKLRDKVKTDPAWAIKIIIKSFDNRADMKRIKAELVPSVLSPSEWNSWSS
RAKDILKTDPMFGNAQDDISTFIVRDRPLSFEEKIYAQFKAEKNFFARVQFFRDYIEMSN
DLDTDYFEDMLSYFVSFLKLTQANEFMIGSYLLVKELSSRYPRLQQHVTMRFADLFGQIK
DNIVPIYLALKDTELRNQLIQNIKTFVSNWPDVYIQLLPYARSAKMLDALESAGQEENLK
RMAMYIVENYKDMREAFIWLARDLKDKPWLKELGIVREKIILTLLHILDITFKEIDSHKD
TTENRKINKLVQNILFKEKDLEEFLEEAPQQVTERVYSMLADIKEVDPAIKLDLKKKISE
RYPNIKFYDGNERLTVSRGLMVTAVKYEEKKHLLGHIMEVDVPANQKEIAFALSLGDLRE
NAEYKAAKEKQDELNSKVGKLKNELDRAQIIGPEHVDASNISFGTKLTLKNLANGEEEIF
TILGPWESDPENQIISYLSPLGKHLLNHKTGETLNFTINERKFSYKVLKIVKAEF*
>SPBDM4_v1_70026|ID:27159484| Tetratricopeptide TPR_2 repeat-containing protein [Uncultured spirochete bdmA 4]
MAYQPQTQSATAADNLAFAYESLKNADIQSADSYLENALKADFEHAEVLLAMKCMAFWKD
QLSKMPETGNLLDRGDFLCASWKKFMLFLTRIGDTSETVRYAYKRFVFGLALDQYLALPD
KEKDALGAALDLRLGRARKATGDIETALIHLERAMQAQRNDACVLAELADAYAMAGEARL
SKALFREAFFLAPQTIEIEFLESEFILKIIENIQELGYSGNDIAEWIPVYAEIWGVFNVK
RELSVAEHNRISASARQLEIELRESPQRRSSLVPRLLNRYFWMADHLKANGDENGLHSVL
LKIKILDQSIHALYVE*
>SPBDM4_v1_70027|ID:27159485|rpe| Ribulose-phosphate 3-epimerase [Uncultured spirochete bdmA 4]
MKEPIIAPSILSADFYDIRAAIQDIESSECPWLHLDIMDGRFVPPITFGNKMVADIRKHT
SLFMDVHLMIVEPERHIHTFIESGADAVTFHVEACIHAHRLVQTIRHAGIKAGISVVPST
PVSAIVPLLESIDQVLVMTVNPGYGGQKLLPFCLAKVDELREIREKNGMNFSIAIDGGIN
LATIQQIALHKPDVLIMGSAFFNAQDKKVFISDVLNVWEHAVENFE*
>SPBDM4_v1_70028|ID:27159486| exported protein of unknown function [Uncultured spirochete bdmA 4]
MKRLAIGCFLIFLNIGFLLYAQANPVALDEFGSAFDMFKRGDFQNAGASFAHLYQTWPED
VLAADSCFMAARAYFNAGDYPASYGFCEEFLQSYPGHSNIPDMEFQRGRILFKMGRFKEA
TLAFNAFLDKYPDSTLYPSAFFWKAESLYCLGDIARALPLFNEIREKWPGSEQASLAAWR
LNVMGLEAREAKYLRLAAFESERNSAGEIVEMQKEAYREQQYLRDYLLLKSLRGRNNFPA
PSASPLNMKYSSRNDKLDMLLDAKKRALDLLISKIKEYLKEIAP*
>SPBDM4_v1_70029|ID:27159487| protein of unknown function [Uncultured spirochete bdmA 4]
MKRAVFFLIMIVVLVEMGAALDIRDGLVRITTDDLTLRPMLYRLVNIAGKSKYEPLWFNG
DPRTSFITISVDNRIYRMGYSQEYQTSQRRIQNGIEIEYRSVTNRVTQRIMFAALAGSRV
ANGFIIELEVENYTSRDMKIMLKEVCDTWLGENTGNHIALASKPKVTDEMLLNSDSKEPY
IISPGQDASIALLFDSDPRPDSVVIANWKRLSDSKFFYDSELMRGFTLSPYSVNDSALGL
YWNEKIVPAKGVVKVESRWLSGGPGSEFISWLTQNYPPKEAEETQAISQKNASPGISSGF
SLDVEAIQALVSRIDSAIENIDNVTDEDLQLILSELEALENSDVSGTPD*
>SPBDM4_v1_70030|ID:27159488|dtd| D-tyrosyl-tRNA(Tyr) deacylase [Uncultured spirochete bdmA 4]
MRAVVQRVSEATVTYVASSSQQNLLDKREQLCGGISNGVLVYIGIGVEDAEEDAEYLADK

IAHLRIFMDDCEKMNLSILDLNYEALVISQFTLLADARKGRRPSYSQAAESSRAKALYEF
FCERLREQGLHVQMGKFQEIMRVRYINEGPVTILLDSKKAF*
>SPBDM4_v1_70031|ID:27159489|ppdK| Pyruvate, phosphate dikinase [Uncultured spirochete bdmA 4]
MPKKKFVYSFGAGVAEGNGSMKELLGGKGAGLHEMTKIGLPVPAGFTITTEVCDLYFKHE
KKWPEGLEKEIQRHLKQLEKTTKKTLGDPRDPLLVSVRSGAPVSMPGMMETILNLGLTDA
SVEGLAAKTGNRRFALDAYRRFIMMYGSTAMGIDREEFDKAFDGVKEKRTRVRLHVSAGQ
KVNDTDVNEEELDELIGVFKKIYREHIKTDFPQDPWEQLKGAIDAVFNSWMAEKAVTYRK
VEKLVGVKGTAVNVVQMVFGNKGETSGTGVCFTRDPNSGDNVFYGDYLINAQGEDVVAGI
RTPIKLAELEKRDPKVFKQLVSIRKKLETHYRDMQDLEFTVEEGKLYMLQCRTGKRSPIA
AFHIAVSMVKEKLITKEEAVQRIKASDIEGIFYPMIDKSNPADLKKAFIVQGIDAVPGAA
TGKVVFNAKAAEEWAAKGEKVILVRKETSPEDVGGMHAAQGILTATGGKTSHAAVVARGW
GKCCVVGCEKLDIDYEKGQFSVGDTVVREGDYITLDGGAGNVYIGQLKLVKPEPPKAYTI
LMSWVDKIRTLKVRTNADTPYDAEKARELGAEGIGLCRTEHMFFDSEERILAIREMIIAD
DLESRKKALAKVLPFQTKDFEGIFKAMNGLPVTIRLIDPPLHEFVPHDEEGQKALADAVG
VPLEKVIQRVNQLHEANPMLGHRGCRLTITYPEILDMQVTAIITAACNMTKKGVKVLPEI
MIPLVIDEKELKILETRTREVADAIIAKSGISLGYMVGTMIEVPRAALLADRIANVAEFF
SFGTNDLTQMTLGLSRDDAGKFLPDYVDEKKAGIFKADPFQSLDQDGVGMLIRMGIEKGR
ATRSKLKVGICGEHGGDPASVKFFHRVGMNYVSSSPFRVPVSRLAAAQAVVEDSLAAKKQ
TKRRGRPPKSGVASAKPSKKTSASKATVTQARRPRGRPRGSKNK*
>SPBDM4_v1_70032|ID:27159490| protein of unknown function [Uncultured spirochete bdmA 4]
MNKKELTNKVFAFFEKLIETTELVKLYINNVIYYSIFMSQ*
>SPBDM4_v1_70033|ID:27159491| conserved exported protein of unknown function [Uncultured spirochete bdmA 4]
MDKRIERMSKFWKVLPNLLLLIVSAFPLTSCIGIDASAKIDAKGAGTLSIEYQISKDFAQ
LGSLESTPMLPLPLSREDIERGLQGVQGVTLKSYSKSERGDNIIVSLMLAFDSPSSLAVY
LDPNGKLAQYQQENGRSHLKLSFGDTVQPLDPQMKAEISEKLKPYRFKFALEAPGAAPEI
SVKNGDFFQVTTSGKKTTLECSMTDIITCDRVPEIDITWQ*
>SPBDM4_v1_70034|ID:27159492|metG| Methionine--tRNA ligase [Uncultured spirochete bdmA 4]
LTAPVCRDIVQTMKRRLITSALPYVNNVPHLGNLIQVLSADVFARFCRLRGYETLYVCGT
DEYGTATETKALEEKVTPRELCDRFHAIHRDIYSWFEIAFDTFGRTSTPEHTEVTQSIFL
DLDRNGYITSQSIDQLYCDTCGRFLADRYVVRGTCPHCGYEGARGDQCEMCGKLLDPMELI
DPKCVTCGGTPRLRSTTHLYIDLPAILPKLEAWMKEASVKGFWANNAVQMTQAWIRDGLH
PRAITRDLKWGIPVPKKGFEDKVFYVWFDAPIGYISMTAKLAKERGFDWKAWWQNPEGVE
LFQFIGKDNIPFHTVIFPSSLLGSGRQWTMLHHMSSSEYLNYEAGKFSKSKGIGVFGTDA
VETGIPADVWRFYIFWNRPETSDYTFTWTDFEEKVNGELIGNLGNLVNRTLTFVSRYYNG
VVPAGEPNGAFWDEVIAREKEVGDHLERADLRDAFRSIFAISDIANKRFQDEEPWKARTS
DPQKAASLMRDLCYVLKDLAIMIHPYMPKAAERLAGFFGLKLGTGGLAWRDLGLKGVLKN
IQNPEMLFQKLEDDRIARLRERYSGSQKEREERETVPAAASPQSSQEPPEVRFHRIIDLR
VARIIKIERHPKADKLYIETLDDGSGKERVIVSGLVPFYKEEDLLGKQIILVNNLKPAKL
RGVESAGMLLAASAEGPEGKELVEVLNAPSATPGDRVFLEASEAETASKPAPADIIDVDT
FFSIPIVARDGYAWIGSQRLKVGSDYIKLANVLNGEIG*
>SPBDM4_v1_70035|ID:27159493| Methicillin resistance protein [Uncultured spirochete bdmA 4]
MKDLPSIQISPITSADDCIPEYVSESFLQTKFWGLFKARTGWRAYACSYRFEDKGIAGHL
IVLRRRFARLFTFLYVPFGAAELIFLSERWEALAALGRAIGKSLGGTDIFIRFDVPWKRS
GGLSHAEKDSPPNSEIRKSGLHKGMDVQVPDTVVLDITRNEDDLLAGMKPKWRYNIRLSQ
KRGVCVKDEGIQGLPTFMNLYQETARRDKIAIHPQSYYKTLFETASEVSSLIEKESLEIP
KPYFSLYVARHGSDALASIIVLHMGENATYLYGASSDKKRNVMPAYALQWHAITEAKKRG
EKTYDFFGIPPEGYDASHPMAGLFLFKTGFGGDIVRRYGACDVGFRPVMYWMFRAGEQLR
ALWHKKIKKNMGKFYHTIFKRRAPAQELERKEQSSSDNQDSEKTNPSNP*
>SPBDM4_v1_70036|ID:27159494| AMMECR1-domain protein [Uncultured spirochete bdmA 4]
VDFSISVDDQKKLLKYARDVIHASFYDHGKVEAPELSATMRCGAFVTLIENGSLRGCIGR
MRSNDPLVATVSEMALAAAFEDPRFPSLKKEELALIDIEITALSPLHPIAPDEVIVGKHG
LLISAWGRSGVLLPQVPTEYGWDRETFLEQVCRKAGLSPDTWKSSLAQLYGFEGFVFSES
*
>SPBDM4_v1_70037|ID:27159495| putative MEMO1 family protein MM_1761 [Uncultured spirochete bdmA 4]

MQTERLTRSPLLSGIFYPSDANQLQSEINMLAAYAKQLPHPACAIVAPHGSFLYSGKIVA
AAWGALIGTEPSLIVIAGPSHLPYEEGVFLPESMQFDIPGTSFSVDANLEDYLLAKIPAL
KKNDLPHLEDHSIEMQLPFAAHFFPGVPILPCIVSGREQGTVDTVVKLLEEIRICVENQG
VLVISSDLAVSDTAEECDSLSKEFIASLSTQKIEPFHGFDASRHSFCGEAAIRAFRAAYP
EARPTLLDYANSSAFNEHNDELVVGYGAISFKR*
>SPBDM4_v1_70038|ID:27159496| putative galactokinase (Galactose kinase) [Uncultured spirochete bdmA 4]
MSAMKDIFAIHQSEYETSPDCASAAPAIVKILGEHTDFTDGLVLAACLSFEVKVAVSFRK
DNSLRFHAADFNERKRANIATLKYKREDRWANHIKSICDYFWSTFDIEPRGLNVTIQSNV
PLGLGLGISSALNMASAYIFKTLYNLDLKQDELAVHAWKAQSTFFEKELPITNYLGITAP
AGRSFSVIDLRLKKRRGIQFLPEPWEMLLTDSKVPRASVDAELEQRTADCNTCLSVLAPG
RNRSIRDIAPRELDELLGIVPERKRRRCLHVLEEVRRVGEAEEALSRQDYVSFGRVVNKS
HASLRSLYEISCPEIDWLTKRALELDGVLCSRLVGQGFGGSTLTILKSEEKEAYHHKLEE
YERIFGFRPIVHEVSTGAGLRILEH*
>SPBDM4_v1_70039|ID:27159497|surE| 5'-nucleotidase SurE [Uncultured spirochete bdmA 4]
MRILLTNDDGIQSEGLHALIDVLSALRDAEGKPLHEIWTVAPEHERSGVSHAMTLKKPTK
IKKLGDFRYSCSGTPADCIIVAGLGVLHEAPDLVISGINKGPNLGTDIIYSGTCGAARQA
ALEGIPAIAMSCATFSDNLEYRGLTSFLTDNLEKLVALWEPDSFININGPSTSCSNLRAV
WATPGKNRYLDNLKCFDGADGYTYCFLAEGRQERTPDVFSDHHAVSQGFIAVSLVEIHPR
AFHDASLNGAPVF*
>SPBDM4_v1_70040|ID:27159498| conserved protein of unknown function [Uncultured spirochete bdmA 4]
MDTETLSKPRCFECIRCNGCCSGQPGYVWLSKKDLDTLSDFLHVSKRKFALDYCKPVDIG
LSYTLSLKEKQNHDCIFLENSGCIVYNARPAQCRTYPFWGAILEEEESWIGEGKACPGIG
RGPAIPSEIIVNAILERRQNPPLSIEKVAELKGLEAEWGT*
>SPBDM4_v1_70041|ID:27159499| Aminodeoxychorismate lyase [Uncultured spirochete bdmA 4]
MGHVMKYHKIKGFVQFFLLSCIILFIVLGILIFVPPFRKDSAEIKIKKGSSLSIVARELY
DARIIPSSKVFVFYMRILGADRTIRTGTYRFAPSLPPFLVAQKLIRGETVAQKVIIPEGR
TASQIAQILESQNIVSKDAFLSAVNDAEFAKKLEIPAASCEGYLFPDTYFFEQESDPQDI
IEQMVSNFRAVIKKIEGSHISALTPQELYQKVVLASIVEREYRLPEDAPLIASVFFNRLE
KKMPLQSCATVVYVLTEHLGRPHPSVVYYNDLRVKDPYNTYINRGLPPGPISNPGKISLS
AVLFPSETNFLYFRLDNASSGKHRFSRTLEEHNEAAIIPKEL*
>SPBDM4_v1_70042|ID:27159500| DNA primase (fragment) [Uncultured spirochete bdmA 4]
MARISPQIRQEILDKTDFLSVYQEHVHLQKKGGSYWGLCPFHSEKTPSFSVSVDTGLFYC
FGCHKGGSVIQFLMDVDKLTYTEAMEELAHRSGVQIHFEESERAGEAEKERQALFELYDR
LSKTFHWFLVNHASGRKGLEVLYKRGLTDVLIEKFSLGYAPANRQWLYSFLKAKGYSDNF
LRHSGLFSKNDQYWPLFADRIMFPISDVKGRVIAFGGRAMDQEGPKYINSPETAVFKKQD
TLFALSQALDSIKSADEAIVCEGYMDVLSFHAAGMTNAVAPLGTAFTSHQAMALRRRASR
VVLCFDSDEAGLHAAERACSIAAGVGLETSVLLPEGGKDASEILEKKGEGELTRMSKNTI
NAGHFLLTRASQLFDISTMEGKAKAVSFLFPFLDALDSDVQRQGYIREIGKALGVGAHAV
EMDYAKAKVSGINRSRNAPSSNS*
>SPBDM4_v1_70043|ID:27159501| protein of unknown function [Uncultured spirochete bdmA 4]
MAAATNTKSDVLAEFDAPGYEFELGAFLERFIPDTFAFA*
>SPBDM4_v1_70044|ID:27159502| protein of unknown function [Uncultured spirochete bdmA 4]
VNLDSDNIDDPRAKDLFSALQEAQKEGIQDIEGILSLCADDAAVRFVREVDASGELKEGL
EKILQDGIAQKRRAILEKERKRLVAQLQTSQSGSSDILQEKMILEDIMKIDGELKASGGD
INE*
>SPBDM4_v1_70045|ID:27159503|rpoD| RNA polymerase sigma factor RpoD [Uncultured spirochete bdmA 4]
MSDIELDPAITKVLEYAKEKKMISFDELSDMLPEQILNSDKMEAIINLLVSSNVKIEEEV
QIEEEPLPKLAAIPSKSKMVANDKEMASDDPVRLYLRDIGRENLLTAEQEVELSRTMEQG
ENIIKNTLRKSGMLIPEFHQIVAKATRKEPKDLSLQKKENTEKIAERRRLNQFYKELVDT
SASDLKGYIELKRWIIAKGGNIFEDQQLKEIRERLLPIVNETDIHPEEITAFSEKFIAAA
KKIIRYRKEQDRIEGILQVVSSRDLRALGRNLTIKEKREEIEQRLGLNAEEIKDRIRQLQ
VNEKKLKDIENNFETPIERIISMAREVNRGRKMMKSAKDKLIKANLRLVVSIAKKYTNRG
MQFFDLVQEGNIGLIKAVEKFEYRKGYKFSTYATWWIRQAITRSISDQARTIRVPVHMIE
QINKVVRESRQLMQKLGREPTDEEVAAQLGWEIDRVKAVKNVAREPVSLDTPIGEDEDSL

LQDFIEDKEVENPAVKTDYNLLQEQLRMVLATLPKREQEVLRMRFGLDEGYSLTLEEVGL
YFNVTRERIRQIEAKALKKLRQFKRSQKLRDYIDH*
>SPBDM4_v1_70046|ID:27159504| conserved protein of unknown function [Uncultured spirochete bdmA 4]
MPMRMDEVFETLRQYQEMLSKRVELEKQLEAMPKTIEIQNQMLSREKKTFVEVREQYELS
EASLKELRQALAEAELKRENAEKQMDGITTQREYDAANKEIREASEKEQELRRRIQEEER
RFADIDDRLHRIEMSVSTLEAEIAQKRAEIDENSREISNDVDKLRTEENDLTQELDEDLK
FKFERIIRSKSGMGIVPIHGVVCTGCHMILPANFVNEVRQADKIVFCPYCSRILYYEETE
ADESMEEILTGSLVDLSNDEESEEAEESESNDESDDASSKSKRGSGRKKKIQDEPLIDND
FLIDTMDE*
>SPBDM4_v1_70047|ID:27159505| protein of unknown function [Uncultured spirochete bdmA 4]
MPHFAAKIAGRPLECISNDTSREMAAVQKAQNAPDAHRTRLTGPPFFDIVICNAQHIFSK
E*
>SPBDM4_v1_70048|ID:27159506| putative Tetratricopeptide repeat domain protein [Uncultured spirochete bdmA 4]
MPNTFFRRNRAHFPKRSLWFIFLLLIIVSVSFLGVYLIIHNQNSKNILSRAELATLWNSG
NIEKTLTESRKATESFPFDEYYLSIKGISSYYWALNAQDEESRQQLFEESVVSLRKALAV
GVPAKMRAQIYYVLAKAYYQKGDPWFDMAERYFLLAKDNGSKEQDIPQYLAIVYAGKKDY
ARAADWFELALKNDASDPLLLSAAITYNNINQKEKSRDLLLRLEASASDAKIRLKAKLLL
AQDSFDAGDISAALQGYQEVLKEDPLNVDAWYGLGLVYSKKNDQLGARAAFRKVVQIDPN
HADARRRLAEKL*
>SPBDM4_v1_70049|ID:27159507|mreB| cell wall structural complex MreBCD, actin-like component MreB [Uncultured spirochete bdmA 4]
VGILDMFSPDIGIDLGTCNTLIYVKGKGIVLNEPSVVAVERGTNRIVAVGTEAKGMLSRT
PTDIIAKRPLRDGVIADPDMTEKMIKYFIQKVFPKNLFFKPRVVVGVPTCITKVEENAVI
DSTYKAGARMVKVIAESLAAAIGAGIPITEPAGHMICDIGGGTTEISVISLKGMVVTNAI
RIGGDEFDDAIVKHIRSIHNLIIGEQTAERLKIEIGNASPERTIEKMEIKGTDAITGLPR
RFEVDSVEIREALMDSVNLIVEEVKKTLGKTPPELAADIVERGIVLSGGGALLKGLPKLI
AKETGVPVILAERPLECVALGAGQYFEIMQRNIGSSSVYESLNL*
>SPBDM4_v1_70050|ID:27159508| putative Cell shape-determining protein MreC [Uncultured spirochete bdmA 4]
MKNSRFHGPTRAVHQKAAICLILSLMILFVSTKTMVRLPDIFKGYVIGGLQKSFSAVSSF
FSRTVGAVSQLRSVQKDYELLLNKMQDYESRERDFAALKEENQRLKELLDLSSLPNTKKM
AAHVIARDPSNTYSSLVIDKGYDDGIRKYMPVIAYQDGLEGLVGKIIEVKASTSILQPLY
DQRFYAAARLAKTRAEGMINGEGFRDSPLSLLYIPKSEMDQLKQGDIVVTSGLDQVFPPE
IMIGRVGNFRLNEFSSSLVIDVQPAIDLSRIEYVFAIDVSSMLSSGKGPGK*
>SPBDM4_v1_70051|ID:27159509| putative Rod shape-determining protein MreD [Uncultured spirochete bdmA 4]
MSSILISSAAAILMLFIQSTWLSHGIFLGIIPDLAMSVLLFSSFINKNGQGIIVSFVVGI
VADMLSASPLGYYAFLFSSCAYLATLLSYTTEKDVFIIPFLLGTGATIVMGFLSRIIALV
FSANIHSYQIFSAEFGVELIMNGLFTVLIFFLLSFVQRFFEHSPRKALP*
>SPBDM4_v1_70052|ID:27159510| Penicillin-binding protein 2 [Uncultured spirochete bdmA 4]
MAVLQKKITRRIRILRILIVALIVVYSINLFSLQILRKKLFVSEAERVSIQSTRIPAPRG
EIYDRSGSVLLAGNIEAYSVYITPSELPASKRKIVVDNLAKLLNISTDDIESKLPDPNSY
SYGRVAVAKNASLAAVTEIAARIDEFPSVSWTTKPLRDYADLGSLSNVIGYVGNITRDEY
KLLYNKGYTLDDLTGKSGVELTYEDLLKGKDGWISKAVDVHGKDISGNAAKIQAPVSGKR
LILSIDTNIQKIAEEALGKRQGSVIVLKPATGEVLAMVTYPYYDSRVFMSDNAGKAYLDL
LNDPQKPMLNRAYQSSYPPASTFKTILTASILEESFIPPDKTIYCPGEISYGGRSWSCWI
KRPGHGSLALEDALAQSCDIYYWTVGRDYVGVENIVSYAQDFGYGKDTGVDLPGENGGLL
PTPSWKEEKYNEPWTPGDTMNLSIGQGYMLASPLQVANMMAMIVNGGVIYKPHVLKEVRD
PGTGALINEIQPEVLHKSSISAKTFDTLRSYLRNVIVNGTAKVPISTKVVQIAGKTGTGE
IGLKDRWHSWFASFGPCDAPPKDQIVVVTMIEASNPWEWWGPYAANVIYQAIYAGQNAQA
AAQTVGVRLEGSSLSGRRE*
>SPBDM4_v1_70053|ID:27159511|mrdB| Rod shape-determining protein RodA [Uncultured spirochete bdmA 4]
MNFQSIRRLFGNIDYYLLFATFFLCAIGIAAIYSAGVDANGMVVSNEYIKQIIWFVIGFF
LLIGAALIDFSRLKDTVWMFFAFTIILLVLTRLAGKVVNGSRSWLGFAGFGIQPAEFAKI
TTVLMLARYLDSAEFDSSFKKLLKSALILSIPLVLILSQPDFGSALVFLPAALVMLTLAD
VDFRYILFGVLSIIGIFLFLSLPLYAANHPNPENIIYILITQDKILFIVFAFILIISIIS

GMGWLKFKKQYYYWLSYSLAILAFSIAGATAAHKILKPYQIERLLVFIDPNIDPKGSGWN
ILQSITAIGSGGFWGKGFLQGTHSHARYIPQQSTDFIFSIIAEEFGFLGCIVLFVLYAVI
FIRCFILIETSKDRFSQFVVGGILGIFTFHFMINIGMAMGIMPVTGIPLYFVSYGGSALW
SGLISIGILTGISARRYTA*
>SPBDM4_v1_70054|ID:27159512| Radical SAM domain protein [Uncultured spirochete bdmA 4]
VNKRFIKPMKDIGAALFQVQKPARYVGGECGAVPPIEQGDSRLRIAVSFPDLYEIGMSNN
ALRIIYSMLNERRDLLACERVFAPAPDFEQLLIQKNIPLYTLESGIPLFNTDVLAFTVGY
ELLATNMLAIMEQGGVPIETARRGEKDPIVIAGGPAITNPLPFARFLDAVWIGEAEAGFV
DLMIEMAEFKKRGADRQEKLKLLSEHPSIWISPLLEKRLGLSHEKHVVRAIFQDFYKTEY
APRFPYPVLNPVHAHGSVEIMRGCPNGCRFCHAGYFYRPQRSKYPEIIKKEVEELVLRKG
YREITLSSLSSGDYPGIVELFDDLNASWSKYKVSFQLPSLRVDSFTLPLLEKISEVRKSG
LTFAVETPLESWQCAINKRVPFEKIAAILREAKAKGFKSAKFYFMVGLPVPERGMREAQE
IVGYIEQIAELERISIHVNVGTFIPKPHTPFEREAQLSEEEALECLQYIRRNLKYRKNVE
VSYHPPFLSILEGLISRGDESVADIIFDAYLKGARLDAWDEYINRDLWRGVLADFNAKRG
EGAWQSFLAQKNEKESLPWRKISLKVTSHWLQKEEQKAQEYNLTQICNEKCTIKCGVCDD
HNKIVSNSILNKEKFSAEMAILDSPGGKSDILNGSSFDDTEEIDMAVIKLIAVWSKRGPA
VLYPLHDVARSFSRTFQISGFQIAFTHGFNPQPKLELSPPLPLGIEGQNEMLLLWINIPK
NRLGEFESFISESQADILSSLNENLAEGLLIKSLGISRYNADRKRTIGSLLRAAAWKYVF
ASEELYIKATDILADYSEDFTLVKDDAVGNEISLIETFSGASQKTLSFYKILKNLFAEEK
PTTNTSESYFRSVERVECYGELEDRGYVRLQDIL*
>SPBDM4_v1_70055|ID:27159513| protein of unknown function [Uncultured spirochete bdmA 4]
MRGQRDKVTFSPRGVAASFIIVMAALLFSCGLPTSEYLYPPAFESLGGVIRLTHKTENID
DAASAALFKGYDIYYRIFDSETEASKSYDELDSSLTSTTIATVAPAKGYYVLVKFGDTNV
PLIPVSDNTYNYFYLNLNTNDGWTITTSTGTTLFHHVLRDKDDSSTADFYLSAQYSSTDQ
DYEGDGISSGNSVYIVFFAVAHGVSSSSIGTDIYSDPKILEPIAYTPGGS*
>SPBDM4_v1_70056|ID:27159514| putative TPR repeat-containing protein [Uncultured spirochete bdmA 4]
VSQQEKQPQKDTNVPKKQTEKKTFSEKLTNFISVHRILIISIGVAVVLAIAGVGIYTAVS
GNIASASSRAMDLADQKLQAWSQETDEQKKADAEKSLVADLDSIAKKWPRTLAAQRALLR
KGAILSQKKDYAEAEKVALDAFARNKKSYAAPLALELAAVSAEEAGNTDAALAHYTALTK
DYLKDNPIAPAALFNLGRLQEGKKDYKAAVTSYNQVISSFGDSDWSMLAKNRVIYLKSQG
LAD*
>SPBDM4_v1_70057|ID:27159515| Transcriptional regulator, NifA subfamily, Fis Family [Uncultured spirochete bdmA 4]
MISAIDSEKFRTLIDINARLNSSYTDFRSLLKTIVESAARLVGAEAASLALFDAPTNSLR
FEIALGPRGAELQGKAIPSSDGIAGWVFHNSRSIIVNDAAHDARFSPAVSKEISYSTNSI
LAVPLKVRDKTEGVIELINKTSHSLFVGDDLEWVELFAVQASIAFDNAKQYERTEHELVY
LQHKVQEEQGWHPLVFSSRVMQDRLDLVKRVAPSDASVLILGESGVGKELIAEQLHLNSR
RNQKPFVRVNCAALPENLLESELFGHVKGAFTDAISNREGRFEAADGGTIFLDEIAELPL
KLQSKLLRVIQQKTFERIGSNETIKADVRIVAATNRDIEKLVQNQEFRSDLYYRLNVLPI
QVPPLRERLDDIPTLAEYFLTKYARETNRGILHFSDSAMECMLSYSWPGNIRELENAVER
AVLIAGGTLITTDDLMIGTKSDAGNERFDGKELKEAIILFKSNYIRAALEQNRWNQTETA
RHLKIQRTYLSRLIKELNILQSKE*
>SPBDM4_v1_70058|ID:27159516| 30S ribosomal protein S1 [Uncultured spirochete bdmA 4]
MAENKEVEMDVTEPSRDDIQTQLQEQYLKSLEGLEEGDLIDGKVVQVAGDTIFIDVGYKS
EGKLSLLEFGDTPPKAGDIVKVILIRKETHSGDIVISKKKADEKLFWKSIANAFKDHLPV
EGTITKEVKGGFEVDLGHGLSGFLPLSKADVQRVEKTAELVGIHAEFYLERLYSDKKINI
ILNRRKLMEEEIERHREAFFQNTQIGDTVKGIVKSFTSFGAFIDLGGFDGLLHINDMSWG
HVTRPKDFVRKGQEIELKVIRLDPEEKRINLSLKHFSPDPWNSFEEKYHVGDVVKGKVTK
LTDYGAFIEVEEGIEGLAHISEFSWVKKVRKPEDMLKAGDVVDCMILGYDIPAGKVSLGL
KQVQPNPWDTINERYPSGTRLKRKIVKVTNAGAFIEMEEGIDGFLHIDDYSWTQKVKNPS
SVLHEGEEIEVIVLESSPEDRNIRLGVKQLSEDPWRSFAKTYKPGSIVEGTVSSVTDFGV
FVKVPGDIEGLIKKQDLVSDRSESYDEALKKYQPDMQVKAVVTDLNPERQKLGLSIRDLV
QKQQREEMSRFIQEDEGEAGYTLGDLLKAKDKEND*
>SPBDM4_v1_70059|ID:27159517|cmk| Cytidylate kinase [Uncultured spirochete bdmA 4]

MKVAIDGPAGCGKSTIARMIADNFGFLYINSGNLYRAVTYLALQEKIGWQDGPGMIALMQ
RHHFDYLPDGSVDIDGKRLSGELRTPQVDAIVSQVSAIPAIRTEVNAIIRSISEGKNVVS
EGRDITTVVFPDAEVKLYLDASAQIRAERRFKERLAQEASGNLSGASSKAASIEDIRKNI
EMRDKLDTQKAVGSLKIAKDAEYLDTSDLTIQQVYEKVYKILRARIAHGRK*
>SPBDM4_v1_70060|ID:27159518| Pseudouridine synthase [Uncultured spirochete bdmA 4]
MREPEEKLIRLHAFLASAGVASRRACEEMIRQGRVVVNGETANIGQKVKGFEKIEVDGQT
ITVSAQKSCRYILLNKPKGYLSSASDPQGRPCAIDLISESVKERLYNVGRLDQWSSGLLL
FTNDGDLASKLMHPSSKVEKEYSVQTDAPLPAGFASNFIRGILYEGIRYRASSVKLLAPD
RALIVLIEGKNREIRRVLEHFGLRARILERIRIGPLQDANLAPGQWRDLTEDEVRALKLI
ANHDNRDEGKRQ*
>SPBDM4_v1_70061|ID:27159519| Transcriptional regulator [Uncultured spirochete bdmA 4]
MNEQMDREAALISAILFLENEPISEEKLAKLSGFSEEQVSLLLQRLCTEFDQPVHGFAPT
RSAGGWILAPKLLFWDQLKDHYGKKNEMKLSRAAMETLAIIAYSQPITRAEIEAIRGVNV
DAMIRLLLSRNLIAELGKRNVPGKPMQYGTTSEFLKYFKLDSIEDLPKLDEIESARFSKP
ETPEG*
>SPBDM4_v1_70062|ID:27159520| ScpA/B protein [Uncultured spirochete bdmA 4]
MMQEFDRDFSKEQIAILSNGRTFHLSDFEGPLDLLLYLIKKNEMNIYDIHLSSITEQFLE
CLNSDSSANLDEISEFYQMAATLLYIKSRSLLPHSDEDLEEIEDPRRTLIDQLIEYHRFK
KLSELMEQREMEVEWFVERSTSQRPLPFVDNPDDDPWIPADSWDLLRTFASMIRHFNSER
IIDLYEEVSINEKIVLINELLSRKGSFRFDDLIAKHASALDLACAFLAVLDATKNRIIRI
RQHRLFGDILIIPYAEAGDPHE*
>SPBDM4_v1_70063|ID:27159521|folK| 2-amino-4-hydroxy-6-hydroxymethyldihydropteridine pyrophosphokinase [Uncultured spirochete bdmA 4]
MMEKVWLGLGSNKGDSPAILRAVLNELDTSLSECRHSGLWRSRARYYEDQEDFFNMAVCG
MTDLNPRELLKKINEIETRFGRNRSKEIKKGPRTIDIDILLYGQKIITEPDLIIPHPGLA
ERKFALLPLLELDYELCHPGMGISIKQLAASLPPQGIYPVNDCRYDGPYSE*
>SPBDM4_v1_70064|ID:27159522|recA| DNA strand exchange and recombination protein with protease and nuclease activity [Uncultured spirochete bdmA 4]
MAKRQTRTAEEPVNEQNSKFVEGGSSISRQGNIDPEVQREREEKLKALEAARLQIEKQFG
QGSLMKLGAHAIARGIEVISSGSILLDEALGIGGYPRGRIIEIYGPESSGKTTLALHAIA
EAQKKGGIAAFIDAEHALDPIYARNLGVDTNELYISQPDNGEQALDITESLIRSGAVDII
VIDSVAALTPQAEIEGEMGDSHVGLQARLMSQALRKLAGTLSKSKTILIFINQIRMKIGV
MFGNPETTTGGNALKFYASVRLEVRRIETIEGGHDEEAIGNKVRVKVVKNKVAPPFRKAE
LEIIFGKGISWSASLLDAAVKCNIIEKKGAWYTSGSEKIGQGRENAKAYLEQFPEEAKKI
EAQVREILFNPDKSKAAEASLGKETQGLSAESSFDQEPAQDESNADNSRIRAEDLF*
>SPBDM4_v1_70065|ID:27159523| conserved membrane protein of unknown function [Uncultured spirochete bdmA 4]
MSRIKAKDLRPLSFLGFRFLFGKPVQGQRYAFHHMRHSTFWGAVLGISVSIVPLYIVLFV
SNGMIQGITDRYLETKTSHLQLSLPFNLSSETRKKIQQDIARLEDVSIASFEVDGIGLAA
SPNGSTSAQIRGLDPSIIKDEGFKKFIQIDGGTIFPEKQNEAVLGRYLARALGVNVGDSV
SLITLRNADEAQMLPKLSIFRVAGIVSSGYRELDANWFIIQSEAALRLLNPSTAYAFIGI
KTKHPYGSGLDSLASEITHKISQSDIPGAQGIGIRTWRDIERSLFASFSSTKSVLILIMA
IAIIVASINLASALTTYVIEHRTEIAILRSFGVSPRQTATIFLLGGAVTGTLGCIAGSAV
GIFISFYVNQILRGAQAAVNFISKLLAPASHPVTLLNPDYYLEHIPISINWNYIILILGI
GFVVSALVSLVPAIKSSHIAPAELIRHEQ*
>SPBDM4_v1_70066|ID:27159524|lolD| outer membrane-specific lipoprotein transporter subunit ; ATP-binding component of ABC superfamily [Uncultured spirochete bdmA 4]
VNEPIIECRDLIKTFSSSAEVLHILDHANFSLEKGNICTILGPSGSGKSTFLAILGSLER
FDSGTVRVGGYDLHTLPEKSLFMFRRNVVGFVFQFHFLLNDFTALENVALPRYMAGISRT
DSWERATRLLDLVGLKNRAHHYPSQLSGGERQRVAIARALINDPPIVLADEPTGNLDTAS
SEEVRSLLQRLPEATNTTLVLVTHDSELATIGNISYLLKGGNFECLA*
>SPBDM4_v1_70067|ID:27159525| putative Releasing system transmembrane [Uncultured spirochete bdmA 4]
MTRTADDQKNTRFLPLLHIAIRWSMTKRKTSHQTGLLFAALGIAAGIVALIVVMSVMNGF
QRQYIDSLLETTSFHIRITPEKNTDLPALSQSLHQNPLVRSVMPFMETNLLAEGLGNRQN

VVRVMWIRPQDLASDSGFCKTLHIPASLAEKSLESGILLGSEAGRILGNSEGSTVTLRGA
LVSPDEGIQQYEIDAPISGFFRSGYYELDAGLAIISREKIAGLSLASAPLVLGVKLKNPE
QVDAFIKTLSHAEGIKSIESWRDYNRSFFSALRTEKIVMFLLVSIIFAVVAINIHYSMRR
TITRKARDLAILSAFGTSQRSISTIFMLEGLIVGVVGAVVGIAVGIPIAQNVDHIINGAI
TLLEGVISLFYRIGIVKNIPDLRLFSPTVFYIDGIPSRVFASDIALIAAFAVIFPAIAVY
LAYRRFKNASPLEVLRSE*
>SPBDM4_v1_70068|ID:27159526|ftsY| Signal recognition particle receptor FtsY [Uncultured spirochete bdmA 4]
MKFAEKIKALFRRNQLSDEVFEELADLLIEGDIGASLAFEVVEALKNSCKKEGISNTEEA
KKKLKDILRPYIKTANFGLDPDKLNIVLILGVNGVGKTTSCANLAFWAQKTSPAKPVILA
AGDTFRAAAIEQLKIHGERLGVRVVAQQHGSDPGAVLWDAIDAARTGNAQLVIADTAGRM
HTRADLLKELEKLDRVVNQKAGNVNYRKLLVLDATTGQNAMRQAEIFHGAVKVDGVIMTK
YDSSSKGGTIIAVSRQFGLSTLFLGTGERYENFEPFDLNVYLDDFVGE*
>SPBDM4_v1_70069|ID:27159527| protein of unknown function [Uncultured spirochete bdmA 4]
MNYVAAQDASIVRNNPKNIAVCFPVRADEPSLSQFSSQAPDEKLSWQFLEAGLAMPQSLP
NGSPYPEAVSLAKLMEESLRARAEIGQIISIDTDTLKALRQGEGEAPIFQAAALQNADVA
VFMMYIIQDSDVQFEALAVTKTGEGASYFSGPRSIFYLSDATNEAVTTFIARKILVPAIQ
IPSDSLSSGQSSTLPSILPAIEPWKDRPKYEQANSRLKIAIGGVAVGLSASFICAGLWQT
YQEAEYRNTAFESAVTASGIATGASVAVTVAFVTSAIWNVVLMLQASH*
>SPBDM4_v1_70070|ID:27159528| Peptide chain release factor 2 (fragment) [Uncultured spirochete bdmA 4]
MYELVREEDRQDMEGDLLASLHDIQARFDKALVIELLSGEADGNDAFLTIHAGSGGTEAC
DWASMLYRMYLRWAERHDFKVETIDFLEAEGGIKSVTIQVSGEYAYGYLKGETGVHRLVR
ISPFDANARRHTSFASVSVMPVLDDSIEIDIRPEDIRIDTYRAGGAGGQKVNKTDSAVRI
THLATGIVVTCQNERSQYKNKDVAMSILKSRLYEYYKVERDKETAKFAGEKKEIAWGSQI
RSYVFQPYTMVKDHRNKFSVGNVQAVMDGEIDPFIEAYLHWQWKGGTTVEEEDEDI*
>SPBDM4_v1_70071|ID:27159529| Aldose 1-epimerase [Uncultured spirochete bdmA 4]
MSASESKIETKPFGILSNGDEVSLFILKAGDFCATFTNWGATWTSFVLPDKKGQSDDILL
GFSTLSGYVGKHPFLGSTVGRFANRIGKARFAMDGKEYRLWANNGQNHLHGGRRGFDKYL
WKYELSAFKGSPAIQFSRTSPDGEEGYPGTLSVELTVNLSESGSLKLRYLAKTDAKTPVN
LTNHAYFNLAGEGKGTILDHELQMHASSYLPVDRELIPTGKEAPVQGTALDFRRTKVIGE
DIAQANGGYDFCYIIDKEFLPLKPFATVREAHTGRCMTVATTLPAVQFYTGNNLAGIAGK
RGSTYDKYAGLCLETEMYLDSPNRPNFPSPFLMPGETWEHETIYSFSINK*
>SPBDM4_v1_70072|ID:27159530| Oxidoreductase [Uncultured spirochete bdmA 4]
MKQETNHGEVAFVTGATSGLGLAIAKSLANAGYTVYGAGRRQAPSEMPANFIYVQTDVTS
DESVQRSAHHVLFQEGHVDLLVCAAGSGISGAIEDIPVDQAKSQFDVNFFGVVRVVQAFL
PALRQQKGGKIIIIGSIAGKTGMPFQAYYSASKFALEGFVESLRYEVRAFNIEACIVEPG
DFRTGFTGARQKIVPDNSFYHDKFEKVIAVQEHDETHGVDPTVAGKRILRLASSRRLPAR
ITIGPMFERFAVWVRRIIPDHWFEAFYRIYYKL*
>SPBDM4_v1_70073|ID:27159531|cmk| Cytidylate kinase [Uncultured spirochete bdmA 4]
MWSKNTMGIIAISGKSGCGNTTVLRLVAEKLGFEPVNYTFRSMAIDMGISFEELLKRAQE
SLEYDRRLDEHQAMLARKGNTVIGSRLAIWMVPEADLKVYLKASHAVRVQRIHAREGGDM
ASIERFTRERDAKDRDRYLTLYNIDIDDISCAHLVIDTERWSASKVAEIIADAYNIAK*
>SPBDM4_v1_70074|ID:27159532| conserved protein of unknown function [Uncultured spirochete bdmA 4]
MKSMTGFAHHTISENGIQGTITLKSYNNRFLDISISLPPSASGIEPYIRDFLGSRISRGK
IECTFRLRKPEASLEDIELHFQSAKILSEQLRSLAEACQLDDRPSLEVLTRFPGIVEMNT
EIDEASLWQVLLPALEETWREFEESRLREGLATQANIMSQMERLKSKLAEIEAQADTLEQ
LVHEQIVARFRDLLAEKYDESRVLQEVAVQLVRLTINEEIERLKAHFEAFQLISAQASCG
KKLDFLCQEMNREVNTIGSKNMLVTISHAVVEMKDALENIREQLRNVE*
>SPBDM4_v1_70075|ID:27159533|murC| UDP-N-acetylmuramate--L-alanine ligase [Uncultured spirochete bdmA 4]
METIEFPGILDGLHIHLVGAKGTGMTALAEILHRRGAFLTGSDVADVFYTDSILHSLNIQ
LFENFEAQHLPRNTDLVIYSAAYSREANPELRAAVQRNIPIFSYPQALGALSLHSRSAGI
AGVHGKTTTTALAGLLMEALSMPATVLAGSAISNFNGRCTLIQGNTYFIAETCEYRKHFL
NFKPSWIVLTSVESDHQDYFPTYESIRDAFVEYVLTLPAGGVLIFCADQKGAVEVADIVL
QKRKDIQFVEYGFLAKGKYKIQDFQISAGKNTFVVGDAPVPIELHVPGRHLVLDAVAAIA
LADSLFEAETSRTFGAGEWRKIAVALSLFRGSKRRSEIIGEFENILILDDYGHHPTAIKT

TIEGIKKFWPHRRLVVDFMSHTYSRTIALQDDFVESLDEADSIVMHKIYSSAREQPIRGF
DGRALFQKLCERRPELIPIEIVPASKENASANPKNAPLKPNKGFAAYSEEPMDARDTLLE
ILQAGDIFLTMGAGDNWKLGKAIAEKLCESHTSQQSKKTEEQHP*
>SPBDM4_v1_70076|ID:27159534| putative 4-alpha-glucanotransferase [Uncultured spirochete bdmA 4]
MQSFRVVIGMYNNLPYNASNALYEQEYQGAWRPFLSGLYKFLSIKSIIHFSGAIFSWLEK
NHPEYMYLLTEMVRRGQIELLGGGFYNPIASLISTQDMTGQIEALSAFIRKTFGKRPSGA
WLYEYAWTASLPAILQNSKIQYTFLPAQYCMCLYPKDIPCFPFASEDHRKIISLFPAFES
RASAGIFEPYEKTLLKLQQAYPQCNTYVIMADGHEIAGSWEESGLESPDVLFERTFAWFQ
KNCLEYDTVTAAQLNKTLKSTSTVYFSQCYSERYKDYCQGTIKQVAMNNPEYIQISKQSV
LDHPLVYALYQKLNFVSTMTGLFRGDKSRKRASLDDIWRAQSGDLYWIGPSGGILLPEAR
LAAYASLIEAEKTIRQNRFHHYLSFDDLNFDGVKEALFRSSVYTCYLRSGTASVSEFDSL
KTGINYACGWNAVQCSTGCFRDSIKEEGGFEKELFAESEGWNLIENAKETMIVAFSHDFI
GKIHNQSISLACRKNYRFENDFFSIDYELVNKSSNALSFRFCTESDIIAMPTLEEHGIKV
FKHRQKQSLDVHTVFSIDSIDGFELSESHGTEKLLIRADMPFSLMGSPNLLQKPSKHQSN
SSKDPSESVIFEGFSMKLGWDFALPAEGTTFFSLSIHLEY*
>SPBDM4_v1_70077|ID:27159535| Acid phosphatase/vanadium-dependent haloperoxidase related protein [Uncultured spirochete bdmA 4]
VEIQSHSFVSLFSNPIFLSGTLSIFLAQVIKALLTVLKRRKLQAKEIAFIMLWKTGGMPS
SHSALVVSLSASIAFVEGFDSLFIVSFFLSLIVIRDAVGVRRSAGLQSRALNLLGKHVSE
RLDIPFTPVKEIHGHRWQEAIVGSLLGLVVAVLICWRFIF*
>SPBDM4_v1_70078|ID:27159536| putative Ribosomal large subunit pseudouridine synthase C [Uncultured spirochete bdmA 4]
MVLGEDDDGRRADRIVRKALRNVPLSTIYRLFRQGNVHVENHSIKPEDRLKKGEIIKISL
PEALIAAKTGEAPDAPPRAISALKSEFNGLLNHEMPQIVWQNEHLIAFHKPRGMLVHDGE
TSLDAYALSVLRDQLPHSVSFSPGPLHRLDRNTSGIIMFSKTRIGAEKFTAAIRNRQIRK
FYISIVQGDALEPLLIQDTLLRDTQKHVSIIDKKGKTASLYIVPLIKNGRYSLLLIELHT
GITHQIRAQLAAHGLPLAGDSKYGGQKTSNIRYYFLHSYCLYLRSQLFGDMPSKIIDPLP
EDFYTMIMTLFDTNPEKLEAGIFQVMKQLLEN*
>SPBDM4_v1_70079|ID:27159537|hflK| Protein HflK [Uncultured spirochete bdmA 4]
MANTAIFKKPPKFNLKWPHVLIIIIVIAVIIFLSTSFVIVDQTEKAVITTFGKYTKTVGA
GLHYKLPFGIQKAYIVKTQVIQTETFGFRTIKPGVVTQYSSQKYPNESTMLTGDLNIVDV
EWIIQYRITNPEAWLFNVTDREKTIRDTSQSVLNTLIGDRTILGVIGPDRQNIQDSAVSM
MNDLFTKYELGIIVTQVQLQNIVPPEGVQAAFEDVNKAIQDMNRLINEGREAYNAEIPKV
QGQAEQTIEIAQGYAAERVNEARGDASRFDAVYAEYRKAPEITKKRLYYEMMEEIFQGQE
NIDLIDKSLQNVLPIKNITQTSSTQEASK*
>SPBDM4_v1_70080|ID:27159538|hflC| Protein HflC [Uncultured spirochete bdmA 4]
MKKIPTIILIVVVLAVVFIVAFGPLFIVQEGEQAVVVRFGKIVAQYTEAGLKWRSPFVDT
VIKYPKKLMSWDGESQRIPTKENQFIYVDTTARWKIVDPVKFYESVNTLESAYGKLDDVI
DSAVRTVISSNYLREAVRSSDEILNASKVETFQTGEVENSSTLQQLAITPTQYEKIEKGR
RELADEMKILVANIVPTFGIEVVDILPRQIKYSDELTESVYQRMIKERNQIAQAFRSYGE
GKKAEWLGKLENEKRSLLSSAYAKGESIKGKADAEASKIYADSFGRDPSFFEFWKAIESY
RKTMPAFNKTLSTGMDYFKYLYSPSGR*
>SPBDM4_v1_70081|ID:27159539| putative Metallophosphoesterase, Calcineurin-like family [Uncultured spirochete bdmA 4]
MADMFLALADIHGETEMLERILKENPHTKGIFVAGDLTNFGKEKEAERIISVFSRVATNA
ALFFVAGNCDTSGARHFFEKQPGYLEQKCFTLAMGRGEQKTPDEIIRIIGCGGGLRHTGL
TPFEISDKELESGLRRAYEHCVTEEHKNAIPLKATTHPLIVLTHTPPRDSFADMRHMKHL
GSAAFASMLYEYEPLLWICGHIHESRGAQWEDRTLVVNPGPAAHGSYAQILVEQKPRGLH
AVAELRAL*
>SPBDM4_v1_70082|ID:27159540|tyrS| Tyrosine--tRNA ligase [Uncultured spirochete bdmA 4]
MNSALKVLQERGFVQQCTDFEGLSGLMDEGPVTFYIGVDPTGPSLHIGHMVPFFALRHLK
DAGHRGVALLGGGTARIGDPSGKTETRKLISYEDIDANTERFVKQLDRFVGFDGVHAFIA
NNKEWLANLNYIEFLRDIGRHFSVNRMLSFEAYRIRMETGLSFIEFNYQLLQSYDFLQLF
KRYNCRLQIGGDDQWGNIVSGIELIRRIEGAECYGLTFPLVTTGDGKKMGKTEKGALFLD

PEMTSPYEFFQYFRNVSDADVERFLRMFTFLPMEECKHLGSMKDAALNEAKERLAWEVTA
LIHGKEEADKALTAARAAFRGDGDATKLPTTQIERARLETGIGVLDLFIESGLAPSKNEA
RRLIQQGGASVNGKKIEDEKQIFSDADLNDGAMMLRAGKKRVARIVLKTE*
>SPBDM4_v1_70083|ID:27159541| Histone family protein DNA-binding protein [Uncultured spirochete bdmA 4]
MPSTGKPLTKVEIINALAKTTGFTKKDVGLILDEFAKLAYSEAKKNKKFTLPGIAIMKIV
HRKAKVGRNPATGQQITIPARNVIKINVAKPCKDAVLGK*
>SPBDM4_v1_70084|ID:27159542|glyQS| Glycine--tRNA ligase [Uncultured spirochete bdmA 4]
MADTAVTMDKIVSLAKRRGFVFQSSEIYGGLSGAWDYGPQGIELKNRIARFWWKEMTQLH
DNIVGIDAAILMHPMVWHASGHVENFTDPLVDCKKCKTRFRADQIPPENLAAKKCPECGG
ELTETRKFNLMFKTTLGPVEDESGLVYLRPETAQGIYVNFKNVQQSNRMKIPFGIAQIGK
AFRNEIVTKNFIFRTCEFEQMEMQFFVKPGTDMEWFEYWREQRWNYYQKLGIDMNKLRWH
RHGPDELAHYAKDAYDIEFEFPMGFKELEGVHNRSDYDLSRHQQFSGKDLQYIDQDNGNE
RYIPNIIETSAGLTRQLLMLLCDSYEEQKVADKGNDDDWRTVLHFHPAVAPITVAVLPLM
KKDGLAELAQEIRAELKEDFVTDYDQSGAIGRRYRRQDEVGTPFCVTIDYQTKEDSTFTI
RDRDSMLQERVPRMRISEYIRHKIKDYKRPERRL*
>SPBDM4_v1_70085|ID:27159543|glnS| fragment of glutaminyl-tRNA synthetase (part 2) [Uncultured spirochete bdmA 4]
MPTIAGMRRRGYSSAGIRDFLGRIGIAKTDSMVDIQLLEHCIREDLNAHARRYMVVLNPV
KLCIENWPKGQVEWLDAVNNPEDPGAGTRKIPFSGTLYIDHDDFREVPPPKYYRLYPENQ
VRLRYGYIVTCTGYEKDSESGEITLIKCRYDPATRGGDAPDNRKVKGTIHWVSAEHSVPF
TARLYDHLFEAERPMEAPEGKTFLDNLSDHSLTVEKNARAEPAVAEIEVGTTVQFERMGY
FCKDPDSTDSGAVFNRTVTLKDTWAKLEKKLQEKA*
>SPBDM4_v1_70086|ID:27159544|glnS| fragment of glutaminyl-tRNA synthetase (part 1) [Uncultured spirochete bdmA 4]
MSESNVKTEYLDFIREAVAEDLRTGRFKEVHTRFPPEPNGWLHIGHAKALFVDFGVAQDF
GGKCNLRMDDTNPEKEDMEYVEAIQRDIKWLGFDWEDRLFFASDYYETLYELACKLIRKG
LAYVDDLDEEQIKEYRGTNVPDKNNITYTPPGRNSPWRDRSPEENLDLFVRMRNGEFPDG
AKTLRAKIDMAHPNLLMRDPVMYRIRREPHYRTGTKWCIYPMYDFAHPLSDAIE*
>SPBDM4_v1_70087|ID:27159545|gltX| Glutamate--tRNA ligase [Uncultured spirochete bdmA 4]
MSVRVRYAPSPTGNQHIGSVRTALINYLFAKSQGGSFILRLEDTDRARYSPEYVQNLYDT
FKWLGFYWDEGPDVGGPVGPYVQSERFDLYRKYAEELVKMDKAYYCFCDSERLEKLRQEQ
IAAKKDEIGYDRHCRSISKEERERNIAEGKSYVIRLKIPLDGTTVFHDSLLGRIEWKNED
ISPDPVLLKSDGFPTYHLANVIDDHLMGITHVMRAQEWIPSAPLHKVMYDAFGWKMPELC
HLPMVLGQDGHKLSKRHGATAVNEFRKAGYLPEALINYIALLGCSYEDGRDIYSLEELVK
LFKIERLNKAPAVFDYQKLEWFNGQYIRQKSDQDLSALVRPYFIEAGLRSKEDPQKDQME
LAAMPLVKERLKFLTDAPAIMAYLYKRLELPPVETYLPKKADAHDAAAFLTEDKKILQEF
GLDDIPFVEEKFRERAIEIGKKLGDLLMPLRVAITYTRVSPPLFESMKILGLGECLERIE
KAIQHLEGA*
>SPBDM4_v1_70088|ID:27159546| membrane protein of unknown function [Uncultured spirochete bdmA 4]
MKSESGPEVESGFLRAFKLFQKIAEIAGVVLWLVVTVLMLILGSETWRSAFEICSIGLLG
ISLFNAISMVAFSLLKRERFHVGHFLILVALILIAVIALALSLFVMGIRLH*
>SPBDM4_v1_70089|ID:27159547| Histidine triad (HIT) protein [Uncultured spirochete bdmA 4]
MEYLFSFNKIAYLKGKRPKGCILCLVRDGSEDVEKLIVHQTELFIVSLNLYPYNPGHMII
FPKRHITDIREYTQEEKKELDELVPECLNALDRYGNPSAFNIGYNMGLSGGASIEHLHLH
IIPRYPREIGIAELVGGSRVLVQDPKDTLVQLKEIFRTLTVAEDVNPGS*
>SPBDM4_v1_70090|ID:27159548| Thiamine diphosphokinase [Uncultured spirochete bdmA 4]
MKALIVTGGDCPSHEIIHMLSEHADFVIAADSGLEACLSAQIEPDFVVGDLDSVQQVSLS
RVSRDKILQFPEDKDYTDTELAIETARNHGAQDIVLAGGGAGRLDHLLAVRALFERKNPV
QEWHTAQESAFFVSARHRLHFATPVPVIVSVFPLSKGASGMHSRGLKWPLDGLEWDCGHF
GVSNKSVESEITIIAGQEPILVILPLGTRVDIFSDG*
>SPBDM4_v1_70091|ID:27159549| tRNA adenylyltransferase/tRNA cytidylyltransferase [Uncultured spirochete bdmA 4]
MHFLVDTIMSLRYTSKPGMRIPNIDPELRKIADRLIQNGKKAYLVGGAVRDAFLKRPVTD
FDIATDATPWECLQLFPRAIPTGVKHGTITVIPRSHKYKIEITTFRTEGTYTDGRHPDNV

AFIADIFQDLSRRDFTINAMAFDLAHTTFLDPFEGKRDLVRRIIRAVGNPLDRFHEDGLR
TIRAIRFATQLGFVIEPATLNAIAECSDSLFKISAERIRDEFSKILGAQKPSVGLQLLLD
TGTIEAIIPELLVCKGVPEPNHGQQDVLAHLLATVDAVIPDNLSEEELLVLRLAALFHDI
GKPVCYKNDNDSISFYKHEIESEKITRKTLQQLKYPNTVIEKVCHLVRHHMFNYEPGWTD
AAVRRFVARVGLQSVKDLFALRLADTIATAGGPFSWPNVSELQSRIDHIVEANQALTLKD
LAVNGDDLAALGIPRTREMGVLLSELLDAALEDPSLNTKEKMLEIAKAKYFSLKR*
>SPBDM4_v1_70092|ID:27159550|rnc| Ribonuclease 3 [Uncultured spirochete bdmA 4]
VKHSSLSQPSGSDITASRRTSLQQFQQLVVISFHNIVLLDIAFTHSSYVNEAHPTRNDNE
RLEFLGDSVLGVCISHILYEKFPDKREGELARMKSVLVSESSLSAVASTLSLADYLLLGK
GEENSGGRQKPAILADAAEALLGAYYLDKGLEAAKALVLRLFADKIEALASTSGKDFKSI
IQEYAQKRGIELPNYKIIKIEGLEHARHFHISCVLDGETFGPLEGHTKKEAEQRAARQAF
EALHAREGETARILDAIIGTNSTGASALD*
>SPBDM4_v1_70093|ID:27159551|acpP| acyl carrier protein (ACP) [Uncultured spirochete bdmA 4]
MDELFEKIKKIIAEKLEVDESKITMDASFRQDLGADSLDTYELVYGIEEELGIQIPDEKA
NEFETVKDAYEFIKTQVK*
>SPBDM4_v1_70094|ID:27159552|coaD| Phosphopantetheine adenylyltransferase [Uncultured spirochete bdmA 4]
MPNAVFAGSFDPPTTGHLDVIESALSIFDTLYVVVAHNPEKEGLFSIDERIEMLNAMNAF
GERVQVCTWEGLVTDFARERHCMVLVRGLRNASELPYESTMAYMNRRLAPEIRTVFFLYD
AKHVDISSSLVRDLVAYKKLPDDIVPEAAAKVLRKILKKRGQPLS*
>SPBDM4_v1_70095|ID:27159553|lnt| Apolipoprotein N-acyltransferase [Uncultured spirochete bdmA 4]
VFVIYYFIMRENGVKAREYAILASRDIGQPPKPFLLRLSWAFFSALLFALAQPNELFLYG
NWLLGLFCLVPLYLALEDTQKIGEASLIGALFGATNHALTSYWLFFYKNFAFWTLGTTTI
AYAVVYAAAIMYGWFMLKHAGIARPVVFALAWAAFEYAKSTGFLGYPWGLLPYSFTNVAI
MLQTADIWGVYGISAFLALCSASIAELFLPQWPKKPGQNRMRLRWVTISAVYGLILLAYG
ANAMSHPVPVRSTVRLLLCQQNTDPWISGENAALESNIRLAKDALKSNEEAGGAKPDLMV
FSESSLQRPFKEYRAWFEKNPPDSPLLPFLQKTDIPLLTGLPVVINWDTYEASNSVGLIY
PSGILGETYAKMHPVPFAEAIPFWEFSWFRTFVQKVIGLERGWVMGEKPVIFSLTLQNRD
AVCSDIRTLPSVKFAAPICFEDAFAGQCRGFVLRGADLLINLTNDSWSRTLSAQIQHYAV
ARFRAIESRKALVRSTNAGVTCVVGADGRNLVELPQFKAESIIVDVPLYASPLTFYMRLG
DWFAWTCVGFVGLLFVWAILREKKIV*
>SPBDM4_v1_70096|ID:27159554| protein of unknown function [Uncultured spirochete bdmA 4]
LSISNYINTEYINKLEDVCDVVQYREHPAFDAVPFVGSIKKHPYDQEKCLVLLLRKQGRM
PWFKEGELIELKTRDVQALDELPSSIDLVGTIINVFRIWVRRRAIAVRFEPFEVTDEEFR
PFDADSISRLLSKRDR*
>SPBDM4_v1_70097|ID:27159555| Radical SAM domain protein (fragment) [Uncultured spirochete bdmA 4]
MEGSNIARYWRLLENQRIQCLLCPHACILSNGERGHCNARENRGDSMVLPNYAWISSIAI
DPVEKKPLRRFMPNTWTYSVGFWHCTMDCPFCQNWEIAHPSRAEKDIVEPQKLIELALAS
GCPSVSFTYSEPTLHIEYAAEAMQIAHRYGLKTILVTNGNVLPEPARDILLHTDATNVDL
KTADPTIYRNTLGGDLKVVQKFIQIAAELCHVEVTSLAVPGVLDSPRQIEEISRFLASVS
TEIPLHITRYHPAWKFSKASLPLEMLHEMGETAKEHLKYVYSYC*
>SPBDM4_v1_70098|ID:27159556| protein of unknown function [Uncultured spirochete bdmA 4]
MSIRIAEFYLLTDFAYFGIVSIADCTVGVAQVVECQTVDLVVVGSIPITHPSPTPLLGFG
TKT*
>SPBDM4_v1_70099|ID:27159558| protein of unknown function [Uncultured spirochete bdmA 4]
LHGGCSSGGRVPDCGSGCRGFDPHHPPEPDTTFRFRDKNLNAFVAQLDRATDFESVGRRF
ESCRTHGFYIGPLAQLAEQQTLNLRVEGSKPSRLIKSGLKNVLGLDEKALFRLVCASHPR
IARVVERQTRQT*
>SPBDM4_v1_70100|ID:27159557| conserved protein of unknown function [Uncultured spirochete bdmA 4]
MRPAGLEPAAYGFEVRRSIQLSYERVQVFVPKPKSGVGLGWVMGIEPTTTRSTVWHSTT*
>SPBDM4_v1_70101|ID:27159559|tig| Trigger factor [Uncultured spirochete bdmA 4]
MEIAEKKIEELELSRVRMTLTVPARDVHAEYAAMMNEYAKDARIDGFRKGHVPVSVLERK
FGPSLKTEAMGRVIEKAVETGLKDVARQPLAYEPPALDGEPPFELDSDFIFSVTYDAYPS
FDLPSLDGIEIEVPIVAIADEDVERELEDIRQRNAIVVEKDGPAETGDIVTADFVEIAED
GSEVSGTARQDFSFELGKNLNVYKFDDEIVGLKTGDEKTFTKTFPADFEYSEYASKTVTI

KVKVTKVKRQDLPALDDELAQDVSEKYKTLEDLKAAVRSQLAESLEARLRRTKENAIIDE
LLKRTNVSIPQSMLAAELAMRWESLKRQMGVDNDERLDMLLSISGKTKEQLLQEWKPLAE
KALSSRILLDKLIEAGAYSATDEEVDAEIVKEAAHTTMSPAEIKTEYEKHGTIEYIKDDI
KTRKFFDAVLSSASVKEAASVSYLDFMKEND*
>SPBDM4_v1_70102|ID:27159560|clpP| proteolytic subunit of ClpA-ClpP and ClpX-ClpP ATP-dependent serine proteases [Uncultured spirochete bdmA 4]
MSEHMSNLVPIVIEQTGIGERSYDIYSRLLKDRIVFIDGEINDLTADLVIAQLLFLESQD
PSRDISIYINSPGGMVTAGLAIYDTIQYIKPDVQTICIGQASSMAALLLACGTHGKRAAL
PSSRILIHQPWGGVSGQASDISLQAREIIRLKKLTIDYFALHSGQPLEKVAQDMERDFYM
SSQEALEYGIIDTIMEPRKHGKK*
>SPBDM4_v1_70103|ID:27159561|clpX| ATPase and specificity subunit of ClpX-ClpP ATP-dependent serine protease [Uncultured spirochete bdmA 4]
MARNKQFDLEQETFCSFCGKSSSYVRKMIAGPGVNICDECVRICSQILDEEEQKVTLDFL
TEIPKPKDIKEFLDMYVIGQEYAKRVLSVAVYNHYKRLVHKVSAPSPDIEIEKSNVLLMG
PTGSGKTLLARTLARKLKVPFAIADATTLTEAGYVGEDVENILLKLIQNAGGNIQAAERG
IVYIDEIDKISRKSENPSITRDVSGEGVQQALLKIIEGTTANVPPQGGRKHPNQEYLRID
TTNILFICGGAFIGLDKIIESRVAVHPMGFGADVKAKHEKNLGQLYAEMQPDDLIKFGLI
PEFVGRLPIKVALDDLSKEELKRIILEPRNALLKQYKASMQLDNVELEFDEAAVDAIAEK
AIAQKTGARGLRAIVEKIMIDLMYSLPSDSNVKKVVLTRDAVEGKGSCIIEYRQKTA*
>SPBDM4_v1_70104|ID:27159562|thiB| Thiamine-binding periplasmic protein [Uncultured spirochete bdmA 4]
MMRTRQVIAIFLLVGALFIPNKLLWAQSNPASAELVIWTYDSFISEWGPAAQIAEAFQKA
YGTKLRFVSKGDGGALLSALLDSTKRPSADIIIGLDDRQLEKALQSGLFVPLSLKNLGDI
QKNLLLDSTNRLVPFDFGQFVFMWDSQSGIEPPRSLEDLTKPLYRKRIIIMDPRTSTPGL
GFLAWTEAVYRDTWENYWKRLSPSVLAMTPSWDTGYGLFTKGEAPLVLSYSTDPAYHKAY
ENTERYKALQFAEGHPMQIEFAGVLASSPNRELAQKFVDFLLSAECQAFLPETQWMFPSN
MNAKLPASFSVVSKYPALTAKVGDMERDPSEAANILISNP*
>SPBDM4_v1_70105|ID:27159563| putative ABC-type transporter, integral membrane subunit [Uncultured spirochete bdmA 4]
MNGENMTRRTSLFLYGALCVILIFSMAPLVAFLKIALSSLWDANKSLGAAGTISSFLSRI
PVSTIVRSLKFTLSQSLASTLLAIAIGLPGAWFAARFRFAGRKALLALAAVPFCFPPILV
ILAFILYYGKEGFLSSVISWFFGPRKQYQGFLYSFWGLVMVHAFYNFPIAVHQISALWIR
IPDTQMEAAKTLGAGKLKAFRTGVLPWLLPGIAQAAGLIFLYCFFSFTTVLVFGGRTGTT
IEVEIYQALRYQSNYPLALTFSVIETFFALLGILMLTHIQGRTRRSLRDFGRTRELTAPK
GIQRFFIIIYGIFILIFFIGPLMSIAIEAFKVPSSMGGTTKFGFGNFERLIKGFQAPLLG
AILSTIKLSGSAALIATIAGIIASLALFFAEKSNKNRAVPRTIEVLQWLPMAVSSAIFAY
GWLFLSTNRVASTALVAAQAVIAWPFVSRAVHASLRAIDPRIREAAKTLGASPFRAFMTV
ELRTILPSIAAAAAFAFSITAGDVNVPLMLGMGEVETLPLLLYRLTAAYRFNEACAAGLV
LGLMTGIVFLLKEKVIDVA*
>SPBDM4_v1_70106|ID:27159564| putative Sulfate-transporting ATPase [Uncultured spirochete bdmA 4]
MSLEVKDLLVVRGTFTLEANFSLERGRVLVLLGPSGCGKTTLLRAIAGLEAIQRGKITLD
GARIDNLPTEKRHMGFVFQDLALFEQMCVRDNIGYSLMIRHENKKYIHEKVMLLAQRFKI
EHLLNRYPSELSGGERQRVAFARALASEPSLILLDEPLSALDAPLRREMRRFIRLQLAEG
HLTAIHVTHDVEEAVDLADEIIVMRDGRMIGHGTIEDLEKRPGSGWLARFMNLGLVLPVK
NICPAGASGALEVETSAGTLMYMAPKINVNTRHLSKNKVSLYAPLNAIDIAGDLGTATIR
ARIVRHIRSASNIAKLVLMFPDVENYYFEFPLKNRDSNISPEGTDIGISINTSLCQLLPE
DPLVFDNKANTSNSLQEPRRLR*
>SPBDM4_v1_70107|ID:27159565| putative Shikimate kinase [Uncultured spirochete bdmA 4]
MKIAFIGMMGSGKTELGRMLAAHYGVHFFDLDHVIEEQYQMKIANLFRDSGEHYFRDIEE
NSLQMLAKGDVPMILGCGGGVVLRDSNRLTLKKYFITVWLDVPLPELQRRLSAEREYRPL
LSSDNWAVDLKNIFQQRRKLYRDTAIIQYTWQQNQSLEESAEIIEKLLTDAKKAKIIMDD
S*
>SPBDM4_v1_70108|ID:27159566| Dipeptidase, Metallo peptidase, MEROPS family M19 [Uncultured spirochete bdmA 4]
MAQQQEDFYVVDAHCDTLMSLTGRSMNKEENKYRDFFTDNSKVHIDLPKLFRGHVACQIM

AIFLEDEQLPNATEEAMLMIEAFDRLLKNSTGGFSLAKNGADVHRAIAEKNVSALLSIEG
AEALGDSLDSLDEFYRRGVRAIGLTWNRRNAFGRGLKAEGDDGLSPLGKQLVERMQDMHM
LVDVSHLSEEGFWEVAEIVRGPFIASHANARTVLDHPRNLTDAQIRAIADHGGAIGCVFV
PYFVTKDPNDCSLDALMLHVDHIVAAGGIESCAMGSDFDGFKPIDACVIEDAGEFHLIYE
ALRRNGYSHAEAQKILGANWLRVFDEVLG*
>SPBDM4_v1_70109|ID:27159567|uvrB| excinulease of nucleotide excision repair, DNA damage recognition component [Uncultured spirochete bdmA 4]
VKPFKVVAPYGPAGDQAQAIDALAGAIRRGERYAALKGVTGSGKTFTMAKVIEAIQKPAL
IISHNKTLSAQLYREFKGFFPDNAVEYFVSYYDYYQPEAYVPSKDLYIEKDSDINEEIDR
MRLAATAALMERRDVVIVATVSCIYGLGSPDLYRDMRLYLDKGQSVDLDAVKRNLISLQY
ERNDMVLERGRFRIRGDVLEIFPAYLEEAYRIELDFDTIIRIRRLNPLTGEATEELEEAV
IYPAKHFVMPENMVQLAVDRIKGELAERHKFFLEHGKYLEAERIKSRTEYDIEMLEEMGY
CSGIENYSAPLSNRKPGERPGVLLDYFPRDFITFIDESHVTLPQIGAMYAGDRSRKLSLV
DYGFRLPCALDNRPLTINEFESIVPQTVYVSATPGEAEIKRSKTLAEQVIRPTGLVDPEI
QVRSTEGQMEDIYARVRQRAQHGERSLIITLTKKMAEELSEYLGGLGLRVRYIHSEIETI
ERVEILTQLRAGEFDVLVGINLLREGIDLPEVSFIAILDADKIGFLRSETSLIQIVGRAA
RNAAGLVVMYADTMSDAMRRAIAETERRRTLQQEYNREHGITPQTIQKKVQDILERHREE
KIEASQDELDVIRRTHNILIPEQRRSLIKEMEKLMFEHAKNLEFEQAAQIRDEIAKLKGL
*
>SPBDM4_v1_70110|ID:27159568| Peptidase S1 and S6 chymotrypsin/Hap [Uncultured spirochete bdmA 4]
MKLYSKTQVGIAVAISVALTALSIAGIIFIFNKPMKSQSPSNFASPIETSHNGTTDNFEP
SGQTGINQTLSAESIPVTNVSQYPGGYSSEEAENISIYEKYNESVVNITTEVLSINWFLD
PVPQSGGSGSGSIIDEQGYILTNNHVVDKAYKLYVSLSDGSRYEAKLVGADSESDLAVIK
FAPEAGKKLKPIPFGTAKNLKVGQKVLAIGNPFGLERTLTTGIISGLGRPIQESNTTILQ
NMIQTDASINPGNSGGPLFNTRGEMIGINTMIYSPSGGSVGIGFAIPVDTAIRIVPQLIK
NGQVRRGWIDMQAIQLFPDLIDYLKQSGKGAPVDSGLLVSTITSGSNAERAGLRGGTTAV
RYGSTVFYIGGDIIVSIDNKPVTSIAQLYSALEDTTPGQQVTVEFYRGSKKMSANITLSE
RKK*
>SPBDM4_v1_70111|ID:27159569| Cob(I)yrinic acid a,c-diamide adenosyltransferase [Uncultured spirochete bdmA 4]
MSIVTKTGDDGTTGLWSDERIGKDSLRVEAYGTIDEFSSALGMARHLCLQDNVLYAIEYV
QRLLFRVGGELASLGVAFDRPINEDDERTIEMKTSEIEERIPLRGFVVPGMTQGSAALDV
ARTIARRAERRVVALSRTESVSPVLLRTLNRLSDFIYMLAREEEAAQGKLTFI*
>SPBDM4_v1_70112|ID:27159570| putative arginyl-tRNA--protein transferase [Uncultured spirochete bdmA 4]
MPQDLGIVQHFQARGTCPYLMDHRESITDYLIFGQEVDPFLYGALIRDGWRRYGKGLYRM
LCPGCRLCIPLRIHAASMHLAGSLRRILLLNRDLLIIPRPPAFIEEHYMLWRNYSLWKHS
CNPDELDEASYCQLFVPWSLIFEYRENNENKRLIALSHVDPLAGGLSSVYFSFAPEAKAR
SPGFFSILAEAYIAAFIKTDKLPLNKEISAKTIFSFYENKKTLKSTRPGADEDIGCQHGS
PWYYYLGFWIPGAPKMDYKARIRPFELAIAESEANIASTWRQYASRDEALAYLHEKRWPG
L*
>SPBDM4_v1_70113|ID:27159571| putative PpiC-type peptidyl-prolyl cis-trans isomerase [Uncultured spirochete bdmA 4]
MASEENKKQTPSNGKASGEKKAENKKNKSSLKRNIKNPFVYIGTIVILVLTIIAFVLIPG
VGGGVSSAGKVPTFGSWNGTPIQYSSDSYFADQVSQINDYLRQQGMNESQSQLYAYQVWR
MAFQNTVIRTAILDSAKRSGMKISEQTIDEEVAKNTQFQVDGKFSLEKYNSTPSATRMSI
RNRIRDDQLIQNYYGDLMSVSPSTGEIDFVASMAKPQRTVQYVNISYADFPQEKTLDWAK
SNESLFRRIGLSKITINTSQKDAEKVLAQIKANSLSFEDAAKSHSKDSYADKGGDMGTKV
FYDLKGEFLNEKDADAVMALKKDEISPVYKESGNTWAFYRVNAEASMPDFSKQSVLDEVK
SYLFDKEKSVMESWALAKADEFASRASKSPFAAAAKEVGLESKAAGPFILNYGKPSFYFY
GQQVGLFQEPYRSLDTELVGAEESETFLTTLFSTAKNAVSKPVVLDASVVVMTVTDDTEG
TDQEISYTKFTYPYFYQTATENQIRGSILSSKLFKDEFNSTFVKLFTPAQK*
>SPBDM4_v1_70114|ID:27159572|lepA| GTP-binding membrane protein [Uncultured spirochete bdmA 4]
MIEPEHIRNFCIIAHIDHGKSTLADRFIEKAHLIDLRKFQDQMLDNMDIERERGITIKSQ
AVSLPYIAKDGQTYMLNLVDTPGHVDFSYEVSRAIAACEGAILVIDATQGVEAQTLSNMY
MALEHDLEIIPVINKIDMPAADVEGVRRQIEKDLGLDGDSALAVSAKFGTGVDELFEAIV

ERVPSPTGRADNPLQALIFDSHYDPYRGAVVHLRLKEGVIKKGMYIRLMSTGAIHEVEEV
GHFRITSVPAEELGAGEVGYCIAGIKTVKEVRVGDTVTGNENPCDSPLPGFKEVKPVVFS
SIYPIDSADYEELRDSIEKLVLNDASLIFEKDHSLALGFGFRCGYLGLLHLEVVQERLER
EYSQSIIMTAPSVRYRLLLKDGTTLFIDNPSEYPDPARIESAEEPYIKAQVITPVDYVGN
VISLCLAKRGTQESFVHLDEKRVELVFEMPLSEVLFDFYDKLKSTSRGYASFDYDVTGYR
PVELAKLDFLINGEPVDALSMLVYRPSAYDRARIVCERLRNNIPRQQYKVAVQGAIGSQI
IARETVNPVRKDVLAKCYGGDITRKRKLLEKQKEGKKKLKMIGNIELGQEVFLSVLRASE
DEEH*
>SPBDM4_v1_70115|ID:27159573| Glutaredoxin [Uncultured spirochete bdmA 4]
MNLQNLPVVTKEGTRNNHDIMVYALSTCGFCKRALAFLDANQFTYKYLYVDLIPIDVKNT
IKQELKERFKENVAFPFVVIDDKKYLVGFIEADWKLTLQV*
>SPBDM4_v1_70116|ID:27159574| Ferredoxin thioredoxin reductase beta chain [Uncultured spirochete bdmA 4]
MANYKTIEQTTVFTQMVANKQGWALNPDKAFYDTLVEGLTKNYNRYGYFLCPCRDTEGSR
EADRPALCPCIWSKPDIAEYGHCYCALYLSKEFVTAGKTPKPIPDRRFENA*
>SPBDM4_v1_70117|ID:27159575| putative cysteine desulfurase [Uncultured spirochete bdmA 4]
MRDVFLDWAATTPADPEILQRSLDLSLEVYANPSSSHWLGRQAKQLLEESRTSLMRALAI
SKGTLVFTGSGSEADHIPLLALVHRLKPVASRKPLHVIVSAIEHSAIDCQIRALEKLGVE
VSWINPDTQGHIDPNQVSARMRKETALISIMGVNNEMGAIQDVSAIGQSIKDAALQSGMK
KPWFHVDAVQMLGKLSMREVPDYADSFAISAHKIRGPRGIGALWLSRPLEAFACGGGQEG
GIRPGTENLFGAFAFRFAVEKASESLAHHAAHAQELESRLIEGISHIEGALLVPRRAPRD
EGYVPSIVSIAFPGLGGETMVRALSDRQIAVSTGSACASNQRRQGRRVLKAMGVPDDISF
SAIRLSTGPLTTPEDIDIFLEHAEDLYRKLKT*
>SPBDM4_v1_70118|ID:27159576|thiI| putative tRNA sulfurtransferase [Uncultured spirochete bdmA 4]
MRTFFLIKPGEIELKLGNRREFIQRLKDQIRRRLKGLHFELEEYPGRFFLNIEEKDSEIA
LFVLQHCPGINGIAQTIKSEKRTERILKAAIEIASKEAARGARRFKVETRRSDKSFPLDS
YGMSVAAADAVLEHFPALSVDVHTPDFVLMVEIRERAYIYSSTAPGPRGLPVGSQGKGLL
LLSGGIDSPVAGYMMAKRGLALESVYFHAYPYTSLEAQQKVERLAARIAIWSGDMSMWIV
PFTEVQQAIARGPREGANTLMLRMAMMQASELIAHRINARAIVTGESLGQVASQTAENMR
LSQNPTSLPVFRPLVGIDKEETIALARRIGTYDISILPYEDCCVLFSPKHPILKPDFDEL
HTYFESLVLGDIIEAAVENAEKKILNFTDALREYGYHIASEQY*
>SPBDM4_v1_70119|ID:27159577| RelA/SpoT domain protein [Uncultured spirochete bdmA 4]
MSDIENIPNKALLSDIYIKNRANYEEALAKTIRDISKLLQRKGIHPTIKSRVKDFESYFA
KKIKVLKNAWDEHKDPLPINDVLAMRIICPFLRDLDEVEATIAKHYDIVEIERKGQDRSF
REFGYESIHVLIRIPDEILPLCAGLERNVIEIQLRTILQEAWAEVEHELVYKAEFTPFDE
PLKRKLAALNATLTLSDIIFQEILDYQNKLNNALKKRREAFYGKIEQTSAQVFGITESLS
GELPSPVRQKKVPAKTGLSAKQGQVQNRWAGLDIDTLLLSALDAHNASDFKTAIAIYTEI
LSRKPEAAIAAVVFKHRGMAYFAQSEYPDALADFTSCLMLDPDCYKALYYRGVVKSIQDE
LRQAIDDFTKALEIHPYHFFSRYRRALCWWQLGDYVQAHADCEIALRIKPENKLAQELQK
KIQENIVKEEI*
>SPBDM4_v1_70120|ID:27159578| protein of unknown function [Uncultured spirochete bdmA 4]
MSAVSDHEHKERYIIALLIIVAVVFIVWRGPTLQFNGMRLYFPDKDGQTLKLEKRPIAPI
GNLEEKAKDIVEELLLGPLSRNLQPVVHVDISLERVVSGKNALFLDFTTEDLPGFSAEYE
TFKAAIEKSLHDTIPGNFRIYLYINGILSR*
>SPBDM4_v1_70121|ID:27159579| Cell wall hydrolase/autolysin [Uncultured spirochete bdmA 4]
MHVCQNRISKLDLNPQSLKATTLISIAIVLMLIFCSVSFAAAQEAPQQDMSLSALVQSLN
ASLEYDPLTHAGTITKEGRSVRFALDVPYVLLDWAIVKKVPSPYETDESLQVKKEFADAL
RQFFEVKPGQSSKYSVKAIVIDPGHGGKDPGAIGEFDDFKLQEKDVNLAIAHRLAELLRI
RYPDRTVLLTRSDDTYPSLEQRVEMANTIKLGETEAIIYISIHSNASFNKNTRGFEVWYL
NPDYRRTVVDEQTAKEKGEDIAPIINTMLEEEFTTESIILAQKVYQRMGKMIGDESPSRG
IRAEEWFVVRNAKMPSILIEVGFVTNEKEALLLSQAGYLRRIADAIYNGVCDFIEHFEQ*
>SPBDM4_v1_70122|ID:27159580|nrdJ| Vitamin B12-dependent ribonucleotide reductase [Uncultured spirochete bdmA 4]
MKIKRLFTKAGHGPYEGIVWEKRRSEIRNPNGASVFSMDAVIVPSFWSPIASDILAQKYF
RKASVPKDKALDWTKYVPTSQASLSDEDIADDSEHDARQVFHRLAYTWCQWARSANYFDS

DEDEKAFYDELCYMLAHQIAAPNSPQWFNTGLFAVYGIEGPAQGHYFVDPSTNQVVKSES
AYQRPQPHACFILDVKDDLVNEGGIMDLVTREARLFKYGSGTGSNFSRLRGANERLSGGG
VSSGLLSFLKIADRSAGAIKSGGTTRRAAKMVILDADHPDVEEFVRWKTEEEYKVACLAA
GSGLLNHYAEAMISAIEDMGAEDENSYLPDRNPLLRRVVKQALDAGAPSSWIHQVLTFYS
QTGKAPRLPLYNTAWESEAYNTVSGQSSNNSVRVSNEFMEAVVNDLDWDLIGRTTQKPFR
TTKARDLWNEIAESAWLCADPGLQFHTTINEWHTCKADGEIRGSNPCSEYMFLDDTACNL
ASLNLLSFYDEKTGTFDDAAYRHAIRLWTIVLEISVVMAQFPSKEIARKSFDYRTLGLGY
ANIGSLLMVMGLPYDSDRGRSVAAALSAILTGESYAQSARMAKEFGPFARYEANKEHMLR
VIRNHRRAAHNAPPGEFEALSVIPKGLDVEFCPRNLSDAAHQCWDEALQLGEKYGFRNAQ
VTAIAPTGTIGLLMDCDTTGVEPDFALVKFKKLAGGGYFKIINTSVPPALHALGYSEGQI
GDIIAYALGKGTLEAAPGIPRQSLLDKGIPEETLQKAEMALQNAISLESCFSPYIVGYDV
MEKLGISKEEADVPTFSLLSRLGYSEKEIEKAELFACGTMSLEGAPHLAEKDLAVFDTAT
PSGKRGTRSISWQAHIAMMAAVQPFVTGSISKTINMPNNVSIDDVKGAYTLAWRSMLKSI
ALYRDGSKLSQPLAALAPGSDLIADSIVALQSNELIAEEPGSGDLTSTAPLQTTPEPELP
GMPFAPRSVRRALPSRRKGYTQKAKIGGHSIFLRTGEYEDGTLGEIFLDMHKEGAAFRSI
LNSFAIAVSLGLQYGVPLEEYVDAFTFTRFEPNGMVSGHDYLKMATSVLDYIFRELAISY
LHRYDLGQVKPEDLIATSTQSIADEGSNAFESGRKQKQDKQGKPKGTRASPASVKASFSG
GGERDQAIMLMRTARQKGYEGDPCPVCGNLTLVRNGTCLKCDTCGSTTGCS*
>SPBDM4_v1_70123|ID:27159581| putative Arginine deiminase [Uncultured spirochete bdmA 4]
MRSVRIESEIGRLRRVIIHNPGPEIEAMTPREAERDLYNDIIPLSAVQQEYIKLRDFLSL
ITTTYELVDLLEACLSDEQDKLQFLKEYSKINPITHIVDDLMAFPAKELARSILTGIIAP
KGSLARYLNPHSFIANPLPNAYFMRDSLAIVGRNIVSAAFTFDVRMAEACITRFIFLHHP
DFQNDGILLDGPEERNRLVTIEGGDIHVLSSNVLAIGISERTTAFAIDRLARRIARATEA
PTTIFAVDLPKERATIHLDMVFTMIDRHAALTYKPVMLSSHRAQVYRLEADSQGEIHYEE
EKSLFDGLQKIGIELEPVLCGGGHSVYQEREQWLSGANSFSFAPGKILMYSCNMYTLEAL
SAHGFAVVPAQDFLDGRADPEKYDRLAVAFDGIELARGGGGARCMTLPVEREQIEA*
>SPBDM4_v1_70124|ID:27159582| conserved protein of unknown function [Uncultured spirochete bdmA 4]
MKQRRIIIIGAAGRDFHNFNTRYRQDESAEVVAFTAAQIPNIDGRKYPAELAGPLYPKGI
PIFSESELSALIKEYNIDECVFSYSDVPYSHVMKVSAIVNAAGASFTLLGPKDTQIKSVK
PVISVCAVRTGAGKSQTSRKIVQMLMKRGLKVVAIRHPMPYGDLVKQKIQRFAKVEDLAY
HKCTIEEMEEYEPHIARGNVIYAGVDYEAILREAEKEADVILWDGGNNDFSFYVPDLQIT
VADPLRAGNEVSFYPGEVSLRTAHVVIINKIDSASPEQIQIVRENIARTNPKAIVIDAAS
PITVDHPEFIRGKRVLCVEDGPTLTHGMMKYGAGIVAARKFQAAQIVDPRPYISGELVQT
FRTYPEIGTLLPAMGYSEVQKRDLEDTINRTDCETVIIATPIDLNRIITIEKPAVRVSYE
LQEIGSPNLEEVLSDFCSQQGLC*
>SPBDM4_v1_70125|ID:27159583| conserved protein of unknown function [Uncultured spirochete bdmA 4]
MEIDAFPKAKEYQDFLEFVFPGSSPSKAPRALLSLKKAGDMKYDPRGETAARLSFFRRLG
IEPGRIVGIELKHSRNVAFIDTREDLRNLLRTRPEGFDGIITTNPSLIPGVTVADCMPIY
LFCKTTGAFGVLHSGWKGTGILKTAAQGMMERYHCRPNDISVVFGPAIGVCCYAVDPERA
ALFRHEFGPSSVREETKEGETHYYIDLLAANMGLAQSLGITHYVAVQTCTSCDDRFSSFR
RDGPQLFTRMLALVLSPFPY*
>SPBDM4_v1_70126|ID:27159584| protein of unknown function [Uncultured spirochete bdmA 4]
MPFIDLNEPYIIDVSHDVAAVFKPCGWHSALHTTGDISMTSWLFEHGDILPASELKNAVH
DQTLPAQNNGVQKWDEHDQFQGELGMLYRLDRDTSGILLFALNRSTMDSMRRAQDNLALK
KRYMFACTESNHELPGSMPRVRYAERAALLDKLYVRQEIDVASYFRPYGPRGARVSCISP
ELVQKPKKRIAKDIYVTKYLSAQNAGVEYHAPLGLPTGTIFIEAEIRKGFRHQIRAHSAW
MGLPILGDSIYGGVYSKRLFLEAFSVTLTDGTRTIAEWKLYNGN*
>SPBDM4_v1_70127|ID:27159585| ABC-type multidrug/protein/lipid transport system, ATPase component [Uncultured spirochete bdmA 4]
MAEFFDTEPVTKGYDAHILRRILSYLKPYKLFAFIALIALALSTAGELLSPTIIQRAVDN
ALVREWYGVDPSIVSTLPKANDLTGITIGARVYYVRTSRLAGIADKERKQWNAQGLLDGEP
SYVFSLESLREKKVLIAQRYPQIVLSESWGVLPSSLLNSLSSDDAETIRAGDNAILGHYA
LLLLLILTVVLLATFTMTWFTNQIGTLIMKDMRLQLYRHVIEQSLAYLSRQPVGRLVTRL
TSDIEVISQFFTDVLSAFIKDATVMVGALVVLFVLNWKLGFVVFATVPLILIASAIVRVK

ARDAFRNQRYWTSKVNSFLSEHISGIDIVKFFVQEKRVAENFGKNNQQLLKANIAEMYVY
ATFRPFVDFMSTLTTALAILAGAVLFLRLEISVGTLIAFINLIAMFYSPIKDLSEKYILL
QSAMASGERVFGLLDTEDKLPEQLPDMRGKDAPLAIRGHIELSHVWFAYKNEEWVLKDIS
FVVNPGEKVAIVGYTGAGKSTIANLLARFWDIQKGDICIDGIPIGQYPLKRIRKFIQPVP
QDVFLFQGTVRENIVLGLDLPQEKLEEAAKAVYAHEFISALPKGYDTPLSEGGLNLSLGQ
RQLISFARVLAHEPSIIILDEATSSIDTETEKLLQKGIEGLLKGHTSIVIAHRLSTIRDA
DRIIVLGQGHAIESGTHDELIAQRGVYWNLYRLQSKEME*
>SPBDM4_v1_70128|ID:27159586| ABC transporter related protein [Uncultured spirochete bdmA 4]
MFKEYVTLLPHLKQYKYRYIAGILCLIAVDASQVLIPRYVQTTIDTIVSGSFQVKEIVHP
LVIMVLLALFISIGRFFWRYFINTASRRIEAEMRDRLFSHILTMSGGFFRKNTTGDLMAR
ATNDIGTIRQATGMGFVALVDGVFMTAMILVAMFANNAAVAAWIIIPLPLITALILLFGK
IVGKLFKRIQDIYGRLSNIAQESLSGVRIVKSFVKEHYFFEKFEVTNTEYKNAIMNLVKT
SGFFFPFITFLSGLSTVLLILFGGNAAIHNKMTPGSIIAMLSYLEMLVWPMMSAGFTVNI
VQRGAASLKRINEILNTEPEIQESPRHVAGKPCGDIEIRELDYRYSGSDMLALKHISVRI
SEASMLGILGKVGSGKSTILKLLPRMLDAGEGHVFIGGIDSCFFGLKELRSIFGMVPQES
FLFSESIRSNILFSASDIGNERFEEVVRIAGLDHDMHLFPDGWDTVVGERGITLSGGQKQ
RIALARALVIDPPVLLLDDALSAVDAETEERILSALLKERRGKTTVIVSHRISTLRNADQ
IIVLDGGEIVQRGTHETLMSEPEGFYARIAALQQLEQESCAACEDAGLKEEAIDG*
>SPBDM4_v1_70129|ID:27159587|rnhA| Ribonuclease H [Uncultured spirochete bdmA 4]
MQGVARHWLVCHSAHMTQQSIVIYTDGGCKGNPGPGGWAYVMRFGTRYRESWGAEPHTTN
NRMELTAVINALSFLKGRLEGTRASCAAKSAVLPAWTGAPVHIFTDSMYVKNGISGWITD
WKRRGWKTAAKKPVMNQDLWQQLDGLCIDLGPHFEWVEGHAGNPDNERCDTLVGLAIEEM
IGQNEQIIHHEAELPASKDDDIL*
>SPBDM4_v1_70130|ID:27159588| conserved exported protein of unknown function [Uncultured spirochete bdmA 4]
MKKRKYFGLMVVAILLSSCASAPSVPEWIRKTPKPDAKYTYFVGSSSAKDSATALNDATS
SLIAGIMQFMGVSISVTSSAEARASLDDYQAQITQTVKTESTGRLAGFEVAEKYIQKDPK
TGQYTVHVLARYETKELLKEKTRIESLFNEKIDAVAIPEQKGDAAASDRRLLDAIRSYAE
AMTAAGGSDIENAQIKLERNAKKASGLAATLKLIVPSSQGASISVGGQLPSFHASLVANK
GGIEQGVSGAPLIITYPRRLASGRIGTSTTQSFTDQNGNAIFEVPAIDIAGNYRVTIQLD
FASISDLFASLPSWALPYSDAVESDLSGIVAYIHYKVISAAKNIPTAIAVYGKNPANAGK
IDLGIFVGSLKEPLIKEGFVILDAEAPEVGENPDIAQLKAAAPPQAQRFALITLDLVSVS
KDGDYFIATTSGSMSVFDLTGGNTLYSASKNAQGMGLSETEAIANALKALGGQTFAKDLL
SALP*
>SPBDM4_v1_70131|ID:27159589| conserved exported protein of unknown function [Uncultured spirochete bdmA 4]
MRVWKVLLTVMIVMLVASCGTKAPLRKGEVLIEDKGTAYGIKTPKWVEQAIIGGYRDIEK
LADYKDKTVFIAQFEAQNLQSAQLLAERMQADTEIARYLSTRVKDAFKGANVADADSKNF
GAYGERFVASVGEAKFSGFRMEADWWVKVQTYTPENKPDKQIYRVIQLWAIEKTMLQKQF
DILFSQMAGTEEPTPEQKRAMDLVQNTVAKDFFGEDK*
>SPBDM4_v1_70132|ID:27159590| conserved protein of unknown function [Uncultured spirochete bdmA 4]
MPGGTQVPSAVPSTPQASTASYGALTPDETLFFQNYINRLSYLVYYDENSGLDPQLAKAA
VSQANRYLIEKSGLSVIDFDQIQKNKKDQMSAYQAETGGSIDIVTYIAQKLNADIYLEID
AKTTSGGGPGAWTGSAQGSMKIFDASTATLLGSISFLSPQTFSPVSADAAMMNAIAGIVW
QSMPKVTEQSKALLSGVTATRGVRYELVLQNTPDAKAVSQFEKNLSKRVREVESLSYAPG
EARFALYAFMPASKIQDAIYEAASAAGFPDCYLLYMRGRSYTFNTGL*
>SPBDM4_v1_70133|ID:27159591| conserved membrane protein of unknown function [Uncultured spirochete bdmA 4]
VKLIHSPTARLSLLVLFSILFLKPTFSQAKDNTLSPPEITGIYFTETGCSHCDSFLYVQK
AKLEHDYGVSIVLETHDILSAAGYEQCVKMLKQKQLNFTVFPVLFIGSNIYRGSSAIEEN
MPQEIEYYLAHGSYRPALHRTNELASNADQSDKAFVASIIPSILAGLLDGINPCAFSTML
FFLSFIALKRRDRRSLAWVGISFILAVFVTYFLIGLGFLGALRKYLSENRFSLYVDIFVS
ALAAIFAVLNVRDAVVATQGRASESLLQMPFFLKRMSHRFIRYFNQLPLYILGAALSGFL
VSVIELACTGQIYLPTIAYMNQSARSSRSIILLLIYNVAFIIPLTLVFVLYFFGLRHERI
RQWYGTHLVLVRVLSALFFIALGVLVWIP*
>SPBDM4_v1_70134|ID:27159592| protein of unknown function [Uncultured spirochete bdmA 4]

VTALAATKARRGKTLWLEKQENTRQNEIQDARPSATPRLVLGADTLVAFQTQGGWITIGK
PANERDAFDMLSMEAGKRHTVFSGLCLLDMTRDQPYVALSVSEVQFSPMTNEDIAWYLSF
GEWRGAAGAYRIQGVGSYFIEEIKGSSSSVMGLPIRELYGILRESGYFASP*
>SPBDM4_v1_70135|ID:27159593|rpsB| 30S ribosomal protein S2 [Uncultured spirochete bdmA 4]
LLHRKEKQNELTIRLFKVGHFPPDFRFSKQDEIKGRGSDYLRSREVYVAVVTMKNLLESG
VHFGHQVKRWDPRMKKYIFAERNGIHIIDLQKTIQAIKEAYDAVQKVVSSGKTVLFVGTK
KQAQSAIQKEAERCEQFYVNNRWLGGMLTNFSTIRKSIQRLKKIERMEVDGTFNSLTKKE
IASLLKEKTKLEKNLGGIKEMTSLPGILFVVDTRKEAIAVAEAQRLGIPIVAIVDTNCNP
EGITYPIPGNDDAIRSISLFTQIIANAVIEAGASEGLKIIETLPTEEEQELAVPAKTEDD
EVEIDVENYADSSVQSEKESEEPAEIETPIVDEDRLYEEK*
>SPBDM4_v1_70136|ID:27159594|tsf| Elongation factor Ts [Uncultured spirochete bdmA 4]
VEIKATDVKALREKTGAGMMDCKKALNESQGDFGAAEKLLREWGMAGVEKRAGRATNEGR
IFVAQTENELALVELACETDFVARNTDFINVGTKIAETALQQKASGPNEGLEARVKEIAS
LIKENIALKRVAYFSAGMNETLHTYLHGEGRIGVVVKFRANDPAAFKNEKVAAFVHDIAL
HVAAFNPMFLDESKVPQAWLSEQKEIFQKQVEMDEKMKSKPAKVLEGILAGKLKKLLAEV
CLVDQGFVRDEKIPVAAALEQVAKETGYQLAIVEYYFAKVGQN*
>SPBDM4_v1_70137|ID:27159595|frr| ribosome recycling factor [Uncultured spirochete bdmA 4]
MESIRAANEERMKKTVSALHDEFNAIRTGRASPALLDKVRVDYYGQKTPLSQVATISVPE
ARLIVIQPWDRSLFSEIEKAILRSDLGLNPSNDGKVLRIAIPPLTEQRRKELVKTARNIA
EQSRVAIRNIRRDGLEELKKLQSAGGVAEDALKKEETEFQKLTDDYIAQIGKILETKEKE
IMEV*
>SPBDM4_v1_70138|ID:27159596|ispU| undecaprenyl pyrophosphate synthase [Uncultured spirochete bdmA 4]
MKEIRVPSHVAIIMDGNGRWAKQRGLARTAGHQEGLEVAKRIVLAARQEKVHFLSLYAFS
TENWKRTTQEVGFLMSLIRIHLTKELDFYRKNKVRVVHSGDRSELPAEVLAEIDRAVEDT
KHHNGLTLNLAINYGGRNEIVRAVRCSLSNLYETGTIGARDAMSKDEVTGAMTEKTIALN
LDHPEIPDPDLIIRTGGEMRLSNFLLWQSAYSELYFSSKYWPDFTPEDFHAALADYAHRE
RRFGDAR*
>SPBDM4_v1_70139|ID:27159597| putative Phosphatidate cytidylyltransferase [Uncultured spirochete bdmA 4]
VNENTRARLLLFFIGIPLFFSVLCCFPFAHFAILVVFLLTVQYLSSLELRSMLSRGGLHD
PGLGVTIAGLVQSAAVYVACVLSVSPSEVGLILFFASLMCLTIELVPLGAKKKALFPGLL
KEAGSIVLVHFYTALLPSLLVLIVSGFPDARNATTTFVLLTIGNDSLAWLFGKYIGKKRN
VVDVSPNKSIAGFIGGTIGSIAGAFLGLGPLAGSWRPLEWNYIILSLTLGMGMAFFVIAG
DLFESALKRSAGAKDSGDIVPGRGGVLDSFDSLYFSAPFFVAFSFLFHLFGL*
>SPBDM4_v1_70140|ID:27159598|dxr| 1-deoxy-D-xylulose 5-phosphate reductoisomerase [Uncultured spirochete bdmA 4]
MPKRVIVLGAAGSIGRQTLDVIRQSLGSEDMLGLAGFSVHSNREMLRQLKNEFPQAVAAW
TGDIAAVPSGTAWAGPEAMARLLSETDADIVVNGIAGASGLSASILALRNKMNLALANKE
SIVMGYRLLKKLADENHCAIIPVDSEHAGLFQLIQRIGRNIISELTITASGGPFRFLPLD
QLPSMTADDACKHPVWKMGRKISIDSATMANKGLELIEASRLFDMPQEHIQVLIHPQSYV
HAMVRTIDGTLYAQISKPDMRLPIHAALYWPKSTPVSFGSANLAGKSLEFYEPQRERYPL
LWIARQALDEGEAPCIAYNAANEIAVGNFDEGNIKFTQIADVVLKTLEKGWNLPISSFED
IFDIDTGARRIAADLMEHYA*
>SPBDM4_v1_70141|ID:27159599| putative zinc metalloprotease TP_0600 [Uncultured spirochete bdmA 4]
MITILLGLIGLSIVVIVHELGHFVIARSVGVEVEAFSVGWGPALFKRKGKKTEWRIGVLP
IGGYCKLKGEDGFRAALDQKLDFIPADKGSYYSAHPLKRVAIAIAGPSFNVLFATLVFVF
VVAIGTTIQTAPNRIVLSSETGAMIQNGMPNPADIAGLETGDVIRAVDGKTIRDYSDLQE
IIASNPGKALSVEVLRNGSVQNLVITPHLDRNSGAGVIGVYAWVDPVIATVKDQSPAAIA
DLRAGDIITEANGKAIHNTVDLMEVFKNASGPVSLMVMRDAEPFPTTIVAKSLEEMGIGF
VSVIRTDKATSLPDAVSKGFKETASTFSLTIKSIGLLFRGVNVFKAVSGPARITYLIGTA
ASEQIKADGLAGLVPVLSFLAYLSVALAITNLLPLPVLDGGLILVFIIEFLRKRALTPQA
LYRFQFIGAAFVLVLFIVATMSDVFFFAGK*
>SPBDM4_v1_70142|ID:27159600|dnaB| Replicative DNA helicase [Uncultured spirochete bdmA 4]
MAGPSLKDTMPLYNGDAEQACLGALLIDPESINSVLKYLRSESFYEPANQEIFESLLAMH
EKGQKPDLITLSEELRSRGSLERVGGSAYVASLASFTPSAANIEYYARIVQEMSTRRRLI

QVSSEMAAMAHEETMDIDRVLDELQAKIFEISQNRRTAEYHSAKEIVTETMLLIEKLSTN
PEAFTGIPSGLTDLDAMTSGFQNSEFIVIGARPSVGKTALALTIAAHASIDLKIPTAFFS
LEMSESAIMLRLISSEAHIPAERIRTGRIRTTDYDSLMEAAARIYEAPLYIVDMPNMKLL
ELRTMARRLVLERGIRILFVDYLTLITHENADLPRWEQISAISRSLKALARELNIPVVAL
SQLKREAEGKQPTLADLRESGSIEQDADLILFLHRDREMTKGHDQQSDQMETDLVVAKQR
NGPIGKTSVWFKSSYAKFVNMERHEHKQ*
>SPBDM4_v1_70143|ID:27159601|rplI| 50S ribosomal protein L9 [Uncultured spirochete bdmA 4]
MKVILNQDISNLGEIGDIKEVAAGYARNYLLPKKLVLVYNEKTVAMLQKRQVEIIAIKEQ
KRLASRSLKEKIEADPLIIAMPAGNNGKLYGAVTNHTIADELLKKGIEVDRKKIEVPGRS
IKSVGNYKVLIRLYEKDEATLHCSVEAQAVAGLEEKKTTEGEAKKHRPRRQRTESEPVTA
EEQAAAFEAAVNRGQLS*
>SPBDM4_v1_70144|ID:27159602| membrane protein of unknown function [Uncultured spirochete bdmA 4]
MDSISSRNSAHHIPAFLSWGVLSGLLYASGFLAMAFLVPIQYVFRKEGKKEGLLSMLISL
VIVGLGNAFRFSDPNIMRLPLLLQTLVSPALLLLAIGSINLVNIDTWKKTVAAAVVLSVI
FGFLLQASIGTEGVQQSIAGMIAQMIDSTGMQSPDISTIAQSYVAPAVSIIFDCFGAALW
LMLAGSWWIGNRLGALKVSTEEKIQEGKSSSFNVPSWLLWPSIAGWTLLLFVLYGHKTGI
LAIIAWNASLAAASWYGLQGFSVISRFFQLKGMQQMTGLVLVLLAVLILLDTKVGLAVAM
LVPVLGVSEVWLQYRIHKGA*
>SPBDM4_v1_70145|ID:27159603|rpsR| 30S ribosomal protein S18 [Uncultured spirochete bdmA 4]
MDEMKDIEEAKASRAEVDQQDMRRDQDDRNGPRKGKGFFRKKVCRFCTQNVKIDYKDADL
LRRYTTERGKILPRRITGTCSKHQRLLAVNIKRARSLALLPYVVK*
>SPBDM4_v1_70146|ID:27159604| Single-stranded DNA-binding protein [Uncultured spirochete bdmA 4]
VSDINVLVLIGRLTRDSELKYTPSGLPICRFSIAVNRSKKQDDQWVDEPHYFDIEFYGKS
AEGLSKYLLKGRQVAVQGELRQDRWEKDGQQRSKVVIVAATIRPLGSPPQGSGDAGGRRY
ASDSVASKPESSNGQTPASPGVDEFTDDIPF*
>SPBDM4_v1_70147|ID:27159605|rpsF| 30S ribosomal protein S6 [Uncultured spirochete bdmA 4]
MRQYELVAVLNSEEDQFKAGKQAVAELLAQYKAIDVKEDDMGDRPLAYPIKKKARAHYVL
YRMSLEPTSIIALERAIKLSPSILKHLVVKIEA*
>SPBDM4_v1_70148|ID:27159606|hisS| Histidine--tRNA ligase [Uncultured spirochete bdmA 4]
MPTIQPRVLKGFRDFLPDIEMARSSLIKKLEQVFVLFGFVPIDTPVLEYADILLGKGGGE
TDKQVYRFRDHGDRDVAMRFDLTVPFARFMAEHVEELYLPFRRYHIAKVWRGENTQRGRY
REFMQCDFDIVGTDAASADADILLTAAKAMTALKVGDFSLRINHRALFNRFLARIDALDK
SVEILRTVDKLEKTGVQATAETLSTLVAPALANDILSFIQKEEGFEKTLNKMFSFCNPSD
VQAQAAQRRLLDIMECVAAAGDEHRVVLDPSITRGLDYYTGMVVETSLAALPEIGSVCSG
GRYDELTSLYTDRKLPGVGASIGLDRLMAALDVLGISAGSDMSAQILIINQGETPIAELH
ALAASLRNAGFACEVFPEPKKTITQYTYAERKHIPLAVFANSSSGPFTIRILSHRENIEI
ADFARLCETARKELHASA*
>SPBDM4_v1_70149|ID:27159607|trmB| tRNA (guanine-N(7)-)-methyltransferase [Uncultured spirochete bdmA 4]
MHEKESGLLDGSIHTFVLRSGRLTEAQRRAIEKFGSKYIVPFAETFLDLNSLFPEKRPLV
LEIGFGMGQATWQIAQARAQFNYLGIEVYTSGVGRLIIDLERHELKNVRIIQHDAIKVLK
NMIPPESLAGIHVFYPDPWPKKRHHKRRLMQDAVVALMAEKLGADGYLYFVTDIEEYASF
AKASLGSCTALKNRFDGYAPHQLWRPETKFENHAKNSGRGTFELFYIKG*
>SPBDM4_v1_70150|ID:27159608| Pre-mRNA-splicing factor ATP-dependent RNA helicase PRP43 (Helicase JA1) [Uncultured spirochete bdmA 4]
MMNPHDLPIYQQKAKILEALAANQVIVVESPTGSGKTTQLPIILHEAGYASNGIIGITQP
RRIATLSVSDFIARQLSTTVPGIVGYKMRFEDATAPNTAIKIMTDGILLQEMKLDPYLTH
YSVIMVDEAHERSLNIDFILGLLKKVLEARSEFRVIVSSATINAEVFSEYFGECPVVRID
APMYPVRVVYDAIKTVPEASPGQGPRPQGPDGGYYRSSDEAIYDKILSIVGRVLDGDAGA
EPGDILIFLPGEKTIKECLQTLMASRWALRLHCIPLYARLGKDEQEKVFAYPPEGKIKVV
ISTNIAETSVTIDGITVVVDSGLAKINYYNPRTFTASLIETPISKASCNQRKGRAGRTRP
GICYRLYSIKDFDSRPLYPTEEIHRTDLSEVLLRMAELGITDFEHFDFITKPPKSAIRGA
IEVLNLLDALNPDRTLTRIGEMMCAFPLLPKLSRMIVEAVLKYPDVISETLIASAFLSTT
SPYVLPPGEETEARAAHHRFRDPMGDFASYLQLYEAFSSARDKTKFCERNYLDERTLREI
VRIKEQLELIVSELGVPIGSGGAISDYLCAVSRGLIQFVCARQGRGMFSSLTAERIQIHP

GSVMFRQDADFIVAGEIVRTTRMYAMSVSPLSKAQLYRISPELAEQLIAIRQKRPAADRN
LRTPKERGRERIAEKSAQGEALHENGRGKRKSEKQRVEVSTGRKLDTISLGGEVFPIEGK
NQAKRTVLLDWERLSKVAPLIGPADEEFGKGLRARVEWGKVQLFNGEKLSTVIKALKWID
IGHDLTANWPQNTNFSLEGRTDSAEQTIAALAKSLEMILHVTKFGKSGKFGFIGLYSDGT
GNFWYQPTRSFSEALNQSLASTELLVDYAEKLSVSASLKEKISALYRRLSDFFNAL*
>SPBDM4_v1_70151|ID:27159609|xseA| Exodeoxyribonuclease 7 large subunit [Uncultured spirochete bdmA 4]
MEFDGALKDFESSLSPLTVSQITSLIRDDLENKYPDITVEGEISNCKTAASGHLYFSLKD
EQAVLQAVMFRRDMLSLSFVPRDGMKVWARGGISVYPGRGQYQLITRSMRKAGLGDILAM
LEERKQRFAKEGLFDESRKKPIPVLPSRIAVVTSPTGAAVRDVIRVLHRRNPKVSILVLP
ALVQGEEAAISIAARIRQANLWNLADVLIVGRGGGSIEDLLPFSEEIVVRAIAESHIPVI
SAVGHETDWALSDYAADLRAPTPSAAAEMACSDINLIYKEITHFETVLAQSMQQALVHAQ
QRLSAVSPRNMEGILVRRHLLLEQQFDTAIETIRQSMQKKMDACSNRLTLASSAVELSNP
RAIMKRGFSVVEKQSRTRPQGQNGEIEIVRSAKTLTAGDEVHILFYEGEADASIQGTVPG
DGRSKEHERL*
>SPBDM4_v1_70152|ID:27159610|xseB| Exodeoxyribonuclease 7 small subunit [Uncultured spirochete bdmA 4]
MKDFEKKIARLEQVAEKMRDSDLPLEKSFSLFEEGVSLARELKGELDTLQGKVEILLNSL
EEMGTEQSTSPRTADFETTEAEQQSGSEKESE*
>SPBDM4_v1_70153|ID:27159611| CTP pyrophosphohydrolase [Uncultured spirochete bdmA 4]
MGSKKGKDTPISLHRSVAGIAVHGELFFIARRRQDASEMSQRWEFPGGKVERKESDEAAL
EREFLEEFNAPIRVLRFLGESVFANKGKARALAAWEISLDPTNIAILNEHSEAAWLPLDT
IANMELADSDRSLIPIVQATLKVNR*
>SPBDM4_v1_70154|ID:27159612| putative Carbohydrate kinase family protein [Uncultured spirochete bdmA 4]
MRPLLSTSQSLELDAFTREVFDFSSDHLMEIAAMRLWQVLKLEIIQKRLNLCQPAALRDP
ERRVSIAAVCGKGDNAGDALAVLRHAKLEGFQNLAAFVPEPDTLKENARLNLRRAEQCGV
EIVRYETAEEGALQGKLLRHDIILDAVLGTGAHGRARGSAARAMQMFEVLDQTQAKNGDK
APCIVAIDIPSGLGDDWQPDYPIVRADATLCIEPMKEALYMPAARSFAGDIVPVGGIFPL
WADAAASNVWLLEHSEVRDFLPPISKWAHKMQRGRVAVLAGSQRGAGAALHCVRGAAAAG
AGYIALYCDEEMFSSYLATVGDVAIVRVLTEDTFLPESWDAIVAGPGWGTDERREEVLDQ
LIHSDTALVLDADAVRLFARRAGQNRDRPRFFKAPVILTPHPGEFRELLPFLDSGESSSA
CNVSETVGQVASLAKKFGIILALRASTTHIAFPDGTCAIFDGSASGLGIAGSGDVLSGLA
GGLLARWIAFSKENGAGKEYEDNKLWHNNLVQAIIGAVLVHGGAGRKLSQTKGWFTPADL
AHACARMTSESKLG*
>SPBDM4_v1_70155|ID:27159613| exported protein of unknown function [Uncultured spirochete bdmA 4]
MNRISRFMSACVLIVLFSGCSASTDEAKTKILFDLGYGKSDSSLDLSGGEKNAVDISLKK
GIFHLLSRGEAKILRISSYGQTLAMWYDPRKGSAPLVLKDVQINDLSAGKELGRFAMQVP
FNDPWPMAADSRQLLYVADNRFIRRFDSDGKELQPLGQEGIGGSMFPMVTALDTLENDDL
AVLCASESETSVYFYDKNAVFLHVLKLNDQTMPVPSSILEKNPDTKGSRIIASLESISPS
YFHGRMVVVLKINYYMEKYDPDSGVTLSIDPVGGWIIGIDAGNGTILDSFPLQSSVNDLG
IDQQLIGAKSNSILSVRWDDKGIGGEVFRYNQKGKVIGKLNFLVPDPEAALVAMIVGDDD
YLYLLGQLPSTLRMYAWKLPSMGK*
>SPBDM4_v1_70156|ID:27159614|secA| Protein translocase subunit SecA [Uncultured spirochete bdmA 4]
MSGNILAALLGSKKEKDIKAVLPILHRINALEPWAFSLSDSDFPAVTNKFRERLAQGETL
DDLLPEAFALVREAARRTLGERPFDVQLVGGIALHQGKIVEMKTGEGKTLSSVTAVYLNA
LTGEGVHVITVNDYLAERDSQWMGQIYKFLGLSVGCILSSMDNAARKISYSRDITYGTNN
EFGFDYLRDNMVWSLDQKVQRGHSYCIIDEIDSILIDEARTPLIISGPADDDTYKVNEVN
RLALSLVEVKKNPETGEYPDEARGELLEGDFKIDEKSKRVMFTSEGMNHIESLLQKRGLI
KGSLFDPESFEFVHYFTQAVRSQRLFSKDVDYVVQENQVQIVDEFTGRILHGRRYSDGLH
EAIEAKERIKIARRNRTLATITFQNYFRLYKKLAGMTGTADTESEEFNKIYNLDVVVIPT
NRPVVRADENDLVYLNEAEKFDAILNEIKTMYAKGQPILVGTVSIEKSEKLSAMLTRNGI
RHEVLNAKNHAREALIIAEAGAKGAVTIATNMAGRGTDIKLGGNPEFRARRKAGTEAPEE
KYREALAREYEIWVHDYEEVQGLGGLCVIGTERHESRRIDNQLRGRSGRQGDPGRSVFFI
SLDDDLMRLFGGENFKQLMSKAGMKPGEPIYHPLLNKSIESAQKKVEQRNFEIRKHLLEY
DDVLNKQRNFIYEQRNSILSDNNLIERVREAADEMLEDQSELLAQAVKDNPSKAIPDFCS
WLFDTFGIDLPAKQAAELYQSGNLTQEVQRILNLDLATKIEAAGTDSLNFFIRFSYLEEI

DEKWLDHLESMEALREAVYLRSYAQKNPLLEYKLEGSDIFESLILSIRHNIASKVFRVKI
KESAQAKETQTPRAPRIEAAQTSHQSVGSFAGETGEAHKESAIAAASAPASATVVRQGQK
IGRNDPCPCGSGKKYKYCHGR*
>SPBDM4_v1_70157|ID:27159615| putative Fibronectin-binding A domain protein [Uncultured spirochete bdmA 4]
MSLNCEEIDRILSEAPLAGTKIQNIYQPSFDSLVLELYGRGALIHYFISIAHNACRLHPL
SAAPPKNERPLRFMECLRSRIRGGTIVRAAQIAKDRIIKIEVVRADENGLPTPLYLYARL
WSGAGNVLLVSKEGAIIDALRRLPSRNEISGETFSLPPSRENVNPLKQYAIRDLGGEGPF
WQRIEHYYSQSTGHLSREVLLAQAHEILGHKKAAIENRLAHLVSKQEEYLQAERFRQIGD
ILIAGYTIETQGTKTFARTFDFYENKELLIEIDSRVNPAQNAATYYEKYHKAKNGIDEIN
AEIHRWKATIRQLDAWFARLDAETDPLVIAQALRKGGAARSKEKPRYPCQSLEYRGWTIL
IGRSGKENDEILRHIVRGSDLWLHARDYAGSYVFIRAQKNKSIPPEVLQAACRLAVYYSK
ARKNLGGDVHTTFVKNLRRAKNGPVGLVIPYLEKNLYIIFTEDQIREILEENQLEEGNSP
*
>SPBDM4_v1_70158|ID:27159616| conserved exported protein of unknown function [Uncultured spirochete bdmA 4]
MKRAQSIFITVLLIISISMVMASPKNKITISVLKGPSGLSSAWMMSELSKTSPDEFEFIT
VSGADMAVAKLLNGEIDAGVLPVNVAAKLYNSGVPIQALAVVGNGMVKFLTTDPTIEGLP
DLKGKTIYIAGQKATPDYIFQYLCARRGLIAGTDYVPVYNLAYPEIAAGLASGKISYAVL
PEPFAAQAILKNPAIKIPIDITKEWQSATNQSDYPMSLFVARSSLVESSPDKIKILIDSY
RASIQKAIQDPSGTGKLAESLGLGISAQIATIAIPISNFVFIEAARATPNIEALLSVFLQ
FDPVSIGGIMPNRSFYAQIQ*
>SPBDM4_v1_70159|ID:27159617| Putative ABC transporter, permease protein (fragment) [Uncultured spirochete bdmA 4]
LLTKASFWSSVAGSFLRVLEAFFLSILFGLVSGTMSGFKPQIKAFMSPFITGVRATPVLA
LILLAMFWFPSSQVPVFSAILMAFPIMHTSTESGVHAADQKLVQMSNLFHVPKSTVFWRL
RLPSALPYLLAGAKNALGLSWKVVVAGEVLSQPRMALGTGMQDARLTLETPSVFAWAITT
ILFCGLSEYFFGVFANKLSLQFQAAQDEETERGKQ*
>SPBDM4_v1_70160|ID:27159618| ABC transporter related protein [Uncultured spirochete bdmA 4]
MSAYVKSLKKSFGSLNVLENISFELKDSSIVSILGPSGCGKTTILNIVSGITKADAGSLV
GFEHKRFSYCFQEPRLLGWLNAEDNLRFALSSLVQSHKDSRDMEMRIERFLREAGLWEFK
RYKPNQLSGGMQQRLALARTFAFPSDMLLLDEAFSAVDLKQKIELMQAFLRLWTDERPTV
IIVTHDIHDALFLADQVVVLSQRPAHVAGTLNIDIPHEKRTFASSELSKHEMELYRLLGF
GHHDNSAQS*
>SPBDM4_v1_70161|ID:27159619| putative Multi-sensor hybrid histidine kinase [Uncultured spirochete bdmA 4]
MPRIAVLEPDSIIALDIAKSIERERLAEVNLFYEVEEILGNQRKLDYELFIVDIGDNQQS
KIDAAIKIHRDAGIYCLVISDHSISASLTKLREAEPLGILVKPFSSRELVANVETALYRA
SMEKRLRDSERRYRNLFAYSLSARCVADPSGSIIERNKAFEASFPATEKIENIKDMFLEE
EEWPKILDSLQRDHVFQKELLTHDFGQGQRDVICNFSFFQEELNKINILCEFIDITESKR
LREELSQSQKLEAIGRLASGIAHDLNNFLTSIMGFLEMVKMEIPEDNAALEDIHGIEKVI
QKTSVLTRQLLGFSRPKSYSPTTVDLRETLNDSRKILKRLIPERILFSFSVPDEPVFANV
DASHIEQILLNLVVNARDALEKTENPRISIQLDLQEDYALIKVKDNGTGIDSRHISKIFE
PFFTTKEDDKGTGLGLSIVKSLTEMNGGQVRVESELGKGTIFSIWIPNLQRTGSFPDTAE
QKEPSSTILDDGCSEILRNKHILIVDDDESILESCKRILERVGADVETCINAGESILLAE
RTEFDLLIADIVLPGIYGTELWARLQKDNRIGACIFMTGYESLQADLSPDTPLLYKPFDA
RTLVEACAKIV*
>SPBDM4_v1_70162|ID:27159620| putative Trypsin domain/PDZ domain protein [Uncultured spirochete bdmA 4]
MKKSHSVLALFVSLGIFIMLNGCVSSPETPSSVDPSQAVAGRIGQLAETDPEQSIAILSD
VFEIQREKGEPLGNSVIGEQALALFRTSADNIVTLFKKAIQENDLIAARRLSFSVSSIIR
YASLYDSLEKMLPREFSAIGAPAMRLQLAFLEAEEKFQKHGYVAGKTSLSAALMAEPGLS
SLSAATNSIENLKSVINTKEPLSYYRSWAERAKENQDSDAEAWFSALASNKDLGSNSVHV
PSDDWLSGAVASVVTVYVDRGFKIQSGYSVPDRVLGTAFQISKGLYLTNYHVIQSEVDPT
YKGYSRISIRPSENPQIKVPAKVIGWDEEMDLALIRSEETAQGTIFLPATMQFQAGDRVF
AVGSPVGLENSVTAGVVSSLSRKIISYGEAAQIDVPVNEGNSGSPLFSANGVLVGMVFAG
LPSFQNINFALPAQWLVASLPALFDGKAAIHARLGLMLGKGSGQNPLVLADLDSSLSAFR
AGDVLTMINDGLSKDIASIQLQLAAIPKDALCYVEAQRNFQPIRRLRRICFTDDASLIPA

WDSIQKNTILEGLLGAKLTQLENSNKVGGLYSVQWVCPAEAADEIGLSENDTIKINKFQL
DRKNKMFILEFSAKSRLSGYFERTIRLEFSSDSATII*
>SPBDM4_v1_70163|ID:27159621|ftsH| protease, ATP-dependent zinc-metallo [Uncultured spirochete bdmA 4]
MSDQNDNRNDNRPELPQAPKGNRAALAVFFSLIMLFGVYLFFGQKENTKEIPYSSFLSYL
DLDEVKYVRIIDQRDIDGALKGKNGAEMPFTTRIPYFDSELMSKLQEKDVSITGAISGTS
PLQILFELTPWIFGFILIWIMMRQMQGNNKAFSFGKSRAKLYNDSGKKITFNDVAGQKEA
KYELMEVVDYLKNPKKFVKMGARIPKGVLLVGMPGTGKTLIAKATAGEANVPFFHMSGSD
FVEMFVGVGASRVRDLFEQGRKHAPCIIFIDELDAVGRTRGSGLGGGHDEREQTLNQMLV
EMDGFDTKDGVIVLAATNRPDVLDPALLRPGRFDRQVVVAMPDVQERADILKIHMSKVPV
SGSVDIDKLSRATPGTSGADLANMVNEATLFAIRKNKEIVEMDDFEDARDKILMGVARKS
LVIADEEKRATAIHESGHALLHYFLPNSDSLHKVTIVPRGRALGLALSLPGKDTYSRTYG
WLYDRIVISYGGYAAEKIVYGQTTTGAAQDIKQATEIARKMVREWGMAEGIGPISLGEEE
EPIFLGREIAQHKDYSEETARKIDAAIQGILNSALEKAMTILKREKDRLLALADKLMQIE
TLEDKDVRELLSLPEGKVVLEEGTVKC*
>SPBDM4_v1_70164|ID:27159622| protein of unknown function [Uncultured spirochete bdmA 4]
MKNLEIVVKKKLFSLDFDREKPVALAFSGGLDSTVLLNILLQIVDNKKLRAIYVCHNLRP
KEELAKEILLIKKTCQIMRVRLTLVYIREGAIAEYAKQARCGIEAAARRFRYHALARTAQ
RWNISTIITAHHADDQIETFLMRLLRGGNLGALAGISPSRAIDKTSGVRVVRPLLQIERR
ELYNYAEKKGLEWSEDSTNEETIFLRNKIRHSLIPYLDSEFPSWKCSVIGYAPQIRNTQA
FLHTCAEKHMHAMQKEYRGRPVLDLNLFKKREAVIRLEILKNFLQNFIYKHHIGYHALQN
LDRAISDGALKAEAGGYEFDLTGGFLQFKGRARFSSAPSTRGISFLLDSYREDQYFLKVQ
APGKYECGAFKLVVAYDSSNTNIPGLAHIVIDESEKIASVKIPISFPFVFRNKKEGDIIY
TEAGIKGIDAIIKKSKIPEGTRHLVPLLEDKYGIAAVLISTFVKGSTLNAVCRNPSLGEN
GEIVYILLLMKGDHIINV*
>SPBDM4_v1_70165|ID:27159623|rplY| 50S ribosomal protein L25 [Uncultured spirochete bdmA 4]
MEHIELKVSPREKLTKGELNKARKSGRVPAQLYGKEIAPMSIFLERDDFVNVARRVTESM
IIDLNLEGKKYAALMKEIQKENISGEILHIDFNLIERGRRIRVKVPLHLVGSAKGVREGG
ILEHAIHDIEVECEPDVLPEKIEIDISGLEVNHALHLRDVAIPEGVKLLTNPETVIAIIK
FARAEVEQPAQPEVAEAEGEVPEQAAAEGTSEAVQK*
>SPBDM4_v1_70166|ID:27159624|spoVG| putative septation protein SpoVG [Uncultured spirochete bdmA 4]
MEVTDIRIRKVSSEGKLKAYVTVTFDECFVVHNVKIIEGKSGMFIAMPSRRTKTGEYKDI
AHPICPDFRSKLQEKILAAYNSDNPSDTSVPEFD*
>SPBDM4_v1_70167|ID:27159625| putative 4-diphosphocytidyl-2-C-methyl-D-erythritol kinase [Uncultured spirochete bdmA 4]
MKGVEIAAFAKINIGLQVFERKPTGYHDIESIFQNVSLADSLRLYINGLGLIVIEGELGG
CPPDNSSLYHAAMAFDAACGGLMCRKGVSIHIEKGIPAGAGLGGAGADAAATLWGLNELF
ETRFSQVELARLGERVASDVPFFLFGGAAIVRGRGERISPISPSTDFGLIVLQPPWTSQT
PEAYMALDSLRDEKSAPHSRPAVIHETSYASCLSDGELETWYRSPIAQWQFKNDFQPVLV
KKHPLYQELFSILKEHGAAYVSLTGSGSCIFGVFESFDRAHKVAEKCKRISSERAAGDQW
HAVNIFTVQPLARSMIVSYIQDCNEDTRSDKERPCYGSN*
>SPBDM4_v1_70168|ID:27159626| protein of unknown function [Uncultured spirochete bdmA 4]
LNLSIELTRSLKSASAFHLREQQAINGTL*
>SPBDM4_v1_70169|ID:27159627| protein of unknown function [Uncultured spirochete bdmA 4]
MNHKTTMTADPQEEFELITLTSGNSADTIYSLRPTVRFDGAYDDEFDYDNDDDFEDDEDL
EDDEDLEEEDDFEDDEDIDEDLDEDEEDFNDEEDFDDEDFEDDDFDYDDDTDE*
>SPBDM4_v1_70170|ID:27159628|rsfS| Ribosomal silencing factor RsfS [Uncultured spirochete bdmA 4]
LDSKAFAYQSAEILAQHNALDVLVMDIREVAGWADYFVLATSTSSTHMRGLQKHIEEFLK
TEDISLLNKPEVSDDQRWLLMDAGNVVIHIMSDEARQFYDLESLWFNAPRFVVTFSEKRS
*
>SPBDM4_v1_70171|ID:27159629| conserved protein of unknown function [Uncultured spirochete bdmA 4]
MSDLKSGMLRRSALPLVLILLVVAIGTFFVVRLMQKGTVDTIIKKDQPMGILFIFEKDEK
PVSNQLLIWYPSRRKAAIMDIPGSMGIILKSADKMSGIDSVYDSRNPGRFVKEISDYLKF
PINGWLTYDEKSLAKTVDLLEGIQIFIPEPVMNNEALKDVSLPGGAVVLDGDKTSQYLSY
SLAADSYSEEINRKQRMLVSLLAQLAQESDIINKNNDIRLIAKEPRSNFSLATREELFRH

FAQIDVDMIITQFISGSFRTFEGRRVLFPYYDGELARDVVSQTAKALGAEDTNAMQKTVV
TIELLNGSGEKGLANSASTLFESYGYQVVSIGNAPSFDYAKTVMYDNDGDKTAFQQIANV
INCKNFGDAAVLPGLRKANITVILGKDFNGRYCVGQ*
>SPBDM4_v1_70172|ID:27159630| conserved protein of unknown function [Uncultured spirochete bdmA 4]
MLSDRFGVDKEASYLAGLSHDMCRELSFEEQEGLVDRHGACIAFLRERPSMKALFSDTVY
KKKMMHGPAAACMLCHEFGMREPDILEAVTLHSIADEKMSDLAKIVYISDKLEPLRNRPH
DADEKLHTLDLDSLFVYTIACVVRWFSETSEPLSPFTADLYSRMLK*
>SPBDM4_v1_70173|ID:27159631|nadD| putative nicotinate-nucleotide adenylyltransferase [Uncultured spirochete bdmA 4]
MRIAIVGGSFNPPHIGHLILAEEVLATRNYDKVLLIPANLPPHKVPQHDPGAKMRLVMLS
ASIEGWQEIIVEPCELQRQGISYTIDTLREIPQKYNCDGKPGLIIGDDLALDFLTAWKNP
EKILDLADLIIAHRLYAEKVNLAYPHRYIDNIIIPISSSLVRERIAQRGAWRSLVMPRVR
ETIETHGLYRNT*
>SPBDM4_v1_70174|ID:27159632|obgE| GTPase involved in cell partioning and DNA repair [Uncultured spirochete bdmA 4]
VIKFADEAIITVASGKGGDGCVAFRREKFIPLGGPAGGDGGRGGDVIFVLKRNLRTLAYL
RYRQVFKAQNGQPGMGKNMHGGDGEDVVIEVPPGTIVRDADTDEILKDFSARRGRDGNSA
SSDEELWFFLKGGKGGWGNTHFKNSVNQAPRFAQPGQPGQERRLKVELSLIADIGLVGFP
NAGKSSLLDYFTNARPKIAPYPFTTKIPNLGVLTVNERDVIIADIPGIIEGAHEGAGLGL
RFLKHVARTACLAFLIDLSEENWRIAFPTLLRELDLFSPELAKKPRILLGTKMDLPEAQE
HFAKFKATFPRENVYGISVFSGFGLDALKQVFFEQVVEYETREEGKESIAERLFDEIEFD
DEGLYE*
>SPBDM4_v1_70175|ID:27159633|rpmA| 50S ribosomal subunit protein L27 [Uncultured spirochete bdmA 4]
MPHKGVNGRDSNPQHLGVKAYASEQISAGTIIVRQRGSRINAGRNVGMGKDDTLFALVDG
SVRFREYKGKKFVDVEPAAQA*
>SPBDM4_v1_70176|ID:27159634|rplU| 50S ribosomal protein L21 [Uncultured spirochete bdmA 4]
MYAVVEINGKQYRAEQGKNLVVDRFSATPGESVVFEKVVLLGGENTAVGTPYVAGAAVKA
TVNEETKGDKVVVFKYMPKKGYRKTQGHRQLYTVLTVNEIVSA*
>SPBDM4_v1_70177|ID:27159635|argS| Arginine--tRNA ligase [Uncultured spirochete bdmA 4]
MEHYVDQWKSIIAAHLNAFAREKGFADYQLSPHEIIAEKPPKPELGDVGFPLFSYAKQFR
MAPAQIAFEIADREHDMPGGNIERDWPGNLKAVGPYLNVYLDRTRAVEYLLAHTEKYSWG
SSPFFNGHKIMVEFSCPNTNKPLHLGHLRNNVLGESLARIMKAAGAEVKKVDLINDRGIH
ICKSMLAYLAYGENRSPEDEGLKSDHFVGKYYVLFNKLKEEDPQAEQKAQELLQKWEAGD
PEVIALWKKMNDWAVEGIMATYKRQQVSFDEFHFEHETYKLGKKEVLDGLARGIFYRGED
GAIWVDLEDLGLGKKVLLRKDGTSIYITQDIGTAIMRHERWSFDELVYVVASEQQYHFKV
LFEVLRRLGYEWAANLYHLSYGLVNLPSGRMKTREGTVVDADDLIDELAKLAAHEIVEKG
RMDAVGDIQSVAEKIALGALHYFLLQVSPNKDMLYNPEQSLSFTGDTGPYIQYMGARASS
ILRKYEQGEGNASRGHPEAALLASDADWPLVRQLMELPSTIEQSARNKDPSILAGYAHAV
ASEFSAWYRDNPVLNNDNANLSASRIALVRAVQSTLRQTSELLCMPFLEAM*
>SPBDM4_v1_70178|ID:27159636| conserved protein of unknown function [Uncultured spirochete bdmA 4]
LITRDELEARLQYLGPALRQEKEGTVLAVTVIPHAPHNVVAGFRNGSLLVKVSAAPEKGK
ANEAVLELIADFLGTAPSNLLLLRGHTSRSKIILLQNMG*
>SPBDM4_v1_70179|ID:27159637|ligA| DNA ligase [Uncultured spirochete bdmA 4]
MRDILRVKYGATALRARELEALIKHNQELYYNGEPEISDEEFDALWDELASIDPGNPLFA
SIGADRSERWPKYRHRMTMGSLSKATDPESFLVWAAKAGYRLYLVQYKLDGASMELQYDD
GRFVRGITRGDGEIGDDITQNVMRMNGVPKKLAEPFTGAVRGEVLMSRNIHATKYADKAN
CRNAANGLMKRKDGKGAEDLDIICYDAVGSLPAGAAFSTEREKISWLQRMGFNVVPTTER
ESPEEVVAYRAKIMDIRAALPYDIDGLVVKGDRIDPKDASKLRPELQIAFKFSPEEAVTT
LIGVEWSESGATVTPIGVVEPVRLAGTTVQRANLANPGMIRAMDLRIRSKVVITKRGEII
PKIESLVENPPDAMPIPIPTRCSLCGTELIDEDTRLYCPNPECPGKAYHRLEKWLSVINI
KDIGSALLKRLFDAGKVVHIPDLYLLTVADLLSFERMGEKSAQKIIRNIKAQSEVSLSQF
LAGFDVEGIGMLMADRLIAAGYDTLDKILLAPWSDFADIDGFAEVTAKALYNGLRLVEGD
MRELVDKGFVRIQTPKSAVSANASPVAGKNFCFTGELTAMKRSEAEKLVRDNGGFVKSGV
TKDLDYLVTNDPASGSEKNRKAQEFSVQIINEAQFLAMLGREVH*

>SPBDM4_v1_70180|ID:27159638| NGN domain-containing protein [Uncultured spirochete bdmA 4]
MRYFAIQIATRREDDWLKRIQPQIKDVKFHKIMKKMYIRRKGKTKLEDAPVFPGYIFFEN
EDDSLPTDIIHDLRHSRFFIRFLPNNESPRSLNNRDSAIIRHFIHFGSLIPPSLVKFDEN
QKIKVIQGPLQGIEGFITKVDRRKHRAKVRLTIAESVMILDLAFEVMEGETIGAGSQDTH
HEKQ*
>SPBDM4_v1_70181|ID:27159639| protein of unknown function [Uncultured spirochete bdmA 4]
MKSNESIPFEQISVPGMLYACMVRAGSDIGQIRAIRTPPLPERYSLITADQLPFRNELWI
GDMSIPLFAARSVSYRGEAVGLIIGPDPLMVEELASTSVVECEEREPFLDWHSFSSSHIA
ARIDFSRRKPAESDRSADKASLGERSTPAKTLEFSSYLNIEPHELTFHSGIGALAEWDYD
KLKLACPTLWPEHVRSCIARMLDASMDDIEISMLQMYDSSELYSWYPSLLAARATAAAWA
LKKPVKLIVSGKREKKFLPCVHGISLHLKSKWSKDTRQLLHVECRFAIPIGAYSIFAQLL
LEKTAHFAAGSIPETPISITGFAIRTDMVPMGALESISAAAIYGMIEAHIARASRAMDSG
LLELSQSTFSKKEFSKNAHKNAEKEIPAVKIARPLLRQTDFYRKYAAYEQIHKRNPGEKE
ATLRAVSFSLAYQNSQALLPAGAHKVQIQLMLDRNLKAVLESDAAYSSERLKTALRLQIS
QNLKIAAAHISFENMQRPHPGSIPLISSSGMAIVSDLARRASDRIQRMRFREALPLAVRT
YSIVKQSETLKKISGSPLRPSIGAAILEVEYDTRTAIMQQVRLDISVFAGKILSRALAKS
TIRSASIEALRSCLVCTYPEQKDHNDIYDSILSQSIINVDIMEDEKASIPRPLGDLAYAL
VLSCFLGVLQQMNDSNRLMLPFRPASSVSVSGDMR*
>SPBDM4_v1_70182|ID:27159640| conserved protein of unknown function [Uncultured spirochete bdmA 4]
MNIEFNLNGSTQKVSITSAERALDVLVRQCNIKSLSGACLEGQCGSCLILIDDRPAYSCI
MPALLLRDRRVETIENFTKTREYSLLYSEINKGGTAVCSSRQNAIMLMGMALVRRTLAPA
QDEIFSTLANAYCDCLAPEDFANAVLNTIQHLATARKTP*
>SPBDM4_v1_70183|ID:27159641| protein of unknown function [Uncultured spirochete bdmA 4]
MTLSEIHYPHNLQELLLLLEREKKVQIIAGGTTFGYIQASRYLSFEPSIAYIQHIPELTS
IHKTERMISFGAACTLSELEHVYPFEHKQTKELMHTIATSAVRNIATIGGHLMYEKRFLT
LWALLACLDAELEFKAFSKTSSKNIWYLANESGLPAIESDKLLTRIRIPLQSIDHLFIKR
IGGSIFPDGDGAYLVSAATVDRSSIANFKLVIAGARAYRDYDAEQRIISAPYPLPTKILQ
NVLRMYAESLRKSNFWNVELLLPLISQALEDMQRSNR*
>SPBDM4_v1_70184|ID:27159642|mfd| Transcription-repair-coupling factor [Uncultured spirochete bdmA 4]
MKPDSLQALFDKISRNSALRMVREQLERGTLPVHIQNSENPLLAFSLVPLVRHTGKKCIV
VLPSDQEAEDFGQDLALTDTKLLLFPSWPGAPYRAIPARSRMFSERTSALAKLAEGDFEI
AVVSARTLAIPVPPPEYMRAHTSLLKVGALFEPTAVSNRLAELGYFRVPSVSLPGEYAMR
GEVLDVYMPGDELAVRVHFDFDRIEKIVRFDPETQSGNEKIGQVHIRPLKEVIWDMTALS
YLRQHSLEFAQSEPRLEQVLEALNAGREMPGEEFWFPLAFDRAYSLIDYADSNTVFVVVA
RERIDSLSETIRKEYASMYRLALHETIVPPPHKIVFNISELLSSVSAAIFCYALKGIDAV
AERISLGAEPPRSYFGNIRLFKEELAGFKRDQYRTWIFASTEPQAERIASLIQDDSITIA
QGPISSGFNLPALKLRVLAEHELFGRRKHVPRSLARAKSAAIESFIELTPGDYVVHVNYG
IGRFNGIERLTVLGLERDYIRIDYAGDEKVFVPIEQANLVQRYIGNEGEAPRLDSLGSKA
WENRKKRVSKSVEELAERLIRIYARRKAARGFVFPPDSDWQIQFEATFPFEETEDQLRCI
EDVKHDMESPKPMDRLVCGDVGFGKTEIAMRACFKAVTAGKQVAFLAPTTILAEQHYENF
LERIGEFPLRVELLSRLIDKKTQNAIIKGLSEGSVDMVIGTHRILQKDVRFKDLGLLVID
EEQRFGVKDKERLKEMKAGIDCLTLTATPIPRTLHMSLLKIRDMSVLQTPPMERQSIQTF
VEEFSLELVARAIRNEMARGGQVFYLHNRIETLPEVEQFIRQLVPEALVESAHGKMDARD
LESIMHRFIHGAFHVLVSTTIIENGIDIPNVNTIIIDRADNYGISQLYQLKGRVGRSDRQ
AYAYLLYPDKRALSELAMKRLQIISDFTELGSGFKIAMKDLEVRGAGNLLGREQSGDIYA
VGFDLYLKLLDEAVSRLSGMGQGEEEPYLELEYSGFIPDGYISIPMIKMEIYKRIASVQS
QEEIDSLHEELIERFGPLPEEALSLLSLAEMRVLCRKLSISSLRERNGIVSVEFSKVSKI
SVEKILRLIRESSGAIRLSPEKPNAIQIQTKAIGLKEKSAYLKERLSFLAG*
>SPBDM4_v1_70185|ID:27159643| protein of unknown function [Uncultured spirochete bdmA 4]
MLVLLLIEENSIANLVLKALHDSEVNALRYRDPLRVLDYIAELEPDAVIVRQKDFPLHEQ
LLAALIQFYKPLQRCKMIVLGKEDAPFSSCTFIKEEAFLKDPSLLLAAFSGATHVSLHGG
SRLVAKAQRMVKE*
>SPBDM4_v1_70186|ID:27159644| Regulatory protein ArsR [Uncultured spirochete bdmA 4]
MDTTPKPCNEKQLLPYAQKYKAIGHPIRLKILCLIAQQDVPCVGDIWRCLDQPQPVVSQH

LAILKRNGIVASEVKKTRRIYSISDPFIKNLIDSMITEICADSDDASTK*
>SPBDM4_v1_70187|ID:27159645|ispG| 4-hydroxy-3-methylbut-2-en-1-yl diphosphate synthase [Uncultured spirochete bdmA 4]
MSPTKAIKVGNVLMGAEWPVSIQTMWKDPLPVFSGYDDPRLPPIIERISDLKRLGCHIIR
FAVPDISAAESLGLLAGRSPMPIVADIHFDWRIALRCMDFPVAKIRINPGNIGAEWKVRE
VAAKAMDKGIPIRIGINGGSLPVDLAQEEHDTVSAALKAAEREIEVLESMHFSDIVVSLK
MNQPDEVVRANEEFASRYPYPLHLGVTEAGPLVAGVVRNTAALVPLLKQGIGATIRVSIS
DSMEQEVLAGKEILACAGKNAHGVRIVSCPRCGRASFDTHAFTSRWMNRLYSIDADISVA
IMGCVVNGPGEARHADLGITGAGNSVLIFKHGEIIRKIDARDADAAFQEELSKLTREKSE
QGRLFARQK*
>SPBDM4_v1_70188|ID:27159646| V-type sodium ATPase, K subunit [Uncultured spirochete bdmA 4]
MNIALIGAACVLGLSATGSGIGAGIAGMAAIGSWKRSYLNNKAASFLLVAFAGAPLTQTI
YGFILMGRIINSSKDPLLLLASGVMSGLAIGMSAVAQGKAAAAGCDAYGETGKGFANYIT
VVGLCETVALFVLAFTFSAI*
>SPBDM4_v1_70189|ID:27159647| putative V-type ATP synthase subunit I 1 [Uncultured spirochete bdmA 4]
MIVPMKKFYLIVLDKDRKDVPKRLRKLGVAHIEELQGSGETYQSLERERSEVESAYFLLQ
NYIGKKSKSGSPKSLSPLEVDGVKSLVQEVSGLKREMDSLNERTNQLVREIERVESWGDV
SAAALVEVNNGSGFSLRFFEAPLKEIASLPKELEYMGLCAPKGKLRIALVLGESEKVPEL
PPSFQEFQLPEFSVSQMRQEIVSVERRKKEIAQAFKDRSEKTPFIEKYLQELESDIVLER
LRSGMPNQEHFAYLQGYVPARDCDKFKKIVAKYGWAIAFDDPSDEENPPTLVENPPAIRI
IDPVFEFLGTVPNYHEYDISFWFLIFFALFVAMIFGDGGYGTLIVLASLGSIVLGKRKGR
HASDAQKLFLVLGIVTVLWGALTASWFGIRFDQLPKMLQKISLNLINGQSPDSESNIKVF
CFIIGLVQLSIAHMKNIRRDFPNLKFLSQIGSLLLLAGMFNAALNLVIDATRFPIRSWAL
WCIAAGFFLVFLFGNWNGKLGPSLLESLKGLIPTFLNTVSVFADIVSYIRLWAVGLAGLA
ISQTVNGMASGILGGAPGILLEFILKLLIAILLLLVSHSLNFMLTVLSVVVHGVRLNMLE
FSGHLGMEWSGYKYDPLREQNRPSAAMESSLEETGV*
>SPBDM4_v1_70190|ID:27159648|atpD| V-type ATP synthase subunit D 1 [Uncultured spirochete bdmA 4]
MAKIKLTKNELKKEKDALKMYQRYLPTLQLKKQQLQVEVRSVQTRLEQLEARKATLEKEY
DVWVAVFSEQQKARTLDGEPLLRISSLKTDTGNIAGVAIPIFQDVEFERLAYDLYATPPW
VDSALTLMEQLLRTGMELEVVSRQGDLLAHELRITTQRVNLFEKVKIPETIADIRKIRIY
LGDQQTAQVVRGKIAKRKVEGAER*
>SPBDM4_v1_70191|ID:27159649|atpB| V-type ATP synthase beta chain [Uncultured spirochete bdmA 4]
MNKIYSRIESIVGNVIAVKAQDIAYGELAQVKTRYGMSLAETIRLDGDMVYLQVFAGGRG
VSTGDEVRFLGHPMQVSFSDNLMGRIFDGAGEPRDGGPVLSDNLITIAGPAVNPYKRIIP
REMIRTGIPMIDVFNTLVKSQKLPIFSVSGEPYNPLLARIAMQAEVDLIVLGGMGLKYDD
YLFFRDTLEQGGALSKTIMFVHTAADPIVECLMVPDISLAVAERFALKGKNVLVLLTDMT
NFADALKEIAITQEQVPSNRGYPGDLYSQLAARYEKAVDFSDAGSITILAVTTMPGDDVT
HPVPDNTGYITEGQFYLHGGHIEPFGSLSRLKQMVNGKTRDDHRSIMDAMIKLFAAYRDT
LEKKSMGFQMSAWDRKLLKYGELFEQRMMDLSVNIPLEKALDQCWGILAECFEPRETGLR
SDLIQKFWVGNE*
>SPBDM4_v1_70192|ID:27159650|atpA| V-type ATP synthase alpha chain [Uncultured spirochete bdmA 4]
MTEGFVTAVNGNMVSVNTDGIVTMNEVAYIETGDRSLKSEVIRIRGDSVQVQVYEITKGI
RVGNKVRFTNDLLSVELGPGLLGQIYDGLQNPLPKIAERVGYFLEPGLYLKALPRDVKWH
FTPLVKEGDLLQRGMAVGWVPELHFKHIIMLPFDFYGEWSITKILPEGDYTIDDVIAEVA
DVKGNKKQVRMWFSWPVKRAVDCYSERLRPTDPLVTKTRIIDTFFPVAKGGTYCIPGPFG
AGKTVLQQVTSRHAEVDIVLIAACGERAGEVVETLKEFPQIIDPKTGRSLMERTVIICNT
SSMPVAAREASVYTAVTIAEYYRQMGLDVLLLADSTSRWAQAMREMSGRLEEIPGEEAFP
AYLESVIAAFYERAGIVKLYDGKTGSVTIGGTISPAGGNFEEPVTQATLKVVGAFHGLSR
ERSDARKYPSIHPLDSWSKYEGIMPKHAVQYAHSFLERGAEVGAMMKVVGEEGTSLEDYV
VYLKSEFLDAVYLQQNSFDPVDASVSPSRQKYVFKLVLRILASRIVFQEKEDARNFFYEL
RQTMLDLNGTPEDSDMFDSLYKKIEQALAERKPEFETRGLKAIRTLE*
>SPBDM4_v1_70193|ID:27159651| conserved protein of unknown function [Uncultured spirochete bdmA 4]
MKRYWYFASTLTSFPFGAPPPISVEEFDVLCSRLVEKDEMEFIRHVEEVRTGEYSDCIEK
SSFLKAYFEWERGVRNALVMLRARANKWDAEQWTRPGSISADALQAAQTVYAASNPLQAE

LAFEHERWDAIERLSSLSAFELDSILAYKMKLLIATRCVSFDKERGREGFKVLYQDIMNA
AAGAASSALDTGVAT*
>SPBDM4_v1_70194|ID:27159652| putative V-type ATP synthase subunit E [Uncultured spirochete bdmA 4]
MEIQVQELLERIRSEGIETAKQQAEEIIQKAQSEASEIVSRAKKEAEEVQDEASRRIDSM
EAASRASLLQASRDTMIALKQSIQRFIDAAISVDVDNAFDEKMAIQVIPEVLKALALNQS
VDTEILLPPALIQKIDASLSARLAKELSKGVTFKPYPSIDAGFRVAQVGSAAQYDFSAES
LAQILSARVNSLLSEYLKEASGSLE*
>SPBDM4_v1_70195|ID:27159653| protein of unknown function [Uncultured spirochete bdmA 4]
MGVEPTSPAWKAGVIAVIRRSRYKIRRFSRHFKKVTLVPLRLLLTGGTVAPECAPSRYDG
RDQNASLFATCPKANPMDEGDLIVWRSSCQVVDKP*
>SPBDM4_v1_70196|ID:27159654| protein of unknown function [Uncultured spirochete bdmA 4]
MTAIRVVHDTSSFSLDFWNISNRIYYVLKNGQSQVENNFYSITF*
>SPBDM4_v1_70197|ID:27159655|mgtB| Magnesium-transporting ATPase, P-type 1 [Uncultured spirochete bdmA 4]
MTKFHLPLSFPQKKGERRNGNISSEEENRLISLCATSIERAFSELDTSPRGLSEKEADRR
LSEFGRNEVSQLKELSFWEDIFERFKSPLVIQLLIIATVSAIVGEATSAIIVGMMILLSV
GLSYILDGRSNREVKSLGKRVQSRTYILRDGTETEIRMSEVVPGDVVLLQAGAIVPADVR
VISAKDFFVSESALTGESMPVEKSANPSSAPVRSALEMPNACFMGTSVTSGTARAVVVTT
GTHTLFGAISKKLSERREETSFDKGVRSFTWLMIRLMLVMVSVVFLIVGLTKGNWLEALL
FALSVAVGLTPEMLPMIVTVNLAKGALAMAKKKVIVKKLPSIQNLGAIDILCTDKTGTLT
QDRVVLEHHVDIIGNKSEEVLNYAYLNSYFQTGLKNLLDRAVIEHVDLNVDECRLVDELP
FDFQRRRMSVVVEYEGDNVLICKGAVEEIYSCCTHYQIDEEIYPLIDMIRADLFEEVQKL
NEEGFRVLGIAYREFTREKMTFTVQDESQLILLGYIAFIDPPKESATEAIQLLTKAGVEV
KVLTGDNGLVTEKVCKDVGIPFSSAITGAELNALDRAAFSTKVKECEVFVKLTPSQKEEI
VRELRKQGHVVGYMGDGINDATALKAADVGISVDSAVDVAKEAADIVLLEKSLLVLEEGI
MEGRRIFANIIKYIRMGASSNFGNMFSVVGASYLLPFLPMQPLQILTNNLLYDFSQTGIP
MDNVDPELVAKPVKWDINNIKRFMVCVGPISSIFDYATFALMWFFFHANGYLDPVLSAAQ
KDTMARLFQTGWFVESLLTQTLIVHIIRTRRIPFFQSRASVPMILTTLTVMAMGVWLPYS
PLATMLGLVPLPGIYWLWISGFLVSYSIITHKVKRWFLRRFEGV*
>SPBDM4_v1_70198|ID:27159656| putative PTS IIA-like nitrogen-regulatory protein PtsN [Uncultured spirochete bdmA 4]
MDSILDALQEGRLFELPENDKNHALQFLAHIIEAFPQIPTGTDIVGNVMAREKSMHTALG
RGWACPHARVEFEEDLMCVVGWSPTGIDYGAPDGIPVSLITMYLVPSNQRNHYLREVSIM
AKVLKSLPETDKLPSIKELNDVRNYLLDLIAASKETIGPDARARMIRLQSKAAMATQPVS
DLSSLIIEPVSIIAGAGLRPLALTQNLELMNFIEGTAGLTERLDADGSYQNGLWRIVRRS
ATAFQGSRTVFDCIALMPAKTAQK*
>SPBDM4_v1_70199|ID:27159657| Translation elongation factor G [Uncultured spirochete bdmA 4]
MSYTTEQIRNIAIIGHGGTGKTTLLEHILFQGGMISKPETVDSGKTVSDYGDDEIARKIS
VRSSLTHVNNDDCKINLIDAPGAGDFVGEAILAIRAAETALLVIDGKSGVQIETIKLWRL
LERHQKPRMVFVTRLDEERANYEAALADIKEKFRAAPVPLTIPMGEGPSFKGVIDVLNRK
AYMRPSSHDQKEQIGEVPTEFADAVESARGQLFEAAAEGTDELMEKYLLEGELTQEDTLS
GLQKALAAGKIIPAFAGAGLANSGASAFIDFVVHSVPSPLFRAPEKAQDQDGNQTEIAID
PSKPASAFVFKTQIDQFSGRLCYIKVMTGSFVPDMDMVITRDAHKERTGKLYAMQGKKLE
EVSSLPAGDLGILAKITTLKTNDTISQPDQLISYIPLRLPSPVHMLAISAINKKEEDKLN
ELLYKAAEEDLTFRVNYNPETKETVIAGMGEQQINMILDKIKAQSKIVAETRVPRVAYRE
TITKNATAEYTHKKQTGGHGQYGRVVFEIEPLERGKGYEFENRIFGGAVSRGFMPGIEKG
IHQAMEAGVLAGYPVVDAKTAIIDGKEHPVDSSEMAFKIAARGAFREAMRQANPVLLEPI
MNLSVFVEEKYLGDVMSDLSGRRGKISGQNPIGGGIVQIDAQVPQAELLRYAIDLRSMTS
GTGSFEIEFSHYAPISGKIAEDVIKAAQAFKTQEAEEE*
>SPBDM4_v1_70200|ID:27159658|nrdR| Transcriptional repressor NrdR [Uncultured spirochete bdmA 4]
MKCPYCGSLEDKVIDSRTLANGEAIRRRRECLSCGLRFTSYERIEEKPLLVIKRDGRREP
FERQKIERGLVRALEKRPVSQMSIENLINEIEDEAAEHAKTSNEISSEELGRMVLQRLYS
LDKVAYIRFASVYRKYDTLDEFIREIERLEKGQTA*
>SPBDM4_v1_70201|ID:27159659| protein of unknown function [Uncultured spirochete bdmA 4]
VFGSLVLYLVDEIFDGHLGHGSFFEGADKTAFNFLPLKRLAPAVTLNHQKRFLLDALVTC

KS*
>SPBDM4_v1_70202|ID:27159660| SPFH domain, Band 7 family protein [Uncultured spirochete bdmA 4]
MSLIYAGFAVLAAWWIVFIVVAIGRKQMPVFLFLLVLFFAWAVPEVLLIAKKPEIATGLL
VVPFIILIQMMRVIPEYQRGVLFRLGRMQKVIQPGFNLILPFSLDIMRKVDMRTFTIDVS
KQEIITRDNVAVIVDAVVYFNVFDPVLAVVKVADYTKSTSLLAQTILRSVLGQHELDEML
AKRAELGQILQKLLDEATDPWGIKVSAVEIKAVELEDTMKRAIARQAEAERERRAKIIAA
EGELQASEKLAQAADVLSKSPSSLQLRYLQTLTEIAVEKNSTIIFPLPMEFLNAVSKLAG
KGAE*
>SPBDM4_v1_70203|ID:27159661| conserved protein of unknown function [Uncultured spirochete bdmA 4]
MNFSEKLKKFMGNSVDASKEILEKAADQAQVWGEMGKLKIEILQLRSKAQTLTARLGTEV
YTLLVEKNEPVIGSSTPEIEPIIKELEELDHVIDEKETLYKSKGGKESDLNEKVKE*
>SPBDM4_v1_70204|ID:27159662| Fmu (Sun) domain protein [Uncultured spirochete bdmA 4]
MAGFRDFDAFYKTLYGDRWESLRRALLGHNDAVGLRFNMKHEIEFLENPPEGVYRMDRAS
VQAAKALHLPSEGTVLDACAAPGGKTLVLAGGSGPQVHIEANELSAERRRRMREVLKAHL
PEAIRSRIRVTGYDAARLCRIRKDWYDAILLDAPCSSERHVLNSPHALSEWTGARTRQLA
VRQWSLLSSCFLMLKPGGCLVYSTCSISRLENDGVMQRLVEKYGDRCNIVNGEWGERTAY
GAIVLPDTSSGAGPLYVAHIHKKA*
>SPBDM4_v1_70205|ID:27159663| HAD-superfamily hydrolase, subfamily IA, variant 3 [Uncultured spirochete bdmA 4]
MTSKVPDLVAFDLDGTLADTIEDIAASVNRVLSQFDLPPHETAAYTQMVGDGFRSLIERA
LPPEKLNDEALLQQVFECAVHEYAEHSLENTRPFPGAIDMLEILTQKDIRLAVLSNKPDA
MSKAIVQSLFGNIPFVAVWGNSPERPRKPNPSALLEICSLAKARPQQSLFIGDSGVDMKT
AKAADMIAIGALYGYRSREELEKAGADFLISSPLELLSLIGMA*
>SPBDM4_v1_70206|ID:27159664|pgcA| Phosphoglucomutase [Uncultured spirochete bdmA 4]
MNREEIIVRAQDYIAHETDSFFSDEVKALLQKGDWQELEDRFYRDLEFGTGGLRGVIGGG
FNRMNTLVVTRATQGLCDYIKEQFPNKPLSACIAYDSRRKSKEFSLATALVFAANGIKAY
LFPSLRPTPELSYAIRMLGADTGIVVTASHNPPQYNGYKAYWNDGSQVVPPHDTGIIEKV
LKVRTVNIIAEEQARAKGLLVNIDEEIDDAYVRMVKAHLMRPELFAQLGKSVNIVYTPLH
GTGAILFERIMRDLGLNVLTVPEQREPNGEFPTVSYPNPEEAAALAMAIELGKKTHADVV
MATDPDADRLGIAVPDGAGGYTLVTGNQLGTLHLDYIALTLKELGKMPPHPAAIRSIVTT
EFQKAIAEKNGIASFECLTGFKWIADLMRRFEHEKYDFIYATEESYGHLIEQEVRDKDGI
SAAALTAEMTLYWRSKGKSLLDRLEDLYREYGYYEEKGLSFYFEGEQGMQIMKGIMDSYR
KEQPQQFGGLAVIRTRDIKTGIERDRNGKQDKVDLPQSDVIQWRLEDETLLTVRPSGTEP
KIKYYILCHGQSGKLPASKEMVQKQIALIEADIRTVIDPHRK*
>SPBDM4_v1_70207|ID:27159665| putative DNA-directed DNA polymerase [Uncultured spirochete bdmA 4]
MTDEKGAPLWTSLFGERREARTPPAPWYKNEAGVWKGLSWPLPACDEEPWEERHYVALDV
ETTGLNAETGNIVEIALVRFFFDNEGALNEEDRFSTLINPAATIPAQASRIHGITNEDVA
NAPLFKDLAKKIADTCNQRVIVGHNVLFDIGFLEKEFWRANIEAEFQECADTFGMAKLAF
PSMPSYNLGKLAFAIGLGSDAQHRALGDALTCMRLFAASIRVLTNNC*
>SPBDM4_v1_70208|ID:27159666| exported protein of unknown function [Uncultured spirochete bdmA 4]
VWFRIKYGLAIVFFIFIVLGLRFAMLALNPYPDTVGSTQSSRNSSSGHDFYENLSMLEPA
RIHQISSQTAGVIAKNTATEGINMGYDSKSLAAMLIPSSGHILAMLIDDKDSEEYQNLDG
VSYPAKL*
>SPBDM4_v1_70209|ID:27159667| protein of unknown function [Uncultured spirochete bdmA 4]
MNTLRSRYESIFDSLYTSLKDYTPSVPPEWLEKDAGYWRGDVTLPGLFTLPRAEDLDYSH
TFALDIFLKNVEYLPGNVQRGPMIHSLSDGIVVAAEGGWIGFPRTSQSLSFIEGGISPKS
GNGVIVYSPDEQRYYLYFHLYNVLVEKGQIIRRGQALGYGGNTGINARKKGGGDHLHLEI
FDARNGNSFRNTQIIALLRESSRKRPAVSSE*
>SPBDM4_v1_70210|ID:27159668| putative Glucokinase [Uncultured spirochete bdmA 4]
MSKLFLVGDVGGTNTNLALAKHDQSGIHLVRKARFSTQQEKSLLAPVERFLEAAREQGFV
DIIDFCCISGAGPVQPDESIQLTNAPWSIQASELSSVLKIPVKLINDFTALSYAVTLLDY
RDTDAVTQLPHPDGSLYEPRDGIMLVIGAGTGLGVGFVDRIGNNIVAFPSEGGHSEMPCF
DDLSHAVHRWLSNKYGYSAGAELVISGQGIANIFEFVCSDEFRVQMLTSDYAVSHAALTT
GLSETAKHILALPRPEWPANIALHAENDVFCTLAMELFGRLYALKAANLASIFLPAGGIW

LAGGISSKNEAWLLKNARFMRWFEKNYAPHIRQFLAHTPVLIVKNYDISLLGAAVAALQL
SAHD*
>SPBDM4_v1_70211|ID:27159669| conserved protein of unknown function [Uncultured spirochete bdmA 4]
MTEQDIYPALRDDMIAKHVDIRLTLDILNRYNSGELDHFAKIIPDGIPPIDGIGVVDIRD
KSPDRVLFKFPKQKTLANLEKMGILPPNGPHHGKSTLFERKERGETSLLFRAKALEEIGL
RILGRCAYGVLNGGSATSYADLKKNRAIDEALFTALGPLFERFAGQCKEMPKGLTPAYIN
PDGAPGANFLELKMRARLLAARQLHLKPATETPFMPLFQMTSTANHEQLMRAYENIQTSP
FLGSLAAEVGLKPSEWLSGIQPMIAAYTHSAEGRPKRIFDRAYGKEHSALALPGGHGQSF
MVLADILRGLQKKGVRYAMLSNIDNIGAYIDPVELAILAISGKPAGFDFSFKTPVDVKGG
ILVRAKDGSRNVVDIGPAIELREVERLEQEGASILFNCATGIFDLDWLVPHLEELTKNLP
VRFSDQNKDAGTYSQAEQVTWEVVSLLPDFTAFAVDKKRRFLAAKMLAEMLLTSGFGIDD
ESVPSQLRETGKALHEGQTWLLEHVYGLRRVNGRWVPRELARY*
>SPBDM4_v1_70212|ID:27159670|lon| Lon protease [Uncultured spirochete bdmA 4]
MSASELIPIDQILPNKLPVITLVGKPIFPGIFTPIMIGKEPDIQLIEQAISADGMIGLVL
QKEDSDESSSTNLFQVGTAAKIIKKINLPDGGINIFISTFKRFKIKKYISKEPPIIAAIN
YLDDANFDTDEVKALTRALISEMKQLSENNPLFSEEMRLNMINIDNPGKIADFIASILNI
EKKEQQDILETLDVRERMEKVLIHISKEKELLRIQKKVQAEINEKIEKSQREYFLREELK
TIKQELGMAPDARSSDYQRFRDKIDSFQFDGEIKEIVEQELEKFNLMEPSSSEYMVTRNW
LDLVCSLPWNNELSSDFDIRRAQKVLEEDHYGLKDVKDRIIEYLAVRKLKKNTRGTILCL
VGPPGVGKTSVGRSVARALGKQFFRFSVGGMRDEAEIKGHRRTYVGALPGKIIQGLKIAK
SRDPVFMIDEIDKMGISYQGDPSSALLEALDPEQNFSFRDHYLDLPFDISNIFFIVTANT
LDTIPRPLLDRMEVIQLPGYVDVEKIEIARHYLIPRSLERSGLAKNQVKYTRDALLAIID
GYAREAGVRNLEKALDKTHRKIAKSIVIENKQEEKFIIDKHSVEKYLGKPVFRDDELKRA
TRPGMAVGLAWTSMGGDTLIIEAMANPGKEGFKLTGQMGSVMQESAGIAYTYVRNIVAQK
YGIEAQFFESRQIHLHIPEGATPKDGPSAGITMATTLLSLVLNKRIKDRLAMTGELSLTG
QVLPIGGLREKTVAAKRNKIKEIIIPAANEKDLEEIPEHVKKGLTFHPVSRMEEVIDIVF
GT*
>SPBDM4_v1_70213|ID:27159671| putative Adenosine kinase [Uncultured spirochete bdmA 4]
VKTVVNSRFLGIGHAMCDISAELDVQTWNAFRAAFSWLASGAPVHLDAAQAHGVFSYLEE
RAAQGQGNISYAAGGSALNAVRVASLLGTKAAFAGCVGNDECGDVIRAGLKSAGVEALLD
TSGGQGSTGMFCTVSPMTETVSGQNATEEAVSERIIIASPSVARRVRDLDFDGFDVEKVE
LIHAEGLLADSPRNLEQLFQRAHRMKKMISIDVVSSEAARRNKDTLKRLIQSYADFVFCN
KSEFEALDVNIANYRSDIVWVIKADRAGVDCFASGQRIHADAPQCTVVDDLGAGDAFAGA
FLSGMLVGLPVDRCLQLGTKAAACALQSRGTEPDEHCLRALSQDILAE*
>SPBDM4_v1_70214|ID:27159672| putative Adenylyl cyclase CyaB [Uncultured spirochete bdmA 4]
VAVQDSAHNVEIELKARIQNKEEIESRLAKFMRLTGNIDKRDEYWEVSGKDPLSSGTFRF
RVRREAGHTTITFKDKTFDGNLEINREFEFGIDNEAAFRAFVERLQGRFVYRKRKTGTRW
ESETGVVAEVVEVASLGLFLEVECVCHTQDAMAQEEAKCQLYDVIDWCDIPRRALEPRPY
SQLLGY*
>SPBDM4_v1_70215|ID:27159673|gpmI| putative 2,3-bisphosphoglycerate-independent phosphoglycerate mutase [Uncultured spirochete bdmA 4]
MVGSLEKNPKWMGREGPVVLVIMDGVGYGTYKEGDAVLAADMRHFRALEATCPATKLKAH
GTAVGLPSDEDMGNSEVGHNAIGCGRVFAQGARLVNLSIESGAMFQGQIWKELVGNVRSN
TSTLHFIGLFSDGNVHSHIEHLKAMLAQAWKDGVSRARIHILLDGRDVGSQSALEYVIPF
EDFLAQLRMQGADYRIASGGGRQYITMDRYGADWQMVKRGWDCHVRGVGRQFAGAEEAIL
AYRKENPSIIDQDIPEFVIVDKGKPIGTIEDGDSVVFYNFRGDRAIEITAAFEQDDFDKF
DRVRRPNVYYAGMMQYDGDLHVPRNFLVSPPAIDRTLGGYLASTGLNSLAISETQKYGHV
TYFFNGNRSGKFSETLEEYVEIPSDRVPFEQRPWMKCAEIADYVIGAIESGKYQFIRLNF
PNGDMVGHTGVFQAVVCGLEGMDIQLGRIKGAVEKAGGVLILTADHGNSDDMYEHDKKTG
NVQHDADGQPKAKTSHSLNPIPCIIYDPKYKGEYKKVLRQGLGISSLAATCIDLLGYVPP
GDYDPSVLEMAYE*
>SPBDM4_v1_70216|ID:27159674|apt| Adenine phosphoribosyltransferase [Uncultured spirochete bdmA 4]
MVNDSAVRSEANGYNLDDAIRKVPDFPKPGILFYDITSILANSDAFQYCIDSMLTLYQET
EFDAVAAIESRGFVFAAPFCYKRDLPLLLVRKKGKLPGKTISCSYDLEYGSAVLEMHVED

IKPGARFLIIDDLIATGGTINATCDMIKRCGAVPVRAFSVIGLPFLNYEEKVKDVGIDTL
IEYFGE*
>SPBDM4_v1_70217|ID:27159675| exported protein of unknown function [Uncultured spirochete bdmA 4]
MLRRGCMLMVALLLAGACFPLHLANAQETGGTTSSSGQTAEGTLQNSESAQTTDIDEENT
TETSENPVSSTNQAALPSFSYKDTKESTVGRALILSIGLFPFTYFYSGIVVDVTRYVSHG
FDSAYAPWSSSVSLTDSEMWTKIAVSSAASVVFGLLGAILK*
>SPBDM4_v1_70218|ID:27159676| Pyridoxal phosphate enzyme, YggS family [Uncultured spirochete bdmA 4]
VDDVSLMAVTKFHPAEAVRSAYLSGIRFFGENRVQEAQAKYPPIRASIKDASLHMIGTLQ
KNKINKALSIFDAIEGIESVETLKAILARTDERQEPVRLYLELHTGEASKAGFPSLDELL
RGCETYAEFLESDVRGAGEATLCGLMTMAPFIEDSATIRKSFRMLAAAKKEIENRFHFSD
FGELSMGMSNDFEIAIEEGSTLVRIGTAIFGERDAHNA*
>SPBDM4_v1_70219|ID:27159677| exported protein of unknown function [Uncultured spirochete bdmA 4]
MKRIKYLALSFLCLAAPMLGALDFQIPEVLGISGFGTTLTEQITAELPGKSHETSPFYGQ
TPRSDIMVESASNNIKAPSNGTIIYVQTEAPVKTIFSFPLGGALAVRHPESYLSLLSGLD
ADQALKFSKQDQTTTQDVPAVKKGETLSGGSGSGVYPPNCFGLRLFDAQSALWVNPVFLA
SWIQDRTSPVIRGAKLTKFDDSGTSPFDLSGAAVKEKRIMCVQGSYRIWVDAFDTIIPGS
SLHSAPYRILVVLDGKSIIDTSFIAANCSEGGLSFLGNPAPSRETLAADGSYNIGQIMLA
RGEHDLQLQVSDYAGNQSSLKTLIIAY*
>SPBDM4_v1_70220|ID:27159678| protein of unknown function [Uncultured spirochete bdmA 4]
MHHQYAMKKRSYSVVLFIAAIFFSLFSVRITRQEDAVEESIFLPFSQAQDVASVPSSRNA
FLSFVGDSALGYVDTAADRLYAQPYQGKAAIDNQGWITYDRLATDVQLHGTDGQHRVLMA
YAYPWFKASWRILVRSDQMGIARFNNQGSILWVKEFSMPITALDASSQVIAVGLLDGSLH
IFDAEGALVFAAQAGFQDIQTIYGIAVSGDSKYIVVLKGFSPQKIETYKQSESSYTRISE
SPLKQQAVLQATMAFAEDDSHVIIARENQLIYYNIKDNYNKQIELQAEHNESLDKDEDVQ
FFALGPIGRATIAVLRIKVPDPLDSEVLILKHGLLRRVAPDAISVSEGQGTLAIVYRDGI
ELLKGWQP*
>SPBDM4_v1_70221|ID:27159679|cdd| Cytidine deaminase [Uncultured spirochete bdmA 4]
MRPEYTSLFKRAYKACEQSYAPYSHFRVGAALLCEDGSVITGVNVENRSFGLTICAERNA
IAAAITAGKTKFKALVIATPDAAYPVSPCGACRQVISEFMPASATVIFGNAEDSYMTSTV
GELLPHDALHDLSDSIHDHPAHA*
>SPBDM4_v1_70222|ID:27159680|recN| DNA repair protein RecN [Uncultured spirochete bdmA 4]
MLEQLSVRNFAIIEQIDFELSQGMTVFSGETGAGKSLIVDALGFLLGARADSSIIREGAP
ECSVSGLFSAENTQEIELWLKEKDIEWVPEDAILLRRILKQNGRSLAWIQNRQVSRAELV
EFTQFLVDIHGQHEHQRLIDPATHIHMLDAFAGLEQELRSYESVYAEWKEALHEYHELLD
QKAKRSQETDYLEFLLKDIISVKPKPDEDEQLAAEEKILSQHEKLFGAVSEAAGLLGAGE
STDVLHALKRVRADIESAGTIDSHLGSLSERLSAAYYELEDISESINEYQASLRFDPARL
ENIEHRLSELQRLKRKYGPLLSDVLNKYERARVTLDTLSHADEDAAKLEKKVKSLKERTL
GAALQLSEKRMAAATSMSSSIKEIIRDLGMPDVQVNLRVARAMDEHGAYRATAQGIDEVE
FYIAPNPGEPERPLSRIASGGELSRFALALKAVLAAHDVVGTLVFDEIDTGIGGQVGVAV
GKYLKQLSQYRQVLCVTHLATIAAQADQQCKVDKYISSGRTSTSLRFLSRQEREVEIARM
LSGSSESEVSRTHAAELLKQSQSDE*
>SPBDM4_v1_70223|ID:27159681|ppnK| putative inorganic polyphosphate/ATP-NAD kinase [Uncultured spirochete bdmA 4]
MSESKSALIFANLNKDNAGLSAQIVQAQLGERGWHADILAFRGPISEHPDFSSYGLLVSL
GGDGTLLYAASFAGPLGIPILPINLGTLGFIAANKMDTWAVTFDGWQRRAIEVSKRLMLK
VQASRGKQILYSGIALNDIVVSSEGNARMIRMCLYVNKECFGHYRADGLIVSTPTGSTAY
NLAAGGPAVHPEISALIINPICPFTLASRPLVIPGTLAVEIAIDETKKTGALLTMDGHEM
ISLKKADMVKIEKYKKYALLIVPPGNAFFSALRTKLGWSGDSDA*
>SPBDM4_v1_70224|ID:27159682| conserved protein of unknown function [Uncultured spirochete bdmA 4]
MISVFSSYITRKDMDLVLSRMVEDAVVDDVFSDRFEKAIKEQFQFEYAIALRSPYYALLK
ALKVCELGAGARIAISALAPSWHRVAVEDIGLTPIILDIEEETLHPSSESIQRADPSAII
LFDALGKLPSSALLDTLSVPVIEDISQTLGNSGQSRVNMPFAYFSVWGLEADSPIATGGG
ALLCARGKRDAQILRALDEALPPELKMTDYNAALGLSQLKSYSQMIGRRKTIREILQAQL
ARTRHSGLRIEDTELWPGYAFPVFAEASAKEIIDYAKKHGVQAELAFASSSAMLEENAEA

ISPSARSIALRCVLFPMHHKLSNQQVDQIGKIIATLP*
>SPBDM4_v1_70227|ID:27159685| protein of unknown function [Uncultured spirochete bdmA 4]
MLESSKKLGYTEGIVSIIVNTLLFGIKIWVGLAASSVAMTADAWHTLSDTLTSLIADGWH
HRSDALASLMIVVGAIAGKYVWWIDGALGIGVSALIVYAAYDIAKTAFNALMGENASPDF
SERIKKIAGEASPELKDIHHIHMHRYGDHLEITLHARLNENTKILDAHNFSTKLEQELRK
ELNADTTVHIEPEK*
>SPBDM4_v1_70228|ID:27159686| Binding-protein-dependent transport systems inner membrane component [Uncultured spirochete bdmA 4]
MRISWIKKGLFFVLVLAIVIPVLFPLLWMLASSFKTQVDIVAWPPRLVFTPTLQNYERVF
REQNFLKYFINSSIVGISAVAFSLIMGLPAAYSIARFKQKRLSIFILVARLMPGISFLMP
WYIIFSRLKLMDSYVALILSHMLITLPIIVWIMSSYFETIPIEMEESAMVDGATRQRAFL
SVVLPIAGPGIVTSVTLSFIFSWNNFMFSQVLSMERTKTLPIAIYNFVSYAEVDWGAVMA
AAVAIMAPAIILTMIFQKYVVKGLTMGAVKG*
>SPBDM4_v1_70229|ID:27159687| Permease component of ABC-type sugar transporter [Uncultured spirochete bdmA 4]
MEPKKIRGSFLERNLHIIFPMPAVIFVVVMMFFPVLYTFFLSFTNWNLTSGMPLSIVMLR
SYQRILREPRFLAALGRTFSFTALAVSVETILGMIIALILNREFKGKGLTKLIMLLPLVS
TPVAIGIAWNLFYDPTIGLANFVLTRLGLPKQAWTGSSSTVIPSLALVDIWQWTPMIALI
LLSGLASLSNEPYESARVDGATEWQIFWRITLPMVTPTLLTALILRTIDALKTFDIIYAM
TGGGPGYSSETLNIMAFKYSFEYFRMGQASVILVFLFLLVLIMSLLIVRMRKSLEL*
>SPBDM4_v1_70230|ID:27159688| Extracellular solute-binding protein family 1 [Uncultured spirochete bdmA 4]
MKSKFMLVILVVLALFSVSAQQKEIRVLLANHPYGDLLKAAIPEYEKATGVKVNVESLQE
SQLTTKLTTEFATRSSTVDVFMTRPLQEGKMFYKNGWYEPLTGYDFSDYPKNALDVATFG
NKAYIVPLVTEWEVLYYRIDLFKKAGLSVPTNFTELEMAAKRLNSSDMAGFASRGKGAAA
VTQLSSYVVYNYGGTYLDKGKAAFDSKEAIDAIRFYGKMLANYGPSGVTNMSWENIMPLFQ
AGKVAMWTDASVFYGQIVDPTKSQVPAENVGIANFPAGPKGNTPFIVVSWGMAIAKQSKN
KALAMDFIKWATSQGLAKRGMLANITMARNSAWKDAEVRSKINTGLIATQEYAAKYGIPY
DRPYMSAVGEARDLIGEVIIESINTAGTSTKLEALAKDKVDAVNGLLEDTGEYGVY*
>SPBDM4_v1_70231|ID:27159689| Transcriptional regulator, RpiR family [Uncultured spirochete bdmA 4]
MKISTRLCTNSAIYYKMADIIGTGMEKPSSSSCLYLIHSLMDTLSEREKRVAEHILEDPA
SAVHPSIEELSESAGVSVSTLVRFVKKLGFKGYQQFRIALASEALAPEAKIYETFVDPGE
DPVGIAFNSARKALEITASMIDRSALTELAARIIRSNCIYLFGVGGSAIVAQDAMHKLIR
TGIRCIGTEDCHMQLMIASQMSEGSTAIVISHTGVNKDTLRIAETAKNAGAFLCVITTYP
RSALARMANMRFISASSGSPSVSEAFSARIAQLALIDSLYIAIMEQLGEQGIDNVEKMRA
AIAKRRM*
>SPBDM4_v1_70232|ID:27159690|gnd| gluconate-6-phosphate dehydrogenase, decarboxylating [Uncultured spirochete bdmA 4]
MRADIGLIGLAVMGENLVLNMENHGFTVAVYNRTIEKVDAFVTGRGYGKHILGAHSPKEF
VAMLKKPRIVMLMVRAGSAVDDTIAQLEPLLEPGDIIIDGGNSNYLDTMRRLSELETKGF
LYVGTGVSGGEEGALNGPSIMPGGSEAAWQHIRPIFQAIAAKVDDGSPCCDWVGPGGAGH
FVKMVHNGIEYGDMQLISEVYHLMRNQLNMTPDEMAEVFDTWNKGELDSYLISITSDILR
YKDEDGSPLIDKILDAAGQKGTGKWTGITALDFSVPVTLIVEAVFARCLSAMKDERVGAA
KLLGKPSGASFDGERAAFIDDLGRALYAAKIISYAQGFMLLREAASEYGWKLSYGSIAMM
WRGGCIIRSRFLGRIKEAFDADSSLSNLVFAPFFAKQVLENEKALRNVVGEAVAAGIPVP
ALSSALEWLDGLRTENLPANLLQAQRDYFGAHTYERVDRPRGQFFHTNWTGHGGSTSASA
YVV*
>SPBDM4_v1_70233|ID:27159691| PfkB domain protein [Uncultured spirochete bdmA 4]
MGALDIIPAEKANLDFLSLGGLVMRMDPGIVPFAFADHYDVHVSGGEYNVAANLARCFGK
RTAIASAMVDYPLGERVEAAAREMKVQGFYKHFKYDGVRGPNIALVWSDRGQGVRPPVVF
YNRANEAAALLKQGDFDFETIFGRGVRWFHSGGIFSALSDTTPKLIIEAMKTAHAHGAIV
SFDLNYRAKLWAAANTGKKPSEVLGEIARHVDVLLGNEEDLQMGLGLPGPDIHSASKLDP
ASFLKTIEQVHAQWPNIEVVATTLREVKSTNHHLWSAVLWIKGQHWLAPTMELDVYDRVG
GGDGFAAGLIYGLLEGFSPEEALRMGWAHGALLTTYPGDTTMASLEQVKTLAQGGSARIQ
R*

>SPBDM4_v1_70234|ID:27159692| Alcohol dehydrogenase GroES domain protein [Uncultured spirochete bdmA 4]
LKALILKEYMHLEIADVPAPQISAPDDVLIRIKATAICGSDVHGIDGSTGRRQPPLTMGH
EAAGLVAAVGPGVKNVATGDRVTFDSTIWCGECHFCRRGEVNLCDNRRVLGVSCDEYRRE
GTFAEYVVVPERIIYRLPESVAFEEAALTEPVSVAMHAFRITNMQPGESAAVVGSGLIGL
LLIQIIRSYSPHLIFAFDTDSQRRAAALRAGADFALDPADPASQKKVLDMTEGRGVDRVF
EAVGASAPIATAVAAARKGGTVTLIGNISPKAEIPLQSVVTREISLIGSCAISGEYPVSL
DLMASHKIDAKSVISAIAPLEEGPLWFERLYRREPGLLKVVLVP*
>SPBDM4_v1_70235|ID:27159693| Oxidoreductase domain protein [Uncultured spirochete bdmA 4]
MNDKIRFGLIGYGKVAALHARALRTAPHCELVSVSGHHQEKRDAFAVQWGLASRNSVEEM
VKEDGIEAVVITTPHPRHYHDAMEAFGAGCHALVEKPLALSVKETEAMIGESLARGRLLS
VISQRRWYPSCQRIRKAIDDGFLGTPALGQLTILGWRDEPYYRSDPWRGSWKHEGGGVIV
NQAPHQFDLLCWYMGEVAEVYGGWANINHPYIEVDDSAVAAVHFRSGGLASIFISNSQRP
GLYAKVHIHGTSGASAGVQTDGGAMFIAGRSGVLEPPYNDIWTIPGQESMRERWQKEDAT
FFSGIDATWYFFAQQEEDFASAILNNGKPAVTGYDGLQVARIIEGIYRANEDKAPVRY*
>SPBDM4_v1_70236|ID:27159694| conserved protein of unknown function [Uncultured spirochete bdmA 4]
MDIYAHEPLGFDGILVHVEADIRTGIPAVEIVGLASTSVREARERARIAMRNAGFEFPQD
RILVNLSPADLPKEGSAYDLPIALKILAKSGQVEDVPARILAMGELTLEGDIRPARGVLP
AVRAAADHGIRSCILPYENAGEAHIVSSVQVWPVRHLSEARQVLIALGKGEAPQRSGRTT
TDRVPDQIQDFDDFRGDERLMRALMVAAAGGHNMFLAGPPGAGKTMAAQRFPSILPELDE
KEAFDTASLYSLWGKTQVQLMRRPPFRAPHHSASLEGMLGGSKPLRPGEVSLAHHGVLFL
DECPEFRRDVLQALREPVEQGYVDIVRAGRVLRFPSDFQLIMAANPCPCGNLGMPGKTCL
CSPEEIRRYWKKLGGPLMDRIDMRIAVTPPEPSRLISASVTPTGLFREKVLSARLHQRSR
LKRACATYGNPRTNARIPPALISDICSIKGSAEKLFLSGMTSYGLSARAGHSILKVARTI
ADLDARTDIGESAIEEAMEYRQFGDGDAVWPI*
>SPBDM4_v1_70237|ID:27159695|pheT| Phenylalanine--tRNA ligase beta subunit [Uncultured spirochete bdmA 4]
MPKIEVNEELFFGLAGKTWSDKEEFEEDLTVAKAELDEWDTSLSADRERTIKIELNDTNR
PDLWSTAGLARQLRMYRTRSIPSYPFFASRKKALKAPYRVVVEKSVKGVRPWLAGLVAKG
KPISDALLRDMIQTQEKLAWNFGRKRKGVSIGIYRIALIEWPVHYRGVDPDKVSFVPLQE
TRTMTLNQILEQHPKGIEYASILKGRPVHPLLTDAKGRVLSYPPIINSADLGAVQVGDAE
IFIEVTGSDYPSVALSSAIMACDLADMGFEIDNVQVDYEYDTPFGSSVVFPYYFQEEVSV
PLDDANRLLGSSLEMADALEALTRMGLCAYSTDGAVIHVAPPEYRNDFLHPFDIVEDIMI
GVGMEKFVPQRPTDFTIGRLTPIERFSRKAKSIMVGLGYQEMIYNYLGSGKDYAEKMQVS
AEGLVKIANPMTENYEYVRNSPLPGLLQTESVSAKASYPHRTFEVGKVVLKAPEVNYGVV
TRQYIGFLTSHAQADYNEIASHVAAFMYLLGKEYTVRESSDSRFVPGRQVDVLVDGARVG
IFGELHPLVLEAFGVMMPCAAGELDLEMLLED*
>SPBDM4_v1_70238|ID:27159696|pheS| Phenylalanine--tRNA ligase alpha subunit [Uncultured spirochete bdmA 4]
MIDQSAARALHPLEVKIILAFSSGDMIDSARVREKLGFKEGQDQQAFSWLVAKGVLEEAD
RQTEVFYELTPFGQEWHDEGTPVRRIFSLLRESGPLSLPDIAQKLGFDQKTVGSAFGALS
KEGVCAMDESRRAVLARPELPQSLLDTEALLDAAAAHNGSLPETSLTPQQKALVAQYAKK
RGAQDAPFRIAERAKVLFRLTPLASEYREAVTALGMTGEELGAITPEMLEKGTWKQGSFR
PYNLQIPPARVIPGRRNPYVEFLDSVKDKLVSLGFEEFDGNMVETEFWNSDALFMPQFHS
ARDIHDVYYIEEPTHAKFIEEPYLSLVAAAHEHGGNTGSRGWQYQFDRDFTRRLILRSQG
TVLSARQLPKAKVPGKYFGIVRCFRYDRVDATHLSDFYQTEGIVLGEDVNLRTLLGFLEM
FAVEVAGAKEVKYVPGYFPFTEPSIEVHIKHPVLGWFELGGSGIFRPEVTRSLGIDVPVL
AWGIGIDRMALMALGLNDLRELFSADLESVRMRRSQYKIQ*
>SPBDM4_v1_70239|ID:27159697| conserved protein of unknown function [Uncultured spirochete bdmA 4]
MYFNELVSSYDRQRRERNIYHDLMRDRVREVLLVGSLYDSFVVESDGVLTEQIYGEYFKL
NLNTIPRVTCAYTEESALDLFREGRFDLIIIMASLDFDMPLRLAASMRRIWPDIPILLMV
TNNSSLAMLDMSRPELAAFNRVFVWNGYSKLFVGMIKYIEDWRNVEIDTRTGEVRVILLI
EDSVRYYSRYLPVLYKVVLRQTQALIEEEHITESYKLMSIRARPKILLASTYEEAHEIFE
TYKPYLLTVITDIRFNRNGVCDENAGYDFLQFAKRELPDLSMLVQSSEPNVKEKAFAIGA
SFIDKNSESLEMELAAFLQANLGFGSFVFRTPDGNEIDHAHNMGEFVQKLNKIPIESLLY
HAEHNHFSAWLMARGEIHLAKILRPYRITDFSTPAELRQFIVRMIDQIRSTRSRGMMSYF
DPEMVKYPRYLCKIADGSVGGKGRGLIFIHSLLENIDFSQYIQGMRISMPNTVFVGIDEF

EHFLESNGLWSWAYYGDNAQDVPPAFVQKSLSPQLLERLRQFLSVSKKPLAVRSSGLFED
MLMVPFSGVYDTYILPNNHPDIDVRLQTLCDAIKLIYASLFSKESRAYFEAANYNLEEER
MAVVIQELVGSPHGAYFYPNAAGVAQSYNYYPVSYVKPDDGLCVTALGLGTYVVGGGAAF
RFCPKYPKLDVLSPEHALESTQRYFHALSLENQAPDLLQGEMASLTELPVGAAEKDRYFP
MLASTWDAADQRFVPGIMIKGHRVIDFANMLKYDAYPFAKAVDMVLDIATRSMGTPVEIE
YALNFDADREEPTLYLLQLKPLIHIDDRVEIDPGSIRKEQCFMLSRGCMGNGRDYDIRAI
VWVDPRKFERSATLEIAAEIEELDSIAREENFNYLLIGPGRWGTRDRWLGVPVSFSQISR
AKAIAEVDLPDFVVESSQGSHFFHNLTTMHIKYMKVSHDSAEDFIDWDWLYAIPPRVRTK
HCALTVLESPMDLRFDGRSGVGAILKPLAAPSGEPSGEPSGEPSGDVSEFEFKAV*
>SPBDM4_v1_70240|ID:27159698|aroF| 3-deoxy-7-phosphoheptulonate synthase [Uncultured spirochete bdmA 4]
VIKNIKLAARTPGQKTTIIDVNGVKVGQGLVIIAGPCSVETERQTIETAIAVKKAGAHML
RGGAFKPRTSPYAFQGLGLKGLKILDKARKETGLPVVTEVVDTRDVSWVGEYADVLQIGA
RNMQNFSLLREAGKSGKPILLKRGMYSTLEEWLNCAEYILAEGNPNVILCERGIRTFETY
TRNTLDLSMIPAVRRESHLPILIDPSHGTGLLPFIEPMSLAAVAAGTDGLEIEVHIDPAS
ALSDKDQQLTIPMFESLMEKLRKLKAFFEESKMYVPGESPDNSTPETKFTLPSAFSEAVD
*
>SPBDM4_v1_70241|ID:27159699| putative 3-dehydroquinate synthase [Uncultured spirochete bdmA 4]
MQTTHSIIRFGPLSEFRELLSPERVVFITSSALREKASEKLPMERESGRWIEVPDGESCK
TFLVLESVYHALLNFDAGPDTVLVAIGGGSVSDLAGFAAHTWKRGIGLILVPTTLLAMID
ASIGGKNAIDIGYAKNAVGSFHLPTSIICDVGWLPSLPEKDLASGMAEAIKHAVLDSEEH
VQFFEQLAETRIPLAELGGKTFEDLIRRSQAVKLRYVNADLYDSHERHALNYGHTFGHAI
ELLTGLPHGFAVAAGMGAANTLAVSRGTLDPVTQGRIGSLLRHFGLPAGIQEAFGMAGRT
MDRRALLELIRADKKRRGNYMDFVLPHGIGDVRIEAIPVDELAEVLQT*
>SPBDM4_v1_70242|ID:27159700|aroC| Chorismate synthase [Uncultured spirochete bdmA 4]
MNTGGRIFRISLYGESHGPAVGVVIDGCPAGIPLSEEDFAVDLARRKSGAEGTTPRIESD
RPAILSGVFRDHTSGAPIHIEFRNENTKSADYADFTRIPRPGHADFSAMTKFAGWQDPRG
SGHFSGRITIGLVAAGVVAKKILNHFAPIVFETKLLEAGGSKNVPEAIRAAKKAGDSIGA
LVEIRVNGVLPGWGEPFFDAAESVIGHYLFAVPAVRGVEFGDGFAAAAMRGSAHNDPFVS
QDGRTARNGAGGINGGITNGNEIVLHVAVKPTSSISVAQHTLDFSRDEMTDLEIQGRHDA
CIALRSAVVLEAATACALADLALTARAESPWESNIPWRKS*
>SPBDM4_v1_70243|ID:27159701| Prephenate dehydratase [Uncultured spirochete bdmA 4]
MNLEDIRTDIDRIDAKILSLLNERMEKALLTRRFKSGTFDPTREQVVLDKVRHSSQCLLD
PQFSVKIYEQMMAESRRIQEASPQTVAFQGEHGAYSEVALRILIPHAATIPCREFSDVFD
GVEKGIYDYGIVPVENTLGGIVGPVNSILIYTTLKIVAAIDMPVRHCLLTVPGADHRELR
TAWSHTQALAQCRNFLQRNHLDSEPYYDTAGAAKALSENRPKGIAAVASKFAGELYGLET
IKEDIQDSPHNRTRFFLISARGNGDEGDKCSAVFTAGNKAGSLFAVLKVFAEEKINLTRI
ESVPDTPGKYAIFIDFEGALASDAVQRAVGKVSKMAEDFKILGCYREMRVEE*
>SPBDM4_v1_70244|ID:27159702| conserved protein of unknown function [Uncultured spirochete bdmA 4]
MKIAILGAGHMGAWLTEELCWHHEVMVHDSDLLKMKYFIKVHRALSIDEFAKFEPELFIN
CVPLGYTLEAFDAVLPLLPKTCIISDIASVKTGFKEYYEQAGRPFVSSHPMFGPTLANIR
DLQEESAIIISESNREGASFFRDFYSSLKIRIFEYSFEEHDKTVAYSLATPFASTMVFAA
CMKKQDAPGTNFKRHLTIAKGLLSEDDRLLTEIMFNPRTIRQLELINSKLTYLTHIIRQR
DYEEMKKFLDSLRANIGE*
>SPBDM4_v1_70245|ID:27159703| Transporter [Uncultured spirochete bdmA 4]
MSTGNRPSNPPQQSAIKIGKKAFLLSAGIILFLMLISGILTIVLPPGEYQRIVQDGKTLV
VNGTYHSVPRPDYPAWRWLTAPIEVLFSQDNLTLITIIIFIFCVGGSIAILEGAGVMEEL
IHKLVRRFSRRKYFLIALVILFFMAISSFIGVYEGMVPMIIFIVPLAISLGWDSLTGLGM
SLLPLAFGFASAVTNPFTIAVAQRIADLPLFSGAWFRIIFFIVVYIIVTMFVILHAKRVE
RDPSSSPTFKEDEAIRASLGEKQSATAQAAAEGEAGSLASPEDSLSKAKTKGLIWFAVCI
GIAMIIVLVTARMPGLSDLAFPIMALLFLIGGIGGGLLAQMKMGSIVKTFLQGAANLAPG
IVLILMAYSVKHIIISGKIMDTILHGAATLIAQAPPLSAAFLVYATTLVMNFFIGSASAK
AFLMMPLLTPLADLVGITRQTAVLAFDFGDGFSNMIFPTNALLLIALSFTVVSYPQWMRW
TWKLQAIMLLVTSIFLGIAVAIHFGPF*
>SPBDM4_v1_70246|ID:27159704|aroA| 3-phosphoshikimate 1-carboxyvinyltransferase [Uncultured spirochete bdmA

4]
METRTMHPAAVGGSIRAPASKSAMQRAIACAILGRGTSRIEGAPVCADSRAALRIAQKLG
AEVREGGGIIEIDGSPFFQKESPRGRGGHRSPERPLEISCGESGLCMRMFSPVVALLEDE
VRLTGEGSLMRRPMHMVESSLNIFGARCASNGGRPPLTIRGPLRSKRATIDAKGSSQLIT
GLLIALPLLPGDSEIEVENLVSAGYLDLTLEMCAHFGVHVEKEEDGATFFIKGGQSYRAS
DIHVEGDWSGGAFLAVAAAIAGREEGLRIRGLSWGSKQPDRAIAEVLSVAGADLRFEGSD
LIVKPGKLVPFSFDATDCPDIFPPLVALACAIRGISRIRGVHRLASKESDRASALRTTFG
TLGVEVSIEGDEMQVSGGHLRGGTVSSWKDHRIAMAAAVAALAAEGEVHIEDTECVSKSW
PGFFEDLASILLH*
>SPBDM4_v1_70247|ID:27159705|gdhA| glutamate dehydrogenase, NADP-specific [Uncultured spirochete bdmA 4]
MNSYIKEVLDGLRARYPWESEFLQASEEVLESITPLIEAEPKYRQQKILERIIEPERVVM
FRVPWVDDKGEYHINRGYRVQFNSAIGPYKGGIRFHPKVTLSTLKFLAFEQIFKNSLTGL
PMGGGKGGSDFDPNGKSDNEIMRFCQSFVTELYRHIGPDTDVPAGDIGVGGREVGYMFGQ
YKRLANEFTGVFTGKGLSFGGSLIRPEATGFGAVYFAEEMLKLKNDSLKGKTVSVSGFGN
VAWGACIKASQLGAKVVTISGPDGYVYDPDGVGTQEKWEYLEEMLIIDRNKVETYVKKFP
KAVFYKGKKPWEQKVDIAMPCAIQNELNGEDAKALIENGVKIVVEVSNMGCTPEAWKLFI
EKKIPFAPGKAANAGGVATSGLEMSQNSMRLAWRAEEVDHRLHEIMMNIHKACMETAVKY
GFEGNYVVGANIAGFKKVADAMIAQGLV*
>SPBDM4_v1_70248|ID:27159706| conserved protein of unknown function [Uncultured spirochete bdmA 4]
MSLFTQFFGARTKTDLPQDESVEEQREMIEGVEHLPEKIVRDVMVPRTDTVCVEDTTTID
EILATLVESGHSRIPVYSGTIDNIVGILYAKDVLAALVKKEPLELKSLMRPPYFVPETKR
IDTLLKEFRRRHVHIAIAVDEYGGTAGIISLEDIIEEIVGEIQDEFDEEAEQIVKIGENA
WRCDARIRLEDINETIGSQLPSGEYDSLAGYVFDLFERIPSAGEQVTAGDLQFSVEAMEG
HRLITIRIDRIDISENDEHGEHVSIS*
>SPBDM4_v1_70249|ID:27159707|ybeY| Endoribonuclease YbeY [Uncultured spirochete bdmA 4]
MNGNEVFYSIIGIEEPIWAQRAADFALSVLDLLEKRDWQVSLTLCDDATIQALNRDYRHI
DSPTDVLSFSLGEFEPVEQGENIYIAGDVVISIPALRRNAAEFEVTEDEELRRLIIHGIL
HLSGMDHEDSSADQSMLELQERLLCQLGGNAIL*
>SPBDM4_v1_70250|ID:27159708| putative 7TM receptor with intracellular metal dependent phosphohydrolase [Uncultured spirochete bdmA 4]
MKKNSEETFLRKVIRWLGSIKPSMWFFVGTIVIAAVGLFLLGPSSIGRSNSLKSVEAGKI
AETDIYAGKDTVYTDKEATQRKILAEERLVLAVFVLDDKITSSIREKTEAFTSYYLQLVN
GESGGADALGLLLSSRFPDFLQPEQYATIVRAKLNSQTFVYVSDIVESLLDKGIVSVPDG
VFDRFNPNYYELVRTLNGREESEQLAVNGMVTLANLGNRLEDEMDARRIPESLKQYVRLL
AKALLKENTFFDQSLSEKRIASARANVEPVTRFVSRNEILVRKGELITPEVYQQIKTIRS
AILVSDVGVLLTDLGLLLIASLLGFLLARIKDLSDFPNDRPSLIFLLLSLLILFYWILAV
QNVLDTKDLSRGALFVPASLFAGLVSLLYGPRTGIFFSLISFFLTGAATNLNAQFMIGIL
LAGVSATLTMRTARTRIMLARAAVLQALVQALLAVVLLFNQKPTAAEVFQAMGLGALNGF
AGGSFILLLLPLFERILDKATQFRLMELADINAPVLKEMLSNAPGTYAHSMNVAHLAETA
AKDVGANPLLARIGAYYHDIGKIDHPEYFTENQKGINRHDEINPTLSASIIRRHVKDGVE
RAREIGLPKEVIDIIGQHHGNSVMEAIINKAKDSPNEADVQSYSYQGERPRSKEAAVVML
ADTVEAASRSLKNPNAAKLEQLVHQLVLHKITNGQLEESELTLHDIDVAEKAFLRILQSQ
MHTRIEYPEQEKQ*
>SPBDM4_v1_70251|ID:27159709|ybeZ| putative enzyme with nucleoside triphosphate hydrolase domain [Uncultured spirochete bdmA 4]
LSPSHIVLEDTQLLAELCGANDYNLSVISSLLGARVLTRGNELFIESEDKKALDLFPRLV
SAIGTSIEEGVPATPELIIALHAELTPEGGQNHSRGPDNAPEEGQSEVSKFLDTCIQIPN
GFGKVFPRSGGQSLYLQGIASHDLVFAIGPAGTGKTYLAMAYALRELMTRSRRKLVLTRP
VVEAGESLGYLPGDLEQKINPYLRPLYDAMEALVPYETIHRLEESRAIEVAPLAYMRGRS
LTNCIVILDEGQNTTKEQMKMFLTRLGERTKAIVTGDITQIDLPKKTESGLVHAAALLEH
IDEVFVSRLDARDVVRSPLVRKIIEAYEKE*
>SPBDM4_v1_70252|ID:27159710| exported protein of unknown function [Uncultured spirochete bdmA 4]
MRRILGILILLHVLLVPMSAQESAVGPASVSAVELASKTLRFRIASATFYELRDMAVEYG
LSAEGSADELRTRLYGHFGFDPVPKTKGDVSMSIEQAANAEYFTLENGTKEIRLQGPLEI

RFVDSQGTVHRIKAQYVVYNRDTREVQATGDVEYTRESKTRTDTYKGQSIAVNLDDYSGV
FVDGSFNMEPTTTESRTLIVHFGSLISRSEDILALADGSLTACDAIDPHYILRAKKVWLF
GSGDWAVVNATLYVGKIPVLWLPFFYYPSKASAFHPVVGFRSRPGGFVQTTTYLSGKQGT
EAQKSSALSITQGALGSFGTYISKTEPSQENTDQPSIAVLADAYSSLGVFAGIRGKTADS
SPINLSWLIGAGVSRSIFYESTGYYSPFDAAGDYASVWNRWYLGSIDLPTRIVANFEASS
KQNVSGLKWQVSLPFYSDPYVEQDFLDRSESYDFFSIIGGTSTSSIGERTSFSQKASLSW
SWRPSASGNGFSFSLSNLSSTLGWKSKYASKSGMNSTQLRLYAVNPQRYFFYPYNARLLD
SNFSLSGTIFQSKNASLVWKNTNAAYVEDRFYNAAWENPQDIDFQSWYWLLGARSDANLN
SSFSVDSAGLTFQVSTGVRGQGQYRPYLYDERTSPTTVHPFRIADYGYNTANWNAGTNIS
WSPLKNSDVFSASRVQYSLSGKIARIDYTGLDGSGTDANPVYDLYWLSWDTDMITDHSVI
ADLAAKLGSSSDHLSFKIALPPLLESYTLQASTSASIVSLGAAYVISRTSTTADLKSTSL
TGNVALKPLKNLRFSTSASWDFDTQTPLSLSTDVTAWSFYARFLAQETDGYVFQSGSWVQ
DGTQYFRPSSVSLSWKPVLRSSPRSSSDKVLWYFETDEAFSLTQNLIQYTNSTFGADFSF
IVKNSAGLSLSFTLSSINTSFWSYYTSLLPTSGDLDPQVYARVFFADLLDSLSIWDTSRL
QSTLFKLQKLSLELSVDAHDWLLSASVDAGPTLVTPASGRPYYQMDVSFSLAVTWKDISA
IKSTVSYSDGTFSQ*
>SPBDM4_v1_70253|ID:27159711|uvrA| ATPase and DNA damage recognition protein of nucleotide excision repair excinuclease UvrABC [Uncultured spirochete bdmA 4]
MDKLIIKGAREHNLKNIDLELPRNKLIVISGLSGSGKSSLAFDTIFAEGQRRYVESLSAY
ARQFLGRMDKPDVDYIEGLSPAISIEQKTTTRNPRSTVGTVTEIYDYLRLLFARIGVPHC
PSCGREIREQSVDQILDVVLSWPSGTKLQVLAPVIRAQKGEHRKVLEDALKQGFLRARVD
GQFIELETPPTLEKQKKHTIELVIDRIRLSEESRRRVAEAVEAALGIADGIVTVIRETDD
QQKEEFFSQKGACPVCGISLPEMEPRLFSFNAPQGACPACGGLGMNMEFDPDLIIPDRSL
SFNEGGCVPYNPQSAWNRSRFEALAHHFHFSLDTPFEELPEKVMQAILYGSDDDIHVRYD
NRDKTGHFEYQSKFPGILADLRRRYMESSSDEIKDWLESFMVEKECEVCHGKRLRPEALA
VTVGARNIYELTCMPVGESVKFFESLRLSETEAQISKLILKEIIARLGFLRNVGLDYLTL
ERKASTLSGGEAQRIRLATQIGSSLVGVLYILDEPSIGLHQRDNQRLIDTLTYLRDLGNT
LIVVEHDEQTLRTADYLVDLGPGAGEHGGYVVAHGTVPEVTANSESLTGQYLAGTLKIEV
PKVRRKGNGAFVALKGARQHNLKNIDVKIPLGKFVCITGVSGSGKSTLLSDVLYPAISNR
IMRSSLREGEYEKILGLEHIDKIINIDQSPIGRTPRSNPATYVGVFTPIRELFASLPESR
ARGWKPGRFSFNVKGGRCEHCQGDGTIKIEMNFLPDVYITCEVCHGKRFNADTLDVRFKG
KNIADVLNMTVEEGSEFFAHIPSIAHKLRTMLDVGLGYIRLGQSALTLSGGEAQRVKLSL
ELARRATGRTLYFLDEPTTGLHFADVRQLLEVIHRLVDSGNTVVMIEHNLDIIKQADWVI
DLGPEGGDRGGTIVAEGTPEEIAACTASYTGQYLRPMLRA*
>SPBDM4_v1_70254|ID:27159712| conserved protein of unknown function [Uncultured spirochete bdmA 4]
MSRCVPHAGKSAEVYGPWPAAAEEGFPPAGAVVGGPARFLVLGTFPSIRSQSRQEYYGHE
RNHFWPLLFSFAAESGLLPLPAEPGQSMWHAGASLAAVSAYEAKIGLAARLQVILWDMVK
SCRRTTSADDALEIVALNDIAALLQAHPEIERIGLNGTRASTLFLKNVVYGCDFRQAREA
LSGVGGRVVLAIAGKTRAIYRLPSTSPVPTPQYRTIEDKWALWQRFFY*
>SPBDM4_v1_70255|ID:27159713|ychF| putative GTP-binding protein [Uncultured spirochete bdmA 4]
MPLNCGIVGLPNVGKSTIFSALTAAKAEAANYPFCTIDPNVGIVPLPDKRLDRLCEIFKP
KRRVPAVVEFVDIAGLVRGASKGEGLGNQFLSHIREVGMIAHVVRCFDDPDVIHVAGRVD
PADDIEVIHTELALADLDSVDKRLDKLSRQLKVQDPNLKKDSEKEMAVLQKIRPALEEGK
PAFTVGLEPEESELARRSFLITLKRELFVCNVDESGAHGNRYVDIVKSIAEKQGSEAVVI
CGKFEAELADIEDPAERAAFLQDIGLEEPALASLAHAAYRLLGLRTFFTGGEDECRAWTI
RAGDKAPTAAGVIHTDFEKGFIKAEVYHYDDLIQYGSEAAVRAAGKMRQEGREYVVQDGD
IMFFKFNV*
>SPBDM4_v1_70256|ID:27159714| putative enzyme [Uncultured spirochete bdmA 4]
MQDLSAYLSANAHNMKKSVIRELLKLTNQPDIISFAGGLPAPEAFPVEELREACDRVFKK
YGDKILQYGTTEGDNDLKAQLVAYESAQGLTLGMQNLLITSASQQALDMLPKIFLDPGDY
VIAGRPTYVGAIQAIQSYQGKVLGIPFSTANDGFDMVELERRYSRALSQKKRIKYIYVIP
DFQNPSGICWSLEKRRALIEFAYQENLFIVEDAPYREIRFLGEPISSIYKLEQQMQNRGI
VINLKTFSKILAPGVRIGWVIAREDVIQKMVVAKQAMDLCTSVLTQKMIAEFMATGKLKN
IVARTCGIYRDKRNFMLEKFDAYMPKRFDLTWTKPEGGLFLWLSLPHYIDTDKMFYKAIE

RKVAYVVGSAFYFDEAETNSMRINFSYSTFEQIEEGVKRLAVTIQEEIEAHEAGPRGQTV
PEDM*
>SPBDM4_v1_70257|ID:27159715| Beta-lactamase domain protein [Uncultured spirochete bdmA 4]
MKVHPVTDRIYALHADIRSDDLFEGIWPIPYGVSLNSYLIKGEKTALIDLVRDWVGAPIE
IKNQLASIGVNLEDIDYLILNHLEPDHTGWLAEFLEINKKVQVLATAKGIDLVKSFYFEH
ERVHAVKNGETLDLGAGQVLQFFETPNVHWPETMMTWAAEGGVLFSCDGFGAFGALGDRV
FDDEISPDEHEFFEAESLRYYANIVASFGIFVKRAIEKVSGLPIKVVAPSHGIIWRAHPE
TIIERYLHYANYLEGPREKEITIVWGSMYGNTERGLKYVIEGIEEEKVPYTIHRVPNENV
SFVLADAYKSEGVVIAMPTYEYRMFPPMAYVLDIFERKHIYQRKALRIGSYGWVGGAKKE
YESKIAVLKWDSIDSAEWAGAPSGDMAQLLRERGRELARRVKGAQ*
>SPBDM4_v1_70258|ID:27159716| protein of unknown function [Uncultured spirochete bdmA 4]
MTEFIETLRSASATVQAIAVSVGGLIGVFATLAFFFMMIVVADRFGKR*
>SPBDM4_v1_70259|ID:27159717|gcdB| Glutaconyl-CoA decarboxylase subunit beta [Uncultured spirochete bdmA 4]
MQDVFIGFTSITWKMLVMYGIGGILIWLAIKKQYEPMLLLPIGFGAILVNLPLSIAWEHE
GVPGFLRILYDAGIANELFPLLIFIAVGAMIDFGPLFRNPLMIFFGAAAQFGIFATMIMA
TLLGFDLKTAASIGIIGAADGPTAIYVANKFARDYLAPISVAAYSYMSLVPIIQPPVIRL
LTTKEERRIHMPLHEKQSPQWLRILFPILVTMTAGIVAPISVPLVGSLMFGNLIRESGVV
ERLSQIAQNELAYLVTLLLGITIGGSMSAEKFLNWNTMLIMAMGLVAFIFDTAGGVLFAK
MVNLFLPRERKINPMIGACGISAFPMSARVVQKMASEEERGNIILMQAIGANVSGQLGSI
LAGGLVLALVPLLVK*
>SPBDM4_v1_70260|ID:27159718|mnmA| tRNA-specific 2-thiouridylase MnmA [Uncultured spirochete bdmA 4]
MNENITVAVGLSGGVDSSLAARLLKEKGYKVIGLTMKIWKGAYKIQEDLKHACFGPGEEE
DIAACERLCKNIGISYNVIDLSEEYEHHVIEYFRKEYLAGRTPNPCIICNRELKFGFLID
RAKGTGLEFDLFATGHYVRTSSIDGTTYLKTARDAAKDQSYFLYGLDSSRLSKIMFPLGE
MTKEETRTAARAYGLEVADKPESQDFVGGGDYAPLFEYDRPEPGDIVDLKGNVLGQHRGL
PFYTIGQRRGLGISIGPEPLYVIALDARQNQVIVGQGKGLFSSGLLSHSFRLQNPQTYGQ
SFACKVKIRQNHKPAAAVVLADSEGDARIDFEIPQRAVAPGQSAVLYSEDGLVLGGGIID
AAIPDEESKSTCLADNA*
>SPBDM4_v1_70261|ID:27159719| conserved protein of unknown function [Uncultured spirochete bdmA 4]
MKTEVLWYLGERSIELREVEIPEPRAHEVLVEIEMCGMCSWDLLSFAGKFGKFHPYPFAA
GHEGVGRVIKVGDNVSSVKVGQRVACHEVPIGTPGGALMARHAIRTEQKVTVIPENTIPL
KYWVVEPVVCIVNGLVYAGIQPGDSIALIGAGYMGLIFVQGLAKTLAKEVVAFDIDEKRL
ALAKEFGASRIVKIEGNAIPEEFRRYFDVIIETAGKPSSIEMALSLAKSGAVIENFAWHH
HMHEFDLDAWHTNAWRILNIQPGVNPHFGDLFPRTIDLMLNGTFSNEKLITHWAPVEEAK
EIFTTALERKDGYMKGAVMFK*
>SPBDM4_v1_70262|ID:27159720|rbsC| D-ribose transporter subunit ; membrane component of ABC superfamily [Uncultured spirochete bdmA 4]
MKVKFVETLKNYGIILAFSLICVGLSILSPVFFTLNNIINVIRQTSIYGIMAVGMTFVIL
TGGIDLSVGSVLAVSGAICAGMLKAGNPIILVILATLGVGVGCGLVTGLFVTKARITPFV
VTLGMMSIARGLALIYTRGYPISGFSPTFRLIGGGYILGIPIPIIIFLVVVIIAYIVLMQ
TKLGRYTYAIGGNEETVRLSGINSDFFKTLVYVISGSTAALSALILTSRLNSAEPIAGTG
YELDVIAAVVIGGASLSGGRGSVWGTFIGALLIGVINNGMNLLGISPYFQQVVKGLIIIG
AVWLDQLRAKEE*
>SPBDM4_v1_70263|ID:27159721|rbsA| fused D-ribose transporter subunits of ABC superfamily: ATP-binding components [Uncultured spirochete bdmA 4]
MDDRVLWMKGISKSFPGVQALKAVDFDLERGEVHALVGENGAGKSTLMKILSGLYKADEG
EIWLRGRKLSSRGIKAMIDAGVSVIYQELNLMRQLSVAENIFIGREPMRHSGLIDWKKMY
LDVQRILEPFNVDINPKTKIYLLSPAYQQVVEIAKALSLNSDILVMDEPTASLTGNEVDK
LFEIIRNLRNSGVSIIYISHRLEEIPQIADRVTVLRDGEKILTKPLGELTTAEIIRHMVG
RTLTEQYPKISVPLGKEILHVENLTKKGYCEDVSFSVRSGEIVGFTGLVGAGRTEIMQTI
YGRMKKDSGRIYIDGKEVQIHNTCDAVRKGIGLIPEERKRQGLVLGLSVFDNVVMTILDK
ESVLGFLKKKKLNIHVEKLVELMEIKTPSTKQLVRYLSGGNQQNIILAKWFLRHCKVYIF
DEPTRGIDVGAKVEIYKLMQNLAKEGAGVIMVSSELPEILNMSDRIEVVFSGSIVKEFQR
EEADSEKVMEYALGLHEHESQQKLRAAGAET*

>SPBDM4_v1_70264|ID:27159722|rbsB| D-ribose-binding protein [Uncultured spirochete bdmA 4]
MKKVVLVALVVIALVPAAVFAQKQIVIGMTVPGLQFPFFVTMKQEAEAAAAKLGVKINFI
DAQNASDKQMAAIETFVSQKVDGILISPLTEDSLVPAIEDAVKAGIPVATVDRKANTNAV
LIHVGADNVEGGRAAARFIIEKLGNKGTVIELEGTPGASAAIDRKKGFDEIIKTSNVKLL
VSQSADFTRSKAQSVMENLMQVYPKFDAVFGANDEMIIGAIEAMLAAGVNPASKVTIGFD
ATTDAFTYMKEGKLNATIDQFPGKQAAMALEALANKIRNNVNPAQKVVYISPLPVTK*
>SPBDM4_v1_70265|ID:27159723| putative HTH-type transcriptional repressor PurR [Uncultured spirochete bdmA 4]
MPSLKDVARLAGVSTSTVSRAINGNIPVSEETLRRVEKAVRDINYKPNLVARGLRIKSTK
LLGLVVPEIQHETFISFIRFTEEAAEAKGYNLIIGSTNSDPDREERFIENLIRRNIDGII
FSRVSDKSHVLKILDKTKIPVVIIDRTLEREDIPSVVMDNYASGKIAAEHLLSLGHEHFA
CITGPLDIAINRDRLAGFTETVHKAGAVLEDKCIYEGNFKFESGKKGIAYFLDTGAQFTA
LWAQNDYMAMGVLNYLSKHSIPVPEAVSVVGLDNIQQSWMMQPALTTIAQPFRDMCSHAV
DIIIDHATNKEDKMGRIQIMLQPELIVRETTAEPPEKCITVN*
>SPBDM4_v1_70266|ID:27159724| conserved protein of unknown function [Uncultured spirochete bdmA 4]
MAQEKQMIEKALNEGKGILRLEPAWVPRSFCIPGRRLKLHPNDYYAYGANRGGIDERWFA
STTKADNGPLTTAFEGLSFVWVEGKHHVLLKDAIEIAGREILGDAVMNAYGGWRAYSKFF
DNQEPLPHHVHHTDKMAANIDQMGKPEAYYFPKQLNNHGGWFPYTFFGLNPGTTKEQIKQ
CLADWDKRDNNILALSRAYMLQPGTGWDVPPGILHAPGSLLTYEPQRASDVFSMFQNIVW
NAYTPKELLQKDIPEDRKDDLDYYVDLLDWDLNTDPLFYEHRFMSPKPVKPIEEMHAEGY
EEYHIVYKTQSFSAKELTILPGRSALIRDKGPYGAIVVQGHGKMGLLDIESPAMIRFGQM
TQDEVFVTEKAAREGVLITNTSKTDELVMLKHFGPEV*
>SPBDM4_v1_70267|ID:27159725| conserved protein of unknown function [Uncultured spirochete bdmA 4]
MITTSAEHIRYFGRNSIVLSNEKVRAVIDDLGGMVPEFSLRKGRGAINAHWIPDFRGNSG
QPWDAATHAPYWKGKILYILAGDFPCSPSFGPDCTVDGTKLPAHGWTANERWAMSSSGIL
ADENVAYAIFSLQSPDRSMPLSYKKYDVVQKDHPVHYSVISIRNHGGSPIAINIAHHNTT
GAPFLQAGCRIYVSAQKFLTPPLHTEFDDTGRLAVGAEFDSLYRAPSRNGKLIDLSVVPG
MIGFTDFVTGAVPKDARLGWSCVVNPVLRLAYVTFFPGPRDIPEKDIPLGFNDLWMQYGG
RNFTPWALSEGGADRTFCLGTENAVGAFANGLEYSRANPILLDSPTIVSIPAGEERNLFY
GTALIELDDSLASEAILNIEAEDDALVLKTTKSSQRILLDGSFSSLRVLIRALSGK*
>SPBDM4_v1_70268|ID:27159726|ulaE| L-ribulose-5-phosphate 3-epimerase UlaE [Uncultured spirochete bdmA 4]
MTQASAEERYSFGIYEKAMPPGLHMREKLQAASRHGYDFMELSIDESDEKLARLNASAQW
KRELADAIRDTNIRIPTMCLSGHRKFPLGSSDQALRVRGMEIMKAAIRTAADLGIRIVQI
AGYDVYYEPSTAETGSFFAENLAKSVEYAACYGVCLAFETMETPFMNTVEKALEFVKSIN
SPYLQLYPDVGNVTNAFQGDTPRVVQDLRCGKGHIVAVHLKETKPGIFREVSYGRGHVDF
DACVAETAKAGARMFTSEFWYSPDVDWEAEMEKSFAFLHSKLERAFSGEI*
>SPBDM4_v1_70269|ID:27159727| Carbohydrate kinase FGGY family protein [Uncultured spirochete bdmA 4]
VKKYFLGIDNGGTVAKAALFDEKGMEVGVASETIPFTSAGGGWAERDMHSMWKANCRIIK
KVLRDSVIAPQKIAGIGCTGHGKGLYLWGKNDTPVYPGIVSTDTRAWEYPLRWQKDGTAA
RVYVKTFQKILACQPVSLLAWFRDHNPDILSRVRWIFEAKDFVRYMLTGEAAAEITDYSG
SGLMNIRDGCFDIGLLKEYGIAECMGALPPLVKSAELAGRISKKAAMATGLVEGTPVAGG
MFDIDACAVASGVIDPENICVVAGTWSINEYVSRFPVTDGSVMMNSLFCLPEYFLIEESS
PTSAGNYEWFIHFFLEKEKAEATASGLSVFEVADRMAAQIAPEECDLLFLPFLFGSNYNP
EARAGFLGFSAAHTRAHMIRAVLEGIAFSHKTHLEKLVTSRRNLMQREAPPQGIPPNPEP
EINEKRTVVRLTGGAAKSRLWVQIFADVFQMPIETVISSEPGALGAAIAAAVACGEYRDL
REAAGYMVHLADRFDPDTGKSDIYEHKYSLYRDMTGSLDQTWSGFKR*
>SPBDM4_v1_70270|ID:27159728|aroE| Shikimate dehydrogenase [Uncultured spirochete bdmA 4]
MPNRNYKSELVGVFGYPVAENPTVVMMEAAFSHLGLNWRYLTIEVRPEDLADAVRGLKAF
NMRGINLTIPHKIEVMRYLDEIAEDARLIGAVNTVINREGCLRGENTDGKGFIQSLKEKA
GEIIPGADVLILGAGGAARAIAVELALAGAAQIGVANRGRDRGETLVRLLNERTQCQARY
IPWDTALRVPEATQILVNATSIGLFPAVKESPHMDYDSIQKGLIVCDVIPNPPDTEFLKR
AAQRGAMTINGLGMLVNQGVIGFRFWTGIDAPREIMIRALSREFTA*
>SPBDM4_v1_70271|ID:27159729| conserved protein of unknown function [Uncultured spirochete bdmA 4]
MSQMTTKTVPDSPPENPLKICVFCGSSLGNHPSYAELAVKLGRTLAERGMVLVYGGGNVG
LMGTVAEAAMNSGGKVIGIIPQRLFDLVEQQELTELLIVKDMHERKALMQKKADAFISLP

GGIGTMEELFEVWSWRYIGYHQKPVAILNIEHYYDRLLEMLRHMVGEGFLHREIYDDLIV
SDNIEAVLEAIHGKARDSGAVPFLKKPERRR*
>SPBDM4_v1_70272|ID:27159730|pncB| Nicotinate phosphoribosyltransferase [Uncultured spirochete bdmA 4]
MNCNYQTALFTDFYELTMAQGYWKRHMTMPVVFDYFFRRHPFGGGYSVFAGLATLMEALE
GFSFSKEDLEYLSTLGLFEDGFLDHLSSFKFKGTIYAAREGEIVFPQEPLVRVEADLISA
QIVEGLILNVLNFQSLIATKTARIWNASGRGAIMEFGLRRAQGADGALSASRAAFIGGAV
GTSNTLAGKEFGIPVLGTMAHSWIMSYPNELEAFEAYAELYPKNTVFLIDTYNSLESGII
NAIKAGKKLIEKGYGFGVRLDSGDIDYLSRMIRGRLDEAGLPDVKIVVSNELNEEIIESL
VDGKAPIDVWGVGTNLVTGGNDAAFAGVYKLSAIDPEGQGRPVMKFSDNPEKSTNPGVKN
LWRLYDQRGAARLDLISCSEEEIQEGFEYLVHHPSADWRQLKIVPARVEPLLFKVMEHGA
RIQGLPDIIHIQAFMKERIQTFDSTYLRLLNPHIYKVSVTDRLFNLKVSLINAFFKQKLS
QG*
>SPBDM4_v1_70273|ID:27159731|pncA| Pyrazinamidase/nicotinamidase [Uncultured spirochete bdmA 4]
VDSALVAVDIQNDFCPGGVLAVPEGDSIVPKVNALLAAYPLSILTQDWHPLMHCSFASTK
SLPPFSLDTSVTPPSILWPDHCVAGSTGADFHPALQTWRARFILRKGMRKTLDSYSAFFE
NDAVTPTGLAGLLSSLGIRRILVCGLATDYCVKATALDARRIGLDVVVVEDAIKGIDANP
GDVANAKEQIRALGCTFASTSELSAKA*
>SPBDM4_v1_70274|ID:27159732| exported protein of unknown function [Uncultured spirochete bdmA 4]
VNLRLVHVGKKKLQCAAIFVLLPLTAALSLDASARSVSGNISLSTSTTGTRAYLSLILPF
DALINASMPLSLDSEILNSASFTAKGKLGGLTLAAGKLKISDAASFLARPDLVSSRGSSL
MYSGNLGSASDPLGISATSSRLSLFLAKGEIYSCGALQYLFLDNPARVAISVGALLDEED
QNSVLHRMKPWLALGSGYSVSNISFLARLHVYPDMEAHRGDFSWLKAAAGRLDISLRASK
QKMKGFLYAETGDFISATGKAASHDAMAQLDYEVDISRIPLFKNFSAGLSVFSKQGAAVP
VMGDTPSFGTLPDPFALKYWPDGGKVHFGIENKEVRTHVFDADVGLSPSFSGTVAREEGQ
WSGKMGFDLDIRSAGPPAPRSPAPQLGLGFALQASCSEQKDASEAEDGDFSGISEYEESD
DTSATARNMDGKFIFDRVLVALRTAFDPFSCSVSTDVPLKHSDSDALRVQIRASAKMPHF
FVEASGTGKFEMGAKQFSIASAHLYVKIPL*
>SPBDM4_v1_70275|ID:27159733| Uncharacterized glutaredoxin-like 8.6 kDa protein in rubredoxin operon [Uncultured spirochete bdmA 4]
MSVTIYTTPSCSYCRMAKDWFRQNGISFTEYNVAADMHRAEEMMHKSGQMGVPVIDVNGR
IIVGFNKLEIERALHR*
>SPBDM4_v1_70276|ID:27159734| conserved protein of unknown function [Uncultured spirochete bdmA 4]
MNQDKAREILEAIEPPPAPFTLVFSGRTNKKVNGLYKPAKSEIVIHNRNFDSENQLIYTA
IHEYAHHILFTRRGGLPMPRPHTQELWALFHELLEKAEQLGLYANVFDTVPEFASLTAEI
KSRCLAGNGSLLLELGRLLVQVEELCRIHHARFEDYVERVLGLPKRTASTAIQAQTLNLD
PSLGWDGLSLVASIRDPEARSKAAEALSRGATPLIAKRTAMPPEDESDDPIETLIAEHKR
LERSIASMTERMHRIEEKLHSVGISEDDIAL*
>SPBDM4_v1_70277|ID:27159735| conserved membrane protein of unknown function [Uncultured spirochete bdmA 4]
MLQMIQILILLAFLLGGALAASLRLAPKPRIVEVSITAALWLLLFSMGFRIGNDPELVHS
IGTIGLLGFVAAVCTIAGSCAAVWLMSRLLPQAGAFQKQKPVIATSFNVALDPGGHKDEK
ATASLVRRMRPPATLLAVVLAGFVTGILLPEAGFNPGIITEWTLNVLLFLIGMQFRQSNI
PLVNMLKSPAVIALPLATAVGSLLGGILLAPLFSLPLPRALALASGFGWYSLSGVLISNL
GDPMLGSSAFLANMIRESLGLLLIPVLGQTRVPAMAISVAGATSMDVSLPLIEQTLGPEA
VPLSFISGAFLSALVPVLVPLFMKL*
>SPBDM4_v1_70278|ID:27159736| putative Dihydroorotate dehydrogenase B (NAD(+)), electron transfer subunit [Uncultured spirochete bdmA 4]
MRQLNAPVVINKAIAPNWVHLAFEWPSDLQEPKPGQFFTVLPAMVELGAGTLLRRPLAFA
GFEPAKQKKNALSGKSSAIVHAIYQIRGPGTKALALQAPGSRIDVIAPLGNSFPWPRQGE
KAIIAGGGIGIGPMLFLAASLFQENLQLGGPEFQLVLGFRSASLIPLRVGFSDGGPMASL
WKELLSRAFFATDDGSEGFRGTVVDALSSLKPKETSPEAWHIYGCGPGPMLASLAKFAKT
HHIAAHFSAEQWMACGVGACHGCVLPSASGDFLRVCADGPVFEADRIDWEAGLR*
>SPBDM4_v1_70279|ID:27159737|pyrD| Dihydroorotate dehydrogenase B (NAD(+)), catalytic subunit [Uncultured spirochete bdmA 4]

MNAEKKQNVFSPPADLDLSVHIGSLAMSNPVGVASGTFGYGEEYDELVRIDALGALYTKA
VTLEPRRGNPAPRLVETPMGLINSIGLANPGVEGFLQEKLPLLRRHRCAIIVNVAGSTED
DYAEVIERIEDHIAVAGMEPGERSGVDGYEINVSCPNVHKGGMSFGIDPVLIGRLTRSLK
DRTRRPLILKLSPNVTDIADIARAAEAGGADAISCINTVVGMVIDTEKLRPAIARGAGGL
SGPAIRPIGIAATYRVSRAVHIPVIGLGGIAHPSDAIQYLLAGAQAVQIGTALFSNPRAP
QEVLDGIIVWMQRHSVHSVCDIRSMLEEPE*
>SPBDM4_v1_70280|ID:27159738|pstB| phosphate transporter subunit ; ATP-binding component of ABC superfamily [Uncultured spirochete bdmA 4]
MMQEARIQTSFILETKDLSVIYGDGNQGVKHVSIGFQERNVTAIIGPSGCGKSTLLRALN
RMNELIPGTHTLGEVWYNDKNIYDASIDPVLIRRHIGMVFQKPNPFPKSVYQNIAWAAKV
HGYSGSMDELVESSLRKAVLWDEVKDKLKKSAFALSGGQQQRLCIARAIALQPDVILMDE
PTSALDPIAASYIEDLISELRNDYTIIIVTHNMQQAGRISDNTAFMLMGDLIEVGPTKKL
FIAPEDERTERYLTGRFG*
>SPBDM4_v1_70281|ID:27159739| Phosphate-specific transport system accessory protein PhoU [Uncultured spirochete bdmA 4]
MPREAFERSIEEINARITAMGAATVDALQKAMYAFCERDLDLAKKIKKADAQIDIQQLEI
DDLVATTMATQQPVAKDLRLLLCAIQMASDLERGADYAAHLAKATKFFAQEPQWRQTEML
EQMANIGASMITGTVNAFIERNALAARQTSLMDDQIDHLHKSVIKEMVVLLSTRPDDAEK
VAKFIQVSGYLERLGDHMTNTCESILYMVEGIHVELNI*
>SPBDM4_v1_70282|ID:27159740|phoB| Phosphate regulon transcriptional regulatory protein PhoB [Uncultured spirochete bdmA 4]
METILVVEDDRDIAQMIATSLGKVGYHVVIQQDAEHAHAFLRDGVVSAILLDLMLPGMDG
FAFIRKLKKDPALSAVPIIIASAKDDDADVVAGLELGAEDYIVKPFSLKVLEARLRAVIR
RHDSSVQSEESGARLQKMGILLDSSRHEVRNENDIVDLSATEFAILELLMKNPGRVFSRE
RLITEIRGGDVAVTERSIDVHILAIRRKLNEKGALVETVRGVGYRFRDE*
>SPBDM4_v1_70283|ID:27159741| putative Histidine kinase [Uncultured spirochete bdmA 4]
MKNTNSRIPMSIRRLLFWGLLGLICSALISLFIADTYNNTLIACAVFLALSIVLFVALFH
LQNAVYQRPLTALASKVGSLALSLSTKSSDTALHEISGTEAHPALEPPSASGQPQDIPDE
FAKIEQNLDLVAENLNAQSREVTELAGRLQSIFDATEEAIVAIRAPNELLAANTAAYRLF
ELPKNPAQTAESFFFKAPPIIGLIDECLREGHALLEQFSMLKEKNEVILTARGRRFRVGN
SDGVVIVINDVTSLKRMELLKKNFVANVSHELRTPVQIVKGYAELLGGAEISEEYRNWAE
VIGHQAARMERIVADLLMLAKLEHDPASWIVREKFFLKSVLAEAARTVRLQYADSGTIEL
DCPEELEAYANPGLIEQAAFNLMANAVQHSGSHEKILVGAVQEENDVVVRVRDFGAGISP
KDLAHIFERFYRADKSRSSASGEGTSRIPSSGGSGLGLAIVKHIALAHGGAVRAESWAGE
GSLFEFRFPNATEDSIAAARPEDPSSIPPSSS*
>SPBDM4_v1_70284|ID:27159742| conserved protein of unknown function [Uncultured spirochete bdmA 4]
MGHEKAFTSERLVVGVLSSDEGAEDAALDAMAKLYGPLCFRSAKEPFLWTKYYCPEMGEN
ILRSYWAFEQLIDPSGLASIKRATDAIELKLAENGRRKVNLDPGMLGTARFCLATTKDRS
HRIPLADGIYVELTLIYERGEFRALPWTYPDWASEPVRRMMAELRTGLLAELRRLNLL*
>SPBDM4_v1_70285|ID:27159743| ABC-type transporter, periplasmic subunit [Uncultured spirochete bdmA 4]
MNSRLSLAIFAIAIILAPLAAQDQETEFVTVYGAQLPELDPQKALFSNEAQIHTALYEGL
FTYDPQSLEPVRALAQSWERSTDKKVYRFTIRENARWSDGTAITAEDFVRSWFRMLDLNA
DYATFFDVIAGAKDYRTGRDRNPEHVGIKALDARTLEVTLLRPVAYFTRLLCHQSFSLIH
PSMLNVEDWRTAIPYPVSGPYKPVSMTDEELVLKKNDQYWDAENVSIPTLRMMFTDDDAL
ATSLFNTGHVHWLDGPGTYEQVLLQTAIQIFPIYSTHYWFFNCRSAPWNDQRVRRALALL
LPWDSIRSSDLYRLPAATLVLPLPGYSKTKGIEKADHEEAMKLLSESGHPNGEGLPGIVI
AFADYERPRSIATAFKTEWEKELGLKVTLKPMSPSSYYDTIGNRADTASFTIAHETWIGD
FADPEAFLQMWTSGAPLNIAGYDDSVYMDYMNQSYSAGDDARMALLAKAETELLQSAACL
PIYHNFAASIIDTDYIQGWYQNALDVHPYKYLRFGMPSVNPNVVRADTAVGALASAAGAS
SSK*
>SPBDM4_v1_70286|ID:27159744| Thymidine kinase [Uncultured spirochete bdmA 4]
MQSNDETGEFLKSLGFPQLNIHTMSRHHDFISAGKRILVIGPMGSGKTEFSARVWRDSRV
ALKKKGAAARLTSSGNVDRRDIFIVRSALDKSRFPDYPEDALAYRGGYERCGDRIGSAKD
SFSLEMLIAENPAVGTWVIDEAAFFDERIAYLMKDEAESRGLCFIYPTLVLNFRREIFNQ

TARLLLETATDIVPLTAYCEHEECLKDSLYTYRYYLVDEQECPALYFDPLIIIGGDRTKH
DGREPNYCTRCDEHHYLPGKEYTFFTLKPLGELAARGNIAPLLAELNALQGNIKDSRLYA
SIESRCTEGQEHEVQMNALKVPLIAERALVYLYAEQNLLSEEQMRFFISELNLDKDYLSQ
RLADNRRPLALYERPVPSASFA*
>SPBDM4_v1_70287|ID:27159745| putative Threonyl/alanyl tRNA synthetase SAD [Uncultured spirochete bdmA 4]
MNARALDERLFYRNPWLSEADAIVMATEWEDGVKPGHVRVLLDTTIFYPEGGGQPPDLGF
IERARVVDVQEIDGQIWHFVEIPPEAGNGGAWLAQKGVRAGASVHLRIDWQRRLYHMQQH
TGQHLLSAVLEQDYGIHTLSFHLGTDYCTIDVSAKNPADLPLEEIEAKVEGWIERDVLVR
IHYCPPEDVAAFRLRKRPPADEAVIRVVEIDGYDWSPCGGTHVERTGQLRALKLLSLERY
KGNVRVYFAAGAHALELLFKAYEEAKQAALLLGAGVGDLSARLRDSMEKTAHLEKKTKKC
AQQRAEAEARLAISQTEPEAVLEFKLEDEDAERGTGLVKAAAVLGRAAIAVSLPDSTVVV
QVVQHGNFPALAELLKEKMTALGGKGGGGATFFRANFASPDQAVLFAGYAKEALGTGRS*
>SPBDM4_v1_70288|ID:27159746|smc| Chromosome partition protein Smc [Uncultured spirochete bdmA 4]
VLFILLKRECLVYLKSLEIFGFKSFADRVRIEFSQGISALLGPNGCGKSNIVDAIKWVVG
EQSAKSLRAESMEDIIFNGTENRRPLSVAEVAITISNDGGALPLDVPEIEVKRRLYRSGE
NEYFINGKQSKLKEVRELFWDTGIGKSAYSVMEQGRIDQVLSSKPEDRRYLFEEAAGITK
HKVRAREAELKLAKTEENMRQIEGIVREVKHSYESLKVQAEKTVQYRQLKDKIFETELDL
YLVRLRQYVRERDRSSAAFDQKKKEREALVQRIDNMSASMSEGLDLVNELEAILVEMQKL
LYGLAVEKNGKENQKTLLAERCRELKTKIEQLQGRDRAIAAKIESLQDEEGEKEAEYAGY
RARIREVEANIHTFDENISAAALSVKANEGTIRQNAEESKEIGQKTAQLRQELDTITERI
AQLVDERLRQSETQIEQRQKLEAQIRQGITEIALSVASRADRLQDYARIATTLSSQERAQ
EIERLSADLRELSSKSKWLEKDFQQYIAIAPSFLNELVAPEGVMTQKRRIDAAISAHAER
LREIDTENQALSARNGDLAKKIEIYRRTLEEARMEKTKIQAQMEAAQDALSVLRREIAGQ
ESYRREIANELAGEQRRLDELDEELGTLEEELAEIEQKGRRLSEDMTELENSISQRTADL
AEKRKESSDLEQKLQAMQQELEDLHMAMAQSETEIRNLKDTFTELYSRDLSAYETRMYEI
RRPIVEIREELASLKSSLSSLGSVNLMAPEEFEEVRQRYEFLTGQYEDMVKARTDLVRIT
DEIRTESAQLFVDTYNRIKKNFHNMFRRLFGGGRAELRLLDADHVLESGIEIYAHPPGKK
LENISLLSGGERTLTAIALLFATYMVKPSPFCFLDEIDAALDEGNIIQLVNLLREFGTAS
QFIVITHNKKTVACADTLLGVTMEESGITKTIAIRVQNENGVVRPVYVRDQPFEEEDIDY
EEGRQLDDKGSMTVTGA*
>SPBDM4_v1_70289|ID:27159747| putative PTS IIA-like nitrogen-regulatory protein PtsN [Uncultured spirochete bdmA 4]
MDDDILTIEEVAKYLRVSERTVYDWAQKGEIPSGKIGTVWRFKKSEIERWVNERLSSNRP
VAFISSVKIQNIISPDRILFLNYPSKRDTLIALSENLSTAPQIKNRQELISEILRREELM
STAIGKGIAIPHVRLNSVTDLVISIGISRCDITDFQTLDDVPVRLLFMIAAAYNQHAYYL
QTLSFFSARLKVVELRDGLLGCKTANEAYDLLIQQDQ*
>SPBDM4_v1_70290|ID:27159748| protein of unknown function [Uncultured spirochete bdmA 4]
MAMRIESVREKASGTLEVAVSGGLMFNFDAKDLQMLGCSFDRGTRTLMAADRTAVTLVGD
ESFDEGAVSVLDRLNALHKARKDALSIVSRAEQASKQLYEKLLKRGYEPEIARTCLLWMC
TERYVDDRRYVTLLLQSHLVRRGQGPDRIKTIAWPRVGLFENPKLILADGFAALEEDSMQ
EAIRKNTANILRRTRTFSRANRGFGRNLCRSSNARGMSGDDSEIEPKNLSFAERRSLLRA
RLRNEGFPSKSVEKFLESWEIEENGKG*
>SPBDM4_v1_70291|ID:27159749|rpsI| 30S ribosomal subunit protein S9 [Uncultured spirochete bdmA 4]
MVKNINLGIGTGRRKCAIARVFLREGSGKIIINQKDLKEYFPVEGLVAQVRKPLLVTDSE
NKFDAYITVIGGGPTGQAGACTHGLARALAQIDPANHVSLKAIGLLTRDPRMVERKKYGQ
RGARRRFQFSKR*
>SPBDM4_v1_70292|ID:27159750|rplM| 50S ribosomal subunit protein L13 [Uncultured spirochete bdmA 4]
MKTVFVTAATAERKWYIIDAAGKPLGRVAVKVASILRGKNKPTFTPSQETGDYVIVVNAD
KVMVTGRKRQNKMYYHHTGFPGGIKDFNFNELIGRNPVSPMEIAIRGMLPKGPLGRKLFK
NAKVYAGPDHPHVAQTPTAIDL*
>SPBDM4_v1_70293|ID:27159751| conserved protein of unknown function [Uncultured spirochete bdmA 4]
MTALLVGIGLIVVAVLMVLPGILGWGNDVLAFLRGALPVLAVLIGALAVFIGIADIKDKV
EEKREEEKEKSENSEGEQEKDVEKKD*
>SPBDM4_v1_70294|ID:27159752| putative enzyme [Uncultured spirochete bdmA 4]

MLPHHSLVMYKTKPAIVVAGDPDRILIHLIDGEERRVRSKDVVLLHNGPISSFPKELPSA
EAEVALELLRDEHPEGLALISWKEFVELAWNDAKPEHIVTAWHSLLDNSAVEILEDGIRI
RSSEEQQKLLEKEKKRKEIVETKAAFIESFRGAWKNHDPALIDKNPLFQQFFDELSRCAR
GFSDESPIARELGIKMTPQAIHETLLATGYWNMAFNPWPERNGCILSSPPAPDAIAERAD
LALERLDLRHLSSYAIDNAWSDDPDDAISTDGERIWIHVADPAAVISPMSKLDSEAMARG
STLYLPEKTVPMLNASLVQTLGLGLSSESKALSFGITLREDGRIAETLIVPSIVKVERLT
YEQADTMFESSPALQKLHGMAEIRAALRRSQSAVDIDFPEVAIRVSDGNPAFLPVPHTRS
ARIVQELMILAGEAAARWAYERNIPFPFAAQDSPVSEKLAGCGLGPSQSLAENYGRRRIM
RAALVSSTCSAHAGLGLSFYSQVTSPLRRYQDLLAHYQIHAILARERNSKHSAPAVRTPA
PLDLETMNERLYLYSQQAAKNHQAERDSRAHWTLVYLSRHRDWKGQGIVLDASAENGQIL
IPEFGYETQMRIPRGIKPDETVILALRRASIPELSASFDILPQSA*
>SPBDM4_v1_70295|ID:27159753| ABC-type transporter, integral membrane subunit [Uncultured spirochete bdmA 4]
MKASQPRKSGTVIAEIVTAIIFLLFLFPFLLVLINSAKTSFEVTQYPLALPTRWGNLIDN
IVKIWTSESVRYPSSLLSSIIITAVSLVLINVFSAMAGWALVRTKTKTSSAIFFIFVASM
VIPFQIVMFPLLSWFRTVTDVTGIRLLRTYQGIILSYIGFGAPLSIFMFHGFIKSIPLEL
EEAATIDGCHKHQIFFKIIFPILTPIQATVLVLNGIWIWNDYLLPLLVLGKGNDVMTFPL
AVSNFAGAFVKQWDLILTAILLAMVPVIIFFLFAQKYIVKGMVAGAIK*
>SPBDM4_v1_70296|ID:27159754| ABC-type transporter, integral membrane subunit [Uncultured spirochete bdmA 4]
MTKKTESRVVFWAFLAPVLFAFIMVMIIPFFLGSYYAFTNWTSSARVDNGLRFVGFKNFT
NSLKDPSFLYSFGITFVVYTILNMIAINVTAFALALLVTGNLRFKNVYRVGFFVPNLIGGI
ILGYIWQFIFNNAVPSLGTIIPALSFLADPDKLILGKNTSALAAMIIVGTWQYAGYIMMI
YVAAIENIPQELIEAAKMDGATPWIRLKAITIPLCAQSFTVTMFLTLVNSFKQFDVNVSL
TSGGPSTMLMGKPILGTELLALNIYNTAFISNKLSEAQARAFVFFLVLAIISLIQVYVNK
KKEIEL*
>SPBDM4_v1_70297|ID:27159755| Extracellular solute-binding protein family 1 [Uncultured spirochete bdmA 4]
MKRIALAFMVLLTATAAFGAGKLTLMQNKPEIDAQLKAYAATWGQKNGVTVVIKSIGGTS
GGMGPQLKADYAAGDMPDIFAFDGLESFKDWEGIILDLSKEPWVSKTSVAFKYNGKVYGF
PVAVEGWGMAYNADLLAKAGIDPKTLNSYDAYKKAFEKLDSMKGELGINAVVSMTASVEM
GWVTAHHNFNSLLSNGLPYGDLSVVNDLLAGKVDMQRLQEYADWVELLFKYADRSILLTG
NYDAQVGAFATGKTVFLHQGNWVDPNLKAANATFKMAFAPHGSMKKTTDGIFVAAPSFYA
INKESKNVALAKKFLNDMVTTTEGQNYMVKEAGMIPAFSGINLNPDGQLSKSVQQWAAAG
KVYSWDQYYFSGDFRDKVLTPIYNQFAGGSITKAQFVDLMTKAFKDNAQK*
>SPBDM4_v1_70298|ID:27159756|rbpA| putative RNA-binding protein RbpA [Uncultured spirochete bdmA 4]
MGKKLYVGNLSYNTNEDSLRNLFQTYGTVESVKVITDRDTGSSKGFGFVEMGTDAEASAA
IAGANGFDLDGRQIKVNEAMDKPRRDNRRY*
>SPBDM4_v1_70299|ID:27159757| Nitroreductase [Uncultured spirochete bdmA 4]
MPLLKSIEMRRSFRALDEKPIQNEILLRLAEAARLTPSWGNKQPWRIVTVTAPSQLTALK
ATLTPGNYWAKKSPAIAAFVTSLDWDGRLDDGRDYALFDLGMAAMAYQLQAVEEGLIAHP
IAGFDAASAKRVLDIPETAILETLIILGFPGDPSGLSEKHLASETSARLRKPVDEIAGFD
RWSDQLVPQRRD*
>SPBDM4_v1_70300|ID:27159758| Aldo/keto reductase [Uncultured spirochete bdmA 4]
MKYRKLGNTGVIVSEVALGTMQFGGTMNMGNLGQEATTKMVKLALDSGINFIDTADVYSR
GESETLLGNALKGIRDEIVLATKARLPMSDNNFNRSGATRLNIMREVEGSLKRLQTDHID
LYQIHGWDSNTPLEETLHTLDDLVRQGKVRYIGLSNYLSWQAAMALSLQERMGLEKYATA
QMHYSLVNRGLEYEFQSFAEYANLGILVWSPLAGGFLAGKYGRTAPAPEGTRFAEAGQFV
PFDKELGYRVVDALKEVAGRHGVNPAQVALAWVLSKPAVSSVIIAARKAENFADDIKAVD
LALSDEDMQLLDKVSDPGVPYPKWMVLQLDTAEDPRPKVLHPERYADGGSWKDLRGTKWS
GAH*
>SPBDM4_v1_70301|ID:27159759| Glycosyltransferase 36 [Uncultured spirochete bdmA 4]
MKRSKRGSGVLLSYFEKREDAESARKKLKRKHFRRITLLSNATDENMRPPGTPFRGRRQK
YGAEDKVPKEDARALLPGETVLVVRASIDTLHVAVTLLREDSDIPPAVFILHDKVSKAPE
KELRRPGVPLSSAQILDHAERLGIEHHIERRPRRSRELLARLDSAYHLTRGTCAELSMAG
RLEQGTSATAEWILDNEYVIESNTRDIRINLPTAFYRRLPTLAVGKSKGLPRIYDLACEL
IANMELRLDRENILAFIDAYQSSCPLAIGELWAIPQMLRIALIESISDFALKVSIELRER

ESADFWAHRLIKANRQGPDQLFSILAKLALRFPSPSPYFATQLIDHLYDEEAVLVFVQGW
LERTYHQPLSGISLREQNRQAKDQISVGNAFTSLRQLALLDWREIFESLSRVEHLLRDEP
CGIYPGMDFDTRDRYRRVVEELSRRSGQAEDQVARQALALAKEAMNGAPCTDERRKHVGT
YLIGEGRQELVRRLHCSESRRYRLLHWVYRNSSTVFFFGLGILTGGPTILLAFSVPGGLA
FGIRVLLALLLALPVSQLALELLNYLVARILPPRVLPKMDFEVSGVPDQFRTLVVVPVLL
GDAQSIRSDVEKLEVRYLANRENNLLFSLFTDYRDADEEHRKDDAELLRVAVESIESLNR
RYGAGHFFLFHRERRWCESERKFIGWERKRGKLEELNGLLAGTRPEGSPELVYVGNKGAL
AAVSFVITLDSDTQLPPGTARRMIETLAHPLNQPRFDEAGKVLAGSYTIIQPRVSPSLPS
TSASLFGRLFADAVGIDPYTKAVSDLYQDLTGEGSYYGKGIYDVRAFNRVLCGRFPEERL
LSHDLIEGAYVRVGLASDIELYDEFPPNYRAASERQHRWIRGDWQIGQWISPRVPVQGKG
LERNPLSRFDRWKLFDNLRRSLLPAANLVLLLASWAVFPQAGWIASLVVAAQLFSHSLAM
CFTMLTTWRGANRLSLSLAAHDILRALADAALLPHQAALGMGAMLRTEYRRHISHRGMLQ
WNSAQAMPGVVPEKSQIPALRMVPVSAGSILTAFALSLWLPASLTCAGPWLAAWSLSPLV
AWLLNLSPRAHKRQSALPQADTKFLREISRRTWRYFDDFVDAGTSWLPPDNYQFSPRNEL
ALRTSPTNIGLWMLSALSAHDFAYLTVDQVVQRLTGTVETLGSLERFKGHLFNWYDVRTR
TPLEPRYVSTVDSGNLLGSLWTLVQGLDELFRQPLLDEKVFSSLQDTGRILWNIMRQGHT
PGGHTGGDRVQTLSGLMKEWEAPPPRFVDMLGLLRQELGKIEASGKAVRPSTDEGAEEAY
WVAQIEKQLSAWLNIVDRYLAWMEILAEKSEEEVAALGPKAAEAVRRDLSRVPSLADLAK
GRVDCIEVLKDVRGAAPQPTVPMCAWLDRLAEAFSKSRWLAGEMLGQGERLIRDCSALSE
QMDMRFLYDPERRLFAIGYDVSESRRDDSFYDLLASESRLGSFVAIARGDVPFEHWFALS
RPYAKLGRHRVLLSWTGTMFEYLMPLLFQKSLMNTLLEDATRGALAAQMDYGRQNQVPWG
ISESAFADMDINRVYQYRAFGVPGLRLKREPEEKIVIAPYATLLALGIAPRESVRNLRDL
VDLGLLDVDGFYESIDFSRQPGQNGERGVVVQTYMAHHQGMSLLSLTNFLFEGSIRRSFR
ADARVRSVEALLQERIPNLPASHYIPTREGERSVQSVVEATPSISKFDTAHTRTPKTQLL
SNGRYSVMVTNAGGGYSQWGTQEITRWRSDPTRDSWGTFFYIHELDSDRLWSPTYWPVGR
KGEDCSASFALDRAIFQRFFNGIHARVEIVVSPEDDVEIRRVTLTNRSLRTRHLDCSSYI
ELSMAPHAADRQHPAFNKLFIQTEALPPQHALLAFRRPREGDAPPLYVAHRFTHEAGGEE
TMRFETDRTRFIGRGRSLANPRGGTEEPQNSEGFVLDPILSLRTSLSLAPGQRVTVSLVI
AAGGTKEQVLGLMGKYADPHAIDRAMDFAWAAAQLELRVLRIQPDDARRFQQLASHLLYP
NRLLRSQAGKIEENTQGQAGLWRYGISGDLPIALVTIGEIRDVGLVGQMLQAHTYWRMHG
LMADLVILAEEAGGYERPLHERLEGLIQAHSTNTGRDKPGGVFLRSADQIPEADLTLLLA
AANIVFMSARGTLAWQVGVPGEMRDQREKPAWKNAVQDPVTPLPFLELPYFNGLGGFTPD
GREYAIYLDADTNTPAPWVNVIANPSFGTLVSEAGSGFTWSGNSQRNRLTAWSNDPVVDP
ASEALYIRDETTGLCWTPTASPIREKAAYRARHGAGYSVFEHNSNGIDQELTVFVPMDKS
GGQPVKLQILRLKNSSARRRTLSLTYYVEWVLGEHSETSRMHVVTAWDDEMQAITARNSF
HPDYGDRIAFAAISPNTDSYCGDRTSFLGRNGSMKSAAAMGYVELSNKTGAGYDPCAALR
LSFELAPGESTEITCMLGQVESREAALALVLAYRKTAAAEAALEGTKAWWDDLLGVVEMH
TPELAADFLINRWLLYQDLSCRIWGRSAFYQSGGAFGFRDQLQDVMALLYASPGLARDHI
LLAASRQFKEGDVQHWWHPPGGAGIRSHISDDLLWLPFVVAQYVRVTGDVGILNTQVSFL
EGPELKEYQREAFFTPAVAAERASLFEHCRRAVARGSTEGPHGLPLMGTGDWNDGMNLVG
AGGKGESVWLAWFLVDVLRGMSEMSEALGLADLSREYLEKREALIGRIERSAWDGEWYLR
AIFDDGTPLGSSMNAEGRIDSLPQSWAFLSGGDTERSRTALESAWKELVHEDEGLVLLFT
PSFDKAVPSPGYIQAYPPGVRENGGQYTHAALWLAMAMARGGDGTRSGQLLRMLNPIEQA
RTPEAVRRYSVEPYAAVADVYRLAGRSGQGGWSWYTGSAAWMYRVWVEEVLGLKLRGDRL
ELDPVIPGWWSGFRVNYRHGDARYEIQVENPENCERGVLWIEMDGLRLGNPLIPLERGPG
DHKILVRMGKS*
>SPBDM4_v1_70302|ID:27159760| membrane protein of unknown function [Uncultured spirochete bdmA 4]
MLFATMKLWLKYAFGIIMGAVLYIALPPSMVHGTPAISFLAEISLKIGGYALILTLAASI
PISVFRLSESHRFSKIDSSTLVFFIISLIIAAGIGLASTLIFRPAPLPLISDSGAMQSLD
PLELIRSTFPSKIFSTFSGAATWFLPLALFMLAFGLALSHDPVMARPILPVLDVISRAAY
LINSFLSEILGIMLIPISLYLFLNLRDTGMLSEYKGILVYSSALTILLVLVIFPIIYRIL
GGKSNPYVLLYRMMGPLLAAAASGSVFFSSGTALRHVSESLGVKRDTNAVILPLALIGGR
TGSVIIVASSFIAMFLSYSKNSPSVGQIALLLIIVPFSVVVGAAGLRSDILVMLSLTCML
FGQGFQNGASLLVPVALPLSICAVILDSAWMMYSVALIAEHHGERTIKKARNFI*

>SPBDM4_v1_70303|ID:27159761| putative PTS IIA-like nitrogen-regulatory protein PtsN [Uncultured spirochete bdmA 4]
MDLENLLKPELVTLDLKGRNKEEIIRELVDLAARSGKVMNKEEAVLRVFERENRMRTGMK
HGIAIPHGKTTAVRELVACVGISKEEVDFDALDRKGCRIFIMTLSPIDKTGPHLQFLAEV
GMLFRNEEKRQALLSAKTPEEVVSILLGNA*
>SPBDM4_v1_70304|ID:27159762|groS| 10 kDa chaperonin [Uncultured spirochete bdmA 4]
MNVKPLGERVLVKIKESESKTAGGIIIPQTAQEKTQTGVVVAVGTDADVIKVKVGDEVMY
DKYAGTQIKMDGAEHLIVKMSDILAVLE*
>SPBDM4_v1_70305|ID:27159763| protein of unknown function [Uncultured spirochete bdmA 4]
LKYKKTNRYGQARVLTFFALKSTIENVLRVRQGVRLIRRKELYSYMDAGGSYDSGTNYRN
ESRANYS*
>SPBDM4_v1_70306|ID:27159764|ftsH| ATP-dependent zinc metalloprotease FtsH [Uncultured spirochete bdmA 4]
MPSKGKNNWSPNQQKKPDKNSNGSDKKPGMPEGPQFRFSLGYVLIALIVVSFFNYFLFKR
DSSVVPYSMFKEKISSGEIKRVEIDSNYYTGYTDLKSSAPSSPLPASAKPGVVYKTVPIS
DPSFTALLDQKGVIYSASPREGSAILSLLLNWVLPFAIMFFLWRSLMGRFMGSNSNVLAF
GQKKATVVAEGDVKTRFTDVAGVDEAKEELVEVVDFLKNPQKYTEIGGKIPKGVLLVGPP
GTGKTLLARAVAGEANVPFFKMSGADFVEMFVGVGAARVRDLFKQAREKAPCIIFIDEID
ALGRSRVSGIMGGNDEREQTLNQLLVEMDGFDSTSGLIIVAATNRPDVLDPAILRPGRFD
RQVLVDRPDMLGREEILKIHSKPIKMDPSVDLSKIARSTPGLVGADLANVVNEAALLAVR
AGRKKVKEEDFQEAIEKVVAGLEKKNRMINPKEKQIVAVHETGHAITAAFTQGADPVRKI
SIVPRGFSALGYTLQMPLEDRYLVTEEELLGQIDVLLGGRAAEELVFHSISTGASNDIAR
STEIAKRMITDYGMSEKFKNVALTKRGAGLPGSQQMADPFATREYSEETQRYIDEKIASI
VDERYNHVVSLLKEKREMLDRISSLLLEKEVIEESEFAALTEESRSEKAIVGAMPVDAAV
SSSSTTESPALPASNS*
>SPBDM4_v1_70307|ID:27159765|fabH| 3-oxoacyl-[acyl-carrier-protein] synthase 3 protein 1 [Uncultured spirochete bdmA 4]
MSVVIRSISSYVPPVRITNEELAARMETTDEWIRSHTGIGARHIAPDGVQTSDMAVSAAQ
SALEKAKIGTSDIDYIIVATATPDYFGFPATACIVQDKLGAYGAAAFDVTAGCTGFIYAL
NIASRMLESSHGRHALVIGADGLSRIVDWNDRSTAVLFGDGAGAAVVSRIDEGGRGCLSF
ILGADGSGAKELYLVQPERTQAFDRQQPVVPVISMNGKKVYDFAVKSITVVIERLLHKTA
YRLEDFSWIVPHQANARIVQAAAKRFSIPMDKIYMNIEEYANTSAASIPLALAEMDEKGL
LKPNDLVMLVGFGAGLTYGAAVVRW*
>SPBDM4_v1_70308|ID:27159766|fabF| 3-oxoacyl-[acyl-carrier-protein] synthase II [Uncultured spirochete bdmA 4]
MQIEVAVTGLGIISPIGTSVDEFWKNCTHGVSGVGPITHFDASKVESKIAAEVKNFDPSQ
WIERKEARKMALFSQYAVAAAAQAWNDAGLPENIESQKPGDKAHIDSDRIAVVVGNGIGG
LDVFTESHAKMLQDGPDRIPPMTIPLMIANEAAANIAMRFGIHGPALTQVTACASGTDAL
GQALDLIRAGRADIVIAGGTEAAITEFAVGAFCRLKALSTSYNDRPQLASRPFDRDRDGF
VMGEGAGILILERLDHVVARNAKIHAHFAGYGASCDAYHLTAPQPEGIFGAKAIELALKD
ADIQPEDIDYYNAHGTGTELNDPMESRMLKTAFGSLASTLKISSTKSMTGHCIAAAGAIE
AIVCIKALETGVLPPTIHLDNPDTENGCDLDYIPNVAQSYAVKTAMSASLGFGGHNGMVI
FKAADA*
>SPBDM4_v1_70309|ID:27159767|fabZ| 3-hydroxyacyl-[acyl-carrier-protein] dehydratase FabZ [Uncultured spirochete bdmA 4]
MNDIESYLPHRPPFLFVDDVYIKDDTIFAEHTFHAEDWFFKGHFPEFPIVPGVLLVESMA
QAGGVGAKLMGIYPISLFMLAKIKEARFKQPVRPGDTIKMEITNVRASTVYLHQRGIGKV
GDTIVTEAEWISIASGVPE*
>SPBDM4_v1_70310|ID:27159768| putative reductase TDE_0597 [Uncultured spirochete bdmA 4]
MILKPMVRNNICINAHPMGCRAEVRLQTGRVKAFRDRLTNSGKNGRGTPKPTHVLVIGCS
TGYGLASRLVAAFGYGADTVGFSYEKEPSSTKPGTPGWYFNREFDALAVEAGLFARTFSD
IDAFSDAAKDKAIELARERGFQYDLIIYSLASPVRTDPETGTLYRSVIKPIGRQYAGRTL
DVFTGKIAEATVQPATEEEIAQTIKVMGGEDWQLWIDALDKAGVLGRDATTLAYSYLGPP
FSWPIYRDGTIGQAKLHLEKTAQALNEHYQDAKGLRAFVSINKAVVTRASAVIPVIPLYI
STLFKVMKERNIHEDCVDQMIRLFAERIYRKGETQIPVDTEGRIRVDDLEMGPSVQNEVS
ARLATVDESNLHKLADPDGFRNDFLRAHGFEVPGVDYEQEVLSFE*

>SPBDM4_v1_70311|ID:27159769| putative Ribosomal RNA small subunit methyltransferase E [Uncultured spirochete bdmA 4]
MNLCILNSSDTLQSLSRKDRRAQHILEVLKKKAGDRFRAGSTDGLVGWASLESITDERIM
LRFIAEHPAPPLRPVRVMLGTVRPIQAARIVKELTTLGVSSIAFFPTELGEKSYTQSNFY
VQKEYYMHAQDGAEQAGNPRLPEIGLVWSLKKALEQVEHIEVSAFKAPLQQGDGIQSADG
TLSPPCATKIVCHPDCAATPLSHMSLSGTPVILAIGTERGWTEHELEQFLQSGFALCALG
DRILKTETAAAVAVSVVLARLGYF*
>SPBDM4_v1_70312|ID:27159770|ziaA| Zinc-transporting ATPase [Uncultured spirochete bdmA 4]
MADKYYLKGLDCAQCANEIETALNKCDDVESATVNFATKTLLIDGVSEEKVRGIVRQLEP
EVELIAANDVAAGDNGAGGESDRAEIAKLVVSAILFVAGSVFHTQLHNTPYSVAEYAVLI
PAYLLVGYPVLKSAALSIVHGRIFNEMFLMAIATLGAIAIHQLPEAVGVMLFYAIGEFLQ
DKAVDKSRRSIVQLMDLRPDFARVVENGASKTISPESVAIGQSVEVRPGERVPLDGEIVE
GSSFVDTSSLTGESVPREVGPGDAVLAGYVNDSGRLVVRVTKEFCQSAVARILDLVENAS
ANKAPTEKFITKFASVYTPVVVVLAALIAVVPPLVIPGATFGDYVYRALVLLVISCPCAL
VVSVPLGYFGGVGSASRHKLLVKGANYIDAMTKIDTVVFDKTGTLTKGVFAVSEVVPHNG
FSGMEVLRYAAGAESVSPHPIARSIREAASAIGAAASVTNIVEKKGYGVVAVVDGHVVMA
GNDRMLHEHKVAHDVCDVGGTVVHVTVDDVYAGYILISDAVKDGAAASIKELRRQGVHKV
VMLTGDGETVAKKVAASLGIDEYYAGLLPQDKVEKLAAIKAQEPAGRKVAFVGDGMNDAP
VLVSSDVGFAMGGLGSDAAIEAADIVVMDDDIGRIPLALRISAFTHSIVMENVVFALVVK
GAFVLLGSVGRADMWEAVVADVGVSLLAVLNSLRAVRYNDKGLSVR*
>SPBDM4_v1_70313|ID:27159771| putative transcriptional regulator [Uncultured spirochete bdmA 4]
MNDDLCSQYSISCSETAPELRASLIDVAGLSELFKAMADETRVKILYLLSKRELCVCDLA
YILETNLPAVSHHLRFLRVLNLVKARREGKMVFYSLADEHVLALIEKAREHYIELT*
>SPBDM4_v1_70314|ID:27159772|asnB| asparagine synthetase B [Uncultured spirochete bdmA 4]
MCGIFTITDIKGDPITLRKRALSHVKRLRHRGPDWSGVYSDKHGIICHERLAIVDVTHGA
QPLIDAGTGRVLSVNGEIYNHRELRAKFLKKPHDFKTESDCEPLLYLYDEMGPEFLKLVN
GIFAFVIYDPRDGDYFIARDHIGINPLYWGRDGEGNLYVASELKAIFDLCPKVEIFPPGH
YYKGSEGKLVKWYEPQWAAPGYIPTGEADLPALRESLVAAVKRQLMSDVPYGLLISGGVD
SSIVAAIAARFAERRVETDGAEQAWWPRIHSFSVGLPDAPDTKYAKMVADYIGSQHHEIN
FTVQEAIDALPDVIWHLETFDVTTIRAGTPMYLMTRKIKAMGIKMILSGEGSDEIFGGYL
YFHKAPNRVEFHDELVRKLSMLHLYDCLRANKVTAAWGVEARVPFLDPEFLDVAMNIDPA
HKMIRKDEGHMEKYILRKAFEGWIPDEILWRQKEQFSDGVGYNWIDSLKEYAEKEVSDSA
MANAAYRFPVKTPPTKEAYLYRSIFEEHFKNPSAVDLVPDGPSIACSTPTAIRWDAAWAN
QADPSGRAVRGVHDKSLV*
>SPBDM4_v1_70315|ID:27159773| putative Predicted iron-sulfur flavoprotein [Uncultured spirochete bdmA 4]
MRSVVLAISGSLRESSFTEKMLDLCIKGLGENQEVHKFYPHKMNIGPCKGCRSCWKPKTP
GECVQKDDFEQILDVYKRADYFLLATPLYFFDLPATVKNVIDRFFIILEPAQIKSVEGGS
THPKRLGRHPKAALISSCGFAEIENFDLLRQHFRLICEHTDWSWSGEILVSEAGAAAAPK
LFDRKYNLIEKAGAELLEGMISQKTTNAIAAPVMPAEDYRRMVTLNFEGGVIAKAKVSLI
AIKAMRDKKKIRE*
>SPBDM4_v1_70316|ID:27159774|pepF| Oligoendopeptidase F homolog [Uncultured spirochete bdmA 4]
MTKTIPERSEISGEHKWNLSSLFKDDAEWERNFAILGAMLPEAEEKKKTDFSASADAFLD
TLRAYEQYLILEERLGYYAQLRAAEDEGNSTARGLFARFVGVSSQGQAAWSWLSPTIQAL
GDDFVAGCLASQTFADYRVFLTKLRRFKPHVLSEAEERLLALQTESAQTAQEAFSVLTNV
DLNFGTVETPEGPKPLTQSTFISFMRMQDRSVRKAAYLQFYSHFEAHQNTLGALYAGSVK
LDKYQAQVRKYPSARTMALFPDNVAEAAYDNLISTVNENLPILHRYYGLRKEILGLEGLR
HYDVYVPLVKEVKASHGYEEAVDIVIEALKPLGEDYVRTLRNGLLGGWVDRYENKGKRSG
AFSAGSFIGEPYILMNYKDDVLDDLFTIAHEGGHSMHSWHSARNNPFMCYNYTIFEAEVA
STFNEQLVFSYLYDHSSDDREKAFLVATRIDDTLATLFRQTMFAEFEKRAHEIAESGEAL
TVDVLRKEYRALLEKYFGPEMAFENVSDMEGLRIPHFYNAFYVVYKYATGLSASIALSEKV
LHGGKAERDAYLDFLKSGGSRFPIESLKLAGVDMSTPEPVEAACRRFAGDVEQLAALLQK
K*
>SPBDM4_v1_70317|ID:27159775| Transcriptional regulator, LacI family [Uncultured spirochete bdmA 4]
VGDGDYYLRQVTKRFDEPMSFVYNSSAMSKITIKDIALRAGVSKTTVSFALNYPDRISKE

TYKRIMAIVEELGYVPNPFARTLTTKRLGAIGLLLPQKIGDIFGNPHLAQVISGIGEECE
QKEFSLAILPLIRGKIIEAARKSYVDGLLTIGVGPDHEVVSLLRKNHIPFVTIDGEESAS
TINIGIDHKSAAREIMRHVLEQGFRQIAILSLEPDTKPSESGHFSIVIQKRLEGFQWALE
ESGLSLDDSRVYMQNCKGSIDGGYESARYLLSRERRPTAIVAMSDIAAIGALLAAKELGL
RVPEDVAVAGFDDIPESVISAPTLTTIHQPAREKGIEAARMVMHMLDGAAGNHIRLPYEL
VVRESTRRT*
>SPBDM4_v1_70318|ID:27159776| protein of unknown function [Uncultured spirochete bdmA 4]
MNCTQKTWARQSASSLDANSNHHPPRRQSFCLVFTPFTHAILMTSLPPAAIRLYYIFTMD
TLTVNIDGLNDISVPAGLSAQDIFERAGLTKGSSEGTAAFAPLAVFVNNELAALSAPINA
NCSLSPVYPDSPMGAEVYRRSLCFLLAMAAREIVPKRRLMVSMAIGNGYYHYFXX
>SPBDM4_v1_80001|ID:27159777| Putative reductase TDE_0597 (fragment) [Uncultured spirochete bdmA 4]
MKERNIHEDCVDQMIRLFAERIYRKGETQIPVDTEGRIRVDDLEMGPSVQNEVSARLATV
DESNLHKLADPDGFRNDFLRAHGFEVPGVDYEQEVLSFE*
>SPBDM4_v1_80002|ID:27159778| putative Ribosomal RNA small subunit methyltransferase E [Uncultured spirochete bdmA 4]
MNLCILNSSDTLQSLSRKDRRAQHILEVLKKKAGDRFRAGSTDGLVGWASLESITDERIM
LRFIAEHPAPPLRPVRVMLGTVRPIQAARIVKELTTLGVSSIAFFPTELGEKSYTQSNFY
VQKEYYMHAQDGAEQAGNPRLPEIGLVWSLKKALEQVEHIEVSAFKAPLQQGDGIQSADG
TLSPPCATKIVCHPDCAATPLSHMSLSGTPVILAIGTERGWTEHELEQFLQSGFALCALG
DRILKTETAAAVAVSVVLARLGYF*
>SPBDM4_v1_80003|ID:27159779|ziaA| Zinc-transporting ATPase [Uncultured spirochete bdmA 4]
MADKYYLKGLDCAQCANKIETALNKCDDVESATVSFATKTLVIAGASEEKARSIIRQVEP
EVELVAANNGSAGSSGNEQEEEGNSRVEIAKLVVSAILFVAGSVFHTQLHNTPYSIAEYA
VLIPAYLLVGYPVLKSAVLSIVHGRVFNEMFLMAIATLGAIAIHQLPEAVGVMLFYAVGE
FLQDKAVDKSRRSIVQLMDLRPDFARVVENGVSKTISPESVAIGQSVEVRPGERVPLDGE
IVEGSSFVDTSSLTGESVPREVGPGDAVLAGYVNDSGRLVVRVTKEFCQSAVARILDLVE
NASANKAPTEKFITKFASVYTPVVVVLAALIAVVPPLVIPGATFGDYVYRALVLLVISCP
CALVVSVPLGYFGGVGSASRHKLLVKGANYIDAMTKIDTVVFDKTGTLTKGVFAVSEVVP
HNGFSGMEVLRYAAGAESVSPHPIARSIREAASAIGAAASVTNIVEKKGYGVVAVVDGHV
VMAGNDRMLHEHKVAHDVCDVGGTVVHVTVDDVYAGYILISDAVKDGAAASIKELRRQGV
HKVVMLTGDGETVAKKVAASLGIDEYYAGLLPQDKVEKLAAIKAQEPAGRKVAFVGDGMN
DAPVLVSSDVGFAMGGLGSDAAIEAADIVVMDDDIGRIPLALRISAFTHSIVMENVVFAL
VVKGAFVLLGSVGRADMWEAVVADVGVSLLAVLNSLRAVRYNDKGLSVR*
>SPBDM4_v1_80004|ID:27159780| putative transcriptional regulator [Uncultured spirochete bdmA 4]
VNDDLCSQYSITCSETAPGLRASLIDVAGLSELFKAMADETRVKILYLLSKRELCVCDLA
YILETNLPAISHHLRFLKALNLVKARREGKMVFYSLADEHVLALIEKAREHYIELT*
>SPBDM4_v1_80005|ID:27159781|asnB| asparagine synthetase B [Uncultured spirochete bdmA 4]
MCGIFTITDIKGDPIALRKRALSHVKRLRHRGPDWSGVYSDKHGIICHERLAIVDVTHGA
QPLIDAGTGRVLSVNGEIYNHRELRAKFLKEPHDFKTESDCEPLLYLYDEMGPEFLKHVN
GIFAFVIYDPRDGDYFIARDHIGINPLYWGRDGEGNLYVASEMKAIFDLCPKVEIFPPGH
YYKGSEGKLVKWYEPVWAEPGYIPTGEADLTALRESLVAAVKRQLMSDVPYGLLISGGVD
SSIVAAIAARFAERRVETDGAEQAWWPRIHSFSVGLPDAPDTKYAKMVADYIGSQHHEIN
FTVQEAIDALPDVIWHLETFDVTTIRAGTPMYLMTRKIKAMGIKMILSGEGSDEIFGGYL
YFHKAPNRVEFHEELVRKLSMLHLYDCLRANKVTAAWGVEARVPFLDPEFLDVAMNIDPA
HKMIRKDEGRMEKYILRKAFEGWIPDEILWRQKEQFSEGVGYNWIDSLKEYAEKEVSDSA
MANAAYRFPVKTPPTKEAYLYRAIFEEHFKNPSTVDLVPDGPSIACSTPTAIRWDAAWAN
QADPSGRAVRGVHDKSLK*
>SPBDM4_v1_80006|ID:27159782| putative Predicted iron-sulfur flavoprotein [Uncultured spirochete bdmA 4]
MRSVVLAISGSLREPSFTEKILDLCIKGLGENQEVHKFYPHKMNIGPCKGCRSCWKPKTP
GECVQKDDFEQILDVYKRADYFLLATPLYFFDLPATVKNVIDRFFIILEPAQIKSDEGGS
THPKRLGRHPKAALISSCGFAEIENFDLLRQHFRLICEHTDWSWSGEILVSEAGAAAAPK
LFDRKYNLIEKAGAELLEGMISQKTTNAIAAPVMPAEDYRRMVTLNFEGGVIAKAKVSLI
AIKAMRDKKKIRE*
>SPBDM4_v1_80007|ID:27159783|pepF| Oligoendopeptidase F homolog [Uncultured spirochete bdmA 4]

MTKTIPERSEISGEHKWNLSSLFKDDAEWERNFAILGAMLPEAEEKKKTDFSASADAFLD
TLRAYEQYLILEERLGYYAQLRAAEDEGNSTARGLFARFVGVSSQGQAAWSWLSPTIQAL
GDDFVAGCLASQTFADYRVFLTKLRRFKPHVLSEAEERLLALQTESAQTAQEAFSVLTNV
DLNFGTVETPEGPKPLTQSTFISFMRMQDRSVRKAAYLQFYSHFEAHQNTLGALYAGSVK
LDKYQAQVRKYPSARTMALFPDNVAEAAYDNLISTVNENLPILHRYYGLRKEILGLEGLR
HYDVYVPLVKEVKASHGYEEAVDIVIEALKPLGEDYVRTLRNGLLGGWVDRYENKGKRSG
AFSAGSFIGEPYILMNYKDDVLDDLFTIAHEGGHSMHSWHSARNNPFMCYNYTIFEAEVA
STFNEQLVFSYLYDHSSDDREKAFLVATRIDDTLATLFRQTMFAEFEKRAHEIAESGEAL
TVDVLRKEYRALLEKYFGPEMAFENVSDMEGLRIPHFYNAFYVYKYATGLSASIALSEKV
LHGGMAERDAYLDFLKSGGSRFPIESLKLAGVDMSTPEPVEAACRRFAGDVEQLAALLQK
K*
>SPBDM4_v1_80008|ID:27159784| Transcriptional regulator, LacI family [Uncultured spirochete bdmA 4]
VGDGDYYLRQVTKRFDEPMSFVYNSSAMSKITIKDIALRAGVSKTTVSFALNYPDRISKE
TYKRIMAIVEELGYVPNPFARTLTTKRLGAIGLLLPQKIGDIFGNPHLAQVISGIGEECE
QKEFSLAILPLIRGKIIEAARKSYVDGLLTIGVGPDHEVVSLLRKNHIPFVTIDGEESAS
TINIGIDHKSAAREIMRHVLEQGFRQIAILSLEPDTKPSESGHFSIVIQKRLEGFQWALE
ESGLSLDDSRVYMQNCKGSIDGGYESARYLLSLERRPTAIVAMSDIAAIGALLAAKELGL
RVPEDVAVAGFDDIPESVISAPTLTTIHQPAREKGIEAARMVMHMLDGAAGNHIRLPYEL
VVRESTRRT*
>SPBDM4_v1_80009|ID:27159785| AAA ATPase [Uncultured spirochete bdmA 4]
MTSLPPAAIRLYYIFTMDTLTVNIDGLNDISVPAGLSAQDIFERAGLTKGSSEGTAAFAP
LAVFVNNELAALSAPINANCSLSPVYPDSPMGAEVYRRSLCFLLAMAAREIVPKRRLMVS
MAIGNGYYHYFDDSEPISPQLLEALSARMRNYIASDLPIRIIMHSWNDALEYFDQSNQAD
TVALMEYINDPFVQLNECDGYRDLHIAPLVPCTGLLSVWELIPYRRGMLLRFPHTKNPYE
MDPFSDIPVLYEIAEEYEQKAKVLNAGSIGALNRINQSRNIQDFVLVAEALQNKKLAAIA
DQIAETSDKTKVILIAGPSSSGKTTSAKKLSVQLRALGFKPIHIELDNYFVDRSRTPLDK
DGKPDYECLEALDVEFLNQQLLDLFDGKEVELPNLDFKSGTRKASGNVISLKNNEILILE
GIHGLNERLTPHIPAENKLKIYVSALTQLNVDDHNRVRTTDYRLLRRMVRDYNFRGHDAR
ATLGMWSSVQRGERLYIFPFQGSANIAFNSALDYELGVLKVFAEPLLRAVKPHHPEYPDA
RRIQAFISRISPISPQYVPSDSILREFIGSSVFKY*
>SPBDM4_v1_80010|ID:27159786| Bacterial extracellular solute-binding protein, family 5 [Uncultured spirochete bdmA 4]
VLAALSVSALGAAPQNSQDYVYVFSTDPRSFNYLNDQRATNIQHIANFVDALLEHDRYGI
LRPALAESWKVNDDFTVWTFDIRQGTKWVTADLETYAEVKAQDWVDAMKYMLDNRSPLTC
LVDGFVRNAGAYLQGKITDFSQVGVKVKGDYVLEYTLERPIPYFDTILTSNAYYPVNGDF
LRSKGVDFGRVDRNAILYNGAYVLSNYASRSVIEYDANPTYWDKDHVYIKHVKYVYSDGK
SPDSLFDNFDAGDYVAAPVYTDNEALFARAQAKYKDYIFRARQDSTTFVYAFNYDRRAYA
SPADPAKGGSPKSDQAKEDTRKAILNRDFRKAIFFGIDRPTILAQRNGDVNKLAAIRNSY
TAPELSFDKAGKDYVKYVEDALKSRNPADFPASFKIDDAQDPYYNPTLAKTYMAKAKAEL
AEQGATFPVELDVAADISYAKGMRMSQSFKAGLEGLFGTDTIKVNIVEMDTDNYDASTCY
ARIGAQSNYDIDNSMGWSPEYGDPYTFLQTLEPIVGALLTPIGLDPVDEGSDKASATAIG
LYDYAHKVEAGNDEYKDSSERFKLFGDAEAQLLDDAVILPYMSFGGAFQVSRVIPYTAPR
AAYGADEYKFKGVIVSDKVISLAEREKYRQDWEKQRQAEYKKLQKQ*
>SPBDM4_v1_80011|ID:27159787| putative amidohydrolase [Uncultured spirochete bdmA 4]
MKIALVQFEPVFGNSDRNVERLIRLCRQNPADLYVLPELAYTGYQFVSIEETRSLADDLA
SPRIHAFRAAARDLDAAIVFGFPEVSGGSIYNASLAVLPDGRDYLYRKTHLFYKETLYFS
PGDTGFFVFEFRGARIGMAICFDWFFPESFRTLALKGADIIAHCSNLVMPYCQKADFAAA
VQNRIFIATVNRVGSEQREEEALTFTGESVLISPRGEYVLRGPVDGEAVLVADINPEDAR
NKSVNRYNDVLAMRRPGMYSL*
>SPBDM4_v1_80012|ID:27159788| NAD-dependent malic enzyme [Uncultured spirochete bdmA 4]
MNLSLSLDNLSEVFPDDLTADQRARAQTIFLKTLSEKAHRFYGGKIQVVPKAGLYGFNWF
NVWYTPGVSKISTTIRENNDQSFALSNRGNLVAVVSDSTRVLGDGDCTPPGGLGVMEGKA
FLMKYLGGVDAIALCIDSRGKDGKPDPEKIIDFVKMLEPSVGAVNLEDISQPNCFKVLDD
LRDACDVPVWHDDAQGTACVTLAGLLNALELAGKKLSEAKIVLLGAGASNTTIARLILAD

GGKGENLIVFDSKGGLHRNRKDIEQDKRYYRKWELCVQTNPQCIETEAEALKGADVLIAL
STPGPDTVKREWVRAMAPKSIVFACANPVPEIWPYAAKEEGAYIVATGRGDFPNQVNNSI
CFPGLLKGALLVRARKITDNMAIRCAHSIAGFAKSRGVSADNIIATMEETEVFAREAADV
AVAAIEEGVARVTDTWENIYAKAKEDILASRALTQDLMGQGHIAPIPERMLQESLDFAVA
EVRKQ*
>SPBDM4_v1_80013|ID:27159789| putative dihydropyrimidine dehydrogenase [NADP+], similar to dihydroorotate dehydrogenase [Uncultured spirochete bdmA 4]
MADLGTYLMGLHLRNPIVIGASGLTNLPDGVEKAAHSGAGAIVLKSLFEEQILAVLGQEM
AGLDIDSYPDAEAFISRTAWEESTDEYLKLIDESKKRVDDVPLIASINCVGPGNWAGFAK
RIEEAGADALELNIAYVPFSPHISGHEIEQKVFSTVKEARLATKLPIMVKLGNNYSSLPH
LVHGLSKEGANAVVLFNRFFRFDFNLNTMRLTGVQPLSSETEYHESLRWTAILYKRAGLE
IVSTTGIHNADAAVKCLLAGANAVELCSIIYQKGWKAIGSILEEMDSLVESLGYSSLDSL
QGRLSAINAEKPEEYLRLQYIKALTGMY*
>SPBDM4_v1_80014|ID:27159790|thrS| Threonine--tRNA ligase [Uncultured spirochete bdmA 4]
MAADIERMRHSFAHVMAEAVQHLYGGVKFGIGPAIEDGFYYDFLFPKPVTDEDLRTIEKE
MRRIMAQNQSFVREEVSREQAREIFRDQPFKLELIEGLEEGTISIYKQGDFVDLCRGPHV
DSTKELKADAFKLKTIAGAYWRGDEKRPMLTRIYAYAFASKADLEAYLHMLEEAERRDHR
KLGKELDLFSTHEEAGPGLIYWHPKGGRVRVELEKWWREEHYKNGYEILFTPHIGKSWLW
ETSGHLGFYKANMYAPMTIDEDDYYIKPMNCPFHMMIYKTSSHSYRDLPLRWAELGTVYR
YERSGVLHGLMRVRGFTQDDAHIICTPEQIEDEILEVLRFSLSMWKTLGFKEIKAYLATR
PADSVGEPERWDQALVSLRKAIDKEGLPYEIDEGGGAFYGPKIDLKVKDAIGREWQMTTI
QFDFNEPERFDMTYVDKDGKQKRPYMVHRALLGSIERFFGVFIEHYAGAFPAWLMPDQVA
VIPVAPTFDEYAKEVTANLRGKGIRAKAMLSEERMNAKIRDAQNQKIPYMVILGQREQDE
RTVSLRLRTGGQENGIALDAFTARALEKIETKALDL*
>SPBDM4_v1_80015|ID:27159791|clpS| ATP-dependent Clp protease adapter protein ClpS [Uncultured spirochete bdmA 4]
MGTKLTETTDTQELTREDVEAKEPEEYRVYLINDDFTTMEFVVSILMTVFHKSLPEATKL
TVEVHRKGRGMAGTYSYDIAITKIQQVHAMARQRGFPLRCTMERA*
>SPBDM4_v1_80016|ID:27159792|clpA| ATPase and specificity subunit of ClpA-ClpP ATP-dependent serine protease, chaperone activity [Uncultured spirochete bdmA 4]
MKVSPEVQAIFNAAYNEAKLRSHEYLMPEHILFAGLSFEKVRNIFENCDADLEQMKRSIE
AYFEQKMPIVRNAEPIQSAGFQAVIERAVMQSQSAGKEEVQIPDLVVSLYDEERTYAGYL
MRKLGIKRLQLLEVLSHGISDEDFGADAFPEEETEERTEEGGKERRAARPGTLERFATDL
TAMATAGKLEPVIGREAEIERTIQVLSRRLKNNPIHVGDAGVGKTAITEGLAQRIAAGDV
PPKLKGYTIWSLDMGALLAGTKFRGDFEERVKKVIDSLLKKEKAILFIDEIHTIIGAGAV
TGSTLDASNLLKPALTSGKLRCIGSTTYDEYNKIFEKDRALSRRFQKIDIIEPTIPETVE
ILKGLRSKYEEYHDVRYSDETLDIAAKLAAQFITERKLPDKAIDVIDEAGALARIRAYKV
NQEAEMQTIDITPHDIEVVIAKIARIPERTVTSSEKDKLATLEASLKQEVFGQDRAIEAV
VKAVKRSRAGFRAPDKPIANFLFVGPTGVGKTELARQLSKHLGVPLHRFDMSEYQEKHTV
SRLIGSPPGYVGYEEGGLLTDAIRKTPNAVVLLDEIEKAHPDIYNILLQIMDYATLTDNQ
GRKADFRNVILIMTSNAGARDIGKPLIGFGDQQVSASALDEAVERAFSPEFRNRLDAVVH
FNNLPMEVIERIVKKAIDEFGAQLAEKNVTLKAEDQVIRFLAERGYSREFGARNIARLVE
DQIKTVFVDEVLFGRLEHGGTALARLENDKIVFDITPRDA*
>SPBDM4_v1_80017|ID:27159793|aat| Leucyl/phenylalanyl-tRNA--protein transferase [Uncultured spirochete bdmA 4]
MRWLTMPEYLDETGEYRFPPATSATQSGIVGYGGNLSPGALLSAYRQGIFPWPSTPALLR
WCSPNPRFIISQGSLYITESARKSLKKALRRGAPYTLTLDKAFSDVINNCATVPRPGQPG
TWIFPNLIQGYTELHRLGYAHSVEVWKNKVLVGGLYGVSIGAAFFGESMFSLESNASKIG
FLSLAATLFERGFTFVDCQVVYTPYLALMGGLEVPRPLYLSTLNHALAAPTLKGNWSGVFP
DFPDTELVISFRKNSETITYHT*
>SPBDM4_v1_80018|ID:27159794| protein of unknown function [Uncultured spirochete bdmA 4]
MKLLFVQLPSQMPDWSSAPANIPLAAGYLASYAESKGLLARREWTILEPEIANFGSDSSI
IASITAREPDIVAFTLYSWNLERSLFVAERLASLLPRARLIAGGPEVVEHMPVTVRTPFH
SLVYGEGEDAFAEVLQDIQEHRPLSSSYSSKDLIDLSKMPNPYLTGALRFDPNFQVHLET

MRGCSEKCSYCYYGKNYKTIRRFPHEQTVDVIRTASEAGVSEIYIMDPNFQSGPDFAGRL
RDIAYANHAHTAIHTELRLEGINEELAGLLKEAGVASVEVGLQSTNPKALEAVHRTFDKK
AFERGAELLQKQKIMIKTGLILGLPYDGYEQVIETFDFLGMQGLGQEAELYPLSLLPGTE
IRERADEYGISAMEIPPYLVTSTHWISYDDTVDAIATFEESFDVEWAMPPAPHFQLFKEG
FVSFIDTRKPENIDWMRLNPEKLSSSITLLVDADDPEILARIVRAARDLRKDNPYSLYQI
VLTSETRIPSEKLVERIRDAFLNPEHYYELANSFSPDPQTSYHTRMFFATKNFTLAYRAL
EEAQDMETMVVLNGREGYNADRLAELLPYVIFDKQALPFDRLYELISIYADFPHMLIEAP
DGLL*
>SPBDM4_v1_80019|ID:27159795| D-isomer specific 2-hydroxyacid dehydrogenase NAD-binding protein [Uncultured spirochete bdmA 4]
MNIGSFMPIHSAWGPVVAQLREEFADHTWLEGLTPESPQISSLDLIIAGRIPSSVITEAV
ALKALFQPFTGVNHLPAEMMVARGVEVYNVHSNAFDVAERALAMTLAFYGRIIEFHNDLK
NEVWHGFWVSRGAEDNWDSIYGRTCAILGTGAIGQELAKLLKAFNCRVYGWRRSKGAEVP
EHFDAVLPTLQEAVNMAEIVFIALPATRETEGLLSRELLMTMKGKFLVNVGRGSIVDEEG
LYEALKTGVLKGAAIDTWYTYPKTGVIGAPSRYPIHRLPNVILSPHVGGSTNQATRKSVE
DTRQNIRAYLRTGHGIWRADLRKMY*
>SPBDM4_v1_80020|ID:27159796| putative Ankyrin repeat protein [Uncultured spirochete bdmA 4]
MQKPKLTKARFLGLIILVVFLLGSCATSPASNKEVTLSDLVIKGDIEGIRKFYSNQEQLN
KADNQGLYPLHYAVMKGDVQIAEILIVLGAKPDNKDLSGKTPLRYAVDRKQADMVKMLVD
RGADPFISDVGGTSPAQVALHSDMGILDAMFNSKNINNTGPDGRTILHYAADALMPKEVD
LLLSKGASVQIKDKADRTALDLVLLYPDKLEAAQIAEKLILKGANPSFPEFSWFAVTVRA
PDYNAVRFANGNTPLHEAISRYQSGFALFLLLNKIDPDLKNLNGEAPLHLAVKAGYVEGA
QLLLQNSANPDILDANQNTPLHLTIPPARRLEMVKLLLSYKANPALMDNQGNTPLHKAVM
QFYAPEIVDTLIQAGAPVNAQNANGDTPLILCARTEHYEYAKSLIEAKADIFKMNQAGEN
ALRIALSKGYKAVDNIVLPSNVNQSDDNSNSVLMTAVSLKAPADVLKLILSKGADPNARN
KAQDSALHLAVRQNFAEQGIVLLDAKADIFQYNARQENPLFLALTAKPAPLEWFFRPNVI
AARDANGDSVVHHAARKNLPDGIAYLIKKGASLNVLNNAAETPLHTAAKYDATDAIQYLV
SAGSSLKAADIKGDIPLQSAVLAGAAKATELLISSGSPVNNQNYSGETALHQSGKNNNAA
IVRLLTANGAQTEIQDSRGFTPLAAAAAYGAYDAAADLLKANALIDTRDNSGSTPLYQAV
SGEHIAIIRLLLVNNADMHALNARGLSPFRLALTKNSQIAAEFYTGRLINKPDSNGDCVL
HILVSNGMSQELFAEALKNGANPNARNAKGDTPLMLALRRNDLNAAVILIKAGSSLFVSN
ADNISPLSVIFTMEPGARNKLFAAAGVNNSDFTGETLLHYAVRANNKDAVADLISLGADR
GAQNRKGETPLDLAQAKGYQEIASLLANK*
>SPBDM4_v1_80021|ID:27159797| RNA polymerase sigma factor [Uncultured spirochete bdmA 4]
MSDQVDCDAEGNSKRPTRRDAGEEPMSELDPLSLYLRQISKYPLLNAEQEKDLGARLDEL
GASVQNSDIELAKNPHDDILAQNRKRVADELRITRELLITSNLRLVVSIAKGYQMRGVNL
LDLIDEGNIGLIEAVNRFDYRRGYRFSTYASWWIRQAIIKCLVDQSRIVRLPIHMLNTIR
RCYASAKELVQELGRDPNAAEIAEKSGISQNKVASAMQFAQGTSSLDLNLDEEKNGSLAD
SIKDEKTPDPFSQAFTATMQELIRYVMYSLSEREQIVLQLRYGLNGEGPKTLEETGRALG
ITRERVRQIQEQALEKIKQRQELSDYTV*
>SPBDM4_v1_80022|ID:27159798| putative LysM domain/M23/M37 peptidase domain protein [Uncultured spirochete bdmA 4]
MPINKAGQYMARRVFVTSILLFSLLSMPLTLCGAEQEYHVIQTGETLYSIAKSYSIPYEM
LANINGITDPSKIRPGIVLLIPQAHIVAKGETYYGIAKKYDISIQELKSANDLSDSYILR
VGDVLVIPEKGSGASQVAQLNPPSNTTASPQPSSVASANSVPPAASAAAGAKGSSAAGAQ
TANAAPSAPAPTTVPAPSAPAPAATPSSTPPTSPASVSSSAPNAKAEASAPRATDALLSW
PVDGKGQYMSGKLEGIMFQTNRGASVKAVASGMVVSAGPSRGFGEVVFIQSKSGFVYVYG
GNESILVKTGENIEPGKTIAKVGLDTKEGSPIAYFFVFRNGQPVDPSLAPRE*
>SPBDM4_v1_80023|ID:27159799| Diphosphate/fructose-6-phosphate1-phosphotransferase [Uncultured spirochete bdmA 4]
MLEQSSLHTARYAYVPKVPEIFLEPVLNIKALPTETVEPEKDREALKRTFPNSYGKPAVK
FIKGEGALEEKAFTVGVILSGGPAAGGHNVIAGLFDALKKANSQSRLLGFKGGPSGLIDG
KYIELTSEIVDQYRNTGGFDIIGSGRTKIETPEQLEKSFENVRQLNLDALVVIGGDDSNT
NAALLSEYFIQKGAPLTVVGVPKTIDGDLKNEWIETSFGFDTAIKVYSELIGNICRDAAS

GRKYWHFIKLMGRSASHIALECAFQTRPNIALISEEVEARHMSLSQIVEIIADAIAARAA
KGENFGVIIIPEGLIEFIPEIKVLIAQINDIIASAGTEYESLSKGETQRAFILSRLPAES
AQLFSNLPESIQTQLCWDRDPHGNVQVSRIETEKLLVEMTSRKLQEMAASGVYKGKFDYQ
THFFGYEGRCAFPSNFDADYCYSLGYDACLLILHRLTGYIAAIRNLSGHPKDWLPGGIPI
VSMMNIEHRHGKEKPVIKKALVDLEGKPFKAFSAVRDKWALSSDYRIPGSIQYFGPPAIT
DAPSIILQLEQHS*
>SPBDM4_v1_80024|ID:27159800| Outer membrane lipoprotein carrier protein LolA [Uncultured spirochete bdmA 4]
VKRTKFAIIAWLVVGFASVAGAGAQTLLTADQFFARLADKYSTVSDYQADVQITAGHQPM
TGSLIFKSPTLLRIDFSQPADQVIVFDGKTLLVYLPQYRAVLRQDAGDQGVSLGSATLAS
KEGLSLLRRNYTIGWEHSPNQEPLDPGSNEQVYRLMLSRKSVSEGYKNIRLSVSADTMLI
RRLEGWTVSNDKIGFDFQNIRLNQGINEDKFGYDAPASANVYNNFLFQ*
>SPBDM4_v1_80025|ID:27159801| protein of unknown function [Uncultured spirochete bdmA 4]
MGKVGSIFRDARLGKGLTLDQVSDETNISKRFLQGIEADNFEGFPGEVYILGFLRNYAEF
LGLDALQIVARYRLNEVPQESGQAESPSPSAEARTPDAPEKVQATEPQKPSSSPLTEASE
KNHVAQAQDLAPAPSPKAAEEESTTQTPSLFSEAEEAENLLSSSSEKKKTAATKKKRRAK
PDAQEKRGKDETTPHALGLKEEASSSGQAPQPQSLSKPEPVKPLKSENTHIQAGRILPFV
LVGIVLIIAAISIIPKLNLPAGTSRTPAEYHAEGLPFEQRLYPQDKVYLPLGDDFISITL
RSIKDKVTFDTPYGALTAGLNEEAVINPSADNERLIATVMDYAPNASQNGALVHFDAKEA
LSQSESPGDILVPAGSGSASGSQGSPAIQPSPSLQGQAEPAVLFRSSGGPHPFYVNISFM
SPVLFRYEADRKEWVEKYYRKGESVTVNAANSITFWTANAQAVKVSVFQSAGKSTELVMG
GPGEIAVQRLSWSNAQGGWAMVAAPLD*
>SPBDM4_v1_80026|ID:27159802|rimO| Ribosomal protein S12 methylthiotransferase RimO [Uncultured spirochete bdmA 4]
MPTFYIDQHGCAKNQVDGEEISARLIDAGFSVVESPENADVIIINTCGFIEDAKKESISA
IVDTKKRWPGKKVIAAGCLSQRYPEVLFSDMPEADGVFGNGDLSLIPGAVEKVVHGTREL
LKTPQQRNMPLVCYPRKAFFDYPGTAHIKITEGCSNHCGYCAIPLIRGELRSRDIADIVS
EAQGLLDRGVFEIVLIGQDLGNYGKDLEGRCMLPDLLSAFAEIKENFRVRVLYIHPDHFP
HEILPIIKGDSRFAPYFDLPFQHASASVLRSMNRQGSTEIYLGLIERIRAELPFAMIRST
FLVGFPGETEADFSALLDFQRAAQLDWLGAFAYSREENTPAYDMKNRVPKSVAQKRKALV
EETQVAITAERLKRFIGMSDHFIVEEAFDHDKLCIGRGWMQAPEVDGVTLIHAAQKPGAI
VEAHIISVNGVDFNAVLEYTATV*
>SPBDM4_v1_80027|ID:27159803| conserved protein of unknown function [Uncultured spirochete bdmA 4]
MISGQIPEYLQGLNPEQLEAVRDEGTRLLILAGAGTGKTRVITTKIAYLVREKHILPESI
LAVTFTNKAANEMRERAMDIEPSCERAIIRTFHSFGAWFMRRNVQAFDLKREFTIYDDDD
SAELVHSIFPTFSKRDCGDYAFLIAKAKDYGLSPDAPNLGFISQHPEFRRIYAAYEERLR
ATGNVDFGDLILMPAQLLEKDEAIRNRTQQRFRVILVDEYQDSNIAQFRLLQQLASPEAM
LCVVGDDDQSIYRFRGAEVRNILGFQTIFPGTKLIKLERNYRSHQSILDLAHSIVAHNAS
RLGKKLLATRSGGTKPKLAILDDQDQEIEYCAYIARQHLQHGGHWRDIAILYRTNAQSLG
FERLFPHYDIPYRIVGALRFYEREEIKDMLAFLAFLANPRDEVSFKRIVNKPARGIGTTS
IEKIVASALQDDSDLLEASHKVIQEVHGKGKDGLRQFISLIGDAKTSLDKGVNDEGNLGM
LGSLVADFAKNSGLIDFYRGKDKISGTQKEENIEELVNAASDSPLSEDGLLEFLDTVMLD
QSQQRNDANADAVTLITMHNTKGLEFPVVIVTGLEQGLFPRNDEEGDDIEEQRRLFYVAV
TRAKEQLYLTACRWRRLHGRLFETVPSRFLTEIDSELYDLWGASGRPAQAAYTHRTPGRE
HYDTTTKAEWAPGMTVYHDEYGNGTVIKVTPSQSAGALVIVKFESGKVAQFFPKYTKKLE
KVEH*
>SPBDM4_v1_80028|ID:27159804| conserved membrane protein of unknown function [Uncultured spirochete bdmA 4]
MNRHRSGSSQLGFFVALVSGISFSTLGIFAEMLYAEGFSVASALAWRFVVASLFLWAVVY
VRMYFRKRFHLRAQDIQGTTEDHVAVRKRFFRVFLLGLIGFSPQAGLFFLTVKILDPGIT
SLFLYLYPSFVFLIGFLLKHQKPGRVQSFALLLSLVGCILTFWKAGSYPTVGLVLGVVVA
IAYAVYLVAGESVLQNVDSLWATAVIMTAAALVYLCILIGGGTFIVPVSLKQILLVVCIA
LFATVMPIVTLFMAMQRIGARDTSIISTVEPVFTNILSMIFFGELMTGRRILGGSLILAG
VIILNRWGYPSRRKAEVAS*
>SPBDM4_v1_80029|ID:27159805| conserved membrane protein of unknown function [Uncultured spirochete bdmA

4]
MSEPKILDYNKNEVRERLLRALNGATKDWTDADLARATGLPLAQINAEMPAISDEYRGRL
KVSEKGDLLYSFPQGLKSRYRGFGPFMRKLWRTVSRGAVATGKVLFKVWILVTFFGYFFL
FIALALVALFGSVALQGGGSRDRGDRRGGGGLGGLWLTTSLFDSMIRLWFYSELFKSSDA
RYRDSYARRERHPLNKAVFSHVFGDGDPNADWNTVLKKAFVAFIQTHKGVITLPEFMAIS
GLKPEEAQDRITRFLVEFEGSPEVSENGALYFFFPSLLVKASDVSVPAASAFPLKKLKRF
SSNDGKMDRTFRWVNIVNLLFGSYYLLNAINVGTNVIIQTAEGVALKGGLTYLYSYTVYL
ASQLGATQPAVLLGWVLGVAPLAFSVLFFGIPLVRSWRLKRENEMLKRENMRKVLYSAVI
ANPRGFDHRTVSLPSEEVRPSAPEAVQEEVRHLAAWSSGEISAEGIWAFKDLEFIQREAE
QVRASIRETDYAPQKTVFDTDEKV*
>SPBDM4_v1_80030|ID:27159806|glgA| Glycogen synthase [Uncultured spirochete bdmA 4]
VKILFISSEVTPFAKSGGLGDAVSSLATALSKLGHEVRILLPRYYFISKKTLRRINGILE
IDSARERHHAFLYTTTMPNSQVKVYFLDCEPLYGRNGIYGYTSTQEFPDNPERFSVLCKA
AFAACRSVGWIPDIMHANDWPAALIPVYLNTKEKNTEFSSTAGVFAIHNLGYQGIYSRDH
FPDLGLGWEYFYGAGFEYYGNINFLKSGIVSAECITTVSPTYAREIQTPNGGFGLDGLLR
QRSRDLVGILNGIDTDVWNTKTDPYLPAHYSSTDLSGKAACKSTLQREFGLPIDPGVPLI
GMVTRLVEQKGISEVFGRTYGCMRKILETIHVQVAVLGSGDAWAEEAIMNYSAQYPQFKG
VIGYNERLAHLIEGGSDFFLMPSRYEPCGLNQMYSLVYGTLPIVHRTGGLADTVVNYNQD
LGEGTGFMFDDLTPQAVYDTVGWAMWAWYNRPQHIQNMRTRAMEQDFSWARSAEEYVRLY
QHAVSLREAR*
>SPBDM4_v1_80031|ID:27159807| exported protein of unknown function [Uncultured spirochete bdmA 4]
MADHIKILPSLILILIAALLLACPIGTPQGMGNVRIQCIVPGDLTGPGAKSAAQGLVGPQ
SGWKIASYRLTFTADNGEVITRTLNRGTGEVALKVGGWACCAEGLSADGSVIVEKQVHVV
AEAGKTINLPISLHLANGKGTLRIVFSPSQTPGTGWKYSVSLVYKGLPGDPSFQGPDDFS
TEVSATEASLNVADLESGYYVLAVQLKDDSSATIAGTTATALILPKQISAGECGISLSDP
SINISVVAPDLELSANAAIAVDRYLNRNRPMIVPLALGPDEAELNVDWYVNGEKIENPQN
EMGQSLPGFRILLGSNEVPNMQTTIKMDALLTETTSGLSQTFPHTSYLALGPTTGWAEWV
QSIDYRAAMGSSVFNASDASNTGTGIQADAKWVATNPAGLIAVAGLDKTSALHLFYSPLG
KEATQGNGSVFTIPSSTGWLRLWRDKIVVEKNERSPDRVSISPDGSLIAIGSSTSNWLRV
YALDGAGEILSKKDIVSTKNGAPAFASTKAMNFSADSKRLFILTNSPEKILVFNTERLAL
GEACSENEFSFESCFVPPPSSSLGMEDMVLLPDGWIAACSSNIARIFFVRYSEAECQFSS
AGLFASGANGESLGDPKAIAFDEEDGLCYILGYSKKLHVFSKPDTSSGYGPLTTLSLPNE
FDKARSLVFLKNHSDAKFLIAGGGASLGIIALDSLGQPLVLSSLDSTPDDYAAISSISNI
ASLGSSIVAAGGTSGLVAMLDIL*
>SPBDM4_v1_80032|ID:27159808|gcvPB| putative glycine dehydrogenase (decarboxylating) subunit 2 [Uncultured spirochete bdmA 4]
MNTMNMRDEPLVYELSRAGRKGCSLPKPDVPEYAVPEDLAREGLDLPELDELTVVRHFTR
LSQKNFSIDTEFYPLGSCTMKYNPKVNEVAASNPGWKNLHPLVGDENAQGALELMWRLQE
GLREVGGFAAVSLQPAAGAHGELSGVLMIRAALRERGAHARKKMLIPDSAHGTNPASCTM
AGFETQTIPSGPDGNIDMAALRAALDETVAGIMITNPNTLGLFERNIEEICRLVHEAGGY
VYGDGANMNALTGIFKPGNSGIDVMHFNLHKTFSTPHGGGGPGAGMVAANKTLAPYLPGP
VAVKNGDGYTLEMPPKSIGRVKAFHGNFGVLVRAYSYLLMTGANGLRRVAENAVLNANYL
KSLVEDVYPVPYKRNCMHEFVAKGTIADGVHTLDIAKRLIDYGFHPPTIYFPLIVQEALM
IEPTETDSKETLDLYADALKKIAMEAVAQPELLHDAPHNAPVARLDEVMAARRPILCYRG
*
>SPBDM4_v1_80033|ID:27159809|gcvPA| putative glycine dehydrogenase (decarboxylating) subunit 1 [Uncultured spirochete bdmA 4]
VPFVPNTDAERAEMLKAVGASSVEELFSDIPELLRIKGLDLSDGVSEIEALNEIDSLAER
NVQTKKMDWFLGAGAYYGYIPAVVPALAGRGEFLTAYTPYQPEVSQGTLQAIFEYQSMAA
KLLGMPVVNASHYDGATALAEAVLMAWKAKENRNRILLPVDLHPEYSQVIATYCTSFNIE
FVQYEGAPESAPVDEDTAAIVIAYPSFSGEVYPIKRAVDRAHEMGALCIVHADPLMCAIM
KSPGEQGADIVAAEGQSLGNPLNFGGPYLGIMGTTEALVRRMPGRIVGETKDKVGKRGFV
LTLSTREQHIRREKAVSNICSNQGLTMLQTCIYLATLGKQGLREVAKQSYDKAHYAAGLI
GGTAGYHVRSSQFFREFLVETPKPAARIVKTLSRRGIMPGLALSRYYPDRPNELLICVTE

MNTRAQIERLAVALKEASK*
>SPBDM4_v1_80034|ID:27159810|gcvH| glycine cleavage complex lipoylprotein [Uncultured spirochete bdmA 4]
MAIDKNARYLESHEYAKPSGEHMIFGISDHAQTELGDVVFVELPAVGKTLSKGAMFGTIE
SVKAASDLYAPVSGTVVEVNEAVKNDPSLVNKDCYGAGWLIKVAPSNASEFDGLLDPAAY
GKAIGEE*
>SPBDM4_v1_80035|ID:27159811|gcvT| Aminomethyltransferase [Uncultured spirochete bdmA 4]
MEEPTNITALHDWHVSHGAKMAAFAGYDMPINYSTGAVGEHHLTRRSVGLFDIDHMGQIE
ITGQGADDFVARMITAKVSDLIPPMARYSLLLDEKGGVIDDLFVYRLPDSWWIVVNASNR
AADLEWFKTHAPSGVKVTDRSDDTYMIAVQGPRAIELIDKVADSPVSSIQRFCWGNIAID
GIPTLFGRTGYTGEDGGELFFPAEKALRLWEFLLQRGEALGIETKPIGLAARDSLRFEAG
MPLHGHEISIEINPIEAGFKWACDFEKEFVGKEALLAIIEEGVSRKLVGIEVFGGVPREG
YEVCSPDGGSIGHCVAGMFCPTVKKYAANAFVAPDFAKAGTNVSVIIRGQPRAAVVIKRP
LYIPAYRR*
>SPBDM4_v1_80036|ID:27159812| putative Lipoprotein LpqB, GerMN domain protein [Uncultured spirochete bdmA 4]
MAARRKKRTSSKTGCLLWIAAFILLLILFLVKFGDIRTAVQKTGFLDALNHAVSKPNSPT
PAPEQPHSPTIQTTEPQQPEESPGPSSAAPSSGQTTPNTPSTSSPAQAPQNIPSSPPESV
PTPAVPAQQEKKTRSAVLYFVQTHDDGSISSQRVKRTIPISDSPIQDTLEILIKGPTESE
LRENLLSLIPSGTKLRGVSVRGSTAIVDFNEAFVYNRYGKEGYMAQIRQIVYTLTEFQNI
TDVQFLIEGKPRAFLTEGIALDKPWARASF*
>SPBDM4_v1_80037|ID:27159813| protein of unknown function [Uncultured spirochete bdmA 4]
VQIHNPYFWQVVFIDREPEKAVFFTVAPNKEQPEYTASLVLDPFARQK*
>SPBDM4_v1_80038|ID:27159814| protein of unknown function [Uncultured spirochete bdmA 4]
MTVGELQEQIFPSAEGLERALKAALRASQTISPALRPRAPGIASPGGLVQLSHQKPCILV
PDLHARPAFIDALLRTEFPDLGEPLHSALEDDRVSLLFLGDILHAEGEEAARRWISAYKR
LAAARDDHAILSPEMDEEMGLSLEALLKVIDLVCRFPNSVFCLKGNHDNVMNAADHGDFP
FYKYADEGRMGALWFQLRYGPDIAQLVRRYELSLPVAAVGENYCASHAEPALPLSRSAII
AYFEHPEVVQALIWTANAEAKEGSVA*
>SPBDM4_v1_80039|ID:27159815| Tetratricopeptide TPR_2 repeat-containing protein [Uncultured spirochete bdmA 4]
MIEKSRRVPGMKPLSRAKSHVASAQKKSRIEDADGQPLSEAFEARKKLALSSIVYIRFEG
PDALARVPEGIDPSIPYPVQLQSPVIKLNPASITIESLLTGMVRVLAWEPNNSNAETYRA
YVKAVRPELFEELIAAGIQKAESKEWTVAEELFFAASGLDPERPEPSINLALMHEEHAKY
LSDSGNEEDAEKEDDLAHKYYQYLLALKETFPPAYYHAAFFFLRKHNYDKAVALLTSYIG
MSDDEERTKRAKSILEKLSSMGYLDTLFKEAYDFIQMGEEQKGLERALQFVDKYPDVWNG
WFLVGWANRRMGKWAEGERAFHAALEKGAEGADVYNELSICEMELGKLDAAKSGLEKALR
IEPENVKIIVNLGALAYRQGRIKEAEGFF*
>SPBDM4_v1_80040|ID:27159816| conserved membrane protein of unknown function [Uncultured spirochete bdmA 4]
MIGFLIKKSFFDGWDNLYLLAALNGVFLLVLLLFVVLPAALGAPTWLIIVLVAIAIVGLS
IWDAAVTDAMYSLADAKSVHFSDIKAALSRSLKLGLIMGGLNLMIVVAVAVALPFYLGQK
AMWGVFAGGVLFWTMLLAMLILQYVPAIFARDGGTPRQAFRTALYLFADNPGFSIFLLVW
RVLTFAISILTMLLAPGPAGTSLASAVAVRLRMKKYRYLKENPEANHRQIPWDDLLLEEK
ELVGKRTLKGMIFPWKD*
>SPBDM4_v1_80041|ID:27159817| Ornithine decarboxylase [Uncultured spirochete bdmA 4]
MHKVCNWSSSELSPCQEACTVKTAYSFPLENFMSREKFERIKEFAKDKETPCLIIDLEVI
RKNYENLQTYLPWSIIYYAVKANPDDAVVALLRDLGSNFDVASRYELDQLLRLGVSPDRM
SFGNTIKKEKDIAYFYEKGVRLFVTDSISDIDKLSRAAPGSKVFFRLLTEGLGADWPLSK
KFGSHPDLARQLMKTAVRFGLEPYGISFHPGSQQRDVGQWSSALAIVSQLFNWARHDLKI
DLKMINMGGGFPANYLEPTDSLQQYAEDIKRFLDNSFGLVWPEKIVIEPGRSMAGDAGVI
VSEIINIAKKSVHERYPWVFLDIGKFGGLIETLEESIKYPIYFEGQGSVEEVILAGPTCD
SMDILYERTPYFMPSSAQIGDKVYILTAGAYTQSYSSVYFNGFPPLKSYILPSV*
>SPBDM4_v1_80042|ID:27159818| Aminotransferase class I and II [Uncultured spirochete bdmA 4]
MNQLAEELNEVLAGTVAERLFSEMGRHMYFPKGIITQGAEASERAYRFNATIGMAYEHGQ

PMMLDALRKELPGLTPTEAVAYAPTGGVLELRKAWKAALYKKNPSLEKKNISLPVVVPGL
TAAVSYICDLFVEPGDTVIVPDLHWPNYRLIIEERKMAAGITYPLFSGNAFNVEALLEGV
RQAGQRRGKAIIVLNFPNNPTGYSLTLEEADRIANGLLEIARGGTDILAIADDAYFGLQY
ENNLLCESMFARLTNLHPNILAVKADGPTKEDYVWGFRMGFVTFGGRDLTDQQYDALTKK
LTGIIRSSVSSSCSLSQNLLLKALTYEGYDAQKAGYRAVLEGRYKAMKKKLASMKLPGGL
EPMPFNSGYFMSFYCEGFSAEALRKKLLDEMGIGTVSLLDRYLRVAFSSVEEKDVDDLCE
AIAQVTAELQA*
>SPBDM4_v1_80043|ID:27159819| conserved exported protein of unknown function [Uncultured spirochete bdmA 4]
MKKILMLAAAFIAVSGIAFAQVDLSKVKDGVYFAEDKSFSSSGWKEQVILEVSKGKIVSA
EWNGISNLPGVADKKTYAAAGKYGMVKGSKIKAEWDAQAKAAEEYLVKTQNVNFNKFDAN
GHTDAISGATLSVKGFFELAQEALKSSPVAKGSYKDGWYYAEEPNFDKSGWKNTVVVTVV
NGSIVDLLWNGISKDPQAKSKYVQSQTGAYKMNAKNGEWYVQADRVAQAIVKAGDPSKLP
VKADGKTDAVSGVSITVPVLPLAVEALKTAK*
>SPBDM4_v1_80044|ID:27159820| GHMP kinase, N-terminal domain protein [Uncultured spirochete bdmA 4]
MDKEYMSNIHTRPQKIPLLTIAELSKNEANRLAARFARLASKLPPTEGSVPQELHYYSSP
GRTELGGNHTDHNNGCVLAASVDLDMLAVVRARNDSIVTLTSSGYAPLSLDMADTGARQD
ERGSPAAIIRGLAGWLSRKGASLPKGFDIAVDSEVPSGSGLSSSAAFELLIAAIFDDIGN
YGFSPVEWAIAGQFAENEYFGKPCGLMDQLACAVGGIVSIDFAEPQRPKIETVSYDFAAH
DLVLAIVNTGSNHEDLTREYASIPQEMKSVAALCEARTLDRVTKSDLLARTPEIRARCGD
RAFLRAWHFVHETRRPGQMQRALARDDIAAYLSLVRESGRSSWMYLQNIQAGDPRRQSLA
LALALSEDLLGDEGAWRVHGGGFAGTVQVYIPKSKFHEFLERMETVFGQDSVRRLNIRPY
GVCRFLM*
>SPBDM4_v1_80045|ID:27159821| Major facilitator superfamily MFS_1 [Uncultured spirochete bdmA 4]
VRTKKGFGPYLGITFLIGFGFFTMGLMDPLYDNYVTIFLSRYIQRMSLVGFFMTIDNILA
IFLIPLVSAWSDRTHTRIGRRMPYILVLLPLTAILFGAIPYAAGVSLAALLTTLILLNVT
KQSVRGPIVALMPDTIPANYRSEANGVINTMGGIASIVGTVGLARLMDLDTTLPLLGATK
DRLPFPLAGIFVVLAVILLFAFVREKEPNPSEAQEQKTPILESFRNVAAQKDKSALYILI
ALFLWFLGYQGVLPFIGKYSVDILKTSTGTAALASGMVGIAYALFAIPSGYIAHRIGRKK
TIRISLLVISILTVILFVHPWITASLPGSLKLGTFWAIMFLFGIFWVSIITNSFPMLWQM
AEWGTIGIYTGLYYTASQAAAILAPILTGFIIDIFGYQGIFLFCTICMLCARFVMGKVTK
GEPGEILNSEER*
>SPBDM4_v1_80046|ID:27159822|ycjV| putative sugar transporter subunit: ATP-binding component of ABC superfamily transporter [Uncultured spirochete bdmA 4]
MAKVELKGIGKVYEGNVRAVTNANITINDKEFVVFVGPSGCGKTTTLRMIAGLEDITEGE
IYIDSKLVNDVPPKDRDIAMVFQNYALYPHMTVYENMAFGLKIRKLPKDEISSRVKEAAR
ILDIDKLLDRKPKQLSGGQRQRVAVGRAIVRKPKVFLFDEPLSNLDAKLRVQMRAELIEL
HDRLQATMIYVTHDQVEAMTMGDKIVVMKDGVVQQIGSPLYLYNDPINKFVAGFIGSPPM
NFMTVNVVEEGGKVLIDEGSFRLAPDEKQAGLLKPYVGKSVYFGIRPEDLLVADAAQSGQ
KFDAKVTVVEPLGSEIHLQATTPTQNMIARIPPHHLHKHGDTITFAPVMAKAIYFDKETE
KSILPVRWNEQEE*
>SPBDM4_v1_80047|ID:27159823| protein of unknown function [Uncultured spirochete bdmA 4]
MMDIRHFANLHGRARTRKAVLILERIEREWRSMDSPSYERSLVYARGLAEFMAISDESPE
EVVLAAQNFLSSKADLQAINRFRHALMAASGQRPADWDFVAEPRPPLSRATSRPGMRVYL
EDIRSPFNVGSVFRTAEALGFEEIILSPECADPLHPRALRTSMGTVERLPWRRAPTSELA
AMEGVFALEVGGIALQEFDFPLPGIMVLGSEELGISRETLGFCRRGCAEIPLVGTNASLN
VATAFGIAGFAWLSL*
>SPBDM4_v1_80048|ID:27159824|rsmI| Ribosomal RNA small subunit methyltransferase I [Uncultured spirochete bdmA 4]
MVATPIGNLGDITLRALETLKSVDTVACEDTRHTLKLLSHFDIHKPLISCHANDERRGAE
RVVSLLDEGKDVAYCSDAGTPGLSDPGALVVREARRRGHKVVPVPGPSAFACLVSAAGVS
GRTFLFDGFLSPKPGKRRTRLKELLLRGESFVLYESPFRVGKLLTDIASIDPNRLVCIGR
ELTKIHEEIIEGTAAELAARFPAGSARGEFCLIVRGLANEPSSGQAGDDL*
>SPBDM4_v1_80049|ID:27159825| conserved membrane protein of unknown function [Uncultured spirochete bdmA 4]

MMKTMNSRKISFLILLLVLFVLVARIIYPFLTVILWSSLLYVFLEPLHKKLKGPEGPHGR
RTLRTYLSAILLAVLGVLIVLVPLAFLTVATGKQLADILKSGVHFFEANADKFKIDPQGQ
IGAALQSFFGDSFDLSNFDLMKELQSFLASAANQILKISTSLIKNVSQFVVTILFIMFTL
YYLLMDGKALGDTFVSMMPIDPEHTRLFMRKLRETGRQLVIGYFLVALFQGLMMFIVSLI
FGFKNNVLLAVLTAISSFIPMFGTSIVWVPMSISIALQGDIVKAIVFLVCAGIVVSGLDN
FIRPMVLGGQLKVHPLLLFFSIVGGLAVFGFNGIILGPLILTIFIAAGELYRALNEEGEN
APKKEESAT*
>SPBDM4_v1_80050|ID:27159826| conserved protein of unknown function [Uncultured spirochete bdmA 4]
MRLVDLHTHSSASDGELAPSALIEKAHALGLSTIALTDHDTVDGIAEAREAAARAGIDFI
SGIEIEIDFEPGEFHLLGYGIDDHAPALVKATERLARARHERNKAILERIAVAGLPLDTV
KIAELSRESYIGRPHLADMLVEARCARNRQDAFDKFLGKGRPFYIKKFCLPLEEALSVIR
AACGIPVVAHPYSLFVSKAMLGELFSRWKEMGIGGIEAYHPTAKYGQCVILERMAREREM
FVTAGSDFHGKTRPACGMGRTAGNMPIEGRFRDELVDALMRFSQTSG*
>SPBDM4_v1_80051|ID:27159827| protein of unknown function [Uncultured spirochete bdmA 4]
MMMVRDTAARNKIAHSMYKIAEFAQNVKRCFFSLRTVRSVSSFHFVSHGIKLEVKEMNTQ
SSPQFLSPERHPRTGGRRTIVYPVLSRRARGLSLGINLFPDRKQCTYDCPYCEVPSFSNP
GLALQPGEVTDALWKFFADDWPMYSARFELKDISLSGNGEPTLSPFLGEALDAAWGTLQG
LAGPCSVAGEVPVVLITNSTGFLRPEVCEMLERFSRRAHLEVWAKLDGGLPYLHRMLSGS
EFAYGRIADAIADFALHVPVKLQTMICRDSRSGALLFDTDGYLETLQSLLQRRAKVRAVQ
LYTVARMPAKPWVEALEDAELQTIARRVRGALPAEIGVECFGRTGYIE*
>SPBDM4_v1_80052|ID:27159828|hisG| ATP phosphoribosyltransferase [Uncultured spirochete bdmA 4]
MTDKGNGKTELRIGLPKGRMQAKVIELFNSAGIPVSVDEREYRPRIGNGKSQASVSKGIE
YEAKLLKPQNIVEMLQAGSRDIGFAGADWVRELGVDLVELLDTGLDPVTIVAAAPEPVTA
VFRASGGDVEAMRAICGRTPIIASEYEGLAKSWIEKNLPGAELVRSYGATEVFPPEDADI
IIDNCATGTTLRINKLEIIDTLMTSSTRLYASKEVMQIPAKSEAADSLILLFRSVLEARR
RVMLEVNVSAELLDQLVAILPCLRAPTVSPLGKGDAFAVKVAAPRDTLASLIPEIKRRGG
TDIVVTQMSQLVP*
>SPBDM4_v1_80053|ID:27159829| putative Histidinol-phosphate transaminase [Uncultured spirochete bdmA 4]
MLRLDANEGRCLLSENDLGEILSPEIARRYPVRGLLEGPLAERLGLPASCVLATAGADDA
IDRAVRTLAGPGGLVMSTHPGFVEFLAAAQRSSAMYASISKDPFGPFPIREICEALREKR
PQLLILSSPDNPSGGVLSPTGLAEIAAACGESGTIFIFDATYGDFSPNTATPADAFAFSN
VLVSGSFSKSRGLAGFRIGYVAGGPQMEGIIGRLAEAGPPFSLSSSAIEAGRRALSIDPD
RVKAFVDEIRREVRELSALLKSMDFLVSESEANFVLIRADEAIPLAAALRRKEIFVRTWQ
GKPGYEDLVRITVPGEAGEFGELTEAIISVGKIMGINKEALA*
>SPBDM4_v1_80054|ID:27159830|hisB| Imidazoleglycerol-phosphate dehydratase [Uncultured spirochete bdmA 4]
MTVQNEKTSVRRATKETDVSLSLAFEPGQIEIDTGIGFFNHMLTQLAFHAGWSLSVTCKG
DLDVDDHHSVEDTAIALGVAFAKLAPSGAGRARFGYAYAPMDEALARAVVDISGRGCCVI
DGTFASPAIGTLSSCNIAHFFQSFAANAGLTLHLDILRGENEQHKAEALFKAAALALRMA
LAPRAGAESGSTKGETAIDTEGAGL*
>SPBDM4_v1_80055|ID:27159831| putative Imidazole glycerol phosphate synthase subunit HisH [Uncultured spirochete bdmA 4]
MSERPKLVIVETGVANLASVRALAERLGMEPLVSANPEEVVRAQTVILPGVGAFGPAMQK
LKDYRLDAALRARVAEGLPTAGICLGMQLFFETSEESEGVDGLGVFQGKVRRLEGGLPLP
QLGWNRIEPDAGARLLQPGWVYFANSYGVRADSFPELLHEDGGAGREPARRGAAGGLFEH
IAAAITTYGGPFVSAIEAWRDDRPSLLLCQFHPELSGVFGTQLFARWLELNGARRPL*
>SPBDM4_v1_80056|ID:27159832|hisF| imidazole glycerol phosphate synthase, catalytic subunit with HisH [Uncultured spirochete bdmA 4]
MSGLAVRIIPCLDFKDGRVVKGVKFENLVDSGDPLERARIYEAQGADELTFLDISATVEG
RKTMAKEVARIRRGIAIPITVGGGIATIDDAARLLDSGADRVSVNSAAVRSPAIIGDIAS
QFGVQCAVVAIDAARNDAMPSGYEVKIAAGAVPTGLDAIAWAREAAIRGAGEILLTSIDR
DGTSLGYECTLLGLVARAVGIPVVASGGASKLEHFLEGYQSGASALLAAGIFHRGEVSIS
EIKKYLSAHSVEVRL*
>SPBDM4_v1_80057|ID:27159833| conserved protein of unknown function [Uncultured spirochete bdmA 4]
MIVPSIDIMNGRAVQLRGGKQPPLDAGNPLELAERYSRVGEFAVVDLDAAMGKGGNRDLI

LTLCKKYSVRVGGGIRSQELAVEYLNAGARFIVLGTAAKPEFLATLPRERLIAALDSRND
TIMVEGWTKPREGKVEDAVTALAPYVAGFLVTFIETEGDQCGIDLERARALIARAPDRKF
TFAGGAAGGGRGTADIAVLDALGADIQAGTSLVLGDLSLADAFAAPLRTDRPDGLWPTSV
CDESGKLLGLVYSSRESLKLALETGKGAYHSRSRGLWIKGESSGNDQRLVRADLDCDRDT
IRFTVRQNGAGFCHLGRRNCFDDGYGLEKLDRTITHRMKEAPQGSYTRRLFSDEALLASK
LREEAQELAQSKTKDEAVAEAADVMYFALVKTISMGGSLADIEAELERRSLRVTRRGGNA
KPDFEGPEGKNEWKSIH*
>SPBDM4_v1_80058|ID:27159834|hisD| Histidinol dehydrogenase [Uncultured spirochete bdmA 4]
MEKHTLKRITIDEIPEPSVRLFDPKVAAVAMAAIEAIRAGGEAALREWAVKFDGLPGDVP
LVISRDEMRAAYESLPHATAELLARAKGRIAAFAAAQRACLAPLDLPAQGGRHGHEFVPV
ERAGCYVPGGAFPLPSSAIMTLVPAKTAGVEEVWCAGPKPTPETLAAAWLAGADGFLRCG
GAHAVAALAFGVAVPKCNVIVGPGGKYVAAAKRLLYGLVGTEAPAGPSELLIIADATANP
EIVAADLLAQAEHSPDAMPALIVTDEKLADRIEEELSSQLSALPEPNRGIARAALGNGFC
CVEPDLGRAVLGANRCAPEHLELATDRPTELARNVRNAGALFLGSGSAEVFGDYGAGPNH
TLPTGGASKFAAGLSVIHFLRARTWLAIDDPSLLIGDTAAFARLEGLEAHARAALIRKRL
QTEP*
>SPBDM4_v1_80059|ID:27159835| Thermostable carboxypeptidase 1 [Uncultured spirochete bdmA 4]
MHKETARLVELDREHSLVVHIGAILGWDQETYMPSKAIEERASQLALLEGLAHQKAVNPE
VGELLSALEAKSDLSDDEKAYVRVVRRDYDRETKLPESFVTEYAKDASISQATWVEAKRN
NDFRAFEPHLARMVELNKERATYLNPGARPYDVLLDLYEPGSTQESIAAVFSTMKSYLVD
ILGKIRSRPQIDDKCTGLQVSARTQERISHYCMKALHYERDRGRLDVSAHPFTTTLGTDD
VRITTRYIEDYFPSSLFSTIHESGHALYEMGIDPNPEFRGTKLADAVSMAVHESQSRLWE
NIIGRSHAFWERNFPVLSALLGEAGDGLDLDSFVKSVNRVSPSLIRTEADEVTYGLHVIA
RFELESALFDGNLSVGDMPAAWQAKYRDLLGIEAPDDRQGCLQDVHWSMGAFGYFPSYAL
GNLYGAQFWATLKKDVPDIETRIAEGETLSVLRWLRANVHVHGSRYTPGELVEKVTGQPL
DPVWFERYLREKYSKIYGF*
>SPBDM4_v1_80060|ID:27159836|recG| ATP-dependent DNA helicase RecG [Uncultured spirochete bdmA 4]
VFLRELTQDLRILRGTGPATLRALANLDVHTVADLLLHLPRDYEDKTKIVPISRFSEGKV
YTTARILRHEWFGFGRMRTLKIVVADDSSEAALLCFNRPFLEQMAPIGSTVQIYGKFQFK
YGEIQSSAFEIKRLDGALNLKIKPEAALSPVYSLSERLSQMALRRMVELALSKFAPHLED
ELPPSLRAAYGFPPKAEAIRAIHFPSSWEAAKRAHDMLAYEELFYFQLGIALRMRSRRAK
VIERRASPGVLARQLRERLPYALTVDQESVLGEIVDDMHKPYPMARLLQGDVGSGKTIVA
ILAALHAVERGGQVAIMAPTELLARQHATIAAHLVEPLGVRLAFVTGSISNAARPPLLAA
LKDGEIDIAIGTHALFSEDVSFKNLELVIIDEQQRFGVLQRIALYKKGRIPDFLMMTATP
IPRSLALTFFGDLQVSTILHLPPGRKPVVTHLAHEDRRHRVYEFTRGKLKEGRQAYFVYP
VISGSERLDLRDAESMASHLAKDVFPEFKVGLLHSRLGDEVKMEVMTKFAAGEISILVAT
TVVEVGVDVPNATVMVIEHAERFGLSALHQLRGRIGRSSHQGYCFLVYSKDLTEEGRLRL
KALYETTDGFTIAEEDLKIRGPGDMLGIEQSGDLRLQIADMRTDFELLKKARHDAFGVIE
SDPALSEPDHEVIRQVLARANPFSEK*
>SPBDM4_v1_80061|ID:27159837| protein of unknown function [Uncultured spirochete bdmA 4]
MKKSEKLILESSNPDEYVSNSLKSRLSPAEKAKLARLWMENTGYTRDDIIRARNRNIYWR
KRKMEGAAERTRRRMEEHDYSQSKNIEWTREHLSEFLTLNRKDMYGRYLHRDWELAAQFE
TSIPSIQYLRRKYNKVRKMLGPAARREKIIDYMSCSELVLQHGGPKSRKRKRSSLPS*
>SPBDM4_v1_80062|ID:27159838| putative Methyltransferase small [Uncultured spirochete bdmA 4]
LSHALFSSFDIDAGTRLLLKEIAHEPGIVNAKRLLDAGCGTGIIGVCLAASCPDMDVVMR
DRDFRAVSFSARNAARNGLAVELRGLGGARLEPCRKRHFSKIKVAERNAVPMIAEAGLLC
EPDGRGPYDVVLSNLPAKAGPLVLAQYFSTVRAELLRDGGIFAFVIVTPLAEQARAWCAE
AGFSLRRTVSTKNHMVAIAQVPEGSSTASPGAASGRWFSRYVRSHVEKRIAGALLAWDGI
QGLSEFDEPSYATTCALSLARKVFAGLLVRRALVIEPGVGIAPLWVQTVLGPQEIVLKSH
DTLALAASSHNLGHAGRHNAVLMLEPFFLQAQLQAGQPSNRPSNAGLEYGGQLAPASTDA
IILFLEDIPKFDIAAYYWPLISKVLKRGGAVVITAESTQTERVARQKPSGFSKSPFAEHK
KGWDAIAFLRD*
>SPBDM4_v1_80063|ID:27159839|efp| Elongation factor P [Uncultured spirochete bdmA 4]
MVRGGDIGIGTCLLINGTPHIVVEREFVSPGKGSAFARVRAKNLKNGNVITQTIKTADFV

EDAQVDLVDCQYQYSDGEFFHFMNNETFDQFEVPLAAHEDRAPYLKDGETYTIVMWEGEP
IDIKIPYKMVFTVAESENYIRGDTVSGATKPVTTETGLVVRVPLFIKQGEKILINTETNE
YVERVNE*
>SPBDM4_v1_80064|ID:27159840| Major facilitator superfamily MFS_1 [Uncultured spirochete bdmA 4]
MTKKQKQILLALMVLVMIILNADGNVMAPTLVTIEAEFGVSDAQVGQMMGMFTIVGAIVS
LLWGYFADKASRKLLFVLAVAIGEIPCLLSAFARSWNSFYVLRMLTGIGVGAAFPLVFSI
LGDIYDEKERPVTTAILATAFGFGNIVGTVVGGYLGPSLGWRIPFILISAPNFILLALFW
VLVPEPQKAASEDATRELVAAGILYPRTIKLSDYAVLFKKKTNLYLLIQGIAGTVPWGAF
FFLNKFLETEKGFDTGTATTIYLIFGIGMTLGTLAGGKFGGMVFKKSDRALPVFCAVTTV
AGALGTFVVIAVDAGFAVLAALGFFTAFFAAMTGPNMKAMLLDVNLPEARGAIFSIFNLT
DSLGTGIGRWVAGLLSATLGLSAALGISTAFWFLCGAVLFAARLIFPPDRISLHREMEAA
AAEMKAAKVSVQE*
>SPBDM4_v1_80065|ID:27159841| Lysine--tRNA ligase [Uncultured spirochete bdmA 4]
MDVEVLQTRSRMVSIVREFFLSRGYLETDTPALAPSLIPESCLEVFATEYVHPTEEHRPL
YLVPSPEIWMKKVIAATGRSVFQLCKAWRNAESTSRIHNPEFTMLEYYGVGLSSGDNIRL
TEELFAALASQETPPRARPPFRVMSMCEAWTEYARADLDALSEPEAMRTACRERGLLVAS
DAAWEDAFNVFFLSLVEPALPRDRPLVLDRYPAGIECLAKDIPGTMYKERWELYVDGIEI
ANCFTEMANPAAVRQYFSSQSAKKLSALVPHRIDDSYASVFEHFPPCSGVAVGFERLMMV
LLGKKSIAEVLLFPFEAFSD*
>SPBDM4_v1_80066|ID:27159842| putative Iron-sulfur protein [Uncultured spirochete bdmA 4]
VTVEEVQGGPPVKLQGEAGARAARWLLFHVQEQGFFSYAILGKEEWETVLLERGFAEEEG
ILETARSDAAGAMLVVALRYAPDVEPEPAAGRAARGLPPLPLPGAGKPVAKVGRFARGNW
YREVIDRLATSAKATATDMVAVGLPFFPPKRWHRFANSRFPEKALAVAAGLGATGRNGLV
IAEASETGGQARGSAAGGGLPSGGVIRAYFHSSARSSAVLLGLMLLPFDLEQAIIPPRHS
PQAMLSLCGNCRRCVDACPSRALHFAPEPASWRASGRVFDRHRCIQHYASISGPLPDFIE
ASWQNQLYGCDLCLEACPYFRPDPDAHVSSGHIGGLFDAKVIAGMEDTQLRALFRGSALD
QRWIEPEALRRDATLAVKNC*
>SPBDM4_v1_80067|ID:27159843|glyA| serine hydroxymethyltransferase [Uncultured spirochete bdmA 4]
MSYIQSMDPELYAAIEAEEGRQRDKLELIASENYTSKAVLEAVGSVLTNKYAEGYPGKRY
YGGCEFVDIAENLARDRAKKLFGADHANVQPHSGSQANMGVLFAALNPGDTIMGMSLAHG
GHLTHGAPVSFSGKFYKVVSYGVNPDTETIDFDQVAYLAREHRPKLIIAGASAYSRIIDF
EAFRRIADEVGAYLMVDMAHIAGIVAVGRHPSPIPVADFTTTTTHKTLRGPRGGMILVGR
DKENNRGIMAPKGDALRKWSEVIDSMIMPGIQGGPLMHVIAGKAVALKEALEPDFAEYID
RVLSNAKVMAQSLTAGGARIVSGGTDNHLMLVDLTPLEITGREAEKWLDEANITVNKNGI
PFDKKSPFVTSGIRVGTPAVTTRGMGPAEMETIATFIMDVLKSKGEAQVVARVKQEVLAL
TKRFPLS*
>SPBDM4_v1_80068|ID:27159844| exported protein of unknown function [Uncultured spirochete bdmA 4]
MKRRSVVIGGALVLLFVTGGALLFYWMLHPALSVIDPVTPLPDDTVSIVGRNFGEAPGEL
LFDNIPLPGHAIQSWSPTYISFKMPSDVDSAVVRVRTTFGFSNPLMLANSSKIPKAAESQ
AVSEFRPNITGVKPTANIQIGKSIVISGSHFGEPEENAAVFFTKIPSISTLETEDLNNFI
KIESASLLVDRWRDTSMAVRVPDGVESGYVFVRTKNGISNTYPVSFSHSLGRTWRGESSK
YVIEQKVILHVTASLPDGRLSLFLPKPATTQNQSAETNIGSGGDHLNADHANWQEFRFGQ
DDASKSKLEITRQFIVDASEIRADINVGSIQAIGAAPPAFLAPYLAADALVPSGDEAIVA
AARTIQRKEKNTFRQVALAAQWVFSNIKLNEQDQGLSDDALPALKNKNGGIRALALIDCA
LLRALGIPAIPVAGFLVAEDMRLIPHYWGEYYLLGIGWIPFDPALATGTVPAGFVPGFSE
RLTYYRGIDNKHIAMSRGYQLLPPVQPEAQKKVKIPWSLSEYDELVQGVSYTSIWNPPEM
APSAPQ*
>SPBDM4_v1_80069|ID:27159845| exported protein of unknown function [Uncultured spirochete bdmA 4]
MRLRTLILVVFSLLSAACAFSQTPPVPATMPAPPQTGAPSSPGQESAPSLTPAAPPNVPV
FQFHFRAAFMLTPAMVAAIQSASLNAQDSKSKSNSFSDRENNPAWEDPILREVSLAAPLG
IKIQGERLVAMIVFIPVELVKKDLTMLVQNQIYARSPDNSIQMNTSVHTVRVPLGALFYY
YPLGGDAKQGAAVAIEILINQK*
>SPBDM4_v1_80070|ID:27159846| conserved protein of unknown function [Uncultured spirochete bdmA 4]
MCPDETLLTAYVDDEVPSPWKERIEMHLGQCERCRSRVAQYRALRSALRVADAIDDSHLQ

EAAQRIQASLDSRMMELSRAPHRKSAFERYPIFYMPGSKRISVPLPVLAASLLLFVFFAG
LAFGFFGLHKNASQAFVLSTKLPADTSANIESLVSTLSQTDPSMFVTIKAPENISQPLPN
TPPVYVIYNTADQKPTVMAVPAQGEAK*
>SPBDM4_v1_80071|ID:27159847| RNA polymerase sigma-24 factor [Uncultured spirochete bdmA 4]
LFKDKDDGQEPDFDRIFVQNIDLLLRIAVRITNDWESAEDVVQDAFGKLIEKRMQFPSDE
DARFWLIRVVKNGAINWSKRKVREFKAYEHWWNAETTQAKTSANAALNGEEQNQNAGTDQ
SAEHELLMEESAQEVRAALMRLPEKLRIVLILKEYEGMNYKEIAKVLGITEGNVKVRAFR
AREALFSLLNEGGSHVSR*
>SPBDM4_v1_80072|ID:27159848|cysS| Cysteine--tRNA ligase [Uncultured spirochete bdmA 4]
MHDVYFFNTMGRSIQRFEPLHDHKVGFYGCGPTVYNFAHIGNLRTYVFEDILVRILRRFG
YEVTHVMNITDVGHLSGDDDTGEDKMVKSARERGKSVLEIAQFYTDAFFRDTERLNINRP
TIVCKATEHIQDMIDLIRRIEANGYTYFAGGNLYFDVSKFDHYGELAGLRLDQLKAGARV
EVDPNKRNPYDFVLWFTKSKFENQALLWDSPWGRGYPGWHIECSAMSMKYLGDSFDIHAG
GVDHIAVHHTNEIAQSEAATGHKWVNYWMHAEFLVLDKGKMSKSSGSFLTLQTLVDKGYD
PLDYRYFCLGGHYRSQLSFSWEGLDQARAARLSLVDRVIALVESAGTPAPLAQLSERATQ
YRAAFDEALADDLAMPRALAQMWTMLKDSEMAPAEELALMLDMDDVLGMGLASAQKREAS
VDAETVRWIEEKIQKRAAAKKARDFATADAIRNELKAKGIQLEDRPDGTAWKLTE*
>SPBDM4_v1_80073|ID:27159849|murB| UDP-N-acetylenolpyruvoylglucosamine reductase [Uncultured spirochete bdmA 4]
MGTLWQIAQKINTEQCSAALGEPMWAHTTFKIGGPADLYVRPRSIRALTEALDRLRSAGL
PVFLLGGGANILVGDRGIRGAVIDMGLLSSIRAGAFGESADSDAEQTRGQIALHAECGAS
VSALCEEALVRGLSGLENFYGMPGSVGGAIYMNARCYEDDISSHIKKITYIDAVGALQEI
AAASLPWSYKRSPFMCGEALAGSTVGAAMFMLVPDNPQRIAGRMRARLADRMQKHHFDYP
SAGSMFKNNRAFGKPTGKILHELGLRGYRIGNAAISSWHANIFINLGGATARDMRALIEH
AQAVVFAATGWHLEPEVLFVGEF*
>SPBDM4_v1_80074|ID:27159850| membrane protein of unknown function [Uncultured spirochete bdmA 4]
MNVIHDPVGIFALLLASVLFSAWLSNVFRFNIAVILLIFGIIAGPEVAGLLDSGAVLQIL
GSIGIVYVFFFAGLSTRISTRFPRHDAYGKIPFNKRSPQFFVWTVIPALTGIAIGFVIGK
SPLQAAAIGIFFASAGIASSLEENYMKFSSLESYGLPGSVSLLVVFVLLALDAVAGGADA
RRCIFVLASGCILAGVIWSLFPRLASIFLRRIKSVGVIEGWFLFFLVFTASYAASFLSIP
NWFTAYIAGIALSSAAMTTSGSGTGRILLKDDIFSPAAFFLMGVSIHISEFSSNTQWLVW
GGIFIAGGLAARMLVAFFSKRIALMKGPMLGLAVPFTTFSLAISWILYSSGIFDAPLFMG
AIALALVTGIISNSLMKKASSAAKVLSSNPTEKAAFVIPNRILIALSKPSSIPHLLELGA
ILHGADNRSPLFPLVVHSPEDSEIAQSAGSETILATAVMKLSGMQKSVLPLNVEAINPGL
GILESAIQKNADTIVIGWNKPPRLAHAFFGNIIDQIVSGSNSMVLVARGRFPWKSSKQIL
AIFPPLVDMHSGYEAALQCIKRFARDSFATLHCFIPKDYEEQLADLWKITFSSINYKIST
FSTWREIPDLFQKAATVHAAIVLVSARPGEASWSPAFERLPHILAENLPEANVLMLYMPS
YNEQREQPSPQPAPPVEVEAASSPQRAEASNAEALLLESVRAGRIQVNMQGSALAEGIYK
IIFSAFPNGEKKQLRALADHCIEMLQRQPIEIEPGVVLLHDRLENIPYPMVCFGAQKTGY
RLSALESPVQIIVLILVPVHQSADEHLRFLADIAALFRMRNLRQRLMDANEPEDLLSYQ*
>SPBDM4_v1_80075|ID:27159851| conserved membrane protein of unknown function [Uncultured spirochete bdmA 4]
MNALLIALGISFLVNALFFVFASIKKTDVVTDLSYGLSFFLTSLGLALVTHVHGFFWLFP
FVAVMLWAARLSFYLFRRILTIKVDHRFDGRREDPVKFAQFWILQAVSTVIIMLPVIIGA
SREPVGFSFLQLLGGLVWLIGLLIEAVADAQKFKFKKNNPDGFVSTGMWSWSRHPNYFGE
MLVWWGLWLYVLPSLQGWENIAVLGPLYITILLRFVSGVPLLEKTASGKYGSLPEYKDYV
SSTHLLFPWPPKSKSANARSSTASIPTIGSLSDEEFAGRWYELGRIPLPVARDWIMTSDV
YEKQPDGTWHVRYEGKPDQDRTRTKVLRQKLKRPDAAAPGEMLVSFLPGIWMKYRAVHMS
SDRQSMLVTSSKMKYLWIMSRNADLPEEEYQTLLSTAASLGFDTRAVQRIPQH*
>SPBDM4_v1_80076|ID:27159852| exported protein of unknown function [Uncultured spirochete bdmA 4]
MKKAREFAMVFVLLIASLSGVSAYDPPKGASLLGRIYSPWTMGSGLSLTERSSGAWNFLL
NPASTAETELPELALSYTGIADMSAGSTQGWGSAGMAAFSLPKSYGVWNGSVRLFSAPGA
MTSMPLGTFVTAGGGFSKKVSSTLSVGASAWLAMGGNGTFGWGLWSDIGAVKELGDLGFL
KNSRLGFVLGGLGKEFNYQTPPVGISAGSTASTGFPAAFTPAIGFSADLLSKYNLGIRAN

VDLRAPSFNDIEAEAGLAFSYRNLLNLRLSMASSLYDIQQKSGRSAWPSITLSGSIPLGK
KGPQNKSVSEIAPAVGFMPLYDSLDAFSVGARLTWGQPDKSPPEVTLELPSTTSGTPVEY
VSPNADGKQDWLELPLTIKDEQRTIAGWLFKIEDRSSGKVVRTITEQSDLPESINSFSTL
KEGFGYSKHSVLVPDVLRWDGKDDQGNLVPEGTYIASFHAWDDLGNTNINYDSCMNVVVD
RSPPTVNAWVMGDEQLFQGNDSLIFSPDGDNNKDIISFRTQGSIEDNWKYEILDAGGNAV
RTMDLKGRSAPRDFVWDGTNDAGKRVPDGYYQFKLSSVDPAGNVGFKTIGVSADKSDWIV
VDTSRPAVRVAADKKAFSPAKEGKTGTIDIDFNLESLNNLVSWKEVIQDQNGKTVWSSSG
DGATPPVSSLTFNGLDASGKAFSDGQYRAGIEMKYRNGYSPIVYSDSFVLDATPPFAEIV
LDDARSVFSPDGDGSRDALTFAFTGSKEDVWNLSIRNEQGDLVYTQQYRDALPDRFVWDG
RDSTGRRIADGKYTLELASTDVAGNEFHTDYSPIVVDTRTPKAAFALNREAFSPNDDGVA
DVLGLNIKLENTEGLLVWNLDVVGTAGTQTAKGEAPRFPLLSGDGTNPPPGALQFDGKSQ
RAPLREGTYHFELALEYENGWSTTQSSPDFIIDNTAPQAQISRSREFFNPRGGAQQSQMI
ISQLGSEEKLWTGELRDAQGTLRRKWEFKNSQPAEILWDGRTADGALLADGIYYYRLLCV
DSAGNSFASEPLKIGIDTASKQSSLSASHMAISPNGDGIQDEMSLSIVVTAPQTVGDWRL
AVLAGQESVMEWKGTGNVPQSITWNGTSLARLSVPDGNYTAVFHAEYPNGDSTEVNLGPL
VVDRAAPKAEAKVESTIFSPNDDGINDTLPIQQTSVPGDDWRGTIVDMAGKEVRGWTWEG
TVQSFEWDGRDNAGNIAADGKYQYKLESKDAAGNAFSYTSPVFEIETEKKAVRLTLSDRA
FSPNGDGVKDVEVFTADVVAPEKVKEYVLQVVAQDGPAALSAVRTWKGETPLGRYEWRGE
TDTQIPAPDGHYATSLKVVYRNGDEAESATGTFLLDRQFPQIDVKVQGSIFSPDGDGRSD
TITITQKSAQGDDWKGTMKDSAGNIARTWTWQAEAKDFVWDGRNASGSAVQDGFYDYTVE
SQDAAGNKTVAGPFHIQVETGRRAVQLRLSDKAVSPNGDGVKDELVINVEADARDRIKQY
SLAIRGKDGAAVRTWTGGIDLAREYRWNGRNDAGAAVPDGDYSVSLEILYLNDALAKDGP
APVSVDRAAPQATVNLSRSIFSPNGDGRADTVEITQNSVYGDRWNGQISSEAGKIVRTWE
WYPMLADIVWDGKDQNGKLVPDGAYYYELRSIDEAGNSFILPRQQIMVDATQKNVSLRVD
PSAFSPNDDGVKDVAYINISAPKPDTLVNYSVSIFSGAKAVQGQAPLRGWKGTTDIRAQY
AWDGMTDAGLKVPDGSYSVYLSLEYANGDLFNFGPMQLVVDTVAPRISLSADSLLFSPNG
DGIKDYITITQSSDPGDDWSGRIRSASGATVRSYSWKGQAKTFTWDGLDSNKRLVPNGTY
TYEVVSVDAAGNSASAVVKGITVDATKPRVFVTASDTGISPNGDGIRDDVSFSLTVENRE
GVESWRFSLLDSKGVERSFFGGSGADVPTRLVWDGRDLQGQVIEDSFTGKLVVKYLKGDV
AEASSGKIVVKIEPPKVDITVSPEYFSPDGDGVDDILTFGIAADKNAGITEWKLEVMETA
VVESSTMQGTGPTRTFMAWNGKGTPPAQITWNGKSSKGELVESATDYPFEFTCWDALGNQ
TKVKGVIAVDVLVIRDGDRLKIRVPSIVFRANHADFIGLDSEIVSRNERVVARVAQILGK
FPDYRVRVEGHGNNVGKMLGYSASRIQQEEVNELIPLSTERAEVVRKMLVDNGVDPRRLS
VEGLGSSEPVVSFTDVQNRWKNRRVEFVLIKNQ*
>SPBDM4_v1_80077|ID:27159853| conserved exported protein of unknown function [Uncultured spirochete bdmA 4]
MGCAKCSIMKALLFSMLIFAVLPSLAEAIEPFPYRLFPSLYRKENFTRIDQNMSPANYQA
SGGFLWDRVPLVANNTIVAYYGSPLSDRMGILGRYPKEKIAEMVKATASEYDQVNGKDGV
IPALYIIYGTCWPGGEIGYLDDKALVEYIKYAQSEGMLVFVDHQIGRYSVHEAMDKILPF
LQYPNVHLAIDPEWRTLSPMKEIGSITAEELNDAQDYMDAYIRQHNIPGIRMLVVHQFAD
KMIQTRREVRSHRDRVILIHTADGFGTPQMKKATYQRNAIALNMPVKGFKLFFKSDFPAA
GFDMPLMSPAEVMQLDPRPSLIIYQ*
>SPBDM4_v1_80078|ID:27159854|ffh| Signal recognition particle protein [Uncultured spirochete bdmA 4]
MLEKISETFTDVFRTISGKASISEKNVEEALERIKIALLEADVNVRVVRRFINGALEEAK
GEKVLRSVTPGQQFIKIVYDRMVALLGDERQDLALKGTDTQSVILLLGLQGSGKTTTAAK
LASLLKKRGRKVLLAACDLVRPAAAEQLAVLADQVGVDVHREDGADAVQVARNALARARR
EGHDVIIVDTAGRLQIDEPLMQELARIRDALSPVESLLVADAMTGQAAVDIAKAFDEKIG
VTGVILTKFDSDTRGGAALSLKSITGKAIKFIGVSERIDGLEPFYPDRIANRILGMGDIV
SLVEKAQEVYDQKEALDLERKISQETFTLEDYLDQIHKMKKMGSVKSMLEMIPGLAGQID
EDRIDLQEMKYEEAILLSMTKKERQNYLIVGPSRRTRIAKGSGTSVAEVNRLLKKFEKSR
SMMKKMVKNKGAMAKMFGGKTP*
>SPBDM4_v1_80079|ID:27159855| Metal dependent phosphohydrolase [Uncultured spirochete bdmA 4]
MTREQAVALWRQWNSDESLWRHALSVEAAMRHFARKYGEDEEYWGLVGLLHDIDYQKYPE
EHLRHAREILAPAGFDDTFIRSVESHGWGICSDVEPTHVMEKVLFATDELTGFIAACAYV
RPSRSVLDMEVKSVKKKWGSAAFAAGVRRSVIEQGAQMLGIPLEELIGETILALRSAAAE

TGLKGNL*
>SPBDM4_v1_80080|ID:27159856| Peptidoglycan glycosyltransferase [Uncultured spirochete bdmA 4]
MENHAQARFHRRMVTLVVFLVIFAGVVCVRYAVLGLEGPEKTGLSENTPIERGRILDRNG
KVLAFDIPKFNLAIRKNEIDPYRITEDLSIVARSLNINADVLERKIRESGQNFVYLAKRL
DVDTVKPLQEKLDKGQLAGFVLEEVNGRIYPEGRLASHLLGFTGEGNKGLEGIEYKYNAE
LSGTQGDKARGDDIYLTIDARLQYALEQITRKAIRDNKAESLFMMAMDVNSGEVLAYVQM
PDFDPNNFGSFREAEREDRMSVYSYEPGSVFKIFSMASILDAGLITPKTKFNCDGAYRRT
LPSGEKIVIKDLHSYGVLDLAGILAKSSNAGAGYASDRISENEFYSRLLNFGFAEKTGIG
SPGENPGSLREPAKWSARTKPTVAIGQEIRVTALQMITAASAVANGGILLKPDTVMRIED
ADGKALYSHETVAVRRAISPETSRAIMTAMESAASLEGTGWRAKVPDVRMAVKTGTAQMI
DLKTKAYSDTDYIASTLGVLPADNPTIAIYVAIVKPMGESYLGGQIAAPVLRDAAESAID
ILGLPRGKSQNTTHGGLVTIQEPAPASIGSKMPDLTGFSKRQLLPLLLRGDLDVTLDGDG
YVVAQSPPPGTAVQGGTRIILRLQ*
>SPBDM4_v1_80081|ID:27159857|lepB| Signal peptidase I [Uncultured spirochete bdmA 4]
MSQAVRHAGKKLDSPALTLILVVVAAFLVRTFILDAAVVEGKSMLPHYRNGRVVLIFKAA
YGIRGVGGKYLVCWKHPPRGDVVAAVRPNSREIVIKRIGEIRGDGGSSEYFLIGDNGIES
IDSRDFGLVTLNAIIGKVVPQR*
>SPBDM4_v1_80082|ID:27159858| protein of unknown function [Uncultured spirochete bdmA 4]
MNSLYQSEDAVLEADKVLNKPLVSVLSGEDILAKAPAKSHKERSGKRRHPLFEKKTPENR
KKRAKNDQPRKDFVIEPFLSPSSVTNITLEAKAPVEIAPAKNRSLLHIVPTGNTRKILLR
AFVVLMIAATMAGASFLAATLISHPHPVIALPNEDSAQEALMAMLEPEPSAPPSGDTALP
PLPSLLVERTYTVRRGDSLQTIARQFGLREDTIISANGLSSKNQLQVGKTLKVPNMNGVY
HTVKRNENLSVISRAYGVDMTRIADANNISSSTIRVGDRLLIPNAKLDASVLRNFYGETF
IWPVRGPISSPFGYRINPFSGQRTFHAAIDIVVNKGTSVKATREGKVADTGYNAVFGNYV
IIRHTDGYQSLYAHLDAILARKGARVNQGEVIGRSGNTGQSTGPHLHFSIFRNGQAVDPR
KYLK*
>SPBDM4_v1_80083|ID:27159859| RNA polymerase sigma factor [Uncultured spirochete bdmA 4]
MAIADSDESLSLYFDSIRKIPLVSRQEESILAGKIAKGDKKALKRLVEGNLRLVVRIAKS
MWTPPHSLLDLIQEGNIGLVKAAERFDAGKNVKFSTYAVWWIRQAISRSLVNTGRQIRLP
HRKEDALRRINAIQMRKSLEISRAPTGKELASEMGCSESEVSAILHMGDQPVALEQAGED
ELSILEVYEDWRYNPEREIEKASISWESTWLLSTLSAREREVVSRRFGLGSGREQSLKAV
GSVVGCSAETVRHIEKKALQSLWQAAASVGLTA*
>SPBDM4_v1_80084|ID:27159860| Divergent polysaccharide deacetylase family [Uncultured spirochete bdmA 4]
VRKKHVHRGFSYALILASAALAAFAASAIINSCTPKESSQKTQKSPYGTPEYVIELPPGY
EDLKVIPQSGQGQKTQPLSGSAPTNESQSTPQGDGTAVARLARPQGPQEEAPRVTGHPTL
IIVIDDVGYNNGELKPFLKLPFPITFAVLPQLPHSTDSVLAIRDAGKEFILHQPMEALGG
NDPGPHAVYLAMDDATIEKTVAENIDSFPYPPAGMNNHMGSAVTRDEKAMEPILKLVKER
GMYYLDSLTAPGTVTVRLCQEMGTPYMERNVFLDNNTDRESILAAIDEGKRIARKNSAAV
MIGHVWSSNLAATLMNIYPELVEEGYSLSTISEYMRMQAEENTGHADSGH*
>SPBDM4_v1_80085|ID:27159861|gcp| O-sialoglycoprotein endopeptidase [Uncultured spirochete bdmA 4]
MRILGIETSCDECAAAVVEDGSRILSNVIMTQIPLHERYQGVVPEVASRAHIEWIMGAVK
RALSEAGIGVASIDGIAATAKPGLIGSLAVGLSFAKALAWSRNLPFKGVNHMLAHLYAPQ
LASHIEYPFLGLLVSGGHTIICRADDFDKITVLGTTIDDAAGEAFDKVAKHYGFGYPGGL
VIDQLAERGDERAFRFPLPSLHKGEHRYDVSYSGLKTAVIHHLPSYMRPGATATPENIAA
SFRKTAIDILLSRLYRAVRDTGIQTVVAGGGVAANAYLRRELGKNDDLRVFFPPLALCGD
NGAMVAGIAWHYFSRGETDGWDIAPSSRVVDFKRR*
>SPBDM4_v1_80086|ID:27159862| putative leucyl aminopeptidase [Uncultured spirochete bdmA 4]
VHSLSETAHIALSQCLELKKNEKILIVTNPDTEQAEIAAALYEQARAMGAQAGLLYQPVK
TQADFAEDEVLRAIEARPDVILSISTEKLGKDKARLAAPLAGADGRSYDHIFNYLLHGIQ
TIRAVWTPGITKDIFVRAVPVDYAAMRDTADRLEALLDEASAVMVRSPRGTDITFSVSGR
KAMRDDGDFRNAGAGGNLPAGEVFISPAIKSAEGTIVFDGSISDIAGDIVIRTPIACTVR
GGYVMGIEGGEEARRLEKALMHGLNMAATLVRDQGMAPELALSYGTNARHLGEFGIGLNP
AARIGGNMLEDEKVMGTIHFAIGANYDEDAPALIHLDGLVKSPTVVLLMPTGEEVAVMEG
GALAV*

>SPBDM4_v1_80087|ID:27159863|lysS| Lysine--tRNA ligase [Uncultured spirochete bdmA 4]
MEQKKTIHWADQTAEKIVQTWGEKDLYTCASGITPSGTVHVGNFREMISVELVVRALRNL
GKNVRFIYSWDDYDVFRKVPLNMPNPELLEKYLRFPITMVPDPWGRDESYARHHEVDVEK
ILPEVGIHPEFLYQANKYRAGTYAKGIRRALEMRGHLKTILDKYRDEDHKIEGEWWPVSV
FCSNCDRDTTQIDGWDGDWGLSYHCESCGHSETGDIRTLKGVKLVWRVDWPMRWEYEKVD
FEPAGKDHHSQGGSFDTSKHVVEDVYGRKPPVTFRYDFIGIKGSPGKMSSSKGKVVDLYE
LLRVYQPEIVRYLFAGTRPNTEFVISFDLDVIKIYEDYDRTERIYWGAEKAKNEDVEALE
RRIYEFSQVGEVPEDMPYQIPFRHLCNLLQIHLGNIDKVISILPDLKPSQEPRVRRRAQC
AWYWITECAPEDFMFSLRQPGERAELSAIEIGALQSLRDDVVAKLETYGGENEVAEAIYA
VAQASGIEPKKLFRASYQALIGKDQGPRLAGFLNAIGKEKVMALLRQY*
>SPBDM4_v1_80088|ID:27159864| Oligoendopeptidase, pepF/M3 family [Uncultured spirochete bdmA 4]
MTGQTAIPEWSLEEVFPGFDSLEYRQAKEEVRTSLETMRRFLLESSPPEGGADFSDWLCE
LLDKLNRLEMLADTLSAYAYARFSTETKSPSVLAELNKIEELLVPATTVQVLFRNLLARH
EDTVRKAIKDDRRFGEYAFILEEALMFQKHQMATELEDLAADLSRSGAEAWSRLQESILA
NASAPWDEASGARKTMVELRNLAYDPDRAVREKAYRLELGIWKTYEIPVAAALNGVKGTV
STLNKRRDWESALDTSLAQARISKATLDALIGVMEESLPMWRRYLRAKARLLGRERLSFF
DIFAPLQQTGSEIPTFGWEEAKDFIVRQFGTFDADMARFAKHAFEHRWIDARPREGKVGG
AFCTHFPAAKQPRVLCSFDGTYSSLITIAHELGHAWHYETIKDRPQLLSQYPMTLAETAS
IFSETLVSNAAMAEIEAGSRMPLVELHLQDACQVIVDILSRFYFERAVFKERDEGELTPD
RLCALMLDAQARTYGDAMREDERHPYMWAVKRHYYIPGLSFYNFPYAFGQLFGMGLYSLY
KKEGPSFAARYRELLRATGSLPAVEVARRAGFDFETPEFWKGAMMAFEPEVAFLEAQGRA
TVQY*
>SPBDM4_v1_80089|ID:27159865|ndk| Nucleoside diphosphate kinase [Uncultured spirochete bdmA 4]
MALERTYAMLKPGVLSRRIAGEIISRIERKGFDIIGFKIMRIPRELAEKHYAEHKDKPFF
GELVDYITSGPVIAMVLEGEQAVKMLRLMCGSTKPEEALPGTIRGDYAMHTNINIIHASD
SPEAATREIGLFFKPEELVCWSDGNKDWI*
>SPBDM4_v1_80090|ID:27159866| exported protein of unknown function [Uncultured spirochete bdmA 4]
MKFFLQNNKTGSTKPIMKLGALGALSAVFFLAISCASVPREVALSGLFDHFEKAPQVLVK
TENNFLRDMAASFNDSTIEALMSVASDQKDAVHKPIDRDRLDKTLMRADMAGIGISWDGN
TSPAIEVVLAGNFPSLLTSLSFSFDNNWERIVGGYAAKNGKLYLRKPSGGQLHFATWAPE
DPPAFSGAAGSMARSSGMLSSDADLTIYLDAKSALVTQMPILDGVTLPFDGIMLSATRDA
ASPAKGSPDARYSTVFQIQMKDEQAARTYKPIIKFMWVLISNKLSSYGVPLSSENSIEQR
GSLFVSQPITMSAQQMIDAMLELSNLDGNTTDKIIANR*
>SPBDM4_v1_80091|ID:27159867| protein of unknown function [Uncultured spirochete bdmA 4]
MSTSGAVSSKLAAFFLFFSVTLLDLLLFIPFGNMLAAQEASATEQPSHNDTQAAPIAPGR
WAGKLVLQSAQETQGGPQSQSAPSSFSFELVVRILAENRGILVDIPEQGMFSYPIDRYSI
DAGRFSFVLDAMGADEGLTFSGNFSSSFVPQGGTQKGGIVGTVRGRTWNGSFYVQKEKAA
SPQGELYIDVPVDGGTLPATLTFPVRTRAVLDANAPAGFPLVILVAGAGKTDRDGNNVDV
PGKTDSLKQMAAMLRARNVGSLRYDRRGTGEAYKLEAPGHMTSFSRHILDLAAVIRAAEA
LPRRGRLIVAGMNEGAWMAMAALNIVGGESPVDGLVVLDASGQSPMETLRQSIESLDPES
REKALEAAQKLIDTGTLIDVPEDLANFFSPNRKDWLATWLAFDPVDTLKKVETPVLFVYG
ERDMQVSREEFSKLAAAKPQAGIRIVPDMNYVLKEVHNEDENYAAFTDPSFKLPALLADL
LASYAKAQPAPQGLLPWSLQ*
>SPBDM4_v1_80092|ID:27159868|adk| adenylate kinase [Uncultured spirochete bdmA 4]
MNIQSMKLIFLGPPGAGKGTISKMVAERLGVPQISTGDMFRAAVKEKTELGLKVQSILAA
GGLVPDELTIAIVKERLSWPDVRQSYILDGFPRTIPQAEALAAFSSLDAAVDFEVRDDLI
LFRLTGRRVCKTCGAIYHAVTKPPKAEGICDLCGEELYTRNDDKEETVKARLSAYHEETE
PLIGYYTKKRLLLTIDGSPDAETVYNTFLSAIGRFLKK*
>SPBDM4_v1_80093|ID:27159869| conserved protein of unknown function [Uncultured spirochete bdmA 4]
MDSSDWAGVPCSRPKWPLHFSHPSVEAVGRKLKRIFDQNASTEYFSCEEIRDYSYYENLL
IEGLEGIDRKMKSGSLKPVELSEYPKIIRADRGKVRACIYIGSFDPFQLTHLAVAVRFLA
SELSSSDFVVVVPEGSPDPAKPLKTDYAFRLSIAKMQIEGIFDPFIKVLDLGMQADTIEI
ARRFIAMHSGLTLELTHLIGSDVLPVAARFIAEDLSIWRKEAQESGVHYLHSTHVVRRGE
DAIDASYFDAIKNEGASAILDPSIVATPSSTDFRTKQAFTIVLPTPSIRDKMEIIFRYHM

HRSWSSDQE*
>SPBDM4_v1_80094|ID:27159870| NADH:ubiquinone oxidoreductase 24 kD subunit [Uncultured spirochete bdmA 4]
MPKAISNVKSQSLDSIIDSWKGRNGNVLGALEQAQQNDPKKYLSEITLREISQKLNVPLS
RVYSVATFYSFFSLKPQGEHVIVVCRGTACHTRGSQVLLNEALARLGIKEFKEEEENSVT
SSDSFATIRTVACFGQCALAPVIMVDGKVISRMTISKLVSLLEKLKKGGEV*
>SPBDM4_v1_80095|ID:27159871| conserved protein of unknown function [Uncultured spirochete bdmA 4]
MTNTLDLGFLAKEGMAHFYGDTPWISVGMGTCGIGSGADKVWEAITEKAENNPVRVRRVG
CFGFCAAEPVVMSWRPGKPVLFFTDVDAQKATRIVKGLSDDASYDKIAKLAEARIEEWDF
RTSKLEFGRGYEHLPTWKELNFFKDQEKIVLRDAGMIDPESIEEYIAVGGYRSLVRALTS
MPPESIIEEVKKSGLRGRGGAGFPTWRKWSLMRQSLIDNPGEGYIICNADEGDPGAYMNR
NEIESDPHMLLEGMLLGAYAMGANRGIVYVRAEYPLAVYRLTKAIEDAREHGLLGKNILG
TKFSFDIDMVTGAGAFVCGEETALISSIEGNAGRPRPRPPFPAQKGIYGRPTSINNVETW
CNIPVIIARGGEWFAGIGTSTSTGTKVFSFVGKVRNTGLVELPLGSTLESVIYGICGGMG
PKKKIKAVQCGGPSGGCVPASLFKTPIDYEHLTELGAIMGSGGMVVMDQDNCMVDVARYF
TSFVVRESCGKCTPCREGTSQMLHILQKVSDGEAAEEDLDTLQDLALTVKDSSLCGLGQT
SANPVLTTLRYFRDEYIQHIKGKRCPAGICENLFIALCESSCPLHMNIPGYLQLLKENRI
EDAFELTLRENPLPGTIGRICHFHCRMRCRRDMLDEPVSQGEIHRYLADTMYKMGREKQI
YAKLIKEKLPSSGKRVAIIGAGPAGLTAASYLVRLGHEVTIYEASNEAGGVLRWGIPAYR
LPKDVLKKEVGFIEKLGVRFVYNTRISEPEQWQRLADSCDAIIVAAGAASELTLGVPGED
AHGVYKACDFLNTLARKEKVRTGGEVVVIGGGNSAIDAARSAMRLGSTVTVVYRRSKTDM
PANEEELKGALEEGIDLICMASPVEILTRQKDGKHFAKAVRIQRMKAGPVDSSGRPTPIP
TDKVEDISCSTVIVAIGEKVEIPGIDSIGVERSKNGRIKVDPFSLVTSNPKVYAIGDATL
GPATAAEAMGQAKTVAEIVDQSLSGKKRFDTLFRRFGYRMEIPLNISKEKMTRAAMLPIN
ARKSNFMEINLGYTGEQARIEANRCLRCDVREHKREPRGTLVSE*
>SPBDM4_v1_80096|ID:27159872| NADP-reducing hydrogenase subunit HndC (fragment) [Uncultured spirochete bdmA 4]
MENLIEVTIDSQTVKVDPSENIVEACARAGVKIPTLCYLKGISQNASCGVCVVEVEGAKS
LVRSCVQKPTPGMKIQTASPRVLRARKTAIELLLANHPSDCLSCVRADTCELHTMANLLE
VRADRFPAYRKYPLPDTTSEGIVRDDKKCILCGR*
>SPBDM4_v1_80097|ID:27159873| NADP-reducing hydrogenase subunit HndC (fragment) [Uncultured spirochete bdmA 4]
VCEETQGVNAIAFTGRGARTRVATFMDRGLAQSACVQCGQCSVVCPTGAITEKDESRDVF
DALRDSKLSVVVQTAPAIRASLGEALGLPAGSLVTGQMVAALRRLGFAKVFDTQFTADLT
IMEEGSELLERLSHGGTTPMITSCSPGWINFIEGFYPDLLGHVSTCKSPQQMFGAVAKTY
YAQAAGIAPDKMRVVSIMPCTAKKYEARRKEMDGAWGWWKEQDPGKVPARPFFDVDWALT
TRELARMIKLAGIEIGHLPEEDFDDPLGRSTGAATIFGTTGGVMEAALRTVYEIVEKKPL
ENIEFTQVRGFESIKSAEVVLGGSPVRVAVAHGLSNARILLDEIRAGKSPYQFIEIMSCP
GGCIGGGGQPVLADIEKKLARSKALYTEDRILPIRKSHENPAVNTLYKDFLGKPLGHLSH
ELLHTSYRARVF*
>SPBDM4_v1_80098|ID:27159874| putative Haloacid dehalogenase domain protein hydrolase [Uncultured spirochete bdmA 4]
VPRQWWTGVLLLLYTFYMQQKIGIRAVVFDVDGTLYPASSLYMRCIDMFIAHPRIVLEFS
AARKELRSLQMQHGYIPQNKEDLHRLQASLMAQHLHIDQPHAQTANRARELMEQLFYLEL
PKRFASIRPYPEVRATIEKIRGSGIRVAALSDLPPQEKIYALGLSDLFERSFCTEDFGVL
KPHPRAFEGMAEALGIDFSEILYVGNNIQYDIEGPKRVGMRAARRGRRCPGADLSFSRWR
ELADWLLTDKG*
>SPBDM4_v1_80099|ID:27159875| conserved protein of unknown function [Uncultured spirochete bdmA 4]
MDMTEREISEHRLYNQFITNRRDQDHVVQNLCGLQAQYEKEALRSLLLRITEKSIEYASL
VKCWTLRGTIHMIHETDLPVFLHTGSLSKYAEEQWRACSSIPMNRKIFFADLILEALQEG
NCTRDALKKRCSEKGMDETESRAYFFNPWGGIIRYLVETGRICYLVGNRSPTYALAPPFV
PLGHDAAWEIIIDRYFSTFGPATMRDASYFLGKSQQFIQKHMAVDRLKTAQCGKKTYFYR
NDAVGKGVPACILLPGFDPLLMAFFKEDSLFFERKDIRNIYSLTGIVYPTVMVRGKIVAR
WKQSGGKIHMAPFKSLPAKEKSLIEKSIEDRGAVDVVWEG*
>SPBDM4_v1_80100|ID:27159876| Death-on-curing family protein [Uncultured spirochete bdmA 4]

MIRFLTLSEVLLIYEDQIRRYGGTYSVRNLALLSSAVYVPQATFEREYLHKTIPEMAAAY
AYHICENHALVDGNKRVALATALVFLDINGYDFACSEEEIYQIMMAVASNKMNKAALTGK
FIQYSKRRIL*
>SPBDM4_v1_80101|ID:27159877| conserved protein of unknown function [Uncultured spirochete bdmA 4]
MVKRLIQHGNSVALVIDKPILELLNVTFDTPFEITTDGRNLILSPQSEGNAAINVIDSLE
KINKKYSNALRRLGK*
>SPBDM4_v1_80102|ID:27159878| putative Ribonuclease Z [Uncultured spirochete bdmA 4]
MTIKILGNGGFYNEGLPYNAMAINGHVLVETPPDILQSLMQQAMRPAQIDTVFISHIHGD
HCFGFPFFFFNWLYWGESDSHYGRGTRLVIIGPEGLGSHLRELMRLAIPPEHPYLRDFDQ
RVQIVEIDEDDVVPVKGNLWFGFRRTKHSLPTFSLIAGERGPDGALPSSSEYLKKALFIY
SSDTSMFEGVRVLLGSGAKLILCDTNGEKENGVHMSPQELMAAAAAQGLSGTGRLRGIHM
SRKMERVGELRFVQPGEEFTI*
>SPBDM4_v1_80103|ID:27159879|dnaE| DNA polymerase III subunit alpha [Uncultured spirochete bdmA 4]
MAEFVHLHNHTDYSLLDGAAPVPRLVKKAKAFGMPGIAITDHGNMFGVMAFEKECHDAGI
NPIIGCEFYVAGGSRLEKTGTENGNKYWHLLLIAENLEGYHNLLKLTSGSFTEGFYYKPR
IDDELLAANSGGLIASTACLAGEIPSLILAGKLDQAVKKIGFYKELFGKDHFYLELQDHG
LDEQRTVNRELIRLSRQLDVPLVATNDLHYVEREDSVAHDILLCIGTNRKRNDPSRMRFP
NDQFYLKSPEEMEALFAEMPEALANTLKINEMARLKIEFPGPLLPDYQVPEGFSAPDEYL
RHIAREGLKTRYPNPGSEVVQRLEYELDVIIKMGFTGYFLIVWDFIHWAKEHGIPVGPGR
GSGAGSIVAYSMRITDIDPLKYDLLFERFLNPERISMPDFDVDFDFERRGEVIDYVAEKY
GHDRVAQIITFGTLKAKAVIKDVARALDIPFDEANQVAKLVPEDPKMSLAKALKMEPKLA
ALMQNPLYSELFSIAAKLENLHRHSSIHAAGIVIGKSALTDYVPLYKDPKTGLVATQYTM
EYLENCGLVKMDFLGLKTLTLIRNTLNLLHKRGIDIEEENIPDSDPKTFKMLCEGKSTSI
FQFESQGMQSVLKRAKPSSIEDLIALNALYRPGPMENIDQFINSKNGKMAITYPHPSLEK
YLKGTYGVIVYQEQVMQVAREVAGYSLGRADLLRRAMGKKKPEILQKEEVPFIEGAVSRG
YSREDAKRIFEILTPFAGYGFNKSHAAAYSVVAYRTAYLKANYPAEFMAANLTNEISNTD
KLTEYISEARQMGLSVLPPDINRSDANFSVDHGQIIYGFLGIKGVGEGIAGAIQEEREAR
GRFVDFIDFLTRLHDAGLNRKTMESLIMAGCFDSLKHGRRQLLNNLERAMEYVEGKRAFE
ASRIGSLFDMEDMGSYPDFVFEPADEFSNQEMLQSEKELLGFFFSAHPMDEYRMIWERSS
TIDMGHLDHASPNREYIAVALLKEFRVHTTQNGRRMAFGQLEDFRGAIDIVIFPDMLEKN
EEAFVKDRVLCVRGVFDNTRRSPSLKIQAILDPESLKKMSWRELHIQLSPALCRTEAGGE
PSGTENSGLATGQLGESGLYQLRDAVYSLHGQCKVIFHLPLSGGGEALIEAGLHTTCSAS
DEDIAFLKRQPAVTEVWRV*
>SPBDM4_v1_80104|ID:27159880| Molybdopterin dehydrogenase FAD-binding protein [Uncultured spirochete bdmA 4]
MEILEYARPRSMDEAFRLIRERHGTAIGGAAWLRTNTKTMALGVDLAGLGLEYIREAGER
IEIGAMTTYRELETSQLLKARFGPLFEATVSHVVGIQLRNIITVGGTVAGRYGFSDLNTT
LCALGAKAVFYPGQTVDLADFIEKGAETPFLLEKILLPASMKASYQQMRISENDFPIINT
ASAWTGSDWRIAVGSRPAATRLCKKAMSLLGDDPYPSDEKIVAAAHAATDELQFGSDIRA
SASYRRETLHVLIRRALMEVRG*
>SPBDM4_v1_80105|ID:27159881|xdhC| Xanthine dehydrogenase iron-sulfur-binding subunit [Uncultured spirochete bdmA 4]
MIITLHINGKEESVECLPGEMLTEVLRRAGYLEVKKGCDTGNCGVCTVLLEGKPVLSCSY
LAIRADSKEVTTIQGLAGEAKKFAQFLTAEGADQCGFCAPGFALTVLSMKKELHDPNEAE
IRHYLAGNLCRCSGYEGQIRAIKKYLEAEHA*
>SPBDM4_v1_80106|ID:27159882| Aldehyde oxidase and xanthine dehydrogenase molybdopterin binding protein [Uncultured spirochete bdmA 4]
MRDYHTPTLPPVDKPLEATEKFNIVAHDVSKVDGEGLVLGRPAYADDLAPQNALHVKLVR
SPHAFARILSIDPSEALALPGVACVLTWKDCPRIPITRAGQGNPEPSPHDRFILDEYVRY
VGDEVAVVAAESEAIAEKAAPLVKVEYEVLKPVLDFEKALDNPIVIHPEPEIHEMFPIGF
EPARNIAAAYHMEIGDVEKEIAASPVAVETTVYTQAQQHVALEPHTAFSYFDVQGRLVIV
TSTQNPWHTRRLLGLAFQMPLRQIRIVKPRIGGGFGGKQHIHVEPYVAMVTMKTGRPARL
ALTRREVFEATFTRHEMRVKVRLGADQNGMLRAIDMQVLSNTGAYGEHALTTFMVAGSKT
LPLYNKAVAVRFGGHVVYTNKVSAGALRGYGAIQGLTGLESAMDELAHKLKMDPVELRRK

NMLHEGETSEVFRIMGEGTEGVAMIIESCKLEECIARGKELIGWDPGNLVREIAPGKVRA
KGMAIAMQGSGIPLVDMGSARIELQDGGFFKLHVGATDLGTGSDTILAQIAAEELGVDMK
DIVIHSSDTDHTPFDVGAYASSTTYVSGSAVLKAARSLKTKLIEAVAEKLGVAPEGIIYK
DKIFRTLDGTKTLSLDDFSYDTLYHDGAKMQTLEATESFTGDKSPPPYLAAFVEIELDVE
TGKVDVVNYVAVADVGTPINPNLTKIQIEGGLLQGIGMALYEDVRYSEAGHMLSHTMMTY
PIPSREDVGRITVELVNSYEPSGPFGAKSAGEIGIDTPPAAIANAIRNAVGIRLTEYPFT
PERVLMAIRAAEKKQRPL*
>SPBDM4_v1_80107|ID:27159883| putative XshC-Cox1-family protein [Uncultured spirochete bdmA 4]
MNELRTWILGRLDRGETVVLAAVTHTSGSTARGTDALLAMDSKGAMAGTVGGGFSEGQTI
DAARQFFVPEGASSHTGQIARDLEFDLTPETNNTQMICGGRLSVHLEKIEPRGSAAQALR
ACFDHVENGAPCAFATMGTSRGRHYLAVGDEKRILFPQTPAQGEKKVLDALLATLEVARP
GFGGAADFALEAGNDPELKAVRVFWLGMAPDPVVYIFGAGHVGVATCDVANLAGFKVVVT
DDRPELLTGERFPRASLLRAIESFERPLTPYGKTPAIDIGPQDCALILTRQPDIDKEMLA
QLLRTDAGYIGLIGSKTKRAGIFAALRNEGFTDADLSRVHSPIGLAIGARTPEEIAVSIV
AEIIAVRAGVLPNSVIPDAETVPKKAAR*
>SPBDM4_v1_80108|ID:27159884| Metal dependent phosphohydrolase (fragment) [Uncultured spirochete bdmA 4]
VSHMNNFEIRRPAAIILAAGYSSRMGATKPIVLIEGTPMLVRAAQTFVNANIGHIIVVTG
FDTENVSELAAAHGMHPVYNSHFSEGMFSSVCAGIGAVPPDFDAVFVLPVDIPFVSPRTV
SAMRDATRDAMRARPIVVPRYGGKAGHPPLLHRTMFDSVGGWHGPDGLKGFFRNHASDIG
YVDVDDRFVLRDIDSRADLEQILMERKST*
>SPBDM4_v1_80109|ID:27159885| Peptidase M20 [Uncultured spirochete bdmA 4]
MNIAEKLSSLVKFRTVSSFRPEEEDEKPFAALIEALPGLYPGAHAALERILVGPRGIVYR
WQGKNPSLKPAIFCAHFDVVPASESDPWDEPPFSGTIRDGFVWGRGTQDIKVQIACILEA
AETLLSAGFVPERTLYFAFGGDEEVGGRRGAGAISKWFSEQNVHASWVIDEGSPVGQGLV
GFVHKPIALIGIAEKGYADIVIEVPGQGGHASMPPRNTALGALSHAIARIEDKPFPARIT
KTTDEFLAALAPHAVSPYKQIFSLRHLLTPVILKAFSATPSTNAMVRTTCAATMAQASPK
ENVLPNLAQAVINVRIMPGTTVAQVIDRFNALIAPYGAKAYAKFPEHTVEPSTESSTSSE
GWNSIVAAISEAFPDAIPAPFLFTAGTDTKHYRGITDDIYRFQPLVQTQADLAAVHNVNE
KVSVENLERCVRFYRALMNQQ*
>SPBDM4_v1_80110|ID:27159886|livF| leucine/isoleucine/valine transporter subunit ; ATP-binding component of ABC superfamily [Uncultured spirochete bdmA 4]
MLEVKNLHVAYGGIRALKGVSLSVRKGEIVTIIGANGAGKSTLLNTISGFLKPVKGSVLY
NGKDLPRRPDLIVKSGICHVPEGRLIFANLSVEDNLLLGAYLRNDKKAVQADMEKVYRIF
PRLKERQSQRAGTLSGGEQQMLAMGRALMTNGEMMLMDEPSLGLAPLLVQTVFDIIEQFR
GLGKTILLVEQNAFKALQVADRAYILEQGRIVKEGPARDLALDPAVKEAYLGSRSRGGGG
D*
>SPBDM4_v1_80111|ID:27159887|livG| leucine/isoleucine/valine transporter subunit ; ATP-binding component of ABC superfamily [Uncultured spirochete bdmA 4]
MTVATLLRVHELTKKFGGLTAVDAVSFDVPEGGIVGLIGPNGAGKTTIFNLITGVYKPTS
GSIEFRGKSIVDLEPFRIADMGITRTFQNIRLFKNLSVFDNVLTACHLNANYSFAEAILR
LPRFRAQEKLLMTKADNLLDIMGLLGYKDLIASNLPYGLQRRLEIVRALALNPSLLLLDE
PAAGMNPDETEQLMRLIARIRNDFKLSVLVIEHHMDLIMGVCEHIVVLNFGEKIAQGSAD
EVSKNPQVIEAYLGTSEEAADA*
>SPBDM4_v1_80112|ID:27159888| Inner-membrane translocator [Uncultured spirochete bdmA 4]
VDWTYIQGILMLAGINMIAVLGLSLLTGFTGLFSFGHAGFMAIGAYVGAWISASPTSTPA
GLGLPFVLAIVGGGVAAGAVSYFIGRISLNLKGDYFCIATLGFGEAIRLIFNNVNRFGGA
RGWPGVPGKSTFLAILVADVVAVLFLANLVKSRHGRNMIAVREEELAAQIAGIDVFRYKM
LSLIISAVYAGVAGVFFAHYMTFIQPKMFSLTKSTELTIIVIFGGLGSISGSVLGALVLT
ALPEILRTFEMWRLVFYGAAVILIMVSRPKGLMGGMELTPSGIRKMVAERNALREAKARL
LAEAQVGEEE*
>SPBDM4_v1_80113|ID:27159889| High-affinity branched-chain amino acid transport system permease protein LivH (TC 3.A.1.4.1) [Uncultured spirochete bdmA 4]
MVQFLQQLINGLSIGSVYALMAVGYSLVYSIMNFSNFAHGGVIMLGAYFGFFGLTLLKLP
FAVAFLLASFGTLLVAIIVERIAYKPLRERKAPFLYFIISAMGASILLENLVIATIGPTF

RTYPEVFSKAPIELGPLAIGRLDLIIFVISAVSLTLLILFIDKTKLGKAIQAASYNVKAC
ALMGVNTDRVILTVFGLGGFLAGIAGVFFGMKYTVYPQIGNITTKSFIAAVFGGLGSLPG
AVLGSIILGIIETFVAGYLSSQFRDLIAFVILIAVLVVRPTGIMGKVTEDKA*
>SPBDM4_v1_80114|ID:27159890| Extracellular ligand-binding receptor (fragment) [Uncultured spirochete bdmA 4]
VGDDYSQGLREFFKVNFEKKGGTIVADESFNSGDVDFRPQLSKIKASNPDVIFMPYFFKE
VALSANQARDLGIKATLLGGDGWPSDQLISMGGKAVEGSYFVNHLDYADPAVQDFKTRYK
AKYGKETELNGFLAHDAVLLFVEGLKKAGKANGEAIAKALEGIDVQGITGRIHISPETHN
PEGKDAAIEKIVDGQYVFQEKYAAQ*
>SPBDM4_v1_80115|ID:27159891| Branched-chain amino acid ABC transporter, amino acid-binding protein (TC 3.A.1.4.1) (fragment) [Uncultured spirochete bdmA 4]
MKKLLFAALAVALVLAGCGGGGKSDVIKVGWLGALTGDQAVWGENELNTVKMLFEEYNAA
GGIEVGGRKYTLEVIGYDNKGDPQESVNVTKRLTGQDKVVAIIGPNSSGNAIPMAPILEK
R*
>SPBDM4_v1_80116|ID:27159892| protein of unknown function [Uncultured spirochete bdmA 4]
MARRHTLDSGSPSAGRLSPHETAQRLESFLARWRESGGRAIDAFALAAGSEDALDLLPDV
PPWVECVGALVTKDRTRVLLWLKSKPSAGESNIYEKISAGVQLPFKRPQWWASRSVRIRY
YVIPPSPAGGADALLSGLEQAHGSLIVLSPPGSGEMLIQISESR*
>SPBDM4_v1_80117|ID:27159893| conserved exported protein of unknown function [Uncultured spirochete bdmA 4]
MKRLLAAWGFLVVFSSVQVHAQTGLELVCSEVVAGEGVTAVRPLSDFFSGLRGTPFDTPV
FVMGDGARGTEGAEAPPCAAIVAGTHANEIAGVLASYWIIERCRVPEGRLVVVPRANASG
ATWSLQDASGPRFLDLGSRTIRYGTRLSNPAQERLADPGSFVPPQASGNFPALAGQEARN
LNRQYPGRQDGNRTAQTAYALTLMLQAEEVRIALDLHEASMTSSLVWSMITRPEHLDAAA
LAALDIEDETGVSFHLEDSRDEFAGYSHWEWGKLGISAFLVETPNPAQPTDDPTIEQLHN
AAAPLAKRVYVHLCAAKALIANAADLLQDAGALRIEGFPSSVEEVGRWLEGTL*
>SPBDM4_v1_80118|ID:27159894| conserved exported protein of unknown function [Uncultured spirochete bdmA 4]
MVRKHRFVARVLSVALMAGIGVLQVCAQGTVPPSLQGSFLQPGYITSAGQTSDAAIVKVL
ANTRLKLGFGYDIAAKPQSIAGAKTLVLVLGASNKGLGAAGLSLEQDIERVKSVIAEAQK
NGVRIISMHTGGTARRGEASNQVIELCVPVSDVVVVVEGGNNDGFFTDLCEKAGVPLVEV
ASISAAGNVLKSLMAQQ*
>SPBDM4_v1_80119|ID:27159895|ggt| Gamma-glutamyltranspeptidase [Uncultured spirochete bdmA 4]
MALQRHEKVARRKRVAMSLLAILAFAFVVAAPLSAADTVKAEVSARNGMVASAQPLASAA
GLEILMAGGNAVDAAVASAFALGVVEPNATGLGGEGMMVIYLADKDVTVAIDYRSMSPLA
DMSKVKFGSTGHVAVAVPGTVAGLCDALQKYGTMSLAEVMAPAIRYARNGFIVSETLAQT
IADRFDPISNNEALLKIVAPEGLPLVAGQIIKNPDLAATMEKIAEGGPDVFYRGEIADAI
ADDMAKNGGYITKADLAAYRAIEREPVRGTYRGYEIVSAPPPVGGISVIEMLNMLENFDL
ASEAPLSPRNVHIMAEVMKRGFADNSAYVGDPAFTDIPVRGLLDKEYAKSRAAEIDLGKM
SPSTKAGTPSEHPSTTHLSVVDRGGNMVALTQTISGFWGACVAVPGTGVILNNEMQNWSS
RGPNAYAPGKRMRTTIAPTIIAKNGVPFVTMGTPGAGRIISTMVILAVDLLDYHMGVQEA
IESPRFYARDTEKDLSIESRMPAETQAWLKSIGYSIKEYPDFDLFFGGAQAILVDPDTGM
MYGGADPRRDGAVFGF*
>SPBDM4_v1_80120|ID:27159896| conserved exported protein of unknown function [Uncultured spirochete bdmA 4]
MRRYFRCFLFGLFLIFIAVPTFSSPFSGAGTILHDVRPGKGITRVTWLSEWSSPLEGTPA
DTRVFIADSGKPGATVFIAGGTHANEIAGILSAVLLIENAVPSAGRLIVIPDMNMSASTW
TESTKVPSWVRIETPNGPRFFKYGARYTDPVHQGAPDPDRYVHPKGGDALEGSEARNLNR
VYPGKPDGTLTERLAWALMSLLKQEGVDIAFDLHEAGPESRLANMIVANPKNLDLAALAV
INLEMDGIRMKLEPSSDAFHGLSHREWGDGTKAYAFLIETPNPAQSTENGNPVEDPQYPL
AGRIATHLASIGAILDAWNMDCPPDKRIEFDALPDWRAVAASGAGAFLNW*
>SPBDM4_v1_80121|ID:27159897| protein of unknown function [Uncultured spirochete bdmA 4]
LGAGSSDTRNFYYSEVLSKVGRVLKEPPFIAAFAYGFNETRANAKPISEATDF*
>SPBDM4_v1_80122|ID:27159898| ABC-type transporter, integral membrane subunit [Uncultured spirochete bdmA 4]
MNELMTVLKKNVREYGMYIALAAIIAVFTILSHGVFISSRNIANLLNQTGYIAVLAVGMT
LVIVIRHIDLSVGFLSGFLGAIAAISLTQWHWPVWLSILFVLGLGICAGLLTALPVAGLG
IPAFVASLAGWLIYRGALMLVTAATGTIIIPNESFNALGNGYIPDLFASNSFLPGIHKTT
LVLGLIATIVFIFGQIADRKRKMQYKFEVLSTGLFVAKLVFMAIILGFISWILAGYNGMS

WTVVIMLAVVLVYDFITRRTVIGRHIYAVGGNPEAAELSGISVRNITFTVFGSMGFLAAL
SGILFAARLKSATPQAGTLFEMDAIAAAYIGGVSASGGIGSISGSLIGALVYMSLMNGMN
LLGTDISLQYVIRGLVLLLAVIFDVITRKRKL*
>SPBDM4_v1_80123|ID:27159899|xylG| fused D-xylose transporter subunits of ABC superfamily: ATP-binding components [Uncultured spirochete bdmA 4]
MSDNILEMRHITKTFPGVRALSDVNFAVRRGEIHCLVGENGAGKSTLMKVLSGLYPHSDF
EGEIIFNGKKKEFNGIHDSEEAGIGIIYQELALVPDMTVYENIMLGHEIRRGMTIDVQEM
IHKAEELLRRVRLSINPSAKVKDLGIGKQQLVVIAKVLSRNVSLLILDEPTAALNEDDCD
NLLNILRILKTQGVTSIMISHKLKEVLSIAETITVLRDGQTICTLDRQKDEVTEQILIKH
MVGRSIENIYPPRTSTHAEEAVLEVKDWSAYDAKLGRKVLNNVNFHVCKGEIVGLSGLVG
SGRTELARSLFGNPDGYIVSGDMVFKGKKQRFTHPRHAIRSGLAYATEDRKRNGLILIKS
IQENISLANLQGVSKNGVINEELDKQNALNFVNSLSIKTPSISQLVMNLSGGNQQKVSVA
KWLFVGPEMLIFDEPTRGIDVGAKYEIYTIMNDLVAKGMSILMISSELPEILGMSDRIYV
VANGTITGELDAKSATQEAVMRLATQY*
>SPBDM4_v1_80124|ID:27159900| Periplasmic sugar-binding protein [Uncultured spirochete bdmA 4]
MKRSLFIAMLFAIVAGIAFADTQVGIVLPTKDEPRWIQDQTRFLDALKSAGFSAKVLFSQ
GDSAKEKANVEALLSENIKVLIICPQDGTAAAAAADLAHKAGVKVISYDRLIRETTSVDY
YVTFDSFQVGKAWGDYLNSKVPAGSKGNNLYLYAGAASDNNSFIFFEGAWSALQPKIADG
TYIVRNSDKATALSKKAALTRDEEAQIIGQVTTNWNFSDAKNKAEANLTAAKKDAKGTVY
VCAPNDGTARAIADAYAADKDVKTYYITGQDAEIASIQYIIDGKQSMTVLKDVRTLVSDA
IAAAVAYMKGQTPPVTNYYNNGKKDVPAKPTAIVVVTQDNVKKEIVDSGYWPADKFTGLK
*
>SPBDM4_v1_80125|ID:27159901|xylF| D-xylose transporter subunit ; periplasmic-binding component of ABC superfamily [Uncultured spirochete bdmA 4]
MREIELRVKRMRHMRRIILVAVSIVCMAASLGLSSCSKSKKPVADRAPMIGFSLDSLVVE
RWRRDVDSFTKAAHDLGADVALRVANQDANTQIAQVRELLNQGIDVLVIIPNDAERLSDV
CREAKRKGVPVLSYDRLVHNADVDLYISFDNEKVGSLQAQAVTEAVPGGNYVIVNGAITD
NNAFMINKGFHAVLDPFIADGRIRLLEEIWPSDWMSDEVRTRFEALLKPGQKIDVVLCGN
DMLAETIISVLSENRMVGKTKVTGQDAELSACQRIAEGSQFATIYKPIDALALKAAGFAV
MMAHGEKIRYDTKIDDGRYKVSYIALEPILVTAKTLYSTVIKDGFHTREDIYRNVKH*
>SPBDM4_v1_80126|ID:27159902| Two component transcriptional regulator, AraC family [Uncultured spirochete bdmA 4]
MKRVLIADDEMPIVNGLSLLFKRYFQGGYTVVGTARSGREAIEKSKALSPEIILMDVQMP
GITGLDAIRELSREGGAKAFILVTAYERFDIAREALSMGVCDYLLKPVSRERLGIALTAA
SSYLDRVQLLNERELEFRDRQQRLVPFVKTAFFVSARQKAGYQSGGSGNDLSLFKEMLQL
SGETGIMGISSFSPSDGAVDAAYERFCSVIQYKTVALAGPLEDRRYCAWFLPLKNEAVPS
QELAAFLAVLQSAFSAQLATGEMKLSYGQPEKLETLGRSWDTALRLFAKGPTSDKWSEEN
AACALDAQFYDEIIEGRFAMAGQALERMLLSLESAEPMKRDILFRIIAALSFAASKLAGG
GVLSEKVYREFMDFSDIERLWAEGASQLFASNVRSRFQTLQQRAAAAGAHSPFVVRALQY
IENHYQEPITLESVAEAIGVSSGHLTRLMSDELKRGFARTLIEYRMQKAKELLKRPNVSI
REVSRLCGYPDANYFARLFRRLTGHTPREYAARVAKGDNSDA*
>SPBDM4_v1_80127|ID:27159903| Signal transduction histidine kinase, LytS [Uncultured spirochete bdmA 4]
MIDLSFLAHLRKPRRRSGLRNRIIVNSVVILLVLVIATSYTAFASFDLARSVELLFRNSI
SMEELRSTLRDTQENLTGYLSAKNSESLKEFIRTSTILSGMTSRLNRENKVDDVFLLEKD
LAFLLDGYLASAEGAVQAKRGRDVAQYVSLYEDARRNASLIEFLSARMDTIHLDQSLKGF
SSYKANISPALVSNGVLLLVAFLLSIALIGRYSYTITEPLARLSVAAEAIGRGEYDHPLP
PYEREDEIRTLHDAFVNMQDSIQRAFAELTRKAELERSLMEERMRVLTYQHRLKDAELLA
LQTQINPHFLYNTLAAGWQLALTEGNEKTAEFLEKLAAFIRYALKPSTRFVLVSEEIECV
RQYIWLLKLRFGERFRFRLEAQDEALGYETPALVLQPLVENAIVHGLKDLEEGGDVIVSA
RLDGETVRFSVSDSGRGMEKDSIALALAAADPEDTTPQHGIGLHNVVRRVALATGGLGRV
EIESELGHGTRVTIILQAGKAV*
>SPBDM4_v1_80128|ID:27159904| conserved exported protein of unknown function [Uncultured spirochete bdmA 4]
MKRAAWRMLPIVVFAIALTGALAVNISLVSRSRNALASTILRNPATVRTARFHVVAILPD
TADPFYTHLIEGLRGESEQQNAALQIFYYPSTAIGEGGSTSEEVSRWFEIALRSKADGIL

LFQPKGMDVARFAQEADSAHIPFVPLAMDAPQAWTRSGVTGDSVSQGKEAGTLVLGLLGS
AARIGIILSSDTSLGYAFNEEPFYRGVEETLKDRPGAVLVAAVREEESILGGEDACARML
TEHPDINAILCIDAKATIGAAQVIIDRGNVGQIVIIGSDEDSEVNRLIEKGVVHASIVRN
AAAMGQSGVALAIGQRIGLRSPEKISVGYHVIPTRGDGP*
>SPBDM4_v1_80129|ID:27159905|glmS| Glutamine--fructose-6-phosphate aminotransferase [isomerizing] [Uncultured spirochete bdmA 4]
MCGIIGYTGPRKTSKILLEGLRRLEYRGYDSAGIAVGRLNAEPRLEIIKSVGKIAELARK
IPEDIDGNWGIGHTRWATHGGVTEANAHPHTDMSGKIAVVHNGIIENYKTLRTMLEKKGV
VFKSETDTEVIPQLIASYYEGDLLAAVLAALQHLEGTYGIACIHADEPGRIVGARNGSPL
IVGVGKDEMFLASDITAMVAYTNRVIYLNDGEVIDITQDSYTITDRHSNALDKKVDEVTW
ELGAIEKSGFMHYMEKEIFEQPESIARAMSGRIDEENAIAKLGGLNLTRRQLADVRRVRI
IAAGTSWHAGMTGSYLLEQAARIPAQAELASELRYRNPVVENGSLWFVVSQSGETADSLY
AMREVQRKGATVLGICNVVGSTIARESDGGVYVHSGPEIAVASTKAFTSQLTVFYLFTLL
MARMRDMSREEGQKFARSLLEIPDMVRDALGKRDLIQAIAKKYCRAKDFLYLGRGILYPI
ALEGALKLKEISYIHAEGYSAGEMKHGPIALISPEVPSVFLVSDDYLHEKTISNMREIKA
RGGPVIAVGVEGDAEAMALADDFIPVKKADPRFYPFSMVVPLQLFAYFCALELDRDVDQP
RNLAKSVTVE*
>SPBDM4_v1_80130|ID:27159906| protein of unknown function [Uncultured spirochete bdmA 4]
MIPYHNHPSKDGTQISHGMMLALFLKLG*
>SPBDM4_v1_80131|ID:27159907| conserved protein of unknown function [Uncultured spirochete bdmA 4]
MPAQEPLFQLFSFPCLGSTMDEARALAREGFTGAAIVRADTQTSGRGRIGGRTWVDAPKL
SLLMTILLPADFKEAKALPLRAGLGVVRALEETAALNETVMIQRDGKPAARSYKFRLKWP
NDVLVQPHPQREPGVRSAAAIPRDGAAYGKLCGILCESAQGRILIGIGMNLRSPPPVRSH
SGLQPASLEETWGCLPPPFDNLDEAAHYVAQQVIDTLSDPQWHSEYERRLWGRGSTLQFL
AGHPQSPERMQGMCVGIDEDGALILNIEGEKKTFASGEIASLRLV*
>SPBDM4_v1_80132|ID:27159908| 2-dehydropantoate 2-reductase [Uncultured spirochete bdmA 4]
MDSIHSTVIVGAGAIGASIAARLVDAGHRVSVSARGERAARYQREGFVVNGRQYFLPVTD
SHGAVPADLVLVAVKNYSLDEAIQEMKPYVGAETIILSLLNGVDAVPKLREAFGPDAVPY
GMILGIDAYRQGNEIQYSAKGQIFCGFEKELAQKNASKLARLEQLFRQSDILFRVPDDIV
REIWFKFMINVSINQWSAVLRAPYQIFQTSSHAIALLERAMKEVIVLSQKIDTGLTEADI
AKAMAVLRTLGADGKTSMLQDVEAHRRTEVEAFAGTMVRLAGAAKVEVPINAMLYEAILA
IEDSYKAQ*
>SPBDM4_v1_80133|ID:27159909|hflX| GTPase HflX [Uncultured spirochete bdmA 4]
MHSNISRMSTLRETERVFLVGVASEDLRKPEARELLDELHGLAQTLGLETVGEILIQLRD
TTPSLLVGTGKAEELAEAADQAGADSIIFDHILSPVQQRNWEKLSSKKVYDRAELIIKIF
ASRALTKEASLQVELAQLQYTLPRLAHSYDDLMRQRGGRYGTKGSGEQKLELDRREIERR
IHEIKDELESVRKERSVQKRRRERAGVPRAAIVGYTNAGKSSLLNALTAASVHAENTLFA
TLDPTTRRLLFAQGQTLLLTDTVGFIRNLPHGLVEAFRSTLEEASQADLLIHVADASDVH
VDAHIATTLQVLCEIGANDVPRILVLNKIDLAERDAAEAFLLRYPGSVAVSAKTGQGLDG
LVEAIQEALTRGMKTYVLRIPHADYSIASLVMREGTVLEEHHDDESTWLHCRIPGRIEKK
VQPYIVDAAGMGAGIFSAKE*
>SPBDM4_v1_80134|ID:27159910| Aminotransferase class I and II [Uncultured spirochete bdmA 4]
MPLQISDLSDAVLHTHYAVRGPIVARAQELEDHGKEIIYCNIGNPQALGQKPLSYVRNVL
AACERPELLKVANHAFLDDVREKARYILEQSRYGIGAYSESKGLRFVRAAISEFIAARDS
HDGVAQASNPEHVYLTDGASKGVQTALRLLIASGNDGILIPIPQYPLYSATITLYGGRQI
GYYLDEESGWGLNEQLLEDAMRRAMKEGIRARAIVVINPGNPTGSVLSEDDMRMVIHFAK
RHNLAILADEVYQENIYKPGLKFISFAHLMSKIEEREVSLFSFHSTSKGFFGECGQRGGY
MEVRNVPEEVIAQITKLQSVALCANLPGQVLTYLMVSPPVAGEPSYRLFDEERNAILSEL
GKRAKMMEEGLNRIPGIHCQPIDGAMYAYPSLSLPAGRTDEEYCMTLLEATGVCVVPGSG
FGQKPGTAHFRTTILPPTSQIEHVIDAIAAFHLQWR*
>SPBDM4_v1_80135|ID:27159911| Diguanylate cyclase with PAS/PAC sensor [Uncultured spirochete bdmA 4]
MNFANDPSELQAMPGLESILQVLHEGVYIVDKERRIQYWNTAAEAITGYSAKTVVGRRCA
DNILRHVSEDGQQLCTSGCPLQATLEDGKTRDLMAYLHHKDGRRLPVHVRSIALRDETGI
PRVLEVFNEISDRGKLLEELETLRQEVLTDPLTKIGNRRYYELNGEARLAAYHAQKIPFG

LLMFDIDHFKAVNDGSGHGAGDSVLKMIAGTISHGLRPLDTVARYGGEEFVVMVPDCTEE
YLSIVGGRIRMLIETSWLELDDGRRLTVTISGGGSMAQPGDTLATLTARADKHLYASKKS
GRNRVIVDA*
>SPBDM4_v1_80136|ID:27159912| RluA family pseudouridine synthase [Uncultured spirochete bdmA 4]
VKKPFSVLYIDTEILAIDKPAGVLAVPDHWDPDVPVAREMLSKDLGKLLPVHRIDKDTTG
VLLYARDEEAHRELNRRFSTRQVEKTYFAIVHGEPEHEAWEVEFPLRADGDRMHRTIIDT
SNGKPAKTRFEVVERFRGFTLVRALPETGRTHQIRVHLTASGLTIVADPLYGDGVPLMLS
ALKRKWKGDAYEERPLVARTALHAAKIAFQHPKSGARLEIEAPLPRDFKAALAQLRKLCS
P*
>SPBDM4_v1_80137|ID:27159913|groL| Cpn60 chaperonin GroEL, large subunit of GroESL [Uncultured spirochete bdmA 4]
MAKQLLFSEDARRKLLVGVETISKAVKVTLGPKGRNVLLDKKFGAPTVTKDGVSVAKEVE
LEDPYENMGAQLLKEVATKTNDIAGDGTTTATVLAYSIVKEGLKSVAAGIDPMALKRGID
QAVTLSVEEIRKNSKEIKEKEEIAHVASVSANNDIEIGTQIADAMDKVGKDGVITVEESK
TMETTIEYVEGMQFDRGYTSPYFVTNRDSMTTVFENPLILIHDKKISNMKDLLPLLEKIA
QTGKPLMIIAEDIEGEALATLIVNHLRGTLNVCAVKAPGFGDRRKAMLEDIAVLTGGEVI
SEELGLKLENTSLNQLGTAKSVKVDKDNTTIINGGGKQKDIQDRIAQIKKQIEETTSDYD
REKLQERLAKLAGGVAVINVGAATEVEMKEKKHRVEDALSATRAAIEEGIVPGGGIALIQ
SALALEKADTSNMTDDERVGFKIVKRALEEPIRQIAENAGIDGSIIADKAKHEKKGVGFD
AAKMEWADMVKSGIIDPAKVTRSALQNAASVAALLLTTECAITDLPEKNPPAPPAGNPGM
GGMDY*
>SPBDM4_v1_80138|ID:27159914| conserved membrane protein of unknown function [Uncultured spirochete bdmA 4]
MSTNEKPTNTNTASTPQTPRRHGQGFGPPGGGPHALMPGEKSRDFKGTMKKLLAYLGPYR
WGLLVVMVLATAATILSIMGPKILGTATTTLFEGVVKKIAHSGDIDFAKIGSILLTVLGL
YVSSAILQYIQGWVVSGIAVKITYTLRKDIMAKINRMPFRAFDNTNHGEILSRMTNDVDT
VNQTLSQSLSQMITSVMTLIGAFVMMLTISWQMTLIALLMIPLSIGVVRFIVKRSQKYFK
IQQDYLGHVNGHVEEIFGAHTVVKAFGGESKAVQTFEELNSTLYQSAWKSQFLSSIMMPL
MNFVGNLGYVAVAVSGGWLAAKQMVTIGDIQAFLQYVRSFTQPITQVANISNVLQQTAAA
AERIFDFLASEEEKQDLAPAKVPEGIKGKVEFRHVHFGYVQGQPILKDFSATVLPGQTVA
IVGPTGAGKTTLVKLLMRFYEPESGEILIDDVPIQELPRSVVRSWFGMVLQDTWLFSGTV
RDNIAYGKHEVREEDIIESAKATHVDHFVRAFPEGYNFVLNEETTNVSQGQKQLLTIARA
MLAKPPMLILDEATSNVDTHTELLIQKAMLTLMQGRTSFVIAHRLSTIRDADLILVMDHG
DIVEQGTHEELLAKCGFYAELYNSQFDESGEAQAACS*
>SPBDM4_v1_80139|ID:27159915| ABC-type multidrug transport system, ATPase and permease component [Uncultured spirochete bdmA 4]
MRTVFRYLKSYWASIILIIVLLFIQANADLTLPDYLSQIVNIGIQQNGIEEDLPQVMRAS
SFEAMGKLLEMEGAGDAFAVLQKVYTHVTPGSAEADALAKKWPLAKTEAVMSLNTGDKAA
TDSARKVFLAEYPKLVVFSQMSGVGTTGSAGGTETSVSTGTAPVAPSNQPGGAQSNAMPG
LPALDSTSPQAILAQIDSMDPLAKSQLVVKGLQAEFAALGVDMSALQTSYILRIGALMLL
ITLVSALATILVGYLGSRMAAGTARDLRSAVFTKVEDFSLAEFDSFSTASLITRSTNDIT
QIQMMIMMGTRMLFYAPIIGVGGVIRALGKASGMWWIIALAVGVLLAIIGLVFAIVVPKF
RIVQKLIDRLNLVVRENLSGMMVIRAFNRQDHEAKRFDKANRDLTGTLLYVTRVMVVLMP
LMTIIMNVVSVSIIWVGSHEVAAGSIRVGDMMAFMQYSMQIFFAFIMMSFMFIMLPRASV
SADRIAQVINTEVSIRDPKTPSQLSSPVHGQIEFRNVRFRYPGAQEDVLHDISFTAKPGT
MTAILGTTGSGKSTLVSLIPRFYDVTEGQVLFDGVDIKSLPLAELRSHIGFVPQKSMLFT
GSIAENIRYGKESASDDELVEALSISQIMSLAEESPEGLEHQISQGGMNVSGGQRQRLSM
ARALVRRAPVYIFDDSFSALDYKTDRAIRSALKTYAKDSVIFLVTQRVAPIRHAEQIIVL
DDGRAVGIGTHEQLMETCEVYRDIALSQLKQEELA*
>SPBDM4_v1_80140|ID:27159916| putative transcriptional regulator, MarR family [Uncultured spirochete bdmA 4]
MMTENIELENALKTWMGQVMQLSMRSFIEYTTETGLSMPQIAVLFLLNGDKHRAVTELGE
EFGVSGAAASQMVEKLVQMGFVERREDSNDRRIRRLLLTTKGKNIVERNIEARQRWMRSF
CSTFSPESARDTARVFRDITEAAKRFETAQAPVPRHNSGI*
>SPBDM4_v1_80141|ID:27159917| Beta-N-acetylhexosaminidase [Uncultured spirochete bdmA 4]

MMACDLVPVPVSFEEKESIFALGPGAGVAAPLALQKEAGLLAQWLSRIGEVGHVGVSVAA
GPAERRHGSAVGGMGSTISLFLDPGMKRAEEYELDIGPEGICIAARDAAGIVRGAASLFQ
LALSQGRMLRAVRIRDWPRFAWRGFMLDCARNFFRVEFIEKLLDLAALHKLNVFHWHLTD
DQAWRLEIPSKPELTDCGAFRQDMRYRIDCRKGGYYSVEDVTRIVEYAAVRHIMVVPEIE
TPGHATAILASHPEFSCLGGQDASVHFEPEDRYGIFEDILCGGNDRVLSFFDEVLDEVCA
MFPGDYVHMGGDEAPKGRWLACPDCQRKMRVLGLIKDDGRYEPEGLQAWFMGELARMLAR
RGKRMIGWDEVVEGCIGKDTLVMSWRGYENGRRAAELGYDVVMCPQTKACYLDHKHLDSP
EEPGHLGVCSVRDSYMFEPLPEGLSVGAASHIIGGQANLWSELLYFGRQAEYMLFPRLCA
LSEVFWSPREKRNFEDFSMRLETHKRRLDALDVLYYKGRLS*
>SPBDM4_v1_80142|ID:27159918|bglA| Beta-glucosidase [Uncultured spirochete bdmA 4]
MKRQDDFPDDFIWGCSTSSYQIEGAVKEDGRGPSIWDTFAHTPGKVTGGHTGDIACDSYH
RWKDDISLLRELNAGAYRFSVSWPRIQPRGKGAPNQVGLDYYSRLVDALIGAGIEPWLEL
FHWDLPQPLEEEGGWRVRDTAMRFEEYAAIMYEHIGDRVCHWTSMNEPWCAAFLGHLTGE
HAPGLRDRQAASRAVHHLLLAHGLAVRAYRQSGLAGEYGLVINPNRPRPATLRPEDVDAS
ERASVERTSLWLDPVFGRGYPADFVERFGDDLPIEAGDMDIIASPLDFIGVNYYNEEAVR
AAAPSADNPYGFEFVPTYQKRTGMDWEIEPQGLRRILEYIAATWPCRSLFVTENGAAFPD
IPGPDGIIRDYDRIEYMRGHIAACGEALAHGVPLKGYFVWSLMDNFEWSFGYTRKFGLVS
IDHITKARRPKLSFYYYRDRIAGFGL*
>SPBDM4_v1_80143|ID:27159919| Binding-protein-dependent transport systems inner membrane component [Uncultured spirochete bdmA 4]
MSEAIRESKLKKALSAIGRVAVYMVMGIFAFMTLYPIFWLIMNSFKTTREFQVSQLALPH
APTLQNYVEAWKMGDFSLLFPNSIIYTLGATAGIIFLSLLAGFAFAKLKSRATKPIYNSF
VIGILLTTQTLMIPLFLEVNLLGIYNTRLAVLLVYIGAGLPIGIYLSTEYIKAIPSAVVE
SARIDGAGFFKIFLKIIIPMSVPVATTLAMLNITSIWNEFALINILVSKTELKSLPLGIY
KFSGSLSTDYGKQFAALTIGMVPMLVFYMIFRKQITKGVAAGAMKG*
>SPBDM4_v1_80144|ID:27159920| ABC-type transporter, integral membrane subunit [Uncultured spirochete bdmA 4]
MAYTNRAHLEEKRAYWAMVTPAFALYLLVMAFPIILSFVLSVSNYSGGKMFGGEKWGFAG
FGAYARVFTDPWFWNALKNNLYIVLISVFGQLPLGFIFAYFIYAKIVKAPNFWQGVLYVP
NIISVIVVGLLWQTIFSPHGPIGEIINYFRVSDFQAQLKGIFNAAGGFSLSDDVIHKILD
LAGPAGQSMFSNPVPELRDLLSSYQGQPMSEVYTALSNLFVQKWSPAFLTKPDIAMVPVL
FVILWMYTGMYLILFLANMQKIDAQIIESARIDGANEGQVMRFIILPALSGTIVNSAILA
ISGSLSSFALIFAMTGGGPSRVTEILSIYMYNNAFLGRPNFPLANAISLIMVIISFVLIA
LTKAVEKRYGGKED*
>SPBDM4_v1_80145|ID:27159921| Extracellular solute-binding protein family 1 [Uncultured spirochete bdmA 4]
MKKGFLVVLVLAIAFGSVVAQEKVTLNVLNYQQADQAGYQEDVAIWQKFQQQNPDITLNM
EVLFNEPYHQKLQAYAAAGTLPDVFYVWPTARSAVIHEKKLAKDLSKLLGPDFLKDFSGA
ATNPNNQLGKYMAMLPQSFTYTSVMYVNKKLLADNGFDLPKTYDDLKKMVPKLKAKGINV
VMLPDKDGWPMQSCLFSTVLGRMAGDSFVDAILAGKAKFTDKPFIDSLKVIDNLYKDGII
AREDNQVGYGEAPGLFVSEKAAIYIDGDWRAGAYITDKASGKALIPPAAQQTDFALLPFP
AIAGEVNPGVLSAIAGTGWAVSGSIPAGSAKEKAAVRLIKYLYSDEVQAIRYSTGAYVPT
RKNVKANVEPLVAKVPVFYADNPKTCYVFDGMFDPAVYNVLNDGLIAIGLGTSTPEKVAA
DIQKAYETWKSSQK*
>SPBDM4_v1_80146|ID:27159922| ROK family protein [Uncultured spirochete bdmA 4]
MPPRGDQWTRTATRIFRTIWRNPNISRIDLADTLGLDKSTVTKQVAYLIDNGLIIEKEEG
EASSRGGRRPIYLAVNKEYGRFIGIELQLGFYEAVVVDLAGNIRATDRGDTHVTRENFAE
TAAEIVDKMQNQSCGDSLLLGVGIGTAGLVDSKKGRVKYSVPLGIDKSLDFAKAMTGKLQ
VPSFIENDANCCAWGELAFNRDEELKDFLFTLVEYRRDETSLLKYGGIGVGFGIVLGGRV
HTGAHGYTGEFRSAFCEGPGELQFSLSRDELMQLRCDKILLGRTVDELARNMAMLINTMD
FQHVYIGGDIEGLDIDLPAILRHRLEENWMYPSPKDVEIRYSSLGDKAVAFGAAGMVFER
LMAERNMPGLAGGTTSDKIRRVS*
>SPBDM4_v1_80147|ID:27159923| Beta-mannosidase [Uncultured spirochete bdmA 4]
VEIIHLAGEWKLVRLADGTVRPMQVPGDIYSALIQAGELPDPYFGENELKAQWPGREDWR
IEHRFWVPDQFLKLGTMRLLAEVVDTIGEVYLNGILVGKSNNMFRRFEADPKPYLHSGEN
TIAVIIRSPEQAAIREAGRLPYPIPASPYPVSSPHRNLVRKAQCMSGWDWGPCLMTGGIY

DDIRLIATDGPLIRSVQTRTEPLGSFDGASSRGPDFSVEVSAEIEVPAPMEVKISCFLDG
KRADAGRFCQAGTTTIRIDMEAKEPKLWWPHGHGEPALHDLHVAAERVTSTYIHRTDPQT
GHASATCLHPHIVHKKIGFRELKVVTEEDSVGRSFKFVVNGKDIFAKGANWIPADALPSR
WTRARIAGLLDSTVEANMNCLRVWGGGRYESDDFYELCDERGIMLWQDCMFSCALYPSST
EFLLNVEAEITYQVKRLSDHPCIALWCGNNEALGAITWYDESKKNPARYIIDYDRLTEGV
LGRVIRSLDPDRCFWPSSPSAGPNDFSDNWHSDERGDMHFWSVWHEGKPFSEYLTVQPRF
CSEFGFQSLPSMRTIASFAPQSEWNISSPVLEAHQKHPRGNSLILETMLRYFRMPKGFRE
LAYLSQVQQAMAIRTAVEFWRSTMPRCMGTLFWQLNDVWPVVSWSSLNHDGSWKILQYEA
KRFYDPVLLALFIKNGTVQAHLVSEASGEHRAKVTLRLLDFHGEPFTIPAGVGGEKGVVE
EGAQASRSSAVERSLIISENSVQHVWAMPLDVLPCKTEEVFLEATLEVPDLDFKTASVLF
LTEPKRCALEDPGLEVALESNDEGALEAKICATAAPAFYVSPELEDAAGQFEDAGFYCAK
GDHRTIRFIPAARQPSEIRAQPSTKTQTHSGLRVYHLQNSFEH*
>SPBDM4_v1_80148|ID:27159924| MATE efflux family protein [Uncultured spirochete bdmA 4]
MMSAPIVTEKNMLNKSIHALARPLRFFGPGPFYREALALAVPVMLQSLITGLVSLVDNFM
VAGLGDAKMAGVNIANQVNFIFMVIINVVSMAGGIFLSQHRGAQNREGMMQSFRFKLVMG
TGISILYFAACQLVPEALLSLMVSNNAEGAVIVAEGARYMRAVSFSFIPLALSGAIGSSF
RDIGEPKVPLIVSTGAAIANTIGNWFLIYGNLGAPRLEVVGAAIATVMARGLEIAAFLFV
LQKRKPAFSFKVKAIFKIDGKFLGGVLSRSAMMFFSETAWVVSETIITALYNRRGGPETV
AGMAAGWSVANLFFLVFPAIHTATNVIVGSTLGAGKLDEGRAKARWIMAGSIVFGVGAGL
AAMLSTAAVPLVFGNLSDAAQHITRSIIIVIGIYLPLWCLLNAQFGVSRAGGDTLMGLLV
DVGVTYAVFIPVAFVIARYTAWGPVALFGIAKLSDIPKAIVAGWWLKKERWVRNLTVSSV
FKAIL*
>SPBDM4_v1_80149|ID:27159925|trxA| Thioredoxin-1 [Uncultured spirochete bdmA 4]
MSTEVILTTENFKQEVLESKIPVLVDFWAEWCMPCKMIGPSVTQIAETYKDKIKVGKLNV
DDQGDIASQYGIISIPTLIVFTEGQVARQKVGAIPRHEIEKLFKDLI*
>SPBDM4_v1_80150|ID:27159926| conserved protein of unknown function [Uncultured spirochete bdmA 4]
MSQKGKIRDQNEARRSMAVSARLCRKSLMLGLLSWIIVSCAPPGIESAVLGDIRPPIVKS
AQLENAREFVLEFDEPVQVREGSFSAEPKNFIVSAESAEGIVNISFEPQPTPGEAVILAG
NVQDLCGNSTHVQVQFKGYNDRPAALLLTEVQTAKNTSKKSPHRDYVELYVKEAGNLGGM
FVQWASSAKTMRYDFPGCEVSAGEVIVLHLAPEGIADERNESGNDLGLSGGIDATPAGRD
FWSDAGGLPDASGAVSVYGREGAAPVDGLFYAESSKTGTVESSRLVALVQELADAGRWHF
DSPPKWENALLWKPSATRPLLRTTTSAYGASAWGVGESGSQSPGFVAR*
>SPBDM4_v1_80151|ID:27159927| protein of unknown function [Uncultured spirochete bdmA 4]
LGLLRFLLWDAVLPLKINAENLGRCFKFTLVKTSSVI*
>SPBDM4_v1_80152|ID:27159928| Integron integrase [Uncultured spirochete bdmA 4]
MGSPYEKTVFKRYLLETCRFRPKGAEWACIWVEQFVRLYPAWRENREHSIEGFGYILSKR
KPENIVNLALQSVRLFMGFMDMNAGDNKAVNAQDSESKNVIPRYDVKKCFQESASPSSGS
DGAQQDEMAAWSAFSAGIMKDVREYIRLRHRSLRTEKTYLGWTRRFLDFIWKRGLVRQKE
GKFCIVADHLRAFLSFLAIEVRVSASTQEQALNALLVLFRMVLHIEIDGLSSVLRAKKRK
RLPVVLSRDEVAALLAHLQQPYRLMASLMYGCGLRLEECLSLRVKDLNFENETIEVRSGK
GGKDRLTVLPGVLKKDLQAYLLELKVGWDKARRRDLPGVFLPEALNKKYTSLSKEWGWYW
LFPAKSPCTNTRTGEVAFWHVHPSVIQKRIHEGIQGAGISKMASAHTLRHSFATHLLEDG
YDIRTIQELLGHSNVQTTMIYTHVAVRNKRGVRSPLENL*
>SPBDM4_v1_80153|ID:27159929| CopG-like domain-containing protein DNA-binding [Uncultured spirochete bdmA 4]
MTSVRLPTDLERKLEMVARKKRTSKTNVIREALENLFTQEETEKDSYELGEEYFGKYGSG
DGSLSTTYKNKLKEKLHGKYHSH*
>SPBDM4_v1_80154|ID:27159930| conserved protein of unknown function [Uncultured spirochete bdmA 4]
MVNTILIDAGPLIALFDKDDKYHAQIKAFIKQGKYRFITTTAVITEVTHMLDFNVDVQID
FFEWMVKDGVVLHEIAQKDISRIIALTKKYRDRPMDFADATLVIAAENTGIKNIISIDAD
FDIYRLPGKVRIENVFKQQ*
>SPBDM4_v1_80155|ID:27159931| protein of unknown function [Uncultured spirochete bdmA 4]
LSRCLLPFCSVHKLEFIEERKTSNERSEDGALTCKHRANLLSQVALTGYARVIREVFGRA
SL*

>SPBDM4_v1_80156|ID:27159932| protein of unknown function [Uncultured spirochete bdmA 4]
MNMDFWGFGFQYGVSAIFSLPFYIKVIREEDRMQRKNEIGAYNEALPKTSRITRA*
>SPBDM4_v1_80157|ID:27159933| protein of unknown function [Uncultured spirochete bdmA 4]
MLSESKQKAIVDIWNNYNSTKVVVKDTKGNDLDNIDELRVVCIEQLKEIVNEYIRSNITV
GQFKTALDSFNKRNNHWGFTATKGQMFFNQLVRSAITDEDSLNKILKDVIKIPADINEAE
RKIDILDKYCQKYFNNAKDKRTVPNPKSVCYFLSYFWQIFNYKKWPIMYTSLIEAFTEIG
IWSDFDSQSNTYKYFLNINNEICVLLSKKANKLIDYWDIEHAFWNYKGNPNKQSKKKVET
PNAQKVITTNIEISEYIIPKISKLVEIGNDKEQSSAKKGYQFEQLVAEAFKQLDFEVEIY
GQGSGRNPDVIIKNREQNTAFLVDAKAYSEGYSLGLDDRAIKEYINTHCPKLMKDGYKKI
GFILVCNSFNSNLDEFINELTWNTEIKRFVLLTSEALLYLVAYKTRDKQSLQTIIDSIVS
IENPITYEKVVSQFEDV*
>SPBDM4_v1_80158|ID:27159934| protein of unknown function [Uncultured spirochete bdmA 4]
VRVTSERALRQQVGAIVASYAAAPLFSLPLSYLGAVSLFYPEAPQEACQERESGSRGCSA
QKPIFEFLFTIRINHRNTEAFLTFKLGFSYLFSFQRRR*
>SPBDM4_v1_80159|ID:27159935| protein of unknown function [Uncultured spirochete bdmA 4]
MNMDFWGFGFQYGVSAIFSLPFYIKVIREEDRMQRKNEIGAYNEALPKTSRITCA*
>SPBDM4_v1_90001|ID:27159936| conserved protein of unknown function [Uncultured spirochete bdmA 4]
MPSLQVRDLPNDIYTQLNYLAEKEHRSLAQETIVLLKEGIITKLGNKERRRKLLEKADHL
NIDGSSFPDPVAMVREDRDR*
>SPBDM4_v1_90002|ID:27159937| conserved protein of unknown function [Uncultured spirochete bdmA 4]
MIVVLDASAAIEIALNREYSTSFREVLRQSDLVLAPDTFPSEITNVFWKYAIYSDMPVET
CEKGIDYCLDLIDDYIETTSLCREVFSESYKNNHSAYDLFYIVTARRYNASLLTRDAKMK
NIADALKINILT*
>SPBDM4_v1_90003|ID:27159938| protein of unknown function [Uncultured spirochete bdmA 4]
VFYAPLSITSAISIFIEYFFILIIYRIPKAERGVHFVIDLYVCFQLLF*
