# Supplementary material for: Fermentative Spirochaetes mediate necromass recycling in anoxic hydrocarbon-contaminated habitats
Source: ISME J. 2018 May 30;12(8):2039–50. doi: 10.1038/s41396-018-0148-3 (PMC6052044; doi:10.1038/s41396-018-0148-3)
Supplement: Supplementary file 11 — Supplementary Database 4 [file 41396_2018_148_MOESM11_ESM.pdf]

Rectinema cohabitans strain HM:

May evolve H_2 during carbohydrate fermentation by two ferredoxin-coupled FeFe A1 hydrogenases
May also enhance H_2 evolution using predicted ferredoxin-coupled FeFe B hydrogenase
May reversibly bifurcate electrons from H_2 to NAD and ferredoxin by multimeric FeFe A3 hydrogenase
May sense H_2 leading to hydrogenase regulation by FeFe C1 and C3 hydrogenases

SPBIB_v1_260013|ID:27163327|hndD| FeFe Group A3 hydrogenase catalytic subunit [Uncultured spirochete bib]
MVNVKVNGIPVQVAEGSTVLEAAKKANVKIPTLCYNPDLSPWASCGICVVKIEGSNKMLRSCCTPVSEGM
SIISNDPDLVQTRKTVIELILSTHPDDCLACPRNQACELQTLAQEFGIREQPYKKMVRDIPQDTSTGSLI
LNPSKCIRCGRCVEVCQEMQGVWAVEFLGRGESIRIAPAADVKLGDSPCIKCGQCSAHCPVGAIYENDQT
KLVWDALMKEGPEAKTCAVQIAPAVRVALAESFGLPPGTDLTGKIYTALRRLGFDAVFDTNFSADLTIME
EGTEFVHRLTGALKAGMQQATADKSMPLITSCCPAWVDYMEKYYPDMIPNFSTAKSPQQMMGAMIKTYWA
AKAKVDPAKIYSVSIMPCTAKKFENSRDESMYSSGYKDVDVTLTTRELARMIKQAGIDFLNLPESEPDSP
LGPYSGAGVIFGATGGVMEAALRTAYFLVTKEELKDVNFTAVRGLSGIKEATVHINGIELRVAAAHQMGN
IATVLDQVRKAREEGRETPWHFIEVMACRGGCIGGGGQPYGATDEVRKLRMRGIYDNDEKQEYRCSHDNP
YIKQIYAEFLQKPASHKAHELLHTQYKERPLYLK*
SPBIB_v1_20025|ID:27161852|hndD| FeFe Group A1 hydrogenase catalytic subunit [Uncultured spirochete bib]
MEEQLIEITIDGQNVRVDPSANIVEACAHAGVKIPTLCYLKGISQNASCGVCVVEVEGAKSLVRSCVQKP
VPGMKIRTSSPRVIRARKTAVELLLANHPDDCLSCIRSDTCELHTIANILEVRADRFPGYKKYPMPDTTS
EGIVRDDSKCILCGRCVAVCEETQGVHAIAFSGRGARTRVSTFLDRGLAQSACVQCGQCSVVCPTGAITE
KDESREVFSAIEDPRLTVVVQTAPAIRASLGEALGLPAGSLVTGQMVAALRRLGFNRVFDTQFTADLTIM
EEGSELLERLSKGGTLPMITSCSPGWINFIEGFYPDLLSHLSTCKSPQQMFGSVAKTYYAKKAGLTPDHM
RVVSIMPCTAKKYEARRKEMDGAWGWWKEQDPGKVPARPFFDVDWALTTRELARMIKLAGIDISRLPEEE
FDDPLGQSTGAGTIFGTTGGVMEAALRTVYELVEKKPLENIEFTKVRGFEAIKTAEVVVGGSPVRVAVAH
GLANARVLLDEIRAGKSPYHFIEIMSCPGGCVGGGGQPVLADLEKKLARSQALYEEDRKLAIRKSHENPA
VRALYQEFLGKPLGHLSHELLHTSYKARVF*
SPBIB_v1_210130|ID:27163022|hndD| FeFe Group A3 hydrogenase catalytic subunit [Uncultured spirochete bib]
MKNVTLTINGKKISVPEHTTVFSAAEKAGISIPALCRHEDLEPKGACGMCIVKIEGQPGYKRSCVTAVEE
GMEVLTSTAEIRDIRRGILELVLAAHPADCLQCIKHGKCELQTLAERFEIRDLRYDRYTRGLPVDRSSFG
IVRDMNKCIGCGRCVQVCNEVQTVASIFFHGRGSNTIVSPAYGTTMGDSVCVNCGQCIVYCPVGALYEKE
AIEEVWKAIDDPDKVVVAQIAPAVRVAIGEEFGLEPGELSIGKLYSALKALGIDVVFDTNFSADLTIVEE
ATEFLERLKESGPFPLITSCSPGWIKFGETFYPELLENVSSCKSPQQMLGALIKTHYAESRGLDRNNIVS
LSIMPCTAKKFEAGRPEMRSSGARDVDYVLTTREIARMIRQVGIDFRNLPDGTPDPLLSRYSGAATIFGA
SGGVMEAALRTAYELATGKALEKVDFVDVRGMTGVKEAEIDVDGTPIKVAVTNGLANARKILDRVAEDKR
RGRSSYHFIEIMACAGGCVGGGGQPIPNTLARRARRIEGLYREDRSLPLRKSHENPEIRALYAEFLGSPG
SEKAHALLHTKYSPREQYQFSE*
SPBIB_v1_290056|ID:27163581| FeFe Group B hydrogenase catalytic subunit [Uncultured spirochete bib]
VPEVFERMRGLYTPCTDIRRKIFVGVTRFVLAGKKPEDIDYLPFDIIDMGKPTYRCCSYRELSIVKQRIR
LAFGLPLIEERDNTPVSAGIGEAFTDRKVISAPLVNVIRAACEKCPEDKVIVTDMCQSCMAHPCSIVCPV
NAISFPNGGKAFIDQKKCVKCMKCVKACPYQSITRMVRPCAAACGVDAIHSDPDGYATIDQDKCVNCGLC
TVSCPFAAISDKTELVQILHQLMGPEDRRPYAILAPSFVGQFGRLASPGAIVAGLRAVGFRGVREVALGA
DCDTLLLAKRLAEKSSPDNPQHKTFLGTSCCPSWVKTARRHFPEFADNIAESFTPMVETAKIIKDQDPSA
RVVFIGPCIAKKAEALEPEMQPYVDHVLTFEELAAIFVAKDIDLAEITPVEFPDEASALGRGYAVAGGVA
TAIAETARQKYGKENIAVARADTLRNCRAMLADIKSGKTSPELVEGMACPGGCVGGPGTLIGLLSARNEV
GKFAGLAPKNPATLTK*
SPBIB_v1_360012|ID:27164068| FeFe Group C3 hydrogenase catalytic subunit [Uncultured spirochete bib]
MNRQVIYTETMRCQDCYKCVRECPVKAIQILDGHARVVEDICILCGHCVMVCPQSAKKVRSDVERVRRLL
ELKPVAVASLAPSFAAEFSGCTAGQLIASIRKLGFAAVSETALGADLVSSAMRNELGQLADNGRNFLIGA
ACPAVVRYVSFYRPDLVPFLSENGSPMIAHARYLRSRFGENALVVFIGPCIAKKKEADESGGLVNAVLTF
AELRQLFEEENIDPSAEKPTSNDTFFPQRPSKGELYPIEGGMIASIKQHGSTDVPCMSFSGMGHIQAALR
DLPDGIPETGIFLELLACEGGCINGPCSDASRGTVSKRMQILLYEKQGESEPFNTEIACQVKHSAAKPVQ

RQVREEEIRAALALVGKKEPSDELNCSGCGYDSCREFAKAIVIGHAEPTMCLSYMRNLAQKKANALLRAM
PSAAVVVNADLKVIECNEPFVRILEKDAQFVAEAKPTLEGADLRKLLPFWERFNDVLHHSPGDAFIESDF
RCGERILHGTIFSIEKQMIAGGLFQDITAPWIQKDRVIKEAKKVIRQNVRTVQKIAYLLGENAAESEAAL
RSIIESFGGEVGQ*
SPBIB_v1_280015|ID:27163481| FeFe Group C1 hydrogenase catalytic subunit [Uncultured spirochete bib]
VNNKTLQNQENNIRTFHSVQLDADLCVGCTTCIKFCPTKAIRVRDGKAKIFEDRCIDCGECIRRCPKGAK
KAISDPLFILDGYDLKVALPAPSLYAQFGTRYSQSDIFAALHHMGFDEVFDVAWGAIVATEITRSILAKP
GPAPRISSACPVIVRLIQQRFPSLIPNLMPILPPSEIAAREARKRLSEISRKIGIFFLSPCTAKVTAVRM
PLGYSQSSIDAVISFSDIFLPLKRALEERKPLSPKKGFKSSVPQASSGHSPILPMMDNLEPGMGWARSDG
ELDALRIPDSVSVDGISNVIDLFEAIENGNIESIQYIEALACPGGCVGGPMAVENPHIARSNMRQRCQKD
NLAAEASSSPMKAIKPPSPKLPEQEELPSYIWTEQVSPKPVLVLDADLSKALEMAEKIDTIHAQLPGIDC
GACGAPDCDCLAEDIVRGFASIEDCRLLEHPYSKGTKENIHSEGLST*
SPBIB_v1_360015|ID:27164071| FeFe Group B hydrogenase catalytic subunit [Uncultured spirochete bib]
MPREDNTTRIRRELLVRLGRLALEGTLADSIDDVPFDMTAEGWETMRCCVHHDRAILRLRSYALLGGDVR
GMDDVRKPLSVLAEGFQNSRCQDGAELGESGRFPLQVLGEACNACAKSRYVVTDACQACVARPCKVNCPK
GAVTVNGRSHIDPDKCVNCGLCEKSCPFHAIVKVPVPCEEVCPTGAISKGEDGIARIDEAKCILCGKCLR
ACPFGAPIEQTQYLEAASWLREQARPLIALVAPATMAQFPVSSGKFMAGLYRLGFAAVAEVAEAACATAE
REAAELRERMHRSEGFLATSCCPSWVLASKALGDVAGHVLHTPSPMAIAAKWARKRYPEARIVFIGPCLA
KRAEARGFPGEDGRKLVDAVLSAEEIGALFTAKDIQLQALEEARAVQLAEQGSSLDLCYGRGFAQSGGVA
ASVQSVLENENSRIEVGSSWTIRTLVIQGLSKQVLALAALWNKQAPDADLVEVMACEGGCIGGPLAIAQA
KSAAVFLQRYMSEPPPEAKVASSSEAVEKAV*

Uncultured spirochete bdmA 4:

May evolve H_2 during carbohydrate fermentation by ferredoxin-coupled FeFe A1 hydrogenases
May reversibly bifurcate electrons from H_2 to NAD and ferredoxin by multimeric FeFe A3 hydrogenase
May sense H_2 leading to hydrogenase regulation by FeFe C1 hydrogenase

SPBDM4_v1_80097|ID:27159873| FeFe Group A1 hydrogenase catalytic subunit (fragment) [Uncultured spirochete bdmA 4]
VCEETQGVNAIAFTGRGARTRVATFMDRGLAQSACVQCGQCSVVCPTGAITEKDESRDVFDALRDSKLSV
VVQTAPAIRASLGEALGLPAGSLVTGQMVAALRRLGFAKVFDTQFTADLTIMEEGSELLERLSHGGTTPM
ITSCSPGWINFIEGFYPDLLGHVSTCKSPQQMFGAVAKTYYAQAAGIAPDKMRVVSIMPCTAKKYEARRK
EMDGAWGWWKEQDPGKVPARPFFDVDWALTTRELARMIKLAGIEIGHLPEEDFDDPLGRSTGAATIFGTT
GGVMEAALRTVYEIVEKKPLENIEFTQVRGFESIKSAEVVLGGSPVRVAVAHGLSNARILLDEIRAGKSP
YQFIEIMSCPGGCIGGGGQPVLADIEKKLARSKALYTEDRILPIRKSHENPAVNTLYKDFLGKPLGHLSH
ELLHTSYRARVF*
SPBDM4_v1_40494|ID:27157799| FeFe Group A3 hydrogenase catalytic subunit [Uncultured spirochete bdmA 4]
MVNVKVNGIPVQVAEGSTMLEAAKKAHVKIPTLCYNPDLSPWAACGICVVKVEGSNKMLRSCCTPVSEGM
SIITNDADLVQTRRTVIELILSTHPDDCLFCPRNQSCELQTLAQEFGIREQPYKKMVRDIPTDTSTNSLI
LNPSKCIRCGRCVEVCQEMQGVWAIEFLGRGESIRIAPAADVKLGDSPCIKCGQCSAHCPVGAIYENDQT
SLVWDALMKEGPDAKICAVQIAPAVRVALAESFGLPPGTDLTGKIYTALRRLGFDAVFDTNFSADLTIME
EGTELVHRLTEALKGGMQQATADHKLPLITSCCPAWVDYMEKYYSDMIPNFSTAKSPQQMMGVMIKTYWA
AKAKVDPAKIYSVSIMPCTAKKFENGRDESMYSSGYKDVDVTLTTRELARMIKQAGIDFLNLPDSQPDSP
LGPYSGAGVIFGATGGVMEAALRTAYFLVTKEELKDVNFTAVRGLSGIKEATVHINGIELRVAAAHQMGN
IATVLEEVRKARAEGRETPWHFIEVMACRGGCIGGGGQPYGATDEVRKLRMRSIYDHDEKSEYRCSHDNP
YIKQIYAEFLEKPGSHRSHELLHTHYVEKPLYLK*
SPBDM4_v1_40759|ID:27158064| FeFe Group C1 hydrogenase catalytic subunit [Uncultured spirochete bdmA 4]
VSFEPANQSPNIFQTFHSVLLDADLCVGCTTCIKFCPTKAIRVRNGKAKIFEDRCIDCGECIRRCPKGAK
KAVSDPLFMMDAYDIKVALPAPSLYAQFGTKYSQSDIFNAIHSAGFDEVFDVAWGALVVTTMTRSILAQE
ALRPRISSACPVIVRLIQQRFPSLIPNLMPILPPSEIAAREARRRLGSLSQKVGIFFLSPCTAKVTSVRT
PLGYEQSAIDAVFSFGDIYASLKRALDPTPSSAPHIDAISSGNASTHHLLPIMNNLEPGMGWARSDGELD
ALHIENAVSVDGISNVIELFEAIENGNIDSIQYIEALACPGGCVGGPMAVENPHIARSTMRQRYQHPFSA
YPGSKTAQDHHRTAKERSKSLSPESSTADKEYLAFKWTRPLPPNPVLVLDTDLSKALQMAEKIEQIRNRL

PGIDCGACGAPDCDAFAEDIVRGLSSIEDCRLLASPPPARNEEENPK*

Uncultured spirochete SA-8:

May evolve H_2 during carbohydrate fermentation by ferredoxin-coupled FeFe B hydrogenases
May reversibly bifurcate electrons from H_2 to NAD and ferredoxin by multimeric FeFe A3 hydrogenase
May sense H_2 leading to hydrogenase regulation by FeFe C1 and C3 hydrogenases

JGI12104J13512_100111416 FeFe Group A3 hydrogenase catalytic subunit [Uncultured spirochete SA-8]
MINCKVNGIPVQVAEGATILEASKKANVKIPTLCYNPDLAPWAACGICVAKIEGSNKMLRACCTPVAEGM
NIITHDPDIVETRKTVIEMILSTHPDDCLACPRNQNCELQTLAQEFGIREQAFPKMLHDLPIDDTTGSIV
LNPEKCVRCGRCVTVCQQMQNVWAIEFLGRGETIRIAPAADAKLGESPCIKCGQCSAHCPVGAIYENDQT
KIVWDALRKTGDDAKTCVVQIAPAVRVALGEAFGLQPGTDLTGKIYTALKRLGFDVVFDTNFAADLTIME
EGTEFVKRLTSALQNGLGTATREKSMPLITSCCPAWVDYMEKYFPDMIPNFSTAKSPQQMMGAMIKTYWA
EKANVRPDKIFSVSIMPCTAKKFETHRDETMCSSGYQDVDVSITTRELARMIKQAGIDLLNLPESEPDSP
LGPYTGAGAIFGATGGVMEAALRTAYYLVTGNELKDVNFTAVRGISGIKEASVHVNGVVLRVAVAHQMGN
IEQVLNEVRKARDEGRDTPWHFIEVMACRGGCIGGGGQPYGATDEVRKLRIRGIYDHDEGKEYRCSHQNP
YIKKVYNEFLEKPGSHKAHELLHTHYTERPLFLK*
JGI12104J13512_10045282 FeFe Group B hydrogenase catalytic subunit [Uncultured spirochete SA-8]
MAKENNAVRLKRRLICEVTSLVLEGKLADEIDSIPYAMTNENWETIQCCIHHDRANLRLRLMSLLGYSTE
GMLDVDKPLRTYAQEALSGKKPDAFPLSLMSAACNGCTKSRYIVTNVCQGCLARPCKVNCPKGAVSIINN
QSHIDPELCVNCGLCEKSCPFHAIIKIPVPCEEVCPVGAIQKGEDGIAKIDESKCILCGKCLSACPFGAP
VEKSEIVHVLSALIHGKKLIAMVAPAAMVQFPFPPGKFIAALEHFGFSKVMEVAEAADRVAEAEAREFAE
RLHENNKVLATSCCPSWVRAAGSMGLEQILSDTPSPMLVAAKTAKAQDPDAFTVFIGPCLAKRWEAHRAS
QNSPLVYAVLTSEEVGAMLMAAGIQVNEENEEDLATAGNASVYGRGFAATQGVTAAVKHALSPSAVEVNS
CIVSGITKDTASQLKKVQESDKPLLIEVMACDGGCINGPCQLTNPKVAGAFLERYKAAAEHTTALKSA*
JGI12104J13512_10009791 FeFe Group C1 hydrogenase catalytic subunit [Uncultured spirochete SA-8]
AAREAKKLVADLDPSLRVGMFFISPCTAKVTAIRMPLGYERSLVDAVFSFQDIFPALKKALSRPSETPSR
FAPLQERIARMRRGHGMDWARSDGEIDGLGIEQAVSVDGIQNVIALLEEIDNGKFSSVPYIEALACPGGC
VGGPMAVANPHVARAAMKHIAAAEKTAAAQSGQSEQPAQAGFGWERELQPKPVFVLDRDMLKALQLAEQM
ELITSQLPGLDCGACGSPDCRALAEDIVKGKAVIEDCLVMMRKKTAFNLTNE*
JGI12104J13512_10045285 FeFe Group C3 hydrogenase catalytic subunit [Uncultured spirochete SA-8]
MDSAQVIYTELTECQDCYKCLRECPVKAIQIVRGHARVLEDRCIHCGNCVEICPQKAKKVRSDLERAKIL
IRLRQKTVLSLAPSFIAEFSGIPMQKLIAGCKKLGFSHVSETSVGADAVSESVSKTIAEQQPPLMLSSAC
PAVVQYVDKYLPEMSGFISTACSPMVAHARIIKQTLGPGTAVVFAGPCIAKKREADASEGAVDVALTFQE
LRQWFCEEGIDPEDAAVRDEDSFFLNQAQDGILYPIEGGMVASIKHLKHSDAHCMTYSGIHQIREAVRGI
VDQYLENENNPGNQKTLFLEMLACEGGCVNGPMVQQSGGTVAKRLRVLSEKEKRTERGRVLSEPVSVQDI
AADLTMPQSIRQKPVAAVAVSEEDIKASLASIGKYSRKDELNCSGCGYDSCQAFAAAMFLGKAEKTMCLS
YTRKLAQKKANALLKAMPSAAVVVDASMHIVECNKPFAELLGTDVMELYALKPSLEGADLRKLLPFWEAF
EQVLMPESQDIVASDFQCNGKIVHGSVFSIEKGLLAGGLFQDITAPWIQKDRVISQARKVMSQNLRTVQK
IAYLLGENAAEAEAALTSIIESFQSERSGDSYKSNKE*

Desulfobacterium naphthalenivorans strain N47:

May couple respiratory H_2 oxidation to sulfate reduction as with other Deltaproteobacteria. High sequence homology and conserved binding motifs to NiFe 1a hydrogenases. However, hydrogenase sequence is highly incomplete maybe due to genome assembly errors or incomplete sequencing.
NiFe 1b hydrogenase: Hydrogenotrophic respiration using sulfate, fumarate, nitrate, metals, and azo compounds as terminal electron acceptors. Enzyme transfers H_2-liberated electrons through cytochromes to terminal reductase.

DESUN47_v1_100151|ID:27211809| NiFe Group 1a hydrogenase catalytic subunit (fragment) [Uncultured Desulfobacterium sp N47]
MKVNLGPVTRIEGHLNIETTVENNKIVDARCMGEMFRGFEVFLQGRSPLDAQQITQRICGVCPYAHAVAS

SYAQESVYKLNVPPNGRIMHNLIQGANHLYDYLLQFYQLAALDFVDITAILKYKG*
DESUN47_v1_100152|ID:27211810| NiFe Group 1b hydrogenase large subunit [Uncultured Desulfobacterium sp N47]
MAKRITIDPITRIEGHLRIEVEVADGKVVNAWSSGQMFRGIEMILKGRDPRDAPLFTQRS
CGVCTYVHYLASVRAIEAAVGVQIPENARILRNLLHGTQYQHDHIIHFYHLHALDWVDIL
SALKADPQKTAGLAENVCQARWGGTAYFKQVQDRIKTFVESGQLGPFNNAYWGHPAYVLS
PEANLMAVSHYLEALRLQAKAAQMHAVFGAKNPHLQSLVVGGVTCAMDLTPDRIAEFLYL
WKETQTFVKNVYLPDVLAIGSFYKDWGALGGTSNFLAWGDFPEGEREPDSLFMPRGLIMN
RDISTVKQAEQDKITEHVAHSWYVGNADLHPFQGQTNPQHGDYNPDDRYSWIKAPRYEGE
PCEVGPLARMLVAYGSGKSTARKLVDDTLTQLSIPVTALFSTLGRTAARALETVLVGDAM
EGWIMKLVENLKSGQDNTYQTWTMPDKAIGCGLNDVPRGSLGHWIEIEDKKIKNYQYVVP
STWNLGPRCSNGKLGPVEQALIGTPVADPKRPLEVLRTVHSFDPCIACAVHMIDPRSNEV
YRIQVL*
